# Inventory of the cichlid olfactory receptor gene repertoires: identification of olfactory genes with more than one coding exon

**DOI:** 10.1186/1471-2164-15-586

**Published:** 2014-07-11

**Authors:** Naoual Azzouzi, Frederique Barloy-Hubler, Francis Galibert

**Affiliations:** Institut Génétique et Développement (UMR 6290) CNRS/Université de Rennes 1, Rennes, France

**Keywords:** Zebrafish, Medaka, Stickleback, Fugu, Tetraodon, Cichlids, Olfactory receptors, Coding exons

## Abstract

**Background:**

To help understand the molecular mechanisms underlying the remarkable phenotypic diversity displayed by cichlids, the genome sequences of *O. niloticus, P. nyererei, H. burtoni, N. brichardi* and *M. zebra* were recently determined. Here, we present the contents of the olfactory receptor (OR) repertoires in the genomes of these five fishes.

**Results:**

We performed an exhaustive TBLASTN search of the five cichlid genomes to identify their OR repertoires as completely as possible. We used as bait a set of ORs described in the literature. The cichlid repertoires thereby extracted contained large numbers of complete genes (*O. niloticus* 158; *H. burtoni* 90; *M. zebra* 102; *N. brichardi* 69; *P. nyererei* 88), a small numbers of pseudogenes and many “edge genes” corresponding to incomplete genes located at the ends of contigs. A phylogenetic tree was constructed and showed these repertoires include a large number of families and subfamilies. It also allowed the identification of a large number of OR analogues between cichlids with very high amino-acid identity (≥99%). Nearly 9% of the full-length cichlid OR genes are composed of several coding exons. This is very unusual for vertebrate OR genes. Nevertheless, the evidence is strong, and includes the donor and acceptor splice junction sequences; also, the positions of these genes in the phylogenetic tree indicate that they constitute subfamilies well apart from non-OR G protein-coupled receptor families.

**Conclusions:**

Cichlid OR repertoires are made up of a larger number of genes and fewer pseudogenes than those in other teleosts except zebrafish. These ORs share all identified properties common to all fish ORs; however, the large number of families and subfamilies, each containing few ORs implies that they have evolved more rapidly. This high level of OR diversity is consistent with the substantial phenotypic diversity that characterizes cichlids.

**Electronic supplementary material:**

The online version of this article (doi:10.1186/1471-2164-15-586) contains supplementary material, which is available to authorized users.

## Background

With more than 2,000 species, the cichlid family is by far the largest fish family. Members of this family occupy all sorts of ecological niches everywhere in the world with a remarkable concentration of species in the great African lakes [1, 2]. Consequently, they constitute a good model for studying evolution and adaptation. Also tilapia, *O. niloticus,* is the second most economically important fish in aquaculture [3]. The complete nucleotide sequences of five cichlid genomes have recently been determined: *O. niloticus, P. nyererei, H. burtoni, N. brichardi* and *M. zebra* [4].

All animal species, whatever their ecological niches, have sophisticated systems to sense the outside world for diverse purposes: to avoid attack by predators, to find food and to select appropriate partners to mate and reproduce. Several of these biological systems are based on volatile and soluble odorant molecules, and such systems involve olfactory receptors (OR), the first components of these systems to be identified [5]. ORs are G protein-coupled receptors (GPCR) [6, 7]. They are found at the cilia membrane of olfactory neurons (OSN) [8–10], which are embedded in the olfactory epithelium. The family of genes encoding ORs is the largest known gene family, with approximately 100 members identified in the genomes of insects and up to around 1,000 in mammals [11–13].

Given the importance of the olfactory system in behaviour, it is believed to be important role in shaping species evolution [14–16]. We therefore tried to identify the complete OR gene repertoires of five members of the cichlid family: *O. niloticus, P. nyererei, H. burtoni, N. brichardi* and *M. zebra.* These species are potentially good models for evolution studies and their genomes were recently sequenced [4].

## Results and discussion

### Cichlid OR repertoires

A comprehensive search of the genome sequences of five fishes belonging to the cichlid family (*Oreochromis niloticus, Pundamilia nyererei, Haplochromis (Astatotilapia) burtoni, Neolaprologus brichardi, Mitriaclima zebra*) was undertaken in order to identify their OR gene repertoires. First, we retrieved 183 fish OR sequences from the literature [17, 18] to construct a query set for TBLASTN searches of each cichlid genome sequence determined by the BROAD Institute. This search, performed with a cut-off of 1e^−50^, identified 820 candidates OR genes distributed over 733 contigs. These candidate genes were checked by TBLASTN against a set of 247 (Additional file 1) non-OR GPCRs to eliminate false positives. The remaining candidate genes were checked with TBLASTX against the fish protein database (NCBI, taxiD: 7898).

Table 1 shows the number of genes identified in each of the five cichlid genomes as well as those of five fish models retrieved from the literature [17, 18], GenBank and ENSEMBL databases and after manual curation as part of this study. Their nucleotide and amino-acid (AA) sequences and position in the genome are provided in supplementary materials (Additional files 2 and 3). In addition to complete and potentially functional genes, we identified a number of pseudogenes, edge genes and gene fragments. Pseudogenes are common to any olfactory repertoires [12, 13, 17–19]. OR pseudogenes, which are not retrogenes arose by gene duplication and their prevalence in vertebrate genomes is thought to be a consequence of both gene duplication and nucleotide misincorporation during DNA replication. They appear to be less numerous in the cichlid family olfactory repertoires than in those of other fish, except zebrafish [17, 18]. Many (33/54) of the pseudogenes we found in the cichlid genomes are due to frameshift mutations, whereas the others are due to a nucleotide misincorporation, changing a sense codon into a stop codon (Table 2). The distribution of pseudogenes appears to be largely random; they are found in many different subfamilies (22 out of 57 – see Table 3), whatever their size. Fragment genes are sequences with substantial similarity to a restricted part of a functional gene. They have been identified in many complete genome sequences [20, 21]. Their significance, if any, is unknown. They may correspond to “dead” genes or more prosaically be artefacts generated by sequencing problems. We also identified another type of gene fragments: they are located at the edges of the contigs and correspond to either the 5’ or the 3’ end of an OR. As such, they potentially correspond to actual OR genes whose sequences were interrupted by genome fragmentation into many contigs. If these genome sequences were completed and the mean contig size, which is currently around 10 Kb were much longer, we suspect these edge genes would become complete genes, pseudogenes or gene fragments.

**Table 1:** **OR genes identified in the five cichlid and five fish model genomes**

	***O. niloticus***	***H. burtoni***	***M. zebra***	***N. brichardi***	***P. nyererei***	***D. rerio***	***G. aculeatus***	***O. Latipes***	***T. rubripes***	***T. nigroviridis***
1 coding exon	146	78	94	62	81	143 [16, 17]	78^(a)^	73^(a)^	40 [16, 17]	42^(a)^
>1 coding exon	12	12	8	7	7					
Pseudo	6	6	11	12	8	10	46	28	54	
	+1f	+3e	+2e	+1e	+3e					
					+1 s					
Edge	100	50	28	36	32					
	+1 s				+1 s					
Fragment	0	1	0	3	0					

^(a)^From a larger set of OR sequences retrieved from ENSEMBL and GENBANK, we characterized a subset of true OR genes by multiple alignment of AA sequences, phylogenic tree construction and BLAST analysis. DNA samples used by the BROAD institute to determine the genomic sequences were for each species extracted from a single fish with 2 N chromosomes.

**Table 2:** **Distribution of pseudogenes in the five cichlids**

	***O. niloticus***	***M. zebra***	***P. nyererei***	***N. brichardi***	***H. burtoni***
Frameshift	4	8	9	6	6
In frame stop codon	3	5	3	7	3

**Table 3:** **Distribution of OR into families and subfamilies**

	Cichlids					Fish models				
	***N. bri.***	***N. bur.***	***P. nye.***	***O. nil.***	***M. zeb.***	***D. rer.***	***G. acu.***	***O. Lat.***	***T. rub.***	***T. nig.***
A1	3(e2,p1)	6(e2,p2,f1)	6(e2,p1)	14(e8,p1)	8(e1,p2)			2		
A2	3(e1,p1)	3(e2,p1)	5(e2)	4(e2)	6(e2)					
A3	1(p1)	2(e1)	3	2(e1)	2(e1)					
A4	1(p1)	2	2	2(e2)	2		1	8		
A5	3(e2,p3)	4(e1)	4(p1)	4(e1)	5(pe1)		10	2		
A6	(e1)	1(p1)	1		1			4		
A7									3	3
A8									1	
B1		1	1	1(e1)	1					
C1	1	1	1	(e1)	1				1	1
D1	3(e2)	4(e6)	7(e3,p3)	11(e14)	7(p2)					
D2							6		2	1
E1	7(e3)	12(e1,pe1)	8(e2,pe1)	13(e5,p2)	7(e7,pe1)		5	3		1
E2	(e1)	2	2	3	2		2		1	3
F1						12				
F2						1				
F3									1	
F4	(pe1)	1	1	1	1		1		1	3
F5	1	1	(p1)	1	1		3		1	4
G1	1	1	1	1	1			1		1
H1						6				
H2	1	1	1	1	1		1	1	2	
H3	2(e2)	5	6	6(e3)	3(e1,p1)		1		2	
H4									5	3
H5	2(e1)	(e3)		4(e4)	4		4	2		
H6	2(e2,p1,f1)	3(e2,p1)	4(e1,p1)	10(e1,p2)	4(e1)		7			
H7									1	2
I1		1	1	3	1		2	2		1
J1						3				
J2	1	(e1)	1	1	1		1	2	1	
J3	1(e1)	(e2)	1(e1)	(e1)	1	3	2	1		1
J4						1				
J5	1	1	1	1	1	2	1	1	1	
K1	1	1	1	1	1		1	1		
K2						6				
K3						6				
K4	1	1(e1)	2	2	1(e1)		2	2	1	
K5	(e1)	1(e2)	2(e1,ps1)	4	2(p1)		1	4	3	
K6								3		
K7				1(e2)						
K8	(e1)							1		
L1						12				
L2	5(e2,f1)	4(e6,p1)	5(e4,pe1)	16(e15,p1)	6(e2)					
M1						3				
M2	(e1)	(e3)	2	(e2)	1		5	2	2	1
N1						2				
N2	2(e2)	2(e1)	2(e1)	1(e3)	2(p1)		11	9	1	1
N3	1(e3,p2)	1(e5,p1)	2(p1)	3(e4)	6(p1)					
N4						11				
N5						12	1			
N6	3	2(pe1)	2(e1)	7(e2)	1(e3)			1		
O1								1		
O2						1				
O3						2				
O4						5				
O5	2	1(e1)	2(e1)	2(e4)	1(e4)		3			
O6						5				
O7	1(e1)	(e1)		(e4)	1		1	2	2	1
O8	1(e1)	(e2)	1	1(e1)	1		1			1
O9		(e1)	(e1)							
P1						1				
P2	1	1	1	1	1		1	1	1	1
P3	2	2	(e2)	4(e1)	2			2		
P4						3				
Q1									1	
Q2	1	1	1	(p1)	1			1		
R1								1		
R2		1(e1)	1		1					
R3									2	4
R4	(e2,p1)	4(e1)	(e2)	9(e8)	3(p1)			3		
R5	1			(e3)						
S1						2				
S2	1(e3,p1)	3(e1)	1(e2)	11(e2)	3(e2,p1)			2		
S3	2	2(e1)	1(e1)	4(e1)	1(e2)			3	1	
S4									1	1
T1						1				
T2						1				
T3						6				
T4		1	(pe1)							
U1	1	1	1	1	1	1				1
V1	1	1	(e2)	1	1					
W1										1
W2								2		
W3	1	1	1		1					
W4										1
W5	1	1(e1)								
W6										1
W7	2	2	2	4(e2)	1			1		1
W8	1	1	1	1	1					
W9	1(e1)	1		(e1)	1			1		
X1		1(e1)	(e1)	(e2)	(e1)					
Y1	1								1	
Z1	1									
Z2								1		
AB1						1				
AB2	(f1)	1		1	(p1)		4			
AC1			(e3)							
AD1										1
AD2						1				
AE1						1				
AE2						2				
AF1						5				
AG1						5				
AG2						3				
AH1						1				
AI1									1	
AJ1						5				
AK						9				
AL						1				
AM						1				
AN										2
**Total**	69	90	88	158	102	**143**	**78**	**73**	**40**	**42**
	**e36, p13, f3**	**e50, p9, f1**	**e33, p12**	**e101, p7**	**e28, p13**					
S/F	**49**	**51**	**47**	**47**	**48**	**37**	**26**	**33**	**26**	**25**
F	**24**	**26**	**25**	**24**	**25**	**22**	**12**	**16**	**17**	**18**

ORs were classified into families and subfamilies according to the phylogenetic tree and the percentage of AA identity calculated by MAFFT alignments. Families (F) were named by letters and subfamilies (S/F) by Arabic numbers (left column). For example, A1 (family A subfamily 1) contains 3 complete genes, 2 edge genes (e2) and 1 pseudogene (p1) from *N. brichardi*. Of the 376 model fish OR (143 zebra fish/*D. rerio,* 78 stickleback/*G. aculeatus*, 73 medaka/*O. latipes*, 40 fugu/*T. rubripes* and 42 tetraodon/*T. nigroviridis* – for more details see Additional file 4) 182 were in subfamilies also containing one of more cichlid ORs.

As shown in this Table the 143 zebrafish ORs are distributed into 37 sub-families and 22 families. A similar number of sub-families was reported by Alioto and Ngai [18] analyzing the same set of ORs, however they described height families only, four of them corresponding to several families in our study. Correspondences between the families in [18] and the families described in this work are as follow: Families A, B, C and G described in [18] correspond to families P, AB1, O and L respectively (this work); Families D [18] correspond to AH, M and N; Family E [18] corresponds to families F, H, AD, AE, AF, AG; Family F [18] corresponds to families K and J; Family H [18] corresponds to families S, T, U, AJ, AK, AL and A.

**Figure 1:**
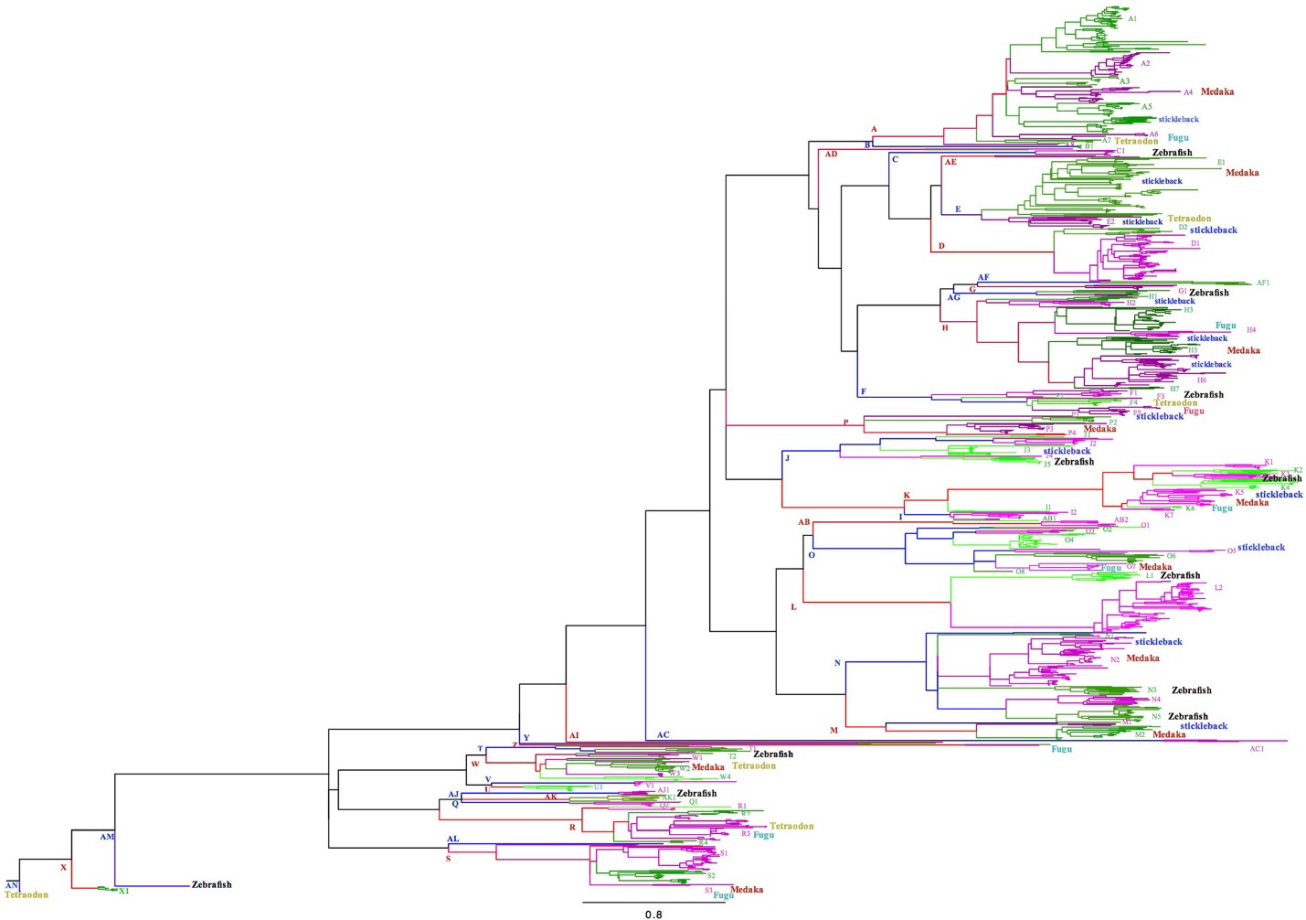
**Phylogenetic tree for the cichlid and fish model ORs (see also Table** 1 **and Additional file** 2 **).** Family names A to AN are alternately coloured in red and blue and similarly sub-families designated by Arabic numbers are coloured in green and purple.

We used MAFFT [22] and PHYML [23] to align the OR AA sequences and construct a phylogenetic tree with the OR repertoires of the five cichlids and the 376 OR known AA sequences identified in the genomes of zebrafish (*Danio rerio*), medaka (*Oryzias latipes),* stickleback (*gasterosteus aculeatus*), takifugu (*takifugu rubripes*) and tetraodon (*Tetraodon nigroviridis*) (Table 1 and Additional files 2 and 4). OR repertoires are usually classified into classes, families and subfamilies according to the percentages of AA identity shared by the different ORs. In this study, we used the same 40% and 60% AA identity thresholds as proposed by Glusman et al. [19]. Each cichlid contains ORs from 24 to 26 families and between 47 and 51 subfamilies. There are, however, only 56 subfamilies in total indicating that most subfamilies are common to the five cichlids (Figure 1, Additional files 2 and 4, Table 3). Compared with the OR cichlid repertoires, four of the fish model repertoires (stickleback, medaka, fugu and tetraodon) have many fewer sub-families (25 to 33) grouped into 12 to 18 families. However the OR zebrafish repertoire appears more similar to the cichlid repertoires with 37 sub-families and 22 families. Thus, among the 507 complete cichlid sequences and the 376 complete model fish sequences, there are 111 subfamilies in all; 37 of these subfamilies contain both cichlid (n = 347) and model fish (n = 182) sequences (Figure 1, Additional file 2 and Table 3). This level of subfamily overlap between the cichlid and the model fish sequences suggests substantial divergence between the cichlid and other teleost repertoires. Of interest regarding the evolution of the Nile tilapia and lake cichlids is the existence of a number of pairs or triplets and even quadruplets genes sharing 99% or more amino-acid sequence identity (Table 4 and Additional file 5). The large number (roughly 50% of each repertoire) of OR genes sharing 99% AA identity observed between *H. burtoni, P. nyereri* and *M. zebra* is in agreement with their close phylogenetic positions [24]. Compared with this, we found fewer paralogous OR pairs except for tilapia for which we identified 7 pairs, 1 triplet and 2 quadruplets of genes with an AA identity above 99%. This last finding is in light of the larger number of ORs present in this species. This observation is in agreement with Nikaido et al. [25] who, by analyzing the expansion of vomeronasal type 2 receptor-like (OlfC) genes in cichlids, noted that recently duplicated paralogs are more variable than orthologs.

**Table 4:** **Distribution of OR gene pairs, triplets and quadruplets sharing a strong percentage level of nucleotide and AA identities**

Pairs		Bur	Zeb	Bri	Nye	Til
	Bur	2	9	0	11	0
	Zeb		0	0	10	0
	Bri			0	1	0
	Nye				0	0
	Til					7
**Triplets**	Bur	Zeb	Nye	37		
	Bur	Bur	Nye	1		
	Til	Til	Til	1		
**Quadruplets**	Bur	Zeb	Nye	Bri	2	
	Bur	Zeb	Nye	Til	1	
	Til	Til	Til	Til	1	

Olfactory receptors sharing 99% of AA identity were identified from the phylogenetic tree. The greatest numbers of pairs or triplets were found between *H. burtoni*, *M. zebra* and *P. nyererei,* in agreement with their closer phylogenetic relatedness. In *O. niloticus* 7 pairs, 1 triplet and 2 quadruplets of paralogous genes were identified consistent with this repertoire having undergone a higher level of duplication. The list of genes is shown in Additional file 5.

### Evolution of the dN/dS ratio

The dN/dS ratio also named KaKs is commonly used to measure the selective pressure exerted on genes during evolution. We used the Nei-Gojobori method modified by Zhang [26] to calculate this ratio for each pair of OR genes from the 14 cichlid OR families containing four or more genes. The mean dN/dS values for these families extend from 0.28 for family G, which includes only one subfamily to 0.50 for family L made of two subfamilies (Table 5a and Additional file 6). These values are clearly above the 0.11 mean value calculated for 1,880 human rodent orthologous gene pairs [27] and similar to the values obtained for medaka and stickleback OR [18]. Although below 1, the theoretical limit between negative and positive evolution trends, the values obtained indicate a tendency for a positive selection favouring OR repertoire diversification as previously noted for other fishes [17, 28] and mammals [29, 30]. However, it is important to note that the different OR pairs behaved very differently. As detailed in Additional file 6, we identified a number of OR gene pairs with only synonymous mutations as in families A, H and W and OR pairs with only non-synonymous mutations as in families A, I and K. Table 5b displays the number of OR pairs with dN/dS ratios above 1. Interestingly, intra-species dN/dS ratios (paralogous comparison) have values that are similar to those found in inter-species values (orthologous comparison) as indicated by a ratio close to 1, suggesting a similar evolution of the five cichlid OR repertoires (Table 5c).

**Table 5:** **dN/dS ratios for the various OR gene pairs identified in 14 families**

Panel a														
Family names		Number of sub-families			Number of genes		Means			Min.		Max.		
Fam A		6			100		0.40±0.09			0.00		>10		
Fam D		1			32		0.44±0.10			0.15		1.30		
Fam E		2			56		0.40±0.11			0.10		2.27		
Fam G		1			5		0.28±0.10			0.18		0.41		
Fam H		4			50		0.41±0.14			0.00		1.14		
Fam I		1			6		0.43±0.29			0.00		>10		
Fam K		4			22		0.29±0.18			0.12		>10		
Fam L		2			36		0.50±0.12			0.04		1.20		
Fam N		5			37		0.39±0.14			0.18		1.79		
Fam O		3			14		0.44±0.10			0.12		0.83		
Fam P		3			15		0.37±0.10			0.07		0.86		
Fam R		4			20		0.43±0.08			0.19		0.88		
Fam S		2			26		0.39±0.09			0.14		1.19		
Fam W		5			24		0.32±0.13			0.00		1.48		
**Panel b**														
**Family names**	**A**	**D**	**E**	**G**	**H**	**I**	**K**	**L**	**N**	**O**	**P**	**R**	**S**	**W**
dN/dS	11	1	4	0	9	2	2	4	5	5	0	0	1	2
dN/dS	2	0	0	0	5	0	2	0	0	0	0	0	0	0
**Panel c**														
**Family D**														
bri/bri		0.436					bri/cich					0.446		
bur/bur		0.385					bur/cich					0.411		
zeb/zeb		0.439					zeb/cich					0.432		
nye/nye		0.422					nye/cich					0.447		
til/til		0.451					til/cich					0.432		
**Family E**														
bri/bri		0.380					bri/cich					0.374		
bur/bur		4.414					bur/cich					0.408		
zeb/zeb		0.378					zeb/cich					0.382		
nye/nye		0.440					nye/cich					0.416		
til/til		0.396					til/cich					0.382		
**Family H**														
bri/bri		0.399					bri/cich					0.407		
bur/bur		0.448					bur/cich					0.367		
zeb/zeb		0.407					zeb/cich					0.399		
nye/nye		0.431					nye/cich					0.398		
til/til		0.414					til/cich					0.399		
**Family L**														
bur/bur		0.503					bur/cich					0.527		
bri/bri		0.507					bri/cich					0.523		
til/til		0.494					til/cich					0.480		
zeb/zeb		0.490					zeb/cich					0.478		
nye/nye		0.464					nye/cich					0.496		
**Family N**														
bur/bur		0.328					bur/cich					0.363		
bri/bri		0.359					bri/cich					0.375		
til/til		0.425					til/cich					0.401		
zeb/zeb		0.446					zeb/cich					0.426		
nye/nye		0.360					nye/cich					0.426		

dN/dS ratios were calculated for each pair of OR genes identified in the 14 families with 4 or more genes (panel a). The numbers of OR pairs per family with a dN/dS ratio above 1 are indicated in panel b. For those in which dS was 0, the dN/dS was arbitrarily given the value >10. In panel c, dN/dS ratios of pairs of paralogous genes (columns 2 and 6) were compared with the ratios of pairs of orthologous genes (columns 4 and 8).

We also calculated the dN/dS ratio of the different OR protein domains (TM regions, internal and external loops) for five families (D, E, H, L and N) selected for their high number of genes. As shown in Table 6 and Additional file 7a to f, the dN/dS values are highly variable along the different parts of the molecules with the TM regions having a tendency to be higher, although this is not always the case (see TM 6 and TM7 of family E). On the other hand, no clear tendency can be drawn for the dN/dS ratio of the internal and external loops, although one should note that the standard deviations are very high in all cases, indicating that the various OR pairs behaved differently.

**Table 6:** **dN/dS ratios for various OR protein domains**

Family D	Entire molecule				
**32 genes**	**0.44±0.10**	TM1	0.28±0.18	IN1	0.36±0.33
		TM2	0.24±0.23	IN2	0.69±0.72
		TM3	0.64±0.46	IN3	0.26±0.26
		TM4	0.83±1.01	OUT1	0.22±0.22
		TM5	0.58±0.39	OUT2	1.14±1.06
		TM6	0.42±0.41	OUT3	0.80±0.56
		TM7	0.36±0.35		
**Family E**	**Entire molecule**				
**56 genes**	**0.40±0.11**	TM1	0.57±0.27	IN1	0.24±0.01
		TM2	0.52±0.48	IN2	0.42±0.37
		TM3	0.73±0.79	IN3	0.32±0.37
		TM4	0.69±0.39	OUT1	0.43±0.24
		TM5	0.70±0.44	OUT2	0.30±0.23
		TM6	0.23±0.21	OUT3	0.41±0.38
		TM7	0.21±0.12		
**Family H**	**Entire molecule**				
**50 genes**	**0.41±0.13**	TM1	0.56±0.47	IN1	0.35±0.24
		TM2	0.24±0.22	IN2	0.23±0.15
		TM3	0.48±0.43	IN3	0.34±0.24
		TM4	0.53±0.39	OUT1	0.39±0.51
		TM5	0.76±0.68	OUT2	0.64±0.36
		TM6	0.84±0.51	OUT3	0.67±0.72
		TM7	0.27±0.29		
**Family L**	**Entire molecule**				
**32 genes**	**0.50±0.12**	TM1	0.56±0.42	IN1	0.23±0.11
		TM2	0.59±0.58	IN2	0.41±0.38
		TM3	0.76±0.43	IN3	0.59±0.33
		TM4	1.48±1.54	OUT1	0.29±0.34
		TM5	0.63±0.36	OUT2	0.35±0.22
		TM6	0.72±0.42	OUT3	0.40±0.34
		TM7	0.56±0.58		
**Family N**	**Entire molecule**				
**32 genes**	**0.39±0.14**	TM1	0.45±0.35	IN1	0.57±0.35
		TM2	0.79±0.37	IN2	0.60±0.55
		TM3	0.68±0.57	IN3	0.41±0.33
		TM4	0.95±0.54	OUT1	0.35±0.27
		TM5	0.56±0.41	OUT2	0.24±0.11
		TM6	0.60±0.41	OUT3	0.40±0.13
		TM7	1.20±1.07		

dN/dS ratios for the 7 TM regions, and the 3 external and 3 internal loops for the 4 largest families were calculated. TM regions and loops were identified with PolyPhobius.

### Conserved amino-acid motifs

**Figure 2:**
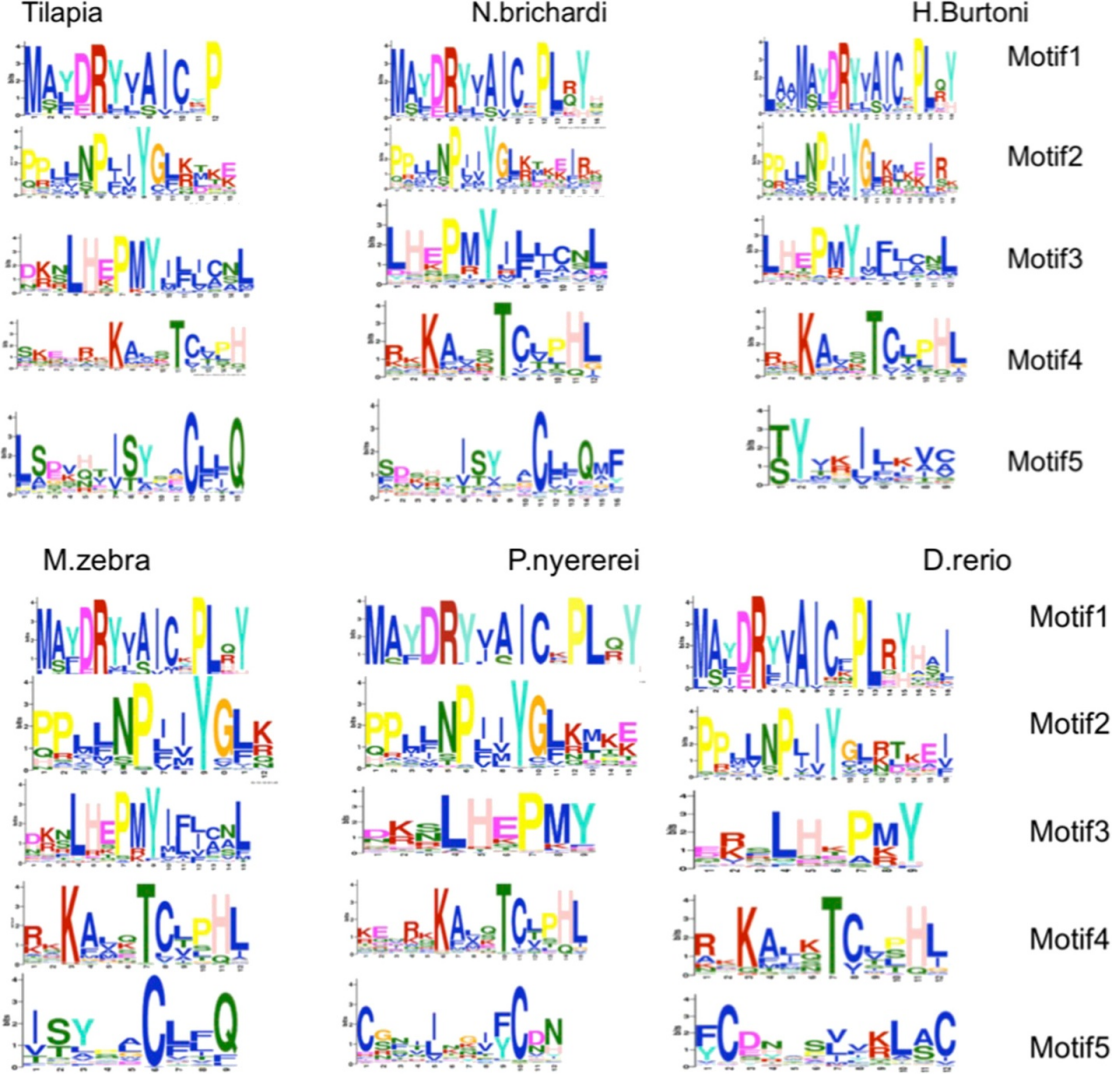
**WebLogo graphical representation of the 5 most significant motifs identified by MEME in cichlid and** ***D. rerio*** **OR repertoires and located at particular positions: motif 1: internal loop 2, motif 2: internal loop 1, motif 3: TM7-intracellular extension and motif 4: internal loop 3.** Motif 5 is not well conserved and its position differs between fish species.

**Figure 3:**
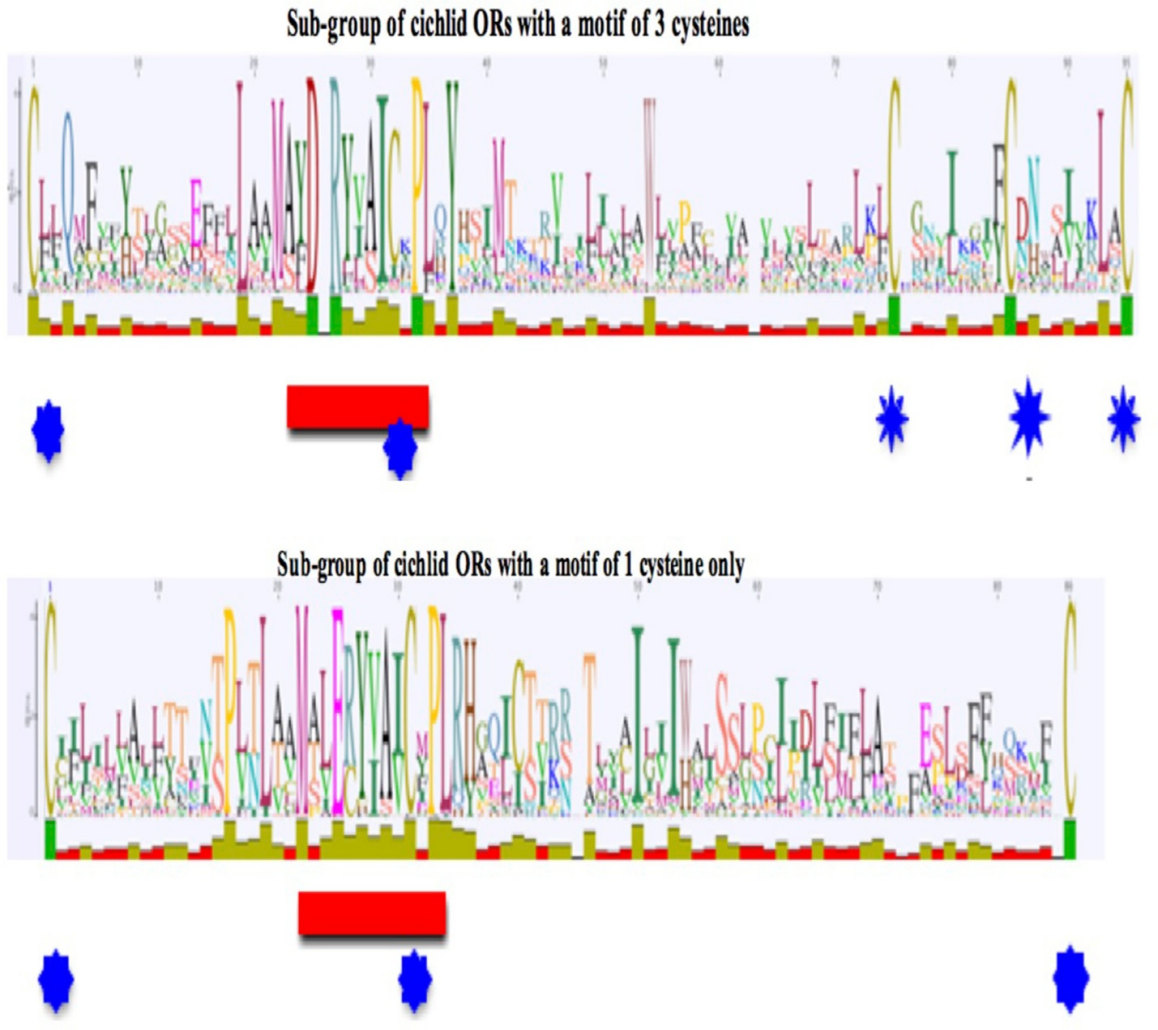
**WebLogo representation of the AA conservation around the MAYDRY motif.** Multiple alignment with MAFFT followed by PHYML clustering revealed two subgroups of cichlid OR: one with a classical MAYDRY motif followed by 3 cysteine residues indicated by a blue star; and a second with an altered MAYDRY motif in which the aspartate residue (D) is replaced by a glutamate residue (E). See Additional file 9 for the genes of each of these two groups and a complete alignment of their sequences.

ORs are GPCRs that belong to the rodhopsin subfamily. They are characterized by a number of AA patterns of which the MAYDRY motif in the internal loop 2 is the most characteristic. These patterns have often been used for mining whole genome sequences for OR identification [12]. We used the MEME program [31] to search for the five best motifs for each of the five cichlids and *D. rerio*. The first four motifs identified for each fish are very similar in both sequence and position between fishes (Figure 2). They are also not very different from those identified in OR mammals, despite the substantial distance from any common ancestor [13, 14, 32, 33]. However, no clear fifth pattern was identified for the fish ORs. ORs are transmembrane proteins. Although no signal peptide has been identified in their sequences using Polyphobius [34], an N-glycosylation site, Asn N Ser/Thr has been detected in all ORs identified to date [18]. OR protein sequences identified in the five cichlid genomes were inspected for the presence of such putative glycosylation sites using NetNGlycserver [35]. For each of them, one to several putative sites were proposed (Additional file 8). But interestingly, in all cases, a site with a very high score was present in close proximity to the extracellular N-terminal part in agreement with the importance of the glycosylation site of this region for the correct expression of the OR and membrane trafficking [36]. OR proteins are also characterized by the presence of cysteine residues located at fixed positions in particular in the extracellular loop 1 and 2 (EC1 and EC2) regions as observed for all *D. rerio* [18] and mouse OR genes [32, 37]. We observed a similar situation for all complete cichlid receptors identified in this study. However, we noted the existence of two subgroups of ORs: one subgroup of ORs with one cysteine residue only in EC2 and one subgroup with three cysteine residues. Interestingly, these two groups have slightly different MAYDRY motifs with an E replacing D in the subgroup with one cysteine residue (Figure 3 and Additional file 9). These two groups differ also by motif 2 located in the cytoplasmic C terminal extremity. Whether these differences affect the recognition and binding of the G alpha subunit and the transduction signal is a matter of interest [38].

Moreover, these feature inspections of AA alignments (Additional file 9) made it possible to identify a number of positions occupied by the same or nearly the same AA, pointing to positions submitted to strong purifying constraint during evolution like, for example, another cysteine residue in the N terminal extension also previously noted [39].

### Spliced OR genes

**Figure 4:**
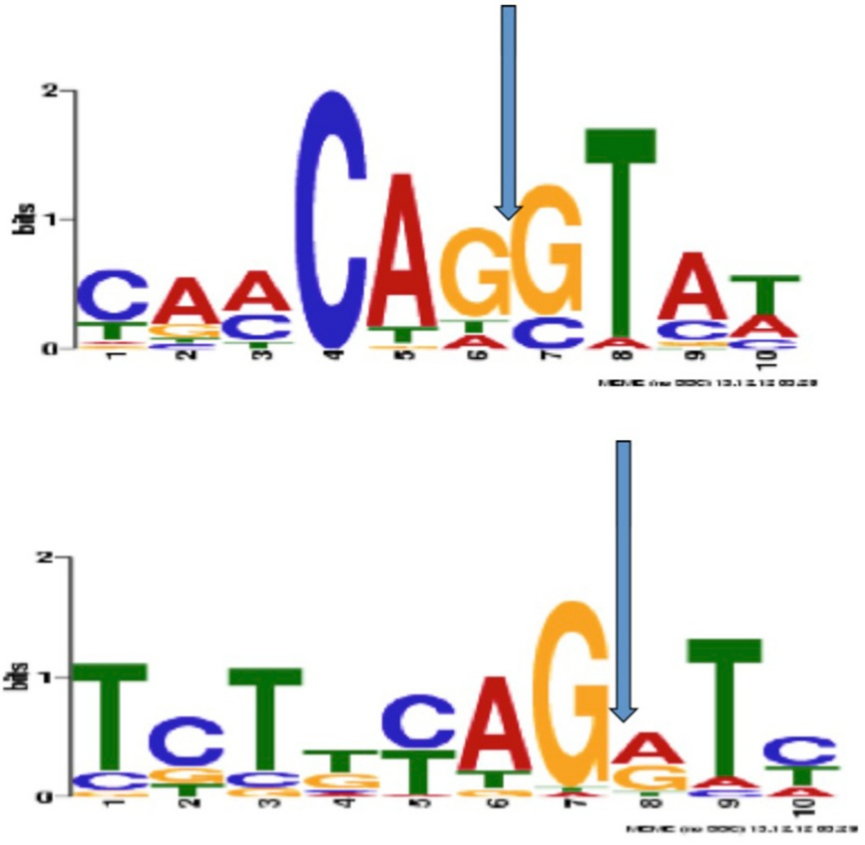
**Sequence logo representation of donor and acceptor splice sites identified in cichlid OR genes aligned onto their cognate contigs and manually corrected using both MAFFT multiple alignment and the FSPLICE tool (Softberry, Fish model).**

**Figure 5:**
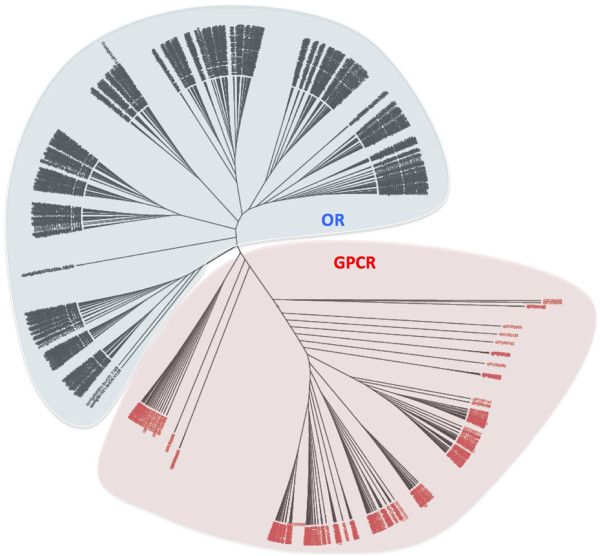
**Phylogenetic tree constructed with the cichlid OR repertoires (in blue) and 247 non-OR class A GPCRs (Additional file** 1 **) (in red).** This tree clearly shows that the cichlid ORs are clearly distinct from the non-OR class A GPCRs.

Our searches for OR genes unexpectedly identified a number of spliced ORs. Among the 507 complete OR genes identified in the five cichlids, 46 ORs (9%) consist of two to four coding exons (Table 1). Proof that these spliced cichlid OR genes are functional will require transcriptional and functional analysis. Nevertheless, there are four types of evidence indicating that they are active OR genes: (i) the splice site junctions, (ii) the intron position, (iii) the BLASTX analysis and (iv) their position within the phylogenetic tree.

i. Exon-intron boundaries. Nucleotide sequences overlapping the exon-intron boundaries were identified by the alignment of the spliced OR sequences with their cognate contig sequences. With the MEME suite, we identified two nucleotide motifs (Figure 4) defining exon boundaries while maintaining the reading frames open through the junction of the adjacent exons. Interestingly, these two motifs are similar to those found at mammalian gene exon/intron boundaries [40]. Most of these donor/acceptor sites were also predicted by the FSPLICE program [41] with the FISH model weight matrix (data not shown).
ii. Introns were in nearly the same positions in all the ORs (Table 7): By comparing the gene nucleotide sequences and the amino acid sequences, we inferred the intron position relative to the 2D OR structure (Additional file 10) and noticed a nearly fixed position (Table 7). In 27 of the 31 OR genes with one intron interrupting the coding frame, the intron is in phase 0 and in phase 2 for the remaining; also for 26, the intron is within the sequence encoding the MAYDRY motif in the first internal loop. Similarly, of the 11 OR genes with two introns within the coding sequence, first intron is in the sequence encoding the extracellular part in five, and in the sequence encoding internal loop 2 in a further five. All 11 OR genes with two introns have their distal intron in the sequence encoding external loop 2.
iii. BLASTX analysis. All proteins identified by TBLASTN search were subjected to a BLASTX search against the non-redundant NCBI protein database. Only proteins giving a strong hit with OR proteins, and no hits or a meaningless hit with other GPCRs, were kept as true ORs.
iv. Phylogenetic analysis. The AA sequences of 507 cichlid ORs and of 247 non OR class A (Additional file 1) GPCRs identified in GenBank were aligned with MAFFT, and a tree constructed with PHYML and drawn with FigTree [42] (Figure 5). All ORs, with one or several coding exons, clearly form a separate branch from the non OR class A GPCRs; this argues for them being true OR genes and not another type of GPCR. Most of the spliced cichlid ORs (39/45) cluster in families or subfamilies not shared by ORs with only one coding exon. Also, the spliced OR genes are grouped according to the number of their coding exons. For example, family W is made up of 24 ORs all with two coding exons, whereas families I and U contain six and five ORs, respectively, all with three coding exons (Table 8). The clustering of the ORs with two or more coding exons into particular families indicates that splice OR genes arose before cichlid speciation and have evolved independently from single coding-exon ORs. However, the possibility that there was horizontal transfer cannot be formally excluded.

**Table 7:** **Intron positions within OR genes**

	OR names	Last codon	Intron phase	Codon position	Intron position
**2 coding exons**	contig034988-NyeORs.A033	GTC.AG	2	159	TM4
	contig050024-NyeORs.W129	CAA.CAG	0	50	IN 1
	contig050025-NyeORs.W131	AAC.AAG	0	50	IN 1
	contig050025-NyeORs.W130	CAC.CAG	0	50	IN 1/TM2
	contig050026-NyeORs.W132	CAC.CAG	0	52	IN 1
	contig090286-BriORs.W112	CAA.CAG	0	50	IN 1
	contig090288-BriORs.W113	CAA.CAG	0	50	IN 1
	contig090291-BriORs.W114	AAC.AA	2	41	TM1
	contig090292-BriORs.W115	CAG.CAG	0	52	IN 1
	contig090301-BriORs.W116	TAT.CAG	0	49	IN 1
	contig046002-ZebORs.K090	AAG.TAT	0	24	N ter
	contig067811-ZebORs.W140	CAA.CAG	0	50	IN 1
	contig062664-ZebORs.W141	AAA.CA	2	43	IN 1
	contig025842-ZebORs.W142	AAA.CAC	0	51	IN 1
	contig025841-ZebORs.W139	AGT.ATC	0	52	IN 1
	contig045454-BurORs.W131	CAA.CAG	0	50	IN 1
	contig066785-BurORs.W148	TAT.CAG	0	49	IN 1
	contig045453-BurORs.W132	CAC.CAG	0	50	IN 1
	contig045452-BurORs.W133	CAC.CAG	0	52	IN 1
	contig045453-BurORs.W134	AAC.AAG	0	50	IN 1
	contig041638-BurORs.W135	AAA.CA	2	43	IN 1
	contig041640-BurORs.V144	CGA.CAC	0	59	IN 1
	contig049605-BurORs.AB153	AAC.AGT	0	77	IN 1
	contig046708-TilORs.K143	AAG.TAT	0	24	N ter
	contig027203-TilORs.W238	CAC.CAG	0	50	IN 1
	contig027204-TilORs.W239	AAC.CGG	0	50	IN 1
	contig027206-TilORs.W240	CAC.CAG	0	50	IN 1
	contig027209-TilORs.W241	CAC.CAG	0	50	IN 1
	contig027202-TilORs.W243	AAA.CAC	0	51	IN 1
	contig046717-TilORs.AB275	TAT.GTG	0	72	TM1
	contig027194-TilORs.V262	CGA.CAC	0	59	IN 1
**3 coding exons**	contig046495-NyeORs.I079	GAG.AGG	0	121	IN 2
		ACA.ATC	0	232	OUT3
	contig051999-NyeORs.U128	TAT.CA	2	15	N ter
		CAC.CAG	0	54	IN 1
	contig090301-BriORs.U109	TAT.CAG	0	16	N ter
		CAG.GAT	0	56	OUT1
	contig025847-ZebORs.U137	TAT.CAG	0	16	N ter
		CAC.CAG	0	54	IN 1
	contig026932-ZebORs.I082	GAC.AG	2	125	IN 2
		GAC.ATC	0	200	OUT2-TM5
	contig048321-BurORs.I076	GAC.AG	2	125	IN 2
		ATC.TAT	0	201	OUT2
	contig041640-BurORs.U130	TAT.CA	2	15	N ter
		CAC.CAG	0	54	IN 1
	contig046690-TilORs.I128	GAC.AG	2	120	IN 2
		AAC.AT	2	194	OUT2-TM5
	contig046694-TilORs.I129	GAC.AG	2	125	IN 2
		ATC.TAT	0	201	OUT2-TM5
	contig046695-TilORs.I130	GAC.AG	2	120	IN 2
		ATC.TAT	0	196	OUT2-TM5
	contig027194-TilORs.U236	TAT.CA	2	15	N ter
		CAC.CAG	0	54	IN 1
**4 coding exons**	contig090302-BriORs.V122	CAC.AG	2	80	IN 1
		CTT.CTG	0	127	OUT1
		GTG.CAG	0	269	TM6
	contig025847-ZebORs.V149	CAC.AG	2	62	IN 1
		CTT.CTG	0	109	OUT1
		GTG.CAG	0	251	TM6
	contig041641-BurORs.T129	CCC.AG	2	48	IN 1
		AAC.AAG	0	96	OUT1
		GTC.CAG	0	184	TM5

*N ter*: Extracellular end, *IN*: Internal loops, *TM*: Transmembrane region, *OUT*: External loops. OR belonging to the different cichlids are alternatively colored.

**Table 8:** **Distribution of OR genes with more than 1 coding exon among the families of OR**

Families	Sub-families	Genes numbers	Genes with >1 coding exon	Number of exons
Fam A	6	100	1	2
Fam K	4	22	2	2
Fam T	1	2	1	4
Fam U	1	5	5	3
Fam V	1	4	4	2 genes/2 exons
				2 genes/4 exons
Fam W	5	24	24	2
Fam AB	1	4	2	2

## Conclusions

The olfactory system enables animals to sense the outside world and contributes to searching for food and sexual partners. It may also help prevent or avoid attack by enemies and predators. As such, olfaction is a vital function. Olfactory receptors (OR) are transmembrane proteins found on the surface of olfactory neurons, and are the first component of the olfactory transduction cascade. They bind odorant molecules inducing a cascade of protein interactions that transform a primary chemical signal into an electrical signal that is conveyed to the brain; there, it is decoded and stored, leading to an appropriate response [14, 43]. Here, we report the identification of repertoires of OR genes in the genomes of five cichlids, recently sequenced by a consortium led by the BROAD Institute [4].

To identify these five olfactory repertoires as completely as possible, we applied the strategy used by Alioto and Ngai [18] for the analysis of the zebrafish OR repertoire. We performed an exhaustive TBLASTN search for sequences corresponding to a set of fish olfactory receptors retrieved from the literature [17, 18]. Although, all OR genes previously found in vertebrates consist of an uninterrupted open reading frame (ORF) [11–14], we did not limit our search to positive hits longer than 700 nucleotides as Alioto and Ngai did [18]. Rather, we retrieved all hits with an e-value cut-off of 1e^−50^. We then checked each of these candidate genes or gene fragments against a set of non-OR non-TAAR class A GPCR sequences. All genes retained as true ORs shared a number of predicted properties characterizing this class of molecule [5]: an extracellular N-terminal extremity, seven hydrophobic transmembrane stretches of 21 to 26 amino-acids each, and an intracellular C-terminal extremity (Additional file 10). They have an N-glycosylation site, Asn N Ser/Thr, near the N-terminus involved in addressing these proteins to the cellular membrane [36].

Characteristic amino-acid motifs have been identified in all ORs. These patterns of AA as well as their localization inside the molecules have often been used as a means for retrieving the corresponding genes from newly determined genome sequences [13]. In the present study, we started with a different perspective that consisted in the characterization of these proteins as actual OR and not in their mining. We identified with MEME, four AA motifs, shared by the five cichlids as well as *D. rerio*. Although, minute variations can be observed when comparing the different motifs between these fishes, they looked very much the same. Interestingly, some striking similarities can be observed with the AA motifs characterizing rat and dog ORs [13]. Finally, we noted the presence of cysteine residues at positions shared by all ORs (Additional file 9). These residues are of prime importance for the correct folding of proteins and mutations changing these cysteine residues into another AA have been shown to often impair their function [44, 45]. Moreover, these alignments enable the identification of regions or amino-acid positions strongly conserved and others highly variable, such as in the ligand binding pocket [46, 47]. The birth and death hypothesis as previously described [48, 49] with a relaxed purifying selection favouring multiple amino-acid changes explains the large repertoire of ORs found in numerous species [11–14] as well as their partition in many families and subfamilies. Their multiplicity, which enables the detection of a large number of odorant molecules, favours the search for food or sexual partners and as such, is a good observer of evolution. Considering the size of the OR repertoires, even in the absence of formal and definitive numbers owing to the non-completeness of the genome sequences, it appears that the numbers of potentially active OR genes found in these five cichlids would be higher than those identified in other teleosts [17, 18], except zebrafish. In addition, fewer pseudogenes were identified in the cichlid genomes, even if one cannot exclude that some of the edge genes could in fact correspond to pseudogenes. But perhaps more importantly from an evolutionary point of view, the OR cichlid repertoires are dispersed in many more sub-families, than most of the teleost repertoires except for zebrafish (Table 3) Nevertheless, despite this substantial variability of OR sequences, there are numerous ORs which share more than 99% AA sequence identity between species (Table 4).

The discovery of a subset of OR genes in the cichlid genomes made of more than one coding exon came as a surprise. While OR genes belong to Class A of the GPCR superfamily within which numerous genes are made of several coding exons, based on their original discovery, it was assumed that vertebrate OR genes were made of two exons, a 5’ non-coding exon and a second exon encompassing an ORF coding for a protein of 300–330 AA [5]. It was due to this belief that several authors mining genome sequences restricted their search to long ORF or eliminated short ORF afterwards [18].

Definitive proof of their status as OR will be provided by functional studies, implying RNA transcription analysis of olfactory epithelium and identification, at least for some of their ligands. Obviously, such studies are out of the scope of the present paper. There are several arguments, in particular the results of the BLAST analysis and their phylogenetic positions (arguments 3 and 4 developed in the Results section) strongly indicate these multi-coding exon genes code actual ORs. Functional studies are required for a conclusive demonstration that these sequences are indeed active OR genes. This will probably involve analysis of RNA transcription in olfactory epithelium and identification, at least for some of them, of their ligands. Such studies are beyond the scope of the present paper. Nevertheless, our work provides several arguments, in particular the results of the BLAST analysis and the phylogenetic positions of the sequences ((iii) and (iv) in the Results section), strongly indicating that these multi-coding exon genes indeed encode true ORs.

We performed a TBLASTN search to determine whether these multi-coding exon genes are specific to the cichlids or whether they had been overlooked during the mining of other fish genomes. We searched the OR fish repertoires in NCBI and ENSEMBL databases with a set of cichlid multi-exon OR gene sequences. We also inspected, one by one, the AA and gene sequences of the medaka, stickleback and zebrafish OR genes in the ENSEMBL database. A number of OR genes made up of two or more coding exons were found in various fish species (Additional file 11). These preliminary findings strongly suggest that ORs in many fishes, and not only cichlids, can be encoded by multi-coding exon genes.

Given the fact that invertebrate [50, 51] and some fish ORs could have more than one coding exon, a more general question would be, why do mammal ORs have only one coding exon and are the only subgroup of GPCRs with this characteristic? Would the peptides, corresponding to one or a subset of exons that made multicoding exon OR genes, have an Additional function lost during mammalian evolution and leading to the loss of these OR genes? Would some RNA transcripts, corresponding to a subset of exons and with no real coding capacity, regulate the expression of their corresponding OR mRNA? These are matters of speculation.

## Methods

The sequences of the five cichlid genomes were determined by the BROAD Institute using DNA samples prepared from a single double-haploid individual of each species, except in the case of *M. zebra,* which was caught in the wild. (http://www.broadinstitute.org). A dataset of 143 zebrafish ORs and 40 takifugu ORs [17, 18] was used as bait for exhaustive TBLASTN searches (http://blast.ncbi.nlm.nih.gov/Blast.cgi). Candidate genes were then compared to a negative dataset of 247 non-OR and non-TAAR animal GPCRs retrieved from the NCBI and ENSEMBL databases (Additional file 1).

TBLASTN results were filtered with a homemade python script so that candidate OR sequences conformed to the following rules: (1) one or more matches with the positive dataset and (2) no match with the negative dataset using an e-value cut-off of 1e^−50^. Selected candidates were re-checked using both BLASTX and BLASTP against the fish protein database (NCBI, taxiD: 7898) using default parameters with a cut-off of 1.e-^100^.

All genes were manually collected, biocurated and translated into protein sequences using Geneious software 6.1 [52]. Incomplete OR genes found at the ends of contigs were annotated as “edges” whereas incomplete OR genes found inside contigs were considered to be “fragments”. Genes with disruptive frameshifts or stop codons were annotated as pseudogenes. For spliced OR genes, predicted sequences and splice sites were manually corrected on the basis of multiple alignment using MAFFT 7 [22] and also by using FSPLICE [41]. The list and sequences of complete cichlid OR genes (spliced or not spliced), pseudogenes, edges and fragments are available as supplementary information (Additional file 2).

Positions of transmembrane domains in selected OR predicted proteins were determined using both TMHMM [53] and PolyPhobius [34].

The deduced AA sequences of all cichlids, zebrafish, sticklebach, tetraodon, takifugu and medaka ORs (Table 1) were aligned using MAFFT 7 with the E-INS version (optimal for sequences with conserved motifs and carrying multiple domains) with default parameters. A classification was proposed based on the estimated relatedness developed by using a bootstrapped maximum-likelihood unrooted tree generated by PHYML (1,000 rounds of bootstrapping) and drawn using FigTree 1.3.1. Thresholds of 40% and 60% AA similarity were used to distinguish between families and subfamilies, respectively, as described by Glusman et al. [19]. The cichlid OR sequences were named according to their phylogenetic positions as follows: Fish Symbol (Bri, Bur, Nye, Til or Zeb for *N. brichardi, H. burtoni, P. nyererei, O. niloticus* and *M. zebra* respectively) then “OR”, then p for pseudogene, e for edge or f for fragment followed by a letter or the family and three digits to designate the gene itself. For example, BRIORe.E041 designates the edge OR gene 041 belonging to family E.

Ratios of non-synonymous to synonymous nucleotide substitutions (ω = dN/dS) were calculated with the method of Nei-Gojobori as modified by Zhang et al. [26] using Perl and python scripts to automate the whole process. These ratios were calculated for both the entire proteins and different subregions (i.e. individual transmembrane domains or loop regions).

Conserved motifs were identified in predicted OR protein sequences with the online program Multiple Expectation Maximization for Motif Elicitation (MEME) online program v.4.9.0 [31]. Potential N-glycosylation sites were detected by NetNGlycserver [35]. Only N-glycosylation sites with a “potential” score > 0.5 and board agreement of “++” or higher) were considered as positive in our analyses.

## Electronic supplementary material

Additional file 1: **Negative data set composed of 247 non-OR GPCRs retrieved from NCBI database.** (PDF 27 KB)

Additional file 2: **Nucleotide and AA sequences of cichlids, tetraodon, medaka and stickleback OR present in the phylogenetic tree shown in Figure** 1 **.** Cichlid ORs are designated by the name of the contig within which they were identified, followed by an acronym indicating the fish species, a capital letter identifying its family, and an Arabic number indicating a particular OR, “s” is for genes with more than 1 coding exon, “p” is for pseudogenes, “e” for edge sequences and “f” for fragments. A sequence can have a combination of more than one of these symbols (for example, see ep). A shorter version of the gene names, from which the contig number is omitted, is found in all the following tables, figures and supplementary materials. Tetraodon, medaka and stickleback sequences correspond to a subset of OR sequences retrieved from NCBI and ENSEMBL databases and validated as true OR through AA multiple alignments and BLAST analysis. (PDF 9 MB)

Additional file 3: **Contigs and scaffolds harbouring ORs.** (PDF 13 MB)

Additional file 4: **Phylogenetic tree constructed from the AA sequences of the cichlid ORs identified in Table** 1 **and Additional file** 2 **and 143 zebrafish, 73 medaka, 78 stickleback, 40 fugu and 42 tetraodon OR AA sequences (Additional file** 2 **).** Fish species are colour coded: *O. niloticus* in red, *M. zebra* in pink, *N. brichardi* in blue, *H. burtoni* in green, *P. nyererei* in orange and fish models in black. (PDF 4 MB)

Additional file 5: **List of pairs, triplets and quadruplets of genes with 99% of identity or more.** (PDF 328 KB)

Additional file 6: **Details of dN/dS ratios for families A, D, E, G, H, I, K, L, N, O, P, R, S, and W.** (PDF 5 MB)

Additional file 7: **a to f. Details of dN/dS ratios of TM regions, external and internal loops for families D, E, H, L and N.** (ZIP 2 KB)

Additional file 8: **N-glycosylation sites as predicted by NetNGly Server for each cichlid OR.** (PDF 9 MB)

Additional file 9: **2C or 3 C groups of OR AA: MAFFT multiple alignments and LOGO presentation.** (PDF 13 MB)

Additional file 10: **2D structure prediction of the cichlid ORs made by PolyPhobius.** (PDF 4 MB)

Additional file 11: **DNA sequences of 6 fish model ORs with more than 1 coding exon.** Exons are indicated by bold letters. (PDF 328 KB)
