# Supplementary material for: Inventory of the cichlid olfactory receptor gene repertoires: identification of olfactory genes with more than one coding exon
Source: BMC Genomics. 2014 Jul 11;15(1):586. doi: 10.1186/1471-2164-15-586 (PMC4122780; doi:10.1186/1471-2164-15-586)
Supplement: Supplementary file 1 — Additional file 1: Negative data set composed of 247 non-OR GPCRs retrieved from NCBI database. (PDF 27 KB) [file 12864_2014_6314_MOESM1_ESM.pdf]

Negative dataset List

gi|1737210|gb|AA_somatostatin
gi|125716860|dbj_taste
gi|3377718|gb|AA_muscarinic
gi|289466095|gb|_CCR5
gi|348546267|
gi|7441593|pir||_cGMP-gated
gi|379656512|gb|_glutathione
gi|379656510|gb|_glutathione
gi|326681390|
gi|365812923|pdb_Chain
gi|301628705|
gi|157875312|pdb_Chain
gi|348536861|
gi|221129418|
gi|348544201|
gi|45387769|ref|_pituitary
gi|221129339|
gi|339648634|gb|_melanocortin
gi|157201143|dbj_growth
gi|339648650|gb|_melanocortin
gi|339648622|gb|_melanocortin
gi|339648602|gb|_melanocortin
gi|339648632|gb|_melanocortin
gi|348526213|
gi|348526207|
gi|325053654|ref_P2Y
gi|339648630|gb|_melanocortin
gi|54261747|ref|_mitochondrial
gi|192451485|ref_lysophosphatidic
gi|254028227|ref_hydroxycarboxylic
gi|339648636|gb|_melanocortin
gi|339648646|gb|_melanocortin
gi|339648600|gb|_melanocortin
gi|339648642|gb|_melanocortin
gi|339648640|gb|_melanocortin
gi|339648598|gb|_melanocortin
gi|339648596|gb|_melanocortin
gi|339648624|gb|_melanocortin
gi|339648648|gb|_melanocortin
gi|339648638|gb|_melanocortin
gi|344253627|gb|_G-protein
gi|344254127|gb|_Metabotropic
gi|347452591|gb|_sphingosine-1-phosphate
gi|68366846|ref|_PREDICTED:
gi|220673316|emb_novel
gi|131888301|ref_trace
gi|348503117|
gi|292618518|
gi|292619868|
gi|148921389|dbj_melanocortin-4
gi|190339544|gb|_Melanocortin
gi|27545307|ref|_melanocortin
gi|190338683|gb|_Melanocortin
gi|190337079|gb|_Mc3r
gi|30725836|ref|_melanocortin
gi|190339542|gb|_Melanocortin
gi|190337067|gb|_Melanocortin
gi|112418837|gb|_Zgc:153137
gi|27545311|ref|_melanocortin
gi|326671433|
gi|348535348|
gi|28827164|gb|A_melanin-concentrating
gi|292618524|
gi|292618526|
gi|348534761|

gi|292619866|
gi|348500202|
gi|348510719|
gi|348500631|
gi|190337212|gb|_Trace
gi|348508390|
gi|190338501|gb|_Trace
gi|348541325|
gi|42741748|gb|A_melanocortin
gi|348514407|
gi|131888140|ref_trace
gi|125848071|
gi|292618477|
gi|292618502|
gi|292618520|
gi|348534757|
gi|348537413|
gi|348534685|
gi|292618500|
gi|292618496|
gi|292618492|
gi|1359759|emb|C_histamine
gi|348516579|
gi|348525466|
gi|292618504|
gi|189524614|
gi|327284105|
gi|292618481|
gi|189524588|
gi|326671427|
gi|189524598|
gi|62203246|gb|A_Melatonin
gi|292618522|
gi|189524594|
gi|189524606|
gi|125828364|
gi|189524612|
gi|189524602|
gi|189524596|
gi|351700872|gb|_N-formyl
gi|348510733|
gi|190337350|gb|_Adenosine
gi|348516796|
gi|118344648|ref_interleukin
gi|348501774|ref|XP_003438444.1|
gi|66911351|gb|A_Apelin
gi|348505216|
gi|348544373|
gi|157787193|ref_KISS1
gi|348532993|
gi|348533930|
gi|62183726|gb|A_mammalian-like
gi|348538276|
gi|348511392|
gi|348533572|
gi|348519793|
gi|348520965|
gi|125805883|
gi|348542007|
gi|348533932|
gi|350276276|ref_apelin
gi|292627430|
gi|348532791|
gi|60416027|gb|A_Zgc:113016
gi|348503524|

gi|189524630|
gi|348516108|
gi|348524536|
gi|348525916|
gi|68370236|ref|_PREDICTED:
gi|45387607|ref|_nociceptin
gi|348515367|
gi|348509571|
gi|348525490|
gi|348507701|
gi|190339650|gb|_Vomeronasal
gi|348535692|
gi|190338699|gb|_Vomeronasal
gi|16945894|gb|A_somatostatin
gi|348516786|
gi|190337360|gb|_Neuropeptide
gi|147903294|ref_high-affinity
gi|292625179|
gi|190337372|gb|_Neuropeptide
gi|190339758|gb|_Neuropeptide
gi|386267899|dbj_vomeronasal
gi|348532935|
gi|190338849|gb|_Oprd1b
gi|190339736|gb|_Neuropeptide
gi|153791315|ref_chemokine
gi|348530587|
gi|126631874|gb|_Zgc:162592
gi|348526584|
gi|190337340|gb|_Neuropeptide
gi|348528168|
gi|190336605|gb|_Opioid
gi|348531627|
gi|213625815|gb|_Coagulation
gi|213626119|gb|_Coagulation
gi|206570747|gb|_somtostatin
gi|6753528|ref|N_prostaglandin
gi|161353479|ref_cannabinoid
gi|348535391|
gi|348531541|
gi|348530922|
gi|292618479|
gi|190338514|gb|_Tmtops
gi|157888794|dbj_growth
gi|190338132|gb|_Adrenergic,
gi|190338962|gb|_Adrenergic,
gi|325296741|ref_octopamine
gi|115647945|
gi|348508725|
gi|347977982|ref_sphingosine-1-phosphate
gi|348535385|
gi|178896|gb|AAA_beta-3-adrenergic
gi|190339846|gb|_Beta-3-adrenergic
gi|348539572|
gi|190338262|gb|_Beta-3-adrenergic
gi|348542772|
gi|348542772|
gi|348531116|
gi|348504978|
gi|348532047|
gi|3421153|emb|C_E6-AP
gi|348505136|
gi|21322666|emb|_alpha2
gi|120300948|ref_orexin
gi|134142085|gb|_hypocretin
gi|348513993|

gi|348512885|
gi|348530360|
gi|190339682|gb|_Adrenergic,
gi|190338512|gb|_Adrenergic,
gi|126304081|
gi|190338372|gb|_Zgc:194773
gi|190338376|gb|_Zgc:194773
gi|89886297|ref|_adenosine
gi|348522181|
gi|348536715|
gi|2340853|emb|C_D1A3
gi|326671147|
gi|190336789|gb|_Adenosine
gi|190339354|gb|_Adenosine
gi|348521146|
gi|1518040|gb|AA_dopamine
gi|316938202|gb|_dopamine
gi|115923283|
gi|46451437|gb|A_type
gi|190337474|gb|_Cholinergic
gi|190339856|gb|_Cholinergic
gi|348530554|
gi|190340094|gb|_Cholinergic
gi|190338621|gb|_Cholinergic
gi|74418612|gb|A_cone-type
gi|113195626|ref_thyrotropin
gi|348533528|
gi|198449216|gb|_muscarinic
gi|190337400|gb|_Adra2b
gi|283362037|dbj_thyrotropin-releasing
gi|348529456|
gi|149046354|gb|_cyclic
gi|348538790|
gi|4826633|emb|C_cyclic
gi|317418829|emb_Histamine
gi|190339810|gb|_G
gi|190338368|gb|_G
gi|348534257|
gi|282765728|gb|_ATP-binding
gi|348527904|
gi|348542983|
gi|348519090|
gi|190336988|gb|_Opsin
gi|348543768|
gi|148726000|emb_novel
gi|348528687|
gi|348538730|
gi|348528681|
gi|326676133|
gi|82752294|gb|A_androgen
gi|224066016|
gi|348509443|
