# Supplementary material for: Inventory of the cichlid olfactory receptor gene repertoires: identification of olfactory genes with more than one coding exon
Source: BMC Genomics. 2014 Jul 11;15(1):586. doi: 10.1186/1471-2164-15-586 (PMC4122780; doi:10.1186/1471-2164-15-586)
Supplement: Supplementary file 2 — Additional file 2: Nucleotide and AA sequences of cichlids, tetraodon, medaka and stickleback OR present in the phylogenetic tree shown in Figure 1 . Cichlid ORs are designated by the name of the contig within which they were identified, followed by an acronym indicating the fish species, a capital letter identifying its family, and an Arabic number indicating a particular OR, “s” is for genes with more than 1 coding exon, “p” is for pseudogenes, “e” for edge sequences and “f” for fragments. A sequence can have a combination of more than one of these symbols (for example, see ep). A shorter version of the gene names, from which the contig number is omitted, is found in all the following tables, figures and supplementary materials. Tetraodon, medaka and stickleback sequences correspond to a subset of OR sequences retrieved from NCBI and ENSEMBL databases and validated as true OR through AA multiple alignments and BLAST analysis. (PDF 9 MB) [file 12864_2014_6314_MOESM2_ESM.pdf]

NetNGlyc 1.0 Server - prediction results

Technical University of Denmark

Asn-Xaa-Ser/Thr sequons in the sequence output below are highlighted in **blue**.
Asparagines predicted to be N-glycosylated are highlighted in **red**.

Output for 'contig068887-BurORe.N116'

```
########################################################################################

    Warning: This sequence may not contain a signal peptide!!

    Proteins without signal peptides are unlikely to be exposed to
    the N-glycosylation machinery and thus may not be glycosylated
    (in vivo) even though they contain potential motifs.

    SignalP-NN euk predictions are as follows:

  # name                   Cmax  pos ?  Ymax  pos ?  Smax  pos ?  Smean ?  D     ?

    SignalP output is explained at http://www.cbs.dtu.dk/services/SignalP/output.html

########################################################################################

Name:  contig068887-BurORe.N116         Length:  304
MEFLNSAVGKNITFVKPAYFIISAFNGIANIRYYFVFLCFIYIFSVVGNTLLMIVIILDHTLRSPKHIGVVNFAFTDLLS      80
SSALMPKLVDIFLFNHHHISYNDCLAFMFFCLTFFAAQAFNLVVLSFDRVLAITYPLHYQMRISHKLILSLIAFFWLLAI     160
TIILTAVGLLTRLSFCDSVVIQSFFCDHGPVYQLGCNDLTPNRVIAHLAPVLVLWVPLACIVGSYCCIGYSLSKTVTCRE     240
RLKALKTCTSHLSLVAIYFLPALFIFTFGSTILPNARTVSLSLATVMPLTLNPIIYGLQTQEIK
..........N.....................................................................      80
................................................................................     160
................................................................................     240
................................................................                     320

(Threshold=0.5)
----------------------------------------------------------------------
SeqName      Position  Potential   Jury    N-Glyc
                                 agreement result
----------------------------------------------------------------------
contig068887-BurORe.N116    11 NITF    0.5222     (6/9)   +
----------------------------------------------------------------------
```

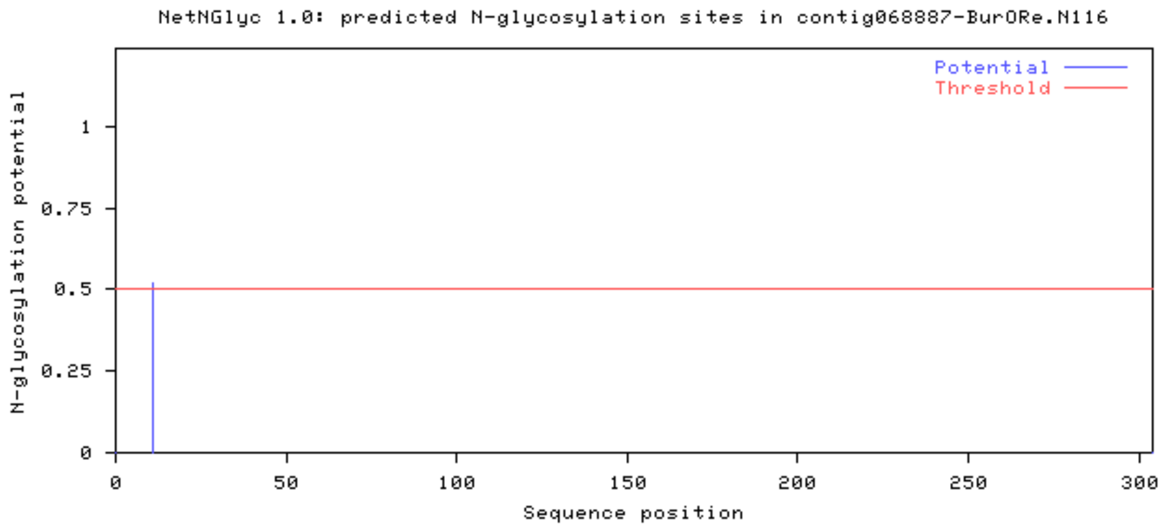

Graphics in PostScript

Output for 'contig067265-BurOR.R141'

```
##########################################################################

    Warning: This sequence may not contain a signal peptide!!

    Proteins without signal peptides are unlikely to be exposed to
    the N-glycosylation machinery and thus may not be glycosylated
    (in vivo) even though they contain potential motifs.

    SignalP-NN euk predictions are as follows:

  # name                  Cmax   pos ?  Ymax   pos ?  Smax   pos ?  Smean ?  D     ?

    SignalP output is explained at http://www.cbs.dtu.dk/services/SignalP/output.html

##########################################################################

Name:  contig067265-BurOR.R141  Length:  317
MFFTNETLTNITVGQQNQLFLEIVFSCIVTTLTCCVFLFINATMLFTLRSKPVFGQTSRYILLYNLLFADTLQMAQSQVM      80
FLLSACRITLLYPICGVLVSLATLLTLISPLTLVAMSLERYVAVCYPLRHATIITFRNTALAVCVVWTISLLNVLIEVVL     160
MLRVRFQDLLHLQMEYSCNKEKLTLDPISDLYAKAFSYFLFVLAAGAFIFSYIGVTVVAQSASTDKASAEKARKTLVLHL     240
VQLGLSVSSTIHNPIFVFIYKTVDSVIVVRIRVVIYLCIIILPRCLSSFIYGLRDHTIRPVLMLNLRCQWKCQPFLX
....N....N.............................N........................................      80
................................................................................     160
................................................................................     240
.............................................................................        320

(Threshold=0.5)
----------------------------------------------------------------------
SeqName      Position  Potential   Jury    N-Glyc
                                agreement  result
----------------------------------------------------------------------
contig067265-BurOR.R141    5 NETL   0.7619     (9/9)    +++
contig067265-BurOR.R141   10 NITV   0.6376     (7/9)    +
contig067265-BurOR.R141   41 NATM   0.6193     (7/9)    +
----------------------------------------------------------------------
```

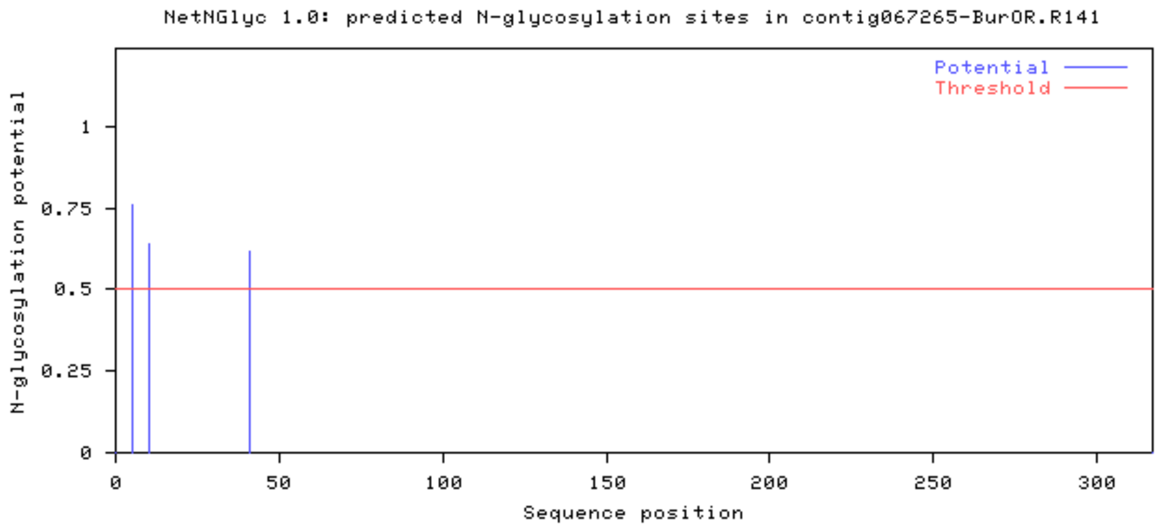

Graphics in PostScript

Output for 'contig067297-BurORe.J078'

```
##########################################################################

    Warning: This sequence may not contain a signal peptide!!

    Proteins without signal peptides are unlikely to be exposed to
    the N-glycosylation machinery and thus may not be glycosylated
    (in vivo) even though they contain potential motifs.

    SignalP-NN euk predictions are as follows:

  # name                Cmax  pos ?  Ymax  pos ?  Smax  pos ?  Smean ?  D     ?

    SignalP output is explained at http://www.cbs.dtu.dk/services/SignalP/output.html

##########################################################################

Name:  contig067297-BurORe.J078         Length:  294
STDIYPAFVFGTLTYLIIMFSNLLVLTVIAMNKKLHKPMFILLFNLPISDIVGATAFFPHLIFSIVAENRLISHHACIFQ      80
AFLIHVYGTGNLLILSAMAYDRYIAICFPLRYTTIMNSHNLMKMIVITWFINLSMMFTLFILLVRFKTCRTNIVDFYCNN     160
QSLVKLICDNTSVNDYYGLGTIFLLMGGPLALILYTYAQILRTCVITNHTDARQKAIQTCATHLIVFLSLQINTVFALIS     240
HRIDSSSPVLRRAFGVSVLIFPPLLDPIIYGLKTKELKQCIVMFLKRNVGLTMX
................................................................................      80
...................................................N...........................N     160
.........N.....................................N................................     240
......................................................                               320

(Threshold=0.5)
----------------------------------------------------------------------
SeqName      Position  Potential   Jury    N-Glyc
                                 agreement result
----------------------------------------------------------------------
contig067297-BurORe.J078   132 NLSM   0.5392     (7/9)    +
contig067297-BurORe.J078   160 NQSL   0.5359     (6/9)    +
contig067297-BurORe.J078   170 NTSV   0.7004     (9/9)    ++
contig067297-BurORe.J078   208 NHTD   0.5127     (4/9)    +
----------------------------------------------------------------------
```

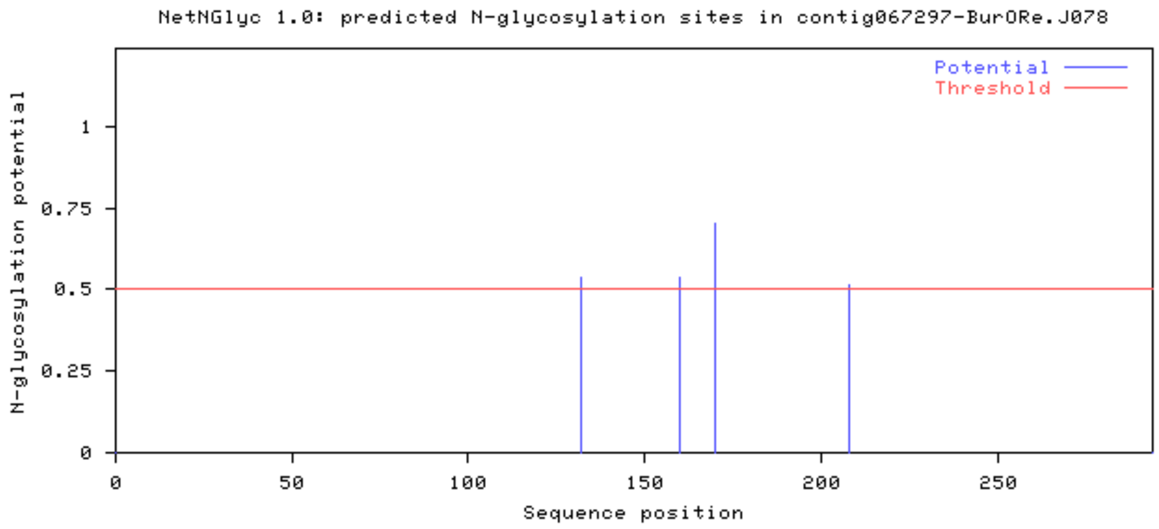

Graphics in PostScript

Output for 'contig056383-BurORe.L094'

```
##########################################################################

    Warning: This sequence may not contain a signal peptide!!

    Proteins without signal peptides are unlikely to be exposed to
    the N-glycosylation machinery and thus may not be glycosylated
    (in vivo) even though they contain potential motifs.

    SignalP-NN euk predictions are as follows:

  # name                 Cmax  pos ?  Ymax  pos ?  Smax  pos ?  Smean ?  D     ?

    SignalP output is explained at http://www.cbs.dtu.dk/services/SignalP/output.html

##########################################################################

Name:  contig056383-BurORe.L094        Length:  123
MSLQNASIKLTYFIIGGFDTVKRPVAVGVVMLITYLLAVFASLVNIIFIVSDKQLHKPMYLLICNLAVVDILYTSSSTPT      80
MIGVLLAGVNTISYVECIIQMYVYQVGATMEMFSLTIMAFDRL
....N...........................................................................      80
...........................................                                          160

(Threshold=0.5)
----------------------------------------------------------------------
SeqName      Position  Potential   Jury    N-Glyc
                                 agreement result
----------------------------------------------------------------------
contig056383-BurORe.L094    5 NASI   0.5856     (6/9)   +
----------------------------------------------------------------------
```

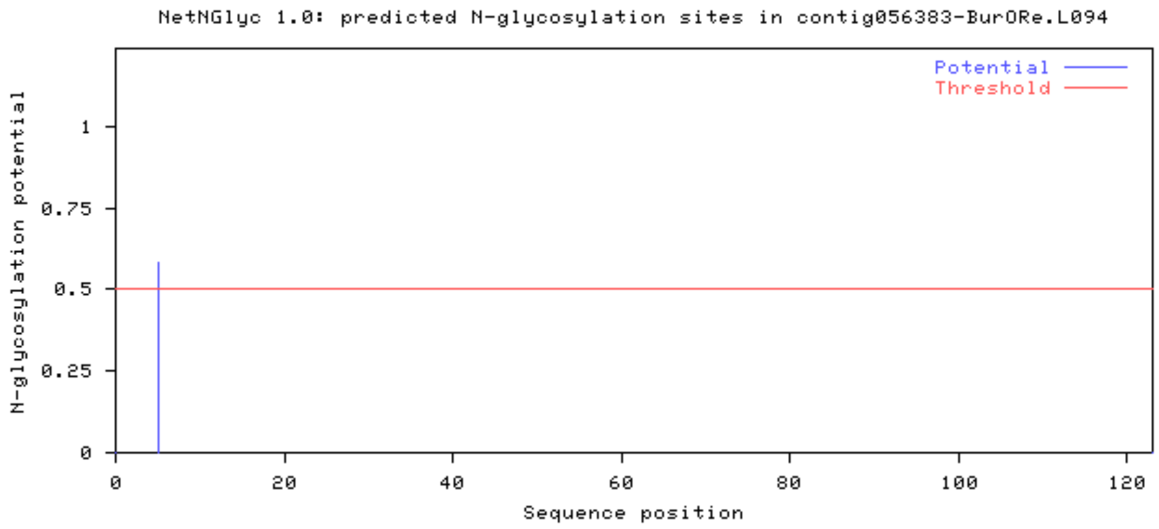

Graphics in PostScript

Output for 'contig068426-BurORe.M106'

```
##########################################################################

    Warning: This sequence may not contain a signal peptide!!

    Proteins without signal peptides are unlikely to be exposed to
    the N-glycosylation machinery and thus may not be glycosylated
    (in vivo) even though they contain potential motifs.

    SignalP-NN euk predictions are as follows:

  # name                  Cmax   pos ?  Ymax   pos ?  Smax   pos ?  Smean ?  D     ?

    SignalP output is explained at http://www.cbs.dtu.dk/services/SignalP/output.html

##########################################################################

Name:  contig068426-BurORe.M106         Length:  314
MIFQRIVLNSSAIIHPPGFYIIGFETFPFISVYFIFLVFVYGVTVLFNILVIYVIASTRCLHTPKFLAVVNLAVIDLFLN      80
TCTIPSMIKIFLIKNNFIPFNLCLLQMYVYYVFVSLESFALAILAYDRLIAICFPLRHNSINTVRSMCCIVSVSWFLVMG     160
VIAFATGIMTRLSFCRSVRVFSYFCDYAPVFRLACNDNTMQWFAASFFSVLLLFVPFTFIFLSYVSILMTVFRMKSVDSR     240
VKALATCAEHLILVAIFYIPLIVIFTVGFFLGVVNPDQRVLSLSLASCIPPCINPIVYSLKTKDIKTRALTLFR
........N.......................................................................      80
................................................................................     160
................................................................................     240
..........................................................................           320

(Threshold=0.5)
----------------------------------------------------------------------
SeqName      Position  Potential   Jury    N-Glyc
                                 agreement result
----------------------------------------------------------------------
contig068426-BurORe.M106    9 NSSA   0.6660     (9/9)    ++
----------------------------------------------------------------------
```

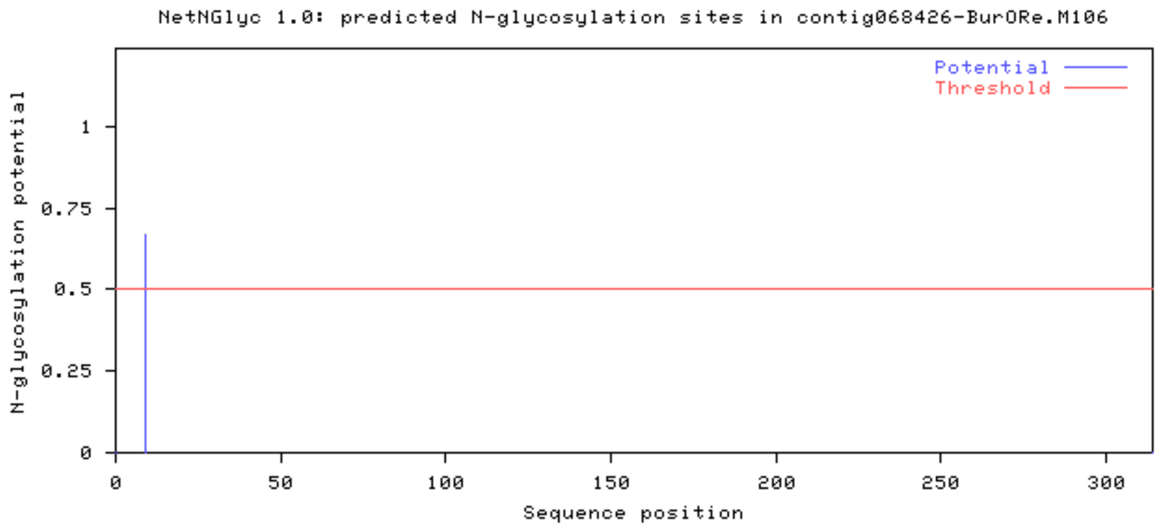

Graphics in PostScript

Output for 'contig006791-BurORe.H070'

```
##########################################################################################

    Warning: This sequence may not contain a signal peptide!!

    Proteins without signal peptides are unlikely to be exposed to
    the N-glycosylation machinery and thus may not be glycosylated
    (in vivo) even though they contain potential motifs.

    SignalP-NN euk predictions are as follows:

  # name                Cmax  pos ?  Ymax  pos ?  Smax  pos ?  Smean ?  D     ?

    SignalP output is explained at http://www.cbs.dtu.dk/services/SignalP/output.html

##########################################################################################

Name:  contig006791-BurORe.H070        Length:  68
NFIAIEFLIISPVMNPLVYGFKLAKIRKRIFTLVHYKTKLLQSKLRVSYFKRRMPFRNILLNIFCCTX
....................................................................                  80

(Threshold=0.5)

No sites predicted in this sequence.
```

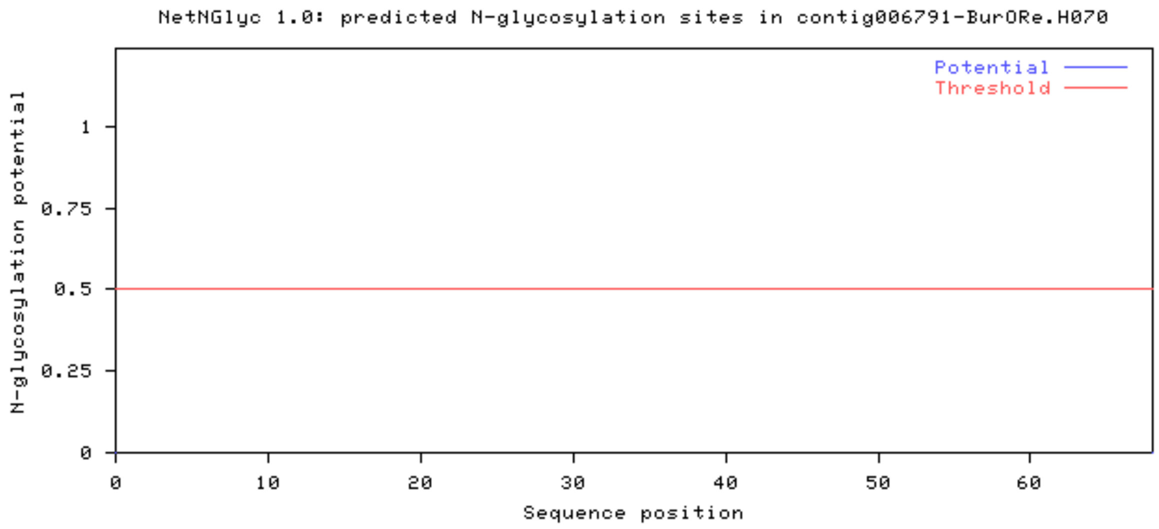

[Graphics in PostScript]

Output for 'contig006793-BurORe.H071'

```
##########################################################################################

    Warning: This sequence may not contain a signal peptide!!

    Proteins without signal peptides are unlikely to be exposed to
    the N-glycosylation machinery and thus may not be glycosylated
    (in vivo) even though they contain potential motifs.

    SignalP-NN euk predictions are as follows:

  # name                 Cmax   pos ?  Ymax   pos ?  Smax   pos ?  Smean ?  D     ?

    SignalP output is explained at http://www.cbs.dtu.dk/services/SignalP/output.html

##########################################################################################

Name:  contig006793-BurORe.H071         Length:  263
MDNVSVVRMFTLSGFNETMNIRLTIFSLTLMYYCMIILINVSLIVLIVLDENLHEPMYILLSSFCINAIYGTTGFYPKFL      80
LDLLSSSQEISYEGCLLQAFIMYSFACCDLSILAVMAFDRYLAICRPLHYHSFMTKRRLSQLVCFSWLTPLCILSINILL     160
TSRLTLCRSKIEKVLCVNWVIVKLACSDTDTLLNSVVSYATIIMYMSHGFFIMWTYMHLIKTSVRSKEDRAKFMQTCVPH     240
LTSLITFLIVILFDLMYMRFGSA
..N............N........................N.......................................      80
................................................................................     160
................................................................................     240
.......................                                                              320

(Threshold=0.5)
----------------------------------------------------------------------
SeqName      Position  Potential   Jury    N-Glyc
                                 agreement result
----------------------------------------------------------------------
contig006793-BurORe.H071    3 NVSV   0.8086     (9/9)   +++
contig006793-BurORe.H071   16 NETM   0.6299     (9/9)   ++
contig006793-BurORe.H071   40 NVSL   0.7702     (9/9)   +++
----------------------------------------------------------------------
```

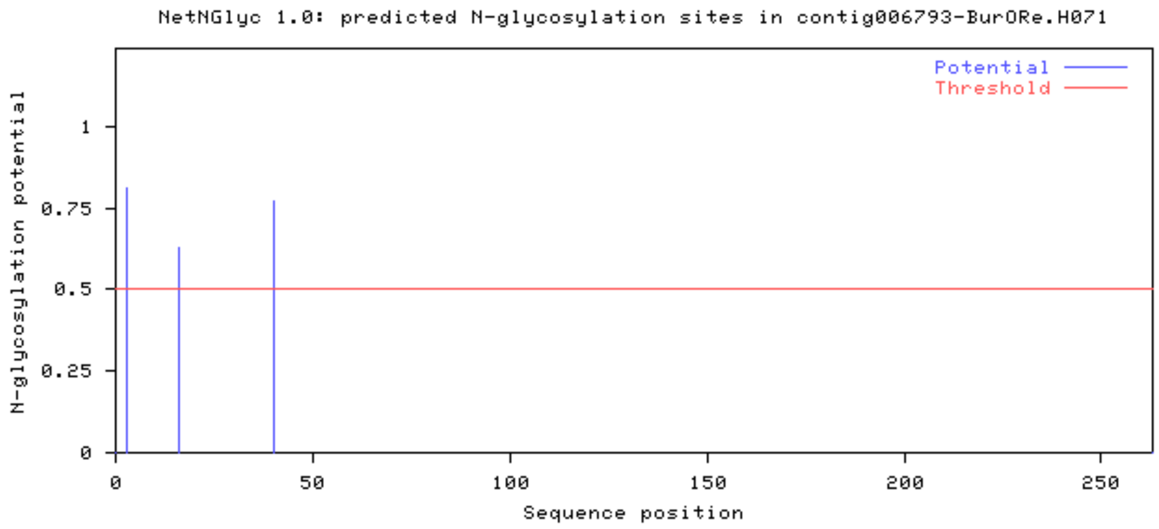

Graphics in PostScript

Output for 'contig006794-BurOR.H061'

```
##########################################################################

   Warning: This sequence may not contain a signal peptide!!

   Proteins without signal peptides are unlikely to be exposed to
   the N-glycosylation machinery and thus may not be glycosylated
   (in vivo) even though they contain potential motifs.

   SignalP-NN euk predictions are as follows:

 # name                Cmax  pos ?  Ymax  pos ?  Smax  pos ?  Smean ?  D     ?

   SignalP output is explained at http://www.cbs.dtu.dk/services/SignalP/output.html

##########################################################################

Name:  contig006794-BurOR.H061  Length:  343
MDNVSIVRSFILSGFNETMNFRVPLFAFTLLYYCMILFFNISLVLLIVLDKNLHEPMYIFLSSFCINTIYGTTGFYPKFL      80
SDLLWSSQTISYEGCLLQAFIIYSFVCCDLSILAVMAFDRYLAICRPLHYHSFMTKRRLSQLVCFSWLTPLCIFAINVLL     160
TSRLKLCGIKIQRVLCVNWLIVKLACPEAETFTNHIAAYATVIIYVSHGFFIMWTYTHLIKTCARSREDREKFMQTCLPH     240
LTSLITFIVVIGFQSIYVQFEYTGFSESLKNFITVEILIIPPFMNPLIYGFKFTKIQNRILTLLKSYKKLTYIKFSLLKK     320
TSVKCSIFLTVKNLLCEPFCCRX
..N............N........................N.......................................      80
................................................................................     160
................................................................................     240
................................................................................     320
.......................                                                              400

(Threshold=0.5)
----------------------------------------------------------------------
SeqName      Position  Potential   Jury    N-Glyc
                                 agreement result
----------------------------------------------------------------------
contig006794-BurOR.H061    3 NVSI   0.7912     (9/9)   +++
contig006794-BurOR.H061   16 NETM   0.6138     (8/9)   +
contig006794-BurOR.H061   40 NISL   0.6980     (9/9)   ++
----------------------------------------------------------------------
```

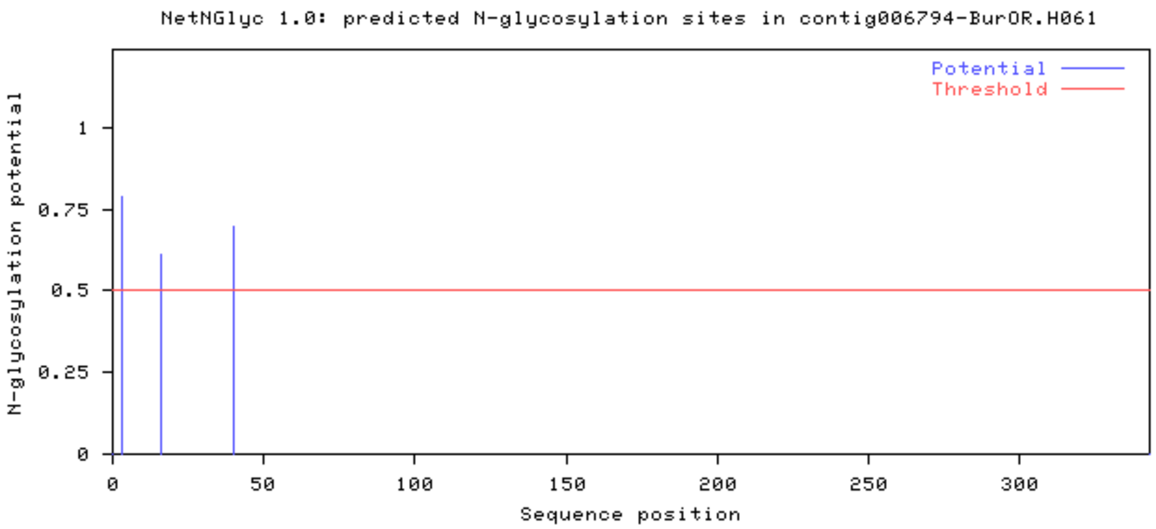

Graphics in PostScript

Output for 'contig009773-BurORs.Q136'

```
##########################################################################################

    Warning: This sequence may not contain a signal peptide!!

    Proteins without signal peptides are unlikely to be exposed to
    the N-glycosylation machinery and thus may not be glycosylated
    (in vivo) even though they contain potential motifs.

    SignalP-NN euk predictions are as follows:

  # name                  Cmax  pos ?  Ymax  pos ?  Smax  pos ?  Smean ?  D     ?

    SignalP output is explained at http://www.cbs.dtu.dk/services/SignalP/output.html

##########################################################################################

Name:  contig009773-BurORs.Q136         Length:  310
MNATTAEFQSLPFQTSVKAALSMLPCFFFLYVNAIMMFALLKKPLLLESSRYILFGHLLMCDSVQLLLTMMLYIFAVMMV      80
RMINYVCVFVSLVAAVTVKMSPLNLAVMSLERYVAVCFPLRHPSFATPRSTGKAIAVMWMAASLDSFIQLFLFVRMEMTI     160
FPMQSFCIRNSVFRLEVYVTLNMAFTILYFVFVSMIIIYTYTAIMITVKSASSRGRHTNKAPKTVLLHLLQLWLYLTSTL     240
FNMINPSMMLKVPPDMAIHAQYVLFVGLIIFPKCLSPLIYGLRDQTLCRVFKYYFTFGFRASVKPSPLSX
.N..............................................................................      80
................................................................................     160
................................................................................     240
......................................................................               320

(Threshold=0.5)
----------------------------------------------------------------------
SeqName      Position  Potential   Jury    N-Glyc
                                 agreement result
----------------------------------------------------------------------
contig009773-BurORs.Q136    2 NATT   0.6720     (8/9)   +
contig009773-BurORs.Q136  245 NPSM   0.3831     (7/9)   -
----------------------------------------------------------------------
```

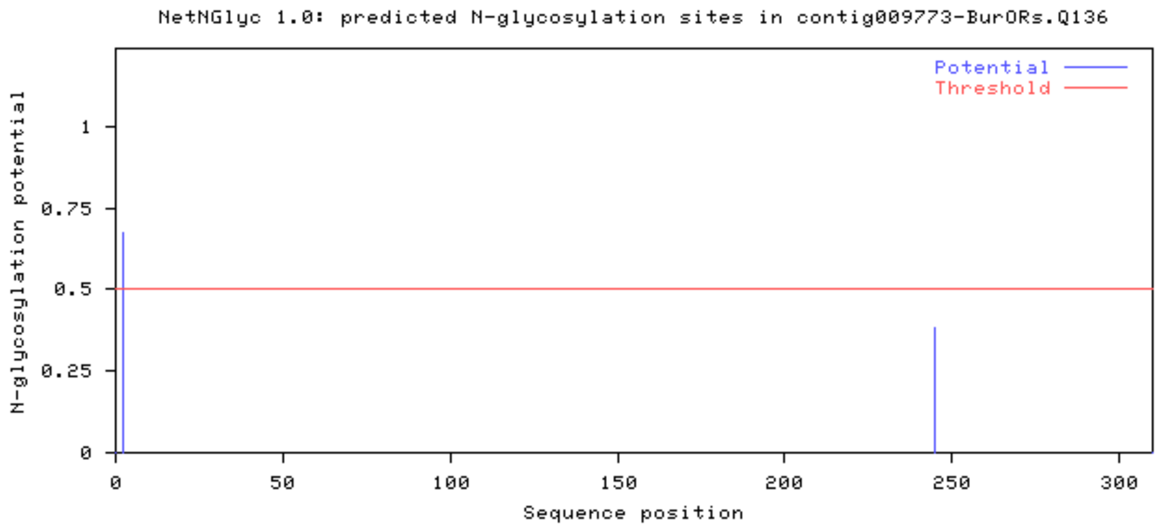

Graphics in PostScript

Output for 'contig009805-BurOR.C031'

```
##########################################################################################

    Warning: This sequence may not contain a signal peptide!!

    Proteins without signal peptides are unlikely to be exposed to
    the N-glycosylation machinery and thus may not be glycosylated
    (in vivo) even though they contain potential motifs.

    SignalP-NN euk predictions are as follows:

  # name                 Cmax  pos ?  Ymax  pos ?  Smax  pos ?  Smean ?  D     ?

    SignalP output is explained at http://www.cbs.dtu.dk/services/SignalP/output.html

##########################################################################################

Name:  contig009805-BurOR.C031  Length:  321
MDNTTAATFKMTAYAVMENYNHGLFSVFFLLYLITLVLNVLLISVIHQNKQLHQPMNVFTCMLSLNEIYGSSALLPAVMA      80
VLVSKTHDVTVKWCMAQAYFLHTYASGEFCILALMGYDRYIAICSPLHYYSIMSYSKTCKLIAFTGLYPFIVFTSFYSLT     160
LQLRFCGKVIPKLYCVNMELVKNSCTNAQYISTVGLAILVLFIVPQLVMIVFSYTHILRVCRTFPKESQANAFRTCVPHL     240
LSLLNYTIASLFEVIQTRFNMSHVAVEAQIFLSLYFIIIPPIANPVLYGLGTQTVRGCIMKLFIKNKVMTTVLAKTLTVG     320
X
..N.............................................................................      80
................................................................................     160
................................................................................     240
....N...........................................................................     320
.                                                                                    400

(Threshold=0.5)
----------------------------------------------------------------------
SeqName      Position  Potential   Jury    N-Glyc
                                agreement  result
----------------------------------------------------------------------
contig009805-BurOR.C031    3 NTTA   0.7210     (9/9)   ++
contig009805-BurOR.C031  245 NYTI   0.6910     (8/9)   +
contig009805-BurOR.C031  260 NMSH   0.4503     (7/9)   -
----------------------------------------------------------------------
```

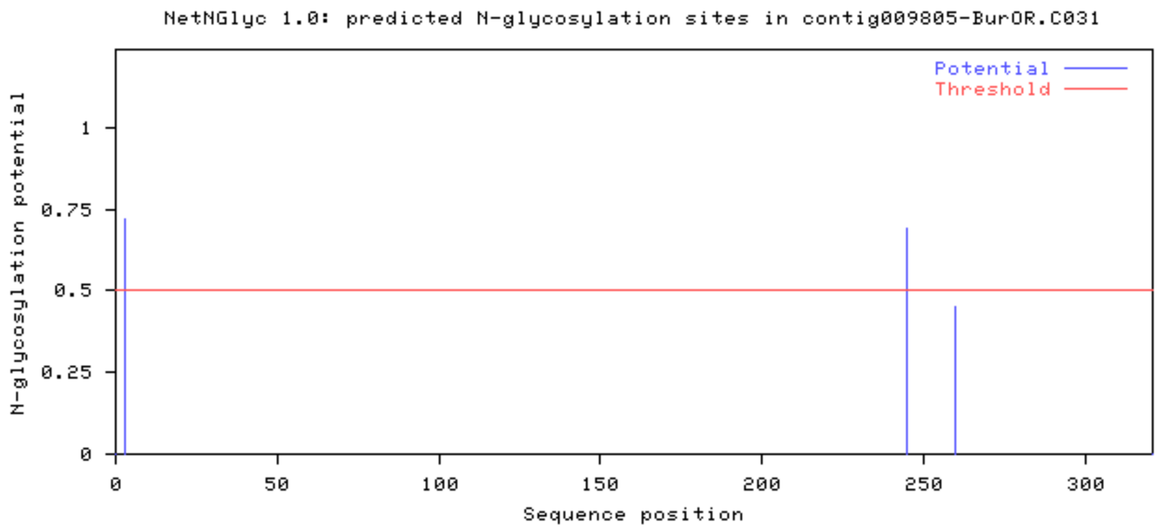

Graphics in PostScript

Output for 'contig013898-BurOR.F058'

```
##########################################################################

    Warning: This sequence may not contain a signal peptide!!

    Proteins without signal peptides are unlikely to be exposed to
    the N-glycosylation machinery and thus may not be glycosylated
    (in vivo) even though they contain potential motifs.

    SignalP-NN euk predictions are as follows:

  # name                 Cmax  pos ?  Ymax  pos ?  Smax  pos ?  Smean ?  D     ?

    SignalP output is explained at http://www.cbs.dtu.dk/services/SignalP/output.html

##########################################################################

Name:  contig013898-BurOR.F058  Length:  310
MENNSHPLYFNLTMFVYIGKFRYPAFVLFLLLYTFIISANLVIIVVISREKTLHEPMYIFIMCLSINSLYGSGGFFFRFL      80
RDLLSDSNLIARSACYTQIYIIIYTYASYELTLLGIMAYDRFVAICQPLHYHSKLTSRVISKLLAFAWIYPAFSVAACVYL     160
ASRLPLCGNKIPKVFCANWPVVKLSCVPTVINNIIGMFVSVTTVFLPLAFVLYTYMRIFLVCRKRSSLFKSKVIQSCLPH     240
IVTFVNYSITVFCDVALSRIDLEELNPFLGIILSLEFVVIPPILNPLMYGLKLPEIRKCILRNLSCLIRX
..N.......N.....................................................................      80
................................................................................     160
................................................................................     240
.....N........................................................N.......     320

(Threshold=0.5)
----------------------------------------------------------------------
SeqName      Position  Potential   Jury    N-Glyc
                                 agreement result
----------------------------------------------------------------------
contig013898-BurOR.F058    3 NNSH   0.5824     (9/9)   ++
contig013898-BurOR.F058   11 NLTM   0.8139     (9/9)   +++
contig013898-BurOR.F058  246 NYSI   0.5721     (7/9)   +
contig013898-BurOR.F058  303 NLSC   0.5138     (5/9)   +
----------------------------------------------------------------------
```

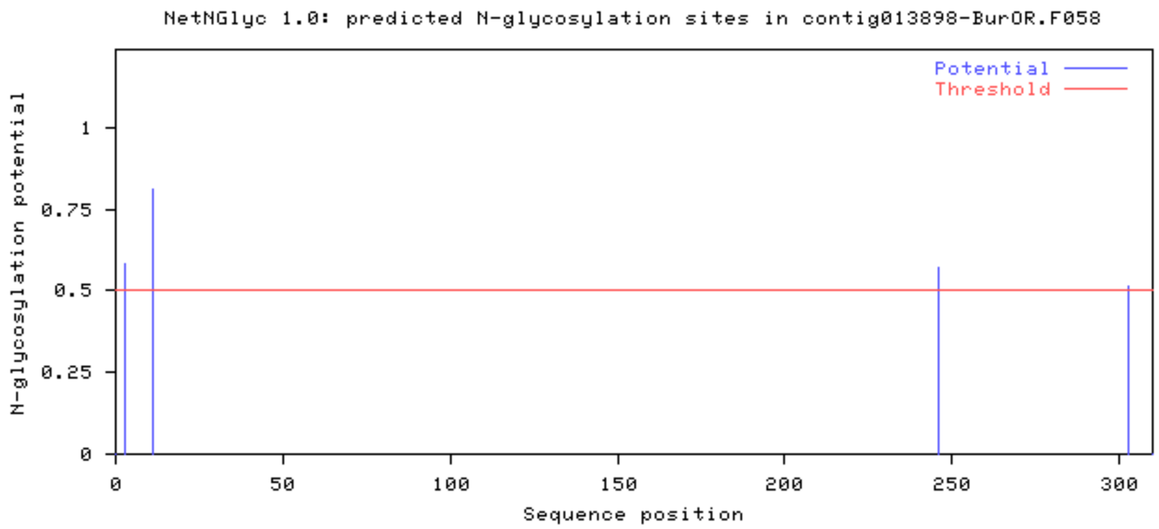

Graphics in PostScript

Output for 'contig014275-BurORe.O102'

```
##########################################################################################

    Warning: This sequence may not contain a signal peptide!!

    Proteins without signal peptides are unlikely to be exposed to
    the N-glycosylation machinery and thus may not be glycosylated
    (in vivo) even though they contain potential motifs.

    SignalP-NN euk predictions are as follows:

  # name                   Cmax  pos ?  Ymax  pos ?  Smax  pos ?  Smean ?  D     ?

    SignalP output is explained at http://www.cbs.dtu.dk/services/SignalP/output.html

##########################################################################################

Name:  contig014275-BurORe.O102         Length:  312
MITNVTRMESFFILGFPGLSPQYYGPISTFLFFVYLAIALGNIFILSFVAYEKSLQKPTYLVFCHLALNDLTFGTVTLPK      80
IMSKYWFDNSVISFYGCFTQMFFVHYLGSVTSFILLVMALDRFVAICIPLRYPVLITNSVISVLCGFAWFIPLPLMIAVV     160
LHALTLPFCKSNIIVQCYCDNISIISQACGDDVRIVVVTSLCLAMFCLLLPLAFILFSYISIIVVIMKMSSSAGRKRTLS     240
TCTSQIFIACLFYLPRCFVYVTNAVGFSFSLDVRIGLILLYSLFPAAVNPVIYCFKTRDIKHMLMKRLKKTK
...N............................................................................      80
................................................................................     160
....................N...........................................................     240
........................................................................             320

(Threshold=0.5)
----------------------------------------------------------------------
SeqName      Position  Potential   Jury    N-Glyc
                                 agreement result
----------------------------------------------------------------------
contig014275-BurORe.O102    4 NVTR   0.7914     (9/9)    +++
contig014275-BurORe.O102  181 NISI   0.6346     (9/9)    ++
----------------------------------------------------------------------
```

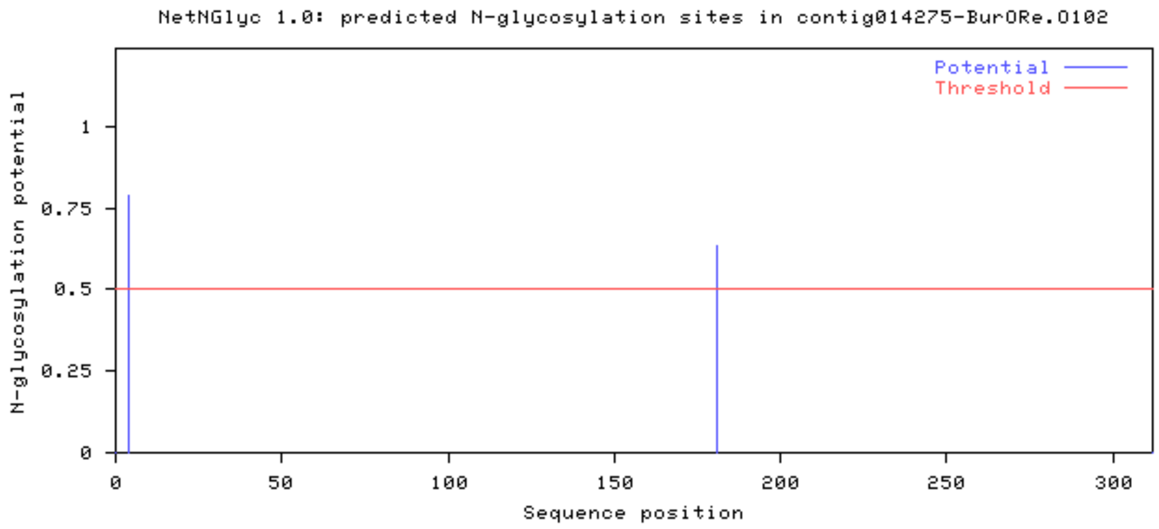

Graphics in PostScript

Output for 'contig014278-BurORe.O101'

```
##########################################################################################

    Warning: This sequence may not contain a signal peptide!!

    Proteins without signal peptides are unlikely to be exposed to
    the N-glycosylation machinery and thus may not be glycosylated
    (in vivo) even though they contain potential motifs.

    SignalP-NN euk predictions are as follows:

  # name                Cmax   pos ?  Ymax   pos ?  Smax   pos ?  Smean ?  D     ?

    SignalP output is explained at http://www.cbs.dtu.dk/services/SignalP/output.html

##########################################################################################

Name:  contig014278-BurORe.0101         Length:  162
MKYTNITTIKEFIIIGFPGLPPEYYGPVSVLLLLVFLAIVIGNGFTIAVIIFERTLHKPIYVIFSNLAMTDICFGVVTLP      80
KIIARYWWNDMITSFGACFTQMYFVHSLGAIQSLNLLMMALDRFVAIWFPFKYPILFTNKAVAIACTMCWVLTFIRLLGI     160
VL
....N...........................................................................      80
................................................................................     160
..                                                                                   240

(Threshold=0.5)
----------------------------------------------------------------------
SeqName      Position  Potential   Jury    N-Glyc
                                 agreement result
----------------------------------------------------------------------
contig014278-BurORe.0101     5 NITT   0.7667     (9/9)    +++
----------------------------------------------------------------------
```

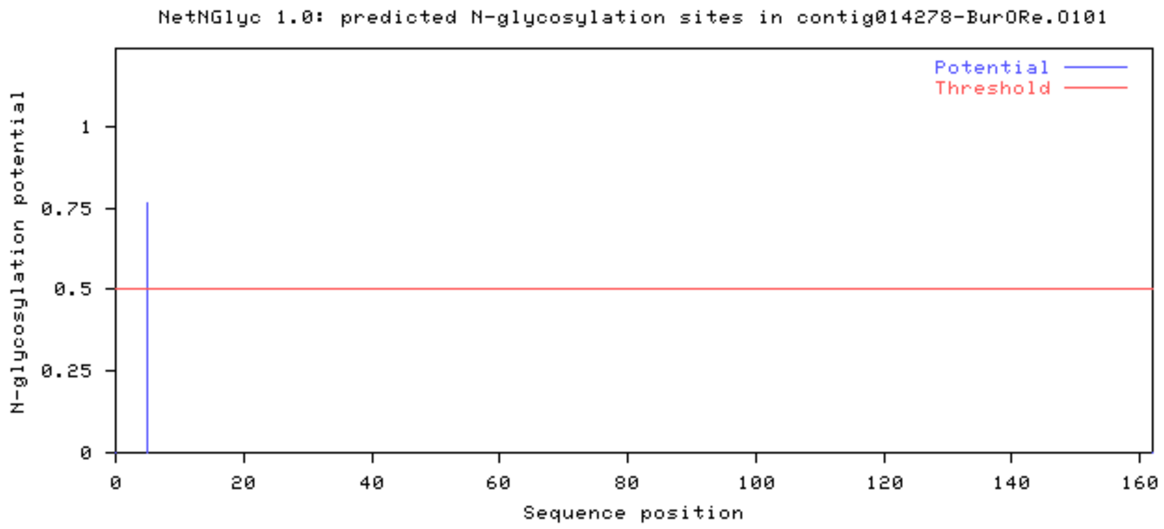

Graphics in PostScript

Output for 'contig014279-BurORe.O103'

```
##########################################################################################

    Warning: This sequence may not contain a signal peptide!!

    Proteins without signal peptides are unlikely to be exposed to
    the N-glycosylation machinery and thus may not be glycosylated
    (in vivo) even though they contain potential motifs.

    SignalP-NN euk predictions are as follows:

  # name                Cmax  pos ?  Ymax  pos ?  Smax  pos ?  Smean ?  D     ?

    SignalP output is explained at http://www.cbs.dtu.dk/services/SignalP/output.html

##########################################################################################

Name:  contig014279-BurORe.0103        Length:  60
GVGFNFNLVIRIIITMMYSLIPAAVNPIIYCFKTKEIKNVLMRRFKKGKVSTGLKTECKX
............................................................                          80

(Threshold=0.5)

No sites predicted in this sequence.
```

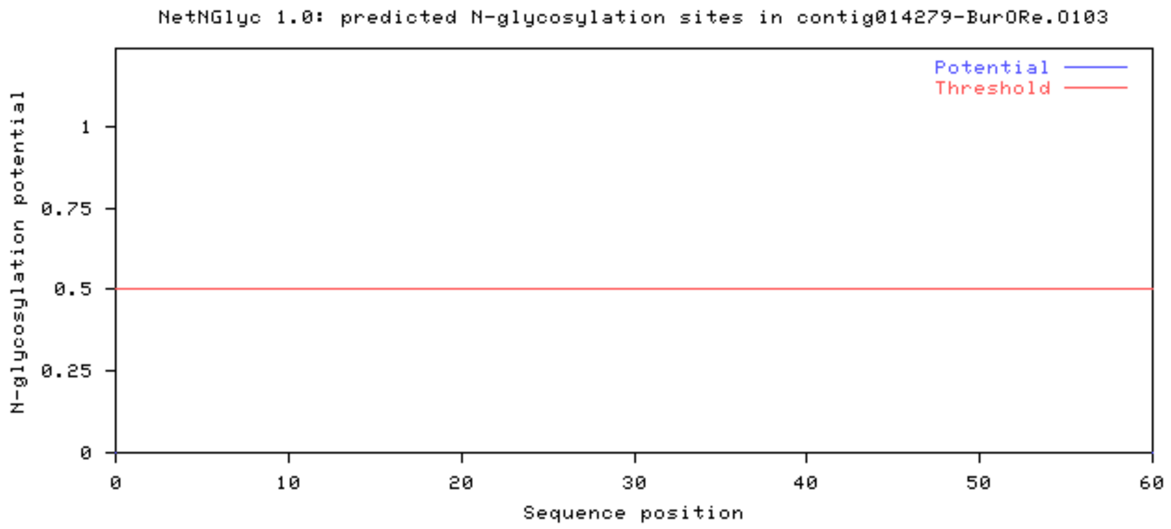

Graphics in PostScript

Output for 'contig014283-BurORe.O100'

```
########################################################################################

    Warning: This sequence may not contain a signal peptide!!

    Proteins without signal peptides are unlikely to be exposed to
    the N-glycosylation machinery and thus may not be glycosylated
    (in vivo) even though they contain potential motifs.

    SignalP-NN euk predictions are as follows:

  # name                 Cmax  pos ?  Ymax  pos ?  Smax  pos ?  Smean ?  D     ?

    SignalP output is explained at http://www.cbs.dtu.dk/services/SignalP/output.html

########################################################################################

Name:  contig014283-BurORe.O100         Length:  234
MITSFGACFTQMYFVHSLGAIQSLNLLMMALDRFVAIWFPFKYPILFTNKAVAIACTMCWVLTFIRLLGIVLHALTLPYC      80
DQNIIMQCYCDHISITRLGCGDGLAYVNSVALANAMVTLLVPLTFIILSYFSVIIAVLRMSQTERRHKVLSTCAPQLFIT     160
CLYYVPRCFVYIANGVGFNFNLVIRIIITMMYSLIPAAVNPIIYCFKTKDIKNVLMRRFKKGKVSTGLKTECKX
................................................................................      80
................................................................................     160
..........................................................................           240

(Threshold=0.5)

No sites predicted in this sequence.
```

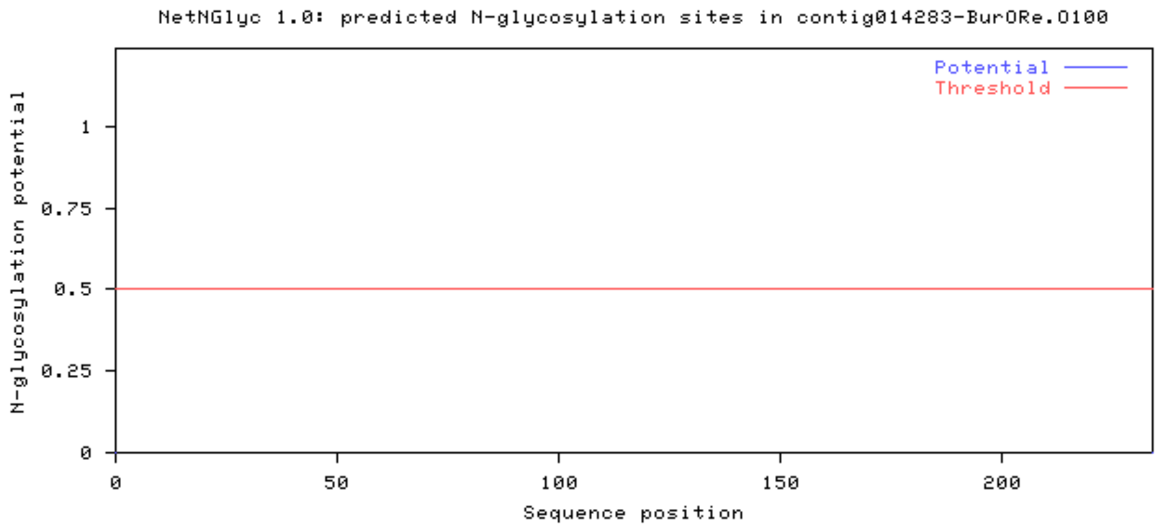

Graphics in PostScript

Output for 'contig017696-BurOR.P118'

```
##########################################################################

    Warning: This sequence may not contain a signal peptide!!

    Proteins without signal peptides are unlikely to be exposed to
    the N-glycosylation machinery and thus may not be glycosylated
    (in vivo) even though they contain potential motifs.

    SignalP-NN euk predictions are as follows:

  # name                    Cmax  pos ?  Ymax  pos ?  Smax  pos ?  Smean ?  D     ?

    SignalP output is explained at http://www.cbs.dtu.dk/services/SignalP/output.html

##########################################################################

Name:  contig017696-BurOR.P118  Length:  332
MLEAPLSRNFSHCTFVLRGFPSLQKHRRLLALPFSASYLLVLLGNSLLVYVICSVERLHSPMYLLICTLCFVDILVVTTI      80
IPNMLLGFLFDWNEISLVGCLTQMFFIHFLSSVESTLLLAMALDRYFAICRPLRYNDIINSSMLVRLVLFTLVRSVSVMA     160
TLVGLAGSLQFCGSNVIQHCYCDHMALVSLACDSTSRSSAAGLAVIICFVGADIPIIFFSYMKILSVVLRSAAAGEDSRK     240
AFHTCSTHLIVMMCFYLVGSITFLSHNLNIPIPTDINNSMGLMYILFPATINPIIYGVRTKEIRDSFLRIFKNRAKKIMT     320
VKVSSAGKEKSX
........N.......................................................................      80
................................................................................     160
................................................................................     240
................................................................................     320
............                                                                         400

(Threshold=0.5)
----------------------------------------------------------------------
SeqName      Position  Potential    Jury    N-Glyc
                                 agreement  result
----------------------------------------------------------------------
contig017696-BurOR.P118     9 NFSH   0.6081     (9/9)   ++
contig017696-BurOR.P118   140 NSSM   0.4438     (7/9)   -
contig017696-BurOR.P118   277 NNSM   0.2283     (9/9)   ---
----------------------------------------------------------------------
```

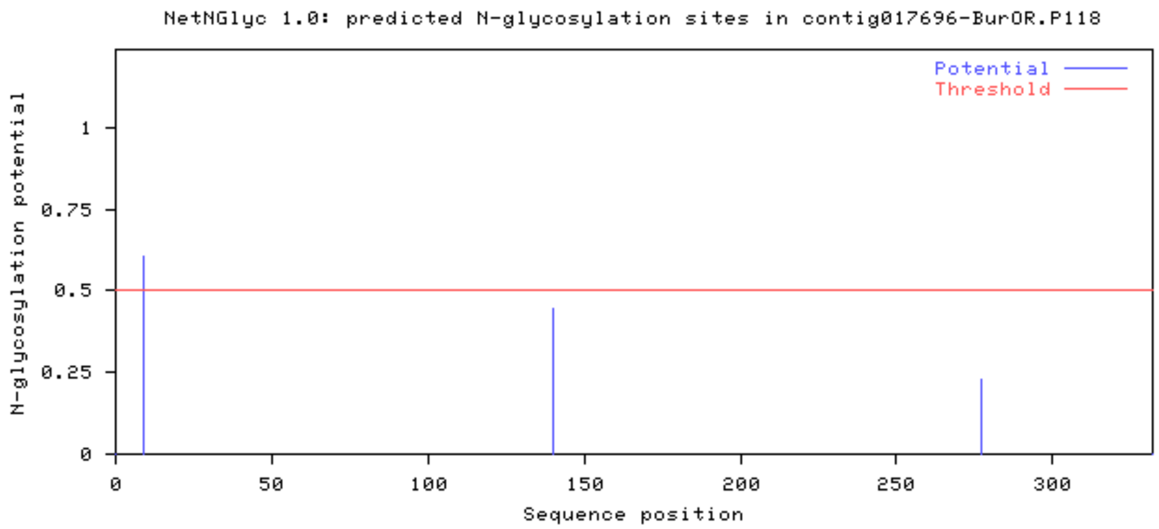

Graphics in PostScript

Output for 'contig017697-BurOR.P119'

```
########################################################################################

    Warning: This sequence may not contain a signal peptide!!

    Proteins without signal peptides are unlikely to be exposed to
    the N-glycosylation machinery and thus may not be glycosylated
    (in vivo) even though they contain potential motifs.

    SignalP-NN euk predictions are as follows:

  # name                 Cmax  pos ?  Ymax  pos ?  Smax  pos ?  Smean ?  D     ?

    SignalP output is explained at http://www.cbs.dtu.dk/services/SignalP/output.html

########################################################################################

Name:  contig017697-BurOR.P119  Length:  310
MEAVKENVSSHKYFFLDGFSELGELRPFLFIPFSFMFVVSLFANSLLVYVIVSQRSLHSPMYILIASMACIDLSLPVFFV      80
PHMLLSFLFDWRGISLIGCLFQMYFIHLLGAFQSTLLLWMALDRYFAICTPLHYQEQMALARFLKFVIPFSIRNMFAVLV     160
VVSLAGKLPFCLRNVINHCFCEHMALVELACGSTTINSLVGLISVFSVPVTDFFLITASYTVIFSSVLSSGKSSAKALHT     240
CVTHIVVMTVSLTIILTAFLSYRIRNGLPAAIRIFFSILYLFFPSCFNPIIYGIRTKEIRQHILNTLRVX
......N.........................................................................      80
................................................................................     160
................................................................................     240
......................................................................               320

(Threshold=0.5)
----------------------------------------------------------------------
SeqName      Position  Potential   Jury    N-Glyc
                                 agreement result
----------------------------------------------------------------------
contig017697-BurOR.P119    7 NVSS   0.7248     (9/9)    ++
----------------------------------------------------------------------
```

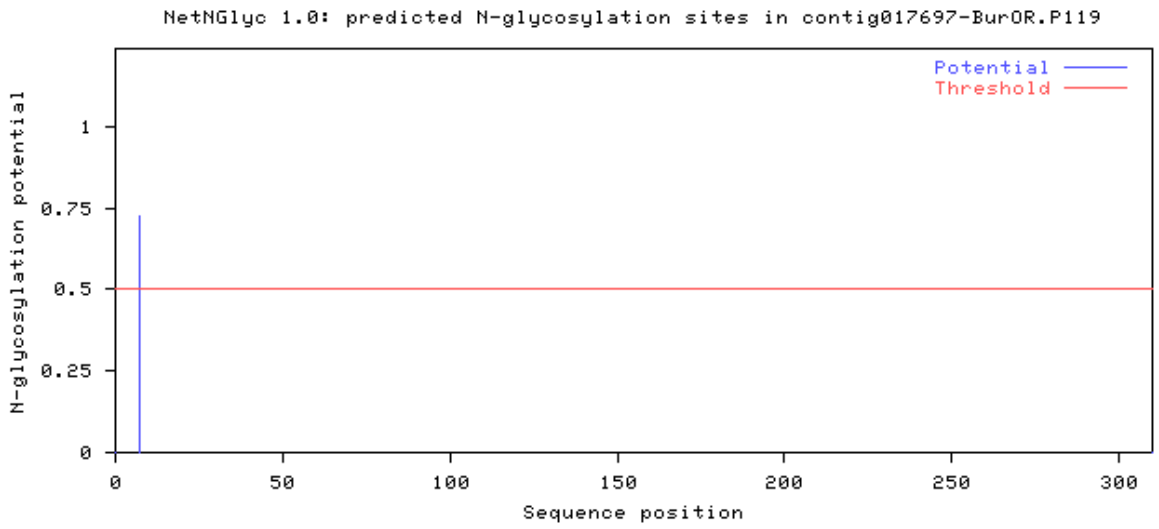

[Graphics in PostScript]

Output for 'contig017698-BurOR.P120'

```
########################################################################################

    Warning: This sequence may not contain a signal peptide!!

    Proteins without signal peptides are unlikely to be exposed to
    the N-glycosylation machinery and thus may not be glycosylated
    (in vivo) even though they contain potential motifs.

    SignalP-NN euk predictions are as follows:

  # name                Cmax  pos ?  Ymax  pos ?  Smax  pos ?  Smean ?  D     ?

    SignalP output is explained at http://www.cbs.dtu.dk/services/SignalP/output.html

########################################################################################

Name:  contig017698-BurOR.P120  Length:  318
MENVSLHTHFTLDGFSELGELRPFLFIPFSFMFVVSLFANSLLVYVIVSQRSLHSPMYILIASMACIDLSLPVFFVPHML      80
LSFLFDWRGISLIGCLVQMHLIHFFGTFQSTLLVWMALDRYFAICTPLYYHNHMILSRFIAFLIPLVVRNVLMITLLVCL     160
AGKLPFCLRNVINHCFCEHMALVELACGSTTINNLVGLMAVFLIPVLDFVFITASYVVIFSSVLKSSRSGVKALHTCITH     240
IMVITVSLILALTAFLSYRIRNGLPAASRVFFSTMYLLFPSCFNPVVYGIRTNEIRQHILKRLTCCHICQMGPVTNKX
..N.............................................................................      80
................................................................................     160
................................................................................     240
..............................................................................       320

(Threshold=0.5)
----------------------------------------------------------------------
SeqName      Position  Potential   Jury    N-Glyc
                                 agreement result
----------------------------------------------------------------------
contig017698-BurOR.P120    3 NVSL   0.7701     (9/9)    +++
----------------------------------------------------------------------
```

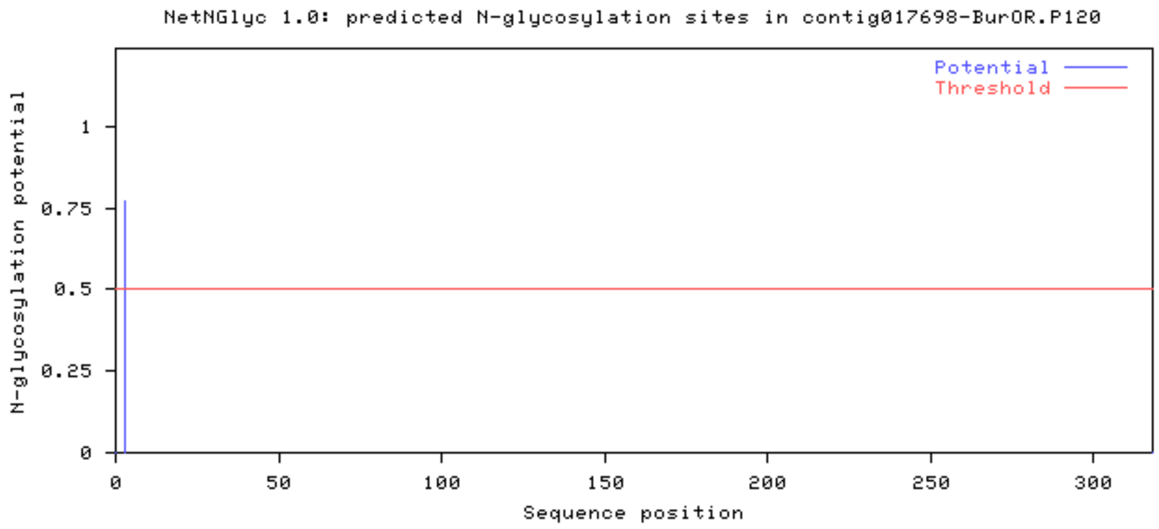

Graphics in PostScript

Output for 'contig017699-BurOR.E042'

```
##########################################################################

   Warning: This sequence may not contain a signal peptide!!

   Proteins without signal peptides are unlikely to be exposed to
   the N-glycosylation machinery and thus may not be glycosylated
   (in vivo) even though they contain potential motifs.

   SignalP-NN euk predictions are as follows:

  # name                Cmax  pos ?  Ymax  pos ?  Smax  pos ?  Smean ?  D     ?

   SignalP output is explained at http://www.cbs.dtu.dk/services/SignalP/output.html

##########################################################################

Name:  contig017699-BurOR.E042  Length:  310
MKNSTHLPDFILGAYFDGGTFRYLYFIIVMSLYVFIFGSNVLLIVVICVNRSLHEPMYMFLCSLFVNELYGSTGLFPSLL      80
VQILSDVHTVSAPLCFLQVFSVYSYGSIEFLNLAVMSYDRYLAICCPLQYNELMTSNKVTKLIVAVWSPPLLVNFLTLPL     160
IVPLKRCGNIINKVYCDNHSIVKLACSDTTLNNIYGLTVSALSVFGPLIVILYTYTRILKVCFSGSKQTRQKAVSTCTPH     240
LASLLNFSFGACFEILQSRFNMNSSPNMLRIFLSLYFLTCPPLFNPLMYGLNLSKIRVTCKNLITHIICX
..N..............................................N..............................      80
................................................................................     160
................................................................................     240
...................................................N..................               320

(Threshold=0.5)
----------------------------------------------------------------------
SeqName      Position  Potential   Jury    N-Glyc
                                  agreement result
----------------------------------------------------------------------
contig017699-BurOR.E042    3 NSTH   0.5550     (5/9)    +
contig017699-BurOR.E042   50 NRSL   0.7492     (9/9)    ++
contig017699-BurOR.E042  178 NHSI   0.4336     (7/9)    -
contig017699-BurOR.E042  246 NFSF   0.4126     (7/9)    -
contig017699-BurOR.E042  263 NSSP   0.1307     (9/9)    ---
contig017699-BurOR.E042  292 NLSK   0.6552     (8/9)    +
----------------------------------------------------------------------
```

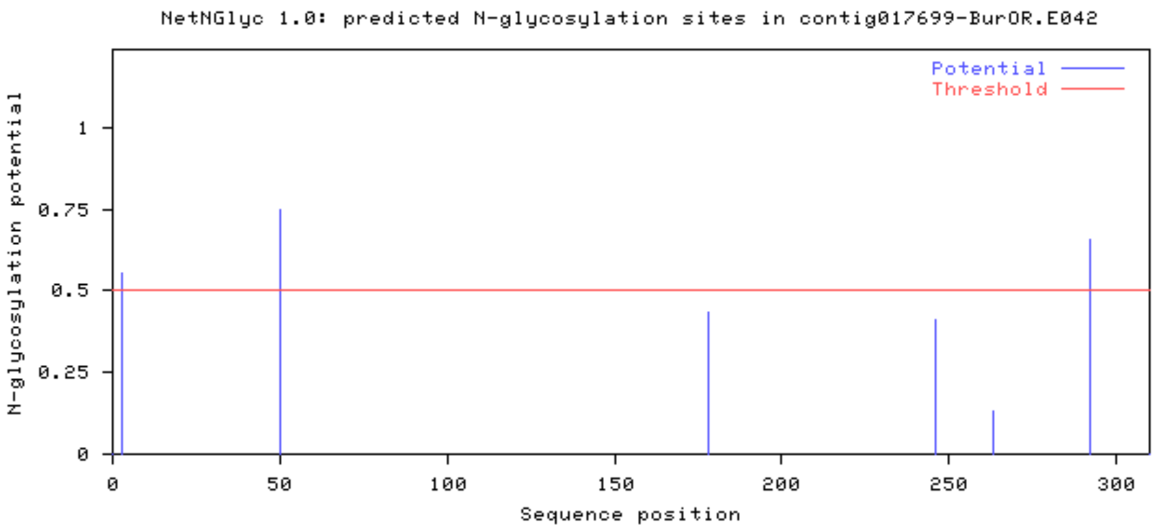

Graphics in PostScript

Output for 'contig017700-BurORe.E056'

```
##########################################################################################

    Warning: This sequence may not contain a signal peptide!!

    Proteins without signal peptides are unlikely to be exposed to
    the N-glycosylation machinery and thus may not be glycosylated
    (in vivo) even though they contain potential motifs.

    SignalP-NN euk predictions are as follows:

  # name                   Cmax  pos ?  Ymax  pos ?  Smax  pos ?  Smean ?  D     ?

    SignalP output is explained at http://www.cbs.dtu.dk/services/SignalP/output.html

##########################################################################################

Name:  contig017700-BurORe.E056         Length:  106
IFGALLFILYTYMKILLVCFSGSDQTRQKAISTCTPHLASILNFSFGASFEILQSRFNMKNVPNMLRIFLSLYFLTCQPL      80
FNPVMYGLKMTKIRNICKSLITNIKX
................................................................................      80
..........................                                                           160

(Threshold=0.5)

No sites predicted in this sequence.
```

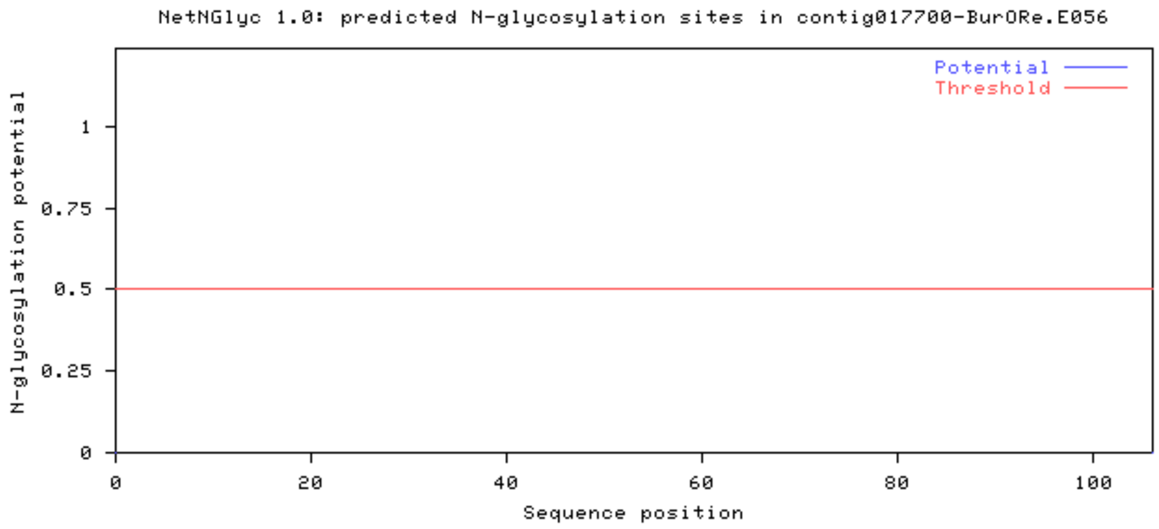

Graphics in PostScript

Output for 'contig028564-BurOR.J077'

```
##############################################################################

    Warning: This sequence may not contain a signal peptide!!

    Proteins without signal peptides are unlikely to be exposed to
    the N-glycosylation machinery and thus may not be glycosylated
    (in vivo) even though they contain potential motifs.

    SignalP-NN euk predictions are as follows:

  # name                    Cmax   pos ?  Ymax   pos ?  Smax   pos ?  Smean ?  D     ?

    SignalP output is explained at http://www.cbs.dtu.dk/services/SignalP/output.html

##############################################################################

Name:  contig028564-BurOR.J077  Length:  312
MENQSFDFSSELTLDPFVIPPGGKYPIFFLGISICIFGISCNLTLLALIILNRNLHKPMYFILFSLPLNDLIGLSAMLPK      80
VLSDIVTETHKIDYHLCVLQAFLLHMYGGGILFILAAMSFDRYVAICMPLRYSSVMTPRFISCIIVLVWGLDFVLIVSLF     160
SLQARLPRCKHVVMNVFCDNPSLLKLTCGNTTVNNIMGLFNTAVIQVVSVSIQAYSYVKILIACVVTRKSETKAKAVNTC     240
VAQLVILFMFEVVATFTILSHRFKNVSVDMQKIMGMLIFTVPPLLNPIVYGLYTNEMRSTLLRVLKNRVSMX
..N......................................N......................................      80
................................................................................     160
...................N.........N..................................................     240
........................N...............................................             320

(Threshold=0.5)
----------------------------------------------------------------------
SeqName      Position  Potential   Jury    N-Glyc
                                 agreement result
----------------------------------------------------------------------
contig028564-BurOR.J077    3 NQSF   0.6051     (9/9)    ++
contig028564-BurOR.J077   42 NLTL   0.6887     (9/9)    ++
contig028564-BurOR.J077  180 NPSL   0.5543     (6/9)    +     WARNING: PRO-X1.
contig028564-BurOR.J077  190 NTTV   0.6812     (9/9)    ++
contig028564-BurOR.J077  265 NVSV   0.5148     (6/9)    +
----------------------------------------------------------------------
```

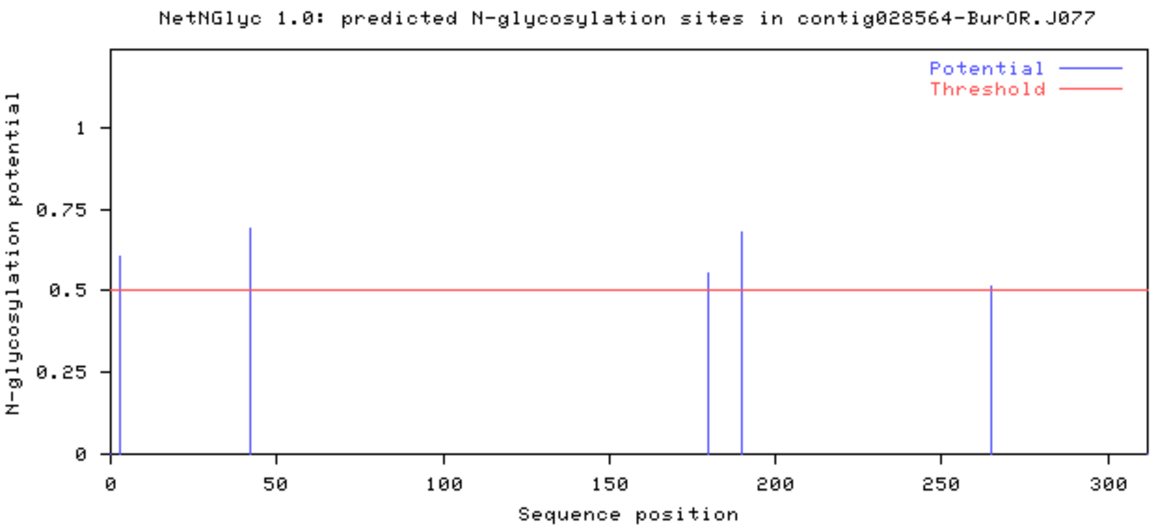

Graphics in PostScript

Output for 'contig028564-BurORe.J079'

```
##########################################################################################

    Warning: This sequence may not contain a signal peptide!!

    Proteins without signal peptides are unlikely to be exposed to
    the N-glycosylation machinery and thus may not be glycosylated
    (in vivo) even though they contain potential motifs.

    SignalP-NN euk predictions are as follows:

  # name                   Cmax  pos ?  Ymax  pos ?  Smax  pos ?  Smean ?  D     ?

    SignalP output is explained at http://www.cbs.dtu.dk/services/SignalP/output.html

##########################################################################################

Name:  contig028564-BurORe.J079        Length:  172
MNNRYNASSFLQINVFNLSSESVFPAFFFATLSYMIILFCNLTLILTIVLNKSLHQPMYLILLNLPINDLIGSSALFPQL      80
IKEILRNSGIMQYSACVAQAFFIHIYAAGTVFNLSAMAYDRYIAICYPLQYSTVMTNAHIMRIITIVWMSCLVLIAVLFF     160
LLLRLPRCRSEM
.....N..........N......................N.........N............................      80
................................................................................     160
............                                                                         240

(Threshold=0.5)
----------------------------------------------------------------------
SeqName       Position  Potential    Jury    N-Glyc
                                   agreement result
----------------------------------------------------------------------
contig028564-BurORe.J079    6 NASS   0.7524     (9/9)   +++
contig028564-BurORe.J079   17 NLSS   0.5060     (6/9)   +
contig028564-BurORe.J079   41 NLTL   0.7098     (9/9)   ++
contig028564-BurORe.J079   51 NKSL   0.7341     (9/9)   ++
contig028564-BurORe.J079  113 NLSA   0.3861     (8/9)   -
----------------------------------------------------------------------
```

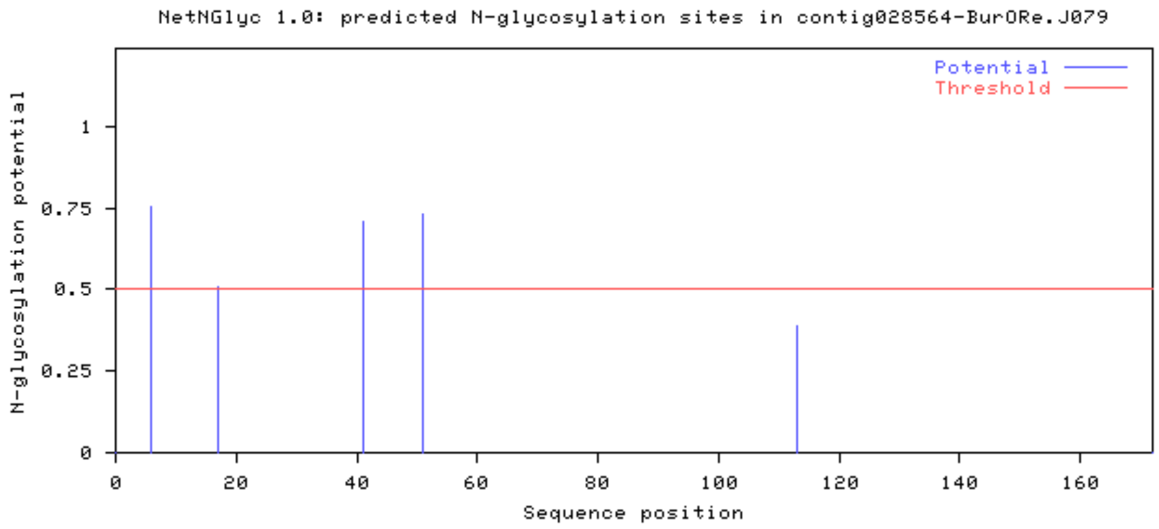

Graphics in PostScript

Output for 'contig028565-BurOR.K080'

```
##########################################################################

    Warning: This sequence may not contain a signal peptide!!

    Proteins without signal peptides are unlikely to be exposed to
    the N-glycosylation machinery and thus may not be glycosylated
    (in vivo) even though they contain potential motifs.

    SignalP-NN euk predictions are as follows:

  # name                 Cmax  pos ?  Ymax  pos ?  Smax  pos ?  Smean ?  D     ?

    SignalP output is explained at http://www.cbs.dtu.dk/services/SignalP/output.html

##########################################################################

Name:  contig028565-BurOR.K080  Length:  312
MEKNKTVSTDILEVQGFDISPQLTYPLFFLLLFVYFTLLFSNIGVLLLIISQKSLHQPMYFLFCNLSVNDLIGNTVLLPQ      80
LMAHILATERFITYKQCVVQAFQSHTFGSASHMILIIMAIDRYVAICHPLRYSSIMTTRTVVGLSAAAWGVSVVLVSILI     160
GLTVRLSRCRSTIQNSYCDNASLFKLSCEDVSVNNIYGLFFTVLLFTSSIASIAATYFRIALICWIKKNKDLNNKALQTC     240
ASHLVLYLIMLWSGFLTIILHRFPNYPDLRKIAYVLFHVVPANLNPIIYGMQTRSLRHKITEILKRKVTPSX
...N............................................................N...............      80
................................................................................     160
...................N............................................................     240
........................................................................             320

(Threshold=0.5)
----------------------------------------------------------------------
SeqName      Position  Potential   Jury    N-Glyc
                                 agreement result
----------------------------------------------------------------------
contig028565-BurOR.K080    4 NKTV   0.7128     (9/9)    ++
contig028565-BurOR.K080   65 NLSV   0.7387     (9/9)    ++
contig028565-BurOR.K080  180 NASL   0.5740     (7/9)    +
----------------------------------------------------------------------
```

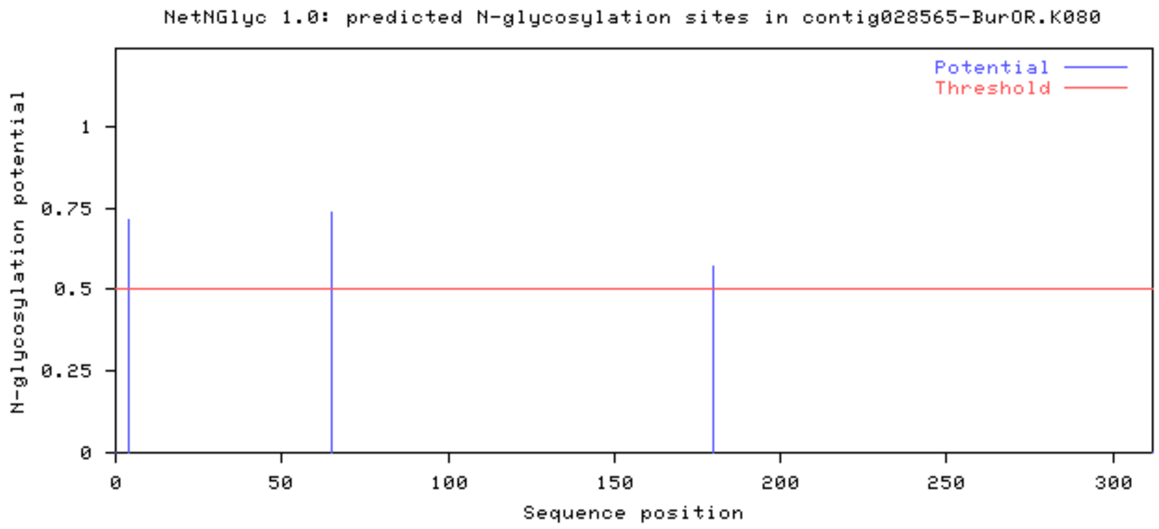

Graphics in PostScript

Output for 'contig028593-BurOR.S121'

```
##########################################################################################

    Warning: This sequence may not contain a signal peptide!!

    Proteins without signal peptides are unlikely to be exposed to
    the N-glycosylation machinery and thus may not be glycosylated
    (in vivo) even though they contain potential motifs.

    SignalP-NN euk predictions are as follows:

  # name                Cmax  pos ?  Ymax  pos ?  Smax  pos ?  Smean ?  D     ?

    SignalP output is explained at http://www.cbs.dtu.dk/services/SignalP/output.html

##########################################################################################

Name:  contig028593-BurOR.S121  Length:  313
MVDNSSFIGVESSRQQLNDQVIIVQVLVGVFLCINTMLIITFFMKDTFYRTMRYILFAVTLLSDCLILILTDLLLILSYF      80
RLSIQVSLCLIMFAVSSVCNFVTPFTLTAMTLERYVAICMPLRHGDLCSTRSALQCILIIHGLSSVPCILILSVFFASVS     160
LSFFTQYRVCSVEMFILRSWQGHLRSAISQFYFLIMCIIIVFSYIQIMKVAKAASGENKKSTHKGLRTVALHAFQLLLCL     240
IQLWCPFIEDAVLQINFMLYVNVRYFNYIMFSLTPRCLSPLIYGLRDDTFFLALRYHVLCHLHRKKSVGVSNX
...N............................................................................      80
................................................................................     160
................................................................................     240
.........................................................................            320

(Threshold=0.5)
----------------------------------------------------------------------
SeqName      Position  Potential   Jury    N-Glyc
                                 agreement result
----------------------------------------------------------------------
contig028593-BurOR.S121    4 NSSF   0.6171     (7/9)   +
----------------------------------------------------------------------
```

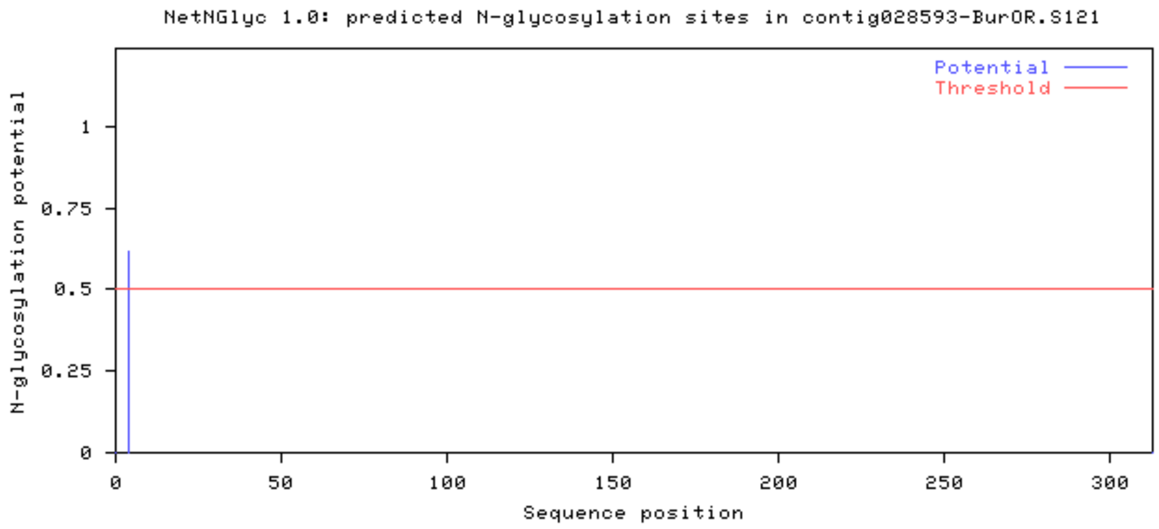

Graphics in PostScript

Output for 'contig028594-BurOR.S122'

```
##########################################################################

    Warning: This sequence may not contain a signal peptide!!

    Proteins without signal peptides are unlikely to be exposed to
    the N-glycosylation machinery and thus may not be glycosylated
    (in vivo) even though they contain potential motifs.

    SignalP-NN euk predictions are as follows:

  # name                Cmax  pos ?  Ymax  pos ?  Smax  pos ?  Smean ?  D     ?

    SignalP output is explained at http://www.cbs.dtu.dk/services/SignalP/output.html

##########################################################################

Name:  contig028594-BurOR.S122  Length:  311
MALNNSVIGGQLAINNINNQVIIVQLLISMFLCINFLLITTFFMKDIFYTTMRYILFAIALLSDSLFLLITNVLLILSYF      80
SFTIQVWLCVIIYIVLSVYTFVTPVTLTAMTLERFVAICVPLRHAELCRTQRALHFILIIHGLSSVPCIVILSIFFASAI     160
TSFYTQSRICAVEMFIFHRWQGHLRSAISQLYFLIMSITIVFSYVQIMKVAKAASGENKKSTWKGLSTVVLHGFQLLLCL     240
FQLWCPFIEAAVLQIDFMLFINVRYFNYITFILAPRCLSPLIYGLRDEMFVNALKYYALCGLYKKHSTAFX
...N............................................................................      80
................................................................................     160
................................................................................     240
.......................................................................             320

(Threshold=0.5)
----------------------------------------------------------------------
SeqName      Position  Potential   Jury    N-Glyc
                                agreement  result
----------------------------------------------------------------------
contig028594-BurOR.S122    4 NNSV   0.7410     (9/9)    ++
----------------------------------------------------------------------
```

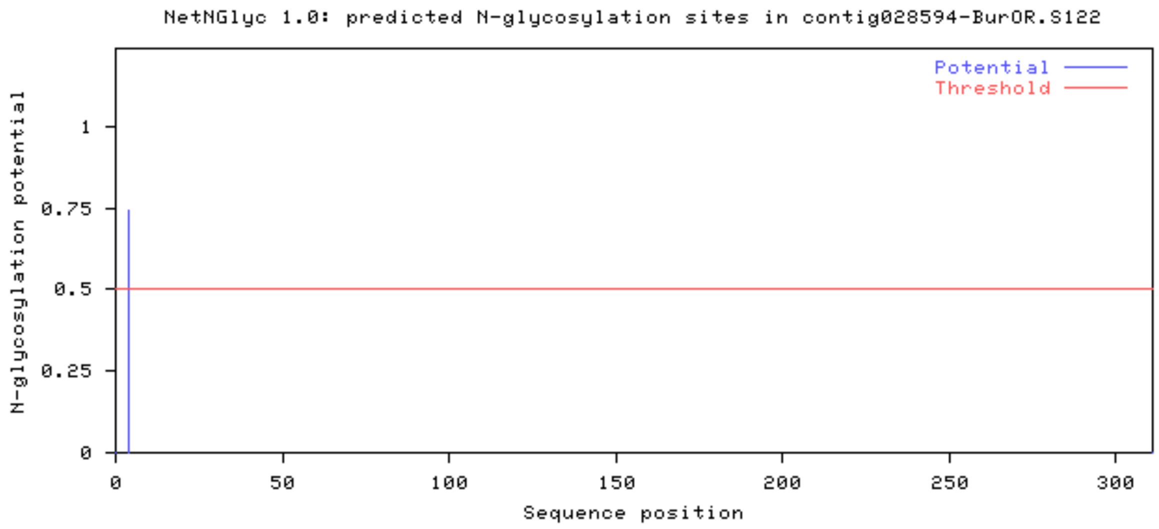

Graphics in PostScript

Output for 'contig028596-BurORe.S126'

```
########################################################################################

    Warning: This sequence may not contain a signal peptide!!

    Proteins without signal peptides are unlikely to be exposed to
    the N-glycosylation machinery and thus may not be glycosylated
    (in vivo) even though they contain potential motifs.

    SignalP-NN euk predictions are as follows:

  # name                Cmax  pos ?  Ymax  pos ?  Smax  pos ?  Smean ?  D     ?

    SignalP output is explained at http://www.cbs.dtu.dk/services/SignalP/output.html

########################################################################################

Name:  contig028596-BurORe.S126         Length:  220
MATNNSVIGGKLAINNINNRVIIVQVLISVFLCINFLLITTFFMKDIFYTTMRYILFAIALLSDSLFLLMTNALLILNYF      80
SFTIQVWLCVIIYIVLSVYTFVTPVTLTAMTLERFVAICVPLRHAELCRTQRALHFILIIHGLSSVPCIVILSIFFASAI     160
TSFYTQSRICAVEMFIFHRWQGHLRSAISQLYFLIMSITIVFSYVQIMKVAKAASGENKK
...N............................................................................      80
................................................................................     160
............................................................                         240

(Threshold=0.5)
----------------------------------------------------------------------
SeqName      Position  Potential   Jury    N-Glyc
                                agreement  result
----------------------------------------------------------------------
contig028596-BurORe.S126     4 NNSV   0.7083     (9/9)   ++
----------------------------------------------------------------------
```

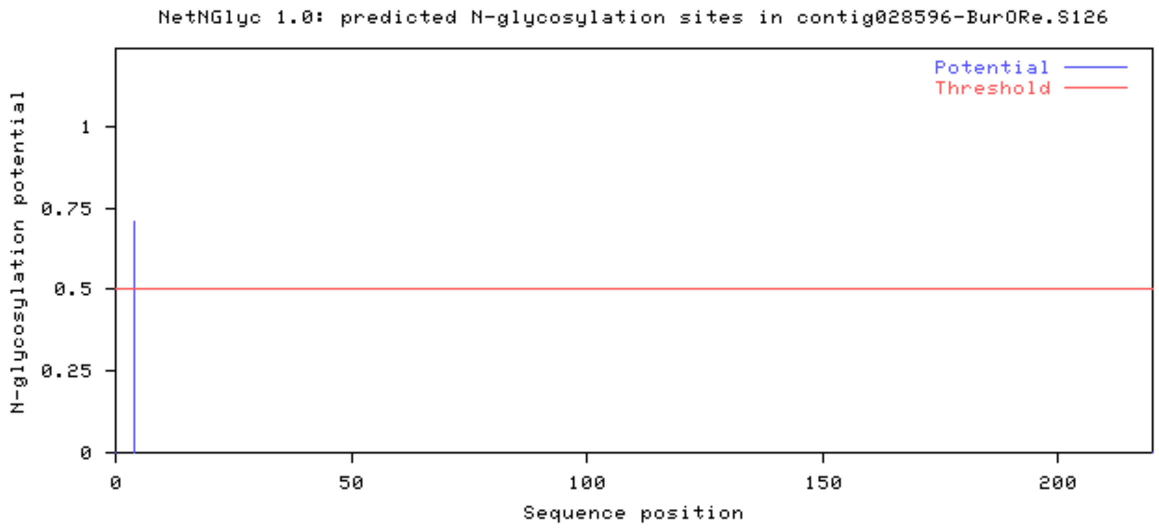

Graphics in PostScript

Output for 'contig032388-BurOR.D032'

```
##########################################################################

    Warning: This sequence may not contain a signal peptide!!

    Proteins without signal peptides are unlikely to be exposed to
    the N-glycosylation machinery and thus may not be glycosylated
    (in vivo) even though they contain potential motifs.

    SignalP-NN euk predictions are as follows:

  # name                Cmax  pos ?  Ymax  pos ?  Smax  pos ?  Smean ?  D     ?

    SignalP output is explained at http://www.cbs.dtu.dk/services/SignalP/output.html

##########################################################################

Name:  contig032388-BurOR.D032  Length:  311
MGNSSEIEVVSFVLAAYANIGALKYMYFVIMLFWYLSICVANTVVIVIIQVDRRLHEPMYILLCSLCVNEINSSTSLYPL      80
LLSQMFSDSHEVTVPWCFLQMCFMYISAPAEFWGLAAMAYDRYISICHPLCYNVIMNTKRVFLLILLVWIFSLVSFILSF     160
SFIFGLKFCRNIVDNVYCDHQLMIRLSCSVSTQSYISEIFFALVSIFIPFTLISVSYFKILAVCRKTSIENKQKAVTTCT     240
PQIVSVSNLFVGCIFHSIDFRFLIARVPDEVRVILPMYVLICQPMLTPFLYGFNLPKIRHSFKRLLFKRKX
..N.............................................................................      80
................................................................................     160
................................................................................     240
.......................................................................              320

(Threshold=0.5)
----------------------------------------------------------------------
SeqName      Position  Potential   Jury    N-Glyc
                                 agreement result
----------------------------------------------------------------------
contig032388-BurOR.D032    3 NSSE   0.7486     (9/9)    ++
contig032388-BurOR.D032   72 NSST   0.3692     (8/9)    -
----------------------------------------------------------------------
```

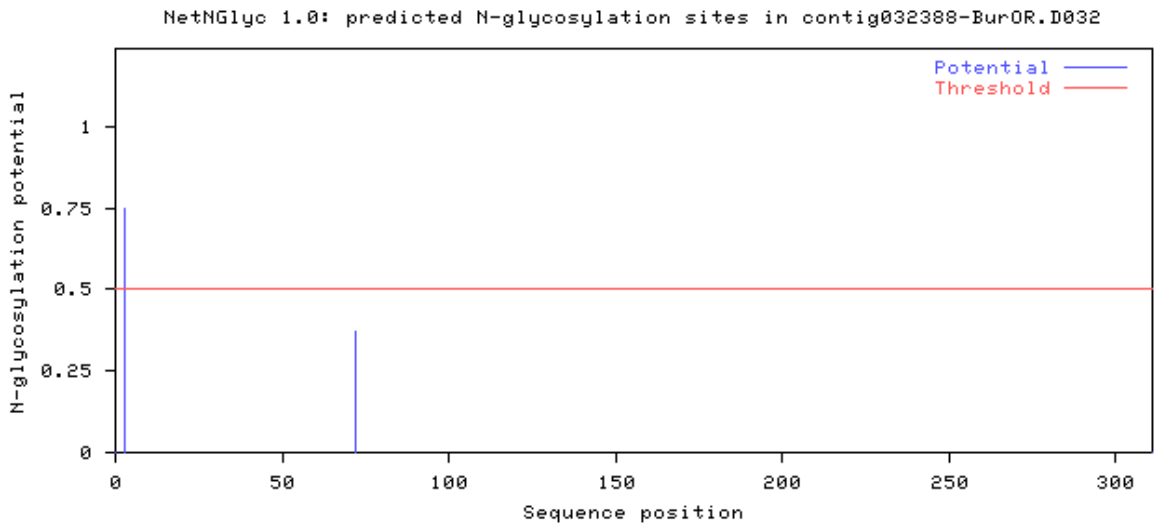

[Graphics in PostScript]

Output for 'contig032388-BurORe.D036'

```
##########################################################################################

    Warning: This sequence may not contain a signal peptide!!

    Proteins without signal peptides are unlikely to be exposed to
    the N-glycosylation machinery and thus may not be glycosylated
    (in vivo) even though they contain potential motifs.

    SignalP-NN euk predictions are as follows:

  # name                Cmax  pos ?  Ymax  pos ?  Smax  pos ?  Smean ?  D     ?

    SignalP output is explained at http://www.cbs.dtu.dk/services/SignalP/output.html

##########################################################################################

Name:  contig032388-BurORe.D036         Length:  305
MENSSEIVSFVLSAYGNVGELKYLYFVIILFWYLSICVANTVLILVIRVDRQLHEPMYILLCNLCVNEINISTSLYPLLL      80
SQMFSDRHEVTVPWCFLQMCFMYTSAPAEFCSLAAMSYDRYISICHPLRYNVIMNTERVFFMILLVWIYSFLSVILSFSF     160
VFSLKFCGNNIENVYCDHQLLIRLSCSLSFHSFISDIFFVFVSIFIPFNLISVSYVKILAICRKTSTENKQKAVTTCTPQ     240
LVSVSNLFVGCIFQSIDSSVIVAQLPHEVNIILSIYLFICQPMLTPFLYGFNLPKIRQSCKRFVF
..N.............................................................................      80
................................................................................     160
................................................................................     240
.................................................................     320

(Threshold=0.5)
----------------------------------------------------------------------
SeqName      Position  Potential   Jury    N-Glyc
                                 agreement result
----------------------------------------------------------------------
contig032388-BurORe.D036    3 NSSE   0.6951     (9/9)   ++
contig032388-BurORe.D036   70 NIST   0.4511     (5/9)   -
----------------------------------------------------------------------
```

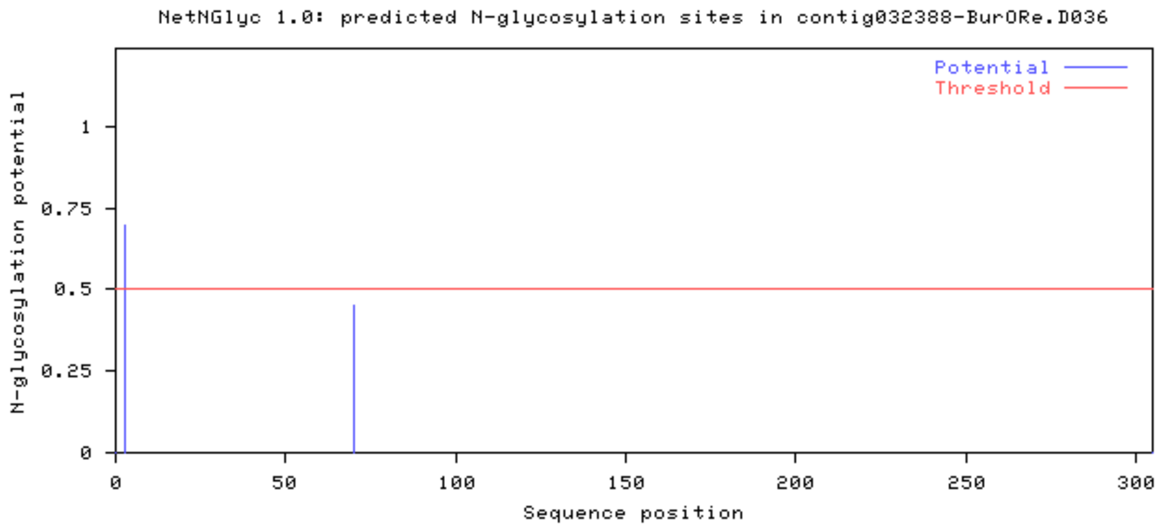

[Graphics in PostScript]

Output for 'contig032389-BurOR.D033'

```
########################################################################################

    Warning: This sequence may not contain a signal peptide!!

    Proteins without signal peptides are unlikely to be exposed to
    the N-glycosylation machinery and thus may not be glycosylated
    (in vivo) even though they contain potential motifs.

    SignalP-NN euk predictions are as follows:

  # name                Cmax  pos ?  Ymax  pos ?  Smax  pos ?  Smean ?  D     ?

    SignalP output is explained at http://www.cbs.dtu.dk/services/SignalP/output.html

########################################################################################

Name:  contig032389-BurOR.D033  Length:  309
MENSSEIVSFVLSAYGNVGDLKYLYLTIILFWYVSICVANIVLIVVIHVDRRLHEPMYILLSNLCVNEINASTSLYPLLL      80
SQMYSDSHEVTLPWCYLQMCCLYTSAPAEFWSLAAMAYDRYISICHPLRYNAIMNTERVLKIILLVWVFSFLIFILSFSF     160
IFSLQFCGNIVDNVYCEHQLIIRLSCSVSVQSSISIIFFVIMSIFIPFSLISVSYVKILTVCRKTSTENKQKAMTTCTPQ     240
IVSVSNLFVGCIFHSINFRLLISQVPDEVNIILPMYMLIFQPMLTPFMYGFNLPKIRQSCKRFLFKRKX
..N.............................................................................      80
................................................................................     160
................................................................................     240
.....................................................................                320

(Threshold=0.5)
----------------------------------------------------------------------
SeqName      Position  Potential   Jury    N-Glyc
                                 agreement result
----------------------------------------------------------------------
contig032389-BurOR.D033    3 NSSE   0.6952     (9/9)   ++
contig032389-BurOR.D033   70 NAST   0.3814     (6/9)   -
----------------------------------------------------------------------
```

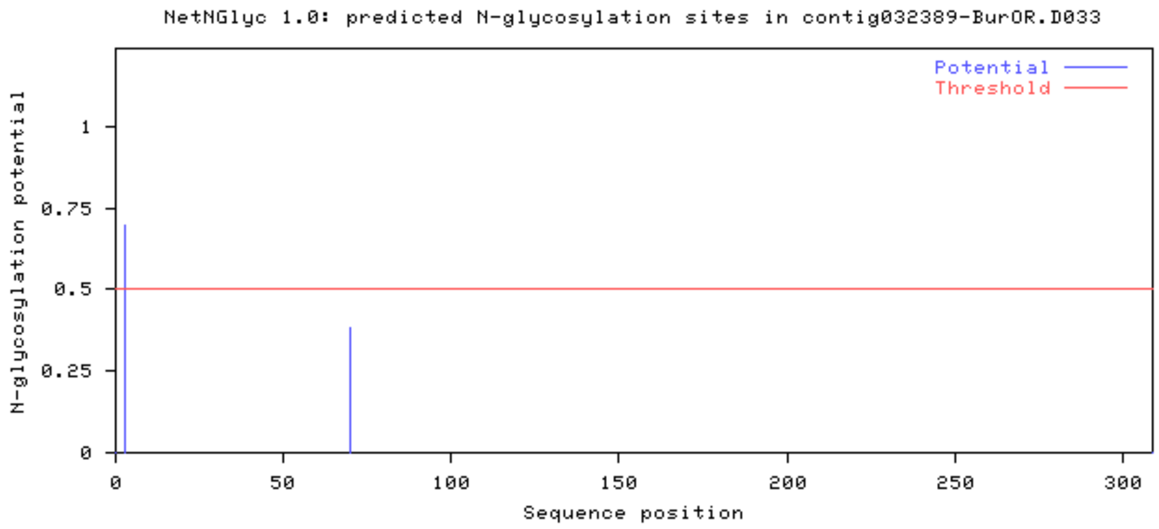

Graphics in PostScript

Output for 'contig032390-BurORe.D037'

```
##########################################################################################

    Warning: This sequence may not contain a signal peptide!!

    Proteins without signal peptides are unlikely to be exposed to
    the N-glycosylation machinery and thus may not be glycosylated
    (in vivo) even though they contain potential motifs.

    SignalP-NN euk predictions are as follows:

  # name                Cmax  pos ?  Ymax  pos ?  Smax  pos ?  Smean ?  D     ?

    SignalP output is explained at http://www.cbs.dtu.dk/services/SignalP/output.html

##########################################################################################

Name:  contig032390-BurORe.D037         Length:  107
SVFIPFSLILVSYMKILRVCRKTSKENKQKAVTTCTPQIISVSNLFFGCIFYFIDFRFVVSQVPDEVRIILPMYVLIFQP      80
MLTPFMYGFNLPKIRQSYQRFLFERKX
................................................................................      80
...........................                                                          160

(Threshold=0.5)

No sites predicted in this sequence.
```

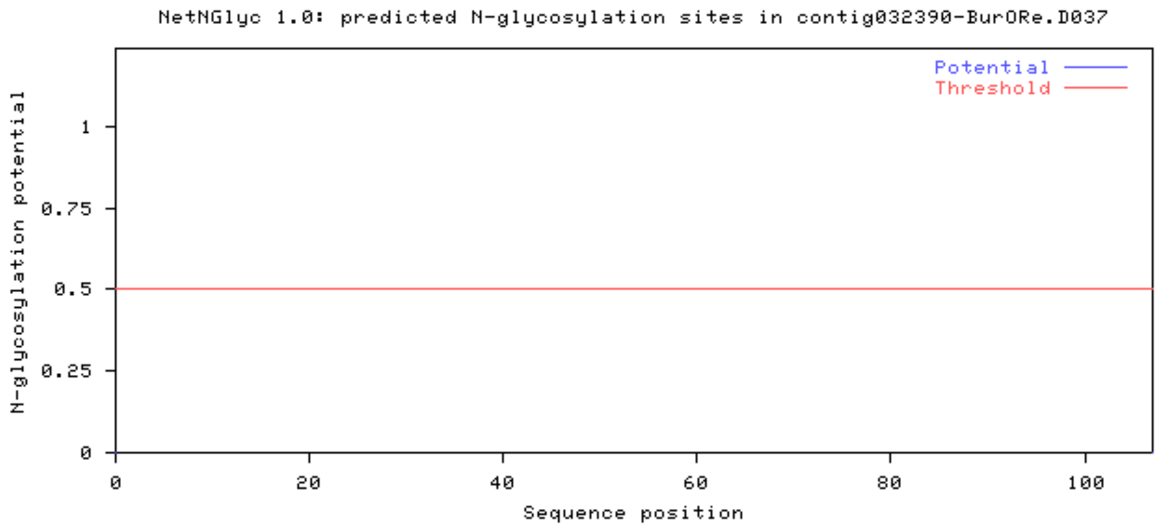

Graphics in PostScript

Output for 'contig032391-BurORe.D038'

```
##########################################################################################

    Warning: This sequence may not contain a signal peptide!!

    Proteins without signal peptides are unlikely to be exposed to
    the N-glycosylation machinery and thus may not be glycosylated
    (in vivo) even though they contain potential motifs.

    SignalP-NN euk predictions are as follows:

  # name                   Cmax  pos ?  Ymax  pos ?  Smax  pos ?  Smean ?  D     ?

    SignalP output is explained at http://www.cbs.dtu.dk/services/SignalP/output.html

##########################################################################################

Name:  contig032391-BurORe.D038         Length:  60
GYIFYFIDFRSVVSQVLDEVHIILPMYVLIFQPMLTPFMYGFNLPKIRQSYQRFLLKRKX
............................................................                          80

(Threshold=0.5)

No sites predicted in this sequence.
```

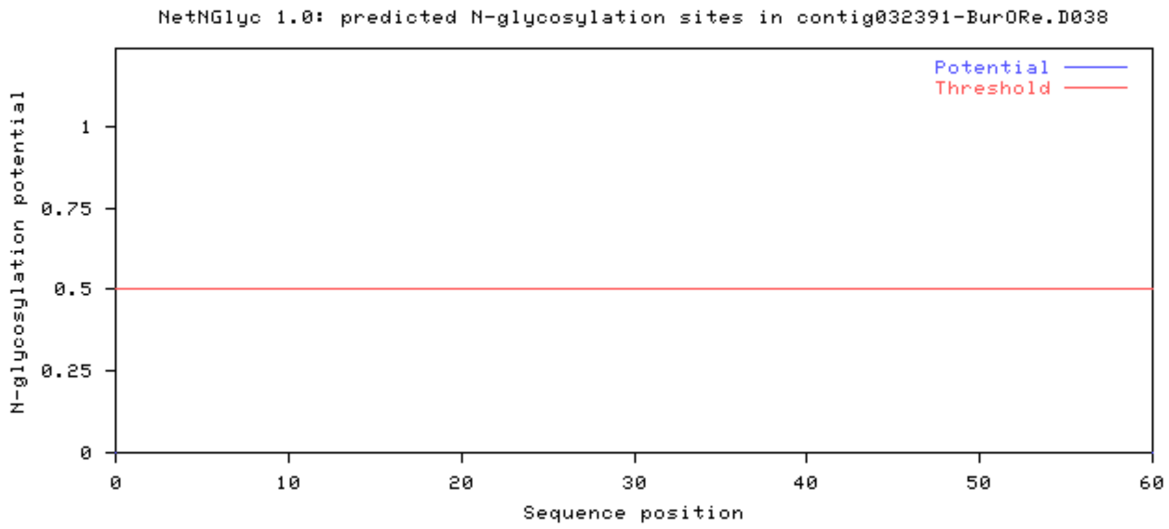

Graphics in PostScript

Output for 'contig032396-BurOR.D034'

```
##########################################################################

    Warning: This sequence may not contain a signal peptide!!

    Proteins without signal peptides are unlikely to be exposed to
    the N-glycosylation machinery and thus may not be glycosylated
    (in vivo) even though they contain potential motifs.

    SignalP-NN euk predictions are as follows:

  # name                  Cmax  pos ?  Ymax  pos ?  Smax  pos ?  Smean ?  D     ?

    SignalP output is explained at http://www.cbs.dtu.dk/services/SignalP/output.html

##########################################################################

Name:  contig032396-BurOR.D034  Length:  309
MGNSSETVSFVLAAYGNIGELKLYLYFIIILVWYFSICVANTVLIVVIRLDRRLHEPMYILLCNLCVNEINASTSLYPLLL      80
SQMFSDSHEVTVPWCFLQMCCMYTSAPAEFCSLAAMAYDRYISICHPLRYNVIMKTERVFLLILLVWVYSFLSFIFSFSF     160
IFSLKFCGNIIHNTYCDHQLIIRLSCSVPIQSFISNISFLLLSVFIPFSLISVSYMKILRVCRKTSKENKQKAVTTCTPQ     240
IICVSNLFVGCIFYFIDIRFLVSQVPDEVRIILPMYLLIFQPMLTPFMYGFKLTKIRQSYQRFLFERKX
..N.............................................................................      80
................................................................................     160
................................................................................     240
.....................................................................                320

(Threshold=0.5)
----------------------------------------------------------------------
SeqName      Position  Potential   Jury    N-Glyc
                                agreement result
----------------------------------------------------------------------
contig032396-BurOR.D034    3 NSSE   0.7408     (9/9)    ++
contig032396-BurOR.D034   70 NAST   0.3703     (6/9)    -
contig032396-BurOR.D034  196 NISF   0.4392     (6/9)    -
----------------------------------------------------------------------
```

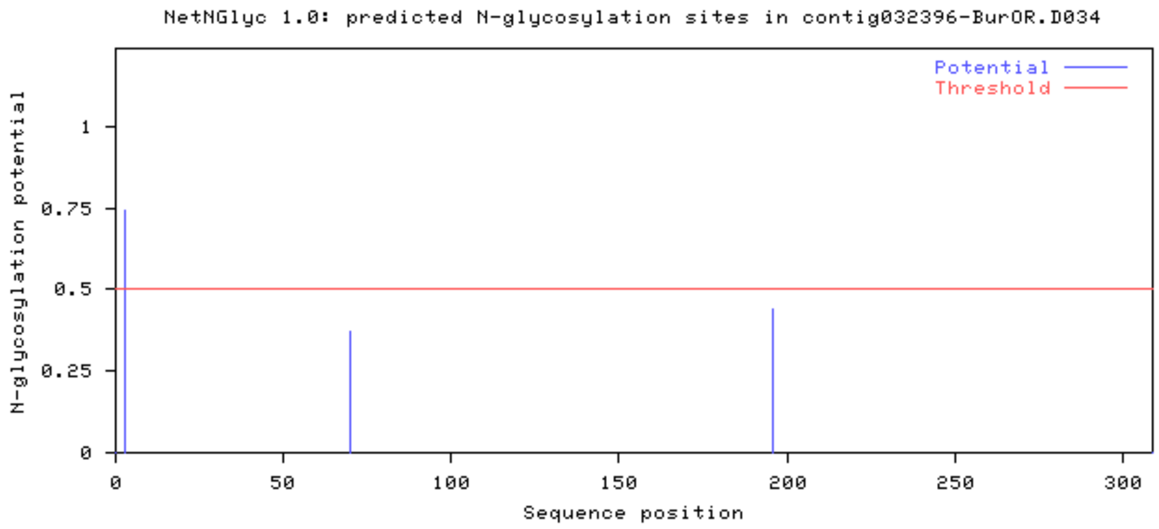

Graphics in PostScript

Output for 'contig036780-BurOR.A001'

```
##############################################################################

   Warning: This sequence may not contain a signal peptide!!

   Proteins without signal peptides are unlikely to be exposed to
   the N-glycosylation machinery and thus may not be glycosylated
   (in vivo) even though they contain potential motifs.

   SignalP-NN euk predictions are as follows:

 # name                 Cmax  pos ?  Ymax  pos ?  Smax  pos ?  Smean ?  D     ?

   SignalP output is explained at http://www.cbs.dtu.dk/services/SignalP/output.html

##############################################################################

Name:  contig036780-BurOR.A001  Length:  309
MELALNVSYITLDGFFQVNKYRYLYFMIMFTLYILILCCNFAIVFLIVVEKSLHEPMYIFIAALLLNSVMLSTVIYPKLL      80
TDFLSKRQIIPYSVCLFQFFMFYSLGGSEFLLLFAMAYDRYVSICKPLQYPMIMTKNTISIFLTLAWIVPSSQVAVVAVL     160
MANKKICNFTFTGIFCNNTIYKLLCVYSKAQTVYDMVVLSNVAILPAVFIFFTYTRILVISYQSCKEVRRKAAQTCLPHL     240
IVLISYLCLCAFDVIVSGLESNFPKIVHSILTLQIVMYPPLFNPIIYGLKMKEISKHLKRLFCAVKKNX
.....N..........................................................................      80
................................................................................     160
.......N........N...............................................................     240
.....................................................................                320

(Threshold=0.5)
----------------------------------------------------------------------
SeqName      Position  Potential   Jury    N-Glyc
                                 agreement result
----------------------------------------------------------------------
contig036780-BurOR.A001    6 NVSY   0.8398     (9/9)   +++
contig036780-BurOR.A001  168 NFTF   0.6488     (8/9)   +
contig036780-BurOR.A001  177 NNTI   0.5013     (4/9)   +
----------------------------------------------------------------------
```

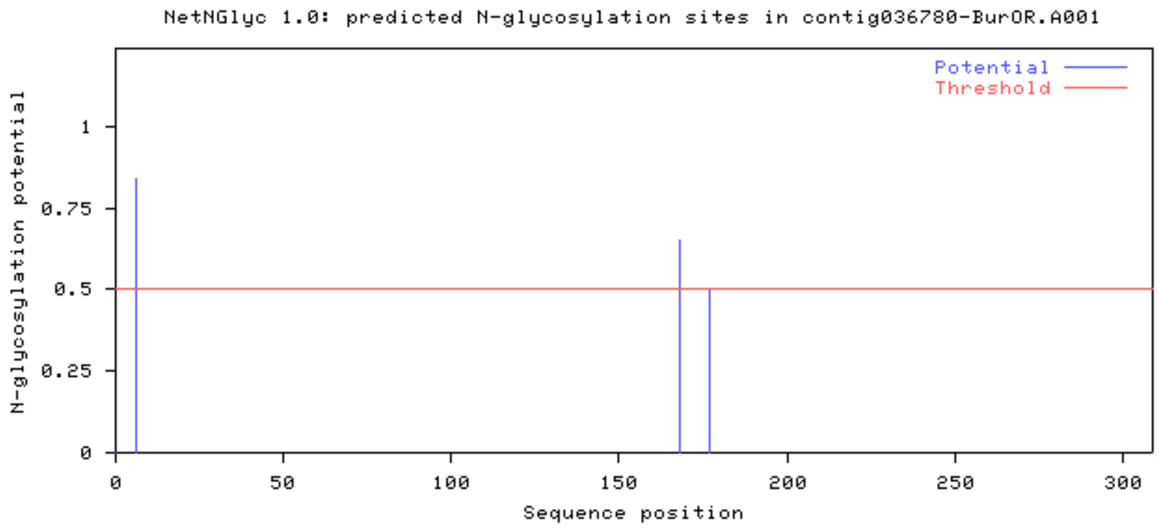

Graphics in PostScript

Output for 'contig036782-BurOR.A002'

```
##########################################################################

   Warning: This sequence may not contain a signal peptide!!

   Proteins without signal peptides are unlikely to be exposed to
   the N-glycosylation machinery and thus may not be glycosylated
   (in vivo) even though they contain potential motifs.

   SignalP-NN euk predictions are as follows:

 # name                Cmax  pos ?  Ymax  pos ?  Smax  pos ?  Smean ?  D     ?

   SignalP output is explained at http://www.cbs.dtu.dk/services/SignalP/output.html

##########################################################################

Name:  contig036782-BurOR.A002  Length:  307
MNADLNTTYVTLGGHVEIHRYRYLYFVIMLTAYILLVCFNVSIICLIVIHKNLHEPMYIFIAALLLNSILFSSNIHPKLL      80
VDFLSEKQIVSYQACLFQVFMFYFLSSSEFLLLSAMAYDRYVSICKPLQYPAIMRTTTVSLLLCFAWLVPACYIVVPVAL     160
NINSKLCSFTLKGIFCNNSLNKLFCVTSNELSIYGVIVLLNLGLFPMLFILFTYTKIIIIAFQSCGDIRRKAVQTCLPHL     240
LVLINYSVLITYDVVVVKLESDFPKTARFVMTLQIITYNPLCNPIIYGLKMKEISNHLKRLLRHMKX
.....N.................................N........................................      80
................................................................................     160
................................................................................     240
....N..............................................................                  320

(Threshold=0.5)
----------------------------------------------------------------------
SeqName      Position  Potential   Jury    N-Glyc
                                 agreement result
----------------------------------------------------------------------
contig036782-BurOR.A002    6 NTTY   0.7240     (9/9)   ++
contig036782-BurOR.A002   40 NVSI   0.7540     (9/9)   +++
contig036782-BurOR.A002  177 NNSL   0.4236     (7/9)   -
contig036782-BurOR.A002  245 NYSV   0.5312     (6/9)   +
----------------------------------------------------------------------
```

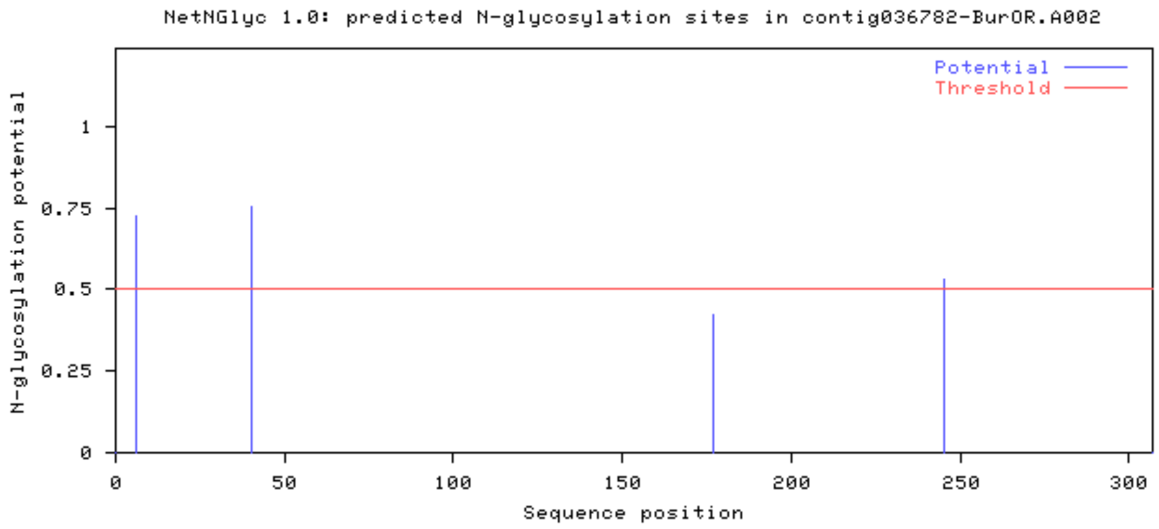

Graphics in PostScript

Output for 'contig036784-BurOR.A003'

```
##########################################################################################

    Warning: This sequence may not contain a signal peptide!!

    Proteins without signal peptides are unlikely to be exposed to
    the N-glycosylation machinery and thus may not be glycosylated
    (in vivo) even though they contain potential motifs.

    SignalP-NN euk predictions are as follows:

  # name                    Cmax  pos ?  Ymax  pos ?  Smax  pos ?  Smean ?  D     ?

    SignalP output is explained at http://www.cbs.dtu.dk/services/SignalP/output.html

##########################################################################################

Name:  contig036784-BurOR.A003  Length:  309
MDDELNVTYITLDGYVELKRCGYLYFLIMVALYVLIIISNSTIVFLICIHRNLHEPMYIFIAALSVNSVVFSTAIYPKLF      80
VDVLSEKQVISFSACQFQHFMYYSIGGSDFLLLSAMAFDRYVSICKPLKYPVIMRQTTINILLFVAWFLPGLQVAVSHAL     160
VLNNKLCNFTLKGIFCNNLLWKLYCESPRTALIYGLIVLLNVAIFPVLFILFTYAKIFLITYRSSRDIQKKAAETCLPHL     240
FVLSIFTTFCAYDVIIGQLEFDFPKTAQLIMTLQVVLYNPLFNPFIYGLKMKEISKHLKRLFCHVRCSX
.....N.................................N........................................      80
................................................................................     160
.......N........................................................................     240
.....................................................................                320

(Threshold=0.5)
----------------------------------------------------------------------
SeqName      Position  Potential   Jury     N-Glyc
                                 agreement  result
----------------------------------------------------------------------
contig036784-BurOR.A003    6 NVTY   0.7911     (9/9)   +++
contig036784-BurOR.A003   40 NSTI   0.7071     (9/9)   ++
contig036784-BurOR.A003  168 NFTL   0.6774     (8/9)   +
----------------------------------------------------------------------
```

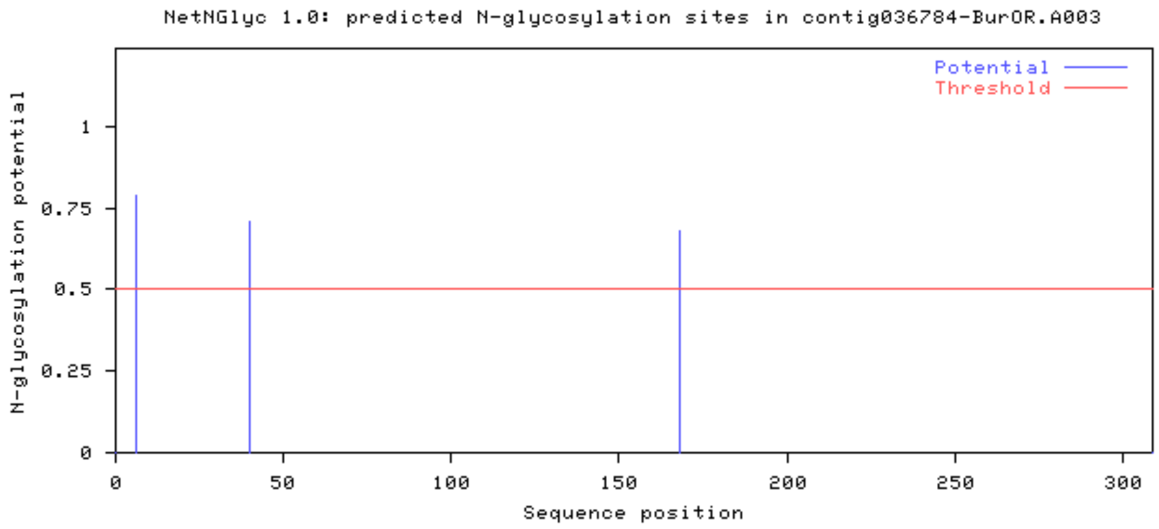

Graphics in PostScript

Output for 'contig036787-BurOR.A004'

```
##########################################################################

   Warning: This sequence may not contain a signal peptide!!

   Proteins without signal peptides are unlikely to be exposed to
   the N-glycosylation machinery and thus may not be glycosylated
   (in vivo) even though they contain potential motifs.

   SignalP-NN euk predictions are as follows:

 # name                  Cmax   pos ?  Ymax   pos ?  Smax   pos ?  Smean ?  D     ?

   SignalP output is explained at http://www.cbs.dtu.dk/services/SignalP/output.html

##########################################################################

Name:  contig036787-BurOR.A004  Length:  337
MEQMHDEFNVTYITFGGHVELEKYKFLYFAIMFTAYILILCSNSTIVCLIRIKKSLHEPMYVFIAALLLNSVVFSTNIYP      80
KLLMDFLSERQITTHSLCRFQGIIYYSLTGSEFFLLASMAYDRYVSISKPLQYHTIMRKTTVTVLLVLAWLLPACQLVPS     160
AVFSNNSQICNFTLNGIFCNNAISKLYCATPKTYYLMYGVFILFNTVFLPLLFIMFTYTKIFIICYRSCREVRKKAAQTC     240
LPHLLVLISFTCLCSYDIIIARLEIDLSQNTRFIMTLQVVLYHPLFNPIVYGLKMKEISQHLRRLFCHRVLCVKTDVGSA     320
VISFVIQVQRPDFSTVX
........N.................................N.....................................      80
................................................................................     160
....N.....N.....................................................................     240
................................................................................     320
.................                                                                    400

(Threshold=0.5)
----------------------------------------------------------------------
SeqName      Position  Potential    Jury    N-Glyc
                                 agreement  result
----------------------------------------------------------------------
contig036787-BurOR.A004    9 NVTY    0.7809     (9/9)    +++
contig036787-BurOR.A004   43 NSTI    0.7286     (9/9)    ++
contig036787-BurOR.A004  165 NNSQ    0.5510     (5/9)    +
contig036787-BurOR.A004  171 NFTL    0.6013     (7/9)    +
----------------------------------------------------------------------
```

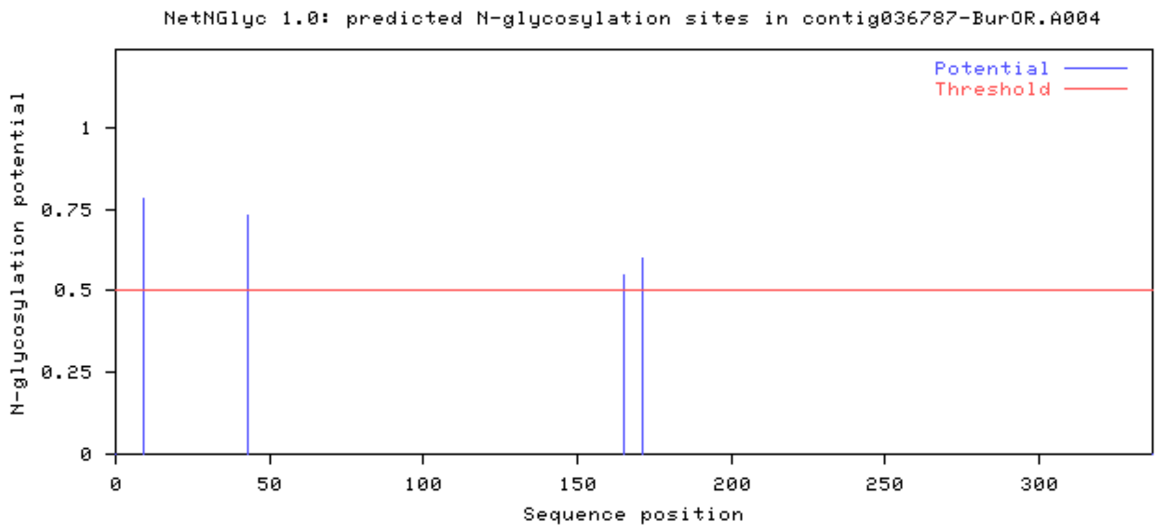

Graphics in PostScript

Output for 'contig037815-BurORe.H072'

```
##########################################################################################

    Warning: This sequence may not contain a signal peptide!!

    Proteins without signal peptides are unlikely to be exposed to
    the N-glycosylation machinery and thus may not be glycosylated
    (in vivo) even though they contain potential motifs.

    SignalP-NN euk predictions are as follows:

  # name                    Cmax  pos ?  Ymax  pos ?  Smax  pos ?  Smean ?  D     ?

    SignalP output is explained at http://www.cbs.dtu.dk/services/SignalP/output.html

##########################################################################################

Name:  contig037815-BurORe.H072         Length:  130
SIVQLACFPAQTIVNGIFANITIIIYALHGGFIVWSYMYIIQTCVKSIENRAKFMQTCVPHLVSLHTFVLIMLLDFTSAR      80
FGSKFLPQVLQNFIAIEFLVIPPVMNPLMYGFKLTKIQKKVFIVILKTKX
...................N............................................................      80
..................................................                                   160

(Threshold=0.5)
----------------------------------------------------------------------
SeqName      Position  Potential   Jury    N-Glyc
                                 agreement result
----------------------------------------------------------------------
contig037815-BurORe.H072    20 NITI   0.7032     (8/9)   +
----------------------------------------------------------------------
```

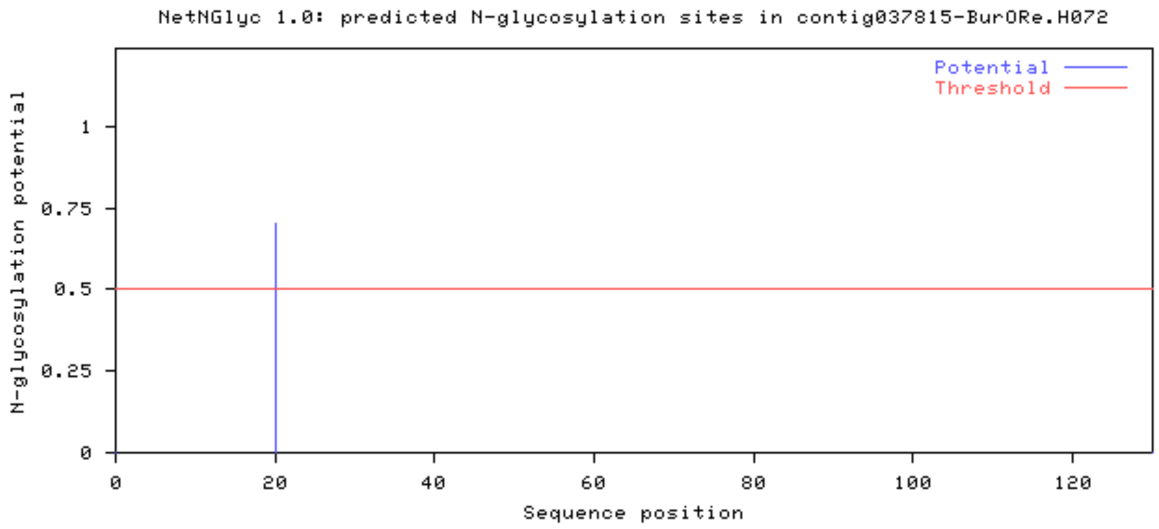

Graphics in PostScript

Output for 'contig037816-BurORe.H073'

```
##########################################################################################

    Warning: This sequence may not contain a signal peptide!!

    Proteins without signal peptides are unlikely to be exposed to
    the N-glycosylation machinery and thus may not be glycosylated
    (in vivo) even though they contain potential motifs.

    SignalP-NN euk predictions are as follows:

  # name                Cmax  pos ?  Ymax  pos ?  Smax  pos ?  Smean ?  D     ?

    SignalP output is explained at http://www.cbs.dtu.dk/services/SignalP/output.html

##########################################################################################

Name:  contig037816-BurORe.H073        Length:  59
MNNVSVITMFFLSGFNETVSHRFVLFFLSLLCYCIICLVNVSLIVIIILDSNLHESMYI
..N............N.......................N...................                          80

(Threshold=0.5)
----------------------------------------------------------------------
SeqName      Position  Potential   Jury    N-Glyc
                                 agreement result
----------------------------------------------------------------------
contig037816-BurORe.H073    3 NVSV   0.7546     (9/9)   +++
contig037816-BurORe.H073   16 NETV   0.6768     (9/9)   ++
contig037816-BurORe.H073   40 NVSL   0.6074     (8/9)   +
----------------------------------------------------------------------
```

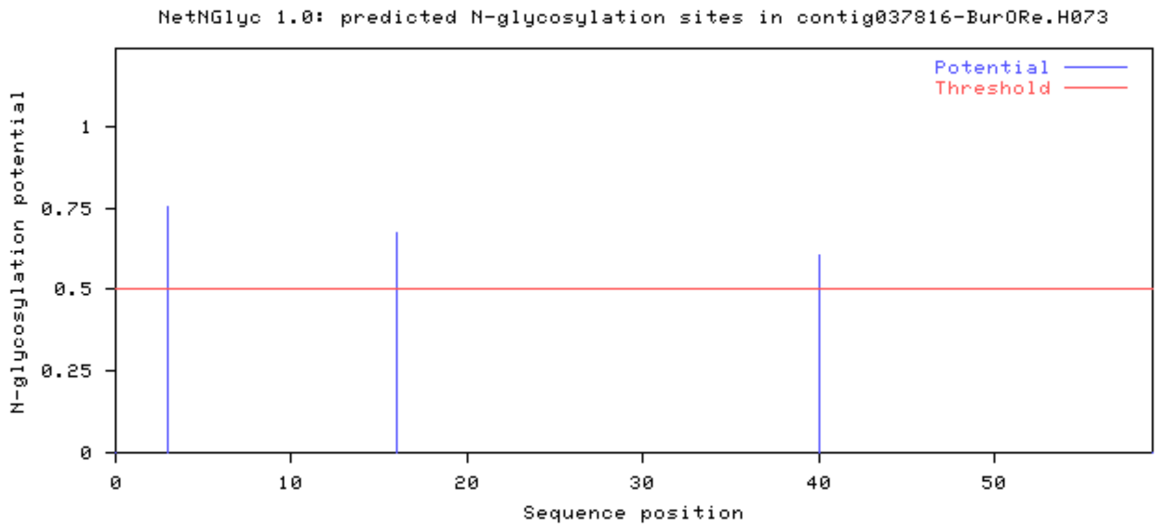

Graphics in PostScript

Output for 'contig037817-BurORe.H074'

```
##########################################################################

    Warning: This sequence may not contain a signal peptide!!

    Proteins without signal peptides are unlikely to be exposed to
    the N-glycosylation machinery and thus may not be glycosylated
    (in vivo) even though they contain potential motifs.

    SignalP-NN euk predictions are as follows:

  # name                Cmax  pos ?  Ymax  pos ?  Smax  pos ?  Smean ?  D     ?

    SignalP output is explained at http://www.cbs.dtu.dk/services/SignalP/output.html

##########################################################################

Name:  contig037817-BurORe.H074        Length:  139
SRLFCVNWSIVQLACFPAQTAINAISANITISIYFLYGVFIVWSYLYIIQTCVRSIENRAKFMQTCVPHLVSLFTFAVTK      80
LLDIINMRLGSKELPQTLQNFAAIEFLVIPPIMNPLIYGFKLTKIRKTICSVVIFKTKX
......N....................N....................................................      80
...........................................................                         160

(Threshold=0.5)
----------------------------------------------------------------------
SeqName      Position  Potential   Jury    N-Glyc
                                 agreement result
----------------------------------------------------------------------
contig037817-BurORe.H074    7 NWSI   0.7557     (9/9)    +++
contig037817-BurORe.H074   28 NITI   0.6833     (9/9)    ++
----------------------------------------------------------------------
```

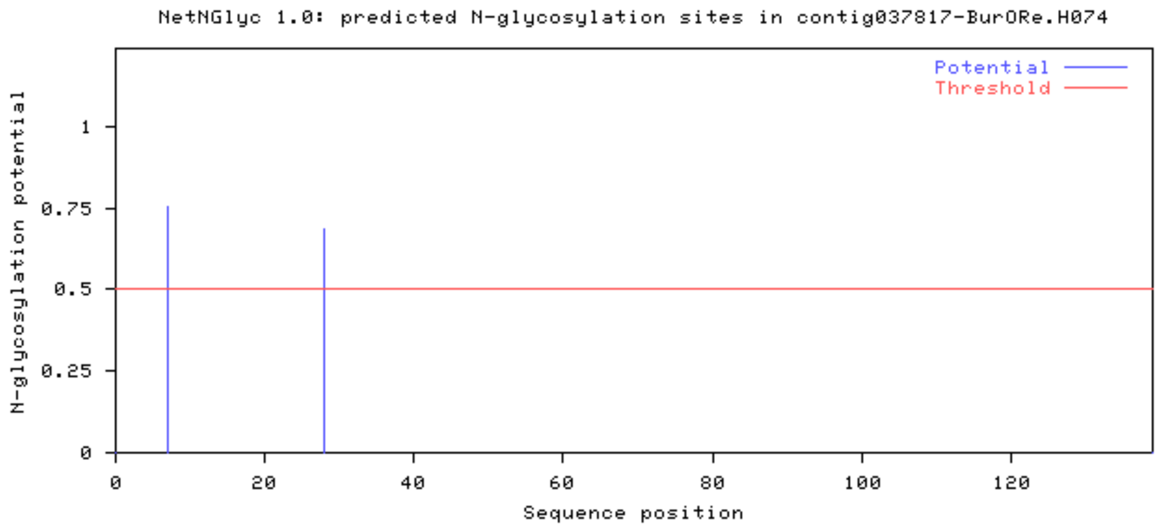

Graphics in PostScript

Output for 'contig040653-BurOR.B030'

```
##########################################################################################

    Warning: This sequence may not contain a signal peptide!!

    Proteins without signal peptides are unlikely to be exposed to
    the N-glycosylation machinery and thus may not be glycosylated
    (in vivo) even though they contain potential motifs.

    SignalP-NN euk predictions are as follows:

  # name                Cmax  pos ?  Ymax  pos ?  Smax  pos ?  Smean ?  D     ?

    SignalP output is explained at http://www.cbs.dtu.dk/services/SignalP/output.html

##########################################################################################

Name:  contig040653-BurOR.B030  Length:  320
MSAGVNVTSLPILVTSVTLDGLAQLSNQRLFFFFFLCAYLFMLCSDSLVVYVICSQRSLHRPMFVFVTAVLMNSVAGSTV      80
FYPKLLVDLLRGVRSVQVTLRVCMCEAWLLYSLGTSSFLLLAAMSFDRYVSICRPLLYTVVMSPATVLALLLLCWLLPVG     160
LVGTAVLLASRLPLCRSQLSRIYCDIYSLVSLSCGGRETLLSEVYNLSVIVATVLLPAIFVLFSYSAVLSVCLRRSRSFS     240
SKALSTCLPHLLVFCNYSVSSGVEVLHRRLQAGSQPTASVLTSIFQVMIPTVFNPVVYGLKVTEIRAQLRRLLGCQRADX     320
.....N..........................................................................      80
................................................................................     160
.............................................N..................................     240
...............N................................................................     320

(Threshold=0.5)
----------------------------------------------------------------------
SeqName      Position  Potential   Jury    N-Glyc
                                agreement result
----------------------------------------------------------------------
contig040653-BurOR.B030    6 NVTS   0.7708     (9/9)   +++
contig040653-BurOR.B030  206 NLSV   0.6110     (7/9)   +
contig040653-BurOR.B030  256 NYSV   0.5472     (7/9)   +
----------------------------------------------------------------------
```

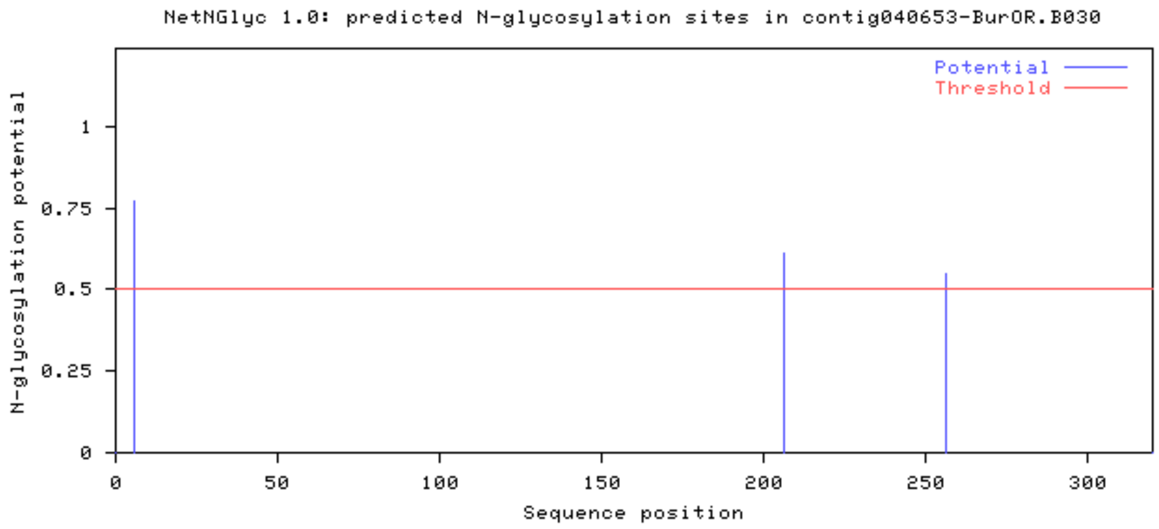

Graphics in PostScript

Output for 'contig041638-BurORs.W135'

```
##########################################################################

    Warning: This sequence may not contain a signal peptide!!

    Proteins without signal peptides are unlikely to be exposed to
    the N-glycosylation machinery and thus may not be glycosylated
    (in vivo) even though they contain potential motifs.

    SignalP-NN euk predictions are as follows:

  # name                  Cmax   pos ?  Ymax   pos ?  Smax   pos ?  Smean ?  D     ?

    SignalP output is explained at http://www.cbs.dtu.dk/services/SignalP/output.html

##########################################################################

Name:  contig041638-BurORs.W135         Length:  304
MNSTTAVYRDSLSTAIIKNLITVGLSASIIYINSSLVHTFKKHQVFNTNPRYILYIHLVIIDILLLIIFTLLQVLSYIIF      80
TLPVPFCIILLLISIICSLNTPMTLAVMAVECHVAICFPLQHSQICTVKNVTVVITVIWVLSSLTILPDLFTILATESRD     160
FFHSRVFCLRETVFRLPELEKKRTISNIVFLVIVWLTLIYTYFRILFAAQAAAANARKARNTVLLHGFQLLLCMLTYVYD     240
LLLNGLTKLFPKGVLTIRYTISVFVHVLPRLVSPLVYGIRDKAFRRYLRKYLFYSPNANVNKSX
.N..............................N...............................................      80
.................................................N..............................     160
................................................................................     240
................................................................                     320

(Threshold=0.5)
----------------------------------------------------------------------
SeqName      Position  Potential   Jury    N-Glyc
                                 agreement result
----------------------------------------------------------------------
contig041638-BurORs.W135     2 NSTT   0.6721     (9/9)   ++
contig041638-BurORs.W135    33 NSSL   0.6696     (9/9)   ++
contig041638-BurORs.W135   130 NVTV   0.6027     (9/9)   ++
contig041638-BurORs.W135   301 NKSX   0.4930     (5/9)   -
----------------------------------------------------------------------
```

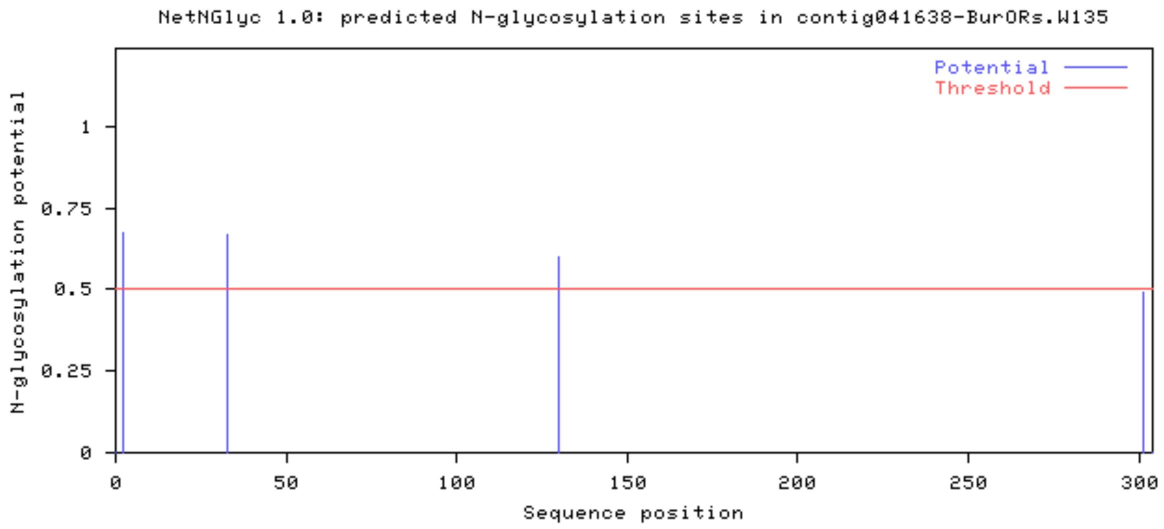

Graphics in PostScript

Output for 'contig041640-BurORs.U130'

```
##########################################################################

    Warning: This sequence may not contain a signal peptide!!

    Proteins without signal peptides are unlikely to be exposed to
    the N-glycosylation machinery and thus may not be glycosylated
    (in vivo) even though they contain potential motifs.

    SignalP-NN euk predictions are as follows:

  # name                  Cmax  pos ?  Ymax  pos ?  Smax  pos ?  Smean ?  D     ?

    SignalP output is explained at http://www.cbs.dtu.dk/services/SignalP/output.html

##########################################################################

Name:  contig041640-BurORs.U130         Length:  328
MSSLLGLRGNMTVPYQLLLIRDTFTTAFVKNLIVVLVWLTLSYINGTLVVTFFRHQTFHDDPRYILFIHMVINDAIQLTV      80
TIMLFILSYIFYKINVAFCCFFILVAVFTTRNTPVNLAAMAIERYIAICEPLRYTQICTVRRTYIVIGMIWFICVAPDIT     160
DLFVTLATESLSFFHESVFCLRQNVFKDPILAYKRQAVDIIYFSCVFLILVVTYLRILFAARALSTDKTSAQKARNTILL     240
HGAQLAMCMLSYVSPSVEVVLHIIFPGRILEIRFANYLIVYILPRFLSPIIYGVRDKKFREYLRMYFLSNRCRNKEKKVT     320
PEDKDHLX
.........N..................................N...................................      80
................................................................................     160
................................................................................     240
................................................................................     320
........                                                                             400

(Threshold=0.5)
----------------------------------------------------------------------
SeqName      Position  Potential    Jury    N-Glyc
                                  agreement result
----------------------------------------------------------------------
contig041640-BurORs.U130   10 NMTV   0.7847     (9/9)    +++
contig041640-BurORs.U130   45 NGTL   0.7593     (9/9)    +++
----------------------------------------------------------------------
```

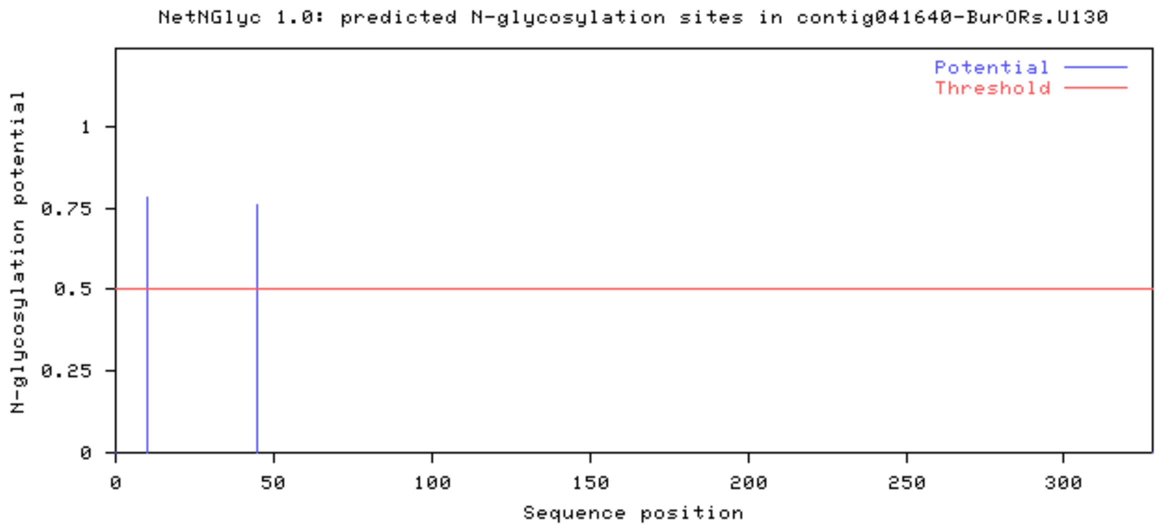

Graphics in PostScript

Output for 'contig041640-BurORs.V144'

```
##########################################################################

   Warning: This sequence may not contain a signal peptide!!

   Proteins without signal peptides are unlikely to be exposed to
   the N-glycosylation machinery and thus may not be glycosylated
   (in vivo) even though they contain potential motifs.

   SignalP-NN euk predictions are as follows:

 # name                Cmax  pos ?  Ymax  pos ?  Smax  pos ?  Smean ?  D     ?

   SignalP output is explained at http://www.cbs.dtu.dk/services/SignalP/output.html

##########################################################################

Name:  contig041640-BurORs.V144        Length:  343
MNSTSTGPLYPLVSNTTSVQTQGVKESFSSILTKNIVAMLVWLVLSIINGSMVHTFQRHSLFYENPRYIMFISMVINDAL      80
QLTLVTALYVISYIFRKIHASVCCLLVIMTAILTTRSTPLILAGMAVERYISICFPLHYSQMCNIPRTLLLICVIVILTI     160
TPPITDLLITIVKEPPSFFHTKIFCDHSLLFRDQSIYYKNCVFDGTYLSFVALALLYTYCKIMLTAQAVSTSLVSVKRAR     240
NTVLLHGVQVLLCMLAFVVPSLQAALISLFPQLSLEIRYIFFLLVYIIPRFLSPMIYGFRDEQFRKYWTRYLSCHEHSVT     320
LLRLASQKMNPQVKQSGNLSSTX
.N............N................................N...............................      80
................................................................................     160
................................................................................     240
................................................................................     320
.................N.....                                                              400

(Threshold=0.5)
----------------------------------------------------------------------
SeqName      Position  Potential   Jury    N-Glyc
                                 agreement result
----------------------------------------------------------------------
contig041640-BurORs.V144    2 NSTS   0.7822     (9/9)   +++
contig041640-BurORs.V144   15 NTTS   0.7447     (9/9)   ++
contig041640-BurORs.V144   49 NGSM   0.6356     (8/9)   +
contig041640-BurORs.V144  338 NLSS   0.6432     (8/9)   +
----------------------------------------------------------------------
```

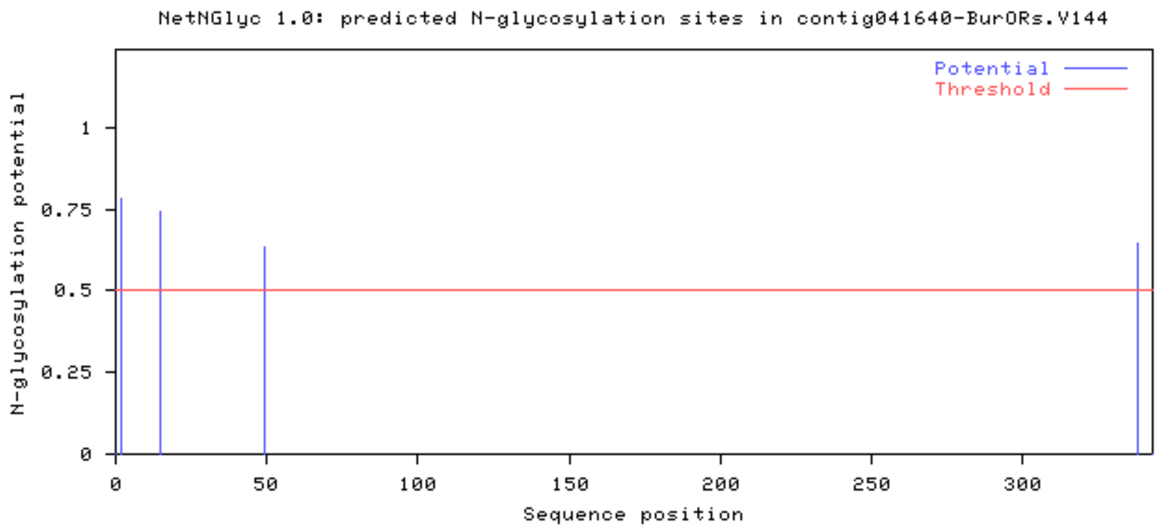

Graphics in PostScript

Output for 'contig041641-BurORs.T129'

```
##########################################################################################

    Warning: This sequence may not contain a signal peptide!!

    Proteins without signal peptides are unlikely to be exposed to
    the N-glycosylation machinery and thus may not be glycosylated
    (in vivo) even though they contain potential motifs.

    SignalP-NN euk predictions are as follows:

  # name                   Cmax  pos ?  Ymax  pos ?  Smax  pos ?  Smean ?  D     ?

    SignalP output is explained at http://www.cbs.dtu.dk/services/SignalP/output.html

##########################################################################################

Name:  contig041641-BurORs.T129         Length:  314
MNTTKRQDSFNDAFTKNFVSFALGFIINYINGMFVYTYFKSVVFQQDPRYVLYIHLVINDMIMLTVTVMLQILTYTTPLS      80
FAPCCVILLISVTANKNSPLNLAGMAVERYIAVCRPLHHSQLCTVQRAYALITLIWAVSFIPSISDVIILLVVQPHSVFT     160
KNVICYPSFVYNTPYHETQSLVVQVLLFSFVFLTLIVTYMKVLCAARAVSSSNQASAKNAHNTILLHGVQLLICMLSYIS     240
PFINLVLVTAWPHKRTIIIFTTFLFTNLLPRLLSPLIYGARDQKLSSHFRVHFGCKGFKSRGKSRKRRVPEKHX
.N..............................................................................      80
................................................................................     160
................................................................................     240
..........................................................................           320

(Threshold=0.5)
----------------------------------------------------------------------
SeqName      Position  Potential   Jury    N-Glyc
                                 agreement result
----------------------------------------------------------------------
contig041641-BurORs.T129    2 NTTK   0.7583     (9/9)    +++
----------------------------------------------------------------------
```

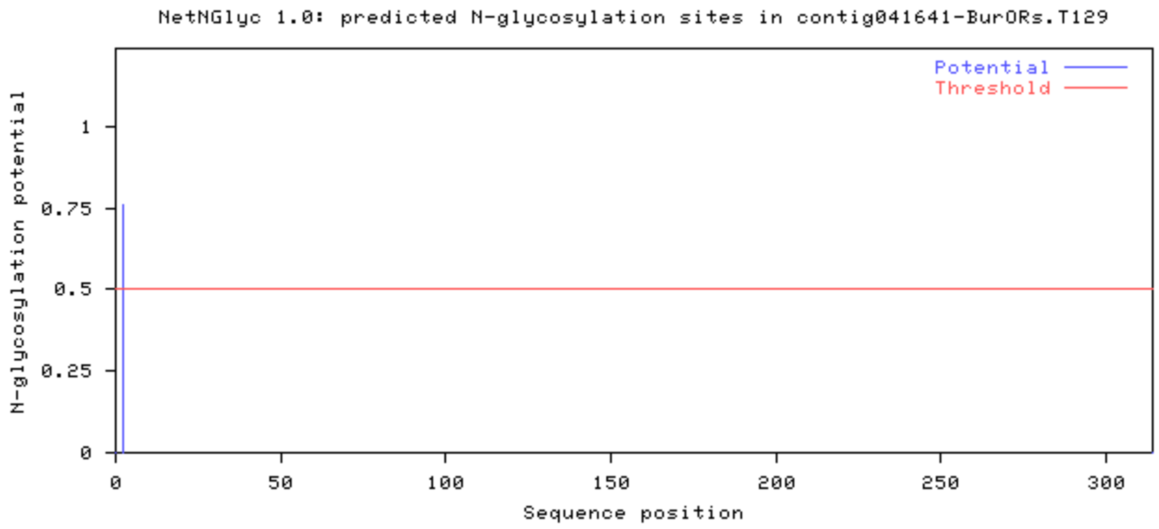

Graphics in PostScript

Output for 'contig042916-BurORe.M104'

```
##########################################################################################

    Warning: This sequence may not contain a signal peptide!!

    Proteins without signal peptides are unlikely to be exposed to
    the N-glycosylation machinery and thus may not be glycosylated
    (in vivo) even though they contain potential motifs.

    SignalP-NN euk predictions are as follows:

  # name                Cmax  pos ?  Ymax  pos ?  Smax  pos ?  Smean ?  D     ?

    SignalP output is explained at http://www.cbs.dtu.dk/services/SignalP/output.html

##########################################################################################

Name:  contig042916-BurORe.M104        Length:  38
SLASCIPPCINPIVYSLKTKDIKTRALTLFRKTKVNAX
......................................                                                80

(Threshold=0.5)

No sites predicted in this sequence.
```

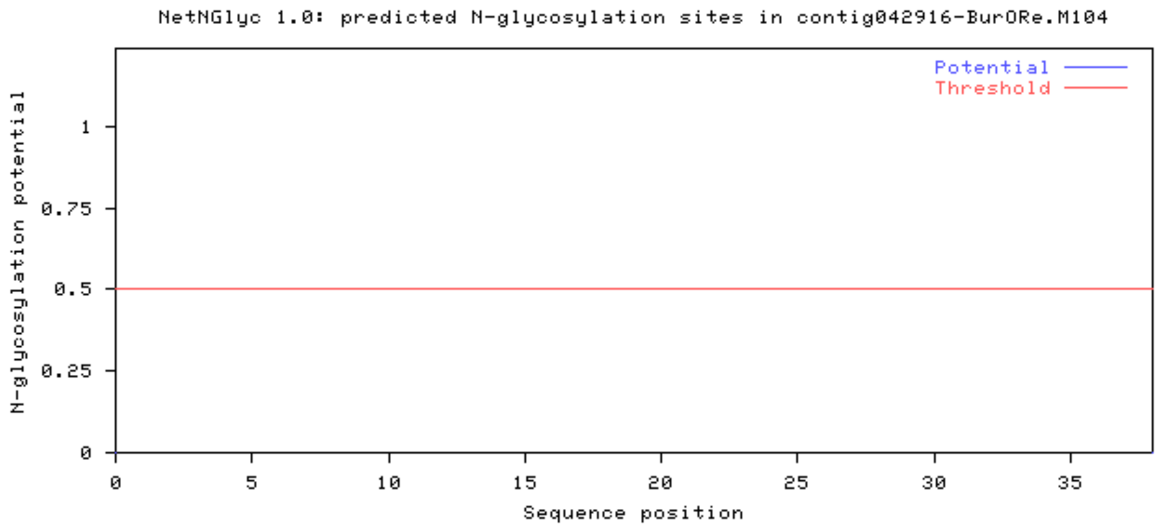

Graphics in PostScript

Output for 'contig042917-BurORe.M105'

```
##########################################################################################

    Warning: This sequence may not contain a signal peptide!!

    Proteins without signal peptides are unlikely to be exposed to
    the N-glycosylation machinery and thus may not be glycosylated
    (in vivo) even though they contain potential motifs.

    SignalP-NN euk predictions are as follows:

  # name                Cmax  pos ?  Ymax  pos ?  Smax  pos ?  Smean ?  D     ?

    SignalP output is explained at http://www.cbs.dtu.dk/services/SignalP/output.html

##########################################################################################

Name:  contig042917-BurORe.M105        Length:  44
SLASCIPPCINPIVYSLKTKDIKTRALTLFRTKVKTKLTKVNAX
............................................                                          80

(Threshold=0.5)

No sites predicted in this sequence.
```

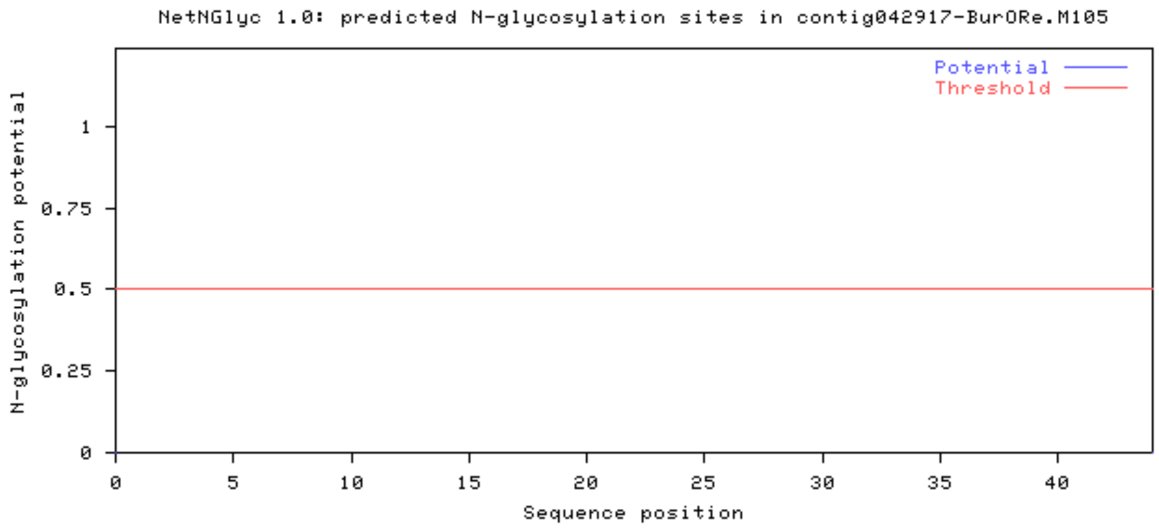

Graphics in PostScript

Output for 'contig042918-BurORep.N117'

```
##########################################################################

    Warning: This sequence may not contain a signal peptide!!

    Proteins without signal peptides are unlikely to be exposed to
    the N-glycosylation machinery and thus may not be glycosylated
    (in vivo) even though they contain potential motifs.

    SignalP-NN euk predictions are as follows:

  # name                    Cmax   pos ?  Ymax   pos ?  Smax   pos ?  Smean ?  D     ?

    SignalP output is explained at http://www.cbs.dtu.dk/services/SignalP/output.html

##########################################################################

Name:  contig042918-BurORep.N117        Length:  213
MAYDRLIAICFPLRYHSIVTEQSITAILLFVWIFFLVSVIATMVGLVNRLSFCRSLVVNSFFCDHGPVYRLACNDTSLNY      80
NMGSALVAILVIIPLIFIIATYVCIFIALSRTTSREERLRALKTCTSHLILVVIFFLPIGITNIAAMTSYIDSNARMINS     160
VLTHTIPALLDPIVYALKTEEVMNAVKKLCKGINLNRMKAKTRPCNHCCIKSX
.........................................................................N......      80
................................................................................     160
.....................................................                               240

(Threshold=0.5)
----------------------------------------------------------------------
SeqName      Position  Potential    Jury    N-Glyc
                                 agreement  result
----------------------------------------------------------------------
contig042918-BurORep.N117    74 NDTS    0.6803     (9/9)    ++
----------------------------------------------------------------------
```

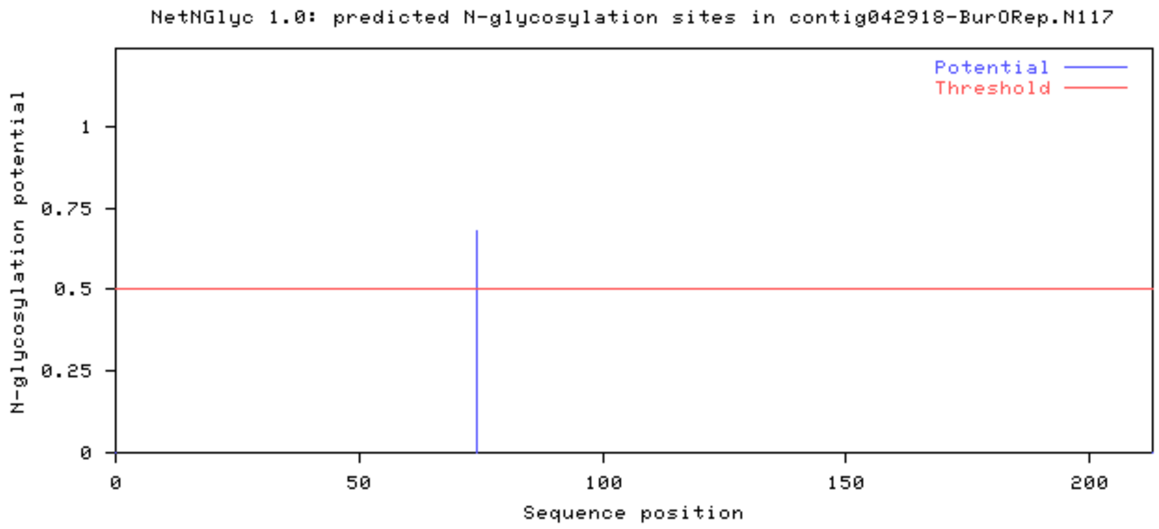

Graphics in PostScript

Output for 'contig042920-BurOR.N107'

```
##########################################################################################

    Warning: This sequence may not contain a signal peptide!!

    Proteins without signal peptides are unlikely to be exposed to
    the N-glycosylation machinery and thus may not be glycosylated
    (in vivo) even though they contain potential motifs.

    SignalP-NN euk predictions are as follows:

  # name                Cmax  pos ?  Ymax  pos ?  Smax  pos ?  Smean ?  D     ?

    SignalP output is explained at http://www.cbs.dtu.dk/services/SignalP/output.html

##########################################################################################

Name:  contig042920-BurOR.N107  Length:  327
MDSEEKATTKSNSTFVRPAIFYLSGFSNIPHVKYFYVFLCFVYIMTVLGNGFLLSLIWLVKTLHTPKYMIVFNMALADLC      80
GSTTLIPKLLDTFLFDRRYIVYEACLSYMFFVFFFGGVQSWTLVTMAYDRLIAICFPLRYHSIVTKTSITSMLLFIWLVM     160
LSLTTLVVGLVNRLSFCDSVVVKSFFCDHGPIYRLACNDPSLNIIMANVVVSIGVFIPLIFIACTYVCISIALSKIASGE     240
ERLKALKTCTSHLILVAILFLPFVGTNIAVWTSYIHPNARIINSTLTHTIPALINPIVYALKTEEVMSAVKKLWKINNIS     320
SPVTKWX
...........N....................................................................      80
................................................................................     160
................................................................................     240
..........................................N..................................N..     320
.......                                                                              400

(Threshold=0.5)
----------------------------------------------------------------------
SeqName      Position  Potential   Jury    N-Glyc
                                 agreement result
----------------------------------------------------------------------
contig042920-BurOR.N107   12 NSTF   0.6204     (9/9)   ++
contig042920-BurOR.N107  283 NSTL   0.5846     (8/9)   +
contig042920-BurOR.N107  318 NISS   0.6381     (7/9)   +
----------------------------------------------------------------------
```

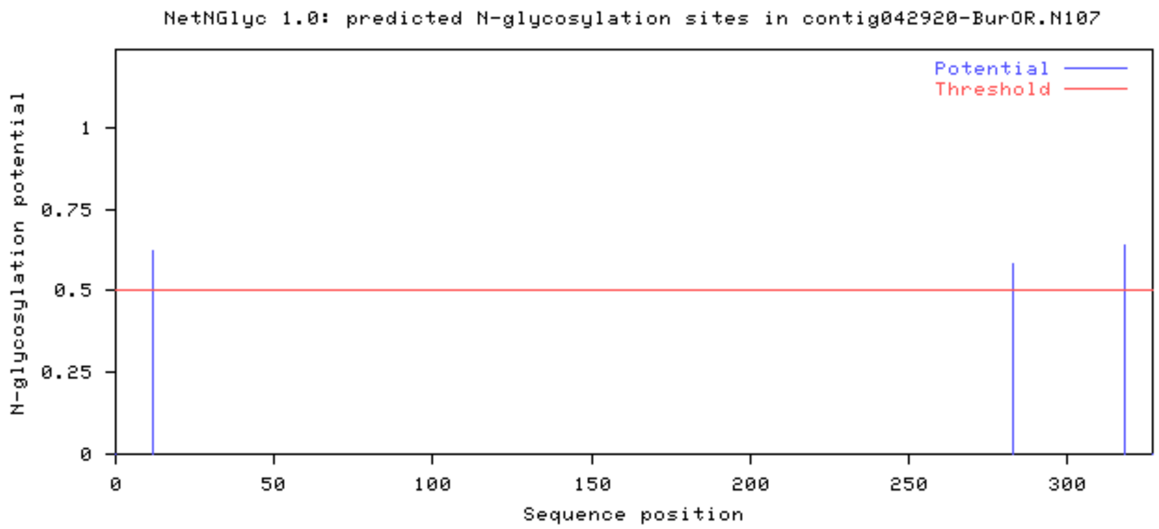

Graphics in PostScript

Output for 'contig042923-BurORe.N115'

```
##########################################################################################

    Warning: This sequence may not contain a signal peptide!!

    Proteins without signal peptides are unlikely to be exposed to
    the N-glycosylation machinery and thus may not be glycosylated
    (in vivo) even though they contain potential motifs.

    SignalP-NN euk predictions are as follows:

  # name                 Cmax  pos ?  Ymax  pos ?  Smax  pos ?  Smean ?  D     ?

    SignalP output is explained at http://www.cbs.dtu.dk/services/SignalP/output.html

##########################################################################################

Name:  contig042923-BurORe.N115         Length:  104
IVGSYCCIGYSLSKTVTCRERLKALKTCTSHLSLVAIYFLPALFIFTFGSTILPNARTVSLSLATVMPLTLNPIIYGLQT      80
QEIKESLKKLLKVKMQFKISANKX
................................................................................      80
........................                                                             160

(Threshold=0.5)

No sites predicted in this sequence.
```

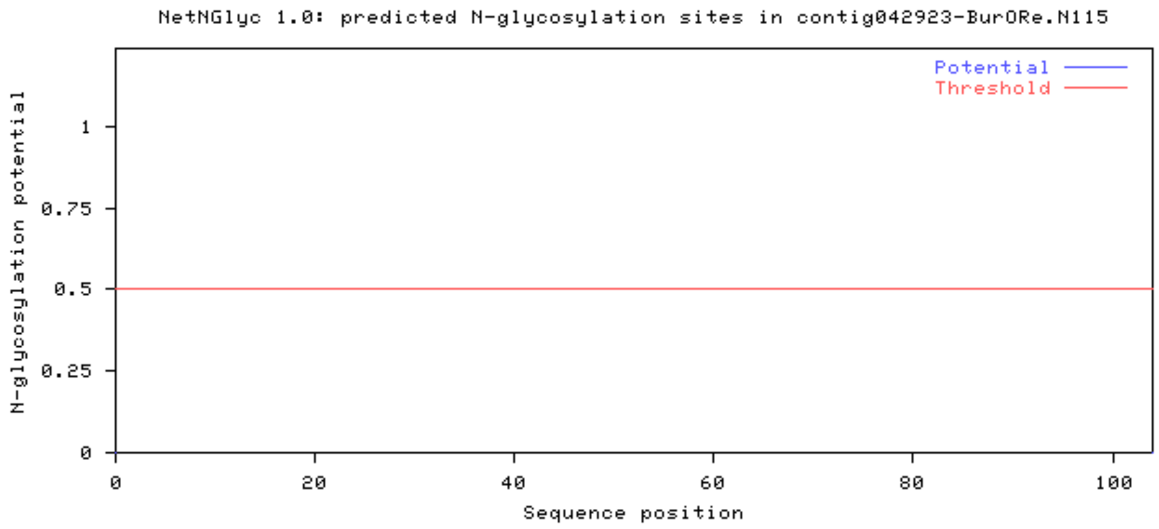

Graphics in PostScript

Output for 'contig042928-BurOR.N108'

```
########################################################################################

    Warning: This sequence may not contain a signal peptide!!

    Proteins without signal peptides are unlikely to be exposed to
    the N-glycosylation machinery and thus may not be glycosylated
    (in vivo) even though they contain potential motifs.

    SignalP-NN euk predictions are as follows:

  # name                Cmax  pos ?  Ymax  pos ?  Smax  pos ?  Smean ?  D     ?

    SignalP output is explained at http://www.cbs.dtu.dk/services/SignalP/output.html

########################################################################################

Name:  contig042928-BurOR.N108  Length:  323
MDFLNSAAEKNTTFVQPANFIISGFVGIPNIRYYFVFLCFIYIFSVVGNTAVMLIIIFDHTLRSPKYIAVFNLAFTDLLS      80
NSALVPKVLDISLFNHHYISYNNCLTFMFFCFTLISMQAFNLVVLSFDRIMAIMYPLHYQMRVSHKIILSLIAFFWLLAV     160
ALTGTAVGLLTRLYFCESVVINSYYCDHGPIYRLSCNDVTPNKTISAWSRAFVLWLPLIFILGSYCCIGYSLSRISTCKE     240
RVKALKTCTGHLSLVAIYFIPILVVYSFGSTMHPNARIVNLSLASVTPPMLNPIIYVFQTAEIKKSLKKLLKAKIQISHR     320
VLX
................................................................................      80
................................................................................     160
.........................................N......................................     240
.......................................N........................................     320
...                                                                                  400

(Threshold=0.5)
----------------------------------------------------------------------
SeqName      Position  Potential   Jury    N-Glyc
                                 agreement result
----------------------------------------------------------------------
contig042928-BurOR.N108   11 NTTF   0.4656     (5/9)   -
contig042928-BurOR.N108  202 NKTI   0.6130     (8/9)   +
contig042928-BurOR.N108  280 NLSL   0.5873     (8/9)   +
----------------------------------------------------------------------
```

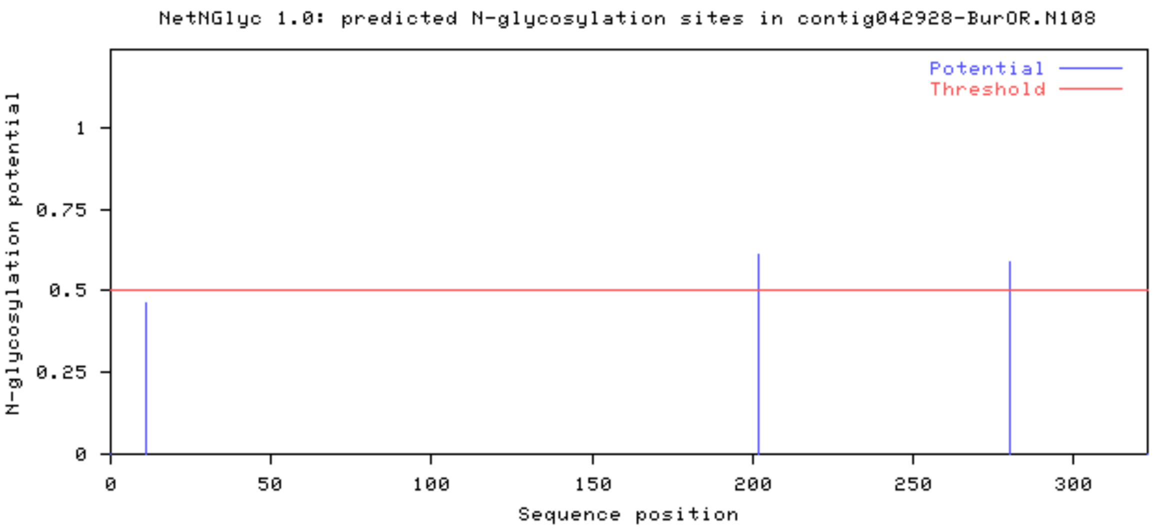

Graphics in PostScript

Output for 'contig043640-BurOR.R137'

```
##########################################################################################

    Warning: This sequence may not contain a signal peptide!!

    Proteins without signal peptides are unlikely to be exposed to
    the N-glycosylation machinery and thus may not be glycosylated
    (in vivo) even though they contain potential motifs.

    SignalP-NN euk predictions are as follows:

  # name                    Cmax  pos ?  Ymax  pos ?  Smax  pos ?  Smean ?  D     ?

    SignalP output is explained at http://www.cbs.dtu.dk/services/SignalP/output.html

##########################################################################################

Name:  contig043640-BurOR.R137  Length:  317
MSSISQTLTNITVGYQALAERVMISTLTTLPTCVFLFINGIMLFTLRSKPVFRETCRYILLYNLLFADTVQLAQSQIHFL      80
LAVLRITVSYPVCTFLVNFTHLTAVISPLTLVVMPLERYVAVCYPLRHATIITIRNTGAAITVIWAISFLNIIIRTLLFL     160
SLFEKLGKVEVKDLCADITILLGTKSDHFDKAFTCIVVVAAGVAVIFSYIGVIVAARSASTDKALAFKARNTLLLNLMQL     240
FFSLSSTIYYPLLVPLSVTVTRIVFVRIQNVFYLLFFILPRCLTSLIYGLRDQTIRPVLIYHLCCQLKCPVVEDKGX
.........N......................................................................      80
.................N..............................................................     160
................................................................................     240
.............................................................................        320

(Threshold=0.5)
----------------------------------------------------------------------
SeqName      Position  Potential   Jury    N-Glyc
                                 agreement result
----------------------------------------------------------------------
contig043640-BurOR.R137   10 NITV   0.6972     (9/9)    ++
contig043640-BurOR.R137   98 NFTH   0.7047     (9/9)    ++
----------------------------------------------------------------------
```

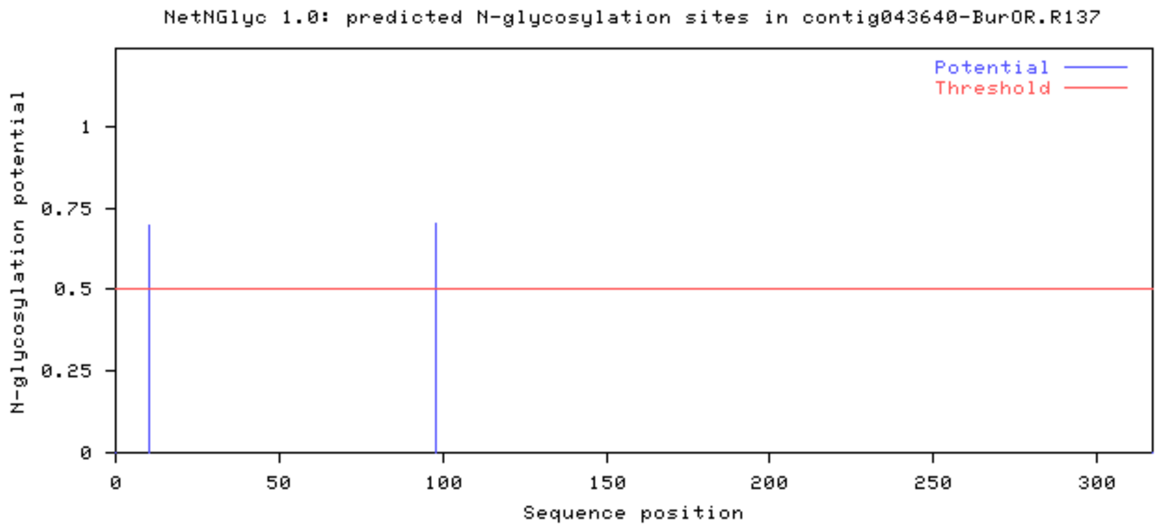

Graphics in PostScript

Output for 'contig043641-BurORe.R143'

```
##########################################################################################

    Warning: This sequence may not contain a signal peptide!!

    Proteins without signal peptides are unlikely to be exposed to
    the N-glycosylation machinery and thus may not be glycosylated
    (in vivo) even though they contain potential motifs.

    SignalP-NN euk predictions are as follows:

  # name                    Cmax   pos ?  Ymax   pos ?  Smax   pos ?  Smean ?  D     ?

    SignalP output is explained at http://www.cbs.dtu.dk/services/SignalP/output.html

##########################################################################################

Name:  contig043641-BurORe.R143         Length:  103
MFFTNETLTNITVGQQNQLFLEIVFSCIVTTLTCCVFLFINATMLFTLRSKPVFGQTSRYILLYNLLFADTLQMAQSQVM      80
FLLSACRITLLYPICGVLVSLAT
....N....N..............................N.......................................      80
.......................                                                              160

(Threshold=0.5)
----------------------------------------------------------------------
SeqName      Position  Potential   Jury    N-Glyc
                                 agreement result
----------------------------------------------------------------------
contig043641-BurORe.R143    5 NETL   0.7620     (9/9)   +++
contig043641-BurORe.R143   10 NITV   0.6327     (7/9)   +
contig043641-BurORe.R143   41 NATM   0.5774     (7/9)   +
----------------------------------------------------------------------
```

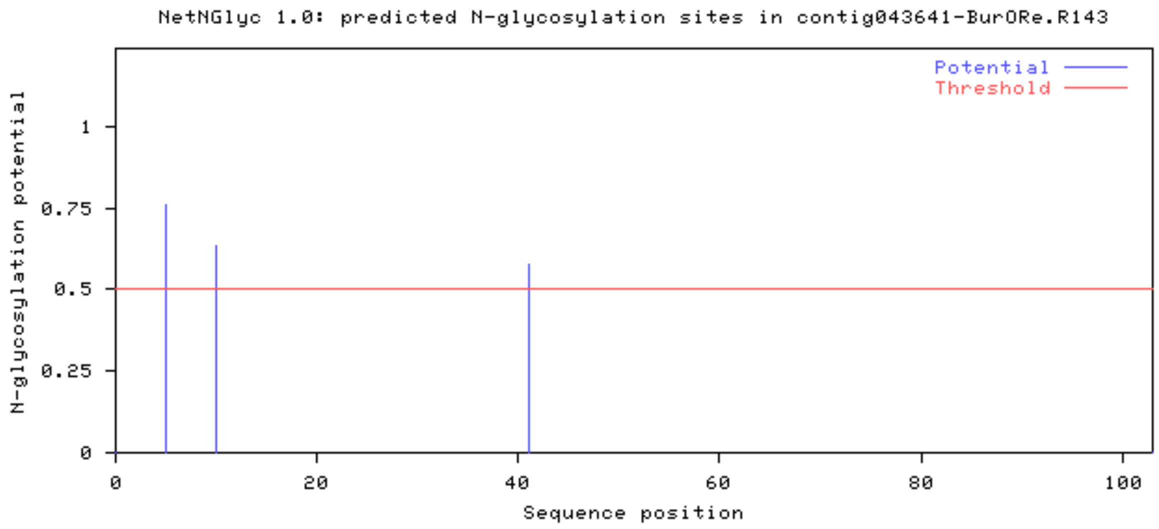

Graphics in PostScript

Output for 'contig045452-BurORs.W133'

```
##########################################################################

    Warning: This sequence may not contain a signal peptide!!

    Proteins without signal peptides are unlikely to be exposed to
    the N-glycosylation machinery and thus may not be glycosylated
    (in vivo) even though they contain potential motifs.

    SignalP-NN euk predictions are as follows:

  # name                Cmax  pos ?  Ymax  pos ?  Smax  pos ?  Smean ?  D     ?

    SignalP output is explained at http://www.cbs.dtu.dk/services/SignalP/output.html

##########################################################################

Name:  contig045452-BurORs.W133         Length:  320
MNSSSYALNASSSLKYRDSPSVAVAKNVIVLALGFTINYINGTLIHTFRKHQVQIFYLNPRYILFIHLVVNDMIQLTSSI      80
SLFVFTYIFYQINVAFCCFLITLAIFTTFNTPINLALMALECYIAICLPLQHAEYCTTKRTYVAIGWIWAMSAVSAMSDI     160
VIILATEPVELFYSTIQCERDNLFRHPIIVKKKEVSYLIFLIGVLLTFMYTYFRIFFAANKAKSAKRESKKARNTILLHG     240
FQLLLSMLTYIATALMQALVRWFPKQPLIVVFISYIIIYIVPRFVSPIVYGLRDKTFKQYLKKYLLCTMKMGNETDFRLX     320
.N......N...............................N.......................................      80
................................................................................     160
................................................................................     240
........................................................................N.......     320

(Threshold=0.5)
----------------------------------------------------------------------
SeqName      Position  Potential   Jury    N-Glyc
                                 agreement result
----------------------------------------------------------------------
contig045452-BurORs.W133     2 NSSS   0.7218     (9/9)   ++
contig045452-BurORs.W133     9 NASS   0.6429     (8/9)   +
contig045452-BurORs.W133    41 NGTL   0.7143     (9/9)   ++
contig045452-BurORs.W133   313 NETD   0.6713     (9/9)   ++
----------------------------------------------------------------------
```

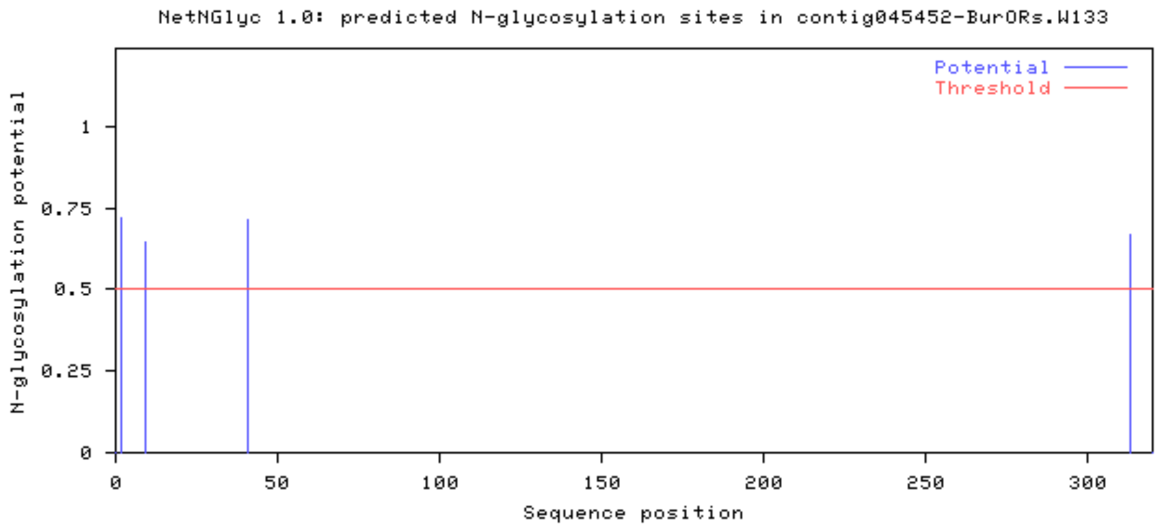

Graphics in PostScript

Output for 'contig045453-BurORs.W132'

```
################################################################################

   Warning: This sequence may not contain a signal peptide!!

   Proteins without signal peptides are unlikely to be exposed to
   the N-glycosylation machinery and thus may not be glycosylated
   (in vivo) even though they contain potential motifs.

   SignalP-NN euk predictions are as follows:

 # name                Cmax  pos ?  Ymax  pos ?  Smax  pos ?  Smean ?  D     ?

   SignalP output is explained at http://www.cbs.dtu.dk/services/SignalP/output.html

################################################################################

Name:  contig045453-BurORs.W132         Length:  313
MNASSGNVTVVLQYRDSFAKAVTKNLIVVFLGISIGYINANLIHTFCKHQIFYKNPRYVLFIHLVINDMVQVMLTAILFI      80
ISYTIYKLNVSVCCIFMLLALFTTENSPLNLACMAVECYIAICFPLRHVQICTVQRTLILISLIWMITTLSVLPDLFITL     160
ATEPLDFFHSRVFCLRNTVFPHPLIIQKRDITYGVFLVIVWVTIIYTYLKILFTAKTASKDAKKAKNTILLHGFQLLLCM     240
ATYAAPHLTNALQKWFPTNYTDSLFVIYVTVQILPRSISPIIYGIRDKTFRKFLKGNLLCKITVHKSDNQNRX
.N....N.........................................................................      80
........N.......................................................................     160
................................................................................     240
..................N......................................................            320

(Threshold=0.5)
----------------------------------------------------------------------
SeqName      Position  Potential   Jury    N-Glyc
                                 agreement result
----------------------------------------------------------------------
contig045453-BurORs.W132    2 NASS   0.6617     (9/9)   ++
contig045453-BurORs.W132    7 NVTV   0.7674     (9/9)   +++
contig045453-BurORs.W132   89 NVSV   0.6073     (8/9)   +
contig045453-BurORs.W132  259 NYTD   0.7026     (9/9)   ++
----------------------------------------------------------------------
```

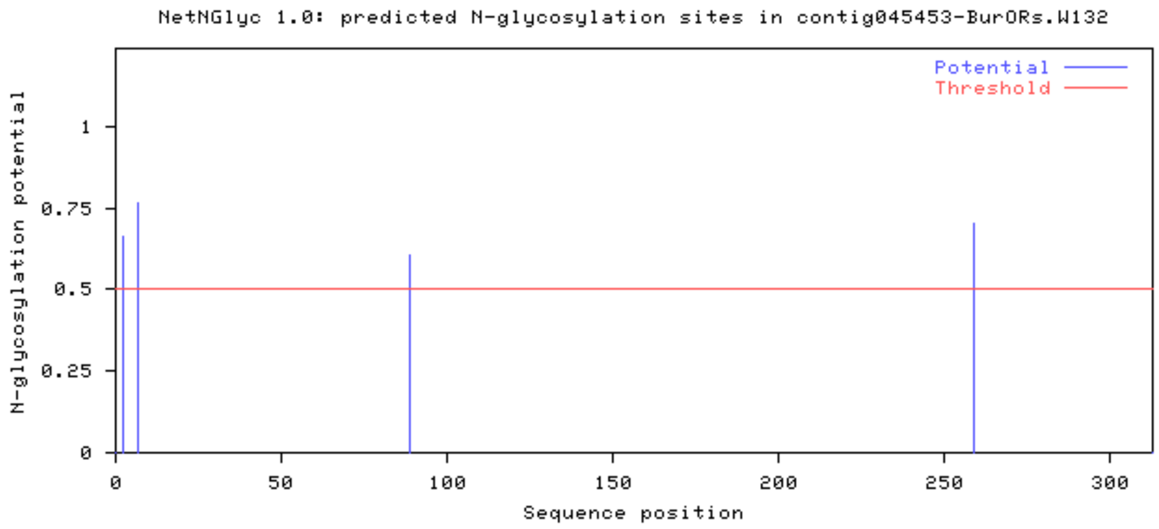

Graphics in PostScript

Output for 'contig045453-BurORs.W134'

```
##########################################################################

    Warning: This sequence may not contain a signal peptide!!

    Proteins without signal peptides are unlikely to be exposed to
    the N-glycosylation machinery and thus may not be glycosylated
    (in vivo) even though they contain potential motifs.

    SignalP-NN euk predictions are as follows:

  # name                Cmax  pos ?  Ymax  pos ?  Smax  pos ?  Smean ?  D     ?

    SignalP output is explained at http://www.cbs.dtu.dk/services/SignalP/output.html

##########################################################################

Name:  contig045453-BurORs.W134        Length:  316
MNLTQVDSNATITLNYWKTFVKAVGKNVTVVVLGITINYINATMIHTFNKYHVQIFRLNPRYILFIHLVFNDIIQLSTSI      80
SLFIFSYAFHTIHVSLCLLLILPAIFTTQNTPLNIAFMAAECCVSVCIPLRYSYICTVKRTYIVIGIIWAISSLSILPDL     160
FILLAVESSQFLQSRVVCNRDTVFRSSYSVKKRDASHTLFLVLVSTLLYTYCQILFVARCADSDTKKARNTILIHGFQV     240
LLCTTVYVQPPLIKLLVYFFPEGLSDINFATFIINQVLPRLGSPIIYGLRDKTFRKFLKRHLCAVNNLPKIIRSSX
.N......N.................N.............N.......................................      80
................................................................................     160
................................................................................     240
............................................................................         320

(Threshold=0.5)
----------------------------------------------------------------------
SeqName      Position  Potential   Jury    N-Glyc
                                 agreement result
----------------------------------------------------------------------
contig045453-BurORs.W134     2 NLTQ   0.8093     (9/9)   +++
contig045453-BurORs.W134     9 NATI   0.6161     (8/9)   +
contig045453-BurORs.W134    27 NVTV   0.7229     (9/9)   ++
contig045453-BurORs.W134    41 NATM   0.5421     (5/9)   +
----------------------------------------------------------------------
```

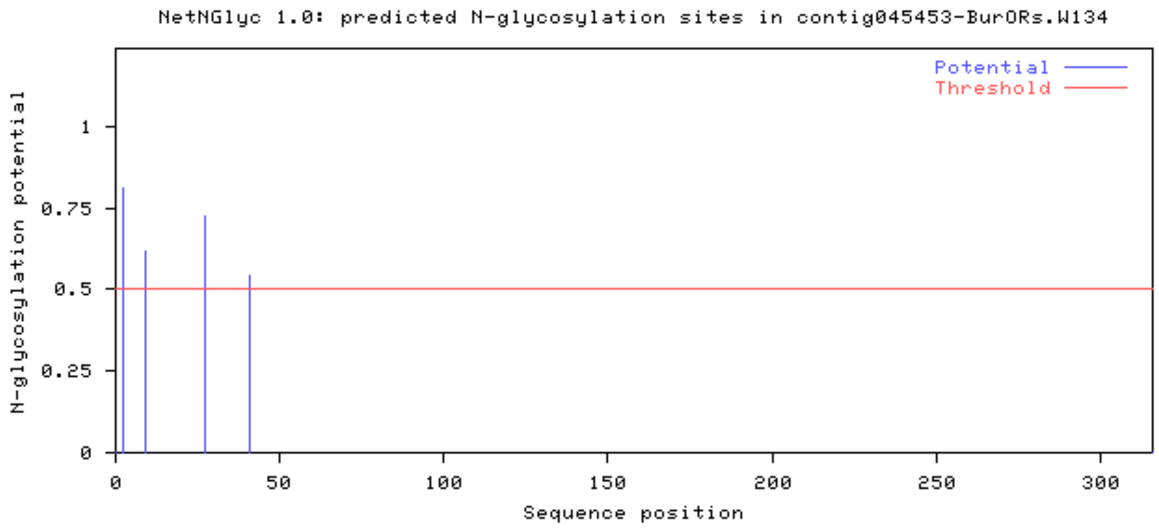

Graphics in PostScript

Output for 'contig045454-BurORs.W131'

```
##########################################################################

    Warning: This sequence may not contain a signal peptide!!

    Proteins without signal peptides are unlikely to be exposed to
    the N-glycosylation machinery and thus may not be glycosylated
    (in vivo) even though they contain potential motifs.

    SignalP-NN euk predictions are as follows:

  # name                Cmax  pos ?  Ymax  pos ?  Smax  pos ?  Smean ?  D     ?

    SignalP output is explained at http://www.cbs.dtu.dk/services/SignalP/output.html

##########################################################################

Name:  contig045454-BurORs.W131        Length:  306
MSASYTNETVVVNYRDAFPKAMVKNVIVVVFCISINYINVALLQTFCKQQVIFYMNPRYILFFHLVLNDMIQVTLTVILF      80
ISSYIFFQINVSVCCVLILLALFATENTPLNLACMAVECYIAICIPLRHVQICTVKRTLMLIGLIWMTSMLSVLPDLFIT     160
LAIEPLDFYNSRVFCLRETVFRNPHIIKKRDITYIVYLVIVWFIIFFTYFKILFTAKAASQDATKARNTIILHGFQVLLC     240
MSIYAEPLLRQVLQQWFPQNYSDSLFACYILFQILPRAISPIVYGVRDKTYRKYLKRYLLCKMSLX
......N.........................................................................      80
.........N......................................................................     160
................................................................................     240
...................N..............................................                   320

(Threshold=0.5)
----------------------------------------------------------------------
SeqName      Position  Potential   Jury    N-Glyc
                                 agreement result
----------------------------------------------------------------------
contig045454-BurORs.W131    7 NETV   0.7383     (9/9)   ++
contig045454-BurORs.W131   90 NVSV   0.6561     (8/9)   +
contig045454-BurORs.W131  260 NYSD   0.6352     (8/9)   +
----------------------------------------------------------------------
```

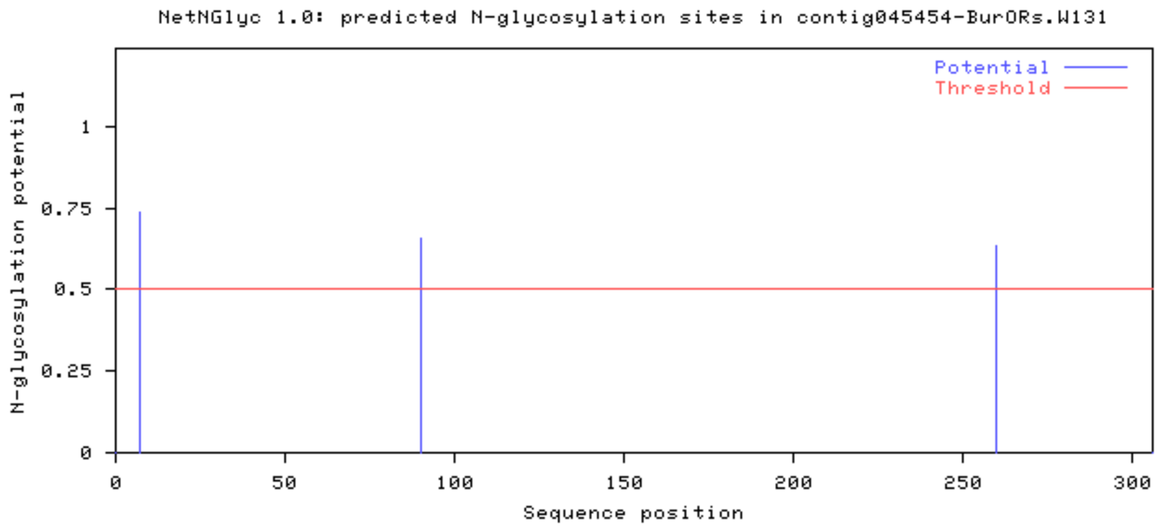

Graphics in PostScript

Output for 'contig048316-BurORe.K083'

```
##########################################################################################

    Warning: This sequence may not contain a signal peptide!!

    Proteins without signal peptides are unlikely to be exposed to
    the N-glycosylation machinery and thus may not be glycosylated
    (in vivo) even though they contain potential motifs.

    SignalP-NN euk predictions are as follows:

  # name                Cmax  pos ?  Ymax  pos ?  Smax  pos ?  Smean ?  D     ?

    SignalP output is explained at http://www.cbs.dtu.dk/services/SignalP/output.html

##########################################################################################

Name:  contig048316-BurORe.K083        Length:  205
MENYTYNSYILQLEGLNTSKDSLYPAFLFLFFSYLFIMIINVGITILIFMNKNLHQPMYLLYCNLPLNDILVTSIVVPRL     80
LIDLMRPPSERLISYYQCVVQAYIAHLVGTTSHTILMIMAYDRYVAICNPFHYVSIMTNKMMIKLTVCAWGVAFVLVGIL    160
LGLTIRLSRCRTLITNPYCDNASLFKLSCESVVINNIYGITFTVA
..N.............N...............................................................     80
................................................................................    160
....................N........................                                       240

(Threshold=0.5)
----------------------------------------------------------------------
SeqName      Position  Potential   Jury    N-Glyc
                                 agreement result
----------------------------------------------------------------------
contig048316-BurORe.K083    3 NYTY   0.7628     (9/9)   +++
contig048316-BurORe.K083   17 NTSK   0.6156     (8/9)   +
contig048316-BurORe.K083  181 NASL   0.5533     (5/9)   +
----------------------------------------------------------------------
```

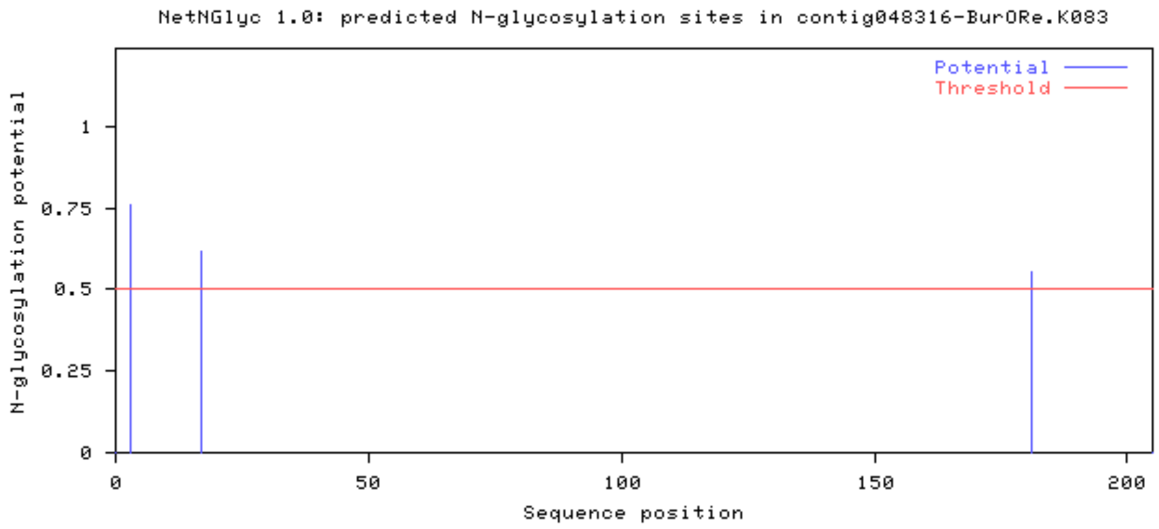

Graphics in PostScript

Output for 'contig048321-BurORs.I076'

```
##########################################################################################

    Warning: This sequence may not contain a signal peptide!!

    Proteins without signal peptides are unlikely to be exposed to
    the N-glycosylation machinery and thus may not be glycosylated
    (in vivo) even though they contain potential motifs.

    SignalP-NN euk predictions are as follows:

  # name                Cmax  pos ?  Ymax  pos ?  Smax  pos ?  Smean ?  D     ?

    SignalP output is explained at http://www.cbs.dtu.dk/services/SignalP/output.html

##########################################################################################

Name:  contig048321-BurORs.I076         Length:  314
MENLTMATPLKQPIVFELEGFYIPPGFGPLLFFLALFTYMLVLLGNGVIVSVIVIDKNLHRPMFVMVCHLLVCDLLGSTA      80
VLPGLMMHFLMGQKRIAYIPAIAQAFSVHTYGLAVQAILGAMAYDRYIAVCEPLRYHAIMTSARLHSCCALAWLLALLPI     160
AVLFSFHMNVPLCGRVILHVYCSNRGILGLACIPTPASDIYGLAMTWTVSTGIFFIIAFSYIRILQVSLKHSRIDTSIRS     240
KAFQTCASHLVVYVLYQIASVIIIVSYRFPSVSENLKKFFSILFIIVPPAINPIIYGLVSKELRSSIIKHFTIX
..N.............................................................................      80
................................................................................     160
................................................................................     240
..........................................................................           320

(Threshold=0.5)
----------------------------------------------------------------------
SeqName      Position  Potential   Jury    N-Glyc
                                 agreement result
----------------------------------------------------------------------
contig048321-BurORs.I076    3 NLTM   0.7366     (9/9)    ++
----------------------------------------------------------------------
```

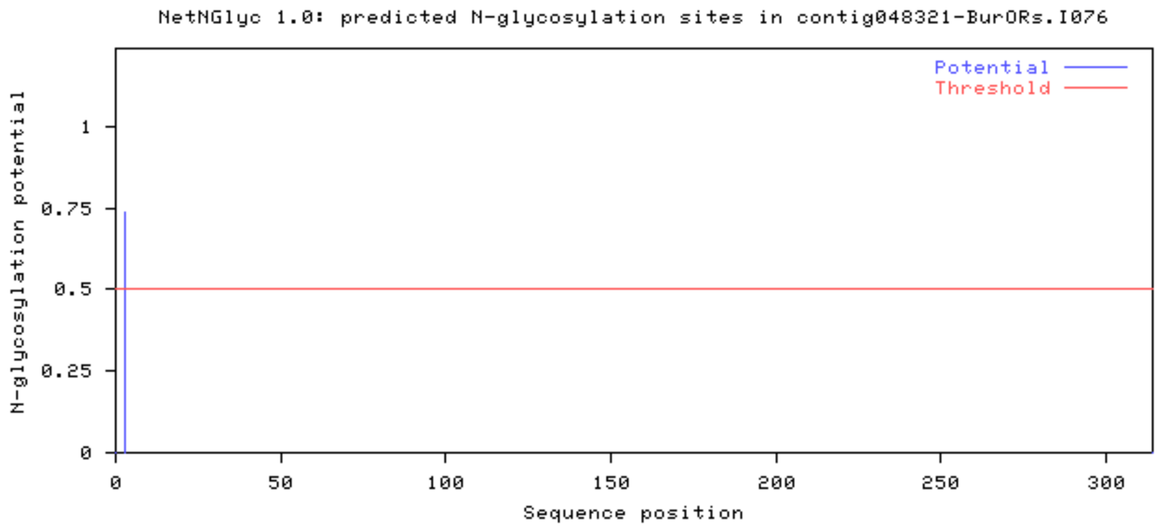

Graphics in PostScript

Output for 'contig048560-BurORp.A028'

```
##############################################################################

    Warning: This sequence may not contain a signal peptide!!

    Proteins without signal peptides are unlikely to be exposed to
    the N-glycosylation machinery and thus may not be glycosylated
    (in vivo) even though they contain potential motifs.

    SignalP-NN euk predictions are as follows:

  # name                 Cmax  pos ?  Ymax  pos ?  Smax  pos ?  Smean ?  D     ?

    SignalP output is explained at http://www.cbs.dtu.dk/services/SignalP/output.html

##############################################################################

Name:  contig048560-BurORp.A028        Length:  321
MDVELNVTLLTLGGFAELHKYRYLYFVVIFTLYILILCFNSTIVFLIWTNENLHEPMYIFIAALLINSVLYSMIIYPKLL      80
SDVLSEKQMISYPLCLFQGLSYCTSVGSEFLLLAAMAYDRYVSICKPLQYPVIMNRITIYVCLILAWLIPAFETSLLGVL     160
YSNVKLCSFTLTGIFFCNTSVYKLQCVPSVAISIYSMVMLINIALLPLLFILFTYIRILRISYHCCREVRKKTVKTCLPH     240
LLVLINFSCFIFFDIIIVRLDSDLSKTLRLTLTFQSILFHPLLNPIIYGLKMNEIFKHIKILLSSLITLVLLPYYQMYGI     320
X
.....N.................................N........................................      80
................................................................................     160
................................................................................     240
.....N..........................................................................     320
.                                                                                    400

(Threshold=0.5)
----------------------------------------------------------------------
SeqName      Position  Potential   Jury    N-Glyc
                                agreement  result
----------------------------------------------------------------------
contig048560-BurORp.A028     6 NVTL   0.7847     (9/9)   +++
contig048560-BurORp.A028    40 NSTI   0.7238     (9/9)   ++
contig048560-BurORp.A028   178 NTSV   0.4714     (5/9)   -
contig048560-BurORp.A028   246 NFSC   0.5428     (5/9)   +
----------------------------------------------------------------------
```

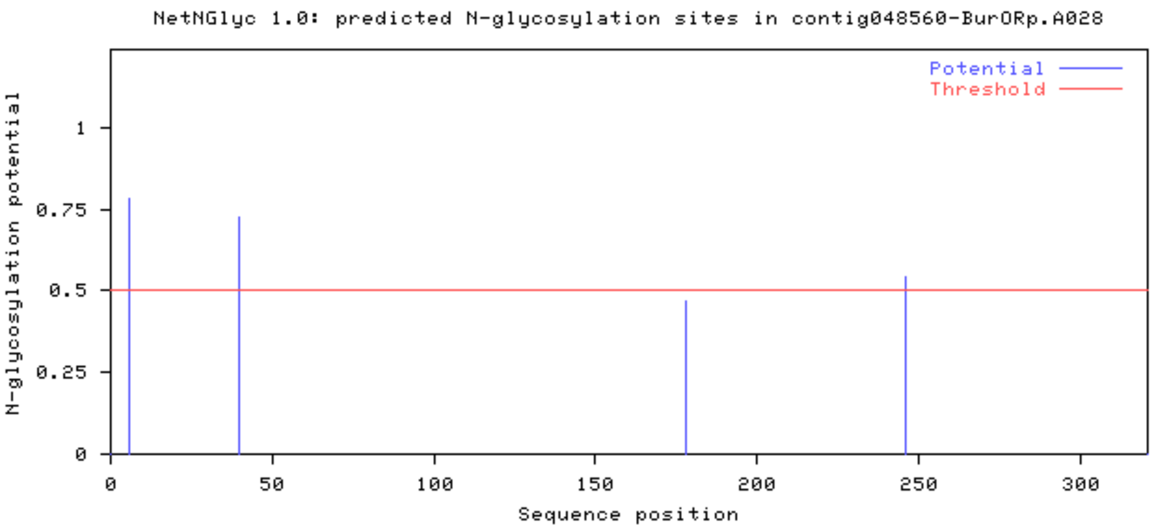

Graphics in PostScript

Output for 'contig048562-BurOR.H062'

```
##########################################################################################

    Warning: This sequence may not contain a signal peptide!!

    Proteins without signal peptides are unlikely to be exposed to
    the N-glycosylation machinery and thus may not be glycosylated
    (in vivo) even though they contain potential motifs.

    SignalP-NN euk predictions are as follows:

  # name                  Cmax  pos ?  Ymax  pos ?  Smax  pos ?  Smean ?  D     ?

    SignalP output is explained at http://www.cbs.dtu.dk/services/SignalP/output.html

##########################################################################################

Name:  contig048562-BurOR.H062  Length:  310
MDNVSTVRIFNLLAFNETANYRAAFFSATLVCYFAIVFLNVTVIMIIVLDESLHEPMYILVCVCCINGLYGSTGFYPKFL      80
IDLLSSSQVISYSECLCQAFVMYSFVCSDTSILAVMAYDRYLAICQPLQYHSVMTKKKLSKLVCFSWLTPFCIFSINIML     160
TDRLIFCGTDIQRLFCVNWLIVKVACPGMDTLVNSAFAYTTLSIYIFHWIFIVWTYIYLVKSCVQSKKDKAKFMQTCVPH     240
LISLVTFFVIVISDLMHMRFASNDVPQSFQNFVAIAVLFIPPVMNPLLYGFKLSKIRNRILVTLHIKRCX
..N............N........................N.......................................      80
................................................................................     160
................................................................................     240
......................................................................               320

(Threshold=0.5)
----------------------------------------------------------------------
SeqName      Position  Potential   Jury    N-Glyc
                                 agreement result
----------------------------------------------------------------------
contig048562-BurOR.H062    3 NVST   0.7628     (9/9)    +++
contig048562-BurOR.H062   16 NETA   0.7288     (9/9)    ++
contig048562-BurOR.H062   40 NVTV   0.7888     (9/9)    +++
----------------------------------------------------------------------
```

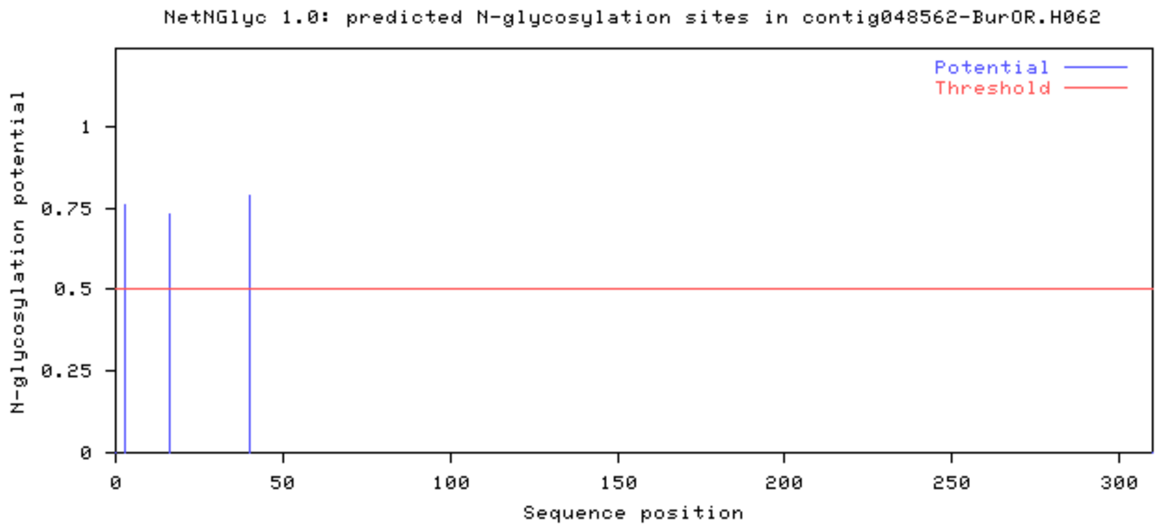

Graphics in PostScript

Output for 'contig048880-BurOR.H063'

```
##########################################################################

    Warning: This sequence may not contain a signal peptide!!

    Proteins without signal peptides are unlikely to be exposed to
    the N-glycosylation machinery and thus may not be glycosylated
    (in vivo) even though they contain potential motifs.

    SignalP-NN euk predictions are as follows:

  # name                Cmax  pos ?  Ymax  pos ?  Smax  pos ?  Smean ?  D    ?

    SignalP output is explained at http://www.cbs.dtu.dk/services/SignalP/output.html

##########################################################################

Name:  contig048880-BurOR.H063  Length:  314
MDNVSVITVFTLSGLSDIANYRVILFVLTLLCYCVIWLVNLTIIVTVIVDKKLHEPMYIFLCNLCFNGLYGTAAFYPKFL      80
YDLLSTTHVISYAGCLLQGFVLHSSVCADFSLLVLMAYDRYVAICRPLVYHSLMTTQKICILVFFAWLIPFYLLFMSTIT     160
TAVLRLCGTHIPRIYCVNWLINNLACSASVARIVIPAFNYTFYIGHVLLVFWSYVHLIKTCQSSKENWNKFMQTCVPHLF     240
SLTVVVLSFLFDMLYMRFGSKEIPKSFENFMAMEIFLIPPIINPLIYGFKLTQIRNRVLNFMRGKSSALRLKSX
..N....................................N........................................      80
................................................................................     160
......................................N.........................................     240
..........................................................................           320

(Threshold=0.5)
----------------------------------------------------------------------
SeqName      Position  Potential    Jury    N-Glyc
                                  agreement result
----------------------------------------------------------------------
contig048880-BurOR.H063    3 NVSV   0.8138     (9/9)    +++
contig048880-BurOR.H063   40 NLTI   0.7719     (9/9)    +++
contig048880-BurOR.H063  199 NYTF   0.5789     (7/9)    +
----------------------------------------------------------------------
```

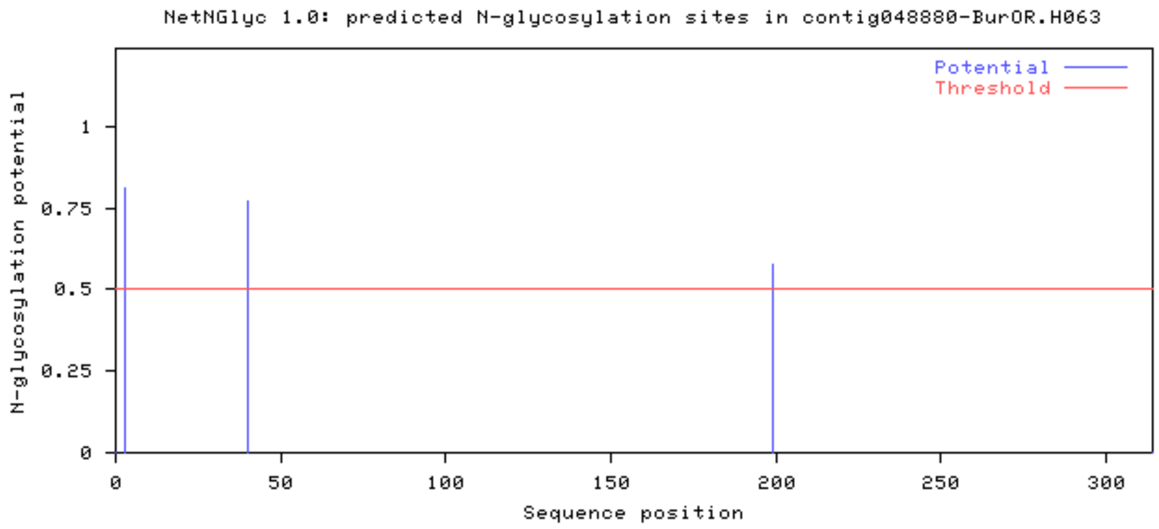

[Graphics in PostScript]

Output for 'contig048881-BurORp.H075'

```
##########################################################################

    Warning: This sequence may not contain a signal peptide!!

    Proteins without signal peptides are unlikely to be exposed to
    the N-glycosylation machinery and thus may not be glycosylated
    (in vivo) even though they contain potential motifs.

    SignalP-NN euk predictions are as follows:

  # name                Cmax  pos ?  Ymax  pos ?  Smax  pos ?  Smean ?  D     ?

    SignalP output is explained at http://www.cbs.dtu.dk/services/SignalP/output.html

##########################################################################

Name:  contig048881-BurORp.H075        Length:  309
MDNVHNVRSFILLGFNETMNFTVPLFSVTLLYYCVILFFNVSLVLLIVLDESLHEPMYIFLSSFCINALYGTTGFYPKFL      80
SDLLSPSQTISHEGCLLQAFIMYSSVCCNSSILAVMAFDRYLAICRPLHYHSFMTKRRLSQLVCFSWLTPLCIFAINVVL     160
TKLKLCGTKIQRVLCLNWLIVKLACPEADTFSNNITAYATVIIYVSHGFFIIWTYTHLIKTCAKSREDRAKFMQTCVPHL     240
ISLITFLSVVVFQFVYMRFDSTDLPQSLQNFIATEFVIIPPLMNPLIYGFKLTKIRNRILGLVCFKRKX
...............N...N...................N........................................      80
............................N...................................................     160
.................................N..............................................     240
.....................................................................                320

(Threshold=0.5)
----------------------------------------------------------------------
SeqName      Position  Potential   Jury    N-Glyc
                                 agreement result
----------------------------------------------------------------------
contig048881-BurORp.H075   16 NETM   0.6571     (9/9)   ++
contig048881-BurORp.H075   20 NFTV   0.7647     (9/9)   +++
contig048881-BurORp.H075   40 NVSL   0.7072     (9/9)   ++
contig048881-BurORp.H075  109 NSSI   0.5243     (6/9)   +
contig048881-BurORp.H075  194 NITA   0.6174     (8/9)   +
----------------------------------------------------------------------
```

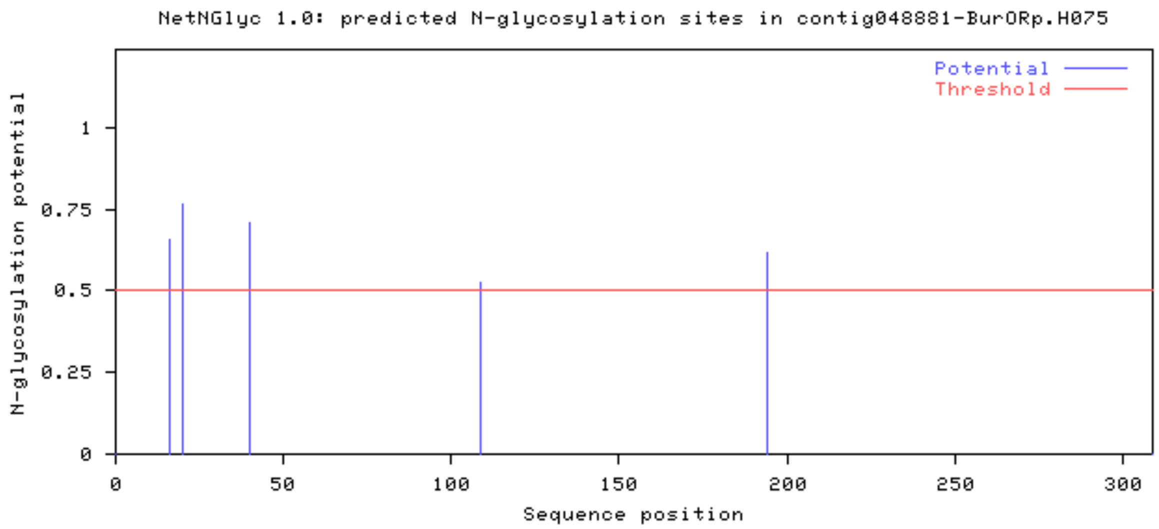

Graphics in PostScript

Output for 'contig048882-BurOR.H064'

```
##########################################################################################

    Warning: This sequence may not contain a signal peptide!!

    Proteins without signal peptides are unlikely to be exposed to
    the N-glycosylation machinery and thus may not be glycosylated
    (in vivo) even though they contain potential motifs.

    SignalP-NN euk predictions are as follows:

  # name                    Cmax  pos ?  Ymax  pos ?  Smax  pos ?  Smean ?  D     ?

    SignalP output is explained at http://www.cbs.dtu.dk/services/SignalP/output.html

##########################################################################################

Name:  contig048882-BurOR.H064  Length:  338
MDNQYNVRSFILSGFNETMNFRVPLFSFTLLCYCVILFINISLVLLIVLDENLHEPMYIFLSSFCINAIYGTTGFYPKFL      80
SDLLSSSQTISYEGCLLQAFIMYSFGYCDLSLLAVMAFDRYLAICRPLHYHSFMTKRRLSQLVCFSWLTPLCIFSINILL     160
TSKLRLCDINIQRVLCLNWLIVKLACPEADTFSNNISAYVIFIFYVSHGFFIMWTYMHLIKTCVRSRDDRLKFMQTCVPH     240
LTSLTTFLSVIVFQYVYLRLDSTDLFQSLQNFIAIEFLIISPVMNPLVYGFKLAKIRKRIFTLVHYKTKLLQSKLRVSYF     320
KRRMPFRNILLNIFCCTX
...............N.......................N........................................      80
................................................................................     160
..................................N.............................................     240
................................................................................     320
..................                                                                   400

(Threshold=0.5)
----------------------------------------------------------------------
SeqName      Position  Potential   Jury    N-Glyc
                                agreement result
----------------------------------------------------------------------
contig048882-BurOR.H064   16 NETM   0.6266     (9/9)   ++
contig048882-BurOR.H064   40 NISL   0.6650     (9/9)   ++
contig048882-BurOR.H064  195 NISA   0.5858     (7/9)   +
----------------------------------------------------------------------
```

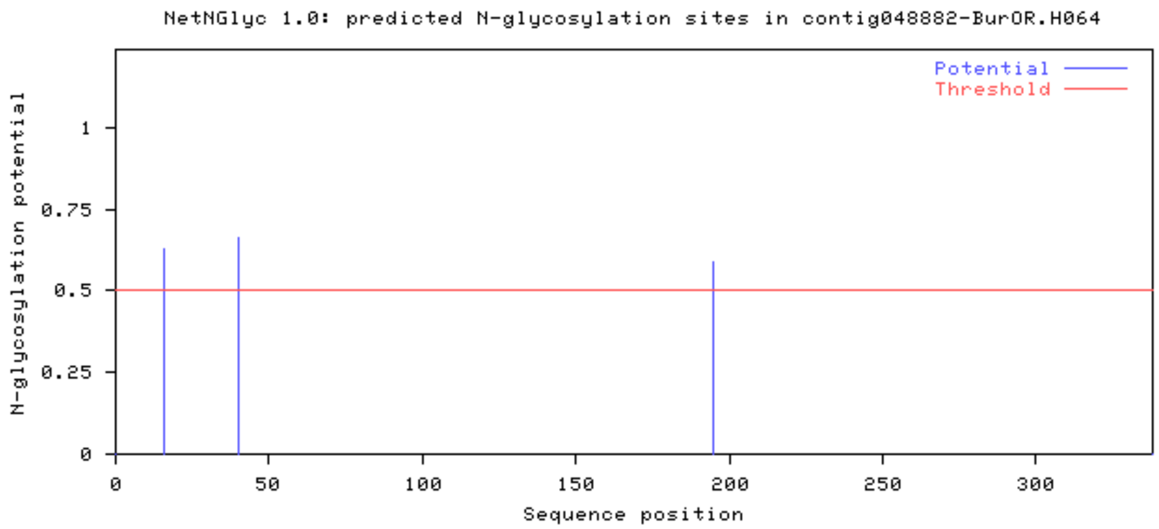

Graphics in PostScript

Output for 'contig049287-BurOR.E043'

```
##########################################################################################

    Warning: This sequence may not contain a signal peptide!!

    Proteins without signal peptides are unlikely to be exposed to
    the N-glycosylation machinery and thus may not be glycosylated
    (in vivo) even though they contain potential motifs.

    SignalP-NN euk predictions are as follows:

  # name                   Cmax  pos ?  Ymax  pos ?  Smax  pos ?  Smean ?  D     ?

    SignalP output is explained at http://www.cbs.dtu.dk/services/SignalP/output.html

##########################################################################################

Name:  contig049287-BurOR.E043  Length:  306
MVNSTVPYFILSTYIYVGSLKYLFFVLIALLYFSIVFVNTSLIVVICVNRSLHEPMYMFLCSLFVNELYGSTGLFPFLLL      80
QILSDVHTVSAPLCFLQIFCLYTYGHVEFCNLAVMSYDRYLAVCYPLHYKSHMTDNKVVILIVVIWLYSFVKFTITLCLT     160
LRLTWCGKIINGLYCHNYLVVKLACSDTNLNNLFGLFGIVITVLVPLLPIFYSYMKILKVCFSGSRQMRRKAVSTCAPHL     240
ASLLNFSFGCLFEILQSRFDTTSVPSALRIFLSLYFLIIQPLLNPIMYGTQMSKIRHVLCYKMSLX
..N...................................N.........N...............................      80
................................................................................     160
................................................................................     240
..................................................................                   320

(Threshold=0.5)
----------------------------------------------------------------------
SeqName      Position  Potential   Jury    N-Glyc
                                 agreement result
----------------------------------------------------------------------
contig049287-BurOR.E043    3 NSTV   0.7899     (9/9)   +++
contig049287-BurOR.E043   39 NTSL   0.6764     (9/9)   ++
contig049287-BurOR.E043   49 NRSL   0.7480     (9/9)   ++
contig049287-BurOR.E043  245 NFSF   0.4448     (7/9)   -
----------------------------------------------------------------------
```

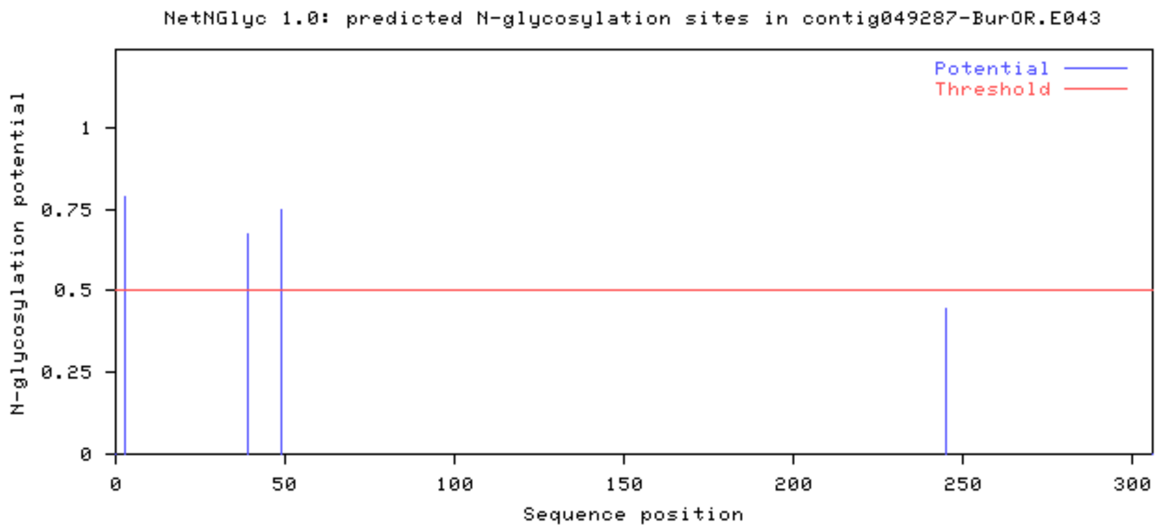

Graphics in PostScript

Output for 'contig049289-BurOR.E044'

```
##########################################################################################

    Warning: This sequence may not contain a signal peptide!!

    Proteins without signal peptides are unlikely to be exposed to
    the N-glycosylation machinery and thus may not be glycosylated
    (in vivo) even though they contain potential motifs.

    SignalP-NN euk predictions are as follows:

  # name                  Cmax  pos ?  Ymax  pos ?  Smax  pos ?  Smean ?  D     ?

    SignalP output is explained at http://www.cbs.dtu.dk/services/SignalP/output.html

##########################################################################################

Name:  contig049289-BurOR.E044  Length:  322
MLNVTTPPLSYFILGGFMNVGSLKFFYFSLTVILYILIIAANTSLIVVICVNRSLHEPMYMFLCSLFVNELYGSTGLFPF      80
LLLQILSDVHTVSAPLCFLQIFCIHTYGSIEVSNLAVMSYDRYLAICCPLQYNTQMTSNNTAVLIVVMWAFSLAKFLIAL     160
SLNLRLRLCGNVLNSLYCQNYLVVRLACSSTKVNNVYGIFDIIMTIIVPTLIILFSYMKILKVCFYGSKQMRQKSLTTCT     240
PQLVSLLNFSFGCCFEIFQSRFDTTGLPAALRIFLSLYFLMMQPLMNPILYGTQMSKIRGVYEHVLSTIMSCGCSKVSQS     320
DX
..N.......................................N.........N...........................      80
..........................................................N.....................     160
................................................................................     240
................................................................................     320
..                                                                                   400

(Threshold=0.5)
----------------------------------------------------------------------
SeqName      Position  Potential   Jury    N-Glyc
                                 agreement result
----------------------------------------------------------------------
contig049289-BurOR.E044    3 NVTT   0.7962     (9/9)   +++
contig049289-BurOR.E044   42 NTSL   0.7264     (9/9)   ++
contig049289-BurOR.E044   52 NRSL   0.7474     (9/9)   ++
contig049289-BurOR.E044  139 NNTA   0.5394     (3/9)   +
contig049289-BurOR.E044  248 NFSF   0.3856     (8/9)   -
```

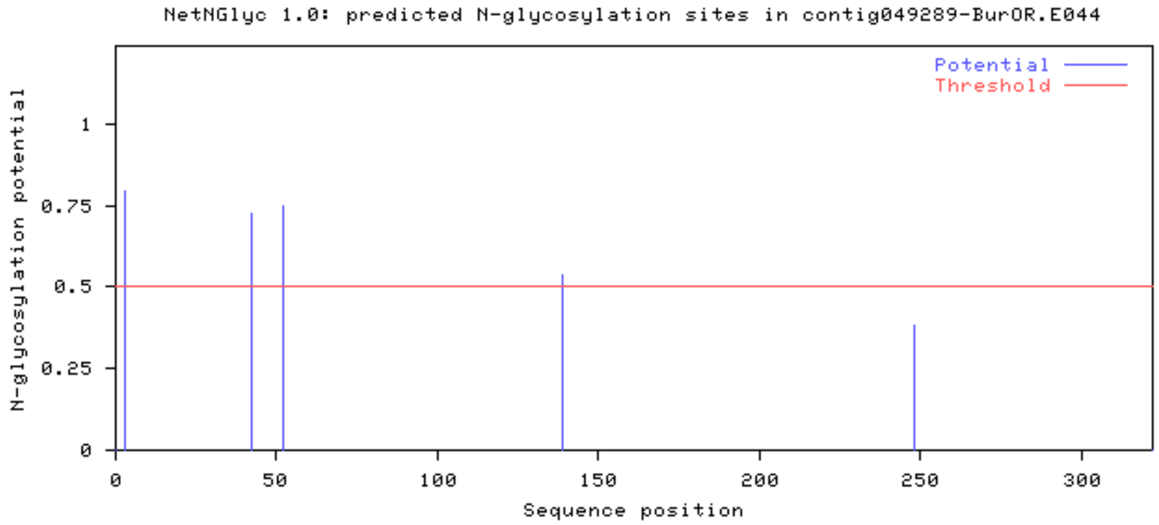

[Graphics in PostScript]

Output for 'contig049295-BurOR.G060'

```
##########################################################################################

    Warning: This sequence may not contain a signal peptide!!

    Proteins without signal peptides are unlikely to be exposed to
    the N-glycosylation machinery and thus may not be glycosylated
    (in vivo) even though they contain potential motifs.

    SignalP-NN euk predictions are as follows:

  # name                    Cmax  pos ?  Ymax  pos ?  Smax  pos ?  Smean ?  D     ?

    SignalP output is explained at http://www.cbs.dtu.dk/services/SignalP/output.html

##########################################################################################

Name:  contig049295-BurOR.G060  Length:  312
MENNFEIVFVLQGLNDSLTNRQIYFAFALMSYLFTVSVNLTLIITISLDKTLHEPIYIFLCSLCFNEICGASSFYPKLLH      80
DLLTNSYVITYTACLGQMFVTYSYIFSEFTSLTVMAYDRYIAICKPLQYRMLMTAQKVAQLLMLTWCFSVFETAVGTVLT     160
ARLPLCGRHIPKIFCTNWEVVKLSCSDLTLNNIYAFMLIFSHLSQTALIMVSYVHLIRAAIRSQADRRKFMQTCLPHLIT     240
LLVFTTSLMFDTMYSRYSGGSTMKALQNALAAQFLVVPPLVNPIIYGLNLQQIRSRMVHRFTHRTGTFRKNX
..............N.......................N.........................................      80
................................................................................     160
................................................................................     240
........................................................................             320

(Threshold=0.5)
----------------------------------------------------------------------
SeqName       Position  Potential    Jury    N-Glyc
                                  agreement result
----------------------------------------------------------------------
contig049295-BurOR.G060   15 NDSL   0.7057     (9/9)   ++
contig049295-BurOR.G060   39 NLTL   0.7857     (9/9)   +++
----------------------------------------------------------------------
```

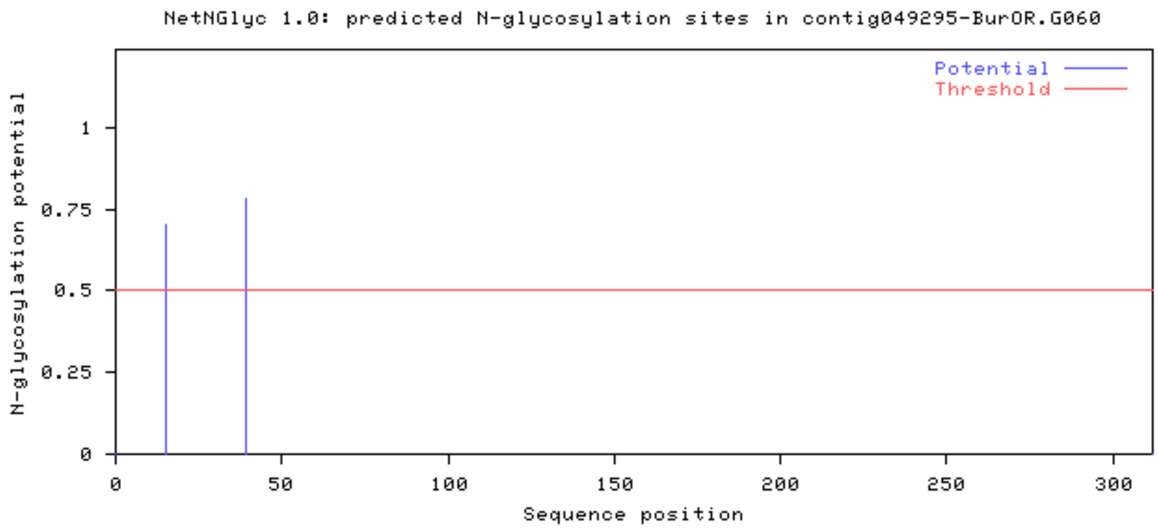

Graphics in PostScript

Output for 'contig049297-BurORep.E057'

```
##########################################################################################

    Warning: This sequence may not contain a signal peptide!!

    Proteins without signal peptides are unlikely to be exposed to
    the N-glycosylation machinery and thus may not be glycosylated
    (in vivo) even though they contain potential motifs.

    SignalP-NN euk predictions are as follows:

  # name                   Cmax  pos ?  Ymax  pos ?  Smax  pos ?  Smean ?  D     ?

    SignalP output is explained at http://www.cbs.dtu.dk/services/SignalP/output.html

##########################################################################################

Name:  contig049297-BurORep.E057        Length:  269
MLIVVICVNRSLHEPMYMFLCSLFVNELYGSTGLFPFLLLQILSDVHTVSAPLCFLQIFSLYSYGSVEFNLAVMSYDRYL      80
AICCPLQYNTRMTSSTVSVLIAVSWIYALLLVAVTVSLSSPLQLCGNIINKVYCDNYAIVKLACSDTTLNNIYGLISTAF     160
TIFLPLTLIFFTYMRILKVCFSGSKQTRQKAVSTCTPHLASLLNFSFGACFEVLQSRFNMNTVPNILRILISLYWLICQP     240
LFNPVLYGLKMSKIRDICKSLIYWKVWII
........N.......................................................................      80
................................................................................     160
................................................................................     240
.............................                                                        320

(Threshold=0.5)
----------------------------------------------------------------------
SeqName      Position  Potential   Jury    N-Glyc
                                 agreement result
----------------------------------------------------------------------
contig049297-BurORep.E057    9 NRSL    0.7590     (9/9)    +++
contig049297-BurORep.E057  204 NFSF    0.4420     (6/9)    -
----------------------------------------------------------------------
```

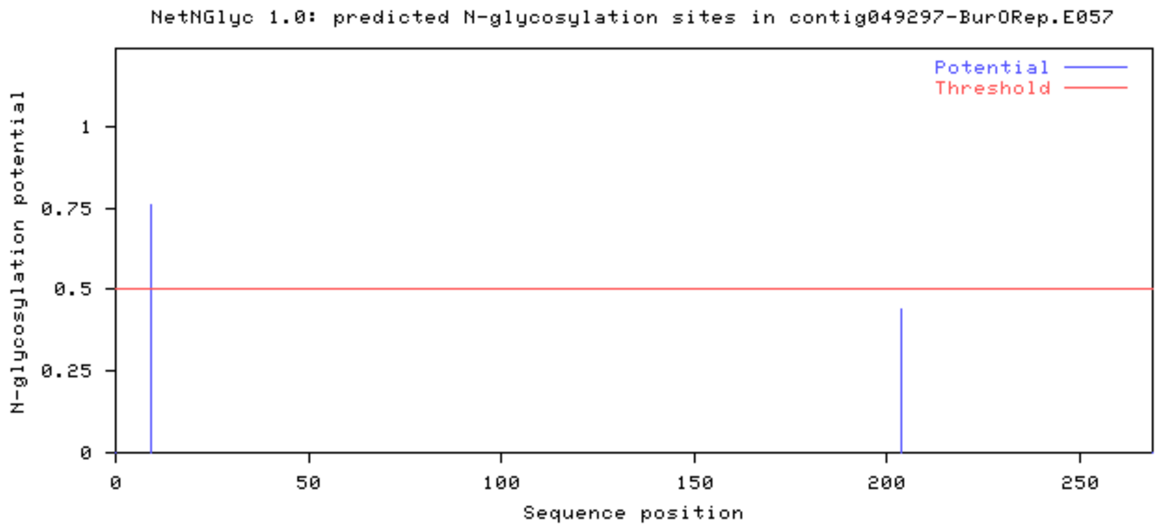

Graphics in PostScript

Output for 'contig049298-BurOR.E045'

```
########################################################################################

    Warning: This sequence may not contain a signal peptide!!

    Proteins without signal peptides are unlikely to be exposed to
    the N-glycosylation machinery and thus may not be glycosylated
    (in vivo) even though they contain potential motifs.

    SignalP-NN euk predictions are as follows:

  # name                  Cmax  pos ?  Ymax  pos ?  Smax  pos ?  Smean ?  D     ?

    SignalP output is explained at http://www.cbs.dtu.dk/services/SignalP/output.html

########################################################################################

Name:  contig049298-BurOR.E045  Length:  314
MINFTEGSYFILGAYFDAGPTKYLFFLLLLSLYSLIICANLLLIVVICVNRSLHEPMYMFLCSLFVNELYGSTGLFPFLL      80
LQILSDVHTVSAPLCFLQIFSLYSYVGVEFLTLAVMSYDRYLAICCPLQYNTRMTSSTVSVLIAVSWIYALLLVAVTVSL     160
SSPLQLCGNIINKVYCDNYAIVKLACSDTTLNNIYGLISTAFTVFVPVTLIFFTYMRILKVCFSGSKQTRQKAVSTCTPH     240
LASLLNFSCSGCFEILQSRFNMNNLPNVLRILISLYWLMCQPLFNPVLYGLKMSKIRDICKCLLYSKVNTILSX
..N..............................................N..............................      80
................................................................................     160
................................................................................     240
.....N....................................................................           320

(Threshold=0.5)
----------------------------------------------------------------------
SeqName      Position  Potential   Jury    N-Glyc
                                 agreement result
----------------------------------------------------------------------
contig049298-BurOR.E045    3 NFTE   0.6487     (9/9)   ++
contig049298-BurOR.E045   50 NRSL   0.7483     (9/9)   ++
contig049298-BurOR.E045  246 NFSC   0.5484     (7/9)   +
----------------------------------------------------------------------
```

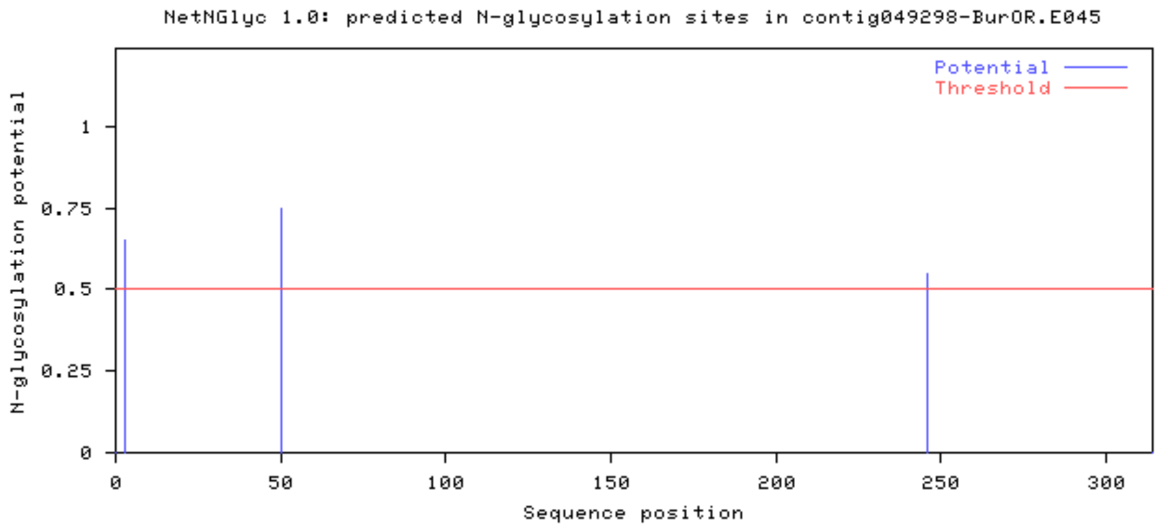

Graphics in PostScript

Output for 'contig049299-BurOR.E046'

```
##########################################################################################

    Warning: This sequence may not contain a signal peptide!!

    Proteins without signal peptides are unlikely to be exposed to
    the N-glycosylation machinery and thus may not be glycosylated
    (in vivo) even though they contain potential motifs.

    SignalP-NN euk predictions are as follows:

  # name                Cmax  pos ?  Ymax  pos ?  Smax  pos ?  Smean ?  D     ?

    SignalP output is explained at http://www.cbs.dtu.dk/services/SignalP/output.html

##########################################################################################

Name:  contig049299-BurOR.E046  Length:  337
MAYDEKRLKCEIVCLQDHSEDTTQGNKIHLIINSSKVSYFTLAAYFDTSTLKYFCFTVVMSLYLLILCANVLLIVVICVN      80
RSLHEPMYMFLCSLFVNELYGSTGLFPFLLLQILSDVHTVSAPLCFLQIFCVFSYVCVEFCILAVMSYDRYLAICCPLQY     160
HTRMTPATVVLLIALSWLYSFLTILALILLIAPLELCGNVINKVYCLNYSIVKLACSETTANNIYGLFITALTVFVPVIL     240
ILCSYVRILKVCFSGSKQTRQKAVSTCTPHLASLLNFSFGVCFEVIQSRFSLSSVHSMVHIVLSLYFLTCQPLFNPVLYG     320
LNMSNIRKRLFAHKRRX
................................N..............................................N      80
................................................................................     160
...............................................N................................     240
................................................................................     320
.N...............                                                                    400

(Threshold=0.5)
----------------------------------------------------------------------
SeqName      Position  Potential   Jury    N-Glyc
                                 agreement result
----------------------------------------------------------------------
contig049299-BurOR.E046   33 NSSK   0.6710     (8/9)    +
contig049299-BurOR.E046   80 NRSL   0.7399     (9/9)    ++
contig049299-BurOR.E046  208 NYSI   0.5956     (8/9)    +
contig049299-BurOR.E046  276 NFSF   0.4402     (7/9)    -
contig049299-BurOR.E046  322 NMSN   0.5659     (4/9)    +
----------------------------------------------------------------------
```

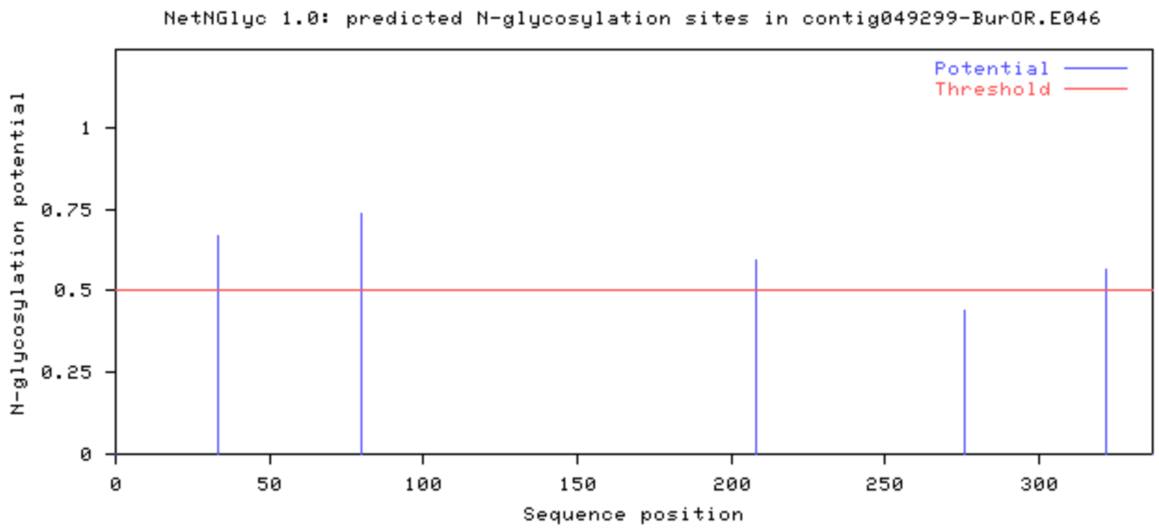

Graphics in PostScript

Output for 'contig049603-BurORe.K086'

```
##########################################################################################

    Warning: This sequence may not contain a signal peptide!!

    Proteins without signal peptides are unlikely to be exposed to
    the N-glycosylation machinery and thus may not be glycosylated
    (in vivo) even though they contain potential motifs.

    SignalP-NN euk predictions are as follows:

  # name                 Cmax  pos ?  Ymax  pos ?  Smax  pos ?  Smean ?  D     ?

    SignalP output is explained at http://www.cbs.dtu.dk/services/SignalP/output.html

##########################################################################################

Name:  contig049603-BurORe.K086        Length:  187
MYILFCNLSINDLFGNSIMIPRLLVDMLRPPSERLISYYECVVQAFTTQMFSTTAHTVLMIMAFDRYVAICNPLCYAAVM      80
TNKMLMKLTVSAWGVSFVLVGILLGLTLRLGRCRTLIKSPYCDNAALFNLSCEDVFINNVYGLTFTVLLFTGSIGSMVLT     160
YTKITVVCLTTKNKSLNNKALKTCSTH
......N.........................................................................      80
................................................N...............................     160
...........................                                                          240

(Threshold=0.5)
----------------------------------------------------------------------
SeqName      Position  Potential    Jury    N-Glyc
                                  agreement result
----------------------------------------------------------------------
contig049603-BurORe.K086    7 NLSI   0.6943     (9/9)   ++
contig049603-BurORe.K086  129 NLSC   0.6050     (7/9)   +
contig049603-BurORe.K086  173 NKSL   0.4486     (6/9)   -
----------------------------------------------------------------------
```

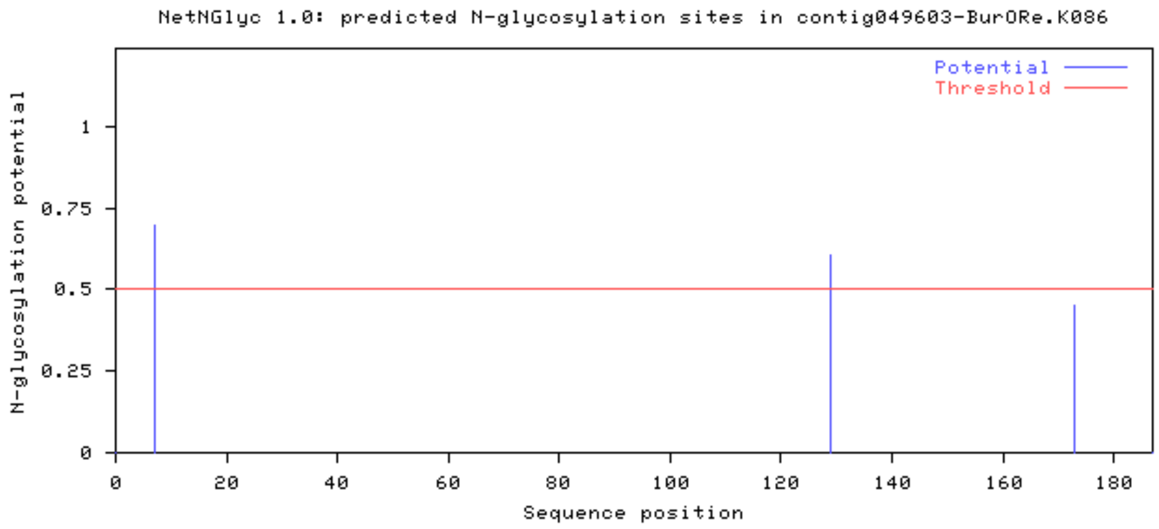

Graphics in PostScript

Output for 'contig049604-BurOR.K081'

```
##########################################################################

    Warning: This sequence may not contain a signal peptide!!

    Proteins without signal peptides are unlikely to be exposed to
    the N-glycosylation machinery and thus may not be glycosylated
    (in vivo) even though they contain potential motifs.

    SignalP-NN euk predictions are as follows:

  # name                Cmax  pos ?  Ymax  pos ?  Smax  pos ?  Smean ?  D     ?

    SignalP output is explained at http://www.cbs.dtu.dk/services/SignalP/output.html

##########################################################################

Name:  contig049604-BurOR.K081  Length:  314
MENYTYNTLTLQLEGLNISVESTYALFLLLLFFYLFIIVANGGIAVLVFMDKNLHQPMYMLFCNLPFNDILGNSIMVPRL      80
LIDILKPPSERFISYYECVVQAFTTHMFGTTSHTVLMIMAFDRYVAICNPLRYASIMTNKMVIKLTVFAWGVAFVLVGIL     160
LGLTVRLSRCRTLITNPYCDNASLFKLSCENVFINNVYGLTFTVVLFTGSMGSIVLTYASITIVCLTSKNKSLNSKALKT     240
CSTHLVVYLIMLLSGMIVIMLHRFPQYSDYRKLCSILFHIIPGSLNPIIYGVQSKEMQKLFAKLLQKKTGPLKX
..N.............N...............................................................      80
................................................................................     160
....................N...........................................................     240
..........................................................................           320

(Threshold=0.5)
----------------------------------------------------------------------
SeqName       Position  Potential   Jury    N-Glyc
                                 agreement  result
----------------------------------------------------------------------
contig049604-BurOR.K081    3 NYTY   0.7730     (9/9)   +++
contig049604-BurOR.K081   17 NISV   0.6960     (9/9)   ++
contig049604-BurOR.K081  181 NASL   0.6140     (7/9)   +
contig049604-BurOR.K081  230 NKSL   0.4033     (7/9)   -
----------------------------------------------------------------------
```

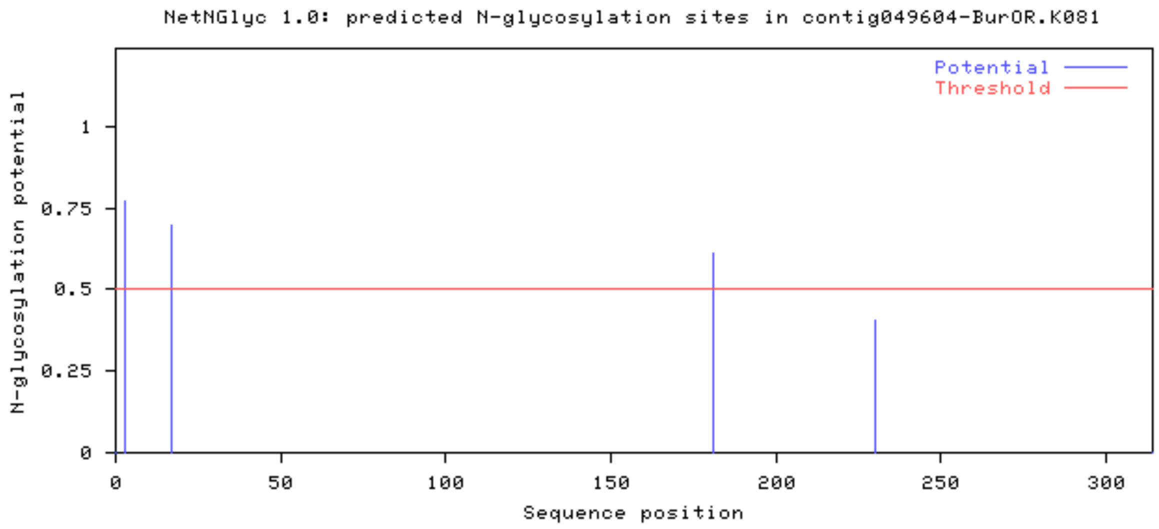

Graphics in PostScript

Output for 'contig049605-BurORs.AB153'

```
##########################################################################################

    Warning: This sequence may not contain a signal peptide!!

    Proteins without signal peptides are unlikely to be exposed to
    the N-glycosylation machinery and thus may not be glycosylated
    (in vivo) even though they contain potential motifs.

    SignalP-NN euk predictions are as follows:

  # name                 Cmax   pos ?  Ymax   pos ?  Smax   pos ?  Smean ?  D     ?

    SignalP output is explained at http://www.cbs.dtu.dk/services/SignalP/output.html

##########################################################################################

Name:  contig049605-BurORs.AB153        Length:  345
MNTDELFFPSDFPTLSTNHRSSSVNETLGLGGVTFFIIQGLTNLDEKKIILFSILLLIYIMVLGGNSIIIYVVQRTVMSF      80
LLQALTDPKLNSPLYFFLCNLSFVDMVYTTTTIPNMLSGLLTDILTISVLGCFLQMYFFIQLSVTGRAILTVMAYDRYVA     160
ICNPLQYNSIMTRPVRLLLVAGAWGFGAICTLPVTVIAFERPYCGPNVVKHAWCDPSSVRRLVCSDTSLDNIVSLLFAMV     240
SLVTTGVFILSSYILIGFSISRMVVAQRLKALRTCSAHLTVVSISYAAASFVYISYRVGNFSSEVKTLLCFLCNVSVQYS     320
VKVLSHPYLFIFLVGKQEIHAEIYX
........................N.......................................................      80
...................N............................................................     160
................................................................................     240
...........................................................N.............N......     320
.........................                                                            400

(Threshold=0.5)
----------------------------------------------------------------------
SeqName      Position  Potential   Jury    N-Glyc
                                agreement  result
----------------------------------------------------------------------
contig049605-BurORs.AB153   25 NETL   0.6289     (8/9)    +
contig049605-BurORs.AB153  100 NLSF   0.6534     (9/9)    ++
contig049605-BurORs.AB153  300 NFSS   0.6208     (8/9)    +
contig049605-BurORs.AB153  314 NVSV   0.6638     (9/9)    ++
----------------------------------------------------------------------
```

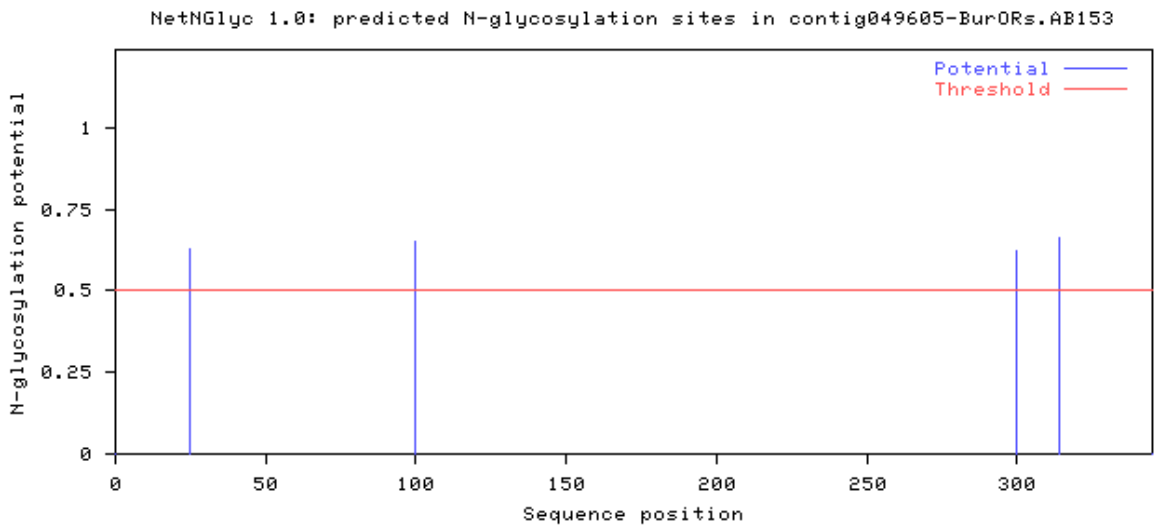

Graphics in PostScript

Output for 'contig049609-BurORe.K084'

```
##########################################################################################

    Warning: This sequence may not contain a signal peptide!!

    Proteins without signal peptides are unlikely to be exposed to
    the N-glycosylation machinery and thus may not be glycosylated
    (in vivo) even though they contain potential motifs.

    SignalP-NN euk predictions are as follows:

  # name                    Cmax  pos ?  Ymax  pos ?  Smax  pos ?  Smean ?  D     ?

    SignalP output is explained at http://www.cbs.dtu.dk/services/SignalP/output.html

##########################################################################################

Name:  contig049609-BurORe.K084         Length:  203
MENQTSDILLLEGLQVSPDASIPAFILLLLIYIFIMFSNIVLVILITLDSSLHQPMYLLFCNMSINDVFGATTIFPRMLR      80
DIFIASSDRYIHYVDCVIQAFCVHIYAGSSHTILMIMAFDRYVAICNPLQYATIMTNWMVVKLSVLAWAVIFVMVTILVG     160
LSVRLSRCRWIIFNPFCDNASLFKLSCESILINNIYGLGYTVL
..N..........................................................N..................      80
................................................................................     160
..................N........................                                          240

(Threshold=0.5)
----------------------------------------------------------------------
SeqName      Position  Potential   Jury    N-Glyc
                                 agreement result
----------------------------------------------------------------------
contig049609-BurORe.K084    3 NQTS    0.7488     (9/9)    ++
contig049609-BurORe.K084   62 NMSI    0.6589     (9/9)    ++
contig049609-BurORe.K084  179 NASL    0.6407     (8/9)    +
----------------------------------------------------------------------
```

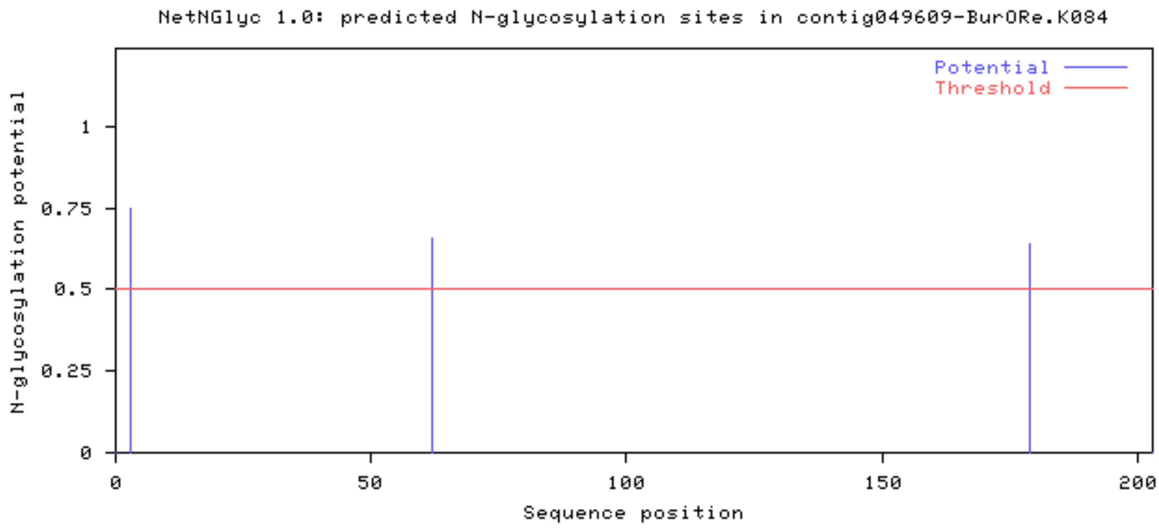

Graphics in PostScript

Output for 'contig049621-BurOR.K082'

```
##########################################################################

    Warning: This sequence may not contain a signal peptide!!

    Proteins without signal peptides are unlikely to be exposed to
    the N-glycosylation machinery and thus may not be glycosylated
    (in vivo) even though they contain potential motifs.

    SignalP-NN euk predictions are as follows:

  # name                Cmax  pos ?  Ymax  pos ?  Smax  pos ?  Smean ?  D     ?

    SignalP output is explained at http://www.cbs.dtu.dk/services/SignalP/output.html

##########################################################################

Name:  contig049621-BurOR.K082  Length:  313
MDNQSLNADILILGGLKVTPKFSIAAFISLLLVYIFIMVANIGLVVLIFMERSLHQPMYLLFCNMSVNEVFGSTIVVPHI      80
LRDLYVSDSERYIHYIVCVVQAFCVNLYGGVCHTILMTMTFDRYMAICNPLRYTIIMTNWMVVKLSVAAWAVVFVMVSIL     160
LSLTIRLSRCRRFIDNVHCDNASLFKLSCEDVVINHVFGLSYSVLLLGSSIGSVTLTYIKIATVCLQSKTKTINSKALQT     240
CATHLTLYIILMFSAFIIIILHRFPHLSDHRKMVSTVGEVALPALNAVIYGLQIKEIRQKIVVLFQRKGHLQX
..N.............................................................N...............      80
................................................................................     160
....................N...........................................................     240
.........................................................................            320

(Threshold=0.5)
----------------------------------------------------------------------
SeqName      Position  Potential   Jury    N-Glyc
                                 agreement result
----------------------------------------------------------------------
contig049621-BurOR.K082    3 NQSL   0.7004     (9/9)   ++
contig049621-BurOR.K082   64 NMSV   0.6972     (9/9)   ++
contig049621-BurOR.K082  181 NASL   0.6081     (8/9)   +
----------------------------------------------------------------------
```

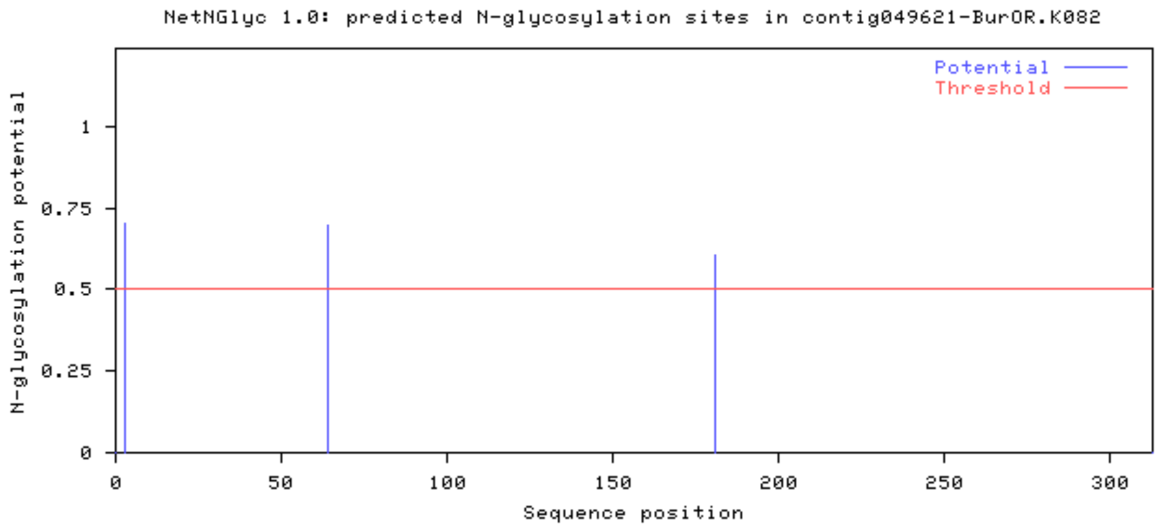

Graphics in PostScript

Output for 'contig051318-BurOR.A005'

```
##########################################################################

    Warning: This sequence may not contain a signal peptide!!

    Proteins without signal peptides are unlikely to be exposed to
    the N-glycosylation machinery and thus may not be glycosylated
    (in vivo) even though they contain potential motifs.

    SignalP-NN euk predictions are as follows:

  # name                   Cmax   pos ?  Ymax   pos ?  Smax   pos ?  Smean ?  D     ?

    SignalP output is explained at http://www.cbs.dtu.dk/services/SignalP/output.html

##########################################################################

Name:  contig051318-BurOR.A005  Length:  314
MDEESNATYLTLDWYTEINKYRYIFFFVMFTLYILIICTNSTILYLIWNHKNLHEPMYIFIAALLLNSVLYSTTVYPKLL     80
IDFSSEKQVTTYSACLFQFFIFYTLVLSEFLLLAAMAYDRYVAICKPLEYQTIMRKTTVGIFLVVAWLVPACQVAVQAIA    160
SAEAKLCDSNIKGIFCNNAVYTLQCERSKLITIFGVVIVLDLAILPMLFIVFTYTKIFIVSHRSCKEIRKKAAETCLPHL    240
LVLLSLSVFFVYDVSIARANPDFPKTTRIIMTLQIMLYQPLLNPFIYGLKMKEISKHLNKLLSQTNIIPCIKTX
.....N.................................N........................................     80
................................................................................    160
................................................................................    240
..........................................................................          320

(Threshold=0.5)
----------------------------------------------------------------------
SeqName      Position  Potential   Jury    N-Glyc
                                 agreement result
----------------------------------------------------------------------
contig051318-BurOR.A005    6 NATY   0.6217     (7/9)    +
contig051318-BurOR.A005   40 NSTI   0.6462     (8/9)    +
----------------------------------------------------------------------
```

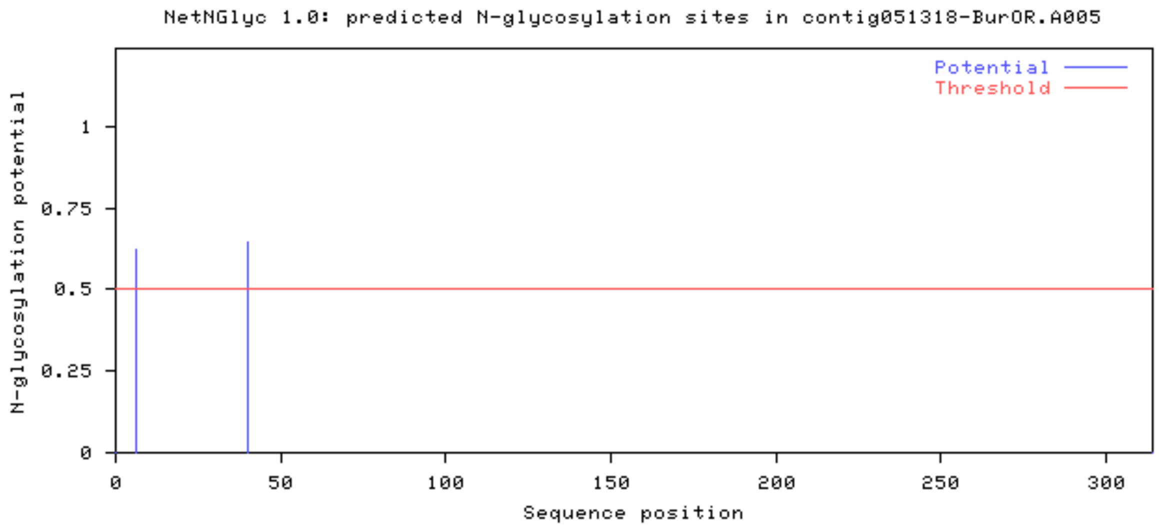

Graphics in PostScript

Output for 'contig051321-BurOR.A006'

```
##########################################################################################

    Warning: This sequence may not contain a signal peptide!!

    Proteins without signal peptides are unlikely to be exposed to
    the N-glycosylation machinery and thus may not be glycosylated
    (in vivo) even though they contain potential motifs.

    SignalP-NN euk predictions are as follows:

  # name                Cmax  pos ?  Ymax  pos ?  Smax  pos ?  Smean ?  D     ?

    SignalP output is explained at http://www.cbs.dtu.dk/services/SignalP/output.html

##########################################################################################

Name:  contig051321-BurOR.A006  Length:  314
MDEESNATYLTLDWYTEINKYRYVFFFVMFTLYILIICTNSTILYLIWIHKNLHEPMYIFIAALLLNSVLYSTTVYPKLL      80
TDFLSEIQVTTYSACLFQFFMFYTLGCSEFLLLAAMAYDRYVAICKPLEYQTIMRKTTVGIFLVMAWLVPACHIAVQAIA     160
SAGAKLCDSNIKGIFCSNAVYTLQCERSRLITIFGVFLLIDLAILPMLFIVYTYTKIFIVSHRSCKEIRKKTAETCLPHL     240
LVLISYSMFFVYDISIARVKSDFPKTTRIIMTLQIMLYQPLLNPFIYGLKMKDISKHLNKLLSQAKTISCIKTX
.....N.................................N........................................      80
................................................................................     160
................................................................................     240
..........................................................................           320

(Threshold=0.5)
----------------------------------------------------------------------
SeqName      Position  Potential   Jury    N-Glyc
                                 agreement result
----------------------------------------------------------------------
contig051321-BurOR.A006    6 NATY   0.6216     (7/9)    +
contig051321-BurOR.A006   40 NSTI   0.6298     (8/9)    +
----------------------------------------------------------------------
```

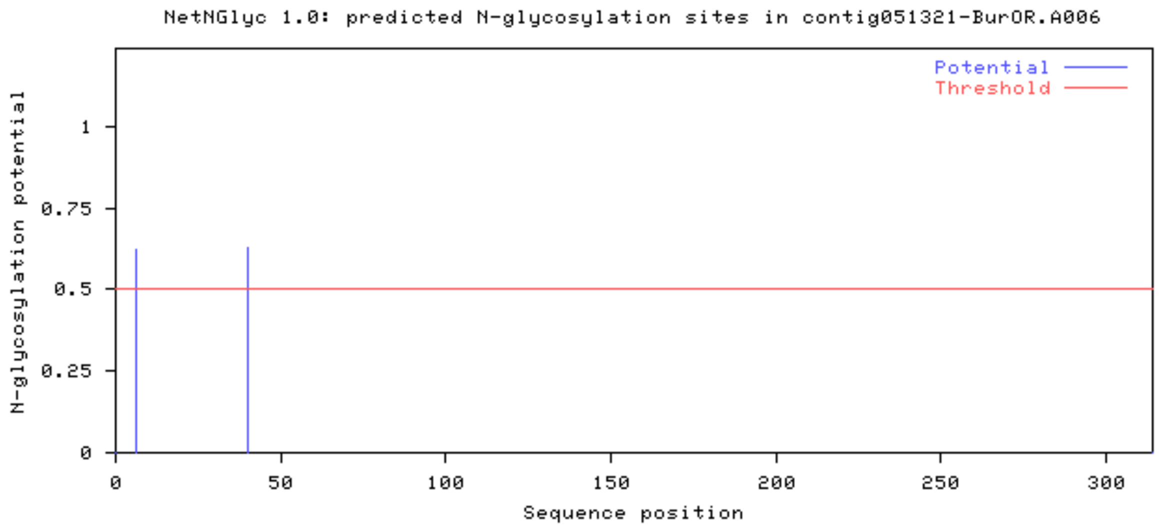

[Graphics in PostScript]

Output for 'contig051559-BurOR.A007'

```
##############################################################################

    Warning: This sequence may not contain a signal peptide!!

    Proteins without signal peptides are unlikely to be exposed to
    the N-glycosylation machinery and thus may not be glycosylated
    (in vivo) even though they contain potential motifs.

    SignalP-NN euk predictions are as follows:

  # name                Cmax  pos ?  Ymax  pos ?  Smax  pos ?  Smean ?  D     ?

    SignalP output is explained at http://www.cbs.dtu.dk/services/SignalP/output.html

##############################################################################

Name:  contig051559-BurOR.A007  Length:  300
MNITYITFGGHVEVEKYRYIYFVIMFMVYGLIICSNSTIVWVIIVQKSLHEPMYIFIAALLVNSVVLSTVIYPKLLIDFL      80
SEKQIILYHACLFQVFMFYVLSSSEFLLLSAMAYDRYVSICKPLQYPTIMRRTRVSIFLILSWFLPAIQIVVPVLRNSIT     160
PLCNFTLKGIFCNNSVNHLYCVTSKELSIYGMVVLFNGALFPMLFILFTYIKIIIVACQSCGNVRKKAAQTCLPHVLVLI     240
NYSCLITYDMVIVRLESEFPKTARFIMTLQFITYNPLCNPIIYGLKMKEISKNLKRLFSX
.N.................................N............................................      80
................................................................................     160
...N............................................................................     240
N...........................................................                         320

(Threshold=0.5)
----------------------------------------------------------------------
SeqName      Position  Potential   Jury    N-Glyc
                                 agreement result
----------------------------------------------------------------------
contig051559-BurOR.A007    2 NITY   0.7926     (9/9)    +++
contig051559-BurOR.A007   36 NSTI   0.7254     (9/9)    ++
contig051559-BurOR.A007  164 NFTL   0.6558     (9/9)    ++
contig051559-BurOR.A007  173 NNSV   0.4574     (6/9)    -
contig051559-BurOR.A007  241 NYSC   0.5027     (4/9)    +
----------------------------------------------------------------------
```

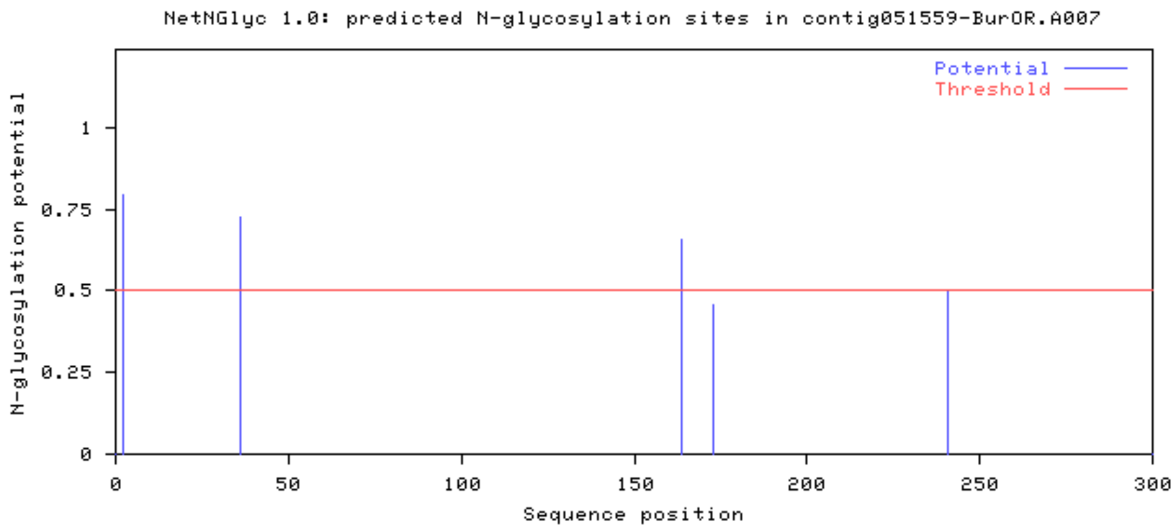

[Graphics in PostScript]

Output for 'contig051559-BurOR.A008'

```
##########################################################################################

    Warning: This sequence may not contain a signal peptide!!

    Proteins without signal peptides are unlikely to be exposed to
    the N-glycosylation machinery and thus may not be glycosylated
    (in vivo) even though they contain potential motifs.

    SignalP-NN euk predictions are as follows:

  # name                 Cmax  pos ?  Ymax  pos ?  Smax  pos ?  Smean ?  D     ?

    SignalP output is explained at http://www.cbs.dtu.dk/services/SignalP/output.html

##########################################################################################

Name:  contig051559-BurOR.A008  Length:  316
MDEVLNATYLTLDGYVEVNKYRYVYFFIFFILYSLIICSNSTIVYIIWIHKNLHEPMYTFIAALLLNCVLYSTTVYPKLL      80
IDFLSERQVTTYSACLLQFFMFYTLGSSEFFLLAAMAYDRYVAICKPLQYQTIMSKTTVSIFLAVAWLVPVCHIAVLTAG     160
SAEATLCNFNLKGIFCNNAVYTLQCVKSRLITVFGVVALIDLVILPMLFIVFTYSNIFILTYQSCKDVRKKALETCLPHL     240
LVLFSFSCLSIYDVSIARVESDFPKTARLIMALQIVLYHPLLNPFIYGLKMKEISKQLKRFFYHAKIISCINSECX
.....N.................................N........................................      80
................................................................................     160
................................................................................     240
............................................................................         320

(Threshold=0.5)
----------------------------------------------------------------------
SeqName      Position  Potential   Jury    N-Glyc
                                 agreement result
----------------------------------------------------------------------
contig051559-BurOR.A008    6 NATY    0.6278     (9/9)    ++
contig051559-BurOR.A008   40 NSTI    0.7253     (9/9)    ++
----------------------------------------------------------------------
```

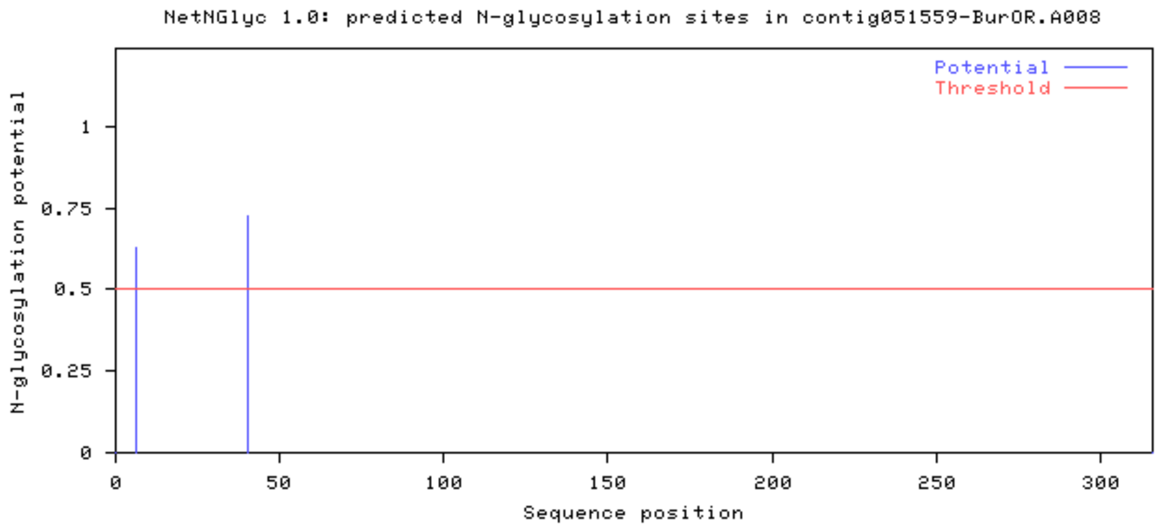

Graphics in PostScript

Output for 'contig051560-BurORe.A019'

```
##########################################################################################

    Warning: This sequence may not contain a signal peptide!!

    Proteins without signal peptides are unlikely to be exposed to
    the N-glycosylation machinery and thus may not be glycosylated
    (in vivo) even though they contain potential motifs.

    SignalP-NN euk predictions are as follows:

  # name                    Cmax  pos ?  Ymax  pos ?  Smax  pos ?  Smean ?  D     ?

    SignalP output is explained at http://www.cbs.dtu.dk/services/SignalP/output.html

##########################################################################################

Name:  contig051560-BurORe.A019         Length:  231
YPKLLSDVLSEKQTISYPLCLFQGFSYYTSAGSEFLLLAAMAYDRYVSICKPLQYPVIMNRITINVFLILAWLIPAFEIA      80
VSFVLYFNIKLCSFTLTGIFCNNSIYRLQCVPSVTISIYGVVTLINIALLPMLFILFTYIRILRISYNCCRETRRKALKT     160
CLPHLLVLINFSCFIVFDSVIIRLDSDLSKTLRLILMFQSILFHPLLNPIIYGLKMNEIFRHIKSLLCQVX
................................................................................      80
................................................................................     160
.........N.............................................................              240

(Threshold=0.5)
----------------------------------------------------------------------
SeqName      Position  Potential   Jury    N-Glyc
                                 agreement result
----------------------------------------------------------------------
contig051560-BurORe.A019  102 NNSI   0.4657     (5/9)   -
contig051560-BurORe.A019  170 NFSC   0.5768     (6/9)   +
----------------------------------------------------------------------
```

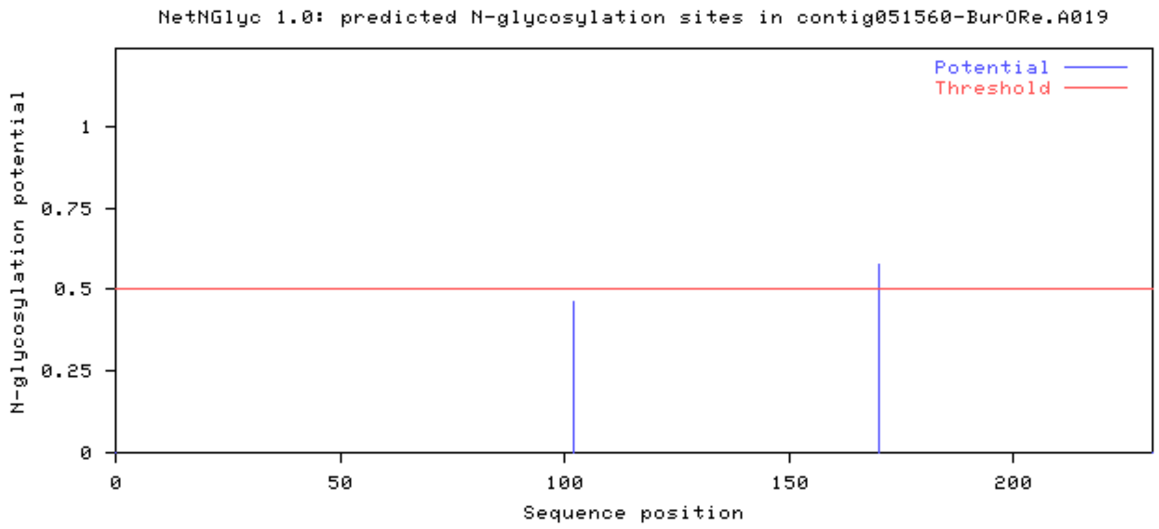

Graphics in PostScript

Output for 'contig051566-BurOR.A009'

```
##########################################################################################

    Warning: This sequence may not contain a signal peptide!!

    Proteins without signal peptides are unlikely to be exposed to
    the N-glycosylation machinery and thus may not be glycosylated
    (in vivo) even though they contain potential motifs.

    SignalP-NN euk predictions are as follows:

  # name                Cmax  pos ?  Ymax  pos ?  Smax  pos ?  Smean ?  D     ?

    SignalP output is explained at http://www.cbs.dtu.dk/services/SignalP/output.html

##########################################################################################

Name:  contig051566-BurOR.A009  Length:  306
MDVELNVTLLTLGGFAELHKYRYLYFVVIFTLYILILCFNTIIVYLIWTHKNLHEPMYIFIAALLINSVLYSMIIYPKLL      80
SDVLSEKQTISYPLCLFQGFSYYTSAVSEFLLLAAMAYDRYVSICKPLQYPVIMNRITIYVCLILAWLIPAFEIAVSFVL     160
YSNVKLCSFTLTAIFCNNSFYRLQCVPSVVISIYGVVMLINMTFLPMLFILFSYIRILRISYSCCRETRRKALKTCLPHL     240
LVLINFSCFFFFDIIIVRLESDLSNTVRLTLTFQSILFHPLLNPIIYGLKVNEIFKHIKMLLCQVX
.....N..........................................................................      80
................................................................................     160
................................................................................     240
....N.............................................................               320

(Threshold=0.5)
----------------------------------------------------------------------
SeqName      Position  Potential   Jury    N-Glyc
                                 agreement result
----------------------------------------------------------------------
contig051566-BurOR.A009    6 NVTL   0.7850     (9/9)    +++
contig051566-BurOR.A009  177 NNSF   0.3830     (9/9)    --
contig051566-BurOR.A009  201 NMTF   0.4056     (6/9)    -
contig051566-BurOR.A009  245 NFSC   0.5666     (6/9)    +
----------------------------------------------------------------------
```

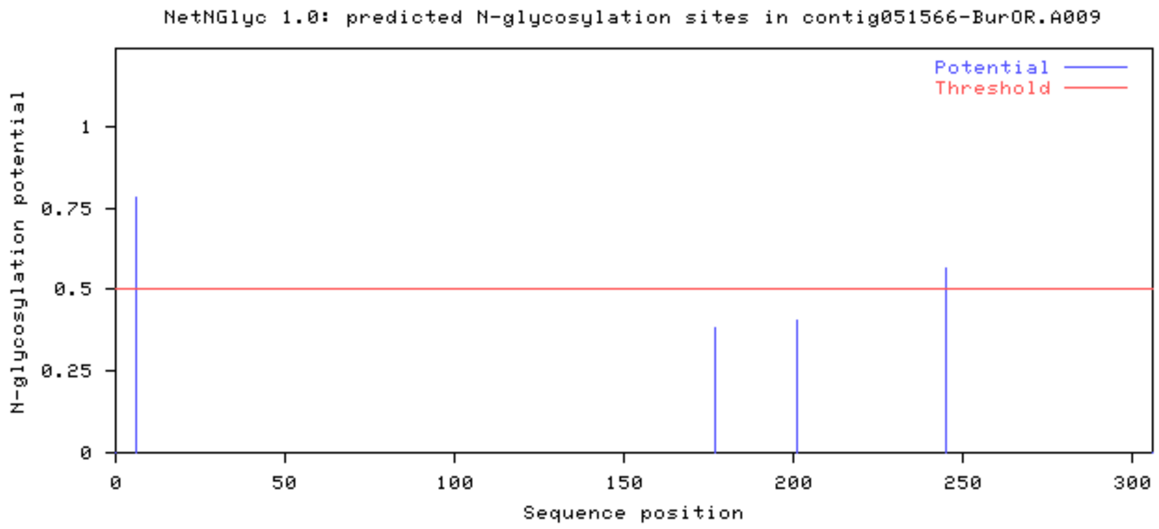

Graphics in PostScript

Output for 'contig051570-BurOR.A010'

```
##########################################################################

   Warning: This sequence may not contain a signal peptide!!

   Proteins without signal peptides are unlikely to be exposed to
   the N-glycosylation machinery and thus may not be glycosylated
   (in vivo) even though they contain potential motifs.

   SignalP-NN euk predictions are as follows:

 # name                Cmax  pos ?  Ymax  pos ?  Smax  pos ?  Smean ?  D     ?

   SignalP output is explained at http://www.cbs.dtu.dk/services/SignalP/output.html

##########################################################################

Name:  contig051570-BurOR.A010  Length:  311
MDEELNTTYVTLDGYIEVNKYRYVYFCIIFTLYIIIICSNSTIVYVIWIHKNLHEPMYIFIAALLLNCLLYSTTIYPKLL      80
IDFLSEKQVITYSACLFQFFIFYTLGSSEFFLLAAMAYDRYVAICKPLEYPTIMNKTTVIIFLVVSWLIPAVHIAIQAIG     160
SAEATLCNFNLKGIFCNNAVYTLLCVKSRLIIVFGVVALIDLIILPVLFIVFTYTNIFIISYQSCKEIRKKAAETCLPHL     240
LVLISISCLSIYDVSIARVESDFPKAARLLMTLQIVLYHPLFNPFIYGLKMKEISKQLKRFFCHARIIVYX
.....N.................................N........................................      80
......................................................N.........................     160
................................................................................     240
.......................................................................              320

(Threshold=0.5)
----------------------------------------------------------------------
SeqName      Position  Potential   Jury    N-Glyc
                                 agreement result
----------------------------------------------------------------------
contig051570-BurOR.A010    6 NTTY   0.7358     (9/9)   ++
contig051570-BurOR.A010   40 NSTI   0.6934     (8/9)   +
contig051570-BurOR.A010  135 NKTT   0.6550     (7/9)   +
----------------------------------------------------------------------
```

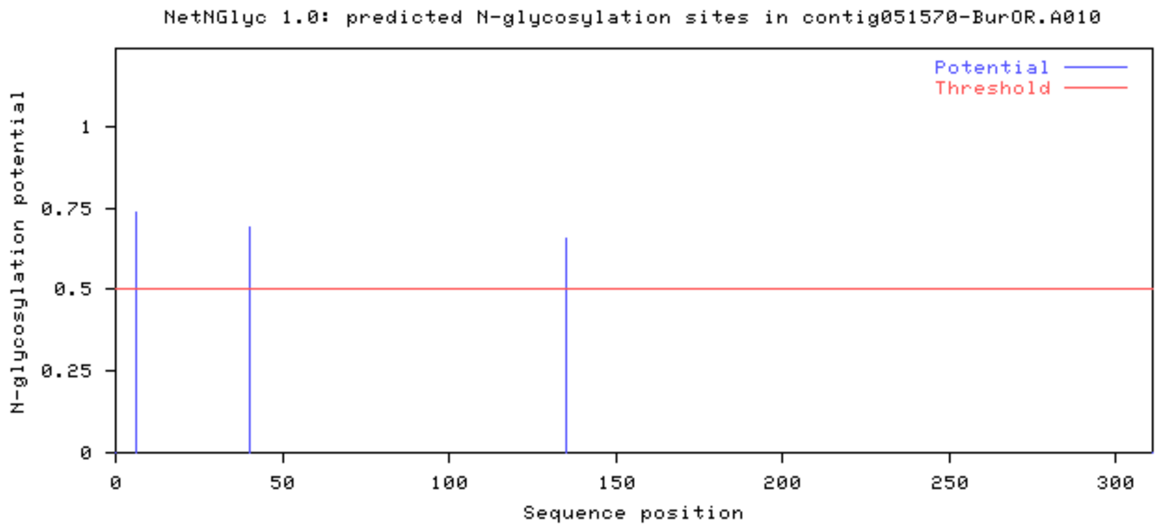

Graphics in PostScript

Output for 'contig051570-BurORe.A020'

```
##########################################################################################

    Warning: This sequence may not contain a signal peptide!!

    Proteins without signal peptides are unlikely to be exposed to
    the N-glycosylation machinery and thus may not be glycosylated
    (in vivo) even though they contain potential motifs.

    SignalP-NN euk predictions are as follows:

  # name                Cmax  pos ?  Ymax  pos ?  Smax  pos ?  Smean ?  D     ?

    SignalP output is explained at http://www.cbs.dtu.dk/services/SignalP/output.html

##########################################################################################

Name:  contig051570-BurORe.A020         Length:  162
MDNKLNLTCITLNGYVEVEKYRYVYFLIFTIYAAVIFSNSTIIRLIVFHQSLHEPMYIFIAVLLINSTFFCTTIYPKFLL      80
DVLSEKQIISHTMCHFQYFVLYTSGASEFLVLAAMAYDRYVSICKPLQYSVIMKKTTISVFLVLAWLVPACQVAGTTVRA     160
LC
.....N................................N..........................N..............      80
................................................................................     160
..                                                                                   240

(Threshold=0.5)
----------------------------------------------------------------------
SeqName      Position  Potential   Jury    N-Glyc
                                 agreement result
----------------------------------------------------------------------
contig051570-BurORe.A020    6 NLTC   0.7574     (9/9)   +++
contig051570-BurORe.A020   39 NSTI   0.6497     (9/9)   ++
contig051570-BurORe.A020   66 NSTF   0.5422     (8/9)   +
----------------------------------------------------------------------
```

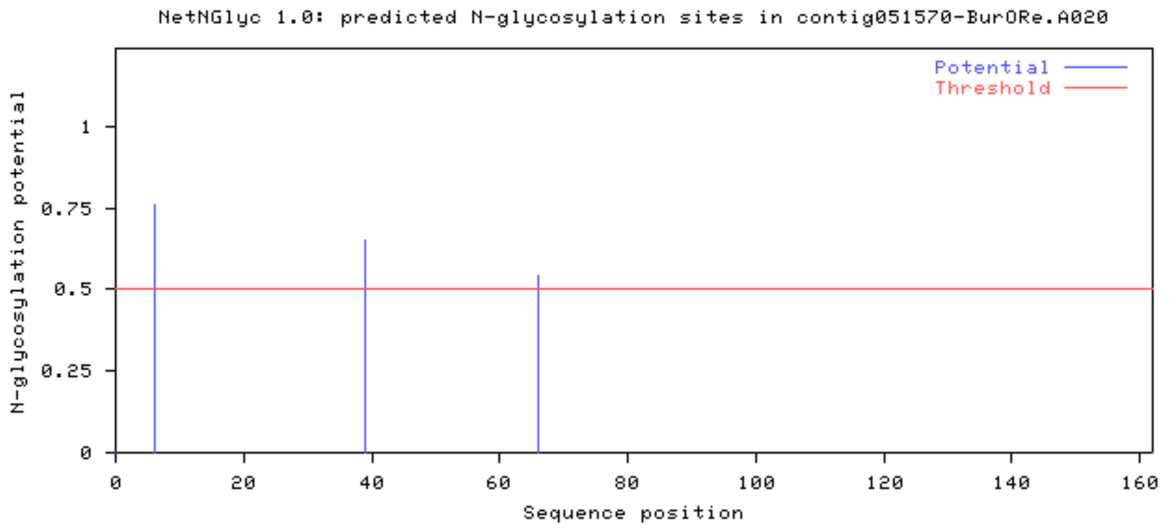

Graphics in PostScript

Output for 'contig051571-BurORe.A021'

```
##########################################################################################

    Warning: This sequence may not contain a signal peptide!!

    Proteins without signal peptides are unlikely to be exposed to
    the N-glycosylation machinery and thus may not be glycosylated
    (in vivo) even though they contain potential motifs.

    SignalP-NN euk predictions are as follows:

  # name                Cmax  pos ?  Ymax  pos ?  Smax  pos ?  Smean ?  D     ?

    SignalP output is explained at http://www.cbs.dtu.dk/services/SignalP/output.html

##########################################################################################

Name:  contig051571-BurORe.A021         Length:  58
MDVELNLTLVTFGGFAELHKYRYLYFVVISTLYILILCFNSTIVCLIWTHKNLHEPMY
.....N.................................N..................                           80

(Threshold=0.5)
----------------------------------------------------------------------
SeqName      Position  Potential   Jury    N-Glyc
                                 agreement result
----------------------------------------------------------------------
contig051571-BurORe.A021    6 NLTL   0.8099     (9/9)   +++
contig051571-BurORe.A021   40 NSTI   0.6327     (8/9)   +
----------------------------------------------------------------------
```

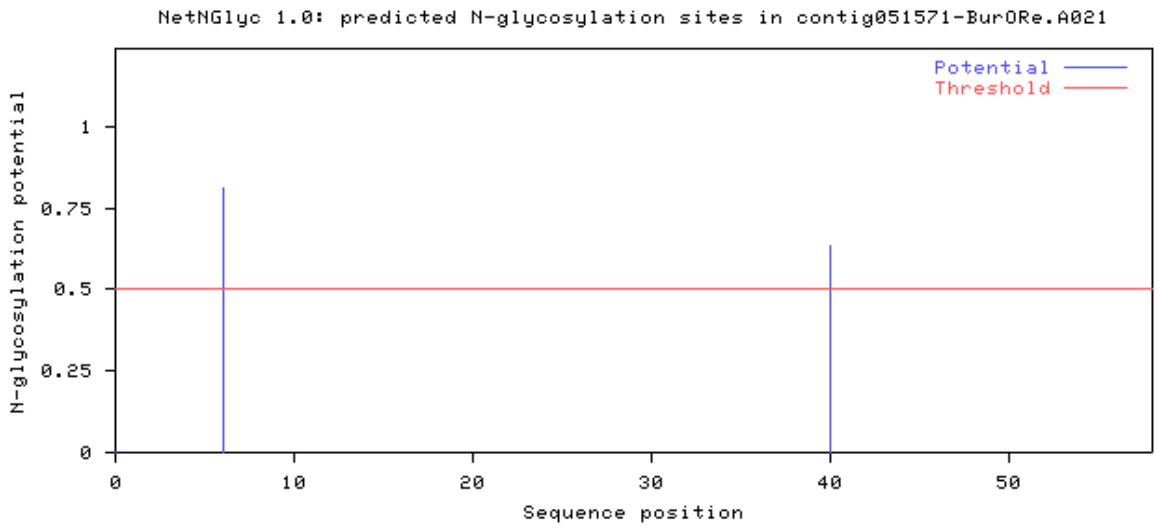

Graphics in PostScript

Output for 'contig051573-BurOR.A011'

```
##########################################################################

    Warning: This sequence may not contain a signal peptide!!

    Proteins without signal peptides are unlikely to be exposed to
    the N-glycosylation machinery and thus may not be glycosylated
    (in vivo) even though they contain potential motifs.

    SignalP-NN euk predictions are as follows:

  # name                    Cmax   pos ?  Ymax   pos ?  Smax   pos ?  Smean ?  D     ?

    SignalP output is explained at http://www.cbs.dtu.dk/services/SignalP/output.html

##########################################################################

Name:  contig051573-BurOR.A011  Length:  307
MDVELNVTLLTLGGFAELHKYRYLYFVIIFTLYILILCFNSTIVFLIWTHKNLHEPMYIFIAALLINSVLYSMIIYPKLL      80
SDVLSEKQMISYPLCLFQGLSYYTSVGSEFLLLAAMAYDRYVSICKPLQYPVIMNRITIYVCLILAWLIPAFEALMLGVL     160
YSNVKLCSFTLTGIFCNNSVHKLQCVPSVAISIYGVVMLINIALLPLLFILFSYIKILKISYQRCREVRKKAVKTCLPHL     240
LVLIHFSCFISFDIIIVRLETDLSKTLRLILTFELILFHPLLNPIIYGLKMNEISKYLKILLCVVKX
.....N.................................N........................................      80
................................................................................     160
................................................................................     240
...................................................................                  320

(Threshold=0.5)
----------------------------------------------------------------------
SeqName      Position  Potential    Jury    N-Glyc
                                 agreement  result
----------------------------------------------------------------------
contig051573-BurOR.A011    6 NVTL   0.7849     (9/9)   +++
contig051573-BurOR.A011   40 NSTI   0.7156     (9/9)   ++
contig051573-BurOR.A011  177 NNSV   0.4207     (7/9)   -
----------------------------------------------------------------------
```

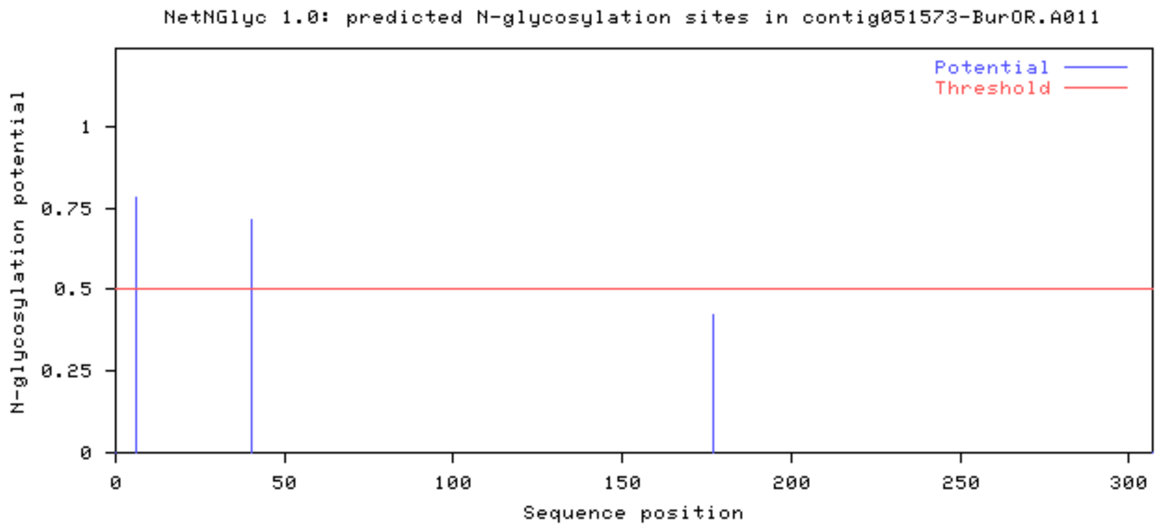

Graphics in PostScript

Output for 'contig052450-BurOR.E055'

```
########################################################################################

    Warning: This sequence may not contain a signal peptide!!

    Proteins without signal peptides are unlikely to be exposed to
    the N-glycosylation machinery and thus may not be glycosylated
    (in vivo) even though they contain potential motifs.

    SignalP-NN euk predictions are as follows:

  # name                Cmax  pos ?  Ymax  pos ?  Smax  pos ?  Smean ?  D     ?

    SignalP output is explained at http://www.cbs.dtu.dk/services/SignalP/output.html

########################################################################################

Name:  contig052450-BurOR.E055  Length:  310
MINTTQVSFFTLTGYFDTGHVTNLCFFVILALYIFIVGSNVLLIVVICVNRSLHEPMYMFLCSLFVNELYGSTGLFPPLL      80
VQILSDVHTVSTGICFLQVFCVHSYGAVEYLNLAIMSYDRYLAICCPLQYNTHMTSKKIAILIAATWFYPCFAMVVLLSL     160
TSPLQFCGNTIYKVYCDTHSVVKLACSDTTVINLYGLLATFSTIFGALLFILYTYMKILLVCFSGSDQTRQKAISTCTPH     240
LASILNFSFGASFEILQSRFNMKNVPNMLRIFLSLYFLTCQPLFNPVMYGLKMTKIRNICKSLITNTHLX
..N..............................................N..............................      80
................................................................................     160
................................................................................     240
......................................................................               320

(Threshold=0.5)
----------------------------------------------------------------------
SeqName      Position  Potential   Jury    N-Glyc
                                agreement result
----------------------------------------------------------------------
contig052450-BurOR.E055    3 NTTQ   0.7369     (9/9)    ++
contig052450-BurOR.E055   50 NRSL   0.7491     (9/9)    ++
contig052450-BurOR.E055  246 NFSF   0.4077     (6/9)    -
----------------------------------------------------------------------
```

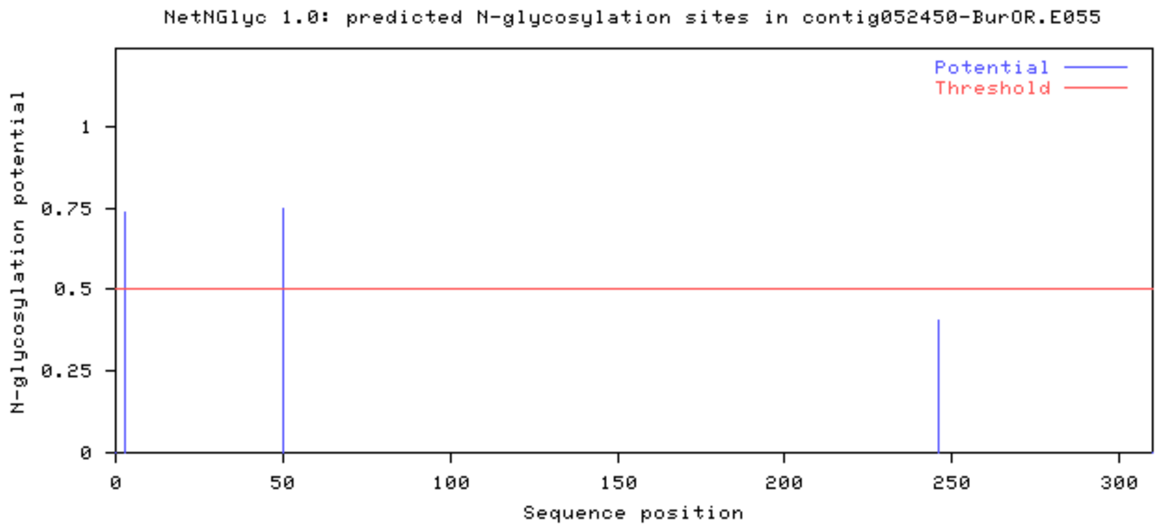

Graphics in PostScript

Output for 'contig052451-BurOR.E047'

```
##########################################################################################

    Warning: This sequence may not contain a signal peptide!!

    Proteins without signal peptides are unlikely to be exposed to
    the N-glycosylation machinery and thus may not be glycosylated
    (in vivo) even though they contain potential motifs.

    SignalP-NN euk predictions are as follows:

  # name                    Cmax   pos ?  Ymax   pos ?  Smax   pos ?  Smean ?  D     ?

    SignalP output is explained at http://www.cbs.dtu.dk/services/SignalP/output.html

##########################################################################################

Name:  contig052451-BurOR.E047  Length:  310
MTNSTQFSHFRLAFLNIGIFKYLFFMLVMCFFISVVCTNVLLIVVICVNRSLHEPMYMFLCSLFVNALYGSTSLFPLLLL      80
HIICDINIISASLCYLQIYCIHCYGSAEYLNLAVMSYDRYLAICFPLHYNTYMTPKRIAILIAITWLYAILACALMISLS     160
STLPLCGNIIDKVYCDNYSVIKLACSDTKALNIYGIIIVLCTVCCPLIFMLYTYIKILRVCSSGSKQVRQKAVTTCSPHL     240
ACVLNFSCGACFEILQSRFNMSGVPILLRIFLSLYWLISQPLLNPLVYGLNMTKIRILCKNLLTLRPLNX
..N.............................................N...............................      80
................................................................................     160
................N...............................................................     240
...................N...............................N..................              320

(Threshold=0.5)
----------------------------------------------------------------------
SeqName      Position  Potential   Jury    N-Glyc
                                 agreement result
----------------------------------------------------------------------
contig052451-BurOR.E047    3 NSTQ   0.7360     (9/9)    ++
contig052451-BurOR.E047   49 NRSL   0.7493     (9/9)    ++
contig052451-BurOR.E047  177 NYSV   0.6687     (9/9)    ++
contig052451-BurOR.E047  245 NFSC   0.3182     (9/9)    ---
contig052451-BurOR.E047  260 NMSG   0.5758     (6/9)    +
contig052451-BurOR.E047  291 NMTK   0.6373     (8/9)    +
----------------------------------------------------------------------
```

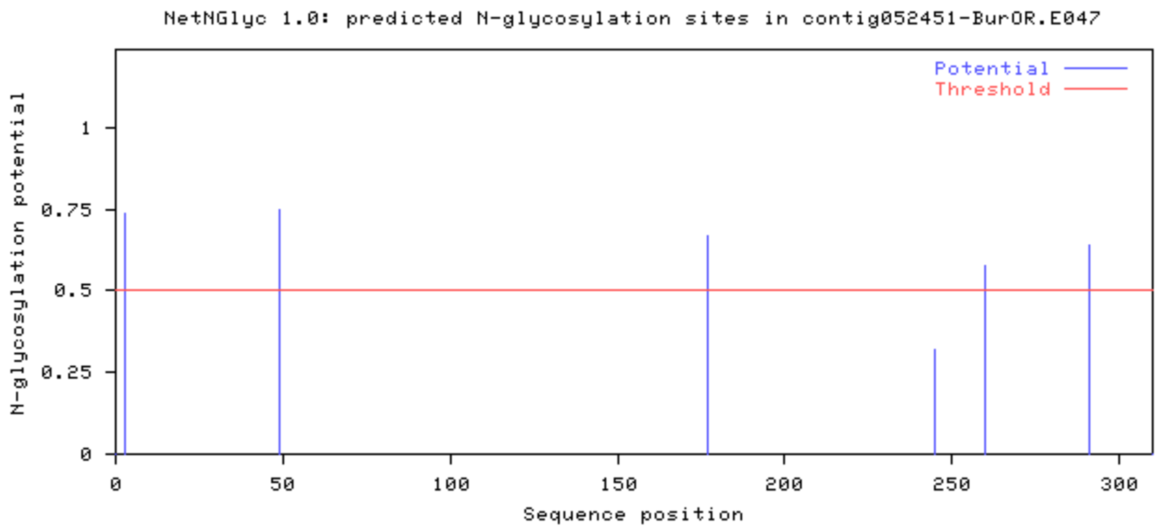

Graphics in PostScript

Output for 'contig052452-BurOR.E048'

```
########################################################################################

    Warning: This sequence may not contain a signal peptide!!

    Proteins without signal peptides are unlikely to be exposed to
    the N-glycosylation machinery and thus may not be glycosylated
    (in vivo) even though they contain potential motifs.

    SignalP-NN euk predictions are as follows:

  # name                  Cmax  pos ?  Ymax  pos ?  Smax  pos ?  Smean ?  D     ?

    SignalP output is explained at http://www.cbs.dtu.dk/services/SignalP/output.html

########################################################################################

Name:  contig052452-BurOR.E048  Length:  320
MINTTQLSFFTLAGYFDTGRVTYLCFIVILALYIFIVGSNVLLIVVICVNRSLHEPMYMFLCSLFVNELYGSTGLFPLLL      80
VQILSDVHTVSASFCFLQIFCVYAYGSIEFSNLAVISYDRYLAICCPLRYHTCMSSSKVSVLIALTWLLTFFAISVLISL     160
SAPLQLCGNIINKVYCDNYSIVKLACSNTTANNIYGILYTFTLVLLVTLIFYTYMRILKVCFSGSKQMRHKAISTCTPHL     240
ASLLNFSCGAFFEIIQNRFDMRQLPNMLRIFLSIYWLTCQPLFNPVIYGLNLTKIRVSCKNVICRMKVFTLFHKSNFKIX     320
..N..............................................N..............................      80
................................................................................     160
.................N.........N....................................................     240
....N.............................................N.............................     320

(Threshold=0.5)
----------------------------------------------------------------------
SeqName      Position  Potential   Jury    N-Glyc
                                 agreement result
----------------------------------------------------------------------
contig052452-BurOR.E048    3 NTTQ    0.6799     (9/9)   ++
contig052452-BurOR.E048   50 NRSL    0.7498     (9/9)   ++
contig052452-BurOR.E048  178 NYSI    0.6013     (8/9)   +
contig052452-BurOR.E048  188 NTTA    0.5963     (8/9)   +
contig052452-BurOR.E048  245 NFSC    0.5501     (6/9)   +
contig052452-BurOR.E048  291 NLTK    0.6826     (9/9)   ++
----------------------------------------------------------------------
```

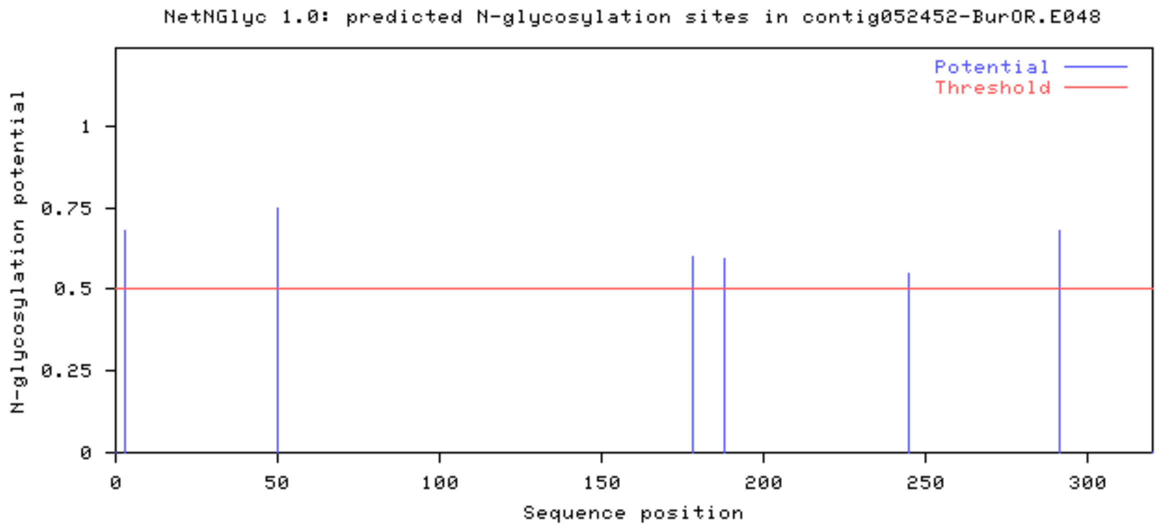

Graphics in PostScript

Output for 'contig052453-BurOR.E049'

```
##########################################################################################

    Warning: This sequence may not contain a signal peptide!!

    Proteins without signal peptides are unlikely to be exposed to
    the N-glycosylation machinery and thus may not be glycosylated
    (in vivo) even though they contain potential motifs.

    SignalP-NN euk predictions are as follows:

  # name                    Cmax  pos ?  Ymax  pos ?  Smax  pos ?  Smean ?  D     ?

    SignalP output is explained at http://www.cbs.dtu.dk/services/SignalP/output.html

##########################################################################################

Name:  contig052453-BurOR.E049  Length:  326
MTNSSQVSYFTLTAYLDSGALKYLYFTVVAFLYIVIVTVNVLLIVVICVNRSLHEPMYMFLCSLFVNELYGSTGLFPFLL      80
LQILSDVHTVSAPLCFLQIFCLYSYANLQLSNLAIMSYDRYLAICFPLQYHTRMSPCKVSMLIVLTWFSSFLVITVLISL     160
SAPLQLCGNIINKVYCDNYSIVNLACSDTTVNNIYGILGAIFITISSVTLILYTYMRILKVCFSGSKQTRQKAVSTCTPH     240
LASLLNFSCGSFFETAQSRSNMKHVPNMVRIFLSLYWLICPPLCNPLLYGLSLTKIRIIYKGLIFVKCRCLMLKLRQGSA     320
LVKRKX
..N.............................................N..............................      80
................................................................................     160
................N...............................................................     240
.....N..........................................................................     320
......                                                                               400

(Threshold=0.5)
----------------------------------------------------------------------
SeqName      Position  Potential   Jury    N-Glyc
                                 agreement result
----------------------------------------------------------------------
contig052453-BurOR.E049    3 NSSQ   0.6903     (9/9)    ++
contig052453-BurOR.E049   50 NRSL   0.7496     (9/9)    ++
contig052453-BurOR.E049  178 NYSI   0.6169     (8/9)    +
```

```
contig052453-BurOR.E049   246 NFSC   0.5354     (6/9)    +
----------------------------------------------------------------------
```

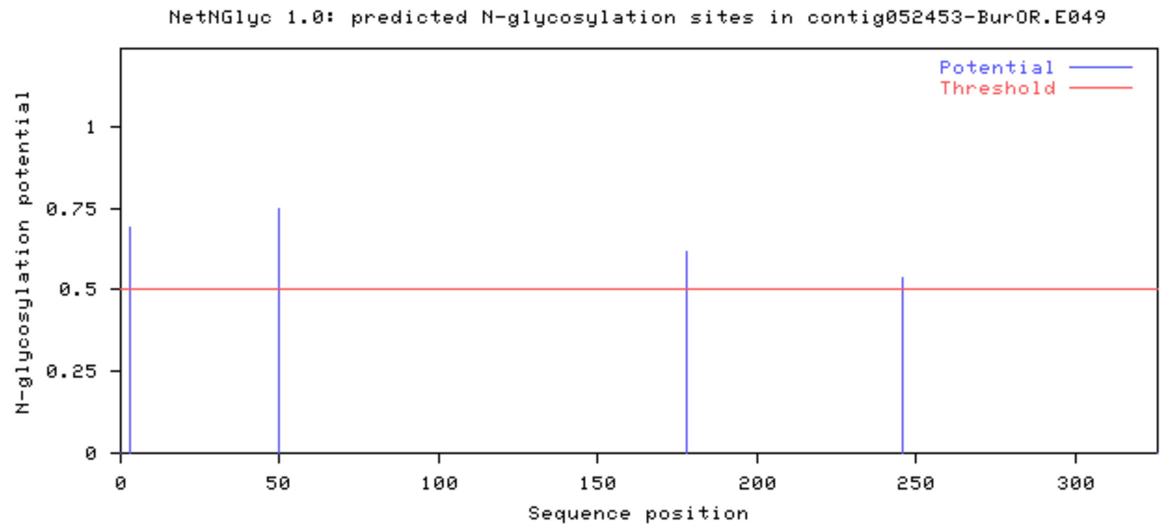

Graphics in PostScript

Output for 'contig052454-BurOR.E050'

```
##########################################################################################

    Warning: This sequence may not contain a signal peptide!!

    Proteins without signal peptides are unlikely to be exposed to
    the N-glycosylation machinery and thus may not be glycosylated
    (in vivo) even though they contain potential motifs.

    SignalP-NN euk predictions are as follows:

  # name                   Cmax  pos ?  Ymax  pos ?  Smax  pos ?  Smean ?  D     ?

    SignalP output is explained at http://www.cbs.dtu.dk/services/SignalP/output.html

##########################################################################################

Name:  contig052454-BurOR.E050  Length:  309
MTNLSQVSYFTFSAYFDTGPFKYLYFTIVMSLYVFIFGSNLLLIVVICVNRSLHEPMYMFLCSLFVNELYGSTGLFPFLL      80
LQILSDVHTVSAPLCFLQIFCLYTYANVEFYNLAIMSYDRYLAICYPLQYHTHMTFNKVAKLIVLTWLFPILNIAVMISL     160
NASLQLCGHTVDTLYCNNYSVVKLACFDTTINNIYGLMYTFTVLIGLALLNLFTYVKILKVCFSGSKQTRQKAVSTCTPH     240
LASLLNFSFGCFFEIVQSRFNLSRAPMILRIFLSIYFLTCQPAFNPVLYGLKLTKIRLICKSLLFGKIX
..N..............................................N..............................      80
................................................................................     160
N................N..............................................................     240
....................N................................................                320

(Threshold=0.5)
----------------------------------------------------------------------
SeqName      Position  Potential   Jury    N-Glyc
                                 agreement result
----------------------------------------------------------------------
contig052454-BurOR.E050    3 NLSQ   0.7482     (9/9)    ++
contig052454-BurOR.E050   50 NRSL   0.7482     (9/9)    ++
contig052454-BurOR.E050  161 NASL   0.6290     (8/9)    +
```

```
contig052454-BurOR.E050  178 NYSV   0.6210     (9/9)   ++
contig052454-BurOR.E050  246 NFSF   0.4718     (7/9)   -
contig052454-BurOR.E050  261 NLSR   0.5961     (9/9)   ++
----------------------------------------------------------------------
```

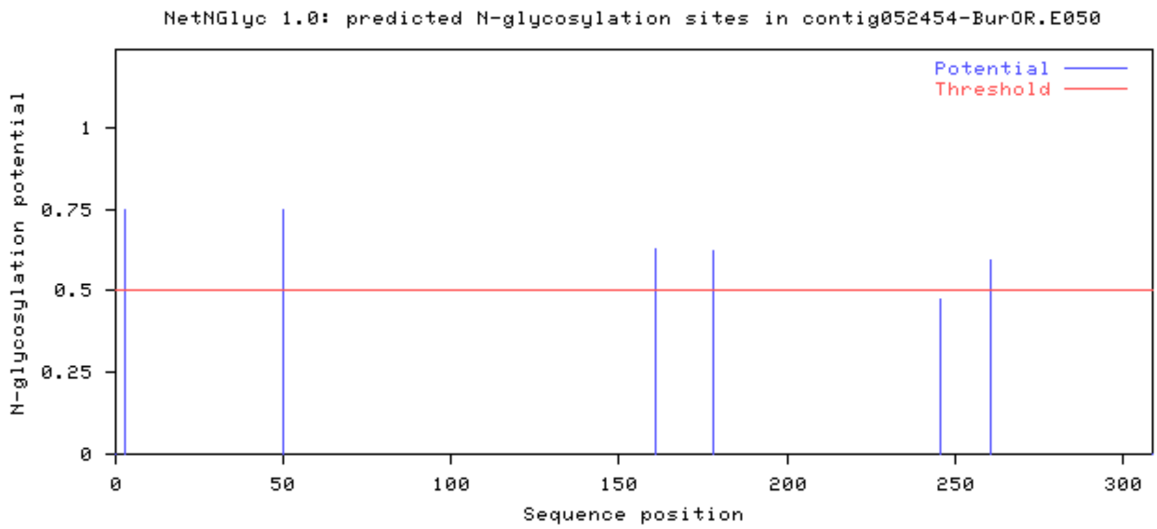

Graphics in PostScript

Output for 'contig052457-BurOR.E051'

```
##########################################################################################

    Warning: This sequence may not contain a signal peptide!!

    Proteins without signal peptides are unlikely to be exposed to
    the N-glycosylation machinery and thus may not be glycosylated
    (in vivo) even though they contain potential motifs.

    SignalP-NN euk predictions are as follows:

  # name                   Cmax  pos ?  Ymax  pos ?  Smax  pos ?  Smean ?  D     ?

    SignalP output is explained at http://www.cbs.dtu.dk/services/SignalP/output.html

##########################################################################################

Name:  contig052457-BurOR.E051  Length:  310
MTVNSSQSSFLVFSAYFDSGHLKYLFFVIVMSLYFLIITANVLLIVVICVNRSLHEPMYMFLCSLFVNELYGSTGLFPLL      80
LLQILSDVHTVSAPLCFLQIFCVHTYGTAELANLAVMSYDRYLAICFPLQYHTRMSPCKVSMLIVLTWFSSFLVITVLIS     160
LSAPLQLCGNIINKVYCDNYSIVKLACSDTTVNNIYGLISSPLVILCPVSLILYTYMRILKICFSGSKHTRQKAVSTCTP     240
HLASLLNFSFGCFFEILQSRFNMNSVPSMLRIFLSLYFLTCQPVFNPLMYGLTLSKISLTCKKLLCADMX
...N..............................................N.............................      80
................................................................................     160
..................N.............................................................     240
......................................................................               320

(Threshold=0.5)
----------------------------------------------------------------------
SeqName      Position  Potential   Jury    N-Glyc
                                 agreement result
----------------------------------------------------------------------
contig052457-BurOR.E051    4 NSSQ   0.6997     (9/9)   ++
```

```
contig052457-BurOR.E051   51 NRSL   0.7483     (9/9)   ++
contig052457-BurOR.E051  179 NYSI   0.5859     (8/9)   +
contig052457-BurOR.E051  247 NFSF   0.4634     (7/9)   -
----------------------------------------------------------------------
```

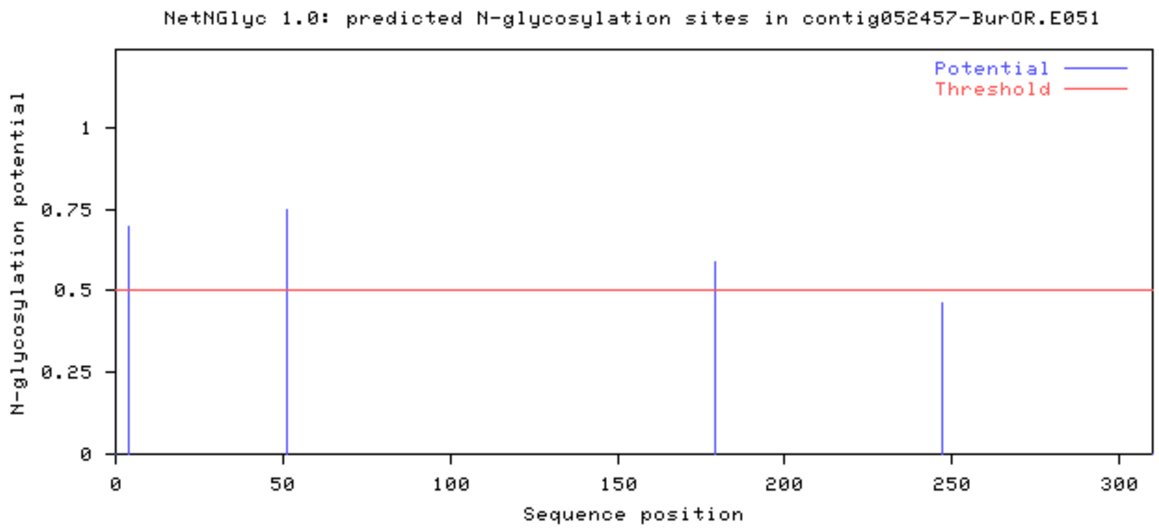

Graphics in PostScript

Output for 'contig052904-BurOR.X128'

```
##########################################################################################

    Warning: This sequence may not contain a signal peptide!!

    Proteins without signal peptides are unlikely to be exposed to
    the N-glycosylation machinery and thus may not be glycosylated
    (in vivo) even though they contain potential motifs.

    SignalP-NN euk predictions are as follows:

  # name                   Cmax  pos ?  Ymax  pos ?  Smax  pos ?  Smean ?  D     ?

    SignalP output is explained at http://www.cbs.dtu.dk/services/SignalP/output.html

##########################################################################################

Name:  contig052904-BurOR.X128  Length:  322
MQNLTELPFNATSQKSLSVIIKVCAVIPFFCTFLYCIVVMLHVFASHRQFLNTPRYILFAHMLINDTLQLLSSVLLFLLV      80
MGQVKFPVFYCFPLLFISTVTYQNTPLILAAMSLERYVAIFYPLQRPAAWRSGRIWIINLCLWLTSCIFLIIEYSIGKQR     160
PAVDVLSTPLLCKNLFVNSSPIQALFRTAVSVMFFAVVAVVIFFTYVRILLETRKLRQDRISVNKAKHTVLLHGFQLLLC     240
MLAFTLPITETLILLYTKWPVEDVTYFCFFCFILIPRFLSPLIYGFRDHSLRGYIGKTFLCCLDTVKPHFRSKQQDSLSA     320
LX
..N......N.....................................................N...............      80
................................................................................     160
................................................................................     240
................................................................................     320
..                                                                                   400

(Threshold=0.5)
----------------------------------------------------------------------
SeqName      Position  Potential   Jury    N-Glyc
                                 agreement result
```

```
----------------------------------------------------------------------
contig052904-BurOR.X128    3 NLTE   0.7553     (9/9)   +++
contig052904-BurOR.X128   10 NATS   0.6986     (8/9)   +
contig052904-BurOR.X128   65 NDTL   0.7207     (9/9)   ++
contig052904-BurOR.X128  178 NSSP   0.1528     (9/9)   ---
----------------------------------------------------------------------
```

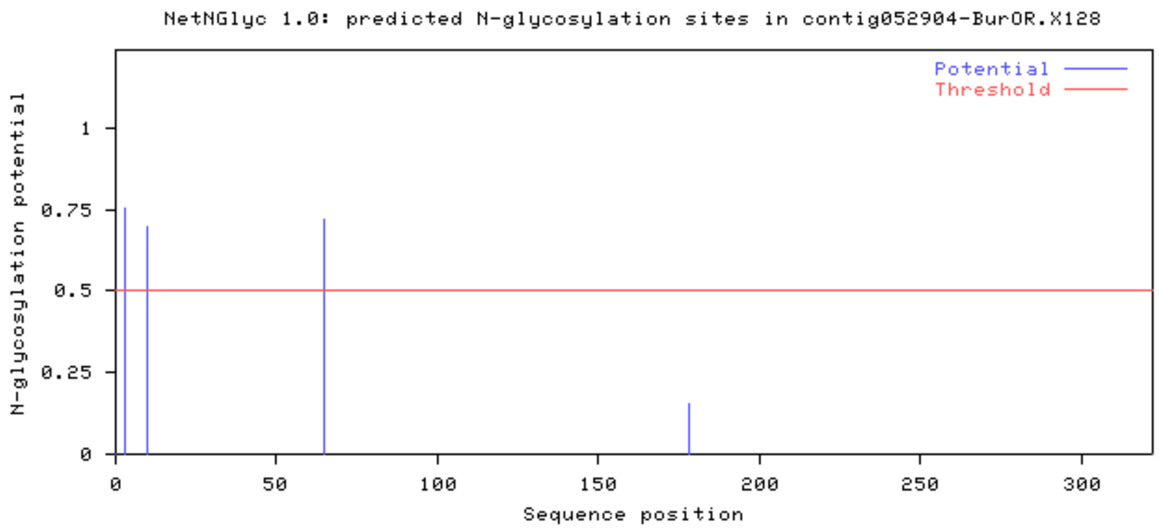

Graphics in PostScript

Output for 'contig053777-BurORe.D039'

```
##########################################################################################

    Warning: This sequence may not contain a signal peptide!!

    Proteins without signal peptides are unlikely to be exposed to
    the N-glycosylation machinery and thus may not be glycosylated
    (in vivo) even though they contain potential motifs.

    SignalP-NN euk predictions are as follows:

  # name                    Cmax  pos ?  Ymax  pos ?  Smax  pos ?  Smean ?  D     ?

    SignalP output is explained at http://www.cbs.dtu.dk/services/SignalP/output.html

##########################################################################################

Name:  contig053777-BurORe.D039         Length:  271
ANTVLIVVIHVDRRLHEPMYILLSNLCVNEINCSTSLYPLLLSQMFSDSHEVTLPWCFLQMCCLYISGPAEFWSLAAMAY      80
DRYIAICHPFSYNVIMNTERVGMLILLVWIFSFVSFLLSFSFIFGLRFCGNIIDNVYCDHQLIIRLSCSVPIQSSISMIF     160
FTLLSIFMPFNLISISYLKILRLCRKTSTENKQKAVTTCTPQIVSVSNLFVGCIFHSIDFSLLAAQIPEEVRIILPMYLL     240
ICQPMLTPFMYGFNLPKIRQACKMLLFKRKX
................................................................................      80
................................................................................     160
................................................................................     240
...............................                                                      320

(Threshold=0.5)

No sites predicted in this sequence.
```

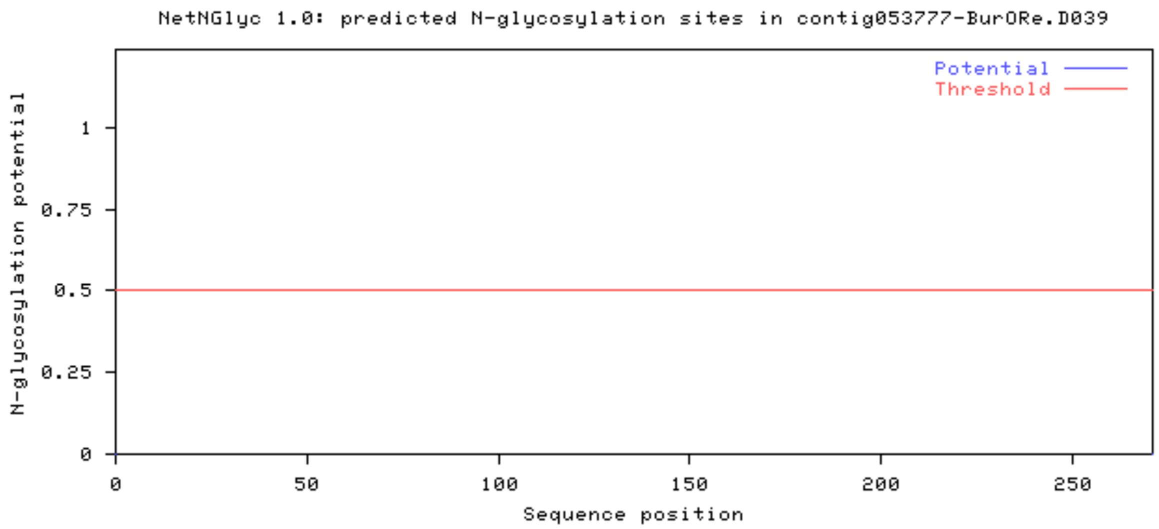

Graphics in PostScript

Output for 'contig053778-BurORe.D040'

```
##########################################################################################

    Warning: This sequence may not contain a signal peptide!!

    Proteins without signal peptides are unlikely to be exposed to
    the N-glycosylation machinery and thus may not be glycosylated
    (in vivo) even though they contain potential motifs.

    SignalP-NN euk predictions are as follows:

  # name                 Cmax  pos ?  Ymax  pos ?  Smax  pos ?  Smean ?  D     ?

    SignalP output is explained at http://www.cbs.dtu.dk/services/SignalP/output.html

##########################################################################################

Name:  contig053778-BurORe.D040        Length:  259
LSNLCVNEINASTSLYPLLLSQMFSDSHEVTVPWCFLQMCCMYTSASAEFCSLAAMAYDRYISICHPLRYNVIMNTGRVF      80
MLILVVWMYSFLSFIFSFSFIFRLMFCGNIVNNVYCDHKLIIRLSCSVLVHSFMSDIFFLLLSIFIPFSLISVSYMKILA     160
VCRKTSTENKQKAVTTCTPQIISVSNLFVRCIFHSTDFSLLAAHVPGEVNIILSIYLLICQPMLTPFMYGFNLPKIRQSR     240
TMLLFKRKSISLFSKRVLX
................................................................................      80
................................................................................     160
................................................................................     240
...................                                                                  320

(Threshold=0.5)

No sites predicted in this sequence.
```

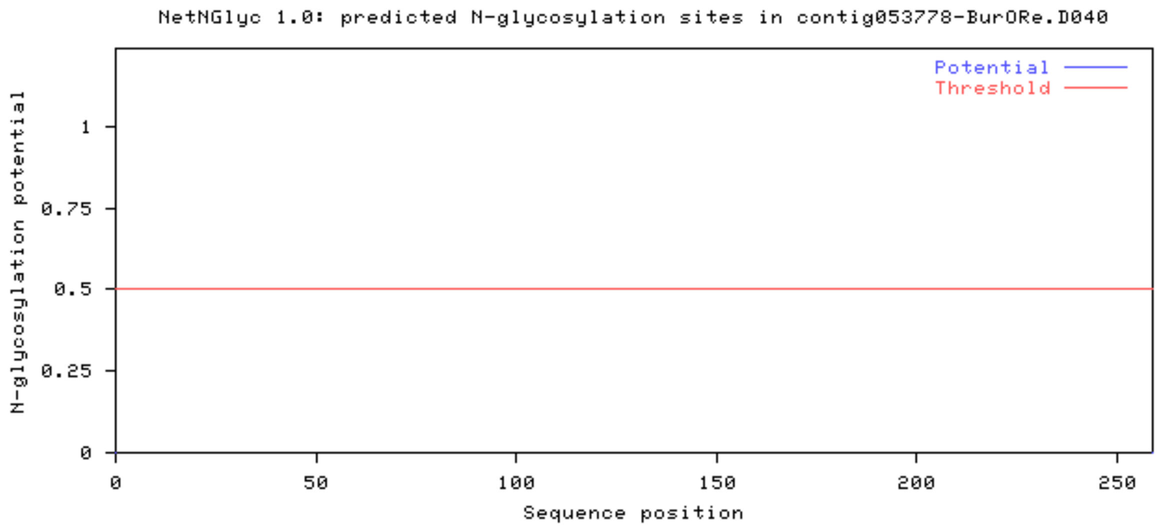

Graphics in PostScript

Output for 'contig053779-BurOR.D035'

```
##########################################################################

    Warning: This sequence may not contain a signal peptide!!

    Proteins without signal peptides are unlikely to be exposed to
    the N-glycosylation machinery and thus may not be glycosylated
    (in vivo) even though they contain potential motifs.

    SignalP-NN euk predictions are as follows:

  # name                Cmax  pos ?  Ymax  pos ?  Smax  pos ?  Smean ?  D     ?

    SignalP output is explained at http://www.cbs.dtu.dk/services/SignalP/output.html

##########################################################################

Name:  contig053779-BurOR.D035  Length:  319
MGNSSETVSFVLAAYGNVGALKYMYFSIILFWYVSICVANTVLIVVIHVDRRLHEPMYILLSNLCVNEINGSTSMYPLLL      80
SQMYSDSHEVTLPWCFLQMCCMYTTASAEFCSLAAMAYDRYISICHPLRYNVIMNTERVFMLILLVWVYSFLIFIFSFSF     160
IFSLKFCGNVVNNVYCDHKLIIRLSCSVSIHSFISDIFFLLLSIFIPFSLISFSYMKILAVCRKTSTENKQKAVTTCTPQ     240
IVSVSNLFVGCIFHAIDSSVLVAQVPGEVRIILSIYLLICQPMLTPFMYGFNLPKIRQSCTMLVFKRKSISLFGKRLFX
..N.............................................................................      80
................................................................................     160
................................................................................     240
...............................................................................      320

(Threshold=0.5)
----------------------------------------------------------------------
SeqName      Position  Potential   Jury    N-Glyc
                                 agreement result
----------------------------------------------------------------------
contig053779-BurOR.D035    3 NSSE   0.7403     (9/9)    ++
contig053779-BurOR.D035   70 NGST   0.4571     (6/9)    -
----------------------------------------------------------------------
```

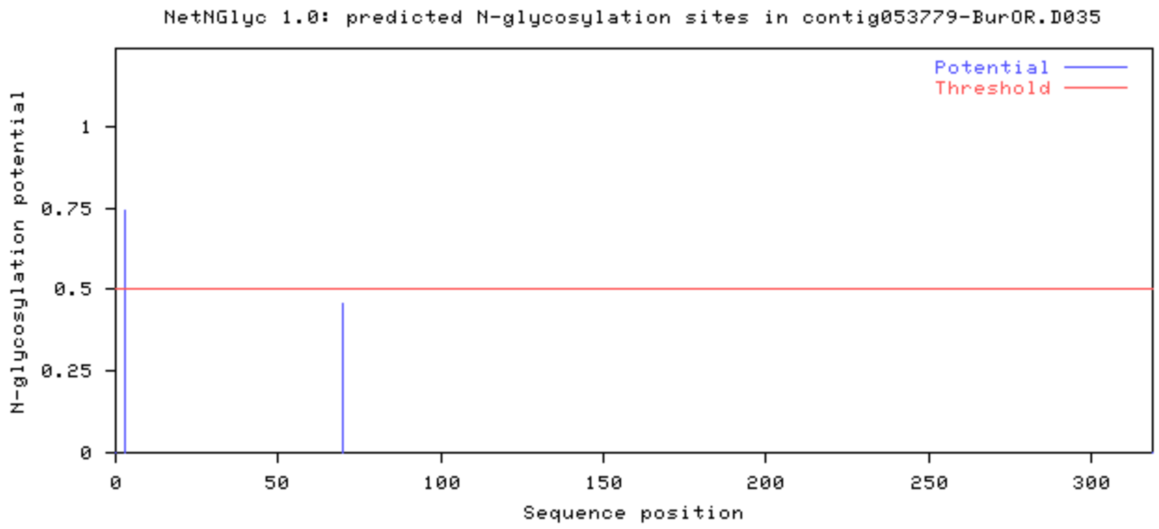

Graphics in PostScript

Output for 'contig053780-BurOR.H065'

```
########################################################################################

    Warning: This sequence may not contain a signal peptide!!

    Proteins without signal peptides are unlikely to be exposed to
    the N-glycosylation machinery and thus may not be glycosylated
    (in vivo) even though they contain potential motifs.

    SignalP-NN euk predictions are as follows:

  # name                    Cmax  pos ?  Ymax  pos ?  Smax  pos ?  Smean ?  D     ?

    SignalP output is explained at http://www.cbs.dtu.dk/services/SignalP/output.html

########################################################################################

Name:  contig053780-BurOR.H065  Length:  314
MNTSSIVVFSLTGFSATVNYRVTLFSLTLLCYFLILMVNISLILTIISDQNLHEPMYIFLCSLCINGLYGTAGFFPRFAF      80
DLLSDTHLISYVGCLLQVFVIYSNAKVDYSTLVLMAYDRYLAICRPLEYHSVMSVRRTVVLVTLSWLVPLCFETLVISLT     160
STLKLCGSNINKLYCENWSIVKLACGSTKVNDIVGLIFITFYCCHVVCIACSYVQLVNVALKSRAGRKKFTQTCAPHLFC     240
LLNVTTALLFDLMFSRYGSASLPQHLKNFMAIEFLIIPPILNPVCYGWVLTKIRRRMIFLCRLAYQRFGVKSQX
.N....................................N.........................................      80
................................................................................     160
................................................................................     240
..N.......................................................................           320

(Threshold=0.5)
----------------------------------------------------------------------
SeqName      Position  Potential   Jury    N-Glyc
                                 agreement result
----------------------------------------------------------------------
contig053780-BurOR.H065    2 NTSS   0.7490     (9/9)    ++
contig053780-BurOR.H065   39 NISL   0.7616     (9/9)    +++
contig053780-BurOR.H065  177 NWSI   0.4820     (5/9)    -
contig053780-BurOR.H065  243 NVTT   0.5967     (9/9)    ++
----------------------------------------------------------------------
```

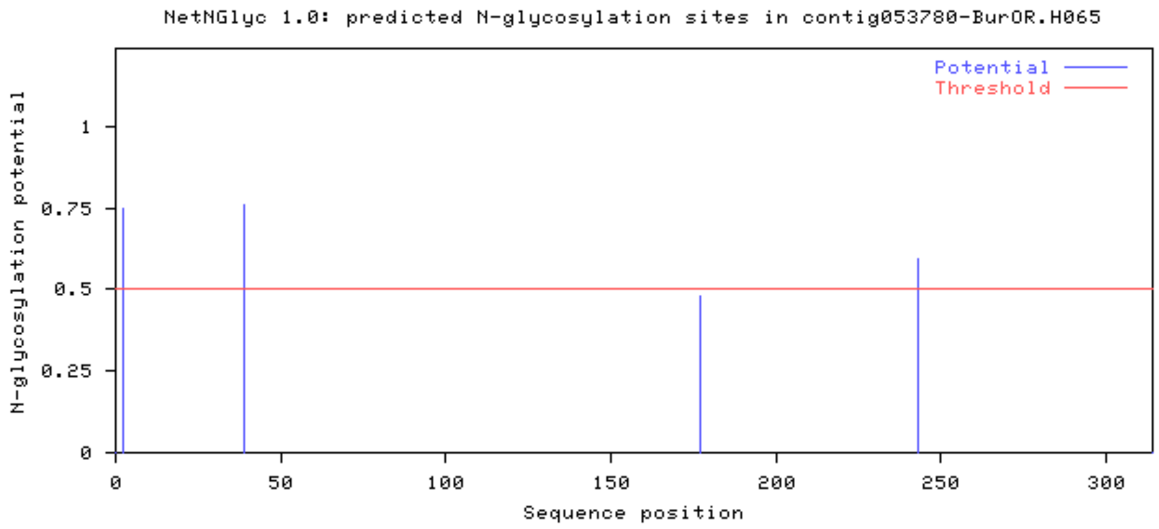

Graphics in PostScript

Output for 'contig053781-BurOR.F059'

```
########################################################################################

    Warning: This sequence may not contain a signal peptide!!

    Proteins without signal peptides are unlikely to be exposed to
    the N-glycosylation machinery and thus may not be glycosylated
    (in vivo) even though they contain potential motifs.

    SignalP-NN euk predictions are as follows:

  # name                 Cmax   pos ?  Ymax   pos ?  Smax   pos ?  Smean ?  D     ?

    SignalP output is explained at http://www.cbs.dtu.dk/services/SignalP/output.html

########################################################################################

Name:  contig053781-BurOR.F059  Length:  305
MENSTLSFYFRFTMFANIGHYRFIAFIFCLLLFIFTIFTNLLMIVVISQQTTLHEPMYIFIACLSVNALYGSSGFFPRFL      80
MDLLSDTHLISRPACFTQIYVIYSYASCELTVLSIMAYDRYIAVCLPLHYHTKMTLETVVKLTALAWIFPAFSLAACLCL     160
SATLPLCGNEIHKVFCANWNVVKLSCVNTAVNNVVGMLLTVATIFLPLFYILYTYLRIVSICWKSSAEFKGKVLESCLPH     240
TISFVIYSIAGFCDVALSRNNLEVTNPFIAVILSLVFVVIPPALNPLVYGLKLPEIRKHILRLFX
..N.............................................................................      80
................................................................................     160
................................................................................     240
.................................................................                    320

(Threshold=0.5)
----------------------------------------------------------------------
SeqName       Position  Potential   Jury    N-Glyc
                                  agreement result
----------------------------------------------------------------------
contig053781-BurOR.F059    3 NSTL   0.7370     (9/9)    ++
----------------------------------------------------------------------
```

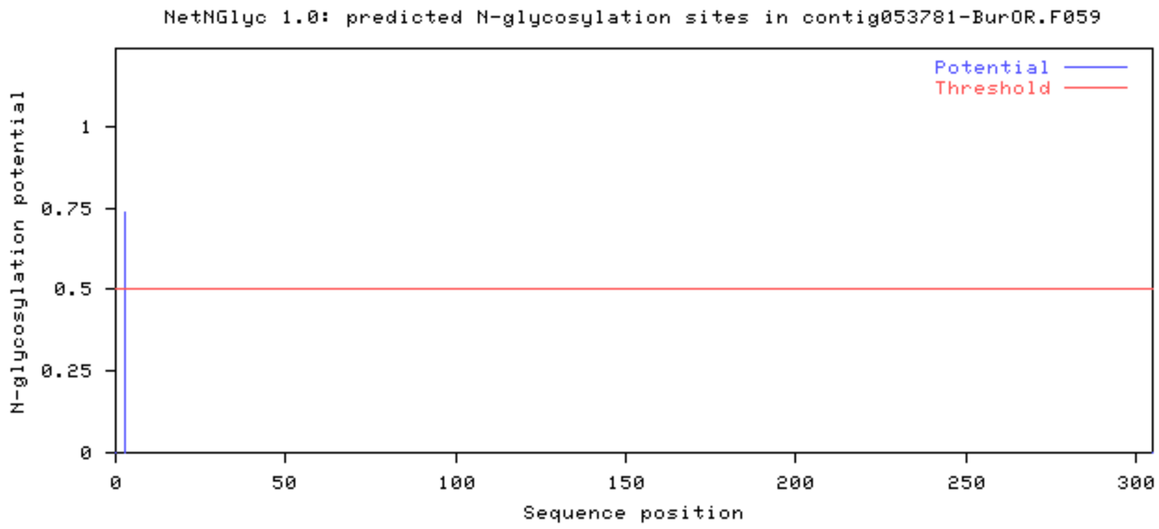

Graphics in PostScript

Output for 'contig053782-BurOR.H066'

```
##########################################################################

    Warning: This sequence may not contain a signal peptide!!

    Proteins without signal peptides are unlikely to be exposed to
    the N-glycosylation machinery and thus may not be glycosylated
    (in vivo) even though they contain potential motifs.

    SignalP-NN euk predictions are as follows:

  # name                  Cmax   pos ?  Ymax   pos ?  Smax   pos ?  Smean ?  D     ?

    SignalP output is explained at http://www.cbs.dtu.dk/services/SignalP/output.html

##########################################################################

Name:  contig053782-BurOR.H066  Length:  314
MDNVSVVTVFTLSGLSGIANYKITIFIFTLLCYCVIWLVNLTIIVTVIVDKSLHEPMYIFLCNLCFNGLYGTAAFYPKFL      80
YDLLSTTHVISYAGCLLQGLMVHSSICTDFSLLALMAYDRYVAICRPLVYHSLMTTQRVCIFVFFAWITPFSLILMSTIT     160
TATSRLCGSHIPRIYCINWLISNLACSASVATIIIPAFNYTFYFGHAVFVFWSYVHLIKTCQSSKENWNKFMQTCVPHLF     240
SLAVVVVSFLFDMLYMRFGSKEIPQSFENFMAMEILFIPPIINPLMYGFKLTKIRKRVLNFICGESSAFILNSX
..N....................................N........................................      80
................................................................................     160
......................................N.........................................     240
..........................................................................           320

(Threshold=0.5)
----------------------------------------------------------------------
SeqName      Position  Potential   Jury    N-Glyc
                                 agreement result
----------------------------------------------------------------------
contig053782-BurOR.H066    3 NVSV   0.8098     (9/9)    +++
contig053782-BurOR.H066   40 NLTI   0.7722     (9/9)    +++
contig053782-BurOR.H066  199 NYTF   0.5938     (8/9)    +
----------------------------------------------------------------------
```

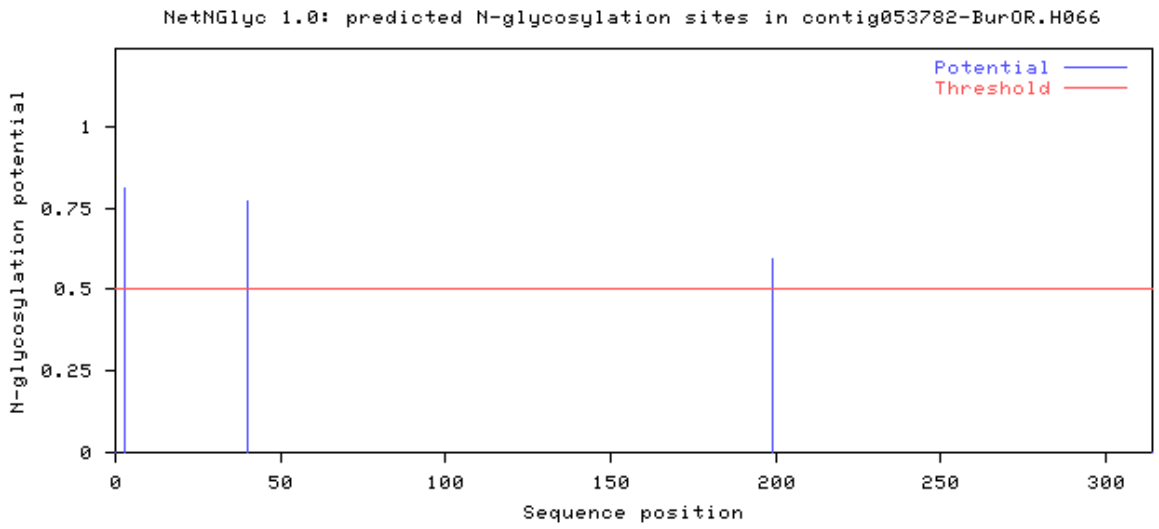

Graphics in PostScript

Output for 'contig053784-BurOR.H067'

```
##########################################################################

    Warning: This sequence may not contain a signal peptide!!

    Proteins without signal peptides are unlikely to be exposed to
    the N-glycosylation machinery and thus may not be glycosylated
    (in vivo) even though they contain potential motifs.

    SignalP-NN euk predictions are as follows:

  # name                    Cmax  pos ?  Ymax  pos ?  Smax  pos ?  Smean ?  D     ?

    SignalP output is explained at http://www.cbs.dtu.dk/services/SignalP/output.html

##########################################################################

Name:  contig053784-BurOR.H067  Length:  314
MDNVSVIAVFTLSGLRDIANYRVILFVLTLLCYCVIWLVNVTIIVTVIVDKKLHEPMYIFLCNLCVNGLYGTAAFYPKFL      80
YDLLSTTHVISYAGCLLQGFVLHSAVAADFSLLALMAYDRYVAICRPLVYHSLMTTQKMSIFVFFAWLIPFYLLLMSTIT     160
TATSRLCGSHIPRIYCVNWLIANLACSASVARIVIPAFNYTFYVGHVVFVFWSYVYLIKTCQSSKENWNKFMQTCVPHLF     240
SLTVVVLSLLFDMLYMRFGSKEIAQNVENFMAMEFLLIPPIMNPLMYGLKLTKIRKRVLNFICGKSSTFRLKSX
..N....................................N........................................      80
................................................................................     160
......................................N.........................................     240
..........................................................................           320

(Threshold=0.5)
----------------------------------------------------------------------
SeqName      Position  Potential   Jury    N-Glyc
                                 agreement result
----------------------------------------------------------------------
contig053784-BurOR.H067     3 NVSV   0.8180     (9/9)   +++
contig053784-BurOR.H067    40 NVTI   0.7899     (9/9)   +++
contig053784-BurOR.H067   199 NYTF   0.6144     (8/9)   +
----------------------------------------------------------------------
```

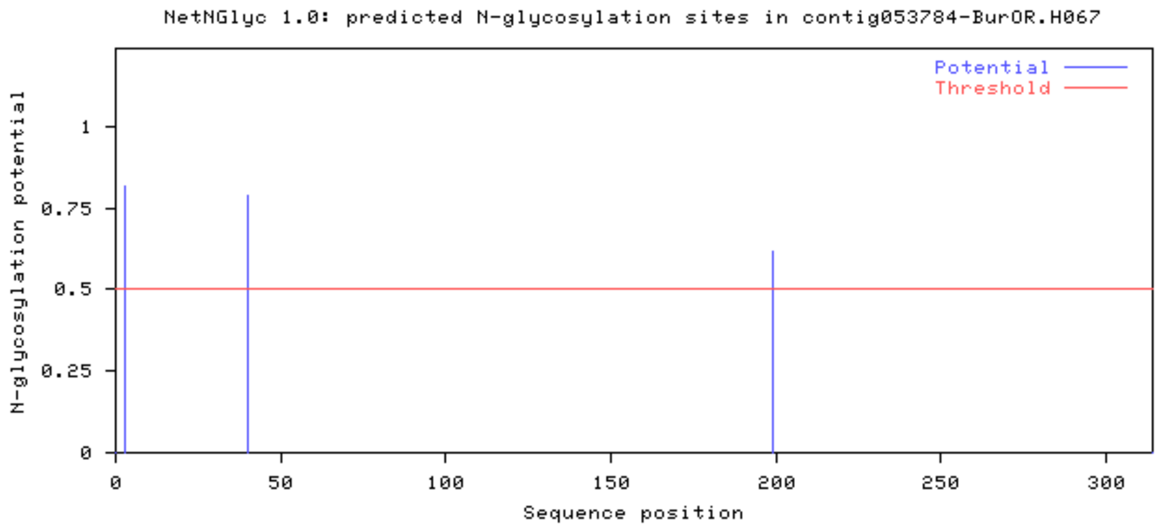

Graphics in PostScript

Output for 'contig053787-BurOR.H068'

```
##########################################################################

    Warning: This sequence may not contain a signal peptide!!

    Proteins without signal peptides are unlikely to be exposed to
    the N-glycosylation machinery and thus may not be glycosylated
    (in vivo) even though they contain potential motifs.

    SignalP-NN euk predictions are as follows:

  # name                    Cmax   pos ?  Ymax   pos ?  Smax   pos ?  Smean ?  D     ?

    SignalP output is explained at http://www.cbs.dtu.dk/services/SignalP/output.html

##########################################################################

Name:  contig053787-BurOR.H068  Length:  314
MDNVSIITVFTLSGLSDIANYRVILFVLTLLCYCVIWLVNVTIIVTVIVDKKLHEPMYIFLCSLCFNGLYGTAAFYPKFL      80
YDLLSTTHVISYAGCFLQGFVLHSAVAADFSLLALMAYDRYVAICRPLVYHSIMTKQRVCILIFFAWIIAFYFLLMSTIT     160
TAILRLCGSHIPKIYCINWLIANLACSASVAKIVIPAFSYTFCFGNVCFVFWSYVHLIKTCQSSKENMGKFMQTCVPHLF     240
SLTVVVVSLLFDLLYMRFGSKEIAQSVQNFMAMEFLLIPPIMNPLMYGFKLTKIRKRVLNFICGKSSAFRFKSX
..N....................................N........................................      80
................................................................................     160
................................................................................     240
..........................................................................           320

(Threshold=0.5)
----------------------------------------------------------------------
SeqName      Position  Potential   Jury    N-Glyc
                                 agreement result
----------------------------------------------------------------------
contig053787-BurOR.H068    3 NVSI   0.8029     (9/9)   +++
contig053787-BurOR.H068   40 NVTI   0.7899     (9/9)   +++
----------------------------------------------------------------------
```

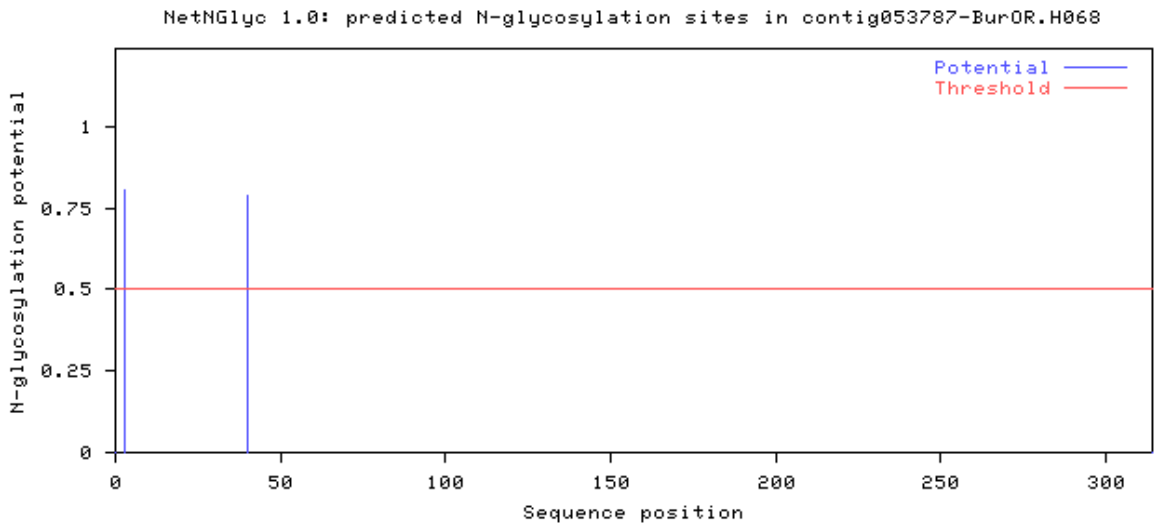

Graphics in PostScript

Output for 'contig053788-BurOR.H069'

```
########################################################################################

    Warning: This sequence may not contain a signal peptide!!

    Proteins without signal peptides are unlikely to be exposed to
    the N-glycosylation machinery and thus may not be glycosylated
    (in vivo) even though they contain potential motifs.

    SignalP-NN euk predictions are as follows:

  # name                Cmax  pos ?  Ymax  pos ?  Smax  pos ?  Smean ?  D     ?

    SignalP output is explained at http://www.cbs.dtu.dk/services/SignalP/output.html

########################################################################################

Name:  contig053788-BurOR.H069  Length:  314
MDNVSVITVFTLAGLSDIANYRVILFVLTLLCYCVIWLVNVTIIVTVIVDKKLHEPMYIFLCNLCVNGLYGTAAFYPKFL      80
YDLLSTTHVISYAGCLLQGFALHSSICADFSLLALMAYDRYVAICRPLVYHSLMTKQKVCIFVFFAWFFPIYLMLLSTIT     160
TAVLRLCGTHIPRIYCINWLINNLACSASAARIVIPAFNYTFYIGHILFVFWTYVHLVKTCQSSKENWNKFMQTCVPHLF     240
SLIVVAVSFLFDMLYVRFGSKEFPQSFENFMAMEILLIPPIINPVMYGFKLTKIRNRVLNFICGISSTLRLKLX
..N....................................N........................................      80
................................................................................     160
......................................N.........................................     240
..........................................................................           320

(Threshold=0.5)
----------------------------------------------------------------------
SeqName      Position  Potential   Jury    N-Glyc
                                 agreement result
----------------------------------------------------------------------
contig053788-BurOR.H069    3 NVSV   0.8144     (9/9)    +++
contig053788-BurOR.H069   40 NVTI   0.7900     (9/9)    +++
contig053788-BurOR.H069  199 NYTF   0.5913     (7/9)    +
----------------------------------------------------------------------
```

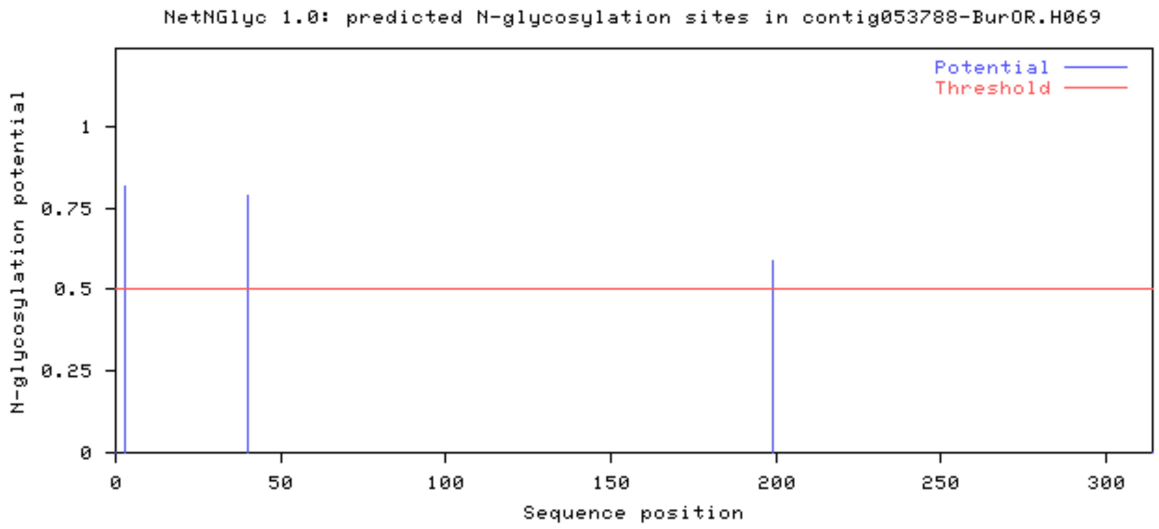

Graphics in PostScript

Output for 'contig054233-BurOR.A012'

```
##########################################################################

    Warning: This sequence may not contain a signal peptide!!

    Proteins without signal peptides are unlikely to be exposed to
    the N-glycosylation machinery and thus may not be glycosylated
    (in vivo) even though they contain potential motifs.

    SignalP-NN euk predictions are as follows:

  # name                Cmax  pos ?  Ymax  pos ?  Smax  pos ?  Smean ?  D    ?

    SignalP output is explained at http://www.cbs.dtu.dk/services/SignalP/output.html

##########################################################################

Name:  contig054233-BurOR.A012  Length:  307
MDVELNVTLLTLGGFAELHKYRYLYFVVIFTLYILILCFNSTIVCLIWTCKNLHEPMYIFIAALLINSVLYSMIIYPKLL      80
SDVLSEKQIISYTLCLFQGFLYYTSAGSEFLLLAAMAYDRYVSICKPLQYPVIMNRITIYVSLVLAWLIPAFETAVSVVL     160
YSEVKLCSFTLTGIFCNNSGYKLQCVTSVAISVYGVVMLINIALLPLLFILFTYIRIVRVSYQSCREVRKKAVKTCLPHL     240
LVLINFSCFIVFDVIIVRLESDLSKTLRLILTFQSILFHPLLNPIIYGLKMNEIFKNLKILFCHVKX
.....N.................................N........................................      80
................................................................................     160
................................................................................     240
....N..............................................................     320

(Threshold=0.5)
----------------------------------------------------------------------
SeqName      Position  Potential   Jury    N-Glyc
                                agreement  result
----------------------------------------------------------------------
contig054233-BurOR.A012    6 NVTL   0.7850     (9/9)   +++
contig054233-BurOR.A012   40 NSTI   0.7134     (9/9)   ++
contig054233-BurOR.A012  177 NNSG   0.3826     (7/9)   -
contig054233-BurOR.A012  245 NFSC   0.5623     (6/9)   +
----------------------------------------------------------------------
```

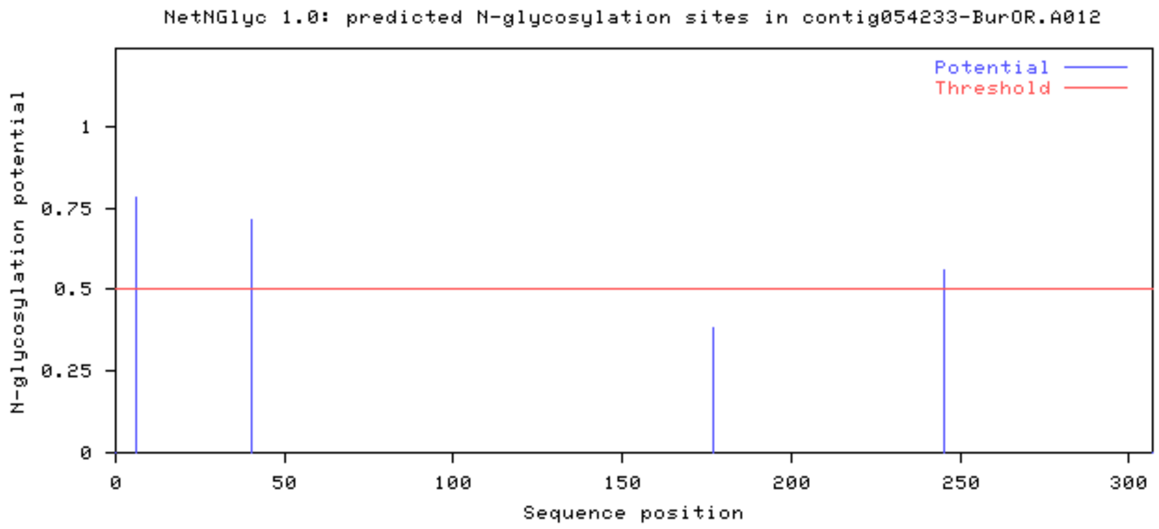

Graphics in PostScript

Output for 'contig054237-BurOR.A013'

```
##########################################################################

    Warning: This sequence may not contain a signal peptide!!

    Proteins without signal peptides are unlikely to be exposed to
    the N-glycosylation machinery and thus may not be glycosylated
    (in vivo) even though they contain potential motifs.

    SignalP-NN euk predictions are as follows:

  # name                 Cmax  pos ?  Ymax  pos ?  Smax  pos ?  Smean ?  D     ?

    SignalP output is explained at http://www.cbs.dtu.dk/services/SignalP/output.html

##########################################################################

Name:  contig054237-BurOR.A013  Length:  316
MDQELNFTYVTLDWYVDINKYRYIFFFIMFALYSLIICTNSTIMYIICIHKNLHEPMYIFIAALLLNSVLYSTTIYPKLL      80
IDFLSDKQVTTYSACLFQFFMFYTLGGSEFFVLAAMAYDRYVAICKPLQYHIIMRKTTVSIFLIIAWLVPACHIAVQAIG     160
SANIKLCDFNIKGIFCNNAVYTLLCERSRLITIFGVVALLDLAVLPMLFIVFTYTKIFIVSYQRCKEIQKKAAETCLPHL     240
LVLISASVFFVYDVSIARVETNFPKTVRIVMTLQVVLYHPIFNPFIYGLKMKKISKHLKRFFSRPQSILALKVNAX
.....N.................................N........................................      80
................................................................................     160
................................................................................     240
............................................................................         320

(Threshold=0.5)
----------------------------------------------------------------------
SeqName      Position  Potential   Jury    N-Glyc
                                agreement result
----------------------------------------------------------------------
contig054237-BurOR.A013    6 NFTY   0.7623     (9/9)    +++
contig054237-BurOR.A013   40 NSTI   0.7281     (9/9)    ++
----------------------------------------------------------------------
```

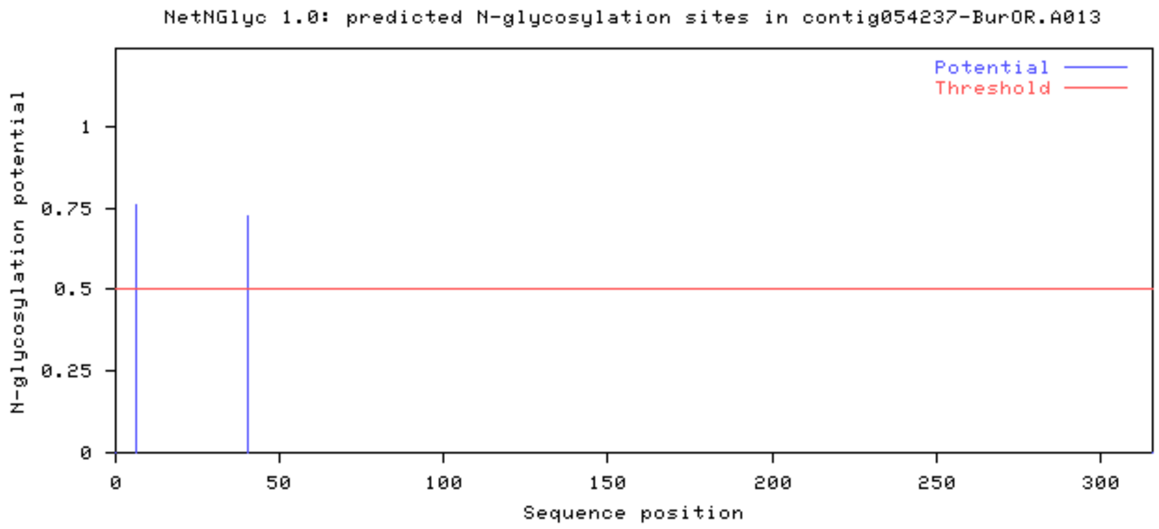

Graphics in PostScript

Output for 'contig054238-BurORep.A022'

```
##########################################################################################

    Warning: This sequence may not contain a signal peptide!!

    Proteins without signal peptides are unlikely to be exposed to
    the N-glycosylation machinery and thus may not be glycosylated
    (in vivo) even though they contain potential motifs.

    SignalP-NN euk predictions are as follows:

  # name                    Cmax   pos ?  Ymax   pos ?  Smax   pos ?  Smean ?  D     ?

    SignalP output is explained at http://www.cbs.dtu.dk/services/SignalP/output.html

##########################################################################################

Name:  contig054238-BurORep.A022        Length:  136
KSIFCNNAIYTLQCVRSRLVTVFGIICYFDFVILPLLFIVFTYTKIFIVSNSRKVIKKKAAETCLPHILVLVSFSCLGVY      80
DVTISVKSDFPKTARFIVSLQLALYQPLFNPLIYGLKMKEISKHLKRLFCPTKIIX
................................................................................      80
........................................................                             160

(Threshold=0.5)

No sites predicted in this sequence.
```

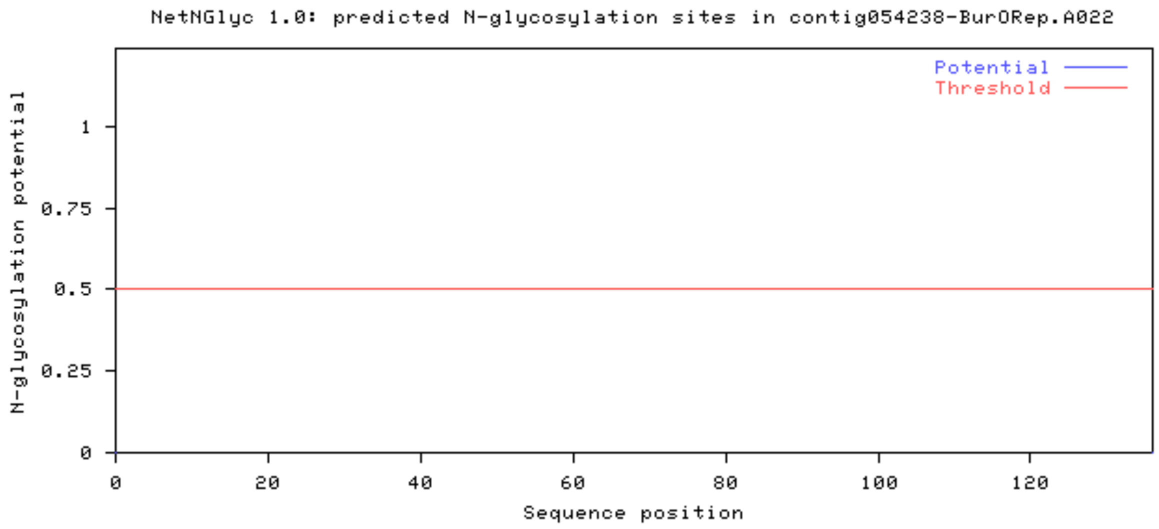

Graphics in PostScript

Output for 'contig054733-BurOR.R138'

```
################################################################################

   Warning: This sequence may not contain a signal peptide!!

   Proteins without signal peptides are unlikely to be exposed to
   the N-glycosylation machinery and thus may not be glycosylated
   (in vivo) even though they contain potential motifs.

   SignalP-NN euk predictions are as follows:

  # name                 Cmax  pos ?  Ymax  pos ?  Smax  pos ?  Smean ?  D     ?

   SignalP output is explained at http://www.cbs.dtu.dk/services/SignalP/output.html

################################################################################

Name:  contig054733-BurOR.R138  Length:  313
MSNVSESYTNMSIEVQYQDLLRVIIVSTLSTVPSFIFLFLNGTMLFTLRSKPVFRDTPRYILLYNLLFADTVQLAQSQVL      80
FLLSIFRVKLPYPVCVCLSLLANLTTGISPLTLSVMPLERYVAVCYPLRYPAIITIRNTGAAIVVIWIISSLNNLTRLIF     160
FFPFEVLKNLQMKDSCSKIALLLGTRSDQYDTAFTCLVFVSAGVAVVFSYIGVILAARLASANKALACKARNTLLLNMMQ     240
LCLSLSSTIYNPLLAALSRTVTMTIFSWVQNVFYLCFIILPRCLSSLVYGLRDQTIRPVLMYHLCCHQKRSQX
..N......N..............................N.......................................      80
......................N..................................................N......     160
................................................................................     240
.........................................................................            320

(Threshold=0.5)
----------------------------------------------------------------------
SeqName      Position  Potential   Jury    N-Glyc
                                 agreement result
----------------------------------------------------------------------
contig054733-BurOR.R138    3 NVSE   0.7628     (9/9)   +++
contig054733-BurOR.R138   10 NMSI   0.6857     (9/9)   ++
contig054733-BurOR.R138   41 NGTM   0.7362     (9/9)   ++
contig054733-BurOR.R138  103 NLTT   0.5889     (7/9)   +
contig054733-BurOR.R138  154 NLTR   0.5738     (7/9)   +
----------------------------------------------------------------------
```

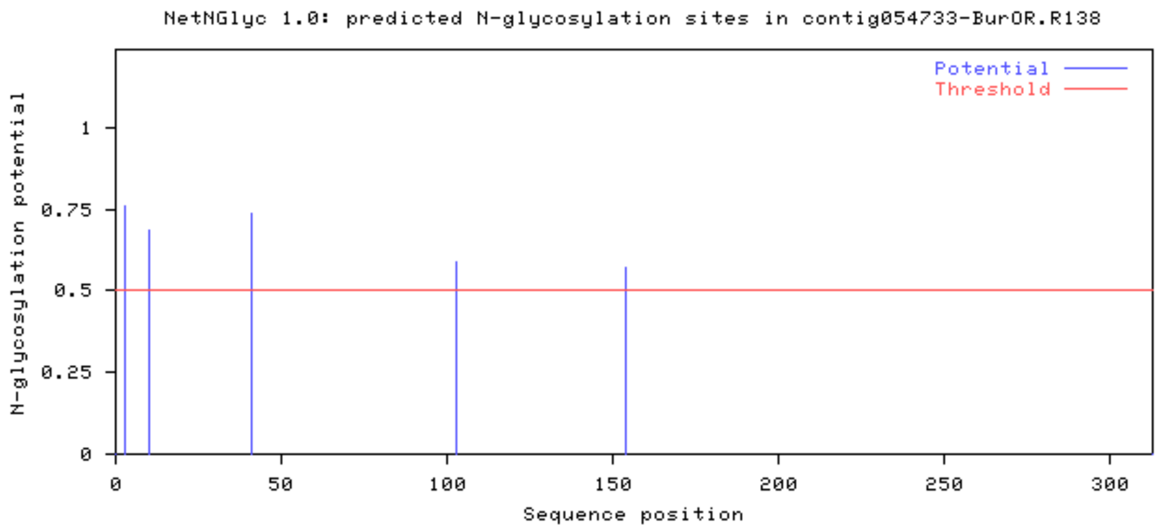

Graphics in PostScript

Output for 'contig055879-BurORe.X147'

```
##########################################################################################

    Warning: This sequence may not contain a signal peptide!!

    Proteins without signal peptides are unlikely to be exposed to
    the N-glycosylation machinery and thus may not be glycosylated
    (in vivo) even though they contain potential motifs.

    SignalP-NN euk predictions are as follows:

  # name                 Cmax  pos ?  Ymax  pos ?  Smax  pos ?  Smean ?  D     ?

    SignalP output is explained at http://www.cbs.dtu.dk/services/SignalP/output.html

##########################################################################################

Name:  contig055879-BurORe.X147         Length:  68
MLYTNWPVEDIAFFNYFCFILIPRFLSPLIYGFRDHSLRGYIGKTFLCCLDTVKPHFRSKQQDSLSAS
....................................................................                  80

(Threshold=0.5)

No sites predicted in this sequence.
```

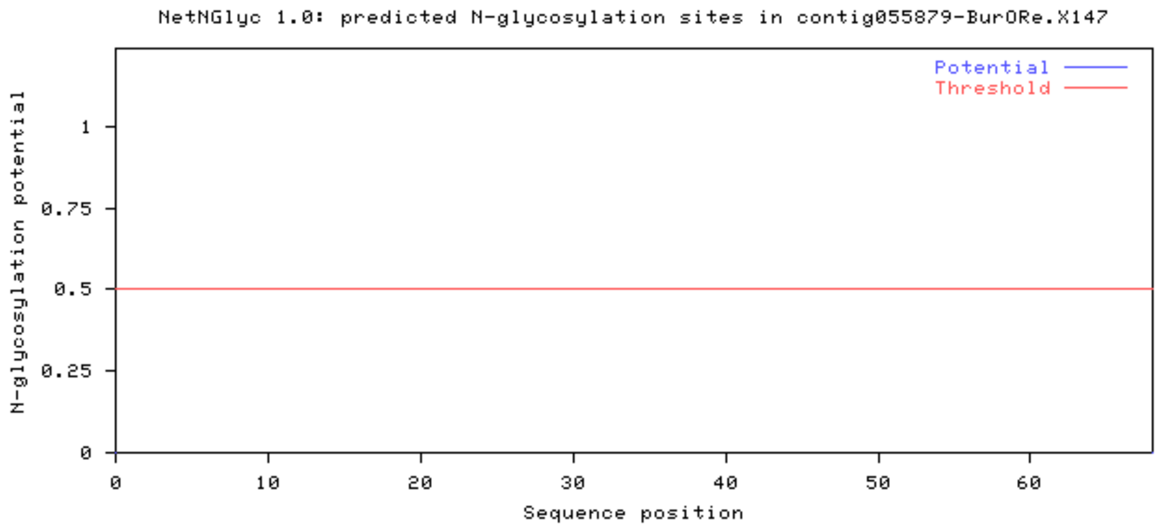

Graphics in PostScript

Output for 'contig055881-BurOR.S123'

```
##########################################################################################

    Warning: This sequence may not contain a signal peptide!!

    Proteins without signal peptides are unlikely to be exposed to
    the N-glycosylation machinery and thus may not be glycosylated
    (in vivo) even though they contain potential motifs.

    SignalP-NN euk predictions are as follows:

  # name                  Cmax  pos ?  Ymax  pos ?  Smax  pos ?  Smean ?  D     ?

    SignalP output is explained at http://www.cbs.dtu.dk/services/SignalP/output.html

##########################################################################################

Name:  contig055881-BurOR.S123  Length:  307
MAGNSSLICAFLHQLLTVRVMIVHTLVIIFLCINMLLIVTFVKKECFHTSARYVLFFVTLLSDSFLLLMTDILLILTRCS      80
VQVWLCIFICLFVLVYSIVTPVTLTAMTLERYVAICMPLRHGQLCSTRSTIYCILIIHGLSSGPCIVIISMFFATASLSF     160
YNQYTICSVEMFMLYRWQDHARSAVSQFYFMTMGITIVFSYVQIMKVAKAASGESKKSTQKGVRTVILHAFQLFLCLVQL     240
WSPFIETAVLQIDVNLFRDFRYFNYVFFSIVPRCLSPLIYGLRDETFFLSLKKLMPTSSCSKKHVIX
...N............................................................................      80
................................................................................     160
................................................................................     240
...................................................................                  320

(Threshold=0.5)
----------------------------------------------------------------------
SeqName      Position  Potential   Jury    N-Glyc
                                 agreement result
----------------------------------------------------------------------
contig055881-BurOR.S123    4 NSSL   0.7858     (9/9)   +++
----------------------------------------------------------------------
```

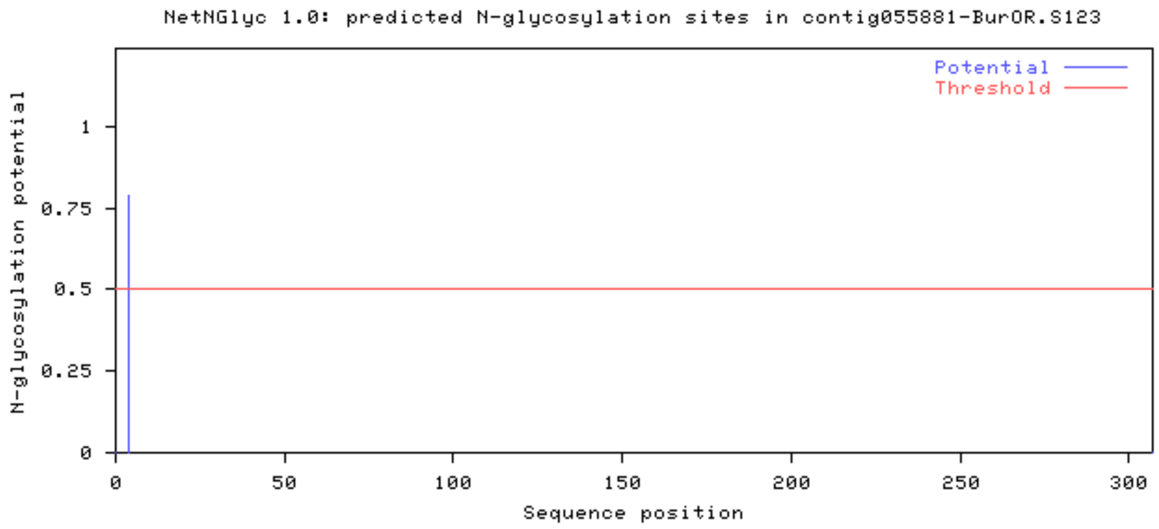

Graphics in PostScript

Output for 'contig055882-BurOR.S124'

```
##########################################################################

    Warning: This sequence may not contain a signal peptide!!

    Proteins without signal peptides are unlikely to be exposed to
    the N-glycosylation machinery and thus may not be glycosylated
    (in vivo) even though they contain potential motifs.

    SignalP-NN euk predictions are as follows:

  # name                Cmax  pos ?  Ymax  pos ?  Smax  pos ?  Smean ?  D     ?

    SignalP output is explained at http://www.cbs.dtu.dk/services/SignalP/output.html

##########################################################################

Name:  contig055882-BurOR.S124  Length:  305
MAGNNSVNCVFFPRPVSYRVIIVEILVIIFLCINMLLIVIFVKKESFHTSARYILFFVTLLSDSVLLLVSDILFILTHFE      80
ITMPVWLCITISVVVLLYFIVTPVTLTAMTLERYVAICMPLRHGQLCSTRSTMYCILIIHVLSSGPCIIILSMFFASGSL     160
KFYKQSMICSGDTFSLYRWQDHVRSAVYQLYFLIMGITIAYSYVQIMKVAKAASGEKKKLTQKGLKTVILHAFQLLLCLI     240
QLWCPFIEIAVLQIDFSLILNVRYFNYIMFNIAPRCLSPLIYGLRDENIFLVLKNLMPTSSCSKX
...N............................................................................      80
................................................................................     160
................................................................................     240
.................................................................                    320

(Threshold=0.5)
----------------------------------------------------------------------
SeqName      Position  Potential   Jury    N-Glyc
                                 agreement result
----------------------------------------------------------------------
contig055882-BurOR.S124    4 NNSV   0.7279     (9/9)    ++
----------------------------------------------------------------------
```

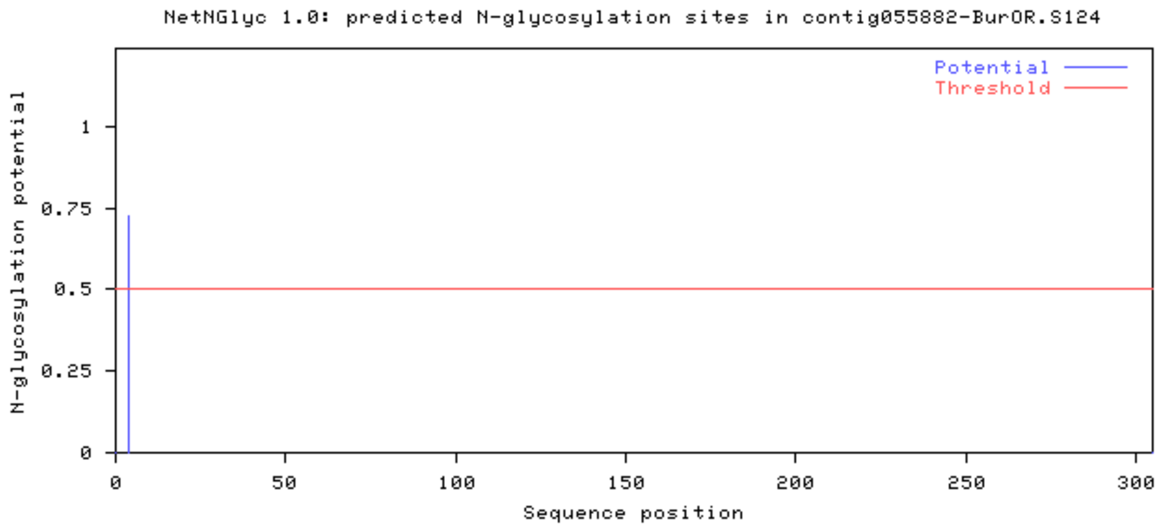

Graphics in PostScript

Output for 'contig055884-BurOR.S125'

```
##########################################################################################

    Warning: This sequence may not contain a signal peptide!!

    Proteins without signal peptides are unlikely to be exposed to
    the N-glycosylation machinery and thus may not be glycosylated
    (in vivo) even though they contain potential motifs.

    SignalP-NN euk predictions are as follows:

  # name                Cmax  pos ?  Ymax  pos ?  Smax  pos ?  Smean ?  D     ?

    SignalP output is explained at http://www.cbs.dtu.dk/services/SignalP/output.html

##########################################################################################

Name:  contig055884-BurOR.S125  Length:  305
MAGNNSVNYVFLQRPVYDRVIIVQILVIIFLCINMLLIVIFVKKECFHTSARYILFFVTLVSDSVLLLVSDVLLVLSQFE      80
FTIQVWLCITISVVVLLYFIVTPVTLTAMTLERYVAICMPLRHGQLCSTRSTMYCILIIHVLSSGPCIIILSMFFASASL     160
KFYKQSMICSGETFTIYRWQDHVRSAVYQFYFLIMGITIAYSYVQIMKVAKAASGEKKKLTHKGLKTVILHAFQLLLCLI     240
QLWCPFIEIALLQIDFSLFRNVRYFNYIMFNIAPRCLSPLIYGLRDENIFLVLKSLMPTSSCSKX
...N............................................................................      80
................................................................................     160
................................................................................     240
.................................................................                    320

(Threshold=0.5)
----------------------------------------------------------------------
SeqName      Position  Potential   Jury    N-Glyc
                                 agreement result
----------------------------------------------------------------------
contig055884-BurOR.S125    4 NNSV   0.7303     (9/9)    ++
----------------------------------------------------------------------
```

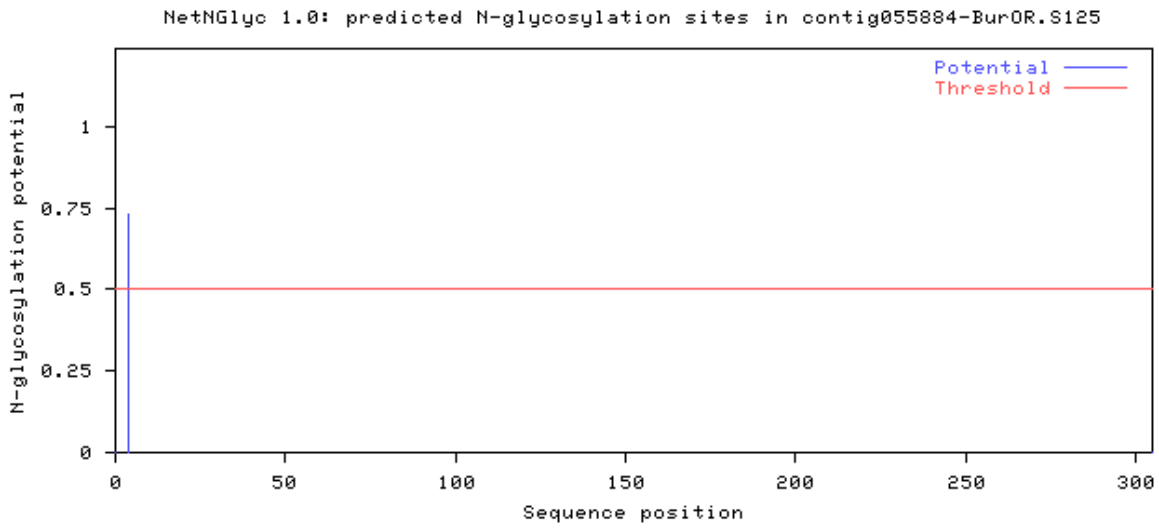

Graphics in PostScript

Output for 'contig055884-BurORe.S127'

```
##########################################################################################

    Warning: This sequence may not contain a signal peptide!!

    Proteins without signal peptides are unlikely to be exposed to
    the N-glycosylation machinery and thus may not be glycosylated
    (in vivo) even though they contain potential motifs.

    SignalP-NN euk predictions are as follows:

  # name                Cmax  pos ?  Ymax  pos ?  Smax  pos ?  Smean ?  D     ?

    SignalP output is explained at http://www.cbs.dtu.dk/services/SignalP/output.html

##########################################################################################

Name:  contig055884-BurORe.S127        Length:  120
MAGNNSVNYVFFQRAISYRVIIVQILVIIFLCINMLLIVVFIKKESFHTSARYILFFVTLLSDSVLLLLSDVLLVLTHFE      80
ITMPVWLCITISVVVLLYFIVTPVTLTAMTLERYVAICMP
...N............................................................................      80
........................................                                             160

(Threshold=0.5)
----------------------------------------------------------------------
SeqName      Position  Potential    Jury    N-Glyc
                                 agreement  result
----------------------------------------------------------------------
contig055884-BurORe.S127    4 NNSV   0.7222     (9/9)    ++
----------------------------------------------------------------------
```

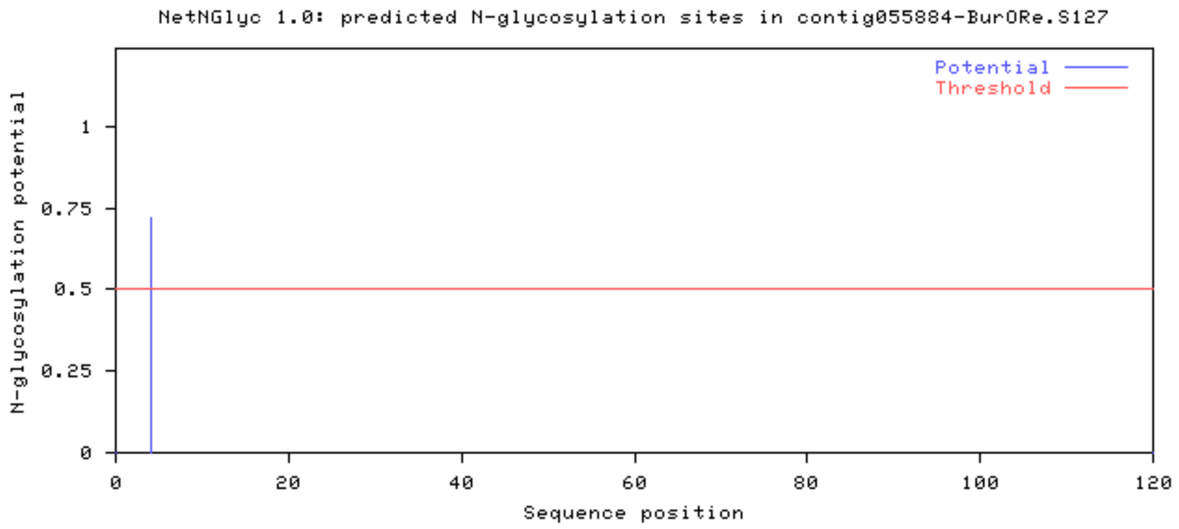

Graphics in PostScript

Output for 'contig056382-BurORe.L095'

```
##########################################################################################

    Warning: This sequence may not contain a signal peptide!!

    Proteins without signal peptides are unlikely to be exposed to
    the N-glycosylation machinery and thus may not be glycosylated
    (in vivo) even though they contain potential motifs.

    SignalP-NN euk predictions are as follows:

  # name                 Cmax  pos ?  Ymax  pos ?  Smax  pos ?  Smean ?  D     ?

    SignalP output is explained at http://www.cbs.dtu.dk/services/SignalP/output.html

##########################################################################################

Name:  contig056382-BurORe.L095         Length:  90
VKILSNNDKKKMGSTLVSHLICVSCLYCPQFIIVILTRFGVVLTLEERQGLLIGTILGPSLVNPFVYFLRTKEIKSKMFK      80
IFRKINTAGX
................................................................................      80
..........                                                                           160

(Threshold=0.5)

No sites predicted in this sequence.
```

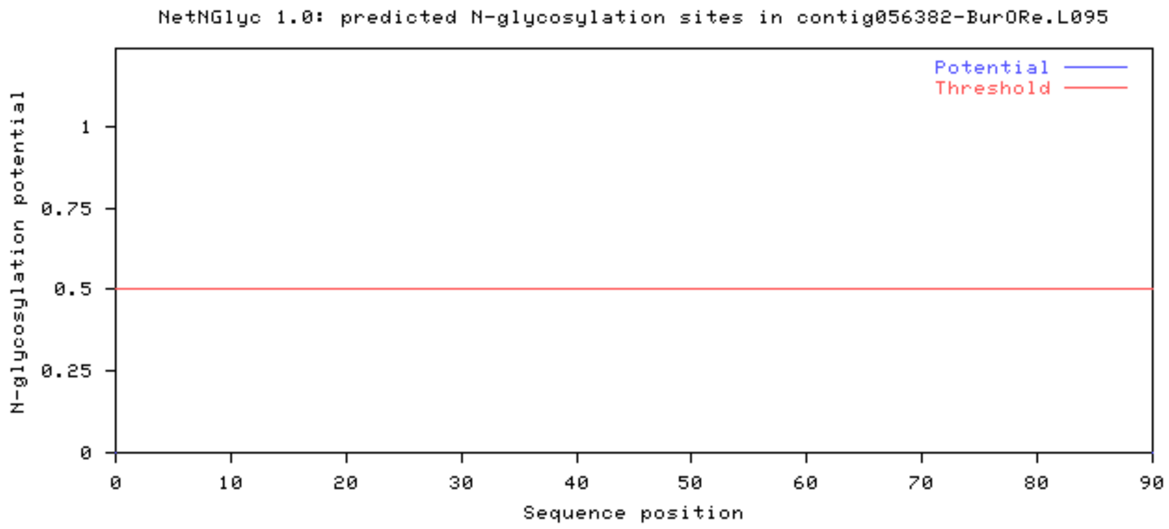

Graphics in PostScript

Output for 'contig056383-BurORe.L096'

```
##########################################################################################

    Warning: This sequence may not contain a signal peptide!!

    Proteins without signal peptides are unlikely to be exposed to
    the N-glycosylation machinery and thus may not be glycosylated
    (in vivo) even though they contain potential motifs.

    SignalP-NN euk predictions are as follows:

  # name                Cmax  pos ?  Ymax  pos ?  Smax  pos ?  Smean ?  D     ?

    SignalP output is explained at http://www.cbs.dtu.dk/services/SignalP/output.html

##########################################################################################

Name:  contig056383-BurORe.L096        Length:  123
MSLQNASIKLTYFIIGGFDTVKRPVAVGVVMLITYLLAVFASLVNIIFIVSDKQLHKPMYLLICNLAVVDILYTSSSTPT      80
MIGVLLAGVNTISYVECIIQMYVYQVGATMEMFSLTIMAFDRL
....N...........................................................................      80
...........................................                                          160

(Threshold=0.5)
----------------------------------------------------------------------
SeqName      Position  Potential   Jury    N-Glyc
                                 agreement result
----------------------------------------------------------------------
contig056383-BurORe.L096    5 NASI   0.5856     (6/9)    +
----------------------------------------------------------------------
```

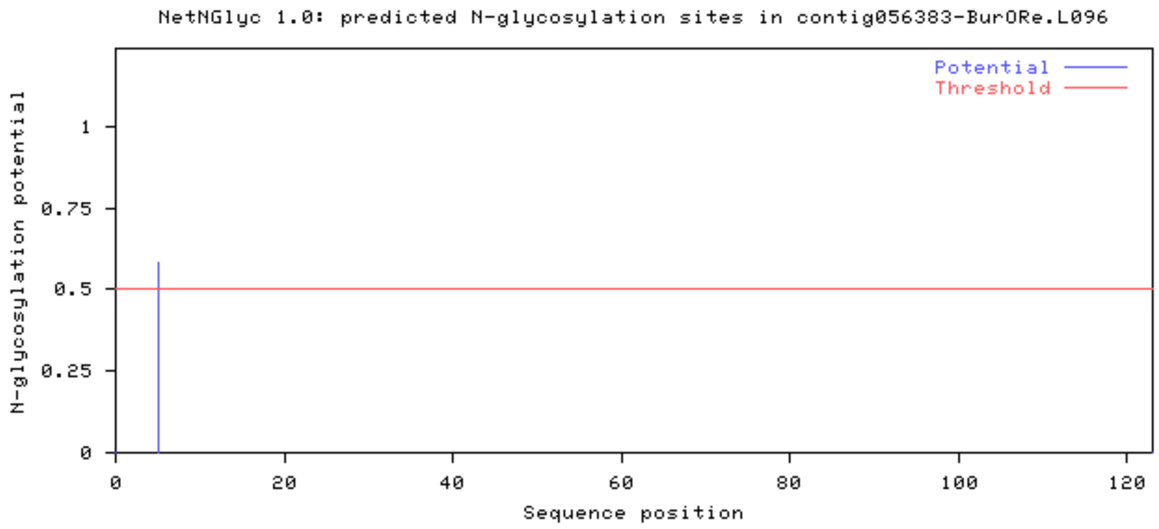

Graphics in PostScript

Output for 'contig056384-BurOR.L087'

```
##########################################################################################

    Warning: This sequence may not contain a signal peptide!!

    Proteins without signal peptides are unlikely to be exposed to
    the N-glycosylation machinery and thus may not be glycosylated
    (in vivo) even though they contain potential motifs.

    SignalP-NN euk predictions are as follows:

  # name                    Cmax   pos ?  Ymax   pos ?  Smax   pos ?  Smean ?  D     ?

    SignalP output is explained at http://www.cbs.dtu.dk/services/SignalP/output.html

##########################################################################################

Name:  contig056384-BurOR.L087  Length:  317
MSSQNTSINVTHFIIGGFDTLSRPIAVGVVILITYLLAVLANMVNIMFIISDKRLHKPMYLLICNLAVVDIMYTSSCSPT      80
MIGVLLAGVNTISYMACLIQMCVFHLGTAMESFVLAVMALDRFIAIIYPFQYHSYLTNTRVLVLTFIVWFVNCFFMCYMP     160
ATAVPLPHCSSRLRYTFCDFAAVIRTTCVNPEKHFNDAAIIAFFILFFTFVFICLSYCGILLFVKLSSNNEKKKMGSTLV     240
SHLICAIVHYCPAFVRIIFTRFGVVLTLEERQGLLIGAVLGPCLVNPFVYFLRTKEIKQKLFKIFKKFYTYITEILX
....N...N.......................................................................      80
................................................................................     160
................................................................................     240
.............................................................................        320

(Threshold=0.5)
----------------------------------------------------------------------
SeqName      Position  Potential   Jury    N-Glyc
                                 agreement result
----------------------------------------------------------------------
contig056384-BurOR.L087    5 NTSI   0.6202     (7/9)    +
contig056384-BurOR.L087    9 NVTH   0.7423     (9/9)    ++
----------------------------------------------------------------------
```

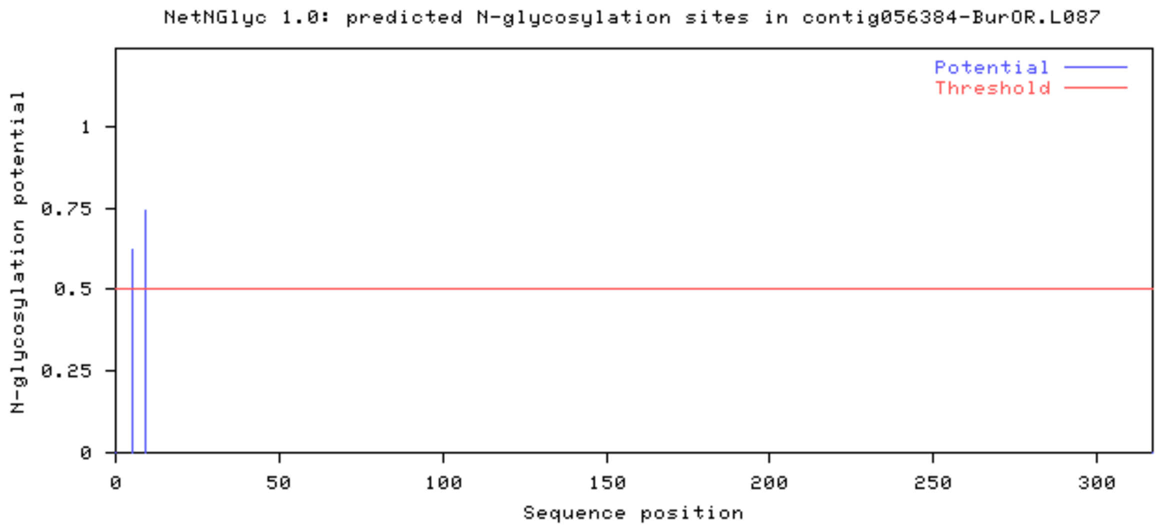

Graphics in PostScript

Output for 'contig056386-BurOR.L088'

```
##########################################################################################

    Warning: This sequence may not contain a signal peptide!!

    Proteins without signal peptides are unlikely to be exposed to
    the N-glycosylation machinery and thus may not be glycosylated
    (in vivo) even though they contain potential motifs.

    SignalP-NN euk predictions are as follows:

  # name                    Cmax  pos ?  Ymax  pos ?  Smax  pos ?  Smean ?  D     ?

    SignalP output is explained at http://www.cbs.dtu.dk/services/SignalP/output.html

##########################################################################################

Name:  contig056386-BurOR.L088  Length:  313
MSSQNASINVTHFIIGGFDTLSRPIAVGVVILIVYLLAVLANMINIMFIISDKRLHKPMYLLICNLAVIDIMYTSSCSPT      80
MIGVLLAGGNTISYVACLIQMCVFHLGTAMESFILAFMALDRFIAIIYPFQYQSYLTNTRVLVLTFIVWFVAWCFVFYMP     160
ATVVPLPHCSSRLKYNFCDFPAVIRTTCVNPEKYFNEVVIFSFFIFFFTFIFICLSYCGILLFVKLSSNNEKRKMGSTLV     240
SHLICVIVYYCPAFVRNIFTRFGVVLTLEERQGLLIGAVLGPCLVNPFVYCLRTKEIKQKFYKIFKKFHTSDX
....N...N.......................................................................      80
................................................................................     160
................................................................................     240
.........................................................................            320

(Threshold=0.5)
----------------------------------------------------------------------
SeqName      Position  Potential   Jury    N-Glyc
                                 agreement result
----------------------------------------------------------------------
contig056386-BurOR.L088    5 NASI   0.6106     (7/9)    +
contig056386-BurOR.L088    9 NVTH   0.7453     (9/9)    ++
----------------------------------------------------------------------
```

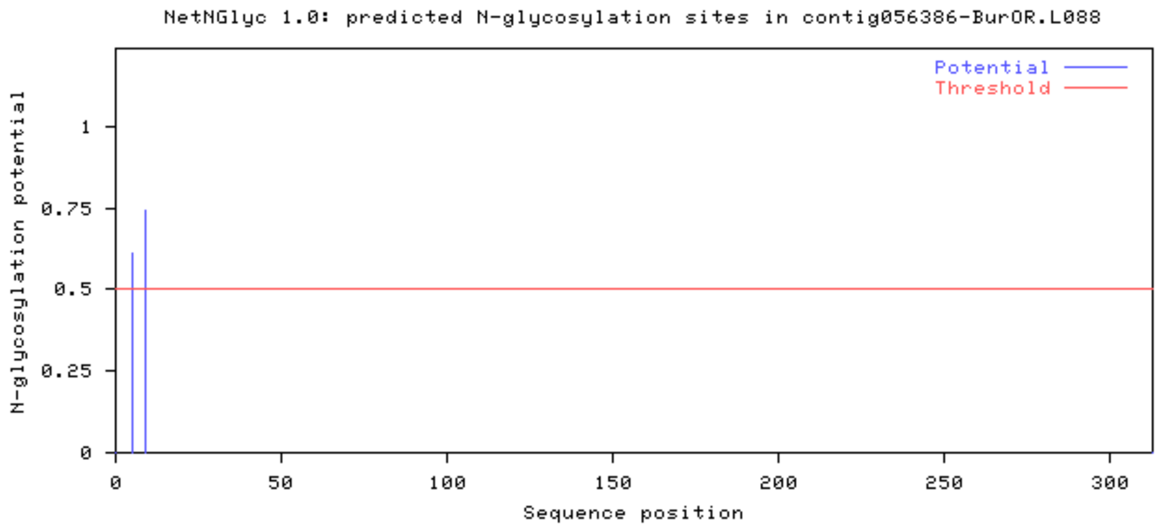

Graphics in PostScript

Output for 'contig057153-BurOR.A014'

```
##########################################################################

    Warning: This sequence may not contain a signal peptide!!

    Proteins without signal peptides are unlikely to be exposed to
    the N-glycosylation machinery and thus may not be glycosylated
    (in vivo) even though they contain potential motifs.

    SignalP-NN euk predictions are as follows:

  # name                 Cmax  pos ?  Ymax  pos ?  Smax  pos ?  Smean ?  D     ?

    SignalP output is explained at http://www.cbs.dtu.dk/services/SignalP/output.html

##########################################################################

Name:  contig057153-BurOR.A014  Length:  309
MNPGLNMTYLILGGHVEVQKYRYLYFMILFTAYILIICCNTSIIYLIVIHKSLHEPMYIFIAALLLNSLFFSTNIYPKLL      80
ADFLSEKQIISYQVCLFQVFIFYSLSCSEFLLLSAMAYDRYVSICKPLQYPTKMRKITVVFLLVLAWLLPACQVAVVIIL     160
NINNKLCNFTLKGIFCNNSLIQLYCVMSRAFSVYGAFVLLNTGLFPMLFIIFTYTKIILTVYRSSGEVKKKAAQTCLPHL     240
FVLINYSCLITYDMIIARLESDFSKTARFLMTLQIITYNPLFNPIIYGLKMKEISKHLQRLLCQSKLNX
.....N.................................N........................................      80
................................................................................     160
.......N........................................................................     240
....N................................................................               320

(Threshold=0.5)
----------------------------------------------------------------------
SeqName      Position  Potential   Jury    N-Glyc
                                 agreement result
----------------------------------------------------------------------
contig057153-BurOR.A014    6 NMTY   0.7297     (9/9)    ++
contig057153-BurOR.A014   40 NTSI   0.6411     (8/9)    +
contig057153-BurOR.A014  168 NFTL   0.6657     (9/9)    ++
contig057153-BurOR.A014  177 NNSL   0.4821     (6/9)    -
contig057153-BurOR.A014  245 NYSC   0.5048     (4/9)    +
----------------------------------------------------------------------
```

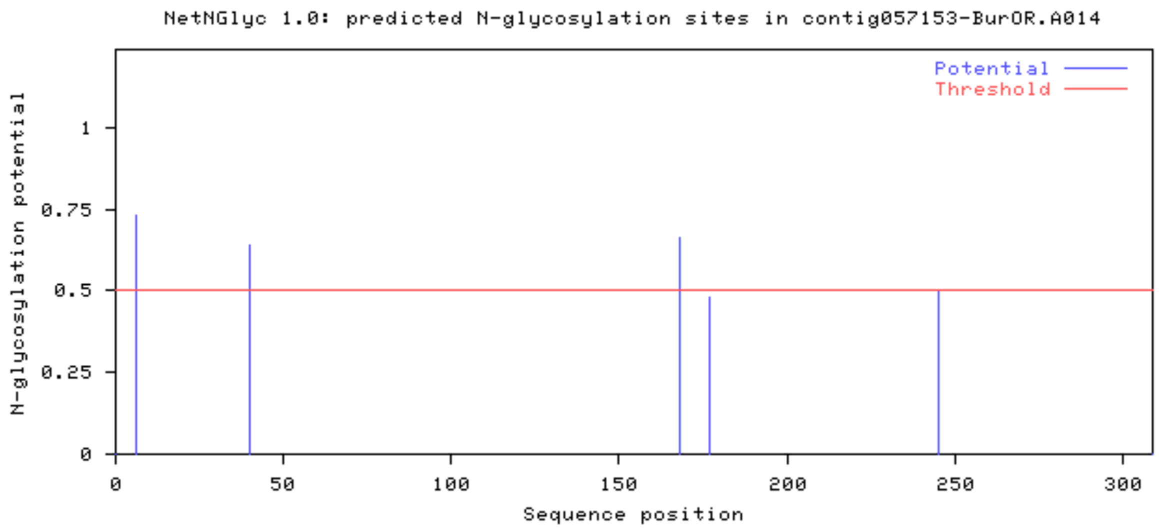

Graphics in PostScript

Output for 'contig057156-BurOR.A015'

```
##########################################################################################

    Warning: This sequence may not contain a signal peptide!!

    Proteins without signal peptides are unlikely to be exposed to
    the N-glycosylation machinery and thus may not be glycosylated
    (in vivo) even though they contain potential motifs.

    SignalP-NN euk predictions are as follows:

  # name                 Cmax  pos ?  Ymax  pos ?  Smax  pos ?  Smean ?  D     ?

    SignalP output is explained at http://www.cbs.dtu.dk/services/SignalP/output.html

##########################################################################################

Name:  contig057156-BurOR.A015  Length:  319
MDQMNDKFNVTYITFGGHVELNKYRFLYFAIMFTAYILILCSNSTILCLIWIKKNLHEPMYIFIAGLLLNSVMFSTNIYP      80
ELLIDFLSDKQITTHSLCSFQAFIYYSLTGSEFFLLAAMAYDRYVSICKPLQYTTIMKKTTIIVLLGLAWLLPACQLVPS     160
VVMSQSYKICSFTLNGIFCNNAISKLYCDTSRTTYIIYGVFILLNTVFLPLLFILFTYTKIFIICYRSCREVRKKAAQTC     240
LPHLLVLVSFSGLCSYDIIVARLEMNLPKVARFILTLQVVLYHPLFNPIVYGLKMKEISKHLTKLFCEAKLNIWQSSCX
........N.................................N.....................................      80
................................................................................     160
................................................................................     240
...............................................................................      320

(Threshold=0.5)
----------------------------------------------------------------------
SeqName      Position  Potential   Jury    N-Glyc
                                 agreement result
----------------------------------------------------------------------
contig057156-BurOR.A015    9 NVTY   0.7640     (9/9)    +++
contig057156-BurOR.A015   43 NSTI   0.6701     (9/9)    ++
----------------------------------------------------------------------
```

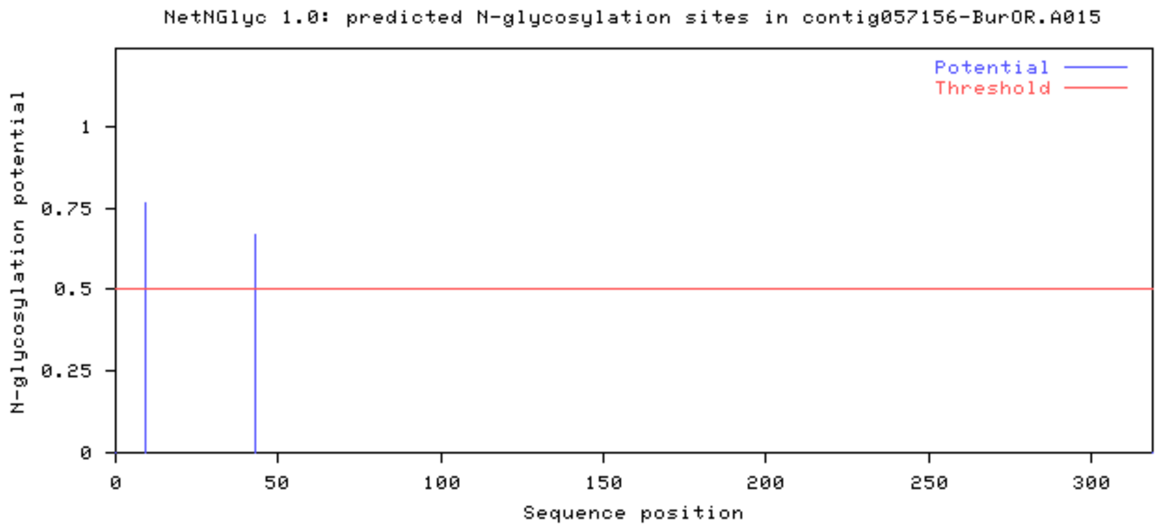

Graphics in PostScript

Output for 'contig057380-BurORe.N112'

```
##########################################################################

    Warning: This sequence may not contain a signal peptide!!

    Proteins without signal peptides are unlikely to be exposed to
    the N-glycosylation machinery and thus may not be glycosylated
    (in vivo) even though they contain potential motifs.

    SignalP-NN euk predictions are as follows:

  # name                 Cmax  pos ?  Ymax  pos ?  Smax  pos ?  Smean ?  D     ?

    SignalP output is explained at http://www.cbs.dtu.dk/services/SignalP/output.html

##########################################################################

Name:  contig057380-BurORe.N112        Length:  83
MKGFKTCTAHLSLVAIYFLPILITFTLGANIEPNARIINLSLTSVFPPMLNPIIYVLQTREIKESLRKLLRIIKHYKIRK      80
VKX
......................................N.........................................      80
...                                                                                   160

(Threshold=0.5)
----------------------------------------------------------------------
SeqName      Position  Potential   Jury    N-Glyc
                                 agreement result
----------------------------------------------------------------------
contig057380-BurORe.N112   39 NLSL   0.6757     (9/9)   ++
----------------------------------------------------------------------
```

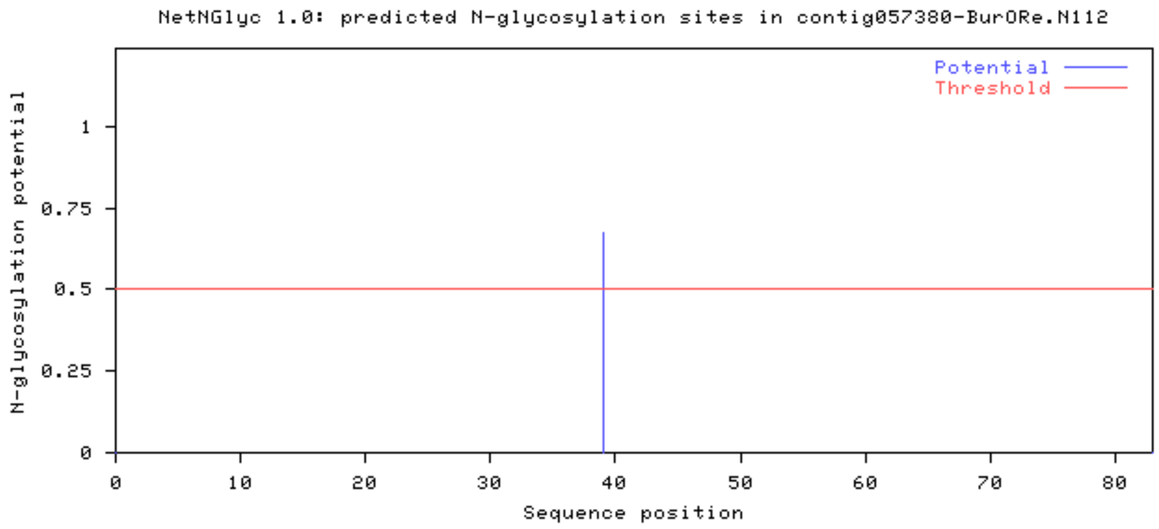

Graphics in PostScript

Output for 'contig057381-BurORp.N152'

##

Warning: This sequence may not contain a signal peptide!!

Proteins without signal peptides are unlikely to be exposed to
the N-glycosylation machinery and thus may not be glycosylated
(in vivo) even though they contain potential motifs.

SignalP-NN euk predictions are as follows:

# name	Cmax	pos	?	Ymax	pos	?	Smax	pos	?	Smean	?	D	?

SignalP output is explained at http://www.cbs.dtu.dk/services/SignalP/output.html

##

```
Name:  contig057381-BurORp.N152        Length:  296
MEFLNSAVEKNTTFVESAYFIISGFIGIPNIRYYFVFLCFIYILAVVGNTLVMIVITLDHMLRSPKYIAVFNLAFTDLLS      80
SSALVPKVVDISLFNRYYISCFWLFLYWLQLAFSQYFLFVSLLLFRATIVIMVLCIVLAAMSFCKSVVIQSYYCDHGPMY     160
RLGCNDVTPNRAIAGLALVIILGFPLAFIVGSYCCIGYSLSKISTFRERVKAFKACTGHLSLVAIYFLPIIFVYVFWPVI     240
HPNARIINLSMTSVMPPMLNPIIYVLQTQEIKESLRRLLIARAHAKLEVFRLNPLX
................................................................................      80
................................................................................     160
................................................................................     240
.......N................................................                              320
```

(Threshold=0.5)

SeqName	Position	Potential	Jury agreement	N-Glyc result
contig057381-BurORp.N152	11 NTTF	0.4961	(4/9)	-
contig057381-BurORp.N152	248 NLSM	0.5436	(5/9)	+

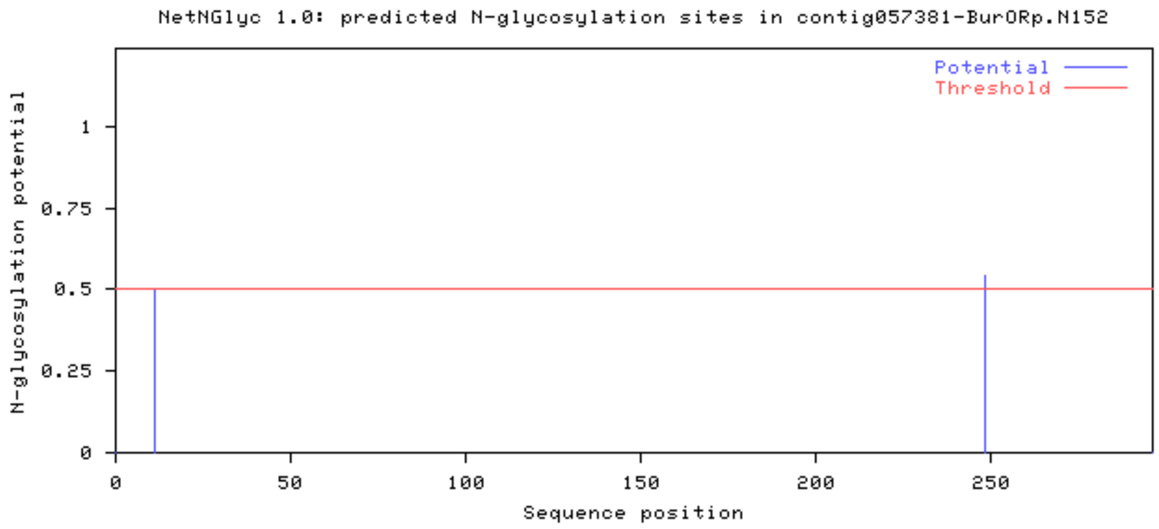

Graphics in PostScript

Output for 'contig057383-BurOR.N109'

```
##########################################################################################

    Warning: This sequence may not contain a signal peptide!!

    Proteins without signal peptides are unlikely to be exposed to
    the N-glycosylation machinery and thus may not be glycosylated
    (in vivo) even though they contain potential motifs.

    SignalP-NN euk predictions are as follows:

  # name                Cmax  pos ?  Ymax  pos ?  Smax  pos ?  Smean ?  D     ?

    SignalP output is explained at http://www.cbs.dtu.dk/services/SignalP/output.html

##########################################################################################

Name:  contig057383-BurOR.N109  Length:  327
MDLFNSALGKNITFLRPAFFIISGFIGIPNIKYYYAFLFFVYIISVLANTAVMAAIYLDHNLRTPKYIAVFNLALVDLLG      80
NSAMVPKVLDIFLFNHPHISYNDCLTFLFFYYVFLSMQALNLVALSYDRVMAIVYPLHYQLKVTHKFMFCLIASFWVFVI     160
IVVLIATGLLTRLSFCESVVIKSFFCDHGQIYRLACNDYTPSDITAWILLVLILWLPLTIVLLSYLSIGYALAKVATFRE     240
RMKGFKTCTAHLSLVAIYFLPILITFTLGANIEPNARIINLSLTSVFPPMLNPIIYVLQTQEIKESLKRFLRITTHYKIS     320
KVKFKKX
..........N.....................................................................      80
................................................................................     160
................................................................................     240
.......................................N........................................     320
.......                                                                              400
```

(Threshold=0.5)

SeqName	Position	Potential	Jury agreement	N-Glyc result
contig057383-BurOR.N109	11 NITF	0.5356	(6/9)	+
contig057383-BurOR.N109	280 NLSL	0.6054	(8/9)	+

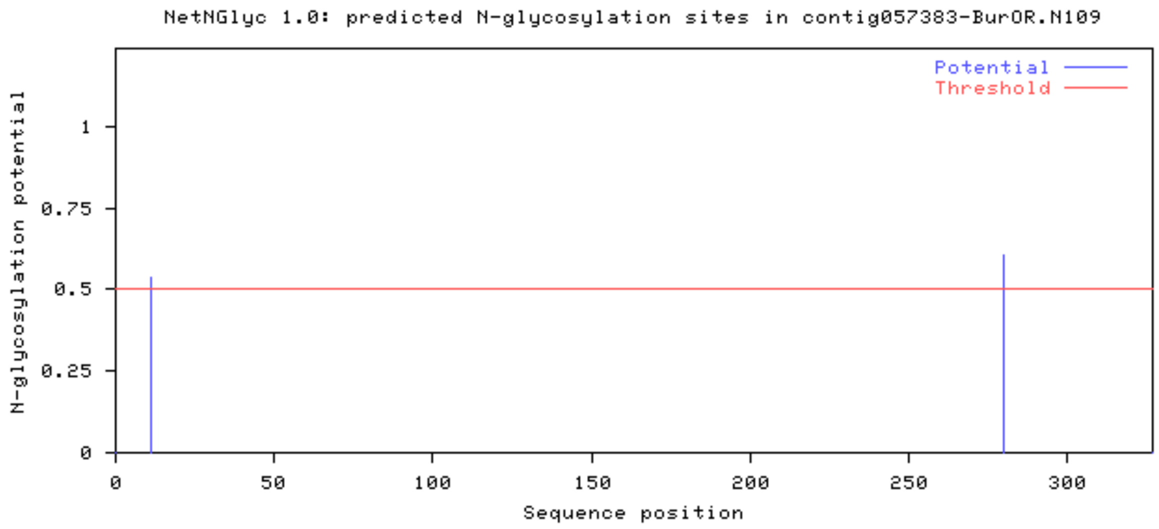

[Graphics in PostScript]

Output for 'contig058162-BurOR.L089'

```
##############################################################################

    Warning: This sequence may not contain a signal peptide!!

    Proteins without signal peptides are unlikely to be exposed to
    the N-glycosylation machinery and thus may not be glycosylated
    (in vivo) even though they contain potential motifs.

    SignalP-NN euk predictions are as follows:

  # name                 Cmax  pos ?  Ymax  pos ?  Smax  pos ?  Smean ?  D     ?

    SignalP output is explained at http://www.cbs.dtu.dk/services/SignalP/output.html

##############################################################################

Name:  contig058162-BurOR.L089  Length:  314
MSLPNASIKVTHFIIGGFDTVKRPIAVGVVMLIIYLLAVIANVLNILFIIFDKRLHKPMYILICNLAVVDILYCCGTTPT      80
MIGVLLAGVNTISYVECFIQMSVFHLVGVMELFALAIMAFDRLIAFSFPFQYHSYLTNTRTLVVTYILWVVASGFVAVMP     160
VTAATLPYCTSRMKYAFCDYAAVIRTTCVDPNYYFNLVSVMMFFLLFFTFSFIGLSYFVIAFLMKFSSNRDKKKMASTCV     240
SHLIVVTCYYSPLFVLIVFTRVGVVLTLEERQGLLIGTILSPSLVNPVVYCFRTKEIKNKIFKIFTKKADILNX
....N...........................................................................      80
................................................................................     160
................................................................................     240
..........................................................................           320

(Threshold=0.5)
----------------------------------------------------------------------
SeqName      Position  Potential   Jury    N-Glyc
                                 agreement result
----------------------------------------------------------------------
contig058162-BurOR.L089    5 NASI   0.6294     (7/9)   +
----------------------------------------------------------------------
```

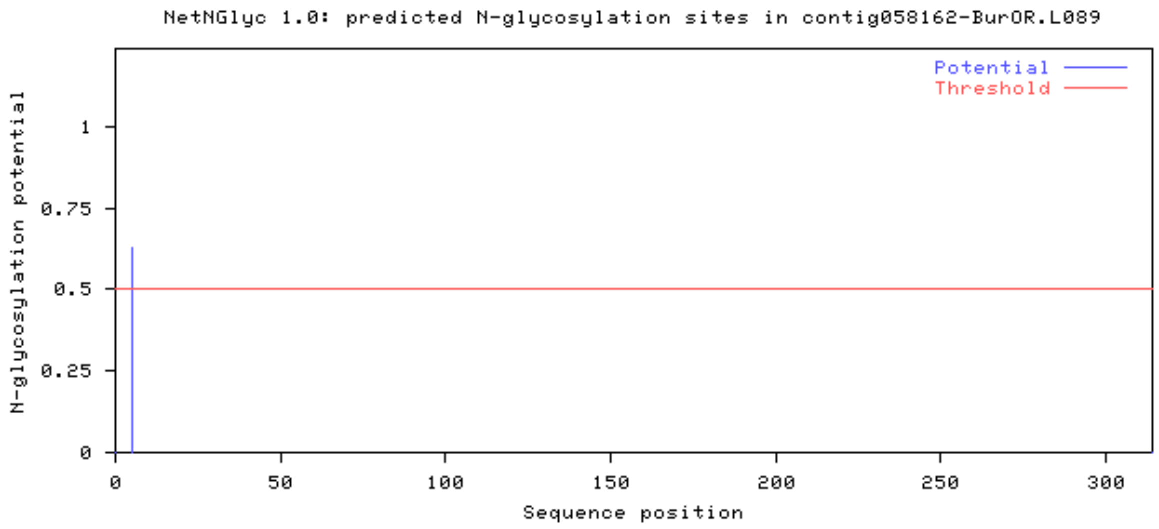

Graphics in PostScript

Output for 'contig058167-BurORe.J146'

```
########################################################################################

    Warning: This sequence may not contain a signal peptide!!

    Proteins without signal peptides are unlikely to be exposed to
    the N-glycosylation machinery and thus may not be glycosylated
    (in vivo) even though they contain potential motifs.

    SignalP-NN euk predictions are as follows:

  # name                Cmax  pos ?  Ymax  pos ?  Smax  pos ?  Smean ?  D     ?

    SignalP output is explained at http://www.cbs.dtu.dk/services/SignalP/output.html

########################################################################################

Name:  contig058167-BurORe.J146         Length:  205
AGTVFNLSAMAYDRYIAICYPLQYSTVMTNAHIMRIITIVWMSCLVLIAVLFFLLLRLPRCRSEMTHVYCDNASLLTLVC      80
ADTTINNIYGLFIVAFSQLVANGIVFYTYLRILITCFRSKRSDTKAKALQTCATHLIVFLLLECLGLFTIISYRLNNVSP     160
HFRRFMGLSTLIFPPTLNPIIYGLKTKEIREKVLNFLKNRMFSSX
.....N.................................................................N........      80
................................................................................     160
.............................................                                         240

(Threshold=0.5)
----------------------------------------------------------------------
SeqName      Position  Potential   Jury    N-Glyc
                                 agreement result
----------------------------------------------------------------------
contig058167-BurORe.J146    6 NLSA   0.5384     (4/9)   +
contig058167-BurORe.J146   72 NASL   0.5042     (5/9)   +
contig058167-BurORe.J146  157 NVSP   0.2237     (9/9)   ---
----------------------------------------------------------------------
```

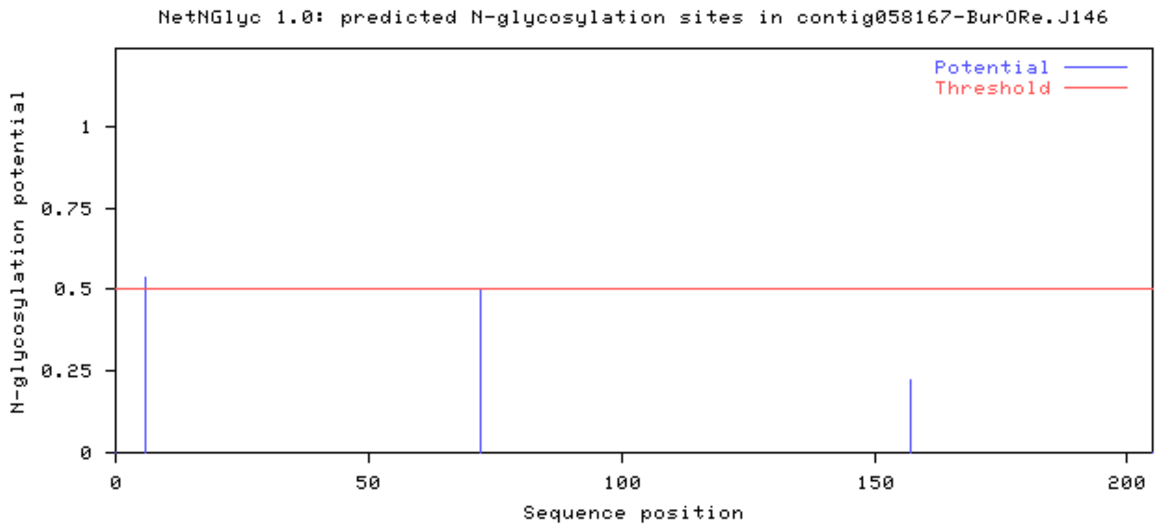

[Graphics in PostScript]

Output for 'contig059247-BurORe.O099'

```
##########################################################################

   Warning: This sequence may not contain a signal peptide!!

   Proteins without signal peptides are unlikely to be exposed to
   the N-glycosylation machinery and thus may not be glycosylated
   (in vivo) even though they contain potential motifs.

   SignalP-NN euk predictions are as follows:

 # name                Cmax  pos ?  Ymax  pos ?  Smax  pos ?  Smean ?  D     ?

   SignalP output is explained at http://www.cbs.dtu.dk/services/SignalP/output.html

##########################################################################

Name:  contig059247-BurORe.O099        Length:  269
IILNLSVCDILFSTTTLPKIISRYWFQSGSISFTACFIQMYFVHYFGTAVAYILFQMALDRYLAICHPLKYSHILTKSNI      80
LILSITAWTVAKASPLMMVIRAYPLPYCASNIITHCFCDHIGITVLACTDRTPYAIPAFVFAMVVLLGPLAFIIFSYGSI     160
LIAVYKIANVQSRMKSLSTCSTQLIIISLYFLPRCFVYLAQNVGITFSADVRIVIIMLYSLAPPMINPLIYCLRAKDMRE     240
SLLKQFCRRIVPEKAQVAAISNSLKTSPX
...N............................................................................      80
................................................................................     160
................................................................................     240
.............................                                                        320

(Threshold=0.5)
----------------------------------------------------------------------
SeqName      Position  Potential   Jury    N-Glyc
                                agreement  result
----------------------------------------------------------------------
contig059247-BurORe.O099    4 NLSV   0.7881     (9/9)    +++
----------------------------------------------------------------------
```

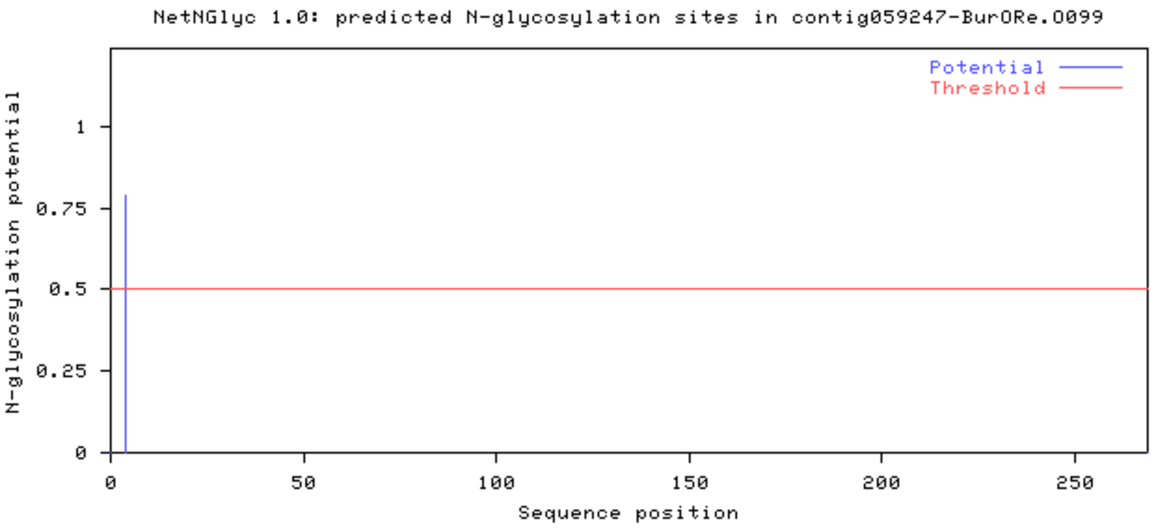

Graphics in PostScript

Output for 'contig059249-BurOR.O098'

```
##########################################################################

    Warning: This sequence may not contain a signal peptide!!

    Proteins without signal peptides are unlikely to be exposed to
    the N-glycosylation machinery and thus may not be glycosylated
    (in vivo) even though they contain potential motifs.

    SignalP-NN euk predictions are as follows:

  # name                    Cmax   pos ?  Ymax   pos ?  Smax   pos ?  Smean ?  D     ?

    SignalP output is explained at http://www.cbs.dtu.dk/services/SignalP/output.html

##########################################################################

Name:  contig059249-BurOR.0098  Length:  326
MPERNHSSVTEFILTGFPGLHQEYYGLVSAVLFFVYLVTLIANATVIFLFATNRSLHKPMYYIILNLSVCDILFSTTTLP      80
KIISRYWFQSGSISFTACFIQMYFVHYLGSVNSFILFQMALDRYLAICYPFRYSLVLTKSNILILSITAWIISKAFPLMM     160
VIRAYPLPYCASNIITHCFCDHIGITVLACTDRTPYATPAFTAAMVTLLGPLAFIIFSYGSILIAVYRIANVQGRLKSLS     240
TCSTQLIIISLYFLPRCFVYLAQNVGITFSADVRIVIIMLYSLPPPMINPLIYCLRAKDMRESLLKQFCKRIVPRKAQVA     320
AISNSX
....N.....................................N.........N...........N..............      80
................................................................................     160
................................................................................     240
................................................................................     320
......                                                                               400

(Threshold=0.5)
----------------------------------------------------------------------
SeqName      Position  Potential   Jury    N-Glyc
                                 agreement result
----------------------------------------------------------------------
contig059249-BurOR.0098    5 NHSS   0.6062     (8/9)    +
contig059249-BurOR.0098   43 NATV   0.7371     (9/9)    ++
contig059249-BurOR.0098   53 NRSL   0.7274     (9/9)    ++
contig059249-BurOR.0098   66 NLSV   0.7956     (9/9)    +++
----------------------------------------------------------------------
```

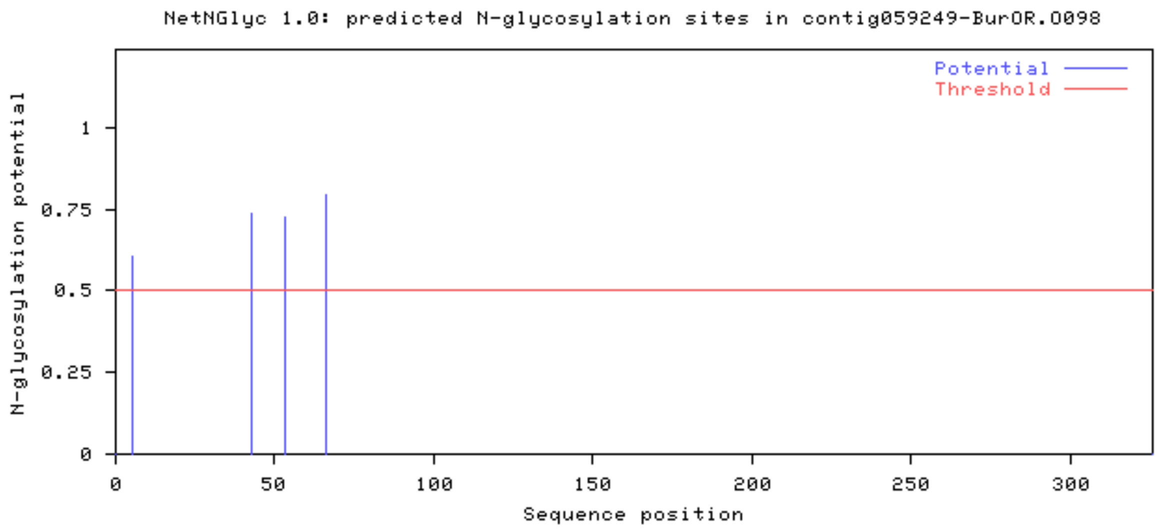

Graphics in PostScript

Output for 'contig059692-BurORp.A029'

```
##########################################################################################

    Warning: This sequence may not contain a signal peptide!!

    Proteins without signal peptides are unlikely to be exposed to
    the N-glycosylation machinery and thus may not be glycosylated
    (in vivo) even though they contain potential motifs.

    SignalP-NN euk predictions are as follows:

  # name                 Cmax  pos ?  Ymax  pos ?  Smax  pos ?  Smean ?  D     ?

    SignalP output is explained at http://www.cbs.dtu.dk/services/SignalP/output.html

##########################################################################################

Name:  contig059692-BurORp.A029        Length:  249
MEKELNVTFLTLDWYTEINKYRYILFFTIFTLYILIICTNSTILYLIFTHKNHHEPMYIFIAALLLNSVLYSTTMYPKLL      80
IDFLSEKQVTTYSFNLSLSVFFMFYTLGGSELPLLTAMAYDRYVAICKPLEYQTIMTKTTVSIFLVLAWLVPACHIAVQA     160
IASAEAKLWDSNIKGIFCNNAVYTLQCQRSRLITVFGVVSLLDLVILPMLFIVFTYTTIFIVSYQSCKEIRKKAAETCLP     240
HLLHCSKKX
.....N.................................N........................................      80
.............N..................................................................     160
................................................................................     240
.........                                                                            320

(Threshold=0.5)
----------------------------------------------------------------------
SeqName       Position  Potential    Jury    N-Glyc
                                   agreement result
----------------------------------------------------------------------
contig059692-BurORp.A029    6 NVTF    0.7858     (9/9)    +++
contig059692-BurORp.A029   40 NSTI    0.6214     (8/9)    +
contig059692-BurORp.A029   95 NLSL    0.6146     (8/9)    +
----------------------------------------------------------------------
```

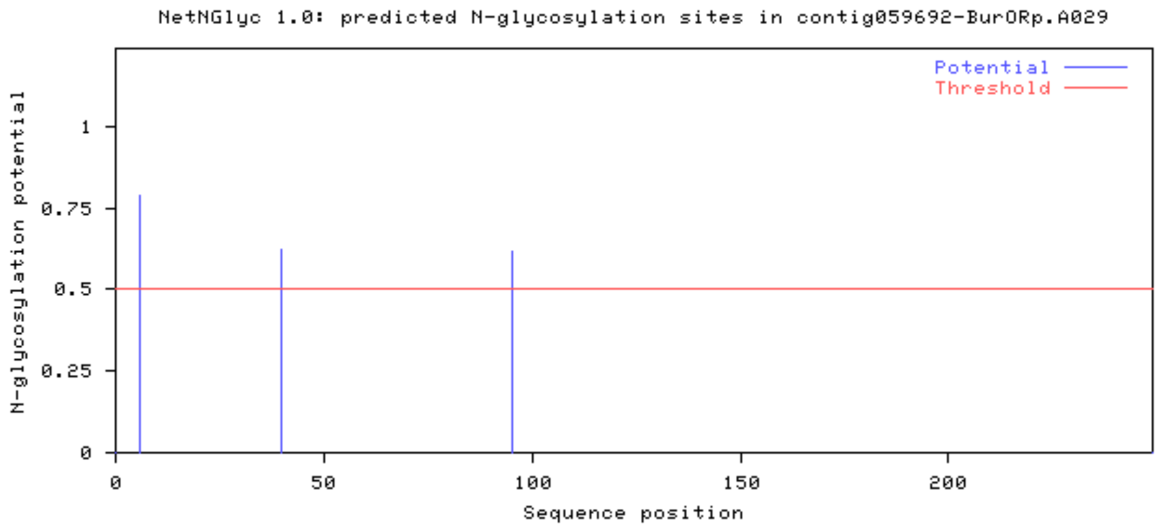

Graphics in PostScript

Output for 'contig059734-BurORp.A026'

```
##########################################################################################

    Warning: This sequence may not contain a signal peptide!!

    Proteins without signal peptides are unlikely to be exposed to
    the N-glycosylation machinery and thus may not be glycosylated
    (in vivo) even though they contain potential motifs.

    SignalP-NN euk predictions are as follows:

  # name                 Cmax  pos ?  Ymax  pos ?  Smax  pos ?  Smean ?  D     ?

    SignalP output is explained at http://www.cbs.dtu.dk/services/SignalP/output.html

##########################################################################################

Name:  contig059734-BurORp.A026         Length:  289
MELALNVSYITLDGFFFYSTIREKETERECRTDRQVTVSGVYTAIQVFFFLQHFSFCKCFLKLQSVWCLRATVGGLFQFF      80
MFYSLGGSEFLLLFAMAYDRYVSICKPLQYPVIMAKNTISIFLTLAWIVPSSQVAVVAVLMANKKKICNFTFTGIFCNNT     160
IYKLLCVHSKAQTVYDMVVLSNVAILPVVFIFFTYTRILVISYQSCKEVRRKAAQTCLPHLIVLISYLCLCAFDVIVSGL     240
ESNFPKILHSILTLQIVYPPLFNPIIYGLKMKEISKHLKRLFCAVKKNX
.....N..........................................................................      80
....................................................................N........N..     160
................................................................................     240
.................................................                                    320

(Threshold=0.5)
----------------------------------------------------------------------
SeqName      Position  Potential   Jury    N-Glyc
                                 agreement result
----------------------------------------------------------------------
contig059734-BurORp.A026    6 NVSY   0.8399     (9/9)   +++
contig059734-BurORp.A026  149 NFTF   0.6459     (9/9)   ++
contig059734-BurORp.A026  158 NNTI   0.5194     (4/9)   +
----------------------------------------------------------------------
```

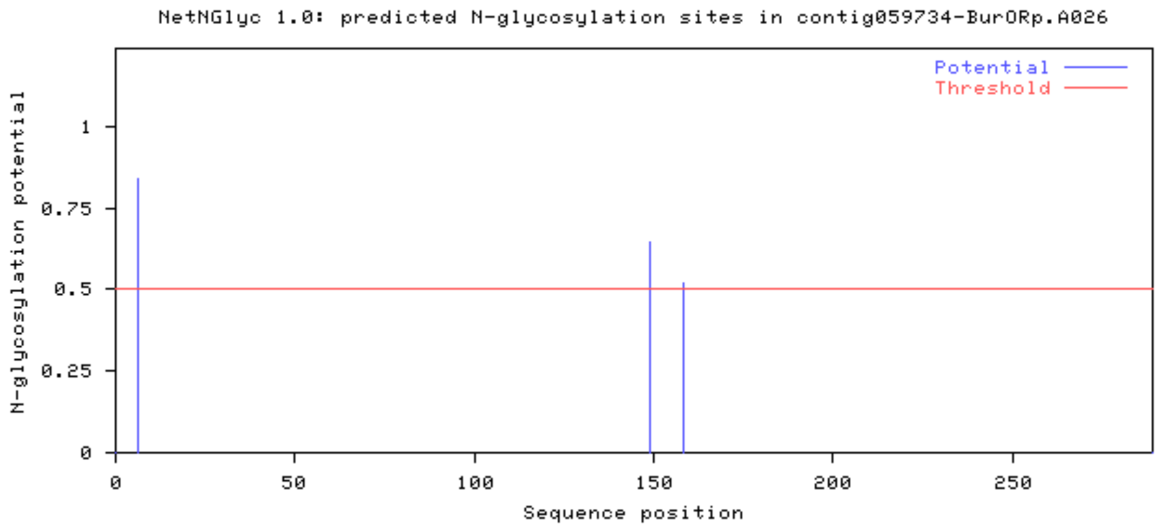

Graphics in PostScript

Output for 'contig059763-BurORe.A023'

```
##########################################################################################

    Warning: This sequence may not contain a signal peptide!!

    Proteins without signal peptides are unlikely to be exposed to
    the N-glycosylation machinery and thus may not be glycosylated
    (in vivo) even though they contain potential motifs.

    SignalP-NN euk predictions are as follows:

  # name                Cmax  pos ?  Ymax  pos ?  Smax  pos ?  Smean ?  D     ?

    SignalP output is explained at http://www.cbs.dtu.dk/services/SignalP/output.html

##########################################################################################

Name:  contig059763-BurORe.A023         Length:  302
IEVNKYRYVYFCIIFTLYIIIICSNSTIVYVIWIHKNLHEPMYIFIAALLLNCLLYSTNIYPKLLIDFLSEKQVITYSAC      80
LFQFFMFYTLGSSEFFLLAAMAYDRYVAICKPLEYPTIMNKTTVIIFLVVSWLIPAVHIAIQAIASAEATLCNFNLKGIF     160
CNNAVYTLHCQRSRLITVFGVVALLDLVILPMIFIVFTYTTIFIVSYQSCKEIRKKAAETCLPHLLVLISISCLSIYDVG     240
IARVESDFPKVARLLMTLQLLLYHPLFNPFIYGLKMKEISKQLKRLFCHATIITCINANVPX
........................N.......................................................      80
.......................................N........................................     160
................................................................................     240
..............................................................                       320

(Threshold=0.5)
----------------------------------------------------------------------
SeqName          Position  Potential   Jury     N-Glyc
                                  agreement  result
----------------------------------------------------------------------
contig059763-BurORe.A023    25 NSTI   0.6996     (8/9)    +
contig059763-BurORe.A023   120 NKTT   0.6602     (7/9)    +
----------------------------------------------------------------------
```

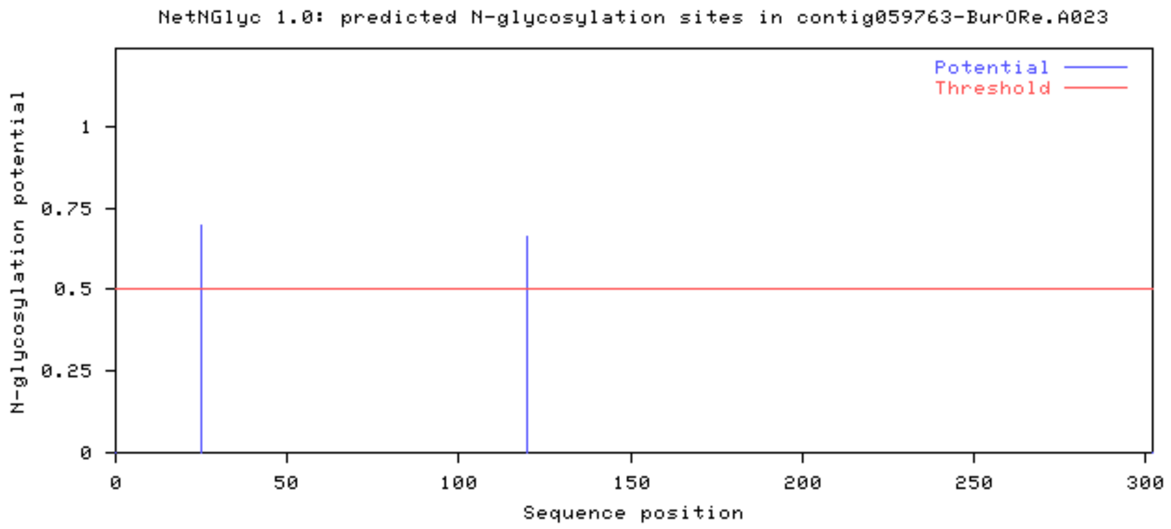

Graphics in PostScript

Output for 'contig059786-BurORe.L091'

```
##########################################################################################

    Warning: This sequence may not contain a signal peptide!!

    Proteins without signal peptides are unlikely to be exposed to
    the N-glycosylation machinery and thus may not be glycosylated
    (in vivo) even though they contain potential motifs.

    SignalP-NN euk predictions are as follows:

  # name                Cmax  pos ?  Ymax  pos ?  Smax  pos ?  Smean ?  D     ?

    SignalP output is explained at http://www.cbs.dtu.dk/services/SignalP/output.html

##########################################################################################

Name:  contig059786-BurORe.L091         Length:  311
SQNTSINVTHFIIGGFDTLSRPIAVGVVILITYLLAVLANMINIMFIISDKRLHKPMYLLICNLAVVDIVYTSSSSPTMI      80
GVLLAGVNTISYVACLIQMCVFNLGTSMESFVLAFMALDRFIAIIYPFQYQRYLTNTRVLVLTFIMWFIAWCFAFYMPAT     160
VVPLPHCSSRLKYSFCDFAAVIRTTCVNPEKYFNEVSIAAFFIFFFTFVFICLSYCGILLFVKLSSNNEKMKMGSTLVSH     240
LICVVVHYCPAFVRIMFTRFGVVLTLEERQGLVIGAVLGPCLVNPFVYCLRTKEIKQKLFKIFKKFNTSDX
..N...N.........................................................................      80
................................................................................     160
................................................................................     240
.......................................................................              320

(Threshold=0.5)
----------------------------------------------------------------------
SeqName      Position  Potential   Jury    N-Glyc
                                 agreement result
----------------------------------------------------------------------
contig059786-BurORe.L091     3 NTSI   0.6121     (9/9)    ++
contig059786-BurORe.L091     7 NVTH   0.7468     (9/9)    ++
contig059786-BurORe.L091   307 NTSD   0.4222     (8/9)    -
----------------------------------------------------------------------
```

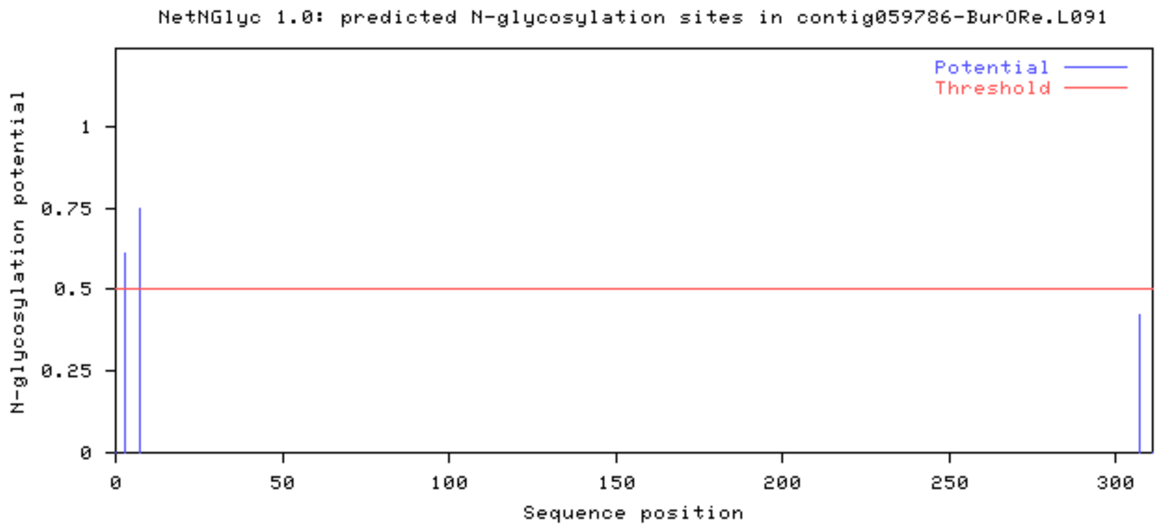

Graphics in PostScript

Output for 'contig060184-BurORe.N113'

```
##########################################################################################

    Warning: This sequence may not contain a signal peptide!!

    Proteins without signal peptides are unlikely to be exposed to
    the N-glycosylation machinery and thus may not be glycosylated
    (in vivo) even though they contain potential motifs.

    SignalP-NN euk predictions are as follows:

  # name                Cmax  pos ?  Ymax  pos ?  Smax  pos ?  Smean ?  D     ?

    SignalP output is explained at http://www.cbs.dtu.dk/services/SignalP/output.html

##########################################################################################

Name:  contig060184-BurORe.N113        Length:  40
MALINSAAENNITFVRPAYFIISGFIGIPNIRYYFVFLCF
..........N.............................                                              80

(Threshold=0.5)
----------------------------------------------------------------------
SeqName      Position  Potential   Jury    N-Glyc
                                agreement result
----------------------------------------------------------------------
contig060184-BurORe.N113   11 NITF   0.6178     (9/9)   ++
----------------------------------------------------------------------
```

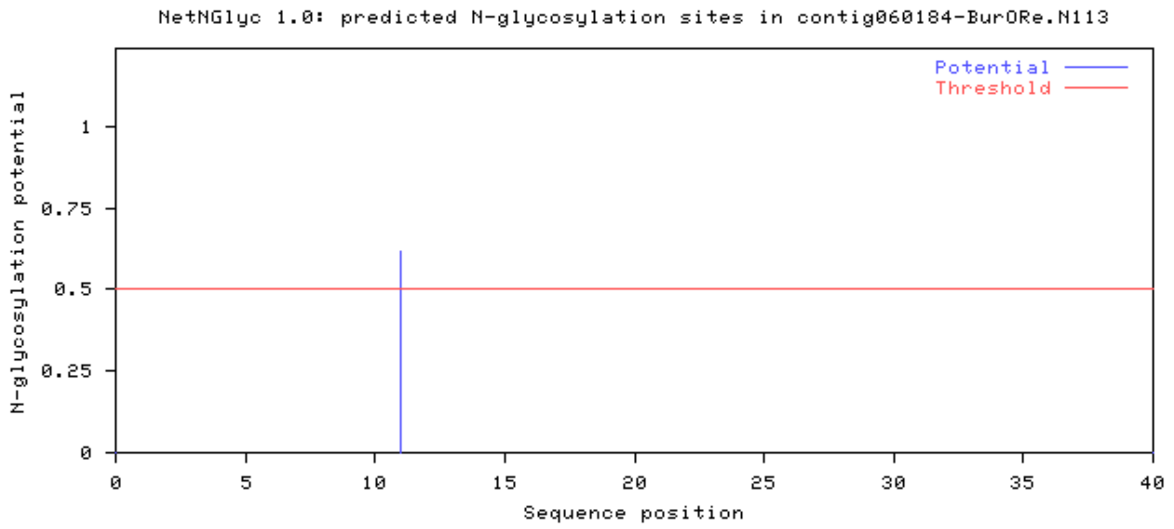

[Graphics in PostScript]

Output for 'contig060631-BurOR.N110'

```
##########################################################################

    Warning: This sequence may not contain a signal peptide!!

    Proteins without signal peptides are unlikely to be exposed to
    the N-glycosylation machinery and thus may not be glycosylated
    (in vivo) even though they contain potential motifs.

    SignalP-NN euk predictions are as follows:

  # name                 Cmax   pos ?  Ymax   pos ?  Smax   pos ?  Smean ?  D     ?

    SignalP output is explained at http://www.cbs.dtu.dk/services/SignalP/output.html

##########################################################################

Name:  contig060631-BurOR.N110  Length:  322
MELFNSALGKNITFVHPAFFIIGGLTGIPNITLYYVFLFFVYIVSVVGNTVVMAVIYLDHNLRTPKYIAVFNLAFVDLFG      80
NTALVPKVLDIFLFGHYYIPYNDCLTFLFFCYTCLSLQSFNLVALSYDRMVAIIFPLHYQVKVTHRFMFSLIASLWVFTI     160
IAVLISVGLLTRLSFCKSVVINSYFCDHGQIYRLACNDHFPSYVIACLYPVIIFWLPLAFILLSYLYIGYTLVKVATLQE     240
GLKALKTCIGHLSLVAIYFIPLLTTFTLMEKIQPNARIINLSLTSVFPPMLNPIIYVLQTQEIKESLKRLLKRRGKSKIT     320
IX
..........N..................N..................................................      80
................................................................................     160
................................................................................     240
.......................................N........................................     320
..                                                                                   400

(Threshold=0.5)
----------------------------------------------------------------------
SeqName      Position  Potential   Jury    N-Glyc
                                 agreement result
----------------------------------------------------------------------
contig060631-BurOR.N110   11 NITF   0.6087     (8/9)   +
contig060631-BurOR.N110   30 NITL   0.8183     (9/9)   +++
contig060631-BurOR.N110  280 NLSL   0.6018     (8/9)   +
----------------------------------------------------------------------
```

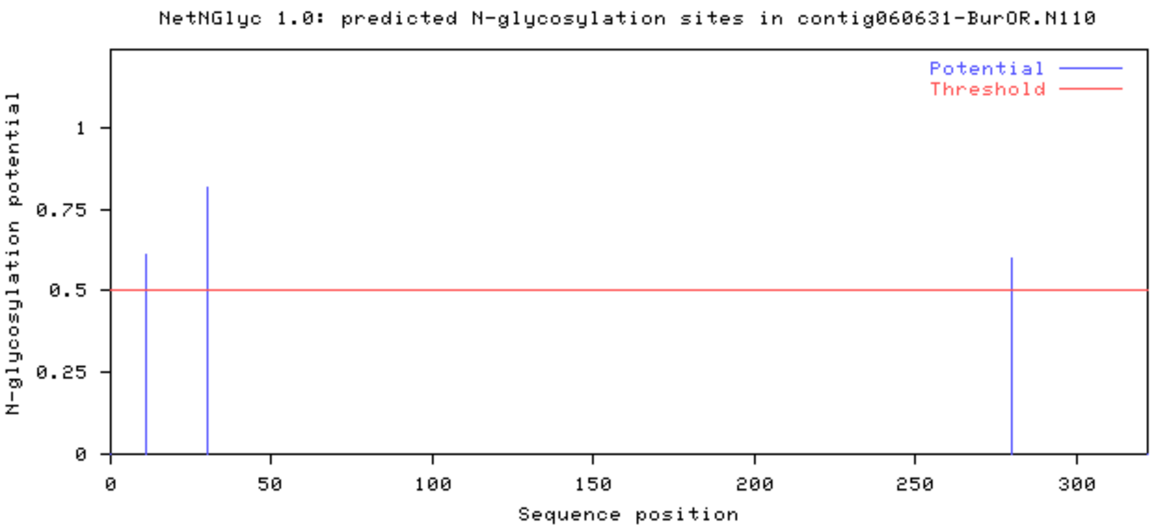

Graphics in PostScript

Output for 'contig061668-BurORe.L092'

```
##########################################################################################

    Warning: This sequence may not contain a signal peptide!!

    Proteins without signal peptides are unlikely to be exposed to
    the N-glycosylation machinery and thus may not be glycosylated
    (in vivo) even though they contain potential motifs.

    SignalP-NN euk predictions are as follows:

  # name                  Cmax  pos ?  Ymax  pos ?  Smax  pos ?  Smean ?  D     ?

    SignalP output is explained at http://www.cbs.dtu.dk/services/SignalP/output.html

##########################################################################################

Name:  contig061668-BurORe.L092         Length:  197
MSLQNASIKLTHFIIGGFDTVKMPVAVGVVMLITYLLAVLASLVNIIFIVSDKQLHKPMYLLICNLAVVDIFYTSSATPT      80
MIGVLLAGVNTISYVECLIQMYVYQVGATMERFSLTIMAFDRLIAIICPLQYHSYLTNTRTVVFTYILWIVACSFVLFTL     160
VTATPLPHCYSRLRYTFCDYAAVMRTTCVDPEKYFNQ
....N...........................................................................      80
................................................................................     160
.....................................                                               240

(Threshold=0.5)
----------------------------------------------------------------------
SeqName      Position  Potential   Jury    N-Glyc
                                 agreement result
----------------------------------------------------------------------
contig061668-BurORe.L092     5 NASI   0.6047     (7/9)    +
----------------------------------------------------------------------
```

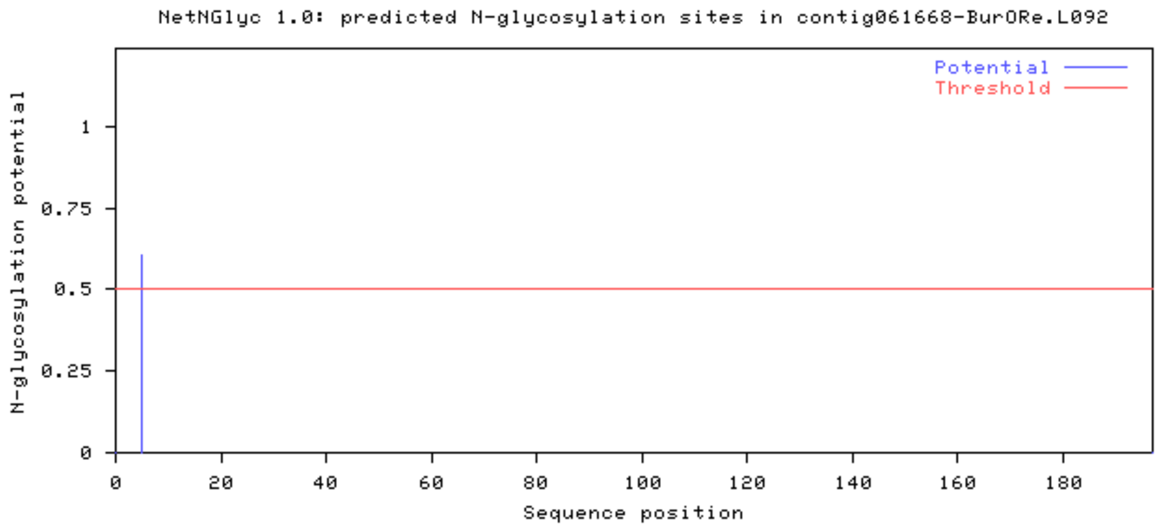

Graphics in PostScript

Output for 'contig062234-BurORe.L093'

```
##########################################################################################

    Warning: This sequence may not contain a signal peptide!!

    Proteins without signal peptides are unlikely to be exposed to
    the N-glycosylation machinery and thus may not be glycosylated
    (in vivo) even though they contain potential motifs.

    SignalP-NN euk predictions are as follows:

  # name                    Cmax   pos ?  Ymax   pos ?  Smax   pos ?  Smean ?  D     ?

    SignalP output is explained at http://www.cbs.dtu.dk/services/SignalP/output.html

##########################################################################################

Name:  contig062234-BurORe.L093         Length:  56
MSLQNASIKLTHFIIGGFDTVKRPVAVGVVMLIIYLLAVAGSLVNIIFIVSDKQLH
....N...................................................                              80

(Threshold=0.5)
----------------------------------------------------------------------
SeqName      Position  Potential    Jury    N-Glyc
                                 agreement result
----------------------------------------------------------------------
contig062234-BurORe.L093    5 NASI   0.5956     (7/9)    +
----------------------------------------------------------------------
```

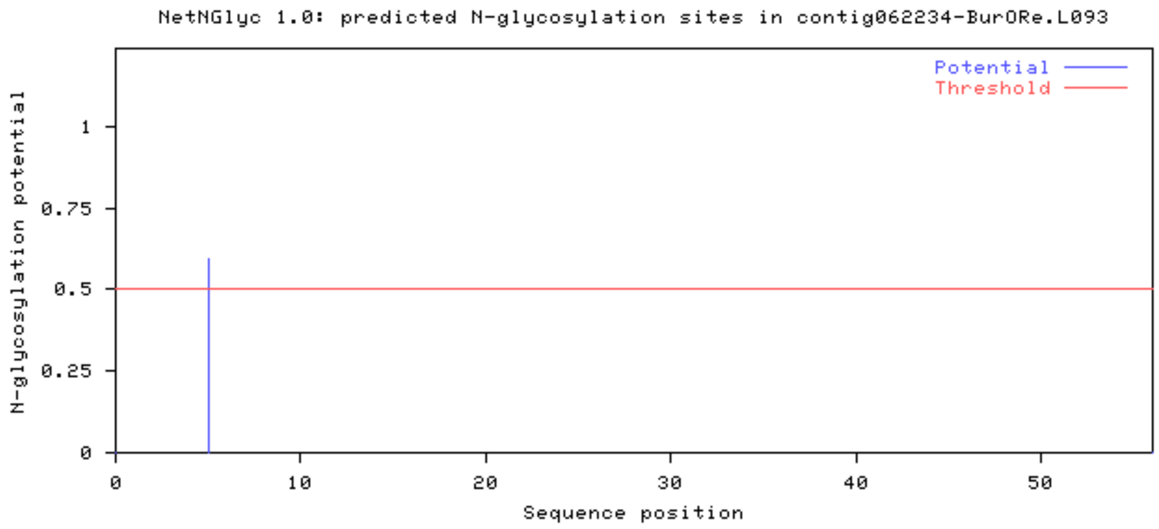

Graphics in PostScript

Output for 'contig063825-BurORe.R151'

```
##########################################################################################

    Warning: This sequence may not contain a signal peptide!!

    Proteins without signal peptides are unlikely to be exposed to
    the N-glycosylation machinery and thus may not be glycosylated
    (in vivo) even though they contain potential motifs.

    SignalP-NN euk predictions are as follows:

  # name                 Cmax  pos ?  Ymax  pos ?  Smax  pos ?  Smean ?  D     ?

    SignalP output is explained at http://www.cbs.dtu.dk/services/SignalP/output.html

##########################################################################################

Name:  contig063825-BurORe.R151         Length:  56
NVFYVCIFLFPRCLSSLIYGIRDQSIRPVLIYYLCCRLKYSVIQPRLKAAIEVECX
........................................................                              80

(Threshold=0.5)

No sites predicted in this sequence.
```

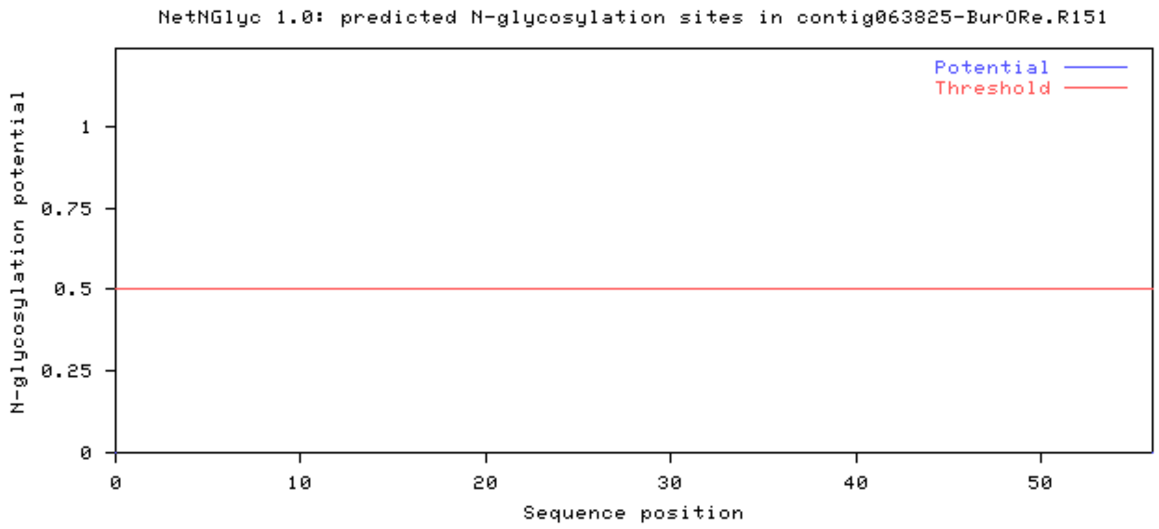

Graphics in PostScript

Output for 'contig064050-BurORe.A024'

```
##########################################################################

    Warning: This sequence may not contain a signal peptide!!

    Proteins without signal peptides are unlikely to be exposed to
    the N-glycosylation machinery and thus may not be glycosylated
    (in vivo) even though they contain potential motifs.

    SignalP-NN euk predictions are as follows:

  # name                    Cmax   pos ?  Ymax   pos ?  Smax   pos ?  Smean ?  D     ?

    SignalP output is explained at http://www.cbs.dtu.dk/services/SignalP/output.html

##########################################################################

Name:  contig064050-BurORe.A024         Length:  162
MAIDNEFNVTYITFGGHIELDKYKFLYFAIMFTAYILILCSNSTIVCLIRIKKSLHEPMYIFIAALLFNSVMFSTNIYPK      80
LLMDFLSEKQITTHSQCSFQGFIYYSLTGSEFFLLASMAYDRYVSISKPLQYHTIMRKTTVTVLLVLTWLLPACQLVPSA     160
VI
.......N.................................N......................................      80
................................................................................     160
..                                                                                   240

(Threshold=0.5)
----------------------------------------------------------------------
SeqName      Position  Potential   Jury    N-Glyc
                                agreement result
----------------------------------------------------------------------
contig064050-BurORe.A024    8 NVTY   0.6873     (9/9)   ++
contig064050-BurORe.A024   42 NSTI   0.7142     (9/9)   ++
----------------------------------------------------------------------
```

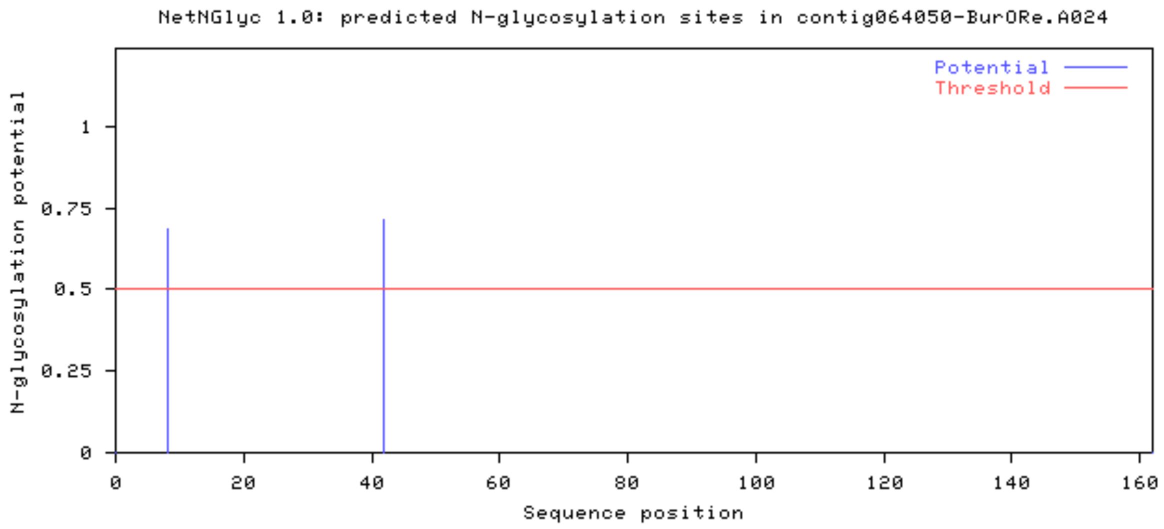

Graphics in PostScript

Output for 'contig064129-BurORe.W149'

```
##########################################################################################

    Warning: This sequence may not contain a signal peptide!!

    Proteins without signal peptides are unlikely to be exposed to
    the N-glycosylation machinery and thus may not be glycosylated
    (in vivo) even though they contain potential motifs.

    SignalP-NN euk predictions are as follows:

  # name                Cmax  pos ?  Ymax  pos ?  Smax  pos ?  Smean ?  D     ?

    SignalP output is explained at http://www.cbs.dtu.dk/services/SignalP/output.html

##########################################################################################

Name:  contig064129-BurORe.W149        Length:  64
IRFASFVIVQIIPRFISPIVYGLRDQTFRKYVRKYLVCSERGITHPENTTTKKASLNVTSATVX
...............................................N........N.......                     80

(Threshold=0.5)
----------------------------------------------------------------------
SeqName      Position  Potential   Jury    N-Glyc
                                 agreement result
----------------------------------------------------------------------
contig064129-BurORe.W149   48 NTTT   0.5018     (5/9)   +
contig064129-BurORe.W149   57 NVTS   0.6446     (8/9)   +
----------------------------------------------------------------------
```

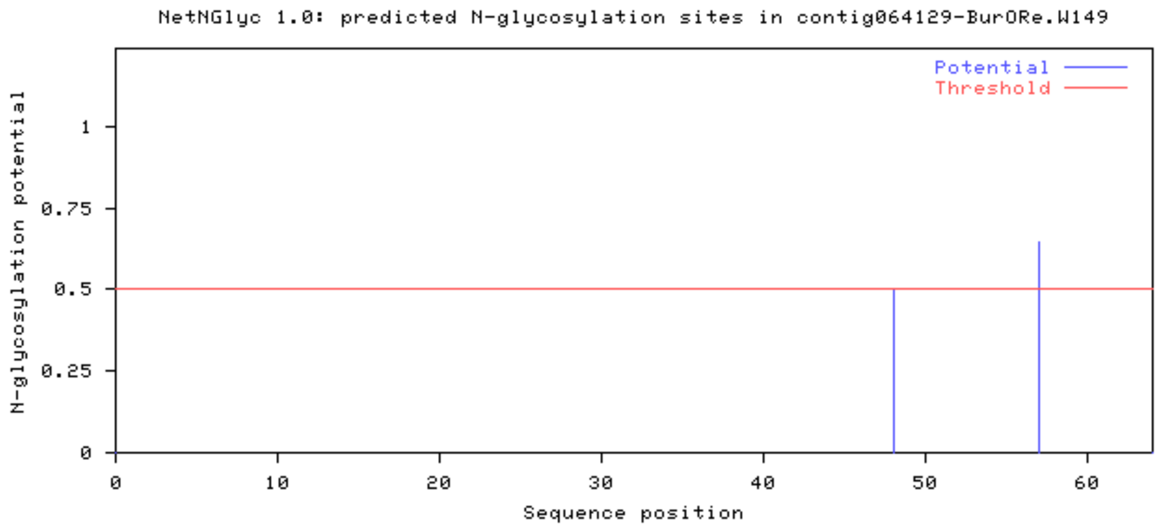

Graphics in PostScript

Output for 'contig064187-BurOR.A016'

```
##########################################################################################

    Warning: This sequence may not contain a signal peptide!!

    Proteins without signal peptides are unlikely to be exposed to
    the N-glycosylation machinery and thus may not be glycosylated
    (in vivo) even though they contain potential motifs.

    SignalP-NN euk predictions are as follows:

  # name                    Cmax   pos ?  Ymax   pos ?  Smax   pos ?  Smean ?  D     ?

    SignalP output is explained at http://www.cbs.dtu.dk/services/SignalP/output.html

##########################################################################################

Name:  contig064187-BurOR.A016  Length:  300
MNLTYITFGGHVEVEKYRYLYFVIMFMVYILIICSNSTIVWLIVVQKSLHEPMYIFIAALLVNSVVLSTVIYPKLLIDFL      80
SEKQIILYQACLFQVFLFYALSCSEFLLLSAMAYDRYVSICKPLQYPSIMRRTRVNIFLLLCWFLPAIQVAVPIAGNANT     160
PLCNFTLKGIFCNNSVNHLYCVNSRELSIYGMVVLFNVALSPMFFILFTYIKIIIVAYQSCGNVRKKAAQTCLPHVLVLM     240
NYSCLLTYDMVIVRLESEFPKTARFIMTLQFVTYNPLCNPIIYGLKMKEISKHLKILFSX
.N.................................N............................................      80
................................................................................     160
...N............................................................................     240
N...........................................................                         320

(Threshold=0.5)
----------------------------------------------------------------------
SeqName      Position  Potential   Jury    N-Glyc
                                 agreement result
----------------------------------------------------------------------
contig064187-BurOR.A016    2 NLTY   0.7966     (9/9)   +++
contig064187-BurOR.A016   36 NSTI   0.6901     (8/9)   +
contig064187-BurOR.A016  164 NFTL   0.6498     (9/9)   ++
contig064187-BurOR.A016  173 NNSV   0.4719     (5/9)   -
contig064187-BurOR.A016  241 NYSC   0.5431     (5/9)   +
----------------------------------------------------------------------
```

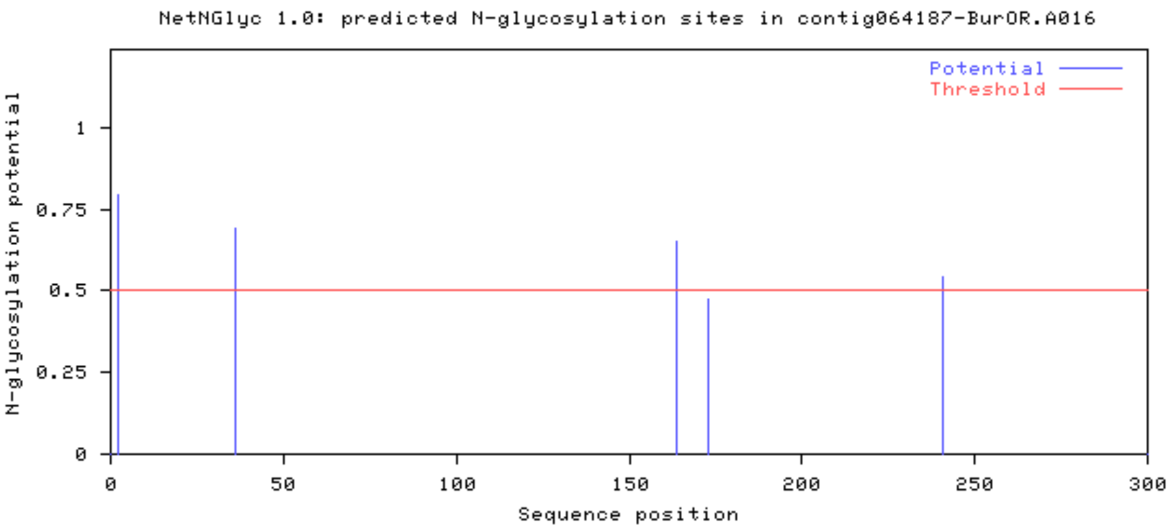

Graphics in PostScript

Output for 'contig064565-BurOR.R139'

```
##########################################################################################

    Warning: This sequence may not contain a signal peptide!!

    Proteins without signal peptides are unlikely to be exposed to
    the N-glycosylation machinery and thus may not be glycosylated
    (in vivo) even though they contain potential motifs.

    SignalP-NN euk predictions are as follows:

  # name                   Cmax  pos ?  Ymax  pos ?  Smax  pos ?  Smean ?  D     ?

    SignalP output is explained at http://www.cbs.dtu.dk/services/SignalP/output.html

##########################################################################################

Name:  contig064565-BurOR.R139  Length:  320
MSDISQTNISVGLHDLERGLLSSLTTLPCCVFVCINVIMLFTLRSKSVFREPCRYILLYNLVLADTLQMAVSQILYMMSI      80
CRITLPYPVCGILVMFANLTNEISPLTLVLMSLERYVAVCYPLRHATIITIRNTEVAIITIWIFCSLNILIRVLLLLEFP     160
FEELQSLQLKRYVCNTFLLFLTPVSHEYDKAYTCFLFVSAFVGVTCSYVGVMLAARSASTDKASARKARNTLLLHLVQLG     240
FSLSSTVNNALLLLTSKTVSNRVSVLIQNALYVLLFILPRCLSALIYGLRDQTIRPILVYNLCCQLKLLAVTAEAKIYPX     320
.......N........................................................................      80
.................N..............................................................     160
................................................................................     240
................................................................................     320

(Threshold=0.5)
----------------------------------------------------------------------
SeqName      Position  Potential   Jury    N-Glyc
                                 agreement result
----------------------------------------------------------------------
contig064565-BurOR.R139    8 NISV   0.6020     (6/9)   +
contig064565-BurOR.R139   98 NLTN   0.6599     (9/9)   ++
----------------------------------------------------------------------
```

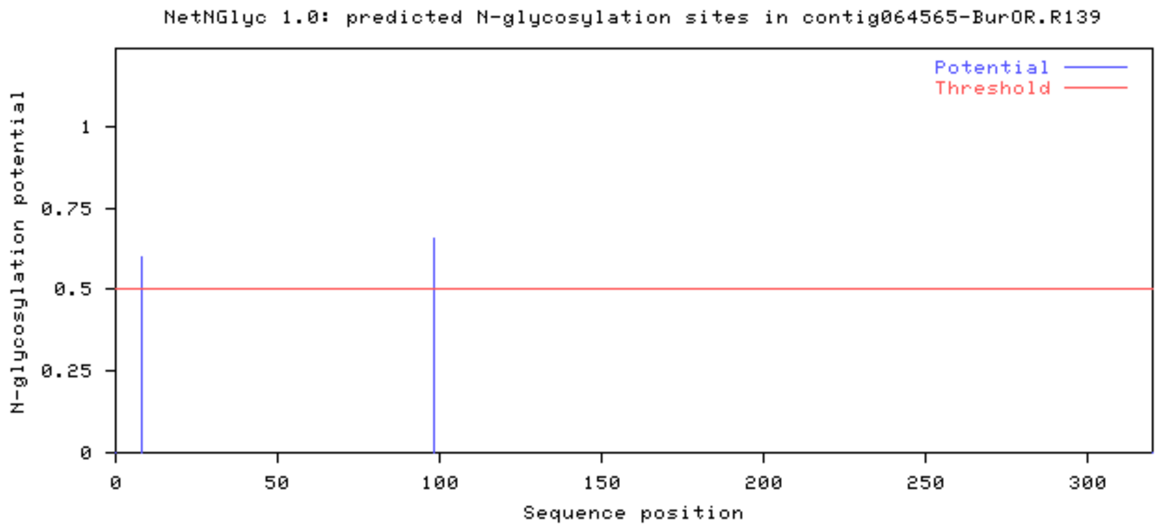

Graphics in PostScript

Output for 'contig064570-BurOR.A017'

```
##############################################################################

    Warning: This sequence may not contain a signal peptide!!

    Proteins without signal peptides are unlikely to be exposed to
    the N-glycosylation machinery and thus may not be glycosylated
    (in vivo) even though they contain potential motifs.

    SignalP-NN euk predictions are as follows:

  # name                   Cmax   pos ?  Ymax   pos ?  Smax   pos ?  Smean ?  D     ?

    SignalP output is explained at http://www.cbs.dtu.dk/services/SignalP/output.html

##############################################################################

Name:  contig064570-BurOR.A017  Length:  304
MDEELNVTYLTLDWYTEINKYRYVFFFVMFTLYILIICTNSTILYLIWIHKNLHEPMYIFIAALLLNSVLYSTTIYPKLL      80
IDFLSEKQVTTYSACLFQFFTFYTLACSEFLLLAAMAYDRYVAICKPLDYQTLMRKTTVGIFLVVAWLVPACHVAVLAIA     160
SAGAKLCDSNIKGIFCNNAVYTLQCERSRLITIFGVVALVDLSILPMLFIVFTYTKLFIVSHRSCKEIRKKAAETCLPHM     240
LVLISYSAFFVYDVSIARVKSDFPKTTRIIMTLQIMLYQPLLNPFIYGLKMKEISKHLNKLLSX
.....N.................................N........................................      80
................................................................................     160
................................................................................     240
................................................................                     320

(Threshold=0.5)
----------------------------------------------------------------------
SeqName      Position  Potential   Jury    N-Glyc
                                agreement  result
----------------------------------------------------------------------
contig064570-BurOR.A017    6 NVTY   0.7651     (9/9)    +++
contig064570-BurOR.A017   40 NSTI   0.6294     (8/9)    +
----------------------------------------------------------------------
```

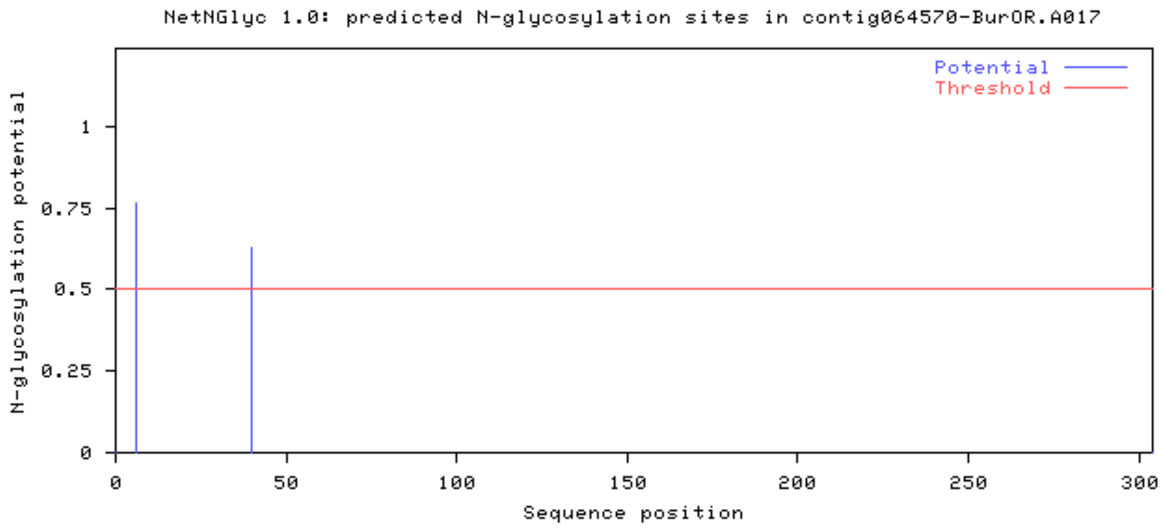

Graphics in PostScript

Output for 'contig064572-BurORe.L154'

```
##########################################################################

    Warning: This sequence may not contain a signal peptide!!

    Proteins without signal peptides are unlikely to be exposed to
    the N-glycosylation machinery and thus may not be glycosylated
    (in vivo) even though they contain potential motifs.

    SignalP-NN euk predictions are as follows:

  # name                Cmax  pos ?  Ymax  pos ?  Smax  pos ?  Smean ?  D     ?

    SignalP output is explained at http://www.cbs.dtu.dk/services/SignalP/output.html

##########################################################################

Name:  contig064572-BurORe.L154        Length:  57
VILTRFGVVLTLEERQGSLIGTILGPSLVNPFVYCLRTKEIKNKIIKIFRKVITAGX
.........................................................                       80

(Threshold=0.5)

No sites predicted in this sequence.
```

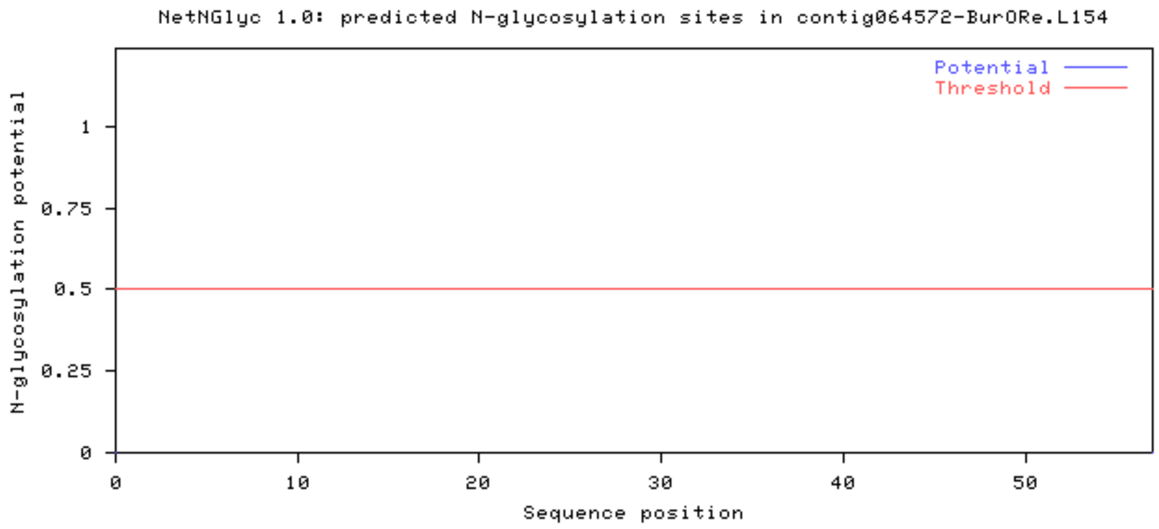

Graphics in PostScript

Output for 'contig064724-BurOR.E052'

```
##########################################################################

   Warning: This sequence may not contain a signal peptide!!

   Proteins without signal peptides are unlikely to be exposed to
   the N-glycosylation machinery and thus may not be glycosylated
   (in vivo) even though they contain potential motifs.

   SignalP-NN euk predictions are as follows:

 # name                 Cmax  pos ?  Ymax  pos ?  Smax  pos ?  Smean ?  D     ?

   SignalP output is explained at http://www.cbs.dtu.dk/services/SignalP/output.html

##########################################################################

Name:  contig064724-BurOR.E052  Length:  326
MEIIRNSTQFSYFTLGAYVDTQMFKYLYFMIILSLYVFTVGSNVLLIVVICVNRSLHEPMYMFLCSLFVNELYGSTGLFP      80
FLLLQILSDVHTVSAPLCFLQVFCIYLYASVEFSNLAAMSYDRYMSICYPLQYHTLMMSNKVALLIAVTWIPPLLAVCVT     160
TCLSASLQLCGNVINKVYCNNHSIIKLGCHGTTVNNIYELTAASVTVCVPLSVILYTYTRILKICFSGSKQTRQKAVSTC     240
TPHLASLLNFSFGVSFEILQSRFDMSHVPNMLRIFLSVYFLTCQPLFNPVMYGLNMSKIRTICKNLLLGYVGKSRILIKI     320
VQIEKX
.....N..............................................N...........................      80
................................................................................     160
................................................................................     240
......................................................N.........................     320
......                                                                               400

(Threshold=0.5)
----------------------------------------------------------------------
SeqName      Position  Potential   Jury    N-Glyc
                                 agreement result
----------------------------------------------------------------------
contig064724-BurOR.E052    6 NSTQ   0.7710     (9/9)    +++
contig064724-BurOR.E052   53 NRSL   0.7487     (9/9)    ++
contig064724-BurOR.E052  181 NHSI   0.3873     (9/9)    --
contig064724-BurOR.E052  249 NFSF   0.4452     (6/9)    -
contig064724-BurOR.E052  295 NMSK   0.6051     (6/9)    +
----------------------------------------------------------------------
```

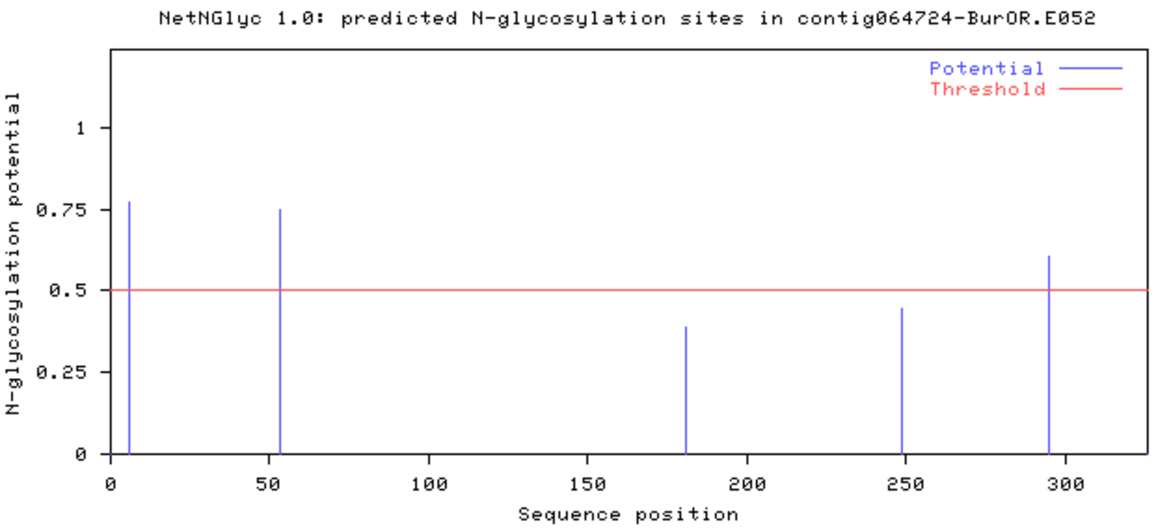

Graphics in PostScript

Output for 'contig064938-BurOR.E053'

```
##########################################################################################

    Warning: This sequence may not contain a signal peptide!!

    Proteins without signal peptides are unlikely to be exposed to
    the N-glycosylation machinery and thus may not be glycosylated
    (in vivo) even though they contain potential motifs.

    SignalP-NN euk predictions are as follows:

  # name                   Cmax  pos ?  Ymax  pos ?  Smax  pos ?  Smean ?  D     ?

    SignalP output is explained at http://www.cbs.dtu.dk/services/SignalP/output.html

##########################################################################################

Name:  contig064938-BurOR.E053  Length:  309
MTNSSQVSYFTLTAYLDSGALKYLYFTVVAFLYIVIVTVNVLLIVVICVNRSLHEPMYMFLCSLFVNELYGSTGLFPLLL      80
LQILSDVHTVSAPLCFLQIFCVHTYGTAELANLVVMSYDRYLAICFPLQYHTRMSPCKVSMLIVLTWFSSFLGITVLISL     160
SAPLQLCGNIINKVYCDNYSVVKLACSDTTVNNIYGLISTSLTTISAVSLILYTYTRILKVCFSGSKQTRQKAVSTCTPH     240
LVSLLNYSCSAFYEIAQSRLNMKHVPNMVRIFLSLYWLICSPLCNPLLYGLSLTKIRIIYKGLILSKVX
..N..............................................N..............................      80
................................................................................     160
.................N..............................................................     240
.....N...............................................................               320

(Threshold=0.5)
----------------------------------------------------------------------
SeqName      Position  Potential   Jury    N-Glyc
                                 agreement result
----------------------------------------------------------------------
contig064938-BurOR.E053    3 NSSQ    0.6908     (9/9)   ++
contig064938-BurOR.E053   50 NRSL    0.7489     (9/9)   ++
contig064938-BurOR.E053  178 NYSV    0.6158     (8/9)   +
contig064938-BurOR.E053  246 NYSC    0.6113     (8/9)   +
----------------------------------------------------------------------
```

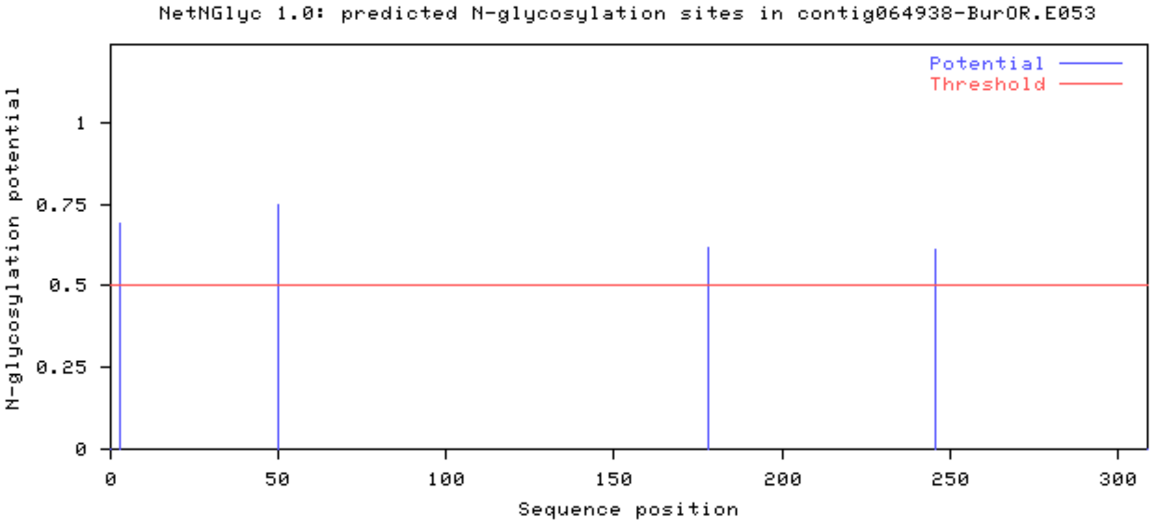

Graphics in PostScript

Output for 'contig065025-BurOR.N111'

```
##########################################################################################

    Warning: This sequence may not contain a signal peptide!!

    Proteins without signal peptides are unlikely to be exposed to
    the N-glycosylation machinery and thus may not be glycosylated
    (in vivo) even though they contain potential motifs.

    SignalP-NN euk predictions are as follows:

  # name                   Cmax  pos ?  Ymax  pos ?  Smax  pos ?  Smean ?  D     ?

    SignalP output is explained at http://www.cbs.dtu.dk/services/SignalP/output.html

##########################################################################################

Name:  contig065025-BurOR.N111  Length:  337
MGTEDKATTMFNNTFVRPEKFYLSGFSNIPHIRYFYAFLCLVYIMTVLGNGFLLSLISLVKTLHTPKYMIVFNMALTDLC      80
GSTALIPKLLDTFLFDRRYILYDACLSYMFFVMFFASVQSWTLVTMAYDRLIAICFPLRYHNIVTETSVAAILLFVWIFL     160
VSVITTMVRLVNRLSFCRSLVVNSFFCDHGPVYRLACNDTSLNYNMASALITILIIIPLIFIIATYVCIFIALSRTTSRK     240
EQIRALKTCTSHLILVVIFFLPIGITNIATRASYIHPNARMINSTLTHTIPALLDPIIYALKTEEVMNAVKKLCKRTYLN     320
CMKAKTRPCNHCCIKSX
...........N....................................................................      80
................................................................................     160
.....................................N..........................................     240
..........................................N.....................................     320
.................                                                                    400

(Threshold=0.5)
----------------------------------------------------------------------
SeqName      Position  Potential   Jury    N-Glyc
                                 agreement result
----------------------------------------------------------------------
contig065025-BurOR.N111   12 NNTF   0.5686     (6/9)   +
contig065025-BurOR.N111  198 NDTS   0.6408     (7/9)   +
contig065025-BurOR.N111  283 NSTL   0.5837     (7/9)   +
----------------------------------------------------------------------
```

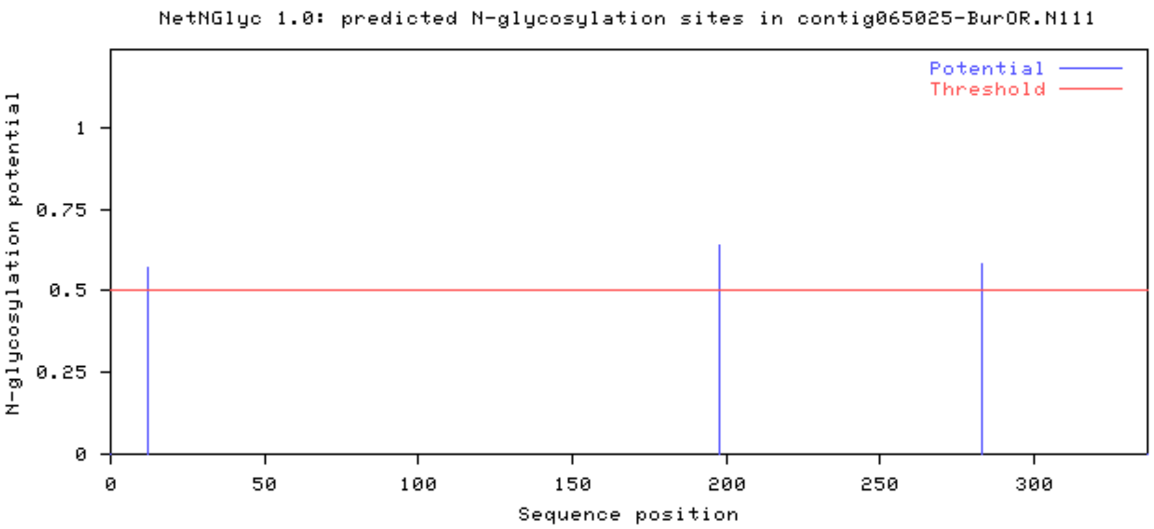

Graphics in PostScript

Output for 'contig065027-BurOR.L090'

```
##########################################################################################

    Warning: This sequence may not contain a signal peptide!!

    Proteins without signal peptides are unlikely to be exposed to
    the N-glycosylation machinery and thus may not be glycosylated
    (in vivo) even though they contain potential motifs.

    SignalP-NN euk predictions are as follows:

  # name                    Cmax  pos ?  Ymax  pos ?  Smax  pos ?  Smean ?  D     ?

    SignalP output is explained at http://www.cbs.dtu.dk/services/SignalP/output.html

##########################################################################################

Name:  contig065027-BurOR.L090  Length:  315
MLLQNTVLSITVKHFIIGGFDTVKRPVAVGVVMLIIYILAIFANMVNILFIIFDKRLHKPMYLLVCNLAVVDIMYTSSAT      80
PTMIGVLLADVKTISYVDCLIQMCVFHLAMVMERFALAIMAFDRLIAIIFPFHYHSYLTNTRTVVLTYILWIIGCGTVVL     160
FPATVIPLPHCTLKLKYTFCDYASIMRTTCVNVDEYFNQSAIWSFFISFFTFTFICISYCGILFCVKLSSNNDKKKMGST     240
VVSHAICVTCFYSPIFIIVILTRVGVVLSLDERQGLLIGNILGPSLVNPFVYCLRTTEIKNKMVKIFKKILRFLX
................................................................................      80
................................................................................     160
................................................................................     240
...........................................................................          320

(Threshold=0.5)

No sites predicted in this sequence.
```

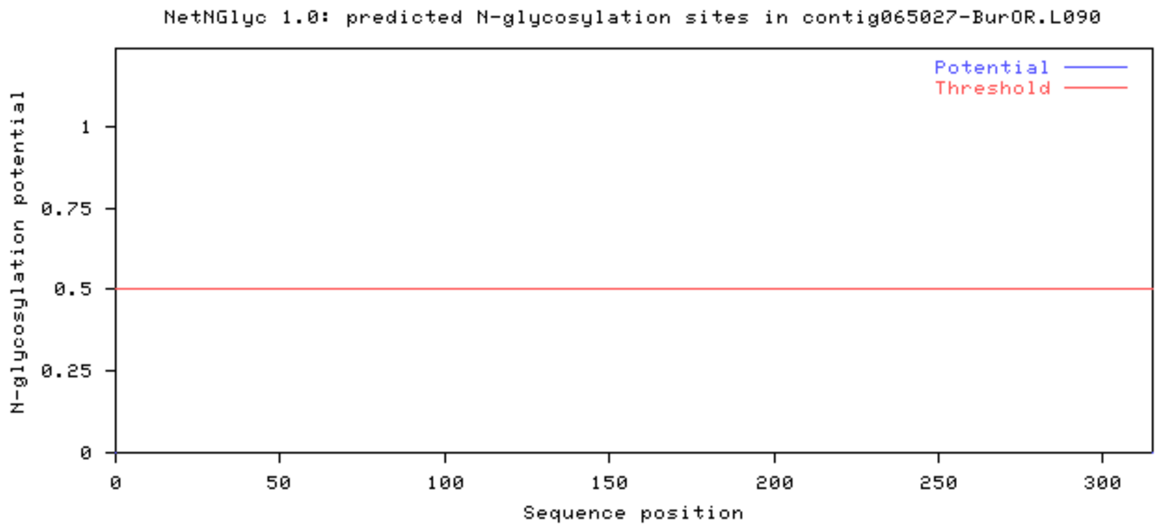

Graphics in PostScript

Output for 'contig065133-BurORe.K085'

```
##########################################################################

    Warning: This sequence may not contain a signal peptide!!

    Proteins without signal peptides are unlikely to be exposed to
    the N-glycosylation machinery and thus may not be glycosylated
    (in vivo) even though they contain potential motifs.

    SignalP-NN euk predictions are as follows:

  # name                   Cmax   pos ?  Ymax   pos ?  Smax   pos ?  Smean ?  D     ?

    SignalP output is explained at http://www.cbs.dtu.dk/services/SignalP/output.html

##########################################################################

Name:  contig065133-BurORe.K085         Length:  138
PYCDNAALFNLSCEDVFINNVYGLTFTVLLFTGSIGSMVLTYTKITVVCLTTKNKSLNNKALKTCSTHVVVYLIFLFSGM      80
SIITLHRFPEYSGSRKIVAVLYHIIPGSLNPIIYGMQSKEIKKFVSNVKLKKVLPLYX
.........N...........................................N..........................      80
..........................................................                           160

(Threshold=0.5)
----------------------------------------------------------------------
SeqName      Position  Potential   Jury    N-Glyc
                                 agreement result
----------------------------------------------------------------------
contig065133-BurORe.K085    10 NLSC    0.7098     (9/9)    ++
contig065133-BurORe.K085    54 NKSL    0.5459     (6/9)    +
----------------------------------------------------------------------
```

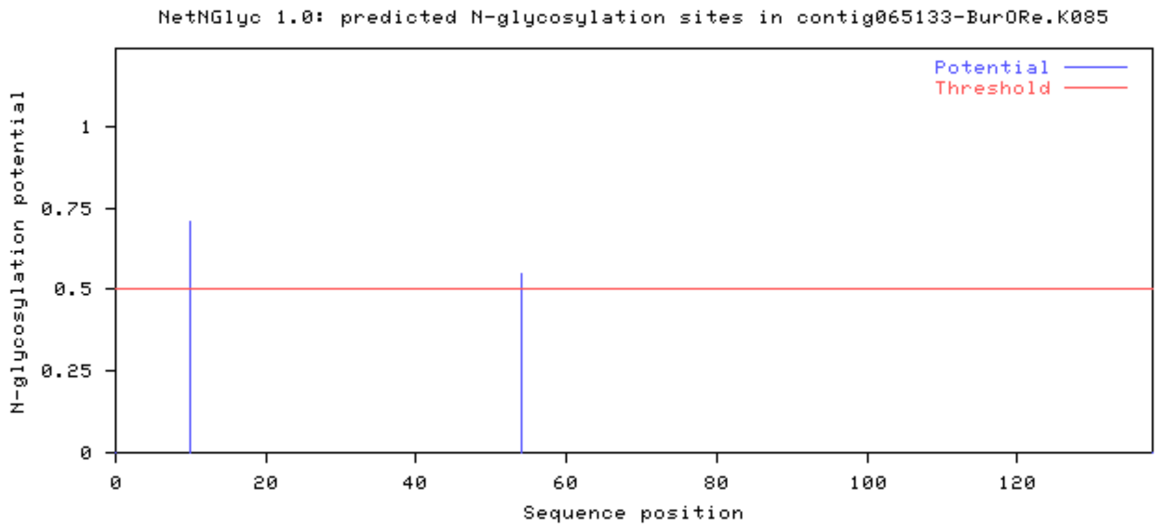

Graphics in PostScript

Output for 'contig065359-BurORp.L097'

```
##############################################################################

    Warning: This sequence may not contain a signal peptide!!

    Proteins without signal peptides are unlikely to be exposed to
    the N-glycosylation machinery and thus may not be glycosylated
    (in vivo) even though they contain potential motifs.

    SignalP-NN euk predictions are as follows:

  # name                    Cmax  pos ?  Ymax  pos ?  Smax  pos ?  Smean ?  D     ?

    SignalP output is explained at http://www.cbs.dtu.dk/services/SignalP/output.html

##############################################################################

Name:  contig065359-BurORp.L097         Length:  305
MSSQNTSINVTHFIIGGFDTLSRPIAVGVVILITYLLAVLANMANIMFIISDKRLHKPMYLLICNLAVVDIMYTSSCSPT      80
MIGVLLAGVNTISVFHLGTSMESFVLAVMALDRFIAIIYPFQYHSYLTNTRVLVLTFIVWFVNWFFMCYIPATVVPLPHC     160
SSRLRYTFCDFAAELRTTCVNPDKYFNEVAIVAFFILFFTFIFICLSYCGILLFVKLSSNNEKKKIGSTLVSHLICVIVH     240
YCPAFVRIIFTRFGVVLTLEERQGLLIGAVLGPCLVNPFVYCLRTKEIKQKKFYKILKMVNTYDX
....N...N.......................................................................      80
................................................................................     160
................................................................................     240
.................................................................                    320

(Threshold=0.5)
----------------------------------------------------------------------
SeqName      Position  Potential   Jury    N-Glyc
                                 agreement result
----------------------------------------------------------------------
contig065359-BurORp.L097    5 NTSI   0.6202     (7/9)   +
contig065359-BurORp.L097    9 NVTH   0.7423     (9/9)   ++
----------------------------------------------------------------------
```

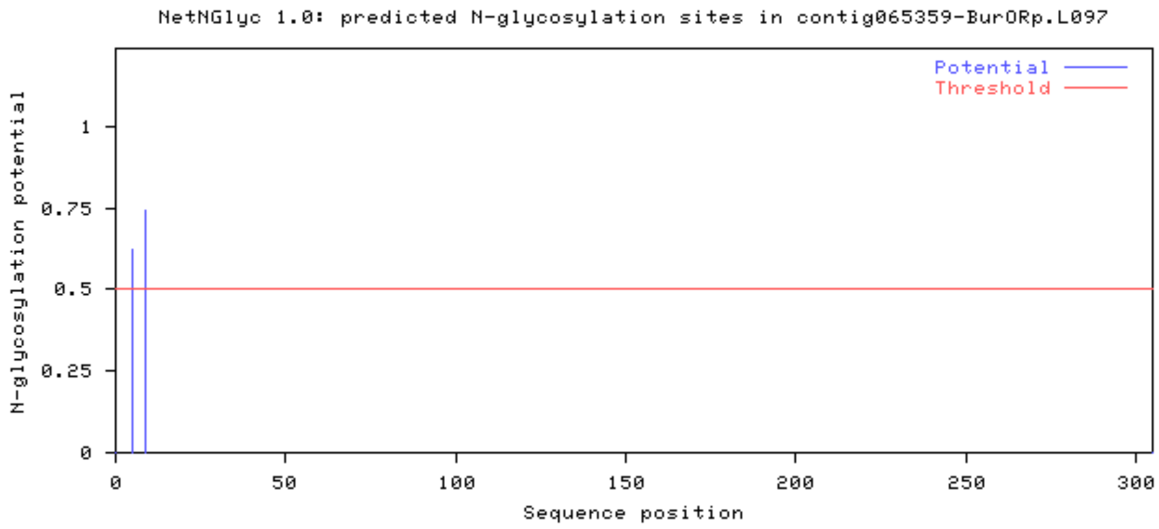

Graphics in PostScript

Output for 'contig065887-BurOR.A018'

```
##########################################################################################

    Warning: This sequence may not contain a signal peptide!!

    Proteins without signal peptides are unlikely to be exposed to
    the N-glycosylation machinery and thus may not be glycosylated
    (in vivo) even though they contain potential motifs.

    SignalP-NN euk predictions are as follows:

  # name                Cmax  pos ?  Ymax  pos ?  Smax  pos ?  Smean ?  D     ?

    SignalP output is explained at http://www.cbs.dtu.dk/services/SignalP/output.html

##########################################################################################

Name:  contig065887-BurOR.A018  Length:  309
MDDELNITHITIDGYVDLKRFGYLYFLIMVALYVLIIISNSVIVFLICIHHNLHEPMYIFIAALSVNSVLLSTVTYPKLF      80
VDVLSEKQIISISACRFQHFMCYSIAGSDFLLLSAMAFDRYVSICKPLKYPVIMRQTTINTLLFLSWFVPGLQIAVLHTL     160
VLNNKLCNFTLKGILCNNSLWKLYCESPRATLIYGLVVMLSVVIFPVFFILFTYAKIFLITYRSSRAIQKKAAETCLPHL     240
FVLSIFTTLCAYDVIIGRLELDFPKTAQLIMTLQVIFYNPLLNPFIYGLKMKEISKHLKRLFCHVRCSX
.....N..........................................................................      80
................................................................................     160
.......N........................................................................     240
.....................................................................                320

(Threshold=0.5)
----------------------------------------------------------------------
SeqName      Position  Potential   Jury    N-Glyc
                                 agreement result
----------------------------------------------------------------------
contig065887-BurOR.A018    6 NITH   0.7698     (9/9)    +++
contig065887-BurOR.A018  168 NFTL   0.6758     (8/9)    +
contig065887-BurOR.A018  177 NNSL   0.4502     (6/9)    -
----------------------------------------------------------------------
```

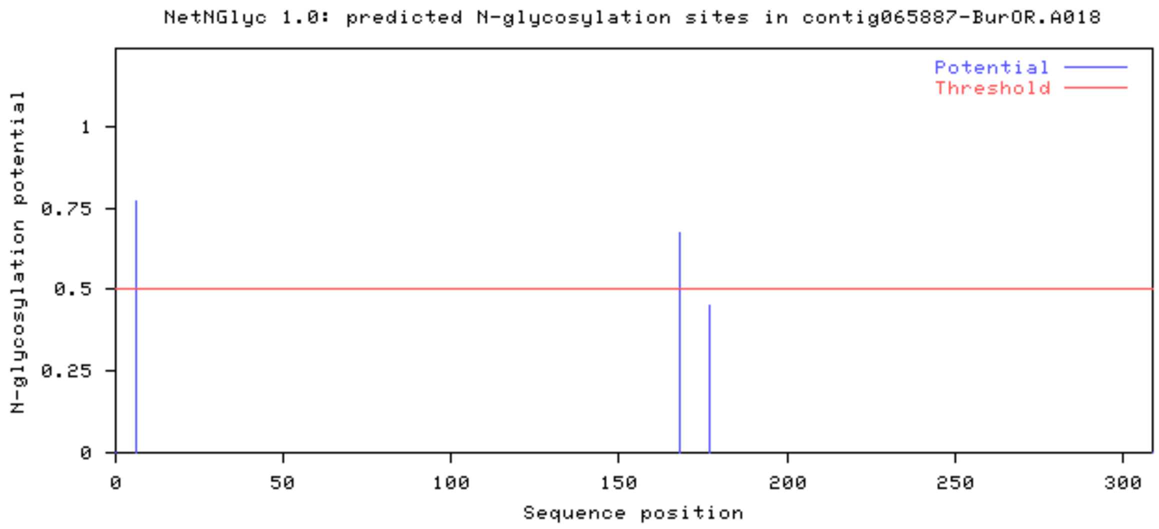

Graphics in PostScript

Output for 'contig066194-BurOR.E054'

```
##########################################################################

   Warning: This sequence may not contain a signal peptide!!

   Proteins without signal peptides are unlikely to be exposed to
   the N-glycosylation machinery and thus may not be glycosylated
   (in vivo) even though they contain potential motifs.

   SignalP-NN euk predictions are as follows:

 # name                Cmax  pos ?  Ymax  pos ?  Smax  pos ?  Smean ?  D     ?

   SignalP output is explained at http://www.cbs.dtu.dk/services/SignalP/output.html

##########################################################################

Name:  contig066194-BurOR.E054  Length:  331
MMINTTEITHFTLSPYFDTGAYGYLYFLIILTSYAAIICANLLLIVVICVNRSLHEPMYMFLCSLFVNELYGSTGLFPFL      80
LLQILSDVHTVSVSFCFLQVFCVYTYACVEFINFVVMSYDRYYAICWPLQYKSCMTLKTVTTLISLTWLLPFIMIVVLIS     160
LSAPLQLCGNVINKVFCGNYAIIKLACSDTRVHNIFGLIYTFISVIIPLVLILYTYVRILKVCFSGSKQTRQKAVSTCTP     240
HLASILNFFFGCCFQILQSRFDTSGVPNVFGILSSLYFLTCQPLFTPLLYGLKMTKIRIACKQLFCGSLTRLFSCSSDIF     320
SKSHQSQSYCX
...N..............................................N.............................      80
................................................................................     160
................................................................................     240
................................................................................     320
...........                                                                           400

(Threshold=0.5)
----------------------------------------------------------------------
SeqName           Position  Potential   Jury     N-Glyc
                                      agreement  result
----------------------------------------------------------------------
contig066194-BurOR.E054    4 NTTE    0.7190     (9/9)    ++
contig066194-BurOR.E054   51 NRSL    0.7488     (9/9)    ++
----------------------------------------------------------------------
```

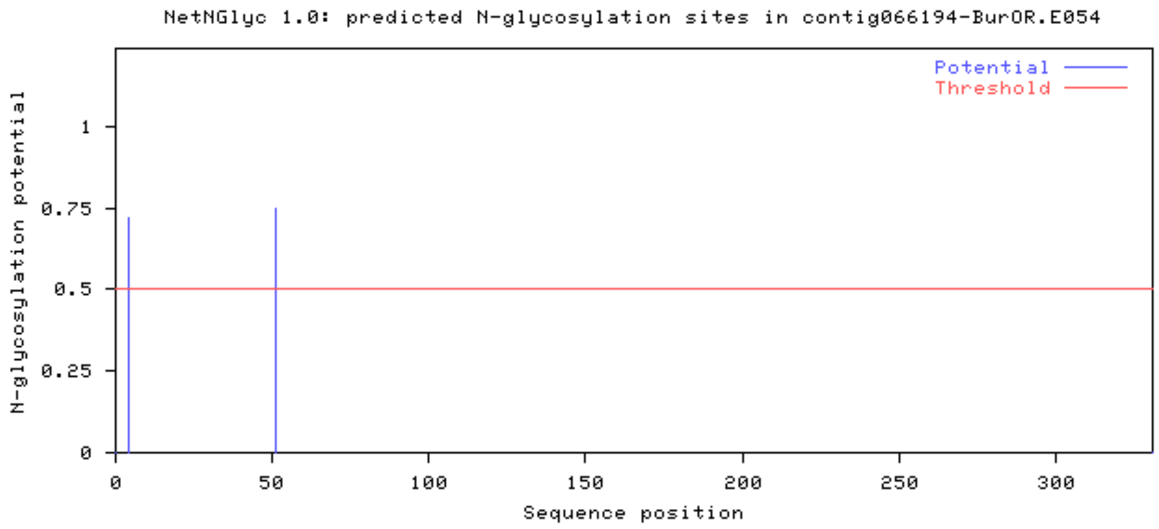

[Graphics in PostScript]

Output for 'contig066785-BurORs.W148'

```
##########################################################################

   Warning: This sequence may not contain a signal peptide!!

   Proteins without signal peptides are unlikely to be exposed to
   the N-glycosylation machinery and thus may not be glycosylated
   (in vivo) even though they contain potential motifs.

   SignalP-NN euk predictions are as follows:

 # name                 Cmax  pos ?  Ymax  pos ?  Smax  pos ?  Smean ?  D     ?

   SignalP output is explained at http://www.cbs.dtu.dk/services/SignalP/output.html

##########################################################################

Name:  contig066785-BurORs.W148         Length:  336
MNLSSVNFSSTLTSYEENPLLTNYIVAAALCITINYINGTLIHTFRKYQVMCGFTVFTLQILHSNPRYILFIHLVINDMM      80
WLFLSTLLLIISYALRIFFVPYCIILLIISIIVSHNSPLNLAGMSIECYIAVCMPLRHGQICTVKKTYILIGLIWAASAL     160
SILPDLFILLATEPLQFFHSKVRCVRDFVFRSTYSLNKRDASHIVCLVVVWLTLIYTYARIMFAAKGLTGDIKKARNTIL     240
LHGFQVLLLMLNYVRNIFERSLFFLFPNRESQIRFASFVIVQIIPRFISPIVYGLRDQTFRKYVRKYLVCSERGITHPEN     320
TTTKKASLNVTSATVX
.N....N..............................N..........................................      80
................................................................................     160
................................................................................     240
................................................................................     320
........N.......                                                                     400

(Threshold=0.5)
----------------------------------------------------------------------
SeqName      Position  Potential    Jury    N-Glyc
                                 agreement  result
----------------------------------------------------------------------
contig066785-BurORs.W148     2 NLSS   0.7897     (9/9)   +++
contig066785-BurORs.W148     7 NFSS   0.7263     (9/9)   ++
contig066785-BurORs.W148    38 NGTL   0.7059     (9/9)   ++
contig066785-BurORs.W148   320 NTTT   0.4568     (6/9)   -
contig066785-BurORs.W148   329 NVTS   0.6237     (8/9)   +
----------------------------------------------------------------------
```

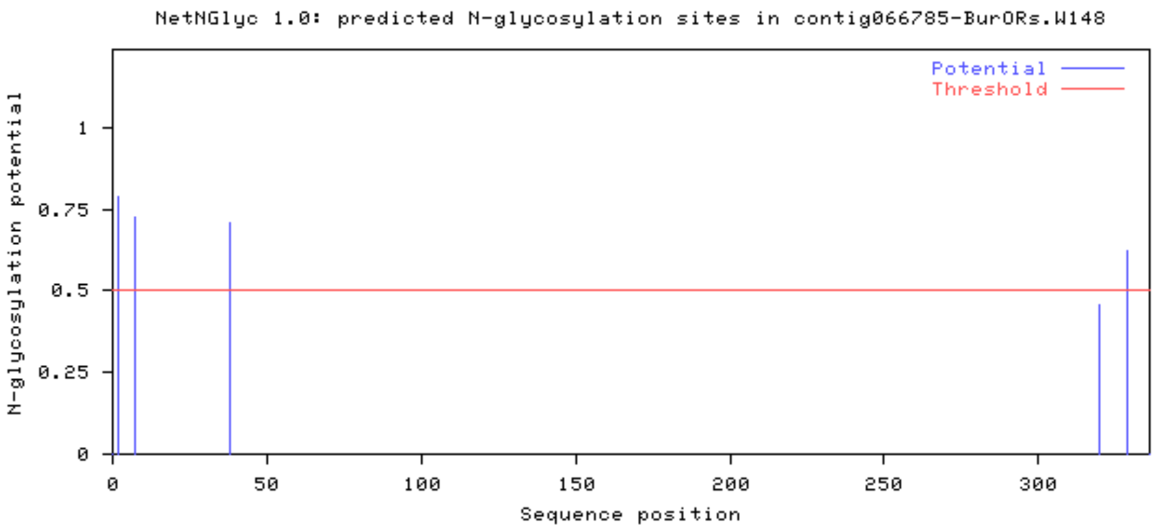

Graphics in PostScript

Output for 'contig066797-BurORe.N114'

```
##########################################################################################

    Warning: This sequence may not contain a signal peptide!!

    Proteins without signal peptides are unlikely to be exposed to
    the N-glycosylation machinery and thus may not be glycosylated
    (in vivo) even though they contain potential motifs.

    SignalP-NN euk predictions are as follows:

  # name                 Cmax  pos ?  Ymax  pos ?  Smax  pos ?  Smean ?  D     ?

    SignalP output is explained at http://www.cbs.dtu.dk/services/SignalP/output.html

##########################################################################################

Name:  contig066797-BurORe.N114         Length:  104
IVGSYCCIGYSLSKTVTCRERLKALKTCTSHLSLVAIYFLPALFIFTFGSTILPNARTVSLSLATVMPLTLNPIIYGLQT      80
QEIKQSLKKLLKVKMQFSISAKKX
................................................................................      80
........................                                                             160

(Threshold=0.5)

No sites predicted in this sequence.
```

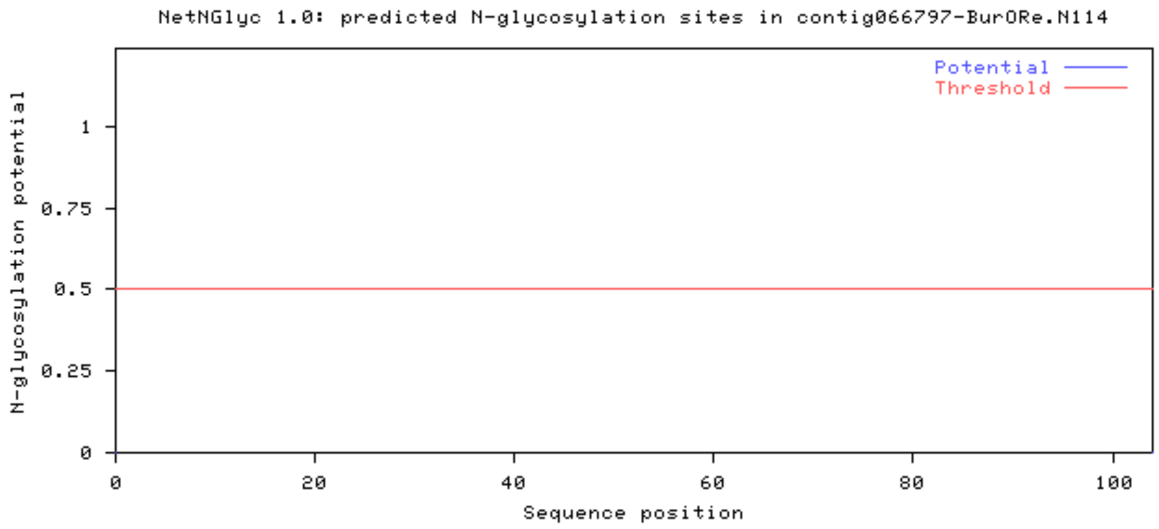

Graphics in PostScript

Output for 'contig066810-BurORe.D041'

```
##########################################################################################

    Warning: This sequence may not contain a signal peptide!!

    Proteins without signal peptides are unlikely to be exposed to
    the N-glycosylation machinery and thus may not be glycosylated
    (in vivo) even though they contain potential motifs.

    SignalP-NN euk predictions are as follows:

  # name                Cmax  pos ?  Ymax  pos ?  Smax  pos ?  Smean ?  D     ?

    SignalP output is explained at http://www.cbs.dtu.dk/services/SignalP/output.html

##########################################################################################

Name:  contig066810-BurORe.D041         Length:  239
ASTSLYPLLLSQMFSDSHEVTVPWCFLQMCCLYTSAPAEFCSLAAMAYDRYISICHPLRYNVIMNTERVFLFILLVWLYS      80
FLSFIFSYSFIFSLKFCGNIIHNAYCDHQLIIRLSCSVPIQSFISNISFLLLSVFIPFSLISVSYMKILRVCRKTSKENK     160
QKAVTTCTPQIICVSNLFVGCIFYFIDFKFLVSQVLDEVRIILPMYPLIFQPMLTPFMYGFKLPKIRQSYQRFLFERKX
................................................................................      80
................................................................................     160
...............................................................................      240

(Threshold=0.5)

No sites predicted in this sequence.
```

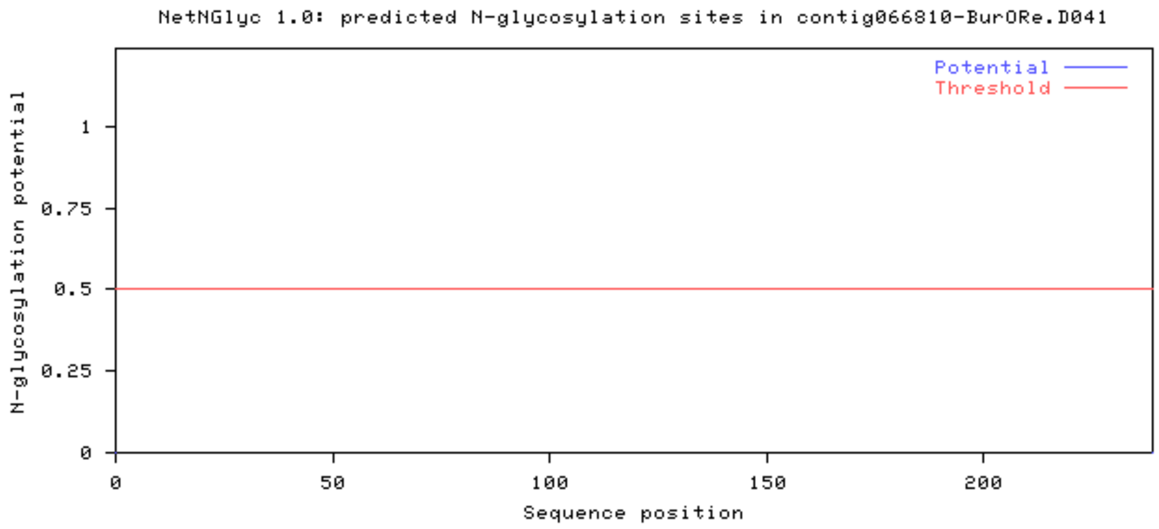

Graphics in PostScript

Output for 'contig067170-BurORe.A145'

```
##########################################################################################

    Warning: This sequence may not contain a signal peptide!!

    Proteins without signal peptides are unlikely to be exposed to
    the N-glycosylation machinery and thus may not be glycosylated
    (in vivo) even though they contain potential motifs.

    SignalP-NN euk predictions are as follows:

  # name                    Cmax   pos ?  Ymax   pos ?  Smax   pos ?  Smean ?  D      ?

    SignalP output is explained at http://www.cbs.dtu.dk/services/SignalP/output.html

##########################################################################################

Name:  contig067170-BurORe.A145         Length:  156
SAEAKLCDSNIKGIFCNNAVYTLQCQRSRLIIIFGVIALLDLVILPMLFIVFTYTTIFIVSYQSCKEIRKKAAETCLPHL      80
LVLISACLFFVYDVSIARVEADFPKTARIVMTLQIVLYHPLFNPFVYGLKMKEISKHLKGLLCQGKITSCIKTGSX
................................................................................      80
............................................................................         160

(Threshold=0.5)

No sites predicted in this sequence.
```

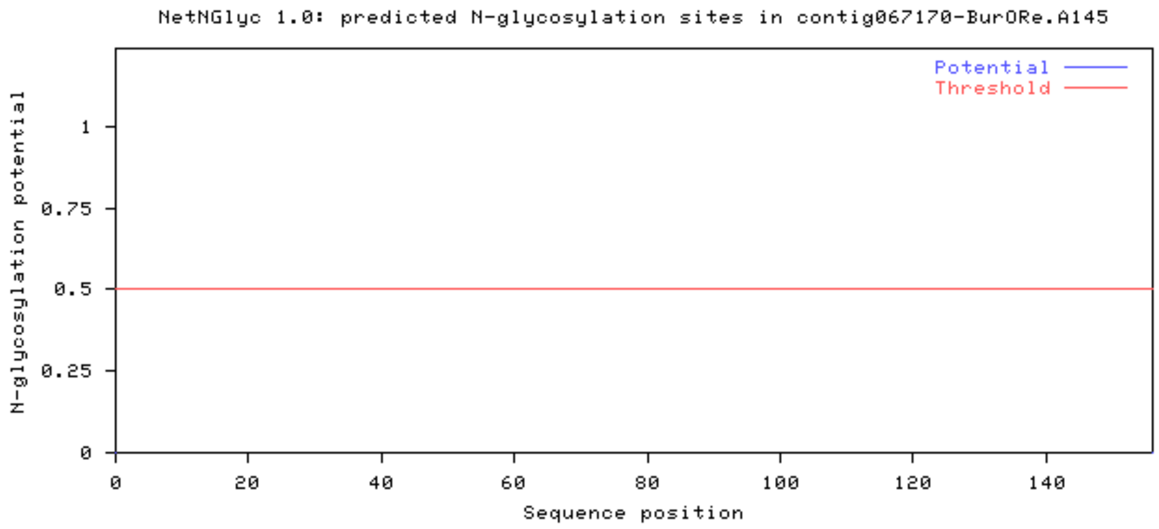

Graphics in PostScript

Output for 'contig067209-BurOR.R140'

```
##########################################################################

    Warning: This sequence may not contain a signal peptide!!

    Proteins without signal peptides are unlikely to be exposed to
    the N-glycosylation machinery and thus may not be glycosylated
    (in vivo) even though they contain potential motifs.

    SignalP-NN euk predictions are as follows:

  # name                   Cmax  pos ?  Ymax  pos ?  Smax  pos ?  Smean ?  D     ?

    SignalP output is explained at http://www.cbs.dtu.dk/services/SignalP/output.html

##########################################################################

Name:  contig067209-BurOR.R140  Length:  328
MLLANLSLTNVSANQQYQGVLERVLFSTLTTLPCCLFLCINGIMLFTLRSKALFCETSRYILLYNLLFADTVQMALSQLL      80
YIIAACRITLTYPVCGFLTMLANLTTVVSPVTLVVMSLERYVAVCYPLRHASIITITNTGVAIIVIWAIGSLNILTRVLL     160
LLEFPFEALDSLQMKDFCSEIAMSGGSMSDDYDKAFTCALFISASVAITCSYIGVIVAARSASTDKASARKACNTLLLHL     240
VQLGLSLSSTICNPLLTALARVLTRIVFVRIHNVFYVCIFLFPRCLSSLIYGIRDQSIRPVLIYYLCCRLKYSVIQPRLK     320
AAIEVECX
....N....N......................................................................      80
......................N.........................................................     160
................................................................................     240
................................................................................     320
........                                                                             400

(Threshold=0.5)
----------------------------------------------------------------------
SeqName      Position  Potential   Jury    N-Glyc
                                 agreement result
----------------------------------------------------------------------
contig067209-BurOR.R140     5 NLSL   0.7997     (9/9)   +++
contig067209-BurOR.R140    10 NVSA   0.6986     (9/9)   ++
contig067209-BurOR.R140   103 NLTT   0.7330     (9/9)   ++
----------------------------------------------------------------------
```

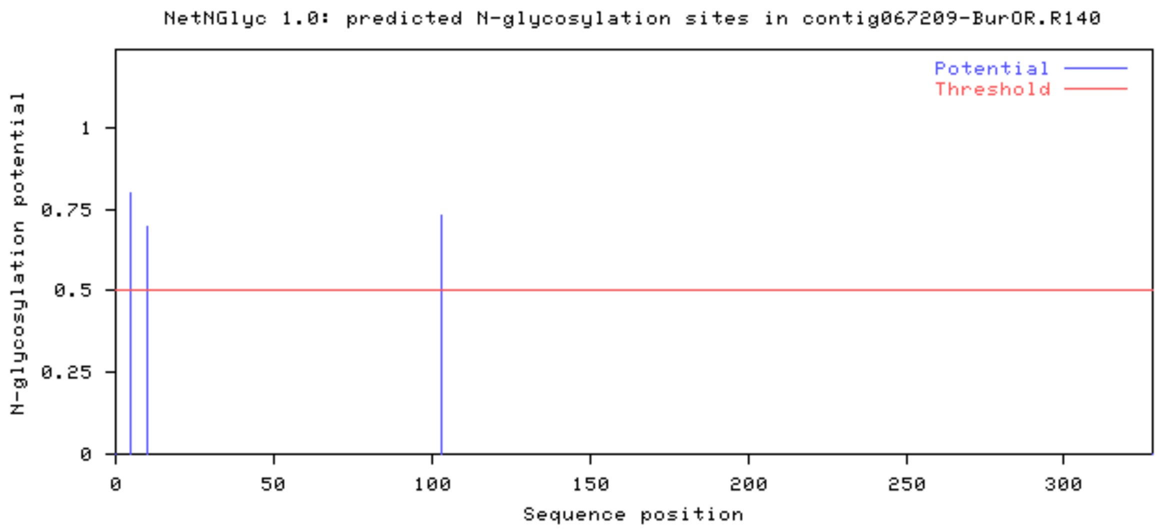

Graphics in PostScript

Output for 'contig067227-BurORe.N150'

```
##########################################################################################

    Warning: This sequence may not contain a signal peptide!!

    Proteins without signal peptides are unlikely to be exposed to
    the N-glycosylation machinery and thus may not be glycosylated
    (in vivo) even though they contain potential motifs.

    SignalP-NN euk predictions are as follows:

  # name                 Cmax  pos ?  Ymax  pos ?  Smax  pos ?  Smean ?  D     ?

    SignalP output is explained at http://www.cbs.dtu.dk/services/SignalP/output.html

##########################################################################################

Name:  contig067227-BurORe.N150        Length:  58
MDLFNSALGKNITFLRPAFFIISGFIGIPNIKYYYAFLFFVYIISVLANTAVMAAIYL
..........N...............................................                          80

(Threshold=0.5)
----------------------------------------------------------------------
SeqName      Position  Potential   Jury    N-Glyc
                                 agreement result
----------------------------------------------------------------------
contig067227-BurORe.N150    11 NITF   0.5146     (5/9)    +
----------------------------------------------------------------------
```

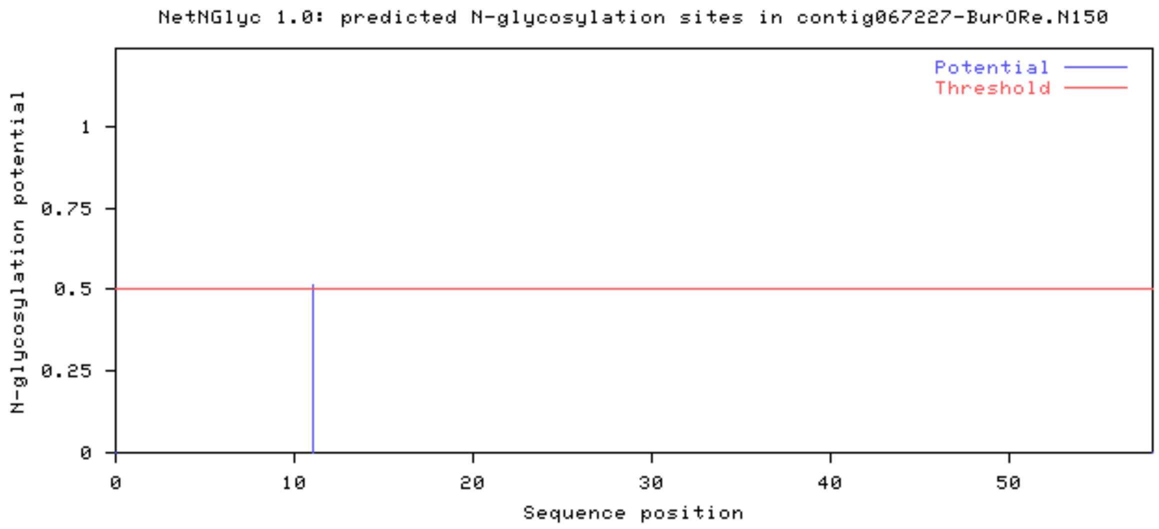

Graphics in PostScript

Explain the output. Go back.

NetNGlyc 1.0 Server - prediction results

Technical University of Denmark

Asn-Xaa-Ser/Thr sequons in the sequence output below are highlighted in **blue**.
Asparagines predicted to be N-glycosylated are highlighted in **red**.

Output for 'contig004252-BriORe.E041'

```
##########################################################################################

    Warning: This sequence may not contain a signal peptide!!

    Proteins without signal peptides are unlikely to be exposed to
    the N-glycosylation machinery and thus may not be glycosylated
    (in vivo) even though they contain potential motifs.

    SignalP-NN euk predictions are as follows:

  # name                   Cmax  pos ?  Ymax  pos ?  Smax  pos ?  Smean ?  D     ?

    SignalP output is explained at http://www.cbs.dtu.dk/services/SignalP/output.html

##########################################################################################

Name:  contig004252-BriORe.E041         Length:  162
SLNLRLRLCGNFLNSLYCQNYPVVRLACSSTKVNNVYGIFDVIMTVIVPTLIILFSYMKILKVCFYGSKQMRQKSLTTCT      80
PQLVSLLNFSFGCCFELFQSRFDTTGLPAALRIFLSLYFLMMQPLMNPILYGTQMSKIRGVYEHVLSSIMSCGCNKVSQS     160
DX
................................................................................      80
................................................................................     160
..                                                                                   240

(Threshold=0.5)

No sites predicted in this sequence.
```

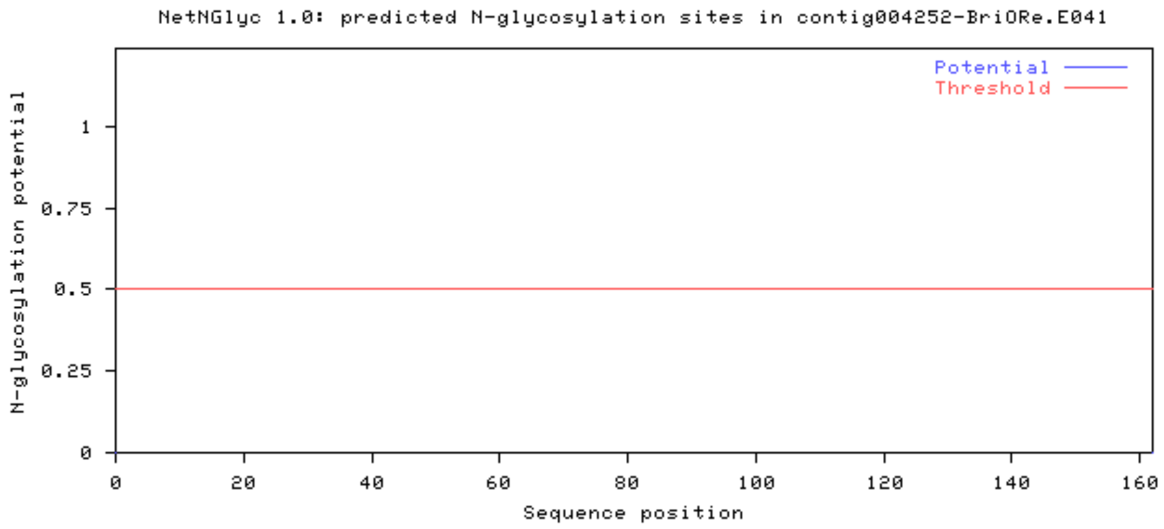

Graphics in PostScript

Output for 'contig004255-BriOR.E036'

```
##########################################################################################

    Warning: This sequence may not contain a signal peptide!!

    Proteins without signal peptides are unlikely to be exposed to
    the N-glycosylation machinery and thus may not be glycosylated
    (in vivo) even though they contain potential motifs.

    SignalP-NN euk predictions are as follows:

  # name                    Cmax   pos ?  Ymax   pos ?  Smax   pos ?  Smean ?  D     ?

    SignalP output is explained at http://www.cbs.dtu.dk/services/SignalP/output.html

##########################################################################################

Name:  contig004255-BriOR.E036  Length:  309
MTNLSQVSYFTFSAYFDTGPFKYLYFTIVMSLYVFIFGSNLLLIVVICVNRSLHEPMYMFLCSLFVNELYGSTGLFPFLL      80
LQILSDVHTVSAPLCFLQIFCLYTYANVEFYNLAIMSYDRYLAICYPLQYHTHMTFNKVAKLIVLTWLFPILNIVVMISL     160
NASLQLCGHTVDALYCNNYSVVKLACFGTTINNIYGLVYTFTVIIGLALLNLFTYVKILKVCFSESKQTRQKAVSTCTPH     240
LASLLNFSFGCFFEIVQSRFNMSRAPMILHIFLSIYFLTCQPVFNPVLYGLKLTKIRLICKSLLFGKIX
..N...............................................N..............................      80
................................................................................     160
N................N...............................................................     240
....................N................................................                320

(Threshold=0.5)
----------------------------------------------------------------------
SeqName      Position  Potential   Jury    N-Glyc
                                agreement result
----------------------------------------------------------------------
contig004255-BriOR.E036    3 NLSQ   0.7482     (9/9)    ++
contig004255-BriOR.E036   50 NRSL   0.7486     (9/9)    ++
contig004255-BriOR.E036  161 NASL   0.6232     (7/9)    +
contig004255-BriOR.E036  178 NYSV   0.6383     (9/9)    ++
contig004255-BriOR.E036  246 NFSF   0.4718     (7/9)    -
contig004255-BriOR.E036  261 NMSR   0.5339     (6/9)    +
----------------------------------------------------------------------
```

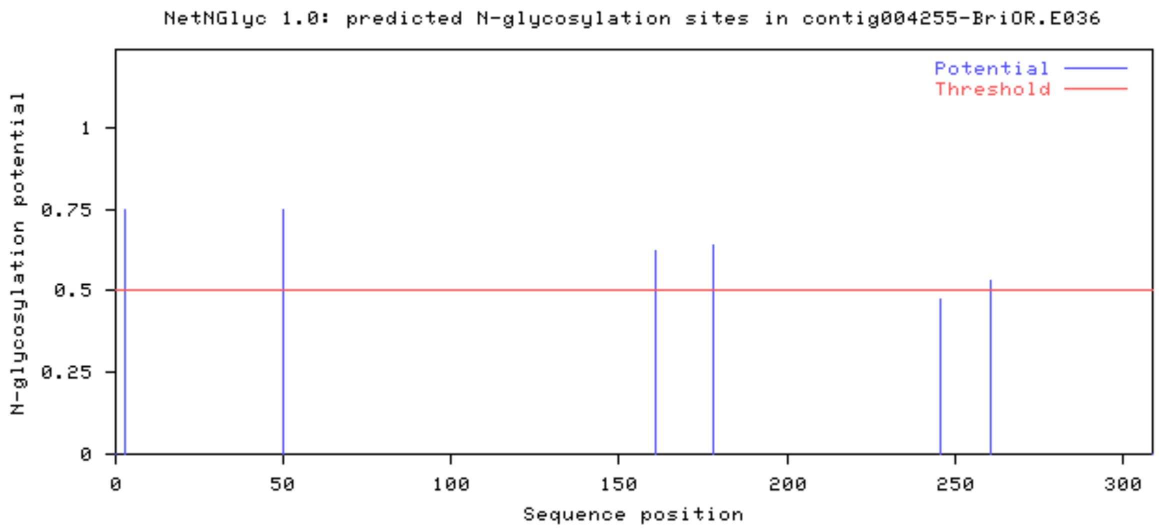

Graphics in PostScript

Output for 'contig004258-BriOR.E037'

```
##########################################################################################

    Warning: This sequence may not contain a signal peptide!!

    Proteins without signal peptides are unlikely to be exposed to
    the N-glycosylation machinery and thus may not be glycosylated
    (in vivo) even though they contain potential motifs.

    SignalP-NN euk predictions are as follows:

  # name                 Cmax  pos ?  Ymax  pos ?  Smax  pos ?  Smean ?  D     ?

    SignalP output is explained at http://www.cbs.dtu.dk/services/SignalP/output.html

##########################################################################################

Name:  contig004258-BriOR.E037  Length:  321
MMINTTQVSFFTLAGYFDTGRVTYLCFIVILALYIFIVGSNVLLIVVICVNRSLHEPMYMFLCSLFVNELYGSTGLFPFL      80
LLQILSDVHTVSASFCFLQIFCVYAYGSIEFSNLAVISYDRYLAICCPLRYHTCMSSSKVSVLIALTWLLTFFAISVLIS     160
LSSHLQLCGNIINKVYCDNYSIVKLACSDTTANNIYGLFYTFTLVLLVTLIFYTYIRILKVCFSGSKQTRHKAVSTCTPH     240
LASLLNFSCGAFFEIIQNRFDMRQLPNMLRIFLSIYWLTCQPLFNPVIYGLNLTKIRVSCKNVIRRMKVFTLCHKSNFKI     320
X
...N.............................................N..............................      80
................................................................................     160
..................N.............................................................     240
.....N.............................................N............................     320
.                                                                                    400

(Threshold=0.5)
----------------------------------------------------------------------
SeqName      Position  Potential   Jury    N-Glyc
                                 agreement result
----------------------------------------------------------------------
contig004258-BriOR.E037    4 NTTQ   0.7372     (9/9)   ++
contig004258-BriOR.E037   51 NRSL   0.7493     (9/9)   ++
contig004258-BriOR.E037  179 NYSI   0.5897     (8/9)   +
contig004258-BriOR.E037  246 NFSC   0.5502     (6/9)   +
contig004258-BriOR.E037  292 NLTK   0.6828     (9/9)   ++
```

```
----------------------------------------------------------------------
```

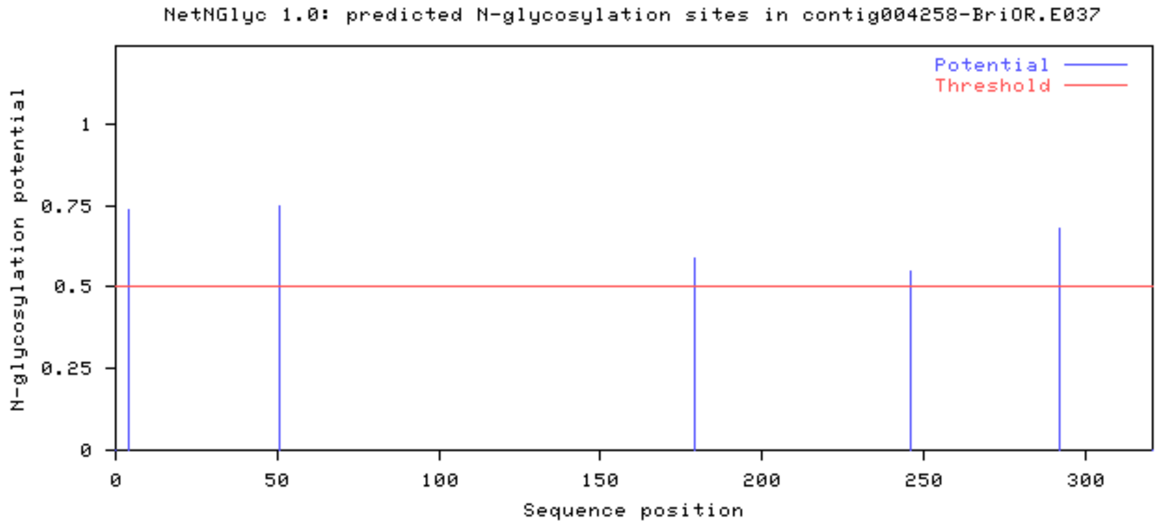

Graphics in PostScript

Output for 'contig004259-BriOR.E038'

```
##########################################################################################

    Warning: This sequence may not contain a signal peptide!!

    Proteins without signal peptides are unlikely to be exposed to
    the N-glycosylation machinery and thus may not be glycosylated
    (in vivo) even though they contain potential motifs.

    SignalP-NN euk predictions are as follows:

  # name                    Cmax  pos ?  Ymax  pos ?  Smax  pos ?  Smean ?  D     ?

    SignalP output is explained at http://www.cbs.dtu.dk/services/SignalP/output.html

##########################################################################################

Name:  contig004259-BriOR.E038  Length:  310
MTNSTQFSHFRLAFLNIGIFKYLFFMLVMCFFISVVCTNVLLIVVICVNRSLHEPMYMFLCSLFVNALYGSTSLIPLLLL      80
HIICDINIISASLCYLQIYCIHCYGSAEYLNLAVMSYDRYLAICFPLQYNTYMTPKRIAILIAITWLYAILACALMISLS     160
STLPLCGNIIDKVYCDNYSVIKLACSGTKALNIYGIIIVLCTICCPLIFMLYTYIKILRVCSSGSKQVRQKAVTTCSPHL     240
ACVLNFSCGACFEILQSRFNMSGVPILLRIFLSLYWLISQPLLNPLVYGLNMTKIRILCKNLLTLRPLNX
..N.............................................N...............................      80
................................................................................     160
................N...............................................................     240
..................N................................N..................               320

(Threshold=0.5)
----------------------------------------------------------------------
SeqName      Position  Potential    Jury    N-Glyc
                                 agreement result
----------------------------------------------------------------------
contig004259-BriOR.E038    3 NSTQ    0.7361     (9/9)    ++
contig004259-BriOR.E038   49 NRSL    0.7493     (9/9)    ++
contig004259-BriOR.E038  177 NYSV    0.6757     (9/9)    ++
contig004259-BriOR.E038  245 NFSC    0.3184     (9/9)    ---
```

```
contig004259-BriOR.E038  260 NMSG   0.5760     (6/9)   +
contig004259-BriOR.E038  291 NMTK   0.6374     (8/9)   +
----------------------------------------------------------------------
```

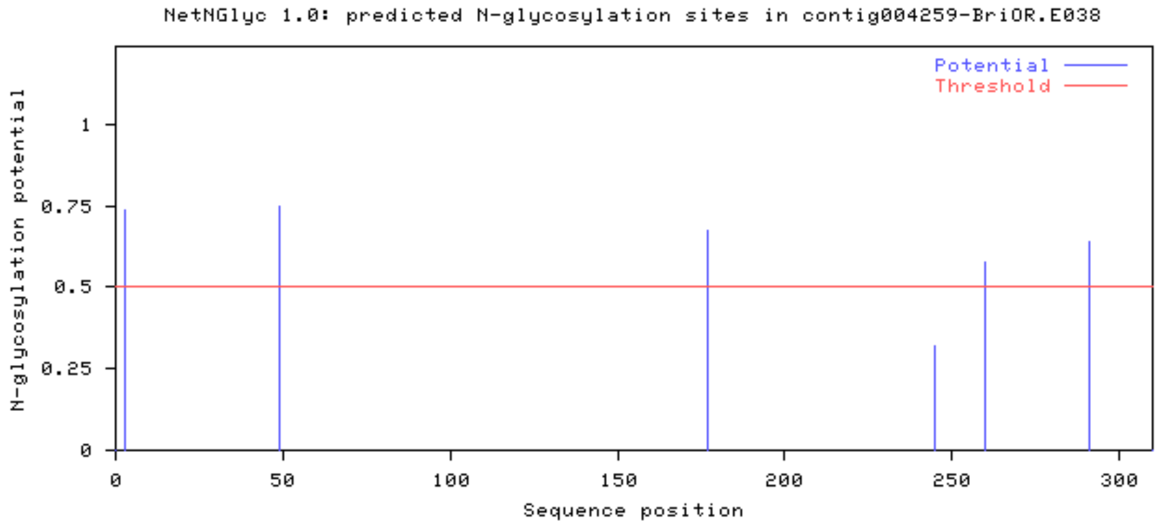

Graphics in PostScript

Output for 'contig004261-BriOR.E034'

```
##########################################################################################

    Warning: This sequence may not contain a signal peptide!!

    Proteins without signal peptides are unlikely to be exposed to
    the N-glycosylation machinery and thus may not be glycosylated
    (in vivo) even though they contain potential motifs.

    SignalP-NN euk predictions are as follows:

  # name                   Cmax  pos ?  Ymax  pos ?  Smax  pos ?  Smean ?  D     ?

    SignalP output is explained at http://www.cbs.dtu.dk/services/SignalP/output.html

##########################################################################################

Name:  contig004261-BriOR.E034  Length:  310
MINTTQVSFFTLTGFFDTGRVTNLCFIVILALYIFIVGSNVLLIVVICVNRSLHEPMYMFLCSLFVNELYGSTGLFPSLL      80
VQILSDVYTVSAPLCFLQVFCVHSYGAVEYLNLAIMSYDRYLAICCPLQYNTHMTSKQIGILIAATWFYSCFAMVVLLSL     160
TSPLQFCGNTISKVYCDTHSVVKLACSDTTVINLYGLLATFSTIFGALLFILYTYMKILLVCFSGSDQTRQKAVSTCTPH     240
LASILNFSFGASFEILQSRFNMKNVPNMLRIFLSLYFLTCQPLFNPVMYGLKMTKIRNICKSLITNTHLX
..N..............................................N..............................      80
................................................................................     160
................................................................................     240
......................................................................               320

(Threshold=0.5)
----------------------------------------------------------------------
SeqName      Position  Potential   Jury    N-Glyc
                                 agreement result
----------------------------------------------------------------------
contig004261-BriOR.E034    3 NTTQ   0.7370     (9/9)   ++
contig004261-BriOR.E034   50 NRSL   0.7494     (9/9)   ++
```

```
contig004261-BriOR.E034   246 NFSF   0.4073     (6/9)   -
----------------------------------------------------------------------
```

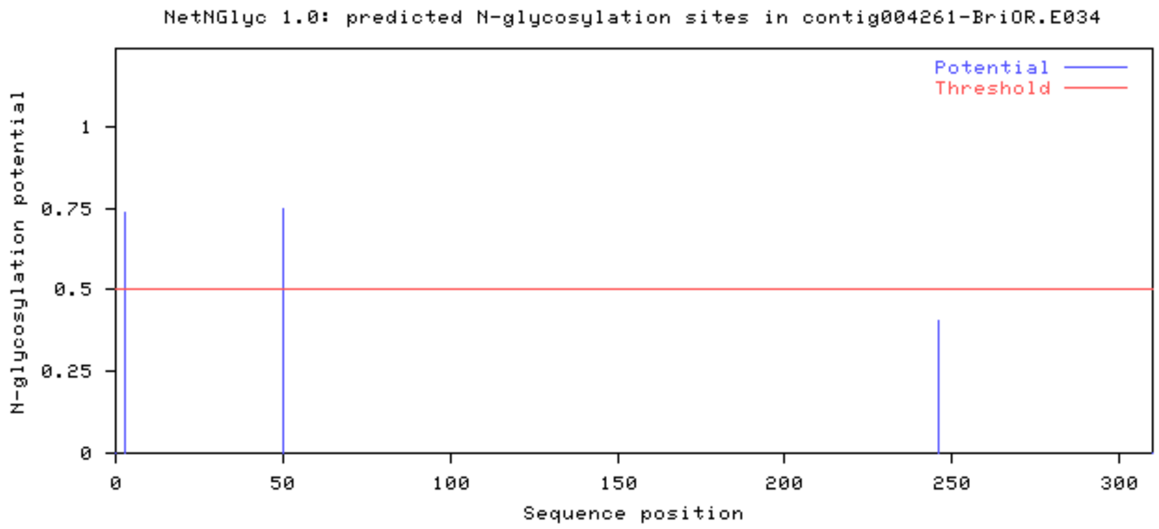

[Graphics in PostScript]

Output for 'contig004265-BriOR.E035'

```
##########################################################################################

    Warning: This sequence may not contain a signal peptide!!

    Proteins without signal peptides are unlikely to be exposed to
    the N-glycosylation machinery and thus may not be glycosylated
    (in vivo) even though they contain potential motifs.

    SignalP-NN euk predictions are as follows:

  # name                   Cmax  pos ?  Ymax  pos ?  Smax  pos ?  Smean ?  D     ?

    SignalP output is explained at http://www.cbs.dtu.dk/services/SignalP/output.html

##########################################################################################

Name:  contig004265-BriOR.E035  Length:  309
MINTTQVSFFTLTGYFDTGLVTNLCFIVILALYIFIVGSNVLLIVVICVNRSLHEPMYMFLCSLFVNELYGSTGLFPSLL      80
VQILSDVHTVSAPLCFLQIFCVHIYGAVEYLNLAIMSYDRYLAICCPLQYNTHMTSKKIGILIAATWFYPCFAMAPLLYL     160
TSPLQLCGNTISKVYCDTHSVVKLACSDTTVINLYGLLATFSTIFGALLFILYTYMKILLVCFSGSDQTRQKAVSTCTPH     240
LASILNFSFGASFEILQSRFNMKNVPNMLRIFLSLYFLTCQPLFNPVMYGLKMTKIRNICKSLITNIKX
..N..............................................N..............................      80
................................................................................     160
................................................................................     240
.....................................................................                320

(Threshold=0.5)
----------------------------------------------------------------------
SeqName      Position  Potential   Jury    N-Glyc
                                 agreement result
----------------------------------------------------------------------
contig004265-BriOR.E035     3 NTTQ   0.7369     (9/9)   ++
contig004265-BriOR.E035    50 NRSL   0.7492     (9/9)   ++
contig004265-BriOR.E035   246 NFSF   0.4068     (6/9)   -
```

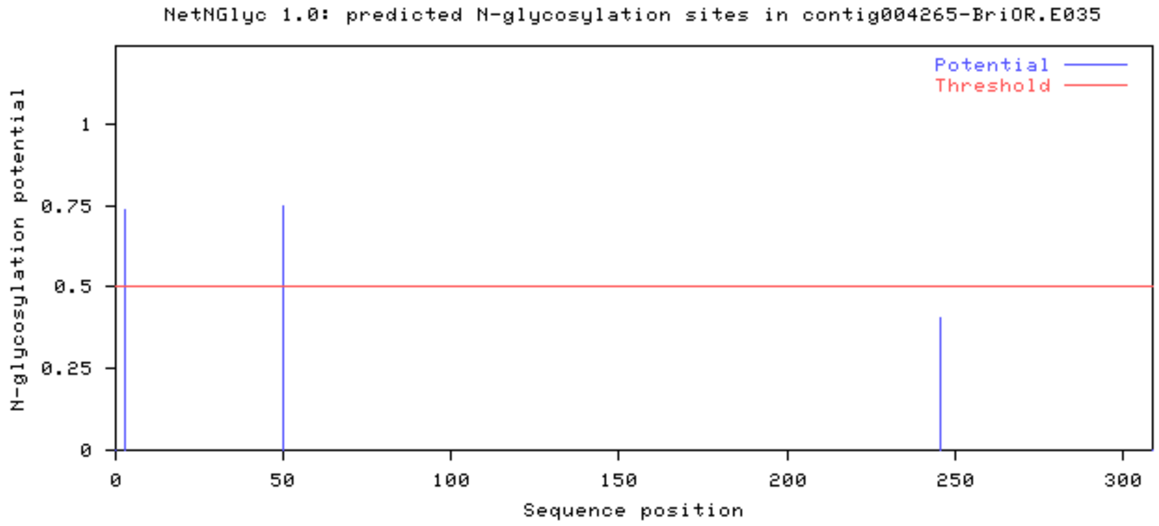

Graphics in PostScript

Output for 'contig004266-BriOR.E039'

```
##########################################################################################

    Warning: This sequence may not contain a signal peptide!!

    Proteins without signal peptides are unlikely to be exposed to
    the N-glycosylation machinery and thus may not be glycosylated
    (in vivo) even though they contain potential motifs.

    SignalP-NN euk predictions are as follows:

  # name                    Cmax  pos ?  Ymax  pos ?  Smax  pos ?  Smean ?  D     ?

    SignalP output is explained at http://www.cbs.dtu.dk/services/SignalP/output.html

##########################################################################################

Name:  contig004266-BriOR.E039  Length:  310
MKNSTQLSDFILGAYFDGGTFRYLYFTIVMSLYVFIFGSNLLLIVVICVNRSLHEPMYMFLCSLFVNELYGSTGLFPFLL      80
LQILSDVHTVSAPLCFLQVFSVYSYGSIEFLNLAVMSYDRYLAICCPLQYNELMTSNKVTKLIVAIWSPPLLVNFLTLPL     160
IVPLKRCGNIINKVYCDNHSIVKLACSDTTLNNIYGLTVSALSVFGPLIVILYTYTRILKVCFSGSKQTRQKAVSTCTPH     240
LASLLNFSFGACFEILQSRFNMNSSPNMLRIFLSLYFLTCPPIFNPLMYGLNLSKIRVTCKNLIAHIICX
..N..............................................N..............................      80
................................................................................     160
................................................................................     240
...................................................N..................               320

(Threshold=0.5)
----------------------------------------------------------------------
SeqName      Position  Potential   Jury    N-Glyc
                                 agreement result
----------------------------------------------------------------------
contig004266-BriOR.E039    3 NSTQ   0.5983     (9/9)   ++
contig004266-BriOR.E039   50 NRSL   0.7483     (9/9)   ++
contig004266-BriOR.E039  178 NHSI   0.4334     (7/9)   -
contig004266-BriOR.E039  246 NFSF   0.4127     (7/9)   -
```

```
contig004266-BriOR.E039  263 NSSP   0.1308     (9/9)   ---
contig004266-BriOR.E039  292 NLSK   0.6518     (8/9)   +
----------------------------------------------------------------------
```

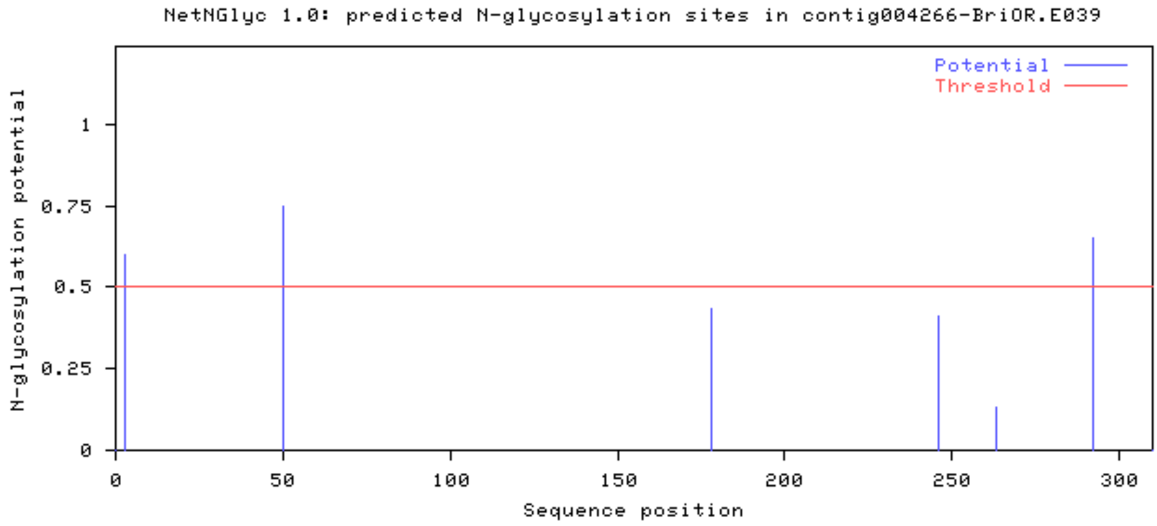

Graphics in PostScript

Output for 'contig004267-BriOR.P099'

```
##########################################################################################

    Warning: This sequence may not contain a signal peptide!!

    Proteins without signal peptides are unlikely to be exposed to
    the N-glycosylation machinery and thus may not be glycosylated
    (in vivo) even though they contain potential motifs.

    SignalP-NN euk predictions are as follows:

  # name                   Cmax  pos ?  Ymax  pos ?  Smax  pos ?  Smean ?  D     ?

    SignalP output is explained at http://www.cbs.dtu.dk/services/SignalP/output.html

##########################################################################################

Name:  contig004267-BriOR.P099  Length:  318
MENVSLHTHFFLEGFSELGELRPFLFIPFSFMFVVSLFANSLLVYVIVSQRSLHSPMYILIASMACIDLSLPVFFVPHML      80
LSFLFDWRGISLIGCLVQMHFIHFFGTFQSTLLVWMALDRYFAICTPLYYHNHMILSRFIAFLLPLVVRNVLMITLFVCL     160
AGKLPFCSRNVINHCFCEHMALVELACGSTTINSLVGLMAVFLIPVLDFVFITASYVVIFSSVLKSSGSGVKALHTCITH     240
IMVITVSLILALTAFLSYRIRNGLPAASRVFFSTMYLLFPSCFNPVVYGIRTNEIRQHILKRLTCCHICQMGPVTKKX
..N.............................................................................      80
................................................................................     160
................................................................................     240
..............................................................................       320

(Threshold=0.5)
----------------------------------------------------------------------
SeqName      Position  Potential   Jury    N-Glyc
                                 agreement result
----------------------------------------------------------------------
contig004267-BriOR.P099    3 NVSL   0.7609     (9/9)   +++
----------------------------------------------------------------------
```

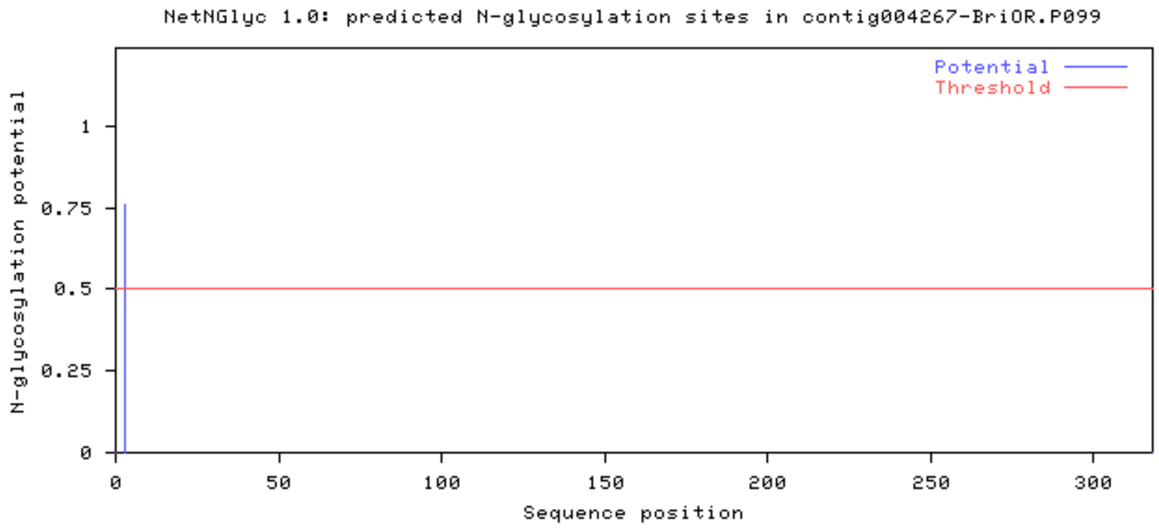

Graphics in PostScript

Output for 'contig004270-BriOR.P100'

```
##########################################################################################

    Warning: This sequence may not contain a signal peptide!!

    Proteins without signal peptides are unlikely to be exposed to
    the N-glycosylation machinery and thus may not be glycosylated
    (in vivo) even though they contain potential motifs.

    SignalP-NN euk predictions are as follows:

  # name                   Cmax  pos ?  Ymax  pos ?  Smax  pos ?  Smean ?  D     ?

    SignalP output is explained at http://www.cbs.dtu.dk/services/SignalP/output.html

##########################################################################################

Name:  contig004270-BriOR.P100  Length:  311
EMEAVKENISSHKYFFLDGFSELGELRPFLFIPFSFMFVVSLFANSLLVYVIVSQRSLHSPMYILIASMACIDLSLPAFF      80
VPHMLLSFLFDWRGISLIGCLVQMYFVHLLGAFQSTLLLWMALDRYFAICTPLHYQEQMALARFLKFVIPSSIRNMFVVL     160
VVVSLAGKLPFCLRNVINHCFCEHMALVELACGSTTINSLVGLISVFSVPVTDFFLITASYTVIFSSVLSSGKSSAKALH     240
TCVTHIVVMTVSLTIVLTAFLSYRVRNGLPAAVRTFFSILYLFFPSCFNPIIYGIRTTEIRQHIINTLRVX
.......N........................................................................      80
................................................................................     160
................................................................................     240
.......................................................................             320

(Threshold=0.5)
----------------------------------------------------------------------
SeqName      Position  Potential   Jury    N-Glyc
                                 agreement result
----------------------------------------------------------------------
contig004270-BriOR.P100    8 NISS   0.6857     (9/9)   ++
----------------------------------------------------------------------
```

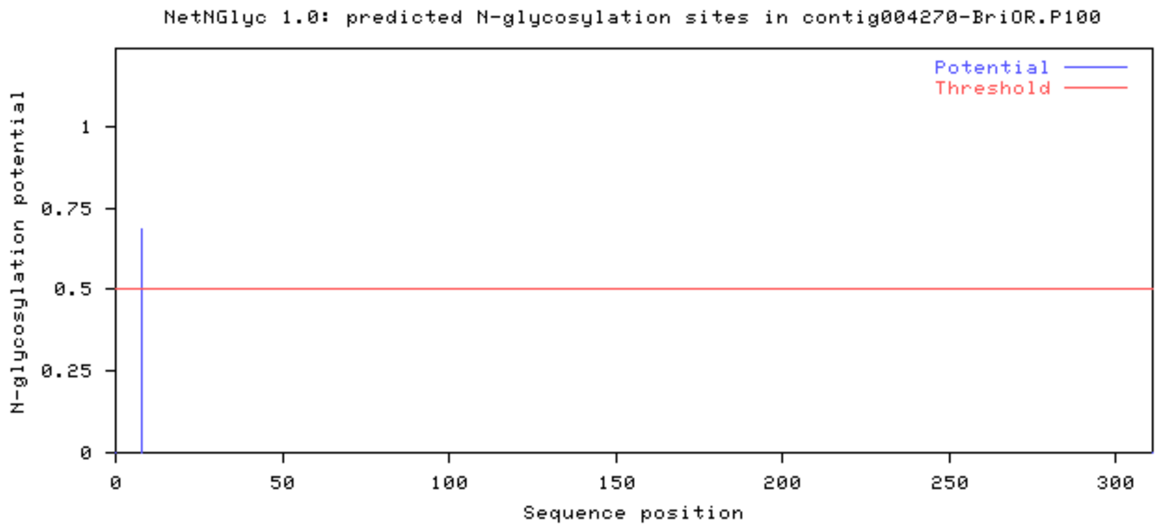

Graphics in PostScript

Output for 'contig004275-BriOR.P101'

```
##########################################################################################

    Warning: This sequence may not contain a signal peptide!!

    Proteins without signal peptides are unlikely to be exposed to
    the N-glycosylation machinery and thus may not be glycosylated
    (in vivo) even though they contain potential motifs.

    SignalP-NN euk predictions are as follows:

  # name                Cmax  pos ?  Ymax  pos ?  Smax  pos ?  Smean ?  D     ?

    SignalP output is explained at http://www.cbs.dtu.dk/services/SignalP/output.html

##########################################################################################

Name:  contig004275-BriOR.P101  Length:  332
MLEAPLSRNFSHSTFVLRGFPSLQKHRRLLALPFSASYLLVLLGNSLLMYIICSVERLHSPMYLLICTLCFVDILVVTTI      80
IPNMLLGFLFNWNEISLVGCLTQMFFIHFLSSVESTLLLAMALDRYFAICRPLRYNEIINSAMLVRLVLFTLVRSVSVMA     160
TLVGLAGSLQFCGSNVIQHCYCDHMALVSLACDSTSRSSAAGLAVIICFVGADIPIIFFSYMKILSVVLRSAAAGEDSRK     240
AFHTCSTHLIVMMCFYLVGSITFLSHNLNIPIPTDINNSMGLMYILFPAAINPVIYGVRTKEIRDSFLRIFKNRAKKIMT     320
VKVSSAGKGKSX
........N.......................................................................      80
................................................................................     160
................................................................................     240
................................................................................     320
............                                                                         400

(Threshold=0.5)
----------------------------------------------------------------------
SeqName      Position  Potential   Jury    N-Glyc
                                agreement  result
----------------------------------------------------------------------
contig004275-BriOR.P101    9 NFSH   0.6399     (9/9)    ++
contig004275-BriOR.P101  277 NNSM   0.2284     (9/9)    ---
----------------------------------------------------------------------
```

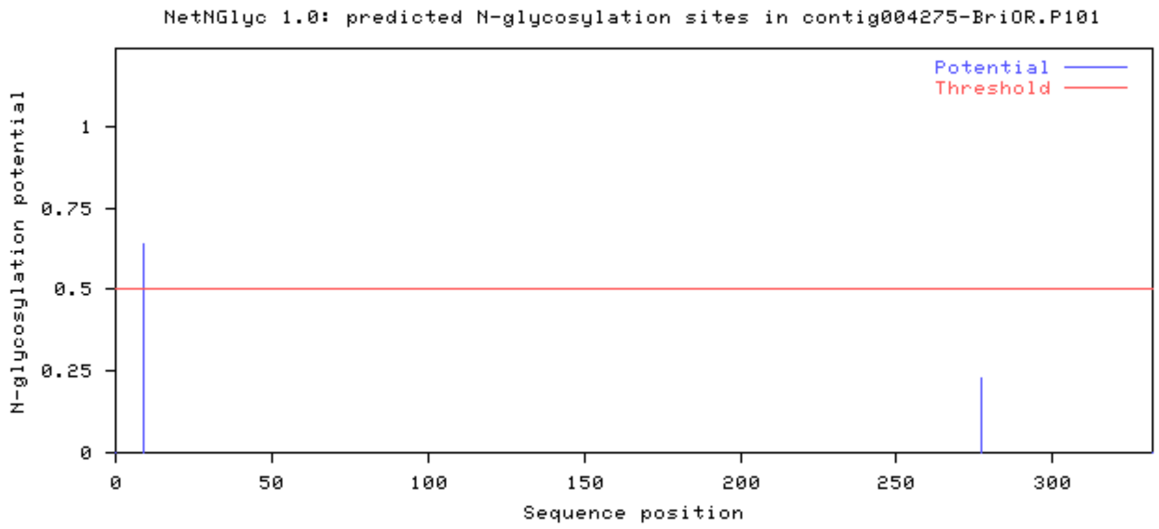

[Graphics in PostScript]

Output for 'contig014340-BriORe.K068'

```
##########################################################################

    Warning: This sequence may not contain a signal peptide!!

    Proteins without signal peptides are unlikely to be exposed to
    the N-glycosylation machinery and thus may not be glycosylated
    (in vivo) even though they contain potential motifs.

    SignalP-NN euk predictions are as follows:

  # name                Cmax  pos ?  Ymax  pos ?  Smax  pos ?  Smean ?  D     ?

    SignalP output is explained at http://www.cbs.dtu.dk/services/SignalP/output.html

##########################################################################

Name:  contig014340-BriORe.K068         Length:  246
MMENYTYNSYILQLEGLNTSKDSLYPAFLFLFFSYLFIMIINVGITILIFMNKNLHQPMYLLFCNLPLNDILVNSIVLPR      80
LLIDLMRPPSERLISYYQCVVQAYIASLIGTTSHTVLMIMAYDRYVAICNPFHYVSIMTNKMMIKLTVCAWGVAFVLVGI     160
LLGLTTRLNRCRTLITNPYCDNASLFKLSCESVVINNIYGITFTVAVYVGSIGAIVLSYTSIAVVCLTSKNKSLNSKALK     240
TCSTHL
...N.............N..............................................................      80
................................................................................     160
.....................N..........................................................     240
......                                                                               320

(Threshold=0.5)
----------------------------------------------------------------------
SeqName         Position  Potential    Jury    N-Glyc
                                    agreement result
----------------------------------------------------------------------
contig014340-BriORe.K068    4 NYTY    0.7621     (9/9)    +++
contig014340-BriORe.K068   18 NTSK    0.6160     (8/9)    +
contig014340-BriORe.K068  182 NASL    0.5778     (6/9)    +
contig014340-BriORe.K068  231 NKSL    0.3573     (9/9)    --
----------------------------------------------------------------------
```

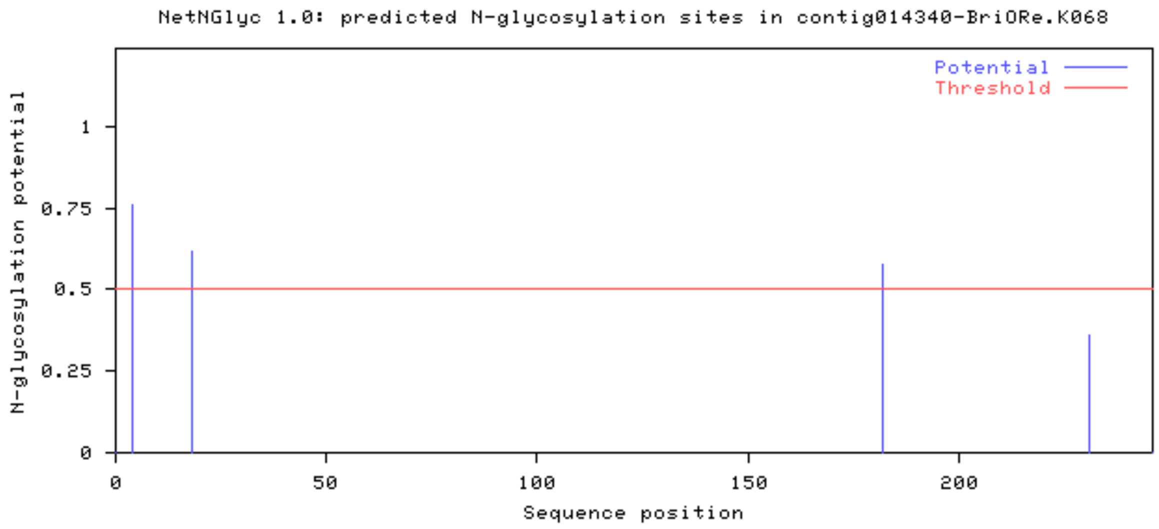

Graphics in PostScript

Output for 'contig014345-BriORe.K124'

```
##########################################################################

    Warning: This sequence may not contain a signal peptide!!

    Proteins without signal peptides are unlikely to be exposed to
    the N-glycosylation machinery and thus may not be glycosylated
    (in vivo) even though they contain potential motifs.

    SignalP-NN euk predictions are as follows:

  # name                Cmax  pos ?  Ymax  pos ?  Smax  pos ?  Smean ?  D     ?

    SignalP output is explained at http://www.cbs.dtu.dk/services/SignalP/output.html

##########################################################################

Name:  contig014345-BriORe.K124         Length:  74
MQNTNNIFTIQLEGLNVPQEATYAVFLFLFFSYLFLIVANVGIAVLVFVDKSLHQPMYILFCNLSINDLFGNSI
..............................................................N...........          80

(Threshold=0.5)
----------------------------------------------------------------------
SeqName      Position  Potential   Jury    N-Glyc
                                  agreement result
----------------------------------------------------------------------
contig014345-BriORe.K124   63 NLSI   0.5359     (5/9)   +
----------------------------------------------------------------------
```

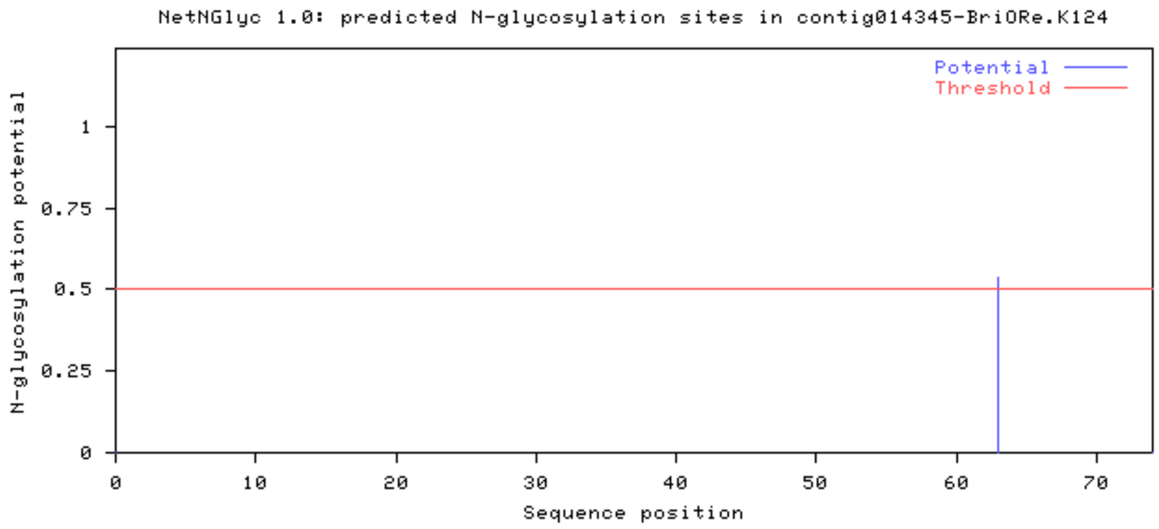

Graphics in PostScript

Output for 'contig014347-BriORf.AB127'

```
##########################################################################

    Warning: This sequence may not contain a signal peptide!!

    Proteins without signal peptides are unlikely to be exposed to
    the N-glycosylation machinery and thus may not be glycosylated
    (in vivo) even though they contain potential motifs.

    SignalP-NN euk predictions are as follows:

  # name                    Cmax   pos ?  Ymax   pos ?  Smax   pos ?  Smean ?  D     ?

    SignalP output is explained at http://www.cbs.dtu.dk/services/SignalP/output.html

##########################################################################

Name:  contig014347-BriORf.AB127        Length:  279
MSFLLQALTDPKLNSPLYFFLWNLSFVDMVYTTTTIPNMLSGLLTDISTISVLGCFLQMFFFIQLSVTGRAILTVMAYDR      80
YVAICNPLQYNSIMTRPVRLLLVAGAWGFGTICTLPASVIAFGRPYCGPNVVKHAWCDPSSVRRLVCGDTSLDNIVSLSF     160
AMVALLTTGVFILSSYILIGFSISRMVVAQRLKAFRTCAAHLTVVSISYAAASFVYISYRVGNFSSEVKTLLCFLCNVSV     240
QYSVKVLSHPSFISIFGRKTRNSCRDLLKQTFTDIFLDQ
......................N.........................................................      80
................................................................................     160
..............................................................N.............N...     240
.......................................                                              320

(Threshold=0.5)
----------------------------------------------------------------------
SeqName      Position  Potential   Jury    N-Glyc
                                 agreement result
----------------------------------------------------------------------
contig014347-BriORf.AB127   23 NLSF   0.7248     (9/9)   ++
contig014347-BriORf.AB127  223 NFSS   0.6340     (8/9)   +
contig014347-BriORf.AB127  237 NVSV   0.6750     (9/9)   ++
----------------------------------------------------------------------
```

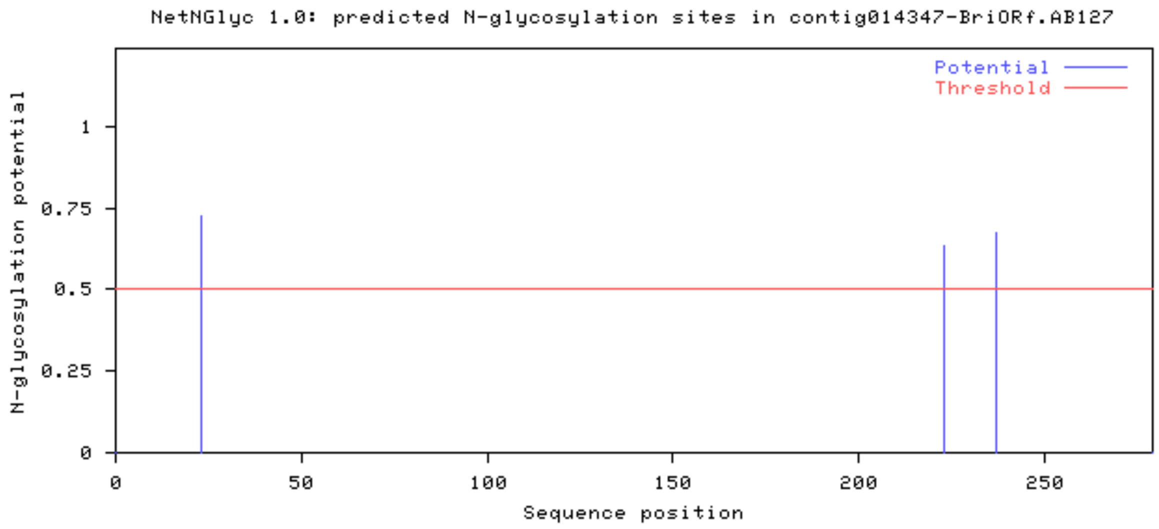

Graphics in PostScript

Output for 'contig014348-BriOR.K066'

```
##########################################################################

    Warning: This sequence may not contain a signal peptide!!

    Proteins without signal peptides are unlikely to be exposed to
    the N-glycosylation machinery and thus may not be glycosylated
    (in vivo) even though they contain potential motifs.

    SignalP-NN euk predictions are as follows:

  # name                 Cmax  pos ?  Ymax  pos ?  Smax  pos ?  Smean ?  D     ?

    SignalP output is explained at http://www.cbs.dtu.dk/services/SignalP/output.html

##########################################################################

Name:  contig014348-BriOR.K066  Length:  315
MENQTSDILLLEGLQVSPDASIPAFILLFLIYIFIMFSNIVLVILITLDSSLHQPMYLLFCNMSINDVFGATTIIPRMLR      80
DIFTPGSDRYIHYVDCVIQAFCVHIYAGSSHTILMIMAFDRYVAICNPLQYATIMTNWMVVKLSVLAWAVIFVMVTILVG     160
LSARLSRCRWIILNPFCDNASLFKLSCESILINNVYGLGYTVLLLGSSIGSVTITYLRIAVVCLSSKSKTLNSRALQTYT     240
THLTMYVIMFVSGIVMVLLHRFPHLSDQRKLASMMFHVVPPALNAVIYGMQIKAVRQKMFIMFTRNTVTVTDGKX
..N..........................................................N..................      80
................................................................................     160
..................N.............................................................     240
...........................................................................          320

(Threshold=0.5)
----------------------------------------------------------------------
SeqName       Position  Potential   Jury    N-Glyc
                                 agreement result
----------------------------------------------------------------------
contig014348-BriOR.K066    3 NQTS   0.7476     (9/9)    ++
contig014348-BriOR.K066   62 NMSI   0.6740     (9/9)    ++
contig014348-BriOR.K066  179 NASL   0.6570     (8/9)    +
----------------------------------------------------------------------
```

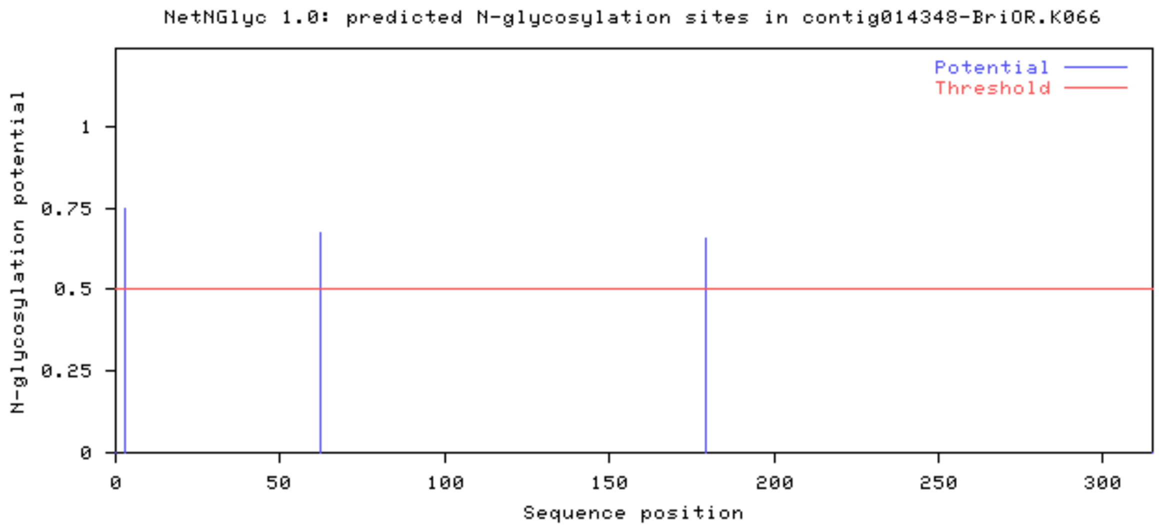

Graphics in PostScript

Output for 'contig014426-BriORp.R121'

```
##########################################################################################

    Warning: This sequence may not contain a signal peptide!!

    Proteins without signal peptides are unlikely to be exposed to
    the N-glycosylation machinery and thus may not be glycosylated
    (in vivo) even though they contain potential motifs.

    SignalP-NN euk predictions are as follows:

  # name                 Cmax  pos ?  Ymax  pos ?  Smax  pos ?  Smean ?  D     ?

    SignalP output is explained at http://www.cbs.dtu.dk/services/SignalP/output.html

##########################################################################################

Name:  contig014426-BriORp.R121         Length:  318
MSDISQTNISVGLHDLERGLLSSLTTLPCCVFFCINVIMLFTLRSKSVFRETCRYILLYNLILADTLQMAVSQILYMMSI      80
CRITLPYPVCGILVMFANLTNEISPLTLVLMSLERYVAVCYPLRHATIITIRNTEVAIIMIWIFCSLNILIRFLLLEFPF     160
EELQSLQLKRYVCNTFLMFLTPVSHEYDKAYTCFLFVSAFVGVTCSYIGVMLAARSASTDKASAQKARNTLLLHLVQLSF     240
SLSSTVNNALLLLTSKTVSNRVSVFIQNALYVLLFILPRCLSALIYGLRDQTIRTILVYNLCCQLKLLAVTAEAKVYP
.......N........................................................................      80
.................N..............................................................     160
................................................................................     240
..............................................................................       320

(Threshold=0.5)
----------------------------------------------------------------------
SeqName      Position  Potential   Jury    N-Glyc
                                 agreement result
----------------------------------------------------------------------
contig014426-BriORp.R121    8 NISV   0.6022     (6/9)   +
contig014426-BriORp.R121   98 NLTN   0.6598     (9/9)   ++
----------------------------------------------------------------------
```

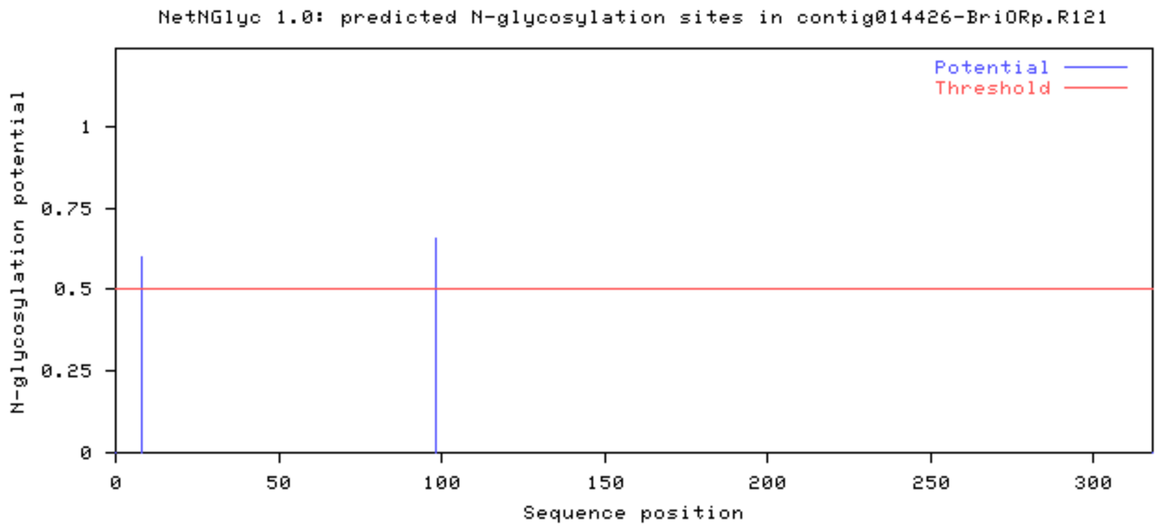

Graphics in PostScript

Output for 'contig033883-BriOR.F045'

```
##########################################################################

   Warning: This sequence may not contain a signal peptide!!

   Proteins without signal peptides are unlikely to be exposed to
   the N-glycosylation machinery and thus may not be glycosylated
   (in vivo) even though they contain potential motifs.

   SignalP-NN euk predictions are as follows:

 # name                  Cmax  pos ?  Ymax  pos ?  Smax  pos ?  Smean ?  D     ?

   SignalP output is explained at http://www.cbs.dtu.dk/services/SignalP/output.html

##########################################################################

Name:  contig033883-BriOR.F045  Length:  310
MENNSHPLYFNLTMFVNIGKFRYPAFVLFLLLYTFIISANLVIIVVISREKTLHEPMYIFIMCLSINSLYGSGGFFFRFL      80
RDLLSDSNLIARSACFTQIYIIYTYASYELTILGIMAYDRFVAICQPLHYHSKLTSRVISKLLAFAWIYPAFSVAACVYL     160
ASRLPLCDNKIPKVFCANWPVVKLSCVPTVINNIIGMFVSVTTVFLPLAFVLYTYMRIFLVCRKRSSLFKSKVIQSCLPH     240
IVTFVNYSITVFCDVSLSRIDLEELNPFLGIILSLEFVVIPPILNPLMYGLKLPEIRKCILRNLPCLIRX
..N.......N.....................................................................      80
................................................................................     160
................................................................................     240
.....N................................................................               320

(Threshold=0.5)
----------------------------------------------------------------------
SeqName      Position  Potential   Jury    N-Glyc
                                 agreement result
----------------------------------------------------------------------
contig033883-BriOR.F045    3 NNSH   0.5828     (9/9)   ++
contig033883-BriOR.F045   11 NLTM   0.8127     (9/9)   +++
contig033883-BriOR.F045  246 NYSI   0.5671     (6/9)   +
----------------------------------------------------------------------
```

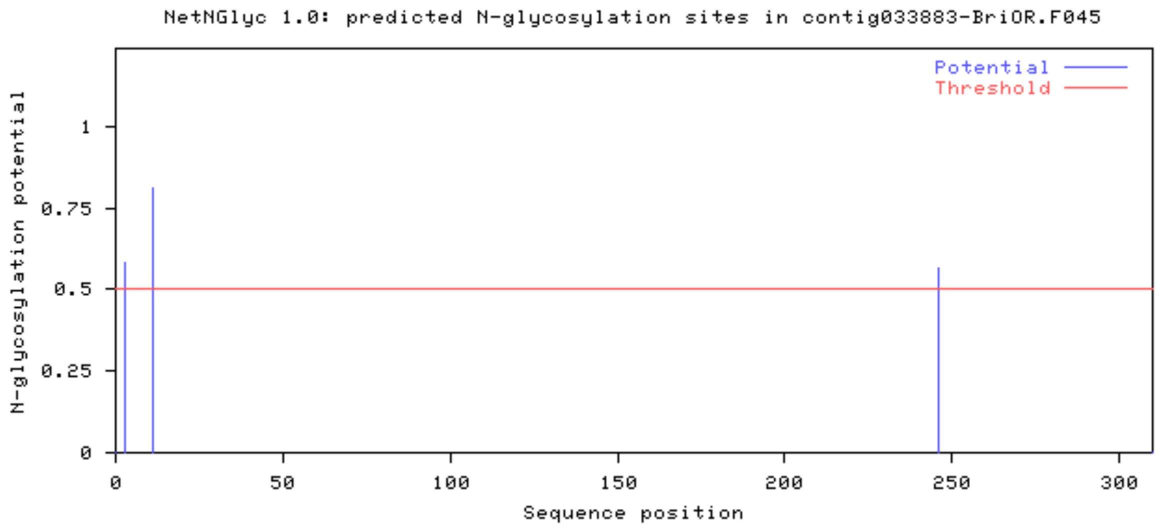

[Graphics in PostScript]

Output for 'contig033889-BriOR.H049'

```
##########################################################################

    Warning: This sequence may not contain a signal peptide!!

    Proteins without signal peptides are unlikely to be exposed to
    the N-glycosylation machinery and thus may not be glycosylated
    (in vivo) even though they contain potential motifs.

    SignalP-NN euk predictions are as follows:

  # name                Cmax  pos ?  Ymax  pos ?  Smax  pos ?  Smean ?  D     ?

    SignalP output is explained at http://www.cbs.dtu.dk/services/SignalP/output.html

##########################################################################

Name:  contig033889-BriOR.H049  Length:  324
MNNVSVITMFVLSGFNETISHRFVLFFLSLLCYCIICLLNVSLIVIIILDRNLHESMYILLCVFCMNALYGTAGFYPRFL      80
WDLLSDVHLISYYGCLIQTKVIFSFVCGELSILALMAYDRYVAICQPLKYHSIMSKQRVIRFACFLWLTTFCIMAVNAFL     160
TSRLKLCSPYISRLFCMNWSIVQLACFPEQTAINAISANIVTIIYLLHGVFIVWSYMYIIQTCVRSIENRAKFMQTCVPH     240
LVSLFTFVVAILTDVISMRLGSKELPRTLQNFVALEFLVIPPIMNPLIYGFKLTKIRKKIYSVVILKRTNFCFIRPENRF     320
THSX
..N............N.......................N........................................      80
................................................................................     160
.................N..............................................................     240
................................................................................     320
....                                                                                 400

(Threshold=0.5)
----------------------------------------------------------------------
SeqName      Position  Potential   Jury    N-Glyc
                                 agreement result
----------------------------------------------------------------------
contig033889-BriOR.H049    3 NVSV   0.7602     (9/9)   +++
contig033889-BriOR.H049   16 NETI   0.6812     (9/9)   ++
contig033889-BriOR.H049   40 NVSL   0.7030     (9/9)   ++
contig033889-BriOR.H049  178 NWSI   0.6432     (8/9)   +
----------------------------------------------------------------------
```

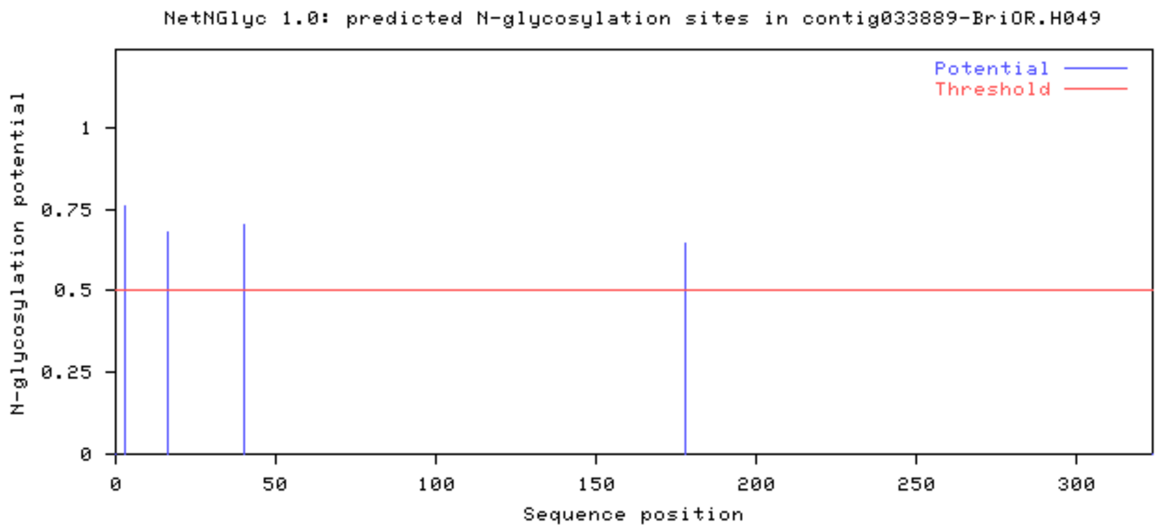

Graphics in PostScript

Output for 'contig033891-BriORe.H056'

```
##########################################################################################

    Warning: This sequence may not contain a signal peptide!!

    Proteins without signal peptides are unlikely to be exposed to
    the N-glycosylation machinery and thus may not be glycosylated
    (in vivo) even though they contain potential motifs.

    SignalP-NN euk predictions are as follows:

  # name                 Cmax  pos ?  Ymax  pos ?  Smax  pos ?  Smean ?  D     ?

    SignalP output is explained at http://www.cbs.dtu.dk/services/SignalP/output.html

##########################################################################################

Name:  contig033891-BriORe.H056         Length:  207
MNNVSIITMFFLSGFNETISHRFVLFFLSLLCYCIICLLNVSLIVIIILHSNLHEPMYILLCVFCINALYGTAGFYPKFL      80
WDLLSDVHLISYYGCLIQNQVIYSSACGELSIPALMAYDRYVAIYQPLKYHSIMSKQRVIRFACFSWFTNFCIVAVNTFL     160
TSRLKLCSPYISRLFCVNWSIVQLACFPAQTTINAISANITISIYFL
..N............N.......................N........................................      80
................................................................................     160
................N...................N........                                        240

(Threshold=0.5)
----------------------------------------------------------------------
SeqName      Position  Potential   Jury    N-Glyc
                                 agreement result
----------------------------------------------------------------------
contig033891-BriORe.H056    3 NVSI   0.7411     (9/9)   ++
contig033891-BriORe.H056   16 NETI   0.6679     (9/9)   ++
contig033891-BriORe.H056   40 NVSL   0.6964     (9/9)   ++
contig033891-BriORe.H056  178 NWSI   0.6290     (8/9)   +
contig033891-BriORe.H056  199 NITI   0.5539     (6/9)   +
----------------------------------------------------------------------
```

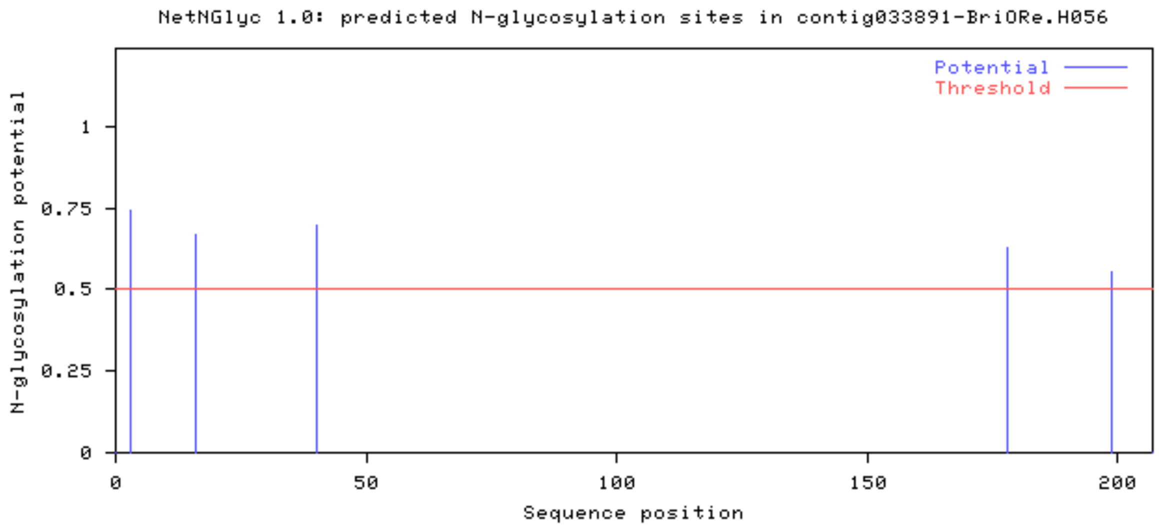

Graphics in PostScript

Output for 'contig042475-BriOR.S102'

```
##########################################################################

   Warning: This sequence may not contain a signal peptide!!

   Proteins without signal peptides are unlikely to be exposed to
   the N-glycosylation machinery and thus may not be glycosylated
   (in vivo) even though they contain potential motifs.

   SignalP-NN euk predictions are as follows:

 # name                 Cmax   pos ?  Ymax   pos ?  Smax   pos ?  Smean ?  D     ?

   SignalP output is explained at http://www.cbs.dtu.dk/services/SignalP/output.html

##########################################################################

Name:  contig042475-BriOR.S102  Length:  311
MATNNSVIGGQLAINNINNRVIIVQVFISVFLCINFLLITTFFMKDIFYTTMRYILFAIALLSDSLYLLITNVLLILNYF      80
SFTIQVWLCVIIYIVLSVYTFVTPITLTAMTLERYVAICVPLRHAELCRTQRALHFILVIHGLSSVPCIVILSIFFASTI     160
TSFYTQSRICSVEMFIFHRWQGHLRSAISQLYFLIMSITIVFSYVQIMKVAKAASGENKKSKWKGLSTVVLHGFQLLLCF     240
IQLWCPFIEAAVLQIDFMLFINVRYFNYITFILAPRCLSPLIYGLRDEMFVNALKYYALCGLYKKYSTAFX
...N............................................................................      80
................................................................................     160
................................................................................     240
.......................................................................              320

(Threshold=0.5)
----------------------------------------------------------------------
SeqName      Position  Potential   Jury    N-Glyc
                                 agreement result
----------------------------------------------------------------------
contig042475-BriOR.S102    4 NNSV   0.7096     (9/9)    ++
----------------------------------------------------------------------
```

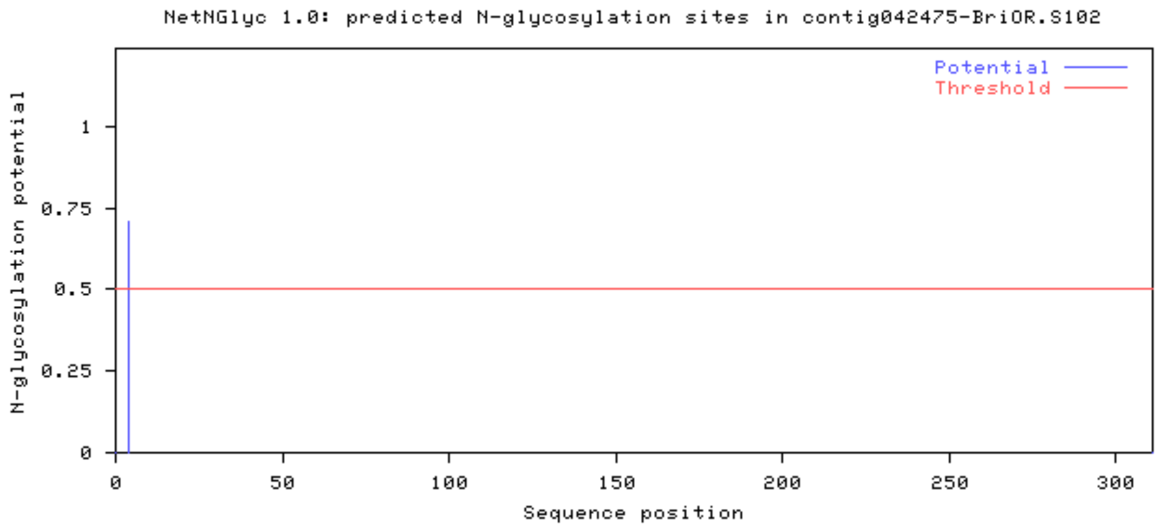

Graphics in PostScript

Output for 'contig042478-BriOR.S103'

```
##########################################################################################

    Warning: This sequence may not contain a signal peptide!!

    Proteins without signal peptides are unlikely to be exposed to
    the N-glycosylation machinery and thus may not be glycosylated
    (in vivo) even though they contain potential motifs.

    SignalP-NN euk predictions are as follows:

  # name                    Cmax  pos ?  Ymax  pos ?  Smax  pos ?  Smean ?  D     ?

    SignalP output is explained at http://www.cbs.dtu.dk/services/SignalP/output.html

##########################################################################################

Name:  contig042478-BriOR.S103  Length:  313
MVDNSSFIGVESSMRQLNDQVIIVQVLVGVFLCINTMLIITFFMKDTFYRTMRYILFAVTLLSDCLILILSDLLLILSYF      80
RLSIQVSLCLIMFGVSSVCNFVTPFTLTAMTLERYVAICMPLRHGELCSTRSALQCILIIHGLSSVPCILILSVFFASVS     160
LSSFTQYRVCSVEMFILRSWQGHLRSAIGQFYFLIMCIIIVFSYIQIMKVAKAASGENKKSTHKGLRTVALHAFQLLLCL     240
IQLWCPFIEAAVLQIDFMLYVNVRYFNYIMFSLTPRCLSPLIYGLRDDKFFLALKYHVLCHLHRKKSVGVSNX
...N............................................................................      80
................................................................................     160
................................................................................     240
.........................................................................            320

(Threshold=0.5)
----------------------------------------------------------------------
SeqName      Position  Potential   Jury    N-Glyc
                                 agreement result
----------------------------------------------------------------------
contig042478-BriOR.S103    4 NSSF   0.6223     (7/9)    +
----------------------------------------------------------------------
```

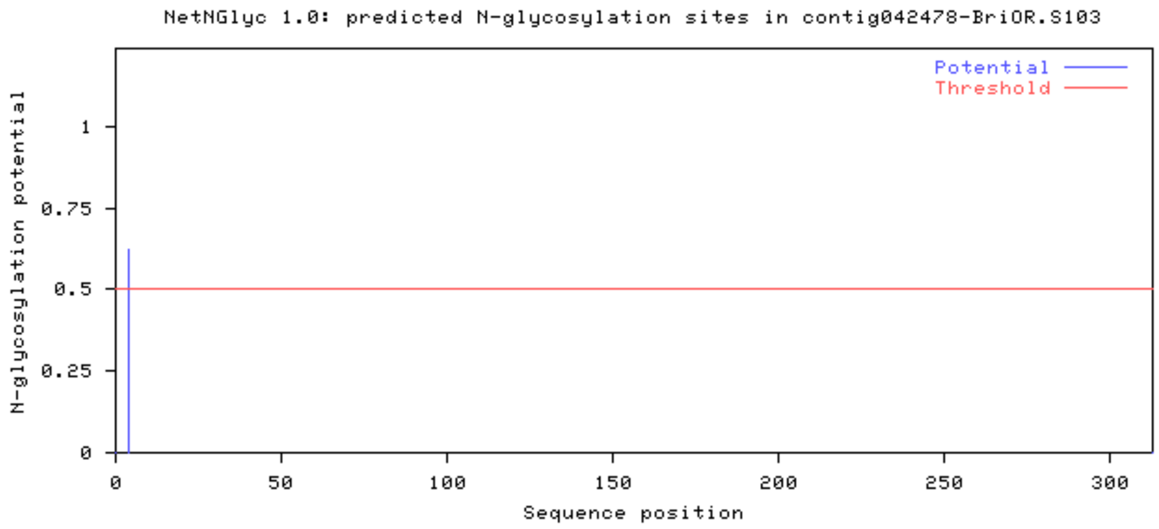

Graphics in PostScript

Output for 'contig042534-BriOR.K067'

```
##########################################################################

    Warning: This sequence may not contain a signal peptide!!

    Proteins without signal peptides are unlikely to be exposed to
    the N-glycosylation machinery and thus may not be glycosylated
    (in vivo) even though they contain potential motifs.

    SignalP-NN euk predictions are as follows:

  # name                    Cmax  pos ?  Ymax  pos ?  Smax  pos ?  Smean ?  D     ?

    SignalP output is explained at http://www.cbs.dtu.dk/services/SignalP/output.html

##########################################################################

Name:  contig042534-BriOR.K067  Length:  312
MEKNETVSTDILEVQGFDISPELTFPLFFLLLFVYFTLLFSNIGVLLLIISQKSLHQPMYFLFCNLSVNDLIGNTVLLPQ      80
LMAHILATERFITYKQCVVQAFQSHTFGSASHMILIIMAIDRYVAICHPLRYSSIMTTRTVVGLSAAAWGVSVVLVSILI     160
GLTVRLSRCRSTIQNSYCDNASLFKLSCEDVSINNIYGLFFTVLLFTSSIASIAATYFRIALICWIKKNKDLNNKALQTC     240
ASHLVLYLIMLWSGFLTIILHRFPNYPDLRKFAYVLFHVVPANLNPIIYGMQTRSLRHKITEILKRKVTPSX
...N............................................................N...............      80
................................................................................     160
...................N............................................................     240
........................................................................             320

(Threshold=0.5)
----------------------------------------------------------------------
SeqName      Position  Potential   Jury    N-Glyc
                                 agreement result
----------------------------------------------------------------------
contig042534-BriOR.K067    4 NETV   0.6623     (9/9)   ++
contig042534-BriOR.K067   65 NLSV   0.7386     (9/9)   ++
contig042534-BriOR.K067  180 NASL   0.5744     (8/9)   +
----------------------------------------------------------------------
```

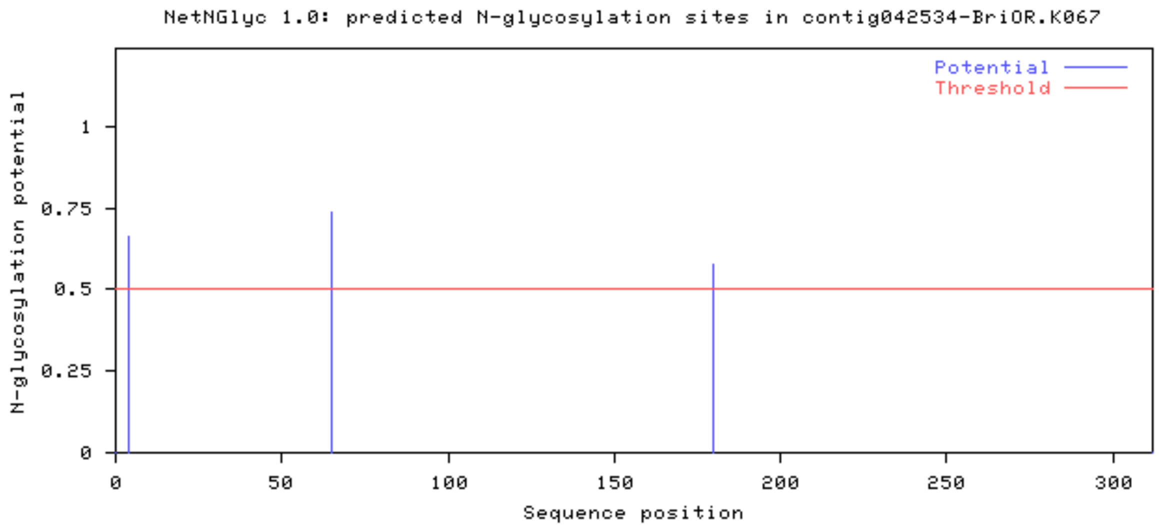

Graphics in PostScript

Output for 'contig042536-BriOR.J063'

```
##########################################################################################

    Warning: This sequence may not contain a signal peptide!!

    Proteins without signal peptides are unlikely to be exposed to
    the N-glycosylation machinery and thus may not be glycosylated
    (in vivo) even though they contain potential motifs.

    SignalP-NN euk predictions are as follows:

  # name                    Cmax   pos ?  Ymax   pos ?  Smax   pos ?  Smean ?  D     ?

    SignalP output is explained at http://www.cbs.dtu.dk/services/SignalP/output.html

##########################################################################################

Name:  contig042536-BriOR.J063  Length:  312
MENQSFDFSSELTLDPFVIPPGGKYPIFFLGISICIFGISCNLTLLALIILNRNLHKPMYFILFSLPLNDLIGLSAMLPK      80
VLSNIVTETHKIDYHLCVLQAFLLHMYGGGILFILAAMSFDRYVAICMPLRYSSVMTPRFISCIIVLVWGLDFVLIASLF     160
SLQARLPRCKHVVMNVFCDNPSLLKLTCGNTTVNNIMGLFNTGVIQVVSVSIQAYSYVKILIACVVTRKSETKAKAVNTC     240
VAQLVILFMFEVVATFTILSHRFKNVSVDMQKIMGMLIFTVPPLLNPIVYGLYTNEIRSTLLRVLKNKVSMX
..N.......................................N.....................................      80
................................................................................     160
...................N.........N..................................................     240
........................N...............................................             320

(Threshold=0.5)
----------------------------------------------------------------------
SeqName      Position  Potential   Jury    N-Glyc
                                agreement  result
----------------------------------------------------------------------
contig042536-BriOR.J063    3 NQSF   0.6052     (9/9)   ++
contig042536-BriOR.J063   42 NLTL   0.6889     (9/9)   ++
contig042536-BriOR.J063  180 NPSL   0.5542     (6/9)   +     WARNING: PRO-X1.
contig042536-BriOR.J063  190 NTTV   0.6811     (9/9)   ++
contig042536-BriOR.J063  265 NVSV   0.5147     (6/9)   +
----------------------------------------------------------------------
```

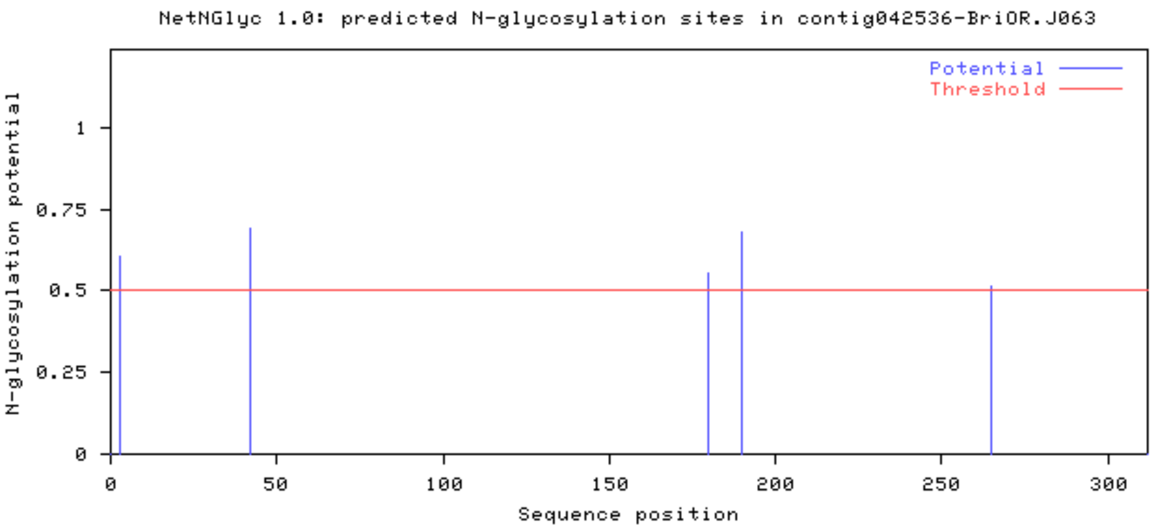

Graphics in PostScript

Output for 'contig042539-BriOR.J064'

```
##########################################################################################

    Warning: This sequence may not contain a signal peptide!!

    Proteins without signal peptides are unlikely to be exposed to
    the N-glycosylation machinery and thus may not be glycosylated
    (in vivo) even though they contain potential motifs.

    SignalP-NN euk predictions are as follows:

  # name                   Cmax  pos ?  Ymax  pos ?  Smax  pos ?  Smean ?  D     ?

    SignalP output is explained at http://www.cbs.dtu.dk/services/SignalP/output.html

##########################################################################################

Name:  contig042539-BriOR.J064  Length:  313
MYTNSSTSSLLTLQTLGLSSTDIYPAFVFGTLTYLIIMFANLLVLTVIAMNKKLHKPMFILLFNLPISDIVGATAFFPHL      80
IFSIVAENRLISHHACIFQAFLIHVYGTGNLLILSVMAYDRYIAICFPLRYTTIMNSHNLMKMIVITWFINLSMMFTLFI     160
LLARFKTCRTNIVDFFCNNQSLVKLICEDTSVNNYYGLATIFLLMGGPLALIVYTYAQILRTCVITNHTDARQKAIQTCA     240
THLIVFLSLQINTVFALISHRIDSSSPVLRRAFGVSVLIFPPFLDPIIYGLKTKELKQCIVMFLKQNVGLTMX
...N............................................................................      80
......................................................................N.........     160
..................N...............................................N.............     240
.........................................................................            320

(Threshold=0.5)
----------------------------------------------------------------------
SeqName       Position  Potential    Jury     N-Glyc
                                   agreement result
----------------------------------------------------------------------
contig042539-BriOR.J064    4 NSST   0.6360     (8/9)   +
contig042539-BriOR.J064  151 NLSM   0.5322     (6/9)   +
contig042539-BriOR.J064  179 NQSL   0.5961     (8/9)   +
contig042539-BriOR.J064  227 NHTD   0.5091     (4/9)   +
----------------------------------------------------------------------
```

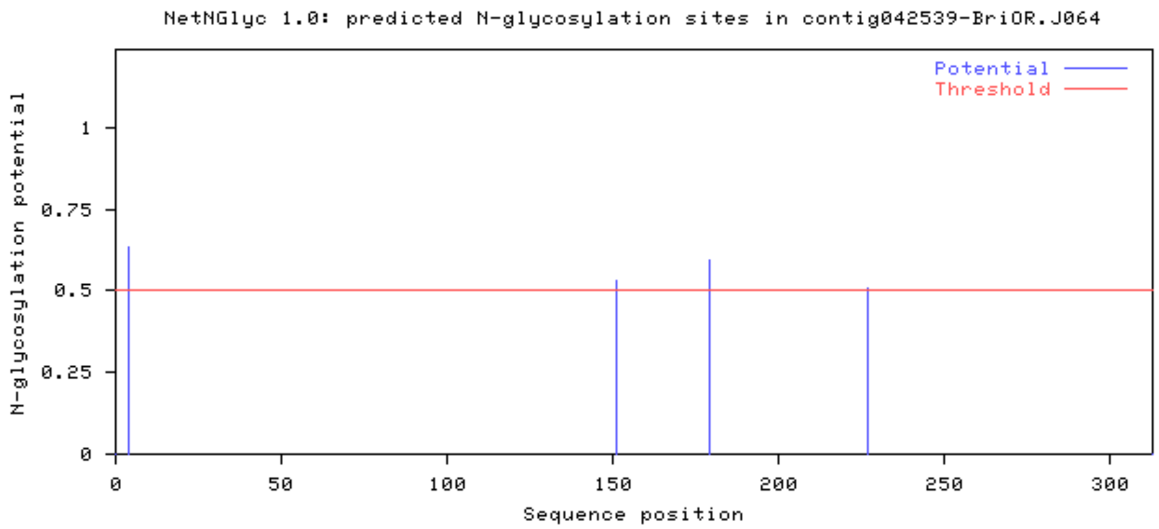

Graphics in PostScript

Output for 'contig042539-BriORe.J065'

```
##########################################################################################

    Warning: This sequence may not contain a signal peptide!!

    Proteins without signal peptides are unlikely to be exposed to
    the N-glycosylation machinery and thus may not be glycosylated
    (in vivo) even though they contain potential motifs.

    SignalP-NN euk predictions are as follows:

  # name                    Cmax  pos ?  Ymax  pos ?  Smax  pos ?  Smean ?  D     ?

    SignalP output is explained at http://www.cbs.dtu.dk/services/SignalP/output.html

##########################################################################################

Name:  contig042539-BriORe.J065        Length:  201
FNLSAMAYDRYIAICYPLQYSTVMTNAHIMRIITIVWMSCLVLIAVLFFLLLRLPRCRSEMTHVYCDNPSLLTLVCADTT      80
INNIYGLLISAVAQLVANGIVFYTYLRILITCFRCKRSDTKAKALQTCATHLIVFLLLECLGLFTIISYRLNNVSPHFRR     160
FMGLSTLIFPPTLNPIIYGLKTKDIREKVLNFLKNRIFSSX
.N.................................................................N............      80
................................................................................     160
.........................................                                            240

(Threshold=0.5)
----------------------------------------------------------------------
SeqName      Position  Potential   Jury    N-Glyc
                                 agreement result
----------------------------------------------------------------------
contig042539-BriORe.J065    2 NLSA   0.6864     (9/9)   ++
contig042539-BriORe.J065   68 NPSL   0.5598     (7/9)   +     WARNING: PRO-X1.
contig042539-BriORe.J065  153 NVSP   0.2240     (9/9)   ---
----------------------------------------------------------------------
```

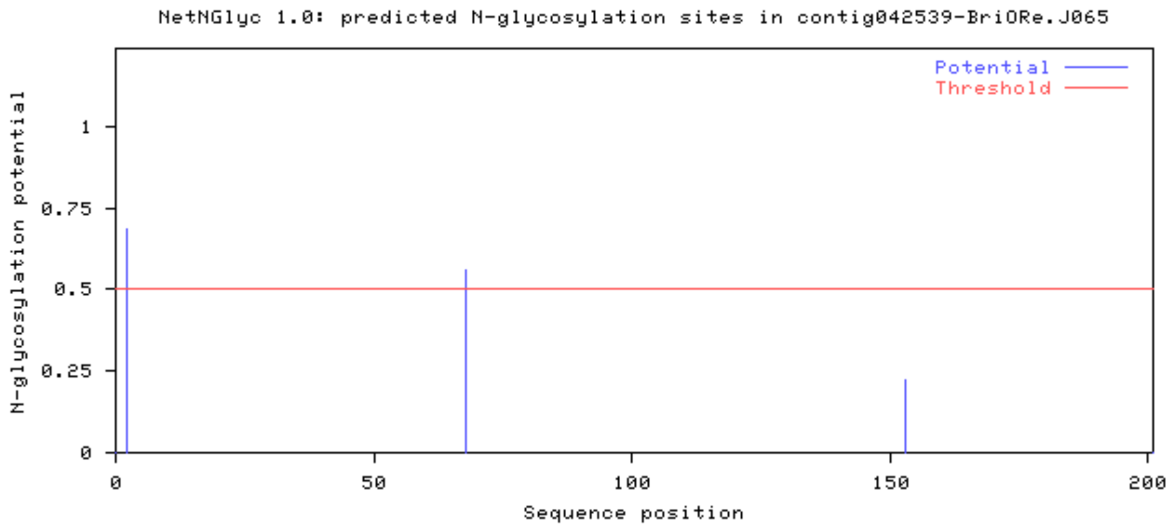

Graphics in PostScript

Output for 'contig042540-BriOR.J123'

```
########################################################################################

    Warning: This sequence may not contain a signal peptide!!

    Proteins without signal peptides are unlikely to be exposed to
    the N-glycosylation machinery and thus may not be glycosylated
    (in vivo) even though they contain potential motifs.

    SignalP-NN euk predictions are as follows:

  # name                    Cmax  pos ?  Ymax  pos ?  Smax  pos ?  Smean ?  D     ?

    SignalP output is explained at http://www.cbs.dtu.dk/services/SignalP/output.html

########################################################################################

Name:  contig042540-BriOR.J123  Length:  312
MNNQYNASSFLQINVFNLSSESVFPAFLFATLSYMIILFCNLTLILTIVLNKSLHQPMYLILLNLPIYDLIGSSALFPQL      80
IKEILRNSGIMQYSACVAQAFFIHIYAAGTVFNLSAMAYDRYIAICYPLQYSTVMTNAHIMRIITIVWMSCLVLIAVLFF     160
LLLRLPRCRSEMTHVYCDNPSLLTLVCADTTINNIYGLLIVALSQLVANGIVFYTYLRILITCFRSKRSDTKAKALQTCA     240
THLIVFLLLECLGLFTIISYRLNNVSPHFRRFMGLSTLIFPPTLNPIIYGLKTKEIREKVLNFLKNRIFSSX
.....N..........N.......................N.........N.............................      80
................................................................................     160
..................N.............................................................     240
........................................................................             320

(Threshold=0.5)
----------------------------------------------------------------------
SeqName      Position  Potential   Jury    N-Glyc
                                 agreement result
----------------------------------------------------------------------
contig042540-BriOR.J123    6 NASS   0.7060     (9/9)    ++
contig042540-BriOR.J123   17 NLSS   0.5106     (6/9)    +
contig042540-BriOR.J123   41 NLTL   0.7211     (9/9)    ++
contig042540-BriOR.J123   51 NKSL   0.7479     (9/9)    ++
contig042540-BriOR.J123  113 NLSA   0.4346     (5/9)    -
contig042540-BriOR.J123  179 NPSL   0.5146     (6/9)    +     WARNING: PRO-X1.
contig042540-BriOR.J123  264 NVSP   0.2140     (9/9)    ---
----------------------------------------------------------------------
```

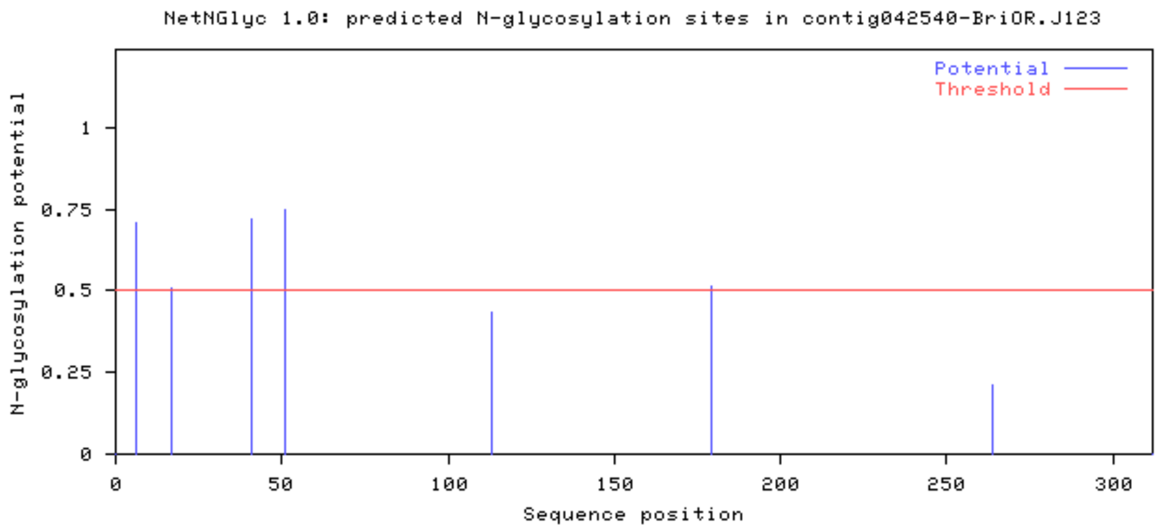

Graphics in PostScript

Output for 'contig042543-BriOR.L069'

```
##########################################################################################

    Warning: This sequence may not contain a signal peptide!!

    Proteins without signal peptides are unlikely to be exposed to
    the N-glycosylation machinery and thus may not be glycosylated
    (in vivo) even though they contain potential motifs.

    SignalP-NN euk predictions are as follows:

  # name                   Cmax  pos ?  Ymax  pos ?  Smax  pos ?  Smean ?  D     ?

    SignalP output is explained at http://www.cbs.dtu.dk/services/SignalP/output.html

##########################################################################################

Name:  contig042543-BriOR.L069  Length:  317
MSLPNASIKVTHFIIGGFDTVKRPIAVGVVMLITYLLAVIANVLNILFIIFDKRLHKPMYILICNLAVVDILYCCSTTPT      80
MIGVLLAGVNTISYVECFIQMSVFHLVGVMELFALAIMAFDRFIAFSFPFQYHSYLTNTRTLGLTYILWVVASGFVAVMP     160
VTAATLPYCTSRMKYAFCDYAAVIRTTCVDPNYYFNLVSVMMFFLLFFTFSFIGLSYFVIAFLMKFSSNRDKKKMASTCV     240
THLIVVTCYYSPLFVLIVFTRVGVVLTLEERQGLLIGTILSPSLVNPVVYCFRTKEIKNKIFTMFTKRLTYPTRFLX
....N...........................................................................      80
................................................................................     160
................................................................................     240
.............................................................................        320

(Threshold=0.5)
----------------------------------------------------------------------
SeqName      Position  Potential   Jury    N-Glyc
                                 agreement result
----------------------------------------------------------------------
contig042543-BriOR.L069    5 NASI   0.6296     (7/9)    +
----------------------------------------------------------------------
```

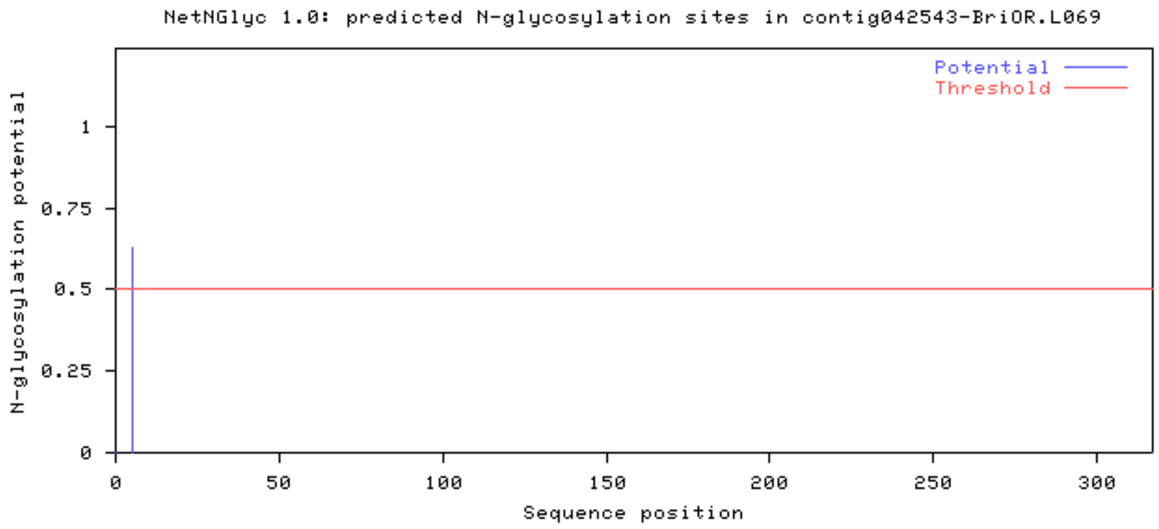

Graphics in PostScript

Output for 'contig042544-BriOR.L070'

```
##########################################################################

    Warning: This sequence may not contain a signal peptide!!

    Proteins without signal peptides are unlikely to be exposed to
    the N-glycosylation machinery and thus may not be glycosylated
    (in vivo) even though they contain potential motifs.

    SignalP-NN euk predictions are as follows:

  # name                 Cmax  pos ?  Ymax  pos ?  Smax  pos ?  Smean ?  D     ?

    SignalP output is explained at http://www.cbs.dtu.dk/services/SignalP/output.html

##########################################################################

Name:  contig042544-BriOR.L070  Length:  315
MSMLLQNASITVKHFIIGGFDTVKRPVAVGVVILIIYILAIFANTANILFIIFDKRLHKPMYLLVCNLAVVDIMYTSSAT      80
PTMIGVLLADVKTISYVDCLIQMCVFHLAMVMERFALAIMAFDRLIAIIFPFHYHCFLTNTRTVVLTYILWIIGCGTVVL     160
FPATVIPLPHCTLKLKYTFCEYAAIMRTTCVNIDEYFNQSAIWSFFISSFTFTFICISYCGILFCVKLSSNNDKKKMGST     240
VVSHAICVTCFYSPIFIIVILSRVGVVLSLDERQGLLMGNILGPSLVNPFVYCLRTTEIKNKMVKIFKKILRFLX
......N.........................................................................      80
................................................................................     160
................................................................................     240
...........................................................................          320

(Threshold=0.5)
----------------------------------------------------------------------
SeqName      Position  Potential   Jury    N-Glyc
                                 agreement result
----------------------------------------------------------------------
contig042544-BriOR.L070    7 NASI   0.7197     (9/9)   ++
contig042544-BriOR.L070  198 NQSA   0.3773     (8/9)   -
----------------------------------------------------------------------
```

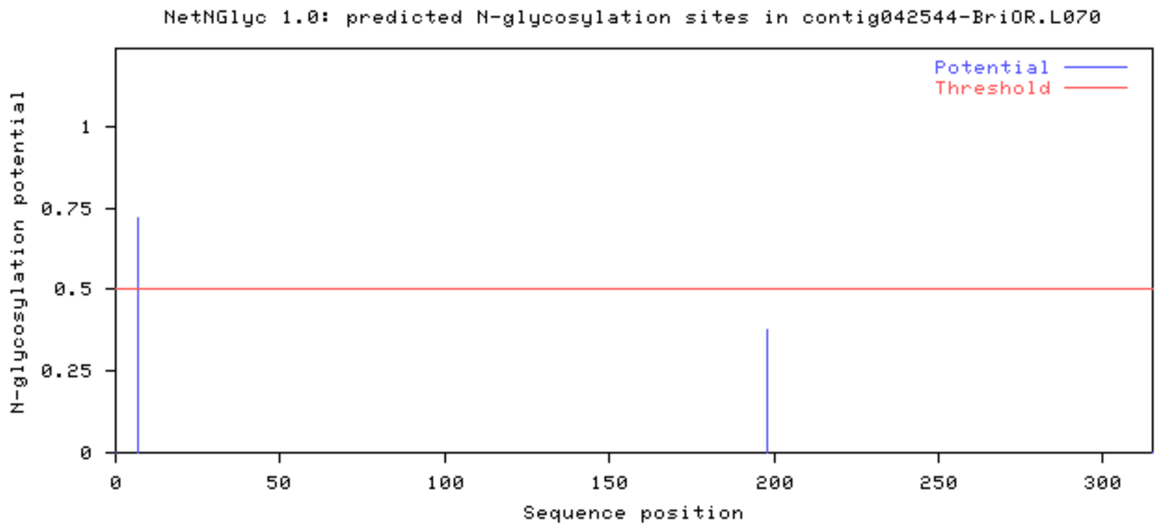

Graphics in PostScript

Output for 'contig042547-BriORe.L076'

```
##########################################################################################

    Warning: This sequence may not contain a signal peptide!!

    Proteins without signal peptides are unlikely to be exposed to
    the N-glycosylation machinery and thus may not be glycosylated
    (in vivo) even though they contain potential motifs.

    SignalP-NN euk predictions are as follows:

  # name                 Cmax  pos ?  Ymax  pos ?  Smax  pos ?  Smean ?  D     ?

    SignalP output is explained at http://www.cbs.dtu.dk/services/SignalP/output.html

##########################################################################################

Name:  contig042547-BriORe.L076         Length:  178
LTNTRIMVLTYILWIVAYSFWASLPATVVPLPHCTLRLRYTFCDAGAVLRTLCVDPQKYFNQGASKLFFLLFFTFVFICL      80
SYCGILFFVKLSSNNDRMKMGSTLVSHLICVICLYCPIFILAILTRFGVVLTLEERQGLSIGTILGPSLVNPFVYFLRTK     160
EIKNKIFKILRKVNTARX
................................................................................      80
................................................................................     160
..................                                                                   240

(Threshold=0.5)

No sites predicted in this sequence.
```

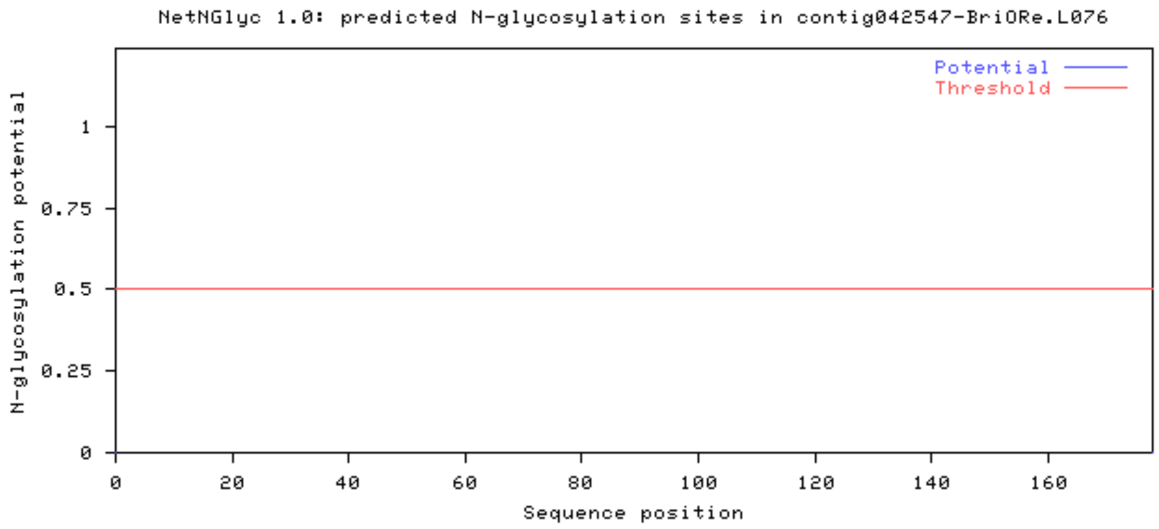

[Graphics in PostScript]

Output for 'contig042552-BriOR.L071'

```
##########################################################################

    Warning: This sequence may not contain a signal peptide!!

    Proteins without signal peptides are unlikely to be exposed to
    the N-glycosylation machinery and thus may not be glycosylated
    (in vivo) even though they contain potential motifs.

    SignalP-NN euk predictions are as follows:

  # name                Cmax  pos ?  Ymax  pos ?  Smax  pos ?  Smean ?  D     ?

    SignalP output is explained at http://www.cbs.dtu.dk/services/SignalP/output.html

##########################################################################

Name:  contig042552-BriOR.L071  Length:  313
MSLQNASIKLTHFIIGGFDTVKRPVAAGVVMLIIYLLAVLASLVNIIFIVSDKQLHKPMYLLICNLAVVDILYTSSATPT      80
MIGVLLAGVNTISYVECIIQMYVYQLGSTMERFSLTIMAFDRLIAIIYPLQYHSYLTITRTLVFTYILWIVACSFVLFPP     160
VTVAPLPHCYSRLRYTFCDYAAVMRTTCVDPEKYFNQVAIISFFLSFFTFTFICLSYCGILFFVKILSNNDKKKMGSTLV     240
SHLICVSCLYCPQFVIVILTRFSVLLALEERQGLLIGTILGPSLVNPFVYCLRTKEIKSKIFKIFRKVNTADX
....N...........................................................................      80
................................................................................     160
................................................................................     240
.........................................................................            320

(Threshold=0.5)
----------------------------------------------------------------------
SeqName      Position  Potential   Jury    N-Glyc
                                 agreement result
----------------------------------------------------------------------
contig042552-BriOR.L071    5 NASI   0.6024     (7/9)   +
----------------------------------------------------------------------
```

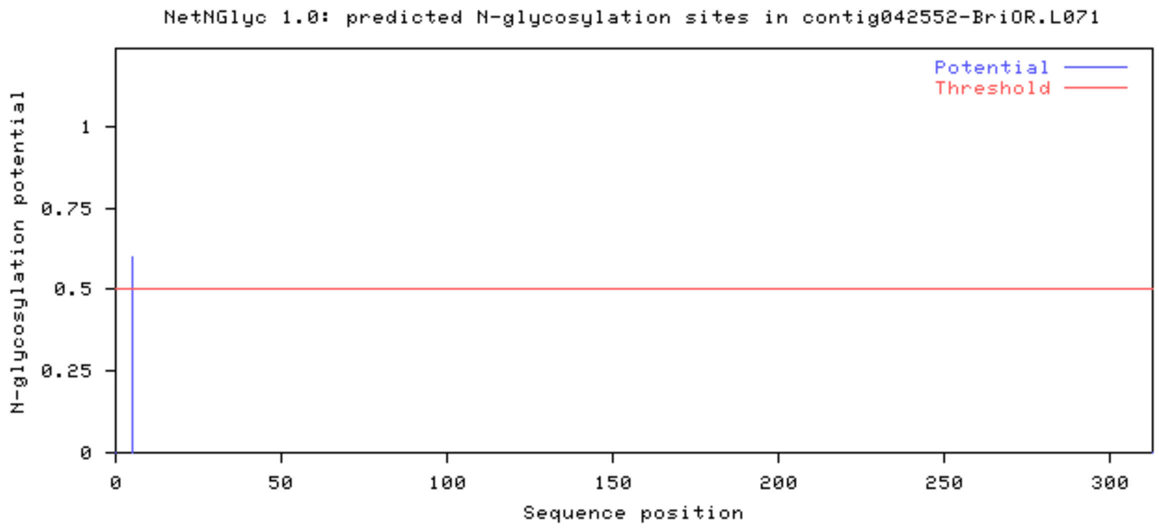

Graphics in PostScript

Output for 'contig042554-BriOR.L072'

```
##########################################################################################

    Warning: This sequence may not contain a signal peptide!!

    Proteins without signal peptides are unlikely to be exposed to
    the N-glycosylation machinery and thus may not be glycosylated
    (in vivo) even though they contain potential motifs.

    SignalP-NN euk predictions are as follows:

  # name                Cmax  pos ?  Ymax  pos ?  Smax  pos ?  Smean ?  D     ?

    SignalP output is explained at http://www.cbs.dtu.dk/services/SignalP/output.html

##########################################################################################

Name:  contig042554-BriOR.L072  Length:  313
MSLQNASINITHFIIGGFDTLSRPIAVGVVILIAYFLAVLANMANIMFIISDKRLHKPMYLLICNLAVVDIMYTSSCSPT      80
MIGVLLAGVNTISYVACLIQMFVFHLGTVMESFVLAVMALDRLIAIIYPFQYHSYLTNTRVLVLTFIVWFVNCFFMCYMP     160
ATVVPLPHCSSRLRYTFCDFAAVLRTTCVNPEKYFNDAAIIAFFIFFFTFVFICLSYCGILLFVKLSSNNEKKKMGSTLV     240
SHLICVIVHFCPPFVRIIFTRFGVVLTLEERQGLLIGAVLGPCLVNPFVYCLRTKEIKQKLFKILKKFHTYDX
....N...N.......................................................................      80
................................................................................     160
................................................................................     240
.........................................................................            320

(Threshold=0.5)
----------------------------------------------------------------------
SeqName      Position  Potential   Jury    N-Glyc
                                agreement  result
----------------------------------------------------------------------
contig042554-BriOR.L072    5 NASI   0.6547     (8/9)    +
contig042554-BriOR.L072    9 NITH   0.6938     (9/9)    ++
----------------------------------------------------------------------
```

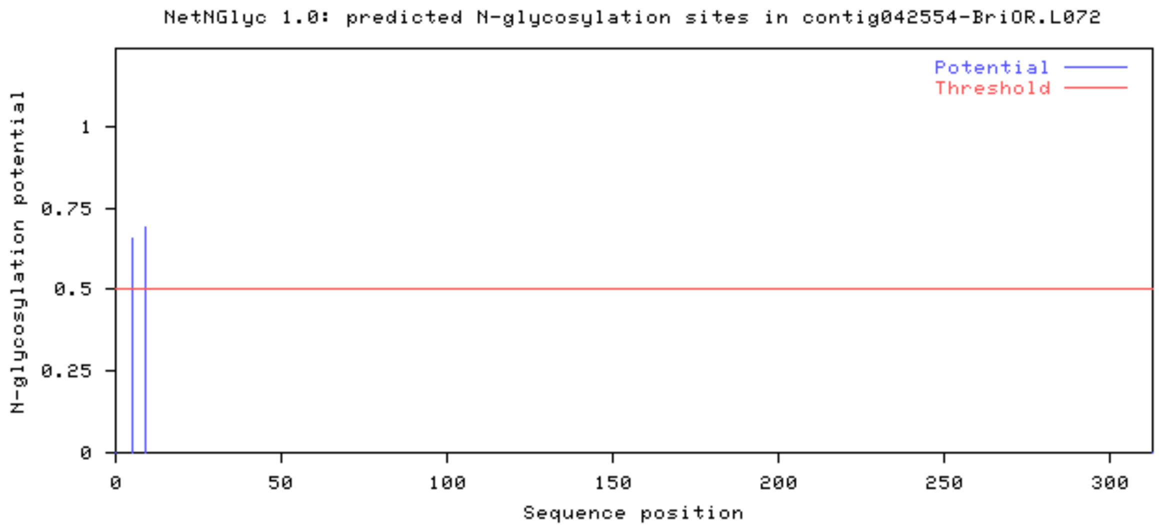

[Graphics in PostScript]

Output for 'contig042554-BriORf.L074'

```
########################################################################################

    Warning: This sequence may not contain a signal peptide!!

    Proteins without signal peptides are unlikely to be exposed to
    the N-glycosylation machinery and thus may not be glycosylated
    (in vivo) even though they contain potential motifs.

    SignalP-NN euk predictions are as follows:

  # name                  Cmax  pos ?  Ymax  pos ?  Smax  pos ?  Smean ?  D     ?

    SignalP output is explained at http://www.cbs.dtu.dk/services/SignalP/output.html

########################################################################################

Name:  contig042554-BriORf.L074         Length:  194
MALDRLIAIIYPFQYHSYLTNTRVLILTFIVWFVNWFFMCYIPATVVPLPHCSSRIRYTFCDFAAVLRTTCVNPDKYFNE      80
VAIVAFLILFFTFIFICLSYCGILLFVKLSSNNEKKKIGSTLVSHLICVIVHYCPAFVRIMFTRLGVVLTLEERQGLLIG     160
AVLGPCLVNPFVYSLRTKEIKNKIFKIFTKKVLX
................................................................................      80
................................................................................     160
..................................                                                   240

(Threshold=0.5)

No sites predicted in this sequence.
```

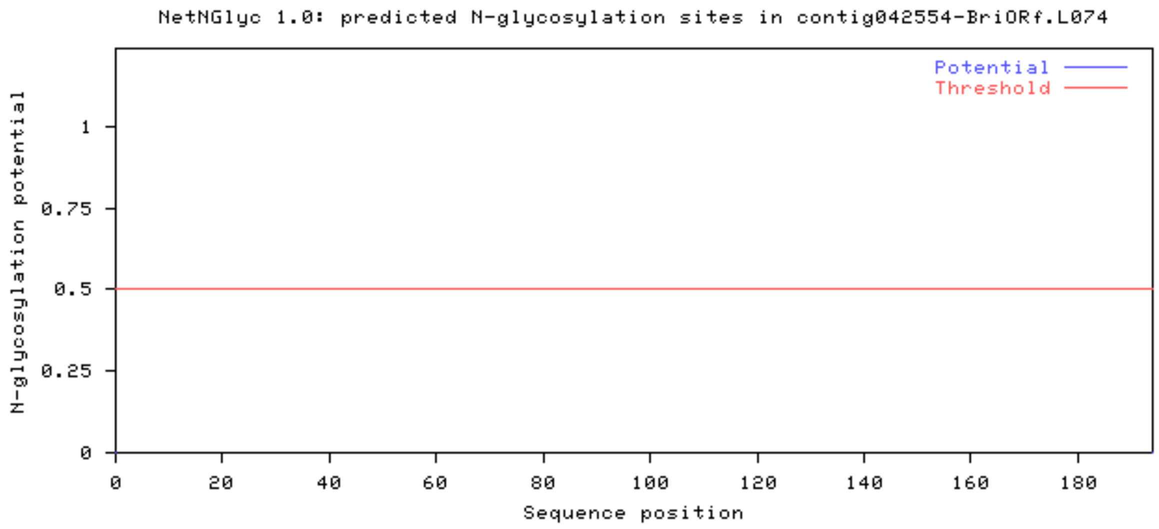

Graphics in PostScript

Output for 'contig042556-BriOR.L073'

```
##########################################################################

    Warning: This sequence may not contain a signal peptide!!

    Proteins without signal peptides are unlikely to be exposed to
    the N-glycosylation machinery and thus may not be glycosylated
    (in vivo) even though they contain potential motifs.

    SignalP-NN euk predictions are as follows:

  # name                    Cmax  pos ?  Ymax  pos ?  Smax  pos ?  Smean ?  D     ?

    SignalP output is explained at http://www.cbs.dtu.dk/services/SignalP/output.html

##########################################################################

Name:  contig042556-BriOR.L073  Length:  313
MSLQNASINVTHFIIGGFDTLSRPIAVGVVILIIYLLAVVANMINIMFIISDKRLHKPMYLLICNLAVVDIVYTSSSSPT      80
MIGVLLAGVNTISYVACLIQMCVFNLGTSMESFVLAFMALDRFIAIIYPFQYQSYLTNTRVLVLTFIMWFVAWCFVFYMP     160
ATVVPLPHCSSRLKYSFCDFAAVIRTTCVNPEKYFNEVSIAAFFIFFFTFVFICLSYCGILLFVKLSSNNEKKKMGSTLV     240
SHLICVIVHYCPAFVRIMFTRVGVVLTLEERQGLLIGAVLGPCLVNPFVYCLRTKEIKQKLFKIFKKFNTSDX
....N...N.......................................................................      80
................................................................................     160
................................................................................     240
.........................................................................            320

(Threshold=0.5)
----------------------------------------------------------------------
SeqName      Position  Potential   Jury    N-Glyc
                                 agreement result
----------------------------------------------------------------------
contig042556-BriOR.L073    5 NASI   0.6716     (8/9)    +
contig042556-BriOR.L073    9 NVTH   0.7233     (9/9)    ++
contig042556-BriOR.L073  309 NTSD   0.4218     (8/9)    -
----------------------------------------------------------------------
```

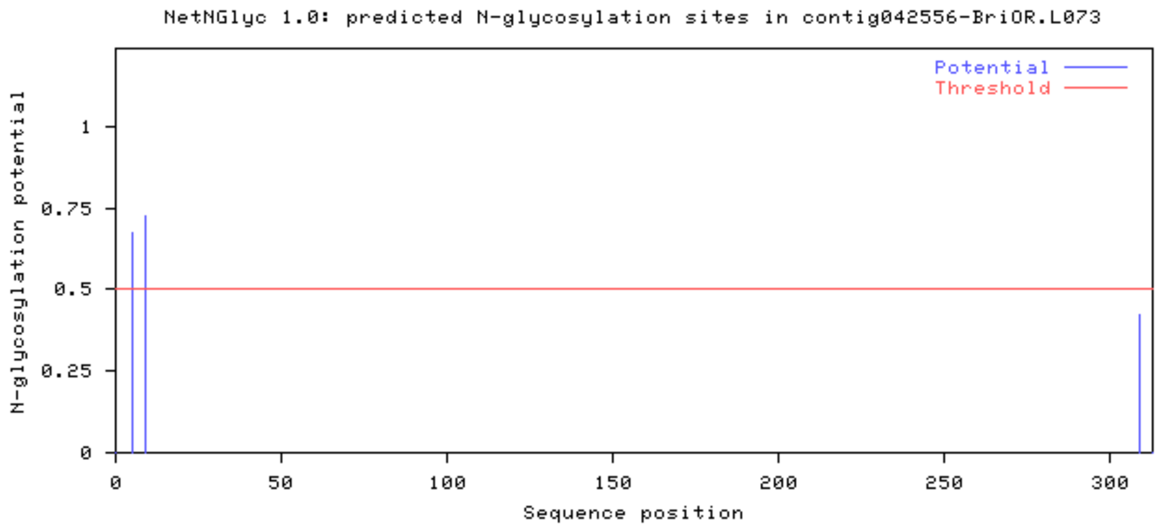

Graphics in PostScript

Output for 'contig042559-BriOR.O077'

```
##########################################################################

    Warning: This sequence may not contain a signal peptide!!

    Proteins without signal peptides are unlikely to be exposed to
    the N-glycosylation machinery and thus may not be glycosylated
    (in vivo) even though they contain potential motifs.

    SignalP-NN euk predictions are as follows:

  # name                 Cmax  pos ?  Ymax  pos ?  Smax  pos ?  Smean ?  D     ?

    SignalP output is explained at http://www.cbs.dtu.dk/services/SignalP/output.html

##########################################################################

Name:  contig042559-BriOR.O077  Length:  331
MPQRNHSVLTEFILTGFPGLHQEYYGLVSAVLFFVYLVTLIANATVIFLIATNRSLHKPMYYIILNLSVCDILFSTTTLP      80
KIISRYWFRSGSISITACFIQMYFVHYLGSVNSFILFQMALDRYLAICHPLRYSHILTKSNILILSITGWIIAKACPLMI     160
VIRAYPLPYCASNTITHCYCDHIGITVLACTDRTPYAIPALVFAMVVLLCPLAFIIFSYCSILIAVYKIANVQGRLKSLS     240
TCSTQLIIISLYYLPRCFVYLASNVGITFSVDVRIVIMLYGLAPPMINPLIYCLRAKDMRESLLKQFFRRIVPEKAQVA     320
AISNSLKTSPX
..........................................N.........N............N..............      80
................................................................................     160
................................................................................     240
................................................................................     320
...........                                                                          400

(Threshold=0.5)
----------------------------------------------------------------------
SeqName      Position  Potential   Jury    N-Glyc
                                 agreement result
----------------------------------------------------------------------
contig042559-BriOR.O077    5 NHSV   0.4837     (4/9)    -
contig042559-BriOR.O077   43 NATV   0.7381     (9/9)    ++
contig042559-BriOR.O077   53 NRSL   0.7400     (9/9)    ++
contig042559-BriOR.O077   66 NLSV   0.7956     (9/9)    +++
----------------------------------------------------------------------
```

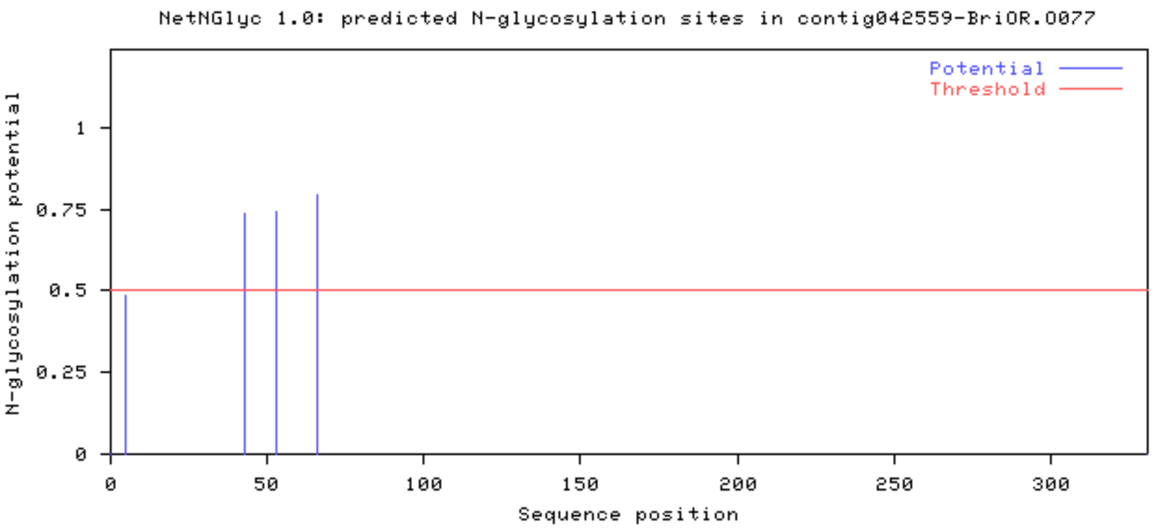

[Graphics in PostScript]

Output for 'contig042560-BriOR.0078'

```
##########################################################################################

    Warning: This sequence may not contain a signal peptide!!

    Proteins without signal peptides are unlikely to be exposed to
    the N-glycosylation machinery and thus may not be glycosylated
    (in vivo) even though they contain potential motifs.

    SignalP-NN euk predictions are as follows:

  # name                 Cmax  pos ?  Ymax  pos ?  Smax  pos ?  Smean ?  D     ?

    SignalP output is explained at http://www.cbs.dtu.dk/services/SignalP/output.html

##########################################################################################

Name:  contig042560-BriOR.0078  Length:  322
MITNVTRMKSFFILGFPGLSPQYYGSISTFLFFVYLAIAVGNIFILSFVSYEKSLQKPTYLVFCHLALNDLTFGTVTLPK      80
IMSKYWFDNSAISFYGCFTQMFFVHYLGSVTSFILLVMALDRFVAICIPLRYPVLITNSVISVLCGFAWFIPLPLMIGVV     160
LHHLTLPFCKSNIIVQCYCDNISIISQACGDDVKSVVVTSLCLAMFCLLLPLAFILFSYISIIVVIMKISSSAGRKRTLS     240
TCTSQIFITCLFYLPRCFVYVANAVGFSFSLDVRIGLILLYSLFPAAVNPVIYCFKTRDIKHMLMKRLKKTKIGLEIKLS     320
PX
...N............................................................................      80
................................................................................     160
....................N...........................................................     240
................................................................................     320
..                                                                                   400

(Threshold=0.5)
----------------------------------------------------------------------
SeqName      Position  Potential   Jury    N-Glyc
                                agreement result
----------------------------------------------------------------------
contig042560-BriOR.0078    4 NVTR    0.7693     (9/9)    +++
contig042560-BriOR.0078  181 NISI    0.6373     (9/9)    ++
----------------------------------------------------------------------
```

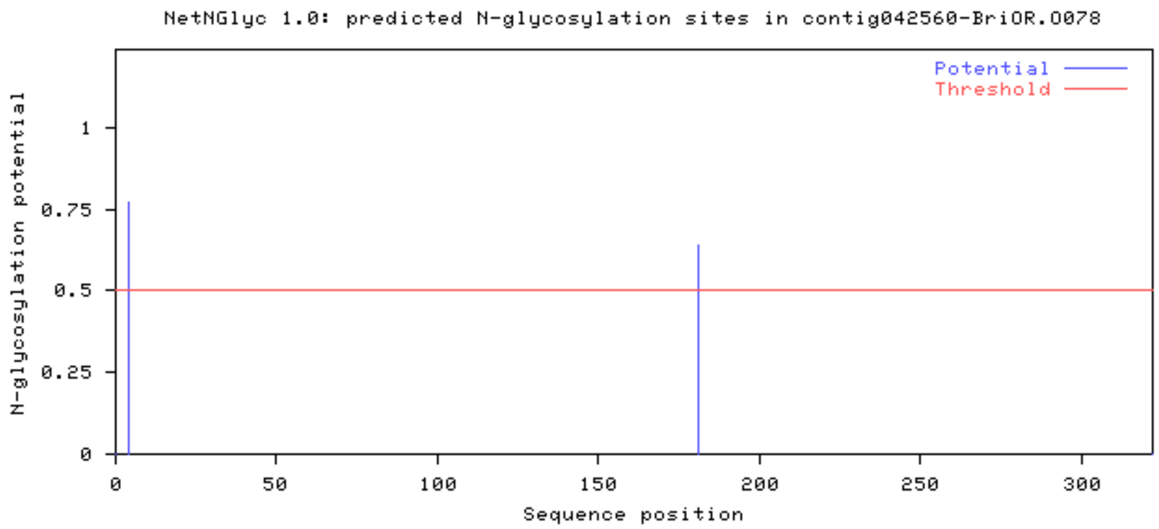

Graphics in PostScript

Output for 'contig042561-BriORe.O082'

```
##########################################################################################

    Warning: This sequence may not contain a signal peptide!!

    Proteins without signal peptides are unlikely to be exposed to
    the N-glycosylation machinery and thus may not be glycosylated
    (in vivo) even though they contain potential motifs.

    SignalP-NN euk predictions are as follows:

  # name                 Cmax  pos ?  Ymax  pos ?  Smax  pos ?  Smean ?  D     ?

    SignalP output is explained at http://www.cbs.dtu.dk/services/SignalP/output.html

##########################################################################################

Name:  contig042561-BriORe.O082        Length:  110
MSFNFTNVTRMKSFFILGFPGLSPQYYGPISTFLFFVYLAIAVGNIFILSFVAYEKSLQKPTYLVFCHLALNDLTFGTVT      80
LPKIMSKYWFDNSVISFYGCFTQMFFVHYL
...N..N.........................................................................      80
..............................                                                        160

(Threshold=0.5)
----------------------------------------------------------------------
SeqName       Position  Potential   Jury    N-Glyc
                                  agreement result
----------------------------------------------------------------------
contig042561-BriORe.O082     4 NFTN    0.7090     (9/9)    ++
contig042561-BriORe.O082     7 NVTR    0.6731     (8/9)    +
----------------------------------------------------------------------
```

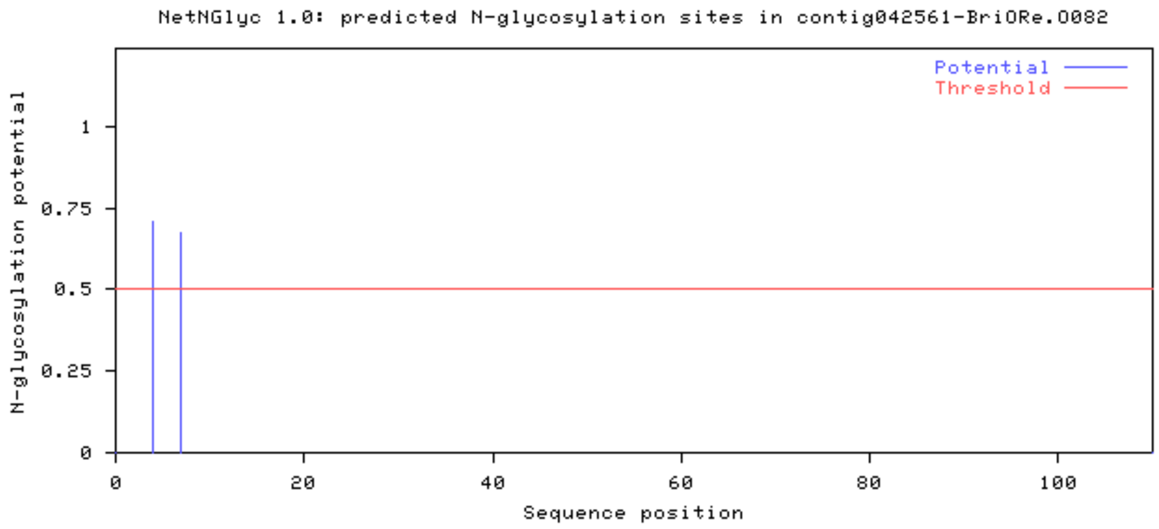

Graphics in PostScript

Output for 'contig042562-BriOR.O079'

```
##########################################################################

    Warning: This sequence may not contain a signal peptide!!

    Proteins without signal peptides are unlikely to be exposed to
    the N-glycosylation machinery and thus may not be glycosylated
    (in vivo) even though they contain potential motifs.

    SignalP-NN euk predictions are as follows:

  # name                    Cmax   pos ?  Ymax   pos ?  Smax   pos ?  Smean ?  D     ?

    SignalP output is explained at http://www.cbs.dtu.dk/services/SignalP/output.html

##########################################################################

Name:  contig042562-BriOR.0079  Length:  324
MKYTNITTIKEFIIIGFPGLPPEYYGPVSVLLLLVFLAIVIGNGFTIAVIIFERTLHKPIYVIFSNLAMTDICFGVVTLP      80
KIIGRYWWNDMITSFGACFTQMYFVHSLGAIQSLNLLMMALDRFVAIWFPFKYPISFTNKTVAIACTMCWVLTFIRLLGI     160
ALHALTLPYCDQNIIMQCYCDLISITRLGCSDEREYVYSVALANAMFTLLVPLTFIILSYFSVIIAVLRMSQTERRHKVL     240
STCAPQLFITCLYYVPRCFVYIANGVGFNFSLVIRIIITMMFSLIPAAVNPIIYCFKTKDIKNVLMRRFKKGKVSTGLKT     320
ECKX
....N...........................................................................      80
..........................................................N.....................     160
................................................................................     240
................................................................................     320
....                                                                                 400

(Threshold=0.5)
----------------------------------------------------------------------
SeqName       Position  Potential   Jury    N-Glyc
                                 agreement  result
----------------------------------------------------------------------
contig042562-BriOR.0079    5 NITT   0.7656     (9/9)   +++
contig042562-BriOR.0079  139 NKTV   0.6400     (9/9)   ++
contig042562-BriOR.0079  269 NFSL   0.4038     (8/9)   -
----------------------------------------------------------------------
```

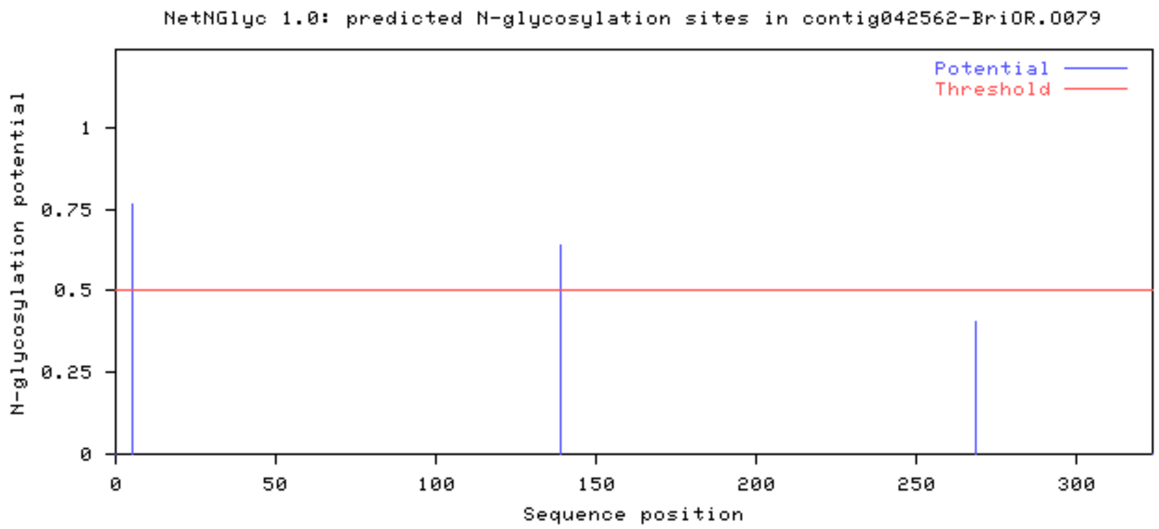

Graphics in PostScript

Output for 'contig042565-BriORe.O081'

```
##########################################################################################

    Warning: This sequence may not contain a signal peptide!!

    Proteins without signal peptides are unlikely to be exposed to
    the N-glycosylation machinery and thus may not be glycosylated
    (in vivo) even though they contain potential motifs.

    SignalP-NN euk predictions are as follows:

  # name                 Cmax  pos ?  Ymax  pos ?  Smax  pos ?  Smean ?  D     ?

    SignalP output is explained at http://www.cbs.dtu.dk/services/SignalP/output.html

##########################################################################################

Name:  contig042565-BriORe.O081        Length:  125
MKYTNITTIKEFIIIGFPGLPPEYYGPVSVLLLLVFLAIVIGNGFTIAVIIFERTLHKPIYVIFSNLAMTDICFGVVTLP      80
KIIGRYWWNDMITSFGACFTQMYFVHSLGAIQSLNLLMMALDRFV
....N...........................................................................      80
.............................................                                         160

(Threshold=0.5)
----------------------------------------------------------------------
SeqName      Position  Potential   Jury    N-Glyc
                                agreement result
----------------------------------------------------------------------
contig042565-BriORe.O081    5 NITT   0.7662     (9/9)    +++
----------------------------------------------------------------------
```

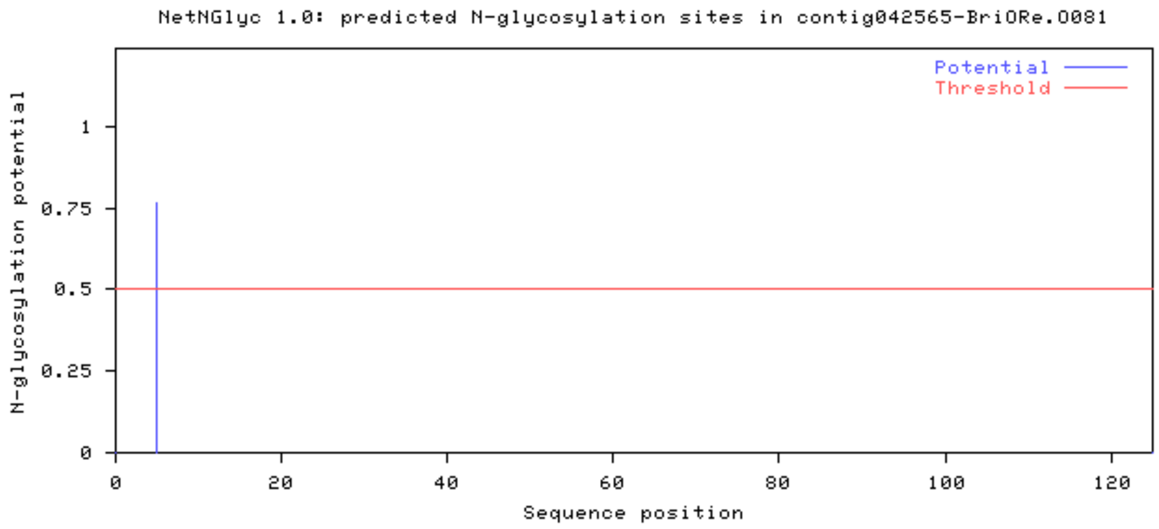

Graphics in PostScript

Output for 'contig049866-BriORp.H062'

```
########################################################################################

    Warning: This sequence may not contain a signal peptide!!

    Proteins without signal peptides are unlikely to be exposed to
    the N-glycosylation machinery and thus may not be glycosylated
    (in vivo) even though they contain potential motifs.

    SignalP-NN euk predictions are as follows:

  # name                Cmax  pos ?  Ymax  pos ?  Smax  pos ?  Smean ?  D     ?

    SignalP output is explained at http://www.cbs.dtu.dk/services/SignalP/output.html

########################################################################################

Name:  contig049866-BriORp.H062         Length:  299
MDNVHNVEVSFLLGFNETMNFRVPLFSVTLLYYCVILFFNVSLVLLIVLDVNLHEPMYIFLSSFCINALYGSTGFYPKFL      80
SDLLSSSQTISHEGCLLQAFIMYSSVCCNSSILAVMAFDRYLAICRPLHYHSFMSKRRLSQLVCFSWLTPLCIFAINVVL     160
TARLKLCGTKIQRVLCLNWLIVKLACPEADTFSNNITAYATVIIYVSHGFFIIWTYIHLIKTCAKSREDRAKFMQTCVPH     240
LTSLITLIFDSTDLPESLQNFIATEFVIIPPIMNPLIYGFKLTKIRNRILGLVCFKRKX
...............N.......................N........................................      80
............................N...................................................     160
..................................N.............................................     240
...........................................................                          320

(Threshold=0.5)
----------------------------------------------------------------------
SeqName       Position  Potential   Jury    N-Glyc
                                  agreement result
----------------------------------------------------------------------
contig049866-BriORp.H062   16 NETM   0.6930     (9/9)   ++
contig049866-BriORp.H062   40 NVSL   0.7068     (9/9)   ++
contig049866-BriORp.H062  109 NSSI   0.5223     (6/9)   +
contig049866-BriORp.H062  195 NITA   0.6136     (8/9)   +
----------------------------------------------------------------------
```

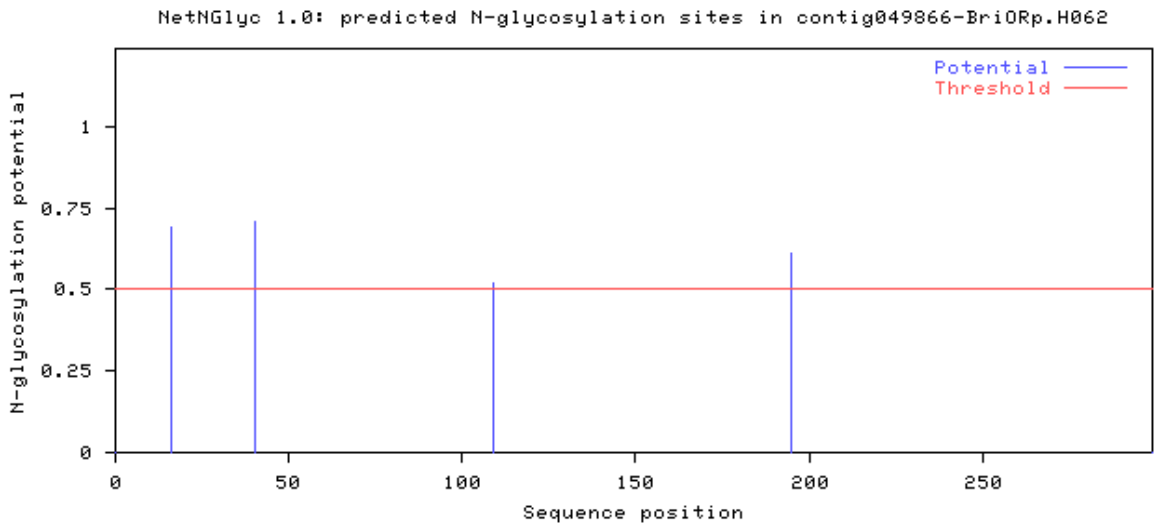

Graphics in PostScript

Output for 'contig049869-BriORe.H126'

```
##########################################################################################

    Warning: This sequence may not contain a signal peptide!!

    Proteins without signal peptides are unlikely to be exposed to
    the N-glycosylation machinery and thus may not be glycosylated
    (in vivo) even though they contain potential motifs.

    SignalP-NN euk predictions are as follows:

  # name                    Cmax   pos ?  Ymax   pos ?  Smax   pos ?  Smean ?  D      ?

    SignalP output is explained at http://www.cbs.dtu.dk/services/SignalP/output.html

##########################################################################################

Name:  contig049869-BriORe.H126         Length:  178
VCFSWLTPLCIFAINILLTTRLKLCGIKIQRVLCLNWLIIKLACPADKTFPNNIIPYAILVIYLSHWLFIIWTYMHLIKT      80
CARSRDDRLKFMQTCVPHLTSLIIFLIVMAIDPVYMEFGSRDLSQTLQNLFSIKFLIIPPVMNPLIYGFKITKIRNRILG     160
LVCFKRQQISLMFVIHKX
................................................................................      80
................................................................................     160
..................                                                                   240

(Threshold=0.5)

No sites predicted in this sequence.
```

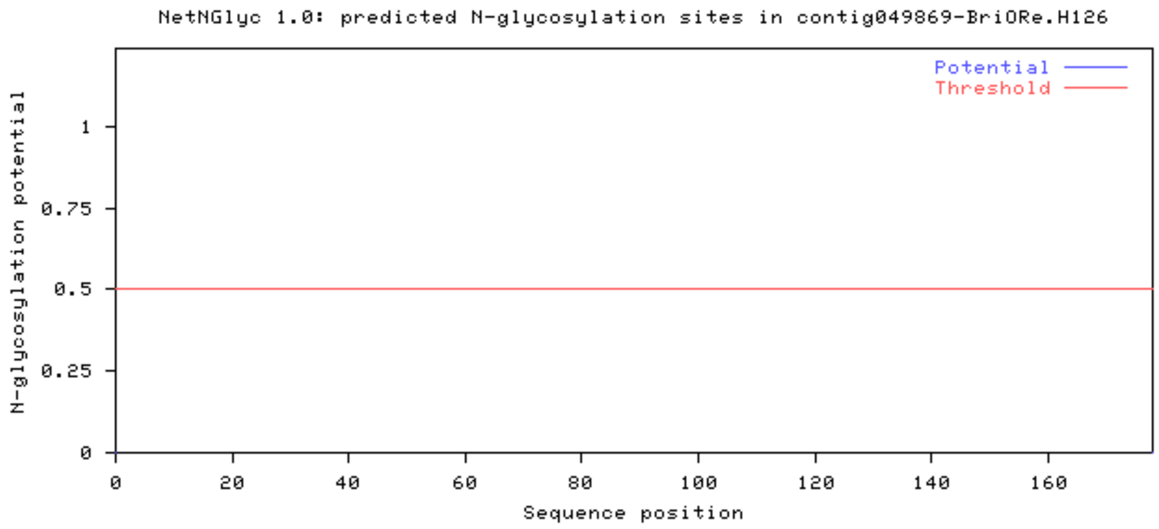

Graphics in PostScript

Output for 'contig049870-BriORf.H060'

```
########################################################################################

    Warning: This sequence may not contain a signal peptide!!

    Proteins without signal peptides are unlikely to be exposed to
    the N-glycosylation machinery and thus may not be glycosylated
    (in vivo) even though they contain potential motifs.

    SignalP-NN euk predictions are as follows:

  # name                Cmax  pos ?  Ymax  pos ?  Smax  pos ?  Smean ?  D     ?

    SignalP output is explained at http://www.cbs.dtu.dk/services/SignalP/output.html

########################################################################################

Name:  contig049870-BriORf.H060         Length:  148
IYLCIYFSIQRVLCLNWLIVKLACPEADTFSNNISSYVTFIFYVSHGFFIMWTYTHLIKTFVRSRDDRLKFMQTCVPHLT      80
SLITFLSVIVFQYVYLRLDSTDLFQSLQNFIAIEFLIIPPVMNPLVYGFKLAKIRKRIFTLVHDKTKX
................................N...............................................      80
....................................................................                 160

(Threshold=0.5)
----------------------------------------------------------------------
SeqName      Position  Potential   Jury    N-Glyc
                                 agreement result
----------------------------------------------------------------------
contig049870-BriORf.H060   33 NISS   0.6978     (8/9)   +
----------------------------------------------------------------------
```

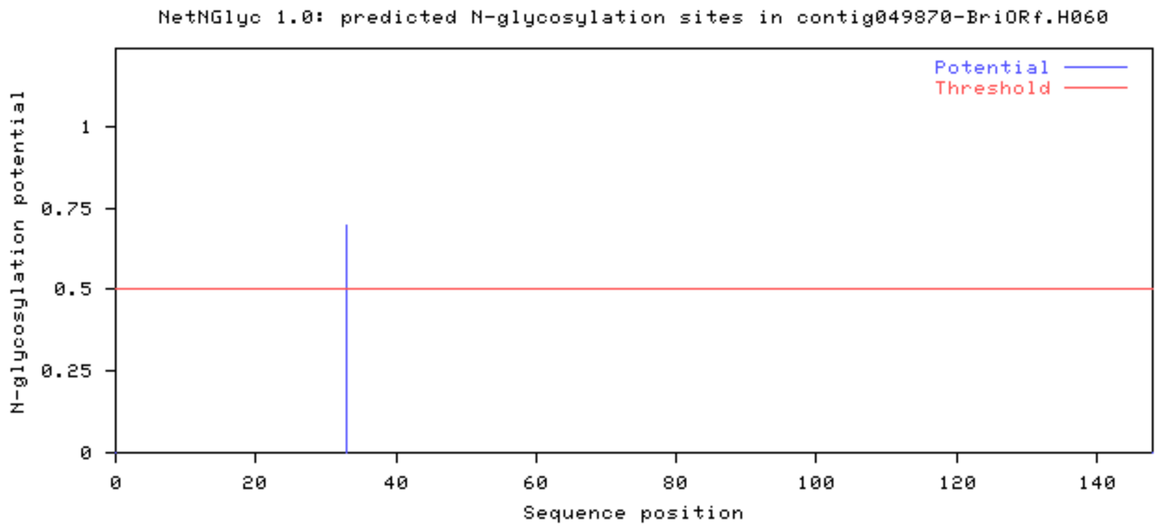

Graphics in PostScript

Output for 'contig049872-BriORe.H057'

```
##########################################################################################

    Warning: This sequence may not contain a signal peptide!!

    Proteins without signal peptides are unlikely to be exposed to
    the N-glycosylation machinery and thus may not be glycosylated
    (in vivo) even though they contain potential motifs.

    SignalP-NN euk predictions are as follows:

  # name                Cmax  pos ?  Ymax  pos ?  Smax  pos ?  Smean ?  D     ?

    SignalP output is explained at http://www.cbs.dtu.dk/services/SignalP/output.html

##########################################################################################

Name:  contig049872-BriORe.H057         Length:  125
MDNVSVVRMFTLSGFNETMNIRLTIFSLTLMYYCMIILINVSLIVIIVLDENLHEPMYILLSSFCINAIYGTTGFYPKFL      80
LDLLLSSQEISYEGCLLQAFIMYSFACCDLSILAVMAFDRYLAIC
..N............N........................N.......................................      80
.............................................                                         160

(Threshold=0.5)
----------------------------------------------------------------------
SeqName      Position  Potential   Jury    N-Glyc
                                 agreement  result
----------------------------------------------------------------------
contig049872-BriORe.H057     3 NVSV   0.8090     (9/9)    +++
contig049872-BriORe.H057    16 NETM   0.6214     (9/9)    ++
contig049872-BriORe.H057    40 NVSL   0.7607     (9/9)    +++
----------------------------------------------------------------------
```

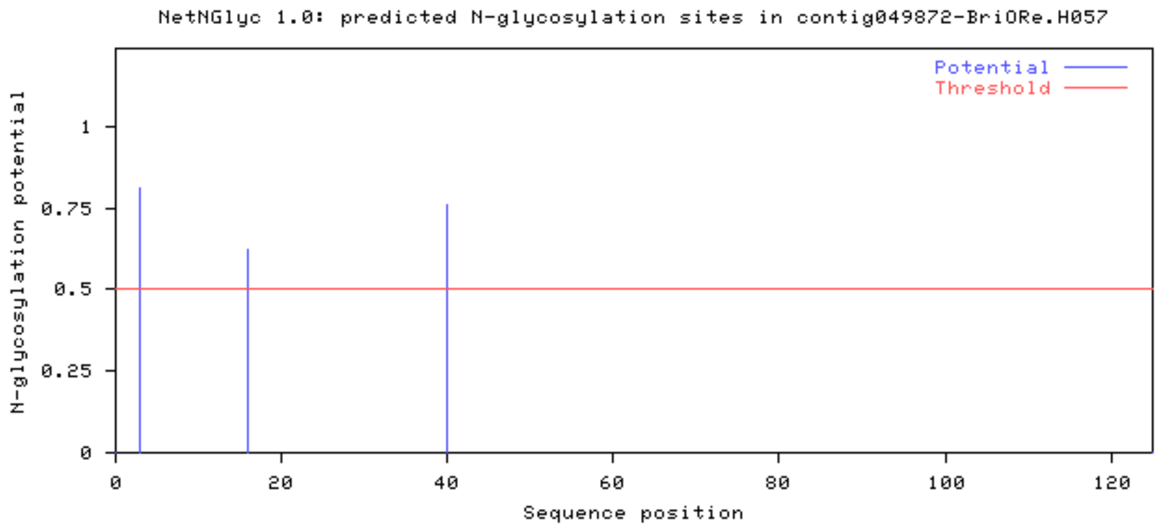

Graphics in PostScript

Output for 'contig049873-BriOR.H050'

```
################################################################################

    Warning: This sequence may not contain a signal peptide!!

    Proteins without signal peptides are unlikely to be exposed to
    the N-glycosylation machinery and thus may not be glycosylated
    (in vivo) even though they contain potential motifs.

    SignalP-NN euk predictions are as follows:

  # name                    Cmax  pos ?  Ymax  pos ?  Smax  pos ?  Smean ?  D     ?

    SignalP output is explained at http://www.cbs.dtu.dk/services/SignalP/output.html

################################################################################

Name:  contig049873-BriOR.H050  Length:  329
MDNVSNVRSFILSGFNETMNFRVPLFTFTLLYYCMILFFNVSLVLLIFLDENLHEPMYIFLSSFCINTIYGTTGFYPKFL      80
SDLLRSSQTISYEGCLLQAFVIYSFVCCDLSILAVMAFDRYLAICRPLHYHSFMTKRRLSQLVCFSWLTPLCIFAINVLL     160
TSRLKLCGINIQRVLCVNWLIVKLACPEAETFTNHIAAYATVIIYVSHGFFIMWTYTHLIKTCARSREDREKFMQTCLPH     240
LTSLITFIVVIGFQSIYVQFEYTGFSESLKNFITVEILIIPPFMNPLIYGFKLTKIQNRILTLLKSYKKLTYIKFSLLKK     320
TRVKCSIFL
..N............N.......................N........................................      80
................................................................................     160
................................................................................     240
................................................................................     320
.........                                                                            400

(Threshold=0.5)
----------------------------------------------------------------------
SeqName      Position  Potential   Jury    N-Glyc
                                 agreement result
----------------------------------------------------------------------
contig049873-BriOR.H050    3 NVSN   0.7936     (9/9)   +++
contig049873-BriOR.H050   16 NETM   0.6270     (9/9)   ++
contig049873-BriOR.H050   40 NVSL   0.7250     (9/9)   ++
----------------------------------------------------------------------
```

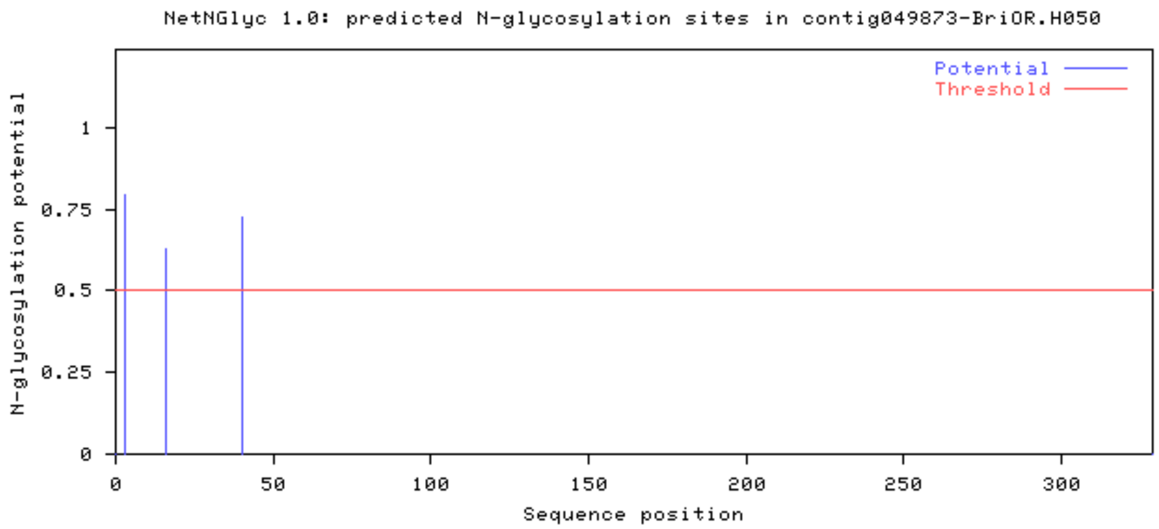

Graphics in PostScript

Output for 'contig054997-BriORe.N090'

```
##########################################################################################

    Warning: This sequence may not contain a signal peptide!!

    Proteins without signal peptides are unlikely to be exposed to
    the N-glycosylation machinery and thus may not be glycosylated
    (in vivo) even though they contain potential motifs.

    SignalP-NN euk predictions are as follows:

  # name                 Cmax  pos ?  Ymax  pos ?  Smax  pos ?  Smean ?  D     ?

    SignalP output is explained at http://www.cbs.dtu.dk/services/SignalP/output.html

##########################################################################################

Name:  contig054997-BriORe.N090        Length:  269
MALINSADENNITFMRPAYFIISGFIGIPNIRYYFVFLCFIYILAVVGNTLVMIVITLDHTLRSPKYIAVFNLAFTDLLS      80
SSSLMPKVLDIFLFNHYYISYNDCLAFMFFSFTFYAMQAFNLVVLSFDRVMAIMYPLHYQMRVSHKLILSLIAFFWLLAI     160
TLILIEVGLLTRLSFCKSVVIQSYFCDHGPIYRLGCNDITPNLAIGRLAPVIFLGFPLAFIVGSYCCIGYSLSKISTFRE     240
RVKAFKTCTGHLSLVAIYFLPVTFVYIFG
..........N.....................................................................      80
................................................................................     160
................................................................................     240
.............................                                                        320

(Threshold=0.5)
----------------------------------------------------------------------
SeqName      Position  Potential   Jury    N-Glyc
                                 agreement result
----------------------------------------------------------------------
contig054997-BriORe.N090   11 NITF   0.6127     (8/9)   +
----------------------------------------------------------------------
```

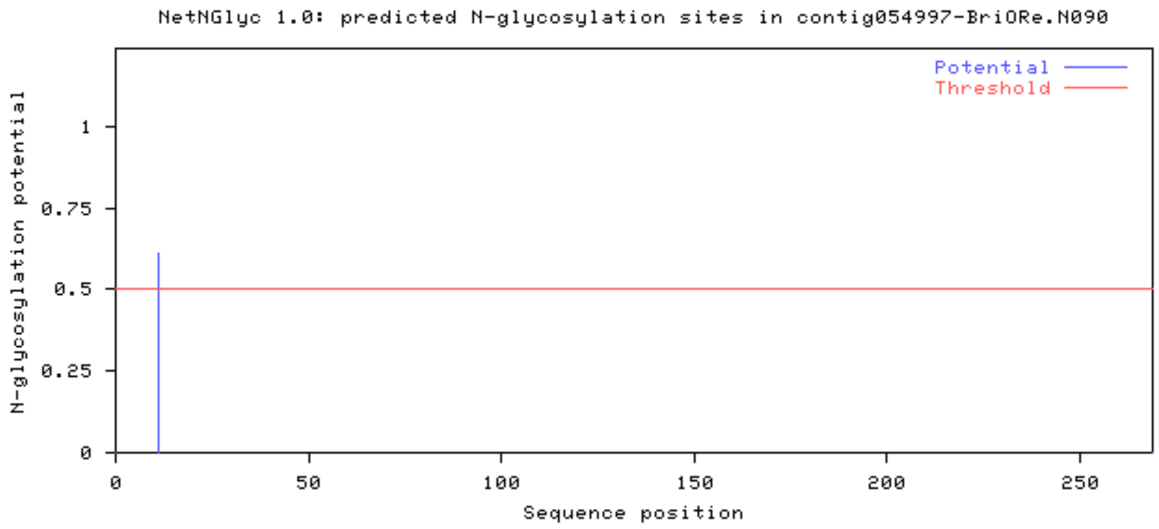

Graphics in PostScript

Output for 'contig055002-BriORp.N091'

```
##########################################################################################

    Warning: This sequence may not contain a signal peptide!!

    Proteins without signal peptides are unlikely to be exposed to
    the N-glycosylation machinery and thus may not be glycosylated
    (in vivo) even though they contain potential motifs.

    SignalP-NN euk predictions are as follows:

  # name                Cmax  pos ?  Ymax  pos ?  Smax  pos ?  Smean ?  D     ?

    SignalP output is explained at http://www.cbs.dtu.dk/services/SignalP/output.html

##########################################################################################

Name:  contig055002-BriORp.N091        Length:  312
MALINSAAENNITFVRPAYFIISGFIGIPNIRYYFVFLCFIYILAVVGNTLVMIVITLDHMLRSPKYIAVFNLAFTDLLS      80
SSSLVPKVVDIFLFNHYYISYNDCLTFMFFCFTLISMQAFNLVVLSFDRVMAIMYPLHYQMRVSHKLILSLITFFWLLAI     160
TLVLIAVGLLTRLSFCKSVVIQSYYCDHGPIYRLGCNDVTPNRAIAGLAPVINLGFPLAFIVGSYCCIGYSLSKTSTFRE     240
RVKAFKTCTGHLLVAIYFLPVTFVYIFGNVIHPNARIISLSISTVLPPMLNPIIYVLQTQEIKESLKKLLKT
..........N.....................................................................      80
................................................................................     160
................................................................................     240
........................................................................             320

(Threshold=0.5)
----------------------------------------------------------------------
SeqName      Position  Potential   Jury    N-Glyc
                                 agreement result
----------------------------------------------------------------------
contig055002-BriORp.N091    11 NITF    0.6511     (9/9)    ++
----------------------------------------------------------------------
```

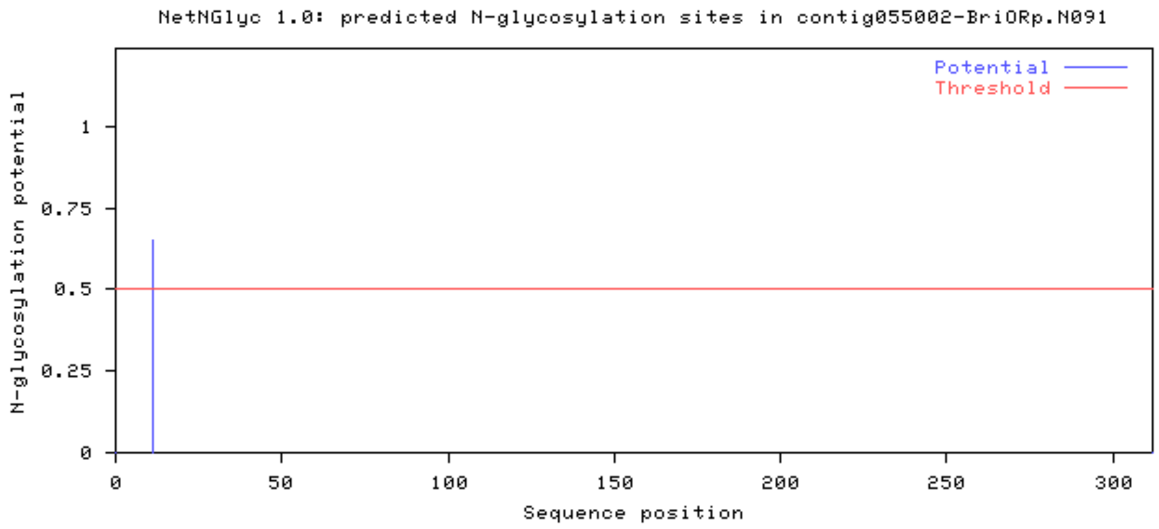

Graphics in PostScript

Output for 'contig055007-BriORe.N092'

```
##########################################################################################

    Warning: This sequence may not contain a signal peptide!!

    Proteins without signal peptides are unlikely to be exposed to
    the N-glycosylation machinery and thus may not be glycosylated
    (in vivo) even though they contain potential motifs.

    SignalP-NN euk predictions are as follows:

  # name                Cmax  pos ?  Ymax  pos ?  Smax  pos ?  Smean ?  D     ?

    SignalP output is explained at http://www.cbs.dtu.dk/services/SignalP/output.html

##########################################################################################

Name:  contig055007-BriORe.N092        Length:  252
LAFTDLLSSSALVPKKVVDIFLFNHYYISYNDCLTFLFFCFTLIFMQGFNLVVMSFDRVMAIMYPLHYQMRVSHKLILSLI      80
AFFWLLAITLILIAVGLLTRLSFCKSVVIQSYYCDHGPMYRLGCNDNTPNHAFAGSALVIILGFPLAFIVGSYCCIGYAL     160
SKISTFRERVKAFKTYTGHLSLVAIYFLPFTFVYMFGGVIHPNARIISLSISTALPPMLNPIIYVLQTQEIKESLNKLLK     240
TRVTSKIATKYX
................................................................................      80
................................................................................     160
................................................................................     240
............                                                                         320

(Threshold=0.5)

No sites predicted in this sequence.
```

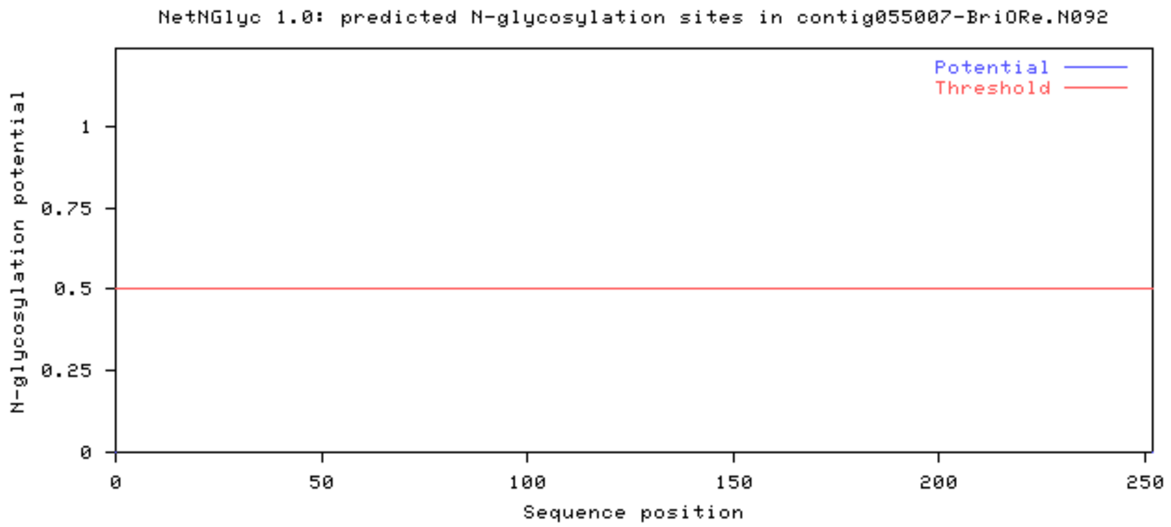

Graphics in PostScript

Output for 'contig114800-BriORp.A020'

```
##########################################################################

   Warning: This sequence may not contain a signal peptide!!

   Proteins without signal peptides are unlikely to be exposed to
   the N-glycosylation machinery and thus may not be glycosylated
   (in vivo) even though they contain potential motifs.

   SignalP-NN euk predictions are as follows:

  # name                Cmax  pos ?  Ymax  pos ?  Smax  pos ?  Smean ?  D     ?

   SignalP output is explained at http://www.cbs.dtu.dk/services/SignalP/output.html

##########################################################################

Name:  contig114800-BriORp.A020         Length:  148
MDNKLNLTYITLNGYVEVEKYRYVYFLIIFTIYAAVIFSNSTIICLILFHQSLHEPMYIFIAALLLQRCREVRKKAAQTC      80
LPHLLVLFNYSFFCTCEVIILRLESDISQTVRLIMTLQVVLYHPLFNPIIYGLKMKKISKHLKRLFCX
.....N.................................N........................................      80
........N...........................................................     160

(Threshold=0.5)
----------------------------------------------------------------------
SeqName      Position  Potential   Jury    N-Glyc
                                 agreement result
----------------------------------------------------------------------
contig114800-BriORp.A020     6 NLTY    0.7540     (9/9)    +++
contig114800-BriORp.A020    40 NSTI    0.6541     (9/9)    ++
contig114800-BriORp.A020    89 NYSF    0.5921     (9/9)    ++
----------------------------------------------------------------------
```

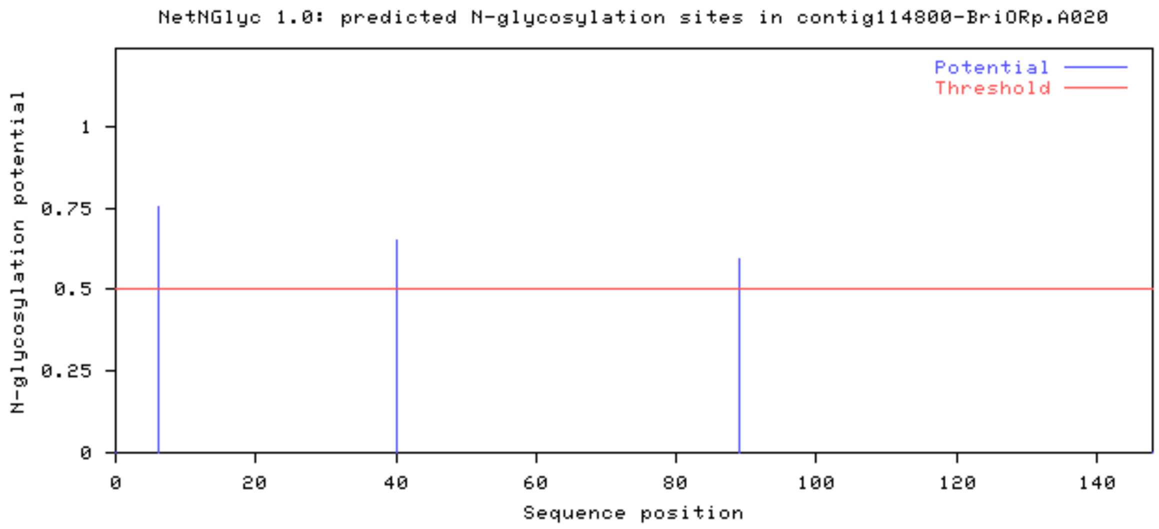

Graphics in PostScript

Output for 'contig060183-BriORp.S128'

```
##########################################################################################

    Warning: This sequence may not contain a signal peptide!!

    Proteins without signal peptides are unlikely to be exposed to
    the N-glycosylation machinery and thus may not be glycosylated
    (in vivo) even though they contain potential motifs.

    SignalP-NN euk predictions are as follows:

  # name                Cmax  pos ?  Ymax  pos ?  Smax  pos ?  Smean ?  D     ?

    SignalP output is explained at http://www.cbs.dtu.dk/services/SignalP/output.html

##########################################################################################

Name:  contig060183-BriORp.S128        Length:  168
MAGNNTFICAFLEPLTDRIMTVQILVIIFLCINMLLIVTFFKKECFYTSACYILFFVTLLSDSFLLLVTDILFILSNFRC      80
SFQVWLCIIISLFVLVYSIVTPITLTAMTLECYVAICMPLRHGQLCFKCSTMYCILIIHGVSSGPYIIIILSMFFVSASLK    160
FYKQYVQE
...N............................................................................      80
................................................................................     160
........                                                                             240

(Threshold=0.5)
----------------------------------------------------------------------
SeqName      Position  Potential   Jury    N-Glyc
                                 agreement result
----------------------------------------------------------------------
contig060183-BriORp.S128    4 NNTF   0.7280     (9/9)    ++
----------------------------------------------------------------------
```

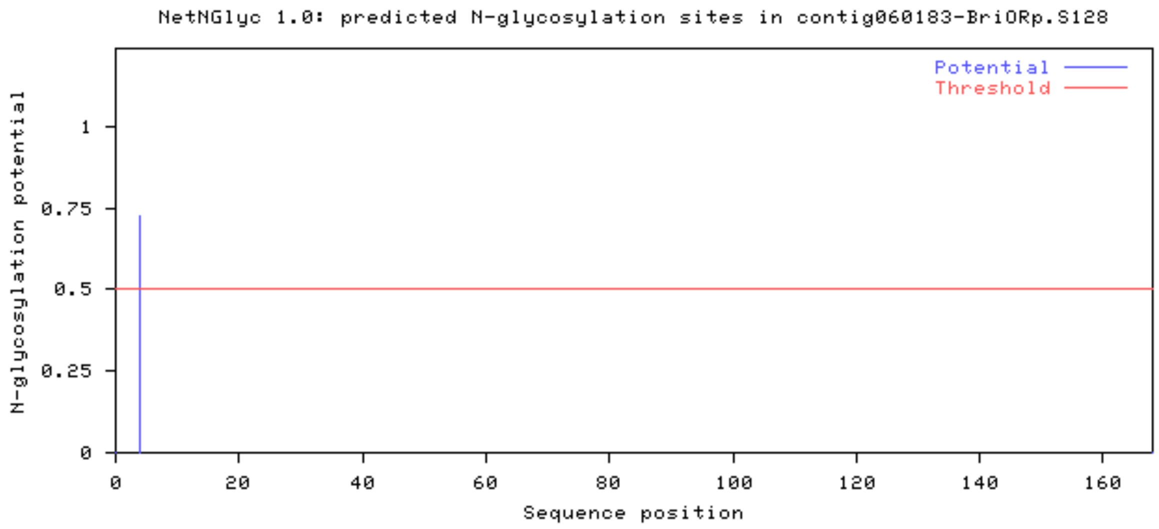

Graphics in PostScript

Output for 'contig060187-BriORe.S108'

```
##########################################################################################

    Warning: This sequence may not contain a signal peptide!!

    Proteins without signal peptides are unlikely to be exposed to
    the N-glycosylation machinery and thus may not be glycosylated
    (in vivo) even though they contain potential motifs.

    SignalP-NN euk predictions are as follows:

  # name                Cmax  pos ?  Ymax  pos ?  Smax  pos ?  Smean ?  D     ?

    SignalP output is explained at http://www.cbs.dtu.dk/services/SignalP/output.html

##########################################################################################

Name:  contig060187-BriORe.S108        Length:  111
MAGNNSVNDVFFQQSISYRVIIVQILVIIFLCINMLLIVIFVKKKSFHTSARYILFFITLLSDSVLLLLSDVLLVLSQFE      80
FTMPVWLCITISVVVLLYFIVTPVTLTAMTL
...N............................................................................      80
...............................                                                      160

(Threshold=0.5)
----------------------------------------------------------------------
SeqName      Position  Potential   Jury    N-Glyc
                                 agreement result
----------------------------------------------------------------------
contig060187-BriORe.S108    4 NNSV   0.7382     (9/9)   ++
----------------------------------------------------------------------
```

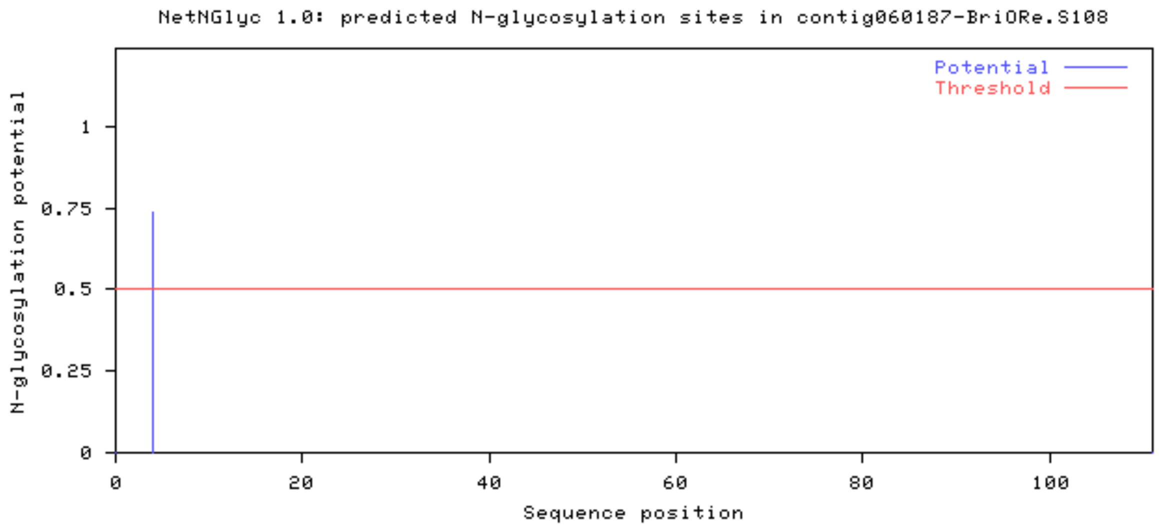

Graphics in PostScript

Output for 'contig060190-BriORe.S105'

```
########################################################################################

    Warning: This sequence may not contain a signal peptide!!

    Proteins without signal peptides are unlikely to be exposed to
    the N-glycosylation machinery and thus may not be glycosylated
    (in vivo) even though they contain potential motifs.

    SignalP-NN euk predictions are as follows:

  # name                  Cmax  pos ?  Ymax  pos ?  Smax  pos ?  Smean ?  D     ?

    SignalP output is explained at http://www.cbs.dtu.dk/services/SignalP/output.html

########################################################################################

Name:  contig060190-BriORe.S105         Length:  294
MAGNNSVNYVFFQRPVNDRVIIVQILVIIFLCINMLLIVIFVKKESFHTSARYILFFVTLLSDSVLLLLSDVLLILTYFE      80
ITIQVWLCITISVVVLLYFIVTPVTLTAMTLERYVAICMPLRHGQLCSTHSTMYCILIIHVLSSGPCIIILSMFFASASL     160
KFYKQSMICSVETFTLFRWQDHVRSAVYQLCFLIMGTTIAYSYVQIMKVAKAASGEKKKLTHKGLKTVILHAFQLLLCLI     240
QLWCPFIEIAVLQIDFILFLNVRYFNYIMFNIAPRCLSPLIYGIRDKNIFLVLK
...N............................................................................      80
................................................................................     160
................................................................................     240
......................................................                               320

(Threshold=0.5)
----------------------------------------------------------------------
SeqName      Position  Potential   Jury    N-Glyc
                                 agreement result
----------------------------------------------------------------------
contig060190-BriORe.S105    4 NNSV   0.7216     (9/9)    ++
----------------------------------------------------------------------
```

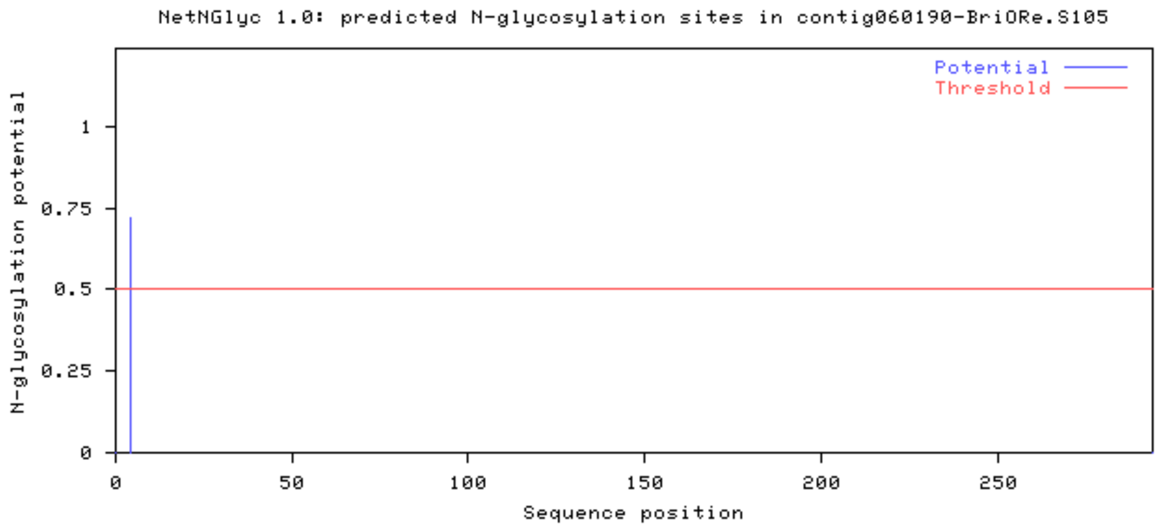

Graphics in PostScript

Output for 'contig060198-BriOR.S104'

```
##########################################################################################

    Warning: This sequence may not contain a signal peptide!!

    Proteins without signal peptides are unlikely to be exposed to
    the N-glycosylation machinery and thus may not be glycosylated
    (in vivo) even though they contain potential motifs.

    SignalP-NN euk predictions are as follows:

  # name                Cmax  pos ?  Ymax  pos ?  Smax  pos ?  Smean ?  D     ?

    SignalP output is explained at http://www.cbs.dtu.dk/services/SignalP/output.html

##########################################################################################

Name:  contig060198-BriOR.S104  Length:  307
MAGNSSIICAFLHQLLTVRVMIVHTLVIIFLCINMLLIVTFVKKECFHTSARYILFFVTLLSDSFLLLMTDILLILTRCS      80
VQVWLCIFICLFVLVYSIVTPVTLTAMTLERYVAICIPLRHGQLCSTRSTMYCILIIHGLSSGPCIVIISMFFATASISF     160
YNQYTICSVEMFMLYRWQDHARSAVSQFYFMIMGITIVFSYVQIMKVAKAASGENKKSTQKGVRTVILHAFQLFLCLVQL     240
WSPFIETAVLQIDVNLFRDFRYFNYVFFSIVPKCLSPLIYGLRDETFFISLKNLMPTSSCSKKHVIX
...N............................................................................      80
................................................................................     160
................................................................................     240
...................................................................                  320

(Threshold=0.5)
----------------------------------------------------------------------
SeqName      Position  Potential   Jury    N-Glyc
                                 agreement result
----------------------------------------------------------------------
contig060198-BriOR.S104    4 NSSI   0.7697     (9/9)    +++
----------------------------------------------------------------------
```

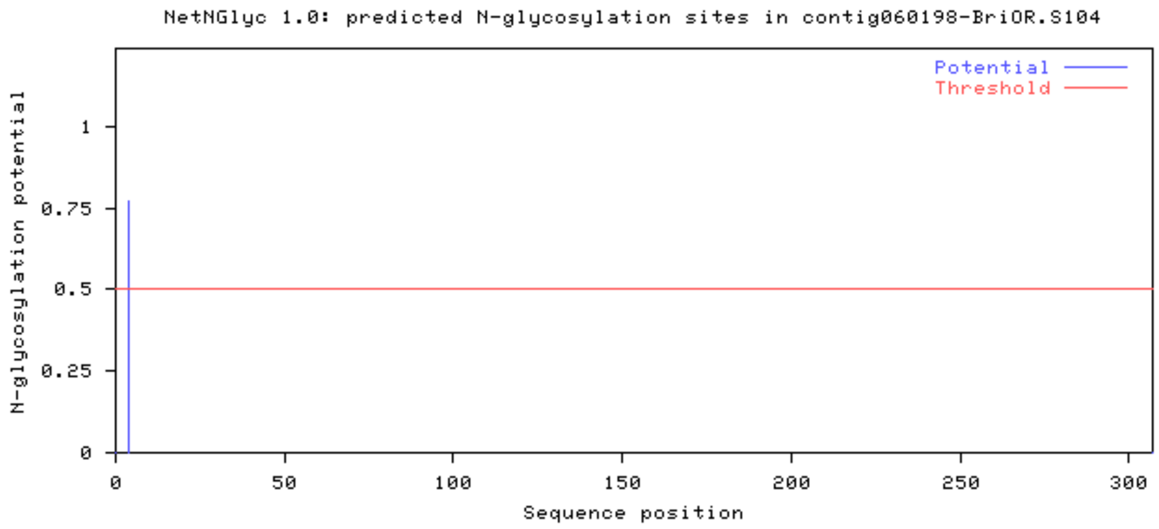

Graphics in PostScript

Output for 'contig061321-BriOR.Y128'

```
##########################################################################################

    Warning: This sequence may not contain a signal peptide!!

    Proteins without signal peptides are unlikely to be exposed to
    the N-glycosylation machinery and thus may not be glycosylated
    (in vivo) even though they contain potential motifs.

    SignalP-NN euk predictions are as follows:

  # name                Cmax  pos ?  Ymax  pos ?  Smax  pos ?  Smean ?  D     ?

    SignalP output is explained at http://www.cbs.dtu.dk/services/SignalP/output.html

##########################################################################################

Name:  contig061321-BriOR.Y128  Length:  312
MSRNNTTEVTSDYTCVRFYVSTISFSVLLFFNLIINWAIVREERLRRHARFVLIFHLLVSALVHLGMSSVFYYQIHLDTR      80
LSRSACMAMITILISSASNILLTLTAMALDRFCAVCHPMRYSSVCNKGHWPWLLGVFTWMVALVIPLSLLFKDSDAPDRG     160
ECGREQLKKGELQKVLFIGLCTLIILYSYVRILVEGRRLGVLNRRNRAGCRTIALHGSQLAVYLLPNFVNFVLSILYKRE     240
FIQRETKELSAVVVFAFFSLAQCVAPVVYGLRKEELLEQLSRRFPCCSRYLKSVLGWTVRANWSHVPYRTRX
...NN...........................................................................      80
................................................................................     160
................................................................................     240
........................................................................             320

(Threshold=0.5)
----------------------------------------------------------------------
SeqName      Position  Potential   Jury    N-Glyc
                                agreement  result
----------------------------------------------------------------------
contig061321-BriOR.Y128    4 NNTT   0.5647     (6/9)   +
contig061321-BriOR.Y128    5 NTTE   0.7359     (9/9)   ++
contig061321-BriOR.Y128  302 NWSH   0.4742     (6/9)   -
----------------------------------------------------------------------
```

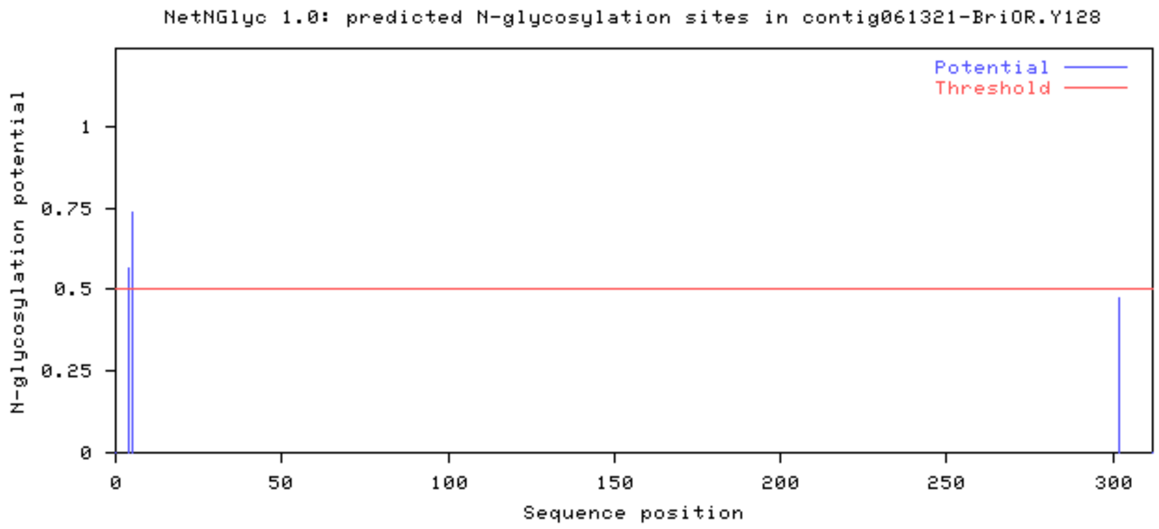

Graphics in PostScript

Output for 'contig063829-BriOR.Q117'

```
##########################################################################

    Warning: This sequence may not contain a signal peptide!!

    Proteins without signal peptides are unlikely to be exposed to
    the N-glycosylation machinery and thus may not be glycosylated
    (in vivo) even though they contain potential motifs.

    SignalP-NN euk predictions are as follows:

  # name                Cmax  pos ?  Ymax  pos ?  Smax  pos ?  Smean ?  D     ?

    SignalP output is explained at http://www.cbs.dtu.dk/services/SignalP/output.html

##########################################################################

Name:  contig063829-BriOR.Q117  Length:  310
MNATTAEFQSSRFQTSVKAALSMLPCFFFLYVNAIMMFALLKKPLLLESSRYILFGHLLICDSVQLLLSMMLYIFAVMTV      80
RMINYVCVFVSLVAAVTVKMSPLNLAVMSLERYVAVCFPLRHPSFATVRSTGKAIAVMWMVASLDSFIQLFLFVRMEKTI     160
FPMQSFCIRNSVFHLEVYVTLNMAFTILYFVFVTMIIIYTYTAIMITVKSASSRGRHTNKAPKTVLLHLVQLWLYLTSTL     240
FNMINPSMMLKVPPDVAIHAQYVLFVGLIIFPKCLSPLIYGLRDQTLCRVFKYYFTFGFRTSVKPSPLSX
.N..............................................................................      80
................................................................................     160
................................................................................     240
......................................................................               320

(Threshold=0.5)
----------------------------------------------------------------------
SeqName      Position  Potential   Jury    N-Glyc
                                 agreement result
----------------------------------------------------------------------
contig063829-BriOR.Q117    2 NATT   0.6731     (8/9)   +
contig063829-BriOR.Q117  245 NPSM   0.3833     (7/9)   -
----------------------------------------------------------------------
```

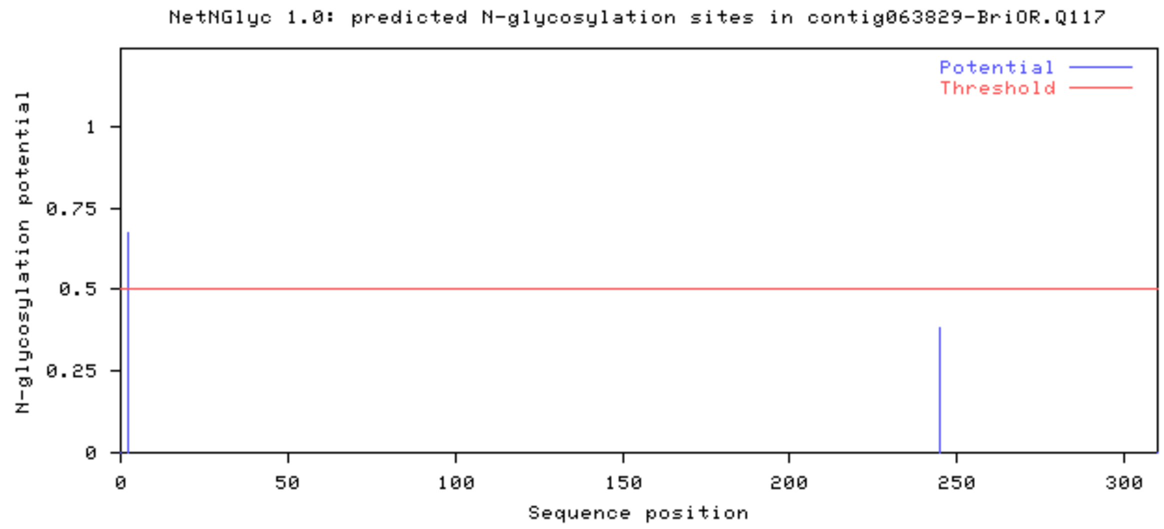

Graphics in PostScript

Output for 'contig063874-BriOR.C026'

```
##########################################################################

    Warning: This sequence may not contain a signal peptide!!

    Proteins without signal peptides are unlikely to be exposed to
    the N-glycosylation machinery and thus may not be glycosylated
    (in vivo) even though they contain potential motifs.

    SignalP-NN euk predictions are as follows:

  # name                Cmax  pos ?  Ymax  pos ?  Smax  pos ?  Smean ?  D     ?

    SignalP output is explained at http://www.cbs.dtu.dk/services/SignalP/output.html

##########################################################################

Name:  contig063874-BriOR.C026  Length:  321
MDNTTAATFKMTAYAVMENYKHGLFSAFFLLYLITLVLNVLLISVIHKNKQLHQPMNVFTCLLSLNEIYGSSALLPAIVA      80
VLVSKTHDVTVKWCMAQAYFLHTYASGEFCILALMGYDRYVAICSPLHYYSIMSYSKTCKLIAFTGLYPFIVFTSFYSLT     160
LQLRFCGKVMPKLYCVNMELVKNSCTNAQYISTVGLAILVLFIVPQLVMIVFSYAHILRVCRTFPKESQANAFRTCFPHL     240
LSLLNYTIASFFEIIQTRFNMSHVAVEAQIFLSLYFIIIPPIANPVLYGLGTQTVRGCIMKLFIKNKVMTTVLAKTLTVG     320
X
..N.............................................................................      80
................................................................................     160
................................................................................     240
....N...........................................................................     320
.                                                                                    400

(Threshold=0.5)
----------------------------------------------------------------------
SeqName      Position  Potential   Jury    N-Glyc
                                 agreement result
----------------------------------------------------------------------
contig063874-BriOR.C026    3 NTTA   0.7209     (9/9)   ++
contig063874-BriOR.C026  245 NYTI   0.6924     (9/9)   ++
contig063874-BriOR.C026  260 NMSH   0.4561     (6/9)   -
----------------------------------------------------------------------
```

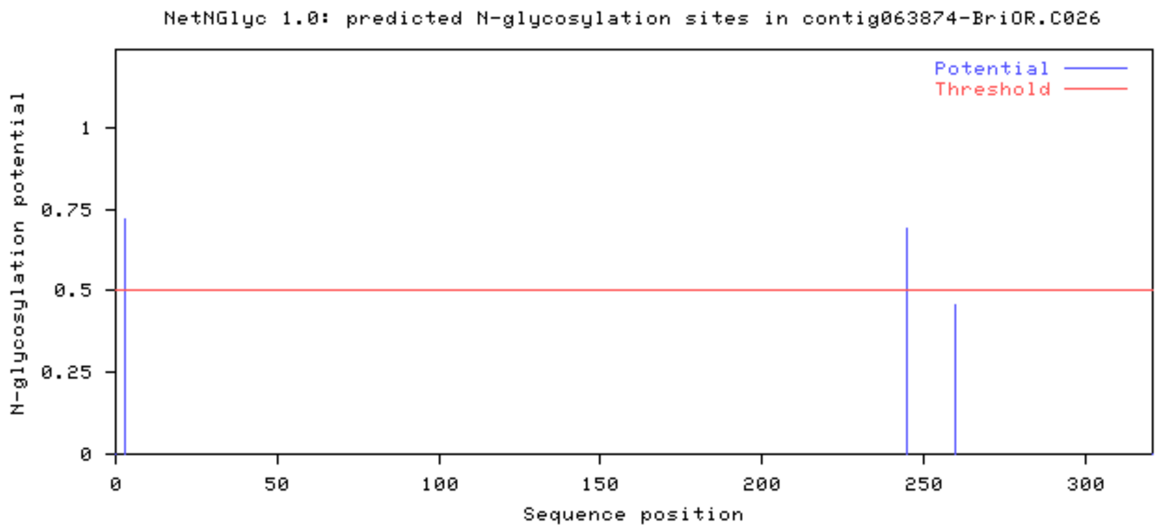

[Graphics in PostScript]

Output for 'contig064802-BriOR.D027'

```
##########################################################################################

    Warning: This sequence may not contain a signal peptide!!

    Proteins without signal peptides are unlikely to be exposed to
    the N-glycosylation machinery and thus may not be glycosylated
    (in vivo) even though they contain potential motifs.

    SignalP-NN euk predictions are as follows:

  # name                  Cmax  pos ?  Ymax  pos ?  Smax  pos ?  Smean ?  D     ?

    SignalP output is explained at http://www.cbs.dtu.dk/services/SignalP/output.html

##########################################################################################

Name:  contig064802-BriOR.D027  Length:  308
MENSSEIVSFVLAAYGNVGELKYLYFVIIMFWYLSICVANTVLIVVIRVDIQLHEPMYILLCNLCVNEINVSTSLYPLLL      80
SQMFSDSHEVTVPWCFLHMFCLYISGPAEFCSLAAMAYDRYISVCRPLRYNVIMNTERVFFMILFVWIYSFLSVILSFSF     160
IFSLKFCGTIIENVYCNHRLILRLSCSLSVYSFLSDIFFLIVSFFIPFTLISVSYVKILAVCRKTSTENKQKAATTCVPQ     240
IVSVSNLFVGSIFQCIDSSVIFAHLPVRIILSIYLFICQPMLTPFLYGFNLPKIRQSCKKLLFKKNQX
..N.............................................................................      80
................................................................................     160
................................................................................     240
....................................................................                 320

(Threshold=0.5)
----------------------------------------------------------------------
SeqName      Position  Potential   Jury    N-Glyc
                                 agreement result
----------------------------------------------------------------------
contig064802-BriOR.D027    3 NSSE   0.6996     (9/9)    ++
contig064802-BriOR.D027   70 NVST   0.4899     (5/9)    -
----------------------------------------------------------------------
```

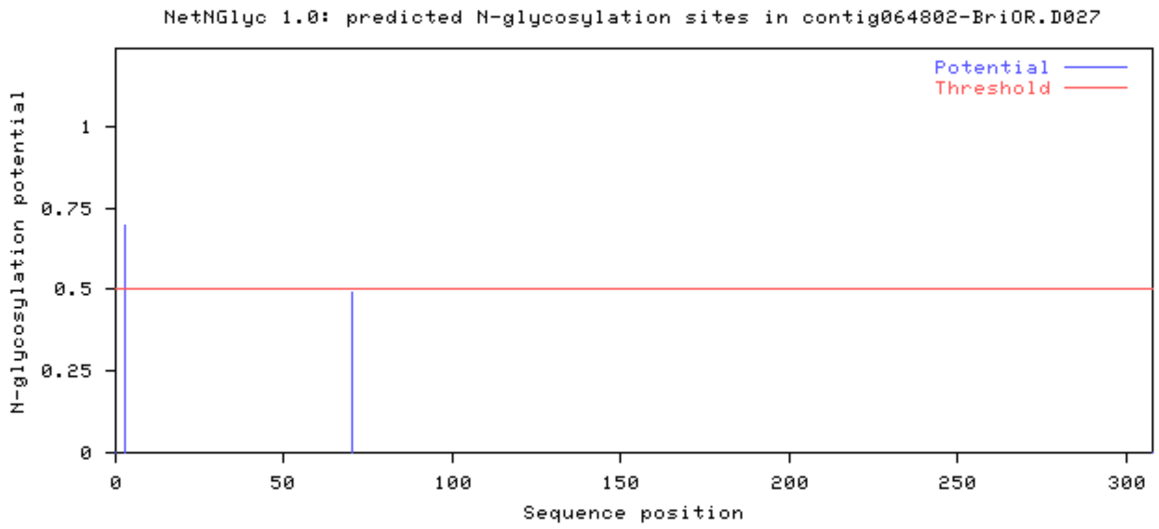

Graphics in PostScript

Output for 'contig064806-BriORe.D032'

```
##########################################################################

    Warning: This sequence may not contain a signal peptide!!

    Proteins without signal peptides are unlikely to be exposed to
    the N-glycosylation machinery and thus may not be glycosylated
    (in vivo) even though they contain potential motifs.

    SignalP-NN euk predictions are as follows:

  # name                Cmax  pos ?  Ymax  pos ?  Smax  pos ?  Smean ?  D     ?

    SignalP output is explained at http://www.cbs.dtu.dk/services/SignalP/output.html

##########################################################################

Name:  contig064806-BriORe.D032        Length:  110
MENSSEIVSFVLSAYGNVGDLKYLYFIMILFWYVSICVANTVLIVVIRVDRQLHEPMYILLSNLCVNEINASTSLYPLLL      80
SQMFSDSHEVTLPWCFLQMCFMYTSAPAEF
..N.............................................................................      80
..............................                                                        160

(Threshold=0.5)
----------------------------------------------------------------------
SeqName      Position  Potential   Jury    N-Glyc
                                agreement  result
----------------------------------------------------------------------
contig064806-BriORe.D032    3 NSSE   0.6970     (9/9)   ++
contig064806-BriORe.D032   70 NAST   0.3184     (8/9)   --
----------------------------------------------------------------------
```

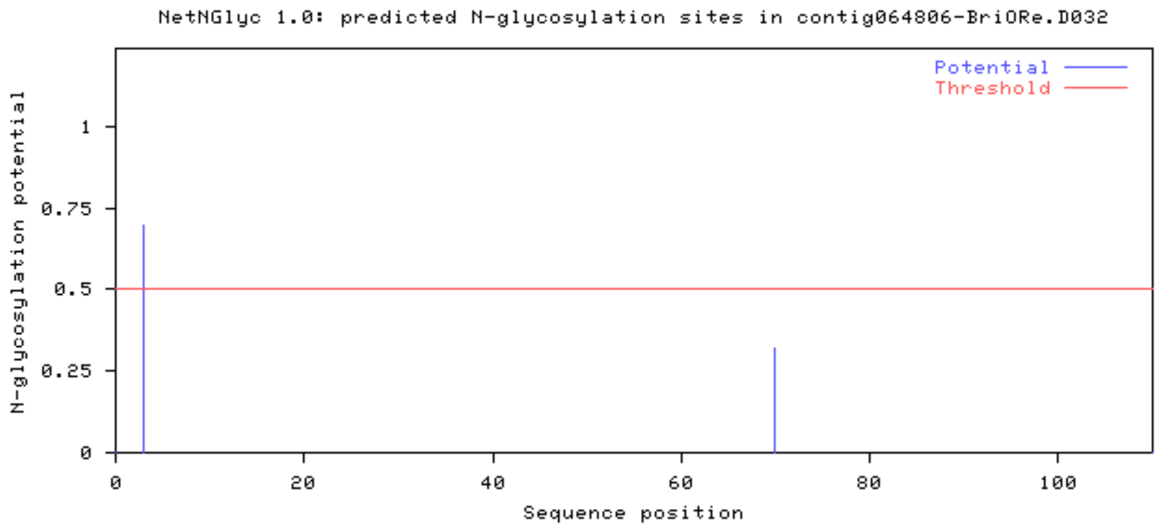

Graphics in PostScript

Output for 'contig064809-BriOR.D028'

```
##########################################################################

    Warning: This sequence may not contain a signal peptide!!

    Proteins without signal peptides are unlikely to be exposed to
    the N-glycosylation machinery and thus may not be glycosylated
    (in vivo) even though they contain potential motifs.

    SignalP-NN euk predictions are as follows:

  # name                   Cmax  pos ?  Ymax  pos ?  Smax  pos ?  Smean ?  D     ?

    SignalP output is explained at http://www.cbs.dtu.dk/services/SignalP/output.html

##########################################################################

Name:  contig064809-BriOR.D028  Length:  309
MGNSSEIVSFVLAAYGNIGELKYLYFIIILIWYLSICVANTVLIVVIRVDRRLHEPMYILLCNLCVNEINASTSLYPLLL      80
SQMFSDSHEVTVPWCFLQMCCMYTSAPAEFCSLAAMAYDRYVSICHPLRYNIIMKTERVFLLILLVWLYSFLSFIFSFSF     160
IFSLKFCGRIIHNTYCDHQLIIRLSCSVPIQSFISNISFLLLSVFIPFSLILVSYMKILRVCRKTSKENKQKAVTTCTPQ     240
IVSVSNLFVGCIFYFIDFKFVVSQVPDEVRIVLPMYLLIFQPMLTPFMYGFKLPKIRQSYQRFLFERKX
..N.............................................................................      80
................................................................................     160
................................................................................     240
.....................................................................                320

(Threshold=0.5)
----------------------------------------------------------------------
SeqName      Position  Potential   Jury    N-Glyc
                                 agreement result
----------------------------------------------------------------------
contig064809-BriOR.D028    3 NSSE   0.7271     (9/9)    ++
contig064809-BriOR.D028   70 NAST   0.3702     (6/9)    -
contig064809-BriOR.D028  196 NISF   0.4391     (6/9)    -
----------------------------------------------------------------------
```

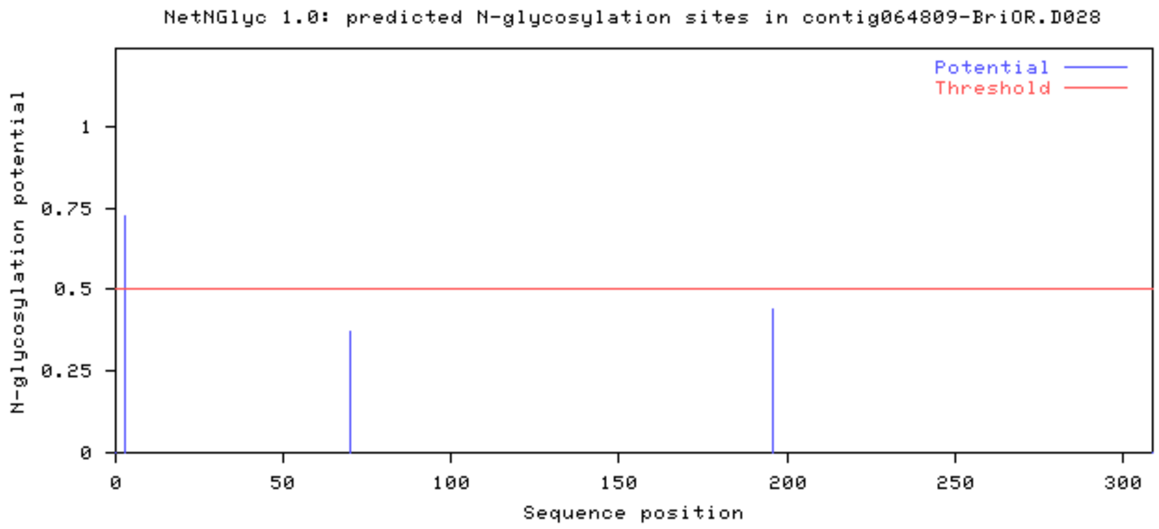

Graphics in PostScript

Output for 'contig064814-BriOR.D029'

```
##########################################################################

    Warning: This sequence may not contain a signal peptide!!

    Proteins without signal peptides are unlikely to be exposed to
    the N-glycosylation machinery and thus may not be glycosylated
    (in vivo) even though they contain potential motifs.

    SignalP-NN euk predictions are as follows:

  # name                  Cmax   pos ?  Ymax   pos ?  Smax   pos ?  Smean ?  D     ?

    SignalP output is explained at http://www.cbs.dtu.dk/services/SignalP/output.html

##########################################################################

Name:  contig064814-BriOR.D029  Length:  312
MQKKGNSSESVSFVLAAYGNVGAFKYPYFIIILFWYVSICVANTVLIVVIHVDRRLHEPMYILLSNLCVNEINCSTSLYP      80
LLLSQMFSDSHEVTVPWCFLQMCCMYISGPAEFWSLAAMAYDRYIAICHPFSYNVIMNTEKVGMLILLVWIFSFVSFLLS     160
FSFIFGLRFCGNIIDNVYCDHQLIIRLSCSVPIQSSISMIFFTLLSIFMPFNLISISYLKILRVCRKTSTENKQKAVTTC     240
TPQIVSVSNLFVGCIFHSIDFSLLAAQIPDEVRITLPMYLLICQPMLTPFMYGFNLPKIRQACKMLLFKRKX
.....N..........................................................................      80
................................................................................     160
................................................................................     240
........................................................................             320

(Threshold=0.5)
----------------------------------------------------------------------
SeqName      Position  Potential   Jury    N-Glyc
                                 agreement result
----------------------------------------------------------------------
contig064814-BriOR.D029    6 NSSE   0.7273     (9/9)   ++
contig064814-BriOR.D029   73 NCST   0.4199     (6/9)   -
----------------------------------------------------------------------
```

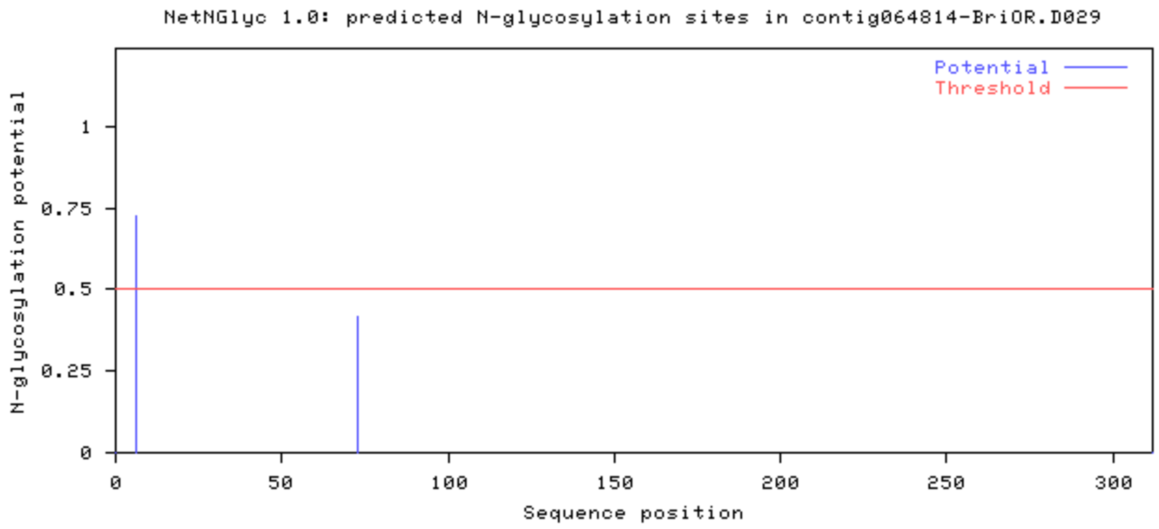

Graphics in PostScript

Output for 'contig064816-BriORpe.F046'

```
########################################################################################

    Warning: This sequence may not contain a signal peptide!!

    Proteins without signal peptides are unlikely to be exposed to
    the N-glycosylation machinery and thus may not be glycosylated
    (in vivo) even though they contain potential motifs.

    SignalP-NN euk predictions are as follows:

  # name                Cmax  pos ?  Ymax  pos ?  Smax  pos ?  Smean ?  D     ?

    SignalP output is explained at http://www.cbs.dtu.dk/services/SignalP/output.html

########################################################################################

Name:  contig064816-BriORpe.F046        Length:  282
FIFCLLLFIFTIFTNLLMIVVISQQTTLHEPLHEYIFIACLSVNALYGSSGFFQRFLMDLLSDTQLISRPACFTQIYVIY      80
SYASCELTVLSIMAYDRYIAVCLPLHYHTKMTLETVVKLTALAWIFPAFSLAACRCLSATLPLCGNEIHKVFCANWNVVK     160
LSCVNTAVNNVVGMLLTVATIFLPLFYSLYTYLRIVSICWKSSAEFRGNVLESCLPHTISFVIYSIAGFCDVALSRNNLE     240
VINPFIAVILSLVFVVIPPALNSLVYGLKLPEIRRHILRLFX
................................................................................      80
................................................................................     160
................................................................................     240
..........................................                                           320

(Threshold=0.5)

No sites predicted in this sequence.
```

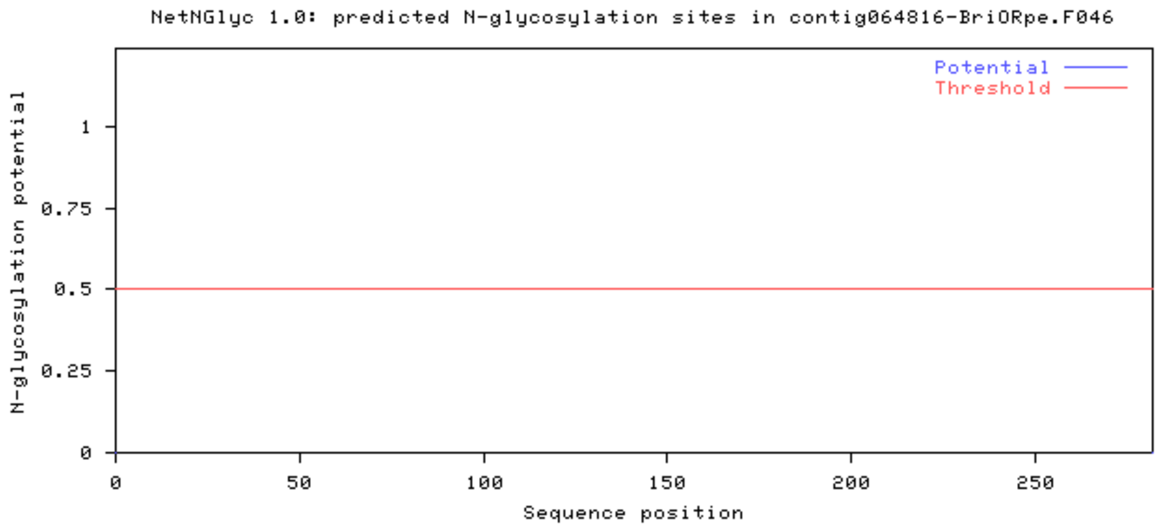

Graphics in PostScript

Output for 'contig064817-BriOR.H051'

```
##########################################################################################

    Warning: This sequence may not contain a signal peptide!!

    Proteins without signal peptides are unlikely to be exposed to
    the N-glycosylation machinery and thus may not be glycosylated
    (in vivo) even though they contain potential motifs.

    SignalP-NN euk predictions are as follows:

  # name                 Cmax   pos ?  Ymax   pos ?  Smax   pos ?  Smean ?  D     ?

    SignalP output is explained at http://www.cbs.dtu.dk/services/SignalP/output.html

##########################################################################################

Name:  contig064817-BriOR.H051  Length:  314
MDNVSVVTVFTLSGLSGIANYKITIFIFTLLCYCVIWLVNLTIIVTVIVDKKLHEPMYIFLCNLCFNGLYGTAAFYPKFL      80
YDLLSTSHVISYAGCLLQGLMVHSSIGTDFSLLALMAYDRYVAICRPLVYHSLMTTQRVCIFVFFAWLTPFCLILMSTIT     160
TATSRLCGSHIPRIYCINWLISNLACSASVATIIIPAFNYTFYFGHAVFVFWSYVHLIKTCQSSKENWNKFMQTCVPHLF     240
SLAVVVVSFLFDMLYMRFGSKEIPQSFENFMAMEILFIPPIINPLMYGFKLTQIRNTVLNFICGKSSAFILRSX
..N....................................N........................................      80
................................................................................     160
......................................N.........................................     240
..........................................................................           320

(Threshold=0.5)
----------------------------------------------------------------------
SeqName      Position  Potential   Jury    N-Glyc
                                 agreement result
----------------------------------------------------------------------
contig064817-BriOR.H051    3 NVSV   0.8098     (9/9)    +++
contig064817-BriOR.H051   40 NLTI   0.7721     (9/9)    +++
contig064817-BriOR.H051  199 NYTF   0.5939     (8/9)    +
----------------------------------------------------------------------
```

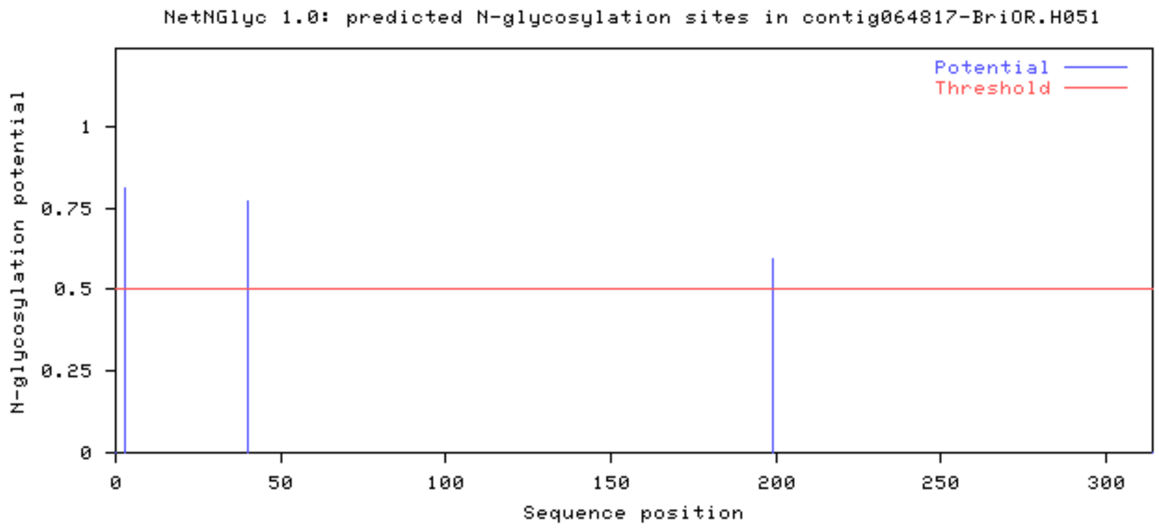

Graphics in PostScript

Output for 'contig064818-BriORe.H061'

```
##########################################################################################

    Warning: This sequence may not contain a signal peptide!!

    Proteins without signal peptides are unlikely to be exposed to
    the N-glycosylation machinery and thus may not be glycosylated
    (in vivo) even though they contain potential motifs.

    SignalP-NN euk predictions are as follows:

  # name                  Cmax  pos ?  Ymax  pos ?  Smax  pos ?  Smean ?  D     ?

    SignalP output is explained at http://www.cbs.dtu.dk/services/SignalP/output.html

##########################################################################################

Name:  contig064818-BriORe.H061         Length:  40
MDNVSIITVFTLSGLSDIANYRVILFVLTLLCYCVIWLVN
..N.....................................                                                80

(Threshold=0.5)
----------------------------------------------------------------------
SeqName       Position  Potential   Jury    N-Glyc
                                  agreement result
----------------------------------------------------------------------
contig064818-BriORe.H061    3 NVSI   0.8001     (9/9)    +++
----------------------------------------------------------------------
```

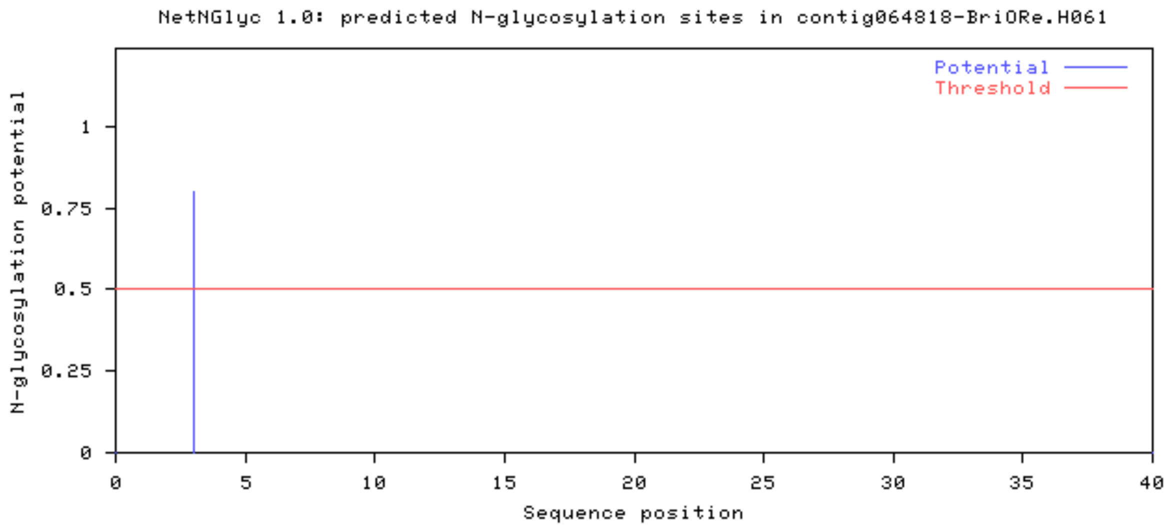

Graphics in PostScript

Output for 'contig064819-BriORe.H059'

```
################################################################################

    Warning: This sequence may not contain a signal peptide!!

    Proteins without signal peptides are unlikely to be exposed to
    the N-glycosylation machinery and thus may not be glycosylated
    (in vivo) even though they contain potential motifs.

    SignalP-NN euk predictions are as follows:

  # name                Cmax  pos ?  Ymax  pos ?  Smax  pos ?  Smean ?  D     ?

    SignalP output is explained at http://www.cbs.dtu.dk/services/SignalP/output.html

################################################################################

Name:  contig064819-BriORe.H059        Length:  245
YGTAAFYPKFLYDLLSTTHVISYAGCFLQGFVLHSSVAADFSLLALMAYDRYVAICRPLVYHSIMTKQKVCILIFFAWNI      80
AVYFLLMSTITTAILRLCGSHIPKIYCINWLIANLACSASVAKIVIPAFSYTFCFGNVCFVFWSYVHLIKTCQSSKENMG     160
KFMQTCVPHLFSLTVVVVSLLFDSLYMRFGSKEIAQSVQNFMAMEFLLIPPIMNPLMYGFKLTKIRKRVLNFICGKSSAF     240
RFKSX
................................................................................      80
................................................................................     160
................................................................................     240
.....                                                                                320

(Threshold=0.5)

No sites predicted in this sequence.
```

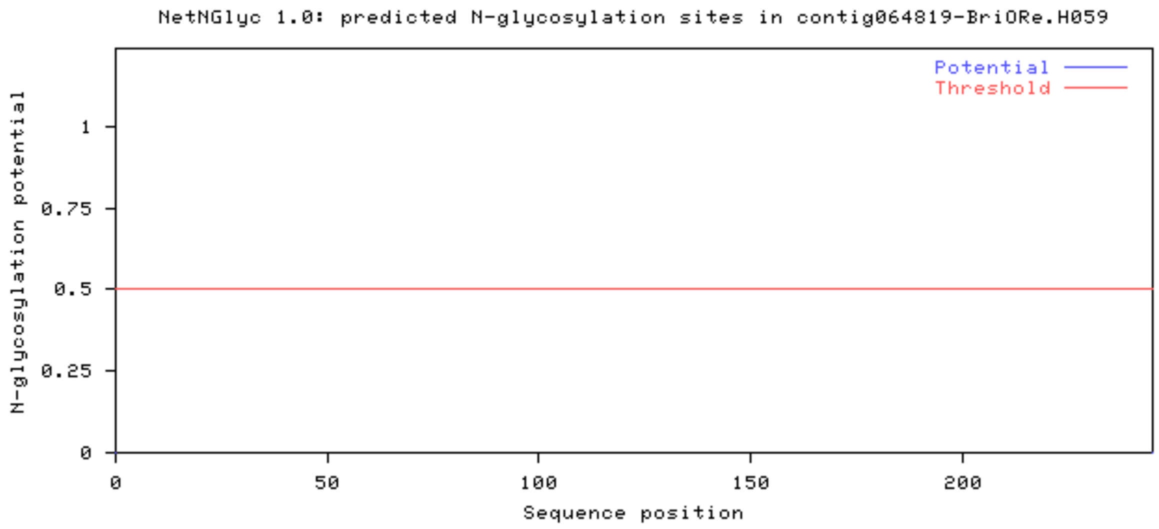

Graphics in PostScript

Output for 'contig064821-BriOR.H052'

```
##########################################################################

    Warning: This sequence may not contain a signal peptide!!

    Proteins without signal peptides are unlikely to be exposed to
    the N-glycosylation machinery and thus may not be glycosylated
    (in vivo) even though they contain potential motifs.

    SignalP-NN euk predictions are as follows:

  # name                Cmax  pos ?  Ymax  pos ?  Smax  pos ?  Smean ?  D     ?

    SignalP output is explained at http://www.cbs.dtu.dk/services/SignalP/output.html

##########################################################################

Name:  contig064821-BriOR.H052  Length:  314
MDNVSVITVFTLAGLSDIENYRAILFVLTLLCYCVIWLVNLTIIVTVIVDKSLHEPMYIFLCNLCVNGLYGTAAFYPKFL      80
YDLLSTTHVISYAGCLLQGFALHSTICADFSLLALMAYDRYVAICRPLVYHSLMTKQKVCIFVFFAWFFPIYLLFLSTIT     160
TAVLRLCGSHIPRIYCINWLINNLACSASVARIVIPAFNYTFYIGHILFVFWSYVHLVKTCQSSKENWNKFMQTCVPHLF     240
SLIVVAVSFLFDMLYVRFGSKEFPQSFENFMAMEILLIPPIINPLMYGFKLTKIRNRVLNFICGKSSALRLKSX
..N....................................N........................................      80
................................................................................     160
......................................N.........................................     240
..........................................................................           320

(Threshold=0.5)
----------------------------------------------------------------------
SeqName      Position  Potential   Jury    N-Glyc
                                 agreement result
----------------------------------------------------------------------
contig064821-BriOR.H052    3 NVSV   0.8143     (9/9)    +++
contig064821-BriOR.H052   40 NLTI   0.7721     (9/9)    +++
contig064821-BriOR.H052  199 NYTF   0.5842     (7/9)    +
----------------------------------------------------------------------
```

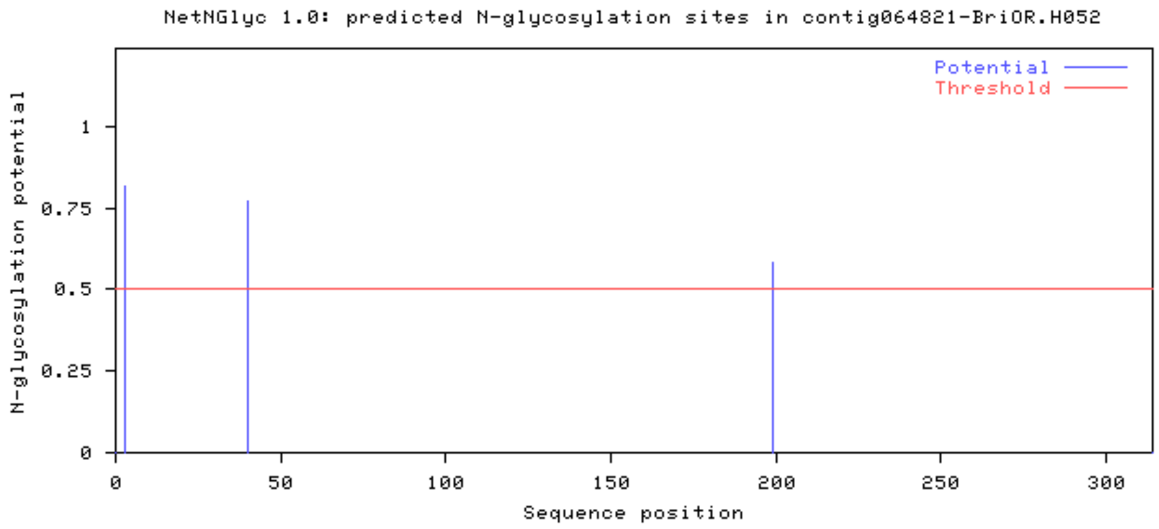

Graphics in PostScript

Output for 'contig073387-BriOR.Z129'

```
##########################################################################

    Warning: This sequence may not contain a signal peptide!!

    Proteins without signal peptides are unlikely to be exposed to
    the N-glycosylation machinery and thus may not be glycosylated
    (in vivo) even though they contain potential motifs.

    SignalP-NN euk predictions are as follows:

  # name                Cmax  pos ?  Ymax  pos ?  Smax  pos ?  Smean ?  D     ?

    SignalP output is explained at http://www.cbs.dtu.dk/services/SignalP/output.html

##########################################################################

Name:  contig073387-BriOR.Z129  Length:  391
MTAVLETGAHNLTEERDSFSQENQTNPHATGQLNNANCLFLSILPEGQAVSVLICLFVLLTALSCFVNAFTLFGLGQSEE      80
FSWQPRFILLKNLIFSDLVQTATFGPAVIHSLIQRRTMAFNGWCYVQYFLGGVSIFCSLVTITCMALERYIYVCHAIRYL     160
PIFTKIRLRGVLGGIWLYSVFIGVSEIVMLHTGRGEDDETATTGLLCEPDVVEQHLGFPRASAVFRKTVGSLTLLLCLLI     240
HAFSFFRMYQVARNAVIPFNAVNVTARNTVLFYCGMLFLQLLPLLLKVASDALWEFRAPVAMMVQSSQSPGLCKVKTTPT     320
ATATALHISLLVMLIVPPCINPLVYGLWSVEMRQALTSRLRSWTERRANERAAERIRLEHVARRNGAQAGX
..........N...........N.........................................................      80
................................................................................     160
................................................................................     240
......................N.........................................................     320
.......................................................................              400

(Threshold=0.5)
----------------------------------------------------------------------
SeqName      Position  Potential   Jury    N-Glyc
                                 agreement result
----------------------------------------------------------------------
contig073387-BriOR.Z129   11 NLTE   0.8619     (9/9)   +++
contig073387-BriOR.Z129   23 NQTN   0.6984     (9/9)   ++
contig073387-BriOR.Z129  263 NVTA   0.7040     (9/9)   ++
----------------------------------------------------------------------
```

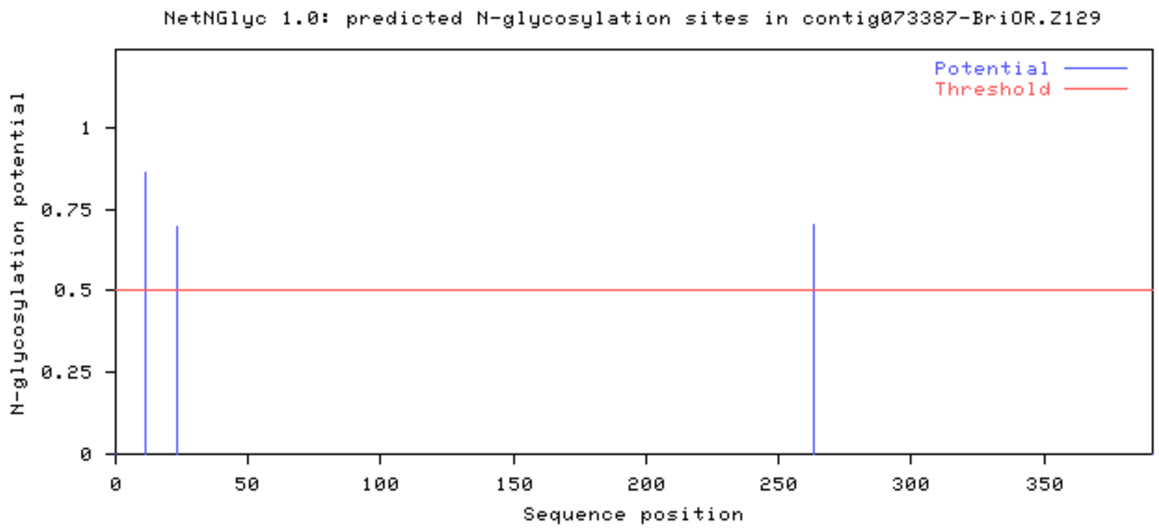

Graphics in PostScript

Output for 'contig082838-BriOR.E040'

```
##########################################################################################

    Warning: This sequence may not contain a signal peptide!!

    Proteins without signal peptides are unlikely to be exposed to
    the N-glycosylation machinery and thus may not be glycosylated
    (in vivo) even though they contain potential motifs.

    SignalP-NN euk predictions are as follows:

  # name                    Cmax  pos ?  Ymax  pos ?  Smax  pos ?  Smean ?  D     ?

    SignalP output is explained at http://www.cbs.dtu.dk/services/SignalP/output.html

##########################################################################################

Name:  contig082838-BriOR.E040  Length:  304
MNSSQVSYFTLTAYLDSGALKYLYFTVVAFLYIVIVTANVLLIVVICVNRSLHEPMYMFLCSLFVNELYGSTGLFPFLLL      80
QILSDVHTVSAPLCFLQIFCLYTYGHVEFCNLAVMSYDRYLAVCYPLHYKSHMTDNKVVIFIVVIWLYSFVKFTITLCLT     160
LHLTWCGKIINGLYCHNYLVVKLACSDTNLNNLFGLFGIVITVLVPLLPIFYSYMKILKVCFSGSRQMRRKAVSTCVPHL     240
ASLLNFSFGCLFEILQSRFDTTSVPSALRIFLSLYFLIIQPLLNPIMYGTQMSKIRHVLCYKMX
.N..............................................N...............................      80
................................................................................     160
................................................................................     240
................................................................                     320

(Threshold=0.5)
----------------------------------------------------------------------
SeqName      Position  Potential   Jury    N-Glyc
                                 agreement result
----------------------------------------------------------------------
contig082838-BriOR.E040    2 NSSQ    0.6940     (9/9)   ++
contig082838-BriOR.E040   49 NRSL    0.7493     (9/9)   ++
contig082838-BriOR.E040  245 NFSF    0.4324     (7/9)   -
----------------------------------------------------------------------
```

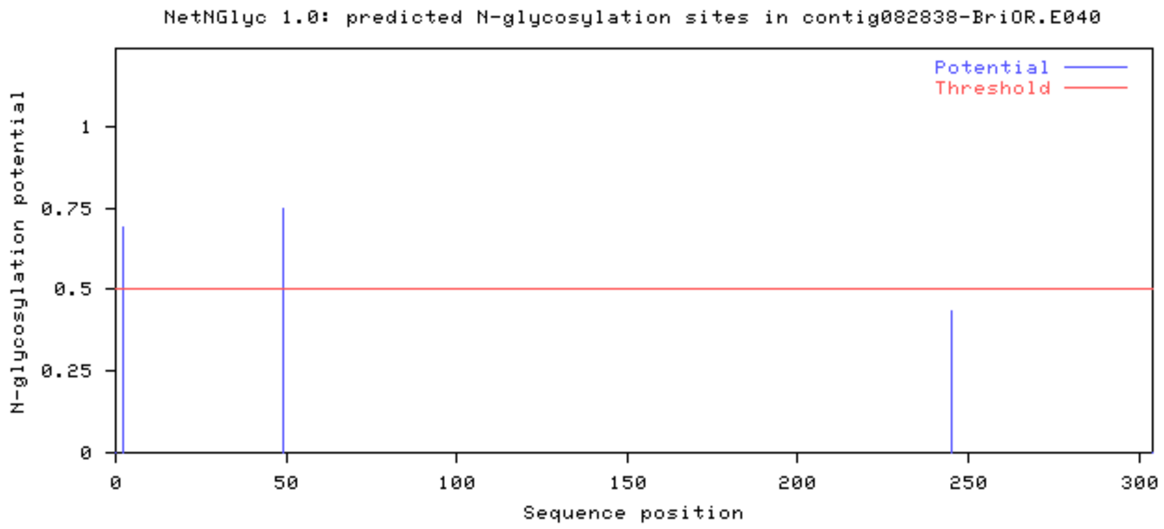

Graphics in PostScript

Output for 'contig082839-BriORe.E042'

```
##########################################################################################

    Warning: This sequence may not contain a signal peptide!!

    Proteins without signal peptides are unlikely to be exposed to
    the N-glycosylation machinery and thus may not be glycosylated
    (in vivo) even though they contain potential motifs.

    SignalP-NN euk predictions are as follows:

  # name                Cmax  pos ?  Ymax  pos ?  Smax  pos ?  Smean ?  D     ?

    SignalP output is explained at http://www.cbs.dtu.dk/services/SignalP/output.html

##########################################################################################

Name:  contig082839-BriORe.E042         Length:  240
YGSTGLFPFLLLQILSDVHTVSAPLCFLQIFCVHTYGTVEFTNVLVMSYDRYLAICFPLQYHTRMSPCKVSMLIVLTWFS      80
SFLGISLLISLSAPLQLCGNIINKVYCDNYSVVKLACSDTTVNNIYGLISTSLTTISAVSLILYTYMRILKVCFSGSKQT     160
RQKAISTCTPHLASLLNYSCSAVFEIAQSRLNMKHVPNMVHILLSLYWLICPPLCNPLLYGLNLTKIRIIYKSLILSKVX     240
................................................................................      80
............................N...................................................     160
................N.............................................N.................     240

(Threshold=0.5)
----------------------------------------------------------------------
SeqName      Position  Potential    Jury    N-Glyc
                                  agreement result
----------------------------------------------------------------------
contig082839-BriORe.E042   109 NYSV   0.6380     (8/9)    +
contig082839-BriORe.E042   177 NYSC   0.6772     (8/9)    +
contig082839-BriORe.E042   223 NLTK   0.7101     (9/9)    ++
----------------------------------------------------------------------
```

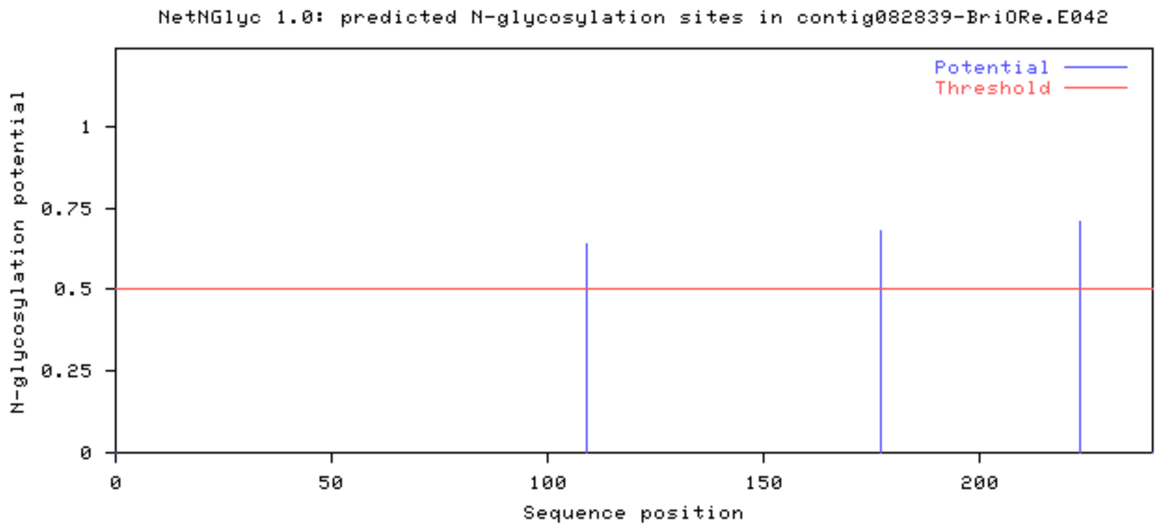

Graphics in PostScript

Output for 'contig084995-BriORe.A012'

```
########################################################################################

    Warning: This sequence may not contain a signal peptide!!

    Proteins without signal peptides are unlikely to be exposed to
    the N-glycosylation machinery and thus may not be glycosylated
    (in vivo) even though they contain potential motifs.

    SignalP-NN euk predictions are as follows:

  # name                Cmax  pos ?  Ymax  pos ?  Smax  pos ?  Smean ?  D     ?

    SignalP output is explained at http://www.cbs.dtu.dk/services/SignalP/output.html

########################################################################################

Name:  contig084995-BriORe.A012         Length:  285
YFMIMFTLYILILCCNFIIVFLIVVEKSLHEPMYIFIAALLLNSVMLSTVIYPKLLTDFLSKRQIISYSVCLFQFFMFYS      80
LGGSEFLLLFAMAYDRYVSICKPLQYPMIMTKNTISIFLTLAWIVPSSQVAVVAVLMANKKICNFTFTGIFCNNTIYKLL     160
CVHSTAQTVYDMVVLSNVAILPAVFIFFTYTRILVISYQSCKEVRRKAAQTCLPHLIVLISYLCFCAFDVIVSGLESNFP     240
KIVHSILTLQIVMYPPLFNPIIYGLKMKEISKHLKRLFCAVKKNX
................................................................................      80
...............................................................N........N.......     160
................................................................................     240
.............................................                                        320

(Threshold=0.5)
----------------------------------------------------------------------
SeqName      Position  Potential   Jury    N-Glyc
                                 agreement result
----------------------------------------------------------------------
contig084995-BriORe.A012  144 NFTF   0.6547     (8/9)   +
contig084995-BriORe.A012  153 NNTI   0.5211     (4/9)   +
----------------------------------------------------------------------
```

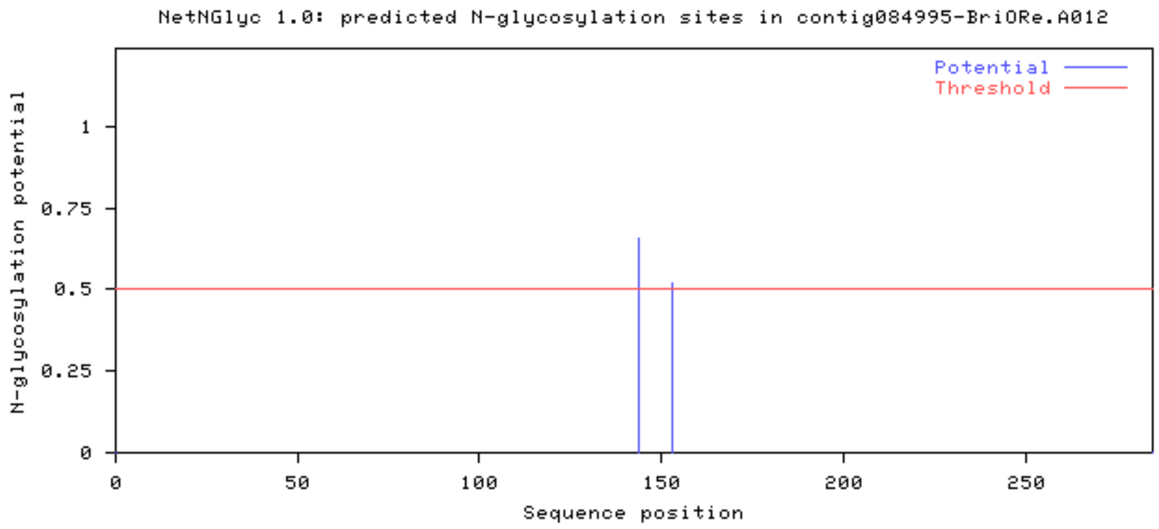

Graphics in PostScript

Output for 'contig084999-BriOR.A001'

```
##########################################################################

    Warning: This sequence may not contain a signal peptide!!

    Proteins without signal peptides are unlikely to be exposed to
    the N-glycosylation machinery and thus may not be glycosylated
    (in vivo) even though they contain potential motifs.

    SignalP-NN euk predictions are as follows:

  # name                 Cmax  pos ?  Ymax  pos ?  Smax  pos ?  Smean ?  D     ?

    SignalP output is explained at http://www.cbs.dtu.dk/services/SignalP/output.html

##########################################################################

Name:  contig084999-BriOR.A001  Length:  307
MNADFNTTYVTLGGHVEIHRYKYLYFVIMLTAYILLICFNVSIICLIVMHKNLHEPMYIFIAALLLNSLLFSTNIYPKLL      80
IDFLSEKQIISYQACLFQAFVFYFLSSSEFLLLSAMAYDRYVSICKPLQYPAIMTTTTVSLLLCFAWLVPACYVVVPVAL     160
NINSKLCSFTLKGIFCNNSLYKLFCVTSNELSIYGVIVLLNLGLFPMLFILFTYTKIIIIAFQSCGDIRRKAVQTCLPHL     240
LVLINYSVLITYDVVIVKLESDFPKTARFVMTLQIITYNPLCNPIIYGLKMKEISNHLKRLLRHMKX
.....N.................................N........................................      80
................................................................................     160
................................................................................     240
....N..............................................................                  320

(Threshold=0.5)
----------------------------------------------------------------------
SeqName      Position  Potential   Jury    N-Glyc
                                 agreement result
----------------------------------------------------------------------
contig084999-BriOR.A001    6 NTTY   0.6674     (9/9)    ++
contig084999-BriOR.A001   40 NVSI   0.7946     (9/9)    +++
contig084999-BriOR.A001  177 NNSL   0.4327     (7/9)    -
contig084999-BriOR.A001  245 NYSV   0.5313     (6/9)    +
----------------------------------------------------------------------
```

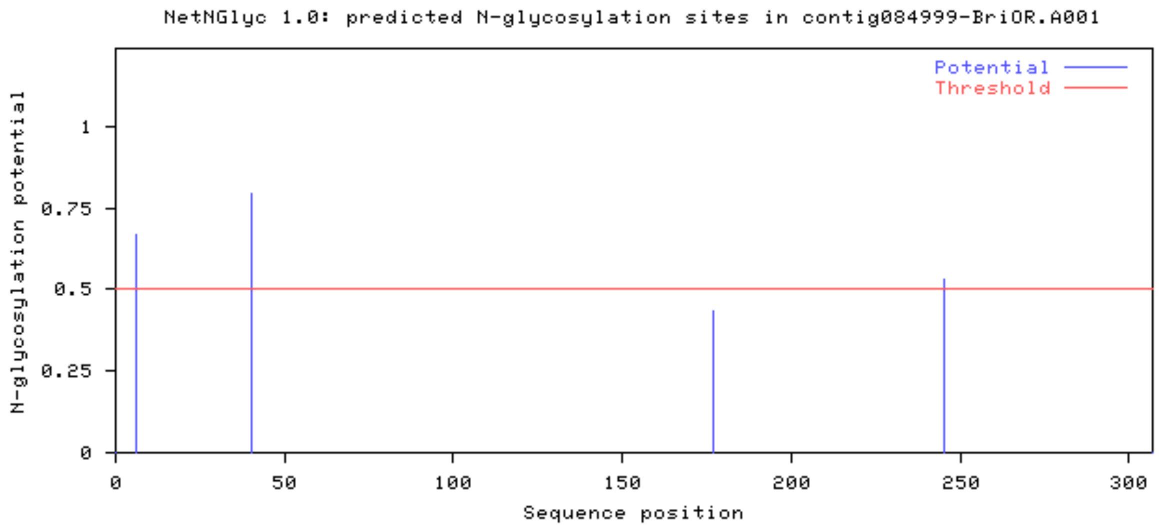

Graphics in PostScript

Output for 'contig084999-BriOR.A002'

```
##########################################################################

    Warning: This sequence may not contain a signal peptide!!

    Proteins without signal peptides are unlikely to be exposed to
    the N-glycosylation machinery and thus may not be glycosylated
    (in vivo) even though they contain potential motifs.

    SignalP-NN euk predictions are as follows:

  # name                 Cmax  pos ?  Ymax  pos ?  Smax  pos ?  Smean ?  D     ?

    SignalP output is explained at http://www.cbs.dtu.dk/services/SignalP/output.html

##########################################################################

Name:  contig084999-BriOR.A002  Length:  328
MAIDNEFNVTYITFGGHIELEKYKFLYFAIMFTAYILILCSNSTIVCLIWIKKSLHEPMYIFIAALLFNSVMFSTNIYPK      80
LLMDFLSEKQITTHSQCSFQGFIYYSLTGSEFFLLASMAYDRYVSISKPLQYHTIMRKATVTVFLVLTWLLPACQLVPSA     160
VIINTSQICRFTLNGIFCNNAISKLYCATPKISYLIYGVFILLNTVFLPLLFILFTYTKIFIICYRSCREVRKKAAQTCL     240
PHLLVLISFSCLCSYDIIITRVEINLSQTARFIMTLQVVLYHPLFNPIIYGLKMKEISQHLRRLFCQSKFKLSVRADAGS     320
AIISFVIX
.......N.................................N......................................      80
................................................................................     160
...N............................................................................     240
........................N.......................................................     320
........                                                                             400

(Threshold=0.5)
----------------------------------------------------------------------
SeqName      Position  Potential   Jury    N-Glyc
                                 agreement result
----------------------------------------------------------------------
contig084999-BriOR.A002    8 NVTY   0.6871     (9/9)   ++
contig084999-BriOR.A002   42 NSTI   0.7384     (9/9)   ++
contig084999-BriOR.A002  164 NTSQ   0.6096     (8/9)   +
contig084999-BriOR.A002  265 NLSQ   0.5974     (5/9)   +
----------------------------------------------------------------------
```

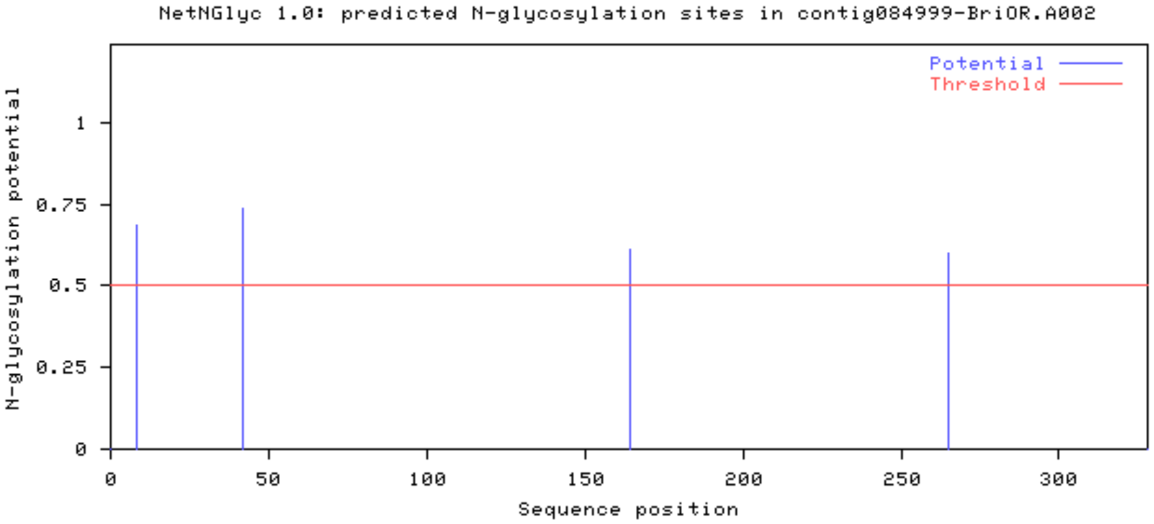

Graphics in PostScript

Output for 'contig084999-BriORp.A021'

```
##########################################################################################

    Warning: This sequence may not contain a signal peptide!!

    Proteins without signal peptides are unlikely to be exposed to
    the N-glycosylation machinery and thus may not be glycosylated
    (in vivo) even though they contain potential motifs.

    SignalP-NN euk predictions are as follows:

  # name                  Cmax  pos ?  Ymax  pos ?  Smax  pos ?  Smean ?  D     ?

    SignalP output is explained at http://www.cbs.dtu.dk/services/SignalP/output.html

##########################################################################################

Name:  contig084999-BriORp.A021         Length:  300
MERMHDEFNVTYITFGGHIELEKYKFLYFVIMFTAYILILCSNSTIVCLIWIKKSLHEPMYVFIAALLLNSVMFSTNIYP      80
KLLMDFLSEKQITTHSLCRFQGIIYYSLTGSEFFLLASMAYDRYMSISKPLQYHTIMRKATVTVLLVLAWLLPACQLVPS     160
AVFSNNLICNFTLNGIFCNNGISKLYCATPKTSYLIYGVFILLNTVFLPLLFILFTYTKIFIICYRSCREVRKKAAQTCS     240
PHLLVLISFTCLCSFDIIIARLEINLSQTTRFIMTLQVVLYHPLFNPIVYGLKMKEISQK
........N................................N.....................................      80
................................................................................     160
.........N......................................................................     240
........................N...................................                         320

(Threshold=0.5)
----------------------------------------------------------------------
SeqName      Position  Potential   Jury    N-Glyc
                                 agreement result
----------------------------------------------------------------------
contig084999-BriORp.A021    9 NVTY    0.7809     (9/9)   +++
contig084999-BriORp.A021   43 NSTI    0.7370     (9/9)   ++
contig084999-BriORp.A021  170 NFTL    0.7033     (9/9)   ++
contig084999-BriORp.A021  265 NLSQ    0.6263     (8/9)   +
----------------------------------------------------------------------
```

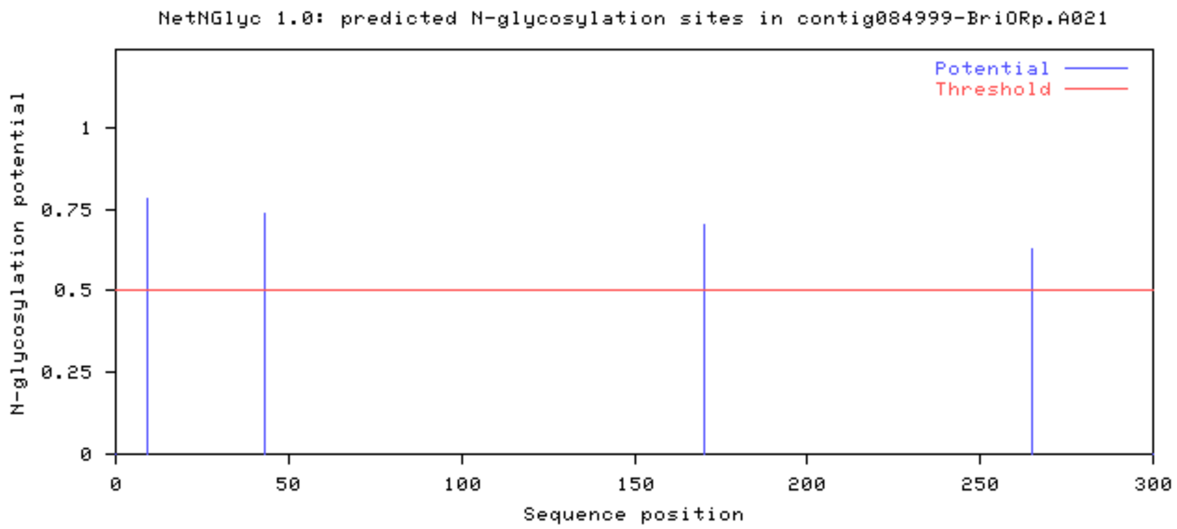

[Graphics in PostScript]

Output for 'contig085000-BriOR.A003'

```
##########################################################################################

    Warning: This sequence may not contain a signal peptide!!

    Proteins without signal peptides are unlikely to be exposed to
    the N-glycosylation machinery and thus may not be glycosylated
    (in vivo) even though they contain potential motifs.

    SignalP-NN euk predictions are as follows:

  # name                   Cmax  pos ?  Ymax  pos ?  Smax  pos ?  Smean ?  D     ?

    SignalP output is explained at http://www.cbs.dtu.dk/services/SignalP/output.html

##########################################################################################

Name:  contig085000-BriOR.A003  Length:  309
MDDELNVTYITLDGYVELKRCGYLYFLIMVALYVLIITSNSTIVFLICIHRNLHEPMYIFIAALSVNSVVFSTAIYPKLF      80
VDVLSEKQVISISACQFQHFMYYSIGGSDFLLLSAMAFDRYVSICKPLKYPVIMRQTTINILLFLAWFLPGLQVAVSHAL     160
VLNYKLCNFTLKGIFCNNSLWKLYCESPRAALIYGLIVLLNVAIFPVLFILFTYAKIFLITYRSSRDIQKKAAETCLPHL     240
FVLSIFTTLCAYDVIIARLEFDFPKIAKLIMTLQVVLYNPLFNPFIYGLKMKEISKHLKRLFCHVRCSX
.....N.................................N........................................      80
................................................................................     160
.......N........................................................................     240
.....................................................................                320

(Threshold=0.5)
----------------------------------------------------------------------
SeqName      Position  Potential   Jury    N-Glyc
                                 agreement result
----------------------------------------------------------------------
contig085000-BriOR.A003    6 NVTY   0.7911     (9/9)    +++
contig085000-BriOR.A003   40 NSTI   0.7129     (9/9)    ++
contig085000-BriOR.A003  168 NFTL   0.7146     (9/9)    ++
contig085000-BriOR.A003  177 NNSL   0.4084     (6/9)    -
----------------------------------------------------------------------
```

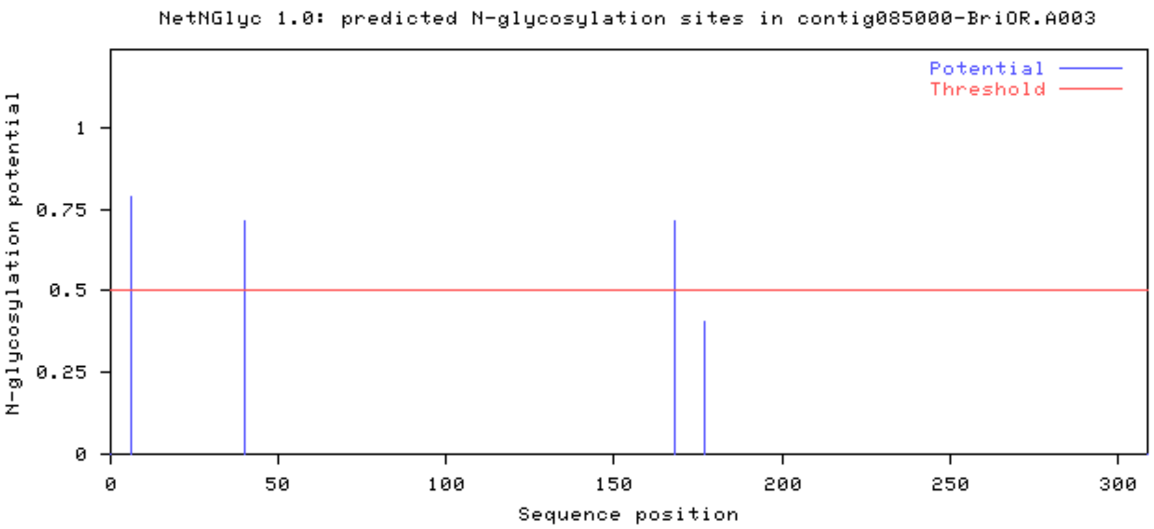

Graphics in PostScript

Output for 'contig085002-BriOR.A004'

```
##########################################################################################

    Warning: This sequence may not contain a signal peptide!!

    Proteins without signal peptides are unlikely to be exposed to
    the N-glycosylation machinery and thus may not be glycosylated
    (in vivo) even though they contain potential motifs.

    SignalP-NN euk predictions are as follows:

  # name                  Cmax  pos ?  Ymax  pos ?  Smax  pos ?  Smean ?  D     ?

    SignalP output is explained at http://www.cbs.dtu.dk/services/SignalP/output.html

##########################################################################################

Name:  contig085002-BriOR.A004  Length:  337
MERMHDEFNVTYITLGGHIELEKYKFLYFVIMFTAYILILCSNSTIVCLIWIKKSLHEPMYVFIAALLLNSVVFSTNIYP      80
KLLMDFLSEKQITTHSLCRFQGIIYYSLTGSEFFLLASMAYDRYVSISQPLQYHTIMRKATVTVLLVLAWLLPACQLVPS     160
AVFSNNSQICNFTLNGIFCNNAISKLYCATPKTSYLMYGVFILFNTVFLPLLFIMFTYTKIFIICYRSCREVRKKAAQTC     240
LPHLLVLISFTCLCSYDIIIARLEINLSQTIRFIMTLQVVLYHPLFNPIVYGLKMKEISQHLRRLFCHCVLCVKTDVRSA     320
VSSFVIQVQRPDLSTVX
........N.................................N.....................................      80
................................................................................     160
....N.....N.....................................................................     240
.........................N......................................................     320
.................                                                                    400

(Threshold=0.5)
----------------------------------------------------------------------
SeqName      Position  Potential   Jury    N-Glyc
                                 agreement result
----------------------------------------------------------------------
contig085002-BriOR.A004    9 NVTY   0.7716     (9/9)   +++
contig085002-BriOR.A004   43 NSTI   0.7384     (9/9)   ++
contig085002-BriOR.A004  165 NNSQ   0.5508     (5/9)   +
contig085002-BriOR.A004  171 NFTL   0.6014     (7/9)   +
contig085002-BriOR.A004  266 NLSQ   0.6598     (9/9)   ++
```

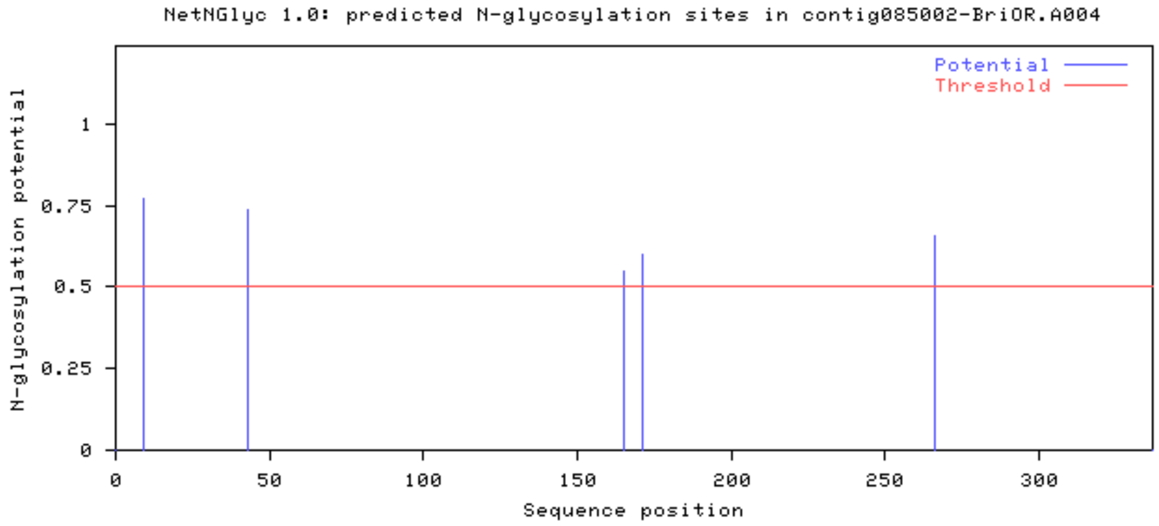

Graphics in PostScript

Output for 'contig085003-BriORp.A022'

```
##########################################################################################

    Warning: This sequence may not contain a signal peptide!!

    Proteins without signal peptides are unlikely to be exposed to
    the N-glycosylation machinery and thus may not be glycosylated
    (in vivo) even though they contain potential motifs.

    SignalP-NN euk predictions are as follows:

  # name                   Cmax  pos ?  Ymax  pos ?  Smax  pos ?  Smean ?  D     ?

    SignalP output is explained at http://www.cbs.dtu.dk/services/SignalP/output.html

##########################################################################################

Name:  contig085003-BriORp.A022         Length:  335
MNPELNMTYLILGGHVEVQKYRYLYFLILFTAYILIICCNTSIIYLIVIHKSLHEPCIFSLQLCYIPFFSALIFIQSYWL      80
ISYMYIFIAALLLNSLFFSTNIYPKLLADFLSEKQIISYQVCLFQVFIFYSLSCSEFLLLSAMAYDRYVSICKPLQYPTI     160
MRKITVVFLLVLAWLLPACQVAVVIILNINNKLCNFTLKGIFCNNSLIQLYCVMSGALSVYGAFVLLNTGLFPMLFIIFT     240
YTKIILTVYQSSGEVKKKAAQACLPHLFVLINYSCLITYDMIIARLESDFSKTARFLMTLQIITYNPLFNPIIYGLKMKE     320
ISKHLQRLFCQSKLN
.....N.................................N........................................      80
................................................................................     160
..................................N.............................................     240
...............................N................................................     320
...............                                                                      400

(Threshold=0.5)
----------------------------------------------------------------------
SeqName      Position  Potential   Jury    N-Glyc
                                 agreement result
----------------------------------------------------------------------
contig085003-BriORp.A022    6 NMTY    0.7443     (9/9)   ++
contig085003-BriORp.A022   40 NTSI    0.6420     (8/9)   +
```

```
contig085003-BriORp.A022  195 NFTL   0.6591     (9/9)   ++
contig085003-BriORp.A022  204 NNSL   0.4744     (6/9)   -
contig085003-BriORp.A022  272 NYSC   0.5266     (5/9)   +
----------------------------------------------------------------------
```

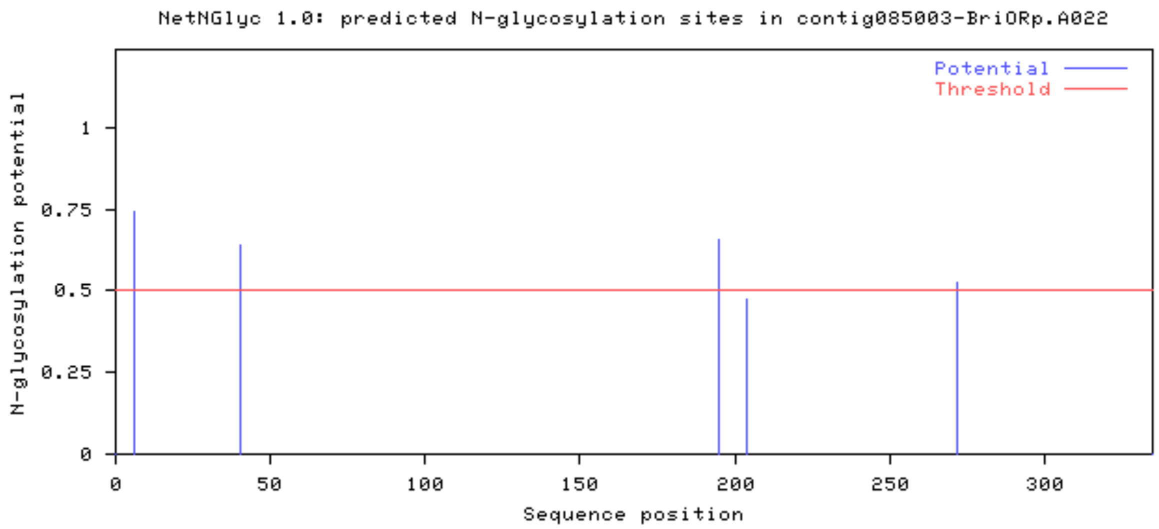

Graphics in PostScript

Output for 'contig085010-BriOR.A005'

```
##########################################################################################

    Warning: This sequence may not contain a signal peptide!!

    Proteins without signal peptides are unlikely to be exposed to
    the N-glycosylation machinery and thus may not be glycosylated
    (in vivo) even though they contain potential motifs.

    SignalP-NN euk predictions are as follows:

  # name                   Cmax  pos ?  Ymax  pos ?  Smax  pos ?  Smean ?  D     ?

    SignalP output is explained at http://www.cbs.dtu.dk/services/SignalP/output.html

##########################################################################################

Name:  contig085010-BriOR.A005  Length:  319
MDQMNDQFNVTYITFGGHVELNKYRFLYFAIMFTAYILILCSNSTILCLIWIKKNLHEPMYIFIAGLLLNSVMFSTNIYP      80
ELLIDFLSDKQITTHSLCSFQAFIYYSLTGSEFFLLAAMAYDRYVAICKPLQYTTIMKKTTIIILLGLAWLLPACQLVPS     160
VVMSQSYKICSFTLNGIFCNNAISKLYCDTSRTTYIIYGVFILLNTVFLPLLFILFTYTKIFMICYQSCREVRKKAAQTC     240
LPHLLVLVSFSGLCSYDIIVARLEMNLPKVARFILTLQVVSYHPLFNPIVYGLKMKEISKHLTKLFCEGKLNIWQSSCX
........N..............................N........................................      80
................................................................................     160
................................................................................     240
...............................................................................      320

(Threshold=0.5)
----------------------------------------------------------------------
SeqName      Position  Potential   Jury    N-Glyc
                                 agreement result
----------------------------------------------------------------------
contig085010-BriOR.A005    9 NVTY   0.7624     (9/9)    +++
```

```
contig085010-BriOR.A005    43 NSTI    0.6700     (9/9)    ++
----------------------------------------------------------------------
```

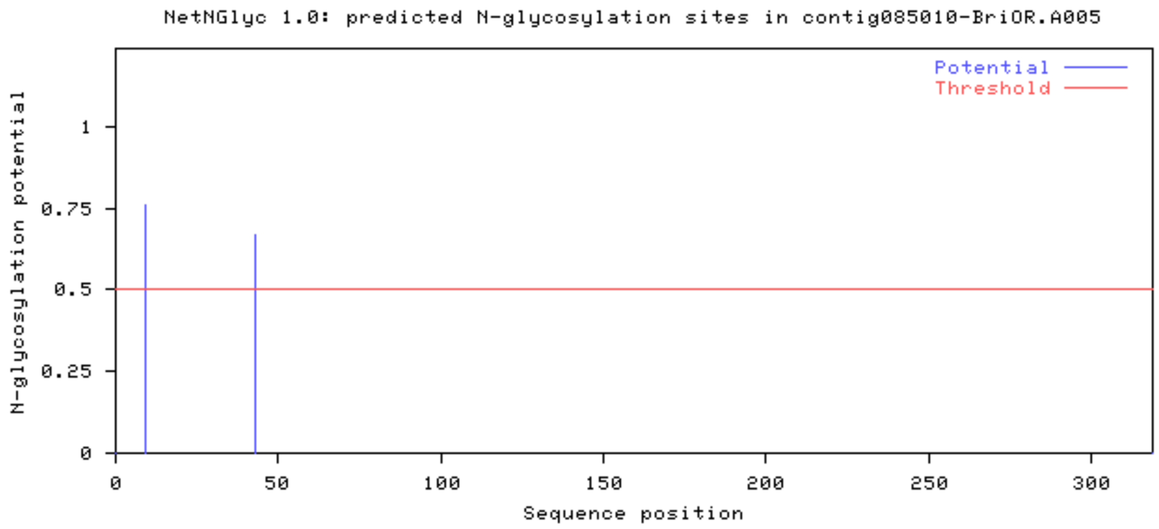

Graphics in PostScript

Output for 'contig085012-BriOR.A006'

```
########################################################################################

    Warning: This sequence may not contain a signal peptide!!

    Proteins without signal peptides are unlikely to be exposed to
    the N-glycosylation machinery and thus may not be glycosylated
    (in vivo) even though they contain potential motifs.

    SignalP-NN euk predictions are as follows:

  # name                   Cmax  pos ?  Ymax  pos ?  Smax  pos ?  Smean ?  D     ?

    SignalP output is explained at http://www.cbs.dtu.dk/services/SignalP/output.html

########################################################################################

Name:  contig085012-BriOR.A006  Length:  315
MDEESNVTYLTLHWYTEINKYRYIFFFVMFTLYILIICTNSTILYLIWIHKNLHEPMYIFIAALLLNSVLYSTTVYPKLL      80
IDFSSEKQVTTYSACLIQFFIFYTLVLSEFLLLAAMAYDRYVAICKPLEYQTIMTKTTVSIFLVVAWLVPACQVAVQAIA     160
SAEAKLCDSNIKGIFCNNAVYTLQCERSKLITIFGVVIVLDLAMLPMLFIVFTYTKIFVVSHRSCKEIRKKAAETCLPHL     240
LVLLSLSVFFVYDVSIARANPDFPKTTRIIMTLQIMLYQPLLNPFIYGLKMKEISKHLNKLLSQANISPKAKVKX
.....N.................................N........................................      80
................................................................................     160
................................................................................     240
...........................................................................          320

(Threshold=0.5)
----------------------------------------------------------------------
SeqName      Position  Potential    Jury    N-Glyc
                                  agreement result
----------------------------------------------------------------------
contig085012-BriOR.A006    6 NVTY    0.7368     (9/9)    ++
contig085012-BriOR.A006   40 NSTI    0.6300     (8/9)    +
contig085012-BriOR.A006  306 NISP    0.1086     (9/9)    ---
```

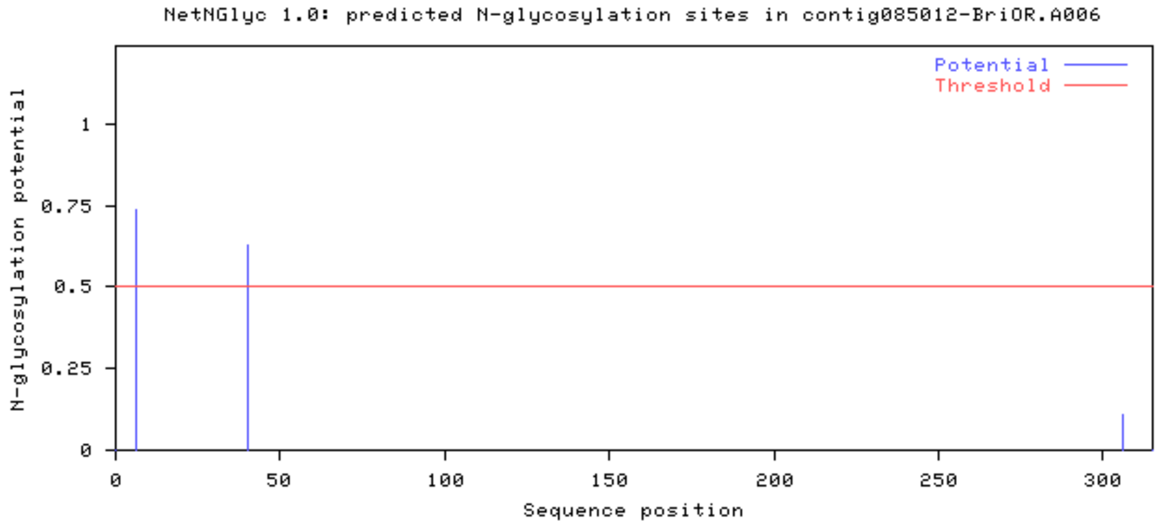

[Graphics in PostScript]

Output for 'contig085018-BriOR.A006'

```
##########################################################################################

    Warning: This sequence may not contain a signal peptide!!

    Proteins without signal peptides are unlikely to be exposed to
    the N-glycosylation machinery and thus may not be glycosylated
    (in vivo) even though they contain potential motifs.

    SignalP-NN euk predictions are as follows:

  # name                    Cmax  pos ?  Ymax  pos ?  Smax  pos ?  Smean ?  D     ?

    SignalP output is explained at http://www.cbs.dtu.dk/services/SignalP/output.html

##########################################################################################

Name:  contig085018-BriOR.A006  Length:  316
MDKELNVTFLTLDWYTEINKYRYIFFFIMFTLYILIICINSIIVYLIWIHKNLHEPMYIFIAALLLNSVLYSTTIYPKLL      80
IDFLSEKQVTTYSACLFQFFMFYTLGGSEFFLLAAMAYDRYVAICKPLQYHIIMRKTTVSISLIIAWLVPVCHIAVVAIA     160
SAEAKLCDSNIKGVFCNNAVYTLQCQRSRLIIVFGVVCLLDLVILPMLFIVFTYTTIFIVSHQSCKEIRKKAAETCLPHL     240
LVLISACLFFVYDVSIARVEADFPKTARIIMTLQIVLYHPLFNPFVYGLKMKKISKHLKGLLCQGKITSCIKTGFX
.....N..........................................................................      80
................................................................................     160
................................................................................     240
............................................................................         320

(Threshold=0.5)
----------------------------------------------------------------------
SeqName      Position  Potential   Jury    N-Glyc
                                 agreement result
----------------------------------------------------------------------
contig085018-BriOR.A006     6 NVTF   0.7646     (9/9)    +++
----------------------------------------------------------------------
```

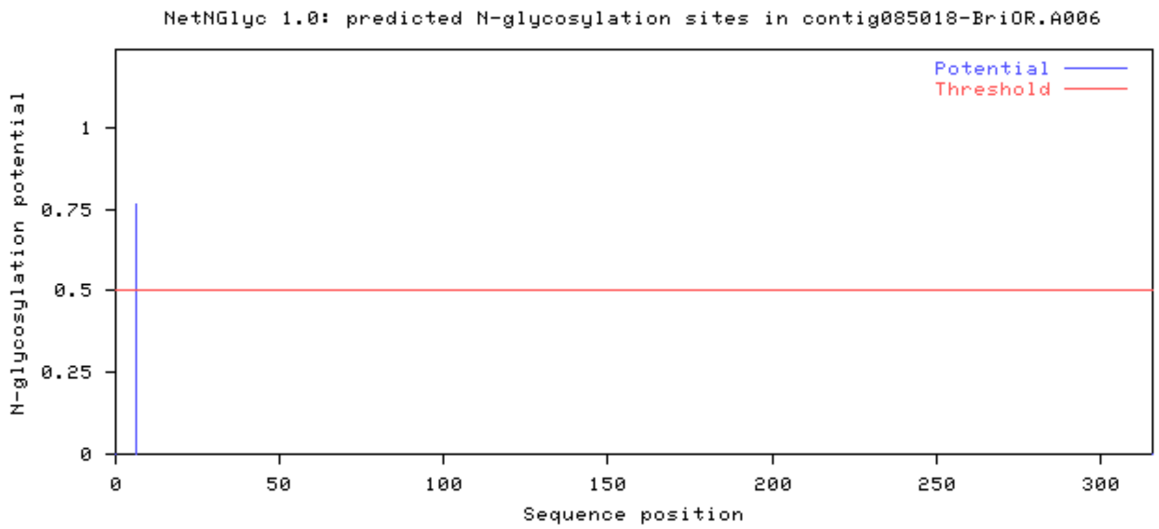

Graphics in PostScript

Output for 'contig085023-BriORp.A023'

```
##########################################################################################

    Warning: This sequence may not contain a signal peptide!!

    Proteins without signal peptides are unlikely to be exposed to
    the N-glycosylation machinery and thus may not be glycosylated
    (in vivo) even though they contain potential motifs.

    SignalP-NN euk predictions are as follows:

  # name                   Cmax  pos ?  Ymax  pos ?  Smax  pos ?  Smean ?  D     ?

    SignalP output is explained at http://www.cbs.dtu.dk/services/SignalP/output.html

##########################################################################################

Name:  contig085023-BriORp.A023         Length:  304
MNLTYITFGGHVEVEKYRYLYFVIMFMVYVLIICSNSTIVWLIIVQKSLHEPMYIFIAALLVNSVILSTVMYPKLLIDFL      80
SEKQIKNRFFLYQACLFQVFLFYALSCSEFLLLSAMAYDSYVSTCKPLQYPTITRRTRVNIFLLCCWFLPAIQVAVPIVG     160
NTNTPLCNFTLKDIFCNNSVNHLYCVNSRELSIYGMVVLFSVALSPMFFILFTYIKIIIVAYQSCGNVRKKAAQTCLPHV     240
LVLINYSCLLTYDMVIVRLESEFPKTARFIMTLQFVTYNPLCNPIIYGLKMKEISKHLKILFSX
.N.................................N............................................      80
................................................................................     160
.......N........................................................................     240
....N...........................................................                     320

(Threshold=0.5)
----------------------------------------------------------------------
SeqName      Position  Potential   Jury    N-Glyc
                                agreement result
----------------------------------------------------------------------
contig085023-BriORp.A023    2 NLTY   0.7966     (9/9)   +++
contig085023-BriORp.A023   36 NSTI   0.6939     (9/9)   ++
contig085023-BriORp.A023  168 NFTL   0.6512     (9/9)   ++
contig085023-BriORp.A023  177 NNSV   0.4741     (5/9)   -
contig085023-BriORp.A023  245 NYSC   0.5037     (5/9)   +
----------------------------------------------------------------------
```

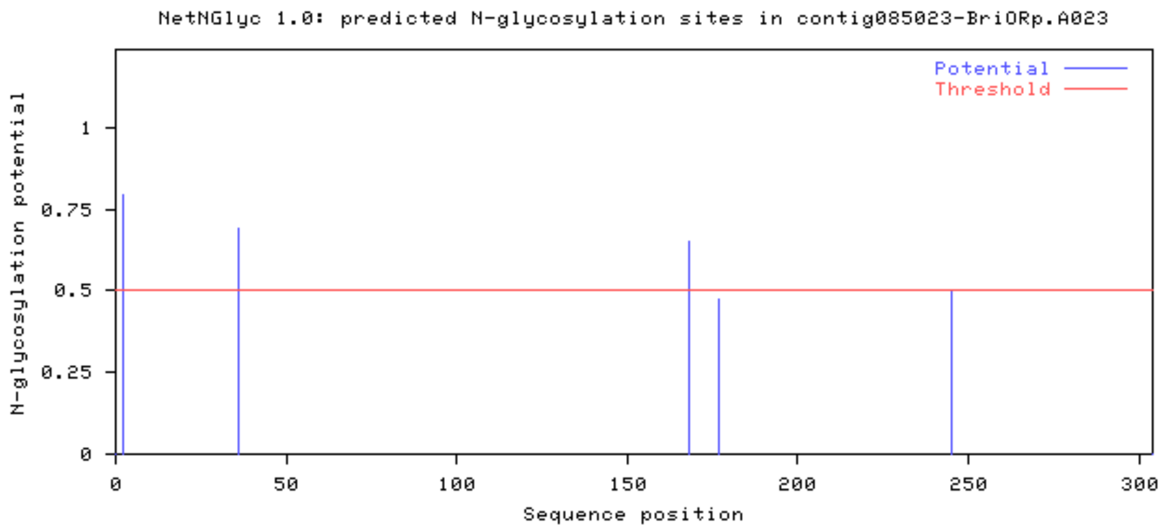

Graphics in PostScript

Output for 'contig085026-BriOR.A008'

```
##########################################################################################

    Warning: This sequence may not contain a signal peptide!!

    Proteins without signal peptides are unlikely to be exposed to
    the N-glycosylation machinery and thus may not be glycosylated
    (in vivo) even though they contain potential motifs.

    SignalP-NN euk predictions are as follows:

  # name                   Cmax  pos ?  Ymax  pos ?  Smax  pos ?  Smean ?  D     ?

    SignalP output is explained at http://www.cbs.dtu.dk/services/SignalP/output.html

##########################################################################################

Name:  contig085026-BriOR.A008  Length:  316
MDQELNFTYVTLDWYVDINKYRYVFFFIMFALYSLIICTNSTIVYIIWIHKNLHEPMYIFIAALLLNSVLYSTTIYPKLL      80
IDFLSEKQVTTYSACLFQFFMFYTLGGSEFFLLAAMAYDRYVAICKPLQYHIIMRKTTVSIFLIIAWLVPACHIAVQAIG     160
SANIKLCDFNIKGIFCNNAVYTLLCERSRLITIFGVVALLDLAVLPMLFIVFTYTKIFIVSYQRCKEIQKKAAETCLPHL     240
LVLISASVFFVYDVSIARVETNFPKAVRIVMTLQVVLYHPIVNPFIYGLKMKKISKHLKRCFPRPQSILELKVNAX
.....N.................................N........................................      80
................................................................................     160
................................................................................     240
............................................................................         320

(Threshold=0.5)
----------------------------------------------------------------------
SeqName       Position  Potential    Jury    N-Glyc
                                   agreement result
----------------------------------------------------------------------
contig085026-BriOR.A008    6 NFTY    0.7623     (9/9)    +++
contig085026-BriOR.A008   40 NSTI    0.7440     (9/9)    ++
----------------------------------------------------------------------
```

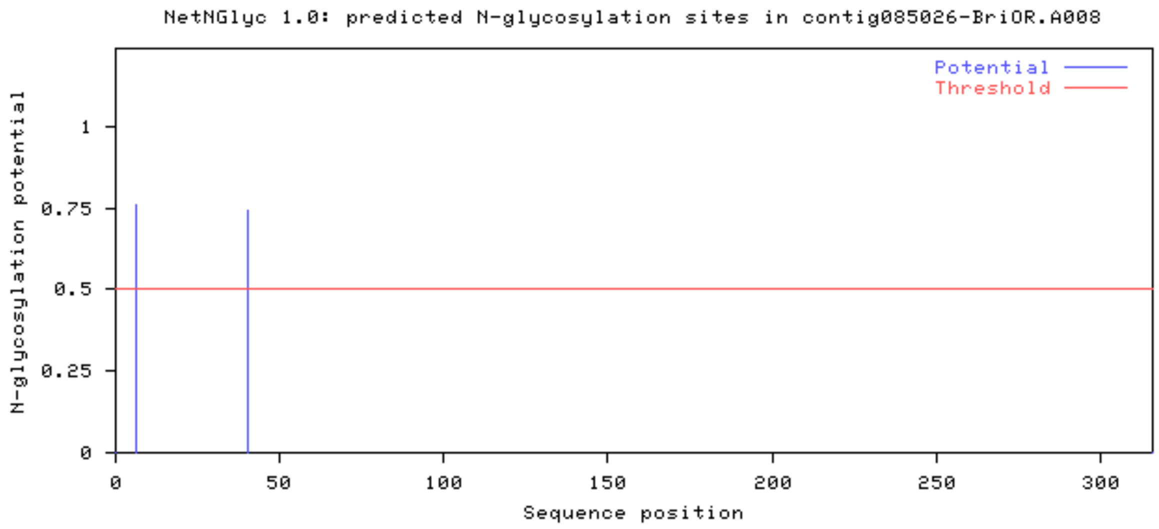

Graphics in PostScript

Output for 'contig085028-BriORp.A024'

```
##########################################################################################

    Warning: This sequence may not contain a signal peptide!!

    Proteins without signal peptides are unlikely to be exposed to
    the N-glycosylation machinery and thus may not be glycosylated
    (in vivo) even though they contain potential motifs.

    SignalP-NN euk predictions are as follows:

  # name                    Cmax   pos ?  Ymax   pos ?  Smax   pos ?  Smean ?  D     ?

    SignalP output is explained at http://www.cbs.dtu.dk/services/SignalP/output.html

##########################################################################################

Name:  contig085028-BriORp.A024         Length:  323
MDEDLNVTYITFDGHVEINKYRYFYFLLMFTLYILITCSNSIIIYLILIHKNLHEPMDTFIAALLLNVAIYATAIYPKLL      80
IDFLSEKQIISYSACLFQFFIIYSLGCSEFVLLAAIAYDRYMAICKPLQYPTIMRKSTVSIFLVIAWLVPIAVQEIGMAK     160
SKLCSFHKSIFCNNTIYTLQCVRSRLVTVFGIICYFDFVILPRLFIVFTYTKIFIVSYHSRKEIKKKAAETCLPHILVLM     240
SFSCLGVYDVTISRVESDFPKTARFIVSLQLALYQPLFNPLIYGLKMKEISKHLKRLFCPIKFFCINKFLISENILNINI     320
INS
.....N..........................................................................      80
................................................................................     160
................................................................................     240
................................................................................     320
...                                                                                  400

(Threshold=0.5)
----------------------------------------------------------------------
SeqName       Position  Potential    Jury    N-Glyc
                                  agreement  result
----------------------------------------------------------------------
contig085028-BriORp.A024     6 NVTY    0.7954     (9/9)    +++
contig085028-BriORp.A024   173 NNTI    0.4962     (5/9)    -
----------------------------------------------------------------------
```

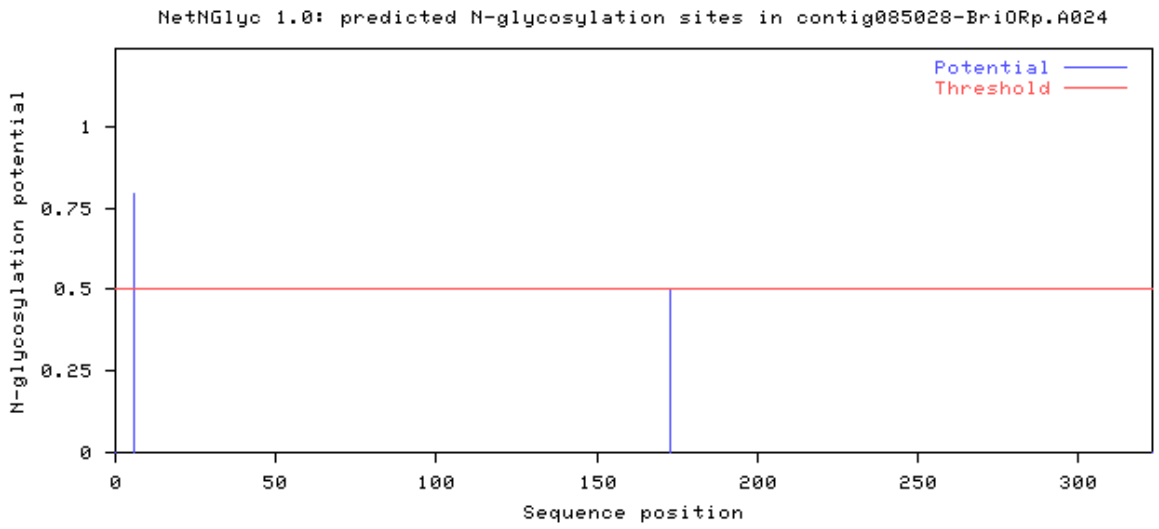

Graphics in PostScript

Output for 'contig085033-BriORe.A013'

```
##########################################################################

    Warning: This sequence may not contain a signal peptide!!

    Proteins without signal peptides are unlikely to be exposed to
    the N-glycosylation machinery and thus may not be glycosylated
    (in vivo) even though they contain potential motifs.

    SignalP-NN euk predictions are as follows:

  # name                 Cmax  pos ?  Ymax  pos ?  Smax  pos ?  Smean ?  D     ?

    SignalP output is explained at http://www.cbs.dtu.dk/services/SignalP/output.html

##########################################################################

Name:  contig085033-BriORe.A013         Length:  222
MNITYITFGGHVEVEKYRYIYFVIMFMVYVLIICSNSTIVWVIIVQKSLHEPMYIFIAALLVNSILLSTVIYPKLLIDFL      80
SEKQIILYHACLFQLFMFYVLSSSEFLLLSAMAYDRYVSICKPLQYPTIMRRTRVSIFLILSWFLPAIQIVVPVLRNSIT     160
PLCNFTLKGIFCNNSVNHLYCVTSKELSIYGMVVLFNVALFPMLFILFTYIKIIIVACQSCG
.N.................................N............................................      80
................................................................................     160
...N..........................................................                       240

(Threshold=0.5)
----------------------------------------------------------------------
SeqName      Position  Potential   Jury     N-Glyc
                                 agreement  result
----------------------------------------------------------------------
contig085033-BriORe.A013     2 NITY   0.7933     (9/9)   +++
contig085033-BriORe.A013    36 NSTI   0.7073     (9/9)   ++
contig085033-BriORe.A013   164 NFTL   0.6262     (9/9)   ++
contig085033-BriORe.A013   173 NNSV   0.4212     (7/9)   -
----------------------------------------------------------------------
```

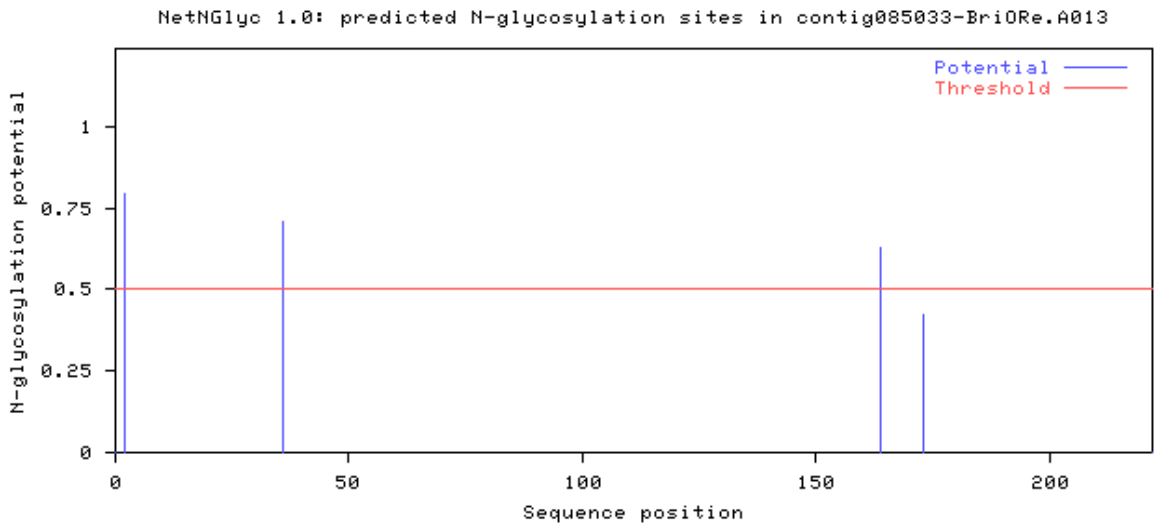

Graphics in PostScript

Output for 'contig085034-BriORe.A019'

```
##########################################################################

    Warning: This sequence may not contain a signal peptide!!

    Proteins without signal peptides are unlikely to be exposed to
    the N-glycosylation machinery and thus may not be glycosylated
    (in vivo) even though they contain potential motifs.

    SignalP-NN euk predictions are as follows:

  # name                Cmax  pos ?  Ymax  pos ?  Smax  pos ?  Smean ?  D     ?

    SignalP output is explained at http://www.cbs.dtu.dk/services/SignalP/output.html

##########################################################################

Name:  contig085034-BriORe.A019         Length:  79
MNITYITFGGYVEVEKYRYIYFVIMFMVYVLIICSNSTIVWVIIVQKSLHEPMYIFIAALLVNSILLSTVIYPKLLIDF
.N.................................N...........................................      80

(Threshold=0.5)
----------------------------------------------------------------------
SeqName      Position  Potential   Jury    N-Glyc
                                agreement  result
----------------------------------------------------------------------
contig085034-BriORe.A019    2 NITY   0.7941     (9/9)    +++
contig085034-BriORe.A019   36 NSTI   0.6657     (9/9)    ++
----------------------------------------------------------------------
```

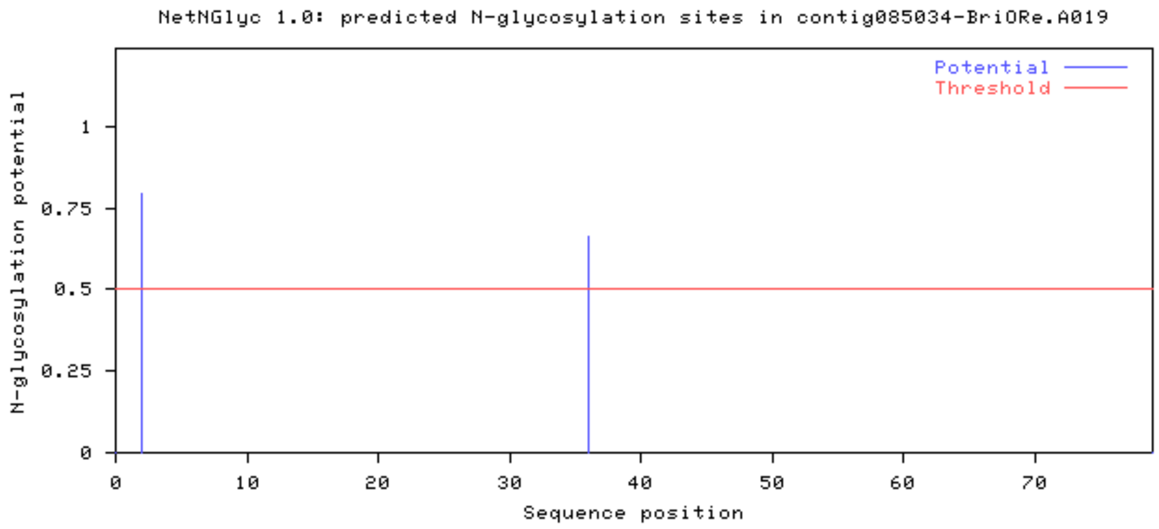

Graphics in PostScript

Output for 'contig090286-BriORs.W112'

```
################################################################################

    Warning: This sequence may not contain a signal peptide!!

    Proteins without signal peptides are unlikely to be exposed to
    the N-glycosylation machinery and thus may not be glycosylated
    (in vivo) even though they contain potential motifs.

    SignalP-NN euk predictions are as follows:

  # name                  Cmax  pos ?  Ymax  pos ?  Smax  pos ?  Smean ?  D     ?

    SignalP output is explained at http://www.cbs.dtu.dk/services/SignalP/output.html

################################################################################

Name:  contig090286-BriORs.W112         Length:  306
MSASYANETVVVNYRDAFSKAMIKNVIVVVLCISINYINVALLQTFCKQQVIFYMNPRYILFFHLVLNDMIQVTLTVVLF      80
ISSYIFFQINVSVCCVLILLALFATENTPLNLACMAVECYIAICIPLRHVQICTVKRTLMLIGLIWMTSMLSVLPDLFIT     160
LAIEPPDFYNSRVFCLRETVFRNPHIIKKRDITYIVYLVIVWFIIFFTYFKILFTAKAASQDATKARNTIILHGFQVLLC     240
MSIYAEPLLRQVLQQWFPQNYSDSLFACYILFQILPRAINPIVYGVRDKTYRKYLKRYLLCKMGPX
......N.........................................................................      80
.........N......................................................................     160
................................................................................     240
...................N..............................................                   320

(Threshold=0.5)
----------------------------------------------------------------------
SeqName      Position  Potential   Jury    N-Glyc
                                 agreement result
----------------------------------------------------------------------
contig090286-BriORs.W112    7 NETV   0.7187     (9/9)   ++
contig090286-BriORs.W112   90 NVSV   0.6560     (8/9)   +
contig090286-BriORs.W112  260 NYSD   0.6354     (8/9)   +
----------------------------------------------------------------------
```

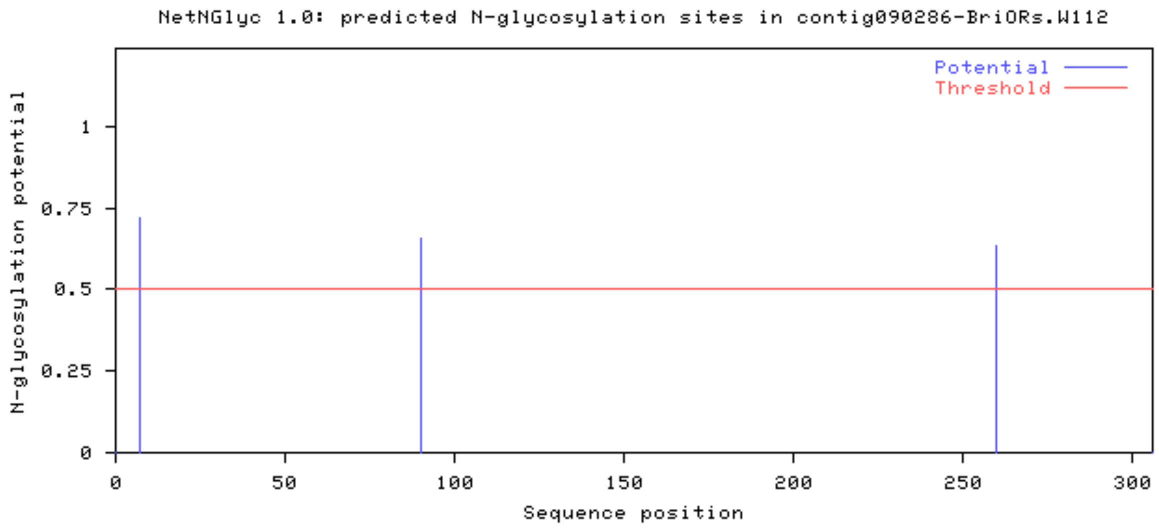

Graphics in PostScript

Output for 'contig090288-BriORs.W113'

```
##########################################################################

    Warning: This sequence may not contain a signal peptide!!

    Proteins without signal peptides are unlikely to be exposed to
    the N-glycosylation machinery and thus may not be glycosylated
    (in vivo) even though they contain potential motifs.

    SignalP-NN euk predictions are as follows:

  # name                Cmax  pos ?  Ymax  pos ?  Smax  pos ?  Smean ?  D     ?

    SignalP output is explained at http://www.cbs.dtu.dk/services/SignalP/output.html

##########################################################################

Name:  contig090288-BriORs.W113         Length:  315
MNASSGNVTVVLQYRDSFAKAVTKNLIVVFLGISISYINVNLIHTFCKHQVQIFYKNPRYVLFIHLVINDMVQVMLTAIL      80
FIISYTIYKLNVSVCSIFTLLALFTTENSPLNLACMAVECYIAICFPLRHVQICTVQRTLILISLIWMSTTLSVLPDLFI     160
TLATEPLDFFHSRVFCLRNIVFPHPLIIQKRDITYGVFLVIVWVTIIYTYLKILFTAKTASKDAKKAKNTILLHGFQLLL     240
CMATYAAPHLTNALQKWFPTNYTDSLFVIYVTVQILPRSISPIIYGIRDKTFRKFLKGNLLCKITVHKSDNQNKX
.N....N.........................................................................      80
..........N.....................................................................     160
................................................................................     240
....................N......................................................          320

(Threshold=0.5)
----------------------------------------------------------------------
SeqName      Position  Potential    Jury    N-Glyc
                                 agreement  result
----------------------------------------------------------------------
contig090288-BriORs.W113    2 NASS   0.6616     (9/9)    ++
contig090288-BriORs.W113    7 NVTV   0.7672     (9/9)    +++
contig090288-BriORs.W113   91 NVSV   0.5760     (7/9)    +
contig090288-BriORs.W113  261 NYTD   0.7023     (9/9)    ++
----------------------------------------------------------------------
```

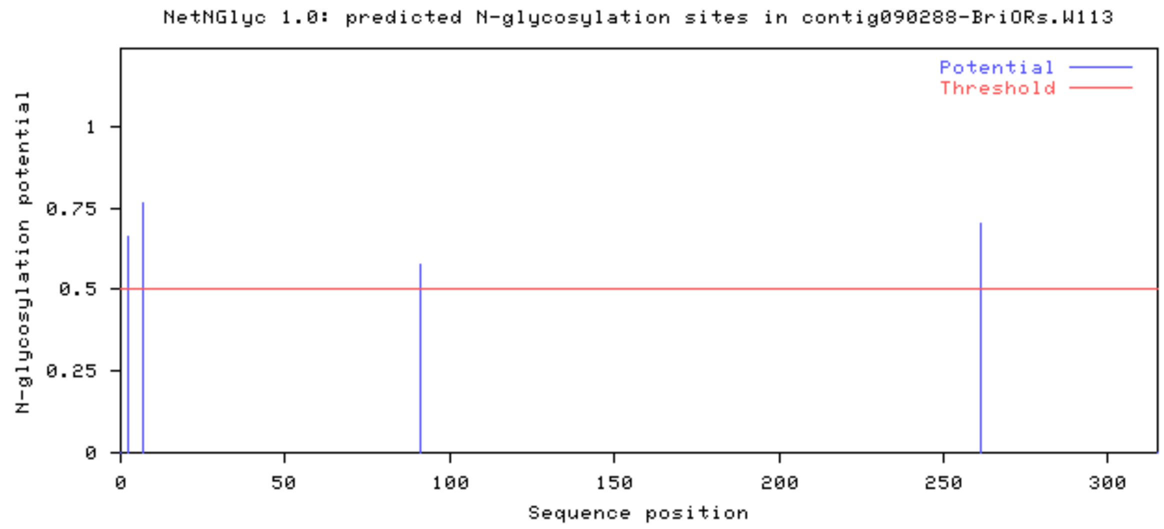

Graphics in PostScript

Output for 'contig090291-BriORs.W114'

```
##########################################################################

    Warning: This sequence may not contain a signal peptide!!

    Proteins without signal peptides are unlikely to be exposed to
    the N-glycosylation machinery and thus may not be glycosylated
    (in vivo) even though they contain potential motifs.

    SignalP-NN euk predictions are as follows:

  # name                Cmax  pos ?  Ymax  pos ?  Smax  pos ?  Smean ?  D     ?

    SignalP output is explained at http://www.cbs.dtu.dk/services/SignalP/output.html

##########################################################################

Name:  contig090291-BriORs.W114         Length:  315
MNLTQVDSNATITLNYWNTFVKAVGRNVTIVVLGITINYINATMIHTFNKYHQIFRLNPRYILFIHLVFNDIIQLSTSIS      80
LFIFSYAFHTIHVSLCLLLILPAIFTTQNTPLNIAFMAAECCVSVCIPLRYSYICTVKRTYIVIGIIWAISSLSILPDLF     160
ILLAVESSQFLQSRVVCNRDTVFRSSYSVKKRDASHTLFLVVVSITLLYTYCRILFVARCADSDTKKARNTILIHGFQVL     240
LCTTVYVQPPLIKLLVYFFPEGLKDINFATFIINQVLPRLGSPIIYGLRDKTFRKFLKRHLCAVNQLPKMIRSSX
.N......N.................N.............N.......................................      80
................................................................................     160
................................................................................     240
...........................................................................          320

(Threshold=0.5)
----------------------------------------------------------------------
SeqName      Position  Potential   Jury    N-Glyc
                                 agreement result
----------------------------------------------------------------------
contig090291-BriORs.W114    2 NLTQ   0.8094     (9/9)   +++
contig090291-BriORs.W114    9 NATI   0.6171     (8/9)   +
contig090291-BriORs.W114   27 NVTI   0.7314     (9/9)   ++
contig090291-BriORs.W114   41 NATM   0.5423     (5/9)   +
----------------------------------------------------------------------
```

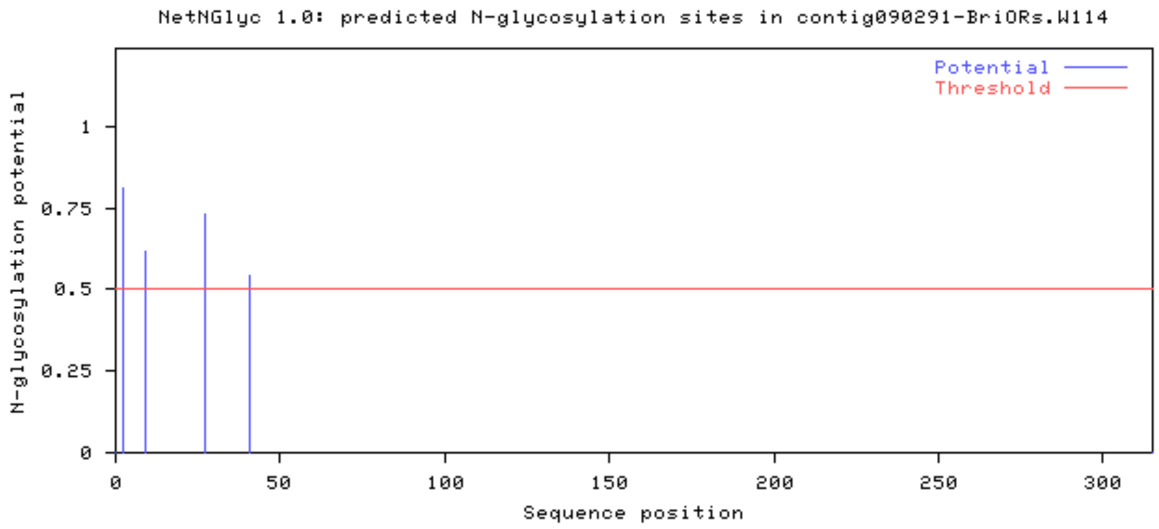

Graphics in PostScript

Output for 'contig090292-BriORs.W115'

```
##########################################################################

    Warning: This sequence may not contain a signal peptide!!

    Proteins without signal peptides are unlikely to be exposed to
    the N-glycosylation machinery and thus may not be glycosylated
    (in vivo) even though they contain potential motifs.

    SignalP-NN euk predictions are as follows:

  # name                Cmax  pos ?  Ymax  pos ?  Smax  pos ?  Smean ?  D     ?

    SignalP output is explained at http://www.cbs.dtu.dk/services/SignalP/output.html

##########################################################################

Name:  contig090292-BriORs.W115         Length:  318
MNSSSYALNASSSLKYRDSPSAAVAKNVIVLALGFTINYINGTLIHTFRKHQIFYLNPRYILFIHLVVNDMIQLTSSISL      80
FVFTYIFYQLNVAFCCFLITLAIFTTFNTPINLALMALECYIAICLPLQHAEHCTTKRTYVAIGWIWAMSAVSAMSDIVI     160
ILATEPVELFYSTIQCERDNLFRHPIIVKKKEVSYLIFLIGVLLTFIYTYFRIFFAANKAKSAKQESKKARNTILLHGFQ     240
LLLSMLTYIATALTQALVRWFPKQYLIVVFISYIIIYIVPRFVSPIVYGLRDKTFKQYLKKYLLCTMKMGNETDFRLX
.N......N...............................N.......................................      80
................................................................................     160
................................................................................     240
......................................................................N.......          320

(Threshold=0.5)
----------------------------------------------------------------------
SeqName       Position  Potential   Jury    N-Glyc
                                  agreement result
----------------------------------------------------------------------
contig090292-BriORs.W115     2 NSSS   0.7220     (9/9)   ++
contig090292-BriORs.W115     9 NASS   0.6427     (8/9)   +
contig090292-BriORs.W115    41 NGTL   0.7142     (9/9)   ++
contig090292-BriORs.W115   311 NETD   0.6714     (9/9)   ++
----------------------------------------------------------------------
```

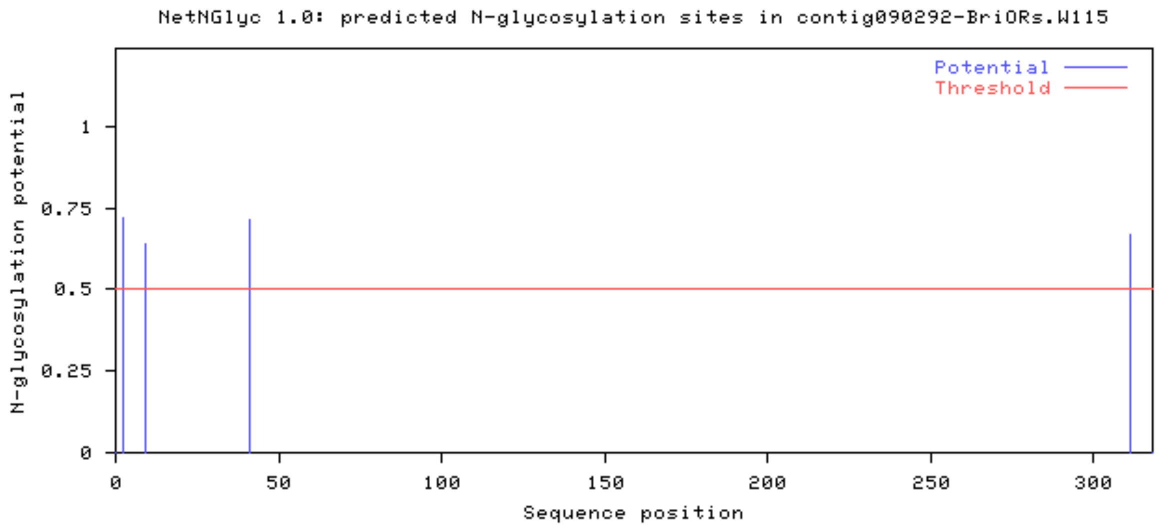

Graphics in PostScript

Output for 'contig090296-BriOR.W110'

```
########################################################################################

    Warning: This sequence may not contain a signal peptide!!

    Proteins without signal peptides are unlikely to be exposed to
    the N-glycosylation machinery and thus may not be glycosylated
    (in vivo) even though they contain potential motifs.

    SignalP-NN euk predictions are as follows:

  # name                Cmax  pos ?  Ymax  pos ?  Smax  pos ?  Smean ?  D     ?

    SignalP output is explained at http://www.cbs.dtu.dk/services/SignalP/output.html

########################################################################################

Name:  contig090296-BriOR.W110  Length:  298
MIFLDVQVWSLKQLFFIHRSMFSGIIHLSCSKSCPSLQVFNTNPRYILYIHLVIIDILLLIMFTLLQVLNYIIFTLPVPF      80
CIILLLISIICSQNNPLTLAVMAVECHIAICFPLQHSQICTVKNVTIVITVIWGLSSLTILPNLFTSLATESRDFFHSRV     160
FCLRKKTFRPPELEKKKNISNIVFLVIVWLTLVYTYFRILFAAQAAAANARKARNTVLLHGFQLLLCMLNYVYDLLLNGL     240
TSLFPKGVLTIRYTISVFVHILPRLVSPLVYGIRDKAFRRYLRKYLFYSPKANVNKSX
................................................................................      80
...........................................N....................................     160
.................N..............................................................     240
..........................................................                           320

(Threshold=0.5)
----------------------------------------------------------------------
SeqName      Position  Potential   Jury    N-Glyc
                                 agreement result
----------------------------------------------------------------------
contig090296-BriOR.W110  124 NVTI   0.5761     (9/9)   ++
contig090296-BriOR.W110  178 NISN   0.6170     (8/9)   +
contig090296-BriOR.W110  295 NKSX   0.4599     (6/9)   -
----------------------------------------------------------------------
```

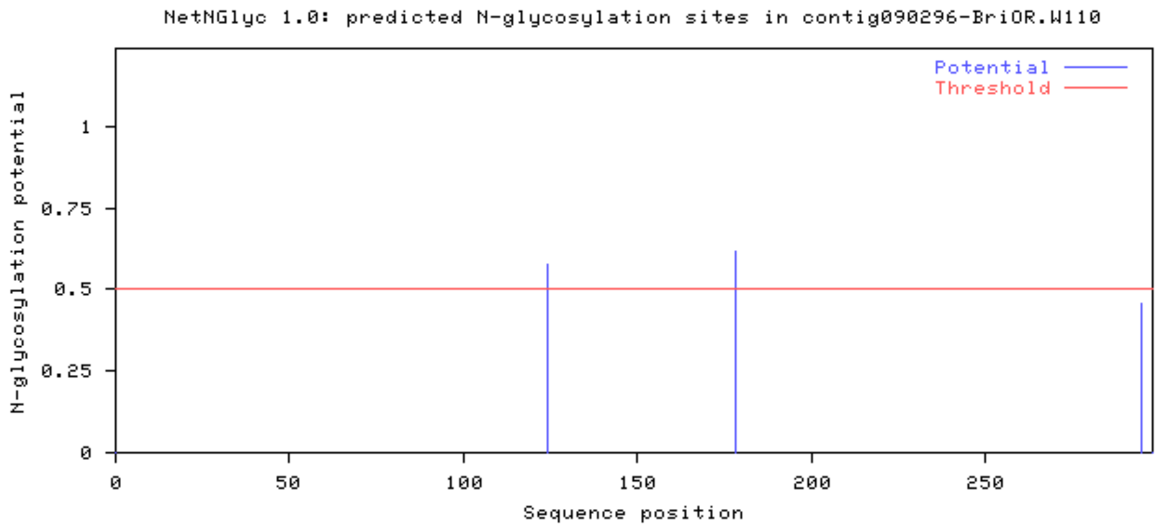

Graphics in PostScript

Output for 'contig090301-BriORs.U109'

```
##########################################################################

    Warning: This sequence may not contain a signal peptide!!

    Proteins without signal peptides are unlikely to be exposed to
    the N-glycosylation machinery and thus may not be glycosylated
    (in vivo) even though they contain potential motifs.

    SignalP-NN euk predictions are as follows:

  # name                Cmax  pos ?  Ymax  pos ?  Smax  pos ?  Smean ?  D    ?

    SignalP output is explained at http://www.cbs.dtu.dk/services/SignalP/output.html

##########################################################################

Name:  contig090301-BriORs.U109         Length:  328
MSSLLGLRGNMTVPYQLLLIRDTFTTAFVKNLIVVLVWLTLSYINATLVVTFFRHQTFHDDPRYILFIHMVINDAIQLTV      80
TIMLFILSYIFYKINVTFCCFFILVAVFTTRNTPVNLAAMAIERYIAICEPLRYTQICTVRRTYVVIGMIWFICVAPDIT     160
DLFVTLATESLSFFHESVFCLRQNVFKDPILAYKRQAFDIIYFSCVFLILVVTYLRILFAARALSTDKTSAQKARNTILL     240
HGAQLAMCMLSYVSPSVEVVLHIIFPGRILEIRFANYLIVYILPRFLSPIIYGVRDKKFREYLKMYYLSNRCRNKEKKVT     320
PEDKNHLX
.........N..................................N...................................      80
.............N..................................................................     160
................................................................................     240
................................................................................     320
........                                                                             400

(Threshold=0.5)
----------------------------------------------------------------------
SeqName      Position  Potential   Jury    N-Glyc
                                agreement  result
----------------------------------------------------------------------
contig090301-BriORs.U109    10 NMTV    0.7846     (9/9)   +++
contig090301-BriORs.U109    45 NATL    0.6906     (8/9)   +
contig090301-BriORs.U109    95 NVTF    0.5571     (8/9)   +
----------------------------------------------------------------------
```

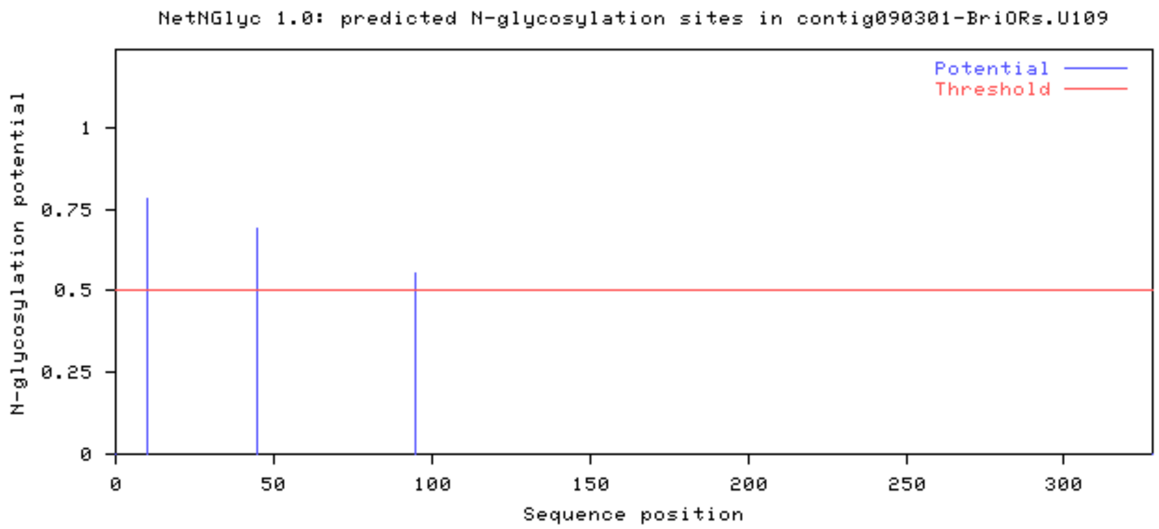

Graphics in PostScript

Output for 'contig090301-BriORs.W116'

```
##########################################################################################

    Warning: This sequence may not contain a signal peptide!!

    Proteins without signal peptides are unlikely to be exposed to
    the N-glycosylation machinery and thus may not be glycosylated
    (in vivo) even though they contain potential motifs.

    SignalP-NN euk predictions are as follows:

  # name                 Cmax  pos ?  Ymax  pos ?  Smax  pos ?  Smean ?  D     ?

    SignalP output is explained at http://www.cbs.dtu.dk/services/SignalP/output.html

##########################################################################################

Name:  contig090301-BriORs.W116        Length:  320
MNLSSVNFSSTLTSYEENPLLTNYIVAATLCITINYINSTLIHTFRKYQVQILHSNPRYILFIHLVINDMMLLFLTTLLL      80
IISYALHIFFVPFCIILLIITVIVTHNSPLNLAGMAMCYIAVCMPLRHGQICTVKKTYILIGLIWAASALSILPDLFILL     160
ATEPLQFFHSKVRCIRDFVFRSTYSLNKRDASHIVCLVMVWLTLIYTYARIIFAAKGLTSDIKKARNTILPHGFQVLLLM     240
LNYVRPICEQSLFFLFPNREIPIQFASFVIVQIIPRFISPIVYGLRDQTFRKYVRRYLVCSERGITHPENTTTLQKGLTX     320
.N....N..............................N..........................................      80
................................................................................     160
................................................................................     240
................................................................................     320

(Threshold=0.5)
----------------------------------------------------------------------
SeqName      Position  Potential   Jury    N-Glyc
                                 agreement result
----------------------------------------------------------------------
contig090301-BriORs.W116    2 NLSS    0.7897     (9/9)   +++
contig090301-BriORs.W116    7 NFSS    0.7264     (9/9)   ++
contig090301-BriORs.W116   38 NSTL    0.6249     (9/9)   ++
contig090301-BriORs.W116  310 NTTT    0.4432     (7/9)   -
----------------------------------------------------------------------
```

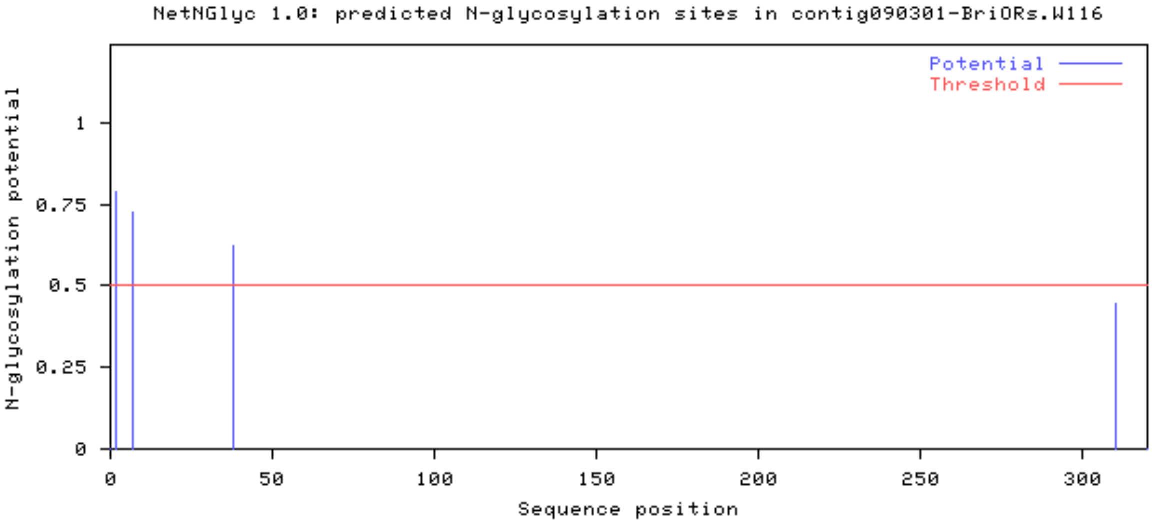

Graphics in PostScript

Output for 'contig090302-BriORs.V122'

```
##########################################################################################

    Warning: This sequence may not contain a signal peptide!!

    Proteins without signal peptides are unlikely to be exposed to
    the N-glycosylation machinery and thus may not be glycosylated
    (in vivo) even though they contain potential motifs.

    SignalP-NN euk predictions are as follows:

  # name                 Cmax  pos ?  Ymax  pos ?  Smax  pos ?  Smean ?  D     ?

    SignalP output is explained at http://www.cbs.dtu.dk/services/SignalP/output.html

##########################################################################################

Name:  contig090302-BriORs.V122         Length:  336
MNSTSTRPLDPLVSNATSVLTEGVKESFSSILTKNIVAMLVWLVLSIINGSMVHTFQRHSLFYENPRYIMFICMVINDAL      80
QLTLVTALYVISYIFRKIHASVCCLLVIMTAILTTRSTPLILAGMAVERYISICFPLRYSHMCNIPRTLLLICVIVILTI     160
TPPITDLLITIVKEPPSFFHTKIFCDHSLLFRDQSIYYKNCVFDGTYLSFVALALLYTYCKIMMAAQAVSTSLASVKRAR     240
NTVLLHGVQVVVPSLQAALISLFPQLSLEIRYIFFLLVYIIPRFLSPMIYGFRDEQFRKYWTRYLSCHEHSMTLLRLASQ     320
KMNPQVKQSGNLSSSTX
.N...........N...............................N..............................      80
................................................................................     160
................................................................................     240
................................................................................     320
..........N.....                                                                     400

(Threshold=0.5)
----------------------------------------------------------------------
SeqName      Position  Potential   Jury    N-Glyc
                                 agreement result
----------------------------------------------------------------------
contig090302-BriORs.V122     2 NSTS   0.7913     (9/9)   +++
contig090302-BriORs.V122    15 NATS   0.7184     (8/9)   +
contig090302-BriORs.V122    49 NGSM   0.6353     (8/9)   +
contig090302-BriORs.V122   331 NLSS   0.6437     (8/9)   +
----------------------------------------------------------------------
```

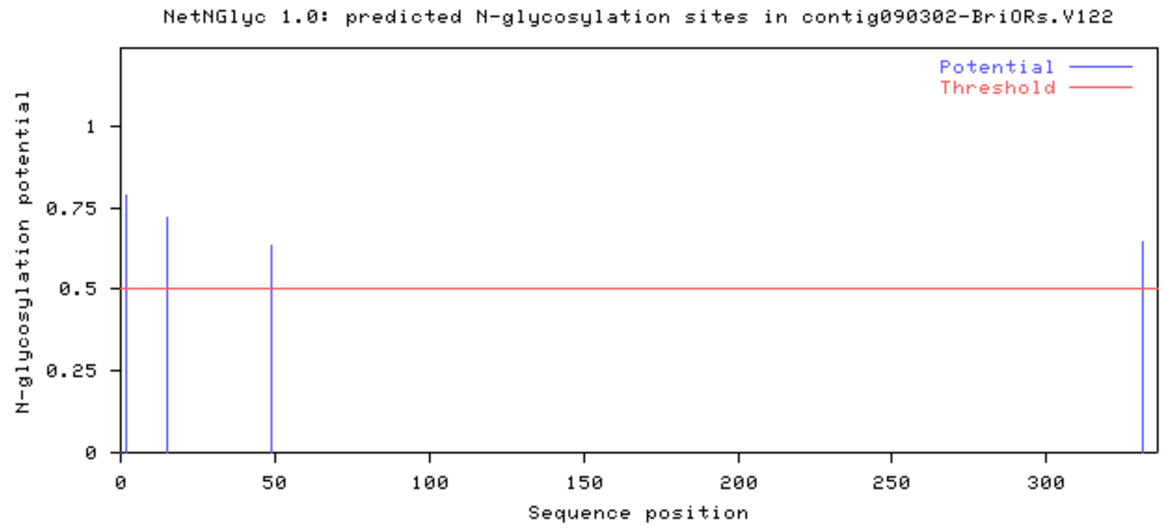

Graphics in PostScript

Output for 'contig093807-BriOR.A009'

```
##########################################################################################

    Warning: This sequence may not contain a signal peptide!!

    Proteins without signal peptides are unlikely to be exposed to
    the N-glycosylation machinery and thus may not be glycosylated
    (in vivo) even though they contain potential motifs.

    SignalP-NN euk predictions are as follows:

  # name                    Cmax  pos ?  Ymax  pos ?  Smax  pos ?  Smean ?  D     ?

    SignalP output is explained at http://www.cbs.dtu.dk/services/SignalP/output.html

##########################################################################################

Name:  contig093807-BriOR.A009  Length:  306
MDVKLNVTLLTLGGFAELHKYRYLYFVVIFTLYILILCFNSTIVYLIWTCKNLHEPMYIFIAALLINSVLYSMIIYPKLL      80
SDVLSEKQTISYPLCLFQGFSYYASAGSEFLLLAAMAYDRYVSICKPLQYPVIMNRITIYMFLILAWLIPAFEIAVSFVL     160
YFNVKLCNFTLTGIFCNNSIYRLQCVPSVTISIYGVVTLINIALLPMFFILFTYIRILRISINCCRETRRKALKTCLPHL     240
LVLINFSCFIVFDSVIIRLDSDLSKTLRLTLTFQSILFHPLLNPIIYGLKMNEIFKHIKTLLCQVX
.....N.................................N........................................      80
................................................................................     160
.......N........................................................................     240
....N.............................................................                   320

(Threshold=0.5)
----------------------------------------------------------------------
SeqName      Position  Potential   Jury    N-Glyc
                                 agreement result
----------------------------------------------------------------------
contig093807-BriOR.A009    6 NVTL   0.7751     (9/9)   +++
contig093807-BriOR.A009   40 NSTI   0.7014     (9/9)   ++
contig093807-BriOR.A009  168 NFTL   0.7139     (9/9)   ++
contig093807-BriOR.A009  177 NNSI   0.4372     (7/9)   -
contig093807-BriOR.A009  245 NFSC   0.5634     (6/9)   +
```

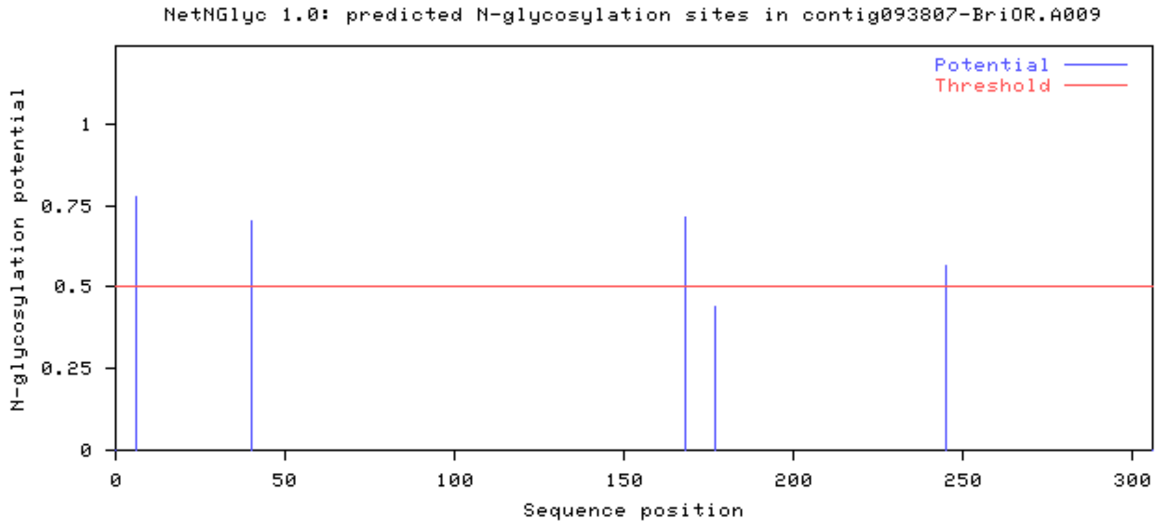

[Graphics in PostScript]

Output for 'contig093808-BriORe.A014'

```
##########################################################################################

    Warning: This sequence may not contain a signal peptide!!

    Proteins without signal peptides are unlikely to be exposed to
    the N-glycosylation machinery and thus may not be glycosylated
    (in vivo) even though they contain potential motifs.

    SignalP-NN euk predictions are as follows:

  # name                    Cmax  pos ?  Ymax  pos ?  Smax  pos ?  Smean ?  D     ?

    SignalP output is explained at http://www.cbs.dtu.dk/services/SignalP/output.html

##########################################################################################

Name:  contig093808-BriORe.A014         Length:  248
LYSTTIYPKLLIDFLSEKQVITYSACLFQFFIFYTLGSSEFFLLAAMAYDRYVAICKPLEYPTIMNKTTVSVFLVVSWLI      80
PAFHIAVQAIGSAEATLCNFNLKGIFCNNAVYTLQCVRSRLIIVFGVVALIDLIILPLLFIVFTYTNIFIISYQSCKEIR     160
KKAAETCLPHLLVLISISCLSIYDVGIARVESDFPKVARLLMTLQLLFYHPLFNPFIYGLKMKEISKQLKRFFCHPRIIT     240
CINANVTX
.................................................................N..............      80
................................................................................     160
................................................................................     240
....N...                                                                             320

(Threshold=0.5)
----------------------------------------------------------------------
SeqName       Position  Potential   Jury    N-Glyc
                                  agreement result
----------------------------------------------------------------------
contig093808-BriORe.A014    66 NKTT    0.6654     (8/9)    +
contig093808-BriORe.A014   245 NVTX    0.5918     (9/9)    ++
----------------------------------------------------------------------
```

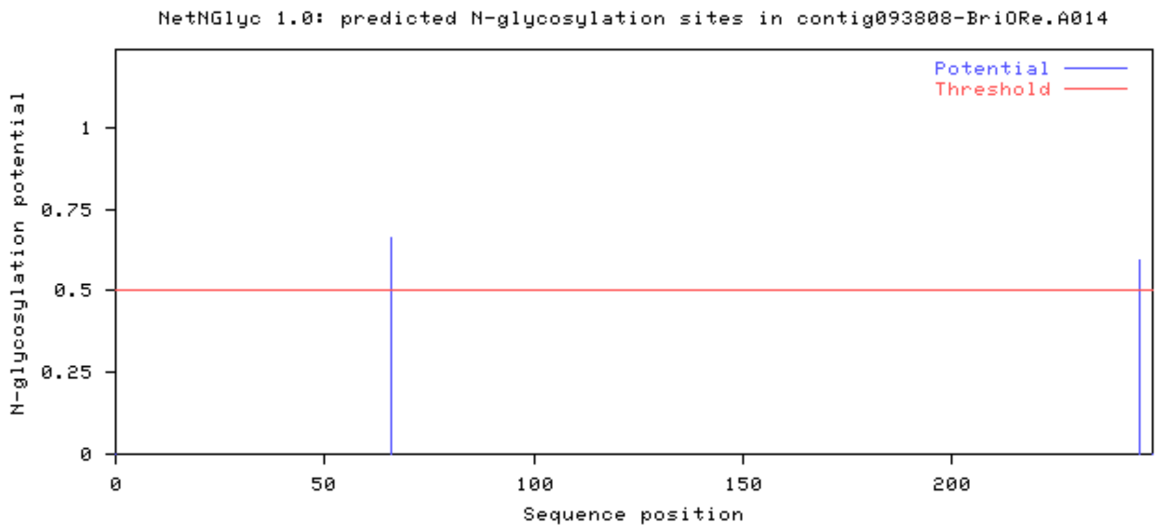

Graphics in PostScript

Output for 'contig093812-BriOR.A010'

```
##########################################################################################

    Warning: This sequence may not contain a signal peptide!!

    Proteins without signal peptides are unlikely to be exposed to
    the N-glycosylation machinery and thus may not be glycosylated
    (in vivo) even though they contain potential motifs.

    SignalP-NN euk predictions are as follows:

  # name                 Cmax  pos ?  Ymax  pos ?  Smax  pos ?  Smean ?  D     ?

    SignalP output is explained at http://www.cbs.dtu.dk/services/SignalP/output.html

##########################################################################################

Name:  contig093812-BriOR.A010  Length:  320
MDVELNVTLVTFGGFAELHKYRYLYFVVISTLYILILCFNSTIVYLIWTHKNLHEPMYIFIAALLINCVLYSMIIYPKLL      80
SDVLSEKQTISYPLCLFQGFLYYTSAGSEFLLLAAMAYDRYVSICKPLQYPVIMNRITIYVCLILAWLIPAFETSVLGVL     160
YSNVKLCSFTLTGIFCNNSLYKLQCVPSVAISIYGMVMLINIALLPMLFILFTYIRIIRISYHCCREVRKKAVKTCLPHL     240
LVLINFSCFIFFDVIIVRLDSDLSKTLRLTLTFQSILFHPLLNPIIYGLKMNAIFKHIKILLSGLITLVLMPYYQMYGIX     320
.....N.................................N........................................      80
................................................................................     160
................................................................................     240
....N...........................................................................     320

(Threshold=0.5)
----------------------------------------------------------------------
SeqName      Position  Potential   Jury    N-Glyc
                                 agreement result
----------------------------------------------------------------------
contig093812-BriOR.A010    6 NVTL   0.8264     (9/9)    +++
contig093812-BriOR.A010   40 NSTI   0.7034     (9/9)    ++
contig093812-BriOR.A010  177 NNSL   0.4230     (7/9)    -
contig093812-BriOR.A010  245 NFSC   0.5691     (6/9)    +
----------------------------------------------------------------------
```

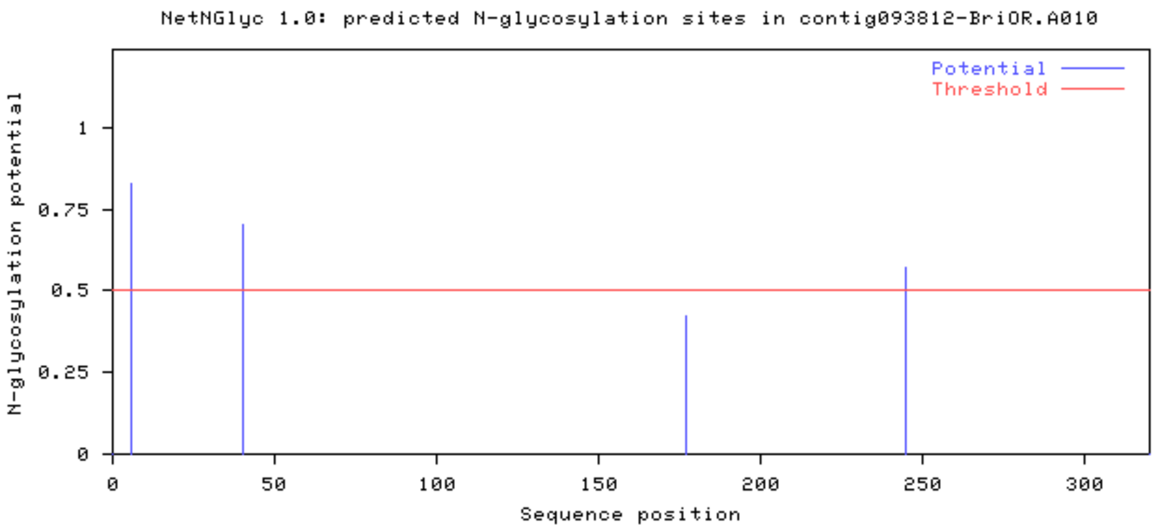

Graphics in PostScript

Output for 'contig093816-BriOR.A011'

```
##########################################################################################

    Warning: This sequence may not contain a signal peptide!!

    Proteins without signal peptides are unlikely to be exposed to
    the N-glycosylation machinery and thus may not be glycosylated
    (in vivo) even though they contain potential motifs.

    SignalP-NN euk predictions are as follows:

  # name                Cmax  pos ?  Ymax  pos ?  Smax  pos ?  Smean ?  D     ?

    SignalP output is explained at http://www.cbs.dtu.dk/services/SignalP/output.html

##########################################################################################

Name:  contig093816-BriOR.A011  Length:  307
MDVELNVTWLTLGGFAELYKYRYLYFVVIFTLYILILCCNSTIVFLIWTHKNLHEPMYIFIAALLINSVLYSMIIYPKLL      80
SDVLFEKQMISYPLCLFQGLSYCTSVGSEFLLLAAMAYDRYVSICKPLQYPVIMNKITIYVCLILAWLIPAFESLMLGVL     160
YSNVKLCSFTLTGIFCNNSVHKLQCVPSVAISIYGVVMLINIALLPLLFILFSYIKILKISYQRCREVRKNAVKTCLPHL     240
LVLIHFSCFISFDIIIVRLETDLSKTLRLILTFQLILFHPLLNPIIYGLKMNEISKHLKILLCLVKX
.....N.................................N........................................      80
................................................................................     160
................................................................................     240
...................................................................                  320

(Threshold=0.5)
----------------------------------------------------------------------
SeqName      Position  Potential   Jury    N-Glyc
                                 agreement result
----------------------------------------------------------------------
contig093816-BriOR.A011    6 NVTW   0.7497     (9/9)   ++
contig093816-BriOR.A011   40 NSTI   0.7237     (9/9)   ++
contig093816-BriOR.A011  177 NNSV   0.4207     (7/9)   -
----------------------------------------------------------------------
```

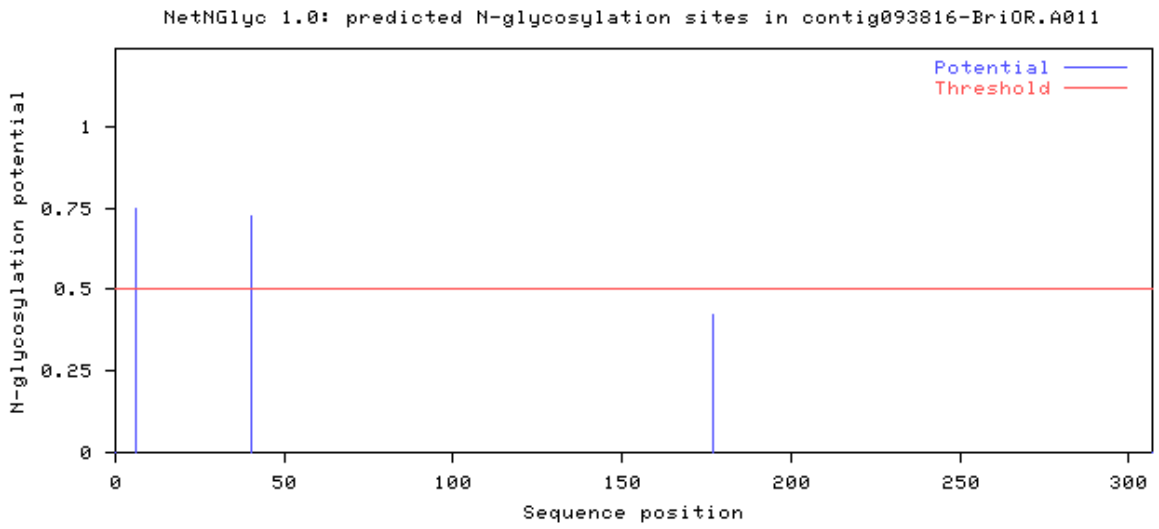

Graphics in PostScript

Output for 'contig093818-BriORp.A025'

```
##########################################################################

    Warning: This sequence may not contain a signal peptide!!

    Proteins without signal peptides are unlikely to be exposed to
    the N-glycosylation machinery and thus may not be glycosylated
    (in vivo) even though they contain potential motifs.

    SignalP-NN euk predictions are as follows:

  # name                 Cmax  pos ?  Ymax  pos ?  Smax  pos ?  Smean ?  D     ?

    SignalP output is explained at http://www.cbs.dtu.dk/services/SignalP/output.html

##########################################################################

Name:  contig093818-BriORp.A025         Length:  298
MNLTYITIGGHVEVEKYRYLYFVIMFMVYVLIICSNSTIVWLIVVQKSLHEPMYIFIAALLVNSVVLSTVIYPKLLIDFL      80
SEKQIILQACLFQVFLFYALSCSEFLLLSAMAYDRYVSICKPLQYPTIMRRTRVNIFLLLCWFLPAIQVAVPIAGNANTP     160
LCNFTLKGIFCNNSVNHLYCVNSRELSIYGMVVLFNVALSPMFFILFTYIKIIIVAYQSCGNVRKKAAQTCLPHVLVLIN     240
YSCLLTYDMVIVRLESEFPKTARFIMTLQFVTYNPLCNPIIYGLKMKEISKHLKILFS
.N.................................N............................................      80
................................................................................     160
..N............................................................................N     240
..........................................................                           320

(Threshold=0.5)
----------------------------------------------------------------------
SeqName      Position  Potential   Jury    N-Glyc
                                agreement result
----------------------------------------------------------------------
contig093818-BriORp.A025     2 NLTY   0.7947     (9/9)   +++
contig093818-BriORp.A025    36 NSTI   0.6928     (9/9)   ++
contig093818-BriORp.A025   163 NFTL   0.6498     (9/9)   ++
contig093818-BriORp.A025   172 NNSV   0.4718     (5/9)   -
contig093818-BriORp.A025   240 NYSC   0.5038     (5/9)   +
----------------------------------------------------------------------
```

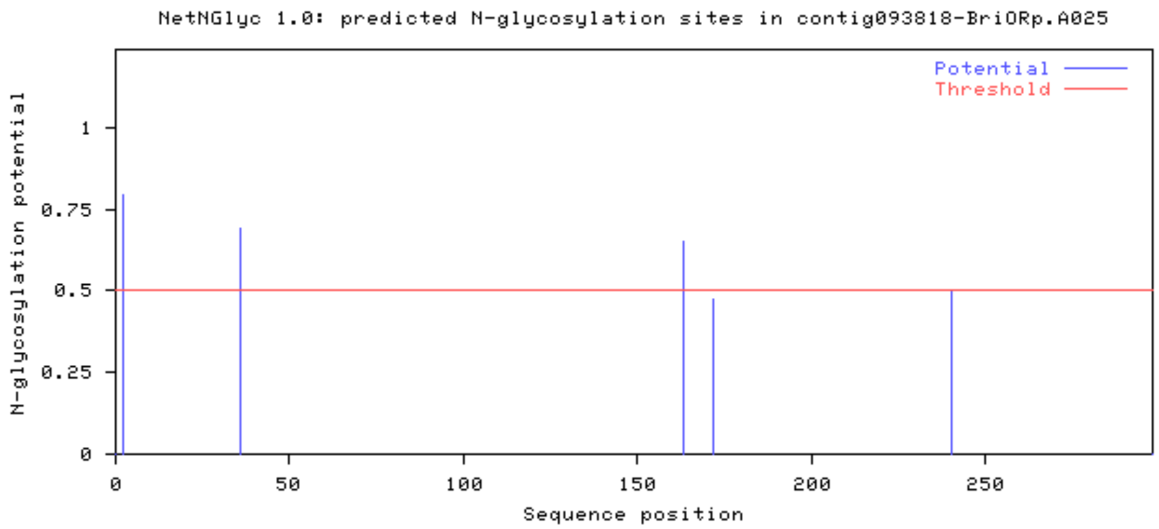

Graphics in PostScript

Output for 'contig093823-BriORe.A015'

```
##########################################################################################

    Warning: This sequence may not contain a signal peptide!!

    Proteins without signal peptides are unlikely to be exposed to
    the N-glycosylation machinery and thus may not be glycosylated
    (in vivo) even though they contain potential motifs.

    SignalP-NN euk predictions are as follows:

  # name                  Cmax  pos ?  Ymax  pos ?  Smax  pos ?  Smean ?  D     ?

    SignalP output is explained at http://www.cbs.dtu.dk/services/SignalP/output.html

##########################################################################################

Name:  contig093823-BriORe.A015        Length:  211
FLLLAAMAYDRYVSICKPLQYPVIMNRITIYVCLILAWLIPAFETSVLGVLYSYVKICSFTLTGIFCNTSVYKLQCVPSV      80
AISIYSMVMLINIALLPLLFILFTYIRILRISYHCSREVRKKAVKTCLPHLLVLINFSCFIFFDIIIVRLDSDLSKTLRL     160
TLTFQSILFHPLLNPIIYGLKMNEIFKHIKILLSSLITLVLLPYYQMYGIX
...................................................................N............      80
.......................................................N........................     160
...................................................                                  240

(Threshold=0.5)
----------------------------------------------------------------------
SeqName      Position  Potential   Jury    N-Glyc
                                 agreement result
----------------------------------------------------------------------
contig093823-BriORe.A015    68 NTSV    0.5371     (7/9)   +
contig093823-BriORe.A015   136 NFSC    0.5674     (5/9)   +
----------------------------------------------------------------------
```

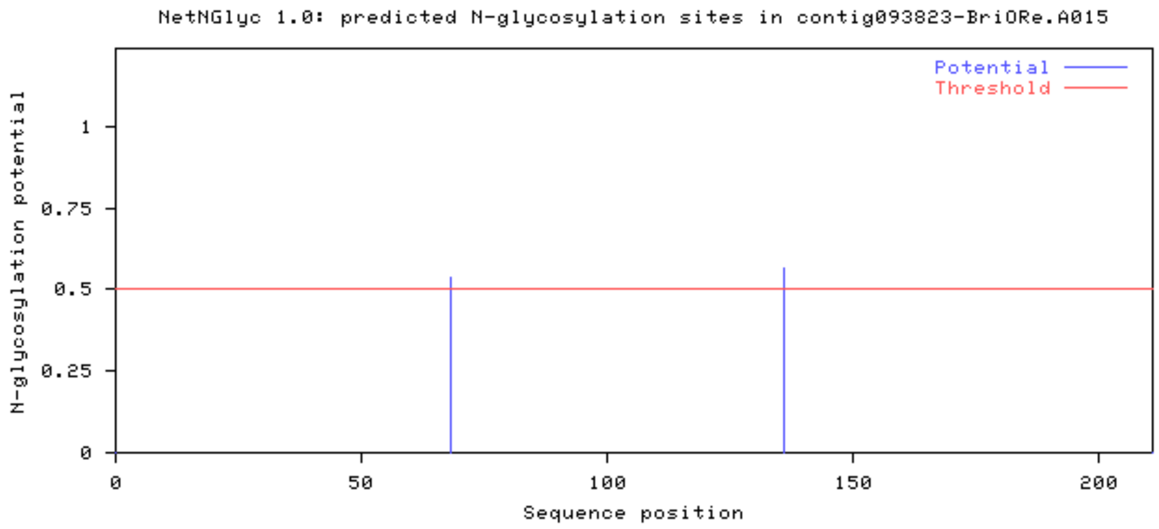

Graphics in PostScript

Output for 'contig093825-BriOR.H053'

```
##########################################################################################

    Warning: This sequence may not contain a signal peptide!!

    Proteins without signal peptides are unlikely to be exposed to
    the N-glycosylation machinery and thus may not be glycosylated
    (in vivo) even though they contain potential motifs.

    SignalP-NN euk predictions are as follows:

  # name                Cmax  pos ?  Ymax  pos ?  Smax  pos ?  Smean ?  D     ?

    SignalP output is explained at http://www.cbs.dtu.dk/services/SignalP/output.html

##########################################################################################

Name:  contig093825-BriOR.H053  Length:  310
MDNVSTVRIFNLLAFNETANYRAALFSATLVCYFAIVFLNVTVIMIIVLDESLHEPMYILVCVCCINGLYGSTGFYPKFL      80
IDLLSSSQVISYSECLCQAFVMYSFVCSDTSILAVMAYDRYLAICQPLEYHSVMTKKKLSKLVCFSWLTPFCIFSINIML     160
TDRLIFCGTDIQRLFCVNWLIVKVACPGMDTLVNSAFAYTTLSIYIFHWIFIVWTYIYLVKSCVQSKKDKAKFMQTCVPH     240
LISLVTFFVIVISDLMHMRFASNDVPQSFQNFVAIAVLFIPPVMNPLLYGFKLSKIRNRILVTLHNKRCX
..N............N.......................N........................................      80
................................................................................     160
................................................................................     240
......................................................................               320

(Threshold=0.5)
----------------------------------------------------------------------
SeqName      Position  Potential   Jury    N-Glyc
                                agreement  result
----------------------------------------------------------------------
contig093825-BriOR.H053    3 NVST   0.7628     (9/9)    +++
contig093825-BriOR.H053   16 NETA   0.7331     (9/9)    ++
contig093825-BriOR.H053   40 NVTV   0.7888     (9/9)    +++
----------------------------------------------------------------------
```

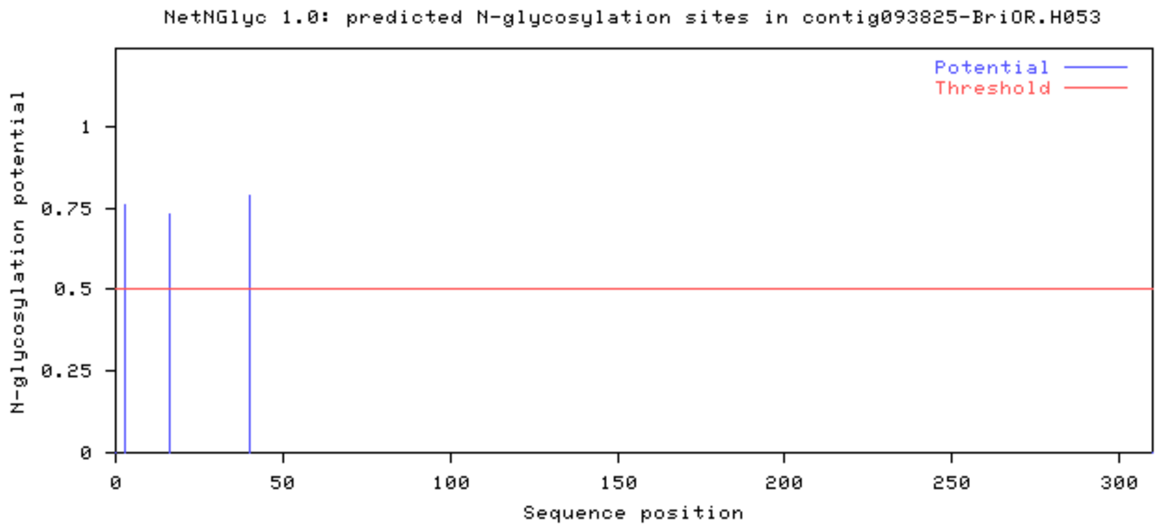

Graphics in PostScript

Output for 'contig094277-BriORe.R119'

```
########################################################################################

    Warning: This sequence may not contain a signal peptide!!

    Proteins without signal peptides are unlikely to be exposed to
    the N-glycosylation machinery and thus may not be glycosylated
    (in vivo) even though they contain potential motifs.

    SignalP-NN euk predictions are as follows:

  # name                    Cmax   pos ?  Ymax   pos ?  Smax   pos ?  Smean ?  D     ?

    SignalP output is explained at http://www.cbs.dtu.dk/services/SignalP/output.html

########################################################################################

Name:  contig094277-BriORe.R119         Length:  280
SFIFLFLNGTMLFTLRSKPVFRDTPRYILLYNLLFADTVQLAQSQVLFLLSIFRVKLPYPVCVCLSLLANLTTGISPLTL      80
SVMPLERYVAVCFPLRYPTIITIRNTGAAIIVIWIISSLNNLTRLIFFFPFEVLKNLQMKDSCSKIALLLGRRSDQYDTA     160
FTCLVFVSAGVAVIFSYIGVILAARLASANKALARRARNTLLLNMMQLCLSLSSTIYNPLLIALSRTVTMTIFLWVQNVF     240
YLCFLILPRCLSSLVYGLRDQTIRPVLMYHLCCHQKRSQX
.......N.............................................................N..........      80
........................................N.......................................     160
................................................................................     240
........................................                                             320

(Threshold=0.5)
----------------------------------------------------------------------
SeqName      Position  Potential   Jury    N-Glyc
                                 agreement result
----------------------------------------------------------------------
contig094277-BriORe.R119    8 NGTM   0.7626     (9/9)   +++
contig094277-BriORe.R119   70 NLTT   0.6032     (8/9)   +
contig094277-BriORe.R119  121 NLTR   0.5782     (7/9)   +
----------------------------------------------------------------------
```

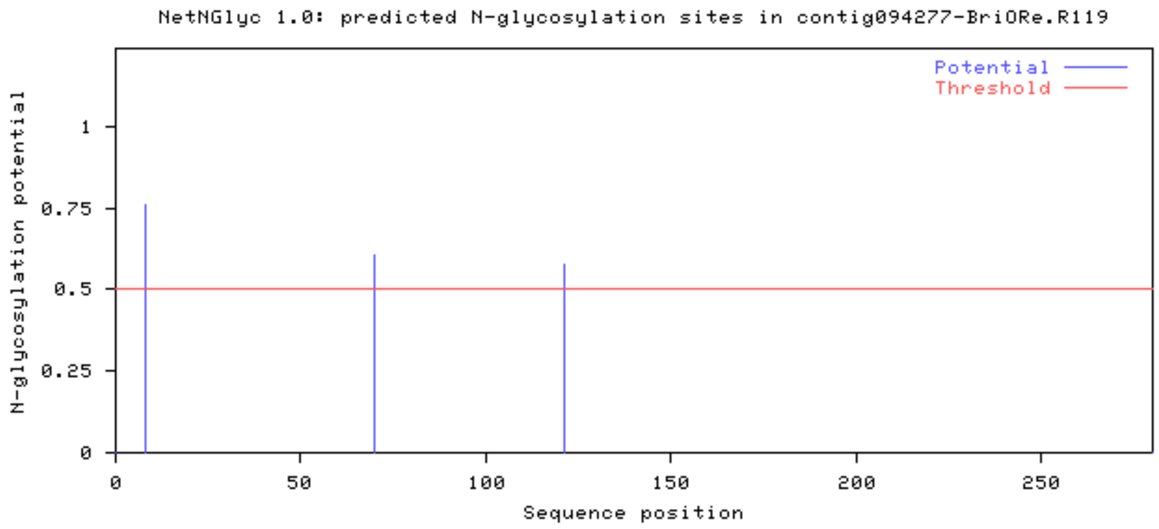

[Graphics in PostScript]

Output for 'contig094278-BriORe.R120'

```
##########################################################################################

    Warning: This sequence may not contain a signal peptide!!

    Proteins without signal peptides are unlikely to be exposed to
    the N-glycosylation machinery and thus may not be glycosylated
    (in vivo) even though they contain potential motifs.

    SignalP-NN euk predictions are as follows:

  # name                    Cmax   pos ?  Ymax   pos ?  Smax   pos ?  Smean ?  D     ?

    SignalP output is explained at http://www.cbs.dtu.dk/services/SignalP/output.html

##########################################################################################

Name:  contig094278-BriORe.R120         Length:  99
ASTDKALAFKARNTLLLNLMQLFLSLSSTIYNPLLVPLLMIVTRIVLVRIQNAFYLFFIILPRCLTSLIYGLRDQTIRPV      80
LIYHLCCRLNCPVVEDKGX
................................................................................      80
...................                                                                  160

(Threshold=0.5)

No sites predicted in this sequence.
```

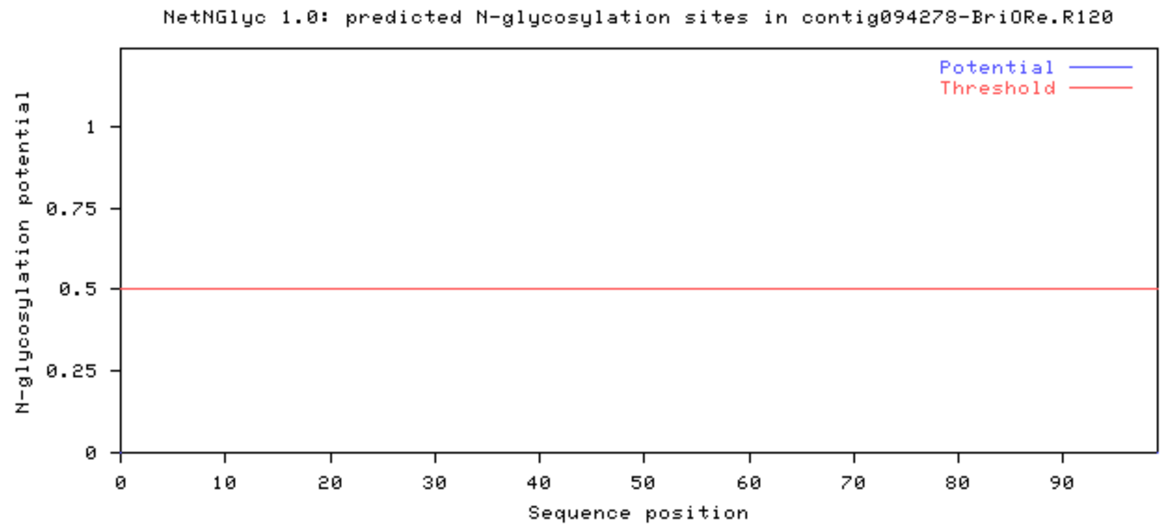

Graphics in PostScript

Output for 'contig094282-BriOR.R118'

```
##########################################################################

   Warning: This sequence may not contain a signal peptide!!

   Proteins without signal peptides are unlikely to be exposed to
   the N-glycosylation machinery and thus may not be glycosylated
   (in vivo) even though they contain potential motifs.

   SignalP-NN euk predictions are as follows:

 # name                Cmax  pos ?  Ymax  pos ?  Smax  pos ?  Smean ?  D     ?

   SignalP output is explained at http://www.cbs.dtu.dk/services/SignalP/output.html

##########################################################################

Name:  contig094282-BriOR.R118  Length:  322
MSYISPSKTNFTVGLQYRGILEVLLFSAPITASCCVLLFINGTMLHILRSKAVFCETACYVLLYNLLFSDTIQMVLSQLL      80
YLLSAFRIRLTYPVCGFLIMLANLTTGISPLTLVVMSLERYVAVCYPLRHASIITIGNTALAIIIVWAVSSLNVFIRLIL     160
LLNFQFEDLESLQIKDFCSNAALLLGPMSDHYDKAYTGVIFVFAGVAVTCSYIGVMIAARSASTDKASARKVRSTLLLHL     240
IQLGLSLSSTIHDPLVTEISKVLDRVTILRIRSILYVSMMLLPRCLSPLIYGIRDQMIRGILMSHLCCRVKLPNSIKLLK     320
HX
.........N..............................N.......................................      80
......................N.........................................................     160
................................................................................     240
................................................................................     320
..                                                                                   400

(Threshold=0.5)
----------------------------------------------------------------------
SeqName      Position  Potential   Jury    N-Glyc
                                agreement  result
----------------------------------------------------------------------
contig094282-BriOR.R118   10 NFTV   0.6413     (8/9)   +
contig094282-BriOR.R118   41 NGTM   0.6972     (9/9)   ++
contig094282-BriOR.R118  103 NLTT   0.5726     (6/9)   +
----------------------------------------------------------------------
```

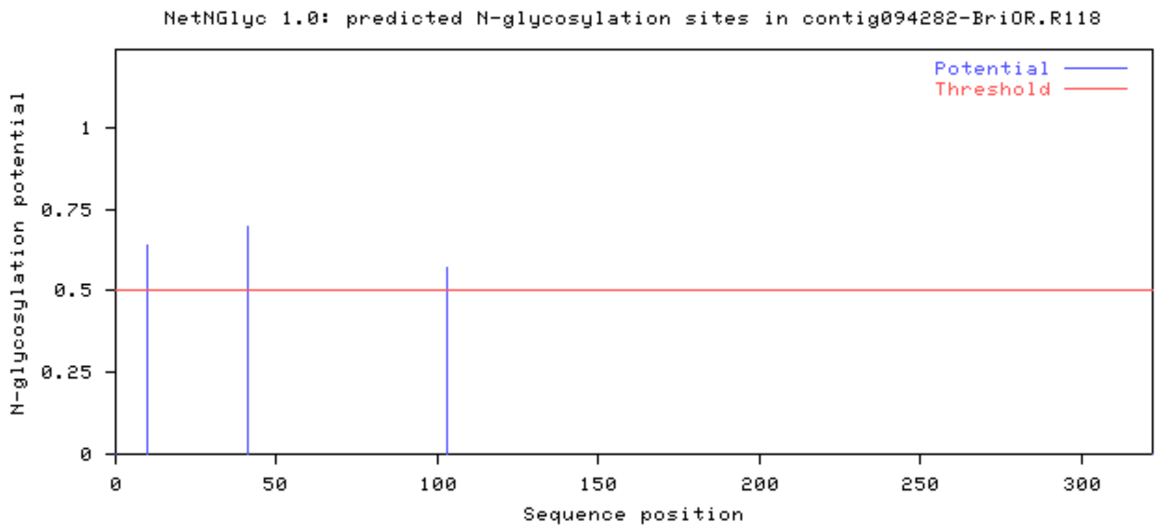

Graphics in PostScript

Output for 'contig096532-BriORe.M083'

```
##########################################################################################

    Warning: This sequence may not contain a signal peptide!!

    Proteins without signal peptides are unlikely to be exposed to
    the N-glycosylation machinery and thus may not be glycosylated
    (in vivo) even though they contain potential motifs.

    SignalP-NN euk predictions are as follows:

  # name                 Cmax  pos ?  Ymax  pos ?  Smax  pos ?  Smean ?  D     ?

    SignalP output is explained at http://www.cbs.dtu.dk/services/SignalP/output.html

##########################################################################################

Name:  contig096532-BriORe.M083         Length:  42
VLSLSLASCIPPCINPIVYSLKTKDIKTRALKLFRKTKVNAX
..........................................                                            80

(Threshold=0.5)

No sites predicted in this sequence.
```

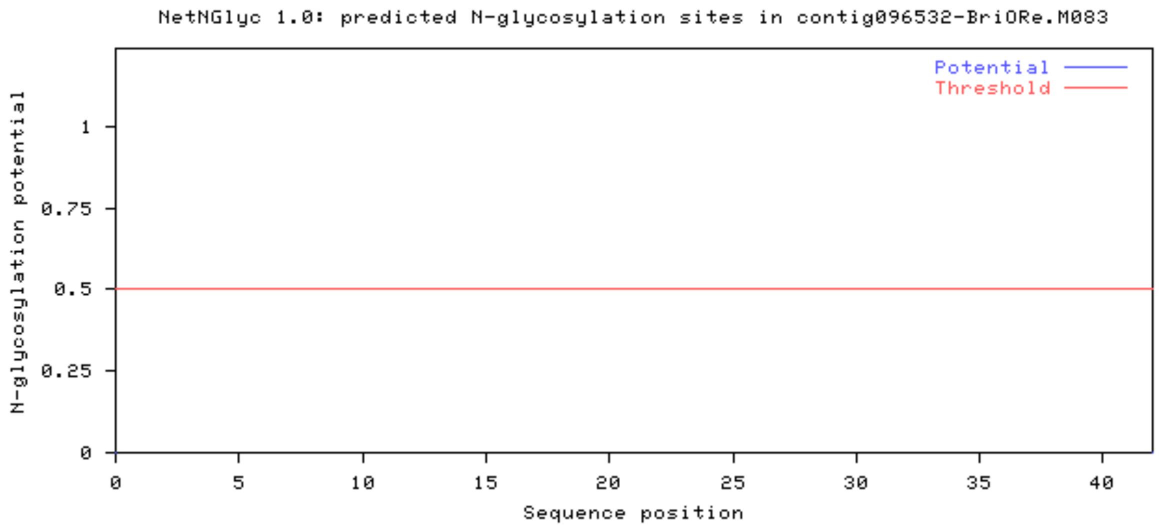

[Graphics in PostScript]

Output for 'contig096535-BriOR.N084'

```
##########################################################################

   Warning: This sequence may not contain a signal peptide!!

   Proteins without signal peptides are unlikely to be exposed to
   the N-glycosylation machinery and thus may not be glycosylated
   (in vivo) even though they contain potential motifs.

   SignalP-NN euk predictions are as follows:

 # name                Cmax  pos ?  Ymax  pos ?  Smax  pos ?  Smean ?  D     ?

   SignalP output is explained at http://www.cbs.dtu.dk/services/SignalP/output.html

##########################################################################

Name:  contig096535-BriOR.N084  Length:  337
MGTEDKATRMFNNTFVRPEKFYLSGFSNIPHIRYFYTFLCLVYIMTVLGNCFLLSLISLVKTLHTPKYMIVFNMALTDLC      80
GSTALIPKLLDTFLFDRRYILYDACLSYMFFVMFFASVQSWTLVTMAYDRLIAICFPLRYHSIVTETSVAAILLFVWIFL     160
VSVIATMVGLVNRVSFCRSLVVNSFFCDHGPVYRLACNDTSLNYNMASALITILIIIPLIFIIATYVCIFIALSRTTSRK     240
ERIRALKTCTSHLILVVMFFLPIGITNIATRASYIHPNARMINSTLTHTIPALLDPIIYALKKEEVMNAVKKLCKRTYLN     320
CMKAKTRPCNHCCIKSX
...........N....................................................................      80
................................................................................     160
.....................................N..........................................     240
..........................................N.....................................     320
.................                                                                    400

(Threshold=0.5)
----------------------------------------------------------------------
SeqName      Position  Potential   Jury    N-Glyc
                                 agreement result
----------------------------------------------------------------------
contig096535-BriOR.N084   12 NNTF   0.6071     (8/9)   +
contig096535-BriOR.N084  198 NDTS   0.6408     (7/9)   +
contig096535-BriOR.N084  283 NSTL   0.5836     (7/9)   +
----------------------------------------------------------------------
```

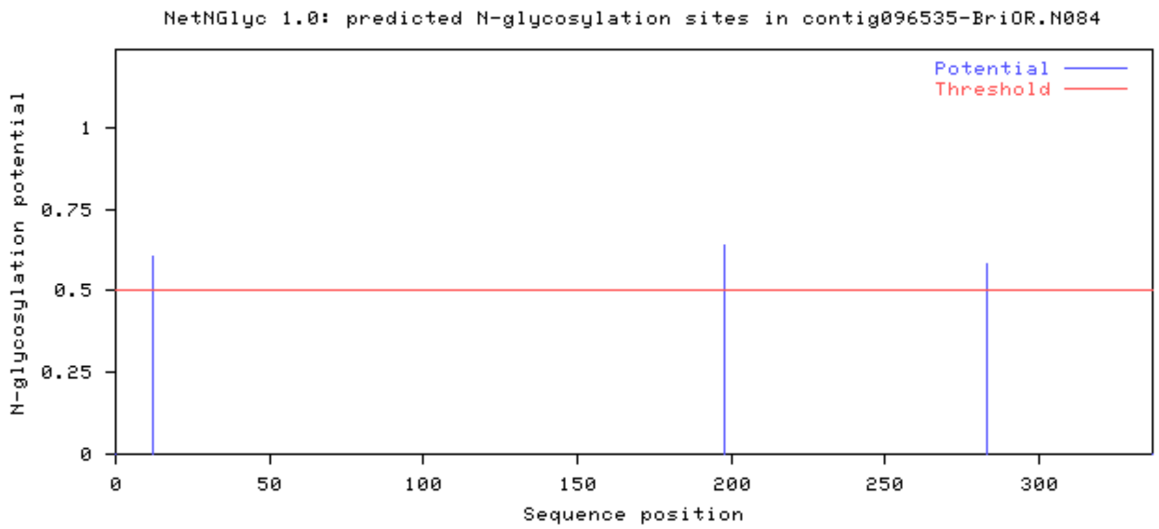

Graphics in PostScript

Output for 'contig096536-BriOR.N085'

```
##########################################################################################

    Warning: This sequence may not contain a signal peptide!!

    Proteins without signal peptides are unlikely to be exposed to
    the N-glycosylation machinery and thus may not be glycosylated
    (in vivo) even though they contain potential motifs.

    SignalP-NN euk predictions are as follows:

  # name                    Cmax  pos ?  Ymax  pos ?  Smax  pos ?  Smean ?  D     ?

    SignalP output is explained at http://www.cbs.dtu.dk/services/SignalP/output.html

##########################################################################################

Name:  contig096536-BriOR.N085  Length:  337
MGAEAKTTTIFNTTFVRPAKFYLSGFSNIPHIRYFYAFLCFVYIMTVLGNGFLLSLISLVKTLHTPKYMIVFNMALTDLC      80
GSTALIPKLLDTFLFDRRYILYDACLSYMFFVIFFASVQSWTLVTMAYDRLIAICFPLRYHSIVTEQSITAILLFVWIFL     160
VSVIATMVGLVNRLSFCRSLVVNSFFCDHGPVYRLACNDTSLNYNMASALVTILILIPLIFIIATYVCIFIALSRTTSRK     240
ERIRALKTCTSHLILVVMFFLPIGITNIAAMASYIDPNARMINSVLTHTIPALLDPIVYALKTEEVMNAVKKLCKRTYLN     320
CMKAKTRPCNHCCIKSX
...........N....................................................................      80
................................................................................     160
.....................................N..........................................     240
................................................................................     320
.................                                                                    400

(Threshold=0.5)
----------------------------------------------------------------------
SeqName      Position  Potential   Jury    N-Glyc
                                 agreement result
----------------------------------------------------------------------
contig096536-BriOR.N085   12 NTTF    0.6069     (6/9)    +
contig096536-BriOR.N085  198 NDTS    0.6408     (7/9)    +
----------------------------------------------------------------------
```

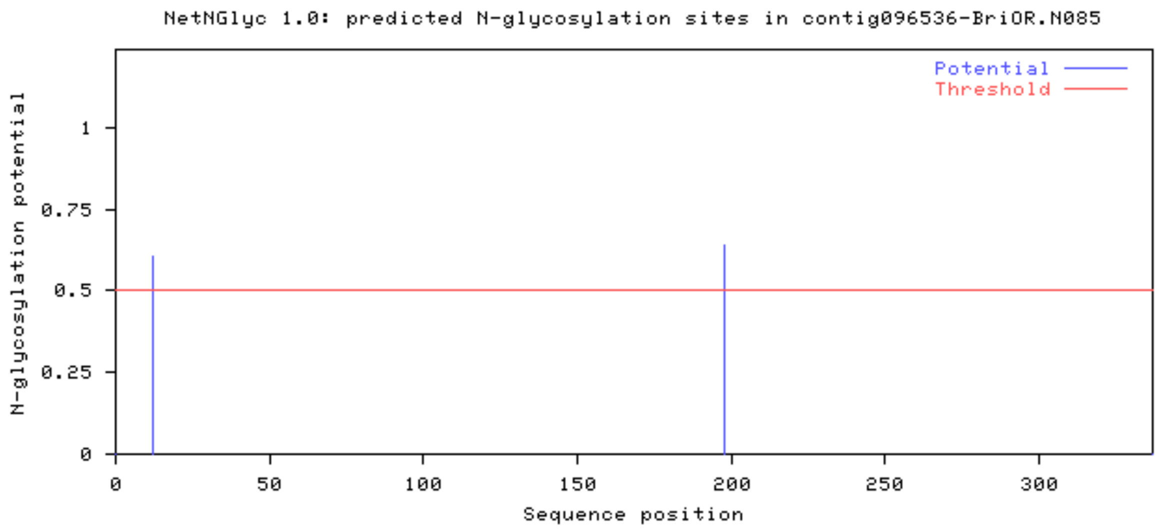

Graphics in PostScript

Output for 'contig096536-BriOR.N086'

```
##########################################################################################

    Warning: This sequence may not contain a signal peptide!!

    Proteins without signal peptides are unlikely to be exposed to
    the N-glycosylation machinery and thus may not be glycosylated
    (in vivo) even though they contain potential motifs.

    SignalP-NN euk predictions are as follows:

  # name                 Cmax  pos ?  Ymax  pos ?  Smax  pos ?  Smean ?  D     ?

    SignalP output is explained at http://www.cbs.dtu.dk/services/SignalP/output.html

##########################################################################################

Name:  contig096536-BriOR.N086  Length:  327
MDSEEKATTKSNSTFVRPEKFYLSGFSNIPHVKYFYVFLCFVYIMTVLGNGFLLSVIWLVKTLHTPKYMIVFNMALADLC      80
GSTALIPKLLDTFLFDRRYIVYEACLSYMFFVFFFGGVQSWTLVTMAYDRLIAICFPLRYHSIVTKTSITSMLLFIWLVT     160
LSLTTLVVGLINRLSFCDSVVVKSFFCDHGPIYRLACNDPSLNIIMANVVVSIGVFIPLIFIACTYVCISIALSKIASGE     240
ERLKALKTCTSHLILVAILFLPFVVTNIAVWTSYIHPNARIINSTLTHTIPALINPIVYALKTEEVMNAVKKLWKINNIS     320
SPVTKWX
...........N....................................................................      80
................................................................................     160
................................................................................     240
..........................................N...................................N..     320
.......                                                                              400

(Threshold=0.5)
----------------------------------------------------------------------
SeqName      Position  Potential   Jury    N-Glyc
                                 agreement result
----------------------------------------------------------------------
contig096536-BriOR.N086   12 NSTF   0.6274     (9/9)   ++
contig096536-BriOR.N086  283 NSTL   0.5847     (8/9)   +
contig096536-BriOR.N086  318 NISS   0.6398     (7/9)   +
----------------------------------------------------------------------
```

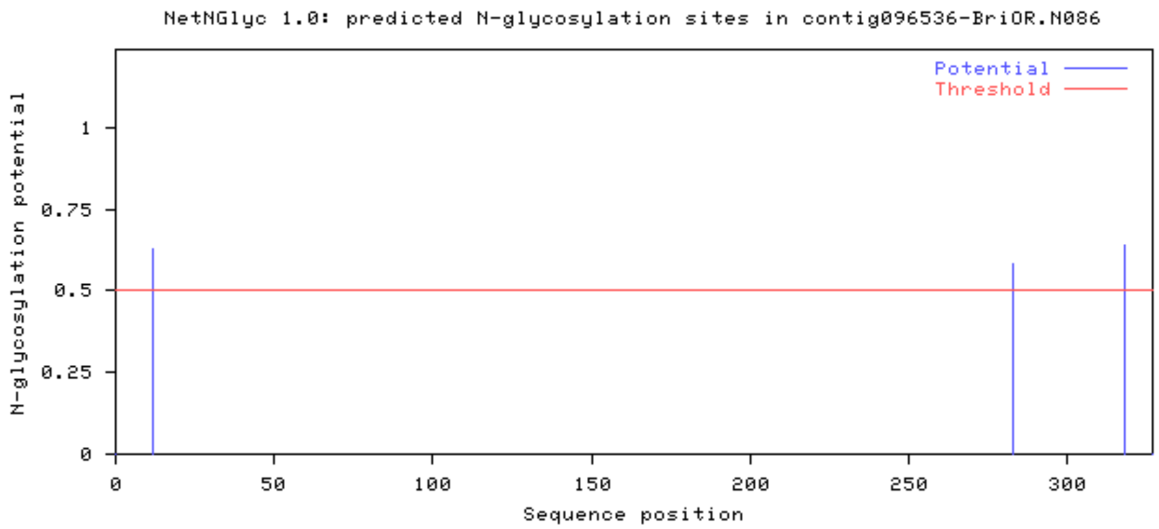

Graphics in PostScript

Output for 'contig096539-BriOR.N087'

```
##########################################################################################

    Warning: This sequence may not contain a signal peptide!!

    Proteins without signal peptides are unlikely to be exposed to
    the N-glycosylation machinery and thus may not be glycosylated
    (in vivo) even though they contain potential motifs.

    SignalP-NN euk predictions are as follows:

  # name                 Cmax  pos ?  Ymax  pos ?  Smax  pos ?  Smean ?  D     ?

    SignalP output is explained at http://www.cbs.dtu.dk/services/SignalP/output.html

##########################################################################################

Name:  contig096539-BriOR.N087  Length:  322
MELFNSALGKNITFVHPKFFIIGGLTGIPNITFYYVFLFFVYIVSVVGNTVVMAVIYLDHNLRTPKYIAVFNLAFVDLFG      80
NTALVPKVLDIFLFGHYYIPYNDCLTFLFFCYTCLSLQSFNLVALSYDRMVAIIFPLHYQVKVTHRFMFSFIASLWVFTI     160
IAVLISVGLLTRLSFCKSVVINSYFCDHGQIYRLACNDHFPSYVIACLYPVIIFWLPLAFILLSYLYIGYTLVKVATLQE     240
GLKAFKTCIGHLSLVAIYFIPLLTTFTLMEKIQPNARIINLSLTSVFPPMLNPIIYVLQTQEIKESLKRLLKRRGKSKIT     320
IX
..........N..................N..................................................      80
................................................................................     160
................................................................................     240
.......................................N........................................     320
..                                                                                   400

(Threshold=0.5)
----------------------------------------------------------------------
SeqName      Position  Potential   Jury    N-Glyc
                                 agreement result
----------------------------------------------------------------------
contig096539-BriOR.N087   11 NITF   0.6004     (8/9)   +
contig096539-BriOR.N087   30 NITF   0.7721     (9/9)   +++
contig096539-BriOR.N087  280 NLSL   0.6020     (8/9)   +
----------------------------------------------------------------------
```

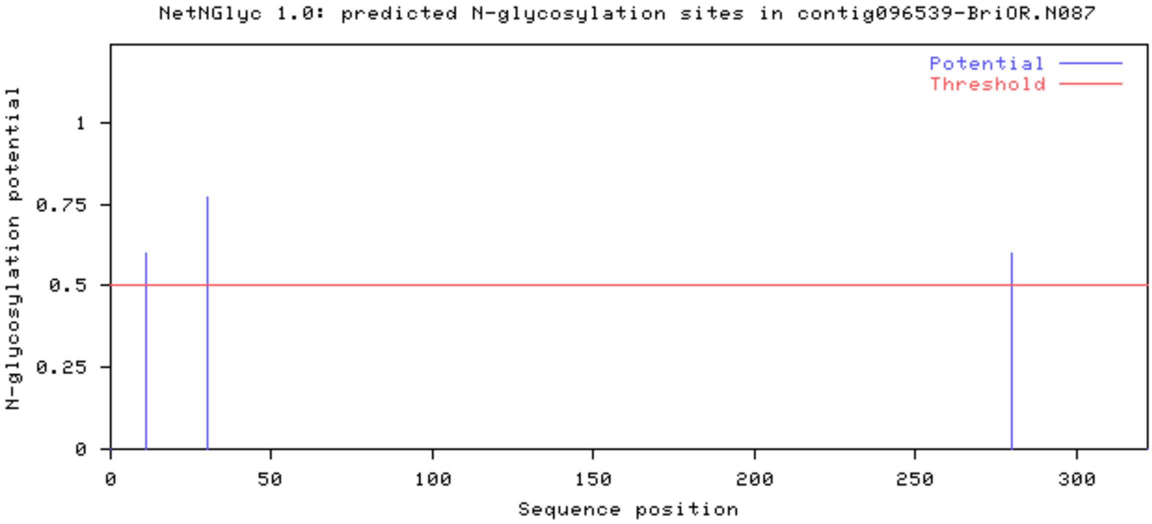

Graphics in PostScript

Output for 'contig096539-BriOR.N088'

```
##########################################################################################

    Warning: This sequence may not contain a signal peptide!!

    Proteins without signal peptides are unlikely to be exposed to
    the N-glycosylation machinery and thus may not be glycosylated
    (in vivo) even though they contain potential motifs.

    SignalP-NN euk predictions are as follows:

  # name                  Cmax  pos ?  Ymax  pos ?  Smax  pos ?  Smean ?  D     ?

    SignalP output is explained at http://www.cbs.dtu.dk/services/SignalP/output.html

##########################################################################################

Name:  contig096539-BriOR.N088  Length:  323
MDFLNSAAEKNTTFVQPANFIISGFVGIPNIRYYFVFLCFIYIFSVVGNTAVMLIIIFDHTLRSPKYIAVFNLAFTDLLS      80
NSALVPKVLEISLFNHHYISYNNCLTFMFFCFTLISMQAFNLVVLSFDRVMAIMYPLHYQMRVSHKIILSLIAFFWLLAM     160
ALTGTAVGLLTRLYFCESVVINSYYCDHGPIYRLGCNDVTPNKTISAWSRAFVLWLPLIFILGSYCCIGYSLSRISTCKE     240
RVKALKTCTGHLSLVAIYFIPILVVYSFGSTMHPNARIVNLSLASVTPPMLNPIIYFLQTAEIKKSLKRLLKAKIQISHR     320
VLX
................................................................................      80
................................................................................     160
.........................................N......................................     240
.......................................N........................................     320
...                                                                                  400

(Threshold=0.5)
----------------------------------------------------------------------
SeqName      Position  Potential   Jury    N-Glyc
                                 agreement result
----------------------------------------------------------------------
contig096539-BriOR.N088   11 NTTF   0.4654     (5/9)   -
contig096539-BriOR.N088  202 NKTI   0.6138     (8/9)   +
contig096539-BriOR.N088  280 NLSL   0.5873     (8/9)   +
----------------------------------------------------------------------
```

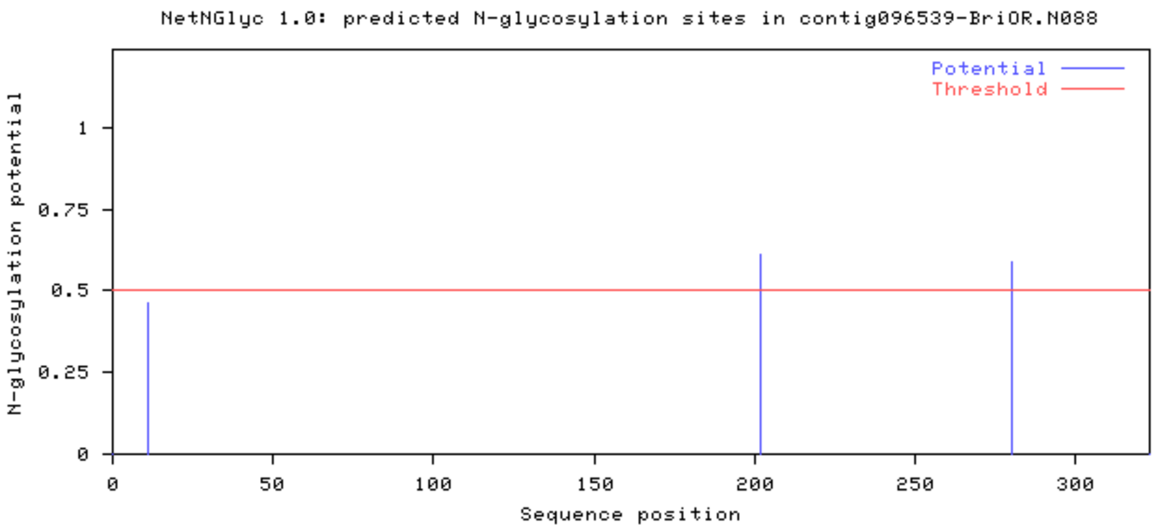

Graphics in PostScript

Output for 'contig096540-BriORe.N093'

```
##########################################################################################

    Warning: This sequence may not contain a signal peptide!!

    Proteins without signal peptides are unlikely to be exposed to
    the N-glycosylation machinery and thus may not be glycosylated
    (in vivo) even though they contain potential motifs.

    SignalP-NN euk predictions are as follows:

  # name                    Cmax  pos ?  Ymax  pos ?  Smax  pos ?  Smean ?  D     ?

    SignalP output is explained at http://www.cbs.dtu.dk/services/SignalP/output.html

##########################################################################################

Name:  contig096540-BriORe.N093         Length:  116
ILWLPLTIVLLSYLSIGYALAKVATVRERMKGFKTCTAHLSLVAIYFLPILITFILHANIEPNARIINLSLTSVFPPMLN      80
PIIYVLQTQEIKESLKKLKGRITTHYKNRKVKFKKX
...................................................................N............      80
....................................                                                 160

(Threshold=0.5)
----------------------------------------------------------------------
SeqName      Position  Potential   Jury    N-Glyc
                                 agreement result
----------------------------------------------------------------------
contig096540-BriORe.N093   68 NLSL   0.6564     (9/9)   ++
----------------------------------------------------------------------
```

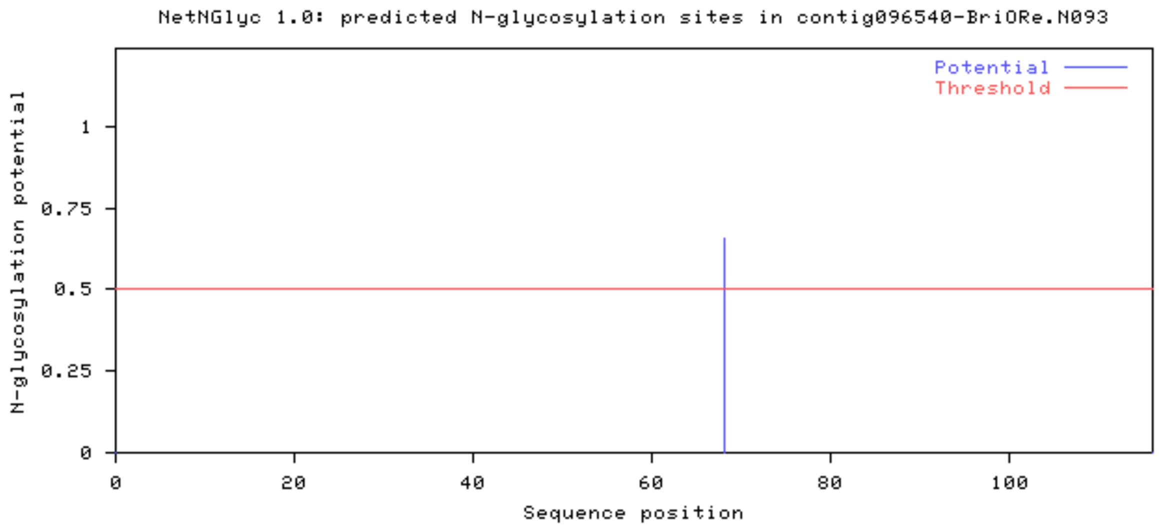

[Graphics in PostScript]

Output for 'contig104344-BriOR.G048'

```
##########################################################################

    Warning: This sequence may not contain a signal peptide!!

    Proteins without signal peptides are unlikely to be exposed to
    the N-glycosylation machinery and thus may not be glycosylated
    (in vivo) even though they contain potential motifs.

    SignalP-NN euk predictions are as follows:

  # name                 Cmax  pos ?  Ymax  pos ?  Smax  pos ?  Smean ?  D     ?

    SignalP output is explained at http://www.cbs.dtu.dk/services/SignalP/output.html

##########################################################################

Name:  contig104344-BriOR.G048  Length:  312
MENNFEIVFVLQGLNDSLTNRQIYFAFALMSYLFTVSVNLTLIITISLDKTLHEPIYIFLCSLCFNEICGASSFYPKLLH      80
DLLTNSYVITYAACLGQMFVTYSYIFSEFTSLTVMAYDRYIAICKPLQYRMLMTAQKVTQLMMLTWCFSVFETAVGAVLT     160
ARLPLCGRHIPKIFCTNWEVVKLSCSDSTLNNIYAFMLIFSHLSQTALIMVSYVHLIRAAVRSQADRRKFMQTCLPHLST     240
LLVFSTSLMFDTMYSRYSGGSTMKALQNALAAQFLVVPPLVNPIIYGLNLQQIRSRMLHRFTHRTGTFRKNX
..............N.......................N.........................................      80
................................................................................     160
................................................................................     240
........................................................................             320

(Threshold=0.5)
----------------------------------------------------------------------
SeqName      Position  Potential   Jury    N-Glyc
                                agreement result
----------------------------------------------------------------------
contig104344-BriOR.G048   15 NDSL   0.7058     (9/9)   ++
contig104344-BriOR.G048   39 NLTL   0.7854     (9/9)   +++
----------------------------------------------------------------------
```

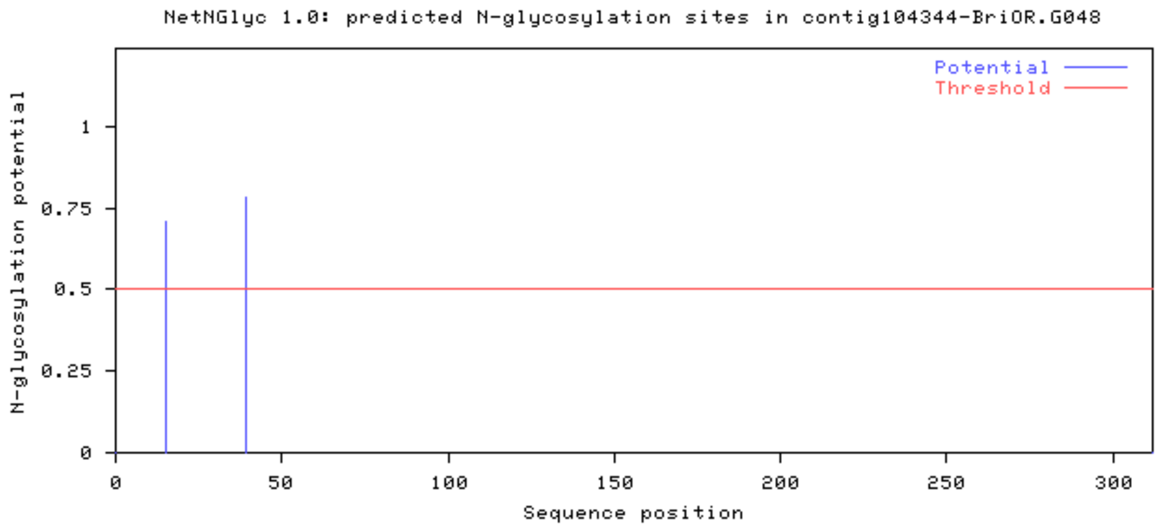

Graphics in PostScript

Output for 'contig106092-BriORe.N094'

```
##########################################################################################

    Warning: This sequence may not contain a signal peptide!!

    Proteins without signal peptides are unlikely to be exposed to
    the N-glycosylation machinery and thus may not be glycosylated
    (in vivo) even though they contain potential motifs.

    SignalP-NN euk predictions are as follows:

  # name                Cmax  pos ?  Ymax  pos ?  Smax  pos ?  Smean ?  D     ?

    SignalP output is explained at http://www.cbs.dtu.dk/services/SignalP/output.html

##########################################################################################

Name:  contig106092-BriORe.N094         Length:  301
GFIGIPNIKYYYAFLFFVYIISVLANTAVMAAIYLDHNLRTPKYIAVFNLALVDLLGNSAMVPKVLHIFLFNHPYISYND      80
CLTFLFFCYIFLSMQALNLVALSYDRVMAIVYPLHYQLKVTHKLMFSLIASFWVSAITSILIAIGLLTRLSFCESVVIKS     160
YFCDHGQMYRLACNDFTPSYVIAKILPALILWLPLTIVLLSYLCICYALAKVATVRERMKGFKTCTAHLSLVAIYFLPIL     240
ITFTLGANIEPNARIINLSLTSVFPPMLNPIIYVLQTREIKESLRKLLRIIKHYKIRKVKX
................................................................................      80
................................................................................     160
................................................................................     240
................N............................................                        320

(Threshold=0.5)
----------------------------------------------------------------------
SeqName       Position  Potential   Jury    N-Glyc
                                  agreement result
----------------------------------------------------------------------
contig106092-BriORe.N094  257 NLSL   0.6064     (8/9)   +
----------------------------------------------------------------------
```

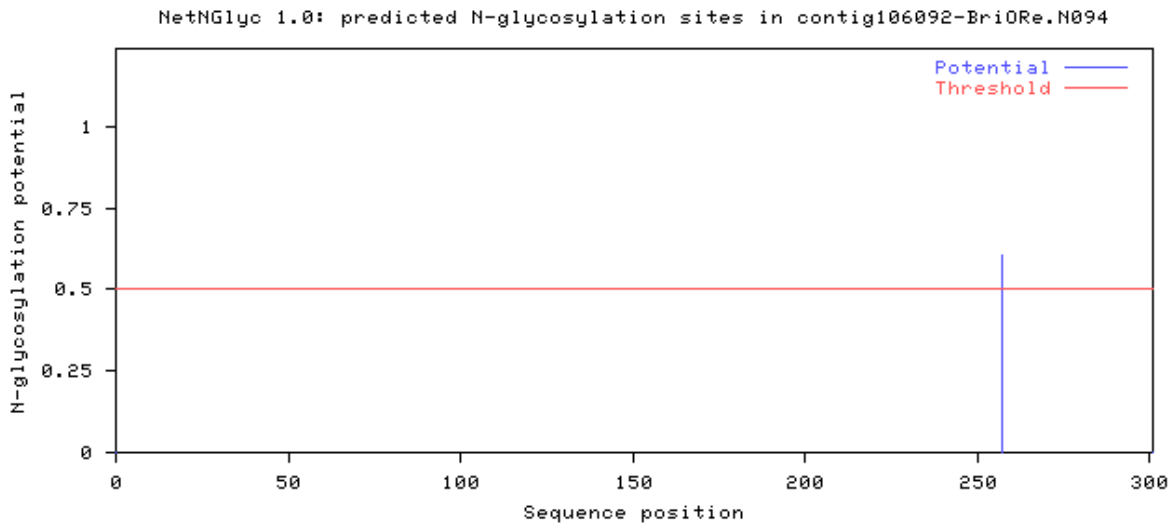

Graphics in PostScript

Output for 'contig106093-BriORp.N098'

```
##############################################################################

    Warning: This sequence may not contain a signal peptide!!

    Proteins without signal peptides are unlikely to be exposed to
    the N-glycosylation machinery and thus may not be glycosylated
    (in vivo) even though they contain potential motifs.

    SignalP-NN euk predictions are as follows:

  # name                Cmax  pos ?  Ymax  pos ?  Smax  pos ?  Smean ?  D     ?

    SignalP output is explained at http://www.cbs.dtu.dk/services/SignalP/output.html

##############################################################################

Name:  contig106093-BriORp.N098        Length:  324
MEFLNSAIENNITFVQSAYFIISGFISIPNIRYYFVFLCFIYIFSVVGNTLVMIVIILDRMLRSPKYIAVFNLAFTDLLS      80
SSALVPKVLDISLFNHYYISYNNCLTFMFFCFTLISMQAFNLVVLSFDRVMAIMYPLHYQMRVTQKLILSLIAFAITLTL     160
VADGLLTRLSFCQSVIIQSYYCDHGPFYLLDCNDATPNHIIAFLAIVLVLGFPLAFIVGGYCCIGYSLSKISTFRERVKA     240
FKTCTGHLSLVAIYFLPIIFVYVFWPVIHPNARIINLSMTSVMPPMLNPIIYALQTQEIKESLRRLLKARVQAKLEVFRL     320
KTLX
..........N.....................................................................      80
................................................................................     160
................................................................................     240
...................................N............................................     320
....                                                                                 400

(Threshold=0.5)
----------------------------------------------------------------------
SeqName      Position  Potential   Jury    N-Glyc
                                agreement result
----------------------------------------------------------------------
contig106093-BriORp.N098   11 NITF   0.6652     (9/9)   ++
contig106093-BriORp.N098  276 NLSM   0.5398     (5/9)   +
----------------------------------------------------------------------
```

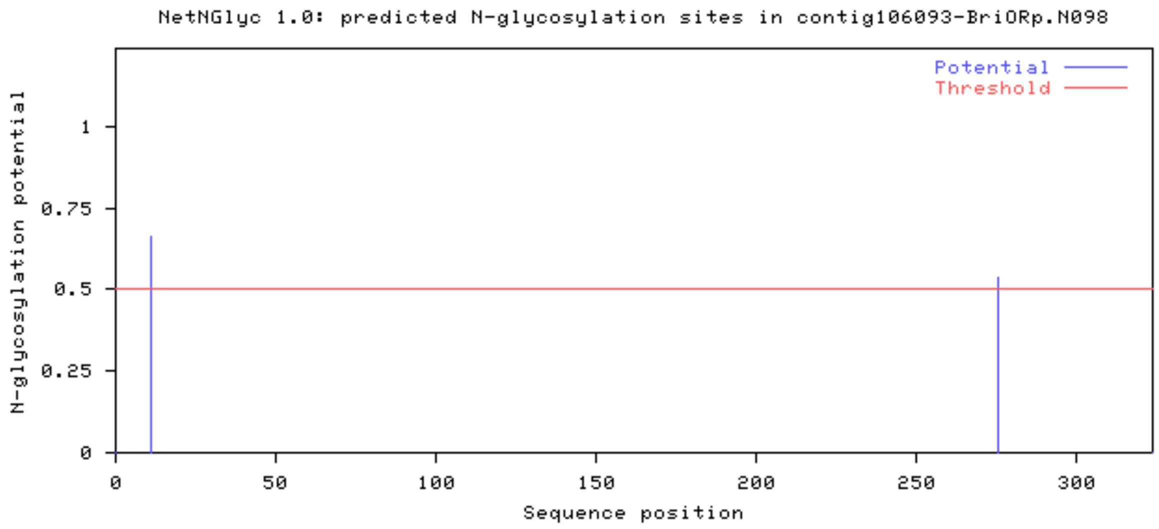

Graphics in PostScript

Output for 'contig106096-BriOR.N089'

```
##############################################################################

   Warning: This sequence may not contain a signal peptide!!

   Proteins without signal peptides are unlikely to be exposed to
   the N-glycosylation machinery and thus may not be glycosylated
   (in vivo) even though they contain potential motifs.

   SignalP-NN euk predictions are as follows:

 # name                Cmax  pos ?  Ymax  pos ?  Smax  pos ?  Smean ?  D     ?

   SignalP output is explained at http://www.cbs.dtu.dk/services/SignalP/output.html

##############################################################################

Name:  contig106096-BriOR.N089  Length:  327
MDLFNSALGKNITFLRPAFFIISGFIGIPNIKYYYAFLFFVYIISVLANTAVMAAIYLDHNLRTPKYIAVFNLALVDLLG      80
NSAMVPKVLHIFLFNHPHIPYNDCLTFLFFCYVFLSMQALNLVALSYDRIMAIVYPLHYQLKVTHKFMFCLIASFWVFVI     160
IVVLIATGLLTRLSFCESVVIKSFFCDHGQIYRLACNDYTPSIIYGWILLVLILWLPLTIVLLSYLSIGYALAKVATVRE     240
RMKGFKTCTAHLSLVAIYFLPILITFTLHANIEPNARIINLSLTSVFPPMLNPIIYVLQTREIKESLKKFLRITTHYKNR     320
KVKFKKX
..........N.....................................................................      80
................................................................................     160
................................................................................     240
.......................................N........................................     320
.......                                                                              400

(Threshold=0.5)
----------------------------------------------------------------------
SeqName      Position  Potential   Jury    N-Glyc
                                 agreement result
----------------------------------------------------------------------
contig106096-BriOR.N089   11 NITF   0.5354     (6/9)    +
contig106096-BriOR.N089  280 NLSL   0.6054     (8/9)    +
----------------------------------------------------------------------
```

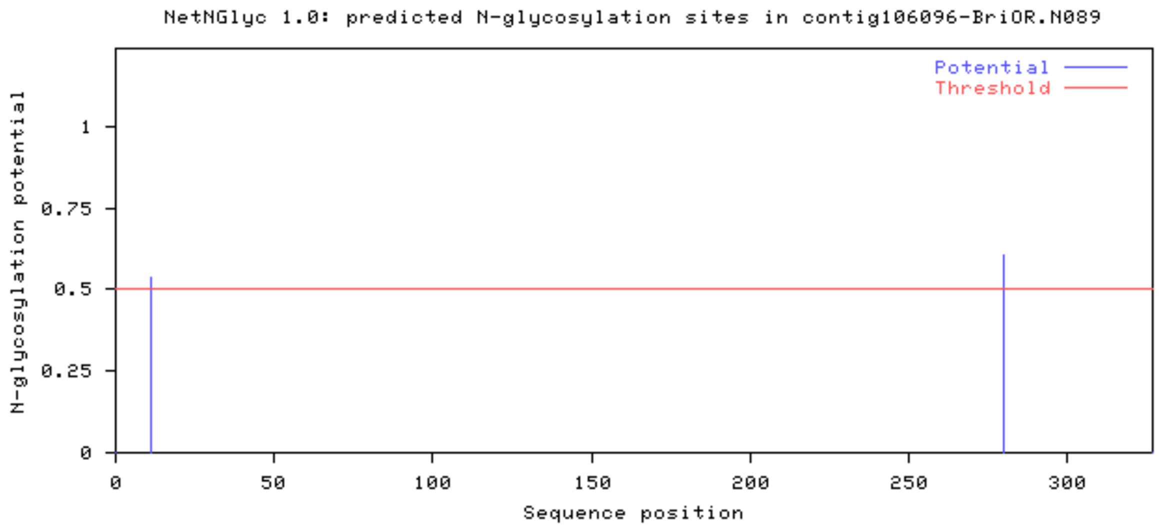

Graphics in PostScript

Output for 'contig106098-BriORe.N125'

```
##########################################################################################

    Warning: This sequence may not contain a signal peptide!!

    Proteins without signal peptides are unlikely to be exposed to
    the N-glycosylation machinery and thus may not be glycosylated
    (in vivo) even though they contain potential motifs.

    SignalP-NN euk predictions are as follows:

  # name                Cmax  pos ?  Ymax  pos ?  Smax  pos ?  Smean ?  D     ?

    SignalP output is explained at http://www.cbs.dtu.dk/services/SignalP/output.html

##########################################################################################

Name:  contig106098-BriORe.N125        Length:  54
VIHPNARIISLSISTVLPPMLNPIIYVLQTQEIKESLKKLLKIRVKSKIATKYX
......................................................                                    80

(Threshold=0.5)

No sites predicted in this sequence.
```

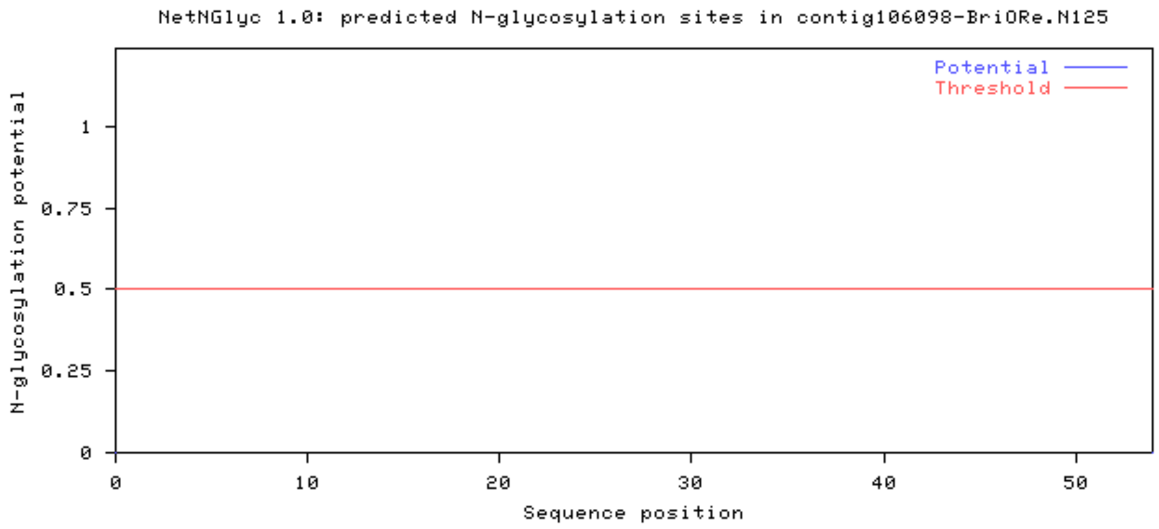

[Graphics in PostScript]

Output for 'contig107605-BriORe.E044'

```
##########################################################################################

    Warning: This sequence may not contain a signal peptide!!

    Proteins without signal peptides are unlikely to be exposed to
    the N-glycosylation machinery and thus may not be glycosylated
    (in vivo) even though they contain potential motifs.

    SignalP-NN euk predictions are as follows:

  # name                    Cmax  pos ?  Ymax  pos ?  Smax  pos ?  Smean ?  D     ?

    SignalP output is explained at http://www.cbs.dtu.dk/services/SignalP/output.html

##########################################################################################

Name:  contig107605-BriORe.E044         Length:  92
FSGSKQMRQKAVSTCTPHLASLLNFSCSGCFEILQSRFNMNTVPNVLRILISLYWLMCQPLFNPVLYGLKMSNIHDICKC      80
LLYSKVSTILSX
.......................N........................................................      80
............                                                                         160

(Threshold=0.5)
----------------------------------------------------------------------
SeqName      Position  Potential   Jury    N-Glyc
                                 agreement result
----------------------------------------------------------------------
contig107605-BriORe.E044    24 NFSC    0.6474     (9/9)   ++
----------------------------------------------------------------------
```

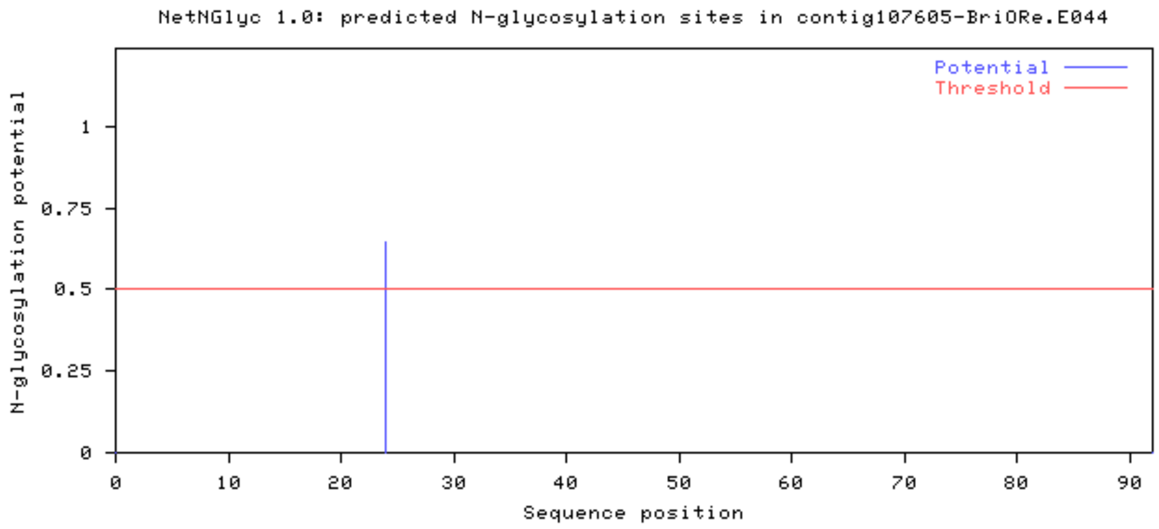

Graphics in PostScript

Output for 'contig107626-BriOR.H054'

```
##########################################################################

   Warning: This sequence may not contain a signal peptide!!

   Proteins without signal peptides are unlikely to be exposed to
   the N-glycosylation machinery and thus may not be glycosylated
   (in vivo) even though they contain potential motifs.

   SignalP-NN euk predictions are as follows:

 # name                 Cmax  pos ?  Ymax  pos ?  Smax  pos ?  Smean ?  D     ?

   SignalP output is explained at http://www.cbs.dtu.dk/services/SignalP/output.html

##########################################################################

Name:  contig107626-BriOR.H054  Length:  314
MNKSSIVVFSLTGFNATVNYRVTLFSLTLLCYFLILMVNISLILTIISDQNLHEPMYIFLCSLCINGLYGTAGFFPRFAL      80
DLLSDTHLISYVGCLLQVFVIYSNAKVDYSTLVLMAYDRYLAICRPLEYHSVMSVRRTAVLVTLSWLVPLCFETLIISLT     160
STLKLCGSNINKLYCENWSIVKLACGSAKVNDIVGLIVITFYCCHAVCIACSYVQLVNAALKSRAGRKKFTQTCVPHLFC     240
LLNVTTALLFDLMFSRFGSASLPQHLKNFMAIEFLIIPPILNPVCYGWVLTKIRRRMIFLCRLAYQRFGVKSQX
.N...........N.......................N..........................................      80
................................................................................     160
................................................................................     240
..N.......................................................................           320

(Threshold=0.5)
----------------------------------------------------------------------
SeqName      Position  Potential   Jury    N-Glyc
                                 agreement result
----------------------------------------------------------------------
contig107626-BriOR.H054    2 NKSS   0.7814     (9/9)    +++
contig107626-BriOR.H054   15 NATV   0.6247     (7/9)    +
contig107626-BriOR.H054   39 NISL   0.7616     (9/9)    +++
contig107626-BriOR.H054  177 NWSI   0.4819     (5/9)    -
contig107626-BriOR.H054  243 NVTT   0.5833     (9/9)    ++
----------------------------------------------------------------------
```

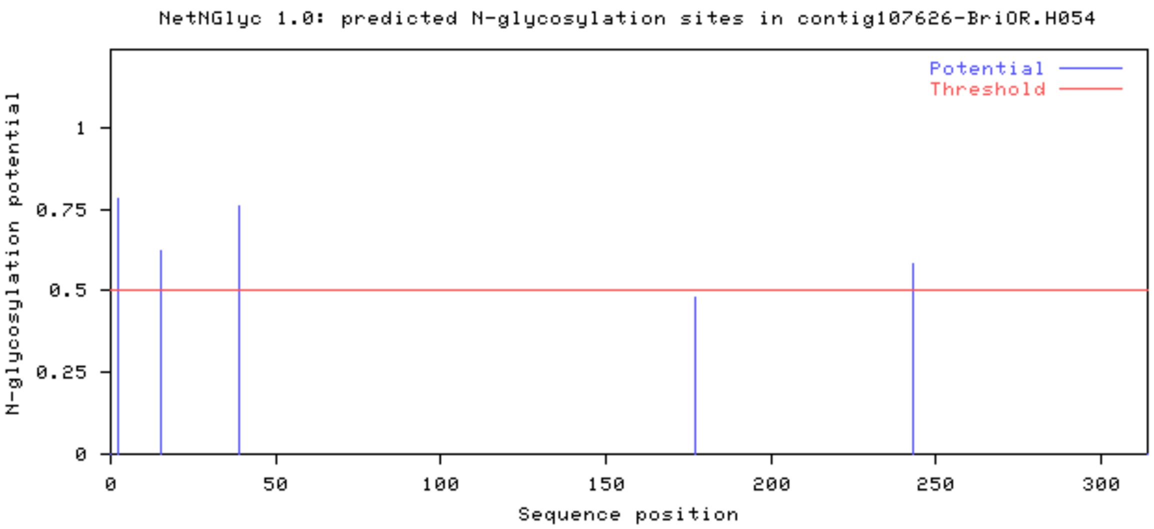

Graphics in PostScript

Output for 'contig107838-BriORe.L075'

```
##########################################################################################

    Warning: This sequence may not contain a signal peptide!!

    Proteins without signal peptides are unlikely to be exposed to
    the N-glycosylation machinery and thus may not be glycosylated
    (in vivo) even though they contain potential motifs.

    SignalP-NN euk predictions are as follows:

  # name                 Cmax  pos ?  Ymax  pos ?  Smax  pos ?  Smean ?  D     ?

    SignalP output is explained at http://www.cbs.dtu.dk/services/SignalP/output.html

##########################################################################################

Name:  contig107838-BriORe.L075         Length:  55
MSLQNASIKLTHFIIGGFDTVKRPVAVGVVMLITYLLAVFANLVNIIFIVSDKQL
....N..................................................                              80

(Threshold=0.5)
----------------------------------------------------------------------
SeqName      Position  Potential   Jury    N-Glyc
                                 agreement result
----------------------------------------------------------------------
contig107838-BriORe.L075    5 NASI   0.5953     (7/9)    +
----------------------------------------------------------------------
```

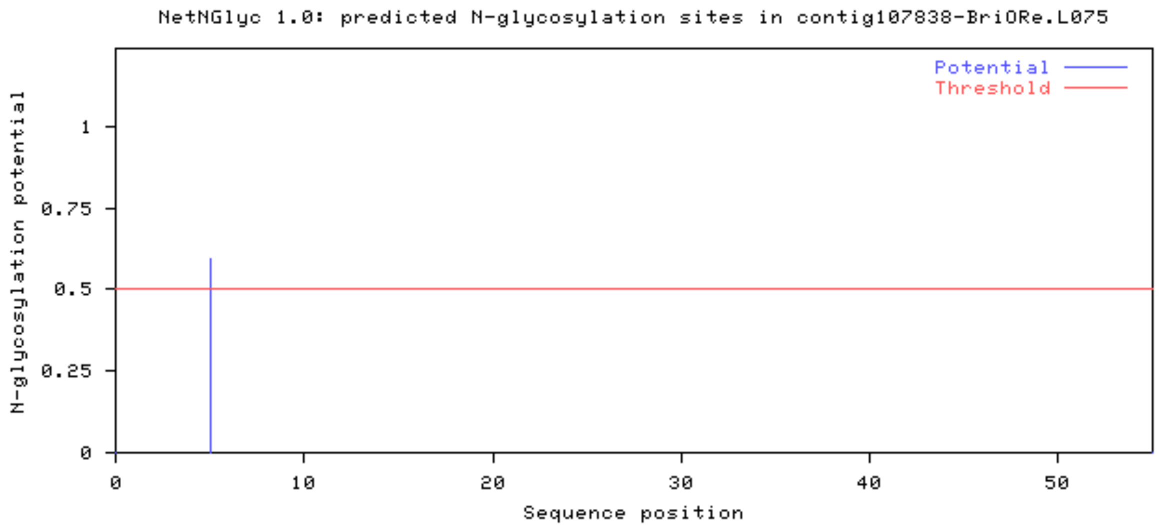

Graphics in PostScript

Output for 'contig109456-BriORp.A018'

```
##########################################################################

   Warning: This sequence may not contain a signal peptide!!

   Proteins without signal peptides are unlikely to be exposed to
   the N-glycosylation machinery and thus may not be glycosylated
   (in vivo) even though they contain potential motifs.

   SignalP-NN euk predictions are as follows:

 # name                Cmax  pos ?  Ymax  pos ?  Smax  pos ?  Smean ?  D     ?

   SignalP output is explained at http://www.cbs.dtu.dk/services/SignalP/output.html

##########################################################################

Name:  contig109456-BriORp.A018        Length:  260
MEYVTVWRDSLSLREPRLHRNHVLSEKQMILYLLCLFQGLSYYTSVGSDFLLLAAMAYDRYVSIYKSLQYPVTMNRITIY      80
VCLILAWRIPAFETSVMGVLYSNVKLCSFSLTGIFCNNSLYKLQCVPSVAISIYGMVMLINIALLPLLFIPFTYINILRT     160
SYHCCREVRKKAVKTCLPHLLVLINFSCFIFLDIIIVRLDSDLSKTLRWTLTFQSILFHPLLNPIIYGLKMNEIFKHIKI     240
LLSSLITLVLLPYYQMYGIX
................................................................................      80
................................................................................     160
........................N.......................................................     240
....................                                                                 320

(Threshold=0.5)
----------------------------------------------------------------------
SeqName      Position  Potential   Jury    N-Glyc
                                 agreement result
----------------------------------------------------------------------
contig109456-BriORp.A018  117 NNSL   0.4459     (7/9)   -
contig109456-BriORp.A018  185 NFSC   0.5629     (5/9)   +
----------------------------------------------------------------------
```

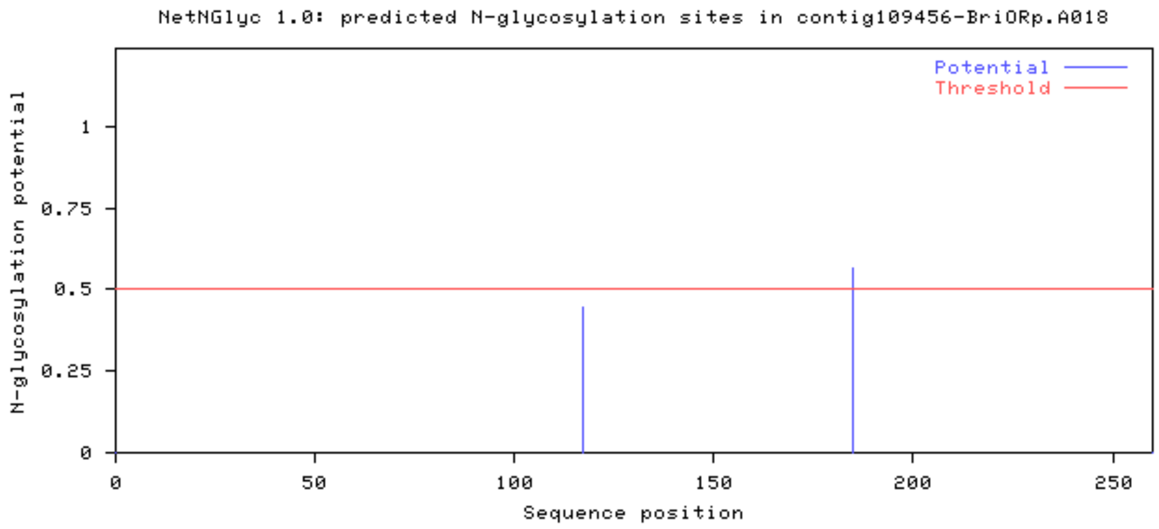

Graphics in PostScript

Output for 'contig110588-BriORe.D030'

```
########################################################################################

    Warning: This sequence may not contain a signal peptide!!

    Proteins without signal peptides are unlikely to be exposed to
    the N-glycosylation machinery and thus may not be glycosylated
    (in vivo) even though they contain potential motifs.

    SignalP-NN euk predictions are as follows:

  # name                Cmax  pos ?  Ymax  pos ?  Smax  pos ?  Smean ?  D     ?

    SignalP output is explained at http://www.cbs.dtu.dk/services/SignalP/output.html

########################################################################################

Name:  contig110588-BriORe.D030         Length:  291
MGNSSETVSFVLAAYGNVGELKHLYFIIILVWYFSICVANTVLIVVICVDRRLHEPMYILLCNLCLSEINGSTSLYPLLL      80
SQMFSDSHEVTVPWCFLQMFCLCTSASVELCSLAAMAYDRYVSICNPFTYNVIMNTERVFLLILLVWVYSFLSFIFTYSF     160
IFSLKFCGNIIHNVYCDHQFLIRLSCPVSIKSFISDISFVLLSAFIPFSLILVSYMKILRVCQKTSKENKQKAVTTCTPQ     240
IISVSNLCVGCICYFIDFKFVVSQVPNEVRIILPMYVLIFQPMLTPFMYGF
..N..................................................................N..........      80
................................................................................     160
................................................................................     240
...................................................                                  320

(Threshold=0.5)
----------------------------------------------------------------------
SeqName      Position  Potential   Jury    N-Glyc
                                agreement  result
----------------------------------------------------------------------
contig110588-BriORe.D030    3 NSSE   0.7409     (9/9)   ++
contig110588-BriORe.D030   70 NGST   0.5752     (5/9)   +
----------------------------------------------------------------------
```

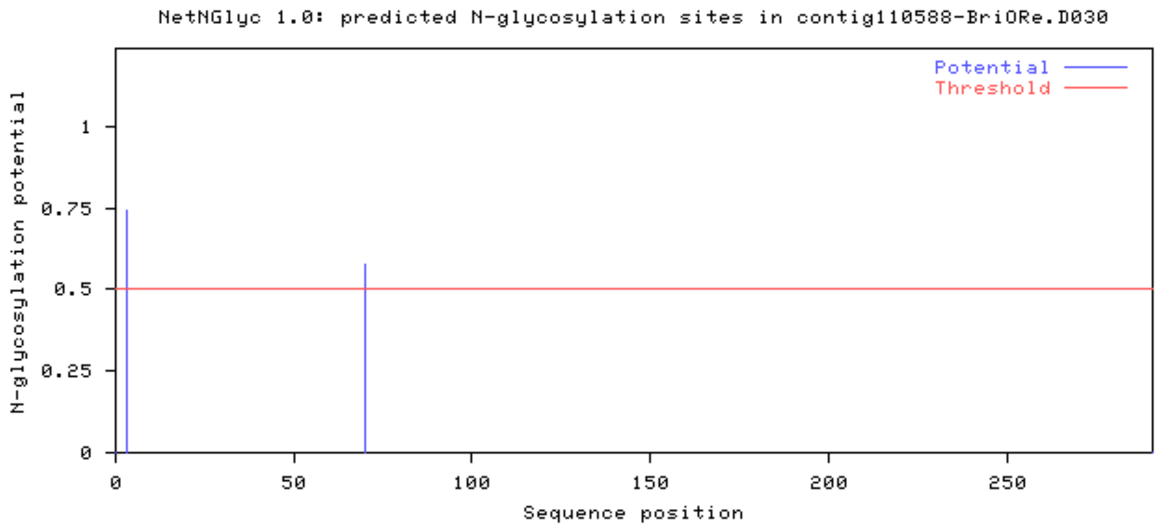

Graphics in PostScript

Output for 'contig110782-BriOR.O080'

```
##########################################################################

   Warning: This sequence may not contain a signal peptide!!

   Proteins without signal peptides are unlikely to be exposed to
   the N-glycosylation machinery and thus may not be glycosylated
   (in vivo) even though they contain potential motifs.

   SignalP-NN euk predictions are as follows:

 # name                Cmax  pos ?  Ymax  pos ?  Smax  pos ?  Smean ?  D     ?

   SignalP output is explained at http://www.cbs.dtu.dk/services/SignalP/output.html

##########################################################################

Name:  contig110782-BriOR.O080  Length:  329
MPERNHSSVTEFILTGFPGLHQEYYGLVSAVLFFVYLITMIANATVIFLFATNQSLHKPMYYIILNLSVCDILFSTTTLP      80
KIISRYWFRSGSISFTACFIQMYFVHYFGTAVAYILFQMALDRYLAICHPLKYSRILTKSNILILSITGWTAAKAFPLMM     160
VIRAYPLPYCASNIITHCFCDHIGITVLACTDRTPYAIPAFVFAMFVLLGPLAFIIFSYCSILIAVHKIASVQGRLKSLS     240
TCSTQLIIISLYFLPRCFVYLAQNVGIKFSADVRIVIIMLYSLAPPMINPLIYCLRAKDMRESLLKVFCRRTIPQKAQVA     320
VINIINHKX
....N.....................................N.........N............N..............      80
................................................................................     160
................................................................................     240
................................................................................     320
.........                                                                            400

(Threshold=0.5)
----------------------------------------------------------------------
SeqName           Position  Potential   Jury     N-Glyc
                                      agreement result
----------------------------------------------------------------------
contig110782-BriOR.O080    5 NHSS   0.6059     (8/9)    +
contig110782-BriOR.O080   43 NATV   0.6882     (7/9)    +
contig110782-BriOR.O080   53 NQSL   0.6770     (9/9)    ++
contig110782-BriOR.O080   66 NLSV   0.7957     (9/9)    +++
----------------------------------------------------------------------
```

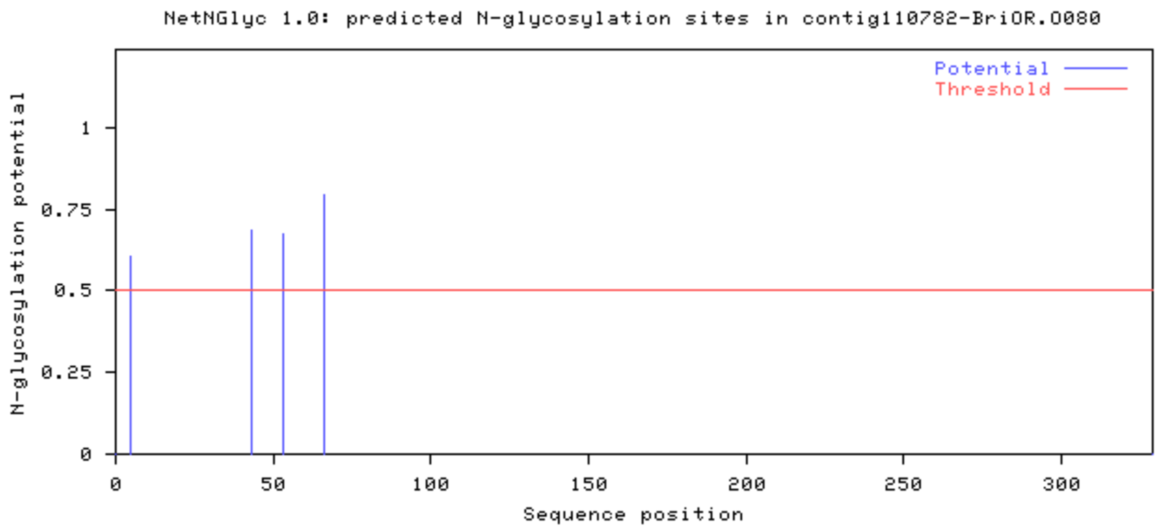

Graphics in PostScript

Output for 'contig111342-BriORe.E043'

```
########################################################################################

    Warning: This sequence may not contain a signal peptide!!

    Proteins without signal peptides are unlikely to be exposed to
    the N-glycosylation machinery and thus may not be glycosylated
    (in vivo) even though they contain potential motifs.

    SignalP-NN euk predictions are as follows:

  # name                 Cmax  pos ?  Ymax  pos ?  Smax  pos ?  Smean ?  D     ?

    SignalP output is explained at http://www.cbs.dtu.dk/services/SignalP/output.html

########################################################################################

Name:  contig111342-BriORe.E043        Length:  191
TTLISLTWLLPFIMIVVLISLSAPLQLCGNVINKVFCGNYAIIKLACSDTRVHNIFGLIYTFISVVIPLVLILYTYVRIL      80
KVCFSGSKQTRQKAVSTCTPHLASILNFFFGCCFQILQSRFDTSGVPNVFGILSSLYFLTCQPLFTALLYGLKMTRIRIA     160
CKQLFCGSLTRLFSCSSDIFSKSHQSQSYCX
................................................................................      80
................................................................................     160
...............................                                                      240

(Threshold=0.5)

No sites predicted in this sequence.
```

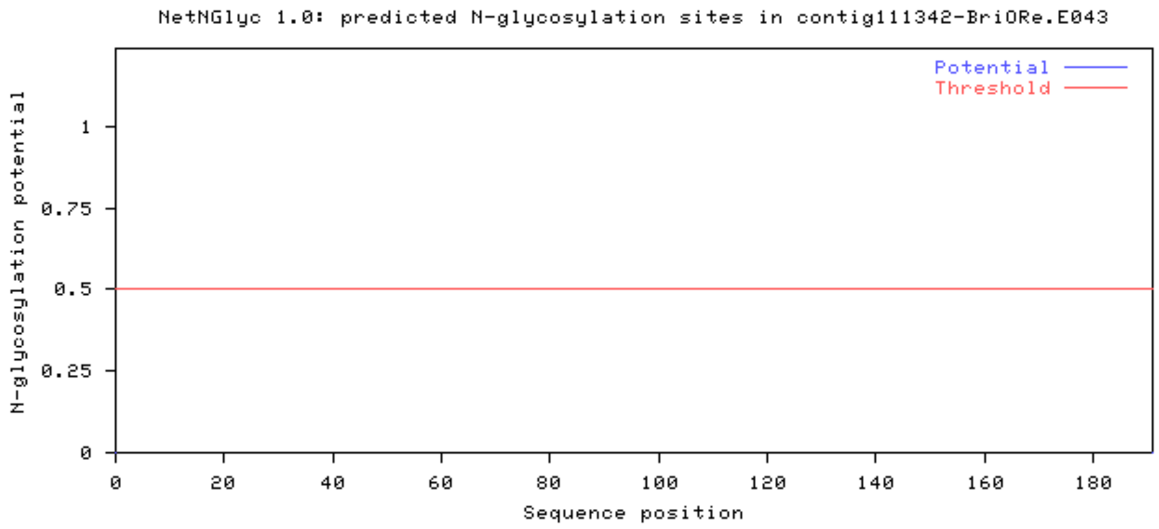

Graphics in PostScript

Output for 'contig116846-BriOR.H055'

```
##############################################################################

    Warning: This sequence may not contain a signal peptide!!

    Proteins without signal peptides are unlikely to be exposed to
    the N-glycosylation machinery and thus may not be glycosylated
    (in vivo) even though they contain potential motifs.

    SignalP-NN euk predictions are as follows:

  # name                    Cmax  pos ?  Ymax  pos ?  Smax  pos ?  Smean ?  D     ?

    SignalP output is explained at http://www.cbs.dtu.dk/services/SignalP/output.html

##############################################################################

Name:  contig116846-BriOR.H055  Length:  324
MNNVSVITMFFLSGFNETISHRFVLFFLSLLCYCIICLVNVSLIVIIILDSNLHESMYILLCVFCINALYGTAGFYPKFL      80
WDLLSNVYLISYYGCLIQTQVIYSFVCGEVLILALMAYDRYVAICQPLKYHSIMSKQRVIRFACFLWLTTVCITATNAFL     160
TSRLKLCSPYLSRLFCVNWIIVQLACFPAQTIVNGIFANITIIIYLLHGVFIVWSYMYIIQTCVKSIENRAKFMQTCVPH     240
LISLFTFVVAILTDIISMRLDSKELPRSLQNFVALEFLIIPPIMNPLIYGFKLTKIRKRLHSVVILKITNFCFIRSENSF     320
THSX
..N............N.......................N........................................      80
................................................................................     160
......................................N.........................................     240
................................................................................     320
....                                                                                 400

(Threshold=0.5)
----------------------------------------------------------------------
SeqName      Position  Potential   Jury    N-Glyc
                                 agreement result
----------------------------------------------------------------------
contig116846-BriOR.H055    3 NVSV   0.7559     (9/9)    +++
contig116846-BriOR.H055   16 NETI   0.6821     (9/9)    ++
contig116846-BriOR.H055   40 NVSL   0.6904     (9/9)    ++
contig116846-BriOR.H055  199 NITI   0.6378     (8/9)    +
----------------------------------------------------------------------
```

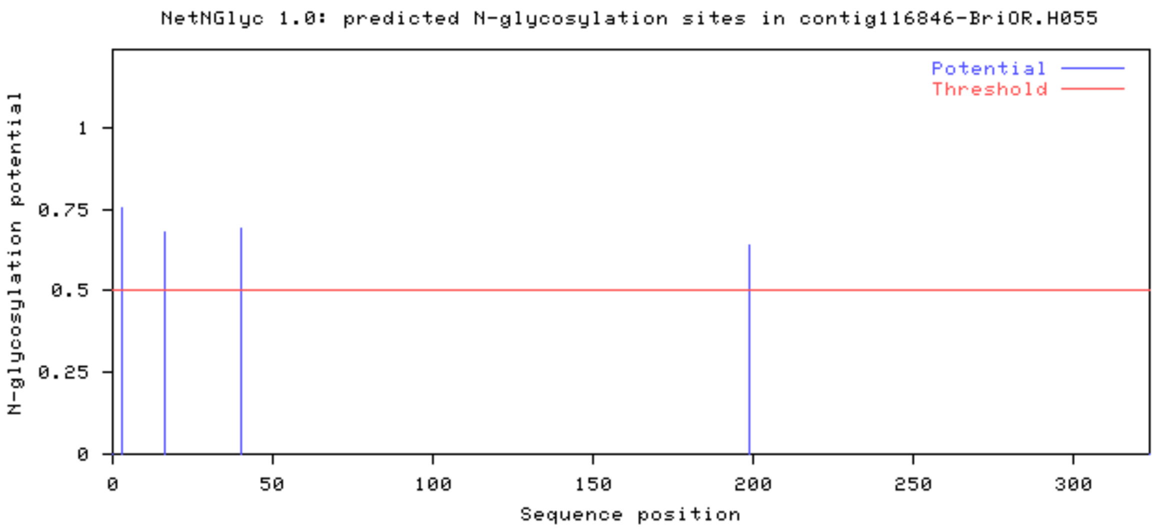

Graphics in PostScript

Output for 'contig117284-BriORe.A016'

```
##########################################################################################

    Warning: This sequence may not contain a signal peptide!!

    Proteins without signal peptides are unlikely to be exposed to
    the N-glycosylation machinery and thus may not be glycosylated
    (in vivo) even though they contain potential motifs.

    SignalP-NN euk predictions are as follows:

  # name                   Cmax  pos ?  Ymax  pos ?  Smax  pos ?  Smean ?  D     ?

    SignalP output is explained at http://www.cbs.dtu.dk/services/SignalP/output.html

##########################################################################################

Name:  contig117284-BriORe.A016         Length:  302
YVEVNKYRYVYFFIFFILYSLIICSNSTIVYIIWIHKNLHEPMYIFIAALLLNCVLYSTTVYPKLLIDFLSEKQVTTYSA      80
CLFQFFMFYTLGSSEFFLLAAMAYDRYVAICKPLQYQTIMSKTTVSIFLAVAWLLPVCHSAVVTAGSAEATLCNFNLKGI     160
FCNNAVYTLQCVKSRLITVFGVVALIDLVILPMLFIVFTYSNIFILTYQSCKEVRKKALETCLPHLLVLFSFSCLSIYDV     240
SIARVESDFPKTARLIMTLQIVLYHPLLNPFIYGLKMKEISKQLKRFFCHDKIISCINSECX
.........................N......................................................      80
................................................................................     160
................................................................................     240
..............................................................                       320

(Threshold=0.5)
----------------------------------------------------------------------
SeqName      Position  Potential   Jury    N-Glyc
                                 agreement result
----------------------------------------------------------------------
contig117284-BriORe.A016   26 NSTI   0.7308     (9/9)    ++
----------------------------------------------------------------------
```

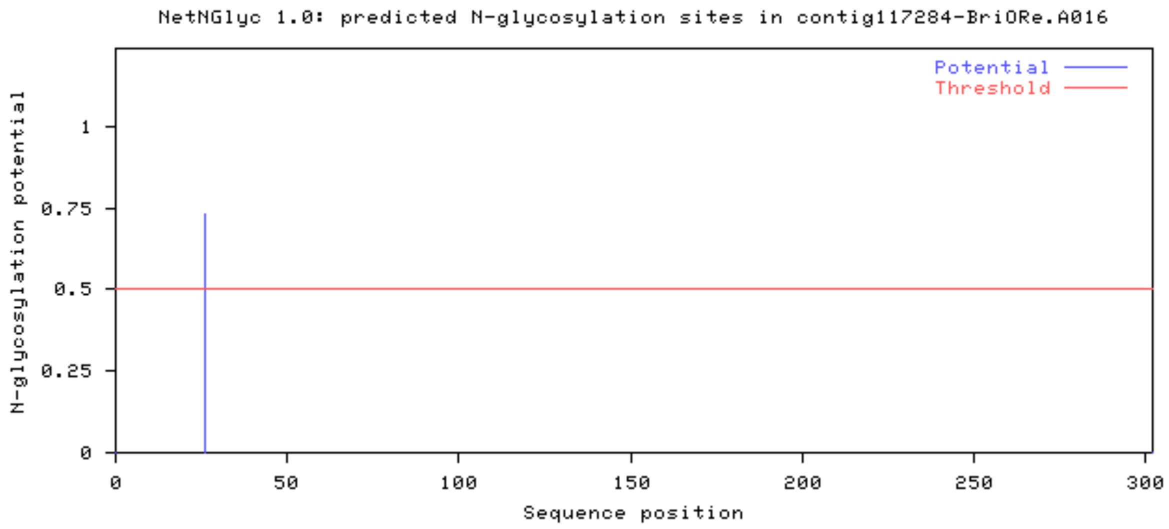

Graphics in PostScript

Output for 'contig117522-BriORe.S106'

```
########################################################################################

    Warning: This sequence may not contain a signal peptide!!

    Proteins without signal peptides are unlikely to be exposed to
    the N-glycosylation machinery and thus may not be glycosylated
    (in vivo) even though they contain potential motifs.

    SignalP-NN euk predictions are as follows:

  # name                Cmax  pos ?  Ymax  pos ?  Smax  pos ?  Smean ?  D     ?

    SignalP output is explained at http://www.cbs.dtu.dk/services/SignalP/output.html

########################################################################################

Name:  contig117522-BriORe.S106        Length:  294
LQRPLTDRVMTVQILVIIFLCINMLLTVTVFKKESFYTSSRYILFFVTLLSDSFLLVTTDILLILTNFRCSLQVWLCIII      80
CLFVIVYSIVTPVTLTAMTLERYVAICMPLRHGQLCSTRSTMYCILIIHGLSSGPCIVIISMFFATASISFYNQYTICSV     160
EMFMLYRWQDHARSAVSQFYFMTMGITIAFSYVQIIKVAKAASGENKRSTQKGLRTVILHAFQLLLCLFQLWTPFIESAV     240
LQIDFNLFLNVRYFNYVIFNLAPKCLSPLIYGLRDEHFFLALKNLMTASSYSKX
................................................................................      80
................................................................................     160
................................................................................     240
......................................................                               320

(Threshold=0.5)

No sites predicted in this sequence.
```

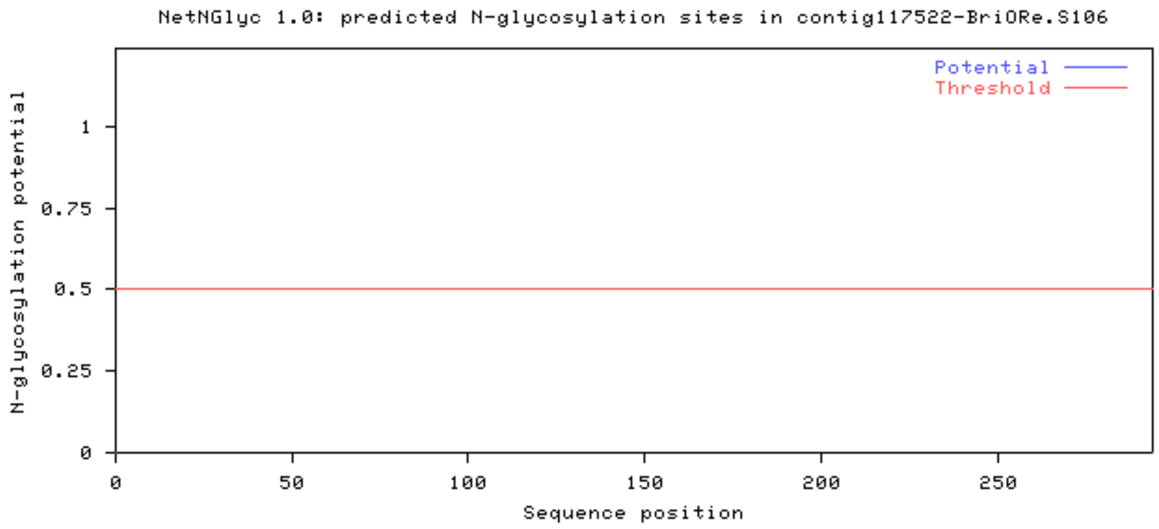

Graphics in PostScript

Output for 'contig117770-BriORe.W111'

```
##########################################################################################

    Warning: This sequence may not contain a signal peptide!!

    Proteins without signal peptides are unlikely to be exposed to
    the N-glycosylation machinery and thus may not be glycosylated
    (in vivo) even though they contain potential motifs.

    SignalP-NN euk predictions are as follows:

  # name                Cmax  pos ?  Ymax  pos ?  Smax  pos ?  Smean ?  D     ?

    SignalP output is explained at http://www.cbs.dtu.dk/services/SignalP/output.html

##########################################################################################

Name:  contig117770-BriORe.W111         Length:  257
TNPRYILYIHLVINDILLLIMFTLLQVLSYIIFTFHVSFCIILRVIAVIPSFNTPMTLAVMAVECYVAICFPLQHSQICT      80
VKNITVVITVIWGLSSLTILPDLFTTLATESRDFFHSRVFCLRETVFRLPELEKKKTISNIVFLVIVWLTLVYTYFRILF     160
AAQAAAANARKARNTVLLHGFQLLLCMLNYVYDLLLNGLTSLFPKGVLTIRYTISVFVHILPRLVSPLVYGIRDKAFRRY     240
LRKYLFYSPKANVNKSX
................................................................................      80
..N.............................................................................     160
................................................................................     240
.................                                                                    320

(Threshold=0.5)
----------------------------------------------------------------------
SeqName      Position  Potential   Jury    N-Glyc
                                 agreement result
----------------------------------------------------------------------
contig117770-BriORe.W111   83 NITV   0.5817     (9/9)   ++
contig117770-BriORe.W111  254 NKSX   0.4620     (6/9)   -
----------------------------------------------------------------------
```

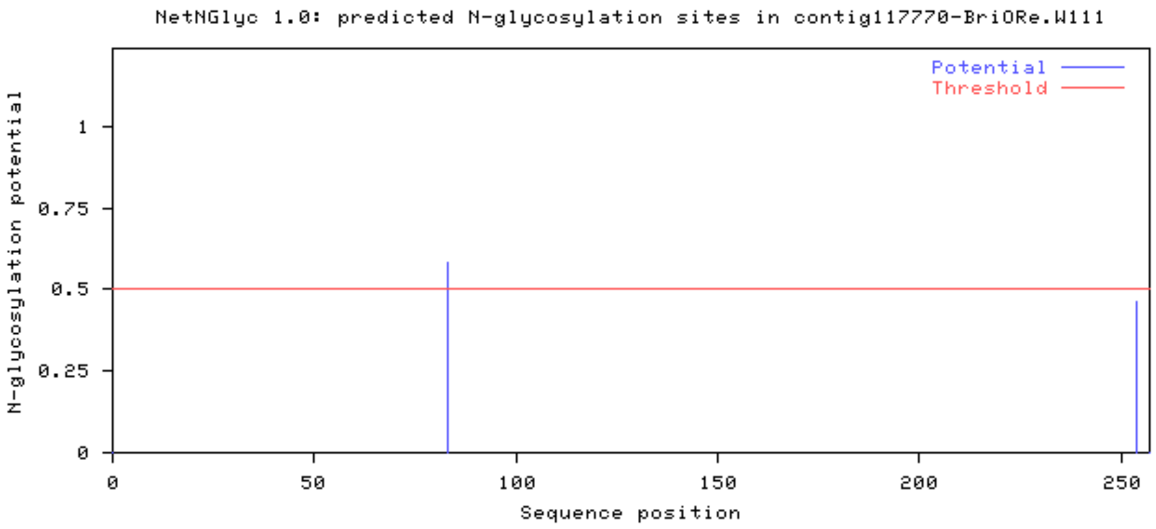

Graphics in PostScript

Explain the output. Go back.

NetNGlyc 1.0 Server - prediction results

Technical University of Denmark

Asn-Xaa-Ser/Thr sequons in the sequence output below are highlighted in **blue**.
Asparagines predicted to be N-glycosylated are highlighted in **red**.

Output for 'contig020980-NyeOR.C035'

```
########################################################################################

    Warning: This sequence may not contain a signal peptide!!

    Proteins without signal peptides are unlikely to be exposed to
    the N-glycosylation machinery and thus may not be glycosylated
    (in vivo) even though they contain potential motifs.

    SignalP-NN euk predictions are as follows:

  # name                Cmax  pos ?  Ymax  pos ?  Smax  pos ?  Smean ?  D     ?

    SignalP output is explained at http://www.cbs.dtu.dk/services/SignalP/output.html

########################################################################################

Name:  contig020980-NyeOR.C035  Length:  321
MDNTTAATFKMTAYAVMENYKHGLFSVFFLLYLITLVLNVLLISVIHQNKQLHQPMNVFTCMLSLNEIYGSSTLLPAVMA      80
VLVSKTHDVTVKWCMAQAYFLHTYASGEFCILALMGYDRYIAICSPLHYYSIMSYSKTCKLIAFTGLYPFIVFTSFYSLT     160
LQLRFCGKVMPKLYCVNMELVKNSCTNAQYISTVGLAILVLFIVPQLVMIVFSYAHILRVCRTFPKESQANAFRTCVPHL     240
LSLLNYTIASLFEVIQTRFNMSHVAVEAQIFLSLYFIIIPTIANPVLYGLGTQTVRGCIMKLFIKNKVMTTVLAKTLTVG     320
X
..N.............................................................................      80
................................................................................     160
................................................................................     240
....N...........................................................................     320
.                                                                                    400

(Threshold=0.5)
----------------------------------------------------------------------
SeqName      Position  Potential   Jury    N-Glyc
                                 agreement result
----------------------------------------------------------------------
contig020980-NyeOR.C035    3 NTTA   0.7209     (9/9)   ++
contig020980-NyeOR.C035  245 NYTI   0.6910     (8/9)   +
contig020980-NyeOR.C035  260 NMSH   0.4502     (7/9)   -
----------------------------------------------------------------------
```

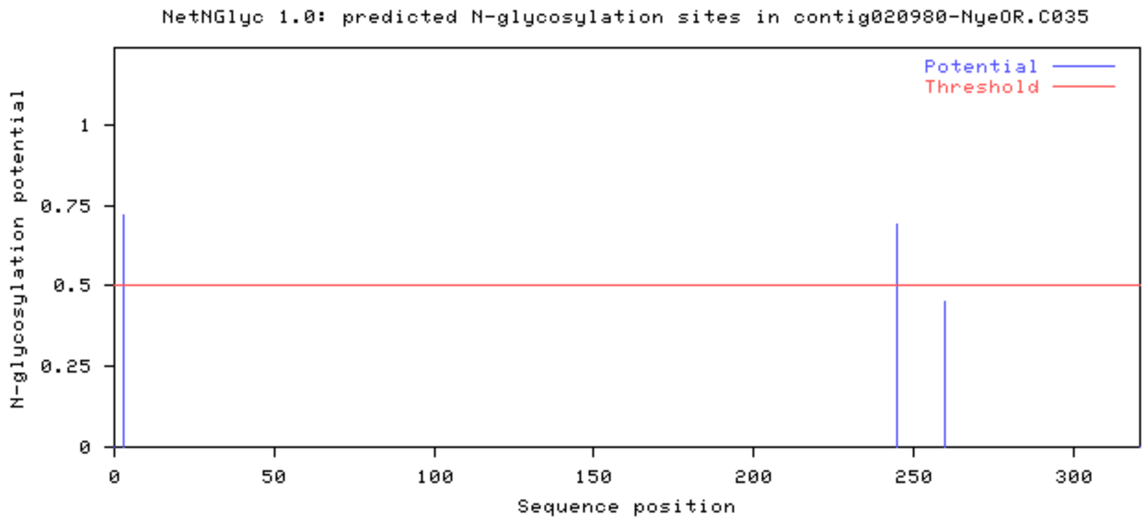

Graphics in PostScript

Output for 'contig021011-NyeOR.Q134'

##

Warning: This sequence may not contain a signal peptide!!

Proteins without signal peptides are unlikely to be exposed to the N-glycosylation machinery and thus may not be glycosylated (in vivo) even though they contain potential motifs.

SignalP-NN euk predictions are as follows:

# name	Cmax	pos	?	Ymax	pos	?	Smax	pos	?	Smean	?	D	?

SignalP output is explained at http://www.cbs.dtu.dk/services/SignalP/output.html

##

Name: contig021011-NyeOR.Q134 Length: 310

MNATTAEFQSLPFQTSVKAALSMLPCFFFLYVNAIMMFALLKKPLLLESSRYILFGHLLMCDSVQLLLTMLLYIFAVMMV 80
RMINYVCVFVSLLAAVTVKMSPLNLAVMSLERYVAVCFPLRHPSFATPRSTGKAIAVMWIVASLDSFIQLFLFVRMEKTI 160
FPMQSFCIRNSVFRLEVYVTLNMAFTILYFVFVSMIIIYTYTAIMITVKSASSRGRHTNKAPKTVLLHLLQLWLYLTSTL 240
FNMINPSMMLKVPPDMAIHAQYVLFVGLIIFPKCLSPLIYGLRDQTLCRVFKYYFTFGFRASVKPSPLSX
.N.. 80
.... 160
.... 240
.... 320

(Threshold=0.5)

SeqName	Position		Potential	Jury agreement	N-Glyc result
contig021011-NyeOR.Q134	2	NATT	0.6722	(8/9)	+
contig021011-NyeOR.Q134	245	NPSM	0.3831	(7/9)	-

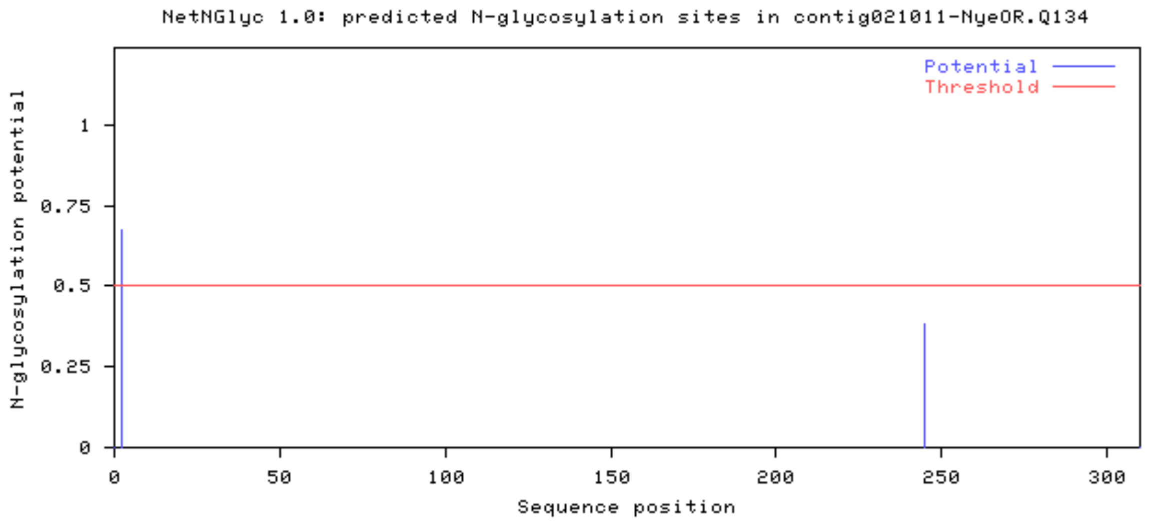

Graphics in PostScript

Output for 'contig021354-NyeOR.O101'

```
##########################################################################################

    Warning: This sequence may not contain a signal peptide!!

    Proteins without signal peptides are unlikely to be exposed to
    the N-glycosylation machinery and thus may not be glycosylated
    (in vivo) even though they contain potential motifs.

    SignalP-NN euk predictions are as follows:

  # name                 Cmax  pos ?  Ymax  pos ?  Smax  pos ?  Smean ?  D     ?

    SignalP output is explained at http://www.cbs.dtu.dk/services/SignalP/output.html

##########################################################################################

Name:  contig021354-NyeOR.O101  Length:  324
MKYTNITTIKEFIIIGFPGLPPEYYGPVSVLLLLVFLAIVIGNGFTIAVIIFERTLHKPIYVIFSNLAMTDICFGVVTLP      80
KIIARYWWNDMITSFGACFTQMYFVHSLGAIQSLNLLMMALDRFVAIWFPFKYPILFTNKAVAIACTMCWVLTFIRLLGI     160
VLLALTLPYCDQNIIMQCYCDLISITRLGCGDGLAYVNSVALANAMFTLLVPLTFIILSYFSVIIAVLRMSQTERRHKVL     240
STCAPQLFITCLFYVPRCFVYLATVLGFNFNLVIRIIITMMYSLIPAAVNPIIYCFKTKDIKNVLMRRFKKGKVSTGLKT     320
ECKX
....N...........................................................................      80
................................................................................     160
................................................................................     240
................................................................................     320
....                                                                                 400

(Threshold=0.5)
----------------------------------------------------------------------
SeqName      Position  Potential   Jury     N-Glyc
                                 agreement result
----------------------------------------------------------------------
contig021354-NyeOR.O101    5 NITT   0.7654     (9/9)    +++
----------------------------------------------------------------------
```

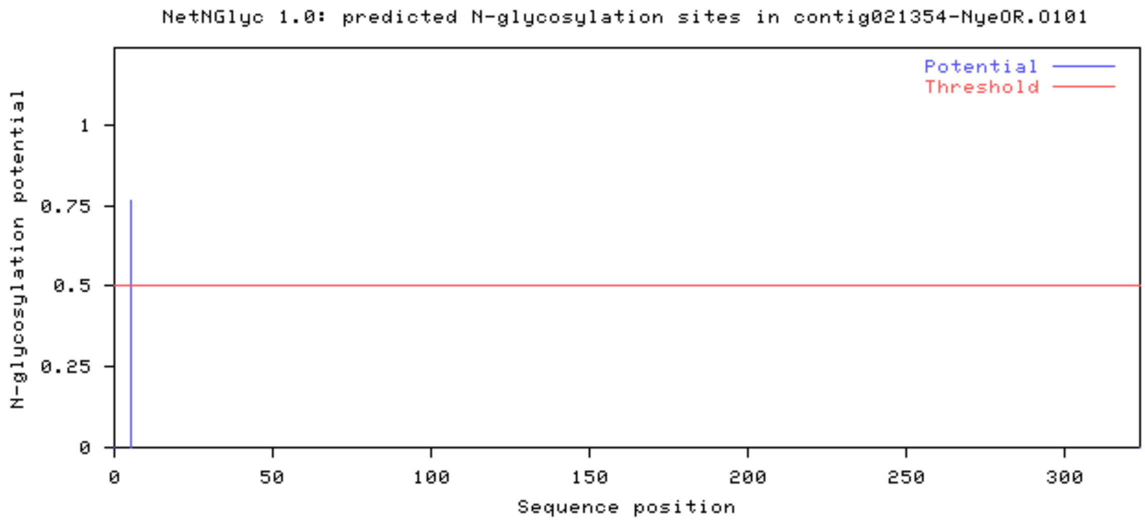

Graphics in PostScript

Output for 'contig021354-NyeORe.O104'

```
##########################################################################################

    Warning: This sequence may not contain a signal peptide!!

    Proteins without signal peptides are unlikely to be exposed to
    the N-glycosylation machinery and thus may not be glycosylated
    (in vivo) even though they contain potential motifs.

    SignalP-NN euk predictions are as follows:

  # name                    Cmax   pos ?  Ymax   pos ?  Smax   pos ?  Smean ?  D      ?

    SignalP output is explained at http://www.cbs.dtu.dk/services/SignalP/output.html

##########################################################################################

Name:  contig021354-NyeORe.O104         Length:  56
NFNLVIRIIITMMYSLIPAAVNPIIYCFKTKEIKNVLMRRFKKGKVSTGLKTECKX
........................................................                              80

(Threshold=0.5)

No sites predicted in this sequence.
```

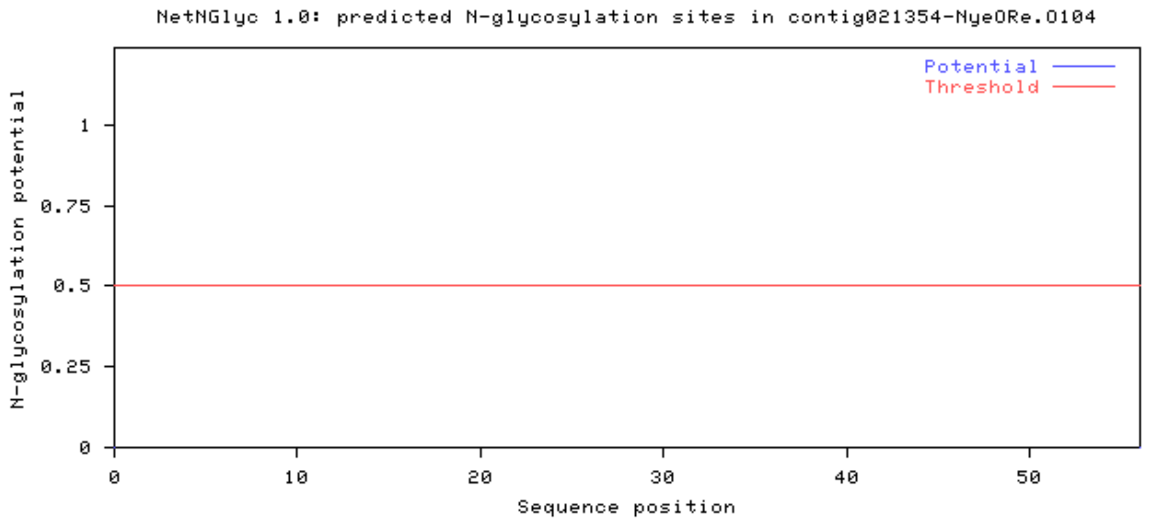

Graphics in PostScript

Output for 'contig021359-NyeOR.O102'

```
##########################################################################################

    Warning: This sequence may not contain a signal peptide!!

    Proteins without signal peptides are unlikely to be exposed to
    the N-glycosylation machinery and thus may not be glycosylated
    (in vivo) even though they contain potential motifs.

    SignalP-NN euk predictions are as follows:

  # name                    Cmax  pos ?  Ymax  pos ?  Smax  pos ?  Smean ?  D     ?

    SignalP output is explained at http://www.cbs.dtu.dk/services/SignalP/output.html

##########################################################################################

Name:  contig021359-NyeOR.O102  Length:  331
MPVENHSSVTEFVLTGFPGLHQEYYGLVSAVLFFVYLITLIANATVIFLFATNHSLHKPMYFIILNLSVCDILFSTTTLP      80
KIISRYWFQSGSISFTACFIQMYFVHYFGTAVAYILFQMALDRYLAICHPLRYSHILTKSNILILSITAWIIAKASPLMM     160
VIRAYPLPYCASNIITHCFCDHIGITVLACTDRTPYAIPAFVFAMVVLLGPLAFIIFSYGSILIAVYKIANVQSRMKSLS     240
TCSTQLIIISLYFLPRCFVYLAQNVGITFSADVRIVIIMLYSLAPPMINPLIYCLRAKDMRESLLKQFCRRIVPEKAKVA     320
AISNSLKTSPX
....N.....................................N.........N............N..............      80
................................................................................     160
................................................................................     240
................................................................................     320
...........                                                                          400

(Threshold=0.5)
----------------------------------------------------------------------
SeqName        Position  Potential   Jury    N-Glyc
                                   agreement result
----------------------------------------------------------------------
contig021359-NyeOR.O102    5 NHSS    0.5881     (7/9)   +
contig021359-NyeOR.O102   43 NATV    0.7127     (8/9)   +
contig021359-NyeOR.O102   53 NHSL    0.6481     (9/9)   ++
contig021359-NyeOR.O102   66 NLSV    0.7707     (9/9)   +++
----------------------------------------------------------------------
```

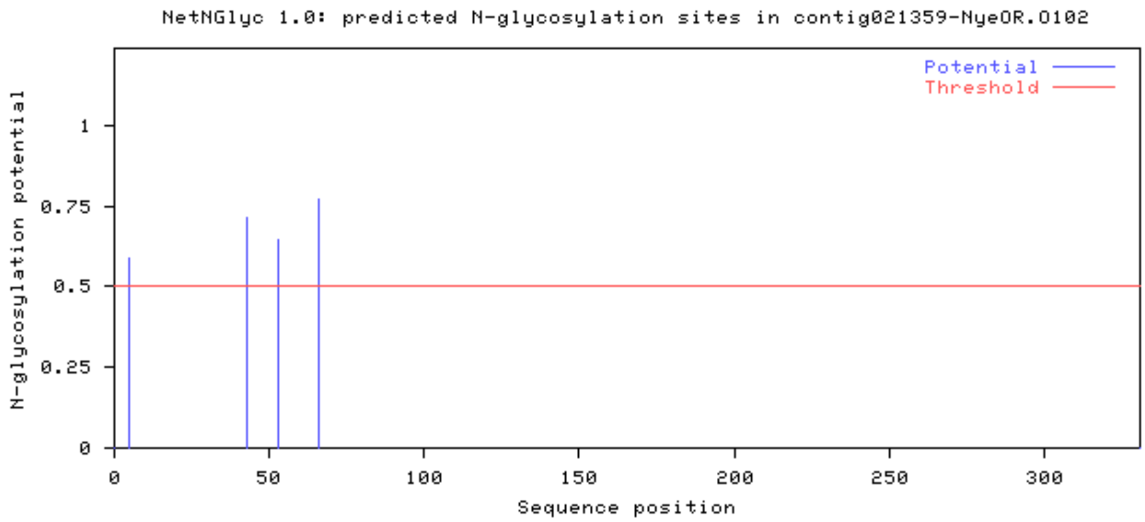

Graphics in PostScript

Output for 'contig023268-NyeOR.P118'

##

Warning: This sequence may not contain a signal peptide!!

Proteins without signal peptides are unlikely to be exposed to
the N-glycosylation machinery and thus may not be glycosylated
(in vivo) even though they contain potential motifs.

SignalP-NN euk predictions are as follows:

\# name Cmax pos ? Ymax pos ? Smax pos ? Smean ? D ?

SignalP output is explained at http://www.cbs.dtu.dk/services/SignalP/output.html

##

```
Name:  contig023268-NyeOR.P118  Length:  332
MLEAPLSRNFSHCTFVLRGFPSLQKHRRLLALPFSASYLLVLLGNSLLVYVICSVERLHSPMYLLICTLCFVDILVVTTI      80
IPNMLLGFLFDWNEISLVGCLTQMFFIHFLSSVESTLLLAMALDRYFAICRPLRYNDIINSSMLVRLVLFTLVRSVSVMA     160
TLVGLAGSLQFCGSNVIQHCYCDHMALVSLACDSTSRSSAAGLAVIICFVGADIPIIFFSYMKILSVVLRSAAAGEDSRK     240
AFHTCSTHLIVMMCFYLVGSITFLSHNLNIPIPTDINNSMGLMYILFPATINPIIYGVRTKEIRDSFLRIFKNRAKKIMT     320
AKVSSAGKEKSX
........N.......................................................................      80
................................................................................     160
................................................................................     240
................................................................................     320
............                                                                         400
```

(Threshold=0.5)

SeqName	Position	Potential	Jury agreement	N-Glyc result
contig023268-NyeOR.P118	9 NFSH	0.6081	(9/9)	++
contig023268-NyeOR.P118	140 NSSM	0.4438	(7/9)	-
contig023268-NyeOR.P118	277 NNSM	0.2284	(9/9)	---

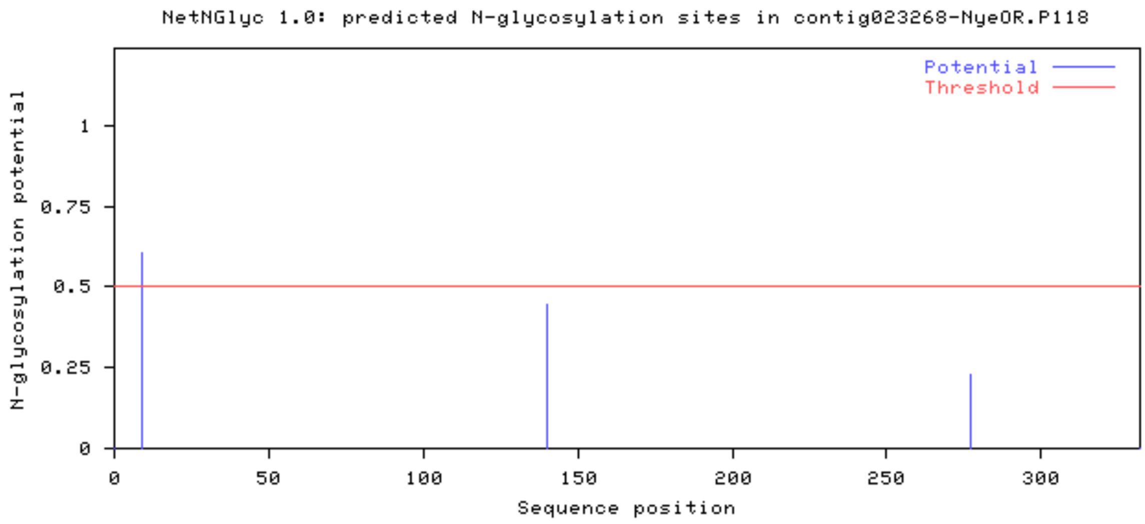

Graphics in PostScript

Output for 'contig023275-NyeORe.P119'

```
##########################################################################################

    Warning: This sequence may not contain a signal peptide!!

    Proteins without signal peptides are unlikely to be exposed to
    the N-glycosylation machinery and thus may not be glycosylated
    (in vivo) even though they contain potential motifs.

    SignalP-NN euk predictions are as follows:

  # name                Cmax  pos ?  Ymax  pos ?  Smax  pos ?  Smean ?  D     ?

    SignalP output is explained at http://www.cbs.dtu.dk/services/SignalP/output.html

##########################################################################################

Name:  contig023275-NyeORe.P119         Length:  223
LVQMHFIHFFGTFQSTLLVWMALDRYFAICTPLYYHNHMILSRFIAFLIPLVVRNVLMITLLVCLAGKLPFCLRNVINHC      80
FCEHMALVELACGSTTINNLVGLMAVFLIPVLDFVFIAASYVVIFSSVLKSSRSGVKALHTCITHIMVITVSLILALTAF     160
LSYRIRNGLPAASRVFFSTMYLLFPSCFNPVVYGIRTNEIRQHILKRLTCCHICQMGPVTNKX
................................................................................      80
................................................................................     160
...............................................................                      240

(Threshold=0.5)

No sites predicted in this sequence.
```

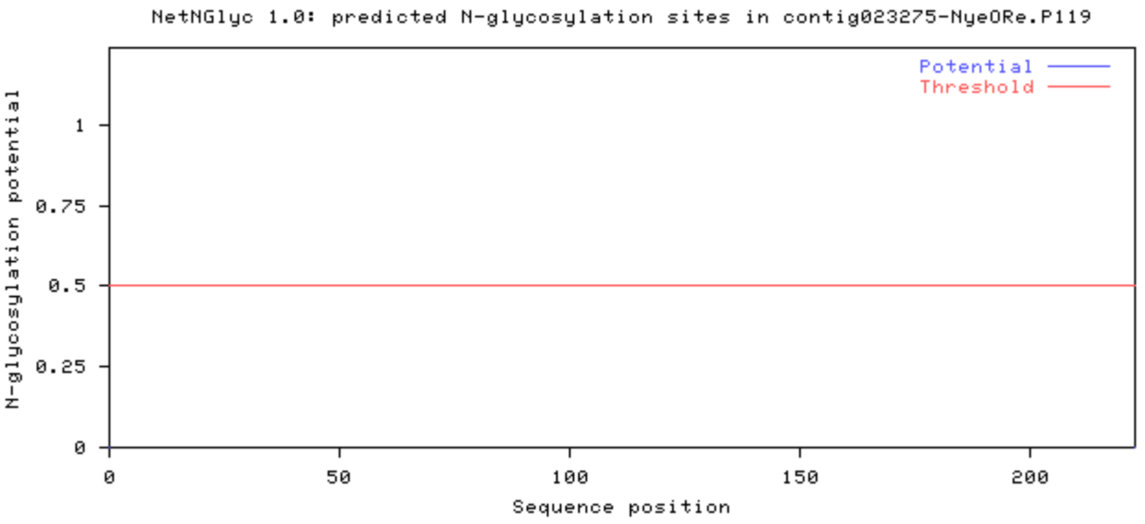

Graphics in PostScript

Output for 'contig023280-NyeOR.E050'

```
##########################################################################################

    Warning: This sequence may not contain a signal peptide!!

    Proteins without signal peptides are unlikely to be exposed to
    the N-glycosylation machinery and thus may not be glycosylated
    (in vivo) even though they contain potential motifs.

    SignalP-NN euk predictions are as follows:

  # name                  Cmax  pos ?  Ymax  pos ?  Smax  pos ?  Smean ?  D     ?

    SignalP output is explained at http://www.cbs.dtu.dk/services/SignalP/output.html

##########################################################################################

Name:  contig023280-NyeOR.E050  Length:  310
MKNSTHLPDFILGAYFDGGTFRYLYFIIVMSLYVFIFGSNVLLIVVICVNKSLHEPMYMFLCSLFVNELYGSTGLFPSLL      80
VQILSDVHTVSAPLCFLQVFSVYSYGSIEFLNLAVMSYDRYLAICCPLQYNELMTSNKVTKLIVAVWSPPLLVNFLTLPL     160
IVPLKRCGNIINKVYCDNHSIVKLACSDTTANNIYGLTVSALSVFGPLIVILYTYMRILKVCFSGSKQTRQKAVSTCTPH     240
LASLLNFSFGACFEILQSRFNMNSSPNMLRIFLSLYFLTCPPLFNPLMYGLNLSKIRVTCKNLITHIICX
..N..............................................N..............................      80
................................................................................     160
................................................................................     240
...................................................N..................               320

(Threshold=0.5)
----------------------------------------------------------------------
SeqName      Position  Potential   Jury    N-Glyc
                                 agreement result
----------------------------------------------------------------------
contig023280-NyeOR.E050    3 NSTH   0.5551     (5/9)   +
contig023280-NyeOR.E050   50 NKSL   0.7630     (9/9)   +++
contig023280-NyeOR.E050  178 NHSI   0.4336     (7/9)   -
contig023280-NyeOR.E050  246 NFSF   0.4126     (7/9)   -
contig023280-NyeOR.E050  263 NSSP   0.1307     (9/9)   ---
contig023280-NyeOR.E050  292 NLSK   0.6554     (8/9)   +
----------------------------------------------------------------------
```

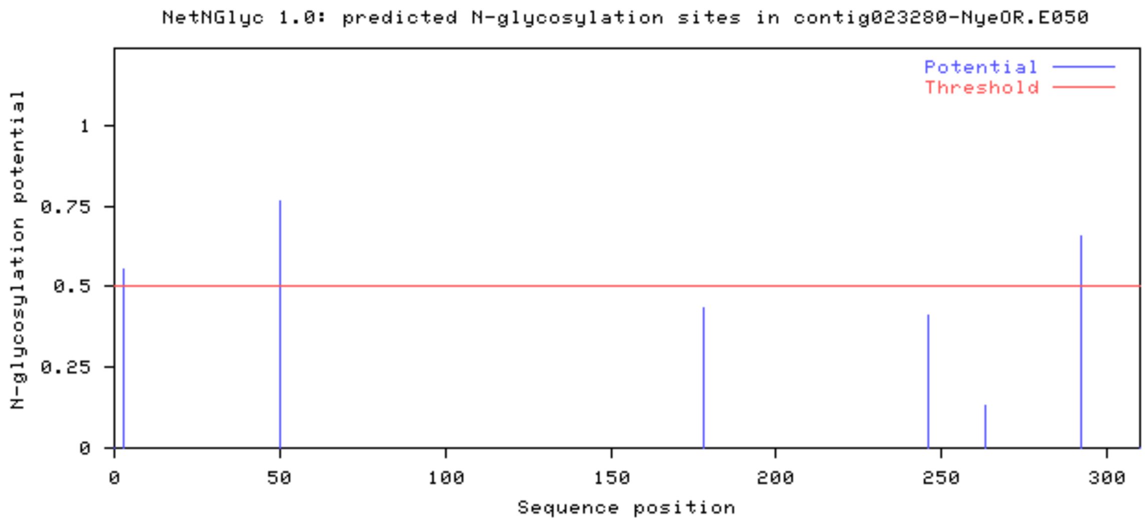

Graphics in PostScript

Output for 'contig023281-NyeORe.P120'

```
##########################################################################################

    Warning: This sequence may not contain a signal peptide!!

    Proteins without signal peptides are unlikely to be exposed to
    the N-glycosylation machinery and thus may not be glycosylated
    (in vivo) even though they contain potential motifs.

    SignalP-NN euk predictions are as follows:

  # name                Cmax  pos ?  Ymax  pos ?  Smax  pos ?  Smean ?  D     ?

    SignalP output is explained at http://www.cbs.dtu.dk/services/SignalP/output.html

##########################################################################################

Name:  contig023281-NyeORe.P120         Length:  69
MENVSLHTHFTLDGFSELGELRPFLFIPFSFMFVVSLFANSLLVYVIVSQRSLHSPMYILIASMACIDL
..N..................................................................                 80

(Threshold=0.5)
----------------------------------------------------------------------
SeqName      Position  Potential   Jury    N-Glyc
                                 agreement result
----------------------------------------------------------------------
contig023281-NyeORe.P120    3 NVSL   0.7700     (9/9)   +++
----------------------------------------------------------------------
```

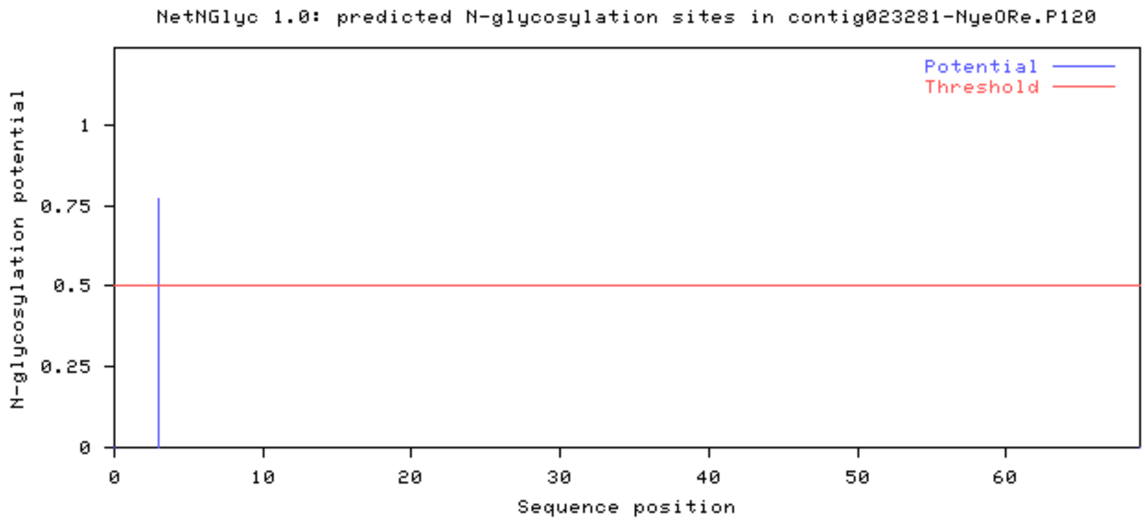

Graphics in PostScript

Output for 'contig023285-NyeORep.E061'

```
##########################################################################################

    Warning: This sequence may not contain a signal peptide!!

    Proteins without signal peptides are unlikely to be exposed to
    the N-glycosylation machinery and thus may not be glycosylated
    (in vivo) even though they contain potential motifs.

    SignalP-NN euk predictions are as follows:

  # name                    Cmax  pos ?  Ymax  pos ?  Smax  pos ?  Smean ?  D     ?

    SignalP output is explained at http://www.cbs.dtu.dk/services/SignalP/output.html

##########################################################################################

Name:  contig023285-NyeORep.E061        Length:  207
MFPFLLLQILSDVHTVSTSICFLQVFCVHIYGAVEYLNLAIMSYDRYLAIRCPLQYNTDMTKKIAILIAATWFYPCFAMV      80
VLLSLTSPLQFCGNTIYKVYCDTHSVVKLACSDTTVINLYGLLATFSMIFGALLFILYTYMKILLVCFSGSDQTRQKAIS     160
TCTPHLASILNFSFGASLNRPIEPFSVNIGTTTSLSPTKVQKDKMLX
................................................................................      80
................................................................................     160
...............................................                                      240

(Threshold=0.5)

No sites predicted in this sequence.
```

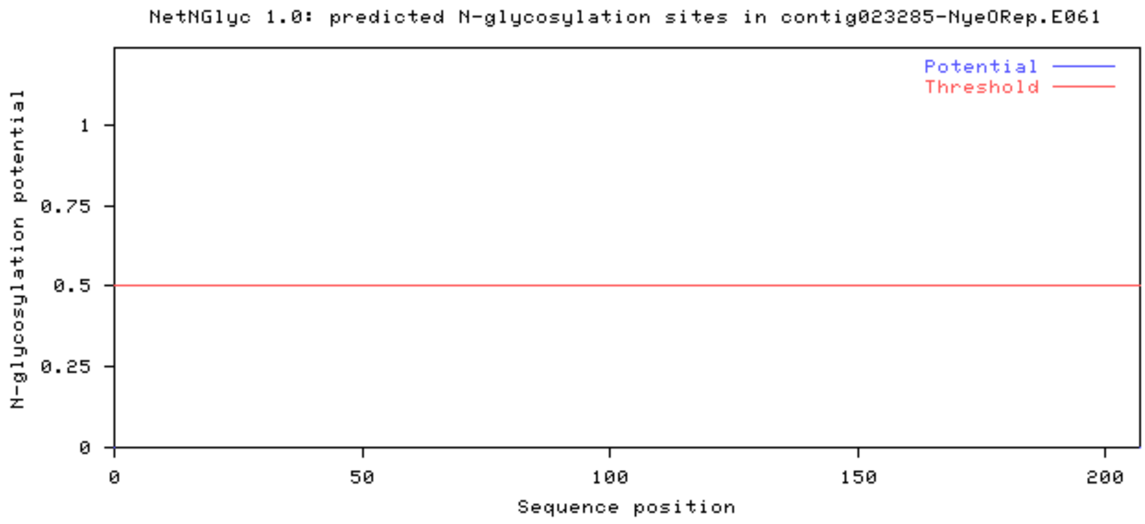

Graphics in PostScript

Output for 'contig029998-NYEORe.AC146'

```
##########################################################################################

    Warning: This sequence may not contain a signal peptide!!

    Proteins without signal peptides are unlikely to be exposed to
    the N-glycosylation machinery and thus may not be glycosylated
    (in vivo) even though they contain potential motifs.

    SignalP-NN euk predictions are as follows:

  # name                 Cmax  pos ?  Ymax  pos ?  Smax  pos ?  Smean ?  D     ?

    SignalP output is explained at http://www.cbs.dtu.dk/services/SignalP/output.html

##########################################################################################

Name:  contig029998-NYEORe.AC146        Length:  172
PGSNVKKWFGQTQQRLCVTAFTLIQINICIIWLTTSPPFPFKNFKEIKDKITLECALGSVVGFWAVLGYIGLLAILCFIF      80
AFLARKLPDNFNEAKFITFSMLIFCAVWITFVPAYVSSPGKFSVAVEIFAILASSFGLLICIFIPKCYIILMKPEKNTKK     160
HMMGKKAPNSLX
................................................................................      80
................................................................................     160
............                                                                         240

(Threshold=0.5)

No sites predicted in this sequence.
```

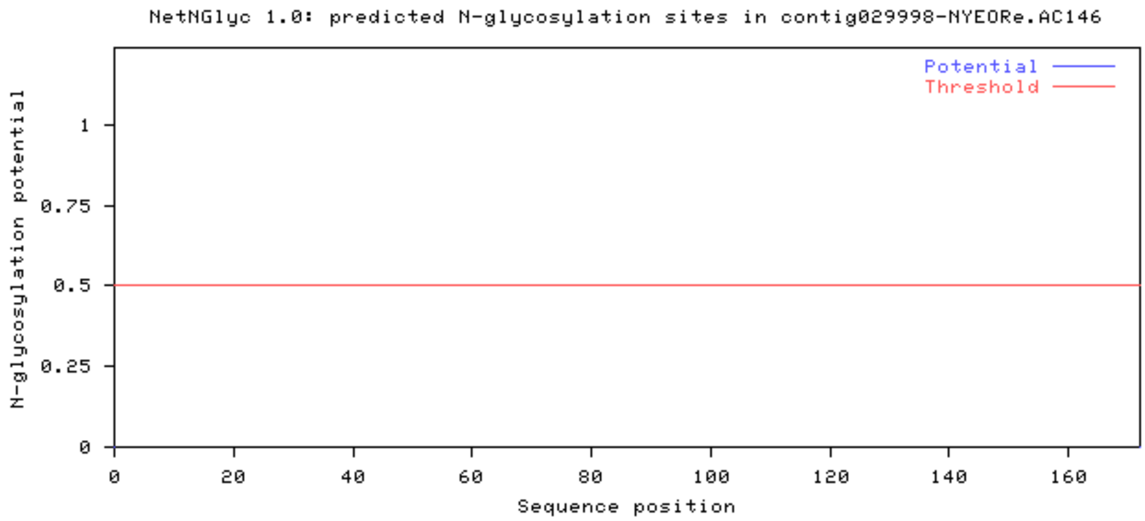

Graphics in PostScript

Output for 'contig034981-NyeOR.A001'

```
##########################################################################################

    Warning: This sequence may not contain a signal peptide!!

    Proteins without signal peptides are unlikely to be exposed to
    the N-glycosylation machinery and thus may not be glycosylated
    (in vivo) even though they contain potential motifs.

    SignalP-NN euk predictions are as follows:

  # name                 Cmax  pos ?  Ymax  pos ?  Smax  pos ?  Smean ?  D     ?

    SignalP output is explained at http://www.cbs.dtu.dk/services/SignalP/output.html

##########################################################################################

Name:  contig034981-NyeOR.A001  Length:  334
MDQELNFTYVTLDWYVGINKYRYVFFFIMFALYSLIICTNSTIMYIICIHKNLHEPMYIFIAALLLNSVLYSTTIYPKLL      80
IDFLSEKQVTTYSACLFQFFMFYTLGGSEFFLLAAMAYDRYVAICKPLQYHIIMRKTTVSIFLIIAWLVPACHIAVQAIG     160
SANIKLCDFNIKGIFCNNAVYTLLCERSRLITIFGVVALLDLAVLPMLFIVFTYTKIFIVSYQRCKEIQKKAAETCLPHL     240
LVLISASVFFVYDVSIARVETNFPKTVRIVMTLQVVLYHPIFNPFIYGLKMKKISKHLKRFFSRPQSILVLKVNAQYNIA     320
VIFWLQLLKTLFIX
.....N.................................N........................................      80
................................................................................     160
................................................................................     240
................................................................................     320
..............                                                                       400

(Threshold=0.5)
----------------------------------------------------------------------
SeqName      Position  Potential   Jury    N-Glyc
                                 agreement result
----------------------------------------------------------------------
contig034981-NyeOR.A001    6 NFTY   0.7620     (9/9)    +++
contig034981-NyeOR.A001   40 NSTI   0.7284     (9/9)    ++
----------------------------------------------------------------------
```

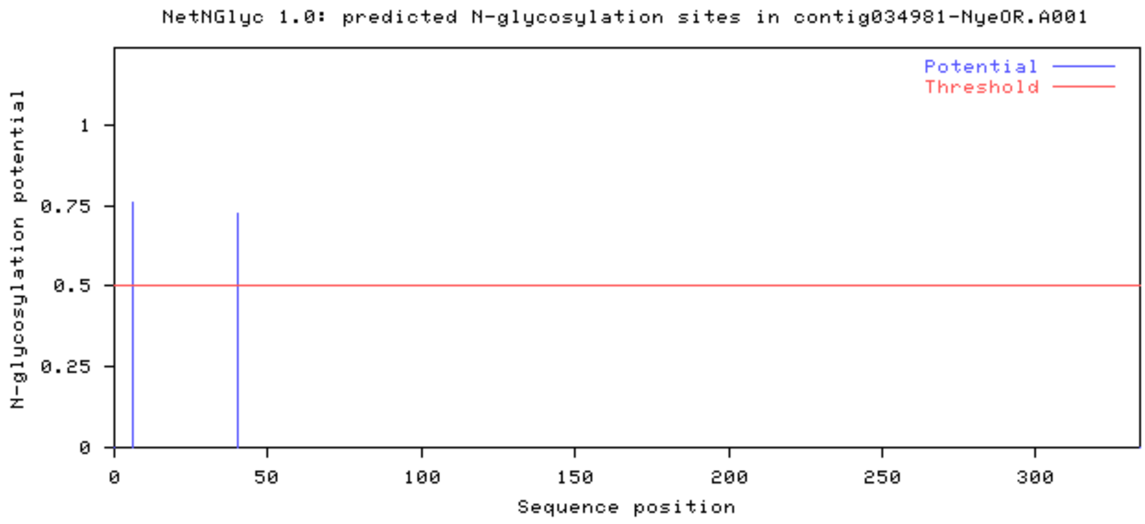

Graphics in PostScript

Output for 'contig034983-NyeOR.A002'

```
##########################################################################################

    Warning: This sequence may not contain a signal peptide!!

    Proteins without signal peptides are unlikely to be exposed to
    the N-glycosylation machinery and thus may not be glycosylated
    (in vivo) even though they contain potential motifs.

    SignalP-NN euk predictions are as follows:

  # name                    Cmax  pos ?  Ymax  pos ?  Smax  pos ?  Smean ?  D     ?

    SignalP output is explained at http://www.cbs.dtu.dk/services/SignalP/output.html

##########################################################################################

Name:  contig034983-NyeOR.A002  Length:  300
MNITYITFGGHVEVEKYRYIYFVIMFMVYGLIICSNSTIVWVIIVQKSLHEPMYIFIAALLVNSVVLSTVIYPKLLIDFL      80
SEKQIILYHACLFQVFMFYVLSSSEFLLLSAMAYDRYVSICKPLQYPTIMRRTRVSIFLIMSWFLPAIQIVVPVLRNSIT     160
PLCNFTLKGIFCNNSVNHLYCVTSKELSIYGMVVLFNGALFPMLFILFTYIKIIIVACQSCGNVRKKAAQTCLPHVLVLI     240
NYSCLVTYDMVIVRLESEFPKTARFIMTLQFITYNPLCNPIIYGLKMKEISKNLKRLFSX
.N.................................N............................................      80
................................................................................     160
...N............................................................................     240
N...........................................................                         320

(Threshold=0.5)
----------------------------------------------------------------------
SeqName      Position  Potential   Jury    N-Glyc
                                 agreement result
----------------------------------------------------------------------
contig034983-NyeOR.A002    2 NITY   0.7927     (9/9)   +++
contig034983-NyeOR.A002   36 NSTI   0.7254     (9/9)   ++
contig034983-NyeOR.A002  164 NFTL   0.6559     (9/9)   ++
contig034983-NyeOR.A002  173 NNSV   0.4574     (6/9)   -
contig034983-NyeOR.A002  241 NYSC   0.5290     (6/9)   +
----------------------------------------------------------------------
```

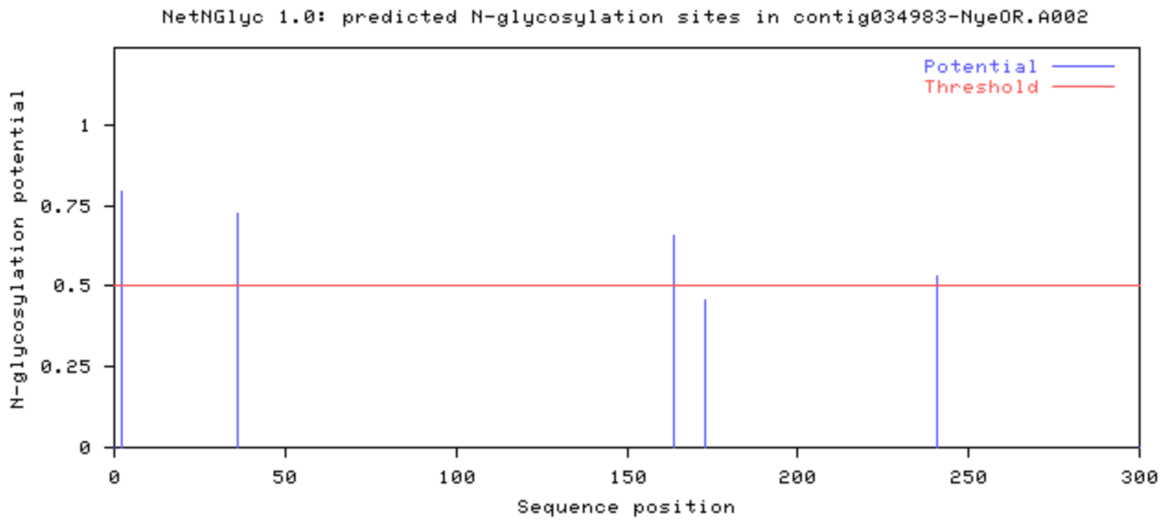

Graphics in PostScript

Output for 'contig034983-NyeOR.A003'

```
##########################################################################################

    Warning: This sequence may not contain a signal peptide!!

    Proteins without signal peptides are unlikely to be exposed to
    the N-glycosylation machinery and thus may not be glycosylated
    (in vivo) even though they contain potential motifs.

    SignalP-NN euk predictions are as follows:

  # name                    Cmax  pos ?  Ymax  pos ?  Smax  pos ?  Smean ?  D     ?

    SignalP output is explained at http://www.cbs.dtu.dk/services/SignalP/output.html

##########################################################################################

Name:  contig034983-NyeOR.A003  Length:  316
MDEVLNATYLTLDGYVEVNKYRYVYFFIFFILYSLIICSNSTIVYIIWIHKNLHEPMYTFIAALLLNCVLYSTTVYPKLL      80
IDFLSERQVTTYSACLFQFFMFYTLGSSEFFLLAAMAYDRYVAICKPLQYQTIMSKTTVSIFLAVAWLVPVCHIAVLTAG     160
SAEATLCNFNLKGIFCNNAVYTLQCVKSRLITVFGVVALIDLVILPMLFIVFTYSNIFILTYQSCKDVRKKALETCLPHL     240
LVLFSFSCLSIYDVSIARVESDFPKTARLIMTLQIVLYHPLLNPFIYGLKMKEISKQLKRFFYHAKIISCINSECX
.....N.................................N........................................      80
................................................................................     160
................................................................................     240
............................................................................         320

(Threshold=0.5)
----------------------------------------------------------------------
SeqName      Position  Potential   Jury    N-Glyc
                                 agreement result
----------------------------------------------------------------------
contig034983-NyeOR.A003    6 NATY   0.6279     (9/9)   ++
contig034983-NyeOR.A003   40 NSTI   0.7253     (9/9)   ++
----------------------------------------------------------------------
```

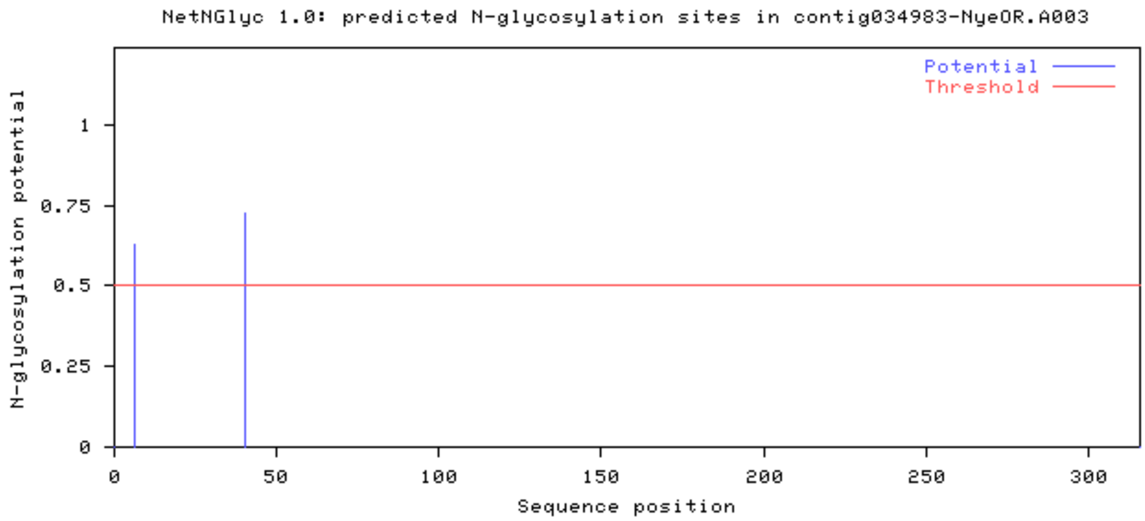

Graphics in PostScript

Output for 'contig034986-NyeORe.A143'

```
##########################################################################################

    Warning: This sequence may not contain a signal peptide!!

    Proteins without signal peptides are unlikely to be exposed to
    the N-glycosylation machinery and thus may not be glycosylated
    (in vivo) even though they contain potential motifs.

    SignalP-NN euk predictions are as follows:

  # name                 Cmax  pos ?  Ymax  pos ?  Smax  pos ?  Smean ?  D     ?

    SignalP output is explained at http://www.cbs.dtu.dk/services/SignalP/output.html

##########################################################################################

Name:  contig034986-NyeORe.A143         Length:  111
MDVKLNVTLLTLGGFAELHKYRYLYFVVIFTLYILILCFNSTIVYLIWTHKNLHEPMYIFIAALLINSVLYSMIIYPKLL      80
SDVLSEKQTISYPLCLFQGFSYYTSAGSEFL
.....N.................................N........................................      80
...............................                                                      160

(Threshold=0.5)
----------------------------------------------------------------------
SeqName      Position  Potential   Jury    N-Glyc
                                agreement result
----------------------------------------------------------------------
contig034986-NyeORe.A143    6 NVTL   0.7748     (9/9)    +++
contig034986-NyeORe.A143   40 NSTI   0.6629     (9/9)    ++
----------------------------------------------------------------------
```

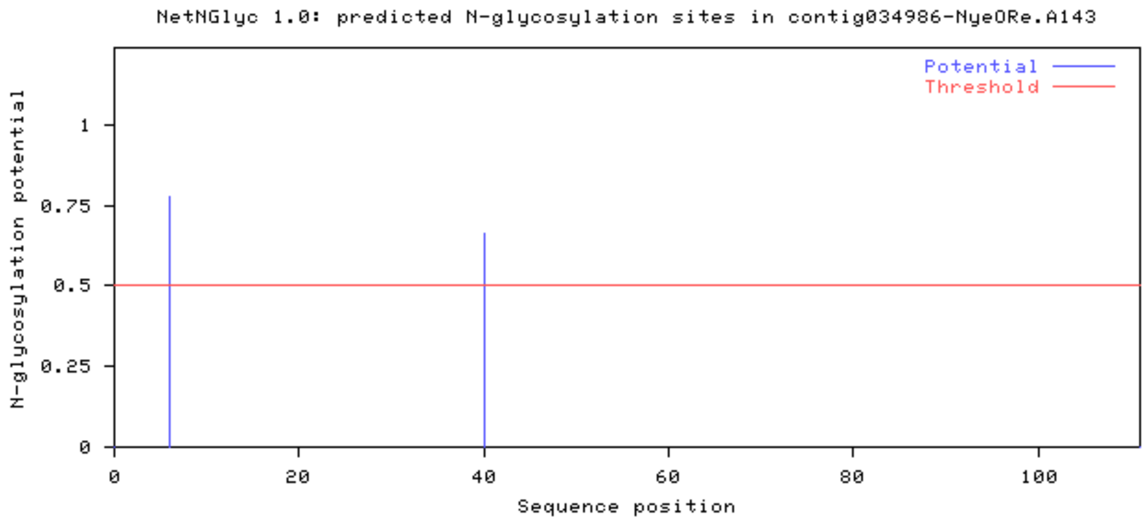

Graphics in PostScript

Output for 'contig034988-NyeOR.A004'

```
##########################################################################################

    Warning: This sequence may not contain a signal peptide!!

    Proteins without signal peptides are unlikely to be exposed to
    the N-glycosylation machinery and thus may not be glycosylated
    (in vivo) even though they contain potential motifs.

    SignalP-NN euk predictions are as follows:

  # name                 Cmax  pos ?  Ymax  pos ?  Smax  pos ?  Smean ?  D     ?

    SignalP output is explained at http://www.cbs.dtu.dk/services/SignalP/output.html

##########################################################################################

Name:  contig034988-NyeOR.A004  Length:  306
MDVELNVTLLTLGGFAELHKYRYLYFVVIFTLYILILCFNSIIVYLIWTCKNLHEPMYIFIAALLINSVLYSMIIYPKLL      80
SDVLSEKQTISYPLCLFQGFSYYTSAVSEFLLLAAMAYDRYVSICKPLQYPVIMNRITIYVCVILAWLIPAFEIAVSFVL     160
YSNVKLCSFTLKAIFCNNSFYRLQCVPSVVISIYGVVMLINMTFLPMLFILFSYIRILRISYSCCRETRRKALKTCLPHL     240
LVLINFSCFFFFDIIIVRLESDLSNTVRLTLTFQSILFHPLLNPIIYGLKVNEIFKHIKMLLCQVX
.....N..........................................................................      80
................................................................................     160
................................................................................     240
....N.............................................................                   320

(Threshold=0.5)
----------------------------------------------------------------------
SeqName      Position  Potential   Jury    N-Glyc
                                 agreement result
----------------------------------------------------------------------
contig034988-NyeOR.A004    6 NVTL    0.7849     (9/9)    +++
contig034988-NyeOR.A004  177 NNSF    0.3941     (9/9)    --
contig034988-NyeOR.A004  201 NMTF    0.4057     (6/9)    -
contig034988-NyeOR.A004  245 NFSC    0.5668     (6/9)    +
----------------------------------------------------------------------
```

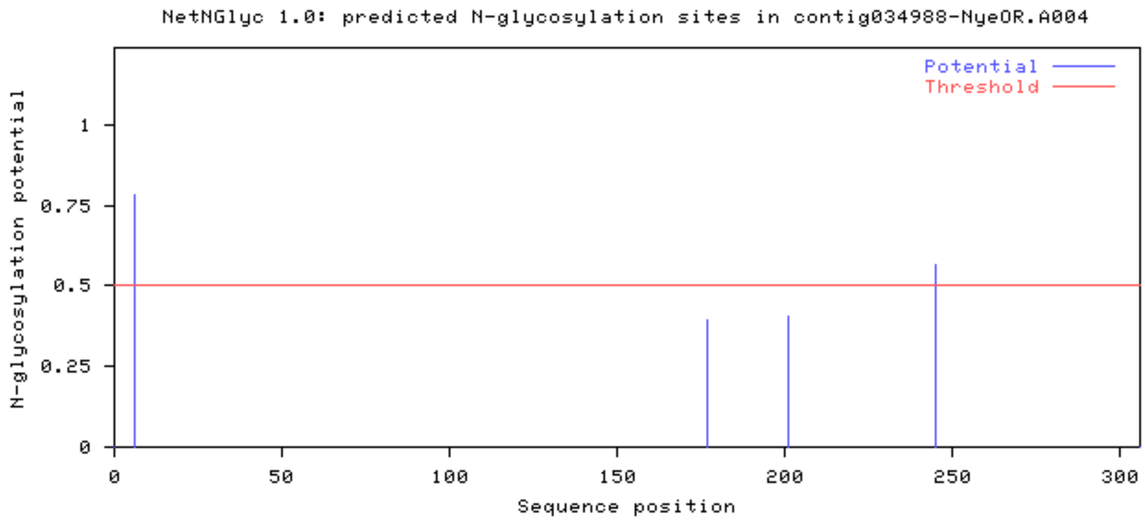

Graphics in PostScript

Output for 'contig034988-NyeOR.A005'

```
##########################################################################################

    Warning: This sequence may not contain a signal peptide!!

    Proteins without signal peptides are unlikely to be exposed to
    the N-glycosylation machinery and thus may not be glycosylated
    (in vivo) even though they contain potential motifs.

    SignalP-NN euk predictions are as follows:

  # name                   Cmax  pos ?  Ymax  pos ?  Smax  pos ?  Smean ?  D     ?

    SignalP output is explained at http://www.cbs.dtu.dk/services/SignalP/output.html

##########################################################################################

Name:  contig034988-NyeOR.A005  Length:  311
MDEELNTTYVTLDGYIEVNKYRYVYFCIIFTLYIIIICSNSTIVYVIWIHKNLHEPMYIFIAALLLNCLLYSTNIYPKLL      80
IDFLSEKQVITYSACLFQFFMFYTLGSSEFFLLAAMAYDRYVAICKPLEYPTIMNKTTVIIFLVVSWLIPAVHIAIQAIG     160
SAEATLCNFNLKGIFCNNAVYTLLCVKSRLIIVFGVVALIDLIILPVLFIVFTYTNIFIISYQSCKEIRKKAAETCLPHL     240
LVLISISCLSIYDVSIARVESDFPKAARLLMTLQIVLYHPLFNPFIYGLKMKEISKQLKRLFCHARIIVYX
.....N.................................N........................................      80
......................................................N.........................     160
................................................................................     240
.......................................................................              320

(Threshold=0.5)
----------------------------------------------------------------------
SeqName      Position  Potential   Jury    N-Glyc
                                 agreement result
----------------------------------------------------------------------
contig034988-NyeOR.A005    6 NTTY    0.7359     (9/9)    ++
contig034988-NyeOR.A005   40 NSTI    0.6933     (8/9)    +
contig034988-NyeOR.A005  135 NKTT    0.6548     (7/9)    +
----------------------------------------------------------------------
```

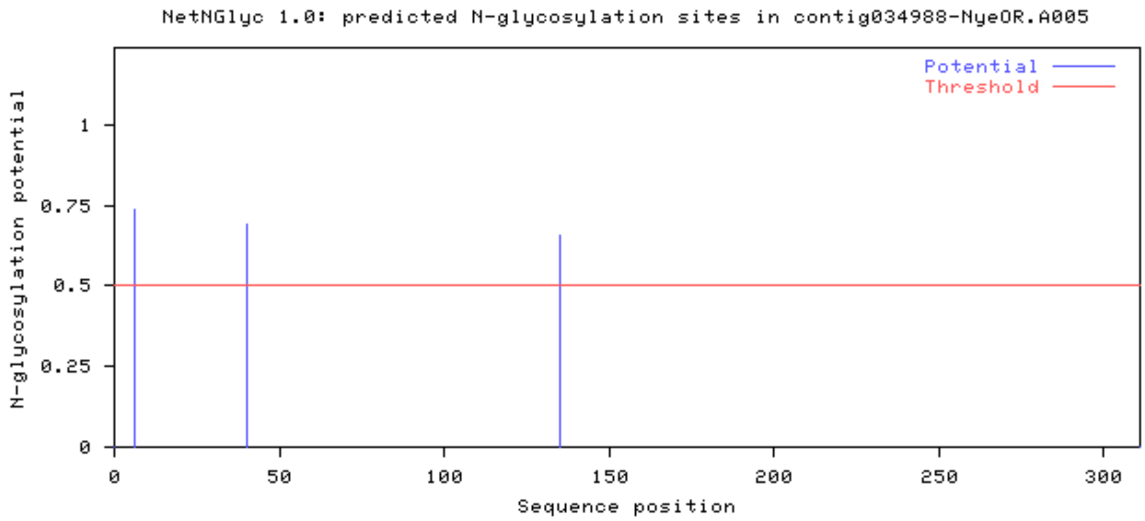

Graphics in PostScript

Output for 'contig034988-NyeOR.A006'

```
##########################################################################################

    Warning: This sequence may not contain a signal peptide!!

    Proteins without signal peptides are unlikely to be exposed to
    the N-glycosylation machinery and thus may not be glycosylated
    (in vivo) even though they contain potential motifs.

    SignalP-NN euk predictions are as follows:

  # name                    Cmax  pos ?  Ymax  pos ?  Smax  pos ?  Smean ?  D     ?

    SignalP output is explained at http://www.cbs.dtu.dk/services/SignalP/output.html

##########################################################################################

Name:  contig034988-NyeOR.A006  Length:  312
MLVQFLIFTNAEMNLTYITFGGHVEVEKYRYLYFVIMFMVYILIICSNSTIVWLIVVQKSLHEPMYIFIAALLVNSVVLS      80
TVIYPKLLIDFLSEKQIILYQACLFQVFLFYALSCSEFLLLSAMAYDRYVSIYKPLQYPSIMRRTRVNIFLLLCWFLPAI     160
QVAVPIAGNANTPLCNFTLKGIFCNNSVNHLHCVNSRELSIYGMVVLFNVALSPMFFILFTYIKIIIVAYQSCGNVRKKA     240
AQTCLPHVLVLMNYSCLLTYDMVIVRLESEFPKTARFIMTLQFVTYNPLCNPIIYGLKMKEISKHLKILFSX
.............N.................................N................................      80
................................................................................     160
...............N................................................................     240
............N...........................................................             320

(Threshold=0.5)
----------------------------------------------------------------------
SeqName      Position  Potential   Jury    N-Glyc
                                 agreement result
----------------------------------------------------------------------
contig034988-NyeOR.A006   14 NLTY   0.7740     (9/9)   +++
contig034988-NyeOR.A006   48 NSTI   0.6849     (8/9)   +
contig034988-NyeOR.A006  176 NFTL   0.6469     (9/9)   ++
contig034988-NyeOR.A006  185 NNSV   0.4843     (5/9)   -
contig034988-NyeOR.A006  253 NYSC   0.5416     (5/9)   +
----------------------------------------------------------------------
```

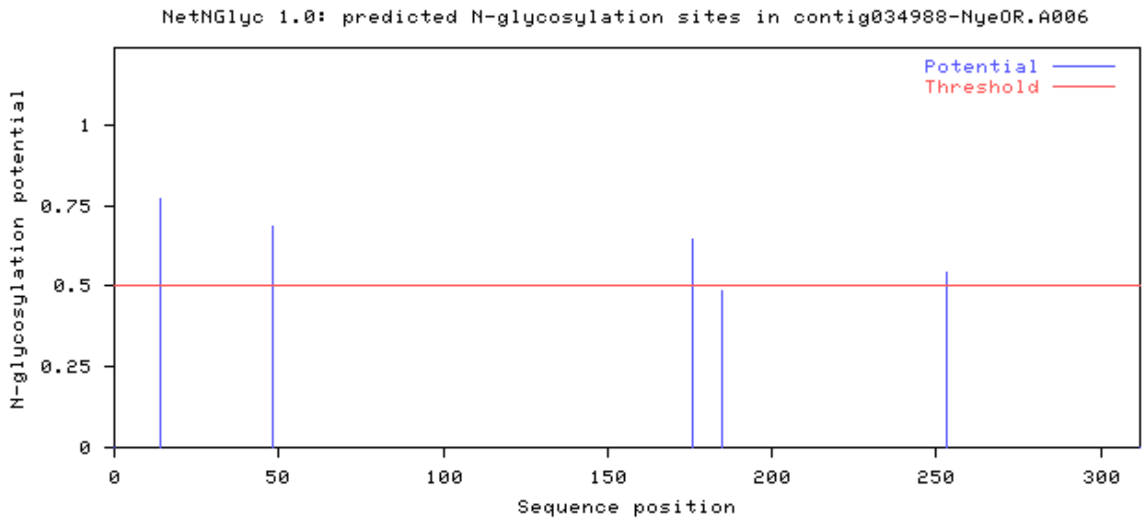

Graphics in PostScript

Output for 'contig034988-NyeORs.A033'

```
##########################################################################################

    Warning: This sequence may not contain a signal peptide!!

    Proteins without signal peptides are unlikely to be exposed to
    the N-glycosylation machinery and thus may not be glycosylated
    (in vivo) even though they contain potential motifs.

    SignalP-NN euk predictions are as follows:

  # name                    Cmax  pos ?  Ymax  pos ?  Smax  pos ?  Smean ?  D     ?

    SignalP output is explained at http://www.cbs.dtu.dk/services/SignalP/output.html

##########################################################################################

Name:  contig034988-NyeORs.A033         Length:  304
MDNKLNLTYITLNGYVEVEKYRYVYFLIIFTIYAAVIFSNSTIIRLIVFHQSLHEPMYIFIAVLLINSTFFCTTIYPKFL      80
LDVLSEKQIISHTMCHFQYFVLYTSGASEFLVLAAMAYDRYVSICKPLQYSVIMKKTTISVFLVLAWLVPACQVAGTTSL     160
SATRKVCNFTLQGIFCNKSVYKLHCVSSRVLAIYGVNVLLNIVFFPMLYIVFTYTKILIISYQSCREVRKKATQTCLPHL     240
LVLFNYSFFCTCEVIILRLESDISQTVRLIMTLQVVLYHPLFNPIIYGLKMKYISKHLKRLFCX
.....N.................................N..........................N.............      80
................................................................................     160
.......N........N...............................................................     240
....N...........................................................                     320

(Threshold=0.5)
----------------------------------------------------------------------
SeqName      Position  Potential   Jury    N-Glyc
                                 agreement result
----------------------------------------------------------------------
contig034988-NyeORs.A033    6 NLTY   0.7541     (9/9)   +++
contig034988-NyeORs.A033   40 NSTI   0.6632     (9/9)   ++
contig034988-NyeORs.A033   67 NSTF   0.5713     (8/9)   +
contig034988-NyeORs.A033  168 NFTL   0.6796     (9/9)   ++
contig034988-NyeORs.A033  177 NKSV   0.5616     (7/9)   +
contig034988-NyeORs.A033  245 NYSF   0.5490     (5/9)   +
----------------------------------------------------------------------
```

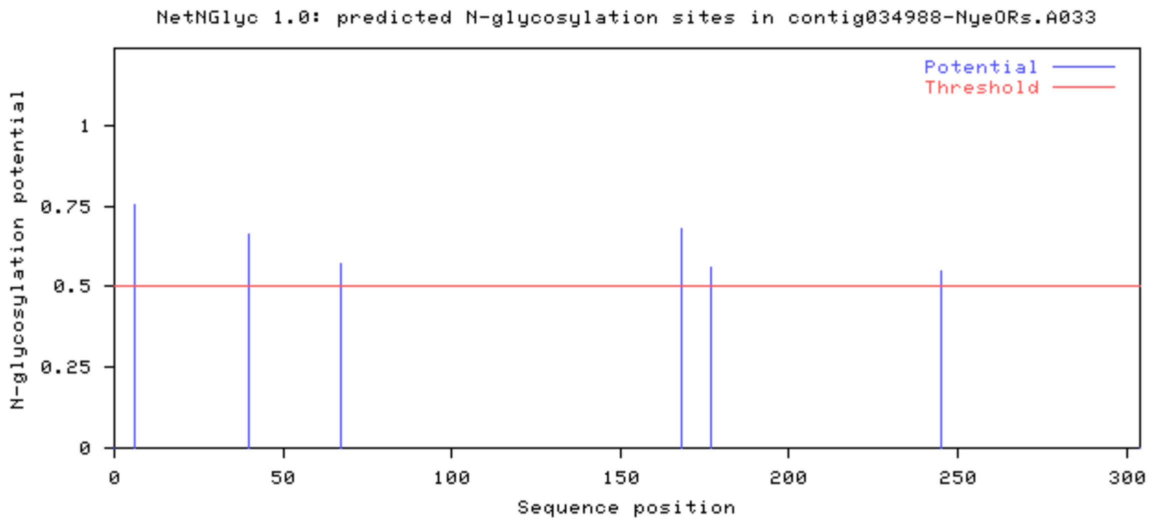

Graphics in PostScript

Output for 'contig034989-NyeORe.A021'

```
##########################################################################################

    Warning: This sequence may not contain a signal peptide!!

    Proteins without signal peptides are unlikely to be exposed to
    the N-glycosylation machinery and thus may not be glycosylated
    (in vivo) even though they contain potential motifs.

    SignalP-NN euk predictions are as follows:

  # name                Cmax  pos ?  Ymax  pos ?  Smax  pos ?  Smean ?  D     ?

    SignalP output is explained at http://www.cbs.dtu.dk/services/SignalP/output.html

##########################################################################################

Name:  contig034989-NyeORe.A021         Length:  200
YDRYVAICKPLEYPTIMNKTTVIIFLVVSWLIPAVHIAIQAIASAEATLCSFNLKGIFCNNAVYTLHCQRSRLITVFGVV      80
ALLDLVILPMIFIVFTYTTIFIVSYQSCKEIRKKAAETCLPHLLVLISISCLSIYDVGIARVESDFPKVARLLMTLQLLL     160
YHPLFNPFIYGLKMKEISKQLKRLFCHATIITCINANVPX
................N...............................................................      80
................................................................................     160
........................................                                             240

(Threshold=0.5)
----------------------------------------------------------------------
SeqName      Position  Potential   Jury    N-Glyc
                                 agreement result
----------------------------------------------------------------------
contig034989-NyeORe.A021   18 NKTT   0.7038     (8/9)    +
----------------------------------------------------------------------
```

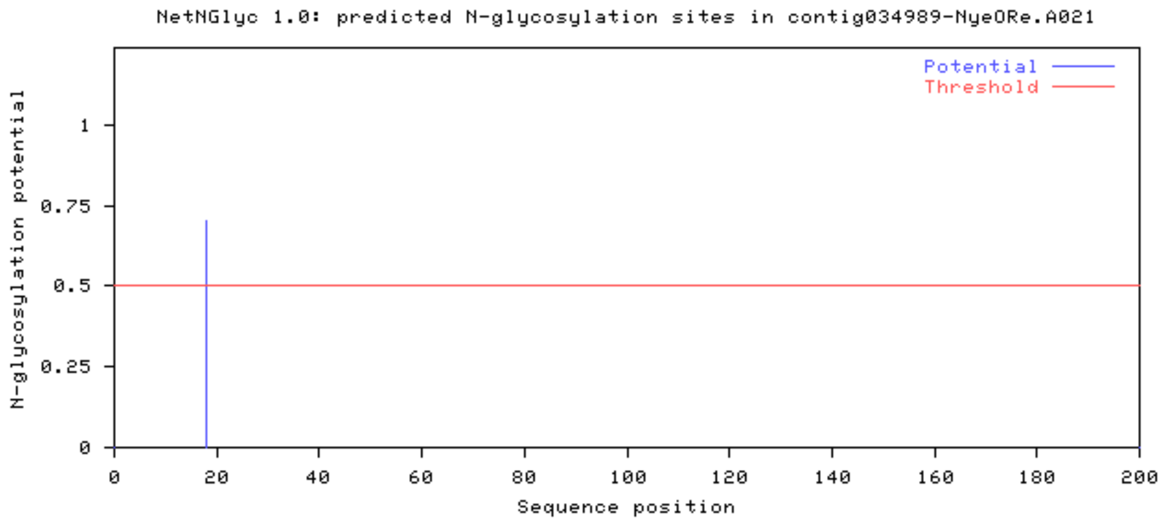

Graphics in PostScript

Output for 'contig034990-NyeOR.A007'

```
##########################################################################################

    Warning: This sequence may not contain a signal peptide!!

    Proteins without signal peptides are unlikely to be exposed to
    the N-glycosylation machinery and thus may not be glycosylated
    (in vivo) even though they contain potential motifs.

    SignalP-NN euk predictions are as follows:

  # name                    Cmax  pos ?  Ymax  pos ?  Smax  pos ?  Smean ?  D     ?

    SignalP output is explained at http://www.cbs.dtu.dk/services/SignalP/output.html

##########################################################################################

Name:  contig034990-NyeOR.A007  Length:  320
MDVELNLTLVTFGGFAELHKYRYLYFVVISTLYILILCFNSTIVCLIWTHKNLHEPMYIFIAALLINSVLYSMIIYPKLL      80
SDVLSEKQMISYPLCLFQGFLYYTSAGSEFLLLAAMAYDRYVSICKPLQYPVIMNRITIYVCLILAWLIPAFETSVLGVL     160
YSNVKLCSFSLTGIFCNNSLYKLQCVPSVAISIYGMVMLINIALLPLLFILFTYIRIIRISYHCCREVRKKAVKTCLPHL     240
LVLINFSCFIFLDIIIVRLDSDLSKTLRLTLTFQSIVFHPLLNPIIYGLKMNEIFKHIKILLSSLITLVLLPYYQMYGIX     320
.....N.................................N........................................      80
................................................................................     160
................................................................................     240
....N...........................................................................     320

(Threshold=0.5)
----------------------------------------------------------------------
SeqName       Position  Potential   Jury    N-Glyc
                                  agreement result
----------------------------------------------------------------------
contig034990-NyeOR.A007    6 NLTL    0.8131     (9/9)   +++
contig034990-NyeOR.A007   40 NSTI    0.7154     (9/9)   ++
contig034990-NyeOR.A007  177 NNSL    0.4251     (7/9)   -
contig034990-NyeOR.A007  245 NFSC    0.5514     (5/9)   +
----------------------------------------------------------------------
```

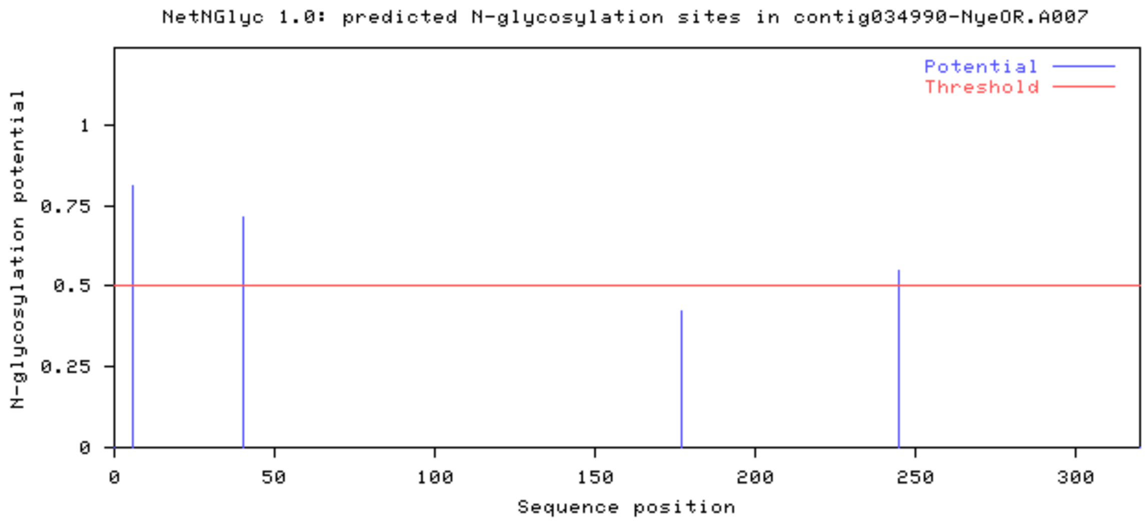

Graphics in PostScript

Output for 'contig034992-NyeORe.A022'

```
##########################################################################################

    Warning: This sequence may not contain a signal peptide!!

    Proteins without signal peptides are unlikely to be exposed to
    the N-glycosylation machinery and thus may not be glycosylated
    (in vivo) even though they contain potential motifs.

    SignalP-NN euk predictions are as follows:

  # name                 Cmax  pos ?  Ymax  pos ?  Smax  pos ?  Smean ?  D     ?

    SignalP output is explained at http://www.cbs.dtu.dk/services/SignalP/output.html

##########################################################################################

Name:  contig034992-NyeORe.A022         Length:  168
YVCLILAWLIPAFEALMLGVLYSNVKLCSFTLTGIFCNNSVHKLQCVPSVAISIYGVVMLINIALLPLLFILFSYIKILK      80
ISYQRCREVRKKAVKTCLPHLLVLIHFSCFISFDIIIVRLETDLSKTLRLILTFELILFHPLLNPIIYGLKMNEISKHLK     160
ILLCLVKX
................................................................................      80
................................................................................     160
........                                                                             240

(Threshold=0.5)

No sites predicted in this sequence.
```

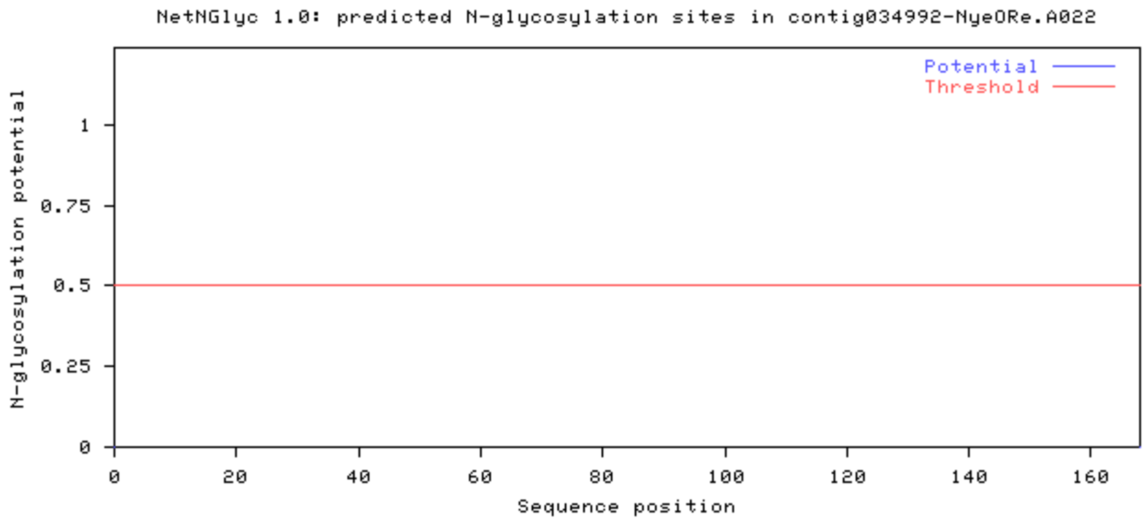

Graphics in PostScript

Output for 'contig034994-NyeOR.A008'

```
########################################################################################

    Warning: This sequence may not contain a signal peptide!!

    Proteins without signal peptides are unlikely to be exposed to
    the N-glycosylation machinery and thus may not be glycosylated
    (in vivo) even though they contain potential motifs.

    SignalP-NN euk predictions are as follows:

  # name                    Cmax  pos ?  Ymax  pos ?  Smax  pos ?  Smean ?  D     ?

    SignalP output is explained at http://www.cbs.dtu.dk/services/SignalP/output.html

########################################################################################

Name:  contig034994-NyeOR.A008  Length:  320
MDVELNVTLLTLGGFAELHKYRYLYFVIIFTLYILILCFNSTIVFLIWTHENLHEPMYIFIAALLINSVLYSMIIYPKLL      80
SDVLSEKQMISYPLCLFQSLSYYTSVGSDFLLLAAMAYDRYVSICKPLQYPFIMNRITIYVCLILAWLIPAFETSVMGVL     160
YSNVKLCSFTLTGIFCNNSLYKLQCVPSVAISIYGVVMLINIAFLPLLFILFTYIRILRISYHCCREVRKKAVKTCLPHL     240
LVLINFSCFIFFDIIIVRLDSDLSKTLRLTLTFQSILFHPLLNPIIYGLKMNEIFKHIKILLSSLITLVLLPYYQMYGIX     320
.....N.................................N........................................      80
................................................................................     160
................................................................................     240
....N...........................................................................     320

(Threshold=0.5)
----------------------------------------------------------------------
SeqName      Position  Potential   Jury    N-Glyc
                                 agreement result
----------------------------------------------------------------------
contig034994-NyeOR.A008    6 NVTL   0.7847     (9/9)   +++
contig034994-NyeOR.A008   40 NSTI   0.7161     (9/9)   ++
contig034994-NyeOR.A008  177 NNSL   0.4224     (7/9)   -
contig034994-NyeOR.A008  245 NFSC   0.5432     (5/9)   +
----------------------------------------------------------------------
```

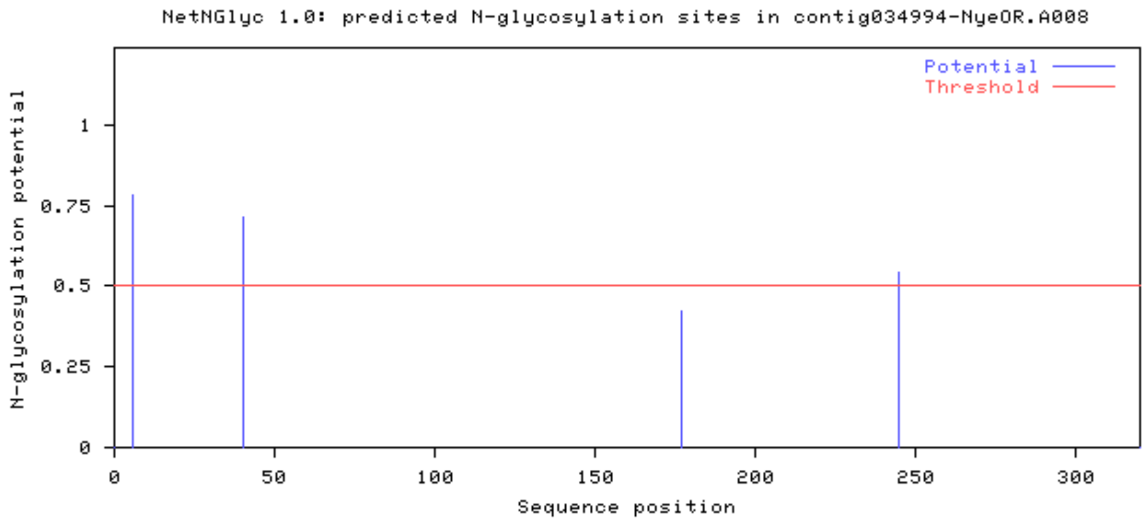

Graphics in PostScript

Output for 'contig034995-NyeOR.A009'

```
##########################################################################################

    Warning: This sequence may not contain a signal peptide!!

    Proteins without signal peptides are unlikely to be exposed to
    the N-glycosylation machinery and thus may not be glycosylated
    (in vivo) even though they contain potential motifs.

    SignalP-NN euk predictions are as follows:

  # name                    Cmax  pos ?  Ymax  pos ?  Smax  pos ?  Smean ?  D     ?

    SignalP output is explained at http://www.cbs.dtu.dk/services/SignalP/output.html

##########################################################################################

Name:  contig034995-NyeOR.A009  Length:  320
MDVELNVTLLTLGGFAELHKYRYLYFVIIFTLYILILCFNSTIVFLIWTHENLHEPMYIFIAALLINSVLYSMIIYPKLL      80
SDVLSEKQMISYPLCLFQGLSYCTSVGSEFLLLAAMAYDRYVSICKPLQYPIIMNRITIYVCLILAWLIPAFETSVLGVL     160
YSNVKLCSFTLTGIFCNTSLYKLQCVPSVAISIYSMVMLINIALLPLLFILFTYIRILRISYHCCREVRKKAVKTCLPHL     240
LVLTNFSCFIFFDIIIVRLDSDLSKTLRLTLTFQSILFHPLLNPIIYGLKMNEIFKHIKILLSSLITLVLLPYYQMYGIX     320
.....N.................................N........................................      80
................................................................................     160
................................................................................     240
....N...........................................................................     320

(Threshold=0.5)
----------------------------------------------------------------------
SeqName      Position  Potential   Jury    N-Glyc
                                 agreement result
----------------------------------------------------------------------
contig034995-NyeOR.A009    6 NVTL    0.7847     (9/9)    +++
contig034995-NyeOR.A009   40 NSTI    0.7163     (9/9)    ++
contig034995-NyeOR.A009  177 NTSL    0.4823     (5/9)    -
contig034995-NyeOR.A009  245 NFSC    0.5731     (5/9)    +
----------------------------------------------------------------------
```

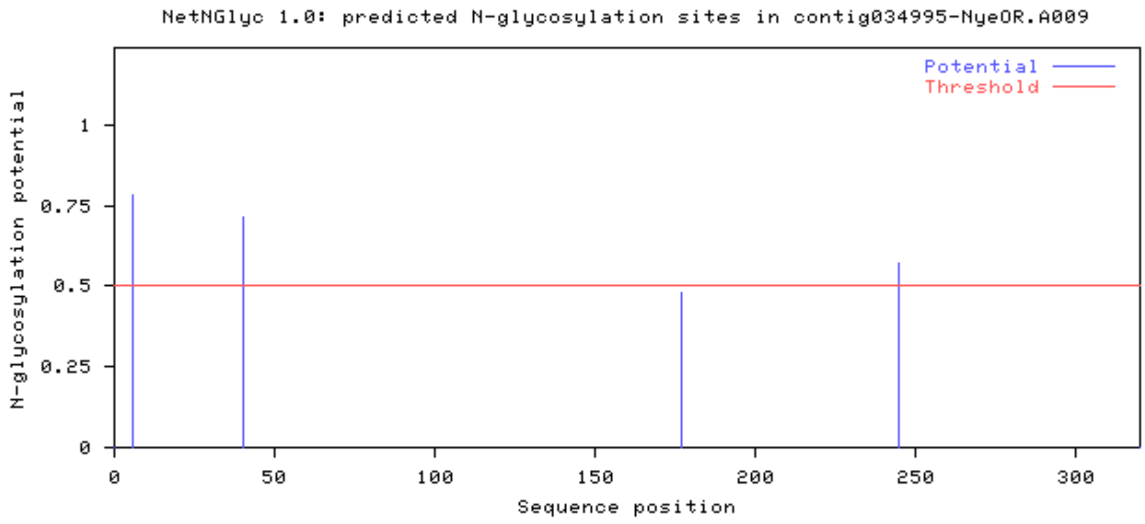

Graphics in PostScript

Output for 'contig034998-NyeOR.H067'

```
########################################################################################

    Warning: This sequence may not contain a signal peptide!!

    Proteins without signal peptides are unlikely to be exposed to
    the N-glycosylation machinery and thus may not be glycosylated
    (in vivo) even though they contain potential motifs.

    SignalP-NN euk predictions are as follows:

  # name                  Cmax  pos ?  Ymax  pos ?  Smax  pos ?  Smean ?  D     ?

    SignalP output is explained at http://www.cbs.dtu.dk/services/SignalP/output.html

########################################################################################

Name:  contig034998-NyeOR.H067  Length:  310
MDNVSTVRIFNLLAFNETANYRAAFFSATLVCYFAIVFLNVTVIMIIVLDESLHEPMYILVCVCCINGLYGSTGFYPKFL      80
IDLLSSSQVISYSECLCQAFVMYSFVCSDTSILAVMAYDRYLAICQPLQYHSVMTKKKLSKLVCFSWLTPFCIFSINIML     160
TDRLIFCGTDIQRLFCVNWLIVKVACPGMDTLVNSAFAYTTLSIYIFHWIFIVWTYIYLVKSCVQSKKDKAKFMQTCVPH     240
LISLVTFFVIVISDLMHMRFASNDVPQSFQNFVAIAVLFIPPVMNPLLYGFKLSKIRNRILVTLHIKRCX
..N............N.......................N........................................      80
................................................................................     160
................................................................................     240
......................................................................               320

(Threshold=0.5)
----------------------------------------------------------------------
SeqName      Position  Potential   Jury    N-Glyc
                                 agreement result
----------------------------------------------------------------------
contig034998-NyeOR.H067    3 NVST   0.7628     (9/9)   +++
contig034998-NyeOR.H067   16 NETA   0.7288     (9/9)   ++
contig034998-NyeOR.H067   40 NVTV   0.7888     (9/9)   +++
----------------------------------------------------------------------
```

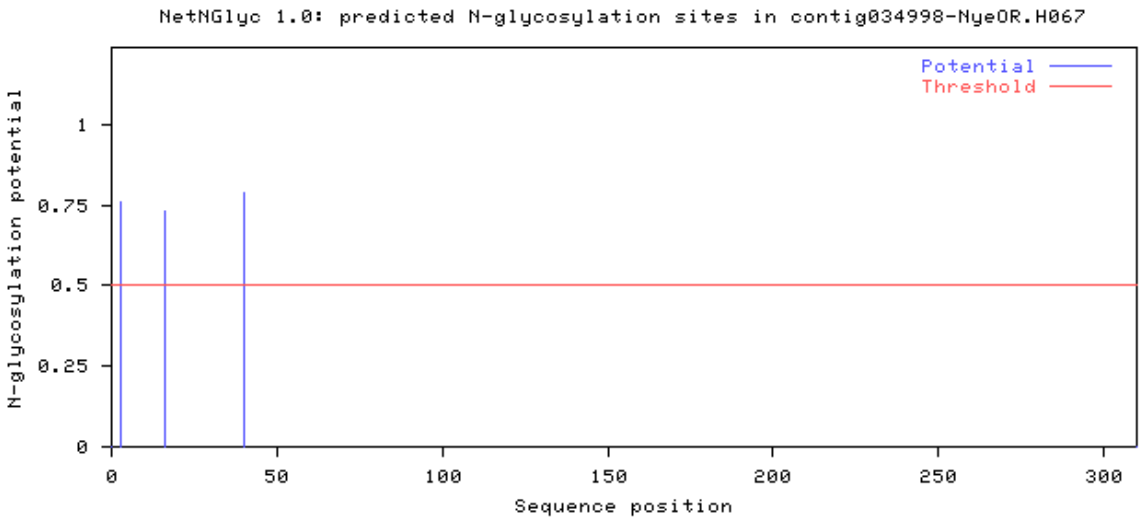

Graphics in PostScript

Output for 'contig035579-NyeOR.H068'

```
##########################################################################################

    Warning: This sequence may not contain a signal peptide!!

    Proteins without signal peptides are unlikely to be exposed to
    the N-glycosylation machinery and thus may not be glycosylated
    (in vivo) even though they contain potential motifs.

    SignalP-NN euk predictions are as follows:

  # name                    Cmax   pos ?  Ymax   pos ?  Smax   pos ?  Smean ?  D     ?

    SignalP output is explained at http://www.cbs.dtu.dk/services/SignalP/output.html

##########################################################################################

Name:  contig035579-NyeOR.H068  Length:  314
MDNVSVITVFTLSGLSDIANYRVILFVLTLLCYCVIWLVNLTIIVTVIVDKKLHEPMYIFLCNLCFNGLYGTAAFYPKFL      80
YDLLSTTHVISYAGCLLQGFVLHSSVCADFSLLVLMAYDRYVAICRPLVYHSLMTTQKICILVFFAWLIPFYLLFMSTIT     160
TAVLRLCGSHIPRIYCVNWLINNLACSASVARIVIPAFSYTFYIGHVLLVFWSYVHLIKTCQSSKDNWNKFMQTCVPHLF     240
SLTVVVLSFLFDMLYMRFGSKEIPQSFENFMAMEIFLIPPIINPLMYGFKLTQIRNRVLNFICGKSSTLRLKSX
..N....................................N........................................      80
................................................................................     160
................................................................................     240
..........................................................................           320

(Threshold=0.5)
----------------------------------------------------------------------
SeqName      Position  Potential   Jury     N-Glyc
                                 agreement result
----------------------------------------------------------------------
contig035579-NyeOR.H068    3 NVSV   0.8138     (9/9)    +++
contig035579-NyeOR.H068   40 NLTI   0.7720     (9/9)    +++
----------------------------------------------------------------------
```

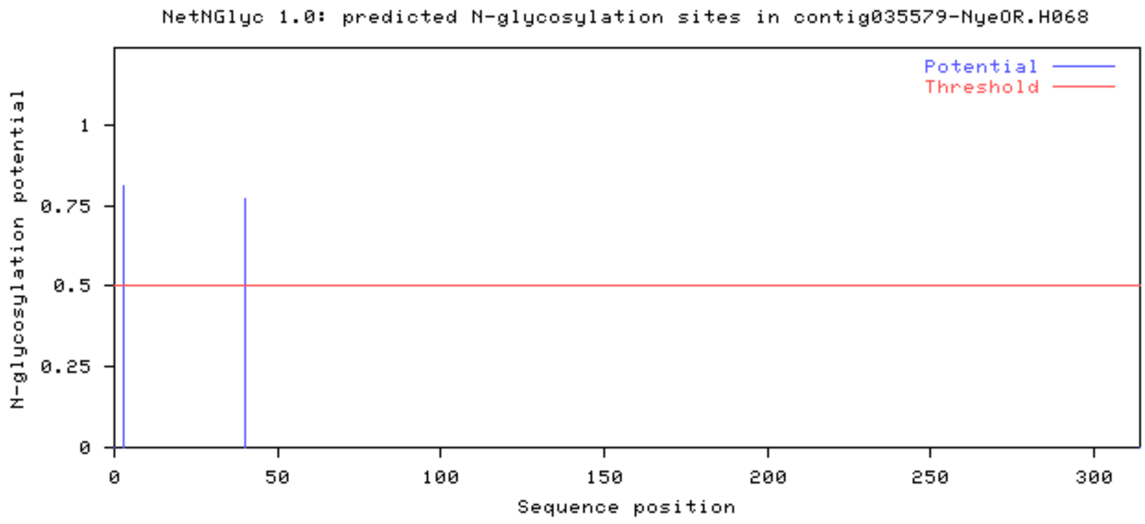

Graphics in PostScript

Output for 'contig035580-NyeOR.H069'

```
##########################################################################################

    Warning: This sequence may not contain a signal peptide!!

    Proteins without signal peptides are unlikely to be exposed to
    the N-glycosylation machinery and thus may not be glycosylated
    (in vivo) even though they contain potential motifs.

    SignalP-NN euk predictions are as follows:

  # name                 Cmax  pos ?  Ymax  pos ?  Smax  pos ?  Smean ?  D     ?

    SignalP output is explained at http://www.cbs.dtu.dk/services/SignalP/output.html

##########################################################################################

Name:  contig035580-NyeOR.H069  Length:  310
MDNVHNVRSFILLGFNETINFRVPLFSVTLLYYCGILFFNVSLVLLIVLDVNLHEPMYIFLSSFCINALYGSTGFYPKFL      80
SDLLSPSQTISHEGCLLQAFIMYSSVCCNSSILAVMAFDRYLAICRPLHYHSFMTKRRLSQLVCFSWLTPFCIFAINVVL     160
TARLKLCGTKIQRVLCLNWLIVKLACPEADTFSNNITAYATVIIYVSHGFFIIWTYTHLIKTCAKSREDRAKFMQTCVPH     240
LTSLITFLSVVVFQFVYMRFDSTDLPQSLQNFIATEFVIIPPLMNPLIYGFKLTKIRNRILGLVCFKRKX
...............N........................N.......................................      80
............................N...................................................     160
..................................N.............................................     240
......................................................................                320

(Threshold=0.5)
----------------------------------------------------------------------
SeqName      Position  Potential   Jury    N-Glyc
                                 agreement result
----------------------------------------------------------------------
contig035580-NyeOR.H069   16 NETI   0.7009     (9/9)   ++
contig035580-NyeOR.H069   40 NVSL   0.7163     (9/9)   ++
contig035580-NyeOR.H069  109 NSSI   0.5247     (6/9)   +
contig035580-NyeOR.H069  195 NITA   0.6174     (8/9)   +
----------------------------------------------------------------------
```

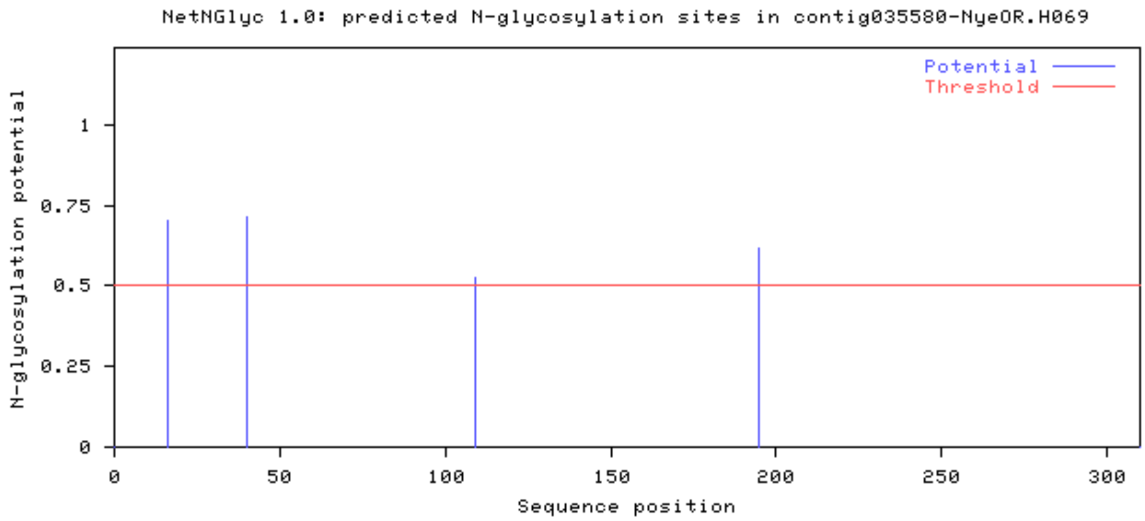

Graphics in PostScript

Output for 'contig035581-NyeORe.H077'

```
##########################################################################################

    Warning: This sequence may not contain a signal peptide!!

    Proteins without signal peptides are unlikely to be exposed to
    the N-glycosylation machinery and thus may not be glycosylated
    (in vivo) even though they contain potential motifs.

    SignalP-NN euk predictions are as follows:

  # name                 Cmax  pos ?  Ymax  pos ?  Smax  pos ?  Smean ?  D     ?

    SignalP output is explained at http://www.cbs.dtu.dk/services/SignalP/output.html

##########################################################################################

Name:  contig035581-NyeORe.H077         Length:  189
MDNVSVVRMFTLSGFNETMNIRLTIFSLTLMYYCMIILINVSLIVLIVLDENLHEPMYILLSSFCINAIYGTTGFYPKFL      80
LDLLSSSQEISYEGCLLQAFIMYSFACCDLSILAVMAFDRYLAICRPLHYHSFMTKKRLSQLVCFSWLTPLCIFSISILL     160
TSRLTLCRSKIEKVFCVNWVIVKLACSDT
..N............N.......................N........................................      80
................................................................................     160
.............................                                                        240

(Threshold=0.5)
----------------------------------------------------------------------
SeqName      Position  Potential   Jury    N-Glyc
                                 agreement result
----------------------------------------------------------------------
contig035581-NyeORe.H077    3 NVSV   0.8088     (9/9)   +++
contig035581-NyeORe.H077   16 NETM   0.6274     (9/9)   ++
contig035581-NyeORe.H077   40 NVSL   0.7651     (9/9)   +++
----------------------------------------------------------------------
```

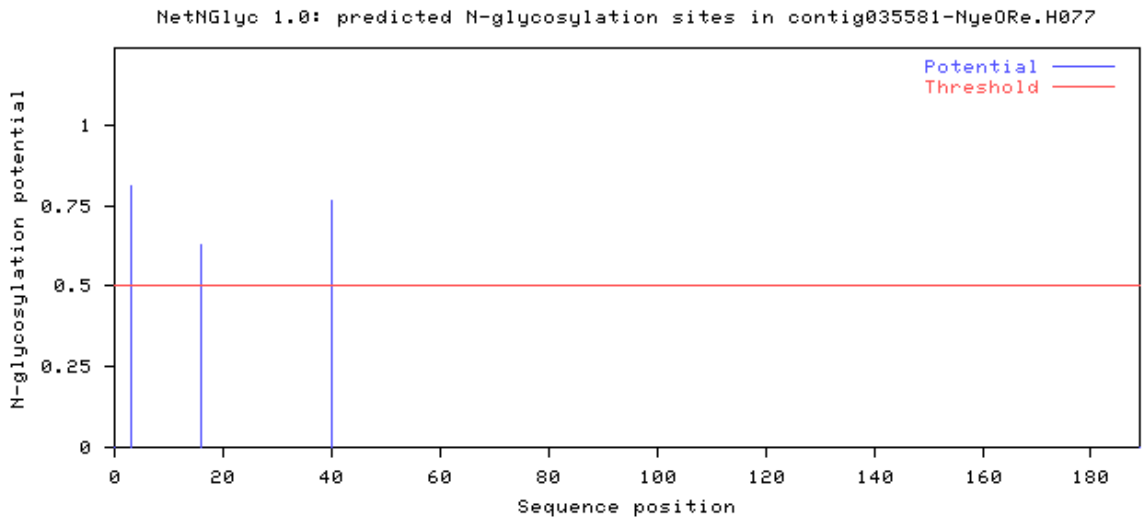

Graphics in PostScript

Output for 'contig035582-NyeOR.H070'

```
##########################################################################################

    Warning: This sequence may not contain a signal peptide!!

    Proteins without signal peptides are unlikely to be exposed to
    the N-glycosylation machinery and thus may not be glycosylated
    (in vivo) even though they contain potential motifs.

    SignalP-NN euk predictions are as follows:

  # name                    Cmax   pos ?  Ymax   pos ?  Smax   pos ?  Smean ?  D      ?

    SignalP output is explained at http://www.cbs.dtu.dk/services/SignalP/output.html

##########################################################################################

Name:  contig035582-NyeOR.H070  Length:  310
MDNVSNVKSFVLLGFNDTMNFTVPLFIITLLHYCVILFFNISLVLLIVLDENLHEPMYIFLSSFCISAIYGTTGFYPKFL      80
SDLLSSFHHISYEGCLLQAFIMYLFPCCDLSILAVMAFDRYLAICRPLHYHSFMTKKRLSQLVCFSWLTPLCILSINILL     160
TSRLTLCRSKIEKVFCVNWVIVKLACSDTDTLLNSVVSYVTIIIYLSHGFFIMWTYMHLIKTSVRSKEDRAKFMQTCVPH     240
LTSLITFLIVILFDLMYMRFGSADLPQSLQNFIAIEFLVIPPVMNPLIYGFKLTKIRNKILSFVYRKQKX
..N............N...N..................N.........................................      80
................................................................................     160
................................................................................     240
......................................................................               320

(Threshold=0.5)
----------------------------------------------------------------------
SeqName      Position  Potential   Jury    N-Glyc
                                 agreement result
----------------------------------------------------------------------
contig035582-NyeOR.H070    3 NVSN    0.7749     (9/9)    +++
contig035582-NyeOR.H070   16 NDTM    0.6647     (9/9)    ++
contig035582-NyeOR.H070   20 NFTV    0.7418     (9/9)    ++
contig035582-NyeOR.H070   40 NISL    0.6719     (9/9)    ++
----------------------------------------------------------------------
```

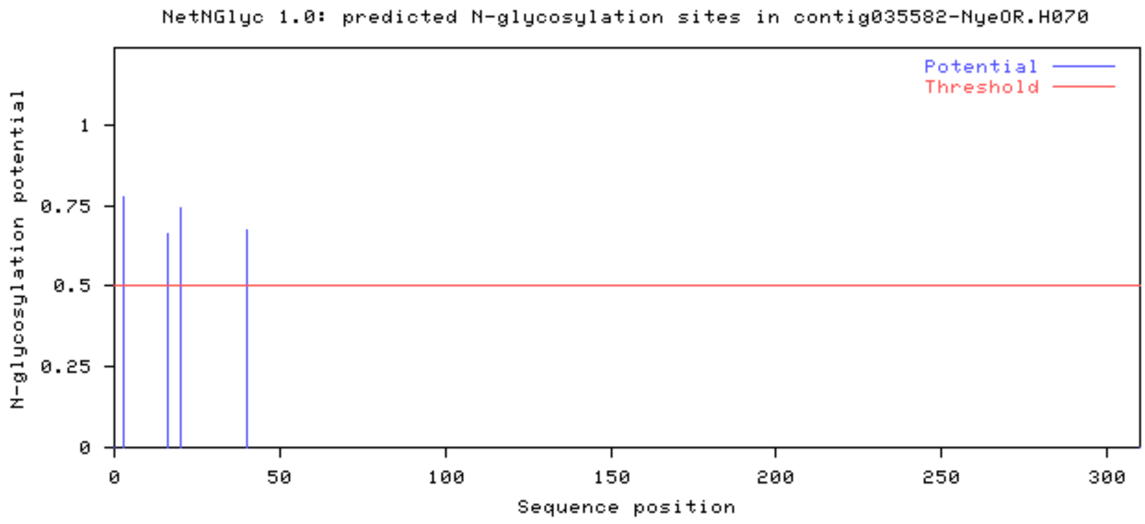

[Graphics in PostScript]

Output for 'contig035583-NyeOR.H071'

##

Warning: This sequence may not contain a signal peptide!!

Proteins without signal peptides are unlikely to be exposed to
the N-glycosylation machinery and thus may not be glycosylated
(in vivo) even though they contain potential motifs.

SignalP-NN euk predictions are as follows:

\# name Cmax pos ? Ymax pos ? Smax pos ? Smean ? D ?

SignalP output is explained at [http://www.cbs.dtu.dk/services/SignalP/output.html]

##

```
Name:  contig035583-NyeOR.H071  Length:  343
MDNVSNVRSFILSGFNETMNFRVPLFAFTLLYYCMILFFNISLVLLIFLDENLHEPMYIFLSSFCINALYGTTGFYPKFL      80
SDLLRSSQTISYEGCLLQAFIIYSFVCCDLSILAVMAFDRYMAICRPLHYHSFMTKRRLSQLVCFSWLTPSCIFAINVLL     160
TSRLKLCGINIRRVLCVNWLIVKLACPEAETFTNHIAAYATVIIYVSHGFFIMWTYTHLIKTCARSREDREKFMQTCLPH     240
LTSLITFIVVIGFQSIYVQFEYTGFSESLKNFITVEILIIPPFMNPLIYGFKLTKIQNRILTLLKSYKKLTYIKFSLLKK     320
TSVKCSIFLRVKNLLCEPFCCRX
..N............N.......................N........................................      80
................................................................................     160
................................................................................     240
................................................................................     320
.......................                                                              400

(Threshold=0.5)
----------------------------------------------------------------------
SeqName       Position  Potential   Jury    N-Glyc
                                 agreement  result
----------------------------------------------------------------------
contig035583-NyeOR.H071    3 NVSN   0.7936     (9/9)    +++
contig035583-NyeOR.H071   16 NETM   0.6269     (9/9)    ++
contig035583-NyeOR.H071   40 NISL   0.6917     (9/9)    ++
----------------------------------------------------------------------
```

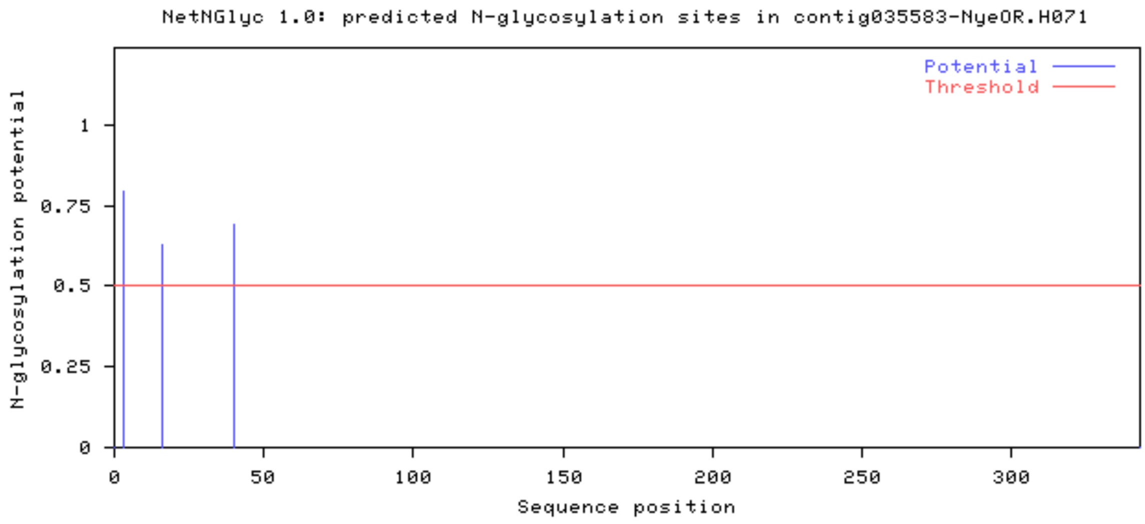

Graphics in PostScript

Output for 'contig038871-NyeOR.S121'

```
##########################################################################################

    Warning: This sequence may not contain a signal peptide!!

    Proteins without signal peptides are unlikely to be exposed to
    the N-glycosylation machinery and thus may not be glycosylated
    (in vivo) even though they contain potential motifs.

    SignalP-NN euk predictions are as follows:

  # name                    Cmax  pos ?  Ymax  pos ?  Smax  pos ?  Smean ?  D     ?

    SignalP output is explained at http://www.cbs.dtu.dk/services/SignalP/output.html

##########################################################################################

Name:  contig038871-NyeOR.S121  Length:  313
MVDNSSFIGVESSMRQLNDQVIIVQVLVGVFLCINTLLIITFFMKDAFYRTMRYILFAVTLLSDCLILILTDLLLILSYF      80
RLSIQVSLCLIMFAVSSVCNFVTPFTLTAMTLERYVAICMPLRHGELCSTRSALHCILIIHGLSSVPCILILSVFFASVS     160
LSFFTQYRVCSVEMFILRSWQGHLRSAISQFYFLIMWIIIVFCYIQIMKVAKAASGENKKSTHKGLRTVALHAFQLLLCL     240
IQLWCPFIEAAVLQIDFMLYVNVRYFNYIMFSLTPRCLSPLIYGLRDDTFFLALRYHVLCHLHRKKSVGVSNX
...N............................................................................      80
................................................................................     160
................................................................................     240
.........................................................................            320

(Threshold=0.5)
----------------------------------------------------------------------
SeqName      Position  Potential   Jury    N-Glyc
                                 agreement result
----------------------------------------------------------------------
contig038871-NyeOR.S121    4 NSSF   0.6224     (7/9)   +
----------------------------------------------------------------------
```

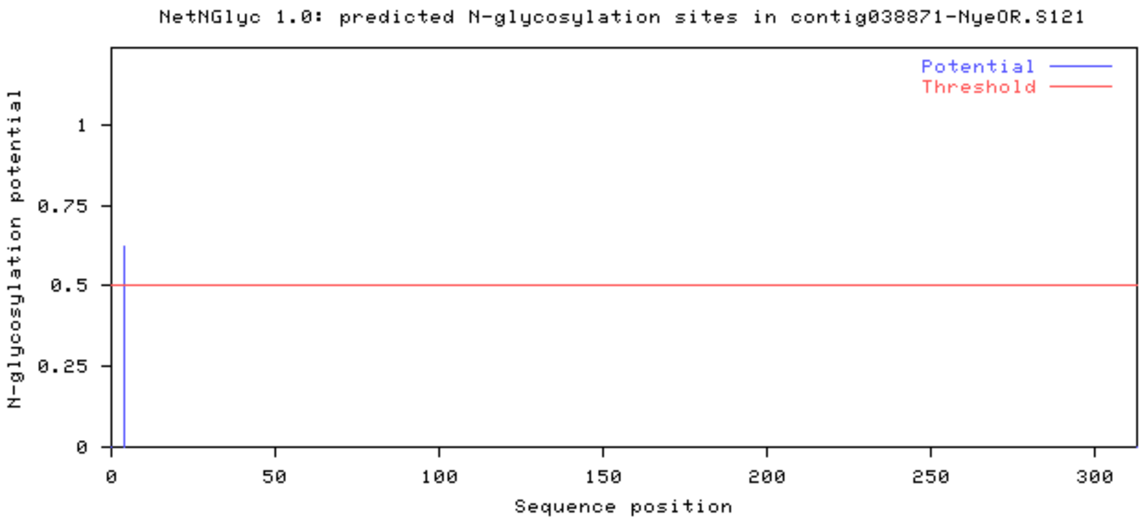

Graphics in PostScript

Output for 'contig039725-NyeOR.H072'

```
##########################################################################################

    Warning: This sequence may not contain a signal peptide!!

    Proteins without signal peptides are unlikely to be exposed to
    the N-glycosylation machinery and thus may not be glycosylated
    (in vivo) even though they contain potential motifs.

    SignalP-NN euk predictions are as follows:

  # name                    Cmax   pos ?  Ymax   pos ?  Smax   pos ?  Smean ?  D     ?

    SignalP output is explained at http://www.cbs.dtu.dk/services/SignalP/output.html

##########################################################################################

Name:  contig039725-NyeOR.H072  Length:  314
MDNVSIITVFTLSGLSDIANYRVILFVLTLLCYCVIWLVNLTIIVTVIVDKKLHEPMYIFLCSLCFNGLYGTAAFYPKFL      80
YDLLSTTHVISYAGCFLQGFVLHSSVAADFSLLALMAYDRYVAICRPLVYHSIMTKQRVCILIFFAWIIAFYFLLMSTIT     160
TAISRLCGSHIPKIYCINWLIANLACSASVAKIVIPAFSYTFCFGNVCFVFWSYVHLIKTCQSSKENMGKFMQTCVPHLF     240
SLTVVVVSLLFDLLYMRFGSKEIAQSVQNFMAMEFLLIPPIMNPLMYGFKLTKIRKRVLNFICGKSSAFRFKSX
..N....................................N........................................      80
................................................................................     160
................................................................................     240
..........................................................................           320

(Threshold=0.5)
----------------------------------------------------------------------
SeqName      Position  Potential   Jury    N-Glyc
                                agreement result
----------------------------------------------------------------------
contig039725-NyeOR.H072    3 NVSI   0.8029     (9/9)   +++
contig039725-NyeOR.H072   40 NLTI   0.7721     (9/9)   +++
----------------------------------------------------------------------
```

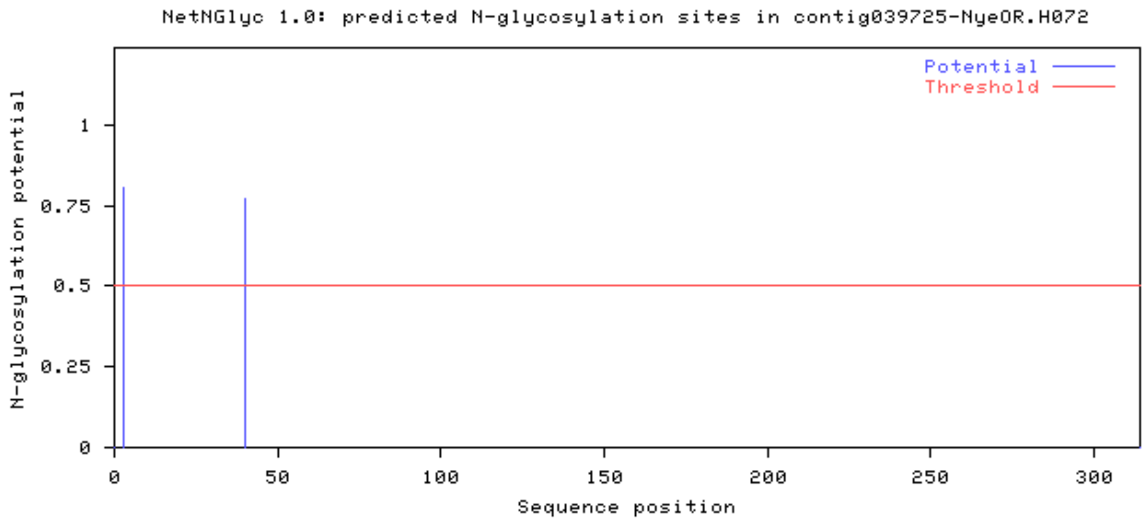

Graphics in PostScript

Output for 'contig039729-NyeOR.F064'

```
##########################################################################################

    Warning: This sequence may not contain a signal peptide!!

    Proteins without signal peptides are unlikely to be exposed to
    the N-glycosylation machinery and thus may not be glycosylated
    (in vivo) even though they contain potential motifs.

    SignalP-NN euk predictions are as follows:

  # name                    Cmax  pos ?  Ymax  pos ?  Smax  pos ?  Smean ?  D     ?

    SignalP output is explained at http://www.cbs.dtu.dk/services/SignalP/output.html

##########################################################################################

Name:  contig039729-NyeOR.F064  Length:  305
MENSTLSFYFRFTMFANIGHYRFIAFIFCLLLFIFTIFTNLLMIVVISQQTTLHEPMYIFIACLSVNALYGSSGFFPRFL      80
MDLLSDTHLISRPACFTQIYVIYSYASCELTVLSIMAYDRYIAVCLPLHYHTKMTLETVVKLTALAWIFPAFSLAACLCL     160
SATLPLCGNEIHKVFCANWNVVKLSCVNTAVNNVVGMLLTVATIFLPLFYILYTYLRIVSICWKSSAEFKGKVLESCLPH     240
TISFVIYSIAGFCDVALSRNNLEVTNPFIAVILSLVFVVIPPALNPLVYGLKLPEIRKHILKLFX
..N.............................................................................      80
................................................................................     160
................................................................................     240
.................................................................                    320

(Threshold=0.5)
----------------------------------------------------------------------
SeqName      Position  Potential   Jury    N-Glyc
                                 agreement result
----------------------------------------------------------------------
contig039729-NyeOR.F064    3 NSTL   0.7370     (9/9)    ++
----------------------------------------------------------------------
```

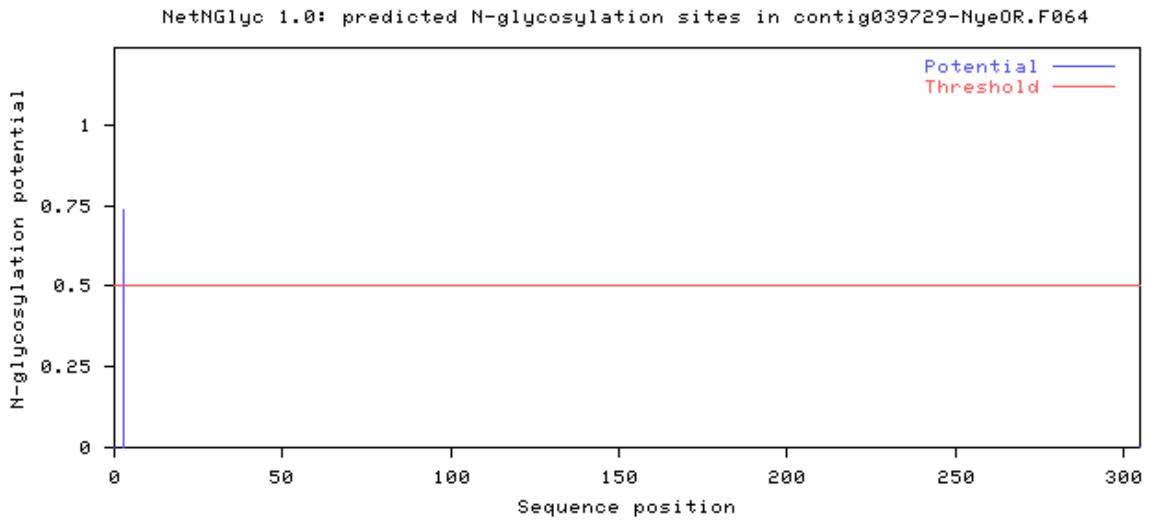

[Graphics in PostScript]

Output for 'contig039729-NyeOR.H073'

```
##########################################################################################

    Warning: This sequence may not contain a signal peptide!!

    Proteins without signal peptides are unlikely to be exposed to
    the N-glycosylation machinery and thus may not be glycosylated
    (in vivo) even though they contain potential motifs.

    SignalP-NN euk predictions are as follows:

  # name                    Cmax  pos ?  Ymax  pos ?  Smax  pos ?  Smean ?  D     ?

    SignalP output is explained at http://www.cbs.dtu.dk/services/SignalP/output.html

##########################################################################################

Name:  contig039729-NyeOR.H073  Length:  314
MDNVSVVTVFTLSGLSGIANYKITIFIFTLLCYCVIWLVNLTIIVTVIVDKSLHEPMYIFLCNLCFNGLYGTAAFYPKLL      80
YDLLSTIHVISYAGCLLQGLMVHSSICTDFSLLALMAYDRYVAICRPLVYHSLMTTQRVCIFVFFAWITPFSLILMSTIT     160
TATSRLCGSHIPRIYCINWLISNLACSASVATIIIPAFNYTFYFGHAVFVFWSYVHLIKTCQSSKENWNKFMQTCVPHLF     240
SLAVVVVSFLFDMLYMRFGSKEIPQSFENFMAMEILFIPPIINPLMYGFKLTKIRKRVLNFICGESSAFILNSX
..N....................................N........................................      80
................................................................................     160
......................................N.........................................     240
..........................................................................           320

(Threshold=0.5)
----------------------------------------------------------------------
SeqName      Position  Potential   Jury    N-Glyc
                                 agreement result
----------------------------------------------------------------------
contig039729-NyeOR.H073    3 NVSV   0.8098     (9/9)   +++
contig039729-NyeOR.H073   40 NLTI   0.7722     (9/9)   +++
contig039729-NyeOR.H073  199 NYTF   0.5938     (8/9)   +
----------------------------------------------------------------------
```

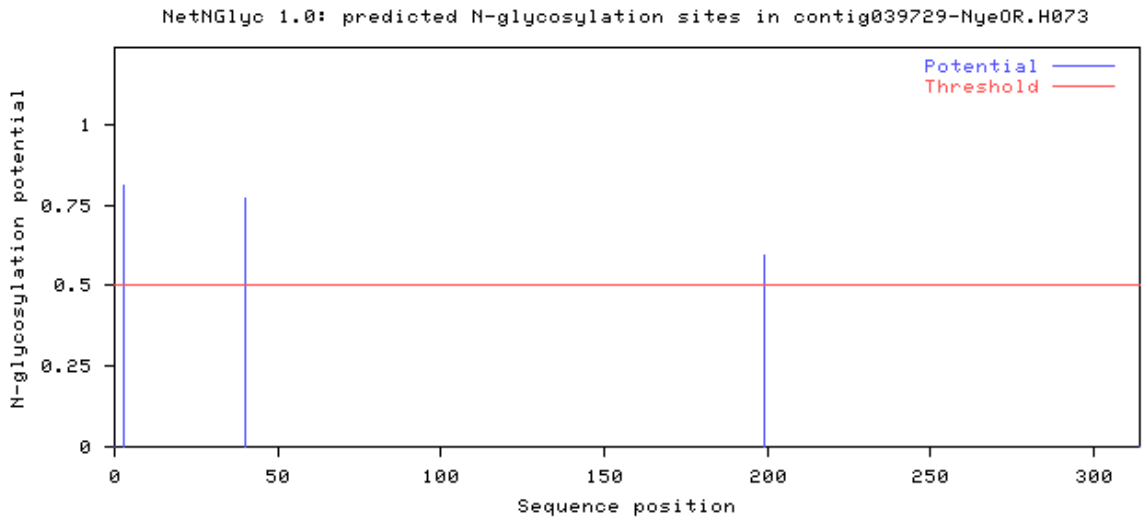

Graphics in PostScript

Output for 'contig039730-NyeOR.D036'

```
##########################################################################################

    Warning: This sequence may not contain a signal peptide!!

    Proteins without signal peptides are unlikely to be exposed to
    the N-glycosylation machinery and thus may not be glycosylated
    (in vivo) even though they contain potential motifs.

    SignalP-NN euk predictions are as follows:

  # name                Cmax  pos ?  Ymax  pos ?  Smax  pos ?  Smean ?  D     ?

    SignalP output is explained at http://www.cbs.dtu.dk/services/SignalP/output.html

##########################################################################################

Name:  contig039730-NyeOR.D036  Length:  319
MGNSSETVSFVLAAYGNVGALKYMYFSIILFWYVSICVANTVLIVVIHVDRRLHEPMYILLSNLCVNEINGSTSMYPLLL      80
SQMFSDSHEVTLPWCFLQMCCMYTTASAEFCSLAAMAYDRYISICHPLRYNITMNTERVFMLILLVWVYSFLIFIFSFSF     160
IFSLKFCGNVVNNVYCDHKLIIRLSCSVSIHSFISDIFFLLLSIFIPFSLISVSYMKILAVCRKTSTENKQKAVTTCTPQ     240
IVSVSNLFVGCIFHAIDSSVLVAQVPGEVRIILSIYLLICQPMLTPFMYGFNLPKIRQSCTMLVFKRKSISLFGKRLFX
..N.............................................................................      80
..................................................N.............................     160
................................................................................     240
...............................................................................      320

(Threshold=0.5)
----------------------------------------------------------------------
SeqName      Position  Potential   Jury    N-Glyc
                                 agreement result
----------------------------------------------------------------------
contig039730-NyeOR.D036    3 NSSE   0.7403     (9/9)    ++
contig039730-NyeOR.D036   70 NGST   0.4571     (6/9)    -
contig039730-NyeOR.D036  131 NITM   0.7782     (9/9)    +++
----------------------------------------------------------------------
```

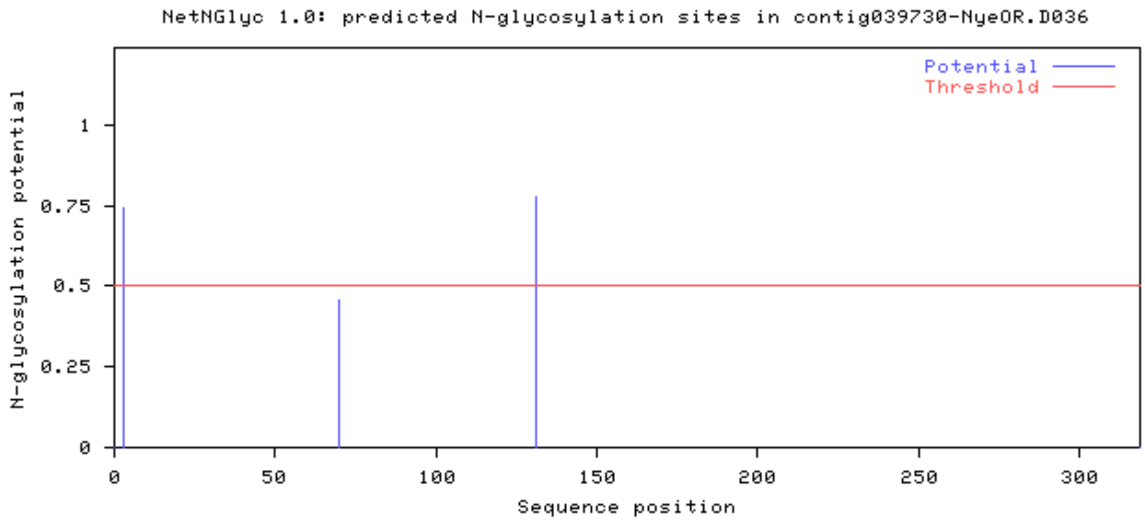

Graphics in PostScript

Output for 'contig039730-NyeOR.D037'

```
##########################################################################################

    Warning: This sequence may not contain a signal peptide!!

    Proteins without signal peptides are unlikely to be exposed to
    the N-glycosylation machinery and thus may not be glycosylated
    (in vivo) even though they contain potential motifs.

    SignalP-NN euk predictions are as follows:

  # name                 Cmax  pos ?  Ymax  pos ?  Smax  pos ?  Smean ?  D     ?

    SignalP output is explained at http://www.cbs.dtu.dk/services/SignalP/output.html

##########################################################################################

Name:  contig039730-NyeOR.D037  Length:  322
MQKIGNSSESVSFVLAAYGNVGAFKYPYFIIILFWYVSICVANTVLIVVIHVDRRLHEPMYILLSNLCVNEINASTSLYP      80
LLLSQMFSDSHEVTLPWCFLQMCCMYTSASAEFCSLAAMAYDRYISICHPLRYNVIMNTERVFMLILVVWMYSFLSFIFS     160
FSFIFRLMFCGNIVNNVYCDHKLIIRLSCSVLVHSFISDIFFLLLSIFIPFSLISVSYMKILAVCRKTSTENKQKAVTTC     240
TPQIISVSNLFVGCIFHSTDFSLLAAHVPGEVNIILSIYLLICQPMLTPFMYGFNLPKIRQSRTMLLFKRKSISLFSKRV     320
LX
.....N..........................................................................      80
................................................................................     160
................................................................................     240
................................................................................     320
..                                                                                   400

(Threshold=0.5)
----------------------------------------------------------------------
SeqName      Position  Potential   Jury    N-Glyc
                                 agreement result
----------------------------------------------------------------------
contig039730-NyeOR.D037    6 NSSE   0.7448     (9/9)   ++
contig039730-NyeOR.D037   73 NAST   0.3812     (6/9)   -
----------------------------------------------------------------------
```

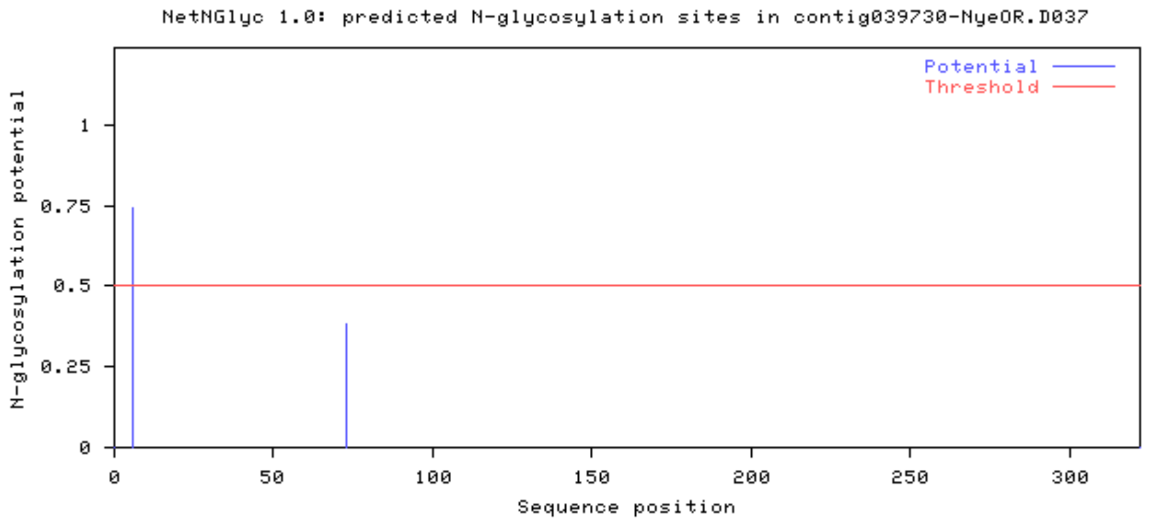

Graphics in PostScript

Output for 'contig039730-NyeOR.H074'

```
##########################################################################################

    Warning: This sequence may not contain a signal peptide!!

    Proteins without signal peptides are unlikely to be exposed to
    the N-glycosylation machinery and thus may not be glycosylated
    (in vivo) even though they contain potential motifs.

    SignalP-NN euk predictions are as follows:

  # name                   Cmax  pos ?  Ymax  pos ?  Smax  pos ?  Smean ?  D     ?

    SignalP output is explained at http://www.cbs.dtu.dk/services/SignalP/output.html

##########################################################################################

Name:  contig039730-NyeOR.H074  Length:  314
MNTSSIVVFSLTGFSATVNYRVTLFSLTLLCYFLILIVNISLILTIISDQNLHEPMYIFLCSLCINGLYGTAGFFPRFAF      80
DLLSDTHLISYVGCLLQVFVIYSNAKVDYSTLVLMAYDRYLAICRPLEYHSVMSVRRTVVLVTLSWLVPLCFETLVISLT     160
STLKLCGSNINKLYCENWSIVKLACGSTKVNDIVGLIFITFYCCHVVCIACSYVQLVNVALKSRAGRKKFTQTCTPHLFC     240
LLNVTTALLFDLMFSRYGSASLPQHLKNFMAIEFLIIPPILNPVCYGWVLTKIRRRMIFLCRLAYQRFGVKSQX
.N....................................N.........................................      80
................................................................................     160
................................................................................     240
..N.......................................................................           320

(Threshold=0.5)
----------------------------------------------------------------------
SeqName      Position  Potential   Jury    N-Glyc
                                 agreement result
----------------------------------------------------------------------
contig039730-NyeOR.H074    2 NTSS   0.7490     (9/9)    ++
contig039730-NyeOR.H074   39 NISL   0.7490     (9/9)    ++
contig039730-NyeOR.H074  177 NWSI   0.4820     (5/9)    -
contig039730-NyeOR.H074  243 NVTT   0.5948     (9/9)    ++
----------------------------------------------------------------------
```

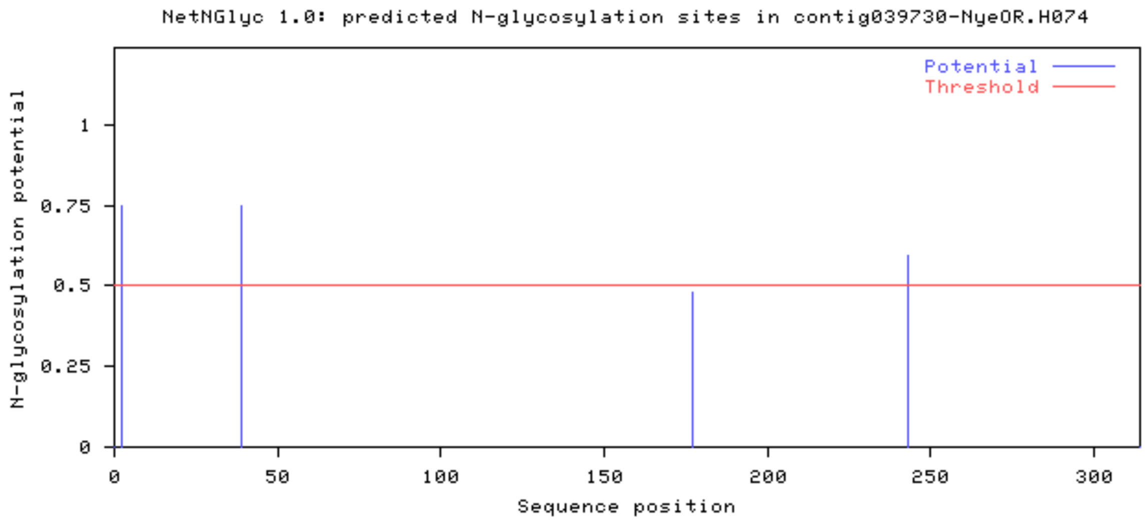

Graphics in PostScript

Output for 'contig039731-NyeOR.D038'

```
##########################################################################################

    Warning: This sequence may not contain a signal peptide!!

    Proteins without signal peptides are unlikely to be exposed to
    the N-glycosylation machinery and thus may not be glycosylated
    (in vivo) even though they contain potential motifs.

    SignalP-NN euk predictions are as follows:

  # name                    Cmax  pos ?  Ymax  pos ?  Smax  pos ?  Smean ?  D     ?

    SignalP output is explained at http://www.cbs.dtu.dk/services/SignalP/output.html

##########################################################################################

Name:  contig039731-NyeOR.D038  Length:  312
MQKIGNSSESVSFVLAAYGNVGAFKYPYFIIILFWYVSICVANTVLIVVIHVDRRLHEPMYILLSNLCVNEINASTSLYP      80
FLLSQMFSDSHEVTLPWCFLQMCCMYTSASAEFCSLAAMAYDRYIAICHPFSYNVIMNTERVVMLILLVWIFSFVSLLLS     160
FSFIFGLRFCGNIIDNVYCDHQLIIRLSCSVPIQSSISMIFFTLLSIFMPFNLISISYLKILRVCRKTSTENKQKAVTTC     240
TPQIVSVSNLFVGCIFHSIDFSLLAAQIPEEVRIILPMYLLICQPMLTPFMYGFNLPKIRQACKMLLFKRKX
.....N..........................................................................      80
................................................................................     160
................................................................................     240
........................................................................             320

(Threshold=0.5)
----------------------------------------------------------------------
SeqName      Position  Potential   Jury    N-Glyc
                                agreement result
----------------------------------------------------------------------
contig039731-NyeOR.D038    6 NSSE   0.7450     (9/9)   ++
contig039731-NyeOR.D038   73 NAST   0.3534     (7/9)   -
----------------------------------------------------------------------
```

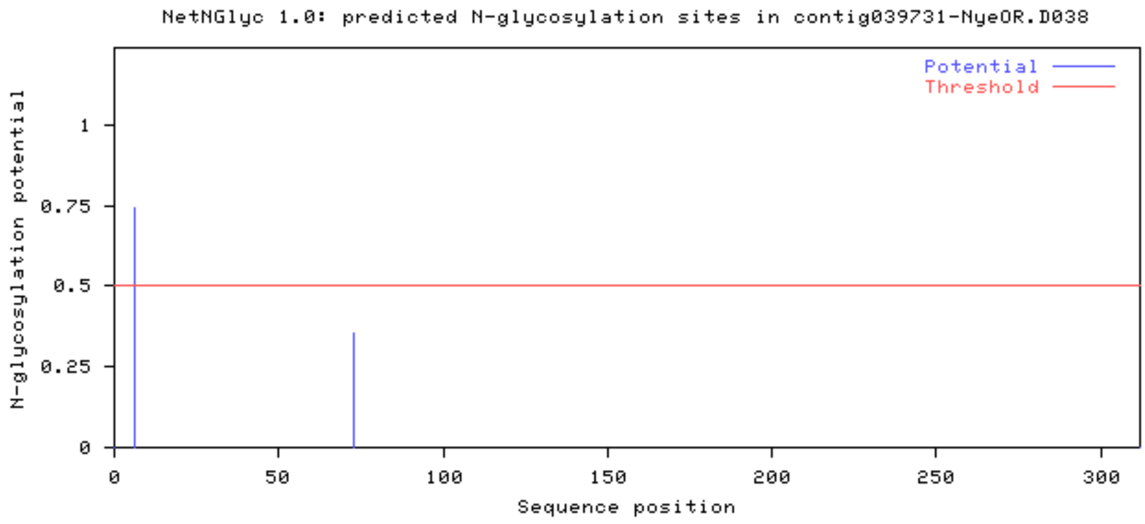

Graphics in PostScript

Output for 'contig039736-NyeORe.D043'

```
########################################################################################

    Warning: This sequence may not contain a signal peptide!!

    Proteins without signal peptides are unlikely to be exposed to
    the N-glycosylation machinery and thus may not be glycosylated
    (in vivo) even though they contain potential motifs.

    SignalP-NN euk predictions are as follows:

  # name                 Cmax  pos ?  Ymax  pos ?  Smax  pos ?  Smean ?  D     ?

    SignalP output is explained at http://www.cbs.dtu.dk/services/SignalP/output.html

########################################################################################

Name:  contig039736-NyeORe.D043         Length:  233
PLLLSQMFSDSHEVTVPWCFLQMCCLYTSAPAEFCSLAAMAYDRYISICHPLRYNIIMNTERVFLFILLVWVYSFLSFIF      80
SYSFIFSLKFCGNIIHNAYCDHQLIIRLSCAVPIQSFISNISFLLLSVFIPFSLILVSYMKILRVCRKTSKENKQKAVTT     160
CTPQIISVSNLFVGCIFYFIDFKFLVSQVPDEIRIILPMYLLIFQPMLTPFMYGFKLPKIRQSYQRFLFERKX
................................................................................      80
................................................................................     160
.........................................................................            240

(Threshold=0.5)

No sites predicted in this sequence.
```

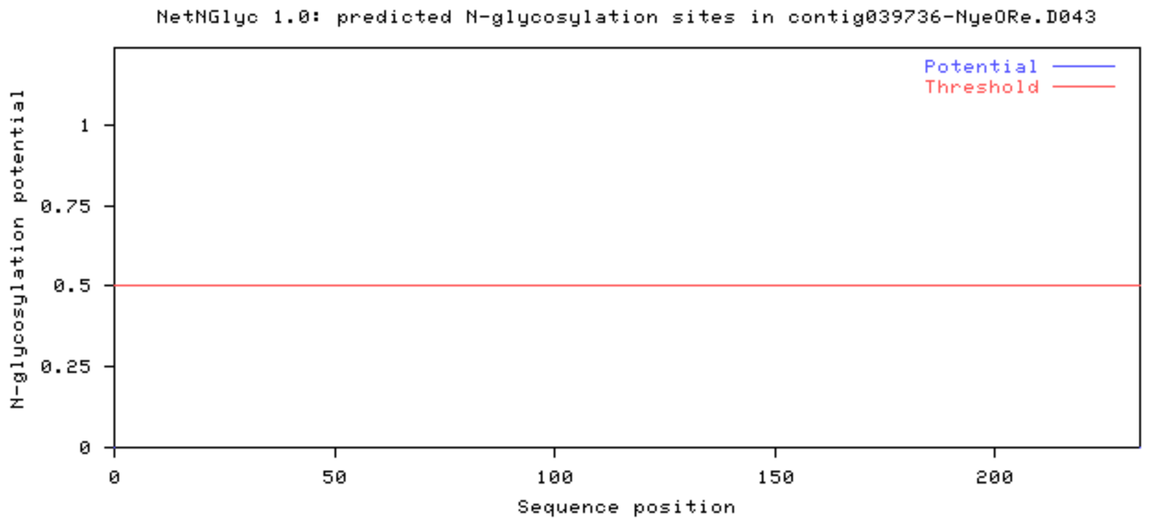

Graphics in PostScript

Output for 'contig039736-NyeORe.D044'

```
##########################################################################################

    Warning: This sequence may not contain a signal peptide!!

    Proteins without signal peptides are unlikely to be exposed to
    the N-glycosylation machinery and thus may not be glycosylated
    (in vivo) even though they contain potential motifs.

    SignalP-NN euk predictions are as follows:

  # name                 Cmax  pos ?  Ymax  pos ?  Smax  pos ?  Smean ?  D     ?

    SignalP output is explained at http://www.cbs.dtu.dk/services/SignalP/output.html

##########################################################################################

Name:  contig039736-NyeORe.D044         Length:  123
MENSSEIVSFVLSAFENVGELKYLYFIIILVWYVSICVANTLLILVIRVDRRLHEPMYILLCNLCVNEINASTSLYPLLL      80
SQMFSDSHEVTVPWCFLQMCCLYTSASVELCGLAAMAYDRYIS
..N.............................................................................      80
...........................................                                          160

(Threshold=0.5)
----------------------------------------------------------------------
SeqName      Position  Potential   Jury    N-Glyc
                                 agreement result
----------------------------------------------------------------------
contig039736-NyeORe.D044    3 NSSE   0.6972     (9/9)   ++
contig039736-NyeORe.D044   70 NAST   0.3170     (9/9)   ---
----------------------------------------------------------------------
```

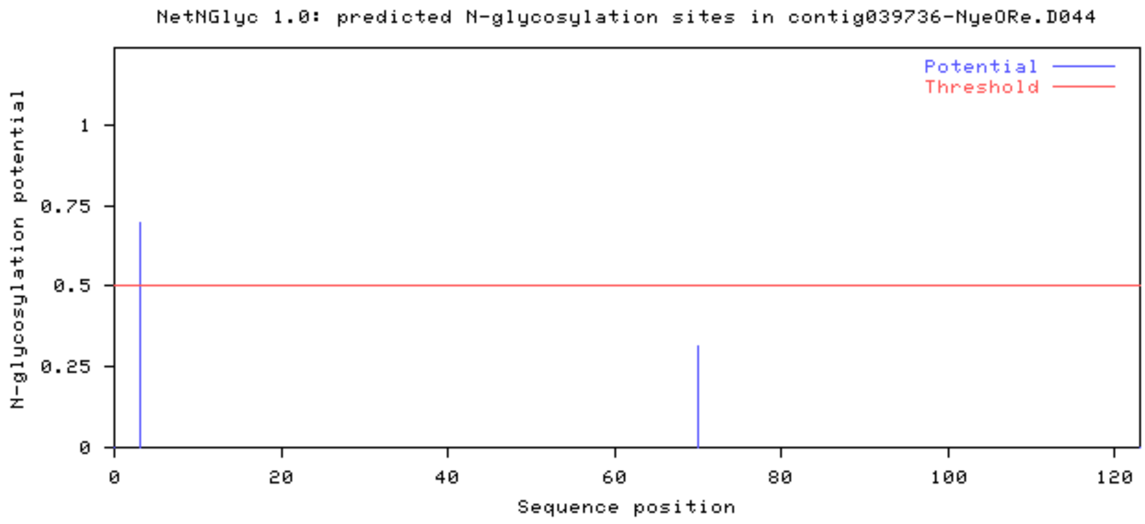

[Graphics in PostScript]

Output for 'contig039737-NyeOR.D039'

```
##########################################################################################

    Warning: This sequence may not contain a signal peptide!!

    Proteins without signal peptides are unlikely to be exposed to
    the N-glycosylation machinery and thus may not be glycosylated
    (in vivo) even though they contain potential motifs.

    SignalP-NN euk predictions are as follows:

  # name                 Cmax  pos ?  Ymax  pos ?  Smax  pos ?  Smean ?  D     ?

    SignalP output is explained at http://www.cbs.dtu.dk/services/SignalP/output.html

##########################################################################################

Name:  contig039737-NyeOR.D039  Length:  309
MGNSSEIVSFVLSAYGNIGELKYLYFIIILVWYLSICVANTVLIVVIRVDRRLHEPMYILLSNLCVNEINASTSLYPLLL      80
SQMFSDSHEVTVPWCFLQMCCLYTSAPAELCSLAAMAYDRYISICHPLRYNIIMNTERVFLMILLVWVYSFLSFIFSFSF     160
IFSLKFCGNIIHSVYCDHQLIIRLSCPVSIQSFISDISFAILSVFIPFSLILVSYMKILGVCRKTSKENKQKAVTTCTPQ     240
IISVSNLFVGYIFYFIDFRSVVSQVPDEVHIILPMYVLIFQPMLTPFMYGFNLPKIRQSYQRFLFERKX
..N.............................................................................      80
................................................................................     160
................................................................................     240
.....................................................................                320

(Threshold=0.5)
----------------------------------------------------------------------
SeqName      Position  Potential   Jury    N-Glyc
                                 agreement result
----------------------------------------------------------------------
contig039737-NyeOR.D039    3 NSSE   0.7241     (9/9)   ++
contig039737-NyeOR.D039   70 NAST   0.3814     (6/9)   -
----------------------------------------------------------------------
```

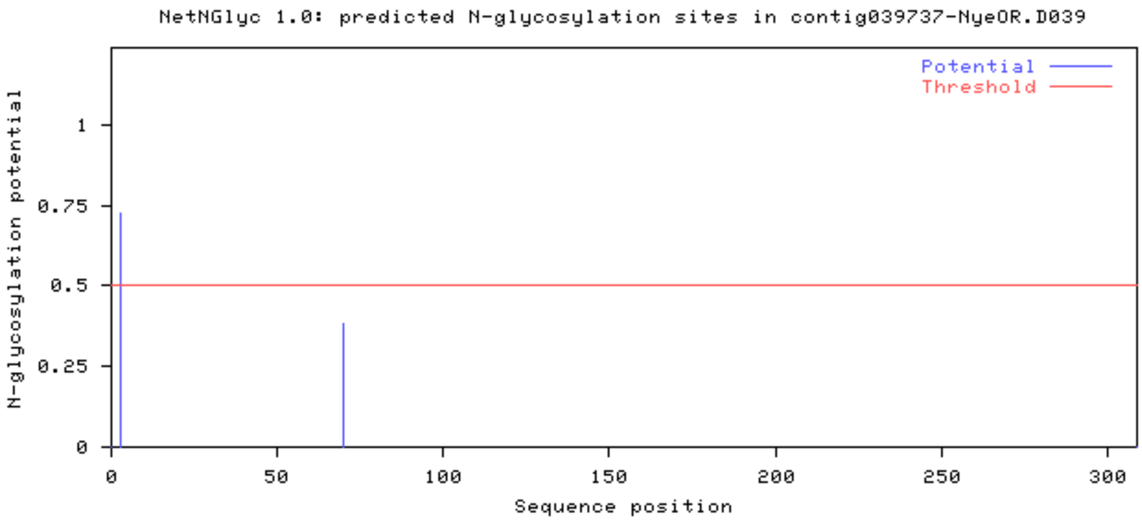

Graphics in PostScript

Output for 'contig039737-NyeOR.D040'

```
##########################################################################################

    Warning: This sequence may not contain a signal peptide!!

    Proteins without signal peptides are unlikely to be exposed to
    the N-glycosylation machinery and thus may not be glycosylated
    (in vivo) even though they contain potential motifs.

    SignalP-NN euk predictions are as follows:

  # name                    Cmax  pos ?  Ymax  pos ?  Smax  pos ?  Smean ?  D     ?

    SignalP output is explained at http://www.cbs.dtu.dk/services/SignalP/output.html

##########################################################################################

Name:  contig039737-NyeOR.D040  Length:  309
MENSSEIVSFVLSAYGNVGDLKYLYLTIILFWYVSICVANIVLIVVIHVDRRLHEPMYILLSNLCVNEINASTSLYPLLL      80
SQMYSDSHEVTLPWCYLQMCCLYTSAPAEFWSLAAMAYDRYISICHPLRYNVIMNTERVLKIILLVWVFSFLIFILSFSF     160
IFSLQFCGNIVDNVYCEHQLIIRLSCSVSVQSSISIIFFVIMSIFIPFSLISVSYVKILTVCRKTSTENKQKAMTTCTPQ     240
IVSVSNLFVGCIFHSINFRLLISQVPDEVNIILPMYMLICQPMLTPFMYGFNLPKIRQSCKRFLFKRKX
..N.............................................................................      80
................................................................................     160
................................................................................     240
.....................................................................               320

(Threshold=0.5)
----------------------------------------------------------------------
SeqName      Position  Potential   Jury    N-Glyc
                                agreement result
----------------------------------------------------------------------
contig039737-NyeOR.D040    3 NSSE   0.6952     (9/9)   ++
contig039737-NyeOR.D040   70 NAST   0.3814     (6/9)   -
----------------------------------------------------------------------
```

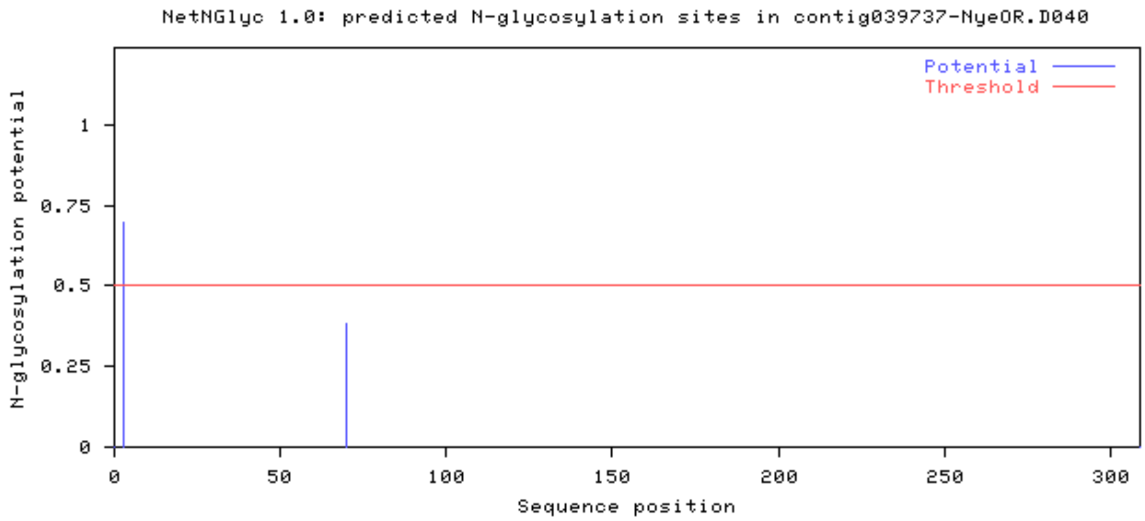

Graphics in PostScript

Output for 'contig039738-NyeOR.D041'

##

Warning: This sequence may not contain a signal peptide!!

Proteins without signal peptides are unlikely to be exposed to
the N-glycosylation machinery and thus may not be glycosylated
(in vivo) even though they contain potential motifs.

SignalP-NN euk predictions are as follows:

# name	Cmax	pos ?	Ymax	pos ?	Smax	pos ?	Smean ?	D	?

SignalP output is explained at http://www.cbs.dtu.dk/services/SignalP/output.html

##

```
Name:  contig039738-NyeOR.D041  Length:  311
MGNSSEIEVVSFVLAAYANIGALKYMYFIIMLFWYLSICVANTVVIVVIQVDRRLHEPMYILLCSLCVNEINSSTSLYPL      80
LLSQMFSDSHEVTVPWCFLQMCFMYISAPAEFWGLAAMAYDRYISICHPLCYNVIMNTKRVFLLILLVWIFSLVSFILSF     160
SFIFGLKFCRNIVENVYCDHQLMIRLSCSVSTQSYISEIFFAIVSIFIPFTLISVSYFKILAVCRKTSIENKQKAVTTCS     240
PQIVSVSNLFVGCIFHSIDFRFLIARVPDEVRIILPMYVLICQPMLTPFLYGFNLPKIRHSFKRLLFKRKX
..N.............................................................................      80
................................................................................     160
................................................................................     240
.......................................................................              320
```

(Threshold=0.5)

SeqName	Position	Potential	Jury agreement	N-Glyc result
contig039738-NyeOR.D041	3 NSSE	0.7486	(9/9)	++
contig039738-NyeOR.D041	72 NSST	0.3689	(8/9)	-

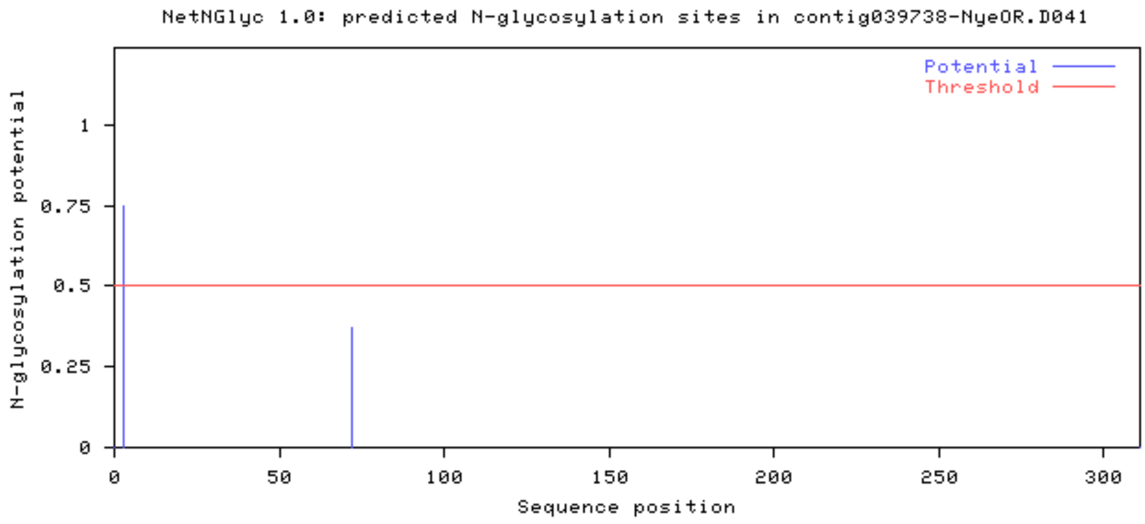

Graphics in PostScript

Output for 'contig039738-NyeOR.D042'

```
##########################################################################################

    Warning: This sequence may not contain a signal peptide!!

    Proteins without signal peptides are unlikely to be exposed to
    the N-glycosylation machinery and thus may not be glycosylated
    (in vivo) even though they contain potential motifs.

    SignalP-NN euk predictions are as follows:

  # name                    Cmax   pos ?  Ymax   pos ?  Smax   pos ?  Smean ?  D     ?

    SignalP output is explained at http://www.cbs.dtu.dk/services/SignalP/output.html

##########################################################################################

Name:  contig039738-NyeOR.D042  Length:  309
MENSSEIMSFVLSAYGNVGELKYLYFVIILFWYLSICVANTVLILVIRVDRQLHEPMYILLCNLCVNEINISTSLYPLLL      80
SQMFSDRHEVTVPWCFLQMCFMYTSAPAEFCSLAAMSYDRYISICHPLRYNVIMNTERVFFMILLVWIYSFLSVILSFSF     160
VFSLKFCGNNIENVYCDHQLLIRLSCSLSFHSFISDIFFVFVSIFIPFNLISVSYVKILAICRKTSTENKQKAVTTCTPQ     240
LVSVSNLFVGCIFQSIDSSVIVAQLPHEVNIILSIYLFICQPMLTPFLYGFNLPKIRQSCKRFVFKKKX
..N.............................................................................      80
................................................................................     160
................................................................................     240
.....................................................................               320

(Threshold=0.5)
----------------------------------------------------------------------
SeqName      Position  Potential   Jury    N-Glyc
                                agreement result
----------------------------------------------------------------------
contig039738-NyeOR.D042    3 NSSE   0.6848     (9/9)   ++
contig039738-NyeOR.D042   70 NIST   0.4514     (5/9)   -
----------------------------------------------------------------------
```

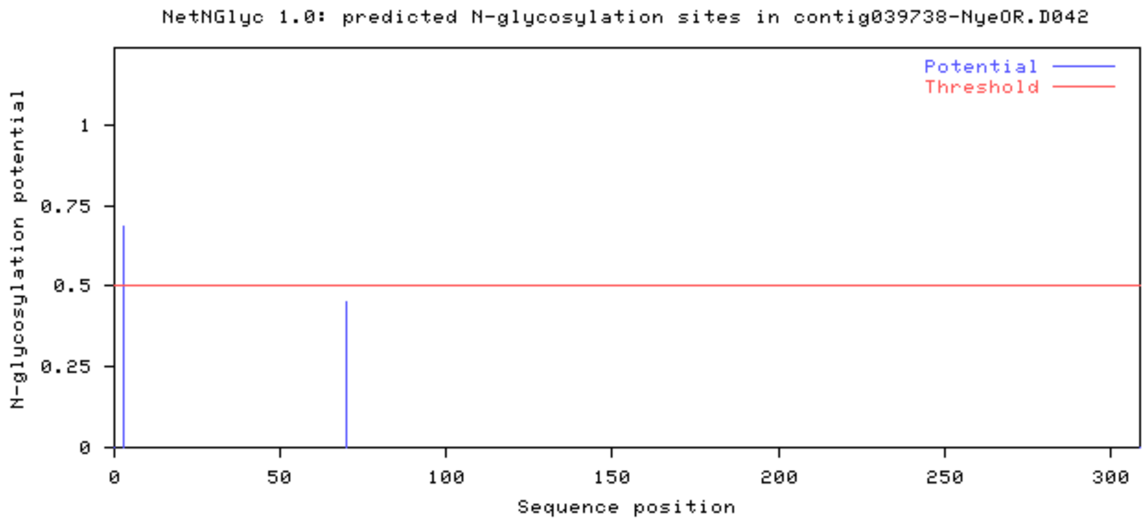

Graphics in PostScript

Output for 'contig039739-NyeORp.D047'

```
##########################################################################################

    Warning: This sequence may not contain a signal peptide!!

    Proteins without signal peptides are unlikely to be exposed to
    the N-glycosylation machinery and thus may not be glycosylated
    (in vivo) even though they contain potential motifs.

    SignalP-NN euk predictions are as follows:

  # name                   Cmax   pos ?  Ymax   pos ?  Smax   pos ?  Smean ?  D     ?

    SignalP output is explained at http://www.cbs.dtu.dk/services/SignalP/output.html

##########################################################################################

Name:  contig039739-NyeORp.D047         Length:  323
MGVGEDSKLSEKLLIREVSPRSFSVKRLEVTRSPVLIVVIRVDIQLHEPMYILLCNLCVNQINVSTSLYPLLLSQMFSDS      80
HEVTLPWCFLHMSCMYTSGPAEFCSLAAMAYDRYISICHPLRYNVIMNTERVFFMILFVWIYSFLSVILSFSFIFSLKFC     160
GTIIENVYCNHRLILRLSCFISVHSFLSDIFFLIVSFFIPFTLISVSYVKILAVCRKTSTENKQKAATTCAPQIVSVSNL     240
FVGSIFHSIDSNFIITQVPNEARIILPMYILIFQPMLTPFLYGFKLPKIRHSCKRLLLHEKINSFVLEHLCSLGDIHQSS     320
YRX
................................................................................      80
................................................................................     160
................................................................................     240
................................................................................     320
...                                                                                  400

(Threshold=0.5)

No sites predicted in this sequence.
```

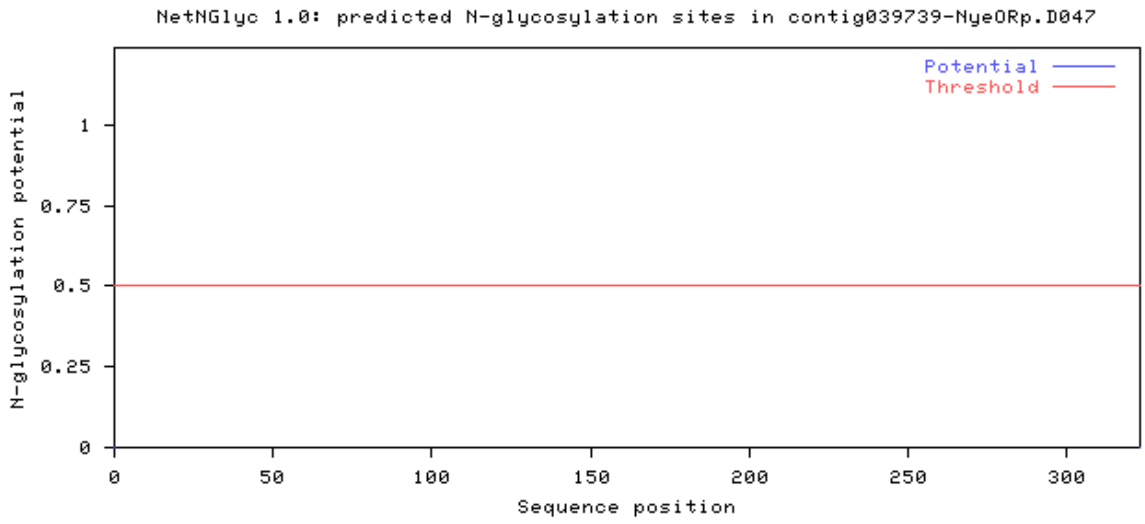

Graphics in PostScript

Output for 'contig040496-NyeORep.L090'

```
##########################################################################################

    Warning: This sequence may not contain a signal peptide!!

    Proteins without signal peptides are unlikely to be exposed to
    the N-glycosylation machinery and thus may not be glycosylated
    (in vivo) even though they contain potential motifs.

    SignalP-NN euk predictions are as follows:

  # name                  Cmax  pos ?  Ymax  pos ?  Smax  pos ?  Smean ?  D     ?

    SignalP output is explained at http://www.cbs.dtu.dk/services/SignalP/output.html

##########################################################################################

Name:  contig040496-NyeORep.L090        Length:  102
MSLQNASVKLTYFIIGGFDTVKRPVAVGVVMLTGSLVNILFIVSDKQLHKPMYLLFCYLAVVDILYTSSATPTMIGVLLA      80
GVNTISYVECLIQMCVFQLGST
....N...........................................................................      80
......................                                                               160

(Threshold=0.5)
----------------------------------------------------------------------
SeqName      Position  Potential   Jury    N-Glyc
                                 agreement result
----------------------------------------------------------------------
contig040496-NyeORep.L090    5 NASV   0.6084     (8/9)    +
----------------------------------------------------------------------
```

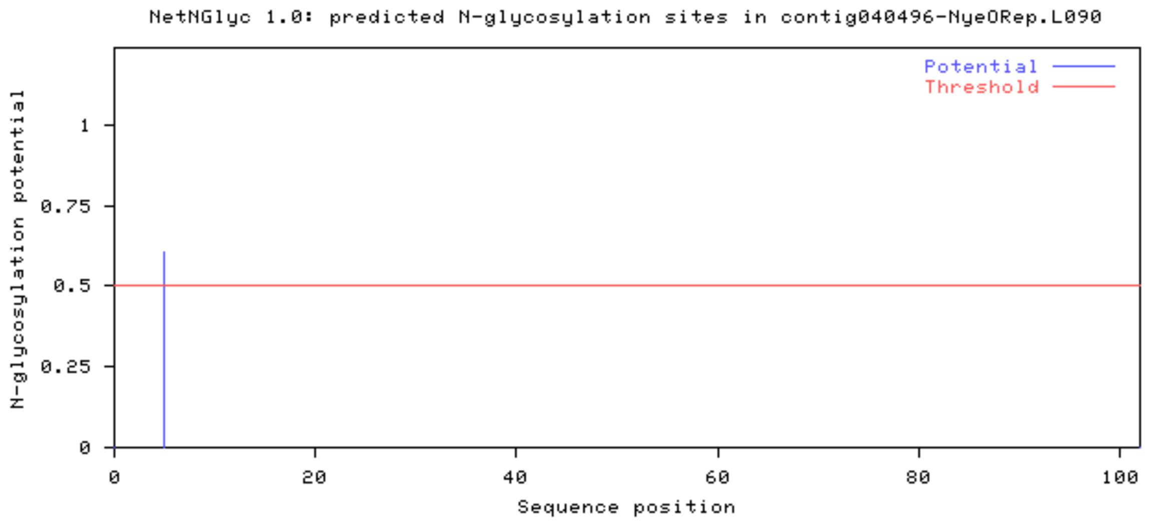

Graphics in PostScript

Output for 'contig040499-NyeORe.L099'

```
##########################################################################################

    Warning: This sequence may not contain a signal peptide!!

    Proteins without signal peptides are unlikely to be exposed to
    the N-glycosylation machinery and thus may not be glycosylated
    (in vivo) even though they contain potential motifs.

    SignalP-NN euk predictions are as follows:

  # name                    Cmax   pos ?  Ymax   pos ?  Smax   pos ?  Smean ?  D     ?

    SignalP output is explained at http://www.cbs.dtu.dk/services/SignalP/output.html

##########################################################################################

Name:  contig040499-NyeORe.L099         Length:  126
CVDPEKYFNQGASRLFFLLFFTFVFICLSYCVILFFVKLSSNNDRMKMGSTLVSHLICVICLYCPIFFHAILTRFGVVLT      80
LEERQGLSIGTILGPSLVNPFVYFLRTKEIKNKIFKILRKANTARX
................................................................................      80
..............................................                                        160

(Threshold=0.5)

No sites predicted in this sequence.
```

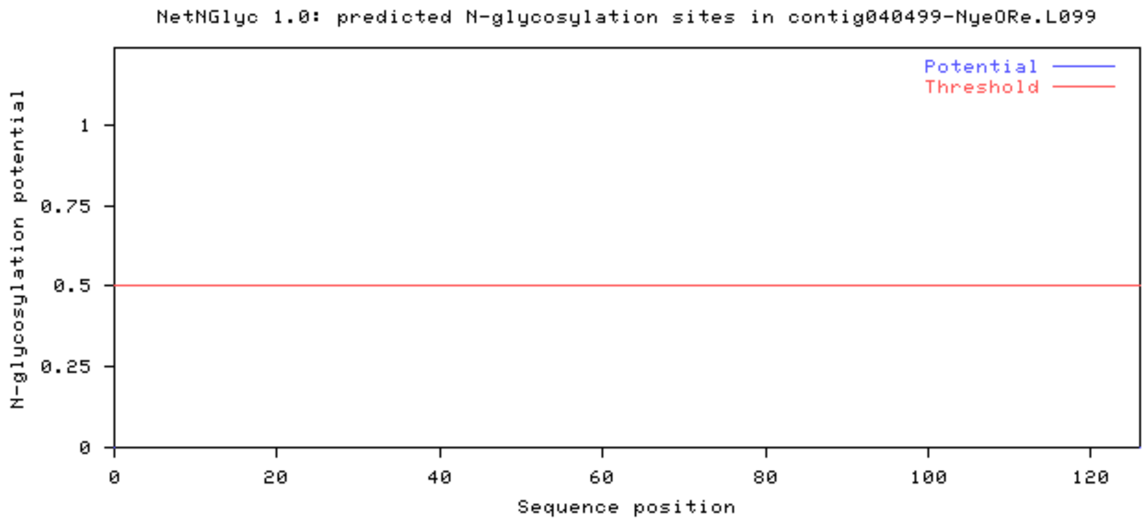

Graphics in PostScript

Output for 'contig040500-NyeORe.L098'

```
##########################################################################################

    Warning: This sequence may not contain a signal peptide!!

    Proteins without signal peptides are unlikely to be exposed to
    the N-glycosylation machinery and thus may not be glycosylated
    (in vivo) even though they contain potential motifs.

    SignalP-NN euk predictions are as follows:

  # name                 Cmax  pos ?  Ymax  pos ?  Smax  pos ?  Smean ?  D     ?

    SignalP output is explained at http://www.cbs.dtu.dk/services/SignalP/output.html

##########################################################################################

Name:  contig040500-NyeORe.L098         Length:  92
MSLQNASTKLTHFIIGGFDTVKGPVAVGVVMLIIYLLAVTGSLVNILFIVSDKQLHKPMYLLICNLAVVDILYTSSATPT      80
MIGVLLAGVNTI
....N...........................................................................      80
............                                                                         160

(Threshold=0.5)
----------------------------------------------------------------------
SeqName      Position  Potential   Jury    N-Glyc
                                agreement result
----------------------------------------------------------------------
contig040500-NyeORe.L098    5 NAST   0.5262     (5/9)   +
----------------------------------------------------------------------
```

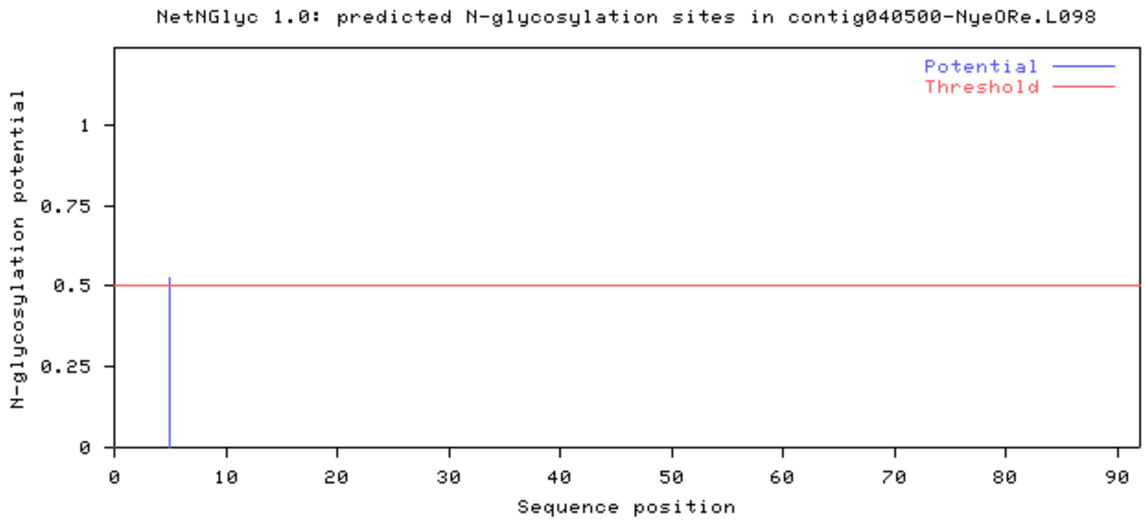

Graphics in PostScript

Output for 'contig040502-NyeOR.L091'

```
##########################################################################################

    Warning: This sequence may not contain a signal peptide!!

    Proteins without signal peptides are unlikely to be exposed to
    the N-glycosylation machinery and thus may not be glycosylated
    (in vivo) even though they contain potential motifs.

    SignalP-NN euk predictions are as follows:

  # name                    Cmax  pos ?  Ymax  pos ?  Smax  pos ?  Smean ?  D     ?

    SignalP output is explained at http://www.cbs.dtu.dk/services/SignalP/output.html

##########################################################################################

Name:  contig040502-NyeOR.L091  Length:  313
MLLQNASITVKHFIIGGFDTVKRPVAVGVVMLIIYILAIFANTVNILFIIFDKRLHKPMYLLVCNLAVVDIMYTSSATPT      80
MIGVLLADVKTISYVDCLIQMCVFHLAMVMERFALAIMAFDRLIAIIFPFHYHSYLTNTRTVVLTYILWIIGCGTVVLFP     160
ATVIPLPHCTLKLKYTFCDYAAIMRTTCVNVDEYFNQSAIWSFFILFFTFTFICISYCGILFCVKLSSNNDKKKMGSTVV     240
SHAICVTCFYSPIFIIVILTRVGVVLSLDERQGLLIGNILGPSLVNPFVYCLRTTEIKNKMVKIFKKILRLLX
....N...........................................................................      80
................................................................................     160
................................................................................     240
.........................................................................            320

(Threshold=0.5)
----------------------------------------------------------------------
SeqName        Position  Potential   Jury    N-Glyc
                                   agreement result
----------------------------------------------------------------------
contig040502-NyeOR.L091    5 NASI    0.7347     (9/9)    ++
contig040502-NyeOR.L091  196 NQSA    0.3958     (7/9)    -
----------------------------------------------------------------------
```

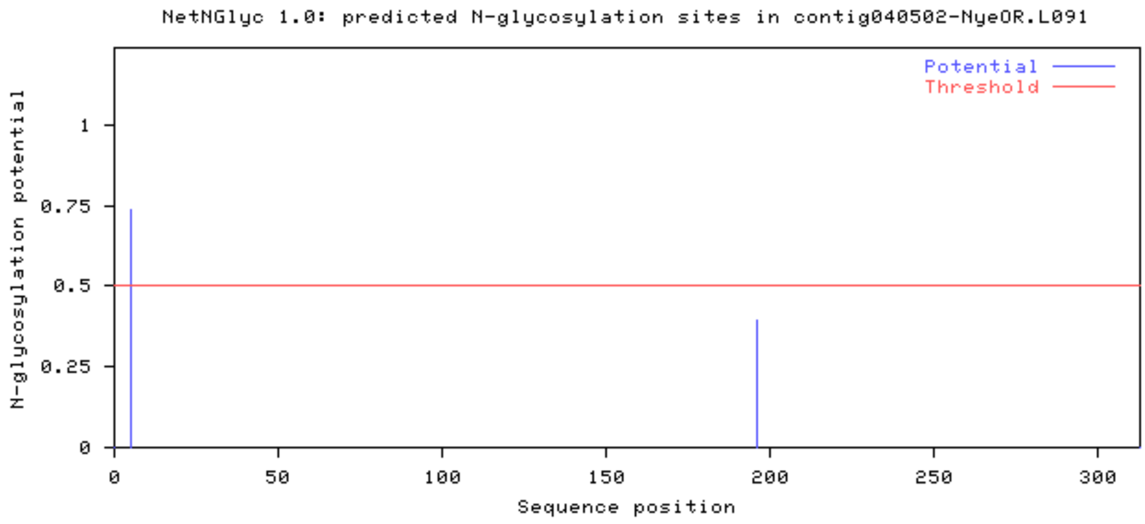

Graphics in PostScript

Output for 'contig040502-NyeOR.L092'

```
########################################################################################

    Warning: This sequence may not contain a signal peptide!!

    Proteins without signal peptides are unlikely to be exposed to
    the N-glycosylation machinery and thus may not be glycosylated
    (in vivo) even though they contain potential motifs.

    SignalP-NN euk predictions are as follows:

  # name                  Cmax  pos ?  Ymax  pos ?  Smax  pos ?  Smean ?  D     ?

    SignalP output is explained at http://www.cbs.dtu.dk/services/SignalP/output.html

########################################################################################

Name:  contig040502-NyeOR.L092  Length:  314
MSLPNASIKVTHFIIGGFDTVKRPIAVGVVMLITYLLAVIANMLNIILIIFDKRLHKPMYILICNLAVVDILYCCSTTPT      80
MIGVLLVGVNTISYVECFIQMSVFHLVGVMELFALAIMAFDRLIAFSFPFQYHSYLTNTRTLVVTYILWVVASGFVAVMP     160
VTAATLPYCTSRMKYAFCDYAAVIRTTCVDPNYYFNLVSVMMFFLLFFTFSFIGLSYFVIAFLMKFSSNRDKKKMASTCV     240
SHLIVVTCYYSPLFVLIVFTRVGVVLTLEERQGLLIGTILSPSLVNPVVYCFRTKEIKNKIFKIFTKKADILNX
....N...........................................................................      80
................................................................................     160
................................................................................     240
..........................................................................           320

(Threshold=0.5)
----------------------------------------------------------------------
SeqName      Position  Potential   Jury    N-Glyc
                                 agreement result
----------------------------------------------------------------------
contig040502-NyeOR.L092    5 NASI   0.6297     (7/9)    +
----------------------------------------------------------------------
```

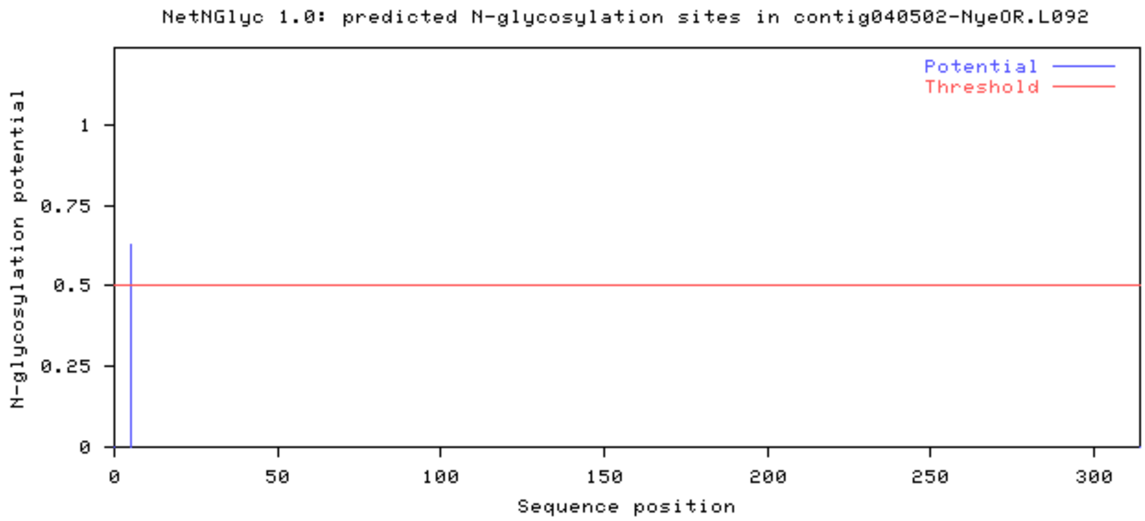

Graphics in PostScript

Output for 'contig040506-NyeOR.J140'

```
##########################################################################################

    Warning: This sequence may not contain a signal peptide!!

    Proteins without signal peptides are unlikely to be exposed to
    the N-glycosylation machinery and thus may not be glycosylated
    (in vivo) even though they contain potential motifs.

    SignalP-NN euk predictions are as follows:

  # name                    Cmax  pos ?  Ymax  pos ?  Smax  pos ?  Smean ?  D     ?

    SignalP output is explained at http://www.cbs.dtu.dk/services/SignalP/output.html

##########################################################################################

Name:  contig040506-NyeOR.J140  Length:  312
MNNRYNTSSFLQINVFNLSSESVLPAFLFATLSYMIILFCNLTLILTIVLNKSLHQPMYLILLNLPMYDLIGSSALFPQL      80
IKEILKNSGIMQYSACVAQAFFIHIYAAGTVFNLSAMAYDRYIAICYPLQYSTVMTNAHIMRIITIVWMSCLVLIAVLFF     160
LLLRLPRCRSEMTHVYCDNPSLLTLVCADTTINNIYGLFIVALSQLVANGIVFYTYLRILITCFRSKRSDTKAKALQTCA     240
THLIVFLLLECLGLFTIISYRLNNVSPHFRRFMGLSTLIFPPTLNPIIYGLKTKEIREKVLNFLKNRMFSSX
.....N..........N.......................N.........N.............................      80
................................................................................     160
..................N.............................................................     240
........................................................................             320

(Threshold=0.5)
----------------------------------------------------------------------
SeqName      Position  Potential   Jury    N-Glyc
                                 agreement result
----------------------------------------------------------------------
contig040506-NyeOR.J140    6 NTSS   0.7650     (9/9)   +++
contig040506-NyeOR.J140   17 NLSS   0.5004     (6/9)   +
contig040506-NyeOR.J140   41 NLTL   0.7212     (9/9)   ++
contig040506-NyeOR.J140   51 NKSL   0.7479     (9/9)   ++
contig040506-NyeOR.J140  113 NLSA   0.4346     (5/9)   -
contig040506-NyeOR.J140  179 NPSL   0.5146     (6/9)   +    WARNING: PRO-X1.
contig040506-NyeOR.J140  264 NVSP   0.2141     (9/9)   ---
----------------------------------------------------------------------
```

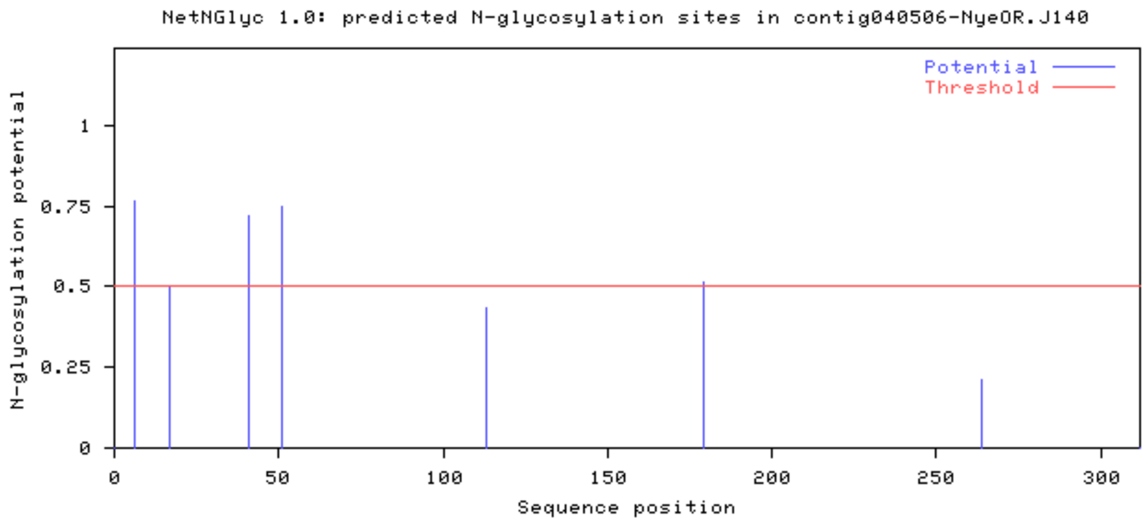

Graphics in PostScript

Output for 'contig040507-NyeOR.J080'

```
##########################################################################################

    Warning: This sequence may not contain a signal peptide!!

    Proteins without signal peptides are unlikely to be exposed to
    the N-glycosylation machinery and thus may not be glycosylated
    (in vivo) even though they contain potential motifs.

    SignalP-NN euk predictions are as follows:

  # name                Cmax  pos ?  Ymax  pos ?  Smax  pos ?  Smean ?  D     ?

    SignalP output is explained at http://www.cbs.dtu.dk/services/SignalP/output.html

##########################################################################################

Name:  contig040507-NyeOR.J080  Length:  312
MENQSFDFSSELTLDPFVIPPGGKYPIFFLGISICIFGISCNLTLLALIIILNRNLHKPMYFILFSLPLNDLIGLSAMLPK      80
VLSDIVTETHKIDYHLCVLQAFLLHMYGGGILFILAAMSFDRYVAICMPLCYSSVMTPRFISCIIVLVWGLDFVLIVSLF     160
SLQARLPRCKHVVMNVFCDNPSLLKLTCGNTTVNNIMGLFNTAVIQVVSVSIQAYSYVKILIACVVTRKSETKAKAVNTC     240
VAQLVILFMFEVVATFTILSHRFKNVSVDMQKIMGMLIFTVPPLLNPIVYGLYTNEIRSTLLRVLKNRVSMX
..N......................................N......................................      80
................................................................................     160
...................N.........N..................................................     240
........................N...............................................             320

(Threshold=0.5)
----------------------------------------------------------------------
SeqName      Position  Potential   Jury    N-Glyc
                                 agreement result
----------------------------------------------------------------------
contig040507-NyeOR.J080    3 NQSF    0.6052     (9/9)    ++
contig040507-NyeOR.J080   42 NLTL    0.6887     (9/9)    ++
contig040507-NyeOR.J080  180 NPSL    0.5543     (6/9)    +    WARNING: PRO-X1.
contig040507-NyeOR.J080  190 NTTV    0.6812     (9/9)    ++
contig040507-NyeOR.J080  265 NVSV    0.5149     (6/9)    +
----------------------------------------------------------------------
```

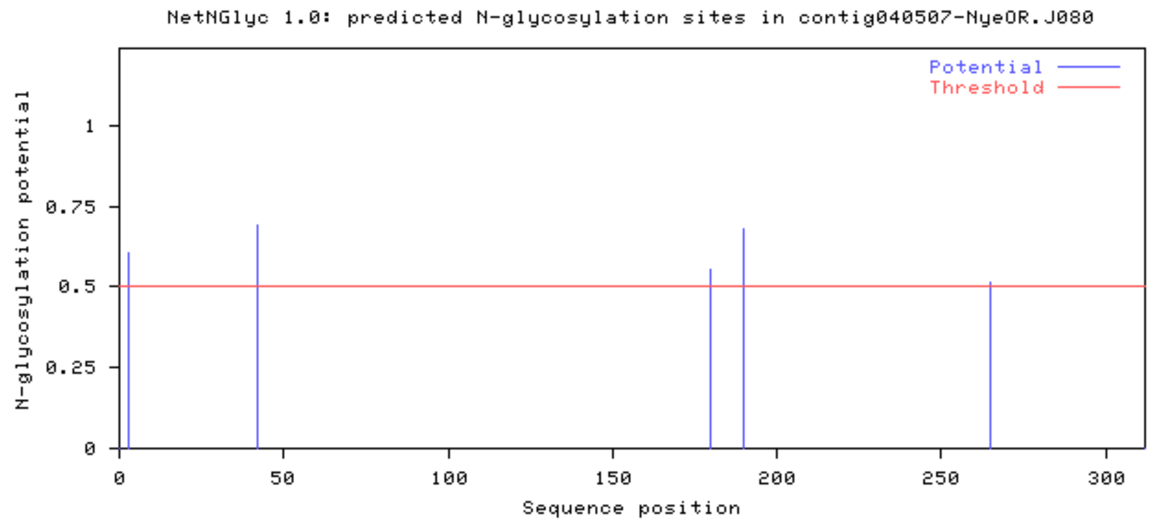

Graphics in PostScript

Output for 'contig040509-NyeOR.K083'

```
##############################################################################

   Warning: This sequence may not contain a signal peptide!!

   Proteins without signal peptides are unlikely to be exposed to
   the N-glycosylation machinery and thus may not be glycosylated
   (in vivo) even though they contain potential motifs.

   SignalP-NN euk predictions are as follows:

 # name                Cmax  pos ?  Ymax  pos ?  Smax  pos ?  Smean ?  D     ?

   SignalP output is explained at http://www.cbs.dtu.dk/services/SignalP/output.html

##############################################################################

Name:  contig040509-NyeOR.K083  Length:  312
MEKNKTVSTDILEVQGFDISPQLTYPLFFLLLFVYFTLLFSNIGVLLLIISQKSLHQPMYFLFCNLSVNDLIGNTVLLPQ      80
LMAHILATERFITYKQCVVQAFQSHTFGSASHMILIIMAIDRYVAICHPLRYSSIMTTRTVVGLSAAAWGVSVVLVSILI     160
GLTVRLSRCRSTIQNSYCDNASLFKLSCEDVSINNIYGLFFTVLLFTSSIASIAATYFRIALICWIKKNKDLNNKALQTC     240
ASHLVLYLIMLWSGFLTIILHRFPNYPDLRKIAYVLFHVVPANLNPIIYGMQMRSLRHKITEILKRKVTPSX
...N............................................................N...............      80
................................................................................     160
...................N............................................................     240
........................................................................             320

(Threshold=0.5)
----------------------------------------------------------------------
SeqName      Position  Potential   Jury    N-Glyc
                                 agreement result
----------------------------------------------------------------------
contig040509-NyeOR.K083    4 NKTV   0.7126     (9/9)   ++
contig040509-NyeOR.K083   65 NLSV   0.7386     (9/9)   ++
contig040509-NyeOR.K083  180 NASL   0.5739     (7/9)   +
----------------------------------------------------------------------
```

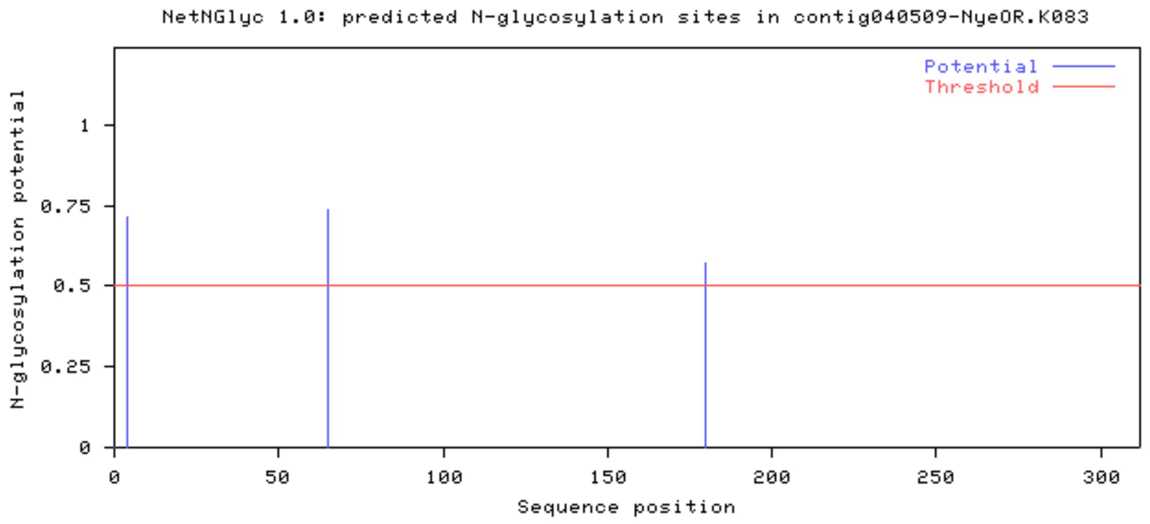

Graphics in PostScript

Output for 'contig041756-NyeOR.H139'

```
##########################################################################################

    Warning: This sequence may not contain a signal peptide!!

    Proteins without signal peptides are unlikely to be exposed to
    the N-glycosylation machinery and thus may not be glycosylated
    (in vivo) even though they contain potential motifs.

    SignalP-NN euk predictions are as follows:

  # name                 Cmax  pos ?  Ymax  pos ?  Smax  pos ?  Smean ?  D     ?

    SignalP output is explained at http://www.cbs.dtu.dk/services/SignalP/output.html

##########################################################################################

Name:  contig041756-NyeOR.H139  Length:  314
MDNVSVITVFTLSGLSDIANYRVILFVLTLLCYCVIWLVNLTIIVTVIVDKKLHEPMYIFLCNLCFNGLYGTAAFYPKFL      80
YDLLSTTHVISYAGCLLQGFVLHSAVAADFSLLALMAYDRYVAICRPLVYHSLMTTQKIYIFVFFAWLIPFYLLLMSTIT     160
TATSRLCGSHIPRIYCVNWLIANLACSASVARIVIPAFNYTFYFGHAVFIFWSYVHLIKTCQSSKENWNKFMQTCVPHLF     240
SLTVVVLSFLFDMLYMRFGSKEIPQSFENFMAMEILLIPPIINPLMYGFKLTQIRNRVLNFICGKSLALRLKSX
..N....................................N........................................      80
................................................................................     160
......................................N.........................................     240
..........................................................................           320

(Threshold=0.5)
----------------------------------------------------------------------
SeqName      Position  Potential   Jury    N-Glyc
                                 agreement result
----------------------------------------------------------------------
contig041756-NyeOR.H139    3 NVSV   0.8138     (9/9)   +++
contig041756-NyeOR.H139   40 NLTI   0.7721     (9/9)   +++
contig041756-NyeOR.H139  199 NYTF   0.6254     (8/9)   +
----------------------------------------------------------------------
```

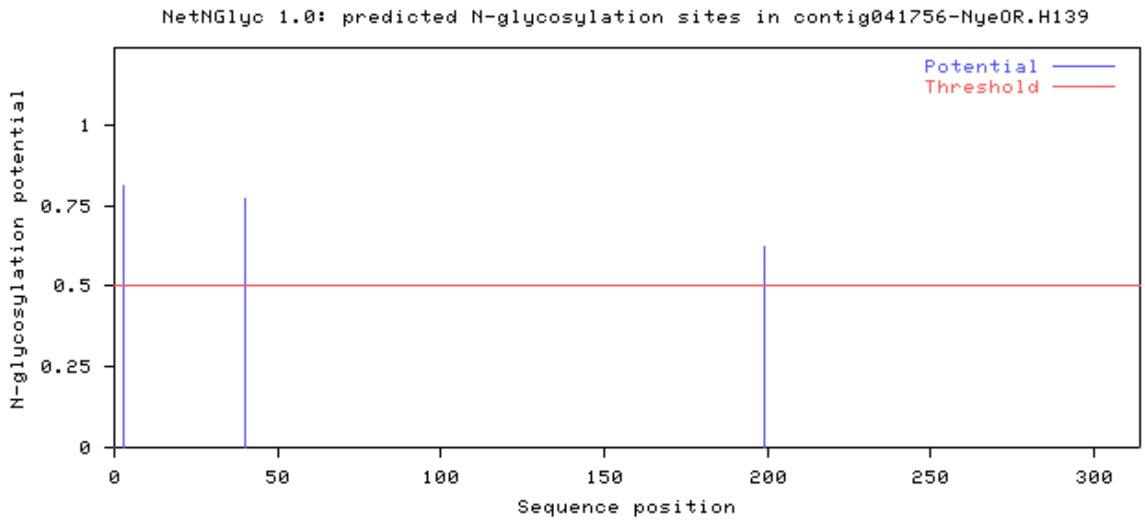

Graphics in PostScript

Output for 'contig041756-NyeOR.H075'

```
##########################################################################################

    Warning: This sequence may not contain a signal peptide!!

    Proteins without signal peptides are unlikely to be exposed to
    the N-glycosylation machinery and thus may not be glycosylated
    (in vivo) even though they contain potential motifs.

    SignalP-NN euk predictions are as follows:

  # name                    Cmax  pos ?  Ymax  pos ?  Smax  pos ?  Smean ?  D     ?

    SignalP output is explained at http://www.cbs.dtu.dk/services/SignalP/output.html

##########################################################################################

Name:  contig041756-NyeOR.H075  Length:  314
MDNVSVITVFTLSGLSDIANYRVILFVLTLLCYCVIWLVNLTIIVTVIVDKKLHEPMYIFLCNLCFNGLYGTAAFYPKFL      80
YDLLSTTHVISYAGCLLQGFVLHSAVAADFSLLALMAYDRYVAICRPLVYHSLMTTQKIYIFVFFAWLIPFYLLLMSTIT     160
TATSRLCGSHIPRIYCVNWLIANLACSASVARIVIPAFNYTFYVGHVVFVFWSYVYLIKTCQSSKENWNKFMQTCVPHLF     240
SLTVVVVSLLFDMLYMRFGSKEIAQNVENFMTMEFLLIPPIMNPLMYGLKLTKIRKRVLNFICGKSSSFRLKSX
..N....................................N........................................      80
................................................................................     160
......................................N.........................................     240
..........................................................................           320

(Threshold=0.5)
----------------------------------------------------------------------
SeqName      Position  Potential   Jury    N-Glyc
                                 agreement result
----------------------------------------------------------------------
contig041756-NyeOR.H075    3 NVSV   0.8139     (9/9)    +++
contig041756-NyeOR.H075   40 NLTI   0.7721     (9/9)    +++
contig041756-NyeOR.H075  199 NYTF   0.6143     (8/9)    +
----------------------------------------------------------------------
```

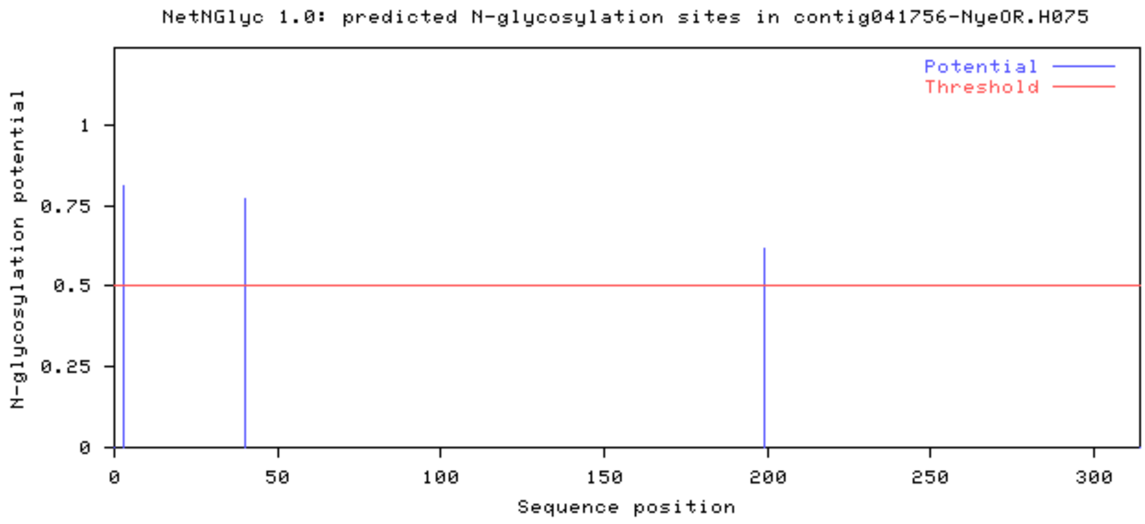

Graphics in PostScript

Output for 'contig041756-NyeORp.H078'

```
##########################################################################################

    Warning: This sequence may not contain a signal peptide!!

    Proteins without signal peptides are unlikely to be exposed to
    the N-glycosylation machinery and thus may not be glycosylated
    (in vivo) even though they contain potential motifs.

    SignalP-NN euk predictions are as follows:

  # name                  Cmax  pos ?  Ymax  pos ?  Smax  pos ?  Smean ?  D     ?

    SignalP output is explained at http://www.cbs.dtu.dk/services/SignalP/output.html

##########################################################################################

Name:  contig041756-NyeORp.H078         Length:  292
MDNVSNVKSFVLLGFNDTMNFRVPLFIITLLHYCVILFFNISLVLLIVLDESLHEPMYIFLSSFCINALYGSTGFYPKFL      80
SDLLSSFHHISYEGCMLQAFIMYSFSSCDLSILAVMAFDRYLAICRPLHYHSFMTMRRLSQLVCFSWLTPFCIFSINIPI     160
TSRVKLCGINIQRVLCLDWLIVKLACYIFHGFFIIWIYTHLIKTCMRSSEDRVKFMQTCVPHLTSLIIFLFVMVFQSIYG     240
RFDSTDSSRIFQNLIAIELLTILPVMNPLIYGFKLTKIRNRILTSVYLNRKX
..N............N.......................N........................................      80
................................................................................     160
................................................................................     240
....................................................                                 320

(Threshold=0.5)
----------------------------------------------------------------------
SeqName      Position  Potential   Jury    N-Glyc
                                 agreement result
----------------------------------------------------------------------
contig041756-NyeORp.H078     3 NVSN   0.7752     (9/9)   +++
contig041756-NyeORp.H078    16 NDTM   0.6704     (9/9)   ++
contig041756-NyeORp.H078    40 NISL   0.6714     (9/9)   ++
----------------------------------------------------------------------
```

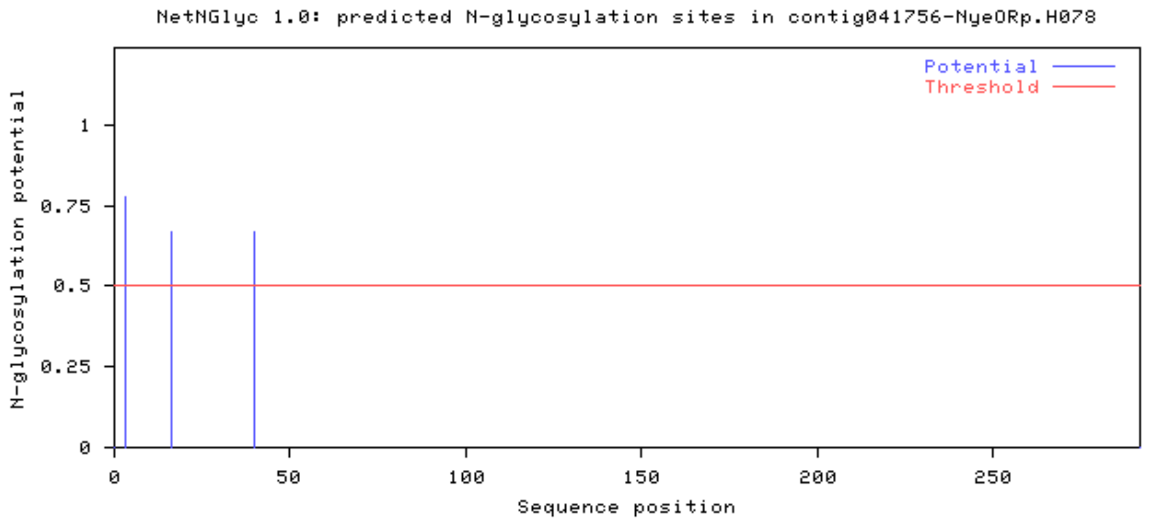

Graphics in PostScript

Output for 'contig041757-NyeOR.H076'

```
##########################################################################################

    Warning: This sequence may not contain a signal peptide!!

    Proteins without signal peptides are unlikely to be exposed to
    the N-glycosylation machinery and thus may not be glycosylated
    (in vivo) even though they contain potential motifs.

    SignalP-NN euk predictions are as follows:

  # name                 Cmax  pos ?  Ymax  pos ?  Smax  pos ?  Smean ?  D     ?

    SignalP output is explained at http://www.cbs.dtu.dk/services/SignalP/output.html

##########################################################################################

Name:  contig041757-NyeOR.H076  Length:  314
MDNVSVITVFTLSGLSDIANYRVILFVLTLLCYCVIWLVNLTIIVTVIVDKKLHEPMYIFLCNLCVNGLYGTAAFYPKFL      80
YDLLSTTHVISYAGCLLQGFALHSSICADFSLLALMAYDRYVAICRPLVYHSLMTKQKVCIFVFFAWFFPIYLMLLSTIT     160
TAVLRLCGTHIPRIYCINWLINNLACSASAARIVIPAFNYTFYIGHILFVFWTYVHLVKTCQSSKENWNKFMQTCVPHLF     240
SLIVVAVSFLFDMLYVRFGSKEFPQSFENFMAMEILLIPPIINPVMYGFKLTKIRNRVLNFICGISSTLRLKLX
..N....................................N........................................      80
................................................................................     160
......................................N.........................................     240
..........................................................................           320

(Threshold=0.5)
----------------------------------------------------------------------
SeqName      Position  Potential   Jury    N-Glyc
                                 agreement result
----------------------------------------------------------------------
contig041757-NyeOR.H076    3 NVSV    0.8138     (9/9)    +++
contig041757-NyeOR.H076   40 NLTI    0.7721     (9/9)    +++
contig041757-NyeOR.H076  199 NYTF    0.5914     (7/9)    +
----------------------------------------------------------------------
```

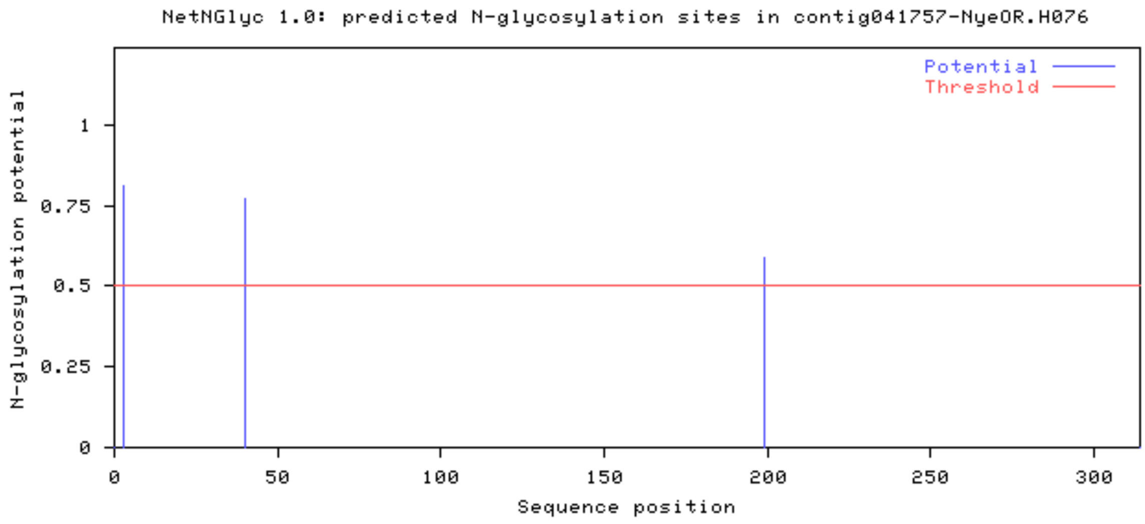

Graphics in PostScript

Output for 'contig044295-NyeOR.R135'

```
##########################################################################################

    Warning: This sequence may not contain a signal peptide!!

    Proteins without signal peptides are unlikely to be exposed to
    the N-glycosylation machinery and thus may not be glycosylated
    (in vivo) even though they contain potential motifs.

    SignalP-NN euk predictions are as follows:

  # name                 Cmax  pos ?  Ymax  pos ?  Smax  pos ?  Smean ?  D     ?

    SignalP output is explained at http://www.cbs.dtu.dk/services/SignalP/output.html

##########################################################################################

Name:  contig044295-NyeOR.R135  Length:  317
MFFTNETLTNITVGQQNQLFLEIVFSCIVTTLTCCVFLFINATMLFTLRSKPVFGQTSRYILLYNLLFADTLQMAQSQVM      80
FLLSACRITLLYPICGVLVSLATLLTLISPLTLVAMSLERYVAVCYPLRHAAIITVRNTALAVCVVWTISLLNVLIEVVL     160
MLRVRFQDLLHLQMEYSCNKEKLTLDPISDLYAKAFSYFLFVLAAGAFIFSYIGVTVVAQSASTDKGSAEKARKTLVLHL     240
VQLGLSVSSTIHNPIFVFIYKTVDSVIVVRIRVVIYLCIIILPRCLSSFIYGLRDHTIRPVLMLNLRCQWKCQPFLX
....N....N..............................N.......................................      80
................................................................................     160
................................................................................     240
.............................................................................        320

(Threshold=0.5)
----------------------------------------------------------------------
SeqName      Position  Potential   Jury    N-Glyc
                                 agreement result
----------------------------------------------------------------------
contig044295-NyeOR.R135    5 NETL    0.7620     (9/9)    +++
contig044295-NyeOR.R135   10 NITV    0.6378     (7/9)    +
contig044295-NyeOR.R135   41 NATM    0.6193     (7/9)    +
----------------------------------------------------------------------
```

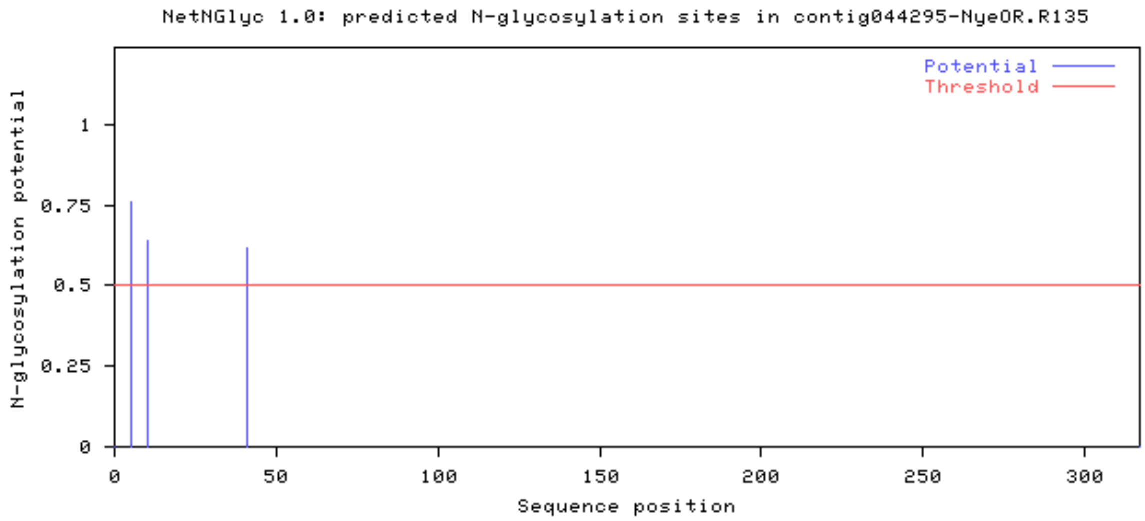

[Graphics in PostScript](#)

Output for 'contig044298-NyeORe.R136'

```
##########################################################################################

    Warning: This sequence may not contain a signal peptide!!

    Proteins without signal peptides are unlikely to be exposed to
    the N-glycosylation machinery and thus may not be glycosylated
    (in vivo) even though they contain potential motifs.

    SignalP-NN euk predictions are as follows:

  # name                  Cmax  pos ?  Ymax  pos ?  Smax  pos ?  Smean ?  D     ?

    SignalP output is explained at http://www.cbs.dtu.dk/services/SignalP/output.html

##########################################################################################

Name:  contig044298-NyeORe.R136         Length:  300
LAERVMISTLTTLPTCVFLFINGIMLFTLRSKPVFRETCRYILLYNLLFADTVQLAQSQIHFLLAVLRITVSYPVCAFLV      80
NFTHLTAVISPLTLVVMPLERYVAVCYPLRHATIITIRNTGAAITVIWAISFLNIIIRTLLFLSLFEKLGKVEVKYLCSE     160
ISILLGTKSDHFDKAFTCIVVVAAGVAVIFSYIGVIVAARSASTDKALAFKARNTLLLNLMQLFLSLSSTIYYPLLVPLS     240
VTVTRIVFVRIQNVFYLLFFILPRCLTSLIYGLRDQTIRPVLIYHLCCQLKCPVVEDKGX
................................................................................      80
N...............................................................................     160
................................................................................     240
............................................................                         320

(Threshold=0.5)
----------------------------------------------------------------------
SeqName      Position  Potential   Jury    N-Glyc
                                agreement result
----------------------------------------------------------------------
contig044298-NyeORe.R136    81 NFTH   0.6727     (9/9)   ++
----------------------------------------------------------------------
```

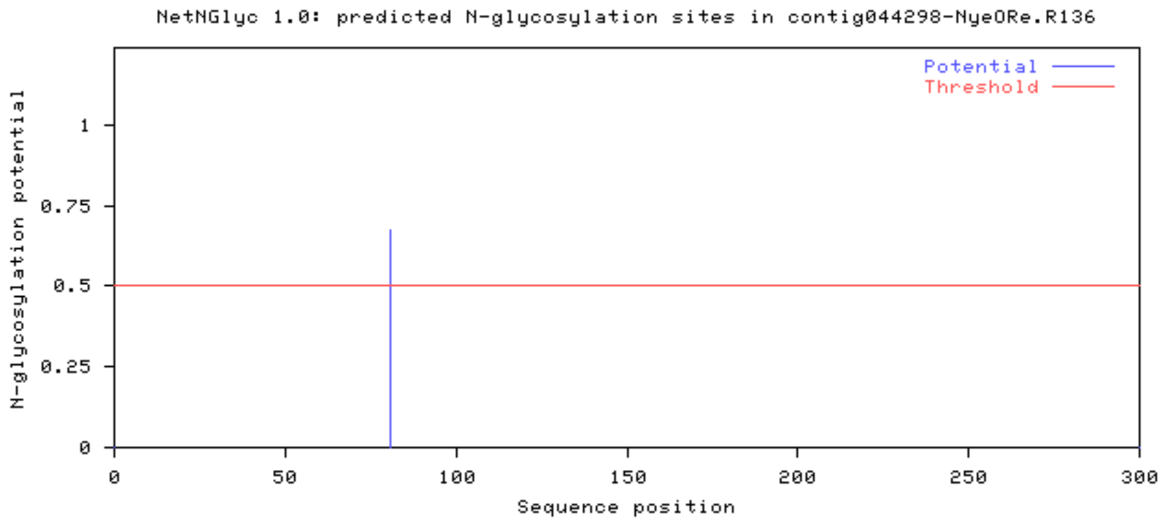

Graphics in PostScript

Output for 'contig044492-NyeOR.B034'

```
##########################################################################################

    Warning: This sequence may not contain a signal peptide!!

    Proteins without signal peptides are unlikely to be exposed to
    the N-glycosylation machinery and thus may not be glycosylated
    (in vivo) even though they contain potential motifs.

    SignalP-NN euk predictions are as follows:

  # name                    Cmax  pos ?  Ymax  pos ?  Smax  pos ?  Smean ?  D     ?

    SignalP output is explained at http://www.cbs.dtu.dk/services/SignalP/output.html

##########################################################################################

Name:  contig044492-NyeOR.B034  Length:  321
MSAGVNVTSLPILVTSVTLDGLAQLSNQRLFFFFFFLCAYLFMLCSDSLVVYVICSQRSLHRPMFVFVTAVLMNSVAGST      80
VFYPKLLVDLLRGVRSVQVTLRVCMCEAWLLYSLGTSSFLLLAAMSFDRYVSICRPLLYTVVMSPATVLALLLLCWLLPV     160
GLVGTAVLLASRLPLCRSQLSRIYCDIYSLVSLSCGGRETLLSEVYNLSVIVATVLLPAIFVLFSYSAVLSVCLQRSRSF     240
SSKALSTCLPHLLVFCNYSVSSGVEVLQRRLQAGSQPTASVLTSIFQVMIPTVFNPVVYGLKVTEIRAQLRRLLGCQRAD     320
X
.....N..........................................................................      80
................................................................................     160
..............................................N.................................     240
................N...............................................................     320
.                                                                                    400

(Threshold=0.5)
----------------------------------------------------------------------
SeqName      Position  Potential   Jury    N-Glyc
                                 agreement result
----------------------------------------------------------------------
contig044492-NyeOR.B034    6 NVTS   0.7708     (9/9)    +++
contig044492-NyeOR.B034  207 NLSV   0.6107     (7/9)    +
contig044492-NyeOR.B034  257 NYSV   0.5469     (7/9)    +
----------------------------------------------------------------------
```

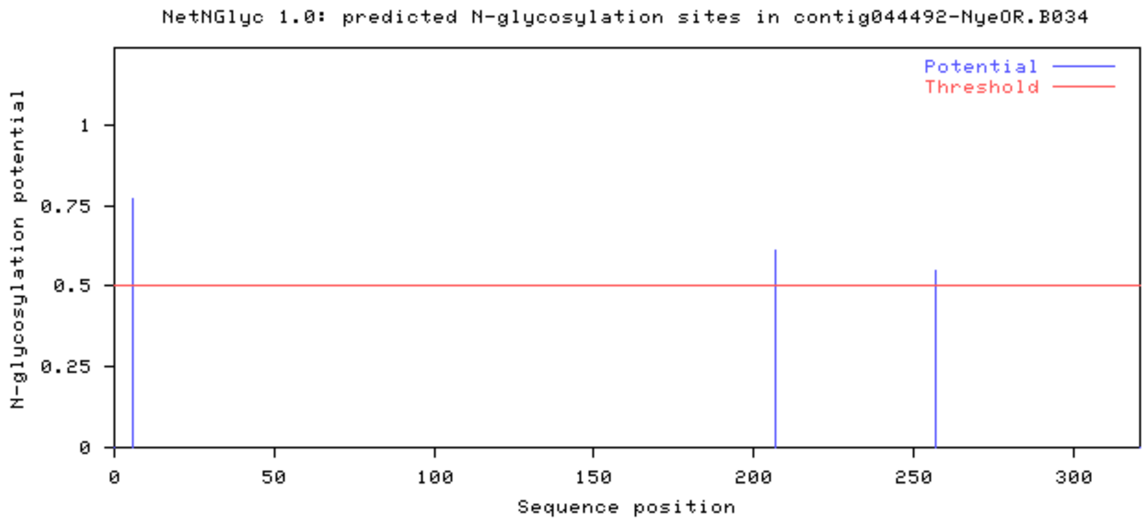

Graphics in PostScript

Output for 'contig046488-NyeOR.K084'

```
##########################################################################################

    Warning: This sequence may not contain a signal peptide!!

    Proteins without signal peptides are unlikely to be exposed to
    the N-glycosylation machinery and thus may not be glycosylated
    (in vivo) even though they contain potential motifs.

    SignalP-NN euk predictions are as follows:

  # name                    Cmax  pos ?  Ymax  pos ?  Smax  pos ?  Smean ?  D     ?

    SignalP output is explained at http://www.cbs.dtu.dk/services/SignalP/output.html

##########################################################################################

Name:  contig046488-NyeOR.K084  Length:  315
MENQTSDILLLEGLQVSPDASIPAFILLLLIYIFIMFSNIVLVILITLDSSLHQPMYLLFCNMSINDVFGATTIIPRMLR      80
DIFIPSSDRYIHYVDCVIQAFCVHIYTGASHTILMIMAFDRYVAICNPLQYATIMTNWMVVKLSVLAWAVIFIMVTILVG     160
LSVRLSRCRWIIFNPFCDNASLFKLSCENILINNIYGLGYTVLLMGSSIGSVTITYLRIAMVCLSSKSKTLNSRALQTYT     240
THLTMYVIMFLSCIVMVLLHRFPHLTDQRKLASMMFHVVPPALNAVIYGMQIKAVRQKMFIMFTRNTVTVTDRKX
..N..........................................................N..................      80
................................................................................     160
..................N.............................................................     240
...........................................................................          320

(Threshold=0.5)
----------------------------------------------------------------------
SeqName      Position  Potential   Jury    N-Glyc
                                 agreement result
----------------------------------------------------------------------
contig046488-NyeOR.K084    3 NQTS   0.7476     (9/9)    ++
contig046488-NyeOR.K084   62 NMSI   0.6738     (9/9)    ++
contig046488-NyeOR.K084  179 NASL   0.6916     (9/9)    ++
----------------------------------------------------------------------
```

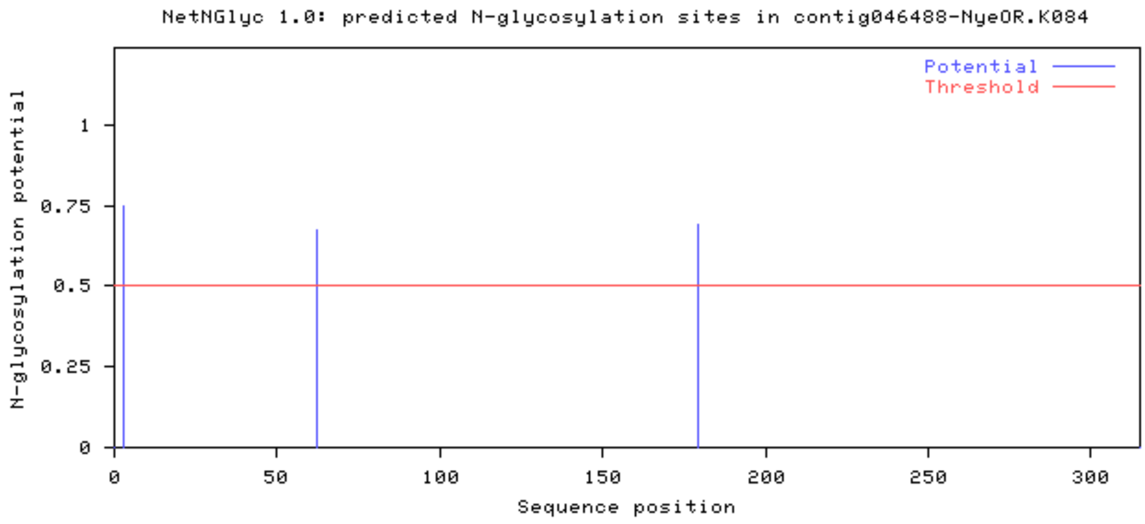

Graphics in PostScript

Output for 'contig046490-NyeOR.K085'

```
##########################################################################################

    Warning: This sequence may not contain a signal peptide!!

    Proteins without signal peptides are unlikely to be exposed to
    the N-glycosylation machinery and thus may not be glycosylated
    (in vivo) even though they contain potential motifs.

    SignalP-NN euk predictions are as follows:

  # name                  Cmax  pos ?  Ymax  pos ?  Smax  pos ?  Smean ?  D     ?

    SignalP output is explained at http://www.cbs.dtu.dk/services/SignalP/output.html

##########################################################################################

Name:  contig046490-NyeOR.K085  Length:  314
MENYTYNILTLQLEGLNISVESTYALFLLLLFFYLFIIVANGGIAVLVFMDKNLHQPMYLLFCNLPFNDILGNSIMVPRL      80
LIDILKPPSERFISYYECVVQAFTTHMFGTTSHTVLMIMAFDRYVAICNPLRYASIMTNKMVIKLTVFAWGVAFVLVGIL     160
LGLTVRLSRCRTLITNPYCDNASLFKLSCENVFINNVYGLTFTVVLFTGSIGSIVLTYASITIVCLTSKNKSLNSKALKT     240
CSTHLVVYLIMLLSGMIVIMLHRFPQYSDYRKLCSILFHIIPGSLNPIIYGVQSKEMQKLFAKLLQKKTGPLKX
..N.............N...............................................................      80
................................................................................     160
....................N...........................................................     240
..........................................................................           320

(Threshold=0.5)
----------------------------------------------------------------------
SeqName      Position  Potential   Jury    N-Glyc
                                 agreement result
----------------------------------------------------------------------
contig046490-NyeOR.K085    3 NYTY   0.7760     (9/9)    +++
contig046490-NyeOR.K085   17 NISV   0.6830     (9/9)    ++
contig046490-NyeOR.K085  181 NASL   0.6141     (7/9)    +
contig046490-NyeOR.K085  230 NKSL   0.4032     (7/9)    -
----------------------------------------------------------------------
```

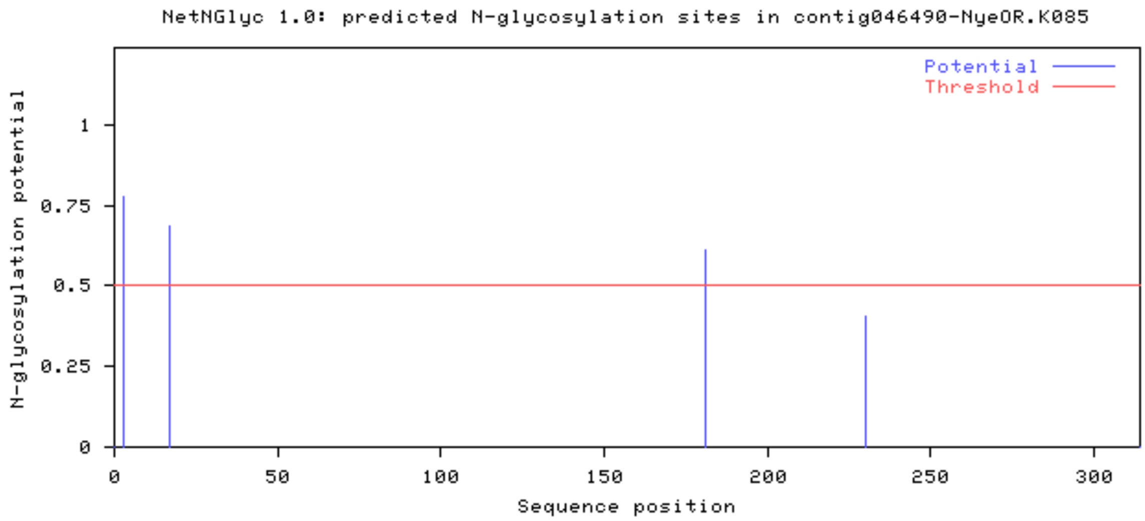

Graphics in PostScript

Output for 'contig046490-NyeORsp.K086'

```
##########################################################################

    Warning: This sequence may not contain a signal peptide!!

    Proteins without signal peptides are unlikely to be exposed to
    the N-glycosylation machinery and thus may not be glycosylated
    (in vivo) even though they contain potential motifs.

    SignalP-NN euk predictions are as follows:

  # name                 Cmax  pos ?  Ymax  pos ?  Smax  pos ?  Smean ?  D     ?

    SignalP output is explained at http://www.cbs.dtu.dk/services/SignalP/output.html

##########################################################################

Name:  contig046490-NyeORsp.K086       Length:  304
MENYMYNSPTLQLQELRIVRTNKYLISSFSFFSYLFLIVANVGIAVLVFVDKSLHQPMYILFCNLSINDLFGNSIMIPRL      80
LVDMLRPPSERLISYYEFVVQAFTTHMFSTTAHTVLMIMAFDRYVAICNPLCYAAVMTNKMLMKLTVSAWGVAFVLVGIL     160
LGLTLRPGRCRTLIKSPYCDNAALFNLSCEDVFINNVYGLTFTVLLFTGSIGSMVLTYTKITVVCLTTKNKSLNNKALKT     240
CSTHLVVYLIFLFSGMSIITLHRFPEYGSRKIVAVLYHIIPGSLNPIIYGMQSKEIKKICIKCX
...............................................................N................      80
................................................................................     160
.........................N......................................................     240
................................................................                     320

(Threshold=0.5)
----------------------------------------------------------------------
SeqName      Position  Potential   Jury    N-Glyc
                                 agreement result
----------------------------------------------------------------------
contig046490-NyeORsp.K086   64 NLSI   0.6490     (9/9)   ++
contig046490-NyeORsp.K086  186 NLSC   0.6164     (8/9)   +
contig046490-NyeORsp.K086  230 NKSL   0.4764     (6/9)   -
----------------------------------------------------------------------
```

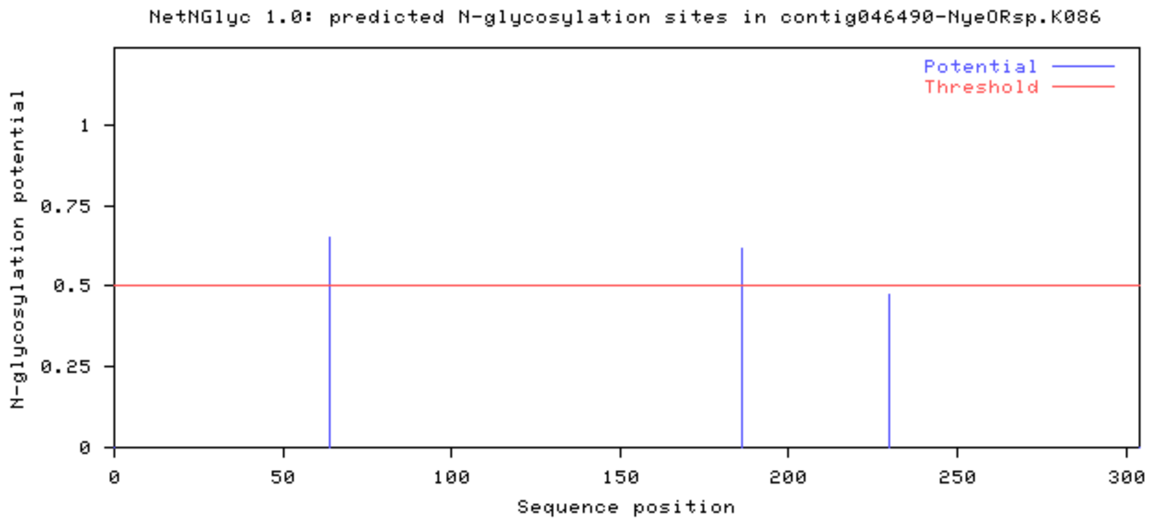

[Graphics in PostScript]

Output for 'contig046491-NyeOR.K087'

```
##########################################################################################

    Warning: This sequence may not contain a signal peptide!!

    Proteins without signal peptides are unlikely to be exposed to
    the N-glycosylation machinery and thus may not be glycosylated
    (in vivo) even though they contain potential motifs.

    SignalP-NN euk predictions are as follows:

  # name                    Cmax  pos ?  Ymax  pos ?  Smax  pos ?  Smean ?  D     ?

    SignalP output is explained at http://www.cbs.dtu.dk/services/SignalP/output.html

##########################################################################################

Name:  contig046491-NyeOR.K087  Length:  314
MENYTYNSYILQLEGLNTSKDSLYPAFLFLFFSYLFIMIINVGITILIFMNKNLHQPMYLLFCNLPLTDILVTSIVVPRL      80
LIDLMRPPSERLISYNQCVVQAYIAHLVGTTSHTVLMIMAYDRYVAICNPFHYVSIMTNKMMIKLTVCAWGVAFVLVGIL     160
LGLTTRLSRCRTLITNPYCDNASLFKLSCESVVINNIYGITFTVAVYVGSIGAMVLSYTSIAVVCLTSKNKSLNSKALKT     240
CSTHLVVYLILTLSGMALITLHRFPQYTEYRKIFAVLFSIFPGSLNPIIYGVQSKDLQKALLKFCVSKKVLASX
..N.............N...............................................................      80
................................................................................     160
....................N...........................................................     240
..........................................................................           320

(Threshold=0.5)
----------------------------------------------------------------------
SeqName      Position  Potential   Jury    N-Glyc
                                 agreement result
----------------------------------------------------------------------
contig046491-NyeOR.K087    3 NYTY   0.7617     (9/9)    +++
contig046491-NyeOR.K087   17 NTSK   0.6181     (8/9)    +
contig046491-NyeOR.K087  181 NASL   0.6051     (7/9)    +
contig046491-NyeOR.K087  230 NKSL   0.3910     (7/9)    -
----------------------------------------------------------------------
```

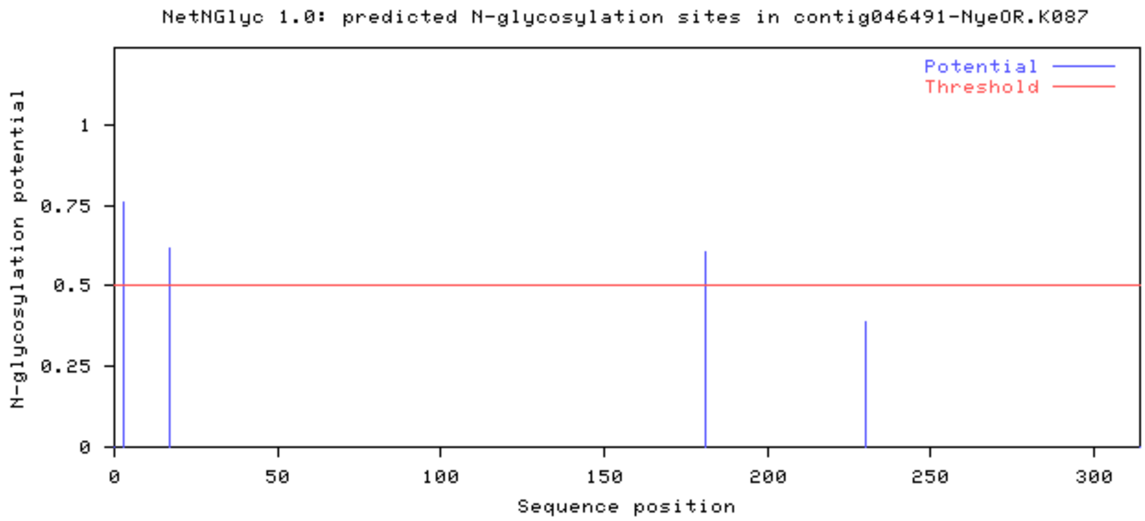

Graphics in PostScript

Output for 'contig046495-NyeORs.I079'

```
##########################################################################################

    Warning: This sequence may not contain a signal peptide!!

    Proteins without signal peptides are unlikely to be exposed to
    the N-glycosylation machinery and thus may not be glycosylated
    (in vivo) even though they contain potential motifs.

    SignalP-NN euk predictions are as follows:

  # name                   Cmax  pos ?  Ymax  pos ?  Smax  pos ?  Smean ?  D     ?

    SignalP output is explained at http://www.cbs.dtu.dk/services/SignalP/output.html

##########################################################################################

Name:  contig046495-NyeORs.I079         Length:  310
MATPLKQLIVFELEGFYIPPGFGPLLFFLALFTYMLVLLGNGVIVSMIVIDKNLHRPMFVMVCHLLVCDLLGSTAVLPGL      80
MMHFLMGQKRIAYIPAIAQAFSVHTYGLAVQAILGAMAYDRRYIAVCEPLRYHAIMTSARLHSCCALAWLLALLPIAVLF     160
SFHMNVPLCGRVILHVYCSNRGILGLACIPTPASDIYGLAMTWTVSTGIFLIIAFSYIRILQVSLKHSRIDTSIRSKAFQ     240
TCASHLVVYVLYQIASVIIIVSYRFPSVSENLKKFFSILFIIVPPAINPIIYGLVSKELRSSIIKHFTIX
................................................................................      80
................................................................................     160
................................................................................     240
......................................................................               320

(Threshold=0.5)

No sites predicted in this sequence.
```

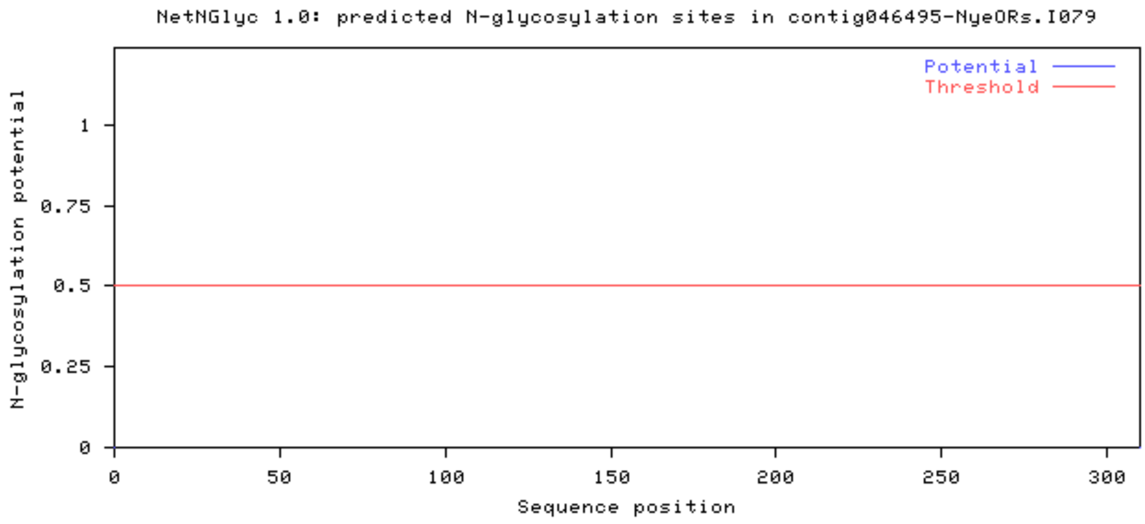

Graphics in PostScript

Output for 'contig048778-NyeOR.M107'

```
##########################################################################################

    Warning: This sequence may not contain a signal peptide!!

    Proteins without signal peptides are unlikely to be exposed to
    the N-glycosylation machinery and thus may not be glycosylated
    (in vivo) even though they contain potential motifs.

    SignalP-NN euk predictions are as follows:

  # name                 Cmax  pos ?  Ymax  pos ?  Smax  pos ?  Smean ?  D     ?

    SignalP output is explained at http://www.cbs.dtu.dk/services/SignalP/output.html

##########################################################################################

Name:  contig048778-NyeOR.M107  Length:  321
MILQRTVLNSSAIIHPPGFYIIGFETFPFISVYFIFLVFVYGVTVLFNILVIYVIASTRRLHTPKFLAVVNLAVIDLFLN      80
TCTIPSMIKIFLIKNNFIPFNLCLLQMYVYYYVFVSLESFALAILAYDRLIAICFPLRQNSINTVQSMCCIISITWFLVMG    160
VIAFTTGIMTRLSFCRSVRVFSYFCDYAPVFRLACNDNTMQWFAASFLSVLLLFVPFTFIFLSYVSILVTVFRMKSVDSR    240
VKALATCVEHLILVAIFYIPLTVIFTIGFFLGVVNPDQRVLSLSLASCIPPCINPIVYSLKTKDIKTRALKLFRKTKVNA    320
X
........N.......................................................................      80
................................................................................     160
................................................................................     240
................................................................................     320
.                                                                                    400

(Threshold=0.5)
----------------------------------------------------------------------
SeqName      Position  Potential   Jury    N-Glyc
                                 agreement result
----------------------------------------------------------------------
contig048778-NyeOR.M107    9 NSSA   0.5519     (6/9)   +
----------------------------------------------------------------------
```

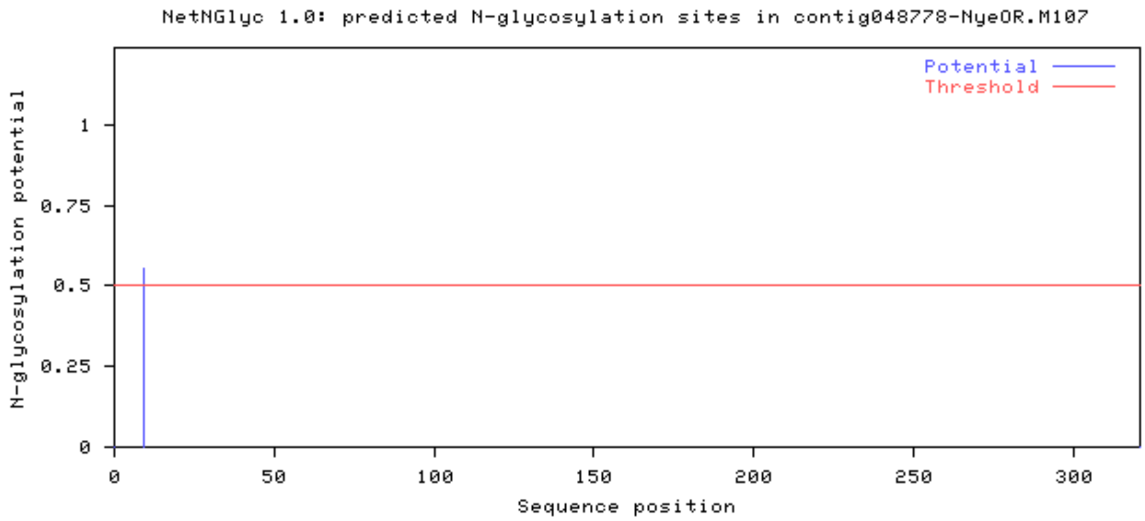

Graphics in PostScript

Output for 'contig048778-NyeOR.M108'

```
##########################################################################################

    Warning: This sequence may not contain a signal peptide!!

    Proteins without signal peptides are unlikely to be exposed to
    the N-glycosylation machinery and thus may not be glycosylated
    (in vivo) even though they contain potential motifs.

    SignalP-NN euk predictions are as follows:

  # name                    Cmax  pos ?  Ymax  pos ?  Smax  pos ?  Smean ?  D     ?

    SignalP output is explained at http://www.cbs.dtu.dk/services/SignalP/output.html

##########################################################################################

Name:  contig048778-NyeOR.M108  Length:  327
MILQRTVLNSSAIIHPPGFYIIGFETFPFISVYFIFLVFVYGVTVLFNILVIYVIASTRRLHTPKFLAVVNLAVIDLFLN      80
TCTIPSMIKIFLIKNNFIPFNLCLLQMYVYYYVFVSLESFALAILAYDRLIAICFPLRHNSINTVRSMCCIVSVSWISNMG     160
ITAFATGIMTRLSFCRSVRVFSYFCDYAPVFRLACNDNTMQWFAASFLSILLLFVPFTFILLSYVSILMTVFRMKSVDSR     240
VKALATCAEHLILVAIFYIPLTVIFTIGFFLGVVNPDQRVLSLSLASCIPPCINPIVYSLKTKDIKTRALTLFRTKVKTK     320
LTKVNAX
........N.......................................................................      80
................................................................................     160
................................................................................     240
................................................................................     320
.......                                                                              400

(Threshold=0.5)
----------------------------------------------------------------------
SeqName      Position  Potential   Jury    N-Glyc
                                agreement result
----------------------------------------------------------------------
contig048778-NyeOR.M108    9 NSSA   0.5514     (6/9)    +
----------------------------------------------------------------------
```

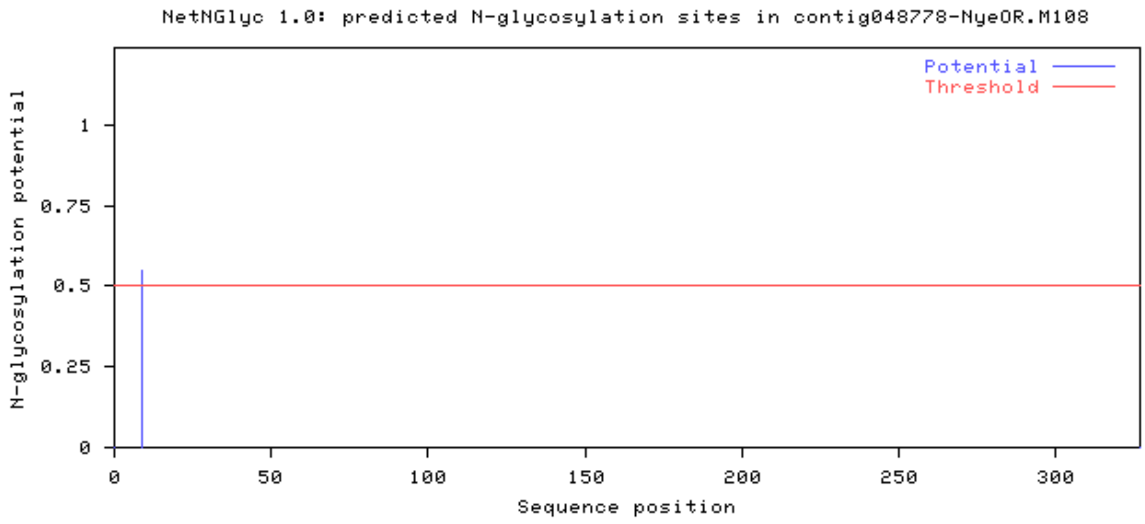

Graphics in PostScript

Output for 'contig049610-NyeORp.F065'

```
##########################################################################################

    Warning: This sequence may not contain a signal peptide!!

    Proteins without signal peptides are unlikely to be exposed to
    the N-glycosylation machinery and thus may not be glycosylated
    (in vivo) even though they contain potential motifs.

    SignalP-NN euk predictions are as follows:

  # name                   Cmax   pos ?  Ymax   pos ?  Smax   pos ?  Smean ?  D     ?

    SignalP output is explained at http://www.cbs.dtu.dk/services/SignalP/output.html

##########################################################################################

Name:  contig049610-NyeORp.F065         Length:  310
MENNSHPLYFNLTMFVYIGKFRYLAFVLFLLLYTFIISANLVIIVVISREKTLHEPMYIFIMCLSINSLYGSGGFFFRFL      80
RDLLSDSNLIARSACYTQIYIIYTYASYELTILGIMAYDRFVAICQPLHYHSKLTSRVISKLLAFAWIYPAFSVAACVYL     160
ASRLPLCGNKIPKVFCANWPVVKLSCVPTVINNIIGMFVSVTTVFLPLAFVLYTYMRIFLVCRKRSSLFKSKVIQSCLPH     240
IVTFVNYSITVFCDVALSRIDLEELNPFLGIILSLEFVVIPPILNPLMYGLKLPEIRKCILRNLSCLIRX
..N.......N.....................................................................      80
................................................................................     160
................................................................................     240
.....N.......................................................N.......                320

(Threshold=0.5)
----------------------------------------------------------------------
SeqName      Position  Potential   Jury    N-Glyc
                                agreement result
----------------------------------------------------------------------
contig049610-NyeORp.F065    3 NNSH   0.5824     (9/9)   ++
contig049610-NyeORp.F065   11 NLTM   0.8139     (9/9)   +++
contig049610-NyeORp.F065  246 NYSI   0.5721     (7/9)   +
contig049610-NyeORp.F065  303 NLSC   0.5138     (5/9)   +
----------------------------------------------------------------------
```

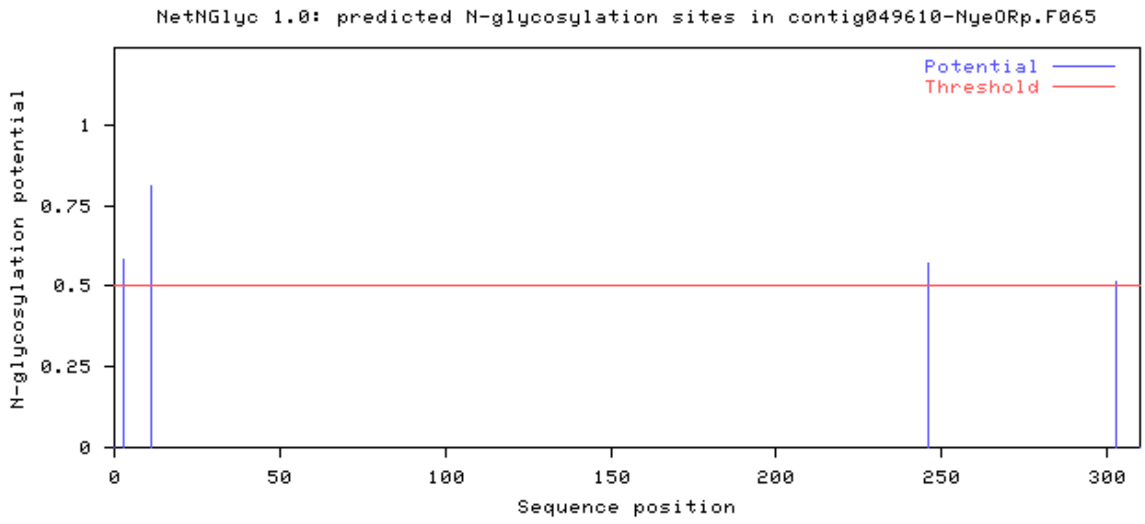

Graphics in PostScript

Output for 'contig050024-NyeORs.W129'

```
##########################################################################################

    Warning: This sequence may not contain a signal peptide!!

    Proteins without signal peptides are unlikely to be exposed to
    the N-glycosylation machinery and thus may not be glycosylated
    (in vivo) even though they contain potential motifs.

    SignalP-NN euk predictions are as follows:

  # name                    Cmax  pos ?  Ymax  pos ?  Smax  pos ?  Smean ?  D     ?

    SignalP output is explained at http://www.cbs.dtu.dk/services/SignalP/output.html

##########################################################################################

Name:  contig050024-NyeORs.W129         Length:  306
MSASYTNETVVVNYRDAFPKAMVKNVIVVVLCISINYINVALLQTFCKQQVIFYMNPRYILFFHLVLNDMIQVTLTVILF      80
ISSYIFFQINVSVCCVLILLALFATENTPLNLACMAVECYIAICIPLRHVQICTVKRTLMLIGLIWMTSMLSVLPDLFIT     160
LAIEPLDFYNSRVFCLRETVFRNPHIIKKRDITYIVYLVIVWFIIFFTYFKILFTAKAASQDATKARNTIILHGFQVLLC     240
MSIYAEPLLRQVLQQWLPQNYSDSLFACYILFQILPRAISPIVYGVRDKTYRKYLKRYLLCKMGPX
......N.........................................................................      80
.........N......................................................................     160
................................................................................     240
...................N..............................................     320

(Threshold=0.5)
----------------------------------------------------------------------
SeqName      Position  Potential   Jury    N-Glyc
                                 agreement result
----------------------------------------------------------------------
contig050024-NyeORs.W129    7 NETV   0.7382     (9/9)   ++
contig050024-NyeORs.W129   90 NVSV   0.6561     (8/9)   +
contig050024-NyeORs.W129  260 NYSD   0.6599     (9/9)   ++
----------------------------------------------------------------------
```

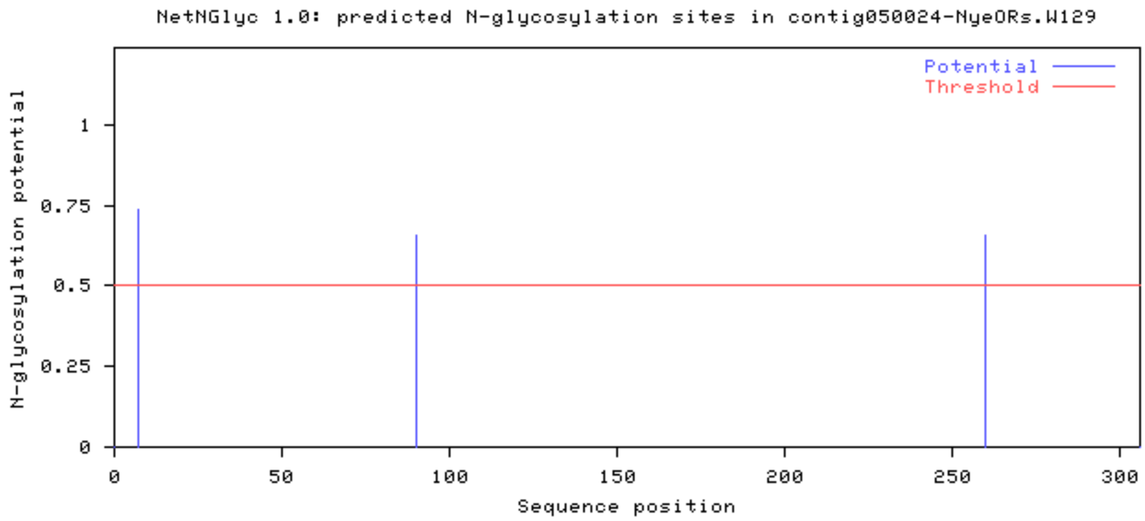

Graphics in PostScript

Output for 'contig050025-NyeORs.W130'

```
##########################################################################################

    Warning: This sequence may not contain a signal peptide!!

    Proteins without signal peptides are unlikely to be exposed to
    the N-glycosylation machinery and thus may not be glycosylated
    (in vivo) even though they contain potential motifs.

    SignalP-NN euk predictions are as follows:

  # name                  Cmax  pos ?  Ymax  pos ?  Smax  pos ?  Smean ?  D     ?

    SignalP output is explained at http://www.cbs.dtu.dk/services/SignalP/output.html

##########################################################################################

Name:  contig050025-NyeORs.W130         Length:  313
MNASSGNVTVVLQYRDSFAKAVTKNLIVVFLGISIGYINANLIHTFCKHQIFYKNPRYVLFIHLVINDMVQVMLTAILFI      80
ISYTIYKLSVSVCCIFMLLALFTTENSPLNLACMAVECYIAICFPLRHVQICTVQRTLILISLIWMITTLSVLPDLFITL     160
ATEPLDFFHSRVFCLRNTVFPHPLIIQKRDITYGVFLVIVWVTIIYTYLKILFTAKTASKDAKKAKNTILLHGFQLLLCM     240
ATYAAPHLTNALQKWFPTNYTDSLFVIYVTVQILPRSISPIIYGIRDKTFRRFLKGNLLCKITVHKSDNQNRX
.N....N.........................................................................      80
................................................................................     160
................................................................................     240
..................N......................................................            320

(Threshold=0.5)
----------------------------------------------------------------------
SeqName      Position  Potential   Jury    N-Glyc
                                 agreement result
----------------------------------------------------------------------
contig050025-NyeORs.W130    2 NASS   0.6618     (9/9)   ++
contig050025-NyeORs.W130    7 NVTV   0.7672     (9/9)   +++
contig050025-NyeORs.W130  259 NYTD   0.7027     (9/9)   ++
----------------------------------------------------------------------
```

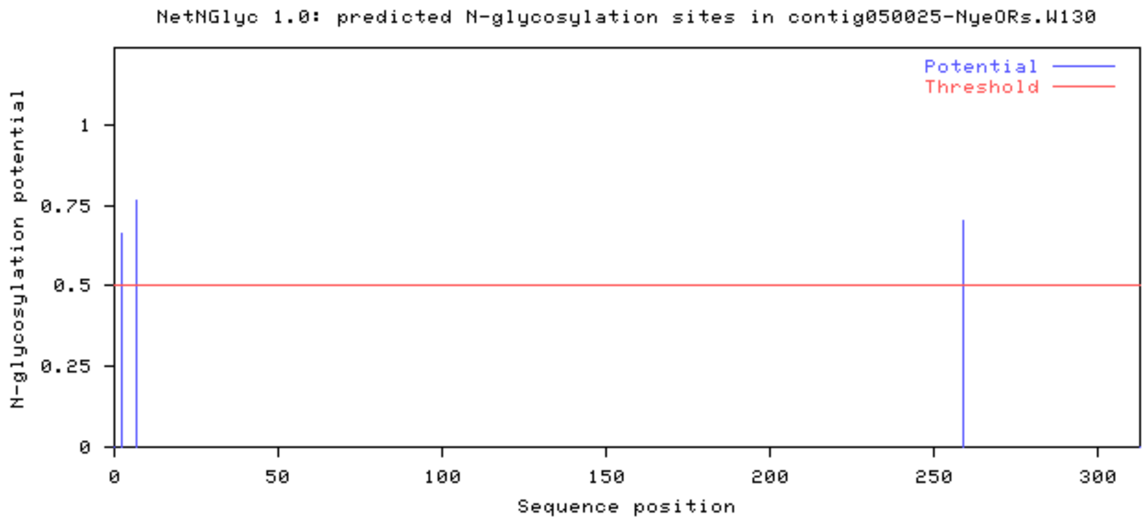

[Graphics in PostScript]

Output for 'contig050025-NyeORs.W131'

```
##########################################################################

    Warning: This sequence may not contain a signal peptide!!

    Proteins without signal peptides are unlikely to be exposed to
    the N-glycosylation machinery and thus may not be glycosylated
    (in vivo) even though they contain potential motifs.

    SignalP-NN euk predictions are as follows:

  # name                 Cmax  pos ?  Ymax  pos ?  Smax  pos ?  Smean ?  D     ?

    SignalP output is explained at http://www.cbs.dtu.dk/services/SignalP/output.html

##########################################################################

Name:  contig050025-NyeORs.W131         Length:  313
MNLTQVDSNATIPLNYWKTFVKAVGKNVTVVVLGITINYINATMIHTFNKQIFRLNPRYILFIHLMFNDIIQLSTSISLF      80
IFSYAFHTIHVSLCLLLILPAIFTTQNTPLNIAFMAAECCVSVCIPLRYSYICTVKRTYIVIGIIWAISSLSILPDLFIL     160
LAVESSQFLQSRVVCNRDTVFRSSYSVKKRDASHTLFLVLVSVTLLYTYCQILFVARCADSDTKKARNTILIHGFQVLLC     240
TTVYVQPPLIKLLVYFFPEGLSDINFATFIINQVLPRLGSPIIYGLRDKTFRKFLKRHLCAVNNLPKIIRSSX
.N......N.................N.............N.......................................      80
................................................................................     160
................................................................................     240
.........................................................................            320

(Threshold=0.5)
----------------------------------------------------------------------
SeqName      Position  Potential   Jury    N-Glyc
                                 agreement result
----------------------------------------------------------------------
contig050025-NyeORs.W131    2 NLTQ   0.8094     (9/9)   +++
contig050025-NyeORs.W131    9 NATI   0.5874     (8/9)   +
contig050025-NyeORs.W131   27 NVTV   0.7228     (9/9)   ++
contig050025-NyeORs.W131   41 NATM   0.5684     (6/9)   +
----------------------------------------------------------------------
```

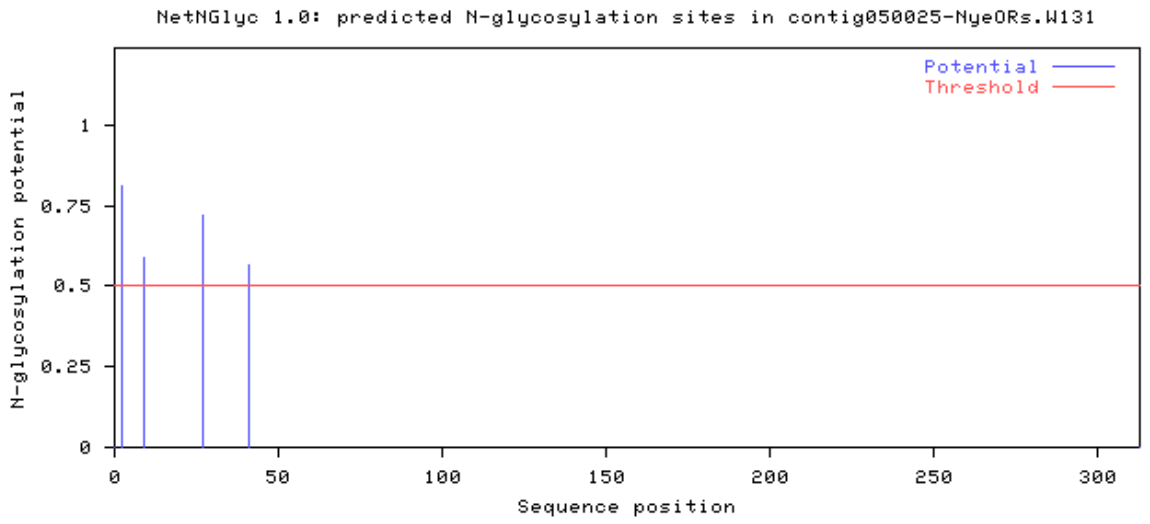

Graphics in PostScript

Output for 'contig050026-NyeORs.W132'

```
##########################################################################################

    Warning: This sequence may not contain a signal peptide!!

    Proteins without signal peptides are unlikely to be exposed to
    the N-glycosylation machinery and thus may not be glycosylated
    (in vivo) even though they contain potential motifs.

    SignalP-NN euk predictions are as follows:

  # name                    Cmax  pos ?  Ymax  pos ?  Smax  pos ?  Smean ?  D     ?

    SignalP output is explained at http://www.cbs.dtu.dk/services/SignalP/output.html

##########################################################################################

Name:  contig050026-NyeORs.W132         Length:  318
MNSSSYALNASSSLKYRDSPSVAVAKNVIVLALGFTINYINGTLIHTFRKHQIFYLNPRYILFIHLVVNDMIQLTSSISL      80
FVFTYIFYQINVAFCCFLITLAIFTTFNTPINLALMALECYIAICLPLQHAEYCTTKRTYVAIGWIWAMSAVSAMSDIVI     160
ILATEPVELFYSTIQCERDNLFRHPIIVKKKEVSYLIFLIGVLLTFMYTYFRIFFAANKAKSAKRESKKARNTILLHGFQ     240
LLLSMLTYIATALMQALVRWFPKQPLIVVFISYIIIYIVPRFVSPIVYGLRDKTFKQYLKKYLLCTMKMGNETDFRLX
.N......N.............................N.........................................      80
................................................................................     160
................................................................................     240
..............................................................N.......               320

(Threshold=0.5)
----------------------------------------------------------------------
SeqName      Position  Potential   Jury    N-Glyc
                                 agreement result
----------------------------------------------------------------------
contig050026-NyeORs.W132    2 NSSS   0.7220     (9/9)   ++
contig050026-NyeORs.W132    9 NASS   0.6429     (8/9)   +
contig050026-NyeORs.W132   41 NGTL   0.7142     (9/9)   ++
contig050026-NyeORs.W132  311 NETD   0.6713     (9/9)   ++
----------------------------------------------------------------------
```

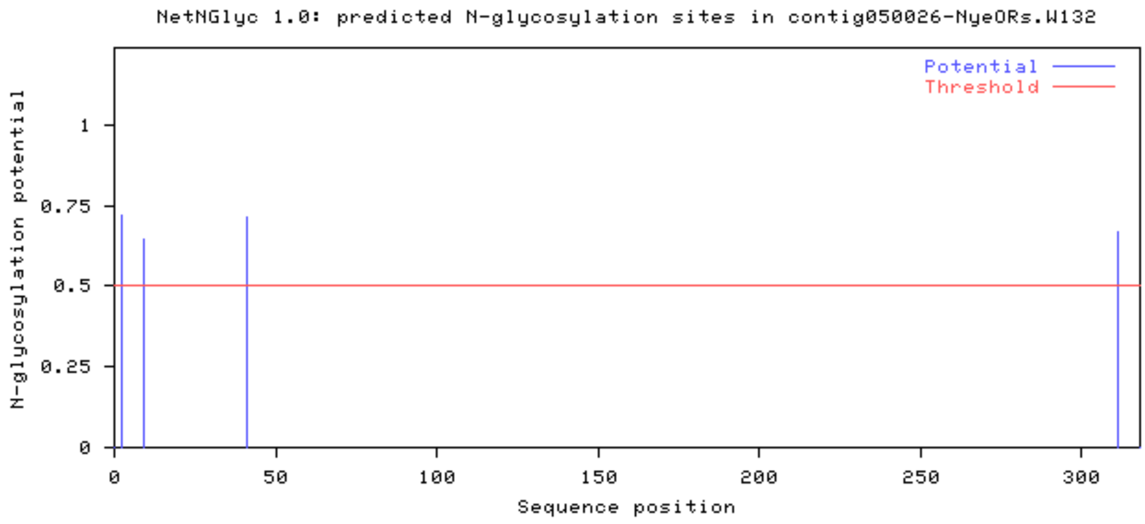

Graphics in PostScript

Output for 'contig051688-NYEORe.AC145'

```
##########################################################################################

    Warning: This sequence may not contain a signal peptide!!

    Proteins without signal peptides are unlikely to be exposed to
    the N-glycosylation machinery and thus may not be glycosylated
    (in vivo) even though they contain potential motifs.

    SignalP-NN euk predictions are as follows:

  # name                    Cmax  pos ?  Ymax  pos ?  Smax  pos ?  Smean ?  D     ?

    SignalP output is explained at http://www.cbs.dtu.dk/services/SignalP/output.html

##########################################################################################

Name:  contig051688-NYEORe.AC145        Length:  117
VECKEPWPPGFYLLLGYIGLLAFVCLLLAFFGRKLPDTFNEAKFISFSMLIFWAVWISFIPAYVSSPAKFSVAVEIFAIL      80
ASSFGLLLCIFVPKCYIILLHPERNIKKGMIGKYQRX
................................................................................      80
.....................................                                                160

(Threshold=0.5)

No sites predicted in this sequence.
```

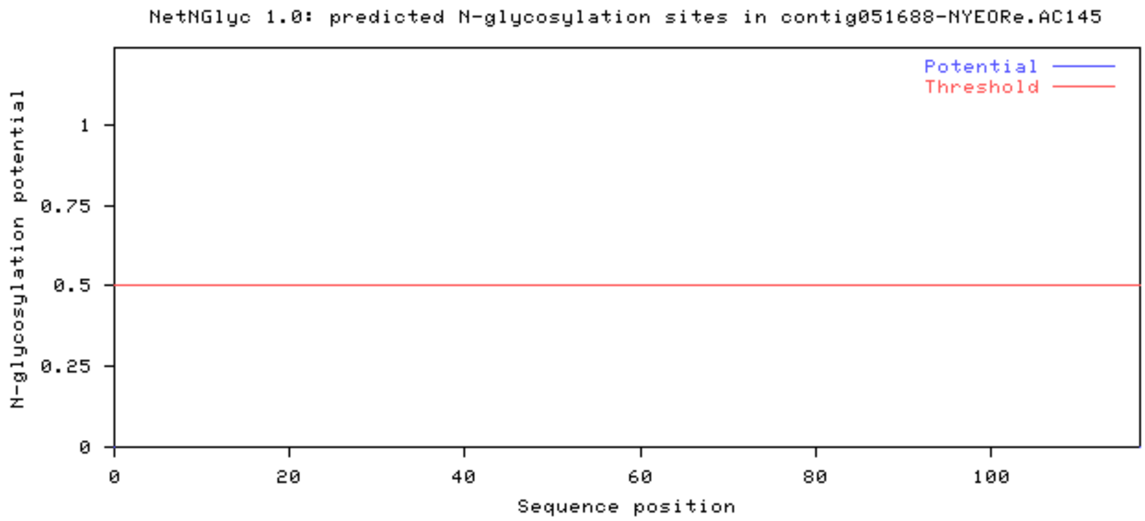

Graphics in PostScript

Output for 'contig051999-NyeORe.V142'

```
##########################################################################################

    Warning: This sequence may not contain a signal peptide!!

    Proteins without signal peptides are unlikely to be exposed to
    the N-glycosylation machinery and thus may not be glycosylated
    (in vivo) even though they contain potential motifs.

    SignalP-NN euk predictions are as follows:

  # name                    Cmax  pos ?  Ymax  pos ?  Smax  pos ?  Smean ?  D     ?

    SignalP output is explained at http://www.cbs.dtu.dk/services/SignalP/output.html

##########################################################################################

Name:  contig051999-NyeORe.V142         Length:  62
MVINDALQLTLVTALYVISYIFRKIHASVCCLLVNHSSISQAHHICFACHVILLWDPGYRPS
..................................N...........................                         80

(Threshold=0.5)
----------------------------------------------------------------------
SeqName      Position  Potential   Jury    N-Glyc
                                 agreement result
----------------------------------------------------------------------
contig051999-NyeORe.V142    35 NHSS    0.6364     (8/9)    +
----------------------------------------------------------------------
```

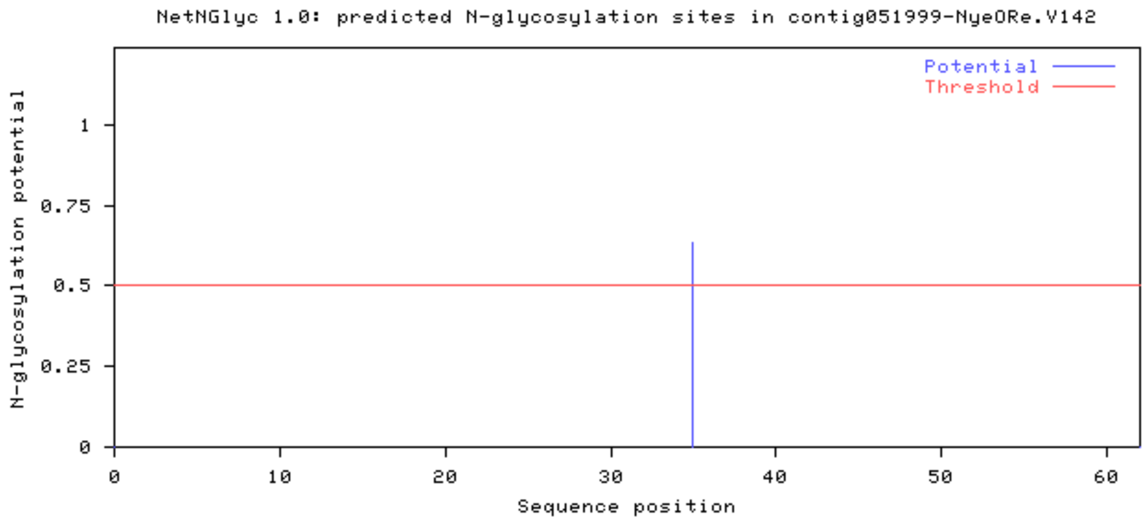

Graphics in PostScript

Output for 'contig051999-NyeORs.U128'

```
##########################################################################################

    Warning: This sequence may not contain a signal peptide!!

    Proteins without signal peptides are unlikely to be exposed to
    the N-glycosylation machinery and thus may not be glycosylated
    (in vivo) even though they contain potential motifs.

    SignalP-NN euk predictions are as follows:

  # name                    Cmax   pos ?  Ymax   pos ?  Smax   pos ?  Smean ?  D     ?

    SignalP output is explained at http://www.cbs.dtu.dk/services/SignalP/output.html

##########################################################################################

Name:  contig051999-NyeORs.U128         Length:  328
MSSLLGLRGNMTIPYQLLLIRDTFTTAFVKNLIVVLVWLTLSYINGTLVVTFFRHQTFHDDPRYILFIHMVINDAIQLTV      80
TIMLFILSYIFYKINVAFCCFFILVAVFTTRNTPVNLAAMAIERYIAICEPLRYTQICTVRRTYIVIGMIWFICVAPDIT     160
DLFVTLATESLSFFHESVFCLRQNVFKDPILAYKRQVFDIIYFSCVFLILVITYLRILFAARACSTDKTSAQKARNTILL     240
HGAQLAMCMLSYVSPSVEVVLHIIFPGRILEIRFANYLIVYILPRFLSPIIYGVRDKKFREYLRMYFLSNRCRNKKKKVT     320
PKDKDHLX
.........N..................................N...................................      80
................................................................................     160
................................................................................     240
................................................................................     320
........                                                                             400

(Threshold=0.5)
----------------------------------------------------------------------
SeqName      Position  Potential   Jury    N-Glyc
                                 agreement result
----------------------------------------------------------------------
contig051999-NyeORs.U128    10 NMTI    0.7688     (9/9)    +++
contig051999-NyeORs.U128    45 NGTL    0.7593     (9/9)    +++
----------------------------------------------------------------------
```

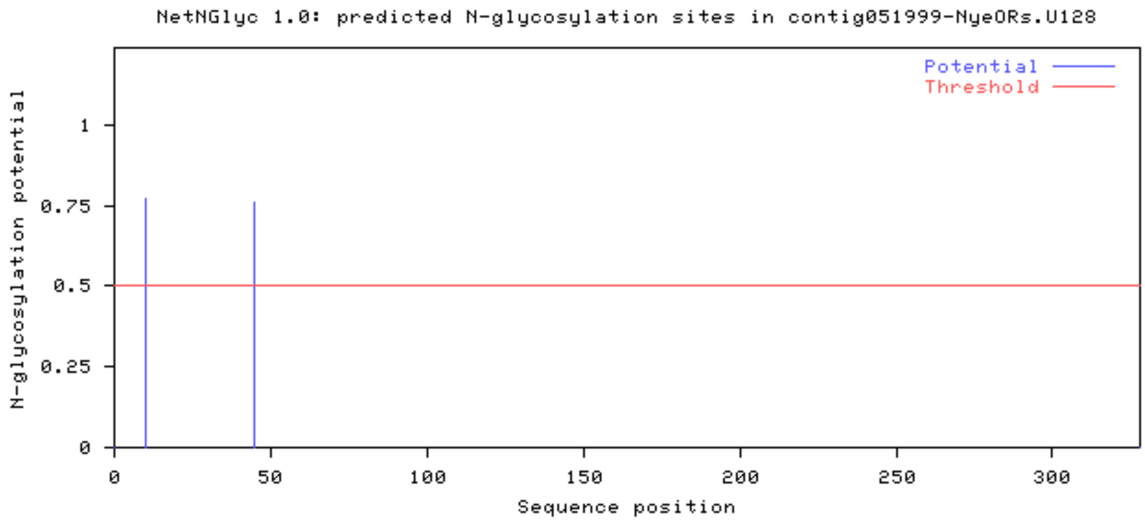

Graphics in PostScript

Output for 'contig052001-NyeORep.T127'

```
##########################################################################################

    Warning: This sequence may not contain a signal peptide!!

    Proteins without signal peptides are unlikely to be exposed to
    the N-glycosylation machinery and thus may not be glycosylated
    (in vivo) even though they contain potential motifs.

    SignalP-NN euk predictions are as follows:

  # name                 Cmax  pos ?  Ymax  pos ?  Smax  pos ?  Smean ?  D     ?

    SignalP output is explained at http://www.cbs.dtu.dk/services/SignalP/output.html

##########################################################################################

Name:  contig052001-NyeORep.T127        Length:  261
MNTTKRQDSFNDAFTKNFVSFALGFIINYINGMFVYTYFKSVVFRQDPRRYVLYIHLVINDMIMLTVTVMLQILTYTAPL      80
SFAPCCVMLLISVTANKNSPLNLAGMAVERYIAVCRPLHHSQLCTVQRAYALIALIWAVSFIPSISDVIILLVVQPLSVF     160
TKNVICYPSFVYNTPYHETQSLVIQVTCSFSTFMAQFKLVDQMNLNSLPQVLLFSFVFLTLIVTYMKVLCAARAVSSSNQ     240
ASAKNAHNTILLHGVQLLICM
.N..............................................................................      80
................................................................................     160
................................................................................     240
.....................                                                                 320

(Threshold=0.5)
----------------------------------------------------------------------
SeqName      Position  Potential   Jury    N-Glyc
                                 agreement result
----------------------------------------------------------------------
contig052001-NyeORep.T127     2 NTTK   0.7591     (9/9)    +++
----------------------------------------------------------------------
```

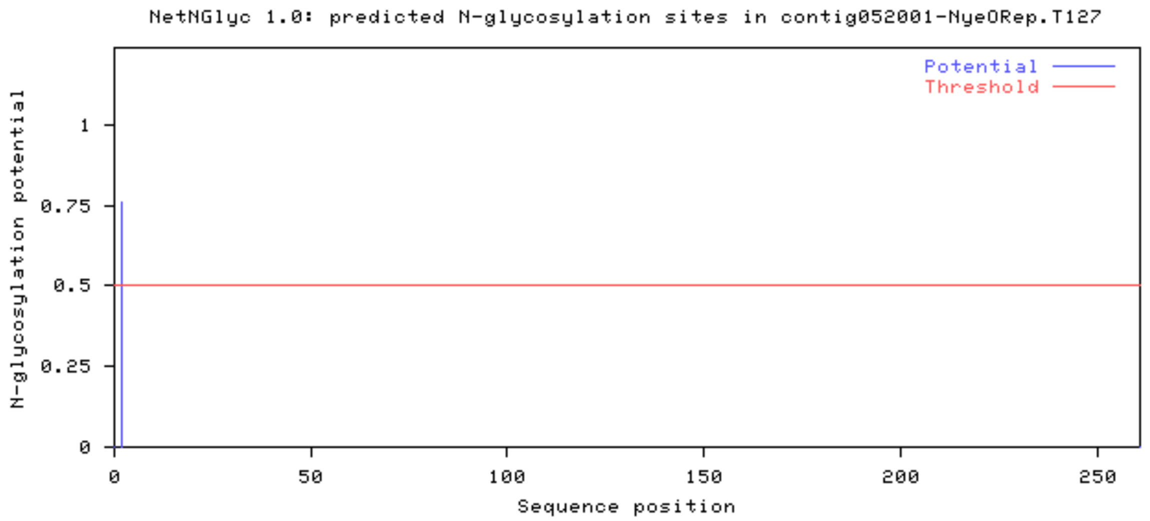

Graphics in PostScript

Output for 'contig053572-NyeOR.E051'

```
##########################################################################################

    Warning: This sequence may not contain a signal peptide!!

    Proteins without signal peptides are unlikely to be exposed to
    the N-glycosylation machinery and thus may not be glycosylated
    (in vivo) even though they contain potential motifs.

    SignalP-NN euk predictions are as follows:

  # name                    Cmax  pos ?  Ymax  pos ?  Smax  pos ?  Smean ?  D     ?

    SignalP output is explained at http://www.cbs.dtu.dk/services/SignalP/output.html

##########################################################################################

Name:  contig053572-NyeOR.E051  Length:  330
MINTTEITHFTLSPYFDTGAYRYLYFLIILTSYAAIICANLLLIVVICVNRSLHEPMYMFLCSLFVNELYGSTGLFPFLL      80
LQILSDVHTVSVSFCFLQVFCVYTYACVEFINFVVMSYDRYYAICWPLQYKSCMTLKTVTTLISLTWLLPFIMIVVLISL     160
SAPLQLCGNVINKVFCGNYAIIKLACSDTRVHNIFGLIYTFISVIIPLVLILYTYVRILKVCFSGSKQTRQKAVSTCTPH     240
LASILNFFFGCCFQILQSRFDTSGVPNVFGILSSLYFLTCQPLFTPLLYGLKMTKIRIACKQLFCGSLTRLFSCSSDIFS     320
KSHQSQSYCX
..N..............................................N..............................      80
................................................................................     160
................................................................................     240
................................................................................     320
..........                                                                           400

(Threshold=0.5)
----------------------------------------------------------------------
SeqName       Position  Potential   Jury    N-Glyc
                                  agreement result
----------------------------------------------------------------------
contig053572-NyeOR.E051    3 NTTE   0.7166     (9/9)    ++
contig053572-NyeOR.E051   50 NRSL   0.7492     (9/9)    ++
----------------------------------------------------------------------
```

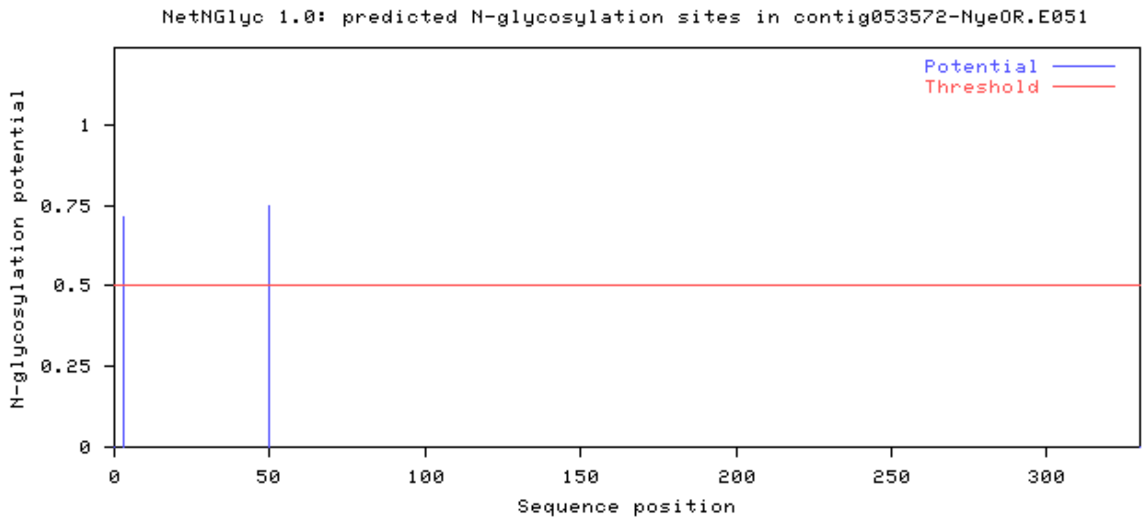

Graphics in PostScript

Output for 'contig053576-NyeOR.E052'

```
##########################################################################################

    Warning: This sequence may not contain a signal peptide!!

    Proteins without signal peptides are unlikely to be exposed to
    the N-glycosylation machinery and thus may not be glycosylated
    (in vivo) even though they contain potential motifs.

    SignalP-NN euk predictions are as follows:

  # name                  Cmax  pos ?  Ymax  pos ?  Smax  pos ?  Smean ?  D     ?

    SignalP output is explained at http://www.cbs.dtu.dk/services/SignalP/output.html

##########################################################################################

Name:  contig053576-NyeOR.E052  Length:  326
MEIIRNSTQFSYFTLGAYVDTQMFKYLYFMIILSLYVFTVGSNVLLIVVICVNRSLHEPMYMFLCSLFVNELYGSTGLFP      80
LLLLQILSDVHTVSAPLCFLQVFSIYLYASVEFSNLAAMSYDRYMSICYPLQYHTLMMSNKVALLIAVTWIPPLLAVCVT     160
TCLSASLQLCGNVINKVYCNNHSIIKLGCHGTTVNNLYELTAASVTVCVPVSVILYTYTRILKICFSGSKQTRQKAVSTC     240
TPHLASLLNFSFGVSFEILQSRFDMSHVPNMLRIFLSVYFLTCQPLFNPVMYGLNMSKIRTICKNLLLGYVGKSRILIKI     320
VQIEKX
.....N..............................................N...........................      80
................................................................................     160
................................................................................     240
......................................................N.........................     320
......                                                                               400

(Threshold=0.5)
----------------------------------------------------------------------
SeqName      Position  Potential   Jury    N-Glyc
                                 agreement result
----------------------------------------------------------------------
contig053576-NyeOR.E052    6 NSTQ   0.7709     (9/9)   +++
contig053576-NyeOR.E052   53 NRSL   0.7489     (9/9)   ++
contig053576-NyeOR.E052  181 NHSI   0.3873     (9/9)   --
contig053576-NyeOR.E052  249 NFSF   0.4451     (6/9)   -
contig053576-NyeOR.E052  295 NMSK   0.6052     (6/9)   +
----------------------------------------------------------------------
```

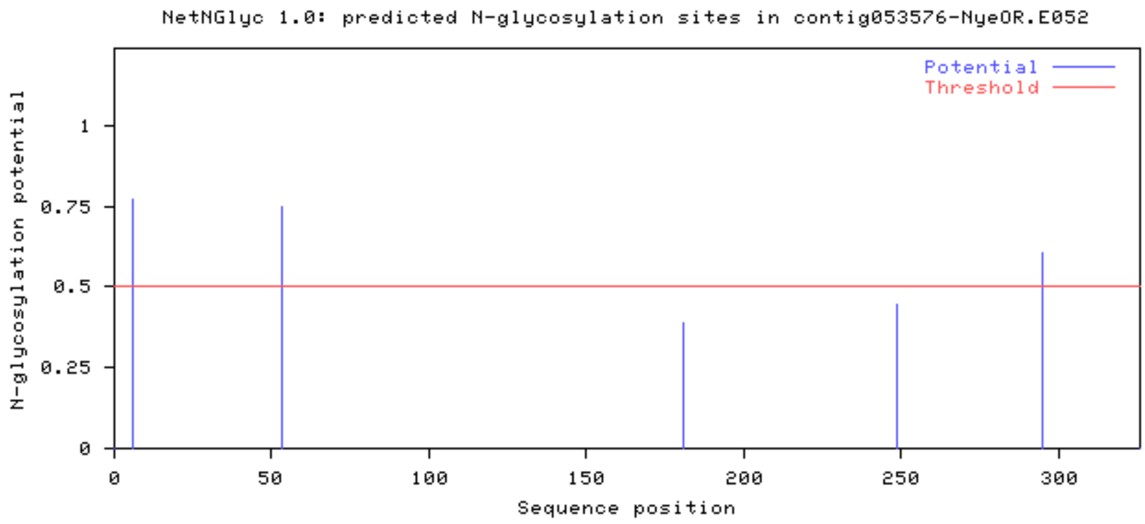

Graphics in PostScript

Output for 'contig053579-NyeOR.E053'

```
##############################################################################

   Warning: This sequence may not contain a signal peptide!!

   Proteins without signal peptides are unlikely to be exposed to
   the N-glycosylation machinery and thus may not be glycosylated
   (in vivo) even though they contain potential motifs.

   SignalP-NN euk predictions are as follows:

 # name                    Cmax  pos ?  Ymax  pos ?  Smax  pos ?  Smean ?  D     ?

   SignalP output is explained at http://www.cbs.dtu.dk/services/SignalP/output.html

##############################################################################

Name:  contig053579-NyeOR.E053  Length:  308
LIINSSKVSYFTLAAYFDTSTLKYFCFTVVMSLYLLILCANVLLIVVICVNRSLHEPMYMFLCSLFVNELYGSTGLFPLL      80
LLQILSDVHTVSAPLCFLQIFCVFSYVCVEFCILAVMSYDRYLAICCPLQYHTRMTPATVVLLIALSWLYSFLTILTLIL     160
LIAPLELCGNVINKVYCLNYSIVKLACSETTANNIYGLFITALTVFVPVILILCSYVRILKVCFSGSKQTRQKAVSTCTP     240
HLASLLNFSFGVCFEVIQSRFSLSSVHSMVHIVLSLYFLTCQPLFNPVLYGLNMSNIRKRLFAHKRRX
...N..............................................N.............................      80
................................................................................     160
..................N.............................................................     240
....................................................N...............                 320

(Threshold=0.5)
----------------------------------------------------------------------
SeqName      Position  Potential   Jury    N-Glyc
                                agreement result
----------------------------------------------------------------------
contig053579-NyeOR.E053    4 NSSK   0.6987     (9/9)   ++
contig053579-NyeOR.E053   51 NRSL   0.7483     (9/9)   ++
contig053579-NyeOR.E053  179 NYSI   0.6027     (8/9)   +
contig053579-NyeOR.E053  247 NFSF   0.4440     (6/9)   -
contig053579-NyeOR.E053  293 NMSN   0.5670     (4/9)   +
----------------------------------------------------------------------
```

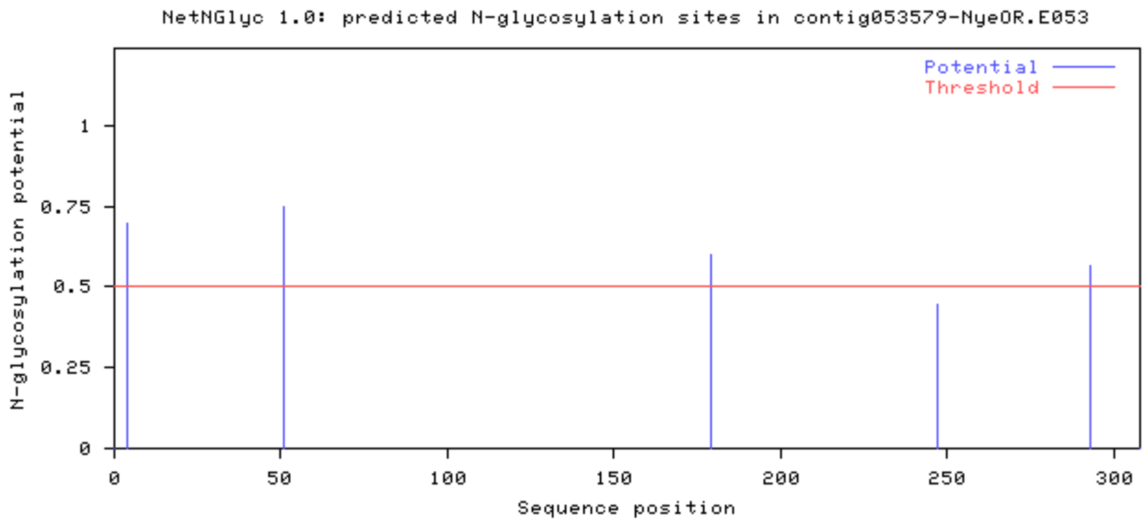

Graphics in PostScript

Output for 'contig053579-NyeOR.E054'

```
########################################################################################

    Warning: This sequence may not contain a signal peptide!!

    Proteins without signal peptides are unlikely to be exposed to
    the N-glycosylation machinery and thus may not be glycosylated
    (in vivo) even though they contain potential motifs.

    SignalP-NN euk predictions are as follows:

  # name                Cmax  pos ?  Ymax  pos ?  Smax  pos ?  Smean ?  D     ?

    SignalP output is explained at http://www.cbs.dtu.dk/services/SignalP/output.html

########################################################################################

Name:  contig053579-NyeOR.E054  Length:  314
MINFTEGSYFILGAYFDAGPTKYLFFLLLLSLYSLIICANVLLIVVICVNRSLHEPMYMFLCSLFVNELYGSTGLFPLLL      80
LQILSDVHAVSAPLCFLQIFSLYSYGSVEFLTLAVMSYDRYLAICCPLQYNTRMTSSTVSVLIAVSWIYALLLVAVTVSL     160
SSPLQLCGNIINKVYCDNYAIVKLACSDTTLNNIYGLISTAFTAFVPVTLIFFTYMRILKVCFSGSKQMRQKAVSTCTPH     240
LASLLNFSCSGCFEILQSRFNMNNLPNVLRILISLYWLMCQPLFNPVLYGLKMSKIRDICKCLLYSKVNTILSX
..N..............................................N..............................      80
................................................................................     160
................................................................................     240
.....N....................................................................           320

(Threshold=0.5)
----------------------------------------------------------------------
SeqName      Position  Potential   Jury    N-Glyc
                                 agreement result
----------------------------------------------------------------------
contig053579-NyeOR.E054    3 NFTE   0.6488     (9/9)   ++
contig053579-NyeOR.E054   50 NRSL   0.7491     (9/9)   ++
contig053579-NyeOR.E054  246 NFSC   0.5482     (7/9)   +
----------------------------------------------------------------------
```

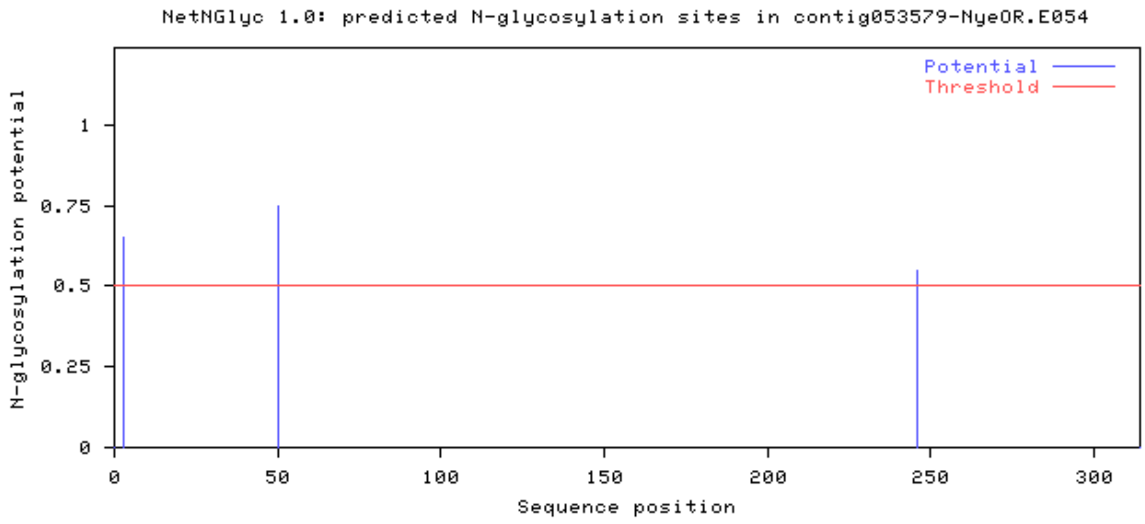

Graphics in PostScript

Output for 'contig053579-NyeOR.E055'

```
##########################################################################################

    Warning: This sequence may not contain a signal peptide!!

    Proteins without signal peptides are unlikely to be exposed to
    the N-glycosylation machinery and thus may not be glycosylated
    (in vivo) even though they contain potential motifs.

    SignalP-NN euk predictions are as follows:

  # name                    Cmax  pos ?  Ymax  pos ?  Smax  pos ?  Smean ?  D     ?

    SignalP output is explained at http://www.cbs.dtu.dk/services/SignalP/output.html

##########################################################################################

Name:  contig053579-NyeOR.E055  Length:  312
MINFTEGSYFILGAYFDAGPTKYLFFLLLLSLYFLIICANVLLIVVICVNRSLHEPMYMFLCSLFVNELYGSTGLFPLLL      80
LQILSDVHAVSAPLCFLQIFCLYSYVGVEFLTLAVMSYDRYLAICCPLQYNTRMTSSTVSVLIAVSWIYALLLVAVTVSL     160
SSPLQLCGNIINKVYCDNYAIVKLACSDTTLNNIYGLISTAFTAFVPVTLIFFTYMRILKVCFSGSKQMRQKAVSTCTPH     240
LASLLNFSCSGCFEILQSRFNMNNLPNVLRILISLYWLMCQQLFNPVLYGLKMSKIYDICKSLLYWKVWIMX
..N...............................................N.............................      80
................................................................................     160
................................................................................     240
.....N..................................................................             320

(Threshold=0.5)
----------------------------------------------------------------------
SeqName      Position  Potential   Jury    N-Glyc
                                 agreement result
----------------------------------------------------------------------
contig053579-NyeOR.E055    3 NFTE    0.6489     (9/9)    ++
contig053579-NyeOR.E055   50 NRSL    0.7492     (9/9)    ++
contig053579-NyeOR.E055  246 NFSC    0.5476     (7/9)    +
----------------------------------------------------------------------
```

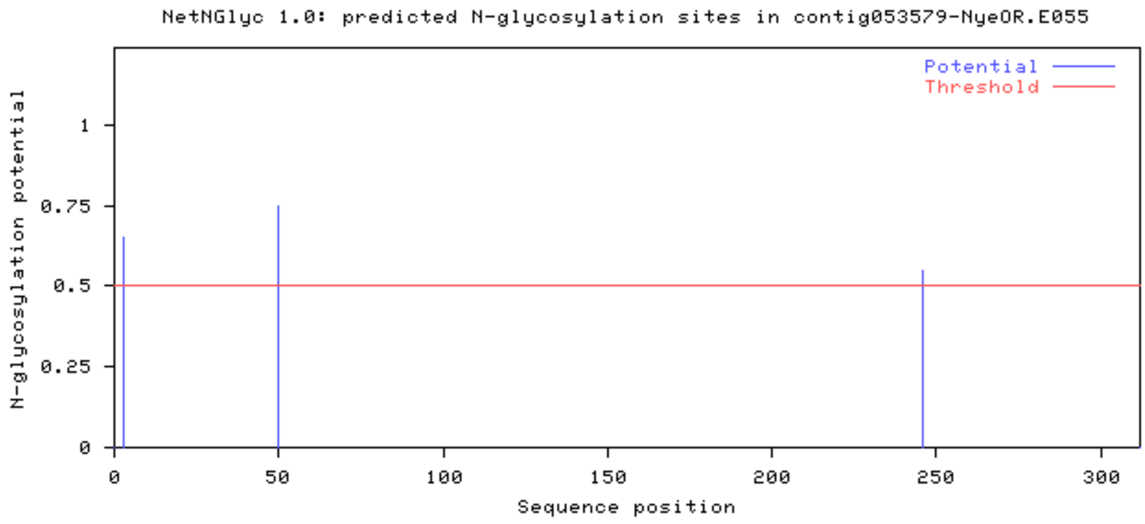

Graphics in PostScript

Output for 'contig053581-NyeOR.G066'

```
##########################################################################################

    Warning: This sequence may not contain a signal peptide!!

    Proteins without signal peptides are unlikely to be exposed to
    the N-glycosylation machinery and thus may not be glycosylated
    (in vivo) even though they contain potential motifs.

    SignalP-NN euk predictions are as follows:

  # name                    Cmax  pos ?  Ymax  pos ?  Smax  pos ?  Smean ?  D     ?

    SignalP output is explained at http://www.cbs.dtu.dk/services/SignalP/output.html

##########################################################################################

Name:  contig053581-NyeOR.G066  Length:  312
MENNFEIVFVLQGLNDSLTNRQIYFAFALMSYLFTVSVNLTLIITISLDKTLHEPIYIFLCSLCFNEICGASSFYPKLLH      80
DLLTNSYVITYTACLGQMFVTYSYIFSEFTSLTVMAYDRYIAICKPLQYRMLMTAQKVAQLLMLTWCFSVFETAVGTVLT     160
ARLPLCGRHIPKIFCTNWEVVKLSCSDSTVNNIYAFMLIFSHLSQTALIMVSYVHLIRAAIRSQADRRKFMQTCLPHLIT     240
LLVFTTSLMFDTMYSRYSGGSTMKALQNALAAQFLVVPPLVNPIIYGLNLQQIRSRMVHRFTHRTGTFRKNX
..............N........................N........................................      80
................................................................................     160
................................................................................     240
........................................................................             320

(Threshold=0.5)
----------------------------------------------------------------------
SeqName      Position  Potential   Jury    N-Glyc
                                 agreement result
----------------------------------------------------------------------
contig053581-NyeOR.G066   15 NDSL   0.7060     (9/9)    ++
contig053581-NyeOR.G066   39 NLTL   0.7856     (9/9)    +++
----------------------------------------------------------------------
```

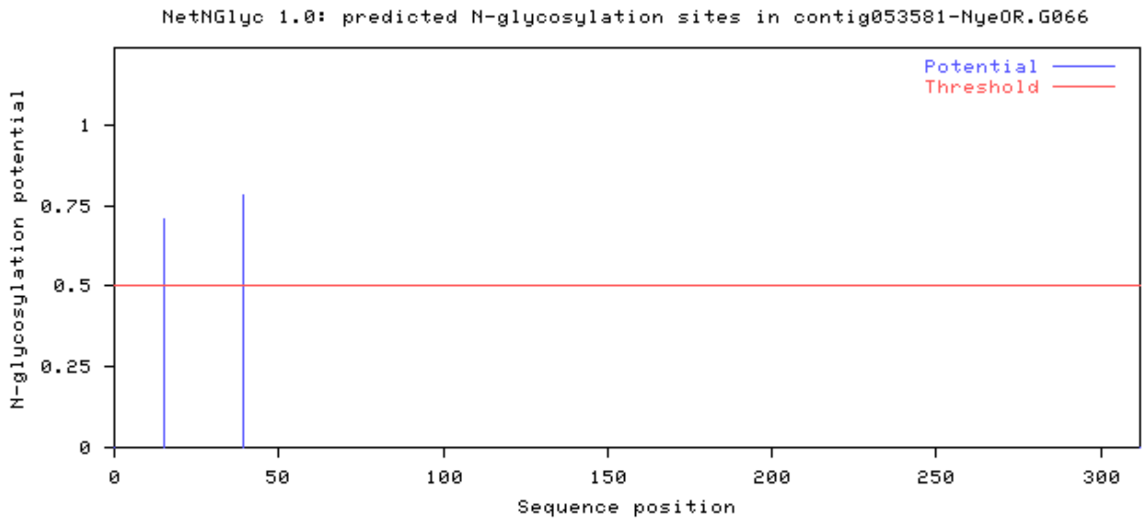

Graphics in PostScript

Output for 'contig053590-NyeOR.E056'

```
##########################################################################################

    Warning: This sequence may not contain a signal peptide!!

    Proteins without signal peptides are unlikely to be exposed to
    the N-glycosylation machinery and thus may not be glycosylated
    (in vivo) even though they contain potential motifs.

    SignalP-NN euk predictions are as follows:

  # name                    Cmax  pos ?  Ymax  pos ?  Smax  pos ?  Smean ?  D     ?

    SignalP output is explained at http://www.cbs.dtu.dk/services/SignalP/output.html

##########################################################################################

Name:  contig053590-NyeOR.E056  Length:  322
MLNVTTPPLSYFILGAFMNVGSLKFFYFSLTVILYILIIAANTSLIVVICVNRSLHEPMYMYLCSLFVNELYGSTGLFPS      80
LLVQILSDVHTVSTPLCFLQIFCIHTYGSIEVSNLAVMSYDRYLAICCPLQYNTQMTSNNTAVLIMVMWAYSLAKFLIAL     160
SLNLRLRLCGNVLNSLYCQNYLVVMLACSSTKVNNVYGIFDIIMTIIVPTLIILFSYMKILKVCFYGSKQTRQKSLTTCT     240
PQLVSLLNFSFGCCFEIFQSRFDTTGLPAALRIFLSLYFLMMQPLMNPILYGTQMSKIRGVYEHVLSSIMSCGCSKVSQS     320
DX
..N.....................................N.........N...........................      80
..........................................................N.....................     160
................................................................................     240
................................................................................     320
..                                                                                  400

(Threshold=0.5)
----------------------------------------------------------------------
SeqName      Position  Potential   Jury    N-Glyc
                                 agreement result
----------------------------------------------------------------------
contig053590-NyeOR.E056    3 NVTT   0.7963     (9/9)   +++
contig053590-NyeOR.E056   42 NTSL   0.7264     (9/9)   ++
contig053590-NyeOR.E056   52 NRSL   0.7338     (9/9)   ++
contig053590-NyeOR.E056  139 NNTA   0.5361     (3/9)   +
contig053590-NyeOR.E056  248 NFSF   0.3858     (8/9)   -
----------------------------------------------------------------------
```

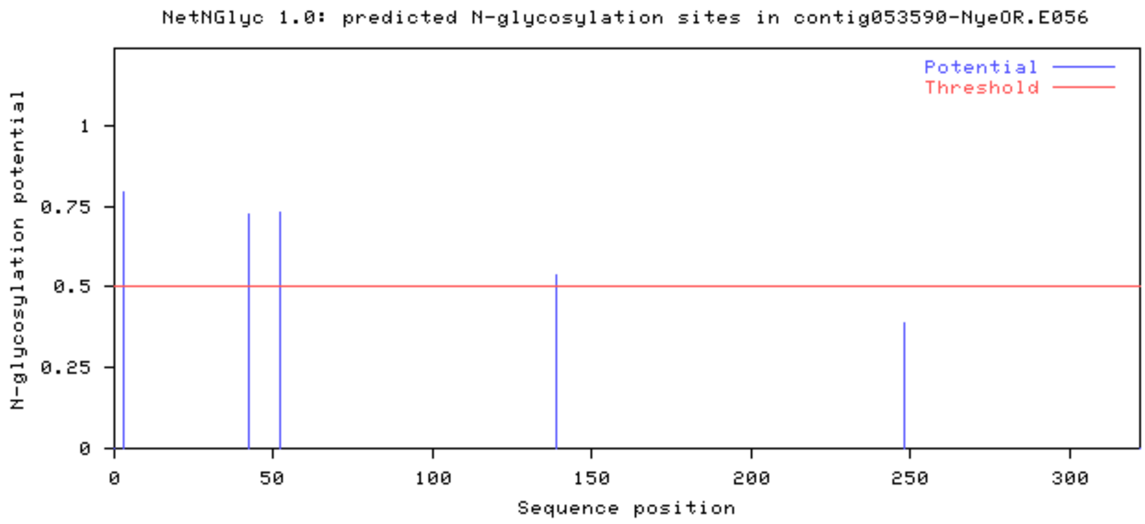

Graphics in PostScript

Output for 'contig053592-NyeOR.E057'

```
##########################################################################################

    Warning: This sequence may not contain a signal peptide!!

    Proteins without signal peptides are unlikely to be exposed to
    the N-glycosylation machinery and thus may not be glycosylated
    (in vivo) even though they contain potential motifs.

    SignalP-NN euk predictions are as follows:

  # name                Cmax  pos ?  Ymax  pos ?  Smax  pos ?  Smean ?  D     ?

    SignalP output is explained at http://www.cbs.dtu.dk/services/SignalP/output.html

##########################################################################################

Name:  contig053592-NyeOR.E057  Length:  306
MVNSTVPYFILSTYIYVGSLKYLFFVLIALLYFSIVFVNTSLIVVICVNRSLHEPMYMFLCSLFVNELYGSTGLFPSLLL      80
QILSDVHTVSAPLCFLQIFCLYTYGHIEFCNLAVMSYDRYLAVCYPLHYKSHMTDNKVVIFIVVIWLYSFVKFTITLCLT     160
LRLTWCGKIINGLYCHNYLVVKLACSDTNLNNLFGLFGIVITVLVPLLPIFYSYMKILKVCFSGSRQMRRKAVSTCAPHL     240
ASLLNFSFGCLFEILQSRFDTTSVPSALRIFLSLYFLIIQPLLNPIMYGTQMSKIRHVLCYKMSLX
..N...................................N.........N...............................      80
................................................................................     160
................................................................................     240
..................................................................                   320

(Threshold=0.5)
----------------------------------------------------------------------
SeqName      Position  Potential   Jury    N-Glyc
                                 agreement result
----------------------------------------------------------------------
contig053592-NyeOR.E057    3 NSTV   0.7900     (9/9)   +++
contig053592-NyeOR.E057   39 NTSL   0.6766     (9/9)   ++
contig053592-NyeOR.E057   49 NRSL   0.7481     (9/9)   ++
contig053592-NyeOR.E057  245 NFSF   0.4448     (7/9)   -
----------------------------------------------------------------------
```

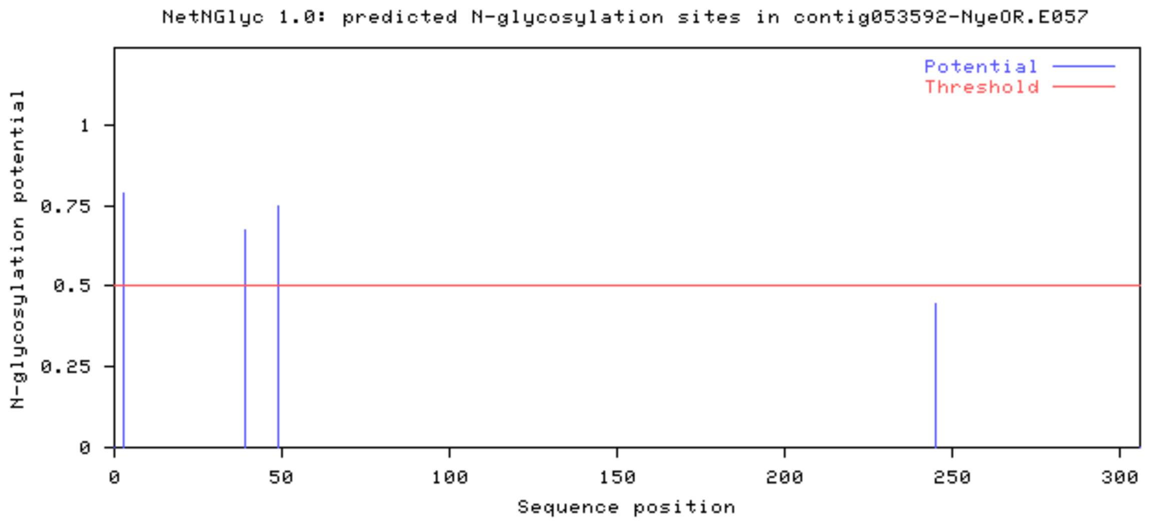

Graphics in PostScript

Output for 'contig054678-NyeOR.A010'

```
##########################################################################################

    Warning: This sequence may not contain a signal peptide!!

    Proteins without signal peptides are unlikely to be exposed to
    the N-glycosylation machinery and thus may not be glycosylated
    (in vivo) even though they contain potential motifs.

    SignalP-NN euk predictions are as follows:

  # name                    Cmax  pos ?  Ymax  pos ?  Smax  pos ?  Smean ?  D     ?

    SignalP output is explained at http://www.cbs.dtu.dk/services/SignalP/output.html

##########################################################################################

Name:  contig054678-NyeOR.A010  Length:  337
MEQMHDESNVTYITFGGHVELEKYKFLYFAIMFTAYILILCSNSTIVCLIWIKKSLHEPMYVFIAALLLNSVVFSTNIYP      80
KLLMDFLSERQITTHSLCRFQGIIYYSLTGSEFFLLASMAYDRYVSISKPLQYHTIMRKTTVTVLLVLAWLLPACQLVPS     160
AVFSNNSQICNFTLNGIFCNNAISKLYCATPKTYYLMYGVFILFNTVFLPLLFIMFTYTKIFIICYRSCREVRKKAAQTC     240
LPHVLVLISFTCLCSYDIIIARLEIDLSQNTRFIMTLQVVLYHPLFNPIVYGLKMKEISQHLRRLFCHRVLCVKTDVGSA     320
VISFVIQVQRPDFSTVX
........N.................................N.....................................      80
................................................................................     160
....N.....N.....................................................................     240
................................................................................     320
.................                                                                    400

(Threshold=0.5)
----------------------------------------------------------------------
SeqName      Position  Potential   Jury    N-Glyc
                                 agreement result
----------------------------------------------------------------------
contig054678-NyeOR.A010    9 NVTY   0.7932     (9/9)   +++
contig054678-NyeOR.A010   43 NSTI   0.7383     (9/9)   ++
contig054678-NyeOR.A010  165 NNSQ   0.5511     (5/9)   +
contig054678-NyeOR.A010  171 NFTL   0.6013     (7/9)   +
----------------------------------------------------------------------
```

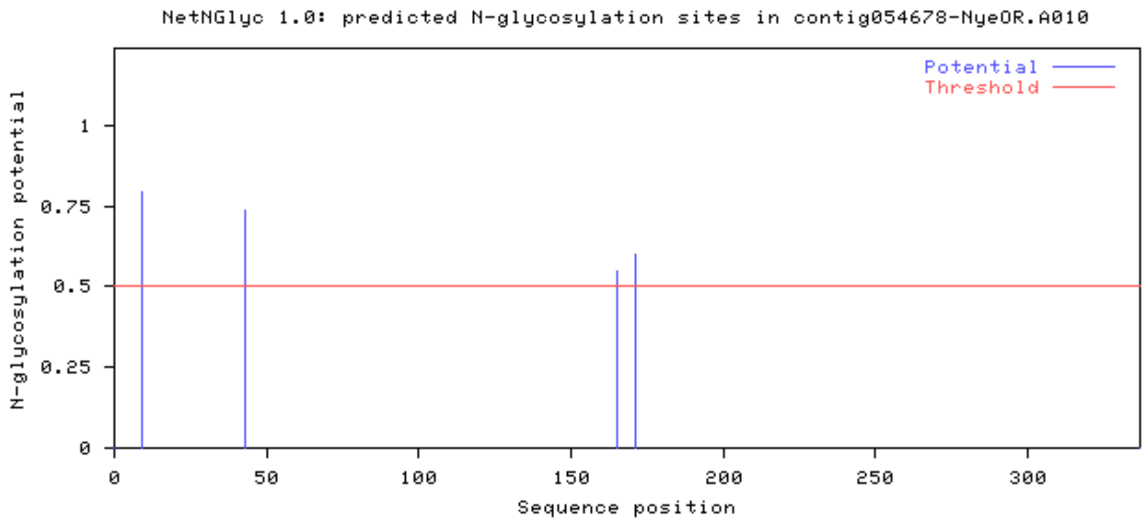

Graphics in PostScript

Output for 'contig054681-NyeOR.A011'

```
##############################################################################

   Warning: This sequence may not contain a signal peptide!!

   Proteins without signal peptides are unlikely to be exposed to
   the N-glycosylation machinery and thus may not be glycosylated
   (in vivo) even though they contain potential motifs.

   SignalP-NN euk predictions are as follows:

 # name                   Cmax  pos ?  Ymax  pos ?  Smax  pos ?  Smean ?  D     ?

   SignalP output is explained at http://www.cbs.dtu.dk/services/SignalP/output.html

##############################################################################

Name:  contig054681-NyeOR.A011  Length:  309
MNPGLNMTYLILGGHVEVQKYRYLYFMILFTAYILIICCNTSIIYLIVIHKSLHEPMYIFIAALLLNSLFFSTNIYPKLL      80
ADFLSEKQIISYQVCLFQVFIFYSLSCSEFLLLSAMAYDRYVSICKPLQYPTKMRKITVVFLLVLAWLLPACQVAVVIIL     160
NINNKLCNFTLKGIFCNNSLIQLYCVMSRALSVYGAFVLLNTGLLPMLFIIFTYTKIILTVYRSSGEVKKKAAQTCLPHL     240
FVLINYSCLITYDMIIARLESDFSKTARFLMTLQIITYNPLFNPIIYGLKMKEISKHLQRLLCQSKLNX
.....N.................................N........................................      80
................................................................................     160
.......N........................................................................     240
....N................................................................                320

(Threshold=0.5)
----------------------------------------------------------------------
SeqName      Position  Potential   Jury    N-Glyc
                                 agreement result
----------------------------------------------------------------------
contig054681-NyeOR.A011    6 NMTY    0.7297     (9/9)   ++
contig054681-NyeOR.A011   40 NTSI    0.6410     (8/9)   +
contig054681-NyeOR.A011  168 NFTL    0.6657     (9/9)   ++
contig054681-NyeOR.A011  177 NNSL    0.4818     (6/9)   -
contig054681-NyeOR.A011  245 NYSC    0.5044     (4/9)   +
----------------------------------------------------------------------
```

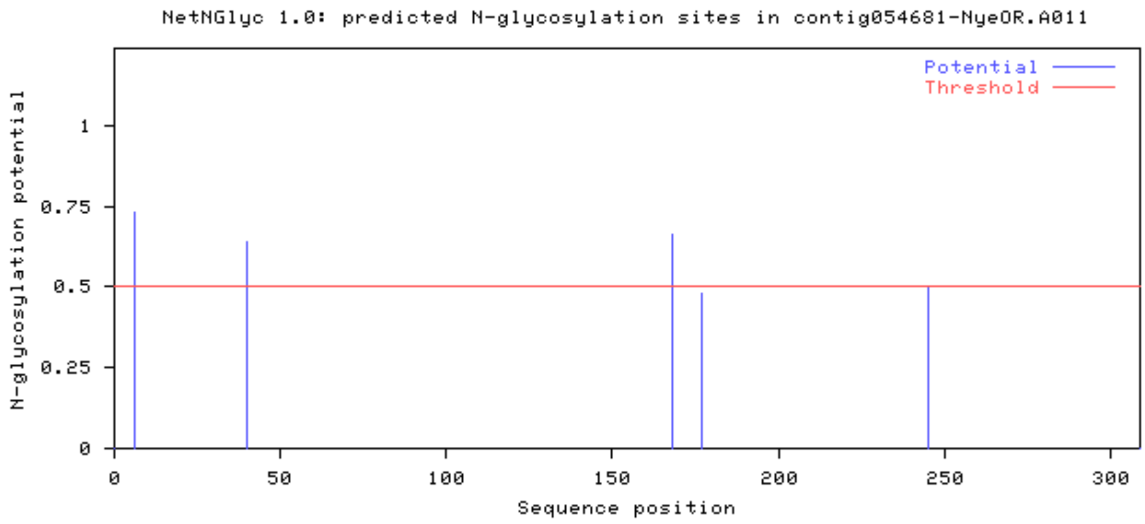

Graphics in PostScript

Output for 'contig054684-NyeOR.A012'

```
##########################################################################

    Warning: This sequence may not contain a signal peptide!!

    Proteins without signal peptides are unlikely to be exposed to
    the N-glycosylation machinery and thus may not be glycosylated
    (in vivo) even though they contain potential motifs.

    SignalP-NN euk predictions are as follows:

  # name                Cmax  pos ?  Ymax  pos ?  Smax  pos ?  Smean ?  D     ?

    SignalP output is explained at http://www.cbs.dtu.dk/services/SignalP/output.html

##########################################################################

Name:  contig054684-NyeOR.A012  Length:  319
MDQMNDKFNVTYITFGGHVELNKYRFLYFAIMFTAYILILCSNSTILCLIWIKKNLHEPMYIFIAGLLLNSVMFSTNIYP      80
ELLIDFLSDKQITTHSLCSFQAFIYYSLTGSEFFLLAAMAYDRYVSICKPLQYTTIMKKTTIIVLLGLAWLLPACQLVPS     160
VVMSQSYKICSFTLNGIFCNNAISKLYCDTSRTTYIIYGVFILLNTVFLPLLFILFTYTKIFIICYRSCREVRKKAAQTC     240
LPHLLVLVSFSGLCSYDIIVARLEMNLPKVARFILTLQVVLYHPLFNPIVYGLKMKEISKHLTKLFCEGKLNIWQSSCX
........N.................................N.....................................      80
................................................................................     160
................................................................................     240
...............................................................................      320

(Threshold=0.5)
----------------------------------------------------------------------
SeqName      Position  Potential   Jury    N-Glyc
                                agreement result
----------------------------------------------------------------------
contig054684-NyeOR.A012    9 NVTY   0.7640     (9/9)    +++
contig054684-NyeOR.A012   43 NSTI   0.6701     (9/9)    ++
----------------------------------------------------------------------
```

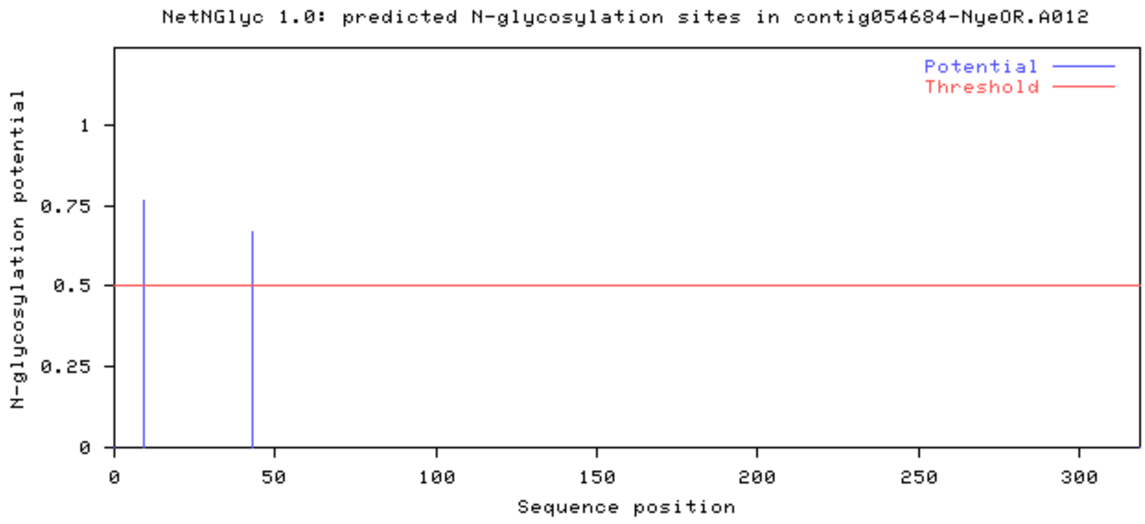

Graphics in PostScript

Output for 'contig054687-NyeOR.A013'

```
##########################################################################################

    Warning: This sequence may not contain a signal peptide!!

    Proteins without signal peptides are unlikely to be exposed to
    the N-glycosylation machinery and thus may not be glycosylated
    (in vivo) even though they contain potential motifs.

    SignalP-NN euk predictions are as follows:

  # name                    Cmax  pos ?  Ymax  pos ?  Smax  pos ?  Smean ?  D     ?

    SignalP output is explained at http://www.cbs.dtu.dk/services/SignalP/output.html

##########################################################################################

Name:  contig054687-NyeOR.A013  Length:  309
MDDELNITHITIDGYVDLKRFGYLYFLIMVALYVLIIISNSVIVFLICIHHNLHEPMYIFIAALSVNSVLLSTVTYPKLF      80
VDVLSEKQIISISACRFQHFMCYSIAGSDFLLLSAMAFDRYVSICKPLKYPVIMRQTTINILLFLSWFVPGLQIAVLHTL     160
VLNNKLCNFTLKGILCNNSLWKLYCESPRATLIYGLVVMLSVVIFPVFFILFTYAKIFLITYRSSRAIQKKAAETCLPHL     240
FVLSIFTTLCAYDVIIGRLELDFPKTAQLIMTLQVIFYNPLLNPFIYGLKMKEISKHLKRLFCHVRCSX
.....N..........................................................................      80
................................................................................     160
.......N........................................................................     240
.....................................................................                320

(Threshold=0.5)
----------------------------------------------------------------------
SeqName      Position  Potential   Jury    N-Glyc
                                 agreement result
----------------------------------------------------------------------
contig054687-NyeOR.A013    6 NITH   0.7698     (9/9)   +++
contig054687-NyeOR.A013  168 NFTL   0.6759     (8/9)   +
contig054687-NyeOR.A013  177 NNSL   0.4501     (6/9)   -
----------------------------------------------------------------------
```

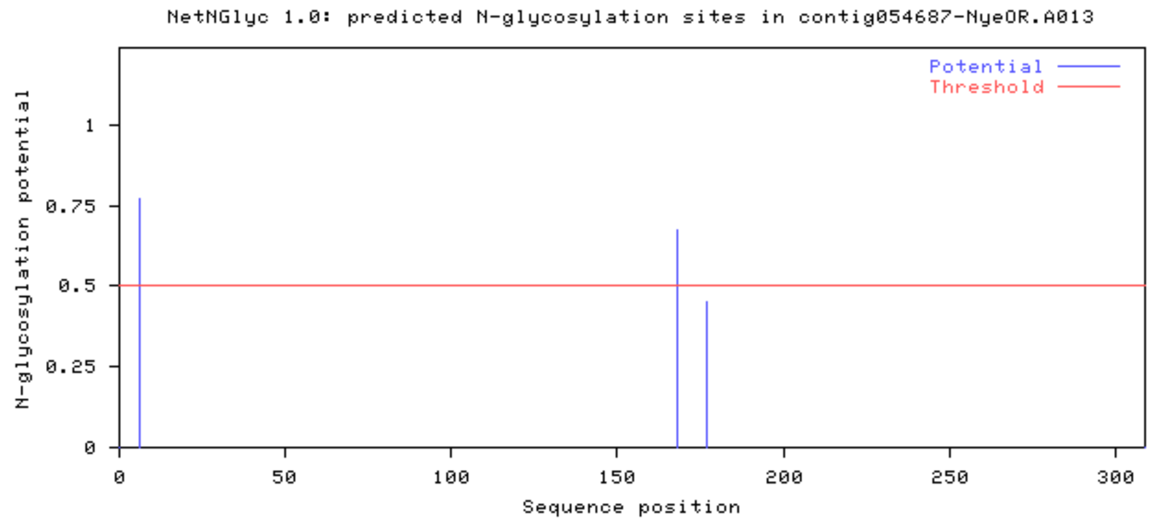

Graphics in PostScript

Output for 'contig054868-NyeOR.A014'

```
##########################################################################################

    Warning: This sequence may not contain a signal peptide!!

    Proteins without signal peptides are unlikely to be exposed to
    the N-glycosylation machinery and thus may not be glycosylated
    (in vivo) even though they contain potential motifs.

    SignalP-NN euk predictions are as follows:

  # name                    Cmax   pos ?  Ymax   pos ?  Smax   pos ?  Smean ?  D      ?

    SignalP output is explained at http://www.cbs.dtu.dk/services/SignalP/output.html

##########################################################################################

Name:  contig054868-NyeOR.A014  Length:  307
MDVELNVTLLTLGGFAELHKYRYLYFVVIFTLYILILCFNSTIVCLIWTCKNLHEPMYIFIAALLINSVLYSMIIYPKLL      80
SDVLSEKQIISYTLCLFQGFLYYTSAGSEFLLLAAMAYDRYVSICKPLQYPVIMTRITIYVSLVLAWLIPAFEIAVSVVL     160
YSEVKLCSFTLTGIFCNNSGYKLQCVTSVAISVYGVVMLINIALLPLLFILFTYIRIVRVSYQSCREVRKKAVKTCLPHL     240
LVLINFSCFIVFDVIIVRLESDLSKTLRLILTFQSILFHPLLNPIIYGLKMNEIFKNLKILFCHVKX
.....N.................................N........................................      80
................................................................................     160
................................................................................     240
....N..............................................................                  320

(Threshold=0.5)
----------------------------------------------------------------------
SeqName      Position  Potential   Jury    N-Glyc
                                 agreement result
----------------------------------------------------------------------
contig054868-NyeOR.A014    6 NVTL   0.7849     (9/9)   +++
contig054868-NyeOR.A014   40 NSTI   0.7134     (9/9)   ++
contig054868-NyeOR.A014  177 NNSG   0.3827     (7/9)   -
contig054868-NyeOR.A014  245 NFSC   0.5624     (6/9)   +
----------------------------------------------------------------------
```

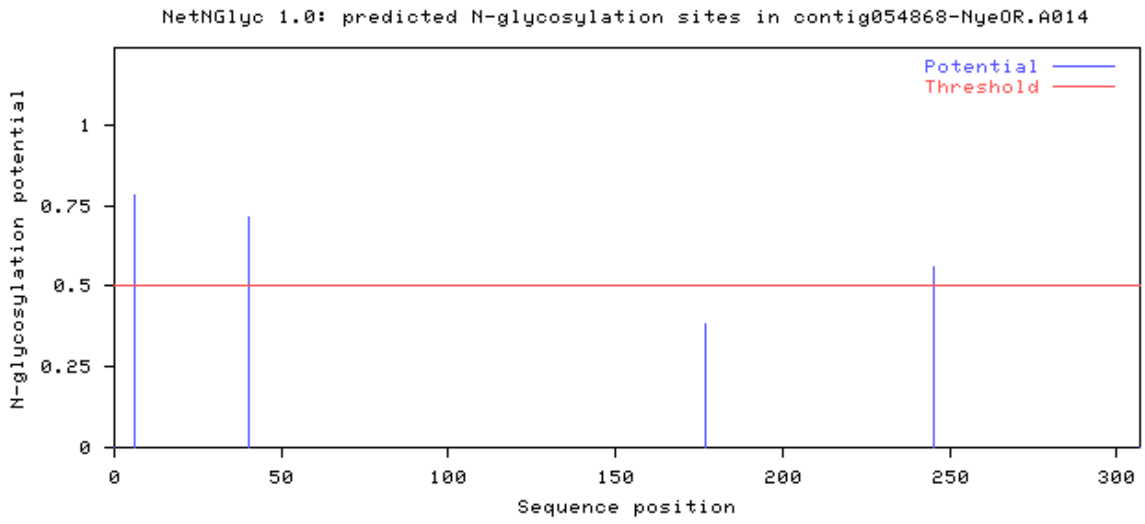

Graphics in PostScript

Output for 'contig055024-NyeORe.R137'

```
##########################################################################

    Warning: This sequence may not contain a signal peptide!!

    Proteins without signal peptides are unlikely to be exposed to
    the N-glycosylation machinery and thus may not be glycosylated
    (in vivo) even though they contain potential motifs.

    SignalP-NN euk predictions are as follows:

  # name                 Cmax  pos ?  Ymax  pos ?  Smax  pos ?  Smean ?  D     ?

    SignalP output is explained at http://www.cbs.dtu.dk/services/SignalP/output.html

##########################################################################

Name:  contig055024-NyeORe.R137         Length:  192
MSNVSESYTNMSIEVQYQDLLRVIIVSTLSTVPSFIFLFLNGTMLFTLRSKLVFRDTPRYILLYNLLFADTVQLAQSQVL      80
FLLSIFRVKLPYPVCVCLSLLANLTTGISPLTLSVMPLERYVAVCYPLRYPAIITIRNTGAAIVVIWIISSLNNLTRLIF     160
FFPFEVLKNLQMKDSCSKIALLLGTRSDQYDT
..N......N..............................N.......................................      80
......................N...................................................N......     160
................................                                                     240

(Threshold=0.5)
----------------------------------------------------------------------
SeqName      Position  Potential   Jury    N-Glyc
                                agreement result
----------------------------------------------------------------------
contig055024-NyeORe.R137    3 NVSE   0.7642     (9/9)   +++
contig055024-NyeORe.R137   10 NMSI   0.6849     (9/9)   ++
contig055024-NyeORe.R137   41 NGTM   0.7278     (9/9)   ++
contig055024-NyeORe.R137  103 NLTT   0.5544     (6/9)   +
contig055024-NyeORe.R137  154 NLTR   0.5267     (7/9)   +
----------------------------------------------------------------------
```

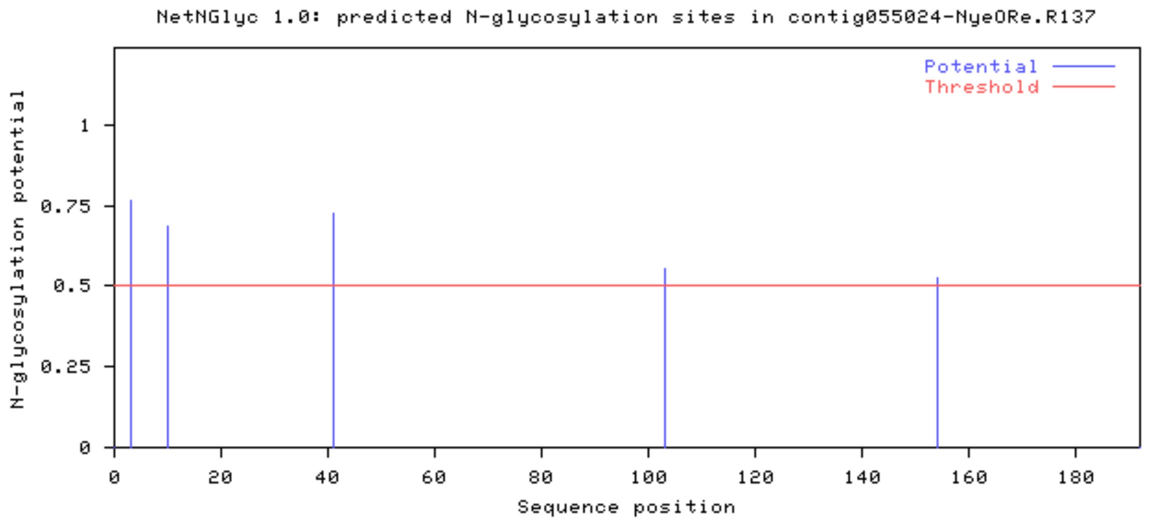

Graphics in PostScript

Output for 'contig055368-NyeORe.X141'

```
##########################################################################################

    Warning: This sequence may not contain a signal peptide!!

    Proteins without signal peptides are unlikely to be exposed to
    the N-glycosylation machinery and thus may not be glycosylated
    (in vivo) even though they contain potential motifs.

    SignalP-NN euk predictions are as follows:

  # name                    Cmax  pos ?  Ymax  pos ?  Smax  pos ?  Smean ?  D     ?

    SignalP output is explained at http://www.cbs.dtu.dk/services/SignalP/output.html

##########################################################################################

Name:  contig055368-NyeORe.X141         Length:  146
INSSPIQALFRTAVSVMFFAVVAVVIFFTYVRILLETRKLRQDRVSVNKAKHTVLLHGFQLLLCMLAFTLPITESLTLLY      80
TKWPVEDVTYFCFFCFILIPRFLSPLIYGFRDHSLRGYIGKTFLCCLDTVKPHFRSKQQDSLSASX
................................................................................      80
..................................................................                   160

(Threshold=0.5)

No sites predicted in this sequence.
```

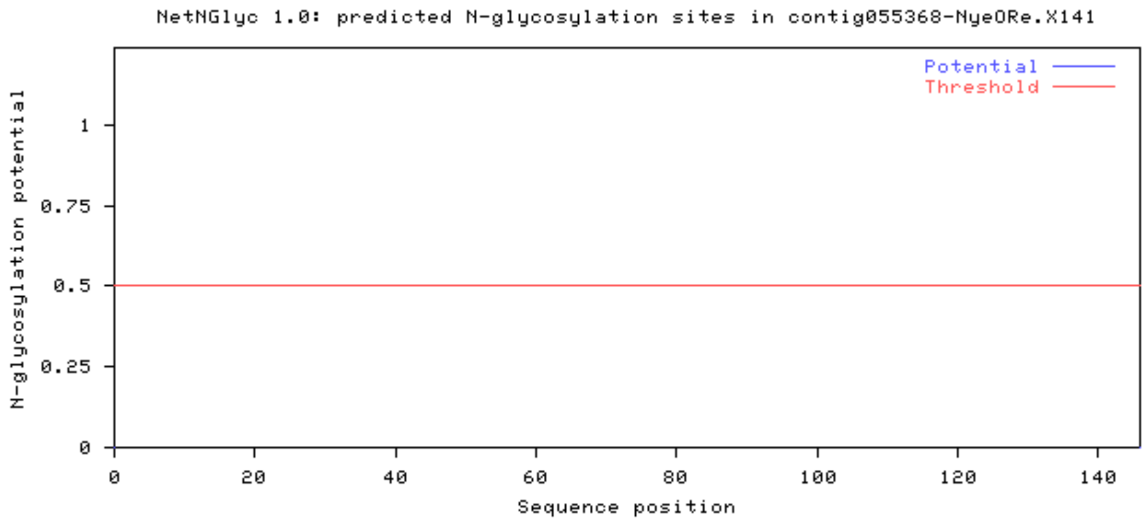

Graphics in PostScript

Output for 'contig055923-NyeORe.N115'

```
##########################################################################################

    Warning: This sequence may not contain a signal peptide!!

    Proteins without signal peptides are unlikely to be exposed to
    the N-glycosylation machinery and thus may not be glycosylated
    (in vivo) even though they contain potential motifs.

    SignalP-NN euk predictions are as follows:

  # name                    Cmax  pos ?  Ymax  pos ?  Smax  pos ?  Smean ?  D     ?

    SignalP output is explained at http://www.cbs.dtu.dk/services/SignalP/output.html

##########################################################################################

Name:  contig055923-NyeORe.N115         Length:  310
PAFFIISGFIGIPNIKYYYAFLFFVYIISVLANTAVMATIYLDHNLRTPKYIAVFNLALVDLLGNSAMVPKILDIFLFNH      80
PHISYNDCLTFLFFCYVFLSMQALNLVALSYDRAMAIVYPLHYQLKVTHKFMFCLIASFWVFVIIVILIATGLLTRLSLC     160
ESVVIKSFFCDHGQIYRLACNDYTPSDITAWILPALILWLPLLFVLLSYLSIGYALAKVATVRERMKGFKTCTAHLSLVA     240
IYFLPILITLRLNIEPNARIINLSLTSVFPPMLNPIIYVLQTQEIKESLKKLKGRITTHYKNRKVKFKKX
................................................................................      80
................................................................................     160
................................................................................     240
.....................N................................................               320

(Threshold=0.5)
----------------------------------------------------------------------
SeqName      Position  Potential   Jury    N-Glyc
                                agreement result
----------------------------------------------------------------------
contig055923-NyeORe.N115   262 NLSL   0.5976     (8/9)   +
----------------------------------------------------------------------
```

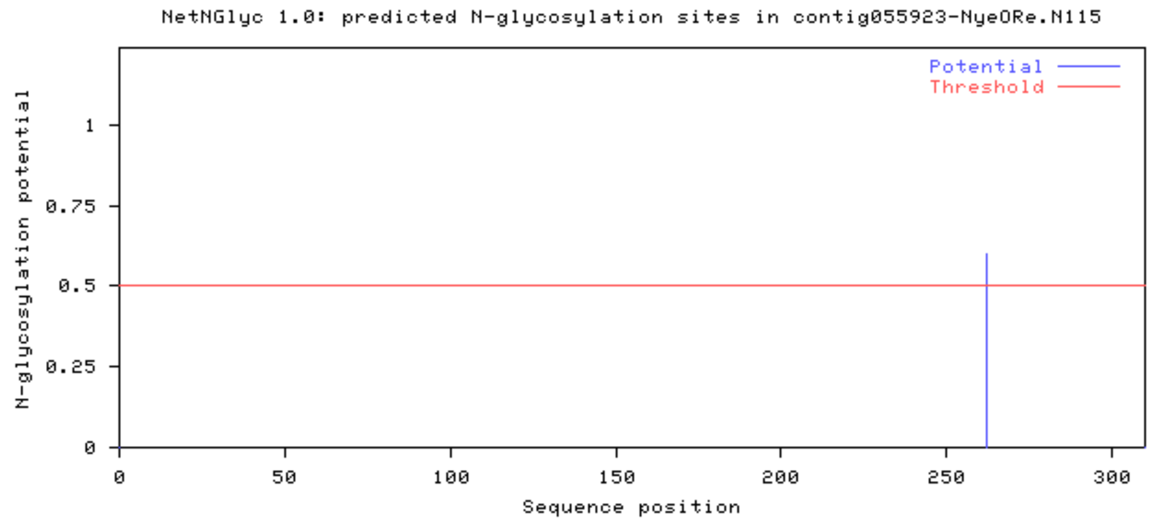

Graphics in PostScript

Output for 'contig055924-NyeOR.N109'

```
##########################################################################################

    Warning: This sequence may not contain a signal peptide!!

    Proteins without signal peptides are unlikely to be exposed to
    the N-glycosylation machinery and thus may not be glycosylated
    (in vivo) even though they contain potential motifs.

    SignalP-NN euk predictions are as follows:

  # name                 Cmax  pos ?  Ymax  pos ?  Smax  pos ?  Smean ?  D     ?

    SignalP output is explained at http://www.cbs.dtu.dk/services/SignalP/output.html

##########################################################################################

Name:  contig055924-NyeOR.N109  Length:  324
MEFLNSAVGKNITFVKPAYFIISTFNGIANIRYYFVFLCFIYIFSVVGNTLLMIVIILDHTLRGPKHIGVVNFAFTDLLS      80
SSALMPKLVDIFLFNHHHISYNDCLAFMFFCLTFFAAQAFNLVVLSFDRVMAIMYPLHYQMRISHKLILSLIAFFWLLAI     160
TIILTAVGLLTRLSFCDSVVIQSFFCDHGPVYRLGCNDLTPNRVIAHLASVLVLWVPLAFIVGSYCCIGYSLSKTVTCRE     240
RLKALKTCTSHLSLVAIYFLPALFIFTFGSTILPNARTISLSLATVMPLTLNPIIYGLQTQEIKESLKKLLKVKMQFKIS     320
AKKX
..........N.....................................................................      80
................................................................................     160
................................................................................     240
................................................................................     320
....                                                                                 400

(Threshold=0.5)
----------------------------------------------------------------------
SeqName      Position  Potential   Jury    N-Glyc
                                 agreement result
----------------------------------------------------------------------
contig055924-NyeOR.N109   11 NITF   0.5221     (6/9)    +
----------------------------------------------------------------------
```

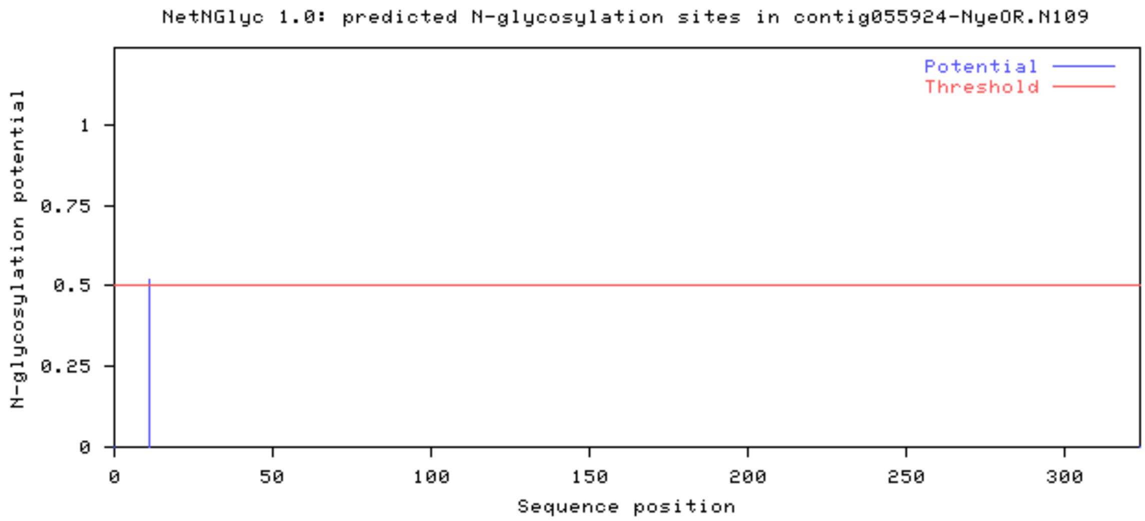

Graphics in PostScript

Output for 'contig055926-NyeOR.N110'

```
##########################################################################################

    Warning: This sequence may not contain a signal peptide!!

    Proteins without signal peptides are unlikely to be exposed to
    the N-glycosylation machinery and thus may not be glycosylated
    (in vivo) even though they contain potential motifs.

    SignalP-NN euk predictions are as follows:

  # name                  Cmax  pos ?  Ymax  pos ?  Smax  pos ?  Smean ?  D     ?

    SignalP output is explained at http://www.cbs.dtu.dk/services/SignalP/output.html

##########################################################################################

Name:  contig055926-NyeOR.N110  Length:  323
MDFLNSAAEKNTTFVQPANFIISGFVGIPNIRYYFVFLCFIYIFSLVGNTAVMLIIIFDHTLRSPKYIAVFNLAFTDLLS      80
NSALVPKVLDISLFNHHYISYNNCLTFMFFCFTLISMQAFNLVVLSFDRIMAIMYPLHYQMRVSHKIILSLIAFFWLLAV     160
ALTGTAVGLLTRLYFCESVVINSYYCDHGPIYRLSCNDVTPNKTISAWSRAFVLWLPLIFILGSYCCIGYSLSRISTCKE     240
RVKALKTCTGHLSLVAIYFIPILVVYSFGSTMHPNARIVNLSLASVTPPMLNPIIYVFQTAEIKKSLKKLLKAKIQISHR     320
VLX
................................................................................      80
................................................................................     160
.........................................N......................................     240
.......................................N........................................     320
...                                                                                  400

(Threshold=0.5)
----------------------------------------------------------------------
SeqName      Position  Potential   Jury    N-Glyc
                                 agreement result
----------------------------------------------------------------------
contig055926-NyeOR.N110   11 NTTF   0.4656     (5/9)   -
contig055926-NyeOR.N110  202 NKTI   0.6129     (8/9)   +
contig055926-NyeOR.N110  280 NLSL   0.5872     (8/9)   +
----------------------------------------------------------------------
```

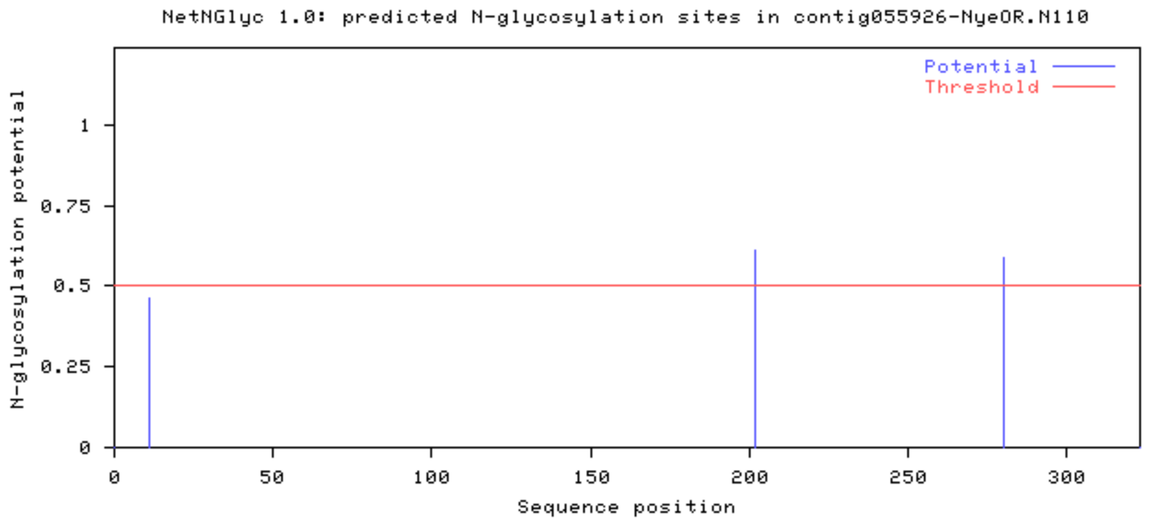

Graphics in PostScript

Output for 'contig055927-NyeOR.N111'

```
##########################################################################################

    Warning: This sequence may not contain a signal peptide!!

    Proteins without signal peptides are unlikely to be exposed to
    the N-glycosylation machinery and thus may not be glycosylated
    (in vivo) even though they contain potential motifs.

    SignalP-NN euk predictions are as follows:

  # name                    Cmax  pos ?  Ymax  pos ?  Smax  pos ?  Smean ?  D     ?

    SignalP output is explained at http://www.cbs.dtu.dk/services/SignalP/output.html

##########################################################################################

Name:  contig055927-NyeOR.N111  Length:  322
MELFNSALGKNITFVHPAFFIIGGLTGIPNITLYYVFLFFVYIVSVVGNTVVMAVIYLDHNLRTPKYIAVFNLAFVDLFG      80
NTALVPKVLDIFLFGHYYIPYNDCLTFLFFCYTCLSLQSFNLVALSYDRMVAIIFPLHYQVKVTHRFMFSLIASLWVFTI     160
IAVLISVGLLTRLSFCKSVVINSYFCDHGQIYRLACNDHFPSYVIACLYPVIIFWLPLAFILLSYLYIGYTLVKVATLQE     240
GLKAFKTCIGHLSLVAIYFIPLLTTFTLMEKIQPNARIINLSLTSVFPPMLNPIIYVLQTQEIKESLKRLLKRRGKSKIT     320
IX
..........N..................N..................................................      80
................................................................................     160
................................................................................     240
.......................................N........................................     320
..                                                                                   400

(Threshold=0.5)
----------------------------------------------------------------------
SeqName      Position  Potential   Jury    N-Glyc
                                 agreement result
----------------------------------------------------------------------
contig055927-NyeOR.N111   11 NITF   0.6087     (8/9)   +
contig055927-NyeOR.N111   30 NITL   0.8183     (9/9)   +++
contig055927-NyeOR.N111  280 NLSL   0.6020     (8/9)   +
----------------------------------------------------------------------
```

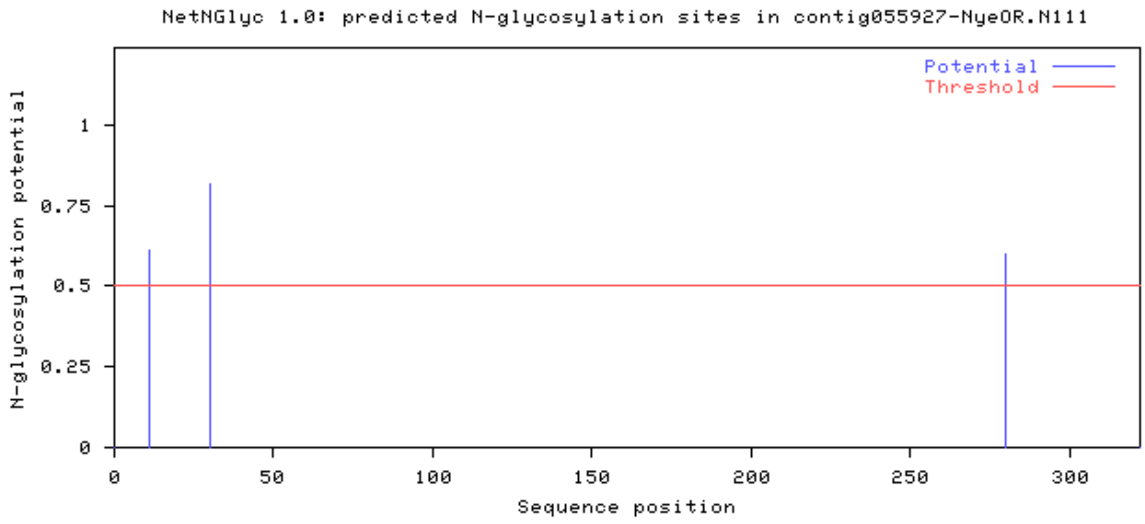

Graphics in PostScript

Output for 'contig055927-NyeOR.N112'

```
##########################################################################################

    Warning: This sequence may not contain a signal peptide!!

    Proteins without signal peptides are unlikely to be exposed to
    the N-glycosylation machinery and thus may not be glycosylated
    (in vivo) even though they contain potential motifs.

    SignalP-NN euk predictions are as follows:

  # name                    Cmax  pos ?  Ymax  pos ?  Smax  pos ?  Smean ?  D     ?

    SignalP output is explained at http://www.cbs.dtu.dk/services/SignalP/output.html

##########################################################################################

Name:  contig055927-NyeOR.N112  Length:  327
MDSEEKATTKSNSTFVRPAIFYLSGFSNIPHVKYFYLFLCFVYIMTVLGNGFLLSLIWLVKTLHTPKYMIVFNMALADLC      80
GSTALIPKLLDTFLFDRRYIVYEACLSYMFFVFFFGGVQSWMLVTMAYDRLIAICFPLRYHSIVTKTSIKSMLLFIWLVM     160
LSLTTLVVGLVNRLSFCDSVVVKSFFCDHGPIYHLACNDPSLNIIMANVVVSIGVFIPLIFIACTYVCISIALSKIASGE     240
ERLKALKTCTSHLILVAILFLPFVGTNIAVWTSYIHPNARIINSTLTHTIPALINPIVYALKTEEVMSAVKKLWKINNIS     320
SPVTKWX
...........N....................................................................      80
................................................................................     160
................................................................................     240
..........................................N..................................N..     320
.......                                                                              400

(Threshold=0.5)
----------------------------------------------------------------------
SeqName      Position  Potential   Jury    N-Glyc
                                 agreement result
----------------------------------------------------------------------
contig055927-NyeOR.N112   12 NSTF   0.6201     (9/9)    ++
contig055927-NyeOR.N112  283 NSTL   0.5844     (8/9)    +
contig055927-NyeOR.N112  318 NISS   0.6380     (7/9)    +
----------------------------------------------------------------------
```

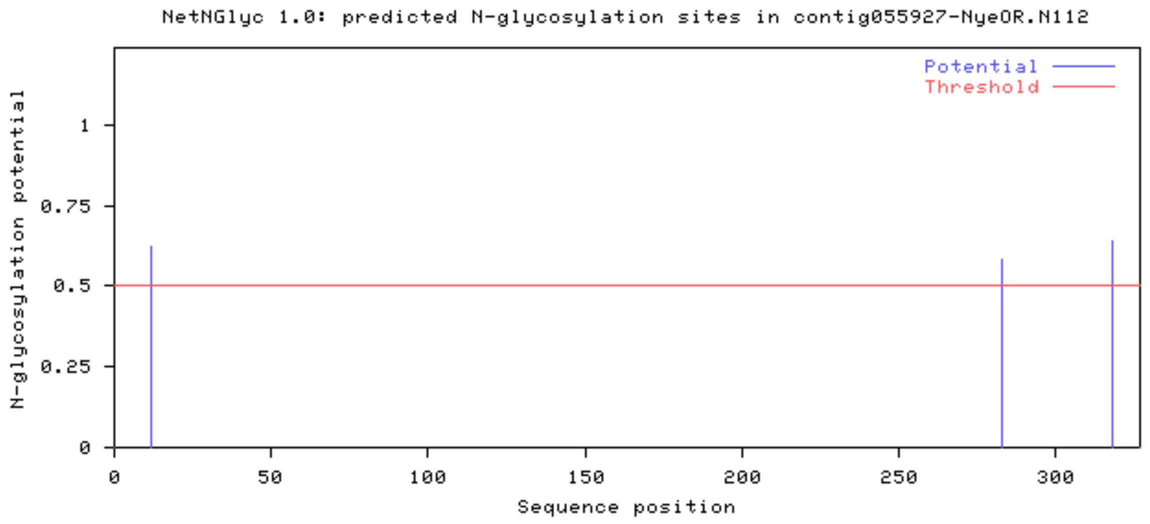

Graphics in PostScript

Output for 'contig055931-NyeORe.N116'

```
##########################################################################################

    Warning: This sequence may not contain a signal peptide!!

    Proteins without signal peptides are unlikely to be exposed to
    the N-glycosylation machinery and thus may not be glycosylated
    (in vivo) even though they contain potential motifs.

    SignalP-NN euk predictions are as follows:

  # name                    Cmax  pos ?  Ymax  pos ?  Smax  pos ?  Smean ?  D     ?

    SignalP output is explained at http://www.cbs.dtu.dk/services/SignalP/output.html

##########################################################################################

Name:  contig055931-NyeORe.N116         Length:  313
MGTEDKATTMFNNTFVRPEKFYLSGFSNIPHIRYYYAFLCLVYILTVLGNGFLLSLIWLVKTLHTPKYMIVFNMALTDLC      80
GSTALIPKLLDTFLLDRRYILYDACLSYMFFVMFFASVQSWTLVTMAYDRLIAICFPLRYHNIVTETSVAAILLFIWIFL     160
VSVIATMVGLVNRLSFCRSLVVNSFFCDHGPVYRLACNDTYLNYNMASALITILIIIPLIFIIATYVCIFIALSRTTSRK     240
EQIRALKTCTSHLILVVIFFLPIGITNIATRASYIHPNARMINSTLTHTIPALLDPIIYALKTEEVMNAVKKL
...........N....................................................................      80
................................................................................     160
.....................................N..........................................     240
..........................................N..............................            320

(Threshold=0.5)
----------------------------------------------------------------------
SeqName      Position  Potential   Jury    N-Glyc
                                 agreement result
----------------------------------------------------------------------
contig055931-NyeORe.N116   12 NNTF   0.5687     (6/9)   +
contig055931-NyeORe.N116  198 NDTY   0.5830     (6/9)   +
contig055931-NyeORe.N116  283 NSTL   0.5723     (6/9)   +
----------------------------------------------------------------------
```

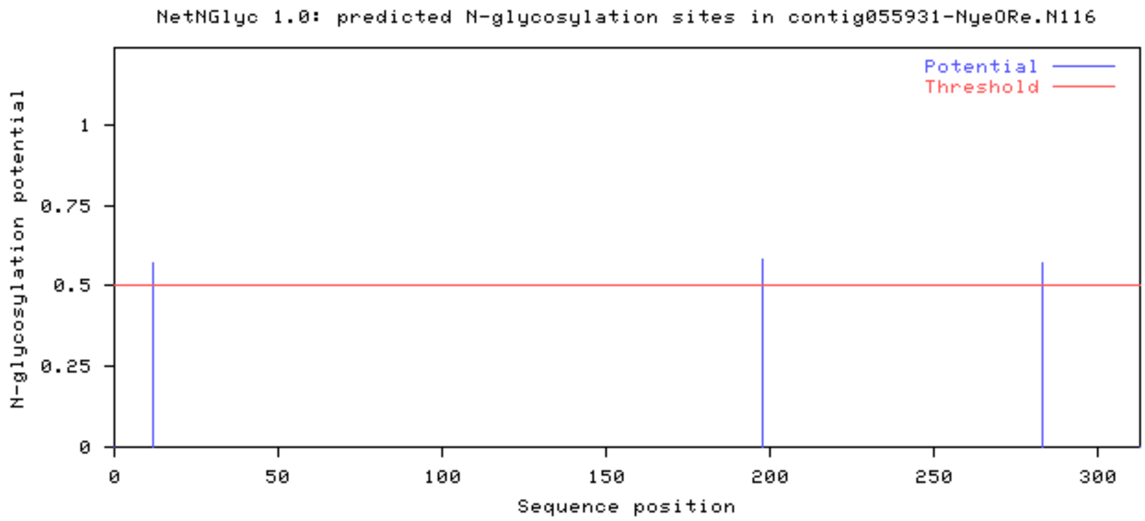

Graphics in PostScript

Output for 'contig055933-NyeOR.N113'

```
########################################################################################

    Warning: This sequence may not contain a signal peptide!!

    Proteins without signal peptides are unlikely to be exposed to
    the N-glycosylation machinery and thus may not be glycosylated
    (in vivo) even though they contain potential motifs.

    SignalP-NN euk predictions are as follows:

  # name                  Cmax  pos ?  Ymax  pos ?  Smax  pos ?  Smean ?  D     ?

    SignalP output is explained at http://www.cbs.dtu.dk/services/SignalP/output.html

########################################################################################

Name:  contig055933-NyeOR.N113  Length:  337
MGAEAKTTTIFNTTFVRPAKFYLSGFSNIPHIRYFYAFLCFVYIMTVLGNGFLLSLISLVKTLHTPKYMIVFNMALADLC      80
GSTALIPKVLDTFLFDRRYILYDACLSYMFFVIFFASVQSWTLVTMAYDRLIAICFPLRYHSIVTEQSITAILLFVWIFL     160
VSVIATMVGLVNRLSFCRSLVVNSFFCDHGPVYRLACNDTSLNYNMASALVAILIIIPLIFIIATYVCIFIALSRTTSRE     240
ERLRALKTCTSHLILVVIFFLPIGITNIAAMTSYIDSNARMINSVLTHTIPASLDPIVYALKTEEVMNAVKKLCKGINLS     320
RMKAKTRPCNHCCIKSX
...........N....................................................................      80
................................................................................     160
.....................................N..........................................     240
.............................................................................N..     320
.................                                                                    400

(Threshold=0.5)
----------------------------------------------------------------------
SeqName      Position  Potential   Jury    N-Glyc
                                 agreement result
----------------------------------------------------------------------
contig055933-NyeOR.N113   12 NTTF   0.6070     (6/9)   +
contig055933-NyeOR.N113  198 NDTS   0.6409     (7/9)   +
contig055933-NyeOR.N113  318 NLSR   0.5328     (5/9)   +
----------------------------------------------------------------------
```

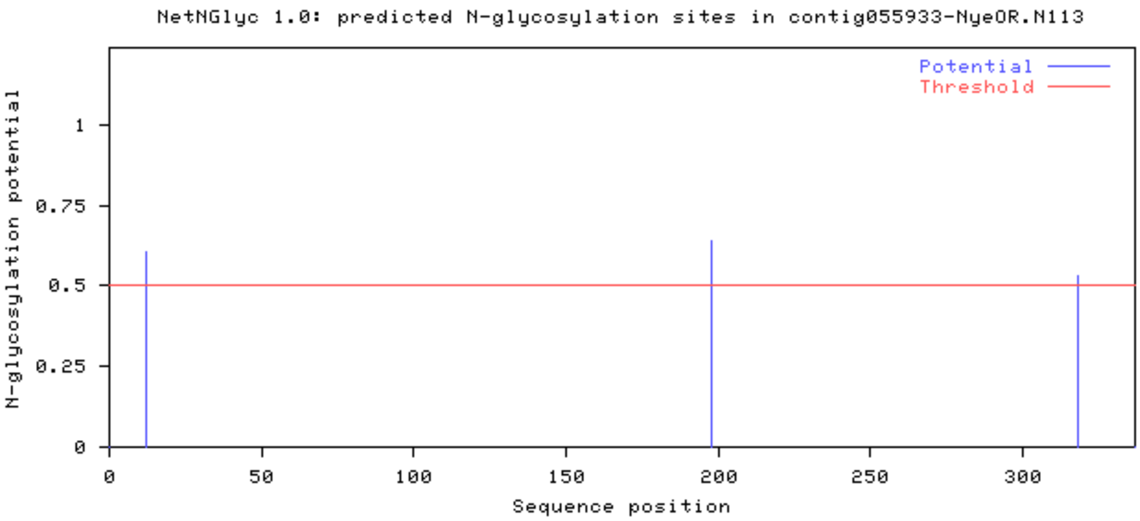

Graphics in PostScript

Output for 'contig056375-NyeOR.A015'

```
##########################################################################################

    Warning: This sequence may not contain a signal peptide!!

    Proteins without signal peptides are unlikely to be exposed to
    the N-glycosylation machinery and thus may not be glycosylated
    (in vivo) even though they contain potential motifs.

    SignalP-NN euk predictions are as follows:

  # name                    Cmax  pos ?  Ymax  pos ?  Smax  pos ?  Smean ?  D     ?

    SignalP output is explained at http://www.cbs.dtu.dk/services/SignalP/output.html

##########################################################################################

Name:  contig056375-NyeOR.A015  Length:  304
MDKVLNVTYLTLDWYTEINKYRYVFFFIMFILFILIICTNSTILYLIWIHKNLHEPMYIFIAALLLNSVLYSTTIYPKLL      80
TDFLSEKQVTTYSACLFQFFTFYTLACSEFLLLAAMAYDRYVAICKPLEYQTIMTKNTVSIFLVVAWLVPACHLAVLAIA     160
SAGAKLCDSNIKGIFCNNAVYTLQCERSRLITIFGVVALVDLSILPMLFIVFTYTKLFIVSHRSCKEIRKKAAETCLPHL     240
LVLISYSAFFVYDVSIARVKSDFPKTTRIIMTLQIMLYQPLLNPFIYGLKMKEISKHLNKLLSX
.....N.................................N........................................      80
................................................................................     160
................................................................................     240
................................................................     320

(Threshold=0.5)
----------------------------------------------------------------------
SeqName      Position  Potential   Jury    N-Glyc
                                 agreement result
----------------------------------------------------------------------
contig056375-NyeOR.A015    6 NVTY   0.7716     (9/9)   +++
contig056375-NyeOR.A015   40 NSTI   0.6063     (8/9)   +
----------------------------------------------------------------------
```

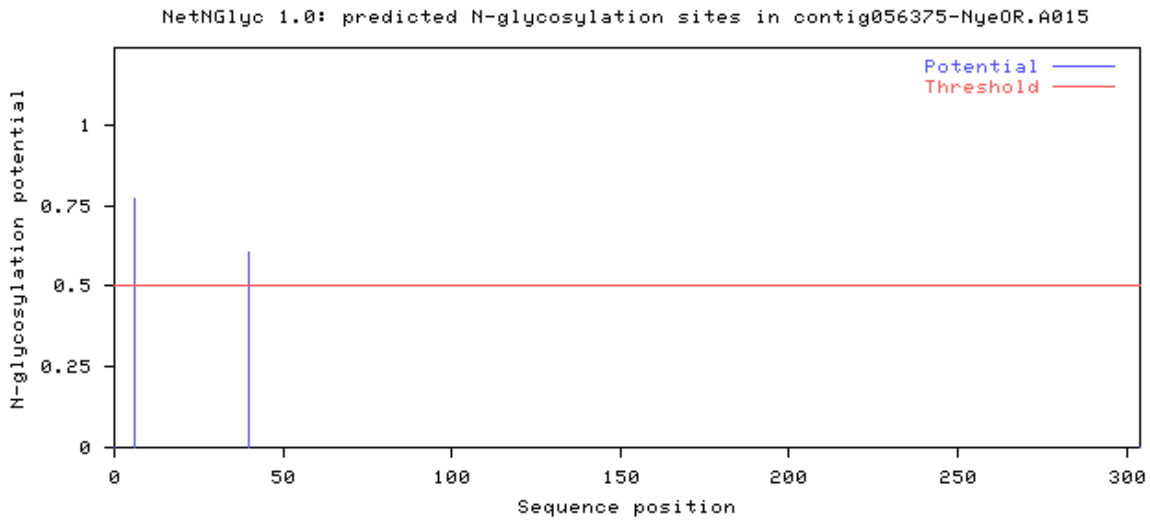

[Graphics in PostScript]

Output for 'contig056375-NyeORp.A031'

```
##########################################################################################

    Warning: This sequence may not contain a signal peptide!!

    Proteins without signal peptides are unlikely to be exposed to
    the N-glycosylation machinery and thus may not be glycosylated
    (in vivo) even though they contain potential motifs.

    SignalP-NN euk predictions are as follows:

  # name                    Cmax  pos ?  Ymax  pos ?  Smax  pos ?  Smean ?  D     ?

    SignalP output is explained at http://www.cbs.dtu.dk/services/SignalP/output.html

##########################################################################################

Name:  contig056375-NyeORp.A031         Length:  311
MDEESNATYLTLDWYTEINKYRYIFFFVMFILYILIICTNSTILYLIWIHKNLHEPMYIFIAALLLNSVLYSTTVYPKLL      80
IDFSSEKQVTTYSACLFQFFIFYTLVLSEFLLLAAMAYDRYVAICKPLEYQTLRKTTVGIFLVVAWLVPACQVAVAIASA     160
EAKLCDSNIKGIFCNNAVYTLQCERSKLITIFGVVIVLDLPMLPMLFIVFTYTKIFIVSHRSCKEIRKKAAETSLPHLLV     240
LLSLSVFFVYDVTARANPDFPKTTRIIMTLQIMLYQPLLNPFIYGLKMKEISKHLNKLLSQANISPCIKTX
.....N.................................N........................................      80
................................................................................     160
................................................................................     240
.......................................................................              320

(Threshold=0.5)
----------------------------------------------------------------------
SeqName      Position  Potential   Jury    N-Glyc
                                 agreement result
----------------------------------------------------------------------
contig056375-NyeORp.A031    6 NATY   0.6219     (7/9)   +
contig056375-NyeORp.A031   40 NSTI   0.6178     (8/9)   +
contig056375-NyeORp.A031  303 NISP   0.1063     (9/9)   ---
----------------------------------------------------------------------
```

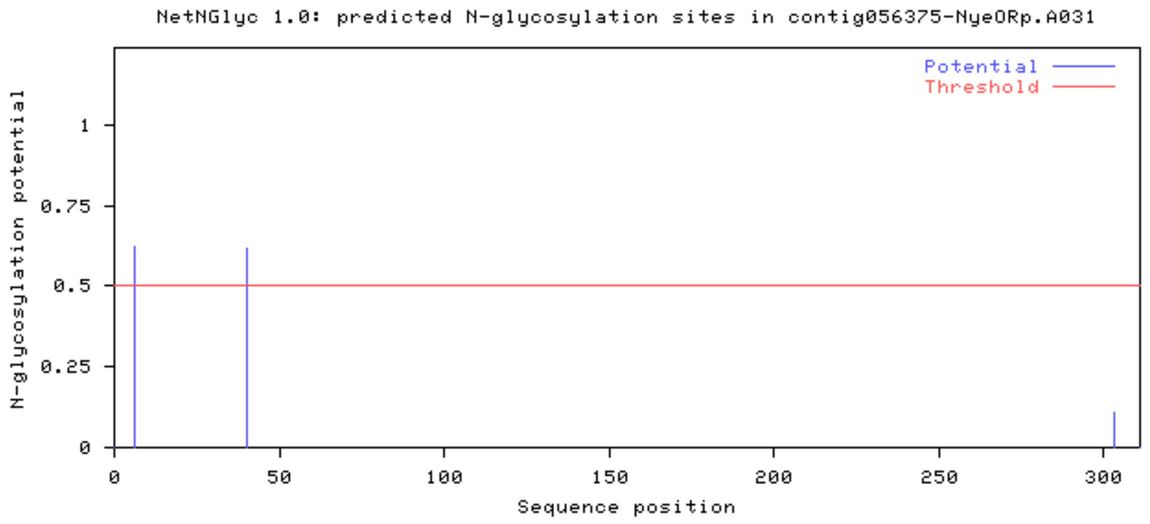

Graphics in PostScript

Output for 'contig056380-NyeOR.A016'

```
##########################################################################################

    Warning: This sequence may not contain a signal peptide!!

    Proteins without signal peptides are unlikely to be exposed to
    the N-glycosylation machinery and thus may not be glycosylated
    (in vivo) even though they contain potential motifs.

    SignalP-NN euk predictions are as follows:

  # name                 Cmax  pos ?  Ymax  pos ?  Smax  pos ?  Smean ?  D     ?

    SignalP output is explained at http://www.cbs.dtu.dk/services/SignalP/output.html

##########################################################################################

Name:  contig056380-NyeOR.A016  Length:  314
MDEESNATYLTLDWYTEINKYRYVFFFVMFTLYILIICTNSTILYLIWIHKNLHEPMYIFIAALLLNSVLYSTTIYPKLL      80
TDFLSEIQVTTYSACLFQFFMFYTLGCSEFLLLAAMAYDRYVAICKPLEYQTIMRKTTVGIFLVMAWLVPACHIAVQAIA     160
SAGAKLCDSNIKGIFCSNAVYTLQCERSRLITVFGVFLLIDLAILPMLFIVYTYTKIFIVSHRSCKEIRKKTAETCLPHL     240
LVLISYSMFFVYDISIARVKSDFPKTTRIIMTLQIMLYQPLLNPFIYGLKMKDISKHLNKLLSQAKIISCIKTX
.....N.................................N........................................      80
................................................................................     160
................................................................................     240
..........................................................................           320

(Threshold=0.5)
----------------------------------------------------------------------
SeqName      Position  Potential   Jury    N-Glyc
                                 agreement result
----------------------------------------------------------------------
contig056380-NyeOR.A016    6 NATY   0.6217     (7/9)    +
contig056380-NyeOR.A016   40 NSTI   0.6299     (8/9)    +
----------------------------------------------------------------------
```

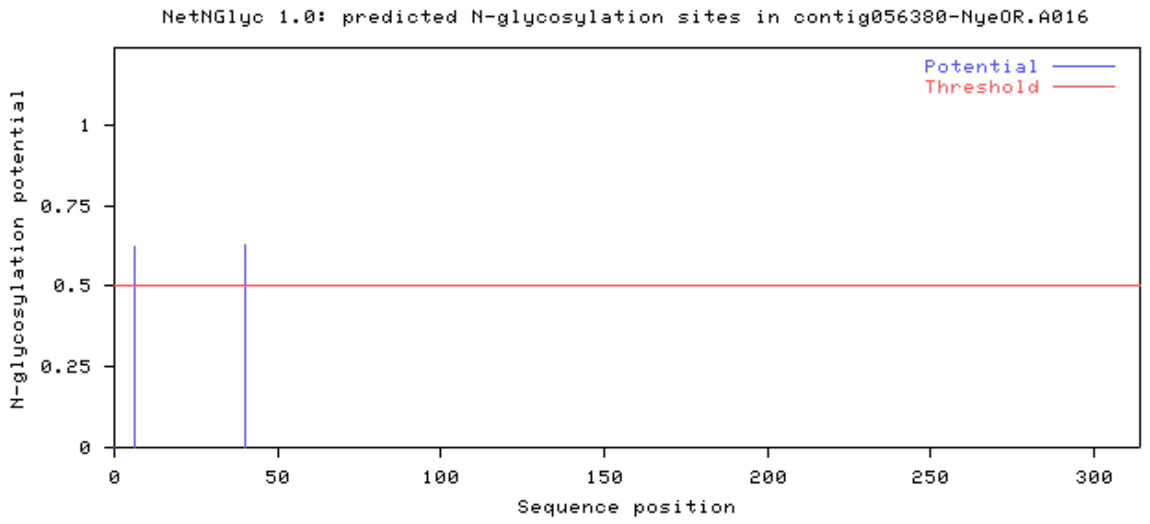

Graphics in PostScript

Output for 'contig056940-NyeOR.L095'

```
##########################################################################################

    Warning: This sequence may not contain a signal peptide!!

    Proteins without signal peptides are unlikely to be exposed to
    the N-glycosylation machinery and thus may not be glycosylated
    (in vivo) even though they contain potential motifs.

    SignalP-NN euk predictions are as follows:

  # name                    Cmax  pos ?  Ymax  pos ?  Smax  pos ?  Smean ?  D     ?

    SignalP output is explained at http://www.cbs.dtu.dk/services/SignalP/output.html

##########################################################################################

Name:  contig056940-NyeOR.L095  Length:  313
MSLQNASINVTHFIIGGFDTLSRPIAVGVVILITYLLAVLANMINIMFIISDKRLHKPMYLLICNLAVVDIVYTSSSSPT      80
MIGVLLAGVNTISYVACLIQMCVFNLGTSMESFVLAFMALDRFIAIIYPFQYQRYLTNTRVLVLTFIMWFIAWCFAFYMP     160
ATVVPLPHCSSRLKYSFCDFAAVIRTTCVNPEKYFNEVSIAAFFIFFFTFVFICLSYCGILLFVKLSSNNEKMKMGSTLV     240
SHLICVVVHYCPAFVRIMFTRFGVVLTLEERQGLVIGAVLGPCLVNPFVYCLRTKEIKQKLFKIFKKFNTSDX
....N...N.......................................................................      80
................................................................................     160
................................................................................     240
.........................................................................            320

(Threshold=0.5)
----------------------------------------------------------------------
SeqName      Position  Potential   Jury    N-Glyc
                                 agreement result
----------------------------------------------------------------------
contig056940-NyeOR.L095    5 NASI   0.6716     (8/9)   +
contig056940-NyeOR.L095    9 NVTH   0.7236     (9/9)   ++
contig056940-NyeOR.L095  309 NTSD   0.4220     (8/9)   -
----------------------------------------------------------------------
```

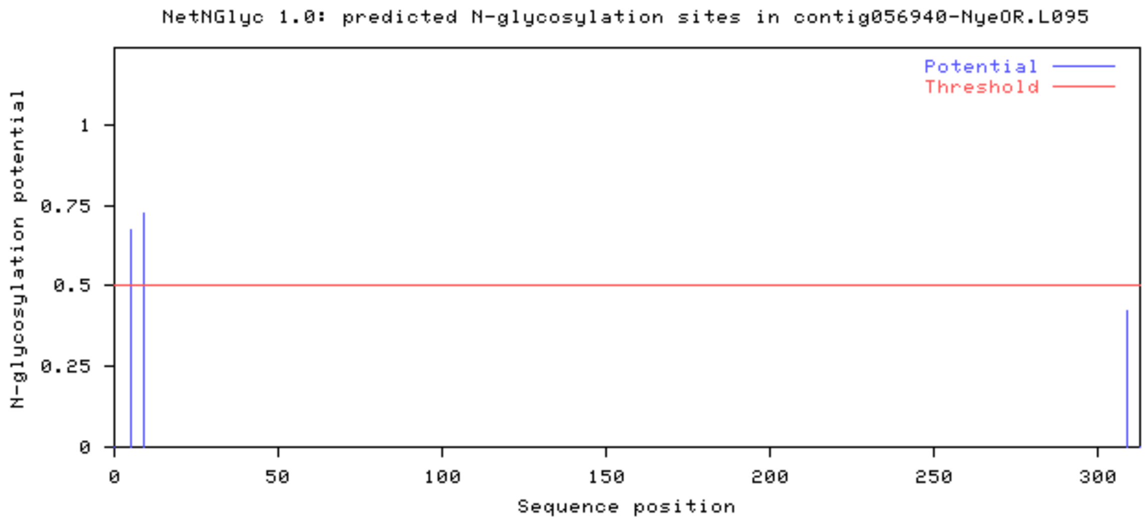

[Graphics in PostScript]

Output for 'contig056942-NyeOR.L093'

```
##########################################################################################

    Warning: This sequence may not contain a signal peptide!!

    Proteins without signal peptides are unlikely to be exposed to
    the N-glycosylation machinery and thus may not be glycosylated
    (in vivo) even though they contain potential motifs.

    SignalP-NN euk predictions are as follows:

  # name                 Cmax  pos ?  Ymax  pos ?  Smax  pos ?  Smean ?  D     ?

    SignalP output is explained at http://www.cbs.dtu.dk/services/SignalP/output.html

##########################################################################################

Name:  contig056942-NyeOR.L093  Length:  313
MSLQNASIKLTYFIIGGFDTVKRPVAVGVVMLITYLLAVFASLVNIIFIVSDKQLHKPMYLLICNLAVVDILYTSSSTPT      80
MIGVLLAGVNTISYMECIIQMYVYQVGATMEMFSLTIMAFDRLIAIIYPLQYHSYLTNTRTLVFTYIVWIVACSFALFPP     160
VTATPLPHCFLRLRYTFCDYGAVMRTTCVNPEKYFNQVAILSFFVSFFTFTFICLSYCGILFFVKISSNNDKKKMGSTLV     240
SHLICVSCLYCPQFILVILTRFGVVLTLEERQGLLIGTILGPSLVNPFVYCLRTKEIKSKMFKIFRKINTAGX
....N...........................................................................      80
................................................................................     160
................................................................................     240
.........................................................................            320

(Threshold=0.5)
----------------------------------------------------------------------
SeqName      Position  Potential   Jury    N-Glyc
                                 agreement result
----------------------------------------------------------------------
contig056942-NyeOR.L093    5 NASI   0.5849     (6/9)    +
----------------------------------------------------------------------
```

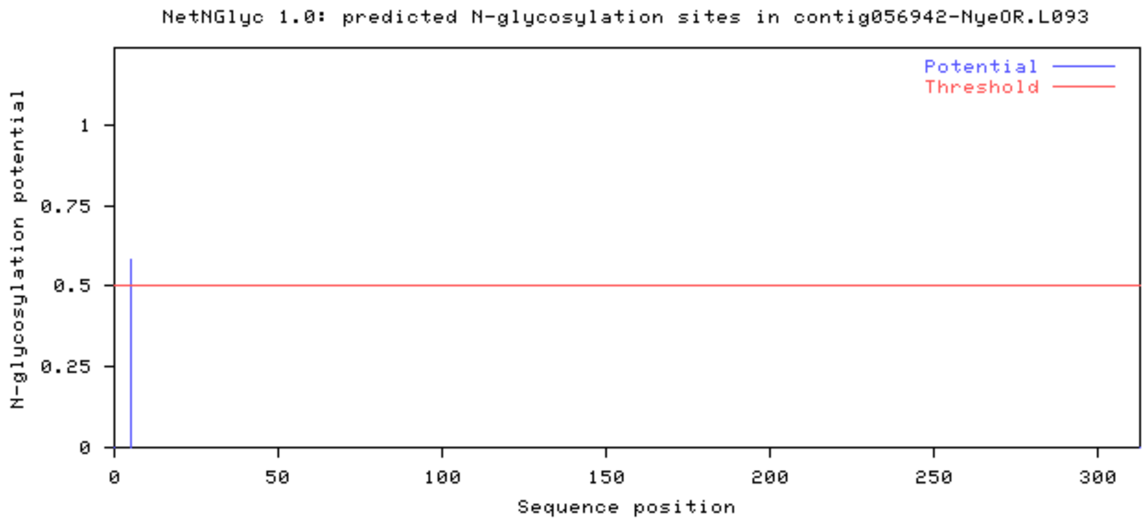

Graphics in PostScript

Output for 'contig056945-NyeORe.L096'

```
##########################################################################################

    Warning: This sequence may not contain a signal peptide!!

    Proteins without signal peptides are unlikely to be exposed to
    the N-glycosylation machinery and thus may not be glycosylated
    (in vivo) even though they contain potential motifs.

    SignalP-NN euk predictions are as follows:

  # name                  Cmax  pos ?  Ymax  pos ?  Smax  pos ?  Smean ?  D     ?

    SignalP output is explained at http://www.cbs.dtu.dk/services/SignalP/output.html

##########################################################################################

Name:  contig056945-NyeORe.L096         Length:  234
TMIGVLLAGVNTISYVECIIQMYVYQVGATMERFSLTIMAFDRLIAIIYPLQYHSYLTITRTLVFTNILWIVACSLVLFT      80
LVTATPLPHCNSRLRYTFCDYAAVMRTTCVNPEKYFNQTAIISFFVSFFTFTFICLSYCGILFFVKKISNNDKKKMGSTL     160
VSHLICVICLYCPLFVIVILTRFGVVLTLEERQGSLIGTILGPSLVNPFVYCLRTKEIKSKIIKIFRKVNTADX
................................................................................      80
....................................N...........................................     160
..........................................................................           240

(Threshold=0.5)
----------------------------------------------------------------------
SeqName      Position  Potential   Jury    N-Glyc
                                 agreement result
----------------------------------------------------------------------
contig056945-NyeORe.L096   117 NQTA   0.5939     (8/9)    +
----------------------------------------------------------------------
```

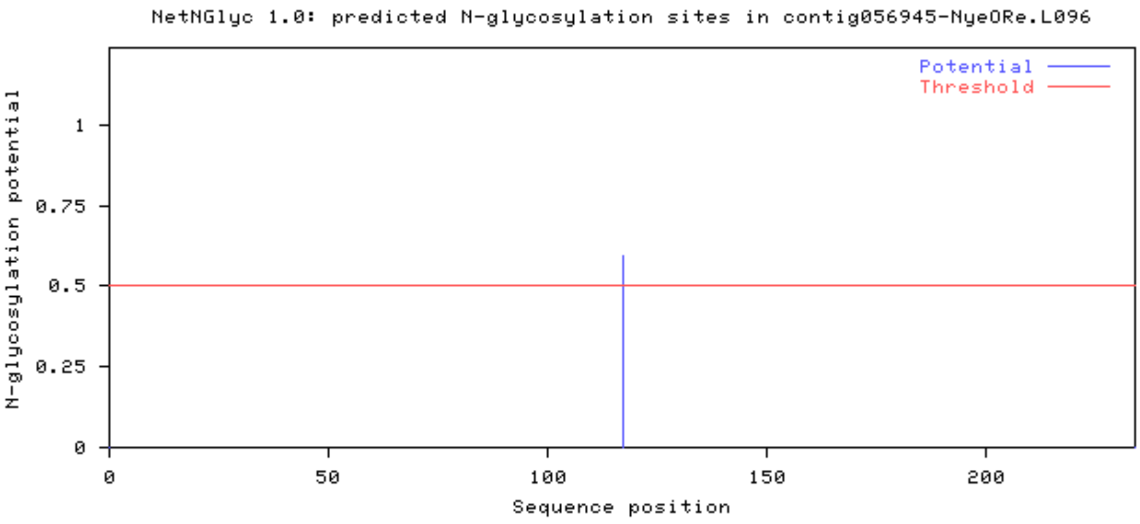

Graphics in PostScript

Output for 'contig057165-NyeOR.A017'

```
##########################################################################################

    Warning: This sequence may not contain a signal peptide!!

    Proteins without signal peptides are unlikely to be exposed to
    the N-glycosylation machinery and thus may not be glycosylated
    (in vivo) even though they contain potential motifs.

    SignalP-NN euk predictions are as follows:

  # name                    Cmax   pos ?  Ymax   pos ?  Smax   pos ?  Smean ?  D     ?

    SignalP output is explained at http://www.cbs.dtu.dk/services/SignalP/output.html

##########################################################################################

Name:  contig057165-NyeOR.A017  Length:  309
MELALNVSYITLDGFFQVNKYRYLYFMIMFTLYILILCCNFAIVFLIVVEKSLHEPMYIFIAALLLNSVMLSTVIYPKLL      80
TDFLSKRQIISYSVCLFQFFMFYSLGGSEFLLLFAMAYDRYVSICKPLQYPMIMTKNTISIFLTLAWIVPSSQVAVVGVL     160
MANKKICNFTFTGIFCNNTIYKLLCVYSKAQTVYDMVVLSNVAILPAVFIFFTYTRILVISYQSCKEVRRKAAQTCLPHL     240
IVLISYLCLCAFDVIVSGLESNFPKIVHSILTLQIVMYPPLFNPIIYGLKMKEISKHLKRLFCAVKKNX
.....N..........................................................................      80
................................................................................     160
.......N........N...............................................................     240
.....................................................................               320

(Threshold=0.5)
----------------------------------------------------------------------
SeqName      Position  Potential   Jury    N-Glyc
                                 agreement result
----------------------------------------------------------------------
contig057165-NyeOR.A017    6 NVSY    0.8398     (9/9)    +++
contig057165-NyeOR.A017  168 NFTF    0.6436     (8/9)    +
contig057165-NyeOR.A017  177 NNTI    0.5012     (4/9)    +
----------------------------------------------------------------------
```

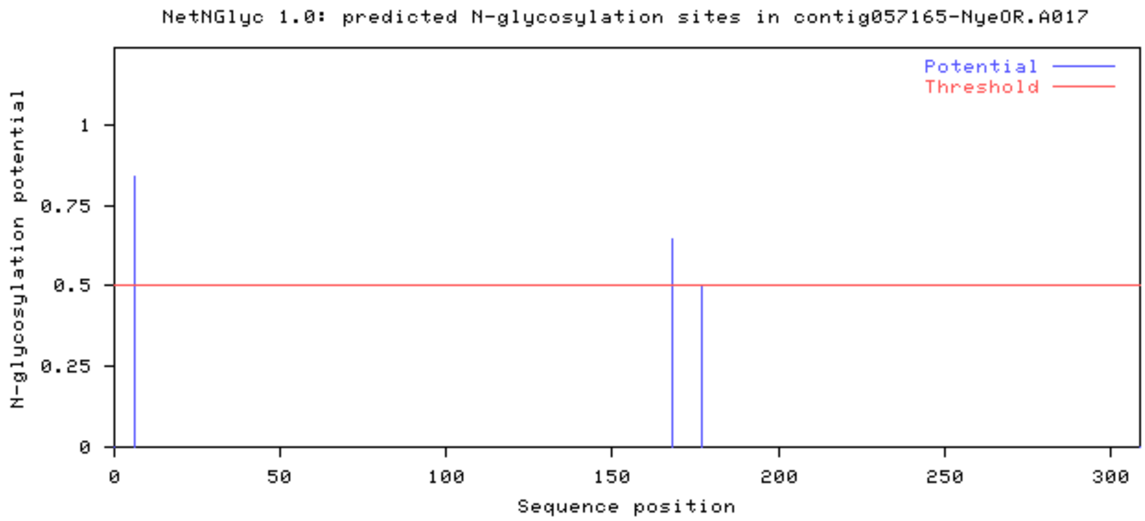

Graphics in PostScript

Output for 'contig057754-NyeOR.A018'

```
##########################################################################################

    Warning: This sequence may not contain a signal peptide!!

    Proteins without signal peptides are unlikely to be exposed to
    the N-glycosylation machinery and thus may not be glycosylated
    (in vivo) even though they contain potential motifs.

    SignalP-NN euk predictions are as follows:

  # name                   Cmax  pos ?  Ymax  pos ?  Smax  pos ?  Smean ?  D     ?

    SignalP output is explained at http://www.cbs.dtu.dk/services/SignalP/output.html

##########################################################################################

Name:  contig057754-NyeOR.A018  Length:  309
MDDELNVTYITLDGYVELKRCGYLYFLIMVALYVLIIISNSTIVFLICIHRNLHEPMYIFIAALSVNSVVFSTAIYPKLF      80
VDVLSEKQVISFSACQFQHFMYYSIGGSDFLLLSAMAFDRYVSICKPLKYPVIMRQTTINILLFLAWFLPGLQVAVSHAL     160
VLNNKLCNFTLKGIFCNNLLWKLYCESPRAALIYGLIVLLNVAIFPVLFILFTYAKIFLITYRSSRDIQKKAAETCLPHL     240
FVLSIFTTFCAYDVIIGQLEFDFPKTAQLIMTLQVVLYNPLFNPFIYGLKMKEISKHLKRLFCHVRCSX
.....N................................N.........................................      80
................................................................................     160
.......N........................................................................     240
.....................................................................               320

(Threshold=0.5)
----------------------------------------------------------------------
SeqName      Position  Potential   Jury    N-Glyc
                                 agreement result
----------------------------------------------------------------------
contig057754-NyeOR.A018    6 NVTY   0.7911     (9/9)   +++
contig057754-NyeOR.A018   40 NSTI   0.7071     (9/9)   ++
contig057754-NyeOR.A018  168 NFTL   0.6774     (8/9)   +
----------------------------------------------------------------------
```

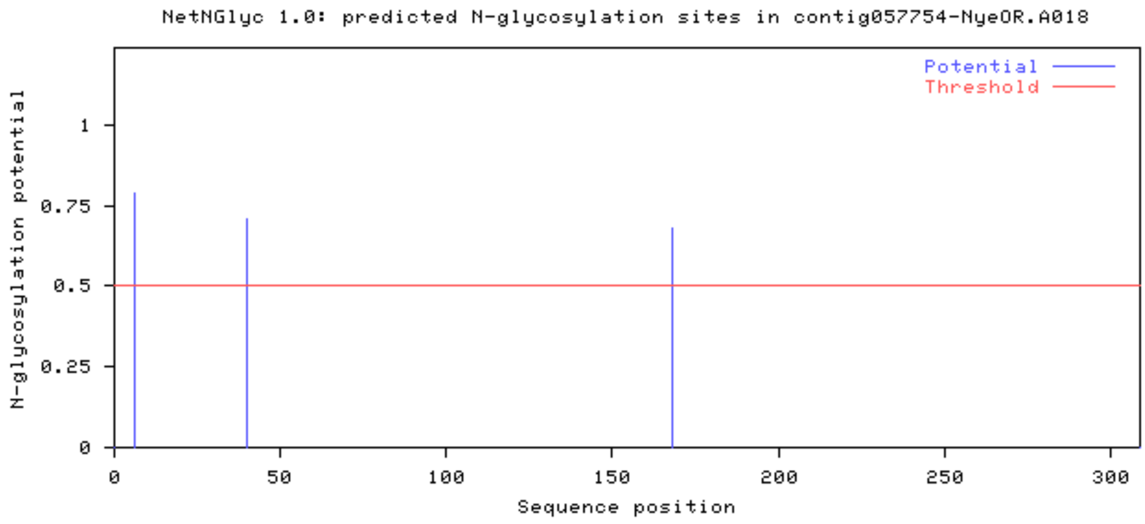

Graphics in PostScript

Output for 'contig057756-NyeOR.A019'

```
##########################################################################################

    Warning: This sequence may not contain a signal peptide!!

    Proteins without signal peptides are unlikely to be exposed to
    the N-glycosylation machinery and thus may not be glycosylated
    (in vivo) even though they contain potential motifs.

    SignalP-NN euk predictions are as follows:

  # name                  Cmax  pos ?  Ymax  pos ?  Smax  pos ?  Smean ?  D     ?

    SignalP output is explained at http://www.cbs.dtu.dk/services/SignalP/output.html

##########################################################################################

Name:  contig057756-NyeOR.A019  Length:  307
MNADLNTTYVTLGGHVEIHRYRYLYFVIMLTAYILLICFNVSIICLIVIHKNLHEPMYIFIAALLLNSILFSSNIHPKLL      80
VDFLSEKQIVSYQACLFQVFMFYFLSSSEFLLLSARAYDRYVSICKPLQYPAIMRTTTVSLLLCFAWLVPACYIVVPVAL     160
NINSKLCSFTLKGIFCNNSLNKLFCVTSNELSIYGVIVLLNLRLFPMLFILFTYTKIIIIAFQSCGDIRRKAVQTCLPHL     240
LVLINYSVLITYDVVIVKLESDFPKTARFVMTLQIITYNPLCNPIIYGLKMKEISNYLKRLLRHMKX
.....N.................................N........................................      80
................................................................................     160
................................................................................     240
....N...........................................................                     320

(Threshold=0.5)
----------------------------------------------------------------------
SeqName      Position  Potential   Jury    N-Glyc
                                 agreement result
----------------------------------------------------------------------
contig057756-NyeOR.A019    6 NTTY   0.7242     (9/9)    ++
contig057756-NyeOR.A019   40 NVSI   0.7863     (9/9)    +++
contig057756-NyeOR.A019  177 NNSL   0.4238     (7/9)    -
contig057756-NyeOR.A019  245 NYSV   0.5312     (6/9)    +
----------------------------------------------------------------------
```

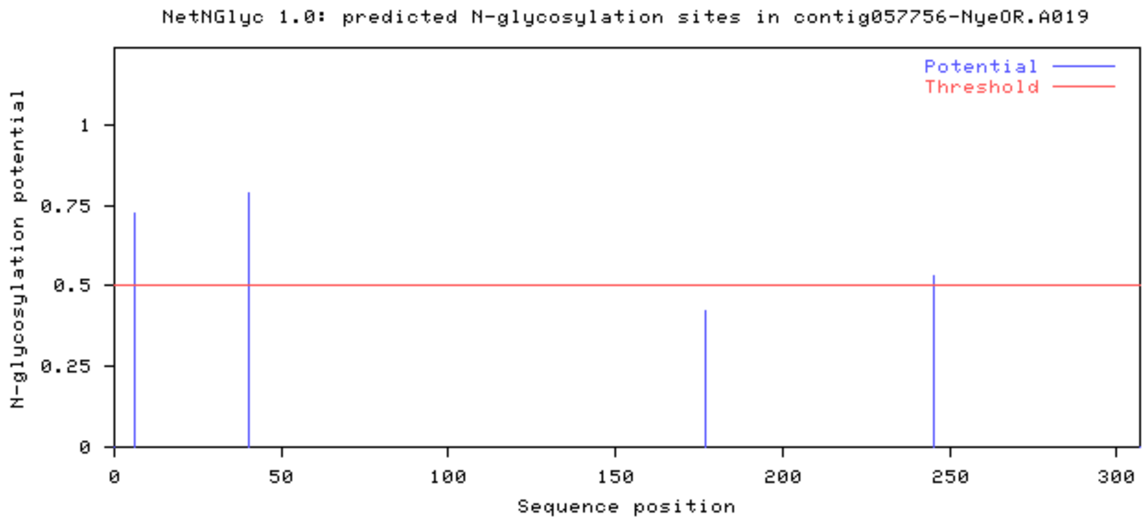

Graphics in PostScript

Output for 'contig057857-NyeORp.A032'

##

Warning: This sequence may not contain a signal peptide!!

Proteins without signal peptides are unlikely to be exposed to the N-glycosylation machinery and thus may not be glycosylated (in vivo) even though they contain potential motifs.

SignalP-NN euk predictions are as follows:

# name Cmax pos ? Ymax pos ? Smax pos ? Smean ? D ?

SignalP output is explained at http://www.cbs.dtu.dk/services/SignalP/output.html

##

```
Name:  contig057857-NyeORp.A032         Length:  328
MAIDNEFNVTYITFGGHIELEKYKFLYFAIMFTAYILILCSNSTIVCLIRIKKSLHEPMYIFIAALLFNSVMFSTNIYPK      80
LLMDFLSEKQITTHSQCSFQGFIYYSLTGSEFFLLASMAYDRYVSISKPLQYHTIMRKTTVTVLLVLTWLLPACQLVPSA     160
VIINTSQICSFTLNGIFCNNAISKLYCATPKISYLIYGVFILLNTVFLPLLFILFTYTKIFIICYRSCREVRKKAAQTCL     240
PHLLVLISFSCLCSYDIIITRVEINLSQTARFIMTLQVVLYHPLFNPIVYGLKMKEISQHLRRLFCQSKFKLSVRADAGS     320
AIISFVIX
.......N.................................N......................................      80
................................................................................     160
...N............................................................................     240
........................N.......................................................     320
........                                                                             400
```

(Threshold=0.5)

SeqName	Position	Potential	Jury agreement	N-Glyc result
contig057857-NyeORp.A032	8 NVTY	0.6870	(9/9)	++
contig057857-NyeORp.A032	42 NSTI	0.7288	(9/9)	++
contig057857-NyeORp.A032	164 NTSQ	0.5962	(8/9)	+
contig057857-NyeORp.A032	265 NLSQ	0.5972	(5/9)	+

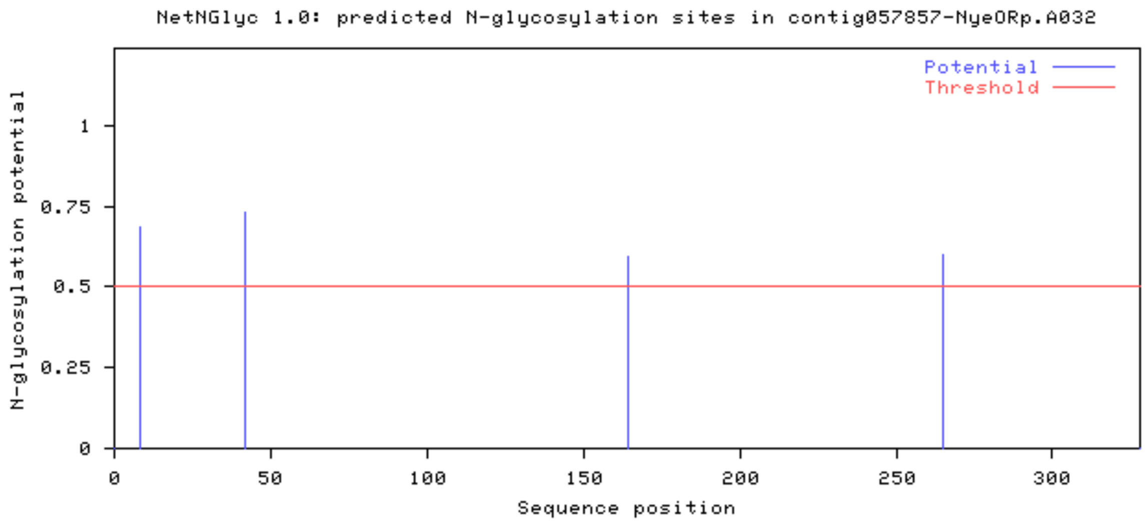

Graphics in PostScript

Output for 'contig059270-NyeOR.S122'

```
##########################################################################################

    Warning: This sequence may not contain a signal peptide!!

    Proteins without signal peptides are unlikely to be exposed to
    the N-glycosylation machinery and thus may not be glycosylated
    (in vivo) even though they contain potential motifs.

    SignalP-NN euk predictions are as follows:

  # name                Cmax  pos ?  Ymax  pos ?  Smax  pos ?  Smean ?  D     ?

    SignalP output is explained at http://www.cbs.dtu.dk/services/SignalP/output.html

##########################################################################################

Name:  contig059270-NyeOR.S122  Length:  307
MAGNSSLICAFLHQLLTVRVMIVHTLVIIFLCINMLLIVTFVKKECFHTSARYILFFVTLLSDSFLLLMTDILLILTRCS      80
VQVWLCIFICLFVLVYSIVTPVTLTAMTLERYVAICMPLRHGQLCSTRSTMYCILIIHGLSSGPCIVIISMFFATASLSF     160
YNQYTICSVEMFMLYRWQDHARSAVSQFYFMTMGITIVFSYVQIMKVAKAASGESKKSTQKGVRTVILHAFQLFLCLVHL     240
WSPFIETAVLQIDVNLFRDFRYFNYVFFSIVPRCLSPLIYGLRDETFFLSLKNLMPTSSCSKKHVIX
...N............................................................................      80
................................................................................     160
................................................................................     240
...................................................................                  320

(Threshold=0.5)
----------------------------------------------------------------------
SeqName      Position  Potential   Jury    N-Glyc
                                 agreement result
----------------------------------------------------------------------
contig059270-NyeOR.S122    4 NSSL   0.7859     (9/9)   +++
----------------------------------------------------------------------
```

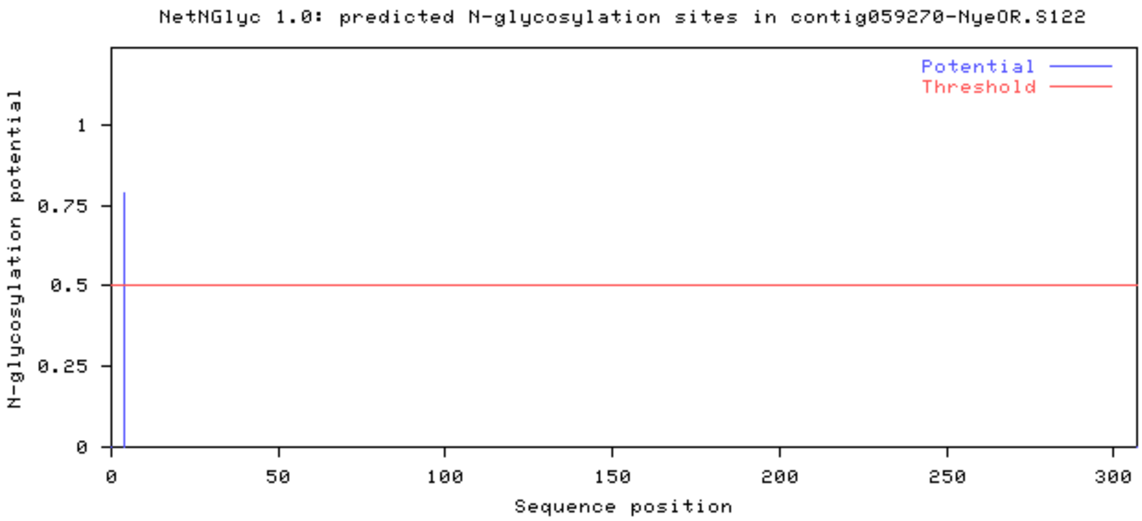

Graphics in PostScript

Output for 'contig059272-NyeORe.S126'

```
##########################################################################################

    Warning: This sequence may not contain a signal peptide!!

    Proteins without signal peptides are unlikely to be exposed to
    the N-glycosylation machinery and thus may not be glycosylated
    (in vivo) even though they contain potential motifs.

    SignalP-NN euk predictions are as follows:

  # name                    Cmax  pos ?  Ymax  pos ?  Smax  pos ?  Smean ?  D     ?

    SignalP output is explained at http://www.cbs.dtu.dk/services/SignalP/output.html

##########################################################################################

Name:  contig059272-NyeORe.S126         Length:  115
YFLIMGITIAYSYVQIMKVAKAASGEKKKLTQKGLKTVILHAFQLLLCLIQLWCPFIEIAVLQIDFSLILNVRYFNYIMF      80
NIAPRCLSPLIYGLRDENIFLVLKNLMPTSSCSKX
................................................................................      80
...................................                                                  160

(Threshold=0.5)

No sites predicted in this sequence.
```

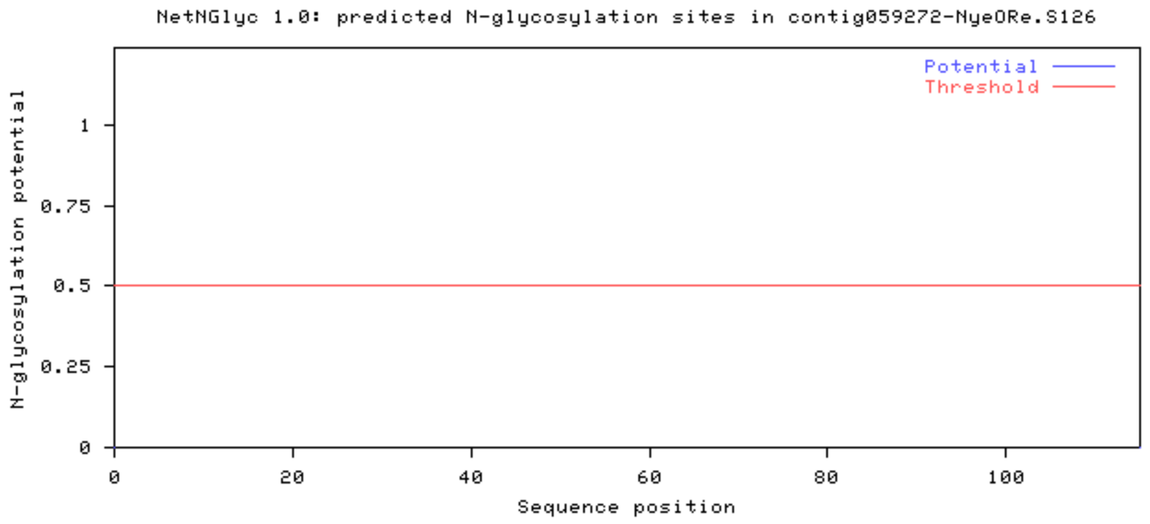

Graphics in PostScript

Output for 'contig059404-NyeOR.E058'

```
##########################################################################################

    Warning: This sequence may not contain a signal peptide!!

    Proteins without signal peptides are unlikely to be exposed to
    the N-glycosylation machinery and thus may not be glycosylated
    (in vivo) even though they contain potential motifs.

    SignalP-NN euk predictions are as follows:

  # name                    Cmax  pos ?  Ymax  pos ?  Smax  pos ?  Smean ?  D     ?

    SignalP output is explained at http://www.cbs.dtu.dk/services/SignalP/output.html

##########################################################################################

Name:  contig059404-NyeOR.E058  Length:  326
MMNSSQVSYFTLTAYLDSGALKYLYFTVVAFLYIVIVTVNVLLIVVICVNRSLHEPMYMFLCSLFVNELYGSTGLFPLLL      80
LQILSDIHTVSAPLCFLQIFCLYSYANLQLSNLAIMSYDRYLAICFPLQYHTRMSPCKVSMFIVLTWFSSFLVITVLISL     160
SAPLQLCGNIINKVYCDNYSIVKLACSDTTVNNIYGILGAIFITISSVTLILYTYMRILKVCFSGSKQTRQKAVSTCTPH     240
LASLLNFSCGSFFESAQSRSNMKHVPNMVRIFLSLYWLICPPLCNPLLYGLSLTKIRIIYKGLIFVKCRCLMLKLRQGSA     320
LVKRKX
..N..............................................N..............................      80
................................................................................     160
.................N..............................................................     240
.....N..........................................................................     320
......                                                                               400

(Threshold=0.5)
----------------------------------------------------------------------
SeqName      Position  Potential   Jury    N-Glyc
                                 agreement result
----------------------------------------------------------------------
contig059404-NyeOR.E058    3 NSSQ    0.6926     (9/9)    ++
contig059404-NyeOR.E058   50 NRSL    0.7497     (9/9)    ++
contig059404-NyeOR.E058  178 NYSI    0.5911     (8/9)    +
contig059404-NyeOR.E058  246 NFSC    0.5401     (6/9)    +
----------------------------------------------------------------------
```

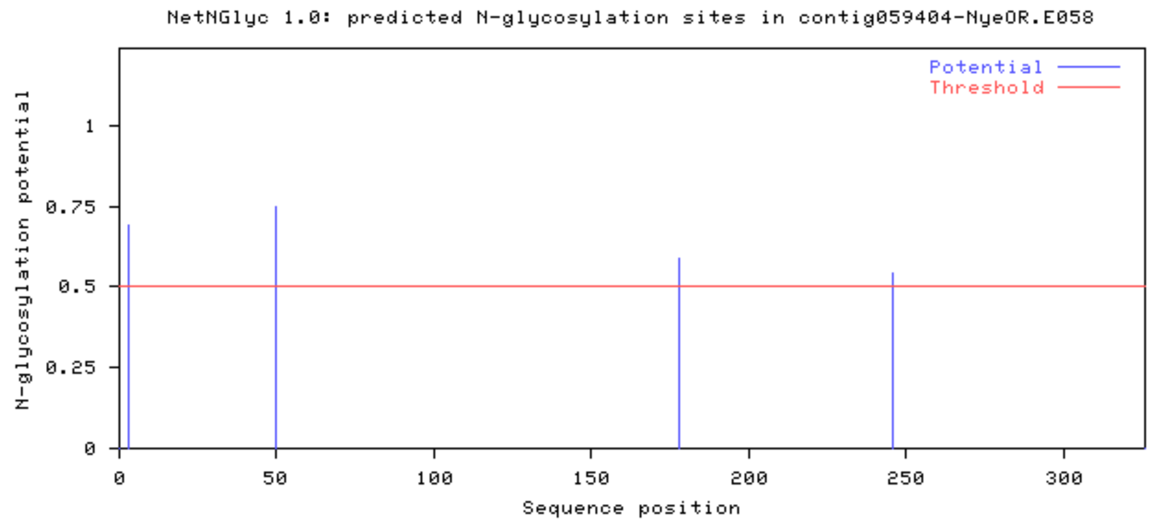

Graphics in PostScript

Output for 'contig060525-NyeOR.K088'

```
##############################################################################

    Warning: This sequence may not contain a signal peptide!!

    Proteins without signal peptides are unlikely to be exposed to
    the N-glycosylation machinery and thus may not be glycosylated
    (in vivo) even though they contain potential motifs.

    SignalP-NN euk predictions are as follows:

  # name                Cmax  pos ?  Ymax  pos ?  Smax  pos ?  Smean ?  D     ?

    SignalP output is explained at http://www.cbs.dtu.dk/services/SignalP/output.html

##############################################################################

Name:  contig060525-NyeOR.K088  Length:  313
MDNQSLNADILILGGLKVTPKFSIAAFIFLLLVYIFIMVANIGLVVLVFMERSLHQPMYLLFCNMSVNEVFGSTIVVPHI      80
LRGLYVSDSERYIHYIVCVVQAFCVNLYGGVSQTILMTMTFDRYMAICNPLRYTIIMTNWMVVKLSVAAWAVVFVMVSIL     160
LSLTIRLSRCRRFIDNVHCDNASLFKLSCEDVVINHVFGLSYSVLLLGSSIGSVTLTYIKIATVCLRSKTKTINSKALQT     240
CATHLTLYIILMFSAFIIIILHRFPHLSDHRKMVSTVGEVALPALNAVIYGLQIKEIRQKIVALFQRKGHLQX
..N...........................................................N................      80
................................................................................     160
....................N...........................................................     240
.........................................................................            320

(Threshold=0.5)
----------------------------------------------------------------------
SeqName      Position  Potential   Jury    N-Glyc
                                 agreement result
----------------------------------------------------------------------
contig060525-NyeOR.K088    3 NQSL   0.7006     (9/9)   ++
contig060525-NyeOR.K088   64 NMSV   0.6973     (9/9)   ++
contig060525-NyeOR.K088  181 NASL   0.6081     (8/9)   +
----------------------------------------------------------------------
```

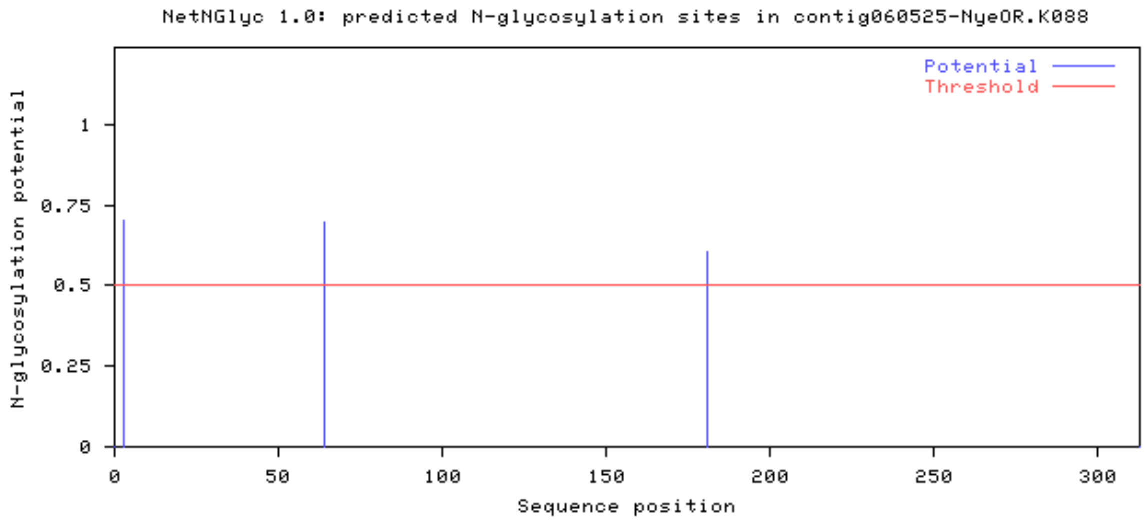

Graphics in PostScript

Output for 'contig060577-NyeORe.S123'

```
##########################################################################################

    Warning: This sequence may not contain a signal peptide!!

    Proteins without signal peptides are unlikely to be exposed to
    the N-glycosylation machinery and thus may not be glycosylated
    (in vivo) even though they contain potential motifs.

    SignalP-NN euk predictions are as follows:

  # name                  Cmax  pos ?  Ymax  pos ?  Smax  pos ?  Smean ?  D     ?

    SignalP output is explained at http://www.cbs.dtu.dk/services/SignalP/output.html

##########################################################################################

Name:  contig060577-NyeORe.S123         Length:  181
MALNNSVIGGQLAINNINNQVIIVQLLILMFLCINLLLITTFFMKDIFYTTMRYILFAIALLSDSLFLLITNVLLILSYF      80
SFTIQVWLCVIIYIVLSVYTFVTPVTLTAMTLERYVAICVPLRHAELCRTQRALHFILIIHGLSSVPCIVILSIFFASAI     160
TSFYTQSRICAVEMFIFHRWQ
...N............................................................................      80
................................................................................     160
.....................                                                                240

(Threshold=0.5)
----------------------------------------------------------------------
SeqName      Position  Potential   Jury    N-Glyc
                                 agreement result
----------------------------------------------------------------------
contig060577-NyeORe.S123     4 NNSV   0.7420     (9/9)    ++
----------------------------------------------------------------------
```

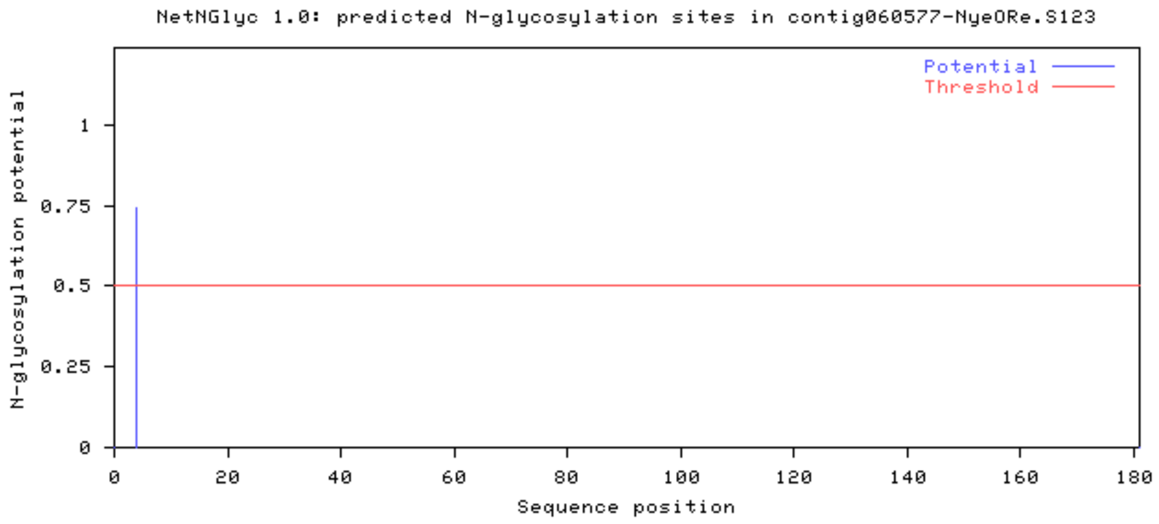

Graphics in PostScript

Output for 'contig060604-NyeORp.N117'

```
##########################################################################

   Warning: This sequence may not contain a signal peptide!!

   Proteins without signal peptides are unlikely to be exposed to
   the N-glycosylation machinery and thus may not be glycosylated
   (in vivo) even though they contain potential motifs.

   SignalP-NN euk predictions are as follows:

 # name                 Cmax  pos ?  Ymax  pos ?  Smax  pos ?  Smean ?  D     ?

   SignalP output is explained at http://www.cbs.dtu.dk/services/SignalP/output.html

##########################################################################

Name:  contig060604-NyeORp.N117         Length:  324
MEFLNSAVEKNTTFVQSAYFIISGFIGIPNIRYYFVFLCFIYIFSVVGNTLVMIVIILDRMLRSPKYIAVFNLAFTDLLS      80
SSALVPKVVDIFLFNHYYISYNICLTFMFFCLTLISMQAFNLVVLSFDRVMAIMYPLHYQMRVTQKLILSLIAFAIILIL     160
IAVGLLTRLSFCKSVVIQSYYCDHGPMYHLGFNDVTPNRAIAGLALLIILGFPLAFIVGSYCCIGYSLSKISTFRERVKA     240
FKACTGHLSLVAIYFLPIIFVYVFRPVIHPNARIINLSMTSVMPPMLNPIIYVLQTQEIKESLRRLLKARAQAKLEVFRL     320
KPLX
................................................................................      80
................................................................................     160
................................................................................     240
...................................N............................................     320
....                                                                                 400

(Threshold=0.5)
----------------------------------------------------------------------
SeqName      Position  Potential   Jury    N-Glyc
                                agreement result
----------------------------------------------------------------------
contig060604-NyeORp.N117    11 NTTF    0.4777     (4/9)   -
contig060604-NyeORp.N117   276 NLSM    0.5397     (5/9)   +
----------------------------------------------------------------------
```

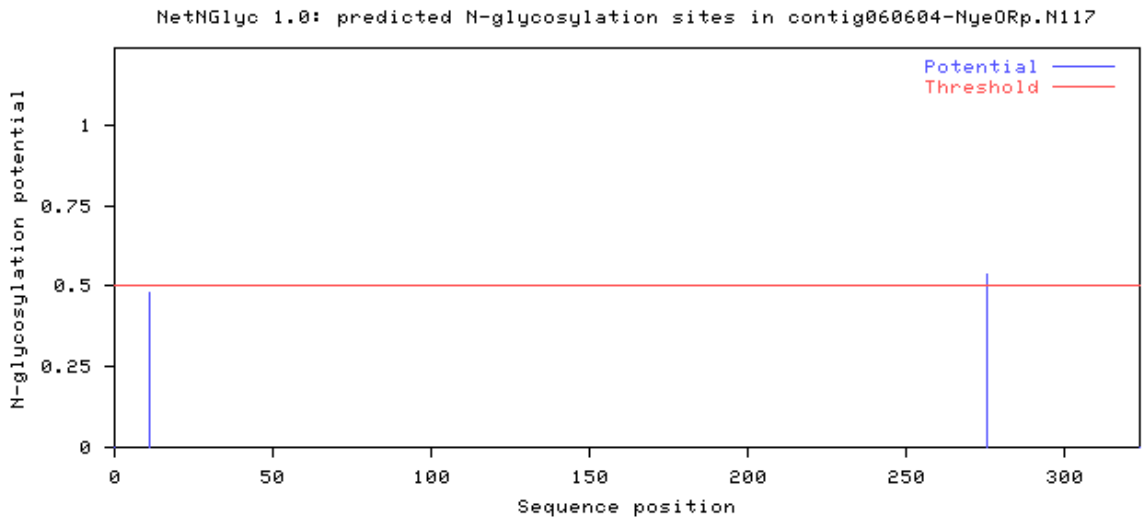

Graphics in PostScript

Output for 'contig061663-NyeOR.N114'

```
##########################################################################################

    Warning: This sequence may not contain a signal peptide!!

    Proteins without signal peptides are unlikely to be exposed to
    the N-glycosylation machinery and thus may not be glycosylated
    (in vivo) even though they contain potential motifs.

    SignalP-NN euk predictions are as follows:

  # name                    Cmax   pos ?  Ymax   pos ?  Smax   pos ?  Smean ?  D     ?

    SignalP output is explained at http://www.cbs.dtu.dk/services/SignalP/output.html

##########################################################################################

Name:  contig061663-NyeOR.N114  Length:  327
MDLFNSALGKNITFLRPAFFIISGFIGIPNIKYYYAFLFFVYIISVLANTAVMAAIYLDHNLRTPKYIAVFNLALVDLLG      80
NSAMVPKVLDIFLFNHPHISYNDCLTFLFFCYVFLSMQALNLVALSYDRVMAIVYPLHYQLKVTHKFMFCLIASFWVFVI     160
IVVLIATGLLTRLSFCESVVIKSFFCDHGQIYRLACNDYTPSDITAWILPALILWLPLTIVLLSYLSIGYALAKVATVRE     240
RMKGFKTCTAHLSLVAIYFLPILITFTLGANIEPNARIINLSLTSVFPPMLNPIIYVLQTQEIKESLKRFLRITTHYKIS     320
KVKFKKX
..........N.....................................................................      80
................................................................................     160
................................................................................     240
.......................................N........................................     320
.......                                                                              400

(Threshold=0.5)
----------------------------------------------------------------------
SeqName      Position  Potential   Jury    N-Glyc
                                 agreement result
----------------------------------------------------------------------
contig061663-NyeOR.N114    11 NITF   0.5354     (6/9)   +
contig061663-NyeOR.N114   280 NLSL   0.6056     (8/9)   +
----------------------------------------------------------------------
```

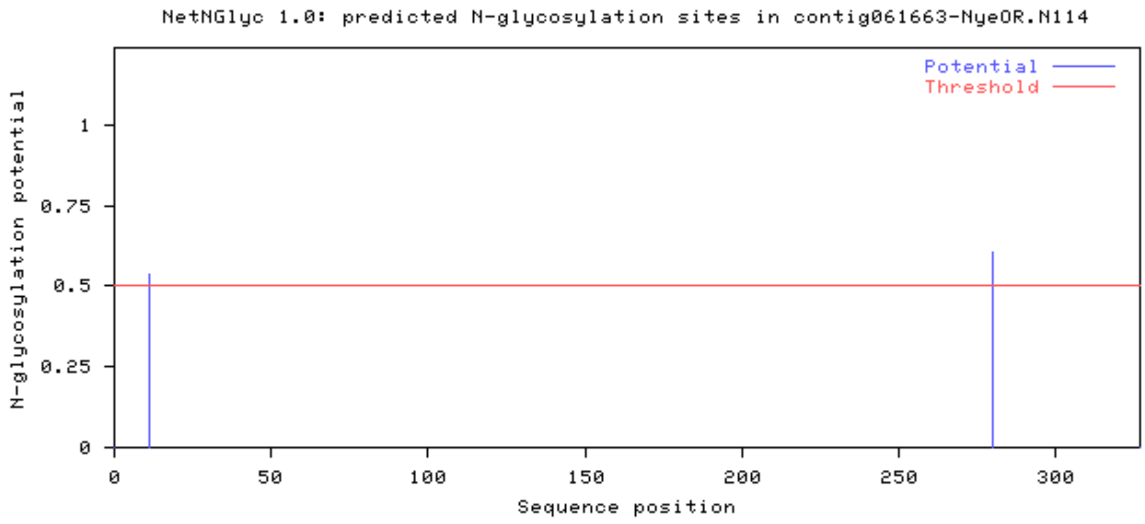

Graphics in PostScript

Output for 'contig061787-NyeORp.D048'

```
##########################################################################################

    Warning: This sequence may not contain a signal peptide!!

    Proteins without signal peptides are unlikely to be exposed to
    the N-glycosylation machinery and thus may not be glycosylated
    (in vivo) even though they contain potential motifs.

    SignalP-NN euk predictions are as follows:

  # name                    Cmax   pos ?  Ymax   pos ?  Smax   pos ?  Smean ?  D      ?

    SignalP output is explained at http://www.cbs.dtu.dk/services/SignalP/output.html

##########################################################################################

Name:  contig061787-NyeORp.D048         Length:  295
MYFVIILFWYLLICVANSTVLIVVIHVDRRLHEPMYILLSNLCDNEINASTSLYPFLLSQMFSDSHEVTLPWCFLQMCCL      80
YTGAPAEFLTAMANDRYVSICHPLRYNVIMKTERVLKIILLVWVFSFLIFILSFSFIFSLKFCGNIVDNVYCDHKLIYKL     160
SCSASVHGSISIIFIVIISYLSVSYVNILRVCRKTIEENKQKAVTTCTPQIVSLSYLFVGCICYSIDFRFLVSLVPDEVR     240
TILAIYLLICQPMLTPFLYGFNLPKIRQSCKSFLFLRKMSLFSKIQSLLSGAETX
................................................................................      80
................................................................................     160
................................................................................     240
.......................................................                              320

(Threshold=0.5)

No sites predicted in this sequence.
```

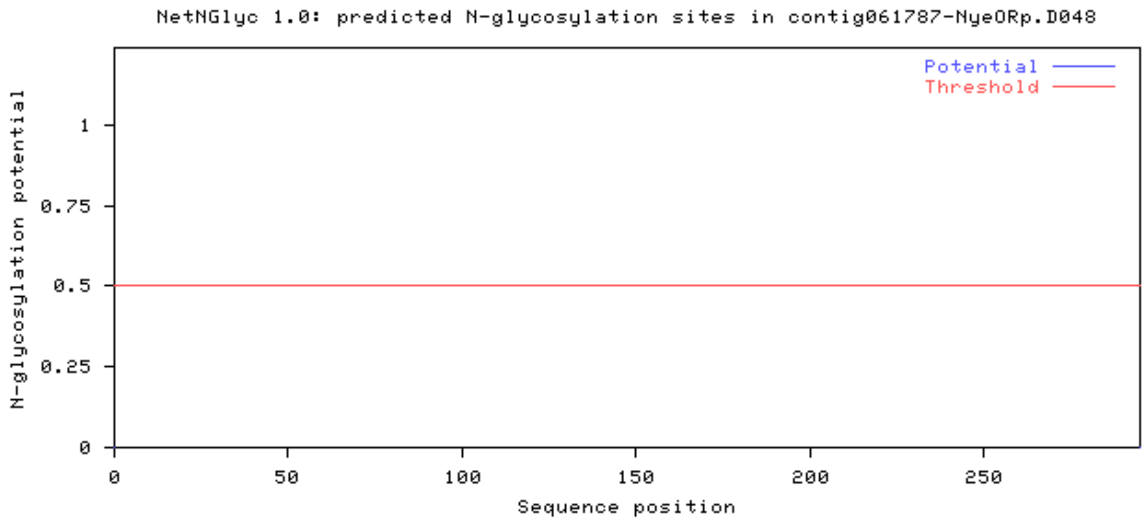

[Graphics in PostScript]

Output for 'contig061788-NyeORe.D045'

```
##########################################################################################

    Warning: This sequence may not contain a signal peptide!!

    Proteins without signal peptides are unlikely to be exposed to
    the N-glycosylation machinery and thus may not be glycosylated
    (in vivo) even though they contain potential motifs.

    SignalP-NN euk predictions are as follows:

  # name                    Cmax  pos ?  Ymax  pos ?  Smax  pos ?  Smean ?  D     ?

    SignalP output is explained at http://www.cbs.dtu.dk/services/SignalP/output.html

##########################################################################################

Name:  contig061788-NyeORe.D045         Length:  225
MGNSSETVSFVLAAYGNLGELKHLYFIIILVWYVSICVANTVLIVVIRLDRRLHEPMYILLCNLCLSEINGSTSLYPLLL      80
SQMFSDSHKVTVPWCFLQMFCLYTSASVEICSLAAMAYDRYIAICNPFTYNVIMNTQRVFLMILLVWVYSFLSVIFSYSF     160
IFSLKFCGNIIHSVYCDHQLIIRLSCAVSIQSVISDISFAIVSAFIPFSLISVSYLKILRVCQKT
..N...................................................................N.........      80
................................................................................     160
.................................................................                    240

(Threshold=0.5)
----------------------------------------------------------------------
SeqName      Position  Potential   Jury    N-Glyc
                                 agreement result
----------------------------------------------------------------------
contig061788-NyeORe.D045    3 NSSE   0.7411     (9/9)    ++
contig061788-NyeORe.D045   70 NGST   0.5641     (5/9)    +
----------------------------------------------------------------------
```

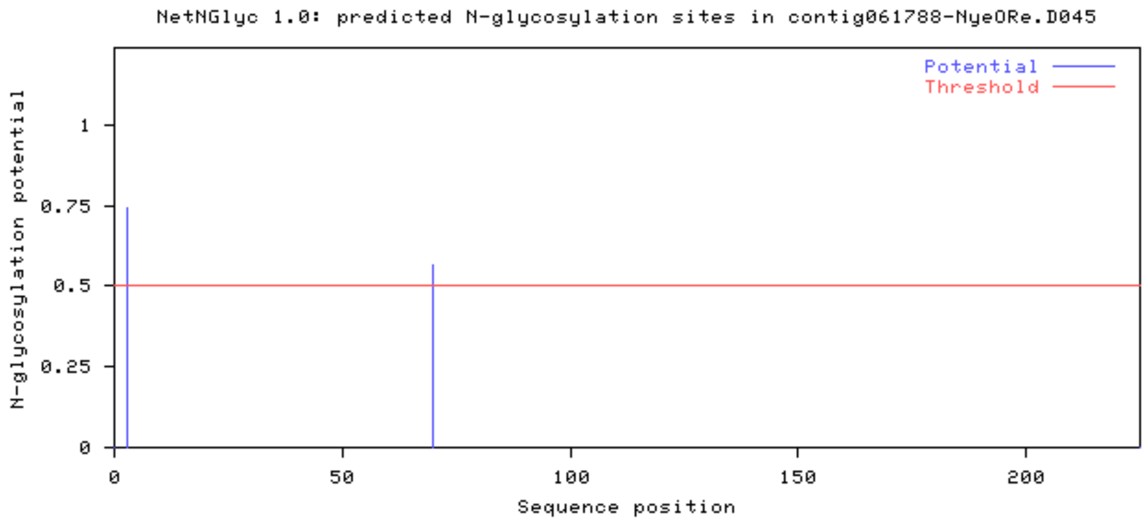

Graphics in PostScript

Output for 'contig062022-NyeORe.E060'

```
##########################################################################################

    Warning: This sequence may not contain a signal peptide!!

    Proteins without signal peptides are unlikely to be exposed to
    the N-glycosylation machinery and thus may not be glycosylated
    (in vivo) even though they contain potential motifs.

    SignalP-NN euk predictions are as follows:

  # name                    Cmax  pos ?  Ymax  pos ?  Smax  pos ?  Smean ?  D     ?

    SignalP output is explained at http://www.cbs.dtu.dk/services/SignalP/output.html

##########################################################################################

Name:  contig062022-NyeORe.E060         Length:  271
SNLLLIVVICVNRSLHEPMYMFLCSLFVNELYGSTGLFPLLLLQILSDVHTVSAPLCFLQIFCLYTYANVEFYNLAIMSY      80
DRYLAICYPLQYHTHMTFNKVAKLIVLTWLFPILNIAVMISLNASLQLCGHTVDTLYCNNYSVVKLACFDTTINNIYGLM     160
YTFTVLIGLALLNLFTYVKILKVCFSGSKQTRQKAVSTCTPHLASLLNFSFGCFFEIVQSRFNLSRAPMILRIFLSIYFL     240
TCQPAFNPVLYGLKLTKIRLICKSLLFGKIX
...........N....................................................................      80
..........................................N.................N...................     160
...............................................................N................     240
...............................                                                      320

(Threshold=0.5)
----------------------------------------------------------------------
SeqName       Position  Potential   Jury    N-Glyc
                                  agreement result
----------------------------------------------------------------------
contig062022-NyeORe.E060   12 NRSL   0.7611     (9/9)   +++
contig062022-NyeORe.E060  123 NASL   0.6403     (8/9)   +
contig062022-NyeORe.E060  140 NYSV   0.6319     (9/9)   ++
contig062022-NyeORe.E060  208 NFSF   0.4772     (6/9)   -
contig062022-NyeORe.E060  223 NLSR   0.6010     (9/9)   ++
----------------------------------------------------------------------
```

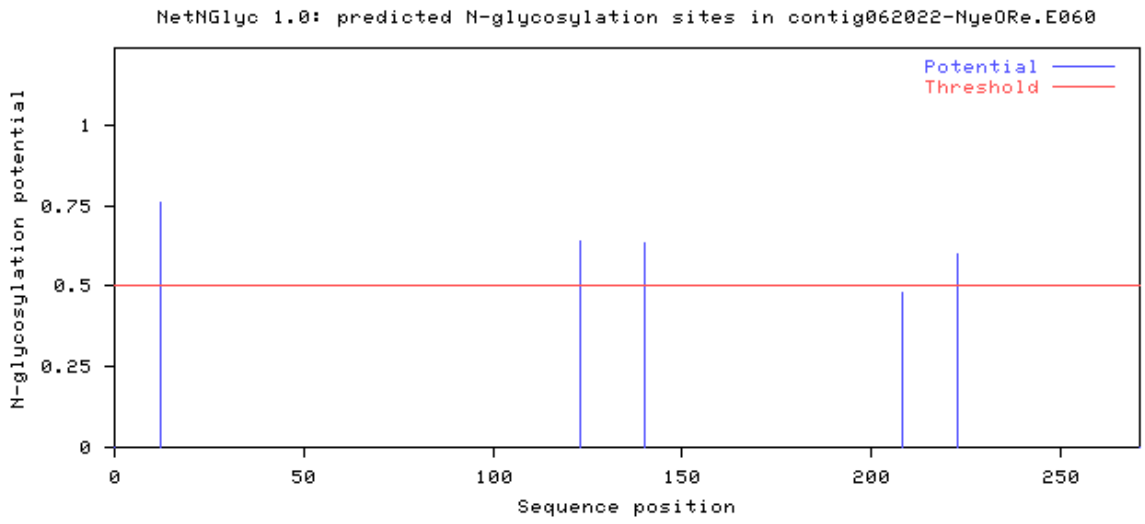

Graphics in PostScript

Output for 'contig062053-NyeOR.O103'

```
##########################################################################################

    Warning: This sequence may not contain a signal peptide!!

    Proteins without signal peptides are unlikely to be exposed to
    the N-glycosylation machinery and thus may not be glycosylated
    (in vivo) even though they contain potential motifs.

    SignalP-NN euk predictions are as follows:

  # name                    Cmax   pos ?  Ymax   pos ?  Smax   pos ?  Smean ?  D     ?

    SignalP output is explained at http://www.cbs.dtu.dk/services/SignalP/output.html

##########################################################################################

Name:  contig062053-NyeOR.O103   Length:  326
MPERNHSSVTEFILTGFPGLHQEYYGLVSAVLFFVYLITLIANATVIFLFATNHSLHKPMYYIILNLSVCDILFSTTTLP      80
KIISRYWFQSGSISFTACFIQMYFVHYLGSVNSFILFQMALDRYLAICYPFRYSLVLTKSNILILSITAWIISKAFPLMM     160
VIRAYPLPYCASNIITHCFCDHIGITVLTCTDRTPYATPAFTAAMVVLLGPLAFIIFSYCSILIAVYKIANVQGRLKSLS     240
TCSTQLIIISLYFLPRCFVYLAQNVGITFSADVRIVIMLYSLAPPMINPLIYCLRAKDMRESLLKQFCKRIVPRKAQVA     320
AISNSX
....N.....................................N.........N............N..............      80
................................................................................     160
................................................................................     240
................................................................................     320
......                                                                               400

(Threshold=0.5)
----------------------------------------------------------------------
SeqName          Position  Potential   Jury    N-Glyc
                                     agreement result
----------------------------------------------------------------------
contig062053-NyeOR.O103    5 NHSS    0.6059     (8/9)    +
contig062053-NyeOR.O103   43 NATV    0.7126     (8/9)    +
contig062053-NyeOR.O103   53 NHSL    0.6530     (9/9)    ++
contig062053-NyeOR.O103   66 NLSV    0.7956     (9/9)    +++
----------------------------------------------------------------------
```

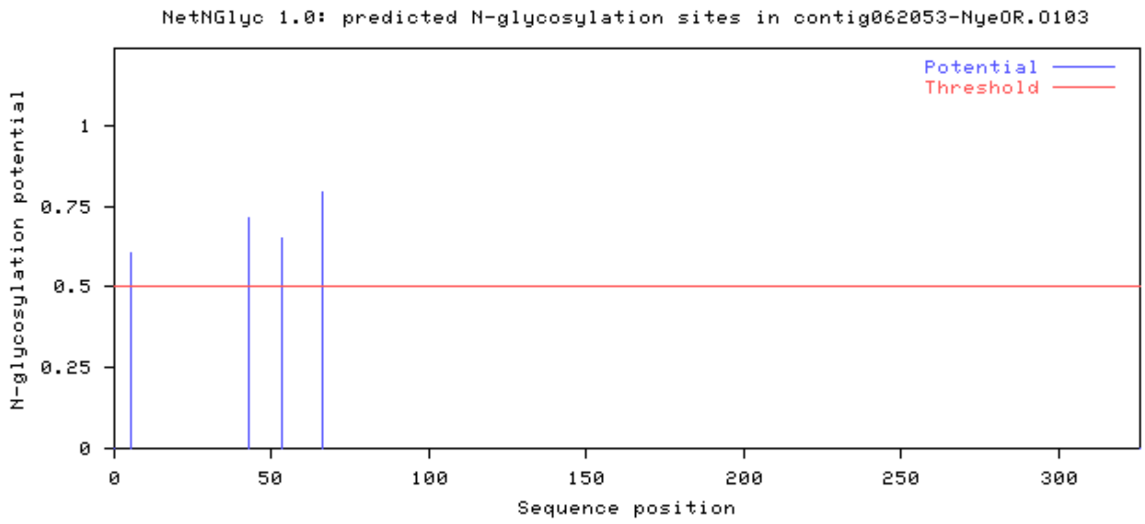

Graphics in PostScript

Output for 'contig062098-NYEORe.AC144'

```
##########################################################################################

    Warning: This sequence may not contain a signal peptide!!

    Proteins without signal peptides are unlikely to be exposed to
    the N-glycosylation machinery and thus may not be glycosylated
    (in vivo) even though they contain potential motifs.

    SignalP-NN euk predictions are as follows:

  # name                Cmax  pos ?  Ymax  pos ?  Smax  pos ?  Smean ?  D     ?

    SignalP output is explained at http://www.cbs.dtu.dk/services/SignalP/output.html

##########################################################################################

Name:  contig062098-NYEORe.AC144        Length:  199
HTAFGVTFALCISCVLGKTIVVVTAFKATFPGNKIAGKFGHAQQRVIICSCTLIQVVICVLWLTLNPPYPHTLFTYSNRM      80
IVLECKTGSEFAFYAVMGNIGILAIICLILAFMARKLPDNFNEAKLITFSLLIFCAVWITFIPAYISSPGKFTVAVEIFA     160
ILSSAFGLLICIFAPKCYIILLKPEKNTKKHVMGKTISX
................................................................................      80
................................................................................     160
.......................................                                              240

(Threshold=0.5)

No sites predicted in this sequence.
```

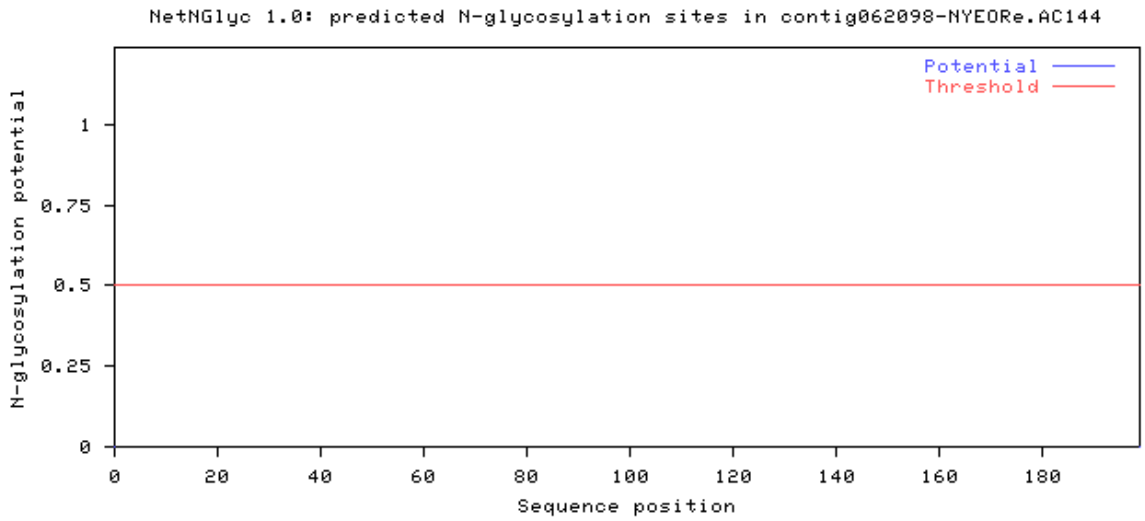

Graphics in PostScript

Output for 'contig062344-NyeOR.A020'

```
##########################################################################################

    Warning: This sequence may not contain a signal peptide!!

    Proteins without signal peptides are unlikely to be exposed to
    the N-glycosylation machinery and thus may not be glycosylated
    (in vivo) even though they contain potential motifs.

    SignalP-NN euk predictions are as follows:

  # name                    Cmax  pos ?  Ymax  pos ?  Smax  pos ?  Smean ?  D     ?

    SignalP output is explained at http://www.cbs.dtu.dk/services/SignalP/output.html

##########################################################################################

Name:  contig062344-NyeOR.A020  Length:  312
MDKELNVTFLTLDWYTEINKYRYIFFFIMFTLYILIICTNSTIVYLIWIHKNLHEPMYIFIAALLLNSVLYSTTIYPKLL      80
IDVLSDKQVTIYSACLFQFFMFYTLGGSEFFLLAAMAYDRYVAICKPLQYHIIMRKTTVSISLIIAWLVPACHIAVLAIA     160
SAEAKLCDSNIKGIFCNNAVYTLQCQRSRLIIIFGVIALLDLVILPMLFIVFTYTTIFIVSYQSCKEIRKKAAETCLPHL     240
LVLISACLFFVYDVSIARVEADFPKTARIVMTLQIVLYHPLFNPFVYGLKMKEISKHLKGLLCQGKNNFLYX
.....N.................................N........................................      80
................................................................................     160
................................................................................     240
........................................................................             320

(Threshold=0.5)
----------------------------------------------------------------------
SeqName      Position  Potential   Jury    N-Glyc
                                 agreement result
----------------------------------------------------------------------
contig062344-NyeOR.A020    6 NVTF    0.7647     (9/9)    +++
contig062344-NyeOR.A020   40 NSTI    0.7026     (9/9)    ++
----------------------------------------------------------------------
```

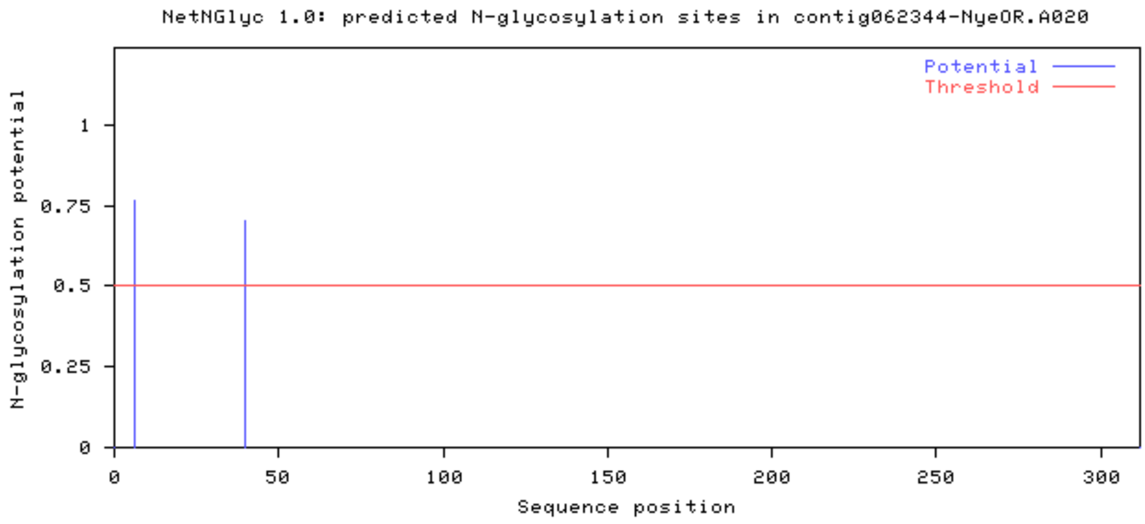

Graphics in PostScript

Output for 'contig062547-NyeOR.J081'

```
##########################################################################################

    Warning: This sequence may not contain a signal peptide!!

    Proteins without signal peptides are unlikely to be exposed to
    the N-glycosylation machinery and thus may not be glycosylated
    (in vivo) even though they contain potential motifs.

    SignalP-NN euk predictions are as follows:

  # name                   Cmax  pos ?  Ymax  pos ?  Smax  pos ?  Smean ?  D     ?

    SignalP output is explained at http://www.cbs.dtu.dk/services/SignalP/output.html

##########################################################################################

Name:  contig062547-NyeOR.J081  Length:  313
MYTNSSTSSLLTLQTLGLSSTDIYPAFVFGTLTYLIIMFSNLLVLTVIAMNKKLHKPMFILLFNLPISDIVGATAFFPHL      80
IFSIVAENRLISHHACIFQAFLIHVYGTGNLLILSAMAYDRYIAICFPLRYTTIMNSHNLMKIIVITWFINLSMMFTLFI     160
LLVRFKTCRTNIVDFYCNNQSLVKLICDDTSVNNYYGLATIFLLMGGPLALILYTYAQILRTCVITNHTDARQKAIQTCA     240
THLIVFLSLQINTVFALISHRIDSSSPVLRRAFGVSVLIFPPFFDPIIYGLKTKELKQCIVMFLKRNVGLTMX
...N............................................................................      80
......................................................................N.........     160
..................N................................................N............     240
.........................................................................            320

(Threshold=0.5)
----------------------------------------------------------------------
SeqName      Position  Potential   Jury    N-Glyc
                                 agreement result
----------------------------------------------------------------------
contig062547-NyeOR.J081    4 NSST   0.6360     (8/9)    +
contig062547-NyeOR.J081  151 NLSM   0.5309     (6/9)    +
contig062547-NyeOR.J081  179 NQSL   0.5177     (5/9)    +
contig062547-NyeOR.J081  227 NHTD   0.5090     (4/9)    +
----------------------------------------------------------------------
```

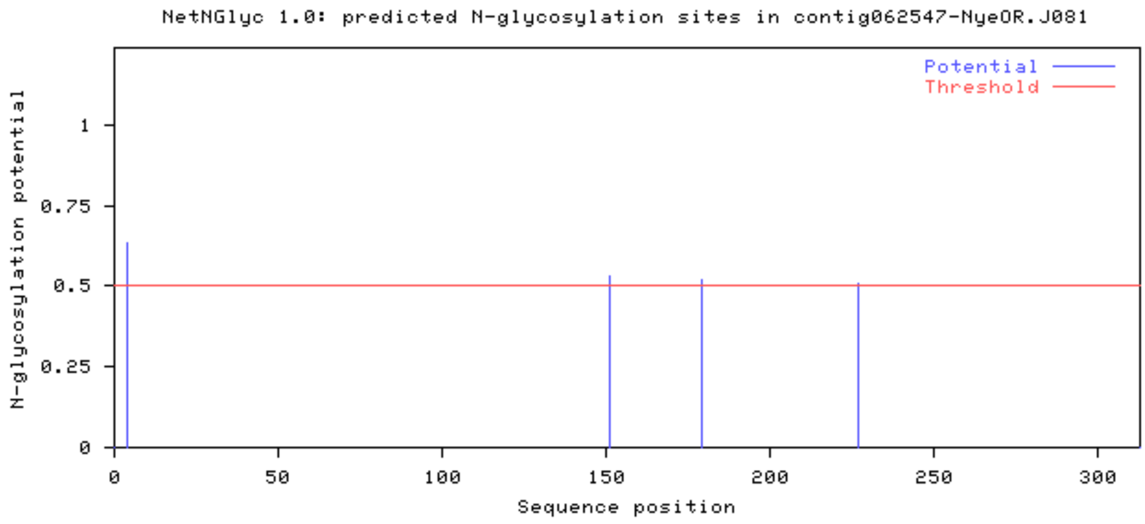

Graphics in PostScript

Output for 'contig062764-NyeORes.V138'

```
##########################################################################################

    Warning: This sequence may not contain a signal peptide!!

    Proteins without signal peptides are unlikely to be exposed to
    the N-glycosylation machinery and thus may not be glycosylated
    (in vivo) even though they contain potential motifs.

    SignalP-NN euk predictions are as follows:

  # name                 Cmax  pos ?  Ymax  pos ?  Smax  pos ?  Smean ?  D     ?

    SignalP output is explained at http://www.cbs.dtu.dk/services/SignalP/output.html

##########################################################################################

Name:  contig062764-NyeORes.V138        Length:  209
MTAILTTRSTPLILAGMAVERYISICFPLHYSQMCNIPRTLLLICVIVILTITPPITDLLITIVKEPPSFFHTKIFCDHS      80
LLFRDQSIYYKNCVFDGTYLSFVALALLYTYCKIMLTAQAVSTSLVSVKRARNTVLLHGVQVLLCMLAFVVPSLQAALIS     160
LFPQLSLEIRYIFFLLVYIIPRFLSPMIYGFRDEQFRKYWTRYLSCHEH
................................................................................      80
................................................................................     160
.................................................                                    240

(Threshold=0.5)

No sites predicted in this sequence.
```

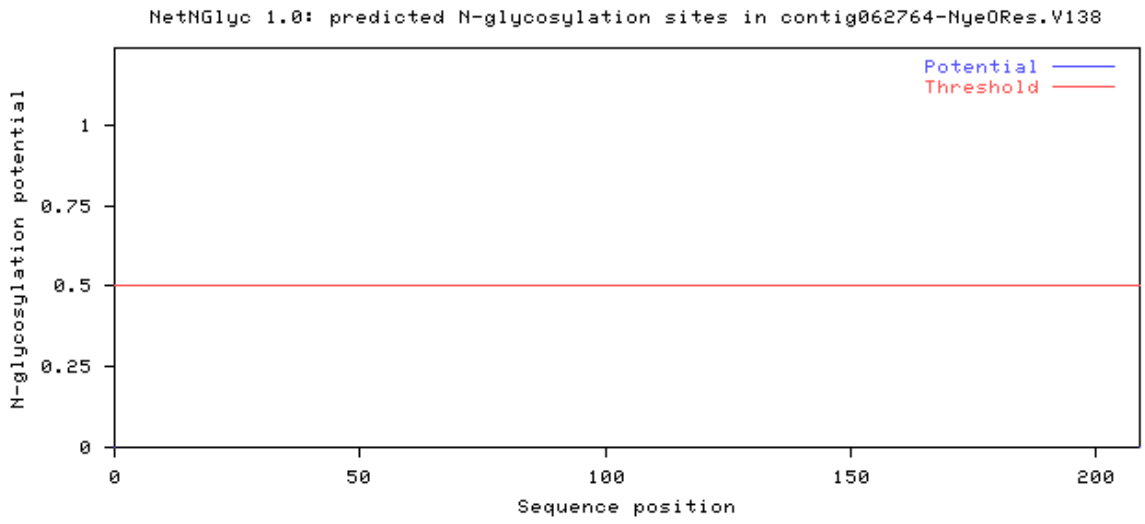

Graphics in PostScript

Output for 'contig062770-NyeOR.E059'

```
##########################################################################################

    Warning: This sequence may not contain a signal peptide!!

    Proteins without signal peptides are unlikely to be exposed to
    the N-glycosylation machinery and thus may not be glycosylated
    (in vivo) even though they contain potential motifs.

    SignalP-NN euk predictions are as follows:

  # name                    Cmax  pos ?  Ymax  pos ?  Smax  pos ?  Smean ?  D     ?

    SignalP output is explained at http://www.cbs.dtu.dk/services/SignalP/output.html

##########################################################################################

Name:  contig062770-NyeOR.E059  Length:  310
MTVNSSQSSFLVFSAYFDSGHLKYLFFVIVMSLYFLIITANVLLIVVICVNRSLHEPMYMFLCSLFVNELYGSTGLFPFL      80
LLQILSDVHTVSAPLCFLQIFCVHTYGTAELANLAVMSYDRYLAICFPLQYHTRMSPCKVSMLIVLTWFSSFLVITVLIS     160
LSAPLQLCGNIINKVYCDNYSIVKLACSDTTVNNIYGLISSPLVILCPVSLILYTYMRILKICFSGSKQTRQKAVSTCTP     240
HLASLLNFSFGCFFEILQSRFNMNSVPSMLRIFLSLYFLTCQPVFNPLMYGLTLSKISLTCKKLLCADMX
...N..............................................N.............................      80
................................................................................     160
..................N.............................................................     240
......................................................................               320

(Threshold=0.5)
----------------------------------------------------------------------
SeqName      Position  Potential   Jury    N-Glyc
                                 agreement result
----------------------------------------------------------------------
contig062770-NyeOR.E059    4 NSSQ   0.6998     (9/9)   ++
contig062770-NyeOR.E059   51 NRSL   0.7483     (9/9)   ++
contig062770-NyeOR.E059  179 NYSI   0.5860     (8/9)   +
contig062770-NyeOR.E059  247 NFSF   0.4634     (7/9)   -
----------------------------------------------------------------------
```

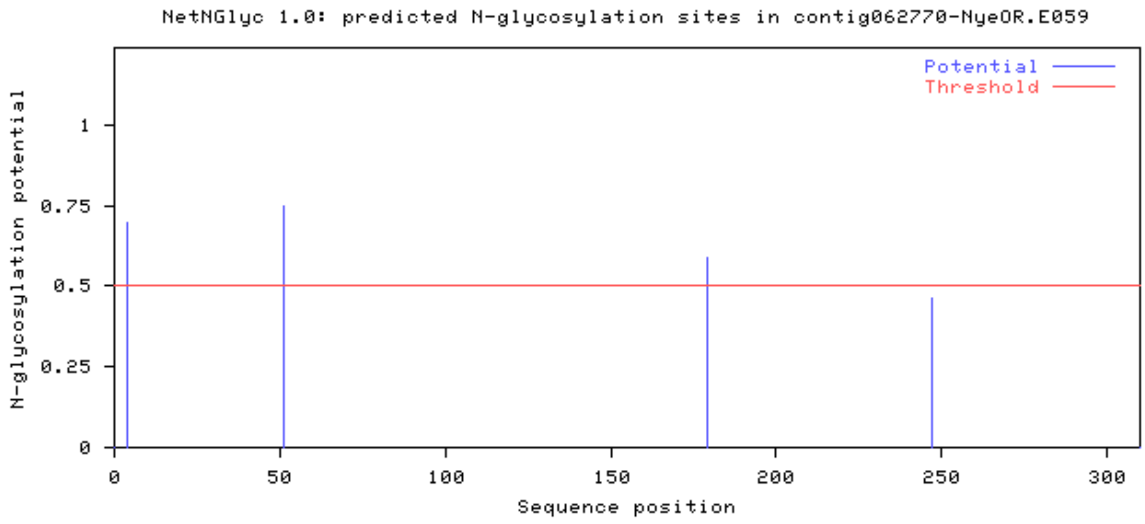

Graphics in PostScript

Output for 'contig062892-NyeORe.S125'

```
##########################################################################################

    Warning: This sequence may not contain a signal peptide!!

    Proteins without signal peptides are unlikely to be exposed to
    the N-glycosylation machinery and thus may not be glycosylated
    (in vivo) even though they contain potential motifs.

    SignalP-NN euk predictions are as follows:

  # name                    Cmax  pos ?  Ymax  pos ?  Smax  pos ?  Smean ?  D     ?

    SignalP output is explained at http://www.cbs.dtu.dk/services/SignalP/output.html

##########################################################################################

Name:  contig062892-NyeORe.S125         Length:  96
AKAASGDKKKLTHKGLKTVILHAFQLLLCLIQLWCPFIETAVLQIDLRLFIDVRYSNYIMFSIAPRCLSPLIYGLRDENF      80
FLVLKSLMPTSSCSKX
................................................................................      80
................                                                                     160

(Threshold=0.5)

No sites predicted in this sequence.
```

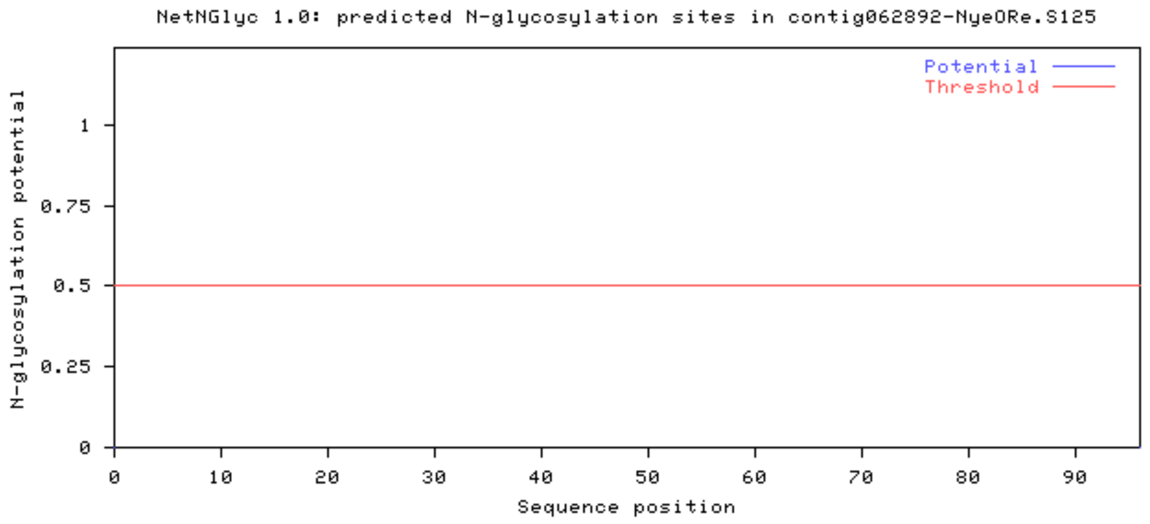

Graphics in PostScript

Output for 'contig063067-NyeORe.J082'

```
##########################################################################################

    Warning: This sequence may not contain a signal peptide!!

    Proteins without signal peptides are unlikely to be exposed to
    the N-glycosylation machinery and thus may not be glycosylated
    (in vivo) even though they contain potential motifs.

    SignalP-NN euk predictions are as follows:

  # name                    Cmax  pos ?  Ymax  pos ?  Smax  pos ?  Smean ?  D     ?

    SignalP output is explained at http://www.cbs.dtu.dk/services/SignalP/output.html

##########################################################################################

Name:  contig063067-NyeORe.J082         Length:  172
MNNRYNTSSFLQINVFNLSSESVFPAFLFATLSYMIILFCNLTLILTIVLNKSLHQPMYLILLNLPINDLIGSSALFPQL      80
IKEILRNSGIMQYSACVAQAFFIHIYAAGTVFNLSAMAYDRYIAICYPLQYSTVMTNAHIMRIITIVWMSCLVLIAVLFF     160
LLLRLPRCRSEM
.....N..................................N.........N............................      80
................................................................................     160
............                                                                         240

(Threshold=0.5)
----------------------------------------------------------------------
SeqName      Position  Potential   Jury    N-Glyc
                                 agreement result
----------------------------------------------------------------------
contig063067-NyeORe.J082    6 NTSS   0.7652     (9/9)   +++
contig063067-NyeORe.J082   17 NLSS   0.4923     (3/9)   -
contig063067-NyeORe.J082   41 NLTL   0.7097     (9/9)   ++
contig063067-NyeORe.J082   51 NKSL   0.7340     (9/9)   ++
contig063067-NyeORe.J082  113 NLSA   0.3859     (8/9)   -
----------------------------------------------------------------------
```

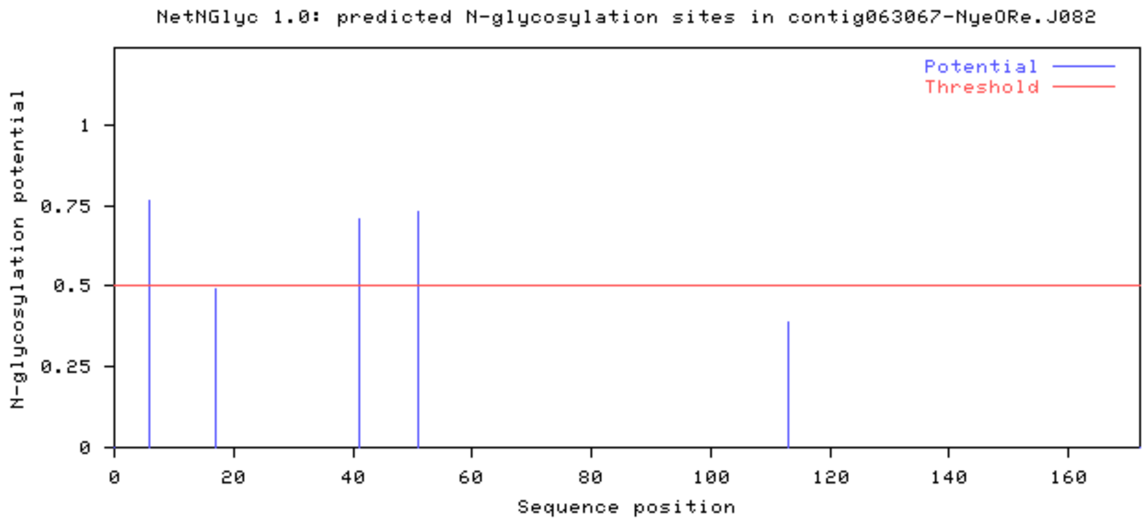

Graphics in PostScript

Output for 'contig063949-NyeORe.L100'

```
########################################################################################

    Warning: This sequence may not contain a signal peptide!!

    Proteins without signal peptides are unlikely to be exposed to
    the N-glycosylation machinery and thus may not be glycosylated
    (in vivo) even though they contain potential motifs.

    SignalP-NN euk predictions are as follows:

  # name                 Cmax  pos ?  Ymax  pos ?  Smax  pos ?  Smean ?  D     ?

    SignalP output is explained at http://www.cbs.dtu.dk/services/SignalP/output.html

########################################################################################

Name:  contig063949-NyeORe.L100         Length:  215
IQMYVYQVGATMERFSLTIMAFDRLIAIIYPLQYHRYLTNTRTLVFTCILWIVACSFVLFTLVTATPLPHCYSRLRYTFC      80
DYGAVMRTTCVDPEKYFNQVAIISFFLSFFTFTFICLSYCGILFFVKILSNNDRKKMGSTLVSHLICVSCLYCPQFIIVI     160
LTRFGVVLTLEERQGLLIGTILAPSLVNPFVYCLRTKEIKSKIIKIFRKVNTAGX
................................................................................      80
................................................................................     160
.......................................................                              240

(Threshold=0.5)

No sites predicted in this sequence.
```

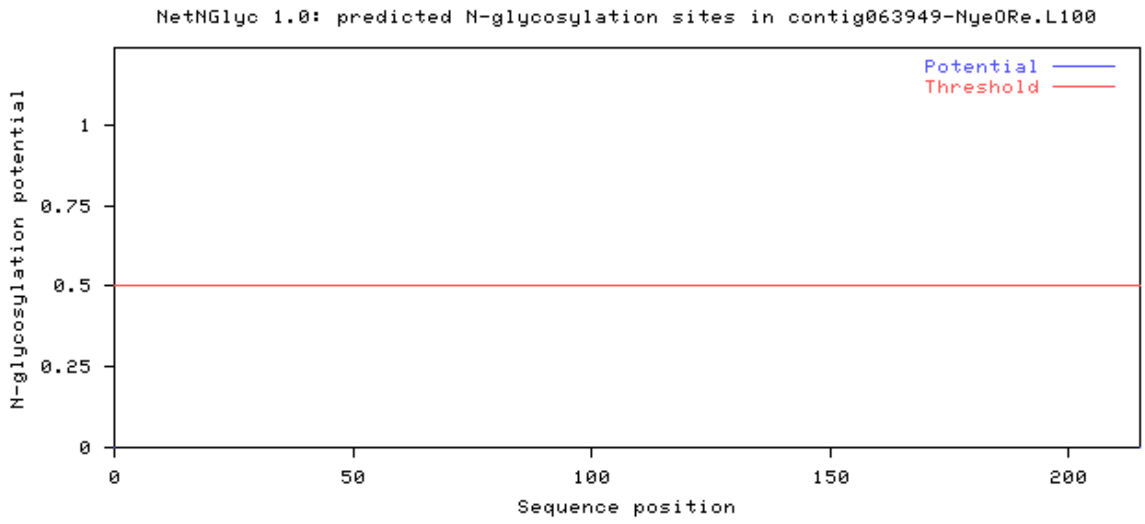

Graphics in PostScript

Output for 'contig064361-NyeOR.L094'

```
##########################################################################################

    Warning: This sequence may not contain a signal peptide!!

    Proteins without signal peptides are unlikely to be exposed to
    the N-glycosylation machinery and thus may not be glycosylated
    (in vivo) even though they contain potential motifs.

    SignalP-NN euk predictions are as follows:

  # name                 Cmax  pos ?  Ymax  pos ?  Smax  pos ?  Smean ?  D     ?

    SignalP output is explained at http://www.cbs.dtu.dk/services/SignalP/output.html

##########################################################################################

Name:  contig064361-NyeOR.L094  Length:  313
MSSQNTSINVTHFIIGGFDTLSRPIAVGVVILITYLLAVLANMANIMFIISDKRLHKPMYLLICNLAVVDIMYTSSCSPT      80
MIGVLLAGVNTISYMACLIQMCVFHLGTAMESFVLAVMALDRFIAIIYPFQYHSYLTNTRVLVLTFIVWFVNCFFMCYMP     160
ATAVPLPHCSSRLRYTFCDFAAVIRTTCVNPEKHFNDAAIIAFFILFFTFVFICLSYCGILLFVKLSSNNEKKKMGSTLV     240
SHLICAIVHYCPAFVRIIFTRFGVVLTVEERQGLLIGAVLGPCLVNPFVYFLRTKEIKQKLFKIFKKFNTSDX
....N...N.......................................................................      80
................................................................................     160
................................................................................     240
.........................................................................            320

(Threshold=0.5)
----------------------------------------------------------------------
SeqName      Position  Potential   Jury    N-Glyc
                                agreement  result
----------------------------------------------------------------------
contig064361-NyeOR.L094    5 NTSI   0.6201     (7/9)   +
contig064361-NyeOR.L094    9 NVTH   0.7423     (9/9)   ++
contig064361-NyeOR.L094  309 NTSD   0.4220     (8/9)   -
----------------------------------------------------------------------
```

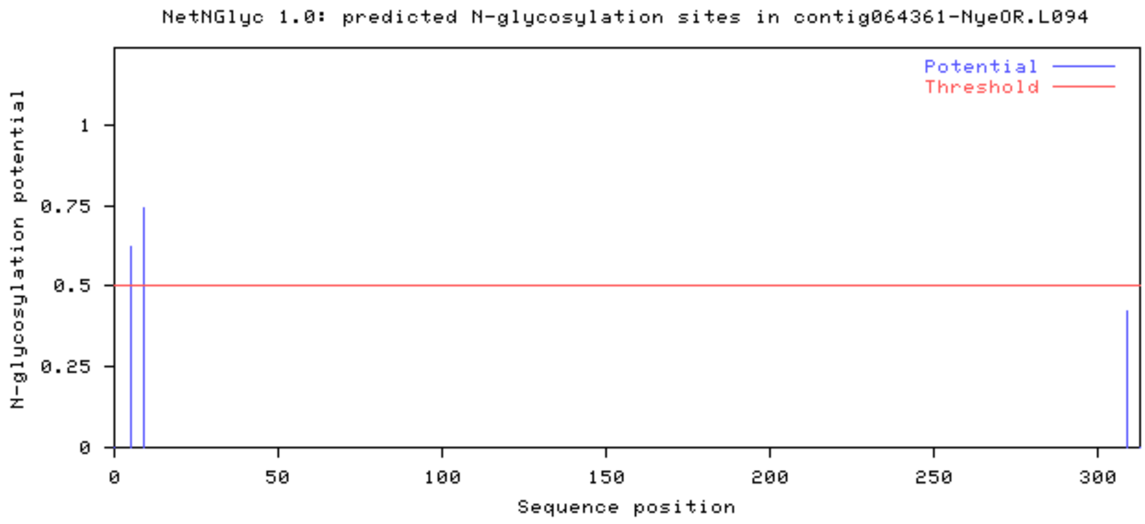

Graphics in PostScript

Output for 'contig064392-NyeORe.A023'

```
##########################################################################################

    Warning: This sequence may not contain a signal peptide!!

    Proteins without signal peptides are unlikely to be exposed to
    the N-glycosylation machinery and thus may not be glycosylated
    (in vivo) even though they contain potential motifs.

    SignalP-NN euk predictions are as follows:

  # name                 Cmax  pos ?  Ymax  pos ?  Smax  pos ?  Smean ?  D     ?

    SignalP output is explained at http://www.cbs.dtu.dk/services/SignalP/output.html

##########################################################################################

Name:  contig064392-NyeORe.A023         Length:  214
FQFFIIYSLGCSEFFLLAAMAYDRYVAICKPLQYPTIMRKSTVSIFLVIAWLVPASNIAVQAIGMAKSKLCSFHLKSIFC      80
NNTIYTLQCVKSGLVTVFGIVSYLDLVVFPILFIVFTYTKIFIVTYRSCKEIKKKAAETCLPHMLVLITFSCLGIYDVIM     160
ARMETDFPKTARLIMTLQLALYQPLFNPFIYGLKMKEISKHLKSLFCPPLLSIX
................................................................................      80
N...............................................................................     160
......................................................                               240

(Threshold=0.5)
----------------------------------------------------------------------
SeqName      Position  Potential   Jury    N-Glyc
                                 agreement result
----------------------------------------------------------------------
contig064392-NyeORe.A023    81 NNTI   0.5550     (7/9)    +
----------------------------------------------------------------------
```

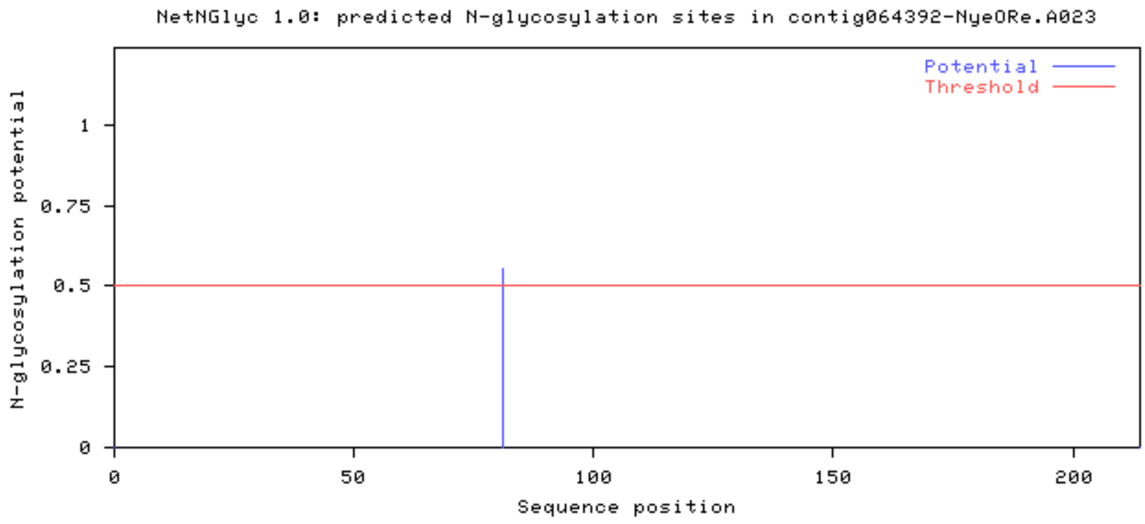

[Graphics in PostScript]

Output for 'contig064435-NyeORe.E062'

```
##########################################################################################

    Warning: This sequence may not contain a signal peptide!!

    Proteins without signal peptides are unlikely to be exposed to
    the N-glycosylation machinery and thus may not be glycosylated
    (in vivo) even though they contain potential motifs.

    SignalP-NN euk predictions are as follows:

  # name                    Cmax   pos ?  Ymax   pos ?  Smax   pos ?  Smean ?  D     ?

    SignalP output is explained at http://www.cbs.dtu.dk/services/SignalP/output.html

##########################################################################################

Name:  contig064435-NyeORe.E062         Length:  134
CDTHSVVKLACSDTTVINLYGLLATFSMIFGALLFILYTYMKILLVCFSGSDQTRQKAVSTCTPHLASILNFSFGASFEI      80
LQSRFDMKNVPNMLRIFLSLYFLTCQPLFNPVMYGLKMTKIRNICKSLITNINX
................................................................................      80
......................................................                               160

(Threshold=0.5)

No sites predicted in this sequence.
```

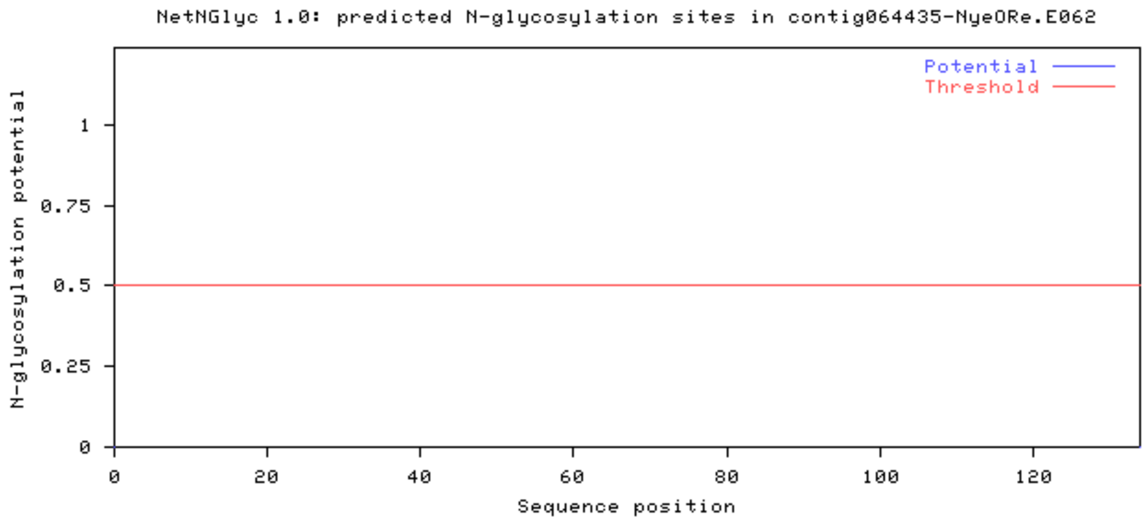

Graphics in PostScript

Output for 'contig064862-NyeORe.L106'

```
##########################################################################################

    Warning: This sequence may not contain a signal peptide!!

    Proteins without signal peptides are unlikely to be exposed to
    the N-glycosylation machinery and thus may not be glycosylated
    (in vivo) even though they contain potential motifs.

    SignalP-NN euk predictions are as follows:

  # name                  Cmax  pos ?  Ymax  pos ?  Smax  pos ?  Smean ?  D     ?

    SignalP output is explained at http://www.cbs.dtu.dk/services/SignalP/output.html

##########################################################################################

Name:  contig064862-NyeORe.L106         Length:  121
MITNVTRMKSFFILGFPGLSPQYYGPISTFLFFVYLAIALGNIFILSFVAYEKSLQKPTYLVFCHLALNDLTFGTVTLPK      80
IMSKYWFDNSVISFYGCFTQMFFVHYLGSVTSFILLVMALD
...N............................................................................      80
.........................................                                            160

(Threshold=0.5)
----------------------------------------------------------------------
SeqName      Position  Potential   Jury    N-Glyc
                                 agreement result
----------------------------------------------------------------------
contig064862-NyeORe.L106     4 NVTR   0.7708     (9/9)    +++
----------------------------------------------------------------------
```

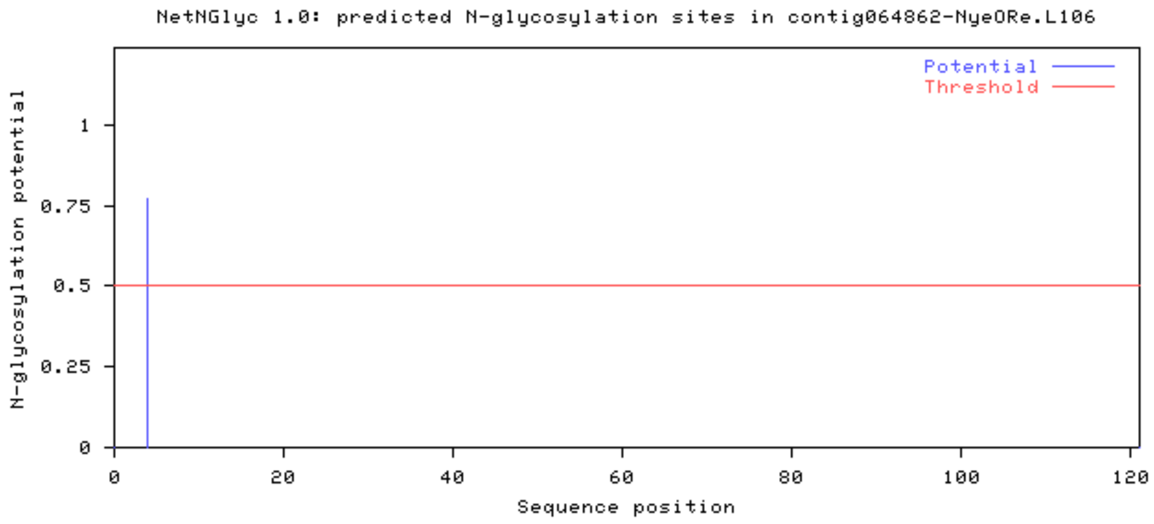

Graphics in PostScript

Output for 'contig065564-NyeORe.O105'

```
##########################################################################################

    Warning: This sequence may not contain a signal peptide!!

    Proteins without signal peptides are unlikely to be exposed to
    the N-glycosylation machinery and thus may not be glycosylated
    (in vivo) even though they contain potential motifs.

    SignalP-NN euk predictions are as follows:

  # name                    Cmax  pos ?  Ymax  pos ?  Smax  pos ?  Smean ?  D     ?

    SignalP output is explained at http://www.cbs.dtu.dk/services/SignalP/output.html

##########################################################################################

Name:  contig065564-NyeORe.O105         Length:  49
MPERNHSVLTEFILTGFPGLHQEYYGLVSAVLFFVYLVTLIANATVIFL
....N..................................N......                                          80

(Threshold=0.5)
----------------------------------------------------------------------
SeqName      Position  Potential   Jury    N-Glyc
                                agreement result
----------------------------------------------------------------------
contig065564-NyeORe.O105     5 NHSV   0.5018     (6/9)    +
contig065564-NyeORe.O105    43 NATV   0.6461     (7/9)    +
----------------------------------------------------------------------
```

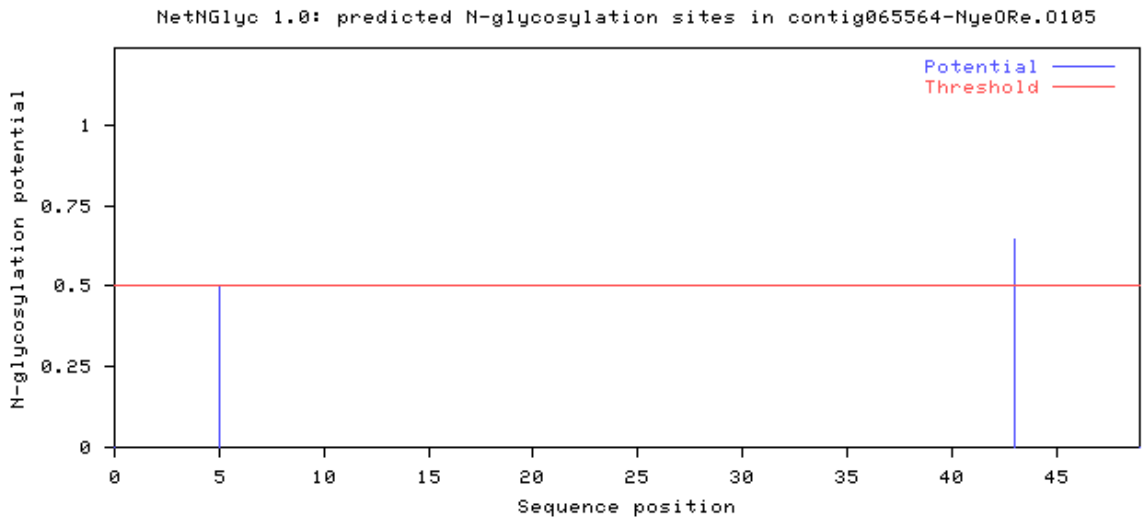

Graphics in PostScript

Output for 'contig066751-NyeORp.D049'

```
##########################################################################################

    Warning: This sequence may not contain a signal peptide!!

    Proteins without signal peptides are unlikely to be exposed to
    the N-glycosylation machinery and thus may not be glycosylated
    (in vivo) even though they contain potential motifs.

    SignalP-NN euk predictions are as follows:

  # name                 Cmax  pos ?  Ymax  pos ?  Smax  pos ?  Smean ?  D     ?

    SignalP output is explained at http://www.cbs.dtu.dk/services/SignalP/output.html

##########################################################################################

Name:  contig066751-NyeORp.D049         Length:  308
MENSSEIVSFVLAAYGNVGELKYLYFVIILFWYLSICVANTVLIVVIRVDIQLHEPMYILLCNLCVNQINVSTSLYPLLL      80
SQMFSDSHEVTLPWCFLHMSSMYISGPAEFCSLAAMAYDRYISICHPLRYNVIMNTERVFFMILFVWIYSFLSVILSFSF     160
IFSLKFCGTIIENVYCNHRLILRLSCFVSVHNFLSDILFLIVSFFIPFTLISVSYVKILAVCRKTSTENKQKAMTTCVPQ     240
IVSVSNLFVGSIFQSIDSSVIVAHLQIHIILSIYIFICQPMLTPFLYGFNLPKIRQSCKKLLFKKKQX
..N.............................................................................      80
................................................................................     160
................................................................................     240
....................................................................                 320

(Threshold=0.5)
----------------------------------------------------------------------
SeqName      Position  Potential   Jury    N-Glyc
                                 agreement result
----------------------------------------------------------------------
contig066751-NyeORp.D049    3 NSSE   0.6997     (9/9)   ++
contig066751-NyeORp.D049   70 NVST   0.4658     (6/9)   -
----------------------------------------------------------------------
```

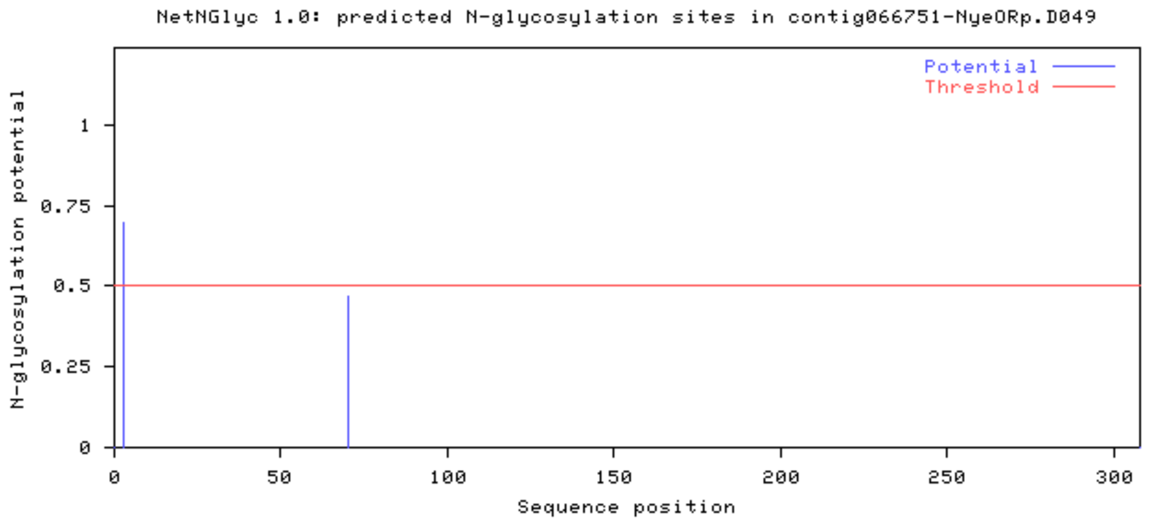

Graphics in PostScript

Output for 'contig067856-NyeORe.K089'

```
##########################################################################

    Warning: This sequence may not contain a signal peptide!!

    Proteins without signal peptides are unlikely to be exposed to
    the N-glycosylation machinery and thus may not be glycosylated
    (in vivo) even though they contain potential motifs.

    SignalP-NN euk predictions are as follows:

  # name                 Cmax  pos ?  Ymax  pos ?  Smax  pos ?  Smean ?  D     ?

    SignalP output is explained at http://www.cbs.dtu.dk/services/SignalP/output.html

##########################################################################

Name:  contig067856-NyeORe.K089         Length:  251
MMENYTYNSYILQLEGLNTSKDSLYPAFLFLFFSYLFIMIINVGITILIFMNKNLHQPMYLLFCNLPLNDILATSIVVPR      80
LLIDLMRPPSERLISYYQCVVQAYIVHLVGTTSHTVLMIMAYDRYVAICNPFHYVSIMTNKMMIKLTVCAWGVAFVLVGI     160
LLGLTTRLSRCRTLITNPYCDNASLFKLSCESVVINNIYGITFTVALYVGSIGAIVLSYTSIAVVCLTSKNKSLNSKALK     240
TCSTHLVVYLI
...N.............N..............................................................      80
................................................................................     160
.....................N..........................................................     240
...........                                                                          320

(Threshold=0.5)
----------------------------------------------------------------------
SeqName       Position  Potential   Jury    N-Glyc
                                  agreement result
----------------------------------------------------------------------
contig067856-NyeORe.K089    4 NYTY   0.7622     (9/9)   +++
contig067856-NyeORe.K089   18 NTSK   0.6162     (8/9)   +
contig067856-NyeORe.K089  182 NASL   0.5806     (6/9)   +
contig067856-NyeORe.K089  231 NKSL   0.3606     (9/9)   --
----------------------------------------------------------------------
```

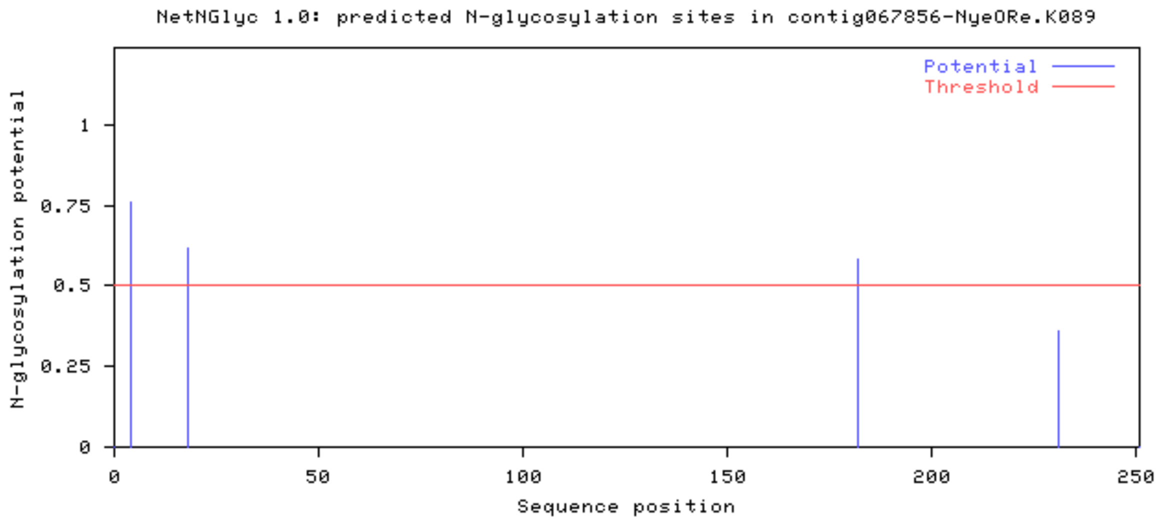

Graphics in PostScript

Explain the output. Go back.

NetNGlyc 1.0 Server - prediction results

Technical University of Denmark

Asn-Xaa-Ser/Thr sequons in the sequence output below are highlighted in **blue**.
Asparagines predicted to be N-glycosylated are highlighted in **red**.

Output for 'contig013326-TilOR.D051'

```
##########################################################################

   Warning: This sequence may not contain a signal peptide!!

   Proteins without signal peptides are unlikely to be exposed to
   the N-glycosylation machinery and thus may not be glycosylated
   (in vivo) even though they contain potential motifs.

   SignalP-NN euk predictions are as follows:

 # name                 Cmax  pos ?  Ymax  pos ?  Smax  pos ?  Smean ?  D     ?

   SignalP output is explained at http://www.cbs.dtu.dk/services/SignalP/output.html

##########################################################################

Name:  contig013326-TilOR.D051  Length:  309
MGNSSETASFVLAAYGNIGQLKYVYFIIILIWYLSICVANTVLIVVIRVDRRLHEPMYMLLNNLCVNEINASTSLYPLLL      80
SQMFSDSHEVTLPWCFLQMCCMYTSGPVEFCSLAAMAYDRYISICHPLRYNVIMKTERVFLLILLVWLYSFLSFIFSFSF     160
IFSLKFCGNIIHSVYCDHQLIIRLSCSFSIQSFISDISFAILSFFIPFSFILVSYLKILRVCLKTSKENKQKAVTTCTPH     240
IISVSNLFVGCIFYFIDFRFLVSQVPVEVRIILPMYVLIFQPMLTPFMYGFNLPKIKQSYQRFLLKRKX
..N.............................................................................      80
................................................................................     160
................................................................................     240
.....................................................................                320

(Threshold=0.5)
----------------------------------------------------------------------
SeqName      Position  Potential   Jury    N-Glyc
                                 agreement result
----------------------------------------------------------------------
contig013326-TilOR.D051    3 NSSE    0.7327     (9/9)   ++
contig013326-TilOR.D051   70 NAST    0.3767     (6/9)   -
----------------------------------------------------------------------
```

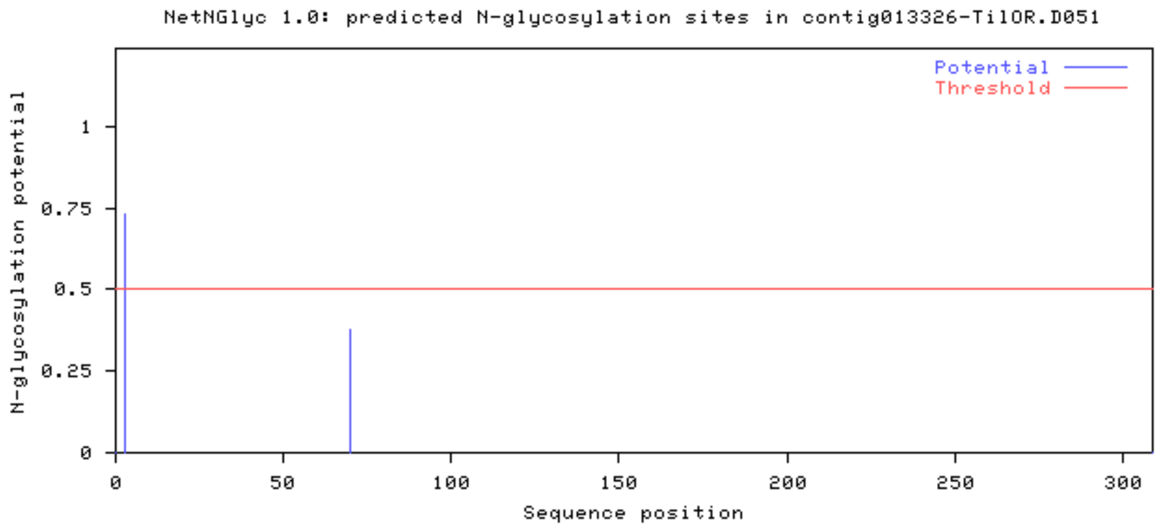

Graphics in PostScript

Output for 'contig046360-TilOR.N197'

```
##########################################################################

   Warning: This sequence may not contain a signal peptide!!

   Proteins without signal peptides are unlikely to be exposed to
   the N-glycosylation machinery and thus may not be glycosylated
   (in vivo) even though they contain potential motifs.

   SignalP-NN euk predictions are as follows:

 # name                Cmax  pos ?  Ymax  pos ?  Smax  pos ?  Smean ?  D     ?

   SignalP output is explained at http://www.cbs.dtu.dk/services/SignalP/output.html

##########################################################################

Name:  contig046360-TilOR.N197  Length:  318
MEFFNSAVGKNITFVKPAYFIISAFNGIVNIRYYFVFLCFIYIFSVVGNTLVMIVIILDHTLKGPKHIGVFNFAFTDLLS      80
SSALMPKLVDIFLFNHHHISYNDCLAFMFFCLAFFAAQAFNLVVLSFDRVMAIMYPLHYQMRMSHKLILSLIAFFWLLVI     160
TIILIAVGLLTRVSFCDSVVIQSYFCDHGPVYRLGCNDLTPNRVIGYLAPVLVLWVPLAFIVGSYCCIGYSLSKTVTCRE     240
RLKALKTCTGHLSLVAIYFLPTLFIFTFGSTIPPNARTVSLSLATVMPLTLNPIIYGLQTQEIKESLKKLLKVKMQFX
..........N.....................................................................      80
................................................................................     160
................................................................................     240
..............................................................................       320

(Threshold=0.5)
----------------------------------------------------------------------
SeqName       Position  Potential   Jury    N-Glyc
                                  agreement result
----------------------------------------------------------------------
contig046360-TilOR.N197    11 NITF   0.5078     (6/9)    +
----------------------------------------------------------------------
```

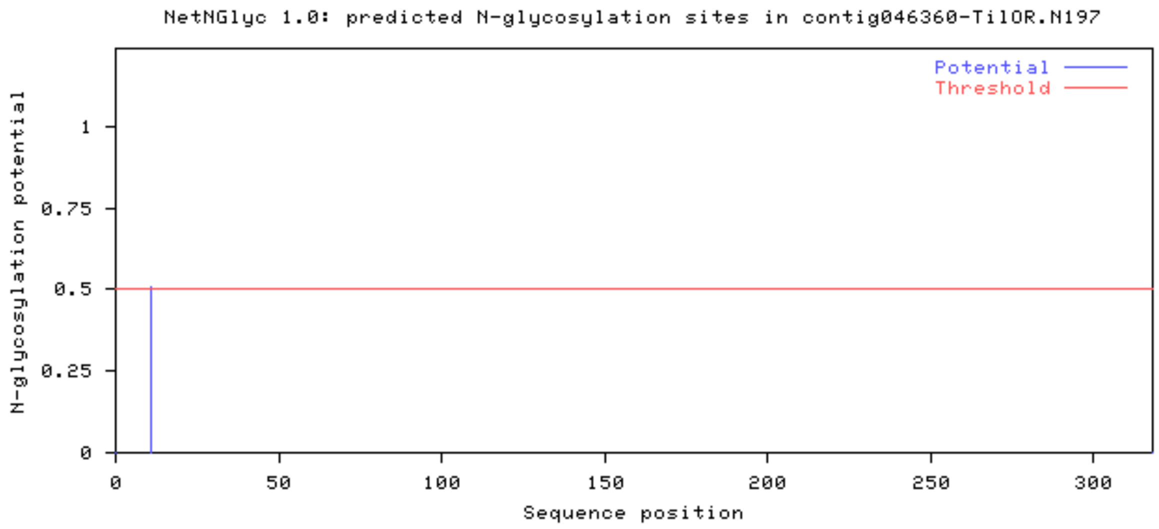

Graphics in PostScript

Output for 'contig013344-TilOR.D058'

```
##########################################################################################

    Warning: This sequence may not contain a signal peptide!!

    Proteins without signal peptides are unlikely to be exposed to
    the N-glycosylation machinery and thus may not be glycosylated
    (in vivo) even though they contain potential motifs.

    SignalP-NN euk predictions are as follows:

  # name                Cmax  pos ?  Ymax  pos ?  Smax  pos ?  Smean ?  D     ?

    SignalP output is explained at http://www.cbs.dtu.dk/services/SignalP/output.html

##########################################################################################

Name:  contig013344-TilOR.D058  Length:  309
MGNSSEIFSFVLAAYGNVGALKYMYFVMILFWYLFICVANTVLIVVIRVDRRLHEPMYILLCNLCVNEINSSTSLYPLLL      80
SQMFSDIHGITLTWCFLQMSCMYTSASVEFCSLAAMAYDRYISICHPFSYNVIMNTERVFLLILLVWIFSFVSFLLSYSF     160
IYSLKFCGDIIDNVYCDHQLIIRLSCSVSIQSSLSIIFFVIMSILLPFTLISVSYLKILAVCRKTSTENKQKAVTTCTPQ     240
IVSVSNLFIGCICYSIDFRFLVAQVPDEVRIILAIYLLICQPILTPFMYGFNLPKIRQSCKMLLFKRKX
..N.............................................................................      80
................................................................................     160
................................................................................     240
.....................................................................                320

(Threshold=0.5)
----------------------------------------------------------------------
SeqName      Position  Potential   Jury    N-Glyc
                                 agreement result
----------------------------------------------------------------------
contig013344-TilOR.D058    3 NSSE   0.7301     (9/9)    ++
contig013344-TilOR.D058   70 NSST   0.3643     (8/9)    -
----------------------------------------------------------------------
```

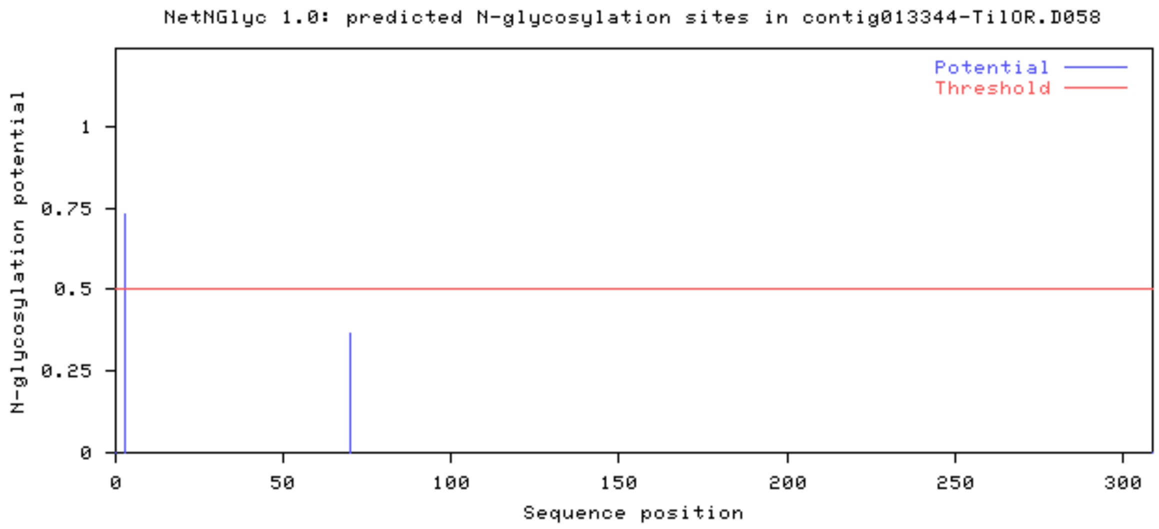

Graphics in PostScript

Output for 'contig004997-TilORe.X235'

```
##########################################################################################

    Warning: This sequence may not contain a signal peptide!!

    Proteins without signal peptides are unlikely to be exposed to
    the N-glycosylation machinery and thus may not be glycosylated
    (in vivo) even though they contain potential motifs.

    SignalP-NN euk predictions are as follows:

  # name                 Cmax  pos ?  Ymax  pos ?  Smax  pos ?  Smean ?  D     ?

    SignalP output is explained at http://www.cbs.dtu.dk/services/SignalP/output.html

##########################################################################################

Name:  contig004997-TilORe.X235         Length:  260
MLINDTLQLLSSVLLFLLVMGQVKFPVFYCFPLLFISTVTYQNTPLILATMSLERYVAIFYPLQRPAAWRSGRIWIINLC      80
LWLISCMFLIIDYSIGKQRPAVDVLFTPVLCKNLVINSSPIQALFRTAVSVMFFAVVAVVIFFTYVRILLETRKLRQDRV     160
SVNKAKHTVLLHGFQLLLCMLAFTLPITETLILLYTKWQVEDITYFCFFCFILIPRFLSPLIYGFRDHSLRGYIGKTFLC     240
CSNAVKPHFRSKQQDSLSAS
...N............................................................................      80
................................................................................     160
................................................................................     240
....................                                                                 320

(Threshold=0.5)
----------------------------------------------------------------------
SeqName      Position  Potential   Jury    N-Glyc
                                 agreement result
----------------------------------------------------------------------
contig004997-TilORe.X235    4 NDTL   0.7486     (9/9)   ++
contig004997-TilORe.X235  117 NSSP   0.1256     (9/9)   ---
----------------------------------------------------------------------
```

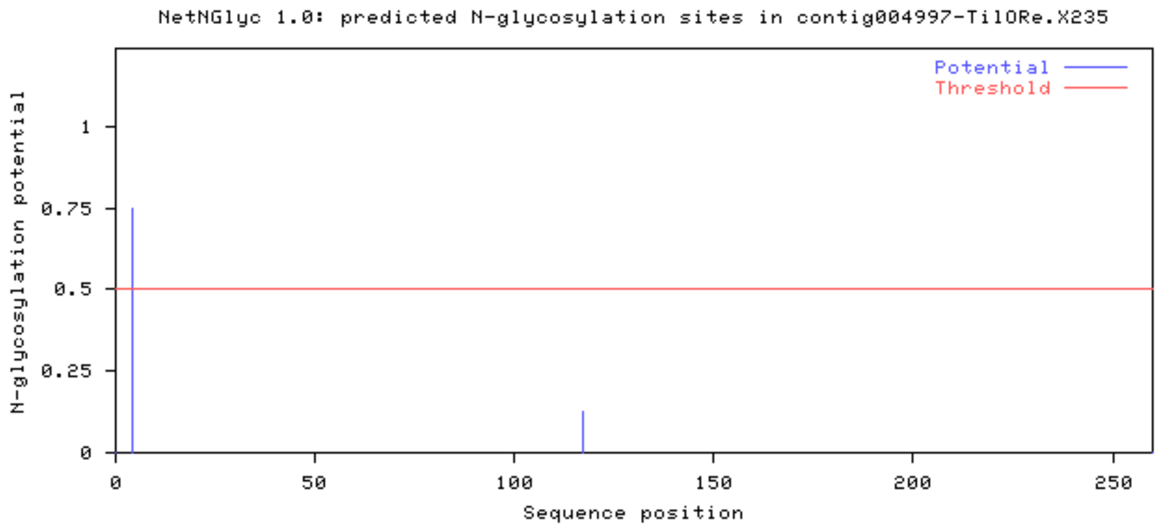

Graphics in PostScript

Output for 'contig004998-TilORe.X234'

```
##########################################################################

    Warning: This sequence may not contain a signal peptide!!

    Proteins without signal peptides are unlikely to be exposed to
    the N-glycosylation machinery and thus may not be glycosylated
    (in vivo) even though they contain potential motifs.

    SignalP-NN euk predictions are as follows:

  # name                    Cmax   pos ?  Ymax   pos ?  Smax   pos ?  Smean ?  D     ?

    SignalP output is explained at http://www.cbs.dtu.dk/services/SignalP/output.html

##########################################################################

Name:  contig004998-TilORe.X234         Length:  260
MLINDTLQLLSSVLLFLLVMGQVKFPVFYCVPLLFISTVTYQNTPLILATMSLERYVAIFYPLQRPAAWRSGRIWIINLC      80
LWLISCMSHIIEYSIGKQRPAVDVLFTPVLCKNILINSSPIQALFRTAMSVMFFAVVAVVIFSTYVRILLETRKLRQDRV     160
SVNKAKHTVLLHGFQLLLCMLAFTLPITESLTLLYTNWPAEDIAFFNYFCFILIPRFLSPLIYGFRDHSLRRYIGKTFLC     240
CSNAVKPHFRSKQQDSLSAS
...N............................................................................      80
................................................................................     160
................................................................................     240
....................                                                                 320

(Threshold=0.5)
----------------------------------------------------------------------
SeqName      Position  Potential   Jury    N-Glyc
                                 agreement result
----------------------------------------------------------------------
contig004998-TilORe.X234     4 NDTL   0.7486     (9/9)   ++
contig004998-TilORe.X234   117 NSSP   0.1464     (9/9)   ---
----------------------------------------------------------------------
```

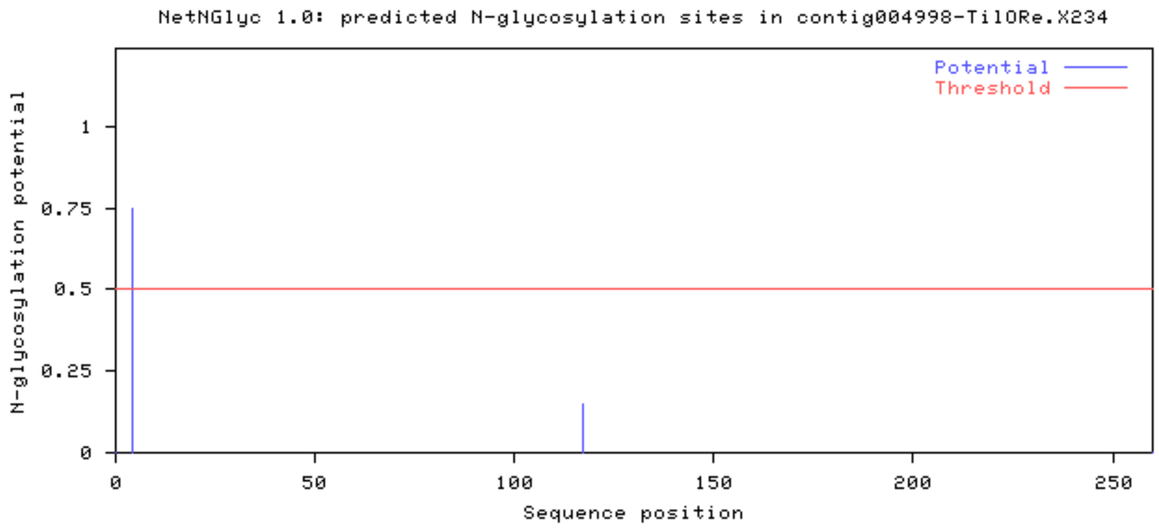

[Graphics in PostScript]

Output for 'contig004999-TilOR.S217'

```
##############################################################################

    Warning: This sequence may not contain a signal peptide!!

    Proteins without signal peptides are unlikely to be exposed to
    the N-glycosylation machinery and thus may not be glycosylated
    (in vivo) even though they contain potential motifs.

    SignalP-NN euk predictions are as follows:

  # name                Cmax  pos ?  Ymax  pos ?  Smax  pos ?  Smean ?  D     ?

    SignalP output is explained at http://www.cbs.dtu.dk/services/SignalP/output.html

##############################################################################

Name:  contig004999-TilOR.S217  Length:  307
MAGNSSLICAFLHQLLTVRVMIVHTLVIIFLCINMLLIVTFVKKECFHTSARYILFFVTLLSDSFLLVMTDILLILTRCS      80
VQVWLCIIITLFVLVYSIVTPVTLTAMTLERYVAICMPLRHGQLCSTRSIKYCILIIHGLSSGPCIVIISMFFATASISF     160
YSQYTICSVEMFMLYRWQDHARSAVSQFYFMIMGITIVFSYVQIMKVAKAASGENKKSTQKGVRTVILHGFQLLLCLVQL     240
WSPFIETAVLQIDVNLFRDFRYFNYVLFSIVPRCLSPLIYGLRDETFFLSLKNLMPTSSCSKKHVIX
...N............................................................................      80
................................................................................     160
................................................................................     240
...................................................................                  320

(Threshold=0.5)
----------------------------------------------------------------------
SeqName      Position  Potential   Jury    N-Glyc
                                 agreement result
----------------------------------------------------------------------
contig004999-TilOR.S217    4 NSSL   0.7857     (9/9)   +++
----------------------------------------------------------------------
```

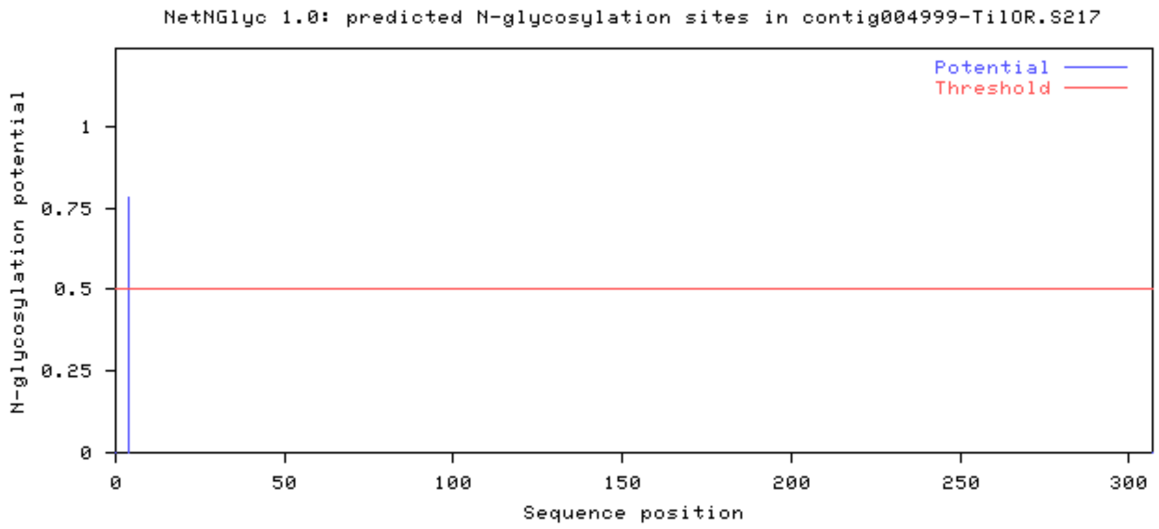

Graphics in PostScript

Output for 'contig005000-TilOR.S218'

```
##############################################################################

    Warning: This sequence may not contain a signal peptide!!

    Proteins without signal peptides are unlikely to be exposed to
    the N-glycosylation machinery and thus may not be glycosylated
    (in vivo) even though they contain potential motifs.

    SignalP-NN euk predictions are as follows:

  # name                Cmax  pos ?  Ymax  pos ?  Smax  pos ?  Smean ?  D     ?

    SignalP output is explained at http://www.cbs.dtu.dk/services/SignalP/output.html

##############################################################################

Name:  contig005000-TilOR.S218  Length:  307
MAGNNTLICVFLQRPLTDRVMTVQILVIIFLCINMLLIVTFFKKESFYTSARYILFFVTLLSDSFLLVTTDILLILTNFR      80
CSVQVWLCIIICLFVIVYSIVTPVTLTAMTLECYVAICMPLRHGQLCSTRSTMYCILIIHGLSSGPCIVIISMFFATASI     160
SFYSQYTICSVEMFMLYRWQDHARSAVSQFYFMIMGITIAFSYVQIMKVAKAASGENKRSTQKGVRTVILHGFQLLLCLV     240
QLWTPIIESAVLQIDFNLFLNVRYFNYVLFSLTPKCLSPLIYGLRDEHFFLALKNLMSASSYSKQTX
...N............................................................................      80
................................................................................     160
................................................................................     240
...................................................................                  320

(Threshold=0.5)
----------------------------------------------------------------------
SeqName      Position  Potential   Jury    N-Glyc
                                 agreement result
----------------------------------------------------------------------
contig005000-TilOR.S218    4 NNTL   0.7952     (9/9)    +++
----------------------------------------------------------------------
```

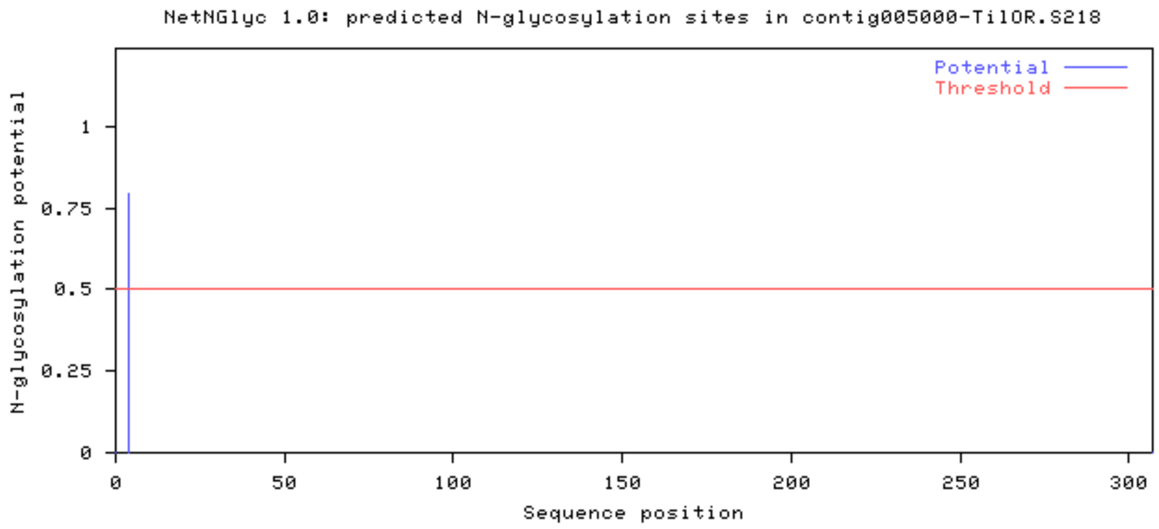

Graphics in PostScript

Output for 'contig005005-TilOR.S219'

```
########################################################################################

    Warning: This sequence may not contain a signal peptide!!

    Proteins without signal peptides are unlikely to be exposed to
    the N-glycosylation machinery and thus may not be glycosylated
    (in vivo) even though they contain potential motifs.

    SignalP-NN euk predictions are as follows:

  # name                Cmax  pos ?  Ymax  pos ?  Smax  pos ?  Smean ?  D     ?

    SignalP output is explained at http://www.cbs.dtu.dk/services/SignalP/output.html

########################################################################################

Name:  contig005005-TilOR.S219  Length:  305
MAGNNSVNDVFFPRPVSYRVIIVEILVIIFLCINMLLIVIFVKKECFHTSARYILFFVTLLSDSVLLLMSDILFILTHFE      80
IAMPVCLCITISVVVLLYFIVTPVTLTAMTLERYVAICMPLRHGQLCSTRSTMYCILIIHGVSSGPCIIILSMFFASASL     160
NFYKQFMICTVDAFTLYRWQDHVRSAVYQFYFLIMGITIAYSYVQIMKVAKAASGEKKKLTHKGLKTVILHGFQLLLCLI     240
QLWCPFIEIAVFQIDFSLILNVRYFNYIMFNIAPRCLSPLIYGLRDENFFLALKNLMPTSSCSKX
...N............................................................................      80
................................................................................     160
................................................................................     240
.................................................................                    320

(Threshold=0.5)
----------------------------------------------------------------------
SeqName      Position  Potential   Jury    N-Glyc
                                 agreement result
----------------------------------------------------------------------
contig005005-TilOR.S219    4 NNSV   0.7314     (9/9)    ++
----------------------------------------------------------------------
```

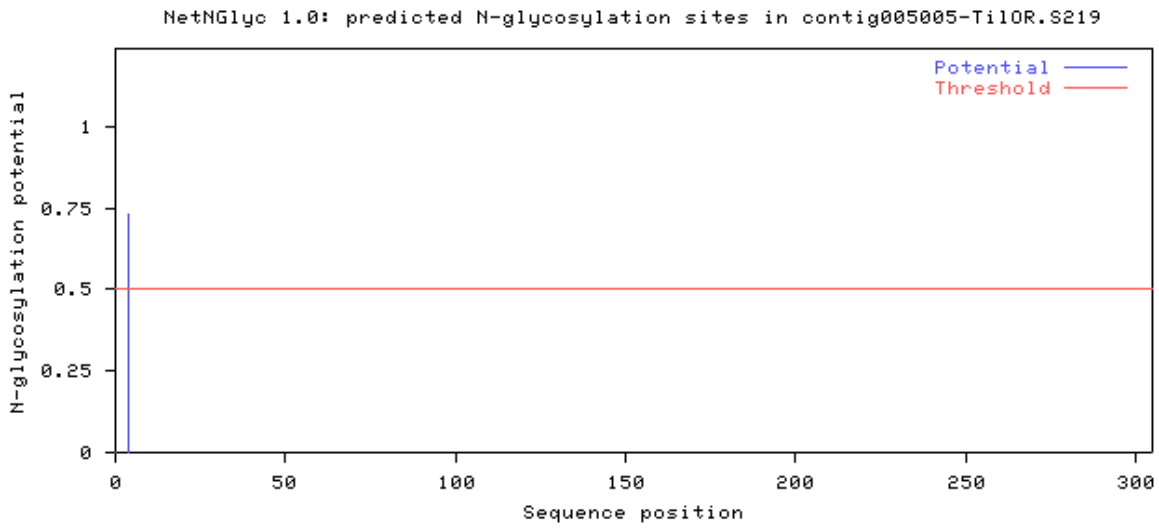

Graphics in PostScript

Output for 'contig005007-TilOR.S220'

```
##########################################################################################

    Warning: This sequence may not contain a signal peptide!!

    Proteins without signal peptides are unlikely to be exposed to
    the N-glycosylation machinery and thus may not be glycosylated
    (in vivo) even though they contain potential motifs.

    SignalP-NN euk predictions are as follows:

  # name                Cmax  pos ?  Ymax  pos ?  Smax  pos ?  Smean ?  D     ?

    SignalP output is explained at http://www.cbs.dtu.dk/services/SignalP/output.html

##########################################################################################

Name:  contig005007-TilOR.S220  Length:  305
MAGNNSVNDVFFPRPVSYRVIIVEILVIIFLCINMLLIVIFVKKECFHTSARYILFFVTLLSDSVLLLVSDILFILTHFE      80
IAMPVWLCITISVVVLLYFIVTPVTLTAMTLERYVAICMPLRHGQLCSTRSTMYCILIIHGVSSGPCIIILSMFFASGSL     160
KFYKQFMVCSVDAFSLYRWQDHVRSAVYQFYFLIMGITIAYSYVQIMKVAKAASGEKKKLTHKGRKTVILHAFQLLLCLI     240
QLWCPFIEIAVLQIDFSLILNVRYFNYIMFSIAPRCLSPLIYGLRDENFFLVLKNLMPTSSCSKX
...N............................................................................      80
................................................................................     160
................................................................................     240
.................................................................                    320

(Threshold=0.5)
----------------------------------------------------------------------
SeqName      Position  Potential   Jury    N-Glyc
                                agreement  result
----------------------------------------------------------------------
contig005007-TilOR.S220    4 NNSV   0.7313     (9/9)    ++
----------------------------------------------------------------------
```

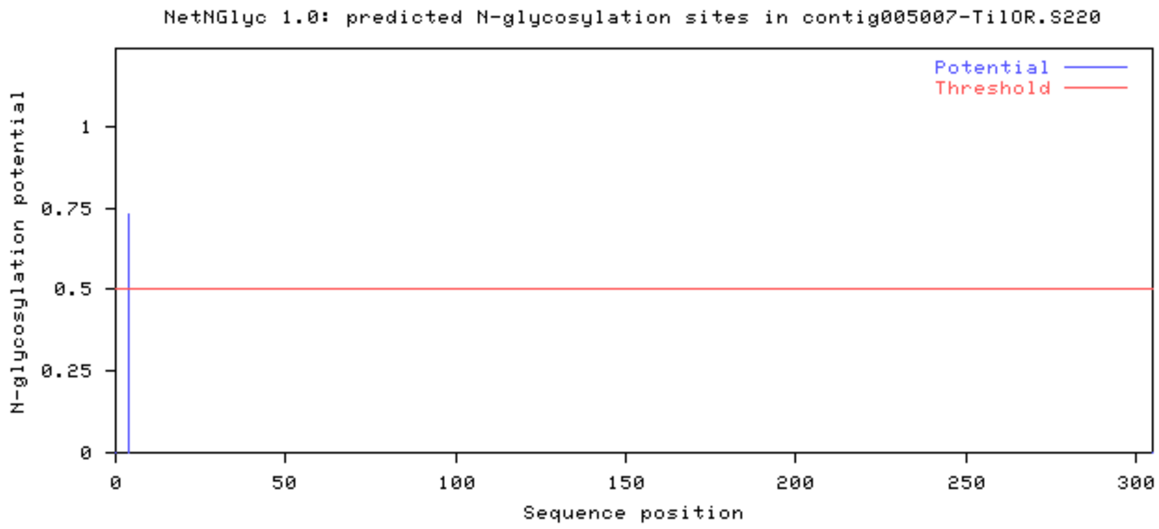

Graphics in PostScript

Output for 'contig009320-TilOR.B045'

```
##############################################################################

    Warning: This sequence may not contain a signal peptide!!

    Proteins without signal peptides are unlikely to be exposed to
    the N-glycosylation machinery and thus may not be glycosylated
    (in vivo) even though they contain potential motifs.

    SignalP-NN euk predictions are as follows:

  # name                    Cmax  pos ?  Ymax  pos ?  Smax  pos ?  Smean ?  D     ?

    SignalP output is explained at http://www.cbs.dtu.dk/services/SignalP/output.html

##############################################################################

Name:  contig009320-TilOR.B045  Length:  321
MSAGVNVTSLPILVTSVTLDGLAQLSNQRLFFFFFFLCAYLFMLCSDSLVVYVICSQRSLHRPMFVFVAAVLMNSVAGST      80
VFYPKLLVDLLRGGRSVQVTLRGCMCEAWLLYSSGTSSFLLLAAMSFDRYVSICRPLLYTVVMSPATVLALLLLCWLLPV     160
GLVGTAVLLASRLPLCRSQLSRIYCDIYSLVSLSCGGRETLLSEVYNLSVIVATVLLPAIFVLFSYSAVLSICLRRSRSF     240
SSKALSTCLPHLLVFCNYSVSSGVEVLQRRLQAGSQPTASVLTSILQVMIPTVFNPVVYGLKVTEIRAQLRRLLGCQRAD     320
X
.....N..........................................................................      80
................................................................................     160
..............................................N.................................     240
................N...............................................................     320
.                                                                                    400

(Threshold=0.5)
----------------------------------------------------------------------
SeqName      Position  Potential   Jury    N-Glyc
                                 agreement result
----------------------------------------------------------------------
contig009320-TilOR.B045    6 NVTS   0.7708     (9/9)   +++
contig009320-TilOR.B045  207 NLSV   0.6106     (7/9)   +
contig009320-TilOR.B045  257 NYSV   0.5469     (7/9)   +
----------------------------------------------------------------------
```

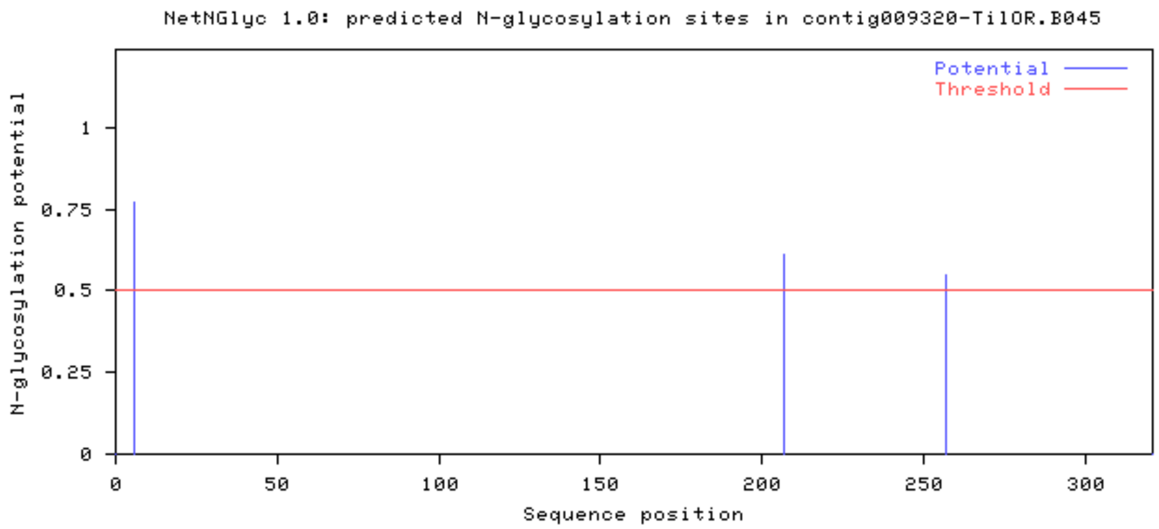

Graphics in PostScript

Output for 'contig009321-TilORe.B046'

```
##########################################################################################

    Warning: This sequence may not contain a signal peptide!!

    Proteins without signal peptides are unlikely to be exposed to
    the N-glycosylation machinery and thus may not be glycosylated
    (in vivo) even though they contain potential motifs.

    SignalP-NN euk predictions are as follows:

  # name                  Cmax  pos ?  Ymax  pos ?  Smax  pos ?  Smean ?  D     ?

    SignalP output is explained at http://www.cbs.dtu.dk/services/SignalP/output.html

##########################################################################################

Name:  contig009321-TilORe.B046         Length:  319
MSAGVNVTSLPILVTSVTLDGLAQLSNQRLFFFFFFLCAYLFMLCSDSLVVYVICSQRSLHRPMFVFVAAVLMNSVAGST      80
VFYPKLLVDLLRGGRSVQVTLRGCMCEAWLLYSSGTSSFLLLAAMSFDRYVSICRPLLYTVVMSPATVLALLLLCWLLPV     160
GLVGTAVLLASRLPLCRSQLSRIYCDIYSLVSLSCGGRETLLSEVYNLSVIVATVLLPAIFVLFSYSAVLSICLRRSRSF     240
SSKALSTCLPHLLVFCNYSVSSGVEVLQRRLQAGSQPTASVLTSILQVMIPTVFNPVVYGLKVTEIRAQLRRLLGCQRA
.....N..........................................................................      80
................................................................................     160
..............................................N.................................     240
................N..............................................................      320

(Threshold=0.5)
----------------------------------------------------------------------
SeqName      Position  Potential   Jury    N-Glyc
                                 agreement result
----------------------------------------------------------------------
contig009321-TilORe.B046    6 NVTS   0.7708     (9/9)   +++
contig009321-TilORe.B046  207 NLSV   0.6102     (7/9)   +
contig009321-TilORe.B046  257 NYSV   0.5464     (7/9)   +
----------------------------------------------------------------------
```

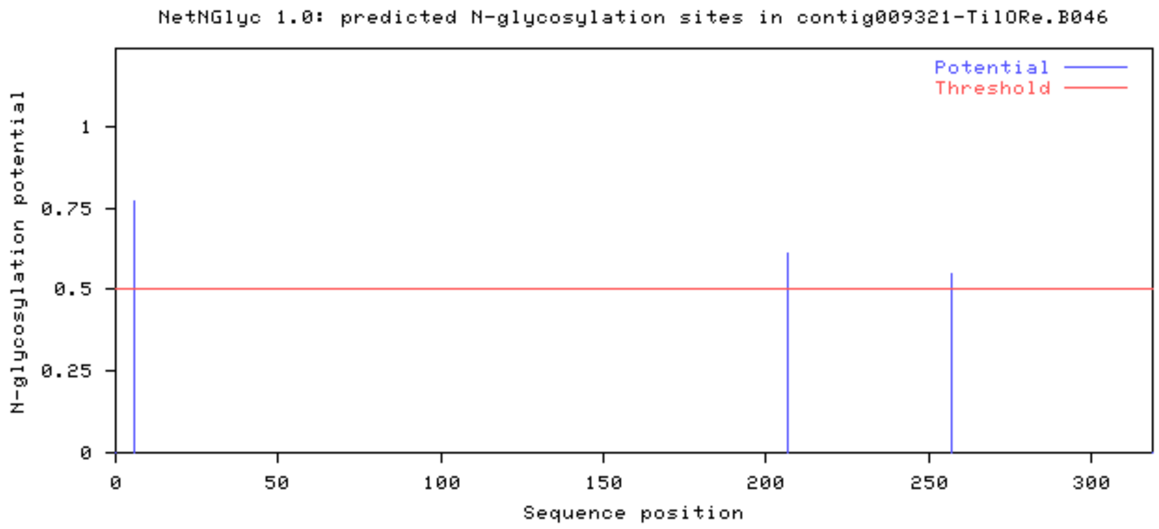

Graphics in PostScript

Output for 'contig009545-TilOR.F097'

```
##########################################################################################

    Warning: This sequence may not contain a signal peptide!!

    Proteins without signal peptides are unlikely to be exposed to
    the N-glycosylation machinery and thus may not be glycosylated
    (in vivo) even though they contain potential motifs.

    SignalP-NN euk predictions are as follows:

  # name                Cmax  pos ?  Ymax  pos ?  Smax  pos ?  Smean ?  D     ?

    SignalP output is explained at http://www.cbs.dtu.dk/services/SignalP/output.html

##########################################################################################

Name:  contig009545-TilOR.F097  Length:  321
MENNSYPLYFNLTMFVNIGKFRYPAFVLFLLLYTFIISANLVIIVVISREKTLHEPMYIFIMCLSINSLYGSGGFFFRFL      80
RDLLSDSNLIARSACFTQIYIIYTYASYELTILGIMAYDRFVAICQPLHYHSKLTSRVISKLLAFAWIYPAFSVAACVYL     160
ASRLPVCGNKIPKVFCANWPVVKLSCVPTVINNIIGMFVSITTVFLPLAFVLYTYMRIFLVCRKRSSVFKSKVIQSCLPH     240
IVTFINYSITVFCDVALSRIDLEELNPFLSIILSLEFVVIPPILNPLMYGLKLPEIRKCILRKLVCLIRYMSHDKENSVK     320
X
..N.......N.....................................................................      80
................................................................................     160
................................................................................     240
.....N..........................................................................     320
.                                                                                    400

(Threshold=0.5)
----------------------------------------------------------------------
SeqName      Position  Potential   Jury    N-Glyc
                                 agreement result
----------------------------------------------------------------------
contig009545-TilOR.F097     3 NNSY   0.5941     (8/9)   +
contig009545-TilOR.F097    11 NLTM   0.8107     (9/9)   +++
contig009545-TilOR.F097   246 NYSI   0.5032     (6/9)   +
----------------------------------------------------------------------
```

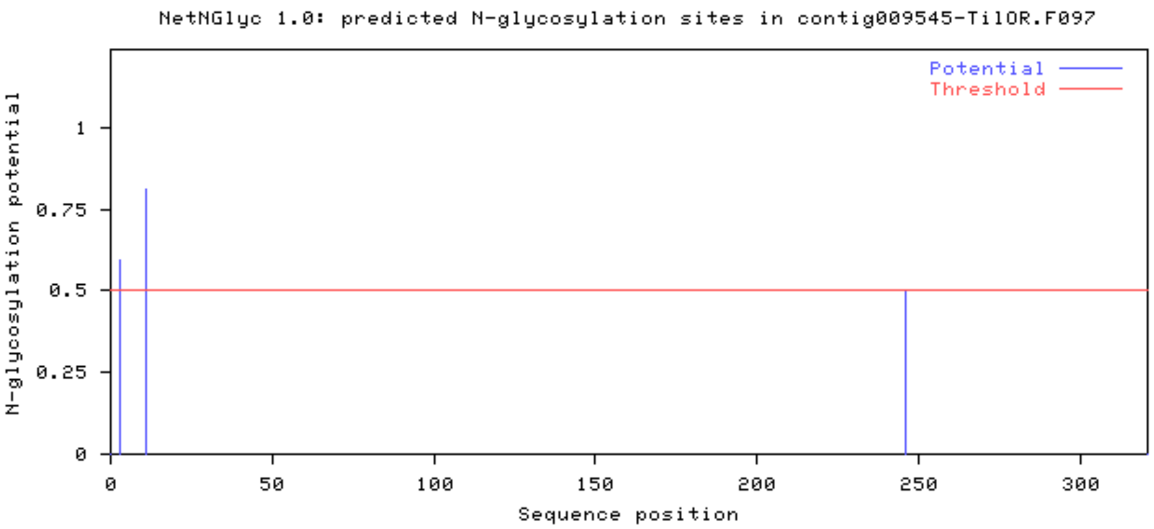

Graphics in PostScript

Output for 'contig009546-TilOR.H100'

```
##########################################################################################

    Warning: This sequence may not contain a signal peptide!!

    Proteins without signal peptides are unlikely to be exposed to
    the N-glycosylation machinery and thus may not be glycosylated
    (in vivo) even though they contain potential motifs.

    SignalP-NN euk predictions are as follows:

  # name                 Cmax  pos ?  Ymax  pos ?  Smax  pos ?  Smean ?  D     ?

    SignalP output is explained at http://www.cbs.dtu.dk/services/SignalP/output.html

##########################################################################################

Name:  contig009546-TilOR.H100  Length:  315
MNNVSVITMFFLSGLNETMNHRFVLFFLSLLCYCIIFLLNLALTVTIILDNNLHEPMYILLCVFCMNTLYGTAGFYPKFL      80
WDLLSPVHVISYYGCLIQALVIYSCGCSDLSILTLMAFDRYVAICQPLKYHSIMSKQRLIRLVCFSWLTPFSIIATNVFL     160
TTRVKLCSPYISRLFCVNWSIVQLACFPAQTKVNGIVANITIIIYVLHGALIVWSYIYLIKTCVNSIENRTKFMQTCVPH     240
LASLLTFVVAILTDVVNIRTDLKNLPQSIQNFVAIEFLIIPPIMNPLIYGFKLTKIRKSICVVIFKISKSSNFIX
..N............N................................................................      80
................................................................................     160
.................N....................N.............................N...........     240
...........................................................................          320

(Threshold=0.5)
----------------------------------------------------------------------
SeqName      Position  Potential   Jury    N-Glyc
                                 agreement result
----------------------------------------------------------------------
contig009546-TilOR.H100    3 NVSV   0.7556     (9/9)    +++
contig009546-TilOR.H100   16 NETM   0.7132     (9/9)    ++
contig009546-TilOR.H100  178 NWSI   0.6747     (8/9)    +
contig009546-TilOR.H100  199 NITI   0.6074     (8/9)    +
contig009546-TilOR.H100  229 NRTK   0.7000     (8/9)    +
----------------------------------------------------------------------
```

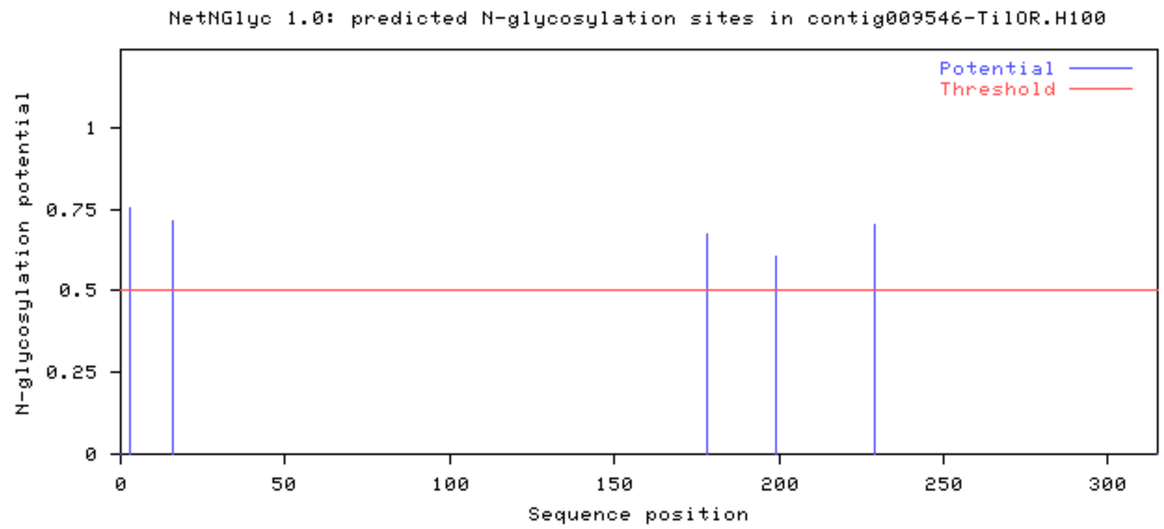

Graphics in PostScript

Output for 'contig009547-TilOR.H101'

```
##########################################################################################

    Warning: This sequence may not contain a signal peptide!!

    Proteins without signal peptides are unlikely to be exposed to
    the N-glycosylation machinery and thus may not be glycosylated
    (in vivo) even though they contain potential motifs.

    SignalP-NN euk predictions are as follows:

  # name                   Cmax  pos ?  Ymax  pos ?  Smax  pos ?  Smean ?  D     ?

    SignalP output is explained at http://www.cbs.dtu.dk/services/SignalP/output.html

##########################################################################################

Name:  contig009547-TilOR.H101  Length:  325
MNNVSVITMFFLSGLNETVNHRFVLFFLSLLCYCIIFLLNLALTVTIILDKNLHEPMYILLCVFCMNALYGTAGFYPKFL      80
WDLLSPVHVISYYDCLIQTHVVYSFACIDVSILTLMAFDRYVAICQPLKYHSFMSKQRVIKVACFSWFTPFCIIAVNIFL     160
TSRLKLCSSYISRLFCVNSVIVTLACSRAETIINNIAAYITITVYVFHGLFIVWSYVYLIKTCVKSTEKRAKFMQTCVPH     240
LLSLLTFLVTILFDVMNIRLGSIVLPQTLQNFVTIEFLVIPPIMNPLIYGFKLTKIKKRICTFITFRISVTPAYAGKELL     320
SFRSX
..N............N................................................................      80
................................................................................     160
................................................................................     240
................................................................................     320
.....                                                                                400

(Threshold=0.5)
----------------------------------------------------------------------
SeqName      Position  Potential   Jury    N-Glyc
                                 agreement result
----------------------------------------------------------------------
contig009547-TilOR.H101    3 NVSV   0.7559     (9/9)    +++
contig009547-TilOR.H101   16 NETV   0.7614     (9/9)    +++
----------------------------------------------------------------------
```

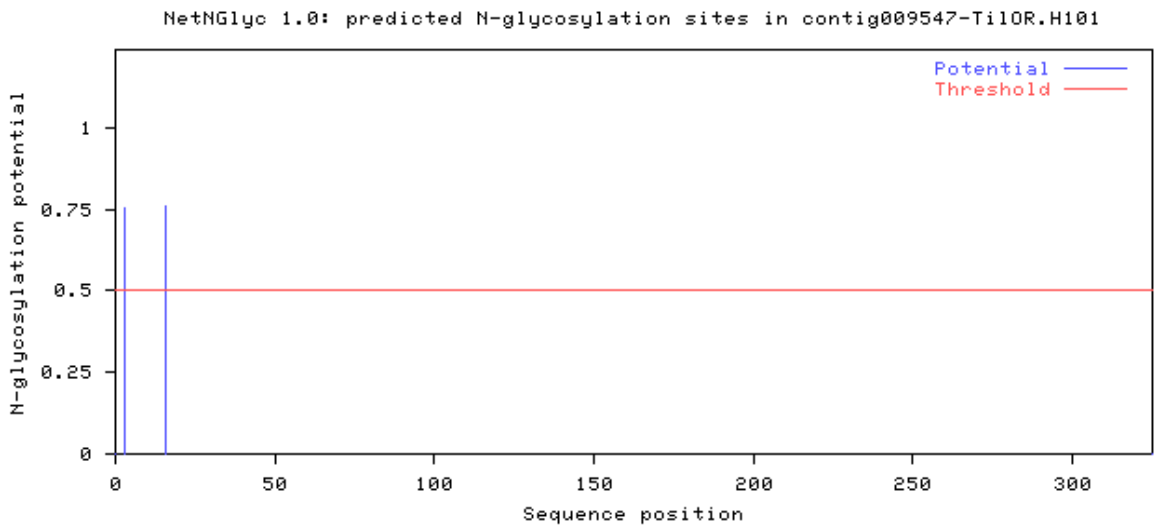

Graphics in PostScript

Output for 'contig009548-TilOR.H102'

```
##########################################################################################

    Warning: This sequence may not contain a signal peptide!!

    Proteins without signal peptides are unlikely to be exposed to
    the N-glycosylation machinery and thus may not be glycosylated
    (in vivo) even though they contain potential motifs.

    SignalP-NN euk predictions are as follows:

  # name                  Cmax  pos ?  Ymax  pos ?  Smax  pos ?  Smean ?  D     ?

    SignalP output is explained at http://www.cbs.dtu.dk/services/SignalP/output.html

##########################################################################################

Name:  contig009548-TilOR.H102  Length:  310
MNNVSVITMFFLSGLNETVNHRFVLFFLSLLCYCIIFLLNLALTVTIILDKNLHEPMYILLCVFCMNALYGTAGFYPKFL      80
WDLLSPVHVISYYDCLIQTHVVYSFACIDVSILTLMAFDRYVAICQPLKYHSFMSKQRVIKVACFSWFTPFCIIAVNIFL     160
TSRLKLCSSYISRLFCVNSVIVTLACSRAETIINNIAAYITITVYVFHGLFIVWSYVYLIKTCVKSTEKRAKFMQTCVPH     240
LLSLLTFLVTILFDVMNIRLGSIVLPQTLQNFVTIEFLVIPPIMNPLIYGFKLTKIKKRICTVMFFIFRX
..N............N................................................................      80
................................................................................     160
................................................................................     240
......................................................................               320

(Threshold=0.5)
----------------------------------------------------------------------
SeqName      Position  Potential   Jury    N-Glyc
                                 agreement result
----------------------------------------------------------------------
contig009548-TilOR.H102    3 NVSV   0.7560     (9/9)    +++
contig009548-TilOR.H102   16 NETV   0.7613     (9/9)    +++
----------------------------------------------------------------------
```

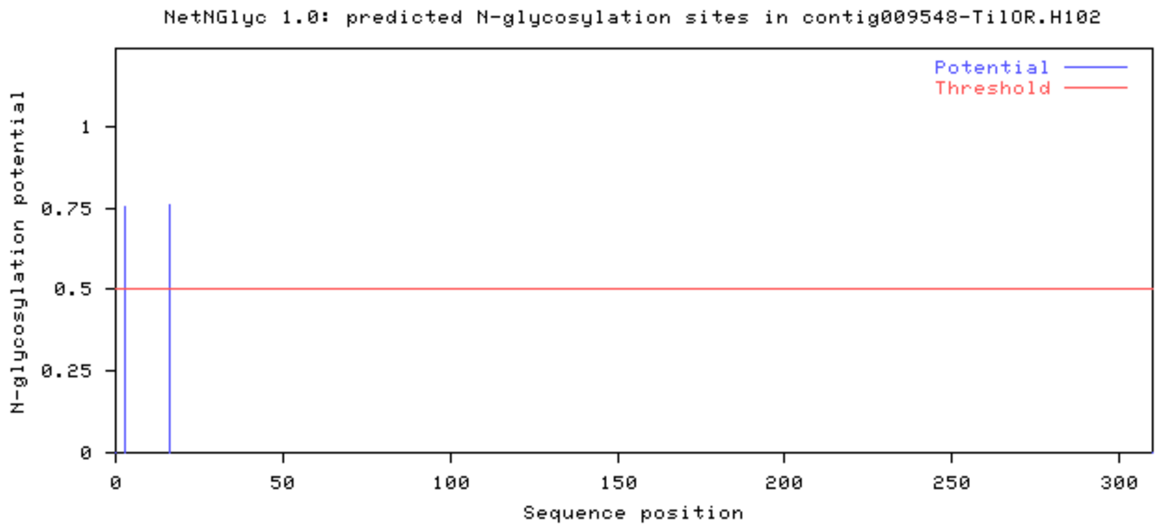

Graphics in PostScript

Output for 'contig009549-TilORe.H264'

```
##########################################################################################

    Warning: This sequence may not contain a signal peptide!!

    Proteins without signal peptides are unlikely to be exposed to
    the N-glycosylation machinery and thus may not be glycosylated
    (in vivo) even though they contain potential motifs.

    SignalP-NN euk predictions are as follows:

  # name                 Cmax  pos ?  Ymax  pos ?  Smax  pos ?  Smean ?  D     ?

    SignalP output is explained at http://www.cbs.dtu.dk/services/SignalP/output.html

##########################################################################################

Name:  contig009549-TilORe.H264         Length:  166
FLTSRLKLCSPYISRLFCVNWSIVQLACFPAQTTINAIVANITISIYFLHGVFIVWSYMYIIQTCVKSIENRAKFMQTCV      80
PHLVSLFTFVVAILTDLINMRLGSKELPRTLQNFIAIEFLVIPPIMNPLIYGFKLTKIQKRIYSLGILKRTNFCFIRSDN     160
SFTHSX
...................N.....................N......................................      80
................................................................................     160
......                                                                               240

(Threshold=0.5)
----------------------------------------------------------------------
SeqName      Position  Potential   Jury    N-Glyc
                                 agreement result
----------------------------------------------------------------------
contig009549-TilORe.H264   20 NWSI   0.7390     (9/9)   ++
contig009549-TilORe.H264   41 NITI   0.6713     (9/9)   ++
----------------------------------------------------------------------
```

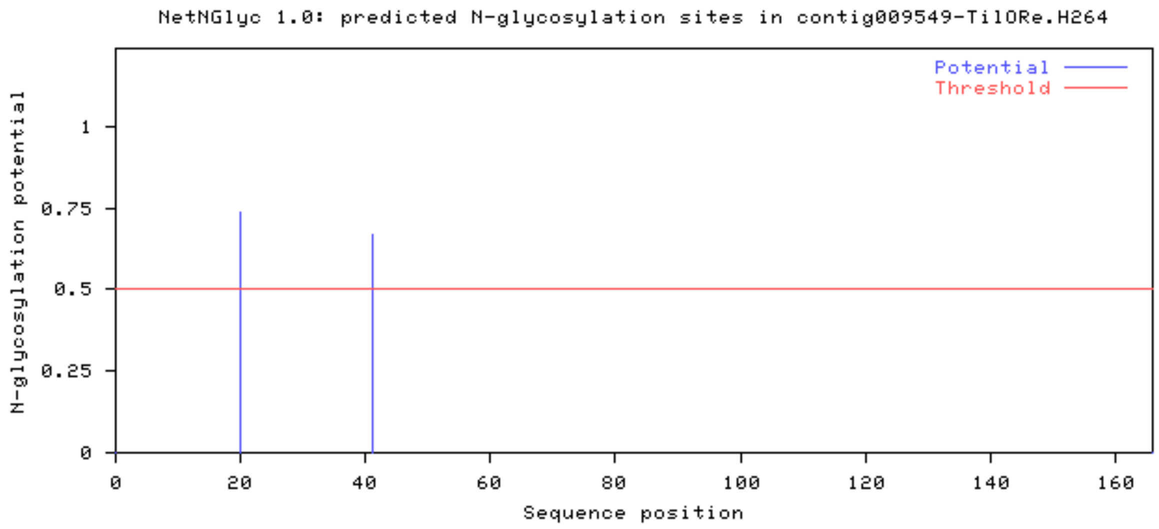

Graphics in PostScript

Output for 'contig009557-TilORe.H120'

```
##########################################################################

    Warning: This sequence may not contain a signal peptide!!

    Proteins without signal peptides are unlikely to be exposed to
    the N-glycosylation machinery and thus may not be glycosylated
    (in vivo) even though they contain potential motifs.

    SignalP-NN euk predictions are as follows:

  # name                 Cmax  pos ?  Ymax  pos ?  Smax  pos ?  Smean ?  D     ?

    SignalP output is explained at http://www.cbs.dtu.dk/services/SignalP/output.html

##########################################################################

Name:  contig009557-TilORe.H120        Length:  54
NMRLGSKELPRTLQNFAAIEFLVIPPIMNPLIYGFKLNKIRKRIYSVVISKTKX
......................................................                          80

(Threshold=0.5)

No sites predicted in this sequence.
```

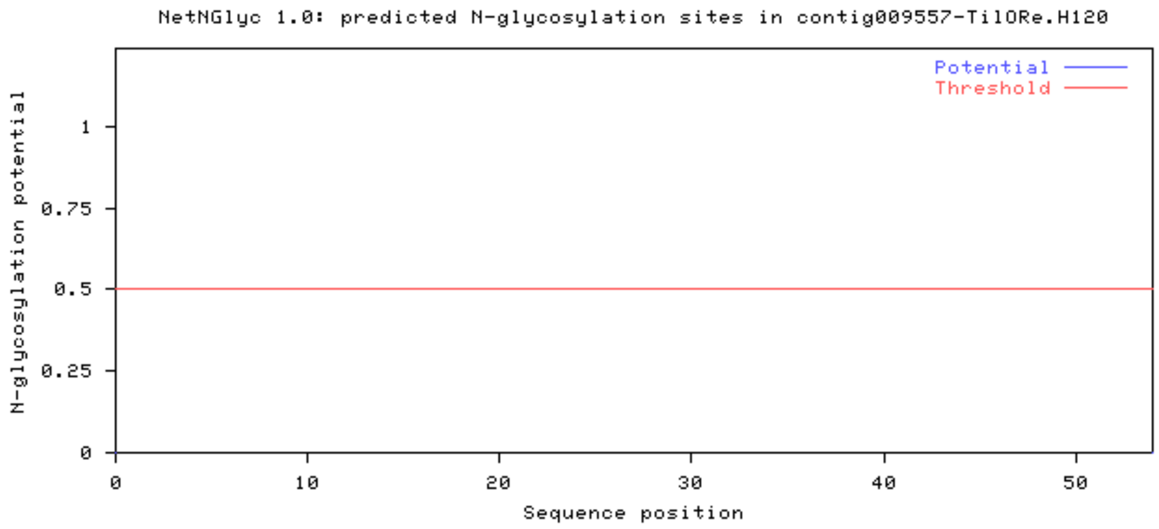

Graphics in PostScript

Output for 'contig009560-TilORe.H265'

```
##########################################################################

    Warning: This sequence may not contain a signal peptide!!

    Proteins without signal peptides are unlikely to be exposed to
    the N-glycosylation machinery and thus may not be glycosylated
    (in vivo) even though they contain potential motifs.

    SignalP-NN euk predictions are as follows:

  # name                 Cmax   pos ?  Ymax   pos ?  Smax   pos ?  Smean ?  D     ?

    SignalP output is explained at http://www.cbs.dtu.dk/services/SignalP/output.html

##########################################################################

Name:  contig009560-TilORe.H265         Length:  173
RVIRFACFSWFTTFCILAVNVFLTSRLKLCSPYISRLFCVNWSIVQLACFPAQTTINAIVANITISIYFLHGVFIVWSYM      80
YIIQTCVKSIENRAKFMQTCVPHLVSLFTFLVTILTDIINMRLGSKELPRTLQNFAAIEFLVIPPIMNPLMYGFKLNKLR     160
KRIYSVVISKTKX
........................................N....................N..................      80
................................................................................     160
.............                                                                        240

(Threshold=0.5)
----------------------------------------------------------------------
SeqName      Position  Potential   Jury    N-Glyc
                                 agreement result
----------------------------------------------------------------------
contig009560-TilORe.H265    41 NWSI   0.7243     (9/9)   ++
contig009560-TilORe.H265    62 NITI   0.6543     (9/9)   ++
----------------------------------------------------------------------
```

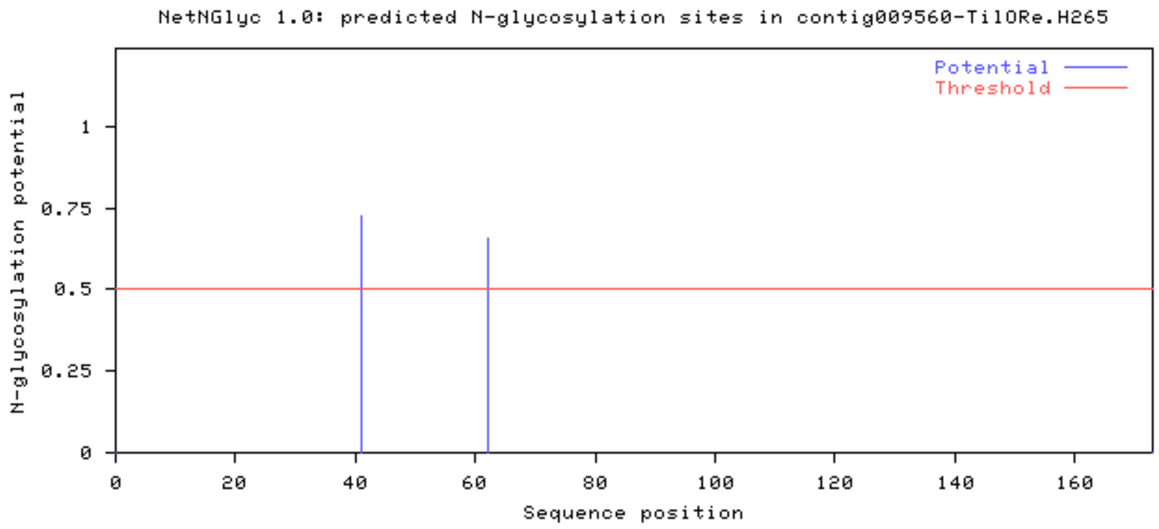

Graphics in PostScript

Output for 'contig009565-TilOR.H126'

```
##############################################################################

    Warning: This sequence may not contain a signal peptide!!

    Proteins without signal peptides are unlikely to be exposed to
    the N-glycosylation machinery and thus may not be glycosylated
    (in vivo) even though they contain potential motifs.

    SignalP-NN euk predictions are as follows:

  # name                    Cmax  pos ?  Ymax  pos ?  Smax  pos ?  Smean ?  D     ?

    SignalP output is explained at http://www.cbs.dtu.dk/services/SignalP/output.html

##############################################################################

Name:  contig009565-TilOR.H126  Length:  280
MLCYCIICLVNVSLIVIIIILDTNLHESMYILLCVFCMNALYGTAGFFPRFLWDLLSDVHLISYYGCLIQTQVVYSSACSE      80
LSILALMAYDRYVAICQPLKYHSIMSKERVIRFACFSWSTTFCIMAVNVFLTSRLKLCGMYISRLFCGNSSIVQLACFPA     160
QTIVNGIVANITIIIYALHGGFIVWSYMYIIQKCVKSIENRAKFMQTCVPHLVSLHTFVVIMLFDFMSMRFGSKVLPQVL     240
QNFIAIEFLVIPPVMNPLMYGFKLTKIQKKVFVVILKTKX
..........N.....................................................................      80
....................................................................N...........     160
.........N......................................................................     240
........................................                                             320

(Threshold=0.5)
----------------------------------------------------------------------
SeqName      Position  Potential   Jury    N-Glyc
                                 agreement result
----------------------------------------------------------------------
contig009565-TilOR.H126   11 NVSL   0.7060     (9/9)   ++
contig009565-TilOR.H126  149 NSSI   0.7029     (9/9)   ++
contig009565-TilOR.H126  170 NITI   0.6119     (8/9)   +
----------------------------------------------------------------------
```

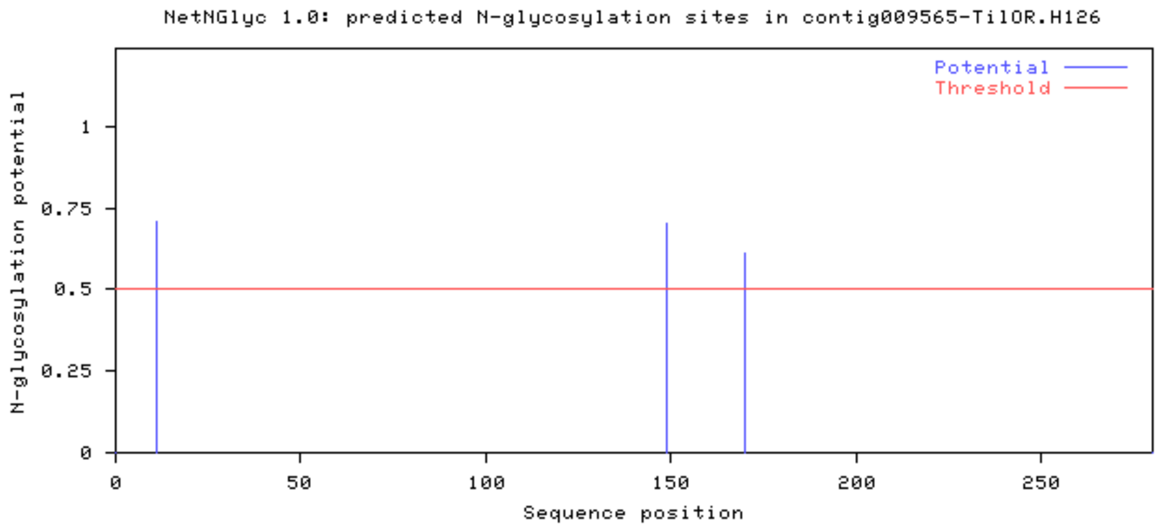

Graphics in PostScript

Output for 'contig013321-TilOR.D048'

```
##############################################################################

    Warning: This sequence may not contain a signal peptide!!

    Proteins without signal peptides are unlikely to be exposed to
    the N-glycosylation machinery and thus may not be glycosylated
    (in vivo) even though they contain potential motifs.

    SignalP-NN euk predictions are as follows:

  # name                Cmax  pos ?  Ymax  pos ?  Smax  pos ?  Smean ?  D     ?

    SignalP output is explained at http://www.cbs.dtu.dk/services/SignalP/output.html

##############################################################################

Name:  contig013321-TilOR.D048  Length:  314
MGNSSKIVSFVLAAYENVGEFKYLYFVIILFWYVSICVANTVLIVVIHVDKRLHEPMYILLCNLCVNEINVSTSLYPLLL      80
SQMFSDSHEVTLPWCFLHMSCMYTSGPAEFCSLAAMAYDRYISICHPLHYNVIMKTKRVFVMILFVWIYSFLSVILSFSF     160
IFSLKFCGNNIENVYCNHRLIIRLSCSLSVHSFISDIFFLIVSIFIPFTLISVSYVKILAVCQKTSTENKQKAVTTCAPQ     240
IVSVSNLFVGSIFHSIDSSFIVTQVPNEVRIILPMYMLIFQPMLTPFLYGFKLPKIRHSCKRLLFKRKSVALFX
..N.............................................................................      80
................................................................................     160
................................................................................     240
..........................................................................           320

(Threshold=0.5)
----------------------------------------------------------------------
SeqName      Position  Potential   Jury    N-Glyc
                                 agreement result
----------------------------------------------------------------------
contig013321-TilOR.D048    3 NSSK   0.7542     (9/9)    +++
contig013321-TilOR.D048   70 NVST   0.4906     (5/9)    -
----------------------------------------------------------------------
```

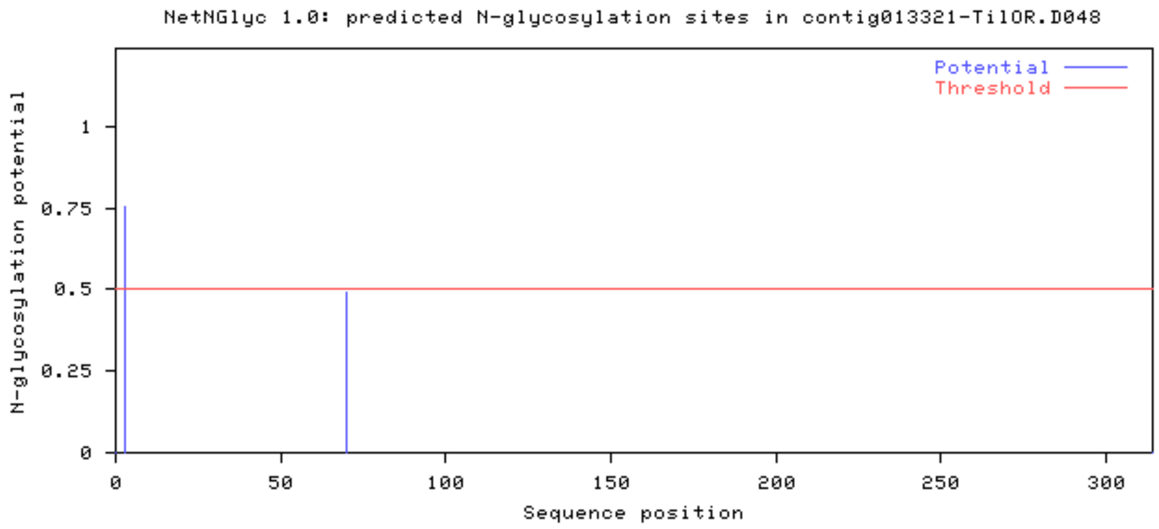

Graphics in PostScript

Output for 'contig013322-TilOR.D049'

```
########################################################################################

    Warning: This sequence may not contain a signal peptide!!

    Proteins without signal peptides are unlikely to be exposed to
    the N-glycosylation machinery and thus may not be glycosylated
    (in vivo) even though they contain potential motifs.

    SignalP-NN euk predictions are as follows:

  # name                    Cmax   pos ?  Ymax   pos ?  Smax   pos ?  Smean ?  D     ?

    SignalP output is explained at http://www.cbs.dtu.dk/services/SignalP/output.html

########################################################################################

Name:  contig013322-TilOR.D049  Length:  309
MENSSEIVSFVLTAYGNVGDLKYLYFVIILFWYLFICVANTVLIVVIRVDRRLHEPMYILLCNLCVNEINISTSLYPLLL      80
SQMFSDSHEVTLPWCFLQMCCMYTSAPAEFCSLAAMAYDRYISVCHPLHYNVIMNTERVFFMILLVWIYSFLSVILSFSF     160
IFSLKFCGNNIENVYCDHQFLIGLSCSLSVHSFIADIFFVFVSIFIPFNLISVSYVKILAICRKTSTENKQKAVTTCTPQ     240
IVSVSNLFVGCIFQSIDSSVIVAQLPHEVNIILSIYLFICQPMLTPFLYGFNLPKIRQSCKRFLFKKKX
..N.............................................................................      80
................................................................................     160
................................................................................     240
.....................................................................                320

(Threshold=0.5)
----------------------------------------------------------------------
SeqName      Position  Potential   Jury    N-Glyc
                                 agreement result
----------------------------------------------------------------------
contig013322-TilOR.D049    3 NSSE   0.6996     (9/9)   ++
contig013322-TilOR.D049   70 NIST   0.4518     (5/9)   -
----------------------------------------------------------------------
```

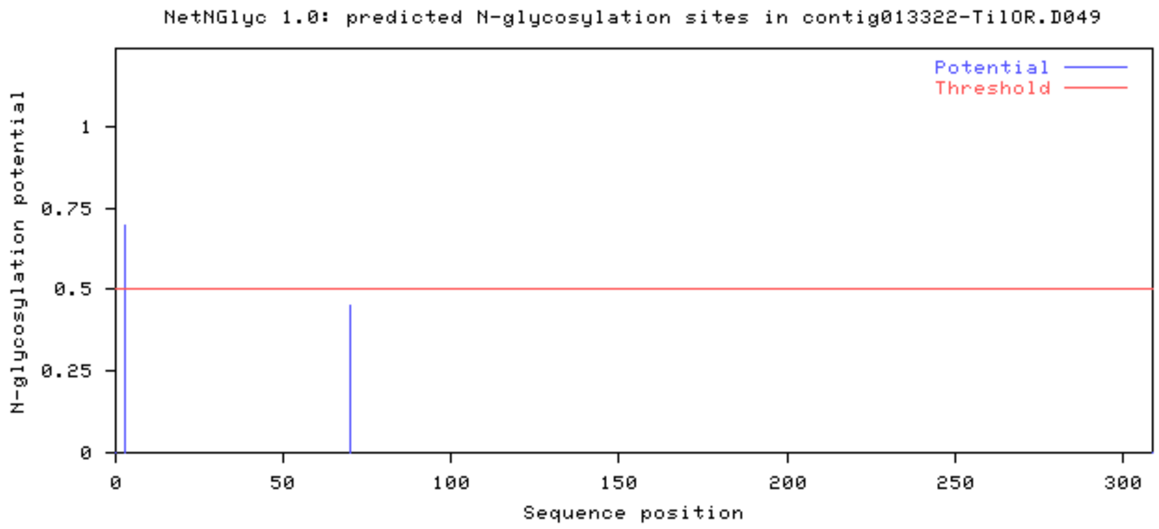

Graphics in PostScript

Output for 'contig013323-TilOR.D050'

```
##########################################################################

   Warning: This sequence may not contain a signal peptide!!

   Proteins without signal peptides are unlikely to be exposed to
   the N-glycosylation machinery and thus may not be glycosylated
   (in vivo) even though they contain potential motifs.

   SignalP-NN euk predictions are as follows:

 # name                 Cmax  pos ?  Ymax  pos ?  Smax  pos ?  Smean ?  D     ?

   SignalP output is explained at http://www.cbs.dtu.dk/services/SignalP/output.html

##########################################################################

Name:  contig013323-TilOR.D050  Length:  311
MGNSSEIEVVSFVLSAYANIGALKYMYFVIMLFWYLSICVANTVVIVVIHVDRRLHEPMYILLCSLCVNEINSSTSLYPL      80
LLSQMFSDSHEVTLPWCFLQMCCMYISAPSEFWGLAAMAYDRYISICHPLRYNVIMNTKRVFLLILLVWIFSLVSFMFSF     160
SFIFSLKFCRNIIDNVYCDHQLMFKLSCSVSTQSYISEIFFAIVSIFIPFTLISVSYFKILAVCRKTSIENKQKAVTTCT     240
PQIVSVSNLFVGCIFHSIDFRLLIARVPDEVRIILPMYILICQPMLTPFLYGFNLPKIRHSFKRLLFNRKX
..N.............................................................................      80
................................................................................     160
................................................................................     240
.......................................................................              320

(Threshold=0.5)
----------------------------------------------------------------------
SeqName      Position  Potential   Jury    N-Glyc
                                 agreement result
----------------------------------------------------------------------
contig013323-TilOR.D050    3 NSSE   0.7487     (9/9)    ++
contig013323-TilOR.D050   72 NSST   0.3689     (8/9)    -
----------------------------------------------------------------------
```

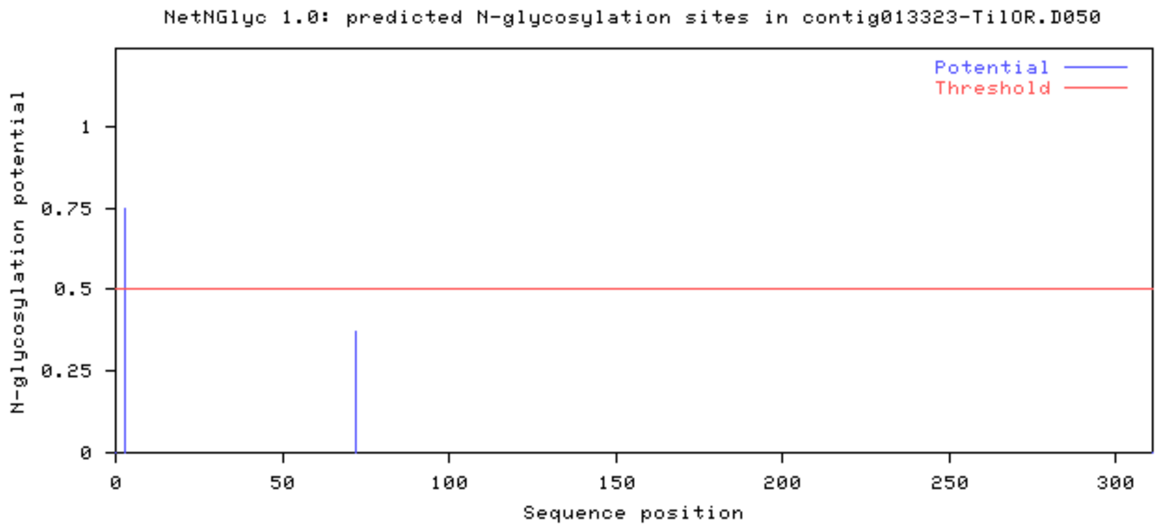

Graphics in PostScript

Output for 'contig013324-TilORe.D059'

```
##########################################################################################

    Warning: This sequence may not contain a signal peptide!!

    Proteins without signal peptides are unlikely to be exposed to
    the N-glycosylation machinery and thus may not be glycosylated
    (in vivo) even though they contain potential motifs.

    SignalP-NN euk predictions are as follows:

  # name                Cmax  pos ?  Ymax  pos ?  Smax  pos ?  Smean ?  D     ?

    SignalP output is explained at http://www.cbs.dtu.dk/services/SignalP/output.html

##########################################################################################

Name:  contig013324-TilORe.D059        Length:  239
ASTSLYPLLLSQMFSDSHEVTLPWCFLQMCCLYTSAPAEFWSLAAMAYDRYISICHPLHYNVIMNTERVLKIILLVWVFS      80
FLIFILSFSFIFSLQFCGNTVDNVYCEHQLIIRLSCSVSVQSSISIIIFVIMSIFIPFSLISVSYVKILTVCRKTSTENK     160
QKAMTTCTPQIVSVSNLFVGCIFHSINFRLLISQVPDEVNIILPMYMLICQPMLTPFMYGFNLPKIRQSCKRFLFKRKX
................................................................................      80
................................................................................     160
...............................................................................      240

(Threshold=0.5)

No sites predicted in this sequence.
```

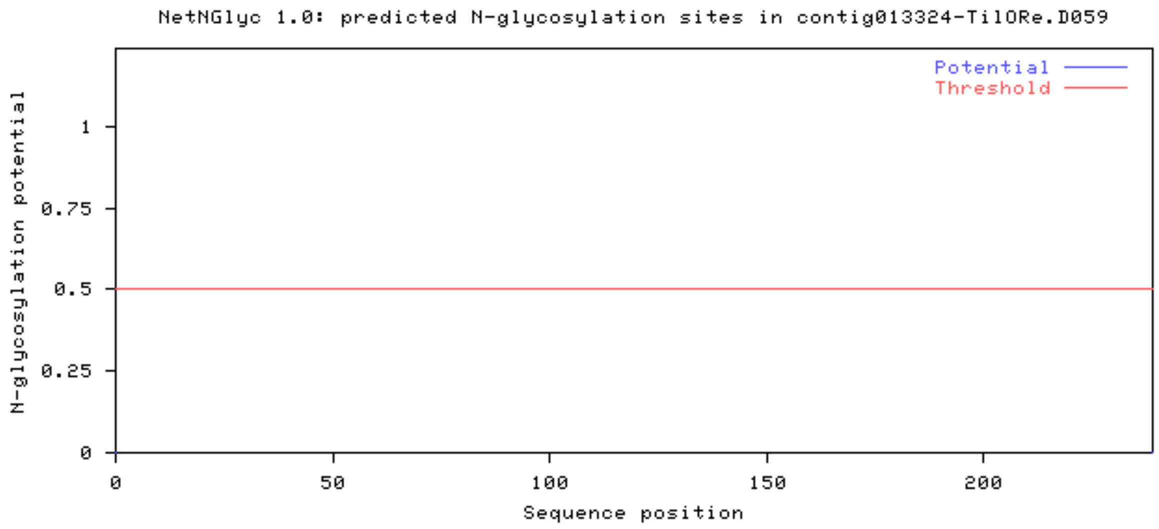

Graphics in PostScript

Output for 'contig013325-TilORe.D067'

```
########################################################################################

    Warning: This sequence may not contain a signal peptide!!

    Proteins without signal peptides are unlikely to be exposed to
    the N-glycosylation machinery and thus may not be glycosylated
    (in vivo) even though they contain potential motifs.

    SignalP-NN euk predictions are as follows:

  # name                Cmax  pos ?  Ymax  pos ?  Smax  pos ?  Smean ?  D     ?

    SignalP output is explained at http://www.cbs.dtu.dk/services/SignalP/output.html

########################################################################################

Name:  contig013325-TilORe.D067         Length:  305
SEIVSFVLAAYGNVGDFKYLYFIIILFWYVSICVANTVLIVVIHVDRRLHEPMYILLSNLCVNEIKAGTSLYPLLLSQMF      80
SDSHEVTLPWCFLQMCSLYTGAPAEFWSLAAMAYDRYISICHPLSYNVIMNTERVFKIILLVWVFSFVIFILSFSFIFSL     160
KFCGNNIENVYCEHQLIIKLSCSGSAQNSISIIIFVIMSIFIPFSLISVSYVKILTVCRKTSTENKQKAMTTCTPQIVSV     240
SNLFVGCIFHSINFRLLISQVPDEVNIILPMYMLICQPILTPFMYGFNLPKIRQSCKRFLFKRKX
................................................................................      80
................................................................................     160
................................................................................     240
.................................................................                    320

(Threshold=0.5)

No sites predicted in this sequence.
```

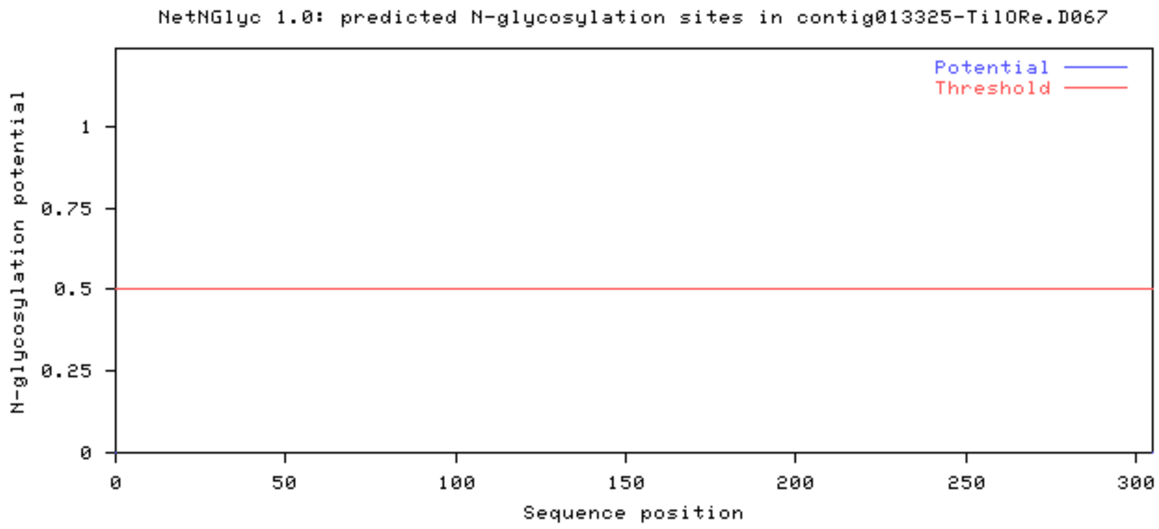

Graphics in PostScript

Output for 'contig013327-TilOR.D052'

```
##############################################################################

    Warning: This sequence may not contain a signal peptide!!

    Proteins without signal peptides are unlikely to be exposed to
    the N-glycosylation machinery and thus may not be glycosylated
    (in vivo) even though they contain potential motifs.

    SignalP-NN euk predictions are as follows:

  # name                    Cmax   pos ?  Ymax   pos ?  Smax   pos ?  Smean ?  D     ?

    SignalP output is explained at http://www.cbs.dtu.dk/services/SignalP/output.html

##############################################################################

Name:  contig013327-TilOR.D052  Length:  309
MENSSEIVSFVLAAFGNVGELKYLYFVIILFWYISICVANTVLIVVIHVDRRLHEPMYILLCNLCVNEINASTSLYPLLL      80
SQMFSDSHEVAVPWCFLQMCCLYTSASVELCSLAAMAYDRYISICHPLRYNVIMNTERVFLMILLVWVYSFLSFIFSFSF     160
IFSLKFCGNVIHNTYCDHQLIIRLSCSVSIQSFISDISFATVSVFIPFSFILVSYMKILTVCLKTSKENKQKAVTTCTPQ     240
IISVSNLFVGCIFYFIDFRFVVSQVPDEVHIILPMYVLIFQPMLTPFMYGFNLPKIRQSYQRFLLKRKX
..N.............................................................................      80
................................................................................     160
................................................................................     240
.....................................................................                320

(Threshold=0.5)
----------------------------------------------------------------------
SeqName      Position  Potential    Jury    N-Glyc
                                 agreement  result
----------------------------------------------------------------------
contig013327-TilOR.D052    3 NSSE   0.6996     (9/9)    ++
contig013327-TilOR.D052   70 NAST   0.3701     (6/9)    -
----------------------------------------------------------------------
```

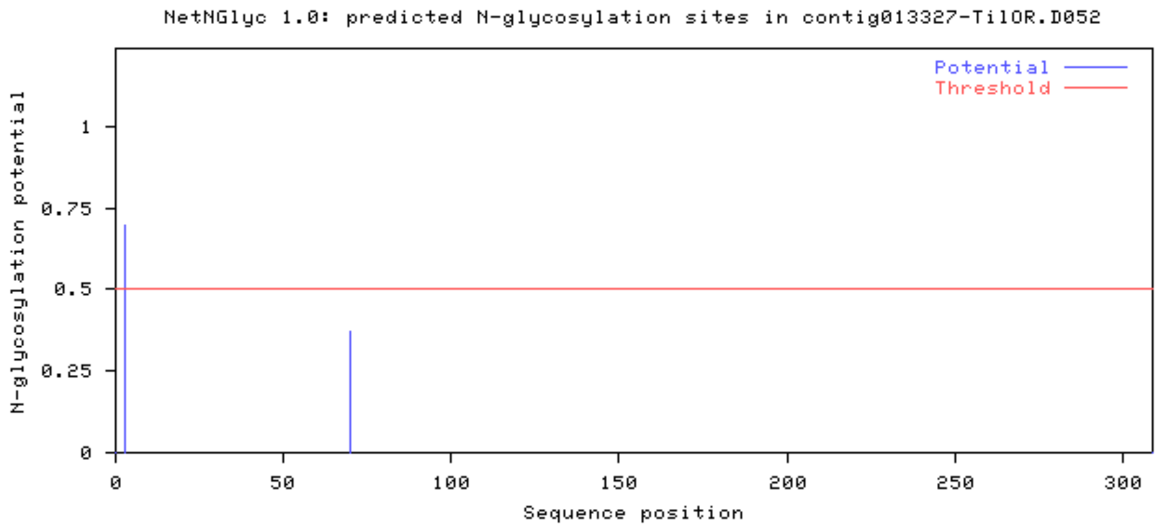

Graphics in PostScript

Output for 'contig013330-TilOR.D053'

```
##########################################################################################

    Warning: This sequence may not contain a signal peptide!!

    Proteins without signal peptides are unlikely to be exposed to
    the N-glycosylation machinery and thus may not be glycosylated
    (in vivo) even though they contain potential motifs.

    SignalP-NN euk predictions are as follows:

  # name                    Cmax   pos ?  Ymax   pos ?  Smax   pos ?  Smean ?  D     ?

    SignalP output is explained at http://www.cbs.dtu.dk/services/SignalP/output.html

##########################################################################################

Name:  contig013330-TilOR.D053  Length:  309
MENSSEIVSFVLSAYGNVGDLKYLYFIIILFWYLSICVANTFLIVVIRVDRRLHEPMYILLSNLCMNEINASTSLYPLLL      80
SQMFSDNHEVTRLWCFLQMCSLYTGAPAEFWSLAAMAYDRYISICHPLRYNVIMNTDRVFLLILLVWVFSFLIFILSFSF     160
IFRLKFCGNIVDNVYCEHQLIIRLSCSVSIYNSISIIFFVIMSIFIPFSLISVSYVKILKVCRKTSKENKQKAVTTCTPQ     240
IVSLSNLFVGCICYSIDFRFLVSLVPDEVRTILAIYLLICQPMLTPFLYGFNLPKIRQSCKSFLFLRKX
..N.............................................................................      80
................................................................................     160
................................................................................     240
.....................................................................                320

(Threshold=0.5)
----------------------------------------------------------------------
SeqName      Position  Potential   Jury    N-Glyc
                                agreement  result
----------------------------------------------------------------------
contig013330-TilOR.D053    3 NSSE   0.6952     (9/9)    ++
contig013330-TilOR.D053   70 NAST   0.4284     (6/9)    -
----------------------------------------------------------------------
```

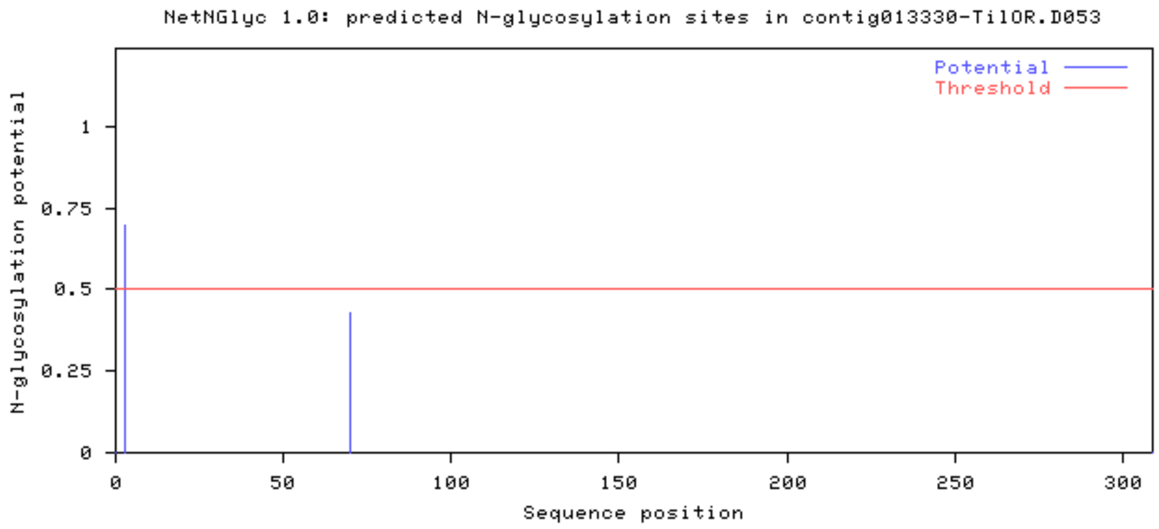

Graphics in PostScript

Output for 'contig013330-TilOR.D054'

```
##########################################################################

    Warning: This sequence may not contain a signal peptide!!

    Proteins without signal peptides are unlikely to be exposed to
    the N-glycosylation machinery and thus may not be glycosylated
    (in vivo) even though they contain potential motifs.

    SignalP-NN euk predictions are as follows:

  # name                 Cmax  pos ?  Ymax  pos ?  Smax  pos ?  Smean ?  D     ?

    SignalP output is explained at http://www.cbs.dtu.dk/services/SignalP/output.html

##########################################################################

Name:  contig013330-TilOR.D054  Length:  316
MGNSSETVSFVLAAYGNVGELKHLYFIIILVWYFSICVANTVLIVIIRVDRRLHEPMYILLCNLCVSEINGSTSLYPLLL      80
SQMFSDSHEVTLSWCFLQMCCLYTSASVELCSLAAMAYDRYISICHPFTYNVIMNTERVFLMILLVWVFSFLSFIFSYSF     160
IFSLKFCGNIIHSVYCDHQLIIRLSCSVSIQSFISDISFAIVSFFIPFSLTLVSYLKILRVCRKTSKENKQKAVTTCTPQ     240
IISVSNLCVGCIFNLIDFRFVVSQVPDEVRIILPMYALIFQPMLTPFMYGFNLPKIRQSYQRFSVKEKINILVQIX
..N...................................................................N.........      80
................................................................................     160
................................................................................     240
............................................................................         320

(Threshold=0.5)
----------------------------------------------------------------------
SeqName      Position  Potential   Jury    N-Glyc
                                 agreement result
----------------------------------------------------------------------
contig013330-TilOR.D054    3 NSSE   0.7404     (9/9)   ++
contig013330-TilOR.D054   70 NGST   0.5177     (4/9)   +
----------------------------------------------------------------------
```

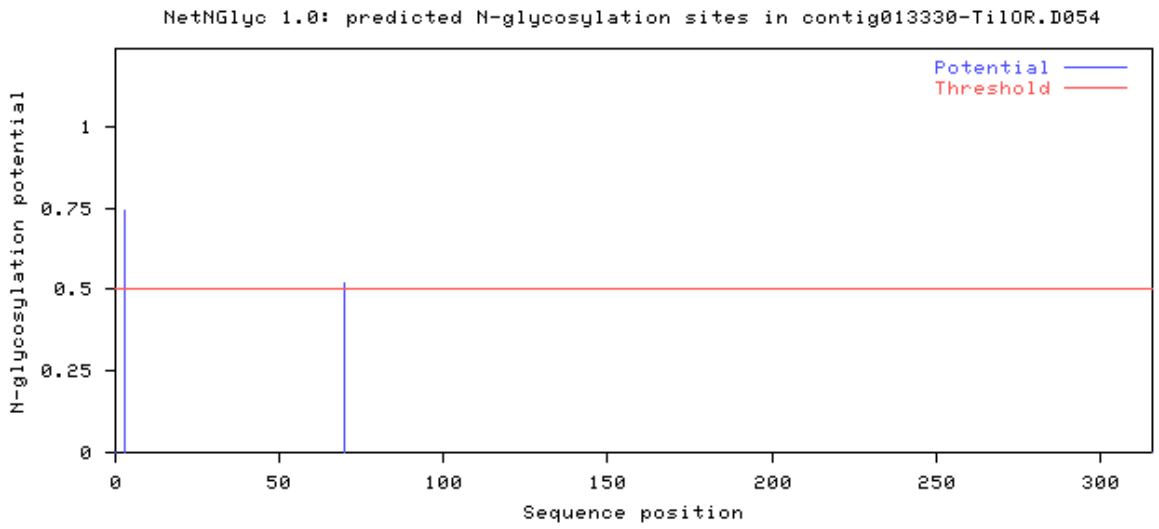

Graphics in PostScript

Output for 'contig013332-TilORe.D061'

```
########################################################################################

    Warning: This sequence may not contain a signal peptide!!

    Proteins without signal peptides are unlikely to be exposed to
    the N-glycosylation machinery and thus may not be glycosylated
    (in vivo) even though they contain potential motifs.

    SignalP-NN euk predictions are as follows:

  # name                    Cmax  pos ?  Ymax  pos ?  Smax  pos ?  Smean ?  D     ?

    SignalP output is explained at http://www.cbs.dtu.dk/services/SignalP/output.html

########################################################################################

Name:  contig013332-TilORe.D061         Length:  283
IIILIWYLSICVANTVLIVVIRVDRRLHEPMYMLLCNLCVNEINASTSLYPLLLSQMFSDSHEVTVPWCFLQMCCMFTCA      80
PAEFCSLAAMAYDRYISICHPFSYDVIMKTERVFLLIMFVWLYSFLSFIFSFSFIFSLKFCGNIIHNAYCDHQLMIRLSC     160
SVPIQSFISNISFLLLSVFIPFSLILVSYLKILRVCRKTSTENKQKAVTTCTPQIVSVSNLFVGCIFYFIDFTFVVSRVP     240
DEVRIILPMYVLIFQPMLTPFMYGFNLPKIRQSYQRFLLKRKX
................................................................................      80
................................................................................     160
................................................................................     240
...........................................                                          320

(Threshold=0.5)

No sites predicted in this sequence.
```

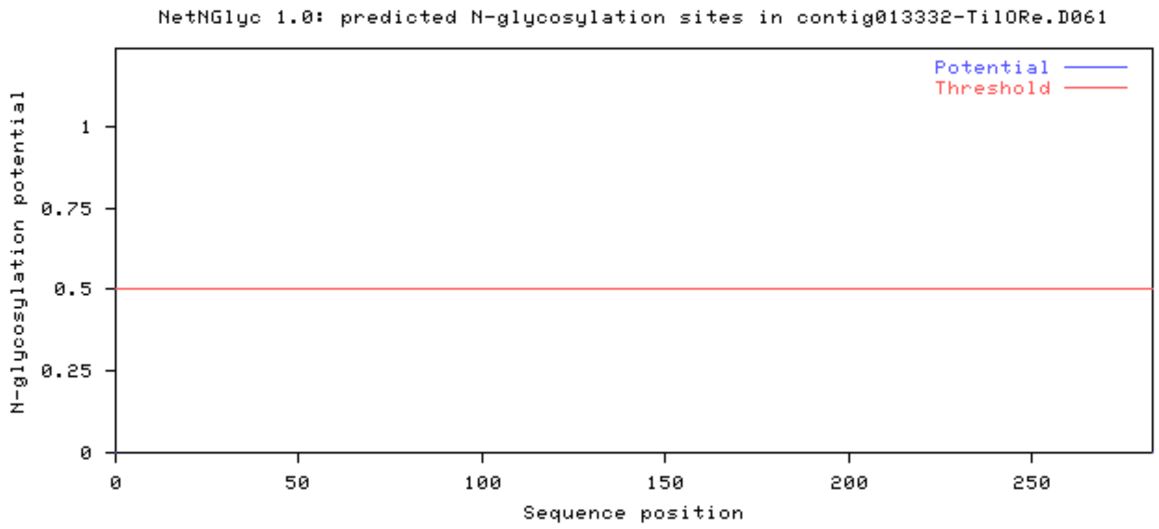

Graphics in PostScript

Output for 'contig013334-TilORe.D062'

```
##########################################################################################

    Warning: This sequence may not contain a signal peptide!!

    Proteins without signal peptides are unlikely to be exposed to
    the N-glycosylation machinery and thus may not be glycosylated
    (in vivo) even though they contain potential motifs.

    SignalP-NN euk predictions are as follows:

  # name                Cmax  pos ?  Ymax  pos ?  Smax  pos ?  Smean ?  D     ?

    SignalP output is explained at http://www.cbs.dtu.dk/services/SignalP/output.html

##########################################################################################

Name:  contig013334-TilORe.D062         Length:  282
IILIWYLSICVANTVLIVVIRVDRRLHEPMYMLLCNLCVNEINASTSLYPLLLSQMFSDSHEVTVPWCFLQMCCMVTCAP     80
AEFCSLAAMAYDRYISICHPFNYNVIMKTERVFLMIMFVWLYSFLSFIFSYSFIFSLKFCGNIIHNAYCDHQLIIRLSCS    160
VPIQSFISNISFLLLSVFIPFSLILVSYLKILRVCRKTSTENKQKAVTTCTPQIVSVSNLFVGCTFYFIDFTFVVSRVPN    240
EVRIILPMYVLIFQPMLTPFMYGFNLPKIRQSYQRFLFKRQX
................................................................................     80
................................................................................    160
................................................................................    240
..........................................                                           320

(Threshold=0.5)

No sites predicted in this sequence.
```

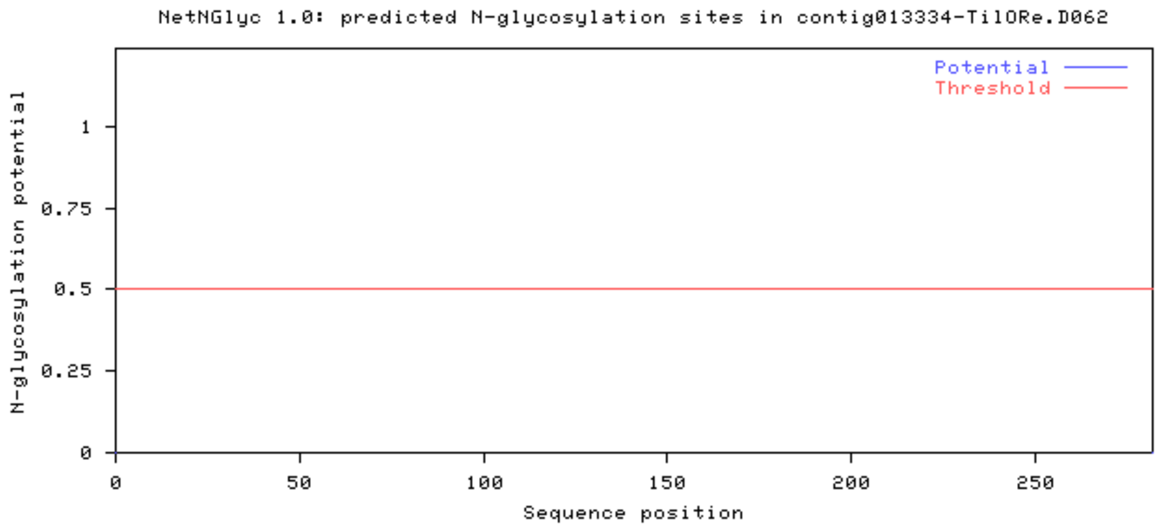

Graphics in PostScript

Output for 'contig013337-TilOR.D055'

```
########################################################################################

    Warning: This sequence may not contain a signal peptide!!

    Proteins without signal peptides are unlikely to be exposed to
    the N-glycosylation machinery and thus may not be glycosylated
    (in vivo) even though they contain potential motifs.

    SignalP-NN euk predictions are as follows:

  # name                    Cmax   pos ?  Ymax   pos ?  Smax   pos ?  Smean ?  D     ?

    SignalP output is explained at http://www.cbs.dtu.dk/services/SignalP/output.html

########################################################################################

Name:  contig013337-TilOR.D055  Length:  316
MGNSSETVSFVLAAYGNVGELKYLYFIIILIWYLSICVANTVLILVIRVDRRLHEPMYILLCNLCVNEINGSTSLYPLLL      80
SQMFSDSHEVTLSWCFLQMCCLYTSASVELCSLAAMAYDRYISICHPFTYNVIMNTERVFLMILLVWVFSFLSFIFSYSF     160
IFSLKFCGNIIHSVYCDHQLIIRLSCSVSIQSFISDISFAIVSFFIPFSLTLVSYLKILRVCRKTSKENKQKAVTTCTPQ     240
IISVSNLCVGCIFNLIDFRFVVSQVPDEVRIILPMYALIFQPMLTPFMYGFNLPKIRQSYQRFSVKEKINILVQIX
..N.............................................................................      80
................................................................................     160
................................................................................     240
............................................................................         320

(Threshold=0.5)
----------------------------------------------------------------------
SeqName      Position  Potential   Jury    N-Glyc
                                 agreement result
----------------------------------------------------------------------
contig013337-TilOR.D055    3 NSSE   0.7404     (9/9)   ++
contig013337-TilOR.D055   70 NGST   0.4433     (6/9)   -
----------------------------------------------------------------------
```

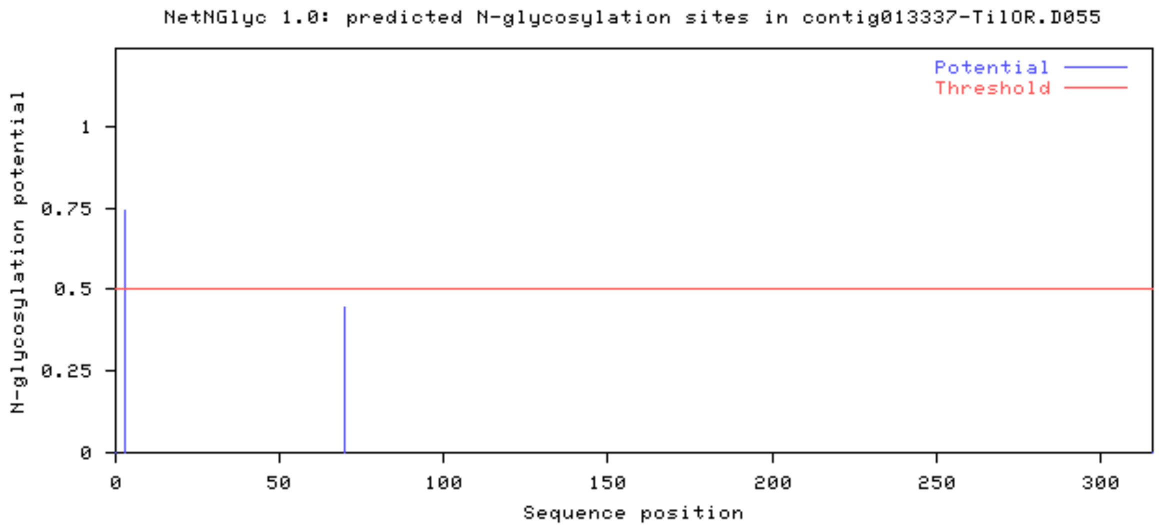

Graphics in PostScript

Output for 'contig013339-TilOR.D056'

```
##########################################################################################

    Warning: This sequence may not contain a signal peptide!!

    Proteins without signal peptides are unlikely to be exposed to
    the N-glycosylation machinery and thus may not be glycosylated
    (in vivo) even though they contain potential motifs.

    SignalP-NN euk predictions are as follows:

  # name                Cmax  pos ?  Ymax  pos ?  Smax  pos ?  Smean ?  D     ?

    SignalP output is explained at http://www.cbs.dtu.dk/services/SignalP/output.html

##########################################################################################

Name:  contig013339-TilOR.D056  Length:  309
MGNSSEIVSFVLSAYGNVGAFKYLYFIIILFWYLSICVANTFLIVVIHVDRRLHEPMYILLSNLCVNEINASTSLYPLLL      80
SQMFSDSHEVTLPWCFLQMCSLYTGAPAEFWSLAAMAYDRYVSICHPLRYNVIMNTERVLKIILLVWVFSFLIFILSFSF     160
IFSLKFCGNIVDNVYCDHKLIIKLSCSASVQGSISIIFFVIMSFFIPFSLISVSYVKILRVCRKTSKENKQKAVTTCTPQ     240
IVSLSNLFVGCICYSIDFRFLVSLVPDEVRTILAIYLLICQPMLTPFLYGFNLPKIRQSCKSFLFLRKX
..N.............................................................................      80
................................................................................     160
................................................................................     240
.....................................................................                320

(Threshold=0.5)
----------------------------------------------------------------------
SeqName      Position  Potential   Jury    N-Glyc
                                 agreement result
----------------------------------------------------------------------
contig013339-TilOR.D056    3 NSSE   0.7239     (9/9)   ++
contig013339-TilOR.D056   70 NAST   0.3816     (6/9)   -
----------------------------------------------------------------------
```

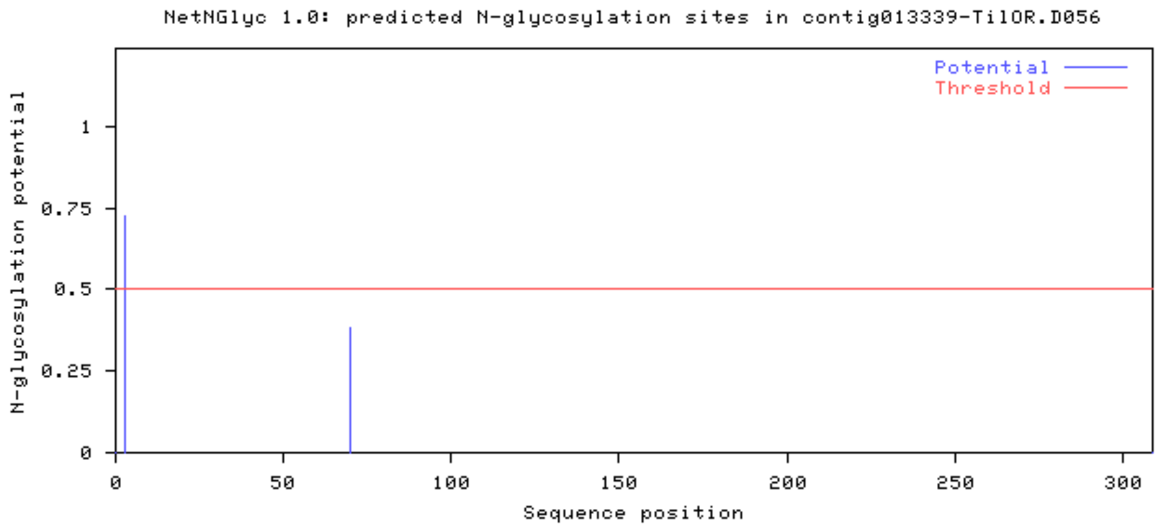

Graphics in PostScript

Output for 'contig013339-TilOR.D057'

```
##########################################################################

    Warning: This sequence may not contain a signal peptide!!

    Proteins without signal peptides are unlikely to be exposed to
    the N-glycosylation machinery and thus may not be glycosylated
    (in vivo) even though they contain potential motifs.

    SignalP-NN euk predictions are as follows:

  # name                  Cmax   pos ?  Ymax   pos ?  Smax   pos ?  Smean ?  D     ?

    SignalP output is explained at http://www.cbs.dtu.dk/services/SignalP/output.html

##########################################################################

Name:  contig013339-TilOR.D057  Length:  309
MGNSSETVSFVLAAYGNVGELKHLYFIIILVWYFSICVANTVLIVIIRVDRRLHEPMYILLCNLCVSEINGSTSLYPLLL      80
SQMFSDSHEVTLSWCFLQMCCLYTSASVELCSLAAMAYDRYISICHPFTYNVIMNTERVFLMILLVWVFSFLSFIFSYSF     160
IFSLKFCGNIIHSVYCDHQLIIRLSCSVSIQSFISDISFAIVSFFLPFSLILVSYLKILRVCRKTSKENKQKAVTTCTPQ     240
IVSVSNLCVGCICYFIDFRFLVSQVPDEVRIILPMYVLIFQPVLTPFMYGFNLPKIRQSYQRFLFERKX
..N.................................................................N..........      80
................................................................................     160
................................................................................     240
.....................................................................                320

(Threshold=0.5)
----------------------------------------------------------------------
SeqName          Position  Potential    Jury    N-Glyc
                                     agreement  result
----------------------------------------------------------------------
contig013339-TilOR.D057    3 NSSE    0.7404     (9/9)    ++
contig013339-TilOR.D057   70 NGST    0.5169     (4/9)    +
----------------------------------------------------------------------
```

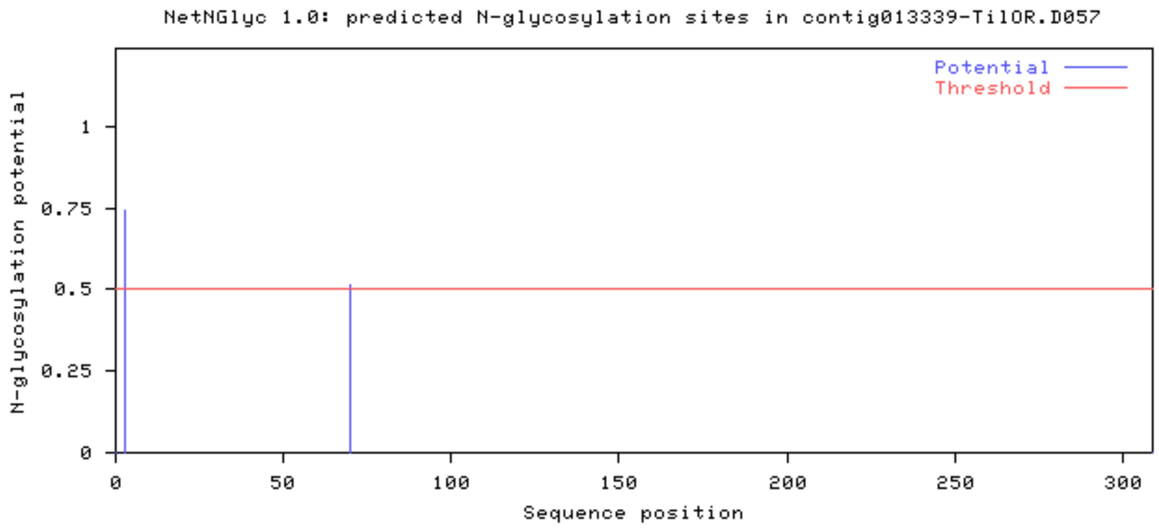

Graphics in PostScript

Output for 'contig013342-TilORe.D063'

```
##########################################################################################

    Warning: This sequence may not contain a signal peptide!!

    Proteins without signal peptides are unlikely to be exposed to
    the N-glycosylation machinery and thus may not be glycosylated
    (in vivo) even though they contain potential motifs.

    SignalP-NN euk predictions are as follows:

  # name                    Cmax   pos ?  Ymax   pos ?  Smax   pos ?  Smean ?  D     ?

    SignalP output is explained at http://www.cbs.dtu.dk/services/SignalP/output.html

##########################################################################################

Name:  contig013342-TilORe.D063         Length:  100
LISASYVKILRVCRKTSTENKQKAVTTCTPQIVSVSNLFVGCICYSIDFRFLVSQVPDEVHIILPIYLLICQPMLTPFLY      80
GFNLPKIRQSCKMLLFKRKX
................................................................................      80
....................                                                                 160

(Threshold=0.5)

No sites predicted in this sequence.
```

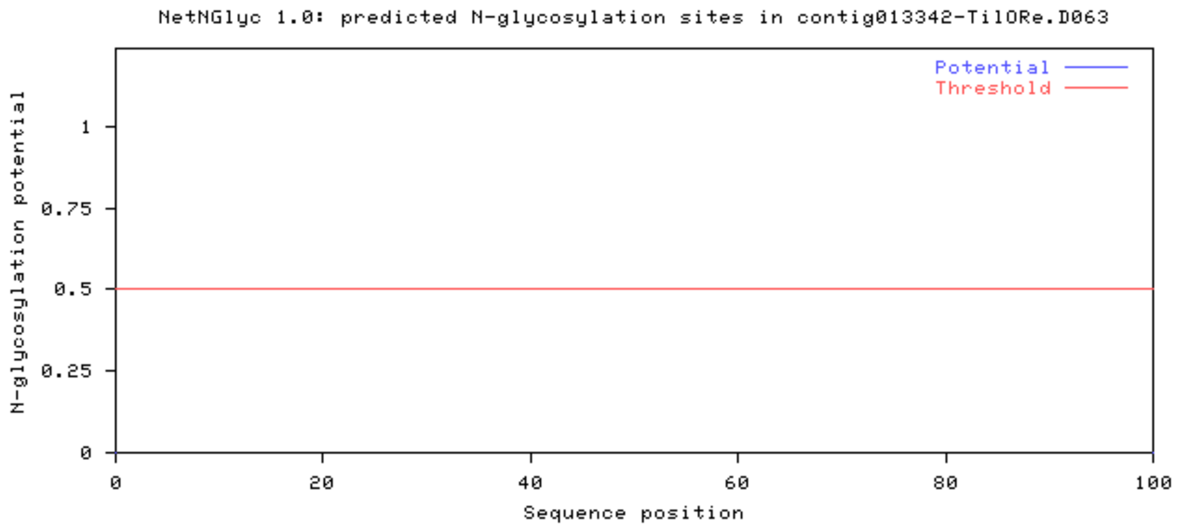

Graphics in PostScript

Output for 'contig013344-TilORe.D066'

```
##########################################################################################

    Warning: This sequence may not contain a signal peptide!!

    Proteins without signal peptides are unlikely to be exposed to
    the N-glycosylation machinery and thus may not be glycosylated
    (in vivo) even though they contain potential motifs.

    SignalP-NN euk predictions are as follows:

  # name                Cmax  pos ?  Ymax  pos ?  Smax  pos ?  Smean ?  D     ?

    SignalP output is explained at http://www.cbs.dtu.dk/services/SignalP/output.html

##########################################################################################

Name:  contig013344-TilORe.D066        Length:  100
LISASYVKILRVCRKTSTENKQKAVTTCTPQIISVSNLFVGCICYSIDFRFLVAQVPDEVRIILPMYLLICQPMLTPFMY      80
GFNLPKIRQSCKMLLFKRKX
................................................................................      80
....................                                                                 160

(Threshold=0.5)

No sites predicted in this sequence.
```

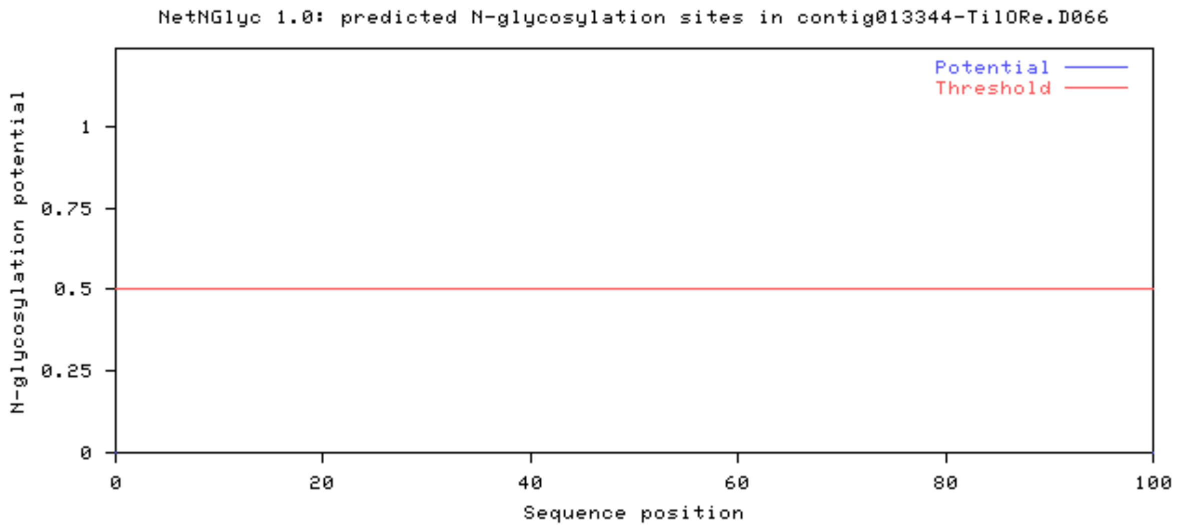

Graphics in PostScript

Output for 'contig013346-TilORe.D064'

```
##########################################################################################

    Warning: This sequence may not contain a signal peptide!!

    Proteins without signal peptides are unlikely to be exposed to
    the N-glycosylation machinery and thus may not be glycosylated
    (in vivo) even though they contain potential motifs.

    SignalP-NN euk predictions are as follows:

  # name                   Cmax  pos ?  Ymax  pos ?  Smax  pos ?  Smean ?  D     ?

    SignalP output is explained at http://www.cbs.dtu.dk/services/SignalP/output.html

##########################################################################################

Name:  contig013346-TilORe.D064         Length:  125
SDIFFVLLSIYLPFTLILVSYMKILAVCRKTSKENKQKAVTTCTPQIVSVSNLFVGCIFHSIDFSLLAAQVPGEVRVILS      80
IYLLICQPMLTPFMYGFNLPKIRQSCTMLLFKRKSISLFGKRVLX
................................................................................      80
.............................................                                        160

(Threshold=0.5)

No sites predicted in this sequence.
```

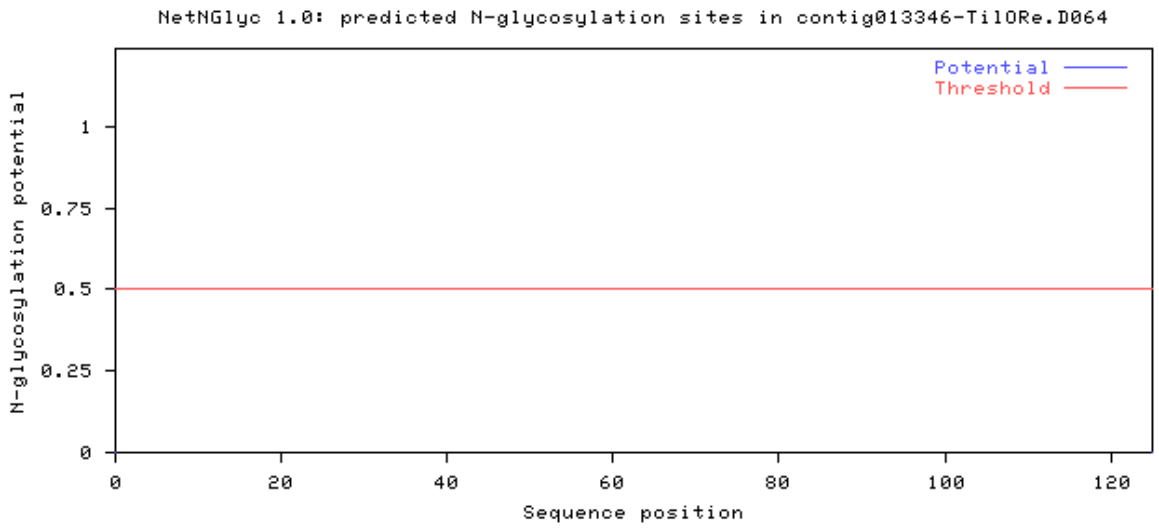

Graphics in PostScript

Output for 'contig013347-TilORe.D065'

```
##########################################################################################

    Warning: This sequence may not contain a signal peptide!!

    Proteins without signal peptides are unlikely to be exposed to
    the N-glycosylation machinery and thus may not be glycosylated
    (in vivo) even though they contain potential motifs.

    SignalP-NN euk predictions are as follows:

  # name                Cmax  pos ?  Ymax  pos ?  Smax  pos ?  Smean ?  D     ?

    SignalP output is explained at http://www.cbs.dtu.dk/services/SignalP/output.html

##########################################################################################

Name:  contig013347-TilORe.D065        Length:  107
VSYMKILAVCRKTSTENKQKAVTTCTPQIVSVSNLFVGCIFHAIDSSVLVAQVPGEVRVILSIYLLICQPMLTPFMYGFN      80
LPKIRQSCTMLLFKKKSISLFGKREFX
................................................................................      80
...........................                                                          160

(Threshold=0.5)

No sites predicted in this sequence.
```

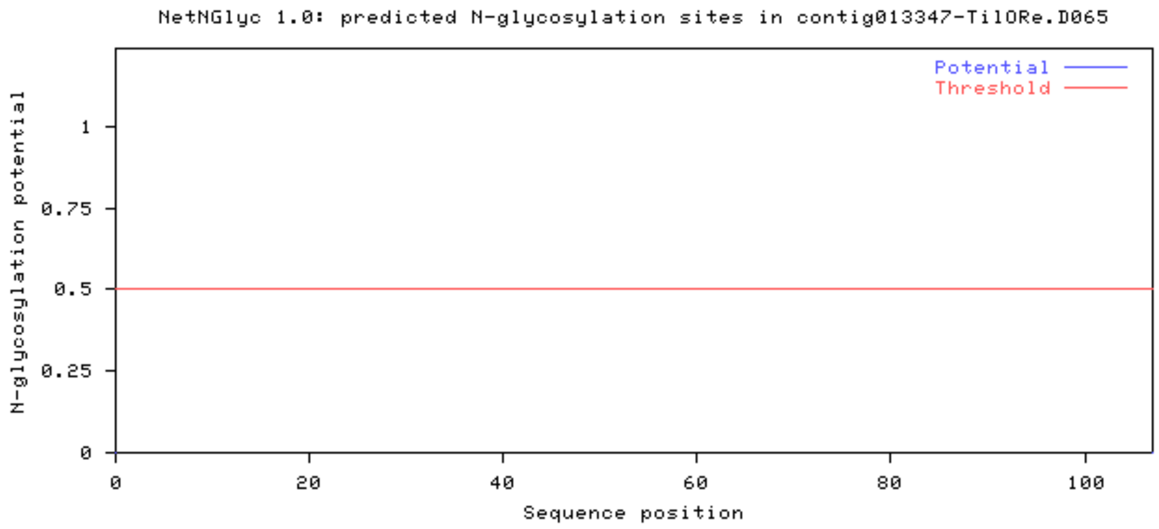

Graphics in PostScript

Output for 'contig013347-TilORe.D068'

```
##########################################################################################

    Warning: This sequence may not contain a signal peptide!!

    Proteins without signal peptides are unlikely to be exposed to
    the N-glycosylation machinery and thus may not be glycosylated
    (in vivo) even though they contain potential motifs.

    SignalP-NN euk predictions are as follows:

  # name                  Cmax  pos ?  Ymax  pos ?  Smax  pos ?  Smean ?  D     ?

    SignalP output is explained at http://www.cbs.dtu.dk/services/SignalP/output.html

##########################################################################################

Name:  contig013347-TilORe.D068        Length:  30
MGNSSETVSFVLAAYGNVGALKYMYFSIIL
..N...........................                                                        80

(Threshold=0.5)
----------------------------------------------------------------------
SeqName      Position  Potential   Jury    N-Glyc
                                 agreement result
----------------------------------------------------------------------
contig013347-TilORe.D068    3 NSSE   0.7350     (9/9)   ++
----------------------------------------------------------------------
```

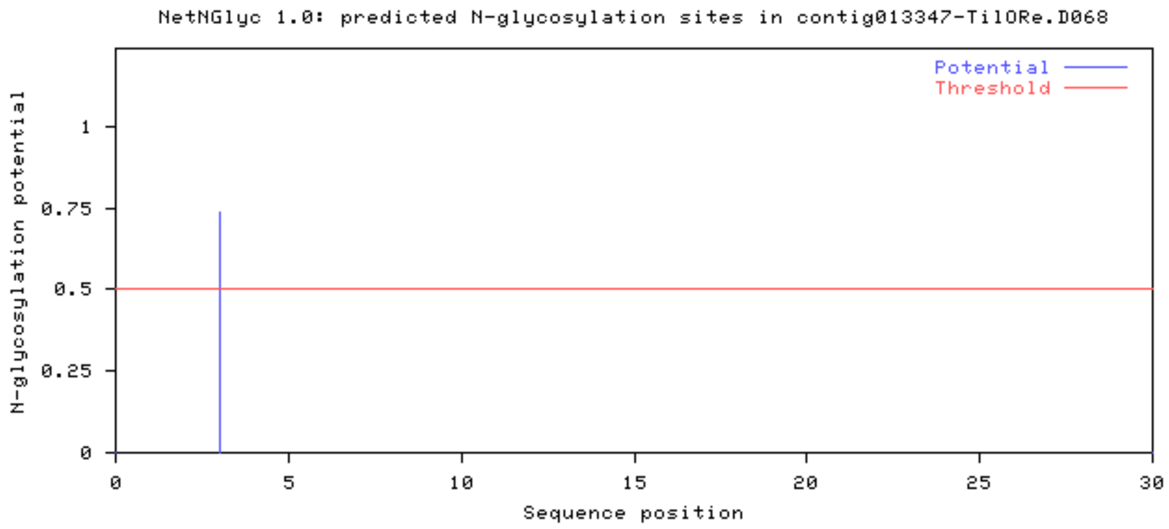

Graphics in PostScript

Output for 'contig013349-TilOR.H103'

```
##########################################################################

    Warning: This sequence may not contain a signal peptide!!

    Proteins without signal peptides are unlikely to be exposed to
    the N-glycosylation machinery and thus may not be glycosylated
    (in vivo) even though they contain potential motifs.

    SignalP-NN euk predictions are as follows:

  # name                Cmax  pos ?  Ymax  pos ?  Smax  pos ?  Smean ?  D     ?

    SignalP output is explained at http://www.cbs.dtu.dk/services/SignalP/output.html

##########################################################################

Name:  contig013349-TilOR.H103  Length:  314
MNTSSTVVFSLTGFSATVNYRVTLFSLTLLCYFLILMVNISLILTIISDQNLHEPMYIFLCSLCINGLYGTAGFFPRFAF      80
DLLSDTHLISYVGCLLQVFVIYSNAKVDYSTLVLMAYDRYVAICRPLEYHSVMSVRRTAVLVTLSWLVPLCFETLVISLT     160
STLKLCGSNINKLYCENWSIVKLACGSTKVNDIVGLILITFYFCHAVCIASSYVQLVNAALKSRAGRKKFTQTCVPHLFC     240
LLNVSTALLFDLMYSRYGSASLPQHLKNFMAIEFLIIPPILNPVCYGLVLTKIRRRMIFLCRQAYQRFGVKSQX
.N....................................N.........................................      80
................................................................................     160
................................................................................     240
..N.......................................................................           320

(Threshold=0.5)
----------------------------------------------------------------------
SeqName      Position  Potential   Jury    N-Glyc
                                 agreement result
----------------------------------------------------------------------
contig013349-TilOR.H103    2 NTSS   0.7593     (9/9)   +++
contig013349-TilOR.H103   39 NISL   0.7616     (9/9)   +++
contig013349-TilOR.H103  177 NWSI   0.4820     (5/9)   -
contig013349-TilOR.H103  243 NVST   0.5093     (7/9)   +
----------------------------------------------------------------------
```

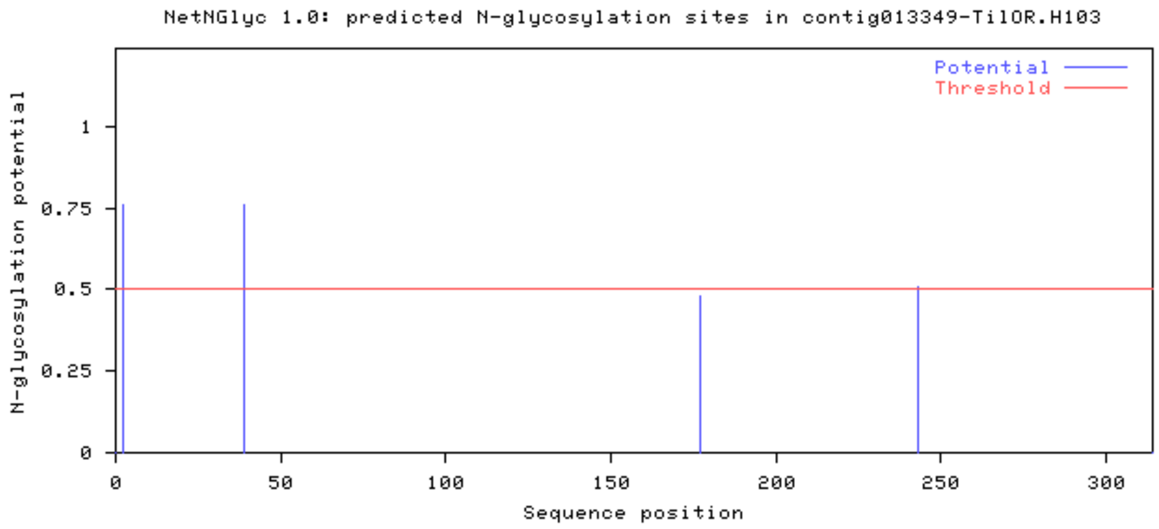

Graphics in PostScript

Output for 'contig013350-TilORe.H123'

```
##########################################################################################

    Warning: This sequence may not contain a signal peptide!!

    Proteins without signal peptides are unlikely to be exposed to
    the N-glycosylation machinery and thus may not be glycosylated
    (in vivo) even though they contain potential motifs.

    SignalP-NN euk predictions are as follows:

  # name                Cmax  pos ?  Ymax  pos ?  Smax  pos ?  Smean ?  D     ?

    SignalP output is explained at http://www.cbs.dtu.dk/services/SignalP/output.html

##########################################################################################

Name:  contig013350-TilORe.H123         Length:  163
MDNVSVVTVFTLSGLGSGIANYRVILFVLTLLCYCVIWLVNLTIIVTVILDKSLHEPMYIFLCNLCFNGLYGTAAFYPKFL      80
YDLLSTTHVISYAGCLLQGFVLHSSVSADCSLLALMAYDRYVAICRPLVYHSLMTTQRVCIFVCFAWLTPFSLILMSTIT     160
TAT
..N.....................................N.......................................      80
................................................................................     160
...                                                                                  240

(Threshold=0.5)
----------------------------------------------------------------------
SeqName      Position  Potential   Jury    N-Glyc
                                agreement result
----------------------------------------------------------------------
contig013350-TilORe.H123    3 NVSV   0.8107     (9/9)    +++
contig013350-TilORe.H123   40 NLTI   0.7579     (9/9)    +++
----------------------------------------------------------------------
```

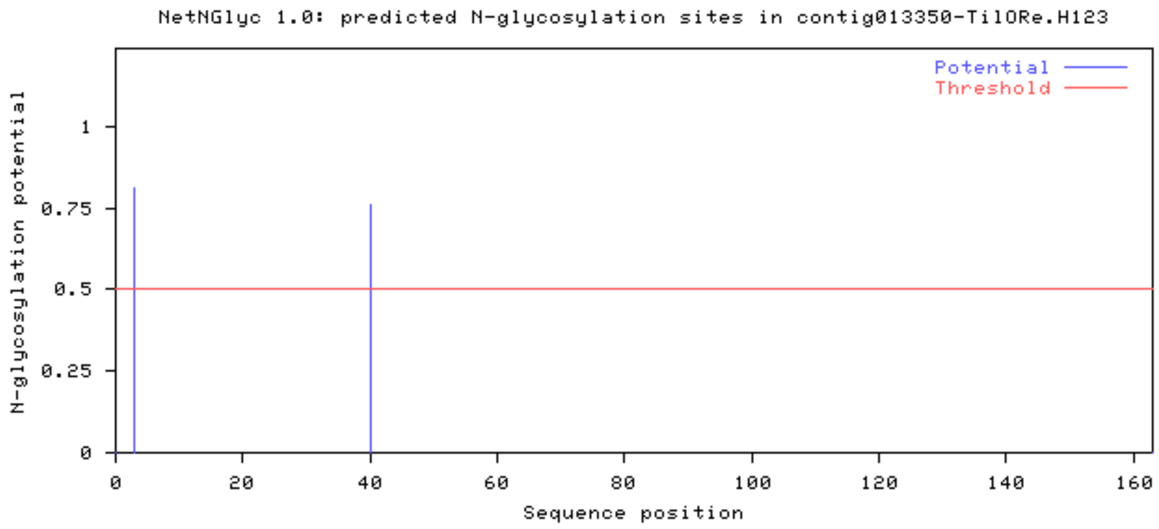

Graphics in PostScript

Output for 'contig013351-TilOR.H104'

```
##########################################################################

    Warning: This sequence may not contain a signal peptide!!

    Proteins without signal peptides are unlikely to be exposed to
    the N-glycosylation machinery and thus may not be glycosylated
    (in vivo) even though they contain potential motifs.

    SignalP-NN euk predictions are as follows:

  # name                 Cmax  pos ?  Ymax  pos ?  Smax  pos ?  Smean ?  D     ?

    SignalP output is explained at http://www.cbs.dtu.dk/services/SignalP/output.html

##########################################################################

Name:  contig013351-TilOR.H104  Length:  314
MDNVSIITVFTLSGLRDIANYRVILFVLTLLCYCVIWLVNVTIIVTVILDKSLHEPMYIFLCSLCFNGLYGTAAFYPKFL      80
YDLLSTTHVISYAGCFLQGFVLHSSVGADFSLLALMAYDRYVAICRPLVYHSLMTKERVCSLIFFAWIIAFYLLFMSTIT     160
TAILRLCGSHIPRIYCINLLIANLACSASVAKIVIPAFSYTFCIGNICFVFWSYVYLIKTCQSSKENMGKFMQTCVPHIF     240
SLTVVVVSLLFDLLYMRFGSKEIPQSVQNFMAMEFLLIPPIMNPLMYGFKLTKIRNRVLNFICGKTSALRFKSX
..N....................................N........................................      80
................................................................................     160
................................................................................     240
..........................................................................           320

(Threshold=0.5)
----------------------------------------------------------------------
SeqName      Position  Potential   Jury    N-Glyc
                                 agreement result
----------------------------------------------------------------------
contig013351-TilOR.H104    3 NVSI   0.8031     (9/9)    +++
contig013351-TilOR.H104   40 NVTI   0.7877     (9/9)    +++
----------------------------------------------------------------------
```

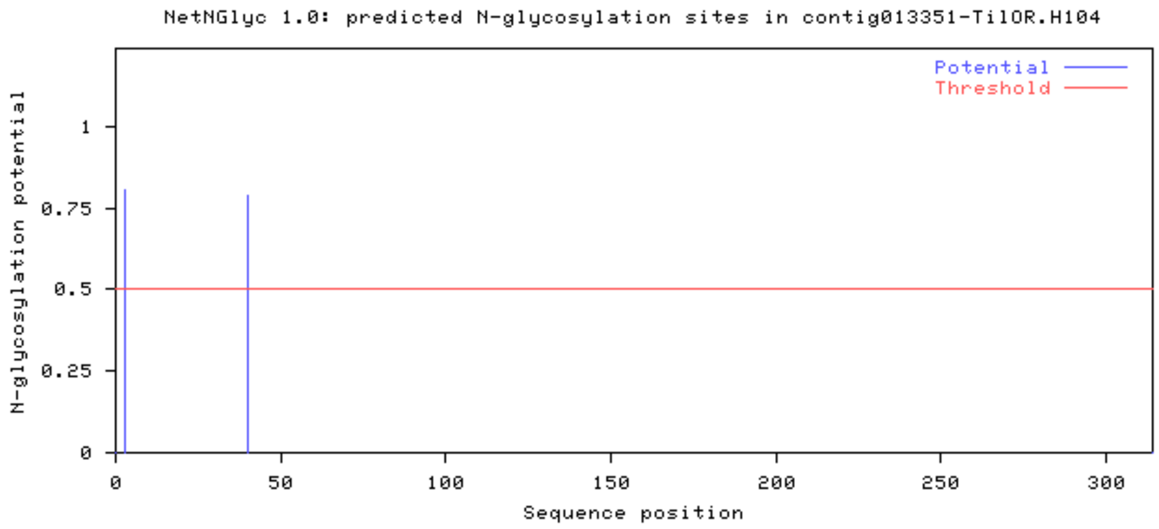

Graphics in PostScript

Output for 'contig013353-TilORe.H124'

```
##########################################################################

    Warning: This sequence may not contain a signal peptide!!

    Proteins without signal peptides are unlikely to be exposed to
    the N-glycosylation machinery and thus may not be glycosylated
    (in vivo) even though they contain potential motifs.

    SignalP-NN euk predictions are as follows:

  # name                 Cmax  pos ?  Ymax  pos ?  Smax  pos ?  Smean ?  D     ?

    SignalP output is explained at http://www.cbs.dtu.dk/services/SignalP/output.html

##########################################################################

Name:  contig013353-TilORe.H124        Length:  262
LHEPMYIFLCNLCVNGLYGTAAFYPKFLYDLLSTTHVISYAGCLLQGFALHSTISADFSLLALMAYDRYVAICRPLVYHS      80
LMTKQRVCIFVFFAWFFPIFLLFLSTITTAVLRLCGSHIPRIYCINWLINNLACSASVARIVIPAFNYTFYIGHILFVFW     160
TYVYLIKTCQSSKENWNKFMQTCVPHLFSLIVVAVSFLFDMLYVRFGSKEFPQSFENFMAMEILLIPPIINPLMYGFKLT     240
KIRNRVLNFICGKSSALRLKSX
................................................................................      80
.................................................................N..............     160
................................................................................     240
......................                                                               320

(Threshold=0.5)
----------------------------------------------------------------------
SeqName      Position  Potential   Jury    N-Glyc
                                 agreement result
----------------------------------------------------------------------
contig013353-TilORe.H124   147 NYTF   0.5993     (8/9)   +
----------------------------------------------------------------------
```

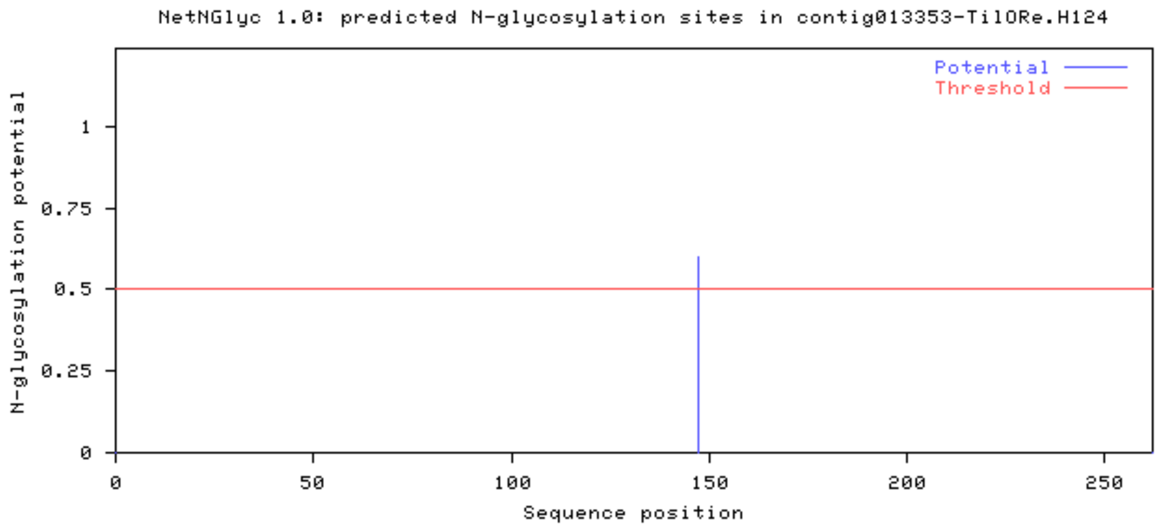

Graphics in PostScript

Output for 'contig013355-TilORe.H121'

```
##########################################################################################

    Warning: This sequence may not contain a signal peptide!!

    Proteins without signal peptides are unlikely to be exposed to
    the N-glycosylation machinery and thus may not be glycosylated
    (in vivo) even though they contain potential motifs.

    SignalP-NN euk predictions are as follows:

  # name                    Cmax   pos ?  Ymax   pos ?  Smax   pos ?  Smean ?  D      ?

    SignalP output is explained at http://www.cbs.dtu.dk/services/SignalP/output.html

##########################################################################################

Name:  contig013355-TilORe.H121         Length:  101
YVHLIKTCQSSKENWNKFMQTCVPHLFCIIVVVVSFLFDMLYLRFGSKEIPQSVQNFMAMEILLIPPIMHPLMYGFKLTK      80
IRNTVLNFICGKSSTFRLKSX
................................................................................      80
.....................                                                                160

(Threshold=0.5)

No sites predicted in this sequence.
```

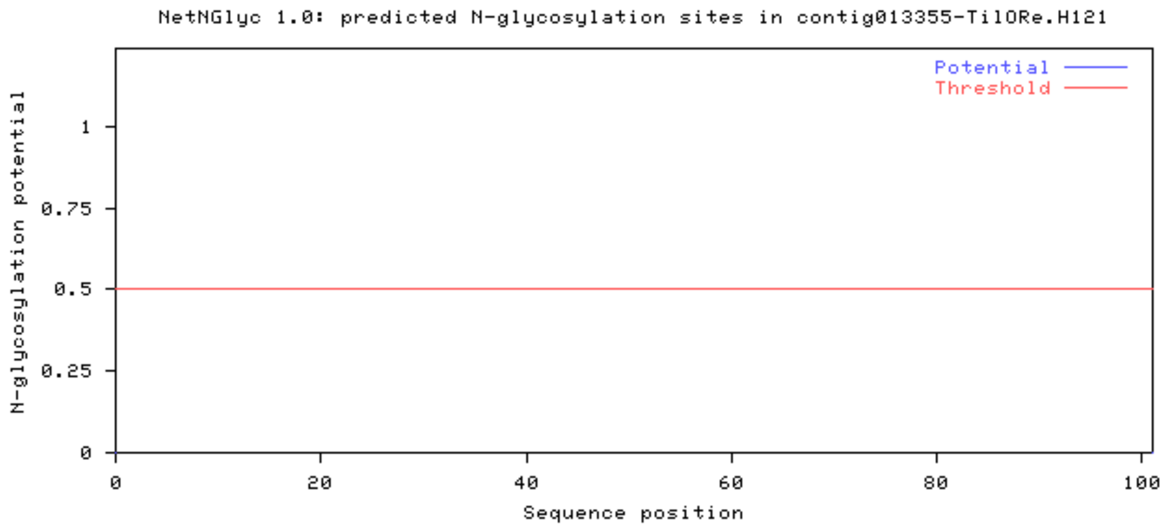

Graphics in PostScript

Output for 'contig013356-TilOR.H105'

```
################################################################################

   Warning: This sequence may not contain a signal peptide!!

   Proteins without signal peptides are unlikely to be exposed to
   the N-glycosylation machinery and thus may not be glycosylated
   (in vivo) even though they contain potential motifs.

   SignalP-NN euk predictions are as follows:

 # name                 Cmax  pos ?  Ymax  pos ?  Smax  pos ?  Smean ?  D     ?

   SignalP output is explained at http://www.cbs.dtu.dk/services/SignalP/output.html

################################################################################

Name:  contig013356-TilOR.H105  Length:  314
MDNVSVITVFTLSGLSDIANYRVILFFLTLLCYSVIWLVNLTIIVTVIVDKSLHEPMYIFLCNLCFNGLYGTAAFYPKFL      80
YDLLSTAHVISYAGCLLQGFALHSTICADFSLLALMAYDRYVAICRPLVYHSLMTKQRVCIFLFFAWFFPFYLLFLSTIT     160
TAVLRLCGSHIPRIYCINWLINNLACTASVARIVIPAFNYTFYIGHILFVFWTYVHLIKTCQSSKENWNKFMQTCVPHLF     240
CIIVVVVSFLFDMLYVRFGSKEFPQSFENFMAMEIVLIPPIINPLIYGFKLTKIRNRVLNFICGKSKALRLKSX
..N....................................N........................................      80
................................................................................     160
......................................N.........................................     240
..........................................................................           320

(Threshold=0.5)
----------------------------------------------------------------------
SeqName      Position  Potential   Jury    N-Glyc
                                 agreement result
----------------------------------------------------------------------
contig013356-TilOR.H105    3 NVSV   0.8139     (9/9)   +++
contig013356-TilOR.H105   40 NLTI   0.7864     (9/9)   +++
contig013356-TilOR.H105  199 NYTF   0.5841     (7/9)   +
----------------------------------------------------------------------
```

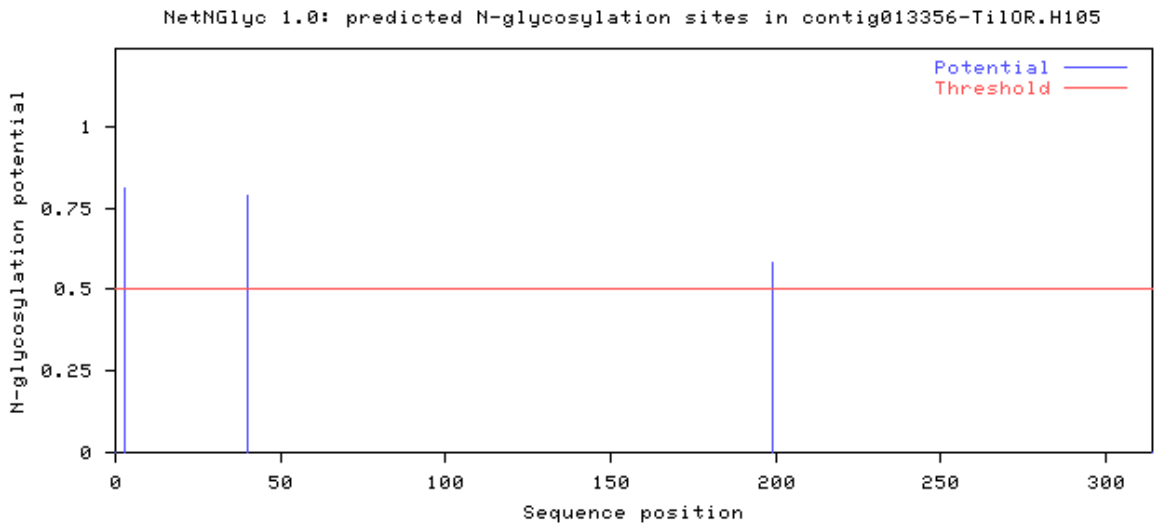

Graphics in PostScript

Output for 'contig013358-TilOR.H106'

```
##########################################################################

    Warning: This sequence may not contain a signal peptide!!

    Proteins without signal peptides are unlikely to be exposed to
    the N-glycosylation machinery and thus may not be glycosylated
    (in vivo) even though they contain potential motifs.

    SignalP-NN euk predictions are as follows:

  # name                 Cmax  pos ?  Ymax  pos ?  Smax  pos ?  Smean ?  D     ?

    SignalP output is explained at http://www.cbs.dtu.dk/services/SignalP/output.html

##########################################################################

Name:  contig013358-TilOR.H106  Length:  314
MDNVSVITVFTLSGLRDIANYRVILFVLTLLCYCVIWLVNLTIILTVIVDKSLHEPMYIFLCNLCFNALYGTAAFYPKFL      80
YDLLSTIHVISYAGCLLQGFALHSSVAADFSLLALMAYDRYVAICRPLVYHSLMTKQKVCIFVFFAWLIPFYLLFMSTIS     160
TAVLRLCGSHIQRIYCVNWLISNLACSPSVAKTVIPAFNYTFYIGHVLFVFWSYVHLIKTCQSSNENWNKFMQTCVPHLF     240
SLTVVVVSLLFDMLYMRFGSKEIPQNVENFMAMEFLLIPPIMNPLMYGLKLTKIRKRVLNFICGKSSTFRLKSX
..N....................................N........................................      80
................................................................................     160
......................................N.........................................     240
..........................................................................           320

(Threshold=0.5)
----------------------------------------------------------------------
SeqName      Position  Potential   Jury    N-Glyc
                                 agreement result
----------------------------------------------------------------------
contig013358-TilOR.H106    3 NVSV   0.8138     (9/9)    +++
contig013358-TilOR.H106   40 NLTI   0.7599     (9/9)    +++
contig013358-TilOR.H106  199 NYTF   0.5714     (7/9)    +
----------------------------------------------------------------------
```

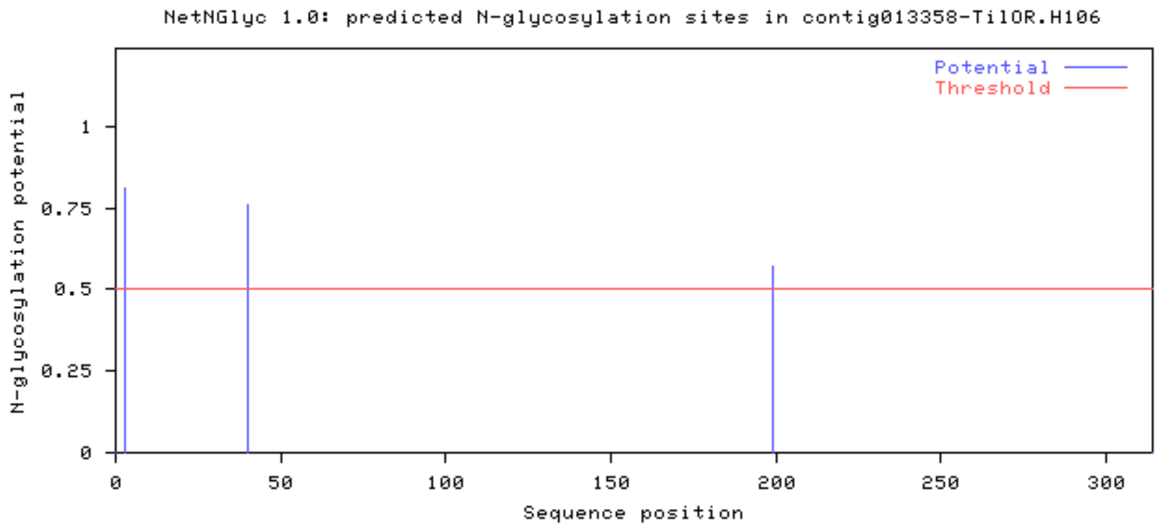

Graphics in PostScript

Output for 'contig013359-TilOR.H107'

```
##########################################################################################

    Warning: This sequence may not contain a signal peptide!!

    Proteins without signal peptides are unlikely to be exposed to
    the N-glycosylation machinery and thus may not be glycosylated
    (in vivo) even though they contain potential motifs.

    SignalP-NN euk predictions are as follows:

  # name                    Cmax   pos ?  Ymax   pos ?  Smax   pos ?  Smean ?  D     ?

    SignalP output is explained at http://www.cbs.dtu.dk/services/SignalP/output.html

##########################################################################################

Name:  contig013359-TilOR.H107  Length:  314
MDNVSIITVFTLSGLSGIANYKITVFIFTLLCYCVIWLVNLTIIVTVIVDKKLHEPMYIFLCNLCFNGLYGTAAFYPKFL      80
YDLLSTTHVISYAGCLLQGFVVHSSVSADFSLLALMAYDRYVAICRPLVYHSLMTKQRVCILIFFAWIIAFYLLFMSTIT     160
TATSRLCGSHIPRIYCINWLISNLACSASVATIIIPAFNYTFYFGHALFIFWSYVHLIKTCQSSKENWNKFMQTCVPHLF     240
SLAVVVVSFLFDMLYMRFGSKEIPQSFENFMAMEILLIPPIINPLMYGFKLTQIRNRVLNFMCGKRSALRLKSX
..N.....................................N.......................................      80
................................................................................     160
......................................N.........................................     240
..........................................................................           320

(Threshold=0.5)
----------------------------------------------------------------------
SeqName      Position  Potential   Jury    N-Glyc
                                  agreement result
----------------------------------------------------------------------
contig013359-TilOR.H107    3 NVSI   0.8031     (9/9)   +++
contig013359-TilOR.H107   40 NLTI   0.7721     (9/9)   +++
contig013359-TilOR.H107  199 NYTF   0.5870     (8/9)   +
----------------------------------------------------------------------
```

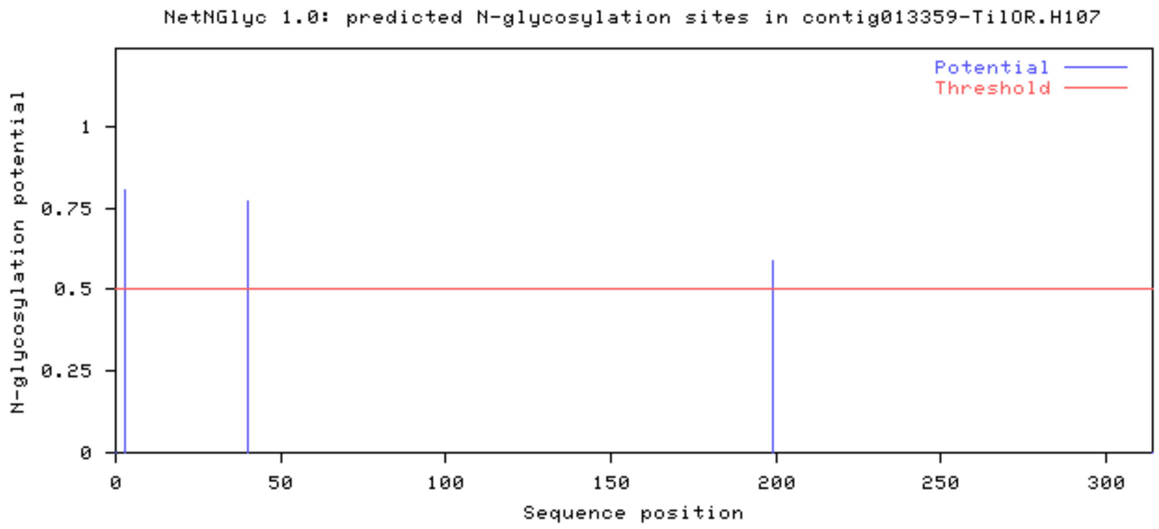

Graphics in PostScript

Output for 'contig013359-TilORp.H125'

```
##########################################################################

    Warning: This sequence may not contain a signal peptide!!

    Proteins without signal peptides are unlikely to be exposed to
    the N-glycosylation machinery and thus may not be glycosylated
    (in vivo) even though they contain potential motifs.

    SignalP-NN euk predictions are as follows:

  # name                    Cmax   pos ?  Ymax   pos ?  Smax   pos ?  Smean ?  D     ?

    SignalP output is explained at http://www.cbs.dtu.dk/services/SignalP/output.html

##########################################################################

Name:  contig013359-TilORp.H125         Length:  370
MDNVHVNVRSFILSGFNETINFRVPLFSVTLLCYCVVLFFNISLVLLIVLDENLHEPMYIFLSSFCINALYGTTGFYPKF      80
LSDLLSSSHRISYEGCLLQAFIMYSFVCSDLSILAVIAFDRYLAICRPLHYHSFMTKRRLSQLVCFSWLTPLCVFSINVL     160
LTTRIKLCGINIQRVLCLNWLIVKLACPEADTFSNNITAYATVIIYVSHGFFIIWTYTHLIKTCVRSREDRVKFMCVPHL     240
TSLITFLSVDFFQFVCMRFDSTDLPQSLHNFIAMEFVIIPPIMNVTVHSRDSSAEQADISKHSLFTTPMENIYNEGGNAG     320
FQGSKGKLCHTHSLLHVQAHTPSRSRKVTGPGRKPGRKPITQEREITQKX
................N.......................N.......................................      80
................................................................................     160
...................................N............................................     240
..........................................N.....................................     320
..................................................                                   400

(Threshold=0.5)
----------------------------------------------------------------------
SeqName      Position  Potential   Jury    N-Glyc
                                 agreement result
----------------------------------------------------------------------
contig013359-TilORp.H125   17 NETI   0.6678     (9/9)   ++
contig013359-TilORp.H125   41 NISL   0.6659     (8/9)   +
contig013359-TilORp.H125  196 NITA   0.6321     (8/9)   +
contig013359-TilORp.H125  284 NVTV   0.6708     (9/9)   ++
----------------------------------------------------------------------
```

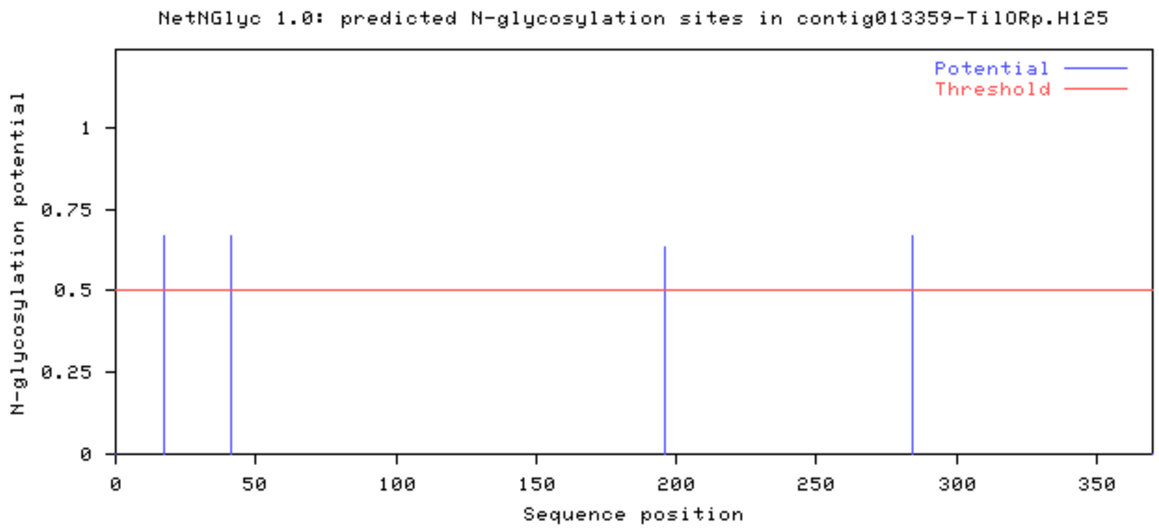

Graphics in PostScript

Output for 'contig013361-TilOR.H108'

```
########################################################################################

    Warning: This sequence may not contain a signal peptide!!

    Proteins without signal peptides are unlikely to be exposed to
    the N-glycosylation machinery and thus may not be glycosylated
    (in vivo) even though they contain potential motifs.

    SignalP-NN euk predictions are as follows:

  # name                   Cmax  pos ?  Ymax  pos ?  Smax  pos ?  Smean ?  D     ?

    SignalP output is explained at http://www.cbs.dtu.dk/services/SignalP/output.html

########################################################################################

Name:  contig013361-TilOR.H108  Length:  310
MDNVSNVISFVLSGFNETMTFRVPLFSCTFLYYCLILFFNISLVLLIVLDANLHEPMYILLSSSCINALYGTTGFYPKFL      80
SDLLLSSQTISYEGCLLQAFIMYSFASCDLSILAVMAFDRYLAICRPLHYHSFMTKRRLSQLVSFSWLTPLCIFTINVLL     160
TSRIKLCGINIQRVLCLNWVIVKLACPEADTLSNNISAYVIVVFYLSHWLFIIWTYIYLIKTCARSSDDRVKFTQTCVPH     240
LISLIIFLTVMAFDSMYLRFGSRDLPQSLQNFITIEFLIIPPVMNPLIYGFKLTKIRDRILSLIYFKRKX
..N............N.......................N........................................      80
................................................................................     160
..................................N.............................................     240
......................................................................               320

(Threshold=0.5)
----------------------------------------------------------------------
SeqName      Position  Potential   Jury    N-Glyc
                                 agreement result
----------------------------------------------------------------------
contig013361-TilOR.H108    3 NVSN   0.7836     (9/9)   +++
contig013361-TilOR.H108   16 NETM   0.6993     (9/9)   ++
contig013361-TilOR.H108   40 NISL   0.6487     (9/9)   ++
contig013361-TilOR.H108  195 NISA   0.6096     (7/9)   +
----------------------------------------------------------------------
```

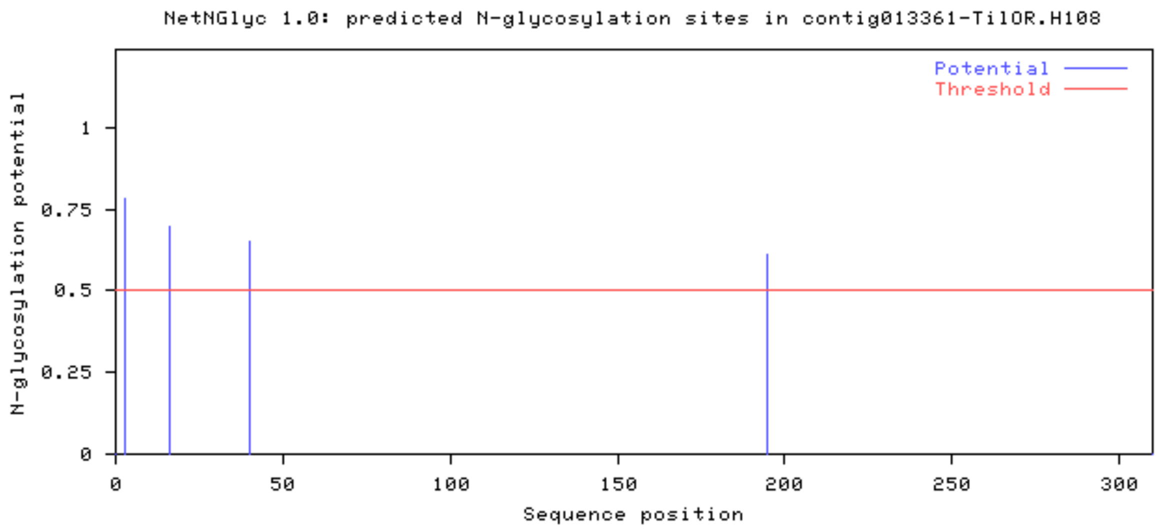

Graphics in PostScript

Output for 'contig013362-TilOR.H109'

```
##########################################################################################

    Warning: This sequence may not contain a signal peptide!!

    Proteins without signal peptides are unlikely to be exposed to
    the N-glycosylation machinery and thus may not be glycosylated
    (in vivo) even though they contain potential motifs.

    SignalP-NN euk predictions are as follows:

  # name                 Cmax  pos ?  Ymax  pos ?  Smax  pos ?  Smean ?  D     ?

    SignalP output is explained at http://www.cbs.dtu.dk/services/SignalP/output.html

##########################################################################################

Name:  contig013362-TilOR.H109  Length:  310
MDNVSNVIRFVLSGFNETMNFSVPLFSITLLYYCVILFVNISLVLLIFFDANLHEPMYILLSSFCVNALYGTTGFYPKFL      80
SDLLSSTYNISYEGCILQAFIMYSFGCCELSILTVMAFDRYLAICRPLHYHSFMTKRRLSQLVCFSWLTPLCMFSINIIL     160
TSRIKLCGINIQRVLCINWVIVKLACPEADTLSNNISAYVIVVFYLSHSLFIIWTYIYLIKTCARSSEDRVKFTQTCVPH     240
LISLIIFLTVMSFDSMYLRYGSRDLPQSLQNFITLEFLIIPPVMNPLMYGFKLTKIRNRILSLIYLKRKX
..N............N...N...................N........................................      80
.......N........................................................................     160
..................................N.............................................     240
......................................................................               320

(Threshold=0.5)
----------------------------------------------------------------------
SeqName      Position  Potential   Jury    N-Glyc
                                 agreement result
----------------------------------------------------------------------
contig013362-TilOR.H109    3 NVSN   0.7912     (9/9)   +++
contig013362-TilOR.H109   16 NETM   0.6417     (9/9)   ++
contig013362-TilOR.H109   20 NFSV   0.7316     (9/9)   ++
contig013362-TilOR.H109   40 NISL   0.7037     (9/9)   ++
contig013362-TilOR.H109   89 NISY   0.6163     (7/9)   +
contig013362-TilOR.H109  195 NISA   0.6097     (8/9)   +
----------------------------------------------------------------------
```

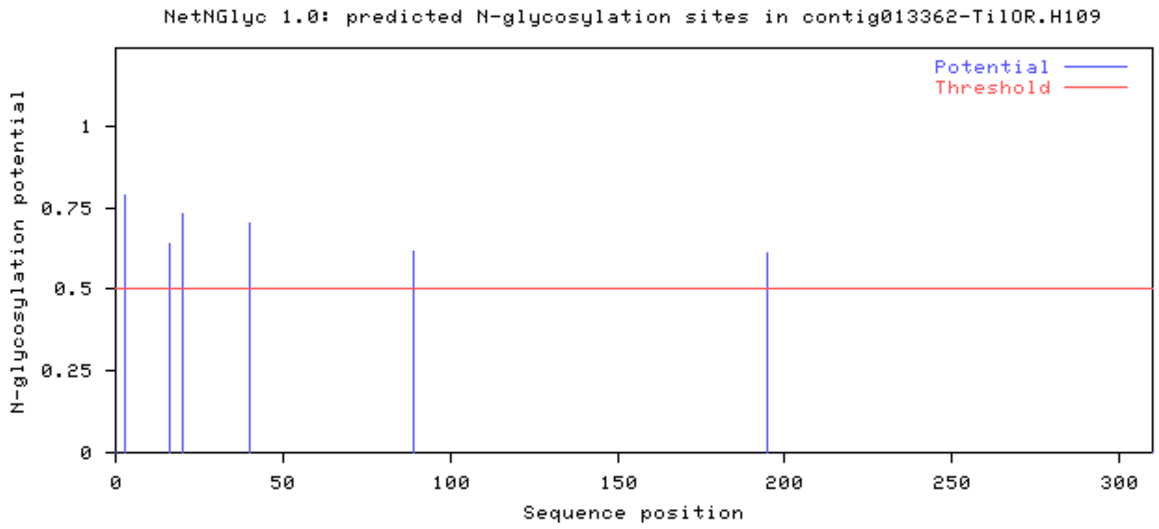

Graphics in PostScript

Output for 'contig013363-TilOR.H110'

```
#######################################################################################

    Warning: This sequence may not contain a signal peptide!!

    Proteins without signal peptides are unlikely to be exposed to
    the N-glycosylation machinery and thus may not be glycosylated
    (in vivo) even though they contain potential motifs.

    SignalP-NN euk predictions are as follows:

  # name                    Cmax  pos ?  Ymax  pos ?  Smax  pos ?  Smean ?  D     ?

    SignalP output is explained at http://www.cbs.dtu.dk/services/SignalP/output.html

#######################################################################################

Name:  contig013363-TilOR.H110  Length:  314
MDNVSVVTVFTLSGLSDITNYKVILFVLTLLCYCVIWLVNLMIIVTVIVDKSLHEPMYIFLCNLCFNGLCGTAAFYPKFL      80
YDLLSTTHVISYAGCLLQGFVVHSSVAADFSLLALMAYDRYVAICRPLVYHSLMTTQKVCIFVFFAWLIPFSLILMSTIT     160
TATSRLCGSHIPRIYCINWLISNLACSASVATIIIPAFNYTFYFGHAVFIFWSYVHLIKTCQSSKENWNKFMQTCVPHLF     240
SLTVVILSFLFDMLYMRFGSKEIPQSFENFMAMEILLIPPIINPLMYGFKLTQIRNRVLNFICGKSSALRLKSX
..N.............................................................................      80
................................................................................     160
......................................N.........................................     240
..........................................................................           320

(Threshold=0.5)
----------------------------------------------------------------------
SeqName      Position  Potential   Jury    N-Glyc
                                 agreement result
----------------------------------------------------------------------
contig013363-TilOR.H110    3 NVSV   0.8098     (9/9)   +++
contig013363-TilOR.H110  199 NYTF   0.5936     (8/9)   +
----------------------------------------------------------------------
```

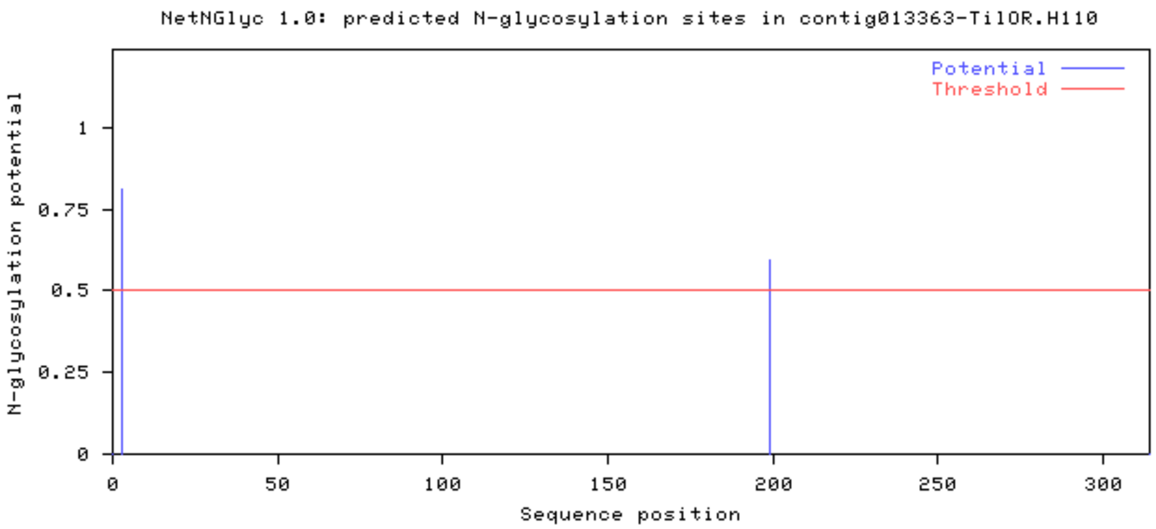

Graphics in PostScript

Output for 'contig013363-TilORe.H122'

```
##########################################################################################

    Warning: This sequence may not contain a signal peptide!!

    Proteins without signal peptides are unlikely to be exposed to
    the N-glycosylation machinery and thus may not be glycosylated
    (in vivo) even though they contain potential motifs.

    SignalP-NN euk predictions are as follows:

  # name                    Cmax  pos ?  Ymax  pos ?  Smax  pos ?  Smean ?  D     ?

    SignalP output is explained at http://www.cbs.dtu.dk/services/SignalP/output.html

##########################################################################################

Name:  contig013363-TilORe.H122        Length:  248
MDNVSNVISFVLSGFNETMTFRVPLFSCTFLYYCLILFFNISLVLLIVLDANLHEPMYILLSSSCINALYGTTGFYPKFL      80
SDLLLSSQTISYEGCLLQAFIMYSFASCDLSILAVMAFDRYLAICRPLHYHSFMTKRRLSQLVSFSWLTPLCIFTINVLL     160
TSRIKLCGINIQRVLCLNWVIVKLACPEADTLSNNISAYVIVVFYLSHSLFIIWTYIYLIKTCARSSEDRVKFTQTCVPH     240
LISLIIFL
..N............N.......................N........................................      80
................................................................................     160
..................................N.............................................     240
........                                                                             320

(Threshold=0.5)
----------------------------------------------------------------------
SeqName      Position  Potential   Jury    N-Glyc
                                 agreement result
----------------------------------------------------------------------
contig013363-TilORe.H122    3 NVSN   0.7840     (9/9)   +++
contig013363-TilORe.H122   16 NETM   0.6986     (9/9)   ++
contig013363-TilORe.H122   40 NISL   0.6451     (9/9)   ++
contig013363-TilORe.H122  195 NISA   0.5848     (6/9)   +
----------------------------------------------------------------------
```

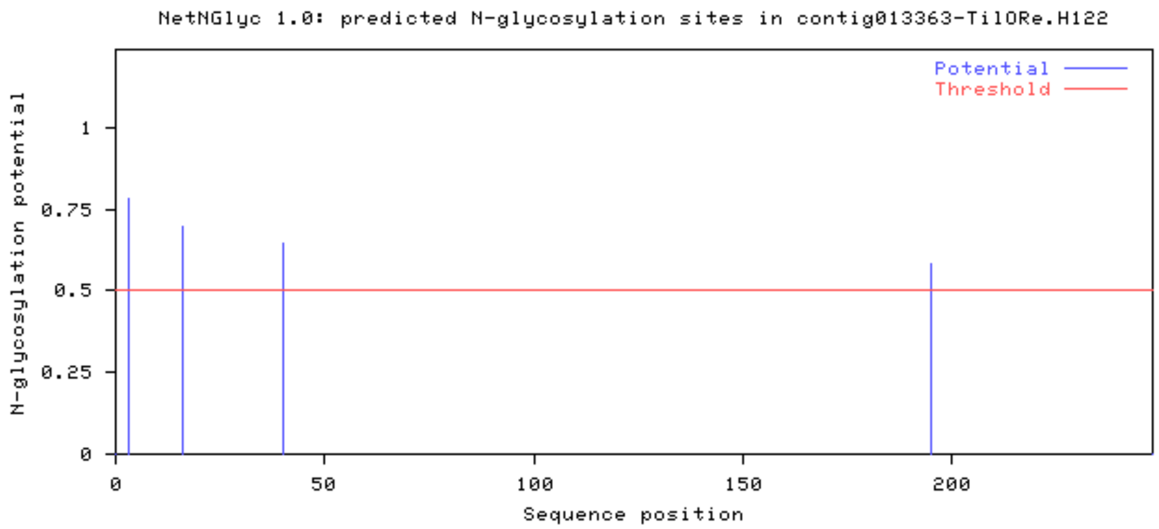

Graphics in PostScript

Output for 'contig013365-TilOR.H111'

```
##########################################################################################

    Warning: This sequence may not contain a signal peptide!!

    Proteins without signal peptides are unlikely to be exposed to
    the N-glycosylation machinery and thus may not be glycosylated
    (in vivo) even though they contain potential motifs.

    SignalP-NN euk predictions are as follows:

  # name                 Cmax  pos ?  Ymax  pos ?  Smax  pos ?  Smean ?  D     ?

    SignalP output is explained at http://www.cbs.dtu.dk/services/SignalP/output.html

##########################################################################################

Name:  contig013365-TilOR.H111  Length:  314
MDNVSVITVFTLSGLSDIANYRVTLFVLTLLCYCVIWLVNLTIIVTVIVDKSLHEPMYIFLCNLCVNGLYGTAAFYPKFL      80
YDLLSTTHVISYAGCLLQGFVLHSSVCADFSLLALMAYDRYVAICRPLLYHSLMTKQRVSIFVFFAWLIPFYLLFMSTIT     160
TAVLRLCGSHIPRIYCINWLISNLACSASVAKIVIPAFTYTFYIGHVLFVFWSYVHLIKTCQSSKENWNKFMQTCVPHVF     240
SLTVVVLSFLFDMLYMRFGSKEIPQGFENFMAMEILLIPPIINPLMYGFKLTQIRNTVLNFICGKSSALRLKLX
..N....................................N........................................      80
................................................................................     160
................................................................................     240
..........................................................................           320

(Threshold=0.5)
----------------------------------------------------------------------
SeqName      Position  Potential   Jury    N-Glyc
                                 agreement result
----------------------------------------------------------------------
contig013365-TilOR.H111    3 NVSV   0.8138     (9/9)   +++
contig013365-TilOR.H111   40 NLTI   0.7720     (9/9)   +++
----------------------------------------------------------------------
```

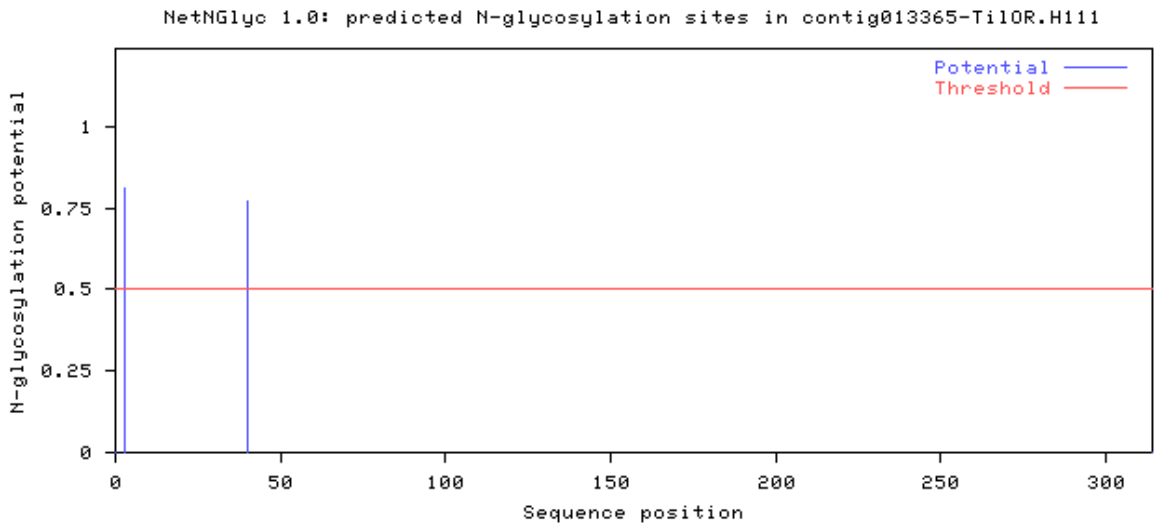

Graphics in PostScript

Output for 'contig013366-TilORp.H127'

```
##########################################################################################

    Warning: This sequence may not contain a signal peptide!!

    Proteins without signal peptides are unlikely to be exposed to
    the N-glycosylation machinery and thus may not be glycosylated
    (in vivo) even though they contain potential motifs.

    SignalP-NN euk predictions are as follows:

  # name                Cmax  pos ?  Ymax  pos ?  Smax  pos ?  Smean ?  D     ?

    SignalP output is explained at http://www.cbs.dtu.dk/services/SignalP/output.html

##########################################################################################

Name:  contig013366-TilORp.H127         Length:  285
MNFRVPLFSVTLLYCVILFFNISVVLLIVLDENLHEPMYILLSSFCINALYGSTGFYPKFLSDLLSPSQTISHEGCLLQA      80
FIMYLFVCCNSSILAVMAFDRYLAICLYYHSFMTKRRLSQLVCFSWLTPFCVFAINVVLTARLKLCGINIQRVLCLNWLI     160
VKLACPEADTFSNNITVIIYVSHGFFIIWTYTHLIKTCTKSREDRVKFMQTCVPHLTSLITFLSVVVFQFVYMQFDSTDL     240
PQSLQNFIATEFVIIPPIMNPLIYGFKLTKIRNRILGFVCFKRKX
....................N...........................................................      80
.........N......................................................................     160
.............N..................................................................     240
.............................................                                         320

(Threshold=0.5)
----------------------------------------------------------------------
SeqName      Position  Potential   Jury    N-Glyc
                                 agreement result
----------------------------------------------------------------------
contig013366-TilORp.H127    21 NISV   0.6773     (9/9)   ++
contig013366-TilORp.H127    90 NSSI   0.6046     (8/9)   +
contig013366-TilORp.H127   174 NITV   0.6948     (8/9)   +
----------------------------------------------------------------------
```

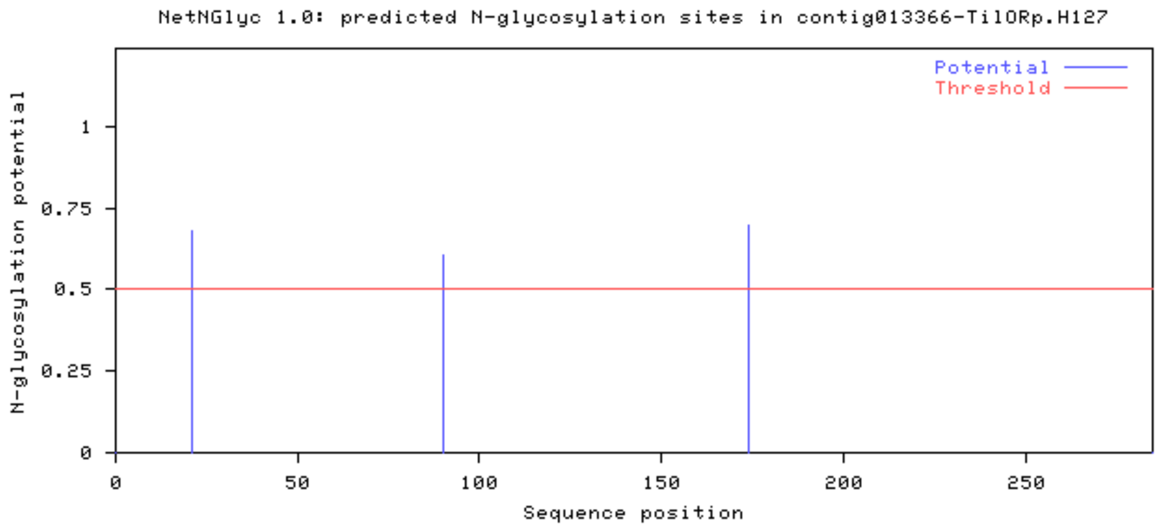

Graphics in PostScript

Output for 'contig013368-TilOR.H112'

```
##########################################################################################

    Warning: This sequence may not contain a signal peptide!!

    Proteins without signal peptides are unlikely to be exposed to
    the N-glycosylation machinery and thus may not be glycosylated
    (in vivo) even though they contain potential motifs.

    SignalP-NN euk predictions are as follows:

  # name                 Cmax  pos ?  Ymax  pos ?  Smax  pos ?  Smean ?  D     ?

    SignalP output is explained at http://www.cbs.dtu.dk/services/SignalP/output.html

##########################################################################################

Name:  contig013368-TilOR.H112  Length:  310
MANQSIERSFILLGFNETMNFRVPLFLLTLLYYCVILFFNISLVLLIVLDTNLHEPMYIFLSSFCINALYGSTGFYPKFL      80
SDLLSSFHGISYEGCLLQAFIMYSFASCDLTILAVMAFDRYLAICRPLHYHSLMTKRRLSQLVCFSWLTPFCIFSINVIL     160
TTRLRLCGLNIQRVLCVNWLIVKLACPEADTFSNNISSYLTIIFYISHGFFIMWTYMHLIKTCVRSREDRVKFMQTCVPH     240
LISLITLLVVMIFDSMYLRFGSRDLPQSLQNFITIEFLIIPPVMNPLIYGFKLTKIRNRILSLIYLKRKX
..N............N.......................N........................................      80
................................................................................     160
..................................N.............................................     240
......................................................................               320

(Threshold=0.5)
----------------------------------------------------------------------
SeqName      Position  Potential   Jury     N-Glyc
                                agreement  result
----------------------------------------------------------------------
contig013368-TilOR.H112    3 NQSI    0.6241     (9/9)    ++
contig013368-TilOR.H112   16 NETM    0.6531     (9/9)    ++
contig013368-TilOR.H112   40 NISL    0.6698     (9/9)    ++
contig013368-TilOR.H112  195 NISS    0.6156     (8/9)    +
----------------------------------------------------------------------
```

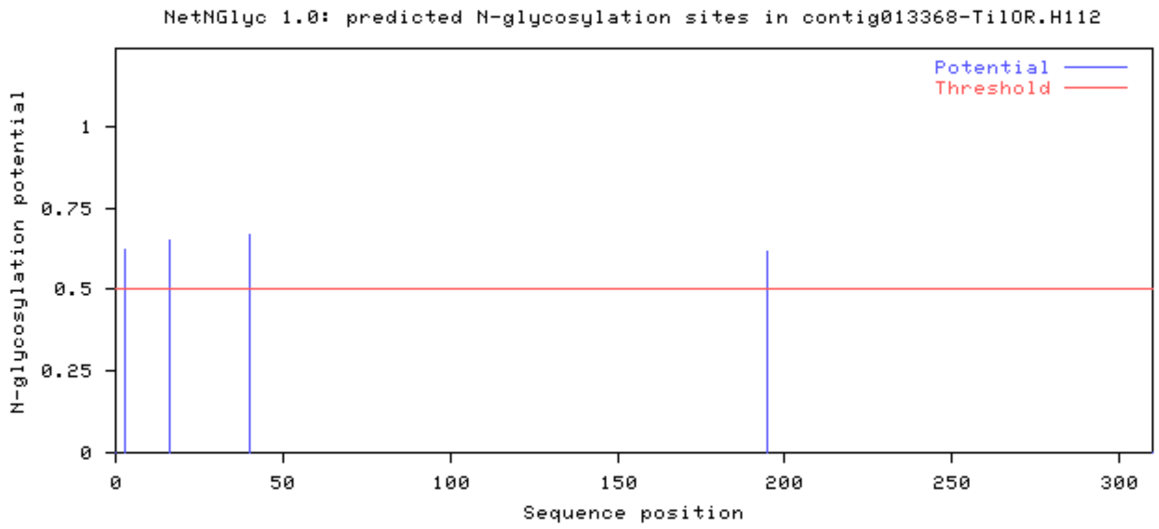

Graphics in PostScript

Output for 'contig013368-TilOR.H113'

```
##########################################################################

    Warning: This sequence may not contain a signal peptide!!

    Proteins without signal peptides are unlikely to be exposed to
    the N-glycosylation machinery and thus may not be glycosylated
    (in vivo) even though they contain potential motifs.

    SignalP-NN euk predictions are as follows:

  # name                    Cmax   pos ?  Ymax   pos ?  Smax   pos ?  Smean ?  D     ?

    SignalP output is explained at http://www.cbs.dtu.dk/services/SignalP/output.html

##########################################################################

Name:  contig013368-TilOR.H113  Length:  319
MDNVSNVRSFILVGFNETTNYRVPLLLATLLYYCVILIINIALILIIVLDENLHEPMYILLSSFCINALYGSTGFYPKFL      80
LDLLSSSHRISYEGCLLQAFIMYSFACSDLSILAVMAFDRYLAICRPLHYHSFMTKRRLSQLVCFSWLTPFCIFFLNILI     160
TSRLKLCDIKIQRVLCLNWLIIKLACPEADTFRKNISAYVTFIAYLSHWLFIVWTYMHLIKTCVRSREDRVKFMQTCVPH     240
LISLMTFLFVLVFDPVYMIFGSTRLPQSLQNFITIEFLIIPPVMNPLIYGFKLTKIRNRILGLVCFKRKSIPSKLRVSX
..N............N................................................................      80
................................................................................     160
..................................N.............................................     240
...............................................................................      320

(Threshold=0.5)
----------------------------------------------------------------------
SeqName      Position  Potential    Jury    N-Glyc
                                agreement  result
----------------------------------------------------------------------
contig013368-TilOR.H113    3 NVSN   0.7952     (9/9)    +++
contig013368-TilOR.H113   16 NETT   0.5424     (5/9)    +
contig013368-TilOR.H113  195 NISA   0.6089     (8/9)    +
----------------------------------------------------------------------
```

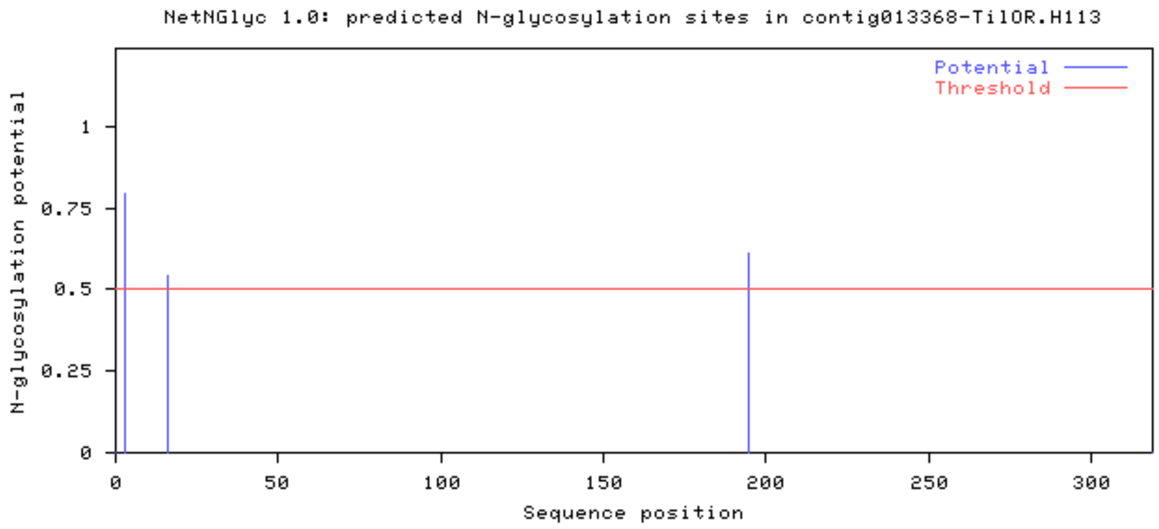

[Graphics in PostScript]

Output for 'contig013369-TilOR.H114'

```
##############################################################################

    Warning: This sequence may not contain a signal peptide!!

    Proteins without signal peptides are unlikely to be exposed to
    the N-glycosylation machinery and thus may not be glycosylated
    (in vivo) even though they contain potential motifs.

    SignalP-NN euk predictions are as follows:

  # name                 Cmax  pos ?  Ymax  pos ?  Smax  pos ?  Smean ?  D     ?

    SignalP output is explained at http://www.cbs.dtu.dk/services/SignalP/output.html

##############################################################################

Name:  contig013369-TilOR.H114  Length:  310
MDNQSNVRSFILSGFNETVNFRVPLFSFTLLCYCVILFINISLVLLIVLDANLHEPMYILLSSFCINAVYGTTGFYPKFL      80
SDLLSSSQTISYEGCLLQAFIMYSFGCCDLSILAVMAFDRYLAICRPLHYHSFMTKRRLSQLVCFSWLTPLCTFSINIVL     160
TSKLRLCGINIQRVLCLNWLIVKLACPEADTFSNNISAYVIFIFYVSHGFFIMWTYMHLIKTCVRSRDDRLKFMQTCVPH     240
LISLITFLSVIVFQYVYLRLDSTDLSQSLQNFIAIEFLIIPPVMNPLVYGFKLAKIRNRIFTLVHYKTKX
..N...........N.......................N.........................................      80
................................................................................     160
..................................N.............................................     240
......................................................................               320

(Threshold=0.5)
----------------------------------------------------------------------
SeqName      Position  Potential   Jury    N-Glyc
                                 agreement result
----------------------------------------------------------------------
contig013369-TilOR.H114    3 NQSN   0.6998     (9/9)   ++
contig013369-TilOR.H114   16 NETV   0.6887     (9/9)   ++
contig013369-TilOR.H114   40 NISL   0.6640     (9/9)   ++
contig013369-TilOR.H114  195 NISA   0.5777     (7/9)   +
----------------------------------------------------------------------
```

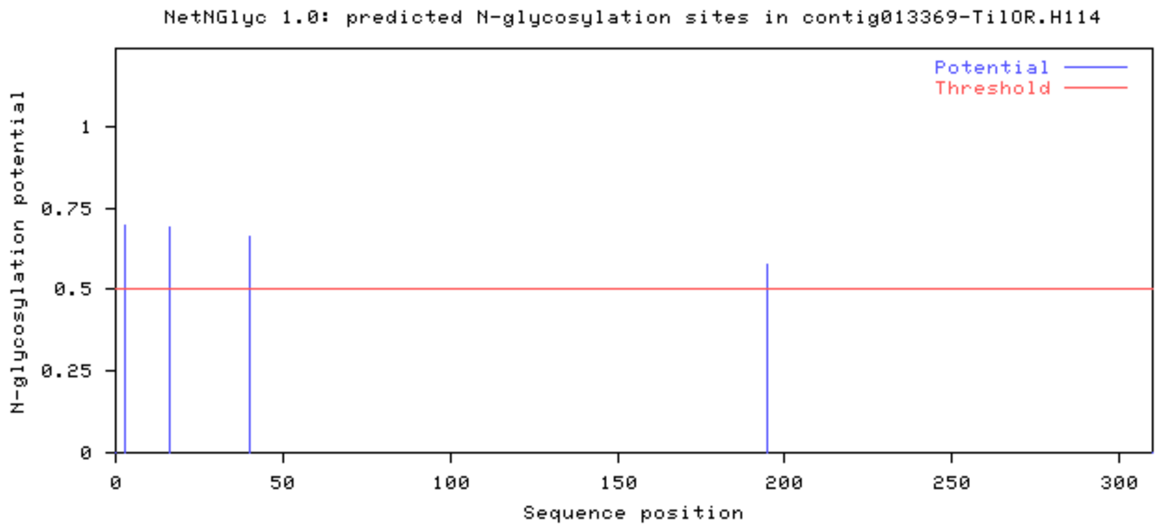

Graphics in PostScript

Output for 'contig013369-TilOR.H115'

```
##########################################################################

    Warning: This sequence may not contain a signal peptide!!

    Proteins without signal peptides are unlikely to be exposed to
    the N-glycosylation machinery and thus may not be glycosylated
    (in vivo) even though they contain potential motifs.

    SignalP-NN euk predictions are as follows:

  # name                    Cmax  pos ?  Ymax  pos ?  Smax  pos ?  Smean ?  D     ?

    SignalP output is explained at http://www.cbs.dtu.dk/services/SignalP/output.html

##########################################################################

Name:  contig013369-TilOR.H115  Length:  310
MANQSNERSFILSGFNETMNFRIPLFLCTLLYYSMILFFSISVVLLIVFDANLHEPMYIFLSSFCINALYGSTGFYPKFL      80
SDLLSSSQRISYEGCLLQAFIMYSFVCSDLSILAVMAFDRYLAICRPLHYHSFMTKRRLSQLVCFSWLTPFCIFSINVIL     160
TTRLRLCGINIQRVLCLNWLIVKLACPEADTFSNNISSYVILVVYLSHWLFIMWTYMHLIKTCVRSREDRVKFMQTCVPH     240
LISLITLLVVMIFDSMYLRFGSRDLPQSLQNFITIEFLIIPPVMNPLIYGFKLTKIRNRILSLIYLKRKX
..N............N................................................................      80
................................................................................     160
..................................N.............................................     240
......................................................................               320

(Threshold=0.5)
----------------------------------------------------------------------
SeqName      Position  Potential   Jury    N-Glyc
                                agreement  result
----------------------------------------------------------------------
contig013369-TilOR.H115    3 NQSN   0.6252     (9/9)    ++
contig013369-TilOR.H115   16 NETM   0.6248     (9/9)    ++
contig013369-TilOR.H115  195 NISS   0.6643     (8/9)    +
----------------------------------------------------------------------
```

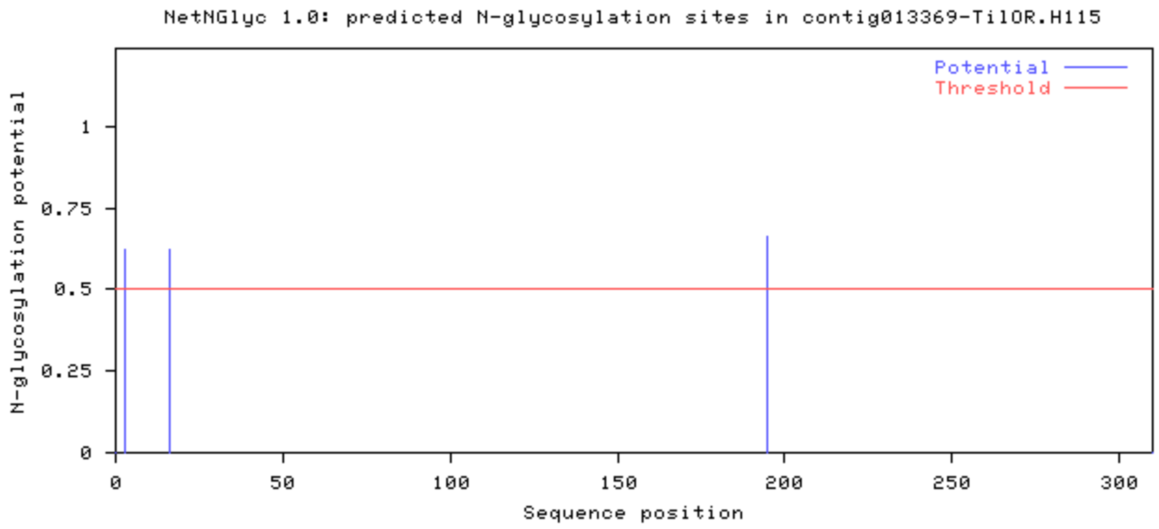

Graphics in PostScript

Output for 'contig013369-TilOR.H116'

```
##########################################################################################

    Warning: This sequence may not contain a signal peptide!!

    Proteins without signal peptides are unlikely to be exposed to
    the N-glycosylation machinery and thus may not be glycosylated
    (in vivo) even though they contain potential motifs.

    SignalP-NN euk predictions are as follows:

  # name                    Cmax  pos ?  Ymax  pos ?  Smax  pos ?  Smean ?  D     ?

    SignalP output is explained at http://www.cbs.dtu.dk/services/SignalP/output.html

##########################################################################################

Name:  contig013369-TilOR.H116  Length:  342
MDNVSNIRSFILSGFNETVNFRVPLFSLTLLYYCVILFFNISLILLIVLDANLHEPMYILLCIFCINAIYGSTGFYPKFL      80
SDLLSSSHRISYEGCLLQAFIMYSFACSDLSILAVMAFDRYLAICRPLHYHSFMTKRRLSQLVCFSWLTPFCIFSINILI     160
TTRLKLCGLNIQRVLCLNWLIVKLACPADKTFPNNISSYVILVVYLSHWLFIIWTYMHLIKTCVRSRDDRLKFMQTCVPH     240
LTSLIIFLIVMAFDPAYVEFGSRDLSQTLQNLFSIEFLIIPPVMNPLIYGFKITKIRNRILGLVCFKRQRISSRFTLLFR     320
NKSEELWKMCITVLDLINITRX
..N............N.......................N........................................      80
................................................................................     160
..................................N.............................................     240
................................................................................     320
N................N....                                                               400

(Threshold=0.5)
----------------------------------------------------------------------
SeqName      Position  Potential   Jury    N-Glyc
                                agreement  result
----------------------------------------------------------------------
contig013369-TilOR.H116    3 NVSN   0.7998     (9/9)   +++
contig013369-TilOR.H116   16 NETV   0.6721     (9/9)   ++
contig013369-TilOR.H116   40 NISL   0.6827     (9/9)   ++
contig013369-TilOR.H116  195 NISS   0.7086     (8/9)   +
contig013369-TilOR.H116  321 NKSE   0.5361     (5/9)   +
contig013369-TilOR.H116  338 NITR   0.5137     (5/9)   +
----------------------------------------------------------------------
```

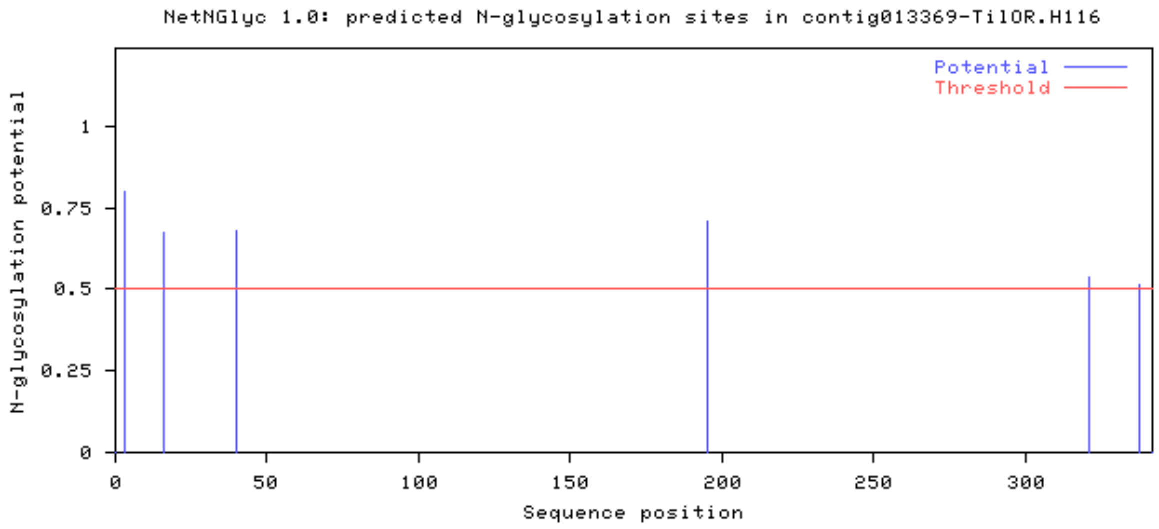

Graphics in PostScript

Output for 'contig013371-TilOR.H117'

```
##########################################################################################

    Warning: This sequence may not contain a signal peptide!!

    Proteins without signal peptides are unlikely to be exposed to
    the N-glycosylation machinery and thus may not be glycosylated
    (in vivo) even though they contain potential motifs.

    SignalP-NN euk predictions are as follows:

  # name                  Cmax  pos ?  Ymax  pos ?  Smax  pos ?  Smean ?  D     ?

    SignalP output is explained at http://www.cbs.dtu.dk/services/SignalP/output.html

##########################################################################################

Name:  contig013371-TilOR.H117  Length:  310
MDNVSVVRIFTLSGFNETMNIRLTIFSLTLMYYCMIILINMSLVVIIVLDENLHEPMYILLSSFCINALYGTTGFYPKFL      80
LDLLSSSQEISYEGCLLQALIMYSFACCDLSILAVMAFDRYLAICRPLHYHSFMTKRRLSQLMCFSWLTPLCIISISILL     160
TSRLTLCRSKVEKVFCVNWVIVKLACSDTDTLLNSVVSYATIIIYMSHGFFIMWTYMHLIKTSVRSKEDRAKFMQTCVPH     240
LTSLITFLIVIVFDLMYMRFGSADLPQSLQNFIAIEFLVIPPVMNPLIYGFKLTKIRNKILSFVYRKQKX
..N............N.......................N........................................      80
................................................................................     160
................................................................................     240
......................................................................               320

(Threshold=0.5)
----------------------------------------------------------------------
SeqName      Position  Potential   Jury    N-Glyc
                                 agreement result
----------------------------------------------------------------------
contig013371-TilOR.H117    3 NVSV   0.8154     (9/9)    +++
contig013371-TilOR.H117   16 NETM   0.6328     (9/9)    ++
contig013371-TilOR.H117   40 NMSL   0.7097     (9/9)    ++
----------------------------------------------------------------------
```

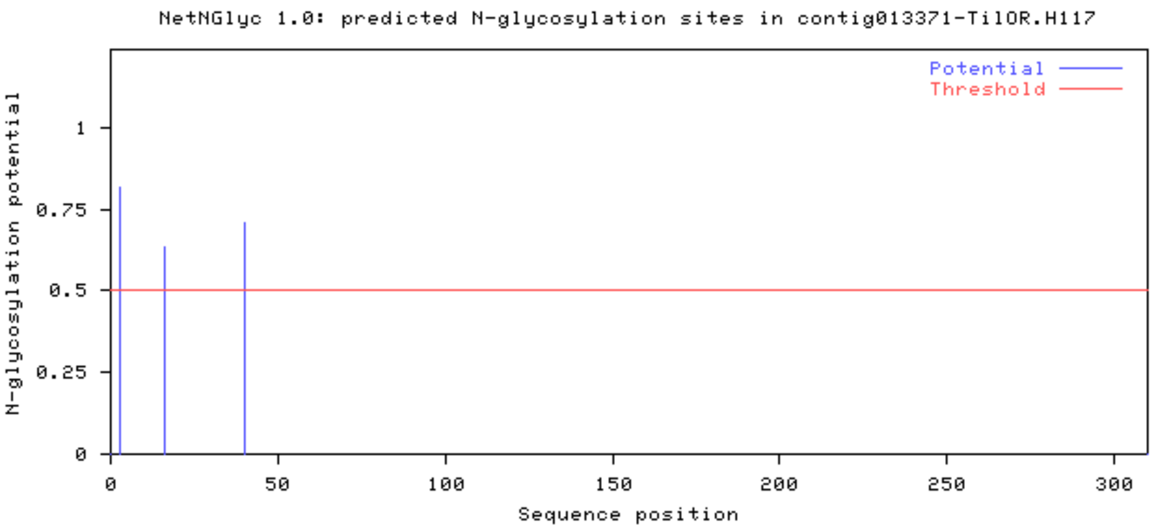

Graphics in PostScript

Output for 'contig013371-TilOR.H118'

```
##########################################################################################

    Warning: This sequence may not contain a signal peptide!!

    Proteins without signal peptides are unlikely to be exposed to
    the N-glycosylation machinery and thus may not be glycosylated
    (in vivo) even though they contain potential motifs.

    SignalP-NN euk predictions are as follows:

  # name                 Cmax  pos ?  Ymax  pos ?  Smax  pos ?  Smean ?  D     ?

    SignalP output is explained at http://www.cbs.dtu.dk/services/SignalP/output.html

##########################################################################################

Name:  contig013371-TilOR.H118  Length:  307
MDNVSNVRSFILSGFNETMNFRVPLFTFTLLYYCMILFINISLVLLIFLDENLHEPMYILLSSFCINAIYGTTGFYPKFL      80
SDLLSSSQRISYEGCLLQAFVIYSFVCCDLSILAVMAFDRYLAICRPLHYHSFMTNRRLSQLVCFSWLTPLCIFAINVLL     160
TSRLKLCGINIRRVLCVNWLIVKLACPEAETFTNNITAYATVIIYVSHGFFIMWTYTHLIKTCARSREDREKFMQTCLPH     240
LTSLITFIVVMGFQSIYVQFEYTGFSESLKNFITVEILIIPPFMNPLMYGFKLTKIRNRIMTLLKSX
..N............N.......................N........................................      80
................................................................................     160
..................................N.............................................     240
...................................................................                  320

(Threshold=0.5)
----------------------------------------------------------------------
SeqName      Position  Potential   Jury    N-Glyc
                                 agreement result
----------------------------------------------------------------------
contig013371-TilOR.H118    3 NVSN   0.7938     (9/9)    +++
contig013371-TilOR.H118   16 NETM   0.6271     (9/9)    ++
contig013371-TilOR.H118   40 NISL   0.6888     (9/9)    ++
contig013371-TilOR.H118  195 NITA   0.6627     (8/9)    +
----------------------------------------------------------------------
```

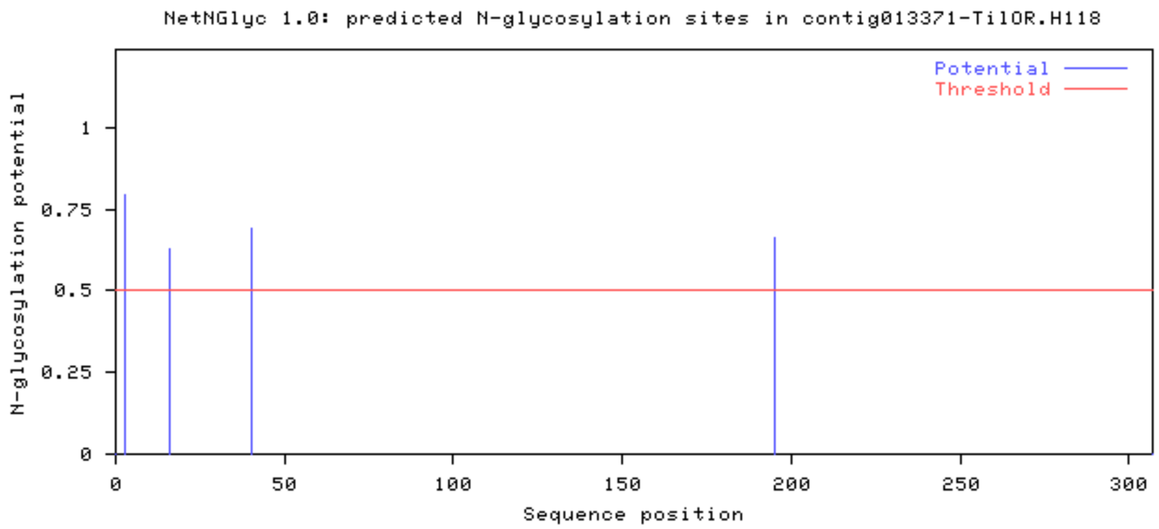

Graphics in PostScript

Output for 'contig022204-TilOR.A001'

```
##########################################################################################

    Warning: This sequence may not contain a signal peptide!!

    Proteins without signal peptides are unlikely to be exposed to
    the N-glycosylation machinery and thus may not be glycosylated
    (in vivo) even though they contain potential motifs.

    SignalP-NN euk predictions are as follows:

  # name                   Cmax  pos ?  Ymax  pos ?  Smax  pos ?  Smean ?  D     ?

    SignalP output is explained at http://www.cbs.dtu.dk/services/SignalP/output.html

##########################################################################################

Name:  contig022204-TilOR.A001  Length:  308
MDDELNVTYITFDGHVEINKYRYVYFFILFTLYILITCSNSIIIYLILIHKNLHEPMYIFIAALLLNVVLYATTIYPKLL      80
IDFLSEKQIISYSACLFQFFIIYSLGCSEFFLLAAMAYDRYVAICKPLQYPTIMRKSTVSIFLVIAWLVPASNIAVQAIG     160
MAKSKLCSFHLKSIFCNNTIYTLQCVRSRLVTVFGIVSYLDLVVFPILFIVFTYTKIFIVTYRSCKEIKKKAAETCLPHM     240
LVLVTFSCLGVYDVIMARMETDFPKTARLIMTLQVAFYHPLFNPFIYGLKMKEISKHLKRLFCPPVSX
.....N..........................................................................      80
................................................................................     160
................................................................................     240
....................................................................                 320

(Threshold=0.5)
----------------------------------------------------------------------
SeqName      Position  Potential   Jury    N-Glyc
                                 agreement result
----------------------------------------------------------------------
contig022204-TilOR.A001    6 NVTY    0.7981     (9/9)    +++
contig022204-TilOR.A001  177 NNTI    0.4913     (4/9)    -
----------------------------------------------------------------------
```

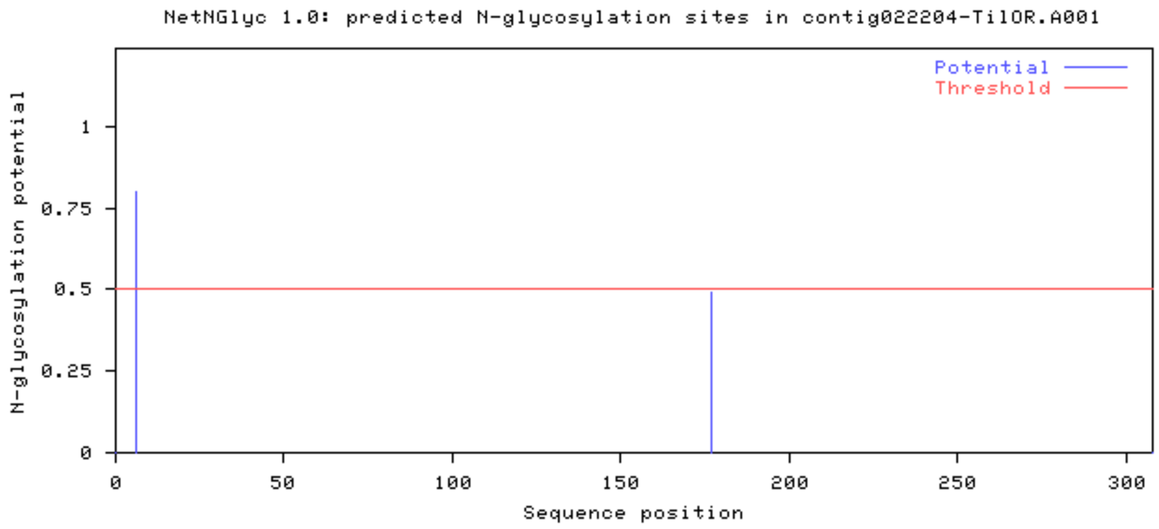

Graphics in PostScript

Output for 'contig022209-TilORe.A035'

```
################################################################################

   Warning: This sequence may not contain a signal peptide!!

   Proteins without signal peptides are unlikely to be exposed to
   the N-glycosylation machinery and thus may not be glycosylated
   (in vivo) even though they contain potential motifs.

   SignalP-NN euk predictions are as follows:

 # name                Cmax  pos ?  Ymax  pos ?  Smax  pos ?  Smean ?  D     ?

   SignalP output is explained at http://www.cbs.dtu.dk/services/SignalP/output.html

################################################################################

Name:  contig022209-TilORe.A035        Length:  147
MDEKINVTYITLDGFVEIDKYRYVYFYIMFIVYILIICCNSTILYLICIHQNLHEPMYIFIAALLLNCALYSTAVYPKYL      80
IDFLSEKQVISYSACLFQYFLFYSLAGSEFFLLAAMAYDRYVAICKPLQYPTIMRKKTVSIFLFIAW
.....N.................................N........................................      80
...................................................................                  160

(Threshold=0.5)
----------------------------------------------------------------------
SeqName      Position  Potential   Jury    N-Glyc
                                 agreement result
----------------------------------------------------------------------
contig022209-TilORe.A035    6 NVTY    0.7557     (9/9)   +++
contig022209-TilORe.A035   40 NSTI    0.5761     (8/9)   +
----------------------------------------------------------------------
```

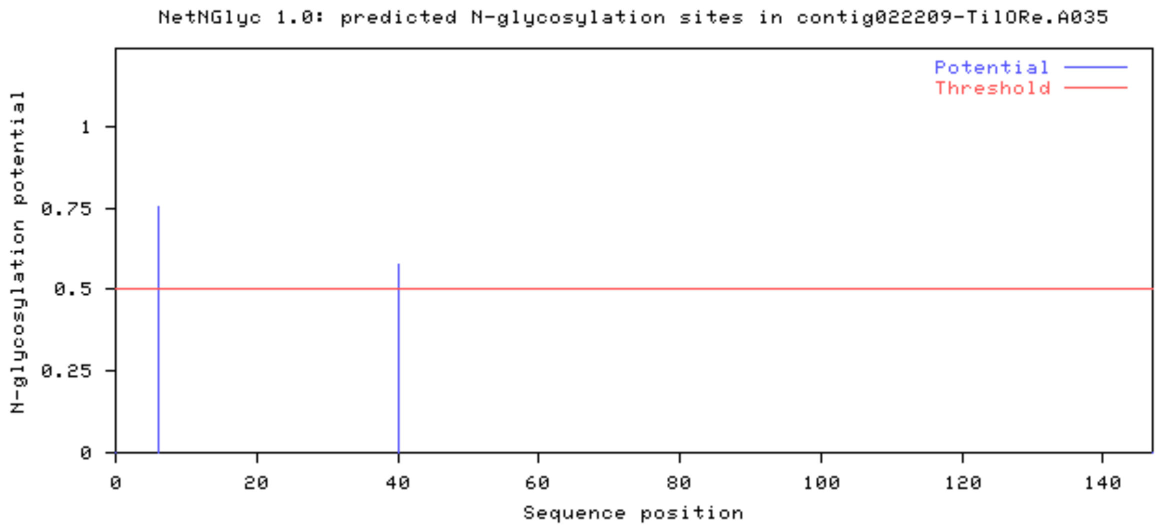

Graphics in PostScript

Output for 'contig022211-TilOR.A003'

```
##################################################################################

    Warning: This sequence may not contain a signal peptide!!

    Proteins without signal peptides are unlikely to be exposed to
    the N-glycosylation machinery and thus may not be glycosylated
    (in vivo) even though they contain potential motifs.

    SignalP-NN euk predictions are as follows:

  # name                Cmax  pos ?  Ymax  pos ?  Smax  pos ?  Smean ?  D     ?

    SignalP output is explained at http://www.cbs.dtu.dk/services/SignalP/output.html

##################################################################################

Name:  contig022211-TilOR.A003  Length:  316
MDQELNFTYVTLDWYVDINKYRYVYFFIMFALYSLIICTNSTIVYIIWIHKNLHEPMYIFIAALLLNSVLYSTTIYPKLL      80
IDFLSEKQVTTYSACLFQFFMFYTLGGSEFFLLAAMAYDRYVAICKPLQYHIIMRKSTVSIFLIIAWVVPGCHIAVQAIG     160
SANIRLCDFNIKGIFCNNAVYTLLCERSRLITIFGVVALLDLAVLPMLFIVFTYTKICIVSYQSCKEIQKKAAETCLPHL     240
LVLISASVFFVYDVSIARVETNFPKTVRIVMTLQVVLYHPIFNPFIYGLKMKQISKHLKRCFHRPRSILVLKVNAX
.....N................................N.........................................      80
................................................................................     160
................................................................................     240
............................................................................         320

(Threshold=0.5)
----------------------------------------------------------------------
SeqName      Position  Potential   Jury    N-Glyc
                                 agreement result
----------------------------------------------------------------------
contig022211-TilOR.A003    6 NFTY   0.7623     (9/9)    +++
contig022211-TilOR.A003   40 NSTI   0.7442     (9/9)    ++
----------------------------------------------------------------------
```

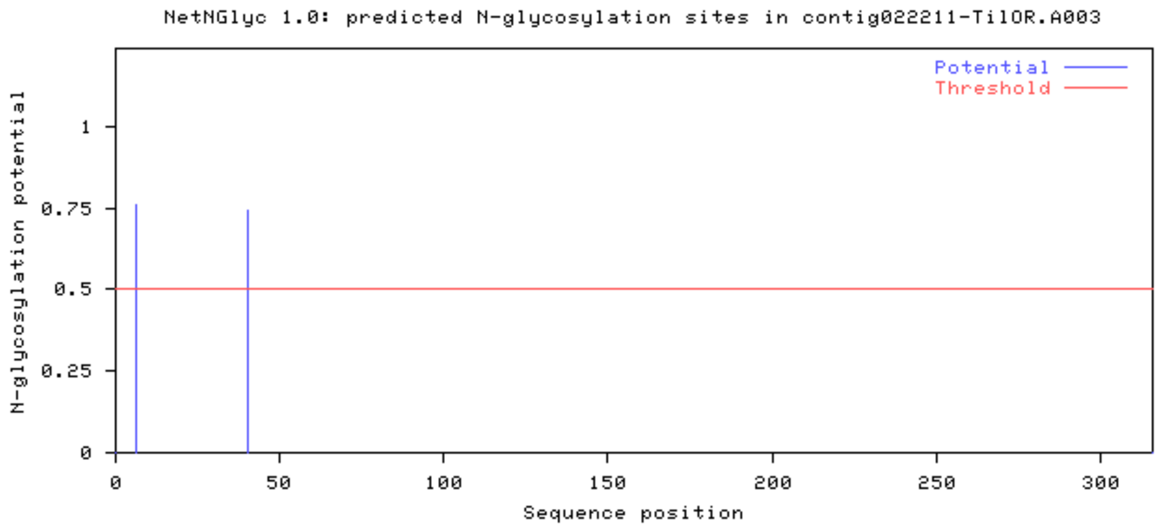

Graphics in PostScript

Output for 'contig022217-TilOR.A004'

```
##########################################################################

    Warning: This sequence may not contain a signal peptide!!

    Proteins without signal peptides are unlikely to be exposed to
    the N-glycosylation machinery and thus may not be glycosylated
    (in vivo) even though they contain potential motifs.

    SignalP-NN euk predictions are as follows:

  # name                    Cmax  pos ?  Ymax  pos ?  Smax  pos ?  Smean ?  D     ?

    SignalP output is explained at http://www.cbs.dtu.dk/services/SignalP/output.html

##########################################################################

Name:  contig022217-TilOR.A004  Length:  316
MDKESNVTFLTLDWYTEISKYRYIFFFIMFTLYILIIYTNSTIVYLIFIHKNLHEPMYIFIAALLLNSVLYSTTVYPKLL      80
IGFLSEKQVTTYSACLFQFFMFYTLGGSEFLLLAAMAYDRYVAICKPLQYQIIMRKTTVSISLIIAWLVPGCHMAVLAIA     160
STEAKLCDFKIKGIFCNNAVYTLQCQRSRLITIFGVVTLLDLAILPMLFIVFTYTKIFIVSHQSCKEIRKKAAETCLPHL     240
LVLISACLFYVYDVSIARVELDFPKTARIIMTLQIVLYHPLFNPFVYGLKMKEISKHLKGLLSQAKFITCIKIGCX
.....N.................................N........................................      80
................................................................................     160
................................................................................     240
............................................................................         320

(Threshold=0.5)
----------------------------------------------------------------------
SeqName      Position  Potential   Jury    N-Glyc
                                 agreement result
----------------------------------------------------------------------
contig022217-TilOR.A004    6 NVTF   0.7299     (9/9)    ++
contig022217-TilOR.A004   40 NSTI   0.6764     (8/9)    +
----------------------------------------------------------------------
```

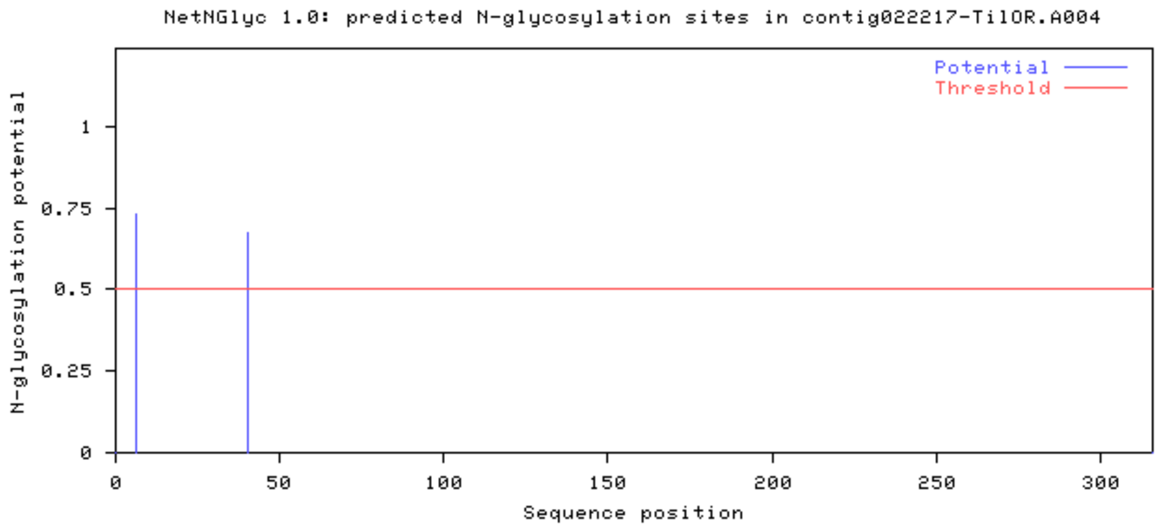

Graphics in PostScript

Output for 'contig022221-TilORe.A034'

```
##########################################################################################

    Warning: This sequence may not contain a signal peptide!!

    Proteins without signal peptides are unlikely to be exposed to
    the N-glycosylation machinery and thus may not be glycosylated
    (in vivo) even though they contain potential motifs.

    SignalP-NN euk predictions are as follows:

  # name                 Cmax  pos ?  Ymax  pos ?  Smax  pos ?  Smean ?  D     ?

    SignalP output is explained at http://www.cbs.dtu.dk/services/SignalP/output.html

##########################################################################################

Name:  contig022221-TilORe.A034         Length:  172
IAWLVPGCHMAVLAIASTEAKLCDFKIKGIFCNNAVYTLQCQRSRLITIFGVVTLLDLVILPMLFIVFTYTKIFIVSYQS      80
CKEIRKKAAETCLPHLLVLISACLFYVYDVSIARVELDFPKTVRIIMTLQIVLYHPLFNPFVYGLKMKEISKHLKGLLCQ     160
GKVIFCIKTGCX
................................................................................      80
................................................................................     160
............                                                                         240

(Threshold=0.5)

No sites predicted in this sequence.
```

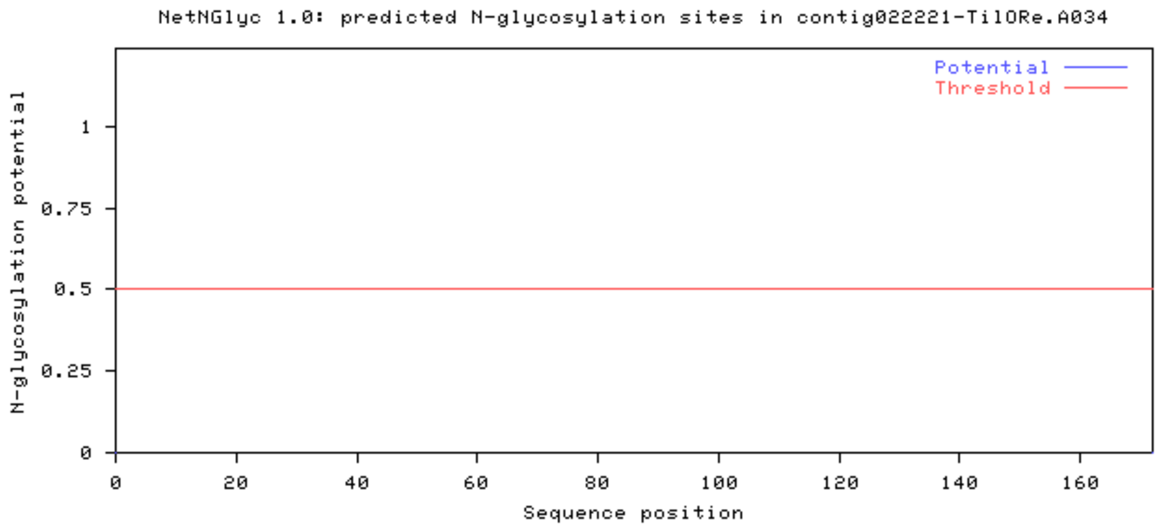

Graphics in PostScript

Output for 'contig022225-TilOR.A005'

```
##########################################################################################

    Warning: This sequence may not contain a signal peptide!!

    Proteins without signal peptides are unlikely to be exposed to
    the N-glycosylation machinery and thus may not be glycosylated
    (in vivo) even though they contain potential motifs.

    SignalP-NN euk predictions are as follows:

  # name                 Cmax  pos ?  Ymax  pos ?  Smax  pos ?  Smean ?  D     ?

    SignalP output is explained at http://www.cbs.dtu.dk/services/SignalP/output.html

##########################################################################################

Name:  contig022225-TilOR.A005  Length:  303
MDEELNVTYVTFDGHVEMNKYRYVYFFIMFTVYILIISSNFIIIYLILTHKNLHEPMYIFIAALLLNVVIYSTTIYPKLL      80
IDFLSEKQIISYSACLFQFYIIYSLGCSEFFLLAAMAYDRYVAICKPLQYPTIMRKSTVSIFLVIAWLVPASSISVQAIG     160
MAKSKLCSFHLKSIFCNNTIYTLQCVRSRLVTVFGIVSYLDLVVFPILFIVFTYTKIFIVTYRSCKEIKKKAAETCLPHM     240
LVLITFSCLGVYDVIMARMETDFPKIARLIMTLQITLYQPLFNPFIYGLKMKEISKHLKRLFX
.....N..........................................................................      80
................................................................................     160
................................................................................     240
...............................................................                      320

(Threshold=0.5)
----------------------------------------------------------------------
SeqName      Position  Potential   Jury    N-Glyc
                                 agreement result
----------------------------------------------------------------------
contig022225-TilOR.A005    6 NVTY   0.8133     (9/9)   +++
contig022225-TilOR.A005  177 NNTI   0.4892     (4/9)   -
----------------------------------------------------------------------
```

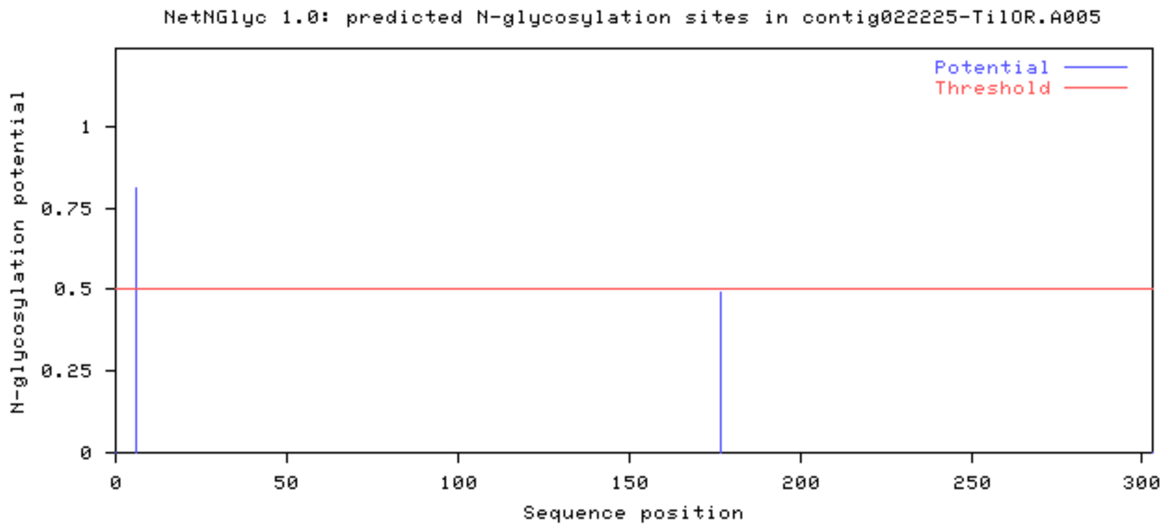

Graphics in PostScript

Output for 'contig022227-TilOR.A006'

```
##########################################################################

    Warning: This sequence may not contain a signal peptide!!

    Proteins without signal peptides are unlikely to be exposed to
    the N-glycosylation machinery and thus may not be glycosylated
    (in vivo) even though they contain potential motifs.

    SignalP-NN euk predictions are as follows:

  # name                Cmax  pos ?  Ymax  pos ?  Smax  pos ?  Smean ?  D     ?

    SignalP output is explained at http://www.cbs.dtu.dk/services/SignalP/output.html

##########################################################################

Name:  contig022227-TilOR.A006  Length:  313
MDDELNVTYIAFDGYVEINKYRYVYFFIIFTVYIVIICSNCIILYLICIHKNLHEPMYIFTAALLLNCVLYTTTIYPKFL      80
IDFLSEKQIISYSACLFQFFTIYSLGSSEFFLLAAMAYDRYVAICKPLQYPTIMRKSTVSIFLVIAWLVPASSISVQAIG     160
MAKSKLCSFHLKSIFCNNTIYTLQCVRSKLVTVFGIVCFFDFVILPLLFIVFTYTKIFIVSYHSCKEIKKKAAETCLPHI     240
LVLVSFSCFGVYDVTIARVESDFPKTARFIVSLQLILYQPLFNPLIYGLKMKEISKHLKRLFCPTKIICCINX
.....N..........................................................................      80
................................................................................     160
................................................................................     240
.........................................................................            320

(Threshold=0.5)
----------------------------------------------------------------------
SeqName      Position  Potential   Jury    N-Glyc
                                agreement  result
----------------------------------------------------------------------
contig022227-TilOR.A006    6 NVTY   0.8030     (9/9)   +++
contig022227-TilOR.A006  177 NNTI   0.4927     (4/9)   -
----------------------------------------------------------------------
```

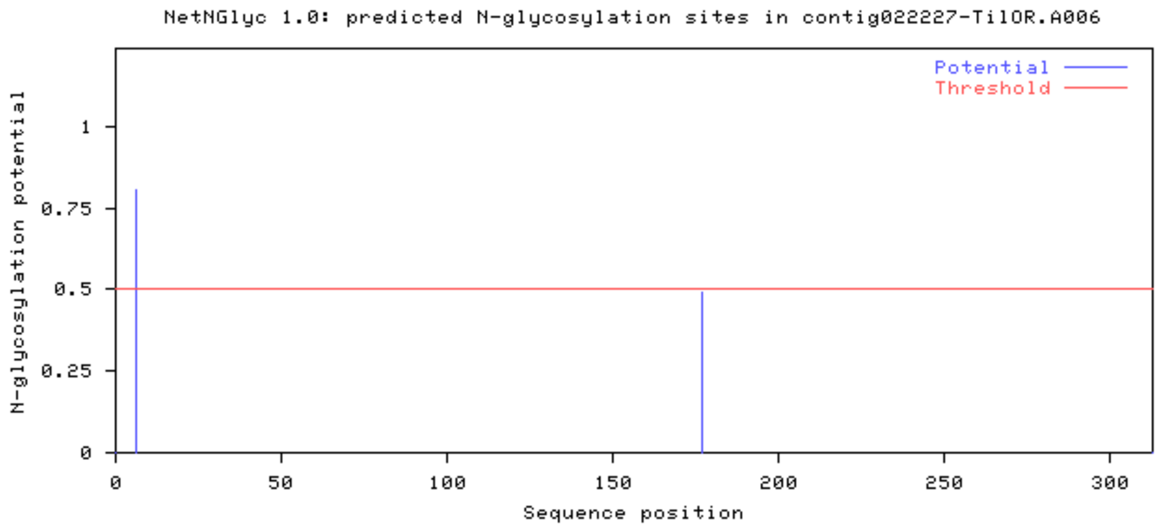

Graphics in PostScript

Output for 'contig022230-TilOR.A007'

```
##########################################################################

   Warning: This sequence may not contain a signal peptide!!

   Proteins without signal peptides are unlikely to be exposed to
   the N-glycosylation machinery and thus may not be glycosylated
   (in vivo) even though they contain potential motifs.

   SignalP-NN euk predictions are as follows:

 # name                Cmax  pos ?  Ymax  pos ?  Smax  pos ?  Smean ?  D     ?

   SignalP output is explained at http://www.cbs.dtu.dk/services/SignalP/output.html

##########################################################################

Name:  contig022230-TilOR.A007  Length:  300
MNITYITFGGHVEVEKYRYIYFVIMFMVYVLIICSNCTIVWLIITHKSLHEPMYIFIAALLVNSVLLSTVIYPKLLIDFL      80
SEKQIILYHACLFQVFMFYVLSSSEFLLLSAMAYDRYVSICKPLQYPTIMRRTTVSIFLLLSWFLPAIQIVVPVLRNSIT     160
PLCNFTLKGIFCNNSVNHLYCVTSKALSIYGMVVLFNGALFPMLFILFTYIKIIIVACQSCGNVRKKAAQTCLPHVLVLI     240
NYSCLVTYDMVIVRLESEFPKTARFIMTLQFVTYNPLCNPIIYGLKMKEISKNLKRLFSX
.N.................................N............................................      80
................................................................................     160
...N............................................................................     240
N...........................................................                         320

(Threshold=0.5)
----------------------------------------------------------------------
SeqName      Position  Potential   Jury    N-Glyc
                                 agreement result
----------------------------------------------------------------------
contig022230-TilOR.A007    2 NITY   0.7926     (9/9)   +++
contig022230-TilOR.A007   36 NCTI   0.7118     (9/9)   ++
contig022230-TilOR.A007  164 NFTL   0.6558     (9/9)   ++
contig022230-TilOR.A007  173 NNSV   0.4574     (6/9)   -
contig022230-TilOR.A007  241 NYSC   0.5286     (6/9)   +
----------------------------------------------------------------------
```

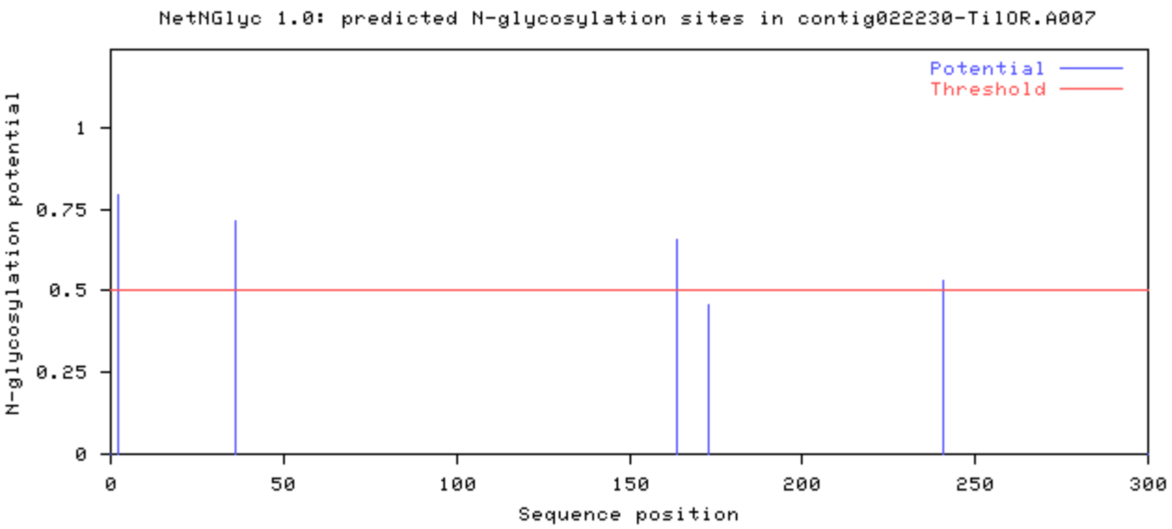

[Graphics in PostScript](#)

Output for 'contig022232-TilOR.A008'

```
##########################################################################################

    Warning: This sequence may not contain a signal peptide!!

    Proteins without signal peptides are unlikely to be exposed to
    the N-glycosylation machinery and thus may not be glycosylated
    (in vivo) even though they contain potential motifs.

    SignalP-NN euk predictions are as follows:

  # name                   Cmax  pos ?  Ymax  pos ?  Smax  pos ?  Smean ?  D     ?

    SignalP output is explained at http://www.cbs.dtu.dk/services/SignalP/output.html

##########################################################################################

Name:  contig022232-TilOR.A008  Length:  306
MDEVLNATYLTLDGYVEVNKYRYVYFFIFFILYSLIICSNSTIVYIIWIHINLHEPMYIFIAALLLNCVLYSTTVYPKLL      80
IDFLSEKQVITYSACLFQFFTFYTLGSSEFFLLAAMAYDRYVAICKPLQYQTIMGKTTVSIFLAVAWLVPACHIVVLTAG     160
SAEATLCNFNLKGIFCNNAVYTLQCVKSRLITVFGVVALIDLVILPMFFIVFTYSNIFILTYQSCKEVRKKAAETCLPHL     240
LVLFSFSCLSIYDVSIARVESDFPKTARLIMTLQIVLYHPLLNPFIYGLKMKDISKQLKRFFYHDX
.....N.................................N........................................      80
................................................................................     160
................................................................................     240
..................................................................                   320

(Threshold=0.5)
----------------------------------------------------------------------
SeqName      Position  Potential   Jury    N-Glyc
                                agreement result
----------------------------------------------------------------------
contig022232-TilOR.A008    6 NATY   0.6281     (9/9)   ++
contig022232-TilOR.A008   40 NSTI   0.7251     (9/9)   ++
----------------------------------------------------------------------
```

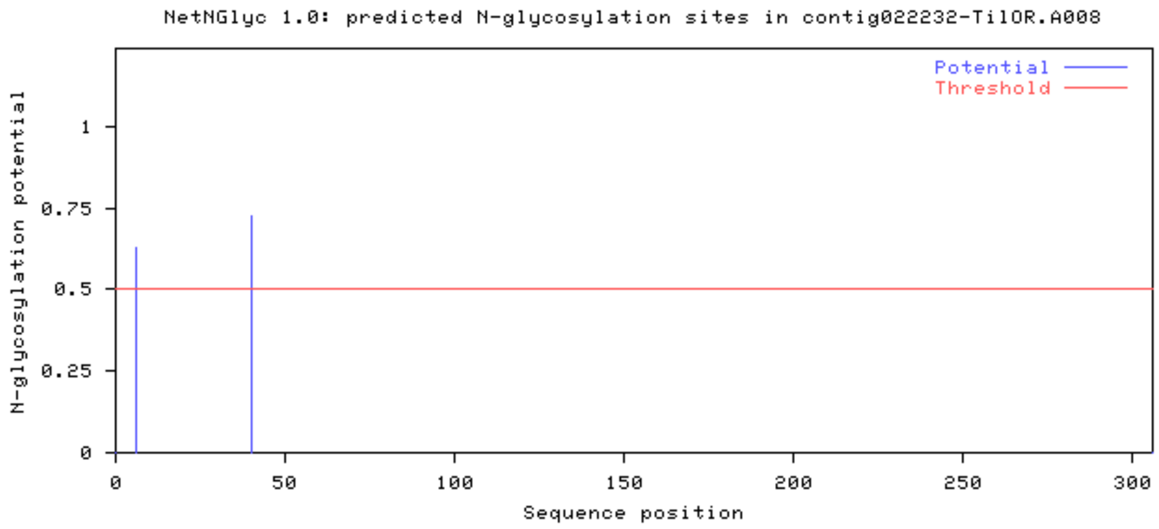

Graphics in PostScript

Output for 'contig022234-TilOR.A009'

```
##########################################################################

    Warning: This sequence may not contain a signal peptide!!

    Proteins without signal peptides are unlikely to be exposed to
    the N-glycosylation machinery and thus may not be glycosylated
    (in vivo) even though they contain potential motifs.

    SignalP-NN euk predictions are as follows:

  # name                 Cmax  pos ?  Ymax  pos ?  Smax  pos ?  Smean ?  D     ?

    SignalP output is explained at http://www.cbs.dtu.dk/services/SignalP/output.html

##########################################################################

Name:  contig022234-TilOR.A009  Length:  320
MDEVLNATYITLDGYVEVNKYRYVYFFIFFILYSLIICSNSTIVYIIWIHKNLHEPMYIFIAALLLNCVLYSTTVYPKLL      80
IDVLSEKQVITYSACIFQLFMFYTLGGSEFFLLAAMAYDRYVAICKPLQYQTIMGKTTVSIFLFIALLIPACHIAVPAIG     160
SAEATLCNVNLKGIFCNNAIYTLQCVRSRLNTVFGVVSLIDLVILPMVFVVFTYTKIFIVSYQSGKEIRKKAAETCLPHL     240
LVLINFSCLSIYDVSIARVESDFPKTARLIMTLQIVLYHPLFNPFIYGIKMKEISKQLKRFFCHDKNHFMKTKFLQFVLX     320
.....N.................................N........................................      80
................................................................................     160
................................................................................     240
................................................................................     320

(Threshold=0.5)
----------------------------------------------------------------------
SeqName      Position  Potential   Jury    N-Glyc
                                 agreement result
----------------------------------------------------------------------
contig022234-TilOR.A009    6 NATY   0.7013     (9/9)    ++
contig022234-TilOR.A009   40 NSTI   0.7259     (9/9)    ++
contig022234-TilOR.A009  245 NFSC   0.4293     (7/9)    -
----------------------------------------------------------------------
```

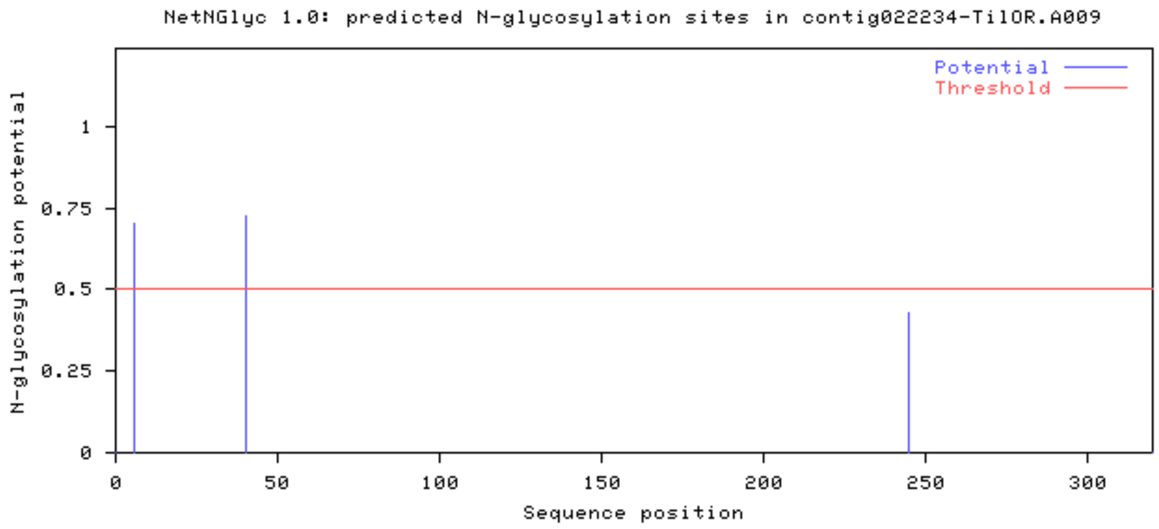

Graphics in PostScript

Output for 'contig022236-TilORe.A027'

```
##########################################################################

    Warning: This sequence may not contain a signal peptide!!

    Proteins without signal peptides are unlikely to be exposed to
    the N-glycosylation machinery and thus may not be glycosylated
    (in vivo) even though they contain potential motifs.

    SignalP-NN euk predictions are as follows:

  # name                Cmax  pos ?  Ymax  pos ?  Smax  pos ?  Smean ?  D     ?

    SignalP output is explained at http://www.cbs.dtu.dk/services/SignalP/output.html

##########################################################################

Name:  contig022236-TilORe.A027         Length:  150
SSLSATRRMCNFTLRGIFCNNSVYKLHCVSSRVLAIYGVIVLLNVAFFPMLYIVFTYTRILIISYHSCREVKKKAAQTCL      80
PHLLVLFNYSLFCAYDIIVLRLESDISQTVGLIMTLQVVLYHPLFNPIIYGLKMKEISKHLKRLFHQVKX
..........N........N............................................................      80
.......N..............................................................               160

(Threshold=0.5)
----------------------------------------------------------------------
SeqName          Position  Potential   Jury     N-Glyc
                                     agreement  result
----------------------------------------------------------------------
contig022236-TilORe.A027   11 NFTL   0.7297     (9/9)    ++
contig022236-TilORe.A027   20 NNSV   0.5666     (5/9)    +
contig022236-TilORe.A027   88 NYSL   0.6657     (9/9)    ++
----------------------------------------------------------------------
```

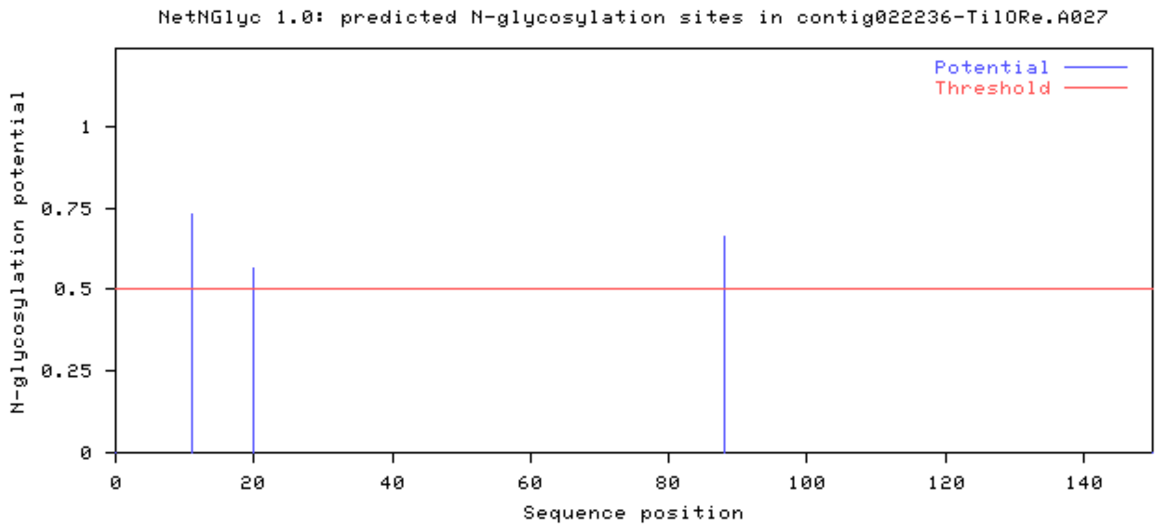

Graphics in PostScript

Output for 'contig022237-TilORe.A028'

```
##########################################################################################

    Warning: This sequence may not contain a signal peptide!!

    Proteins without signal peptides are unlikely to be exposed to
    the N-glycosylation machinery and thus may not be glycosylated
    (in vivo) even though they contain potential motifs.

    SignalP-NN euk predictions are as follows:

  # name                Cmax  pos ?  Ymax  pos ?  Smax  pos ?  Smean ?  D     ?

    SignalP output is explained at http://www.cbs.dtu.dk/services/SignalP/output.html

##########################################################################################

Name:  contig022237-TilORe.A028         Length:  97
MENKLNLTYITLNGYVEVEKYRYVYFVIIFTIYALVIFSNSTIVYLIVIHQSLHEPMYIFIAALLINSIFCSTTIYPKLL      80
FDVLSEKQIISHTMCHF
.....N................................N.........................................      80
.................                                                                    160

(Threshold=0.5)
----------------------------------------------------------------------
SeqName      Position  Potential   Jury    N-Glyc
                                 agreement result
----------------------------------------------------------------------
contig022237-TilORe.A028     6 NLTY   0.7722     (9/9)   +++
contig022237-TilORe.A028    40 NSTI   0.5842     (8/9)   +
----------------------------------------------------------------------
```

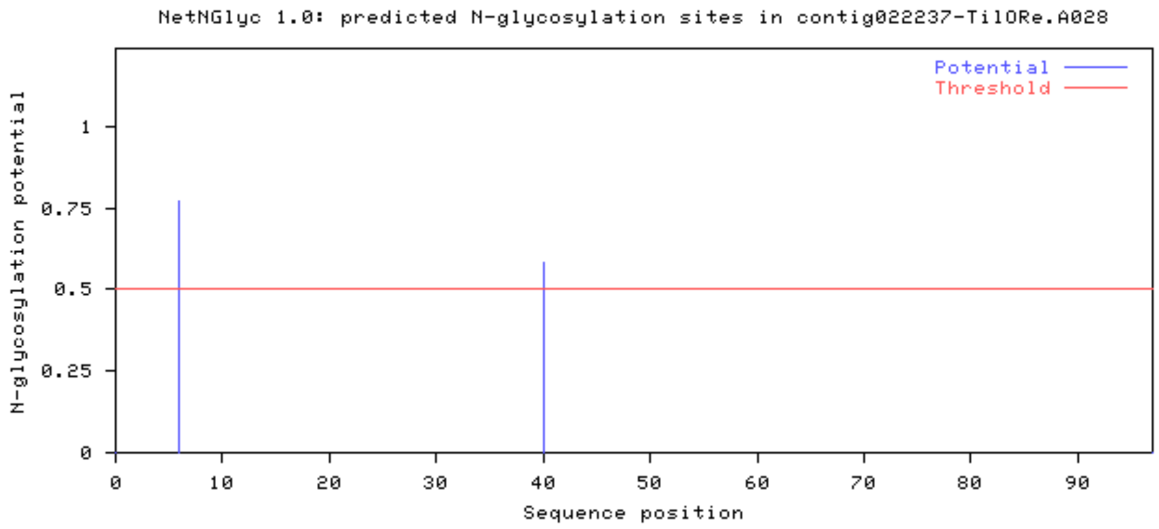

Graphics in PostScript

Output for 'contig022238-TilOR.A010'

```
##########################################################################

    Warning: This sequence may not contain a signal peptide!!

    Proteins without signal peptides are unlikely to be exposed to
    the N-glycosylation machinery and thus may not be glycosylated
    (in vivo) even though they contain potential motifs.

    SignalP-NN euk predictions are as follows:

  # name                    Cmax  pos ?  Ymax  pos ?  Smax  pos ?  Smean ?  D     ?

    SignalP output is explained at http://www.cbs.dtu.dk/services/SignalP/output.html

##########################################################################

Name:  contig022238-TilOR.A010  Length:  306
MDVKLNVTLLTLGGFAELHKYRYLYFVVIFTLYILILCCNSTIVCLIWTHKNLHEPMYIFIAALLINSVLYSMIIYPKLL      80
SDVLSEKQTISYPLCLFQGFSYYASAGSEFLLLAAMAYDRYVSICKPLQYPVIMNRITIYVCLILAWLIPAFEIAVSFVL     160
YFNVKLCSFTLTGIFCNNSIYRLQCVPSVVISIYGVVTLINVALLPMLFILFTYIRILRISYNCCRETRRKALKTCLPHL     240
LVLINFSCFIVFDSVIIRLDSDLSKTLRLTLTFQSILFHPLLNPIIYGLKMNEIFKHIQTLLCQVX
.....N.................................N........................................      80
................................................................................     160
................................................................................     240
....N.............................................................                   320

(Threshold=0.5)
----------------------------------------------------------------------
SeqName      Position  Potential   Jury    N-Glyc
                                agreement  result
----------------------------------------------------------------------
contig022238-TilOR.A010    6 NVTL   0.7749     (9/9)   +++
contig022238-TilOR.A010   40 NSTI   0.7140     (9/9)   ++
contig022238-TilOR.A010  177 NNSI   0.4376     (7/9)   -
contig022238-TilOR.A010  245 NFSC   0.5633     (6/9)   +
----------------------------------------------------------------------
```

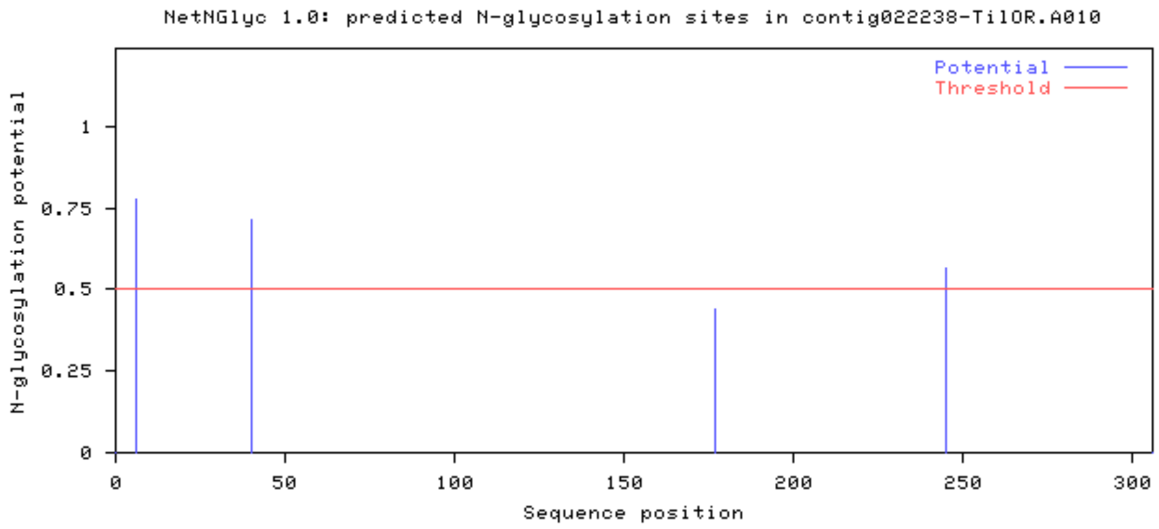

Graphics in PostScript

Output for 'contig022241-TilOR.A011'

```
##############################################################################

    Warning: This sequence may not contain a signal peptide!!

    Proteins without signal peptides are unlikely to be exposed to
    the N-glycosylation machinery and thus may not be glycosylated
    (in vivo) even though they contain potential motifs.

    SignalP-NN euk predictions are as follows:

  # name                  Cmax   pos ?  Ymax   pos ?  Smax   pos ?  Smean ?  D     ?

    SignalP output is explained at http://www.cbs.dtu.dk/services/SignalP/output.html

##############################################################################

Name:  contig022241-TilOR.A011  Length:  307
MDKELNVTFLTLDWYTEINNYRYILFFIMFTLYILIICTNSTIVYLIFTHKNLHEPMYIFIAALLLNSVLYSTTIYPKLL      80
IDFLSEKQVTTYSACLFQFFMFYTLGGSELPLLAAMAYDRYVAICKPLQYHIIMRKTTVSIFLVVAWLVPACHIAVLVIA     160
SAEAKLCDSNIKGIFCNNAVYTLQCQRSRLITVFGVVTLLDIVILPMLFIVFTYTTIFIVSHQSCKEIRKKAAETCLPHL     240
LVLISDCLFFMYDVSIARVELDFPKTVRIIMTLQIVLYHPLFNPVVYGLKMKEISKHLKGLLCQAFX
.....N.................................N........................................      80
................................................................................     160
................................................................................     240
...................................................................                  320

(Threshold=0.5)
----------------------------------------------------------------------
SeqName      Position  Potential   Jury    N-Glyc
                                 agreement result
----------------------------------------------------------------------
contig022241-TilOR.A011    6 NVTF   0.7647     (9/9)   +++
contig022241-TilOR.A011   40 NSTI   0.6966     (8/9)   +
----------------------------------------------------------------------
```

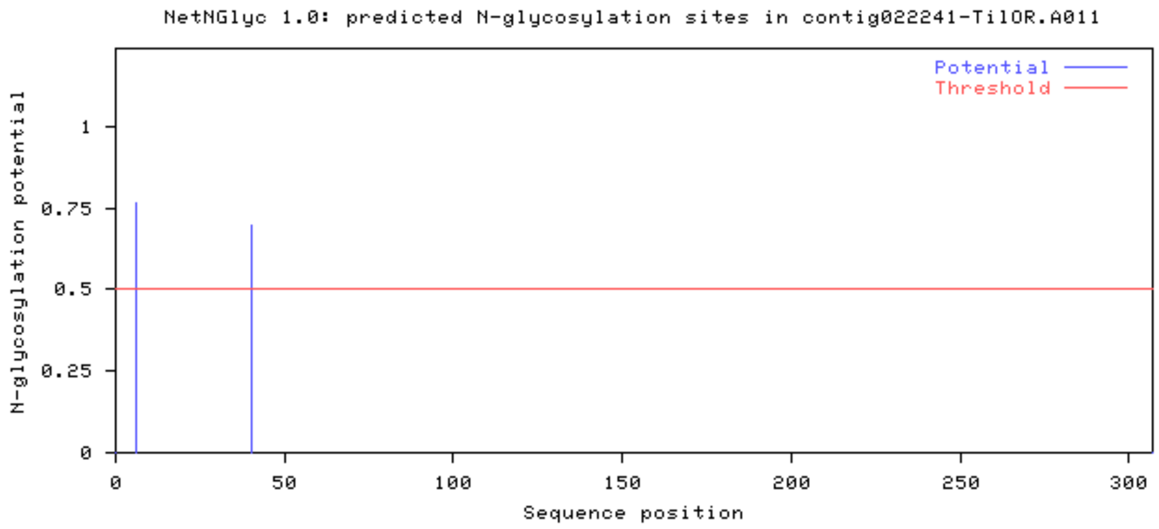

Graphics in PostScript

Output for 'contig022245-TilOR.A012'

```
##########################################################################

    Warning: This sequence may not contain a signal peptide!!

    Proteins without signal peptides are unlikely to be exposed to
    the N-glycosylation machinery and thus may not be glycosylated
    (in vivo) even though they contain potential motifs.

    SignalP-NN euk predictions are as follows:

  # name                 Cmax  pos ?  Ymax  pos ?  Smax  pos ?  Smean ?  D     ?

    SignalP output is explained at http://www.cbs.dtu.dk/services/SignalP/output.html

##########################################################################

Name:  contig022245-TilOR.A012  Length:  316
MDKELNVTFLTLDWYTEINNYRYILFFIMFTLYILIICTNSTIVYLIFTHKNLHEPMYIFIVALLLNSVLYSTTVYPKLL      80
IDFLSEKQVTTYSACLFQFFIFYTLGGSELPLLAAMAYDRYVAICKPLEYQTIMTKTTVSIFLVVAWLVPACHVAVQAIA     160
SAEAKLCDSNIKGIFCNNAVYTLQCQRSRLITIFGVVGLLDLAILPMLFIVFTYTIIFIVSYQSCKEIRKKAAETCLPHL     240
LVLISASVFFVYDISIARVELGFPKTVRVIMTLQIVLYHPLFNPFVYGLKMKEISKHLKKLLCQGKIISCIKIGCX
.....N.................................N........................................      80
................................................................................     160
................................................................................     240
............................................................................         320

(Threshold=0.5)
----------------------------------------------------------------------
SeqName      Position  Potential   Jury    N-Glyc
                                agreement  result
----------------------------------------------------------------------
contig022245-TilOR.A012    6 NVTF   0.7647     (9/9)   +++
contig022245-TilOR.A012   40 NSTI   0.6972     (8/9)   +
----------------------------------------------------------------------
```

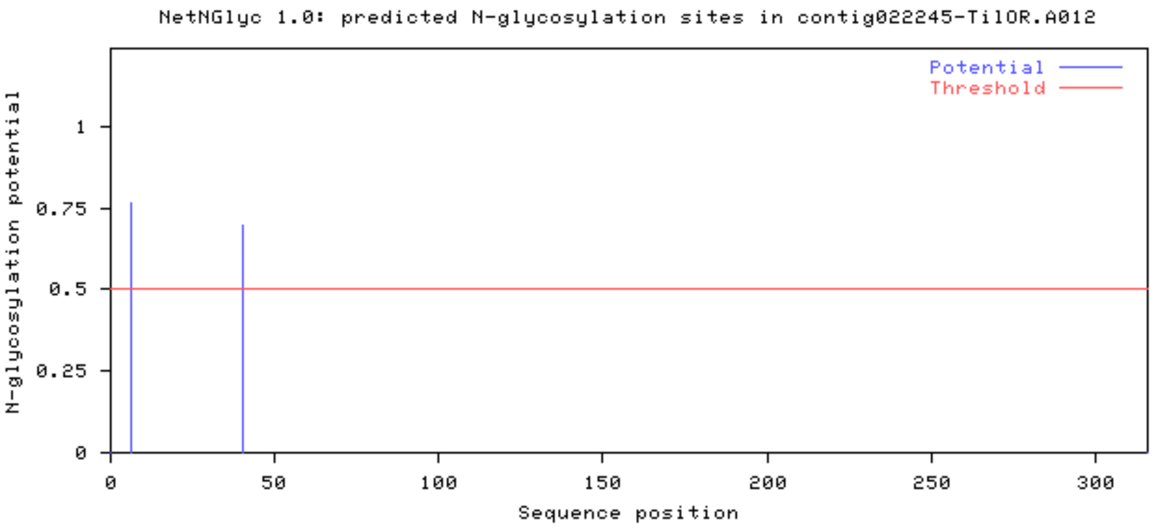

Graphics in PostScript

Output for 'contig022251-TilOR.A013'

```
##############################################################################

    Warning: This sequence may not contain a signal peptide!!

    Proteins without signal peptides are unlikely to be exposed to
    the N-glycosylation machinery and thus may not be glycosylated
    (in vivo) even though they contain potential motifs.

    SignalP-NN euk predictions are as follows:

  # name                 Cmax  pos ?  Ymax  pos ?  Smax  pos ?  Smean ?  D     ?

    SignalP output is explained at http://www.cbs.dtu.dk/services/SignalP/output.html

##############################################################################

Name:  contig022251-TilOR.A013  Length:  314
MDEELNVTHLTLDWYTEVNKYRYVFFFIMFILYSLIICTNSIILYLIWIHKNLHEPMYIFIAALLLNSVLYSTTVYPKLL      80
IDFLSEKQVTTYSACLFQFFMFYTLVCSEFLLLAAMAYDRYVAICKPLEYQTIMRKTTVSIFLVVAWLVPACHVAVLAIA     160
SAEAKLCDSNIKGIFCNNAVYTLQCERSRLITIFGVVALLDLVILPMLFIVFTYTKIFIVSHRSCKEIRKKAAETCLPHL     240
LVLLSLSVFFVYDVSIARANPDFPKTTRIIMTLQIMLYQPLLNPFIYGLKMKEISKHLNKLLSQANISPCIKTX
.....N..........................................................................      80
................................................................................     160
................................................................................     240
..........................................................................           320

(Threshold=0.5)
----------------------------------------------------------------------
SeqName      Position  Potential   Jury    N-Glyc
                                 agreement result
----------------------------------------------------------------------
contig022251-TilOR.A013    6 NVTH   0.7521     (9/9)    +++
contig022251-TilOR.A013  306 NISP   0.1063     (9/9)    ---
----------------------------------------------------------------------
```

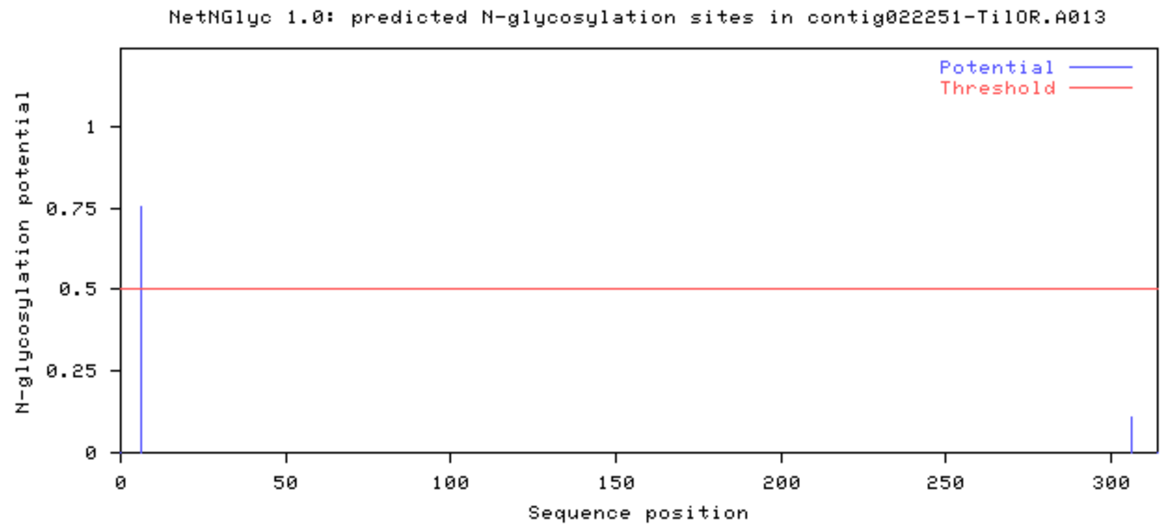

Graphics in PostScript

Output for 'contig022256-TilORe.A037'

```
##########################################################################

    Warning: This sequence may not contain a signal peptide!!

    Proteins without signal peptides are unlikely to be exposed to
    the N-glycosylation machinery and thus may not be glycosylated
    (in vivo) even though they contain potential motifs.

    SignalP-NN euk predictions are as follows:

  # name                Cmax  pos ?  Ymax  pos ?  Smax  pos ?  Smean ?  D     ?

    SignalP output is explained at http://www.cbs.dtu.dk/services/SignalP/output.html

##########################################################################

Name:  contig022256-TilORe.A037        Length:  243
MDEESNVTYLTLDWYTEINKYRYVFFSVMFTLYILIICTNSTILYLIWIHKNLHEPMYIFIAALLLNSVLYSTTVYPKLL      80
IDFLSEKQVTTYSVCLFQFFMFYTLVLSEFLLLAAMAYDRYVAICKPLEYQTIMRKTTVRIFLVVAWLVPACHIAVQAIA     160
SAEAKLCDFNIKGIFCNNAVYTLQCERSRLITIFGVVIVLDLSILPMFFIVFTYTKIFIVSHRSCKEIRKKTAETCLPHM     240
LVL
.....N.................................N........................................      80
................................................................................     160
................................................................................     240
...                                                                                  320

(Threshold=0.5)
----------------------------------------------------------------------
SeqName        Position  Potential    Jury     N-Glyc
                                    agreement  result
----------------------------------------------------------------------
contig022256-TilORe.A037    6 NVTY   0.7339     (9/9)    ++
contig022256-TilORe.A037   40 NSTI   0.6256     (8/9)    +
----------------------------------------------------------------------
```

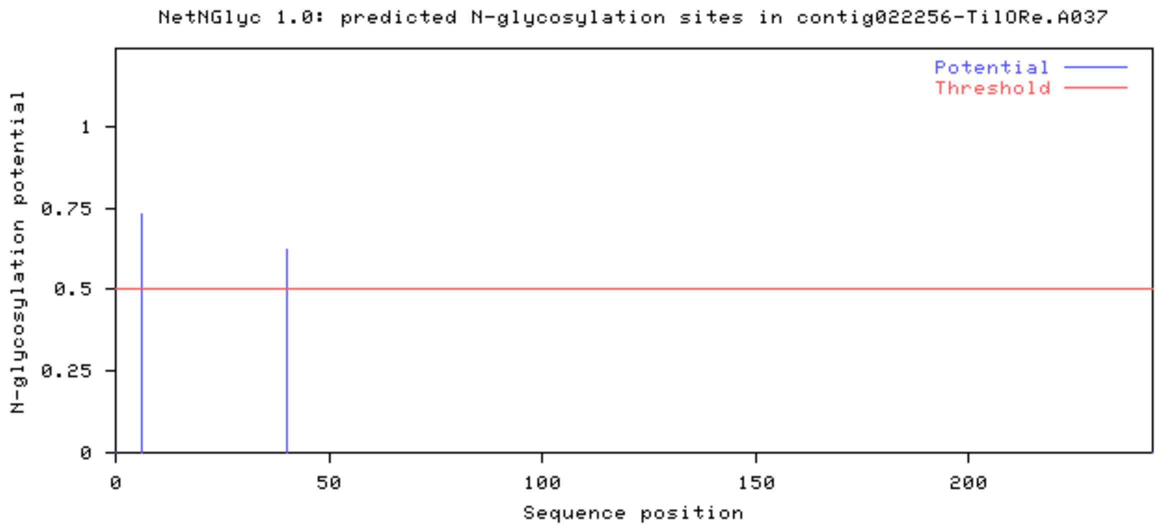

Graphics in PostScript

Output for 'contig022257-TilORe.A038'

```
##########################################################################################

    Warning: This sequence may not contain a signal peptide!!

    Proteins without signal peptides are unlikely to be exposed to
    the N-glycosylation machinery and thus may not be glycosylated
    (in vivo) even though they contain potential motifs.

    SignalP-NN euk predictions are as follows:

  # name                Cmax  pos ?  Ymax  pos ?  Smax  pos ?  Smean ?  D     ?

    SignalP output is explained at http://www.cbs.dtu.dk/services/SignalP/output.html

##########################################################################################

Name:  contig022257-TilORe.A038         Length:  123
TIFGVVIVLDLSILPMFFIVFTYTKIFIVSHRSCKEIRKKTAETCLPHMLVLISYSVFFVYDVSIARVKSDFPKTTRIIM      80
TLQIMLYQPLLNPFIYGLKMKEISKHLKNLLSQANISPCIKTX
................................................................................      80
...........................................                                          160

(Threshold=0.5)

No sites predicted in this sequence.
```

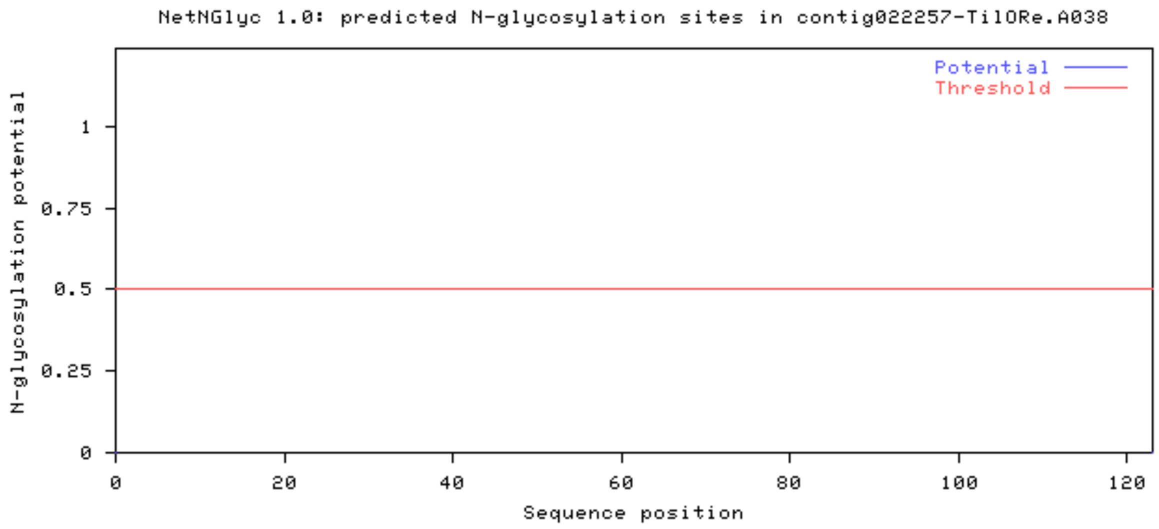

Graphics in PostScript

Output for 'contig022259-TilOR.A014'

```
##########################################################################

    Warning: This sequence may not contain a signal peptide!!

    Proteins without signal peptides are unlikely to be exposed to
    the N-glycosylation machinery and thus may not be glycosylated
    (in vivo) even though they contain potential motifs.

    SignalP-NN euk predictions are as follows:

  # name                    Cmax   pos ?  Ymax   pos ?  Smax   pos ?  Smean ?  D     ?

    SignalP output is explained at http://www.cbs.dtu.dk/services/SignalP/output.html

##########################################################################

Name:  contig022259-TilOR.A014  Length:  314
MDEELNVTYLTLDWYTEINKYRYVFFFIMFTLYILIICTNSTILYLIWIHKNLHEPMYIFIAALLLNSVLYSTTVYPKLL      80
TDFLSEKQVTTYSACLFQFFMFYTLGCSEFLLLAAMAYDRYVAICKPLQYQTIMRKTTVSIFLIIAWLVPACHIAVQAIA     160
SAEAKLCDSNIKGIFCNNAVYTLQCERSRLITIFGVVALVDLSILPMLFIVFTYTKIFIVSHRSCKEIRKKAAETCLPHM     240
LVLIGYSIFFVYDVSIARVKSDFPKPTRIIMTLQIMLYHPLFNPLIYGLKMKEISKHLKKLLSQAKIFPCIKTX
.....N.................................N........................................      80
................................................................................     160
................................................................................     240
..........................................................................           320

(Threshold=0.5)
----------------------------------------------------------------------
SeqName      Position  Potential   Jury    N-Glyc
                                 agreement result
----------------------------------------------------------------------
contig022259-TilOR.A014    6 NVTY   0.7652     (9/9)   +++
contig022259-TilOR.A014   40 NSTI   0.6299     (8/9)   +
----------------------------------------------------------------------
```

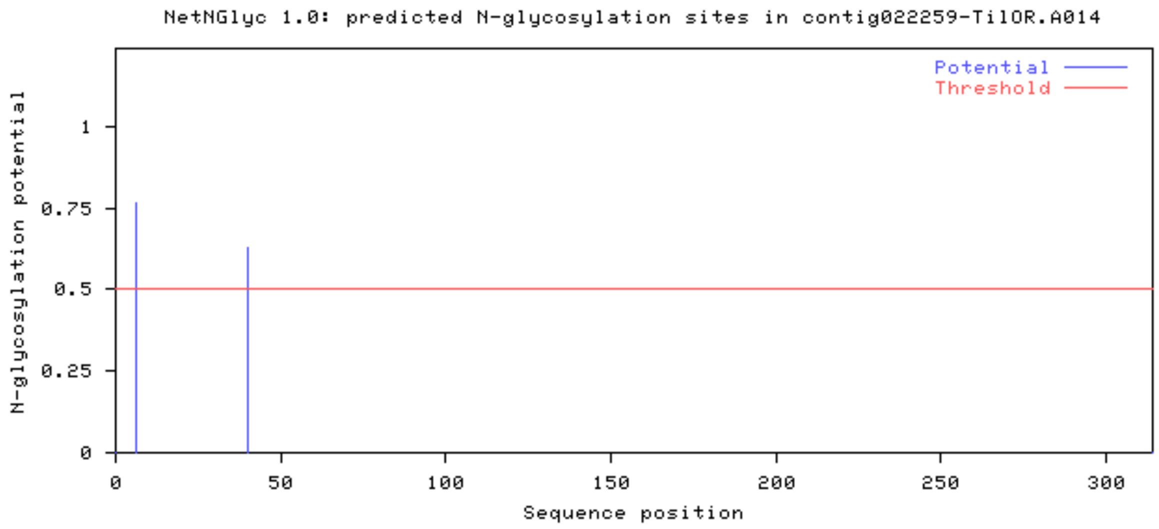

Graphics in PostScript

Output for 'contig022259-TilOR.A015'

```
##########################################################################

    Warning: This sequence may not contain a signal peptide!!

    Proteins without signal peptides are unlikely to be exposed to
    the N-glycosylation machinery and thus may not be glycosylated
    (in vivo) even though they contain potential motifs.

    SignalP-NN euk predictions are as follows:

  # name                 Cmax  pos ?  Ymax  pos ?  Smax  pos ?  Smean ?  D     ?

    SignalP output is explained at http://www.cbs.dtu.dk/services/SignalP/output.html

##########################################################################

Name:  contig022259-TilOR.A015  Length:  314
MDEESNVTYLTLDWYTEINKYRYVFFFIMFTLYILIICTNSTILYLIWIHKNLHEPMYIFIAALLLNSVLYSTTVYPKLL      80
IDFLSEKQVTTYSVCLFQFFMFYTLGCSEFLLLAAMAYDRYVAICKPLEYQTIMRKTTVSIFLVMAWFVPACHIGVQAIA     160
SAEAKLCHSNIKGIFCNNAVYTLQCERSRLITIFGVFLLIDLAVLPMLFIVYTYTKIFIVSHRSCKEIRKKTAETCLPHM     240
LVLISYSAFFVYDISIARVKSDFPKTTRIIMTLQIMLYQPLLNPFIYGLKMKDISKHLNKLLSQAKIIPCIKTX
.....N.................................N........................................      80
................................................................................     160
................................................................................     240
..........................................................................           320

(Threshold=0.5)
----------------------------------------------------------------------
SeqName      Position  Potential   Jury    N-Glyc
                                 agreement result
----------------------------------------------------------------------
contig022259-TilOR.A015    6 NVTY   0.7328     (9/9)   ++
contig022259-TilOR.A015   40 NSTI   0.6298     (8/9)   +
----------------------------------------------------------------------
```

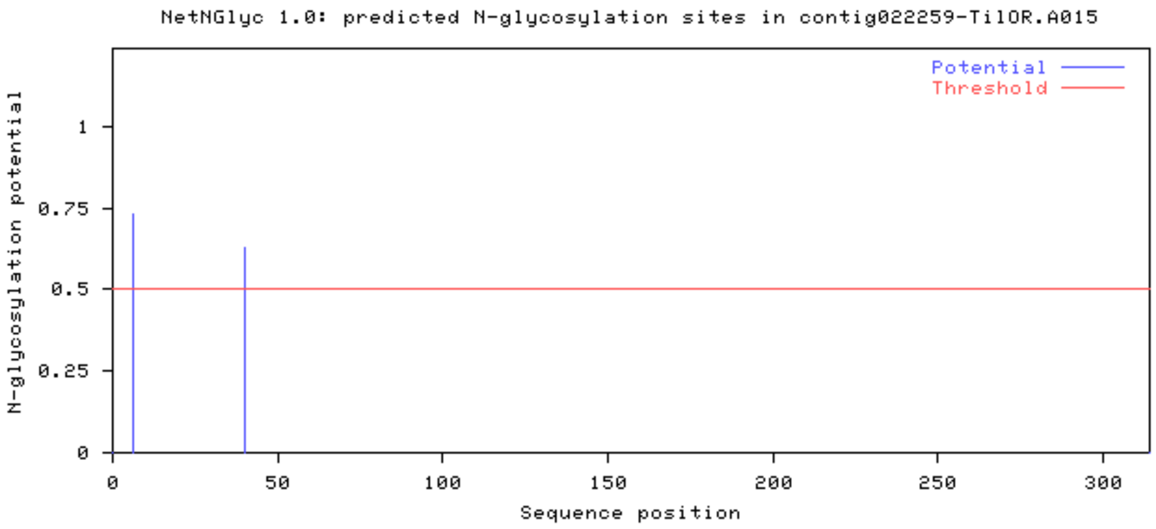

Graphics in PostScript

Output for 'contig022264-TilOR.A016'

```
##########################################################################################

    Warning: This sequence may not contain a signal peptide!!

    Proteins without signal peptides are unlikely to be exposed to
    the N-glycosylation machinery and thus may not be glycosylated
    (in vivo) even though they contain potential motifs.

    SignalP-NN euk predictions are as follows:

  # name                 Cmax  pos ?  Ymax  pos ?  Smax  pos ?  Smean ?  D     ?

    SignalP output is explained at http://www.cbs.dtu.dk/services/SignalP/output.html

##########################################################################################

Name:  contig022264-TilOR.A016  Length:  309
MDDELNATYITLDGYVDLKRFGYLYFLIMVALYVLIIISNSVIVFLICIHHNLHEPMYIFIAALSVNSVLLSTVTYPKLF      80
VDVLSEKQVITFSACRFQHFMCYSIAGSDILLLSAMAFDRYVSICKPLKYPDIMRQTTINILLFLSWFVPGLQVAVLHAL     160
VPNKKLCNFTLKGILCNNSLWKLYCESPRATLIYGLIAMLNVVIFPVFFILFTYAKIFLITYRSSRAIQKKAAETCLPHL     240
FVLSIFTTLCAYDVIIGRLELDFPKTAQLIMTVQVIFYNPLFNPFIYGLKMKEISKHLKRLFCRVRCSX
.....N..........................................................................      80
................................................................................     160
.......N........................................................................     240
.....................................................................                320

(Threshold=0.5)
----------------------------------------------------------------------
SeqName      Position  Potential   Jury    N-Glyc
                                 agreement result
----------------------------------------------------------------------
contig022264-TilOR.A016    6 NATY   0.7060     (9/9)    ++
contig022264-TilOR.A016  168 NFTL   0.6889     (9/9)    ++
contig022264-TilOR.A016  177 NNSL   0.4504     (6/9)    -
----------------------------------------------------------------------
```

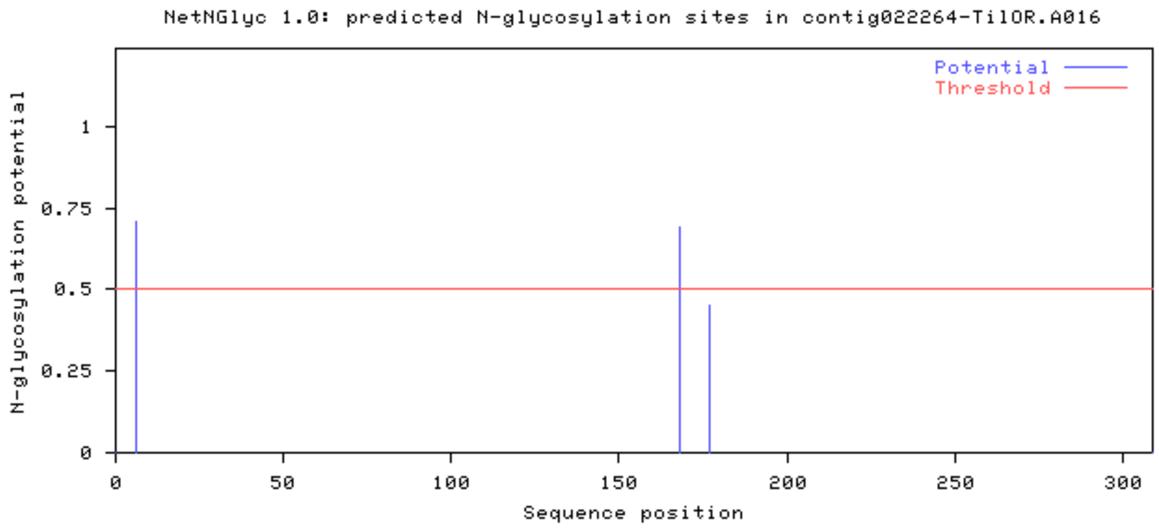

Graphics in PostScript

Output for 'contig022265-TilOR.A017'

```
##############################################################################

   Warning: This sequence may not contain a signal peptide!!

   Proteins without signal peptides are unlikely to be exposed to
   the N-glycosylation machinery and thus may not be glycosylated
   (in vivo) even though they contain potential motifs.

   SignalP-NN euk predictions are as follows:

 # name                Cmax  pos ?  Ymax  pos ?  Smax  pos ?  Smean ?  D     ?

   SignalP output is explained at http://www.cbs.dtu.dk/services/SignalP/output.html

##############################################################################

Name:  contig022265-TilOR.A017  Length:  319
MDEMNDKFNVTYITFVGHVELNKYRFLYFAIMFTAYILILCSNSTILCLIWIKKSLHEPMYIFIAGLLLNSVMFSTNIYP      80
ELLIDFLSDKQITTHSLCSFQAFIYYSLTGSEFFLLAAMAYDRYVSICKPLQYTSIMKKTTIIILLGLAWLLPACQLVPS     160
VVMSQSYKICNFTLNGIFCNNAISKLYCDTSRTTYIIYGVFILLNTIFLPLLFILFTYTKIFIICYRSCREVRKKAAQTC     240
LPHLLILVSFSCFCSYDIIIARLEMNLPNVARFILTLQVVLYHPLFNPIVYGLKMKEISKHLKKLFCEGKLNIWQSSCX
........N.................................N.....................................      80
................................................................................     160
..........N.....................................................................     240
...............................................................................      320

(Threshold=0.5)
----------------------------------------------------------------------
SeqName      Position  Potential   Jury    N-Glyc
                                 agreement result
----------------------------------------------------------------------
contig022265-TilOR.A017    9 NVTY   0.7618     (9/9)    +++
contig022265-TilOR.A017   43 NSTI   0.6699     (9/9)    ++
contig022265-TilOR.A017  171 NFTL   0.7597     (9/9)    +++
----------------------------------------------------------------------
```

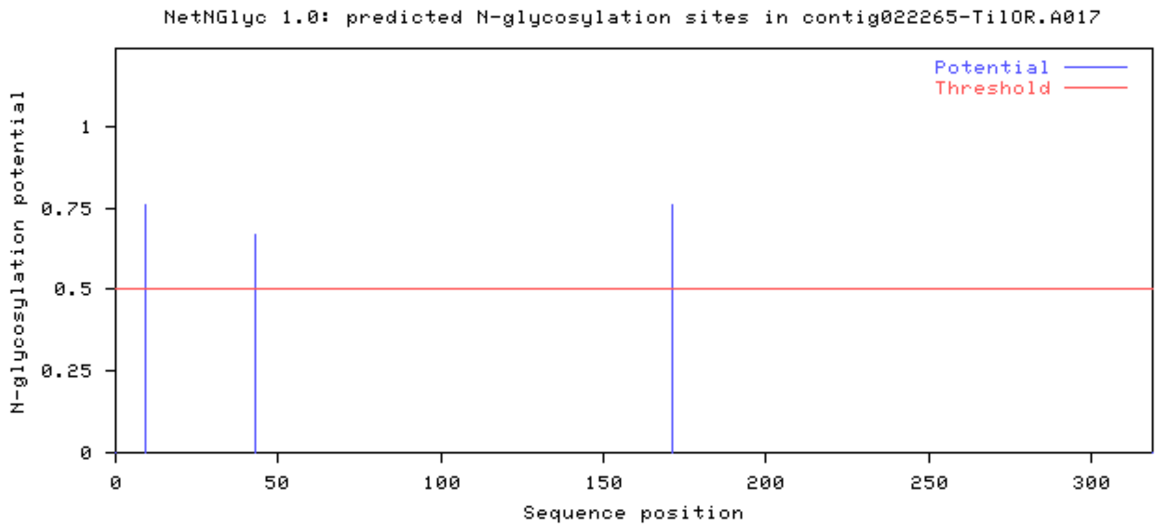

Graphics in PostScript

Output for 'contig022266-TilOR.A018'

```
##########################################################################

   Warning: This sequence may not contain a signal peptide!!

   Proteins without signal peptides are unlikely to be exposed to
   the N-glycosylation machinery and thus may not be glycosylated
   (in vivo) even though they contain potential motifs.

   SignalP-NN euk predictions are as follows:

  # name                Cmax  pos ?  Ymax  pos ?  Smax  pos ?  Smean ?  D     ?

   SignalP output is explained at http://www.cbs.dtu.dk/services/SignalP/output.html

##########################################################################

Name:  contig022266-TilOR.A018  Length:  309
MNPGLNMTYLILGGHVEVQKYRYLYFFILFTAYILIICCNTSIIYLTVIHKSLHEPMYIFIAALLLNSLFFSTNIYPRLL      80
LDFVSEKQIISYPVCLFQVFMFYSLSCSEFLLLSAMAYDRYVSICKPLQYPTIMRKITVVFLLVLAWLLPACQVAVPIIL     160
NINNKLCNFTLKGIFCNNSLIQLYCVMSRALSVYGAFVLLNTGLFPMLFIIFTYTKIILTVYRSSGEVKKKAAQTCLPHL     240
LVLINYSCLITYDMIIARMESDFSKTARFVMTVQIITYNPLFNPIIYGLKLKEISKHLQRLLCQSKLTX
.....N.................................N........................................      80
................................................................................     160
.......N........................................................................     240
.....................................................................                320

(Threshold=0.5)
----------------------------------------------------------------------
SeqName      Position  Potential   Jury    N-Glyc
                                  agreement result
----------------------------------------------------------------------
contig022266-TilOR.A018    6 NMTY   0.7301     (9/9)   ++
contig022266-TilOR.A018   40 NTSI   0.6390     (8/9)   +
contig022266-TilOR.A018  168 NFTL   0.6657     (9/9)   ++
contig022266-TilOR.A018  177 NNSL   0.4818     (6/9)   -
contig022266-TilOR.A018  245 NYSC   0.4894     (5/9)   -
----------------------------------------------------------------------
```

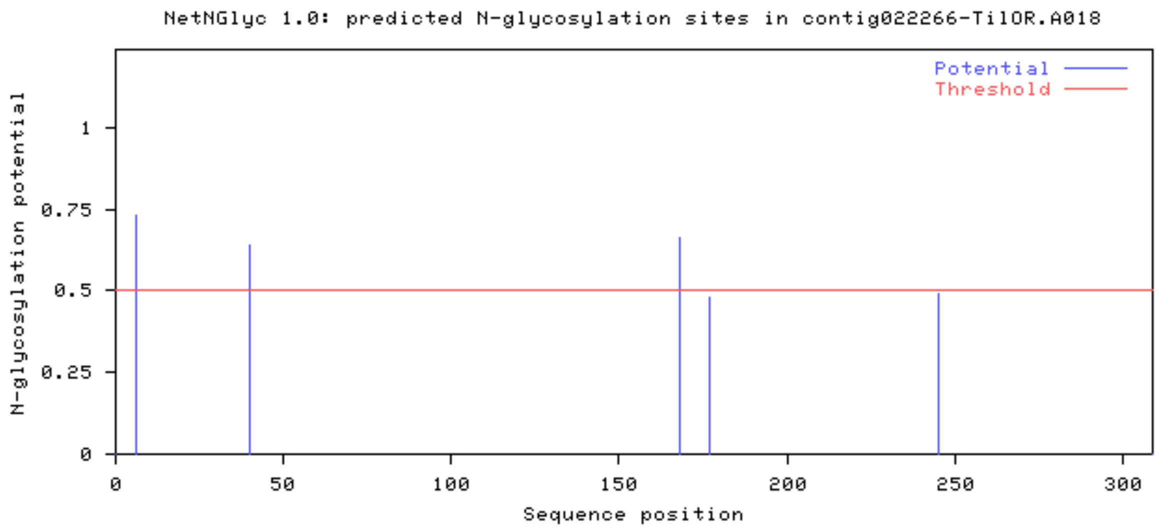

Graphics in PostScript

Output for 'contig022266-TilORe.A032'

```
##########################################################################################

    Warning: This sequence may not contain a signal peptide!!

    Proteins without signal peptides are unlikely to be exposed to
    the N-glycosylation machinery and thus may not be glycosylated
    (in vivo) even though they contain potential motifs.

    SignalP-NN euk predictions are as follows:

  # name                 Cmax  pos ?  Ymax  pos ?  Smax  pos ?  Smean ?  D     ?

    SignalP output is explained at http://www.cbs.dtu.dk/services/SignalP/output.html

##########################################################################################

Name:  contig022266-TilORe.A032         Length:  65
MNDEFNETYITFGGHIELEKYKFLYFVIMFTAYILILCSNSTIVCLIWIKKSLHEPMYVFIAALL
.....N................................N........................                     80

(Threshold=0.5)
----------------------------------------------------------------------
SeqName       Position  Potential   Jury    N-Glyc
                                  agreement result
----------------------------------------------------------------------
contig022266-TilORe.A032    6 NETY   0.6972     (9/9)   ++
contig022266-TilORe.A032   40 NSTI   0.6738     (9/9)   ++
----------------------------------------------------------------------
```

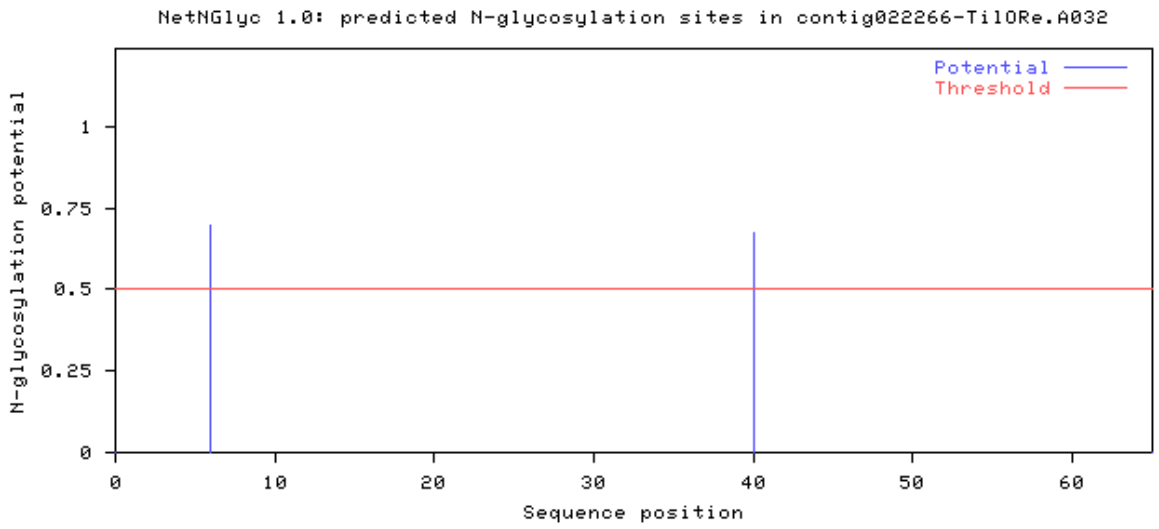

Graphics in PostScript

Output for 'contig022268-TilOR.A019'

```
##########################################################################

    Warning: This sequence may not contain a signal peptide!!

    Proteins without signal peptides are unlikely to be exposed to
    the N-glycosylation machinery and thus may not be glycosylated
    (in vivo) even though they contain potential motifs.

    SignalP-NN euk predictions are as follows:

  # name                 Cmax  pos ?  Ymax  pos ?  Smax  pos ?  Smean ?  D     ?

    SignalP output is explained at http://www.cbs.dtu.dk/services/SignalP/output.html

##########################################################################

Name:  contig022268-TilOR.A019  Length:  328
MAIDNEFNETYITFGGHIELEKYKFLYFVIMFTAYILILCSNSTIVCLIWIKKSLHEPMYVFIAALLLNSVMFSTNIYPK      80
LLMDFLSEKQITTHSQCSFQGFIYYSLTGSEFFLLASMAYDRYVSISKPLQYHTIMRKTTVKILLVLTWLLPACQLVPSA     160
VIRNNSQICNFTLNGIFCNNAISKLYCATPKTSYLIYGVFILINTVFLPLLFILFTYTKIFIICYQSCREVRRKAAQTCL     240
PHLLVLISFSCLCSYDIIITRLEINLSQTARFIMTLQVVLYHPLFNPVVYGLKMKEISQHLRRLFCQSKFKLSVRADARS     320
AIISFVIX
.......N.................................N......................................      80
................................................................................     160
...N.....N......................................................................     240
........................N.......................................................     320
........                                                                             400

(Threshold=0.5)
----------------------------------------------------------------------
SeqName       Position  Potential   Jury    N-Glyc
                                  agreement result
----------------------------------------------------------------------
contig022268-TilOR.A019    8 NETY   0.5661     (5/9)   +
contig022268-TilOR.A019   42 NSTI   0.7384     (9/9)   ++
contig022268-TilOR.A019  164 NNSQ   0.5440     (6/9)   +
contig022268-TilOR.A019  170 NFTL   0.5941     (6/9)   +
contig022268-TilOR.A019  265 NLSQ   0.6592     (9/9)   ++
----------------------------------------------------------------------
```

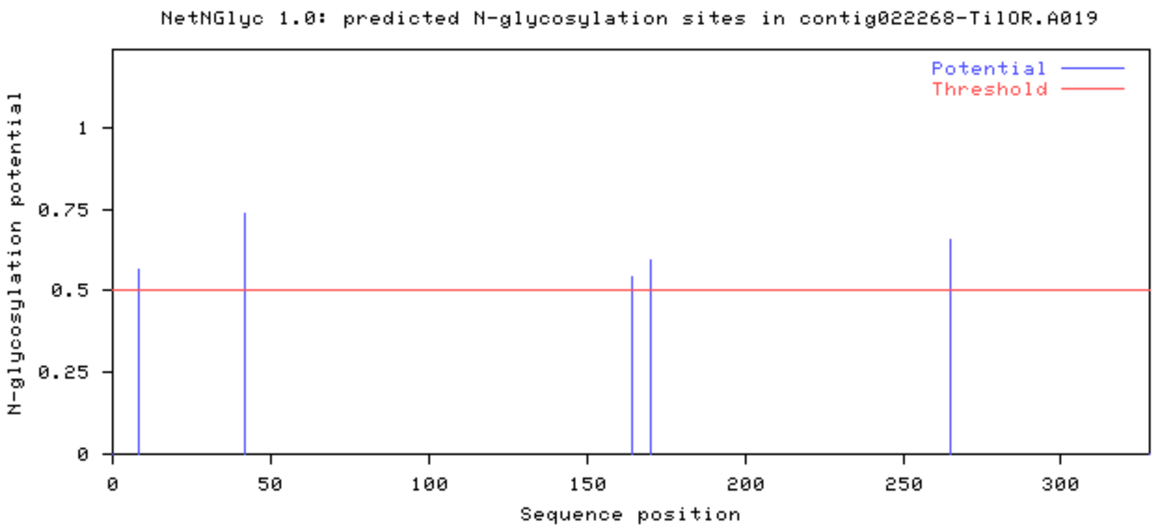

Graphics in PostScript

Output for 'contig022268-TilOR.A020'

```
##########################################################################################

    Warning: This sequence may not contain a signal peptide!!

    Proteins without signal peptides are unlikely to be exposed to
    the N-glycosylation machinery and thus may not be glycosylated
    (in vivo) even though they contain potential motifs.

    SignalP-NN euk predictions are as follows:

  # name                   Cmax  pos ?  Ymax  pos ?  Smax  pos ?  Smean ?  D     ?

    SignalP output is explained at http://www.cbs.dtu.dk/services/SignalP/output.html

##########################################################################################

Name:  contig022268-TilOR.A020  Length:  307
MNADLNMTYVTLGGHVEIHRYRYLYFVIMLTAYILLICFNVSIVCLIVIHKNLHEPMYIFIAALLLNSLLFSTNIYPKLL      80
IDFLSEKQIISYQACLFQAFMFYFLSSSEFLLLSAMAYDRYVSICKPLKYQIIMRTTTVSFLLIFAWLVPACYVVVPIAL     160
NINSKLCSFTLKGIFCNNSASKLLCVSSRALSIYGVILLFNLGLFPTLFIIFTYTKIIIIAYQSCGDVRRKAAQTCLPHL     240
LVLINYSILITYDVVIVKLESDFSKTARFIMTLQVIIYNPLCNPIIYGLKMKDISKHLKRLFVKKSX
.....N.................................N........................................      80
................................................................................     160
................................................................................     240
....N..............................................................                   320

(Threshold=0.5)
----------------------------------------------------------------------
SeqName      Position  Potential   Jury    N-Glyc
                                agreement result
----------------------------------------------------------------------
contig022268-TilOR.A020    6 NMTY   0.7463     (9/9)   ++
contig022268-TilOR.A020   40 NVSI   0.7799     (9/9)   +++
contig022268-TilOR.A020  177 NNSA   0.3716     (8/9)   -
contig022268-TilOR.A020  245 NYSI   0.5020     (6/9)   +
----------------------------------------------------------------------
```

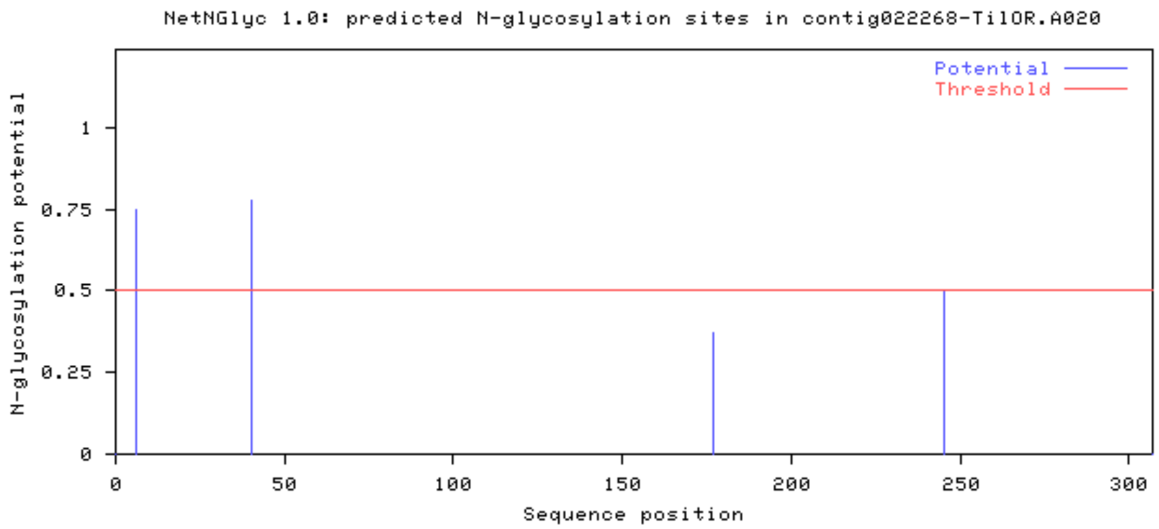

Graphics in PostScript

Output for 'contig022269-TilORe.A029'

```
##########################################################################################

    Warning: This sequence may not contain a signal peptide!!

    Proteins without signal peptides are unlikely to be exposed to
    the N-glycosylation machinery and thus may not be glycosylated
    (in vivo) even though they contain potential motifs.

    SignalP-NN euk predictions are as follows:

  # name                    Cmax  pos ?  Ymax  pos ?  Smax  pos ?  Smean ?  D     ?

    SignalP output is explained at http://www.cbs.dtu.dk/services/SignalP/output.html

##########################################################################################

Name:  contig022269-TilORe.A029        Length:  170
MTYVTLGGHVEIHRYRYLYFVILLTAYILLICFNVSIICLIVIHKNLHEPMYIFIAALLLNSLLFSTNIHPKLLVDFLSD      80
KQIVSYQACLFQIFMFYFLSSSEFLLLSAMAYDRYVSICKPLQYPAIMTTTTVSLLLCFAWLVPACYVVVPIALNINSKL     160
CSFTLKGNFC
.................................N..............................................      80
................................................................................     160
..........                                                                           240

(Threshold=0.5)
----------------------------------------------------------------------
SeqName      Position  Potential   Jury    N-Glyc
                                 agreement result
----------------------------------------------------------------------
contig022269-TilORe.A029   34 NVSI   0.7817     (9/9)    +++
----------------------------------------------------------------------
```

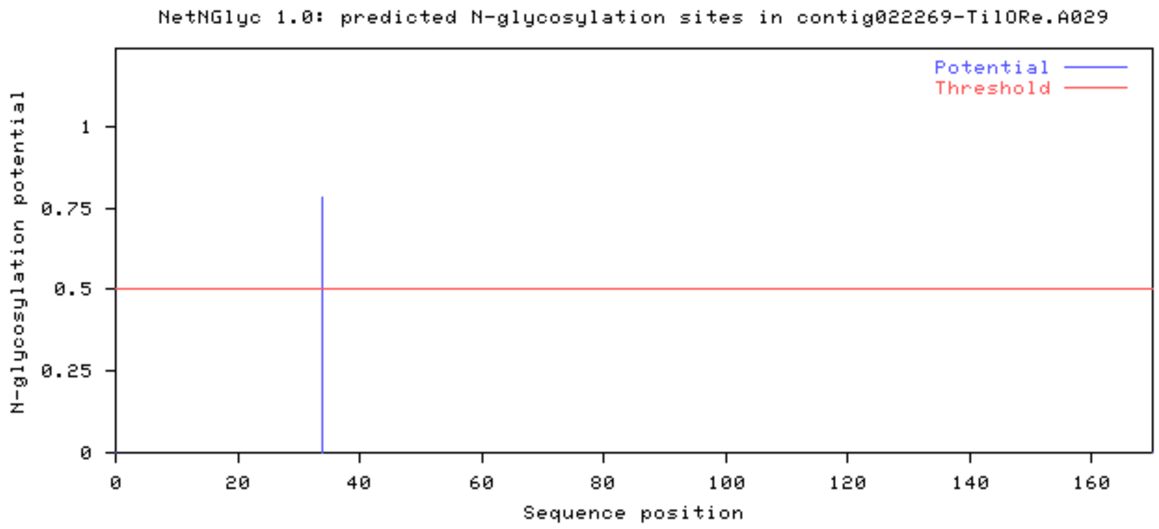

Graphics in PostScript

Output for 'contig022270-TilORe.A030'

```
##########################################################################################

    Warning: This sequence may not contain a signal peptide!!

    Proteins without signal peptides are unlikely to be exposed to
    the N-glycosylation machinery and thus may not be glycosylated
    (in vivo) even though they contain potential motifs.

    SignalP-NN euk predictions are as follows:

  # name                Cmax  pos ?  Ymax  pos ?  Smax  pos ?  Smean ?  D     ?

    SignalP output is explained at http://www.cbs.dtu.dk/services/SignalP/output.html

##########################################################################################

Name:  contig022270-TilORe.A030        Length:  67
MLVLMNCSVLITYDVVIVKLESDFPKTARFVMTLQIITYNPLCNPIIYGLKMKEISKHLKRLLRHMK
.....N.............................................................                   80

(Threshold=0.5)
----------------------------------------------------------------------
SeqName      Position  Potential   Jury    N-Glyc
                                 agreement result
----------------------------------------------------------------------
contig022270-TilORe.A030    6 NCSV   0.7034     (8/9)   +
----------------------------------------------------------------------
```

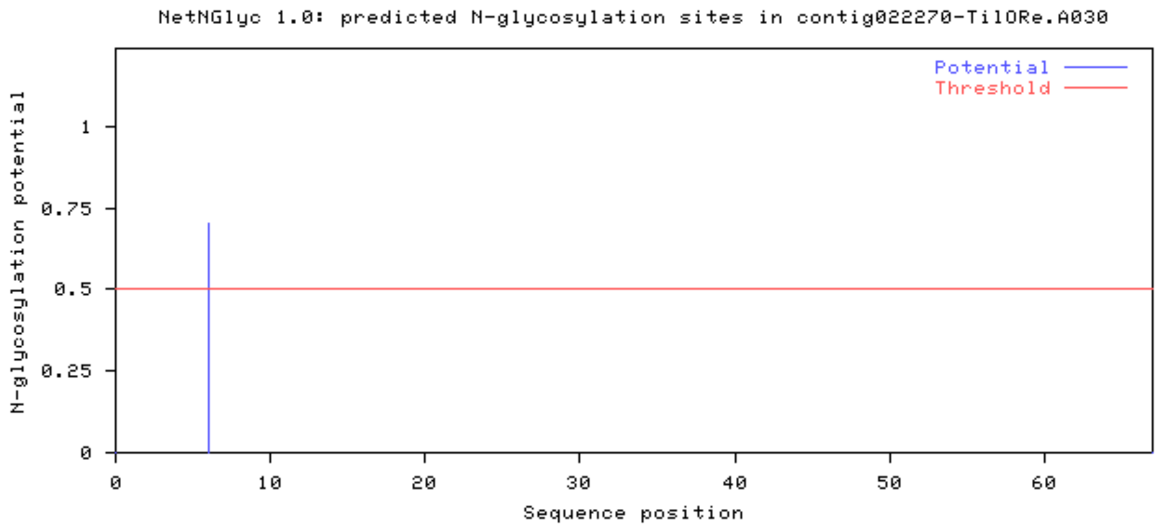

Graphics in PostScript

Output for 'contig023711-TilORe.L163'

```
########################################################################################

    Warning: This sequence may not contain a signal peptide!!

    Proteins without signal peptides are unlikely to be exposed to
    the N-glycosylation machinery and thus may not be glycosylated
    (in vivo) even though they contain potential motifs.

    SignalP-NN euk predictions are as follows:

  # name                    Cmax   pos ?  Ymax   pos ?  Smax   pos ?  Smean ?  D     ?

    SignalP output is explained at http://www.cbs.dtu.dk/services/SignalP/output.html

########################################################################################

Name:  contig023711-TilORe.L163         Length:  235
PTMIGVLLAGVNTISYVECLIQMCVFTLGTSMESFVLAVMALDRFIAIIYPFQYHSYLTNTRVVVLTFILWFVNWCFMCY      80
MPATVVPLPHCSSRLKYSFCDFAAIIRTTCVNPEKYFNEAAIIMFFILFFTFVFICLTYCGILLFVKLSSNNEKKKIGST     160
LVSHLICVIVHYCPAFVRIIFTRFGVVLTLEERQGLVIGAVLGPCLVNPFVYCLRTKEIKQKLFKIFKRFHTSEX
................................................................................      80
................................................................................     160
...........................................................................          240

(Threshold=0.5)

No sites predicted in this sequence.
```

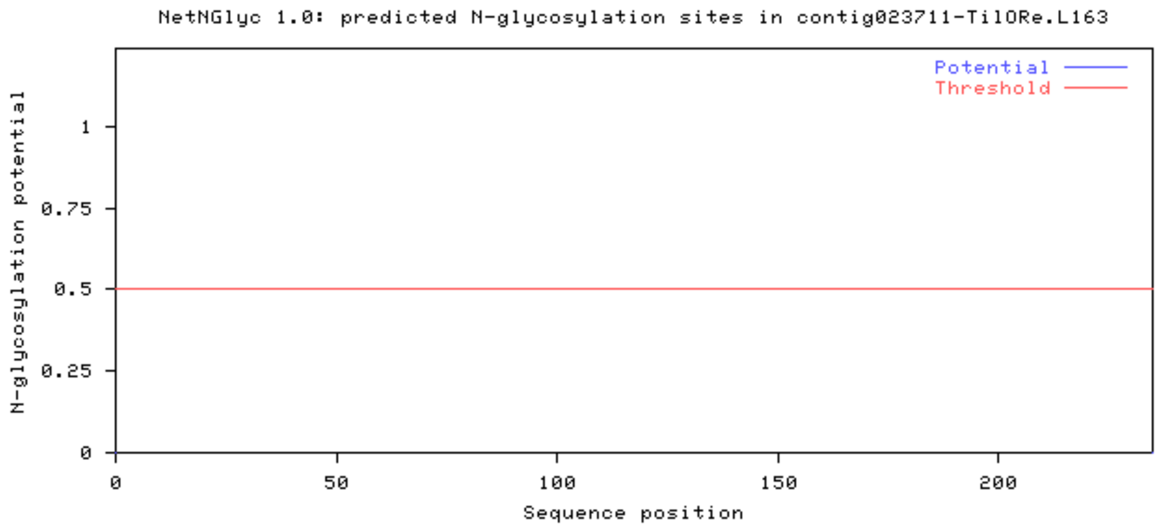

[Graphics in PostScript]

Output for 'contig023712-TilORe.L164'

```
##########################################################################################

    Warning: This sequence may not contain a signal peptide!!

    Proteins without signal peptides are unlikely to be exposed to
    the N-glycosylation machinery and thus may not be glycosylated
    (in vivo) even though they contain potential motifs.

    SignalP-NN euk predictions are as follows:

  # name                    Cmax  pos ?  Ymax  pos ?  Smax  pos ?  Smean ?  D     ?

    SignalP output is explained at http://www.cbs.dtu.dk/services/SignalP/output.html

##########################################################################################

Name:  contig023712-TilORe.L164         Length:  310
MSLQNASINVTHFIIGGFDTLSRPTAVGVVILITYLLAVLANIANIMFIISDKRLHKPMYLLICNLAVVDIVYTSSCSPT      80
MIGVLLAGVNTISYVECLIQMCVFTLGTSMESFVLAVMALDRFIAIIYPFQYHSYLTNTRVVVLTFILWFVNWCFMCYMP     160
ATVVPLPHCSSRLKYSFCDFAAIIRTTCVNPEKYFNEAAIIMFFILFFTFVFICLTYCGILLFVKLSSNNEKKKIGSTLV     240
SHLICVIVHYCPAFVRIIFTRFGVVLTLEERQGLVIGAVLGPCLVNPFVVYCLRTKEIKQKLFKIFKRFHT
....N...N.......................................................................      80
................................................................................     160
................................................................................     240
.......................................................................              320

(Threshold=0.5)
----------------------------------------------------------------------
SeqName      Position  Potential   Jury     N-Glyc
                                 agreement  result
----------------------------------------------------------------------
contig023712-TilORe.L164    5 NASI   0.6718     (8/9)   +
contig023712-TilORe.L164    9 NVTH   0.7236     (9/9)   ++
----------------------------------------------------------------------
```

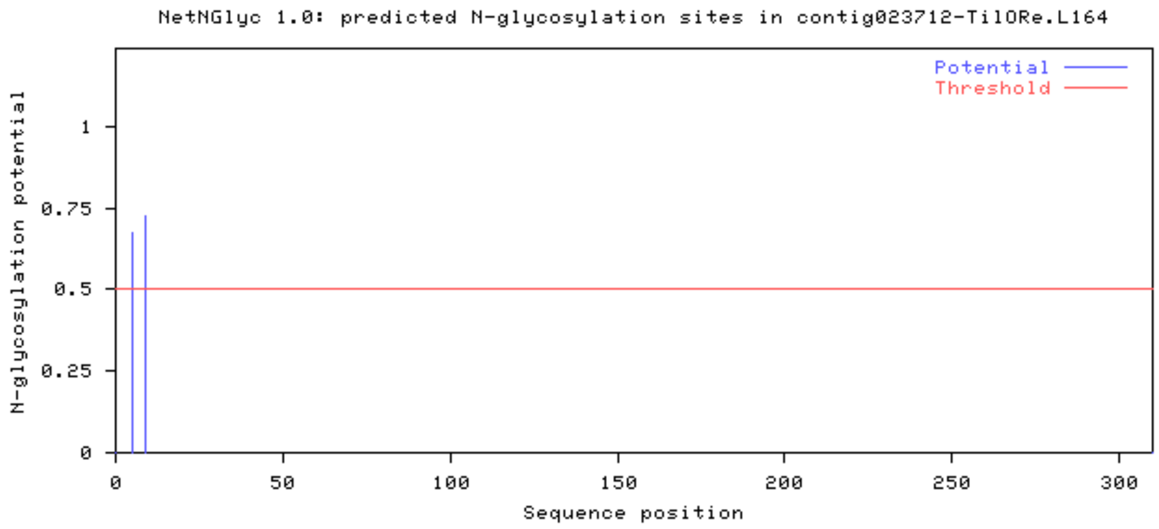

Graphics in PostScript

Output for 'contig023714-TilORe.L173'

```
##########################################################################################

    Warning: This sequence may not contain a signal peptide!!

    Proteins without signal peptides are unlikely to be exposed to
    the N-glycosylation machinery and thus may not be glycosylated
    (in vivo) even though they contain potential motifs.

    SignalP-NN euk predictions are as follows:

  # name                    Cmax  pos ?  Ymax  pos ?  Smax  pos ?  Smean ?  D     ?

    SignalP output is explained at http://www.cbs.dtu.dk/services/SignalP/output.html

##########################################################################################

Name:  contig023714-TilORe.L173         Length:  193
DRFIAIIYPFQYHSYLTNTRVLVLTFILWFVAWCFVCYMPATVVPLTHCSSRLKYSFCDFAAVIRTTCVNPEKYFNEVAI      80
VTFFIFFFTLVFICLSYCAILLFVKLSSNNEKKKMGSTLVSHLICVTVNYCPAFVRIVFTRFGVVLTLEERQGLLIGAVL     160
GPCLVNPFVYSLRTKEIKQKFYKIFKKFHTSDX
................................................................................      80
................................................................................     160
.................................                                                    240

(Threshold=0.5)

No sites predicted in this sequence.
```

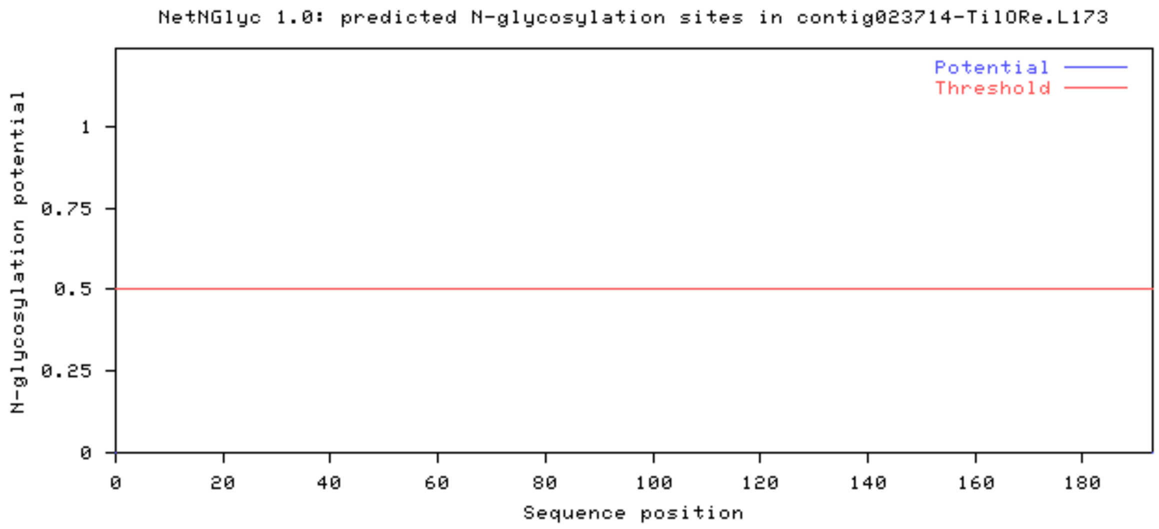

Graphics in PostScript

Output for 'contig023716-TilOR.L144'

```
##########################################################################################

    Warning: This sequence may not contain a signal peptide!!

    Proteins without signal peptides are unlikely to be exposed to
    the N-glycosylation machinery and thus may not be glycosylated
    (in vivo) even though they contain potential motifs.

    SignalP-NN euk predictions are as follows:

  # name                Cmax  pos ?  Ymax  pos ?  Smax  pos ?  Smean ?  D     ?

    SignalP output is explained at http://www.cbs.dtu.dk/services/SignalP/output.html

##########################################################################################

Name:  contig023716-TilOR.L144  Length:  313
MSLQNASMKVTHFIINGFDTVKSPVAVGVVILIIYLVSIVTNLLNILFVISDKRLHKPMYILICNLAIVDIMYISSASPT      80
MVGVLVAGVNTISYLECLIQMFVFHLGGMMERFALAIMAFDRLVAIIYPFQYHSYLKNTRILLYTYISWVVACGFVVVLP     160
ATVAPLPHCYSSLRYSFCDYAAVVRTTCVDPTYFFNIGAIIYSFLLFLTFIFICLSYCVILIFAKLSSSNDKKKIGSTLV     240
SHLICVICHYCPAFVIIVLTRLGVVLTLDERQGLLIANILGPSVVNPFVYCLRTKEIKHKIFMLFKKLQVLRX
....N...........................................................................      80
................................................................................     160
................................................................................     240
.........................................................................            320

(Threshold=0.5)
----------------------------------------------------------------------
SeqName      Position  Potential   Jury    N-Glyc
                                 agreement result
----------------------------------------------------------------------
contig023716-TilOR.L144     5 NASM   0.5826     (7/9)    +
----------------------------------------------------------------------
```

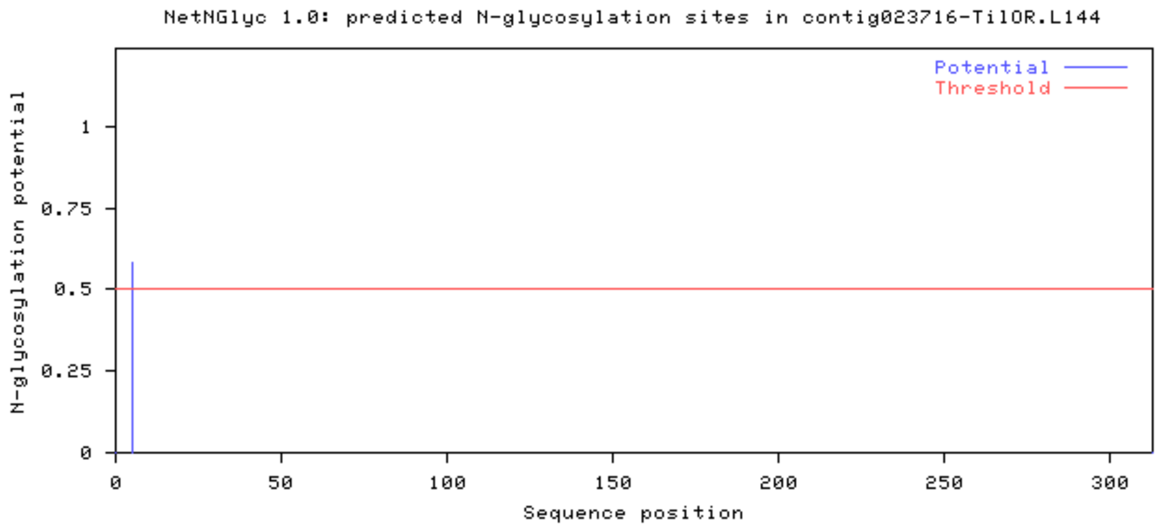

Graphics in PostScript

Output for 'contig023717-TilOR.O175'

```
##########################################################################

    Warning: This sequence may not contain a signal peptide!!

    Proteins without signal peptides are unlikely to be exposed to
    the N-glycosylation machinery and thus may not be glycosylated
    (in vivo) even though they contain potential motifs.

    SignalP-NN euk predictions are as follows:

  # name                Cmax  pos ?  Ymax  pos ?  Smax  pos ?  Smean ?  D     ?

    SignalP output is explained at http://www.cbs.dtu.dk/services/SignalP/output.html

##########################################################################

Name:  contig023717-TilOR.O175  Length:  326
MPERNHSSVTEFVLTGFPGLHQEYYGLVSALLFFVYLVTMIANATVIFLFATNRSLHKPMYYIILNLSVCDILFSTTTLP      80
KIISRYWFQSGSISFTACFIQMYFVHYLGSVNSFILFQMALDRYLAICHPFRYSHILTKSNILILSITAWIIAKAFPLMM     160
VIRAYPLPYCASNIITHCFCDHIGITVLACTDRAPYSIPAFAAAMVTLLGPLAFILFSYCSIIIAVYKIANVQGRLKSLS     240
TCSTQLIIISLYFLPRCFVYLASNVGITFSADVRIVIIMLYSLAPPMINPLIYCLRAKDMRESLLKQFCKRIVPQKAQVA     320
AISNSX
....N.....................................N.........N............N..............      80
................................................................................     160
................................................................................     240
................................................................................     320
......                                                                               400

(Threshold=0.5)
----------------------------------------------------------------------
SeqName      Position  Potential   Jury    N-Glyc
                                 agreement result
----------------------------------------------------------------------
contig023717-TilOR.O175    5 NHSS   0.6061     (8/9)   +
contig023717-TilOR.O175   43 NATV   0.7146     (8/9)   +
contig023717-TilOR.O175   53 NRSL   0.7274     (9/9)   ++
contig023717-TilOR.O175   66 NLSV   0.7954     (9/9)   +++
----------------------------------------------------------------------
```

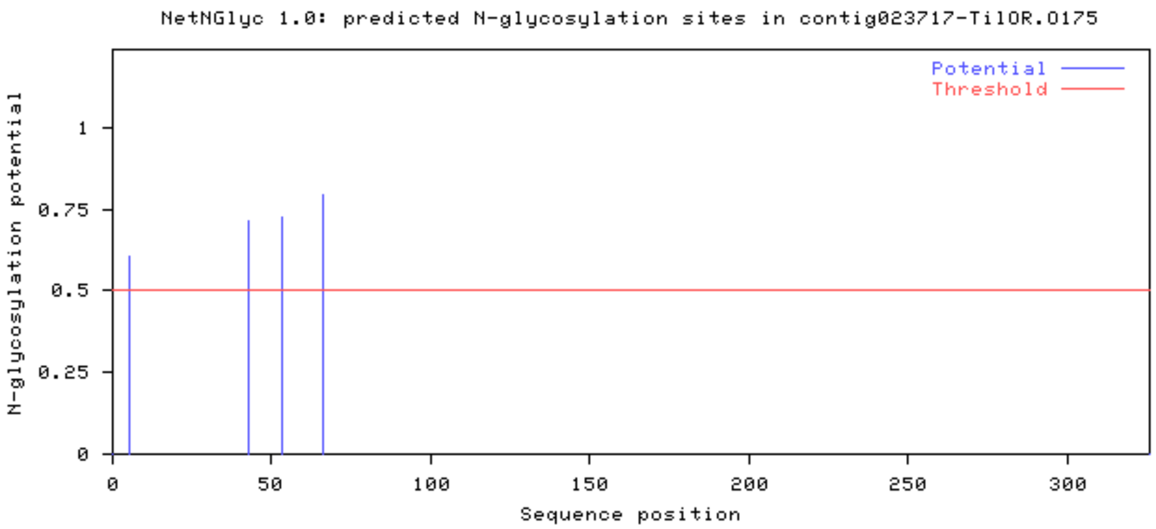

Graphics in PostScript

Output for 'contig023717-TilORe.O183'

```
##########################################################################################

    Warning: This sequence may not contain a signal peptide!!

    Proteins without signal peptides are unlikely to be exposed to
    the N-glycosylation machinery and thus may not be glycosylated
    (in vivo) even though they contain potential motifs.

    SignalP-NN euk predictions are as follows:

  # name                    Cmax   pos ?  Ymax   pos ?  Smax   pos ?  Smean ?  D     ?

    SignalP output is explained at http://www.cbs.dtu.dk/services/SignalP/output.html

##########################################################################################

Name:  contig023717-TilORe.O183         Length:  75
MPVENHSSVTEFVLTGFPGLHQEYYGLVSALLFFVYLITMIANVTVIFLFATNRSLHKPMYYIILNLSVCDILFS
....N......................................N.........N............N.........          80

(Threshold=0.5)
----------------------------------------------------------------------
SeqName      Position  Potential    Jury    N-Glyc
                                  agreement result
----------------------------------------------------------------------
contig023717-TilORe.O183    5 NHSS   0.5858     (7/9)    +
contig023717-TilORe.O183   43 NVTV   0.7386     (9/9)    ++
contig023717-TilORe.O183   53 NRSL   0.6438     (8/9)    +
contig023717-TilORe.O183   66 NLSV   0.7259     (9/9)    ++
----------------------------------------------------------------------
```

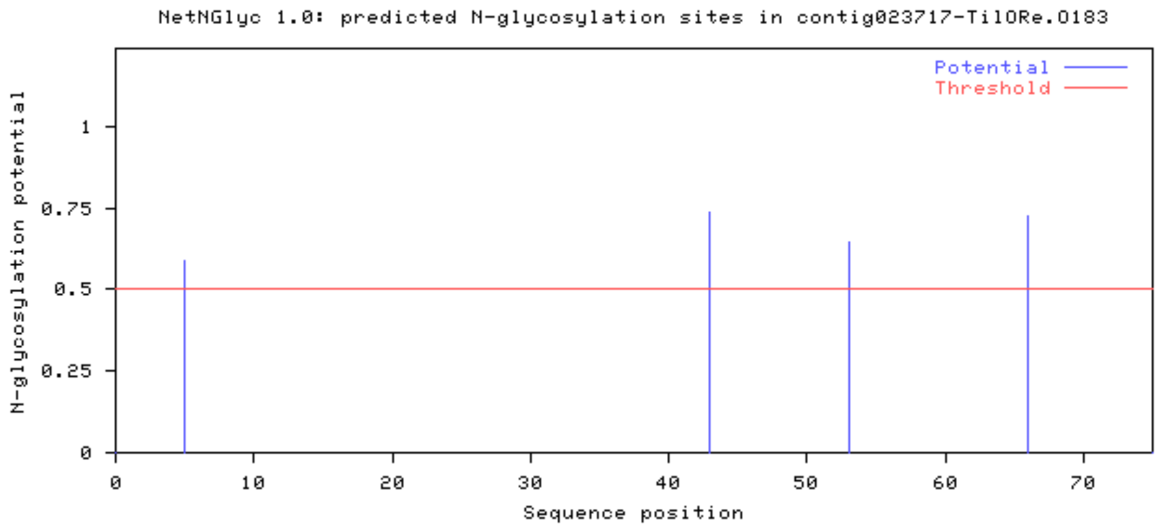

Graphics in PostScript

Output for 'contig023719-TilORe.O178'

```
##########################################################################################

    Warning: This sequence may not contain a signal peptide!!

    Proteins without signal peptides are unlikely to be exposed to
    the N-glycosylation machinery and thus may not be glycosylated
    (in vivo) even though they contain potential motifs.

    SignalP-NN euk predictions are as follows:

  # name                 Cmax   pos ?  Ymax   pos ?  Smax   pos ?  Smean ?  D     ?

    SignalP output is explained at http://www.cbs.dtu.dk/services/SignalP/output.html

##########################################################################################

Name:  contig023719-TilORe.0178         Length:  107
MPERNYSVLTEFILTGFPGLHQEYYGLVSAVLFFVYLITMIANVTVIFLFATNRSLHKPMYYIILNLSVCDILFSTTTLP      80
KIISRYWFQSGSISFTACFIQMYFVHY
....N.....................................N.........N...........N.............      80
...........................                                                         160

(Threshold=0.5)
----------------------------------------------------------------------
SeqName      Position  Potential   Jury    N-Glyc
                                 agreement result
----------------------------------------------------------------------
contig023719-TilORe.0178    5 NYSV   0.6598     (9/9)   ++
contig023719-TilORe.0178   43 NVTV   0.7561     (9/9)   +++
contig023719-TilORe.0178   53 NRSL   0.6774     (9/9)   ++
contig023719-TilORe.0178   66 NLSV   0.7586     (9/9)   +++
----------------------------------------------------------------------
```

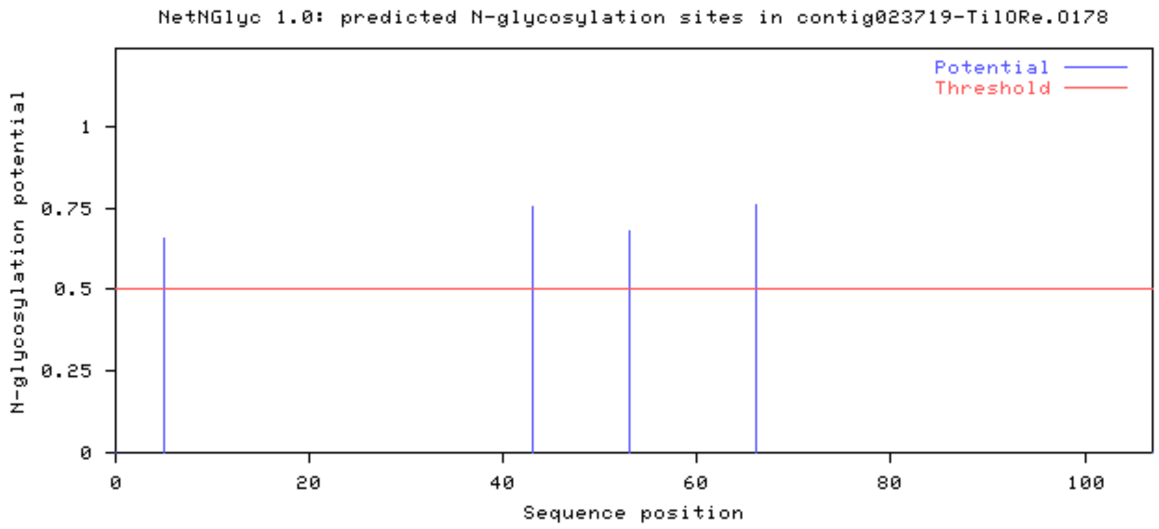

Graphics in PostScript

Output for 'contig023720-TilORe.O185'

```
##########################################################################################

    Warning: This sequence may not contain a signal peptide!!

    Proteins without signal peptides are unlikely to be exposed to
    the N-glycosylation machinery and thus may not be glycosylated
    (in vivo) even though they contain potential motifs.

    SignalP-NN euk predictions are as follows:

  # name                    Cmax   pos ?  Ymax   pos ?  Smax   pos ?  Smean ?  D     ?

    SignalP output is explained at http://www.cbs.dtu.dk/services/SignalP/output.html

##########################################################################################

Name:  contig023720-TilORe.0185         Length:  103
IAVYKIANVQGRLKSLSTCSTQLIIISLYYLPRCFVYLASNVGITFSADVRIVIIMLYSLAPPMINPLIYCLRAKDMRDS      80
LLKHICGRTIPRKAQVAAISNSX
................................................................................      80
.......................                                                               160

(Threshold=0.5)

No sites predicted in this sequence.
```

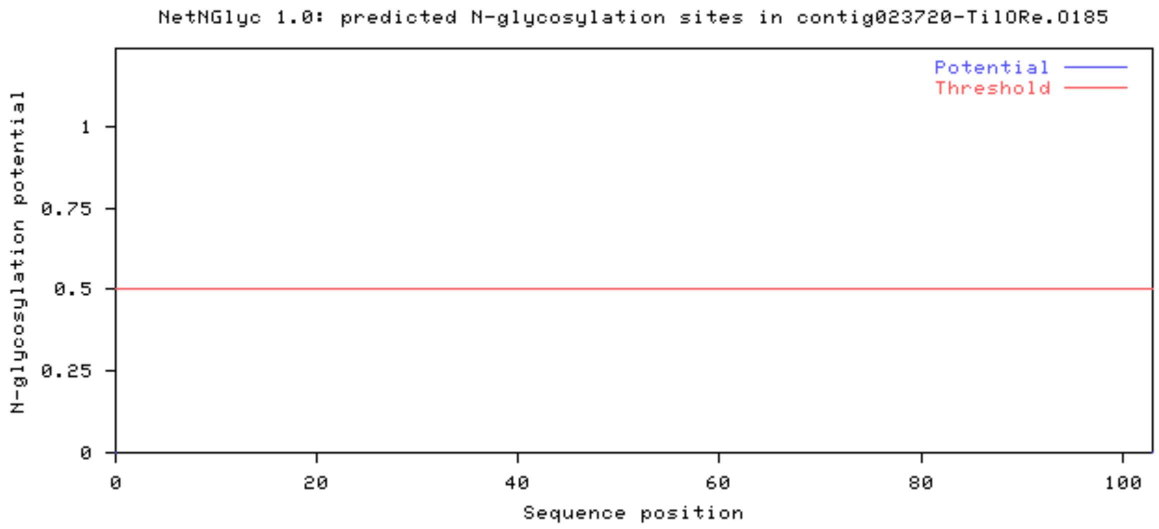

Graphics in PostScript

Output for 'contig023722-TilORe.O184'

```
##########################################################################################

    Warning: This sequence may not contain a signal peptide!!

    Proteins without signal peptides are unlikely to be exposed to
    the N-glycosylation machinery and thus may not be glycosylated
    (in vivo) even though they contain potential motifs.

    SignalP-NN euk predictions are as follows:

  # name                    Cmax   pos ?  Ymax   pos ?  Smax   pos ?  Smean ?  D     ?

    SignalP output is explained at http://www.cbs.dtu.dk/services/SignalP/output.html

##########################################################################################

Name:  contig023722-TilORe.O184          Length:  291
MPERNHSSVTEFILTGFPGLHQEYYGLVSALLFFVYLVTMIANVTVIFLIATNRSLHKPMYYIILNLSVCDILFSTTTLP      80
KIISRYWFQSGSISFTACFIQMYFVHYLGTVNSYILFQMALDRYLAICHPLRYSRVLTKSNILILSITAWITAKTSNLML     160
VIRAYPLPYCASNIITHCFCDHIGITVLACTERAPYSIPAFAGAMVTLLGPLAFIIFSYCSIIIAVYKIANVQGRLKSLS     240
TCSTQLIIISLYYLPRCFVYLASNVGITFSADVRIVIIMLYSLAPPMINPL
....N.....................................N.........N............N..............      80
................................................................................     160
................................................................................     240
...................................................                                  320

(Threshold=0.5)
----------------------------------------------------------------------
```

SeqName	Position	Potential	Jury agreement	N-Glyc result
contig023722-TilORe.O184	5 NHSS	0.6069	(8/9)	+
contig023722-TilORe.O184	43 NVTV	0.7957	(9/9)	+++
contig023722-TilORe.O184	53 NRSL	0.7316	(9/9)	++
contig023722-TilORe.O184	66 NLSV	0.7940	(9/9)	+++

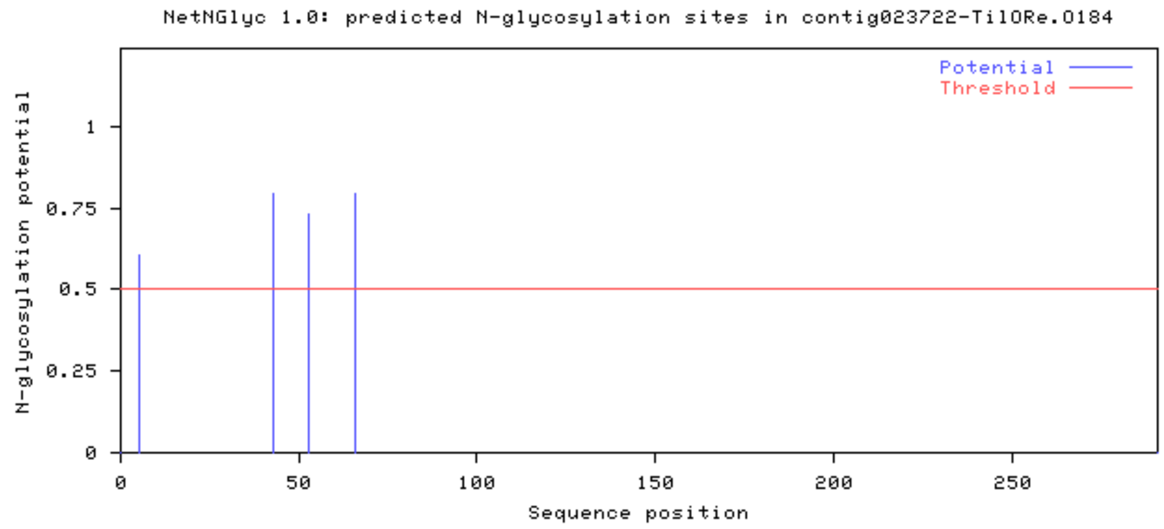

Graphics in PostScript

Output for 'contig023723-TilORe.O179'

```
##########################################################################################

    Warning: This sequence may not contain a signal peptide!!

    Proteins without signal peptides are unlikely to be exposed to
    the N-glycosylation machinery and thus may not be glycosylated
    (in vivo) even though they contain potential motifs.

    SignalP-NN euk predictions are as follows:

  # name                 Cmax  pos ?  Ymax  pos ?  Smax  pos ?  Smean ?  D     ?

    SignalP output is explained at http://www.cbs.dtu.dk/services/SignalP/output.html

##########################################################################################

Name:  contig023723-TilORe.O179        Length:  73
LPRCFVYLASNVGITFSADVRIVIIMLYSLAPPMINPLIYCLRAKDMRDSLLKQFCRRTVPRKPQVAAISNSX
.........................................................................           80

(Threshold=0.5)

No sites predicted in this sequence.
```

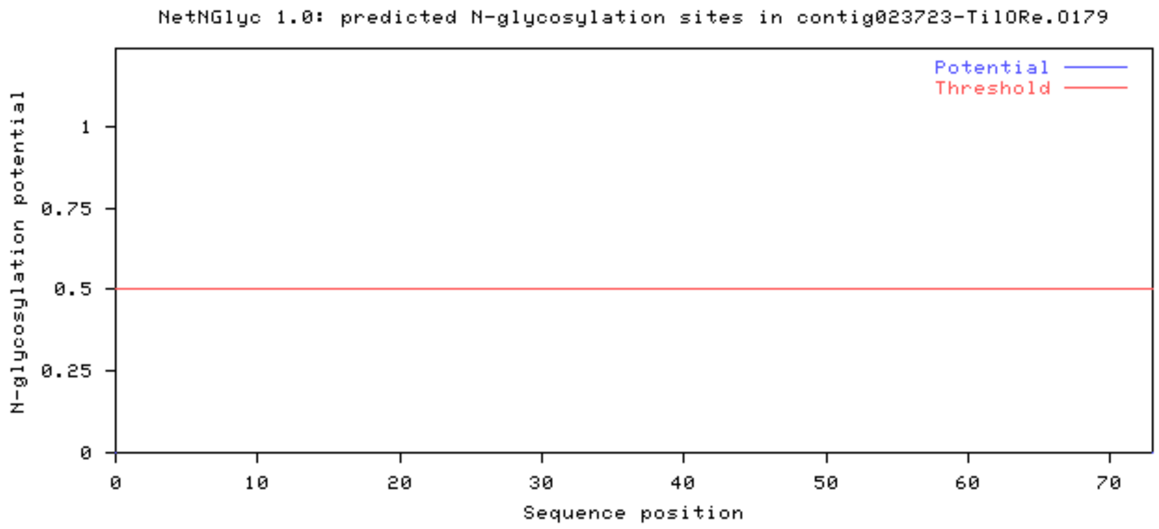

Graphics in PostScript

Output for 'contig023724-TilOR.O176'

```
##########################################################################

    Warning: This sequence may not contain a signal peptide!!

    Proteins without signal peptides are unlikely to be exposed to
    the N-glycosylation machinery and thus may not be glycosylated
    (in vivo) even though they contain potential motifs.

    SignalP-NN euk predictions are as follows:

  # name                Cmax  pos ?  Ymax  pos ?  Smax  pos ?  Smean ?  D     ?

    SignalP output is explained at http://www.cbs.dtu.dk/services/SignalP/output.html

##########################################################################

Name:  contig023724-TilOR.O176  Length:  326
MPERNHSVLTEFVLTGFPGLHQEYYGLVSALLFFVYLVTMIANVTVVFLIATNRSLHKTMYYIILNLSVCDILFSTTTLP      80
KIISRYWFQSGSISFTGCFIQMYFVHYLGTVNSYILFQMALDRYLAICHPLRYSRVLTKSNILILSITAWIIAKASPLMT     160
VIRAYPLPYCASNIITHCFCDHIGITVLACTDRAPYAIPAFVFAMVTLLGPLAFIIFSYCSIIIAVYKIANVQGRLKSLS     240
TCSTQLIIISLYYLPRCFVYLASNVGITFSADVRIVIMLYSLAPPMINPLIYCLRAKDMRESLLKQFCKKNIPQKAEVA     320
AISNSX
....N.....................................N.........N............N..............      80
................................................................................     160
................................................................................     240
................................................................................     320
......                                                                               400

(Threshold=0.5)
----------------------------------------------------------------------
SeqName       Position  Potential    Jury    N-Glyc
                                  agreement  result
----------------------------------------------------------------------
contig023724-TilOR.O176    5 NHSV   0.5099     (6/9)    +
contig023724-TilOR.O176   43 NVTV   0.7919     (9/9)    +++
contig023724-TilOR.O176   53 NRSL   0.7350     (9/9)    ++
contig023724-TilOR.O176   66 NLSV   0.7957     (9/9)    +++
----------------------------------------------------------------------
```

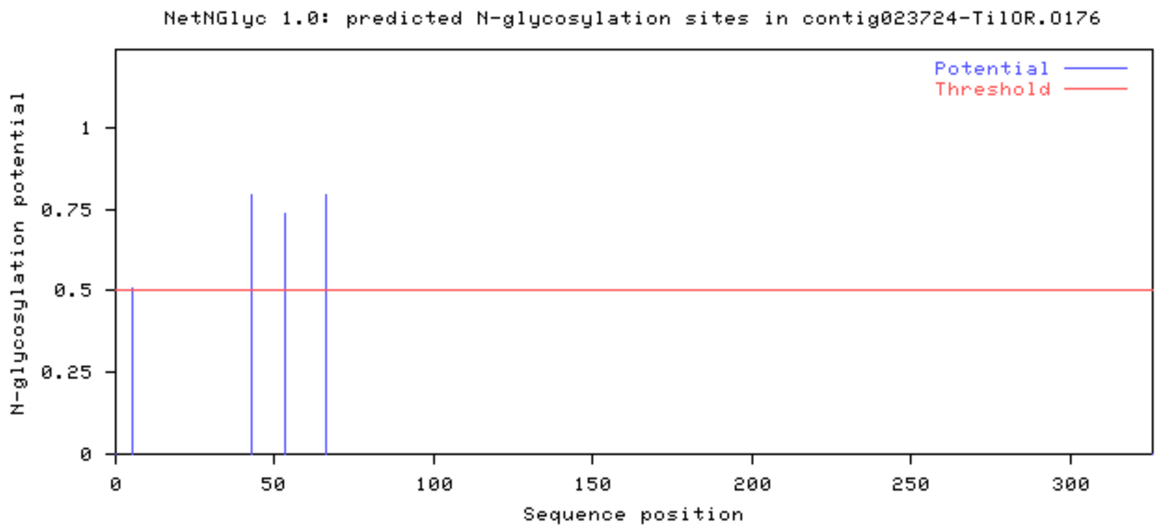

Graphics in PostScript

Output for 'contig023726-TilORe.O180'

```
##########################################################################################

    Warning: This sequence may not contain a signal peptide!!

    Proteins without signal peptides are unlikely to be exposed to
    the N-glycosylation machinery and thus may not be glycosylated
    (in vivo) even though they contain potential motifs.

    SignalP-NN euk predictions are as follows:

  # name                 Cmax  pos ?  Ymax  pos ?  Smax  pos ?  Smean ?  D     ?

    SignalP output is explained at http://www.cbs.dtu.dk/services/SignalP/output.html

##########################################################################################

Name:  contig023726-TilORe.O180        Length:  81
MITNVTRMKSFFILGFPGLSPQYYGTISTFLLFVYLAIAVGNIFILSFVAYEKSLQKPTYLVFCHLALNDLTFGTVTLPK      80
I
...N................................................................................      80
.                                                                                    160

(Threshold=0.5)
----------------------------------------------------------------------
SeqName      Position  Potential   Jury    N-Glyc
                                 agreement result
----------------------------------------------------------------------
contig023726-TilORe.O180     4 NVTR    0.7698     (9/9)    +++
----------------------------------------------------------------------
```

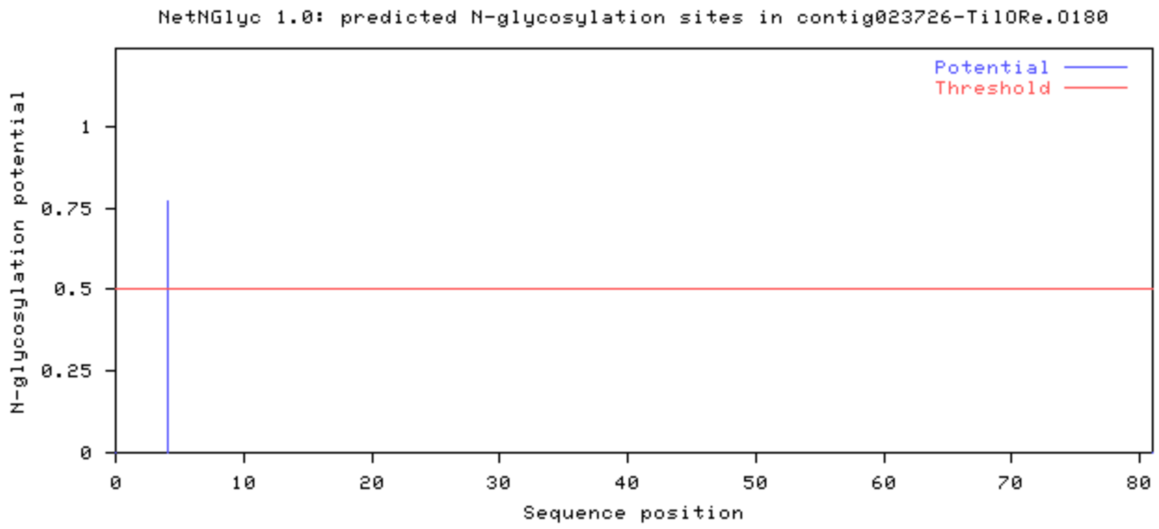

Graphics in PostScript

Output for 'contig023727-TilORe.O268'

```
##########################################################################################

    Warning: This sequence may not contain a signal peptide!!

    Proteins without signal peptides are unlikely to be exposed to
    the N-glycosylation machinery and thus may not be glycosylated
    (in vivo) even though they contain potential motifs.

    SignalP-NN euk predictions are as follows:

  # name                    Cmax   pos ?  Ymax   pos ?  Smax   pos ?  Smean ?  D     ?

    SignalP output is explained at http://www.cbs.dtu.dk/services/SignalP/output.html

##########################################################################################

Name:  contig023727-TilORe.0268         Length:  92
SSAGRKRTLSTCTSQIFITCLFYLPRCFVYVANAVGFSFSLDVRIGLILLYSLFPAAVNPVIYCFKTHDIKHMLMKKLKK      80
TKIGLQIKLSLX
................................................................................      80
............                                                                         160

(Threshold=0.5)

No sites predicted in this sequence.
```

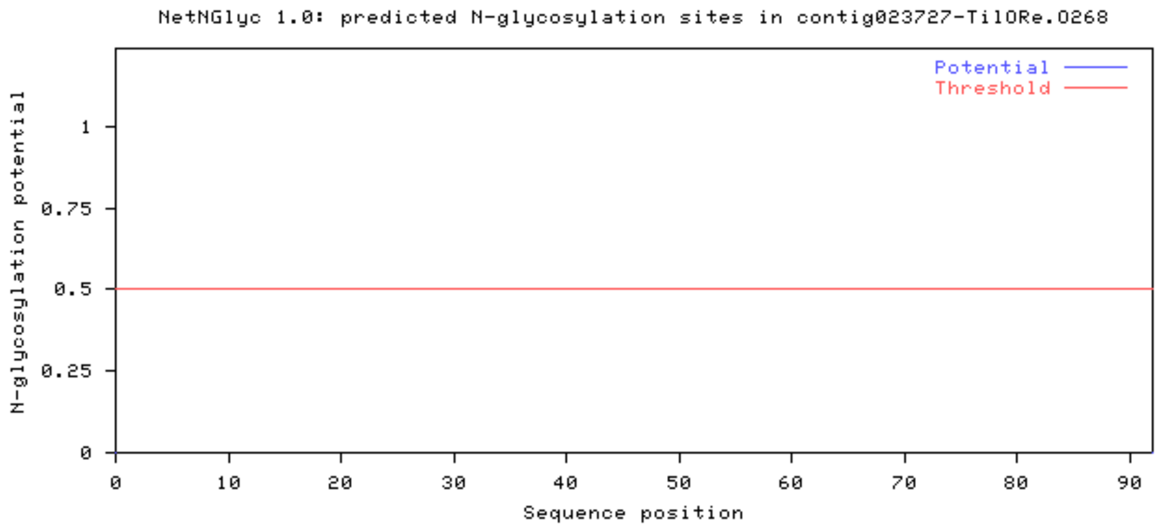

Graphics in PostScript

Output for 'contig023727-TilORe.O181'

```
##########################################################################

    Warning: This sequence may not contain a signal peptide!!

    Proteins without signal peptides are unlikely to be exposed to
    the N-glycosylation machinery and thus may not be glycosylated
    (in vivo) even though they contain potential motifs.

    SignalP-NN euk predictions are as follows:

  # name                Cmax  pos ?  Ymax  pos ?  Smax  pos ?  Smean ?  D     ?

    SignalP output is explained at http://www.cbs.dtu.dk/services/SignalP/output.html

##########################################################################

Name:  contig023727-TilORe.O181         Length:  83
MSFNFTNVTRMKSFFILGFPGLSPQYYGTISTFLFFVYLAIAVGNIFILSFVVYEKSLQKPTYLVFCHLALNDLTFGTVT      80
LPK
...N..N.........................................................................      80
...                                                                                  160

(Threshold=0.5)
----------------------------------------------------------------------
SeqName      Position  Potential   Jury    N-Glyc
                                agreement  result
----------------------------------------------------------------------
contig023727-TilORe.O181    4 NFTN   0.7078     (9/9)   ++
contig023727-TilORe.O181    7 NVTR   0.6707     (8/9)   +
----------------------------------------------------------------------
```

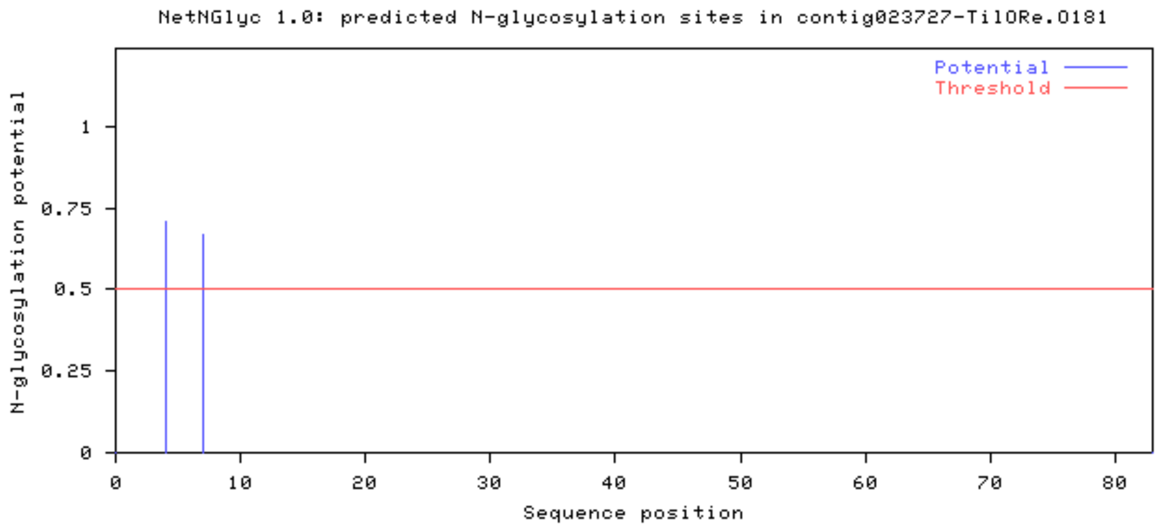

Graphics in PostScript

Output for 'contig023728-TilORe.O267'

```
##########################################################################################

    Warning: This sequence may not contain a signal peptide!!

    Proteins without signal peptides are unlikely to be exposed to
    the N-glycosylation machinery and thus may not be glycosylated
    (in vivo) even though they contain potential motifs.

    SignalP-NN euk predictions are as follows:

  # name                Cmax  pos ?  Ymax  pos ?  Smax  pos ?  Smean ?  D     ?

    SignalP output is explained at http://www.cbs.dtu.dk/services/SignalP/output.html

##########################################################################################

Name:  contig023728-TilORe.O267         Length:  88
SSAGRKRTLSTCTSQIFITCLFYLPRCFVYVTYTVGFSFSLDVRIGLILLYSLFPAAVNPVIYCFKTHDIKHIMMKKLKK      80
TKIELKLX
................................................................................      80
........                                                                             160

(Threshold=0.5)

No sites predicted in this sequence.
```

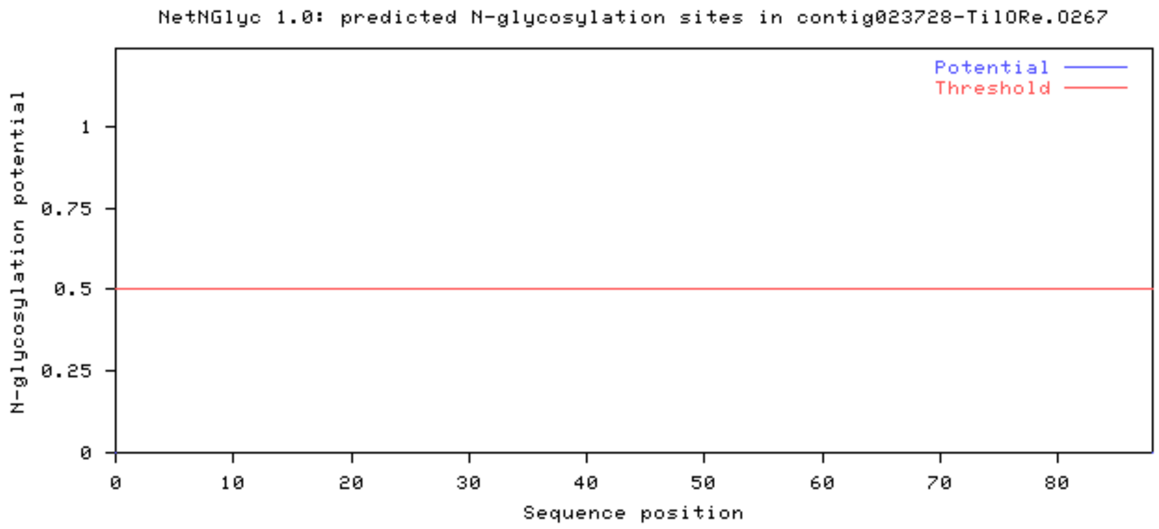

Graphics in PostScript

Output for 'contig023730-TilORe.O182'

```
########################################################################################

    Warning: This sequence may not contain a signal peptide!!

    Proteins without signal peptides are unlikely to be exposed to
    the N-glycosylation machinery and thus may not be glycosylated
    (in vivo) even though they contain potential motifs.

    SignalP-NN euk predictions are as follows:

  # name                Cmax  pos ?  Ymax  pos ?  Smax  pos ?  Smean ?  D     ?

    SignalP output is explained at http://www.cbs.dtu.dk/services/SignalP/output.html

########################################################################################

Name:  contig023730-TilORe.O182         Length:  316
IKEFIIIGFPGLPPEYYGPVSVLLLLVFLAIVIGNGFTIAVIIFERTLHKPIYVIFSNLAMTDICFGVVTLPKIIARYWW      80
NDMITSFGACFTQMYFVHSLGAIQSLNLLMMALDRFVAIWFPFKYPVLFTNKAVAIACTMCWVLTFIRLLGIVLHALTLP     160
YCDQNIIMQCYCDHISITRLGCSDEREYVYSVALANAMVTLLFPLTLIILSYFSVIIAVLRMSQTERRHKVLSTCAPQLF     240
ITCLYYVPRCFVYLANGVGFNFNLVIRIIITMLYSLIPAVVNPMIYCFKTKEIKNVLMQRFKKRKVSTGLKTDCKX
................................................................................      80
................................................................................     160
................................................................................     240
............................................................................         320

(Threshold=0.5)

No sites predicted in this sequence.
```

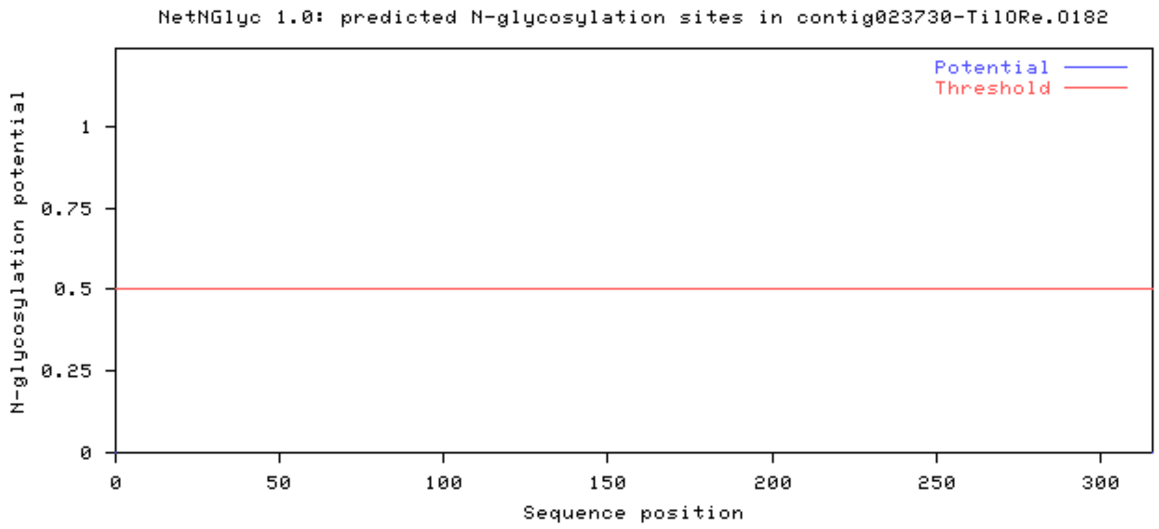

Graphics in PostScript

Output for 'contig023731-TilOR.O177'

```
##########################################################################

    Warning: This sequence may not contain a signal peptide!!

    Proteins without signal peptides are unlikely to be exposed to
    the N-glycosylation machinery and thus may not be glycosylated
    (in vivo) even though they contain potential motifs.

    SignalP-NN euk predictions are as follows:

  # name                Cmax  pos ?  Ymax  pos ?  Smax  pos ?  Smean ?  D     ?

    SignalP output is explained at http://www.cbs.dtu.dk/services/SignalP/output.html

##########################################################################

Name:  contig023731-TilOR.0177  Length:  324
MKYTNITTIKEFIIIGFPGLPPEYYGPVSVLLLLVFLAIVIGNGFTIAVIIFERTLHKPIYVIFSNLAMTDICFGVVTLP      80
KIIARYWWNDMITSFGACFTQMYFVHSLGAIQSLNLLMMALDRFVAIWFPFKYPVLFTNKAVAIACTMCWVLTFIRLLGI     160
VLHALTLPYCDQNIIMQCYCDHISITRLGCSDEREYVNSVALANAMVTLLVPLTFIILSYFSVIIAVLRMSQTERRHKVL     240
STCAPQLFITCLYYVPRCFVYLANVLGFNFSLVIRIIITMLYSLIPAAVNPMIYCFKTKEIKNVLMRRFKKRKVSTGLKT     320
DCKX
....N...........................................................................      80
................................................................................     160
................................................................................     240
................................................................................     320
....                                                                                 400

(Threshold=0.5)
----------------------------------------------------------------------
SeqName      Position  Potential   Jury    N-Glyc
                                 agreement result
----------------------------------------------------------------------
contig023731-TilOR.0177     5 NITT   0.7654     (9/9)    +++
contig023731-TilOR.0177   269 NFSL   0.4380     (6/9)    -
----------------------------------------------------------------------
```

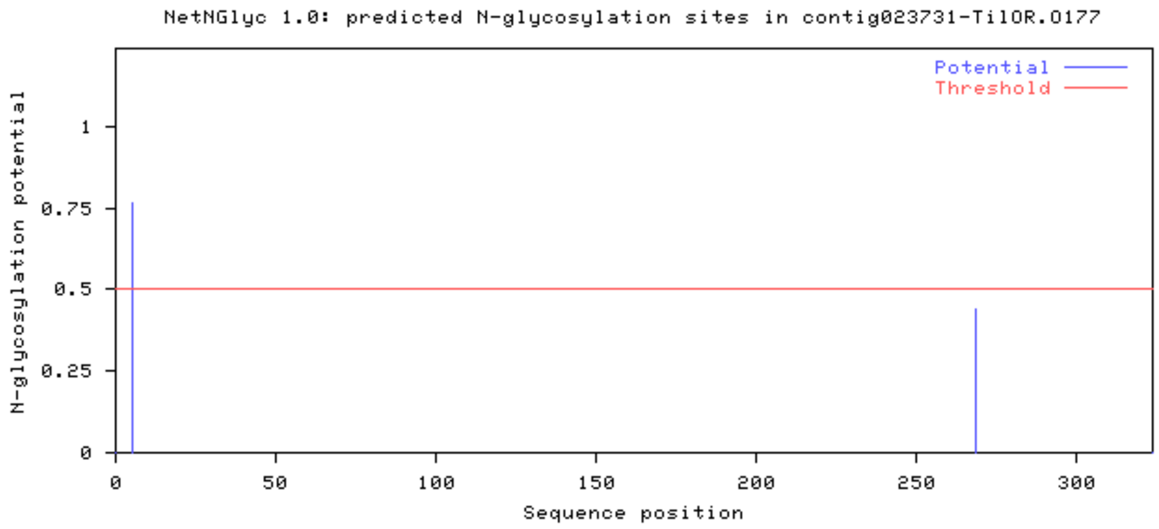

Graphics in PostScript

Output for 'contig027194-TilORs.U236'

```
##########################################################################

    Warning: This sequence may not contain a signal peptide!!

    Proteins without signal peptides are unlikely to be exposed to
    the N-glycosylation machinery and thus may not be glycosylated
    (in vivo) even though they contain potential motifs.

    SignalP-NN euk predictions are as follows:

  # name                Cmax  pos ?  Ymax  pos ?  Smax  pos ?  Smean ?  D     ?

    SignalP output is explained at http://www.cbs.dtu.dk/services/SignalP/output.html

##########################################################################

Name:  contig027194-TilORs.U236         Length:  326
MSSLLGLRGNMTVPYQLIRDTFTTAFVKNLIVVLVWLTLSYINGTLVVTFFRHQTFYDDPRYILFIHMVINDAIQLTVTI      80
MLFILSYIFYKINVAFCCFFILVAVFTTRNTPVNLAAMAIERYIAICEPLRYTQICTVRRTYIVIGMIWFICVAPDITDL     160
FVTLATESLSFFHESVFCLRQNVFKDPILAYKRQVFDIIYFSCVFLILVVTYLRILFAARALSTDKTSAQKARNTILLHG     240
AQLAMCMLSYVSPSVEVVLHIIFPGRILEIRFANYLIVYILPRFLSPIIYGVRDKKFREYLRMYFLSNRCRNKERKVTPE     320
DKDHLX
.........N................................N.....................................      80
................................................................................     160
................................................................................     240
................................................................................     320
......                                                                               400

(Threshold=0.5)
----------------------------------------------------------------------
SeqName      Position  Potential   Jury    N-Glyc
                                 agreement result
----------------------------------------------------------------------
contig027194-TilORs.U236   10 NMTV   0.7831     (9/9)   +++
contig027194-TilORs.U236   43 NGTL   0.7602     (9/9)   +++
----------------------------------------------------------------------
```

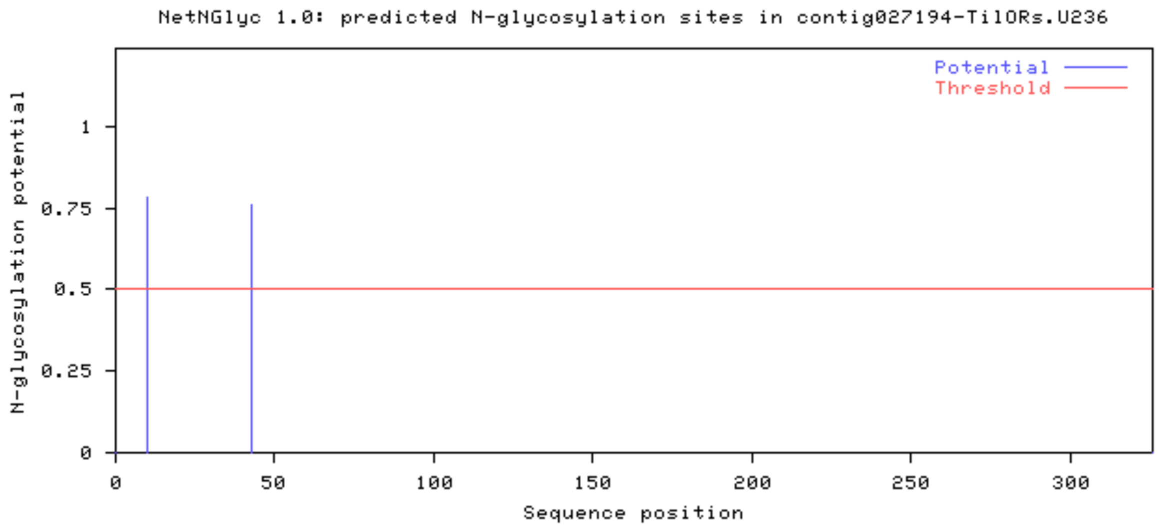

[Graphics in PostScript]

Output for 'contig027194-TilORs.V262'

```
##############################################################################

    Warning: This sequence may not contain a signal peptide!!

    Proteins without signal peptides are unlikely to be exposed to
    the N-glycosylation machinery and thus may not be glycosylated
    (in vivo) even though they contain potential motifs.

    SignalP-NN euk predictions are as follows:

  # name                 Cmax  pos ?  Ymax  pos ?  Smax  pos ?  Smean ?  D     ?

    SignalP output is explained at http://www.cbs.dtu.dk/services/SignalP/output.html

##############################################################################

Name:  contig027194-TilORs.V262         Length:  335
MNSTSSGPLDPLVSNITSVQTEGVKESFSSILTKNIVAMLVWLVLSIINGSMMHTFQRHSLFYENPRYIMFICMVINDAL      80
QLTLVTALYVISYIFRKIHASVCCLLVIMTAILTTRSTPLILAGMAVERYISICFPLHYTQMCNIPRTLLLICVIFILTV     160
TPPITDLLITIVKEPPSFFHTKIFCDHSLLFRDQSIYYKNCVFDGTYLSFVALTLLYTYCKIMLTAQAVSTSLVSVKRAR     240
NTVLLHGVQLLLCMLAFVVPSLQAALISLFPQLGLEIRYIFFLLVYIIPRFLSPMIYGFRDEQFRKYWTRYLSCHEHSMT     320
LLRLASQKMNPQVKX
.N............N.................................N...............................      80
................................................................................     160
................................................................................     240
................................................................................     320
...............                                                                      400

(Threshold=0.5)
----------------------------------------------------------------------
SeqName       Position  Potential   Jury    N-Glyc
                                  agreement result
----------------------------------------------------------------------
contig027194-TilORs.V262     2 NSTS   0.7468     (9/9)   ++
contig027194-TilORs.V262    15 NITS   0.7867     (9/9)   +++
contig027194-TilORs.V262    49 NGSM   0.6123     (8/9)   +
----------------------------------------------------------------------
```

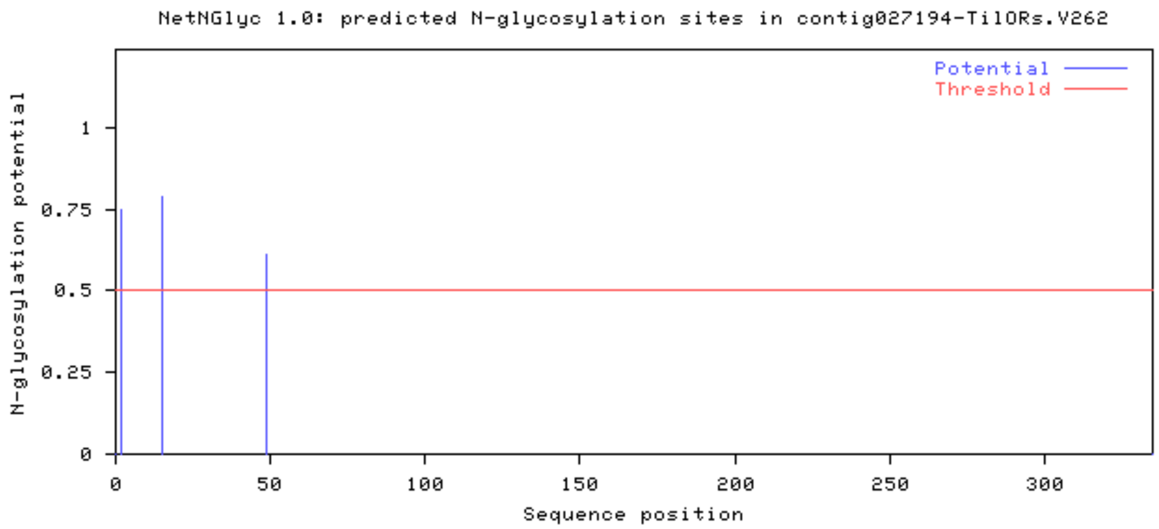

[Graphics in PostScript]

Output for 'contig027198-TilORe.W271'

```
##########################################################################################

    Warning: This sequence may not contain a signal peptide!!

    Proteins without signal peptides are unlikely to be exposed to
    the N-glycosylation machinery and thus may not be glycosylated
    (in vivo) even though they contain potential motifs.

    SignalP-NN euk predictions are as follows:

  # name                 Cmax  pos ?  Ymax  pos ?  Smax  pos ?  Smean ?  D     ?

    SignalP output is explained at http://www.cbs.dtu.dk/services/SignalP/output.html

##########################################################################################

Name:  contig027198-TilORe.W271         Length:  120
SNIVFLVIVWLTLVYTYFRILFTAQAAAANARKARNTVLLHGFQLLLCMLTYVYDLLLNGLTSLFPSGVLTIRYTISILV      80
HVLPRIISPIVYGIRDKMFRKYLKKYLFHTKNANALVHRX
................................................................................      80
........................................                                             160

(Threshold=0.5)

No sites predicted in this sequence.
```

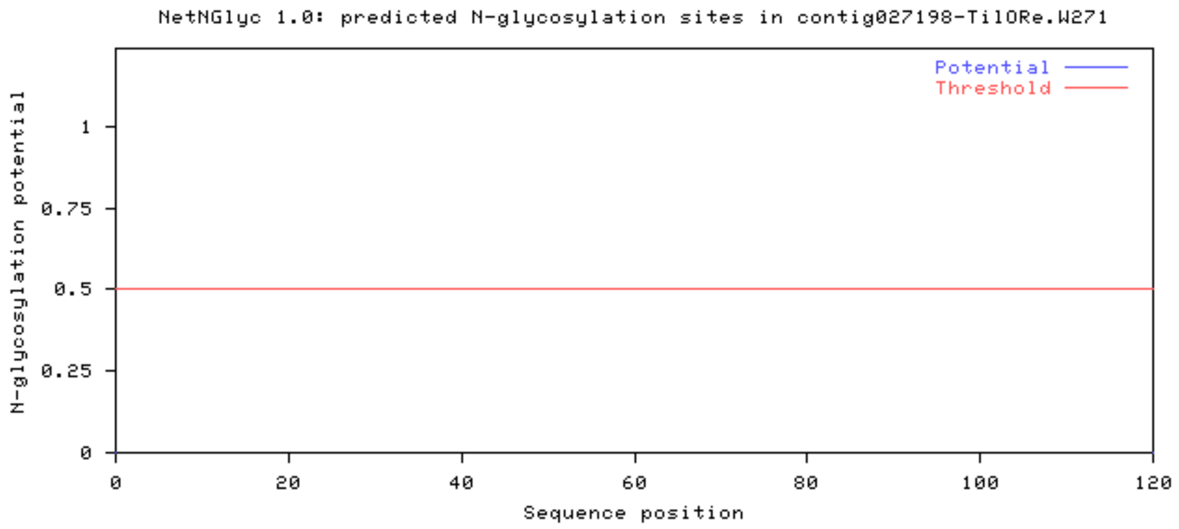

Graphics in PostScript

Output for 'contig027202-TilORs.W243'

```
##########################################################################################

    Warning: This sequence may not contain a signal peptide!!

    Proteins without signal peptides are unlikely to be exposed to
    the N-glycosylation machinery and thus may not be glycosylated
    (in vivo) even though they contain potential motifs.

    SignalP-NN euk predictions are as follows:

  # name                  Cmax  pos ?  Ymax  pos ?  Smax  pos ?  Smean ?  D     ?

    SignalP output is explained at http://www.cbs.dtu.dk/services/SignalP/output.html

##########################################################################################

Name:  contig027202-TilORs.W243         Length:  318
MNSSAYAFNASSSLKYRDSPSAVVAKNVIVLTLGFTINYINGTLIHTFRKHQIFYLNPRYILFIHLVVNDMIQLTSSISL      80
FVFTYIFYQINVAFCCFLITLAIFTTFNTPINLALMALECYIAICLPLQHAEYCTTKRTYVAIGWIWAMSAVSAMSDIVI     160
ILATEPVELFYSTIQCERDNLFRHPIIVKKKEVSYLIFLIGVLLTFFYTYFRIFFAANKAKSAERESKKARNTILLHGFQ     240
LLLSMLTYIATVLTQTLVRWFPKQYLIVVFISYIIIYIVPRFVSPIVYGLRDKTFKQYLKKYLLCTMKMGNETDFRLX
.N......N...............................N.......................................      80
................................................................................     160
................................................................................     240
......................................................................N.......        320

(Threshold=0.5)
----------------------------------------------------------------------
SeqName      Position  Potential   Jury    N-Glyc
                                 agreement result
----------------------------------------------------------------------
contig027202-TilORs.W243    2 NSSA   0.6753     (7/9)   +
contig027202-TilORs.W243    9 NASS   0.6116     (8/9)   +
contig027202-TilORs.W243   41 NGTL   0.7080     (9/9)   ++
contig027202-TilORs.W243  311 NETD   0.6717     (9/9)   ++
----------------------------------------------------------------------
```

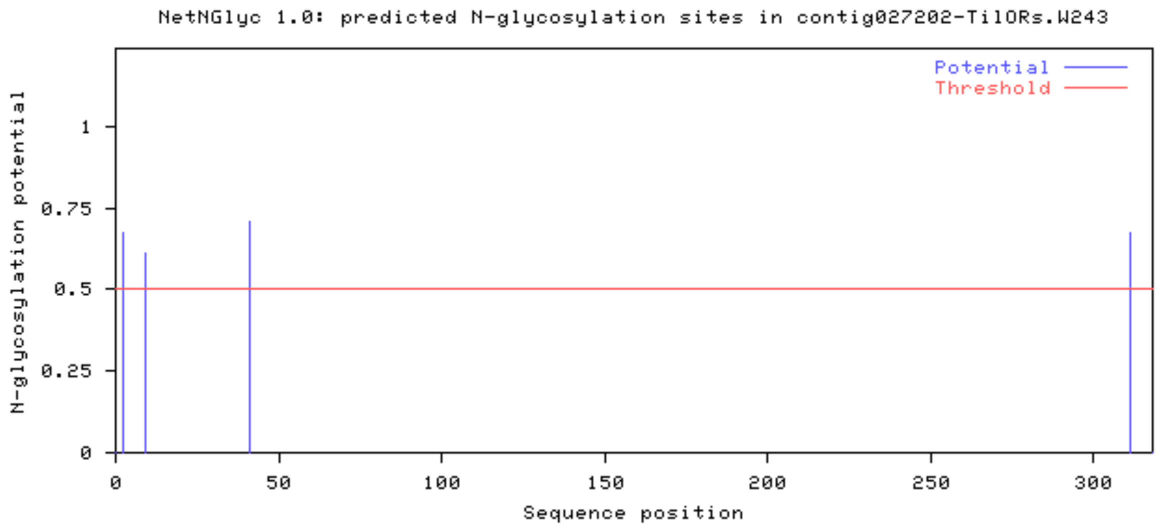

[Graphics in PostScript]

Output for 'contig027203-TilORs.W238'

```
##############################################################################

    Warning: This sequence may not contain a signal peptide!!

    Proteins without signal peptides are unlikely to be exposed to
    the N-glycosylation machinery and thus may not be glycosylated
    (in vivo) even though they contain potential motifs.

    SignalP-NN euk predictions are as follows:

  # name                Cmax  pos ?  Ymax  pos ?  Smax  pos ?  Smean ?  D     ?

    SignalP output is explained at http://www.cbs.dtu.dk/services/SignalP/output.html

##############################################################################

Name:  contig027203-TilORs.W238         Length:  313
MNASSANVTVVLQYRDSFPKAVTKNLIVVVLGISISYVNATLIHTFCKHQIFYKNPRYVLFIHLVINDMVQVMLTAILFI      80
ISYTIYKLNVSVCCIFMLLALFTTENSPLNLACMAVECYIAICFPLRHVQICTVQRTLILISLIWMTTTLSVLPDLFITL     160
ATEPLDFFHSRVFCLRNTVFPHPLIIQKRDITYAVFLVIVWVTIIYTYLKILFTAKTASKDAKKAKNTILLHGFQLLLCM     240
ATYAAPHVTNALQKWFPTNYTDSLFVVYVTVQILPRSISPIIYGIRDKTFRKFLKGNLLCKITVHKSDRQNKX
.N....N...............................N.........................................      80
........N.......................................................................     160
................................................................................     240
..................N......................................................            320

(Threshold=0.5)
----------------------------------------------------------------------
SeqName      Position  Potential   Jury    N-Glyc
                                 agreement result
----------------------------------------------------------------------
contig027203-TilORs.W238    2 NASS   0.6924     (9/9)   ++
contig027203-TilORs.W238    7 NVTV   0.7204     (9/9)   ++
contig027203-TilORs.W238   39 NATL   0.7478     (9/9)   ++
contig027203-TilORs.W238   89 NVSV   0.6076     (8/9)   +
contig027203-TilORs.W238  259 NYTD   0.7022     (9/9)   ++
----------------------------------------------------------------------
```

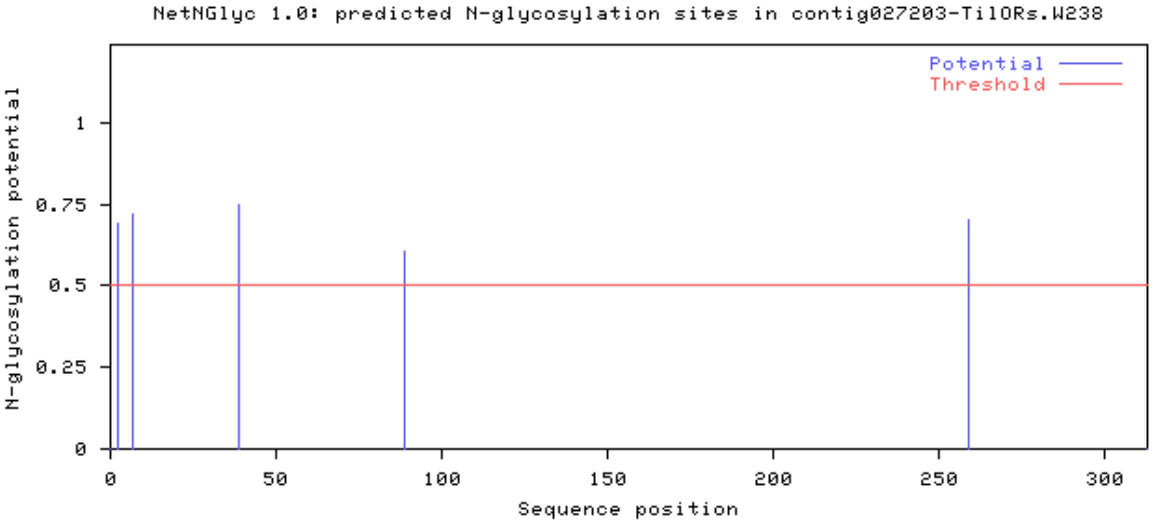

Graphics in PostScript

Output for 'contig027204-TilORs.W239'

```
##########################################################################################

    Warning: This sequence may not contain a signal peptide!!

    Proteins without signal peptides are unlikely to be exposed to
    the N-glycosylation machinery and thus may not be glycosylated
    (in vivo) even though they contain potential motifs.

    SignalP-NN euk predictions are as follows:

  # name                 Cmax  pos ?  Ymax  pos ?  Smax  pos ?  Smean ?  D     ?

    SignalP output is explained at http://www.cbs.dtu.dk/services/SignalP/output.html

##########################################################################################

Name:  contig027204-TilORs.W239        Length:  305
MSTSSTNQTVAINYRDALTNFVPKNLIVVVLCISINYINVALMQTFSKNRIFYMNPRYILFFHLVLNDMIQVTLTVILFV      80
SSYIFFQINVSVCCVLILLALFATENTPLNLACMAVECYIAICIPLRHVQICTVKRTLMLIGLIWMTSMLSVLPDLFITL     160
AIVPLGFFRSRVFCLRETVFRNPHIIKKRDITYIVYLVIVWFIIFFTYFKVLFTAKTASQDATKARNTIILHGFQVLLCM     240
SMYAEPLLKQALLQWFPQYYSDSLFACYIIFQILPRAISPIVYGVRDKTYRKYLKRYLLCKMSSX
......N.........................................................................      80
........N.......................................................................     160
................................................................................     240
.................................................................                    320

(Threshold=0.5)
----------------------------------------------------------------------
SeqName      Position  Potential   Jury    N-Glyc
                                 agreement result
----------------------------------------------------------------------
contig027204-TilORs.W239    7 NQTV   0.6488     (9/9)   ++
contig027204-TilORs.W239   89 NVSV   0.6708     (8/9)   +
----------------------------------------------------------------------
```

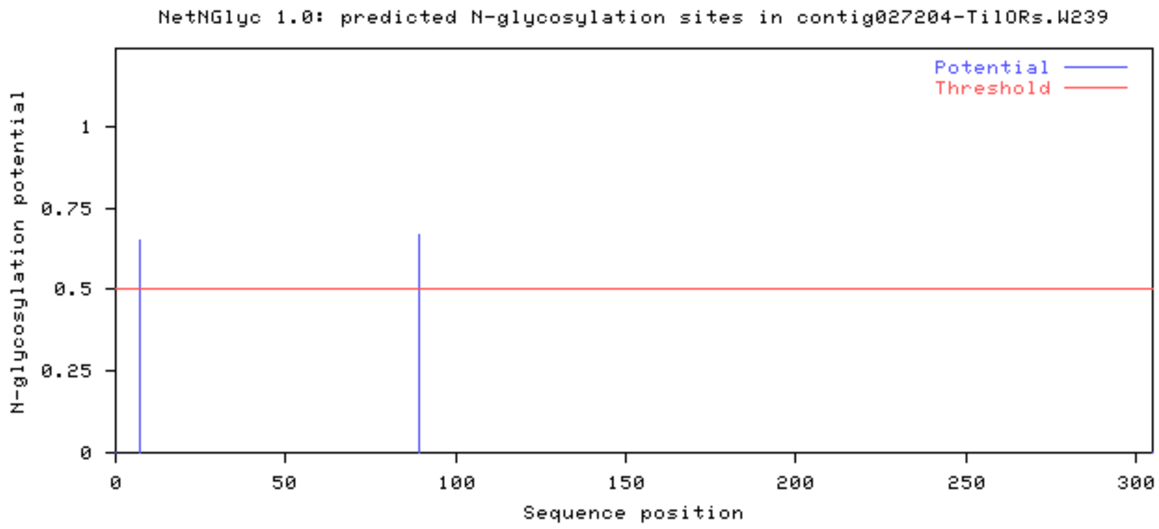

Graphics in PostScript

Output for 'contig027205-TilORes.W242'

```
##########################################################################################

    Warning: This sequence may not contain a signal peptide!!

    Proteins without signal peptides are unlikely to be exposed to
    the N-glycosylation machinery and thus may not be glycosylated
    (in vivo) even though they contain potential motifs.

    SignalP-NN euk predictions are as follows:

  # name                Cmax  pos ?  Ymax  pos ?  Smax  pos ?  Smean ?  D     ?

    SignalP output is explained at http://www.cbs.dtu.dk/services/SignalP/output.html

##########################################################################################

Name:  contig027205-TilORes.W242        Length:  299
MSTSSTNQTVAINYRDALTNFVPKNLIVVVLCISINYINVALMQTFSKNRIFYMNPRYILFFHLVLNDMIQVTLTVILFV      80
SSYIFFQINVSVCCVLILLALFATENTPLNLACMAVECYIAICIPLRHVQICTVKRTLMLIGLIWMTSMLSVLPDLFITL     160
ATVPLGFFSSRVFCLRETVFQNPHIIKKRDITYIVYLVIVWFIIFFTYFKILFTAKTASQDATKARNTIILHGFQVLLCM     240
SMYAEPLLKQALLQWFPQYYSNSLFACYIIFQILPRAISPIVYGVRDKTYRKYLKRYLL
......N.........................................................................      80
........N.......................................................................     160
................................................................................     240
...........................................................                          320

(Threshold=0.5)
----------------------------------------------------------------------
SeqName      Position  Potential   Jury    N-Glyc
                                 agreement result
----------------------------------------------------------------------
contig027205-TilORes.W242    7 NQTV    0.6491     (9/9)    ++
contig027205-TilORes.W242   89 NVSV    0.6703     (8/9)    +
----------------------------------------------------------------------
```

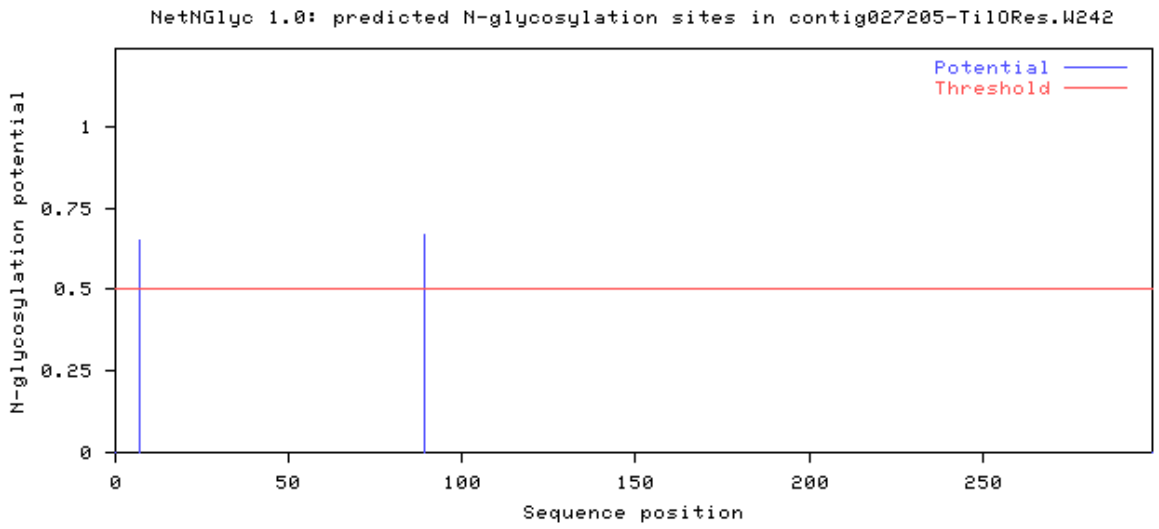

Graphics in PostScript

Output for 'contig027206-TilORs.W240'

```
##########################################################################

    Warning: This sequence may not contain a signal peptide!!

    Proteins without signal peptides are unlikely to be exposed to
    the N-glycosylation machinery and thus may not be glycosylated
    (in vivo) even though they contain potential motifs.

    SignalP-NN euk predictions are as follows:

  # name                Cmax  pos ?  Ymax  pos ?  Smax  pos ?  Smean ?  D     ?

    SignalP output is explained at http://www.cbs.dtu.dk/services/SignalP/output.html

##########################################################################

Name:  contig027206-TilORs.W240         Length:  305
MSTSYINETVVENYRDAFTKALFKNVIVVVLCISINYINVALIQTFCKHQIFYMNPRYILFFHLVLNDMIQVTLTVILFV      80
SSYIFFQINVSVCCVLILLALFATENTPLNLACMAVECYIAICIPLRHVQICTVKRTLMLIGLIWMTSMLSVLPDLFITL     160
AIEPLDFYHSRVFCLRETVFRNPHIIKKRDITYIVYLVIVWFIIFFTYFKVLFTAKTASQDATKARNTIILHGFQVLLCM     240
SIYAEPLLKQALQQWFPKNYSDSLFACYIIFQILPRAISPIVYGVRDKTYRKYLKRYLLCKMSPX
......N.........................................................................      80
........N.......................................................................     160
................................................................................     240
..................N..............................................                    320

(Threshold=0.5)
----------------------------------------------------------------------
SeqName      Position  Potential   Jury    N-Glyc
                                 agreement result
----------------------------------------------------------------------
contig027206-TilORs.W240    7 NETV   0.7422     (9/9)   ++
contig027206-TilORs.W240   89 NVSV   0.6710     (8/9)   +
contig027206-TilORs.W240  259 NYSD   0.5763     (6/9)   +
----------------------------------------------------------------------
```

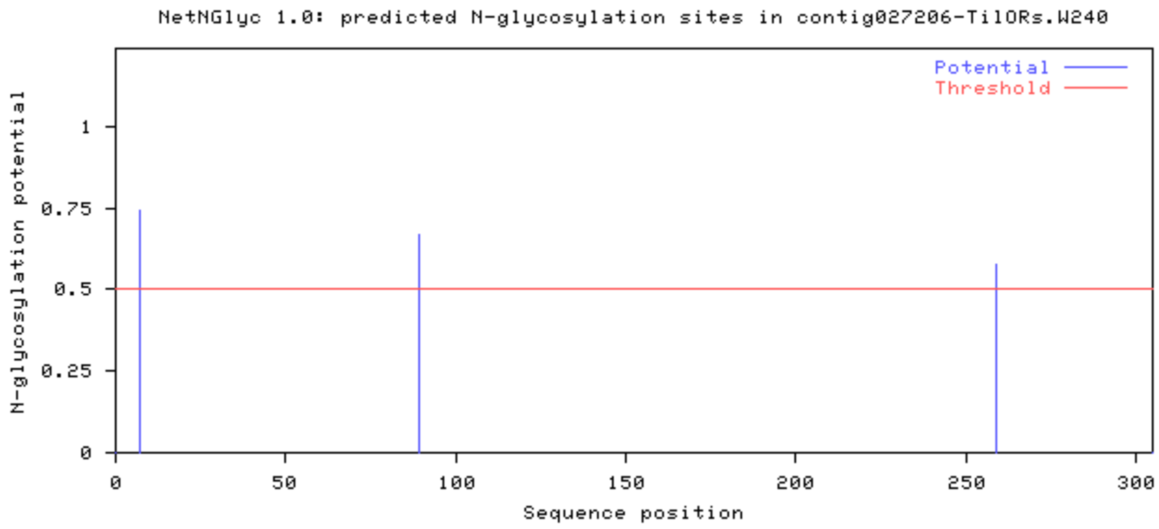

Graphics in PostScript

Output for 'contig027209-TilORs.W241'

```
##########################################################################################

    Warning: This sequence may not contain a signal peptide!!

    Proteins without signal peptides are unlikely to be exposed to
    the N-glycosylation machinery and thus may not be glycosylated
    (in vivo) even though they contain potential motifs.

    SignalP-NN euk predictions are as follows:

  # name                 Cmax  pos ?  Ymax  pos ?  Smax  pos ?  Smean ?  D     ?

    SignalP output is explained at http://www.cbs.dtu.dk/services/SignalP/output.html

##########################################################################################

Name:  contig027209-TilORs.W241         Length:  305
MSTSYINETVVENYRDAFTKALFKNVIVVVLCISINYINVALIQTFCKHQIFYMNPRYILFFHLVLNDMIQVTLTVILFV      80
SSYIFFQINVSVCCVLILLALFATENTPLNLACMAVECYIAICIPLRHVQICTVKRTLMLIGLIWMTSMLSVLPDLFISL     160
ATVPLGFFSSRVFCLRETVFQNPHIIKKRDITYIVYLVIVWFIIFFTYFKVLFTAKTASQDATKARNTIILHGFQVLLCM     240
SMYAEPLLRQVLLQWFPQYYSDSLFACYILFQILPRAISPIVYGVRDKTYRKYLKRYLLCKMSPX
......N.........................................................................      80
........N.......................................................................     160
................................................................................     240
.................................................................                    320

(Threshold=0.5)
----------------------------------------------------------------------
SeqName      Position  Potential   Jury    N-Glyc
                                 agreement result
----------------------------------------------------------------------
contig027209-TilORs.W241    7 NETV   0.7421     (9/9)   ++
contig027209-TilORs.W241   89 NVSV   0.6711     (8/9)   +
----------------------------------------------------------------------
```

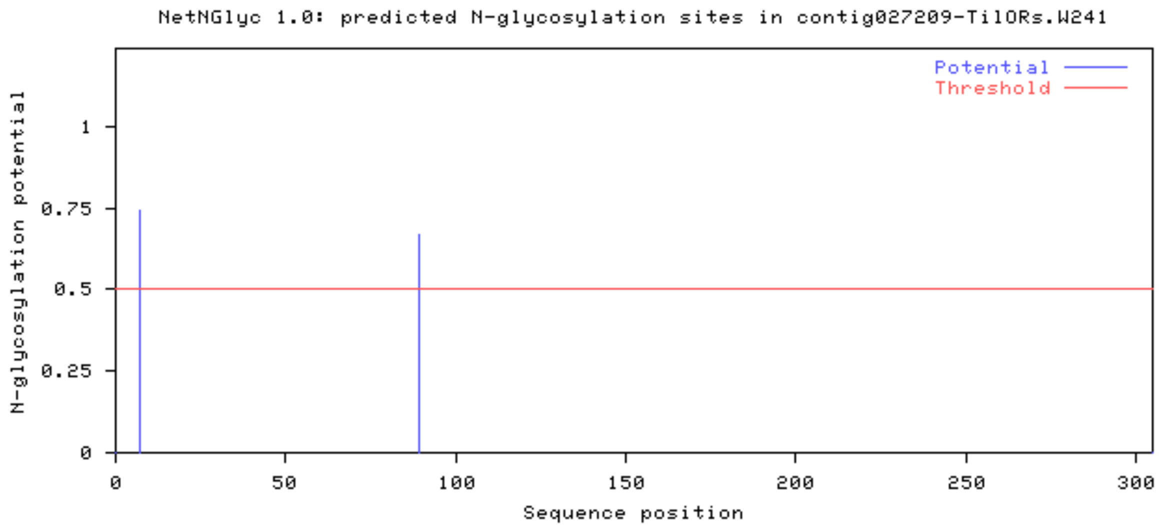

Graphics in PostScript

Output for 'contig028593-TilORe.R254'

```
##########################################################################################

    Warning: This sequence may not contain a signal peptide!!

    Proteins without signal peptides are unlikely to be exposed to
    the N-glycosylation machinery and thus may not be glycosylated
    (in vivo) even though they contain potential motifs.

    SignalP-NN euk predictions are as follows:

  # name                Cmax  pos ?  Ymax  pos ?  Smax  pos ?  Smean ?  D     ?

    SignalP output is explained at http://www.cbs.dtu.dk/services/SignalP/output.html

##########################################################################################

Name:  contig028593-TilORe.R254         Length:  213
MLANLTTGISPLTLVVMSLERYVAVCYPLRHASIVTIRKTALAIIIVWAVSSLNVFIRLILLLNFPFEDLESLQMKDFCS      80
NTPLLLGPMSDHYDKAYTWVLFVSAGVAVTCSYIGVMIAARSASTDKVSACNVRNTLLLHLIQLGLSLSSTIYDPLVTEI     160
SKVLDRVTVLRIWSILYVFMILFPRCLSPLIYGIRDQMIRGILMYHLCCQVKL
...N............................................................................      80
................................................................................     160
.....................................................                                240

(Threshold=0.5)
----------------------------------------------------------------------
SeqName      Position  Potential   Jury     N-Glyc
                                 agreement  result
----------------------------------------------------------------------
contig028593-TilORe.R254     4 NLTT   0.6839     (9/9)   ++
----------------------------------------------------------------------
```

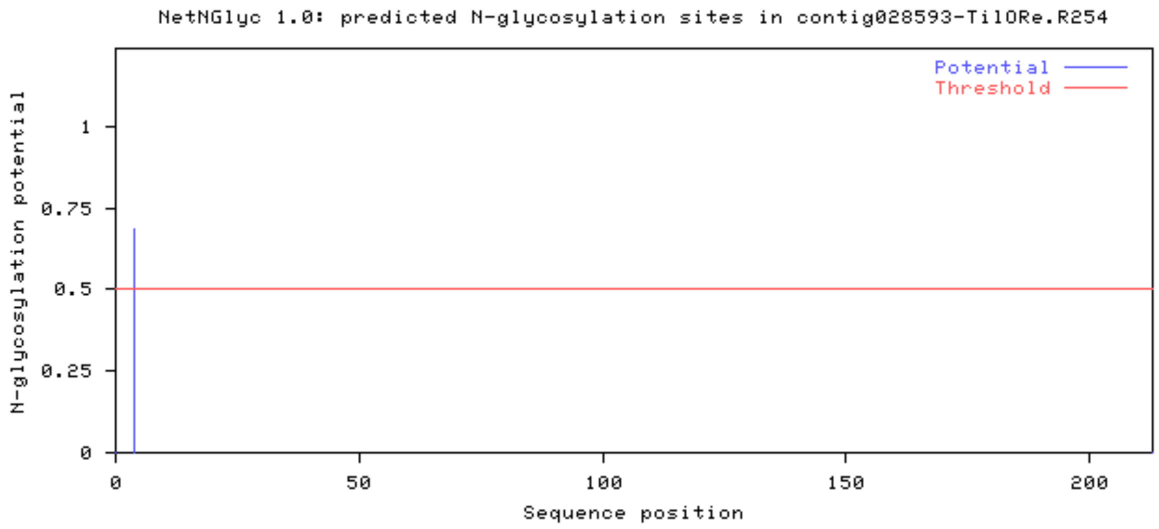

Graphics in PostScript

Output for 'contig028604-TilORe.R255'

```
##########################################################################

    Warning: This sequence may not contain a signal peptide!!

    Proteins without signal peptides are unlikely to be exposed to
    the N-glycosylation machinery and thus may not be glycosylated
    (in vivo) even though they contain potential motifs.

    SignalP-NN euk predictions are as follows:

  # name                 Cmax  pos ?  Ymax  pos ?  Smax  pos ?  Smean ?  D     ?

    SignalP output is explained at http://www.cbs.dtu.dk/services/SignalP/output.html

##########################################################################

Name:  contig028604-TilORe.R255         Length:  222
MLANLTTGISPLTLVVMSLERYVAVCYPLRHASIVTIRKTALAIIIVWAVSSLNVFIRLILLLNFPFEDLESLQMKDFCS      80
NTPLLLGPMSDHYDKAYTWVLFVSAGVAVTCSYIGVMIAARSASTDKVSACNVRNTLLLHLIQLGLSLSSTIYDPLVTEI     160
SKVLDRVTVLRIWSILYVFMILFPRCLSPLIYGIRDQMIRGILMYHLCCQVKLPNSIKLLKY
...N............................................................................      80
................................................................................     160
..............................................................                       240

(Threshold=0.5)
----------------------------------------------------------------------
SeqName      Position  Potential   Jury    N-Glyc
                                 agreement result
----------------------------------------------------------------------
contig028604-TilORe.R255    4 NLTT   0.6838     (9/9)   ++
----------------------------------------------------------------------
```

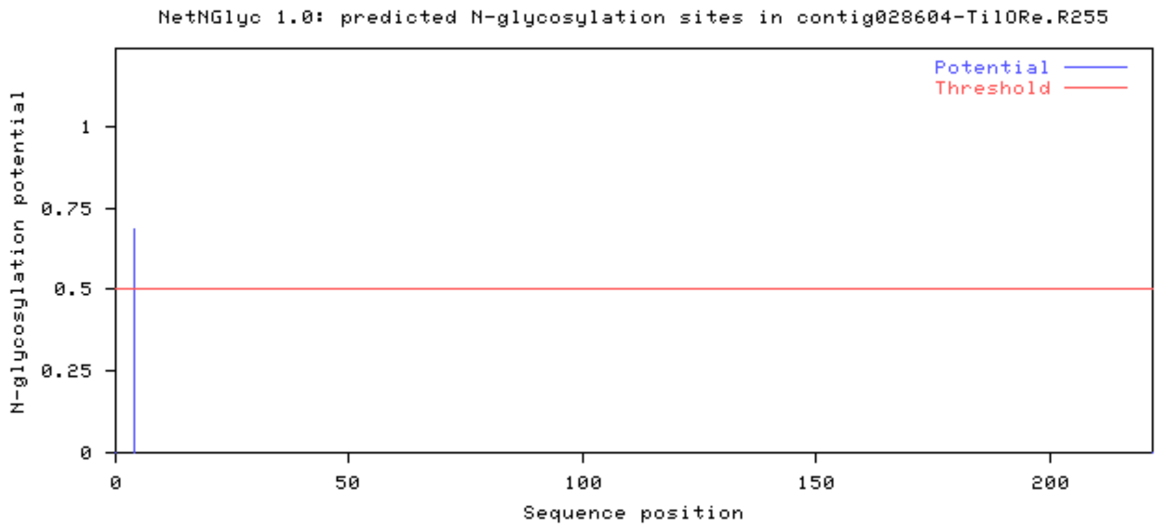

Graphics in PostScript

Output for 'contig028605-TilORe.R276'

```
##########################################################################################

    Warning: This sequence may not contain a signal peptide!!

    Proteins without signal peptides are unlikely to be exposed to
    the N-glycosylation machinery and thus may not be glycosylated
    (in vivo) even though they contain potential motifs.

    SignalP-NN euk predictions are as follows:

  # name                    Cmax   pos ?  Ymax   pos ?  Smax   pos ?  Smean ?  D     ?

    SignalP output is explained at http://www.cbs.dtu.dk/services/SignalP/output.html

##########################################################################################

Name:  contig028605-TilORe.R276         Length:  58
MSYVTPSKTNFTVGLQYRGTLEVLLFSAPITTSCCVFLLVNGTMLHILRSKAVFRETA
.........N..............................N.................                           80

(Threshold=0.5)
----------------------------------------------------------------------
SeqName      Position  Potential   Jury    N-Glyc
                                agreement  result
----------------------------------------------------------------------
contig028605-TilORe.R276   10 NFTV   0.6276     (8/9)    +
contig028605-TilORe.R276   41 NGTM   0.7116     (9/9)    ++
----------------------------------------------------------------------
```

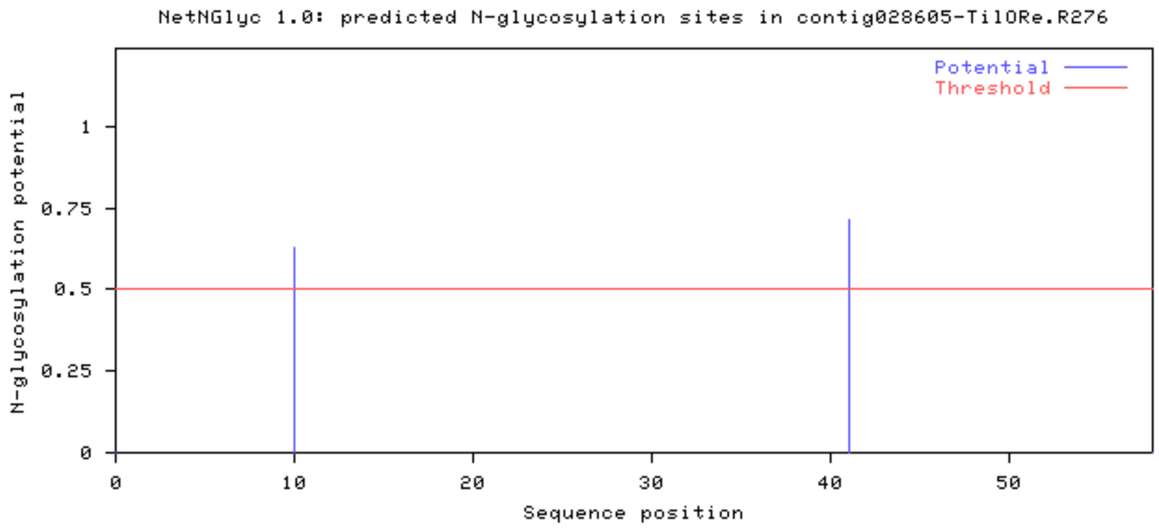

[Graphics in PostScript]

Output for 'contig028607-TilOR.R245'

```
##########################################################################

    Warning: This sequence may not contain a signal peptide!!

    Proteins without signal peptides are unlikely to be exposed to
    the N-glycosylation machinery and thus may not be glycosylated
    (in vivo) even though they contain potential motifs.

    SignalP-NN euk predictions are as follows:

  # name                    Cmax   pos ?  Ymax   pos ?  Smax   pos ?  Smean ?  D     ?

    SignalP output is explained at http://www.cbs.dtu.dk/services/SignalP/output.html

##########################################################################

Name:  contig028607-TilOR.R245  Length:  326
MLLANLSLTNVTANQQYQGVLERVLFSTLTTLPCCVFLFINGIMLFTLRSKALFCETSRYILLYNLLFADTVQMALSQLL      80
YIIATSRITLTYPVCGFLTMLANLTTVVSPLTLVVMSLERYVAVCYPLRHATIITITNTGVAIIAIWAIGSLNILTRVLL     160
LLEFPFEALDSLQMKDFCSDIAMFVGSMSDDYDKAFTCVLFISASVAITCSYIGVIVAARSASTDKASAHKALNTLLLHL     240
VQLGLSLSSTIYNPLLTALARVLTRIVFVRIQNAFYVCIFIFPRCLSSLIYGIRDQSIRPVLIYHLCCRLKYSVIQPRLN     320
FHPRLX
....N....N......................................................................      80
......................N.........................................................     160
................................................................................     240
................................................................................     320
......                                                                               400

(Threshold=0.5)
----------------------------------------------------------------------
SeqName      Position  Potential   Jury    N-Glyc
                                 agreement result
----------------------------------------------------------------------
contig028607-TilOR.R245    5 NLSL   0.7994     (9/9)    +++
contig028607-TilOR.R245   10 NVTA   0.7507     (9/9)    +++
contig028607-TilOR.R245  103 NLTT   0.7351     (9/9)    ++
----------------------------------------------------------------------
```

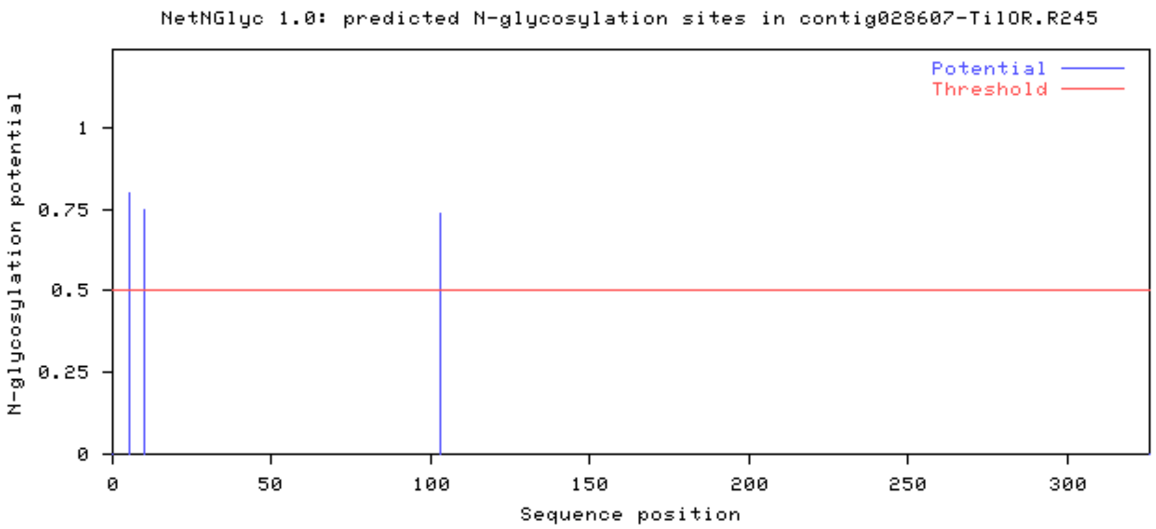

Graphics in PostScript

Output for 'contig028611-TilOR.R246'

```
##########################################################################################

    Warning: This sequence may not contain a signal peptide!!

    Proteins without signal peptides are unlikely to be exposed to
    the N-glycosylation machinery and thus may not be glycosylated
    (in vivo) even though they contain potential motifs.

    SignalP-NN euk predictions are as follows:

  # name                  Cmax  pos ?  Ymax  pos ?  Smax  pos ?  Smean ?  D     ?

    SignalP output is explained at http://www.cbs.dtu.dk/services/SignalP/output.html

##########################################################################################

Name:  contig028611-TilOR.R246  Length:  312
MSLSNQTLTNVTANLQYLGVLEIVLFFTLSTMSCCIFLFINGIMLFTLRSKILFCETSRYILLYNLLFADTVQMALSQLL      80
YIIATSRITLTYPVCGFLTMLANLTTVVSPLTLVVMSLERYVAVCYPLRHATIITITNTGVAIIAIWAIGSLNILTRVLL     160
LLEFPFEALDSLQMKDFCSDIAMFVGSMSDDYDKAFTCVLFISASVAITCSYIGVIVAARSASTDKASAHKALNTLLLHL     240
VQLGLNLSSTIHNPLLTALARVLKRIVFVRIQVVFYVCIFLLPRCLSSLIYGIRDQSIRPVLMQHLCCRLRX
....N....N......................................................................      80
......................N.........................................................     160
................................................................................     240
.....N..................................................................             320

(Threshold=0.5)
----------------------------------------------------------------------
SeqName      Position  Potential   Jury    N-Glyc
                                 agreement result
----------------------------------------------------------------------
contig028611-TilOR.R246    5 NQTL   0.7749     (9/9)    +++
contig028611-TilOR.R246   10 NVTA   0.7268     (9/9)    ++
contig028611-TilOR.R246  103 NLTT   0.7338     (9/9)    ++
contig028611-TilOR.R246  246 NLSS   0.6912     (8/9)    +
----------------------------------------------------------------------
```

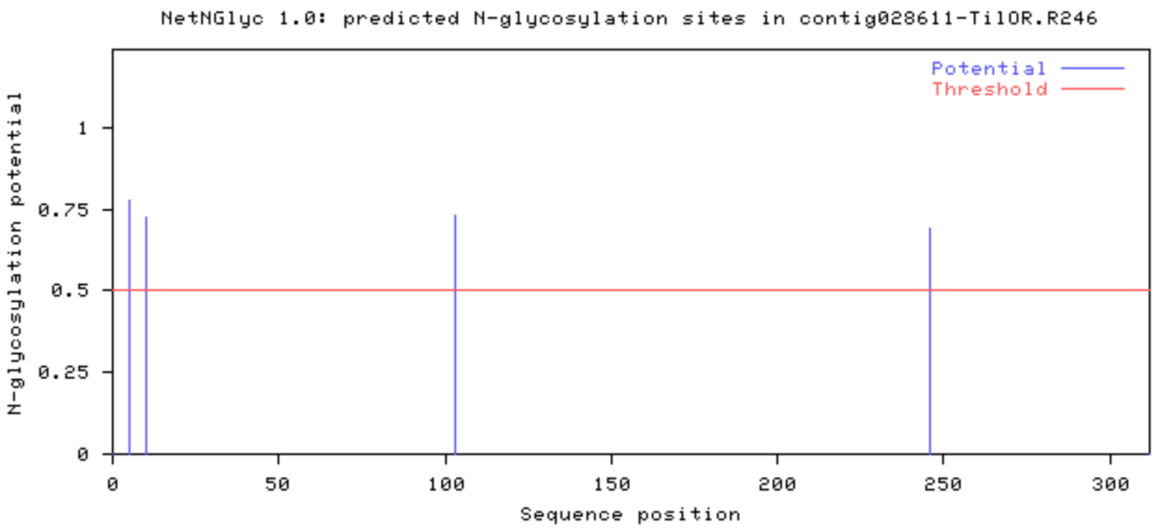

Graphics in PostScript

Output for 'contig028614-TilOR.R247'

```
##########################################################################################

    Warning: This sequence may not contain a signal peptide!!

    Proteins without signal peptides are unlikely to be exposed to
    the N-glycosylation machinery and thus may not be glycosylated
    (in vivo) even though they contain potential motifs.

    SignalP-NN euk predictions are as follows:

  # name                    Cmax  pos ?  Ymax  pos ?  Smax  pos ?  Smean ?  D     ?

    SignalP output is explained at http://www.cbs.dtu.dk/services/SignalP/output.html

##########################################################################################

Name:  contig028614-TilOR.R247   Length:  317
MSSISQTLTNITVGYQALEERVMISTLTTLPTCVFLFINSIMLFTLRSKPVFRETCRYILLYNLLFADTVQLAQSQIHFL      80
LAVLRIRISYPVCTFLVNFTQLTAVISPLTLVVMPLERYVAVCYPLRHGTIITIRNTGAAIIVIWAISFLNIIIRTLLFL     160
ALFEKLDKIEVKYLCSEIGILLGSKSDHFDKAFTCIVFVAAGVAVIFSYIGVIVAARSASTDKALAIKARNTLLLNLFQL     240
ILSLSSTIYYPLLVPLSVTVTRIVLVRIQNVFYLFFIILPRCLTSLTYGLRDQTIRPVLIYHLCCRLKCPVVEDKGX
.........N......................................................................      80
.................N..............................................................     160
................................................................................     240
.............................................................................        320

(Threshold=0.5)
----------------------------------------------------------------------
SeqName      Position  Potential   Jury    N-Glyc
                                 agreement result
----------------------------------------------------------------------
contig028614-TilOR.R247   10 NITV   0.6960     (9/9)   ++
contig028614-TilOR.R247   98 NFTQ   0.7397     (9/9)   ++
----------------------------------------------------------------------
```

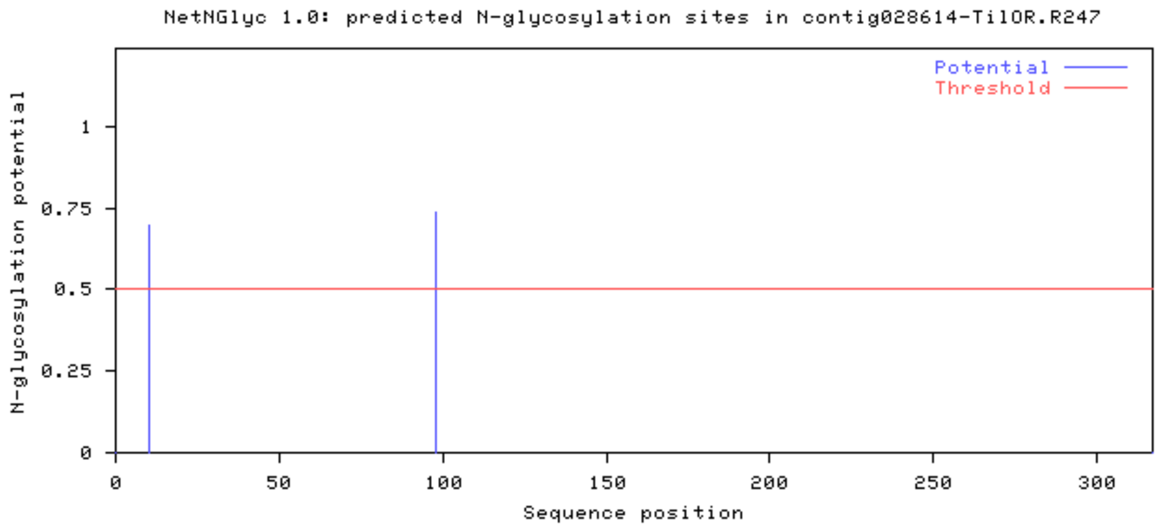

[Graphics in PostScript]

Output for 'contig028617-TilOR.R248'

```
##########################################################################################

    Warning: This sequence may not contain a signal peptide!!

    Proteins without signal peptides are unlikely to be exposed to
    the N-glycosylation machinery and thus may not be glycosylated
    (in vivo) even though they contain potential motifs.

    SignalP-NN euk predictions are as follows:

  # name                Cmax  pos ?  Ymax  pos ?  Smax  pos ?  Smean ?  D     ?

    SignalP output is explained at http://www.cbs.dtu.dk/services/SignalP/output.html

##########################################################################################

Name:  contig028617-TilOR.R248  Length:  312
MSLSNQTLTNVTANLQYLGVLEIVLFFTLSTMSCCIFLFINGIMLFTLRSKILFCETSRYILLYNLLFADTVQMALSQLL      80
YIIATSRITLTYPVCGFLTMLANLTTVVSPLTLVVMSLERYVAVCYPLRHATIITITNTGVAIIAIWAIGSLNILTRVLL     160
LLEFPFEALDSLQMKDFCSDIAMFVGSMSDDYDKAFTCVLFISASVAITCSYIGVIVAARSASTDKASAHKALNTLLLHL     240
VQLGLSLSSTIYNPLLTALARVLTRIVFVRIQVVFYVCIFLLPRCLSSLIYGIRDQSIRPVLMQHLCCRLRX
....N....N......................................................................      80
......................N.........................................................     160
................................................................................     240
........................................................................             320

(Threshold=0.5)
----------------------------------------------------------------------
SeqName      Position  Potential   Jury    N-Glyc
                                agreement  result
----------------------------------------------------------------------
contig028617-TilOR.R248     5 NQTL   0.7749     (9/9)    +++
contig028617-TilOR.R248    10 NVTA   0.7268     (9/9)    ++
contig028617-TilOR.R248   103 NLTT   0.7338     (9/9)    ++
----------------------------------------------------------------------
```

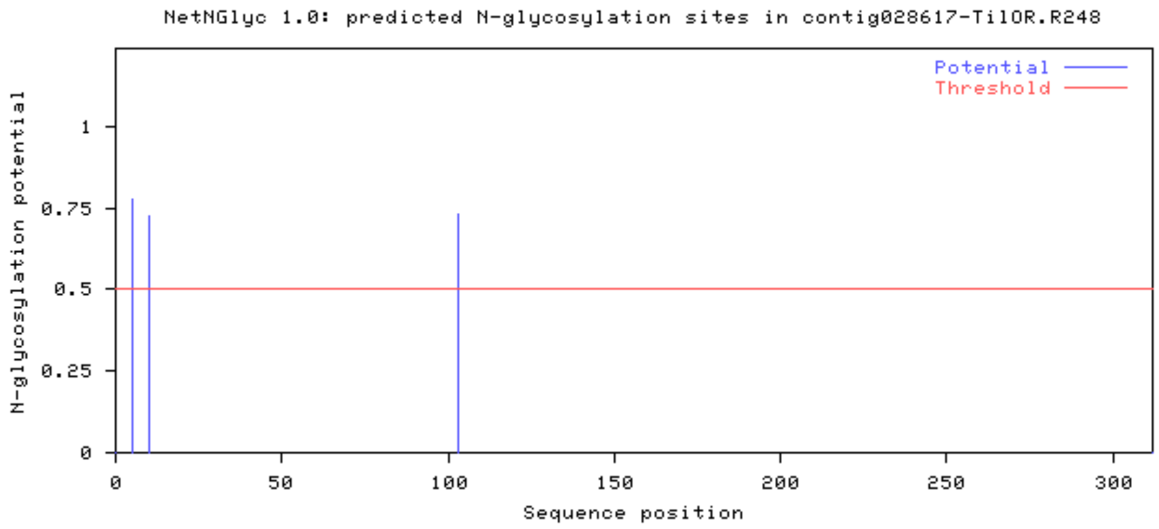

[Graphics in PostScript]

Output for 'contig028619-TilOR.R249'

```
##############################################################################

    Warning: This sequence may not contain a signal peptide!!

    Proteins without signal peptides are unlikely to be exposed to
    the N-glycosylation machinery and thus may not be glycosylated
    (in vivo) even though they contain potential motifs.

    SignalP-NN euk predictions are as follows:

  # name                  Cmax  pos ?  Ymax  pos ?  Smax  pos ?  Smean ?  D     ?

    SignalP output is explained at http://www.cbs.dtu.dk/services/SignalP/output.html

##############################################################################

Name:  contig028619-TilOR.R249  Length:  317
MTSTSQTLTNITVQAPGLLERVMISTLTTLPTCVFLFINSIMLFTLRSKPVFRETCRYILLYNLLFADTVQLAQSQIHFL      80
LAVLRIRISYPVCTFLVNFTQLTAVISPLTLVVMPLERYVAVCYPLRHGTIITIRNTGAAIIVIWAISFLNIIIRTLLFL     160
ALFEELGDLEVKDFCGDIAILLGSKSDHFDKAFTCIVFVAAGVAVIFSYIGVIVAARSASTDKALAIKARNTLLLNLFQL     240
ILSLSSTIYNPLLVPLLMIVTRIVLVRIQNAFYLFFIILPRCLTSLIYGLRDQTIRPVLIYHLCCQLKCPVVEDKGX
.........N......................................................................      80
.................N..............................................................     160
................................................................................     240
.............................................................................        320

(Threshold=0.5)
----------------------------------------------------------------------
SeqName      Position  Potential    Jury    N-Glyc
                                 agreement result
----------------------------------------------------------------------
contig028619-TilOR.R249   10 NITV   0.7742     (9/9)    +++
contig028619-TilOR.R249   98 NFTQ   0.7398     (9/9)    ++
----------------------------------------------------------------------
```

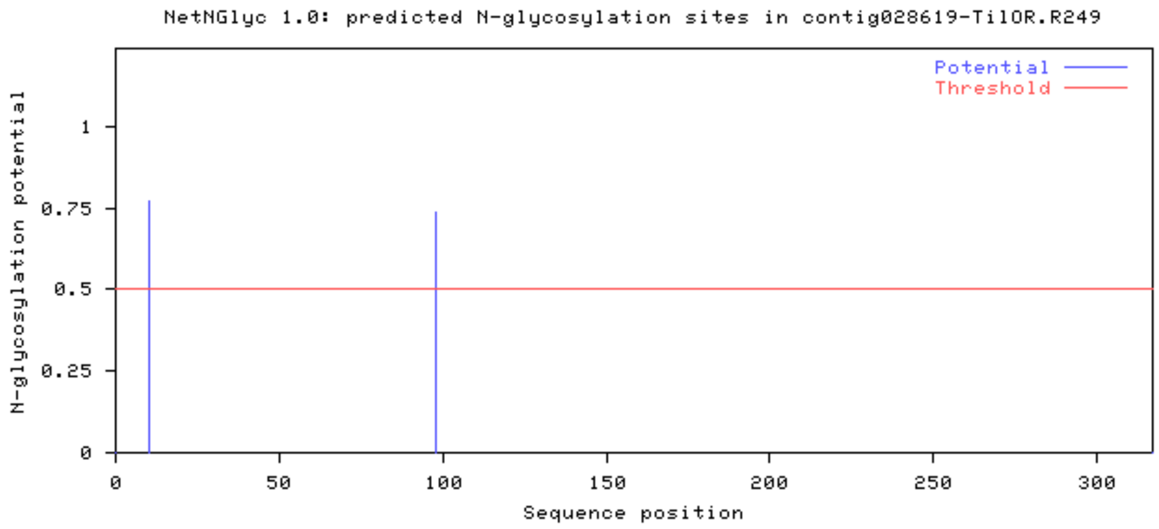

Graphics in PostScript

Output for 'contig028622-TilORe.R261'

```
##########################################################################################

    Warning: This sequence may not contain a signal peptide!!

    Proteins without signal peptides are unlikely to be exposed to
    the N-glycosylation machinery and thus may not be glycosylated
    (in vivo) even though they contain potential motifs.

    SignalP-NN euk predictions are as follows:

  # name                    Cmax   pos ?  Ymax   pos ?  Smax   pos ?  Smean ?  D      ?

    SignalP output is explained at http://www.cbs.dtu.dk/services/SignalP/output.html

##########################################################################################

Name:  contig028622-TilORe.R261         Length:  98
MSNVSQSYTNMSIEVQYQELLRVIIISTLSTVPSFIFLFINGTMLFTLRSKPVFRDTPRYILLYNLLFADTVQLAQSQVL      80
FLLSIFRVKLPYPVCGFL
..N......N.............................N........................................      80
..................                                                                   160

(Threshold=0.5)
----------------------------------------------------------------------
SeqName      Position  Potential   Jury    N-Glyc
                                 agreement result
----------------------------------------------------------------------
contig028622-TilORe.R261    3 NVSQ   0.7746     (9/9)   +++
contig028622-TilORe.R261   10 NMSI   0.6659     (9/9)   ++
contig028622-TilORe.R261   41 NGTM   0.6294     (9/9)   ++
----------------------------------------------------------------------
```

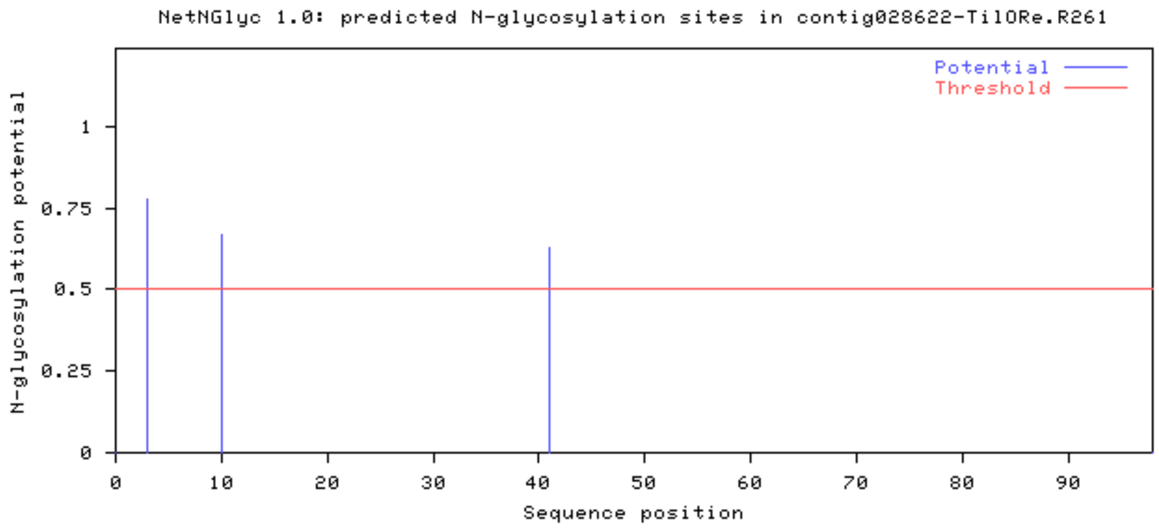

Graphics in PostScript

Output for 'contig028623-TilORe.R276'

```
##########################################################################################

    Warning: This sequence may not contain a signal peptide!!

    Proteins without signal peptides are unlikely to be exposed to
    the N-glycosylation machinery and thus may not be glycosylated
    (in vivo) even though they contain potential motifs.

    SignalP-NN euk predictions are as follows:

  # name                    Cmax   pos ?  Ymax   pos ?  Smax   pos ?  Smean ?  D     ?

    SignalP output is explained at http://www.cbs.dtu.dk/services/SignalP/output.html

##########################################################################################

Name:  contig028623-TilORe.R276         Length:  41
FYVCFIILPRCLSSLIYGLRDQTIRPVLMYHLCCYQKLSQX
.........................................                                             80

(Threshold=0.5)

No sites predicted in this sequence.
```

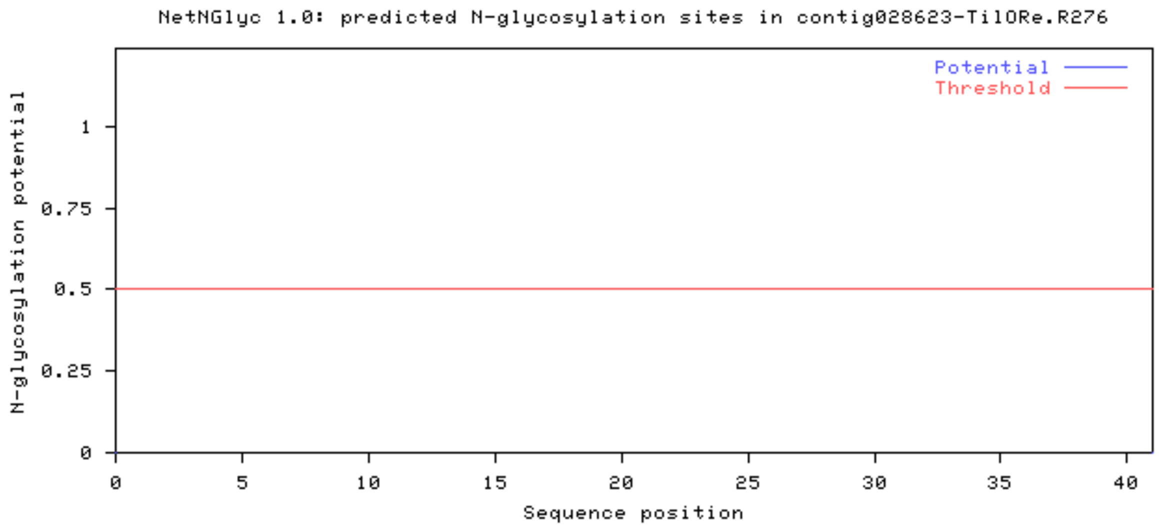

Graphics in PostScript

Output for 'contig028630-TilORe.R257'

```
##########################################################################

    Warning: This sequence may not contain a signal peptide!!

    Proteins without signal peptides are unlikely to be exposed to
    the N-glycosylation machinery and thus may not be glycosylated
    (in vivo) even though they contain potential motifs.

    SignalP-NN euk predictions are as follows:

  # name                 Cmax  pos ?  Ymax  pos ?  Smax  pos ?  Smean ?  D     ?

    SignalP output is explained at http://www.cbs.dtu.dk/services/SignalP/output.html

##########################################################################

Name:  contig028630-TilORe.R257         Length:  262
MSNVSQSYTNMSIEVQYQELLRVIIISTLSTVPSFIFLFINGTMLFTLRSKPVFRDTPRYILLYNLLFADTVQLAQSQVL      80
FLLSIFRVKLPYPVCGFLSSLANLTTGISPLTLSVMPLERYVAVCYPLRYPTIITIRNTGAAIIVIWIISSLNNLTRLIF     160
FFPFEMLKNLQVKDRCSNIALLLGTKSDQYDTAFTCLVFVSAGVAVIFSYIGVILAARSASANKALARKARNTLLLNMMQ     240
LCLSLCSTINNPLLIALSRTVT
..N......N.............................N........................................      80
.....................N...................................................N......     160
................................................................................     240
......................                                                               320

(Threshold=0.5)
----------------------------------------------------------------------
SeqName      Position  Potential    Jury    N-Glyc
                                 agreement  result
----------------------------------------------------------------------
contig028630-TilORe.R257     3 NVSQ   0.7744     (9/9)   +++
contig028630-TilORe.R257    10 NMSI   0.6723     (9/9)   ++
contig028630-TilORe.R257    41 NGTM   0.6696     (9/9)   ++
contig028630-TilORe.R257   103 NLTT   0.5748     (7/9)   +
contig028630-TilORe.R257   154 NLTR   0.5556     (7/9)   +
----------------------------------------------------------------------
```

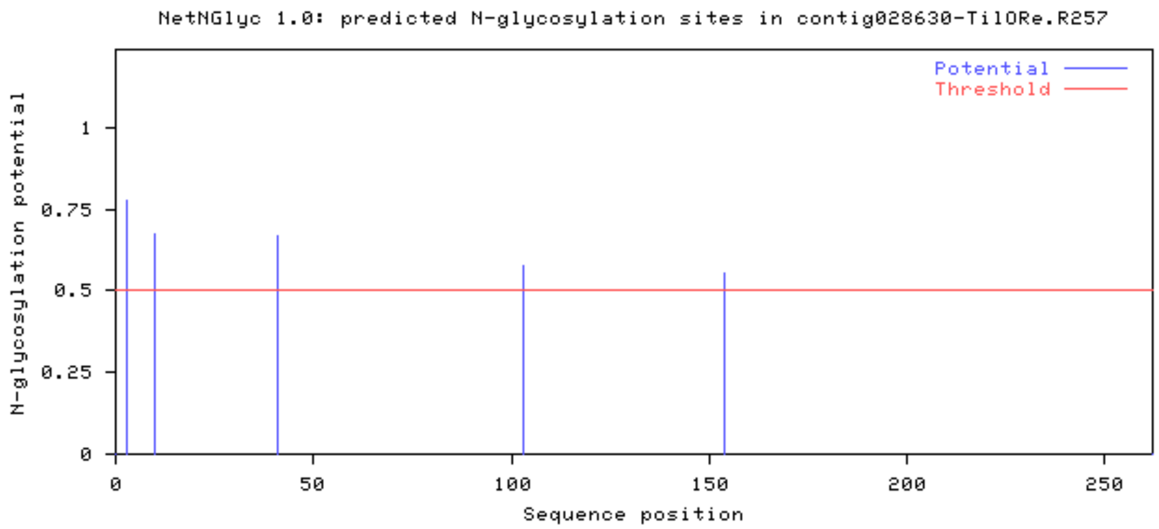

Graphics in PostScript

Output for 'contig028631-TilORe.R274'

```
##########################################################################################

    Warning: This sequence may not contain a signal peptide!!

    Proteins without signal peptides are unlikely to be exposed to
    the N-glycosylation machinery and thus may not be glycosylated
    (in vivo) even though they contain potential motifs.

    SignalP-NN euk predictions are as follows:

  # name                 Cmax  pos ?  Ymax  pos ?  Smax  pos ?  Smean ?  D     ?

    SignalP output is explained at http://www.cbs.dtu.dk/services/SignalP/output.html

##########################################################################################

Name:  contig028631-TilORe.R274         Length:  41
FYVCFIILPRCLSSLIYGLRDQTIRPVLMYHLCCYQKLSQX
.........................................                                            80

(Threshold=0.5)

No sites predicted in this sequence.
```

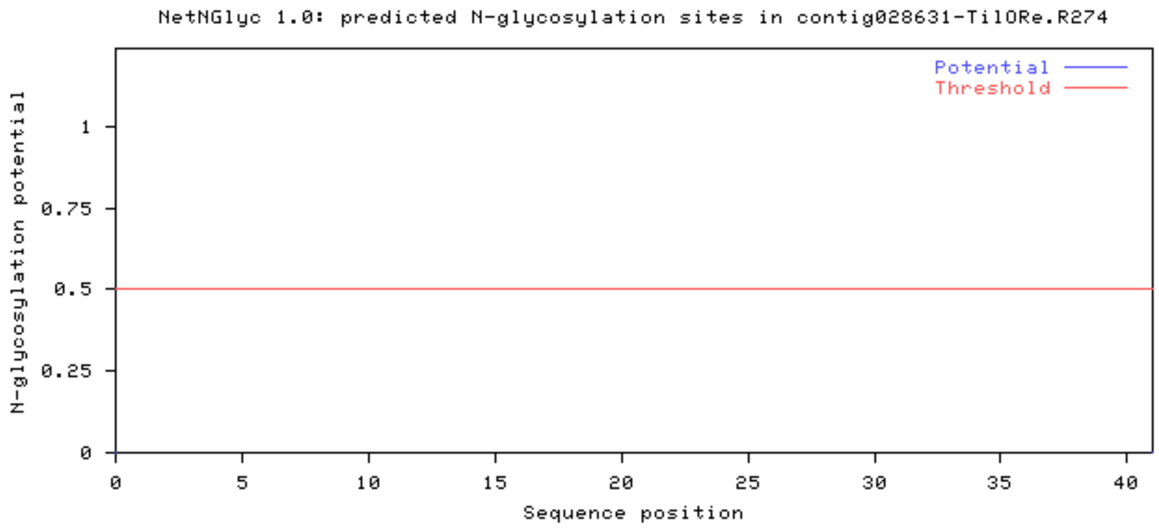

Graphics in PostScript

Output for 'contig028633-TilORe.R260'

```
##########################################################################################

    Warning: This sequence may not contain a signal peptide!!

    Proteins without signal peptides are unlikely to be exposed to
    the N-glycosylation machinery and thus may not be glycosylated
    (in vivo) even though they contain potential motifs.

    SignalP-NN euk predictions are as follows:

  # name                Cmax  pos ?  Ymax  pos ?  Smax  pos ?  Smean ?  D    ?

    SignalP output is explained at http://www.cbs.dtu.dk/services/SignalP/output.html

##########################################################################################

Name:  contig028633-TilORe.R260        Length:  98
MSNVSQSYTNMSIEVQYQELLRVIIISTLSTVPSFIFLFINGTMLFTLRSKPVFRDTPRYILLYNLLFADTVQLAQSQVL      80
FLLSIFRVKLPYPVCGFL
..N......N.............................N........................................      80
..................                                                                    160

(Threshold=0.5)
----------------------------------------------------------------------
SeqName      Position  Potential   Jury    N-Glyc
                                 agreement result
----------------------------------------------------------------------
contig028633-TilORe.R260    3 NVSQ   0.7746     (9/9)   +++
contig028633-TilORe.R260   10 NMSI   0.6659     (9/9)   ++
contig028633-TilORe.R260   41 NGTM   0.6294     (9/9)   ++
----------------------------------------------------------------------
```

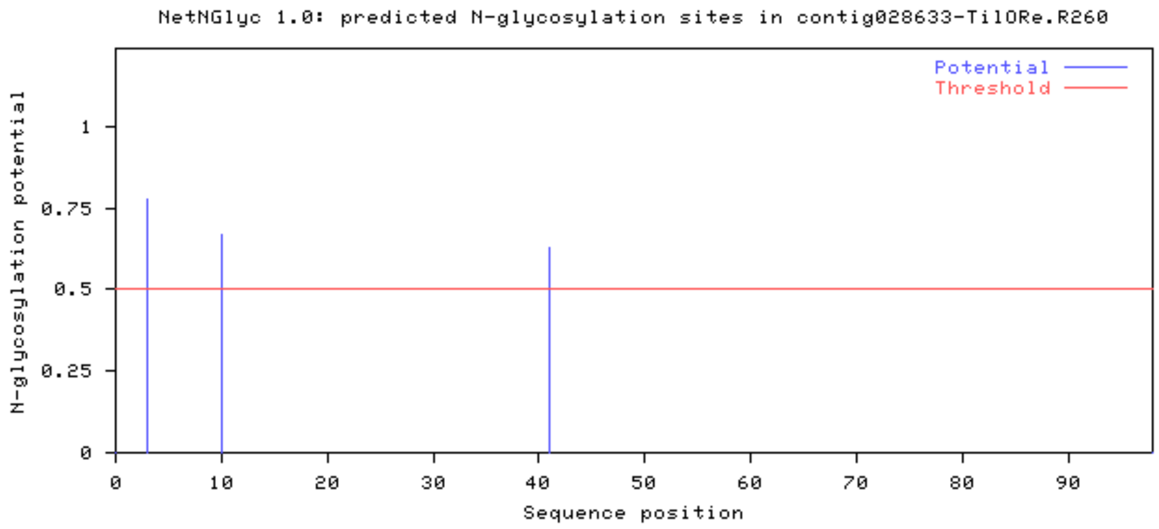

Graphics in PostScript

Output for 'contig028635-TilORe.R259'

```
##########################################################################################

    Warning: This sequence may not contain a signal peptide!!

    Proteins without signal peptides are unlikely to be exposed to
    the N-glycosylation machinery and thus may not be glycosylated
    (in vivo) even though they contain potential motifs.

    SignalP-NN euk predictions are as follows:

  # name                 Cmax  pos ?  Ymax  pos ?  Smax  pos ?  Smean ?  D     ?

    SignalP output is explained at http://www.cbs.dtu.dk/services/SignalP/output.html

##########################################################################################

Name:  contig028635-TilORe.R259         Length:  99
MSNVSQSYTNMSIEVQYQELLRVIIISTLSTVPSFIFLFINGTMLFTLRSKPVFRDTPRYILLYNLLFADTVQLAQSQVL      80
FLLSIFRVKLPYPVCGFLS
..N......N............................N....................................      80
...................                                                              160

(Threshold=0.5)
----------------------------------------------------------------------
SeqName      Position  Potential   Jury    N-Glyc
                                agreement  result
----------------------------------------------------------------------
contig028635-TilORe.R259     3 NVSQ   0.7746     (9/9)   +++
contig028635-TilORe.R259    10 NMSI   0.6660     (9/9)   ++
contig028635-TilORe.R259    41 NGTM   0.6300     (9/9)   ++
----------------------------------------------------------------------
```

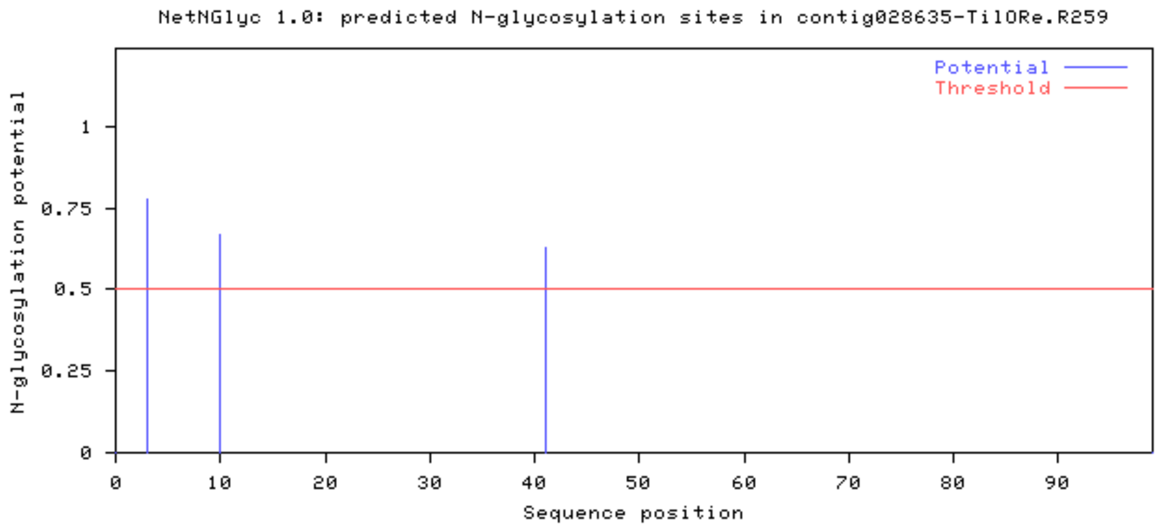

Graphics in PostScript

Output for 'contig028636-TilORe.R256'

```
##########################################################################################

    Warning: This sequence may not contain a signal peptide!!

    Proteins without signal peptides are unlikely to be exposed to
    the N-glycosylation machinery and thus may not be glycosylated
    (in vivo) even though they contain potential motifs.

    SignalP-NN euk predictions are as follows:

  # name                 Cmax   pos ?  Ymax   pos ?  Smax   pos ?  Smean ?  D     ?

    SignalP output is explained at http://www.cbs.dtu.dk/services/SignalP/output.html

##########################################################################################

Name:  contig028636-TilORe.R256         Length:  209
TTGISPLTLSVMPLERYVAVCYPLRYPTIITIRNTGAAIIVIWIISSLNNLTRLIFFFPFEILKNLQTKELCSKIALLLG      80
TKSDQYDTAFTCLVFVSAGVAVIFSYIAVILAARSASANKALARKARNTLLLNMMQLCLSLSSTVYRPFRMALSRTVTMT     160
IFSWVQNVFYVCFIILPRCLSSLIYGLRDQTIRPVLMYHLCCYQKLSQX
.................................................N..............................      80
................................................................................     160
.................................................                                    240

(Threshold=0.5)
----------------------------------------------------------------------
SeqName      Position  Potential   Jury    N-Glyc
                                 agreement result
----------------------------------------------------------------------
contig028636-TilORe.R256    50 NLTR   0.6080     (7/9)   +
----------------------------------------------------------------------
```

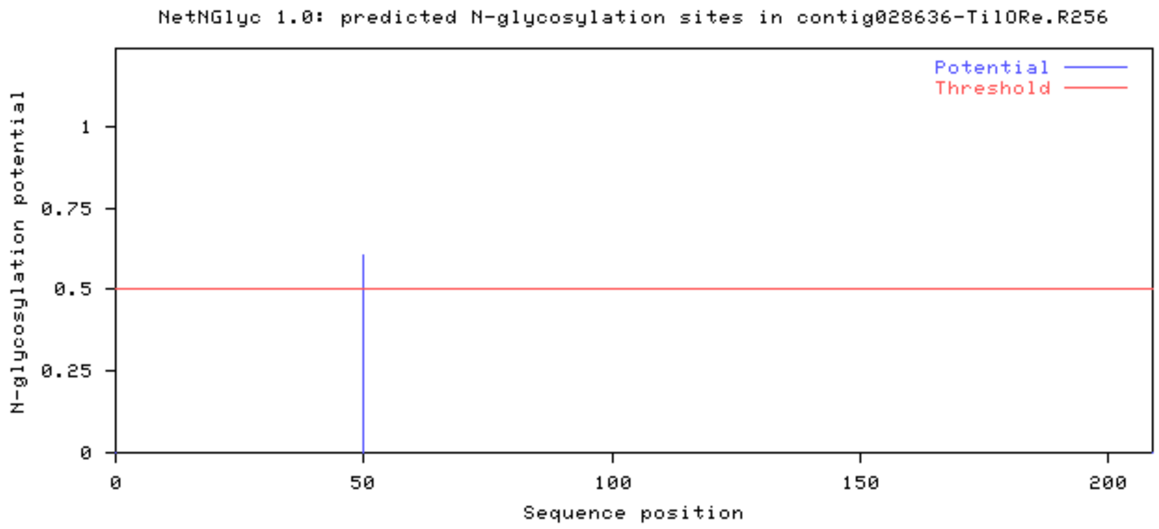

Graphics in PostScript

Output for 'contig028637-TilOR.R250'

```
##########################################################################################

    Warning: This sequence may not contain a signal peptide!!

    Proteins without signal peptides are unlikely to be exposed to
    the N-glycosylation machinery and thus may not be glycosylated
    (in vivo) even though they contain potential motifs.

    SignalP-NN euk predictions are as follows:

  # name                Cmax  pos ?  Ymax  pos ?  Smax  pos ?  Smean ?  D     ?

    SignalP output is explained at http://www.cbs.dtu.dk/services/SignalP/output.html

##########################################################################################

Name:  contig028637-TilOR.R250  Length:  315
MSNVSQIYSDFNFEVQYQRLLRIVIISALSTLPACVFLFVNGIMLFMLRRKRVFRETCRYILLYNLLFADTAQLAQTQLI      80
FLLSVCQIQLPFSVCAILILLANLTGRITPLTLVVMPLERYVAVCYPLRHATIITIRNTRVVIIVIWVVSSLNNLTRILL     160
LFEFLFENVKNLQVSDYCSNVDIILGSKSEQYDTLYTCCLFVSAGVAVIFSYIGVIVAARLASTDKGLAQKARNTLLLNV     240
IQLCLSLSATIYHPLLRALSRTVARTVFLWVQNVFYVCFIIFPRCLTSLIYGLRDQTIGPVLINHLCCRMKATVX
..N.............................................................................      80
......................N...................................................N.....     160
................................................................................     240
...........................................................................          320

(Threshold=0.5)
----------------------------------------------------------------------
SeqName      Position  Potential   Jury    N-Glyc
                                 agreement result
----------------------------------------------------------------------
contig028637-TilOR.R250    3 NVSQ   0.7829     (9/9)   +++
contig028637-TilOR.R250  103 NLTG   0.6837     (9/9)   ++
contig028637-TilOR.R250  154 NLTR   0.6583     (8/9)   +
----------------------------------------------------------------------
```

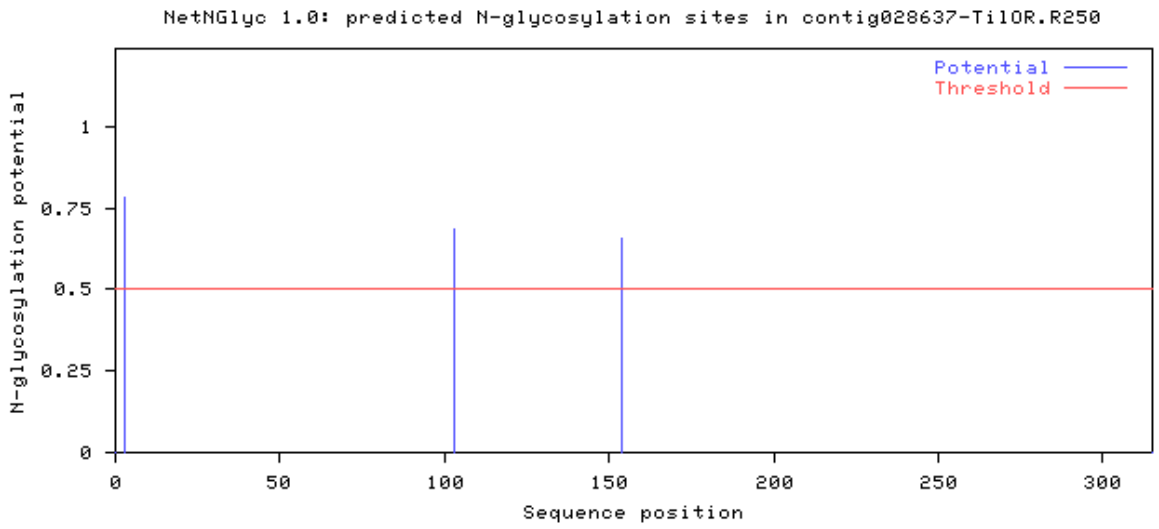

Graphics in PostScript

Output for 'contig028639-TilOR.R251'

```
########################################################################################

   Warning: This sequence may not contain a signal peptide!!

   Proteins without signal peptides are unlikely to be exposed to
   the N-glycosylation machinery and thus may not be glycosylated
   (in vivo) even though they contain potential motifs.

   SignalP-NN euk predictions are as follows:

 # name                Cmax  pos ?  Ymax  pos ?  Smax  pos ?  Smean ?  D     ?

   SignalP output is explained at http://www.cbs.dtu.dk/services/SignalP/output.html

########################################################################################

Name:  contig028639-TilOR.R251  Length:  310
MLTVSRSNITDAFQYPDFLRIMIISTLTTFPSFIFLFINGTMLFTLRSKPVFHETPRYILLYNLLFAETVQLAQSQVLFL      80
LSVSQVKLFYPVCGFLLFFTSLTTVISPLTLVVMPLERYVAVCYPLRHPTIITIRNTVVGVIVTWAVSSVNILIRGLLVV     160
KVLLKLENVNVKDHCYKIDILLGLNTDYYDKAFTCVIFVSAALAIIFSYIGVVAAARSASADKGLARKARNTLLLNLVQL     240
CLSLCATIYRPLLTALSTTVTMTAFSWIQNVFYVCLVVLPRFLTSLVYGLRDQTIRPVLMYHLCCHCLKX
.......N..............................N.........................................      80
................................................................................     160
................................................................................     240
......................................................................               320

(Threshold=0.5)
----------------------------------------------------------------------
SeqName      Position  Potential   Jury    N-Glyc
                                 agreement result
----------------------------------------------------------------------
contig028639-TilOR.R251    8 NITD   0.7231     (8/9)    +
contig028639-TilOR.R251   39 NGTM   0.6702     (9/9)    ++
----------------------------------------------------------------------
```

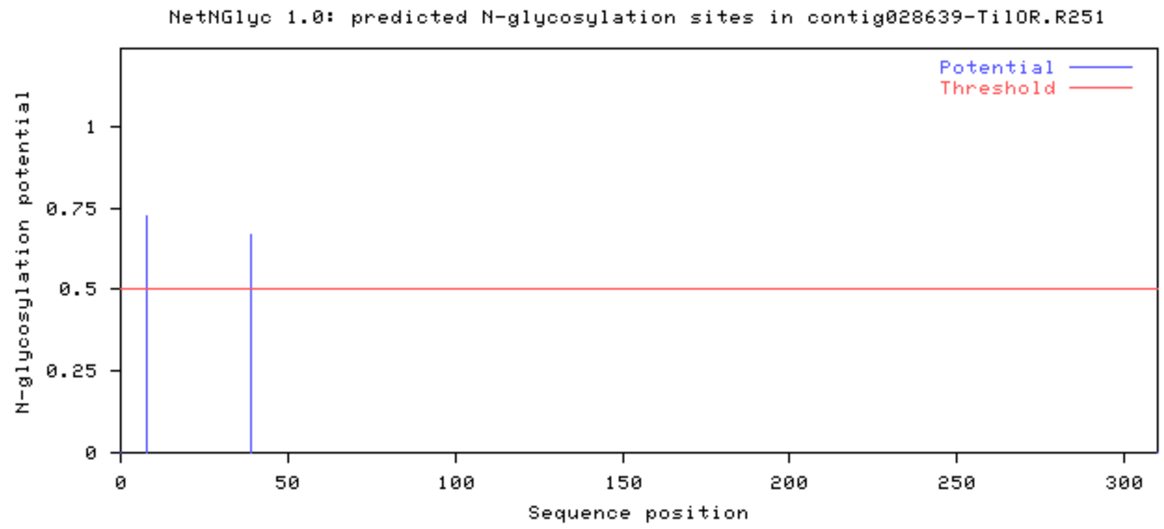

Graphics in PostScript

Output for 'contig028641-TilOR.R253'

```
##########################################################################################

    Warning: This sequence may not contain a signal peptide!!

    Proteins without signal peptides are unlikely to be exposed to
    the N-glycosylation machinery and thus may not be glycosylated
    (in vivo) even though they contain potential motifs.

    SignalP-NN euk predictions are as follows:

  # name                Cmax  pos ?  Ymax  pos ?  Smax  pos ?  Smean ?  D     ?

    SignalP output is explained at http://www.cbs.dtu.dk/services/SignalP/output.html

##########################################################################################

Name:  contig028641-TilOR.R253  Length:  321
MPDISQSQTNISVGLHDLERGLLSSLTTLPCCVFFCINVIMLFTLRSKSVFRETCRYILLYNLILADTLQMAVSQILYMM      80
SICRITLPYPVCGILVMFANLTNEISPLTLVLMSLERYVAVCYPLRHATIITIRNTEVAIIMIWIFCSLNILIRVLLLLE     160
FPFEELQSLQMKDFCSTFLMFLTPVSHEYDKAYSCFLFVSAFVGVTCSYIGVMLAARLASTDKASARKARNTLLLHLVQL     240
GFSLSSTVNNALLLVTSKTVSRRVLVVVQIVFYVFLIILPRCLSALIYGLRDQTIRPILVYNLCCQLKLTVVTRKAKVSP     320
X
.........N......................................................................      80
...................N............................................................     160
................................................................................     240
................................................................................     320
.                                                                                    400

(Threshold=0.5)
----------------------------------------------------------------------
SeqName      Position  Potential   Jury    N-Glyc
                                 agreement result
----------------------------------------------------------------------
contig028641-TilOR.R253   10 NISV   0.6044     (7/9)   +
contig028641-TilOR.R253  100 NLTN   0.6590     (9/9)   ++
----------------------------------------------------------------------
```

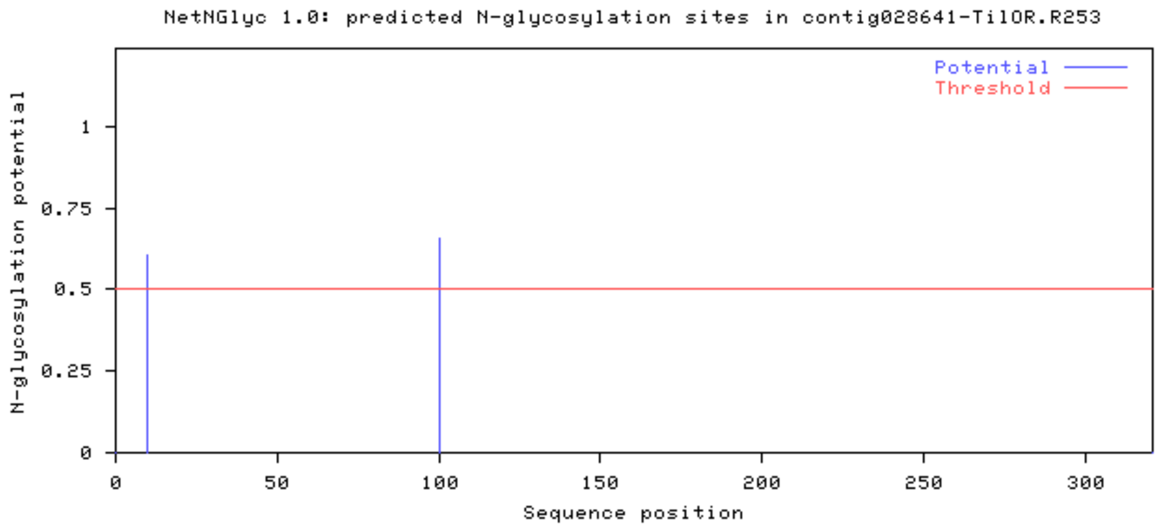

Graphics in PostScript

Output for 'contig028644-TilOR.R252'

```
##########################################################################

    Warning: This sequence may not contain a signal peptide!!

    Proteins without signal peptides are unlikely to be exposed to
    the N-glycosylation machinery and thus may not be glycosylated
    (in vivo) even though they contain potential motifs.

    SignalP-NN euk predictions are as follows:

  # name                Cmax  pos ?  Ymax  pos ?  Smax  pos ?  Smean ?  D     ?

    SignalP output is explained at http://www.cbs.dtu.dk/services/SignalP/output.html

##########################################################################

Name:  contig028644-TilOR.R252  Length:  321
MPDISQSQTNISVGLHDLERGLLSSLTTLPCCVFFCINVIMLFTLRSKSVFRETCRYILLYNLILADTLQMAVSQILYMM      80
SICRITLPYPVCGILVMFANLTNEISPLTLVLMSLERYVAVCYPLRHATIITIRNTEVAIIMIWIFCSLNILIRVLLLLE     160
FPFEELQSLQMKDFCSTFLMFLTPVSHEYDKAYSCFLFVSAFVGVTCSYIGVMLAARLASTDKASARKARNTLLLHLVQL     240
GFSLSSTVNNALLLVTSKTVSRRVLVVVQIVFYVFLIILPRCLSALIYGLRDQTIRPILVYNLCCQLKLTVVTRKAKVSP     320
X
.........N......................................................................      80
...................N............................................................     160
................................................................................     240
................................................................................     320
.                                                                                    400

(Threshold=0.5)
----------------------------------------------------------------------
SeqName      Position  Potential   Jury    N-Glyc
                                 agreement result
----------------------------------------------------------------------
contig028644-TilOR.R252   10 NISV   0.6044     (7/9)    +
contig028644-TilOR.R252  100 NLTN   0.6590     (9/9)    ++
----------------------------------------------------------------------
```

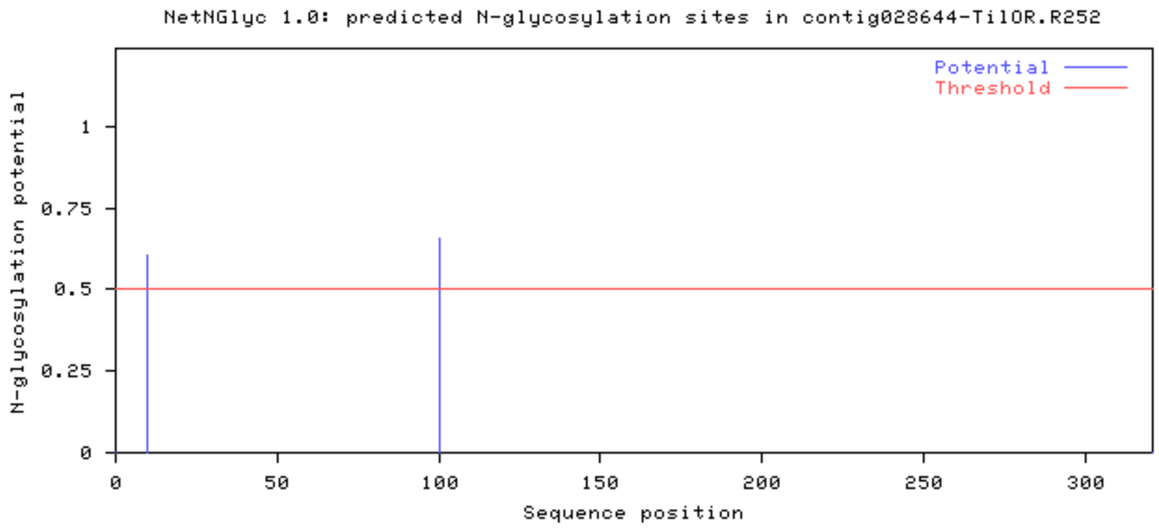

Graphics in PostScript

Output for 'contig039415-TilOR.S221'

```
################################################################################

   Warning: This sequence may not contain a signal peptide!!

   Proteins without signal peptides are unlikely to be exposed to
   the N-glycosylation machinery and thus may not be glycosylated
   (in vivo) even though they contain potential motifs.

   SignalP-NN euk predictions are as follows:

 # name                Cmax  pos ?  Ymax  pos ?  Smax  pos ?  Smean ?  D     ?

   SignalP output is explained at http://www.cbs.dtu.dk/services/SignalP/output.html

################################################################################

Name:  contig039415-TilOR.S221  Length:  304
MAGNNSVNDVFLQRPVYDRVIIVQILVIIFLCINMSLIVIFVKKECFHTSARYILFFVTLLSDSVLLLVSDVLLVLSQFE      80
FTIQVWLCITISVVVLLYFIVTPVTLTAMTLERYVAICMPLRHGQLCSTRSTMYCILVIHGVSSGPCIIILSMFFASSSL     160
KFYKQSIICSGETFSLYRWQDHVRSAVYQFYFFIMGITIAYSYVQIMKVAKAASGEKKKLTHKGLKTVILHAFQLLLCLI     240
QLWCPFIEIAVLQIDFTLFLNVRYFNYIMFNIASRCLSPLIYGLRDENFFLVLKNLMPTSSCSX
...N.............................N..............................................      80
................................................................................     160
................................................................................     240
................................................................                     320

(Threshold=0.5)
----------------------------------------------------------------------
SeqName      Position  Potential   Jury    N-Glyc
                                 agreement result
----------------------------------------------------------------------
contig039415-TilOR.S221    4 NNSV   0.7400     (9/9)   ++
contig039415-TilOR.S221   34 NMSL   0.7349     (9/9)   ++
----------------------------------------------------------------------
```

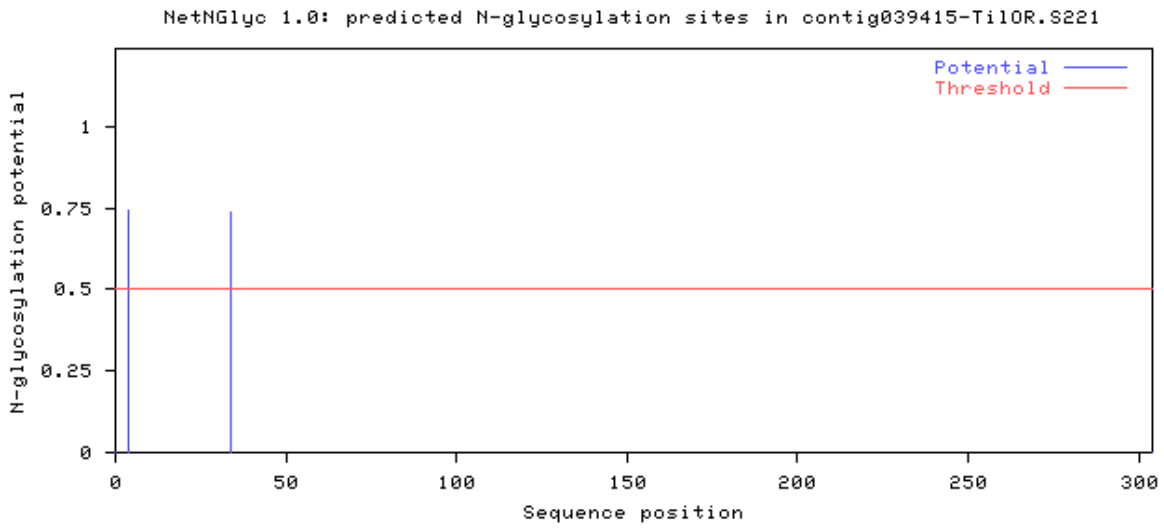

Graphics in PostScript

Output for 'contig039416-TilOR.S222'

```
##############################################################################

    Warning: This sequence may not contain a signal peptide!!

    Proteins without signal peptides are unlikely to be exposed to
    the N-glycosylation machinery and thus may not be glycosylated
    (in vivo) even though they contain potential motifs.

    SignalP-NN euk predictions are as follows:

  # name                Cmax  pos ?  Ymax  pos ?  Smax  pos ?  Smean ?  D     ?

    SignalP output is explained at http://www.cbs.dtu.dk/services/SignalP/output.html

##############################################################################

Name:  contig039416-TilOR.S222  Length:  305
MAGNNTVICAFLERPLTDRIITVQILVIIFLCVNMLLIVTFFKKECFYTSARYILFFVTLLSDSFLLLMTDILLILTTFR      80
CSMQVWLCIIVCFFVLVYSIVTPVTLTTMTLERYVAICMPLRHGQTCSTRNTKYCILIIHGLSSGPCIVVISTFFSTASI     160
SFYKQYAICSVEMFMLYRWQDHVRSAVNQFYFFIMGITIIFSYAEIMKVAKAASGENKKSTQKGLRTVILHGFQLLLCLF     240
QLWSPFIETAVFQIDSNLFRNFRYFNYVLFNIVPRCLSPLIYGLRDEKFFLVLKNLLPASSNSEX
...N............................................................................      80
................................................................................     160
................................................................................     240
.................................................................                    320

(Threshold=0.5)
----------------------------------------------------------------------
SeqName      Position  Potential   Jury    N-Glyc
                                 agreement result
----------------------------------------------------------------------
contig039416-TilOR.S222    4 NNTV   0.7886     (9/9)    +++
----------------------------------------------------------------------
```

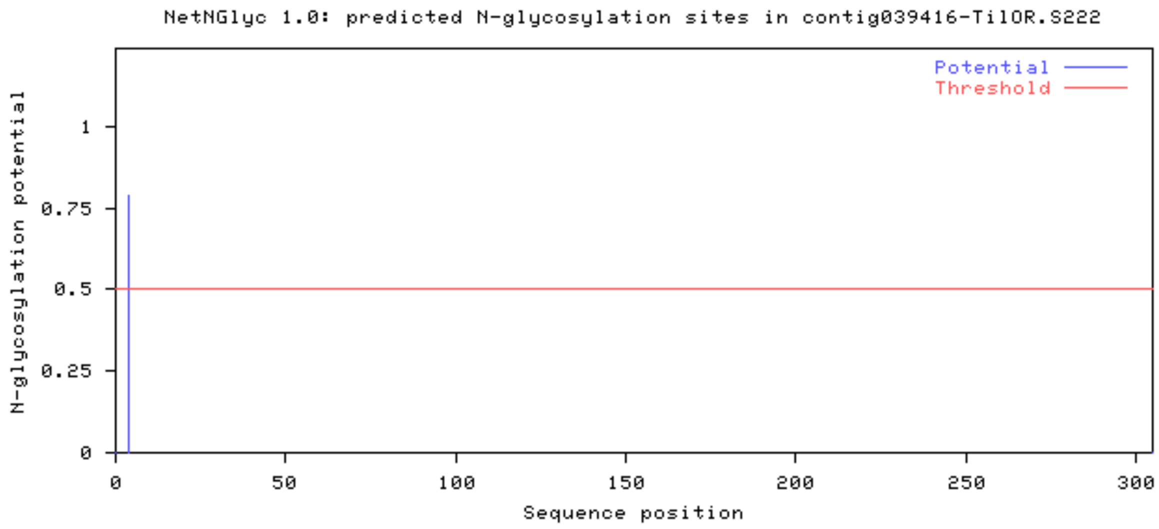

Graphics in PostScript

Output for 'contig039416-TilOR.S223'

```
##############################################################################

   Warning: This sequence may not contain a signal peptide!!

   Proteins without signal peptides are unlikely to be exposed to
   the N-glycosylation machinery and thus may not be glycosylated
   (in vivo) even though they contain potential motifs.

   SignalP-NN euk predictions are as follows:

 # name                 Cmax  pos ?  Ymax  pos ?  Smax  pos ?  Smean ?  D     ?

   SignalP output is explained at http://www.cbs.dtu.dk/services/SignalP/output.html

##############################################################################

Name:  contig039416-TilOR.S223  Length:  305
MAGNNSVNDVFLQRPVYDRVIIVQILVIIFLCINMSLIVIFVKKECFHTSARYILFFVTLLSDSVLLLVSDVLLVLSQFE      80
FTIQVWLCITISVVVLLYFIVTPVTLTAMTLERYVAICMPLRHGQLCSTRSTMYCILIIHLLSSGPCIIILSMFFASASL     160
NFYKQSMICSGETFTLYRWQDHVRSAVYQFYFLIMGTTIAYSYVQIMKVAKAASGEKKKLTHKGLKTVILHAFQLLLCLI     240
QLWCPFIEIALLQIDFSLFRNVRYFNYIMFNIAPRCLSPLIYGLRDENFFLVLKSLMPTSSCSKX
...N.............................N..............................................      80
................................................................................     160
................................................................................     240
.................................................................                    320

(Threshold=0.5)
----------------------------------------------------------------------
SeqName      Position  Potential   Jury    N-Glyc
                                agreement  result
----------------------------------------------------------------------
contig039416-TilOR.S223    4 NNSV   0.7398     (9/9)    ++
contig039416-TilOR.S223   34 NMSL   0.7350     (9/9)    ++
----------------------------------------------------------------------
```

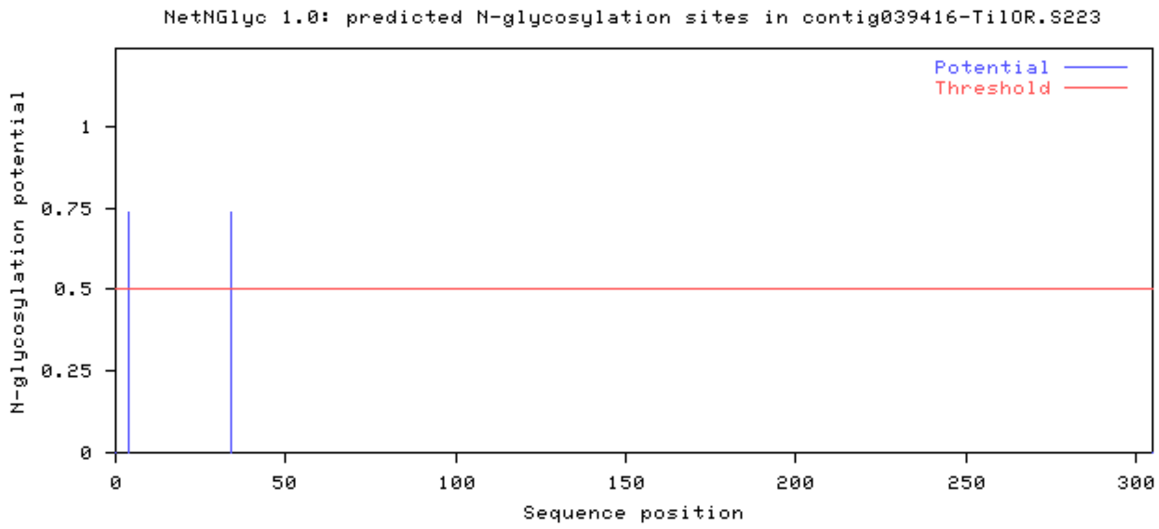

Graphics in PostScript

Output for 'contig039419-TilOR.S224'

```
##########################################################################################

    Warning: This sequence may not contain a signal peptide!!

    Proteins without signal peptides are unlikely to be exposed to
    the N-glycosylation machinery and thus may not be glycosylated
    (in vivo) even though they contain potential motifs.

    SignalP-NN euk predictions are as follows:

  # name                Cmax  pos ?  Ymax  pos ?  Smax  pos ?  Smean ?  D     ?

    SignalP output is explained at http://www.cbs.dtu.dk/services/SignalP/output.html

##########################################################################################

Name:  contig039419-TilOR.S224  Length:  305
MVGNNSVNDVFLQQPVNDRVIIVQILVIIFLCINMLLIVIFVKKECFHTSARYILFFVTLLSDSVLLLVSDVLLILTNFE      80
FTMPVWLCIAISGVVFLYFIVTPVTLTAMTLERYVAICMPLRHGQLCSTRSTMYCILIIHLLSSGPCIIILSMFFASASL     160
NFYKQSMICSVEAFTLYRWQDHVRSAVYQFYFLIMGTTIAYSYVQIMKVAKAASGEKKKLTHKGRKTVILHAFQLLLCLI     240
QLWRPFIETPLLQIDFSLFRNVRFFNYIMFNIAPRCLSPLIYGLRDRKISLVLKSLMPTSSCSKX
...N............................................................................      80
................................................................................     160
................................................................................     240
.................................................................                    320

(Threshold=0.5)
----------------------------------------------------------------------
SeqName      Position  Potential   Jury    N-Glyc
                                 agreement result
----------------------------------------------------------------------
contig039419-TilOR.S224    4 NNSV   0.6543     (9/9)    ++
----------------------------------------------------------------------
```

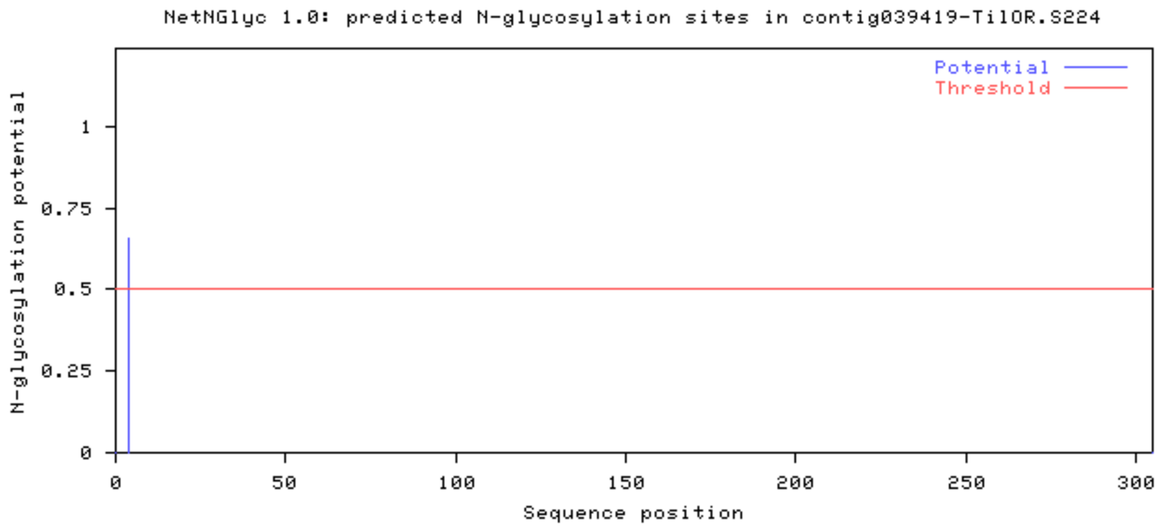

[Graphics in PostScript]

Output for 'contig039425-TilOR.S225'

```
##########################################################################

   Warning: This sequence may not contain a signal peptide!!

   Proteins without signal peptides are unlikely to be exposed to
   the N-glycosylation machinery and thus may not be glycosylated
   (in vivo) even though they contain potential motifs.

   SignalP-NN euk predictions are as follows:

 # name                 Cmax  pos ?  Ymax  pos ?  Smax  pos ?  Smean ?  D     ?

   SignalP output is explained at http://www.cbs.dtu.dk/services/SignalP/output.html

##########################################################################

Name:  contig039425-TilOR.S225  Length:  305
MAGNNSVNDVLLQRPVNDRVIIVQILVIIFLCINMLLIMIFVKKESFHTSARYILFFVTLLSDSVLLLVSDVLLILTYFE      80
FPIQVWLCIILTIFVVMYSSVTPVTLTAMTLERYVAICMPLRHGQLCSTRSTMYCILIIHVLSSGPCIIILSMFFASASL     160
NFYKQSMICSVEAFTFYRWQDHVRSAVYQFYFLIMGITIAYSYVQIMKVAKAASGDKKKLTHKGLKTVILHAFQLLLCLI     240
QLWCPFIETAVLQIDFRLFINVRYSNYIMFNIAPRCLSPLIYGLRDENFFLVLKRFMPTSSCSKX
...N............................................................................      80
................................................................................     160
................................................................................     240
.................................................................                    320

(Threshold=0.5)
----------------------------------------------------------------------
SeqName      Position  Potential   Jury    N-Glyc
                                 agreement result
----------------------------------------------------------------------
contig039425-TilOR.S225    4 NNSV   0.7411     (9/9)    ++
----------------------------------------------------------------------
```

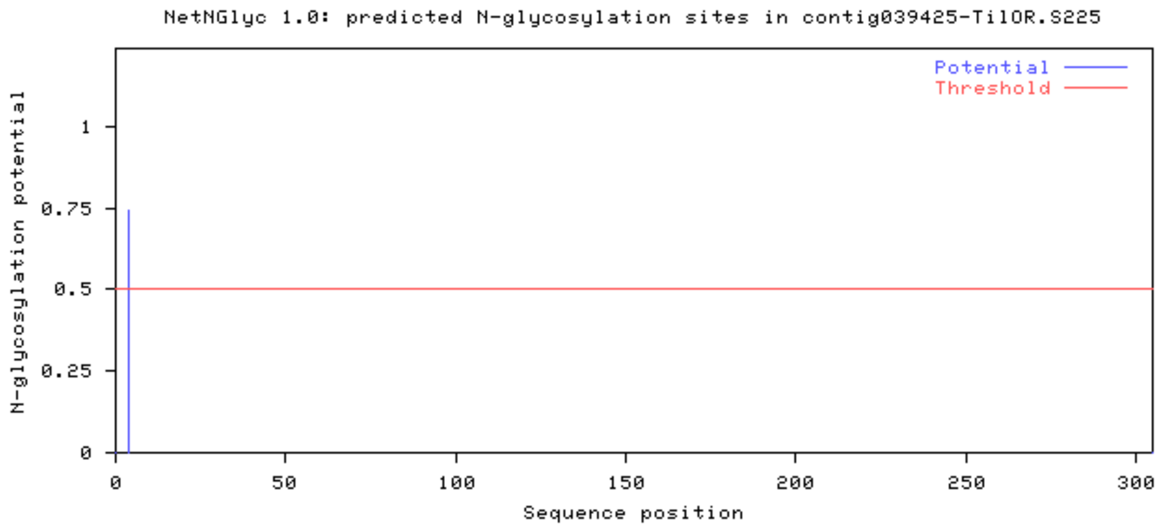

[Graphics in PostScript]

Output for 'contig039426-TilOR.S226'

```
##########################################################################################

    Warning: This sequence may not contain a signal peptide!!

    Proteins without signal peptides are unlikely to be exposed to
    the N-glycosylation machinery and thus may not be glycosylated
    (in vivo) even though they contain potential motifs.

    SignalP-NN euk predictions are as follows:

  # name                Cmax  pos ?  Ymax  pos ?  Smax  pos ?  Smean ?  D     ?

    SignalP output is explained at http://www.cbs.dtu.dk/services/SignalP/output.html

##########################################################################################

Name:  contig039426-TilOR.S226  Length:  305
MAGNNSVNDVLLQRPVDDRVIIVQILVIIFLCINMLLIMIFIKKESFHTSARYILFFVTLLSDSVLLLVSDVLLILTHFE      80
FTIQVWLCIILTIFVVMYSFVTPVTLTAMTLERYVAICMPLRHGQLCSTRSTMYCILIIHGVSFGPCIIILSMFFASASL     160
NFYKQSMICSVEAFTLYRWQDHVRSAVFQFYFLIMGITIAYSYVQIMKVAKAASGEKKKLTHKGLKTVILHAFQLLLCLI     240
QLWCPFIETAVLQIDLRLFVNVRYSNYIMFNIAPRCLSPLIYGLRDENFFLVLKSLMPTSSCSKX
...N............................................................................      80
................................................................................     160
................................................................................     240
.................................................................                    320

(Threshold=0.5)
----------------------------------------------------------------------
SeqName      Position  Potential   Jury    N-Glyc
                                 agreement result
----------------------------------------------------------------------
contig039426-TilOR.S226    4 NNSV   0.7412     (9/9)   ++
----------------------------------------------------------------------
```

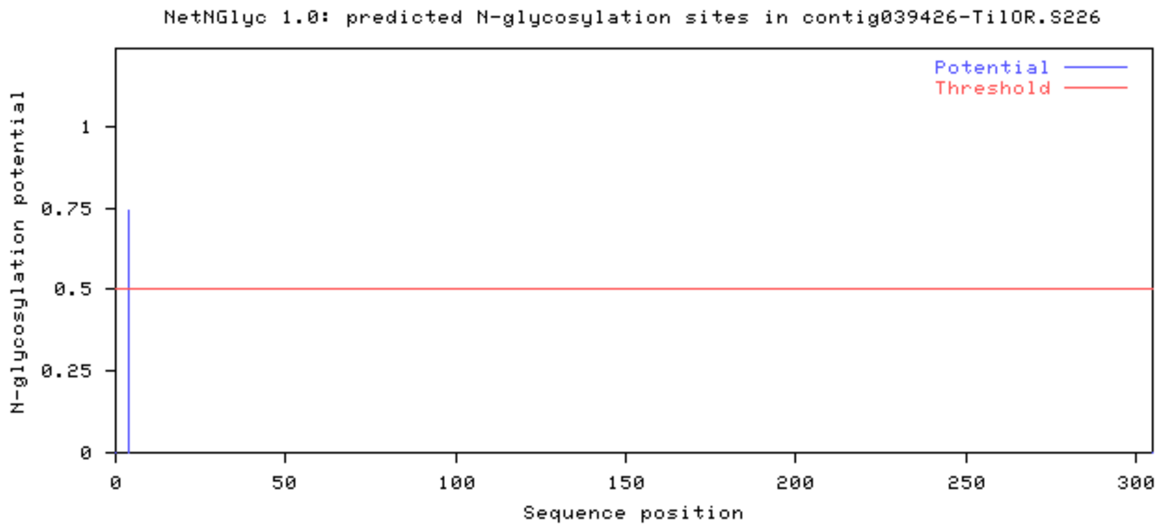

Graphics in PostScript

Output for 'contig039428-TilOR.S227'

```
##########################################################################

    Warning: This sequence may not contain a signal peptide!!

    Proteins without signal peptides are unlikely to be exposed to
    the N-glycosylation machinery and thus may not be glycosylated
    (in vivo) even though they contain potential motifs.

    SignalP-NN euk predictions are as follows:

  # name                 Cmax  pos ?  Ymax  pos ?  Smax  pos ?  Smean ?  D     ?

    SignalP output is explained at http://www.cbs.dtu.dk/services/SignalP/output.html

##########################################################################

Name:  contig039428-TilOR.S227  Length:  304
MADNNSVNNVFLQRPNDRMIIVQILVIIFLCINMLLILTFIKKESFHTSARYILFSVTLLSDSLLLFVSDILVILTYFQF      80
TIPVWLCIILTIFVVMYTFVTPVTLTAMTLERYVAICMPLRHGQLCSTRSTMYCILIIHVVSFGPCIIILSMFFAFASLK     160
FYKQSMICSVEAFTLYRWQDHVRSAVFQFYFLIMGITIAYSYVQIMKVAKAASGDKKKLTHKGLKTVILHAFQLLLCLIQ     240
LWCPFIETAVLQIDLRLFVNVRYSNYIMFSIAPRCLSPLIYGLRDENFFLVLKSLMPTSSCSKX
...N............................................................................      80
................................................................................     160
................................................................................     240
................................................................                     320

(Threshold=0.5)
----------------------------------------------------------------------
SeqName      Position  Potential   Jury    N-Glyc
                                 agreement result
----------------------------------------------------------------------
contig039428-TilOR.S227    4 NNSV   0.7287     (9/9)    ++
----------------------------------------------------------------------
```

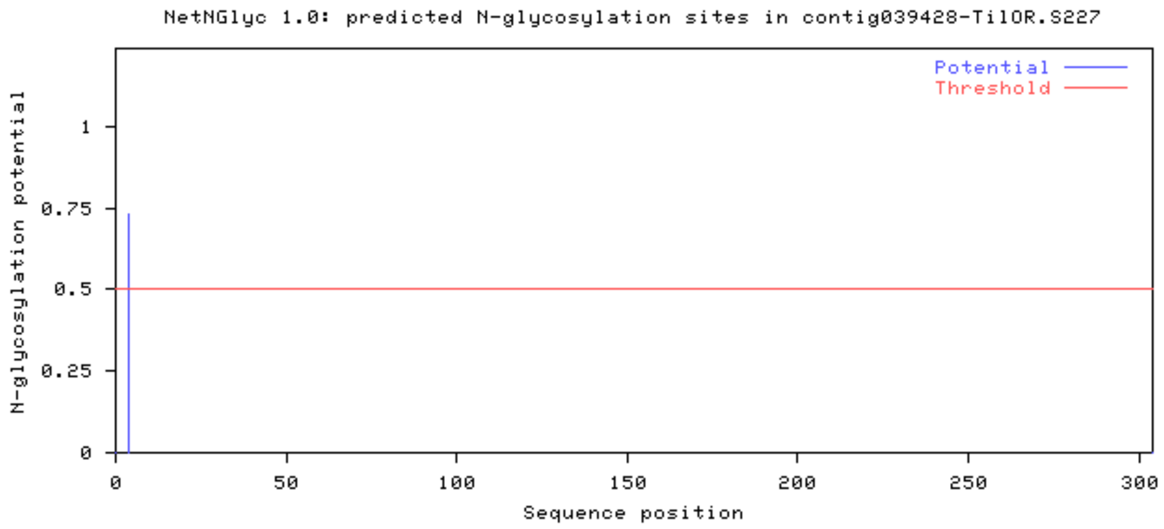

Graphics in PostScript

Output for 'contig039429-TilORe.S232'

```
##########################################################################################

    Warning: This sequence may not contain a signal peptide!!

    Proteins without signal peptides are unlikely to be exposed to
    the N-glycosylation machinery and thus may not be glycosylated
    (in vivo) even though they contain potential motifs.

    SignalP-NN euk predictions are as follows:

  # name                Cmax  pos ?  Ymax  pos ?  Smax  pos ?  Smean ?  D     ?

    SignalP output is explained at http://www.cbs.dtu.dk/services/SignalP/output.html

##########################################################################################

Name:  contig039429-TilORe.S232        Length:  115
IGTFFSKEFFYTTTRYMFFAVTLMSDSFMLIMSNILVLLNFFHLPIQFYQCVIIYIPLVIFTFVTPITLTAMTLERYVAI      80
CMPLRHAELCSTCNTVHIIIIIHGLSSVPMVDGGX
................................................................................      80
...................................                                                  160

(Threshold=0.5)

No sites predicted in this sequence.
```

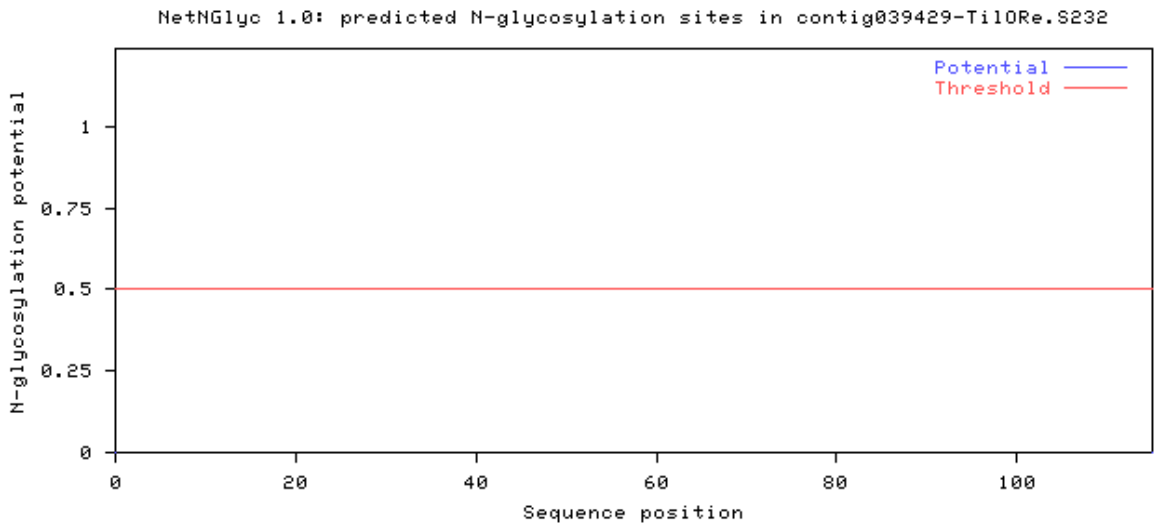

Graphics in PostScript

Output for 'contig039435-TilOR.S228'

```
##########################################################################################

    Warning: This sequence may not contain a signal peptide!!

    Proteins without signal peptides are unlikely to be exposed to
    the N-glycosylation machinery and thus may not be glycosylated
    (in vivo) even though they contain potential motifs.

    SignalP-NN euk predictions are as follows:

  # name                 Cmax  pos ?  Ymax  pos ?  Smax  pos ?  Smean ?  D     ?

    SignalP output is explained at http://www.cbs.dtu.dk/services/SignalP/output.html

##########################################################################################

Name:  contig039435-TilOR.S228  Length:  310
MVFNYSLIEGKMPGTKLSDRVILVQVFVSVFLFINVLQITTFFMKQFFYTVMRYILFAITLLSDCLFLIITNTTLLLGHF      80
TITIQMWLCVIIFIVLSLYTFITPVTLTAMTLERYVAICMPMRHAELCSTQRALQFILIIHSLSSVPCIVVLFMFFASAN     160
PSFYFQGRVCSAEMLIIYRWQAHVRSAVSQFYFLIMSTTIVFSYVQIMRVAKAASGENKKSTHKGLRTVVLHGFQLFLCL     240
IQMWCPIIEDAVFQIDLNVFSTVRYFNYITFILAPRCLSPLIYGLRDEKFFYALKYSTLNGLFKVQISFX
...N...................................................................N........      80
................................................................................     160
................................................................................     240
......................................................................               320

(Threshold=0.5)
----------------------------------------------------------------------
SeqName      Position  Potential   Jury    N-Glyc
                                agreement result
----------------------------------------------------------------------
contig039435-TilOR.S228    4 NYSL   0.7237     (9/9)    ++
contig039435-TilOR.S228   72 NTTL   0.6447     (8/9)    +
contig039435-TilOR.S228  160 NPSF   0.4500     (6/9)    -
----------------------------------------------------------------------
```

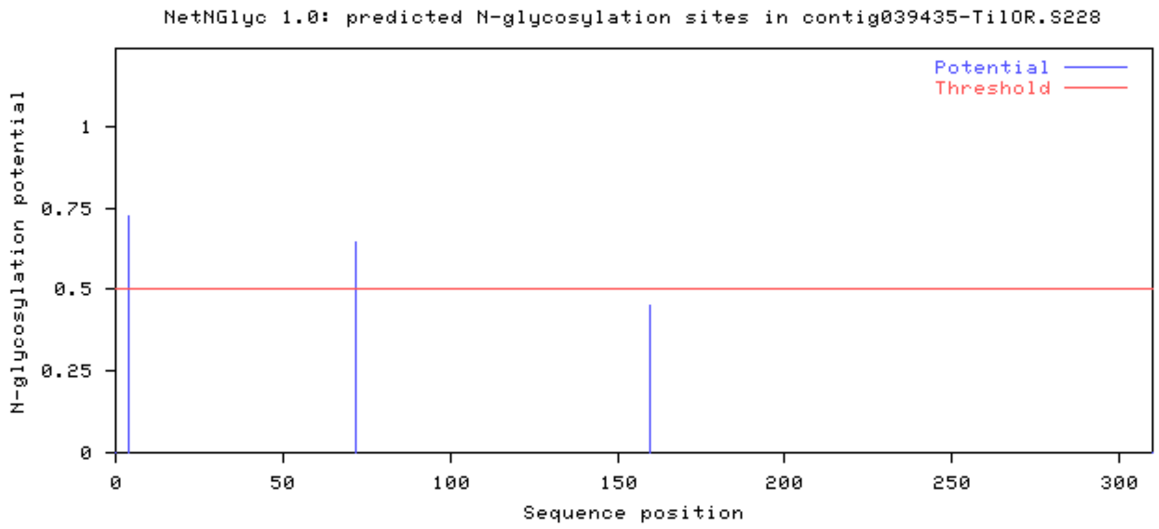

Graphics in PostScript

Output for 'contig039435-TilOR.S229'

```
##########################################################################################

    Warning: This sequence may not contain a signal peptide!!

    Proteins without signal peptides are unlikely to be exposed to
    the N-glycosylation machinery and thus may not be glycosylated
    (in vivo) even though they contain potential motifs.

    SignalP-NN euk predictions are as follows:

  # name                    Cmax  pos ?  Ymax  pos ?  Smax  pos ?  Smean ?  D     ?

    SignalP output is explained at http://www.cbs.dtu.dk/services/SignalP/output.html

##########################################################################################

Name:  contig039435-TilOR.S229  Length:  316
MALNNSVIGGQLAINNINNQVIIVQVFISMFLCINCLLITTFFMKDIFYTTMRYILFAIALLSDSLFLLITNVLLILSYF      80
SFTIQVWLCVIIYIVLSVYTFVTPVTLTAMTLERYVAICIPLRHAELCRTQRALHFILIIHGLSSVPCIVILSIFFASAI     160
PSFYTQSRICAVEMFIFHRWQGHLRSAISQLFFLIMSITIVFSYVQIMKVAKAASGENKKSTWKGLSTVVLHGFQLLLCL     240
IQLWCPFIEAAVLQIDFMLFINVRYFNYITFILAPRCLSPLIYGLRDEMFVNALKYYALCGLYKKHSTACSFIRFX
...N............................................................................      80
................................................................................     160
................................................................................     240
............................................................................         320

(Threshold=0.5)
----------------------------------------------------------------------
SeqName      Position  Potential   Jury    N-Glyc
                                 agreement result
----------------------------------------------------------------------
contig039435-TilOR.S229    4 NNSV   0.7407     (9/9)    ++
----------------------------------------------------------------------
```

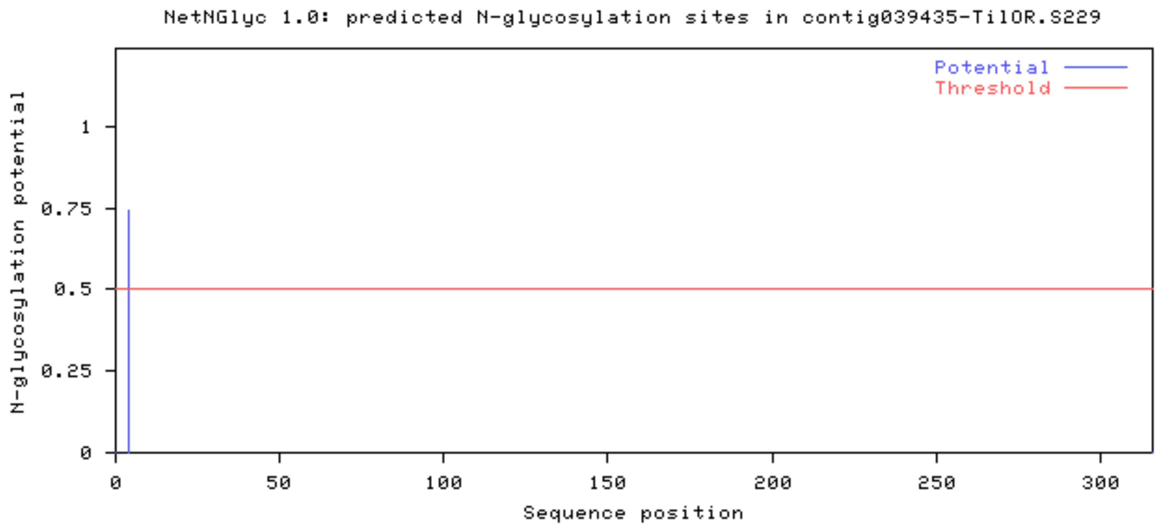

Graphics in PostScript

Output for 'contig039436-TilOR.S230'

```
##############################################################################

    Warning: This sequence may not contain a signal peptide!!

    Proteins without signal peptides are unlikely to be exposed to
    the N-glycosylation machinery and thus may not be glycosylated
    (in vivo) even though they contain potential motifs.

    SignalP-NN euk predictions are as follows:

  # name                 Cmax  pos ?  Ymax  pos ?  Smax  pos ?  Smean ?  D     ?

    SignalP output is explained at http://www.cbs.dtu.dk/services/SignalP/output.html

##############################################################################

Name:  contig039436-TilOR.S230  Length:  311
MASNNSVIGGQLAINNINNRVIIVQVFISMFLCINCLLITTFFMKDIFYTTMRYILFAIALLSDSLFLLITNVLLILNYF      80
SFTIQVWLCVIIYIVLAVYTFVTPVTLTAMTLERYVAICIPLRHAELCRTQRALHFILIIHGLSSVPCIVILSIFFASAI     160
PSFYTQSRICAVEMFIFHRWQGHLRSAISQLFFLIMSITIVFSYVQIMKVAKAASGENKKSTWKGLSTVVLHGFQLLLCL     240
IQLWCPFIEAAVLQIDFMLFINVRYFNYITFILAPRCLSPLIYGLRDEMFVNALKYYALCGLYKKHSTAFX
...N............................................................................      80
................................................................................     160
................................................................................     240
.......................................................................              320

(Threshold=0.5)
----------------------------------------------------------------------
SeqName      Position  Potential   Jury    N-Glyc
                                 agreement result
----------------------------------------------------------------------
contig039436-TilOR.S230    4 NNSV   0.7072     (9/9)    ++
----------------------------------------------------------------------
```

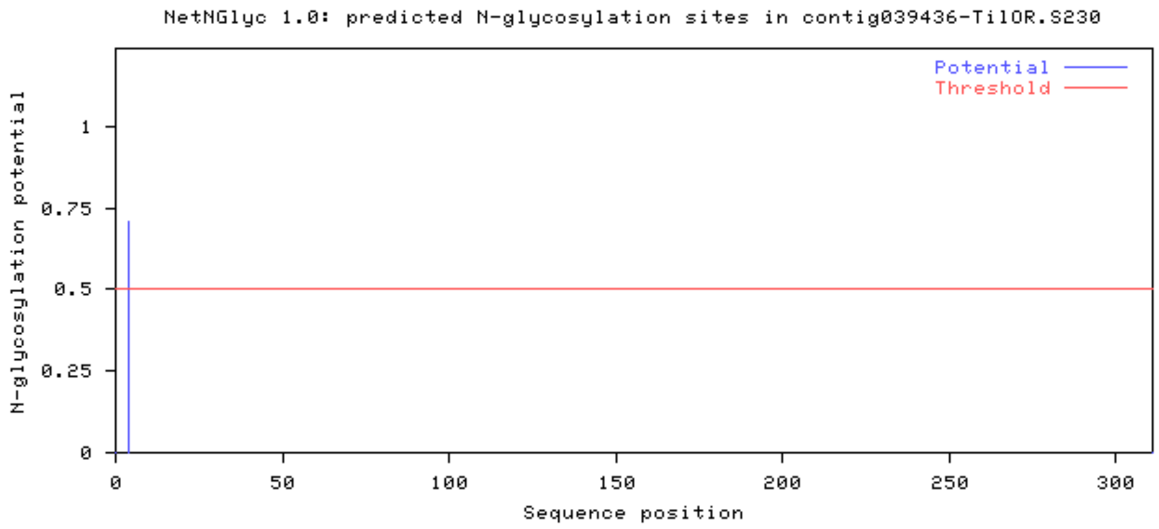

Graphics in PostScript

Output for 'contig039437-TilOR.S231'

```
##########################################################################

    Warning: This sequence may not contain a signal peptide!!

    Proteins without signal peptides are unlikely to be exposed to
    the N-glycosylation machinery and thus may not be glycosylated
    (in vivo) even though they contain potential motifs.

    SignalP-NN euk predictions are as follows:

  # name                   Cmax  pos ?  Ymax  pos ?  Smax  pos ?  Smean ?  D     ?

    SignalP output is explained at http://www.cbs.dtu.dk/services/SignalP/output.html

##########################################################################

Name:  contig039437-TilOR.S231  Length:  313
MADNSSFIGGESSVRQINDQVIIVQVLVGIFLCINTLLIITFFMKDTFYRTMRYILFAVTLLSDCLILILTDLLLILNYF      80
RLSIQLSLCLIMFAVSSVCNFVTPFTLTAMTLERYVAICMPLRHGELCSTRSALQCILIIHGLSSVPCILILSVFFASVS     160
LSFFKQYQFCFGQTFIIRSWQGHLRSAISQFYFLIMCVIVVFSYIRIMKVAKAASGENKKSTHKGLRTVALHAFQLLLCL     240
IQLWCPFIEDAVLEINFVLYLNVRYFNYIMFSLAPRCLSPLIYGLRDDKFFLALKYHVLCPLYRKKSVAFSNX
...N............................................................................      80
................................................................................     160
................................................................................     240
.........................................................................            320

(Threshold=0.5)
----------------------------------------------------------------------
SeqName      Position  Potential   Jury    N-Glyc
                                 agreement result
----------------------------------------------------------------------
contig039437-TilOR.S231    4 NSSF   0.7049     (9/9)    ++
----------------------------------------------------------------------
```

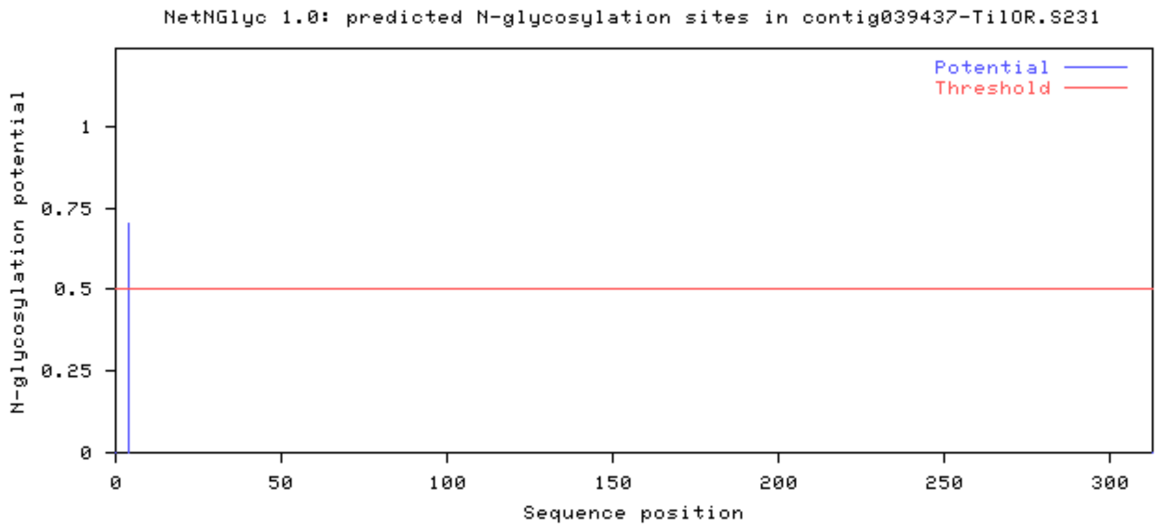

Graphics in PostScript

Output for 'contig039437-TilORe.S233'

```
##########################################################################################

    Warning: This sequence may not contain a signal peptide!!

    Proteins without signal peptides are unlikely to be exposed to
    the N-glycosylation machinery and thus may not be glycosylated
    (in vivo) even though they contain potential motifs.

    SignalP-NN euk predictions are as follows:

  # name                Cmax  pos ?  Ymax  pos ?  Smax  pos ?  Smean ?  D     ?

    SignalP output is explained at http://www.cbs.dtu.dk/services/SignalP/output.html

##########################################################################################

Name:  contig039437-TilORe.S233        Length:  216
LCTFTLTAMTLERYVAICMPLRHGELCSTRSALQCILIIHGLSSVPCILILSVFFASVSLSFFKPYRFCFVEMFILRSWQ      80
GHLRSAISQFYFLIMCVIVVFSYIRIMKVAKAASGENKKSTHKGLRTVALHAFQLLLCLIQLWCPFIEAAVLEIDFVLFV     160
NVRYFNYIMFSLAPRCLSPLIYGLRDDKFFLVLKYHVLCPLYRKKKQLGFLTNNKX
................................................................................      80
................................................................................     160
........................................................                             240

(Threshold=0.5)

No sites predicted in this sequence.
```

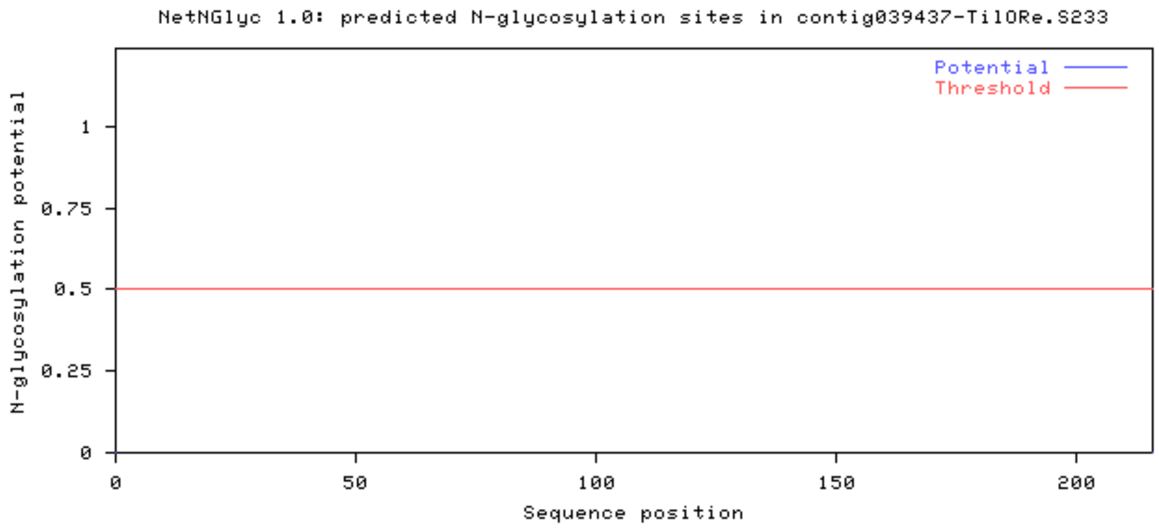

Graphics in PostScript

Output for 'contig039450-TilOR.J131'

```
##########################################################################

    Warning: This sequence may not contain a signal peptide!!

    Proteins without signal peptides are unlikely to be exposed to
    the N-glycosylation machinery and thus may not be glycosylated
    (in vivo) even though they contain potential motifs.

    SignalP-NN euk predictions are as follows:

  # name                   Cmax   pos ?  Ymax   pos ?  Smax   pos ?  Smean ?  D     ?

    SignalP output is explained at http://www.cbs.dtu.dk/services/SignalP/output.html

##########################################################################

Name:  contig039450-TilOR.J131  Length:  312
MENQSFEFSSELTLDPFVIPPGGKYPIFFLGIFIYIFGISCNLTLLALIILNRNLHKPMYFILFSLPLNDLIGLSAMLPK      80
VLSDIVTETHKIHYLLCVLQAFLLHMYGGGILFILAAMSFDRYVAICMPLRYSSVMTPRVTSCIIALVWGLNFVLIVSLF     160
SLQTRLPRCKYAVLNVFCDNPSLLKLTCGNTTVNNIIGLFNTAVIQVVSISIQAYSYVKILITCVVTRKSEAKAKAVNTC     240
VAQLVILVMFEVVGTFTILSHRFKNVSVDMQKIMGMLIFIVPPLLNPIVYGLYTKEIRSTLLRVLKNRVSIX
..N.......................................N.....................................      80
................................................................................     160
...................N.........N..................................................     240
........................N...............................................             320

(Threshold=0.5)
----------------------------------------------------------------------
SeqName      Position  Potential    Jury    N-Glyc
                                  agreement result
----------------------------------------------------------------------
contig039450-TilOR.J131    3 NQSF   0.6019     (8/9)   +
contig039450-TilOR.J131   42 NLTL   0.6774     (9/9)   ++
contig039450-TilOR.J131  180 NPSL   0.5446     (6/9)   +     WARNING: PRO-X1.
contig039450-TilOR.J131  190 NTTV   0.6813     (9/9)   ++
contig039450-TilOR.J131  265 NVSV   0.5147     (6/9)   +
----------------------------------------------------------------------
```

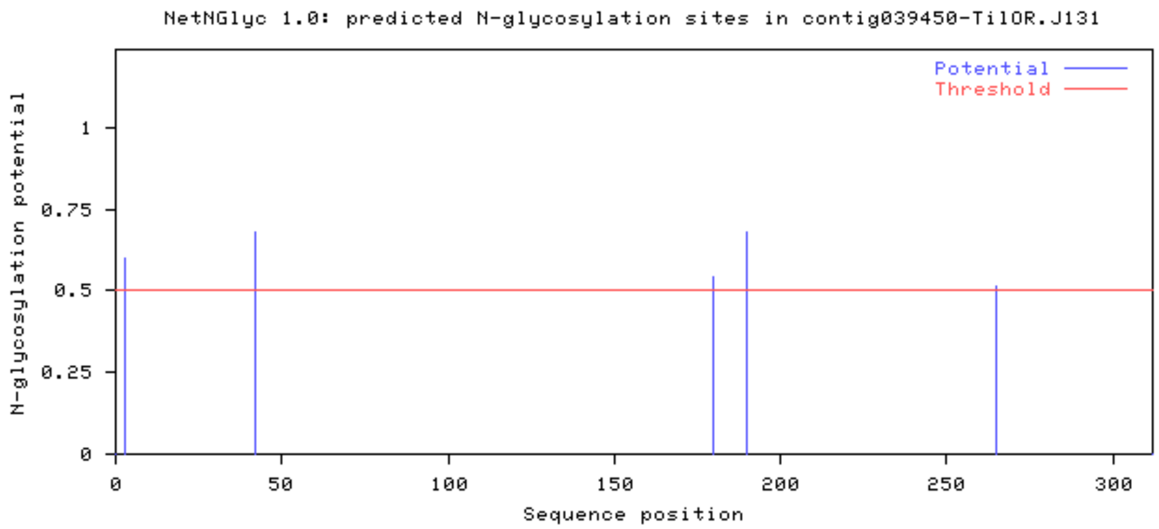

Graphics in PostScript

Output for 'contig039450-TilOR.K133'

```
##########################################################################################

    Warning: This sequence may not contain a signal peptide!!

    Proteins without signal peptides are unlikely to be exposed to
    the N-glycosylation machinery and thus may not be glycosylated
    (in vivo) even though they contain potential motifs.

    SignalP-NN euk predictions are as follows:

  # name                 Cmax  pos ?  Ymax  pos ?  Smax  pos ?  Smean ?  D     ?

    SignalP output is explained at http://www.cbs.dtu.dk/services/SignalP/output.html

##########################################################################################

Name:  contig039450-TilOR.K133  Length:  312
MEKNKTVSTDILEVQGFDISPESTYPLFFLLLFVYFTLLFSNIGVLVLIISQKSLHQPMYFLFCNLSVNDLIGNTVLLPQ      80
LMAHILATERFITYKQCVVQAFQSHTFGSASHMILIIMAIDRYVAICHPLRYSSIMTTRTVVGLSAAAWGVSVVLVSILI     160
GLTVRLSRCRSTIQNAYCDNASLFKLSCEDVSINNIYGLFFTVLLFTSSIASIAATYFRIALICWIKKNKDLNNRALQTC     240
ASHLVLYLIMLWSGFLTIILHRFPNYPDLRKIAYVLFHVVPANLNPIIYGMQTRSLRHKITEILKRKVTPSX
...N............................................................N...............      80
................................................................................     160
...................N............................................................     240
........................................................................             320

(Threshold=0.5)
----------------------------------------------------------------------
SeqName      Position  Potential   Jury    N-Glyc
                                 agreement result
----------------------------------------------------------------------
contig039450-TilOR.K133    4 NKTV   0.7128     (9/9)    ++
contig039450-TilOR.K133   65 NLSV   0.7388     (9/9)    ++
contig039450-TilOR.K133  180 NASL   0.6132     (8/9)    +
----------------------------------------------------------------------
```

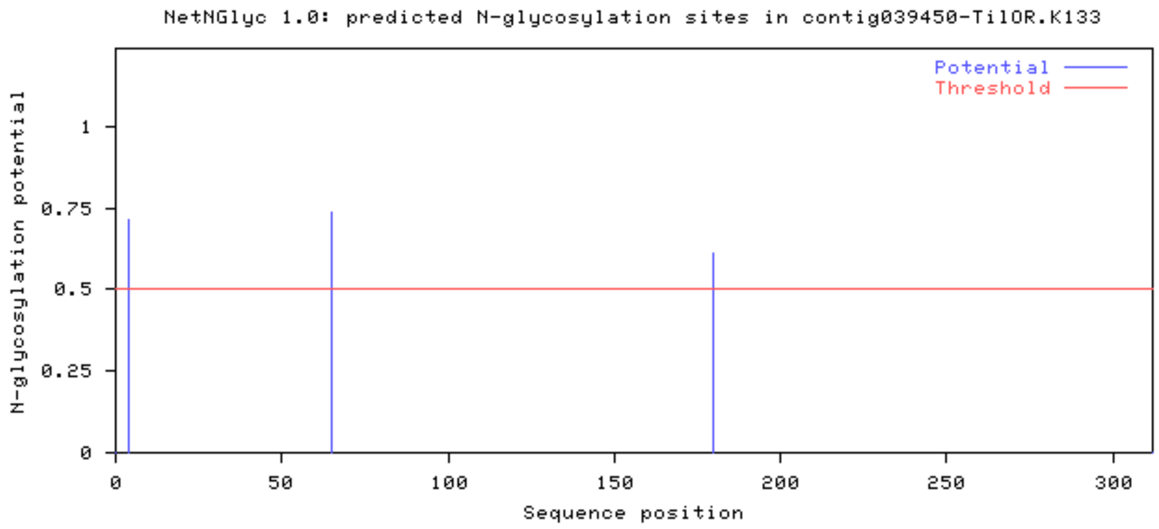

[Graphics in PostScript]

Output for 'contig039451-TilOR.J266'

```
##########################################################################################

    Warning: This sequence may not contain a signal peptide!!

    Proteins without signal peptides are unlikely to be exposed to
    the N-glycosylation machinery and thus may not be glycosylated
    (in vivo) even though they contain potential motifs.

    SignalP-NN euk predictions are as follows:

  # name                    Cmax   pos ?  Ymax   pos ?  Smax   pos ?  Smean ?  D     ?

    SignalP output is explained at http://www.cbs.dtu.dk/services/SignalP/output.html

##########################################################################################

Name:  contig039451-TilOR.J266  Length:  313
MYTNSSTSSLLKLQTLGLSSTDIYPAFVFGTLTYLIIMFSNLLVLTVIAMNKKLHKPMFILLFNLPISDIVGATAFFPHL      80
IFSIVTENRLISHHACIFQAFLIHVYGTGNLLILSVMAYDRYIAICFPLRYTTIMNSHNLMKMIVITWFINLSMMFTLFI     160
LLARFKTCRTNIVDFYCNNQSLVKLICADTSVNNYYGLATIFVLMGGPLSLIVYTYAQILRTCVLTNHTDARQKAIQTCG     240
THLIVFLSLQINTVFALISHRIESSSPVLRRAFGLSVLIFPPFLDPIIYGLKTKELKQCIVMFLKQNVGFTKX
...N............................................................................      80
......................................................................N.........     160
..................N...............................................N.............     240
..........................................................................            320

(Threshold=0.5)
----------------------------------------------------------------------
SeqName      Position  Potential   Jury    N-Glyc
                                 agreement result
----------------------------------------------------------------------
contig039451-TilOR.J266    4 NSST   0.6331     (8/9)   +
contig039451-TilOR.J266  151 NLSM   0.5320     (6/9)   +
contig039451-TilOR.J266  179 NQSL   0.5257     (6/9)   +
contig039451-TilOR.J266  227 NHTD   0.5306     (5/9)   +
----------------------------------------------------------------------
```

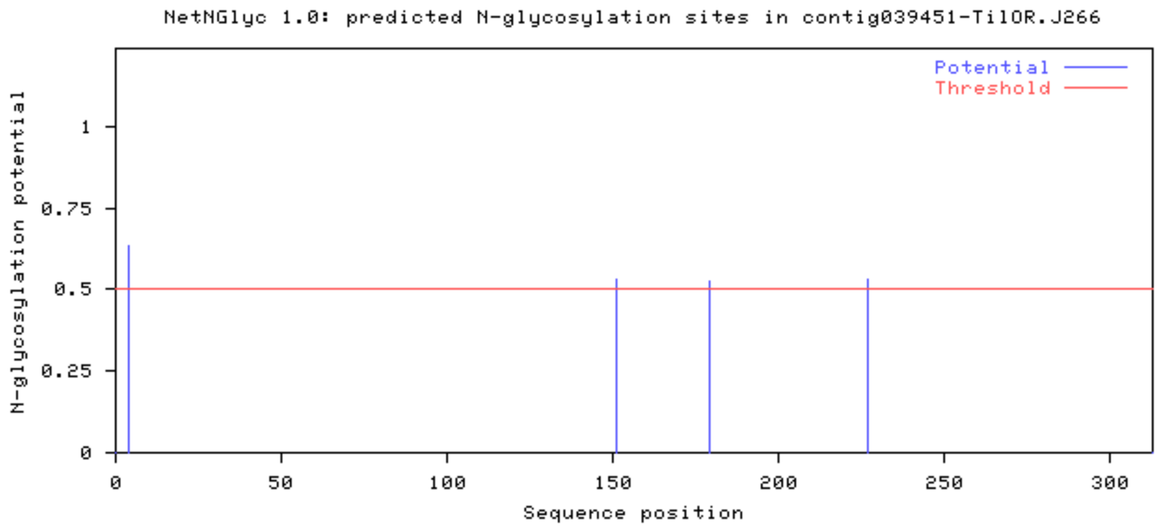

Graphics in PostScript

Output for 'contig039453-TilORe.J132'

```
##########################################################################

   Warning: This sequence may not contain a signal peptide!!

   Proteins without signal peptides are unlikely to be exposed to
   the N-glycosylation machinery and thus may not be glycosylated
   (in vivo) even though they contain potential motifs.

   SignalP-NN euk predictions are as follows:

 # name                Cmax  pos ?  Ymax  pos ?  Smax  pos ?  Smean ?  D     ?

   SignalP output is explained at http://www.cbs.dtu.dk/services/SignalP/output.html

##########################################################################

Name:  contig039453-TilORe.J132        Length:  84
MYNQYNTSSFLQMKGFNLSSESVIPAFLFATLSYMIILFCNLTLILTIVLNKSLHQPMYLILLNLPINDLIGSSALFPQL      80
IKEI
.....N..........N.......................N.........N.............................      80
....                                                                                 160

(Threshold=0.5)
----------------------------------------------------------------------
SeqName      Position  Potential   Jury    N-Glyc
                                 agreement result
----------------------------------------------------------------------
contig039453-TilORe.J132    6 NTSS   0.7450     (9/9)   ++
contig039453-TilORe.J132   17 NLSS   0.6640     (8/9)   +
contig039453-TilORe.J132   41 NLTL   0.6753     (8/9)   +
contig039453-TilORe.J132   51 NKSL   0.6930     (9/9)   ++
----------------------------------------------------------------------
```

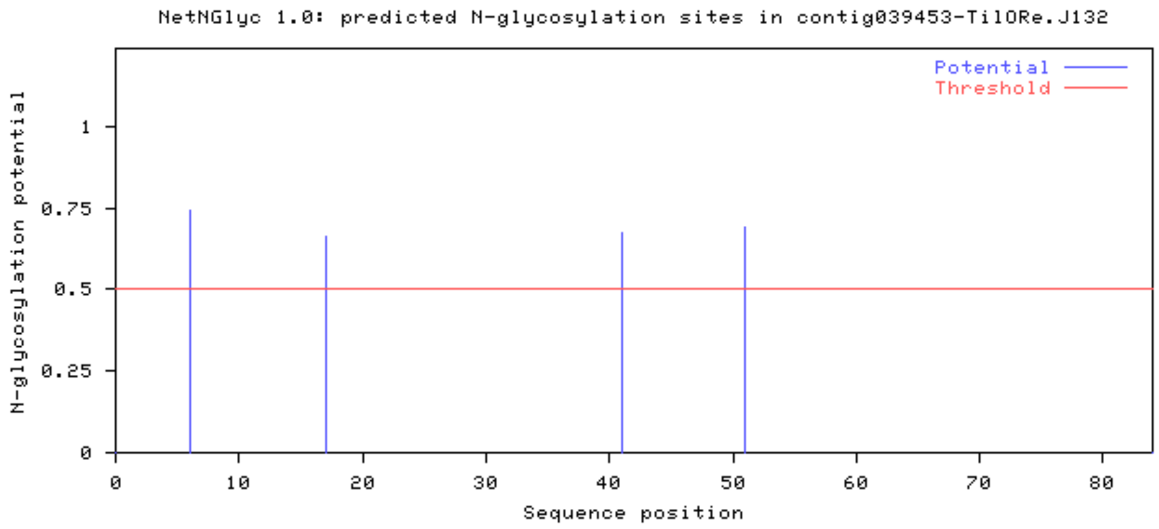

Graphics in PostScript

Output for 'contig039460-TilOR.L145'

```
##########################################################################################

    Warning: This sequence may not contain a signal peptide!!

    Proteins without signal peptides are unlikely to be exposed to
    the N-glycosylation machinery and thus may not be glycosylated
    (in vivo) even though they contain potential motifs.

    SignalP-NN euk predictions are as follows:

  # name                Cmax  pos ?  Ymax  pos ?  Smax  pos ?  Smean ?  D     ?

    SignalP output is explained at http://www.cbs.dtu.dk/services/SignalP/output.html

##########################################################################################

Name:  contig039460-TilOR.L145  Length:  314
MSLPNASIKVTHFIIGGFDTVKRPIAVGVVMLITYLLAVIANVLNILFIIFDKRLHKPMYLLICNLAVVDLMYCCSTTPT      80
MIGVLLAGVNTISYVECFIQMSVFHLVGVMELFALAIMAFDRLIAFSFPFQYYSYLTNTRTLVVTYILWVVACGFVAVMP     160
VTAATLPYCTSRMKYAFCDYAAVIRTTCVDPNYYFNLVSVMMFFLLFFTFSFICLSYFVIAFFMKVSSNRDKTKMASTCV     240
SHLIVVTCYYSPLFVLVLLTRVGVVLTLEERQGLLIGTILSPSLVNPVVYCFRTKEIKNKIFKIFTKKADISDX
....N...........................................................................      80
................................................................................     160
................................................................................     240
..........................................................................           320

(Threshold=0.5)
----------------------------------------------------------------------
SeqName      Position  Potential   Jury    N-Glyc
                                 agreement result
----------------------------------------------------------------------
contig039460-TilOR.L145    5 NASI   0.6294     (7/9)   +
----------------------------------------------------------------------
```

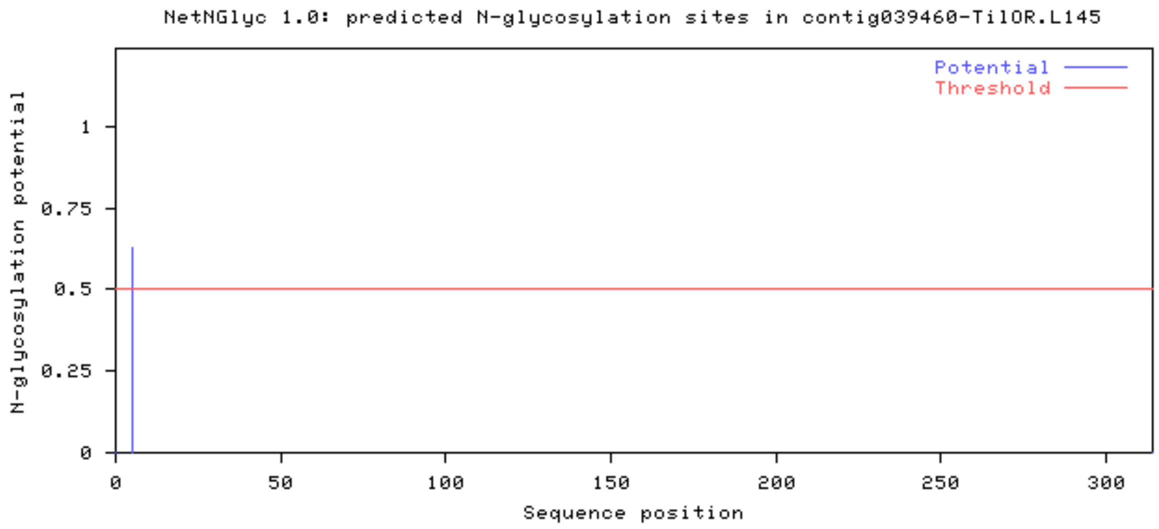

Graphics in PostScript

Output for 'contig039460-TilOR.L146'

```
##############################################################################

    Warning: This sequence may not contain a signal peptide!!

    Proteins without signal peptides are unlikely to be exposed to
    the N-glycosylation machinery and thus may not be glycosylated
    (in vivo) even though they contain potential motifs.

    SignalP-NN euk predictions are as follows:

  # name                Cmax  pos ?  Ymax  pos ?  Smax  pos ?  Smean ?  D     ?

    SignalP output is explained at http://www.cbs.dtu.dk/services/SignalP/output.html

##############################################################################

Name:  contig039460-TilOR.L146  Length:  311
MSLQNTSIKVTHFIIGGFDTVKRPVAVGVVMLITYLLAVIANVLNIVFIIFDKRLHKPMYLLICNLAVVDLLYTSSTCPT      80
MIGVLLAGVNTISYVDCFIQMCIFNWVEVMAFFALAFMAFDRLIAIIYPFQYHSYMTNTRTLVVTYILWVVSLGVSAVMP     160
VTAATLPYCTSRMKYAFCDYAAVMRTTCVDPNFYFNIVSVMLFFILFFTFSFICLSYVVIAFFMKFSSNRDKTKMASTCV     240
SHLIVVTCYYSPLFVLIVFTRVGVVLTLEERQGLLIGNILGPAVVNPFVYCFRTKEIKNKIFKIFTKNGIX
....N...........................................................................      80
................................................................................     160
................................................................................     240
.......................................................................              320

(Threshold=0.5)
----------------------------------------------------------------------
SeqName      Position  Potential   Jury    N-Glyc
                                 agreement result
----------------------------------------------------------------------
contig039460-TilOR.L146    5 NTSI   0.6330     (9/9)   ++
----------------------------------------------------------------------
```

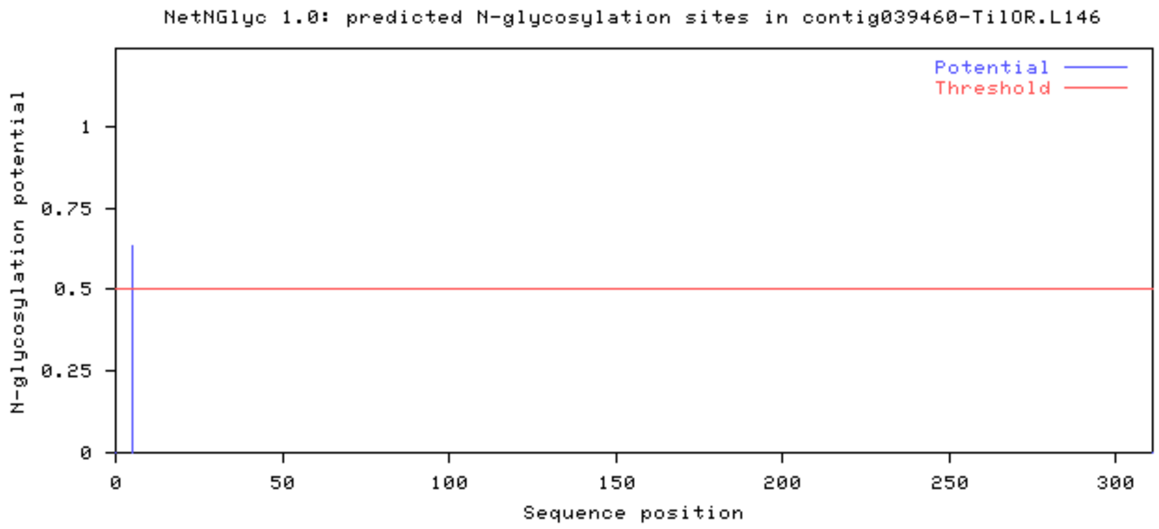

Graphics in PostScript

Output for 'contig039461-TilOR.L147'

```
##########################################################################

    Warning: This sequence may not contain a signal peptide!!

    Proteins without signal peptides are unlikely to be exposed to
    the N-glycosylation machinery and thus may not be glycosylated
    (in vivo) even though they contain potential motifs.

    SignalP-NN euk predictions are as follows:

  # name                  Cmax   pos ?  Ymax   pos ?  Smax   pos ?  Smean ?  D     ?

    SignalP output is explained at http://www.cbs.dtu.dk/services/SignalP/output.html

##########################################################################

Name:  contig039461-TilOR.L147  Length:  311
MYLQNASINVTHFIIGGFDTVIRPEAIGVVMLIIYILVVLANTVNILFIIFDKRLHKPMYLLVCNLAVVDIIYTSSTSPT      80
MIGVLLAGVNTISYVECIIQMFVFHLGAVMERLALAIMALDRLIAIICPFQYHSYLTNTHIVFLTYILWFVGSGFAALAP     160
AVVVRLPHCYSRLKYTFCDYAAVLRTTCADPNYYLNLGAIILFFILFFTLIFICLSYCVILIFVKLSNNDKKKMGGTLLS     240
HLICVTCHYCPAFIIIVLTRLGVALSLEERQGLLIGTILGPSLVNPFVYSLRTTEIKTKIFRILKNGWHVX
....N...N.......................................................................      80
................................................................................     160
................................................................................     240
.......................................................................             320

(Threshold=0.5)
----------------------------------------------------------------------
SeqName      Position  Potential   Jury    N-Glyc
                                 agreement result
----------------------------------------------------------------------
contig039461-TilOR.L147    5 NASI   0.6240     (9/9)    ++
contig039461-TilOR.L147    9 NVTH   0.7200     (9/9)    ++
----------------------------------------------------------------------
```

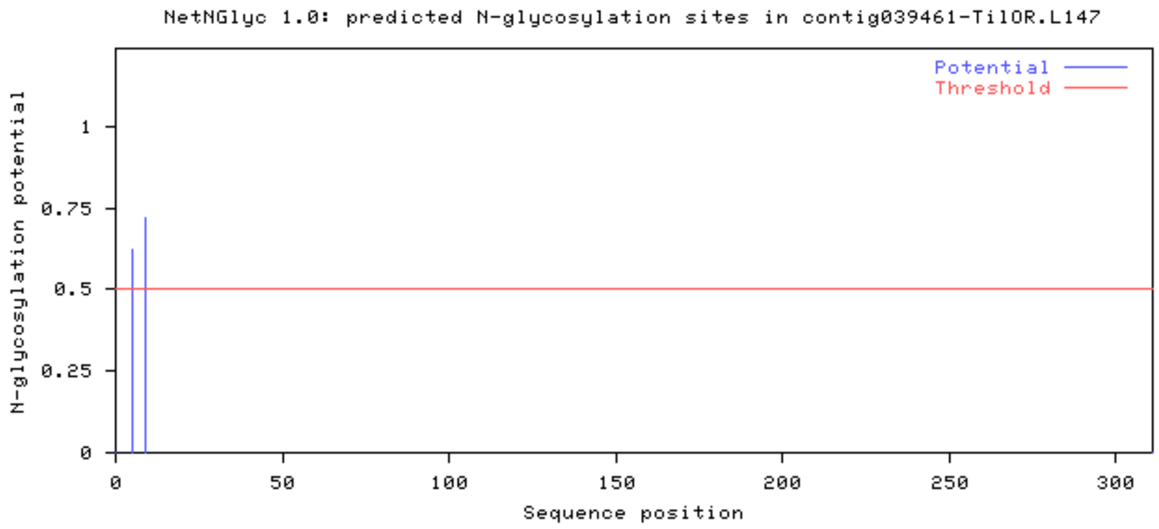

Graphics in PostScript

Output for 'contig039461-TilOR.L148'

```
########################################################################################

    Warning: This sequence may not contain a signal peptide!!

    Proteins without signal peptides are unlikely to be exposed to
    the N-glycosylation machinery and thus may not be glycosylated
    (in vivo) even though they contain potential motifs.

    SignalP-NN euk predictions are as follows:

  # name                   Cmax   pos ?  Ymax   pos ?  Smax   pos ?  Smean ?  D     ?

    SignalP output is explained at http://www.cbs.dtu.dk/services/SignalP/output.html

########################################################################################

Name:  contig039461-TilOR.L148  Length:  313
MSLQNASIKLTHFIIGGFDTVKRPVAVGVVMLITYLLAVTGSLVNIIFIVSDKQLHKPMYLLICNLAVVDILYTSSSTPT      80
MIRVLLAGVNTISYVECLIQMCVFQLGGIMELFSLTIMAFDRLIAIIYPLRYHSYLTNTRIMVLTYILWIVASNLVAVLP     160
AIVVPLPHCSLRLRYAFCDAAAVIRTTCVDAEKFFDLGAILSFFLFFFTFTFICLSYCMILFFVKLSSNNDRRKMGSTLV     240
SHLICVICLYCPMFVIGILTRFGVVLSLEERQGLLIGTILGPSLVNPLVYCFRTKEIKNKIVKIFRKVNTAGX
....N...........................................................................      80
................................................................................     160
................................................................................     240
.........................................................................            320

(Threshold=0.5)
----------------------------------------------------------------------
SeqName      Position  Potential   Jury    N-Glyc
                                agreement result
----------------------------------------------------------------------
contig039461-TilOR.L148    5 NASI   0.6026     (7/9)   +
----------------------------------------------------------------------
```

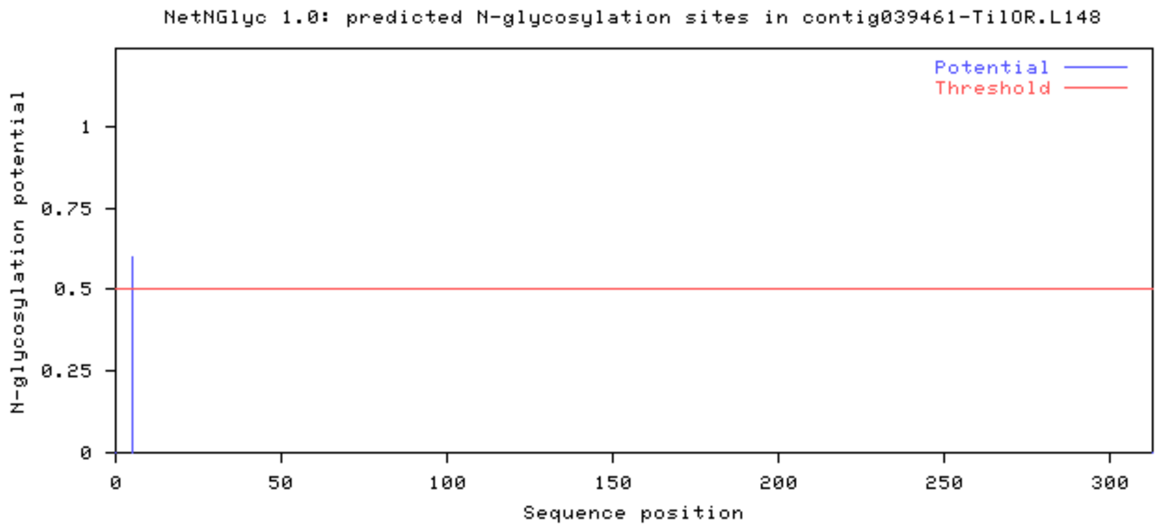

Graphics in PostScript

Output for 'contig039462-TilOR.L149'

```
##########################################################################

    Warning: This sequence may not contain a signal peptide!!

    Proteins without signal peptides are unlikely to be exposed to
    the N-glycosylation machinery and thus may not be glycosylated
    (in vivo) even though they contain potential motifs.

    SignalP-NN euk predictions are as follows:

  # name                Cmax  pos ?  Ymax  pos ?  Smax  pos ?  Smean ?  D     ?

    SignalP output is explained at http://www.cbs.dtu.dk/services/SignalP/output.html

##########################################################################

Name:  contig039462-TilOR.L149  Length:  313
MSLPNASIKVTHFIIGGFDTVNRPIAVGVVILIIYILAVFANTVNILFIIFDKRLHKPMYLLVCNLAVVDIIYTSSATPT      80
MIGVLLADVNTISYVECLIQMCVFHLAMVMERFALAIMAFDRLIAIIFPFHYHSYLTNTRTVFLTYILWIIGCGTVVLFP     160
ATVIPLPHCTLRLKYNFCDYAAIMRTTCVNVDEYFNQSAIWSFFILFFTFTFICLSYCGILFFVKLSSNNDKKKMGSTLV     240
SHAICVTCFYSPIFIIVILTRFGVVLSLDERQGLLIGNILGPSLVNPFVYCLRTTEIKNKIVKIFKQILRFLX
....N...........................................................................      80
................................................................................     160
................................................................................     240
.........................................................................            320

(Threshold=0.5)
----------------------------------------------------------------------
SeqName      Position  Potential   Jury    N-Glyc
                                 agreement result
----------------------------------------------------------------------
contig039462-TilOR.L149    5 NASI   0.6294     (7/9)   +
contig039462-TilOR.L149  196 NQSA   0.3959     (7/9)   -
----------------------------------------------------------------------
```

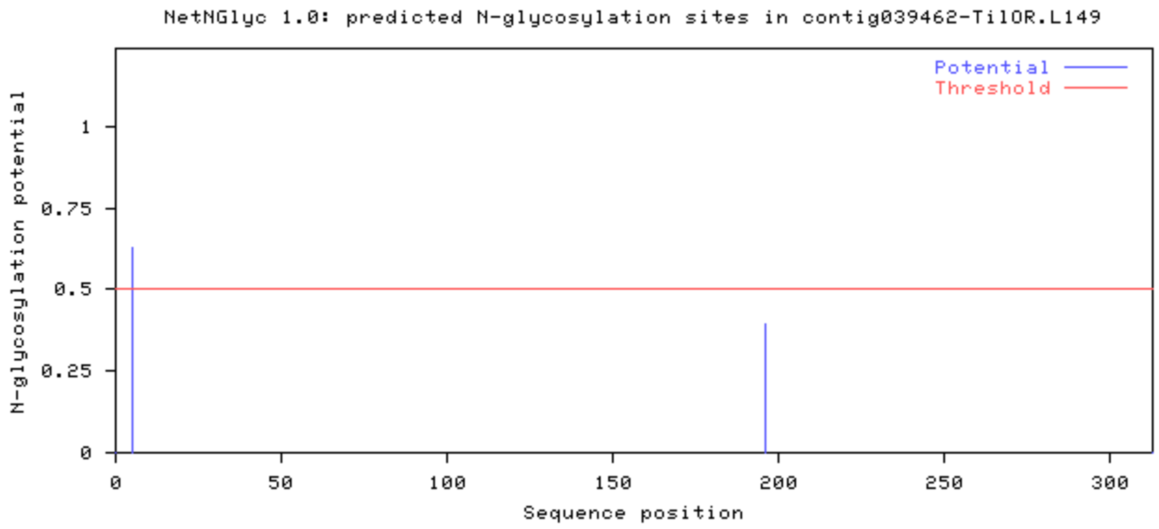

Graphics in PostScript

Output for 'contig039465-TilOR.L150'

```
##########################################################################

    Warning: This sequence may not contain a signal peptide!!

    Proteins without signal peptides are unlikely to be exposed to
    the N-glycosylation machinery and thus may not be glycosylated
    (in vivo) even though they contain potential motifs.

    SignalP-NN euk predictions are as follows:

  # name                  Cmax   pos ?  Ymax   pos ?  Smax   pos ?  Smean ?  D     ?

    SignalP output is explained at http://www.cbs.dtu.dk/services/SignalP/output.html

##########################################################################

Name:  contig039465-TilOR.L150  Length:  313
MSLQNASIKLTHFIIGGFDTVKRPVAVGVGMLITYLLAVTGSLVNIIFIISDKQLHKPMYLLICNLAVVDILYTSSSTPT      80
MIGVLLAGVNTISYVECLIQMCVYQLGATMELFSLTIMAFDRLIAIIYPLQYHSYLTNTRIMVLTYILWIVGSGFVAVLP     160
ATVTPLPHCSLRLKYTFCDYAAVIRTTCVDPEKYFNLGAILSFFVLFFTFIFICLSYCVILFFVKLSSNNDRKKMGSTLV     240
SHLICVICLYCPMFVIGILTRFGVVLTLEERQGLIIGIILGPTLVNPLVYCLRTKEIKNKIVKIFRKVNTVGX
....N...........................................................................      80
................................................................................     160
................................................................................     240
.........................................................................            320

(Threshold=0.5)
----------------------------------------------------------------------
SeqName      Position  Potential   Jury    N-Glyc
                                 agreement result
----------------------------------------------------------------------
contig039465-TilOR.L150    5 NASI   0.6024     (7/9)    +
----------------------------------------------------------------------
```

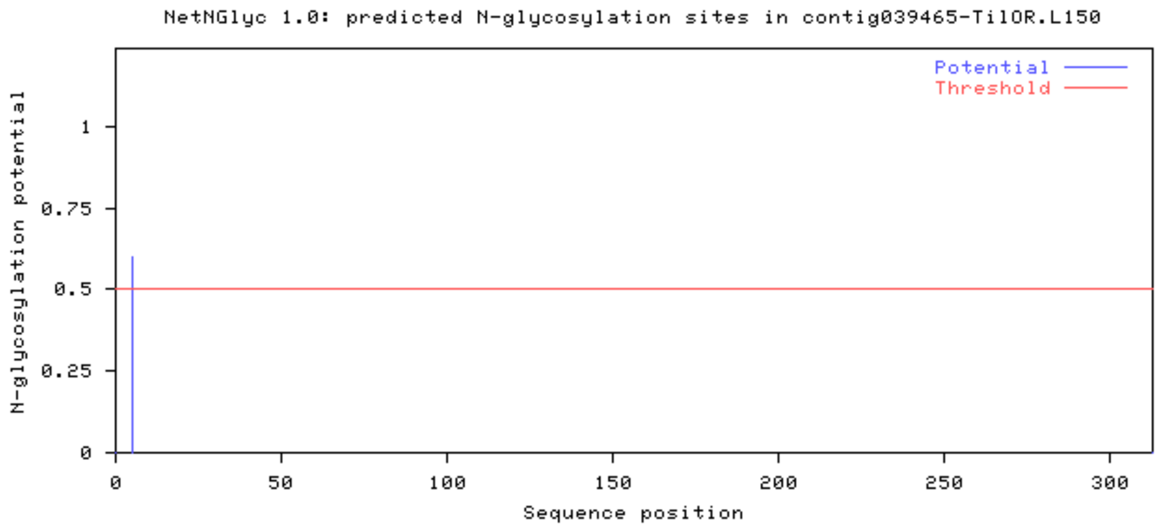

Graphics in PostScript

Output for 'contig039467-TilORe.L160'

```
##########################################################################################

    Warning: This sequence may not contain a signal peptide!!

    Proteins without signal peptides are unlikely to be exposed to
    the N-glycosylation machinery and thus may not be glycosylated
    (in vivo) even though they contain potential motifs.

    SignalP-NN euk predictions are as follows:

  # name                  Cmax   pos ?  Ymax   pos ?  Smax   pos ?  Smean ?  D     ?

    SignalP output is explained at http://www.cbs.dtu.dk/services/SignalP/output.html

##########################################################################################

Name:  contig039467-TilORe.L160         Length:  65
MSLQNASIKLTHFIIGGFDTVKRPVALGVVMLIAYLLAVTGSLVNIIFIVSDKQLHKPMYLLICN
....N............................................................                    80

(Threshold=0.5)
----------------------------------------------------------------------
SeqName      Position  Potential    Jury    N-Glyc
                                 agreement result
----------------------------------------------------------------------
contig039467-TilORe.L160    5 NASI   0.5972     (7/9)    +
----------------------------------------------------------------------
```

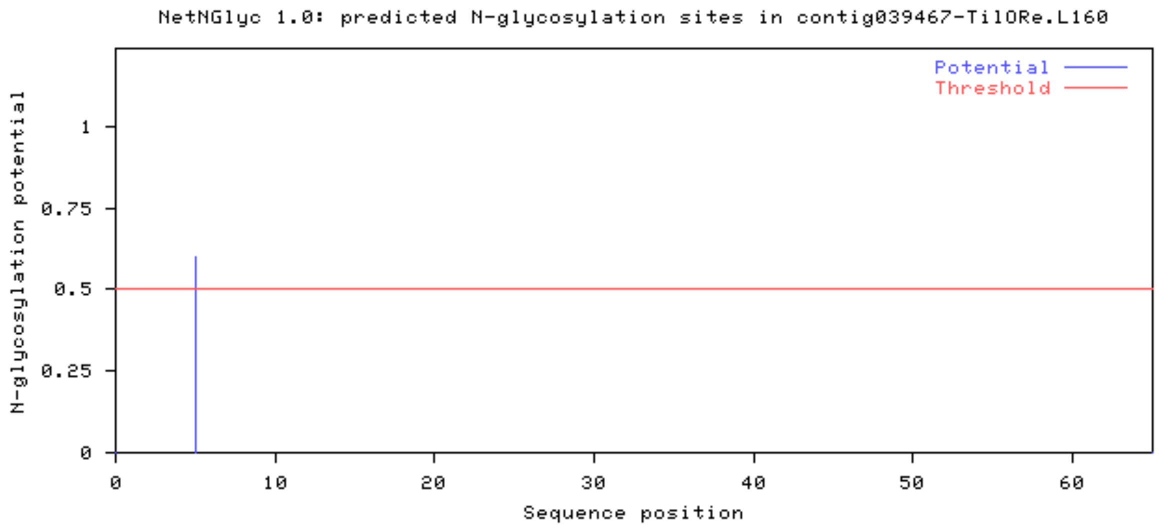

Graphics in PostScript

Output for 'contig039469-TilOR.L151'

```
##########################################################################

    Warning: This sequence may not contain a signal peptide!!

    Proteins without signal peptides are unlikely to be exposed to
    the N-glycosylation machinery and thus may not be glycosylated
    (in vivo) even though they contain potential motifs.

    SignalP-NN euk predictions are as follows:

  # name                    Cmax   pos ?  Ymax   pos ?  Smax   pos ?  Smean ?  D     ?

    SignalP output is explained at http://www.cbs.dtu.dk/services/SignalP/output.html

##########################################################################

Name:  contig039469-TilOR.L151  Length:  313
MSLQNASINVTHFIIGGFDTLSRPIAVGVVILIMYLLAVFANMANIMFIISDKRLHKPMYLLICNLAVVDIMYTSSCNPT      80
MIGVLLAGVNTISYVECLIQMCVFTLGTSMESFVLAVMALDRFIAIIYPFQYHSYLTNTRVLVLTFILWFVAWYFVCYMP     160
ATVVPFPHCSSKLKYSFCDFAAVIRTTCVNPEKYFNEGATIAFFFFFFTFVFICLSYCGILLYVKLPSNNEKKKMGSTLV     240
SHLICVIVHYCPAFVRIIFTRFGVVLTLEENQGLVIGAVLGPCLVNPFVYCLRTKEIKQKLFKIFKKFHTSDX
....N...N.....................................................................N..      80
................................................................................     160
................................................................................     240
..........................................................................           320

(Threshold=0.5)
----------------------------------------------------------------------
SeqName      Position  Potential   Jury    N-Glyc
                                 agreement result
----------------------------------------------------------------------
contig039469-TilOR.L151    5 NASI   0.6719     (8/9)    +
contig039469-TilOR.L151    9 NVTH   0.7238     (9/9)    ++
contig039469-TilOR.L151   78 NPTM   0.7054     (9/9)    ++    WARNING: PRO-X1.
----------------------------------------------------------------------
```

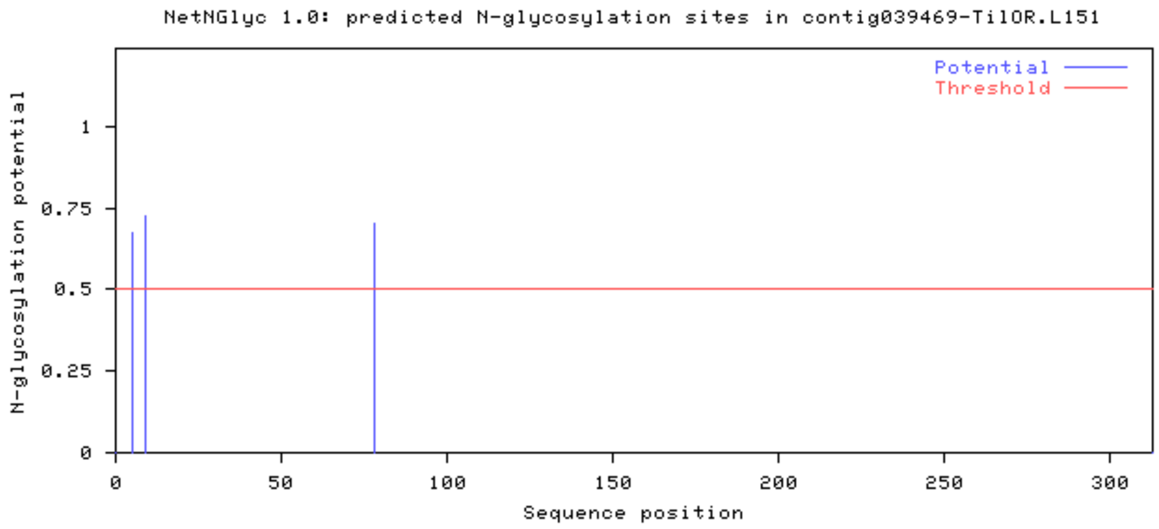

Graphics in PostScript

Output for 'contig039469-TilOR.L152'

```
##########################################################################################

    Warning: This sequence may not contain a signal peptide!!

    Proteins without signal peptides are unlikely to be exposed to
    the N-glycosylation machinery and thus may not be glycosylated
    (in vivo) even though they contain potential motifs.

    SignalP-NN euk predictions are as follows:

  # name                Cmax  pos ?  Ymax  pos ?  Smax  pos ?  Smean ?  D     ?

    SignalP output is explained at http://www.cbs.dtu.dk/services/SignalP/output.html

##########################################################################################

Name:  contig039469-TilOR.L152  Length:  322
MSLQNASIKLTYFIIGGFDTVKRPVAVGVVMLITYLLAVFASMVNIIFIVSDKQLHKPMYLLICNLAVVDIFYTSSATPT      80
MIGVLLAGVNTISYVECLIQMYVFQVGGTMEMFSLTIMGFDRLIAIICPLQYHSYLTNTRILIFTYILWIVACSFVLFTL     160
VTLAPLPHCYSRLRYTFCDYAAVIRTTCVNPEKYFNQIAIISFFLSFSTFTFICLSYCGILFFVKISSNNDKKKMGSTLV     240
SHLICVICVYCPLVIIVILTRFGVVLTLEERQGLLIGTILGPSLVNPFVYCLRTKEIKIRIFKIFRKVNTLRPELFHGMH     320
FX
....N...........................................................................      80
................................................................................     160
................................................................................     240
................................................................................     320
..                                                                                   400

(Threshold=0.5)
----------------------------------------------------------------------
SeqName      Position  Potential   Jury    N-Glyc
                                 agreement result
----------------------------------------------------------------------
contig039469-TilOR.L152    5 NASI   0.5848     (6/9)    +
----------------------------------------------------------------------
```

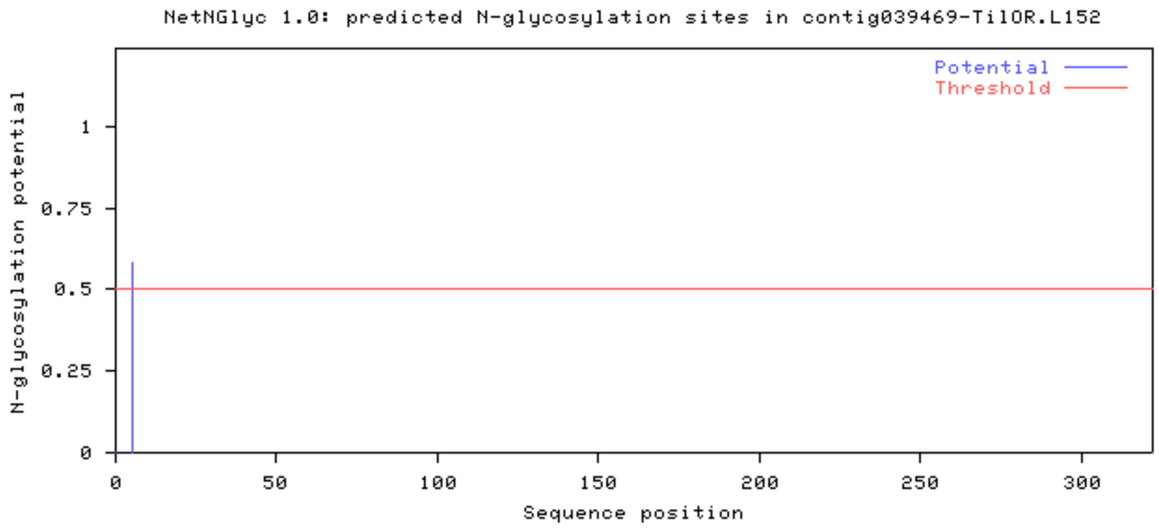

Graphics in PostScript

Output for 'contig039472-TilORe.L168'

```
##########################################################################################

    Warning: This sequence may not contain a signal peptide!!

    Proteins without signal peptides are unlikely to be exposed to
    the N-glycosylation machinery and thus may not be glycosylated
    (in vivo) even though they contain potential motifs.

    SignalP-NN euk predictions are as follows:

  # name                 Cmax  pos ?  Ymax  pos ?  Smax  pos ?  Smean ?  D     ?

    SignalP output is explained at http://www.cbs.dtu.dk/services/SignalP/output.html

##########################################################################################

Name:  contig039472-TilORe.L168        Length:  191
LIAIICPLQYHSYLTNTRTLVFTYILWIVACSFVLFTLVTATPLPHCYSRLRYTFCDYAAVMRATCVNPEKYFTQTAIIS      80
FFVSFSTFTFICLSYCGILFFVKILSNNDKKKMGSTLVSHLICVSCLYGPQFVIVILTRFGVVLTLEERQGLLIGTILGP     160
PLVNPFVYCLRTKEIKSKIFMIFRKINTAGX
................................................................................      80
................................................................................     160
...............................                                                      240

(Threshold=0.5)

No sites predicted in this sequence.
```

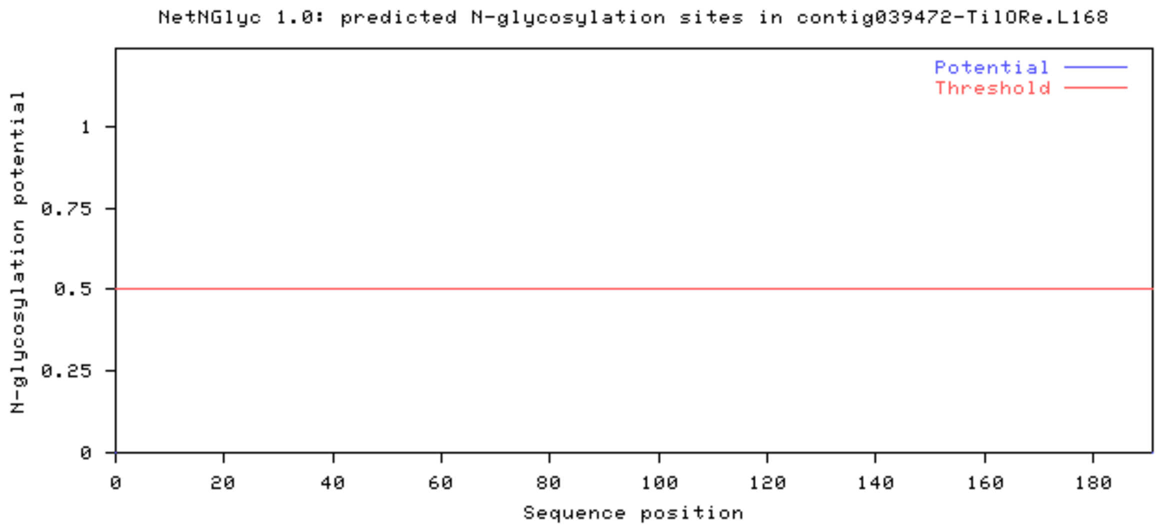

Graphics in PostScript

Output for 'contig039474-TilORe.L169'

```
##########################################################################################

    Warning: This sequence may not contain a signal peptide!!

    Proteins without signal peptides are unlikely to be exposed to
    the N-glycosylation machinery and thus may not be glycosylated
    (in vivo) even though they contain potential motifs.

    SignalP-NN euk predictions are as follows:

  # name                Cmax  pos ?  Ymax  pos ?  Smax  pos ?  Smean ?  D     ?

    SignalP output is explained at http://www.cbs.dtu.dk/services/SignalP/output.html

##########################################################################################

Name:  contig039474-TilORe.L169        Length:  207
MSLQNASIKLTHFVIGGFDTVKRPVAVGVVMLITYLLAVFASSVNIIFIVSDKQLHKPMYLLICNLAVVDIFYTSSVTPT      80
MIGVLLAGVNTISYVECLIQMYVYQVGATMEMFSLTIMAFDRLIAIICPLQYHSYLTNTRTLVFTYILWIVACSFVLFTL     160
VTATPLPLCNSRLRYTFCDYAAVMRATCVNPEKYFNQVAILSFFVSF
....N...........................................................................      80
................................................................................     160
...............................................                                      240

(Threshold=0.5)
----------------------------------------------------------------------
SeqName      Position  Potential   Jury    N-Glyc
                                 agreement result
----------------------------------------------------------------------
contig039474-TilORe.L169    5 NASI   0.6233     (7/9)   +
----------------------------------------------------------------------
```

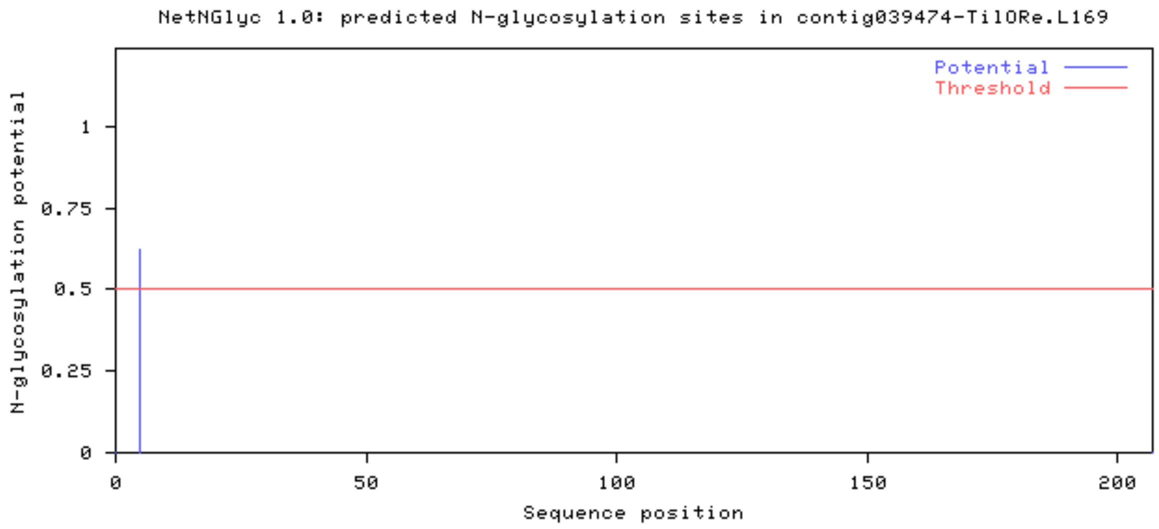

Graphics in PostScript

Output for 'contig039481-TilOR.L153'

```
##########################################################################################

    Warning: This sequence may not contain a signal peptide!!

    Proteins without signal peptides are unlikely to be exposed to
    the N-glycosylation machinery and thus may not be glycosylated
    (in vivo) even though they contain potential motifs.

    SignalP-NN euk predictions are as follows:

  # name                    Cmax   pos ?  Ymax   pos ?  Smax   pos ?  Smean ?  D     ?

    SignalP output is explained at http://www.cbs.dtu.dk/services/SignalP/output.html

##########################################################################################

Name:  contig039481-TilOR.L153  Length:  313
MSLQNASINVTHFIIGGFDTLSRPIAVGVVILITYLLAVFANMANIMFIISDKRLHKPMYLLICNLAVVDIMYTSSCSPT      80
VIGVLLAGVNTISYVECLIQMFVFHLGTAMESFVLAVMALDRLIAIMYPFQYHSYLTNTRVLLLTFIVWFVNWFFMCYMP     160
ATVVPLPHCSSRLRYTFCDFAAVLRTTCVNPEKYFNEAATIAFFILFFTFVFICLSYCGILLFVKLSSNNEKKKMGSTLV     240
SHLICAIIHYCPAFVRIIFTRFGVVLTLEERQGLLIGAVLGPCLVNPFVYCLRTKEIKHKLSKILKKFHTYDX
....N...N.......................................................................      80
................................................................................     160
................................................................................     240
.........................................................................            320

(Threshold=0.5)
----------------------------------------------------------------------
SeqName      Position  Potential   Jury    N-Glyc
                                 agreement result
----------------------------------------------------------------------
contig039481-TilOR.L153    5 NASI   0.6716     (8/9)    +
contig039481-TilOR.L153    9 NVTH   0.7236     (9/9)    ++
----------------------------------------------------------------------
```

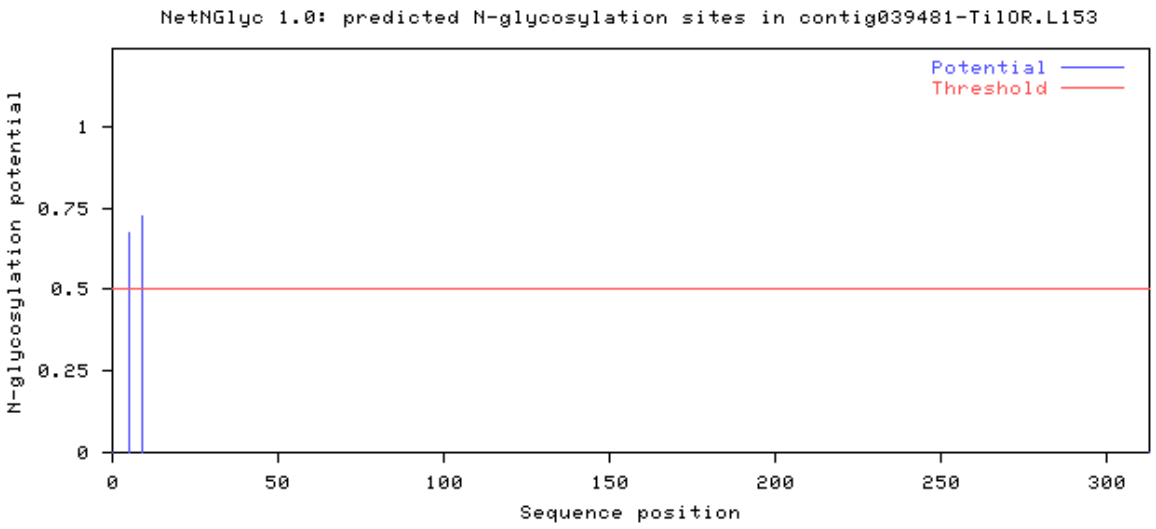

Graphics in PostScript

Output for 'contig039484-TilOR.L154'

```
##########################################################################################

    Warning: This sequence may not contain a signal peptide!!

    Proteins without signal peptides are unlikely to be exposed to
    the N-glycosylation machinery and thus may not be glycosylated
    (in vivo) even though they contain potential motifs.

    SignalP-NN euk predictions are as follows:

  # name                 Cmax  pos ?  Ymax  pos ?  Smax  pos ?  Smean ?  D     ?

    SignalP output is explained at http://www.cbs.dtu.dk/services/SignalP/output.html

##########################################################################################

Name:  contig039484-TilOR.L154  Length:  313
MSLQNASINVTHFIIGGFDTLSRPIAVGVAILITYLLAVLANMANIMFIISDKRLHKPMYLLICNLAIVDIVYTSSSSPT      80
MIGVLLAGVNTISYVECLIQMCVFNLGTAMESFVLAVMALDRLIAIIYPFQYHTYLTNTRVLVLTLIMWFAVAWCFVFYMP     160
ATVVPLPHCSSRLKYSFCDFAAIIRTTCVNPEKYFNEVAIAAFFILFFTFVFICLSYCGILIFVKLSSNNEKKKMGSTLV     240
SHLICVIVHYCPAFVRIIFTRFGVVLTLEERQGLVIGAVLGPCLVNPFVYCLRTKEIKQKLFKIFKKFNTSDX
....N...N.......................................................................      80
................................................................................     160
................................................................................     240
.........................................................................            320

(Threshold=0.5)
----------------------------------------------------------------------
SeqName      Position  Potential   Jury    N-Glyc
                                 agreement result
----------------------------------------------------------------------
contig039484-TilOR.L154    5 NASI   0.6714     (8/9)   +
contig039484-TilOR.L154    9 NVTH   0.7234     (9/9)   ++
contig039484-TilOR.L154  309 NTSD   0.4219     (8/9)   -
----------------------------------------------------------------------
```

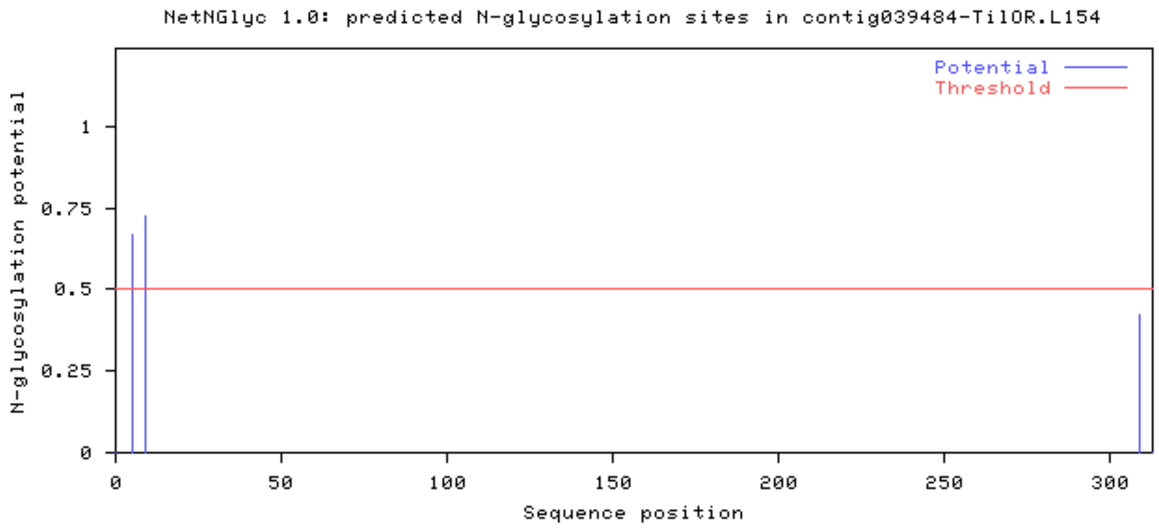

Graphics in PostScript

Output for 'contig039487-TilORp.L174'

```
##########################################################################################

    Warning: This sequence may not contain a signal peptide!!

    Proteins without signal peptides are unlikely to be exposed to
    the N-glycosylation machinery and thus may not be glycosylated
    (in vivo) even though they contain potential motifs.

    SignalP-NN euk predictions are as follows:

  # name                Cmax  pos ?  Ymax  pos ?  Smax  pos ?  Smean ?  D     ?

    SignalP output is explained at http://www.cbs.dtu.dk/services/SignalP/output.html

##########################################################################################

Name:  contig039487-TilORp.L174         Length:  313
MSSQNASINVTHFIIGGFDTLSRPIAVGVVILIIYLLAVLANMANIMFIISDKRLHKPMYLMICNLAVVDIVYTSSCSPT      80
MIGVLLAGVNTVSYVECLIQMCAFTLGTSMESFVLAFMALDRFIAIIYPFQYHSYLTNTRVLILTFIMWFVNWCFMCYMP     160
ATVVPLPHCSSRLIYSFCDFAAIIRTTCVNPEKYFNEAAIIMFFILFFTFIFISLSYCGILLFVKLSSNNGKKKMGSTLV     240
SHLICVILHYCPAFVHIIFTRFGVVLTLEERQGLVIGAVLGPCLVNPFVYCLRTKEIKQKLLKIFTHLTKILX
....N...N.......................................................................      80
................................................................................     160
................................................................................     240
.........................................................................            320

(Threshold=0.5)
----------------------------------------------------------------------
SeqName      Position  Potential   Jury    N-Glyc
                                 agreement result
----------------------------------------------------------------------
contig039487-TilORp.L174     5 NASI   0.6104     (7/9)    +
contig039487-TilORp.L174     9 NVTH   0.7450     (9/9)    ++
----------------------------------------------------------------------
```

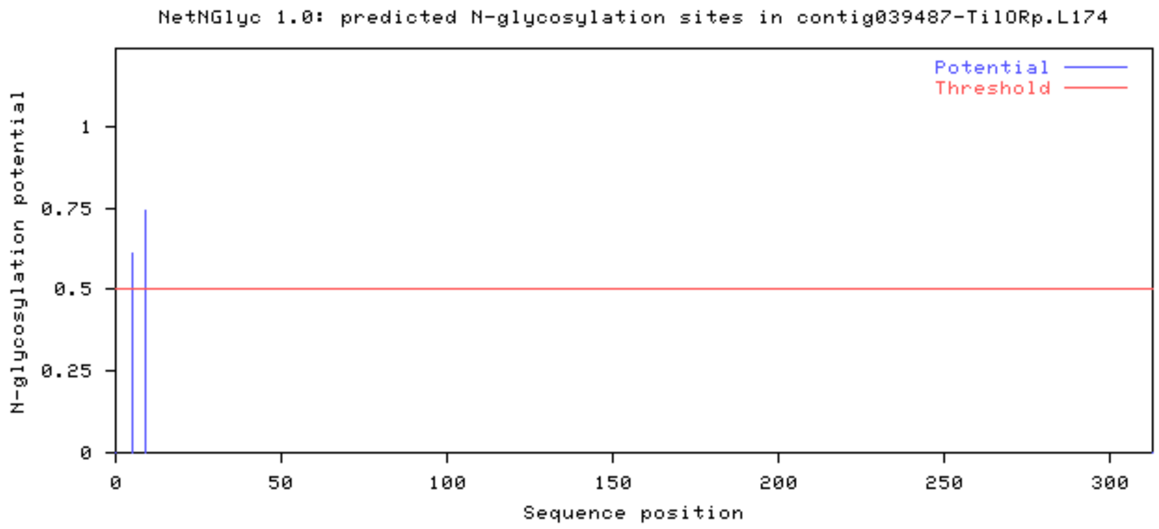

Graphics in PostScript

Output for 'contig041950-TilORe.A039'

```
##########################################################################################

    Warning: This sequence may not contain a signal peptide!!

    Proteins without signal peptides are unlikely to be exposed to
    the N-glycosylation machinery and thus may not be glycosylated
    (in vivo) even though they contain potential motifs.

    SignalP-NN euk predictions are as follows:

  # name                   Cmax   pos ?  Ymax   pos ?  Smax   pos ?  Smean ?  D     ?

    SignalP output is explained at http://www.cbs.dtu.dk/services/SignalP/output.html

##########################################################################################

Name:  contig041950-TilORe.A039         Length:  248
MDEELNTTYVTLDGYIEVNKYRYVYFCIIFTLYIIIICSNSTIVYVIWIHKNLHEPMYIFIAALLLNCLLCITSVYPKLL      80
IDFLSEKQVITYSACLFQFFIFYTLGSSELFLLAAMAYDRYVAICKPLEYPTIMNKTTVSIFLVVSWLIPAFHIAVQAIG     160
SAEATLCNFNLKGIFCNNAVYTLQCVRSRLIIVFGVVALIDLIILPLLFIVFTYTNIFIISYQSCKEIRKKAAETCLPHL     240
LVLISISC
.....N.................................N........................................      80
......................................................N.........................     160
................................................................................     240
........                                                                             320

(Threshold=0.5)
----------------------------------------------------------------------
SeqName      Position  Potential    Jury    N-Glyc
                                 agreement result
----------------------------------------------------------------------
contig041950-TilORe.A039    6 NTTY    0.7366     (9/9)   ++
contig041950-TilORe.A039   40 NSTI    0.6897     (8/9)   +
contig041950-TilORe.A039  135 NKTT    0.6257     (7/9)   +
----------------------------------------------------------------------
```

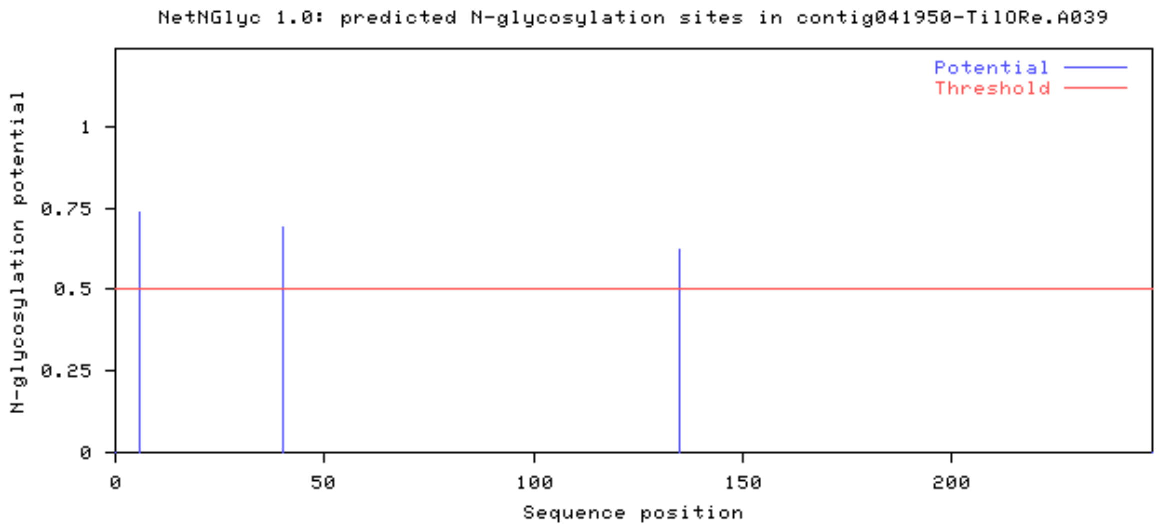

Graphics in PostScript

Output for 'contig041951-TilOR.A021'

```
##########################################################################################

    Warning: This sequence may not contain a signal peptide!!

    Proteins without signal peptides are unlikely to be exposed to
    the N-glycosylation machinery and thus may not be glycosylated
    (in vivo) even though they contain potential motifs.

    SignalP-NN euk predictions are as follows:

  # name                Cmax  pos ?  Ymax  pos ?  Smax  pos ?  Smean ?  D     ?

    SignalP output is explained at http://www.cbs.dtu.dk/services/SignalP/output.html

##########################################################################################

Name:  contig041951-TilOR.A021  Length:  307
MDNKLNLTYITLNGYVEVEKYRYVYFLIICTIYAAVIFSNSTIIYLIVIHQSLHEPMYIFIAALLINSIFYCTTIYPKFL      80
FDVLSEKQIVSHTMCHFQYFVLYTSGASEFLLLAAMAYDRYVSICKPLQYPVIMKKTTISVFLVLAWLVPACQVAGTTSL     160
SATRRMCNFTLRGIFCNNSIYKLHCVSSRVLSIYGVILLLNIVFFPMLYIVFTYSKILVISYQRCREVRKKAAQTCLPHL     240
LVLFNYSFFCTCEVIILRLESDISQTVRLIMTLQVVLYHPLFNPIIYGLKMKEISKHLKRLFCQEKX
.....N.................................N........................................      80
................................................................................     160
.......N........................................................................     240
....N..............................................................                 320

(Threshold=0.5)
----------------------------------------------------------------------
SeqName      Position  Potential   Jury    N-Glyc
                                 agreement result
----------------------------------------------------------------------
contig041951-TilOR.A021    6 NLTY   0.7540     (9/9)   +++
contig041951-TilOR.A021   40 NSTI   0.6574     (9/9)   ++
contig041951-TilOR.A021  168 NFTL   0.6488     (9/9)   ++
contig041951-TilOR.A021  177 NNSI   0.4476     (7/9)   -
contig041951-TilOR.A021  245 NYSF   0.5503     (5/9)   +
----------------------------------------------------------------------
```

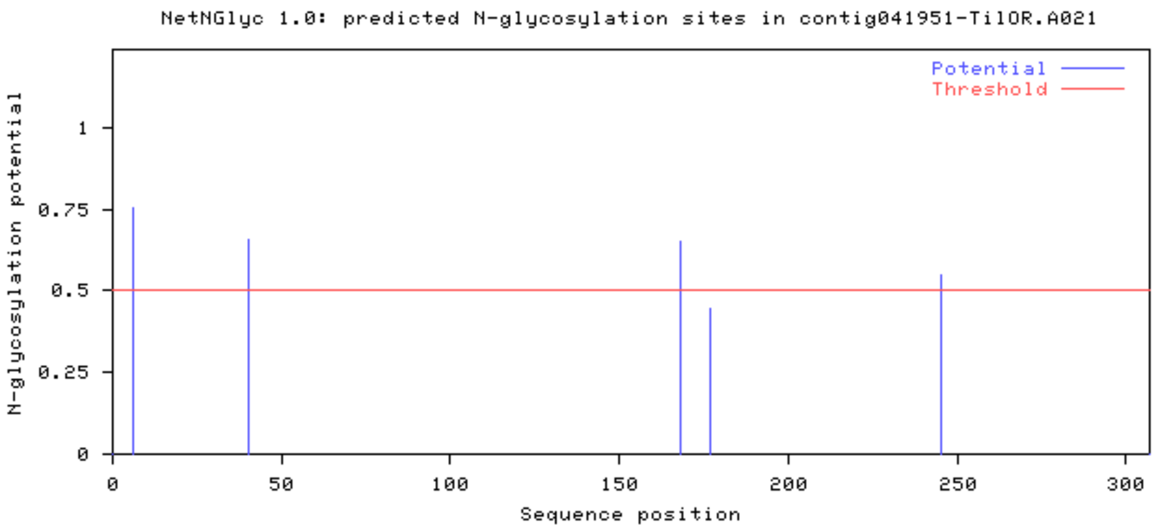

Graphics in PostScript

Output for 'contig041951-TilOR.A022'

```
##########################################################################################

    Warning: This sequence may not contain a signal peptide!!

    Proteins without signal peptides are unlikely to be exposed to
    the N-glycosylation machinery and thus may not be glycosylated
    (in vivo) even though they contain potential motifs.

    SignalP-NN euk predictions are as follows:

  # name                 Cmax  pos ?  Ymax  pos ?  Smax  pos ?  Smean ?  D     ?

    SignalP output is explained at http://www.cbs.dtu.dk/services/SignalP/output.html

##########################################################################################

Name:  contig041951-TilOR.A022  Length:  300
MNATYITFGGHVEVEKYRYLYFVVMFMVYVLIICSNSTIVWLIIVQKSLHEPMYIFIAALLVNSVVLSTVIYPKLLIDFL      80
SEKQIILYQACLFQVFLFYALSCSEFLLLSAMAYDRYVSICKPLQYPSIMRRTRVNIFLVLCWFLPAIQVAVPIAGNANT     160
PLCNFTLNGIFCNNSVNRLYCVNSRELSIYGMVVLFNVALSPMFFILFTYIKIIIVAYQSCGNVRKKAAQTCLPHVLVLI     240
NYSCLLTYDMVIVRLESEFPKTARFIMTLQFVTYNPLCNPIIYGLKMKEISKHLKILFSX
.N.................................N............................................      80
................................................................................     160
...N............................................................................     240
N...........................................................                         320

(Threshold=0.5)
----------------------------------------------------------------------
SeqName      Position  Potential   Jury    N-Glyc
                                 agreement result
----------------------------------------------------------------------
contig041951-TilOR.A022    2 NATY   0.7406     (9/9)    ++
contig041951-TilOR.A022   36 NSTI   0.6939     (9/9)    ++
contig041951-TilOR.A022  164 NFTL   0.6937     (9/9)    ++
contig041951-TilOR.A022  173 NNSV   0.4582     (5/9)    -
contig041951-TilOR.A022  241 NYSC   0.5044     (5/9)    +
----------------------------------------------------------------------
```

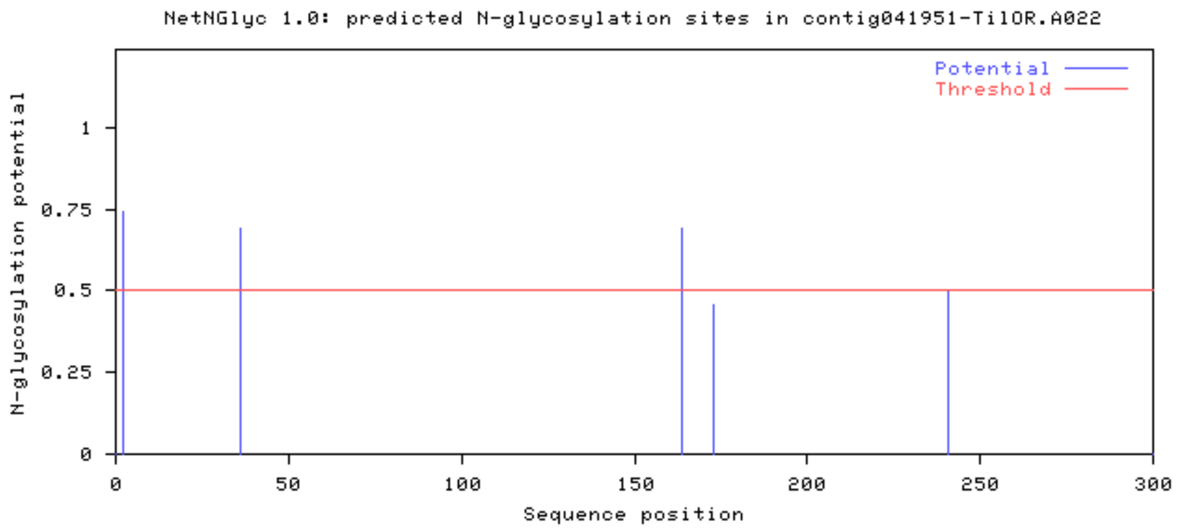

Graphics in PostScript

Output for 'contig041952-TilOR.A023'

```
##########################################################################################

    Warning: This sequence may not contain a signal peptide!!

    Proteins without signal peptides are unlikely to be exposed to
    the N-glycosylation machinery and thus may not be glycosylated
    (in vivo) even though they contain potential motifs.

    SignalP-NN euk predictions are as follows:

  # name                   Cmax  pos ?  Ymax  pos ?  Smax  pos ?  Smean ?  D     ?

    SignalP output is explained at http://www.cbs.dtu.dk/services/SignalP/output.html

##########################################################################################

Name:  contig041952-TilOR.A023  Length:  327
MDVELNVTLVTFGGFAELHKYRYLYFVVIFTLYILILCCNSTIVCLIWTHKNLHEPMYIFIAALLINSVLYSMTIYPKLL      80
SDVLSEKQIISYPLCVFQGFSYYTSGGSEFLLLAAMAYDRYVSICKPLQYPVIMNRITIYVCLILAWLIPAFETSVLGVL     160
YSNVKLCSFTLTGIFCNNSIYKLQCVPSVAISIYGVVMFINLVLLPMLFILFTYIRILRISYHCCRETRRKALKTCLPHL     240
LVLVNFSCFIFFDVIIVRLDSDLSKTLRLTLTFQSILFHPLLNPIIYGLKMNEISKHLKILLCQVYTGITAPLSNVWHII     320
CNCNANX
.....N................................N.........................................      80
................................................................................     160
................................................................................     240
....N...........................................................................     320
.......                                                                              400

(Threshold=0.5)
----------------------------------------------------------------------
SeqName      Position  Potential   Jury    N-Glyc
                                 agreement result
----------------------------------------------------------------------
contig041952-TilOR.A023    6 NVTL   0.8262     (9/9)   +++
contig041952-TilOR.A023   40 NSTI   0.7149     (9/9)   ++
contig041952-TilOR.A023  177 NNSI   0.4011     (8/9)   -
contig041952-TilOR.A023  245 NFSC   0.6381     (7/9)   +
----------------------------------------------------------------------
```

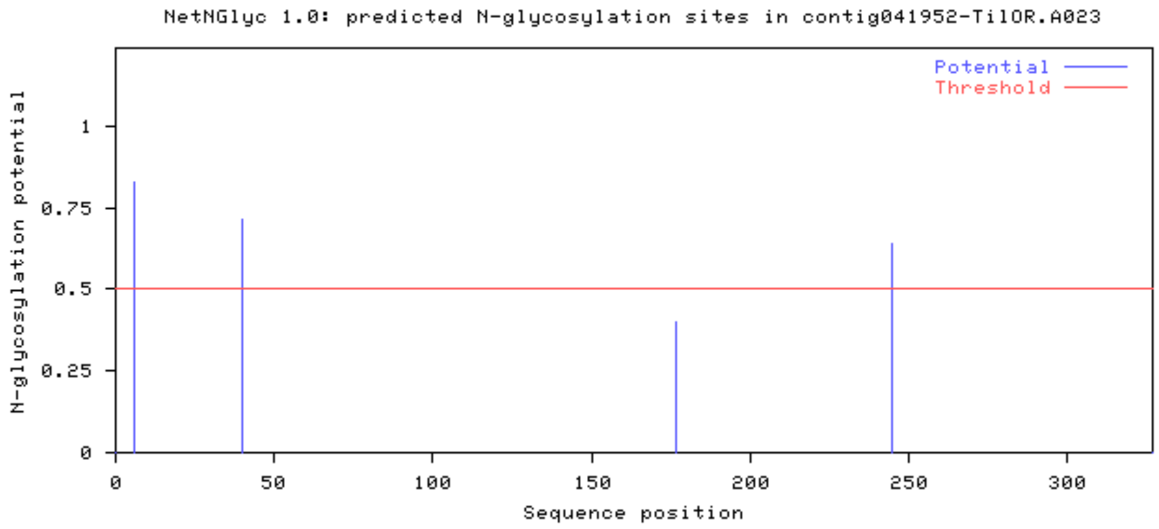

Graphics in PostScript

Output for 'contig041954-TilORe.A033'

```
##########################################################################################

    Warning: This sequence may not contain a signal peptide!!

    Proteins without signal peptides are unlikely to be exposed to
    the N-glycosylation machinery and thus may not be glycosylated
    (in vivo) even though they contain potential motifs.

    SignalP-NN euk predictions are as follows:

  # name                 Cmax  pos ?  Ymax  pos ?  Smax  pos ?  Smean ?  D     ?

    SignalP output is explained at http://www.cbs.dtu.dk/services/SignalP/output.html

##########################################################################################

Name:  contig041954-TilORe.A033         Length:  152
MDVKLNVTLLLTLGGFAELHKYRYLYFVVIFTLYILILCCNSTIVCLICSHKNLHEPMYIFIAALLINSVLYSMIIYPKLL      80
SDVLSQKQMISYPLCLFQGLSYYTSVGSEFLLLAAMAYDRYVSICKPLQYPVIMNRITIYVCLILAWLIPAF
.....N..................................N.......................................      80
........................................................................             160

(Threshold=0.5)
----------------------------------------------------------------------
SeqName      Position  Potential   Jury    N-Glyc
                                agreement  result
----------------------------------------------------------------------
contig041954-TilORe.A033    6 NVTL    0.7754     (9/9)    +++
contig041954-TilORe.A033   40 NSTI    0.7054     (9/9)    ++
----------------------------------------------------------------------
```

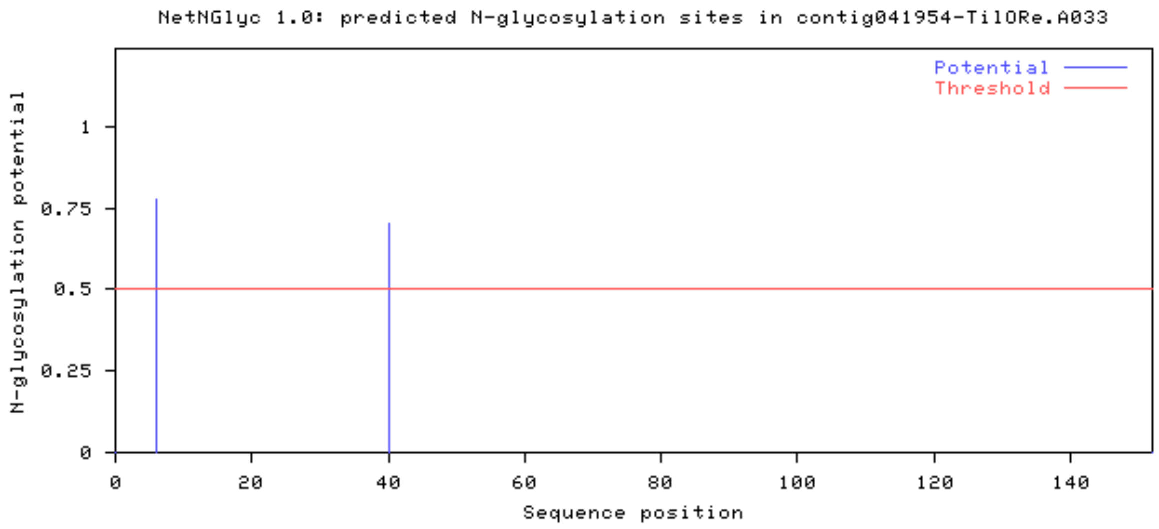

Graphics in PostScript

Output for 'contig041955-TilOR.H119'

```
##########################################################################

    Warning: This sequence may not contain a signal peptide!!

    Proteins without signal peptides are unlikely to be exposed to
    the N-glycosylation machinery and thus may not be glycosylated
    (in vivo) even though they contain potential motifs.

    SignalP-NN euk predictions are as follows:

  # name                Cmax  pos ?  Ymax  pos ?  Smax  pos ?  Smean ?  D     ?

    SignalP output is explained at http://www.cbs.dtu.dk/services/SignalP/output.html

##########################################################################

Name:  contig041955-TilOR.H119  Length:  310
MDNVSTVSIFNLLAFNDTVNHRAALFSVTLVCYFAILFLNVTVILIIVLDESLHEPMYILVCVCCINGLYGSTGFYPKFL      80
IDLLSSSQVISYTGCLCQAFVMYSFVCSDTSILAVMAYDRYLAICQPLQYHSVMTKKKLSKLVCYSWLTPFCIFSINIVL     160
TDRLIFCGTDIQRLFCVNWLIVKVACPGMDTLVNSAYAYTTLSIYIFHWIFIVWTYIYLVKSCVQSKKDKAKFMQTCVPH     240
LISLVTFFVIVISDLMHMRFASNDVPQSFLNFVAIAVLFIPPVMNPLLYGFKLTKIRNRILVALHIKRCX
..N............N.......................N........................................      80
................................................................................     160
................................................................................     240
......................................................................               320

(Threshold=0.5)
----------------------------------------------------------------------
SeqName      Position  Potential   Jury    N-Glyc
                                 agreement result
----------------------------------------------------------------------
contig041955-TilOR.H119    3 NVST   0.7421     (9/9)    ++
contig041955-TilOR.H119   16 NDTV   0.7621     (9/9)    +++
contig041955-TilOR.H119   40 NVTV   0.8147     (9/9)    +++
----------------------------------------------------------------------
```

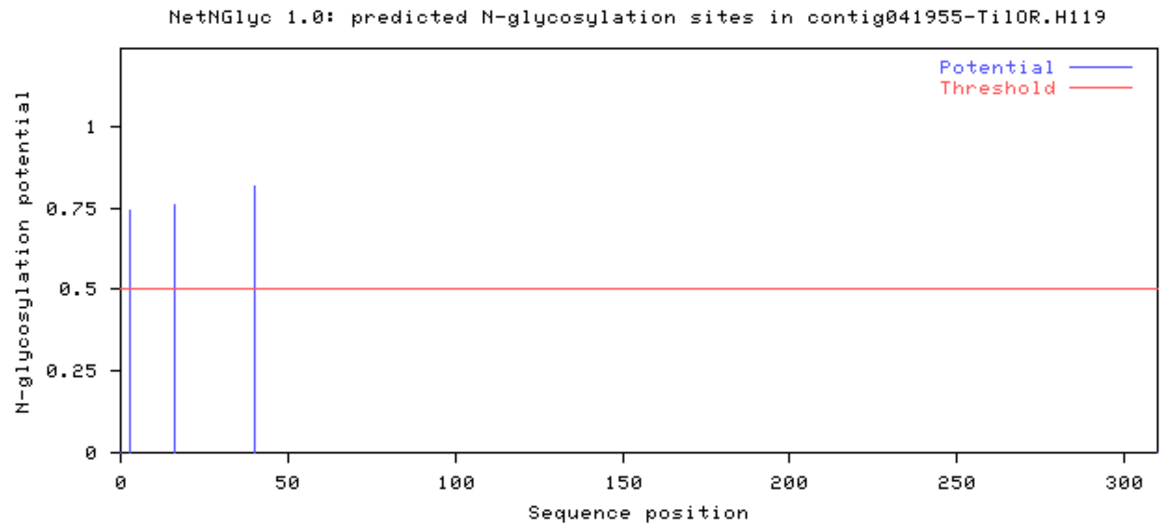

Graphics in PostScript

Output for 'contig042801-TilORe.C047'

```
##########################################################################################

    Warning: This sequence may not contain a signal peptide!!

    Proteins without signal peptides are unlikely to be exposed to
    the N-glycosylation machinery and thus may not be glycosylated
    (in vivo) even though they contain potential motifs.

    SignalP-NN euk predictions are as follows:

  # name                    Cmax   pos ?  Ymax   pos ?  Smax   pos ?  Smean ?  D     ?

    SignalP output is explained at http://www.cbs.dtu.dk/services/SignalP/output.html

##########################################################################################

Name:  contig042801-TilORe.C047         Length:  245
AIMAVLVSKTHDVTVKWCMAQVYFLHTYASGEFCILALMGYDRYIAICSPLHYYSIMSYSKTCKLIAFTGLYPFIVFTSF      80
YSLTLQLRFCGKVMPKLYCVNMELVKNSCTNAQYISTVGLAIIPLFIVPQLVMIVFSYTHILRVCRTLPKESQANAFRTC     160
VPHLLSLLNYTIASLFEIIQTRFNMSHVAVEARIFLSLYFVIIPPIANPVLYGLGTQTVRGCIMKLFIKNKVMTTVLAKT     240
LTVGX
................................................................................      80
................................................................................     160
........N.......................................................................     240
.....                                                                                320

(Threshold=0.5)
----------------------------------------------------------------------
SeqName      Position  Potential   Jury    N-Glyc
                                 agreement result
----------------------------------------------------------------------
contig042801-TilORe.C047  169 NYTI   0.6831     (9/9)   ++
contig042801-TilORe.C047  184 NMSH   0.4749     (5/9)   -
----------------------------------------------------------------------
```

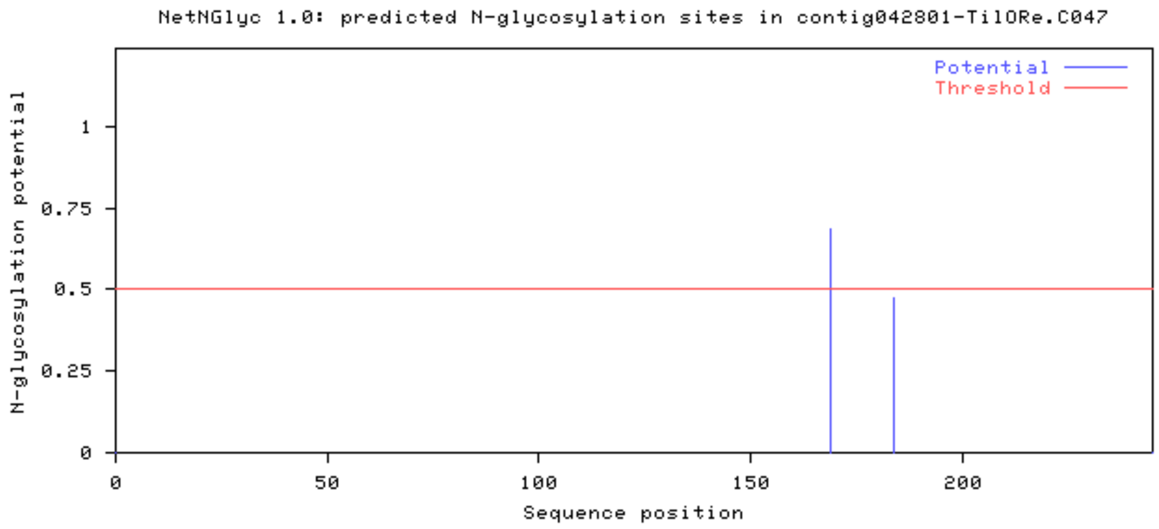

Graphics in PostScript

Output for 'contig042818-TilORp.Q244'

```
##########################################################################################

    Warning: This sequence may not contain a signal peptide!!

    Proteins without signal peptides are unlikely to be exposed to
    the N-glycosylation machinery and thus may not be glycosylated
    (in vivo) even though they contain potential motifs.

    SignalP-NN euk predictions are as follows:

  # name                  Cmax   pos ?  Ymax   pos ?  Smax   pos ?  Smean ?  D     ?

    SignalP output is explained at http://www.cbs.dtu.dk/services/SignalP/output.html

##########################################################################################

Name:  contig042818-TilORp.Q244         Length:  298
MNGTTAEFQSLLFQTSVKAALSMLPCFLLPVCMMFALLKKPLLLESSRYILFGHLLICDSVQLLMTVMLYIFAVTMVRMI      80
NYVCVFVSLVAAVTFKKSPLNLAVMSLERYVAVCFPLRHPSFATPRSTGKAIAVMWMVASLDSFIQLFLFVRMEKTSFPM     160
QSFCIRNSVFRLEVYVTLNMAFTILYFVFVSMIIIYTYTAIMITVKLASSRGCHTNKAQLWLYLTSTLFNTINPSMMLKV     240
PPDVAIHVQYVLFVGLIIFPKCLSPLIYGLRDQTLCHVFKYYFTFGFKTSVKPSPLSX
.N..............................................................................      80
................................................................................     160
................................................................................     240
..........................................................                           320

(Threshold=0.5)
----------------------------------------------------------------------
SeqName      Position  Potential   Jury    N-Glyc
                                 agreement result
----------------------------------------------------------------------
contig042818-TilORp.Q244    2 NGTT   0.7404     (9/9)   ++
contig042818-TilORp.Q244  233 NPSM   0.3854     (8/9)   -
----------------------------------------------------------------------
```

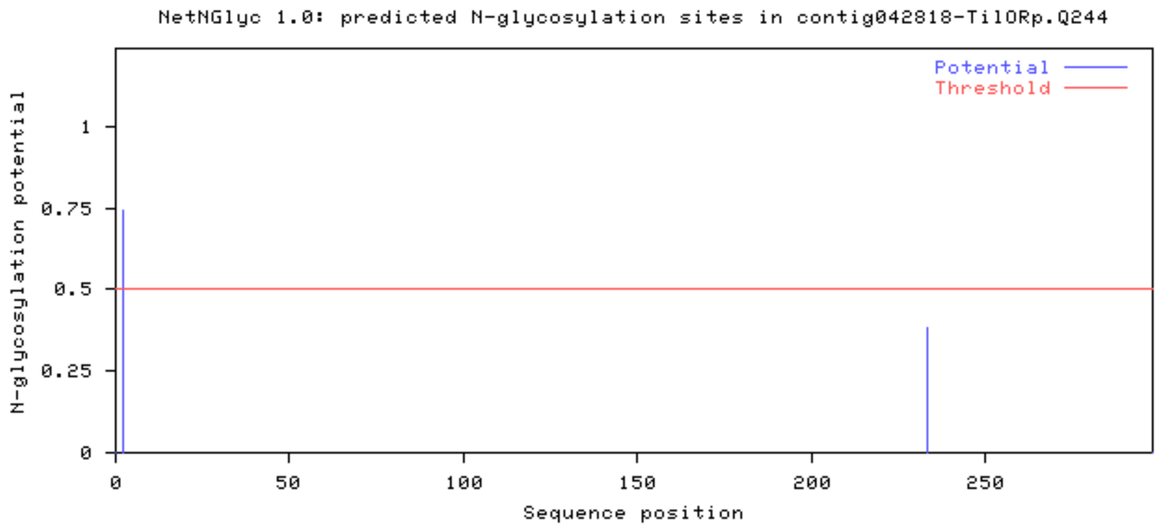

Graphics in PostScript

Output for 'contig046338-TilORe.M186'

```
##########################################################################################

    Warning: This sequence may not contain a signal peptide!!

    Proteins without signal peptides are unlikely to be exposed to
    the N-glycosylation machinery and thus may not be glycosylated
    (in vivo) even though they contain potential motifs.

    SignalP-NN euk predictions are as follows:

  # name                Cmax  pos ?  Ymax  pos ?  Smax  pos ?  Smean ?  D     ?

    SignalP output is explained at http://www.cbs.dtu.dk/services/SignalP/output.html

##########################################################################################

Name:  contig046338-TilORe.M186         Length:  56
TIGFFLGVVNPDQRVLSLSLASCIPPCINPIVYSLKTKEIKTGALKLFRKTKVIAX
........................................................                              80

(Threshold=0.5)

No sites predicted in this sequence.
```

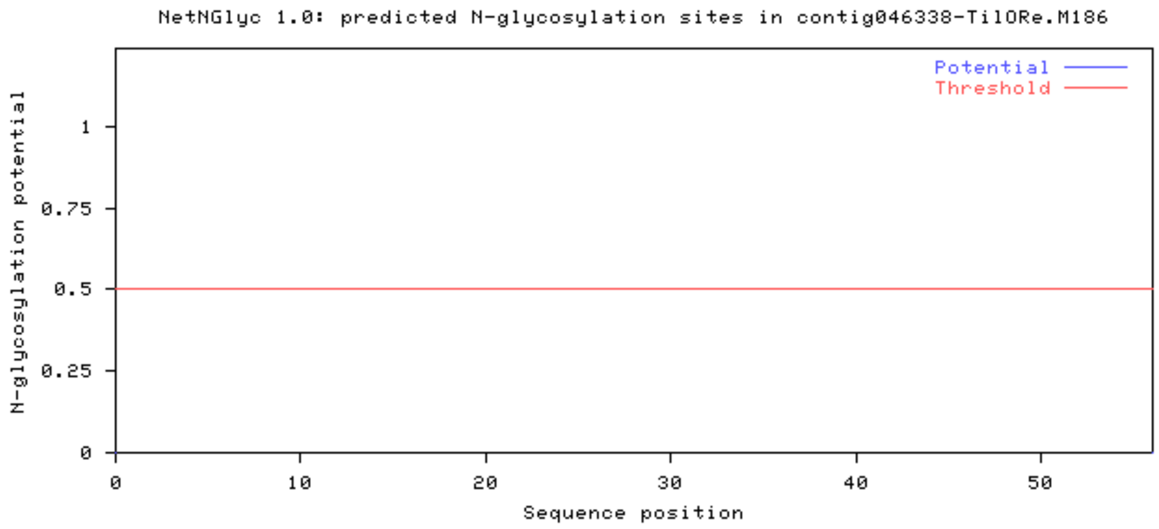

Graphics in PostScript

Output for 'contig046340-TilOR.N188'

```
##########################################################################

    Warning: This sequence may not contain a signal peptide!!

    Proteins without signal peptides are unlikely to be exposed to
    the N-glycosylation machinery and thus may not be glycosylated
    (in vivo) even though they contain potential motifs.

    SignalP-NN euk predictions are as follows:

  # name                    Cmax   pos ?  Ymax   pos ?  Smax   pos ?  Smean ?  D     ?

    SignalP output is explained at http://www.cbs.dtu.dk/services/SignalP/output.html

##########################################################################

Name:  contig046340-TilOR.N188  Length:  337
MGTEDKATTMFNNTFVRPEKFYLSGFSNIPHIRYYYAFLCFVYIMTVLGNGFLLSVIWLMKTLHTPKYMIVFNMALTDLC      80
GSTALIPKLLDTFLFDRRYIIYEACLSYMFFVMFFASVQSWTLVTMAYDRLIAICFPLRYHSIVTETSVAAILLFVWIFL     160
VSVIATMVGLLNRLSFCRSLVVNSFFCDHGPVYRLACNDTSLNYNMSSALITILIIIPLIFIIASYVCIFIALSRTTSRK     240
ERLRALKTCTSHLILVVMFFLPIGITNIATRASYIHPNARMINSTLTHTIPALLDPIVYALKTEEVMNAVKKLCKRIYLN     320
RMKAKTRPCNHCYIKSX
...........N....................................................................      80
................................................................................     160
.....................................N......N...................................     240
..........................................N.....................................     320
.................                                                                    400

(Threshold=0.5)
----------------------------------------------------------------------
SeqName      Position  Potential   Jury    N-Glyc
                                 agreement result
----------------------------------------------------------------------
contig046340-TilOR.N188   12 NNTF   0.5687     (6/9)   +
contig046340-TilOR.N188  198 NDTS   0.6424     (7/9)   +
contig046340-TilOR.N188  205 NMSS   0.6854     (8/9)   +
contig046340-TilOR.N188  283 NSTL   0.5838     (7/9)   +
----------------------------------------------------------------------
```

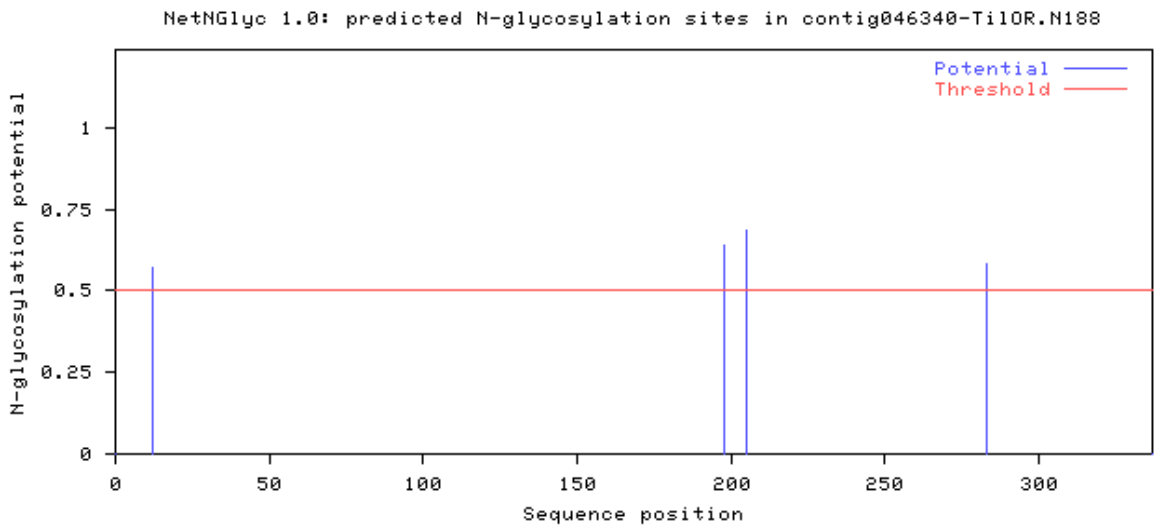

Graphics in PostScript

Output for 'contig046340-TilORe.M187'

```
##########################################################################################

    Warning: This sequence may not contain a signal peptide!!

    Proteins without signal peptides are unlikely to be exposed to
    the N-glycosylation machinery and thus may not be glycosylated
    (in vivo) even though they contain potential motifs.

    SignalP-NN euk predictions are as follows:

  # name                 Cmax  pos ?  Ymax  pos ?  Smax  pos ?  Smean ?  D     ?

    SignalP output is explained at http://www.cbs.dtu.dk/services/SignalP/output.html

##########################################################################################

Name:  contig046340-TilORe.M187         Length:  53
FFLGVVNPDQRVLSLSLASCIPPCINPIVYSLKTKDIKTGALTLFRTTKVIAX
.....................................................                                   80

(Threshold=0.5)

No sites predicted in this sequence.
```

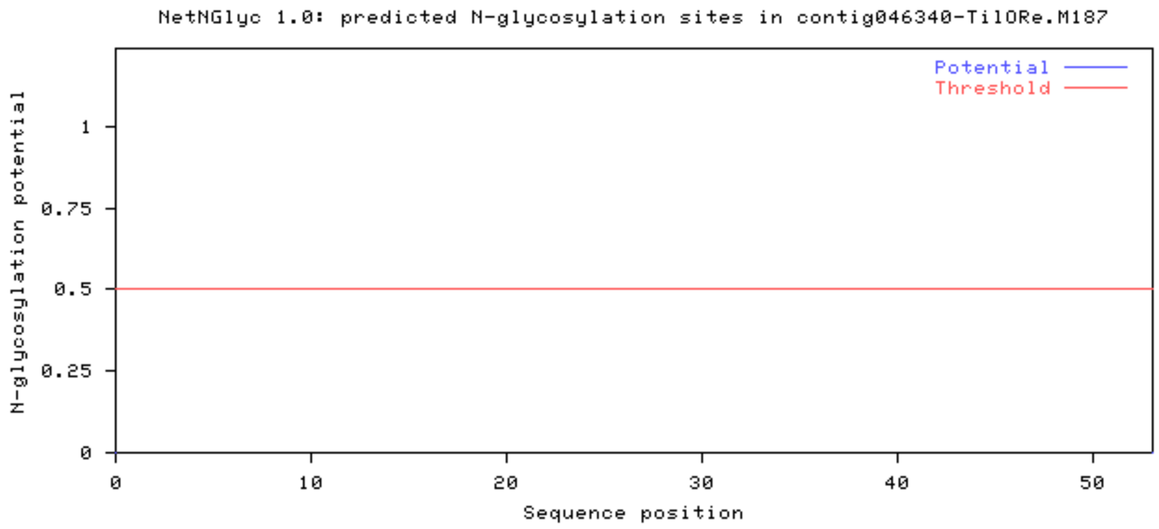

Graphics in PostScript

Output for 'contig046342-TilORe.N199'

```
##########################################################################################

    Warning: This sequence may not contain a signal peptide!!

    Proteins without signal peptides are unlikely to be exposed to
    the N-glycosylation machinery and thus may not be glycosylated
    (in vivo) even though they contain potential motifs.

    SignalP-NN euk predictions are as follows:

  # name                Cmax  pos ?  Ymax  pos ?  Smax  pos ?  Smean ?  D     ?

    SignalP output is explained at http://www.cbs.dtu.dk/services/SignalP/output.html

##########################################################################################

Name:  contig046342-TilORe.N199         Length:  85
MLYSILLFLCLGKQIFAMGAEDKTITIFNITFVHPAKFYLSGFSNIPHIRYFYIFLCFVYIMTVLGNGFLLSVIWLVKTL      80
HTPKY
............................N...................................................      80
.....                                                                                160

(Threshold=0.5)
----------------------------------------------------------------------
SeqName      Position  Potential   Jury    N-Glyc
                                 agreement result
----------------------------------------------------------------------
contig046342-TilORe.N199   29 NITF   0.5598     (6/9)   +
----------------------------------------------------------------------
```

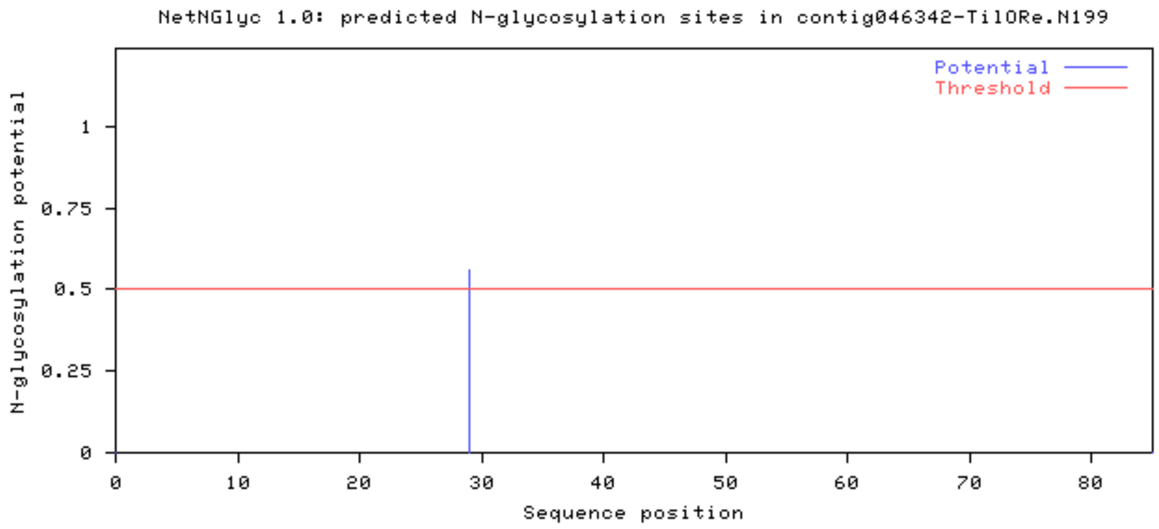

Graphics in PostScript

Output for 'contig046343-TilORe.N200'

```
##########################################################################################

    Warning: This sequence may not contain a signal peptide!!

    Proteins without signal peptides are unlikely to be exposed to
    the N-glycosylation machinery and thus may not be glycosylated
    (in vivo) even though they contain potential motifs.

    SignalP-NN euk predictions are as follows:

  # name                 Cmax  pos ?  Ymax  pos ?  Smax  pos ?  Smean ?  D     ?

    SignalP output is explained at http://www.cbs.dtu.dk/services/SignalP/output.html

##########################################################################################

Name:  contig046343-TilORe.N200         Length:  85
MFYSILLFLCLGKQIFAMGTEDKTTTIFNNTFVRPEKFYLSGFSNIPHIRYYYAFLCFVYIMTVLGNGFLLSVIWLVKTL      80
HTPKY
............................N...................................................      80
.....                                                                                160

(Threshold=0.5)
----------------------------------------------------------------------
SeqName      Position  Potential   Jury    N-Glyc
                                 agreement result
----------------------------------------------------------------------
contig046343-TilORe.N200   29 NNTF   0.5149     (6/9)   +
----------------------------------------------------------------------
```

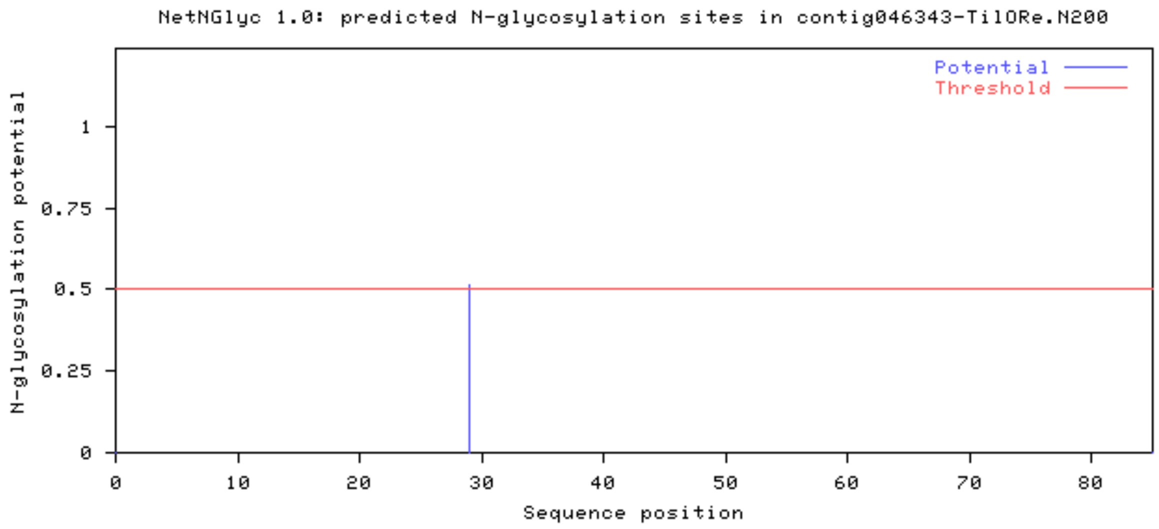

Graphics in PostScript

Output for 'contig046344-TilOR.N189'

```
##########################################################################

    Warning: This sequence may not contain a signal peptide!!

    Proteins without signal peptides are unlikely to be exposed to
    the N-glycosylation machinery and thus may not be glycosylated
    (in vivo) even though they contain potential motifs.

    SignalP-NN euk predictions are as follows:

  # name                Cmax  pos ?  Ymax  pos ?  Smax  pos ?  Smean ?  D     ?

    SignalP output is explained at http://www.cbs.dtu.dk/services/SignalP/output.html

##########################################################################

Name:  contig046344-TilOR.N189  Length:  328
MGSEDQATTMFNNTFVRPAKFYISGFSNIPHIRYFYVFLCFVYIMTVLGNGFLLLVIWLVKTLHTPKYMIVFNMALTDLC      80
GSTALIPKVLDTFLFDRRYIIYDACLSYMFFVMFFGSVQSWTLVIMAYDRLIAICLPLRYHNIVTETSITAILLFVWCFF     160
VIAIATMIGLVNRLSFCRSLVINSFFCDHGPTFRLACNDTTVNFNLVISLIIIILIIPLAFIMATYICISIALSRTAFRE     240
KRIRALKTCTSHLILVAIFFLPWTGTNLAAVTSYIHPNARMINSALTHTIPPLLNPIIYALKTEEVMNAIKKLCKRHYFS     320
NKKKGKTX
...........N....................................................................      80
................................................................................     160
.....................................N..........................................     240
................................................................................     320
........                                                                             400

(Threshold=0.5)
----------------------------------------------------------------------
SeqName      Position  Potential   Jury    N-Glyc
                                 agreement result
----------------------------------------------------------------------
contig046344-TilOR.N189   12 NNTF   0.5684     (6/9)   +
contig046344-TilOR.N189  198 NDTT   0.6422     (9/9)   ++
----------------------------------------------------------------------
```

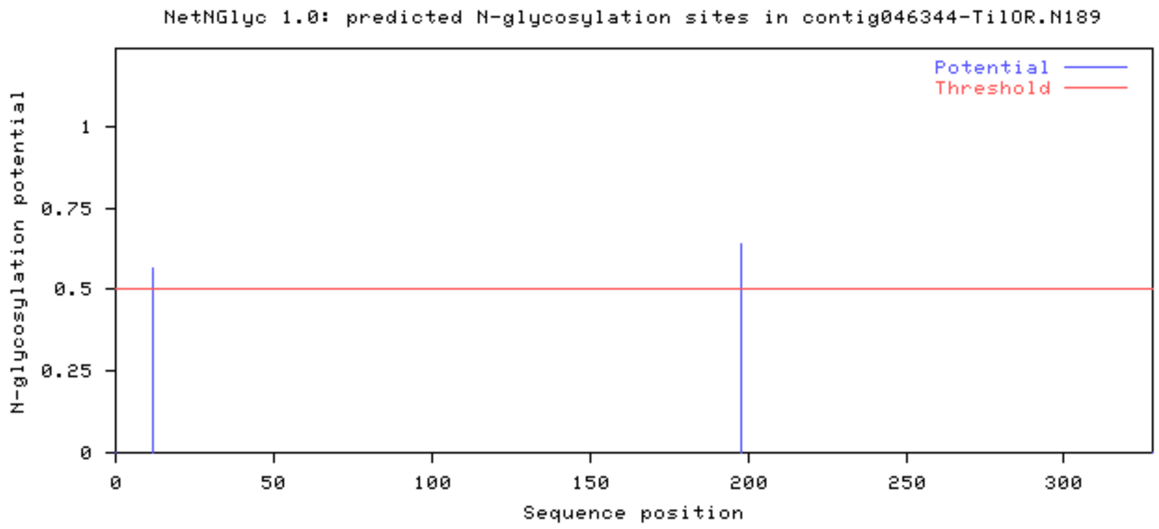

Graphics in PostScript

Output for 'contig046347-TilOR.N190'

```
##########################################################################################

    Warning: This sequence may not contain a signal peptide!!

    Proteins without signal peptides are unlikely to be exposed to
    the N-glycosylation machinery and thus may not be glycosylated
    (in vivo) even though they contain potential motifs.

    SignalP-NN euk predictions are as follows:

  # name                 Cmax  pos ?  Ymax  pos ?  Smax  pos ?  Smean ?  D     ?

    SignalP output is explained at http://www.cbs.dtu.dk/services/SignalP/output.html

##########################################################################################

Name:  contig046347-TilOR.N190  Length:  324
MGLEQKLIIGFNATFVHPGRFYLGGFSDMAHDNYYYVFLGFVYIFTVVGNVLFILIIFLIKTLHTPKYMIVFSLALTDLC      80
GSTALIPKLLDTFLFDRRYILYEACLSYMFFVLFFASIQSWTLVIMAYDRFIAIWFPLRYHSIVTKTSIAAMLAFEWLLI     160
TTIVASTVGLIDRLSFCGSFVVKSFFCDHGPVFYLACNDTSLNRIMAYVALVSFICIPLILIAFTYVCISIALSRIASGE     240
ERLKALKTCTSHVILVAVFFLPLVGTNIAAVASYIHPNARIINSTLTYTIPALLNPIIYALKTEEVMNAVKKLWKKKTFI     320
NTAX
...........N....................................................................      80
................................................................................     160
.....................................N..........................................     240
..........................................N.....................................     320
....                                                                                 400

(Threshold=0.5)
----------------------------------------------------------------------
SeqName      Position  Potential   Jury    N-Glyc
                                 agreement result
----------------------------------------------------------------------
contig046347-TilOR.N190   12 NATF   0.5632     (6/9)    +
contig046347-TilOR.N190  198 NDTS   0.7052     (9/9)    ++
contig046347-TilOR.N190  283 NSTL   0.5692     (8/9)    +
----------------------------------------------------------------------
```

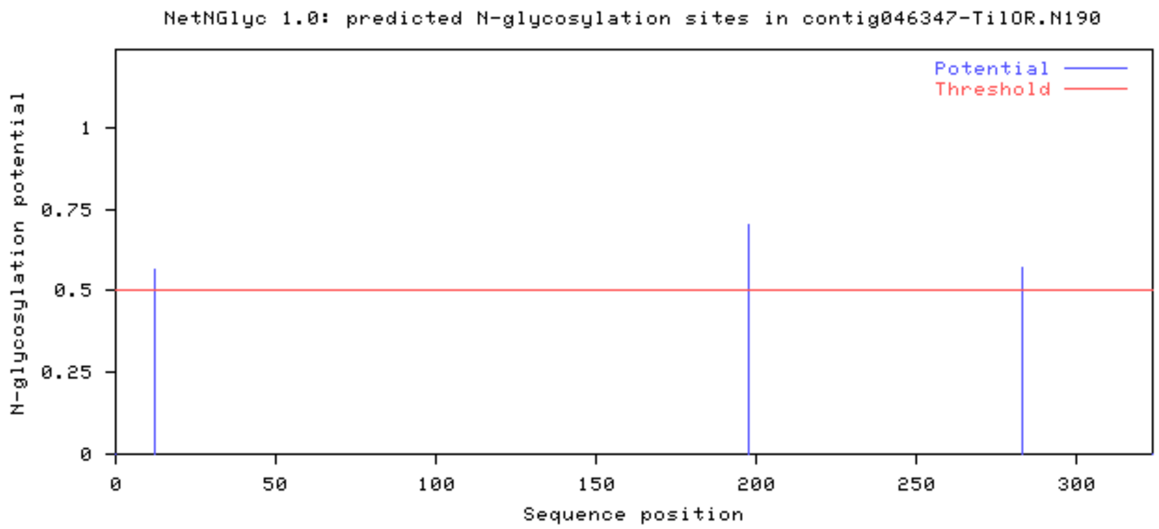

Graphics in PostScript

Output for 'contig046350-TilOR.N191'

```
########################################################################################

    Warning: This sequence may not contain a signal peptide!!

    Proteins without signal peptides are unlikely to be exposed to
    the N-glycosylation machinery and thus may not be glycosylated
    (in vivo) even though they contain potential motifs.

    SignalP-NN euk predictions are as follows:

  # name                  Cmax  pos ?  Ymax  pos ?  Smax  pos ?  Smean ?  D     ?

    SignalP output is explained at http://www.cbs.dtu.dk/services/SignalP/output.html

########################################################################################

Name:  contig046350-TilOR.N191  Length:  319
MFNTTFIRPAKFYLGGFSNIPHIRYFYIFLCFVYIVTVLGNGFLLSVIWLVKTLHTPKYMIVFNMALTDLCGSTALIPKV      80
LDTFLFDRRYIVYEACLSYMFFVIFFASVQSWTLVTMAYDRLIAICLPLRYHNIVTETSITAILLFVWCFFVSAIATMVG     160
LLNRLSFCSSLVINSFFCDHGPVYRLACNDISINQNLASAIVSIILIIPLAFIVATYICISIAVSRTAFREERLRALKTC     240
TSHLILVAIFFLPWTGTNVAAVTSYIHPNARMINSALTHTIPPLLNPIIYALKTEEVMNAIKKLCKRHYFSNKKKGKTX
..N.............................................................................      80
................................................................................     160
................................................................................     240
...............................................................................      320

(Threshold=0.5)
----------------------------------------------------------------------
SeqName      Position  Potential   Jury    N-Glyc
                                 agreement result
----------------------------------------------------------------------
contig046350-TilOR.N191    3 NTTF   0.6722     (9/9)    ++
----------------------------------------------------------------------
```

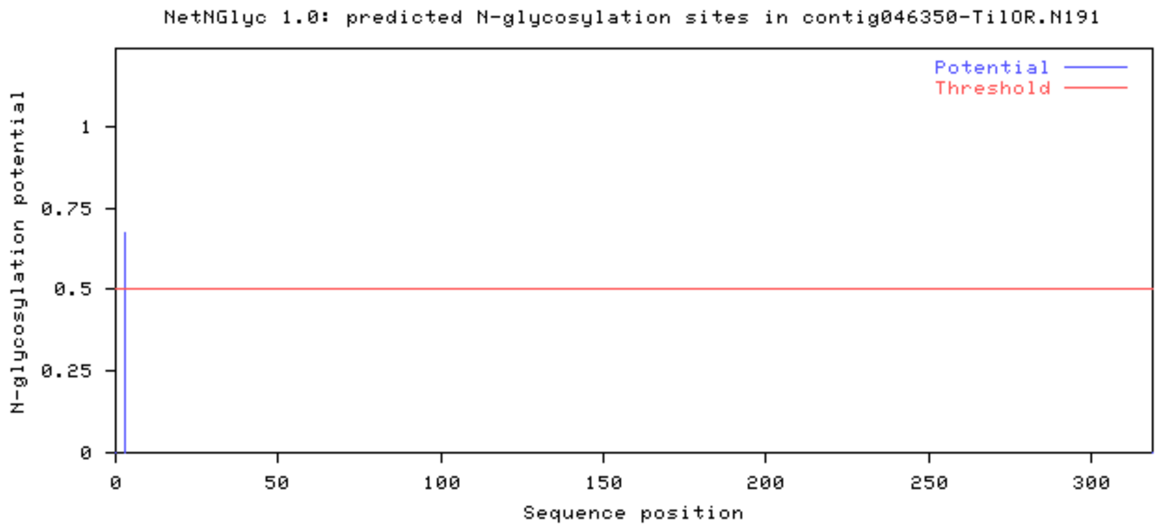

Graphics in PostScript

Output for 'contig046351-TilOR.N192'

```
##########################################################################################

    Warning: This sequence may not contain a signal peptide!!

    Proteins without signal peptides are unlikely to be exposed to
    the N-glycosylation machinery and thus may not be glycosylated
    (in vivo) even though they contain potential motifs.

    SignalP-NN euk predictions are as follows:

  # name                 Cmax  pos ?  Ymax  pos ?  Smax  pos ?  Smean ?  D     ?

    SignalP output is explained at http://www.cbs.dtu.dk/services/SignalP/output.html

##########################################################################################

Name:  contig046351-TilOR.N192  Length:  327
MGSEDQATTMFNNTFVRPEKFYLSGFSNIPHIRYFYVFLCFVYIMTVLGNGFLLLVIWLVKTLHTPKYMIVFNMALTDLC      80
GSTALIPKVLDTFLFDRRYIFYDPCLGYMFFVVFFASMQSWTLVAMAYDRLIAICLPLRYHSIVTEASIAAVLLFVWIFL     160
VSVIATMVGLINHLSFCRSLVINSFYCDHGPVFRLACNDTSVNFNLAISLVIIILIIPLAFIVVTYVYISIALRRTTSRE     240
ERVRALKTCTSHLILVVMFFLPVGITNIVAWASYIDPNARMINYTLTHTIPPLLNPIVYALKTEEVKNAIKKLCKRNNVS     320
NTVTKCX
...........N....................................................................      80
................................................................................     160
.....................................N..........................................     240
..........................................N..................................N..     320
.......                                                                              400

(Threshold=0.5)
----------------------------------------------------------------------
SeqName      Position  Potential   Jury    N-Glyc
                                 agreement result
----------------------------------------------------------------------
contig046351-TilOR.N192   12 NNTF   0.5668     (6/9)   +
contig046351-TilOR.N192  198 NDTS   0.7217     (9/9)   ++
contig046351-TilOR.N192  283 NYTL   0.6698     (9/9)   ++
contig046351-TilOR.N192  318 NVSN   0.7088     (8/9)   +
----------------------------------------------------------------------
```

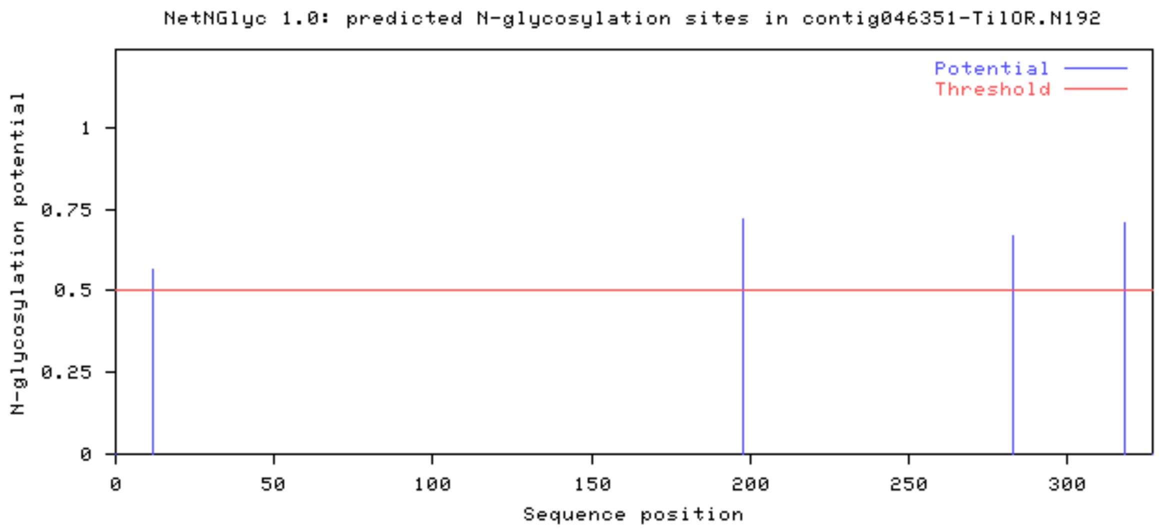

Graphics in PostScript

Output for 'contig046352-TilOR.N193'

```
##########################################################################################

    Warning: This sequence may not contain a signal peptide!!

    Proteins without signal peptides are unlikely to be exposed to
    the N-glycosylation machinery and thus may not be glycosylated
    (in vivo) even though they contain potential motifs.

    SignalP-NN euk predictions are as follows:

  # name                 Cmax  pos ?  Ymax  pos ?  Smax  pos ?  Smean ?  D     ?

    SignalP output is explained at http://www.cbs.dtu.dk/services/SignalP/output.html

##########################################################################################

Name:  contig046352-TilOR.N193  Length:  324
MGLEQKLIIGFNATFVHPGRFYLGGFSDMAHDNYYYIFLCFVYIFTVVGNVLLILIIFLIKTLHTPKYLIVFNLALTDLC      80
GSTALIPKLLDTFLFDRRYILYEACLSYMFFVLFFASIQSWTLVIMAYDRFIAICFPLRYHSIVTKTSIAAMLAFEWVLI     160
TSIMASTVGLIDRLSFCGSFVVKSFFCDHGPVFYLACNDTSLNRIMAYVALVSFICIPLILIAFTYVCISIALSRIASGE     240
ERLKALKTCTSHVILVAVFFLPLVGTNIAAVASYIHPNARIINSTLAYTIPALLNPIIYALKTEEVMNAVKKLWKKTPFI     320
NTAX
...........N....................................................................      80
................................................................................     160
.....................................N..........................................     240
................................................................................     320
....                                                                                 400

(Threshold=0.5)
----------------------------------------------------------------------
SeqName      Position  Potential   Jury    N-Glyc
                                 agreement result
----------------------------------------------------------------------
contig046352-TilOR.N193   12 NATF   0.5633     (6/9)   +
contig046352-TilOR.N193  198 NDTS   0.7052     (9/9)   ++
contig046352-TilOR.N193  283 NSTL   0.4889     (4/9)   -
----------------------------------------------------------------------
```

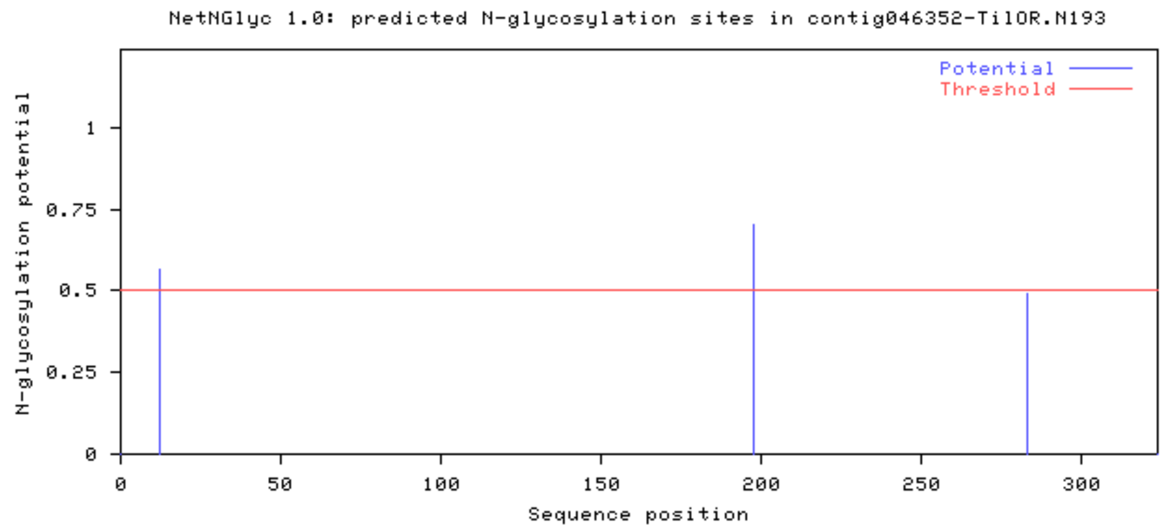

Graphics in PostScript

Output for 'contig046352-TilOR.N194'

```
##########################################################################################

    Warning: This sequence may not contain a signal peptide!!

    Proteins without signal peptides are unlikely to be exposed to
    the N-glycosylation machinery and thus may not be glycosylated
    (in vivo) even though they contain potential motifs.

    SignalP-NN euk predictions are as follows:

  # name                   Cmax  pos ?  Ymax  pos ?  Smax  pos ?  Smean ?  D     ?

    SignalP output is explained at http://www.cbs.dtu.dk/services/SignalP/output.html

##########################################################################################

Name:  contig046352-TilOR.N194  Length:  327
MDSDEEATTKFNNTFVRPAKFYLSGFSNIPHVKYFYVFLCFVYIMTVLGNGFLLSVIWLVKTLHTPKYMIVFNMALTDLC      80
GSTALIPKLLDTFLFDRRYIVYEACLSYMFFVLFFGGVQSWTLVTMAYDRLIAICFPLRYHSIVTKTAITSMLLFIWLLM     160
LSLTTLVVGLINRLSFCDSVVVKSFFCDHGPIYRLACNDPSLNIIMANVGVSIGVFIPLIFIACTYVCISIALSKIASGE     240
ERLKALKTCTSHLILVAILFLPFVGTNIAVWASYIHPNARIINSTLTHTIPALINPIVYALKTEEVMNAVKKLWKKKKIS     320
SGVTKCX
...........N....................................................................      80
................................................................................     160
................................................................................     240
..........................................N.....................................     320
.......                                                                              400

(Threshold=0.5)
----------------------------------------------------------------------
SeqName      Position  Potential   Jury    N-Glyc
                                 agreement result
----------------------------------------------------------------------
contig046352-TilOR.N194   12 NNTF   0.5388     (6/9)   +
contig046352-TilOR.N194  283 NSTL   0.5844     (8/9)   +
----------------------------------------------------------------------
```

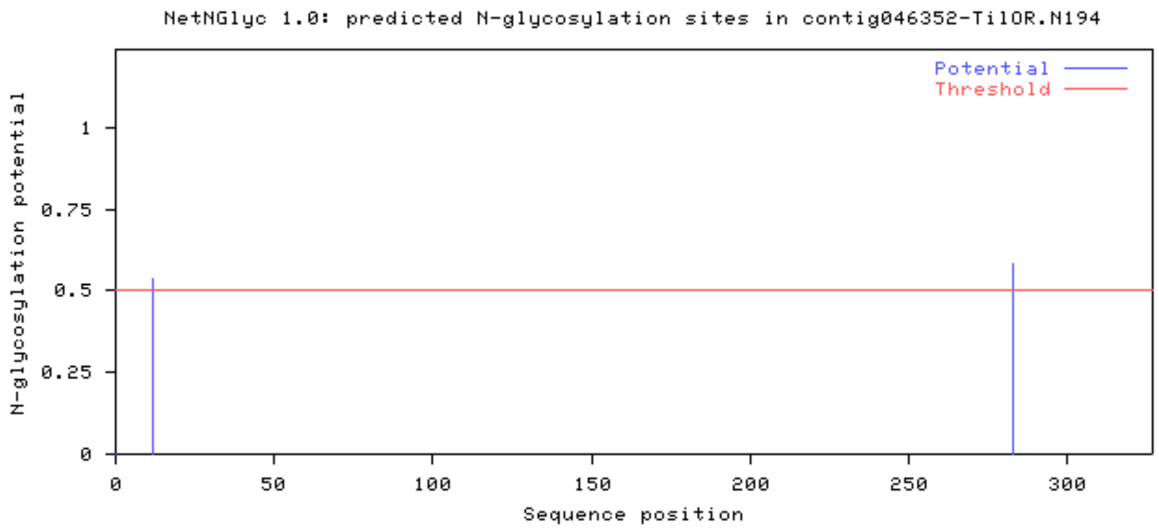

Graphics in PostScript

Output for 'contig046353-TilOR.N195'

```
##########################################################################################

    Warning: This sequence may not contain a signal peptide!!

    Proteins without signal peptides are unlikely to be exposed to
    the N-glycosylation machinery and thus may not be glycosylated
    (in vivo) even though they contain potential motifs.

    SignalP-NN euk predictions are as follows:

  # name                 Cmax  pos ?  Ymax  pos ?  Smax  pos ?  Smean ?  D     ?

    SignalP output is explained at http://www.cbs.dtu.dk/services/SignalP/output.html

##########################################################################################

Name:  contig046353-TilOR.N195  Length:  322
MELFNSALGKNITFVHPAFFIIGGLTGIPNITFYYVFLFFVYIVSVVGNTVVMAVICLDHNLRTPKYIAVFNLAFVDLFG      80
NTALVPKVLDIFLFGHYFIPYNDCLTFLFFCYTCLSLQSFNLVALSYDRMVAIIFPLHYQVKVTHRFMFSLIASLWVFTI     160
IAVLIAVGLLTRLSFCKSVVINSYFCDHGQIYRLACNDHFPSYVIACLYPVIIFWLPLAFILLSYLYIGYTLVKVATLQE     240
GLKAFKTCIGHLSLVAIYFIPLLTTFTLMEKIQPNARIINLSLTSVFPPMLNPIIYVLQTQEIKESLKRLLKRRGKSKIT     320
IX
..........N..................N..................................................      80
................................................................................     160
................................................................................     240
.......................................N........................................     320
..                                                                                   400

(Threshold=0.5)
----------------------------------------------------------------------
SeqName      Position  Potential   Jury    N-Glyc
                                 agreement result
----------------------------------------------------------------------
contig046353-TilOR.N195   11 NITF   0.6088     (8/9)   +
contig046353-TilOR.N195   30 NITF   0.7719     (9/9)   +++
contig046353-TilOR.N195  280 NLSL   0.6021     (8/9)   +
----------------------------------------------------------------------
```

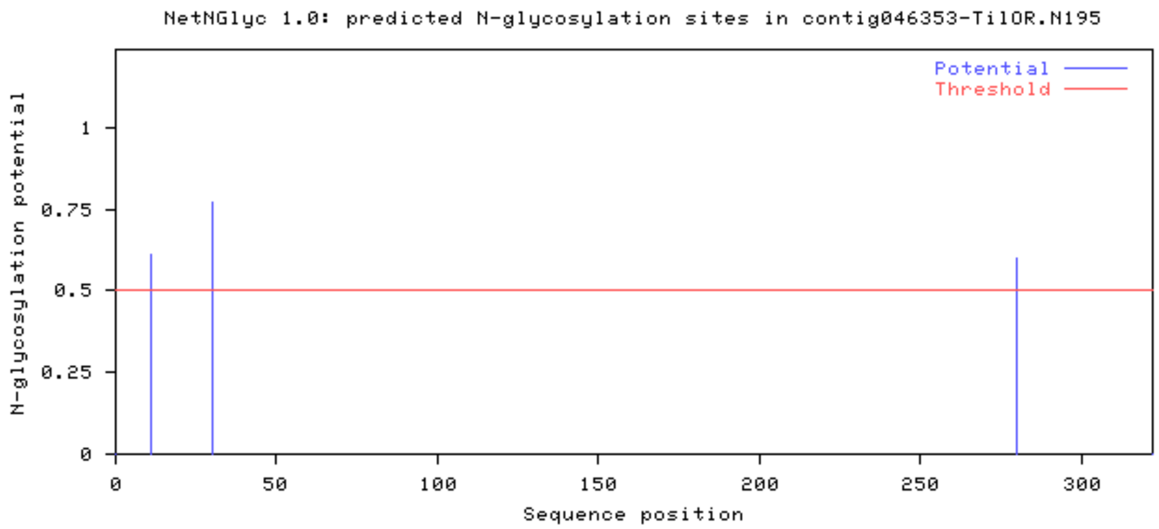

Graphics in PostScript

Output for 'contig046356-TilOR.N196'

```
##########################################################################################

    Warning: This sequence may not contain a signal peptide!!

    Proteins without signal peptides are unlikely to be exposed to
    the N-glycosylation machinery and thus may not be glycosylated
    (in vivo) even though they contain potential motifs.

    SignalP-NN euk predictions are as follows:

  # name                   Cmax  pos ?  Ymax  pos ?  Smax  pos ?  Smean ?  D     ?

    SignalP output is explained at http://www.cbs.dtu.dk/services/SignalP/output.html

##########################################################################################

Name:  contig046356-TilOR.N196  Length:  330
MDFLNSAAEKNTTFVRPANFIISGFVGIPNIRYYFVFLCFIYIFSVVGNTAVMLIIIFDHTLRSPKYIAVFNLAFTDLLS      80
NSALVPKVLDISLFNHHYISYNNCLTFMFFCFTLISMQAFNLVVLSFDRIMAIMYPLHYQMRVSHKIILSLITFFWLLAI     160
ALTGTAVGLLTRLHFCESVVINSYYCDHGPIYRLGCNDVTPNKTISAWSRAFVLWLPLIFVLGSYCCIGYSLSRISTCKE     240
RVKALKTCTGHLSLVAIYFIPILVVYSFGSTIHPNARIVNLSLASVMPPMLNPIIYVLQTAEIKKSLKRLLRAKIQICHT     320
ECCSLMKHMX
................................................................................      80
................................................................................     160
.........................................N......................................     240
.......................................N........................................     320
..........                                                                           400

(Threshold=0.5)
----------------------------------------------------------------------
SeqName      Position  Potential   Jury    N-Glyc
                                 agreement result
----------------------------------------------------------------------
contig046356-TilOR.N196   11 NTTF   0.4802     (5/9)   -
contig046356-TilOR.N196  202 NKTI   0.6154     (8/9)   +
contig046356-TilOR.N196  280 NLSL   0.5906     (8/9)   +
----------------------------------------------------------------------
```

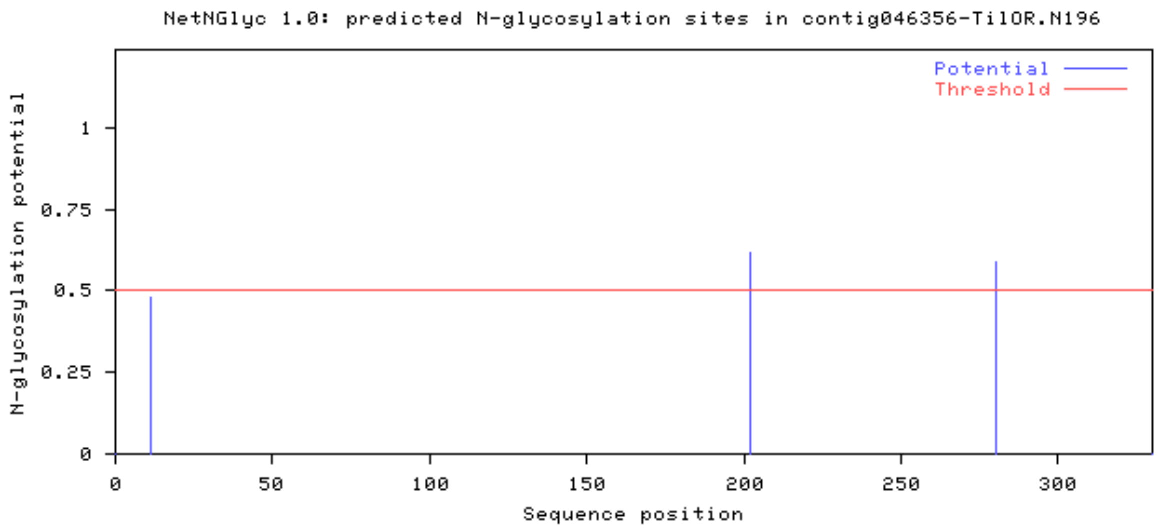

Graphics in PostScript

Output for 'contig013329-TilORe.D209'

```
##########################################################################################

    Warning: This sequence may not contain a signal peptide!!

    Proteins without signal peptides are unlikely to be exposed to
    the N-glycosylation machinery and thus may not be glycosylated
    (in vivo) even though they contain potential motifs.

    SignalP-NN euk predictions are as follows:

  # name                 Cmax  pos ?  Ymax  pos ?  Smax  pos ?  Smean ?  D     ?

    SignalP output is explained at http://www.cbs.dtu.dk/services/SignalP/output.html

##########################################################################################

Name:  contig013329-TilORe.D209         Length:  82
ENKQKAVTTCTPQIVSLSNLFVGCICYSIDFRFLFSQVPDEVRIILAIYLLICQPMLTPFMYGFNLPKIRQSCKRLLFKR      80
KX
................................................................................      80
..                                                                                   160

(Threshold=0.5)

No sites predicted in this sequence.
```

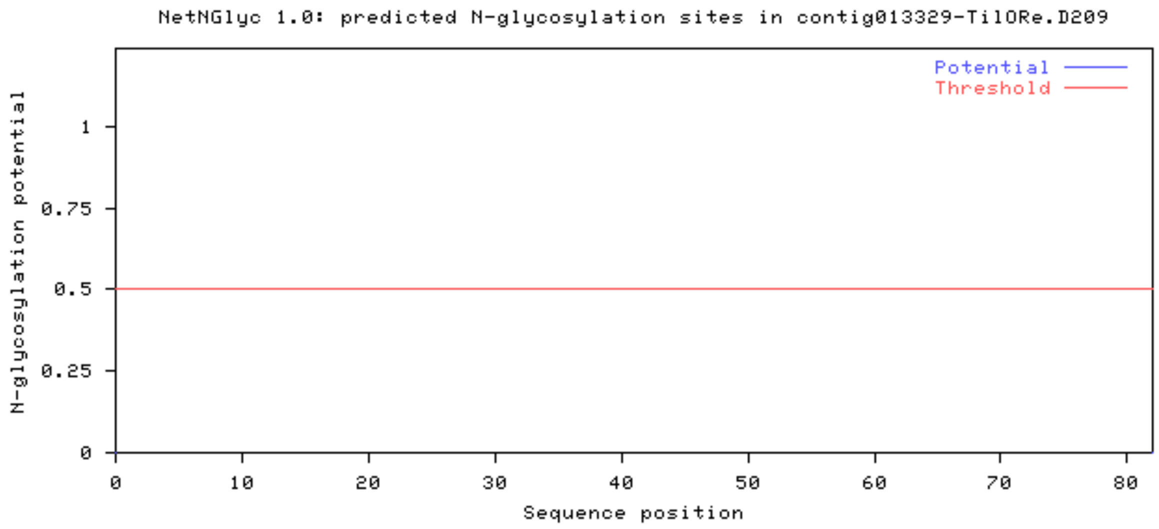

Graphics in PostScript

Output for 'contig046360-TilORe.N208'

```
##########################################################################

    Warning: This sequence may not contain a signal peptide!!

    Proteins without signal peptides are unlikely to be exposed to
    the N-glycosylation machinery and thus may not be glycosylated
    (in vivo) even though they contain potential motifs.

    SignalP-NN euk predictions are as follows:

  # name                Cmax  pos ?  Ymax  pos ?  Smax  pos ?  Smean ?  D     ?

    SignalP output is explained at http://www.cbs.dtu.dk/services/SignalP/output.html

##########################################################################

Name:  contig046360-TilORe.N208        Length:  273
IYLDHNLRTPKYIAVFNLALVDLLGNSAMVPKVLDIFLFNHPHISYNDCLTFLFFCYVFLSMQALNLVALSYDRVMAIVY      80
PLHYQLKLTHKLMFSLIASFWVFVIIVVLIATGLLTRLSFCESVVIKSYFCDHGQIYRLACNDFTPSDITAWILPALILW     160
LPLTIVLLSYLIIGYALAKVATVRERMKGFKTCTAHLSLVAIYFLPILITFTLRANIEPNARIINLSLTSVFPPMLNPII     240
YVLQTREIKESLKKLQGRIITHYKNRKVKFKKX
................................................................................      80
................................................................................     160
................................................................N...............     240
.................................                                                    320

(Threshold=0.5)
----------------------------------------------------------------------
SeqName      Position  Potential   Jury    N-Glyc
                                 agreement result
----------------------------------------------------------------------
contig046360-TilORe.N208  225 NLSL   0.6123     (9/9)   ++
----------------------------------------------------------------------
```

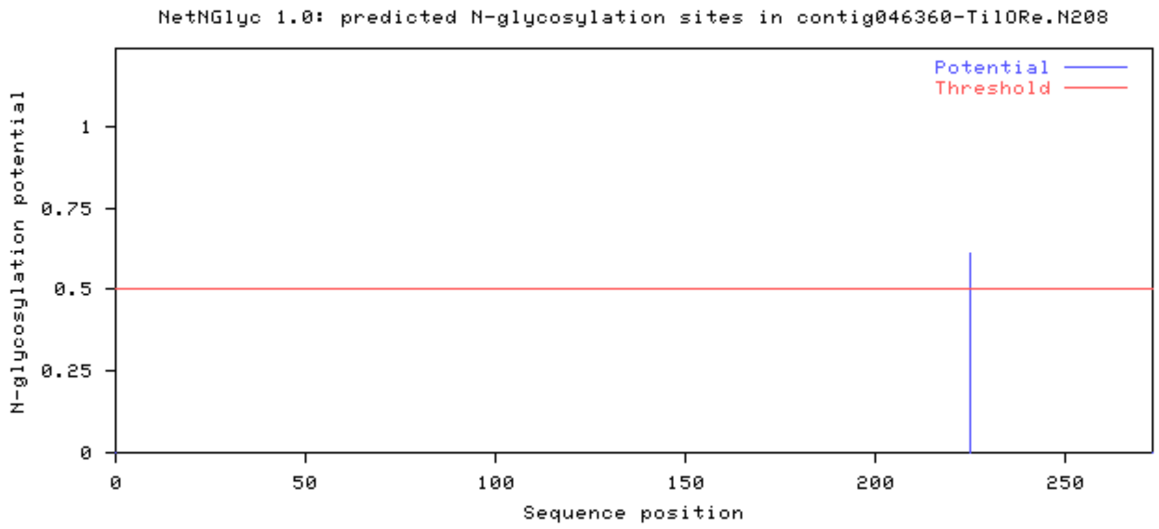

Graphics in PostScript

Output for 'contig046367-TilORe.N201'

```
##########################################################################

    Warning: This sequence may not contain a signal peptide!!

    Proteins without signal peptides are unlikely to be exposed to
    the N-glycosylation machinery and thus may not be glycosylated
    (in vivo) even though they contain potential motifs.

    SignalP-NN euk predictions are as follows:

  # name                Cmax  pos ?  Ymax  pos ?  Smax  pos ?  Smean ?  D     ?

    SignalP output is explained at http://www.cbs.dtu.dk/services/SignalP/output.html

##########################################################################

Name:  contig046367-TilORe.N201         Length:  325
MALINSAAENNITFVQPAYFIISGFIGIPNIRYYFVFLCFIYILAVVGNTLVILVITLDHMLRSPKYIAVFNLAFTDLLS      80
SSALVPKVVDIFLFNHYHISYNDCLTFMFFCFTSIFMQAFNLVVLSFDRVMAIMYPLHYQMRVTHKLILSLIAFFWLLAI     160
TLILIAVGLLTRLSFCQSVIIQSYFCDHGPMYRLGCNDVTPNYIFAVLTIVLVLGFPLAFIVGSYCCIGYSLSKISTFRE     240
RVKAFKTCTGHLSLVAIYFLPVIFVYAFGPVIHPNARIINLSIATVLPPMLNPIIYVLQTQEIKESLRRLLKARVQAKLE     320
VFRFK
..........N.....................................................................      80
................................................................................     160
................................................................................     240
.......................................N........................................     320
.....                                                                               400

(Threshold=0.5)
----------------------------------------------------------------------
SeqName      Position  Potential   Jury    N-Glyc
                                agreement  result
----------------------------------------------------------------------
contig046367-TilORe.N201    11 NITF   0.6379     (9/9)   ++
contig046367-TilORe.N201   280 NLSI   0.5192     (4/9)   +
----------------------------------------------------------------------
```

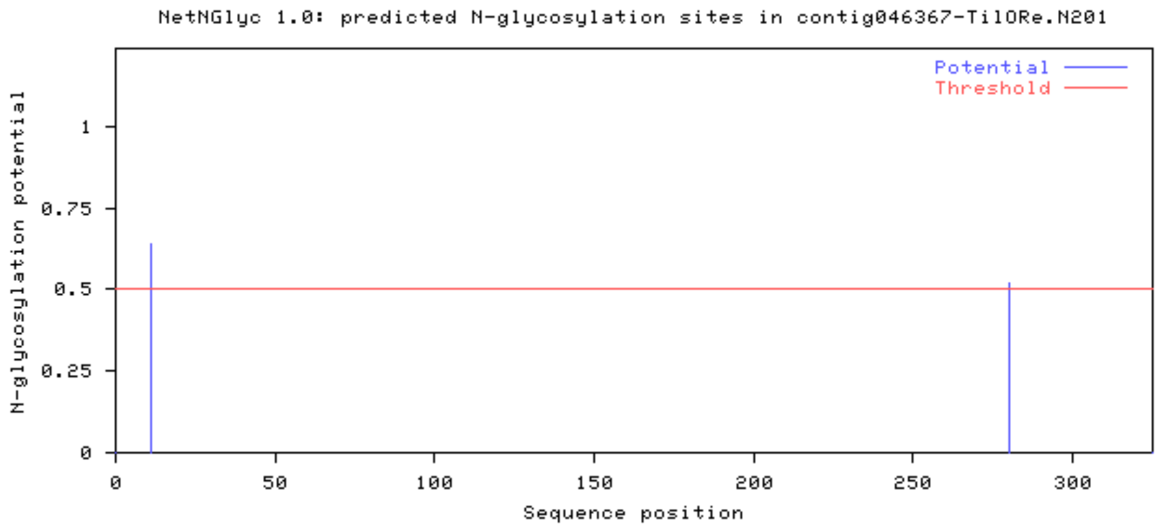

Graphics in PostScript

Output for 'contig046369-TilORe.N207'

```
##########################################################################

   Warning: This sequence may not contain a signal peptide!!

   Proteins without signal peptides are unlikely to be exposed to
   the N-glycosylation machinery and thus may not be glycosylated
   (in vivo) even though they contain potential motifs.

   SignalP-NN euk predictions are as follows:

  # name                 Cmax  pos ?  Ymax  pos ?  Smax  pos ?  Smean ?  D     ?

   SignalP output is explained at http://www.cbs.dtu.dk/services/SignalP/output.html

##########################################################################

Name:  contig046369-TilORe.N207         Length:  117
MDFFNSALGKNITFLRPAFFIISGFIGIPNIKYYYAFLVFVYIISVLANTAVMAAIYLDHNLRTPKYIAVFNLALVDLLG      80
NSAMVPKVLDIFLFNHPHISYNDCLTFLFFCYVFLSM
..........N.....................................................................      80
.....................................                                                 160

(Threshold=0.5)
----------------------------------------------------------------------
SeqName      Position  Potential   Jury    N-Glyc
                                 agreement result
----------------------------------------------------------------------
contig046369-TilORe.N207    11 NITF   0.5193     (6/9)   +
----------------------------------------------------------------------
```

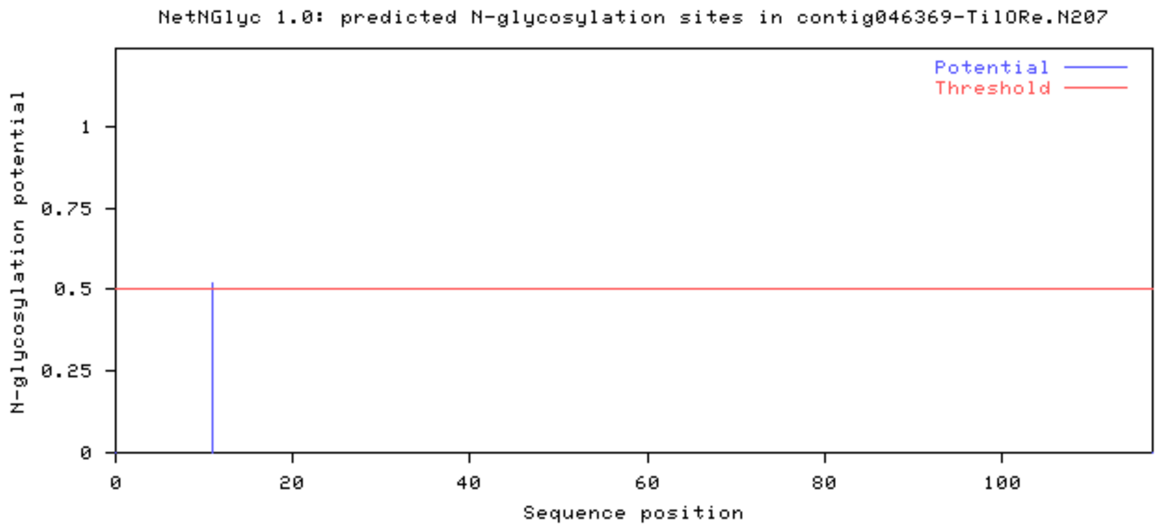

Graphics in PostScript

Output for 'contig046373-TilORe.N204'

```
##########################################################################################

    Warning: This sequence may not contain a signal peptide!!

    Proteins without signal peptides are unlikely to be exposed to
    the N-glycosylation machinery and thus may not be glycosylated
    (in vivo) even though they contain potential motifs.

    SignalP-NN euk predictions are as follows:

  # name                 Cmax  pos ?  Ymax  pos ?  Smax  pos ?  Smean ?  D     ?

    SignalP output is explained at http://www.cbs.dtu.dk/services/SignalP/output.html

##########################################################################################

Name:  contig046373-TilORe.N204        Length:  261
MEFLNSAVEKNTTFVQSAYFIISGFIGIPNIRYYFVFLCFIYIFSVVGNSLVMIVIILDRMLRSPKYIAVFNLALTDLLS      80
SCALVPKVLDISLFNHYYISYNNCLTFMFFCFTLISMQAFNLVVLSFDRVMAIMYPLHYQMRVSHKVILSLIAFFFLLAI     160
TLTLIAVGLLTRLSFCKSVIIQSYYCDHGPMYRLGCNDVTPNYIIGFLAIILVLAFPLAFIVGSYCCIGYSLSKISTFRE     240
RVKAFKTCTGHLSLVAIYFLP
................................................................................      80
................................................................................     160
................................................................................     240
.....................                                                                320

(Threshold=0.5)

No sites predicted in this sequence.
```

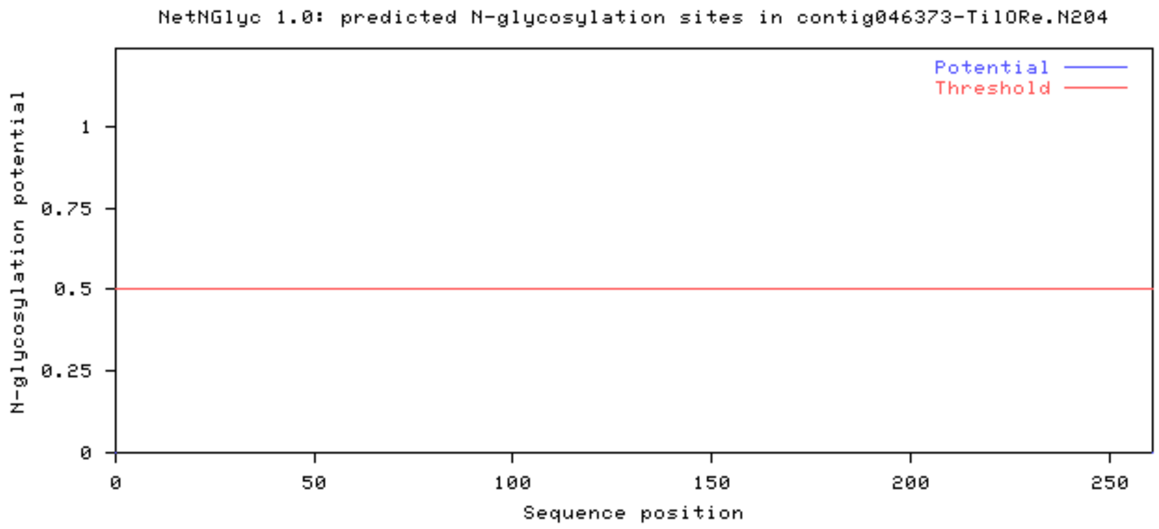

Graphics in PostScript

Output for 'contig046375-TilORe.N202'

```
##########################################################################

    Warning: This sequence may not contain a signal peptide!!

    Proteins without signal peptides are unlikely to be exposed to
    the N-glycosylation machinery and thus may not be glycosylated
    (in vivo) even though they contain potential motifs.

    SignalP-NN euk predictions are as follows:

  # name                 Cmax  pos ?  Ymax  pos ?  Smax  pos ?  Smean ?  D     ?

    SignalP output is explained at http://www.cbs.dtu.dk/services/SignalP/output.html

##########################################################################

Name:  contig046375-TilORe.N202        Length:  268
MDLFNSALGKNITFLRPAFFIISGFIGIPNINYYYAFLVFVYIMSVLANTAVMAAIYLDHNLRTPKYIAVFNLALVDLLG      80
NSAMVPKVLDIFLFNHPHISYNDCLTFLFFCYVFLSMQALNLVALSYDRVMAIVYPLHYQLKVTHRFMFSLIASFWVFVI     160
IVVLIATGLLTRLSFCESVVIKSYFCDHGQMYRLACNDYTPSDITAWILLVLILWLPLSIVLLSYLCICYALAKVATVRE     240
RMKGFKTCTAHLSLVAIYFLPILITFTL
..........N.....................................................................      80
................................................................................     160
................................................................................     240
............................                                                         320

(Threshold=0.5)
----------------------------------------------------------------------
SeqName      Position  Potential   Jury    N-Glyc
                                 agreement result
----------------------------------------------------------------------
contig046375-TilORe.N202    11 NITF   0.5357     (6/9)   +
----------------------------------------------------------------------
```

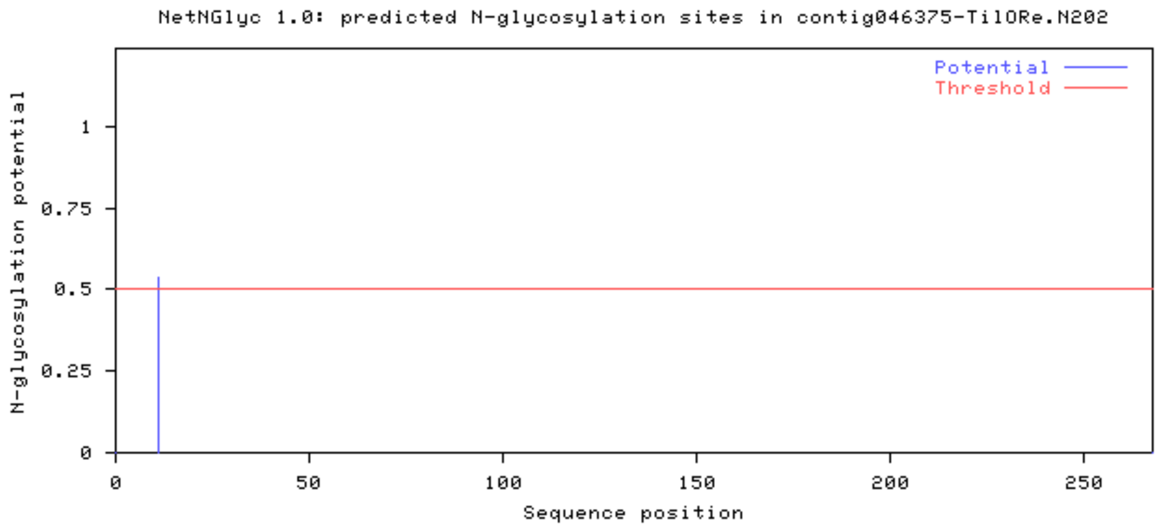

Graphics in PostScript

Output for 'contig046383-TilORe.N205'

```
##########################################################################################

    Warning: This sequence may not contain a signal peptide!!

    Proteins without signal peptides are unlikely to be exposed to
    the N-glycosylation machinery and thus may not be glycosylated
    (in vivo) even though they contain potential motifs.

    SignalP-NN euk predictions are as follows:

  # name                Cmax  pos ?  Ymax  pos ?  Smax  pos ?  Smean ?  D     ?

    SignalP output is explained at http://www.cbs.dtu.dk/services/SignalP/output.html

##########################################################################################

Name:  contig046383-TilORe.N205         Length:  123
CDHGPMYRLGCNDVTPNRTIAVMAPILILGFPLAFIVGSYCCIGYFLSKISTNRERMKAFKTCTGHLSLVAIYFLPITFV      80
YIFGNVIHPNARIISLSITTVLPPMLNPIIYVLFDLRRSKSHX
................N...............................................................      80
...........................................                                          160

(Threshold=0.5)
----------------------------------------------------------------------
SeqName      Position  Potential   Jury    N-Glyc
                                 agreement result
----------------------------------------------------------------------
contig046383-TilORe.N205    17 NRTI    0.6256     (8/9)    +
----------------------------------------------------------------------
```

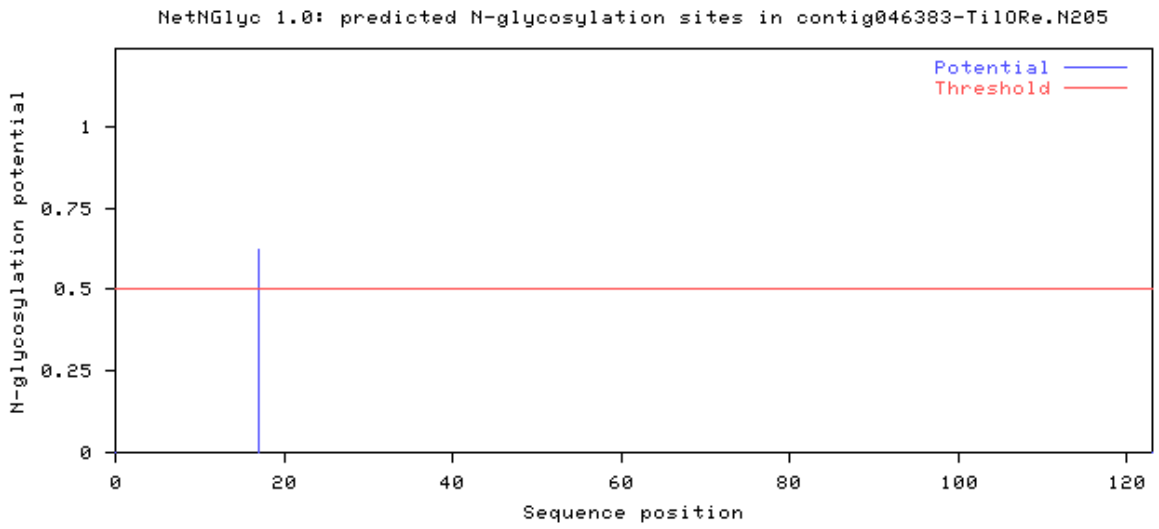

Graphics in PostScript

Output for 'contig046384-TilORe.N203'

```
##########################################################################################

    Warning: This sequence may not contain a signal peptide!!

    Proteins without signal peptides are unlikely to be exposed to
    the N-glycosylation machinery and thus may not be glycosylated
    (in vivo) even though they contain potential motifs.

    SignalP-NN euk predictions are as follows:

  # name                    Cmax   pos ?  Ymax   pos ?  Smax   pos ?  Smean ?  D     ?

    SignalP output is explained at http://www.cbs.dtu.dk/services/SignalP/output.html

##########################################################################################

Name:  contig046384-TilORe.N203         Length:  52
MEFINSAAENNITFVRPAYFIISGFIGIPNIRYYFVFLCFIYILAVVGNTLV
..........N.........................................                                  80

(Threshold=0.5)
----------------------------------------------------------------------
SeqName      Position  Potential   Jury    N-Glyc
                                 agreement result
----------------------------------------------------------------------
contig046384-TilORe.N203    11 NITF   0.6152     (9/9)   ++
----------------------------------------------------------------------
```

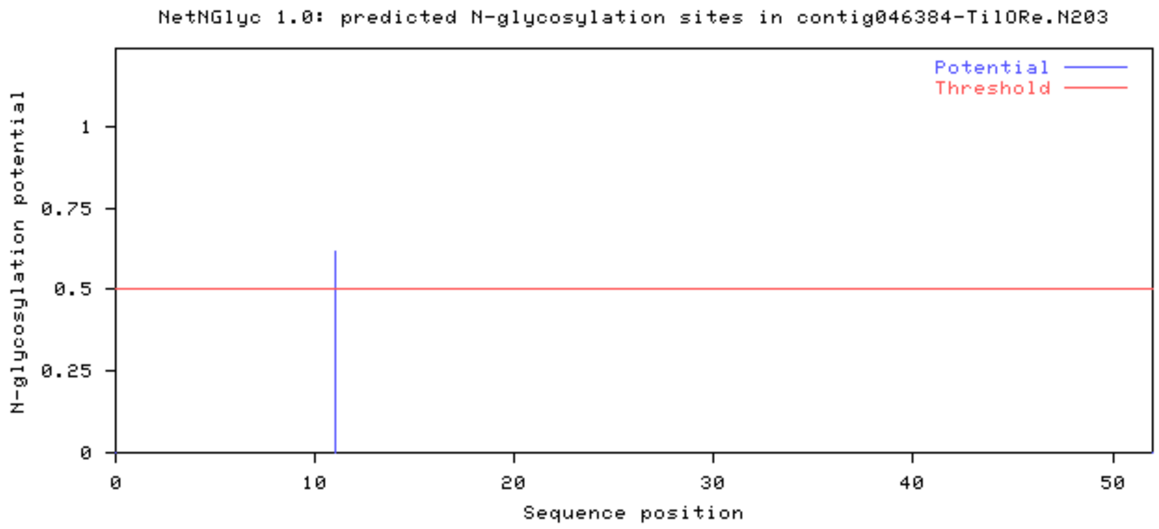

Graphics in PostScript

Output for 'contig046690-TilORs.I128'

```
##########################################################################################

    Warning: This sequence may not contain a signal peptide!!

    Proteins without signal peptides are unlikely to be exposed to
    the N-glycosylation machinery and thus may not be glycosylated
    (in vivo) even though they contain potential motifs.

    SignalP-NN euk predictions are as follows:

  # name                 Cmax  pos ?  Ymax  pos ?  Smax  pos ?  Smean ?  D     ?

    SignalP output is explained at http://www.cbs.dtu.dk/services/SignalP/output.html

##########################################################################################

Name:  contig046690-TilORs.I128         Length:  311
MVTPLKQPIVFELEGFYIPPGFGPLLFFLALFTYMVVLLGNGVIVYVIVMDKNLHRPMFVMVCHLLVCDLLGSTAVLPGL      80
MMHFLMGQKRIAYIPAIAQAFSVHTYGVAVQTVLGAMAYDRYIAVCEPLRYHAIMTSARLHSCCALAWFIALVVIAVLFG     160
FHMNVPLCGRTIHHVYCSNQSILSLACIPTPVSNIYGLSLTWIVNTGIFLIIAFSYIRILSASLRQSGVHSKAFQTCASH     240
LLVYVLYQIAVLIIVISYRFPSASQNLKKFFSILFIIVPPAINPIIYGLVSKDLRSNIIKQFTTQVCHKHX
................................................................................      80
................................................................................     160
................................................................................     240
.......................................................................              320

(Threshold=0.5)

No sites predicted in this sequence.
```

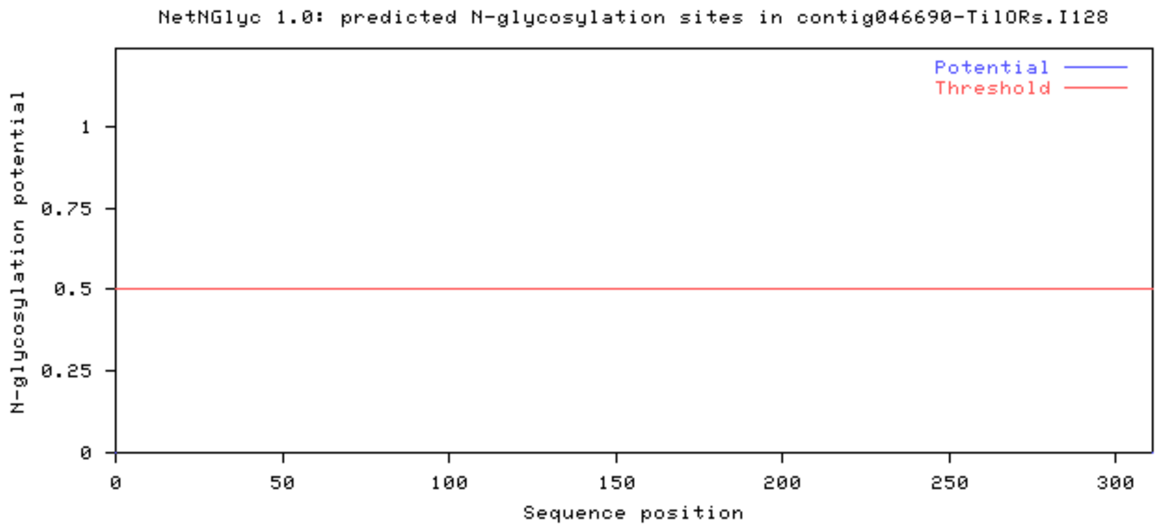

Graphics in PostScript

Output for 'contig046694-TilORs.I129'

```
##########################################################################

    Warning: This sequence may not contain a signal peptide!!

    Proteins without signal peptides are unlikely to be exposed to
    the N-glycosylation machinery and thus may not be glycosylated
    (in vivo) even though they contain potential motifs.

    SignalP-NN euk predictions are as follows:

  # name                 Cmax  pos ?  Ymax  pos ?  Smax  pos ?  Smean ?  D     ?

    SignalP output is explained at http://www.cbs.dtu.dk/services/SignalP/output.html

##########################################################################

Name:  contig046694-TilORs.I129          Length:  314
MENLTMVTPLKQPIVFELEGFYIPPGFGPLLFFLALFTYMVVLLGNGVIVYVIVMDKNLHRPMFVMVCHLLVCDLLGSTA      80
VLPGLMMHFLMERKRIAYIPAIAQAFSVHTYGVAVQTVLGVMAYDRYIAVCEPLRYHAIMTSARLHSCCALAWLLALLLI     160
AVLFGFHMNVPLCGRVILHVYCSNRGILGLACIPTPASNIYGLAMTWTVSTGIFLIIAFSYIRILHVSLKQGRINTSIRS     240
KAFQTCASHLVVYVLYQIASVIIIVSYRFPSVSENLKKFFSILFIIVPPAINPIIYGLVSKELRSSIIKHFTIX
..N.............................................................................      80
................................................................................     160
..........................................................................N.....     240
..........................................................................           320

(Threshold=0.5)
----------------------------------------------------------------------
SeqName      Position  Potential   Jury    N-Glyc
                                agreement result
----------------------------------------------------------------------
contig046694-TilORs.I129    3 NLTM   0.7616     (9/9)   +++
contig046694-TilORs.I129  235 NTSI   0.5043     (5/9)   +
----------------------------------------------------------------------
```

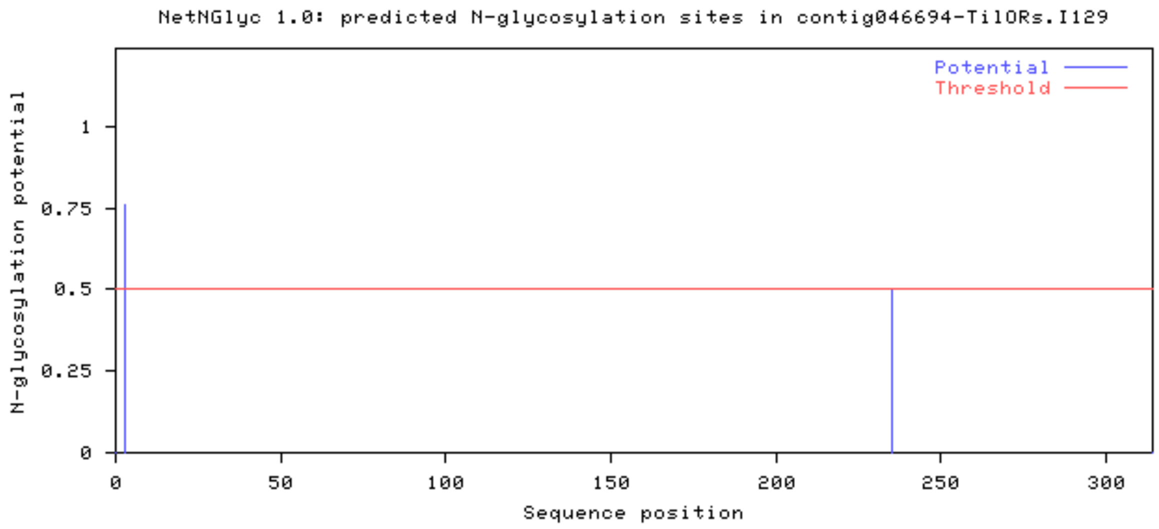

Graphics in PostScript

Output for 'contig046695-TilORs.I130'

```
##########################################################################

    Warning: This sequence may not contain a signal peptide!!

    Proteins without signal peptides are unlikely to be exposed to
    the N-glycosylation machinery and thus may not be glycosylated
    (in vivo) even though they contain potential motifs.

    SignalP-NN euk predictions are as follows:

  # name                Cmax  pos ?  Ymax  pos ?  Smax  pos ?  Smean ?  D     ?

    SignalP output is explained at http://www.cbs.dtu.dk/services/SignalP/output.html

##########################################################################

Name:  contig046695-TilORs.I130        Length:  316
MLTPLKQPIVFELEGFYIPPGFGPLLFFLALLTYLVVLLGNGVVVFVIVNDKNLHRPMFVMVCHLLVCDLLGATTVLPRI      80
MMHFLTGQKKIAYIPAITQAFFMHTYGVAVQTILAAMAYDRYIAVCEPLRYYAIMTSARLHSCCALAWFLAVVLIAVLFG     160
FHMNVPLCGRIIQHVYCSNRGILGLACIPTPTSDIYGLSMTWSVSTGMFLIIAFSYIRILCASVKQGRTDSRIRSKAFQT     240
CASHLVVYVLFEIASLTIIVSYRFPLLSQNIKKFLSILFIIVPPAINPIIYGVVSKDLRMSIIRLVTIQASHRSRX
................................................................................      80
................................................................................     160
................................................................................     240
............................................................................         320

(Threshold=0.5)

No sites predicted in this sequence.
```

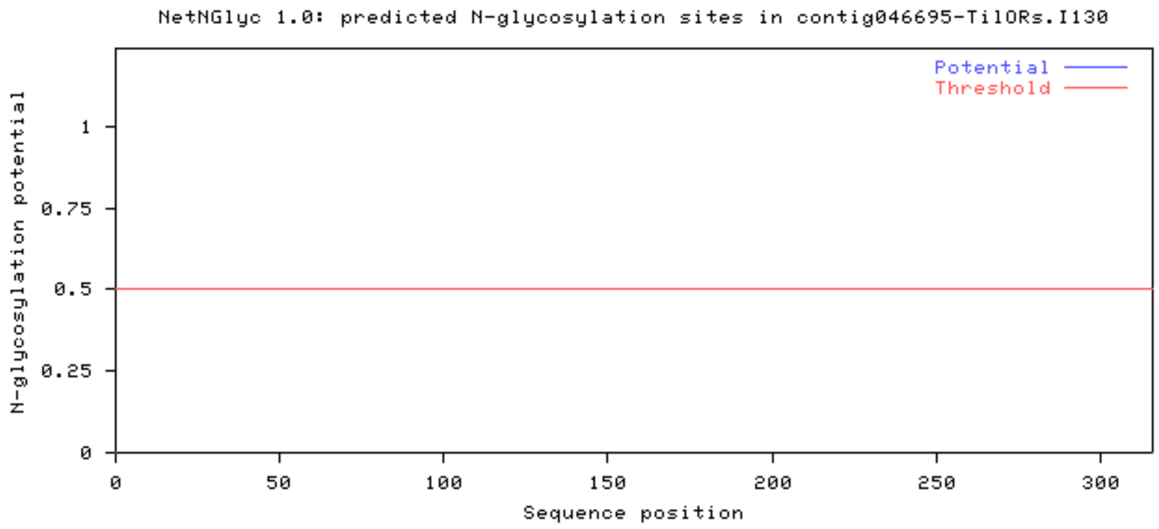

Graphics in PostScript

Output for 'contig046699-TilOR.K134'

```
##########################################################################################

    Warning: This sequence may not contain a signal peptide!!

    Proteins without signal peptides are unlikely to be exposed to
    the N-glycosylation machinery and thus may not be glycosylated
    (in vivo) even though they contain potential motifs.

    SignalP-NN euk predictions are as follows:

  # name                Cmax  pos ?  Ymax  pos ?  Smax  pos ?  Smean ?  D     ?

    SignalP output is explained at http://www.cbs.dtu.dk/services/SignalP/output.html

##########################################################################################

Name:  contig046699-TilOR.K134  Length:  313
MENYTYNSLTLQLEGFKVSEDLMYPVFFLFLTSYILSMFMNIGILIVIMIDKNLKQPMYRLFCNLSVSDIIGITQIVPRL      80
IADLLRPPSERLISYYECAFQAFVTQLFATTSHTVLMVMAFDRYVAICNPLRYTAIMSNKMVIKLTVLAWGVAFVLVVIL     160
ISLTVRLSTCRTIINNPFCDNASLFKLSCESVLINNVYGLTFSVVLLTSSIGSIVLTYANITVICLMSNNKSLNSKALKT     240
CSTHLVVYLIMLFSGMIAIALHRFPQYSDYRKLSAILFVIVPGSLNPVIYGVQSKEIRTFLYEKFHSKKCLFX
..N............................................................N................      80
................................................................................     160
....................N......................................N....................     240
.........................................................................            320

(Threshold=0.5)
----------------------------------------------------------------------
SeqName      Position  Potential   Jury     N-Glyc
                                 agreement  result
----------------------------------------------------------------------
contig046699-TilOR.K134    3 NYTY   0.7667     (9/9)   +++
contig046699-TilOR.K134   64 NLSV   0.7177     (9/9)   ++
contig046699-TilOR.K134  181 NASL   0.6537     (9/9)   ++
contig046699-TilOR.K134  220 NITV   0.7061     (9/9)   ++
contig046699-TilOR.K134  230 NKSL   0.4977     (4/9)   -
----------------------------------------------------------------------
```

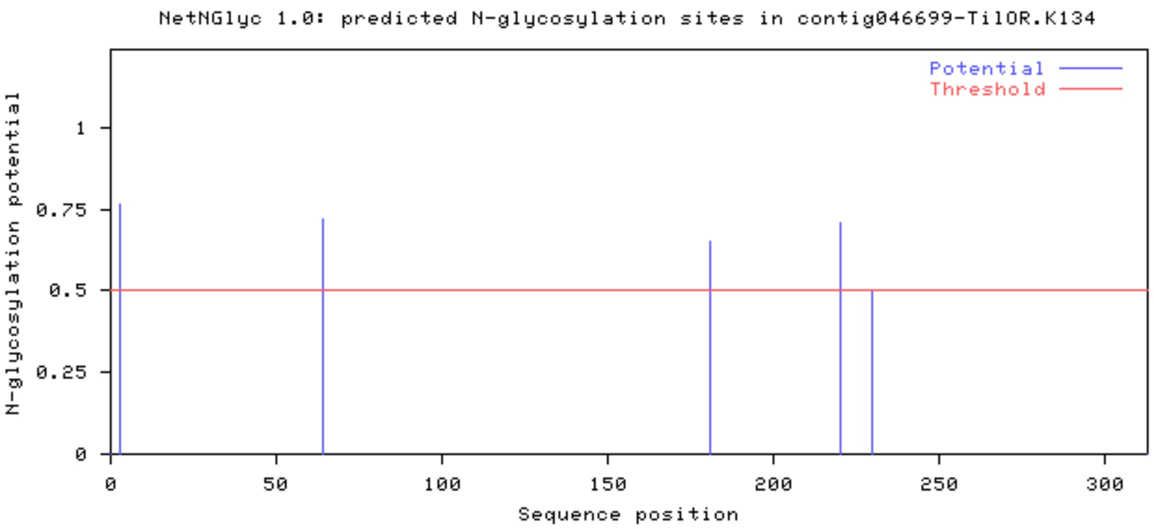

Graphics in PostScript

Output for 'contig046700-TilORe.K141'

```
##########################################################################################

    Warning: This sequence may not contain a signal peptide!!

    Proteins without signal peptides are unlikely to be exposed to
    the N-glycosylation machinery and thus may not be glycosylated
    (in vivo) even though they contain potential motifs.

    SignalP-NN euk predictions are as follows:

  # name                 Cmax  pos ?  Ymax  pos ?  Smax  pos ?  Smean ?  D     ?

    SignalP output is explained at http://www.cbs.dtu.dk/services/SignalP/output.html

##########################################################################################

Name:  contig046700-TilORe.K141         Length:  140
MENYTYNSLTLQMEGLKVSKDSVYTVCFLFLTSYIFIIIMNIGISLLIVLDKNLKQPMYLLFCNLSVSDIIGSTQIVPRL      80
IADLLRPPSERLISYYECVVQAFTTQLFGTTSHTVLMVMAFDRYVAICNPLRYTAIMSNK
..N.............................................................N...............      80
............................................................                         160

(Threshold=0.5)
----------------------------------------------------------------------
SeqName      Position  Potential   Jury    N-Glyc
                                 agreement result
----------------------------------------------------------------------
contig046700-TilORe.K141    3 NYTY    0.7689     (9/9)    +++
contig046700-TilORe.K141   64 NLSV    0.7078     (9/9)    ++
----------------------------------------------------------------------
```

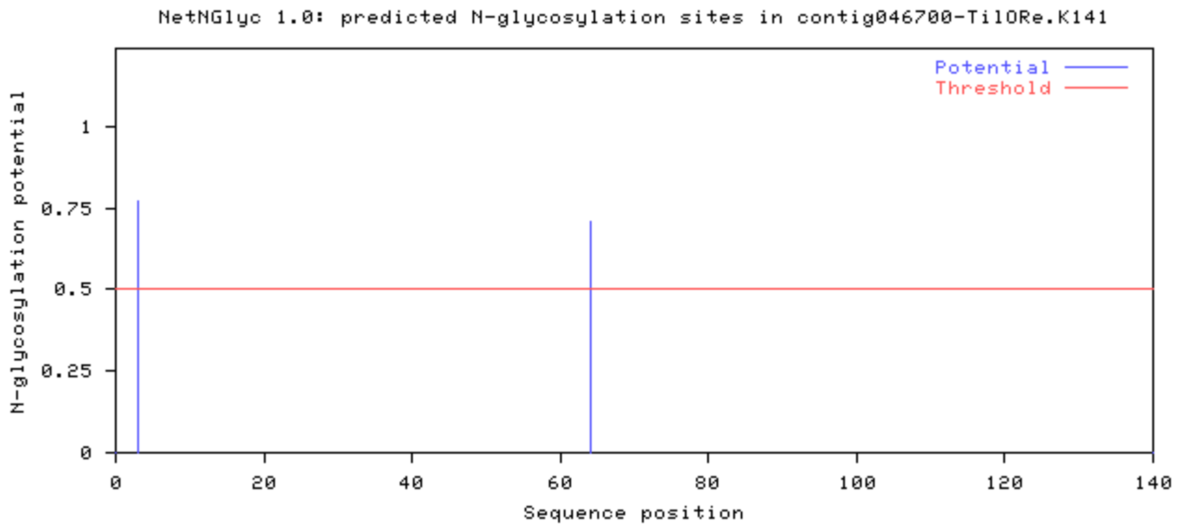

Graphics in PostScript

Output for 'contig046701-TilORe.K140'

```
##########################################################################

   Warning: This sequence may not contain a signal peptide!!

   Proteins without signal peptides are unlikely to be exposed to
   the N-glycosylation machinery and thus may not be glycosylated
   (in vivo) even though they contain potential motifs.

   SignalP-NN euk predictions are as follows:

 # name                Cmax  pos ?  Ymax  pos ?  Smax  pos ?  Smean ?  D     ?

   SignalP output is explained at http://www.cbs.dtu.dk/services/SignalP/output.html

##########################################################################

Name:  contig046701-TilORe.K140        Length:  188
ICNPLRYTAIMSNKMVIKLTVLAWGVAFVLVGILISLTVRLSTCRTIINNPFCDNASLFKLSCENVIINNIYGLTFTVVL      80
LTSSIGTVVLTYSKITVVCLISKNKSLNSKALKTCSTHLVVYLIMLFSGMIVIMLHRFPDYTDYRNLSAILFVIVPGSLN     160
PVIYGVQSKEIRTFLYEKFHSKKCLPKX
......................................................N.........................      80
.......................N..........................................N.............     160
............................                                                        240

(Threshold=0.5)
----------------------------------------------------------------------
SeqName      Position  Potential   Jury    N-Glyc
                                 agreement result
----------------------------------------------------------------------
contig046701-TilORe.K140   55 NASL   0.7051     (9/9)   ++
contig046701-TilORe.K140  104 NKSL   0.5313     (5/9)   +
contig046701-TilORe.K140  146 NLSA   0.6373     (7/9)   +
----------------------------------------------------------------------
```

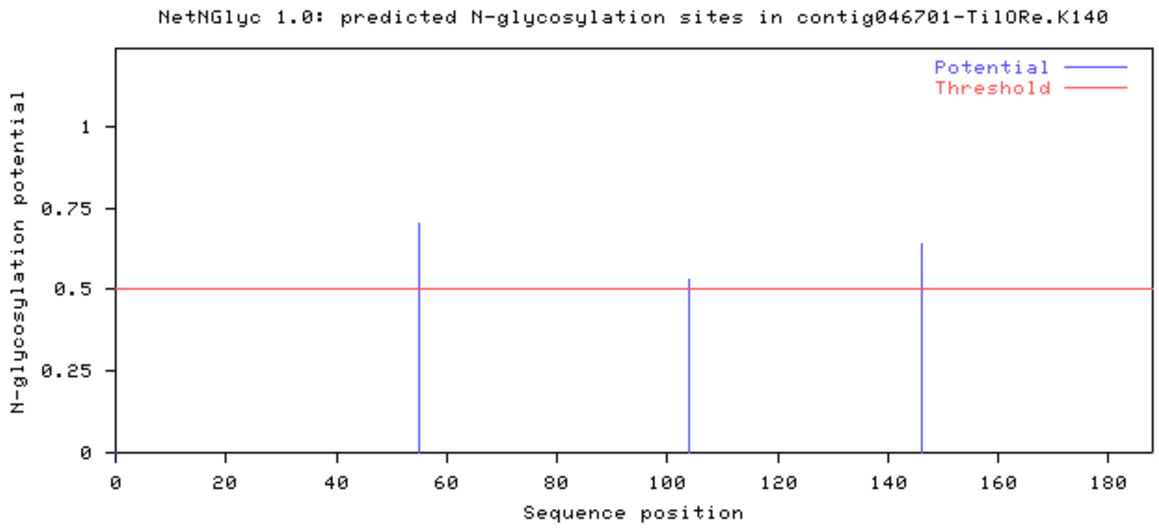

Graphics in PostScript

Output for 'contig046706-TilOR.K135'

```
##########################################################################

    Warning: This sequence may not contain a signal peptide!!

    Proteins without signal peptides are unlikely to be exposed to
    the N-glycosylation machinery and thus may not be glycosylated
    (in vivo) even though they contain potential motifs.

    SignalP-NN euk predictions are as follows:

  # name                Cmax  pos ?  Ymax  pos ?  Smax  pos ?  Smean ?  D     ?

    SignalP output is explained at http://www.cbs.dtu.dk/services/SignalP/output.html

##########################################################################

Name:  contig046706-TilOR.K135  Length:  315
MMENYTYNSYTLQLEGLNISKDSLYPALLFLFFSYLFIMILNVGITILIFINKNLHQPMYLLYCNLPLNDILANSIVVPR      80
LLIDLMRPPSERLISYYQCVVQAYIAHLVGTTSHTVLMIMAYDRYVAICNPLHYASTMTNKMVIKLTVCAWGVAFVLVGI     160
LLGLTIRLSRCRTLITNPYCDNASLFKLSCESVVINNIYGITFTVAVYVGSIGAIVLSYTNIAVVCLTSKNKSLNSKALK     240
TCSTHLVVYLIMTFSGMTLITLHRFPQYSEYRKLSALLFRIVPGSLNPIIYGVQSKELQKALLKFYVSKKVLASX
...N.............N..............................................................      80
................................................................................     160
.....................N..........................................................     240
...........................................................................          320

(Threshold=0.5)
----------------------------------------------------------------------
SeqName      Position  Potential   Jury    N-Glyc
                                agreement  result
----------------------------------------------------------------------
contig046706-TilOR.K135    4 NYTY   0.7607     (9/9)   +++
contig046706-TilOR.K135   18 NISK   0.6761     (8/9)   +
contig046706-TilOR.K135  182 NASL   0.6047     (7/9)   +
contig046706-TilOR.K135  231 NKSL   0.3950     (7/9)   -
----------------------------------------------------------------------
```

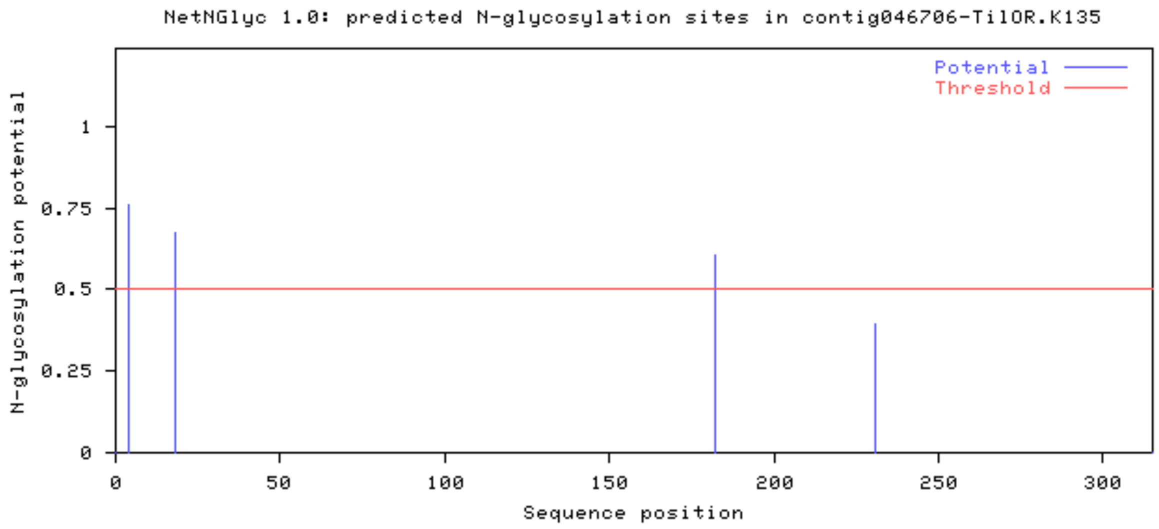

Graphics in PostScript

Output for 'contig046708-TilORs.K143'

```
################################################################################

    Warning: This sequence may not contain a signal peptide!!

    Proteins without signal peptides are unlikely to be exposed to
    the N-glycosylation machinery and thus may not be glycosylated
    (in vivo) even though they contain potential motifs.

    SignalP-NN euk predictions are as follows:

  # name                Cmax  pos ?  Ymax  pos ?  Smax  pos ?  Smean ?  D     ?

    SignalP output is explained at http://www.cbs.dtu.dk/services/SignalP/output.html

################################################################################

Name:  contig046708-TilORs.K143         Length:  313
MENYMYNSPTLQLQELRIVRTNKPMFLFLFFSYLFLIVANVGIAVLVFVDKSLHQPMYILFCNLSINDLFGNSIMIPHLL      80
VDMLRPPSERLISYYECVVQAFTTHMFSTTAHTVLMIMAFDRYVAICNPLRYAAVMTNKMLTKLTVSAWGVAFVLVGILL     160
GLTLRLGRCRTLIKSPYCDNAALFNLSCEDVFINNVYGLTFTVLLFTGSIGSMVLTYTKITVVCLTTKNKSLNNKALKTC     240
STHLVVYLIFLFSGMSIITLHRFPEYSESRKIVAVLYHIIPGSLNPIIYGMQSKEIKKFVSKVKLKKVLPLYX
..............................................................N.................      80
................................................................................     160
........................N.......................................................     240
.........................................................................            320

(Threshold=0.5)
----------------------------------------------------------------------
SeqName      Position  Potential   Jury    N-Glyc
                                 agreement result
----------------------------------------------------------------------
contig046708-TilORs.K143   63 NLSI   0.6501     (9/9)   ++
contig046708-TilORs.K143  185 NLSC   0.6196     (8/9)   +
contig046708-TilORs.K143  229 NKSL   0.4806     (6/9)   -
----------------------------------------------------------------------
```

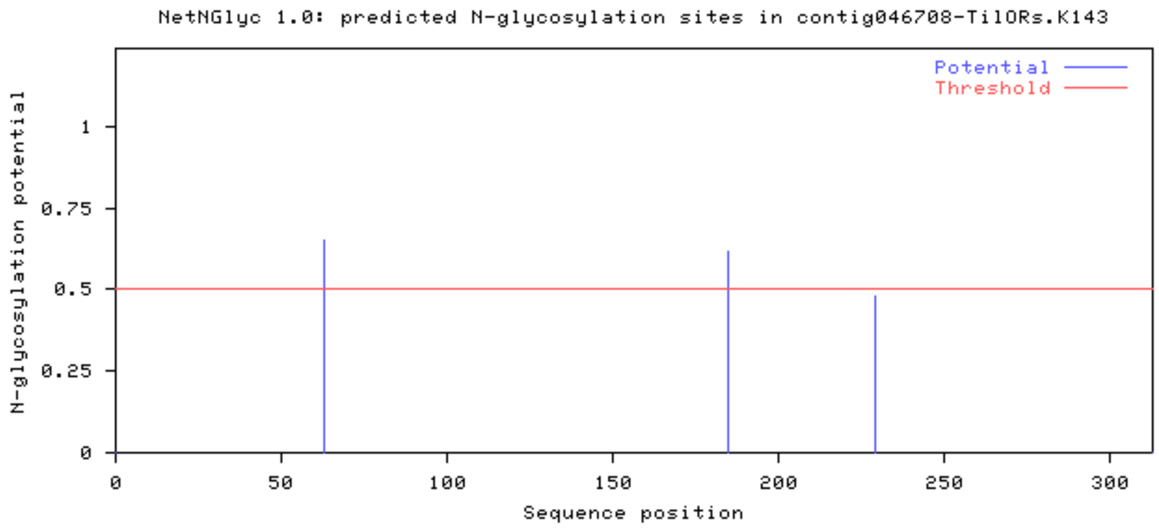

Graphics in PostScript

Output for 'Contig046714_contig046713-TilOR.K136'

```
##########################################################################

   Warning: This sequence may not contain a signal peptide!!

   Proteins without signal peptides are unlikely to be exposed to
   the N-glycosylation machinery and thus may not be glycosylated
   (in vivo) even though they contain potential motifs.

   SignalP-NN euk predictions are as follows:

 # name                 Cmax  pos ?  Ymax  pos ?  Smax  pos ?  Smean ?  D     ?

   SignalP output is explained at http://www.cbs.dtu.dk/services/SignalP/output.html

##########################################################################

Name:  Contig046714_contig046713-TilOR.K136     Length:  319
MENYTYNTLTLQLEWLNISVESTYALFLLLLFFYLFIMVANMSIAVLVFMDKNLHQPMYLLFCNLPFNDILGNSIMVPRL      80
LVDMLKPPSERFISYYECVVQAFTTHMFGATSHTVLMIMAFDRYVAICNPLRYASIMTNKMVIKLTVFAWGVAFVLVGIL     160
LGLTVRLSRCRTLITNPYCDNASLFKLSCENVFINNVYGLTFTVVLFTGSIGSIVLTYARITIVCLTSKNKSLNSKALKT     240
CSTHLVVYMIMLLSGMSLIALHRFPQYSEYRKLCSILFHILPGSLNPIIYGVQSREIQRFLSKVIENYAIKITNFIFTX
..N.............N...............................................................      80
................................................................................     160
....................N...........................................................     240
...............................................................................      320

(Threshold=0.5)
----------------------------------------------------------------------
SeqName      Position  Potential   Jury    N-Glyc
                                 agreement result
----------------------------------------------------------------------
Contig046714_contig046713-TilOR.K136    3 NYTY   0.7729     (9/9)   +++
Contig046714_contig046713-TilOR.K136   17 NISV   0.7223     (9/9)   ++
Contig046714_contig046713-TilOR.K136   41 NMSI   0.4852     (5/9)   -
Contig046714_contig046713-TilOR.K136  181 NASL   0.6158     (7/9)   +
Contig046714_contig046713-TilOR.K136  230 NKSL   0.4144     (7/9)   -
----------------------------------------------------------------------
```

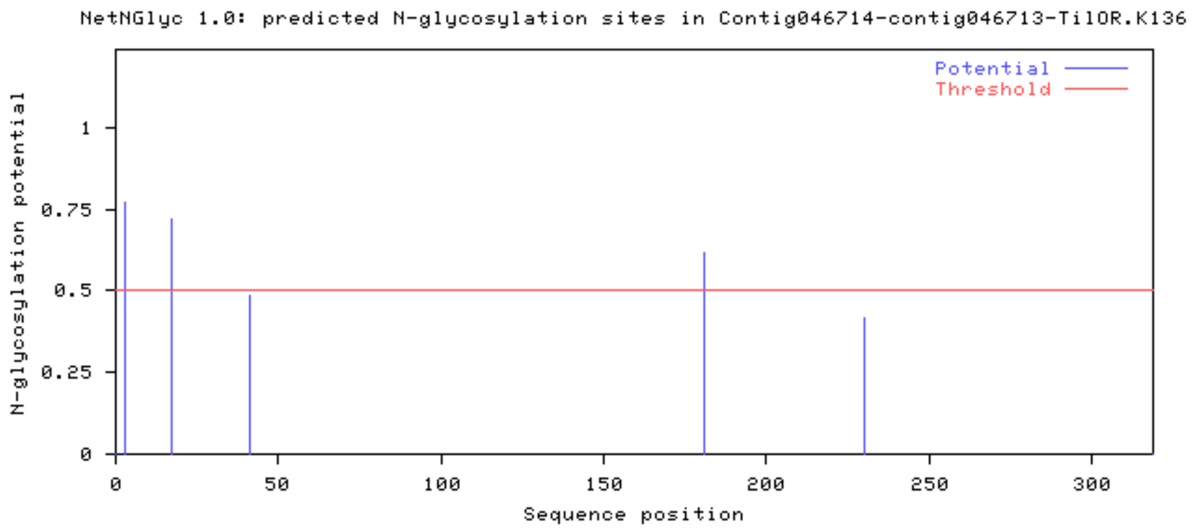

Graphics in PostScript

Output for 'contig046717-TilORs.AB275'

```
##########################################################################################

    Warning: This sequence may not contain a signal peptide!!

    Proteins without signal peptides are unlikely to be exposed to
    the N-glycosylation machinery and thus may not be glycosylated
    (in vivo) even though they contain potential motifs.

    SignalP-NN euk predictions are as follows:

  # name                   Cmax   pos ?  Ymax   pos ?  Smax   pos ?  Smean ?  D     ?

    SignalP output is explained at http://www.cbs.dtu.dk/services/SignalP/output.html

##########################################################################################

Name:  contig046717-TilORs.AB275        Length:  363
MNTDGLFFPSDFPPLSTNHRSSSANGTLELSGVTFFIIQGLTNLDEKKIILFSILLLIYIMILGGNSIIIYVFVDMVYTT      80
TTIPNMLSGLLTDILTISVLGCFLQMYFFIQLSVTGRAILTVMAYDRYVAICTPLQYNSIMTRPVRLLLVAGAWGFGAVC     160
TLPATVIAFERPYCGPNVVKHAWCDPSSVRRLVCGDTSLDNIVSLSFAMVALLTTGILILSSYILIGVSISRMVVAQRLK     240
AFRTCAAHLTVVSISYAAASFVYISYRVGNFSPEVQTLLYFLRNVSVQYSVKVLSHPSFISIFGRKTRNTCRDLLKQANP     320
KWKYSIWTFLMSCRYSFSTFILHLFLFSRTRTLIDRLSCKLPX
........................N.......................................................      80
................................................................................     160
................................................................................     240
...........................................N....................................     320
...........................................                                          400

(Threshold=0.5)
----------------------------------------------------------------------
SeqName      Position  Potential    Jury    N-Glyc
                                 agreement result
----------------------------------------------------------------------
contig046717-TilORs.AB275   25 NGTL    0.6999     (9/9)    ++
contig046717-TilORs.AB275  270 NFSP    0.1374     (9/9)    ---
contig046717-TilORs.AB275  284 NVSV    0.7277     (9/9)    ++
----------------------------------------------------------------------
```

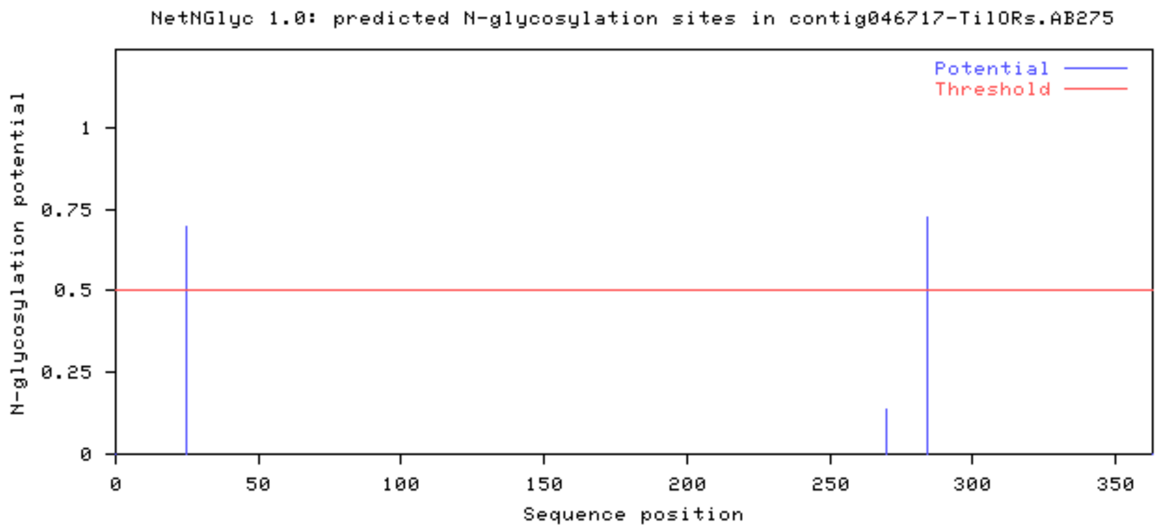

Graphics in PostScript

Output for 'contig046718-TilOR.K137'

```
##########################################################################################

    Warning: This sequence may not contain a signal peptide!!

    Proteins without signal peptides are unlikely to be exposed to
    the N-glycosylation machinery and thus may not be glycosylated
    (in vivo) even though they contain potential motifs.

    SignalP-NN euk predictions are as follows:

  # name                    Cmax  pos ?  Ymax  pos ?  Smax  pos ?  Smean ?  D     ?

    SignalP output is explained at http://www.cbs.dtu.dk/services/SignalP/output.html

##########################################################################################

Name:  contig046718-TilOR.K137  Length:  315
MENQTLDILLLEGLQVSSNASIPAFVLLLLIYILIMFSNIVLVILITLDSSLHQPMYLLFCNMSINDVFGATTIIPRMLR      80
DIFIPSSDRYIHYVDCVIQAFCVHIYAGASHTILMIMAFDRYVAICNPLHYATIMTNWMVVKLSVLAWAVIFVMVTILVG     160
LSVRLSRCRWIILNPFCDNASLFKLSCESILINNIYGLGYTVLLLGSSLGSVAITYLRIAMVCLSSKSKTLNSRALQTYT     240
THLTMYVIMFVSGIVMVLLHRFPHLTDQRKLASMMFHVVPPALNAVIYGMQIKAVRQKLFIMFMRNTVTVTEGKX
..N...............N..........................................N..................      80
................................................................................     160
..................N.............................................................     240
...........................................................................          320

(Threshold=0.5)
----------------------------------------------------------------------
SeqName      Position  Potential   Jury    N-Glyc
                                 agreement result
----------------------------------------------------------------------
contig046718-TilOR.K137    3 NQTL   0.7386     (9/9)    ++
contig046718-TilOR.K137   19 NASI   0.6323     (7/9)    +
contig046718-TilOR.K137   62 NMSI   0.6740     (9/9)    ++
contig046718-TilOR.K137  179 NASL   0.6568     (8/9)    +
----------------------------------------------------------------------
```

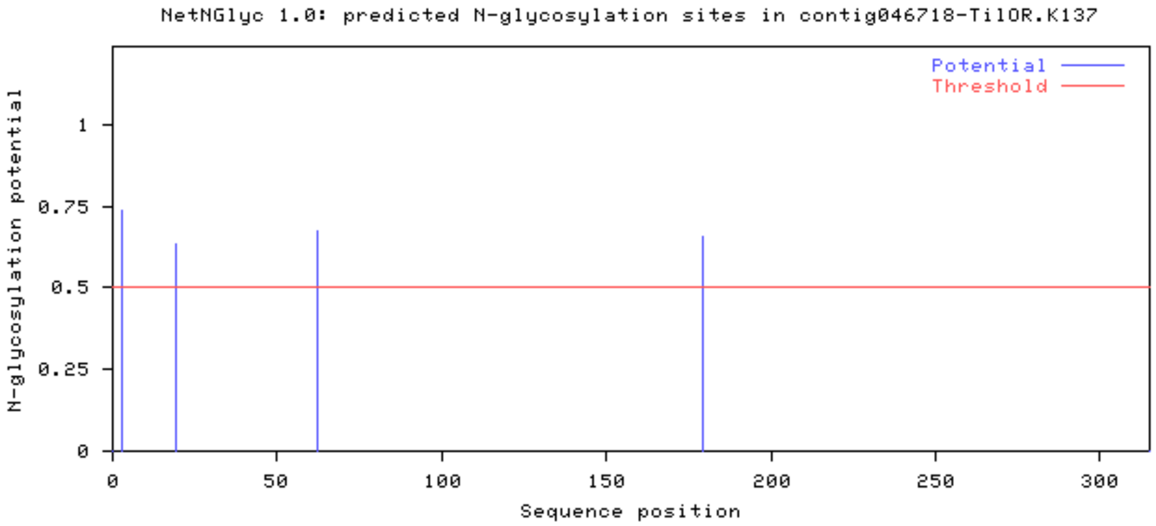

Graphics in PostScript

Output for 'contig046723-TilOR.K138'

```
##########################################################################################

    Warning: This sequence may not contain a signal peptide!!

    Proteins without signal peptides are unlikely to be exposed to
    the N-glycosylation machinery and thus may not be glycosylated
    (in vivo) even though they contain potential motifs.

    SignalP-NN euk predictions are as follows:

  # name                 Cmax  pos ?  Ymax  pos ?  Smax  pos ?  Smean ?  D     ?

    SignalP output is explained at http://www.cbs.dtu.dk/services/SignalP/output.html

##########################################################################################

Name:  contig046723-TilOR.K138  Length:  313
MDNQSLNADILILEGLKVTPRSSIAAFIFLLLMYIFIMVANIGLVVLIFMERSLHQPMYLLFCNMSVNEVFGATIVVPHI      80
LRDLFVSDSERYIHYIVCVVQAFCVNLYGGVCHTILMTMTFDRYMAICNPLRYTIIMTNWMVVKLSVAAWAVVFVMVSIL     160
LSLTIRLSRCRRFIDNAHCDNASLFKLSCEDVVINHAFGLSYSVLLLGSSIGSVTLTYIKIATVCLRSKTKTINSKALQT     240
CATHFTLYIILMFSAFIIIILHRFPHLSDHRKMVSTVGEVALPALNAVIYGLQIKEIRQKIVAFFQRKGHLQX
..N............................................................N................      80
................................................................................     160
....................N...........................................................     240
.........................................................................            320

(Threshold=0.5)
----------------------------------------------------------------------
SeqName      Position  Potential   Jury    N-Glyc
                                agreement result
----------------------------------------------------------------------
contig046723-TilOR.K138    3 NQSL   0.7008     (9/9)   ++
contig046723-TilOR.K138   64 NMSV   0.6942     (9/9)   ++
contig046723-TilOR.K138  181 NASL   0.6550     (9/9)   ++
----------------------------------------------------------------------
```

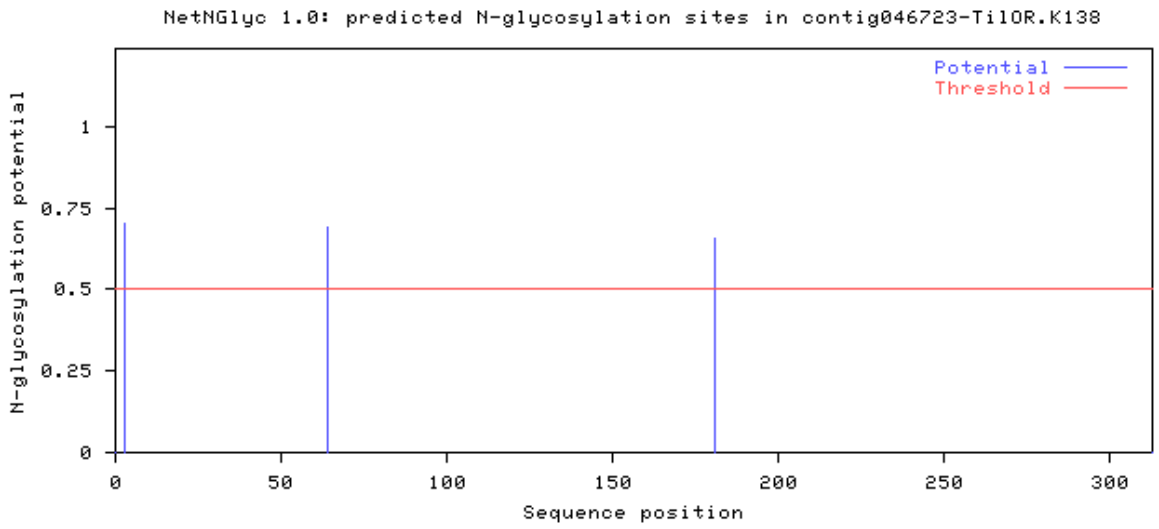

[Graphics in PostScript]

Output for 'contig046724-TilOR.K139'

```
##########################################################################################

    Warning: This sequence may not contain a signal peptide!!

    Proteins without signal peptides are unlikely to be exposed to
    the N-glycosylation machinery and thus may not be glycosylated
    (in vivo) even though they contain potential motifs.

    SignalP-NN euk predictions are as follows:

  # name                   Cmax  pos ?  Ymax  pos ?  Smax  pos ?  Smean ?  D     ?

    SignalP output is explained at http://www.cbs.dtu.dk/services/SignalP/output.html

##########################################################################################

Name:  contig046724-TilOR.K139  Length:  315
MENYTFNSFTLQLEGLKVSEVSKYPLFFFSLFSYILIMTSNIGIVVLVFIDKNLHRPMYLLFCNLPFNDVVGNSIMMPRL      80
LSDILLPPSERLISYHECLIQAFTTHMYGTTSHTVLMIMAFDRYVAICNPLRYASIMTNKMVIKLTVLAWGVAFVLVGVL     160
LGLTIRLSRCRTLITNPFCDNASLFKLSCDSVVINNIYGLTFTVVLFTGSIGTIVLTYTQIMVVCVTSKNTSLNSKALKT     240
CSTHLLVYMIMLFTGILVIALHRFPQYSDYRKMCAILFHIIPGSLNPIIYGVQSKEIQKFLSKLLHFTKILPSKX
..N.............................................................................      80
................................................................................     160
....................N...........................................................     240
...........................................................................          320

(Threshold=0.5)
----------------------------------------------------------------------
SeqName      Position  Potential   Jury    N-Glyc
                                 agreement result
----------------------------------------------------------------------
contig046724-TilOR.K139    3 NYTF   0.7396     (9/9)   ++
contig046724-TilOR.K139  181 NASL   0.6587     (8/9)   +
contig046724-TilOR.K139  230 NTSL   0.2816     (9/9)   ---
----------------------------------------------------------------------
```

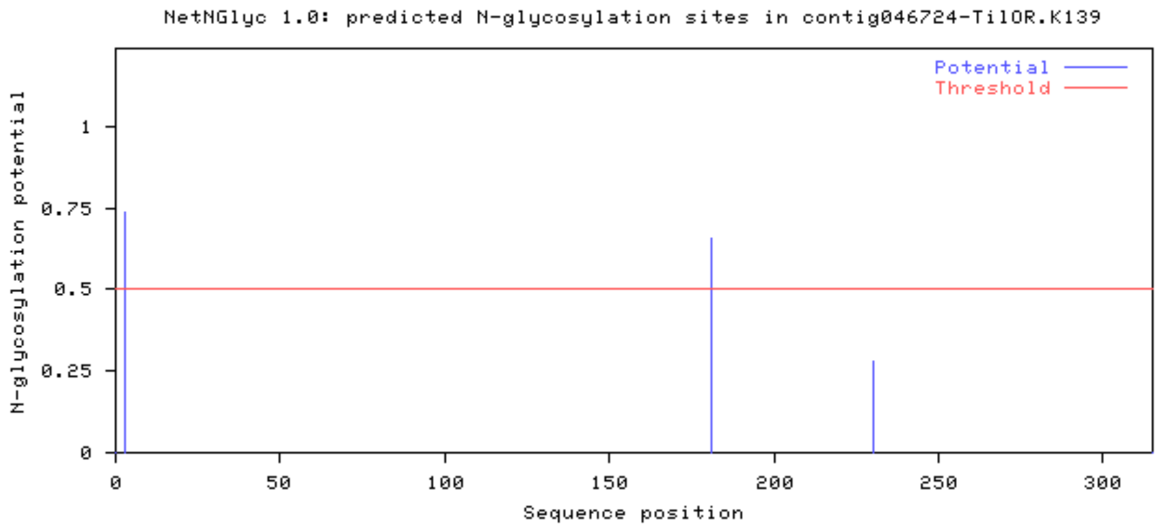

Graphics in PostScript

Output for 'contig047709-TilORe.E095'

```
##############################################################################

    Warning: This sequence may not contain a signal peptide!!

    Proteins without signal peptides are unlikely to be exposed to
    the N-glycosylation machinery and thus may not be glycosylated
    (in vivo) even though they contain potential motifs.

    SignalP-NN euk predictions are as follows:

  # name                Cmax  pos ?  Ymax  pos ?  Smax  pos ?  Smean ?  D     ?

    SignalP output is explained at http://www.cbs.dtu.dk/services/SignalP/output.html

##############################################################################

Name:  contig047709-TilORe.E095         Length:  242
VNELYGSTGLFPFLLLQILSDVHTVSAPLCFLQIFCVFSYVCVEFCILAVMSYDRYLAICCPLQYHTRMTPATVVLLIAL      80
SWLYSFLTILTLILLIAPLELCGNVINKVYCLNYSIVKLACSETTANNIYGLFITALTVFVPVILILCSYVRILKVCFSG     160
SKQTRQKAVSTCTPHLSSLLNFSFGVCFEVIQSRFSLSSMHSMVHIVLSLYFLTCQPLFNPVLYGLNMSNIRKRLFAHKR     240
RX
................................................................................      80
................................N...............................................     160
..................................................................N.............     240
..                                                                                   320

(Threshold=0.5)
----------------------------------------------------------------------
SeqName      Position  Potential   Jury    N-Glyc
                                 agreement result
----------------------------------------------------------------------
contig047709-TilORe.E095  113 NYSI   0.6238     (8/9)   +
contig047709-TilORe.E095  181 NFSF   0.4163     (6/9)   -
contig047709-TilORe.E095  227 NMSN   0.5711     (4/9)   +
----------------------------------------------------------------------
```

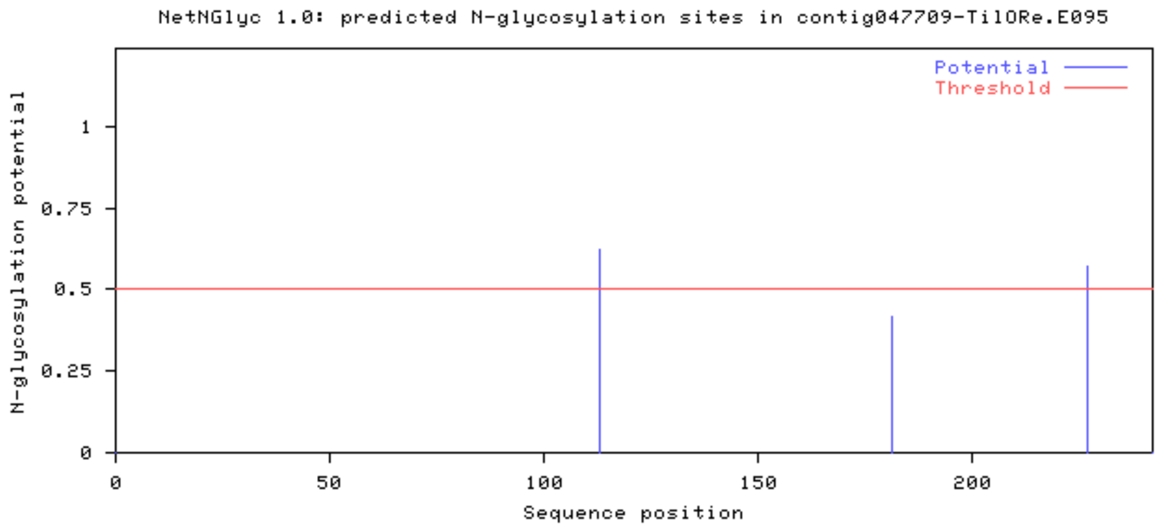

[Graphics in PostScript]

Output for 'contig047714-TilOR.G099'

```
##########################################################################

    Warning: This sequence may not contain a signal peptide!!

    Proteins without signal peptides are unlikely to be exposed to
    the N-glycosylation machinery and thus may not be glycosylated
    (in vivo) even though they contain potential motifs.

    SignalP-NN euk predictions are as follows:

  # name                   Cmax  pos ?  Ymax  pos ?  Smax  pos ?  Smean ?  D     ?

    SignalP output is explained at http://www.cbs.dtu.dk/services/SignalP/output.html

##########################################################################

Name:  contig047714-TilOR.G099  Length:  312
MENNFEIVFVLQGLNNSLANRQIYFAFALMSYLFTVSVNLTLIITISLDKTLHEPIYIFLCSLCFNEICGASSFYPKLLH      80
DLLTNSYVITYTACLGQMFVSYSYIFSEFTGLTVMAYDRYIAICKPLQYRMLMTAQKVAQLLMLTWCFSVFETAVGTVLT     160
ARLPLCRRHIPKIFCTNWEVVKLSCSDSTINNVYAFMLIFSHLSQATLIMVSYVHLIQAAIRSQADRRKFMQTCLPHLIT     240
LLFFTTSLMFDTMYSRYSGGSTMKALQNALAAQFLVVPPLVNPIIYGMNLQQIRSRMLQRFTHRTGIFRKNX
..............N.......................N.........................................      80
................................................................................     160
................................................................................     240
........................................................................             320

(Threshold=0.5)
----------------------------------------------------------------------
SeqName      Position  Potential   Jury    N-Glyc
                                 agreement result
----------------------------------------------------------------------
contig047714-TilOR.G099   15 NNSL   0.6054     (9/9)    ++
contig047714-TilOR.G099   39 NLTL   0.7857     (9/9)    +++
----------------------------------------------------------------------
```

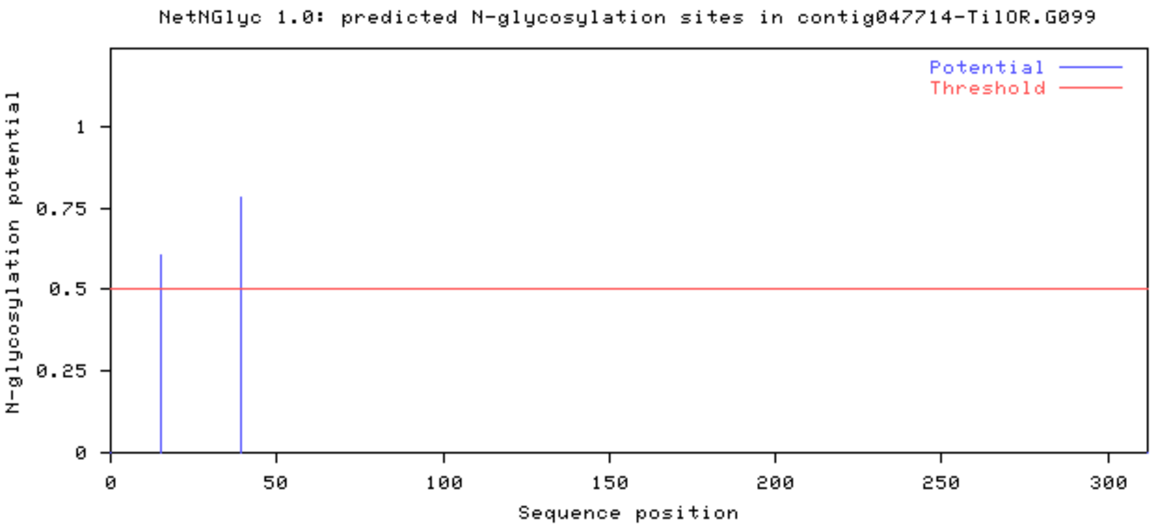

[Graphics in PostScript]

Output for 'contig047725-TilOR.E074'

```
##########################################################################################

    Warning: This sequence may not contain a signal peptide!!

    Proteins without signal peptides are unlikely to be exposed to
    the N-glycosylation machinery and thus may not be glycosylated
    (in vivo) even though they contain potential motifs.

    SignalP-NN euk predictions are as follows:

  # name                 Cmax  pos ?  Ymax  pos ?  Smax  pos ?  Smean ?  D     ?

    SignalP output is explained at http://www.cbs.dtu.dk/services/SignalP/output.html

##########################################################################################

Name:  contig047725-TilOR.E074  Length:  322
MLNVTTPPLSYFILGAFMNVGSLKFFYLLLTLILYILIIAANTFLIVVICVNRSLHEPMYMFLCSLFVNELYGSTGLFPF      80
LLLQILSDVHTVSAPLCFLQIFCIHTYGSVEVSNLAIMSYDRYLAICCPLQYNTRMTSNSTAVLIMVTWAYSLVKFLIAL     160
SLNLRLRLCGNFLNSLYCQNYLVVRLACSGTKVNNVYGIFDIIITIIVPTLIILFSYMKILKVCFYGSKQMRQKSLTTCT     240
PQLVSLLNFSFGCCFEIFQSRFDTTGLPGALRIFLSLYFLMMQPLMNPILYGTQMSKIRGVYEHVLSSKVSCRCSKVSQS     320
DX
..N................................................N............................      80
..........................................................N.....................     160
................................................................................     240
................................................................................     320
..                                                                                   400

(Threshold=0.5)
----------------------------------------------------------------------
SeqName      Position  Potential   Jury    N-Glyc
                                 agreement result
----------------------------------------------------------------------
contig047725-TilOR.E074    3 NVTT   0.7962     (9/9)   +++
contig047725-TilOR.E074   52 NRSL   0.7391     (9/9)   ++
contig047725-TilOR.E074  139 NSTA   0.5957     (7/9)   +
contig047725-TilOR.E074  248 NFSF   0.3859     (8/9)   -
----------------------------------------------------------------------
```

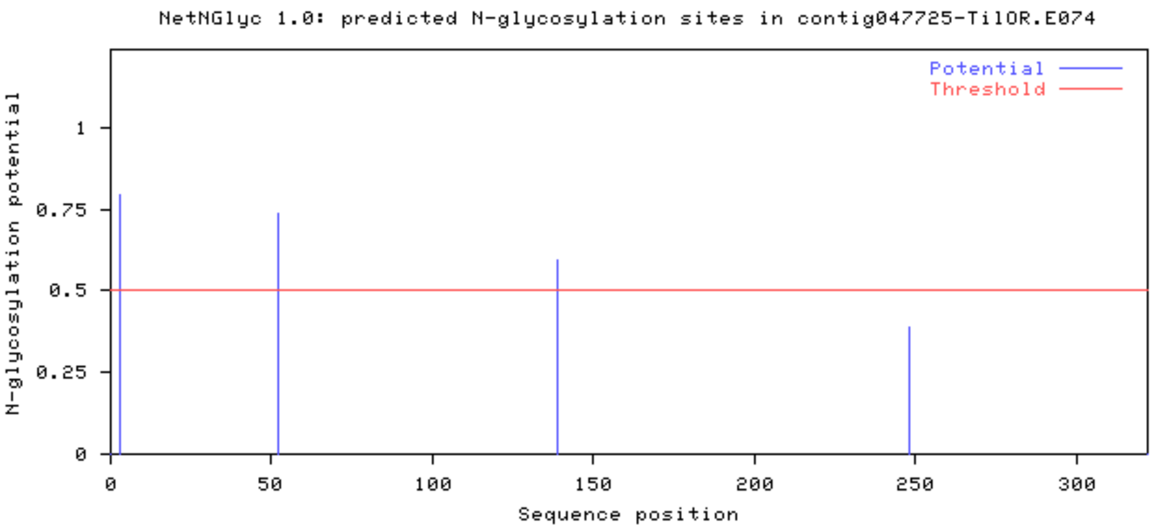

Graphics in PostScript

Output for 'contig047726-TilOR.E075'

```
##########################################################################################

    Warning: This sequence may not contain a signal peptide!!

    Proteins without signal peptides are unlikely to be exposed to
    the N-glycosylation machinery and thus may not be glycosylated
    (in vivo) even though they contain potential motifs.

    SignalP-NN euk predictions are as follows:

  # name                 Cmax  pos ?  Ymax  pos ?  Smax  pos ?  Smean ?  D     ?

    SignalP output is explained at http://www.cbs.dtu.dk/services/SignalP/output.html

##########################################################################################

Name:  contig047726-TilOR.E075  Length:  304
MVNSTVPYFILSTYIYVGSLKYLFFVLIALLYFSIVFVNTSLIVVICVNRSLHEPMYMFLCSLFVNELYGSTGLFPFLLL      80
QILSDVHTVSAPLCFLQIFCLYTYGHVEFCNLAVMSYDRYLAVCYPLHYKSHMTDNKVVIFIVVIWLYSFVKFTITLCLT     160
LRLTWCGKIINGLYCHNYLVVKLACSDTNLNNLFGLFGTVITVLVPLLPIFYSYMKILKVCFSGSREMRCKAVSTCAPHL     240
ASLLNFSFGCLFEILQSRFDTTSVPSALRIFLSLYFLIIQPLLNPIMYGTQMSKIRHVLCYKMX
..N...................................N.........N...............................      80
................................................................................     160
................................................................................     240
................................................................                     320

(Threshold=0.5)
----------------------------------------------------------------------
SeqName      Position  Potential   Jury    N-Glyc
                                 agreement result
----------------------------------------------------------------------
contig047726-TilOR.E075    3 NSTV   0.7904     (9/9)   +++
contig047726-TilOR.E075   39 NTSL   0.6764     (9/9)   ++
contig047726-TilOR.E075   49 NRSL   0.7482     (9/9)   ++
contig047726-TilOR.E075  245 NFSF   0.4441     (7/9)   -
----------------------------------------------------------------------
```

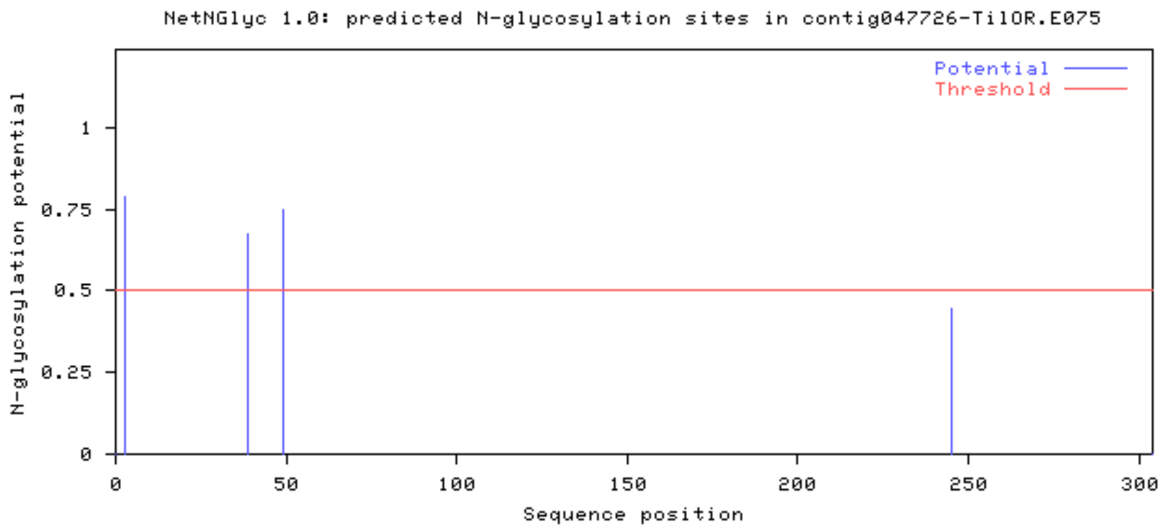

Graphics in PostScript

Output for 'contig047729-TilOR.E076'

```
##########################################################################################

    Warning: This sequence may not contain a signal peptide!!

    Proteins without signal peptides are unlikely to be exposed to
    the N-glycosylation machinery and thus may not be glycosylated
    (in vivo) even though they contain potential motifs.

    SignalP-NN euk predictions are as follows:

  # name                 Cmax  pos ?  Ymax  pos ?  Smax  pos ?  Smean ?  D     ?

    SignalP output is explained at http://www.cbs.dtu.dk/services/SignalP/output.html

##########################################################################################

Name:  contig047729-TilOR.E076  Length:  308
MTVNSSQSSFFVFSAYFDCGHLKYLFFVIVMSLYFLTITANVLLIVVICVNRSLHEPMYMFLCSLFVNELYGSTGLFPSL      80
LIQILSDVHTVSAPLCFLQVFSVYSYGSVEFASLAVMSYDRYLAICYPLEYNMRTTSCKVAMLIALSWLSPFLAIVVLMS     160
LLVPLQLCGNIINKVYCDNYSIVKLACSDITVNNIYGLIATCLTILCPVSLILYTYMRILKICFSGSKQTRQKAVSTCTP     240
HLASLLNFSFGCFFEILQSRFNMSSVPSMLRIFLSLYFLTCQPLFNPLMYGLTLSKISLTCKKTTLCX
...N.............................................N..............................      80
................................................................................     160
..................N.............................................................     240
.....................N..............................................                 320

(Threshold=0.5)
----------------------------------------------------------------------
SeqName      Position  Potential   Jury    N-Glyc
                                agreement result
----------------------------------------------------------------------
contig047729-TilOR.E076    4 NSSQ   0.6927     (9/9)   ++
contig047729-TilOR.E076   51 NRSL   0.7483     (9/9)   ++
contig047729-TilOR.E076  179 NYSI   0.5854     (8/9)   +
contig047729-TilOR.E076  247 NFSF   0.4627     (7/9)   -
contig047729-TilOR.E076  262 NMSS   0.6076     (8/9)   +
----------------------------------------------------------------------
```

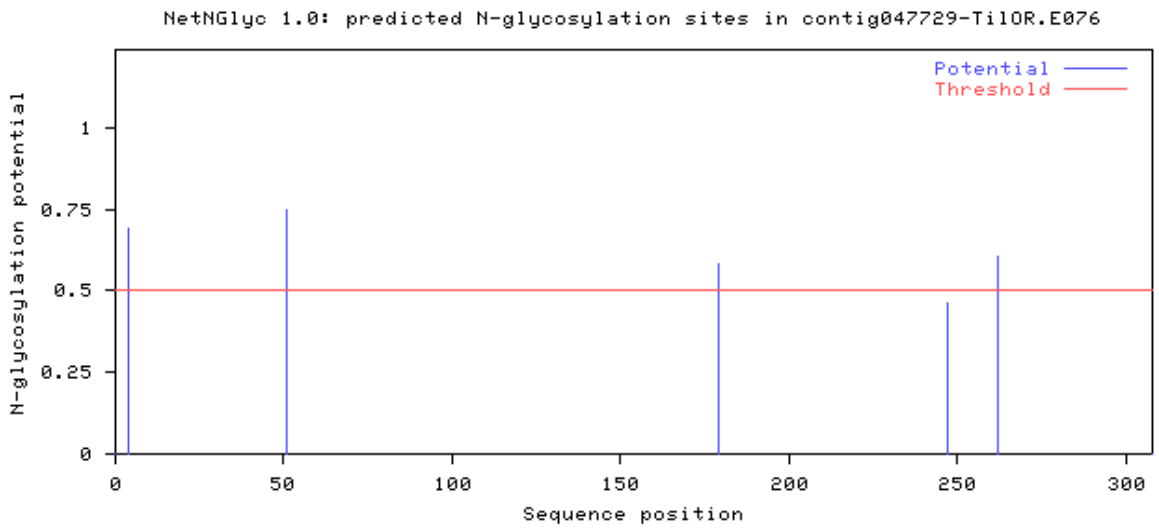

Graphics in PostScript

Output for 'contig047734-TilOR.E077'

```
##########################################################################################

    Warning: This sequence may not contain a signal peptide!!

    Proteins without signal peptides are unlikely to be exposed to
    the N-glycosylation machinery and thus may not be glycosylated
    (in vivo) even though they contain potential motifs.

    SignalP-NN euk predictions are as follows:

  # name                   Cmax  pos ?  Ymax  pos ?  Smax  pos ?  Smean ?  D     ?

    SignalP output is explained at http://www.cbs.dtu.dk/services/SignalP/output.html

##########################################################################################

Name:  contig047734-TilOR.E077  Length:  309
MMNSSQVSYFTLTAYLDSGALKYLYFTVVVFLYIVIVTANVLLIVVICVNRSLHEPMYMFLCSLFVNELYGSTGLFPFLL      80
LQILSDVHTVSAPLCFLQIFCLHTYANAQLTNLAIMSYDRYLAICCPLQYHTRMSSSKVSMLIALTWLYPFLAITLVISL     160
SAPLQLCGNIINKVYCDNNSIVKLACSDTTANNIYGLIATTLTTISSVSLILYTYMRILKVCFSGSKQTRQKAISTCTPH     240
LASLLNFSCGAFFETAQSRFNMKHVPNRVRIFLSLYWLICPPLCNPLLYGLNLTKIRIIYKGLILSKVX
..N..............................................N..............................      80
................................................................................     160
................................................................................     240
.....N.........................................N.................                    320

(Threshold=0.5)
----------------------------------------------------------------------
SeqName      Position  Potential    Jury    N-Glyc
                                  agreement result
----------------------------------------------------------------------
contig047734-TilOR.E077    3 NSSQ   0.6929     (9/9)   ++
contig047734-TilOR.E077   50 NRSL   0.7490     (9/9)   ++
contig047734-TilOR.E077  178 NNSI   0.4251     (7/9)   -
contig047734-TilOR.E077  246 NFSC   0.5578     (6/9)   +
contig047734-TilOR.E077  292 NLTK   0.7153     (9/9)   ++
----------------------------------------------------------------------
```

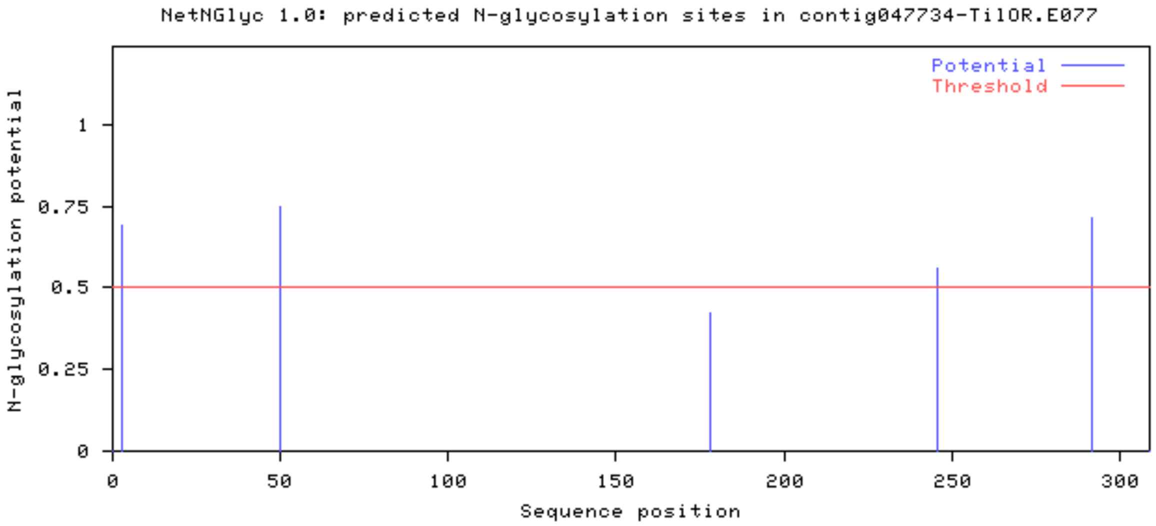

Graphics in PostScript

Output for 'contig047735-TilORfp.E094'

```
##########################################################################################

    Warning: This sequence may not contain a signal peptide!!

    Proteins without signal peptides are unlikely to be exposed to
    the N-glycosylation machinery and thus may not be glycosylated
    (in vivo) even though they contain potential motifs.

    SignalP-NN euk predictions are as follows:

  # name                    Cmax  pos ?  Ymax  pos ?  Smax  pos ?  Smean ?  D     ?

    SignalP output is explained at http://www.cbs.dtu.dk/services/SignalP/output.html

##########################################################################################

Name:  contig047735-TilORfp.E094        Length:  175
MTNLSQVSYFIFSAYFDTGPFKYLYFTIVMSLYVFIFGSNLLLIVVICVNRSLHEPMYMFLCSLFVNELYGSTGLFPFLL      80
LQILSDVHTVSAPLCFLQIFCLYTYANIEFYNLAIMSYDRYLAICYPLQQGSSISVHEGRCPCRFISPWVNTPESHDLIT     160
RPLENFKTCEGNLSI
..N.............................................N...............................      80
................................................................................     160
...............                                                                      240

(Threshold=0.5)
----------------------------------------------------------------------
SeqName      Position  Potential   Jury    N-Glyc
                                 agreement result
----------------------------------------------------------------------
contig047735-TilORfp.E094    3 NLSQ   0.7377     (9/9)   ++
contig047735-TilORfp.E094   50 NRSL   0.7368     (9/9)   ++
contig047735-TilORfp.E094  172 NLSI   0.4790     (5/9)   -
----------------------------------------------------------------------
```

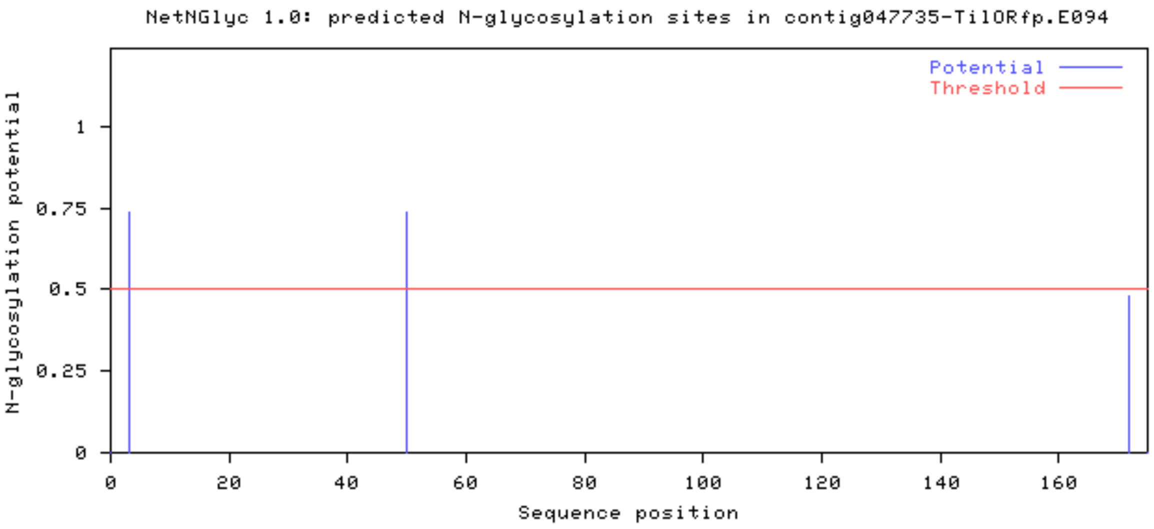

Graphics in PostScript

Output for 'contig047736-TilORe.E091'

```
##########################################################################################

    Warning: This sequence may not contain a signal peptide!!

    Proteins without signal peptides are unlikely to be exposed to
    the N-glycosylation machinery and thus may not be glycosylated
    (in vivo) even though they contain potential motifs.

    SignalP-NN euk predictions are as follows:

  # name                Cmax  pos ?  Ymax  pos ?  Smax  pos ?  Smean ?  D     ?

    SignalP output is explained at http://www.cbs.dtu.dk/services/SignalP/output.html

##########################################################################################

Name:  contig047736-TilORe.E091         Length:  205
WIKDTSKTCRDTGPRGLRLGTPALQYHTHMTLNKVAKLIVLTWLFPILNIVVMISLNASLQLCGHTVDTLYCNNYSVMKL      80
ACFDTTVNNIYGLVYTFTVLIGLAFLNLFTYVKILKVCFSGSKQTRQKAISTCTPHLASLLNFSFGCFFEIVQSRFNMSR     160
APMILRVFLSIYFLTCQPVFNPVLYGLKLTKIQLICKSLLFGKIX
........................................................N................N......      80
................................................................................N...     160
.............................................                                      240

(Threshold=0.5)
----------------------------------------------------------------------
SeqName       Position  Potential   Jury    N-Glyc
                                 agreement result
----------------------------------------------------------------------
contig047736-TilORe.E091   57 NASL   0.6633     (8/9)   +
contig047736-TilORe.E091   74 NYSV   0.6360     (8/9)   +
contig047736-TilORe.E091  142 NFSF   0.4931     (5/9)   -
contig047736-TilORe.E091  157 NMSR   0.5606     (6/9)   +
----------------------------------------------------------------------
```

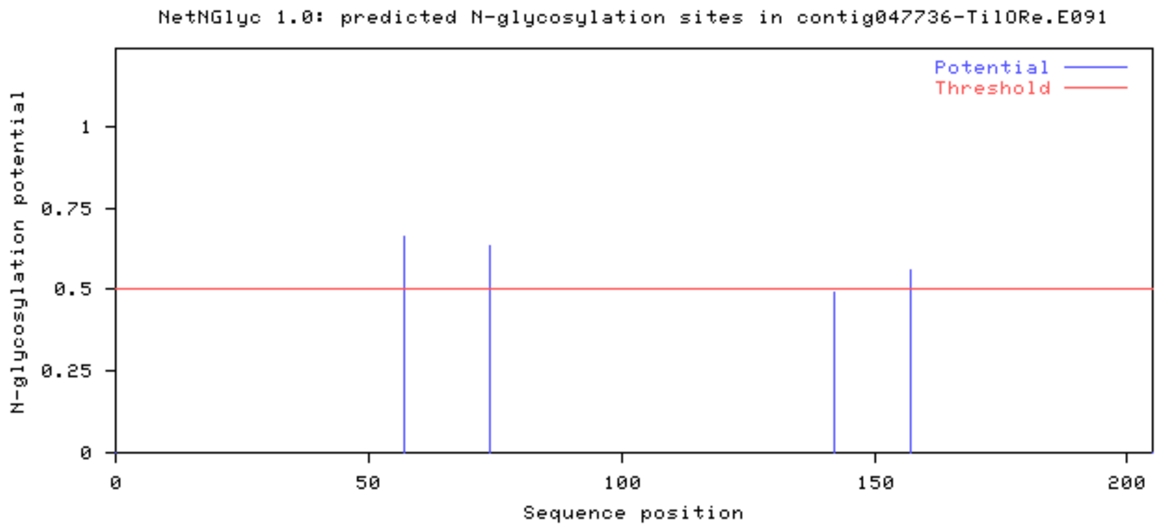

[Graphics in PostScript]

Output for 'contig047738-TilORp.E096'

```
########################################################################################

    Warning: This sequence may not contain a signal peptide!!

    Proteins without signal peptides are unlikely to be exposed to
    the N-glycosylation machinery and thus may not be glycosylated
    (in vivo) even though they contain potential motifs.

    SignalP-NN euk predictions are as follows:

  # name                Cmax  pos ?  Ymax  pos ?  Smax  pos ?  Smean ?  D     ?

    SignalP output is explained at http://www.cbs.dtu.dk/services/SignalP/output.html

########################################################################################

Name:  contig047738-TilORp.E096         Length:  328
MSNSTKFSYLTLSAYFDTMHFQYFCFMFTSVYIFFIITANVLLIVVICVNRSLNEPMYMFLCSLFVNELYGSTGLFPFLL      80
LQILSDVHTVSVSFCFLQVFCLYSYACVEFFDLAIMSYDRYLAISCPLQYHTLMTNTVLITLTWMYASVLCFVPPLLSAP     160
LQLCGNIIDKVYCDNYSITKLVCSDTTLINIYGLISAFVTFGCLVSIILYTYMWILQVCFSGSKQTQQKAVSTCMPHLAS     240
LLNLSFGIFFEIVQNRFNMKHVPDLLRIFLSLYWLTFQPLFNPILYGLNLTKVRIQCKTLIFGKMQMLDVETVVYFFKFN     320
KLKKKSEX
..N..............................................N..............................      80
................................................................................     160
..............N.................................................................     240
..N.............................................N...............................     320
........                                                                             400

(Threshold=0.5)
----------------------------------------------------------------------
SeqName      Position  Potential   Jury    N-Glyc
                                agreement  result
----------------------------------------------------------------------
contig047738-TilORp.E096    3 NSTK   0.7704     (9/9)   +++
contig047738-TilORp.E096   50 NRSL   0.7488     (9/9)   ++
contig047738-TilORp.E096  175 NYSI   0.6706     (9/9)   ++
contig047738-TilORp.E096  243 NLSF   0.5104     (5/9)   +
contig047738-TilORp.E096  289 NLTK   0.6938     (9/9)   ++
----------------------------------------------------------------------
```

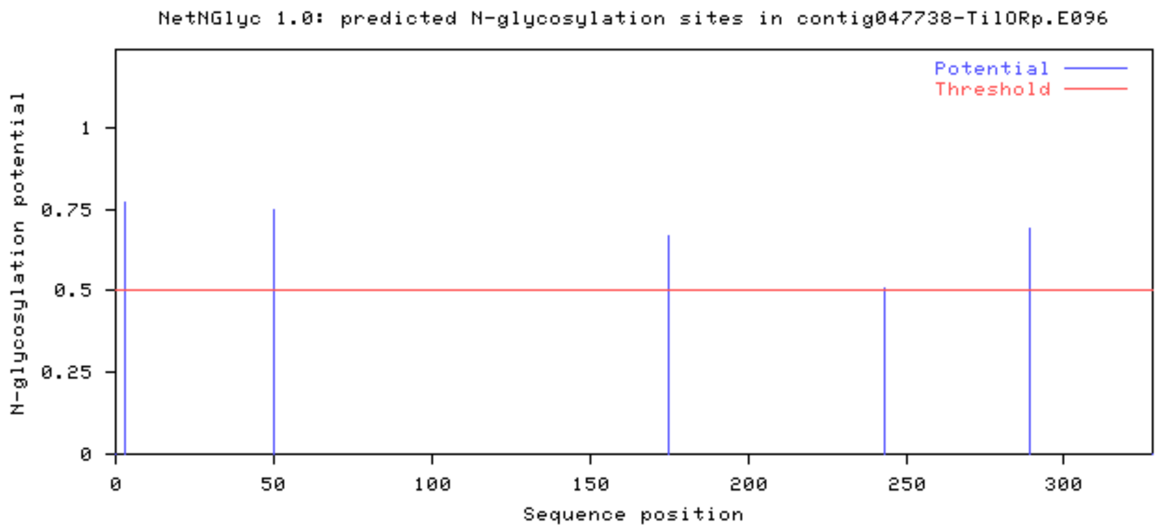

Graphics in PostScript

Output for 'contig047817-TilORe.E092'

```
################################################################################

   Warning: This sequence may not contain a signal peptide!!

   Proteins without signal peptides are unlikely to be exposed to
   the N-glycosylation machinery and thus may not be glycosylated
   (in vivo) even though they contain potential motifs.

   SignalP-NN euk predictions are as follows:

 # name                 Cmax  pos ?  Ymax  pos ?  Smax  pos ?  Smean ?  D     ?

   SignalP output is explained at http://www.cbs.dtu.dk/services/SignalP/output.html

################################################################################

Name:  contig047817-TilORe.E092        Length:  300
MQLLRQLFCEMICVKAGMRTKSNSTQVLYFTLTAYFDTGLFKYLYFISVMSLYMFTVDSNVLLIVVICVNRSLHEPMYMF      80
LCSLFVNELYGSTGLFPFLLLQILSDVHTVSAPLCFLQVYCLCVYGSVEFFILGVMSYLAICYPLQYNTRMTCCKVVVIT     160
AFMWFYSTLTTAVVICLSASLQLCGNIIHRVYCHNSILNLICSDTPVNNVLGYLFSLFLGLMLLIIYSYMRILKVCFSGS     240
KQTRQKAVSTCAPHLASLINFHPSIHLHPLYPRPGRGGSSLSREAQTSLSPATSSSLSGG
......................N..............................................N..........      80
................................................................................     160
................................................................................     240
............................................................                         320

(Threshold=0.5)
----------------------------------------------------------------------
SeqName      Position  Potential   Jury    N-Glyc
                                 agreement result
----------------------------------------------------------------------
contig047817-TilORe.E092    23 NSTQ    0.6983     (9/9)    ++
contig047817-TilORe.E092    70 NRSL    0.7412     (9/9)    ++
----------------------------------------------------------------------
```

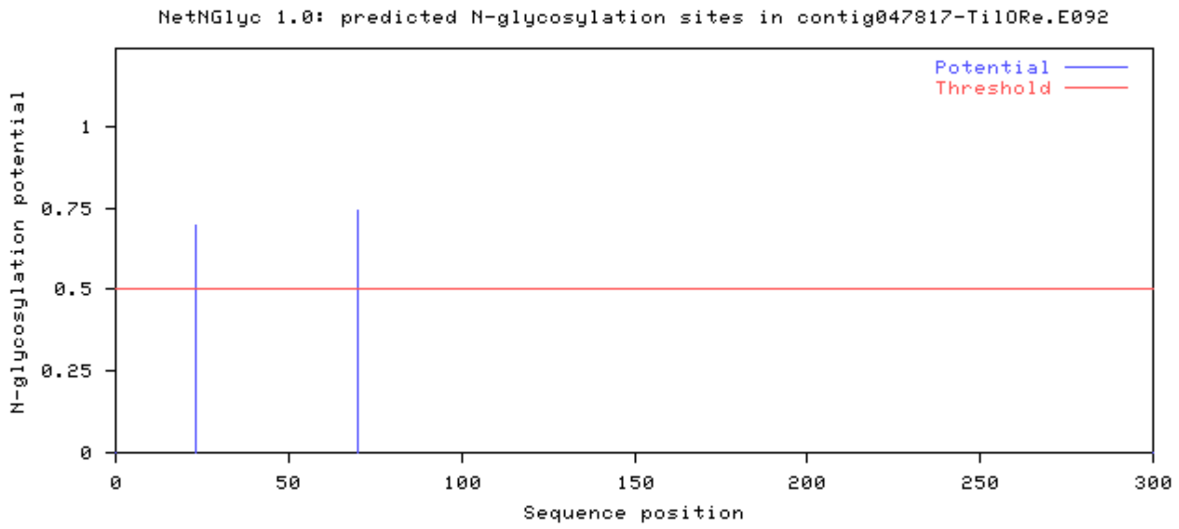

Graphics in PostScript

Output for 'contig047820-TilOR.E078'

```
##########################################################################

    Warning: This sequence may not contain a signal peptide!!

    Proteins without signal peptides are unlikely to be exposed to
    the N-glycosylation machinery and thus may not be glycosylated
    (in vivo) even though they contain potential motifs.

    SignalP-NN euk predictions are as follows:

  # name                   Cmax  pos ?  Ymax  pos ?  Smax  pos ?  Smean ?  D     ?

    SignalP output is explained at http://www.cbs.dtu.dk/services/SignalP/output.html

##########################################################################

Name:  contig047820-TilOR.E078  Length:  309
MNGTRISDFTLGAYFDTGVYKSLYFLIVLTVYIVILCANVLLIVVICVNRSLHEPMYMFLCSLFVNELYGSTGLFPFLLL      80
QILSDVHTVSAPLCFLQIFCVYTYASVEFFNLAVMSFDRYLAICCPLQYRTRMTDNKVAMLTALAWFYSFLVNASILSSL     160
TVSLQLCRGIINKLYCDNYYIIKLACSDTTISSDFGRVHMFTVIFGLILLILYSYMRIFKVCFYGSKQTRQKAISTCTPH     240
LASLFNFSCGAFFGIVQSSLNMNTLPTMFRIFLSLYYLTCQPLVNPLLYGLKMSQIRMLYKSLLFWKKX
.N..............................................N...............................      80
........................................................................N.......     160
................................................................................     240
.....................................................................                320

(Threshold=0.5)
----------------------------------------------------------------------
SeqName      Position  Potential   Jury    N-Glyc
                                 agreement result
----------------------------------------------------------------------
contig047820-TilOR.E078    2 NGTR   0.7903     (9/9)    +++
contig047820-TilOR.E078   49 NRSL   0.7494     (9/9)    ++
contig047820-TilOR.E078  153 NASI   0.5329     (4/9)    +
contig047820-TilOR.E078  246 NFSC   0.4708     (7/9)    -
----------------------------------------------------------------------
```

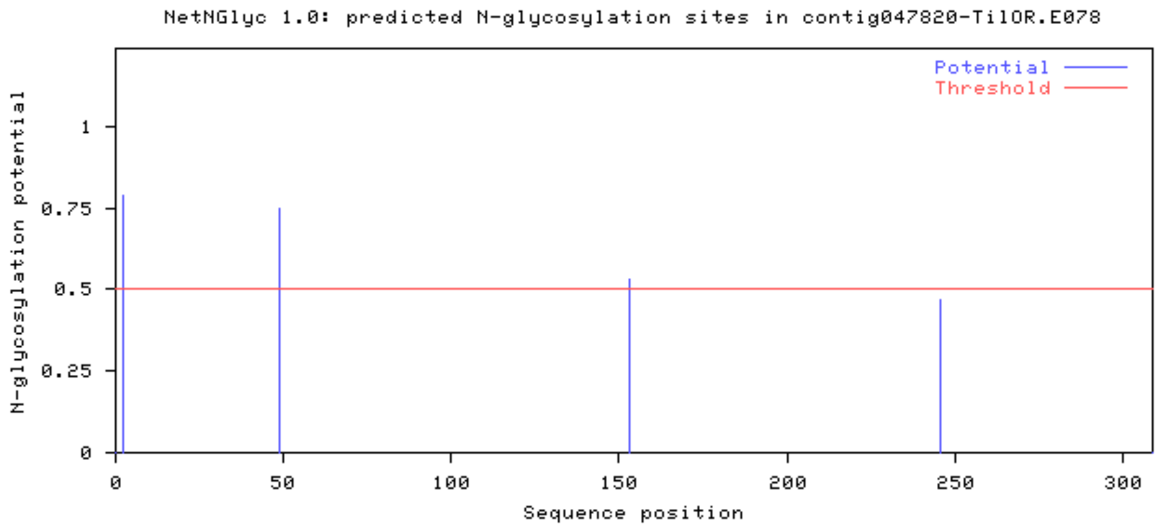

Graphics in PostScript

Output for 'contig047825-TilOR.E079'

```
##########################################################################

    Warning: This sequence may not contain a signal peptide!!

    Proteins without signal peptides are unlikely to be exposed to
    the N-glycosylation machinery and thus may not be glycosylated
    (in vivo) even though they contain potential motifs.

    SignalP-NN euk predictions are as follows:

  # name                 Cmax  pos ?  Ymax  pos ?  Smax  pos ?  Smean ?  D     ?

    SignalP output is explained at http://www.cbs.dtu.dk/services/SignalP/output.html

##########################################################################

Name:  contig047825-TilOR.E079  Length:  330
MINATEITHFTLSPYFDTGAYRYLYFLIIQISYAAIICANLLLIVVICVNRSLHEPMYMFLCSLFVNELYGSTGLFPFLL      80
LQILSDVHTVSISFCFLQVFCVYTYACVEFINLVIMSYDRYYAICWPLQYKSYMTQKTVTMLISLTWLLPFIMIVVLISL     160
SAPLQLCGNVINKVFCGNYAIIKLACSDTRVHNIFGLIYTFISVIIPLVLILYTYVRILKVCFSGSKQTRQKAVSTCTPH     240
LASILNFFFGCCFQILQSRFDTNGVPNVFGILSSLYFLTCQPLFTPLLYGLKMTKIRIACKQLFCGPLTRLFSCSSDIFS     320
KSHQSQTYCX
..N..............................................N..............................      80
................................................................................     160
................................................................................     240
................................................................................     320
..........                                                                           400

(Threshold=0.5)
----------------------------------------------------------------------
SeqName      Position  Potential   Jury    N-Glyc
                                 agreement result
----------------------------------------------------------------------
contig047825-TilOR.E079    3 NATE   0.7098     (9/9)   ++
contig047825-TilOR.E079   50 NRSL   0.7491     (9/9)   ++
----------------------------------------------------------------------
```

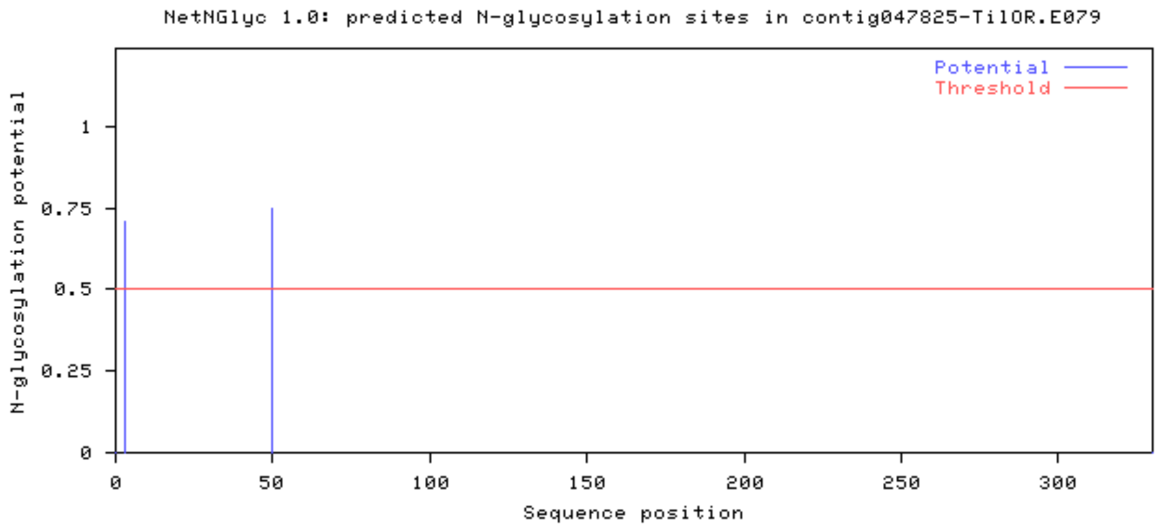

Graphics in PostScript

Output for 'contig047826-TilOR.E080'

```
##########################################################################

    Warning: This sequence may not contain a signal peptide!!

    Proteins without signal peptides are unlikely to be exposed to
    the N-glycosylation machinery and thus may not be glycosylated
    (in vivo) even though they contain potential motifs.

    SignalP-NN euk predictions are as follows:

  # name                Cmax  pos ?  Ymax  pos ?  Smax  pos ?  Smean ?  D     ?

    SignalP output is explained at http://www.cbs.dtu.dk/services/SignalP/output.html

##########################################################################

Name:  contig047826-TilOR.E080  Length:  326
MEIIRNSTQFSYFTLGAYVDTQMFKHLYFIIILSLYMFTVGSNVLLIVVICVNRSLHEPMYMFLCSLFVNELYGSTGLFP      80
LLLLQILSDVHTVSAPLCFLQVFCIYLYASVEFSNLAAMSYDRYMSICYPLQYHTLMTSNKVALLIAVTWIPPLLAVCVT     160
TCLSASLQLCGNVINKVYCNNHSIIKLGCHSTTVNNLYELTAASVTVCVPVSVILYTYTRILKICFSGSKQTRQKAVSTC     240
TPHLASLLNFSFGVSFEILQSRFDMGHVPNMLRIFLSVYFLTCQPLFNPVMYGLNMSKIRTICKNLLLGSVGKSRIFIKI     320
VQIEKX
.....N..............................................N...........................      80
................................................................................     160
................................................................................     240
......................................................N.........................     320
......                                                                               400

(Threshold=0.5)
----------------------------------------------------------------------
SeqName      Position  Potential    Jury     N-Glyc
                                  agreement  result
----------------------------------------------------------------------
contig047826-TilOR.E080    6 NSTQ   0.7710     (9/9)    +++
contig047826-TilOR.E080   53 NRSL   0.7487     (9/9)    ++
contig047826-TilOR.E080  181 NHSI   0.3713     (9/9)    --
contig047826-TilOR.E080  249 NFSF   0.4451     (6/9)    -
contig047826-TilOR.E080  295 NMSK   0.6051     (6/9)    +
----------------------------------------------------------------------
```

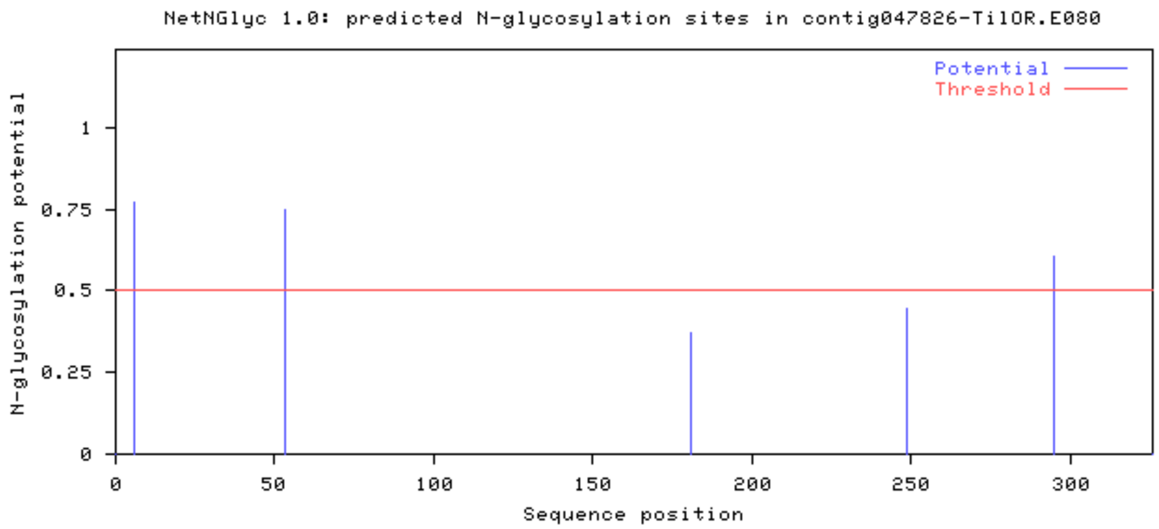

Graphics in PostScript

Output for 'contig047829-TilOR.E081'

```
##########################################################################################

    Warning: This sequence may not contain a signal peptide!!

    Proteins without signal peptides are unlikely to be exposed to
    the N-glycosylation machinery and thus may not be glycosylated
    (in vivo) even though they contain potential motifs.

    SignalP-NN euk predictions are as follows:

  # name                 Cmax  pos ?  Ymax  pos ?  Smax  pos ?  Smean ?  D     ?

    SignalP output is explained at http://www.cbs.dtu.dk/services/SignalP/output.html

##########################################################################################

Name:  contig047829-TilOR.E081  Length:  311
MKTNSTNSTKVLYFTLTAYFDTGLFKYLYFIIALSLYAFIVGSNVLLIVVICVNRSLHEPMYMFLCSLFVNELYGSTGLF      80
PFLLLQILSDVHTVSAPLCFLQVFCVFTYAGVGLLNLAVMSYDRYLAICYPLQYRTLMTMWKVAFLIAVTWLPRFLVVVI     160
ITSLSASLQLCGNIINKVYCDNQSIVKLACSDTTVTNISGVIVSALTIFVPVILIFYSYMRILKVCFSGSKQTRQKAVST     240
CTPHLVSLLNFSFGSFLEVLQSRFNNNNVPNMLKIFLSLYFLACQPLFNPVLYGLKMSSIRKSLFLLKSRX
...N..N..............................................N..........................      80
................................................................................     160
....................................N...........................................     240
.......................................................................              320

(Threshold=0.5)
----------------------------------------------------------------------
SeqName      Position  Potential   Jury    N-Glyc
                                 agreement result
----------------------------------------------------------------------
contig047829-TilOR.E081    4 NSTN   0.7024     (9/9)   ++
contig047829-TilOR.E081    7 NSTK   0.6981     (9/9)   ++
contig047829-TilOR.E081   54 NRSL   0.7477     (9/9)   ++
contig047829-TilOR.E081  182 NQSI   0.4644     (4/9)   -
contig047829-TilOR.E081  197 NISG   0.6040     (6/9)   +
contig047829-TilOR.E081  250 NFSF   0.4099     (8/9)   -
----------------------------------------------------------------------
```

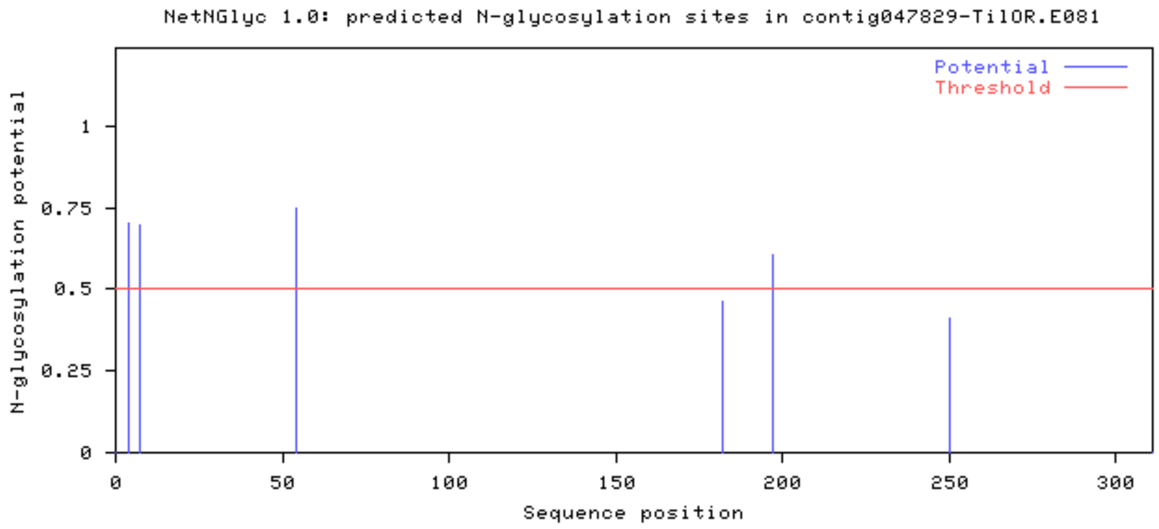

Graphics in PostScript

Output for 'contig047832-TilOR.E082'

```
##########################################################################################

    Warning: This sequence may not contain a signal peptide!!

    Proteins without signal peptides are unlikely to be exposed to
    the N-glycosylation machinery and thus may not be glycosylated
    (in vivo) even though they contain potential motifs.

    SignalP-NN euk predictions are as follows:

  # name                   Cmax  pos ?  Ymax  pos ?  Smax  pos ?  Smean ?  D     ?

    SignalP output is explained at http://www.cbs.dtu.dk/services/SignalP/output.html

##########################################################################################

Name:  contig047832-TilOR.E082  Length:  309
MTNFTEGSYFILGAYFDAGPTKYLFFLLLLSLYSLIICANVLLIVVICVNRSLHEPMYMFLCSLFVNELYGSTGLFPSLL      80
VQILSDVHTVSAPLCFLQIFCLYSYGSVEFYNLATMSYDRYLAICCPLQYNTRMTSNTVSVLIAVSWIYALLLVAVTVSL     160
SSPLQLCGNIINKVYCDNYAIVKLACSDTTLNNIYGLISTAFTTFVPLTLIFFTYMRILKVCFSGSKQTRQKAVSTCTPH     240
LASLLNFSFGACFEVLQSRFNMNTVPNILRILISLYWLICQPLFNPVLYGLKMSKIYDICKSLLYWKVX
..N..............................................N..............................      80
................................................................................     160
................................................................................     240
.....................................................................                320

(Threshold=0.5)
----------------------------------------------------------------------
SeqName      Position  Potential   Jury    N-Glyc
                                 agreement result
----------------------------------------------------------------------
contig047832-TilOR.E082    3 NFTE   0.6752     (9/9)   ++
contig047832-TilOR.E082   50 NRSL   0.7491     (9/9)   ++
contig047832-TilOR.E082  246 NFSF   0.4348     (7/9)   -
----------------------------------------------------------------------
```

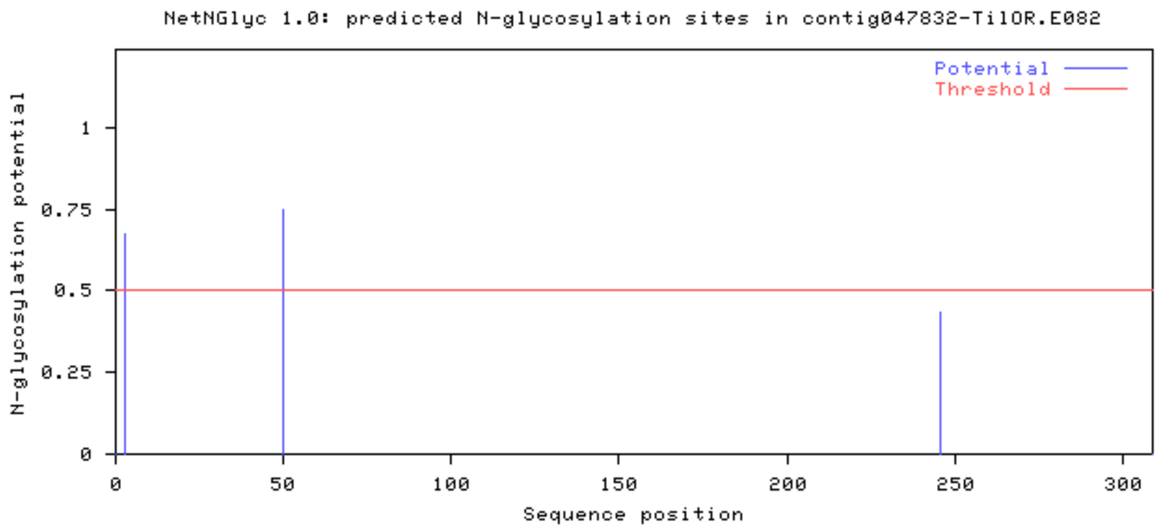

Graphics in PostScript

Output for 'contig047833-TilOR.E083'

```
##########################################################################################

    Warning: This sequence may not contain a signal peptide!!

    Proteins without signal peptides are unlikely to be exposed to
    the N-glycosylation machinery and thus may not be glycosylated
    (in vivo) even though they contain potential motifs.

    SignalP-NN euk predictions are as follows:

  # name                 Cmax  pos ?  Ymax  pos ?  Smax  pos ?  Smean ?  D     ?

    SignalP output is explained at http://www.cbs.dtu.dk/services/SignalP/output.html

##########################################################################################

Name:  contig047833-TilOR.E083  Length:  314
MTNFTEGSYFILGAYFDAGPTKYLFFLLLLSLYSLILCANLLLIVVICVNRSLHEPMYMFLCSLFVNELYGSTGLFPLLL      80
LQILSDVHTVPAPLCFLQIFCLYSYVGVEFLTLAIMSYDRYLAICCPLQYNTRMTSNTVSVLIAVSWIYALLLVAVTVSL     160
SSPLQLCGNIINKVYCDNYAIVKLACSDTTLNNIYGLISTAFTVFVPLTLIFFTYMRILKVCFSGSKQTRQKAVSTCTPH     240
LASLLNFSCSGCFEILQSRFNMNNLPNVLRILISLYWLICQPLFNPVLYGLKMSKIRDICKCLLYSKVNTILSX
..N..............................................N..............................      80
................................................................................     160
................................................................................     240
.....N....................................................................           320

(Threshold=0.5)
----------------------------------------------------------------------
SeqName       Position  Potential   Jury    N-Glyc
                                 agreement result
----------------------------------------------------------------------
contig047833-TilOR.E083    3 NFTE   0.6750     (9/9)    ++
contig047833-TilOR.E083   50 NRSL   0.7483     (9/9)    ++
contig047833-TilOR.E083  246 NFSC   0.5483     (7/9)    +
----------------------------------------------------------------------
```

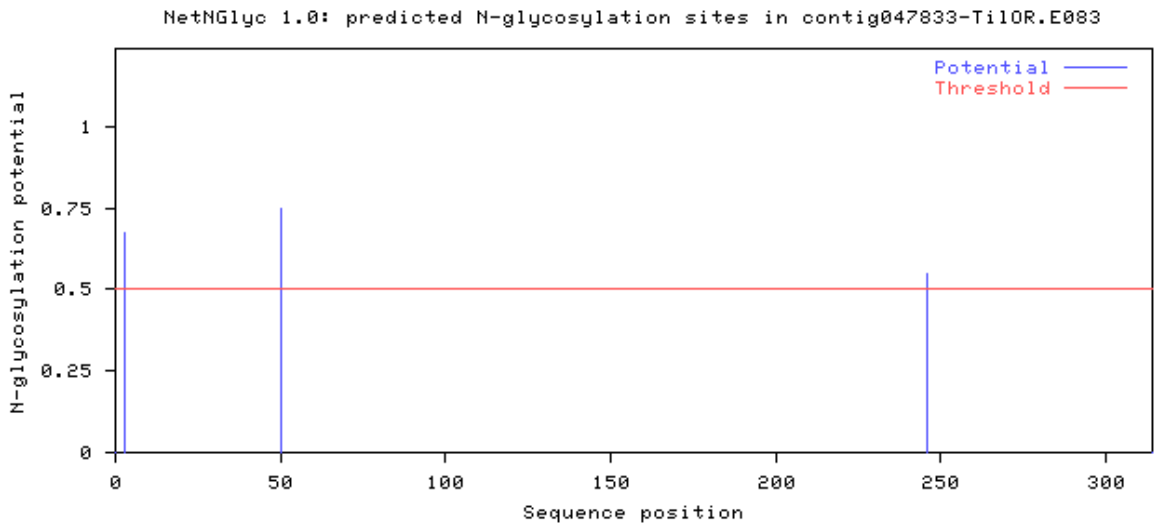

Graphics in PostScript

Output for 'contig047834-TilOR.E084'

```
##########################################################################################

    Warning: This sequence may not contain a signal peptide!!

    Proteins without signal peptides are unlikely to be exposed to
    the N-glycosylation machinery and thus may not be glycosylated
    (in vivo) even though they contain potential motifs.

    SignalP-NN euk predictions are as follows:

  # name                 Cmax  pos ?  Ymax  pos ?  Smax  pos ?  Smean ?  D     ?

    SignalP output is explained at http://www.cbs.dtu.dk/services/SignalP/output.html

##########################################################################################

Name:  contig047834-TilOR.E084  Length:  316
MLNVTSPPLSYFILGGYMDVGSFKLSYFSLTTVLYIMIIAANTFLIVVICVNRGLHEPMYMFLCSLFVNELYGSTGLFPF      80
LLIQILSDVHTVPAPLCFLQVYCLYTYAVIEFCILAVMSYDRYLAVCCPLQYNAIMTYNKVFLFIISVCLFSFINMLIGL     160
SLTLRLTLCGNTINSLYCSNYLIVKLACSGTKVNNIYGLFNTAVSILVPLLPVLYSYIKILIICFSRSKQARRKAINTCA     240
PHLASLLSFYFGCLFEILQSRFDMSSVPSTLRIFLFLYFLIIQPLFNPIMYGTQMSKIRKACKHVFCIKGFTGYRX
..N.............................................................................      80
................................................................................     160
................................................................................     240
............................................................................         320

(Threshold=0.5)
----------------------------------------------------------------------
SeqName      Position  Potential   Jury    N-Glyc
                                 agreement result
----------------------------------------------------------------------
contig047834-TilOR.E084    3 NVTS   0.8390     (9/9)    +++
----------------------------------------------------------------------
```

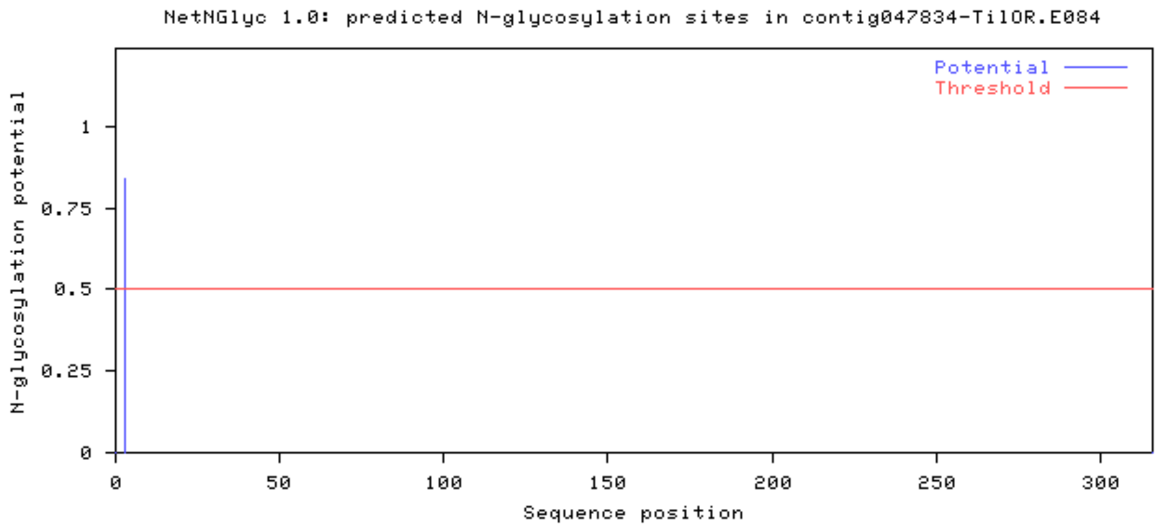

Graphics in PostScript

Output for 'contig049155-TilORe.L161'

```
##########################################################################################

    Warning: This sequence may not contain a signal peptide!!

    Proteins without signal peptides are unlikely to be exposed to
    the N-glycosylation machinery and thus may not be glycosylated
    (in vivo) even though they contain potential motifs.

    SignalP-NN euk predictions are as follows:

  # name                    Cmax   pos ?  Ymax   pos ?  Smax   pos ?  Smean ?  D      ?

    SignalP output is explained at http://www.cbs.dtu.dk/services/SignalP/output.html

##########################################################################################

Name:  contig049155-TilORe.L161         Length:  91
FVKILSNNDKKKMGSTLVNHLICVSCLYGPQFVIVILTRFGVVLTLEERHGLLIGTILGLPLVNPFVYCLRTKEIKSKIF      80
MIFRKVNTAGX
................................................................................      80
...........                                                                          160

(Threshold=0.5)

No sites predicted in this sequence.
```

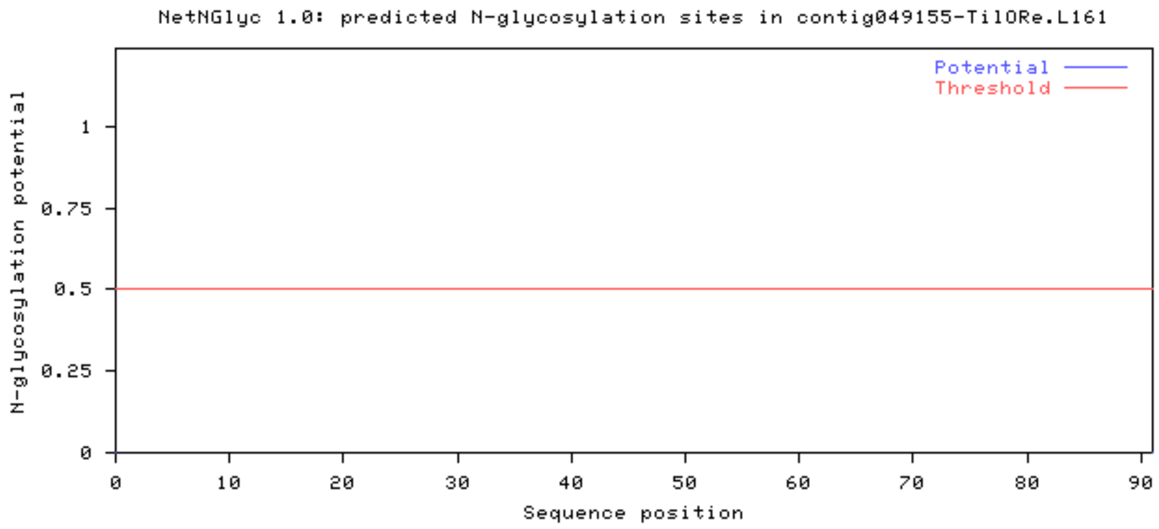

Graphics in PostScript

Output for 'contig049156-TilORe.L162'

```
##########################################################################################

    Warning: This sequence may not contain a signal peptide!!

    Proteins without signal peptides are unlikely to be exposed to
    the N-glycosylation machinery and thus may not be glycosylated
    (in vivo) even though they contain potential motifs.

    SignalP-NN euk predictions are as follows:

  # name                 Cmax  pos ?  Ymax  pos ?  Smax  pos ?  Smean ?  D     ?

    SignalP output is explained at http://www.cbs.dtu.dk/services/SignalP/output.html

##########################################################################################

Name:  contig049156-TilORe.L162         Length:  76
MSLQNTSIKLTYFIIGGFDTVKRPVAVGVVMLITYLLAVFASLVNIIFIVSDKQLHKPMYLLICNLAVVDILYTSS
....N.......................................................................          80

(Threshold=0.5)
----------------------------------------------------------------------
SeqName      Position  Potential   Jury    N-Glyc
                                 agreement result
----------------------------------------------------------------------
contig049156-TilORe.L162    5 NTSI   0.5892     (8/9)   +
----------------------------------------------------------------------
```

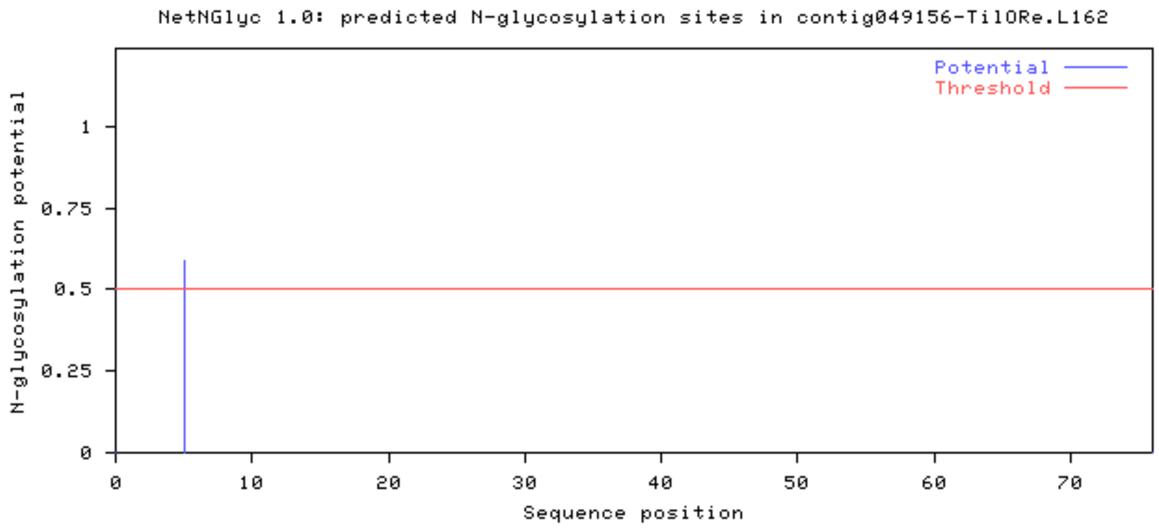

[Graphics in PostScript]

Output for 'contig050080-TilOR.N198'

```
##########################################################################################

    Warning: This sequence may not contain a signal peptide!!

    Proteins without signal peptides are unlikely to be exposed to
    the N-glycosylation machinery and thus may not be glycosylated
    (in vivo) even though they contain potential motifs.

    SignalP-NN euk predictions are as follows:

  # name                Cmax  pos ?  Ymax  pos ?  Smax  pos ?  Smean ?  D     ?

    SignalP output is explained at http://www.cbs.dtu.dk/services/SignalP/output.html

##########################################################################################

Name:  contig050080-TilOR.N198  Length:  323
MAFINLAVENITFVRPAYFIISGFIGIPNIRYYFVFLCFIYILAVVGTTLVMTVITLDHTLRSPKYVAVFNLAFTDLLSS      80
SSLMPKVLDIFLLSHYYISYNDCMTFMFFSFTFYAMQAFNLVVLSYDRVMAIMYPLHYQMRVSHKLILSMIAFFWLLAIT     160
LMLIEVGLLTRLSFCKSVVIQSYFCDHGPMYRLGCNDITPNRAIAGLAPVIILGFPLAFIVGSYCCIGYSLSKISTYRER     240
VKAFKTCTGHLSLVAIYFLPITFVYIFGNVIHPNARIISLSITTALPPMLNPIIYVLQTQEIKESLKKLFQTRAQFRVAA     320
KYX
.........N......................................................................      80
................................................................................     160
................................................................................     240
................................................................................     320
...                                                                                  400

(Threshold=0.5)
----------------------------------------------------------------------
SeqName      Position  Potential   Jury    N-Glyc
                                 agreement result
----------------------------------------------------------------------
contig050080-TilOR.N198   10 NITF   0.6152     (8/9)    +
----------------------------------------------------------------------
```

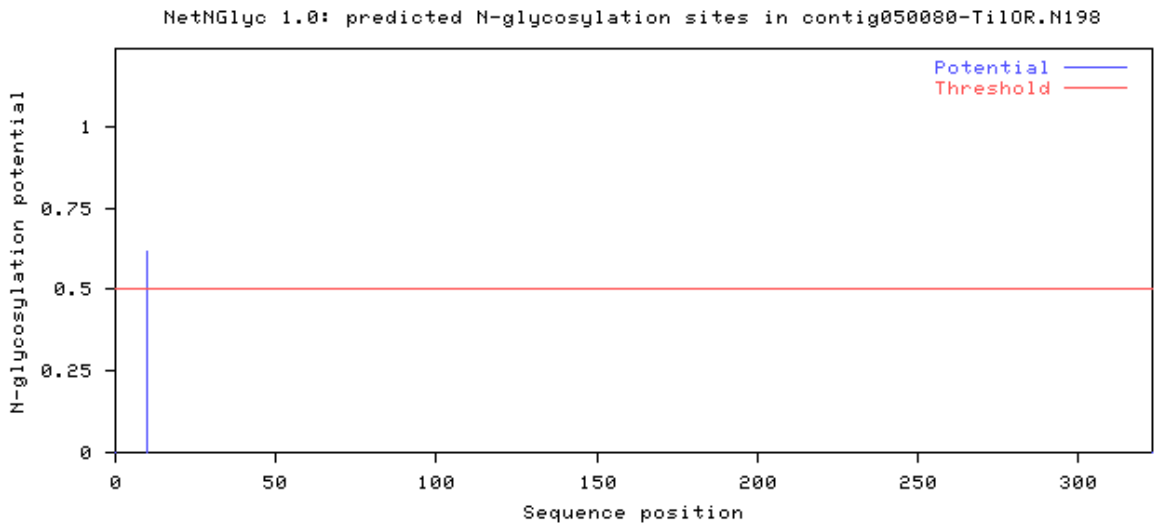

[Graphics in PostScript]

Output for 'contig065241-TilORe.P216'

```
########################################################################################

    Warning: This sequence may not contain a signal peptide!!

    Proteins without signal peptides are unlikely to be exposed to
    the N-glycosylation machinery and thus may not be glycosylated
    (in vivo) even though they contain potential motifs.

    SignalP-NN euk predictions are as follows:

  # name                Cmax  pos ?  Ymax  pos ?  Smax  pos ?  Smean ?  D     ?

    SignalP output is explained at http://www.cbs.dtu.dk/services/SignalP/output.html

########################################################################################

Name:  contig065241-TilORe.P216         Length:  274
MFVVSLFANSLLVYVIVSQRSLHSPMYILIASMACIDLILPIFFLPHMLLSFLFDWRGISLIGCLVQMYFIHFFGTFQST      80
LLLWMALDRYFAICTPLYYQEQVTLAKFLKFVIPSSIRNMFVVLVVVSLAGKLPFCLRNVINHCFCEHMALVELACGSTI     160
INSLVGLISVFLVPVTDFFLVTASYTVIFSSVLTSGKSNAKAFQTCVTHIVVIIVNLTIVLTAFLSYRIRNGIPSAVRIF     240
FSILYVLFPCCFNPIIYGIRTTEIRQHILKTLKI
................................................................................      80
................................................................................     160
.......................................................N........................     240
..................................                                                   320

(Threshold=0.5)
----------------------------------------------------------------------
SeqName      Position  Potential   Jury    N-Glyc
                                 agreement result
----------------------------------------------------------------------
contig065241-TilORe.P216  216 NLTI   0.6993     (9/9)   ++
----------------------------------------------------------------------
```

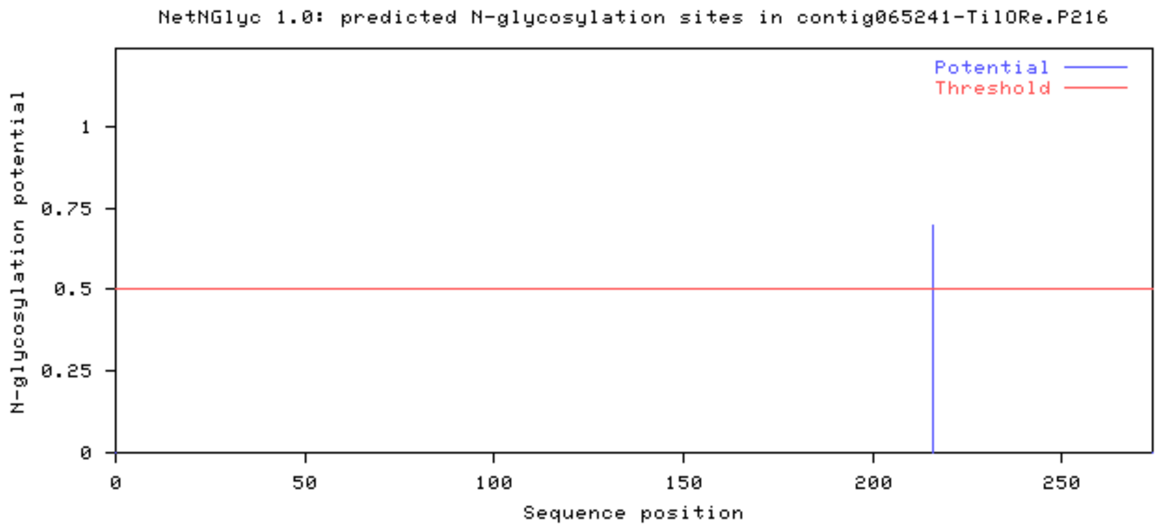

Graphics in PostScript

Output for 'contig065247-TilOR.P211'

```
##########################################################################

   Warning: This sequence may not contain a signal peptide!!

   Proteins without signal peptides are unlikely to be exposed to
   the N-glycosylation machinery and thus may not be glycosylated
   (in vivo) even though they contain potential motifs.

   SignalP-NN euk predictions are as follows:

 # name                Cmax  pos ?  Ymax  pos ?  Smax  pos ?  Smean ?  D     ?

   SignalP output is explained at http://www.cbs.dtu.dk/services/SignalP/output.html

##########################################################################

Name:  contig065247-TilOR.P211  Length:  310
MEPVTENISSHKYFFLVGFSELGELRPFLFIPFSLMFVVSLFANSLLVYVIVSQRSLHSPMYILIASMACIDLILPIFFL      80
PHMLLSFLFDWRGISLIGCLVQMYFIHFFGTFQSTLLVWMALDRYFAICTPLYYQEQMTLAKFLKFVIPLFIRNMFVVLV     160
VVSLAGKLPFCLRNVINHCFCEHMALVELACGSTIINSLVGLISVFLVPVTDFFLVTASYIIVFNSVLRLKKSSAKALHT     240
CVTHIVVITVNLSIVLIALLSYRIRNGLPSAIRIFFSIMYLLFPTCFNPIIYGIRTTEIRQHILKTLKIX
......N.........................................................................      80
................................................................................     160
................................................................................     240
..........N...........................................................               320

(Threshold=0.5)
----------------------------------------------------------------------
SeqName      Position  Potential   Jury    N-Glyc
                                 agreement result
----------------------------------------------------------------------
contig065247-TilOR.P211    7 NISS   0.7181     (9/9)    ++
contig065247-TilOR.P211  251 NLSI   0.6674     (9/9)    ++
----------------------------------------------------------------------
```

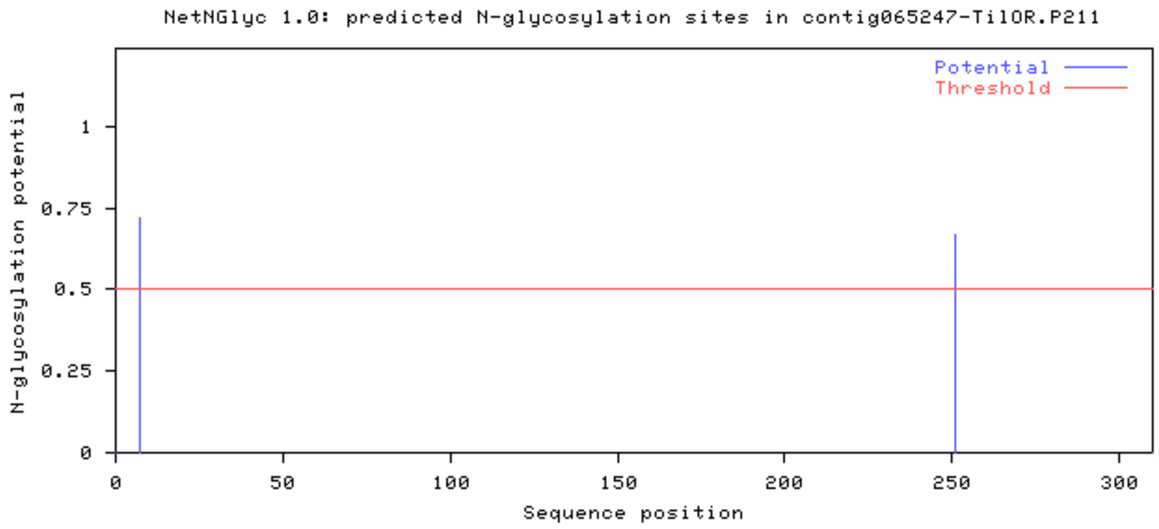

[Graphics in PostScript]

Output for 'contig065253-TilOR.P212'

```
########################################################################################

    Warning: This sequence may not contain a signal peptide!!

    Proteins without signal peptides are unlikely to be exposed to
    the N-glycosylation machinery and thus may not be glycosylated
    (in vivo) even though they contain potential motifs.

    SignalP-NN euk predictions are as follows:

  # name                Cmax  pos ?  Ymax  pos ?  Smax  pos ?  Smean ?  D     ?

    SignalP output is explained at http://www.cbs.dtu.dk/services/SignalP/output.html

########################################################################################

Name:  contig065253-TilOR.P212  Length:  310
MEPVKENISSHKYFFLVGFSELGELRPFLFIPFSVMFVVSLFANSLLVYVIVSQRSLHSPMYILIASMACIDLSLPVFFV      80
PHMLLSFLFDWRGISLGACLVQMYFVHLLGAFQSTLLLWMALDRYFAICTPLYYQEQMALEKFLKFVIPLFIRNMFVVLV     160
VVSLAGKLPFCLRNVINHCFCEHMALVELACGSTIINSLVGLISVISVPVTDFFLITASYTVIFSSVLSSGKSSAKALHT     240
CVTHIVVMTVSLTIVLTAFLSYRIRNGIPSAIRIFFSIMYLFFPSCFNPIIYGIRTTEIRQHILNTLKIX
......N.........................................................................      80
................................................................................     160
................................................................................     240
......................................................................               320

(Threshold=0.5)
----------------------------------------------------------------------
SeqName      Position  Potential   Jury    N-Glyc
                                 agreement result
----------------------------------------------------------------------
contig065253-TilOR.P212    7 NISS   0.7094     (9/9)    ++
----------------------------------------------------------------------
```

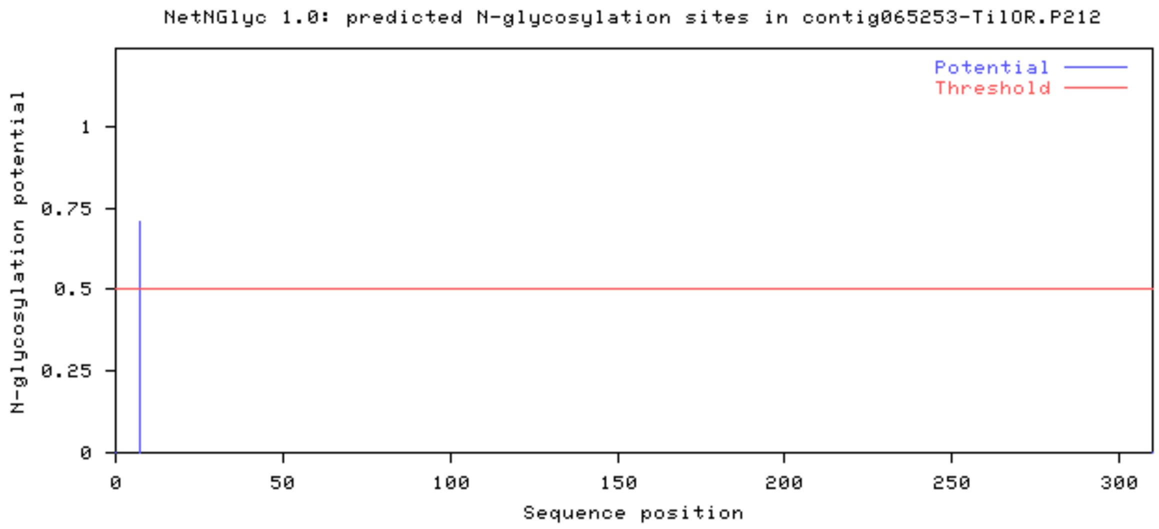

Graphics in PostScript

Output for 'contig065254-TilOR.P213'

```
##########################################################################################

    Warning: This sequence may not contain a signal peptide!!

    Proteins without signal peptides are unlikely to be exposed to
    the N-glycosylation machinery and thus may not be glycosylated
    (in vivo) even though they contain potential motifs.

    SignalP-NN euk predictions are as follows:

  # name                Cmax  pos ?  Ymax  pos ?  Smax  pos ?  Smean ?  D     ?

    SignalP output is explained at http://www.cbs.dtu.dk/services/SignalP/output.html

##########################################################################################

Name:  contig065254-TilOR.P213  Length:  332
MLEAPLSRNFSHSTFVLRGFPSLQKHRRLLALPFSASYLLVLLGNSLLVYVIRSVESLHSPMYLLICTLCFVDMLVVTTI      80
IPNMLLGFLFNWNEISLVGCLTQMFFTHFMSSVESTLLLAMALDRYVAICRPLHYNEIINSSMLVRLVLFTLVRSVSIMA     160
TLVGLAGSLQFCGSNVIQHCYCDHMALVSLACDSTSRSGAAGLAVIICFVGLDIPIIFFSYMKILSVVLRSAAAGEDRRK     240
AFHTCSTHLIVMMCFYLVGSITFLSHNLNIAIPTDINNSMGLMYILFPATINPIIYGVRTKEIRNGFLRIFKNRAKKIMT     320
VKVSSAGKGKSX
........N.......................................................................      80
................................................................................     160
................................................................................     240
................................................................................     320
............                                                                         400

(Threshold=0.5)
----------------------------------------------------------------------
SeqName      Position  Potential   Jury    N-Glyc
                                 agreement result
----------------------------------------------------------------------
contig065254-TilOR.P213    9 NFSH   0.6402     (9/9)   ++
contig065254-TilOR.P213  140 NSSM   0.4670     (5/9)   -
contig065254-TilOR.P213  277 NNSM   0.2172     (9/9)   ---
----------------------------------------------------------------------
```

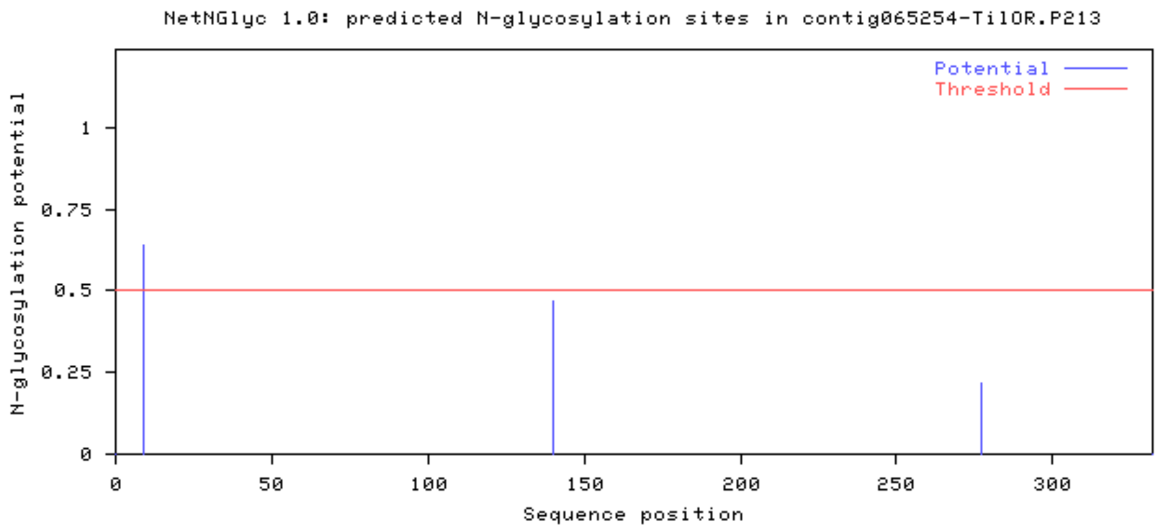

Graphics in PostScript

Output for 'contig065444-TilOR.P214'

```
##########################################################################################

    Warning: This sequence may not contain a signal peptide!!

    Proteins without signal peptides are unlikely to be exposed to
    the N-glycosylation machinery and thus may not be glycosylated
    (in vivo) even though they contain potential motifs.

    SignalP-NN euk predictions are as follows:

  # name                 Cmax  pos ?  Ymax  pos ?  Smax  pos ?  Smean ?  D     ?

    SignalP output is explained at http://www.cbs.dtu.dk/services/SignalP/output.html

##########################################################################################

Name:  contig065444-TilOR.P214  Length:  318
MENISLHTHFILDGFSELGELRPFLFIPFSFMFVVSLFANSLLLYIILSQRSLHSPMCILIAGMACIDLSLPVFFVPHML      80
LSFLFDWRGISLIGCLVQMHFIHFFGTFQSTLLVWMALDRYFAICTPLYYHNHMILSRFIVFLLPLVVRNVLMITLFVSL     160
AGKLPFCLRNVINHCFCEHMALVELACGSTTTNNLVGLMAVFLIPVLDFVFIAAFYVVIFSSVLRSSRSGVKALHTCITH     240
IVVITVSLILALTAFLSYRIKNGLPAASRVFFSTMYLLFPSCFNPVIYGIRTTEIRQHIRKTLTCCHICKMGSVTNKX
..N.............................................................................      80
................................................................................     160
................................................................................     240
..............................................................................     320

(Threshold=0.5)
----------------------------------------------------------------------
SeqName      Position  Potential   Jury    N-Glyc
                                 agreement result
----------------------------------------------------------------------
contig065444-TilOR.P214    3 NISL   0.7339     (9/9)    ++
----------------------------------------------------------------------
```

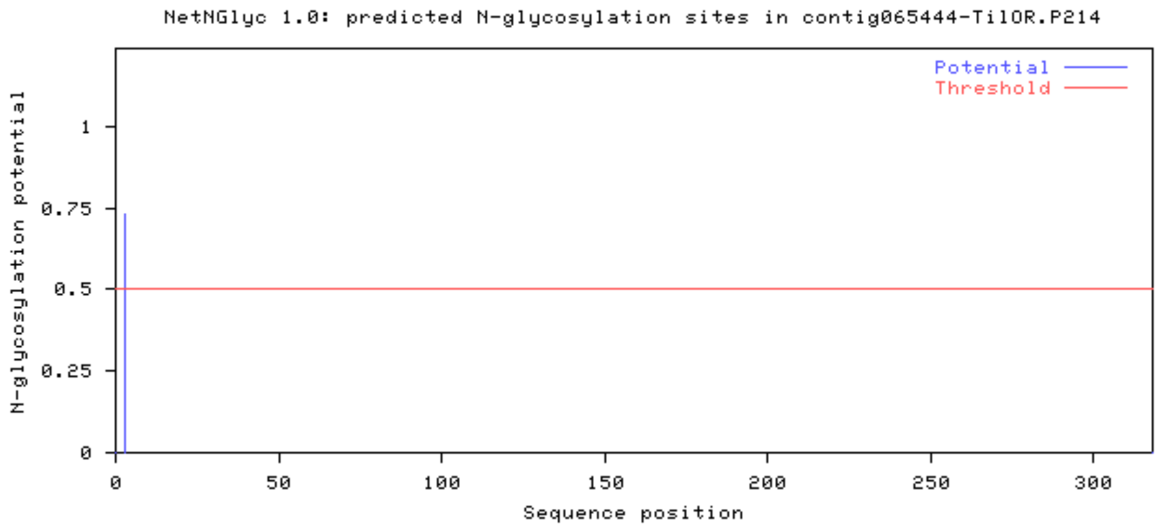

Graphics in PostScript

Output for 'contig065449-TilOR.P215'

```
##########################################################################

    Warning: This sequence may not contain a signal peptide!!

    Proteins without signal peptides are unlikely to be exposed to
    the N-glycosylation machinery and thus may not be glycosylated
    (in vivo) even though they contain potential motifs.

    SignalP-NN euk predictions are as follows:

  # name                Cmax  pos ?  Ymax  pos ?  Smax  pos ?  Smean ?  D     ?

    SignalP output is explained at http://www.cbs.dtu.dk/services/SignalP/output.html

##########################################################################

Name:  contig065449-TilOR.P215  Length:  310
MEPVKENISSHKYFFLVGFGELGELRPFLFIPFSLMFVVSLFANSLLVYVIVSQRSLHSPMYILIASMACIDLSLPVFFV      80
PHMLLSFLFDWRGISLGACLVQMYFVHLLGAFQSTLLLWMALDRYFAICTPLYYQEQMALAKFLKFVIPLFIRNMFVVLV     160
VVSLAGKLPFCLRNVINHCFCEHMALVELACGSTIINSVAGLITVFSVPVTDFFLITASYTVIFSSVLSSGKASAKALHT     240
CVTHIVVMTVSLTIILTAFLSYRIRNSLPTAIRIFFSIMYLLFPSFFNPIIYGIRTTEIRQHILKTLKIX
......N.........................................................................      80
................................................................................     160
................................................................................     240
......................................................................               320

(Threshold=0.5)
----------------------------------------------------------------------
SeqName      Position  Potential   Jury    N-Glyc
                                 agreement result
----------------------------------------------------------------------
contig065449-TilOR.P215    7 NISS   0.7094     (9/9)    ++
----------------------------------------------------------------------
```

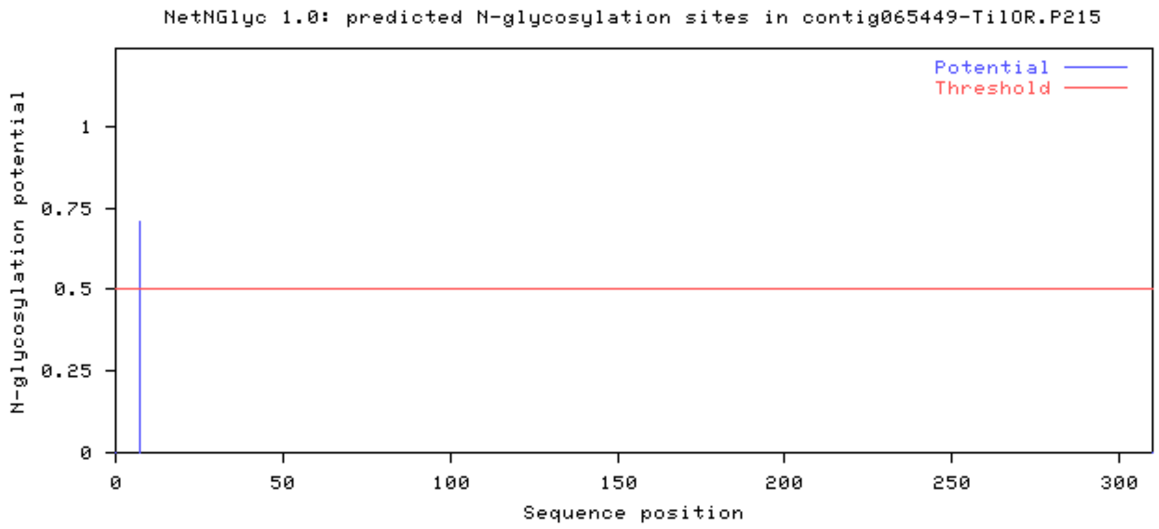

[Graphics in PostScript]

Output for 'contig065453-TilOR.E087'

```
########################################################################################

    Warning: This sequence may not contain a signal peptide!!

    Proteins without signal peptides are unlikely to be exposed to
    the N-glycosylation machinery and thus may not be glycosylated
    (in vivo) even though they contain potential motifs.

    SignalP-NN euk predictions are as follows:

  # name                    Cmax   pos ?  Ymax   pos ?  Smax   pos ?  Smean ?  D     ?

    SignalP output is explained at http://www.cbs.dtu.dk/services/SignalP/output.html

########################################################################################

Name:  contig065453-TilOR.E087  Length:  310
MKNSTQLSDFILGAYFDGGPFRSLYFTIVMSLYVFIFGSNLLLIVVICVNRSLHEPMYMFLCSLFVNELYGSTGLFPSLL      80
VQILSDVHTISADICFLQVFCVHSYGAVEYLNLAIMSYDRYLAICCPLQYSTHMTSKKIGILIAVTWFYPCFAMVVLLSL     160
TSPLQLCGNTIYKVYCDTHSVVKLACSDTTVINLYGLLATFSTIFGALLFILYTYMKILLVCFSGSDQTRQKAVSTCTPH     240
LASILNFSFGASFEILQSRFNMKNVPNMVRIFLSLYFLTCQPLFNPVMYGLKMTKIRNICKSLITNTHLX
..N..............................................N..............................      80
................................................................................     160
................................................................................     240
......................................................................               320

(Threshold=0.5)
----------------------------------------------------------------------
SeqName      Position  Potential   Jury    N-Glyc
                                 agreement result
----------------------------------------------------------------------
contig065453-TilOR.E087    3 NSTQ   0.5984     (9/9)    ++
contig065453-TilOR.E087   50 NRSL   0.7484     (9/9)    ++
contig065453-TilOR.E087  246 NFSF   0.4071     (6/9)    -
----------------------------------------------------------------------
```

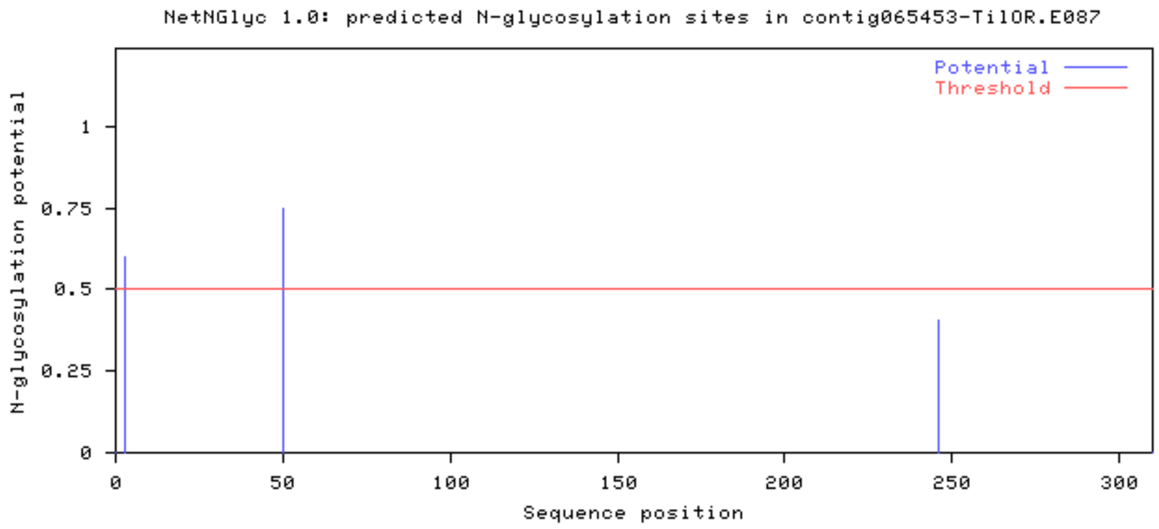

Graphics in PostScript

Output for 'contig065454-TilOR.E088'

```
##########################################################################################

    Warning: This sequence may not contain a signal peptide!!

    Proteins without signal peptides are unlikely to be exposed to
    the N-glycosylation machinery and thus may not be glycosylated
    (in vivo) even though they contain potential motifs.

    SignalP-NN euk predictions are as follows:

  # name                    Cmax   pos ?  Ymax   pos ?  Smax   pos ?  Smean ?  D     ?

    SignalP output is explained at http://www.cbs.dtu.dk/services/SignalP/output.html

##########################################################################################

Name:  contig065454-TilOR.E088  Length:  310
MLNTTQASYFTLAGYFDTGHVTNLCFIVILALYIFIVGSNVLLIVVICVNRSLHEPMYMFLCSLFVNELYGSTGLFPSLL      80
VQILSDVHTISADICFLQVFCVHTYGAVEYLNLAIMSYDRYLAICCPLQYSTHMTSKKIGILIAVTWFYPCFAMVVLLSL     160
TSPLQLCGNTIYKVYCDTHSVVKLACSDTTVINLYGLLATFSTIFGALLFILYTYMKILLVCFSGSDQTRQKAVSTCTPH     240
LASILNFSFGASFEILQSRFNMKNVPNMVRIFLSLYFLTCQPLFNPVMYGLKMTKIRNICKSLITNTHLX
..N..............................................N..............................      80
................................................................................     160
................................................................................     240
......................................................................               320

(Threshold=0.5)
----------------------------------------------------------------------
SeqName       Position  Potential   Jury     N-Glyc
                                  agreement  result
----------------------------------------------------------------------
contig065454-TilOR.E088    3 NTTQ   0.7431     (9/9)    ++
contig065454-TilOR.E088   50 NRSL   0.7493     (9/9)    ++
contig065454-TilOR.E088  246 NFSF   0.4073     (6/9)    -
----------------------------------------------------------------------
```

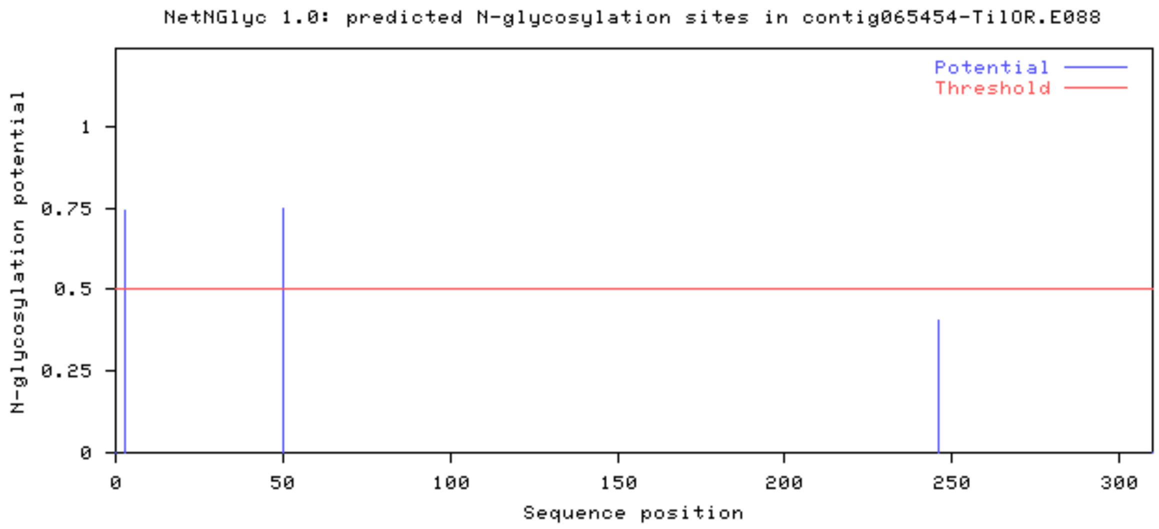

Graphics in PostScript

Output for 'contig065454-TilOR.E089'

```
##########################################################################################

    Warning: This sequence may not contain a signal peptide!!

    Proteins without signal peptides are unlikely to be exposed to
    the N-glycosylation machinery and thus may not be glycosylated
    (in vivo) even though they contain potential motifs.

    SignalP-NN euk predictions are as follows:

  # name                Cmax  pos ?  Ymax  pos ?  Smax  pos ?  Smean ?  D     ?

    SignalP output is explained at http://www.cbs.dtu.dk/services/SignalP/output.html

##########################################################################################

Name:  contig065454-TilOR.E089  Length:  309
MLNTTQASYFTLAGYFDTGHVTNLCFIVILALYIFIVGSNVLLIVVICVNRSLHEPMYMFLCSLFVNELYGSTGLFPSLL      80
VQILSDVHTVSADICFLQVFCVHLYGAVEYLNLAIMSYDRYLAICCPLQYSTHMTSKKIGILIAVTWFYPCFAMAPLLYL     160
TSRLQLCGNTIYKVYCDTHSVVKLACSDTTVINLYGLLATFSTIFGALLFILYTYMKILLVCFSGSDQTRQKAVSTCTPH     240
LASILNFSFGASFEILQSRFNMKNVPNMVRIFLSLYFLTCQPLFNPVMYGLKMTKIRNICKSLISNIKX
..N..............................................N..............................      80
................................................................................     160
................................................................................     240
.....................................................................                320

(Threshold=0.5)
----------------------------------------------------------------------
SeqName      Position  Potential   Jury    N-Glyc
                                 agreement result
----------------------------------------------------------------------
contig065454-TilOR.E089    3 NTTQ   0.7433     (9/9)    ++
contig065454-TilOR.E089   50 NRSL   0.7493     (9/9)    ++
contig065454-TilOR.E089  246 NFSF   0.4070     (6/9)    -
----------------------------------------------------------------------
```

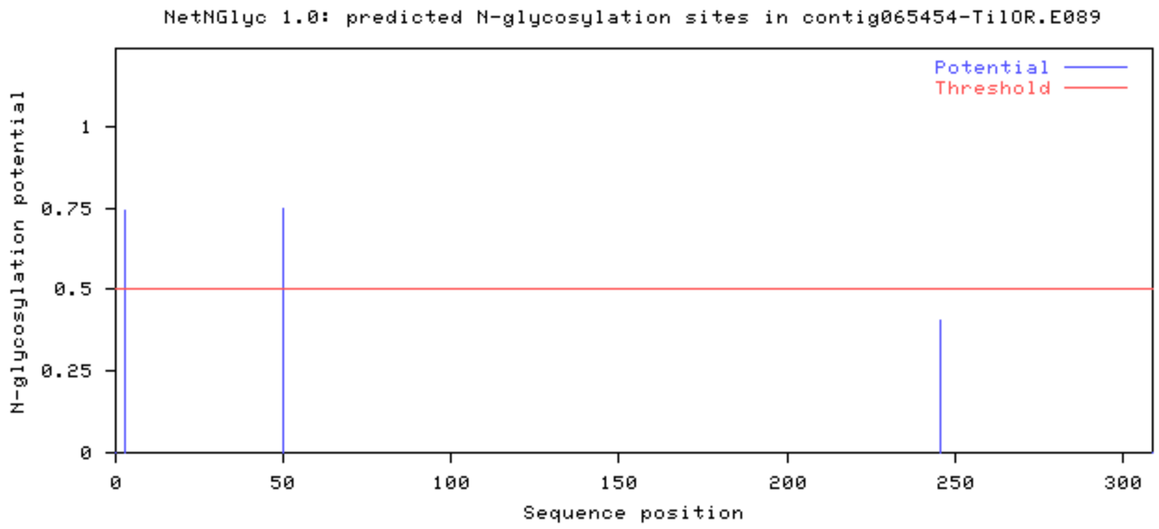

Graphics in PostScript

Output for 'contig065455-TilOR.E085'

```
##########################################################################################

    Warning: This sequence may not contain a signal peptide!!

    Proteins without signal peptides are unlikely to be exposed to
    the N-glycosylation machinery and thus may not be glycosylated
    (in vivo) even though they contain potential motifs.

    SignalP-NN euk predictions are as follows:

  # name                    Cmax   pos ?  Ymax   pos ?  Smax   pos ?  Smean ?  D     ?

    SignalP output is explained at http://www.cbs.dtu.dk/services/SignalP/output.html

##########################################################################################

Name:  contig065455-TilOR.E085  Length:  313
MVIMTNSTQFSHFRLAFLNIGIFKYLFFMLVICFYISVVCTNVLLIVVICVNRSLHEPMYMFLCSLFVNALYGSTGLFPS      80
LLLHIICDINIISASLCYLQIYCIHCYGSAEYLNLAVMSYDRYLAICFPLQYNTYMTPKRIAILIAITWLYAILACALMI     160
SLSSTLPLCGNIINKVYCDNYSVIKLACSDTKALNIYGIIIVLCTVCCPLVFMLYTYIKILRVCSSGSKQVRQKAISTCS     240
PHLACVLNFSCGACFEILQSRFNMSGVPILLRIFLSLYWLISQPLINPLVYGLNMTKIRILCKNLLTLRPLNX
.....N.............................................N............................      80
................................................................................     160
...................N............................................................     240
......................N..............................N...................            320

(Threshold=0.5)
----------------------------------------------------------------------
SeqName       Position  Potential   Jury    N-Glyc
                                  agreement result
----------------------------------------------------------------------
contig065455-TilOR.E085    6 NSTQ   0.7502     (9/9)    +++
contig065455-TilOR.E085   52 NRSL   0.7483     (9/9)    ++
contig065455-TilOR.E085  180 NYSV   0.6321     (9/9)    ++
contig065455-TilOR.E085  248 NFSC   0.3181     (9/9)    ---
contig065455-TilOR.E085  263 NMSG   0.5757     (6/9)    +
contig065455-TilOR.E085  294 NMTK   0.6332     (8/9)    +
----------------------------------------------------------------------
```

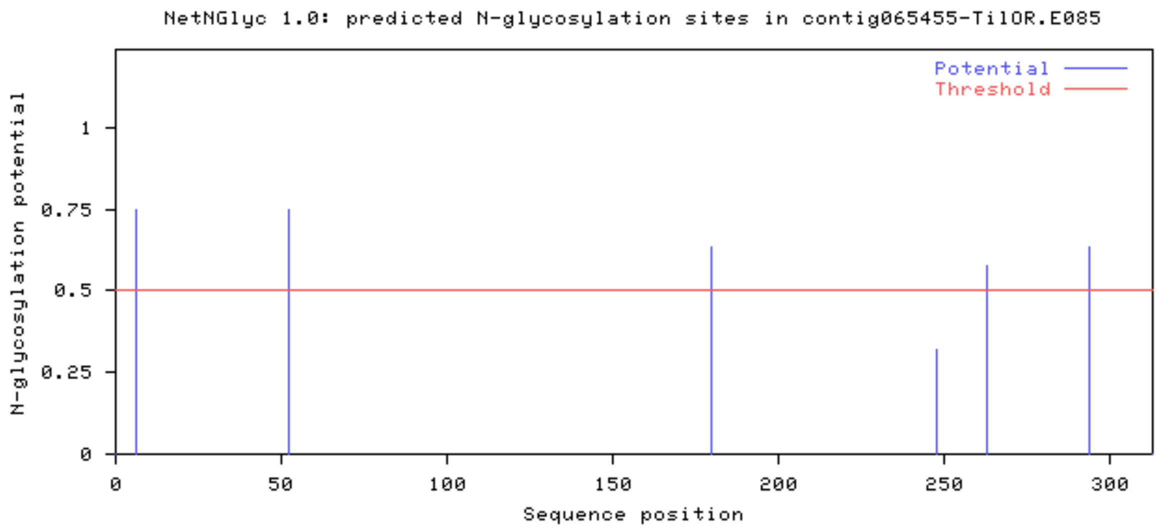

Graphics in PostScript

Output for 'contig065458-TilOR.E086'

```
##########################################################################################

    Warning: This sequence may not contain a signal peptide!!

    Proteins without signal peptides are unlikely to be exposed to
    the N-glycosylation machinery and thus may not be glycosylated
    (in vivo) even though they contain potential motifs.

    SignalP-NN euk predictions are as follows:

  # name                 Cmax  pos ?  Ymax  pos ?  Smax  pos ?  Smean ?  D     ?

    SignalP output is explained at http://www.cbs.dtu.dk/services/SignalP/output.html

##########################################################################################

Name:  contig065458-TilOR.E086  Length:  321
MTINTTQVSYFTLAGYFDTGRVTYLCFIVILALYIFIVGSNVLLIVVICVNRSLHEPMYLFLCSLFVNELYGSTGLFPFL      80
LLQILSDVHTVSASFCFLQIFCFYAYGSIEFSNLAVISYDRYLAICCPLRYHTCMSSSKVSVLIALTWLLTFLAVGVLIS     160
LSAPLQLCGNIINKVYCDNYSIVKLACSDTKVNNIYGILYTLTLVVLVTLIFYTYMRILKVCFSGSKQTRHKAISTCTPH     240
LASLLNFSCGAFFEIIQNRFDMRQLPNMLRIFLSIYWLTCQPLFSPVIYGLNLTKIRVSCKNVIGRMKVFTLCHKLNFKI     320
X
...N.............................................N..............................      80
................................................................................     160
..................N.............................................................     240
.....N.............................................N............................     320
.                                                                                    400

(Threshold=0.5)
----------------------------------------------------------------------
SeqName      Position  Potential   Jury    N-Glyc
                                 agreement result
----------------------------------------------------------------------
contig065458-TilOR.E086    4 NTTQ   0.7078     (9/9)   ++
contig065458-TilOR.E086   51 NRSL   0.7464     (9/9)   ++
contig065458-TilOR.E086  179 NYSI   0.5896     (8/9)   +
contig065458-TilOR.E086  246 NFSC   0.5499     (6/9)   +
contig065458-TilOR.E086  292 NLTK   0.6830     (9/9)   ++
```

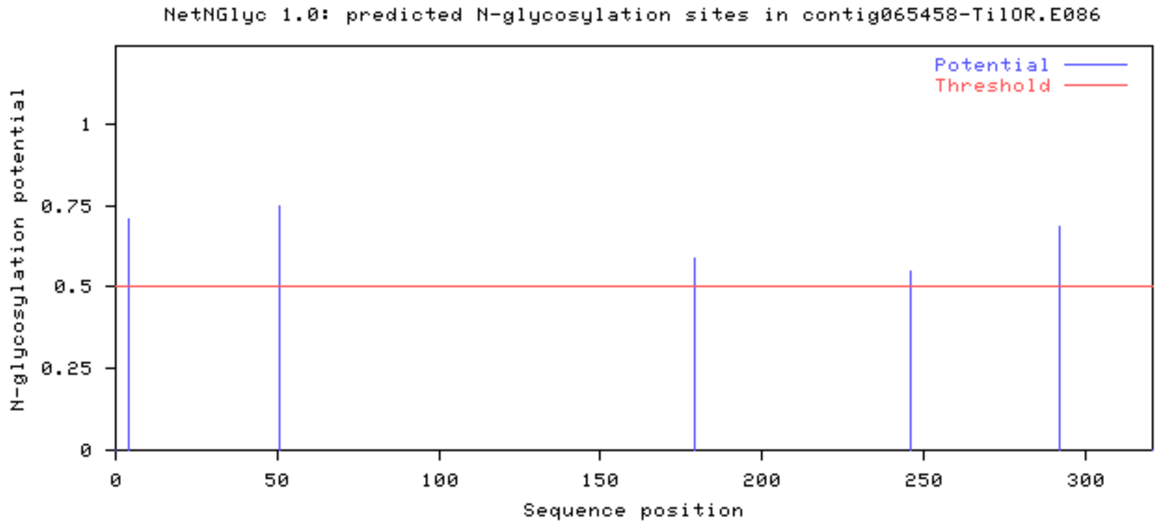

Graphics in PostScript

Output for 'contig065459-TilORe.E093'

##

Warning: This sequence may not contain a signal peptide!!

Proteins without signal peptides are unlikely to be exposed to the N-glycosylation machinery and thus may not be glycosylated (in vivo) even though they contain potential motifs.

SignalP-NN euk predictions are as follows:

# name	Cmax	pos	?	Ymax	pos	?	Smax	pos	?	Smean	?	D	?

SignalP output is explained at http://www.cbs.dtu.dk/services/SignalP/output.html

##

```
Name:  contig065459-TilORe.E093        Length:  281
VICVNRSLHEPMYMFLCSLFVNELYGSTGLFPFLLLQILSDVHTVSAPLCFLQIFCLYTYANLQLSNLAIMSYDRYLAIC      80
CPLHYHTRMSSSKVSMLIALMWLSPFLAISVVISLSAPLQLCGNIINKVYCDNNSIVKLACSDTTANNVYGVLGAIFITI     160
SSVSLILYTYMRILKVCFSGSKQTRQKAISTCTPHLASLLNFSCGSFFETAQNRSNMNHVPNMLRTLLSLYWLICPPLFN     240
PLLYGLNLTKIRIICKGLIFVKCRCLMLKFRQGSALVKRKX
....N...........................................................................      80
................................................................................     160
........................................N...........N...........................     240
......N..................................                                            320
```

(Threshold=0.5)

SeqName	Position		Potential	Jury agreement	N-Glyc result
contig065459-TilORe.E093	5	NRSL	0.7733	(9/9)	+++
contig065459-TilORe.E093	133	NNSI	0.4444	(7/9)	-
contig065459-TilORe.E093	201	NFSC	0.5436	(6/9)	+
contig065459-TilORe.E093	213	NRSN	0.5750	(8/9)	+

```
contig065459-TilORe.E093   247 NLTK   0.7283     (9/9)   ++
---------------------------------------------------------------------
```

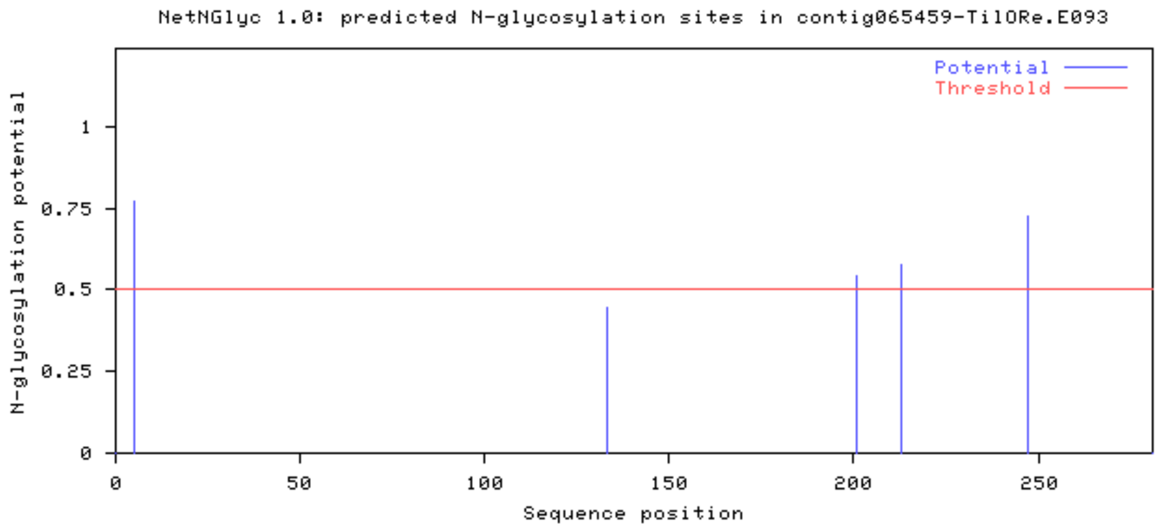

Graphics in PostScript

Output for 'contig068421-TilORe.L166'

```
##########################################################################################

    Warning: This sequence may not contain a signal peptide!!

    Proteins without signal peptides are unlikely to be exposed to
    the N-glycosylation machinery and thus may not be glycosylated
    (in vivo) even though they contain potential motifs.

    SignalP-NN euk predictions are as follows:

  # name                 Cmax  pos ?  Ymax  pos ?  Smax  pos ?  Smean ?  D     ?

    SignalP output is explained at http://www.cbs.dtu.dk/services/SignalP/output.html

##########################################################################################

Name:  contig068421-TilORe.L166         Length:  105
TFTFICLSYCGILFFVKILSNNDKKKMGSTLVSHLICVSCLYCPQFVIVILTRFGVVLTLEERQGLLIGTILGPSLVNPF      80
VYCLRTKEIKSKIFKIFRKVTTAGX
................................................................................      80
.........................                                                            160

(Threshold=0.5)

No sites predicted in this sequence.
```

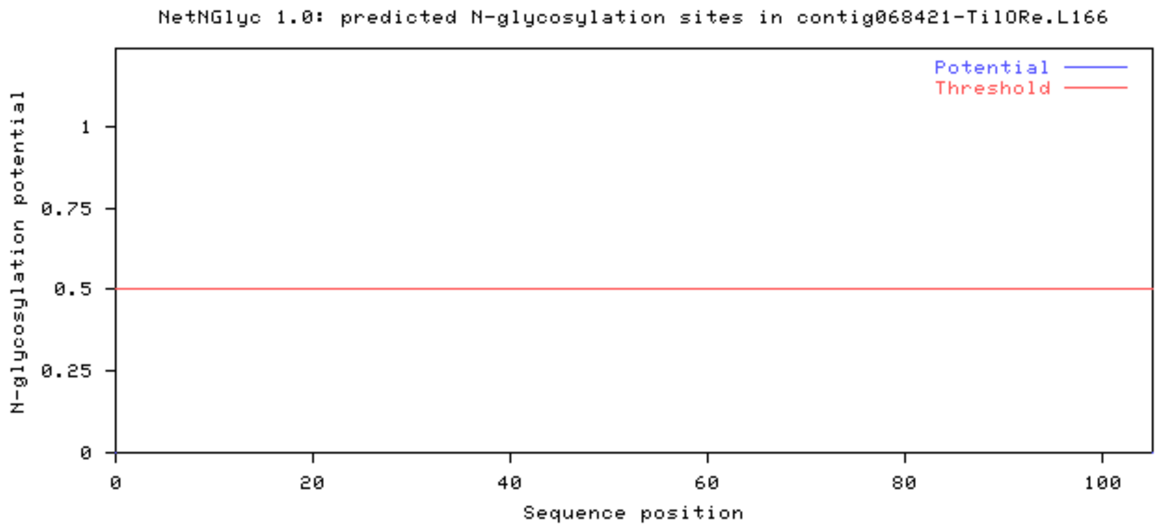

Graphics in PostScript

Output for 'contig068422-TilORe.L171'

```
##########################################################################################

    Warning: This sequence may not contain a signal peptide!!

    Proteins without signal peptides are unlikely to be exposed to
    the N-glycosylation machinery and thus may not be glycosylated
    (in vivo) even though they contain potential motifs.

    SignalP-NN euk predictions are as follows:

  # name                Cmax  pos ?  Ymax  pos ?  Smax  pos ?  Smean ?  D     ?

    SignalP output is explained at http://www.cbs.dtu.dk/services/SignalP/output.html

##########################################################################################

Name:  contig068422-TilORe.L171        Length:  162
MSLQNASIKLTYFIIGGFDTVKRPVAVGVVMLITYLLAVFASMVNIIFIVSDKQLHKPMYLLICSLAFVDILYTSSATPT      80
MIGVLLAGVNTISYVECLIQMYVYQVGATMEMFSLTIMAFDRLIAIICPLQYHSYLTNTRTLVFTYIVWIVACSFALFPP     160
VT
....N...........................................................................      80
................................................................................     160
..                                                                                   240

(Threshold=0.5)
----------------------------------------------------------------------
SeqName        Position  Potential    Jury    N-Glyc
                                   agreement  result
----------------------------------------------------------------------
contig068422-TilORe.L171    5 NASI   0.5862     (6/9)    +
----------------------------------------------------------------------
```

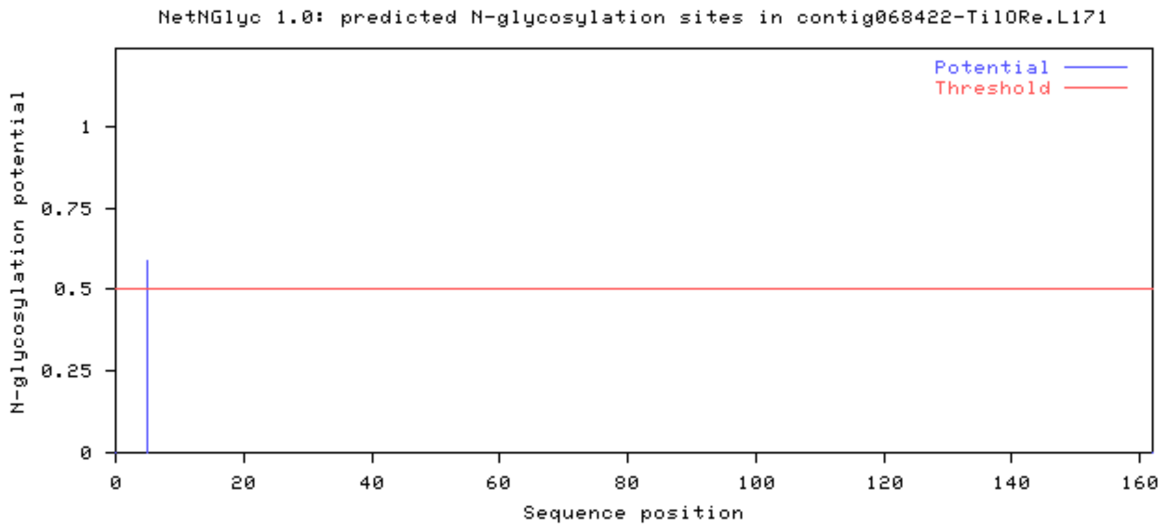

Graphics in PostScript

Output for 'contig068425-TilOR.L155'

```
##########################################################################

    Warning: This sequence may not contain a signal peptide!!

    Proteins without signal peptides are unlikely to be exposed to
    the N-glycosylation machinery and thus may not be glycosylated
    (in vivo) even though they contain potential motifs.

    SignalP-NN euk predictions are as follows:

  # name                 Cmax  pos ?  Ymax  pos ?  Smax  pos ?  Smean ?  D     ?

    SignalP output is explained at http://www.cbs.dtu.dk/services/SignalP/output.html

##########################################################################

Name:  contig068425-TilOR.L155  Length:  313
MSSQNASVNVTHFIIGGFDTLSRPIAVGVVILITYFLAVLANMINIMFIISDKRLHKPMYLMICNLAVVDIVYTSSCSPT      80
MIGVLLAGVNTISYVECLIQMCVFNLGTSMESFVLAIMALDRFIAIIYPFQYHSYLTNTRVLVLTFIVWFIAWCFVCYMP     160
AAVVPLPHCSSRLKYSFCDFAAVLRTTCVNPEKYFNEVSIAAFFIFFFTFVFICLSYCGILLFVKLSSNNEKKKMGSTLV     240
SHLICVIVHYCPAFVRIIFTRFGVVLTLEERQGLVIGAVLGPCLVNPFVYCLRTKEIKQKLFKIFKTFHTYDX
....N...N.......................................................................      80
................................................................................     160
................................................................................     240
.........................................................................            320

(Threshold=0.5)
----------------------------------------------------------------------
SeqName      Position  Potential   Jury    N-Glyc
                                 agreement result
----------------------------------------------------------------------
contig068425-TilOR.L155    5 NASV   0.6328     (7/9)   +
contig068425-TilOR.L155    9 NVTH   0.7873     (9/9)   +++
----------------------------------------------------------------------
```

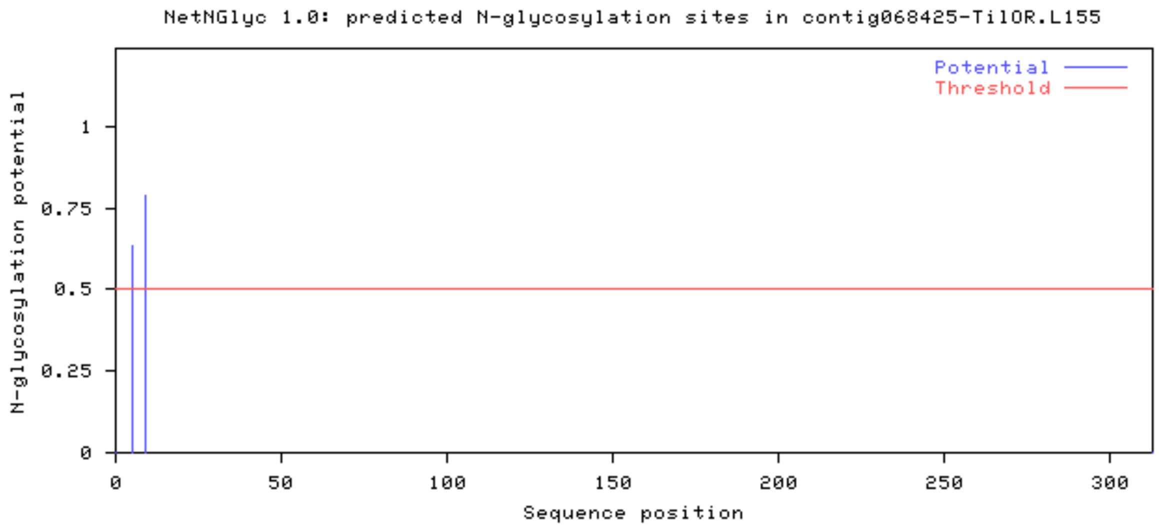

Graphics in PostScript

Output for 'contig068521-TilOR.L156'

```
##########################################################################

    Warning: This sequence may not contain a signal peptide!!

    Proteins without signal peptides are unlikely to be exposed to
    the N-glycosylation machinery and thus may not be glycosylated
    (in vivo) even though they contain potential motifs.

    SignalP-NN euk predictions are as follows:

  # name                Cmax  pos ?  Ymax  pos ?  Smax  pos ?  Smean ?  D     ?

    SignalP output is explained at http://www.cbs.dtu.dk/services/SignalP/output.html

##########################################################################

Name:  contig068521-TilOR.L156  Length:  313
MSLQNASINVTHFIIGGFDTLSRPIAVGVVILITYLLAVLANIANIMFIISDKRLHKPMYLLICNLAVVDIMYTSSCSPT      80
MIGVLLAGVNTISYVECLIQMCVFHLGTVMESFVLAFMALDRFIAIIYPFHYHSYLTNTRVVVLTFILWFVASCFVCYMP     160
ATVVPLPHCSSRLKYSFCDFPALIRTTCVNPEKYFNEVAIVSFFIFFFTLVFICLSYCAILLFVKLSSYNEKKKMGSTLV     240
SHLICVIVYYCPAFVRSIFTRFGVVLTLEERQGLLIGAVLGPCLVNPFVYCLRTKEIKQKLFKIFKKFNTSEX
....N...N.......................................................................      80
................................................................................     160
................................................................................     240
.........................................................................            320

(Threshold=0.5)
----------------------------------------------------------------------
SeqName      Position  Potential   Jury    N-Glyc
                                agreement result
----------------------------------------------------------------------
contig068521-TilOR.L156    5 NASI   0.6714     (8/9)   +
contig068521-TilOR.L156    9 NVTH   0.7234     (9/9)   ++
contig068521-TilOR.L156  309 NTSE   0.4157     (7/9)   -
----------------------------------------------------------------------
```

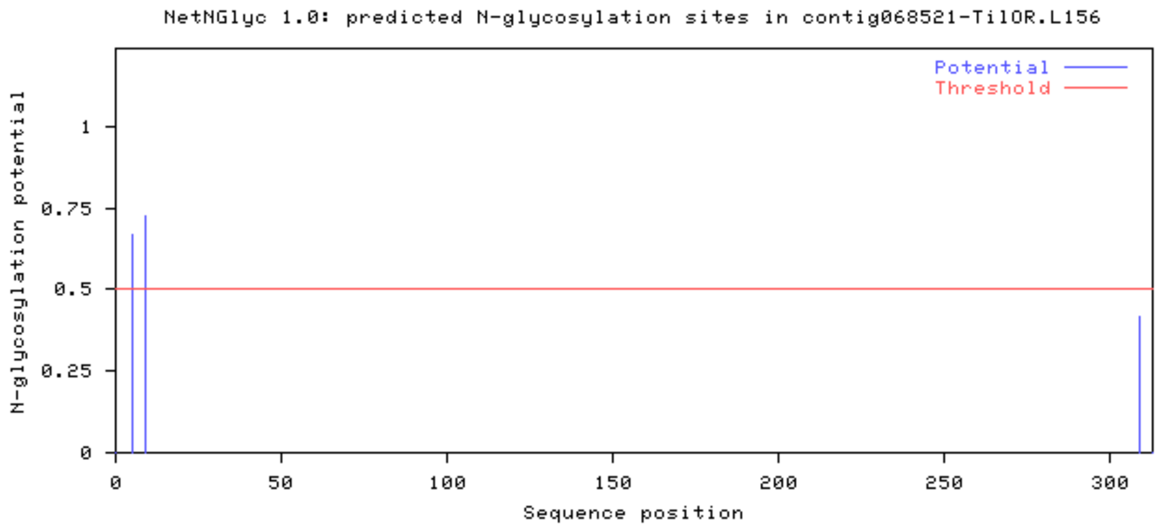

[Graphics in PostScript]

Output for 'contig068527-TilOR.L157'

```
##########################################################################################

    Warning: This sequence may not contain a signal peptide!!

    Proteins without signal peptides are unlikely to be exposed to
    the N-glycosylation machinery and thus may not be glycosylated
    (in vivo) even though they contain potential motifs.

    SignalP-NN euk predictions are as follows:

  # name                Cmax  pos ?  Ymax  pos ?  Smax  pos ?  Smean ?  D     ?

    SignalP output is explained at http://www.cbs.dtu.dk/services/SignalP/output.html

##########################################################################################

Name:  contig068527-TilOR.L157  Length:  313
MSLQNASIKLTHFIIGGFDTVKRPVAVGVVMLITYLLAVFASLVNIIFIVSDKQLHKPMYLLICNLAVVDIFYTSSATPT      80
MIGVLLAGVNTVSYVECLIQMYVYQVGATMERFSLTIMAFDRLIAIIYPLQYHSYLTNTRIMVLTYILWIVACSFVLFTL     160
VTATPLPHCYSRLRYTFCDYAAVMRTTCVNPEKYFNQIAIISFFLSFSTFTFICLSYCGILFFVKILSNNDKKKMGSTLV     240
SHLICVSCLYGPQFVIVILTRFGVVLTLEERQGLLIGTILGPPLVNPFVYCLRTKEIKSKIFMIVRKVNTAGX
....N...........................................................................      80
................................................................................     160
................................................................................     240
.........................................................................            320

(Threshold=0.5)
----------------------------------------------------------------------
SeqName      Position  Potential   Jury    N-Glyc
                                 agreement result
----------------------------------------------------------------------
contig068527-TilOR.L157    5 NASI   0.6030     (7/9)   +
----------------------------------------------------------------------
```

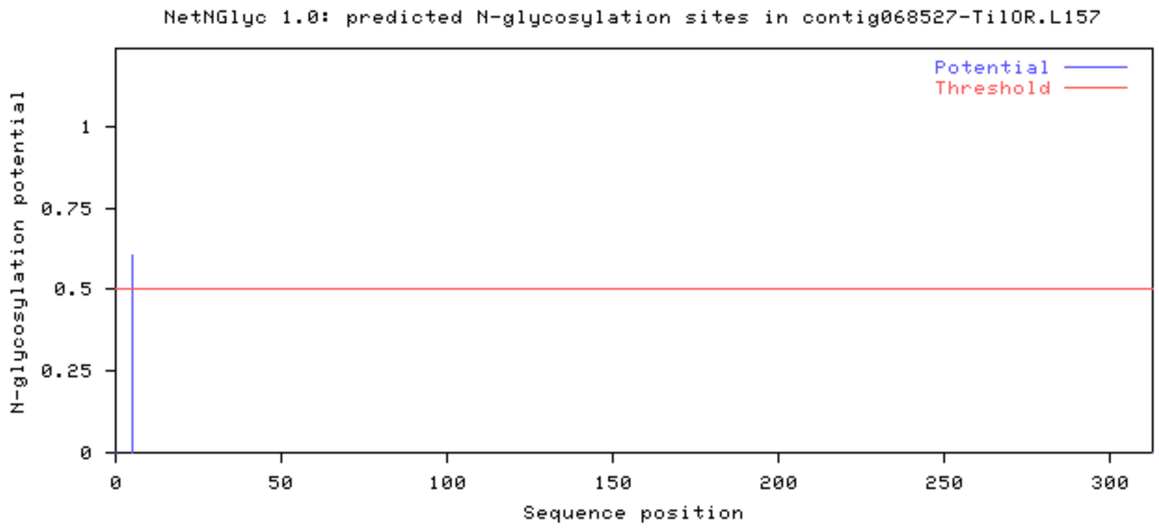

Graphics in PostScript

Output for 'contig068532-TilORe.L167'

```
##########################################################################################

    Warning: This sequence may not contain a signal peptide!!

    Proteins without signal peptides are unlikely to be exposed to
    the N-glycosylation machinery and thus may not be glycosylated
    (in vivo) even though they contain potential motifs.

    SignalP-NN euk predictions are as follows:

  # name                    Cmax   pos ?  Ymax   pos ?  Smax   pos ?  Smean ?  D     ?

    SignalP output is explained at http://www.cbs.dtu.dk/services/SignalP/output.html

##########################################################################################

Name:  contig068532-TilORe.L167         Length:  115
MSLQNASINVTHFIIGGFDTLNRPIAVGVFILIIYLLAVLANMANIMFIVSDKSLHKPMYLLICNLAVVDIMYTSSCNPT      80
MIGVLLAGVNTISYVECLIQMCVFHLGTVMESFVL
....N...N.....................................................................N..      80
...................................                                                  160

(Threshold=0.5)
----------------------------------------------------------------------
SeqName      Position  Potential   Jury    N-Glyc
                                 agreement result
----------------------------------------------------------------------
contig068532-TilORe.L167     5 NASI   0.6710     (8/9)    +
contig068532-TilORe.L167     9 NVTH   0.7197     (9/9)    ++
contig068532-TilORe.L167    78 NPTM   0.6460     (7/9)    +     WARNING: PRO-X1.
----------------------------------------------------------------------
```

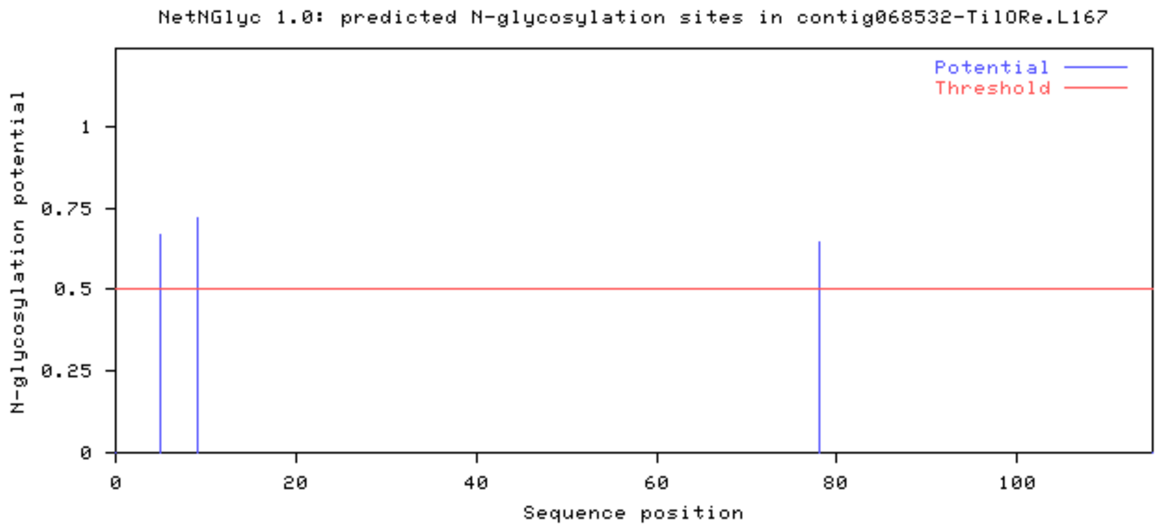

Graphics in PostScript

Output for 'contig068538-TilORe.L269'

```
##########################################################################################

    Warning: This sequence may not contain a signal peptide!!

    Proteins without signal peptides are unlikely to be exposed to
    the N-glycosylation machinery and thus may not be glycosylated
    (in vivo) even though they contain potential motifs.

    SignalP-NN euk predictions are as follows:

  # name                    Cmax   pos ?  Ymax   pos ?  Smax   pos ?  Smean ?  D      ?

    SignalP output is explained at http://www.cbs.dtu.dk/services/SignalP/output.html

##########################################################################################

Name:  contig068538-TilORe.L269         Length:  83
MIGVLLAGVNTISYVECLIQMCAFTLGTLMESFPLAIMALDRLIAIIYPFQYHSYLTNTRVLVLTFIVCFIAWCFVYYMS      80
CVG
................................................................................      80
...                                                                                  160

(Threshold=0.5)

No sites predicted in this sequence.
```

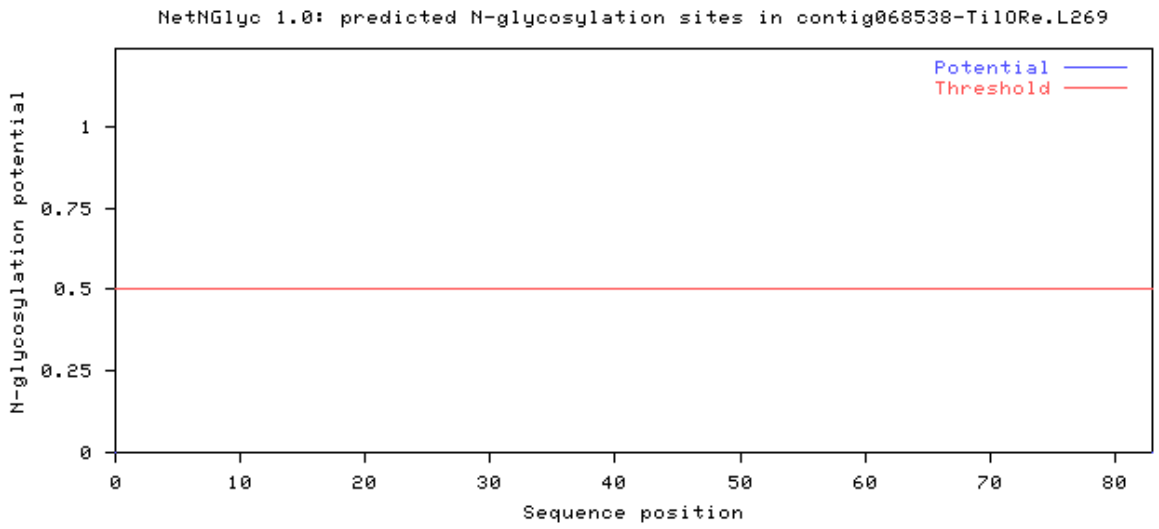

Graphics in PostScript

Output for 'contig068539-TilOR.L158'

```
##########################################################################################

    Warning: This sequence may not contain a signal peptide!!

    Proteins without signal peptides are unlikely to be exposed to
    the N-glycosylation machinery and thus may not be glycosylated
    (in vivo) even though they contain potential motifs.

    SignalP-NN euk predictions are as follows:

  # name                Cmax  pos ?  Ymax  pos ?  Smax  pos ?  Smean ?  D     ?

    SignalP output is explained at http://www.cbs.dtu.dk/services/SignalP/output.html

##########################################################################################

Name:  contig068539-TilOR.L158  Length:  313
MSLQNTSINVTHFIIGGFDTLSRPIAVGVAILITYLLAVLANMANIMFIISDKRLHKPMYLLICNLAVVDIVYTSSSSPT      80
MIGVLLAGVNTISYVECLIQMCAFTLGILMESFVLAIMALDRFIAIIYPFQYHSYLINTRVLVLTFVLWFIAWCFVCYMP     160
VTLVSLPHCSSKLKYNFCEFAAIIETTCVNPEKYFNEVATIAFFIFFFTFVFICLSYCGILLYVKLLSNNEKKKMGSTLV     240
SHLICVIVHYCPAFVRIIFTRFGVVLTLEERQGLIIGAVLGPCLVNPFVYCLRTKEIKQKLFNIFKKLNTYDX
....N...N.......................................................................      80
................................................................................     160
................................................................................     240
.........................................................................            320

(Threshold=0.5)
----------------------------------------------------------------------
SeqName      Position  Potential   Jury    N-Glyc
                                 agreement result
----------------------------------------------------------------------
contig068539-TilOR.L158    5 NTSI   0.6794     (9/9)    ++
contig068539-TilOR.L158    9 NVTH   0.7193     (9/9)    ++
----------------------------------------------------------------------
```

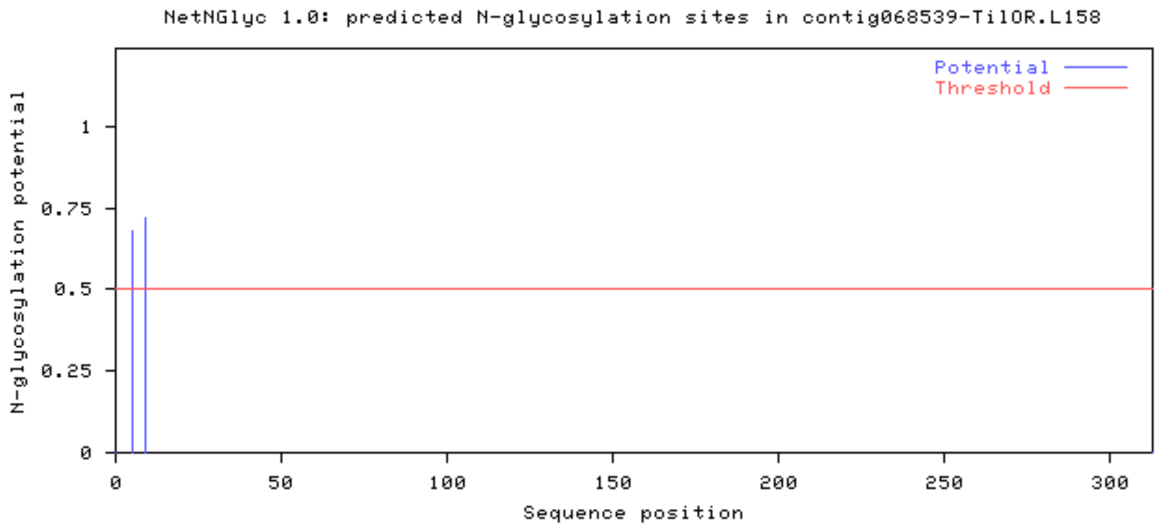

[Graphics in PostScript]

Output for 'contig068541-TilORe.L170'

```
##########################################################################################

    Warning: This sequence may not contain a signal peptide!!

    Proteins without signal peptides are unlikely to be exposed to
    the N-glycosylation machinery and thus may not be glycosylated
    (in vivo) even though they contain potential motifs.

    SignalP-NN euk predictions are as follows:

  # name                    Cmax   pos ?  Ymax   pos ?  Smax   pos ?  Smean ?  D     ?

    SignalP output is explained at http://www.cbs.dtu.dk/services/SignalP/output.html

##########################################################################################

Name:  contig068541-TilORe.L170         Length:  240
TPTMIGVLLAGVNTISYVECLIQMYVYQVGATMEMFSLTIMAFDRLIAIICPLQYHSYLTNTRTLVFTYIVWIVACSFGL      80
FPPVTATPLPHCYSRLRYTFCDYAAVMRTTCVNPEKYFNQTAIIYFFVSFSTFTFICLSYCGILFFVKILSNNDKKKMGS     160
TLVSHLICVSCLYGPQFVIVILTRFGVALTLEERQGLLIGTILGPSLVNPFVYCLRTKEIKSKIFKIFKTHSWLESCKLX     240
................................................................................      80
......................................N.........................................     160
................................................................................     240

(Threshold=0.5)
----------------------------------------------------------------------
SeqName      Position  Potential    Jury    N-Glyc
                                 agreement result
----------------------------------------------------------------------
contig068541-TilORe.L170  119 NQTA   0.5981     (7/9)   +
----------------------------------------------------------------------
```

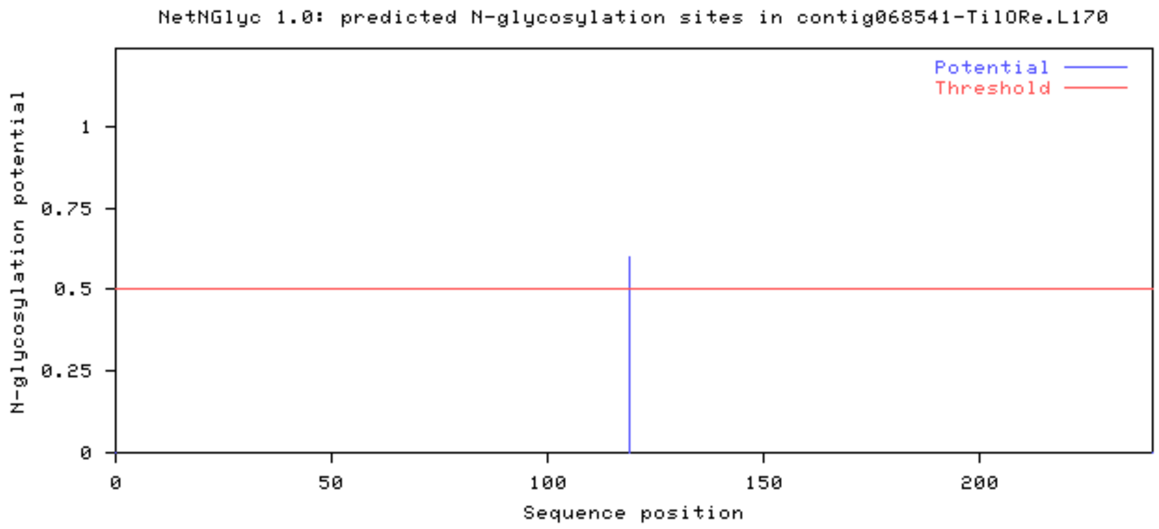

Graphics in PostScript

Output for 'contig070882-TilORe.A031'

```
##########################################################################################

    Warning: This sequence may not contain a signal peptide!!

    Proteins without signal peptides are unlikely to be exposed to
    the N-glycosylation machinery and thus may not be glycosylated
    (in vivo) even though they contain potential motifs.

    SignalP-NN euk predictions are as follows:

  # name                  Cmax   pos ?  Ymax   pos ?  Smax   pos ?  Smean ?  D     ?

    SignalP output is explained at http://www.cbs.dtu.dk/services/SignalP/output.html

##########################################################################################

Name:  contig070882-TilORe.A031         Length:  70
MDEELNTTYVTLDGYIEVNKYRYVYFCIIFTLYIIIICSNSTIVYVIWIHKNLHEPMYIFIAALLLNCLL
.....N.................................N..............................                80

(Threshold=0.5)
----------------------------------------------------------------------
SeqName      Position  Potential   Jury    N-Glyc
                                 agreement result
----------------------------------------------------------------------
contig070882-TilORe.A031     6 NTTY    0.7323     (9/9)    ++
contig070882-TilORe.A031    40 NSTI    0.6309     (8/9)    +
----------------------------------------------------------------------
```

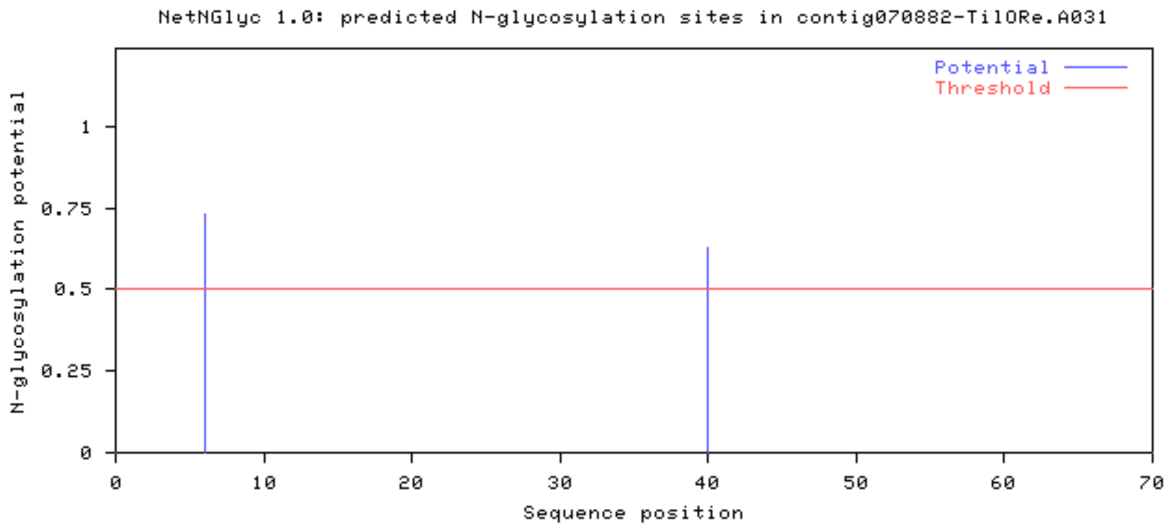

Graphics in PostScript

Output for 'contig070883-TilORe.A040'

```
##########################################################################

    Warning: This sequence may not contain a signal peptide!!

    Proteins without signal peptides are unlikely to be exposed to
    the N-glycosylation machinery and thus may not be glycosylated
    (in vivo) even though they contain potential motifs.

    SignalP-NN euk predictions are as follows:

  # name                 Cmax  pos ?  Ymax  pos ?  Smax  pos ?  Smean ?  D     ?

    SignalP output is explained at http://www.cbs.dtu.dk/services/SignalP/output.html

##########################################################################

Name:  contig070883-TilORe.A040         Length:  239
KLLIDFLSEKQVITYSACLFQFFMFYTLGSSEFFLLAAMAYDRYVAICKPLEYPTIMNKTTVSIFLVVSWLIPAFHVAVQ      80
AIGSAEATLCNFNLKGIFCNNAVYTLQCVRSRLIIVFGVVALIDLTILPLLFIVFTYTNIFIISYQSCKEIRKKAAETCL     160
PHLLVLISISCLSIYDICIARVESDFPKIARLLMTLQIVLYHPLFNPFIYGLKMKEISKQLKRFFCHARIITCINYECX
.........................................................N......................      80
................................................................................     160
...............................................................................      240

(Threshold=0.5)
----------------------------------------------------------------------
SeqName      Position  Potential    Jury    N-Glyc
                                 agreement  result
----------------------------------------------------------------------
contig070883-TilORe.A040    58 NKTT    0.6706     (8/9)    +
----------------------------------------------------------------------
```

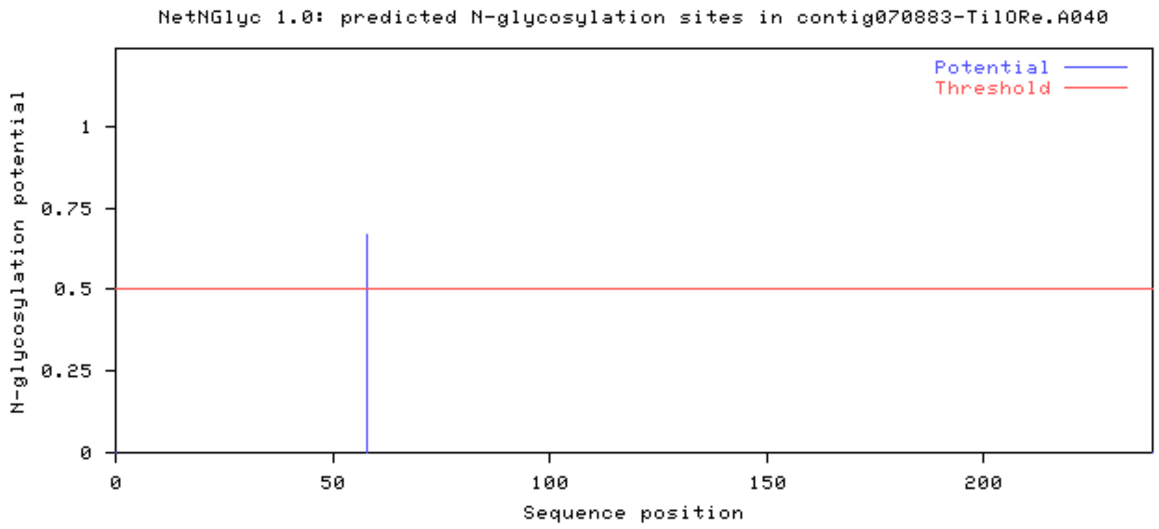

Graphics in PostScript

Output for 'contig070885-TilOR.A024'

```
##########################################################################################

    Warning: This sequence may not contain a signal peptide!!

    Proteins without signal peptides are unlikely to be exposed to
    the N-glycosylation machinery and thus may not be glycosylated
    (in vivo) even though they contain potential motifs.

    SignalP-NN euk predictions are as follows:

  # name                 Cmax  pos ?  Ymax  pos ?  Smax  pos ?  Smean ?  D     ?

    SignalP output is explained at http://www.cbs.dtu.dk/services/SignalP/output.html

##########################################################################################

Name:  contig070885-TilOR.A024  Length:  309
MDEELNTTYVTLDGYIEVNKYRYVYFCIIFTLYIIIICSNSTIMYVIWIHKNLHEPMYIFIAALLLNCLLYSTTIYPKLL      80
IDFLSEKQVITYSACLFQFFIFYTLGSSEFFLLAAMAYDRYVAICKPLEYPTIMNKTTVSIFLVVSWLIPAFHIAVQAIA     160
SAEATLCNFNLKGIFCNNAVYTLQCVRSRLIIVFGVVALIDLIILPLLFIVFTYTNIFIISYQSCKEIRKKAAETCLPHL     240
LVLISISCLSIYDVSIARVESDFPKIARLLMTLQIVLYHPLFNPFIYGLKMKEISKQLKRFFCHARIIX
.....N.................................N........................................      80
......................................................N.........................     160
................................................................................     240
.....................................................................                320

(Threshold=0.5)
----------------------------------------------------------------------
SeqName      Position  Potential   Jury    N-Glyc
                                agreement  result
----------------------------------------------------------------------
contig070885-TilOR.A024    6 NTTY   0.7359     (9/9)    ++
contig070885-TilOR.A024   40 NSTI   0.6720     (8/9)    +
contig070885-TilOR.A024  135 NKTT   0.6409     (7/9)    +
----------------------------------------------------------------------
```

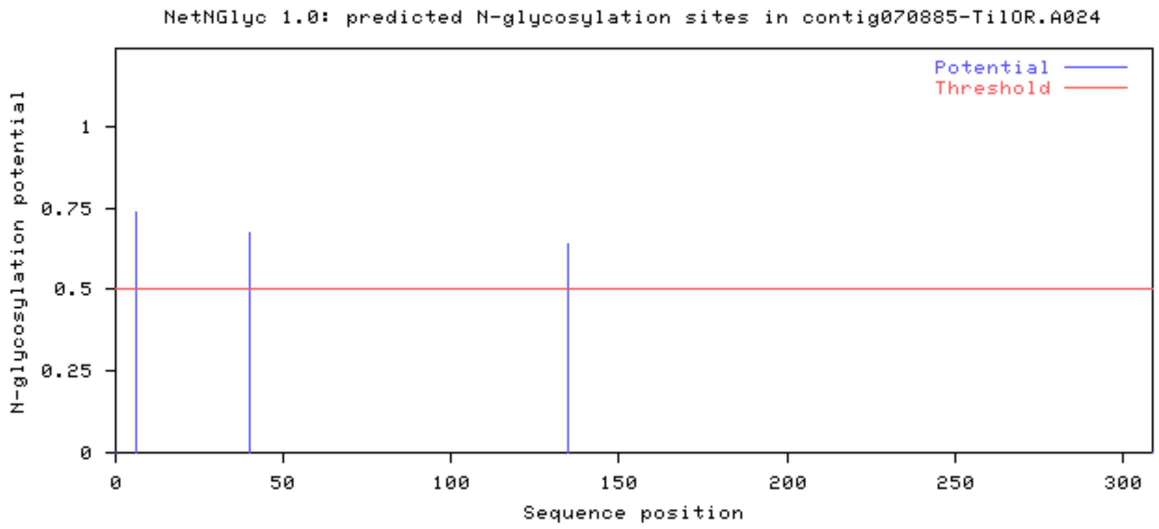

Graphics in PostScript

Output for 'contig070886-TilOR.A025'

```
##########################################################################

    Warning: This sequence may not contain a signal peptide!!

    Proteins without signal peptides are unlikely to be exposed to
    the N-glycosylation machinery and thus may not be glycosylated
    (in vivo) even though they contain potential motifs.

    SignalP-NN euk predictions are as follows:

  # name                Cmax  pos ?  Ymax  pos ?  Smax  pos ?  Smean ?  D     ?

    SignalP output is explained at http://www.cbs.dtu.dk/services/SignalP/output.html

##########################################################################

Name:  contig070886-TilOR.A025  Length:  306
MDVELNVTLLTLGGFAELHKYRYLYFVVIFTLYILILCCNSTIVCLIWTHKNLHEPMYIFIAALLINSVLYSMTIYPKLL      80
SDVLSEKQTISYPLCLFQGFSYYTSAVSEFLLLAAMAYDRYVSICKPLQYPVIMNRITIYVCLILAWLVPAFEIAVSFVL     160
YSNVKLCSFTLTGIFCNNSFYRLQCVPSVVISIYGVVMLINIALLPMLFILFSYIRILRISYNCCRETRRKALKTCLPHL     240
LVLINFSCFFFFDIIIVRLESDLSNTLRLTLTFQSILFHPLLNPIIYGLKMNEIFKHIKILLCQVX
.....N.................................N........................................      80
................................................................................     160
................................................................................     240
....N.............................................................                   320

(Threshold=0.5)
----------------------------------------------------------------------
SeqName      Position  Potential   Jury    N-Glyc
                                 agreement result
----------------------------------------------------------------------
contig070886-TilOR.A025    6 NVTL   0.7849     (9/9)   +++
contig070886-TilOR.A025   40 NSTI   0.7141     (9/9)   ++
contig070886-TilOR.A025  177 NNSF   0.3558     (9/9)   --
contig070886-TilOR.A025  245 NFSC   0.5663     (6/9)   +
----------------------------------------------------------------------
```

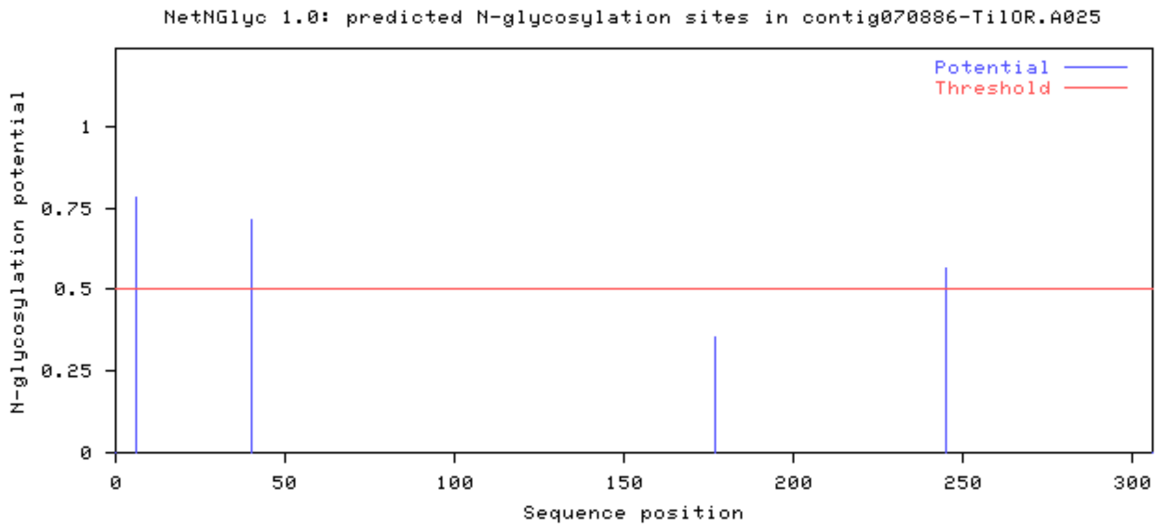

Graphics in PostScript

Output for 'contig072645-TilOR.L159'

```
########################################################################################

    Warning: This sequence may not contain a signal peptide!!

    Proteins without signal peptides are unlikely to be exposed to
    the N-glycosylation machinery and thus may not be glycosylated
    (in vivo) even though they contain potential motifs.

    SignalP-NN euk predictions are as follows:

  # name                 Cmax  pos ?  Ymax  pos ?  Smax  pos ?  Smean ?  D     ?

    SignalP output is explained at http://www.cbs.dtu.dk/services/SignalP/output.html

########################################################################################

Name:  contig072645-TilOR.L159  Length:  313
MSLQNASINVRHFIIGGFDTLSRPIAVGVVILIAYFLAVLANIANIMFIISDKRLHKPMYLLICNLAVVDIMYTSSCSPT      80
MIGVLLAGVNTISYVACLIQMCVFHLGTSMESFVLAVMALDRFIAIIYPFQYHSYLTNTRVLVLTFIVWFVNWFFVFYLP     160
ATVVPLPHCSSRLRYTFCDFAAVLRTTCVNPDKYFNEVAIVTFFILFFTFVFICLSYCGILLFVKLSSNNEKKKMGSTLV     240
SHLICVIVHYCPAFVRIIFTRFGVVLTFEERQGLLIGAVLGPCLVNPFVYCLRTKEIKQKLLKIFKKFHTYDX
....N...........................................................................      80
................................................................................     160
................................................................................     240
.........................................................................            320

(Threshold=0.5)
----------------------------------------------------------------------
SeqName      Position  Potential   Jury    N-Glyc
                                 agreement result
----------------------------------------------------------------------
contig072645-TilOR.L159    5 NASI   0.6778     (8/9)   +
----------------------------------------------------------------------
```

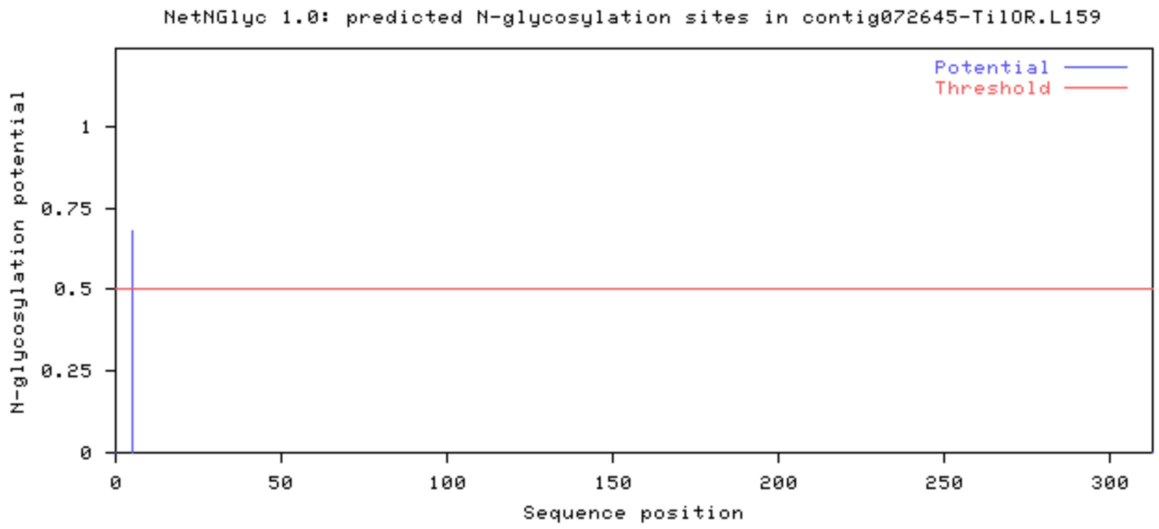

Graphics in PostScript

Output for 'contig072817-TilORe.R258'

```
##########################################################################################

    Warning: This sequence may not contain a signal peptide!!

    Proteins without signal peptides are unlikely to be exposed to
    the N-glycosylation machinery and thus may not be glycosylated
    (in vivo) even though they contain potential motifs.

    SignalP-NN euk predictions are as follows:

  # name                    Cmax   pos ?  Ymax   pos ?  Smax   pos ?  Smean ?  D     ?

    SignalP output is explained at http://www.cbs.dtu.dk/services/SignalP/output.html

##########################################################################################

Name:  contig072817-TilORe.R258        Length:  73
MTSTSQTLTNITVQAPGLLERVMISTLTTLPTCVFLFINSIMLFTLRSKPVFRETCRYILLYNLLFADTVQLA
.........N...............................................................             80

(Threshold=0.5)
----------------------------------------------------------------------
SeqName      Position  Potential   Jury    N-Glyc
                                 agreement result
----------------------------------------------------------------------
contig072817-TilORe.R258   10 NITV   0.7676     (9/9)   +++
----------------------------------------------------------------------
```

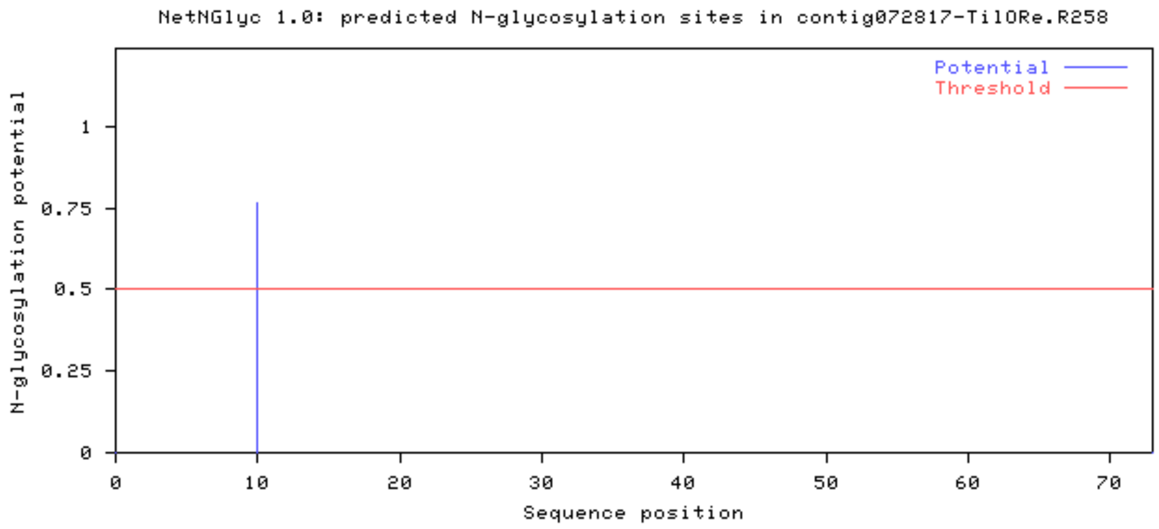

Graphics in PostScript

Output for 'contig072998-TilORp.A044'

```
##########################################################################################

    Warning: This sequence may not contain a signal peptide!!

    Proteins without signal peptides are unlikely to be exposed to
    the N-glycosylation machinery and thus may not be glycosylated
    (in vivo) even though they contain potential motifs.

    SignalP-NN euk predictions are as follows:

  # name                    Cmax   pos ?  Ymax   pos ?  Smax   pos ?  Smean ?  D     ?

    SignalP output is explained at http://www.cbs.dtu.dk/services/SignalP/output.html

##########################################################################################

Name:  contig072998-TilORp.A044          Length:  312
MDESNVTYLTLDWYTEINKYRYIFFFIMFSLYILIICTNSIILYLIWIHKNLHEPMYIFIAALLLNSVLYSTTVYPKLLI      80
DFSSEKQVTTYSACLFQFFIFYTLVSEFLLLAAMAYDRYVAICKPLEYQTIMRKTTVSIFLVVAWLVPACHVAVQAIASA     160
EAKLCDSNIKGIFCNNAVYTLQCERSRLITIFGVVALLDLAILPMLFIVFTYTKIFIVSHRSCKEIRKKAAETCLPHLLV     240
LISHSAFFVYDVSIARAKPDFPKTTRIIMTLQIMLYQPLLNPFIYGLKMKEISKHLNKLLSQANISPCIKTX
....N...........................................................................      80
................................................................................     160
................................................................................     240
........................................................................             320

(Threshold=0.5)
----------------------------------------------------------------------
SeqName      Position  Potential   Jury    N-Glyc
                                 agreement result
----------------------------------------------------------------------
contig072998-TilORp.A044    5 NVTY   0.7049     (9/9)   ++
contig072998-TilORp.A044  304 NISP   0.1064     (9/9)   ---
----------------------------------------------------------------------
```

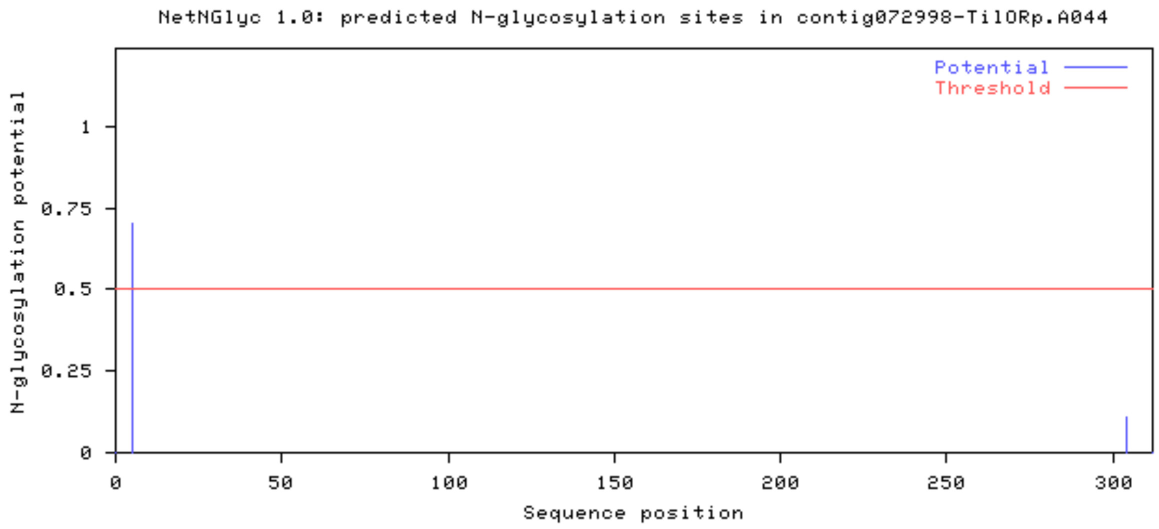

Graphics in PostScript

Output for 'contig073309-TilOR.A026'

```
##########################################################################################

    Warning: This sequence may not contain a signal peptide!!

    Proteins without signal peptides are unlikely to be exposed to
    the N-glycosylation machinery and thus may not be glycosylated
    (in vivo) even though they contain potential motifs.

    SignalP-NN euk predictions are as follows:

  # name                 Cmax   pos ?  Ymax   pos ?  Smax   pos ?  Smean ?  D     ?

    SignalP output is explained at http://www.cbs.dtu.dk/services/SignalP/output.html

##########################################################################################

Name:  contig073309-TilOR.A026  Length:   312
MDVELNVTLLTLGGFAELHKYRYLYFVVTFTLYILILCFNSTIVCLIWTHTNLHEPMYIFIAALLINSVLYSMTIYPKLL      80
SDVLSEKQIISYPLCLFQGFLYYTSAGSEFLLLAAMAYDRYVSICKPLQYAVIMNRITIYVSLVLAWIIPAFEIAVSVVL     160
YSNVKLCSFTLTGIFCNNSGYKLQCVQSVAISIYGVVMLINIALLPMLFILFTYIRIVRISYQSCREVRKKAVKTCLPHL     240
LVLINFSCFIVFDVVIVRLESDLSKTLRLILTFQSILFHPLLNPIIYGLKMNEIFKSLKILFCHVTVAGNIX
.....N.................................N........................................      80
................................................................................     160
................................................................................     240
....N...................................................................             320

(Threshold=0.5)
----------------------------------------------------------------------
SeqName      Position  Potential   Jury    N-Glyc
                                 agreement result
----------------------------------------------------------------------
contig073309-TilOR.A026    6 NVTL   0.7850     (9/9)   +++
contig073309-TilOR.A026   40 NSTI   0.7070     (9/9)   ++
contig073309-TilOR.A026  177 NNSG   0.3968     (7/9)   -
contig073309-TilOR.A026  245 NFSC   0.5724     (6/9)   +
----------------------------------------------------------------------
```

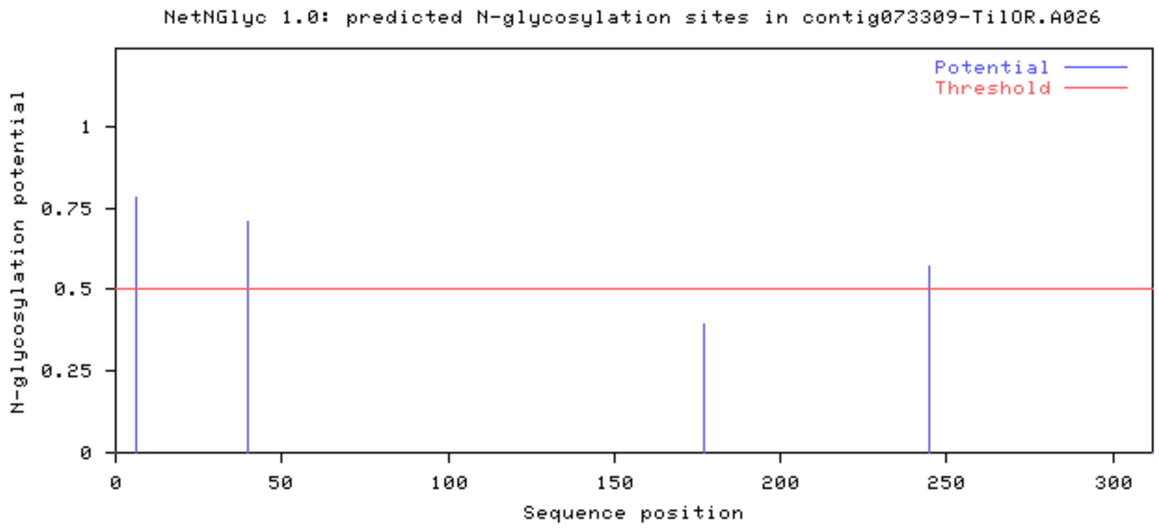

Graphics in PostScript

Output for 'contig073741-TilORe.S270'

```
##########################################################################################

    Warning: This sequence may not contain a signal peptide!!

    Proteins without signal peptides are unlikely to be exposed to
    the N-glycosylation machinery and thus may not be glycosylated
    (in vivo) even though they contain potential motifs.

    SignalP-NN euk predictions are as follows:

  # name                Cmax  pos ?  Ymax  pos ?  Smax  pos ?  Smean ?  D     ?

    SignalP output is explained at http://www.cbs.dtu.dk/services/SignalP/output.html

##########################################################################################

Name:  contig073741-TilORe.S270        Length:  101
IMGTTIAYSYVQIMKVAKAASGEKKKLTHKGLKTVILHAFQLLLCLIQLWCPFIETAVLQIDFRLFINVRYSNYIMFNIA      80
PRCLSPLIYGLRDENFFLVLK
................................................................................      80
.....................                                                                160

(Threshold=0.5)

No sites predicted in this sequence.
```

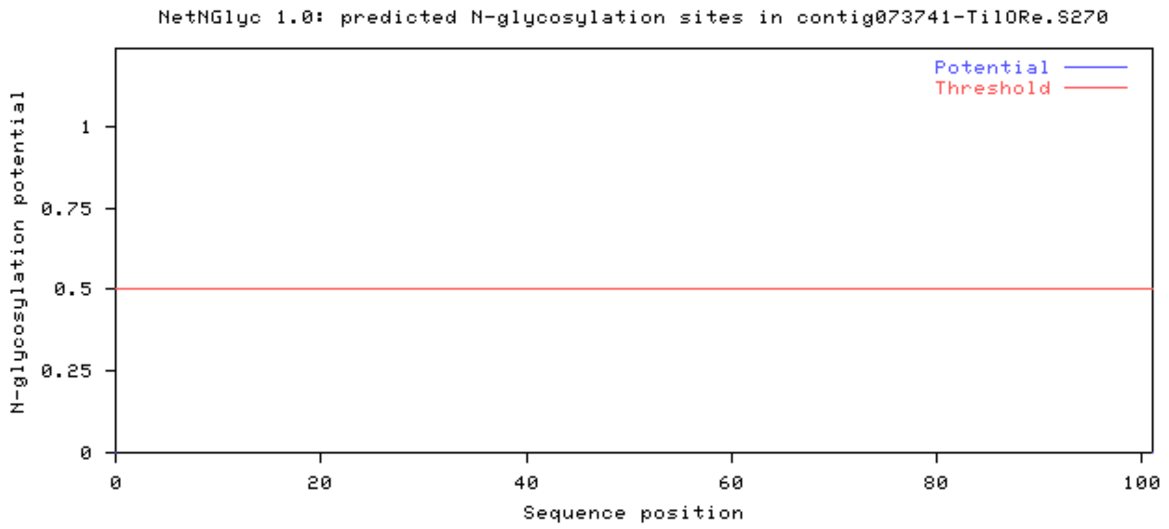

Graphics in PostScript

Output for 'contig074021-TilORe.A036'

```
##########################################################################

    Warning: This sequence may not contain a signal peptide!!

    Proteins without signal peptides are unlikely to be exposed to
    the N-glycosylation machinery and thus may not be glycosylated
    (in vivo) even though they contain potential motifs.

    SignalP-NN euk predictions are as follows:

  # name                Cmax  pos ?  Ymax  pos ?  Smax  pos ?  Smean ?  D     ?

    SignalP output is explained at http://www.cbs.dtu.dk/services/SignalP/output.html

##########################################################################

Name:  contig074021-TilORe.A036         Length:  152
MDVELNVTLVTFGGFAELHKYRYLYFVVIFTLYILILCCNSTIVCLIWTHKNLHEPMYIFIAALLINSVLYSMTIYPKLL      80
SDVLSEKQIISYPLCLFQGFLYYTSAGSEFLLLAAMAYDRYVSICKPLQYPVIMNRITIYVCLILAWLIPAF
.....N.................................N........................................      80
........................................................................             160

(Threshold=0.5)
----------------------------------------------------------------------
SeqName      Position  Potential   Jury    N-Glyc
                                agreement  result
----------------------------------------------------------------------
contig074021-TilORe.A036     6 NVTL    0.8269     (9/9)   +++
contig074021-TilORe.A036    40 NSTI    0.6979     (9/9)   ++
----------------------------------------------------------------------
```

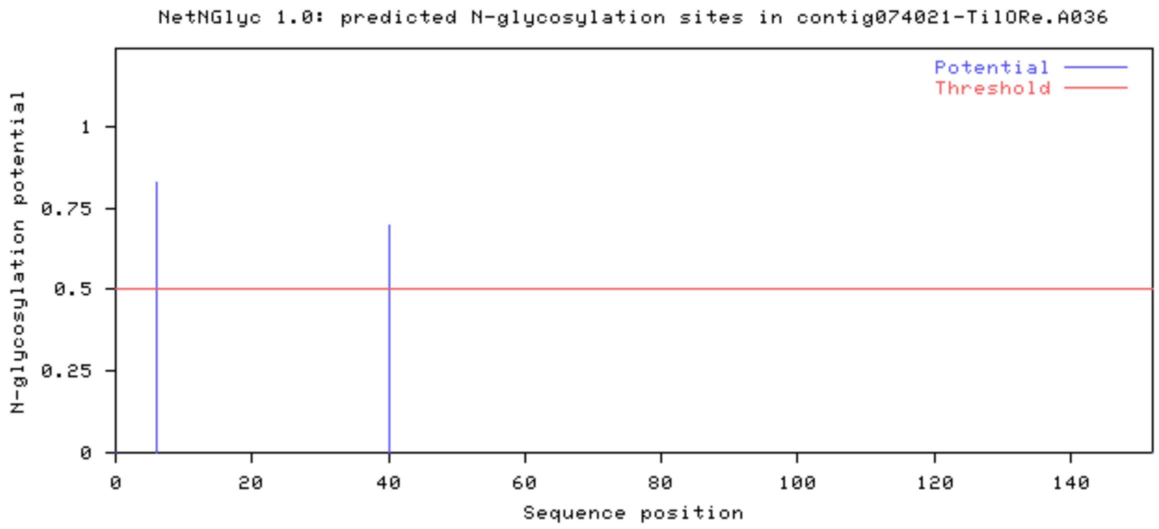

Graphics in PostScript

Output for 'contig074369-TilORe.E090'

```
##########################################################################################

    Warning: This sequence may not contain a signal peptide!!

    Proteins without signal peptides are unlikely to be exposed to
    the N-glycosylation machinery and thus may not be glycosylated
    (in vivo) even though they contain potential motifs.

    SignalP-NN euk predictions are as follows:

  # name                    Cmax   pos ?  Ymax   pos ?  Smax   pos ?  Smean ?  D     ?

    SignalP output is explained at http://www.cbs.dtu.dk/services/SignalP/output.html

##########################################################################################

Name:  contig074369-TilORe.E090         Length:  242
VNELYGSTGLFPFLLLQILSDVHTVSAPLCFLQIFCVFSYVCVEFCILAVMSYDRYLAICCPLQYHTRMTPATVVLLIAL      80
SWLYSFLTILTLILLIAPLELCGNVINKVYCLNYSIVKLACSETTANNIYGLFITALTVFVPVILILCSYVRILKVCFSG     160
SKQTRQKAVSTCTPHLSSLLNFSFGVCFEVIQSRFSLSSMHSMVHIVLSLYFLTCQPLFNPVLYGLNMSNIRKRLFAHKR     240
RX
................................................................................      80
................................N...............................................     160
..................................................................N.............     240
..                                                                                   320

(Threshold=0.5)
----------------------------------------------------------------------
SeqName      Position  Potential   Jury    N-Glyc
                                agreement  result
----------------------------------------------------------------------
contig074369-TilORe.E090  113 NYSI   0.6238     (8/9)   +
contig074369-TilORe.E090  181 NFSF   0.4163     (6/9)   -
contig074369-TilORe.E090  227 NMSN   0.5711     (4/9)   +
----------------------------------------------------------------------
```

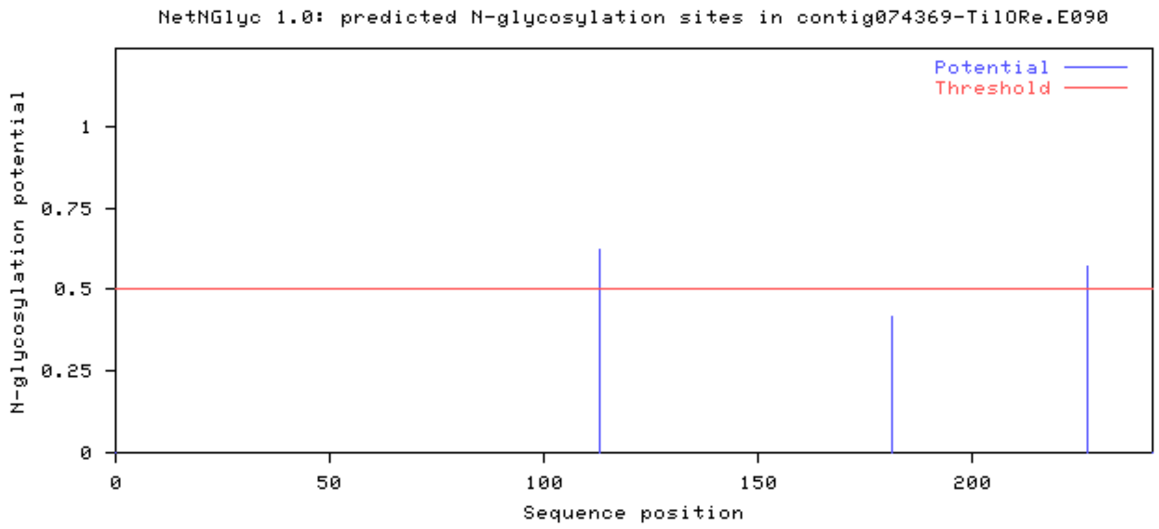

Graphics in PostScript

Output for 'contig074640-TilOR.A002'

```
##########################################################################

    Warning: This sequence may not contain a signal peptide!!

    Proteins without signal peptides are unlikely to be exposed to
    the N-glycosylation machinery and thus may not be glycosylated
    (in vivo) even though they contain potential motifs.

    SignalP-NN euk predictions are as follows:

  # name                 Cmax  pos ?  Ymax  pos ?  Smax  pos ?  Smean ?  D     ?

    SignalP output is explained at http://www.cbs.dtu.dk/services/SignalP/output.html

##########################################################################

Name:  contig074640-TilOR.A002  Length:  309
MDEKINVTYITLDGFVEIDKYRYVYFYIMFIVYILIICCNSTILYLICIHQNLHEPMYIFIAALLLNCALYSTAVYPKYL      80
IDFLSEKQVISYSACLFQYFLFYSLAGSEFFLLAAMAYDRYVAICKPLQYPTIMRKKTVSIFLFIAWLVPAFHITIPAIG     160
SAKAKLCSFYLNETFCNNRIYTLQCLRSEFFAAFGLVCLLDLGILPLLFILYTYTKIFLMSYRSCKEIRKKAAETCLPHL     240
SVLISFSCLGGYDVVITRVESDFPKTARLIMTLQLALYHPLFNPFVYGLKMKEISKHLKRLFSPPPKIX
.....N.................................N........................................      80
................................................................................     160
...........N....................................................................     240
.....................................................................                320

(Threshold=0.5)
----------------------------------------------------------------------
SeqName      Position  Potential   Jury    N-Glyc
                                agreement  result
----------------------------------------------------------------------
contig074640-TilOR.A002    6 NVTY   0.7550     (9/9)    +++
contig074640-TilOR.A002   40 NSTI   0.5953     (8/9)    +
contig074640-TilOR.A002  172 NETF   0.5539     (5/9)    +
----------------------------------------------------------------------
```

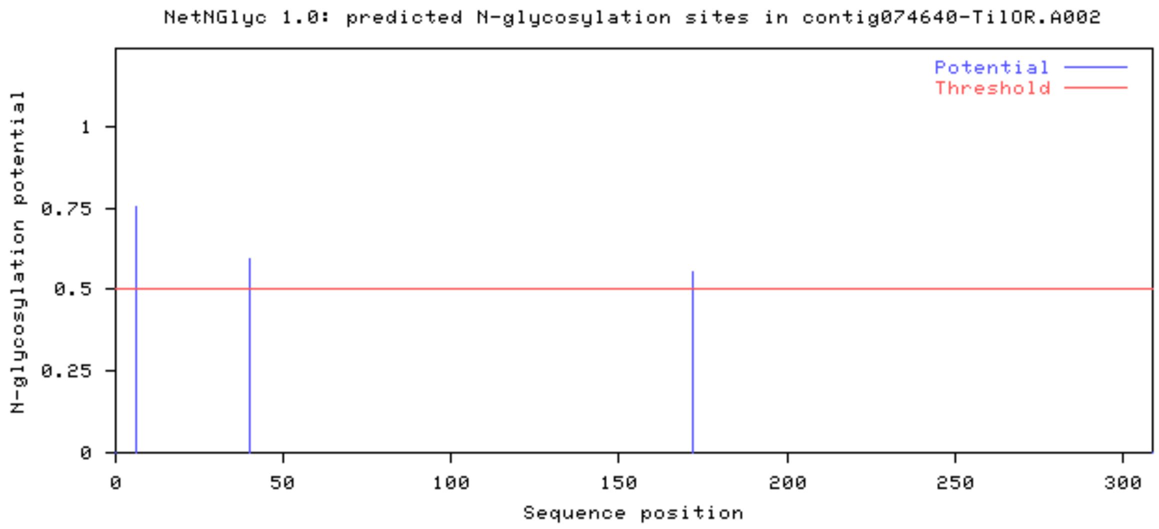

Graphics in PostScript

Output for 'contig075516-TilORe.D073'

```
##########################################################################################

    Warning: This sequence may not contain a signal peptide!!

    Proteins without signal peptides are unlikely to be exposed to
    the N-glycosylation machinery and thus may not be glycosylated
    (in vivo) even though they contain potential motifs.

    SignalP-NN euk predictions are as follows:

  # name                Cmax  pos ?  Ymax  pos ?  Smax  pos ?  Smean ?  D     ?

    SignalP output is explained at http://www.cbs.dtu.dk/services/SignalP/output.html

##########################################################################################

Name:  contig075516-TilORe.D073        Length:  62
MFVGCTFYFIDFTFVVSRVPNEVRIILPMYVLIFQPMLTPFMYGFNLPKIRQSYQRFLFKRQ
..............................................................                  80

(Threshold=0.5)

No sites predicted in this sequence.
```

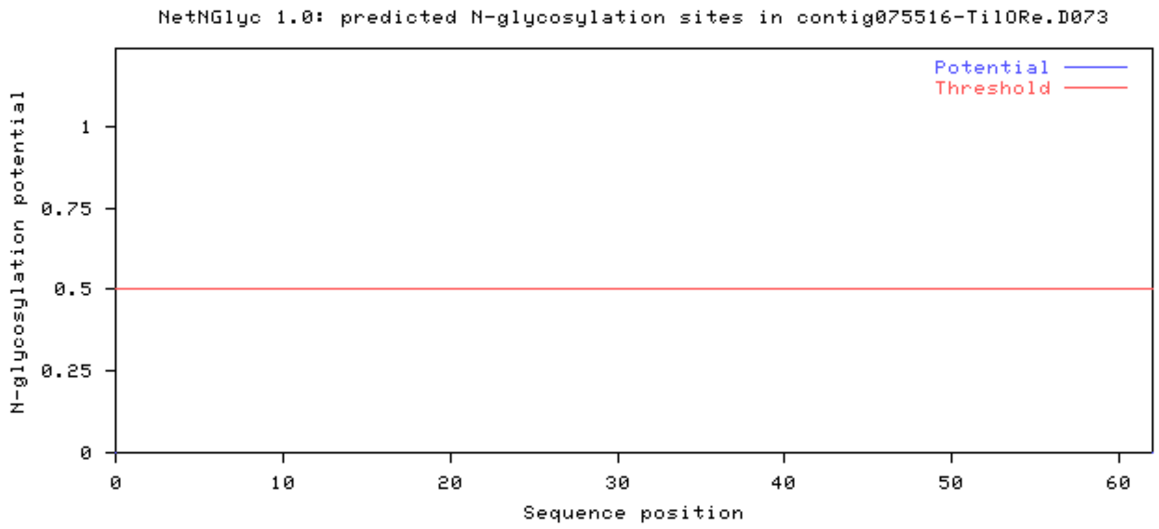

Graphics in PostScript

Output for 'contig075517-TilORe.D072'

```
##########################################################################################

    Warning: This sequence may not contain a signal peptide!!

    Proteins without signal peptides are unlikely to be exposed to
    the N-glycosylation machinery and thus may not be glycosylated
    (in vivo) even though they contain potential motifs.

    SignalP-NN euk predictions are as follows:

  # name                 Cmax  pos ?  Ymax  pos ?  Smax  pos ?  Smean ?  D     ?

    SignalP output is explained at http://www.cbs.dtu.dk/services/SignalP/output.html

##########################################################################################

Name:  contig075517-TilORe.D072        Length:  62
MFVGCTFYFIDFTFVVSRVPNEVRIILPMYVLIFQPMLTPFMYGFNLPKIRQSYQRFLFKRQ
..............................................................                        80

(Threshold=0.5)

No sites predicted in this sequence.
```

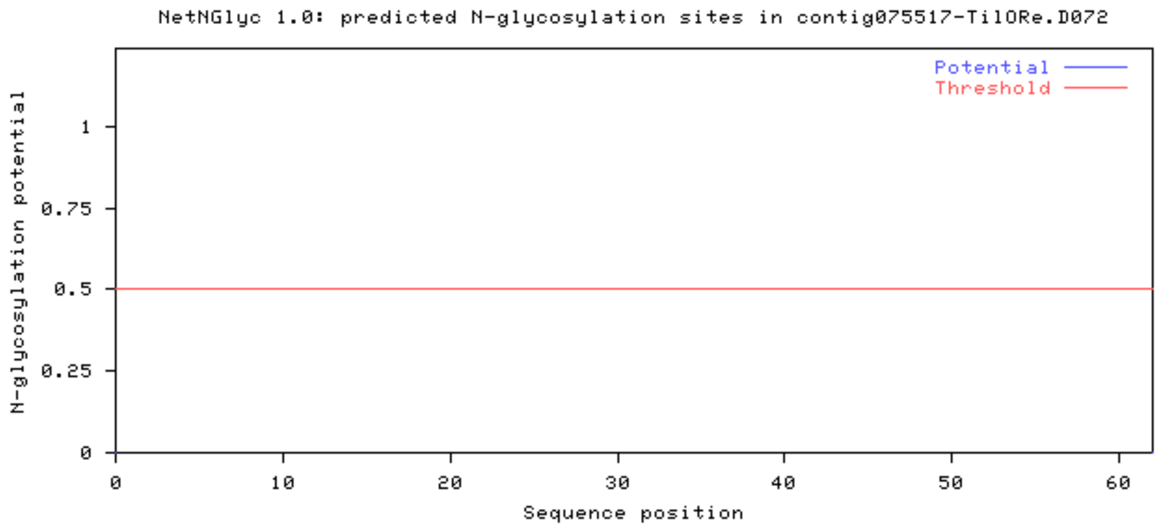

Graphics in PostScript

Output for 'contig075518-TilORe.D071'

```
##########################################################################################

    Warning: This sequence may not contain a signal peptide!!

    Proteins without signal peptides are unlikely to be exposed to
    the N-glycosylation machinery and thus may not be glycosylated
    (in vivo) even though they contain potential motifs.

    SignalP-NN euk predictions are as follows:

  # name                Cmax  pos ?  Ymax  pos ?  Smax  pos ?  Smean ?  D     ?

    SignalP output is explained at http://www.cbs.dtu.dk/services/SignalP/output.html

##########################################################################################

Name:  contig075518-TilORe.D071        Length:  62
MFVGCTFYFIDFTFVVSRVPNEVRIILPMYVLIFQPMLTPFMYGFNLPKIRQSYQRFLFKRQ
..............................................................                   80

(Threshold=0.5)

No sites predicted in this sequence.
```

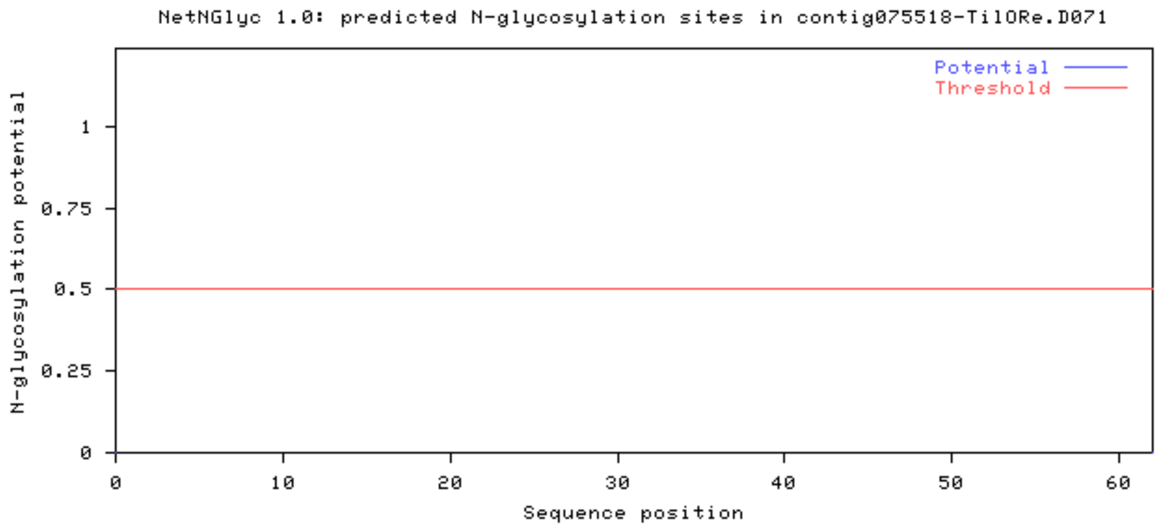

Graphics in PostScript

Output for 'contig075546-TilORe.D070'

```
##########################################################################

    Warning: This sequence may not contain a signal peptide!!

    Proteins without signal peptides are unlikely to be exposed to
    the N-glycosylation machinery and thus may not be glycosylated
    (in vivo) even though they contain potential motifs.

    SignalP-NN euk predictions are as follows:

  # name                   Cmax  pos ?  Ymax  pos ?  Smax  pos ?  Smean ?  D     ?

    SignalP output is explained at http://www.cbs.dtu.dk/services/SignalP/output.html

##########################################################################

Name:  contig075546-TilORe.D070        Length:  62
MFVGCTFYFIDFTFVVSRVPNEVRIILPMYVLIFQPMLTPFMYGFNLPKIRQSYQRFLFKRQ
..............................................................                    80

(Threshold=0.5)

No sites predicted in this sequence.
```

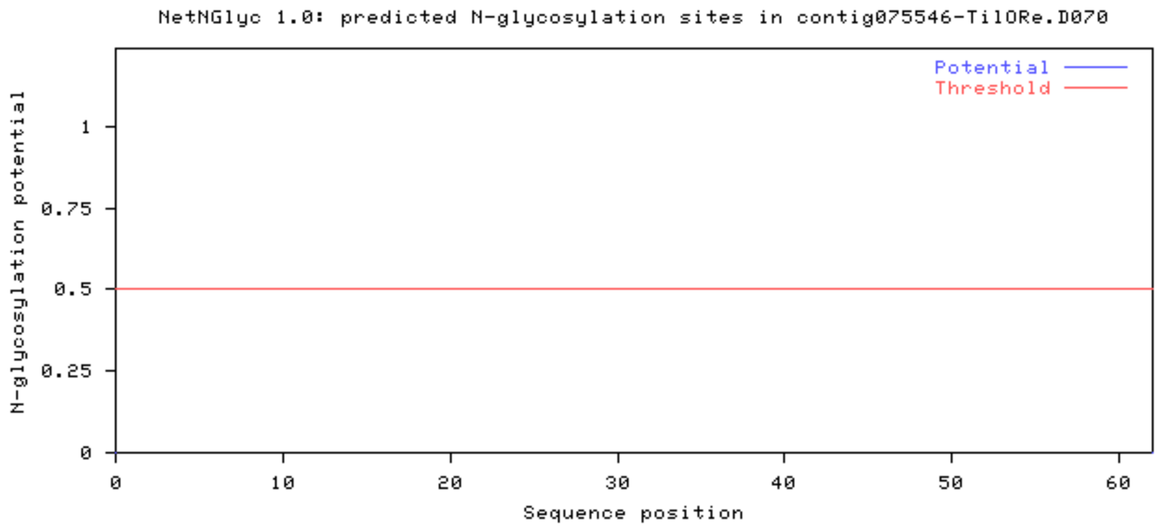

Graphics in PostScript

Output for 'contig075822-TilOR.F098'

```
##########################################################################################

    Warning: This sequence may not contain a signal peptide!!

    Proteins without signal peptides are unlikely to be exposed to
    the N-glycosylation machinery and thus may not be glycosylated
    (in vivo) even though they contain potential motifs.

    SignalP-NN euk predictions are as follows:

  # name                Cmax  pos ?  Ymax  pos ?  Smax  pos ?  Smean ?  D     ?

    SignalP output is explained at http://www.cbs.dtu.dk/services/SignalP/output.html

##########################################################################################

Name:  contig075822-TilOR.F098  Length:  305
MENSTLSFYFRFTMFANIGHYRFMAFIFCLLLFIFTIFTNLLMIVVISQQTTLHEPMYIFIACLSVNALYGSSGFFPRFL      80
MDLLSDTHLISRPACFTQIYVIYSYASCELTVLSIMAYDRYIAVCLPLHYHTKMTLKTVVKLTALAWILPAFSLAACLCL     160
SATLPLCGNEIHKVFCANWNVVKLSCVNTAVNNVVGMLLTVATIFLPLFYILYTYLRIVAICWKSSAEFKGKVLESCLPH     240
TISFVIYSIAGFCDVALSRNNLEVINPFIAVILSLVFVVIPPALNPLVYGLKLPEIRKHILRLFX
..N.............................................................................      80
................................................................................     160
................................................................................     240
.................................................................                    320

(Threshold=0.5)
----------------------------------------------------------------------
SeqName         Position  Potential    Jury     N-Glyc
                                     agreement  result
----------------------------------------------------------------------
contig075822-TilOR.F098    3 NSTL   0.7370     (9/9)    ++
----------------------------------------------------------------------
```

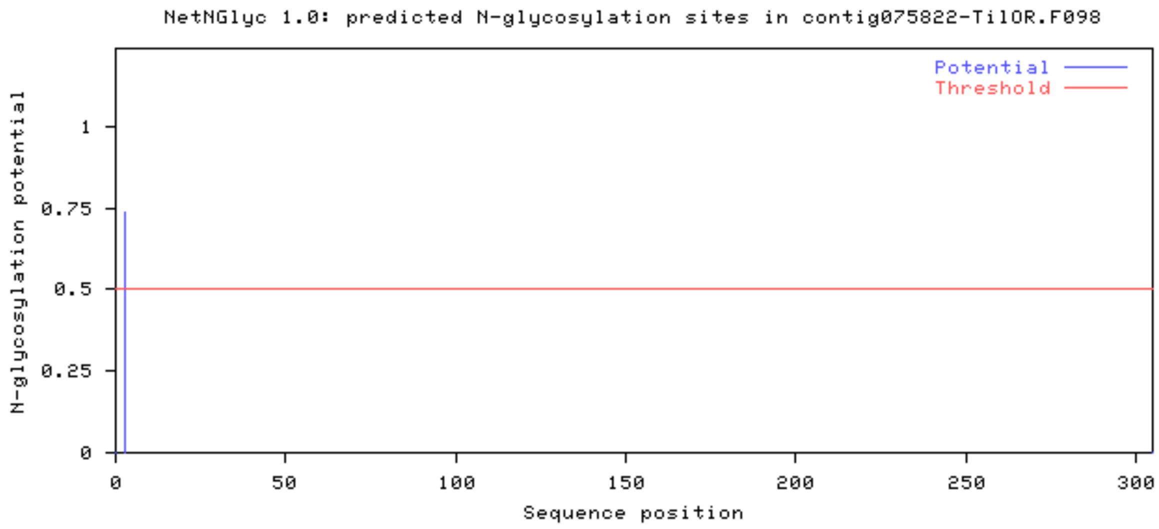

Graphics in PostScript

Output for 'contig076257-TilORe.L165'

```
##########################################################################

    Warning: This sequence may not contain a signal peptide!!

    Proteins without signal peptides are unlikely to be exposed to
    the N-glycosylation machinery and thus may not be glycosylated
    (in vivo) even though they contain potential motifs.

    SignalP-NN euk predictions are as follows:

  # name                Cmax  pos ?  Ymax  pos ?  Smax  pos ?  Smean ?  D     ?

    SignalP output is explained at http://www.cbs.dtu.dk/services/SignalP/output.html

##########################################################################

Name:  contig076257-TilORe.L165        Length:  220
MSLQNASINVTHFIIGGFDTLKRPIAVGVFILITYLLAVLANMANIMFIISDKRLHKPMYLLICNLAVVDIMYTSSCNPT      80
MIGVLLAGVNTISYVECLIQMCVFHLGTVMESFVLAFMALDRFIAIIYPFQYHSYLTNTHVPVLTFILWFVAWCSTCYMP     160
AIVIPLPHCSSRLRYTFCDFPAVLRTTCVNPDKYFNEGAIIAFFIFFFTFVFICLSYCGI
....N...N.....................................................................N..      80
................................................................................     160
............................................................                          240

(Threshold=0.5)
----------------------------------------------------------------------
SeqName      Position  Potential    Jury    N-Glyc
                                  agreement result
----------------------------------------------------------------------
contig076257-TilORe.L165     5 NASI    0.6728     (8/9)    +
contig076257-TilORe.L165     9 NVTH    0.7237     (9/9)    ++
contig076257-TilORe.L165    78 NPTM    0.6930     (9/9)    ++    WARNING: PRO-X1.
----------------------------------------------------------------------
```

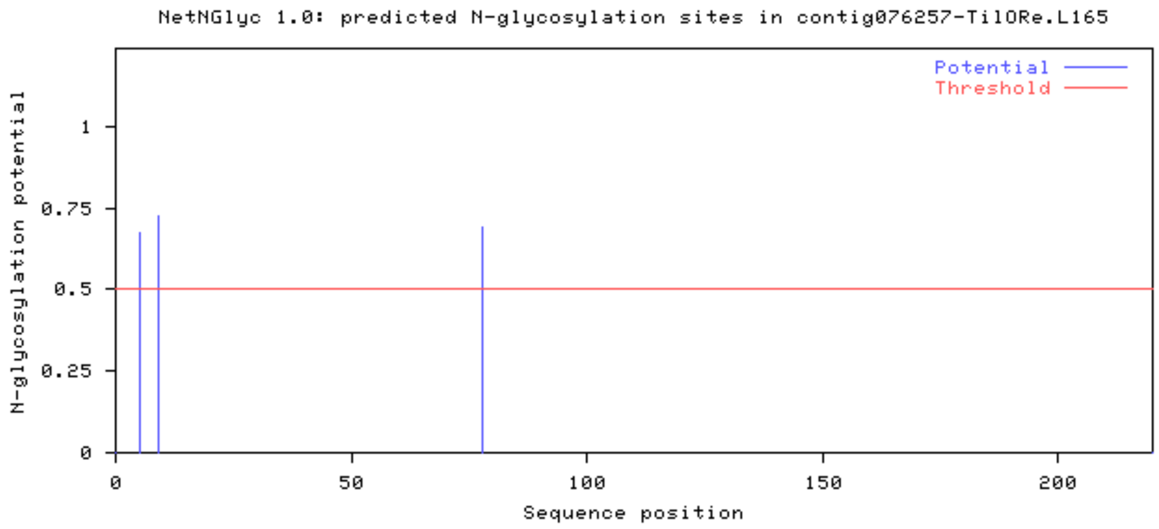

Graphics in PostScript

Output for 'contig077116-TilORe.H263'

```
##########################################################################################

    Warning: This sequence may not contain a signal peptide!!

    Proteins without signal peptides are unlikely to be exposed to
    the N-glycosylation machinery and thus may not be glycosylated
    (in vivo) even though they contain potential motifs.

    SignalP-NN euk predictions are as follows:

  # name                Cmax  pos ?  Ymax  pos ?  Smax  pos ?  Smean ?  D     ?

    SignalP output is explained at http://www.cbs.dtu.dk/services/SignalP/output.html

##########################################################################################

Name:  contig077116-TilORe.H263        Length:  67
MRLGSKELPRTLQNFAAIEFLVIPPIMNPLIYGFKLTKIRKRIYSLVIFKRTNFCFIRSDNSFTHSX
...................................................................                   80

(Threshold=0.5)

No sites predicted in this sequence.
```

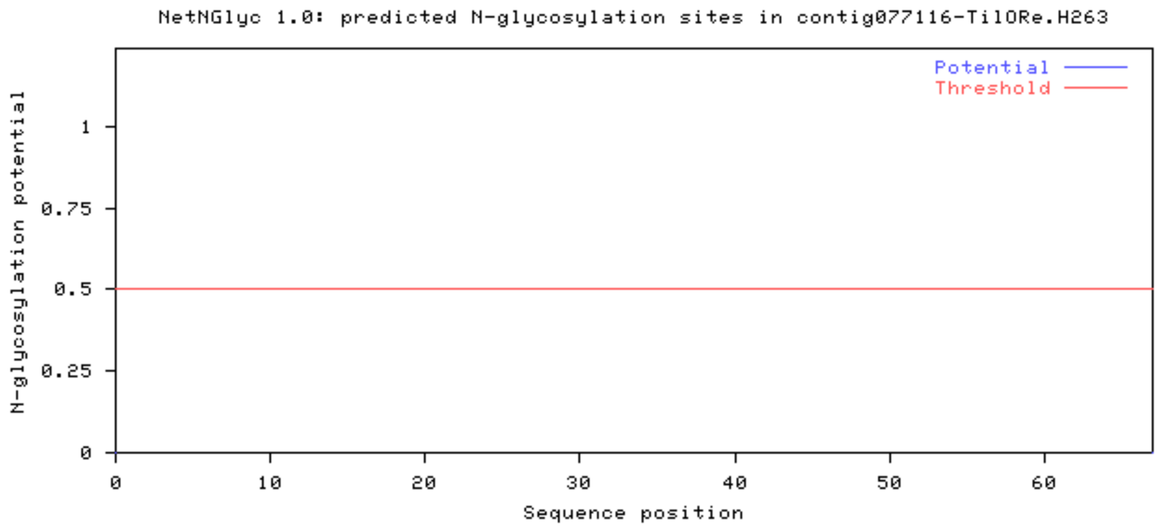

Graphics in PostScript

Output for 'contig077534-TilORe.L273'

```
##########################################################################################

    Warning: This sequence may not contain a signal peptide!!

    Proteins without signal peptides are unlikely to be exposed to
    the N-glycosylation machinery and thus may not be glycosylated
    (in vivo) even though they contain potential motifs.

    SignalP-NN euk predictions are as follows:

  # name                Cmax  pos ?  Ymax  pos ?  Smax  pos ?  Smean ?  D     ?

    SignalP output is explained at http://www.cbs.dtu.dk/services/SignalP/output.html

##########################################################################################

Name:  contig077534-TilORe.L273        Length:  231
GVLLAGVNTISYVECLIQMCVFHLGTVMESFVLAFMALDRFIAIIYPFHYHSYLTNTRVVVLTFILWFVASCFVCYMPAT      80
VVPLPHCSSRLKYSFCDFPALIRTTCVNPEKYFNEVAIVSFFIFFFTLVFICLSYCAILLFVKLSSYNEKKKMGSTLVSH     160
LICVIVYYCPAFVRSIFTRFGVVLTLEERQGLLIGAVLGPCLVNPFVYCLRTKEIKQKLFKIFKKFNTSEX
................................................................................      80
................................................................................     160
.......................................................................              240

(Threshold=0.5)

No sites predicted in this sequence.
```

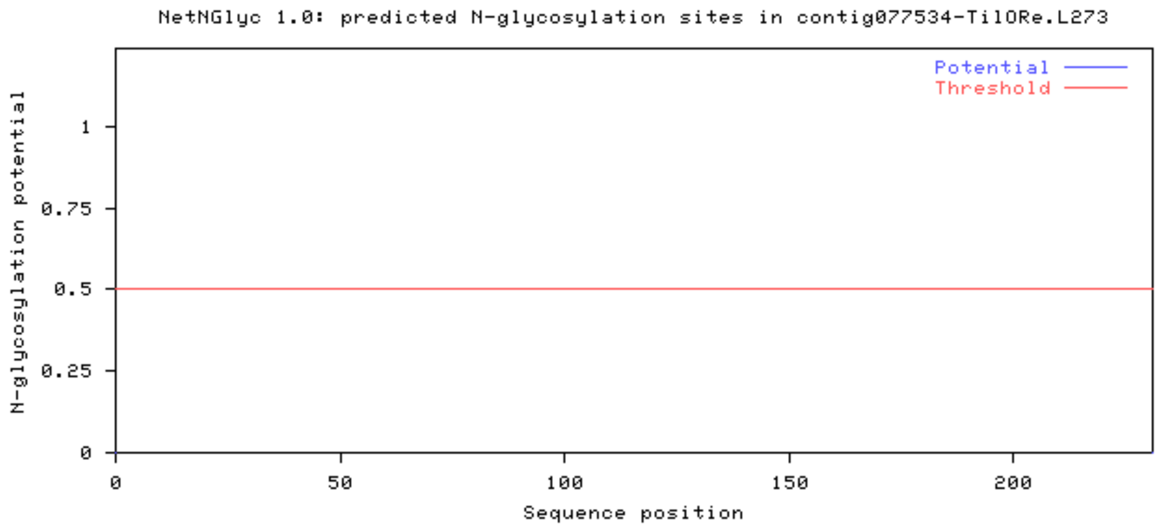

Graphics in PostScript

Output for 'contig077635-TilORe.W272'

```
##########################################################################################

    Warning: This sequence may not contain a signal peptide!!

    Proteins without signal peptides are unlikely to be exposed to
    the N-glycosylation machinery and thus may not be glycosylated
    (in vivo) even though they contain potential motifs.

    SignalP-NN euk predictions are as follows:

  # name                    Cmax  pos ?  Ymax  pos ?  Smax  pos ?  Smean ?  D     ?

    SignalP output is explained at http://www.cbs.dtu.dk/services/SignalP/output.html

##########################################################################################

Name:  contig077635-TilORe.W272         Length:  64
LWKKHLLKQALQQWFPKNFSDSLFACYIIFQILPRAISSIVYGVRDKTYRKYLKRYLLCKMSPX
.................N..............................................                      80

(Threshold=0.5)
----------------------------------------------------------------------
SeqName      Position  Potential   Jury    N-Glyc
                                 agreement result
----------------------------------------------------------------------
contig077635-TilORe.W272    18 NFSD   0.6206     (9/9)    ++
----------------------------------------------------------------------
```

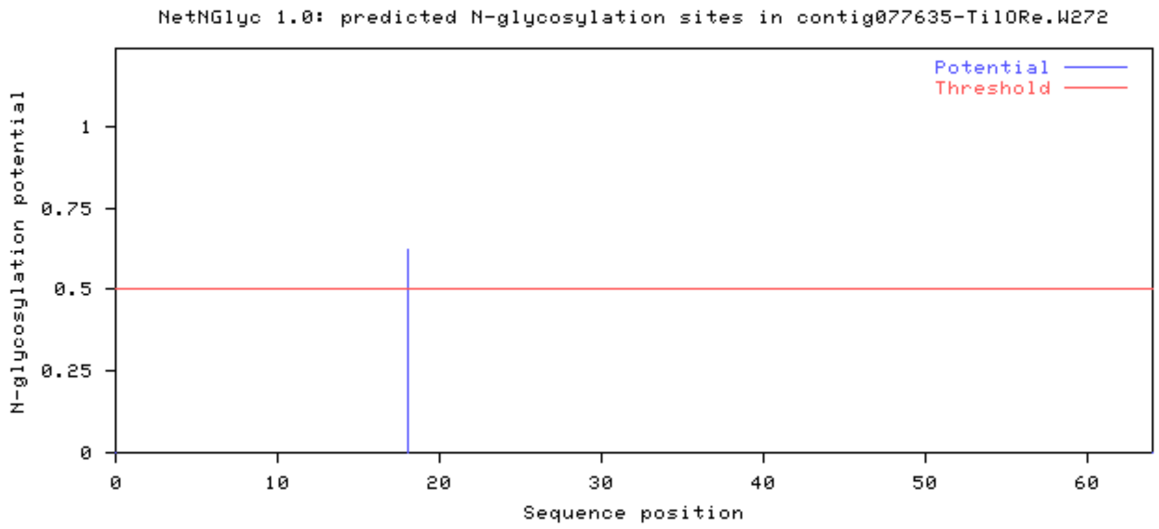

Graphics in PostScript

Explain the output. Go back.

NetNGlyc 1.0 Server - prediction results

Technical University of Denmark

Asn-Xaa-Ser/Thr sequons in the sequence output below are highlighted in **blue**.
Asparagines predicted to be N-glycosylated are highlighted in **red**.

Output for 'contig057403-ZebOR.H077'

```
##########################################################################################

    Warning: This sequence may not contain a signal peptide!!

    Proteins without signal peptides are unlikely to be exposed to
    the N-glycosylation machinery and thus may be glycosylated
    (in vivo) even though they contain potential motifs.

    SignalP-NN euk predictions are as follows:

  # name                Cmax  pos ?  Ymax  pos ?  Smax  pos ?  Smean ?  D     ?

    SignalP output is explained at http://www.cbs.dtu.dk/services/SignalP/output.html

##########################################################################################

Name:  contig057403-ZebOR.H077  Length:  343
MDNVSNVRSFILSGFNETMNFRVPLFAFTLLYYCMILFFNISLVLLIFLDENLHEPMYIFLSSFCINAIYGTTGFYPKFL      80
SDLLRSSQTISYEGCLLQAFVIYSFVCCDLSILAVMAFDRYLAICRPLHYHSFMTKRRLSQLVCFSWLTPLCIFAINVLL     160
TSRLKLCGINIRRVLCVNWLIVKLACPEAETFTNHITACATVIIYVSHGFFIMWTYTHLIKTCARSREDREKFMPTCLPH     240
LTSLITFIVVIGFQSIYVQFEYTGFSESLKNFITVEILIIPPFMNPLIYGFKLTKIQNRILTLLKSYKKLTYIKFSLLKK     320
TSVKCSIFLRVKNLLCEPFCCRX
..N............N........................N.......................................      80
................................................................................     160
................................................................................     240
................................................................................     320
.......................                                                              400

(Threshold=0.5)
----------------------------------------------------------------------
SeqName      Position  Potential   Jury    N-Glyc
                                 agreement result
----------------------------------------------------------------------
contig057403-ZebOR.H077    3 NVSN    0.7936     (9/9)   +++
contig057403-ZebOR.H077   16 NETM    0.6270     (9/9)   ++
contig057403-ZebOR.H077   40 NISL    0.6916     (9/9)   ++
----------------------------------------------------------------------
```

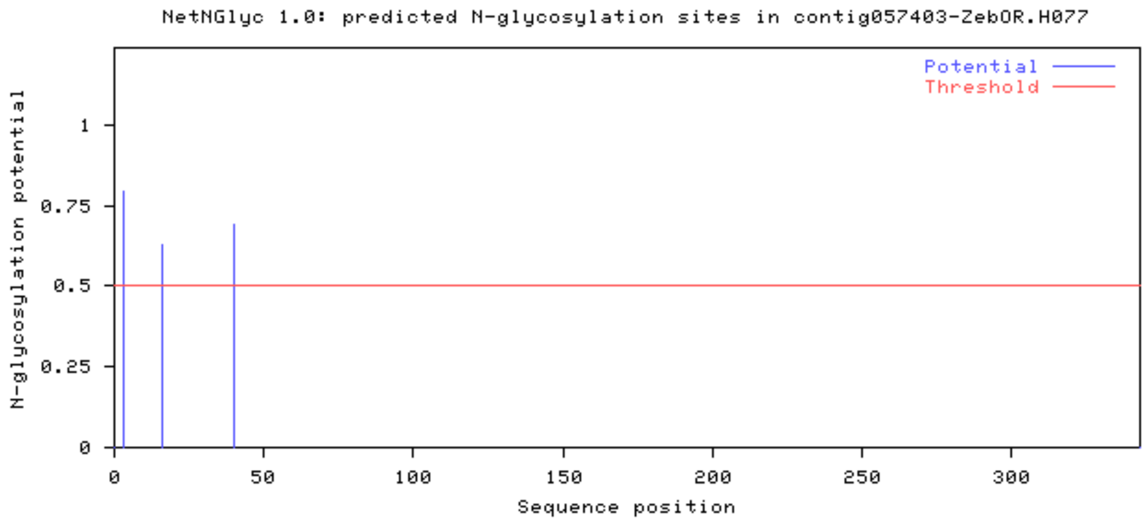

Graphics in PostScript

Output for 'contig046002-ZebOR.K087'

```
##########################################################################################

    Warning: This sequence may not contain a signal peptide!!

    Proteins without signal peptides are unlikely to be exposed to
    the N-glycosylation machinery and thus may not be glycosylated
    (in vivo) even though they contain potential motifs.

    SignalP-NN euk predictions are as follows:

  # name                  Cmax  pos ?  Ymax  pos ?  Smax  pos ?  Smean ?  D     ?

    SignalP output is explained at http://www.cbs.dtu.dk/services/SignalP/output.html

##########################################################################################

Name:  contig046002-ZebOR.K087  Length:  314
MENYTYNILTLQLEGLNISVESTYALFLLLLFFYLFIIVANGGIVVLVFMDKNLHQPMYMLFCNLPFNDILGNSIMVPRL      80
LIDILKPPSERFISYYECVVQAFTTHMFGTTSHTVLMIMAFDRYVAICNPLRYASIMTNKMVIKLTVFAWGVAFVLVGIL     160
LGLTVRLSRCRTLITNPYCDNASLFKLSCENVFINNVYGLTFTVVLFTGSIGSIVLTYASITIVCLTSKNKSLNSKALKT     240
CSTHLVVYLIMLLSGMIVIMLHRFPQYSDYRKLCSILFHIIPGSLNPIIYGVQSKEMQKLFAKLLQKKTGPLKX
..N.............N...............................................................      80
................................................................................     160
....................N...........................................................     240
..........................................................................           320

(Threshold=0.5)
----------------------------------------------------------------------
SeqName      Position  Potential   Jury    N-Glyc
                                agreement result
----------------------------------------------------------------------
contig046002-ZebOR.K087    3 NYTY   0.7761     (9/9)   +++
contig046002-ZebOR.K087   17 NISV   0.6830     (9/9)   ++
contig046002-ZebOR.K087  181 NASL   0.6140     (7/9)   +
contig046002-ZebOR.K087  230 NKSL   0.4032     (7/9)   -
----------------------------------------------------------------------
```

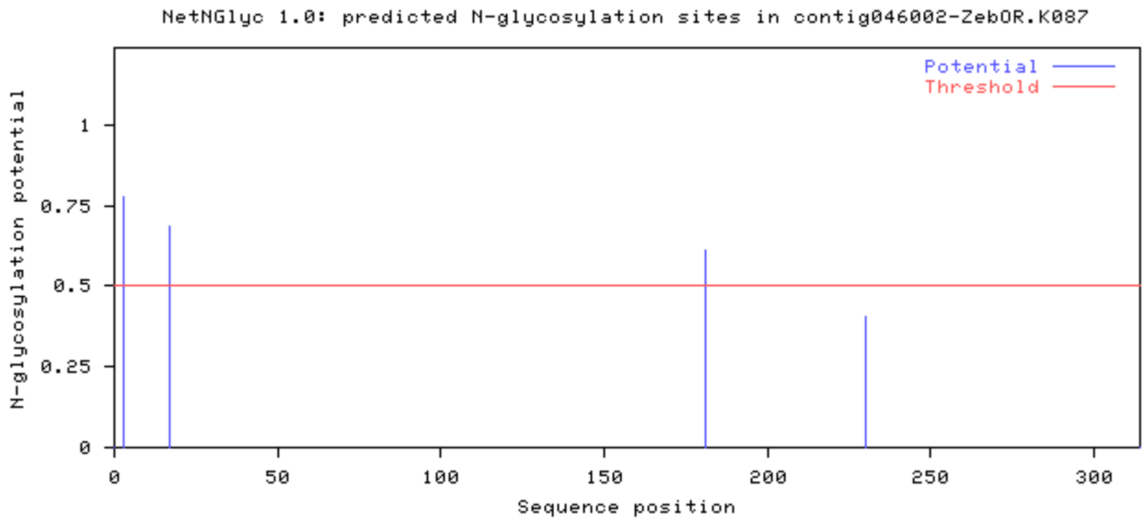

Graphics in PostScript

Output for 'contig017736-ZebOR.S127'

```
##########################################################################################

    Warning: This sequence may not contain a signal peptide!!

    Proteins without signal peptides are unlikely to be exposed to
    the N-glycosylation machinery and thus may not be glycosylated
    (in vivo) even though they contain potential motifs.

    SignalP-NN euk predictions are as follows:

  # name                 Cmax  pos ?  Ymax  pos ?  Smax  pos ?  Smean ?  D     ?

    SignalP output is explained at http://www.cbs.dtu.dk/services/SignalP/output.html

##########################################################################################

Name:  contig017736-ZebOR.S127  Length:  305
MAGNNSVNDVFLQRPVYDRVIIVQILVIIFLCINMLLIVIFIKKESFHTSARYTLFFVTLLSDSVLLLVSDVLLVLSQFE      80
FTIQVWLCITISVVVLLYFIVTPVTLTAMTLERYVAICMPLRHGQLCSTRSTMYCILIIHVLSSGPCIIILSMFFASASL     160
KFYKQSMICSGETFTIYRWQDHVRSAVYQFYFLIMGITIAYSYVQIMKVAKAASGDKKKLTHKGLKTVILHAFQLLLCLI     240
QLWCPFIEIALLQIDFSLFRNVRYFNYIVFNIAPRCLSPLIYGLRDENIFLVLKSLMPTSSCSKX
...N............................................................................      80
................................................................................     160
................................................................................     240
.................................................................                    320

(Threshold=0.5)
----------------------------------------------------------------------
SeqName      Position  Potential   Jury    N-Glyc
                                 agreement result
----------------------------------------------------------------------
contig017736-ZebOR.S127    4 NNSV   0.7398     (9/9)    ++
----------------------------------------------------------------------
```

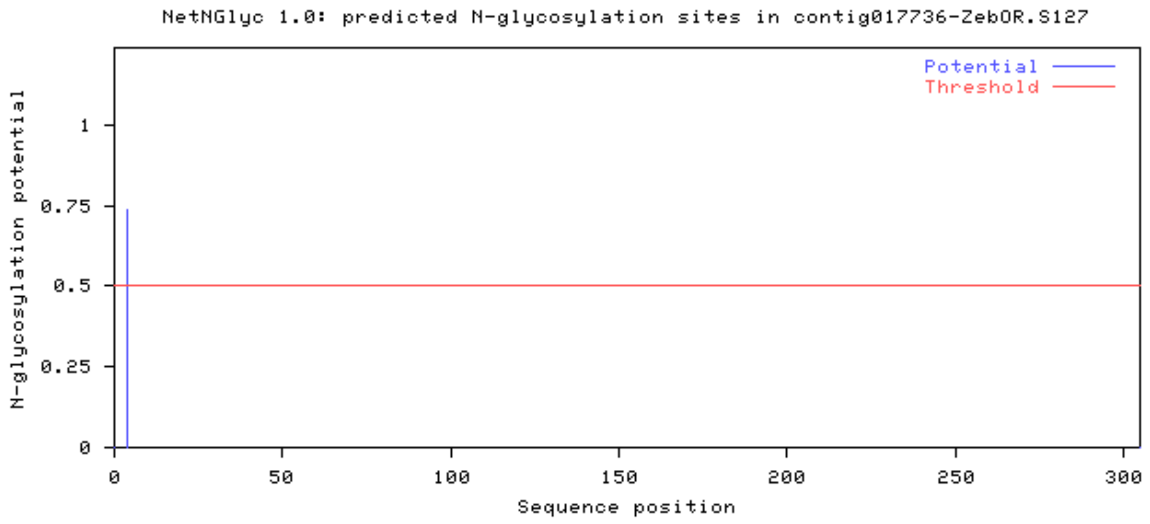

Graphics in PostScript

Output for 'contig046010-ZebOR.K088'

```
##########################################################################################

    Warning: This sequence may not contain a signal peptide!!

    Proteins without signal peptides are unlikely to be exposed to
    the N-glycosylation machinery and thus may not be glycosylated
    (in vivo) even though they contain potential motifs.

    SignalP-NN euk predictions are as follows:

  # name                  Cmax   pos ?  Ymax   pos ?  Smax   pos ?  Smean ?  D     ?

    SignalP output is explained at http://www.cbs.dtu.dk/services/SignalP/output.html

##########################################################################################

Name:  contig046010-ZebOR.K088  Length:  313
MDNQSLNADILIVGGLKVTPKFSIAAFISLLLVYIFIMVANIGLVVLIFMERSLHQPMYLLFCNMSVNEVFGSTIVVPHI      80
LRDLYVSDSERYIHYIVCVVQAFCVNLYGGVCHTILMTMTFDRYMAICNPLRYTIIMTNWMVVKLSVAAWAVVFVMVSIL     160
LSLTIRLSRCRRFIDNVHCDNASLFKLSCEDVVINHVFGLSYSVLLLGSSIGSVTLTYIKIATVCLRSKTKTINSKALQT     240
CATHLTLYIILMFSAFIIIILHRFPHLSDHRKMVSTVGEVALPALNAVIYGLQIKEIRQKIVALFQRKGHLQX
..N............................................................N................      80
................................................................................     160
....................N...........................................................     240
.........................................................................            320

(Threshold=0.5)
----------------------------------------------------------------------
SeqName      Position  Potential   Jury    N-Glyc
                                 agreement result
----------------------------------------------------------------------
contig046010-ZebOR.K088    3 NQSL   0.7056     (9/9)   ++
contig046010-ZebOR.K088   64 NMSV   0.6973     (9/9)   ++
contig046010-ZebOR.K088  181 NASL   0.6080     (8/9)   +
----------------------------------------------------------------------
```

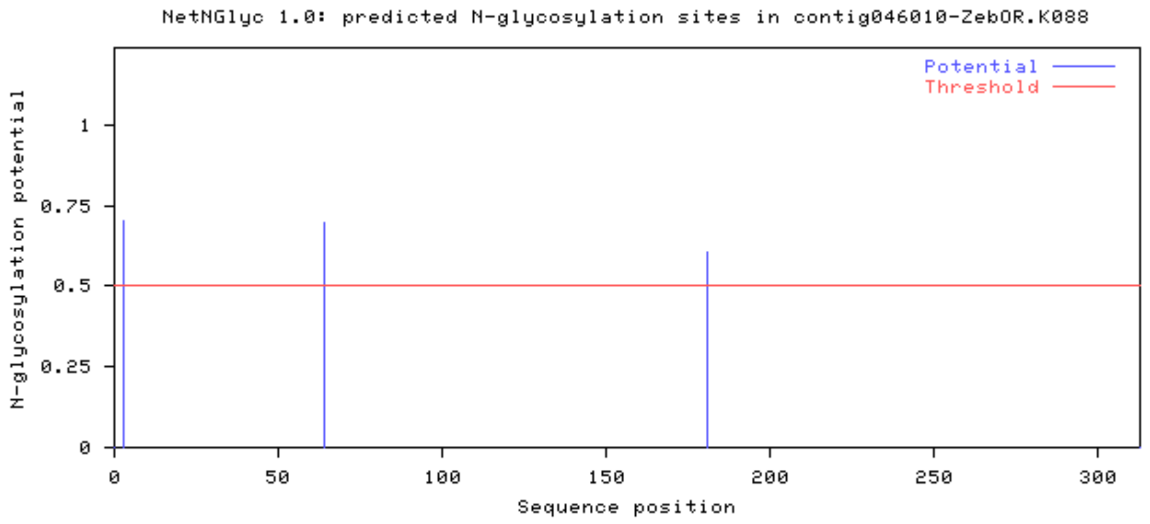

Graphics in PostScript

Output for 'contig010712-ZebOR.N108'

```
##########################################################################################

    Warning: This sequence may not contain a signal peptide!!

    Proteins without signal peptides are unlikely to be exposed to
    the N-glycosylation machinery and thus may not be glycosylated
    (in vivo) even though they contain potential motifs.

    SignalP-NN euk predictions are as follows:

  # name                 Cmax  pos ?  Ymax  pos ?  Smax  pos ?  Smean ?  D     ?

    SignalP output is explained at http://www.cbs.dtu.dk/services/SignalP/output.html

##########################################################################################

Name:  contig010712-ZebOR.N108  Length:  324
MALINTAAENNITFVRPAYFIISGFIGIPNIRYYFVFLCFIYILAVVGNTLVMLVITLDHMLRSPKYIAVFNLAFTDLLS      80
SSALMPKVVDIFLLNHYYISYNDCLTFMFFCSTFYAMQTFNLVVLSFDRVMAIMYPLHYQMRVSHKLILTLIAFFWLLAT     160
TLILIVVGLLTRLSFCKSVVIQSYFCDHGPMYRLGCNDITPNRAIAGLASVIILGFPLAFIVGSYCCIGYSLSKISTFRE     240
RVKAFKTCTGHLSLVAIYFLPFTFVYIFGSVIHPNARIISLSISTVLPPMLNPIIYVLQTQEIKESLKKLLQTRAQFRIA     320
EKYX
..........N.....................................................................      80
................................................................................     160
................................................................................     240
................................................................................     320
....                                                                                 400

(Threshold=0.5)
----------------------------------------------------------------------
SeqName      Position  Potential   Jury    N-Glyc
                                 agreement result
----------------------------------------------------------------------
contig010712-ZebOR.N108   11 NITF   0.6459     (9/9)    ++
----------------------------------------------------------------------
```

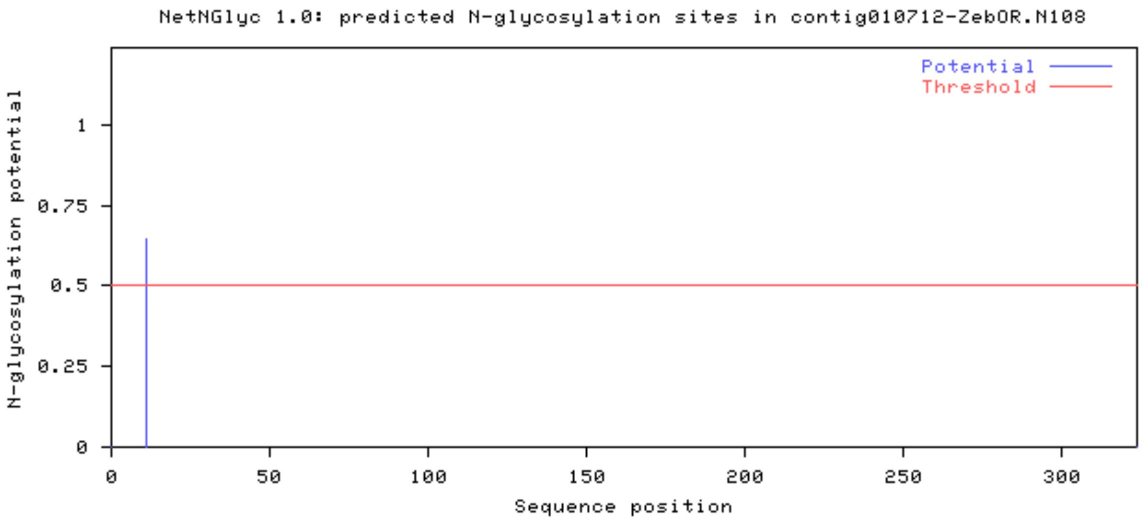

Graphics in PostScript

Output for 'contig010714-ZebOR.N109'

```
##########################################################################################

    Warning: This sequence may not contain a signal peptide!!

    Proteins without signal peptides are unlikely to be exposed to
    the N-glycosylation machinery and thus may not be glycosylated
    (in vivo) even though they contain potential motifs.

    SignalP-NN euk predictions are as follows:

  # name                   Cmax  pos ?  Ymax  pos ?  Smax  pos ?  Smean ?  D     ?

    SignalP output is explained at http://www.cbs.dtu.dk/services/SignalP/output.html

##########################################################################################

Name:  contig010714-ZebOR.N109  Length:  324
MALINSAAENNITFVRPAYFIISGFIGIPNIRYYFVFLCFIYILAVVGNTLVMIVITLDHMLRSPKYIAVFNLAFTDLLS      80
SSALVPKVVDIFLFNHYYISYNACLTFMFFCFTFISMQAFNLVVLSFDRVMAIMYPLHYQMRVSHKLILSLIAFFWLLAI     160
TLILIAAGLLTRLSFCKSVVIQSYYCDHGPMYRLGCNDNTPNRAIARSTPVIILGFPLAFIMGSYCCIGYSLSKISTFRE     240
RVKAFKTYTGHLSLVAIYFLPFTFVYIFGSVIHPNARIISLSMSTVLPPMLNPIIYVLQTQEIKQSLKKLLKTRVTSKIA     320
TKYX
..........N.....................................................................      80
................................................................................     160
................................................................................     240
................................................................................     320
....                                                                                 400

(Threshold=0.5)
----------------------------------------------------------------------
SeqName      Position  Potential   Jury    N-Glyc
                                 agreement result
----------------------------------------------------------------------
contig010714-ZebOR.N109    11 NITF   0.6510     (9/9)    ++
----------------------------------------------------------------------
```

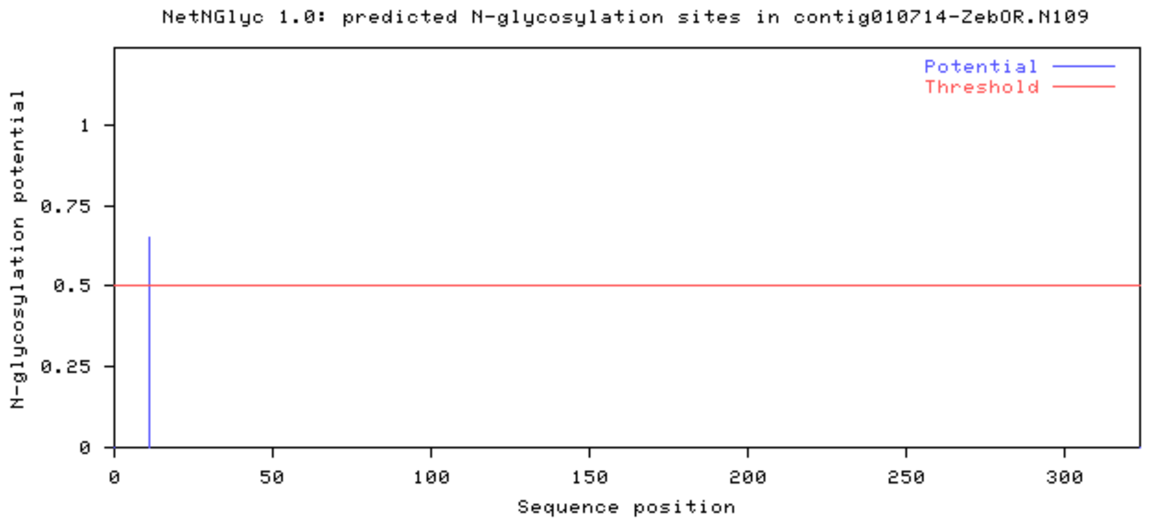

Graphics in PostScript

Output for 'contig010718-ZebOR.N110'

```
##########################################################################################

    Warning: This sequence may not contain a signal peptide!!

    Proteins without signal peptides are unlikely to be exposed to
    the N-glycosylation machinery and thus may not be glycosylated
    (in vivo) even though they contain potential motifs.

    SignalP-NN euk predictions are as follows:

  # name                   Cmax  pos ?  Ymax  pos ?  Smax  pos ?  Smean ?  D     ?

    SignalP output is explained at http://www.cbs.dtu.dk/services/SignalP/output.html

##########################################################################################

Name:  contig010718-ZebOR.N110  Length:  324
MALINSAAENNITFVQPAYFIISGFIGIPNIRYYFVFLCFIYILAVVGNTLVMIVITLDRMLRSPKYIAVFNLAFTDLLS      80
SSALVPKVVDIFLFNHYYVSYNDCLTFMFFCFTFIFMQGFNLVVLSFDRVMAIMYPLHYQMRVSHKLILSLIAFFWLLAI     160
TLILIAVGLLTRLSFCKSVVIQSYYCDHGPMYRLGCNDNTPNHAFAGSALVIILGFPLAFIVGSYCCIGYSLSKISTFRE     240
RVKAFKTYTGHLSLVAIYFLPFTFVYMFGSVIHPNARIISLSMSTVLPPMLNPIIYVLQTQEIKESLKKLLKTRVTSKIA     320
TKYX
..........N.....................................................................      80
................................................................................     160
................................................................................     240
................................................................................     320
....                                                                                 400

(Threshold=0.5)
----------------------------------------------------------------------
SeqName      Position  Potential   Jury    N-Glyc
                                 agreement result
----------------------------------------------------------------------
contig010718-ZebOR.N110    11 NITF    0.6380     (9/9)    ++
----------------------------------------------------------------------
```

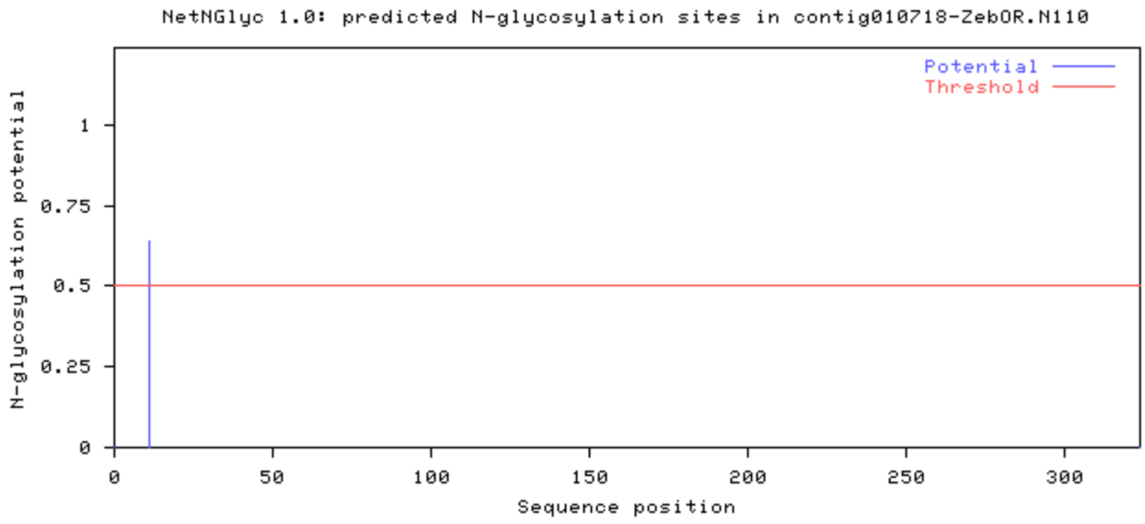

Graphics in PostScript

Output for 'contig010720-ZebORp.N121'

```
##########################################################################################

    Warning: This sequence may not contain a signal peptide!!

    Proteins without signal peptides are unlikely to be exposed to
    the N-glycosylation machinery and thus may not be glycosylated
    (in vivo) even though they contain potential motifs.

    SignalP-NN euk predictions are as follows:

  # name                    Cmax   pos ?  Ymax   pos ?  Smax   pos ?  Smean ?  D      ?

    SignalP output is explained at http://www.cbs.dtu.dk/services/SignalP/output.html

##########################################################################################

Name:  contig010720-ZebORp.N121         Length:  327
MDLFNSALGKNITFLRPAFFIISGFIGIPNIKYYYAFLFFVYIISVLANTAVMAAIYLDHNLRTPKYIAVFNLALVDLLG      80
NSAMVPKVLDIFLFNHPPISYNDCLTFLFFCYVFLSMQALNLVALSYDRVMAIVYPLHYQLKVTHKFMFCLIASFWVFVI     160
IVVLIATGLLTRLSFCESVVIKSFFCDHGQIYRLACNDYTPSDITAWILPALILWLPLTIVLLSYLCICYALAKVATVRE     240
RMKGFKTCTAHLSLVAIYFLPILITFTLGANIEPNARIINLSLTSVFPPMLNPIIYVLQTQEIKESLKRFLRITTHYKIS     320
KVKFKKX
..........N.....................................................................      80
................................................................................     160
................................................................................     240
.......................................N........................................     320
.......                                                                              400

(Threshold=0.5)
----------------------------------------------------------------------
SeqName      Position  Potential   Jury    N-Glyc
                                agreement result
----------------------------------------------------------------------
contig010720-ZebORp.N121   11 NITF   0.5352     (6/9)   +
contig010720-ZebORp.N121  280 NLSL   0.6052     (8/9)   +
----------------------------------------------------------------------
```

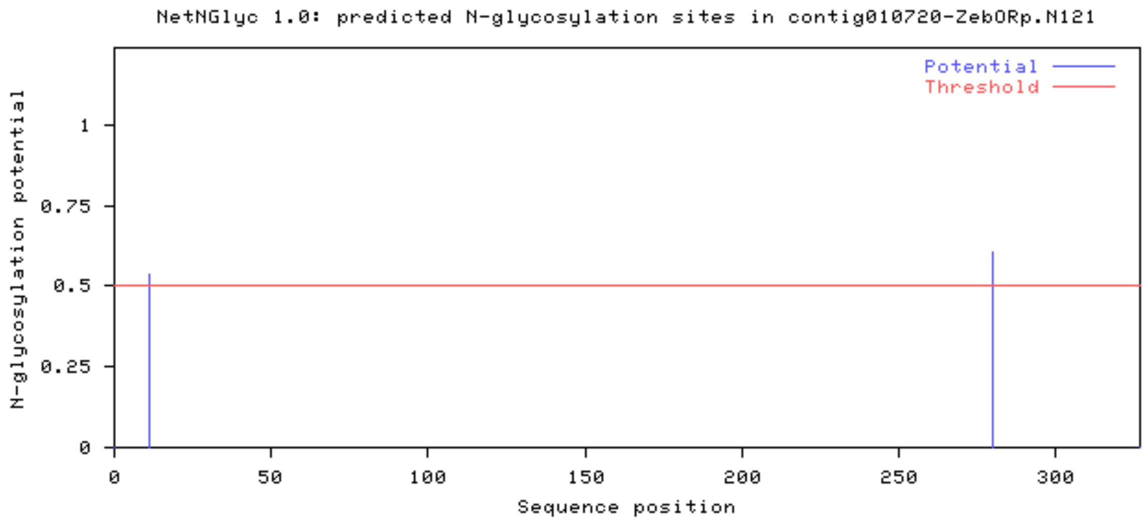

Graphics in PostScript

Output for 'contig010722-ZebOR.N111'

```
##########################################################################################

    Warning: This sequence may not contain a signal peptide!!

    Proteins without signal peptides are unlikely to be exposed to
    the N-glycosylation machinery and thus may not be glycosylated
    (in vivo) even though they contain potential motifs.

    SignalP-NN euk predictions are as follows:

  # name                    Cmax   pos ?  Ymax   pos ?  Smax   pos ?  Smean ?  D     ?

    SignalP output is explained at http://www.cbs.dtu.dk/services/SignalP/output.html

##########################################################################################

Name:  contig010722-ZebOR.N111  Length:  324
MDFFNSALGKNITFFRPAFFIISGFIGIPNIKYYYAFLFFVYIISVLANTAVMATIYLDHNLRTPKYIAVFNLALVDLLG      80
NSAMVPKVLDIFLFNHPHISYNDCLTFLFFCYIFLSLQALNLVALSYDRVMAIVYPLHYQLKVTHKLMFCLIASFWVSAI     160
TFILIAIGLLTRLSFCESVVIKSYFCDHGQMYRLACNDYTPSYVIAKILPALILWLPLTIVLLSYLCICYALAKVATVRE     240
RTKGFKTCTAHLSLVAIYFLPILITFTLGANIEPNARIINLSLTSVFPPMLNPIIYVLQTREIKESLRKLLRIIKHYKIR     320
KVKX
..........N.....................................................................      80
................................................................................     160
................................................................................     240
.......................................N........................................     320
....                                                                                 400

(Threshold=0.5)
----------------------------------------------------------------------
SeqName      Position  Potential   Jury    N-Glyc
                                 agreement result
----------------------------------------------------------------------
contig010722-ZebOR.N111   11 NITF   0.6267     (8/9)   +
contig010722-ZebOR.N111  280 NLSL   0.6041     (8/9)   +
----------------------------------------------------------------------
```

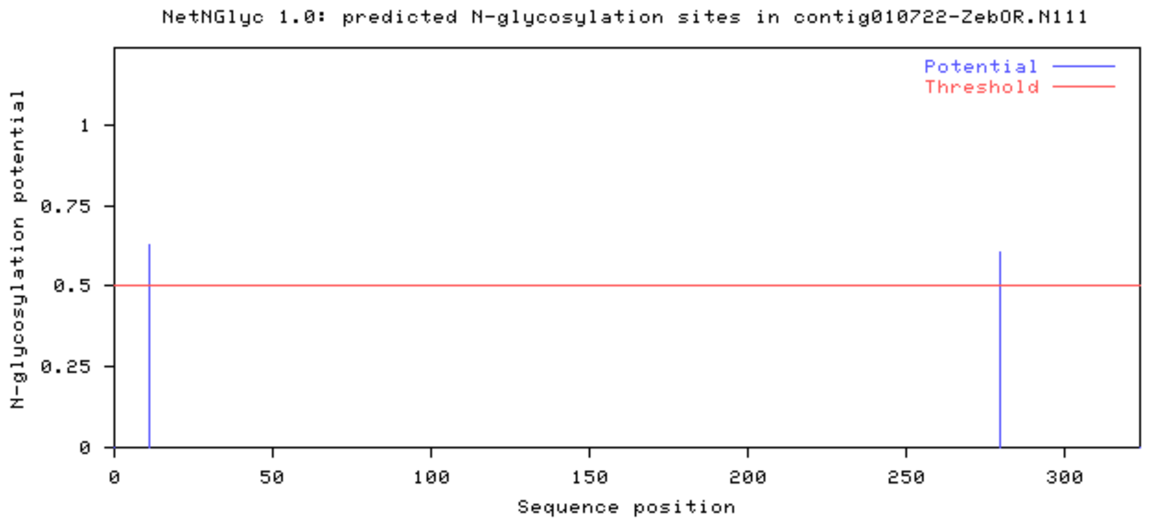

Graphics in PostScript

Output for 'contig010722-ZebORp.N122'

```
##########################################################################################

    Warning: This sequence may not contain a signal peptide!!

    Proteins without signal peptides are unlikely to be exposed to
    the N-glycosylation machinery and thus may not be glycosylated
    (in vivo) even though they contain potential motifs.

    SignalP-NN euk predictions are as follows:

  # name                    Cmax  pos ?  Ymax  pos ?  Smax  pos ?  Smean ?  D     ?

    SignalP output is explained at http://www.cbs.dtu.dk/services/SignalP/output.html

##########################################################################################

Name:  contig010722-ZebORp.N122         Length:  324
MEFLNSAVEKNTTFVQSAYFIISGFIGIPNIRYYFVFLCFIYIFSVVGNTLVMIVIILDRMLRSPKYITVFNLAFTDLLS      80
SSALVPKVLDISLFNHYYISYNICLTFMFFCFTLISMQAFNLVVLSFDRVMAIMYPLHYQMRVTQKLILSLIALAITLTL     160
VAVGLLTRLSFCQSVIIQSYYCDHGPMYRLGCNDATPNYIIAFLAIVLVLGFPLAFIVGSYCCIGYSLSNISTFRERVKA     240
FKACTGHLSLVAIYFLPIIFVYVFWPVIHPNARIINLSMTSVMPPMLNPIIYVLQTQEIKESLRRLLKARAQAKLEVFRL     320
KPLX
................................................................................      80
................................................................................     160
.....................................................................N..........     240
...................................N............................................     320
....                                                                                 400

(Threshold=0.5)
----------------------------------------------------------------------
SeqName        Position  Potential   Jury    N-Glyc
                                   agreement result
----------------------------------------------------------------------
contig010722-ZebORp.N122   11 NTTF   0.4779     (4/9)   -
contig010722-ZebORp.N122  230 NIST   0.6731     (7/9)   +
contig010722-ZebORp.N122  276 NLSM   0.5399     (5/9)   +
----------------------------------------------------------------------
```

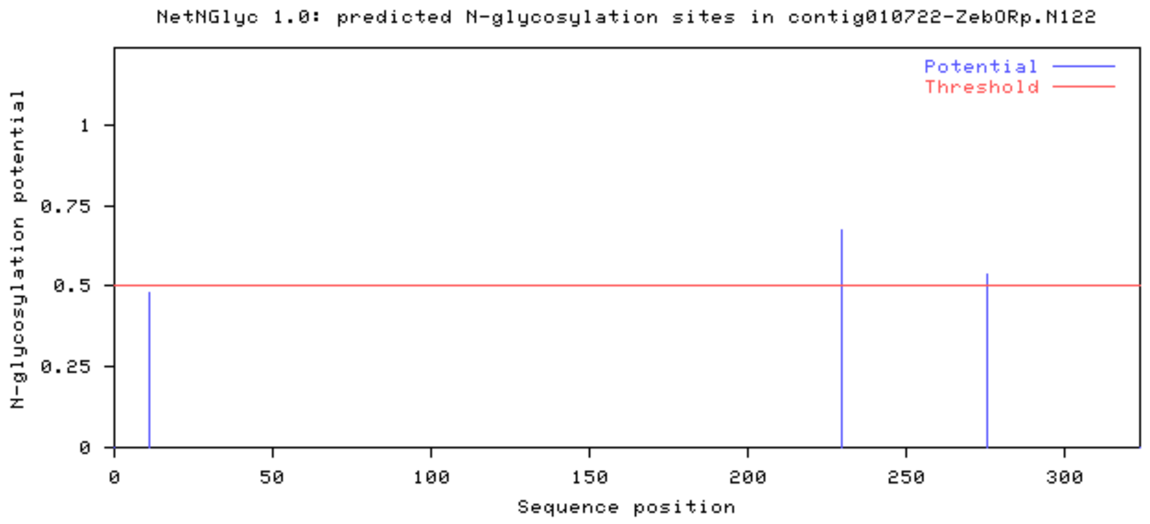

Graphics in PostScript

Output for 'contig010725-ZebOR.N112'

```
########################################################################################

    Warning: This sequence may not contain a signal peptide!!

    Proteins without signal peptides are unlikely to be exposed to
    the N-glycosylation machinery and thus may not be glycosylated
    (in vivo) even though they contain potential motifs.

    SignalP-NN euk predictions are as follows:

  # name                 Cmax  pos ?  Ymax  pos ?  Smax  pos ?  Smean ?  D     ?

    SignalP output is explained at http://www.cbs.dtu.dk/services/SignalP/output.html

########################################################################################

Name:  contig010725-ZebOR.N112  Length:  324
MEFLNSAVGKNITFVKPAYFIISAFNGIANIRYYFVFLCFIYIFSVVGNTLLMIGIILDHTLRGPKHIGVVNFAFTDLLS      80
SSALMPKLVDIFLFNHHHISYNDCLAFMFFCLTFFAAQAFNLVVLSFDRVLAIMYPLHYQMRISHKLILSLIAFFWLLAI     160
TIILTAVGLLTRLSFCDSVVIQSFFCDHGPVYRLGCNDLTPNRVIAHLASVLVLWVPLAFIVGSYCCIGYSLSKTVTCRE     240
RLKALKTCTSHLSLVAIYFLPALFIFTFGSTILPNARTVSLSLATVMPLTLNPIIYGLQTQEIKESLKKLLKVKMQFKIS     320
AKKX
..........N.....................................................................      80
................................................................................     160
................................................................................     240
................................................................................     320
....                                                                                 400

(Threshold=0.5)
----------------------------------------------------------------------
SeqName      Position  Potential   Jury    N-Glyc
                                 agreement result
----------------------------------------------------------------------
contig010725-ZebOR.N112   11 NITF   0.5220     (6/9)   +
----------------------------------------------------------------------
```

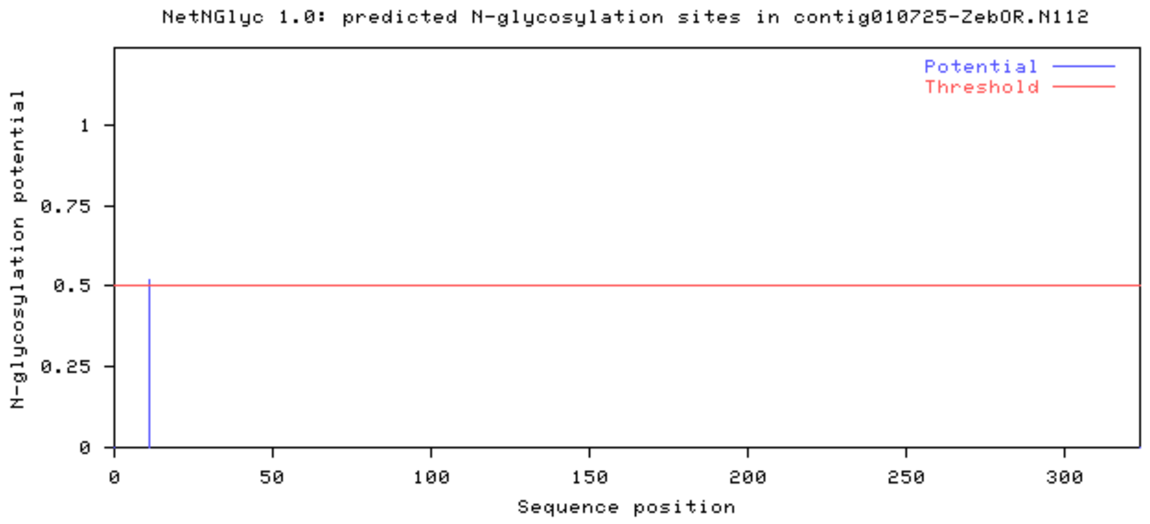

Graphics in PostScript

Output for 'contig010726-ZebOR.N113'

```
##########################################################################################

    Warning: This sequence may not contain a signal peptide!!

    Proteins without signal peptides are unlikely to be exposed to
    the N-glycosylation machinery and thus may not be glycosylated
    (in vivo) even though they contain potential motifs.

    SignalP-NN euk predictions are as follows:

  # name                  Cmax  pos ?  Ymax  pos ?  Smax  pos ?  Smean ?  D     ?

    SignalP output is explained at http://www.cbs.dtu.dk/services/SignalP/output.html

##########################################################################################

Name:  contig010726-ZebOR.N113  Length:  324
MEFLNSAVGKNITFVKPAYFIISAFNGIANIRYYFVFLCFIYIFSVVGNTLLMIGIILDHTLRGPKHIGVVNFAFTDLLS      80
SSALMPKLVDIFLFNHHHISYNDCLAFMFFCLTFFAAQAFNLVVLSFDRVMAIMYPLHYQMRISHKLILSLIAFFWLLAI     160
TIILTAVGLLTRLSFCDSVIIQSYFCDHGPVYRLGCNDLTPNRVIAHLAPVLVLWVPLAFIVGSYCCIGYSLSKTATCRE     240
RLKALKTCTSHLSLVAIYFLPALFIFTFGSTILPNARTVSLSLATVMPLTLNPIIYGLQTQEIKESLKKLLKVKMQFKIS     320
ANKX
..........N.....................................................................      80
................................................................................     160
................................................................................     240
................................................................................     320
....                                                                                 400

(Threshold=0.5)
----------------------------------------------------------------------
SeqName      Position  Potential   Jury    N-Glyc
                                 agreement result
----------------------------------------------------------------------
contig010726-ZebOR.N113   11 NITF   0.5220     (6/9)   +
----------------------------------------------------------------------
```

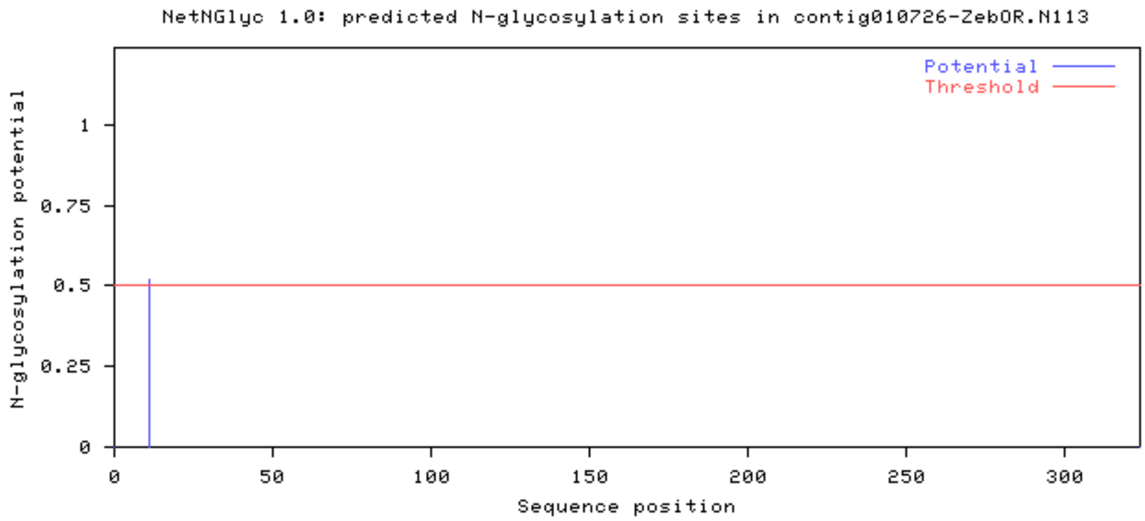

Graphics in PostScript

Output for 'contig010727-ZebOR.N114'

```
##########################################################################################

    Warning: This sequence may not contain a signal peptide!!

    Proteins without signal peptides are unlikely to be exposed to
    the N-glycosylation machinery and thus may not be glycosylated
    (in vivo) even though they contain potential motifs.

    SignalP-NN euk predictions are as follows:

  # name                    Cmax  pos ?  Ymax  pos ?  Smax  pos ?  Smean ?  D     ?

    SignalP output is explained at http://www.cbs.dtu.dk/services/SignalP/output.html

##########################################################################################

Name:  contig010727-ZebOR.N114  Length:  327
MDSEEKATTKSNSTFVRPAIFYLSGFSNIPHVKYFYVFLCFVYIMTVLGNGFLLSLIWLVKTLHTPKYMIVFNMALTDLC      80
GSTALIPKLLDTFLFDRRYIVYEACLSYMFFVFFFGGVQSWTLVTMAYDRLIAICFPLRYHSIVTKTSITSMLLFIWLVM     160
LSLTTLVVGLINRLSFCDSVVVKSFFCDHGPIYRLACNDPSLNIIMANVVVSIGVFIPLIFISCTYVCISIALSKIASGE     240
ERLKALKTCTSHLILVAILFLPFVGTNIAVWTSYIHPNARIINSTLTHTLPALINPIVYALKTEEVMNAVKKLWKINNIR     320
SPVTKWX
...........N....................................................................      80
................................................................................     160
................................................................................     240
..........................................N.....................................     320
.......                                                                              400

(Threshold=0.5)
----------------------------------------------------------------------
SeqName      Position  Potential   Jury    N-Glyc
                                 agreement result
----------------------------------------------------------------------
contig010727-ZebOR.N114    12 NSTF   0.6203     (9/9)    ++
contig010727-ZebOR.N114   283 NSTL   0.5880     (8/9)    +
----------------------------------------------------------------------
```

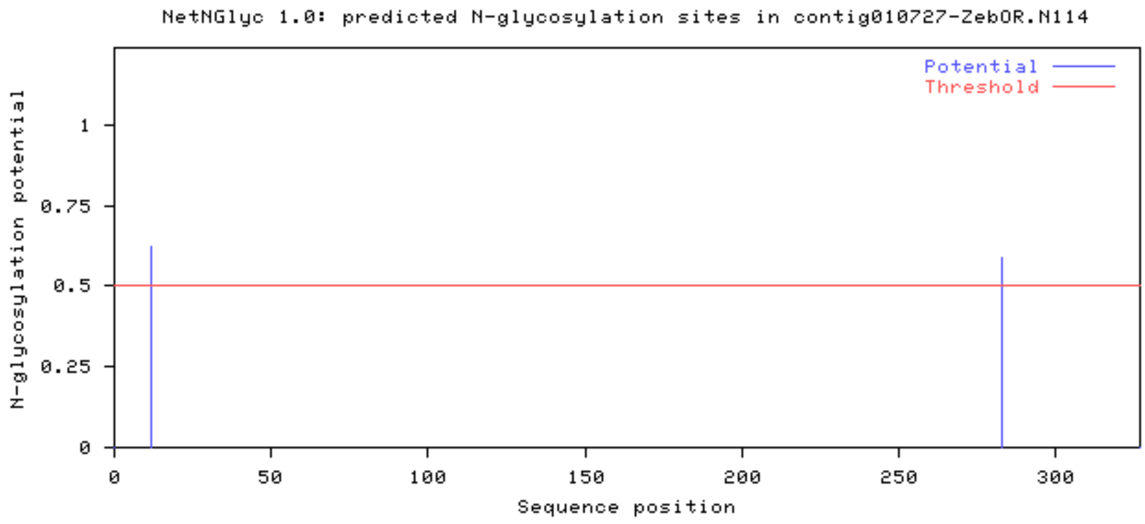

Graphics in PostScript

Output for 'contig010733-ZebORe.N117'

```
##########################################################################################

    Warning: This sequence may not contain a signal peptide!!

    Proteins without signal peptides are unlikely to be exposed to
    the N-glycosylation machinery and thus may not be glycosylated
    (in vivo) even though they contain potential motifs.

    SignalP-NN euk predictions are as follows:

  # name                    Cmax  pos ?  Ymax  pos ?  Smax  pos ?  Smean ?  D     ?

    SignalP output is explained at http://www.cbs.dtu.dk/services/SignalP/output.html

##########################################################################################

Name:  contig010733-ZebORe.N117         Length:  245
FLFDRRYILYDACLSYMFFVIFFASVQSWTLVTMAYDRLIAICFPLRYHSIVTEQSITAILLFVWIFLVSVIATMVGLVN      80
RLSFCRSLVVNSFFCDHGPVYRLACNDTSLNYNMASALVAILIIIPLIFIIATYVCIFIALSRTTSREERLRALKTCTSH     160
LILVVIFFLPIGITNIAAMTSYIDPNARMINSTLTHTIPALLDPIVYALKTEEVMNAVKKLCKRTYLNRMKAKTRPCNHC     240
CIKSX
................................................................................      80
.........................N......................................................     160
..............................N.................................................     240
.....                                                                                320

(Threshold=0.5)
----------------------------------------------------------------------
SeqName      Position  Potential   Jury    N-Glyc
                                 agreement result
----------------------------------------------------------------------
contig010733-ZebORe.N117   106 NDTS   0.6668     (9/9)   ++
contig010733-ZebORe.N117   191 NSTL   0.5999     (7/9)   +
----------------------------------------------------------------------
```

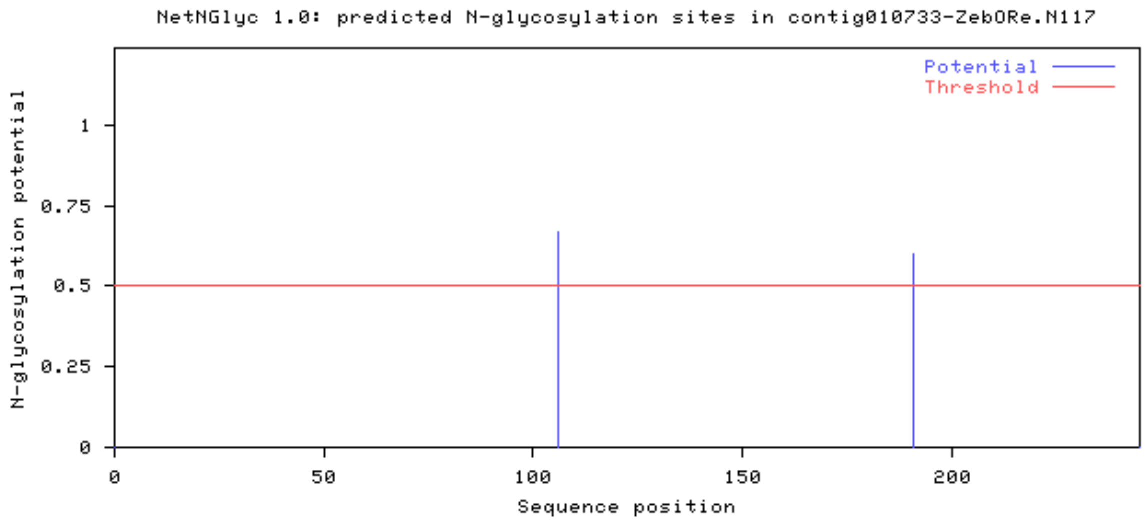

Graphics in PostScript

Output for 'contig010734-ZebOR.M107'

```
##########################################################################################

    Warning: This sequence may not contain a signal peptide!!

    Proteins without signal peptides are unlikely to be exposed to
    the N-glycosylation machinery and thus may not be glycosylated
    (in vivo) even though they contain potential motifs.

    SignalP-NN euk predictions are as follows:

  # name                  Cmax  pos ?  Ymax  pos ?  Smax  pos ?  Smean ?  D     ?

    SignalP output is explained at http://www.cbs.dtu.dk/services/SignalP/output.html

##########################################################################################

Name:  contig010734-ZebOR.M107  Length:  327
MIFQRAVLNSSAIIHPPGFYIIGFETFPFISVYFIFLVFVYGVTVLFNILVIYVIASTRRLHTPKFLAVVNLAVIDLFLN      80
TCTIPSMIKIFLIKNNFIPFNLCLLQMYVYYVFVSLESFALAILAYDRLIAICFPLRQNSINTVRSMCCIVSVSWFLVMG     160
VIGFATGIMTRLSFCRSVRVFSYFCDYAPVFRLACNDNTMQWFAASFFSVLLLFFPFTFIFLSYVSILITVFRMKSVDSR     240
VKALATCAEHLILVAIFYIPLIVIFTVGFFLGVVHPDQRVLSLSLASCIPPCINPVVYSLKTKDIKTRALTLFRTKVKTK     320
LTKVNAX
........N.......................................................................      80
................................................................................     160
................................................................................     240
................................................................................     320
.......                                                                              400

(Threshold=0.5)
----------------------------------------------------------------------
SeqName      Position  Potential   Jury    N-Glyc
                                 agreement result
----------------------------------------------------------------------
contig010734-ZebOR.M107    9 NSSA   0.5947     (7/9)    +
----------------------------------------------------------------------
```

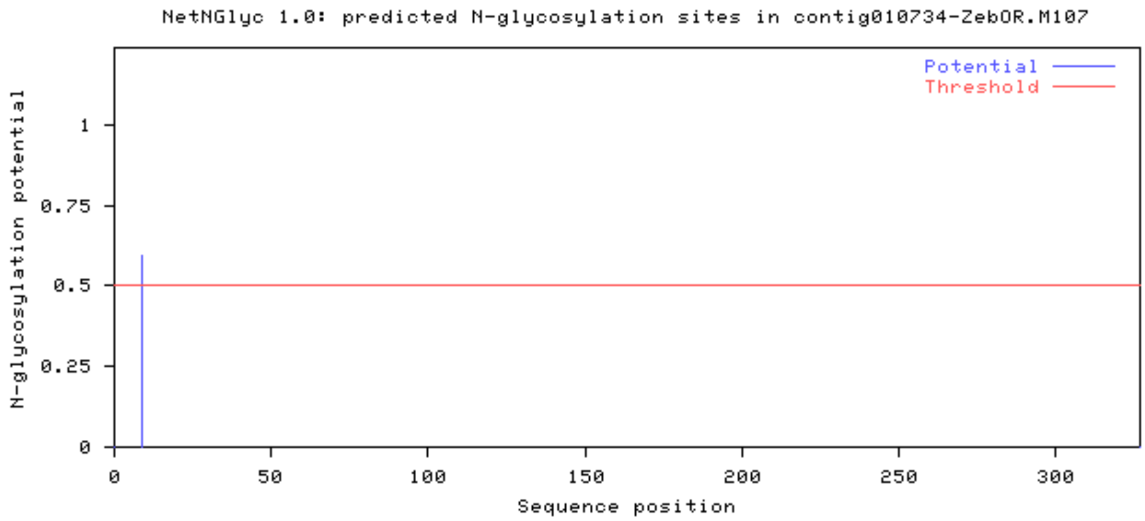

Graphics in PostScript

Output for 'contig014047-ZebOR.D036'

```
##########################################################################################

    Warning: This sequence may not contain a signal peptide!!

    Proteins without signal peptides are unlikely to be exposed to
    the N-glycosylation machinery and thus may not be glycosylated
    (in vivo) even though they contain potential motifs.

    SignalP-NN euk predictions are as follows:

  # name                    Cmax   pos ?  Ymax   pos ?  Smax   pos ?  Smean ?  D      ?

    SignalP output is explained at http://www.cbs.dtu.dk/services/SignalP/output.html

##########################################################################################

Name:  contig014047-ZebOR.D036  Length:  308
MENSSEIVSFVLAAYGNVGELKYLYFVIILFWYLSICVANTVLIVVIRVDIQLHEPMYILLCNLCVNQINVSTSLYPLLL      80
SQMFSDSHEVTLPWCFLHMSSMYISGPAEFCSLAAMAYDRYISICHPLRYNVIMNTERVFFMILFVWIYSFLSVILSFSF     160
IFSLKFCGTIIENVYCNHRLILRLSCFVSVHNFLSDIFFLFVSFFIPFTLISVSYVKILAVCRKTSTENKQKAVTTCVPQ     240
IVSVSNLFVGSIFQCIDSSVIVARLPVRIILSIYLFICQPMLTPFLYGFNLPKIRQSCKKLLFKKKQX
..N.............................................................................      80
................................................................................     160
................................................................................     240
....................................................................                 320

(Threshold=0.5)
----------------------------------------------------------------------
SeqName      Position  Potential   Jury    N-Glyc
                                agreement result
----------------------------------------------------------------------
contig014047-ZebOR.D036    3 NSSE   0.6997     (9/9)    ++
contig014047-ZebOR.D036   70 NVST   0.4661     (6/9)    -
----------------------------------------------------------------------
```

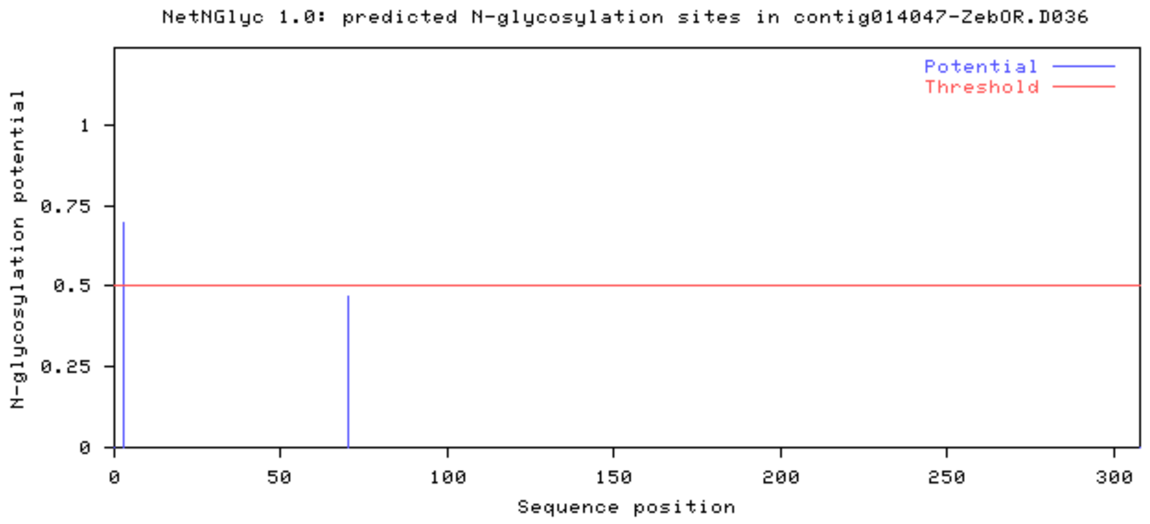

[Graphics in PostScript]

Output for 'contig014048-ZebORp.D043'

```
########################################################################################

    Warning: This sequence may not contain a signal peptide!!

    Proteins without signal peptides are unlikely to be exposed to
    the N-glycosylation machinery and thus may not be glycosylated
    (in vivo) even though they contain potential motifs.

    SignalP-NN euk predictions are as follows:

  # name                 Cmax  pos ?  Ymax  pos ?  Smax  pos ?  Smean ?  D     ?

    SignalP output is explained at http://www.cbs.dtu.dk/services/SignalP/output.html

########################################################################################

Name:  contig014048-ZebORp.D043         Length:  279
MTVLSICVANTVLILVIHVDRQLHEPMYILLCNLCVNEINISTSLYPLLLSQMFSDRHEVTVPWCFLQMCFMYTSAPAEF      80
CSLAAMSYDRYISICHPLRYNVIMNTERVFFMILLVWIYSFLSVILSFSFVFSLKFCGNNIENVYCDHQLLIRLSCSLSF     160
HSFISDIFFVFVSIFIPFNLISVSYVKILAICRKTSRENKQKAVTTCTPQLVSVSNLFVGCIFQSIDSSVIVAQLPHEVN     240
IILSIYLFICQPMLTPFLYGFNLPKIRQSCKRFVFKKKX
................................................................................      80
................................................................................     160
................................................................................     240
.......................................                                              320

(Threshold=0.5)

No sites predicted in this sequence.
```

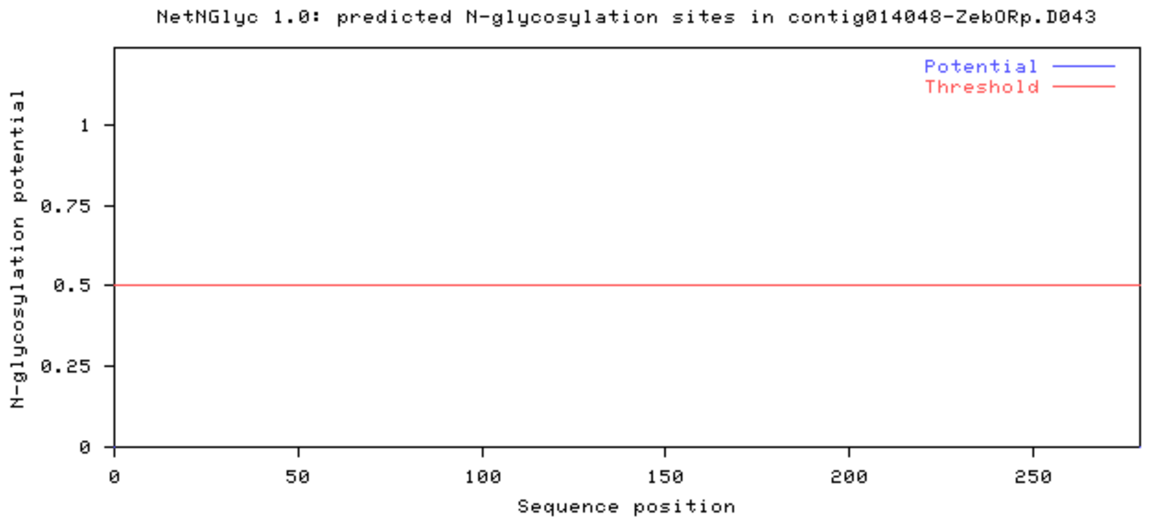

Graphics in PostScript

Output for 'contig014049-ZebOR.D038'

```
##########################################################################################

    Warning: This sequence may not contain a signal peptide!!

    Proteins without signal peptides are unlikely to be exposed to
    the N-glycosylation machinery and thus may not be glycosylated
    (in vivo) even though they contain potential motifs.

    SignalP-NN euk predictions are as follows:

  # name                  Cmax  pos ?  Ymax  pos ?  Smax  pos ?  Smean ?  D     ?

    SignalP output is explained at http://www.cbs.dtu.dk/services/SignalP/output.html

##########################################################################################

Name:  contig014049-ZebOR.D038  Length:  309
MENSSEIVSFVLSAFENVGELKYLYFVIILVWYVSICVANTVLIVVIRVDRRLHEPMYILLCNLCVNEINASTSLYPLLL      80
SQMFSDSHEVTLPWCFLQMCCLYTSASVELCSLAAMAYDRYISICHPLRYNVIMKAERVFLLILLVWVYSFLSFIFSFSF     160
IFSLKFCENIIHNVYCDHQLIIRLSCSVSIQSFISDISFATVSVFIPFSLILVSYMKILRVCRKTSKENKQKAVTTCTPQ     240
IISVSNLFVGYIFYFIDFRSVVSQVLDEVHIILPMYVLIFQPMLTPFMYGFNLPKIRQSYQRFLLKRKX
..N.............................................................................      80
................................................................................     160
................................................................................     240
.....................................................................                320

(Threshold=0.5)
----------------------------------------------------------------------
SeqName      Position  Potential   Jury    N-Glyc
                                 agreement result
----------------------------------------------------------------------
contig014049-ZebOR.D038    3 NSSE    0.6951     (9/9)   ++
contig014049-ZebOR.D038   70 NAST    0.3700     (6/9)   -
----------------------------------------------------------------------
```

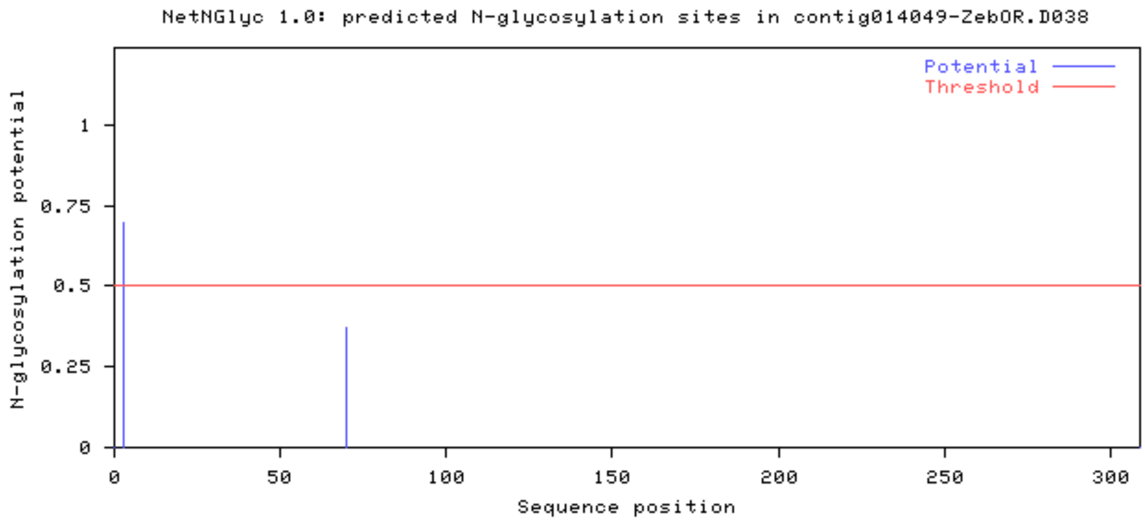

Graphics in PostScript

Output for 'contig014049-ZebOR.D037'

```
##########################################################################################

    Warning: This sequence may not contain a signal peptide!!

    Proteins without signal peptides are unlikely to be exposed to
    the N-glycosylation machinery and thus may not be glycosylated
    (in vivo) even though they contain potential motifs.

    SignalP-NN euk predictions are as follows:

  # name                    Cmax  pos ?  Ymax  pos ?  Smax  pos ?  Smean ?  D     ?

    SignalP output is explained at http://www.cbs.dtu.dk/services/SignalP/output.html

##########################################################################################

Name:  contig014049-ZebOR.D037   Length:  309
MGNSSEIVSFVLSAYGNIGELKYLYFIIILVWYLSICVANTVLIVVIRVNRRLHEPMYILLSNLCVNEINASTSLYPLLL      80
SQMFSDSHEVTVPWCFLQMCFMYISAPAEFWGLAAMAYDRYISICHPLCYNVIMNTKRVFLLILLVWIFSLVSFILSFSF     160
IFGLKFCRNIVENVYCDHQLMIRLSCSVSTQSYISEIFFAIVSIFIPFTLISVSYFKILAVCRKTSIENKQKAVTTCTPQ     240
IVSVSNLFVGGIFHSIDFRFLIARVPDEVRIILPMYVLICQPMLTPFLYGFNLPKIRHSFKRLLFKRKX
..N.............................................................................      80
................................................................................     160
................................................................................     240
.....................................................................               320

(Threshold=0.5)
----------------------------------------------------------------------
SeqName      Position  Potential   Jury    N-Glyc
                                 agreement result
----------------------------------------------------------------------
contig014049-ZebOR.D037    3 NSSE    0.7241     (9/9)    ++
contig014049-ZebOR.D037   70 NAST    0.3817     (6/9)    -
----------------------------------------------------------------------
```

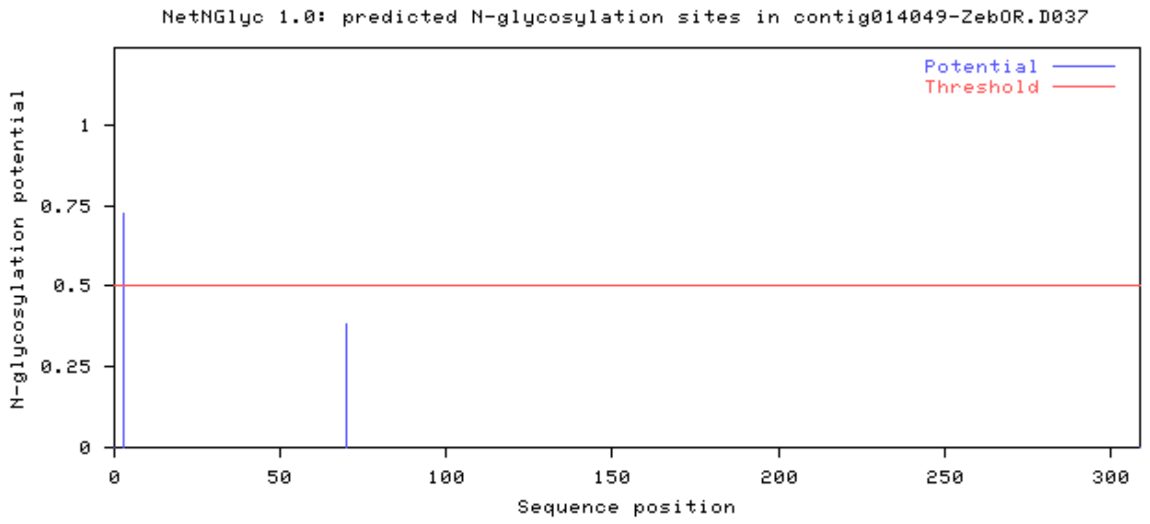

Graphics in PostScript

Output for 'contig014050-ZebOR.D039'

```
##########################################################################################

    Warning: This sequence may not contain a signal peptide!!

    Proteins without signal peptides are unlikely to be exposed to
    the N-glycosylation machinery and thus may not be glycosylated
    (in vivo) even though they contain potential motifs.

    SignalP-NN euk predictions are as follows:

  # name                    Cmax   pos ?  Ymax   pos ?  Smax   pos ?  Smean ?  D     ?

    SignalP output is explained at http://www.cbs.dtu.dk/services/SignalP/output.html

##########################################################################################

Name:  contig014050-ZebOR.D039  Length:  309
MENSSEIVSFVLAAYGNIGGLKYLYFIIILVWYLSICVANTVLIVVIRVDRRLHEPMYILLCNLCVNEINASTSLYPLLL      80
SQMFSDSHEVTVPWCFLQMCCLYTSAPAEFCSLAAMAYDRYISICHPLRYNVIMNTERVFLLILLVWLYSFLSFIFSFSF     160
IFSLKFCGNIIHNAYCDHQLIIRLSCAVPIQSFISNISFLLLSVFIPFSLISVTYMKILRVCRKTSKENKQKAVTTCTPQ     240
IISVSNLFVGCIFYFIDFKFLVSQVPDEVRIILPMYFLIFQPMLTPFMYGFKLPKIRQSYQRFLFERKX
..N.............................................................................      80
................................................................................     160
................................................................................     240
.....................................................................                320

(Threshold=0.5)
----------------------------------------------------------------------
SeqName      Position  Potential   Jury    N-Glyc
                                 agreement result
----------------------------------------------------------------------
contig014050-ZebOR.D039    3 NSSE   0.6996     (9/9)   ++
contig014050-ZebOR.D039   70 NAST   0.3702     (6/9)   -
contig014050-ZebOR.D039  196 NISF   0.4480     (6/9)   -
----------------------------------------------------------------------
```

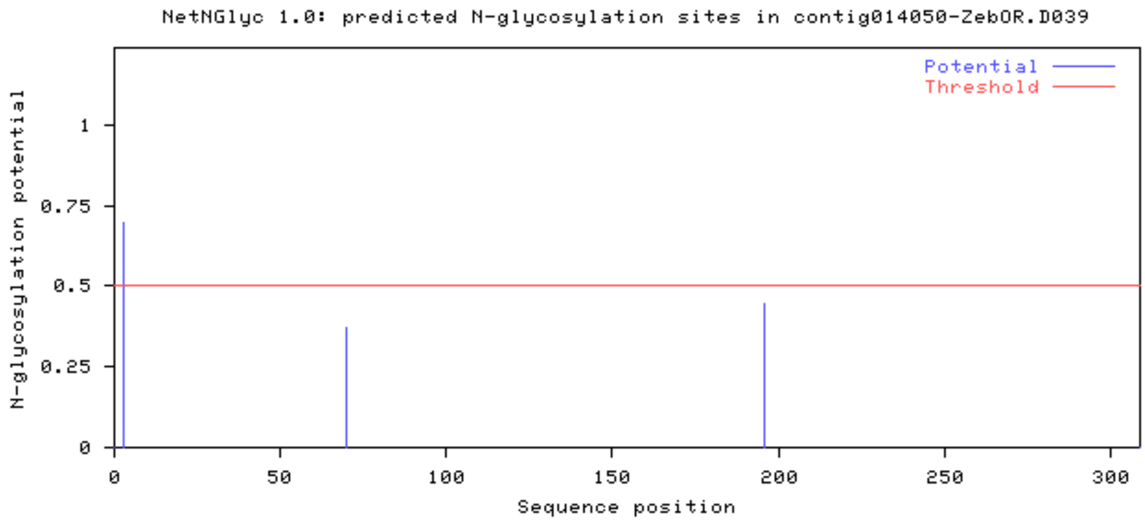

[Graphics in PostScript]

Output for 'contig014051-ZebORp.D044'

```
########################################################################################

    Warning: This sequence may not contain a signal peptide!!

    Proteins without signal peptides are unlikely to be exposed to
    the N-glycosylation machinery and thus may not be glycosylated
    (in vivo) even though they contain potential motifs.

    SignalP-NN euk predictions are as follows:

  # name                Cmax  pos ?  Ymax  pos ?  Smax  pos ?  Smean ?  D     ?

    SignalP output is explained at http://www.cbs.dtu.dk/services/SignalP/output.html

########################################################################################

Name:  contig014051-ZebORp.D044         Length:  294
NVAALKYMYFVIILFWYLLICVANTVLIVVIHVDRRLHEPMYILLSNLCENEINGSTSLYPFLLSQMFLDSHEVTLPWCF      80
LQMCCLYTGAPAEFNSCVVYTQVHLLSLTAMANDRYVSICHPLHYNVIMKTEIILLVWVFSFLIFILSFSFIFSLKFCGN     160
IVDNVYCDHKLIYKLSCSASVQGFISIIFFVIISYLSVSYVKILRVCRKTIQENKQKAVTTCTHQIVSVSNLFVGCICYS     240
IDFRFLVSLVPDEVRTILAIYLLICQPMLTPFMYGFNLPKIRQSCQSFLFLRKX
................................................................................      80
................................................................................     160
................................................................................     240
......................................................                               320

(Threshold=0.5)

No sites predicted in this sequence.
```

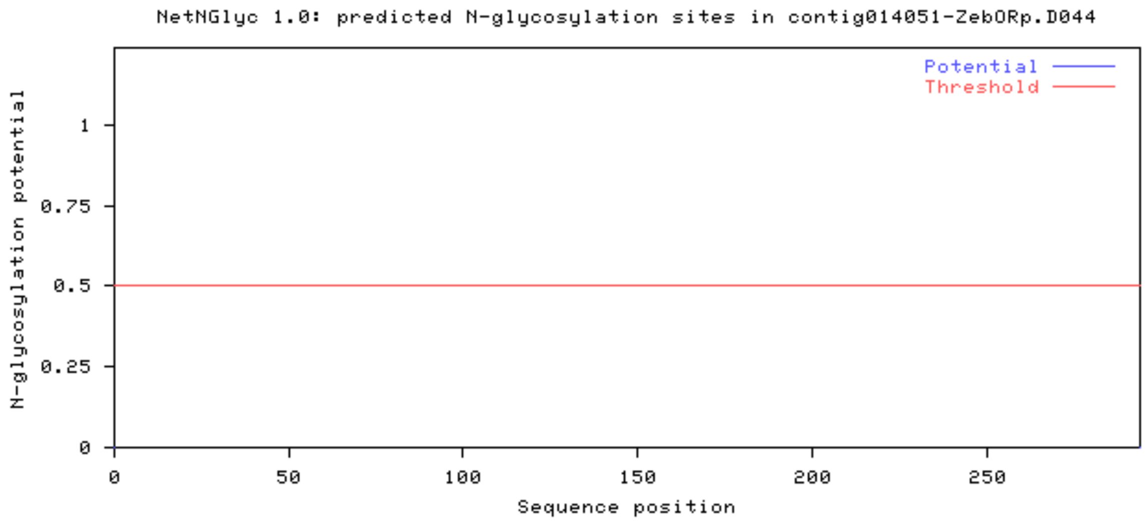

Graphics in PostScript

Output for 'contig014051-ZebOR.D040'

```
##########################################################################################

    Warning: This sequence may not contain a signal peptide!!

    Proteins without signal peptides are unlikely to be exposed to
    the N-glycosylation machinery and thus may not be glycosylated
    (in vivo) even though they contain potential motifs.

    SignalP-NN euk predictions are as follows:

  # name                 Cmax  pos ?  Ymax  pos ?  Smax  pos ?  Smean ?  D     ?

    SignalP output is explained at http://www.cbs.dtu.dk/services/SignalP/output.html

##########################################################################################

Name:  contig014051-ZebOR.D040  Length:  309
MGNSSKTVSFVLAAYGNVGELKHLYFIIILIWYFSICVANTVLIVVIRLDRRLHEPMYILLCNLCLSEINGSTSLYPLLL      80
SQMFSDSHEVTVPWCFLQMFCLYTSASVEICSLAAMAYDRYISICNPFTYNVIMNTDRVFLMILLVWVYSFLSVIFSYSF     160
IFSLKFCGNIIHSVYCDHQLIIRLSCAVPIQSFISDISFAIVSVFIPFSLISFSYLRILRVCQKTSKENKQKAVTTCTPQ     240
IISVSNLCVGCICYFIDFRFLVSRVPDEVRIILPMYVLIFQPMLTPFMYGFNLPKIRQSYQRFLFERKX
..N...................................................................N.........      80
................................................................................     160
................................................................................     240
.....................................................................                320

(Threshold=0.5)
----------------------------------------------------------------------
SeqName       Position  Potential   Jury     N-Glyc
                                  agreement result
----------------------------------------------------------------------
contig014051-ZebOR.D040    3 NSSK   0.7661     (9/9)    +++
contig014051-ZebOR.D040   70 NGST   0.5776     (5/9)    +
----------------------------------------------------------------------
```

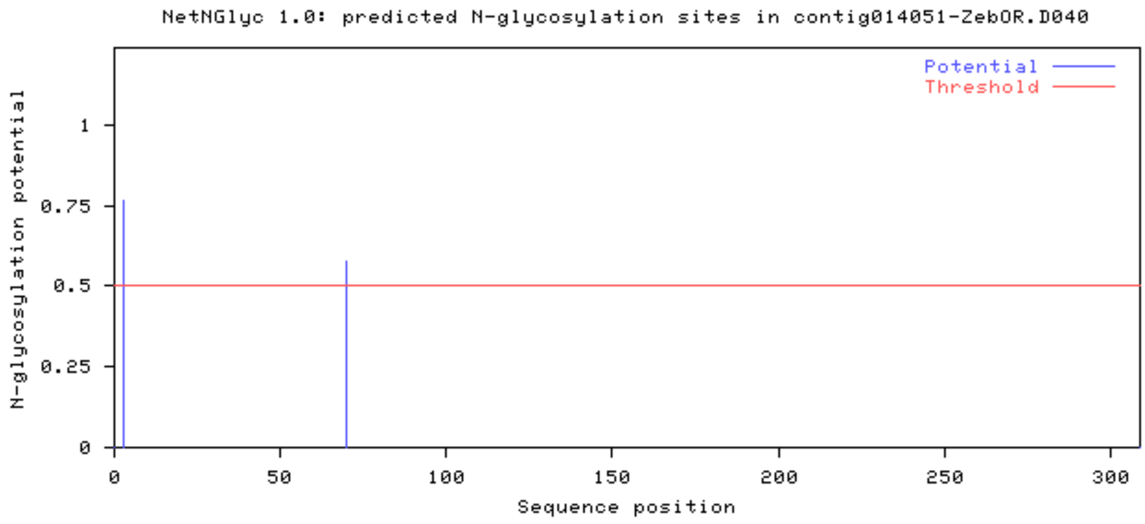

Graphics in PostScript

Output for 'contig014054-ZebOR.D041'

```
##########################################################################################

    Warning: This sequence may not contain a signal peptide!!

    Proteins without signal peptides are unlikely to be exposed to
    the N-glycosylation machinery and thus may not be glycosylated
    (in vivo) even though they contain potential motifs.

    SignalP-NN euk predictions are as follows:

  # name                 Cmax  pos ?  Ymax  pos ?  Smax  pos ?  Smean ?  D     ?

    SignalP output is explained at http://www.cbs.dtu.dk/services/SignalP/output.html

##########################################################################################

Name:  contig014054-ZebOR.D041  Length:  312
MQKIGNSSESVSFVLAAYGNVGAFKYPYFIIILFWYVSICVANTVLIVVIHVDRRLHEPMYILLSNLCVNEINASTALYP      80
LLLSQMFSDSHEVTLPWCFLQMCCMYISGSAEFCSLTAMAYDRYIAICHPFSYNVIMNTERVGMLILLVWIFSFVSFLLS     160
FSFIFGLRFCGNIIDNVYCDHQLIIRLSCSVPIQSSISMIFFTLLSIFMPFNLISISYLKILRVCRKTSTENKQKAVTTC     240
TPQIVSVSNLFVGCIFHSIDFSLLAPQIPDEVRIILPMYLLICQPMLTPFMYGFNLPKIRQACKMLLFKRKX
.....N..........................................................................      80
................................................................................     160
................................................................................     240
........................................................................             320

(Threshold=0.5)
----------------------------------------------------------------------
SeqName      Position  Potential   Jury    N-Glyc
                                 agreement result
----------------------------------------------------------------------
contig014054-ZebOR.D041    6 NSSE   0.7448     (9/9)   ++
contig014054-ZebOR.D041   73 NAST   0.3760     (6/9)   -
----------------------------------------------------------------------
```

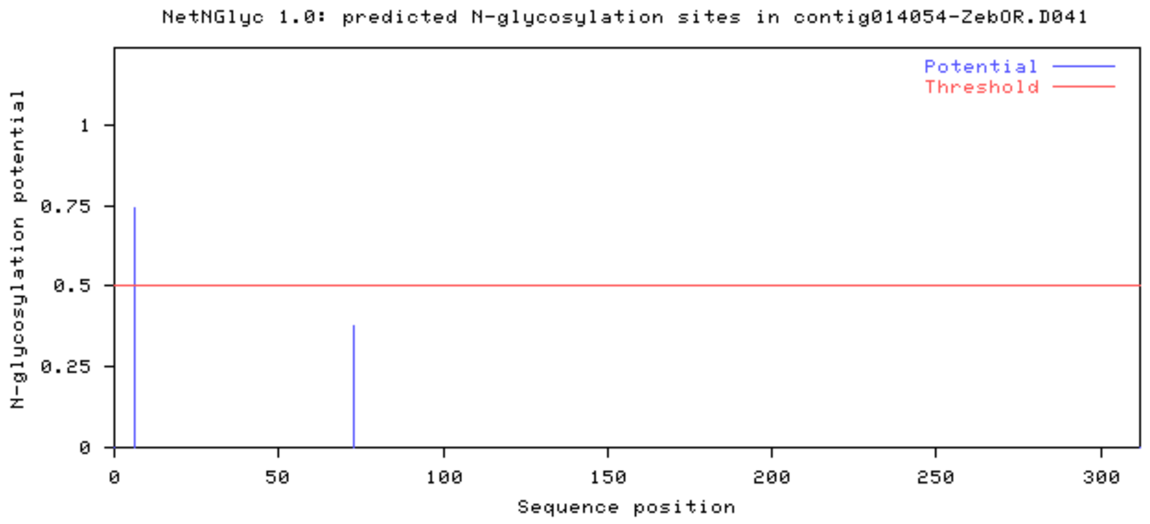

Graphics in PostScript

Output for 'contig014054-ZebOR.D042'

```
##########################################################################################

    Warning: This sequence may not contain a signal peptide!!

    Proteins without signal peptides are unlikely to be exposed to
    the N-glycosylation machinery and thus may not be glycosylated
    (in vivo) even though they contain potential motifs.

    SignalP-NN euk predictions are as follows:

  # name                    Cmax   pos ?  Ymax   pos ?  Smax   pos ?  Smean ?  D     ?

    SignalP output is explained at http://www.cbs.dtu.dk/services/SignalP/output.html

##########################################################################################

Name:  contig014054-ZebOR.D042  Length:  319
MGNSSETVSFVLAAYGNVGALKYMYFSIILFWYVSICVANTVLIVVIHVDRRLHEPMYILLSNLCVNEINGSTSMYPLLL      80
SQMFSDSHEVTLPWCFLQMCCMYTTASAEFCSLAAMAYDRYISICHPLRYNVIMNTERVFILILLVWVYSFLIFIFSFSF     160
IFSLTFCGNVVNNVYCDHKLIIQLSCSVSVHSFISDIFFLLLSIFIPFSLISVSYMKILAVCRKTSTENKQKAVTTCTPQ     240
IVSVSNLFVGCIFHAIDSSVLAAHVPGEVRIILSIYLLICQPMLTPFMYGFNLPKIRQSCTMLVFKRKSMSLFKKRLFX
..N.............................................................................      80
................................................................................     160
................................................................................     240
...............................................................................      320

(Threshold=0.5)
----------------------------------------------------------------------
SeqName       Position  Potential   Jury    N-Glyc
                                  agreement result
----------------------------------------------------------------------
contig014054-ZebOR.D042    3 NSSE   0.7402     (9/9)   ++
contig014054-ZebOR.D042   70 NGST   0.4570     (6/9)   -
----------------------------------------------------------------------
```

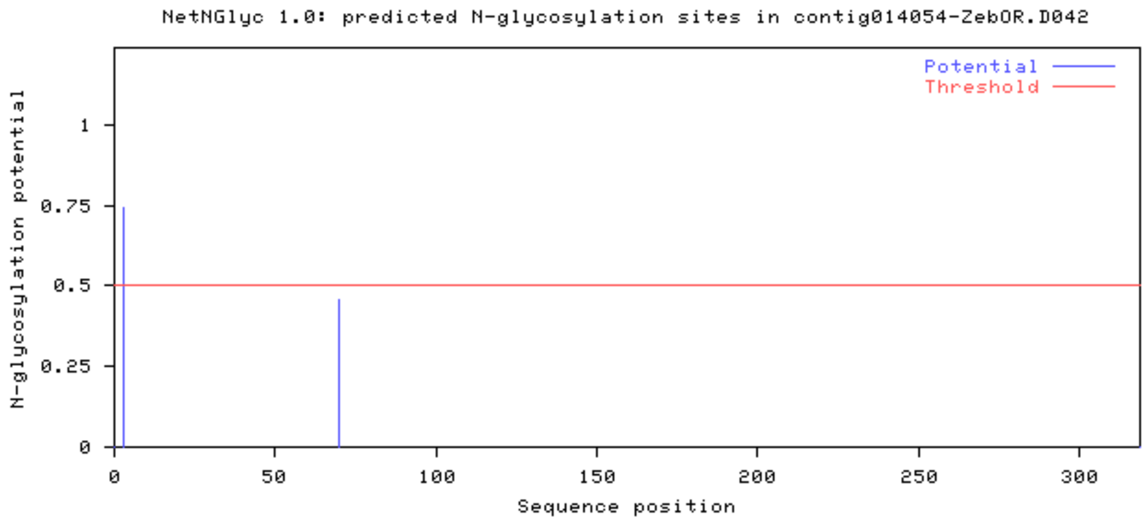

Graphics in PostScript

Output for 'contig014055-ZebOR.H066'

##

Warning: This sequence may not contain a signal peptide!!

Proteins without signal peptides are unlikely to be exposed to
the N-glycosylation machinery and thus may not be glycosylated
(in vivo) even though they contain potential motifs.

SignalP-NN euk predictions are as follows:

\# name Cmax pos ? Ymax pos ? Smax pos ? Smean ? D ?

SignalP output is explained at http://www.cbs.dtu.dk/services/SignalP/output.html

##

Name: contig014055-ZebOR.H066 Length: 314

```
MNTSSIVVFSLTGFSATVNYRVTLFSLTLLCYFLILMVNIALILTIISDQNLHEPMYIFLCSLCINGLYGTAGFFPRFAF      80
DLLSDTHLISYVGCLLQVFVIYSNAKVDYSTLVLMAYDRYLAICRPLEYHSVMSVRRTVVLVTLSWLVPLCFETLVISLT     160
STLKLCGSNINKLYCENWSIVKLACGSTKVNDIVGLIFITFYCCHVVCIACSYVQLVNVALKSRAGRKKFTQTCAPHLFC     240
LLNVTTALLFDLMFSRYGSASLPQHLKNFMAIEFLIIPPILNPVCYGWVLTKIRRRMIFLCRLAYQRFGVKSQX
.N..............................................................................      80
................................................................................     160
................................................................................     240
..N.......................................................................           320
```

(Threshold=0.5)

SeqName	Position	Potential	Jury agreement	N-Glyc result
contig014055-ZebOR.H066	2 NTSS	0.7489	(9/9)	++
contig014055-ZebOR.H066	177 NWSI	0.4820	(5/9)	-
contig014055-ZebOR.H066	243 NVTT	0.5970	(9/9)	++

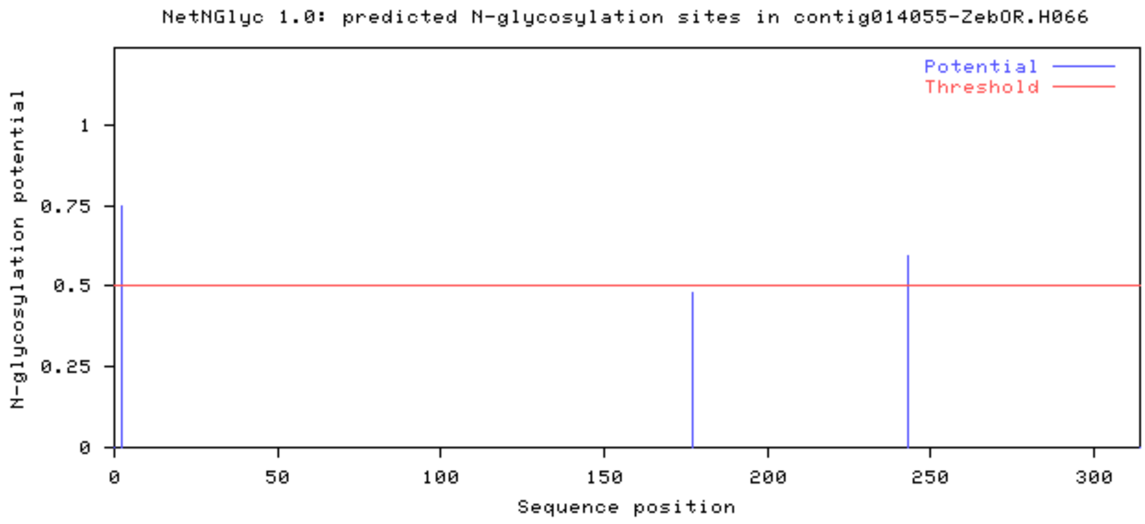

Graphics in PostScript

Output for 'contig014056-ZebOR.F063'

```
##########################################################################################

    Warning: This sequence may not contain a signal peptide!!

    Proteins without signal peptides are unlikely to be exposed to
    the N-glycosylation machinery and thus may not be glycosylated
    (in vivo) even though they contain potential motifs.

    SignalP-NN euk predictions are as follows:

  # name                    Cmax  pos ?  Ymax  pos ?  Smax  pos ?  Smean ?  D     ?

    SignalP output is explained at http://www.cbs.dtu.dk/services/SignalP/output.html

##########################################################################################

Name:  contig014056-ZebOR.F063  Length:  305
MENSTLSFYFRFTMFANIGHYRFIAFIFCLLLFIFTIFTNLLMIVVISQQTTLHEPMYIFIACLSVNALYGSSGFFPRFL      80
MDLLSDTHLISRPACFTQIYVIYSYASCELTVLSIMAYDRYIAVCLPLHYHTKMTLETVVKLTELAWIFPAFSLAACLCL     160
SATLPLCGNEIHKVFCANWNVVKLSCVNTAVNNVVGMLLTVATIFLPLFYILYTYLRIVSICWRSSAEFKGKVLESCLPH     240
TISFVIYSIAGFCDVALSRNNLEVTNPFIAVILSLVFVVIPPALNPLVYGLKLPEIRKHILRLFX
..N.............................................................................      80
................................................................................     160
................................................................................     240
.................................................................                    320

(Threshold=0.5)
----------------------------------------------------------------------
SeqName      Position  Potential   Jury    N-Glyc
                                 agreement result
----------------------------------------------------------------------
contig014056-ZebOR.F063    3 NSTL   0.7371     (9/9)    ++
----------------------------------------------------------------------
```

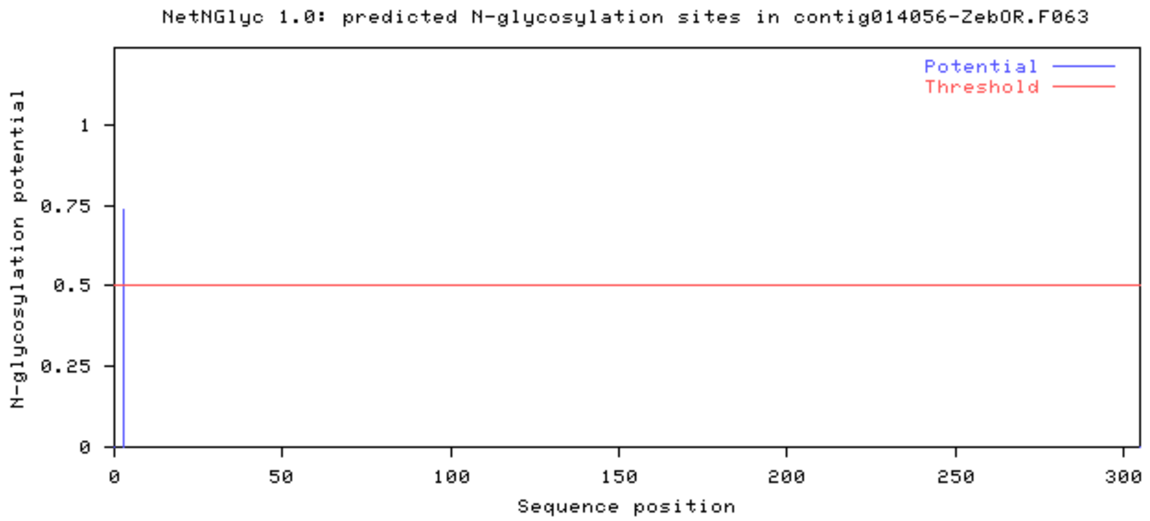

Graphics in PostScript

Output for 'contig014057-ZebOR.H067'

```
##########################################################################################

    Warning: This sequence may not contain a signal peptide!!

    Proteins without signal peptides are unlikely to be exposed to
    the N-glycosylation machinery and thus may not be glycosylated
    (in vivo) even though they contain potential motifs.

    SignalP-NN euk predictions are as follows:

  # name                 Cmax  pos ?  Ymax  pos ?  Smax  pos ?  Smean ?  D     ?

    SignalP output is explained at http://www.cbs.dtu.dk/services/SignalP/output.html

##########################################################################################

Name:  contig014057-ZebOR.H067  Length:  314
MDNVSVVTVFTLSGLSGIANYKITIFIFTLLCYCVIWLVNLTIIVTVIVDKSLHEPMYIFLCNLCFNGLYGTAAFYPKFL      80
YDLLSTTHVISYAGCLLQGLMVHSSICTDFSLLALMAYDRYVAICRPLVYHSLMTTQRVCIFVFFAWITPFYLILMSTIT     160
TATSRLCGSHIPRIYCINWLISNLACSASVATIIIPAFNYTFYFGHAVFVFWSYVHLIKTCQSSKENWNKFMQTCVPHLF     240
SLAVVVVSFLFDMLYMRFGSKEIPQSFENFMAMEILFIPPIINPLMYGFKLTQIRNRVLNFICGKSSAFILKSX
..N....................................N........................................      80
................................................................................     160
......................................N.........................................     240
..........................................................................           320

(Threshold=0.5)
----------------------------------------------------------------------
SeqName      Position  Potential   Jury    N-Glyc
                                 agreement result
----------------------------------------------------------------------
contig014057-ZebOR.H067    3 NVSV    0.8098     (9/9)    +++
contig014057-ZebOR.H067   40 NLTI    0.7723     (9/9)    +++
contig014057-ZebOR.H067  199 NYTF    0.5938     (8/9)    +
----------------------------------------------------------------------
```

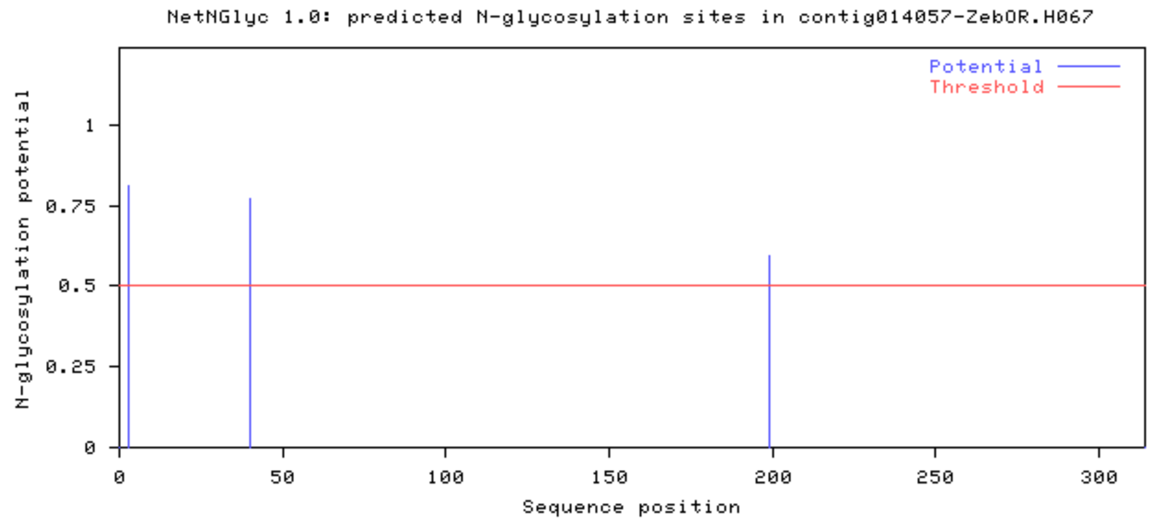

Graphics in PostScript

Output for 'contig014059-ZebOR.H068'

```
##########################################################################################

    Warning: This sequence may not contain a signal peptide!!

    Proteins without signal peptides are unlikely to be exposed to
    the N-glycosylation machinery and thus may not be glycosylated
    (in vivo) even though they contain potential motifs.

    SignalP-NN euk predictions are as follows:

  # name                  Cmax  pos ?  Ymax  pos ?  Smax  pos ?  Smean ?  D     ?

    SignalP output is explained at http://www.cbs.dtu.dk/services/SignalP/output.html

##########################################################################################

Name:  contig014059-ZebOR.H068  Length:  314
MDNVSIITVFTLSGLSDIANYRVILFVLTLLCYCVIWLVNVTIIVTVIVDKKLHEPMYIFLCSLCFNGLYGTAAFYPKFL      80
YDLLSTTHVISYAGCFLQGFVLHSSVAADFSLLALMAYDRYVAICRPLVYHSIMTKQRVCILIFFAWIIAFYFLLMSTIT     160
TAILRLCGSHIPKIYCINWLIANLACSASVAKIVIPAFSYTFCFGNVCFVFWSYVHLIKTCQSSKENMGKFMQTCVPHLF     240
SLTVVVVSLLFDMLYMRFGSKQIAQSVQNFMAMEFLLIPPIMNPLMYGFKLTKIRKRVLNFICGKSSAFRFKSX
..N....................................N........................................      80
................................................................................     160
................................................................................     240
..........................................................................           320

(Threshold=0.5)
----------------------------------------------------------------------
SeqName      Position  Potential   Jury    N-Glyc
                                 agreement result
----------------------------------------------------------------------
contig014059-ZebOR.H068    3 NVSI   0.8029     (9/9)    +++
contig014059-ZebOR.H068   40 NVTI   0.7897     (9/9)    +++
----------------------------------------------------------------------
```

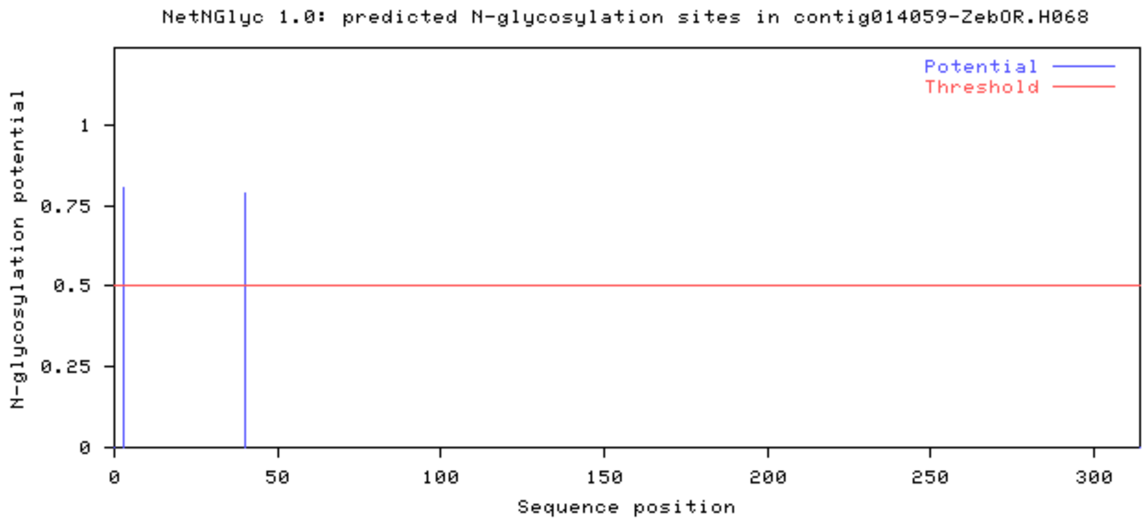

Graphics in PostScript

Output for 'contig014060-ZebOR.H069'

```
##########################################################################################

    Warning: This sequence may not contain a signal peptide!!

    Proteins without signal peptides are unlikely to be exposed to
    the N-glycosylation machinery and thus may not be glycosylated
    (in vivo) even though they contain potential motifs.

    SignalP-NN euk predictions are as follows:

  # name                 Cmax  pos ?  Ymax  pos ?  Smax  pos ?  Smean ?  D     ?

    SignalP output is explained at http://www.cbs.dtu.dk/services/SignalP/output.html

##########################################################################################

Name:  contig014060-ZebOR.H069  Length:  314
MDNVSVIAVFTLSGLRDIANYRVILFVLTLLCYCVIWLVNVTIIVTVIVDKKLHEPMYIFLCNLCVNGLYGTAAFYPKFL      80
YDLLSTTHVISYAGCLLQGFVLHSAVAADFSLLALMAYDRYVAICRPLVYHSLMTTQKISIFVFFAWLIPFYLLLMSTIT     160
TATSRLCGSHIPRIYCVNWLIANLACSASVARNVIPAFNYTFYVGHVVFVFWSYVYLIKTCQSSKENWNKFMQTCVPHLF     240
SLTVVVVSLLFDMLYMRFGSKEIAQNVENFMAMEFLLIPPIMNPLMYGLKLTKIRKRVLNFICGKSSTFRLKSX
..N....................................N........................................      80
................................................................................     160
......................................N.........................................     240
..........................................................................           320

(Threshold=0.5)
----------------------------------------------------------------------
SeqName      Position  Potential   Jury    N-Glyc
                                 agreement result
----------------------------------------------------------------------
contig014060-ZebOR.H069    3 NVSV   0.8180     (9/9)    +++
contig014060-ZebOR.H069   40 NVTI   0.7900     (9/9)    +++
contig014060-ZebOR.H069  199 NYTF   0.5933     (7/9)    +
----------------------------------------------------------------------
```

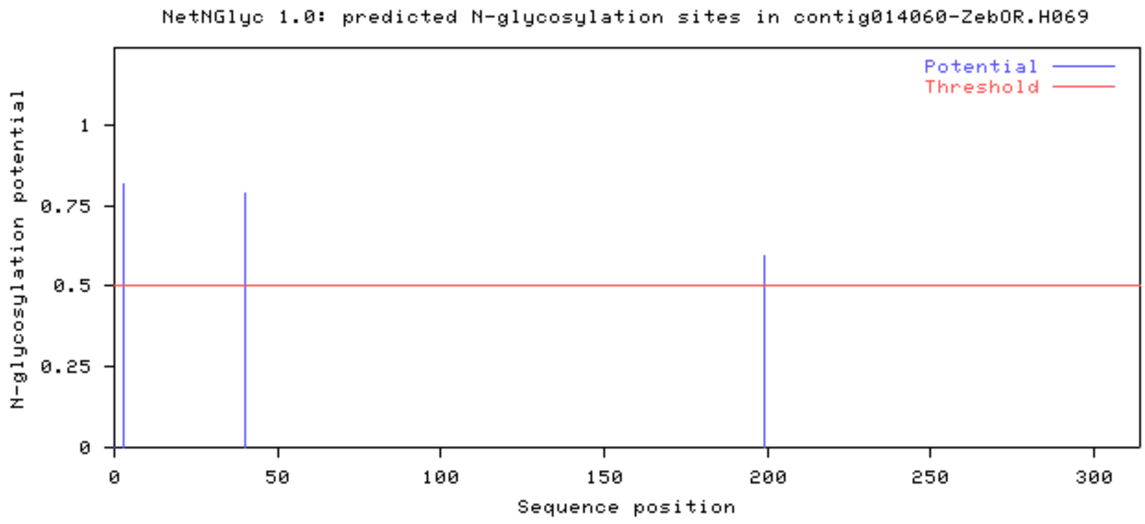

Graphics in PostScript

Output for 'contig014064-ZebORe.H078'

```
##########################################################################################

    Warning: This sequence may not contain a signal peptide!!

    Proteins without signal peptides are unlikely to be exposed to
    the N-glycosylation machinery and thus may not be glycosylated
    (in vivo) even though they contain potential motifs.

    SignalP-NN euk predictions are as follows:

  # name                    Cmax  pos ?  Ymax  pos ?  Smax  pos ?  Smean ?  D     ?

    SignalP output is explained at http://www.cbs.dtu.dk/services/SignalP/output.html

##########################################################################################

Name:  contig014064-ZebORe.H078         Length:  106
FIFWSYVHLIKTCQSSKENWNKFMQTCVPHLFSLTVVVLSFLFDMLYMRFGSKEIAQSFENFMAMEILLIPPIINPLMYG      80
FKLTKIRKRVLNFICGKSLALRLKSX
................................................................................      80
..........................                                                           160

(Threshold=0.5)

No sites predicted in this sequence.
```

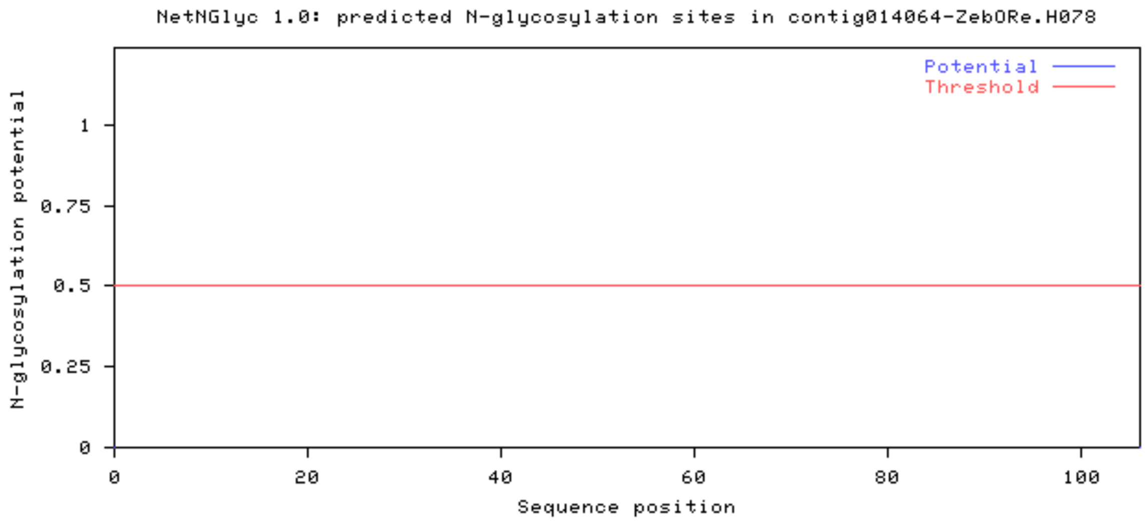

Graphics in PostScript

Output for 'contig014064-ZebORe.H079'

```
##########################################################################################

    Warning: This sequence may not contain a signal peptide!!

    Proteins without signal peptides are unlikely to be exposed to
    the N-glycosylation machinery and thus may not be glycosylated
    (in vivo) even though they contain potential motifs.

    SignalP-NN euk predictions are as follows:

  # name                    Cmax   pos ?  Ymax   pos ?  Smax   pos ?  Smean ?  D      ?

    SignalP output is explained at http://www.cbs.dtu.dk/services/SignalP/output.html

##########################################################################################

Name:  contig014064-ZebORe.H079          Length:  61
MDNVSNVKSFVLLGFNDTMNFRVPLFIITLLHYCVILFFNISLVLLIVLDESLHEPMYIFL
..N...........N.......................N....................                          80

(Threshold=0.5)
----------------------------------------------------------------------
SeqName      Position  Potential   Jury    N-Glyc
                                 agreement result
----------------------------------------------------------------------
contig014064-ZebORe.H079    3 NVSN   0.7740     (9/9)   +++
contig014064-ZebORe.H079   16 NDTM   0.6424     (8/9)   +
contig014064-ZebORe.H079   40 NISL   0.5900     (8/9)   +
----------------------------------------------------------------------
```

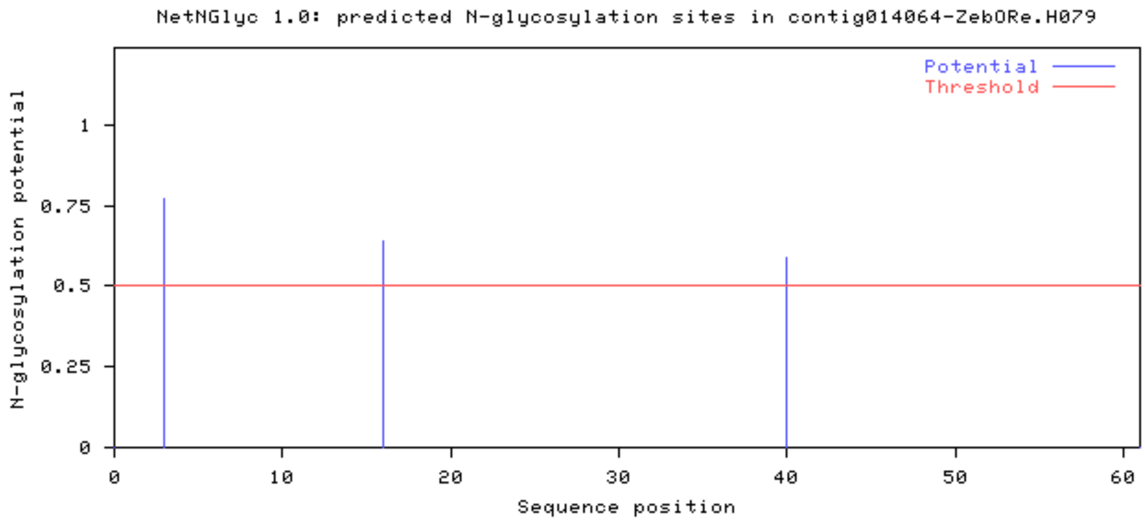

Graphics in PostScript

Output for 'contig017729-ZebORe.X150'

```
##########################################################################################

    Warning: This sequence may not contain a signal peptide!!

    Proteins without signal peptides are unlikely to be exposed to
    the N-glycosylation machinery and thus may not be glycosylated
    (in vivo) even though they contain potential motifs.

    SignalP-NN euk predictions are as follows:

  # name                    Cmax  pos ?  Ymax  pos ?  Smax  pos ?  Smean ?  D     ?

    SignalP output is explained at http://www.cbs.dtu.dk/services/SignalP/output.html

##########################################################################################

Name:  contig017729-ZebORe.X150         Length:  146
INSSPIQALFRTAVSVMFFAVVAVVIFFTYVRILLETRKLRQDRVSVNKAKHTVLLHGFQLLLCMLAFTLPITESLILLY      80
TNWPVEDVAFFCFFCFILIPRFLSPLIYGFRDHSLRGYIGKTFLCCLDTVKPHFRSKQQDSLSALX
................................................................................      80
..................................................................                   160

(Threshold=0.5)

No sites predicted in this sequence.
```

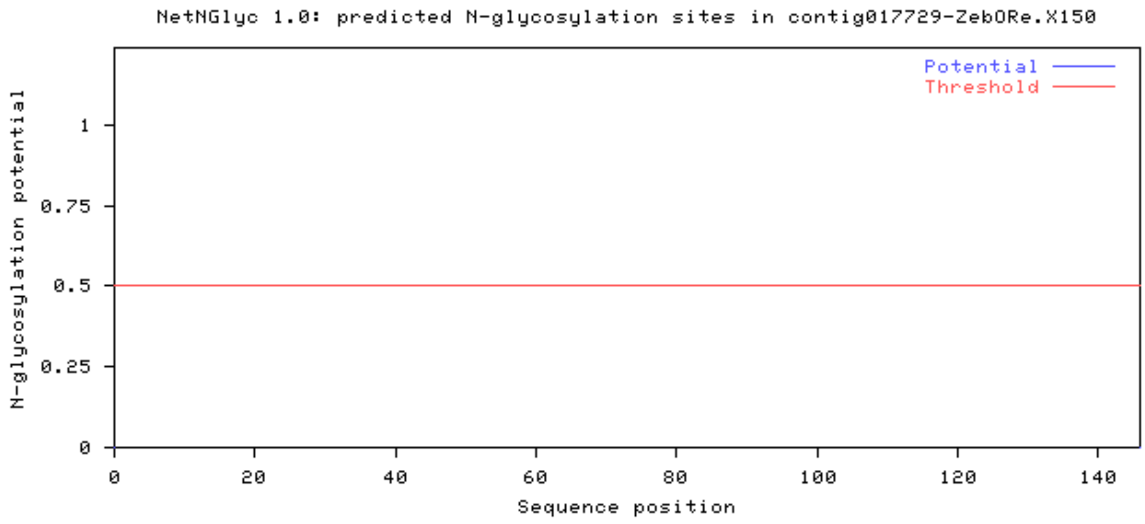

Graphics in PostScript

Output for 'contig017733-ZebOR.S126'

```
##########################################################################################

    Warning: This sequence may not contain a signal peptide!!

    Proteins without signal peptides are unlikely to be exposed to
    the N-glycosylation machinery and thus may not be glycosylated
    (in vivo) even though they contain potential motifs.

    SignalP-NN euk predictions are as follows:

  # name                 Cmax  pos ?  Ymax  pos ?  Smax  pos ?  Smean ?  D     ?

    SignalP output is explained at http://www.cbs.dtu.dk/services/SignalP/output.html

##########################################################################################

Name:  contig017733-ZebOR.S126  Length:  307
MAGNSSLICAFLHQLLTVRVMIVHTLVIIFLCINMLLIVTFVKKECFHTSARYILFFVTLLSDSFLLLMTDILLILTRCS      80
VQVWLCIFICLFVLVYSIVTPVTLTAMTLERYVAICMPLRHGQLCSTRSTMYCILIIHGLSSGPCIVIISMFFATASLSF     160
YNQYTICSVEMFMLYRWQDHARSAVSQFYFMTMGITIVFSYVQIMKVAKAASGESKKSTQKGVRTVILHAFQLFLCLVQL     240
WSPFIETAVLQIDVNLFRDFRYFNYVFFSIVPRCLSPLIYGLRDETFFLSLKNLMPTSSCSKKHVIX
...N............................................................................      80
................................................................................     160
................................................................................     240
...................................................................                  320

(Threshold=0.5)
----------------------------------------------------------------------
SeqName      Position  Potential   Jury    N-Glyc
                                 agreement result
----------------------------------------------------------------------
contig017733-ZebOR.S126    4 NSSL   0.7859     (9/9)    +++
----------------------------------------------------------------------
```

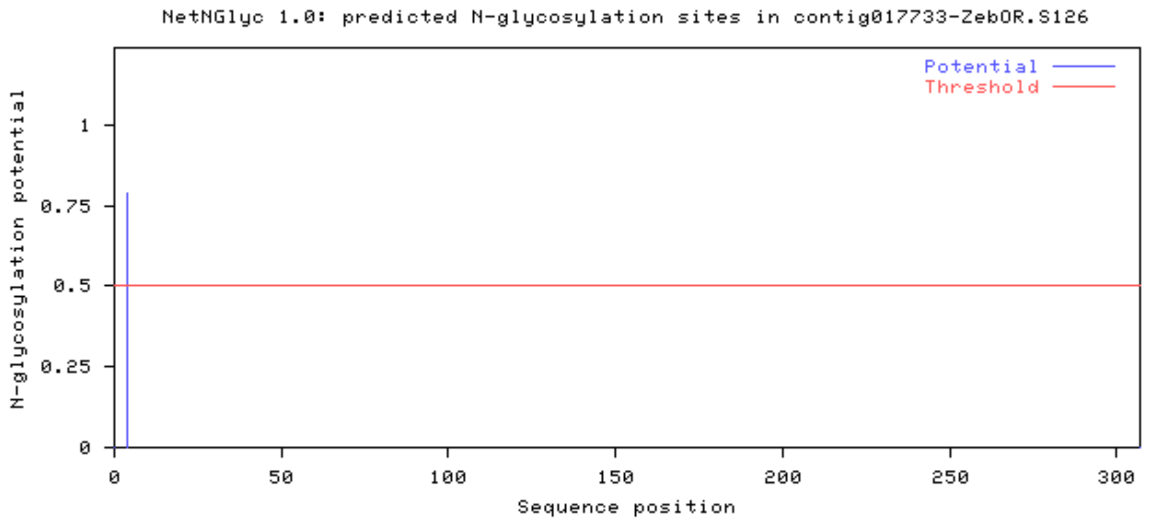

Graphics in PostScript

Output for 'contig017734-ZebORp.S135'

```
##########################################################################################

    Warning: This sequence may not contain a signal peptide!!

    Proteins without signal peptides are unlikely to be exposed to
    the N-glycosylation machinery and thus may not be glycosylated
    (in vivo) even though they contain potential motifs.

    SignalP-NN euk predictions are as follows:

  # name                    Cmax  pos ?  Ymax  pos ?  Smax  pos ?  Smean ?  D     ?

    SignalP output is explained at http://www.cbs.dtu.dk/services/SignalP/output.html

##########################################################################################

Name:  contig017734-ZebORp.S135         Length:  304
MAGNNSVNYGFFQRSISYRVIIVQILVIIFLCINMLLIVIFVKKESFHTSARYILFFVTLLSDSVLLLLSDVLLVLTHFE      80
ITMPVWLCITISVVVLLYFIVTPVTLTAMTLERYVAICMPLRHGQLCSTRSTMYCILIIHGVSSGPCIIILSMFFASASL     160
KFYKQSIICSGETFSLYRWQDHVRSAVQLYFLIMGITIAYSYVQIMKVAKAASGEKKKLTQKGLKTVILHAFQLLLCLIQ     240
LWCPFIEIAVLQIDFTLFLNVRYFNYIVFNIAPRCLSPLIYGLRDENIFLVLKKLMPTSSYSKX
...N............................................................................      80
................................................................................     160
................................................................................     240
................................................................                     320

(Threshold=0.5)
----------------------------------------------------------------------
SeqName      Position  Potential   Jury    N-Glyc
                                agreement result
----------------------------------------------------------------------
contig017734-ZebORp.S135    4 NNSV   0.7002     (9/9)   ++
----------------------------------------------------------------------
```

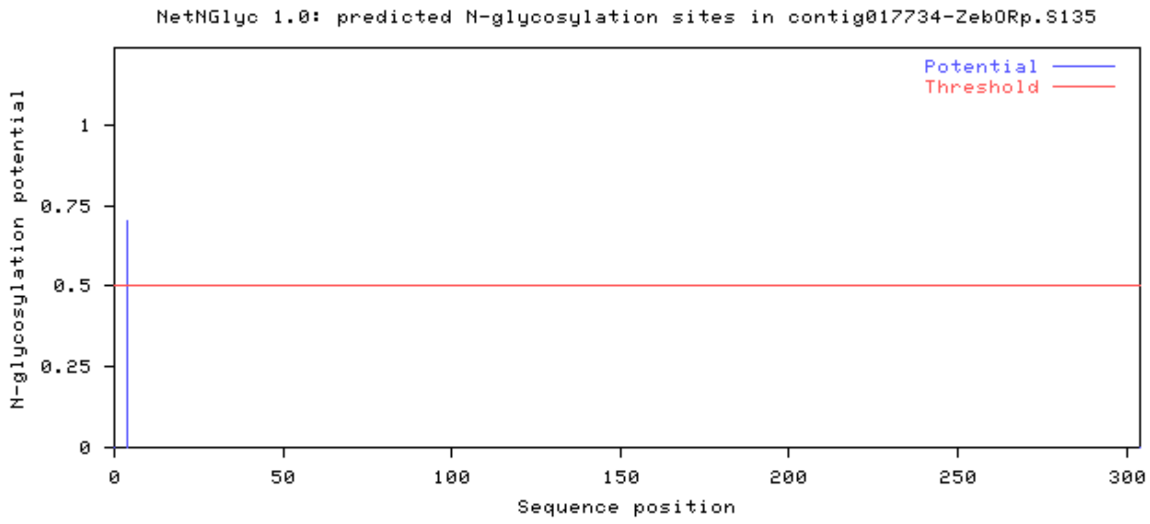

Graphics in PostScript

Output for 'contig017740-ZebORe.S133'

```
##########################################################################################

    Warning: This sequence may not contain a signal peptide!!

    Proteins without signal peptides are unlikely to be exposed to
    the N-glycosylation machinery and thus may not be glycosylated
    (in vivo) even though they contain potential motifs.

    SignalP-NN euk predictions are as follows:

  # name                    Cmax   pos ?  Ymax   pos ?  Smax   pos ?  Smean ?  D     ?

    SignalP output is explained at http://www.cbs.dtu.dk/services/SignalP/output.html

##########################################################################################

Name:  contig017740-ZebORe.S133         Length:  119
MSVWLCITISVVVLLYFIVTPVTLTAMTLERYVAICMPLRHGQLWSTRSTMYCILIIHGVSSGPCIIILSMFFASASLKF      80
YKQSMICNVEAFTLYRWQDHVRSAVYQCYFLIMGITIAY
................................................................................      80
.......................................                                              160

(Threshold=0.5)

No sites predicted in this sequence.
```

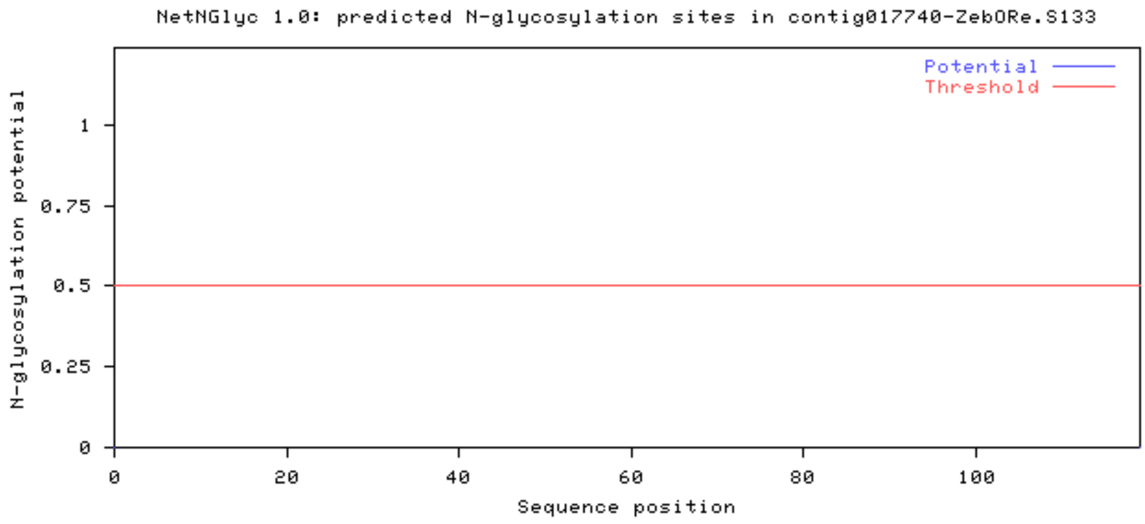

[Graphics in PostScript]

Output for 'contig017743-ZebOR.S128'

```
##########################################################################################

    Warning: This sequence may not contain a signal peptide!!

    Proteins without signal peptides are unlikely to be exposed to
    the N-glycosylation machinery and thus may not be glycosylated
    (in vivo) even though they contain potential motifs.

    SignalP-NN euk predictions are as follows:

  # name                 Cmax  pos ?  Ymax  pos ?  Smax  pos ?  Smean ?  D     ?

    SignalP output is explained at http://www.cbs.dtu.dk/services/SignalP/output.html

##########################################################################################

Name:  contig017743-ZebOR.S128  Length:  304
MADNNSVNNVFLQRPNDRMIIVQILVIIFLCINMLLILTFIKKESFHTSARYILFSVTLLSDSILLFVSDILVILTYYQF      80
TIQVWLCIISTIFVLMYTFVTPVTLTAMTLECYVAICMPLRHGQLCSTRSTMYCILIIHVLSSGPCIIILSMFFASASLK     160
FYKQSMICSVEAFTLYRWQDHVRSAVYQCYFLIMGITIAYSYVQIMKVAKAASGDKKKLTHKGLKTVILHAFQLLLCLIQ     240
LWCPFIETAVLQIDFRLFIDVRYSNYIMFSIAPRCLSPLIYGLRDEHFFLVLKSLMPTSSCSKX
...N............................................................................      80
................................................................................     160
................................................................................     240
................................................................                     320

(Threshold=0.5)
----------------------------------------------------------------------
SeqName      Position  Potential   Jury    N-Glyc
                                 agreement result
----------------------------------------------------------------------
contig017743-ZebOR.S128    4 NNSV   0.7286     (9/9)   ++
----------------------------------------------------------------------
```

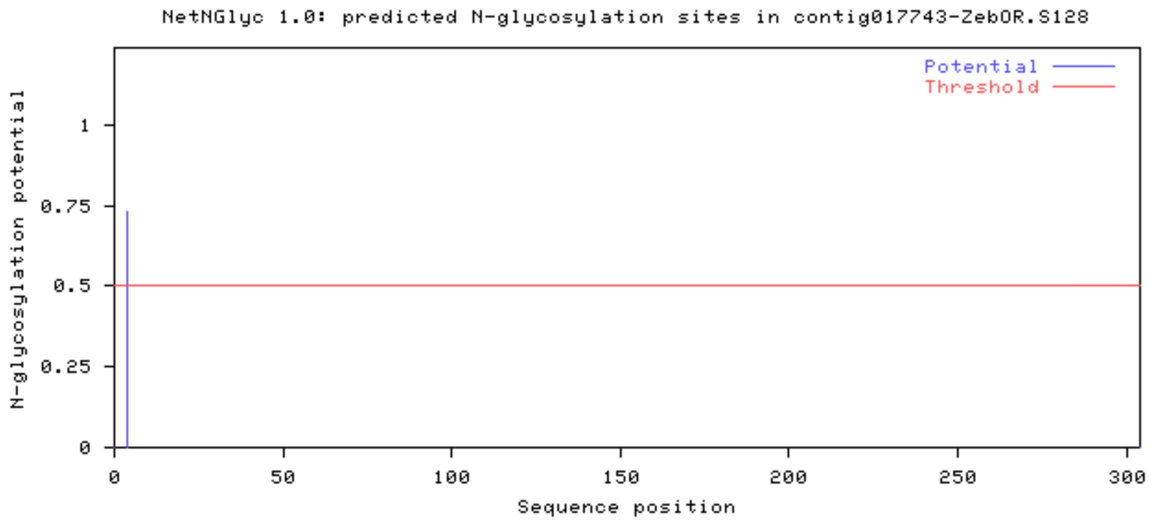

Graphics in PostScript

Output for 'contig017746-ZebORe.S130'

```
##########################################################################################

    Warning: This sequence may not contain a signal peptide!!

    Proteins without signal peptides are unlikely to be exposed to
    the N-glycosylation machinery and thus may not be glycosylated
    (in vivo) even though they contain potential motifs.

    SignalP-NN euk predictions are as follows:

  # name                    Cmax  pos ?  Ymax  pos ?  Smax  pos ?  Smean ?  D     ?

    SignalP output is explained at http://www.cbs.dtu.dk/services/SignalP/output.html

##########################################################################################

Name:  contig017746-ZebORe.S130         Length:  181
MATNNSVIGGQLAINNINNRVIIVQVLISVFLCINFLLITTFFMKDIFYTTMRYILFAIALSSDSLFLLMTNVLLILNYF      80
SFTIQVWLCVIIYIVLSVYTFVTPVTLTAMTLERYVAICVPLRHAELCRTQRALHFILIIHGLSSVPCIVILSIFFASAI     160
TSFYTQSRICAVEMFIFHRWQ
...N............................................................................      80
................................................................................     160
.....................                                                                240

(Threshold=0.5)
----------------------------------------------------------------------
SeqName      Position  Potential   Jury    N-Glyc
                                 agreement result
----------------------------------------------------------------------
contig017746-ZebORe.S130    4 NNSV   0.7107     (9/9)    ++
----------------------------------------------------------------------
```

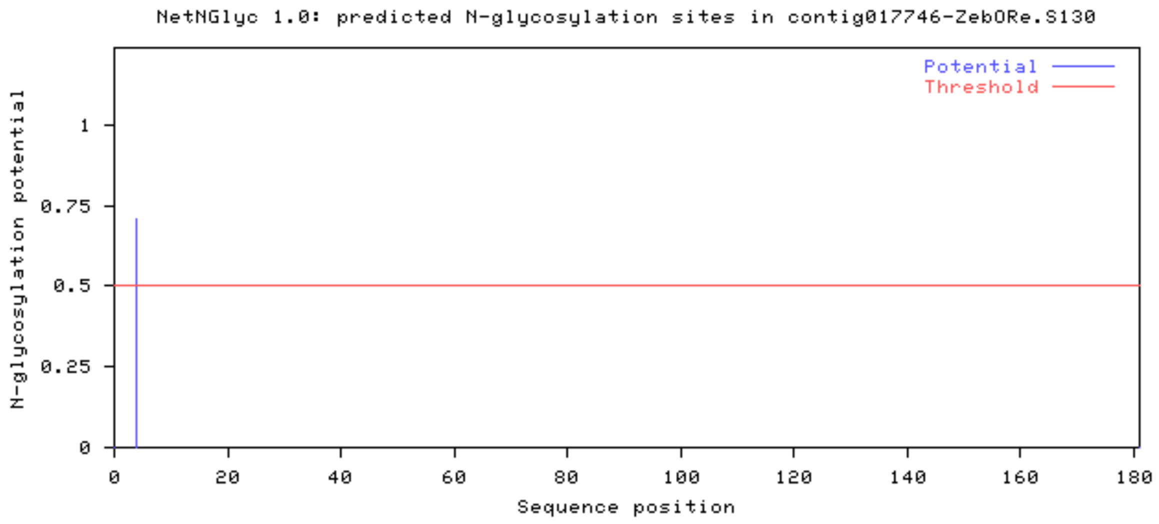

Graphics in PostScript

Output for 'contig017750-ZebORe.S132'

```
##########################################################################################

    Warning: This sequence may not contain a signal peptide!!

    Proteins without signal peptides are unlikely to be exposed to
    the N-glycosylation machinery and thus may not be glycosylated
    (in vivo) even though they contain potential motifs.

    SignalP-NN euk predictions are as follows:

  # name                    Cmax  pos ?  Ymax  pos ?  Smax  pos ?  Smean ?  D     ?

    SignalP output is explained at http://www.cbs.dtu.dk/services/SignalP/output.html

##########################################################################################

Name:  contig017750-ZebORe.S132         Length:  97
ENKKSTHKGLRTVALHAFQLLLCLIQLWCPFIEAAVLQIDFMLYVNVRYFNYIMFSLTPRCLSPLIYGLRDDKFFLALRY      80
HVLCHLHRKKSVGVSNX
................................................................................      80
.................                                                                    160

(Threshold=0.5)

No sites predicted in this sequence.
```

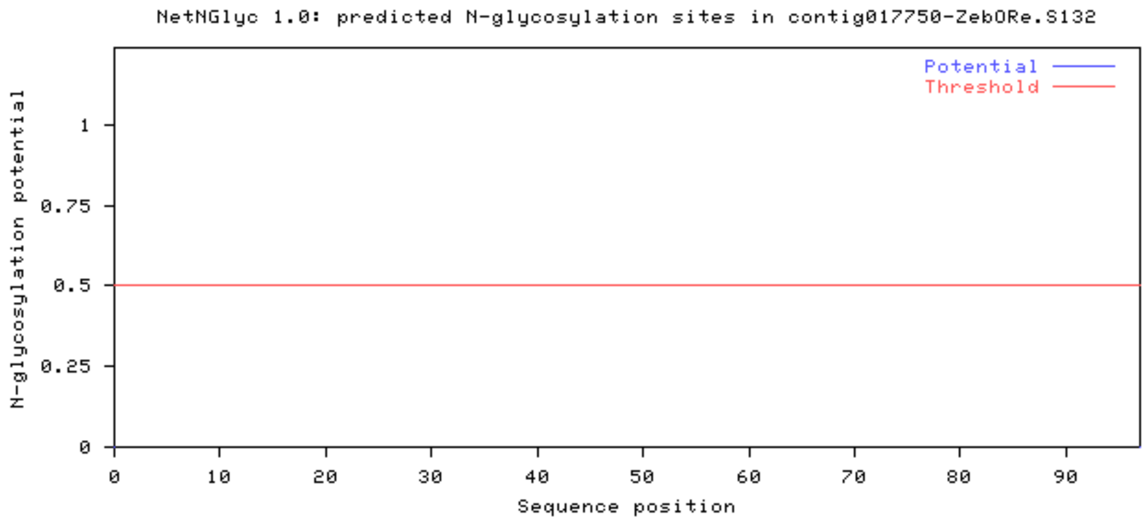

Graphics in PostScript

Output for 'contig017778-ZebOR.K086'

```
##########################################################################################

    Warning: This sequence may not contain a signal peptide!!

    Proteins without signal peptides are unlikely to be exposed to
    the N-glycosylation machinery and thus may not be glycosylated
    (in vivo) even though they contain potential motifs.

    SignalP-NN euk predictions are as follows:

  # name                 Cmax  pos ?  Ymax  pos ?  Smax  pos ?  Smean ?  D     ?

    SignalP output is explained at http://www.cbs.dtu.dk/services/SignalP/output.html

##########################################################################################

Name:  contig017778-ZebOR.K086  Length:  312
MEKNKTVSTDILEVQGFDISPQLTYPLFFLLLFVYFTLLFSNIGVLLLIISQKSLHQPMYFLFCNLSVNDLIGNTVLLPQ      80
LMAHILATERFITYKQCVVQAFQSHTFGSASHMILIIMAIDRYVAICHPLRYSSIMTTRTVVGLSAAAWGVSVVLVSILI     160
GLTVRLSRCRSTIQNSYCDNASLFKLSCEDVSINNIYGLFFTVLLFTSSIASIAATYFRIALICWIKKNKDLNNKALQTC     240
ASHLVLYLIMLWSGFLTIILHRFPNYPDLRKIAYVLFHVVPANLNPIIYGMQTRSLRHKITEILKRKVTPSX
...N............................................................N...............      80
................................................................................     160
...................N............................................................     240
........................................................................             320

(Threshold=0.5)
----------------------------------------------------------------------
SeqName      Position  Potential   Jury    N-Glyc
                                agreement result
----------------------------------------------------------------------
contig017778-ZebOR.K086    4 NKTV    0.7126     (9/9)    ++
contig017778-ZebOR.K086   65 NLSV    0.7387     (9/9)    ++
contig017778-ZebOR.K086  180 NASL    0.5739     (7/9)    +
----------------------------------------------------------------------
```

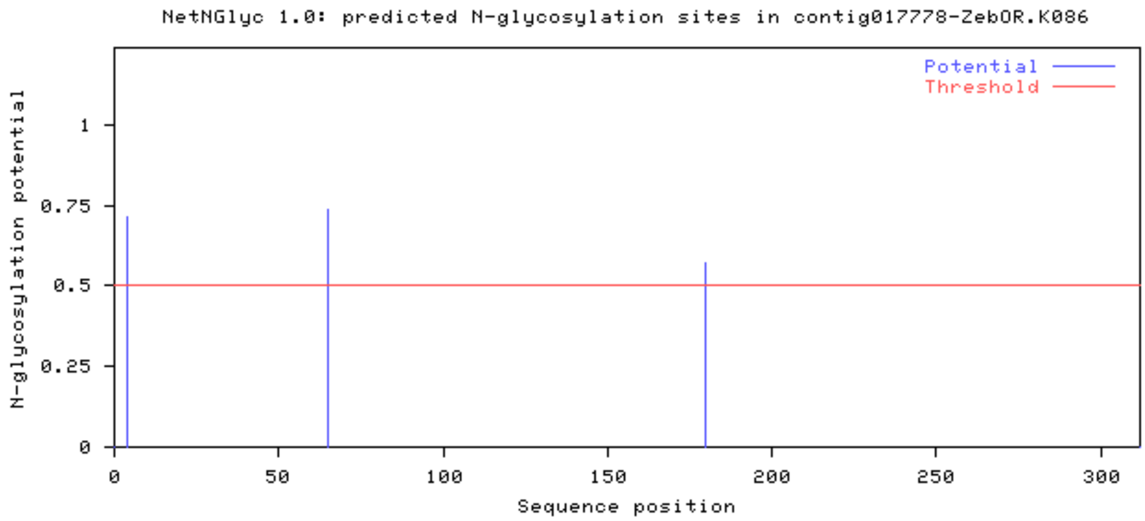

Graphics in PostScript

Output for 'contig017781-ZebOR.J083'

##

Warning: This sequence may not contain a signal peptide!!

Proteins without signal peptides are unlikely to be exposed to
the N-glycosylation machinery and thus may not be glycosylated
(in vivo) even though they contain potential motifs.

SignalP-NN euk predictions are as follows:

# name	Cmax	pos ?	Ymax	pos ?	Smax	pos ?	Smean ?	D	?

SignalP output is explained at http://www.cbs.dtu.dk/services/SignalP/output.html

##

```
Name:  contig017781-ZebOR.J083  Length:  312
MENQSFDFSSELTLDPFVIPPGGKYPIFFLGISICIFGISCNLTLLALIIILNRNLHKPMYFILFSLPLNDLIGLSAMLPK      80
VLSDIVTETHKIDYHLCVLQAFLLHMYGGGILFILAAMSFDRYVAICMPLRYSSVMTPRFISCIIVLVWGLDFVLIVSLF     160
SLQARLPRCKHVVMNVFCDNPSLLKLTCGNTTVNNIMGLFNTAVIQVVSVSIQAYSYVKILIACVVTRKSETKAKAVNTC     240
VAQLVILFMFEVVATFTILSHRFKNVSVDMQKIMGMLIFTVPPLLNPIVYGLYTNEIRSTLLRVLKNRVSMX
..N.....................................N.......................................      80
................................................................................     160
...................N.........N..................................................     240
........................N...............................................             320
```

(Threshold=0.5)

SeqName	Position	Potential	Jury agreement	N-Glyc result	
contig017781-ZebOR.J083	3 NQSF	0.6052	(9/9)	++	
contig017781-ZebOR.J083	42 NLTL	0.6887	(9/9)	++	
contig017781-ZebOR.J083	180 NPSL	0.5544	(6/9)	+	WARNING: PRO-X1.
contig017781-ZebOR.J083	190 NTTV	0.6813	(9/9)	++	
contig017781-ZebOR.J083	265 NVSV	0.5149	(6/9)	+	

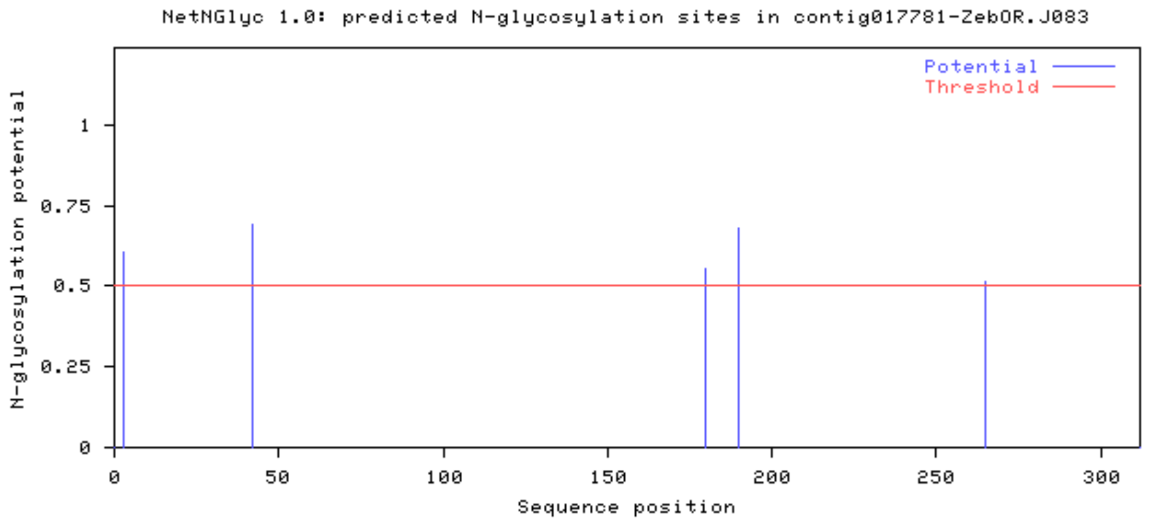

Graphics in PostScript

Output for 'contig017781-ZebOR.J084'

```
##########################################################################################

    Warning: This sequence may not contain a signal peptide!!

    Proteins without signal peptides are unlikely to be exposed to
    the N-glycosylation machinery and thus may not be glycosylated
    (in vivo) even though they contain potential motifs.

    SignalP-NN euk predictions are as follows:

  # name                    Cmax  pos ?  Ymax  pos ?  Smax  pos ?  Smean ?  D     ?

    SignalP output is explained at http://www.cbs.dtu.dk/services/SignalP/output.html

##########################################################################################

Name:  contig017781-ZebOR.J084  Length:  312
MNNRYNTSSFLQINVFNLSSESVFPAFLFATLSYMIILFCNLTLILTIVLNKSLHQPMYLILLNLPINDLIGSSALFPQL      80
IKEILRNSGIMQYSACVAQAFFIHIYAAGTVFNLSAMAYDRYIAICYPLQYSTVMTNAHIMRIITIVWMSCLVLIAVLFF     160
LLLRLPRCRSEMTNPYCDNPSLLTLVCADTTINNIYGLLISALSQLVANGIVFYTYLRILITCFRSKRSDTKAKALQTCA     240
THLIVFLLLECLGLFTIISYRLNNVSPHFRRFMGLSTLIFPPTLNPIIYGLKTKEIREKVLNFLKNRMFSSX
.....N..................................N.........N.............................      80
................................................................................     160
..................N.............................................................     240
........................................................................             320

(Threshold=0.5)
----------------------------------------------------------------------
SeqName      Position  Potential   Jury    N-Glyc
                                 agreement result
----------------------------------------------------------------------
contig017781-ZebOR.J084    6 NTSS   0.7649     (9/9)   +++
contig017781-ZebOR.J084   17 NLSS   0.4970     (3/9)   -
contig017781-ZebOR.J084   41 NLTL   0.7211     (9/9)   ++
contig017781-ZebOR.J084   51 NKSL   0.7480     (9/9)   ++
contig017781-ZebOR.J084  113 NLSA   0.4347     (5/9)   -
contig017781-ZebOR.J084  179 NPSL   0.5584     (5/9)   +     WARNING: PRO-X1.
contig017781-ZebOR.J084  264 NVSP   0.2140     (9/9)   ---
----------------------------------------------------------------------
```

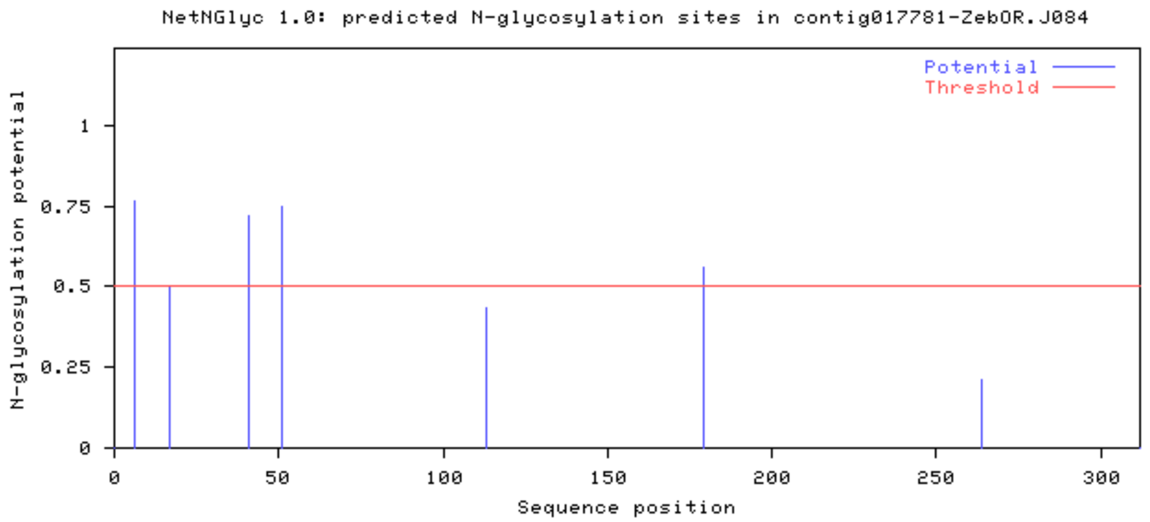

Graphics in PostScript

Output for 'contig017782-ZebOR.J085'

```
##############################################################################

   Warning: This sequence may not contain a signal peptide!!

   Proteins without signal peptides are unlikely to be exposed to
   the N-glycosylation machinery and thus may not be glycosylated
   (in vivo) even though they contain potential motifs.

   SignalP-NN euk predictions are as follows:

 # name                Cmax  pos ?  Ymax  pos ?  Smax  pos ?  Smean ?  D     ?

   SignalP output is explained at http://www.cbs.dtu.dk/services/SignalP/output.html

##############################################################################

Name:  contig017782-ZebOR.J085  Length:  313
MYTNSSTSSLLTLQSLGLSSTDIYPAFVFGTLTYLIIMFSNLLVLTVIAMNKKLHKPMFILLFNLPISDIVGATAFFPHL      80
IFSIVAENRLISHHACIFQAFLIHVYGTGNLLILSAMAYDRYIAICFPLRYTTIMNSHNLMKMIVITWFINLSMMFTLFI     160
LLVRFKTCRTNIVDFYCNNQSLVKLICDDTSVNNYYGLATIFLLMGGPLALILYTYAQILRTCVITNHTDARQKAIQTCA     240
THLIVFLSLQINTVFALISHRIDSSSPVLRRAFGVSVLIFPPFFDPIIYGLKTKELKQCIVMFLKRNVGLTMX
...N............................................................................      80
......................................................................N.........     160
..................N...............................................N.............     240
.........................................................................            320

(Threshold=0.5)
----------------------------------------------------------------------
SeqName      Position  Potential   Jury    N-Glyc
                                 agreement result
----------------------------------------------------------------------
contig017782-ZebOR.J085    4 NSST   0.6361     (8/9)   +
contig017782-ZebOR.J085  151 NLSM   0.5319     (6/9)   +
contig017782-ZebOR.J085  179 NQSL   0.5174     (5/9)   +
contig017782-ZebOR.J085  227 NHTD   0.5090     (4/9)   +
----------------------------------------------------------------------
```

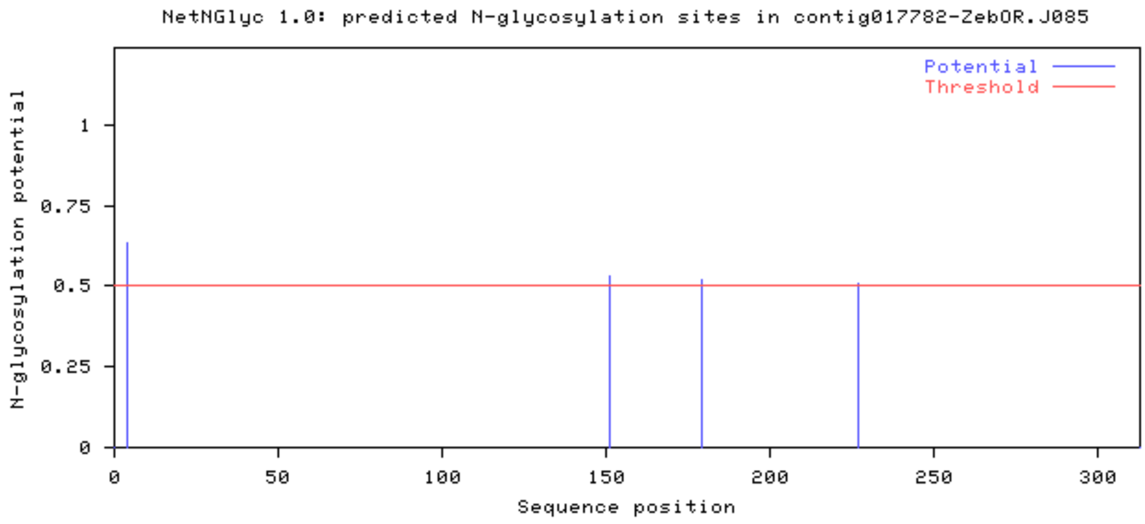

Graphics in PostScript

Output for 'contig017786-ZebOR.L091'

```
##########################################################################################

    Warning: This sequence may not contain a signal peptide!!

    Proteins without signal peptides are unlikely to be exposed to
    the N-glycosylation machinery and thus may not be glycosylated
    (in vivo) even though they contain potential motifs.

    SignalP-NN euk predictions are as follows:

  # name                  Cmax  pos ?  Ymax  pos ?  Smax  pos ?  Smean ?  D     ?

    SignalP output is explained at http://www.cbs.dtu.dk/services/SignalP/output.html

##########################################################################################

Name:  contig017786-ZebOR.L091  Length:  314
MSLPNASIKVTHFIIGGFDTVKRPIAVGVVMLITYLLAVIANVLNILFIIFDKRLHKPMYILICNLAVVDILYCCSTTPT      80
MIGVLLAGVNTISYVECFIQMSVFHLVGVMELFALAIMAFDRLIAFSFPFQYHSYLTNTRTLVVTYILWVVASGFVAVMP     160
VTAATLPYCTSRMKYAFCDYAAVIRTTCVDPNYYFNLVSVMMFFLLFFTFSFIGLSYFVIAFLMKFSSNRDKKKMASTCV     240
SHLIVVTCYYSPLFVLIVFTRVGVVLTLEECQGLLIGTILSPSLVNPVVYCFRTKEIKNKIFKIFTKKADILNX
....N...........................................................................      80
................................................................................     160
................................................................................     240
..........................................................................           320

(Threshold=0.5)
----------------------------------------------------------------------
SeqName      Position  Potential   Jury    N-Glyc
                                 agreement result
----------------------------------------------------------------------
contig017786-ZebOR.L091    5 NASI   0.6294     (7/9)   +
----------------------------------------------------------------------
```

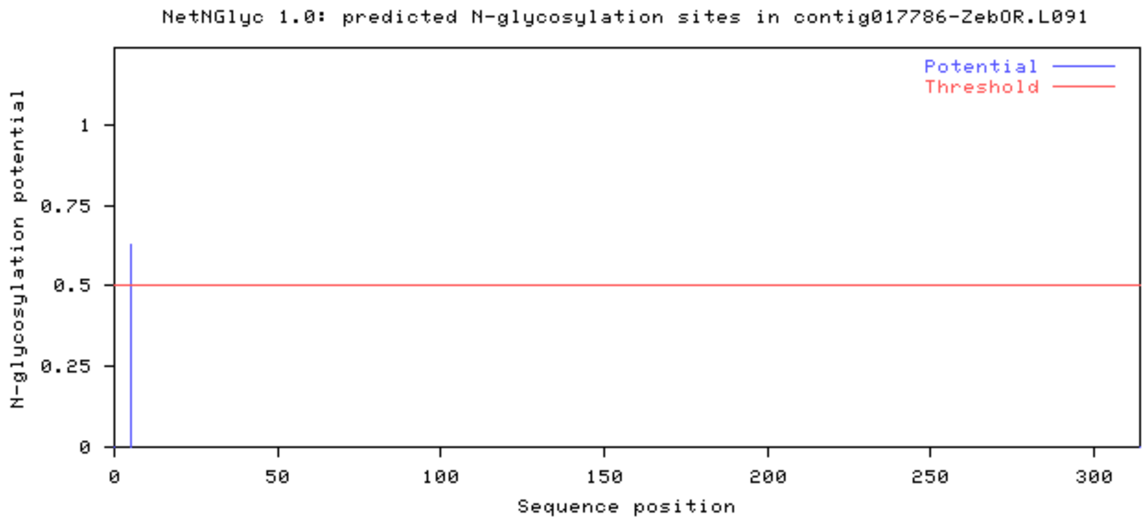

Graphics in PostScript

Output for 'contig017787-ZebOR.L092'

```
##########################################################################################

    Warning: This sequence may not contain a signal peptide!!

    Proteins without signal peptides are unlikely to be exposed to
    the N-glycosylation machinery and thus may not be glycosylated
    (in vivo) even though they contain potential motifs.

    SignalP-NN euk predictions are as follows:

  # name                   Cmax  pos ?  Ymax  pos ?  Smax  pos ?  Smean ?  D     ?

    SignalP output is explained at http://www.cbs.dtu.dk/services/SignalP/output.html

##########################################################################################

Name:  contig017787-ZebOR.L092  Length:  313
MLLQNASITVKHFIIGGFDTVKRPVAVGVVMLIIYILAIFANTVNILFIIFDKRLHKPMYLLVCNLAVVDIMYTSSATPT      80
MIGVLLADVKTILYVDCLIQMCVFHLAMVMERFALAIMAFDRLIAIIFPFHYHSYLTNTRTVVLTYILWIIGCGTVVLFP     160
ATVIPLPHCTLKLKYTFCDYAAIMRTTCVNVDEYFNQSAIWSFFILFFTFTFICISYCGILFCVKLSSNNDKKKMGSTVV     240
SHAICVTCFYSPIFIIVILTRVGVVLSLDERQGLLIGNILGPSLVNPFVYCLRTTEIKNKMVKIFKKILRLLX
....N...........................................................................      80
................................................................................     160
................................................................................     240
.........................................................................            320

(Threshold=0.5)
----------------------------------------------------------------------
SeqName      Position  Potential   Jury    N-Glyc
                                agreement  result
----------------------------------------------------------------------
contig017787-ZebOR.L092    5 NASI    0.7347     (9/9)   ++
contig017787-ZebOR.L092  196 NQSA    0.3956     (7/9)   -
----------------------------------------------------------------------
```

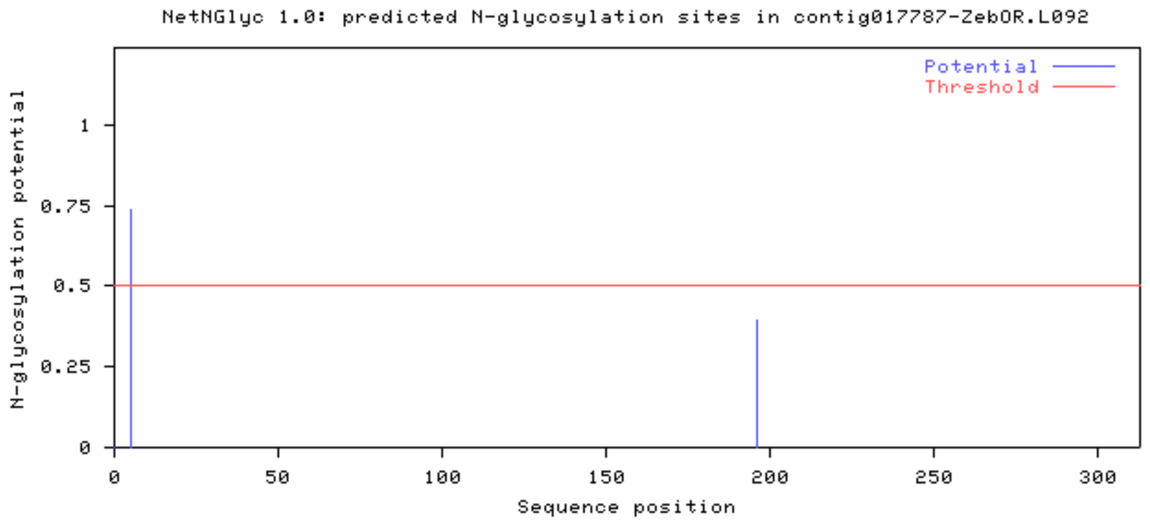

Graphics in PostScript

Output for 'contig018434-ZebOR.H070'

```
##########################################################################################

    Warning: This sequence may not contain a signal peptide!!

    Proteins without signal peptides are unlikely to be exposed to
    the N-glycosylation machinery and thus may not be glycosylated
    (in vivo) even though they contain potential motifs.

    SignalP-NN euk predictions are as follows:

  # name                   Cmax  pos ?  Ymax  pos ?  Smax  pos ?  Smean ?  D     ?

    SignalP output is explained at http://www.cbs.dtu.dk/services/SignalP/output.html

##########################################################################################

Name:  contig018434-ZebOR.H070  Length:  309
MNNVSVITMFFLSGFNKTISHRFVLFFLSLLCYCIICLVNVSLIVIIILDSNLHESMYILLCVFCINALYGTAGFFPKFL      80
WDLLSDVHLISYYGCLIQTQVIYSFVCGELSILALMAYDRYVAICQPLKYHSIMSKQRVIRFACLLWSTTFCIMAVNVFL     160
TFRLKLCSPYISRLFCVNSSIVQLACFPAQTIVNGIFANITIIIYALHGVFIVWSYMYIIQTCVKSIENRAKFMQTCVPH     240
LVSLHTFVLIMLLDSTSARFGSKFLPQVLQNFIAIEFLVIPPVMNPLMYGFKLTKIQKKVFIVILKTKX
..N............N......................N.........................................      80
................................................................................     160
.................N....................N.........................................     240
.....................................................................                320

(Threshold=0.5)
----------------------------------------------------------------------
SeqName      Position  Potential   Jury    N-Glyc
                                 agreement result
----------------------------------------------------------------------
contig018434-ZebOR.H070    3 NVSV   0.7560     (9/9)    +++
contig018434-ZebOR.H070   16 NKTI   0.7237     (9/9)    ++
contig018434-ZebOR.H070   40 NVSL   0.6898     (9/9)    ++
contig018434-ZebOR.H070  178 NSSI   0.6973     (9/9)    ++
contig018434-ZebOR.H070  199 NITI   0.6327     (8/9)    +
----------------------------------------------------------------------
```

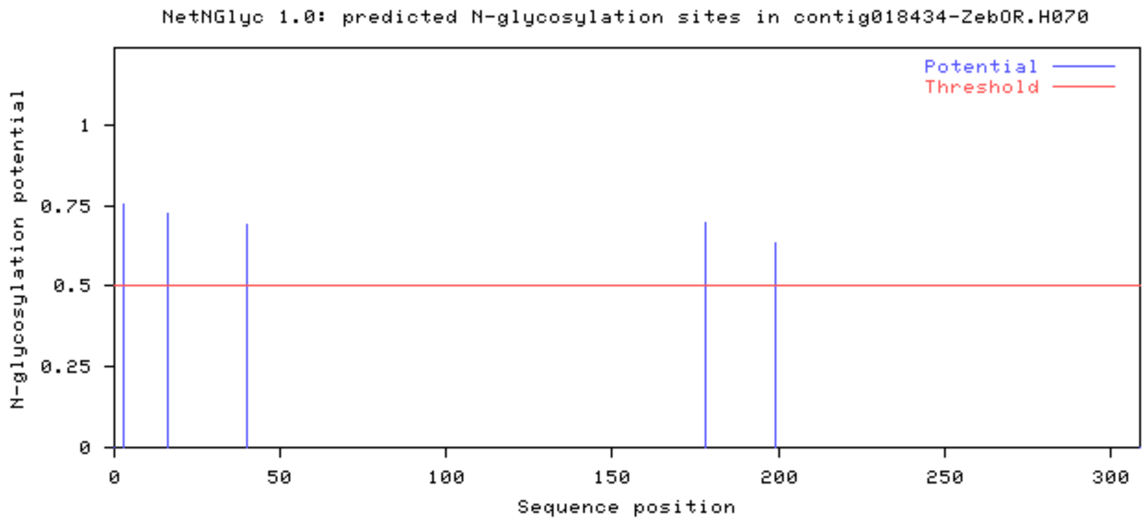

Graphics in PostScript

Output for 'contig018434-ZebOR.H071'

```
##########################################################################################

    Warning: This sequence may not contain a signal peptide!!

    Proteins without signal peptides are unlikely to be exposed to
    the N-glycosylation machinery and thus may not be glycosylated
    (in vivo) even though they contain potential motifs.

    SignalP-NN euk predictions are as follows:

  # name                    Cmax  pos ?  Ymax  pos ?  Smax  pos ?  Smean ?  D     ?

    SignalP output is explained at http://www.cbs.dtu.dk/services/SignalP/output.html

##########################################################################################

Name:  contig018434-ZebOR.H071  Length:  310
MNNVSVITMFFLSGFNETVSHRFVLFSLSLLCYCIICLLNVSLIVIIILHSNLHEPMYILLCVFCINALYGTAGFYPKFL      80
WDLLSDVHLISYYGCLIQTQVIYSSACGELSIPALMAYDRYVAICQPLKYHSIMSKQRVIRFAWFLWFTNFCIVAVNTFL     160
TSRLKLCSPYLSRLFCVNWSIVQLACFPAQTAINAISANITISIYFLYGVFIVWSYLYIIQTCVRSIENRAKFMQTCVPH     240
LVSLFTFAVTKLLDITNMRLGSKELPQTVQNFAAIEFLVIPPIMNPLIYGFKLTKIRKTICSVVIFKTKX
..N............N.......................N........................................      80
................................................................................     160
.................N....................N.........................................     240
......................................................................               320

(Threshold=0.5)
----------------------------------------------------------------------
SeqName      Position  Potential   Jury     N-Glyc
                                 agreement result
----------------------------------------------------------------------
contig018434-ZebOR.H071    3 NVSV   0.7560     (9/9)    +++
contig018434-ZebOR.H071   16 NETV   0.7039     (9/9)    ++
contig018434-ZebOR.H071   40 NVSL   0.7034     (9/9)    ++
contig018434-ZebOR.H071  178 NWSI   0.6654     (8/9)    +
contig018434-ZebOR.H071  199 NITI   0.6098     (6/9)    +
----------------------------------------------------------------------
```

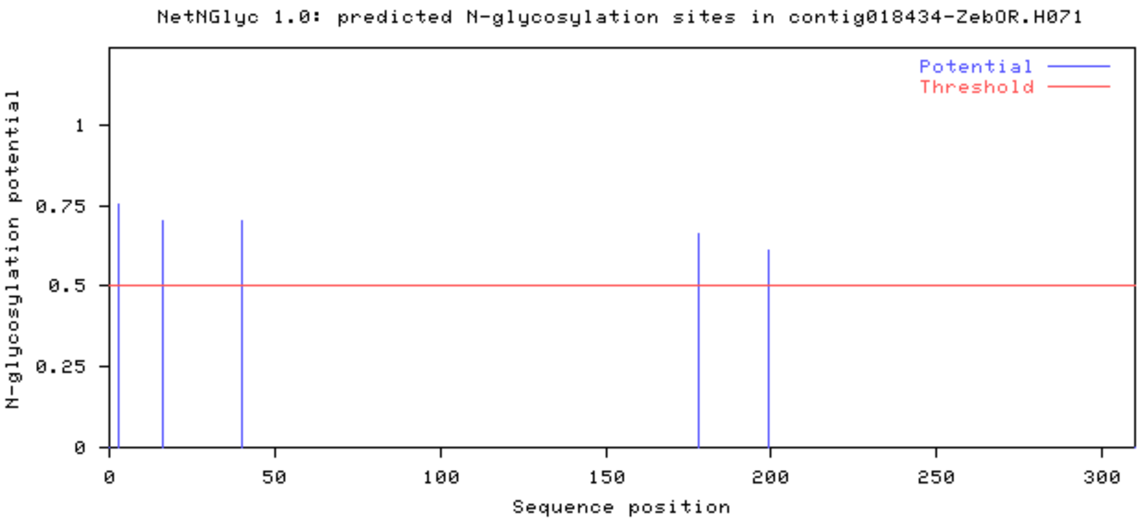

[Graphics in PostScript]

Output for 'contig018437-ZebOR.H072'

```
##########################################################################################

    Warning: This sequence may not contain a signal peptide!!

    Proteins without signal peptides are unlikely to be exposed to
    the N-glycosylation machinery and thus may not be glycosylated
    (in vivo) even though they contain potential motifs.

    SignalP-NN euk predictions are as follows:

  # name                    Cmax  pos ?  Ymax  pos ?  Smax  pos ?  Smean ?  D     ?

    SignalP output is explained at http://www.cbs.dtu.dk/services/SignalP/output.html

##########################################################################################

Name:  contig018437-ZebOR.H072  Length:  324
MNNVSVITTFFLSGFNETVSHRFVLFFLSLLCYCIICLVNVSLIVIIILDSNLHESMYILLCVFCINALYGTAGFFPKFL      80
WDLLSDVHLISYYGCLIQTKVIYSFLCGELSILALMAYDRYVAICQPLKYHSIMSKQRVIRFACFLWFTNFCIMAVNAFL     160
TSRLKLCSPYLSTLFCVNWIIVQLACFPAQTIVNGIFANITIIIYLLHGVFIVWSYLYIIQTCVKSIENRAKFMQTCVPH     240
LISLFTFVVAVLTDVISMRLGSKELPRTLQNFVAIEVLVIPPIMNPLIYGFKLTKIRKKIYSVVILKGTNFCFIQSENSF     320
THSX
..N............N.......................N.....................................      80
................................................................................     160
......................................N.........................................     240
................................................................................     320
....                                                                                 400

(Threshold=0.5)
----------------------------------------------------------------------
SeqName      Position  Potential   Jury    N-Glyc
                                 agreement result
----------------------------------------------------------------------
contig018437-ZebOR.H072    3 NVSV   0.7582     (9/9)    +++
contig018437-ZebOR.H072   16 NETV   0.7024     (9/9)    ++
contig018437-ZebOR.H072   40 NVSL   0.6904     (9/9)    ++
contig018437-ZebOR.H072  199 NITI   0.6379     (8/9)    +
----------------------------------------------------------------------
```

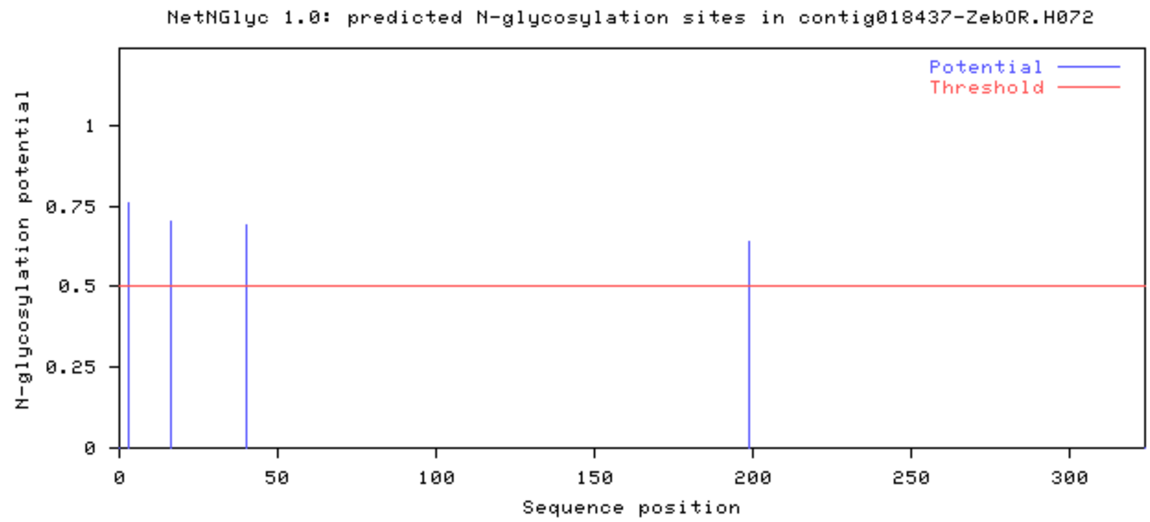

Graphics in PostScript

Output for 'contig020427-ZebOR.O099'

```
##########################################################################################

    Warning: This sequence may not contain a signal peptide!!

    Proteins without signal peptides are unlikely to be exposed to
    the N-glycosylation machinery and thus may not be glycosylated
    (in vivo) even though they contain potential motifs.

    SignalP-NN euk predictions are as follows:

  # name                    Cmax  pos ?  Ymax  pos ?  Smax  pos ?  Smean ?  D     ?

    SignalP output is explained at http://www.cbs.dtu.dk/services/SignalP/output.html

##########################################################################################

Name:  contig020427-ZebOR.O099  Length:  324
MKYTNITTIKEFIIIGFPGLPPEYYGPVSVLLLLVFLAIVIGNGFTIAVIIFERTLHKPIYVIFSNLAMTDICFGVVTLP      80
KIIARYWWNDMITSFGACFTQMYFVHSLGAIQSLNLLMMALDRFVAIWFPFKYPILFTNKAVAIACTMCWVLTFIRLLGI     160
VLHALTLPYCDQNIIMQCYCDHISITRLGCGDGLAYVNSVALANAMVTLLVPLTFIILSYFSVIIAVLRMSQTERRHKVL     240
STCAPQLFITCLYYVPRCFVYLANVLGFNFNLVIRIIITMMYSLIPAAVNPIIYCFKTKDIKNVLMRRFKKGKVSTGLKT     320
ECKX
....N...........................................................................      80
................................................................................     160
................................................................................     240
................................................................................     320
....                                                                                 400

(Threshold=0.5)
----------------------------------------------------------------------
SeqName      Position  Potential   Jury    N-Glyc
                                 agreement result
----------------------------------------------------------------------
contig020427-ZebOR.O099     5 NITT    0.7654     (9/9)    +++
----------------------------------------------------------------------
```

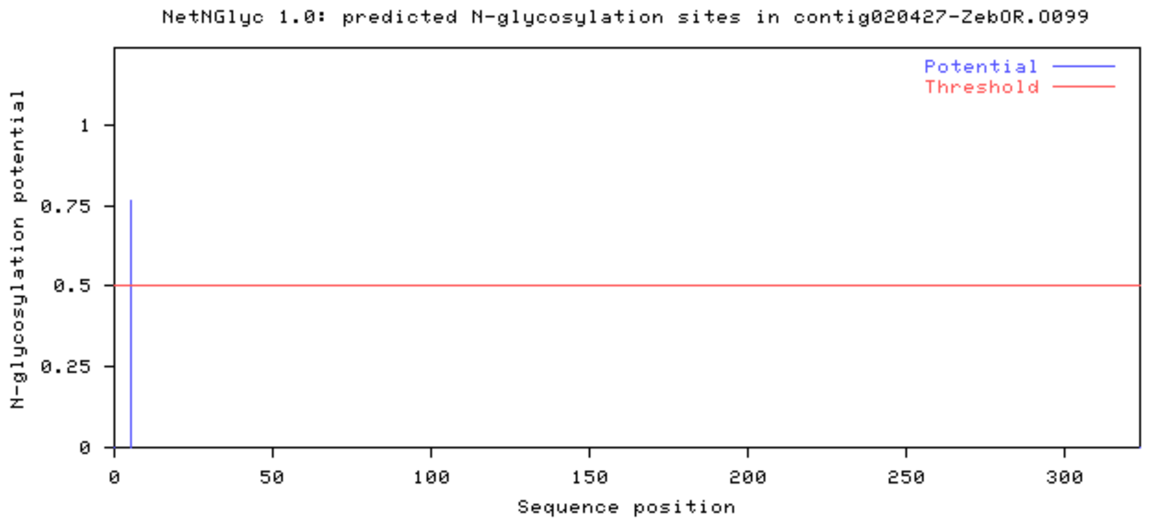

Graphics in PostScript

Output for 'contig020430-ZebOR.O100'

```
##########################################################################################

    Warning: This sequence may not contain a signal peptide!!

    Proteins without signal peptides are unlikely to be exposed to
    the N-glycosylation machinery and thus may not be glycosylated
    (in vivo) even though they contain potential motifs.

    SignalP-NN euk predictions are as follows:

  # name                    Cmax   pos ?  Ymax   pos ?  Smax   pos ?  Smean ?  D     ?

    SignalP output is explained at http://www.cbs.dtu.dk/services/SignalP/output.html

##########################################################################################

Name:  contig020430-ZebOR.O100  Length:  322
MITNVTRMKSFFILGFPGLSPQYYGPISTFLFFVYLAIALGNIFILSFVAYEKSLQKPTYLVFCHLALNDLTFGTVTLPK      80
IMSKYWFDNSVISFYGCFTQMFFVHYLGSVTSFILLVMALDRFVAICIPLRYPVLITNSVISVLCGFAWFIPLPLMIAVV     160
LHALTLPFCKSNIIVQCYCDHISIVSQACGDDVRIVVVTSLCLAMFCLLLPLAFILFSYISIIVVIMKMSSSAGRKRTLS     240
TCTSQIFITCLFYLPRCFVYVAYAFGFSFSLDVRIGLILLYSLFPAAVNPVIYCFKTRDIKHMLMKRLKKTKIGLEIKLA     320
LX
...N............................................................................      80
................................................................................     160
................................................................................     240
................................................................................     320
..                                                                                   400

(Threshold=0.5)
----------------------------------------------------------------------
SeqName      Position  Potential    Jury    N-Glyc
                                agreement result
----------------------------------------------------------------------
contig020430-ZebOR.O100    4 NVTR   0.7693     (9/9)    +++
----------------------------------------------------------------------
```

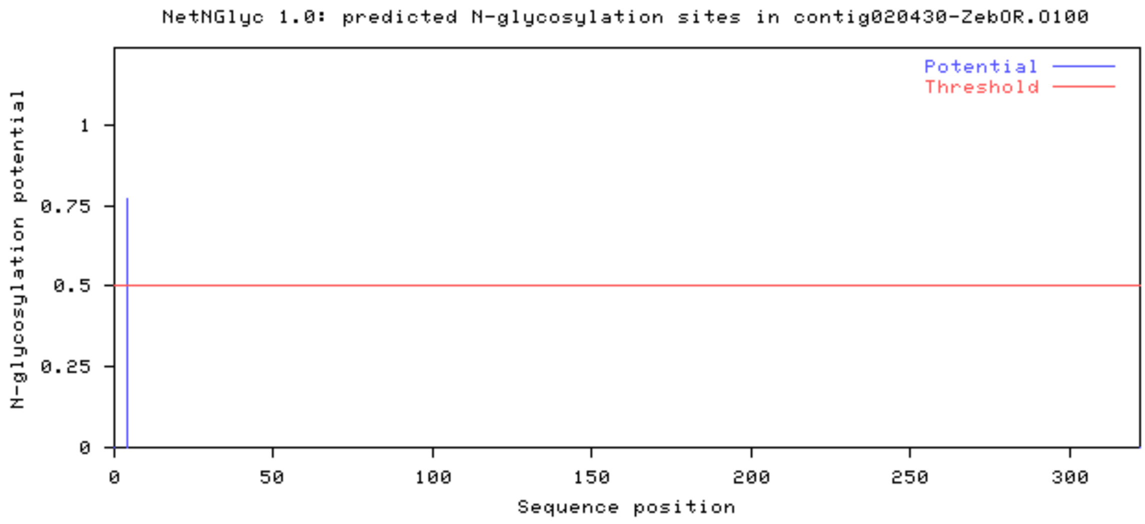

Graphics in PostScript

Output for 'contig020431-ZebOR.O101'

```
##########################################################################################

    Warning: This sequence may not contain a signal peptide!!

    Proteins without signal peptides are unlikely to be exposed to
    the N-glycosylation machinery and thus may not be glycosylated
    (in vivo) even though they contain potential motifs.

    SignalP-NN euk predictions are as follows:

  # name                    Cmax  pos ?  Ymax  pos ?  Smax  pos ?  Smean ?  D     ?

    SignalP output is explained at http://www.cbs.dtu.dk/services/SignalP/output.html

##########################################################################################

Name:  contig020431-ZebOR.O101  Length:  331
MPVENHSSVTEFVLTGFPGLHQEYYGLVSAVLFFVYLITLIANATVIFLFATNHSLHKPMYFIILNLSVCDILFSTTTLP      80
KIISRYWFQSGSISFTACFIQMYFVHYFGTAVAYILFQMALDRYLAICHPLRYSHILTKSNILILSITAWIIAKASPLMM     160
VIRAYPLPYCASNIITHCFCDHIGITVLACTDRTPYAIPAFVFAMVVLLGPLAFIIFSYGSILIAVYKIANVQSRMKSLS     240
TCSTQLIIISLYFLPRCFVYLAQNVGITFSADVRIVIIMLYSLAPPMINPLIYCLRAKDMRESLLKQFCRRIVPEKAQVA     320
AISNSLKTSPX
....N.....................................N.........N...........N..............      80
................................................................................     160
................................................................................     240
................................................................................     320
...........                                                                          400

(Threshold=0.5)
----------------------------------------------------------------------
SeqName      Position  Potential   Jury    N-Glyc
                                 agreement result
----------------------------------------------------------------------
contig020431-ZebOR.O101    5 NHSS    0.5881     (7/9)    +
contig020431-ZebOR.O101   43 NATV    0.7127     (8/9)    +
contig020431-ZebOR.O101   53 NHSL    0.6481     (9/9)    ++
contig020431-ZebOR.O101   66 NLSV    0.7706     (9/9)    +++
----------------------------------------------------------------------
```

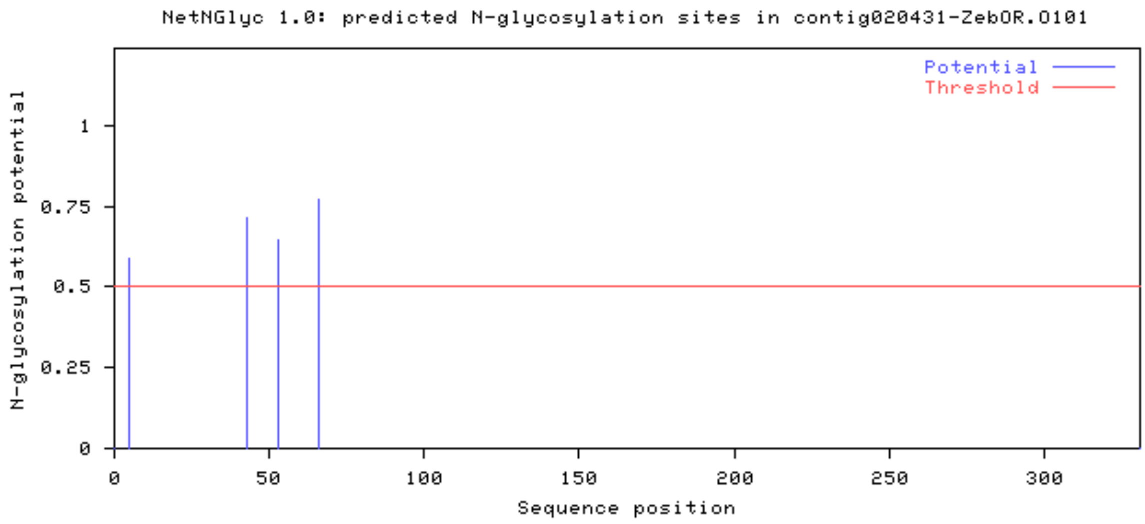

Graphics in PostScript

Output for 'contig020433-ZebORe.O104'

```
##########################################################################################

    Warning: This sequence may not contain a signal peptide!!

    Proteins without signal peptides are unlikely to be exposed to
    the N-glycosylation machinery and thus may not be glycosylated
    (in vivo) even though they contain potential motifs.

    SignalP-NN euk predictions are as follows:

  # name                 Cmax  pos ?  Ymax  pos ?  Smax  pos ?  Smean ?  D     ?

    SignalP output is explained at http://www.cbs.dtu.dk/services/SignalP/output.html

##########################################################################################

Name:  contig020433-ZebORe.O104         Length:  65
AQNVGITFSADVRIVIIMLYSLAPPMINPLIYCLRAKDMRESLLKQFCKRLVPRKAHVAAISNSX
.................................................................                       80

(Threshold=0.5)

No sites predicted in this sequence.
```

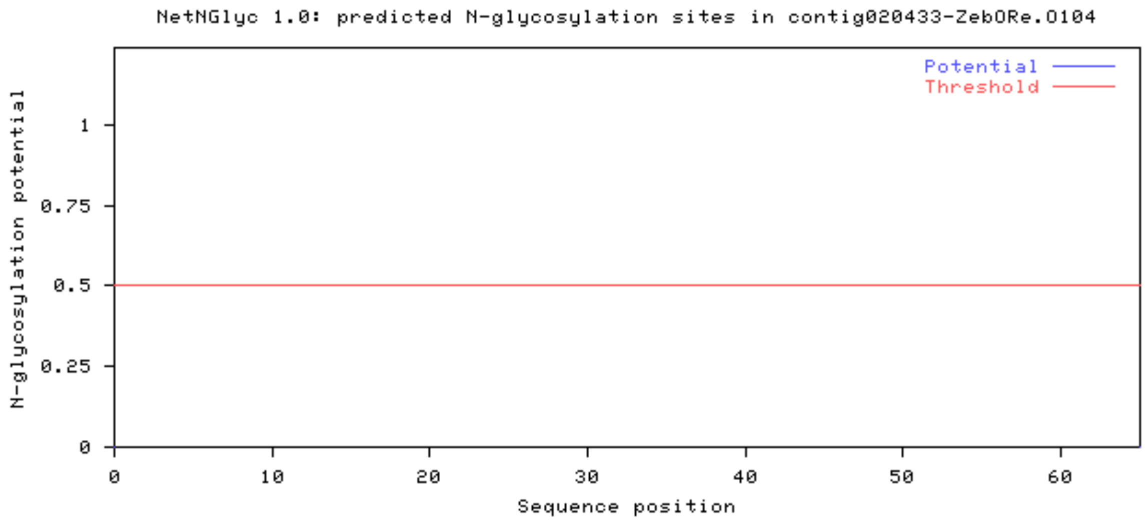

[Graphics in PostScript]

Output for 'contig020434-ZebORe.O102'

```
##########################################################################################

    Warning: This sequence may not contain a signal peptide!!

    Proteins without signal peptides are unlikely to be exposed to
    the N-glycosylation machinery and thus may not be glycosylated
    (in vivo) even though they contain potential motifs.

    SignalP-NN euk predictions are as follows:

  # name                    Cmax  pos ?  Ymax  pos ?  Smax  pos ?  Smean ?  D     ?

    SignalP output is explained at http://www.cbs.dtu.dk/services/SignalP/output.html

##########################################################################################

Name:  contig020434-ZebORe.0102         Length:  292
MPERNHSVLTEFILTGFPGLHQEYYGLVSAVLFFVYSVTLIANATVIFLFATNRSLHKPMYYIILNLSVCDILFSTTTLP      80
KIISRYWFQSGSISFTACFIQMYFVHYFGTAVAYILFQMALDRYLAICHPLKYSRILTKSNILILSITGWIVSKASPLML     160
VIRAYALPYCASNIITHCFCDHIGITVLACTDRTPYAIPAFVFAMVVLLGPLAFIIFSYCSILIAVYKIASVQGRLKSLS     240
TCSTQLIIISLYFLPRCFVYLAQNVGITFSADVRIVIIMLYSLAPPMINPLI
....N......................................N.........N...........N..............      80
................................................................................     160
................................................................................     240
....................................................                                 320

(Threshold=0.5)
----------------------------------------------------------------------
SeqName      Position  Potential   Jury    N-Glyc
                                agreement result
----------------------------------------------------------------------
contig020434-ZebORe.0102    5 NHSV   0.5108     (6/9)   +
contig020434-ZebORe.0102   43 NATV   0.7534     (9/9)   +++
contig020434-ZebORe.0102   53 NRSL   0.7257     (9/9)   ++
contig020434-ZebORe.0102   66 NLSV   0.7942     (9/9)   +++
----------------------------------------------------------------------
```

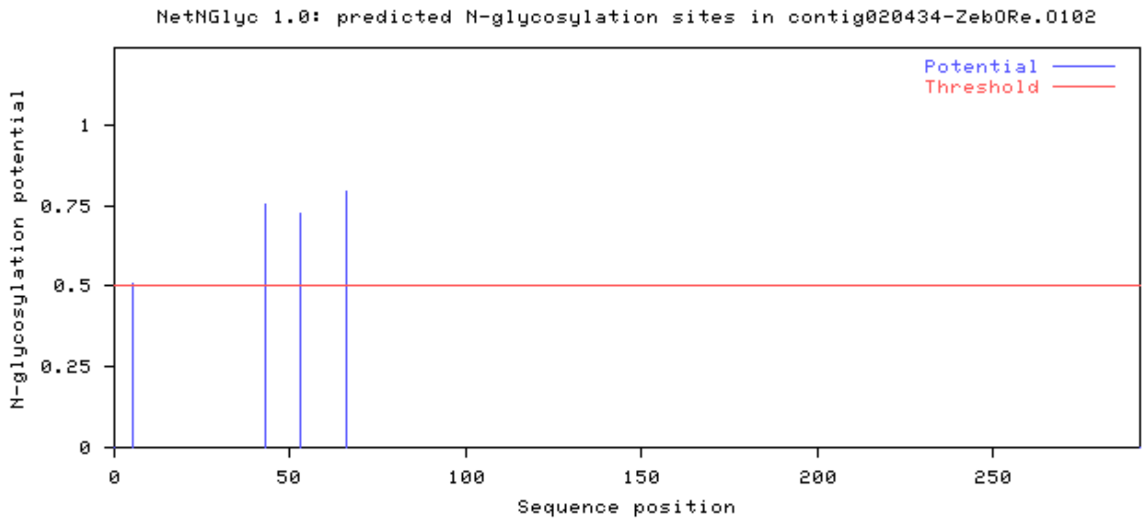

Graphics in PostScript

Output for 'contig020434-ZebORe.O103'

```
##########################################################################################

    Warning: This sequence may not contain a signal peptide!!

    Proteins without signal peptides are unlikely to be exposed to
    the N-glycosylation machinery and thus may not be glycosylated
    (in vivo) even though they contain potential motifs.

    SignalP-NN euk predictions are as follows:

  # name                    Cmax  pos ?  Ymax  pos ?  Smax  pos ?  Smean ?  D     ?

    SignalP output is explained at http://www.cbs.dtu.dk/services/SignalP/output.html

##########################################################################################

Name:  contig020434-ZebORe.O103         Length:  68
AQNVGITFSADVRIVIIMLYSLAPPMINPLIYCLRAKDMRESLLKVFCRRTIPQKAQVAVINIINHKX
....................................................................                  80

(Threshold=0.5)

No sites predicted in this sequence.
```

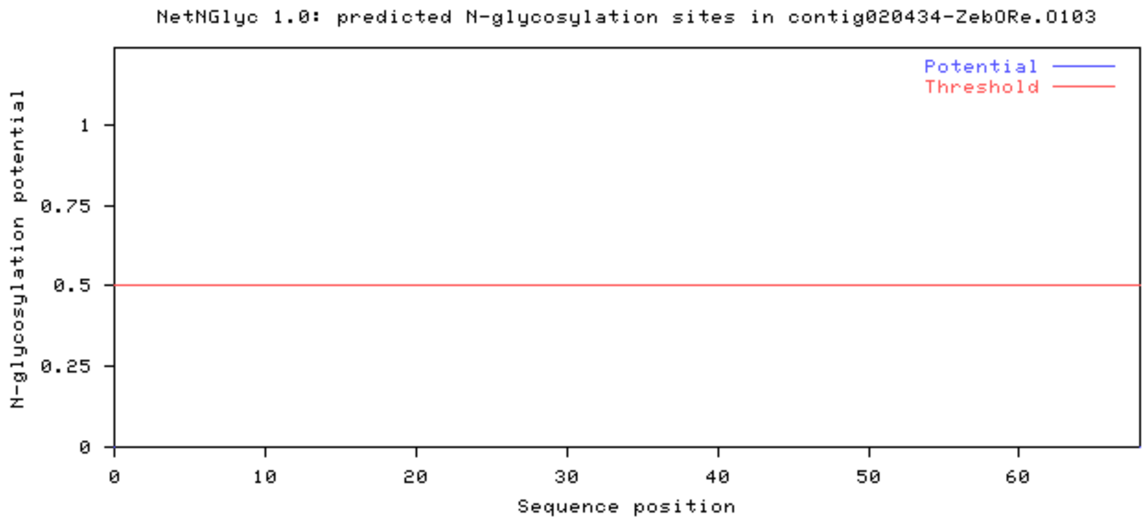

Graphics in PostScript

Output for 'contig020435-ZebORe.O105'

```
##########################################################################################

    Warning: This sequence may not contain a signal peptide!!

    Proteins without signal peptides are unlikely to be exposed to
    the N-glycosylation machinery and thus may not be glycosylated
    (in vivo) even though they contain potential motifs.

    SignalP-NN euk predictions are as follows:

  # name                    Cmax   pos ?  Ymax   pos ?  Smax   pos ?  Smean ?  D      ?

    SignalP output is explained at http://www.cbs.dtu.dk/services/SignalP/output.html

##########################################################################################

Name:  contig020435-ZebORe.0105         Length:  85
MPERNHSSVTEFILTGFAGLHQEYYGLVSAVLFFVYLITLIANAKVIFLFATNHSLHKPMYYIILNLSVCDILFSTTTLP      80
KIISR
....N................................................N............N..............      80
.....                                                                                160

(Threshold=0.5)
----------------------------------------------------------------------
SeqName      Position  Potential    Jury    N-Glyc
                                 agreement result
----------------------------------------------------------------------
contig020435-ZebORe.0105    5 NHSS   0.6042     (8/9)    +
contig020435-ZebORe.0105   53 NHSL   0.5730     (7/9)    +
contig020435-ZebORe.0105   66 NLSV   0.7402     (9/9)    ++
----------------------------------------------------------------------
```

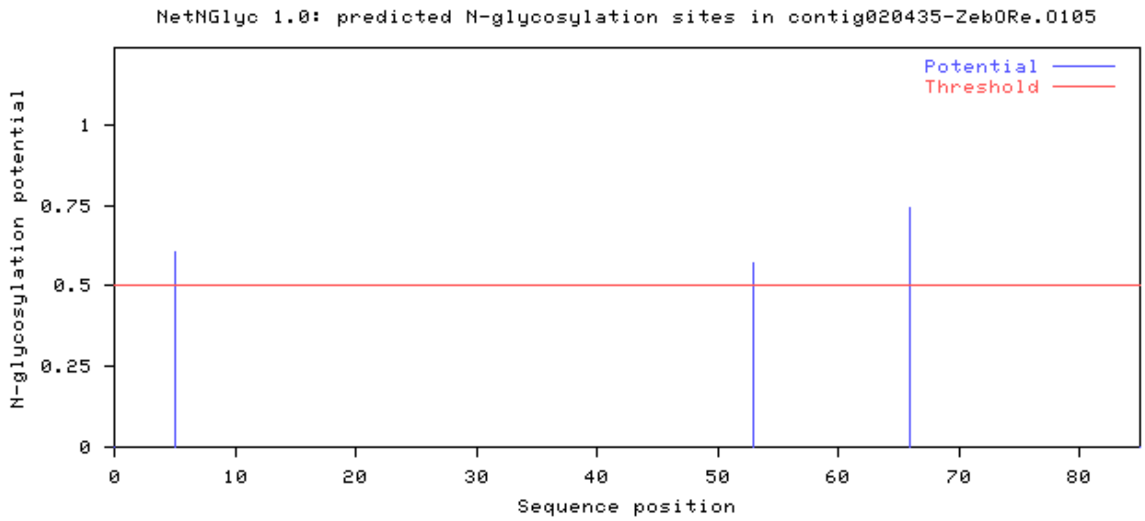

[Graphics in PostScript]

Output for 'contig020437-ZebOR.L093'

```
########################################################################################

    Warning: This sequence may not contain a signal peptide!!

    Proteins without signal peptides are unlikely to be exposed to
    the N-glycosylation machinery and thus may not be glycosylated
    (in vivo) even though they contain potential motifs.

    SignalP-NN euk predictions are as follows:

  # name                 Cmax  pos ?  Ymax  pos ?  Smax  pos ?  Smean ?  D     ?

    SignalP output is explained at http://www.cbs.dtu.dk/services/SignalP/output.html

########################################################################################

Name:  contig020437-ZebOR.L093  Length:  313
MSLQNASINVTHFIIGGFDTLSRPIAVGVVILIVYLLAVLANMINIMFIISDKRLHKPMYLLICNLAVVDIMYTSSCSPT      80
MIGVLLAGVNTISYMACLIQMCVGHLGTAMESFVLAIMALDRFIAIIYPFQYQSYLTNTRVLVLTFIVWFVAWCFVFYMP     160
ATVVLLPHCSSRLRYTFCDFAAVLRTTCVNPEKYFNEVAIVSFFIFFFTFIFICLSYCGILLFVKLSSNNEKKKMGSTLV     240
SHLICVFVHYCPAFVRNIFTRFGVVLTLEERQGLLIGAVLGPCLVNPFVYFLRTKEIKQKFYKIFKKFHTSDX
....N...N..............................................................................      80
................................................................................     160
................................................................................     240
.........................................................................            320

(Threshold=0.5)
----------------------------------------------------------------------
SeqName      Position  Potential   Jury    N-Glyc
                                 agreement result
----------------------------------------------------------------------
contig020437-ZebOR.L093    5 NASI   0.6717     (8/9)   +
contig020437-ZebOR.L093    9 NVTH   0.7234     (9/9)   ++
----------------------------------------------------------------------
```

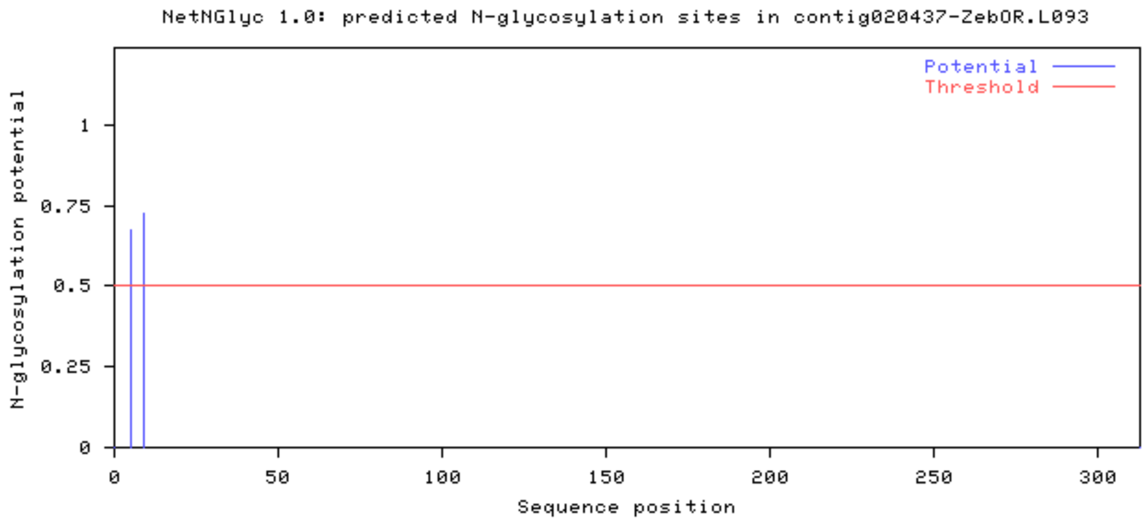

Graphics in PostScript

Output for 'contig020440-ZebOR.L094'

```
##########################################################################################

    Warning: This sequence may not contain a signal peptide!!

    Proteins without signal peptides are unlikely to be exposed to
    the N-glycosylation machinery and thus may not be glycosylated
    (in vivo) even though they contain potential motifs.

    SignalP-NN euk predictions are as follows:

  # name                 Cmax  pos ?  Ymax  pos ?  Smax  pos ?  Smean ?  D     ?

    SignalP output is explained at http://www.cbs.dtu.dk/services/SignalP/output.html

##########################################################################################

Name:  contig020440-ZebOR.L094  Length:  313
MSLQNASINVTHFIIGGFDTLSRPIAVGVVILITYLLAVLANMINIMFIISDKRLHKPMYLLICNLAVVDIVYTSSSSPT      80
MIGVLLAGVNTISYVACLIQMCVFNLGTSMESFVLAFMALDRFIAIIYPFQYQRYLTNTRVLVLTFIMWFIAWCFAFYMP     160
ATVVPLPHCSSRLKYSFCDFAAVIRTTCVNPEKYFNEVSIAAFFIFFFTFIFICLSYCGILLFVKLSSNNEKMKMGSTLV     240
SHLICVVVHYCPAFVRIMFTRFGVVLTLEERQGLVIGAVLGPCLVNPFVYCLRTKEIKQKLFKIFKKFNTSDX
....N...N.......................................................................      80
................................................................................     160
................................................................................     240
.........................................................................            320

(Threshold=0.5)
----------------------------------------------------------------------
SeqName      Position  Potential   Jury    N-Glyc
                                 agreement result
----------------------------------------------------------------------
contig020440-ZebOR.L094    5 NASI   0.6714     (8/9)   +
contig020440-ZebOR.L094    9 NVTH   0.7236     (9/9)   ++
contig020440-ZebOR.L094  309 NTSD   0.4221     (8/9)   -
----------------------------------------------------------------------
```

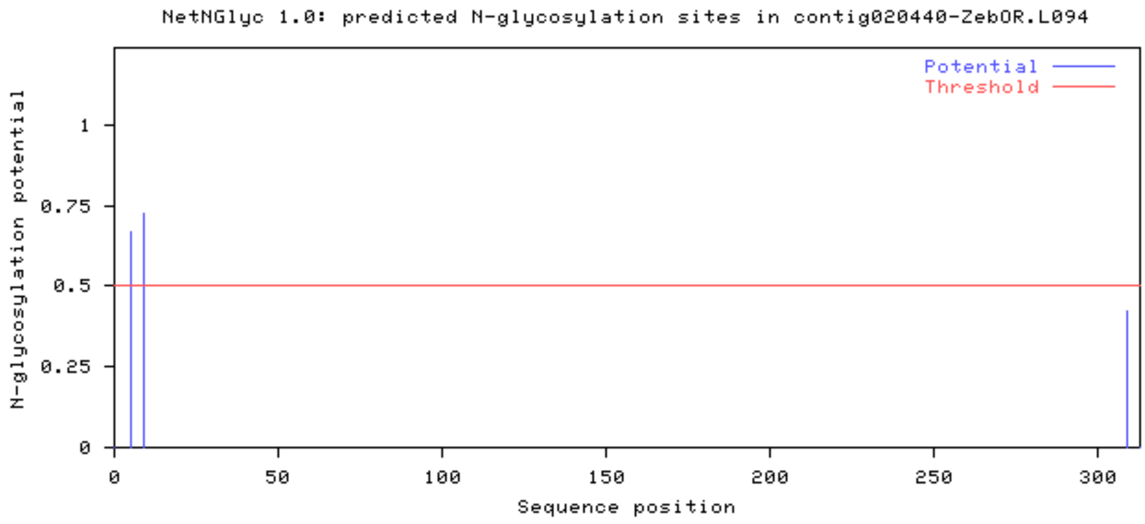

Graphics in PostScript

Output for 'contig020442-ZebOR.L095'

```
##########################################################################################

    Warning: This sequence may not contain a signal peptide!!

    Proteins without signal peptides are unlikely to be exposed to
    the N-glycosylation machinery and thus may not be glycosylated
    (in vivo) even though they contain potential motifs.

    SignalP-NN euk predictions are as follows:

  # name                 Cmax  pos ?  Ymax  pos ?  Smax  pos ?  Smean ?  D     ?

    SignalP output is explained at http://www.cbs.dtu.dk/services/SignalP/output.html

##########################################################################################

Name:  contig020442-ZebOR.L095  Length:  313
MSSQNTSINVTHFIIGGFDTLSRPIAVGVVILITYLLAVLANMANIMFIIADKRLHKPMYLLICNLAVVDIMYTSSCSPT      80
MIGVLLAGVNTISYMACLIQMCVFHLGTAMESFVLAVMALDRFIAIIYPFQYHSYLTNTRVLVLTFIVWFVNCFFMCYMP     160
ATAVPLPHCSSRLRYTFCDFAAVIRTTCVNPEKHFNDAAIIAFFILFFTFVFICLSYCGILLFVKLSSNNEKKKMGSTLV     240
SHLICAIVHYCPAFVRIIFTRFGVVLTLEERQGLLIGAVLGPCLVNPFVYFLRTKEIKQKLFKIFKKFYTYYX
....N...N.......................................................................      80
................................................................................     160
................................................................................     240
.........................................................................            320

(Threshold=0.5)
----------------------------------------------------------------------
SeqName      Position  Potential   Jury    N-Glyc
                                 agreement result
----------------------------------------------------------------------
contig020442-ZebOR.L095    5 NTSI   0.6202     (7/9)    +
contig020442-ZebOR.L095    9 NVTH   0.7423     (9/9)    ++
----------------------------------------------------------------------
```

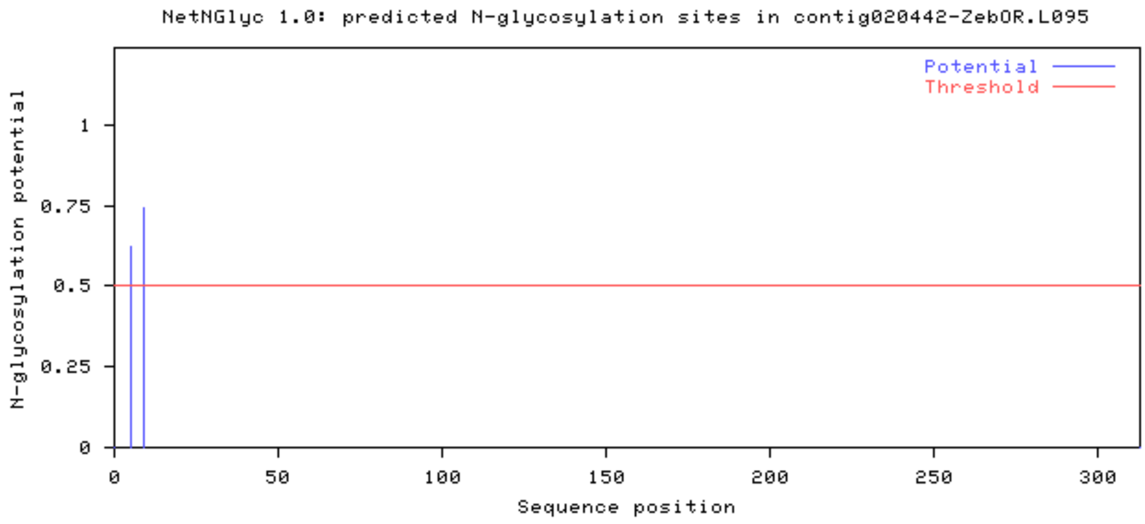

Graphics in PostScript

Output for 'contig020445-ZebOR.L096'

```
##########################################################################################

    Warning: This sequence may not contain a signal peptide!!

    Proteins without signal peptides are unlikely to be exposed to
    the N-glycosylation machinery and thus may not be glycosylated
    (in vivo) even though they contain potential motifs.

    SignalP-NN euk predictions are as follows:

  # name                    Cmax  pos ?  Ymax  pos ?  Smax  pos ?  Smean ?  D     ?

    SignalP output is explained at http://www.cbs.dtu.dk/services/SignalP/output.html

##########################################################################################

Name:  contig020445-ZebOR.L096  Length:  313
MSLQNASIKLTYFIIGGFDTVKRPVAVGVVMLITYLLAVFASLVNIIFIVSDKQLHKPMYLLICNLAVVDILYTSSSTPT      80
MIGVLLAGVNTISYVECIIQMYVYQVGATMEMFSLTIMAFDRLIAIIYPLQYHSYLTNTRTLVFTYIVWIVACSFALFPP     160
VTATPLPHCFLRLRYTFCDYGAVMRTTCVNPEKYFNQVAILSFFVSFFTFTFICLSYCGILFFVKILSNNDKKKMGSTLV     240
SHLICVSCLYCPQFIIVILTRFGVVLTLEERQGLLIGTILGPSLVNPFVYCLRTKEIKSKMFKIFRKINTAGX
....N...........................................................................      80
................................................................................     160
................................................................................     240
.........................................................................           320

(Threshold=0.5)
----------------------------------------------------------------------
SeqName      Position  Potential   Jury    N-Glyc
                                 agreement result
----------------------------------------------------------------------
contig020445-ZebOR.L096    5 NASI    0.5849     (6/9)    +
----------------------------------------------------------------------
```

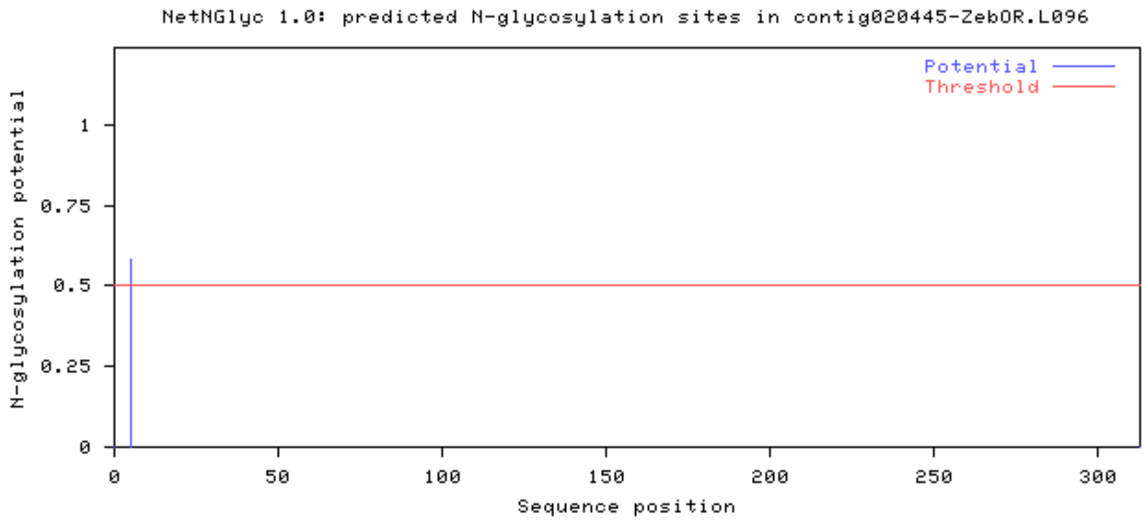

Graphics in PostScript

Output for 'contig025224-ZebOR.C035'

```
##########################################################################################

    Warning: This sequence may not contain a signal peptide!!

    Proteins without signal peptides are unlikely to be exposed to
    the N-glycosylation machinery and thus may not be glycosylated
    (in vivo) even though they contain potential motifs.

    SignalP-NN euk predictions are as follows:

  # name                Cmax  pos ?  Ymax  pos ?  Smax  pos ?  Smean ?  D     ?

    SignalP output is explained at http://www.cbs.dtu.dk/services/SignalP/output.html

##########################################################################################

Name:  contig025224-ZebOR.C035  Length:  321
MDNTTAATFKMTAYAVMENYKHGLFSVFFLLYLITLVLNVLLISVIHQNKQLHQPMNVFTCMLSLNEIYGSSALLPAVMA      80
VLVSKTHDVTVKWCMAQAYFLHTYASGEFCILALMGYDRYIAICSPLHYYSIMSYSKTCKLIAFTGLYPFIVFTSFYSLT     160
LQLRFCGKVMPKLYCVNMELVKNSCRNAQYISTVGLAILVLFIVPQLVMIVFSYTHILRVCRTFPKESQANAFRTCVPHL     240
LSLLNYTIASLFEVIQTRFNMSHVAVEAQIFLSLYFIIIPPIANPVLYGLGTQTVRGCIMKLFIKNKVMTTVLAKTLTVG     320
X
..N.............................................................................      80
................................................................................     160
................................................................................     240
....N...........................................................................     320
.                                                                                    400

(Threshold=0.5)
----------------------------------------------------------------------
SeqName      Position  Potential   Jury    N-Glyc
                                 agreement result
----------------------------------------------------------------------
contig025224-ZebOR.C035    3 NTTA   0.7208     (9/9)   ++
contig025224-ZebOR.C035  245 NYTI   0.6910     (8/9)   +
contig025224-ZebOR.C035  260 NMSH   0.4503     (7/9)   -
----------------------------------------------------------------------
```

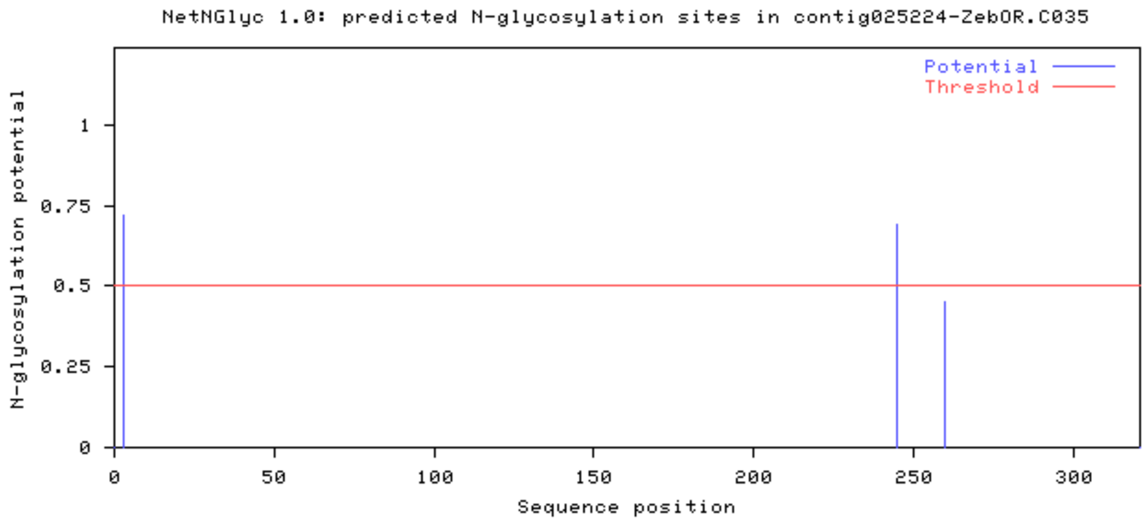

Graphics in PostScript

Output for 'contig025439-ZebOR.E045'

```
##########################################################################################

    Warning: This sequence may not contain a signal peptide!!

    Proteins without signal peptides are unlikely to be exposed to
    the N-glycosylation machinery and thus may not be glycosylated
    (in vivo) even though they contain potential motifs.

    SignalP-NN euk predictions are as follows:

  # name                  Cmax  pos ?  Ymax  pos ?  Smax  pos ?  Smean ?  D     ?

    SignalP output is explained at http://www.cbs.dtu.dk/services/SignalP/output.html

##########################################################################################

Name:  contig025439-ZebOR.E045  Length:  309
MTNLSQVSYFTFSAYFDTGPFKYLYFTIVMSLYVFIFGSNLLLIVVICVNRSLHEPMYMFLCSLFVNELYGSTGLFPFLL      80
LQILSDVHTVSAPLCFLQIFCLYTYANVEFYNLAIMSYDRYLAICCPLQYHTHMTFNKVAKLIVLTWLFPILNIVVMISL     160
NASLQLCGHTVDTLYCNNYSVVKLACFDTTINNIYGLMYTFTVLIGLALLNLFTYVKILKVCFSGSKQTRQKAVSTCTPH     240
LASLLNFSFGCFFEIVQSRFNLSRAPMILRIFLSIYFLTCQPAFNPVLYGLKLTKIRLICKSLLFGKIX
..N..............................................N..............................      80
................................................................................     160
N................N..............................................................     240
....................N................................................               320

(Threshold=0.5)
----------------------------------------------------------------------
SeqName      Position  Potential   Jury    N-Glyc
                                agreement result
----------------------------------------------------------------------
contig025439-ZebOR.E045    3 NLSQ    0.7482     (9/9)   ++
contig025439-ZebOR.E045   50 NRSL    0.7482     (9/9)   ++
contig025439-ZebOR.E045  161 NASL    0.6231     (7/9)   +
contig025439-ZebOR.E045  178 NYSV    0.6209     (9/9)   ++
contig025439-ZebOR.E045  246 NFSF    0.4717     (7/9)   -
contig025439-ZebOR.E045  261 NLSR    0.5960     (9/9)   ++
----------------------------------------------------------------------
```

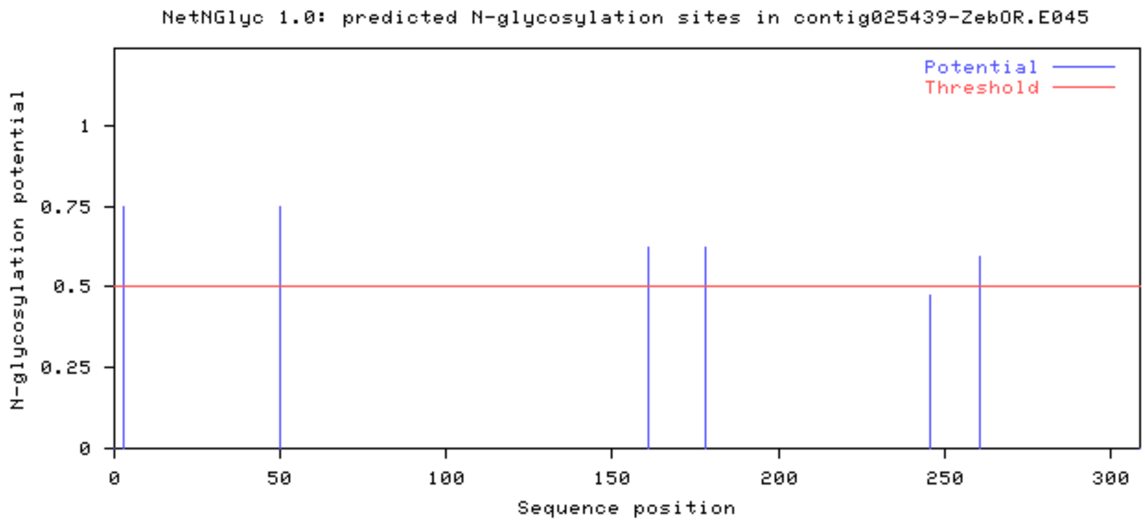

Graphics in PostScript

Output for 'contig025443-ZebOR.E046'

```
##########################################################################################

    Warning: This sequence may not contain a signal peptide!!

    Proteins without signal peptides are unlikely to be exposed to
    the N-glycosylation machinery and thus may not be glycosylated
    (in vivo) even though they contain potential motifs.

    SignalP-NN euk predictions are as follows:

  # name                Cmax  pos ?  Ymax  pos ?  Smax  pos ?  Smean ?  D     ?

    SignalP output is explained at http://www.cbs.dtu.dk/services/SignalP/output.html

##########################################################################################

Name:  contig025443-ZebOR.E046  Length:  320
MINTTQLSFFTLAGYFDTGRVTYLCFFVILALYIFIVGSNVLLIVVICVNRSLHEPMYMFLCSLFVNELYGSTGLFPSLL      80
VQILSDVHTVSASFCFLQIFCVYAYGSIEFSNLAVISYDRYLAICCPLRYHTCMSSSKVSVLIALTWLLTFFAISVLISL     160
SAPLQLCGNIINKVYCDNYSIVKLACSDTTANNIYGILYTFTLVLLVTLIFYTYMRILKVCFSGSKQMRHKAISTCTPHL     240
ASLLNFSCGAFFEIIQNRFDMRQLPNMLRIFLSLYWLTCQPLFNPVIYGLNLTKIRVSCKNVICRMKVFTLCHKSNFKIX     320
..N..............................................N..............................      80
................................................................................     160
.................N..............................................................     240
....N..............................................N............................     320

(Threshold=0.5)
----------------------------------------------------------------------
SeqName      Position  Potential   Jury    N-Glyc
                                agreement result
----------------------------------------------------------------------
contig025443-ZebOR.E046    3 NTTQ    0.6800     (9/9)    ++
contig025443-ZebOR.E046   50 NRSL    0.7496     (9/9)    ++
contig025443-ZebOR.E046  178 NYSI    0.5900     (8/9)    +
contig025443-ZebOR.E046  245 NFSC    0.5503     (6/9)    +
contig025443-ZebOR.E046  291 NLTK    0.6827     (9/9)    ++
----------------------------------------------------------------------
```

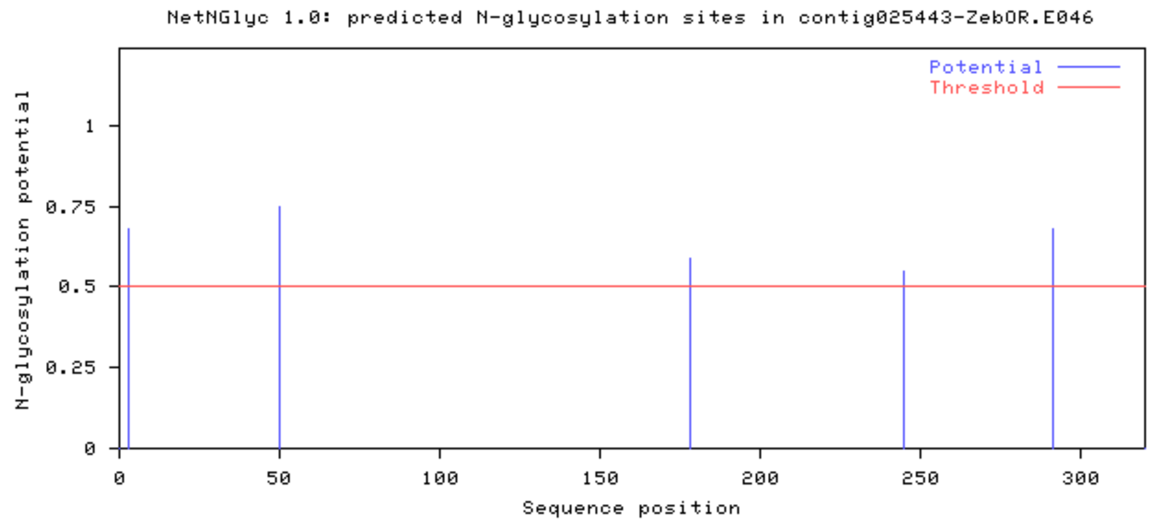

Graphics in PostScript

Output for 'contig025446-ZebORe.E061'

```
########################################################################################

    Warning: This sequence may not contain a signal peptide!!

    Proteins without signal peptides are unlikely to be exposed to
    the N-glycosylation machinery and thus may not be glycosylated
    (in vivo) even though they contain potential motifs.

    SignalP-NN euk predictions are as follows:

  # name                Cmax  pos ?  Ymax  pos ?  Smax  pos ?  Smean ?  D     ?

    SignalP output is explained at http://www.cbs.dtu.dk/services/SignalP/output.html

########################################################################################

Name:  contig025446-ZebORe.E061         Length:  216
ICFLQVFCVHSYGAVEYLNLAIMSYDRYLAICCPLQYNTHMTSKKIAILIAATWFYPCFAMAPLLYLTSPLQLCGNTIYK      80
VYCDTHSVVKLACSDTTVINLYGLLATFSTIFGALLFILYTYMKILLVCFSGSDQTRQKAISTCTPHLASILNFSFGASF     160
EILQSRFNMKNVPNMLRIFLSLYFLTCQPLFNPVMYGLKMTKIRNICKSLITNIKX
................................................................................      80
................................................................................     160
........................................................                             240

(Threshold=0.5)

No sites predicted in this sequence.
```

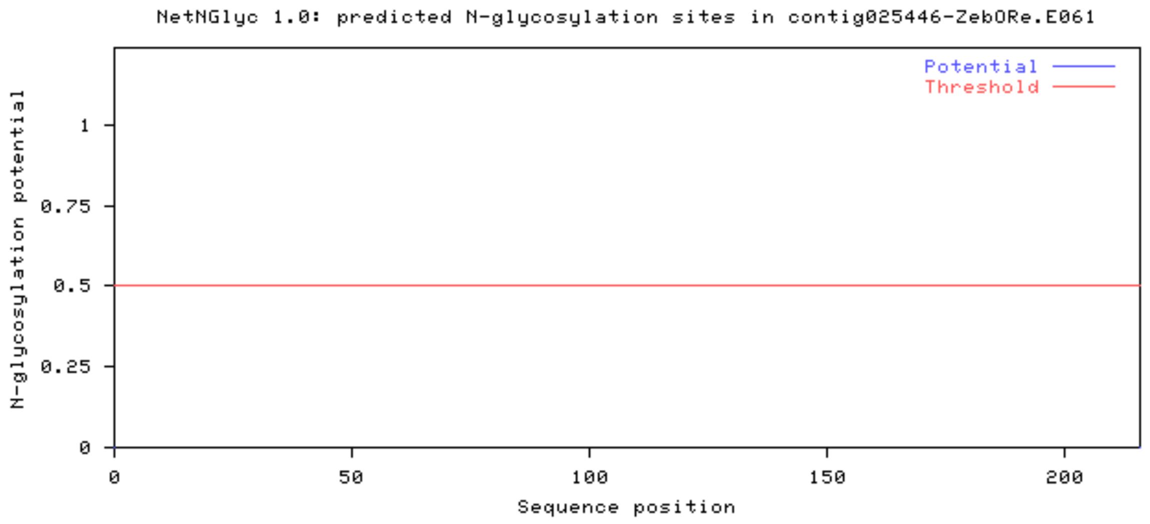

Graphics in PostScript

Output for 'contig025447-ZebOR.E047'

```
##########################################################################################

    Warning: This sequence may not contain a signal peptide!!

    Proteins without signal peptides are unlikely to be exposed to
    the N-glycosylation machinery and thus may not be glycosylated
    (in vivo) even though they contain potential motifs.

    SignalP-NN euk predictions are as follows:

  # name                    Cmax  pos ?  Ymax  pos ?  Smax  pos ?  Smean ?  D     ?

    SignalP output is explained at http://www.cbs.dtu.dk/services/SignalP/output.html

##########################################################################################

Name:  contig025447-ZebOR.E047  Length:  310
MKNSTQLSDFILGAYFDGGTFRYLYFTIVMSLYVFIFGSNLLLIVVICVNRSLHEPMYMFLCSLFVNELYGSTGLFPFLL      80
VQILSDVHTVSAPLCFLQVFSVYSYGSIEFLNLAVMSYDRYLAICCPLQYNKLMTSNKVTKLIVAVWSPPLFLNFLTLPL     160
IVPLKRCGNIINKVYCDNHSIVKLACSDTTLNNIYGLTVCALSVFGPLIVILYTYMRILKVCFSGSKQTRQKAVSTCTPH     240
LASLLNFSFGACFEILQSRFNMNSSPNMLRIFLSLYFLTCPPLFNPLMYGLNLSKIRVTCKNLITHIICX
..N..............................................N..............................      80
................................................................................     160
................................................................................     240
...................................................N..................                320

(Threshold=0.5)
----------------------------------------------------------------------
SeqName      Position  Potential   Jury    N-Glyc
                                 agreement result
----------------------------------------------------------------------
contig025447-ZebOR.E047    3 NSTQ   0.5981     (9/9)   ++
contig025447-ZebOR.E047   50 NRSL   0.7480     (9/9)   ++
contig025447-ZebOR.E047  178 NHSI   0.4333     (7/9)   -
contig025447-ZebOR.E047  246 NFSF   0.4126     (7/9)   -
contig025447-ZebOR.E047  263 NSSP   0.1308     (9/9)   ---
contig025447-ZebOR.E047  292 NLSK   0.6552     (8/9)   +
----------------------------------------------------------------------
```

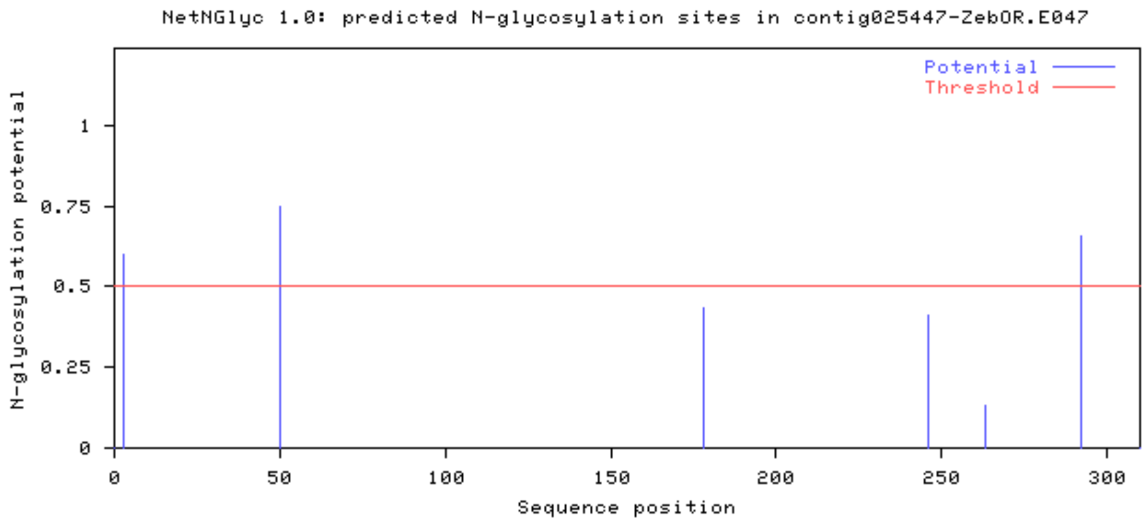

Graphics in PostScript

Output for 'contig025452-ZebOR.P123'

```
##########################################################################

    Warning: This sequence may not contain a signal peptide!!

    Proteins without signal peptides are unlikely to be exposed to
    the N-glycosylation machinery and thus may not be glycosylated
    (in vivo) even though they contain potential motifs.

    SignalP-NN euk predictions are as follows:

  # name                Cmax  pos ?  Ymax  pos ?  Smax  pos ?  Smean ?  D     ?

    SignalP output is explained at http://www.cbs.dtu.dk/services/SignalP/output.html

##########################################################################

Name:  contig025452-ZebOR.P123  Length:  318
MENVSLHTHFILDGFSELGELRPFLFIPFSFMFVVSLFANSLLVYVIVSQRSLHSPMYILIASMACIDLSLPVFFVPHML      80
LSFLFDWRGISLIGCLVQMHFIHFFGTFQSTLLVWMALDRYFAICTPLYYHNHMILSRFIAFLIPLVVRNVLMITLLVCL     160
AGKLPFCFRNVINHCFCEHMALVELACGSTTINNLVGLMAVFLIPVLDFVFIAASYVVIFSSVLKSSRSGVKALHTCITH     240
IMVITVSLILALTAFLSYRIRNGLPAASRVFFSTMYLLFPSCFNPVVYGIRTNEIRQHILKRLTCCHICQMSPVTNKX
..N.............................................................................      80
................................................................................     160
................................................................................     240
..............................................................................       320

(Threshold=0.5)
----------------------------------------------------------------------
SeqName      Position  Potential   Jury    N-Glyc
                                 agreement result
----------------------------------------------------------------------
contig025452-ZebOR.P123    3 NVSL    0.7638     (9/9)    +++
----------------------------------------------------------------------
```

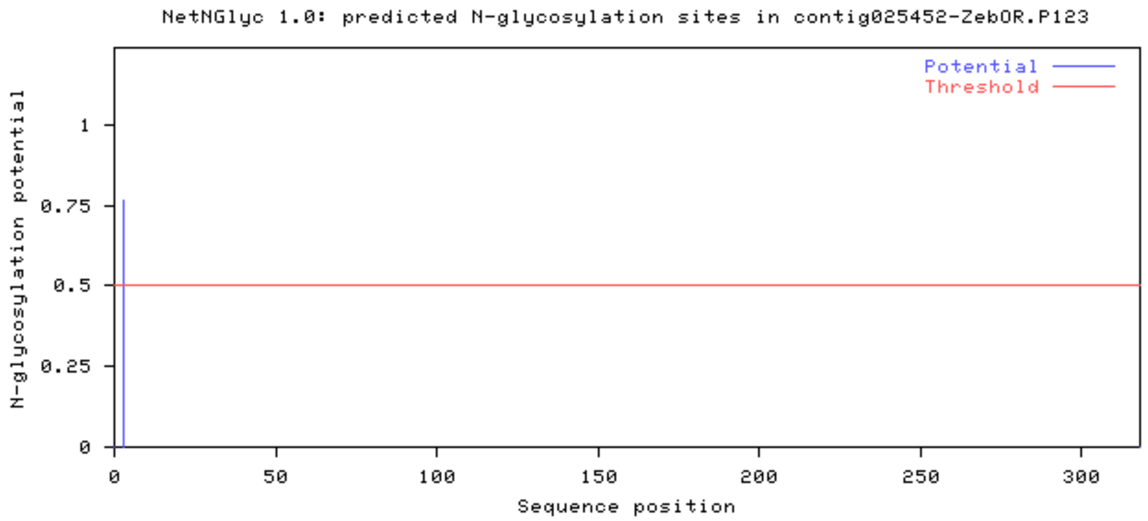

[Graphics in PostScript]

Output for 'contig025453-ZebOR.P124'

```
##########################################################################################

    Warning: This sequence may not contain a signal peptide!!

    Proteins without signal peptides are unlikely to be exposed to
    the N-glycosylation machinery and thus may not be glycosylated
    (in vivo) even though they contain potential motifs.

    SignalP-NN euk predictions are as follows:

  # name                 Cmax  pos ?  Ymax  pos ?  Smax  pos ?  Smean ?  D     ?

    SignalP output is explained at http://www.cbs.dtu.dk/services/SignalP/output.html

##########################################################################################

Name:  contig025453-ZebOR.P124  Length:  310
MEAVKENISSHKYFFLDGFSELGELRPFLFIPFSFMFVVSLFANSLLVYVIVSQRSLHSPMYILIASMACIDLSLPVFFV      80
PHMLLSFLFDWRGISLIGCLVQMYFIHLLGAFQSTLLLWMALDRYFAICTPLHYQEQMALARFLKFVIPFSIRNMFAVLV     160
VVSLAGKLPFCLRNVINHCFCEHMALVELACGSTTINSLVGLISVFSVPVTDFFLITASYTVIFSSVLSSGKSSAKALHT     240
CVTHIVVMTVSLTIILTAFLSYRIRNSLPAAIRIFFSILYLFFPSCFNPIIYGIRTKEIRQHILNTLRVX
......N.........................................................................      80
................................................................................     160
................................................................................     240
......................................................................              320

(Threshold=0.5)
----------------------------------------------------------------------
SeqName      Position  Potential   Jury    N-Glyc
                                 agreement result
----------------------------------------------------------------------
contig025453-ZebOR.P124    7 NISS    0.6872     (9/9)    ++
----------------------------------------------------------------------
```

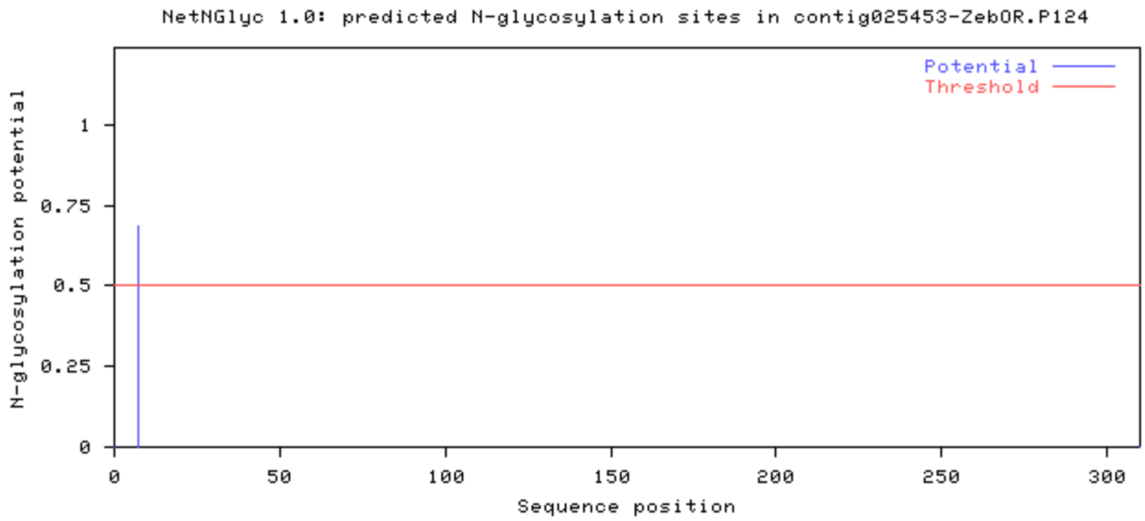

Graphics in PostScript

Output for 'contig025458-ZebOR.P125'

```
##########################################################################################

    Warning: This sequence may not contain a signal peptide!!

    Proteins without signal peptides are unlikely to be exposed to
    the N-glycosylation machinery and thus may not be glycosylated
    (in vivo) even though they contain potential motifs.

    SignalP-NN euk predictions are as follows:

  # name                 Cmax  pos ?  Ymax  pos ?  Smax  pos ?  Smean ?  D     ?

    SignalP output is explained at http://www.cbs.dtu.dk/services/SignalP/output.html

##########################################################################################

Name:  contig025458-ZebOR.P125  Length:  332
MLEAPLSRNFSHCTFVLRGFPSLQKHRRLLALPFSASYLLVLLGNSLLVYVICSVERLHSPMYLLICTLCFVDILVVTTI      80
IPNMLLGFLFDWNEISLVGCLTQMFFIHFLSSVESTLLLAMALDRYFAICRPLRYNEIINSSMLVRLVLFTLVRSVSIMA     160
TLVGLAGSLQFCGSNVIQHCYCDHMALVSLACDSTSRSSAAGLAVIICFVGADIPIIFFSYMKILSVVLRSAAAGEDSRK     240
AFHTCSTHLIVMMCFYLVGSITFLSHNLNIPIPTDINNSMGLMYILFPATINPIIYGVRTKEIRDSFLRIFKNRAKKIMT     320
AKVSSAGKEKSX
........N.......................................................................      80
................................................................................     160
................................................................................     240
................................................................................     320
............                                                                         400

(Threshold=0.5)
----------------------------------------------------------------------
SeqName      Position  Potential   Jury    N-Glyc
                                 agreement result
----------------------------------------------------------------------
contig025458-ZebOR.P125    9 NFSH    0.6082     (9/9)   ++
contig025458-ZebOR.P125  140 NSSM    0.4788     (5/9)   -
contig025458-ZebOR.P125  277 NNSM    0.2283     (9/9)   ---
----------------------------------------------------------------------
```

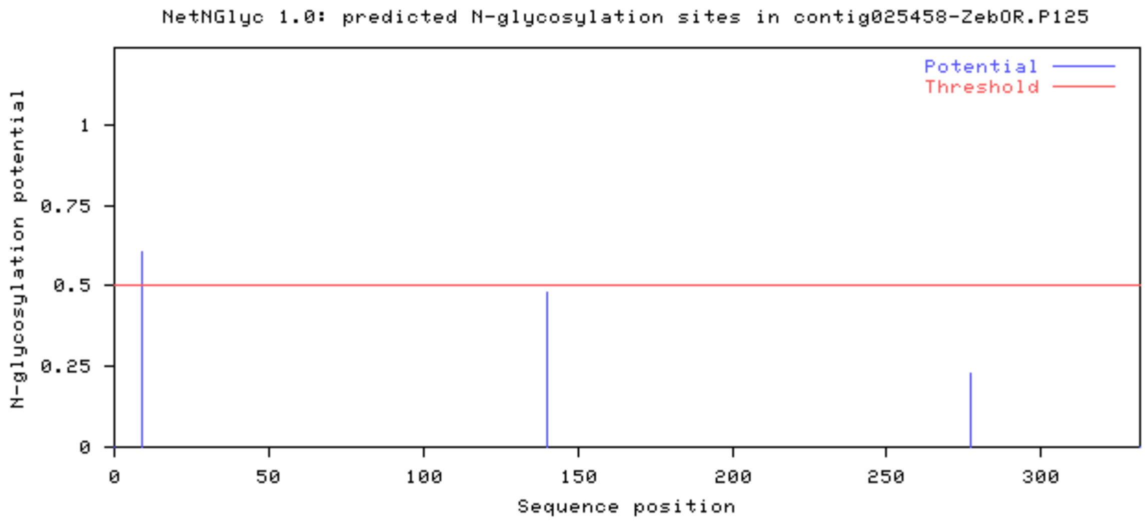

Graphics in PostScript

Output for 'contig025841-ZebORs.W139'

```
##########################################################################

   Warning: This sequence may not contain a signal peptide!!

   Proteins without signal peptides are unlikely to be exposed to
   the N-glycosylation machinery and thus may not be glycosylated
   (in vivo) even though they contain potential motifs.

   SignalP-NN euk predictions are as follows:

 # name                 Cmax  pos ?  Ymax  pos ?  Smax  pos ?  Smean ?  D     ?

   SignalP output is explained at http://www.cbs.dtu.dk/services/SignalP/output.html

##########################################################################

Name:  contig025841-ZebORs.W139         Length:  315
MNLTQVDSNATITLNYWKTFVKAVGKNVTVVVLGITINYINATMIHTFNKYHQIFKLNPRYILFIHLVLNDIIQLSTSIS      80
LFIFSYAFHTIHVSLCLLLILPAIFTTQNTPLNIAFMAAECCVSVCIPLRYSYICTVKRTYIVIGIIWAISSLSILPDLF     160
ILLAVESSQFLQSRVVCNRDSVFRSSYSVKKRDASHTLFLVLVSVTLLYTYCQILFVARCADSDTKKARNTILIHGFQVL     240
LCTTVYVQPPLIKLLVYFFPEGLSDINFATFVINQVLPRLGSPIIYGLRDKTFRKFLKRHLCAVNNLPKIIRSSX
.N......N.................N.............N.......................................      80
................................................................................     160
................................................................................     240
...........................................................................          320

(Threshold=0.5)
----------------------------------------------------------------------
SeqName        Position  Potential   Jury    N-Glyc
                                   agreement result
----------------------------------------------------------------------
contig025841-ZebORs.W139    2 NLTQ   0.8093     (9/9)   +++
contig025841-ZebORs.W139    9 NATI   0.6162     (8/9)   +
contig025841-ZebORs.W139   27 NVTV   0.7229     (9/9)   ++
contig025841-ZebORs.W139   41 NATM   0.5420     (5/9)   +
----------------------------------------------------------------------
```

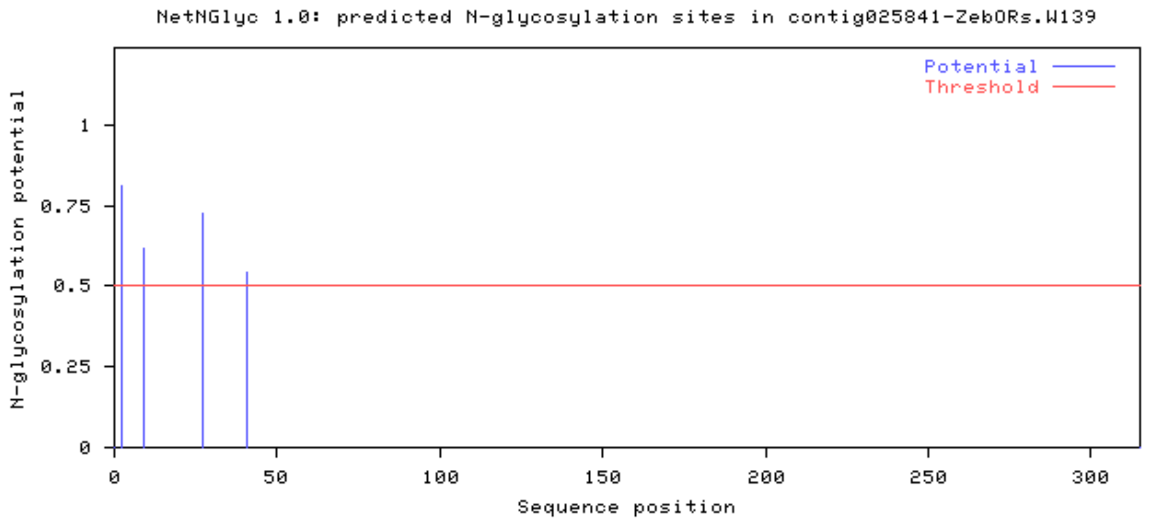

Graphics in PostScript

Output for 'contig025842-ZebORs.W142'

```
##########################################################################################

    Warning: This sequence may not contain a signal peptide!!

    Proteins without signal peptides are unlikely to be exposed to
    the N-glycosylation machinery and thus may not be glycosylated
    (in vivo) even though they contain potential motifs.

    SignalP-NN euk predictions are as follows:

  # name                    Cmax  pos ?  Ymax  pos ?  Smax  pos ?  Smean ?  D     ?

    SignalP output is explained at http://www.cbs.dtu.dk/services/SignalP/output.html

##########################################################################################

Name:  contig025842-ZebORs.W142         Length:  318
MNSSSYALNASSSLNYRDSPSAAVAKNVIVLALGFTINYINGTLIHTFRKHQIFYLNPRYILFIHLVVNDMIQLTSSISL      80
FVFTYIFYQINVAFCCFLITLAIFTTFNTPINLALMALECYIAICLPLQHAEYCTTKRTYVAIGWIWAMSAVSALSDIVI     160
ILATEPVELFYSTIQCERDNLFRHPIIVKKKEVSYLIFLIGVLLTFMYTYFRIFFAANKAKSAKRESKKARNTILLHSFQ     240
LLLSMLTYIATALMQALVRWFPKQYLIVVFISYIIIYIVPRFVSPIVYGLRDKTFKQYLKKYLLCTMKMGNETDFRLX
.N......N...............................N.......................................      80
................................................................................     160
................................................................................     240
......................................................................N.......       320

(Threshold=0.5)
----------------------------------------------------------------------
SeqName      Position  Potential   Jury    N-Glyc
                                 agreement result
----------------------------------------------------------------------
contig025842-ZebORs.W142     2 NSSS   0.7219     (9/9)   ++
contig025842-ZebORs.W142     9 NASS   0.6336     (8/9)   +
contig025842-ZebORs.W142    41 NGTL   0.7140     (9/9)   ++
contig025842-ZebORs.W142   311 NETD   0.6714     (9/9)   ++
----------------------------------------------------------------------
```

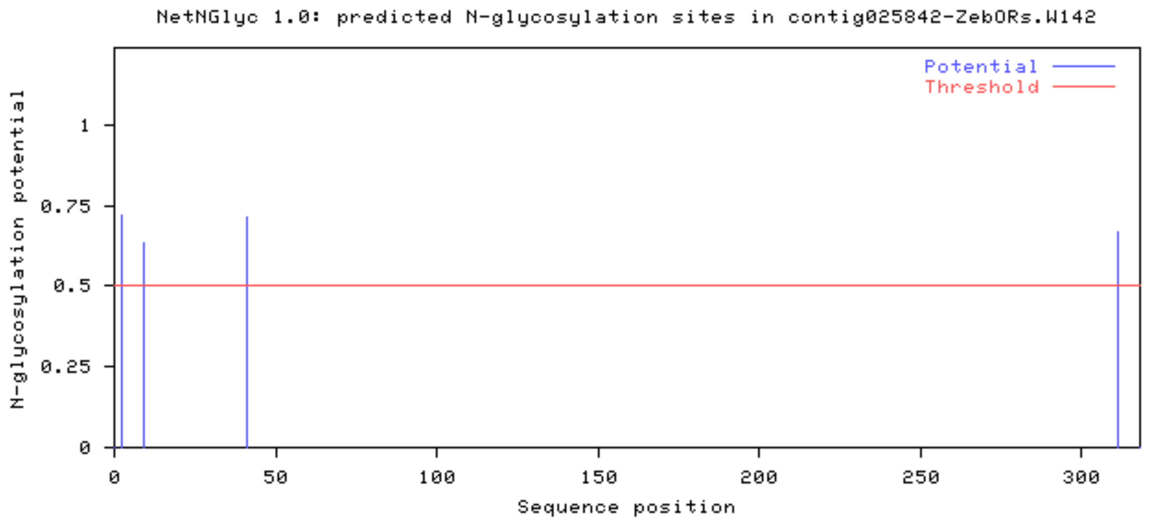

Graphics in PostScript

Output for 'contig025847-ZebORs.U137'

```
##########################################################################################

    Warning: This sequence may not contain a signal peptide!!

    Proteins without signal peptides are unlikely to be exposed to
    the N-glycosylation machinery and thus may not be glycosylated
    (in vivo) even though they contain potential motifs.

    SignalP-NN euk predictions are as follows:

  # name                 Cmax  pos ?  Ymax  pos ?  Smax  pos ?  Smean ?  D     ?

    SignalP output is explained at http://www.cbs.dtu.dk/services/SignalP/output.html

##########################################################################################

Name:  contig025847-ZebORs.U137         Length:  330
MSSLLGLRGNMTVPYQVIRDTFTTAFVKNLIVVLVWLTLSYINGTLVVTFFRHQFLLQTFHDDPRYILFIHMVINDAIQL      80
TVTIMLFILSYIFYKINVAFCCFFILVAVFTTRNTPVNLAAMAIERYIAICEPLRYTQICTVRRTYIVIGMIWFICVAPD     160
ITDLFVTLATESLSFFHESVFCLRQNVFKDPILAYKRQAVDIIYFSCVFLILVVTYLRILFAARALSTDKTSAQKARNTI     240
LLHGAQLAMCMLSYVSPSVEVVLHIIFPSRILKIRFANYLIVYILPRFLSPIIYGVRDKKFREYLRMDFLSNRCRNKVKK     320
VTPEDKDHLX
.........N................................N.....................................      80
................................................................................     160
................................................................................     240
................................................................................     320
..........                                                                           400

(Threshold=0.5)
----------------------------------------------------------------------
SeqName      Position  Potential   Jury    N-Glyc
                                 agreement result
----------------------------------------------------------------------
contig025847-ZebORs.U137    10 NMTV   0.7828     (9/9)    +++
contig025847-ZebORs.U137    43 NGTL   0.7600     (9/9)    +++
----------------------------------------------------------------------
```

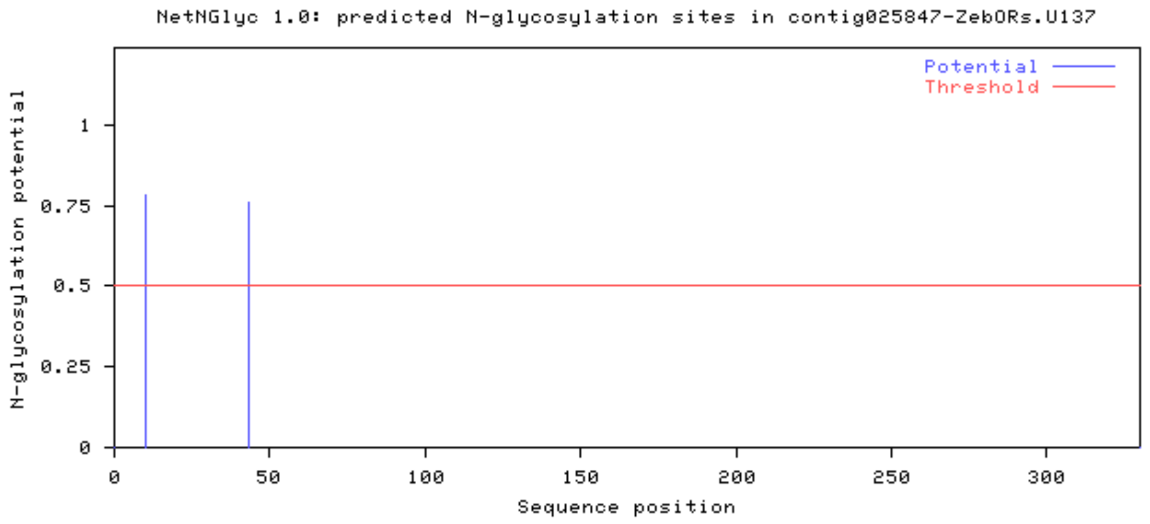

Graphics in PostScript

Output for 'contig025847-ZebORs.V149'

```
##########################################################################

    Warning: This sequence may not contain a signal peptide!!

    Proteins without signal peptides are unlikely to be exposed to
    the N-glycosylation machinery and thus may not be glycosylated
    (in vivo) even though they contain potential motifs.

    SignalP-NN euk predictions are as follows:

  # name                 Cmax  pos ?  Ymax  pos ?  Smax  pos ?  Smean ?  D     ?

    SignalP output is explained at http://www.cbs.dtu.dk/services/SignalP/output.html

##########################################################################

Name:  contig025847-ZebORs.V149         Length:  339
MLVMNSTSTGPLDPLVSNTTSVQTQGVKESFSSILTKNIVAMLVWLVLSIINGSMVHTFQRHSLFYENPRYIMFISMVIN      80
DALQLTLVTALYVISYIFRKIHASVCCLLVIMTAVLTTRSTPLILAGMAVERYISICFPLHYSQMCNIPRTLLLICVIVI     160
LTITPPITDLLITIVKEPPSFFHTKIFCDHSLLFRDQSIYYKNCVFDGTYLSFVALALLYTYCKIMLTAQAVSTSLVSVK     240
RARNTVLLHGVQVVVPSLQAALISLFPQLSLEIRYIFFLLVYIIPRFLSPMIYGFRDEQFRKYWTRYLSCHEHSVTLLRL     320
ASQKMNPQVKQSGNLSSTX
....N............N.................................N............................      80
................................................................................     160
................................................................................     240
................................................................................     320
.............N.....                                                                  400

(Threshold=0.5)
----------------------------------------------------------------------
SeqName        Position  Potential   Jury    N-Glyc
                                  agreement result
----------------------------------------------------------------------
contig025847-ZebORs.V149    5 NSTS   0.7686     (9/9)   +++
contig025847-ZebORs.V149   18 NTTS   0.7452     (9/9)   ++
contig025847-ZebORs.V149   52 NGSM   0.6339     (8/9)   +
contig025847-ZebORs.V149  334 NLSS   0.6434     (8/9)   +
----------------------------------------------------------------------
```

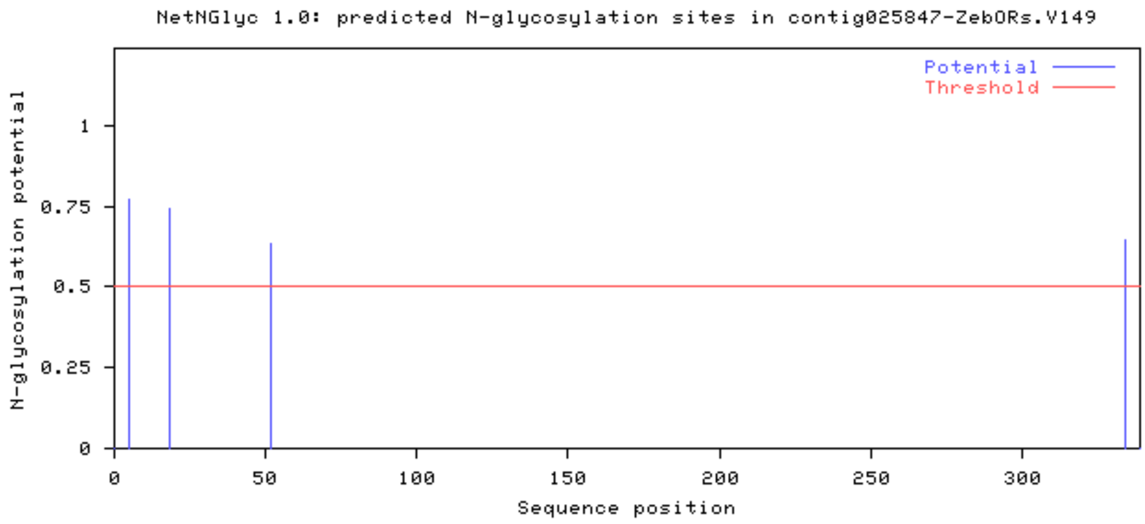

Graphics in PostScript

Output for 'contig026932-ZebORs.I082'

```
##########################################################################################

    Warning: This sequence may not contain a signal peptide!!

    Proteins without signal peptides are unlikely to be exposed to
    the N-glycosylation machinery and thus may not be glycosylated
    (in vivo) even though they contain potential motifs.

    SignalP-NN euk predictions are as follows:

  # name                 Cmax  pos ?  Ymax  pos ?  Smax  pos ?  Smean ?  D     ?

    SignalP output is explained at http://www.cbs.dtu.dk/services/SignalP/output.html

##########################################################################################

Name:  contig026932-ZebORs.I082         Length:  313
MENLTMATPLKQPIVFELEGFYIPPGFGPLLFFLALFTYMLVLLGNGVIVSVIVIDKNLHRPMFVMVCHLLVCDLLGSTA      80
VLPGLMMHFLMGQKRIAYIPAIAQAFSVHTYGLAVQAILGVMAYDRYIAVCEPLRYHAIMTSAWLHSCCALAWLLALLPI     160
AVLFSFHMNVPLCGRVILHVYCSNRGILGLACIPTPASDIGLAMTWTVSTGIFLIIAFSYIRILQVSLKHSRIDTSIRSK     240
AFQTCASHLVVYVLYQIASVIIIVSYRFPSVSENLKKFFSILFIIVPPAINPIIYGLVSKELRSSIIKHFTIX
..N.............................................................................      80
................................................................................     160
................................................................................     240
.........................................................................            320

(Threshold=0.5)
----------------------------------------------------------------------
SeqName      Position  Potential   Jury    N-Glyc
                                agreement result
----------------------------------------------------------------------
contig026932-ZebORs.I082     3 NLTM   0.7364     (9/9)   ++
----------------------------------------------------------------------
```

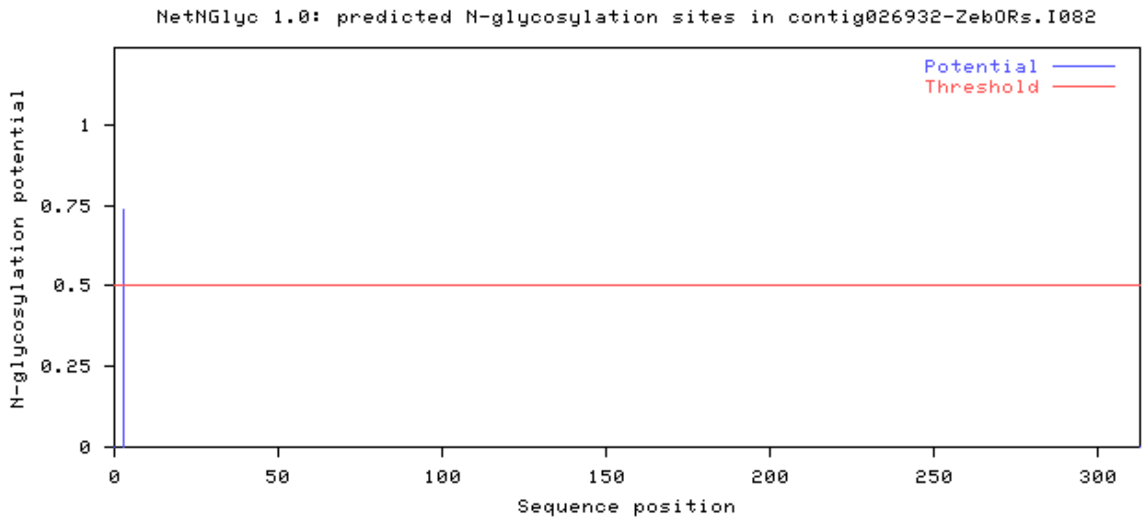

Graphics in PostScript

Output for 'contig030011-ZebOR.H073'

```
##########################################################################################

    Warning: This sequence may not contain a signal peptide!!

    Proteins without signal peptides are unlikely to be exposed to
    the N-glycosylation machinery and thus may not be glycosylated
    (in vivo) even though they contain potential motifs.

    SignalP-NN euk predictions are as follows:

  # name                    Cmax  pos ?  Ymax  pos ?  Smax  pos ?  Smean ?  D     ?

    SignalP output is explained at http://www.cbs.dtu.dk/services/SignalP/output.html

##########################################################################################

Name:  contig030011-ZebOR.H073  Length:  324
MNNVSVITMFFLSGFNETVSHRFVLFFLSLLCYCIICLLNVSLIVIIILDSNLHESMYILLCVFCINALYGTAGFYPKFL      80
WDLLSDVHLISYYGCLIQTKVIFSFVCGELSILALMAYDRYVAICQPLKYHSIMSKQRVIRFACFLWLTTFCIMAVNVFL     160
TSRLKLCSPYISRLFCMNWSIVQLACFPEQTAINAISANIVTIIYLLHGVFIVWSYMYIIQTCVKSIENRAKFMQTCVPH     240
LVSLFTFVVAVLTDVISMRLGSKELPRSLQNFVALEFLVIPPIMNPLIYGFKLTKIRKKIYSVVILKRTNFCFIRSENSF     320
THSX
..N............N.......................N........................................      80
................................................................................     160
.................N..............................................................     240
................................................................................     320
....                                                                                 400

(Threshold=0.5)
----------------------------------------------------------------------
SeqName       Position  Potential    Jury     N-Glyc
                                   agreement result
----------------------------------------------------------------------
contig030011-ZebOR.H073    3 NVSV   0.7557     (9/9)    +++
contig030011-ZebOR.H073   16 NETV   0.7037     (9/9)    ++
contig030011-ZebOR.H073   40 NVSL   0.7032     (9/9)    ++
contig030011-ZebOR.H073  178 NWSI   0.6432     (8/9)    +
----------------------------------------------------------------------
```

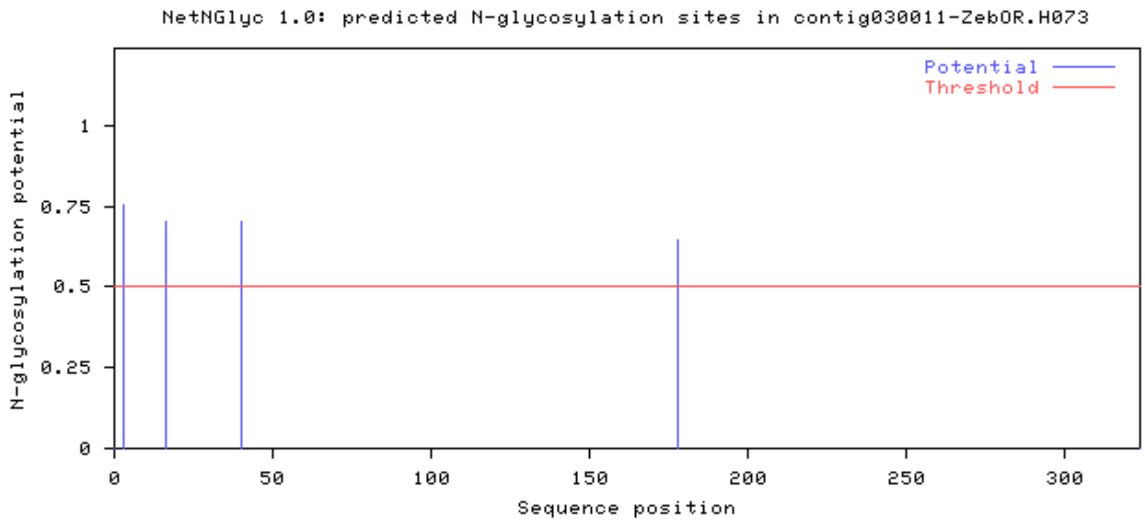

Graphics in PostScript

Output for 'contig030552-ZebOR.A001'

```
##########################################################################

   Warning: This sequence may not contain a signal peptide!!

   Proteins without signal peptides are unlikely to be exposed to
   the N-glycosylation machinery and thus may not be glycosylated
   (in vivo) even though they contain potential motifs.

   SignalP-NN euk predictions are as follows:

 # name                Cmax  pos ?  Ymax  pos ?  Smax  pos ?  Smean ?  D     ?

   SignalP output is explained at http://www.cbs.dtu.dk/services/SignalP/output.html

##########################################################################

Name:  contig030552-ZebOR.A001  Length:  309
MELALNVSYITLDGFFQVNKYRYLYFMIMFTLYILILCCNFAIVFLIVVEKSLHEPMYIFIAALLLNSVMLSTVIYPKLL      80
TDFLSKRQIISYSVCLFQFFMFYSLGGSEFLLLFAMAYDRYVSICKPLQYPMIMTKNTISIFLTLAWIVPSSQVAVVAVL     160
MANKKICNFTFTGIFCNNTIYKLLCVYSKAQTVYDMVVLSNVAILPAVFIFFTYTRILVISYQSCKEVRRKAAQTCLPHL     240
IVLISYLCLCAFDVIVSGLESNFPKIVHSILTLQIVMYPPLFNPIIYGLKMKEISKHLKRLFCAVKKNX
.....N..........................................................................      80
................................................................................     160
.......N........N...............................................................     240
.....................................................................                320

(Threshold=0.5)
----------------------------------------------------------------------
SeqName      Position  Potential   Jury    N-Glyc
                                 agreement result
----------------------------------------------------------------------
contig030552-ZebOR.A001    6 NVSY    0.8398     (9/9)    +++
contig030552-ZebOR.A001  168 NFTF    0.6488     (8/9)    +
contig030552-ZebOR.A001  177 NNTI    0.5013     (4/9)    +
----------------------------------------------------------------------
```

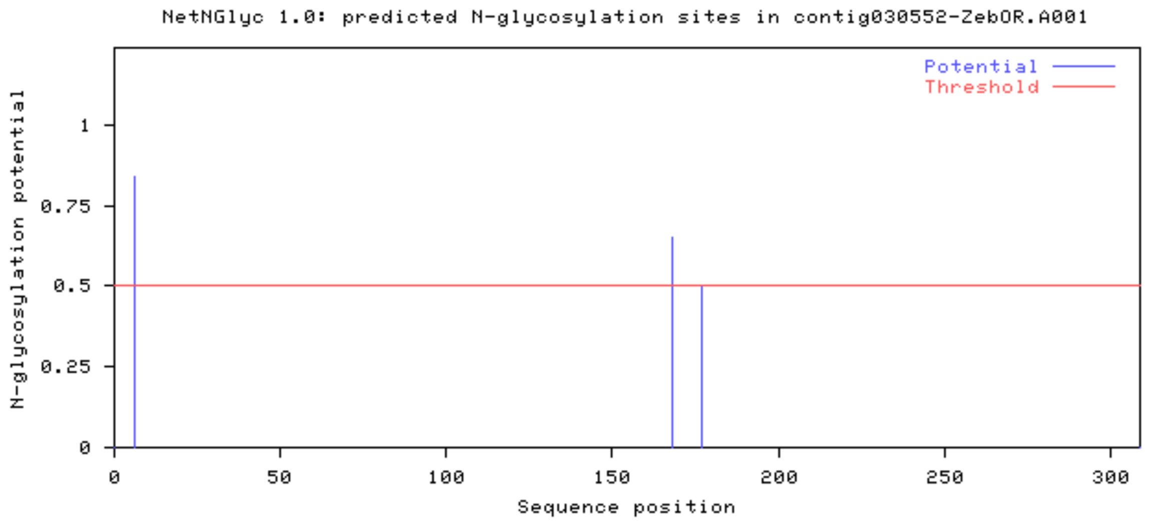

Graphics in PostScript

Output for 'contig030553-ZebOR.A002'

```
##########################################################################################

    Warning: This sequence may not contain a signal peptide!!

    Proteins without signal peptides are unlikely to be exposed to
    the N-glycosylation machinery and thus may not be glycosylated
    (in vivo) even though they contain potential motifs.

    SignalP-NN euk predictions are as follows:

  # name                   Cmax  pos ?  Ymax  pos ?  Smax  pos ?  Smean ?  D     ?

    SignalP output is explained at http://www.cbs.dtu.dk/services/SignalP/output.html

##########################################################################################

Name:  contig030553-ZebOR.A002  Length:  307
MNADLNTTYVTLGGHVEIHRYRYLYFVIMLTAYILLICFNVSIICLIVIHKNLHEPMYIFIAALLLNSILFSSNIHPKLL      80
VDFLSEKQIVSYQACLFQVFMFYFLSSSEFLLLSAMAYDRYVSICKPLQYPAIMRTTTVSLLLCFAWLVPACYIVVPVAL     160
NINSKLCSFTLKGIFCNNSLNKLFCVTSNELSIYGVIVLLNLGLFPMLFILFTYTKIIIIAFQSCGDIRRKAVQTCLPHL     240
LVLINYSVLITYDVVIVKLESDFPKTARFVMTLQIITYNPLCNPIIYGLKMKEISNHLKRLLRHMKX
.....N.................................N........................................      80
................................................................................     160
................................................................................     240
....N..............................................................                  320

(Threshold=0.5)
----------------------------------------------------------------------
SeqName      Position  Potential   Jury    N-Glyc
                                 agreement result
----------------------------------------------------------------------
contig030553-ZebOR.A002    6 NTTY   0.7239     (9/9)    ++
contig030553-ZebOR.A002   40 NVSI   0.7863     (9/9)    +++
contig030553-ZebOR.A002  177 NNSL   0.4236     (7/9)    -
contig030553-ZebOR.A002  245 NYSV   0.5312     (6/9)    +
----------------------------------------------------------------------
```

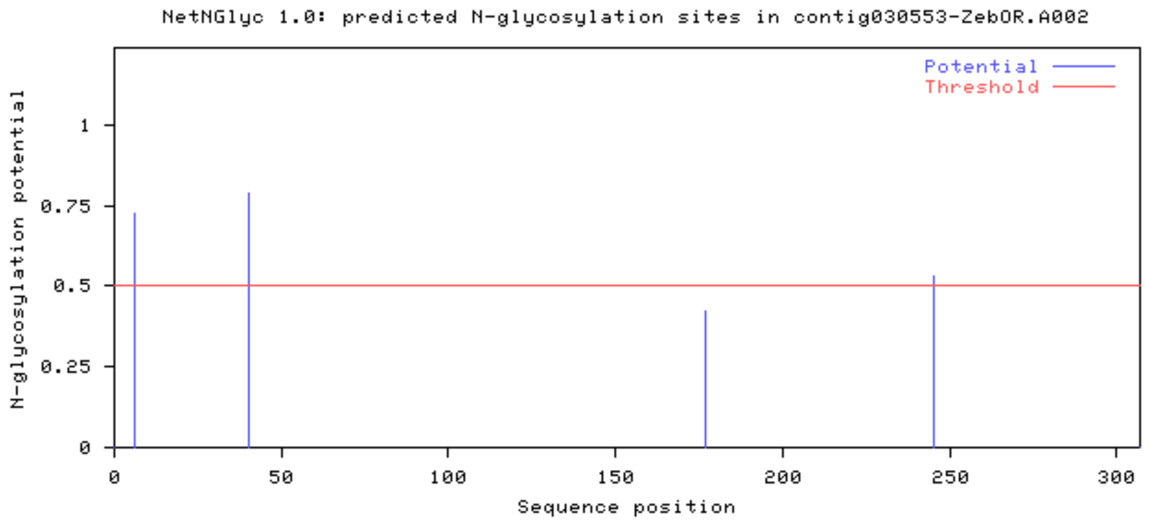

Graphics in PostScript

Output for 'contig030553-ZebOR.A003'

```
##########################################################################################

    Warning: This sequence may not contain a signal peptide!!

    Proteins without signal peptides are unlikely to be exposed to
    the N-glycosylation machinery and thus may not be glycosylated
    (in vivo) even though they contain potential motifs.

    SignalP-NN euk predictions are as follows:

  # name                    Cmax  pos ?  Ymax  pos ?  Smax  pos ?  Smean ?  D     ?

    SignalP output is explained at http://www.cbs.dtu.dk/services/SignalP/output.html

##########################################################################################

Name:  contig030553-ZebOR.A003  Length:  309
MDDELNVTYITLDGYVELKRCGYLYFLIMVALYVLIIISNSTIVFLICIHRNLHEPMYIFIAALSVNSVVFSTAIYPKLF      80
VDVLSEKQVISFSACQFQHFMYYSIGGSDFLLLSAMAFDRYVSICKPLKYPVIMRQTTINILLFLAWFLPGLQVAVSHAL     160
VLNNKLCNFTLKGIFCNNLLWKLYCESPRAAFIYGLIVLLNVAIFPVLFILFTYAKIFLITYRSSRDIQKKAAETCLPHL     240
FVLSIFTTFCAYDVIIGQLEFDFPKTAQLIMTLQVVLYNPLFNPFIYGLKMKEISKHLKRLFCHVRCSX
.....N................................N.........................................      80
................................................................................     160
.......N........................................................................     240
.....................................................................               320

(Threshold=0.5)
----------------------------------------------------------------------
SeqName      Position  Potential   Jury    N-Glyc
                                 agreement result
----------------------------------------------------------------------
contig030553-ZebOR.A003    6 NVTY    0.7912     (9/9)    +++
contig030553-ZebOR.A003   40 NSTI    0.7070     (9/9)    ++
contig030553-ZebOR.A003  168 NFTL    0.6776     (8/9)    +
----------------------------------------------------------------------
```

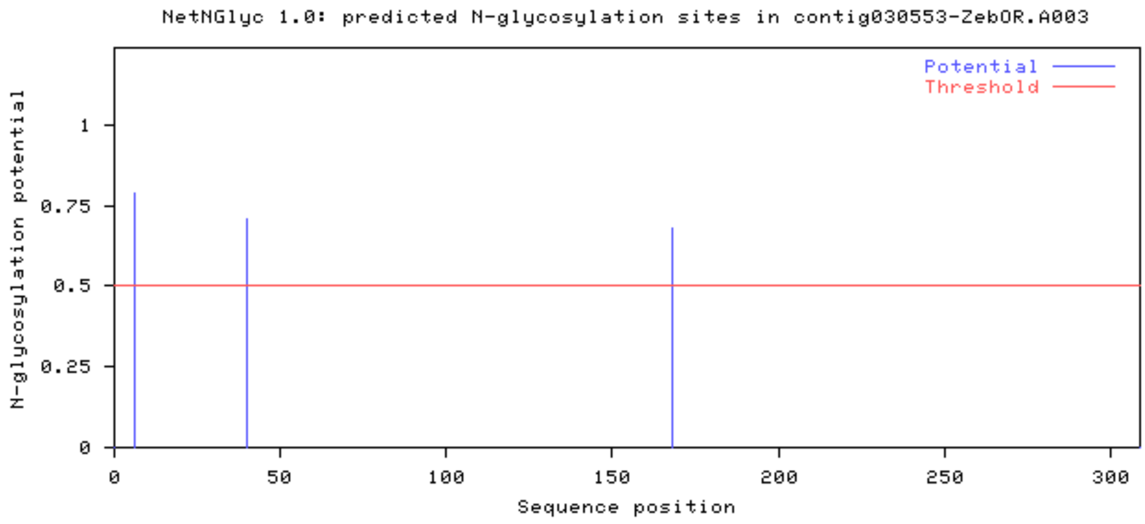

Graphics in PostScript

Output for 'contig030554-ZebOR.A004'

```
##########################################################################################

    Warning: This sequence may not contain a signal peptide!!

    Proteins without signal peptides are unlikely to be exposed to
    the N-glycosylation machinery and thus may not be glycosylated
    (in vivo) even though they contain potential motifs.

    SignalP-NN euk predictions are as follows:

  # name                  Cmax  pos ?  Ymax  pos ?  Smax  pos ?  Smean ?  D     ?

    SignalP output is explained at http://www.cbs.dtu.dk/services/SignalP/output.html

##########################################################################################

Name:  contig030554-ZebOR.A004  Length:  328
MAIDNEFNVTYITFGGHIELEKYKFLYFAIMFTAYILILCSNSTIVCLIRIKKSLHEPMYIFIAALLFNSVMFSTNIYPK      80
LLMDFLSERQITTHLLCSFQGFIYYSLTGSEFFLLASMAYDRYVSISKPLQYHTIMRKTTVTVLLVLTWLLPACQLVPSA     160
VIINTSQICSFTLNGIFCNNAISKLYCATPKISYLIYGVFILLNTVFLPLLFILFTYTKIFIICYRSCREVRKKAAQTCL     240
PHLLVLISFSCLCSYDIIITRVEINLSQTARFIMTLQVVLYHPLFNPIVYGLKMKEISQHLRRLFCQSKFKLSVRADAGS     320
AIISFAIX
.......N.................................N......................................      80
................................................................................     160
...N............................................................................     240
........................N.......................................................     320
........                                                                             400

(Threshold=0.5)
----------------------------------------------------------------------
SeqName      Position  Potential   Jury    N-Glyc
                                 agreement result
----------------------------------------------------------------------
contig030554-ZebOR.A004    8 NVTY   0.6870     (9/9)   ++
contig030554-ZebOR.A004   42 NSTI   0.7286     (9/9)   ++
contig030554-ZebOR.A004  164 NTSQ   0.5964     (8/9)   +
contig030554-ZebOR.A004  265 NLSQ   0.5972     (5/9)   +
----------------------------------------------------------------------
```

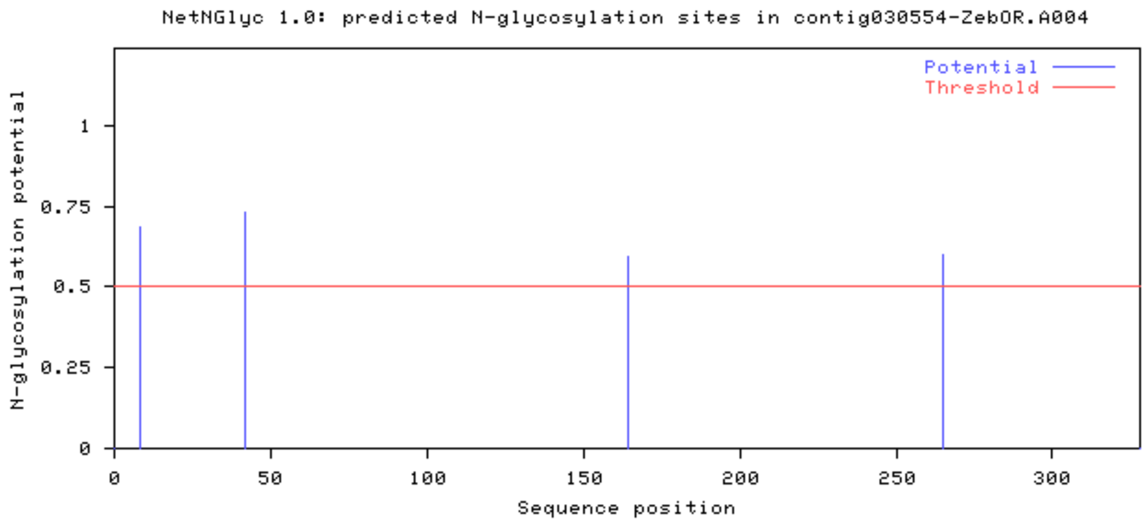

Graphics in PostScript

Output for 'contig030555-ZebORpe.A031'

```
##########################################################################

    Warning: This sequence may not contain a signal peptide!!

    Proteins without signal peptides are unlikely to be exposed to
    the N-glycosylation machinery and thus may not be glycosylated
    (in vivo) even though they contain potential motifs.

    SignalP-NN euk predictions are as follows:

  # name                Cmax  pos ?  Ymax  pos ?  Smax  pos ?  Smean ?  D     ?

    SignalP output is explained at http://www.cbs.dtu.dk/services/SignalP/output.html

##########################################################################

Name:  contig030555-ZebORpe.A031        Length:  327
KFNVTDITFGGHIELEKYKFLYFAIMFTAYILILCSNSTIVCLIWIKKSLHEPMYIFIAALLFNSVMFSTNIYPKLLMDF      80
LSERQITTHLLCSFQGFIYYSLTGSEFFLLASMAYDRYVSISKPLQYNTILYSIIKAAVKILLVLCLLVPSAVIINTSQI     160
CSFTLNGIFCNNAISKLYCVTVETPYLMYGVLILLNTLFLPLFFILFTYTKIFIICYRSCREVRKKAAQTCLPHLLVLIS     240
FSCLCSYDRIITVEIDLSQTARFIITLQVVLCHPLFNPIVYGLKMKEISQHLRRLFCHCVLCVKTNVGSAVISFVIQVQR     320
PDFSPEX
..N.................................N...........................................      80
...........................................................................N....     160
................................................................................     240
................................................................................     320
.......                                                                              400

(Threshold=0.5)
----------------------------------------------------------------------
SeqName      Position  Potential   Jury    N-Glyc
                                 agreement result
----------------------------------------------------------------------
contig030555-ZebORpe.A031    3 NVTD   0.8013     (9/9)   +++
contig030555-ZebORpe.A031   37 NSTI   0.7402     (9/9)   ++
contig030555-ZebORpe.A031  156 NTSQ   0.5988     (8/9)   +
----------------------------------------------------------------------
```

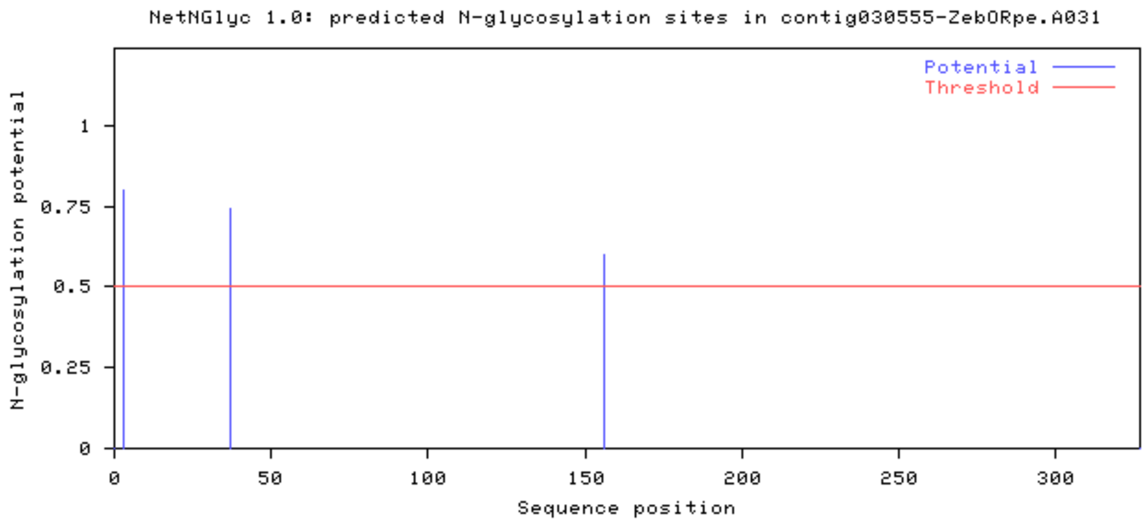

Graphics in PostScript

Output for 'contig030556-ZebOR.A005'

```
##########################################################################

    Warning: This sequence may not contain a signal peptide!!

    Proteins without signal peptides are unlikely to be exposed to
    the N-glycosylation machinery and thus may not be glycosylated
    (in vivo) even though they contain potential motifs.

    SignalP-NN euk predictions are as follows:

  # name                Cmax  pos ?  Ymax  pos ?  Smax  pos ?  Smean ?  D     ?

    SignalP output is explained at http://www.cbs.dtu.dk/services/SignalP/output.html

##########################################################################

Name:  contig030556-ZebOR.A005  Length:  337
MEQMHDEFNVTYITFGGHVELEKYKFLYFAIMFTAYILILCSNSTIVCLIWIKKSLHEPMYVFIAALLLNSVVFSTNIYP      80
KLLMDFLSERQITTHSLCRFQGIIYYSLTGSEFFLLASMAYDRYVSISKPLQYHTIMRKTTVTVLLVLAWLLPACQLVPS     160
AVFSNNSQICSFTLNGIFCNNAISKLYCATPKTYYLMYGVFILFNTVFLPLLFIMFTYTKIFIICYRSCREVRKKAAQTC     240
LPHLLVLISFTCLCSYDIIIARLEIDLSQNTRFIMTLQVVLYHPLFNPIVYGLKMKEISQHLRRLFCHRVLCVKTDVGSA     320
VISFVIQVQRPDFSTVX
........N................................N......................................      80
................................................................................     160
....N...........................................................................     240
................................................................................     320
.................                                                                    400

(Threshold=0.5)
----------------------------------------------------------------------
SeqName      Position  Potential   Jury    N-Glyc
                                 agreement result
----------------------------------------------------------------------
contig030556-ZebOR.A005    9 NVTY   0.7809     (9/9)   +++
contig030556-ZebOR.A005   43 NSTI   0.7383     (9/9)   ++
contig030556-ZebOR.A005  165 NNSQ   0.5563     (5/9)   +
----------------------------------------------------------------------
```

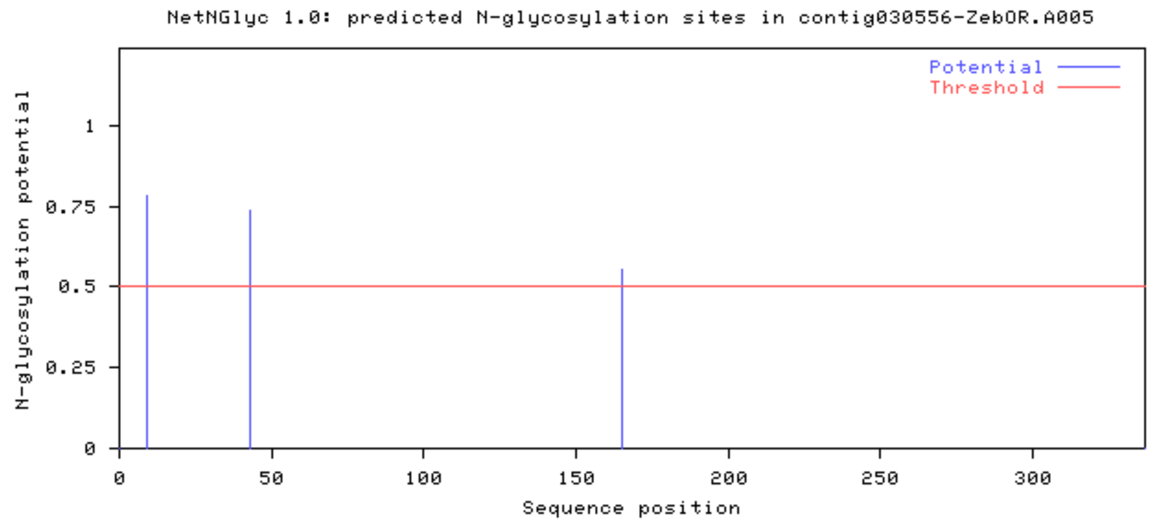

[Graphics in PostScript]

Output for 'contig030557-ZebOR.A006'

```
##########################################################################

    Warning: This sequence may not contain a signal peptide!!

    Proteins without signal peptides are unlikely to be exposed to
    the N-glycosylation machinery and thus may not be glycosylated
    (in vivo) even though they contain potential motifs.

    SignalP-NN euk predictions are as follows:

  # name                Cmax  pos ?  Ymax  pos ?  Smax  pos ?  Smean ?  D     ?

    SignalP output is explained at http://www.cbs.dtu.dk/services/SignalP/output.html

##########################################################################

Name:  contig030557-ZebOR.A006  Length:  309
MNPGLNMTYLILGGHVEVQKYRYLYFMILFTAYILIICCNTSIIYLIVIHKSLHEPMYIFIAALLLNSLFFSTNIYPKLL      80
ADFLSEKQIISYQVCLFQVFIFYSLSCSEFLLLSAMAYDRYVSICKPLQYPTIMRKITVVFLLVLAWLLPACQVAVVIIL     160
NINNKLCNFTLKGIFCNNSLIQLYCVMSRALSVYGAFVLLNTGLFPMLFIIFTYTKIILTVYRSSGEVKKKAAQTCLPHL     240
FVLINYSCLITYDMIIARLESDFSKTARFLMTLQIITYNPLFNPIIYGLKMKEISKHLQRLLCQSKLNX
.....N.................................N........................................      80
................................................................................     160
.......N........................................................................     240
....N................................................................                320

(Threshold=0.5)
----------------------------------------------------------------------
SeqName      Position  Potential   Jury    N-Glyc
                                 agreement result
----------------------------------------------------------------------
contig030557-ZebOR.A006    6 NMTY   0.7301     (9/9)    ++
contig030557-ZebOR.A006   40 NTSI   0.6411     (8/9)    +
contig030557-ZebOR.A006  168 NFTL   0.6657     (9/9)    ++
contig030557-ZebOR.A006  177 NNSL   0.4821     (6/9)    -
contig030557-ZebOR.A006  245 NYSC   0.5046     (4/9)    +
----------------------------------------------------------------------
```

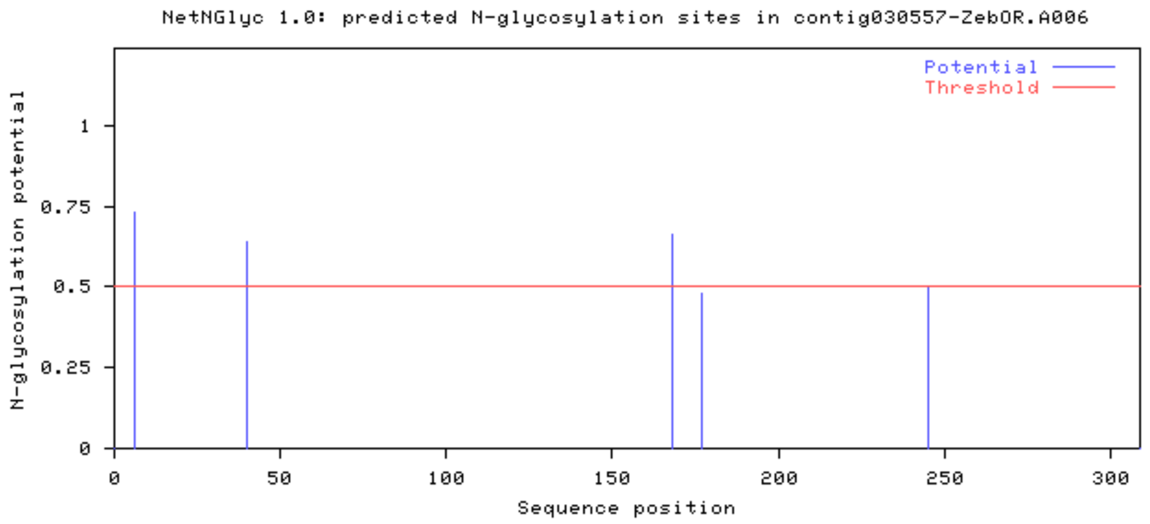

Graphics in PostScript

Output for 'contig030560-ZebOR.A007'

```
##########################################################################

    Warning: This sequence may not contain a signal peptide!!

    Proteins without signal peptides are unlikely to be exposed to
    the N-glycosylation machinery and thus may not be glycosylated
    (in vivo) even though they contain potential motifs.

    SignalP-NN euk predictions are as follows:

  # name                Cmax  pos ?  Ymax  pos ?  Smax  pos ?  Smean ?  D     ?

    SignalP output is explained at http://www.cbs.dtu.dk/services/SignalP/output.html

##########################################################################

Name:  contig030560-ZebOR.A007   Length:  319
MDQMNDKFNVTYITFGGHVELNKYRFLYFAIMFTAYTLILCSNSTILCLIWIKKNLHEPMYIFIAGLLLNSVMFSTNIYP      80
ELLIDFLSDKQITTHSLCSFQAFIYYSLTGSEFFLLAAMAYDRYVSICKPLQYTTIMKKTTIIVLLGLAWLLPACQLVPS     160
VVMSQSYKICSFTLNGIFCNNAISKLYCDTSRTTYIIYGVFILLNTVFLPLLFILFTYTKIFIICYRSCREVRKKAAQTC     240
LPHLLVLVSFSGLCSYDIIVARLEMNLPKVARFILTLQVVLYHPLFNPIVYGLKMKEISKHLTKLFCEGKLNIWQSSCX
........N.................................N.....................................      80
................................................................................     160
................................................................................     240
...............................................................................      320

(Threshold=0.5)
----------------------------------------------------------------------
SeqName        Position  Potential    Jury     N-Glyc
                                    agreement result

----------------------------------------------------------------------
contig030560-ZebOR.A007    9 NVTY    0.7641     (9/9)    +++
contig030560-ZebOR.A007   43 NSTI    0.6553     (9/9)    ++
----------------------------------------------------------------------
```

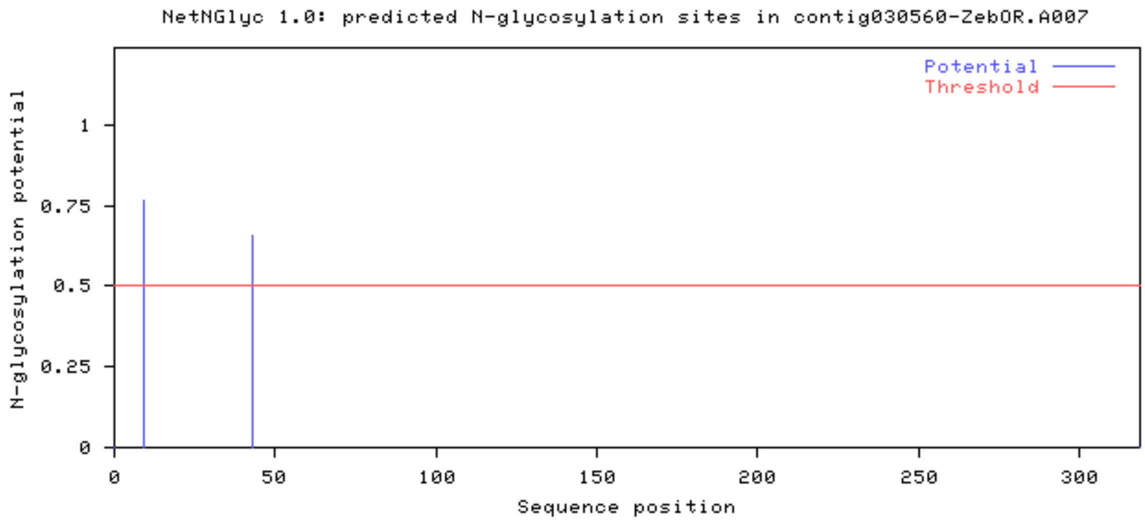

Graphics in PostScript

Output for 'contig030566-ZebOR.A008'

```
##########################################################################################

    Warning: This sequence may not contain a signal peptide!!

    Proteins without signal peptides are unlikely to be exposed to
    the N-glycosylation machinery and thus may not be glycosylated
    (in vivo) even though they contain potential motifs.

    SignalP-NN euk predictions are as follows:

  # name                    Cmax  pos ?  Ymax  pos ?  Smax  pos ?  Smean ?  D     ?

    SignalP output is explained at http://www.cbs.dtu.dk/services/SignalP/output.html

##########################################################################################

Name:  contig030566-ZebOR.A008  Length:  309
MDDELNITHITIDGYVDLKRFGYLYFLIMVALYVLIIISNSVIVFLICIHHNLHEPMYIFIAALSVNSVLLSTVTYPKLF      80
VDVLSEKQIISISACRFQHFMCYSIAGSDFLLLSAMAFDRYVSICKPLKYPVIMRQTTINTLLFLSWFVPGLQIAVLHTL     160
VLNNKLCNFTLKGILCNNSLWKLYCESPRATLIYGLVVMLSVVIFPVFFILFTYAKIFLITYRSSRAIQKKAAETCLPHL     240
FVLSIFTTLCAYDVIIGRLELDFPKTAQLIMTLQVIFYNPLLNPFIYGLKMKEISKHLKRLFCHVRCSX
.....N..........................................................................      80
................................................................................     160
.......N........................................................................     240
.....................................................................                320

(Threshold=0.5)
----------------------------------------------------------------------
SeqName      Position  Potential   Jury    N-Glyc
                                 agreement result
----------------------------------------------------------------------
contig030566-ZebOR.A008    6 NITH   0.7698     (9/9)   +++
contig030566-ZebOR.A008  168 NFTL   0.6758     (8/9)   +
contig030566-ZebOR.A008  177 NNSL   0.4502     (6/9)   -
----------------------------------------------------------------------
```

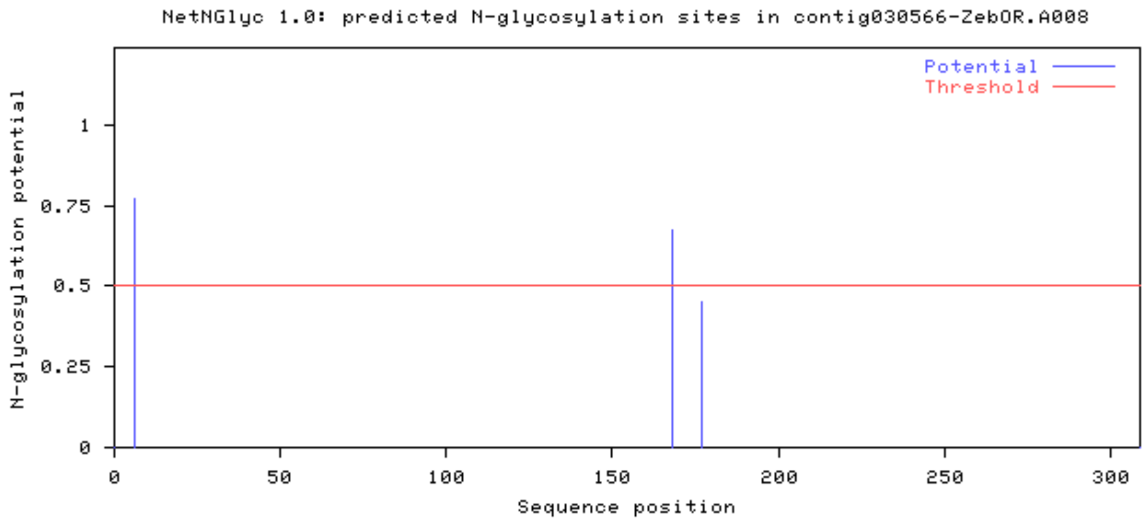

Graphics in PostScript

Output for 'contig030572-ZebOR.A009'

```
##########################################################################################

    Warning: This sequence may not contain a signal peptide!!

    Proteins without signal peptides are unlikely to be exposed to
    the N-glycosylation machinery and thus may not be glycosylated
    (in vivo) even though they contain potential motifs.

    SignalP-NN euk predictions are as follows:

  # name                 Cmax  pos ?  Ymax  pos ?  Smax  pos ?  Smean ?  D     ?

    SignalP output is explained at http://www.cbs.dtu.dk/services/SignalP/output.html

##########################################################################################

Name:  contig030572-ZebOR.A009  Length:  314
MDEESNATYLTLDWYTEINKYRYIFFFVMFTLYILIICTNSTILYLIWNHKNLHEPMYIFIAALLLNSVLYSTTVYPKLL      80
IDFSSEKQVTTYSACLFQFFIFYTLVLSEFLLLAAMAYDRYVAICKPLEYQTIMRKTTVGIFLVVAWLVPACQVAVQAIA     160
SAEAKLCDSNIKGIFCNNAVYTLQCERSKLITIFGVVIVLDLAILPMLFIVFTYTKIFIVSHRSCKEIRKKAAETCLPHL     240
LVLLSLSVFFVYDVSIARANPDFPKTTRIIMTLQIMLYQPLLNPFIYGLKMKEISKHLNKLLSQTNISPCIKTX
.....N.................................N........................................      80
................................................................................     160
................................................................................     240
..........................................................................           320

(Threshold=0.5)
----------------------------------------------------------------------
SeqName      Position  Potential   Jury    N-Glyc
                                 agreement result
----------------------------------------------------------------------
contig030572-ZebOR.A009    6 NATY   0.6218     (7/9)   +
contig030572-ZebOR.A009   40 NSTI   0.6462     (8/9)   +
contig030572-ZebOR.A009  306 NISP   0.1124     (9/9)   ---
----------------------------------------------------------------------
```

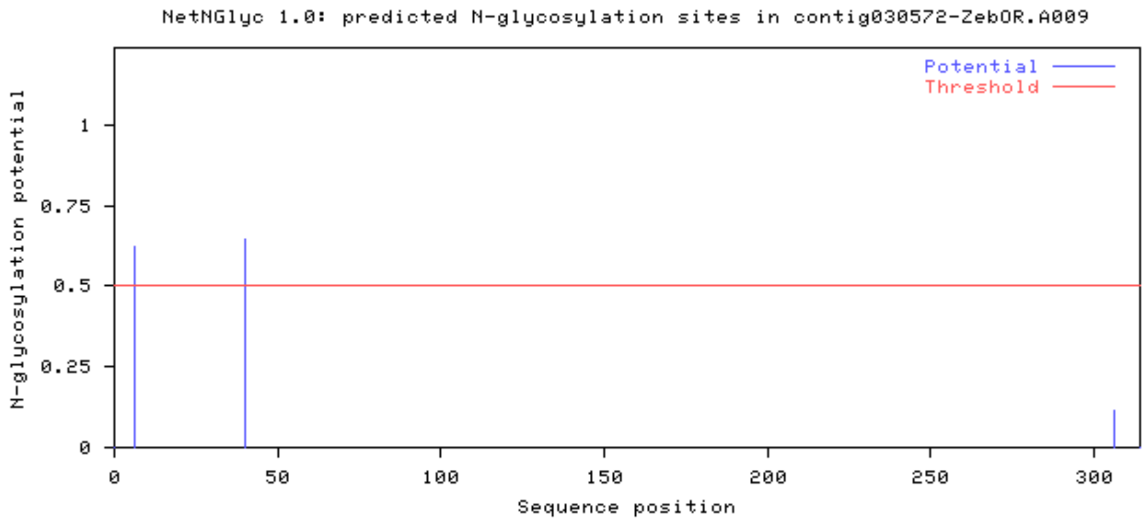

Graphics in PostScript

Output for 'contig030576-ZebOR.A010'

```
##########################################################################################

    Warning: This sequence may not contain a signal peptide!!

    Proteins without signal peptides are unlikely to be exposed to
    the N-glycosylation machinery and thus may not be glycosylated
    (in vivo) even though they contain potential motifs.

    SignalP-NN euk predictions are as follows:

  # name                    Cmax  pos ?  Ymax  pos ?  Smax  pos ?  Smean ?  D     ?

    SignalP output is explained at http://www.cbs.dtu.dk/services/SignalP/output.html

##########################################################################################

Name:  contig030576-ZebOR.A010  Length:  316
MDKELNVTFLTLDWYTEINKYRYIFFFIMFTLYILIICTNSTIVYLIWIHKNLHEPMYIFIAALLLNSVLYSTTIYPKLL      80
IDVLSDKQVTTYSACLFQFFMFYTLGGSEFFLLAAMAYDRYVAICKPLQYHIIMRKTTVSISLIIAWLVPACHIAVLAIA     160
SAEAKLCDSNIKGIFCNNAVYSLQCQRSRLIIIFGVIALLDLVILPMLFIVFTYTTIFIVSCQSCKEIRKKAAETCLPHL     240
LVLISACLFFVYDVSIARVEADFPKTARIIMTLQIVLYHPLFNPFVYGLKMKEISKHLKGLLCQGKITSCIKTGSX
.....N.................................N........................................      80
................................................................................     160
................................................................................     240
............................................................................         320

(Threshold=0.5)
----------------------------------------------------------------------
SeqName      Position  Potential   Jury    N-Glyc
                                 agreement result
----------------------------------------------------------------------
contig030576-ZebOR.A010    6 NVTF    0.7646     (9/9)    +++
contig030576-ZebOR.A010   40 NSTI    0.7028     (9/9)    ++
----------------------------------------------------------------------
```

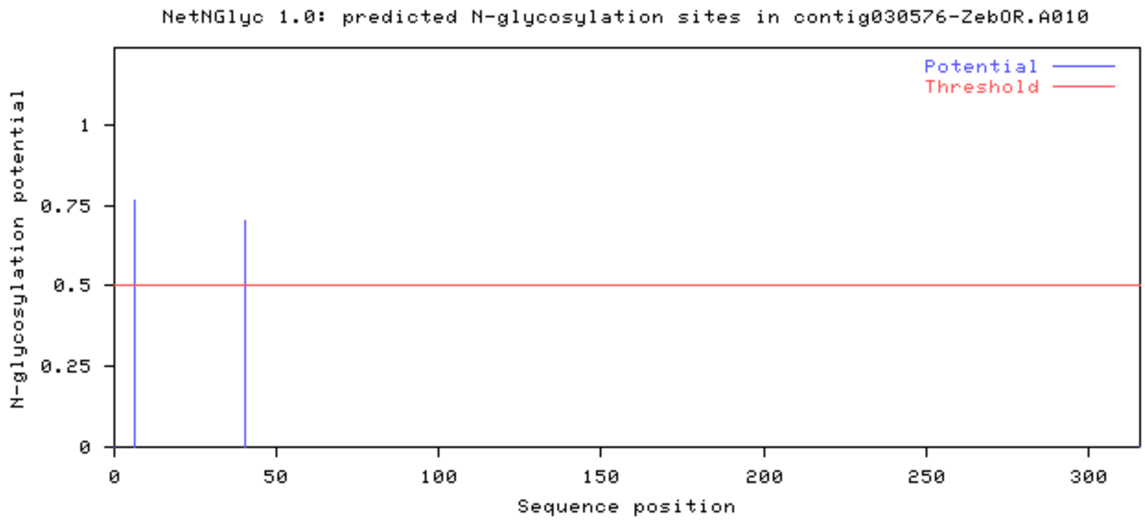

Graphics in PostScript

Output for 'contig046002-ZebORs.K090'

```
##########################################################################################

    Warning: This sequence may not contain a signal peptide!!

    Proteins without signal peptides are unlikely to be exposed to
    the N-glycosylation machinery and thus may not be glycosylated
    (in vivo) even though they contain potential motifs.

    SignalP-NN euk predictions are as follows:

  # name                 Cmax  pos ?  Ymax  pos ?  Smax  pos ?  Smean ?  D     ?

    SignalP output is explained at http://www.cbs.dtu.dk/services/SignalP/output.html

##########################################################################################

Name:  contig046002-ZebORs.K090         Length:  307
MENYMYNSPTLQLQELRIVRTNKYLISSFSFFSYLFLIVANVGIAVLVFVDKSLHQPMYILFCNLSINDLFGNSIMIPRL      80
LVDMLRPPSERLISYYECVVQAFTTHMFSTTAHTVLMIMAFDRYVAICNPLRYAAVMTNKMLMKLTVSAWGVAFVLVGIL     160
LGLTLRPGRCRTLIKSPYCDNAALFNLSCEDVFINNVYGLTFTVLLFTGSISSMVLTYTKITVVCLTTKNKSLNNKALKT     240
CSTHLVVYLIFLFSGMSIITLHCFPEYSGSRKIVAVLYHIIPGSLNPIIYGMQSKEIKKNLYQKLSX
...............................................................N................      80
................................................................................     160
.........................N......................................................     240
...................................................................                  320

(Threshold=0.5)
----------------------------------------------------------------------
SeqName      Position  Potential   Jury    N-Glyc
                                 agreement result
----------------------------------------------------------------------
contig046002-ZebORs.K090   64 NLSI   0.6490     (9/9)   ++
contig046002-ZebORs.K090  186 NLSC   0.6170     (8/9)   +
contig046002-ZebORs.K090  230 NKSL   0.4776     (6/9)   -
----------------------------------------------------------------------
```

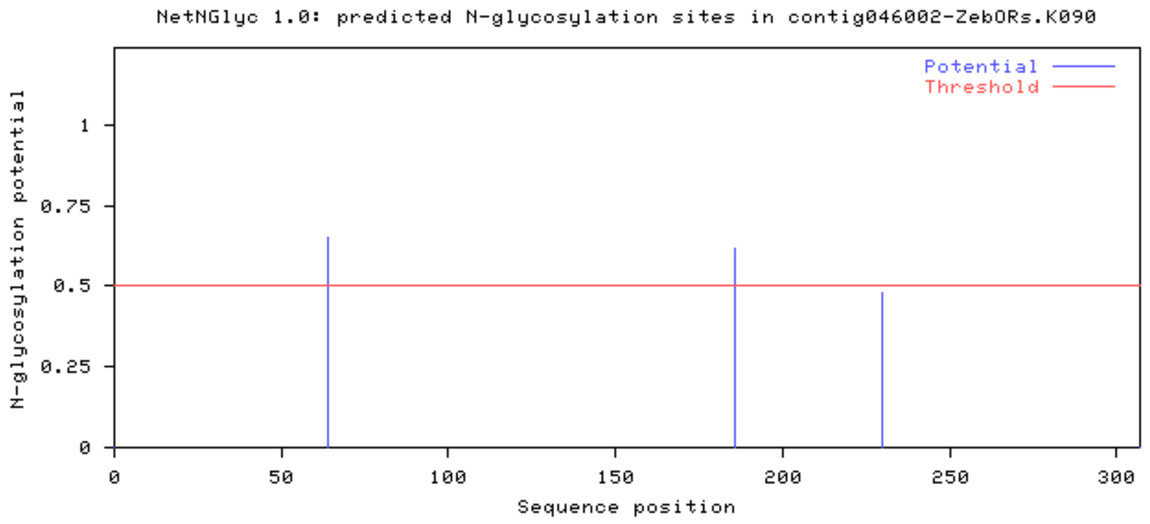

Graphics in PostScript

Output for 'contig046004-ZebORp.AB044'

```
########################################################################################

    Warning: This sequence may not contain a signal peptide!!

    Proteins without signal peptides are unlikely to be exposed to
    the N-glycosylation machinery and thus may not be glycosylated
    (in vivo) even though they contain potential motifs.

    SignalP-NN euk predictions are as follows:

  # name                  Cmax  pos ?  Ymax  pos ?  Smax  pos ?  Smean ?  D     ?

    SignalP output is explained at http://www.cbs.dtu.dk/services/SignalP/output.html

########################################################################################

Name:  contig046004-ZebORp.AB044        Length:  331
MSSFFPSDFPTLSTNHRSSSVNETLGLGGVTFFIIQGLTSLDEKKIILFSILLLIYIMVLGGNSIIIYVALTDPKLNSPL      80
YFFLCNLSFVDMVYTTTTIPNMLSGLLTDILTISVLGCFLQMYFFIQLTVTGRAILTVMAYDRYVAICNPLQYNSIMTRP     160
VRLLLVAGAWGFGAICTLPVTVIAFERPYCGPNVVKHAWCDPSSVRRLVCSDTSLDNIVSLLFAMVSLVTTSVFILSSYI     240
LIGFSISRMVVAQRLKALRTCSAHLTVVSISYAAASFVYISYRVGNFSSEVKTLLCFLCNVSVQYSVKVLSHLYLFIFLV     320
GKQEIHAEIYX
.....................N..........................................................      80
.....N..........................................................................     160
................................................................................     240
.............................................N.............N....................     320
...........                                                                          400

(Threshold=0.5)
----------------------------------------------------------------------
SeqName      Position  Potential   Jury    N-Glyc
                                 agreement result
----------------------------------------------------------------------
contig046004-ZebORp.AB044   22 NETL   0.6302     (8/9)   +
contig046004-ZebORp.AB044   86 NLSF   0.6588     (9/9)   ++
contig046004-ZebORp.AB044  286 NFSS   0.6219     (8/9)   +
contig046004-ZebORp.AB044  300 NVSV   0.6647     (9/9)   ++
----------------------------------------------------------------------
```

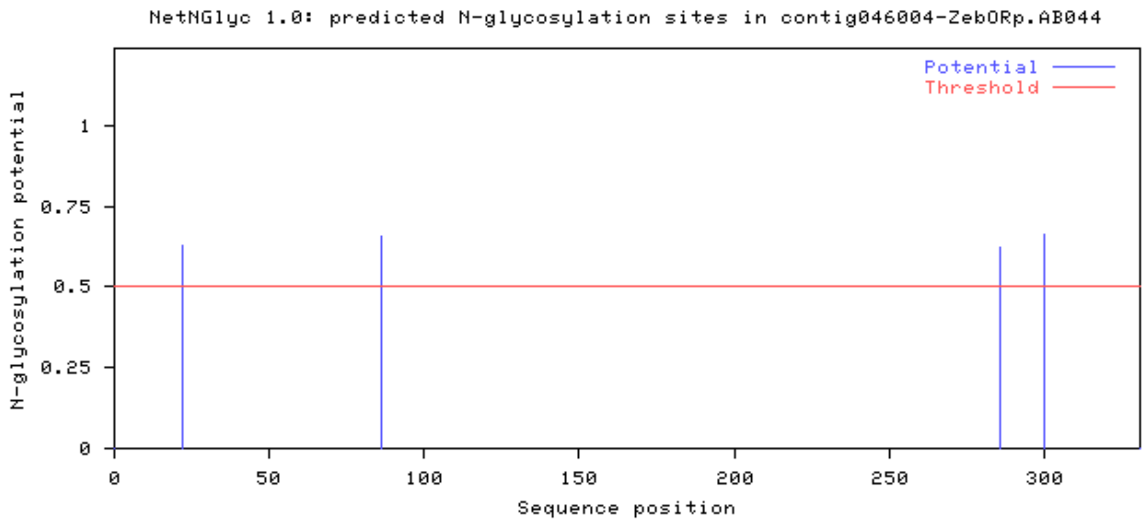

Graphics in PostScript

Output for 'contig046010-ZebORp.K089'

```
##############################################################################

    Warning: This sequence may not contain a signal peptide!!

    Proteins without signal peptides are unlikely to be exposed to
    the N-glycosylation machinery and thus may not be glycosylated
    (in vivo) even though they contain potential motifs.

    SignalP-NN euk predictions are as follows:

  # name                  Cmax  pos ?  Ymax  pos ?  Smax  pos ?  Smean ?  D     ?

    SignalP output is explained at http://www.cbs.dtu.dk/services/SignalP/output.html

##############################################################################

Name:  contig046010-ZebORp.K089         Length:  253
MENYTFNSFTLQLEGLKVSEVSKYPIFFFFLFLFSYILIMTSNIGIVLLVFIDQNLHHHLSAPVCFQIFCCLHLSASPVI      80
MSVLFRLLPHIIMSPRLLSDILLPPSERLTSYHECLIQAFTTHMYVTTSHIVLMIMAFDRYVAICNPLRYASIMTNKMMI     160
ELTVLAWGVAFVLVGVLLGLTIWLSCRTMITNPFCDSLFKLSCDSVVINNVYGLTFTVILFTGSVTIVLTYTQITVDCVT     240
SKNKSLNSKALTL
..N.............................................................................      80
................................................................................     160
................................................................................     240
.............                                                                        320

(Threshold=0.5)
----------------------------------------------------------------------
SeqName      Position  Potential   Jury    N-Glyc
                                 agreement result
----------------------------------------------------------------------
contig046010-ZebORp.K089    3 NYTF   0.7402     (9/9)   ++
contig046010-ZebORp.K089  243 NKSL   0.3132     (9/9)   ---
----------------------------------------------------------------------
```

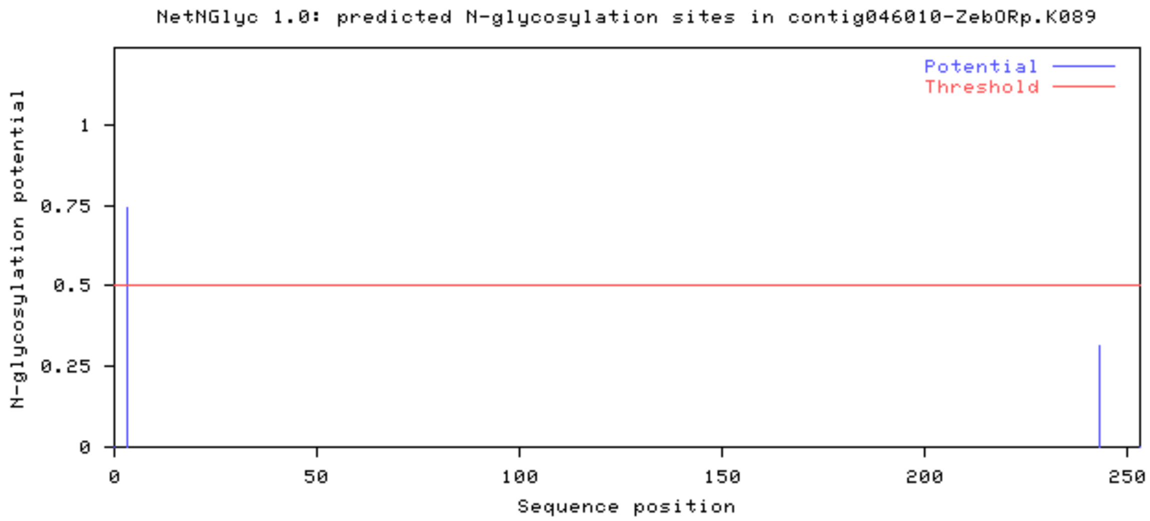

Graphics in PostScript

Output for 'contig046037-ZebORp.R148'

```
##########################################################################################

    Warning: This sequence may not contain a signal peptide!!

    Proteins without signal peptides are unlikely to be exposed to
    the N-glycosylation machinery and thus may not be glycosylated
    (in vivo) even though they contain potential motifs.

    SignalP-NN euk predictions are as follows:

  # name                    Cmax   pos ?  Ymax   pos ?  Smax   pos ?  Smean ?  D      ?

    SignalP output is explained at http://www.cbs.dtu.dk/services/SignalP/output.html

##########################################################################################

Name:  contig046037-ZebORp.R148         Length:  324
MSDISQTNISVGLHDLERGLLSSLTTLPCCVFFCINVIMLFTLRSKSVFRETCRYILLYNLVLADTLQMAVSQILYMMFF      80
CRITLPYPVCGILVMFANLTNEISPLTLVLMSLERYVAVCYPLRHATIITIRNTEVSIITIWIFCSLNILIRVLLLLEFP     160
FEELQRLQLKRYVCNTFLMFLTPVSHEYDKAYTCFLFVSAFVGVTCSYVGVMLAARSASTDKASAGKARNTLLLHLVQLG     240
FSLSSTVNNALLLLTSKTVSHRVSLLIQNALYVLLFILPRCLPQMSGALIYGLRDQTIRPILVYNLCCQLKLLAVTAGAK     320
IYPX
.......N........................................................................      80
.................N..............................................................     160
................................................................................     240
................................................................................     320
....                                                                                 400

(Threshold=0.5)
----------------------------------------------------------------------
SeqName      Position  Potential   Jury    N-Glyc
                                 agreement result
----------------------------------------------------------------------
contig046037-ZebORp.R148     8 NISV   0.6020     (6/9)   +
contig046037-ZebORp.R148    98 NLTN   0.6604     (9/9)   ++
----------------------------------------------------------------------
```

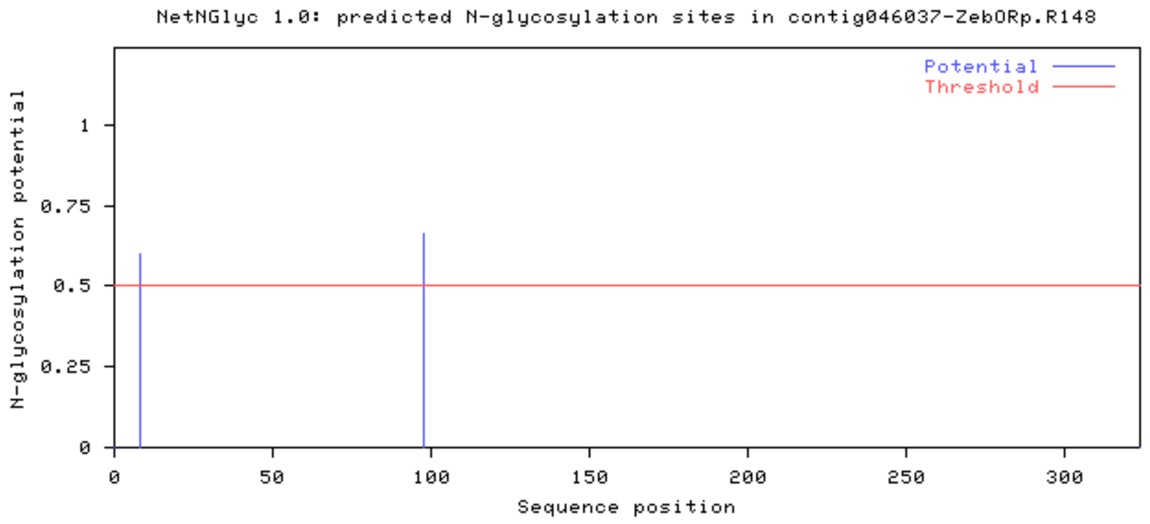

Graphics in PostScript

Output for 'contig046040-ZebOR.R144'

```
##########################################################################################

    Warning: This sequence may not contain a signal peptide!!

    Proteins without signal peptides are unlikely to be exposed to
    the N-glycosylation machinery and thus may not be glycosylated
    (in vivo) even though they contain potential motifs.

    SignalP-NN euk predictions are as follows:

  # name                    Cmax  pos ?  Ymax  pos ?  Smax  pos ?  Smean ?  D     ?

    SignalP output is explained at http://www.cbs.dtu.dk/services/SignalP/output.html

##########################################################################################

Name:  contig046040-ZebOR.R144  Length:  313
MSNVSQSYTNMSFEVQYQDLLRVIIVSTLSTVPSFTFLFLNGTMLFTLRSKPVFRDTPRYILLYNLLFADTVQLAQSQVL      80
FLLSIFRVKLPYPVCVCLSLLANLTTGISPLTLSVMPLERYVAVCYPLRYPTIITIRNTGAAIVVIWIISSLNNLTRVIF     160
FFPFEVLKNLQIKDSCSKIALLLGTRSDQYDTAFTCLVFVSAGVAVVFSYIGVILAARLASANKALARKARNTLLLNMMQ     240
LCLSLSSTIYNPLLAALSRTITMTIFLWVQNVFYLCFIILPRCLSSLVYGLRDQTIRPVLMYHLCCHQKRSQX
..N......N.............................N....................................      80
......................N...................................................N.....     160
................................................................................     240
.........................................................................            320

(Threshold=0.5)
----------------------------------------------------------------------
SeqName      Position  Potential   Jury    N-Glyc
                                 agreement result
----------------------------------------------------------------------
contig046040-ZebOR.R144    3 NVSQ   0.7751     (9/9)    +++
contig046040-ZebOR.R144   10 NMSF   0.5882     (7/9)    +
contig046040-ZebOR.R144   41 NGTM   0.7466     (9/9)    ++
contig046040-ZebOR.R144  103 NLTT   0.5888     (7/9)    +
contig046040-ZebOR.R144  154 NLTR   0.6351     (8/9)    +
----------------------------------------------------------------------
```

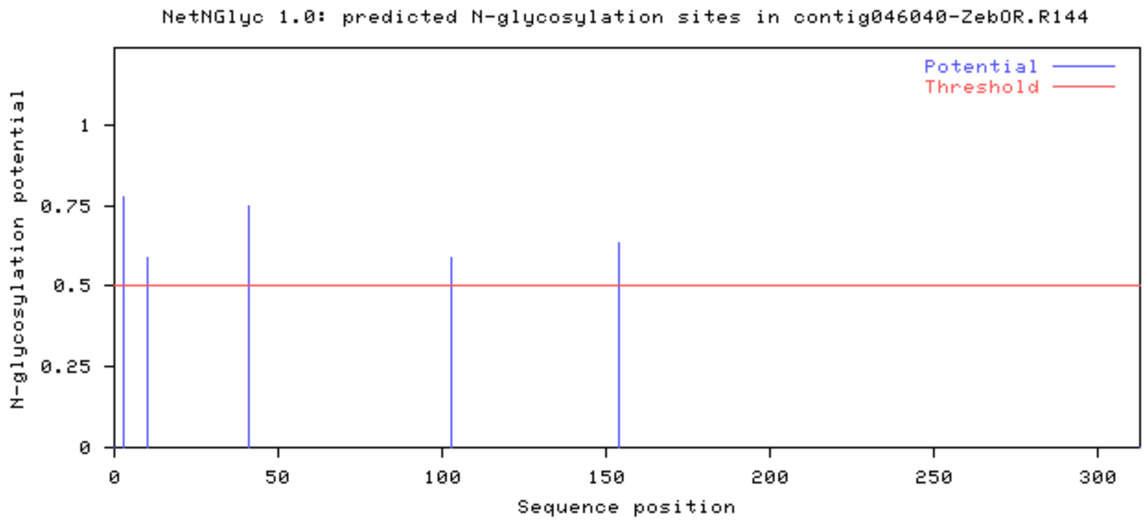

Graphics in PostScript

Output for 'contig046042-ZebOR.R145'

```
##########################################################################################

    Warning: This sequence may not contain a signal peptide!!

    Proteins without signal peptides are unlikely to be exposed to
    the N-glycosylation machinery and thus may not be glycosylated
    (in vivo) even though they contain potential motifs.

    SignalP-NN euk predictions are as follows:

  # name                    Cmax  pos ?  Ymax  pos ?  Smax  pos ?  Smean ?  D     ?

    SignalP output is explained at http://www.cbs.dtu.dk/services/SignalP/output.html

##########################################################################################

Name:  contig046042-ZebOR.R145  Length:  317
MTSTSQTLTNITVQSPGLAERVMISTLTTLPTCVFLFINGIMLFTLRSKPVFRETCRYILLYNLLFADTVQLAQSQIHFL      80
LAVLRITVSYPVCTFLVNFTHLTSVISPLTLVVTPLERYVAVCYPLRHATIIITIRNTGAAITVIWAISFLNIIIRTLLFL     160
SLFEELGDLEVKGFCGDIAILLGTKSDRFDKAFTCIVVVAAGVAVIFSYIGVIVAARSASTDKALAFKARNTLLLNLMQL     240
FLSLSSTIYYPLLVPLLMIVTRIVLVRIQNVFYLLFIIVPRCLTSLIYGLRDQTIRPVLIYHLCCRLKCPVAEDKGX
.........N......................................................................      80
.................N..............................................................     160
................................................................................     240
.............................................................................        320

(Threshold=0.5)
----------------------------------------------------------------------
SeqName      Position  Potential   Jury    N-Glyc
                                 agreement result
----------------------------------------------------------------------
contig046042-ZebOR.R145   10 NITV   0.7526     (9/9)    +++
contig046042-ZebOR.R145   98 NFTH   0.6942     (9/9)    ++
----------------------------------------------------------------------
```

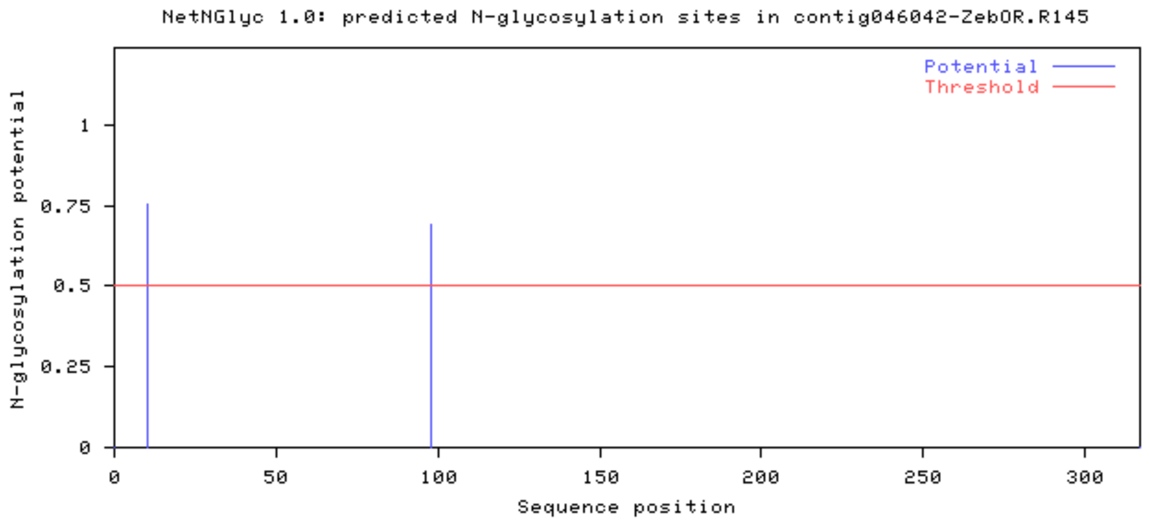

Graphics in PostScript

Output for 'contig046048-ZebOR.R146'

```
##########################################################################################

    Warning: This sequence may not contain a signal peptide!!

    Proteins without signal peptides are unlikely to be exposed to
    the N-glycosylation machinery and thus may not be glycosylated
    (in vivo) even though they contain potential motifs.

    SignalP-NN euk predictions are as follows:

  # name                    Cmax  pos ?  Ymax  pos ?  Smax  pos ?  Smean ?  D     ?

    SignalP output is explained at http://www.cbs.dtu.dk/services/SignalP/output.html

##########################################################################################

Name:  contig046048-ZebOR.R146  Length:  317
MSSTNETLTNITVGQQNQLFLEIVFSCIVTTLTCCVFLFINATMLFTLRSKPVFGQTSRYILLYNLLFADTLQMAQSQLM      80
FLLSACRITLLYPICGVLVSLATLLTLISPLTLVAMSLERYVAVCYPLRHATIITVRNTALAVCVVWTLSLLNVLIEVVL     160
MLRVRFQDLLHLQMEYSCNKEKLTLDPISDLYAKAFSYFLFVLAAGAFIFSYIGVTVVAQSASTDKASAEKARKTLVLHL     240
VQLGLSVSSTIHNPIFVFIYKTVDSVIVVRIRVVIYLCIIILPRCLSSFIYGLRDRTIRPVLMLNLRCQWKCQPFLX
....N....N.............................N........................................      80
................................................................................     160
................................................................................     240
.............................................................................        320

(Threshold=0.5)
----------------------------------------------------------------------
SeqName      Position  Potential   Jury    N-Glyc
                                 agreement result
----------------------------------------------------------------------
contig046048-ZebOR.R146    5 NETL   0.7403     (9/9)   ++
contig046048-ZebOR.R146   10 NITV   0.6742     (8/9)   +
contig046048-ZebOR.R146   41 NATM   0.6189     (7/9)   +
----------------------------------------------------------------------
```

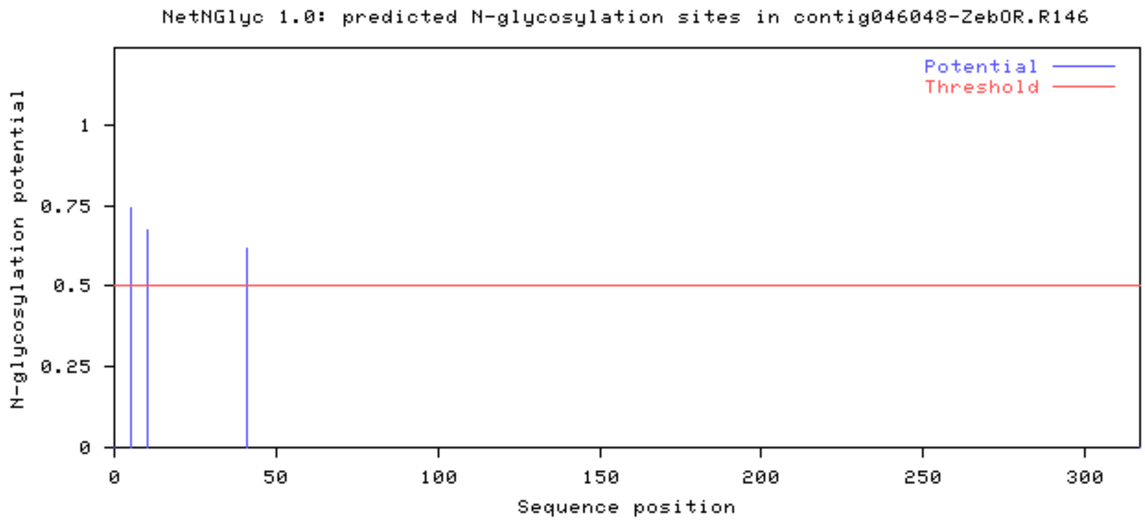

Graphics in PostScript

Output for 'contig047492-ZebOR.H074'

```
##########################################################################################

    Warning: This sequence may not contain a signal peptide!!

    Proteins without signal peptides are unlikely to be exposed to
    the N-glycosylation machinery and thus may not be glycosylated
    (in vivo) even though they contain potential motifs.

    SignalP-NN euk predictions are as follows:

  # name                 Cmax  pos ?  Ymax  pos ?  Smax  pos ?  Smean ?  D     ?

    SignalP output is explained at http://www.cbs.dtu.dk/services/SignalP/output.html

##########################################################################################

Name:  contig047492-ZebOR.H074  Length:  310
MDNVSTVRIFNLLAFNETANYRAAFFSATLVCYFAIVFLNVTVIMIIVLDESLHEPMYILVCVCCINGLYGSTGFYPKFL      80
IDLLSSSQVISYSECLCQAFVMYSFVCSDTSILAVMAYDRYLAICQPLQYHSVMTKKKLSKLVCFSWLTPFCIFSININML     160
TDRLIFCGTDIQRLFCVNWLIVKVACPGMDTLVNSAFAYATLSIYIFHWIFIVWTYIYLVKSCVQSKKDKAKFMQTCVPH     240
LISLVTFFVIVISDLMHMRFASNDVPQSFQNFVAIAVLFIPPVMNPLLYGFKLSKIRNRILVTLHIKRCX
..N............N.......................N........................................      80
................................................................................     160
................................................................................     240
......................................................................               320

(Threshold=0.5)
----------------------------------------------------------------------
SeqName      Position  Potential   Jury    N-Glyc
                                 agreement result
----------------------------------------------------------------------
contig047492-ZebOR.H074    3 NVST   0.7628     (9/9)    +++
contig047492-ZebOR.H074   16 NETA   0.7288     (9/9)    ++
contig047492-ZebOR.H074   40 NVTV   0.7888     (9/9)    +++
----------------------------------------------------------------------
```

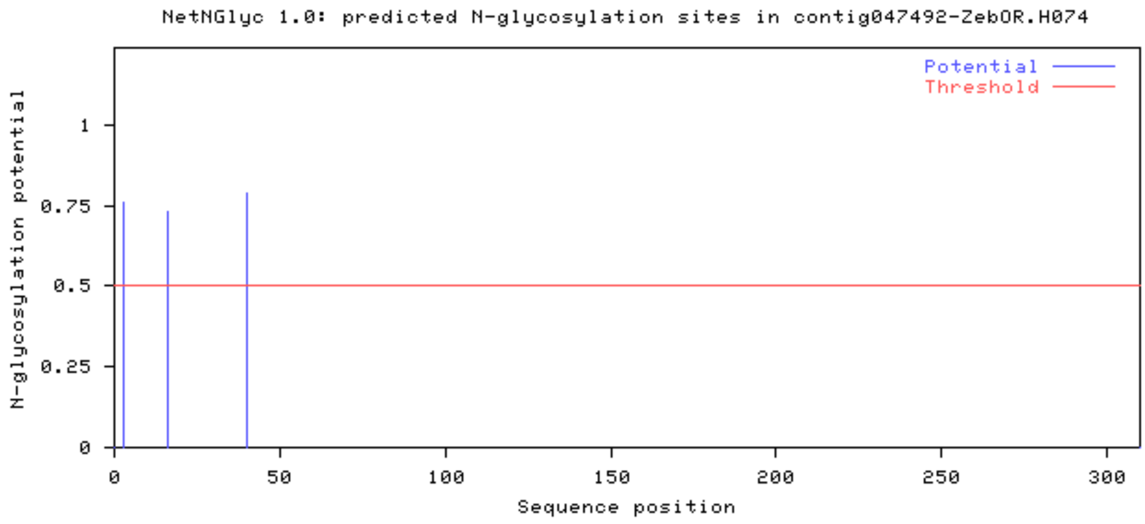

Graphics in PostScript

Output for 'contig047494-ZebORe.A029'

```
##########################################################################################

    Warning: This sequence may not contain a signal peptide!!

    Proteins without signal peptides are unlikely to be exposed to
    the N-glycosylation machinery and thus may not be glycosylated
    (in vivo) even though they contain potential motifs.

    SignalP-NN euk predictions are as follows:

  # name                    Cmax  pos ?  Ymax  pos ?  Smax  pos ?  Smean ?  D     ?

    SignalP output is explained at http://www.cbs.dtu.dk/services/SignalP/output.html

##########################################################################################

Name:  contig047494-ZebORe.A029         Length:  50
MDVELNVTLLTLGGFAELHKYRYLYFVIIFTLYILILCFNSTIVFLIWTH
.....N..................................N.........                                  80

(Threshold=0.5)
----------------------------------------------------------------------
SeqName      Position  Potential   Jury    N-Glyc
                                 agreement result
----------------------------------------------------------------------
contig047494-ZebORe.A029    6 NVTL   0.7799     (9/9)   +++
contig047494-ZebORe.A029   40 NSTI   0.6147     (8/9)   +
----------------------------------------------------------------------
```

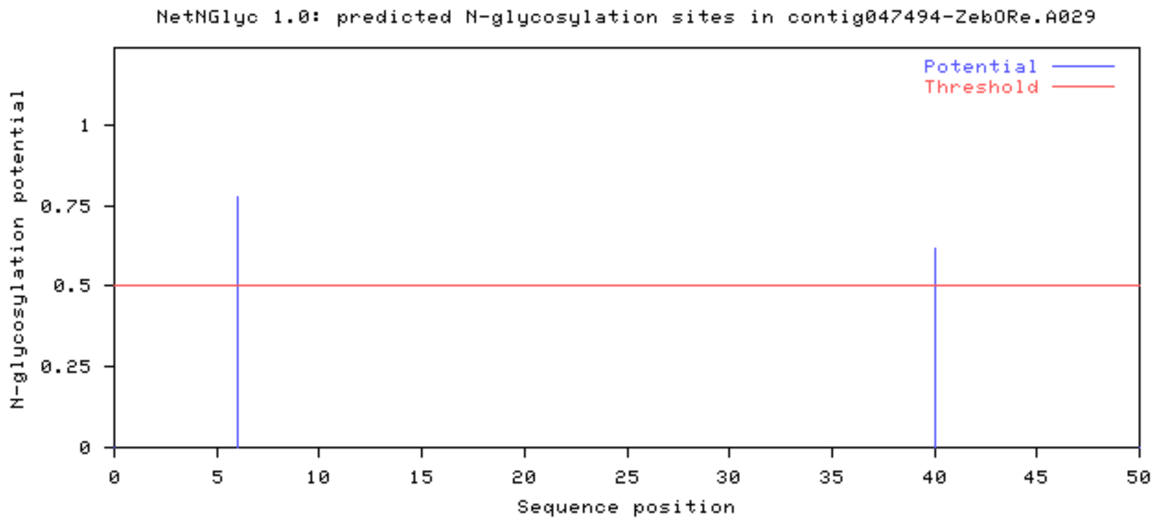

Graphics in PostScript

Output for 'contig047497-ZebOR.A011'

```
########################################################################################

    Warning: This sequence may not contain a signal peptide!!

    Proteins without signal peptides are unlikely to be exposed to
    the N-glycosylation machinery and thus may not be glycosylated
    (in vivo) even though they contain potential motifs.

    SignalP-NN euk predictions are as follows:

  # name                    Cmax  pos ?  Ymax  pos ?  Smax  pos ?  Smean ?  D     ?

    SignalP output is explained at http://www.cbs.dtu.dk/services/SignalP/output.html

########################################################################################

Name:  contig047497-ZebOR.A011  Length:  320
MDVELNVTLLTLGGFAELHKYRYLYFVIIFTLYILILCFNSTIVCLIWTHKNLHEPMYIFIAALLINSVLYSMIIYPKLL      80
SDVLSEKQMISYPLCLFQGLSYYTSVGSEFLLLAAMAYDRYVSICKPLQYPVIMNRITIYVCLILAWLIPAFETSVMGGL     160
YSNVKLCSFSLTGIFCNNSLYKLQCVPSVAISIYGVVMLIRIALLPLLFILFTYIRILRISYHCCREVRKKAVKTCLPHL     240
LVLTNFSCFIFFDIIIVRLDSDLSKTLRLTLTFQSIVFHPLLNPIIYGLKMNEIFKHIKILLSSLITLVLLPYYQMYGIX     320
.....N.................................N........................................      80
................................................................................     160
................................................................................     240
....N...........................................................................     320

(Threshold=0.5)
----------------------------------------------------------------------
SeqName      Position  Potential   Jury    N-Glyc
                                 agreement result
----------------------------------------------------------------------
contig047497-ZebOR.A011    6 NVTL    0.7848     (9/9)   +++
contig047497-ZebOR.A011   40 NSTI    0.7070     (9/9)   ++
contig047497-ZebOR.A011  177 NNSL    0.4252     (7/9)   -
contig047497-ZebOR.A011  245 NFSC    0.5732     (5/9)   +
----------------------------------------------------------------------
```

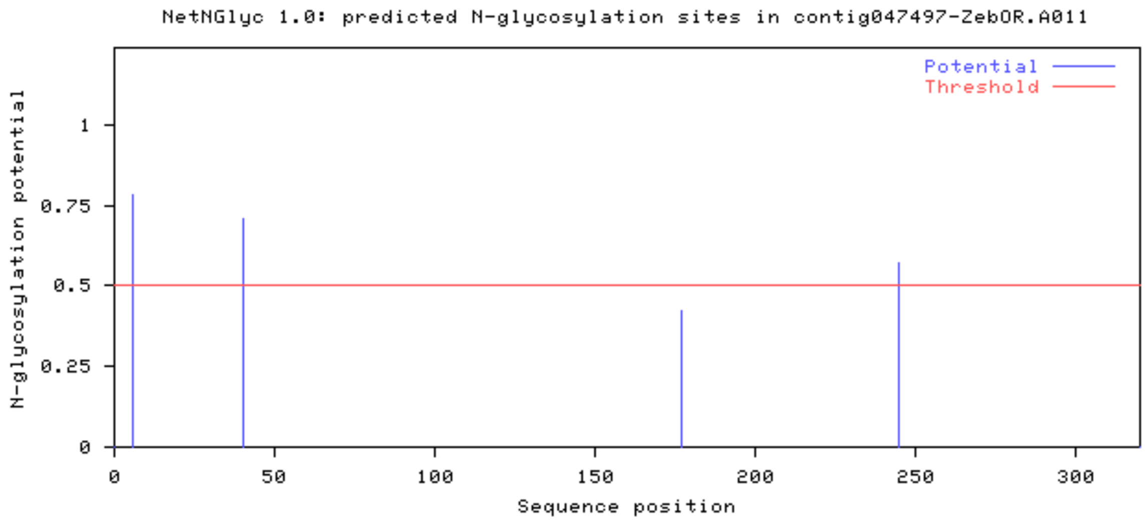

Graphics in PostScript

Output for 'contig047499-ZebOR.A012'

```
##########################################################################################

    Warning: This sequence may not contain a signal peptide!!

    Proteins without signal peptides are unlikely to be exposed to
    the N-glycosylation machinery and thus may not be glycosylated
    (in vivo) even though they contain potential motifs.

    SignalP-NN euk predictions are as follows:

  # name                  Cmax  pos ?  Ymax  pos ?  Smax  pos ?  Smean ?  D     ?

    SignalP output is explained at http://www.cbs.dtu.dk/services/SignalP/output.html

##########################################################################################

Name:  contig047499-ZebOR.A012  Length:  320
MDVELNLTLVTFGGFAELHKYRYLYFVVISTLYILILCFNSTIVFLIWTHKNLHEPMYIFIAALLINSVLYSMIIYPKLL      80
SDVLSEKQTISYPLCLFQGFLYYTSGGSEFLLLAAMAYDRYVSICKPLQYPVIMNRITIYVCLILAWLIPAFETSVLGVL     160
YSNVKLCSFTLTGIFCNNSLYKLQCVPSVAISIYGMVTLINIALLPLLFILFTYIRIIRISYHCCREVRKKAVKTCLPHL     240
LVLINFSCFIFLDIIIVRLDSDLSKTLRLTLTFQSIVFHPLLNPIIYGLKMNEIFKHIKILLSSLITLVLLPYYQMYGIX     320
.....N.................................N........................................      80
................................................................................     160
................................................................................     240
....N...........................................................................     320

(Threshold=0.5)
----------------------------------------------------------------------
SeqName      Position  Potential   Jury    N-Glyc
                                 agreement result
----------------------------------------------------------------------
contig047499-ZebOR.A012    6 NLTL    0.8133     (9/9)   +++
contig047499-ZebOR.A012   40 NSTI    0.7226     (9/9)   ++
contig047499-ZebOR.A012  177 NNSL    0.4228     (7/9)   -
contig047499-ZebOR.A012  245 NFSC    0.5516     (5/9)   +
----------------------------------------------------------------------
```

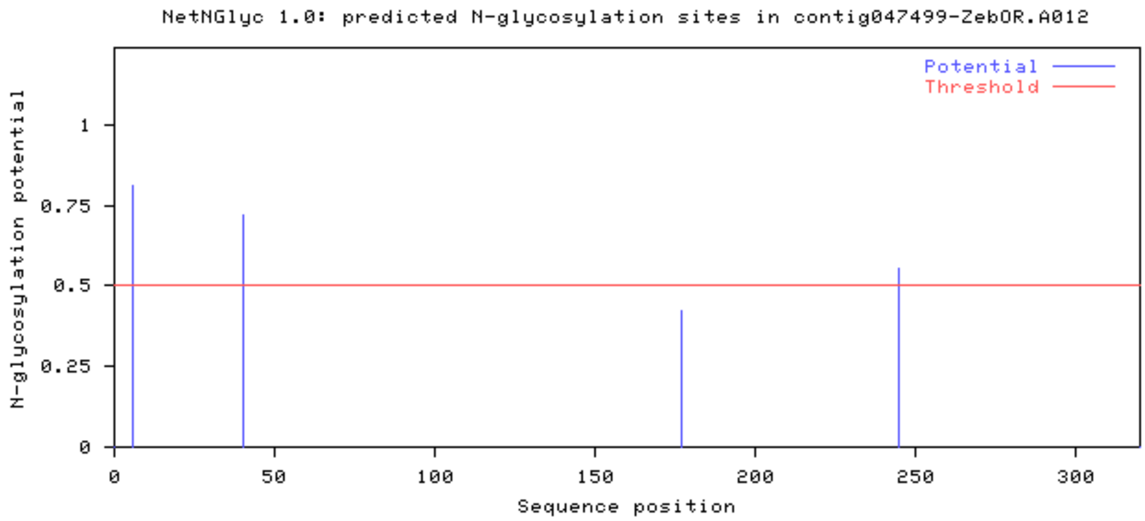

Graphics in PostScript

Output for 'contig047503-ZebOR.A013'

```
##########################################################################################

    Warning: This sequence may not contain a signal peptide!!

    Proteins without signal peptides are unlikely to be exposed to
    the N-glycosylation machinery and thus may not be glycosylated
    (in vivo) even though they contain potential motifs.

    SignalP-NN euk predictions are as follows:

  # name                    Cmax  pos ?  Ymax  pos ?  Smax  pos ?  Smean ?  D     ?

    SignalP output is explained at http://www.cbs.dtu.dk/services/SignalP/output.html

##########################################################################################

Name:  contig047503-ZebOR.A013  Length:  317
MDEELNTTYVTLDGYIEVNKYRYVYFCIIFTLYIIIICSNSTIVYVIWIHKNLHEPMYIFIAALLLNCLLYSTTIYPKLL      80
IDFLSEKQVITYSACLFQFFMFYTLGSSEFFLLAAMAYDRYVAICKPLEYPTIMNKTTVIIFLVVSWLIPAVHIAIQAIA     160
SAEATLCNFNLKGIFCNNAVYTLHCQRSRLITVFGVVALLDLVILPMIFIVFTYTTIFIVSYQSCKEIRKKAAETCLPHL     240
LVLISISCLSIYDVGIARVESDFPKVARLLMTLQLLLYHPLFNPFIYGLKMKEISKQLKRFFCHATIITCINANVPX
.....N.................................N........................................      80
......................................................N.........................     160
................................................................................     240
.............................................................................        320

(Threshold=0.5)
----------------------------------------------------------------------
SeqName      Position  Potential   Jury    N-Glyc
                                agreement result
----------------------------------------------------------------------
contig047503-ZebOR.A013    6 NTTY    0.7358     (9/9)    ++
contig047503-ZebOR.A013   40 NSTI    0.6937     (8/9)    +
contig047503-ZebOR.A013  135 NKTT    0.6558     (7/9)    +
----------------------------------------------------------------------
```

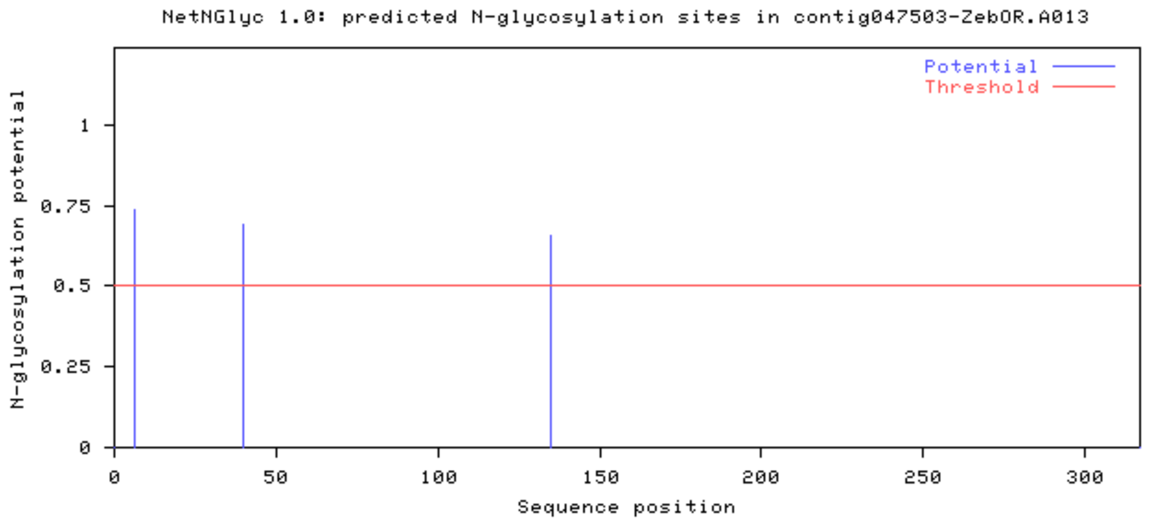

Graphics in PostScript

Output for 'contig047506-ZebOR.A014'

```
##########################################################################################

    Warning: This sequence may not contain a signal peptide!!

    Proteins without signal peptides are unlikely to be exposed to
    the N-glycosylation machinery and thus may not be glycosylated
    (in vivo) even though they contain potential motifs.

    SignalP-NN euk predictions are as follows:

  # name                  Cmax  pos ?  Ymax  pos ?  Smax  pos ?  Smean ?  D     ?

    SignalP output is explained at http://www.cbs.dtu.dk/services/SignalP/output.html

##########################################################################################

Name:  contig047506-ZebOR.A014  Length:  300
MNLTYITFGGHVEVEKYRYLYFVIMFMVYILIICSNSTIVWLIVVQKSLHEPMYIFIAALLVNSVVLSTVIYPKLLIDFL      80
SEKQIILYQACLFQVFLFYALSCSEFLLLSAMAYDRYVSICKPLQYPSIMRRTRVNIFLLLCWFLPAIQVAVPIAGNANT     160
PLCNFTLKGIFCNNSANHLYCVNSRELSIYGMVVLFNVALSPMFFILFTYIKIIIVAYQSCGNVRKKAAQTCLPHVLVLM     240
NYSCLLTYDMVIVRLESEFPKTARFIMTLQFVTYNPLCNPIIYGLKMKEISKHLKILFSX
.N.................................N............................................      80
................................................................................     160
...N............................................................................     240
N...........................................................                         320

(Threshold=0.5)
----------------------------------------------------------------------
SeqName      Position  Potential   Jury    N-Glyc
                                 agreement result
----------------------------------------------------------------------
contig047506-ZebOR.A014    2 NLTY   0.7966     (9/9)    +++
contig047506-ZebOR.A014   36 NSTI   0.6902     (8/9)    +
contig047506-ZebOR.A014  164 NFTL   0.6499     (9/9)    ++
contig047506-ZebOR.A014  173 NNSA   0.4152     (6/9)    -
contig047506-ZebOR.A014  241 NYSC   0.5433     (5/9)    +
----------------------------------------------------------------------
```

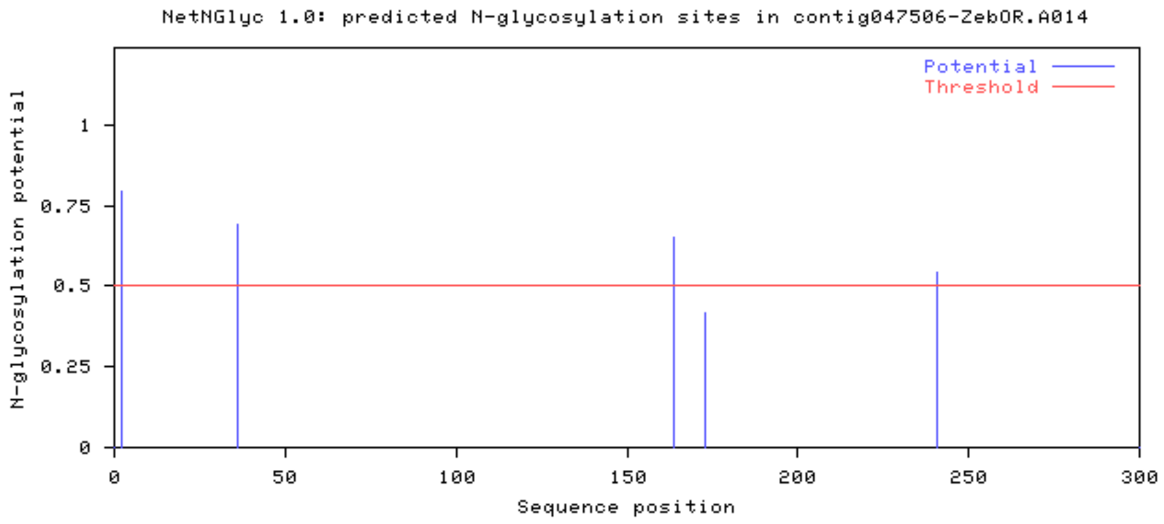

Graphics in PostScript

Output for 'contig047508-ZebOR.A015'

```
##########################################################################################

    Warning: This sequence may not contain a signal peptide!!

    Proteins without signal peptides are unlikely to be exposed to
    the N-glycosylation machinery and thus may not be glycosylated
    (in vivo) even though they contain potential motifs.

    SignalP-NN euk predictions are as follows:

  # name                 Cmax  pos ?  Ymax  pos ?  Smax  pos ?  Smean ?  D     ?

    SignalP output is explained at http://www.cbs.dtu.dk/services/SignalP/output.html

##########################################################################################

Name:  contig047508-ZebOR.A015  Length:  311
MDEELNTTYVTLDGYIEVNKYRYVYFCIIFTLYIIIICSNSTIVYVIWIHKNLHEPMYIFIAALLLNCLLYSTTIYPKLL      80
IDFLSEKQVITYSACLFQFFIFYTLGSSEFFLLAAMAYDRYVAICKPLEYPTIMNKTTVIIFLVVSWLIPAVHIAIQAIG     160
SAEATLCNFNLKGIFCNNAVYTLLCVKSRLIIVFGVVALIDLIILPVLFIVFTYTNIFIISYQSCKEIRKKAAETCLPHL     240
LVLISISCLSIYDVSIARVESDFPKAARLLMTLQIVLYHPLFNPFIYGLKMKEISKQLKRFFCHARIIVYX
.....N.................................N........................................      80
......................................................N.........................     160
................................................................................     240
.......................................................................              320

(Threshold=0.5)
----------------------------------------------------------------------
SeqName      Position  Potential   Jury    N-Glyc
                                 agreement result
----------------------------------------------------------------------
contig047508-ZebOR.A015    6 NTTY   0.7358     (9/9)    ++
contig047508-ZebOR.A015   40 NSTI   0.6934     (8/9)    +
contig047508-ZebOR.A015  135 NKTT   0.6550     (7/9)    +
----------------------------------------------------------------------
```

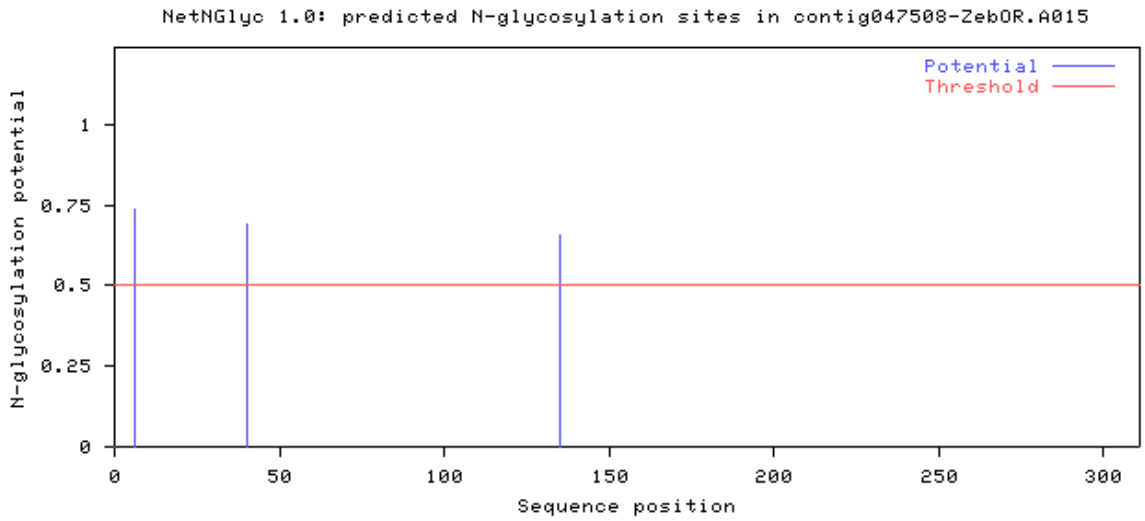

Graphics in PostScript

Output for 'contig047508-ZebOR.A016'

```
##########################################################################################

    Warning: This sequence may not contain a signal peptide!!

    Proteins without signal peptides are unlikely to be exposed to
    the N-glycosylation machinery and thus may not be glycosylated
    (in vivo) even though they contain potential motifs.

    SignalP-NN euk predictions are as follows:

  # name                   Cmax  pos ?  Ymax  pos ?  Smax  pos ?  Smean ?  D     ?

    SignalP output is explained at http://www.cbs.dtu.dk/services/SignalP/output.html

##########################################################################################

Name:  contig047508-ZebOR.A016  Length:  306
MDVELNVTLLTLGGFAELHKYRYLYFVVIFTLYILILCFNSIIVYLIWTCKNLHEPMYIFIAALLINSVLYSMIIYPKLL      80
SDVLSEKQTISYPLCLFQGFSYYTSAVSEFLLLAAMAYDRYVSICKPLQYPVIMNRITIYVCVILAWLIPAFEIAVSFVL     160
YSNVKLCSFTLTAIFCNNSFYRLQCVPSVVISIYGVVMLINMTFLPMLFILFSYIRILRISYSCCRETRRKALKTCLPHL     240
LVLINFSCFFFFDIIIVRLESDLSNTVRLTLTFQSILFHPLLNPIIYGLKVNEIFKHIKMLLCQVX
.....N..........................................................................      80
................................................................................     160
................................................................................     240
....N.............................................................                  320

(Threshold=0.5)
----------------------------------------------------------------------
SeqName      Position  Potential   Jury    N-Glyc
                                 agreement result
----------------------------------------------------------------------
contig047508-ZebOR.A016    6 NVTL    0.7849     (9/9)   +++
contig047508-ZebOR.A016  177 NNSF    0.3830     (9/9)   --
contig047508-ZebOR.A016  201 NMTF    0.4057     (6/9)   -
contig047508-ZebOR.A016  245 NFSC    0.5668     (6/9)   +
----------------------------------------------------------------------
```

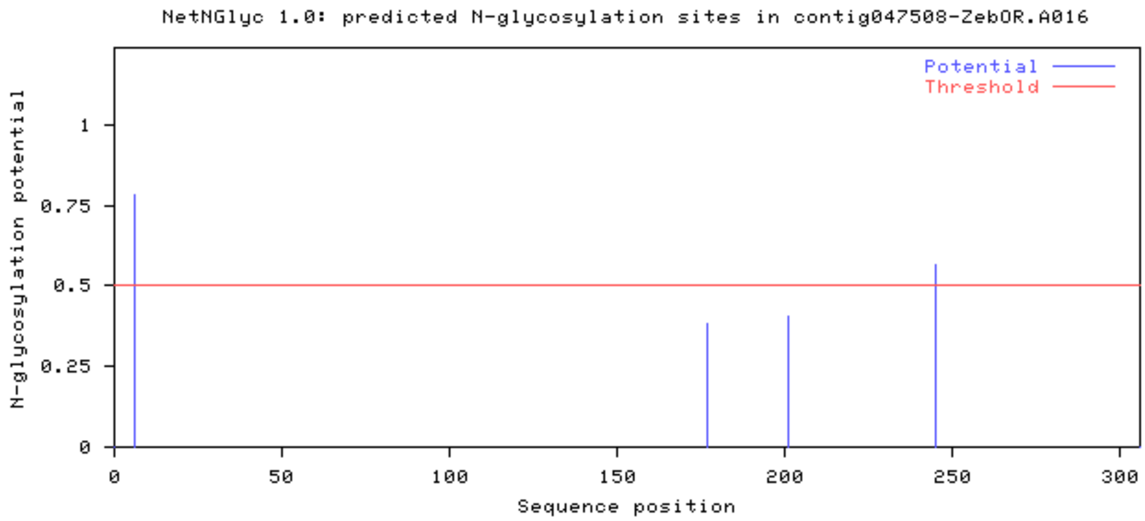

Graphics in PostScript

Output for 'contig047508-ZebORe.A026'

```
##########################################################################################

    Warning: This sequence may not contain a signal peptide!!

    Proteins without signal peptides are unlikely to be exposed to
    the N-glycosylation machinery and thus may not be glycosylated
    (in vivo) even though they contain potential motifs.

    SignalP-NN euk predictions are as follows:

  # name                    Cmax  pos ?  Ymax  pos ?  Smax  pos ?  Smean ?  D     ?

    SignalP output is explained at http://www.cbs.dtu.dk/services/SignalP/output.html

##########################################################################################

Name:  contig047508-ZebORe.A026        Length:  71
MDNKLNLTYITLNGYVEVEKYRYVYFLIIFTIYAAVIFSNSTIIRLIVFHQSLHEPMYIFIAVLLINSTFF
.....N................................N..............................               80

(Threshold=0.5)
----------------------------------------------------------------------
SeqName      Position  Potential   Jury    N-Glyc
                                 agreement result
----------------------------------------------------------------------
contig047508-ZebORe.A026    6 NLTY   0.7512     (9/9)   +++
contig047508-ZebORe.A026   40 NSTI   0.5973     (8/9)   +
contig047508-ZebORe.A026   67 NSTF   0.4141     (5/9)   -
----------------------------------------------------------------------
```

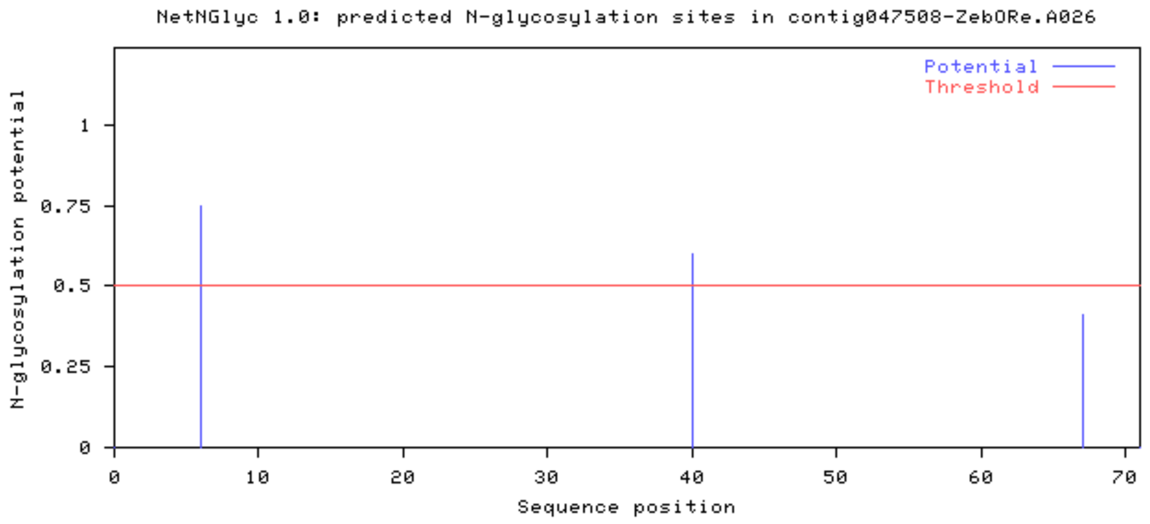

Graphics in PostScript

Output for 'contig047514-ZebOR.A017'

```
##########################################################################################

    Warning: This sequence may not contain a signal peptide!!

    Proteins without signal peptides are unlikely to be exposed to
    the N-glycosylation machinery and thus may not be glycosylated
    (in vivo) even though they contain potential motifs.

    SignalP-NN euk predictions are as follows:

  # name                    Cmax  pos ?  Ymax  pos ?  Smax  pos ?  Smean ?  D     ?

    SignalP output is explained at http://www.cbs.dtu.dk/services/SignalP/output.html

##########################################################################################

Name:  contig047514-ZebOR.A017  Length:  306
MDVKLNVTLLTLGGFAELHKYRYLYFVVIFTLYILILCFNSTIVYLIWTHKNLHEPMYIFIAALLINSVLYSMIIYPKLL      80
SDVLSEKQTISYPLCLFQGFSYYTSAGSEFLLLAAMAYDRYVSICKPLQYPVIMNRITINVFLILAWLIPAFEIAVSFVL     160
YFNIKLCSFTLTGIFCNNSIYRLQCVPSVTISIYGVVTLINIALLPMLFILFTYIRILRISYNCCRETRRKALKTCLPHL     240
LVLINFSCFIVFDSVIIRLDSDLSKTLRLTLTFQSILFHPLLNPIIYGLKMNEIFRHIKSLLCQVX
.....N.................................N........................................      80
................................................................................     160
................................................................................     240
....N.............................................................                   320

(Threshold=0.5)
----------------------------------------------------------------------
SeqName      Position  Potential   Jury    N-Glyc
                                 agreement result
----------------------------------------------------------------------
contig047514-ZebOR.A017    6 NVTL   0.7750     (9/9)   +++
contig047514-ZebOR.A017   40 NSTI   0.6940     (9/9)   ++
contig047514-ZebOR.A017  177 NNSI   0.4374     (7/9)   -
contig047514-ZebOR.A017  245 NFSC   0.5634     (6/9)   +
----------------------------------------------------------------------
```

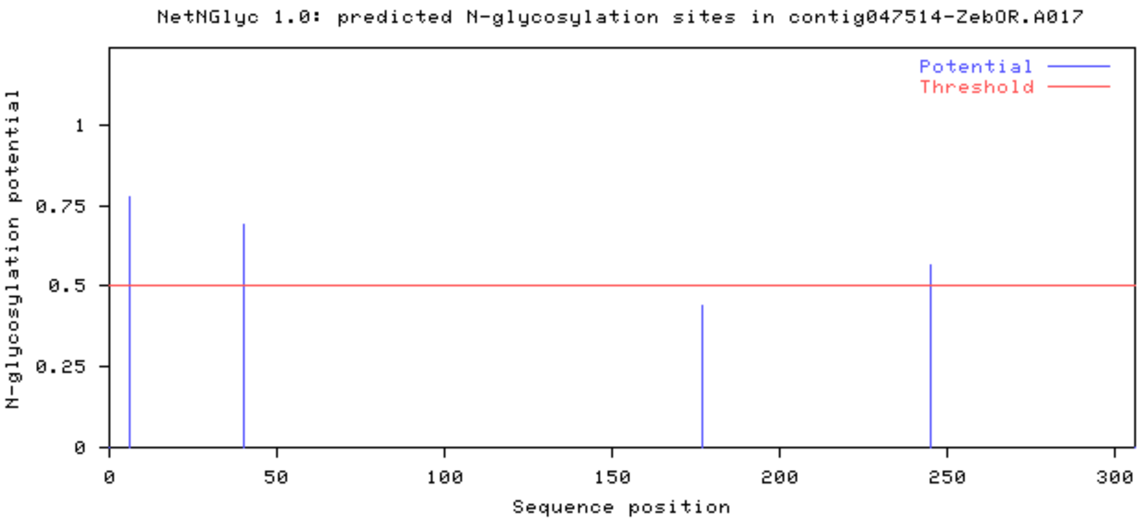

Graphics in PostScript

Output for 'contig047515-ZebOR.A018'

```
##########################################################################################

    Warning: This sequence may not contain a signal peptide!!

    Proteins without signal peptides are unlikely to be exposed to
    the N-glycosylation machinery and thus may not be glycosylated
    (in vivo) even though they contain potential motifs.

    SignalP-NN euk predictions are as follows:

  # name                    Cmax  pos ?  Ymax  pos ?  Smax  pos ?  Smean ?  D     ?

    SignalP output is explained at http://www.cbs.dtu.dk/services/SignalP/output.html

##########################################################################################

Name:  contig047515-ZebOR.A018  Length:  316
MDEVLNATYLTLDGYVEVNKYRYVYFFIFFILYSLIICSNSTIVYIIWIHKNLHEPMYTFIAALLLNCVLYSTTVYPKLL      80
IDFLSEKQVTTYSACLFQFFMFYTLGSSEFFLLAAMAYDRYVAICKPLQYQTIMSKTTVSIFLAVAWLVPVCHIAVLTAG     160
SAEATLCNFNLKGIFCNNAVYTLQCVKSRLITVFGVVALIDLVILPMLFIVFTYSNIFILTYQSCKDVRKKALETCLPHL     240
LVLFSFSCLSIYDVSIARVESDFPKTARLIMTLQIVLYHPLLNPFIYGLKMKEISKQLKRFFYHAKIISCINSECX
.....N.................................N........................................      80
................................................................................     160
................................................................................     240
............................................................................         320

(Threshold=0.5)
----------------------------------------------------------------------
SeqName      Position  Potential   Jury    N-Glyc
                                 agreement result
----------------------------------------------------------------------
contig047515-ZebOR.A018    6 NATY    0.6279     (9/9)    ++
contig047515-ZebOR.A018   40 NSTI    0.7253     (9/9)    ++
----------------------------------------------------------------------
```

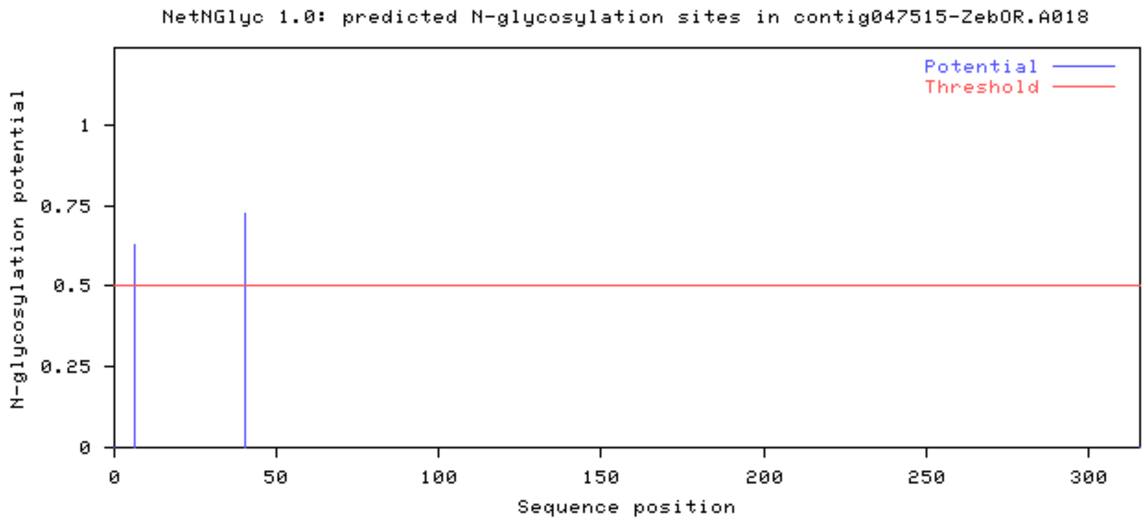

Graphics in PostScript

Output for 'contig047515-ZebOR.A019'

```
##########################################################################################

    Warning: This sequence may not contain a signal peptide!!

    Proteins without signal peptides are unlikely to be exposed to
    the N-glycosylation machinery and thus may not be glycosylated
    (in vivo) even though they contain potential motifs.

    SignalP-NN euk predictions are as follows:

  # name                    Cmax  pos ?  Ymax  pos ?  Smax  pos ?  Smean ?  D     ?

    SignalP output is explained at http://www.cbs.dtu.dk/services/SignalP/output.html

##########################################################################################

Name:  contig047515-ZebOR.A019  Length:  300
MNITYITFGGHVEVEKYRYIYFVIMFMVYGLIICSNSTIVWVIIVQKSLHEPMYIFIAALLVNSVVLSTVIYPKLLIDFL      80
SEKQIILYHACLFQVFMFYVLSSSEFLLLSAMAYDRYVSICKPLQYPTIMRRTRVSIFLIMSWFLPAIQIVVPVLRNSIT     160
PLCNFTLKGIFCNNSVNHLYCVTSKELSIYGMVVLFNGALFPMLFILFTYIKIIIVACQSCGNVRKKAAQTCLPHVLVLI     240
NYSCLVTYDMVIVRLESEFPKTARFIMTLQFITYNPLCNPIIYGLKMKEISKNLKRLFSX
.N.................................N............................................      80
................................................................................     160
...N............................................................................     240
N...........................................................                         320

(Threshold=0.5)
----------------------------------------------------------------------
SeqName      Position  Potential   Jury    N-Glyc
                                 agreement result
----------------------------------------------------------------------
contig047515-ZebOR.A019    2 NITY   0.7927     (9/9)    +++
contig047515-ZebOR.A019   36 NSTI   0.7254     (9/9)    ++
contig047515-ZebOR.A019  164 NFTL   0.6559     (9/9)    ++
contig047515-ZebOR.A019  173 NNSV   0.4574     (6/9)    -
contig047515-ZebOR.A019  241 NYSC   0.5290     (6/9)    +
----------------------------------------------------------------------
```

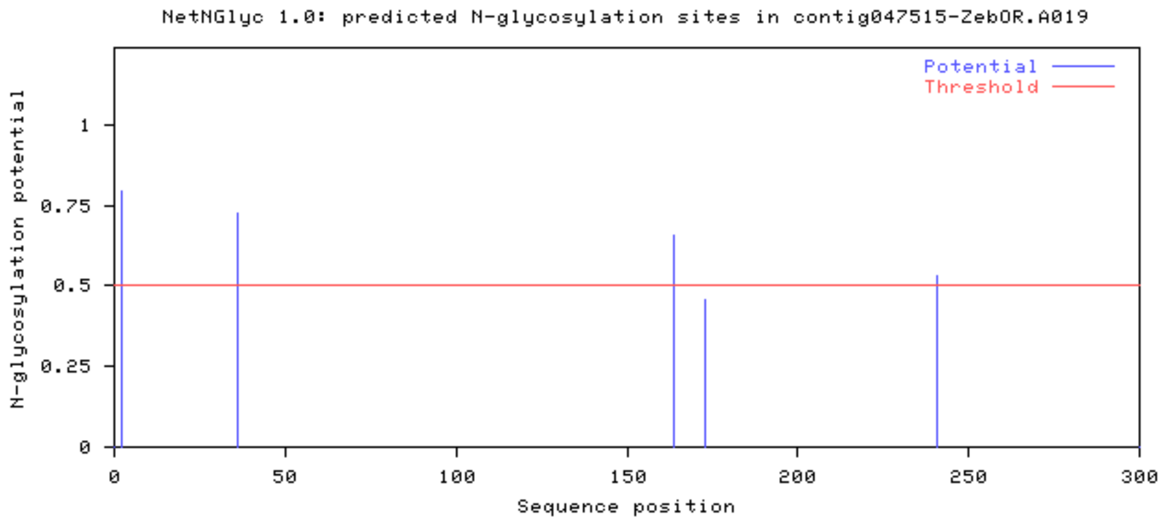

Graphics in PostScript

Output for 'contig047518-ZebORp.A032'

##

Warning: This sequence may not contain a signal peptide!!

Proteins without signal peptides are unlikely to be exposed to the N-glycosylation machinery and thus may not be glycosylated (in vivo) even though they contain potential motifs.

SignalP-NN euk predictions are as follows:

```
 # name                    Cmax  pos ?  Ymax  pos ?  Smax  pos ?  Smean ?  D     ?
```

SignalP output is explained at http://www.cbs.dtu.dk/services/SignalP/output.html

##

```
Name:  contig047518-ZebORp.A032         Length:  207
KTLMDEELNVTYISFDRHVEINYRYFYFFIMFTLYILITCSNSIIIYLILIHKNLHKPMYTFIAALLLNVVIYATTIYPK      80
LLIDFLSKKQIISYSACLFQFSIIYSLGSSEFVLLAAIAYDRYMAICKPLQYPTIMRKSTVSIFLVIAWLVPVFVTVRHG     160
KPWGVWGRRTQARCSMYKQCVYLQWWNKTTINPRAFPDSSVVSLREX
........N.......................................................................      80
................................................................................     160
..........................N....................                                     240
```

(Threshold=0.5)

SeqName	Position		Potential	Jury agreement	N-Glyc result
contig047518-ZebORp.A032	9	NVTY	0.7980	(9/9)	+++
contig047518-ZebORp.A032	187	NKTT	0.6240	(8/9)	+

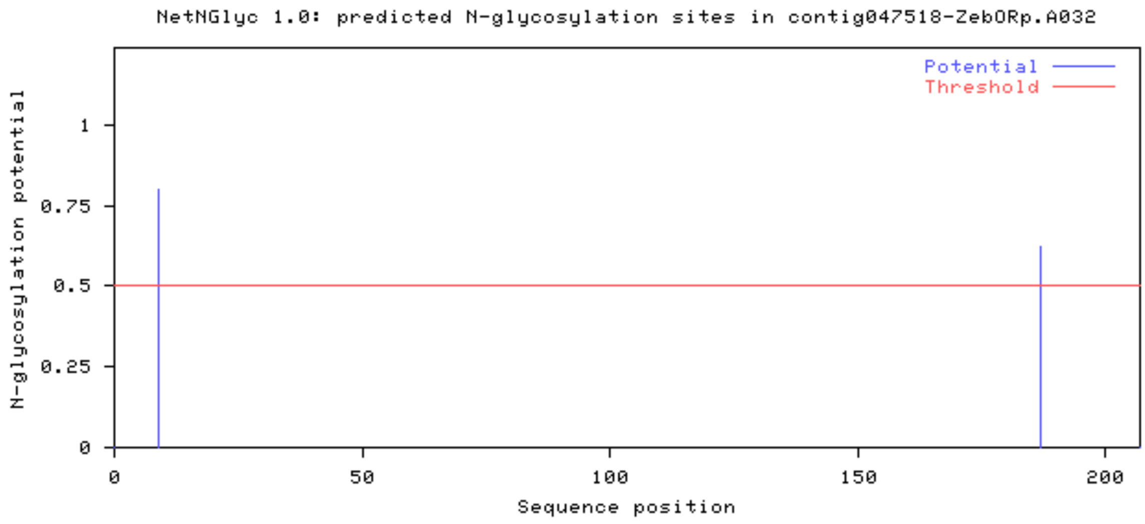

Graphics in PostScript

Output for 'contig047520-ZebORp.A033'

```
##########################################################################################

    Warning: This sequence may not contain a signal peptide!!

    Proteins without signal peptides are unlikely to be exposed to
    the N-glycosylation machinery and thus may not be glycosylated
    (in vivo) even though they contain potential motifs.

    SignalP-NN euk predictions are as follows:

  # name                 Cmax  pos ?  Ymax  pos ?  Smax  pos ?  Smean ?  D     ?

    SignalP output is explained at http://www.cbs.dtu.dk/services/SignalP/output.html

##########################################################################################

Name:  contig047520-ZebORp.A033         Length:  272
AYFSRDCNGCVTDANGAKKGALYSTAVYPKYLNDFLSEKQVISYSACLFQYFLFYSLACSEFFLLAAMGYDRYVAICKSL      80
QYPTIMRKKDCEYFPVHSFACACFYMTIQAIGSAKAKLCSFYLNETFCNNRIYTFQCVRSELFAAFALVCLLDLGILPLL     160
FILYTYTKIFLMSYRSCKEIRKKAAETCLPHLTVLITFSCLGCYDVVIARVESDFPKTVRLIMTLQLALYHPLFNPFVYG     240
LKMKEIFKHLKSLLSPPAKKKKKFDALILNCX
................................................................................      80
...........................................N....................................     160
................................................................................     240
................................                                                     320

(Threshold=0.5)
----------------------------------------------------------------------
SeqName      Position  Potential   Jury    N-Glyc
                                 agreement result
----------------------------------------------------------------------
contig047520-ZebORp.A033  124 NETF   0.5728     (7/9)   +
----------------------------------------------------------------------
```

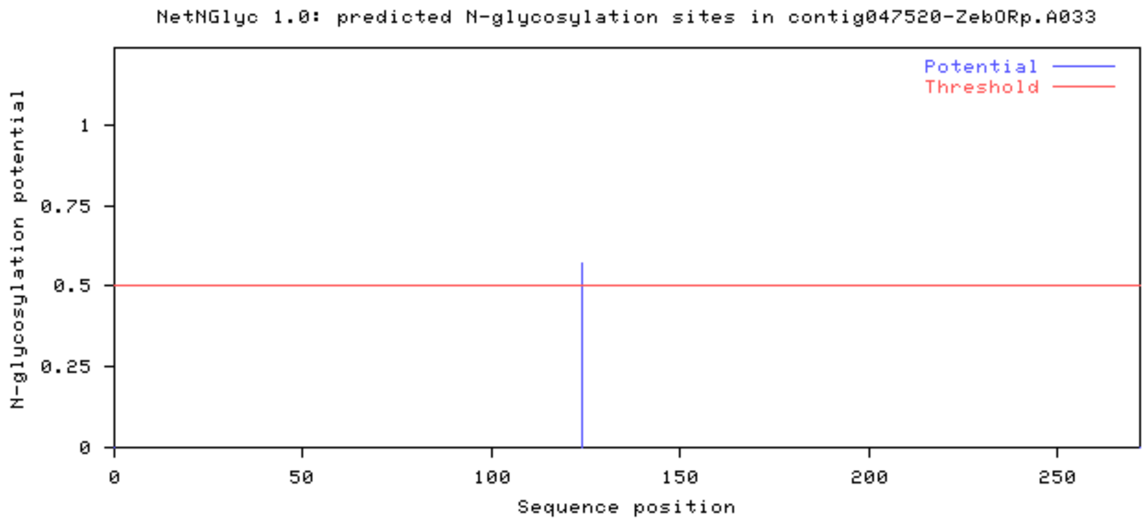

Graphics in PostScript

Output for 'contig047521-ZebOR.A020'

```
##########################################################################################

    Warning: This sequence may not contain a signal peptide!!

    Proteins without signal peptides are unlikely to be exposed to
    the N-glycosylation machinery and thus may not be glycosylated
    (in vivo) even though they contain potential motifs.

    SignalP-NN euk predictions are as follows:

  # name                    Cmax  pos ?  Ymax  pos ?  Smax  pos ?  Smean ?  D     ?

    SignalP output is explained at http://www.cbs.dtu.dk/services/SignalP/output.html

##########################################################################################

Name:  contig047521-ZebOR.A020  Length:  316
MDQELNFTYVTLDWYVDINKYRYVFFFIMFALYSLIICTNSTIMYIICIHKNLHEPMYIFIAALLLNSVLYSTTIYPKLL      80
IDFLSEKQVTTYSACLFQFFMFYTLGGSEFFLLAAMAYDRYVAICKPLQYHIIMRKTTVSIFLIIAWLVPACHIAVQAIG     160
SANIKLCDFNIKGIFCNNAVYTLLCERSRLITIFGVVALLDLAVLPMLFIVFTYTKIFIVSYQRCKEIQKKAAETCLPHL     240
LVLISASVFFVYDVSIARVETNFPKTVRIVMTLQVVLYHPIFNPFIYGLKMKKISKHLKRFFSRPQSILVLKVNAX
.....N.................................N........................................      80
................................................................................     160
................................................................................     240
............................................................................         320

(Threshold=0.5)
----------------------------------------------------------------------
SeqName      Position  Potential   Jury    N-Glyc
                                 agreement result
----------------------------------------------------------------------
contig047521-ZebOR.A020    6 NFTY    0.7623     (9/9)    +++
contig047521-ZebOR.A020   40 NSTI    0.7280     (9/9)    ++
----------------------------------------------------------------------
```

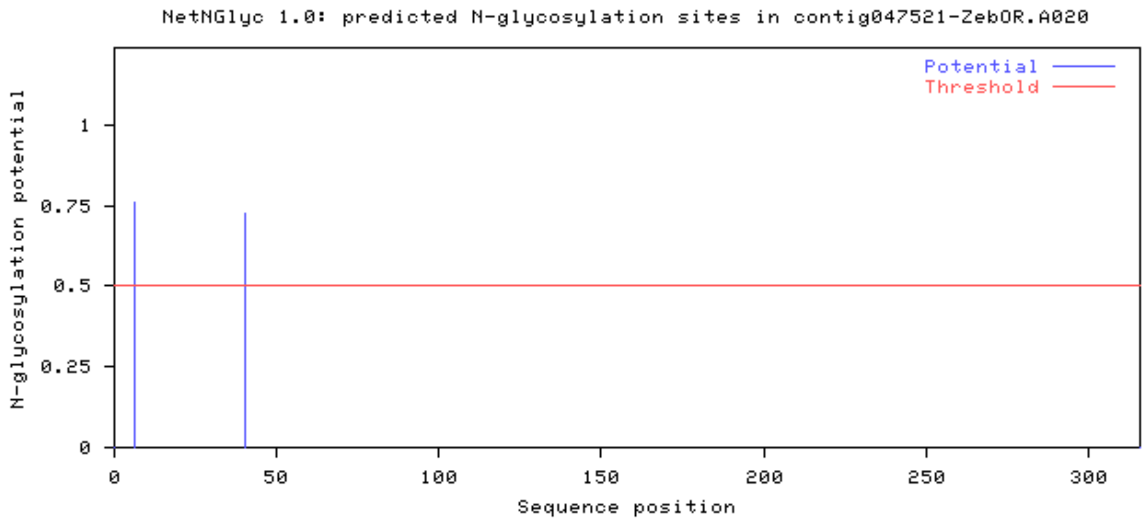

Graphics in PostScript

Output for 'contig047523-ZebOR.A022'

```
##########################################################################################

    Warning: This sequence may not contain a signal peptide!!

    Proteins without signal peptides are unlikely to be exposed to
    the N-glycosylation machinery and thus may not be glycosylated
    (in vivo) even though they contain potential motifs.

    SignalP-NN euk predictions are as follows:

  # name                  Cmax  pos ?  Ymax  pos ?  Smax  pos ?  Smean ?  D     ?

    SignalP output is explained at http://www.cbs.dtu.dk/services/SignalP/output.html

##########################################################################################

Name:  contig047523-ZebOR.A022  Length:  307
MDVELNVTLLTLGGFAELHKYRYLYFVVIFTLYILILCFNSTIVCLIWTCKNLHEPMYIFIAALLINSVLYSMIIYPKLL      80
SDVLSEKQIISYTLCLFQGFLYYTSAGSEFLLLAAMAYDRYVSICKPLQYPVIMNRITIYVSLVLAWLIPAFETAVSVVL     160
YSEVKLCSFTLTGIFCNNSGYKLQCVTSVAISVYGVVMLINIALLPLLFILFTYIRIVRVSYQSCREVRKKAVKTCLPHL     240
LVLINFSCFIVFDVIIVRLESDLSKTLRLILTFQSILFHPLLNPIIYGLKMNEIFKNLKILFCHVKX
.....N.................................N........................................      80
................................................................................     160
................................................................................     240
....N..............................................................                  320

(Threshold=0.5)
----------------------------------------------------------------------
SeqName      Position  Potential   Jury    N-Glyc
                                 agreement result
----------------------------------------------------------------------
contig047523-ZebOR.A022    6 NVTL    0.7850     (9/9)   +++
contig047523-ZebOR.A022   40 NSTI    0.7134     (9/9)   ++
contig047523-ZebOR.A022  177 NNSG    0.3826     (7/9)   -
contig047523-ZebOR.A022  245 NFSC    0.5623     (6/9)   +
----------------------------------------------------------------------
```

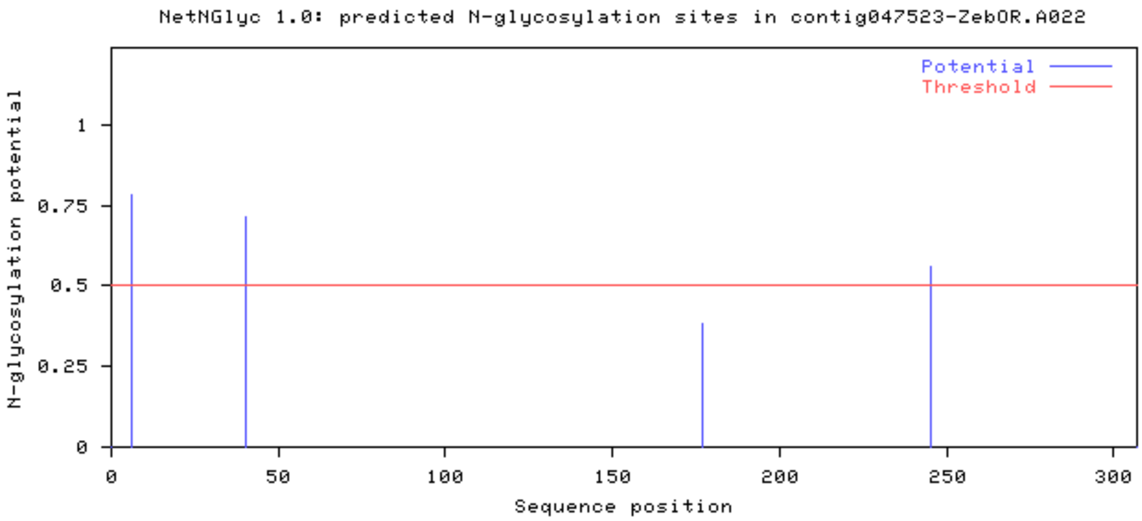

Graphics in PostScript

Output for 'contig047526-ZebOR.A021'

```
##########################################################################################

    Warning: This sequence may not contain a signal peptide!!

    Proteins without signal peptides are unlikely to be exposed to
    the N-glycosylation machinery and thus may not be glycosylated
    (in vivo) even though they contain potential motifs.

    SignalP-NN euk predictions are as follows:

  # name                  Cmax  pos ?  Ymax  pos ?  Smax  pos ?  Smean ?  D     ?

    SignalP output is explained at http://www.cbs.dtu.dk/services/SignalP/output.html

##########################################################################################

Name:  contig047526-ZebOR.A021  Length:  306
MDVELNVTLLTLGGFAELHKYRYLYFVIIFTLYILILCFNSTIVYLIWTCKNLHEPMYIFIAALLINSVLYSMIIYPKLL      80
SDVLFEKQTISYPLCLFQGFSYYTSAGSEFLLLAAMAYDRYVSICKPLQYPVIMNRITIYVCVILAWLIPAFEIAVSFVL     160
YSNVKLCSFTLTGIFCNNSFYRLQCVPSVVISVYGVVMLINVAFLPMLFILFSYIRILRISYSCCRETRRKALKTCLPHL     240
LVLINFSCFFFFDIIIVRLESDLSNTVRLTLTFQSILFHPLLNPIIYGLKVNEIFKHIKMLLCQVX
.....N.................................N........................................      80
................................................................................     160
................................................................................     240
....N.............................................................                  320

(Threshold=0.5)
----------------------------------------------------------------------
SeqName      Position  Potential   Jury    N-Glyc
                                 agreement result
----------------------------------------------------------------------
contig047526-ZebOR.A021    6 NVTL   0.7851     (9/9)    +++
contig047526-ZebOR.A021   40 NSTI   0.7014     (9/9)    ++
contig047526-ZebOR.A021  177 NNSF   0.3559     (9/9)    --
contig047526-ZebOR.A021  245 NFSC   0.5668     (6/9)    +
----------------------------------------------------------------------
```

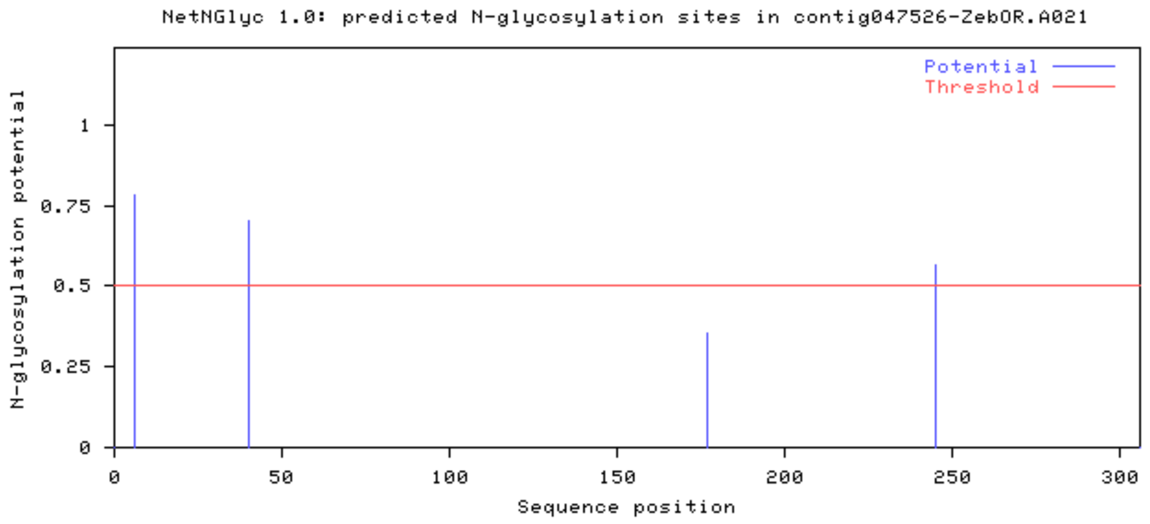

Graphics in PostScript

Output for 'contig048235-ZebORe.E062'

##

Warning: This sequence may not contain a signal peptide!!

Proteins without signal peptides are unlikely to be exposed to
the N-glycosylation machinery and thus may not be glycosylated
(in vivo) even though they contain potential motifs.

SignalP-NN euk predictions are as follows:

\# name Cmax pos ? Ymax pos ? Smax pos ? Smean ? D ?

SignalP output is explained at http://www.cbs.dtu.dk/services/SignalP/output.html

##

Name: contig048235-ZebORe.E062 Length: 104
ALLFILYTYMKILLVCFSGSDQTRQKAISTCTPHLASILNFSFGASFEILQSRFNMKNVPNMLRIFLSLYFLTCQPLFNP 80
VMYGLKMTKIRNICKSLITNTHLX
.... 80
.... 160

(Threshold=0.5)

No sites predicted in this sequence.

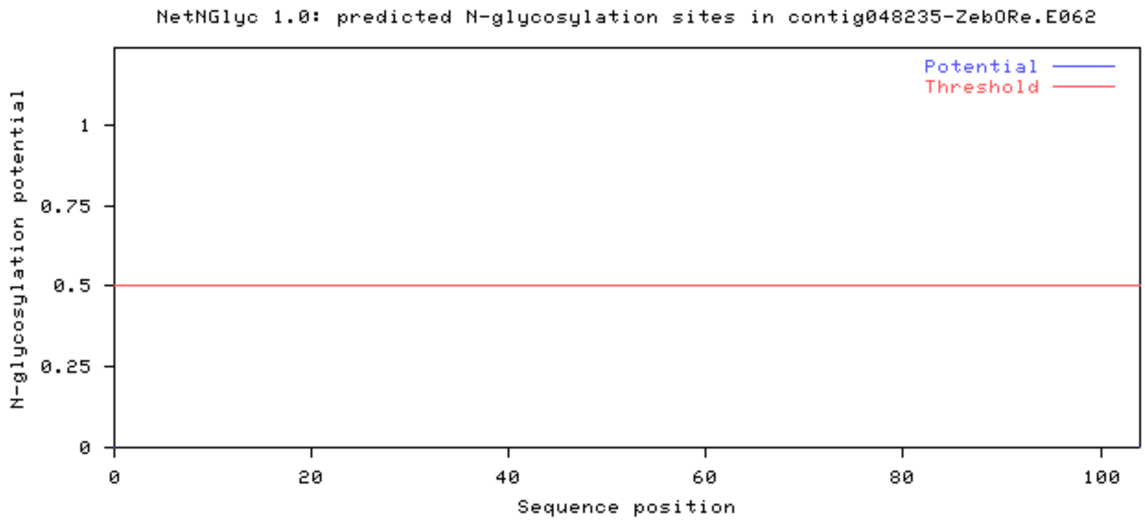

Graphics in PostScript

Output for 'contig048237-ZebOR.G065'

```
##########################################################################################

    Warning: This sequence may not contain a signal peptide!!

    Proteins without signal peptides are unlikely to be exposed to
    the N-glycosylation machinery and thus may not be glycosylated
    (in vivo) even though they contain potential motifs.

    SignalP-NN euk predictions are as follows:

  # name                  Cmax  pos ?  Ymax  pos ?  Smax  pos ?  Smean ?  D     ?

    SignalP output is explained at http://www.cbs.dtu.dk/services/SignalP/output.html

##########################################################################################

Name:  contig048237-ZebOR.G065  Length:  312
MENNFEIVFVLQGLNDSLTNRQIYFAFALMSYLFTVSVNLTLIITISLDKTLHEPIYIFLCSLCFNEICGASSFYPKLLH      80
DLLTNSYVITYTACLGQMFVTYSYIFSECTSLTVMAYDRYIAICKPLQYRMLMTAQKVAQLLMLTWCFSVFETAVGTVLT     160
ARLPLCGRHIPKIFCTNWEVVKLSCSDSTLNNIYAFMLIFSHLSQTALIMVSYVHLIRAAIRSQADRRKFMQTCLPHLIT     240
LLVFTTSLMFDTMYSRYSGGSTMKALQNALAAQFLVVPPLVNPIIYGLNLQQIRSRMVHRFTHRTGTFRKNX
..............N........................N........................................      80
................................................................................     160
................................................................................     240
........................................................................             320

(Threshold=0.5)
----------------------------------------------------------------------
SeqName      Position  Potential   Jury    N-Glyc
                                agreement result
----------------------------------------------------------------------
contig048237-ZebOR.G065   15 NDSL   0.7059     (9/9)   ++
contig048237-ZebOR.G065   39 NLTL   0.7856     (9/9)   +++
----------------------------------------------------------------------
```

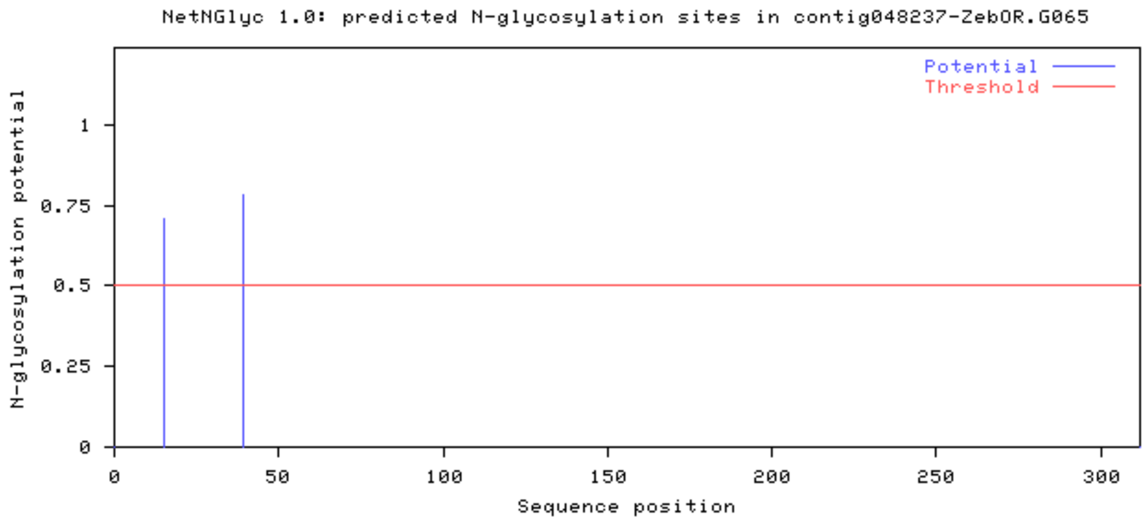

Graphics in PostScript

Output for 'contig048238-ZebORe.E054'

```
##########################################################################################

    Warning: This sequence may not contain a signal peptide!!

    Proteins without signal peptides are unlikely to be exposed to
    the N-glycosylation machinery and thus may not be glycosylated
    (in vivo) even though they contain potential motifs.

    SignalP-NN euk predictions are as follows:

  # name                 Cmax  pos ?  Ymax  pos ?  Smax  pos ?  Smean ?  D     ?

    SignalP output is explained at http://www.cbs.dtu.dk/services/SignalP/output.html

##########################################################################################

Name:  contig048238-ZebORe.E054         Length:  195
VMSYDRYLAICFPLQYHTRMSPCKVSMLIVLTWFSSFLGITLLISLSAPLQLCGNIINKVYCDNYSVVKLACSDTTVNNI      80
CGLISTSLTTISSVTLILYTYMRILKVCFSGSKQTRQKAISTCTPHLASLLNYSCSAFFEIAQSRLNMKHVPNMVRIFLS     160
LYWLICPPLCNPLLYGLSLTKIRIIYKGLILSKVX
...............................................................N................      80
...................................................N............................     160
...................................                                                  240

(Threshold=0.5)
----------------------------------------------------------------------
SeqName      Position  Potential   Jury    N-Glyc
                                 agreement result
----------------------------------------------------------------------
contig048238-ZebORe.E054   64 NYSV   0.6600     (9/9)   ++
contig048238-ZebORe.E054  132 NYSC   0.6877     (8/9)   +
----------------------------------------------------------------------
```

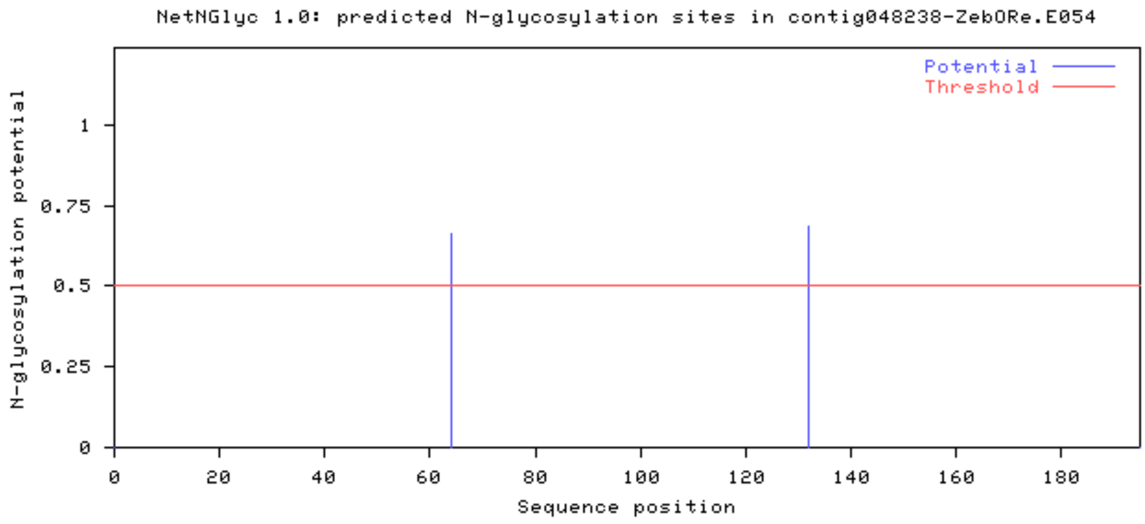

Graphics in PostScript

Output for 'contig048239-ZebOR.E048'

```
##########################################################################################

    Warning: This sequence may not contain a signal peptide!!

    Proteins without signal peptides are unlikely to be exposed to
    the N-glycosylation machinery and thus may not be glycosylated
    (in vivo) even though they contain potential motifs.

    SignalP-NN euk predictions are as follows:

  # name                    Cmax  pos ?  Ymax  pos ?  Smax  pos ?  Smean ?  D     ?

    SignalP output is explained at http://www.cbs.dtu.dk/services/SignalP/output.html

##########################################################################################

Name:  contig048239-ZebOR.E048  Length:  310
MTVNSSQSSFLVFSAYFDSGHLKYLFFVIVMSLYFLIITANVLLIVVICVNRSLHEPMYMFLCSLFVNELYGSTGLFPFL      80
LLQILSDVHTVSAPLCFLQIFCVHTYGTAELANLAVMSYDRYLAICFPLQYHTRMSPCKVSMLIVLTWFSSFLVITVLIS     160
LSAPLQLCGNIINKVYCDNYSIVKLACSDTTVNNIYGLISTPLVILCPVSLILYTYMRILKICFSGSKQTRQKAVSTCTP     240
HLASLLNFSFGCFFEILQSRFNMNSVPSMLRIFLSLYFLTCQPVFNPLMYGLTLSKISLTCKKLLCADMX
...N.............................................N..............................      80
................................................................................     160
..................N.............................................................     240
......................................................................               320

(Threshold=0.5)
----------------------------------------------------------------------
SeqName      Position  Potential   Jury    N-Glyc
                                agreement  result
----------------------------------------------------------------------
contig048239-ZebOR.E048    4 NSSQ   0.6998     (9/9)   ++
contig048239-ZebOR.E048   51 NRSL   0.7483     (9/9)   ++
contig048239-ZebOR.E048  179 NYSI   0.5860     (8/9)   +
contig048239-ZebOR.E048  247 NFSF   0.4634     (7/9)   -
----------------------------------------------------------------------
```

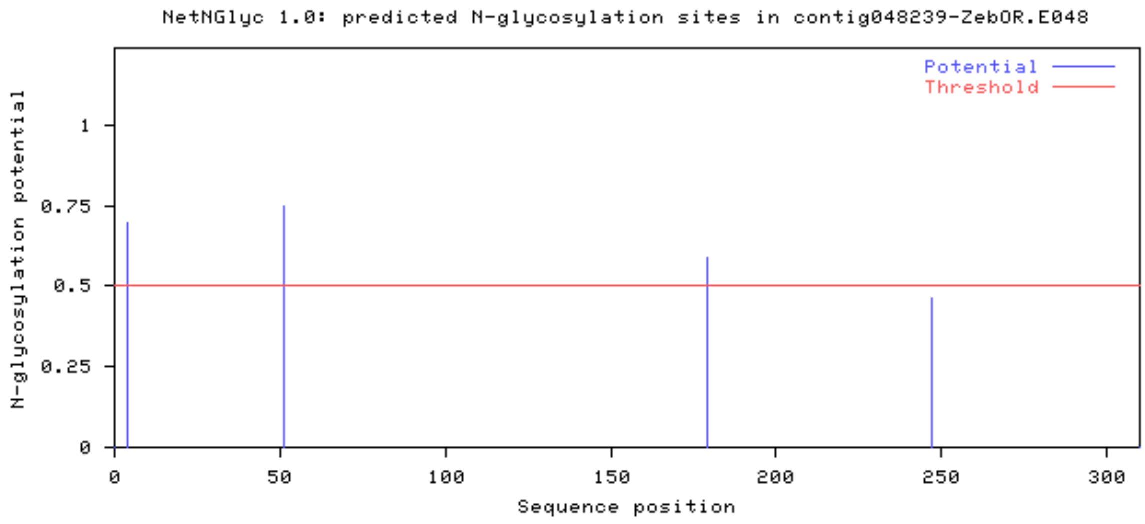

Graphics in PostScript

Output for 'contig048239-ZebORe.E056'

```
########################################################################################

    Warning: This sequence may not contain a signal peptide!!

    Proteins without signal peptides are unlikely to be exposed to
    the N-glycosylation machinery and thus may not be glycosylated
    (in vivo) even though they contain potential motifs.

    SignalP-NN euk predictions are as follows:

  # name                   Cmax   pos ?  Ymax   pos ?  Smax   pos ?  Smean ?  D     ?

    SignalP output is explained at http://www.cbs.dtu.dk/services/SignalP/output.html

########################################################################################

Name:  contig048239-ZebORe.E056        Length:  38
MMNSSQVSYFTLTAYLDSGALKYLYFTVVAFLYIVIVT
..N...................................                                          80

(Threshold=0.5)
----------------------------------------------------------------------
SeqName      Position  Potential    Jury    N-Glyc
                                 agreement result
----------------------------------------------------------------------
contig048239-ZebORe.E056    3 NSSQ    0.6893     (9/9)    ++
----------------------------------------------------------------------
```

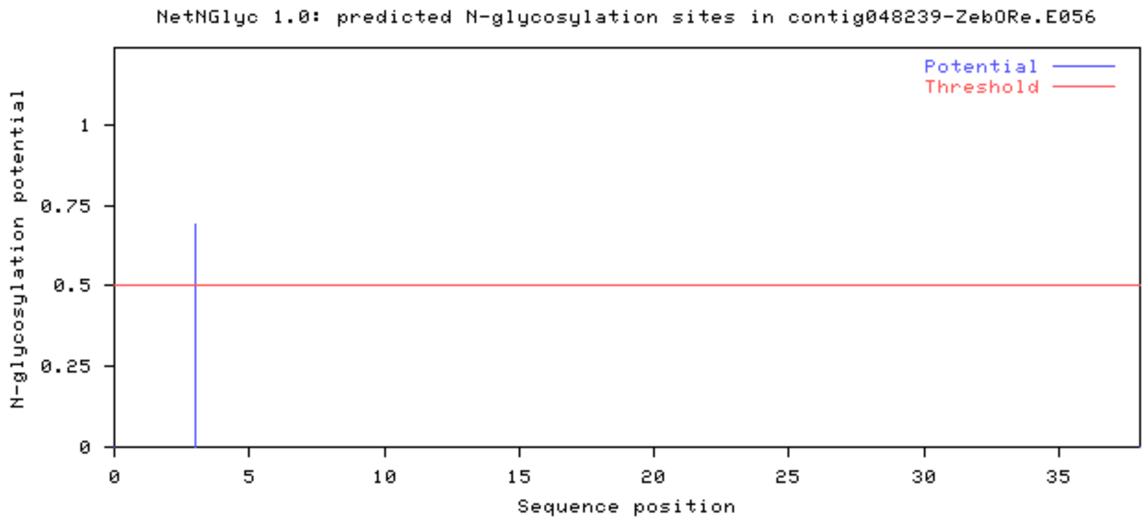

Graphics in PostScript

Output for 'contig048242-ZebOR.E049'

```
##########################################################################################

    Warning: This sequence may not contain a signal peptide!!

    Proteins without signal peptides are unlikely to be exposed to
    the N-glycosylation machinery and thus may not be glycosylated
    (in vivo) even though they contain potential motifs.

    SignalP-NN euk predictions are as follows:

  # name                    Cmax   pos ?   Ymax   pos ?   Smax   pos ?   Smean ?   D     ?

    SignalP output is explained at http://www.cbs.dtu.dk/services/SignalP/output.html

##########################################################################################

Name:  contig048242-ZebOR.E049  Length:  306
MVNSTVPYFILSTYINVGSLKYLFFVLIALLYFSIVFVNTSLIVVICVNRSLHEPMYMFLCSLFVNELYGSAGLFPFLLL      80
QILSDVHTVSAPLCFLQIFCLYTYGHVEFCNLAVMSYDRYLAVCYPLHYKSHMTDNKVVIFIVVIWLYSFVKFTITLCLT     160
LRLTWCGKIINGLYCHNYLVVKLACSDTNLNNLFGLFGIVITVLVPLLPIFYSYMKILKVCFSGSRQMRRKAVSTCAPHL     240
ASLLNFSFGCLFEILQSRFDTTSVPSALRIFLSLYFLIIQPLLNPIMYGTQMSKIRHVLCYKMSLX
..N...................................N.........N...............................      80
................................................................................     160
................................................................................     240
..................................................................                   320

(Threshold=0.5)
----------------------------------------------------------------------
SeqName      Position  Potential   Jury    N-Glyc
                                 agreement result
----------------------------------------------------------------------
contig048242-ZebOR.E049    3 NSTV   0.7900     (9/9)   +++
contig048242-ZebOR.E049   39 NTSL   0.6763     (9/9)   ++
contig048242-ZebOR.E049   49 NRSL   0.7481     (9/9)   ++
contig048242-ZebOR.E049  245 NFSF   0.4447     (7/9)   -
----------------------------------------------------------------------
```

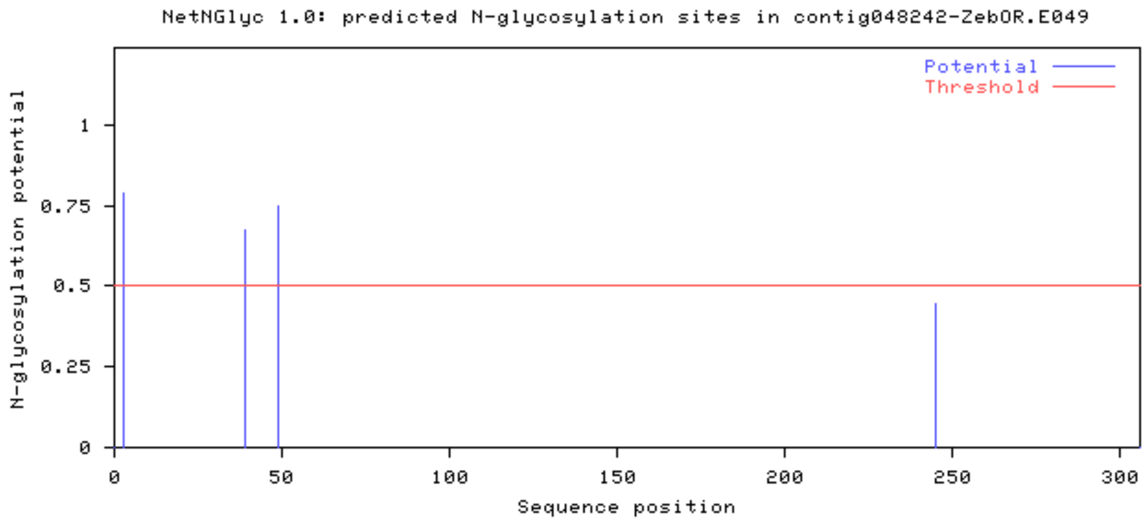

Graphics in PostScript

Output for 'contig048243-ZebOR.E050'

```
##########################################################################################

    Warning: This sequence may not contain a signal peptide!!

    Proteins without signal peptides are unlikely to be exposed to
    the N-glycosylation machinery and thus may not be glycosylated
    (in vivo) even though they contain potential motifs.

    SignalP-NN euk predictions are as follows:

  # name                    Cmax  pos ?  Ymax  pos ?  Smax  pos ?  Smean ?  D     ?

    SignalP output is explained at http://www.cbs.dtu.dk/services/SignalP/output.html

##########################################################################################

Name:  contig048243-ZebOR.E050  Length:  322
MLNVTTPPLSYFILGGFMNVGSLKFFYFSLTVILYILIIAANTSLIVVICVNRSLHEPMYMFLCSLFVNELYGSAGLFPF      80
LLLQILSDVHTVSAPLCFLQIFCIHTYGSIEVSNLAVMSYDRYLAICCPLQYNTQMTSNNTAVLIMVMWAYSLAKFLIAL     160
SLNLRLRLCGNVLNSLYCQNYLVVRLACSSTKVNNVYGIFDIIMTIIVPTLIILFSYMKILKVCFYGSKQTRQKSLTTCT     240
PQLVSLLNFSCGCCFEIFQSRFDTTGLPAALRIFLSLYFLMMQPLMNPILYGTQMSKIRGVYEHVLSSIMSCGCSKVSQS     320
DX
..N......................................N.........N............................      80
.........................................................N......................     160
................................................................................     240
................................................................................     320
..                                                                                   400

(Threshold=0.5)
----------------------------------------------------------------------
SeqName      Position  Potential   Jury    N-Glyc
                                 agreement result
----------------------------------------------------------------------
contig048243-ZebOR.E050    3 NVTT   0.7962     (9/9)    +++
contig048243-ZebOR.E050   42 NTSL   0.7263     (9/9)    ++
contig048243-ZebOR.E050   52 NRSL   0.7474     (9/9)    ++
contig048243-ZebOR.E050  139 NNTA   0.5361     (3/9)    +
contig048243-ZebOR.E050  248 NFSC   0.4680     (5/9)    -
----------------------------------------------------------------------
```

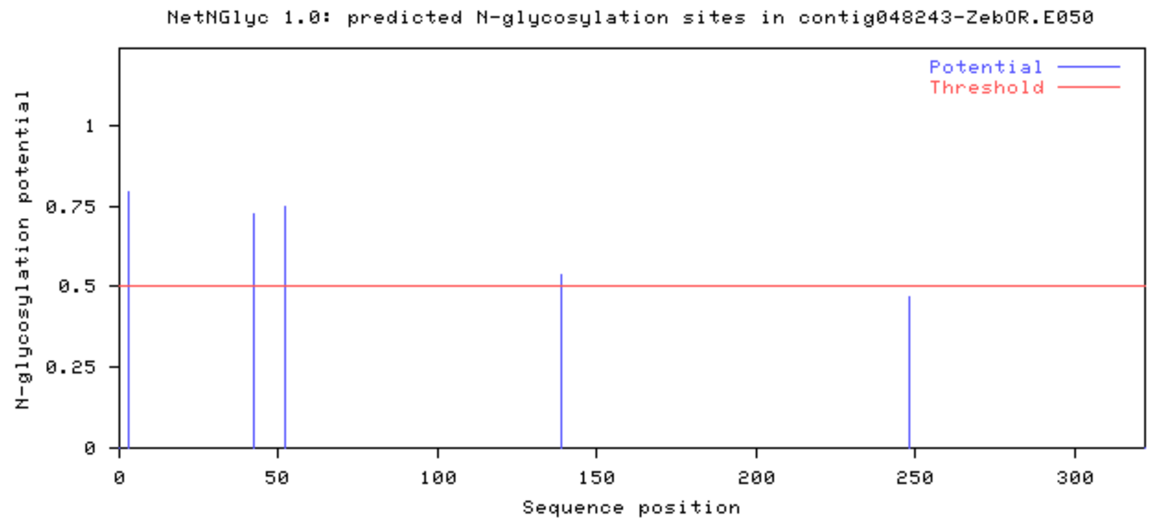

Graphics in PostScript

Output for 'contig048260-ZebOR.E051'

```
##########################################################################

   Warning: This sequence may not contain a signal peptide!!

   Proteins without signal peptides are unlikely to be exposed to
   the N-glycosylation machinery and thus may not be glycosylated
   (in vivo) even though they contain potential motifs.

   SignalP-NN euk predictions are as follows:

 # name                 Cmax  pos ?  Ymax  pos ?  Smax  pos ?  Smean ?  D     ?

   SignalP output is explained at http://www.cbs.dtu.dk/services/SignalP/output.html

##########################################################################

Name:  contig048260-ZebOR.E051   Length:  314
MINFTEGSYFILGAYFDAGPTKYLFFLLLLSLYSLIICANLLLIVVICVNRSLHEPMYMFLCSLFVNELYGSTGLFPLLL      80
LQILSDVHTVSAPLCFLQIFSLYSYVVVEFLTLAVMSYDRYLAICCPLQYNMRMTSSTVSVLIAVSWIYALLLVAVTVSL     160
SSPLQLCGNIINKVYCDNYAIVKLACSDTTLNNIYGLISTAFTAFVPVTLIFFTYMRILKVCFSGSKQTRQKAVSTCTPH     240
LASLLNFSCSGCFEIVQSRFNMNNVPNVLRILISLYWLMCQPLFNPVLYGLKMSKIRDICKSLLYSKVNTILSX
..N..............................................N..............................      80
................................................................................     160
................................................................................     240
.....N....................................................................           320

(Threshold=0.5)
----------------------------------------------------------------------
SeqName      Position  Potential   Jury    N-Glyc
                                agreement result
----------------------------------------------------------------------
contig048260-ZebOR.E051    3 NFTE    0.6487     (9/9)   ++
contig048260-ZebOR.E051   50 NRSL    0.7482     (9/9)   ++
contig048260-ZebOR.E051  246 NFSC    0.5554     (7/9)   +
----------------------------------------------------------------------
```

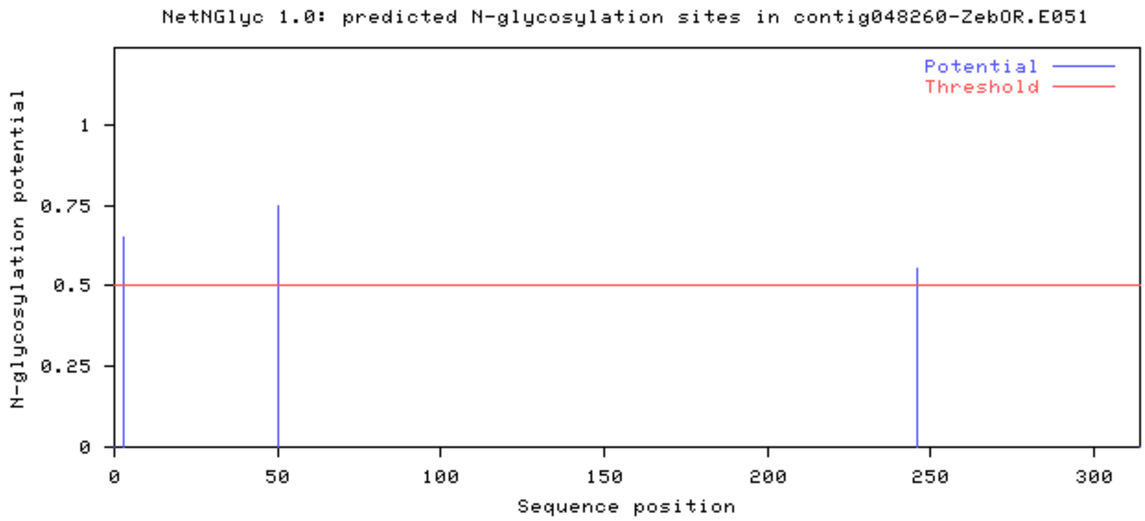

Graphics in PostScript

Output for 'contig048260-ZebORe.E055'

```
##########################################################################################

    Warning: This sequence may not contain a signal peptide!!

    Proteins without signal peptides are unlikely to be exposed to
    the N-glycosylation machinery and thus may not be glycosylated
    (in vivo) even though they contain potential motifs.

    SignalP-NN euk predictions are as follows:

  # name                    Cmax   pos ?  Ymax   pos ?  Smax   pos ?  Smean ?  D     ?

    SignalP output is explained at http://www.cbs.dtu.dk/services/SignalP/output.html

##########################################################################################

Name:  contig048260-ZebORe.E055         Length:  95
ILKVCFSGSKQTRQKAVSTCTPHLASLLNFSFGACFEVLQSRFNMNTVPNILRILISLYWLICQPLFNPVLYGLKMSKIY      80
DICKSLLYWKVWIMX
............................N...................................................      80
...............                                                                     160

(Threshold=0.5)
----------------------------------------------------------------------
SeqName      Position  Potential   Jury    N-Glyc
                                 agreement result
----------------------------------------------------------------------
contig048260-ZebORe.E055    29 NFSF   0.5300     (6/9)    +
----------------------------------------------------------------------
```

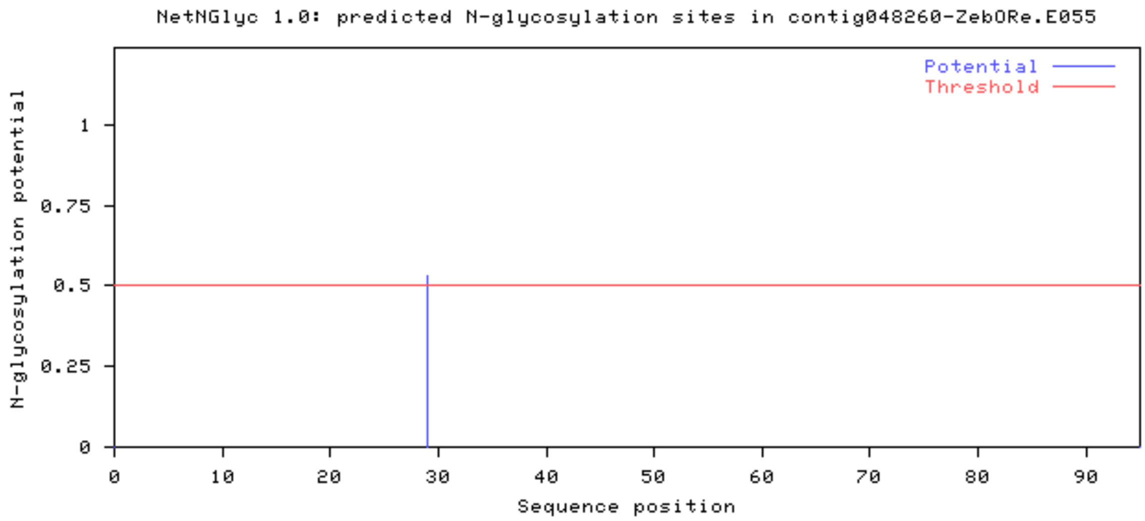

Graphics in PostScript

Output for 'contig048262-ZebORe.E060'

```
##########################################################################################

    Warning: This sequence may not contain a signal peptide!!

    Proteins without signal peptides are unlikely to be exposed to
    the N-glycosylation machinery and thus may not be glycosylated
    (in vivo) even though they contain potential motifs.

    SignalP-NN euk predictions are as follows:

  # name                  Cmax  pos ?  Ymax  pos ?  Smax  pos ?  Smean ?  D     ?

    SignalP output is explained at http://www.cbs.dtu.dk/services/SignalP/output.html

##########################################################################################

Name:  contig048262-ZebORe.E060         Length:  192
MSYDRYLAICCPLQYHTRMTPATVVLLIALSWLYSFLTILALILLIAPLELCGNVINKVYCLNYSIVKLACSETTANNIY      80
GLFITALTVFVPVILILCSYVRILKVCFSGSKQTRQKAVSTCTPHLSSLLNFSFGVCFEVIQSRFSLSSVHSMVHIVLSL     160
YFLTCQPLFNPVLYGLNMSNIRKRLFAHKRRX
..............................................................N.................      80
................................................................................     160
................N...............                                                     240

(Threshold=0.5)
----------------------------------------------------------------------
SeqName      Position  Potential   Jury    N-Glyc
                                 agreement result
----------------------------------------------------------------------
contig048262-ZebORe.E060   63 NYSI   0.6478     (8/9)   +
contig048262-ZebORe.E060  131 NFSF   0.4287     (5/9)   -
contig048262-ZebORe.E060  177 NMSN   0.5754     (4/9)   +
----------------------------------------------------------------------
```

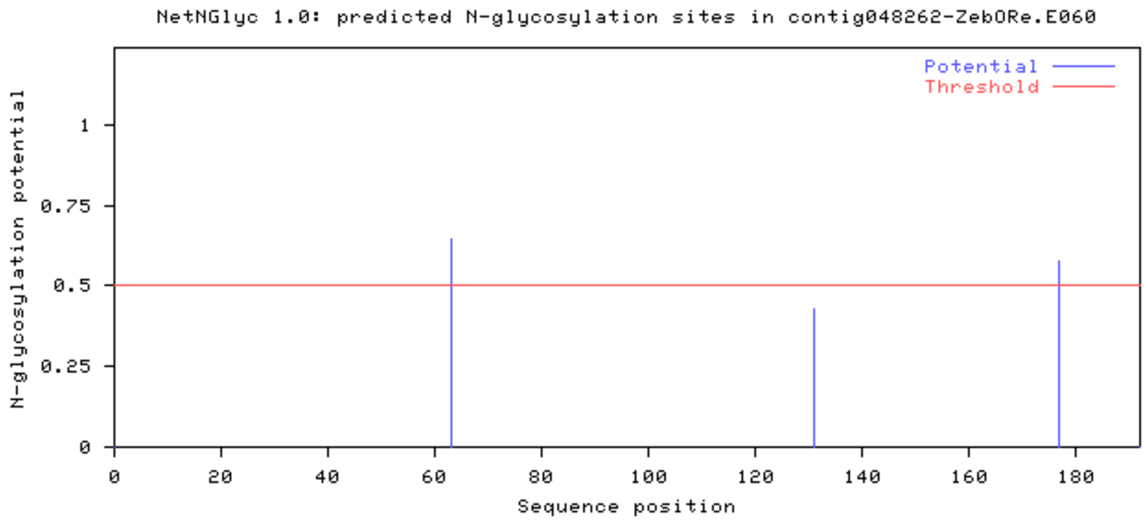

Graphics in PostScript

Output for 'contig048263-ZebOR.E052'

```
##########################################################################################

    Warning: This sequence may not contain a signal peptide!!

    Proteins without signal peptides are unlikely to be exposed to
    the N-glycosylation machinery and thus may not be glycosylated
    (in vivo) even though they contain potential motifs.

    SignalP-NN euk predictions are as follows:

  # name                 Cmax  pos ?  Ymax  pos ?  Smax  pos ?  Smean ?  D     ?

    SignalP output is explained at http://www.cbs.dtu.dk/services/SignalP/output.html

##########################################################################################

Name:  contig048263-ZebOR.E052  Length:  326
MEIIRNSTQFSYFTLGAYVDTQMFKYLYFMIILSLYVFTVGSNVLLIVVICVNRSLHEPMYMFLCSLFVNELYGSTGLFP      80
FLLVQILSDVHTVSAPLCFLQVFSIYLYGSVEFSNLAAMSYDRYMSICYPLQYHTLMMSNKVALLIAVTWIPPLLAVCVT     160
TCLSASLQLCGNVINKVYCNNHSIIKLGCHGATVNNLYELTAASVTVCVPVSVILYTYTRILKICFSGSKQTRQKAVSTC     240
TPHLASLLNFSFGVSFEILQSRFDMSHVPNMLRIFLSVYFLTCQPLFNPVMYGLNMSKIRTICKNLLLGYVGKSRILIKI     320
VQIEKX
.....N..............................................N...........................      80
................................................................................     160
................................................................................     240
......................................................N.........................     320
......                                                                               400

(Threshold=0.5)
----------------------------------------------------------------------
SeqName      Position  Potential   Jury    N-Glyc
                                 agreement result
----------------------------------------------------------------------
contig048263-ZebOR.E052    6 NSTQ    0.7710     (9/9)    +++
contig048263-ZebOR.E052   53 NRSL    0.7489     (9/9)    ++
contig048263-ZebOR.E052  181 NHSI    0.3874     (9/9)    --
contig048263-ZebOR.E052  249 NFSF    0.4453     (6/9)    -
contig048263-ZebOR.E052  295 NMSK    0.6053     (6/9)    +
----------------------------------------------------------------------
```

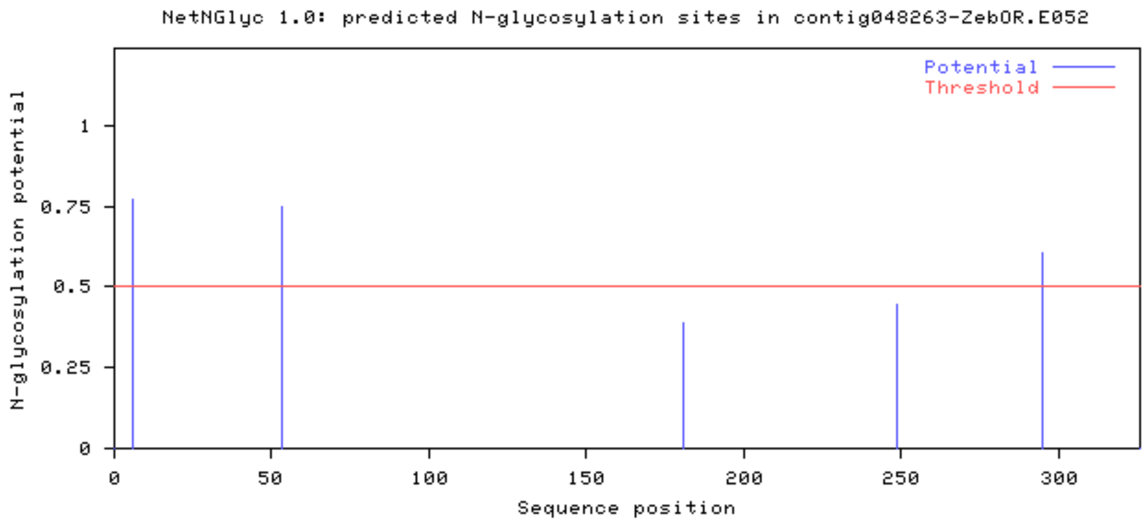

Graphics in PostScript

Output for 'contig050422-ZebOR.Q143'

```
##########################################################################

    Warning: This sequence may not contain a signal peptide!!

    Proteins without signal peptides are unlikely to be exposed to
    the N-glycosylation machinery and thus may not be glycosylated
    (in vivo) even though they contain potential motifs.

    SignalP-NN euk predictions are as follows:

  # name                Cmax  pos ?  Ymax  pos ?  Smax  pos ?  Smean ?  D     ?

    SignalP output is explained at http://www.cbs.dtu.dk/services/SignalP/output.html

##########################################################################

Name:  contig050422-ZebOR.Q143  Length:  310
MNATTAEFQSLPFQTSVKAALSMLPCFFFLYVNAIMMFALLKKPLLLESSRYILFGHLLMCDSVQLLLTMLLYIFAVMMV      80
RMINYVCVFVSLLAAVTVKMSPLNLAVMSLERYVAVCFPLRHPSFATPRSTGKAIAVMWIVASLDSFIQLFLFVRMEKTI     160
FPMQSFCIRNSVFRLEVYVTLNMAFTILYFVFVSMIIIYTYTAIMITVKSASSRGRHTNKAPKTVLLHLLQLWLYLTSTL     240
FNMINPSMMLKVPPDMAIHAQYVLFVGLIIFPKCLSPLIYSLRDQTLCRVFKYYFTFGFRASVKPSPLSX
.N..............................................................................      80
................................................................................     160
................................................................................     240
......................................................................               320

(Threshold=0.5)
----------------------------------------------------------------------
SeqName       Position  Potential   Jury    N-Glyc
                                  agreement result
----------------------------------------------------------------------
contig050422-ZebOR.Q143     2 NATT   0.6719     (8/9)   +
contig050422-ZebOR.Q143   245 NPSM   0.3831     (7/9)   -
----------------------------------------------------------------------
```

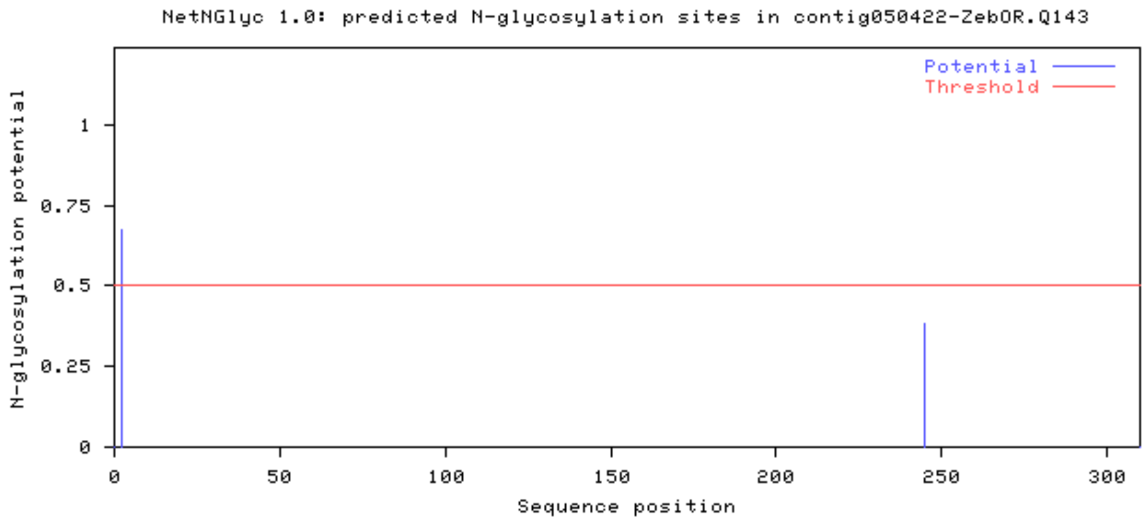

Graphics in PostScript

Output for 'contig053886-ZebOR.B034'

```
##########################################################################################

    Warning: This sequence may not contain a signal peptide!!

    Proteins without signal peptides are unlikely to be exposed to
    the N-glycosylation machinery and thus may not be glycosylated
    (in vivo) even though they contain potential motifs.

    SignalP-NN euk predictions are as follows:

  # name                 Cmax  pos ?  Ymax  pos ?  Smax  pos ?  Smean ?  D     ?

    SignalP output is explained at http://www.cbs.dtu.dk/services/SignalP/output.html

##########################################################################################

Name:  contig053886-ZebOR.B034   Length:  320
MSAGVNVTSLPILVTSVTLDGLAQLSNQRLFFFFFLCAYLFMLCSDSLVVYVICSQRSLHHPMFVFVTAVLMNSVAGSTV      80
FYPKLLVDLLRGVRSVQVTLRVCMCEAWLLYSLGTSSFLLLAAMSFDRYVSICRPLLYTVVMSPATVLALLLLCWLLPVG     160
LVGTAVLLASRLPLCRSQLSRIYCDIYSLVSLSCGGRETLLSEVYNLSVIVATVLLPAIFVLFSYSAVLSVCLQRSRSFS     240
SKALSTCLPHLLVFCNYSVSSGVEVLQRRLQAGSQPTASVLTSIFQVMIPTVFNPVVYGLKVTEIRAQLRRLLGCQRADX     320
.....N..........................................................................      80
................................................................................     160
.............................................N..................................     240
...............N................................................................     320

(Threshold=0.5)
----------------------------------------------------------------------
SeqName      Position  Potential   Jury    N-Glyc
                                 agreement result
----------------------------------------------------------------------
contig053886-ZebOR.B034    6 NVTS   0.7708     (9/9)   +++
contig053886-ZebOR.B034  206 NLSV   0.6109     (7/9)   +
contig053886-ZebOR.B034  256 NYSV   0.5470     (7/9)   +
----------------------------------------------------------------------
```

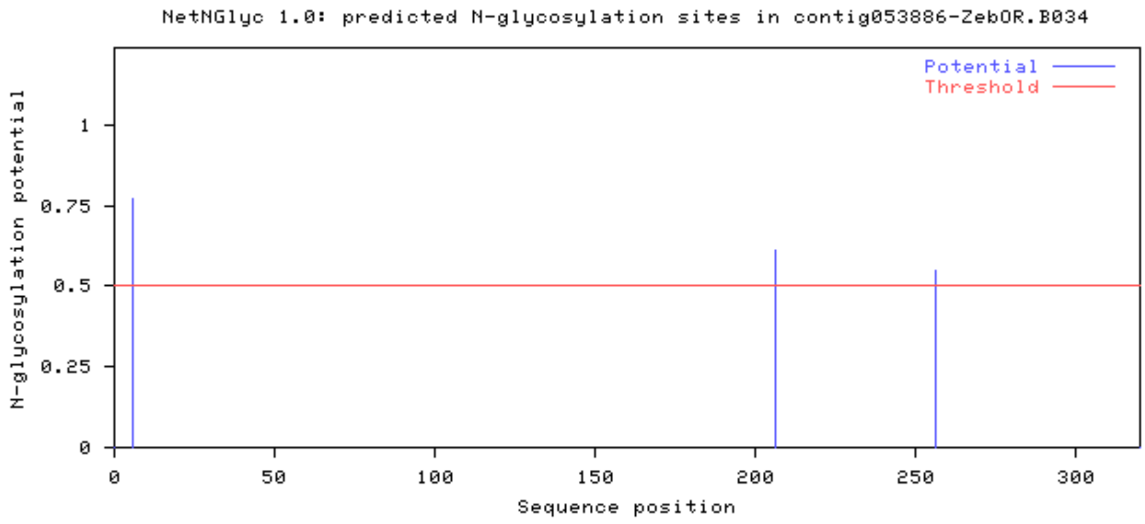

Graphics in PostScript

Output for 'contig057399-ZebORp.H081'

```
##########################################################################################

    Warning: This sequence may not contain a signal peptide!!

    Proteins without signal peptides are unlikely to be exposed to
    the N-glycosylation machinery and thus may not be glycosylated
    (in vivo) even though they contain potential motifs.

    SignalP-NN euk predictions are as follows:

  # name                 Cmax  pos ?  Ymax  pos ?  Smax  pos ?  Smean ?  D     ?

    SignalP output is explained at http://www.cbs.dtu.dk/services/SignalP/output.html

##########################################################################################

Name:  contig057399-ZebORp.H081         Length:  314
MDNVSVITVFTLSGLRDIANYRVILFVLTLLCYCVIWLVNLTIIVTVIVDKSLHEPMYIFLCNLCFNGLYGTAAFYPKFL      80
YDLLSTTHVISYAGCLLQGFVLHSSVCADFSLLVLMAYDRYVAICRPLVYHSLMTTQKICILVFFAWLIPIYLLFMSTIT     160
TAVLRLCGSHIPRIYCVNWLINNLACSASVARIVIPAFNYTFYIGHVLLVFWSYVHLIKTCQSSKDNWNKFMQTCVPHLF     240
SLTVVVLSFLFDMLYMRFGSKDLAQSFENFMAMEIFLIPPIINPLMYGFKLTQIRNRVLNFICGKSSALRLKSX
..N....................................N........................................      80
................................................................................     160
.......................................N........................................     240
..........................................................................           320

(Threshold=0.5)
----------------------------------------------------------------------
SeqName      Position  Potential   Jury    N-Glyc
                                 agreement result
----------------------------------------------------------------------
contig057399-ZebORp.H081    3 NVSV   0.8138     (9/9)    +++
contig057399-ZebORp.H081   40 NLTI   0.7720     (9/9)    +++
contig057399-ZebORp.H081  199 NYTF   0.5784     (7/9)    +
----------------------------------------------------------------------
```

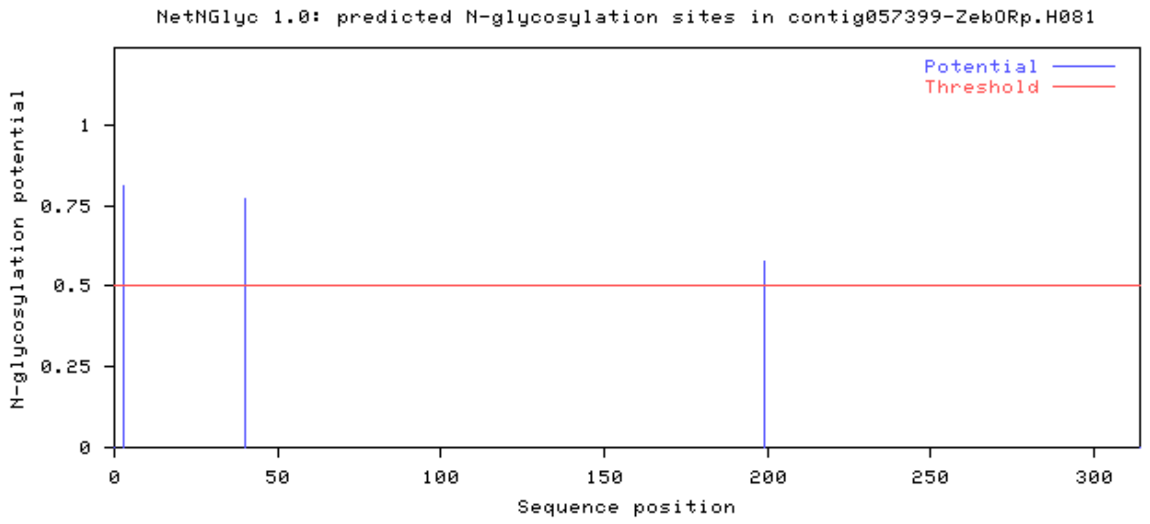

Graphics in PostScript

Output for 'contig057400-ZebOR.H075'

```
##########################################################################################

    Warning: This sequence may not contain a signal peptide!!

    Proteins without signal peptides are unlikely to be exposed to
    the N-glycosylation machinery and thus may not be glycosylated
    (in vivo) even though they contain potential motifs.

    SignalP-NN euk predictions are as follows:

  # name                    Cmax  pos ?  Ymax  pos ?  Smax  pos ?  Smean ?  D     ?

    SignalP output is explained at http://www.cbs.dtu.dk/services/SignalP/output.html

##########################################################################################

Name:  contig057400-ZebOR.H075  Length:  310
MDNVHNVRSFILLGFNETINFRVPLFSVTLLYYCVILFFNVSLVLLIVLDENLHEPMYIFLSSFCINALYGSTGFYPKFL      80
SDLLLPSQTISHEGCLLQAFIMYSSVCCNSSILAVMAFDRYLAICRPLHYHSFMTKRRLSQLVCFSWLTPFCIFAINVVL     160
TARLKLCGTKIQRVLCLNWLIVKLACPEADTFSNNITAYATVIIYVSHGFFIIWTYTHLIKTCAKSREDRAKFMQTCVPH     240
LTSLITSLSVAVFQFVYMRFDSTDLPQSLQNFIATEFVIIPPLMNPLIYGFKLTKIRNRILGLVCFKRKX
...............N.......................N........................................      80
............................N...................................................     160
..................................N.............................................     240
......................................................................               320

(Threshold=0.5)
----------------------------------------------------------------------
SeqName      Position  Potential   Jury    N-Glyc
                                agreement result
----------------------------------------------------------------------
contig057400-ZebOR.H075   16 NETI   0.7009     (9/9)   ++
contig057400-ZebOR.H075   40 NVSL   0.7074     (9/9)   ++
contig057400-ZebOR.H075  109 NSSI   0.5244     (6/9)   +
contig057400-ZebOR.H075  195 NITA   0.6174     (8/9)   +
----------------------------------------------------------------------
```

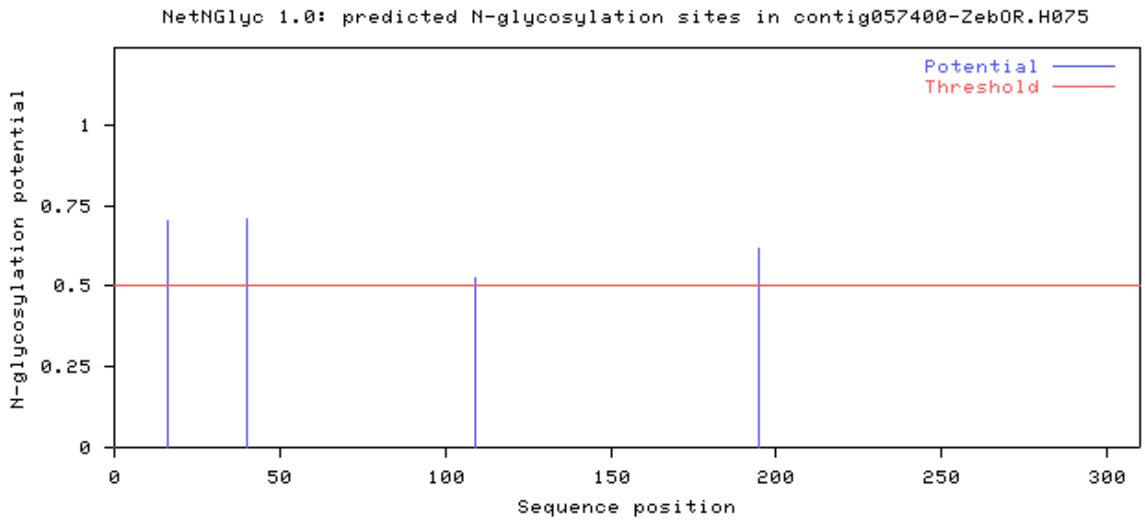

Graphics in PostScript

Output for 'contig057403-ZebOR.H076'

```
##########################################################################################

    Warning: This sequence may not contain a signal peptide!!

    Proteins without signal peptides are unlikely to be exposed to
    the N-glycosylation machinery and thus may not be glycosylated
    (in vivo) even though they contain potential motifs.

    SignalP-NN euk predictions are as follows:

  # name                    Cmax  pos ?  Ymax  pos ?  Smax  pos ?  Smean ?  D     ?

    SignalP output is explained at http://www.cbs.dtu.dk/services/SignalP/output.html

##########################################################################################

Name:  contig057403-ZebOR.H076  Length:  310
MDNVSVVRMFTLSGFNETMNIRLTIFSLTLMYYCMIILINVSLIVLIVLDENLHEPMYILLSSFCINEIYGTTGFYPKFL      80
LDLLSSSQEISYEGCLLQAFIMYSFACCDLSILAVMAFDRYLAICRPLHYHSFMTKRRLSQLVCFSWLTPLCIFSISILL     160
TSRLTLCRSKIEKVFCVNWVIVKLACSDTDTLLNSVVSYATIIMYISHGFFIMWTYMHLIKTSVTSKEDRAKFMQTCVPH     240
LTSLITFLIVILFDLMYMRFGSADLPQSLQNFIAIEFLVIPPVMNPLIYGFKLTKIRNKILSFVYRKQKX
..N............N.......................N........................................      80
................................................................................     160
................................................................................     240
......................................................................               320

(Threshold=0.5)
```

SeqName	Position	Potential	Jury agreement	N-Glyc result
contig057403-ZebOR.H076	3 NVSV	0.8082	(9/9)	+++
contig057403-ZebOR.H076	16 NETM	0.6306	(9/9)	++
contig057403-ZebOR.H076	40 NVSL	0.7719	(9/9)	+++

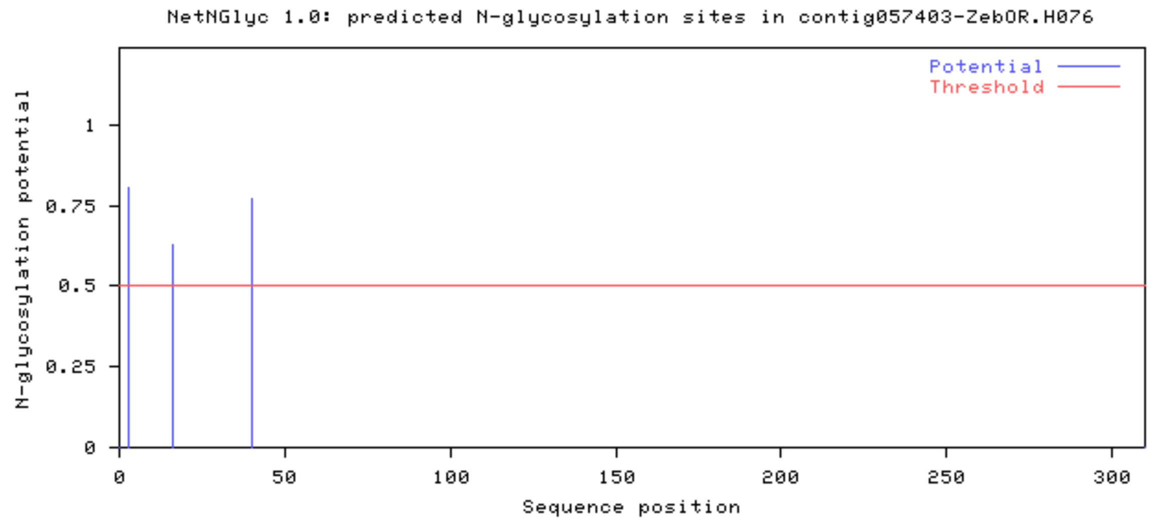

Graphics in PostScript

Output for 'contig062094-ZebOR.A023'

```
##########################################################################################

    Warning: This sequence may not contain a signal peptide!!

    Proteins without signal peptides are unlikely to be exposed to
    the N-glycosylation machinery and thus may not be glycosylated
    (in vivo) even though they contain potential motifs.

    SignalP-NN euk predictions are as follows:

  # name                    Cmax   pos ?  Ymax   pos ?  Smax   pos ?  Smean ?  D     ?

    SignalP output is explained at http://www.cbs.dtu.dk/services/SignalP/output.html

##########################################################################################

Name:  contig062094-ZebOR.A023  Length:  314
MDEESNATYLTLDWYTEINKYRYVFFFVMFTLYILIICTNSTILYLIWIHKNLHEPMYIFIAALLLNSVLYSTTIYPKLL      80
TDFLSEIQVTTYSACLFQFFMFYTLGCSEFLLLAAMAYDRYVAICKPLEYQTIMRTTTVGIFLVMAWLVPACHIAVQAIA     160
SAEAKLCDSNIKGIFCSNAVYTLQCERSRLITIFGVFLLIDLAILPMLFIVYTYTKIFIVSHRSCKEIRKKTAETCLPHM     240
LVLISYSMFFVYDISIARVKSDFPKTTRIIMTLQIMLYQPLLNPFIYGLKMKDISKHLNKLLSQAKIISCIKTX
.....N.................................N........................................      80
................................................................................     160
................................................................................     240
..........................................................................           320

(Threshold=0.5)
----------------------------------------------------------------------
SeqName      Position  Potential   Jury    N-Glyc
                                 agreement result
----------------------------------------------------------------------
contig062094-ZebOR.A023    6 NATY   0.6218     (7/9)   +
contig062094-ZebOR.A023   40 NSTI   0.6299     (8/9)   +
----------------------------------------------------------------------
```

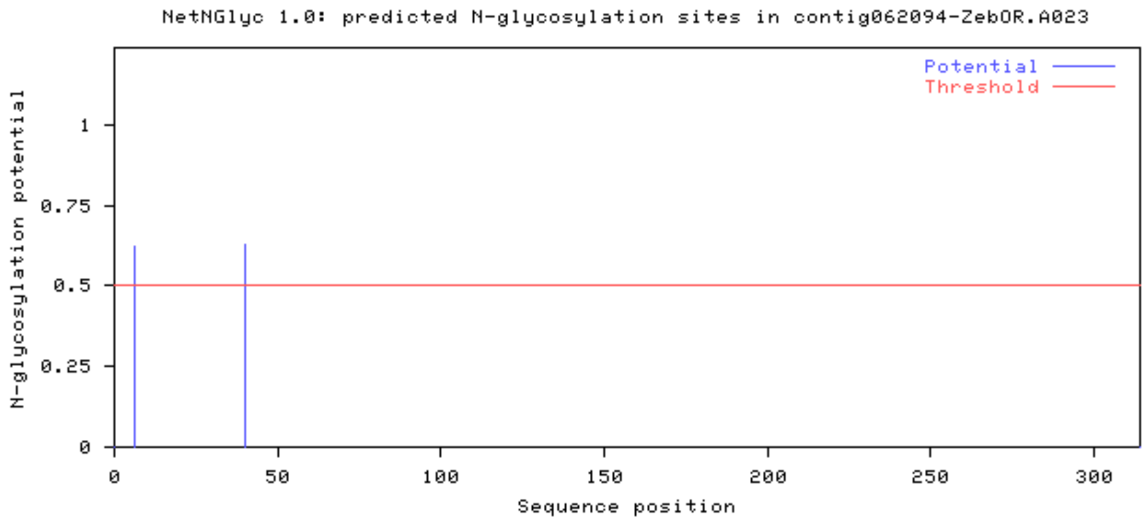

Graphics in PostScript

Output for 'contig062095-ZebOR.A024'

```
##########################################################################################

    Warning: This sequence may not contain a signal peptide!!

    Proteins without signal peptides are unlikely to be exposed to
    the N-glycosylation machinery and thus may not be glycosylated
    (in vivo) even though they contain potential motifs.

    SignalP-NN euk predictions are as follows:

  # name                    Cmax  pos ?  Ymax  pos ?  Smax  pos ?  Smean ?  D     ?

    SignalP output is explained at http://www.cbs.dtu.dk/services/SignalP/output.html

##########################################################################################

Name:  contig062095-ZebOR.A024  Length:  304
MDEELNVTYLTLDWYTEMNKYRYVFFFIMFTLFILIICTNSTILYLIWIHKNLHEPMYIFIAALLLNSVLYSTTIYPKLL      80
IDFLSEKQVTTYSACLFQFFTFYTLACSEFLLLAAMAYDRYVAICKPLEYQTLMRKTTVGIFLVVAWLVPACHVAVLAIA     160
SAGAKLCDSNIKGIFCNNAVYTLQCERSRLITIFGVVALVDLSILPMLFIVFTYTKLFIVSHRSCKEIRKKAAETCLPHL     240
LVLISYSAFFVYDVSIARVKSDFPKTTRIIMTLQIMLYQPLLNPFIYGLKMKEISKHLNKLLSX
.....N................................N.........................................      80
................................................................................     160
................................................................................     240
................................................................                     320

(Threshold=0.5)
----------------------------------------------------------------------
SeqName      Position  Potential   Jury    N-Glyc
                                 agreement result
----------------------------------------------------------------------
contig062095-ZebOR.A024    6 NVTY    0.7650     (9/9)    +++
contig062095-ZebOR.A024   40 NSTI    0.6140     (8/9)    +
----------------------------------------------------------------------
```

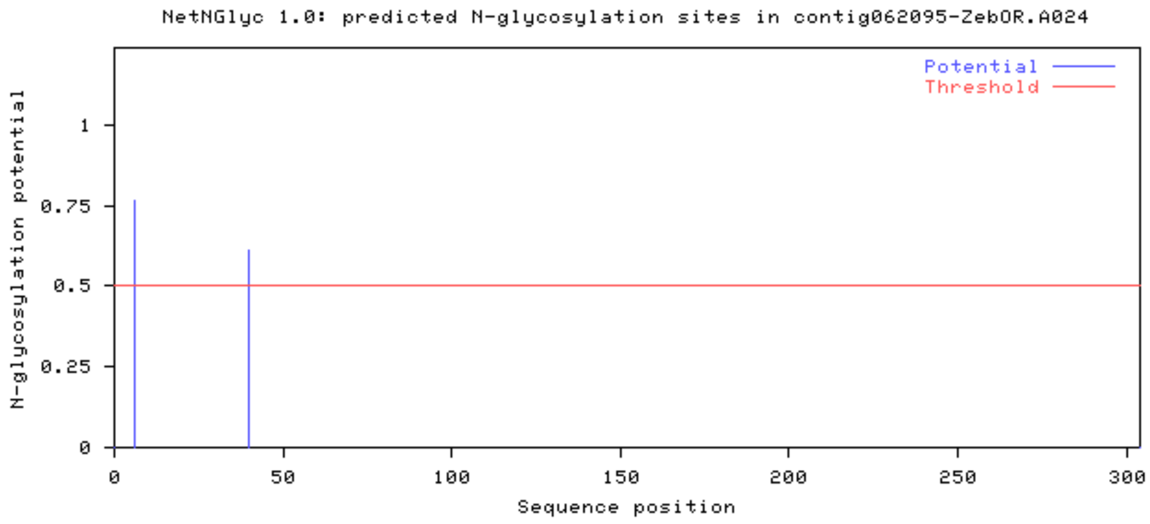

Graphics in PostScript

Output for 'contig062664-ZebORs.W141'

```
##########################################################################################

    Warning: This sequence may not contain a signal peptide!!

    Proteins without signal peptides are unlikely to be exposed to
    the N-glycosylation machinery and thus may not be glycosylated
    (in vivo) even though they contain potential motifs.

    SignalP-NN euk predictions are as follows:

  # name                    Cmax   pos ?  Ymax   pos ?  Smax   pos ?  Smean ?  D     ?

    SignalP output is explained at http://www.cbs.dtu.dk/services/SignalP/output.html

##########################################################################################

Name:  contig062664-ZebORs.W141         Length:  304
MNSTTAVYQDSLSTAIIKNLITVGLSVSIIYINSSLVHTFKKHQVFNTNPRYILYIHLVIIDILLLIIFTLLQVLSYIIF      80
TLPVPFCIILLLISIICSLNTPLTLAVMAVECHVAICFPLQHSQICTVKNVTVVITVIWMLSSLTILPDLFTIMATESRD     160
FFHSRVFCLRETVFRLPELEKKRTISNIVFLVIVWLTLVYTYFRILFAAQAAAANARKARNTVLLHGFQLLLCMLTYVYD     240
LLLNGLTKLFPKGVLTIRYTVSVFVHVLPRLVSPLVYGIRDKAFRRYLRKYLFYSPNANVNKSX
.N..............................N...............................................      80
.................................................N..............................     160
................................................................................     240
................................................................                     320

(Threshold=0.5)
----------------------------------------------------------------------
SeqName      Position  Potential   Jury    N-Glyc
                                agreement result
----------------------------------------------------------------------
contig062664-ZebORs.W141    2 NSTT   0.6638     (9/9)   ++
contig062664-ZebORs.W141   33 NSSL   0.6626     (9/9)   ++
contig062664-ZebORs.W141  130 NVTV   0.5968     (9/9)   ++
contig062664-ZebORs.W141  301 NKSX   0.4931     (5/9)   -
----------------------------------------------------------------------
```

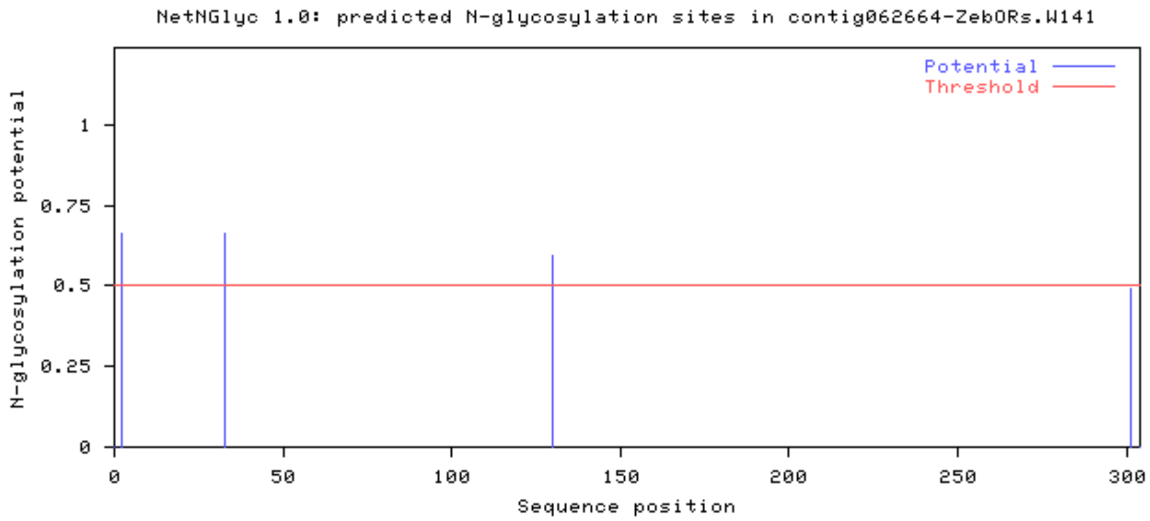

Graphics in PostScript

Output for 'contig063018-ZebOR.E053'

```
##########################################################################################

    Warning: This sequence may not contain a signal peptide!!

    Proteins without signal peptides are unlikely to be exposed to
    the N-glycosylation machinery and thus may not be glycosylated
    (in vivo) even though they contain potential motifs.

    SignalP-NN euk predictions are as follows:

  # name                    Cmax  pos ?  Ymax  pos ?  Smax  pos ?  Smean ?  D     ?

    SignalP output is explained at http://www.cbs.dtu.dk/services/SignalP/output.html

##########################################################################################

Name:  contig063018-ZebOR.E053  Length:  306
MTNSTQFSHFRLAFLNIGIFKYLFFMLVMCFFVSVVCTNVLLIVVICVNRSLHEPMYMFLCNLFVNALYGSTSLFPLLLL      80
HIICDINIISASLCYLQIYCIHCYGSAEYLNLAVMSYDRYLAICFPLQYNTYMTPKRIAILIAITWLYAILACALMISLS     160
STLPLCGNIIDKVYCDNYSVIKLACSDTKALNIIVLCTVCCPLIFMLYTYIKILRVCSSGSKQMRQKAVTTCSPHLACVL     240
NFSCGACFEILQSRFNMSGIPILLRIFLSLYWLISQPLLNPLVYGLNMTKIRILCKNLLTLRPLNX
..N.............................................N...............................      80
................................................................................     160
................N...............................................................     240
...............N..............................N...................                   320

(Threshold=0.5)
----------------------------------------------------------------------
SeqName      Position  Potential   Jury    N-Glyc
                                 agreement result
----------------------------------------------------------------------
contig063018-ZebOR.E053    3 NSTQ    0.7361     (9/9)   ++
contig063018-ZebOR.E053   49 NRSL    0.7490     (9/9)   ++
contig063018-ZebOR.E053  177 NYSV    0.6674     (9/9)   ++
contig063018-ZebOR.E053  241 NFSC    0.3189     (9/9)   ---
contig063018-ZebOR.E053  256 NMSG    0.5896     (6/9)   +
contig063018-ZebOR.E053  287 NMTK    0.6374     (8/9)   +
----------------------------------------------------------------------
```

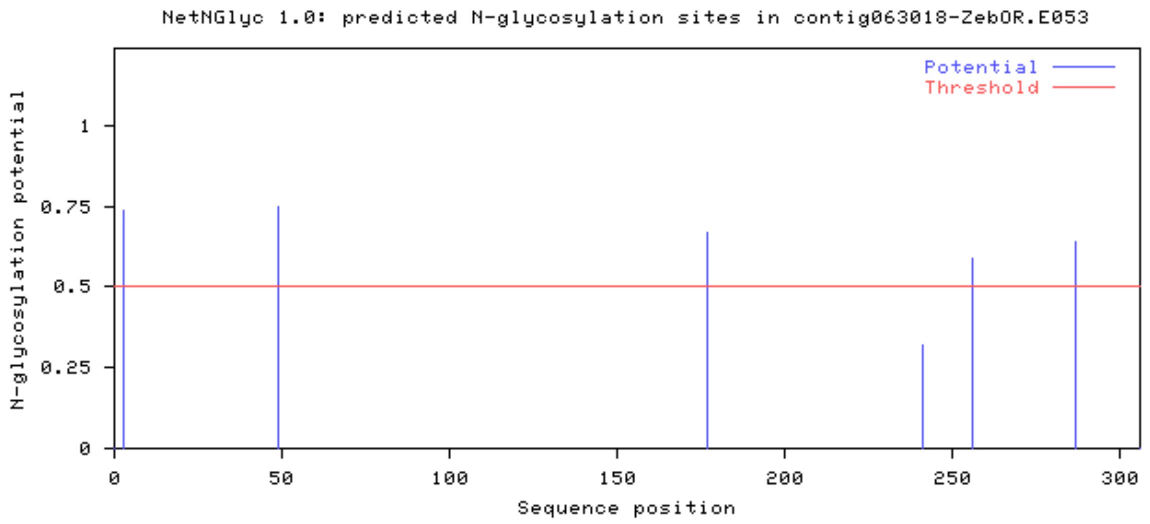

Graphics in PostScript

Output for 'contig063324-ZebORe.K151'

```
##########################################################################################

    Warning: This sequence may not contain a signal peptide!!

    Proteins without signal peptides are unlikely to be exposed to
    the N-glycosylation machinery and thus may not be glycosylated
    (in vivo) even though they contain potential motifs.

    SignalP-NN euk predictions are as follows:

  # name                 Cmax  pos ?  Ymax  pos ?  Smax  pos ?  Smean ?  D     ?

    SignalP output is explained at http://www.cbs.dtu.dk/services/SignalP/output.html

##########################################################################################

Name:  contig063324-ZebORe.K151         Length:  172
MSVLAWAVIFIMVTILVGLSVRLSRCRWIIFNPFCDNASLFKLSCESILINNIYGLGYTVLLLGSSLGSVIITYLRIAMV      80
CLSSKSKTLNSRALQTYTTHLTMFVIMFLSCIVMVLLHRFPHLTDQRKLASMMFHVVPPALNAVIYGMQIKAVRQKMFIM     160
FTRNTVTVTDGK
....................................N...........................................      80
................................................................................     160
............                                                                         240

(Threshold=0.5)
----------------------------------------------------------------------
SeqName      Position  Potential   Jury    N-Glyc
                                 agreement result
----------------------------------------------------------------------
contig063324-ZebORe.K151    37 NASL    0.7354     (9/9)    ++
----------------------------------------------------------------------
```

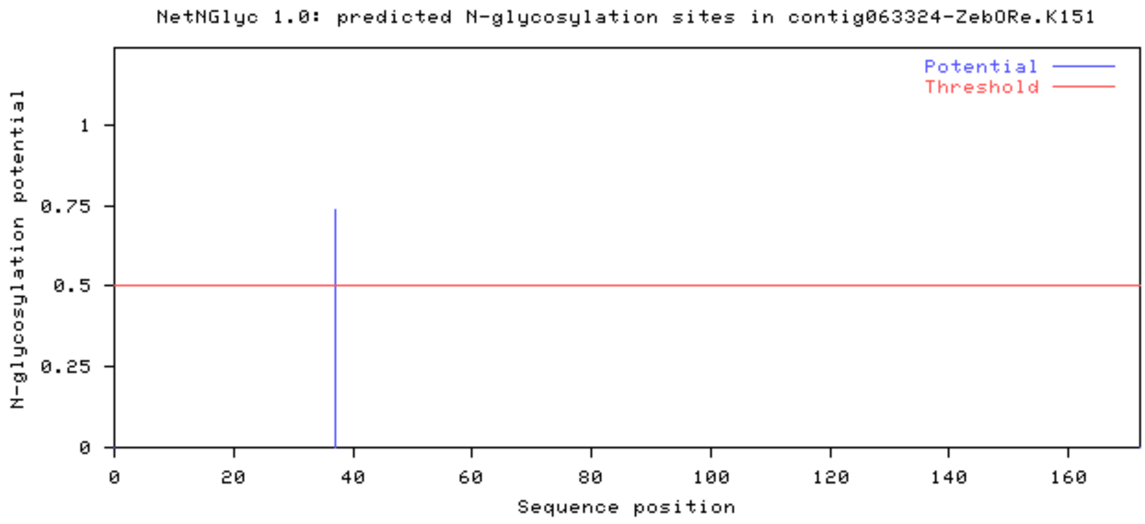

Graphics in PostScript

Output for 'contig064097-ZebOR.N115'

```
########################################################################################

    Warning: This sequence may not contain a signal peptide!!

    Proteins without signal peptides are unlikely to be exposed to
    the N-glycosylation machinery and thus may not be glycosylated
    (in vivo) even though they contain potential motifs.

    SignalP-NN euk predictions are as follows:

  # name                 Cmax  pos ?  Ymax  pos ?  Smax  pos ?  Smean ?  D     ?

    SignalP output is explained at http://www.cbs.dtu.dk/services/SignalP/output.html

########################################################################################

Name:  contig064097-ZebOR.N115  Length:  322
MELFNSALGKNITFVHPAFFIIGGLTGIPNITLYYVFLFFVYIVSVVGNTVVMAVIYLDHNLRTPKYIAVFNLAFVDLFG      80
NTALVPNVLDIFLFGHYYIPYNDCLTFLFFCYTCLSLQSFNLVALSYDRMVAIIFPLHYQVKVTHRFMFSLIASLWVFTI     160
IAVLISVGLLTRLSFCKSVVINSYFCDHGQIYRLACNDHFPSYVIACLYPVIIFWLPLAFILLSYLYIGYTLVKVATLQE     240
GLKAFKTCIGHLSLVAIYFIPLLTTFTLMEKIQPNARIINLSLTSVFPPMLNPIIYVLQTQEIKESLKRLLKRRGKSKIT     320
IX
..........N..................N..................................................      80
................................................................................     160
................................................................................     240
.......................................N........................................     320
..                                                                                   400

(Threshold=0.5)
----------------------------------------------------------------------
SeqName      Position  Potential   Jury    N-Glyc
                                 agreement result
----------------------------------------------------------------------
contig064097-ZebOR.N115   11 NITF   0.6087     (8/9)   +
contig064097-ZebOR.N115   30 NITL   0.8183     (9/9)   +++
contig064097-ZebOR.N115  280 NLSL   0.6020     (8/9)   +
----------------------------------------------------------------------
```

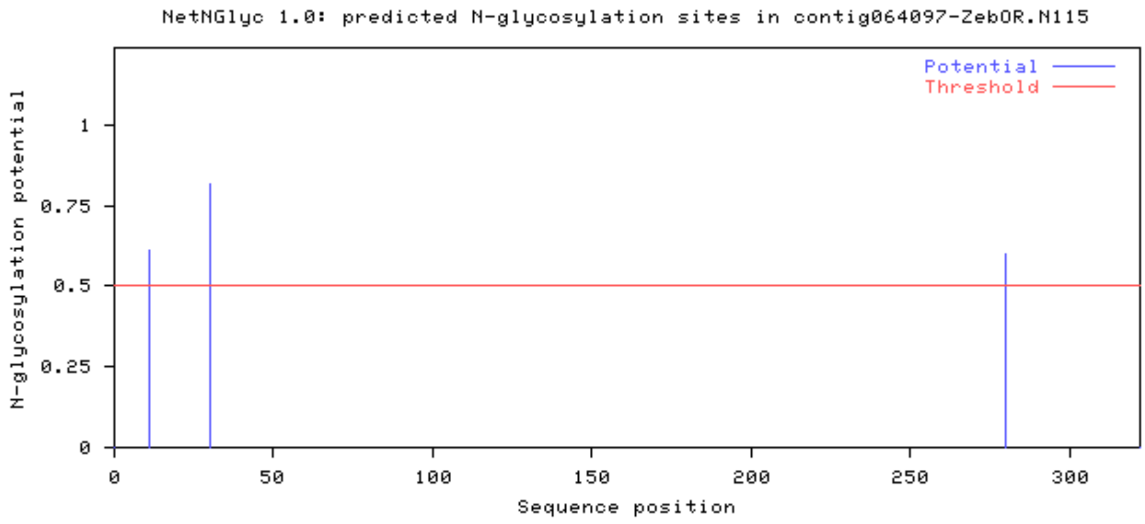

Graphics in PostScript

Output for 'contig064098-ZebOR.N116'

```
##########################################################################################

    Warning: This sequence may not contain a signal peptide!!

    Proteins without signal peptides are unlikely to be exposed to
    the N-glycosylation machinery and thus may not be glycosylated
    (in vivo) even though they contain potential motifs.

    SignalP-NN euk predictions are as follows:

  # name                 Cmax  pos ?  Ymax  pos ?  Smax  pos ?  Smean ?  D     ?

    SignalP output is explained at http://www.cbs.dtu.dk/services/SignalP/output.html

##########################################################################################

Name:  contig064098-ZebOR.N116  Length:  323
MDFLNSAAEKNTTFVQPANFIISGFVGIPNIRYYFVFLCFIYIFSVVGNTAVMLIIIFDHTLRSPKYIAVFNLAFTDLLS      80
NSALVPKVLDISLFNHHYISYHNCLTFMFFCFTLISMQAFNLVVLSFDRIMAIMYPLHYQMRASHKIILSLIAFFWLLAI     160
ALTGTAVGLLTRLYFCESVVINSYYCDHGPIYRLSCNDVTPNKTISAWSRAFVLWLPLIFILGSYCCIGYSLSRISTCKE     240
RVKALKTCTGHLSLVAIYFIPILVVYSFGSTMHPNARIVNLSLASVTPPMLNPIIYVFQTAEIKKSLKKLLKAKIQISHR     320
VLX
................................................................................      80
................................................................................     160
.........................................N......................................     240
.......................................N........................................     320
...                                                                                  400

(Threshold=0.5)
----------------------------------------------------------------------
SeqName      Position  Potential   Jury    N-Glyc
                                 agreement result
----------------------------------------------------------------------
contig064098-ZebOR.N116   11 NTTF   0.4656     (5/9)   -
contig064098-ZebOR.N116  202 NKTI   0.6128     (8/9)   +
contig064098-ZebOR.N116  280 NLSL   0.5873     (8/9)   +
----------------------------------------------------------------------
```

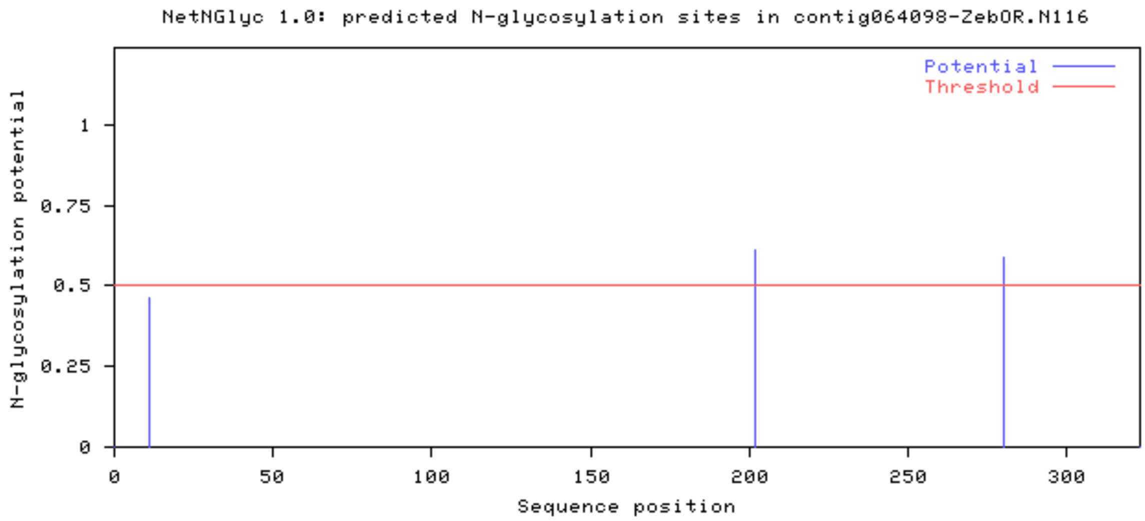

Graphics in PostScript

Output for 'contig064392-ZebORe.A030'

```
##########################################################################################

    Warning: This sequence may not contain a signal peptide!!

    Proteins without signal peptides are unlikely to be exposed to
    the N-glycosylation machinery and thus may not be glycosylated
    (in vivo) even though they contain potential motifs.

    SignalP-NN euk predictions are as follows:

  # name                 Cmax  pos ?  Ymax  pos ?  Smax  pos ?  Smean ?  D     ?

    SignalP output is explained at http://www.cbs.dtu.dk/services/SignalP/output.html

##########################################################################################

Name:  contig064392-ZebORe.A030         Length:  214
FQFFIIYSLGCSEFFLLAAMAYDRYVAICKPLQYPTIMRKSTVSIFLVIAWLVPASNIAVQAIGMAKSKLCSFHLKSIFC      80
NNTIYTLQCVKSGLVTVFGIVSYLDLVVFPILFIVFTYTKIFIVTYRSCKEIKKKAAETCLPHMLVLITFSCLGIYDVIM     160
ARMETDFPKTARLIMTLQLALYQPLFNPFIYGLKMKEISKHLKSLFCPPLLSIX
................................................................................      80
N...............................................................................     160
......................................................                               240

(Threshold=0.5)
----------------------------------------------------------------------
SeqName      Position  Potential    Jury    N-Glyc
                                  agreement result
----------------------------------------------------------------------
contig064392-ZebORe.A030    81 NNTI    0.5550     (7/9)    +
----------------------------------------------------------------------
```

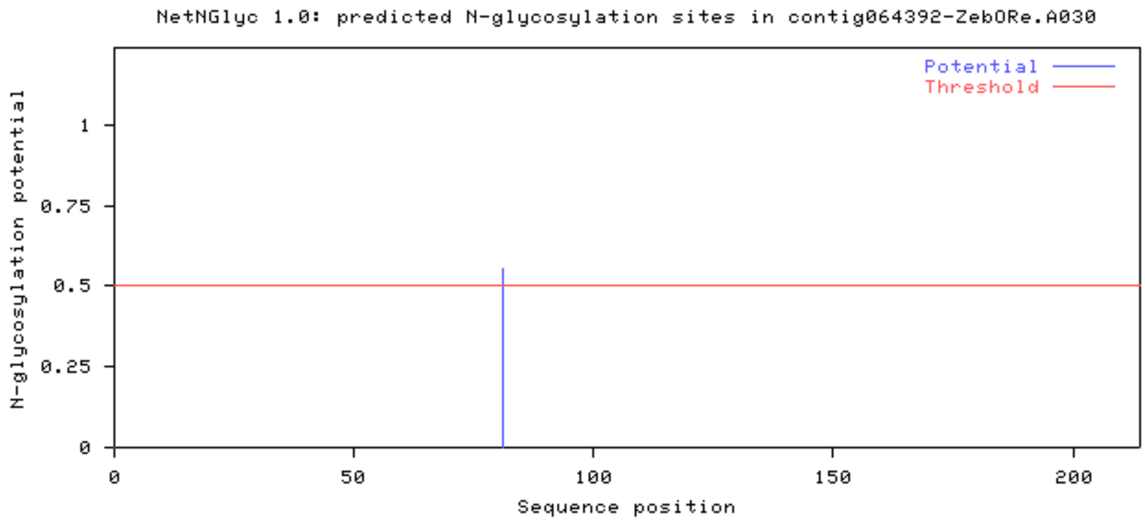

Graphics in PostScript

Output for 'contig064861-ZebORe.S131'

```
##########################################################################################

    Warning: This sequence may not contain a signal peptide!!

    Proteins without signal peptides are unlikely to be exposed to
    the N-glycosylation machinery and thus may not be glycosylated
    (in vivo) even though they contain potential motifs.

    SignalP-NN euk predictions are as follows:

  # name                 Cmax  pos ?  Ymax  pos ?  Smax  pos ?  Smean ?  D     ?

    SignalP output is explained at http://www.cbs.dtu.dk/services/SignalP/output.html

##########################################################################################

Name:  contig064861-ZebORe.S131         Length:  218
MCITISVVVLLYFIVTPVTLTAMTLERYVAICMPLRHGQLCSTRSTMYCILIIHGVSSGPCIIILSMFFASGSLKFYKQS      80
MICSGDTFSLYRWQDHVRSAVYQLYFLIMGITIAYSYVQIMKVAKAASGEKKKLTQKGLKTVILHAFQLLLCLIQLWCPF     160
IEIAVLQIDFSLILNVRYFNYIMFNIAPRCLSPLIYGLRDENIFLVLKNLMPTSSCSK
................................................................................      80
................................................................................     160
..........................................................                           240

(Threshold=0.5)

No sites predicted in this sequence.
```

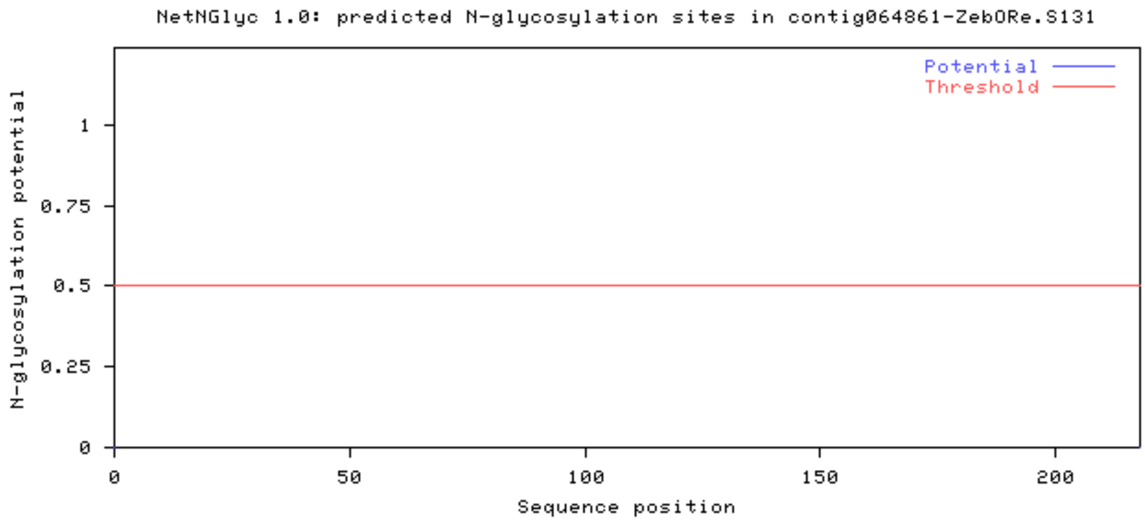

Graphics in PostScript

Output for 'contig065193-ZebOR.R147'

```
##########################################################################################

    Warning: This sequence may not contain a signal peptide!!

    Proteins without signal peptides are unlikely to be exposed to
    the N-glycosylation machinery and thus may not be glycosylated
    (in vivo) even though they contain potential motifs.

    SignalP-NN euk predictions are as follows:

  # name                  Cmax  pos ?  Ymax  pos ?  Smax  pos ?  Smean ?  D     ?

    SignalP output is explained at http://www.cbs.dtu.dk/services/SignalP/output.html

##########################################################################################

Name:  contig065193-ZebOR.R147  Length:  328
MLLANLSLTNVTANQQYQGVLERVLFSTLTTLPCCLFLCINGIMLFTLRSKALFCETSRYILLYNLLFADTVQMALSQLL      80
YIIAACRITLTYPVCGFLTMLANLTTVVSPVTLVVMSLERYVAVCYPLRHASIITITNTGVAIIVIWAIGSLNILTRVLL     160
LLEFPFGALDSLQMKDFCSEIAMSGGSMSDDYDKAFTCALFISASVAITCSYIGVIVAARSASTDKASARKACNTLLLHL     240
VQLGLSLSSTICNPLLTALARVLTRIVFVRIQNVFYVCIFLFPRCLSSLIYGIRDQSIRPVLIYYLCCRLKYSVIQPRLK     320
AAIKVECX
....N....N......................................................................      80
......................N.........................................................     160
................................................................................     240
................................................................................     320
........                                                                             400

(Threshold=0.5)
----------------------------------------------------------------------
SeqName      Position  Potential   Jury    N-Glyc
                                 agreement result
----------------------------------------------------------------------
contig065193-ZebOR.R147    5 NLSL   0.7992     (9/9)   +++
contig065193-ZebOR.R147   10 NVTA   0.7510     (9/9)   +++
contig065193-ZebOR.R147  103 NLTT   0.7330     (9/9)   ++
----------------------------------------------------------------------
```

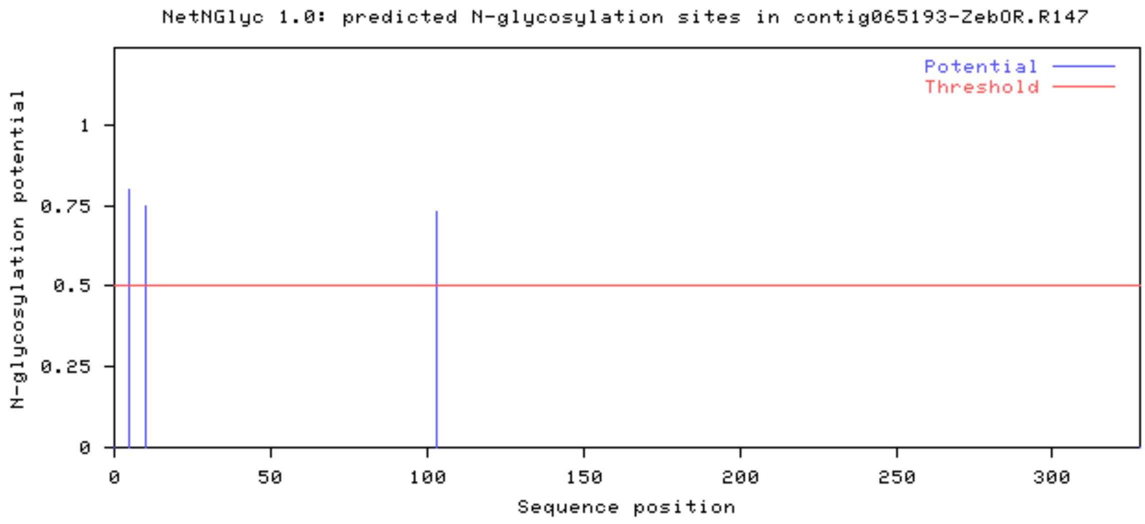

Graphics in PostScript

Output for 'contig065407-ZebORe.N118'

```
##########################################################################################

    Warning: This sequence may not contain a signal peptide!!

    Proteins without signal peptides are unlikely to be exposed to
    the N-glycosylation machinery and thus may not be glycosylated
    (in vivo) even though they contain potential motifs.

    SignalP-NN euk predictions are as follows:

  # name                    Cmax   pos ?  Ymax   pos ?  Smax   pos ?  Smean ?  D     ?

    SignalP output is explained at http://www.cbs.dtu.dk/services/SignalP/output.html

##########################################################################################

Name:  contig065407-ZebORe.N118         Length:  140
NDTSLNYNMASALITILIIIPLIFIIATYVCIFIALSRTTSRKERIRALKTCTSHLILVVIFFLPIGITNIATRASYIHP      80
NARMINSTLTHTIPALLDPIIYALKTEEVMIAVKKLCKRTYLNCMKAKTRPSNHCCIKSX
N...............................................................................      80
.....N......................................................                         160

(Threshold=0.5)
----------------------------------------------------------------------
SeqName      Position  Potential    Jury    N-Glyc
                                 agreement result
----------------------------------------------------------------------
contig065407-ZebORe.N118     1 NDTS    0.6953     (9/9)    ++
contig065407-ZebORe.N118    86 NSTL    0.6289     (8/9)    +
----------------------------------------------------------------------
```

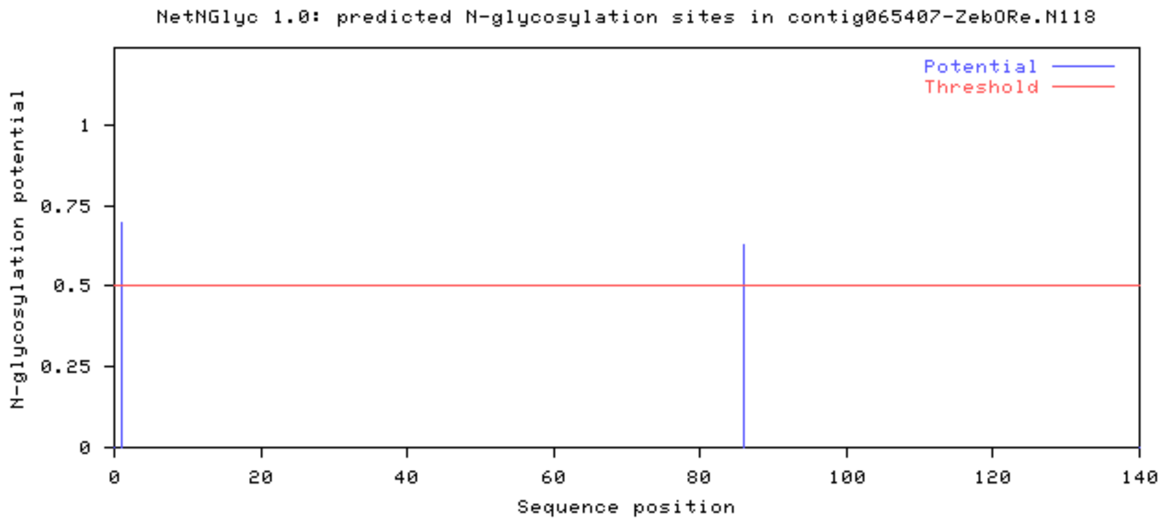

Graphics in PostScript

Output for 'contig065408-ZebORe.N119'

```
##########################################################################################

    Warning: This sequence may not contain a signal peptide!!

    Proteins without signal peptides are unlikely to be exposed to
    the N-glycosylation machinery and thus may not be glycosylated
    (in vivo) even though they contain potential motifs.

    SignalP-NN euk predictions are as follows:

  # name                 Cmax  pos ?  Ymax  pos ?  Smax  pos ?  Smean ?  D     ?

    SignalP output is explained at http://www.cbs.dtu.dk/services/SignalP/output.html

##########################################################################################

Name:  contig065408-ZebORe.N119         Length:  195
MGTEDKATTMFNNTFVRPAKFYLSGFSNIPHIRYYYAFLCFVYIMTVLGNGFLLSVIWLVKTLHTPKYMIVFNMALTDLC      80
GSTALIPKLLDTFLFDRRYILYDACLSYMFFVIFFASVQSWTLVTMAYDRLIAICFPLRYHSIVTETSVAAILLFWIFL     160
VSVIATMVGLVNRLSFCRSLVVNSFFCDHGPVYRL
...........N....................................................................      80
................................................................................     160
...................................                                                  240

(Threshold=0.5)
----------------------------------------------------------------------
SeqName      Position  Potential   Jury    N-Glyc
                                 agreement result
----------------------------------------------------------------------
contig065408-ZebORe.N119   12 NNTF   0.5691     (6/9)   +
----------------------------------------------------------------------
```

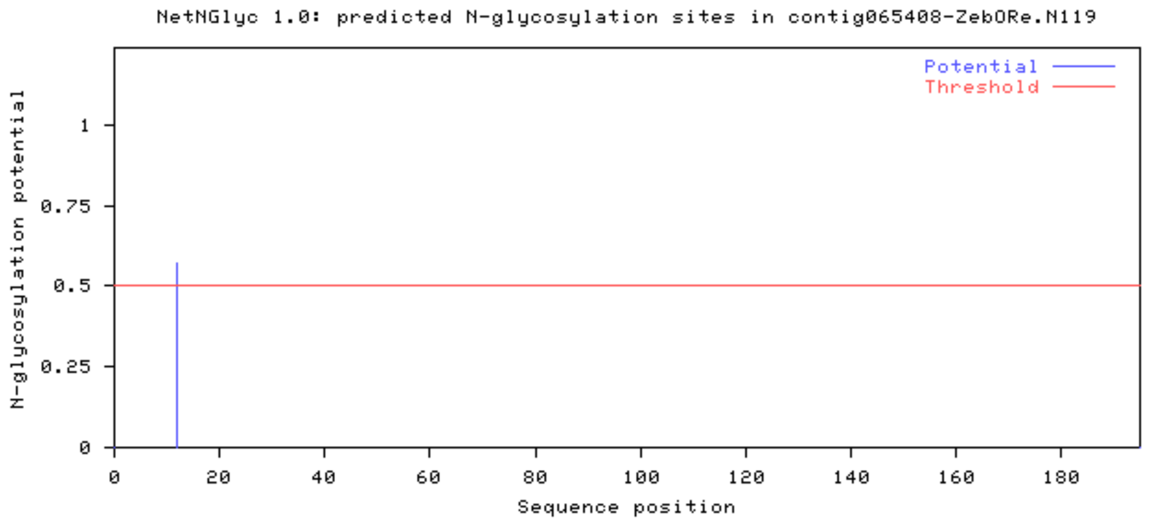

Graphics in PostScript

Output for 'contig066327-ZebOR.F064'

```
##########################################################################################

    Warning: This sequence may not contain a signal peptide!!

    Proteins without signal peptides are unlikely to be exposed to
    the N-glycosylation machinery and thus may not be glycosylated
    (in vivo) even though they contain potential motifs.

    SignalP-NN euk predictions are as follows:

  # name                    Cmax  pos ?  Ymax  pos ?  Smax  pos ?  Smean ?  D     ?

    SignalP output is explained at http://www.cbs.dtu.dk/services/SignalP/output.html

##########################################################################################

Name:  contig066327-ZebOR.F064  Length:  310
MENNSHPLYFNLTMFVYIGKFRYPAFVLFLLLYTFIISANLVIIVVISREKTLHEPMYIFIMCLSINSLYGSGGFFFRFL      80
RDLLSDSNLIAHSACYTQIYIIYTYASYELTILGIMAYDRFVAICQPLHYHSKLTSRVISKLLAFAWIYPAFSVAACVYL     160
ASRLPLCGNKIPKVFCANWPVVKLSCVPTVINNIIGMFVSVTTVFLPLAFVLYTYMQIFLVCRKRSSLFKSKVIQSCLPH     240
IVTFVNYSITVFCDVALSRIDLEELNPFLGIILSLEFVVIPPILNPLMYGLKLAEIRKCILRNLSCLIRX
..N.......N.....................................................................      80
................................................................................     160
................................................................................     240
.....N.........................................................N.......              320

(Threshold=0.5)
----------------------------------------------------------------------
SeqName      Position  Potential   Jury    N-Glyc
                                 agreement result
----------------------------------------------------------------------
contig066327-ZebOR.F064    3 NNSH   0.5829     (9/9)   ++
contig066327-ZebOR.F064   11 NLTM   0.8139     (9/9)   +++
contig066327-ZebOR.F064  246 NYSI   0.5722     (7/9)   +
contig066327-ZebOR.F064  303 NLSC   0.5276     (6/9)   +
----------------------------------------------------------------------
```

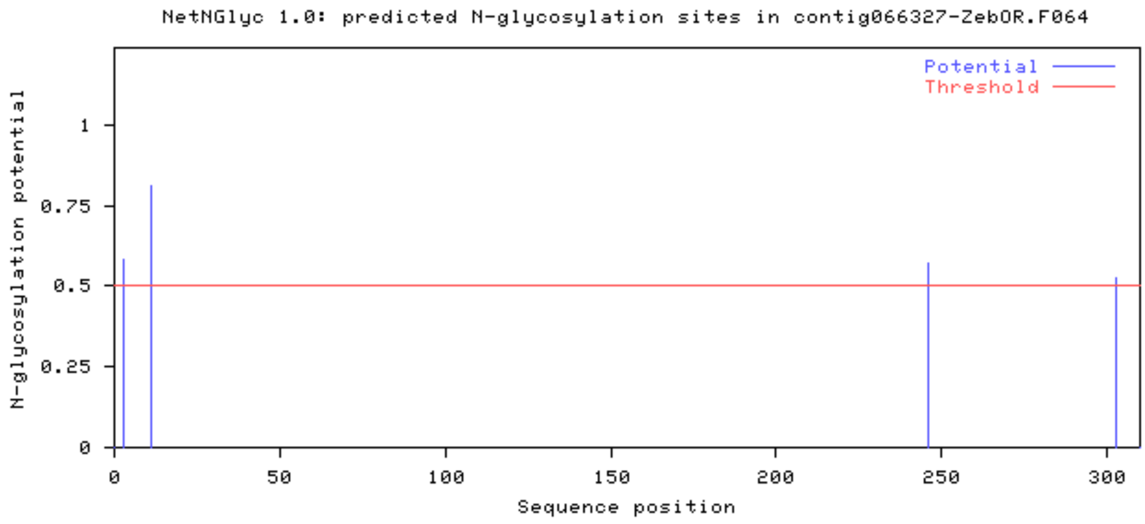

Graphics in PostScript

Output for 'contig067121-ZebORe.L097'

```
##########################################################################################

    Warning: This sequence may not contain a signal peptide!!

    Proteins without signal peptides are unlikely to be exposed to
    the N-glycosylation machinery and thus may not be glycosylated
    (in vivo) even though they contain potential motifs.

    SignalP-NN euk predictions are as follows:

  # name                 Cmax  pos ?  Ymax  pos ?  Smax  pos ?  Smean ?  D     ?

    SignalP output is explained at http://www.cbs.dtu.dk/services/SignalP/output.html

##########################################################################################

Name:  contig067121-ZebORe.L097         Length:  149
MSLQNASIKLTHFIIGGFDTVKMPVAVGVVMLITYLLAVLASLVNIIFIVSDKQLHKPMYLLICNLAVVDIFYTSTATPT      80
MIGVLLAGVNTISYVECLIQMYVYQVGVTMERFSLTIMAFDRLIAIIYPLQYHSYLTITRTLVFTYILW
....N...........................................................................      80
.....................................................................               160

(Threshold=0.5)
----------------------------------------------------------------------
SeqName      Position  Potential    Jury    N-Glyc
                                 agreement result
----------------------------------------------------------------------
contig067121-ZebORe.L097     5 NASI    0.6037     (7/9)    +
----------------------------------------------------------------------
```

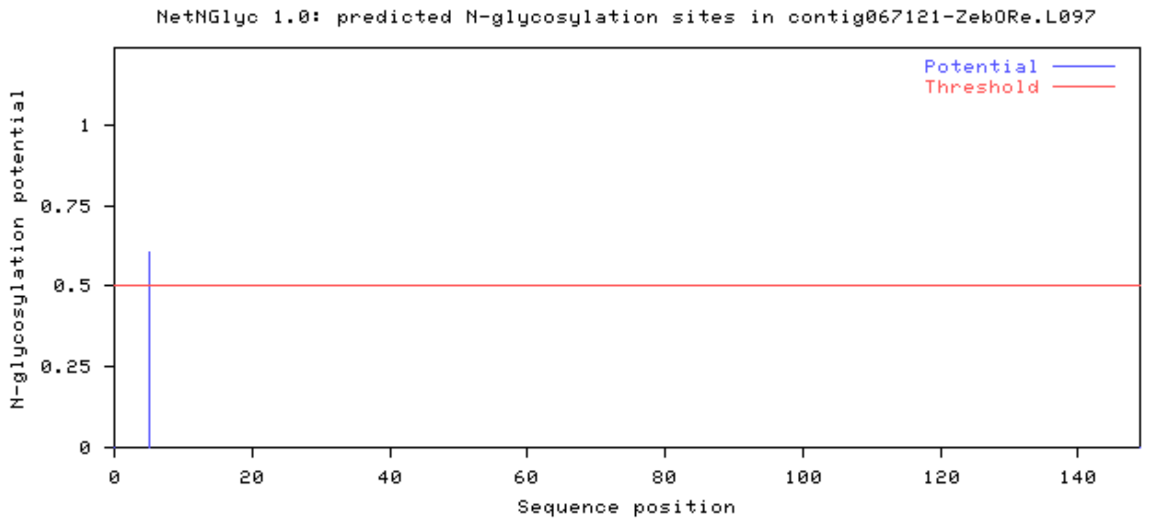

Graphics in PostScript

Output for 'contig067811-ZebORs.W140'

```
##########################################################################################

    Warning: This sequence may not contain a signal peptide!!

    Proteins without signal peptides are unlikely to be exposed to
    the N-glycosylation machinery and thus may not be glycosylated
    (in vivo) even though they contain potential motifs.

    SignalP-NN euk predictions are as follows:

  # name                 Cmax  pos ?  Ymax  pos ?  Smax  pos ?  Smean ?  D     ?

    SignalP output is explained at http://www.cbs.dtu.dk/services/SignalP/output.html

##########################################################################################

Name:  contig067811-ZebORs.W140         Length:  305
MSASYTNETVVVNYRDAFPKAMVKNVIVVVLCISINYINVALLQTFCKQQIFYMNPRYILFFHLVLNDMIQVTLTVILFI      80
SSYIFFQINVSVCCVLILLALFATENTPLNLACMAVECYIAICIPLRHVQICTVKRTLMLIGLIWMTSMLSVLPDLFITL     160
AIEPLDFYNSRVFCLRETVFRNPHIIKKRDITYIVYLVIVWFIIFFTYFKILFTAKAASQDATKARNTIILHGFQVLLCM     240
SIYAEPLLRQVLQQWFPQNYSDSLFACYILFQILPRAISPIVYGVRDKTYRKYLKRYLLCKMGPX
......N.........................................................................      80
........N.......................................................................     160
................................................................................     240
..................N..............................................                    320

(Threshold=0.5)
----------------------------------------------------------------------
SeqName      Position  Potential   Jury    N-Glyc
                                 agreement result
----------------------------------------------------------------------
contig067811-ZebORs.W140    7 NETV   0.7383     (9/9)   ++
contig067811-ZebORs.W140   89 NVSV   0.6564     (8/9)   +
contig067811-ZebORs.W140  259 NYSD   0.6356     (8/9)   +
----------------------------------------------------------------------
```

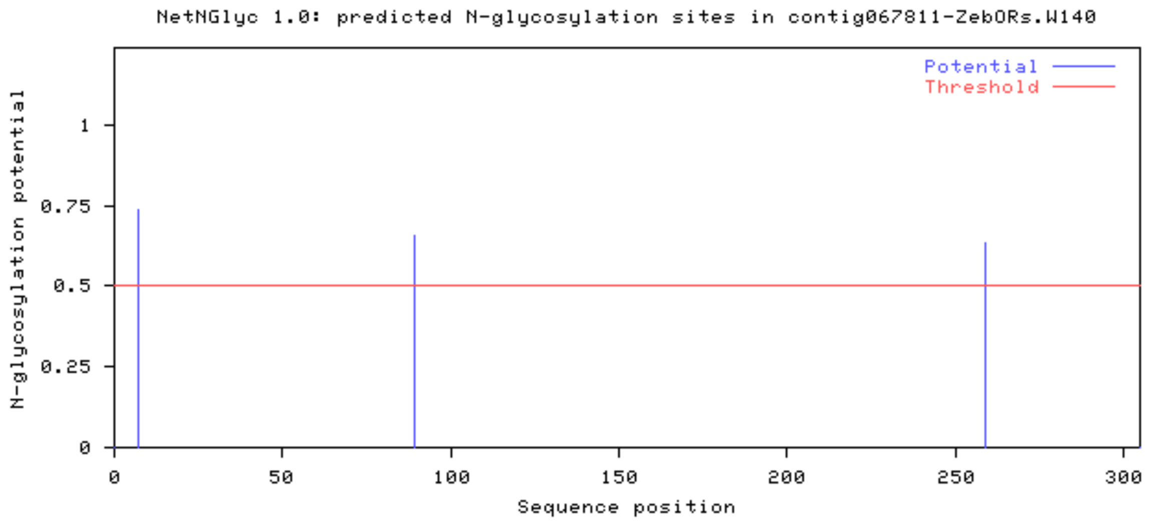

Graphics in PostScript

Output for 'contig068037-ZebORe.A025'

```
##########################################################################################

    Warning: This sequence may not contain a signal peptide!!

    Proteins without signal peptides are unlikely to be exposed to
    the N-glycosylation machinery and thus may not be glycosylated
    (in vivo) even though they contain potential motifs.

    SignalP-NN euk predictions are as follows:

  # name                 Cmax  pos ?  Ymax  pos ?  Smax  pos ?  Smean ?  D     ?

    SignalP output is explained at http://www.cbs.dtu.dk/services/SignalP/output.html

##########################################################################################

Name:  contig068037-ZebORe.A025         Length:  204
TSVGSEFLLLAAMAYDRYVSICKPLQYPVIMNRITIYVCLILAWLIPAFEALMLGVLYSNVKLCSFTLTGIFCNNSVHKL      80
QCVPSVAISIYGVVMLINIALLPLLFILFSYIKILKISYQRCREVRKKAVKTCLPHLLVLIHFSCFISFDIIIVRLETDL     160
SKTLRLILTFELILFHPLLNPIIYGLKMNEISKHLKILLCLVKX
................................................................................      80
................................................................................     160
............................................                                         240

(Threshold=0.5)

No sites predicted in this sequence.
```

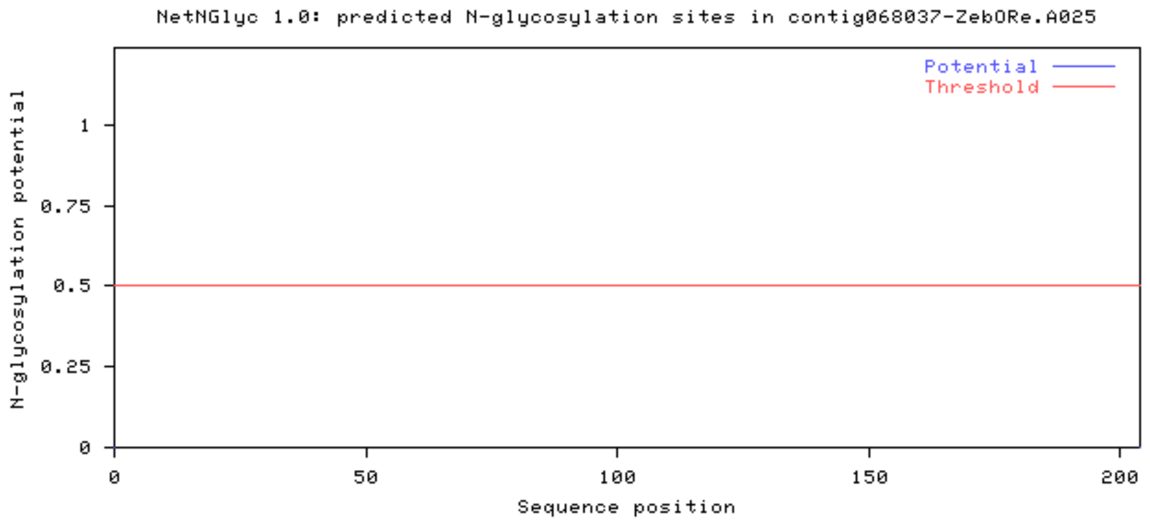

Graphics in PostScript

Output for 'contig068054-ZebOR.S129'

```
##########################################################################################

    Warning: This sequence may not contain a signal peptide!!

    Proteins without signal peptides are unlikely to be exposed to
    the N-glycosylation machinery and thus may not be glycosylated
    (in vivo) even though they contain potential motifs.

    SignalP-NN euk predictions are as follows:

  # name                 Cmax  pos ?  Ymax  pos ?  Smax  pos ?  Smean ?  D     ?

    SignalP output is explained at http://www.cbs.dtu.dk/services/SignalP/output.html

##########################################################################################

Name:  contig068054-ZebOR.S129  Length:  307
MVDNSSFIGVSSMRQLNDQVIIVQVLVGVFLCINTMLIITFFMKDTFYRTMRYILFAVTLLSDCLILILTDLLLILTYFH      80
LSIQVSLCLIMFAVSSVCNFVTPFTLTAMTLERYVASCMPLRHGELCSTRSALQCILIIHGLSSVPCILILSVFFASVSL     160
SFFTQYRVCFGQTFIIRSWQGHLRSAISQFYFLIMCIIIVFSYIQIMKVAKAASGENKKSTHKGLRTVALHAFQLLLCLI     240
QLWCPFIEAAVLQIDFMLYVNVRYFNYIMFSLTPRCLSPLIYGLRDDKFFLALRYHVLCPLFRKNNX
...N............................................................................      80
................................................................................     160
................................................................................     240
...................................................................                  320

(Threshold=0.5)
----------------------------------------------------------------------
SeqName      Position  Potential   Jury    N-Glyc
                                agreement result
----------------------------------------------------------------------
contig068054-ZebOR.S129    4 NSSF   0.6268     (7/9)   +
----------------------------------------------------------------------
```

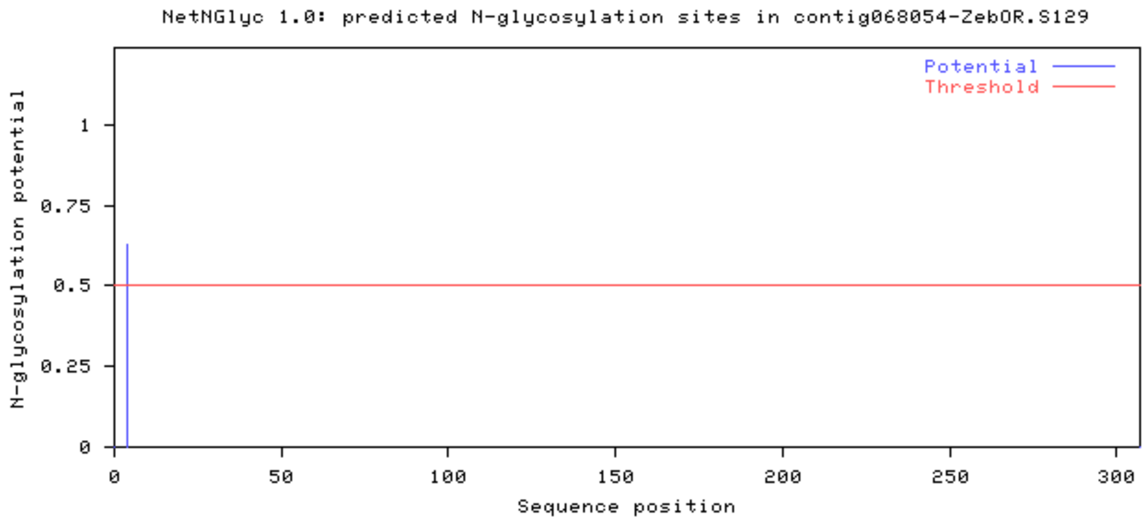

Graphics in PostScript

Output for 'contig069298-ZebORe.E058'

```
##########################################################################################

    Warning: This sequence may not contain a signal peptide!!

    Proteins without signal peptides are unlikely to be exposed to
    the N-glycosylation machinery and thus may not be glycosylated
    (in vivo) even though they contain potential motifs.

    SignalP-NN euk predictions are as follows:

  # name                  Cmax  pos ?  Ymax  pos ?  Smax  pos ?  Smean ?  D     ?

    SignalP output is explained at http://www.cbs.dtu.dk/services/SignalP/output.html

##########################################################################################

Name:  contig069298-ZebORe.E058         Length:  252
LLLQILSDVHTVSVSFCFLQVFCVYTYACVEFINFVVMSYDRYYAICWPLQYKSCMTLKTVTSLISLTWLLPFIMIVVLI      80
SLSAPLQLCGNVINKVFCGNYAIIKLACSDTRVHNIFGLIYTFISVIIPLVLILYTYVRILKVCFSGSKQTRQKAVSTCT     160
PHLASISNFFFGCCFQILQSRFDTSGVPNVFGILSSLYFLTCQPLFTPLLYGLKMTKIRIACKQLFCGSLTRLFSCSSDI     240
FSKSHQSQSYCX
................................................................................      80
................................................................................     160
................................................................................     240
............                                                                         320

(Threshold=0.5)

No sites predicted in this sequence.
```

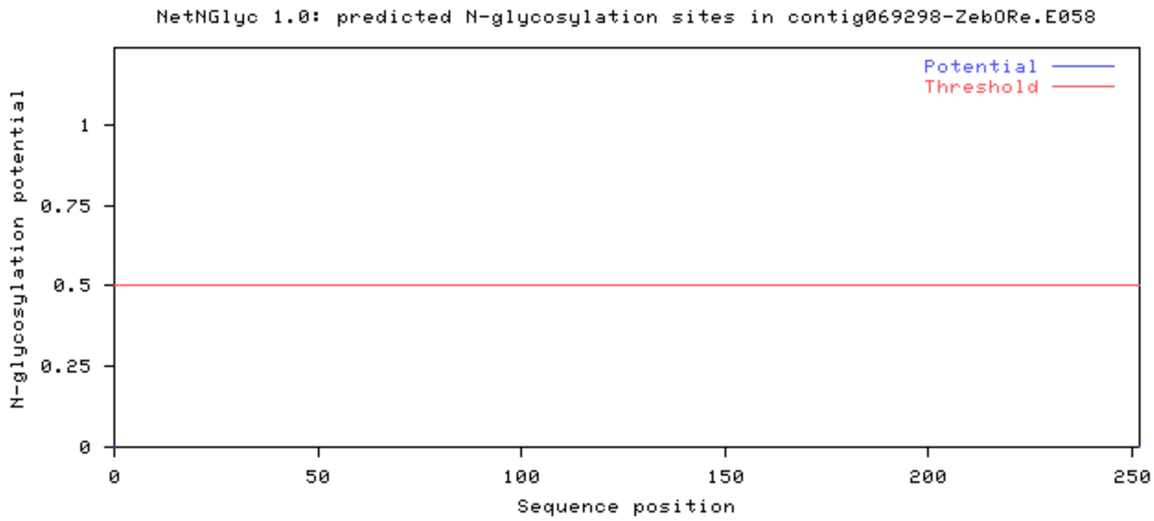

Graphics in PostScript

Output for 'contig069603-ZebORep.E057'

```
##########################################################################################

    Warning: This sequence may not contain a signal peptide!!

    Proteins without signal peptides are unlikely to be exposed to
    the N-glycosylation machinery and thus may not be glycosylated
    (in vivo) even though they contain potential motifs.

    SignalP-NN euk predictions are as follows:

  # name                    Cmax   pos ?  Ymax   pos ?  Smax   pos ?  Smean ?  D     ?

    SignalP output is explained at http://www.cbs.dtu.dk/services/SignalP/output.html

##########################################################################################

Name:  contig069603-ZebORep.E057        Length:  223
RYLYFLIILTSYAAIICANLLLIVVICVNRSLHEPMYMFLCSLFVNELYGSTGLFPFLLLQILSDVHTVSVSFCFLQVFC      80
VYTYACVEFINFVVMSYDRYYAICWPLQYKSCMTLKTVTSLISLTWLLPFIMIVVLISLSAPLQLCGNVINKVFCGNYAI     160
IKLACSDTRVHNIFGLIYTFISVIIPLVLILYTYVRILKVCFSGSKQTRQKAVSTCTPHLASI
............................N...................................................      80
................................................................................     160
...............................................................                      240

(Threshold=0.5)
----------------------------------------------------------------------
SeqName      Position  Potential   Jury    N-Glyc
                                 agreement result
----------------------------------------------------------------------
contig069603-ZebORep.E057    29 NRSL   0.7532     (9/9)   +++
----------------------------------------------------------------------
```

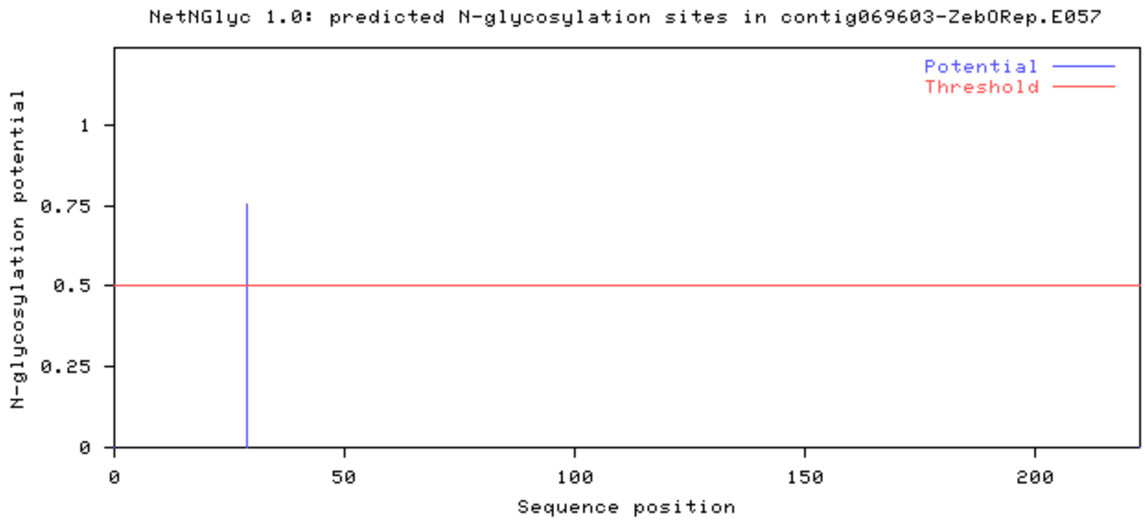

Graphics in PostScript

Output for 'contig069781-ZebORe.L098'

```
##########################################################################################

    Warning: This sequence may not contain a signal peptide!!

    Proteins without signal peptides are unlikely to be exposed to
    the N-glycosylation machinery and thus may not be glycosylated
    (in vivo) even though they contain potential motifs.

    SignalP-NN euk predictions are as follows:

  # name                    Cmax  pos ?  Ymax  pos ?  Smax  pos ?  Smean ?  D     ?

    SignalP output is explained at http://www.cbs.dtu.dk/services/SignalP/output.html

##########################################################################################

Name:  contig069781-ZebORe.L098         Length:  94
ILFFVKLSSNNDRMKMGSTLVSHLICVICLYCPIFIHAILTRFGVVLTLEERQGLSIGTILGPSLVNPFVYFLRTKEIKS      80
KIFKILRKANTARX
................................................................................      80
..............                                                                       160

(Threshold=0.5)

No sites predicted in this sequence.
```

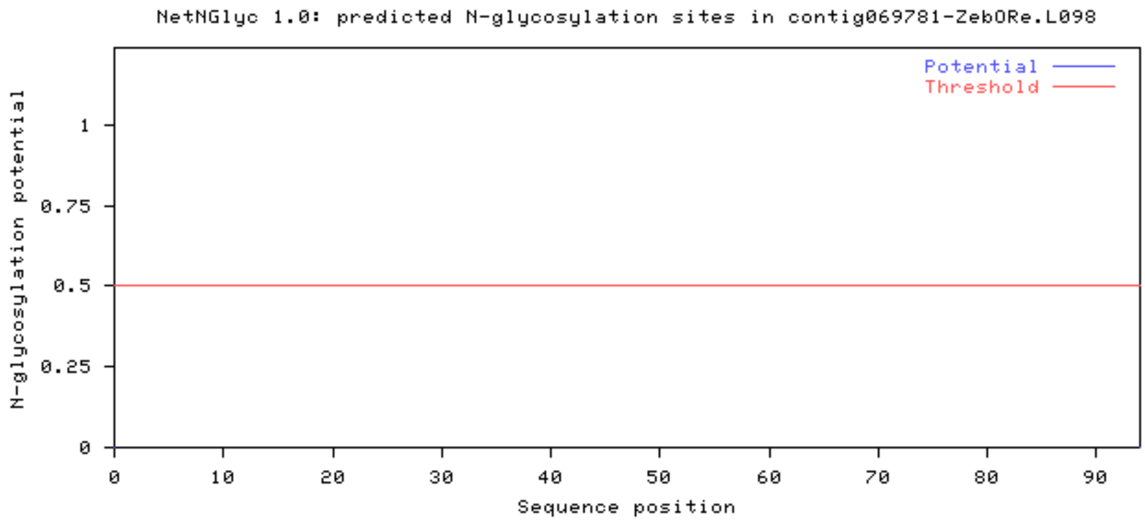

Graphics in PostScript

Explain the output. Go back.
