# Supplementary material for: Inventory of the cichlid olfactory receptor gene repertoires: identification of olfactory genes with more than one coding exon
Source: BMC Genomics. 2014 Jul 11;15(1):586. doi: 10.1186/1471-2164-15-586 (PMC4122780; doi:10.1186/1471-2164-15-586)
Supplement: Supplementary file 3 — Additional file 3: Contigs and scaffolds harbouring ORs. (PDF 13 MB) [file 12864_2014_6314_MOESM3_ESM.pdf]

Cichlid Olfactory Receptors

Group 2C

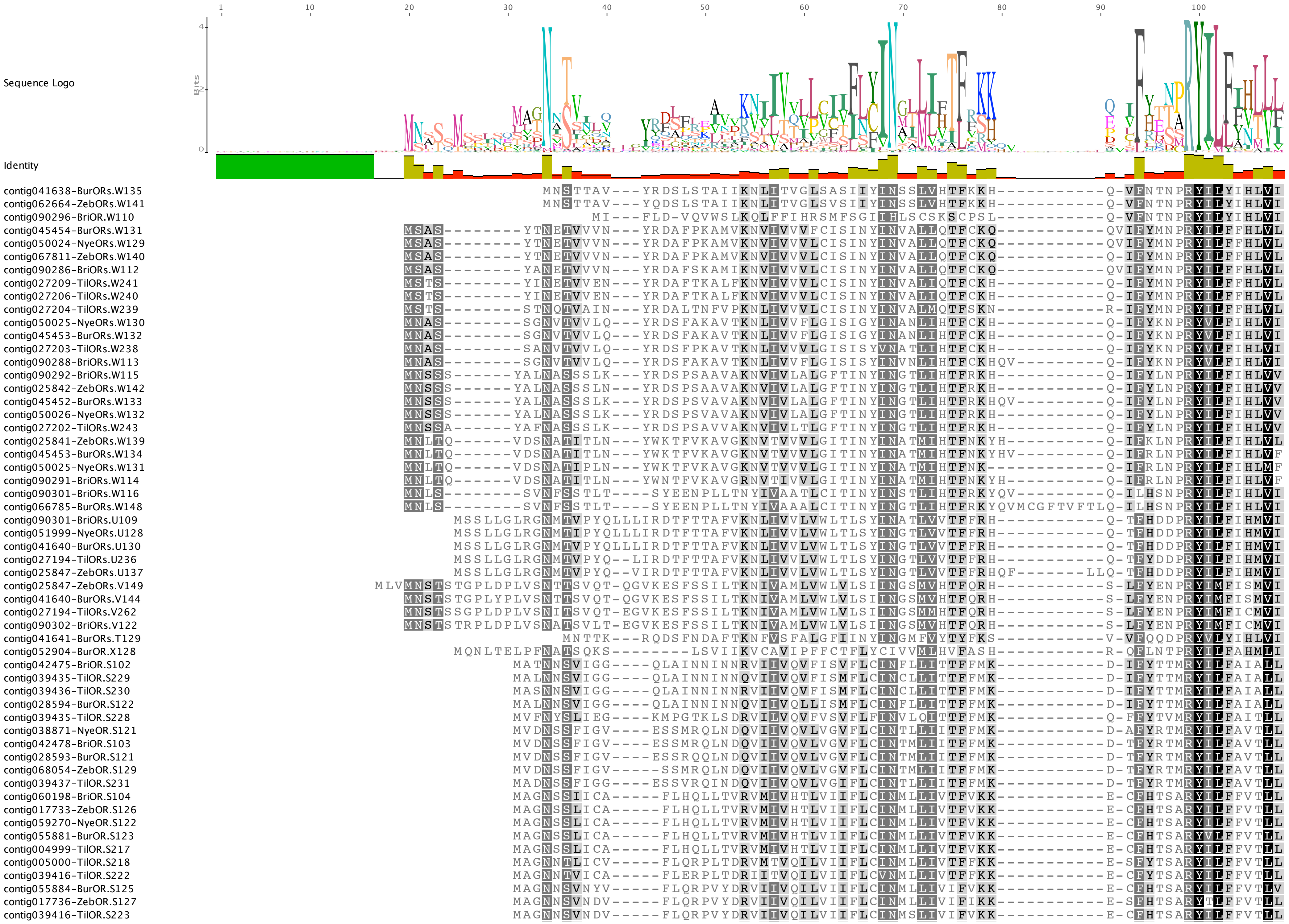

Sequence Logo
Identity
Bits
1
10
20
30
40
50
60
70
80
90
100
contig041638-BurORs.W135
contig062664-ZebORs.W141
contig090296-BriOR.W110
contig045454-BurORs.W131
contig050024-NyeORs.W129
contig067811-ZebORs.W140
contig090286-BriORs.W112
contig027209-TilORs.W241
contig027206-TilORs.W240
contig027204-TilORs.W239
contig050025-NyeORs.W130
contig045453-BurORs.W132
contig027203-TilORs.W238
contig090288-BriORs.W113
contig090292-BriORs.W115
contig025842-ZebORs.W142
contig045452-BurORs.W133
contig050026-NyeORs.W132
contig027202-TilORs.W243
contig025841-ZebORs.W139
contig045453-BurORs.W134
contig050025-NyeORs.W131
contig090291-BriORs.W114
contig090301-BriORs.W116
contig066785-BurORs.W148
contig090301-BriORs.U109
contig051999-NyeORs.U128
contig041640-BurORs.U130
contig027194-TilORs.U236
contig025847-ZebORs.U137
contig025847-ZebORs.V149
contig041640-BurORs.V144
contig027194-TilORs.V262
contig090302-BriORs.V122
contig041641-BurORs.T129
contig052904-BurOR.X128
contig042475-BriOR.S102
contig039435-TilOR.S229
contig039436-TilOR.S230
contig028594-BurOR.S122
contig039435-TilOR.S228
contig038871-NyeOR.S121
contig042478-BriOR.S103
contig028593-BurOR.S121
contig068054-ZebOR.S129
contig039437-TilOR.S231
contig060198-BriOR.S104
contig017733-ZebOR.S126
contig059270-NyeOR.S122
contig055881-BurOR.S123
contig004999-TilOR.S217
contig005000-TilOR.S218
contig039416-TilOR.S222
contig055884-BurOR.S125
contig017736-ZebOR.S127
contig039416-TilOR.S223

```
contig039419-TilOR.S224      MVGNNSVNDV-----FLQQPVNDRVIIVQILVIIFLCINMLLIVIFVKK-------------E-CFHTSARYILFFVTLL
contig055882-BurOR.S124      MAGNNSVNCV-----FFPRPVSYRVIIVEILVIIFLCINMLLIVIFVKK-------------E-SFHTSARYILFFVTLL
contig005005-TilOR.S219      MAGNNSVNDV-----FFPRPVSYRVIIVEILVIIFLCINMLLIVIFVKK-------------E-CFHTSARYILFFVTLL
contig005007-TilOR.S220      MAGNNSVNDV-----FFPRPVSYRVIIVEILVIIFLCINMLLIVIFVKK-------------E-CFHTSARYILFFVTLL
contig039428-TilOR.S227      MADNNSVNNV-----FLQRP-NDRMIIVQILVIIFLCINMLLILTFIKK-------------E-SFHTSARYILFSVTLL
contig017743-ZebOR.S128      MADNNSVNNV-----FLQRP-NDRMIIVQILVIIFLCINMLLILTFIKK-------------E-SFHTSARYILFSVTLL
contig039426-TilOR.S226      MAGNNSVNDV-----LLQRPVDDRVIIVQILVIIFLCINMLLIMIFIKK-------------E-SFHTSARYILFFVTLL
contig039425-TilOR.S225      MAGNNSVNDV-----LLQRPVNDRVIIVQILVIIFLCINMLLIMIFVKK-------------E-SFHTSARYILFFVTLL
contig009773-BurORs.Q136     MNATTAEF----QSLPFQTSVKAALSMLPCFFFLYVNAIMMFALLKK-------------P-LLLESSRYILFGHLLM
contig021011-NyeOR.Q134      MNATTAEF----QSLPFQTSVKAALSMLPCFFFLYVNAIMMFALLKK-------------P-LLLESSRYILFGHLLM
contig050422-ZebOR.Q143      MNATTAEF----QSLPFQTSVKAALSMLPCFFFLYVNAIMMFALLKK-------------P-LLLESSRYILFGHLLM
contig063829-BriOR.Q117      MNATTAEF----QSSRFQTSVKAALSMLPCFFFLYVNAIMMFALLKK-------------P-LLLESSRYILFGHLLI
contig028611-TilOR.R246      MSLSNQTLTNVTANLQ----YLGVLEIVLFFTLSTMSCCIFLFINGIMLFTLRSK-------------I-LFCETSRYILLYNLLF
contig028617-TilOR.R248      MSLSNQTLTNVTANLQ----YLGVLEIVLFFTLSTMSCCIFLFINGIMLFTLRSK-------------I-LFCETSRYILLYNLLF
contig028607-TilOR.R245      MLLANLSLTNVTANQQ----YQGVLERVLFSTLTTLPCCVFLFINGIMLFTLRSK-------------A-LFCETSRYILLYNLLF
contig065193-ZebOR.R147      MLLANLSLTNVTANQQ----YQGVLERVLFSTLTTLPCCLFLCINGIMLFTLRSK-------------A-LFCETSRYILLYNLLF
contig067209-BurOR.R140      MLLANLSLTNVSANQQ----YQGVLERVLFSTLTTLPCCLFLCINGIMLFTLRSK-------------A-LFCETSRYILLYNLLF
contig094282-BriOR.R118      MSYISPSKTNFTVGLQ----YRGILEVLLFSAPITASCCVLLFINGTMLHILRSK-------------A-VFCETACYVLLYNLLF
contig043640-BurOR.R137      MSSISQTLTNITVG------YQALAERVMISTLTTLPTCVFLFINGIMLFTLRSK-------------P-VFRETCRYILLYNLLF
contig046042-ZebOR.R145      MTSTSQTLTNITVQ------SPGLAERVMISTLTTLPTCVFLFINGIMLFTLRSK-------------P-VFRETCRYILLYNLLF
contig028619-TilOR.R249      MTSTSQTLTNITVQ------APGLLERVMISTLTTLPTCVFLFINSIMLFTLRSK-------------P-VFRETCRYILLYNLLF
contig028614-TilOR.R247      MSSISQTLTNITVG------YQALEERVMISTLTTLPTCVFLFINSIMLFTLRSK-------------P-VFRETCRYILLYNLLF
contig028639-TilOR.R251      MLTV--SRSNITDAFQ----YPDFLRIMIISTLTTFPSFIFLFINGTMLFTLRSK-------------P-VFHETPRYILLYNLLF
contig028637-TilOR.R250      MSNVSQIYSDFNFEVQ----YQRLLRIVIISALSTLPACVFLFVNGIMLFMLRRK-------------R-VFRETCRYILLYNLLF
contig046040-ZebOR.R144      MSNVSQSYTNMSFEVQ----YQDLLRVIIVSTLSTVPSFTFLFLNGTMLFTLRSK-------------P-VFRDTPRYILLYNLLF
contig054733-BurOR.R138      MSNVSESYTNMSIEVQ----YQDLLRVIIVSTLSTVPSFIFLFLNGTMLFTLRSK-------------P-VFRDTPRYILLYNLLF
contig028641-TilOR.R253      MPDISQSQTNISVGLH-------DLERGLLSSLTTLPCCVFFCINVIMLFTLRSK-------------S-VFRETCRYILLYNLIL
contig028644-TilOR.R252      MPDISQSQTNISVGLH-------DLERGLLSSLTTLPCCVFFCINVIMLFTLRSK-------------S-VFRETCRYILLYNLIL
contig064565-BurOR.R139      MSDI--SQTNISVGLH-------DLERGLLSSLTTLPCCVFVCINVIMLFTLRSK-------------S-VFREPCRYILLYNLVL
contig046048-ZebOR.R146      MSSTNETLTNITVGQQ----NQLFLEIVFSCIVTTLTCCVFLFINATMLFTLRSK-------------P-VFGQTSRYILLYNLLF
contig067265-BurOR.R141      MFFTNETLTNITVGQQ----NQLFLEIVFSCIVTTLTCCVFLFINATMLFTLRSK-------------P-VFGQTSRYILLYNLLF
contig044295-NyeOR.R135      MFFTNETLTNITVGQQ----NQLFLEIVFSCIVTTLTCCVFLFINATMLFTLRSK-------------P-VFGQTSRYILLYNLLF
contig061321-BriOR.Y128      MSRNNTTEVT-------SDYTCVRFYVSTISFSVLLFFNLIINWAIVRE-------------E-RLRRHARFVLIFHLLV
contig073387-BriOR.Z129      MTAVLETGAHNLTEERDSFSQENQTNPHATGQLNNANCLF-LSILPEGQAVSVLICLFVLLTALSCFVNAFTLFGLGQS-------------E-EFSWQPRFILLKNLIF
```

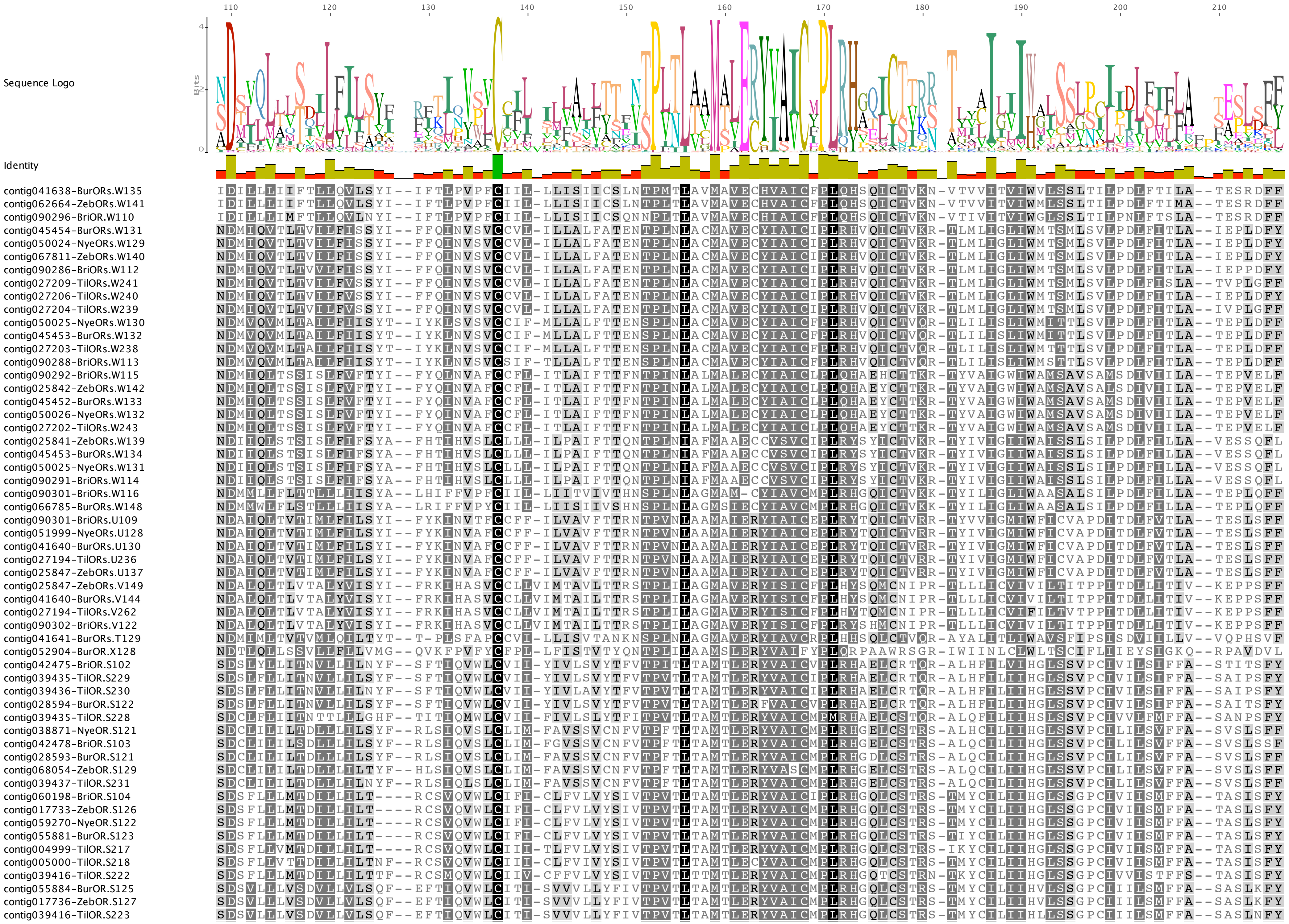
110
120
130
140
150
160
170
180
190
200
210
Sequence Logo
Bits
4
2
0
Identity
contig041638-BurORs.W135
contig062664-ZebORs.W141
contig090296-BriOR.W110
contig045454-BurORs.W131
contig050024-NyeORs.W129
contig067811-ZebORs.W140
contig090286-BriORs.W112
contig027209-TilORs.W241
contig027206-TilORs.W240
contig027204-TilORs.W239
contig050025-NyeORs.W130
contig045453-BurORs.W132
contig027203-TilORs.W238
contig090288-BriORs.W113
contig090292-BriORs.W115
contig025842-ZebORs.W142
contig045452-BurORs.W133
contig050026-NyeORs.W132
contig027202-TilORs.W243
contig025841-ZebORs.W139
contig045453-BurORs.W134
contig050025-NyeORs.W131
contig090291-BriORs.W114
contig090301-BriORs.W116
contig066785-BurORs.W148
contig090301-BriORs.U109
contig051999-NyeORs.U128
contig041640-BurORs.U130
contig027194-TilORs.U236
contig025847-ZebORs.U137
contig025847-ZebORs.V149
contig041640-BurORs.V144
contig027194-TilORs.V262
contig090302-BriORs.V122
contig041641-BurORs.T129
contig052904-BurOR.X128
contig042475-BriOR.S102
contig039435-TilOR.S229
contig039436-TilOR.S230
contig028594-BurOR.S122
contig039435-TilOR.S228
contig038871-NyeOR.S121
contig042478-BriOR.S103
contig028593-BurOR.S121
contig068054-ZebOR.S129
contig039437-TilOR.S231
contig060198-BriOR.S104
contig017733-ZebOR.S126
contig059270-NyeOR.S122
contig055881-BurOR.S123
contig004999-TilOR.S217
contig005000-TilOR.S218
contig039416-TilOR.S222
contig055884-BurOR.S125
contig017736-ZebOR.S127
contig039416-TilOR.S223

```
contig039419-TilOR.S224    SDSVLLLVSDVLLILTNF--EFTMPVWLCIAI-SGVVFLYFIVTPVTLTAMTLERYVAICMPLRHGQLCSTRS-TMYCILIIHLLSSGPCIIILSMFFA--SASLNFY
contig055882-BurOR.S124    SDSVLLLVSDILFILTHF--EITMPVWLCITI-SVVVLLYFIVTPVTLTAMTLERYVAICMPLRHGQLCSTRS-TMYCILIIHVLSSGPCIIILSMFFA--SGSLKFY
contig005005-TilOR.S219    SDSVLLLMSDILFILTHF--EIAMPVCLCITI-SVVVLLYFIVTPVTLTAMTLERYVAICMPLRHGQLCSTRS-TMYCILIIHGVSSGPCIIILSMFFA--SASLNFY
contig005007-TilOR.S220    SDSVLLLVSDILFILTHF--EIAMPVWLCITI-SVVVLLYFIVTPVTLTAMTLERYVAICMPLRHGQLCSTRS-TMYCILIIHGVSSGPCIIILSMFFA--SGSLKFY
contig039428-TilOR.S227    SDSLLLFVSDILVILTYF--QFTIPVWLCIIL-TIFVVMYTFVTPVTLTAMTLERYVAICMPLRHGQLCSTRS-TMYCILIIHVVSFGPCIIILSMFFA--FASLKFY
contig017743-ZebOR.S128    SDSILLFVSDILVILTYY--QFTIQVWLCIIS-TIFVLMYTFVTPVTLTAMTLECYVAICMPLRHGQLCSTRS-TMYCILIIHVLSSGPCIIILSMFFA--SASLKFY
contig039426-TilOR.S226    SDSVLLLVSDVLLILTHF--EFTIQVWLCIIL-TIFVVMYSFVTPVTLTAMTLERYVAICMPLRHGQLCSTRS-TMYCILIIHGVSFGPCIIILSMFFA--SASLNFY
contig039425-TilOR.S225    SDSVLLLVSDVLLILTYF--EFPIQVWLCIIL-TIFVVMYSSVTPVTLTAMTLERYVAICMPLRHGQLCSTRS-TMYCILIIHVLSSGPCIIILSMFFA--SASLNFY
contig009773-BurORs.Q136   CDSVQLLLTMMLYIFAVM--MVRMINYVCVFV-SLVAAVTVKMSPLNLAVMSLERYVAVCFPLRHPSFATPRS-TGKAIAVMWMAASLDSFIQLFLFVR---MEMTIF
contig021011-NyeOR.Q134    CDSVQLLLTMLLYIFAVM--MVRMINYVCVFV-SLLAAVTVKMSPLNLAVMSLERYVAVCFPLRHPSFATPRS-TGKAIAVMWIVASLDSFIQLFLFVR---MEKTIF
contig050422-ZebOR.Q143    CDSVQLLLTMLLYIFAVM--MVRMINYVCVFV-SLLAAVTVKMSPLNLAVMSLERYVAVCFPLRHPSFATPRS-TGKAIAVMWIVASLDSFIQLFLFVR---MEKTIF
contig063829-BriOR.Q117    CDSVQLLLSMMLYIFAVM--TVRMINYVCVFV-SLVAAVTVKMSPLNLAVMSLERYVAVCFPLRHPSFATVRS-TGKAIAVMWMVASLDSFIQLFLFVR---MEKTIF
contig028611-TilOR.R246    ADTVQMALSQLLYIIATS--RITLTYPVCGFL-TMLANLTTVVSPLTLVVMSLERYVAVCYPLRHATIITITN-TGVAIIAIWAIGSLNILTRVLLLLEFPFEALDSL
contig028617-TilOR.R248    ADTVQMALSQLLYIIATS--RITLTYPVCGFL-TMLANLTTVVSPLTLVVMSLERYVAVCYPLRHATIITITN-TGVAIIAIWAIGSLNILTRVLLLLEFPFEALDSL
contig028607-TilOR.R245    ADTVQMALSQLLYIIATS--RITLTYPVCGFL-TMLANLTTVVSPLTLVVMSLERYVAVCYPLRHATIITITN-TGVAIIAIWAIGSLNILTRVLLLLEFPFEALDSL
contig065193-ZebOR.R147    ADTVQMALSQLLYIIAAC--RITLTYPVCGFL-TMLANLTTVVSPVTLVVMSLERYVAVCYPLRHASIITITN-TGVAIIVIWAIGSLNILTRVLLLLEFPFGALDSL
contig067209-BurOR.R140    ADTVQMALSQLLYIIAAC--RITLTYPVCGFL-TMLANLTTVVSPVTLVVMSLERYVAVCYPLRHASIITITN-TGVAIIVIWAIGSLNILTRVLLLLEFPFEALDSL
contig094282-BriOR.R118    SDTIQMVLSQLLYLLSAF--RIRLTYPVCGFL-IMLANLTTGISPLTLVVMSLERYVAVCYPLRHASIITIGN-TALAIIIVWAVSSLNVFIRLILLLNFQFEDLESL
contig043640-BurOR.R137    ADTVQLAQSQIHFLLAVL--RITVSYPVCTFL-VNFTHLTAVISPLTLVVMPLERYVAVCYPLRHATIITIRN-TGAAITVIWAISFLNIIIRTLLFLSL-FEKLGKV
contig046042-ZebOR.R145    ADTVQLAQSQIHFLLAVL--RITVSYPVCTFL-VNFTHLTSVISPLTLVVTPLERYVAVCYPLRHATIITIRN-TGAAITVIWAISFLNIIIRTLLFLSL-FEELGDL
contig028619-TilOR.R249    ADTVQLAQSQIHFLLAVL--RIRISYPVCTFL-VNFTQLTAVISPLTLVVMPLERYVAVCYPLRHGTIITIRN-TGAAIIVIWAISFLNIIIRTLLFLAL-FEELGDL
contig028614-TilOR.R247    ADTVQLAQSQIHFLLAVL--RIRISYPVCTFL-VNFTQLTAVISPLTLVVMPLERYVAVCYPLRHGTIITIRN-TGAAIIVIWAISFLNIIIRTLLFLAL-FEKLDKI
contig028639-TilOR.R251    AETVQLAQSQVLFLLSVS--QVKLFYPVCGFL-LFFTSLTTVISPLTLVVMPLERYVAVCYPLRHPTIITIRN-TVVGVIVTWAVSSVNILIRGLLVVKV-LLKLENV
contig028637-TilOR.R250    ADTAQLAQTQLIFLLSVC--QIQLPFSVCAIL-ILLANLTGRITPLTLVVMPLERYVAVCYPLRHATIITIRN-TRVVIIVIWVVSSLNNLTRILLLFEFLFENVKNL
contig046040-ZebOR.R144    ADTVQLAQSQVLFLLSIF--RVKLPYPVCVCL-SLLANLTTGISPLTLSVMPLERYVAVCYPLRYPTIITIRN-TGAAIVVIWIISSLNNLTRVIFFFP--FEVLKNL
contig054733-BurOR.R138    ADTVQLAQSQVLFLLSIF--RVKLPYPVCVCL-SLLANLTTGISPLTLSVMPLERYVAVCYPLRYPAIITIRN-TGAAIVVIWIISSLNNLTRLIFFFP--FEVLKNL
contig028641-TilOR.R253    ADTLQMAVSQILYMMSIC--RITLPYPVCGIL-VMFANLTNEISPLTLVLMSLERYVAVCYPLRHATIITIRN-TEVAIIMIWIFCSLNILIRVLLLLEFPFEELQSL
contig028644-TilOR.R252    ADTLQMAVSQILYMMSIC--RITLPYPVCGIL-VMFANLTNEISPLTLVLMSLERYVAVCYPLRHATIITIRN-TEVAIIMIWIFCSLNILIRVLLLLEFPFEELQSL
contig064565-BurOR.R139    ADTLQMAVSQILYMMSIC--RITLPYPVCGIL-VMFANLTNEISPLTLVLMSLERYVAVCYPLRHATIITIRN-TEVAIITIWIFCSLNILIRVLLLLEFPFEELQSL
contig046048-ZebOR.R146    ADTLQMAQSQLMFLLSAC--RITLLYPICGVL-VSLATLLTLISPLTLVAMSLERYVAVCYPLRHATIITVRN-TALAVCVVWTLSLLNVLIEVVLMLRVRFQDLLHL
contig067265-BurOR.R141    ADTLQMAQSQVMFLLSAC--RITLLYPICGVL-VSLATLLTLISPLTLVAMSLERYVAVCYPLRHATIITFRN-TALAVCVVWTISLLNVLIEVVLMLRVRFQDLLHL
contig044295-NyeOR.R135    ADTLQMAQSQVMFLLSAC--RITLLYPICGVL-VSLATLLTLISPLTLVAMSLERYVAVCYPLRHAAIITVRN-TALAVCVVWTISLLNVLIEVVLMLRVRFQDLLHL
contig061321-BriOR.Y128    SALVHLGMSSVFYYQIHL--DTRLSRSACMAM-ITILISSASNILLTLTAMALDRFCAVCHPMRYSSVCNKGHWPWLLGVFTWMVALVIPLSLLFKDSD--------A
contig073387-BriOR.Z129    SDLVQTATFGPAVIHSLIQRRTMAFNGWCYVQ-YFLGGVSIFCSLVTITCMALERYIYVCHAIRYLPIFTKIR-LRGVLGGIWLYSVFIGVSEIVMLHTGRGEDDETA
```

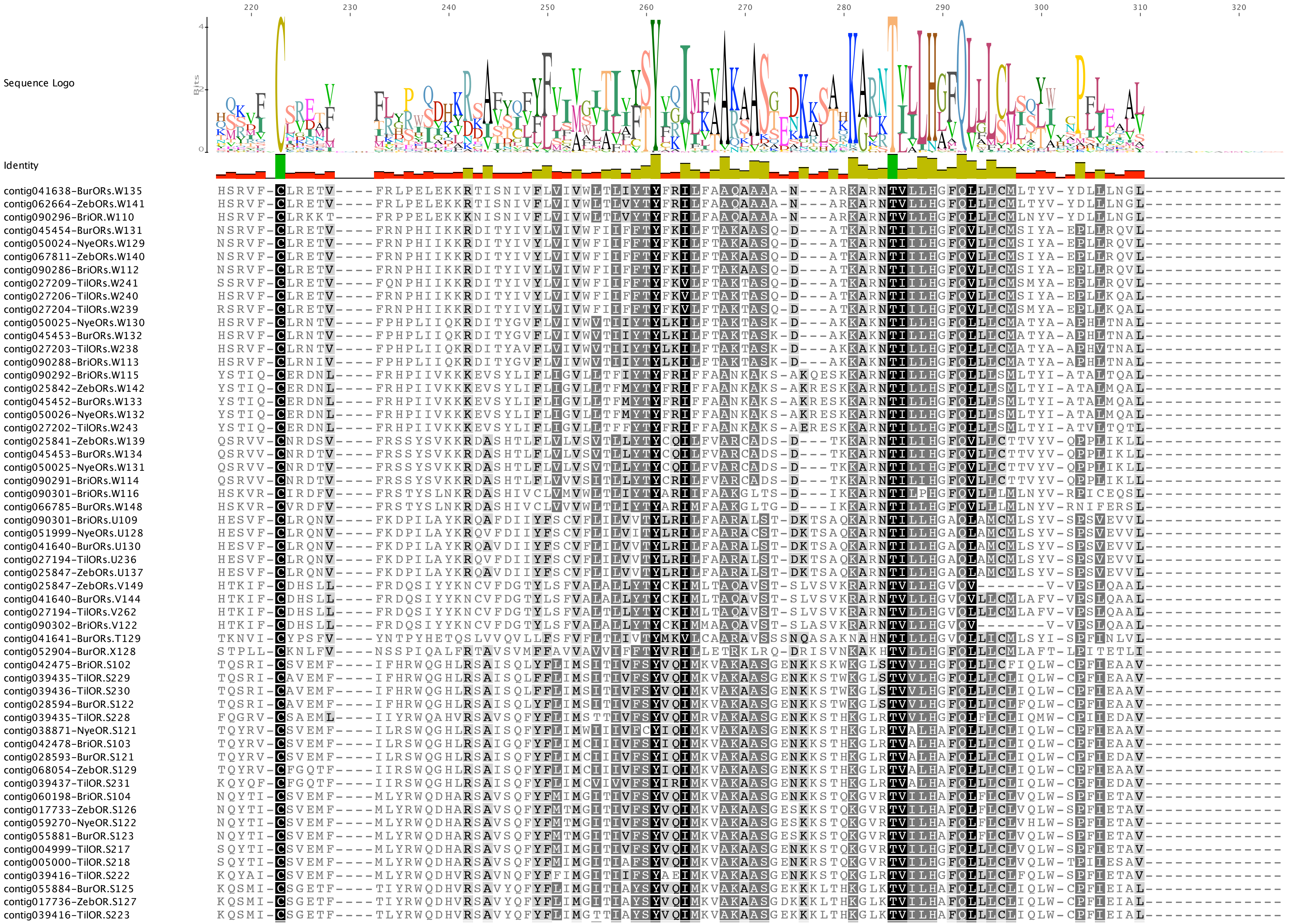

220
230
240
250
260
270
280
290
300
310
320
Sequence Logo
Bits
Identity
contig041638-BurORs.W135
contig062664-ZebORs.W141
contig090296-BriOR.W110
contig045454-BurORs.W131
contig050024-NyeORs.W129
contig067811-ZebORs.W140
contig090286-BriORs.W112
contig027209-TilORs.W241
contig027206-TilORs.W240
contig027204-TilORs.W239
contig050025-NyeORs.W130
contig045453-BurORs.W132
contig027203-TilORs.W238
contig090288-BriORs.W113
contig090292-BriORs.W115
contig025842-ZebORs.W142
contig045452-BurORs.W133
contig050026-NyeORs.W132
contig027202-TilORs.W243
contig025841-ZebORs.W139
contig045453-BurORs.W134
contig050025-NyeORs.W131
contig090291-BriORs.W114
contig090301-BriORs.W116
contig066785-BurORs.W148
contig090301-BriORs.U109
contig051999-NyeORs.U128
contig041640-BurORs.U130
contig027194-TilORs.U236
contig025847-ZebORs.U137
contig025847-ZebORs.V149
contig041640-BurORs.V144
contig027194-TilORs.V262
contig090302-BriORs.V122
contig041641-BurORs.T129
contig052904-BurOR.X128
contig042475-BriOR.S102
contig039435-TilOR.S229
contig039436-TilOR.S230
contig028594-BurOR.S122
contig039435-TilOR.S228
contig038871-NyeOR.S121
contig042478-BriOR.S103
contig028593-BurOR.S121
contig068054-ZebOR.S129
contig039437-TilOR.S231
contig060198-BriOR.S104
contig017733-ZebOR.S126
contig059270-NyeOR.S122
contig055881-BurOR.S123
contig004999-TilOR.S217
contig005000-TilOR.S218
contig039416-TilOR.S222
contig055884-BurOR.S125
contig017736-ZebOR.S127
contig039416-TilOR.S223

```
contig039419-TilOR.S224     KQSMI-CSVEAF----TLYRWQDHVRSAVYQFYFLIMGTTIAYSYVQIMKVAKAASGEKKKLTHKGRKTVILHAFQLLLCLIQLW-RPFIETPL---------------
contig055882-BurOR.S124     KQSMI-CSGDTF----SLYRWQDHVRSAVYQLYFLIMGITIAYSYVQIMKVAKAASGEKKKLTQKGLKTVILHAFQLLLCLIQLW-CPFIEIAV---------------
contig005005-TilOR.S219     KQFMI-CTVDAF----TLYRWQDHVRSAVYQFYFLIMGITIAYSYVQIMKVAKAASGEKKKLTHKGLKTVILHGFQLLLCLIQLW-CPFIEIAV---------------
contig005007-TilOR.S220     KQFMV-CSVDAF----SLYRWQDHVRSAVYQFYFLIMGITIAYSYVQIMKVAKAASGEKKKLTHKGRKTVILHAFQLLLCLIQLW-CPFIEIAV---------------
contig039428-TilOR.S227     KQSMI-CSVEAF----TLYRWQDHVRSAVFQFYFLIMGITIAYSYVQIMKVAKAASGDKKKLTHKGLKTVILHAFQLLLCLIQLW-CPFIETAV---------------
contig017743-ZebOR.S128     KQSMI-CSVEAF----TLYRWQDHVRSAVYQCYFLIMGITIAYSYVQIMKVAKAASGDKKKLTHKGLKTVILHAFQLLLCLIQLW-CPFIETAV---------------
contig039426-TilOR.S226     KQSMI-CSVEAF----TLYRWQDHVRSAVFQFYFLIMGITIAYSYVQIMKVAKAASGEKKKLTHKGLKTVILHAFQLLLCLIQLW-CPFIETAV---------------
contig039425-TilOR.S225     KQSMI-CSVEAF----TFYRWQDHVRSAVYQFYFLIMGITIAYSYVQIMKVAKAASGDKKKLTHKGLKTVILHAFQLLLCLIQLW-CPFIETAV---------------
contig009773-BurORs.Q136    PMQSF-CIRNSV----FRLEVYVTLNMAFTILYFVFVSMIIIYTYTAIMITVKSASS-RGRHTNKAPKTVLLHLLQLWLYLTSTLFNMINPSMM---------------
contig021011-NyeOR.Q134     PMQSF-CIRNSV----FRLEVYVTLNMAFTILYFVFVSMIIIYTYTAIMITVKSASS-RGRHTNKAPKTVLLHLLQLWLYLTSTLFNMINPSMM---------------
contig050422-ZebOR.Q143     PMQSF-CIRNSV----FRLEVYVTLNMAFTILYFVFVSMIIIYTYTAIMITVKSASS-RGRHTNKAPKTVLLHLLQLWLYLTSTLFNMINPSMM---------------
contig063829-BriOR.Q117     PMQSF-CIRNSV----FHLEVYVTLNMAFTILYFVFVTMIIIYTYTAIMITVKSASS-RGRHTNKAPKTVLLHLVQLWLYLTSTLFNMINPSMM---------------
contig028611-TilOR.R246     QMKDF-CSDIAM----FVGSMSDDYDKAFTCVLFISASVAITCSYIGVIVAARSAST-DKASAHKALNTLLLHLVQLGLNLSSTIHNPLLTALA---------------
contig028617-TilOR.R248     QMKDF-CSDIAM----FVGSMSDDYDKAFTCVLFISASVAITCSYIGVIVAARSAST-DKASAHKALNTLLLHLVQLGLSLSSTIYNPLLTALA---------------
contig028607-TilOR.R245     QMKDF-CSDIAM----FVGSMSDDYDKAFTCVLFISASVAITCSYIGVIVAARSAST-DKASAHKALNTLLLHLVQLGLSLSSTIYNPLLTALA---------------
contig065193-ZebOR.R147     QMKDF-CSEIAM----SGGSMSDDYDKAFTCALFISASVAITCSYIGVIVAARSAST-DKASARKACNTLLLHLVQLGLSLSSTICNPLLTALA---------------
contig067209-BurOR.R140     QMKDF-CSEIAM----SGGSMSDDYDKAFTCALFISASVAITCSYIGVIVAARSAST-DKASARKACNTLLLHLVQLGLSLSSTICNPLLTALA---------------
contig094282-BriOR.R118     QIKDF-CSNAAL----LLGPMSDHYDKAYTGVIFVFAGVAVTCSYIGVMIAARSAST-DKASARKVRSTLLLHLIQLGLSLSSTIHDPLVTEIS---------------
contig043640-BurOR.R137     EVKDL-CADITI----LLGTKSDHFDKAFTCIVVVAAGVAVIFSYIGVIVAARSAST-DKALAFKARNTLLLNLMQLFFSLSSTIYYPLLVPLS---------------
contig046042-ZebOR.R145     EVKGF-CGDIAI----LLGTKSDRFDKAFTCIVVVAAGVAVIFSYIGVIVAARSAST-DKALAFKARNTLLLNLMQLFLSLSSTIYYPLLVPLL---------------
contig028619-TilOR.R249     EVKDF-CGDIAI----LLGSKSDHFDKAFTCIVFVAAGVAVIFSYIGVIVAARSAST-DKALAIKARNTLLLNLFQLILSLSSTIYNPLLVPLL---------------
contig028614-TilOR.R247     EVKYL-CSEIGI----LLGSKSDHFDKAFTCIVFVAAGVAVIFSYIGVIVAARSAST-DKALAIKARNTLLLNLFQLILSLSSTIYYPLLVPLS---------------
contig028639-TilOR.R251     NVKDH-CYKIDI----LLGLNTDYYDKAFTCVIFVSAALAIIFSYIGVVAAARSASA-DKGLARKARNTLLLNLVQLCLSLCATIYRPLLTALS---------------
contig028637-TilOR.R250     QVSDY-CSNVDI----ILGSKSEQYDTLYTCCLFVSAGVAVIFSYIGVIVAARLAST-DKGLAQKARNTLLLNVIQLCLSLSATIYHPLLRALS---------------
contig046040-ZebOR.R144     QIKDS-CSKIAL----LLGTRSDQYDTAFTCLVFVSAGVAVVFSYIGVILAARLASA-NKALARKARNTLLLNMMQLCLSLSSTIYNPLLAALS---------------
contig054733-BurOR.R138     QMKDS-CSKIAL----LLGTRSDQYDTAFTCLVFVSAGVAVVFSYIGVILAARLASA-NKALACKARNTLLLNMMQLCLSLSSTIYNPLLAALS---------------
contig028641-TilOR.R253     QMKDF-CSTFLM----FLTPVSHEYDKAYSCFLFVSAFVGVTCSYIGVMLAARLAST-DKASARKARNTLLLHLVQLGFSLSSTVNNALLLVTS---------------
contig028644-TilOR.R252     QMKDF-CSTFLM----FLTPVSHEYDKAYSCFLFVSAFVGVTCSYIGVMLAARLAST-DKASARKARNTLLLHLVQLGFSLSSTVNNALLLVTS---------------
contig064565-BurOR.R139     QLKRYVCNTFLL----FLTPVSHEYDKAYTCFLFVSAFVGVTCSYVGVMLAARSAST-DKASARKARNTLLLHLVQLGFSLSSTVNNALLLLTS---------------
contig046048-ZebOR.R146     QMEYS-CNKEKL----TLDPISDLYAKAFSYFLFVLAAGAFIFSYIGVTVVAQSAST-DKASAEKARKTLVLHLVQLGLSVSSTIHNPIFVFIY---------------
contig067265-BurOR.R141     QMEYS-CNKEKL----TLDPISDLYAKAFSYFLFVLAAGAFIFSYIGVTVVAQSAST-DKASAEKARKTLVLHLVQLGLSVSSTIHNPIFVFIY---------------
contig044295-NyeOR.R135     QMEYS-CNKEKL----TLDPISDLYAKAFSYFLFVLAAGAFIFSYIGVTVVAQSAST-DKGSAEKARKTLVLHLVQLGLSVSSTIHNPIFVFIY---------------
contig061321-BriOR.Y128     PDRGE-CGREQL----KKGELQ-------KVLFIGLCTLIILYSYVRILVEGRRLGV-LNRRNRAGCRTIALHGSQLAVYLLPNFVNFVLSILY---------------
contig073387-BriOR.Z129     TTGLL-CEPDVVEQHLGFPRASAVFRKTVGSLTLLLCLLIHAFSFFRMYQVARNAVIPFNAVNVTARNTVLFYCGMLFLQLLPLL-LKVASDALWEFRAPVAMMVQSS
```

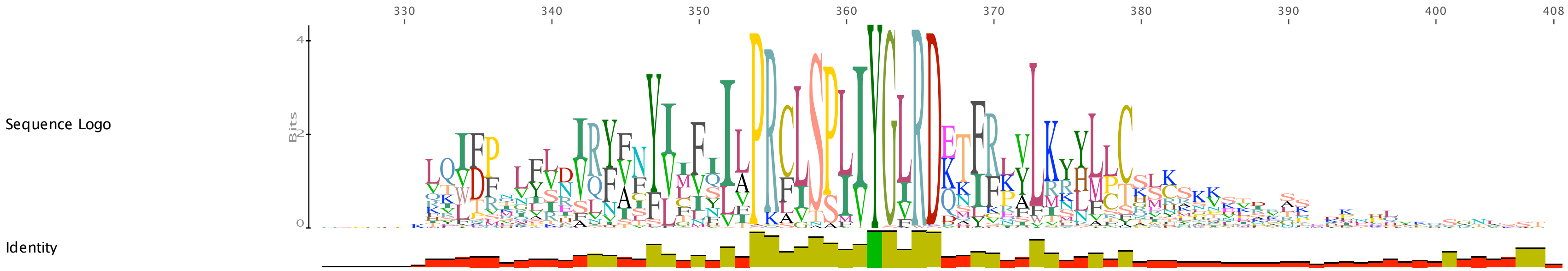


```
contig041638-BurORs.W135   -------TKLFPKGVLTIRYTISVFVHVLPRLVSPLVYGIRDKAFRRYLRKYLFYSPNANVNKSX
contig062664-ZebORs.W141   -------TKLFPKGVLTIRYTVSVFVHVLPRLVSPLVYGIRDKAFRRYLRKYLFYSPNANVNKSX
contig090296-BriOR.W110    -------TSLFPKGVLTIRYTISVFVHILPRLVSPLVYGIRDKAFRRYLRKYLFYSPKANVNKSX
contig045454-BurORs.W131   -------QQWFPQNYSDSLFACYILFQILPRAISPIVYGVRDKTYRKYLKRYLLCKMSLX
contig050024-NyeORs.W129   -------QQWLPQNYSDSLFACYILFQILPRAISPIVYGVRDKTYRKYLKRYLLCKMGPX
contig067811-ZebORs.W140   -------QQWFPQNYSDSLFACYILFQILPRAISPIVYGVRDKTYRKYLKRYLLCKMGPX
contig090286-BriORs.W112   -------QQWFPQNYSDSLFACYILFQILPRAINPIVYGVRDKTYRKYLKRYLLCKMGPX
contig027209-TilORs.W241   -------LQWFPQYYSDSLFACYILFQILPRAISPIVYGVRDKTYRKYLKRYLLCKMSPX
contig027206-TilORs.W240   -------QQWFPKNYSDSLFACYIIFQILPRAISPIVYGVRDKTYRKYLKRYLLCKMSPX
contig027204-TilORs.W239   -------LQWFPQYYSDSLFACYIIFQILPRAISPIVYGVRDKTYRKYLKRYLLCKMSSX
contig050025-NyeORs.W130   -------QKWFPTNYTDSLFVIYVTVQILPRSISPIIYGIRDKTFRRFLKGNLLCKITVHKSDNQNRX
contig045453-BurORs.W132   -------QKWFPTNYTDSLFVIYVTVQILPRSISPIIYGIRDKTFRKFLKGNLLCKITVHKSDNQNRX
contig027203-TilORs.W238   -------QKWFPTNYTDSLFVVYVTVQILPRSISPIIYGIRDKTFRKFLKGNLLCKITVHKSDRQNKX
contig090288-BriORs.W113   -------QKWFPTNYTDSLFVIYVTVQILPRSISPIIYGIRDKTFRKFLKGNLLCKITVHKSDNQNKX
contig090292-BriORs.W115   -------VRWFPKQYLIVVFISYIIIYIVPRFVSPIVYGLRDKTFKQYLKKYLLCTMKMGNETDFRLX
contig025842-ZebORs.W142   -------VRWFPKQYLIVVFISYIIIYIVPRFVSPIVYGLRDKTFKQYLKKYLLCTMKMGNETDFRLX
contig045452-BurORs.W133   -------VRWFPKQYLIVVFISYIIIYIVPRFVSPIVYGLRDKTFKQYLKKYLLCTMKMGNETDFRLX
contig050026-NyeORs.W132   -------VRWFPKQPLIVVFISYIIIYIVPRFVSPIVYGLRDKTFKQYLKKYLLCTMKMGNETDFRLX
contig027202-TilORs.W243   -------VRWFPKQYLIVVFISYIIIYIVPRFVSPIVYGLRDKTFKQYLKKYLLCTMKMGNETDFRLX
contig025841-ZebORs.W139   -------VYFFPEGLSDINFATFVINQVLPRLGSPIIYGLRDKTFRKFLKRHL-CAVNNLPKIIRSSX
contig045453-BurORs.W134   -------VYFFPEGLSDINFATFIINQVLPRLGSPIIYGLRDKTFRKFLKRHL-CAVNNLPKIIRSSX
contig050025-NyeORs.W131   -------VYFFPEGLSDINFATFIINQVLPRLGSPIIYGLRDKTFRKFLKRHL-CAVNNLPKIIRSSX
contig090291-BriORs.W114   -------VYFFPEGLKDINFATFIINQVLPRLGSPIIYGLRDKTFRKFLKRHL-CAVNQLPKMIRSSX
contig090301-BriORs.W116   -------FFLFPNREIPIQFASFVIVQIIPRFISPIVYGLRDQTFRKYVRRYLVCSERGITHPENTTTLQKGLTX
contig066785-BurORs.W148   -------FFLFPNRESQIRFASFVIVQIIPRFISPIVYGLRDQTFRKYVRKYLVCSERGITHPENTTTKKASLNVTSATVX
contig090301-BriORs.U109   -------HIIFPGRILEIRFANYLIVYILPRFLSPIIYGVRDKKFREYLKMYYLSNRCRNKEKKVTPEDKNHLX
contig051999-NyeORs.U128   -------HIIFPGRILEIRFANYLIVYILPRFLSPIIYGVRDKKFREYLRMYFLSNRCRNKKKKVTPKDKDHLX
contig041640-BurORs.U130   -------HIIFPGRILEIRFANYLIVYILPRFLSPIIYGVRDKKFREYLRMYFLSNRCRNKEKKVTPEDKDHLX
contig027194-TilORs.U236   -------HIIFPGRILEIRFANYLIVYILPRFLSPIIYGVRDKKFREYLRMYFLSNRCRNKERKVTPEDKDHLX
contig025847-ZebORs.U137   -------HIIFPSRILKIRFANYLIVYILPRFLSPIIYGVRDKKFREYLRMDFLSNRCRNKVKKVTPEDKDHLX
contig025847-ZebORs.V149   -------ISLFPQLSLEIRYIFFLLVYIIPRFLSPMIYGFRDEQFRKYWTRYLSCHEHSVTLLRLASQKMNPQVKQSGNLSSTX
contig041640-BurORs.V144   -------ISLFPQLSLEIRYIFFLLVYIIPRFLSPMIYGFRDEQFRKYWTRYLSCHEHSVTLLRLASQKMNPQVKQSGNLSSTX
contig027194-TilORs.V262   -------ISLFPQLGLEIRYIFFLLVYIIPRFLSPMIYGFRDEQFRKYWTRYLSCHEHSMTLLRLASQKMNPQVKX
contig090302-BriORs.V122   -------ISLFPQLSLEIRYIFFLLVYIIPRFLSPMIYGFRDEQFRKYWTRYLSCHEHSMTLLRLASQKMNPQVKQSGNLSSTX
contig041641-BurORs.T129   -------VTAWPHKRTIIIFTTFLFTNLLPRLLSPLIYGARDQKLSSHFRVHFGCKGFKSRGKSRKRRVPEKHX
contig052904-BurOR.X128    -------LLYTKWPVEDVTYFCFFCFILIPRFLSPLIYGFRDHSLRGYIGKTFLCCLDTVKPHFRSKQQDSLSALX
contig042475-BriOR.S102    -------LQIDFMLFINVRYFNYITFILAPRCLSPLIYGLRDEMFVNALKYYALCGLYKKYSTA-----FX
contig039435-TilOR.S229    -------LQIDFMLFINVRYFNYITFILAPRCLSPLIYGLRDEMFVNALKYYALCGLYKKHSTACSFIRFX
contig039436-TilOR.S230    -------LQIDFMLFINVRYFNYITFILAPRCLSPLIYGLRDEMFVNALKYYALCGLYKKHSTA-----FX
contig028594-BurOR.S122    -------LQIDFMLFINVRYFNYITFILAPRCLSPLIYGLRDEMFVNALKYYALCGLYKKHSTA-----FX
contig039435-TilOR.S228    -------FQIDLNVFSTVRYFNYITFILAPRCLSPLIYGLRDEKFFYALKYSTLNGLFKVQISFX
contig038871-NyeOR.S121    -------LQIDFMLYVNVRYFNYIMFSLTPRCLSPLIYGLRDDTFFLALRYHVLCHLHRKKSVGVSNX
contig042478-BriOR.S103    -------LQIDFMLYVNVRYFNYIMFSLTPRCLSPLIYGLRDDKFFLALKYHVLCHLHRKKSVGVSNX
contig028593-BurOR.S121    -------LQINFMLYVNVRYFNYIMFSLTPRCLSPLIYGLRDDTFFLALRYHVLCHLHRKKSVGVSNX
contig068054-ZebOR.S129    -------LQIDFMLYVNVRYFNYIMFSLTPRCLSPLIYGLRDDKFFLALRYHVLCPLFRKNNX
contig039437-TilOR.S231    -------LEINFVLYLNVRYFNYIMFSLAPRCLSPLIYGLRDDKFFLALKYHVLCPLYRKKSVAFSNX
contig060198-BriOR.S104    -------LQIDVNLFRDFRYFNYVFFSIVPKCLSPLIYGLRDETFFISLKNLMPTSSCSKKHVIX
contig017733-ZebOR.S126    -------LQIDVNLFRDFRYFNYVFFSIVPRCLSPLIYGLRDETFFLSLKNLMPTSSCSKKHVIX
contig059270-NyeOR.S122    -------LQIDVNLFRDFRYFNYVFFSIVPRCLSPLIYGLRDETFFLSLKNLMPTSSCSKKHVIX
contig055881-BurOR.S123    -------LQIDVNLFRDFRYFNYVFFSIVPRCLSPLIYGLRDETFFLSLKKLMPTSSCSKKHVIX
contig004999-TilOR.S217    -------LQIDVNLFRDFRYFNYVLFSIVPRCLSPLIYGLRDETFFLSLKNLMPTSSCSKKHVIX
contig005000-TilOR.S218    -------LQIDFNLFLNVRYFNYVLFSLTPKCLSPLIYGLRDEHFFLALKNLMSASSYSKQTX
contig039416-TilOR.S222    -------FQIDSNLFRNFRYFNYVLFNIVPRCLSPLIYGLRDEKFFLVLKNLLPASSNSEX
contig055884-BurOR.S125    -------LQIDFSLFRNVRYFNYIMFNIAPRCLSPLIYGLRDENIFLVLKSLMPTSSCSKX
contig017736-ZebOR.S127    -------LQIDFSLFRNVRYFNYIVFNIAPRCLSPLIYGLRDENIFLVLKSLMPTSSCSKX
contig039416-TilOR.S223    -------LQIDFSLFRNVRYFNYIMFNIAPRCLSPLIYGLRDENFFLVLKSLMPTSSCSKX
```

```
contig039419-TilOR.S224     -------LQIDFSLFRNVRFFNYIMFNIAPRCLSPLIYGLRDRKISLVLKSLMPTSSCSKX
contig055882-BurOR.S124     -------LQIDFSLILNVRYFNYIMFNIAPRCLSPLIYGLRDENIFLVLKNLMPTSSCSKX
contig005005-TilOR.S219     -------FQIDFSLILNVRYFNYIMFNIAPRCLSPLIYGLRDENFFLALKNLMPTSSCSKX
contig005007-TilOR.S220     -------LQIDFSLILNVRYFNYIMFSIAPRCLSPLIYGLRDENFFLVLKNLMPTSSCSKX
contig039428-TilOR.S227     -------LQIDLRLFVNVRYSNYIMFSIAPRCLSPLIYGLRDENFFLVLKSLMPTSSCSKX
contig017743-ZebOR.S128     -------LQIDFRLFIDVRYSNYIMFSIAPRCLSPLIYGLRDEHFFLVLKSLMPTSSCSKX
contig039426-TilOR.S226     -------LQIDLRLFVNVRYSNYIMFNIAPRCLSPLIYGLRDENFFLVLKSLMPTSSCSKX
contig039425-TilOR.S225     -------LQIDFRLFINVRYSNYIMFNIAPRCLSPLIYGLRDENFFLVLKRFMPTSSCSKX
contig009773-BurORs.Q136    -------LKVPPDMAIHAQYVLFVGLIIFPKCLSPLIYGLRDQTLCRVFKYYFTFGFRASVKPSPLSX
contig021011-NyeOR.Q134     -------LKVPPDMAIHAQYVLFVGLIIFPKCLSPLIYGLRDQTLCRVFKYYFTFGFRASVKPSPLSX
contig050422-ZebOR.Q143     -------LKVPPDMAIHAQYVLFVGLIIFPKCLSPLIYSLRDQTLCRVFKYYFTFGFRASVKPSPLSX
contig063829-BriOR.Q117     -------LKVPPDVAIHAQYVLFVGLIIFPKCLSPLIYGLRDQTLCRVFKYYFTFGFRTSVKPSPLSX
contig028611-TilOR.R246     -------RVLKRIVFVRIQVVFYVCIFLLPRCLSSLIYGIRDQSIRPVLMQHLCCRLRX
contig028617-TilOR.R248     -------RVLTRIVFVRIQVVFYVCIFLLPRCLSSLIYGIRDQSIRPVLMQHLCCRLRX
contig028607-TilOR.R245     -------RVLTRIVFVRIQNAFYVCIFIFPRCLSSLIYGIRDQSIRPVLIYHLCCRLKYSVIQPRLNFHPRLX
contig065193-ZebOR.R147     -------RVLTRIVFVRIQNVFYVCIFLFPRCLSSLIYGIRDQSIRPVLIYYLCCRLKYSVIQPRLKAAIKVECX
contig067209-BurOR.R140     -------RVLTRIVFVRIHNVFYVCIFLFPRCLSSLIYGIRDQSIRPVLIYYLCCRLKYSVIQPRLKAAIEVECX
contig094282-BriOR.R118     -------KVLDRVTILRIRSILYVSMMLLPRCLSPLIYGIRDQMIRGILMSHLCCRVKLPNSIKLLKHX
contig043640-BurOR.R137     -------VTVTRIVFVRIQNVFYLLFFILPRCLTSLIYGLRDQTIRPVLIYHLCCQLKCPVVEDKGX
contig046042-ZebOR.R145     -------MIVTRIVLVRIQNVFYLLFIIVPRCLTSLIYGLRDQTIRPVLIYHLCCRLKCPVAEDKGX
contig028619-TilOR.R249     -------MIVTRIVLVRIQNAFYLFFIILPRCLTSLIYGLRDQTIRPVLIYHLCCQLKCPVVEDKGX
contig028614-TilOR.R247     -------VTVTRIVLVRIQNVFYLFFIILPRCLTSLTYGLRDQTIRPVLIYHLCCRLKCPVVEDKGX
contig028639-TilOR.R251     -------TTVTMTAFSWIQNVFYVCLVVLPRFLTSLVYGLRDQTIRPVLMYHLCCHCLKX
contig028637-TilOR.R250     -------RTVARTVFLWVQNVFYVCFIIFPRCLTSLIYGLRDQTIGPVLINHLCCRMKATVX
contig046040-ZebOR.R144     -------RTITMTIFLWVQNVFYLCFIILPRCLSSLVYGLRDQTIRPVLMYHLCCHQKRSQX
contig054733-BurOR.R138     -------RTVTMTIFSWVQNVFYLCFIILPRCLSSLVYGLRDQTIRPVLMYHLCCHQKRSQX
contig028641-TilOR.R253     -------KTVSRRVLVVVQIVFYVFLIILPRCLSALIYGLRDQTIRPILVYNLCCQLKLTVVTRKAKVSPX
contig028644-TilOR.R252     -------KTVSRRVLVVVQIVFYVFLIILPRCLSALIYGLRDQTIRPILVYNLCCQLKLTVVTRKAKVSPX
contig064565-BurOR.R139     -------KTVSNRVSVLIQNALYVLLFILPRCLSALIYGLRDQTIRPILVYNLCCQLKLLAVTAEAKIYPX
contig046048-ZebOR.R146     -------KTVDSVIVVRIRVVIYLCIIILPRCLSSFIYGLRDRTIRPVLMLNLRCQWKCQPFLX
contig067265-BurOR.R141     -------KTVDSVIVVRIRVVIYLCIIILPRCLSSFIYGLRDHTIRPVLMLNLRCQWKCQPFLX
contig044295-NyeOR.R135     -------KTVDSVIVVRIRVVIYLCIIILPRCLSSFIYGLRDHTIRPVLMLNLRCQWKCQPFLX
contig061321-BriOR.Y128     ------KREFIQRETKELSAVVVFAFFSLAQCVAPVVYGLRKEELLEQLSRRFPCCSRYLKSVLGWTVRANWSHVPYRTRX
contig073387-BriOR.Z129     QSPGLCKVKTTPTATATALHISLLVMLIVPPCINPLVYGLWSVEMRQALTSRLRSWTERRANERAAERIRLEHVARRNGAQAGX
```

Cichlid Olfactory Receptors

Group 3C

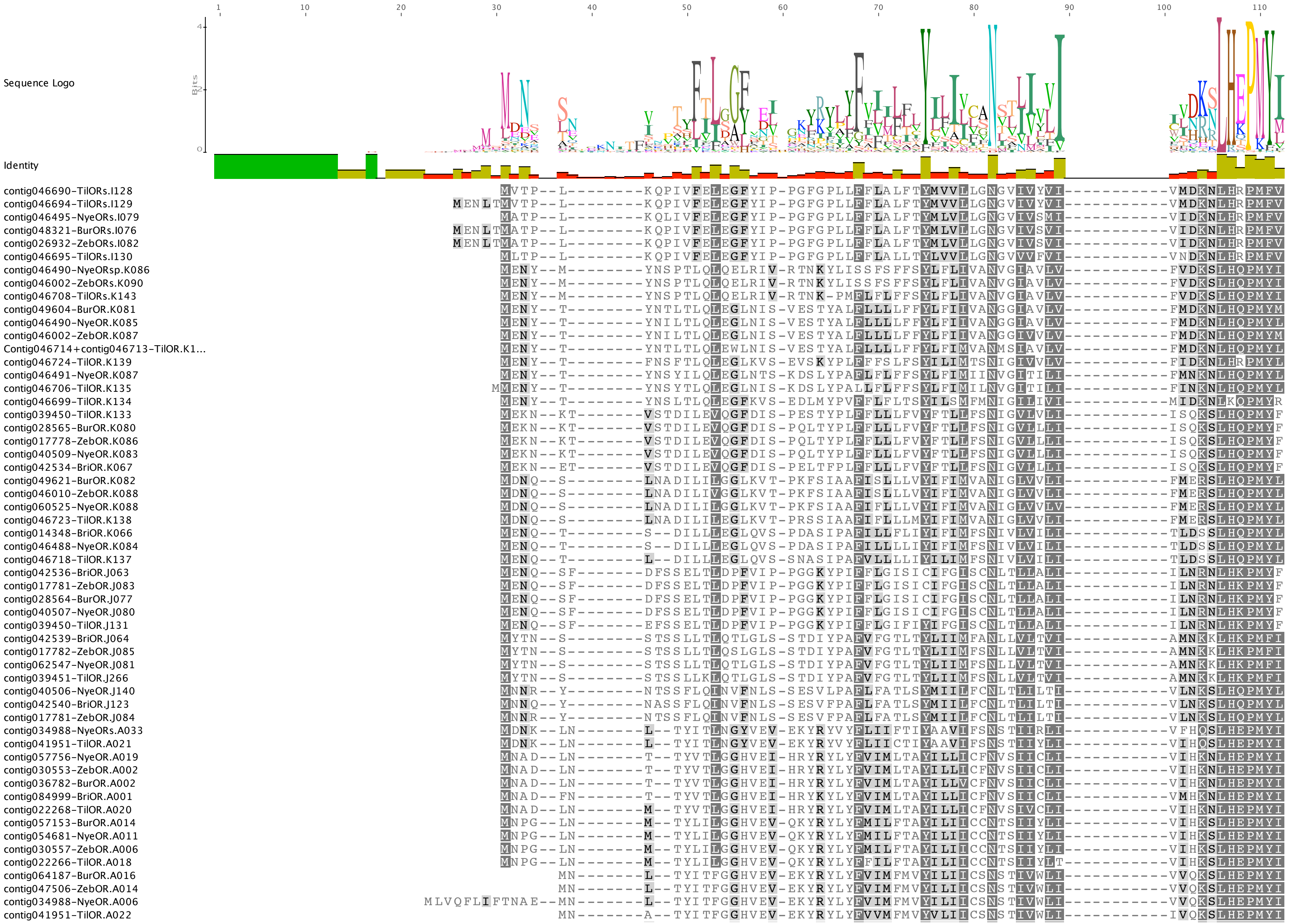
Sequence Logo
Identity
contig046690-TilORs.I128
contig046694-TilORs.I129
contig046495-NyeORs.I079
contig048321-BurORs.I076
contig026932-ZebORs.I082
contig046695-TilORs.I130
contig046490-NyeORsp.K086
contig046002-ZebORs.K090
contig046708-TilORs.K143
contig049604-BurOR.K081
contig046490-NyeOR.K085
contig046002-ZebOR.K087
Contig046714+contig046713-TilOR.K1...
contig046724-TilOR.K139
contig046491-NyeOR.K087
contig046706-TilOR.K135
contig046699-TilOR.K134
contig039450-TilOR.K133
contig028565-BurOR.K080
contig017778-ZebOR.K086
contig040509-NyeOR.K083
contig042534-BriOR.K067
contig049621-BurOR.K082
contig046010-ZebOR.K088
contig060525-NyeOR.K088
contig046723-TilOR.K138
contig014348-BriOR.K066
contig046488-NyeOR.K084
contig046718-TilOR.K137
contig042536-BriOR.J063
contig017781-ZebOR.J083
contig028564-BurOR.J077
contig040507-NyeOR.J080
contig039450-TilOR.J131
contig042539-BriOR.J064
contig017782-ZebOR.J085
contig062547-NyeOR.J081
contig039451-TilOR.J266
contig040506-NyeOR.J140
contig042540-BriOR.J123
contig017781-ZebOR.J084
contig034988-NyeORs.A033
contig041951-TilOR.A021
contig057756-NyeOR.A019
contig030553-ZebOR.A002
contig036782-BurOR.A002
contig084999-BriOR.A001
contig022268-TilOR.A020
contig057153-BurOR.A014
contig054681-NyeOR.A011
contig030557-ZebOR.A006
contig022266-TilOR.A018
contig064187-BurOR.A016
contig047506-ZebOR.A014
contig034988-NyeOR.A006
contig041951-TilOR.A022

```
contig034983-NyeOR.A002            MN-------I--TYITFGGHVEV-EKYRYIYFVIMFMVYGLIICSNSTIVWVI-----------IVQKSLHEPMYI
contig047515-ZebOR.A019            MN-------I--TYITFGGHVEV-EKYRYIYFVIMFMVYGLIICSNSTIVWVI-----------IVQKSLHEPMYI
contig051559-BurOR.A007            MN-------I--TYITFGGHVEV-EKYRYIYFVIMFMVYGLIICSNSTIVWVI-----------IVQKSLHEPMYI
contig022259-TilOR.A015        MDEE--SN-------V--TYLTLDWYTEI-NKYRYVFFFIMFTLYILIICTNSTILYLI-----------WIHKNLHEPMYI
contig051321-BurOR.A006        MDEE--SN-------A--TYLTLDWYTEI-NKYRYVFFFVMFTLYILIICTNSTILYLI-----------WIHKNLHEPMYI
contig056380-NyeOR.A016        MDEE--SN-------A--TYLTLDWYTEI-NKYRYVFFFVMFTLYILIICTNSTILYLI-----------WIHKNLHEPMYI
contig062094-ZebOR.A023        MDEE--SN-------A--TYLTLDWYTEI-NKYRYVFFFVMFTLYILIICTNSTILYLI-----------WIHKNLHEPMYI
contig056375-NyeOR.A015        MDKV--LN-------V--TYLTLDWYTEI-NKYRYVFFFIMFILFILIICTNSTILYLI-----------WIHKNLHEPMYI
contig064570-BurOR.A017        MDEE--LN-------V--TYLTLDWYTEI-NKYRYVFFFVMFTLYILIICTNSTILYLI-----------WIHKNLHEPMYI
contig062095-ZebOR.A024        MDEE--LN-------V--TYLTLDWYTEM-NKYRYVFFFIMFTLFILIICTNSTILYLI-----------WIHKNLHEPMYI
contig022259-TilOR.A014        MDEE--LN-------V--TYLTLDWYTEI-NKYRYVFFFIMFTLYILIICTNSTILYLI-----------WIHKNLHEPMYI
contig051318-BurOR.A005        MDEE--SN-------A--TYLTLDWYTEI-NKYRYIFFFVMFTLYILIICTNSTILYLI-----------WNHKNLHEPMYI
contig030572-ZebOR.A009        MDEE--SN-------A--TYLTLDWYTEI-NKYRYIFFFVMFTLYILIICTNSTILYLI-----------WNHKNLHEPMYI
contig085012-BriOR.A006        MDEE--SN-------V--TYLTLHWYTEI-NKYRYIFFFVMFTLYILIICTNSTILYLI-----------WIHKNLHEPMYI
contig022251-TilOR.A013        MDEE--LN-------V--THLTLDWYTEV-NKYRYVFFFIMFILYSLIICTNSIILYLI-----------WIHKNLHEPMYI
contig030576-ZebOR.A010        MDKE--LN-------V--TFLTLDWYTEI-NKYRYIFFFIMFTLYILIICTNSTIVYLI-----------WIHKNLHEPMYI
contig085018-BriOR.A006        MDKE--LN-------V--TFLTLDWYTEI-NKYRYIFFFIMFTLYILIICINSIIVYLI-----------WIHKNLHEPMYI
contig062344-NyeOR.A020        MDKE--LN-------V--TFLTLDWYTEI-NKYRYIFFFIMFTLYILIICTNSTIVYLI-----------WIHKNLHEPMYI
contig022241-TilOR.A011        MDKE--LN-------V--TFLTLDWYTEI-NNYRYILFFIMFTLYILIICTNSTIVYLI-----------FTHKNLHEPMYI
contig022245-TilOR.A012        MDKE--LN-------V--TFLTLDWYTEI-NNYRYILFFIMFTLYILIICTNSTIVYLI-----------FTHKNLHEPMYI
contig022217-TilOR.A004        MDKE--SN-------V--TFLTLDWYTEI-SKYRYIFFFIMFTLYILIIYTNSTIVYLI-----------FIHKNLHEPMYI
contig047521-ZebOR.A020        MDQE--LN-------F--TYVTLDWYVDI-NKYRYVFFFIMFALYSLIICTNSTIMYII-----------CIHKNLHEPMYI
contig054237-BurOR.A013        MDQE--LN-------F--TYVTLDWYVDI-NKYRYIFFFIMFALYSLIICTNSTIMYII-----------CIHKNLHEPMYI
contig034981-NyeOR.A001        MDQE--LN-------F--TYVTLDWYVGI-NKYRYVFFFIMFALYSLIICTNSTIMYII-----------CIHKNLHEPMYI
contig085026-BriOR.A008        MDQE--LN-------F--TYVTLDWYVDI-NKYRYVFFFIMFALYSLIICTNSTIVYII-----------WIHKNLHEPMYI
contig022211-TilOR.A003        MDQE--LN-------F--TYVTLDWYVDI-NKYRYVYFFIMFALYSLIICTNSTIVYII-----------WIHKNLHEPMYI
contig034988-NyeOR.A005        MDEE--LN-------T--TYVTLDGYIEV-NKYRYVYFCIIFTLYIIIICSNSTIVYVI-----------WIHKNLHEPMYI
contig047508-ZebOR.A015        MDEE--LN-------T--TYVTLDGYIEV-NKYRYVYFCIIFTLYIIIICSNSTIVYVI-----------WIHKNLHEPMYI
contig051570-BurOR.A010        MDEE--LN-------T--TYVTLDGYIEV-NKYRYVYFCIIFTLYIIIICSNSTIVYVI-----------WIHKNLHEPMYI
contig070885-TilOR.A024        MDEE--LN-------T--TYVTLDGYIEV-NKYRYVYFCIIFTLYIIIICSNSTIMYVI-----------WIHKNLHEPMYI
contig047503-ZebOR.A013        MDEE--LN-------T--TYVTLDGYIEV-NKYRYVYFCIIFTLYIIIICSNSTIVYVI-----------WIHKNLHEPMYI
contig051559-BurOR.A008        MDEV--LN-------A--TYLTLDGYVEV-NKYRYVYFFIFFILYSLIICSNSTIVYII-----------WIHKNLHEPMYT
contig047515-ZebOR.A018        MDEV--LN-------A--TYLTLDGYVEV-NKYRYVYFFIFFILYSLIICSNSTIVYII-----------WIHKNLHEPMYT
contig034983-NyeOR.A003        MDEV--LN-------A--TYLTLDGYVEV-NKYRYVYFFIFFILYSLIICSNSTIVYII-----------WIHKNLHEPMYT
contig022232-TilOR.A008        MDEV--LN-------A--TYLTLDGYVEV-NKYRYVYFFIFFILYSLIICSNSTIVYII-----------WIHINLHEPMYI
contig022234-TilOR.A009        MDEV--LN-------A--TYITLDGYVEV-NKYRYVYFFIFFILYSLIICSNSTIVYII-----------WIHKNLHEPMYI
contig022204-TilOR.A001        MDDE--LN-------V--TYITFDGHVEI-NKYRYVYFFILFTLYILITCSNSIIIYLI-----------LIHKNLHEPMYI
contig022225-TilOR.A005        MDEE--LN-------V--TYVTFDGHVEM-NKYRYVYFFIMFTVYILIISSNFIIIYLI-----------LTHKNLHEPMYI
contig022227-TilOR.A006        MDDE--LN-------V--TYIAFDGYVEI-NKYRYVYFFIIFTVYIVIICSNCIILYLI-----------CIHKNLHEPMYI
contig074640-TilOR.A002        MDEK--IN-------V--TYITLDGFVEI-DKYRYVYFYIMFIVYILIICCNSTILYLI-----------CIHQNLHEPMYI
contig057165-NyeOR.A017        MELA--LN-------V--SYITLDGFFQV-NKYRYLYFMIMFTLYILILCCNFAIVFLI-----------VVEKSLHEPMYI
contig030552-ZebOR.A001        MELA--LN-------V--SYITLDGFFQV-NKYRYLYFMIMFTLYILILCCNFAIVFLI-----------VVEKSLHEPMYI
contig036780-BurOR.A001        MELA--LN-------V--SYITLDGFFQV-NKYRYLYFMIMFTLYILILCCNFAIVFLI-----------VVEKSLHEPMYI
contig054684-NyeOR.A012     MDQMNDK--FN-------V--TYITFGGHVEL-NKYRFLYFAIMFTAYILILCSNSTILCLI-----------WIKKNLHEPMYI
contig057156-BurOR.A015     MDQMNDK--FN-------V--TYITFGGHVEL-NKYRFLYFAIMFTAYILILCSNSTILCLI-----------WIKKNLHEPMYI
contig030560-ZebOR.A007     MDQMNDK--FN-------V--TYITFGGHVEL-NKYRFLYFAIMFTAYTLILCSNSTILCLI-----------WIKKNLHEPMYI
contig085010-BriOR.A005     MDQMNDQ--FN-------V--TYITFGGHVEL-NKYRFLYFAIMFTAYILILCSNSTILCLI-----------WIKKNLHEPMYI
contig022265-TilOR.A017     MDEMNDK--FN-------V--TYITFVGHVEL-NKYRFLYFAIMFTAYILILCSNSTILCLI-----------WIKKSLHEPMYI
contig054678-NyeOR.A010     MEQMHDE--SN-------V--TYITFGGHVEL-EKYKFLYFAIMFTAYILILCSNSTIVCLI-----------WIKKSLHEPMYV
contig030556-ZebOR.A005     MEQMHDE--FN-------V--TYITFGGHVEL-EKYKFLYFAIMFTAYILILCSNSTIVCLI-----------WIKKSLHEPMYV
contig036787-BurOR.A004     MEQMHDE--FN-------V--TYITFGGHVEL-EKYKFLYFAIMFTAYILILCSNSTIVCLI-----------RIKKSLHEPMYV
contig085002-BriOR.A004     MERMHDE--FN-------V--TYITLGGHIEL-EKYKFLYFVIMFTAYILILCSNSTIVCLI-----------WIKKSLHEPMYV
contig030554-ZebOR.A004      MAIDNE--FN-------V--TYITFGGHIEL-EKYKFLYFAIMFTAYILILCSNSTIVCLI-----------RIKKSLHEPMYI
contig084999-BriOR.A002      MAIDNE--FN-------V--TYITFGGHIEL-EKYKFLYFAIMFTAYILILCSNSTIVCLI-----------WIKKSLHEPMYI
contig022268-TilOR.A019      MAIDNE--FN-------E--TYITFGGHIEL-EKYKFLYFVIMFTAYILILCSNSTIVCLI-----------WIKKSLHEPMYV
contig047514-ZebOR.A017        MDVK--LN-------V--TLLTLGGFAEL-HKYRYLYFVVIFTLYILILCFNSTIVYLI-----------WTHKNLHEPMYI
contig093807-BriOR.A009        MDVK--LN-------V--TLLTLGGFAEL-HKYRYLYFVVIFTLYILILCFNSTIVYLI-----------WTCKNLHEPMYI
contig022238-TilOR.A010        MDVK--LN-------V--TLLTLGGFAEL-HKYRYLYFVVIFTLYILILCCNSTIVCLI-----------WTHKNLHEPMYI
contig034988-NyeOR.A004        MDVE--LN-------V--TLLTLGGFAEL-HKYRYLYFVVIFTLYILILCFNSIIVYLI-----------WTCKNLHEPMYI
contig047508-ZebOR.A016        MDVE--LN-------V--TLLTLGGFAEL-HKYRYLYFVVIFTLYILILCFNSIIVYLI-----------WTCKNLHEPMYI
contig051566-BurOR.A009        MDVE--LN-------V--TLLTLGGFAEL-HKYRYLYFVVIFTLYILILCFNTIIVYLI-----------WTHKNLHEPMYI
contig047526-ZebOR.A021        MDVE--LN-------V--TLLTLGGFAEL-HKYRYLYFVIIFTLYILILCFNSTIVYLI-----------WTCKNLHEPMYI
contig070886-TilOR.A025        MDVE--LN-------V--TLLTLGGFAEL-HKYRYLYFVVIFTLYILILCCNSTIVCLI-----------WTHKNLHEPMYI
contig041952-TilOR.A023        MDVE--LN-------V--TLVTFGGFAEL-HKYRYLYFVVIFTLYILILCCNSTIVCLI-----------WTHKNLHEPMYI
contig034990-NyeOR.A007        MDVE--LN-------L--TLVTFGGFAEL-HKYRYLYFVVISTLYILILCFNSTIVCLI-----------WTHKNLHEPMYI
contig047499-ZebOR.A012        MDVE--LN-------L--TLVTFGGFAEL-HKYRYLYFVVISTLYILILCFNSTIVFLI-----------WTHKNLHEPMYI
contig093812-BriOR.A010        MDVE--LN-------V--TLVTFGGFAEL-HKYRYLYFVVISTLYILILCFNSTIVYLI-----------WTHKNLHEPMYI
contig047497-ZebOR.A011        MDVE--LN-------V--TLLTLGGFAEL-HKYRYLYFVIIFTLYILILCFNSTIVCLI-----------WTHKNLHEPMYI
contig034995-NyeOR.A009        MDVE--LN-------V--TLLTLGGFAEL-HKYRYLYFVIIFTLYILILCFNSTIVFLI-----------WTHENLHEPMYI
```

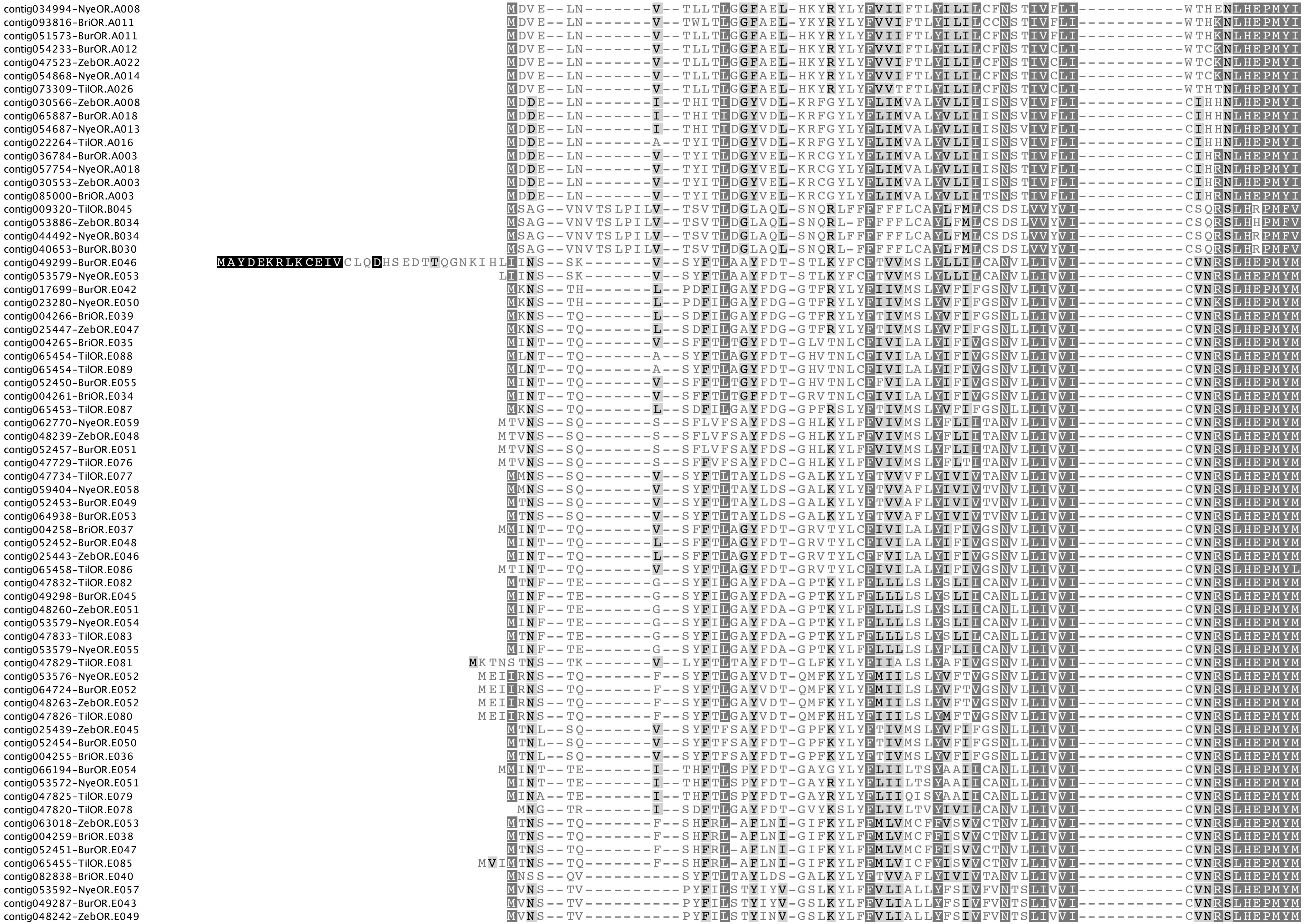
contig034994-NyeOR.A008
contig093816-BriOR.A011
contig051573-BurOR.A011
contig054233-BurOR.A012
contig047523-ZebOR.A022
contig054868-NyeOR.A014
contig073309-TilOR.A026
contig030566-ZebOR.A008
contig065887-BurOR.A018
contig054687-NyeOR.A013
contig022264-TilOR.A016
contig036784-BurOR.A003
contig057754-NyeOR.A018
contig030553-ZebOR.A003
contig085000-BriOR.A003
contig009320-TilOR.B045
contig053886-ZebOR.B034
contig044492-NyeOR.B034
contig040653-BurOR.B030
contig049299-BurOR.E046
contig053579-NyeOR.E053
contig017699-BurOR.E042
contig023280-NyeOR.E050
contig004266-BriOR.E039
contig025447-ZebOR.E047
contig004265-BriOR.E035
contig065454-TilOR.E088
contig065454-TilOR.E089
contig052450-BurOR.E055
contig004261-BriOR.E034
contig065453-TilOR.E087
contig062770-NyeOR.E059
contig048239-ZebOR.E048
contig052457-BurOR.E051
contig047729-TilOR.E076
contig047734-TilOR.E077
contig059404-NyeOR.E058
contig052453-BurOR.E049
contig064938-BurOR.E053
contig004258-BriOR.E037
contig052452-BurOR.E048
contig025443-ZebOR.E046
contig065458-TilOR.E086
contig047832-TilOR.E082
contig049298-BurOR.E045
contig048260-ZebOR.E051
contig053579-NyeOR.E054
contig047833-TilOR.E083
contig053579-NyeOR.E055
contig047829-TilOR.E081
contig053576-NyeOR.E052
contig064724-BurOR.E052
contig048263-ZebOR.E052
contig047826-TilOR.E080
contig025439-ZebOR.E045
contig052454-BurOR.E050
contig004255-BriOR.E036
contig066194-BurOR.E054
contig053572-NyeOR.E051
contig047825-TilOR.E079
contig047820-TilOR.E078
contig063018-ZebOR.E053
contig004259-BriOR.E038
contig052451-BurOR.E047
contig065455-TilOR.E085
contig082838-BriOR.E040
contig053592-NyeOR.E057
contig049287-BurOR.E043
contig048242-ZebOR.E049

```
contig047726-TilOR.E075        MVNS--TV----------PYFILSTYIYV-GSLKYLFFVLIALLYFSIVFVNTSLIVVI-----------CVNRSLHEPMYM
contig053590-NyeOR.E056        MLNV--TT-------PPLSYFILGAFMNV-GSLKFFYFSLTVILYILIIAANTSLIVVI-----------CVNRSLHEPMYM
contig049289-BurOR.E044        MLNV--TT-------PPLSYFILGGFMNV-GSLKFFYFSLTVILYILIIAANTSLIVVI-----------CVNRSLHEPMYM
contig048243-ZebOR.E050        MLNV--TT-------PPLSYFILGGFMNV-GSLKFFYFSLTVILYILIIAANTSLIVVI-----------CVNRSLHEPMYM
contig047725-TilOR.E074        MLNV--TT-------PPLSYFILGAFMNV-GSLKFFYLLLTLILYILIIAANTFLIVVI-----------CVNRSLHEPMYM
contig047834-TilOR.E084        MLNV--TS-------PPLSYFILGGYMDV-GSFKLSYFSLTTVLYIMIIAANTFLIVVI-----------CVNRGLHEPMYM
contig014049-ZebOR.D038        MENS--SE-------I--VSFVLSAFENV-GELKYLYFVIILVWYVSICVANTVLIVVI-----------RVDRRLHEPMYI
contig013327-TilOR.D052        MENS--SE-------I--VSFVLAAFGNV-GELKYLYFVIILFWYISICVANTVLIVVI-----------HVDRRLHEPMYI
contig039737-NyeOR.D039        MGNS--SE-------I--VSFVLSAYGNI-GELKYLYFIIILVWYLSICVANTVLIVVI-----------RVDRRLHEPMYI
contig032396-BurOR.D034        MGNS--SE-------T--VSFVLAAYGNI-GELKYLYFIIILVWYFSICVANTVLIVVI-----------RLDRRLHEPMYI
contig064809-BriOR.D028        MGNS--SE-------I--VSFVLAAYGNI-GELKYLYFIIILIWYLSICVANTVLIVVI-----------RVDRRLHEPMYI
contig014050-ZebOR.D039        MENS--SE-------I--VSFVLAAYGNI-GGLKYLYFIIILVWYLSICVANTVLIVVI-----------RVDRRLHEPMYI
contig013326-TilOR.D051        MGNS--SE-------T--ASFVLAAYGNI-GQLKYVYFIIILIWYLSICVANTVLIVVI-----------RVDRRLHEPMYM
contig014051-ZebOR.D040        MGNS--SK-------T--VSFVLAAYGNV-GELKHLYFIIILIWYFSICVANTVLIVVI-----------RLDRRLHEPMYI
contig013337-TilOR.D055        MGNS--SE-------T--VSFVLAAYGNV-GELKYLYFIIILIWYLSICVANTVLILVI-----------RVDRRLHEPMYI
contig013330-TilOR.D054        MGNS--SE-------T--VSFVLAAYGNV-GELKHLYFIIILVWYFSICVANTVLIVII-----------RVDRRLHEPMYI
contig013339-TilOR.D057        MGNS--SE-------T--VSFVLAAYGNV-GELKHLYFIIILVWYFSICVANTVLIVII-----------RVDRRLHEPMYI
contig032389-BurOR.D033        MENS--SE-------I--VSFVLSAYGNV-GDLKYLYLTIILFWYVSICVANIVLIVVI-----------HVDRRLHEPMYI
contig039737-NyeOR.D040        MENS--SE-------I--VSFVLSAYGNV-GDLKYLYLTIILFWYVSICVANIVLIVVI-----------HVDRRLHEPMYI
contig013339-TilOR.D056        MGNS--SE-------I--VSFVLSAYGNV-GAFKYLYFIIILFWYLSICVANTFLIVVI-----------HVDRRLHEPMYI
contig013330-TilOR.D053        MENS--SE-------I--VSFVLSAYGNV-GDLKYLYFIIILFWYLSICVANTFLIVVI-----------RVDRRLHEPMYI
contig064814-BriOR.D029    MQKKGNS--SE-------S--VSFVLAAYGNV-GAFKYPYFIIILFWYVSICVANTVLIVVI-----------HVDRRLHEPMYI
contig014054-ZebOR.D041    MQKIGNS--SE-------S--VSFVLAAYGNV-GAFKYPYFIIILFWYVSICVANTVLIVVI-----------HVDRRLHEPMYI
contig039731-NyeOR.D038    MQKIGNS--SE-------S--VSFVLAAYGNV-GAFKYPYFIIILFWYVSICVANTVLIVVI-----------HVDRRLHEPMYI
contig014054-ZebOR.D042        MGNS--SE-------T--VSFVLAAYGNV-GALKYMYFSIILFWYVSICVANTVLIVVI-----------HVDRRLHEPMYI
contig039730-NyeOR.D036        MGNS--SE-------T--VSFVLAAYGNV-GALKYMYFSIILFWYVSICVANTVLIVVI-----------HVDRRLHEPMYI
contig053779-BurOR.D035        MGNS--SE-------T--VSFVLAAYGNV-GALKYMYFSIILFWYVSICVANTVLIVVI-----------HVDRRLHEPMYI
contig039730-NyeOR.D037    MQKIGNS--SE-------S--VSFVLAAYGNV-GAFKYPYFIIILFWYVSICVANTVLIVVI-----------HVDRRLHEPMYI
contig013344-TilOR.D058        MGNS--SE-------I--FSFVLAAYGNV-GALKYMYFVMILFWYLFICVANTVLIVVI-----------RVDRRLHEPMYI
contig014049-ZebOR.D037        MGNS--SE-------I--VSFVLSAYGNI-GELKYLYFIIILVWYLSICVANTVLIVVI-----------RVNRRLHEPMYI
contig032388-BurOR.D032        MGNS--SE-------IEVVSFVLAAYANI-GALKYMYFVIMLFWYLSICVANTVVIVII-----------QVDRRLHEPMYI
contig039738-NyeOR.D041        MGNS--SE-------IEVVSFVLAAYANI-GALKYMYFIIMLFWYLSICVANTVVIVVI-----------QVDRRLHEPMYI
contig013323-TilOR.D050        MGNS--SE-------IEVVSFVLSAYANI-GALKYMYFVIMLFWYLSICVANTVVIVVI-----------HVDRRLHEPMYI
contig014047-ZebOR.D036        MENS--SE-------I--VSFVLAAYGNV-GELKYLYFVIILFWYLSICVANTVLIVVI-----------RVDIQLHEPMYI
contig064802-BriOR.D027        MENS--SE-------I--VSFVLAAYGNV-GELKYLYFVIIMFWYLSICVANTVLIVVI-----------RVDIQLHEPMYI
contig013322-TilOR.D049        MENS--SE-------I--VSFVLTAYGNV-GDLKYLYFVIILFWYLFICVANTVLIVVI-----------RVDRRLHEPMYI
contig039738-NyeOR.D042        MENS--SE-------I--MSFVLSAYGNV-GELKYLYFVIILFWYLSICVANTVLILVI-----------RVDRQLHEPMYI
contig013321-TilOR.D048        MGNS--SK-------I--VSFVLAAYENV-GEFKYLYFVIILFWYVSICVANTVLIVVI-----------HVDKRLHEPMYI
contig066327-ZebOR.F064        MENN--SH-------P--LYFNLTMFVYI-GKFRYPAFVLFLLLYTFIISANLVIIVVI-----------SREKTLHEPMYI
contig013898-BurOR.F058        MENN--SH-------P--LYFNLTMFVYI-GKFRYPAFVLFLLLYTFIISANLVIIVVI-----------SREKTLHEPMYI
contig033883-BriOR.F045        MENN--SH-------P--LYFNLTMFVNI-GKFRYPAFVLFLLLYTFIISANLVIIVVI-----------SREKTLHEPMYI
contig009545-TilOR.F097        MENN--SY-------P--LYFNLTMFVNI-GKFRYPAFVLFLLLYTFIISANLVIIVVI-----------SREKTLHEPMYI
contig039729-NyeOR.F064        MENS--TL-------S--FYFRFTMFANI-GHYRFIAFIFCLLLFIFTIFTNLLMIVVI-----------SQQTTLHEPMYI
contig053781-BurOR.F059        MENS--TL-------S--FYFRFTMFANI-GHYRFIAFIFCLLLFIFTIFTNLLMIVVI-----------SQQTTLHEPMYI
contig014056-ZebOR.F063        MENS--TL-------S--FYFRFTMFANI-GHYRFIAFIFCLLLFIFTIFTNLLMIVVI-----------SQQTTLHEPMYI
contig075822-TilOR.F098        MENS--TL-------S--FYFRFTMFANI-GHYRFMAFIFCLLLFIFTIFTNLLMIVVI-----------SQQTTLHEPMYI
contig025224-ZebOR.C035        MDNT--TA----------ATFKMTAYAVM-ENYKHGLFSVFFLLYLITLVLNVLLISVI-----------HQNKQLHQPMNV
contig009805-BurOR.C031        MDNT--TA----------ATFKMTAYAVM-ENYNHGLFSVFFLLYLITLVLNVLLISVI-----------HQNKQLHQPMNV
contig020980-NyeOR.C035        MDNT--TA----------ATFKMTAYAVM-ENYKHGLFSVFFLLYLITLVLNVLLISVI-----------HQNKQLHQPMNV
contig063874-BriOR.C026        MDNT--TA----------ATFKMTAYAVM-ENYKHGLFSAFFLLYLITLVLNVLLISVI-----------HKNKQLHQPMNV
contig048237-ZebOR.G065        MENN--FE----------IVFVLQGLNDS-LTNRQIYFAFALMSYLFTVSVNLTLIITI-----------SLDKTLHEPIYI
contig049295-BurOR.G060        MENN--FE----------IVFVLQGLNDS-LTNRQIYFAFALMSYLFTVSVNLTLIITI-----------SLDKTLHEPIYI
contig053581-NyeOR.G066        MENN--FE----------IVFVLQGLNDS-LTNRQIYFAFALMSYLFTVSVNLTLIITI-----------SLDKTLHEPIYI
contig104344-BriOR.G048        MENN--FE----------IVFVLQGLNDS-LTNRQIYFAFALMSYLFTVSVNLTLIITI-----------SLDKTLHEPIYI
contig047714-TilOR.G099        MENN--FE----------IVFVLQGLNNS-LANRQIYFAFALMSYLFTVSVNLTLIITI-----------SLDKTLHEPIYI
contig057403-ZebOR.H076        MDNV--SV-------V--RMFTLSGFNET-MNIRLTIFSLTLMYYCMIILINVSLIVLI-----------VLDENLHEPMYI
contig013371-TilOR.H117        MDNV--SV-------V--RIFTLSGFNET-MNIRLTIFSLTLMYYCMIILINMSLVVII-----------VLDENLHEPMYI
contig035582-NyeOR.H070        MDNV--SN-------V--KSFVLLGFNDT-MNFTVPLFIITLLHYCVILFFNISLVLLI-----------VLDENLHEPMYI
contig013361-TilOR.H108        MDNV--SN-------V--ISFVLSGFNET-MTFRVPLFSCTFLYYCLILFFNISLVLLI-----------VLDANLHEPMYI
contig013362-TilOR.H109        MDNV--SN-------V--IRFVLSGFNET-MNFSVPLFSITLLYYCVILFVNISLVLLI-----------FFDANLHEPMYI
contig013368-TilOR.H112        MANQ--SI-------E--RSFILLGFNET-MNFRVPLFLLTLLYYCVILFFNISLVLLI-----------VLDTNLHEPMYI
contig013369-TilOR.H115        MANQ--SN-------E--RSFILSGFNET-MNFRIPLFLCTLLYYSMILFFSISVVLLI-----------VFDANLHEPMYI
contig013369-TilOR.H116        MDNV--SN-------I--RSFILSGFNET-VNFRVPLFSLTLLYYCVILFFNISLILLI-----------VLDANLHEPMYI
contig013369-TilOR.H114        MDNQ--SN-------V--RSFILSGFNET-VNFRVPLFSFTLLCYCVILFINISLVLLI-----------VLDANLHEPMYI
contig048882-BurOR.H064        MDNQ--YN-------V--RSFILSGFNET-MNFRVPLFSFTLLCYCVILFINISLVLLI-----------VLDENLHEPMYI
contig035580-NyeOR.H069        MDNV--HN-------V--RSFILLGFNET-INFRVPLFSVTLLYYCGILFFNVSLVLLI-----------VLDVNLHEPMYI
contig057400-ZebOR.H075        MDNV--HN-------V--RSFILLGFNET-INFRVPLFSVTLLYYCVILFFNVSLVLLI-----------VLDENLHEPMYI
contig013368-TilOR.H113        MDNV--SN-------V--RSFILVGFNET-TNYRVPLLLATLLYYCVILIINIALILII-----------VLDENLHEPMYI
contig006794-BurOR.H061        MDNV--SI-------V--RSFILSGFNET-MNFRVPLFAFTLLYYCMILFFNISLVLLI-----------VLDKNLHEPMYI
```

```
contig035583-NyeOR.H071     MDNV--SN-------V--RSFILSGFNET-MNFRVPLFAFTLLYYCMILFFNISLVLLI-----------FLDENLHEPMYI
contig057403-ZebOR.H077     MDNV--SN-------V--RSFILSGFNET-MNFRVPLFAFTLLYYCMILFFNISLVLLI-----------FLDENLHEPMYI
contig049873-BriOR.H050     MDNV--SN-------V--RSFILSGFNET-MNFRVPLFTFTLLYYCMILFFNVSLVLLI-----------FLDENLHEPMYI
contig013371-TilOR.H118     MDNV--SN-------V--RSFILSGFNET-MNFRVPLFTFTLLYYCMILFINISLVLLI-----------FLDENLHEPMYI
contig093825-BriOR.H053     MDNV--ST-------V--RIFNLLAFNET-ANYRAALFSATLVCYFAIVFLNVTVIMII-----------VLDESLHEPMYI
contig048562-BurOR.H062     MDNV--ST-------V--RIFNLLAFNET-ANYRAAFFSATLVCYFAIVFLNVTVIMII-----------VLDESLHEPMYI
contig034998-NyeOR.H067     MDNV--ST-------V--RIFNLLAFNET-ANYRAAFFSATLVCYFAIVFLNVTVIMII-----------VLDESLHEPMYI
contig047492-ZebOR.H074     MDNV--ST-------V--RIFNLLAFNET-ANYRAAFFSATLVCYFAIVFLNVTVIMII-----------VLDESLHEPMYI
contig041955-TilOR.H119     MDNV--ST-------V--SIFNLLAFNDT-VNHRAALFSVTLVCYFAILFLNVTVILII-----------VLDESLHEPMYI
contig116846-BriOR.H055     MNNV--SV-------I--TMFFLSGFNET-ISHRFVLFFLSLLCYCIICLVNVSLIVII-----------ILDSNLHESMYI
contig033889-BriOR.H049     MNNV--SV-------I--TMFVLSGFNET-ISHRFVLFFLSLLCYCIICLLNVSLIVII-----------ILDRNLHESMYI
contig030011-ZebOR.H073     MNNV--SV-------I--TMFFLSGFNET-VSHRFVLFFLSLLCYCIICLLNVSLIVII-----------ILDSNLHESMYI
contig018437-ZebOR.H072     MNNV--SV-------I--TTFFLSGFNET-VSHRFVLFFLSLLCYCIICLVNVSLIVII-----------ILDSNLHESMYI
contig018434-ZebOR.H070     MNNV--SV-------I--TMFFLSGFNKT-ISHRFVLFFLSLLCYCIICLVNVSLIVII-----------ILDSNLHESMYI
contig009565-TilOR.H126                                                MLCYCIICLVNVSLIVII-----------ILDTNLHESMYI
contig018434-ZebOR.H071     MNNV--SV-------I--TMFFLSGFNET-VSHRFVLFSLSLLCYCIICLLNVSLIVII-----------ILHSNLHEPMYI
contig009547-TilOR.H101     MNNV--SV-------I--TMFFLSGLNET-VNHRFVLFFLSLLCYCIIFLLNLALTVTI-----------ILDKNLHEPMYI
contig009548-TilOR.H102     MNNV--SV-------I--TMFFLSGLNET-VNHRFVLFFLSLLCYCIIFLLNLALTVTI-----------ILDKNLHEPMYI
contig009546-TilOR.H100     MNNV--SV-------I--TMFFLSGLNET-MNHRFVLFFLSLLCYCIIFLLNLALTVTI-----------ILDNNLHEPMYI
contig014060-ZebOR.H069     MDNV--SV-------I--AVFTLSGLRDI-ANYRVILFVLTLLCYCVIWLVNVTIIVTV-----------IVDKKLHEPMYI
contig053784-BurOR.H067     MDNV--SV-------I--AVFTLSGLRDI-ANYRVILFVLTLLCYCVIWLVNVTIIVTV-----------IVDKKLHEPMYI
contig041756-NyeOR.H075     MDNV--SV-------I--TVFTLSGLSDI-ANYRVILFVLTLLCYCVIWLVNLTIIVTV-----------IVDKKLHEPMYI
contig041756-NyeOR.H139     MDNV--SV-------I--TVFTLSGLSDI-ANYRVILFVLTLLCYCVIWLVNLTIIVTV-----------IVDKKLHEPMYI
contig013363-TilOR.H110     MDNV--SV-------V--TVFTLSGLSDI-TNYKVILFVLTLLCYCVIWLVNLMIIVTV-----------IVDKSLHEPMYI
contig035579-NyeOR.H068     MDNV--SV-------I--TVFTLSGLSDI-ANYRVILFVLTLLCYCVIWLVNLTIIVTV-----------IVDKKLHEPMYI
contig048880-BurOR.H063     MDNV--SV-------I--TVFTLSGLSDI-ANYRVILFVLTLLCYCVIWLVNLTIIVTV-----------IVDKKLHEPMYI
contig013365-TilOR.H111     MDNV--SV-------I--TVFTLSGLSDI-ANYRVTLFVLTLLCYCVIWLVNLTIIVTV-----------IVDKSLHEPMYI
contig053782-BurOR.H066     MDNV--SV-------V--TVFTLSGLSGI-ANYKITIFIFTLLCYCVIWLVNLTIIVTV-----------IVDKSLHEPMYI
contig039729-NyeOR.H073     MDNV--SV-------V--TVFTLSGLSGI-ANYKITIFIFTLLCYCVIWLVNLTIIVTV-----------IVDKSLHEPMYI
contig014057-ZebOR.H067     MDNV--SV-------V--TVFTLSGLSGI-ANYKITIFIFTLLCYCVIWLVNLTIIVTV-----------IVDKSLHEPMYI
contig064817-BriOR.H051     MDNV--SV-------V--TVFTLSGLSGI-ANYKITIFIFTLLCYCVIWLVNLTIIVTV-----------IVDKKLHEPMYI
contig013359-TilOR.H107     MDNV--SI-------I--TVFTLSGLSGI-ANYKITVFIFTLLCYCVIWLVNLTIIVTV-----------IVDKKLHEPMYI
contig064821-BriOR.H052     MDNV--SV-------I--TVFTLAGLSDI-ENYRAILFVLTLLCYCVIWLVNLTIIVTV-----------IVDKSLHEPMYI
contig053788-BurOR.H069     MDNV--SV-------I--TVFTLAGLSDI-ANYRVILFVLTLLCYCVIWLVNVTIIVTV-----------IVDKKLHEPMYI
contig041757-NyeOR.H076     MDNV--SV-------I--TVFTLSGLSDI-ANYRVILFVLTLLCYCVIWLVNLTIIVTV-----------IVDKKLHEPMYI
contig013356-TilOR.H105     MDNV--SV-------I--TVFTLSGLSDI-ANYRVILFFLTLLCYSVIWLVNLTIIVTV-----------IVDKSLHEPMYI
contig013358-TilOR.H106     MDNV--SV-------I--TVFTLSGLRDI-ANYRVILFVLTLLCYCVIWLVNLTIILTV-----------IVDKSLHEPMYI
contig013351-TilOR.H104     MDNV--SI-------I--TVFTLSGLRDI-ANYRVILFVLTLLCYCVIWLVNVTIIVTV-----------ILDKSLHEPMYI
contig014059-ZebOR.H068     MDNV--SI-------I--TVFTLSGLSDI-ANYRVILFVLTLLCYCVIWLVNVTIIVTV-----------IVDKKLHEPMYI
contig053787-BurOR.H068     MDNV--SI-------I--TVFTLSGLSDI-ANYRVILFVLTLLCYCVIWLVNVTIIVTV-----------IVDKKLHEPMYI
contig039725-NyeOR.H072     MDNV--SI-------I--TVFTLSGLSDI-ANYRVILFVLTLLCYCVIWLVNLTIIVTV-----------IVDKKLHEPMYI
contig039730-NyeOR.H074     MNTS--SI----------VVFSLTGFSAT-VNYRVTLFSLTLLCYFLILIVNISLILTI-----------ISDQNLHEPMYI
contig014055-ZebOR.H066     MNTS--SI----------VVFSLTGFSAT-VNYRVTLFSLTLLCYFLILMVNIALILTI-----------ISDQNLHEPMYI
contig053780-BurOR.H065     MNTS--SI----------VVFSLTGFSAT-VNYRVTLFSLTLLCYFLILMVNISLILTI-----------ISDQNLHEPMYI
contig107626-BriOR.H054     MNKS--SI----------VVFSLTGFNAT-VNYRVTLFSLTLLCYFLILMVNISLILTI-----------ISDQNLHEPMYI
contig013349-TilOR.H103     MNTS--ST----------VVFSLTGFSAT-VNYRVTLFSLTLLCYFLILMVNISLILTI-----------ISDQNLHEPMYI
contig106096-BriOR.N089   MDLFNS--AL-GKNITFLRPAFFIISGFIGI--PNIKYYYAFLFFVYIISVLANTAVMAAI-----------YLDHNLRTPKYI
contig061663-NyeOR.N114   MDLFNS--AL-GKNITFLRPAFFIISGFIGI--PNIKYYYAFLFFVYIISVLANTAVMAAI-----------YLDHNLRTPKYI
contig057383-BurOR.N109   MDLFNS--AL-GKNITFLRPAFFIISGFIGI--PNIKYYYAFLFFVYIISVLANTAVMAAI-----------YLDHNLRTPKYI
contig010722-ZebOR.N111   MDFFNS--AL-GKNITFFRPAFFIISGFIGI--PNIKYYYAFLFFVYIISVLANTAVMATI-----------YLDHNLRTPKYI
contig096539-BriOR.N087   MELFNS--AL-GKNITFVHPKFFIIGGLTGI--PNITFYYVFLFFVYIVSVVGNTVVMAVI-----------YLDHNLRTPKYI
contig064097-ZebOR.N115   MELFNS--AL-GKNITFVHPAFFIIGGLTGI--PNITLYYVFLFFVYIVSVVGNTVVMAVI-----------YLDHNLRTPKYI
contig055927-NyeOR.N111   MELFNS--AL-GKNITFVHPAFFIIGGLTGI--PNITLYYVFLFFVYIVSVVGNTVVMAVI-----------YLDHNLRTPKYI
contig060631-BurOR.N110   MELFNS--AL-GKNITFVHPAFFIIGGLTGI--PNITLYYVFLFFVYIVSVVGNTVVMAVI-----------YLDHNLRTPKYI
contig046353-TilOR.N195   MELFNS--AL-GKNITFVHPAFFIIGGLTGI--PNITFYYVFLFFVYIVSVVGNTVVMAVI-----------CLDHNLRTPKYI
contig055926-NyeOR.N110   MDFLNS--AA-EKNTTFVQPANFIISGFVGI--PNIRYYFVFLCFIYIFSLVGNTAVMLII-----------IFDHTLRSPKYI
contig042928-BurOR.N108   MDFLNS--AA-EKNTTFVQPANFIISGFVGI--PNIRYYFVFLCFIYIFSVVGNTAVMLII-----------IFDHTLRSPKYI
contig064098-ZebOR.N116   MDFLNS--AA-EKNTTFVQPANFIISGFVGI--PNIRYYFVFLCFIYIFSVVGNTAVMLII-----------IFDHTLRSPKYI
contig096539-BriOR.N088   MDFLNS--AA-EKNTTFVQPANFIISGFVGI--PNIRYYFVFLCFIYIFSVVGNTAVMLII-----------IFDHTLRSPKYI
contig046356-TilOR.N196   MDFLNS--AA-EKNTTFVRPANFIISGFVGI--PNIRYYFVFLCFIYIFSVVGNTAVMLII-----------IFDHTLRSPKYI
contig055924-NyeOR.N109   MEFLNS--AV-GKNITFVKPAYFIISTFNGI--ANIRYYFVFLCFIYIFSVVGNTLLMIVI-----------ILDHTLRGPKHI
contig010725-ZebOR.N112   MEFLNS--AV-GKNITFVKPAYFIISAFNGI--ANIRYYFVFLCFIYIFSVVGNTLLMIGI-----------ILDHTLRGPKHI
contig010726-ZebOR.N113   MEFLNS--AV-GKNITFVKPAYFIISAFNGI--ANIRYYFVFLCFIYIFSVVGNTLLMIGI-----------ILDHTLRGPKHI
contig046360-TilOR.N197   MEFFNS--AV-GKNITFVKPAYFIISAFNGI--VNIRYYFVFLCFIYIFSVVGNTLVMIVI-----------ILDHTLKGPKHI
contig010714-ZebOR.N109   MALINS--AA-ENNITFVRPAYFIISGFIGI--PNIRYYFVFLCFIYILAVVGNTLVMIVI-----------TLDHMLRSPKYI
contig010718-ZebOR.N110   MALINS--AA-ENNITFVQPAYFIISGFIGI--PNIRYYFVFLCFIYILAVVGNTLVMIVI-----------TLDRMLRSPKYI
contig010712-ZebOR.N108   MALINT--AA-ENNITFVRPAYFIISGFIGI--PNIRYYFVFLCFIYILAVVGNTLVMLVI-----------TLDHMLRSPKYI
contig050080-TilOR.N198   MAFINL--AV-E-NITFVRPAYFIISGFIGI--PNIRYYFVFLCFIYILAVVGTTLVMTVI-----------TLDHTLRSPKYV
contig046352-TilOR.N193   MGLEQK--LIIGFNATFVHPGRFYLGGFSDM--AHDNYYYIFLCFVYIFTVVGNVLLILII-----------FLIKTLHTPKYL
```

```
contig046347-TilOR.N190      MGLEQK--LIIGFNATFVHPGRFYLGGFSDM--AHDNYYYVFLGFVYIFTVVGNVLFILII------------FLIKTLHTPKYM
contig096536-BriOR.N085      MGAEAK--TTTIFNTTFVRPAKFYLSGFSNI--PHIRYFYAFLCFVYIMTVLGNGFLLSLI------------SLVKTLHTPKYM
contig055933-NyeOR.N113      MGAEAK--TTTIFNTTFVRPAKFYLSGFSNI--PHIRYFYAFLCFVYIMTVLGNGFLLSLI------------SLVKTLHTPKYM
contig065025-BurOR.N111      MGTEDK--ATTMFNNTFVRPEKFYLSGFSNI--PHIRYFYAFLCLVYIMTVLGNGFLLSLI------------SLVKTLHTPKYM
contig096535-BriOR.N084      MGTEDK--ATRMFNNTFVRPEKFYLSGFSNI--PHIRYFYTFLCLVYIMTVLGNCFLLSLI------------SLVKTLHTPKYM
contig046340-TilOR.N188      MGTEDK--ATTMFNNTFVRPEKFYLSGFSNI--PHIRYYYAFLCFVYIMTVLGNGFLLSVI------------WLMKTLHTPKYM
contig046351-TilOR.N192      MGSEDQ--ATTMFNNTFVRPEKFYLSGFSNI--PHIRYFYVFLCFVYIMTVLGNGFLLLVI------------WLVKTLHTPKYM
contig046350-TilOR.N191                MFNTTFIRPAKFYLGGFSNI--PHIRYFYIFLCFVYIVTVLGNGFLLSVI------------WLVKTLHTPKYM
contig046344-TilOR.N189      MGSEDQ--ATTMFNNTFVRPAKFYISGFSNI--PHIRYFYVFLCFVYIMTVLGNGFLLLVI------------WLVKTLHTPKYM
contig010727-ZebOR.N114      MDSEEK--ATTKSNSTFVRPAIFYLSGFSNI--PHVKYFYVFLCFVYIMTVLGNGFLLSLI------------WLVKTLHTPKYM
contig042920-BurOR.N107      MDSEEK--ATTKSNSTFVRPAIFYLSGFSNI--PHVKYFYVFLCFVYIMTVLGNGFLLSLI------------WLVKTLHTPKYM
contig055927-NyeOR.N112      MDSEEK--ATTKSNSTFVRPAIFYLSGFSNI--PHVKYFYLFLCFVYIMTVLGNGFLLSLI------------WLVKTLHTPKYM
contig096536-BriOR.N086      MDSEEK--ATTKSNSTFVRPEKFYLSGFSNI--PHVKYFYVFLCFVYIMTVLGNGFLLSVI------------WLVKTLHTPKYM
contig046352-TilOR.N194      MDSDEE--ATTKFNNTFVRPAKFYLSGFSNI--PHVKYFYVFLCFVYIMTVLGNGFLLSVI------------WLVKTLHTPKYM
contig048778-NyeOR.M107      MILQRT--VL--NSSAIIHPPGFYIIGFETF--PFISVYFIFLVFVYGVTVLFNILVIYVI------------ASTRRLHTPKFL
contig010734-ZebOR.M107      MIFQRA--VL--NSSAIIHPPGFYIIGFETF--PFISVYFIFLVFVYGVTVLFNILVIYVI------------ASTRRLHTPKFL
contig048778-NyeOR.M108      MILQRT--VL--NSSAIIHPPGFYIIGFETF--PFISVYFIFLVFVYGVTVLFNILVIYVI------------ASTRRLHTPKFL
contig039460-TilOR.L145      MSLPNA--S---------IKVTHFIIGGFDTV--KRPIAVGVVMLITYLLAVIANVLNILFI------------IFDKRLHKPMYL
contig017786-ZebOR.L091      MSLPNA--S---------IKVTHFIIGGFDTV--KRPIAVGVVMLITYLLAVIANVLNILFI------------IFDKRLHKPMYI
contig058162-BurOR.L089      MSLPNA--S---------IKVTHFIIGGFDTV--KRPIAVGVVMLIIYLLAVIANVLNILFI------------IFDKRLHKPMYI
contig040502-NyeOR.L092      MSLPNA--S---------IKVTHFIIGGFDTV--KRPIAVGVVMLITYLLAVIANMLNIILI------------IFDKRLHKPMYI
contig042543-BriOR.L069      MSLPNA--S---------IKVTHFIIGGFDTV--KRPIAVGVVMLITYLLAVIANVLNILFI------------IFDKRLHKPMYI
contig039460-TilOR.L146      MSLQNT--S---------IKVTHFIIGGFDTV--KRPVAVGVVMLITYLLAVIANVLNIVFI------------IFDKRLHKPMYL
contig039465-TilOR.L150      MSLQNA--S---------IKLTHFIIGGFDTV--KRPVAVGVGMLITYLLAVTGSLVNIIFI------------ISDKQLHKPMYL
contig039461-TilOR.L148      MSLQNA--S---------IKLTHFIIGGFDTV--KRPVAVGVVMLITYLLAVTGSLVNIIFI------------VSDKQLHKPMYL
contig042552-BriOR.L071      MSLQNA--S---------IKLTHFIIGGFDTV--KRPVAAGVVMLIIYLLAVLASLVNIIFI------------VSDKQLHKPMYL
contig020445-ZebOR.L096      MSLQNA--S---------IKLTYFIIGGFDTV--KRPVAVGVVMLITYLLAVFASLVNIIFI------------VSDKQLHKPMYL
contig056942-NyeOR.L093      MSLQNA--S---------IKLTYFIIGGFDTV--KRPVAVGVVMLITYLLAVFASLVNIIFI------------VSDKQLHKPMYL
contig068527-TilOR.L157      MSLQNA--S---------IKLTHFIIGGFDTV--KRPVAVGVVMLITYLLAVFASLVNIIFI------------VSDKQLHKPMYL
contig039469-TilOR.L152      MSLQNA--S---------IKLTYFIIGGFDTV--KRPVAVGVVMLITYLLAVFASMVNIIFI------------VSDKQLHKPMYL
contig064361-NyeOR.L094      MSSQNT--S---------INVTHFIIGGFDTL--SRPIAVGVVILITYLLAVLANMANIMFI------------ISDKRLHKPMYL
contig056384-BurOR.L087      MSSQNT--S---------INVTHFIIGGFDTL--SRPIAVGVVILITYLLAVLANMVNIMFI------------ISDKRLHKPMYL
contig020442-ZebOR.L095      MSSQNT--S---------INVTHFIIGGFDTL--SRPIAVGVVILITYLLAVLANMANIMFI------------IADKRLHKPMYL
contig042554-BriOR.L072      MSLQNA--S---------INITHFIIGGFDTL--SRPIAVGVVILIAYFLAVLANMANIMFI------------ISDKRLHKPMYL
contig039481-TilOR.L153      MSLQNA--S---------INVTHFIIGGFDTL--SRPIAVGVVILITYLLAVFANMANIMFI------------ISDKRLHKPMYL
contig072645-TilOR.L159      MSLQNA--S---------INVRHFIIGGFDTL--SRPIAVGVVILIAYFLAVLANIANIMFI------------ISDKRLHKPMYL
contig056940-NyeOR.L095      MSLQNA--S---------INVTHFIIGGFDTL--SRPIAVGVVILITYLLAVLANMINIMFI------------ISDKRLHKPMYL
contig020440-ZebOR.L094      MSLQNA--S---------INVTHFIIGGFDTL--SRPIAVGVVILITYLLAVLANMINIMFI------------ISDKRLHKPMYL
contig042556-BriOR.L073      MSLQNA--S---------INVTHFIIGGFDTL--SRPIAVGVVILIIYLLAVVANMINIMFI------------ISDKRLHKPMYL
contig039484-TilOR.L154      MSLQNA--S---------INVTHFIIGGFDTL--SRPIAVGVAILITYLLAVLANMANIMFI------------ISDKRLHKPMYL
contig068425-TilOR.L155      MSSQNA--S---------VNVTHFIIGGFDTL--SRPIAVGVVILITYFLAVLANMINIMFI------------ISDKRLHKPMYL
contig020437-ZebOR.L093      MSLQNA--S---------INVTHFIIGGFDTL--SRPIAVGVVILIVYLLAVLANMINIMFI------------ISDKRLHKPMYL
contig056386-BurOR.L088      MSSQNA--S---------INVTHFIIGGFDTL--SRPIAVGVVILIVYLLAVLANMINIMFI------------ISDKRLHKPMYL
contig039469-TilOR.L151      MSLQNA--S---------INVTHFIIGGFDTL--SRPIAVGVVILIMYLLAVFANMANIMFI------------ISDKRLHKPMYL
contig068521-TilOR.L156      MSLQNA--S---------INVTHFIIGGFDTL--SRPIAVGVVILITYLLAVLANIANIMFI------------ISDKRLHKPMYL
contig068539-TilOR.L158      MSLQNT--S---------INVTHFIIGGFDTL--SRPIAVGVAILITYLLAVLANMANIMFI------------ISDKRLHKPMYL
contig042544-BriOR.L070    MSMLLQNA--S---------ITVKHFIIGGFDTV--KRPVAVGVVILIIYILAIFANTANILFI------------IFDKRLHKPMYL
contig065027-BurOR.L090      MLLQNTVLS---------ITVKHFIIGGFDTV--KRPVAVGVVMLIIYILAIFANMVNILFI------------IFDKRLHKPMYL
contig017787-ZebOR.L092      MLLQNA--S---------ITVKHFIIGGFDTV--KRPVAVGVVMLIIYILAIFANTVNILFI------------IFDKRLHKPMYL
contig040502-NyeOR.L091      MLLQNA--S---------ITVKHFIIGGFDTV--KRPVAVGVVMLIIYILAIFANTVNILFI------------IFDKRLHKPMYL
contig039462-TilOR.L149      MSLPNA--S---------IKVTHFIIGGFDTV--NRPIAVGVVILIIYILAVFANTVNILFI------------IFDKRLHKPMYL
contig023716-TilOR.L144      MSLQNA--S---------MKVTHFIINGFDTV--KSPVAVGVVILIIYLVSIVTNLLNILFV------------ISDKRLHKPMYI
contig039461-TilOR.L147      MYLQNA--S---------INVTHFIIGGFDTV--IRPEAIGVVMLIIYILVVLANTVNILFI------------IFDKRLHKPMYL
contig017698-BurOR.P120        MENV------------SLHTHFTLDGFSEL-GELRPFLFIPFSFMFVVSLFANSLLVYVI------------VSQRSLHSPMYI
contig025452-ZebOR.P123        MENV------------SLHTHFILDGFSEL-GELRPFLFIPFSFMFVVSLFANSLLVYVI------------VSQRSLHSPMYI
contig004267-BriOR.P099        MENV------------SLHTHFFLEGFSEL-GELRPFLFIPFSFMFVVSLFANSLLVYVI------------VSQRSLHSPMYI
contig065444-TilOR.P214        MENI------------SLHTHFILDGFSEL-GELRPFLFIPFSFMFVVSLFANSLLLYII------------LSQRSLHSPMCI
contig065247-TilOR.P211    MEPVTENI------------SSHKYFFLVGFSEL-GELRPFLFIPFSLMFVVSLFANSLLVYVI------------VSQRSLHSPMYI
contig004270-BriOR.P100   EMEAVKENI------------SSHKYFFLDGFSEL-GELRPFLFIPFSFMFVVSLFANSLLVYVI------------VSQRSLHSPMYI
contig017697-BurOR.P119    MEAVKENV------------SSHKYFFLDGFSEL-GELRPFLFIPFSFMFVVSLFANSLLVYVI------------VSQRSLHSPMYI
contig025453-ZebOR.P124    MEAVKENI------------SSHKYFFLDGFSEL-GELRPFLFIPFSFMFVVSLFANSLLVYVI------------VSQRSLHSPMYI
contig065449-TilOR.P215    MEPVKENI------------SSHKYFFLVGFGEL-GELRPFLFIPFSLMFVVSLFANSLLVYVI------------VSQRSLHSPMYI
contig065253-TilOR.P212    MEPVKENI------------SSHKYFFLVGFSEL-GELRPFLFIPFSVMFVVSLFANSLLVYVI------------VSQRSLHSPMYI
contig065254-TilOR.P213   MLEAPLSRN------------FSHSTFVLRGFPSL-QKHRRLLALPFSASYLLVLLGNSLLVYVI------------RSVESLHSPMYL
contig023268-NyeOR.P118   MLEAPLSRN------------FSHCTFVLRGFPSL-QKHRRLLALPFSASYLLVLLGNSLLVYVI------------CSVERLHSPMYL
contig017696-BurOR.P118   MLEAPLSRN------------FSHCTFVLRGFPSL-QKHRRLLALPFSASYLLVLLGNSLLVYVI------------CSVERLHSPMYL
contig025458-ZebOR.P125   MLEAPLSRN------------FSHCTFVLRGFPSL-QKHRRLLALPFSASYLLVLLGNSLLVYVI------------CSVERLHSPMYL
contig004275-BriOR.P101   MLEAPLSRN------------FSHSTFVLRGFPSL-QKHRRLLALPFSASYLLVLLGNSLLMYII------------CSVERLHSPMYL
contig020431-ZebOR.O101      MPVENH------------SSVTEFVLTGFPGLHQEYYGLVSAVLFFVYLITLIANATVIFLF------------ATNHSLHKPMYF
```

```
contig021359-NyeOR.O102                   MPVENH-----------SSVTEFVLTGFPGLHQEYYGLVSAVLFFVYLITLIANATVIFLF-----------ATNHSLHKPMYF
contig059249-BurOR.O098                   MPERNH-----------SSVTEFILTGFPGLHQEYYGLVSAVLFFVYLVTLIANATVIFLF-----------ATNRSLHKPMYY
contig062053-NyeOR.O103                   MPERNH-----------SSVTEFILTGFPGLHQEYYGLVSAVLFFVYLITLIANATVIFLF-----------ATNHSLHKPMYY
contig023717-TilOR.O175                   MPERNH-----------SSVTEFVLTGFPGLHQEYYGLVSALLFFVYLVTMIANATVIFLF-----------ATNRSLHKPMYY
contig023724-TilOR.O176                   MPERNH-----------SVLTEFVLTGFPGLHQEYYGLVSALLFFVYLVTMIANVTVVFLI-----------ATNRSLHKTMYY
contig042559-BriOR.O077                   MPQRNH-----------SVLTEFILTGFPGLHQEYYGLVSAVLFFVYLVTLIANATVIFLI-----------ATNRSLHKPMYY
contig110782-BriOR.O080                   MPERNH-----------SSVTEFILTGFPGLHQEYYGLVSAVLFFVYLITMIANATVIFLF-----------ATNQSLHKPMYY
contig020430-ZebOR.O100                    MITNV-----------TRMKSFFILGFPGLSPQYYGPISTFLFFVYLAIALGNIFILSFV-----------AYEKSLQKPTYL
contig042560-BriOR.O078                    MITNV-----------TRMKSFFILGFPGLSPQYYGSISTFLFFVYLAIAVGNIFILSFV-----------SYEKSLQKPTYL
contig021354-NyeOR.O101                   MKYTNI-----------TTIKEFIIIGFPGLPPEYYGPVSVLLLLVFLAIVIGNGFTIAVI-----------IFERTLHKPIYV
contig020427-ZebOR.O099                   MKYTNI-----------TTIKEFIIIGFPGLPPEYYGPVSVLLLLVFLAIVIGNGFTIAVI-----------IFERTLHKPIYV
contig023731-TilOR.O177                   MKYTNI-----------TTIKEFIIIGFPGLPPEYYGPVSVLLLLVFLAIVIGNGFTIAVI-----------IFERTLHKPIYV
contig042562-BriOR.O079                   MKYTNI-----------TTIKEFIIIGFPGLPPEYYGPVSVLLLLVFLAIVIGNGFTIAVI-----------IFERTLHKPIYV
contig049605-BurORs.AB153  MNTDELFFPSDFPTLSTNHRS--SSVNETLGLGGVTFFIIQGLTNL-DEKKIILFSILLLIYIMVLGGNSIIIYVVQRTVMSFLLQALTDPKLNSPLYF
contig046717-TilORs.AB275  MNTDGLFFPSDFPPLSTNHRS--SSANGTLELSGVTFFIIQGLTNL-DEKKIILFSILLLIYIMILGGNSIII------------------------YV
```

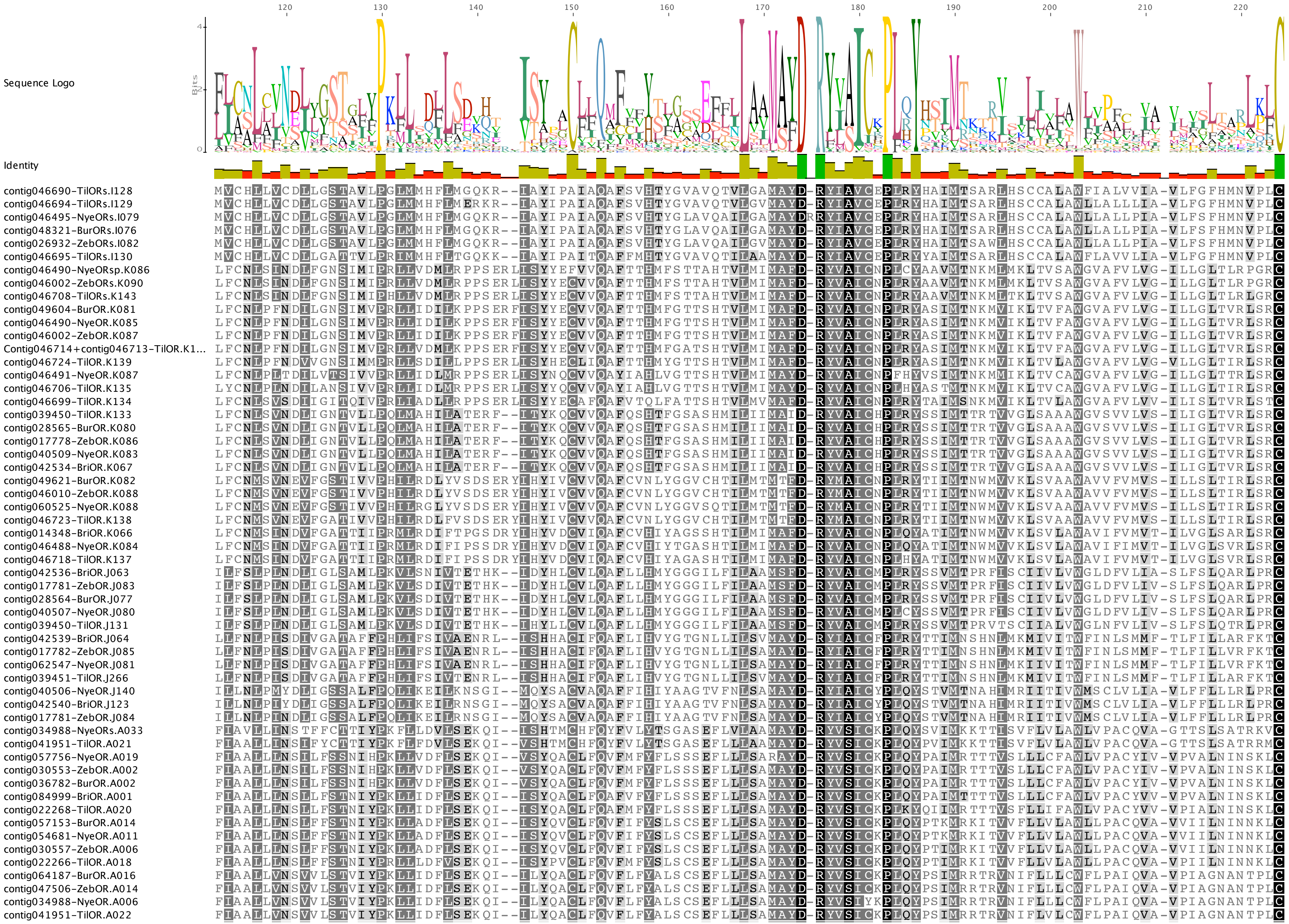

120
130
140
150
160
170
180
190
200
210
220
Sequence Logo
Bits
Identity
contig046690-TilORs.I128
contig046694-TilORs.I129
contig046495-NyeORs.I079
contig048321-BurORs.I076
contig026932-ZebORs.I082
contig046695-TilORs.I130
contig046490-NyeORsp.K086
contig046002-ZebORs.K090
contig046708-TilORs.K143
contig049604-BurOR.K081
contig046490-NyeOR.K085
contig046002-ZebOR.K087
Contig046714+contig046713-TilOR.K1...
contig046724-TilOR.K139
contig046491-NyeOR.K087
contig046706-TilOR.K135
contig046699-TilOR.K134
contig039450-TilOR.K133
contig028565-BurOR.K080
contig017778-ZebOR.K086
contig040509-NyeOR.K083
contig042534-BriOR.K067
contig049621-BurOR.K082
contig046010-ZebOR.K088
contig060525-NyeOR.K088
contig046723-TilOR.K138
contig014348-BriOR.K066
contig046488-NyeOR.K084
contig046718-TilOR.K137
contig042536-BriOR.J063
contig017781-ZebOR.J083
contig028564-BurOR.J077
contig040507-NyeOR.J080
contig039450-TilOR.J131
contig042539-BriOR.J064
contig017782-ZebOR.J085
contig062547-NyeOR.J081
contig039451-TilOR.J266
contig040506-NyeOR.J140
contig042540-BriOR.J123
contig017781-ZebOR.J084
contig034988-NyeORs.A033
contig041951-TilOR.A021
contig057756-NyeOR.A019
contig030553-ZebOR.A002
contig036782-BurOR.A002
contig084999-BriOR.A001
contig022268-TilOR.A020
contig057153-BurOR.A014
contig054681-NyeOR.A011
contig030557-ZebOR.A006
contig022266-TilOR.A018
contig064187-BurOR.A016
contig047506-ZebOR.A014
contig034988-NyeOR.A006
contig041951-TilOR.A022

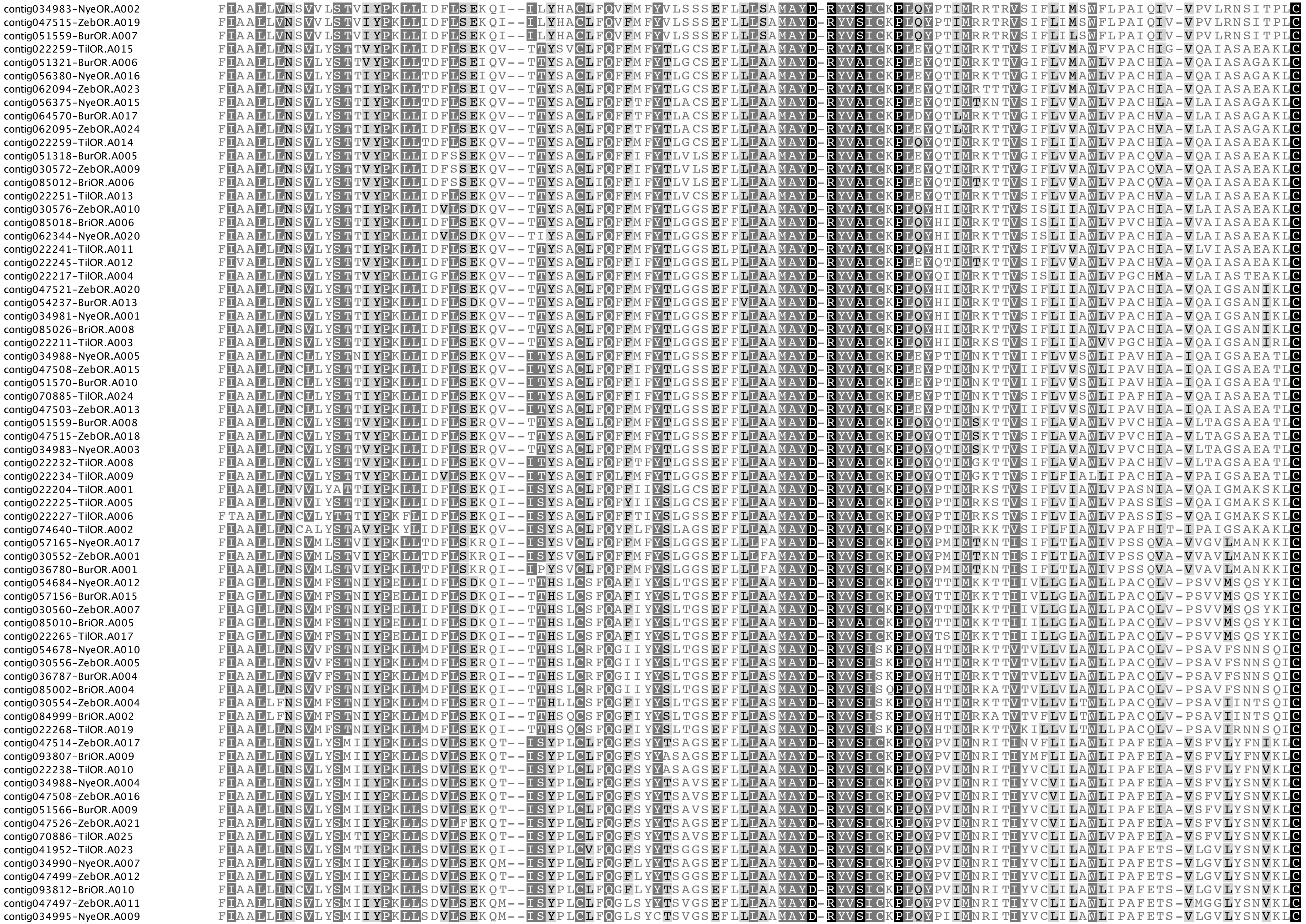
contig034983-NyeOR.A002
contig047515-ZebOR.A019
contig051559-BurOR.A007
contig022259-TilOR.A015
contig051321-BurOR.A006
contig056380-NyeOR.A016
contig062094-ZebOR.A023
contig056375-NyeOR.A015
contig064570-BurOR.A017
contig062095-ZebOR.A024
contig022259-TilOR.A014
contig051318-BurOR.A005
contig030572-ZebOR.A009
contig085012-BriOR.A006
contig022251-TilOR.A013
contig030576-ZebOR.A010
contig085018-BriOR.A006
contig062344-NyeOR.A020
contig022241-TilOR.A011
contig022245-TilOR.A012
contig022217-TilOR.A004
contig047521-ZebOR.A020
contig054237-BurOR.A013
contig034981-NyeOR.A001
contig085026-BriOR.A008
contig022211-TilOR.A003
contig034988-NyeOR.A005
contig047508-ZebOR.A015
contig051570-BurOR.A010
contig070885-TilOR.A024
contig047503-ZebOR.A013
contig051559-BurOR.A008
contig047515-ZebOR.A018
contig034983-NyeOR.A003
contig022232-TilOR.A008
contig022234-TilOR.A009
contig022204-TilOR.A001
contig022225-TilOR.A005
contig022227-TilOR.A006
contig074640-TilOR.A002
contig057165-NyeOR.A017
contig030552-ZebOR.A001
contig036780-BurOR.A001
contig054684-NyeOR.A012
contig057156-BurOR.A015
contig030560-ZebOR.A007
contig085010-BriOR.A005
contig022265-TilOR.A017
contig054678-NyeOR.A010
contig030556-ZebOR.A005
contig036787-BurOR.A004
contig085002-BriOR.A004
contig030554-ZebOR.A004
contig084999-BriOR.A002
contig022268-TilOR.A019
contig047514-ZebOR.A017
contig093807-BriOR.A009
contig022238-TilOR.A010
contig034988-NyeOR.A004
contig047508-ZebOR.A016
contig051566-BurOR.A009
contig047526-ZebOR.A021
contig070886-TilOR.A025
contig041952-TilOR.A023
contig034990-NyeOR.A007
contig047499-ZebOR.A012
contig093812-BriOR.A010
contig047497-ZebOR.A011
contig034995-NyeOR.A009

```
contig034994-NyeOR.A008   FIAALLINSVLYSMIIYPKLLSDVLSEKQM--ISYPLCLFQSLSYYTSVGSDFLLAAMAYD-RYVSICKPLQYPFIMNRITIYVCLILAWLIPAFETS-VMGVLYSNVKLC
contig093816-BriOR.A011   FIAALLINSVLYSMIIYPKLLSDVLFEKQM--ISYPLCLFQGLSYCTSVGSEFLLAAMAYD-RYVSICKPLQYPVIMNKITIYVCLILAWLIPAFESL-MLGVLYSNVKLC
contig051573-BurOR.A011   FIAALLINSVLYSMIIYPKLLSDVLSEKQM--ISYPLCLFQGLSYYTSVGSEFLLAAMAYD-RYVSICKPLQYPVIMNRITIYVCLILAWLIPAFEAL-MLGVLYSNVKLC
contig054233-BurOR.A012   FIAALLINSVLYSMIIYPKLLSDVLSEKQI--ISYTLCLFQGFLYYTSAGSEFLLAAMAYD-RYVSICKPLQYPVIMNRITIYVSLVLAWLIPAFETA-VSVVLYSEVKLC
contig047523-ZebOR.A022   FIAALLINSVLYSMIIYPKLLSDVLSEKQI--ISYTLCLFQGFLYYTSAGSEFLLAAMAYD-RYVSICKPLQYPVIMNRITIYVSLVLAWLIPAFETA-VSVVLYSEVKLC
contig054868-NyeOR.A014   FIAALLINSVLYSMIIYPKLLSDVLSEKQI--ISYTLCLFQGFLYYTSAGSEFLLAAMAYD-RYVSICKPLQYPVIMTRITIYVSLVLAWLIPAFEIA-VSVVLYSEVKLC
contig073309-TilOR.A026   FIAALLINSVLYSMTIYPKLLSDVLSEKQI--ISYPLCLFQGFLYYTSAGSEFLLAAMAYD-RYVSICKPLQYAVIMNRITIYVSLVLAWLIPAFEIA-VSVVLYSNVKLC
contig030566-ZebOR.A008   FIAALSVNSVLLSTVTYPKLFVDVLSEKQI--ISISACRFQHFMCYSIAGSDFLLSAMAFD-RYVSICKPLKYPVIMRQTTINTLLFLSWFVPGLQIA-VLHTLVLNNKLC
contig065887-BurOR.A018   FIAALSVNSVLLSTVTYPKLFVDVLSEKQI--ISISACRFQHFMCYSIAGSDFLLSAMAFD-RYVSICKPLKYPVIMRQTTINTLLFLSWFVPGLQIA-VLHTLVLNNKLC
contig054687-NyeOR.A013   FIAALSVNSVLLSTVTYPKLFVDVLSEKQI--ISISACRFQHFMCYSIAGSDFLLSAMAFD-RYVSICKPLKYPVIMRQTTINILLFLSWFVPGLQIA-VLHTLVLNNKLC
contig022264-TilOR.A016   FIAALSVNSVLLSTVTYPKLFVDVLSEKQV--ITFSACRFQHFMCYSIAGSDILLSAMAFD-RYVSICKPLKYPDIMRQTTINILLFLSWFVPGLQVA-VLHALVPNKKLC
contig036784-BurOR.A003   FIAALSVNSVVFSTAIYPKLFVDVLSEKQV--ISFSACQFQHFMYYSIGGSDFLLSAMAFD-RYVSICKPLKYPVIMRQTTINILLFVAWFLPGLQVA-VSHALVLNNKLC
contig057754-NyeOR.A018   FIAALSVNSVVFSTAIYPKLFVDVLSEKQV--ISFSACQFQHFMYYSIGGSDFLLSAMAFD-RYVSICKPLKYPVIMRQTTINILLFLAWFLPGLQVA-VSHALVLNNKLC
contig030553-ZebOR.A003   FIAALSVNSVVFSTAIYPKLFVDVLSEKQV--ISFSACQFQHFMYYSIGGSDFLLSAMAFD-RYVSICKPLKYPVIMRQTTINILLFLAWFLPGLQVA-VSHALVLNNKLC
contig085000-BriOR.A003   FIAALSVNSVVFSTAIYPKLFVDVLSEKQV--ISISACQFQHFMYYSIGGSDFLLSAMAFD-RYVSICKPLKYPVIMRQTTINILLFLAWFLPGLQVA-VSHALVLNYKLC
contig009320-TilOR.B045   FVAAVLMNSVAGSTVFYPKLLVDLLRGGRSVQVTLRGCMCEAWLLYSSGTSSFLLAAMSFD-RYVSICRPLLYTVVMSPATVLALLLLCWLLPVGLVG-TAVLLASRLPLC
contig053886-ZebOR.B034   FVTAVLMNSVAGSTVFYPKLLVDLLRGVRSVQVTLRVCMCEAWLLYSLGTSSFLLAAMSFD-RYVSICRPLLYTVVMSPATVLALLLLCWLLPVGLVG-TAVLLASRLPLC
contig044492-NyeOR.B034   FVTAVLMNSVAGSTVFYPKLLVDLLRGVRSVQVTLRVCMCEAWLLYSLGTSSFLLAAMSFD-RYVSICRPLLYTVVMSPATVLALLLLCWLLPVGLVG-TAVLLASRLPLC
contig040653-BurOR.B030   FVTAVLMNSVAGSTVFYPKLLVDLLRGVRSVQVTLRVCMCEAWLLYSLGTSSFLLAAMSFD-RYVSICRPLLYTVVMSPATVLALLLLCWLLPVGLVG-TAVLLASRLPLC
contig049299-BurOR.E046   FLCSLFVNELYGSTGLFPFLLLQILSDVHT--VSAPLCFLQIFCVFSYVCVEFCILAVMSYD-RYLAICCPLQYHTRMTPATVVLLIALSWLYSFLTIL-ALILLIAPLELC
contig053579-NyeOR.E053   FLCSLFVNELYGSTGLFPLLLLQILSDVHT--VSAPLCFLQIFCVFSYVCVEFCILAVMSYD-RYLAICCPLQYHTRMTPATVVLLIALSWLYSFLTIL-TLILLIAPLELC
contig017699-BurOR.E042   FLCSLFVNELYGSTGLFPSLLVQILSDVHT--VSAPLCFLQVFSVYSYGSIEFLNLAVMSYD-RYLAICCPLQYNELMTSNKVTKLIVAVWSPPLLVNF-LTLPLIVPLKRC
contig023280-NyeOR.E050   FLCSLFVNELYGSTGLFPSLLVQILSDVHT--VSAPLCFLQVFSVYSYGSIEFLNLAVMSYD-RYLAICCPLQYNELMTSNKVTKLIVAVWSPPLLVNF-LTLPLIVPLKRC
contig004266-BriOR.E039   FLCSLFVNELYGSTGLFPFLLVQILSDVHT--VSAPLCFLQVFSVYSYGSIEFLNLAVMSYD-RYLAICCPLQYNELMTSNKVTKLIVAIWSPPLLVNF-LTLPLIVPLKRC
contig025447-ZebOR.E047   FLCSLFVNELYGSTGLFPFLLVQILSDVHT--VSAPLCFLQVFSVYSYGSIEFLNLAVMSYD-RYLAICCPLQYNKLMTSNKVTKLIVAVWSPPLFLNF-LTLPLIVPLKRC
contig004265-BriOR.E035   FLCSLFVNELYGSTGLFPSLLVQILSDVHT--VSAPLCFLQIFCVHIYGAVEYLNLAIMSYD-RYLAICCPLQYNTHMTSKKIGILIAATWFYPCFAMA-PLLYLTSPLQLC
contig065454-TilOR.E088   FLCSLFVNELYGSTGLFPSLLVQILSDVHT--ISADICFLQVFCVHTYGAVEYLNLAIMSYD-RYLAICCPLQYSTHMTSKKIGILIAVTWFYPCFAMV-VLLSLTSPLQLC
contig065454-TilOR.E089   FLCSLFVNELYGSTGLFPSLLVQILSDVHT--VSADICFLQVFCVHLYGAVEYLNLAIMSYD-RYLAICCPLQYSTHMTSKKIGILIAVTWFYPCFAMA-PLLYLTSRLQLC
contig052450-BurOR.E055   FLCSLFVNELYGSTGLFPPLLVQILSDVHT--VSTGICFLQVFCVHSYGAVEYLNLAIMSYD-RYLAICCPLQYNTHMTSKKIAILIAATWFYPCFAMV-VLLSLTSPLQFC
contig004261-BriOR.E034   FLCSLFVNELYGSTGLFPSLLVQILSDVYT--VSAPLCFLQVFCVHSYGAVEYLNLAIMSYD-RYLAICCPLQYNTHMTSKQIGILIAATWFYSCFAMV-VLLSLTSPLQFC
contig065453-TilOR.E087   FLCSLFVNELYGSTGLFPSLLVQILSDVHT--ISADICFLQVFCVHSYGAVEYLNLAIMSYD-RYLAICCPLQYSTHMTSKKIGILIAVTWFYPCFAMV-VLLSLTSPLQLC
contig062770-NyeOR.E059   FLCSLFVNELYGSTGLFPFLLLQILSDVHT--VSAPLCFLQIFCVHTYGTAELANLAVMSYD-RYLAICFPLQYHTRMSPCKVSMLIVLTWFSSFLVIT-VLISLSAPLQLC
contig048239-ZebOR.E048   FLCSLFVNELYGSTGLFPFLLLQILSDVHT--VSAPLCFLQIFCVHTYGTAELANLAVMSYD-RYLAICFPLQYHTRMSPCKVSMLIVLTWFSSFLVIT-VLISLSAPLQLC
contig052457-BurOR.E051   FLCSLFVNELYGSTGLFPLLLLQILSDVHT--VSAPLCFLQIFCVHTYGTAELANLAVMSYD-RYLAICFPLQYHTRMSPCKVSMLIVLTWFSSFLVIT-VLISLSAPLQLC
contig047729-TilOR.E076   FLCSLFVNELYGSTGLFPSLLIQILSDVHT--VSAPLCFLQVFSVYSYGSVEFASLAVMSYD-RYLAICYPLEYNMRTTSCKVAMLIALSWLSPFLAIV-VLMSLLVPLQLC
contig047734-TilOR.E077   FLCSLFVNELYGSTGLFPFLLLQILSDVHT--VSAPLCFLQIFCLHTYANAQLTNLAIMSYD-RYLAICCPLQYHTRMSSSKVSMLIALTWLYPFLAIT-LVISLSAPLQLC
contig059404-NyeOR.E058   FLCSLFVNELYGSTGLFPLLLLQILSDIHT--VSAPLCFLQIFCLYSYANLQLSNLAIMSYD-RYLAICFPLQYHTRMSPCKVSMFIVLTWFSSFLVIT-VLISLSAPLQLC
contig052453-BurOR.E049   FLCSLFVNELYGSTGLFPLLLLQILSDVHT--VSAPLCFLQIFCLYSYANLQLSNLAIMSYD-RYLAICFPLQYHTRMSPCKVSMLIVLTWFSSFLVIT-VLISLSAPLQLC
contig064938-BurOR.E053   FLCSLFVNELYGSTGLFPLLLLQILSDVHT--VSAPLCFLQIFCVHTYGTAELANLVVMSYD-RYLAICFPLQYHTRMSPCKVSMLIVLTWFSSFLGIT-VLISLSAPLQLC
contig004258-BriOR.E037   FLCSLFVNELYGSTGLFPFLLLQILSDVHT--VSASFCFLQIFCVYAYGSIEFSNLAVISYD-RYLAICCPLRYHTCMSSSKVSVLIALTWLLTFFAIS-VLISLSSHLQLC
contig052452-BurOR.E048   FLCSLFVNELYGSTGLFPLLLVQILSDVHT--VSASFCFLQIFCVYAYGSIEFSNLAVISYD-RYLAICCPLRYHTCMSSSKVSVLIALTWLLTFFAIS-VLISLSAPLQLC
contig025443-ZebOR.E046   FLCSLFVNELYGSTGLFPSLLVQILSDVHT--VSASFCFLQIFCVYAYGSIEFSNLAVISYD-RYLAICCPLRYHTCMSSSKVSVLIALTWLLTFFAIS-VLISLSAPLQLC
contig065458-TilOR.E086   FLCSLFVNELYGSTGLFPLLLLQILSDVHT--VSASFCFLQIFCFYAYGSIEFSNLAVISYD-RYLAICCPLRYHTCMSSSKVSVLIALTWLLTFLAVG-VLISLSAPLQLC
contig047832-TilOR.E082   FLCSLFVNELYGSTGLFPSLLVQILSDVHT--VSAPLCFLQIFCLYSYGSVEFYNLATMSYD-RYLAICCPLQYNTRMTSNTVSVLIAVSWIYALLVA-VTVSLSSPLQLC
contig049298-BurOR.E045   FLCSLFVNELYGSTGLFPFLLLQILSDVHT--VSAPLCFLQIFSLYSYVGVEFLTLAVMSYD-RYLAICCPLQYNTRMTSSTVSVLIAVSWIYALLVA-VTVSLSSPLQLC
contig048260-ZebOR.E051   FLCSLFVNELYGSTGLFPLLLLQILSDVHT--VSAPLCFLQIFSLYSYVVVEFLTLAVMSYD-RYLAICCPLQYNMRMTSSTVSVLIAVSWIYALLVA-VTVSLSSPLQLC
contig053579-NyeOR.E054   FLCSLFVNELYGSTGLFPLLLLQILSDVHA--VSAPLCFLQIFSLYSYGSVEFLTLAVMSYD-RYLAICCPLQYNTRMTSSTVSVLIAVSWIYALLVA-VTVSLSSPLQLC
contig047833-TilOR.E083   FLCSLFVNELYGSTGLFPLLLLQILSDVHT--VPAPLCFLQIFCLYSYVGVEFLTLAIMSYD-RYLAICCPLQYNTRMTSNTVSVLIAVSWIYALLVA-VTVSLSSPLQLC
contig053579-NyeOR.E055   FLCSLFVNELYGSTGLFPLLLLQILSDVHA--VSAPLCFLQIFCLYSYVGVEFLTLAVMSYD-RYLAICCPLQYNTRMTSSTVSVLIAVSWIYALLVA-VTVSLSSPLQLC
contig047829-TilOR.E081   FLCSLFVNELYGSTGLFPFLLLQILSDVHT--VSAPLCFLQVFCVFTYAGVGLLNLAVMSYD-RYLAICYPLQYRTLMTMWKVAFLIAVTWLPRFLVVV-IITSLSASLQLC
contig053576-NyeOR.E052   FLCSLFVNELYGSTGLFPLLLLQILSDVHT--VSAPLCFLQVFSIYLYASVEFSNLAAMSYD-RYMSICYPLQYHTLMMSNKVALLIAVTWIPPLLAVC-VTTCLSASLQLC
contig064724-BurOR.E052   FLCSLFVNELYGSTGLFPLLLLQILSDVHT--VSAPLCFLQVFCIYLYASVEFSNLAAMSYD-RYMSICYPLQYHTLMMSNKVALLIAVTWIPPLLAVC-VTTCLSASLQLC
contig048263-ZebOR.E052   FLCSLFVNELYGSTGLFPFLLVQILSDVHT--VSAPLCFLQVFSIYLYGSVEFSNLAAMSYD-RYMSICYPLQYHTLMMSNKVALLIAVTWIPPLLAVC-VTTCLSASLQLC
contig047826-TilOR.E080   FLCSLFVNELYGSTGLFPLLLLQILSDVHT--VSAPLCFLQVFCIYLYASVEFSNLAAMSYD-RYMSICYPLQYHTLMTSNKVALLIAVTWIPPLLAVC-VTTCLSASLQLC
contig025439-ZebOR.E045   FLCSLFVNELYGSTGLFPFLLLQILSDVHT--VSAPLCFLQIFCLYTYANVEFYNLAIMSYD-RYLAICYPLQYHTHMTFNKVAKLIVLTWLFPILNIV-VMISLNASLQLC
contig052454-BurOR.E050   FLCSLFVNELYGSTGLFPFLLLQILSDVHT--VSAPLCFLQIFCLYTYANVEFYNLAIMSYD-RYLAICYPLQYHTHMTFNKVAKLIVLTWLFPILNIA-VMISLNASLQLC
contig004255-BriOR.E036   FLCSLFVNELYGSTGLFPFLLLQILSDVHT--VSAPLCFLQIFCLYTYANVEFYNLAIMSYD-RYLAICYPLQYHTHMTFNKVAKLIVLTWLFPILNIV-VMISLNASLQLC
contig066194-BurOR.E054   FLCSLFVNELYGSTGLFPFLLLQILSDVHT--VSVSFCFLQVFCVYTYACVEFINFVVMSYD-RYYAICWPLQYKSCMTLKTVTTLISLTWLLPFIMIV-VLISLSAPLQLC
contig053572-NyeOR.E051   FLCSLFVNELYGSTGLFPFLLLQILSDVHT--VSVSFCFLQVFCVYTYACVEFINFVVMSYD-RYYAICWPLQYKSCMTLKTVTTLISLTWLLPFIMIV-VLISLSAPLQLC
contig047825-TilOR.E079   FLCSLFVNELYGSTGLFPFLLLQILSDVHT--VSISFCFLQVFCVYTYACVEFINLVIMSYD-RYYAICWPLQYKSYMTQKTVTMLISLTWLLPFIMIV-VLISLSAPLQLC
contig047820-TilOR.E078   FLCSLFVNELYGSTGLFPFLLLQILSDVHT--VSAPLCFLQIFCVYTYASVEFFNLAVMSFD-RYLAICCPLQYRTRMTDNKVAMLTALAWFYSFLVNASILSSLTVSLQLC
contig063018-ZebOR.E053   FLCNLFVNALYGSTSLFPLLLLHIICDINI--ISASLCYLQIYCIHCYGSAEYLNLAVMSYD-RYLAICFPLQYNTYMTPKRIAILIAITWLYAILACA-LMISLSSTLPLC
contig004259-BriOR.E038   FLCSLFVNALYGSTSLIPLLLLHIICDINI--ISASLCYLQIYCIHCYGSAEYLNLAVMSYD-RYLAICFPLQYNTYMTPKRIAILIAITWLYAILACA-LMISLSSTLPLC
contig052451-BurOR.E047   FLCSLFVNALYGSTSLFPLLLLHIICDINI--ISASLCYLQIYCIHCYGSAEYLNLAVMSYD-RYLAICFPLHYNTYMTPKRIAILIAITWLYAILACA-LMISLSSTLPLC
contig065455-TilOR.E085   FLCSLFVNALYGSTGLFPSLLLHIICDINI--ISASLCYLQIYCIHCYGSAEYLNLAVMSYD-RYLAICFPLQYNTYMTPKRIAILIAITWLYAILACA-LMISLSSTLPLC
contig082838-BriOR.E040   FLCSLFVNELYGSTGLFPFLLLQILSDVHT--VSAPLCFLQIFCLYTYGHVEFCNLAVMSYD-RYLAVCYPLHYKSHMTDNKVVIFIVVIWLYSFVKFT-ITLCLTLHLTWC
contig053592-NyeOR.E057   FLCSLFVNELYGSTGLFPSLLLQILSDVHT--VSAPLCFLQIFCLYTYGHIEFCNLAVMSYD-RYLAVCYPLHYKSHMTDNKVVIFIVVIWLYSFVKFT-ITLCLTLRLTWC
contig049287-BurOR.E043   FLCSLFVNELYGSTGLFPFLLLQILSDVHT--VSAPLCFLQIFCLYTYGHVEFCNLAVMSYD-RYLAVCYPLHYKSHMTDNKVVILIVVIWLYSFVKFT-ITLCLTLRLTWC
contig048242-ZebOR.E049   FLCSLFVNELYGSAGLFPFLLLQILSDVHT--VSAPLCFLQIFCLYTYGHVEFCNLAVMSYD-RYLAVCYPLHYKSHMTDNKVVIFIVVIWLYSFVKFT-ITLCLTLRLTWC
```

```
contig047726-TilOR.E075   FLCSLFVNELYGSTGLFPFLLLQILSDVHT--VSAPLCFLQIFCLYTYGHVEFCNLAVMSYD-RYLAVCYPLHYKSHMTDNKVVIFIVVIWLYSFVKFT-ITLCLTLRLTWC
contig053590-NyeOR.E056   YLCSLFVNELYGSTGLFPSLLVQILSDVHT--VSTPLCFLQIFCIHTYGSIEVSNLAVMSYD-RYLAICCPLQYNTQMTSNNTAVLIMVMWAYSLAKFL-IALSLNLRLRLC
contig049289-BurOR.E044   FLCSLFVNELYGSTGLFPFLLLQILSDVHT--VSAPLCFLQIFCIHTYGSIEVSNLAVMSYD-RYLAICCPLQYNTQMTSNNTAVLIVVMWAFSLAKFL-IALSLNLRLRLC
contig048243-ZebOR.E050   FLCSLFVNELYGSAGLFPFLLLQILSDVHT--VSAPLCFLQIFCIHTYGSIEVSNLAVMSYD-RYLAICCPLQYNTQMTSNNTAVLIMVMWAYSLAKFL-IALSLNLRLRLC
contig047725-TilOR.E074   FLCSLFVNELYGSTGLFPFLLLQILSDVHT--VSAPLCFLQIFCIHTYGSVEVSNLAIMSYD-RYLAICCPLQYNTRMTSNSTAVLIMVTWAYSLVKFL-IALSLNLRLRLC
contig047834-TilOR.E084   FLCSLFVNELYGSTGLFPFLLIQILSDVHT--VPAPLCFLQVYCLYTYAVIEFCILAVMSYD-RYLAVCCPLQYNAIMTYNKVFLFIISVCLFSFINML-IGLSLTLRLTLC
contig014049-ZebOR.D038   LLCNLCVNEINASTSLYPLLLSQMFSDSHE--VTLPWCFLQMCCLYTSASVELCSLAAMAYD-RYISICHPLRYNVIMKAERVFLLILLVWVYSFLSFI-FSFSFIFSLKFC
contig013327-TilOR.D052   LLCNLCVNEINASTSLYPLLLSQMFSDSHE--VAVPWCFLQMCCLYTSASVELCSLAAMAYD-RYISICHPLRYNVIMNTERVFLMILLVWVYSFLSFI-FSFSFIFSLKFC
contig039737-NyeOR.D039   LLSNLCVNEINASTSLYPLLLSQMFSDSHE--VTVPWCFLQMCCLYTSAPAELCSLAAMAYD-RYISICHPLRYNIIMNTERVFLMILLVWVYSFLSFI-FSFSFIFSLKFC
contig032396-BurOR.D034   LLCNLCVNEINASTSLYPLLLSQMFSDSHE--VTVPWCFLQMCCMYTSAPAEFCSLAAMAYD-RYISICHPLRYNVIMKTERVFLLILLVWVYSFLSFI-FSFSFIFSLKFC
contig064809-BriOR.D028   LLCNLCVNEINASTSLYPLLLSQMFSDSHE--VTVPWCFLQMCCMYTSAPAEFCSLAAMAYD-RYVSICHPLRYNIIMKTERVFLLILLVWLYSFLSFI-FSFSFIFSLKFC
contig014050-ZebOR.D039   LLCNLCVNEINASTSLYPLLLSQMFSDSHE--VTVPWCFLQMCCLYTSAPAEFCSLAAMAYD-RYISICHPLRYNVIMNTERVFLLILLVWLYSFLSFI-FSFSFIFSLKFC
contig013326-TilOR.D051   LLNNLCVNEINASTSLYPLLLSQMFSDSHE--VTLPWCFLQMCCMYTSGPVEFCSLAAMAYD-RYISICHPLRYNVIMKTERVFLLILLVWLYSFLSFI-FSFSFIFSLKFC
contig014051-ZebOR.D040   LLCNLCLSEINGSTSLYPLLLSQMFSDSHE--VTVPWCFLQMFCLYTSASVEICSLAAMAYD-RYISICNPFTYNVIMNTDRVFLMILLVWVYSFLSVI-FSYSFIFSLKFC
contig013337-TilOR.D055   LLCNLCVNEINGSTSLYPLLLSQMFSDSHE--VTLSWCFLQMCCLYTSASVELCSLAAMAYD-RYISICHPFTYNVIMNTERVFLMILLVWVFSFLSFI-FSYSFIFSLKFC
contig013330-TilOR.D054   LLCNLCVSEINGSTSLYPLLLSQMFSDSHE--VTLSWCFLQMCCLYTSASVELCSLAAMAYD-RYISICHPFTYNVIMNTERVFLMILLVWVFSFLSFI-FSYSFIFSLKFC
contig013339-TilOR.D057   LLCNLCVSEINGSTSLYPLLLSQMFSDSHE--VTLSWCFLQMCCLYTSASVELCSLAAMAYD-RYISICHPFTYNVIMNTERVFLMILLVWVFSFLSFI-FSYSFIFSLKFC
contig032389-BurOR.D033   LLSNLCVNEINASTSLYPLLLSQMYSDSHE--VTLPWCYLQMCCLYTSAPAEFWSLAAMAYD-RYISICHPLRYNAIMNTERVLKIILLVWVFSFLIFI-LSFSFIFSLQFC
contig039737-NyeOR.D040   LLSNLCVNEINASTSLYPLLLSQMYSDSHE--VTLPWCYLQMCCLYTSAPAEFWSLAAMAYD-RYISICHPLRYNVIMNTERVLKIILLVWVFSFLIFI-LSFSFIFSLQFC
contig013339-TilOR.D056   LLSNLCVNEINASTSLYPLLLSQMFSDSHE--VTLPWCFLQMCSLYTGAPAEFWSLAAMAYD-RYVSICHPLRYNVIMNTERVLKIILLVWVFSFLIFI-LSFSFIFSLKFC
contig013330-TilOR.D053   LLSNLCMNEINASTSLYPLLLSQMFSDNHE--VTRLWCFLQMCSLYTGAPAEFWSLAAMAYD-RYISICHPLRYNVIMNTDRVFLLILLVWVFSFLIFI-LSFSFIFRLKFC
contig064814-BriOR.D029   LLSNLCVNEINCSTSLYPLLLSQMFSDSHE--VTVPWCFLQMCCMYISGPAEFWSLAAMAYD-RYIAICHPFSYNVIMNTEKVGMLILLVWIFSFVSFL-LSFSFIFGLRFC
contig014054-ZebOR.D041   LLSNLCVNEINASTALYPLLLSQMFSDSHE--VTLPWCFLQMCCMYISGSAEFCSLTAMAYD-RYIAICHPFSYNVIMNTERVGMLILLVWIFSFVSFL-LSFSFIFGLRFC
contig039731-NyeOR.D038   LLSNLCVNEINASTSLYPFLLSQMFSDSHE--VTLPWCFLQMCCMYTSASAEFCSLAAMAYD-RYIAICHPFSYNVIMNTERVVMLILLVWIFSFVSLL-LSFSFIFGLRFC
contig014054-ZebOR.D042   LLSNLCVNEINGSTSMYPLLLSQMFSDSHE--VTLPWCFLQMCCMYTTASAEFCSLAAMAYD-RYISICHPLRYNVIMNTERVFILILLVWVYSFLIFI-FSFSFIFSLTFC
contig039730-NyeOR.D036   LLSNLCVNEINGSTSMYPLLLSQMFSDSHE--VTLPWCFLQMCCMYTTASAEFCSLAAMAYD-RYISICHPLRYNITMNTERVFMLILLVWVYSFLIFI-FSFSFIFSLKFC
contig053779-BurOR.D035   LLSNLCVNEINGSTSMYPLLLSQMYSDSHE--VTLPWCFLQMCCMYTTASAEFCSLAAMAYD-RYISICHPLRYNVIMNTERVFMLILLVWVYSFLIFI-FSFSFIFSLKFC
contig039730-NyeOR.D037   LLSNLCVNEINASTSLYPLLLSQMFSDSHE--VTLPWCFLQMCCMYTSASAEFCSLAAMAYD-RYISICHPLRYNVIMNTERVFMLILVVWMYSFLSFI-FSFSFIFRLMFC
contig013344-TilOR.D058   LLCNLCVNEINSSTSLYPLLLSQMFSDIHG--ITLTWCFLQMSCMYTSASVEFCSLAAMAYD-RYISICHPFSYNVIMNTERVFLLILLVWIFSFVSFL-LSYSFIYSLKFC
contig014049-ZebOR.D037   LLSNLCVNEINASTSLYPLLLSQMFSDSHE--VTVPWCFLQMCFMYISAPAEFWGLAAMAYD-RYISICHPLCYNVIMNTKRVFLLILLVWIFSLVSFI-LSFSFIFGLKFC
contig032388-BurOR.D032   LLCSLCVNEINSSTSLYPLLLSQMFSDSHE--VTVPWCFLQMCFMYISAPAEFWGLAAMAYD-RYISICHPLCYNVIMNTKRVFLLILLVWIFSLVSFI-LSFSFIFGLKFC
contig039738-NyeOR.D041   LLCSLCVNEINSSTSLYPLLLSQMFSDSHE--VTVPWCFLQMCFMYISAPAEFWGLAAMAYD-RYISICHPLCYNVIMNTKRVFLLILLVWIFSLVSFI-LSFSFIFGLKFC
contig013323-TilOR.D050   LLCSLCVNEINSSTSLYPLLLSQMFSDSHE--VTLPWCFLQMCCMYISAPSEFWGLAAMAYD-RYISICHPLRYNVIMNTKRVFLLILLVWIFSLVSFM-FSFSFIFSLKFC
contig014047-ZebOR.D036   LLCNLCVNQINVSTSLYPLLLSQMFSDSHE--VTLPWCFLHMSSMYISGPAEFCSLAAMAYD-RYISICHPLRYNVIMNTERVFFMILFVWIYSFLSVI-LSFSFIFSLKFC
contig064802-BriOR.D027   LLCNLCVNEINVSTSLYPLLLSQMFSDSHE--VTVPWCFLHMFCLYISGPAEFCSLAAMAYD-RYISVCRPLRYNVIMNTERVFFMILFVWIYSFLSVI-LSFSFIFSLKFC
contig013322-TilOR.D049   LLCNLCVNEINISTSLYPLLLSQMFSDSHE--VTLPWCFLQMCCMYTSAPAEFCSLAAMAYD-RYISVCHPLHYNVIMNTERVFFMILLVWIYSFLSVI-LSFSFIFSLKFC
contig039738-NyeOR.D042   LLCNLCVNEINISTSLYPLLLSQMFSDRHE--VTVPWCFLQMCFMYTSAPAEFCSLAAMSYD-RYISICHPLRYNVIMNTERVFFMILLVWIYSFLSVI-LSFSFVFSLKFC
contig013321-TilOR.D048   LLCNLCVNEINVSTSLYPLLLSQMFSDSHE--VTLPWCFLHMSCMYTSGPAEFCSLAAMAYD-RYISICHPLHYNVIMKTKRVFVMILFVWIYSFLSVI-LSFSFIFSLKFC
contig066327-ZebOR.F064   FIMCLSINSLYGSGGFFRFLRDLLSDSNL--IAHSACYTQIYIIYTYASYELTILGIMAYD-RFVAICQPLHYHSKLTSRVISKLLAFAWIYPAFSVA-ACVYLASRLPLC
contig013898-BurOR.F058   FIMCLSINSLYGSGGFFRFLRDLLSDSNL--IARSACYTQIYIIYTYASYELTLLGIMAYD-RFVAICQPLHYHSKLTSRVISKLLAFAWIYPAFSVA-ACVYLASRLPLC
contig033883-BriOR.F045   FIMCLSINSLYGSGGFFRFLRDLLSDSNL--IARSACFTQIYIIYTYASYELTILGIMAYD-RFVAICQPLHYHSKLTSRVISKLLAFAWIYPAFSVA-ACVYLASRLPLC
contig009545-TilOR.F097   FIMCLSINSLYGSGGFFRFLRDLLSDSNL--IARSACFTQIYIIYTYASYELTILGIMAYD-RFVAICQPLHYHSKLTSRVISKLLAFAWIYPAFSVA-ACVYLASRLPVC
contig039729-NyeOR.F064   FIACLSVNALYGSSGFFPRFLMDLLSDTHL--ISRPACFTQIYVIYSYASCELTVLSIMAYD-RYIAVCLPLHYHTKMTLETVVKLTALAWIFPAFSLA-ACLCLSATLPLC
contig053781-BurOR.F059   FIACLSVNALYGSSGFFPRFLMDLLSDTHL--ISRPACFTQIYVIYSYASCELTVLSIMAYD-RYIAVCLPLHYHTKMTLETVVKLTALAWIFPAFSLA-ACLCLSATLPLC
contig014056-ZebOR.F063   FIACLSVNALYGSSGFFPRFLMDLLSDTHL--ISRPACFTQIYVIYSYASCELTVLSIMAYD-RYIAVCLPLHYHTKMTLETVVKLTELAWIFPAFSLA-ACLCLSATLPLC
contig075822-TilOR.F098   FIACLSVNALYGSSGFFPRFLMDLLSDTHL--ISRPACFTQIYVIYSYASCELTVLSIMAYD-RYIAVCLPLHYHTKMTLKTVVKLTALAWILPAFSLA-ACLCLSATLPLC
contig025224-ZebOR.C035   FTCMLSLNEIYGSSALLPAVMAVLVSKTHD--VTVKWCMAQAYFLHTYASGEFCILALMGYD-RYIAICSPLHYYSIMSYSKTCKLIAFTGLYPFIVFT-SFYSLTLQLRFC
contig009805-BurOR.C031   FTCMLSLNEIYGSSALLPAVMAVLVSKTHD--VTVKWCMAQAYFLHTYASGEFCILALMGYD-RYIAICSPLHYYSIMSYSKTCKLIAFTGLYPFIVFT-SFYSLTLQLRFC
contig020980-NyeOR.C035   FTCMLSLNEIYGSSTLLPAVMAVLVSKTHD--VTVKWCMAQAYFLHTYASGEFCILALMGYD-RYIAICSPLHYYSIMSYSKTCKLIAFTGLYPFIVFT-SFYSLTLQLRFC
contig063874-BriOR.C026   FTCLLSLNEIYGSSALLPAIVAVLVSKTHD--VTVKWCMAQAYFLHTYASGEFCILALMGYD-RYVAICSPLHYYSIMSYSKTCKLIAFTGLYPFIVFT-SFYSLTLQLRFC
contig048237-ZebOR.G065   FLCSLCFNEICGASSFYPKLLHDLLTNSYV--ITYTACLGQMFVTYSYIFSECTSLTVMAYD-RYIAICKPLQYRMLMTAQKVAQLLMLTWCFSVFETA-VGTVLTARLPLC
contig049295-BurOR.G060   FLCSLCFNEICGASSFYPKLLHDLLTNSYV--ITYTACLGQMFVTYSYIFSEFTSLTVMAYD-RYIAICKPLQYRMLMTAQKVAQLLMLTWCFSVFETA-VGTVLTARLPLC
contig053581-NyeOR.G066   FLCSLCFNEICGASSFYPKLLHDLLTNSYV--ITYTACLGQMFVTYSYIFSEFTSLTVMAYD-RYIAICKPLQYRMLMTAQKVAQLLMLTWCFSVFETA-VGTVLTARLPLC
contig104344-BriOR.G048   FLCSLCFNEICGASSFYPKLLHDLLTNSYV--ITYAACLGQMFVTYSYIFSEFTSLTVMAYD-RYIAICKPLQYRMLMTAQKVTQLMMLTWCFSVFETA-VGAVLTARLPLC
contig047714-TilOR.G099   FLCSLCFNEICGASSFYPKLLHDLLTNSYV--ITYTACLGQMFVSYSYIFSEFTGLTVMAYD-RYIAICKPLQYRMLMTAQKVAQLLMLTWCFSVFETA-VGTVLTARLPLC
contig057403-ZebOR.H076   LLSSFCINEIYGTTGFYPKFLLDLLSSSQE--ISYEGCLLQAFIMYSFACCDLSILAVMAFD-RYLAICRPLHYHSFMTKRRLSQLVCFSWLTPLCIFS-ISILLTSRLTLC
contig013371-TilOR.H117   LLSSFCINALYGTTGFYPKFLLDLLSSSQE--ISYEGCLLQALIMYSFACCDLSILAVMAFD-RYLAICRPLHYHSFMTKRRLSQLMCFSWLTPLCIIS-ISILLTSRLTLC
contig035582-NyeOR.H070   FLSSFCISALYGTTGFYPKFLSDLLSSFHH--ISYEGCLLQAFIMYLFPCCDLSILAVMAFD-RYLAICRPLHYHSFMTKKRLSQLVCFSWLTPLCILS-INILLTSRLTLC
contig013361-TilOR.H108   LLSSSCINALYGTTGFYPKFLSDLLLSSQT--ISYEGCLLQAFIMYSFASCDLSILAVMAFD-RYLAICRPLHYHSFMTKRRLSQLVSFSWLTPLCIFT-INVLLTSRIKLC
contig013362-TilOR.H109   LLSSFCVNALYGTTGFYPKFLSDLLSSTYN--ISYEGCILQAFIMYSFGCCELSILTVMAFD-RYLAICRPLHYHSFMTKRRLSQLVCFSWLTPLCMFS-INIILTSRIKLC
contig013368-TilOR.H112   FLSSFCINALYGSTGFYPKFLSDLLSSFHG--ISYEGCLLQAFIMYSFASCDLTILAVMAFD-RYLAICRPLHYHSLMTKRRLSQLVCFSWLTPFCIFS-INVILTTRLRLC
contig013369-TilOR.H115   FLSSFCINALYGSTGFYPKFLSDLLSSSQR--ISYEGCLLQAFIMYSFVCSDLSILAVMAFD-RYLAICRPLHYHSFMTKRRLSQLVCFSWLTPFCIFS-INVILTTRLRLC
contig013369-TilOR.H116   LLCIFCINAIYGSTGFYPKFLSDLLSSSHR--ISYEGCLLQAFIMYSFACSDLSILAVMAFD-RYLAICRPLHYHSFMTKRRLSQLVCFSWLTPFCIFS-INILITTRLKLC
contig013369-TilOR.H114   LLSSFCINAVYGTTGFYPKFLSDLLSSSQT--ISYEGCLLQAFIMYSFGCCDLSILAVMAFD-RYLAICRPLHYHSFMTKRRLSQLVCFSWLTPLCTFS-INIVLTSKLRLC
contig048882-BurOR.H064   FLSSFCINAIYGTTGFYPKFLSDLLSSSQT--ISYEGCLLQAFIMYSFGYCDLSLLAVMAFD-RYLAICRPLHYHSFMTKRRLSQLVCFSWLTPLCIFS-INILLTSKLRLC
contig035580-NyeOR.H069   FLSSFCINALYGSTGFYPKFLSDLLSPSQT--ISHEGCLLQAFIMYSSVCCNSSILAVMAFD-RYLAICRPLHYHSFMTKRRLSQLVCFSWLTPFCIFA-INVVLTARLKLC
contig057400-ZebOR.H075   FLSSFCINALYGSTGFYPKFLSDLLLPSQT--ISHEGCLLQAFIMYSSVCCNSSILAVMAFD-RYLAICRPLHYHSFMTKRRLSQLVCFSWLTPFCIFA-INVVLTARLKLC
contig013368-TilOR.H113   LLSSFCINALYGSTGFYPKFLLDLLSSSHR--ISYEGCLLQAFIMYSFACSDLSILAVMAFD-RYLAICRPLHYHSFMTKRRLSQLVCFSWLTPFCIFF-LNILITSRLKLC
contig006794-BurOR.H061   FLSSFCINTIYGTTGFYPKFLSDLLWSSQT--ISYEGCLLQAFIIYSFVCCDLSILAVMAFD-RYLAICRPLHYHSFMTKRRLSQLVCFSWLTPLCIFA-INVLLTSRLKLC
```

```
contig035583-NyeOR.H071   FLSSFCINALYGTTGFYPKFLSDLLRSSQT--ISYEGCLLQAFIIYSFVCCDLSILAVMAFD-RYMAICRPLHYHSFMTKRRLSQLVCFSWLTPSCIFA-INVLLTSRLKLC
contig057403-ZebOR.H077   FLSSFCINAIYGTTGFYPKFLSDLLRSSQT--ISYEGCLLQAFVIYSFVCCDLSILAVMAFD-RYLAICRPLHYHSFMTKRRLSQLVCFSWLTPLCIFA-INVLLTSRLKLC
contig049873-BriOR.H050   FLSSFCINTIYGTTGFYPKFLSDLLRSSQT--ISYEGCLLQAFVIYSFVCCDLSILAVMAFD-RYLAICRPLHYHSFMTKRRLSQLVCFSWLTPLCIFA-INVLLTSRLKLC
contig013371-TilOR.H118   LLSSFCINAIYGTTGFYPKFLSDLLSSSQR--ISYEGCLLQAFVIYSFVCCDLSILAVMAFD-RYLAICRPLHYHSFMTNRRLSQLVCFSWLTPLCIFA-INVLLTSRLKLC
contig093825-BriOR.H053   LVCVCCINGLYGSTGFYPKFLIDLLSSSQV--ISYSECLCQAFVMYSFVCSDTSILAVMAYD-RYLAICQPLEYHSVMTKKKLSKLVCFSWLTPFCIFS-INIMLTDRLIFC
contig048562-BurOR.H062   LVCVCCINGLYGSTGFYPKFLIDLLSSSQV--ISYSECLCQAFVMYSFVCSDTSILAVMAYD-RYLAICQPLQYHSVMTKKKLSKLVCFSWLTPFCIFS-INIMLTDRLIFC
contig034998-NyeOR.H067   LVCVCCINGLYGSTGFYPKFLIDLLSSSQV--ISYSECLCQAFVMYSFVCSDTSILAVMAYD-RYLAICQPLQYHSVMTKKKLSKLVCFSWLTPFCIFS-INIMLTDRLIFC
contig047492-ZebOR.H074   LVCVCCINGLYGSTGFYPKFLIDLLSSSQV--ISYSECLCQAFVMYSFVCSDTSILAVMAYD-RYLAICQPLQYHSVMTKKKLSKLVCFSWLTPFCIFS-INIMLTDRLIFC
contig041955-TilOR.H119   LVCVCCINGLYGSTGFYPKFLIDLLSSSQV--ISYTGCLCQAFVMYSFVCSDTSILAVMAYD-RYLAICQPLQYHSVMTKKKLSKLVCYSWLTPFCIFS-INIVLTDRLIFC
contig116846-BriOR.H055   LLCVFCINALYGTAGFYPKFLWDLLSNVYL--ISYYGCLIQTQVIYSFVCGEVLILALMAYD-RYVAICQPLKYHSIMSKQRVIRFACFLWLTTVCITA-TNAFLTSRLKLC
contig033889-BriOR.H049   LLCVFCMNALYGTAGFYPRFLWDLLSDVHL--ISYYGCLIQTKVIFSFVCGELSILALMAYD-RYVAICQPLKYHSIMSKQRVIRFACFLWLTTFCIMA-VNAFLTSRLKLC
contig030011-ZebOR.H073   LLCVFCINALYGTAGFYPKFLWDLLSDVHL--ISYYGCLIQTKVIFSFVCGELSILALMAYD-RYVAICQPLKYHSIMSKQRVIRFACFLWLTTFCIMA-VNVFLTSRLKLC
contig018437-ZebOR.H072   LLCVFCINALYGTAGFFPKFLWDLLSDVHL--ISYYGCLIQTKVIYSFLCGELSILALMAYD-RYVAICQPLKYHSIMSKQRVIRFACFLWFTNFCIMA-VNAFLTSRLKLC
contig018434-ZebOR.H070   LLCVFCINALYGTAGFFPKFLWDLLSDVHL--ISYYGCLIQTQVIYSFVCGELSILALMAYD-RYVAICQPLKYHSIMSKQRVIRFACLLWSTTFCIMA-VNVFLTFRLKLC
contig009565-TilOR.H126   LLCVFCMNALYGTAGFFPRFLWDLLSDVHL--ISYYGCLIQTQVVYSSACSELSILALMAYD-RYVAICQPLKYHSIMSKERVIRFACFSWSTTFCIMA-VNVFLTSRLKLC
contig018434-ZebOR.H071   LLCVFCINALYGTAGFYPKFLWDLLSDVHL--ISYYGCLIQTQVIYSSACGELSIPALMAYD-RYVAICQPLKYHSIMSKQRVIRFAWFLWFTNFCIVA-VNTFLTSRLKLC
contig009547-TilOR.H101   LLCVFCMNALYGTAGFYPKFLWDLLSPVHV--ISYYDCLIQTHVVYSFACIDVSILTLMAFD-RYVAICQPLKYHSFMSKQRVIKVACFSWFTPFCIIA-VNIFLTSRLKLC
contig009548-TilOR.H102   LLCVFCMNALYGTAGFYPKFLWDLLSPVHV--ISYYDCLIQTHVVYSFACIDVSILTLMAFD-RYVAICQPLKYHSFMSKQRVIKVACFSWFTPFCIIA-VNIFLTSRLKLC
contig009546-TilOR.H100   LLCVFCMNTLYGTAGFYPKFLWDLLSPVHV--ISYYGCLIQALVIYSCGCSDLSILTLMAFD-RYVAICQPLKYHSIMSKQRLIRLVCFSWLTPFSIIA-TNVFLTTRVKLC
contig014060-ZebOR.H069   FLCNLCVNGLYGTAAFYPKFLYDLLSTTHV--ISYAGCLLQGFVLHSAVAADFSLLALMAYD-RYVAICRPLVYHSLMTTQKISIFVFFAWLIPFYLLL-MSTITTATSRLC
contig053784-BurOR.H067   FLCNLCVNGLYGTAAFYPKFLYDLLSTTHV--ISYAGCLLQGFVLHSAVAADFSLLALMAYD-RYVAICRPLVYHSLMTTQKMSIFVFFAWLIPFYLLL-MSTITTATSRLC
contig041756-NyeOR.H075   FLCNLCFNGLYGTAAFYPKFLYDLLSTTHV--ISYAGCLLQGFVLHSAVAADFSLLALMAYD-RYVAICRPLVYHSLMTTQKIYIFVFFAWLIPFYLLL-MSTITTATSRLC
contig041756-NyeOR.H139   FLCNLCFNGLYGTAAFYPKFLYDLLSTTHV--ISYAGCLLQGFVLHSAVAADFSLLALMAYD-RYVAICRPLVYHSLMTTQKIYIFVFFAWLIPFYLLL-MSTITTATSRLC
contig013363-TilOR.H110   FLCNLCFNGLCGTAAFYPKFLYDLLSTTHV--ISYAGCLLQGFVVHSSVAADFSLLALMAYD-RYVAICRPLVYHSLMTTQKVCIFVFFAWLIPFSLIL-MSTITTATSRLC
contig035579-NyeOR.H068   FLCNLCFNGLYGTAAFYPKFLYDLLSTTHV--ISYAGCLLQGFVLHSSVCADFSLLVLMAYD-RYVAICRPLVYHSLMTTQKICILVFFAWLIPFYLLF-MSTITTAVLRLC
contig048880-BurOR.H063   FLCNLCFNGLYGTAAFYPKFLYDLLSTTHV--ISYAGCLLQGFVLHSSVCADFSLLVLMAYD-RYVAICRPLVYHSLMTTQKICILVFFAWLIPFYLLF-MSTITTAVLRLC
contig013365-TilOR.H111   FLCNLCVNGLYGTAAFYPKFLYDLLSTTHV--ISYAGCLLQGFVLHSSVCADFSLLALMAYD-RYVAICRPLLYHSLMTKQRVSIFVFFAWLIPFYLLF-MSTITTAVLRLC
contig053782-BurOR.H066   FLCNLCFNGLYGTAAFYPKFLYDLLSTTHV--ISYAGCLLQGLMVHSSICTDFSLLALMAYD-RYVAICRPLVYHSLMTTQRVCIFVFFAWITPFSLIL-MSTITTATSRLC
contig039729-NyeOR.H073   FLCNLCFNGLYGTAAFYPKLLYDLLSTIHV--ISYAGCLLQGLMVHSSICTDFSLLALMAYD-RYVAICRPLVYHSLMTTQRVCIFVFFAWITPFSLIL-MSTITTATSRLC
contig014057-ZebOR.H067   FLCNLCFNGLYGTAAFYPKFLYDLLSTTHV--ISYAGCLLQGLMVHSSICTDFSLLALMAYD-RYVAICRPLVYHSLMTTQRVCIFVFFAWITPFYLIL-MSTITTATSRLC
contig064817-BriOR.H051   FLCNLCFNGLYGTAAFYPKFLYDLLSTSHV--ISYAGCLLQGLMVHSSIGTDFSLLALMAYD-RYVAICRPLVYHSLMTTQRVCIFVFFAWLTPFCLIL-MSTITTATSRLC
contig013359-TilOR.H107   FLCNLCFNGLYGTAAFYPKFLYDLLSTTHV--ISYAGCLLQGFVVHSSVSADFSLLALMAYD-RYVAICRPLVYHSLMTKQRVCILIFFAWIIAFYLLF-MSTITTATSRLC
contig064821-BriOR.H052   FLCNLCVNGLYGTAAFYPKFLYDLLSTTHV--ISYAGCLLQGFALHSTICADFSLLALMAYD-RYVAICRPLVYHSLMTKQKVCIFVFFAWFFPIYLLF-LSTITTAVLRLC
contig053788-BurOR.H069   FLCNLCVNGLYGTAAFYPKFLYDLLSTTHV--ISYAGCLLQGFALHSSICADFSLLALMAYD-RYVAICRPLVYHSLMTKQKVCIFVFFAWFFPIYLML-LSTITTAVLRLC
contig041757-NyeOR.H076   FLCNLCVNGLYGTAAFYPKFLYDLLSTTHV--ISYAGCLLQGFALHSSICADFSLLALMAYD-RYVAICRPLVYHSLMTKQKVCIFVFFAWFFPIYLML-LSTITTAVLRLC
contig013356-TilOR.H105   FLCNLCFNGLYGTAAFYPKFLYDLLSTAHV--ISYAGCLLQGFALHSTICADFSLLALMAYD-RYVAICRPLVYHSLMTKQRVCIFLFFAWFFPFYLLF-LSTITTAVLRLC
contig013358-TilOR.H106   FLCNLCFNALYGTAAFYPKFLYDLLSTIHV--ISYAGCLLQGFALHSSVAADFSLLALMAYD-RYVAICRPLVYHSLMTKQKVCIFVFFAWLIPFYLLF-MSTISTAVLRLC
contig013351-TilOR.H104   FLCSLCFNGLYGTAAFYPKFLYDLLSTTHV--ISYAGCFLQGFVLHSSVGADFSLLALMAYD-RYVAICRPLVYHSLMTKERVCSLIFFAWIIAFYLLF-MSTITTAILRLC
contig014059-ZebOR.H068   FLCSLCFNGLYGTAAFYPKFLYDLLSTTHV--ISYAGCFLQGFVLHSSVAADFSLLALMAYD-RYVAICRPLVYHSIMTKQRVCILIFFAWIIAFYFLL-MSTITTAILRLC
contig053787-BurOR.H068   FLCSLCFNGLYGTAAFYPKFLYDLLSTTHV--ISYAGCFLQGFVLHSAVAADFSLLALMAYD-RYVAICRPLVYHSIMTKQRVCILIFFAWIIAFYFLL-MSTITTAILRLC
contig039725-NyeOR.H072   FLCSLCFNGLYGTAAFYPKFLYDLLSTTHV--ISYAGCFLQGFVLHSSVAADFSLLALMAYD-RYVAICRPLVYHSIMTKQRVCILIFFAWIIAFYFLL-MSTITTAISRLC
contig039730-NyeOR.H074   FLCSLCINGLYGTAGFFPRFAFDLLSDTHL--ISYVGCLLQVFVIYSNAKVDYSTLVLMAYD-RYLAICRPLEYHSVMSVRRTVVLVTLSWLVPLCFET-LVISLTSTLKLC
contig014055-ZebOR.H066   FLCSLCINGLYGTAGFFPRFAFDLLSDTHL--ISYVGCLLQVFVIYSNAKVDYSTLVLMAYD-RYLAICRPLEYHSVMSVRRTVVLVTLSWLVPLCFET-LVISLTSTLKLC
contig053780-BurOR.H065   FLCSLCINGLYGTAGFFPRFAFDLLSDTHL--ISYVGCLLQVFVIYSNAKVDYSTLVLMAYD-RYLAICRPLEYHSVMSVRRTVVLVTLSWLVPLCFET-LVISLTSTLKLC
contig107626-BriOR.H054   FLCSLCINGLYGTAGFFPRFALDLLSDTHL--ISYVGCLLQVFVIYSNAKVDYSTLVLMAYD-RYLAICRPLEYHSVMSVRRTAVLVTLSWLVPLCFET-LIISLTSTLKLC
contig013349-TilOR.H103   FLCSLCINGLYGTAGFFPRFAFDLLSDTHL--ISYVGCLLQVFVIYSNAKVDYSTLVLMAYD-RYVAICRPLEYHSVMSVRRTAVLVTLSWLVPLCFET-LVISLTSTLKLC
contig106096-BriOR.N089   AVFNLALVDLLGNSAMVPKVLHIFLFNHPH--IPYNDCLTFLFFCYVFLSMQALNLVALSYD-RIMAIVYPLHYQLKVTHKFMFCLIASFWVFVIIVVL-IATGLLTRLSFC
contig061663-NyeOR.N114   AVFNLALVDLLGNSAMVPKVLDIFLFNHPH--ISYNDCLTFLFFCYVFLSMQALNLVALSYD-RVMAIVYPLHYQLKVTHKFMFCLIASFWVFVIIVVL-IATGLLTRLSFC
contig057383-BurOR.N109   AVFNLALVDLLGNSAMVPKVLDIFLFNHPH--ISYNDCLTFLFFYYVFLSMQALNLVALSYD-RVMAIVYPLHYQLKVTHKFMFCLIASFWVFVIIVVL-IATGLLTRLSFC
contig010722-ZebOR.N111   AVFNLALVDLLGNSAMVPKVLDIFLFNHPH--ISYNDCLTFLFFCYIFLSLQALNLVALSYD-RVMAIVYPLHYQLKVTHKLMFCLIASFWVSAITFIL-IAIGLLTRLSFC
contig096539-BriOR.N087   AVFNLAFVDLFGNTALVPKVLDIFLFGHYY--IPYNDCLTFLFFCYTCLSLQSFNLVALSYD-RMVAIIFPLHYQVKVTHRFMFSFIASLWVFTIIAVL-ISVGLLTRLSFC
contig064097-ZebOR.N115   AVFNLAFVDLFGNTALVPNVLDIFLFGHYY--IPYNDCLTFLFFCYTCLSLQSFNLVALSYD-RMVAIIFPLHYQVKVTHRFMFSLIASLWVFTIIAVL-ISVGLLTRLSFC
contig055927-NyeOR.N111   AVFNLAFVDLFGNTALVPKVLDIFLFGHYY--IPYNDCLTFLFFCYTCLSLQSFNLVALSYD-RMVAIIFPLHYQVKVTHRFMFSLIASLWVFTIIAVL-ISVGLLTRLSFC
contig060631-BurOR.N110   AVFNLAFVDLFGNTALVPKVLDIFLFGHYY--IPYNDCLTFLFFCYTCLSLQSFNLVALSYD-RMVAIIFPLHYQVKVTHRFMFSLIASLWVFTIIAVL-ISVGLLTRLSFC
contig046353-TilOR.N195   AVFNLAFVDLFGNTALVPKVLDIFLFGHYF--IPYNDCLTFLFFCYTCLSLQSFNLVALSYD-RMVAIIFPLHYQVKVTHRFMFSLIASLWVFTIIAVL-IAVGLLTRLSFC
contig055926-NyeOR.N110   AVFNLAFTDLLSNSALVPKVLDISLFNHHY--ISYNNCLTFMFFCFTLISMQAFNLVVLSFD-RIMAIMYPLHYQMRVSHKIILSLIAFFWLLAVALTG-TAVGLLTRLYFC
contig042928-BurOR.N108   AVFNLAFTDLLSNSALVPKVLDISLFNHHY--ISYNNCLTFMFFCFTLISMQAFNLVVLSFD-RIMAIMYPLHYQMRVSHKIILSLIAFFWLLAVALTG-TAVGLLTRLYFC
contig064098-ZebOR.N116   AVFNLAFTDLLSNSALVPKVLDISLFNHHY--ISYHNCLTFMFFCFTLISMQAFNLVVLSFD-RIMAIMYPLHYQMRASHKIILSLIAFFWLLAIALTG-TAVGLLTRLYFC
contig096539-BriOR.N088   AVFNLAFTDLLSNSALVPKVLEISLFNHHY--ISYNNCLTFMFFCFTLISMQAFNLVVLSFD-RVMAIMYPLHYQMRVSHKIILSLIAFFWLLAMALTG-TAVGLLTRLYFC
contig046356-TilOR.N196   AVFNLAFTDLLSNSALVPKVLDISLFNHHY--ISYNNCLTFMFFCFTLISMQAFNLVVLSFD-RIMAIMYPLHYQMRVSHKIILSLITFFWLLAIALTG-TAVGLLTRLHFC
contig055924-NyeOR.N109   GVVNFAFTDLLSSSALMPKLVDIFLFNHHH--ISYNDCLAFMFFCLTFFAAQAFNLVVLSFD-RVMAIMYPLHYQMRISHKLILSLIAFFWLLAITIIL-TAVGLLTRLSFC
contig010725-ZebOR.N112   GVVNFAFTDLLSSSALMPKLVDIFLFNHHH--ISYNDCLAFMFFCLTFFAAQAFNLVVLSFD-RVLAIMYPLHYQMRISHKLILSLIAFFWLLAITIIL-TAVGLLTRLSFC
contig010726-ZebOR.N113   GVVNFAFTDLLSSSALMPKLVDIFLFNHHH--ISYNDCLAFMFFCLTFFAAQAFNLVVLSFD-RVMAIMYPLHYQMRISHKLILSLIAFFWLLAITIIL-TAVGLLTRLSFC
contig046360-TilOR.N197   GVFNFAFTDLLSSSALMPKLVDIFLFNHHH--ISYNDCLAFMFFCLAFFAAQAFNLVVLSFD-RVMAIMYPLHYQMRMSHKLILSLIAFFWLLVITIIL-IAVGLLTRVSFC
contig010714-ZebOR.N109   AVFNLAFTDLLSSSALVPKVVDIFLFNHYY--ISYNACLTFMFFCFTFISMQAFNLVVLSFD-RVMAIMYPLHYQMRVSHKLILSLIAFFWLLAITLIL-IAAGLLTRLSFC
contig010718-ZebOR.N110   AVFNLAFTDLLSSSALVPKVVDIFLFNHYY--VSYNDCLTFMFFCFTFIFMQGFNLVVLSFD-RVMAIMYPLHYQMRVSHKLILSLIAFFWLLAITLIL-IAVGLLTRLSFC
contig010712-ZebOR.N108   AVFNLAFTDLLSSSALMPKVVDIFLLNHYY--ISYNDCLTFMFFCSTFYAMQTFNLVVLSFD-RVMAIMYPLHYQMRVSHKLILTLIAFFWLLATTLIL-IVVGLLTRLSFC
contig050080-TilOR.N198   AVFNLAFTDLLSSSSLMPKVLDIFLLSHYY--ISYNDCMTFMFFSFTFYAMQAFNLVVLSYD-RVMAIMYPLHYQMRVSHKLILSMIAFFWLLAITLML-IEVGLLTRLSFC
contig046352-TilOR.N193   IVFNLALTDLCGSTALIPKLLDTFLFDRRY--ILYEACLSYMFFVLFFASIQSWTLVIMAYD-RFIAICFPLRYHSIVTKTSIAAMLAFEWVLITSIMA-STVGLIDRLSFC
```

```
contig046347-TilOR.N190   IVFSLALTDLCGSTALIPKLLDTFLFDRRY--ILYEACLSYMFFVLFFASIQSWTLVIMAYD-RFIAIWFPLRYHSIVTKTSIAAMLAFEWLLITTIVA-STVGLIDRLSFC
contig096536-BriOR.N085   IVFNMALTDLCGSTALIPKLLDTFLFDRRY--ILYDACLSYMFFVIFFASVQSWTLVTMAYD-RLIAICFPLRYHSIVTEQSITAILLFVWIFLVSVIA-TMVGLVNRLSFC
contig055933-NyeOR.N113   IVFNMALADLCGSTALIPKVLDTFLFDRRY--ILYDACLSYMFFVIFFASVQSWTLVTMAYD-RLIAICFPLRYHSIVTEQSITAILLFVWIFLVSVIA-TMVGLVNRLSFC
contig065025-BurOR.N111   IVFNMALTDLCGSTALIPKLLDTFLFDRRY--ILYDACLSYMFFVMFFASVQSWTLVTMAYD-RLIAICFPLRYHNIVTETSVAAILLFVWIFLVSVIT-TMVRLVNRLSFC
contig096535-BriOR.N084   IVFNMALTDLCGSTALIPKLLDTFLFDRRY--ILYDACLSYMFFVMFFASVQSWTLVTMAYD-RLIAICFPLRYHSIVTETSVAAILLFVWIFLVSVIA-TMVGLVNRVSFC
contig046340-TilOR.N188   IVFNMALTDLCGSTALIPKLLDTFLFDRRY--IIYEACLSYMFFVMFFASVQSWTLVTMAYD-RLIAICFPLRYHSIVTETSVAAILLFVWIFLVSVIA-TMVGLLNRLSFC
contig046351-TilOR.N192   IVFNMALTDLCGSTALIPKVLDTFLFDRRY--IFYDPCLGYMFFVVFFASMQSWTLVAMAYD-RLIAICLPLRYHSIVTEASIAAVLLFVWIFLVSVIA-TMVGLINHLSFC
contig046350-TilOR.N191   IVFNMALTDLCGSTALIPKVLDTFLFDRRY--IVYEACLSYMFFVIFFASVQSWTLVTMAYD-RLIAICLPLRYHNIVTETSITAILLFVWCFFVSAIA-TMVGLLNRLSFC
contig046344-TilOR.N189   IVFNMALTDLCGSTALIPKVLDTFLFDRRY--IIYDACLSYMFFVMFFGSVQSWTLVIMAYD-RLIAICLPLRYHNIVTETSITAILLFVWCFFVIAIA-TMIGLVNRLSFC
contig010727-ZebOR.N114   IVFNMALTDLCGSTALIPKLLDTFLFDRRY--IVYEACLSYMFFVFFFGGVQSWTLVTMAYD-RLIAICFPLRYHSIVTKTSITSMLLFIWLVMLSLTT-LVVGLINRLSFC
contig042920-BurOR.N107   IVFNMALADLCGSTTLIPKLLDTFLFDRRY--IVYEACLSYMFFVFFFGGVQSWTLVTMAYD-RLIAICFPLRYHSIVTKTSITSMLLFIWLVMLSLTT-LVVGLVNRLSFC
contig055927-NyeOR.N112   IVFNMALADLCGSTALIPKLLDTFLFDRRY--IVYEACLSYMFFVFFFGGVQSWMLVTMAYD-RLIAICFPLRYHSIVTKTSIKSMLLFIWLVMLSLTT-LVVGLVNRLSFC
contig096536-BriOR.N086   IVFNMALADLCGSTALIPKLLDTFLFDRRY--IVYEACLSYMFFVFFFGGVQSWTLVTMAYD-RLIAICFPLRYHSIVTKTSITSMLLFIWLVTLSLTT-LVVGLINRLSFC
contig046352-TilOR.N194   IVFNMALTDLCGSTALIPKLLDTFLFDRRY--IVYEACLSYMFFVLFFGGVQSWTLVTMAYD-RLIAICFPLRYHSIVTKTAITSMLLFIWLLMLSLTT-LVVGLINRLSFC
contig048778-NyeOR.M107   AVVNLAVIDLFLNTCTIPSMIKIFLIKNNF--IPFNLCLLQMYVYYVFVSLESFALAILAYD-RLIAICFPLRQNSINTVQSMCCIISITWFLVMGVIA-FTTGIMTRLSFC
contig010734-ZebOR.M107   AVVNLAVIDLFLNTCTIPSMIKIFLIKNNF--IPFNLCLLQMYVYYVFVSLESFALAILAYD-RLIAICFPLRQNSINTVRSMCCIVSVSWFLVMGVIG-FATGIMTRLSFC
contig048778-NyeOR.M108   AVVNLAVIDLFLNTCTIPSMIKIFLIKNNF--IPFNLCLLQMYVYYVFVSLESFALAILAYD-RLIAICFPLRHNSINTVRSMCCIVSVSWISNMGITA-FATGIMTRLSFC
contig039460-TilOR.L145   LICNLAVVDLMYCCSTTPTMIGVLLAGVNT--ISYVECFIQMSVFHLVGVMELFALAIMAFD-RLIAFSFPFQYYSYLTNTRTLVVTYILWVVACGFVA-VMPVTAATLPYC
contig017786-ZebOR.L091   LICNLAVVDILYCCSTTPTMIGVLLAGVNT--ISYVECFIQMSVFHLVGVMELFALAIMAFD-RLIAFSFPFQYHSYLTNTRTLVVTYILWVVASGFVA-VMPVTAATLPYC
contig058162-BurOR.L089   LICNLAVVDILYCCGTTPTMIGVLLAGVNT--ISYVECFIQMSVFHLVGVMELFALAIMAFD-RLIAFSFPFQYHSYLTNTRTLVVTYILWVVASGFVA-VMPVTAATLPYC
contig040502-NyeOR.L092   LICNLAVVDILYCCSTTPTMIGVLLVGVNT--ISYVECFIQMSVFHLVGVMELFALAIMAFD-RLIAFSFPFQYHSYLTNTRTLVVTYILWVVASGFVA-VMPVTAATLPYC
contig042543-BriOR.L069   LICNLAVVDILYCCSTTPTMIGVLLAGVNT--ISYVECFIQMSVFHLVGVMELFALAIMAFD-RFIAFSFPFQYHSYLTNTRTLGLTYILWVVASGFVA-VMPVTAATLPYC
contig039460-TilOR.L146   LICNLAVVDLLYTSSTCPTMIGVLLAGVNT--ISYVDCFIQMCIFNWVEVMAFFALAFMAFD-RLIAIIYPFQYHSYMTNTRTLVVTYILWVVSLGVSA-VMPVTAATLPYC
contig039465-TilOR.L150   LICNLAVVDILYTSSSTPTMIGVLLAGVNT--ISYVECLIQMCVYQLGATMELFSLTIMAFD-RLIAIIYPLQYHSYLTNTRIMVLTYILWIVGSGFVA-VLPATVTPLPHC
contig039461-TilOR.L148   LICNLAVVDILYTSSSTPTMIRVLLAGVNT--ISYVECLIQMCVFQLGGIMELFSLTIMAFD-RLIAIIYPLRYHSYLTNTRIMVLTYILWIVASNLVA-VLPAIVVPLPHC
contig042552-BriOR.L071   LICNLAVVDILYTSSATPTMIGVLLAGVNT--ISYVECIIQMYVYQLGSTMERFSLTIMAFD-RLIAIIYPLQYHSYLTITRTLVFTYILWIVACSFVL-FPPVTVAPLPHC
contig020445-ZebOR.L096   LICNLAVVDILYTSSSTPTMIGVLLAGVNT--ISYVECIIQMYVYQVGATMEMFSLTIMAFD-RLIAIIYPLQYHSYLTNTRTLVFTYIVWIVACSFAL-FPPVTATPLPHC
contig056942-NyeOR.L093   LICNLAVVDILYTSSSTPTMIGVLLAGVNT--ISYMECIIQMYVYQVGATMEMFSLTIMAFD-RLIAIIYPLQYHSYLTNTRTLVFTYIVWIVACSFAL-FPPVTATPLPHC
contig068527-TilOR.L157   LICNLAVVDIFYTSSATPTMIGVLLAGVNT--VSYVECLIQMYVYQVGATMERFSLTIMAFD-RLIAIIYPLQYHSYLTNTRIMVLTYILWIVACSFVL-FTLVTATPLPHC
contig039469-TilOR.L152   LICNLAVVDIFYTSSATPTMIGVLLAGVNT--ISYVECLIQMYVFQVGGTMEMFSLTIMGFD-RLIAIICPLQYHSYLTNTRILIFTYILWIVACSFVL-FTLVTLAPLPHC
contig064361-NyeOR.L094   LICNLAVVDIMYTSSCSPTMIGVLLAGVNT--ISYMACLIQMCVFHLGTAMESFVLAVMALD-RFIAIIYPFQYHSYLTNTRVLVLTFIVWFVNCFFMC-YMPATAVPLPHC
contig056384-BurOR.L087   LICNLAVVDIMYTSSCSPTMIGVLLAGVNT--ISYMACLIQMCVFHLGTAMESFVLAVMALD-RFIAIIYPFQYHSYLTNTRVLVLTFIVWFVNCFFMC-YMPATAVPLPHC
contig020442-ZebOR.L095   LICNLAVVDIMYTSSCSPTMIGVLLAGVNT--ISYMACLIQMCVFHLGTAMESFVLAVMALD-RFIAIIYPFQYHSYLTNTRVLVLTFIVWFVNCFFMC-YMPATAVPLPHC
contig042554-BriOR.L072   LICNLAVVDIMYTSSCSPTMIGVLLAGVNT--ISYVACLIQMFVFHLGTVMESFVLAVMALD-RLIAIIYPFQYHSYLTNTRVLVLTFIVWFVNCFFMC-YMPATVVPLPHC
contig039481-TilOR.L153   LICNLAVVDIMYTSSCSPTVIGVLLAGVNT--ISYVECLIQMFVFHLGTAMESFVLAVMALD-RLIAIMYPFQYHSYLTNTRVLLLTFIVWFVNWFFMC-YMPATVVPLPHC
contig072645-TilOR.L159   LICNLAVVDIMYTSSCSPTMIGVLLAGVNT--ISYVACLIQMCVFHLGTSMESFVLAVMALD-RFIAIIYPFQYHSYLTNTRVLVLTFIVWFVNWFFVF-YLPATVVPLPHC
contig056940-NyeOR.L095   LICNLAVVDIVYTSSSSPTMIGVLLAGVNT--ISYVACLIQMCVFNLGTSMESFVLAFMALD-RFIAIIYPFQYQRYLTNTRVLVLTFIMWFIAWCFAF-YMPATVVPLPHC
contig020440-ZebOR.L094   LICNLAVVDIVYTSSSSPTMIGVLLAGVNT--ISYVACLIQMCVFNLGTSMESFVLAFMALD-RFIAIIYPFQYQRYLTNTRVLVLTFIMWFIAWCFAF-YMPATVVPLPHC
contig042556-BriOR.L073   LICNLAVVDIVYTSSSSPTMIGVLLAGVNT--ISYVACLIQMCVFNLGTSMESFVLAFMALD-RFIAIIYPFQYQSYLTNTRVLVLTFIMWFVAWCFVF-YMPATVVPLPHC
contig039484-TilOR.L154   LICNLAIVDIVYTSSSSPTMIGVLLAGVNT--ISYVECLIQMCVFNLGTAMESFVLAVMALD-RLIAIIYPFQYHTYLTNTRVLVLTLIMWFVAWCFVF-YMPATVVPLPHC
contig068425-TilOR.L155   MICNLAVVDIVYTSSCSPTMIGVLLAGVNT--ISYVECLIQMCVFNLGTSMESFVLAIMALD-RFIAIIYPFQYHSYLTNTRVLVLTFIVWFIAWCFVC-YMPAAVVPLPHC
contig020437-ZebOR.L093   LICNLAVVDIMYTSSCSPTMIGVLLAGVNT--ISYMACLIQMCVGHLGTAMESFVLAIMALD-RFIAIIYPFQYQSYLTNTRVLVLTFIVWFVAWCFVF-YMPATVVLLPHC
contig056386-BurOR.L088   LICNLAVIDIMYTSSCSPTMIGVLLAGGNT--ISYVACLIQMCVFHLGTAMESFILAFMALD-RFIAIIYPFQYQSYLTNTRVLVLTFIVWFVAWCFVF-YMPATVVPLPHC
contig039469-TilOR.L151   LICNLAVVDIMYTSSCNPTMIGVLLAGVNT--ISYVECLIQMCVFTLGTSMESFVLAVMALD-RFIAIIYPFQYHSYLTNTRVLVLTFILWFVAWYFVC-YMPATVVPFPHC
contig068521-TilOR.L156   LICNLAVVDIMYTSSCSPTMIGVLLAGVNT--ISYVECLIQMCVFHLGTVMESFVLAFMALD-RFIAIIYPFHYHSYLTNTRVVVLTFILWFVASCFVC-YMPATVVPLPHC
contig068539-TilOR.L158   LICNLAVVDIVYTSSSSPTMIGVLLAGVNT--ISYVECLIQMCAFTLGILMESFVLAIMALD-RFIAIIYPFQYHSYLINTRVLVLTFVLWFIAWCFVC-YMPVTLVSLPHC
contig042544-BriOR.L070   LVCNLAVVDIMYTSSATPTMIGVLLADVKT--ISYVDCLIQMCVFHLAMVMERFALAIMAFD-RLIAIIFPFHYHCFLTNTRTVVLTYILWIIGCGTVV-LFPATVIPLPHC
contig065027-BurOR.L090   LVCNLAVVDIMYTSSATPTMIGVLLADVKT--ISYVDCLIQMCVFHLAMVMERFALAIMAFD-RLIAIIFPFHYHSYLTNTRTVVLTYILWIIGCGTVV-LFPATVIPLPHC
contig017787-ZebOR.L092   LVCNLAVVDIMYTSSATPTMIGVLLADVKT--ILYVDCLIQMCVFHLAMVMERFALAIMAFD-RLIAIIFPFHYHSYLTNTRTVVLTYILWIIGCGTVV-LFPATVIPLPHC
contig040502-NyeOR.L091   LVCNLAVVDIMYTSSATPTMIGVLLADVKT--ISYVDCLIQMCVFHLAMVMERFALAIMAFD-RLIAIIFPFHYHSYLTNTRTVVLTYILWIIGCGTVV-LFPATVIPLPHC
contig039462-TilOR.L149   LVCNLAVVDIIYTSSATPTMIGVLLADVNT--ISYVECLIQMCVFHLAMVMERFALAIMAFD-RLIAIIFPFHYHSYLTNTRTVFLTYILWIIGCGTVV-LFPATVIPLPHC
contig023716-TilOR.L144   LICNLAIVDIMYISSASPTMVGVLVAGVNT--ISYLECLIQMFVFHLGGMMERFALAIMAFD-RLVAIIYPFQYHSYLKNTRILLYTYISWVVACGFVV-VLPATVAPLPHC
contig039461-TilOR.L147   LVCNLAVVDIIYTSSTSPTMIGVLLAGVNT--ISYVECIIQMFVFHLGAVMERLALAIMALD-RLIAIICPFQYHSYLTNTHIVFLTYILWFVGSGFAA-LAPAVVVRLPHC
contig017698-BurOR.P120   LIASMACIDLSLPVFFVPHMLLSFLFDWRG--ISLIGCLVQMHLIHFFGTFQSTLLVWMALD-RYFAICTPLYYHNHMILSRFIAFLIPLVVRNVLMIT-LLVCLAGKLPFC
contig025452-ZebOR.P123   LIASMACIDLSLPVFFVPHMLLSFLFDWRG--ISLIGCLVQMHFIHFFGTFQSTLLVWMALD-RYFAICTPLYYHNHMILSRFIAFLIPLVVRNVLMIT-LLVCLAGKLPFC
contig004267-BriOR.P099   LIASMACIDLSLPVFFVPHMLLSFLFDWRG--ISLIGCLVQMHFIHFFGTFQSTLLVWMALD-RYFAICTPLYYHNHMILSRFIAFLLPLVVRNVLMIT-LFVCLAGKLPFC
contig065444-TilOR.P214   LIAGMACIDLSLPVFFVPHMLLSFLFDWRG--ISLIGCLVQMHFIHFFGTFQSTLLVWMALD-RYFAICTPLYYHNHMILSRFIVFLLPLVVRNVLMIT-LFVSLAGKLPFC
contig065247-TilOR.P211   LIASMACIDLILPIFFLPHMLLSFLFDWRG--ISLIGCLVQMYFIHFFGTFQSTLLVWMALD-RYFAICTPLYYQEQMTLAKFLKFVIPLFIRNMFVVL-VVVSLAGKLPFC
contig004270-BriOR.P100   LIASMACIDLSLPAFFVPHMLLSFLFDWRG--ISLIGCLVQMYFVHLLGAFQSTLLLWMALD-RYFAICTPLHYQEQMALARFLKFVIPSSIRNMFVVL-VVVSLAGKLPFC
contig017697-BurOR.P119   LIASMACIDLSLPVFFVPHMLLSFLFDWRG--ISLIGCLFQMYFIHLLGAFQSTLLLWMALD-RYFAICTPLHYQEQMALARFLKFVIPFSIRNMFAVL-VVVSLAGKLPFC
contig025453-ZebOR.P124   LIASMACIDLSLPVFFVPHMLLSFLFDWRG--ISLIGCLVQMYFIHLLGAFQSTLLLWMALD-RYFAICTPLHYQEQMALARFLKFVIPFSIRNMFAVL-VVVSLAGKLPFC
contig065449-TilOR.P215   LIASMACIDLSLPVFFVPHMLLSFLFDWRG--ISLGACLVQMYFVHLLGAFQSTLLLWMALD-RYFAICTPLYYQEQMALAKFLKFVIPLFIRNMFVVL-VVVSLAGKLPFC
contig065253-TilOR.P212   LIASMACIDLSLPVFFVPHMLLSFLFDWRG--ISLGACLVQMYFVHLLGAFQSTLLLWMALD-RYFAICTPLYYQEQMALEKFLKFVIPLFIRNMFVVL-VVVSLAGKLPFC
contig065254-TilOR.P213   LICTLCFVDMLVVTTIIPNMLLGFLFNWNE--ISLVGCLTQMFFTHFMSSVESTLLLAMALD-RYVAICRPLHYNEIINSSMLVRLVLFTLVRSVSIMA-TLVGLAGSLQFC
contig023268-NyeOR.P118   LICTLCFVDILVVTTIIPNMLLGFLFDWNE--ISLVGCLTQMFFIHFLSSVESTLLLAMALD-RYFAICRPLRYNDIINSSMLVRLVLFTLVRSVSVMA-TLVGLAGSLQFC
contig017696-BurOR.P118   LICTLCFVDILVVTTIIPNMLLGFLFDWNE--ISLVGCLTQMFFIHFLSSVESTLLLAMALD-RYFAICRPLRYNDIINSSMLVRLVLFTLVRSVSVMA-TLVGLAGSLQFC
contig025458-ZebOR.P125   LICTLCFVDILVVTTIIPNMLLGFLFDWNE--ISLVGCLTQMFFIHFLSSVESTLLLAMALD-RYFAICRPLRYNEIINSSMLVRLVLFTLVRSVSIMA-TLVGLAGSLQFC
contig004275-BriOR.P101   LICTLCFVDILVVTTIIPNMLLGFLFNWNE--ISLVGCLTQMFFIHFLSSVESTLLLAMALD-RYFAICRPLRYNEIINSAMLVRLVLFTLVRSVSVMA-TLVGLAGSLQFC
contig020431-ZebOR.O101   IILNLSVCDILFSTTTLPKIISRYWFQSGS--ISFTACFIQMYFVHYFGTAVAYILFQMALD-RYLAICHPLRYSHILTKSNILILSITAWIIAKASPL-MMVIRAYPLPYC
```

```
contig021359-NyeOR.O102     IILNLSVCDILFSTTTLPKIISRYWFQSGS--ISFTACFIQMYFVHYFGTAVAYILFQMALD-RYLAICHPLRYSHILTKSNILILSITAWIIAKASPL-MMVIRAYPLPYC
contig059249-BurOR.O098     IILNLSVCDILFSTTTLPKIISRYWFQSGS--ISFTACFIQMYFVHYLGSVNSFILFQMALD-RYLAICYPFRYSLVLTKSNILILSITAWIISKAFPL-MMVIRAYPLPYC
contig062053-NyeOR.O103     IILNLSVCDILFSTTTLPKIISRYWFQSGS--ISFTACFIQMYFVHYLGSVNSFILFQMALD-RYLAICYPFRYSLVLTKSNILILSITAWIISKAFPL-MMVIRAYPLPYC
contig023717-TilOR.O175     IILNLSVCDILFSTTTLPKIISRYWFQSGS--ISFTACFIQMYFVHYLGSVNSFILFQMALD-RYLAICHPFRYSHILTKSNILILSITAWIIAKAFPL-MMVIRAYPLPYC
contig023724-TilOR.O176     IILNLSVCDILFSTTTLPKIISRYWFQSGS--ISFTGCFIQMYFVHYLGTVNSYILFQMALD-RYLAICHPLRYSRVLTKSNILILSITAWIIAKASPL-MTVIRAYPLPYC
contig042559-BriOR.O077     IILNLSVCDILFSTTTLPKIISRYWFRSGS--ISITACFIQMYFVHYLGSVNSFILFQMALD-RYLAICHPLRYSHILTKSNILILSITGWIIAKACPL-MIVIRAYPLPYC
contig110782-BriOR.O080     IILNLSVCDILFSTTTLPKIISRYWFRSGS--ISFTACFIQMYFVHYFGTAVAYILFQMALD-RYLAICHPLKYSRILTKSNILILSITGWTAAKAFPL-MMVIRAYPLPYC
contig020430-ZebOR.O100     VFCHLALNDLTFGTVTLPKIMSKYWFDNSV--ISFYGCFTQMFFVHYLGSVTSFILLVMALD-RFVAICIPLRYPVLITNSVISVLCGFAWFIPLPLMI-AVVLHALTLPFC
contig042560-BriOR.O078     VFCHLALNDLTFGTVTLPKIMSKYWFDNSA--ISFYGCFTQMFFVHYLGSVTSFILLVMALD-RFVAICIPLRYPVLITNSVISVLCGFAWFIPLPLMI-GVVLHHLTLPFC
contig021354-NyeOR.O101     IFSNLAMTDICFGVVTLPKIIARYWWNDMI--TSFGACFTQMYFVHSLGAIQSLNLLMMALD-RFVAIWFPFKYPILFTNKAVAIACTMCWVLTFIRLL-GIVLLALTLPYC
contig020427-ZebOR.O099     IFSNLAMTDICFGVVTLPKIIARYWWNDMI--TSFGACFTQMYFVHSLGAIQSLNLLMMALD-RFVAIWFPFKYPILFTNKAVAIACTMCWVLTFIRLL-GIVLHALTLPYC
contig023731-TilOR.O177     IFSNLAMTDICFGVVTLPKIIARYWWNDMI--TSFGACFTQMYFVHSLGAIQSLNLLMMALD-RFVAIWFPFKYPVLFTNKAVAIACTMCWVLTFIRLL-GIVLHALTLPYC
contig042562-BriOR.O079     IFSNLAMTDICFGVVTLPKIIGRYWWNDMI--TSFGACFTQMYFVHSLGAIQSLNLLMMALD-RFVAIWFPFKYPISFTNKTVAIACTMCWVLTFIRLL-GIALHALTLPYC
contig049605-BurORs.AB153   FLCNLSFVDMVYTTTTIPNMLSGLLTDILT--ISVLGCFLQMYFFIQLSVTGRAILTVMAYD-RYVAICNPLQYNSIMTRPVRLLLVAGAWGFGAICTL-PVTVIAFERPYC
contig046717-TilORs.AB275   FV------DMVYTTTTIPNMLSGLLTDILT--ISVLGCFLQMYFFIQLSVTGRAILTVMAYD-RYVAICTPLQYNSIMTRPVRLLLVAGAWGFGAVCTL-PATVIAFERPYC
```

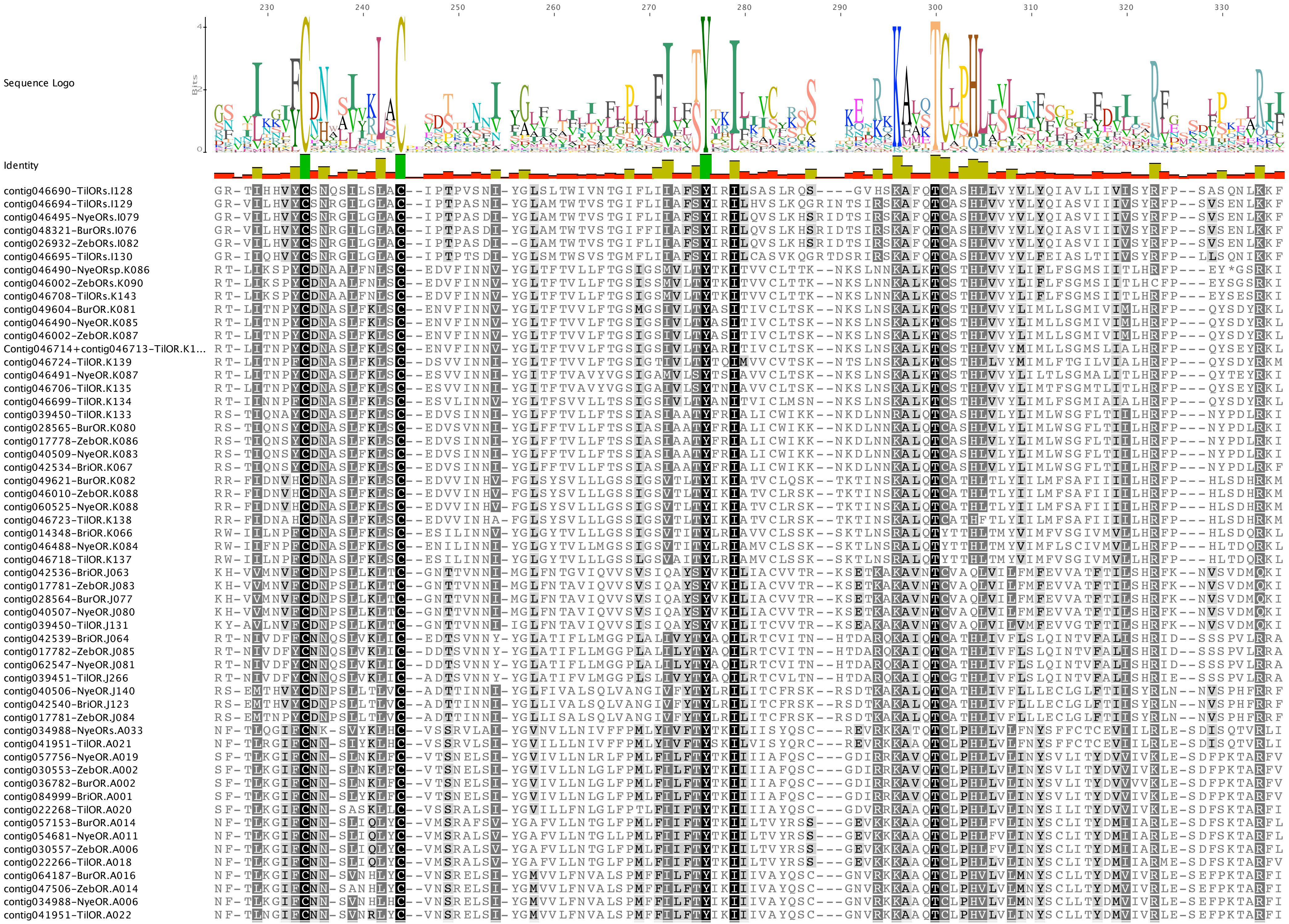
230
240
250
260
270
280
290
300
310
320
330
Sequence Logo
Bits
Identity
contig046690-TilORs.I128
contig046694-TilORs.I129
contig046495-NyeORs.I079
contig048321-BurORs.I076
contig026932-ZebORs.I082
contig046695-TilORs.I130
contig046490-NyeORsp.K086
contig046002-ZebORs.K090
contig046708-TilORs.K143
contig049604-BurOR.K081
contig046490-NyeOR.K085
contig046002-ZebOR.K087
Contig046714+contig046713-TilOR.K1...
contig046724-TilOR.K139
contig046491-NyeOR.K087
contig046706-TilOR.K135
contig046699-TilOR.K134
contig039450-TilOR.K133
contig028565-BurOR.K080
contig017778-ZebOR.K086
contig040509-NyeOR.K083
contig042534-BriOR.K067
contig049621-BurOR.K082
contig046010-ZebOR.K088
contig060525-NyeOR.K088
contig046723-TilOR.K138
contig014348-BriOR.K066
contig046488-NyeOR.K084
contig046718-TilOR.K137
contig042536-BriOR.J063
contig017781-ZebOR.J083
contig028564-BurOR.J077
contig040507-NyeOR.J080
contig039450-TilOR.J131
contig042539-BriOR.J064
contig017782-ZebOR.J085
contig062547-NyeOR.J081
contig039451-TilOR.J266
contig040506-NyeOR.J140
contig042540-BriOR.J123
contig017781-ZebOR.J084
contig034988-NyeORs.A033
contig041951-TilOR.A021
contig057756-NyeOR.A019
contig030553-ZebOR.A002
contig036782-BurOR.A002
contig084999-BriOR.A001
contig022268-TilOR.A020
contig057153-BurOR.A014
contig054681-NyeOR.A011
contig030557-ZebOR.A006
contig022266-TilOR.A018
contig064187-BurOR.A016
contig047506-ZebOR.A014
contig034988-NyeOR.A006
contig041951-TilOR.A022

```
contig034983-NyeOR.A002   NF-TLKGIFCNN-SVNHLYC--VTSKELSI-YGMVVLFNGALFPMLFILFTYIKIIIVACQSC---GNVRKKAAQTCLPHVLVLINYSCLVTYDMVIVRLE-SEFPKTARFI
contig047515-ZebOR.A019   NF-TLKGIFCNN-SVNHLYC--VTSKELSI-YGMVVLFNGALFPMLFILFTYIKIIIVACQSC---GNVRKKAAQTCLPHVLVLINYSCLVTYDMVIVRLE-SEFPKTARFI
contig051559-BurOR.A007   NF-TLKGIFCNN-SVNHLYC--VTSKELSI-YGMVVLFNGALFPMLFILFTYIKIIIVACQSC---GNVRKKAAQTCLPHVLVLINYSCLITYDMVIVRLE-SEFPKTARFI
contig022259-TilOR.A015   HS-NIKGIFCNN-AVYTLQC--ERSRLITI-FGVFLLIDLAVLPMLFIVYTYTKIFIVSHRSC---KEIRKKTAETCLPHMLVLISYSAFFVYDISIARVK-SDFPKTTRII
contig051321-BurOR.A006   DS-NIKGIFCSN-AVYTLQC--ERSRLITI-FGVFLLIDLAILPMLFIVYTYTKIFIVSHRSC---KEIRKKTAETCLPHLLVLISYSMFFVYDISIARVK-SDFPKTTRII
contig056380-NyeOR.A016   DS-NIKGIFCSN-AVYTLQC--ERSRLITV-FGVFLLIDLAILPMLFIVYTYTKIFIVSHRSC---KEIRKKTAETCLPHLLVLISYSMFFVYDISIARVK-SDFPKTTRII
contig062094-ZebOR.A023   DS-NIKGIFCSN-AVYTLQC--ERSRLITI-FGVFLLIDLAILPMLFIVYTYTKIFIVSHRSC---KEIRKKTAETCLPHMLVLISYSMFFVYDISIARVK-SDFPKTTRII
contig056375-NyeOR.A015   DS-NIKGIFCNN-AVYTLQC--ERSRLITI-FGVVALVDLSILPMLFIVFTYTKLFIVSHRSC---KEIRKKAAETCLPHLLVLISYSAFFVYDVSIARVK-SDFPKTTRII
contig064570-BurOR.A017   DS-NIKGIFCNN-AVYTLQC--ERSRLITI-FGVVALVDLSILPMLFIVFTYTKLFIVSHRSC---KEIRKKAAETCLPHMLVLISYSAFFVYDVSIARVK-SDFPKTTRII
contig062095-ZebOR.A024   DS-NIKGIFCNN-AVYTLQC--ERSRLITI-FGVVALVDLSILPMLFIVFTYTKLFIVSHRSC---KEIRKKAAETCLPHLLVLISYSAFFVYDVSIARVK-SDFPKTTRII
contig022259-TilOR.A014   DS-NIKGIFCNN-AVYTLQC--ERSRLITI-FGVVALVDLSILPMLFIVFTYTKIFIVSHRSC---KEIRKKAAETCLPHMLVLIGYSIFFVYDVSIARVK-SDFPKPTRII
contig051318-BurOR.A005   DS-NIKGIFCNN-AVYTLQC--ERSKLITI-FGVVIVLDLAILPMLFIVFTYTKIFIVSHRSC---KEIRKKAAETCLPHLLVLLSLSVFFVYDVSIARAN-PDFPKTTRII
contig030572-ZebOR.A009   DS-NIKGIFCNN-AVYTLQC--ERSKLITI-FGVVIVLDLAILPMLFIVFTYTKIFIVSHRSC---KEIRKKAAETCLPHLLVLLSLSVFFVYDVSIARAN-PDFPKTTRII
contig085012-BriOR.A006   DS-NIKGIFCNN-AVYTLQC--ERSKLITI-FGVVIVLDLAMLPMLFIVFTYTKIFVVSHRSC---KEIRKKAAETCLPHLLVLLSLSVFFVYDVSIARAN-PDFPKTTRII
contig022251-TilOR.A013   DS-NIKGIFCNN-AVYTLQC--ERSRLITI-FGVVALLDLVILPMLFIVFTYTKIFIVSHRSC---KEIRKKAAETCLPHLLVLLSLSVFFVYDVSIARAN-PDFPKTTRII
contig030576-ZebOR.A010   DS-NIKGIFCNN-AVYSLQC--QRSRLIII-FGVIALLDLVILPMLFIVFTYTTIFIVSCQSC---KEIRKKAAETCLPHLLVLISACLFFVYDVSIARVE-ADFPKTARII
contig085018-BriOR.A006   DS-NIKGVFCNN-AVYTLQC--QRSRLIIV-FGVVCLLDLVILPMLFIVFTYTTIFIVSHQSC---KEIRKKAAETCLPHLLVLISACLFFVYDVSIARVE-ADFPKTARII
contig062344-NyeOR.A020   DS-NIKGIFCNN-AVYTLQC--QRSRLIII-FGVIALLDLVILPMLFIVFTYTTIFIVSYQSC---KEIRKKAAETCLPHLLVLISACLFFVYDVSIARVE-ADFPKTARIV
contig022241-TilOR.A011   DS-NIKGIFCNN-AVYTLQC--QRSRLITV-FGVVTLLDIVILPMLFIVFTYTTIFIVSHQSC---KEIRKKAAETCLPHLLVLISDCLFFMYDVSIARVE-LDFPKTVRII
contig022245-TilOR.A012   DS-NIKGIFCNN-AVYTLQC--QRSRLITI-FGVVGLLDLAILPMLFIVFTYTIIFIVSYQSC---KEIRKKAAETCLPHLLVLISASVFFVYDISIARVE-LGFPKTVRVI
contig022217-TilOR.A004   DF-KIKGIFCNN-AVYTLQC--QRSRLITI-FGVVTLLDLAILPMLFIVFTYTKIFIVSHQSC---KEIRKKAAETCLPHLLVLISACLFYVYDVSIARVE-LDFPKTARII
contig047521-ZebOR.A020   DF-NIKGIFCNN-AVYTLLC--ERSRLITI-FGVVALLDLAVLPMLFIVFTYTKIFIVSYQRC---KEIQKKAAETCLPHLLVLISASVFFVYDVSIARVE-TNFPKTVRIV
contig054237-BurOR.A013   DF-NIKGIFCNN-AVYTLLC--ERSRLITI-FGVVALLDLAVLPMLFIVFTYTKIFIVSYQRC---KEIQKKAAETCLPHLLVLISASVFFVYDVSIARVE-TNFPKTVRIV
contig034981-NyeOR.A001   DF-NIKGIFCNN-AVYTLLC--ERSRLITI-FGVVALLDLAVLPMLFIVFTYTKIFIVSYQRC---KEIQKKAAETCLPHLLVLISASVFFVYDVSIARVE-TNFPKTVRIV
contig085026-BriOR.A008   DF-NIKGIFCNN-AVYTLLC--ERSRLITI-FGVVALLDLAVLPMLFIVFTYTKIFIVSYQRC---KEIQKKAAETCLPHLLVLISASVFFVYDVSIARVE-TNFPKAVRIV
contig022211-TilOR.A003   DF-NIKGIFCNN-AVYTLLC--ERSRLITI-FGVVALLDLAVLPMLFIVFTYTKICIVSYQSC---KEIQKKAAETCLPHLLVLISASVFFVYDVSIARVE-TNFPKTVRIV
contig034988-NyeOR.A005   NF-NLKGIFCNN-AVYTLLC--VKSRLIIV-FGVVALIDLIILPVLFIVFTYTNIFIISYQSC---KEIRKKAAETCLPHLLVLISISCLSIYDVSIARVE-SDFPKAARLL
contig047508-ZebOR.A015   NF-NLKGIFCNN-AVYTLLC--VKSRLIIV-FGVVALIDLIILPVLFIVFTYTNIFIISYQSC---KEIRKKAAETCLPHLLVLISISCLSIYDVSIARVE-SDFPKAARLL
contig051570-BurOR.A010   NF-NLKGIFCNN-AVYTLLC--VKSRLIIV-FGVVALIDLIILPVLFIVFTYTNIFIISYQSC---KEIRKKAAETCLPHLLVLISISCLSIYDVSIARVE-SDFPKAARLL
contig070885-TilOR.A024   NF-NLKGIFCNN-AVYTLQC--VRSRLIIV-FGVVALIDLIILPLLFIVFTYTNIFIISYQSC---KEIRKKAAETCLPHLLVLISISCLSIYDVSIARVE-SDFPKIARLL
contig047503-ZebOR.A013   NF-NLKGIFCNN-AVYTLHC--QRSRLITV-FGVVALLDLVILPMIFIVFTYTTIFIVSYQSC---KEIRKKAAETCLPHLLVLISISCLSIYDVGIARVE-SDFPKVARLL
contig051559-BurOR.A008   NF-NLKGIFCNN-AVYTLQC--VKSRLITV-FGVVALIDLVILPMLFIVFTYSNIFILTYQSC---KDVRKKALETCLPHLLVLFSFSCLSIYDVSIARVE-SDFPKTARLI
contig047515-ZebOR.A018   NF-NLKGIFCNN-AVYTLQC--VKSRLITV-FGVVALIDLVILPMLFIVFTYSNIFILTYQSC---KDVRKKALETCLPHLLVLFSFSCLSIYDVSIARVE-SDFPKTARLI
contig034983-NyeOR.A003   NF-NLKGIFCNN-AVYTLQC--VKSRLITV-FGVVALIDLVILPMLFIVFTYSNIFILTYQSC---KDVRKKALETCLPHLLVLFSFSCLSIYDVSIARVE-SDFPKTARLI
contig022232-TilOR.A008   NF-NLKGIFCNN-AVYTLQC--VKSRLITV-FGVVALIDLVILPMFFIVFTYSNIFILTYQSC---KEVRKKAAETCLPHLLVLFSFSCLSIYDVSIARVE-SDFPKTARLI
contig022234-TilOR.A009   NV-NLKGIFCNN-AIYTLQC--VRSRLNTV-FGVVSLIDLVILPMVFVVFTYTKIFIVSYQSG---KEIRKKAAETCLPHLLVLINFSCLSIYDVSIARVE-SDFPKTARLI
contig022204-TilOR.A001   SF-HLKSIFCNN-TIYTLQC--VRSRLVTV-FGIVSYLDLVVFPILFIVFTYTKIFIVTYRSC---KEIKKKAAETCLPHMLVLVTFSCLGVYDVIMARME-TDFPKTARLI
contig022225-TilOR.A005   SF-HLKSIFCNN-TIYTLQC--VRSRLVTV-FGIVSYLDLVVFPILFIVFTYTKIFIVTYRSC---KEIKKKAAETCLPHMLVLITFSCLGVYDVIMARME-TDFPKIARLI
contig022227-TilOR.A006   SF-HLKSIFCNN-TIYTLQC--VRSKLVTV-FGIVCFFDFVILPLLFIVFTYTKIFIVSYHSC---KEIKKKAAETCLPHILVLVSFSCFGVYDVTIARVE-SDFPKTARFI
contig074640-TilOR.A002   SF-YLNETFCNN-RIYTLQC--LRSEFFAA-FGLVCLLDLGILPLLFILYTYTKIFLMSYRSC---KEIRKKAAETCLPHLSVLISFSCLGGYDVVITRVE-SDFPKTARLI
contig057165-NyeOR.A017   NF-TFTGIFCNN-TIYKLLC--VYSKAQTV-YDMVVLSNVAILPAVFIFFTYTRILVISYQSC---KEVRRKAAQTCLPHLIVLISYLCLCAFDVIVSGLE-SNFPKIVHSI
contig030552-ZebOR.A001   NF-TFTGIFCNN-TIYKLLC--VYSKAQTV-YDMVVLSNVAILPAVFIFFTYTRILVISYQSC---KEVRRKAAQTCLPHLIVLISYLCLCAFDVIVSGLE-SNFPKIVHSI
contig036780-BurOR.A001   NF-TFTGIFCNN-TIYKLLC--VYSKAQTV-YDMVVLSNVAILPAVFIFFTYTRILVISYQSC---KEVRRKAAQTCLPHLIVLISYLCLCAFDVIVSGLE-SNFPKIVHSI
contig054684-NyeOR.A012   SF-TLNGIFCNN-AISKLYC--DTSRTTYIIYGVFILLNTVFLPLLFILFTYTKIFIICYRSC---REVRKKAAQTCLPHLLVLVSFSGLCSYDIIVARLE-MNLPKVARFI
contig057156-BurOR.A015   SF-TLNGIFCNN-AISKLYC--DTSRTTYIIYGVFILLNTVFLPLLFILFTYTKIFIICYRSC---REVRKKAAQTCLPHLLVLVSFSGLCSYDIIVARLE-MNLPKVARFI
contig030560-ZebOR.A007   SF-TLNGIFCNN-AISKLYC--DTSRTTYIIYGVFILLNTVFLPLLFILFTYTKIFIICYRSC---REVRKKAAQTCLPHLLVLVSFSGLCSYDIIVARLE-MNLPKVARFI
contig085010-BriOR.A005   SF-TLNGIFCNN-AISKLYC--DTSRTTYIIYGVFILLNTVFLPLLFILFTYTKIFMICYQSC---REVRKKAAQTCLPHLLVLVSFSGLCSYDIIVARLE-MNLPKVARFI
contig022265-TilOR.A017   NF-TLNGIFCNN-AISKLYC--DTSRTTYIIYGVFILLNTIFLPLLFILFTYTKIFIICYRSC---REVRKKAAQTCLPHLLILVSFSCFCSYDIIIARLE-MNLPNVARFI
contig054678-NyeOR.A010   NF-TLNGIFCNN-AISKLYC--ATPKTYYLMYGVFILFNTVFLPLLFIMFTYTKIFIICYRSC---REVRKKAAQTCLPHVLVLISFTCLCSYDIIIARLE-IDLSQNTRFI
contig030556-ZebOR.A005   SF-TLNGIFCNN-AISKLYC--ATPKTYYLMYGVFILFNTVFLPLLFIMFTYTKIFIICYRSC---REVRKKAAQTCLPHLLVLISFTCLCSYDIIIARLE-IDLSQNTRFI
contig036787-BurOR.A004   NF-TLNGIFCNN-AISKLYC--ATPKTYYLMYGVFILFNTVFLPLLFIMFTYTKIFIICYRSC---REVRKKAAQTCLPHLLVLISFTCLCSYDIIIARLE-IDLSQNTRFI
contig085002-BriOR.A004   NF-TLNGIFCNN-AISKLYC--ATPKTSYLMYGVFILFNTVFLPLLFIMFTYTKIFIICYRSC---REVRKKAAQTCLPHLLVLISFTCLCSYDIIIARLE-INLSQTIRFI
contig030554-ZebOR.A004   SF-TLNGIFCNN-AISKLYC--ATPKISYLIYGVFILLNTVFLPLLFILFTYTKIFIICYRSC---REVRKKAAQTCLPHLLVLISFSCLCSYDIIITRVE-INLSQTARFI
contig084999-BriOR.A002   RF-TLNGIFCNN-AISKLYC--ATPKISYLIYGVFILLNTVFLPLLFILFTYTKIFIICYRSC---REVRKKAAQTCLPHLLVLISFSCLCSYDIIITRVE-INLSQTARFI
contig022268-TilOR.A019   NF-TLNGIFCNN-AISKLYC--ATPKTSYLIYGVFILINTVFLPLLFILFTYTKIFIICYQSC---REVRRKAAQTCLPHLLVLISFSCLCSYDIIITRLE-INLSQTARFI
contig047514-ZebOR.A017   SF-TLTGIFCNN-SIYRLQC--VPSVTISI-YGVVTLINIALLPMLFILFTYIRILRISYNCC---RETRRKALKTCLPHLLVLINFSCFIVFDSVIIRLD-SDLSKTLRLT
contig093807-BriOR.A009   NF-TLTGIFCNN-SIYRLQC--VPSVTISI-YGVVTLINIALLPMFFILFTYIRILRISINCC---RETRRKALKTCLPHLLVLINFSCFIVFDSVIIRLD-SDLSKTLRLT
contig022238-TilOR.A010   SF-TLTGIFCNN-SIYRLQC--VPSVVISI-YGVVTLINVALLPMLFILFTYIRILRISYNCC---RETRRKALKTCLPHLLVLINFSCFIVFDSVIIRLD-SDLSKTLRLT
contig034988-NyeOR.A004   SF-TLKAIFCNN-SFYRLQC--VPSVVISI-YGVVMLINMTFLPMLFILFSYIRILRISYSCC---RETRRKALKTCLPHLLVLINFSCFFFFDIIIVRLE-SDLSNTVRLT
contig047508-ZebOR.A016   SF-TLTAIFCNN-SFYRLQC--VPSVVISI-YGVVMLINMTFLPMLFILFSYIRILRISYSCC---RETRRKALKTCLPHLLVLINFSCFFFFDIIIVRLE-SDLSNTVRLT
contig051566-BurOR.A009   SF-TLTAIFCNN-SFYRLQC--VPSVVISI-YGVVMLINMTFLPMLFILFSYIRILRISYSCC---RETRRKALKTCLPHLLVLINFSCFFFFDIIIVRLE-SDLSNTVRLT
contig047526-ZebOR.A021   SF-TLTGIFCNN-SFYRLQC--VPSVVISV-YGVVMLINVAFLPMLFILFSYIRILRISYSCC---RETRRKALKTCLPHLLVLINFSCFFFFDIIIVRLE-SDLSNTVRLT
contig070886-TilOR.A025   SF-TLTGIFCNN-SFYRLQC--VPSVVISI-YGVVMLINIALLPMLFILFSYIRILRISYNCC---RETRRKALKTCLPHLLVLINFSCFFFFDIIIVRLE-SDLSNTLRLT
contig041952-TilOR.A023   SF-TLTGIFCNN-SIYKLQC--VPSVAISI-YGVVMFINLVLLPMLFILFTYIRILRISYHCC---RETRRKALKTCLPHLLVLVNFSCFIFFDVIIVRLD-SDLSKTLRLT
contig034990-NyeOR.A007   SF-SLTGIFCNN-SLYKLQC--VPSVAISI-YGMVMLINIALLPLLFILFTYIRIIRISYHCC---REVRKKAVKTCLPHLLVLINFSCFIFLDIIIVRLD-SDLSKTLRLT
contig047499-ZebOR.A012   SF-TLTGIFCNN-SLYKLQC--VPSVAISI-YGMVTLINIALLPLLFILFTYIRIIRISYHCC---REVRKKAVKTCLPHLLVLINFSCFIFLDIIIVRLD-SDLSKTLRLT
contig093812-BriOR.A010   SF-TLTGIFCNN-SLYKLQC--VPSVAISI-YGMVMLINIALLPMLFILFTYIRIIRISYHCC---REVRKKAVKTCLPHLLVLINFSCFIFFDVIIVRLD-SDLSKTLRLT
contig047497-ZebOR.A011   SF-SLTGIFCNN-SLYKLQC--VPSVAISI-YGVVMLIRIALLPLLFILFTYIRILRISYHCC---REVRKKAVKTCLPHLLVLTNFSCFIFFDIIIVRLD-SDLSKTLRLT
contig034995-NyeOR.A009   SF-TLTGIFCNT-SLYKLQC--VPSVAISI-YSMVMLINIALLPLLFILFTYIRILRISYHCC---REVRKKAVKTCLPHLLVLTNFSCFIFFDIIIVRLD-SDLSKTLRLT
```

```
contig034994-NyeOR.A008  SF-TLTGIFCNN-SLYKLQC--VPSVAISI-YGVVMLINIAFLPLLFILFTYIRILRISYHCC---REVRKKAVKTCLPHLLVLINFSCFIFFDIIVRLD-SDLSKTLRLT
contig093816-BriOR.A011  SF-TLTGIFCNN-SVHKLQC--VPSVAISI-YGVVMLINIALLPLLFILFSYIKILKISYQRC---REVRKNAVKTCLPHLLVLIHFSCFISFDIIVRLE-TDLSKTLRLI
contig051573-BurOR.A011  SF-TLTGIFCNN-SVHKLQC--VPSVAISI-YGVVMLINIALLPLLFILFSYIKILKISYQRC---REVRKKAVKTCLPHLLVLIHFSCFISFDIIVRLE-TDLSKTLRLI
contig054233-BurOR.A012  SF-TLTGIFCNN-SGYKLQC--VTSVAISV-YGVVMLINIALLPLLFILFTYIRIVRVSYQSC---REVRKKAVKTCLPHLLVLINFSCFIVFDVIIVRLE-SDLSKTLRLI
contig047523-ZebOR.A022  SF-TLTGIFCNN-SGYKLQC--VTSVAISV-YGVVMLINIALLPLLFILFTYIRIVRVSYQSC---REVRKKAVKTCLPHLLVLINFSCFIVFDVIIVRLE-SDLSKTLRLI
contig054868-NyeOR.A014  SF-TLTGIFCNN-SGYKLQC--VTSVAISV-YGVVMLINIALLPLLFILFTYIRIVRVSYQSC---REVRKKAVKTCLPHLLVLINFSCFIVFDVIIVRLE-SDLSKTLRLI
contig073309-TilOR.A026  SF-TLTGIFCNN-SGYKLQC--VQSVAISI-YGVVMLINIALLPMLFILFTYIRIVRISYQSC---REVRKKAVKTCLPHLLVLINFSCFIVFDVVIVRLE-SDLSKTLRLI
contig030566-ZebOR.A008  NF-TLKGILCNN-SLWKLYC--ESPRATLI-YGLVVMLSVVIFPVFFILFTYAKIFLITYRSS---RAIQKKAAETCLPHLFVLSIFTTLCAYDVIIGRLE-LDFPKTAQLI
contig065887-BurOR.A018  NF-TLKGILCNN-SLWKLYC--ESPRATLI-YGLVVMLSVVIFPVFFILFTYAKIFLITYRSS---RAIQKKAAETCLPHLFVLSIFTTLCAYDVIIGRLE-LDFPKTAQLI
contig054687-NyeOR.A013  NF-TLKGILCNN-SLWKLYC--ESPRATLI-YGLVVMLSVVIFPVFFILFTYAKIFLITYRSS---RAIQKKAAETCLPHLFVLSIFTTLCAYDVIIGRLE-LDFPKTAQLI
contig022264-TilOR.A016  NF-TLKGILCNN-SLWKLYC--ESPRATLI-YGLIAMLNVVIFPVFFILFTYAKIFLITYRSS---RAIQKKAAETCLPHLFVLSIFTTLCAYDVIIGRLE-LDFPKTAQLI
contig036784-BurOR.A003  NF-TLKGIFCNN-LLWKLYC--ESPRTALI-YGLIVLLNVAIFPVLFILFTYAKIFLITYRSS---RDIQKKAAETCLPHLFVLSIFTTFCAYDVIIGQLE-FDFPKTAQLI
contig057754-NyeOR.A018  NF-TLKGIFCNN-LLWKLYC--ESPRAALI-YGLIVLLNVAIFPVLFILFTYAKIFLITYRSS---RDIQKKAAETCLPHLFVLSIFTTFCAYDVIIGQLE-FDFPKTAQLI
contig030553-ZebOR.A003  NF-TLKGIFCNN-LLWKLYC--ESPRAAFI-YGLIVLLNVAIFPVLFILFTYAKIFLITYRSS---RDIQKKAAETCLPHLFVLSIFTTFCAYDVIIGQLE-FDFPKTAQLI
contig085000-BriOR.A003  NF-TLKGIFCNN-SLWKLYC--ESPRAALI-YGLIVLLNVAIFPVLFILFTYAKIFLITYRSS---RDIQKKAAETCLPHLFVLSIFTTFCAYDVIIARLE-FDFPKIAKLI
contig009320-TilOR.B045  RS-QLSRIYCDIYSLVSLSCGGRETLLSEV-YNLSVIVATVLLPAIFVLFSYSAVLSICLRRS---RSFSSKALSTCLPHLLVFCNYSVSSGVEVLQRRLQ-AGSQPTASVL
contig053886-ZebOR.B034  RS-QLSRIYCDIYSLVSLSCGGRETLLSEV-YNLSVIVATVLLPAIFVLFSYSAVLSVCLQRS---RSFSSKALSTCLPHLLVFCNYSVSSGVEVLQRRLQ-AGSQPTASVL
contig044492-NyeOR.B034  RS-QLSRIYCDIYSLVSLSCGGRETLLSEV-YNLSVIVATVLLPAIFVLFSYSAVLSVCLQRS---RSFSSKALSTCLPHLLVFCNYSVSSGVEVLQRRLQ-AGSQPTASVL
contig040653-BurOR.B030  RS-QLSRIYCDIYSLVSLSCGGRETLLSEV-YNLSVIVATVLLPAIFVLFSYSAVLSVCLRRS---RSFSSKALSTCLPHLLVFCNYSVSSGVEVLHRRLQ-AGSQPTASVL
contig049299-BurOR.E046  GN-VINKVYCLNYSIVKLAC--SETTANNI-YGLFITALTVFVPVILILCSYVRILKVCFSGS---KQTRQKAVSTCTPHLASLLNFSFGVCFEVIQSRFSLSSVHSMVHIV
contig053579-NyeOR.E053  GN-VINKVYCLNYSIVKLAC--SETTANNI-YGLFITALTVFVPVILILCSYVRILKVCFSGS---KQTRQKAVSTCTPHLASLLNFSFGVCFEVIQSRFSLSSVHSMVHIV
contig017699-BurOR.E042  GN-IINKVYCDNHSIVKLAC--SDTTLNNI-YGLTVSALSVFGPLIVILYTYTRILKVCFSGS---KQTRQKAVSTCTPHLASLLNFSFGACFEILQSRFNMNSSPNMLRIF
contig023280-NyeOR.E050  GN-IINKVYCDNHSIVKLAC--SDTTANNI-YGLTVSALSVFGPLIVILYTYTRILKVCFSGS---KQTRQKAVSTCTPHLASLLNFSFGACFEILQSRFNMNSSPNMLRIF
contig004266-BriOR.E039  GN-IINKVYCDNHSIVKLAC--SDTTLNNI-YGLTVSALSVFGPLIVILYTYTRILKVCFSGS---KQTRQKAVSTCTPHLASLLNFSFGACFEILQSRFNMNSSPNMLRIF
contig025447-ZebOR.E047  GN-IINKVYCDNHSIVKLAC--SDTTLNNI-YGLTVCALSVFGPLIVILYTYMRILKVCFSGS---KQTRQKAVSTCTPHLASLLNFSFGACFEILQSRFNMNSSPNMLRIF
contig004265-BriOR.E035  GN-TISKVYCDTHSVVKLAC--SDTTVINL-YGLLATFSTIFGALLFILYTYMKILLVCFSGS---DQTRQKAVSTCTPHLASILNFSFGASFEILQSRFNMKNVPNMLRIF
contig065454-TilOR.E088  GN-TIYKVYCDTHSVVKLAC--SDTTVINL-YGLLATFSTIFGALLFILYTYMKILLVCFSGS---DQTRQKAVSTCTPHLASILNFSFGASFEILQSRFNMKNVPNMVRIF
contig065454-TilOR.E089  GN-TIYKVYCDTHSVVKLAC--SDTTVINL-YGLLATFSTIFGALLFILYTYMKILLVCFSGS---DQTRQKAVSTCTPHLASILNFSFGASFEILQSRFNMKNVPNMVRIF
contig052450-BurOR.E055  GN-TIYKVYCDTHSVVKLAC--SDTTVINL-YGLLATFSTIFGALLFILYTYMKILLVCFSGS---DQTRQKAISTCTPHLASILNFSFGASFEILQSRFNMKNVPNMLRIF
contig004261-BriOR.E034  GN-TISKVYCDTHSVVKLAC--SDTTVINL-YGLLATFSTIFGALLFILYTYMKILLVCFSGS---DQTRQKAVSTCTPHLASILNFSFGASFEILQSRFNMKNVPNMLRIF
contig065453-TilOR.E087  GN-TIYKVYCDTHSVVKLAC--SDTTVINL-YGLLATFSTIFGALLFILYTYMKILLVCFSGS---DQTRQKAVSTCTPHLASILNFSFGASFEILQSRFNMKNVPNMVRIF
contig062770-NyeOR.E059  GN-IINKVYCDNYSIVKLAC--SDTTVNNI-YGLISSPLVILCPVSLILYTYMRILKICFSGS---KQTRQKAVSTCTPHLASLLNFSFGCFFEILQSRFNMNSVPSMLRIF
contig048239-ZebOR.E048  GN-IINKVYCDNYSIVKLAC--SDTTVNNI-YGLISTPLVILCPVSLILYTYMRILKICFSGS---KQTRQKAVSTCTPHLASLLNFSFGCFFEILQSRFNMNSVPSMLRIF
contig052457-BurOR.E051  GN-IINKVYCDNYSIVKLAC--SDTTVNNI-YGLISSPLVILCPVSLILYTYMRILKICFSGS---KHTRQKAVSTCTPHLASLLNFSFGCFFEILQSRFNMNSVPSMLRIF
contig047729-TilOR.E076  GN-IINKVYCDNYSIVKLAC--SDITVNNI-YGLIATCLTILCPVSLILYTYMRILKICFSGS---KQTRQKAVSTCTPHLASLLNFSFGCFFEILQSRFNMSSVPSMLRIF
contig047734-TilOR.E077  GN-IINKVYCDNNSIVKLAC--SDTTANNI-YGLIATTLTTISSVSLILYTYMRILKVCFSGS---KQTRQKAISTCTPHLASLLNFSCGAFFETAQSRFNMKHVPNRVRIF
contig059404-NyeOR.E058  GN-IINKVYCDNYSIVKLAC--SDTTVNNI-YGILGAIFITISSVTLILYTYMRILKVCFSGS---KQTRQKAVSTCTPHLASLLNFSCGSFFESAQSRSNMKHVPNMVRIF
contig052453-BurOR.E049  GN-IINKVYCDNYSIVNLAC--SDTTVNNI-YGILGAIFITISSVTLILYTYMRILKVCFSGS---KQTRQKAVSTCTPHLASLLNFSCGSFFETAQSRSNMKHVPNMVRIF
contig064938-BurOR.E053  GN-IINKVYCDNYSVVKLAC--SDTTVNNI-YGLISTSLTTISAVSLILYTYTRILKVCFSGS---KQTRQKAVSTCTPHLVSLLNYSCSAFYEIAQSRLNMKHVPNMVRIF
contig004258-BriOR.E037  GN-IINKVYCDNYSIVKLAC--SDTTANNI-YGLFYT-FTLVLLVTLIFYTYIRILKVCFSGS---KQTRHKAVSTCTPHLASLLNFSCGAFFEIIQNRFDMRQLPNMLRIF
contig052452-BurOR.E048  GN-IINKVYCDNYSIVKLAC--SNTTANNI-YGILYT-FTLVLLVTLIFYTYMRILKVCFSGS---KQMRHKAISTCTPHLASLLNFSCGAFFEIIQNRFDMRQLPNMLRIF
contig025443-ZebOR.E046  GN-IINKVYCDNYSIVKLAC--SDTTANNI-YGILYT-FTLVLLVTLIFYTYMRILKVCFSGS---KQMRHKAISTCTPHLASLLNFSCGAFFEIIQNRFDMRQLPNMLRIF
contig065458-TilOR.E086  GN-IINKVYCDNYSIVKLAC--SDTKVNNI-YGILYT-LTLVVLVTLIFYTYMRILKVCFSGS---KQTRHKAISTCTPHLASLLNFSCGAFFEIIQNRFDMRQLPNMLRIF
contig047832-TilOR.E082  GN-IINKVYCDNYAIVKLAC--SDTTLNNI-YGLISTAFTTFVPLTLIFFTYMRILKVCFSGS---KQTRQKAVSTCTPHLASLLNFSFGACFEVLQSRFNMNTVPNILRIL
contig049298-BurOR.E045  GN-IINKVYCDNYAIVKLAC--SDTTLNNI-YGLISTAFTVFVPVTLIFFTYMRILKVCFSGS---KQTRQKAVSTCTPHLASLLNFSCSGCFEILQSRFNMNNLPNVLRIL
contig048260-ZebOR.E051  GN-IINKVYCDNYAIVKLAC--SDTTLNNI-YGLISTAFTAFVPVTLIFFTYMRILKVCFSGS---KQTRQKAVSTCTPHLASLLNFSCSGCFEIVQSRFNMNNVPNVLRIL
contig053579-NyeOR.E054  GN-IINKVYCDNYAIVKLAC--SDTTLNNI-YGLISTAFTAFVPVTLIFFTYMRILKVCFSGS---KQMRQKAVSTCTPHLASLLNFSCSGCFEILQSRFNMNNLPNVLRIL
contig047833-TilOR.E083  GN-IINKVYCDNYAIVKLAC--SDTTLNNI-YGLISTAFTVFVPLTLIFFTYMRILKVCFSGS---KQTRQKAVSTCTPHLASLLNFSCSGCFEILQSRFNMNNLPNVLRIL
contig053579-NyeOR.E055  GN-IINKVYCDNYAIVKLAC--SDTTLNNI-YGLISTAFTAFVPVTLIFFTYMRILKVCFSGS---KQMRQKAVSTCTPHLASLLNFSCSGCFEILQSRFNMNNLPNVLRIL
contig047829-TilOR.E081  GN-IINKVYCDNQSIVKLAC--SDTTVTNI-SGVIVSALTIFVPVTLILFSYMRILKVCFSGS---KQTRQKAVSTCTPHLVSLLNFSFGSFLEVLQSRFNNNVPNMLKIF
contig053576-NyeOR.E052  GN-VINKVYCNNHSIIKLGC--HGTTVNNL-YELTAASVTVCVPVSVILYTYTRILKICFSGS---KQTRQKAVSTCTPHLASLLNFSFGVSFEILQSRFDMSHVPNMLRIF
contig064724-BurOR.E052  GN-VINKVYCNNHSIIKLGC--HGTTVNNI-YELTAASVTVCVPLSVILYTYTRILKICFSGS---KQTRQKAVSTCTPHLASLLNFSFGVSFEILQSRFDMSHVPNMLRIF
contig048263-ZebOR.E052  GN-VINKVYCNNHSIIKLGC--HGATVNNL-YELTAASVTVCVPVSVILYTYTRILRICFSGS---KQTRQKAVSTCTPHLASLLNFSFGVSFEILQSRFDMSHVPNMLRIF
contig047826-TilOR.E080  GN-VINKVYCNNHSIIKLGC--HSTTVNNL-YELTAASVTVCVPVSVILYTYTRILKICFSGS---KQTRQKAVSTCTPHLASLLNFSFGVSFEILQSRFDMGHVPNMLRIF
contig025439-ZebOR.E045  GH-TVDTLYCNNYSVVKLAC--FDTTINNI-YGLMYTFTVLIGLALLNLFTYVKILKVCFSGS---KQTRQKAVSTCTPHLASLLNFSFGCFFEIVQSRFNLSRAPMILRIF
contig052454-BurOR.E050  GH-TVDTLYCNNYSVVKLAC--FDTTINNI-YGLMYTFTVLIGLALLNLFTYVKILKVCFSGS---KQTRQKAVSTCTPHLASLLNFSFGCFFEIVQSRFNLSRAPMILRIF
contig004255-BriOR.E036  GH-TVDALYCNNYSVVKLAC--FGTTINNI-YGLVYTFTVIIGLALLNLFTYVKILKVCFSES---KQTRQKAVSTCTPHLASLLNFSFGCFFEIVQSRFNMSRAPMILHIF
contig066194-BurOR.E054  GN-VINKVFCGNYAIIKLAC--SDTRVHNI-FGLIYTFISVIIPLVLILYTYVRILKVCFSGS---KQTRQKAVSTCTPHLASILNFFFGCCFQILQSRFDTSGVPNVFGIL
contig053572-NyeOR.E051  GN-VINKVFCGNYAIIKLAC--SDTRVHNI-FGLIYTFISVIIPLVLILYTYVRILKVCFSGS---KQTRQKAVSTCTPHLASILNFFFGCCFQILQSRFDTSGVPNVFGIL
contig047825-TilOR.E079  GN-VINKVFCGNYAIIKLAC--SDTRVHNI-FGLIYTFISVIIPLVLILYTYVRILKVCFSGS---KQTRQKAVSTCTPHLASILNFFFGCCFQILQSRFDTNGVPNVFGIL
contig047820-TilOR.E078  RG-IINKLYCDNYYIIKLAC--SDTTISSD-FGRVHMFTVIFGLILLILYSYMRIFKVCFYGS---KQTRQKAISTCTPHLASLFNFSCGAFFGIVQSSLNMNTLPTMFRIF
contig063018-ZebOR.E053  GN-IIDKVYCDNYSVIKLAC--SDTKALNI-----IVLCTVCCPLIFMLYTYIKILRVCSSGS---KQMRQKAVTTCSPHLACVLNFSCGACFEILQSRFNMSGIPILLRIF
contig004259-BriOR.E038  GN-IIDKVYCDNYSVIKLAC--SGTKALNI-YGIIIVLCTICCPLIFMLYTYIKILRVCSSGS---KQVRQKAVTTCSPHLACVLNFSCGACFEILQSRFNMSGVPILLRIF
contig052451-BurOR.E047  GN-IIDKVYCDNYSVIKLAC--SDTKALNI-YGIIIVLCTVCCPLIFMLYTYIKILRVCSSGS---KQVRQKAVTTCSPHLACVLNFSCGACFEILQSRFNMSGVPILLRIF
contig065455-TilOR.E085  GN-IINKVYCDNYSVIKLAC--SDTKALNI-YGIIIVLCTVCCPLVFMLYTYIKILRVCSSGS---KQVRQKAISTCSPHLACVLNFSCGACFEILQSRFNMSGVPILLRIF
contig082838-BriOR.E040  GK-IINGLYCHNYLVVKLAC--SDTNLNNL-FGLFGIVITVLVPLLPIFYSYMKILKVCFSGS---RQMRRKAVSTCVPHLASLLNFSFGCLFEILQSRFDTTSVPSALRIF
contig053592-NyeOR.E057  GK-IINGLYCHNYLVVKLAC--SDTNLNNL-FGLFGIVITVLVPLLPIFYSYMKILKVCFSGS---RQMRRKAVSTCAPHLASLLNFSFGCLFEILQSRFDTTSVPSALRIF
contig049287-BurOR.E043  GK-IINGLYCHNYLVVKLAC--SDTNLNNL-FGLFGIVITVLVPLLPIFYSYMKILKVCFSGS---RQMRRKAVSTCAPHLASLLNFSFGCLFEILQSRFDTTSVPSALRIF
contig048242-ZebOR.E049  GK-IINGLYCHNYLVVKLAC--SDTNLNNL-FGLFGIVITVLVPLLPIFYSYMKILKVCFSGS---RQMRRKAVSTCAPHLASLLNFSFGCLFEILQSRFDTTSVPSALRIF
```

```
contig047726-TilOR.E075   GK-IINGLYCHNYLVVKLAC--SDTNLNNL-FGLFGTVITVLVPLLPIFYSYMKILKVCFSGS---REMRCKAVSTCAPHLASLLNFSFGCLFEILQSRFDTTSVPSALRIF
contig053590-NyeOR.E056   GN-VLNSLYCQNYLVVMLAC--SSTKVNNV-YGIFDIIMTIIVPTLIILFSYMKILKVCFYGS---KQTRQKSLTTCTPQLVSLLNFSFGCCFEIFQSRFDTTGLPAALRIF
contig049289-BurOR.E044   GN-VLNSLYCQNYLVVRLAC--SSTKVNNV-YGIFDIIMTIIVPTLIILFSYMKILKVCFYGS---KQMRQKSLTTCTPQLVSLLNFSFGCCFEIFQSRFDTTGLPAALRIF
contig048243-ZebOR.E050   GN-VLNSLYCQNYLVVRLAC--SSTKVNNV-YGIFDIIMTIIVPTLIILFSYMKILKVCFYGS---KQTRQKSLTTCTPQLVSLLNFSCGCCFEIFQSRFDTTGLPAALRIF
contig047725-TilOR.E074   GN-FLNSLYCQNYLVVRLAC--SGTKVNNV-YGIFDIIITIIVPTLIILFSYMKILKVCFYGS---KQMRQKSLTTCTPQLVSLLNFSFGCCFEIFQSRFDTTGLPGALRIF
contig047834-TilOR.E084   GN-TINSLYCSNYLIVKLAC--SGTKVNNI-YGLFNTAVSILVPLLPVLYSYIKILIICFSRS---KQARRKAINTCAPHLASLLSFYFGCLFEILQSRFDMSSVPSTLRIF
contig014049-ZebOR.D038   EN-IIHNVYCDHQLIIRLSC--SVSIQSFI-SDISFATVSVFIPFSLILVSYMKILRVCRKTS---KENKQKAVTTCTPQIISVSNLFVGYIFYFIDFRSVVSQVLDEVHII
contig013327-TilOR.D052   GN-VIHNTYCDHQLIIRLSC--SVSIQSFI-SDISFATVSVFIPFSFILVSYMKILTVCLKTS---KENKQKAVTTCTPQIISVSNLFVGCIFYFIDFRFVVSQVPDEVHII
contig039737-NyeOR.D039   GN-IIHSVYCDHQLIIRLSC--PVSIQSFI-SDISFAILSVFIPFSLILVSYMKILGVCRKTS---KENKQKAVTTCTPQIISVSNLFVGYIFYFIDFRSVVSQVPDEVHII
contig032396-BurOR.D034   GN-IIHNTYCDHQLIIRLSC--SVPIQSFI-SNISFLLLSVFIPFSLILSVSYMKILRVCRKTS---KENKQKAVTTCTPQIICVSNLFVGCIFYFIDIRFLVSQVPDEVRII
contig064809-BriOR.D028   GR-IIHNTYCDHQLIIRLSC--SVPIQSFI-SNISFLLLSVFIPFSLILVSYMKILRVCRKTS---KENKQKAVTTCTPQIISVSNLFVGCIFYFIDFKFVVSQVPDEVRIV
contig014050-ZebOR.D039   GN-IIHNAYCDHQLIIRLSC--AVPIQSFI-SNISFLLLSVFIPFSLISVTYMKILRVCRKTS---KENKQKAVTTCTPQIISVSNLFVGCIFYFIDFKFLVSQVPDEVRII
contig013326-TilOR.D051   GN-IIHSVYCDHQLIIRLSC--SFSIQSFI-SDISFAILSFFIPFSFILVSYLKILRVCLKTS---KENKQKAVTTCTPHIISVSNLFVGCIFYFIDFRFLVSQVPVEVRII
contig014051-ZebOR.D040   GN-IIHSVYCDHQLIIRLSC--AVPIQSFI-SDISFAIVSVFIPFSLISFSYLRILRVCQKTS---KENKQKAVTTCTPQIISVSNLCVGCICYFIDFRFLVSRVPDEVRII
contig013337-TilOR.D055   GN-IIHSVYCDHQLIIRLSC--SVSIQSFI-SDISFAIVSFFIPFSLTLVSYLKILRVCRKTS---KENKQKAVTTCTPQIISVSNLCVGCIFNLIDFRFVVSQVPDEVRII
contig013330-TilOR.D054   GN-IIHSVYCDHQLIIRLSC--SVSIQSFI-SDISFAIVSFFIPFSLTLVSYLKILRVCRKTS---KENKQKAVTTCTPQIISVSNLCVGCIFNLIDFRFVVSQVPDEVRII
contig013339-TilOR.D057   GN-IIHSVYCDHQLIIRLSC--SVSIQSFI-SDISFAIVSFFLPFSLILVSYLKILRVCRKTS---KENKQKAVTTCTPQIVSVSNLCVGCICYFIDFRFLVSQVPDEVRII
contig032389-BurOR.D033   GN-IVDNVYCEHQLIIRLSC--SVSVQSSI-SIIFFVIMSIFIPFSLISVSYVKILTVCRKTS---TENKQKAMTTCTPQIVSVSNLFVGCIFHSINFRLLISQVPDEVNII
contig039737-NyeOR.D040   GN-IVDNVYCEHQLIIRLSC--SVSVQSSI-SIIFFVIMSIFIPFSLISVSYVKILTVCRKTS---TENKQKAMTTCTPQIVSVSNLFVGCIFHSINFRLLISQVPDEVNII
contig013339-TilOR.D056   GN-IVDNVYCDHKLIIKLSC--SASVQGSI-SIIFFVIMSFFIPFSLISVSYVKILRVCRKTS---KENKQKAVTTCTPQIVSLSNLFVGCICYSIDFRFLVSLVPDEVRTI
contig013330-TilOR.D053   GN-IVDNVYCEHQLIIRLSC--SVSIYNSI-SIIFFVIMSIFIPFSLISVSYVKILKVCRKTS---KENKQKAVTTCTPQIVSLSNLFVGCICYSIDFRFLVSLVPDEVRTI
contig064814-BriOR.D029   GN-IIDNVYCDHQLIIRLSC--SVPIQSSI-SMIFFTLLSIFMPFNLISISYLKILRVCRKTS---TENKQKAVTTCTPQIVSVSNLFVGCIFHSIDFSLLAAQIPDEVRIT
contig014054-ZebOR.D041   GN-IIDNVYCDHQLIIRLSC--SVPIQSSI-SMIFFTLLSIFMPFNLISISYLKILRVCRKTS---TENKQKAVTTCTPQIVSVSNLFVGCIFHSIDFSLLAPQIPDEVRII
contig039731-NyeOR.D038   GN-IIDNVYCDHQLIIRLSC--SVPIQSSI-SMIFFTLLSIFMPFNLISISYLKILRVCRKTS---TENKQKAVTTCTPQIVSVSNLFVGCIFHSIDFSLLAAQIPEEVRII
contig014054-ZebOR.D042   GN-VVNNVYCDHKLIIQLSC--SVSVHSFI-SDIFFLLLSIFIPFSLISVSYMKILAVCRKTS---TENKQKAVTTCTPQIVSVSNLFVGCIFHAIDSSVLAAHVPGEVRII
contig039730-NyeOR.D036   GN-VVNNVYCDHKLIIRLSC--SVSIHSFI-SDIFFLLLSIFIPFSLISVSYMKILAVCRKTS---TENKQKAVTTCTPQIVSVSNLFVGCIFHAIDSSVLVAQVPGEVRII
contig053779-BurOR.D035   GN-VVNNVYCDHKLIIRLSC--SVSIHSFI-SDIFFLLLSIFIPFSLISFSYMKILAVCRKTS---TENKQKAVTTCTPQIVSVSNLFVGCIFHAIDSSVLVAQVPGEVRII
contig039730-NyeOR.D037   GN-IVNNVYCDHKLIIRLSC--SVLVHSFI-SDIFFLLLSIFIPFSLISVSYMKILAVCRKTS---TENKQKAVTTCTPQIISVSNLFVGCIFHSTDFSLLAAHVPGEVNII
contig013344-TilOR.D058   GD-IIDNVYCDHQLIIRLSC--SVSIQSSL-SIIFFVIMSILLPFTLISVSYLKILAVCRKTS---TENKQKAVTTCTPQIVSVSNLFIGCICYSIDFRFLVAQVPDEVRII
contig014049-ZebOR.D037   RN-IVENVYCDHQLMIRLSC--SVSTQSYI-SEIFFAIVSIFIPFTLISVSYFKILAVCRKTS---IENKQKAVTTCTPQIVSVSNLFVGGIFHSIDFRFLIARVPDEVRII
contig032388-BurOR.D032   RN-IVDNVYCDHQLMIRLSC--SVSTQSYI-SEIFFALVSIFIPFTLISVSYFKILAVCRKTS---IENKQKAVTTCTPQIVSVSNLFVGCIFHSIDFRFLIARVPDEVRVI
contig039738-NyeOR.D041   RN-IVENVYCDHQLMIRLSC--SVSTQSYI-SEIFFAIVSIFIPFTLISVSYFKILAVCRKTS---IENKQKAVTTCSPQIVSVSNLFVGCIFHSIDFRFLIARVPDEVRII
contig013323-TilOR.D050   RN-IIDNVYCDHQLMFKLSC--SVSTQSYI-SEIFFAIVSIFIPFTLISVSYFKILAVCRKTS---IENKQKAVTTCTPQIVSVSNLFVGCIFHSIDFRLLIARVPDEVRII
contig014047-ZebOR.D036   GT-IIENVYCNHRLILRLSC--FVSVHNFL-SDIFFLFVSFFIPFTLISVSYVKILAVCRKTS---TENKQKAVTTCVPQIVSVSNLFVGSIFQCIDSSVIVARLP--VRII
contig064802-BriOR.D027   GT-IIENVYCNHRLILRLSC--SLSVYSFL-SDIFFLIVSFFIPFTLISVSYVKILAVCRKTS---TENKQKAATTCVPQIVSVSNLFVGSIFQCIDSSVIFAHLP--VRII
contig013322-TilOR.D049   GN-NIENVYCDHQFLIGLSC--SLSVHSFI-ADIFVFVSIFIPFNLISVSYVKILAICRKTS---TENKQKAVTTCTPQIVSVSNLFVGCIFQSIDSSVIVAQLPHEVNII
contig039738-NyeOR.D042   GN-NIENVYCDHQLIIRLSC--SLSFHSFI-SDIFFVFVSIFIPFNLISVSYVKILAICRKTS---TENKQKAVTTCTPQIVSVSNLFVGCIFQSIDSSVIVAQLPHEVNII
contig013321-TilOR.D048   GN-NIENVYCNHRLIIRLSC--SLSVHSFI-SDIFFLIVSIFIPFTLISVSYVKILAVCQKTS---TENKQKAVTTCAPQIVSVSNLFVGSIFHSIDSSFIVTQVPNEVRII
contig066327-ZebOR.F064   GN-KIPKVFCANWPVVKLSC--VPTVINNI-IGMFVSVTTVFLPLAFVLYTYMQIFLVCRKRS---SLFKSKVIQSCLPHIVTFVNYSITVFCDVALSRIDLEELNPFLGII
contig013898-BurOR.F058   GN-KIPKVFCANWPVVKLSC--VPTVINNI-IGMFVSVTTVFLPLAFVLYTYMRIFLVCRKRS---SLFKSKVIQSCLPHIVTFVNYSITVFCDVALSRIDLEELNPFLGII
contig033883-BriOR.F045   DN-KIPKVFCANWPVVKLSC--VPTVINNI-IGMFVSVTTVFLPLAFVLYTYMRIFLVCRKRS---SLFKSKVIQSCLPHIVTFVNYSITVFCDVALSRIDLEELNPFLGII
contig009545-TilOR.F097   GN-KIPKVFCANWPVVKLSC--VPTVINNI-IGMFVSITTVFLPLAFVLYTYMRIFLVCRKRS---SVFKSKVIQSCLPHIVTFINYSITVFCDVALSRIDLEELNPFLSII
contig039729-NyeOR.F064   GN-EIHKVFCANWNVVKLSC--VNTAVNNV-VGMLLTVATIFLPLFYILYTYLRIVSICWKSS---AEFKGKVLESCLPHTISFVIYSIAGFCDVALSRNNLEVTNPFIAVI
contig053781-BurOR.F059   GN-EIHKVFCANWNVVKLSC--VNTAVNNV-VGMLLTVATIFLPLFYILYTYLRIVSICWKSS---AEFKGKVLESCLPHTISFVIYSIAGFCDVALSRNNLEVTNPFIAVI
contig014056-ZebOR.F063   GN-EIHKVFCANWNVVKLSC--VNTAVNNV-VGMLLTVATIFLPLFYILYTYLRIVSICWRSS---AEFKGKVLESCLPHTISFVIYSIAGFCDVALSRNNLEVTNPFIAVI
contig075822-TilOR.F098   GN-EIHKVFCANWNVVKLSC--VNTAVNNV-VGMLLTVATIFLPLFYILYTYLRIVAICWKSS---AEFKGKVLESCLPHTISFVIYSIAGFCDVALSRNNLEVINPFIAVI
contig025224-ZebOR.C035   GK-VMPKLYCVNMELVKNSC--RNAQYIST-VGLAILVLFIVPQLVMIVFSYTHILRVCRTFP---KESQANAFRTCVPHLLSLLNYTIASLFEVIQTRFNMSHVAVEAQIF
contig009805-BurOR.C031   GK-VIPKLYCVNMELVKNSC--TNAQYIST-VGLAILVLFIVPQLVMIVFSYTHILRVCRTFP---KESQANAFRTCVPHLLSLLNYTIASLFEVIQTRFNMSHVAVEAQIF
contig020980-NyeOR.C035   GK-VMPKLYCVNMELVKNSC--TNAQYIST-VGLAILVLFIVPQLVMIVFSYAHILRVCRTFP---KESQANAFRTCVPHLLSLLNYTIASLFEVIQTRFNMSHVAVEAQIF
contig063874-BriOR.C026   GK-VMPKLYCVNMELVKNSC--TNAQYIST-VGLAILVLFIVPQLVMIVFSYAHILRVCRTFP---KESQANAFRTCVPHLLSLLNYTIASFFEIIQTRFNMSHVAVEAQIF
contig048237-ZebOR.G065   GR-HIPKIFCTNWEVVKLSC--SDSTLNNI-YAFM-LIFSHLSQTALIMVSYVHLIRAAIR-S---QADRRKFMQTCLPHLITLLVFTTSLMFDTMYSRYSGGSTMKALQNA
contig049295-BurOR.G060   GR-HIPKIFCTNWEVVKLSC--SDLTLNNI-YAFM-LIFSHLSQTALIMVSYVHLIRAAIR-S---QADRRKFMQTCLPHLITLLVFTTSLMFDTMYSRYSGGSTMKALQNA
contig053581-NyeOR.G066   GR-HIPKIFCTNWEVVKLSC--SDSTVNNI-YAFM-LIFSHLSQTALIMVSYVHLIRAAIR-S---QADRRKFMQTCLPHLITLLVFTTSLMFDTMYSRYSGGSTMKALQNA
contig104344-BriOR.G048   GR-HIPKIFCTNWEVVKLSC--SDSTLNNI-YAFM-LIFSHLSQTALIMVSYVHLIRAAVR-S---QADRRKFMQTCLPHLSTLLVFSTSLMFDTMYSRYSGGSTMKALQNA
contig047714-TilOR.G099   RR-HIPKIFCTNWEVVKLSC--SDSTINNV-YAFM-LIFSHLSQATLIMVSYVHLIQAAIR-S---QADRRKFMQTCLPHLITLLFFTTSLMFDTMYSRYSGGSTMKALQNA
contig057403-ZebOR.H076   RS-KIEKVFCVNWVIVKLACSDTDTLLNSV-VSYA-TIIMYISHGFFIMWTYMHLIKTSVT-S---KEDRAKFMQTCVPHLTSLITFLIVILFDLMYMRFGSADLPQSLQNF
contig013371-TilOR.H117   RS-KVEKVFCVNWVIVKLACSDTDTLLNSV-VSYA-TIIIYMSHGFFIMWTYMHLIKTSVR-S---KEDRAKFMQTCVPHLTSLITFLIVIVFDLMYMRFGSADLPQSLQNF
contig035582-NyeOR.H070   RS-KIEKVFCVNWVIVKLACSDTDTLLNSV-VSYV-TIIIYLSHGFFIMWTYMHLIKTSVR-S---KEDRAKFMQTCVPHLTSLITFLIVILFDLMYMRFGSADLPQSLQNF
contig013361-TilOR.H108   GI-NIQRVLCLNWVIVKLACPEADTLSNNI-SAYV-IVVFYLSHWLFIIWTYIYLIKTCAR-S---SDDRVKFTQTCFPHLISLIIFLTVMAFDSMYLRFGSRDLPQSLQNF
contig013362-TilOR.H109   GI-NIQRVLCLNWVIVKLACPEADTLSNNI-SAYV-IVVFYLSHSLFIIWTYIYLIKTCAR-S---SEDRVKFTQTCVPHLISLIIFLTVMSFDSMYLRYGSRDLPQSLQNF
contig013368-TilOR.H112   GL-NIQRVLCVNWLIVKLACPEADTFSNNI-SSYL-TIIFYISHGFFIMWTYMHLIKTCVR-S---REDRVKFMQTCVPHLISLITLLVVMIFDSMYLRFGSRDLPQSLQNF
contig013369-TilOR.H115   GI-NIQRVLCLNWLIVKLACPEADTFSNNI-SSYV-ILVVYLSHWLFIMWTYMHLIKTCVR-S---REDRVKFMQTCVPHLISLITLLVVMIFDSMYLRFGSRDLPQSLQNF
contig013369-TilOR.H116   GL-NIQRVLCLNWLIVKLACPADKTFPNNI-SSYV-ILVVYLSHWLFIIWTYMHLIKTCVR-S---RDDRLKFMQTCVPHLTSLIIFLIVMAFDPAYVEFGSRDLSQTLQNL
contig013369-TilOR.H114   GI-NIQRVLCLNWLIVKLACPEADTFSNNI-SAYV-IFIFYVSHGFFIMWTYMHLIKTCVR-S---RDDRLKFMQTCVPHLTSLIIFLSVIVFQYVYLRFGSRDLSQSLQNF
contig048882-BurOR.H064   DI-NIQRVLCLNWLIVKLACPEADTFSNNI-SAYV-IFIFYVSHGFFIMWTYMHLIKTCVR-S---RDDRLKFMQTCVPHLTSLTTFLSVIVFQYVYLRLDSTDLFQSLQNF
contig035580-NyeOR.H069   GT-KIQRVLCLNWLIVKLACPEADTFSNNI-TAYA-TVIIYVSHGFFIIWTYTHLIKTCAK-S---REDRAKFMQTCVPHLTSLITFLSVVVFQFVYMRFDSTDLPQSLQNF
contig057400-ZebOR.H075   GT-KIQRVLCLNWLIVKLACPEADTFSNNI-TAYA-TVIIYVSHGFFIIWTYTHLIKTCAK-S---REDRAKFMQTCVPHLTSLITSLSVAVFQFVYMRFDSTDLPQSLQNF
contig013368-TilOR.H113   DI-KIQRVLCLNWLIIKLACPEADTFRKNI-SAYV-TFIAYLSHWLFIVWTYMHLIKTCVR-S---REDRVKFMQTCVPHLISLMTFLFVLVFDPVYMIFGSTRLPQSLQNF
contig006794-BurOR.H061   GI-KIQRVLCVNWLIVKLACPEAETFTNHI-AAYA-TVIIYVSHGFFIMWTYTHLIKTCAR-S---EDREKFMQTCLPHLTSLITFIVVIGFQSIYVQFEYTGFSESLKNF
```

contig035583-NyeOR.H071	GI-NIRRVLCVNWLIVKLACPEAETFTNHI-AAYA-TVIIYVSHGFFIMWTYTHLIKTCAR-S---REDREKFMQTCLPHLTSLITFIVVIGFQSIYVQFEYTGFSESLKNF
contig057403-ZebOR.H077	GI-NIRRVLCVNWLIVKLACPEAETFTNHI-TACA-TVIIYVSHGFFIMWTYTHLIKTCAR-S---REDREKFMPTCLPHLTSLITFIVVIGFQSIYVQFEYTGFSESLKNF
contig049873-BriOR.H050	GI-NIQRVLCVNWLIVKLACPEAETFTNHI-AAYA-TVIIYVSHGFFIMWTYTHLIKTCAR-S---REDREKFMQTCLPHLTSLITFIVVIGFQSIYVQFEYTGFSESLKNF
contig013371-TilOR.H118	GI-NIRRVLCVNWLIVKLACPEAETFTNNI-TAYA-TVIIYVSHGFFIMWTYTHLIKTCAR-S---REDREKFMQTCLPHLTSLITFIVVMGFQSIYVQFEYTGFSESLKNF
contig093825-BriOR.H053	GT-DIQRLFCVNWLIVKVACPGMDTLVNSA-FAYT-TLSIYIFHWIFIVWTYIYLVKSCVQ-S---KKDKAKFMQTCVPHLISLVTFFVIVISDLMHMRFASNDVPQSFQNF
contig048562-BurOR.H062	GT-DIQRLFCVNWLIVKVACPGMDTLVNSA-FAYT-TLSIYIFHWIFIVWTYIYLVKSCVQ-S---KKDKAKFMQTCVPHLISLVTFFVIVISDLMHMRFASNDVPQSFQNF
contig034998-NyeOR.H067	GT-DIQRLFCVNWLIVKVACPGMDTLVNSA-FAYT-TLSIYIFHWIFIVWTYIYLVKSCVQ-S---KKDKAKFMQTCVPHLISLVTFFVIVISDLMHMRFASNDVPQSFQNF
contig047492-ZebOR.H074	GT-DIQRLFCVNWLIVKVACPGMDTLVNSA-FAYA-TLSIYIFHWIFIVWTYIYLVKSCVQ-S---KKDKAKFMQTCVPHLISLVTFFVIVISDLMHMRFASNDVPQSFQNF
contig041955-TilOR.H119	GT-DIQRLFCVNWLIVKVACPGMDTLVNSA-YAYT-TLSIYIFHWIFIVWTYIYLVKSCVQ-S---KKDKAKFMQTCVPHLISLVTFFVIVISDLMHMRFASNDVPQSFLNF
contig116846-BriOR.H055	SP-YLSRLFCVNWIIVQLACFPAQTIVNGI-FANI-TIIIYLLHGVFIVWSYMYIIQTCVK-S---IENRAKFMQTCVPHLISLFTFVVAILTDIISMRLDSKELPRSLQNF
contig033889-BriOR.H049	SP-YISRLFCMNWSIVQLACFPEQTAINAI-SANI-VTIIYLLHGVFIVWSYMYIIQTCVR-S---IENRAKFMQTCVPHLVSLFTFVVAILTDVISMRLGSKELPRTLQNF
contig030011-ZebOR.H073	SP-YISRLFCMNWSIVQLACFPEQTAINAI-SANI-VTIIYLLHGVFIVWSYMYIIQTCVK-S---IENRAKFMQTCVPHLVSLFTFVVAVLTDVISMRLGSKELPRSLQNF
contig018437-ZebOR.H072	SP-YLSTLFCVNWIIVQLACFPAQTIVNGI-FANI-TIIIYLLHGVFIVWSYLYIIQTCVK-S---IENRAKFMQTCVPHLISLFTFVVAVLTDVISMRLGSKELPRTLQNF
contig018434-ZebOR.H070	SP-YISRLFCVNSSIVQLACFPAQTIVNGI-FANI-TIIIYALHGVFIVWSYMYIIQTCVK-S---IENRAKFMQTCVPHLVSLHTFVVIMLLDSTSARFGSKFLPQVLQNF
contig009565-TilOR.H126	GM-YISRLFCGNSSIVQLACFPAQTIVNGI-VANI-TIIIYALHGGFIVWSYMYIIQKCVK-S---IENRAKFMQTCVPHLVSLHTFVVIMLFDFMSMRFGSKVLPQVLQNF
contig018434-ZebOR.H071	SP-YLSRLFCVNWSIVQLACFPAQTAINAI-SANI-TISIYFLYGVFIVWSYLYIIQTCVR-S---IENRAKFMQTCVPHLVSLFTFAVTKLLDITNMRLGSKELPQTVQNF
contig009547-TilOR.H101	SS-YISRLFCVNSVIVTLACSRAETIINNI-AAYI-TITVYVFHGLFIVWSYVYLIKTCVK-S---TEKRAKFMQTCVPHLLSLLTFLVTILFDVMNIRLGSIVLPQTLQNF
contig009548-TilOR.H102	SS-YISRLFCVNSVIVTLACSRAETIINNI-AAYI-TITVYVFHGLFIVWSYVYLIKTCVK-S---TEKRAKFMQTCVPHLLSLLTFLVTILFDVMNIRLGSIVLPQTLQNF
contig009546-TilOR.H100	SP-YISRLFCVNWSIVQLACFPAQTKVNGI-VANI-TIIIYVLHGALIVWSYIYLIKTCVN-S---IENRTKFMQTCVPHLASLLTFVVAILTDVVNIRLTDLKNLPQSIQNF
contig014060-ZebOR.H069	GS-HIPRIYCVNWLIANLAC--SASVARNV-IPAF-NYTFYVGHVVFVFWSYVYLIKTCQS-S---KENWNKFMQTCVPHLFSLTVVVVSLLFDMLYMRFGSKEIAQNVENF
contig053784-BurOR.H067	GS-HIPRIYCVNWLIANLAC--SASVARIV-IPAF-NYTFYVGHVVFVFWSYVYLIKTCQS-S---KENWNKFMQTCVPHLFSLTVVVLSLLFDMLYMRFGSKEIAQNVENF
contig041756-NyeOR.H075	GS-HIPRIYCVNWLIANLAC--SASVARIV-IPAF-NYTFYVGHVVFVFWSYVYLIKTCQS-S---KENWNKFMQTCVPHLFSLTVVVVSLLFDMLYMRFGSKEIAQNVENF
contig041756-NyeOR.H139	GS-HIPRIYCVNWLIANLAC--SASVARIV-IPAF-NYTFYFGHAVFIFWSYVHLIKTCQS-S---KENWNKFMQTCVPHLFSLTVVVVSLLFDMLYMRFGSKEIPQSFENF
contig013363-TilOR.H110	GS-HIPRIYCINWLISNLAC--SASVATII-IPAF-NYTFYFGHAVFIFWSYVHLIKTCQS-S---KENWNKFMQTCVPHLFSLTVVILSFLFDMLYMRFGSKEIPQSFENF
contig035579-NyeOR.H068	GS-HIPRIYCVNWLINNLAC--SASVARIV-IPAF-SYTFYIGHVLLVFWSYVHLIKTCQS-S---KDNWNKFMQTCVPHLFSLTVVVLSFLFDMLYMRFGSKEIPQSFENF
contig048880-BurOR.H063	GT-HIPRIYCVNWLINNLAC--SASVARIV-IPAF-NYTFYIGHVLLVFWSYVHLIKTCQS-S---KENWNKFMQTCVPHLFSLTVVVLSFLFDMLYMRFGSKEIPKSFENF
contig013365-TilOR.H111	GS-HIPRIYCINWLISNLAC--SASVAKIV-IPAF-TYTFYIGHVLFVFWSYVHLIKTCQS-S---KENWNKFMQTCVPHVFSLTVVVLSFLFDMLYMRFGSKEIPQGFENF
contig053782-BurOR.H066	GS-HIPRIYCINWLISNLAC--SASVATII-IPAF-NYTFYFGHAVFVFWSYVHLIKTCQS-S---KENWNKFMQTCVPHLFSLAVVVVSFLFDMLYMRFGSKEIPQSFENF
contig039729-NyeOR.H073	GS-HIPRIYCINWLISNLAC--SASVATII-IPAF-NYTFYFGHAVFVFWSYVHLIKTCQS-S---KENWNKFMQTCVPHLFSLAVVVVSFLFDMLYMRFGSKEIPQSFENF
contig014057-ZebOR.H067	GS-HIPRIYCINWLISNLAC--SASVATII-IPAF-NYTFYFGHAVFVFWSYVHLIKTCQS-S---KENWNKFMQTCVPHLFSLAVVVVSFLFDMLYMRFGSKEIPQSFENF
contig064817-BriOR.H051	GS-HIPRIYCINWLISNLAC--SASVATII-IPAF-NYTFYFGHAVFVFWSYVHLIKTCQS-S---KENWNKFMQTCVPHLFSLAVVVVSFLFDMLYMRFGSKEIPQSFENF
contig013359-TilOR.H107	GS-HIPRIYCINWLISNLAC--SASVATII-IPAF-NYTFYFGHALFVFWSYVHLIKTCQS-S---KENWNKFMQTCVPHLFSLAVVVVSFLFDMLYMRFGSKEIPQSFENF
contig064821-BriOR.H052	GS-HIPRIYCINWLINNLAC--SASVARIV-IPAF-NYTFYIGHILFVFWSYVHLVKTCQS-S---KENWNKFMQTCVPHLFSLIVVAVSFLFDMLYVRFGSKEFPQSFENF
contig053788-BurOR.H069	GT-HIPRIYCINWLINNLAC--SASAARIV-IPAF-NYTFYIGHILFVFWTYVHLVKTCQS-S---KENWNKFMQTCVPHLFSLIVVAVSFLFDMLYVRFGSKEFPQSFENF
contig041757-NyeOR.H076	GT-HIPRIYCINWLINNLAC--SASAARIV-IPAF-NYTFYIGHILFVFWTYVHLVKTCQS-S---KENWNKFMQTCVPHLFSLIVVAVSFLFDMLYVRFGSKEFPQSFENF
contig013356-TilOR.H105	GS-HIPRIYCINWLINNLAC--TASVARTV-IPAF-NYTFYIGHILFVFWTYVHLIKTCQS-S---KENWNKFMQTCVPHLFCIIVVVVSFLFDMLYMRFGSKEFPQSFENF
contig013358-TilOR.H106	GS-HIQRIYCVNWLISNLAC--SPSVAKTV-IPAF-NYTFYIGHVLFVFWSYVHLIKTCQS-S---NENWNKFMQTCVPHLFSLTVVVVSLLFDMLYMRFGSKEIPQNVENF
contig013351-TilOR.H104	GS-HIPRIYCINLLIANLAC--SASVAKIV-IPAF-SYTFCIGNICFVFWSYVYLIKTCQS-S---KENMGKFMQTCVPHLFSLTVVVVSLLFDLLYMRFGSKEIPQSVQNF
contig014059-ZebOR.H068	GS-HIPKIYCINWLIANLAC--SASVAKIV-IPAF-SYTFCFGNVCFVFWSYVHLIKTCQS-S---KENMGKFMQTCVPHLFSLTVVVVSLLFDMLYMRFGSKQIAQSVQNF
contig053787-BurOR.H068	GS-HIPKIYCINWLIANLAC--SASVAKIV-IPAF-SYTFCFGNVCFVFWSYVHLIKTCQS-S---KENMGKFMQTCVPHLFSLTVVVVSLLFDLLYMRFGSKEIAQSVQNF
contig039725-NyeOR.H072	GS-HIPKIYCINWLIANLAC--SASVAKIV-IPAF-SYTFCFGNVCFVFWSYVHLIKTCQS-S---KENMGKFMQTCVPHLFSLTVVVVSLLFDLLYMRFGSKEIAQSVQNF
contig039730-NyeOR.H074	GS-NINKLYCENWSIVKLAC--GSTKVNDI-VGLI-FITFYCCHVVCIACSYVQLVNVALK-S---RAGRKKFTQTCTPHLFCLLNVTTALLFDLMFSRYGSASLPQHLKNF
contig014055-ZebOR.H066	GS-NINKLYCENWSIVKLAC--GSTKVNDI-VGLI-FITFYCCHVVCIACSYVQLVNVALK-S---RAGRKKFTQTCAPHLFCLLNVTTALLFDLMFSRYGSASLPQHLKNF
contig053780-BurOR.H065	GS-NINKLYCENWSIVKLAC--GSTKVNDI-VGLI-FITFYCCHVVCIACSYVQLVNVALK-S---RAGRKKFTQTCAPHLFCLLNVTTALLFDLMFSRYGSASLPQHLKNF
contig107626-BriOR.H054	GS-NINKLYCENWSIVKLAC--GSAKVNDI-VGLI-VITFYCCHAVCIACSYVQLVNAALK-S---RAGRKKFTQTCVPHLFCLLNVTTALLFDLMFSRFGSASLPQHLKNF
contig013349-TilOR.H103	GS-NINKLYCENWSIVKLAC--GSTKVNDI-VGLI-LITFYFCHAVCIASSYVQLVNAALK-S---RAGRKKFTQTCVPHLFCLLNVSTALLFDLMYSRYGSASLPQHLKNF
contig106096-BriOR.N089	ESVVIKSFFCDHGQIYRLAC--NDYTPSII-YGWILLVLILWLPLTIVLLSYLSIGYALAKVA--TVRERMKGFKTCTAHLSLVAIYFLPILITFTLH----ANIEPNARII
contig061663-NyeOR.N114	ESVVIKSFFCDHGQIYRLAC--NDYTPSDI-TAWILPALILWLPLTIVLLSYLSIGYALAKVA--TVRERMKGFKTCTAHLSLVAIYFLPILITFTLG----ANIEPNARII
contig057383-BurOR.N109	ESVVIKSFFCDHGQIYRLAC--NDYTPSDI-TAWILLVLILWLPLTIVLLSYLSIGYALAKVA--TFRERMKGFKTCTAHLSLVAIYFLPILITFTLG----ANIEPNARII
contig010722-ZebOR.N111	ESVVIKSYFCDHGQMYRLAC--NDYTPSYV-IAKILPALILWLPLTIVLLSYLCICYALAKVA--TVRERTKGFKTCTAHLSLVAIYFLPILITFTLG----ANIEPNARII
contig096539-BriOR.N087	KSVVINSYFCDHGQIYRLAC--NDHFPSYV-IACLYPVIIFWLPLAFILLSYLYIGYTLVKVA--TLQEGLKAFKTCIGHLSLVAIYFIPLLTTFTLM----EKIQPNARII
contig064097-ZebOR.N115	KSVVINSYFCDHGQIYRLAC--NDHFPSYV-IACLYPVIIFWLPLAFILLSYLYIGYTLVKVA--TLQEGLKAFKTCIGHLSLVAIYFIPLLTTFTLM----EKIQPNARII
contig055927-NyeOR.N111	KSVVINSYFCDHGQIYRLAC--NDHFPSYV-IACLYPVIIFWLPLAFILLSYLYIGYTLVKVA--TLQEGLKAFKTCIGHLSLVAIYFIPLLTTFTLM----EKIQPNARII
contig060631-BurOR.N110	KSVVINSYFCDHGQIYRLAC--NDHFPSYV-IACLYPVIIFWLPLAFILLSYLYIGYTLVKVA--TLQEGLKALKTCIGHLSLVAIYFIPLLTTFTLM----EKIQPNARII
contig046353-TilOR.N195	KSVVINSYFCDHGQIYRLAC--NDHFPSYV-IACLYPVIIFWLPLAFILLSYLYIGYTLVKVA--TLQEGLKALKTCIGHLSLVAIYFIPLLTTFTLM----EKIQPNARII
contig055926-NyeOR.N110	ESVVINSYYCDHGPIYRLSC--NDVTPNKT-ISAWSRAFVLWLPLIFILGSYCCIGYSLSRIS--TCKERVKALKTCTGHLSLVAIYFIPILVVYSFG----STMHPNARIV
contig042928-BurOR.N108	ESVVINSYYCDHGPIYRLSC--NDVTPNKT-ISAWSRAFVLWLPLIFILGSYCCIGYSLSRIS--TCKERVKALKTCTGHLSLVAIYFIPILVVYSFG----STMHPNARIV
contig064098-ZebOR.N116	ESVVINSYYCDHGPIYRLSC--NDVTPNKT-ISAWSRAFVLWLPLIFILGSYCCIGYSLSRIS--TCKERVKALKTCTGHLSLVAIYFIPILVVYSFG----STMHPNARIV
contig096539-BriOR.N088	ESVVINSYYCDHGPIYRLGC--NDVTPNKT-ISAWSRAFVLWLPLIFILGSYCCIGYSLSRIS--TCKERVKALKTCTGHLSLVAIYFIPILVVYSFG----STMHPNARIV
contig046356-TilOR.N196	ESVVINSYYCDHGPIYRLGC--NDVTPNKT-ISAWSRAFVLWLPLIFVLGSYCCIGYSLSRIS--TCKERVKALKTCTGHLSLVAIYFIPILVVYSFG----STIHPNARIV
contig055924-NyeOR.N109	DSVVIQSFFCDHGPVYRLGC--NDLTPNRV-IAHLASVLVLWVPLAFIVGSYCCIGYSLSKTV--TCRERLKALKTCTSHLSLVAIYFLPALFIFTFG----STILPNARTI
contig010725-ZebOR.N112	DSVVIQSFFCDHGPVYRLGC--NDLTPNRV-IAHLASVLVLWVPLAFIVGSYCCIGYSLSKTV--TCRERLKALKTCTSHLSLVAIYFLPALFIFTFG----STILPNARTV
contig010726-ZebOR.N113	DSVIIQSYFCDHGPVYRLGC--NDLTPNRV-IAHLAPVLVLWVPLAFIVGSYCCIGYSLSKTA--TCRERLKALKTCTSHLSLVAIYFLPALFIFTFG----STILPNARTV
contig046360-TilOR.N197	DSVVIQSYFCDHGPVYRLGC--NDLTPNRV-IGYLAPVLVLWVPLAFIVGSYCCIGYSLSKTV--TCRERLKALKTCTGHLSLVAIYFLPTLFIFTFG----STIPPNARTV
contig010714-ZebOR.N109	KSVVIQSYYCDHGPMYRLGC--NDNTPNRA-IARSTPVIILGFPLAFIMGSYCCIGYSLSKIS--TFRERVKAFKTYTGHLSLVAIYFLPFTFVYIFG----SVIHPNARII
contig010718-ZebOR.N110	KSVVIQSYYCDHGPMYRLGC--NDNTPNHA-FAGSALVIILGFPLAFIVGSYCCIGYSLSKIS--TFRERVKAFKTYTGHLSLVAIYFLPFTFVYMFG----SVIHPNARII
contig010712-ZebOR.N108	KSVVIQSYFCDHGPMYRLGC--NDITPNRA-IAGLASVIILGFPLAFIVGSYCCIGYSLSKIS--TFRERVKAFKTCTGHLSLVAIYFLPFTFVYIFG----SVIHPNARII
contig050080-TilOR.N198	KSVVIQSYFCDHGPMYRLGC--NDITPNRA-IAGLAPVIILGFPLAFIVGSYCCIGYSLSKIS--TYRERVKAFKTCTGHLSLVAIYFLPITFVYIFG----NVIHPNARII
contig046352-TilOR.N193	GSFVVKSFFCDHGPVFYLAC--NDTSLNRI-MAYVALVSFICIPLILIAFTYVCISIALSRIA--SGEERLKALKTCTSHVILVAVFFLPLVGTNIAAVA--SYIHPNARII

contig046347-TilOR.N190	GSFVVKSFFCDHGPVFYLAC--NDTSLNRI-MAYVALVSFICIPLILIAFTYVCISIALSRIA--SGEERLKALKTCTSHVILVAVFFLPLVGTNIAAVA--SYIHPNARII
contig096536-BriOR.N085	RSLVVNSFFCDHGPVYRLAC--NDTSLNYN-MASALVTILILIPLIFIIATYVCIFIALSRTT--SRKERIRALKTCTSHLILVVMFFLPIGITNIAAMA--SYIDPNARMI
contig055933-NyeOR.N113	RSLVVNSFFCDHGPVYRLAC--NDTSLNYN-MASALVAILIIIPLIFIIATYVCIFIALSRTT--SREERLRALKTCTSHLILVVIFFLPIGITNIAAMT--SYIDSNARMI
contig065025-BurOR.N111	RSLVVNSFFCDHGPVYRLAC--NDTSLNYN-MASALITILIIIPLIFIIATYVCIFIALSRTT--SRKEQIRALKTCTSHLILVVIFFLPIGITNIATRA--SYIHPNARMI
contig096535-BriOR.N084	RSLVVNSFFCDHGPVYRLAC--NDTSLNYN-MASALITILIIIPLIFIIATYVCIFIALSRTT--SRKERIRALKTCTSHLILVVMFFLPIGITNIATRA--SYIHPNARMI
contig046340-TilOR.N188	RSLVVNSFFCDHGPVYRLAC--NDTSLNYN-MSSALITILIIIPLIFIIASYVCIFIALSRTT--SRKERLRALKTCTSHLILVVMFFLPIGITNIATRA--SYIHPNARMI
contig046351-TilOR.N192	RSLVINSFYCDHGPVFRLAC--NDTSVNFN-LAISLVIIILIIPLAFIVVTYVYISIALRRTT--SREERVRALKTCTSHLILVVMFFLPVGITNIVAWA--SYIDPNARMI
contig046350-TilOR.N191	SSLVINSFFCDHGPVYRLAC--NDISINQN-LASAIVSIILIIPLAFIVATYICISIAVSRTA--FREERLRALKTCTSHLILVAIFFLPWTGTNVAAVT--SYIHPNARMI
contig046344-TilOR.N189	RSLVINSFFCDHGPTFRLAC--NDTTVNFN-LVISLIIIILIIPLAFIMATYICISIALSRTA--FREKRIRALKTCTSHLILVAIFFLPWTGTNLAAVT--SYIHPNARMI
contig010727-ZebOR.N114	DSVVVKSFFCDHGPIYRLAC--NDPSLNII-MANVVVSIGVFIPLIFISCTYVCISIALSKIA--SGEERLKALKTCTSHLILVAILFLPFVGTNIAVWT--SYIHPNARII
contig042920-BurOR.N107	DSVVVKSFFCDHGPIYRLAC--NDPSLNII-MANVVVSIGVFIPLIFIACTYVCISIALSKIA--SGEERLKALKTCTSHLILVAILFLPFVGTNIAVWT--SYIHPNARII
contig055927-NyeOR.N112	DSVVVKSFFCDHGPIYHLAC--NDPSLNII-MANVVVSIGVFIPLIFIACTYVCISIALSKIA--SGEERLKALKTCTSHLILVAILFLPFVGTNIAVWT--SYIHPNARII
contig096536-BriOR.N086	DSVVVKSFFCDHGPIYRLAC--NDPSLNII-MANVVVSIGVFIPLIFIACTYVCISIALSKIA--SGEERLKALKTCTSHLILVAILFLPFVVTNIAVWT--SYIHPNARII
contig046352-TilOR.N194	DSVVVKSFFCDHGPIYRLAC--NDPSLNII-MANVGVSIGVFIPLIFIACTYVCISIALSKIA--SGEERLKALKTCTSHLILVAILFLPFVGTNIAVWA--SYIHPNARII
contig048778-NyeOR.M107	RSVRVFSYFCDYAPVFRLAC--NDNTMQWF-AASFLSVLLLFVPFTFIFLSYVSILVTVFRMK--SVDSRVKALATCVEHLILVAIFYIPLTVIFTIGFFL-GVVNPDQRVL
contig010734-ZebOR.M107	RSVRVFSYFCDYAPVFRLAC--NDNTMQWF-AASFFSVLLLFFPFTFIFLSYVSILITVFRMK--SVDSRVKALATCAEHLILVAIFYIPLIVIFTVGFFL-GVVHPDQRVL
contig048778-NyeOR.M108	RSVRVFSYFCDYAPVFRLAC--NDNTMQWF-AASFLSILLLFVPFTFILLSYVSILMTVFRMK--SVDSRVKALATCAEHLILVAIFYIPLTVIFTIGFFL-GVVNPDQRVL
contig039460-TilOR.L145	TS-RMKYAFCDYAAVIRTTC--VDPNYYFN-LVSVMMFFLLFFTFSFICLSYFVIAFFMKVSS---NRDKTKMASTCVSHLIVVTCYYSPLFVLVLLTRVG-VVLTLEERQG
contig017786-ZebOR.L091	TS-RMKYAFCDYAAVIRTTC--VDPNYYFN-LVSVMMFFLLFFTFSFIGLSYFVIAFLMKFSS---NRDKKKMASTCVSHLIVVTCYYSPLFVLIVFTRVG-VVLTLEECQG
contig058162-BurOR.L089	TS-RMKYAFCDYAAVIRTTC--VDPNYYFN-LVSVMMFFLLFFTFSFIGLSYFVIAFLMKFSS---NRDKKKMASTCVSHLIVVTCYYSPLFVLIVFTRVG-VVLTLEERQG
contig040502-NyeOR.L092	TS-RMKYAFCDYAAVIRTTC--VDPNYYFN-LVSVMMFFLLFFTFSFIGLSYFVIAFLMKFSS---NRDKKKMASTCVSHLIVVTCYYSPLFVLIVFTRVG-VVLTLEERQG
contig042543-BriOR.L069	TS-RMKYAFCDYAAVIRTTC--VDPNYYFN-LVSVMMFFLLFFTFSFIGLSYFVIAFLMKFSS---NRDKKKMASTCVTHLIVVTCYYSPLFVLIVFTRVG-VVLTLEERQG
contig039460-TilOR.L146	TS-RMKYAFCDYAAVMRTTC--VDPNFYFN-IVSVMLFFILFFTFSFICLSYVVIAFFMKFSS---NRDKTKMASTCVSHLIVVTCYYSPLFVLIVFTRVG-VVLTLEERQG
contig039465-TilOR.L150	SL-RLKYTFCDYAAVIRTTC--VDPEKYFN-LGAILSFFVLFFTFIFICLSYCVILFFVKLSS---NNDRKKMGSTLVSHLICVICLYCPMFVIGILTRFG-VVLTLEERQG
contig039461-TilOR.L148	SL-RLRYAFCDAAAVIRTTC--VDAEKFFD-LGAILSFFLFFFTFTFICLSYCMILFFVKLSS---NNDRRKMGSTLVSHLICVICLYCPMFVIGILTRFG-VVLSLEERQG
contig042552-BriOR.L071	YS-RLRYTFCDYAAVMRTTC--VDPEKYFN-QVAIISFFLSFFTFTFICLSYCGILFFVKILS---NNDKKKMGSTLVSHLICVSCLYCPQFVIVILTRFS-VLLALEERQG
contig020445-ZebOR.L096	FL-RLRYTFCDYGAVMRTTC--VNPEKYFN-QVAILSFFVSFFTFTFICLSYCGILFFVKILS---NNDKKKMGSTLVSHLICVSCLYCPQFIIVILTRFG-VVLTLEERQG
contig056942-NyeOR.L093	FL-RLRYTFCDYGAVMRTTC--VNPEKYFN-QVAILSFFVSFFTFTFICLSYCGILFFVKISS---NNDKKKMGSTLVSHLICVSCLYCPQFILVILTRFG-VVLTLEERQG
contig068527-TilOR.L157	YS-RLRYTFCDYAAVMRTTC--VNPEKYFN-QIAIISFFLSFSTFTFICLSYCGILFFVKILS---NNDKKKMGSTLVSHLICVSCLYGPQFVIVILTRFG-VVLTLEERQG
contig039469-TilOR.L152	YS-RLRYTFCDYAAVIRTTC--VNPEKYFN-QIAIISFFLSFSTFTFICLSYCGILFFVKISS---NNDKKKMGSTLVSHLICVICVYCPLVIIVILTRFG-VVLTLEERQG
contig064361-NyeOR.L094	SS-RLRYTFCDFAAVIRTTC--VNPEKHFN-DAAIIAFFILFFTFVFICLSYCGILLFVKLSS---NNEKKKMGSTLVSHLICAIVHYCPAFVRIIFTRFG-VVLTVEERQG
contig056384-BurOR.L087	SS-RLRYTFCDFAAVIRTTC--VNPEKHFN-DAAIIAFFILFFTFVFICLSYCGILLFVKLSS---NNEKKKMGSTLVSHLICAIVHYCPAFVRIIFTRFG-VVLTLEERQG
contig020442-ZebOR.L095	SS-RLRYTFCDFAAVIRTTC--VNPEKHFN-DAAIIAFFILFFTFVFICLSYCGILLFVKLSS---NNEKKKMGSTLVSHLICAIVHYCPAFVRIIFTRFG-VVLTLEERQG
contig042554-BriOR.L072	SS-RLRYTFCDFAAVLRTTC--VNPEKYFN-DAAIIAFFIFFFTFVFICLSYCGILLFVKLSS---NNEKKKMGSTLVSHLICVIVHFCPPFVRIIFTRFG-VVLTLEERQG
contig039481-TilOR.L153	SS-RLRYTFCDFAAVLRTTC--VNPEKYFN-EAATIAFFILFFTFVFICLSYCGILLFVKLSS---NNEKKKMGSTLVSHLICAIIHYCPAFVRIIFTRFG-VVLTLEERQG
contig072645-TilOR.L159	SS-RLRYTFCDFAAVLRTTC--VNPDKYFN-EVAIVTFFILFFTFVFICLSYCGILLFVKLSS---NNEKKKMGSTLVSHLICVIVHYCPAFVRIIFTRFG-VVLTFEERQG
contig056940-NyeOR.L095	SS-RLKYSFCDFAAVIRTTC--VNPEKYFN-EVSIAAFFIFFFTFVFICLSYCGILLFVKLSS---NNEKMKMGSTLVSHLICVVVHYCPAFVRIMFTRFG-VVLTLEERQG
contig020440-ZebOR.L094	SS-RLKYSFCDFAAVIRTTC--VNPEKYFN-EVSIAAFFIFFFTFIFICLSYCGILLFVKLSS---NNEKMKMGSTLVSHLICVVVHYCPAFVRIMFTRFG-VVLTLEERQG
contig042556-BriOR.L073	SS-RLKYSFCDFAAVIRTTC--VNPEKYFN-EVSIAAFFIFFFTFVFICLSYCGILLFVKLSS---NNEKKKMGSTLVSHLICVIVHYCPAFVRIMFTRVG-VVLTLEERQG
contig039484-TilOR.L154	SS-RLKYSFCDFAAIIRTTC--VNPEKYFN-EVAIAAFFILFFTFVFICLSYCGILIFVKLSS---NNEKKKMGSTLVSHLICVIVHYCPAFVRIIFTRFG-VVLTLEERQG
contig068425-TilOR.L155	SS-RLKYSFCDFAAVLRTTC--VNPEKYFN-EVSIAAFFIFFFTFVFICLSYCGILLFVKLSS---NNEKKKMGSTLVSHLICVIVHYCPAFVRIIFTRFG-VVLTLEERQG
contig020437-ZebOR.L093	SS-RLRYTFCDFAAVLRTTC--VNPEKYFN-EVAIVSFFIFFFTFIFICLSYCGILLFVKLSS---NNEKKKMGSTLVSHLICVFVHYCPAFVRNIFTRFG-VVLTLEERQG
contig056386-BurOR.L088	SS-RLKYNFCDFPAVIRTTC--VNPEKYFN-EVVIFSFFIFFFTFIFICLSYCGILLFVKLSS---NNEKRKMGSTLVSHLICVIVYYCPAFVRNIFTRFG-VVLTLEERQG
contig039469-TilOR.L151	SS-KLKYSFCDFAAVIRTTC--VNPEKYFN-EGATIAFFFFFFTFVFICLSYCGILLYVKLPS---NNEKKKMGSTLVSHLICVIVHYCPAFVRIIFTRFG-VVLTLEENQG
contig068521-TilOR.L156	SS-RLKYSFCDFPALIRTTC--VNPEKYFN-EVAIVSFFIFFFTLVFICLSYCAILLFVKLSS---YNEKKKMGSTLVSHLICVIVYYCPAFVRSIFTRFG-VVLTLEERQG
contig068539-TilOR.L158	SS-KLKYNFCEFAAIIETTC--VNPEKYFN-EVATIAFFIFFFTFVFICLSYCGILLYVKLLS---NNEKKKMGSTLVSHLICVIVHYCPAFVRIIFTRFG-VVLTLEERQG
contig042544-BriOR.L070	TL-KLKYTFCEYAAIMRTTC--VNIDEYFN-QSAIWSFFISSFTFTFICISYCGILFCVKLSS---NNDKKKMGSTVVSHAICVTCFYSPIFIIVILSRVG-VVLSLDERQG
contig065027-BurOR.L090	TL-KLKYTFCDYASIMRTTC--VNVDEYFN-QSAIWSFFISFFTFTFICISYCGILFCVKLSS---NNDKKKMGSTVVSHAICVTCFYSPIFIIVILTRVG-VVLSLDERQG
contig017787-ZebOR.L092	TL-KLKYTFCDYAAIMRTTC--VNVDEYFN-QSAIWSFFILFFTFTFICISYCGILFCVKLSS---NNDKKKMGSTVVSHAICVTCFYSPIFIIVILTRVG-VVLSLDERQG
contig040502-NyeOR.L091	TL-KLKYTFCDYAAIMRTTC--VNVDEYFN-QSAIWSFFILFFTFTFICISYCGILFCVKLSS---NNDKKKMGSTVVSHAICVTCFYSPIFIIVILTRVG-VVLSLDERQG
contig039462-TilOR.L149	TL-RLKYNFCDYAAIMRTTC--VNVDEYFN-QSAIWSFFILFFTFTFICLSYCGILFFVKLSS---NNDKKKMGSTLVSHAICVTCFYSPIFIIVILTRFG-VVLSLDERQG
contig023716-TilOR.L144	YS-SLRYSFCDYAAVVRTTC--VDPTYFFN-IGAIIYSFLLFLTFIFICLSYCVILIFAKLSS---SNDKKKIGSTLVSHLICVICHYCPAFVIIVLTRLG-VVLTLDERQG
contig039461-TilOR.L147	YS-RLKYTFCDYAAVLRTTC--ADPNYYLN-LGAIILFFILFFTLIFICLSYCVILIFVKLS----NNDKKKMGGTLLSHLICVTCHYCPAFIIIVLTRLG-VALSLEERQG
contig017698-BurOR.P120	LRNVINHCFCEHMALVELAC--GSTTINNL-VGLMAVFLIPVLDFVFITASYVVIFSSVLKSS----RSGVKALHTCITHIMVITVSLILALTAFLSYRIR-NGLPAASRVF
contig025452-ZebOR.P123	FRNVINHCFCEHMALVELAC--GSTTINNL-VGLMAVFLIPVLDFVFIAASYVVIFSSVLKSS----RSGVKALHTCITHIMVITVSLILALTAFLSYRIR-NGLPAASRVF
contig004267-BriOR.P099	SRNVINHCFCEHMALVELAC--GSTTINSL-VGLMAVFLIPVLDFVFITASYVVIFSSVLKSS----GSGVKALHTCITHIMVITVSLILALTAFLSYRIR-NGLPAASRVF
contig065444-TilOR.P214	LRNVINHCFCEHMALVELAC--GSTTTNNL-VGLMAVFLIPVLDFVFIAAFYVVIFSSVLRSS----RSGVKALHTCITHIVVITVSLILALTAFLSYRIK-NGLPAASRVF
contig065247-TilOR.P211	LRNVINHCFCEHMALVELAC--GSTIINSL-VGLISVFLVPVTDFFLVTASYIIVFNSVLRLK----KSSAKALHTCVTHIVVITVNLSIVLIALLSYRIR-NGLPSAIRIF
contig004270-BriOR.P100	LRNVINHCFCEHMALVELAC--GSTTINSL-VGLISVFSVPVTDFFLITASYTVIFSSVLSSG----KSSAKALHTCVTHIVVMTVSLTIVLTAFLSYRVR-NGLPAAVRTF
contig017697-BurOR.P119	LRNVINHCFCEHMALVELAC--GSTTINSL-VGLISVFSVPVTDFFLITASYTVIFSSVLSSG----KSSAKALHTCVTHIVVMTVSLTIILTAFLSYRIR-NGLPAAIRIF
contig025453-ZebOR.P124	LRNVINHCFCEHMALVELAC--GSTTINSL-VGLISVFSVPVTDFFLITASYTVIFSSVLSSG----KSSAKALHTCVTHIVVMTVSLTIILTAFLSYRIR-NSLPAAIRIF
contig065449-TilOR.P215	LRNVINHCFCEHMALVELAC--GSTIINSV-AGLITVFSVPVTDFFLITASYTVIFSSVLSSG----KASAKALHTCVTHIVVMTVSLTIILTAFLSYRIR-NSLPTAIRIF
contig065253-TilOR.P212	LRNVINHCFCEHMALVELAC--GSTIINSL-VGLISVISVPVTDFFLITASYTVIFSSVLSSG----KSSAKALHTCVTHIVVMTVSLTIVLTAFLSYRIR-NGIPSAIRIF
contig065254-TilOR.P213	GSNVIQHCYCDHMALVSLAC--DSTSRSGA-AGLAVIICFVGLDIPIIFFSYMKILSVVLRSA-AAGEDRRKAFHTCSTHLIVMMCFYLVGSITFLSHNLN-IAIPTDINNS
contig023268-NyeOR.P118	GSNVIQHCYCDHMALVSLAC--DSTSRSSA-AGLAVIICFVGADIPIIFFSYMKILSVVLRSA-AAGEDSRKAFHTCSTHLIVMMCFYLVGSITFLSHNLN-IPIPTDINNS
contig017696-BurOR.P118	GSNVIQHCYCDHMALVSLAC--DSTSRSSA-AGLAVIICFVGADIPIIFFSYMKILSVVLRSA-AAGEDSRKAFHTCSTHLIVMMCFYLVGSITFLSHNLN-IPIPTDINNS
contig025458-ZebOR.P125	GSNVIQHCYCDHMALVSLAC--DSTSRSSA-AGLAVIICFVGADIPIIFFSYMKILSVVLRSA-AAGEDSRKAFHTCSTHLIVMMCFYLVGSITFLSHNLN-IPIPTDINNS
contig004275-BriOR.P101	GSNVIQHCYCDHMALVSLAC--DSTSRSSA-AGLAVIICFVGADIPIIFFSYMKILSVVLRSA-AAGEDSRKAFHTCSTHLIVMMCFYLVGSITFLSHNLN-IPIPTDINNS
contig020431-ZebOR.O101	ASNIITHCFCDHIGITVLAC--TDRTPYAI-PAFVFAMVVLLGPLAFIIFSYGSILIAVYKIA--NVQSRMKSLSTCSTQLIIISLYFLPRCFVYLAQNVG-ITFSADVRIV

```
contig021359-NyeOR.O102    ASNIITHCFCDHIGITVLAC--TDRTPYAI-PAFVFAMVVLLGPLAFIIFSYGSILIAVYKIA--NVQSRMKSLSTCSTQLIIISLYFLPRCFVYLAQNVG-ITFSADVRIV
contig059249-BurOR.O098    ASNIITHCFCDHIGITVLAC--TDRTPYAT-PAFTAAMVTLLGPLAFIIFSYGSILIAVYRIA--NVQGRLKSLSTCSTQLIIISLYFLPRCFVYLAQNVG-ITFSADVRIV
contig062053-NyeOR.O103    ASNIITHCFCDHIGITVLTC--TDRTPYAT-PAFTAAMVVLLGPLAFIIFSYCSILIAVYKIA--NVQGRLKSLSTCSTQLIIISLYFLPRCFVYLAQNVG-ITFSADVRIV
contig023717-TilOR.O175    ASNIITHCFCDHIGITVLAC--TDRAPYSI-PAFAAAMVTLLGPLAFILFSYCSIIIAVYKIA--NVQGRLKSLSTCSTQLIIISLYFLPRCFVYLASNVG-ITFSADVRIV
contig023724-TilOR.O176    ASNIITHCFCDHIGITVLAC--TDRAPYAI-PAFVFAMVTLLGPLAFIIFSYCSIIIAVYKIA--NVQGRLKSLSTCSTQLIIISLYYLPRCFVYLASNVG-ITFSADVRIV
contig042559-BriOR.O077    ASNTITHCYCDHIGITVLAC--TDRTPYAI-PALVFAMVVLLCPLAFIIFSYCSILIAVYKIA--NVQGRLKSLSTCSTQLIIISLYYLPRCFVYLASNVG-ITFSVDVRIV
contig110782-BriOR.O080    ASNIITHCFCDHIGITVLAC--TDRTPYAI-PAFVFAMFVLLGPLAFIIFSYCSILIAVHKIA--SVQGRLKSLSTCSTQLIIISLYFLPRCFVYLAQNVG-IKFSADVRIV
contig020430-ZebOR.O100    KSNIIVQCYCDHISIVSQAC--GDDVRIVVVTSLCLAMFCLLLPLAFILFSYISIIVVIMKMS--SSAGRKRTLSTCTSQIFITCLFYLPRCFVYVAYAFG-FSFSLDVRIG
contig042560-BriOR.O078    KSNIIVQCYCDNISIISQAC--GDDVKSVVVTSLCLAMFCLLLPLAFILFSYISIIVVIMKIS--SSAGRKRTLSTCTSQIFITCLFYLPRCFVYVANAVG-FSFSLDVRIG
contig021354-NyeOR.O101    DQNIIMQCYCDLISITRLGC--GDGLAYVNSVALANAMFTLLVPLTFIILSYFSVIIAVLRMS--QTERRHKVLSTCAPQLFITCLFYVPRCFVYLATVLG-FNFNLVIRII
contig020427-ZebOR.O099    DQNIIMQCYCDHISITRLGC--GDGLAYVNSVALANAMVTLLVPLTFIILSYFSVIIAVLRMS--QTERRHKVLSTCAPQLFITCLYYVPRCFVYLANVLG-FNFNLVIRII
contig023731-TilOR.O177    DQNIIMQCYCDHISITRLGC--SDEREYVNSVALANAMVTLLVPLTFIILSYFSVIIAVLRMS--QTERRHKVLSTCAPQLFITCLYYVPRCFVYLANVLG-FNFSLVIRII
contig042562-BriOR.O079    DQNIIMQCYCDLISITRLGC--SDEREYVYSVALANAMFTLLVPLTFIILSYFSVIIAVLRMS--QTERRHKVLSTCAPQLFITCLYYVPRCFVYIANGVG-FNFSLVIRII
contig049605-BurORs.AB153  GPNVVKHAWCDPSSVRRLVC--SDTSLDNI-VSLLFAMVSLVTTGVFILSSYILIGFSISRMV---VAQRLKALRTCSAHLTVVSISYAAASFVYISYRVG--NFSSEVKTL
contig046717-TilORs.AB275  GPNVVKHAWCDPSSVRRLVC--GDTSLDNI-VSLSFAMVALLTTGILILSSYILIGVSISRMV---VAQRLKAFRTCAAHLTVVSISYAAASFVYISYRVG--NFSPEVQTL
```

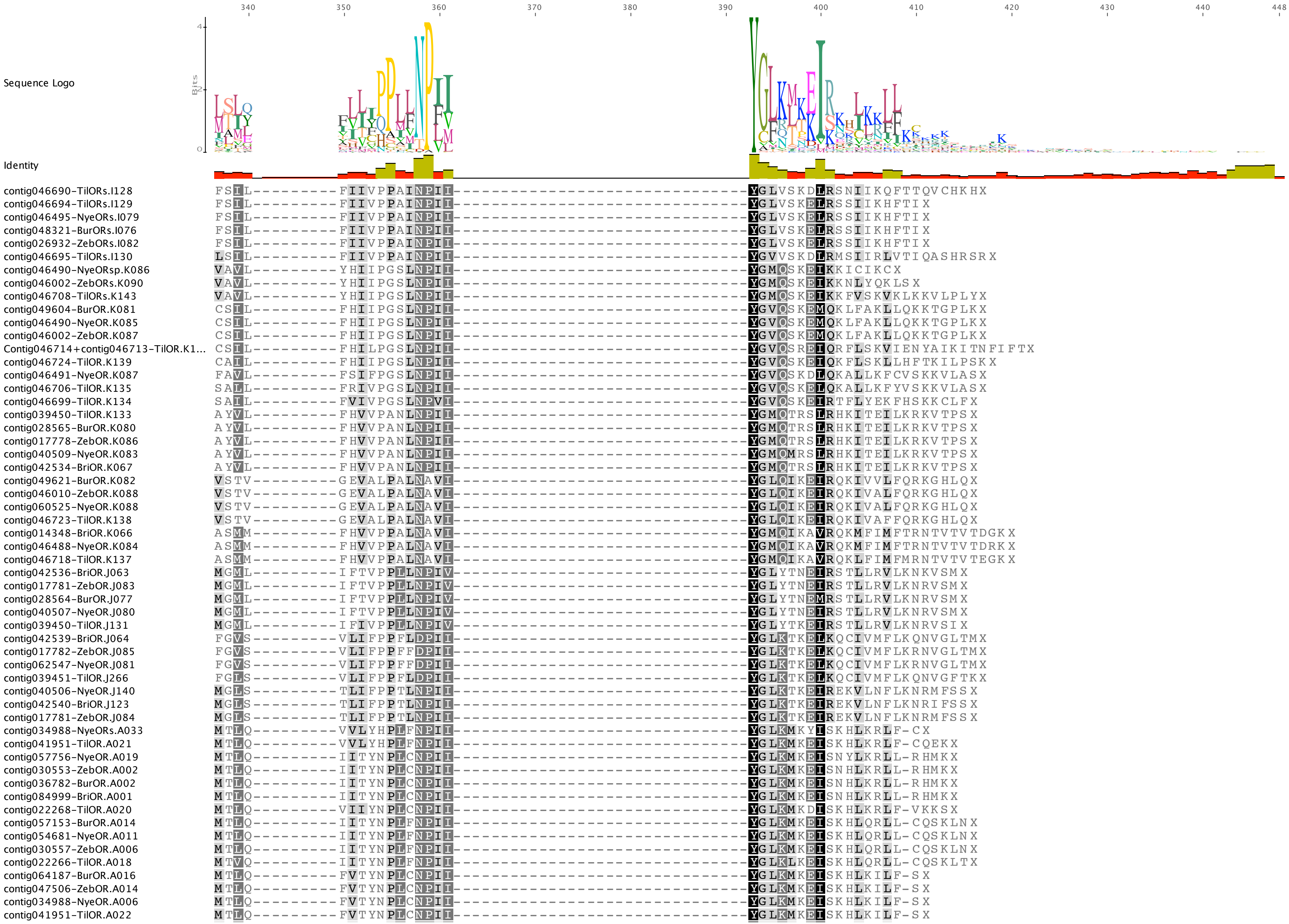

Sequence Logo
Bits
Identity
340 350 360 370 380 390 400 410 420 430 440 448
contig046690-TilORs.I128 FSIL---------FIIVPPAINPII-------------------------------YGLVSKDLRSNIIKQFTTQVCHKHX
contig046694-TilORs.I129 FSIL---------FIIVPPAINPII-------------------------------YGLVSKELRSSIIKHFTIX
contig046495-NyeORs.I079 FSIL---------FIIVPPAINPII-------------------------------YGLVSKELRSSIIKHFTIX
contig048321-BurORs.I076 FSIL---------FIIVPPAINPII-------------------------------YGLVSKELRSSIIKHFTIX
contig026932-ZebORs.I082 FSIL---------FIIVPPAINPII-------------------------------YGLVSKELRSSIIKHFTIX
contig046695-TilORs.I130 LSIL---------FIIVPPAINPII-------------------------------YGVVSKDLRMSIIRLVTIQASHRSRX
contig046490-NyeORsp.K086 VAVL---------YHIIPGSLNPII-------------------------------YGMQSKEIKKICIKCX
contig046002-ZebORs.K090 VAVL---------YHIIPGSLNPII-------------------------------YGMQSKEIKKNLYQKLSX
contig046708-TilORs.K143 VAVL---------YHIIPGSLNPII-------------------------------YGMQSKEIKKFVSKVKLKKVLPLYX
contig049604-BurOR.K081 CSIL---------FHIIPGSLNPII-------------------------------YGVQSKEMQKLFAKLLQKKTGPLKX
contig046490-NyeOR.K085 CSIL---------FHIIPGSLNPII-------------------------------YGVQSKEMQKLFAKLLQKKTGPLKX
contig046002-ZebOR.K087 CSIL---------FHIIPGSLNPII-------------------------------YGVQSKEMQKLFAKLLQKKTGPLKX
Contig046714+contig046713-TilOR.K1… CSIL---------FHILPGSLNPII-------------------------------YGVQSREIQRFLSKVIENYAIKITNFIFTX
contig046724-TilOR.K139 CAIL---------FHIIPGSLNPII-------------------------------YGVQSKEIQKFLSKLLHFTKILPSKX
contig046491-NyeOR.K087 FAVL---------FSIFPGSLNPII-------------------------------YGVQSKDLQKALLKFCVSKKVLASX
contig046706-TilOR.K135 SALL---------FRIVPGSLNPII-------------------------------YGVQSKELQKALLKFYVSKKVLASX
contig046699-TilOR.K134 SAIL---------FVIVPGSLNPVI-------------------------------YGVQSKEIRTFLYEKFHSKKCLFX
contig039450-TilOR.K133 AYVL---------FHVVPANLNPII-------------------------------YGMQTRSLRHKITEILKRKVTPSX
contig028565-BurOR.K080 AYVL---------FHVVPANLNPII-------------------------------YGMQTRSLRHKITEILKRKVTPSX
contig017778-ZebOR.K086 AYVL---------FHVVPANLNPII-------------------------------YGMQTRSLRHKITEILKRKVTPSX
contig040509-NyeOR.K083 AYVL---------FHVVPANLNPII-------------------------------YGMQMRSLRHKITEILKRKVTPSX
contig042534-BriOR.K067 AYVL---------FHVVPANLNPII-------------------------------YGMQTRSLRHKITEILKRKVTPSX
contig049621-BurOR.K082 VSTV---------GEVALPALNAVI-------------------------------YGLQIKEIRQKIVVLFQRKGHLQX
contig046010-ZebOR.K088 VSTV---------GEVALPALNAVI-------------------------------YGLQIKEIRQKIVALFQRKGHLQX
contig060525-NyeOR.K088 VSTV---------GEVALPALNAVI-------------------------------YGLQIKEIRQKIVALFQRKGHLQX
contig046723-TilOR.K138 VSTV---------GEVALPALNAVI-------------------------------YGLQIKEIRQKIVAFFQRKGHLQX
contig014348-BriOR.K066 ASMM---------FHVVPPALNAVI-------------------------------YGMQIKAVRQKMFIMFTRNTVTVTDGKX
contig046488-NyeOR.K084 ASMM---------FHVVPPALNAVI-------------------------------YGMQIKAVRQKMFIMFTRNTVTVTDRKX
contig046718-TilOR.K137 ASMM---------FHVVPPALNAVI-------------------------------YGMQIKAVRQKLFIMFMRNTVTVTEGKX
contig042536-BriOR.J063 MGML---------IFTVPPLLNPIV-------------------------------YGLYTNEIRSTLLRVLKNKVSMX
contig017781-ZebOR.J083 MGML---------IFTVPPLLNPIV-------------------------------YGLYTNEIRSTLLRVLKNRVSMX
contig028564-BurOR.J077 MGML---------IFTVPPLLNPIV-------------------------------YGLYTNEMRSTLLRVLKNRVSMX
contig040507-NyeOR.J080 MGML---------IFTVPPLLNPIV-------------------------------YGLYTNEIRSTLLRVLKNRVSMX
contig039450-TilOR.J131 MGML---------IFIVPPLLNPIV-------------------------------YGLYTKEIRSTLLRVLKNRVSIX
contig042539-BriOR.J064 FGVS---------VLIFPPFLDPII-------------------------------YGLKTKELKQCIVMFLKQNVGLTMX
contig017782-ZebOR.J085 FGVS---------VLIFPPFFDPII-------------------------------YGLKTKELKQCIVMFLKRNVGLTMX
contig062547-NyeOR.J081 FGVS---------VLIFPPFFDPII-------------------------------YGLKTKELKQCIVMFLKRNVGLTMX
contig039451-TilOR.J266 FGLS---------VLIFPPFLDPII-------------------------------YGLKTKELKQCIVMFLKQNVGFTKX
contig040506-NyeOR.J140 MGLS---------TLIFPPTLNPII-------------------------------YGLKTKEIREKVLNFLKNRMFSSX
contig042540-BriOR.J123 MGLS---------TLIFPPTLNPII-------------------------------YGLKTKEIREKVLNFLKNRIFSSX
contig017781-ZebOR.J084 MGLS---------TLIFPPTLNPII-------------------------------YGLKTKEIREKVLNFLKNRMFSSX
contig034988-NyeORs.A033 MTLQ---------VVLYHPLFNPII-------------------------------YGLKMKYISKHLKRLF-CX
contig041951-TilOR.A021 MTLQ---------VVLYHPLFNPII-------------------------------YGLKMKEISKHLKRLF-CQEKX
contig057756-NyeOR.A019 MTLQ---------IITYNPLCNPII-------------------------------YGLKMKEISNYLKRLL-RHMKX
contig030553-ZebOR.A002 MTLQ---------IITYNPLCNPII-------------------------------YGLKMKEISNHLKRLL-RHMKX
contig036782-BurOR.A002 MTLQ---------IITYNPLCNPII-------------------------------YGLKMKEISNHLKRLL-RHMKX
contig084999-BriOR.A001 MTLQ---------IITYNPLCNPII-------------------------------YGLKMKEISNHLKRLL-RHMKX
contig022268-TilOR.A020 MTLQ---------VIIYNPLCNPII-------------------------------YGLKMKDISKHLKRLF-VKKSX
contig057153-BurOR.A014 MTLQ---------IITYNPLFNPII-------------------------------YGLKMKEISKHLQRLL-CQSKLNX
contig054681-NyeOR.A011 MTLQ---------IITYNPLFNPII-------------------------------YGLKMKEISKHLQRLL-CQSKLNX
contig030557-ZebOR.A006 MTLQ---------IITYNPLFNPII-------------------------------YGLKMKEISKHLQRLL-CQSKLNX
contig022266-TilOR.A018 MTVQ---------IITYNPLFNPII-------------------------------YGLKLKEISKHLQRLL-CQSKLTX
contig064187-BurOR.A016 MTLQ---------FVTYNPLCNPII-------------------------------YGLKMKEISKHLKILF-SX
contig047506-ZebOR.A014 MTLQ---------FVTYNPLCNPII-------------------------------YGLKMKEISKHLKILF-SX
contig034988-NyeOR.A006 MTLQ---------FVTYNPLCNPII-------------------------------YGLKMKEISKHLKILF-SX
contig041951-TilOR.A022 MTLQ---------FVTYNPLCNPII-------------------------------YGLKMKEISKHLKILF-SX

```
contig034983-NyeOR.A002   MTLQ---------FITYNPLCNPII------------------------------YGLKMKEISKNLKRLF-SX
contig047515-ZebOR.A019   MTLQ---------FITYNPLCNPII------------------------------YGLKMKEISKNLKRLF-SX
contig051559-BurOR.A007   MTLQ---------FITYNPLCNPII------------------------------YGLKMKEISKNLKRLF-SX
contig022259-TilOR.A015   MTLQ---------IMLYQPLLNPFI------------------------------YGLKMKDISKHLNKLL-SQAKIIPCIKTX
contig051321-BurOR.A006   MTLQ---------IMLYQPLLNPFI------------------------------YGLKMKDISKHLNKLL-SQAKTISCIKTX
contig056380-NyeOR.A016   MTLQ---------IMLYQPLLNPFI------------------------------YGLKMKDISKHLNKLL-SQAKIISCIKTX
contig062094-ZebOR.A023   MTLQ---------IMLYQPLLNPFI------------------------------YGLKMKDISKHLNKLL-SQAKIISCIKTX
contig056375-NyeOR.A015   MTLQ---------IMLYQPLLNPFI------------------------------YGLKMKEISKHLNKLL-SX
contig064570-BurOR.A017   MTLQ---------IMLYQPLLNPFI------------------------------YGLKMKEISKHLNKLL-SX
contig062095-ZebOR.A024   MTLQ---------IMLYQPLLNPFI------------------------------YGLKMKEISKHLNKLL-SX
contig022259-TilOR.A014   MTLQ---------IMLYHPLFNPLI------------------------------YGLKMKEISKHLKKLL-SQAKIFPCIKTX
contig051318-BurOR.A005   MTLQ---------IMLYQPLLNPFI------------------------------YGLKMKEISKHLNKLL-SQTNIIPCIKTX
contig030572-ZebOR.A009   MTLQ---------IMLYQPLLNPFI------------------------------YGLKMKEISKHLNKLL-SQTNISPCIKTX
contig085012-BriOR.A006   MTLQ---------IMLYQPLLNPFI------------------------------YGLKMKEISKHLNKLL-SQANISPKAKVKX
contig022251-TilOR.A013   MTLQ---------IMLYQPLLNPFI------------------------------YGLKMKEISKHLNKLL-SQANISPCIKTX
contig030576-ZebOR.A010   MTLQ---------IVLYHPLFNPFV------------------------------YGLKMKEISKHLKGLL-CQGKITSCIKTGSX
contig085018-BriOR.A006   MTLQ---------IVLYHPLFNPFV------------------------------YGLKMKKISKHLKGLL-CQGKITSCIKTGFX
contig062344-NyeOR.A020   MTLQ---------IVLYHPLFNPFV------------------------------YGLKMKEISKHLKGLL-CQGKNNFLYX
contig022241-TilOR.A011   MTLQ---------IVLYHPLFNPVV------------------------------YGLKMKEISKHLKGLL-CQAFX
contig022245-TilOR.A012   MTLQ---------IVLYHPLFNPFV------------------------------YGLKMKEISKHLKKLL-CQGKIISCIKIGCX
contig022217-TilOR.A004   MTLQ---------IVLYHPLFNPFV------------------------------YGLKMKEISKHLKGLL-SQAKFITCIKIGCX
contig047521-ZebOR.A020   MTLQ---------VVLYHPIFNPFI------------------------------YGLKMKKISKHLKRFF-SRPQSILVLKVNAX
contig054237-BurOR.A013   MTLQ---------VVLYHPIFNPFI------------------------------YGLKMKKISKHLKRFF-SRPQSILALKVNAX
contig034981-NyeOR.A001   MTLQ---------VVLYHPIFNPFI------------------------------YGLKMKKISKHLKRFF-SRPQSILVLKVNAQYNIAVIFWLQLLKTLFIX
contig085026-BriOR.A008   MTLQ---------VVLYHPIVNPFI------------------------------YGLKMKKISKHLKRCF-PRPQSILELKVNAX
contig022211-TilOR.A003   MTLQ---------VVLYHPIFNPFI------------------------------YGLKMKQISKHLKRCF-HRPRSILVLKVNAX
contig034988-NyeOR.A005   MTLQ---------IVLYHPLFNPFI------------------------------YGLKMKEISKQLKRLF-CHARIIVYX
contig047508-ZebOR.A015   MTLQ---------IVLYHPLFNPFI------------------------------YGLKMKEISKQLKRFF-CHARIIVYX
contig051570-BurOR.A010   MTLQ---------IVLYHPLFNPFI------------------------------YGLKMKEISKQLKRFF-CHARIIVYX
contig070885-TilOR.A024   MTLQ---------IVLYHPLFNPFI------------------------------YGLKMKEISKQLKRFF-CHARIIX
contig047503-ZebOR.A013   MTLQ---------LLLYHPLFNPFI------------------------------YGLKMKEISKQLKRFF-CHATIITCINANVPX
contig051559-BurOR.A008   MALQ---------IVLYHPLLNPFI------------------------------YGLKMKEISKQLKRFF-YHAKIISCINSECX
contig047515-ZebOR.A018   MTLQ---------IVLYHPLLNPFI------------------------------YGLKMKEISKQLKRFF-YHAKIISCINSECX
contig034983-NyeOR.A003   MTLQ---------IVLYHPLLNPFI------------------------------YGLKMKEISKQLKRFF-YHAKIISCINSECX
contig022232-TilOR.A008   MTLQ---------IVLYHPLLNPFI------------------------------YGLKMKDISKQLKRFF-YHDX
contig022234-TilOR.A009   MTLQ---------IVLYHPLFNPFI------------------------------YGIKMKEISKQLKRFF-CHDKNHFMKTKFLQFVLX
contig022204-TilOR.A001   MTLQ---------VAFYHPLFNPFI------------------------------YGLKMKEISKHLKRLF-CPPVSX
contig022225-TilOR.A005   MTLQ---------ITLYQPLFNPFI------------------------------YGLKMKEISKHLKRLF-X
contig022227-TilOR.A006   VSLQ---------LILYQPLFNPLI------------------------------YGLKMKEISKHLKRLF-CPTKIICCINX
contig074640-TilOR.A002   MTLQ---------LALYHPLFNPFV------------------------------YGLKMKEISKHLKRLF-SPPPKIX
contig057165-NyeOR.A017   LTLQ---------IVMYPPLFNPII------------------------------YGLKMKEISKHLKRLF-CAVKKNX
contig030552-ZebOR.A001   LTLQ---------IVMYPPLFNPII------------------------------YGLKMKEISKHLKRLF-CAVKKNX
contig036780-BurOR.A001   LTLQ---------IVMYPPLFNPII------------------------------YGLKMKEISKHLKRLF-CAVKKNX
contig054684-NyeOR.A012   LTLQ---------VVLYHPLFNPIV------------------------------YGLKMKEISKHLTKLF-CEGKLNIWQSSCX
contig057156-BurOR.A015   LTLQ---------VVLYHPLFNPIV------------------------------YGLKMKEISKHLTKLF-CEAKLNIWQSSCX
contig030560-ZebOR.A007   LTLQ---------VVLYHPLFNPIV------------------------------YGLKMKEISKHLTKLF-CEGKLNIWQSSCX
contig085010-BriOR.A005   LTLQ---------VVSYHPLFNPIV------------------------------YGLKMKEISKHLTKLF-CEGKLNIWQSSCX
contig022265-TilOR.A017   LTLQ---------VVLYHPLFNPIV------------------------------YGLKMKEISKHLKKLF-CEGKLNIWQSSCX
contig054678-NyeOR.A010   MTLQ---------VVLYHPLFNPIV------------------------------YGLKMKEISQHLRRLF-CH--RVLCVKTDVGSAVISFVIQVQRPDFSTVX
contig030556-ZebOR.A005   MTLQ---------VVLYHPLFNPIV------------------------------YGLKMKEISQHLRRLF-CH--RVLCVKTDVGSAVISFVIQVQRPDFSTVX
contig036787-BurOR.A004   MTLQ---------VVLYHPLFNPIV------------------------------YGLKMKEISQHLRRLF-CH--RVLCVKTDVGSAVISFVIQVQRPDFSTVX
contig085002-BriOR.A004   MTLQ---------VVLYHPLFNPIV------------------------------YGLKMKEISQHLRRLF-CH--CVLCVKTDVRSAVSSFVIQVQRPDLSTVX
contig030554-ZebOR.A004   MTLQ---------VVLYHPLFNPIV------------------------------YGLKMKEISQHLRRLF-CQSKFKLSVRADAGSAIISFAIX
contig084999-BriOR.A002   MTLQ---------VVLYHPLFNPII------------------------------YGLKMKEISQHLRRLF-CQSKFKLSVRADAGSAIISFVIX
contig022268-TilOR.A019   MTLQ---------VVLYHPLFNPVV------------------------------YGLKMKEISQHLRRLF-CQSKFKLSVRADARSAIISFVIX
contig047514-ZebOR.A017   LTFQ---------SILFHPLLNPII------------------------------YGLKMNEIFRHIKSLL-CQVX
contig093807-BriOR.A009   LTFQ---------SILFHPLLNPII------------------------------YGLKMNEIFKHIKTLL-CQVX
contig022238-TilOR.A010   LTFQ---------SILFHPLLNPII------------------------------YGLKMNEIFKHIQTLL-CQVX
contig034988-NyeOR.A004   LTFQ---------SILFHPLLNPII------------------------------YGLKVNEIFKHIKMLL-CQVX
contig047508-ZebOR.A016   LTFQ---------SILFHPLLNPII------------------------------YGLKVNEIFKHIKMLL-CQVX
contig051566-BurOR.A009   LTFQ---------SILFHPLLNPII------------------------------YGLKVNEIFKHIKMLL-CQVX
contig047526-ZebOR.A021   LTFQ---------SILFHPLLNPII------------------------------YGLKVNEIFKHIKMLL-CQVX
contig070886-TilOR.A025   LTFQ---------SILFHPLLNPII------------------------------YGLKMNEIFKHIKILL-CQVX
contig041952-TilOR.A023   LTFQ---------SILFHPLLNPII------------------------------YGLKMNEISKHLKILL-CQVYTGITAPLSNVWHIICNCNANX
contig034990-NyeOR.A007   LTFQ---------SIVFHPLLNPII------------------------------YGLKMNEIFKHIKILL-SSLITLVLLPYYQMYGIX
contig047499-ZebOR.A012   LTFQ---------SIVFHPLLNPII------------------------------YGLKMNEIFKHIKILL-SSLITLVLLPYYQMYGIX
contig093812-BriOR.A010   LTFQ---------SILFHPLLNPII------------------------------YGLKMNAIFKHIKILL-SGLITLVLMPYYQMYGIX
contig047497-ZebOR.A011   LTFQ---------SIVFHPLLNPII------------------------------YGLKMNEIFKHIKILL-SSLITLVLLPYYQMYGIX
contig034995-NyeOR.A009   LTFQ---------SILFHPLLNPII------------------------------YGLKMNEIFKHIKILL-SSLITLVLLPYYQMYGIX
```

```
contig034994-NyeOR.A008   LTFQ---------SILFHPLLNPII-----------------------------YGLKMNEIFKHIKILL-SSLITLVLLPYYQMYGIX
contig093816-BriOR.A011   LTFQ---------LILFHPLLNPII-----------------------------YGLKMNEISKHLKILL-CLVKX
contig051573-BurOR.A011   LTFE---------LILFHPLLNPII-----------------------------YGLKMNEISKYLKILL-CVVKX
contig054233-BurOR.A012   LTFQ---------SILFHPLLNPII-----------------------------YGLKMNEIFKNLKILF-CHVKX
contig047523-ZebOR.A022   LTFQ---------SILFHPLLNPII-----------------------------YGLKMNEIFKNLKILF-CHVKX
contig054868-NyeOR.A014   LTFQ---------SILFHPLLNPII-----------------------------YGLKMNEIFKNLKILF-CHVKX
contig073309-TilOR.A026   LTFQ---------SILFHPLLNPII-----------------------------YGLKMNEIFKSLKILF-CHVTVAGNIX
contig030566-ZebOR.A008   MTLQ---------VIFYNPLLNPFI-----------------------------YGLKMKEISKHLKRLF-CHVRCSX
contig065887-BurOR.A018   MTLQ---------VIFYNPLLNPFI-----------------------------YGLKMKEISKHLKRLF-CHVRCSX
contig054687-NyeOR.A013   MTLQ---------VIFYNPLLNPFI-----------------------------YGLKMKEISKHLKRLF-CHVRCSX
contig022264-TilOR.A016   MTVQ---------VIFYNPLFNPFI-----------------------------YGLKMKEISKHLKRLF-CRVRCSX
contig036784-BurOR.A003   MTLQ---------VVLYNPLFNPFI-----------------------------YGLKMKEISKHLKRLF-CHVRCSX
contig057754-NyeOR.A018   MTLQ---------VVLYNPLFNPFI-----------------------------YGLKMKEISKHLKRLF-CHVRCSX
contig030553-ZebOR.A003   MTLQ---------VVLYNPLFNPFI-----------------------------YGLKMKEISKHLKRLF-CHVRCSX
contig085000-BriOR.A003   MTLQ---------VVLYNPLFNPFI-----------------------------YGLKMKEISKHLKRLF-CHVRCSX
contig009320-TilOR.B045   TSIL---------QVMIPTVFNPVV-----------------------------YGLKVTEIRAQLRRLLGCQRADX
contig053886-ZebOR.B034   TSIF---------QVMIPTVFNPVV-----------------------------YGLKVTEIRAQLRRLLGCQRADX
contig044492-NyeOR.B034   TSIF---------QVMIPTVFNPVV-----------------------------YGLKVTEIRAQLRRLLGCQRADX
contig040653-BurOR.B030   TSIF---------QVMIPTVFNPVV-----------------------------YGLKVTEIRAQLRRLLGCQRADX
contig049299-BurOR.E046   LSLY---------FLTCQPLFNPVL-----------------------------YGLNMSNIR---KRLF-AHKRRX
contig053579-NyeOR.E053   LSLY---------FLTCQPLFNPVL-----------------------------YGLNMSNIR---KRLF-AHKRRX
contig017699-BurOR.E042   LSLY---------FLTCPPLFNPLM-----------------------------YGLNLSKIRVTCKNLI-THIICX
contig023280-NyeOR.E050   LSLY---------FLTCPPLFNPLM-----------------------------YGLNLSKIRVTCKNLI-THIICX
contig004266-BriOR.E039   LSLY---------FLTCPPIFNPLM-----------------------------YGLNLSKIRVTCKNLI-AHIICX
contig025447-ZebOR.E047   LSLY---------FLTCPPLFNPLM-----------------------------YGLNLSKIRVTCKNLI-THIICX
contig004265-BriOR.E035   LSLY---------FLTCQPLFNPVM-----------------------------YGLKMTKIRNICKSLI-TNIKX
contig065454-TilOR.E088   LSLY---------FLTCQPLFNPVM-----------------------------YGLKMTKIRNICKSLI-TNTHLX
contig065454-TilOR.E089   LSLY---------FLTCQPLFNPVM-----------------------------YGLKMTKIRNICKSLI-SNIKX
contig052450-BurOR.E055   LSLY---------FLTCQPLFNPVM-----------------------------YGLKMTKIRNICKSLI-TNTHLX
contig004261-BriOR.E034   LSLY---------FLTCQPLFNPVM-----------------------------YGLKMTKIRNICKSLI-TNTHLX
contig065453-TilOR.E087   LSLY---------FLTCQPLFNPVM-----------------------------YGLKMTKIRNICKSLI-TNTHLX
contig062770-NyeOR.E059   LSLY---------FLTCQPVFNPLM-----------------------------YGLTLSKISLTCKKLL-CADMX
contig048239-ZebOR.E048   LSLY---------FLTCQPVFNPLM-----------------------------YGLTLSKISLTCKKLL-CADMX
contig052457-BurOR.E051   LSLY---------FLTCQPVFNPLM-----------------------------YGLTLSKISLTCKKLL-CADMX
contig047729-TilOR.E076   LSLY---------FLTCQPLFNPLM-----------------------------YGLTLSKISLTCKKTT-LCX
contig047734-TilOR.E077   LSLY---------WLICPPLCNPLL-----------------------------YGLNLTKIRIIYKGLI-LSKVX
contig059404-NyeOR.E058   LSLY---------WLICPPLCNPLL-----------------------------YGLSLTKIRIIYKGLI-FVKCRCLMLKLRQGSALVKRKX
contig052453-BurOR.E049   LSLY---------WLICPPLCNPLL-----------------------------YGLSLTKIRIIYKGLI-FVKCRCLMLKLRQGSALVKRKX
contig064938-BurOR.E053   LSLY---------WLICSPLCNPLL-----------------------------YGLSLTKIRIIYKGLI-LSKVX
contig004258-BriOR.E037   LSIY---------WLTCQPLFNPVI-----------------------------YGLNLTKIRVSCKNVI-RRMKVFTLCHKSNFKIX
contig052452-BurOR.E048   LSIY---------WLTCQPLFNPVI-----------------------------YGLNLTKIRVSCKNVI-CRMKVFTLFHKSNFKIX
contig025443-ZebOR.E046   LSIY---------WLTCQPLFNPVI-----------------------------YGLNLTKIRVSCKNVI-CRMKVFTLCHKSNFKIX
contig065458-TilOR.E086   LSIY---------WLTCQPLFSPVI-----------------------------YGLNLTKIRVSCKNVI-GRMKVFTLCHKLNFKIX
contig047832-TilOR.E082   ISLY---------WLICQPLFNPVL-----------------------------YGLKMSKIYDICKSLL-YWKVX
contig049298-BurOR.E045   ISLY---------WLMCQPLFNPVL-----------------------------YGLKMSKIRDICKCLL-YSKVNTILSX
contig048260-ZebOR.E051   ISLY---------WLMCQPLFNPVL-----------------------------YGLKMSKIRDICKSLL-YSKVNTILSX
contig053579-NyeOR.E054   ISLY---------WLMCQPLFNPVL-----------------------------YGLKMSKIRDICKCLL-YSKVNTILSX
contig047833-TilOR.E083   ISLY---------WLICQPLFNPVL-----------------------------YGLKMSKIRDICKCLL-YSKVNTILSX
contig053579-NyeOR.E055   ISLY---------WLMCQQLFNPVL-----------------------------YGLKMSKIYDICKSLL-YWKVWIMX
contig047829-TilOR.E081   LSLY---------FLACQPLFNPVL-----------------------------YGLKMSSIR---KSLFLLKSRX
contig053576-NyeOR.E052   LSVY---------FLTCQPLFNPVM-----------------------------YGLNMSKIRTICKNLL-LGYVGKSRILIKIVQIEKX
contig064724-BurOR.E052   LSVY---------FLTCQPLFNPVM-----------------------------YGLNMSKIRTICKNLL-LGYVGKSRILIKIVQIEKX
contig048263-ZebOR.E052   LSVY---------FLTCQPLFNPVM-----------------------------YGLNMSKIRTICKNLL-LGYVGKSRILIKIVQIEKX
contig047826-TilOR.E080   LSVY---------FLTCQPLFNPVM-----------------------------YGLNMSKIRTICKNLL-LGSVGKSRIFIKIVQIEKX
contig025439-ZebOR.E045   LSIY---------FLTCQPAFNPVL-----------------------------YGLKLTKIRLICKSLL-FGKIX
contig052454-BurOR.E050   LSIY---------FLTCQPAFNPVL-----------------------------YGLKLTKIRLICKSLL-FGKIX
contig004255-BriOR.E036   LSIY---------FLTCQPVFNPVL-----------------------------YGLKLTKIRLICKSLL-FGKIX
contig066194-BurOR.E054   SSLY---------FLTCQPLFTPLL-----------------------------YGLKMTKIRIACKQLF-CGSLTRLFSCSSDIFSKSHQSQSYCX
contig053572-NyeOR.E051   SSLY---------FLTCQPLFTPLL-----------------------------YGLKMTKIRIACKQLF-CGSLTRLFSCSSDIFSKSHQSQSYCX
contig047825-TilOR.E079   SSLY---------FLTCQPLFTPLL-----------------------------YGLKMTKIRIACKQLF-CGPLTRLFSCSSDIFSKSHQSQTYCX
contig047820-TilOR.E078   LSLY---------YLTCQPLVNPLL-----------------------------YGLKMSQIRMLYKSLL-FWKKX
contig063018-ZebOR.E053   LSLY---------WLISQPLLNPLV-----------------------------YGLNMTKIRILCKNLL-TLRPLNX
contig004259-BriOR.E038   LSLY---------WLISQPLLNPLV-----------------------------YGLNMTKIRILCKNLL-TLRPLNX
contig052451-BurOR.E047   LSLY---------WLISQPLLNPLV-----------------------------YGLNMTKIRILCKNLL-TLRPLNX
contig065455-TilOR.E085   LSLY---------WLISQPLINPLV-----------------------------YGLNMTKIRILCKNLL-TLRPLNX
contig082838-BriOR.E040   LSLY---------FLIIQPLLNPIM-----------------------------YGTQMSKIRHVLCYKMX
contig053592-NyeOR.E057   LSLY---------FLIIQPLLNPIM-----------------------------YGTQMSKIRHVLCYKMSLX
contig049287-BurOR.E043   LSLY---------FLIIQPLLNPIM-----------------------------YGTQMSKIRHVLCYKMSLX
contig048242-ZebOR.E049   LSLY---------FLIIQPLLNPIM-----------------------------YGTQMSKIRHVLCYKMSLX
```

```
contig047726-TilOR.E075  LSLY---------FLIIQPLLNPIM-------------------------------YGTQMSKIRHVLCYKMX
contig053590-NyeOR.E056  LSLY---------FLMMQPLMNPIL-------------------------------YGTQMSKIRGVYEHVL-SSIMSCGCSKVSQSDX
contig049289-BurOR.E044  LSLY---------FLMMQPLMNPIL-------------------------------YGTQMSKIRGVYEHVL-STIMSCGCSKVSQSDX
contig048243-ZebOR.E050  LSLY---------FLMMQPLMNPIL-------------------------------YGTQMSKIRGVYEHVL-SSIMSCGCSKVSQSDX
contig047725-TilOR.E074  LSLY---------FLMMQPLMNPIL-------------------------------YGTQMSKIRGVYEHVL-SSKVSCRCSKVSQSDX
contig047834-TilOR.E084  LFLY---------FLIIQPLFNPIM-------------------------------YGTQMSKIRKACKHVFCIKGFTGYRX
contig014049-ZebOR.D038  LPMY---------VLIFQPMLTPFM-------------------------------YGFNLPKIRQSYQRFL-LKRKX
contig013327-TilOR.D052  LPMY---------VLIFQPMLTPFM-------------------------------YGFNLPKIRQSYQRFL-LKRKX
contig039737-NyeOR.D039  LPMY---------VLIFQPMLTPFM-------------------------------YGFNLPKIRQSYQRFL-FERKX
contig032396-BurOR.D034  LPMY---------LLIFQPMLTPFM-------------------------------YGFKLTKIRQSYQRFL-FERKX
contig064809-BriOR.D028  LPMY---------LLIFQPMLTPFM-------------------------------YGFKLPKIRQSYQRFL-FERKX
contig014050-ZebOR.D039  LPMY---------FLIFQPMLTPFM-------------------------------YGFKLPKIRQSYQRFL-FERKX
contig013326-TilOR.D051  LPMY---------VLIFQPMLTPFM-------------------------------YGFNLPKIKQSYQRFL-LKRKX
contig014051-ZebOR.D040  LPMY---------VLIFQPMLTPFM-------------------------------YGFNLPKIRQSYQRFL-FERKX
contig013337-TilOR.D055  LPMY---------ALIFQPMLTPFM-------------------------------YGFNLPKIRQSYQRFS-VKEKINILVQIX
contig013330-TilOR.D054  LPMY---------ALIFQPMLTPFM-------------------------------YGFNLPKIRQSYQRFS-VKEKINILVQIX
contig013339-TilOR.D057  LPMY---------VLIFQPVLTPFM-------------------------------YGFNLPKIRQSYQRFL-FERKX
contig032389-BurOR.D033  LPMY---------MLIFQPMLTPFM-------------------------------YGFNLPKIRQSCKRFL-FKRKX
contig039737-NyeOR.D040  LPMY---------MLICQPMLTPFM-------------------------------YGFNLPKIRQSCKRFL-FKRKX
contig013339-TilOR.D056  LAIY---------LLICQPMLTPFL-------------------------------YGFNLPKIRQSCKSFL-FLRKX
contig013330-TilOR.D053  LAIY---------LLICQPMLTPFL-------------------------------YGFNLPKIRQSCKSFL-FLRKX
contig064814-BriOR.D029  LPMY---------LLICQPMLTPFM-------------------------------YGFNLPKIRQACKMLL-FKRKX
contig014054-ZebOR.D041  LPMY---------LLICQPMLTPFM-------------------------------YGFNLPKIRQACKMLL-FKRKX
contig039731-NyeOR.D038  LPMY---------LLICQPMLTPFM-------------------------------YGFNLPKIRQACKMLL-FKRKX
contig014054-ZebOR.D042  LSIY---------LLICQPMLTPFM-------------------------------YGFNLPKIRQSCTMLV-FKRKSMSLFKKRLFX
contig039730-NyeOR.D036  LSIY---------LLICQPMLTPFM-------------------------------YGFNLPKIRQSCTMLV-FKRKSISLFGKRLFX
contig053779-BurOR.D035  LSIY---------LLICQPMLTPFM-------------------------------YGFNLPKIRQSCTMLV-FKRKSISLFGKRLFX
contig039730-NyeOR.D037  LSIY---------LLICQPMLTPFM-------------------------------YGFNLPKIRQSRTMLL-FKRKSISLFSKRVLX
contig013344-TilOR.D058  LAIY---------LLICQPILTPFM-------------------------------YGFNLPKIRQSCKMLL-FKRKX
contig014049-ZebOR.D037  LPMY---------VLICQPMLTPFL-------------------------------YGFNLPKIRHSFKRLL-FKRKX
contig032388-BurOR.D032  LPMY---------VLICQPMLTPFL-------------------------------YGFNLPKIRHSFKRLL-FKRKX
contig039738-NyeOR.D041  LPMY---------VLICQPMLTPFL-------------------------------YGFNLPKIRHSFKRLL-FKRKX
contig013323-TilOR.D050  LPMY---------ILICQPMLTPFL-------------------------------YGFNLPKIRHSFKRLL-FNRKX
contig014047-ZebOR.D036  LSIY---------LFICQPMLTPFL-------------------------------YGFNLPKIRQSCKKLL-FKKKQX
contig064802-BriOR.D027  LSIY---------LFICQPMLTPFL-------------------------------YGFNLPKIRQSCKKLL-FKKNQX
contig013322-TilOR.D049  LSIY---------LFICQPMLTPFL-------------------------------YGFNLPKIRQSCKRFL-FKKKX
contig039738-NyeOR.D042  LSIY---------LFICQPMLTPFL-------------------------------YGFNLPKIRQSCKRFV-FKKKX
contig013321-TilOR.D048  LPMY---------MLIFQPMLTPFL-------------------------------YGFKLPKIRHSCKRLL-FKRKSVALFX
contig066327-ZebOR.F064  LSLE---------FVVIPPILNPLM-------------------------------YGLKLAEIRKCILRNLSCLIRX
contig013898-BurOR.F058  LSLE---------FVVIPPILNPLM-------------------------------YGLKLPEIRKCILRNLSCLIRX
contig033883-BriOR.F045  LSLE---------FVVIPPILNPLM-------------------------------YGLKLPEIRKCILRNLPCLIRX
contig009545-TilOR.F097  LSLE---------FVVIPPILNPLM-------------------------------YGLKLPEIRKCILRKLVCLIRYMSHDKENSVKX
contig039729-NyeOR.F064  LSLV---------FVVIPPALNPLV-------------------------------YGLKLPEIRKHILKLF-X
contig053781-BurOR.F059  LSLV---------FVVIPPALNPLV-------------------------------YGLKLPEIRKHILRLF-X
contig014056-ZebOR.F063  LSLV---------FVVIPPALNPLV-------------------------------YGLKLPEIRKHILRLF-X
contig075822-TilOR.F098  LSLV---------FVVIPPALNPLV-------------------------------YGLKLPEIRKHILRLF-X
contig025224-ZebOR.C035  LSLY---------FIIIPPIANPVL-------------------------------YGLGTQTVRGCIMKLF-IKNKVMTTVLAKTLTVGX
contig009805-BurOR.C031  LSLY---------FIIIPPIANPVL-------------------------------YGLGTQTVRGCIMKLF-IKNKVMTTVLAKTLTVGX
contig020980-NyeOR.C035  LSLY---------FIIIPTIANPVL-------------------------------YGLGTQTVRGCIMKLF-IKNKVMTTVLAKTLTVGX
contig063874-BriOR.C026  LSLY---------FIIIPPIANPVL-------------------------------YGLGTQTVRGCIMKLF-IKNKVMTTVLAKTLTVGX
contig048237-ZebOR.G065  LAAQ---------FLVVPPLVNPII-------------------------------YGLNLQQIRSRMVHRF-THRTGTFRKNX
contig049295-BurOR.G060  LAAQ---------FLVVPPLVNPII-------------------------------YGLNLQQIRSRMVHRF-THRTGTFRKNX
contig053581-NyeOR.G066  LAAQ---------FLVVPPLVNPII-------------------------------YGLNLQQIRSRMVHRF-THRTGTFRKNX
contig104344-BriOR.G048  LAAQ---------FLVVPPLVNPII-------------------------------YGLNLQQIRSRMLHRF-THRTGTFRKNX
contig047714-TilOR.G099  LAAQ---------FLVVPPLVNPII-------------------------------YGMNLQQIRSRMLQRF-THRTGIFRKNX
contig057403-ZebOR.H076  IAIE---------FLVIPPVMNPLI-------------------------------YGFKLTKIRNKILSFV-YRKQKX
contig013371-TilOR.H117  IAIE---------FLVIPPVMNPLI-------------------------------YGFKLTKIRNKILSFV-YRKQKX
contig035582-NyeOR.H070  IAIE---------FLVIPPVMNPLI-------------------------------YGFKLTKIRNKILSFV-YRKQKX
contig013361-TilOR.H108  ITIE---------FLIIPPVMNPLI-------------------------------YGFKLTKIRDRILSLI-YFKRKX
contig013362-TilOR.H109  ITIE---------FLIIPPVMNPLM-------------------------------YGFKLTKIRNRILSLI-YLKRKX
contig013368-TilOR.H112  ITIE---------FLIIPPVMNPLI-------------------------------YGFKLTKIRNRILSLI-YLKRKX
contig013369-TilOR.H115  ITIE---------FLIIPPVMNPLI-------------------------------YGFKLTKIRNRILSLI-YLKRKX
contig013369-TilOR.H116  FSIE---------FLIIPPVMNPLI-------------------------------YGFKITKIRNRILGLV-CFKRQRISSRFTLLFRNKSEELWKMCITVLDLINITRX
contig013369-TilOR.H114  IAIE---------FLIIPPVMNPLV-------------------------------YGFKLAKIRNRIFTLV-HYKTKX
contig048882-BurOR.H064  IAIE---------FLIISPVMNPLV-------------------------------YGFKLAKIRKRIFTLV-HYKTKLLQSKLRVSYFKRRMPFRNILLNIFCCTX
contig035580-NyeOR.H069  IATE---------FVIIPPLMNPLI-------------------------------YGFKLTKIRNRILGLV-CFKRKX
contig057400-ZebOR.H075  IATE---------FVIIPPLMNPLI-------------------------------YGFKLTKIRNRILGLV-CFKRKX
contig013368-TilOR.H113  ITIE---------FLIIPPVMNPLI-------------------------------YGFKLTKIRNRILGLV-CFKRKSIPSKLRVSX
contig006794-BurOR.H061  ITVE---------ILIIPPFMNPLI-------------------------------YGFKFTKIQNRILTLL-KSYKKLTYIKFSLLKKTSVKCSIFLTVKNLLCEPFCCRX
```

```
contig035583-NyeOR.H071  ITVE---------ILIIPPFMNPLI------------------------------YGFKLTKIQNRILTLL-KSYKKLTYIKFSLLKKTSVKCSIFLRVKNLLCEPFCCRX
contig057403-ZebOR.H077  ITVE---------ILIIPPFMNPLI------------------------------YGFKLTKIQNRILTLL-KSYKKLTYIKFSLLKKTSVKCSIFLRVKNLLCEPFCCRX
contig049873-BriOR.H050  ITVE---------ILIIPPFMNPLI------------------------------YGFKLTKIQNRILTLL-KSYKKLTYIKFSLLKKTRVKCSIFL
contig013371-TilOR.H118  ITVE---------ILIIPPFMNPLM------------------------------YGFKLTKIRNRIMTLL-KSX
contig093825-BriOR.H053  VAIA---------VLFIPPVMNPLL------------------------------YGFKLSKIRNRILVTL-HNKRCX
contig048562-BurOR.H062  VAIA---------VLFIPPVMNPLL------------------------------YGFKLSKIRNRILVTL-HIKRCX
contig034998-NyeOR.H067  VAIA---------VLFIPPVMNPLL------------------------------YGFKLSKIRNRILVTL-HIKRCX
contig047492-ZebOR.H074  VAIA---------VLFIPPVMNPLL------------------------------YGFKLSKIRNRILVTL-HIKRCX
contig041955-TilOR.H119  VAIA---------VLFIPPVMNPLL------------------------------YGFKLTKIRNRILVAL-HIKRCX
contig116846-BriOR.H055  VALE---------FLIIPPIMNPLI------------------------------YGFKLTKIRKRLHSVV-ILKITNFCFIRSENSFTHSX
contig033889-BriOR.H049  VALE---------FLVIPPIMNPLI------------------------------YGFKLTKIRKKIYSVV-ILKRTNFCFIRPENRFTHSX
contig030011-ZebOR.H073  VALE---------FLVIPPIMNPLI------------------------------YGFKLTKIRKKIYSVV-ILKRTNFCFIRSENSFTHSX
contig018437-ZebOR.H072  VAIE---------VLVIPPIMNPLI------------------------------YGFKLTKIRKKIYSVV-ILKGTNFCFIQSENSFTHSX
contig018434-ZebOR.H070  IAIE---------FLVIPPVMNPLM------------------------------YGFKLTKIQKKVFIVI--LKTKX
contig009565-TilOR.H126  IAIE---------FLVIPPVMNPLM------------------------------YGFKLTKIQKKVFVVI--LKTKX
contig018434-ZebOR.H071  AAIE---------FLVIPPIMNPLI------------------------------YGFKLTKIRKTICSVV-IFKTKX
contig009547-TilOR.H101  VTIE---------FLVIPPIMNPLI------------------------------YGFKLTKIKKRICTFI-TFRISVTPAYAGKELLSFRSX
contig009548-TilOR.H102  VTIE---------FLVIPPIMNPLI------------------------------YGFKLTKIKKRICTVM-FFIFRX
contig009546-TilOR.H100  VAIE---------FLIIPPIMNPLI------------------------------YGFKLTKIRKSICVVI--FKISKSSNFIX
contig014060-ZebOR.H069  MAME---------FLLIPPIMNPLM------------------------------YGLKLTKIRKRVLNFI-CGKSSTFRLKSX
contig053784-BurOR.H067  MAME---------FLLIPPIMNPLM------------------------------YGLKLTKIRKRVLNFI-CGKSSTFRLKSX
contig041756-NyeOR.H075  MTME---------FLLIPPIMNPLM------------------------------YGLKLTKIRKRVLNFI-CGKSSSFRLKSX
contig041756-NyeOR.H139  MAME---------ILLIPPIINPLM------------------------------YGFKLTQIRNRVLNFI-CGKSLALRLKSX
contig013363-TilOR.H110  MAME---------ILLIPPIINPLM------------------------------YGFKLTQIRNRVLNFI-CGKSSALRLKSX
contig035579-NyeOR.H068  MAME---------IFLIPPIINPLM------------------------------YGFKLTQIRNRVLNFI-CGKSSTLRLKSX
contig048880-BurOR.H063  MAME---------IFLIPPIINPLI------------------------------YGFKLTQIRNRVLNFM-RGKSSALRLKSX
contig013365-TilOR.H111  MAME---------ILLIPPIINPLM------------------------------YGFKLTQIRNTVLNFI-CGKSSALRLKLX
contig053782-BurOR.H066  MAME---------ILFIPPIINPLM------------------------------YGFKLTKIRKRVLNFI-CGESSAFILNSX
contig039729-NyeOR.H073  MAME---------ILFIPPIINPLM------------------------------YGFKLTKIRKRVLNFI-CGESSAFILNSX
contig014057-ZebOR.H067  MAME---------ILFIPPIINPLM------------------------------YGFKLTQIRNRVLNFI-CGKSSAFILKSX
contig064817-BriOR.H051  MAME---------ILFIPPIINPLM------------------------------YGFKLTQIRNTVLNFI-CGKSSAFILRSX
contig013359-TilOR.H107  MAME---------ILLIPPIINPLM------------------------------YGFKLTQIRNRVLNFM-CGKRSALRLKSX
contig064821-BriOR.H052  MAME---------ILLIPPIINPLM------------------------------YGFKLTKIRNRVLNFI-CGKSSALRLKSX
contig053788-BurOR.H069  MAME---------ILLIPPIINPVM------------------------------YGFKLTKIRNRVLNFI-CGISSTLRLKLX
contig041757-NyeOR.H076  MAME---------ILLIPPIINPVM------------------------------YGFKLTKIRNRVLNFI-CGISSTLRLKLX
contig013356-TilOR.H105  MAME---------IVLIPPIINPLI------------------------------YGFKLTKIRNRVLNFI-CGKSKALRLKSX
contig013358-TilOR.H106  MAME---------FLLIPPIMNPLM------------------------------YGLKLTKIRKRVLNFI-CGKSSTFRLKSX
contig013351-TilOR.H104  MAME---------FLLIPPIMNPLM------------------------------YGFKLTKIRNRVLNFI-CGKTSALRFKSX
contig014059-ZebOR.H068  MAME---------FLLIPPIMNPLM------------------------------YGFKLTKIRKRVLNFI-CGKSSAFRFKSX
contig053787-BurOR.H068  MAME---------FLLIPPIMNPLM------------------------------YGFKLTKIRKRVLNFI-CGKSSAFRFKSX
contig039725-NyeOR.H072  MAME---------FLLIPPIMNPLM------------------------------YGFKLTKIRKRVLNFI-CGKSSAFRFKSX
contig039730-NyeOR.H074  MAIE---------FLIIPPILNPVC------------------------------YGWVLTKIRRRMIFLC-RLAYQRFGVKSQX
contig014055-ZebOR.H066  MAIE---------FLIIPPILNPVC------------------------------YGWVLTKIRRRMIFLC-RLAYQRFGVKSQX
contig053780-BurOR.H065  MAIE---------FLIIPPILNPVC------------------------------YGWVLTKIRRRMIFLC-RLAYQRFGVKSQX
contig107626-BriOR.H054  MAIE---------FLIIPPILNPVC------------------------------YGWVLTKIRRRMIFLC-RLAYQRFGVKSQX
contig013349-TilOR.H103  MAIE---------FLIIPPILNPVC------------------------------YGLVLTKIRRRMIFLC-RQAYQRFGVKSQX
contig106096-BriOR.N089  NLSL---------TSVFPPMLNPII------------------------------YVLQTREIKESLKKFLRITTHYKNRKVKFKKX
contig061663-NyeOR.N114  NLSL---------TSVFPPMLNPII------------------------------YVLQTQEIKESLKRFLRITTHYKISKVKFKKX
contig057383-BurOR.N109  NLSL---------TSVFPPMLNPII------------------------------YVLQTQEIKESLKRFLRITTHYKISKVKFKKX
contig010722-ZebOR.N111  NLSL---------TSVFPPMLNPII------------------------------YVLQTREIKESLRKLLRIIKHYKIRKVKX
contig096539-BriOR.N087  NLSL---------TSVFPPMLNPII------------------------------YVLQTQEIKESLKRLLKRRGKSKITIX
contig064097-ZebOR.N115  NLSL---------TSVFPPMLNPII------------------------------YVLQTQEIKESLKRLLKRRGKSKITIX
contig055927-NyeOR.N111  NLSL---------TSVFPPMLNPII------------------------------YVLQTQEIKESLKRLLKRRGKSKITIX
contig060631-BurOR.N110  NLSL---------TSVFPPMLNPII------------------------------YVLQTQEIKESLKRLLKRRGKSKITIX
contig046353-TilOR.N195  NLSL---------TSVFPPMLNPII------------------------------YVLQTQEIKESLKRLLKRRGKSKITIX
contig055926-NyeOR.N110  NLSL---------ASVTPPMLNPII------------------------------YVFQTAEIKKSLKKLLKAKIQISHRVLX
contig042928-BurOR.N108  NLSL---------ASVTPPMLNPII------------------------------YVFQTAEIKKSLKKLLKAKIQISHRVLX
contig064098-ZebOR.N116  NLSL---------ASVTPPMLNPII------------------------------YVFQTAEIKKSLKKLLKAKIQISHRVLX
contig096539-BriOR.N088  NLSL---------ASVTPPMLNPII------------------------------YFLQTAEIKKSLKRLLKAKIQISHRVLX
contig046356-TilOR.N196  NLSL---------ASVMPPMLNPII------------------------------YVLQTAEIKKSLKRLLRAKIQICHTECCSLMKHMX
contig055924-NyeOR.N109  SLSL---------ATVMPLTLNPII------------------------------YGLQTQEIKESLKKLLKVKMQFKISAKKX
contig010725-ZebOR.N112  SLSL---------ATVMPLTLNPII------------------------------YGLQTQEIKESLKKLLKVKMQFKISAKKX
contig010726-ZebOR.N113  SLSL---------ATVMPLTLNPII------------------------------YGLQTQEIKESLKKLLKVKMQFKISANKX
contig046360-TilOR.N197  SLSL---------ATVMPLTLNPII------------------------------YGLQTQEIKESLKKLLKVKMQFX
contig010714-ZebOR.N109  SLSM---------STVLPPMLNPII------------------------------YVLQTQEIKQSLKKLLKTRVTSKIATKYX
contig010718-ZebOR.N110  SLSM---------STVLPPMLNPII------------------------------YVLQTQEIKESLKKLLKTRVTSKIATKYX
contig010712-ZebOR.N108  SLSI---------STVLPPMLNPII------------------------------YVLQTQEIKESLKKLLQTRAQFRIAEKYX
contig050080-TilOR.N198  SLSI---------TTALPPMLNPII------------------------------YVLQTQEIKESLKKLFQTRAQFRVAAKYX
contig046352-TilOR.N193  NSTL---------AYTIPALLNPII------------------------------YALKTEEVMNAVKKLWKKTPFINTAX
```

```
contig046347-TilOR.N190   NSTL---------TYTIPALLNPII------------------------------YALKTEEVMNAVKKLWKKKTFINTAX
contig096536-BriOR.N085   NSVL---------THTIPALLDPIV------------------------------YALKTEEVMNAVKKLCKRTYLNCMKAKTRPCNHCCIKSX
contig055933-NyeOR.N113   NSVL---------THTIPASLDPIV------------------------------YALKTEEVMNAVKKLCKGINLSRMKAKTRPCNHCCIKSX
contig065025-BurOR.N111   NSTL---------THTIPALLDPII------------------------------YALKTEEVMNAVKKLCKRTYLNCMKAKTRPCNHCCIKSX
contig096535-BriOR.N084   NSTL---------THTIPALLDPII------------------------------YALKKEEVMNAVKKLCKRTYLNCMKAKTRPCNHCCIKSX
contig046340-TilOR.N188   NSTL---------THTIPALLDPIV------------------------------YALKTEEVMNAVKKLCKRIYLNRMKAKTRPCNHCYIKSX
contig046351-TilOR.N192   NYTL---------THTIPPLLNPIV------------------------------YALKTEEVKNAIKKLCKRNNVSNTVTKCX
contig046350-TilOR.N191   NSAL---------THTIPPLLNPII------------------------------YALKTEEVMNAIKKLCKRHYFSNKKKGKTX
contig046344-TilOR.N189   NSAL---------THTIPPLLNPII------------------------------YALKTEEVMNAIKKLCKRHYFSNKKKGKTX
contig010727-ZebOR.N114   NSTL---------THTLPALINPIV------------------------------YALKTEEVMNAVKKLWKINNIRSPVTKWX
contig042920-BurOR.N107   NSTL---------THTIPALINPIV------------------------------YALKTEEVMSAVKKLWKINNISSPVTKWX
contig055927-NyeOR.N112   NSTL---------THTIPALINPIV------------------------------YALKTEEVMSAVKKLWKINNISSPVTKWX
contig096536-BriOR.N086   NSTL---------THTIPALINPIV------------------------------YALKTEEVMNAVKKLWKINNISSPVTKWX
contig046352-TilOR.N194   NSTL---------THTIPALINPIV------------------------------YALKTEEVMNAVKKLWKKKISSGVTKCX
contig048778-NyeOR.M107   SLSL---------ASCIPPCINPIV------------------------------YSLKTKDIKTRALKLFRK------TKVNAX
contig010734-ZebOR.M107   SLSL---------ASCIPPCINPVV------------------------------YSLKTKDIKTRALTLFRTKVKTKLTKVNAX
contig048778-NyeOR.M108   SLSL---------ASCIPPCINPIV------------------------------YSLKTKDIKTRALTLFRTKVKTKLTKVNAX
contig039460-TilOR.L145   LLIG---------TILSPSLVNPVV------------------------------YCFRTKEIKNKIFKIFTKKADISDX
contig017786-ZebOR.L091   LLIG---------TILSPSLVNPVV------------------------------YCFRTKEIKNKIFKIFTKKADILNX
contig058162-BurOR.L089   LLIG---------TILSPSLVNPVV------------------------------YCFRTKEIKNKIFKIFTKKADILNX
contig040502-NyeOR.L092   LLIG---------TILSPSLVNPVV------------------------------YCFRTKEIKNKIFKIFTKKADILNX
contig042543-BriOR.L069   LLIG---------TILSPSLVNPVV------------------------------YCFRTKEIKNKIFTMFTKRLTYPTRFLX
contig039460-TilOR.L146   LLIG---------NILGPAVVNKFV------------------------------YCFRTKEIKNKIFKIFTKNGIX
contig039465-TilOR.L150   LLIG---------IILGPTLVNPLV------------------------------YCLRTKEIKNKIVKIFRKVNTVGX
contig039461-TilOR.L148   LLIG---------TILGPSLVNPLV------------------------------YCFRTKEIKNKIVKIFRKVNTAGX
contig042552-BriOR.L071   LLIG---------TILGPSLVNPFV------------------------------YCLRTKEIKSKIFKIFRKVNTADX
contig020445-ZebOR.L096   LLIG---------TILGPSLVNPFV------------------------------YCLRTKEIKSKMFKIFRKINTAGX
contig056942-NyeOR.L093   LLIG---------TILGPSLVNPFV------------------------------YCLRTKEIKSKMFKIFRKINTAGX
contig068527-TilOR.L157   LLIG---------TILGPPLVNPFV------------------------------YCLRTKEIKSKIFMIVRKVNTAGX
contig039469-TilOR.L152   LLIG---------TILGPSLVNPFV------------------------------YCLRTKEIKIRIFKIFRKVNTLRPELFHGMHFX
contig064361-NyeOR.L094   LLIG---------AVLGPCLVNPFV------------------------------YFLRTKEIKQKLFKIFKKFNTSDX
contig056384-BurOR.L087   LLIG---------AVLGPCLVNPFV------------------------------YFLRTKEIKQKLFKIFKKFYTYITEILX
contig020442-ZebOR.L095   LLIG---------AVLGPCLVNPFV------------------------------YFLRTKEIKQKLFKIFKKFYTYYX
contig042554-BriOR.L072   LLIG---------AVLGPCLVNPFV------------------------------YCLRTKEIKQKLFKILKKFHTYDX
contig039481-TilOR.L153   LLIG---------AVLGPCLVNPFV------------------------------YCLRTKEIKHKLSKILKKFHTYDX
contig072645-TilOR.L159   LLIG---------AVLGPCLVNPFV------------------------------YCLRTKEIKQKLLKIFKKFHTYDX
contig056940-NyeOR.L095   LVIG---------AVLGPCLVNPFV------------------------------YCLRTKEIKQKLFKIFKKFNTSDX
contig020440-ZebOR.L094   LVIG---------AVLGPCLVNPFV------------------------------YCLRTKEIKQKLFKIFKKFNTSDX
contig042556-BriOR.L073   LLIG---------AVLGPCLVNPFV------------------------------YCLRTKEIKQKLFKIFKKFNTSDX
contig039484-TilOR.L154   LVIG---------AVLGPCLVNPFV------------------------------YCLRTKEIKQKLFKIFKKFNTSDX
contig068425-TilOR.L155   LVIG---------AVLGPCLVNPFV------------------------------YCLRTKEIKQKLFKIFKTFHTYDX
contig020437-ZebOR.L093   LLIG---------AVLGPCLVNPFV------------------------------YFLRTKEIKQKFYKIFKKFHTSDX
contig056386-BurOR.L088   LLIG---------AVLGPCLVNPFV------------------------------YCLRTKEIKQKFYKIFKKFHTSDX
contig039469-TilOR.L151   LVIG---------AVLGPCLVNPFV------------------------------YCLRTKEIKQKLFKIFKKFHTSDX
contig068521-TilOR.L156   LLIG---------AVLGPCLVNPFV------------------------------YCLRTKEIKQKLFKIFKKFNTSEX
contig068539-TilOR.L158   LLIG---------AVLGPCLVNPFV------------------------------YCLRTKEIKQKLFNIFKKLNTYDX
contig042544-BriOR.L070   LLMG---------NILGPSLVNPFV------------------------------YCLRTTEIKNKMVKIFKKILRFLX
contig065027-BurOR.L090   LLIG---------NILGPSLVNPFV------------------------------YCLRTTEIKNKMVKIFKKILRFLX
contig017787-ZebOR.L092   LLIG---------NILGPSLVNPFV------------------------------YCLRTTEIKNKMVKIFKKILRLLX
contig040502-NyeOR.L091   LLIG---------NILGPSLVNPFV------------------------------YCLRTTEIKNKMVKIFKKILRLLX
contig039462-TilOR.L149   LLIG---------NILGPSLVNPFV------------------------------YCLRTTEIKNKIVKIFKQILRFLX
contig023716-TilOR.L144   LLIA---------NILGPSVVNPFV------------------------------YCLRTKEIKHKIFMLFKKLQVLRX
contig039461-TilOR.L147   LLIG---------TILGPSLVNPFV------------------------------YSLRTTEIKTKIFRILKNGWHVX
contig017698-BurOR.P120   FSTM---------YLLFPSCFNPVV------------------------------YGIRTNEIRQHILKRLTCCHICQMGPVTNKX
contig025452-ZebOR.P123   FSTM---------YLLFPSCFNPVV------------------------------YGIRTNEIRQHILKRLTCCHICQMSPVTNKX
contig004267-BriOR.P099   FSTM---------YLLFPSCFNPVV------------------------------YGIRTNEIRQHILKRLTCCHICQMGPVTKKX
contig065444-TilOR.P214   FSTM---------YLLFPSCFNPVI------------------------------YGIRTTEIRQHIRKTLTCCHICKMGSVTNKX
contig065247-TilOR.P211   FSIM---------YLLFPTCFNPII------------------------------YGIRTTEIRQHILKTLKIX
contig004270-BriOR.P100   FSIL---------YLFFPSCFNPII------------------------------YGIRTKEIRQHIINTLRVX
contig017697-BurOR.P119   FSIL---------YLFFPSCFNPII------------------------------YGIRTKEIRQHILNTLRVX
contig025453-ZebOR.P124   FSIL---------YLFFPSCFNPII------------------------------YGIRTKEIRQHILNTLRVX
contig065449-TilOR.P215   FSIM---------YLLFPSFFNPII------------------------------YGIRTTEIRQHILKTLKIX
contig065253-TilOR.P212   FSIM---------YLFFPSCFNPII------------------------------YGIRTTEIRQHILNTLKIX
contig065254-TilOR.P213   MGLM---------YILFPATINPII------------------------------YGVRTKEIRNGFLRIFKNRAKKIMTVKVSSAGKGKSX
contig023268-NyeOR.P118   MGLM---------YILFPATINPII------------------------------YGVRTKEIRDSFLRIFKNRAKKIMTAKVSSAGKEKSX
contig017696-BurOR.P118   MGLM---------YILFPATINPII------------------------------YGVRTKEIRDSFLRIFKNRAKKIMTVKVSSAGKEKSX
contig025458-ZebOR.P125   MGLM---------YILFPATINPII------------------------------YGVRTKEIRDSFLRIFKNRAKKIMTAKVSSAGKEKSX
contig004275-BriOR.P101   MGLM---------YILFPAAINPVI------------------------------YGVRTKEIRDSFLRIFKNRAKKIMTVKVSSAGKGKSX
contig020431-ZebOR.O101   IIML---------YSLAPPMINPLI------------------------------YCLRAKDMRESLLKQFCRRIVPEKAQVAAISNSLKTSPX
```

```
contig021359-NyeOR.O102    IIML---------YSLAPPMINPLI-------------------------------YCLRAKDMRESLLKQFCRRIVPEKAKVAAISNSLKTSPX
contig059249-BurOR.O098    IIML---------YSLPPPMINPLI-------------------------------YCLRAKDMRESLLKQFCKRIVPRKAQVAAISNSX
contig062053-NyeOR.O103    IIML---------YSLAPPMINPLI-------------------------------YCLRAKDMRESLLKQFCKRIVPRKAQVAAISNSX
contig023717-TilOR.O175    IIML---------YSLAPPMINPLI-------------------------------YCLRAKDMRESLLKQFCKRIVPQKAQVAAISNSX
contig023724-TilOR.O176    IIML---------YSLAPPMINPLI-------------------------------YCLRAKDMRESLLKQFCKKNIPQKAEVAAISNSX
contig042559-BriOR.O077    IIML---------YGLAPPMINPLI-------------------------------YCLRAKDMRESLLKQFFRRIVPEKAQVAAISNSLKTSPX
contig110782-BriOR.O080    IIML---------YSLAPPMINPLI-------------------------------YCLRAKDMRESLLKVFCRRTIPQKAQVAVINIINHKX
contig020430-ZebOR.O100    LILL---------YSLFPAAVNPVI-------------------------------YCFKTRDIKHMLMKRLKKTKIGLEIKLALX
contig042560-BriOR.O078    LILL---------YSLFPAAVNPVI-------------------------------YCFKTRDIKHMLMKRLKKTKIGLEIKLSPX
contig021354-NyeOR.O101    ITMM---------YSLIPAAVNPII-------------------------------YCFKTKDIKNVLMRRFKKGKVSTGLKTECKX
contig020427-ZebOR.O099    ITMM---------YSLIPAAVNPII-------------------------------YCFKTKDIKNVLMRRFKKGKVSTGLKTECKX
contig023731-TilOR.O177    ITML---------YSLIPAAVNPMI-------------------------------YCFKTKEIKNVLMRRFKKRKVSTGLKTDCKX
contig042562-BriOR.O079    ITMM---------FSLIPAAVNPII-------------------------------YCFKTKDIKNVLMRRFKKGKVSTGLKTECKX
contig049605-BurORs.AB153  LCFLCNVSVQYSVKVLSHPYL--FI-------------------------------FLVGKQEIHAEIYX
contig046717-TilORs.AB275  LYFLRNVSVQYSVKVLSHPSFISIFGRKTRNTCRDLLKQANPKWKYSIWTFLMSCRYSFSTFILHLFLFSRTRTLIDRLSCKLPX
```
