# Supplementary material for: Inventory of the cichlid olfactory receptor gene repertoires: identification of olfactory genes with more than one coding exon
Source: BMC Genomics. 2014 Jul 11;15(1):586. doi: 10.1186/1471-2164-15-586 (PMC4122780; doi:10.1186/1471-2164-15-586)
Supplement: Supplementary file 4 — Additional file 4: Phylogenetic tree constructed from the AA sequences of the cichlid ORs identified in Table 1 and Additional file 2 and 143 zebrafish, 73 medaka, 78 stickleback, 40 fugu and 42 tetraodon OR AA sequences (Additional file 2 ). Fish species are colour coded: O. niloticus in red, M. zebra in pink, N. brichardi in blue, H. burtoni in green, P. nyererei in orange and fish models in black. (PDF 4 MB) [file 12864_2014_6314_MOESM4_ESM.pdf]

PolyPhobius prediction

Prediction of contig046690-TilORs.I128

```
ID   contig046690-TilORs.I128
FT   TOPO_DOM      1      23       NON CYTOPLASMIC.
FT   TRANSMEM     24      49
FT   TOPO_DOM     50      58       CYTOPLASMIC.
FT   TRANSMEM     59      81
FT   TOPO_DOM     82      96       NON CYTOPLASMIC.
FT   TRANSMEM     97     119
FT   TOPO_DOM    120     139       CYTOPLASMIC.
FT   TRANSMEM    140     162
FT   TOPO_DOM    163     194       NON CYTOPLASMIC.
FT   TRANSMEM    195     217
FT   TOPO_DOM    218     235       CYTOPLASMIC.
FT   TRANSMEM    236     258
FT   TOPO_DOM    259     268       NON CYTOPLASMIC.
FT   TRANSMEM    269     289
FT   TOPO_DOM    290     311       CYTOPLASMIC.
//
```

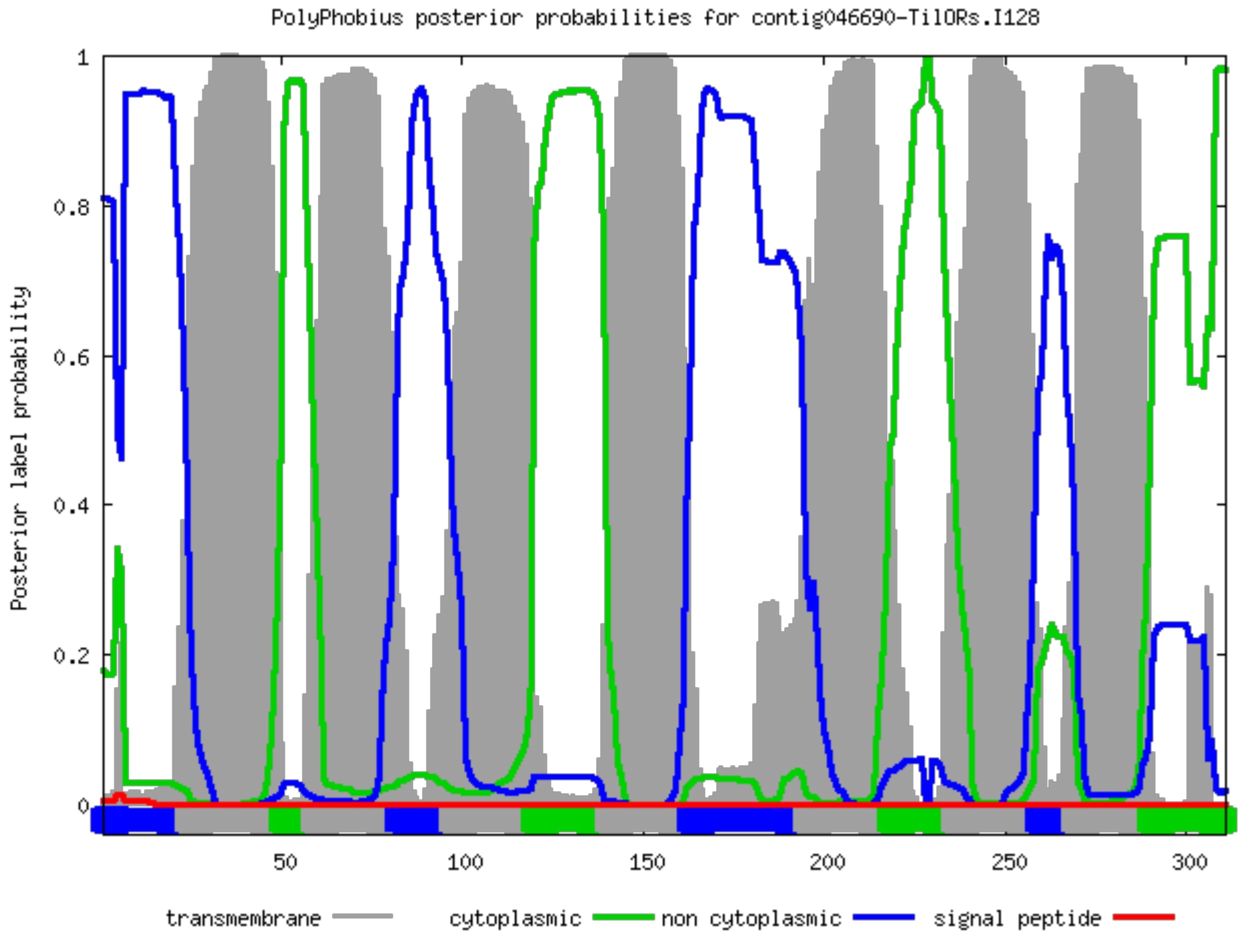

The prediction is based on an alignment. The probability data used in the plot is found here, and the gnuplot script is here.

Prediction of contig046490-NyeORsp.K086

```
ID    contig046490-NyeORsp.K086
FT    TOPO_DOM       1      24       NON CYTOPLASMIC.
FT    TRANSMEM      25      50
FT    TOPO_DOM      51      58       CYTOPLASMIC.
FT    TRANSMEM      59      81
FT    TOPO_DOM      82     100       NON CYTOPLASMIC.
FT    TRANSMEM     101     121
FT    TOPO_DOM     122     141       CYTOPLASMIC.
FT    TRANSMEM     142     165
FT    TOPO_DOM     166     196       NON CYTOPLASMIC.
FT    TRANSMEM     197     224
FT    TOPO_DOM     225     243       CYTOPLASMIC.
FT    TRANSMEM     244     264
FT    TOPO_DOM     265     269       NON CYTOPLASMIC.
FT    TRANSMEM     270     291
FT    TOPO_DOM     292     304       CYTOPLASMIC.
//
```

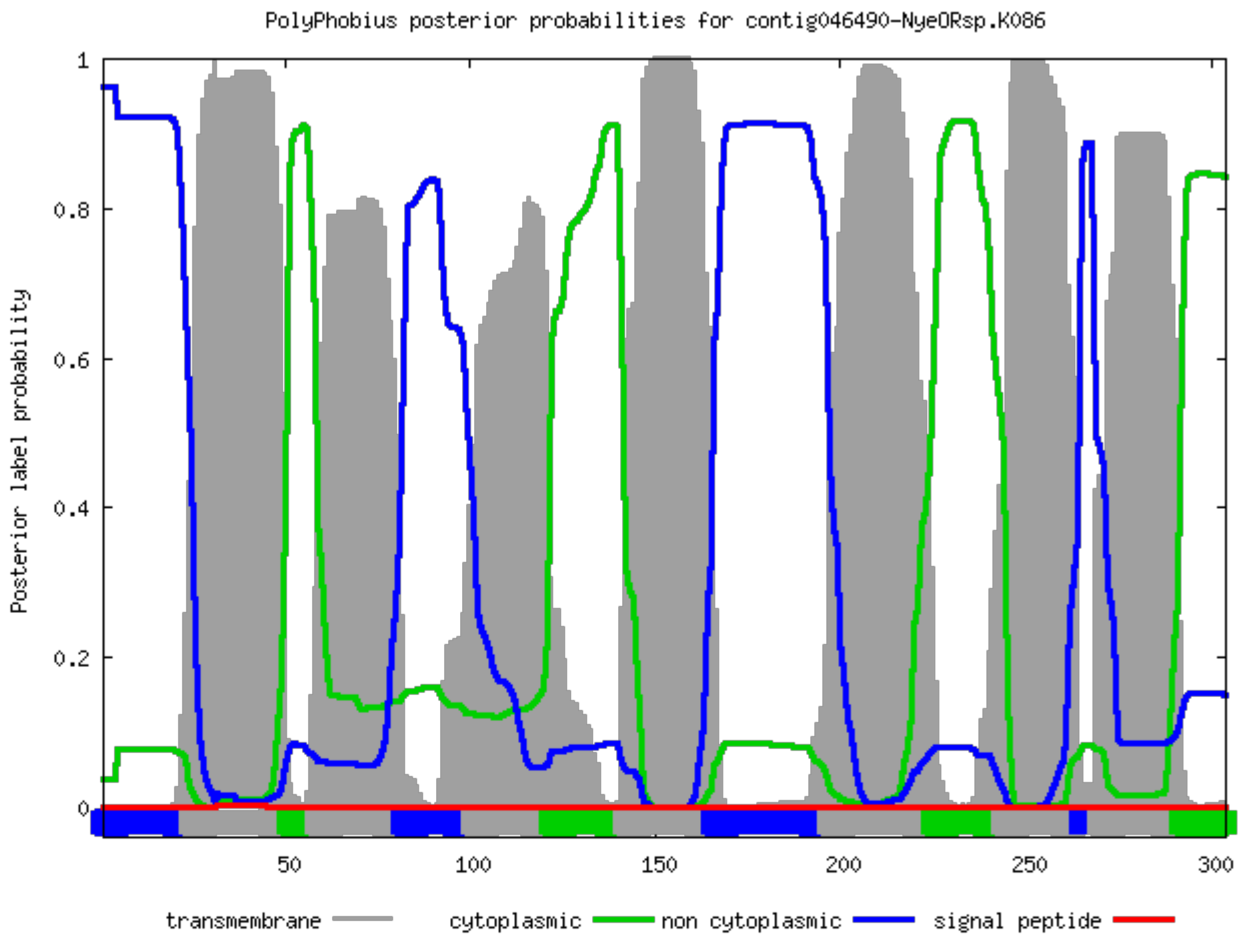

The prediction is based on an alignment. The probability data used in the plot is found here, and the gnuplot script is here.

Prediction of contig041638-BurORs.W135

```
ID    contig041638-BurORs.W135
FT    TOPO_DOM       1      18       NON CYTOPLASMIC.
```

```
FT   TRANSMEM     19     40
FT   TOPO_DOM     41     51       CYTOPLASMIC.
FT   TRANSMEM     52     75
FT   TOPO_DOM     76     77       NON CYTOPLASMIC.
FT   TRANSMEM     78    110
FT   TOPO_DOM    111    131       CYTOPLASMIC.
FT   TRANSMEM    132    155
FT   TOPO_DOM    156    185       NON CYTOPLASMIC.
FT   TRANSMEM    186    207
FT   TOPO_DOM    208    222       CYTOPLASMIC.
FT   TRANSMEM    223    242
FT   TOPO_DOM    243    257       NON CYTOPLASMIC.
FT   TRANSMEM    258    279
FT   TOPO_DOM    280    304       CYTOPLASMIC.
//
```

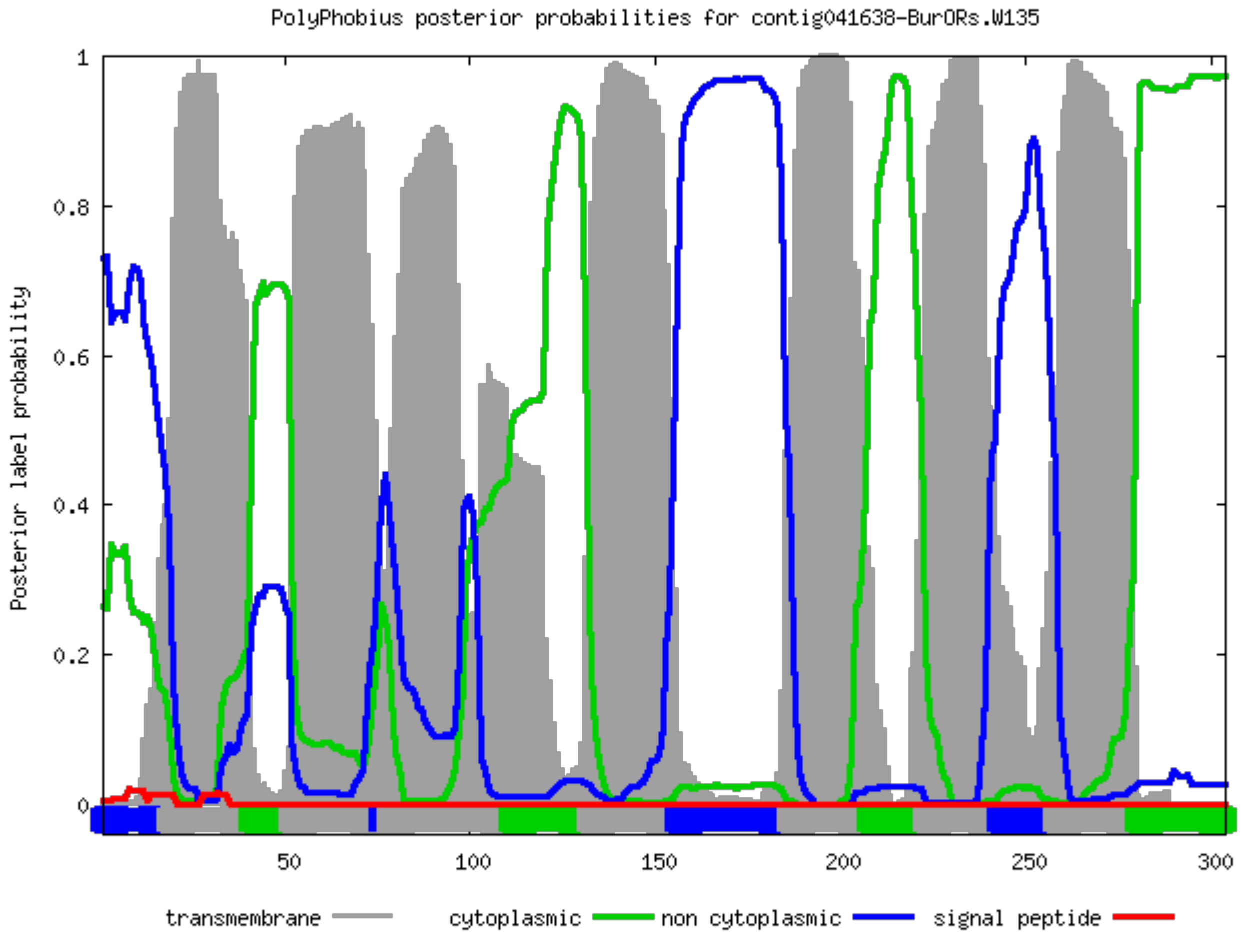

The prediction is based on an alignment. The probability data used in the plot is found here, and the gnuplot script is here.

Prediction of contig045454-BurORs.W131

```
ID   contig045454-BurORs.W131
FT   TOPO_DOM      1     24       NON CYTOPLASMIC.
FT   TRANSMEM     25     46
FT   TOPO_DOM     47     58       CYTOPLASMIC.
FT   TRANSMEM     59     82
FT   TOPO_DOM     83     85       NON CYTOPLASMIC.
FT   TRANSMEM     86    105
```

```
FT   TOPO_DOM     106     138       CYTOPLASMIC.
FT   TRANSMEM     139     162
FT   TOPO_DOM     163     192       NON CYTOPLASMIC.
FT   TRANSMEM     193     214
FT   TOPO_DOM     215     229       CYTOPLASMIC.
FT   TRANSMEM     230     248
FT   TOPO_DOM     249     264       NON CYTOPLASMIC.
FT   TRANSMEM     265     286
FT   TOPO_DOM     287     306       CYTOPLASMIC.
//
```

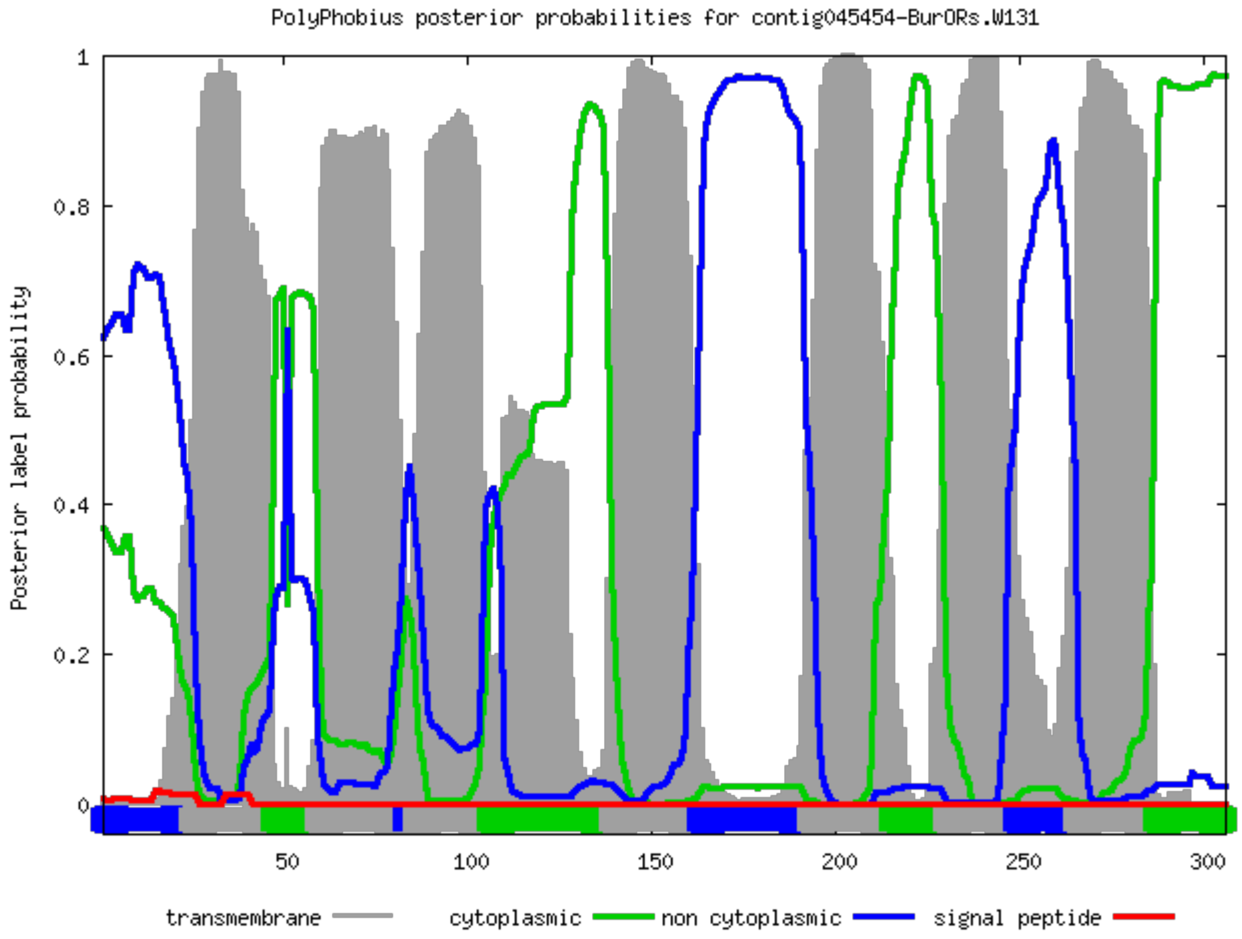

The prediction is based on an alignment. The probability data used in the plot is found here, and the gnuplot script is here.

Prediction of contig090286-BriORs.W112

```
ID   contig090286-BriORs.W112
FT   TOPO_DOM       1      24       NON CYTOPLASMIC.
FT   TRANSMEM      25      46
FT   TOPO_DOM      47      58       CYTOPLASMIC.
FT   TRANSMEM      59      82
FT   TOPO_DOM      83      85       NON CYTOPLASMIC.
FT   TRANSMEM      86     105
FT   TOPO_DOM     106     138       CYTOPLASMIC.
FT   TRANSMEM     139     162
FT   TOPO_DOM     163     192       NON CYTOPLASMIC.
FT   TRANSMEM     193     214
FT   TOPO_DOM     215     229       CYTOPLASMIC.
```

```
FT   TRANSMEM    230    248
FT   TOPO_DOM    249    264       NON CYTOPLASMIC.
FT   TRANSMEM    265    286
FT   TOPO_DOM    287    306       CYTOPLASMIC.
//
```

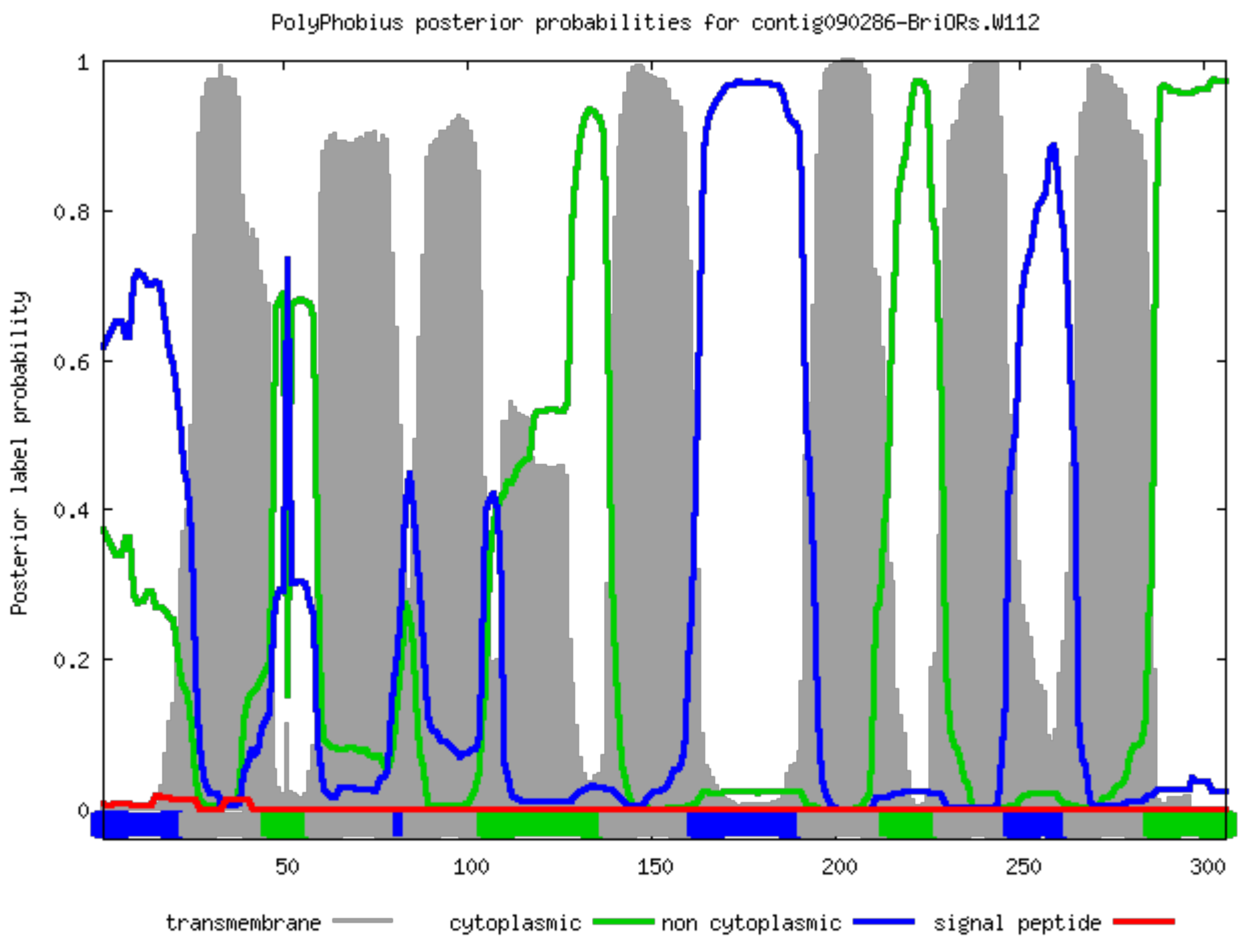

The prediction is based on an alignment. The probability data used in the plot is found here, and the gnuplot script is here.

Prediction of contig046694-TilORs.I129

```
ID   contig046694-TilORs.I129
FT   TOPO_DOM      1     28       NON CYTOPLASMIC.
FT   TRANSMEM     29     55
FT   TOPO_DOM     56     63       CYTOPLASMIC.
FT   TRANSMEM     64     86
FT   TOPO_DOM     87    101       NON CYTOPLASMIC.
FT   TRANSMEM    102    124
FT   TOPO_DOM    125    144       CYTOPLASMIC.
FT   TRANSMEM    145    167
FT   TOPO_DOM    168    199       NON CYTOPLASMIC.
FT   TRANSMEM    200    224
FT   TOPO_DOM    225    245       CYTOPLASMIC.
FT   TRANSMEM    246    267
FT   TOPO_DOM    268    278       NON CYTOPLASMIC.
FT   TRANSMEM    279    299
FT   TOPO_DOM    300    314       CYTOPLASMIC.
//
```

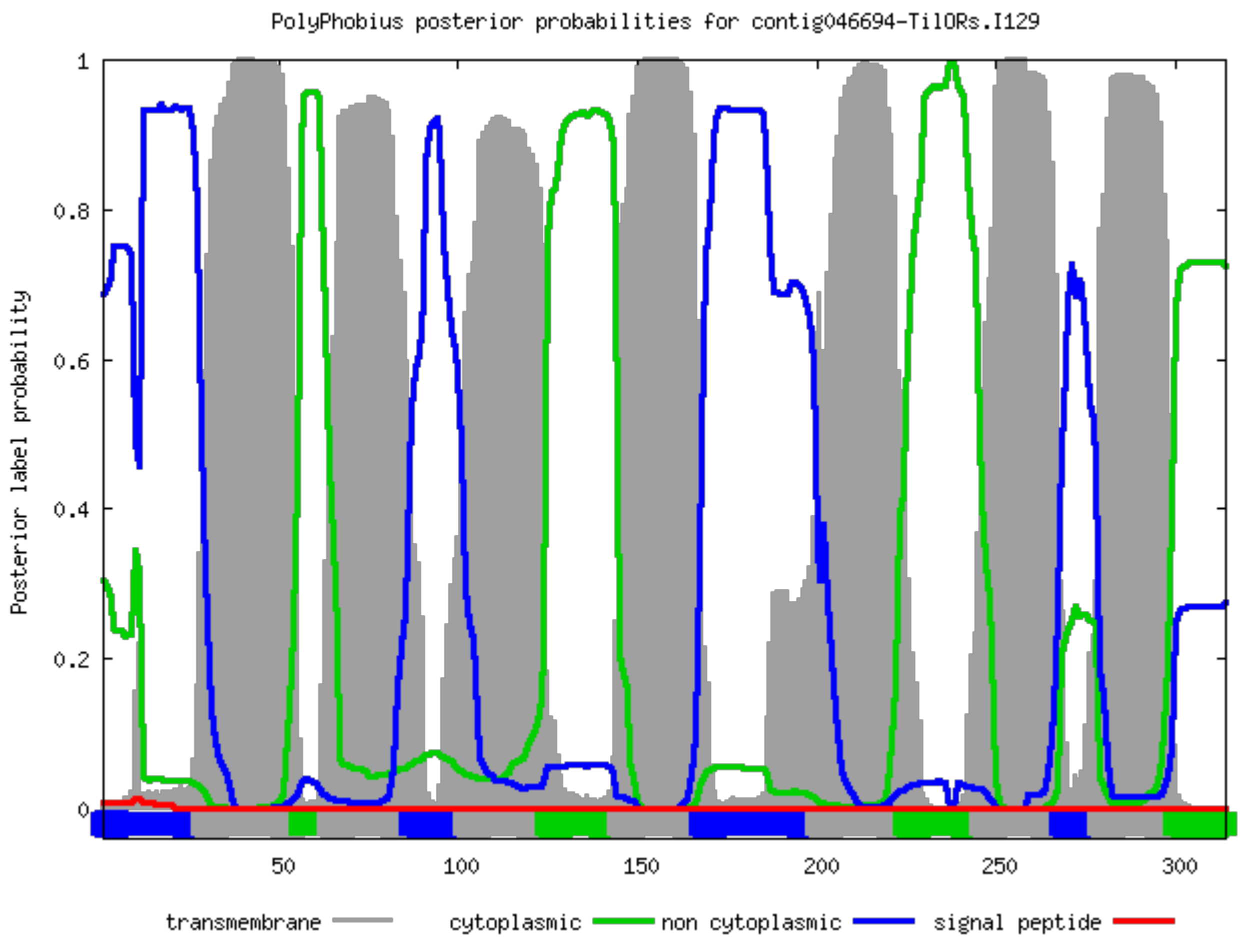

The prediction is based on an alignment. The probability data used in the plot is found here, and the gnuplot script is here.

Prediction of contig027209-TilORs.W241

```
ID   contig027209-TilORs.W241
FT   TOPO_DOM      1     24       NON CYTOPLASMIC.
FT   TRANSMEM     25     46
FT   TOPO_DOM     47     57       CYTOPLASMIC.
FT   TRANSMEM     58     81
FT   TOPO_DOM     82     84       NON CYTOPLASMIC.
FT   TRANSMEM     85    116
FT   TOPO_DOM    117    137       CYTOPLASMIC.
FT   TRANSMEM    138    161
FT   TOPO_DOM    162    191       NON CYTOPLASMIC.
FT   TRANSMEM    192    213
FT   TOPO_DOM    214    228       CYTOPLASMIC.
FT   TRANSMEM    229    247
FT   TOPO_DOM    248    263       NON CYTOPLASMIC.
FT   TRANSMEM    264    285
FT   TOPO_DOM    286    305       CYTOPLASMIC.
//
```

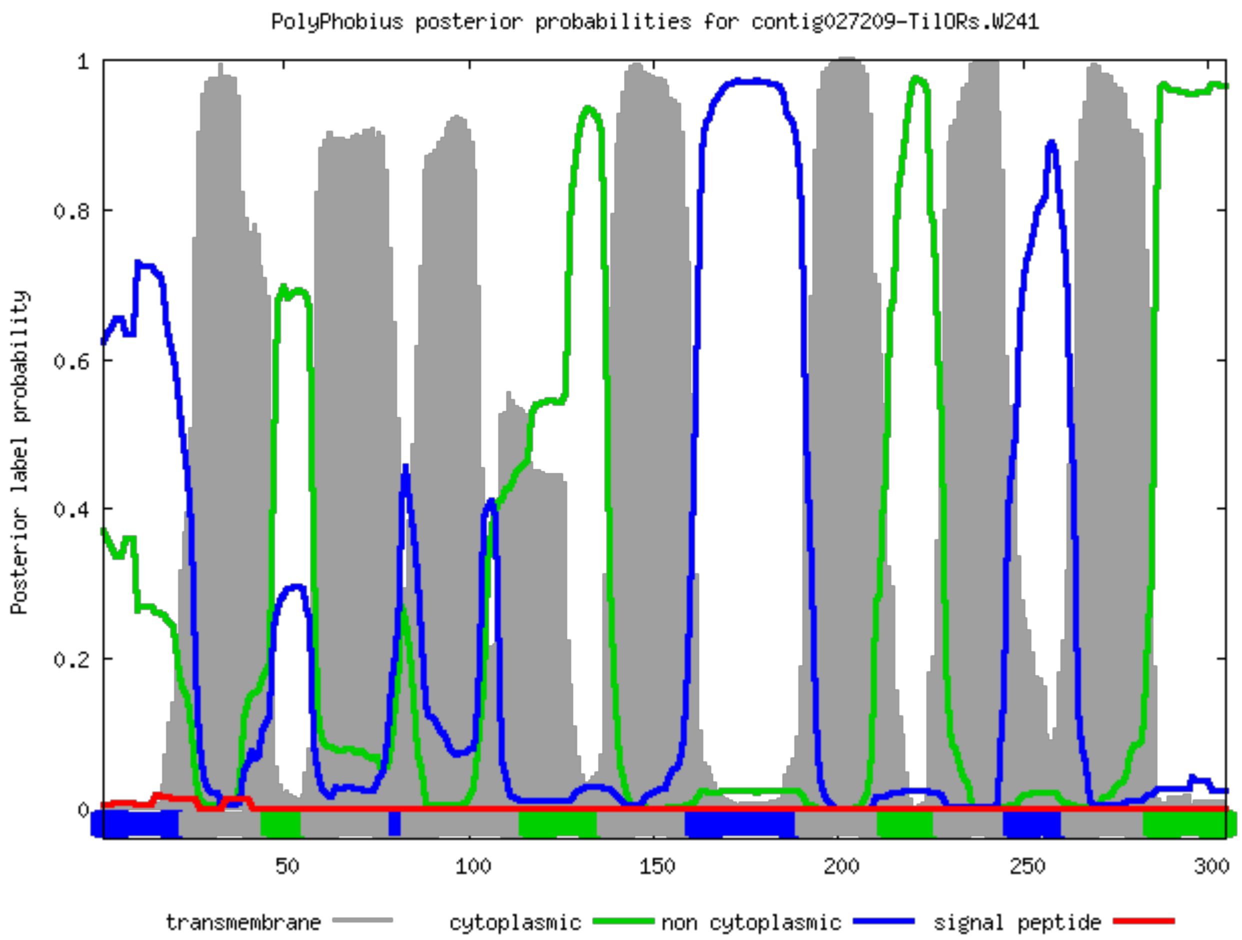

The prediction is based on an alignment. The probability data used in the plot is found here, and the gnuplot script is here.

Prediction of contig046495-NyeORs.I079

```
ID   contig046495-NyeORs.I079
FT   TOPO_DOM      1      23       NON CYTOPLASMIC.
FT   TRANSMEM     24      49
FT   TOPO_DOM     50      59       CYTOPLASMIC.
FT   TRANSMEM     60      81
FT   TOPO_DOM     82      96       NON CYTOPLASMIC.
FT   TRANSMEM     97     119
FT   TOPO_DOM    120     140       CYTOPLASMIC.
FT   TRANSMEM    141     163
FT   TOPO_DOM    164     195       NON CYTOPLASMIC.
FT   TRANSMEM    196     219
FT   TOPO_DOM    220     240       CYTOPLASMIC.
FT   TRANSMEM    241     263
FT   TOPO_DOM    264     273       NON CYTOPLASMIC.
FT   TRANSMEM    274     294
FT   TOPO_DOM    295     310       CYTOPLASMIC.
//
```

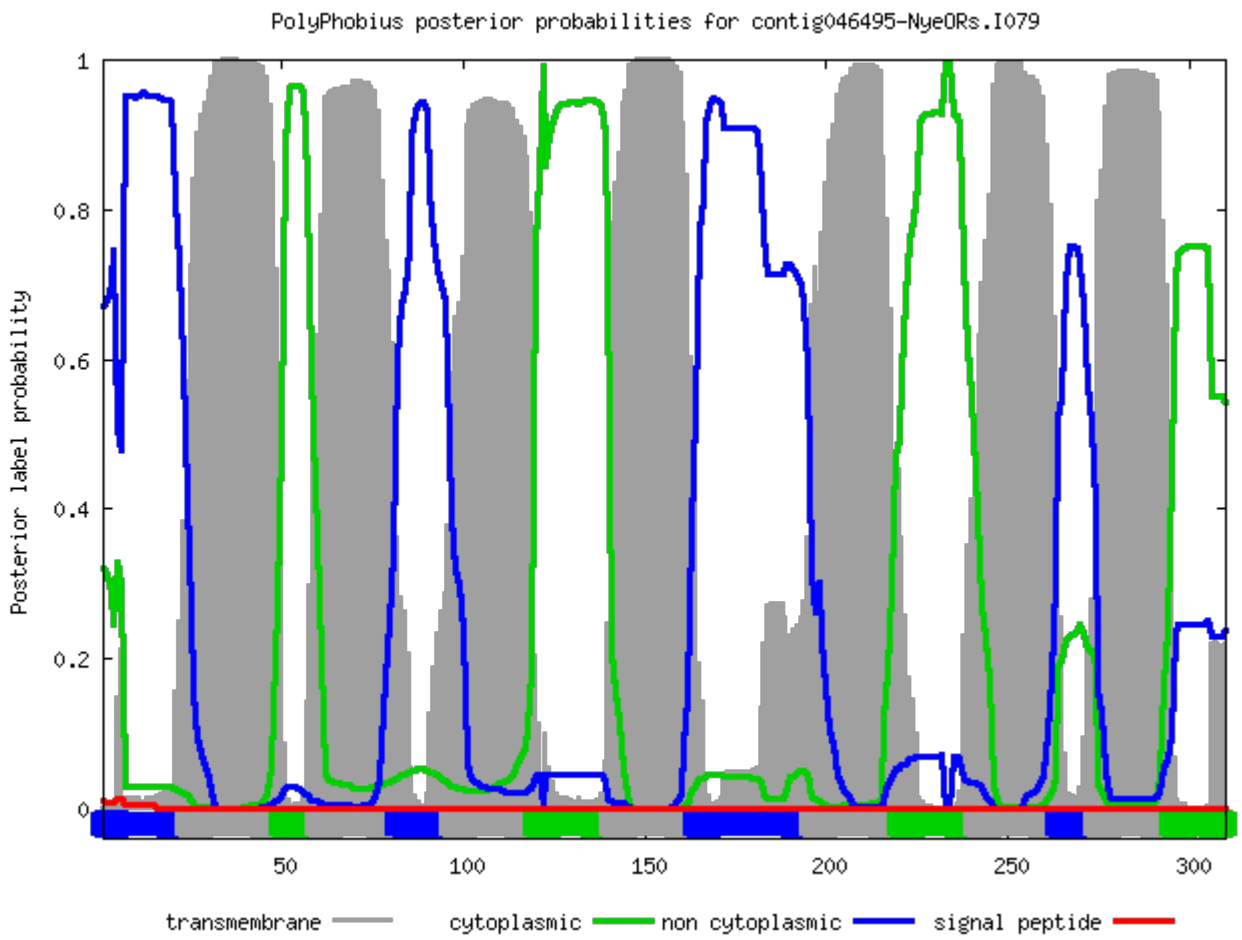

The prediction is based on an alignment. The probability data used in the plot is found here, and the gnuplot script is here.

Prediction of contig062664-ZebORs.W141

```
ID   contig062664-ZebORs.W141
FT   TOPO_DOM      1     17       NON CYTOPLASMIC.
FT   TRANSMEM     18     40
FT   TOPO_DOM     41     51       CYTOPLASMIC.
FT   TRANSMEM     52     75
FT   TOPO_DOM     76     77       NON CYTOPLASMIC.
FT   TRANSMEM     78    110
FT   TOPO_DOM    111    131       CYTOPLASMIC.
FT   TRANSMEM    132    155
FT   TOPO_DOM    156    185       NON CYTOPLASMIC.
FT   TRANSMEM    186    207
FT   TOPO_DOM    208    222       CYTOPLASMIC.
FT   TRANSMEM    223    241
FT   TOPO_DOM    242    257       NON CYTOPLASMIC.
FT   TRANSMEM    258    279
FT   TOPO_DOM    280    304       CYTOPLASMIC.
//
```

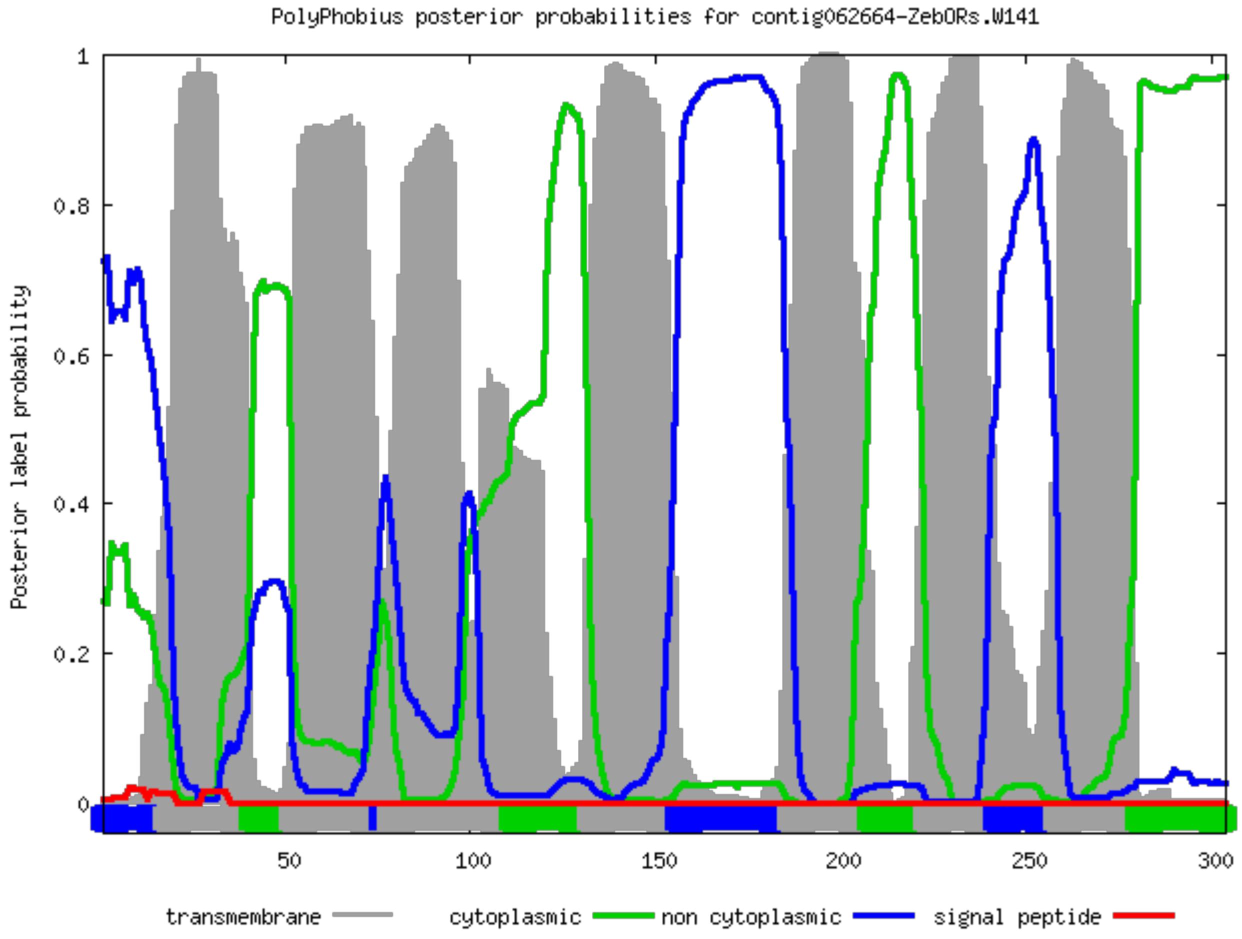

The prediction is based on an alignment. The probability data used in the plot is found here, and the gnuplot script is here.

Prediction of contig050025-NyeORs.W130

```
ID    contig050025-NyeORs.W130
FT    TOPO_DOM      1     24       NON CYTOPLASMIC.
FT    TRANSMEM     25     46
FT    TOPO_DOM     47     57       CYTOPLASMIC.
FT    TRANSMEM     58     81
FT    TOPO_DOM     82     84       NON CYTOPLASMIC.
FT    TRANSMEM     85    117
FT    TOPO_DOM    118    137       CYTOPLASMIC.
FT    TRANSMEM    138    161
FT    TOPO_DOM    162    191       NON CYTOPLASMIC.
FT    TRANSMEM    192    213
FT    TOPO_DOM    214    228       CYTOPLASMIC.
FT    TRANSMEM    229    247
FT    TOPO_DOM    248    263       NON CYTOPLASMIC.
FT    TRANSMEM    264    285
FT    TOPO_DOM    286    313       CYTOPLASMIC.
//
```

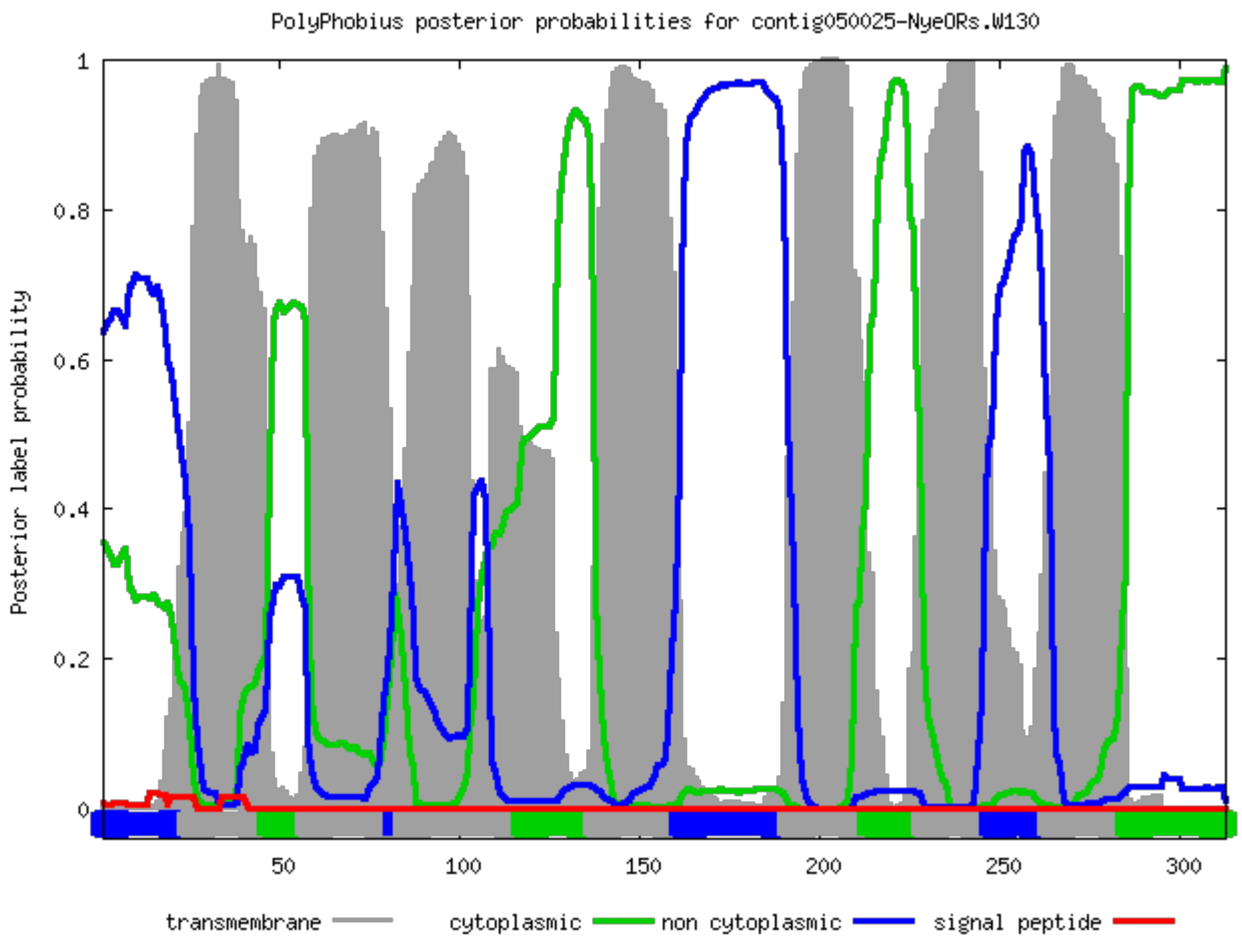

The prediction is based on an alignment. The probability data used in the plot is found here, and the gnuplot script is here.

Prediction of contig025841-ZebORs.W139

```
ID   contig025841-ZebORs.W139
FT   TOPO_DOM      1     26       NON CYTOPLASMIC.
FT   TRANSMEM     27     48
FT   TOPO_DOM     49     60       CYTOPLASMIC.
FT   TRANSMEM     61     84
FT   TOPO_DOM     85     87       NON CYTOPLASMIC.
FT   TRANSMEM     88    119
FT   TOPO_DOM    120    140       CYTOPLASMIC.
FT   TRANSMEM    141    164
FT   TOPO_DOM    165    194       NON CYTOPLASMIC.
FT   TRANSMEM    195    216
FT   TOPO_DOM    217    231       CYTOPLASMIC.
FT   TRANSMEM    232    251
FT   TOPO_DOM    252    266       NON CYTOPLASMIC.
FT   TRANSMEM    267    288
FT   TOPO_DOM    289    315       CYTOPLASMIC.
//
```

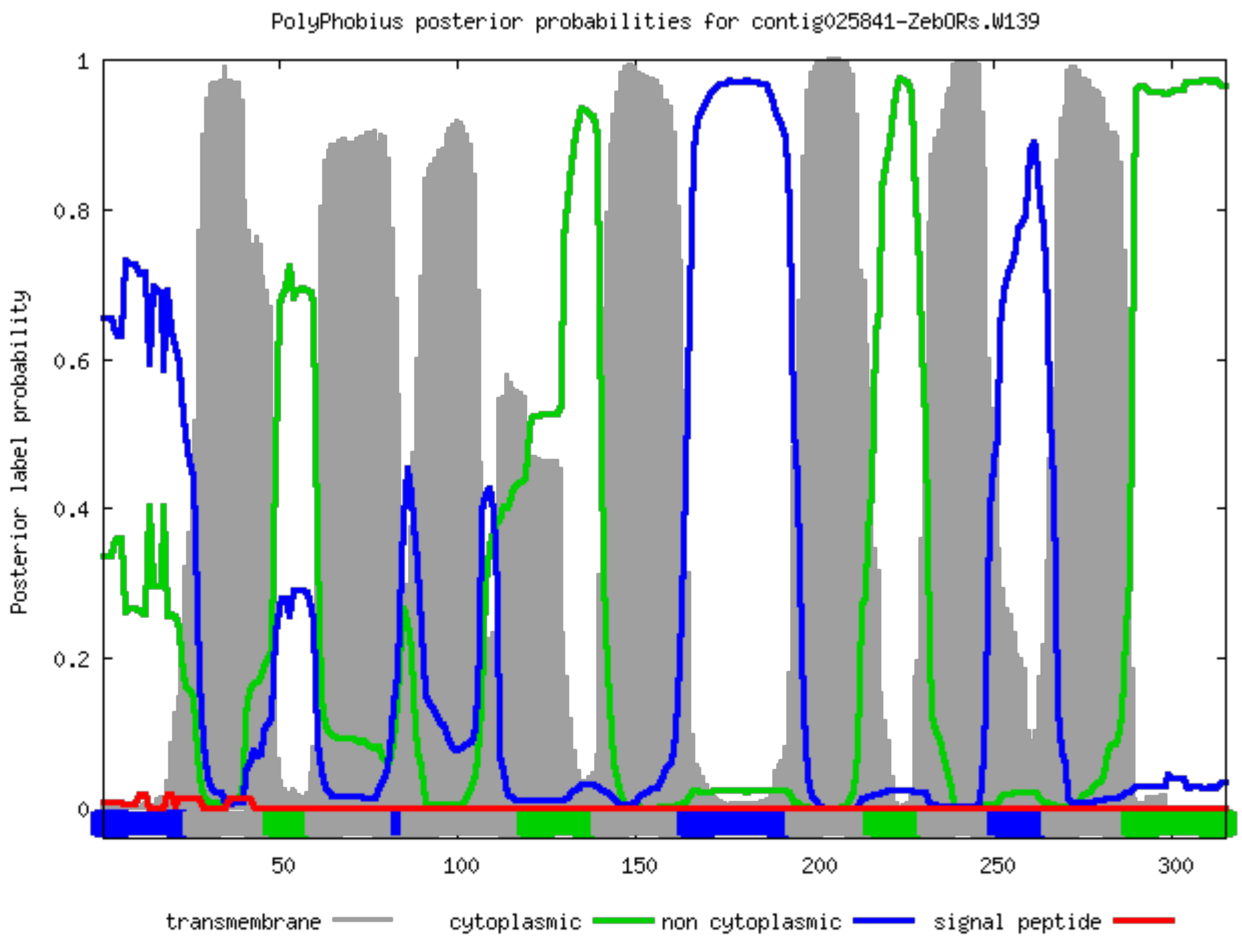

The prediction is based on an alignment. The probability data used in the plot is found here, and the gnuplot script is here.

Prediction of contig090292-BriORs.W115

```
ID   contig090292-BriORs.W115
FT   TOPO_DOM      1     26       NON CYTOPLASMIC.
FT   TRANSMEM     27     48
FT   TOPO_DOM     49     59       CYTOPLASMIC.
FT   TRANSMEM     60     83
FT   TOPO_DOM     84     86       NON CYTOPLASMIC.
FT   TRANSMEM     87    118
FT   TOPO_DOM    119    139       CYTOPLASMIC.
FT   TRANSMEM    140    163
FT   TOPO_DOM    164    193       NON CYTOPLASMIC.
FT   TRANSMEM    194    215
FT   TOPO_DOM    216    233       CYTOPLASMIC.
FT   TRANSMEM    234    253
FT   TOPO_DOM    254    268       NON CYTOPLASMIC.
FT   TRANSMEM    269    290
FT   TOPO_DOM    291    318       CYTOPLASMIC.
//
```

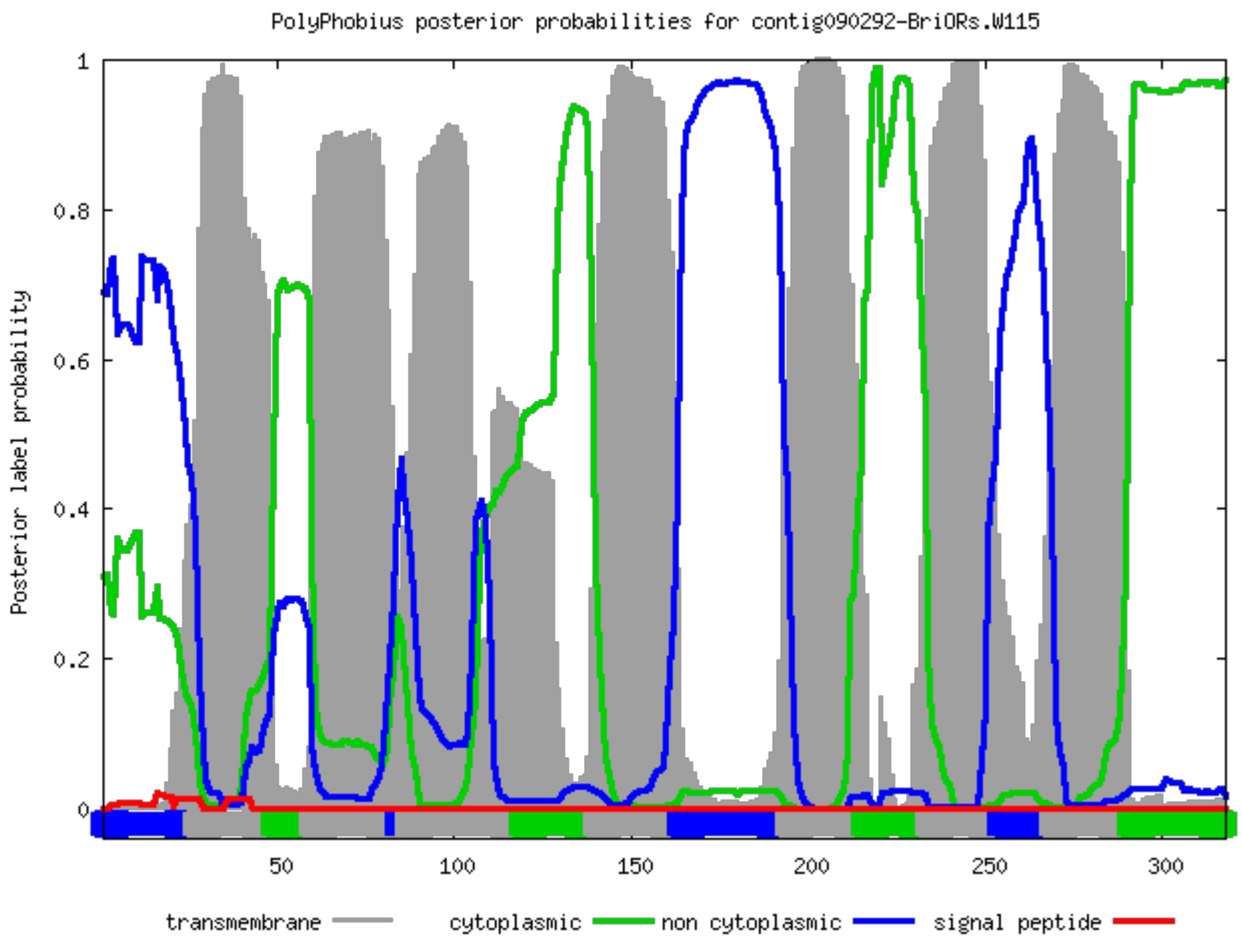

The prediction is based on an alignment. The probability data used in the plot is found here, and the gnuplot script is here.

Prediction of contig048321-BurORs.I076

```
ID   contig048321-BurORs.I076
FT   TOPO_DOM      1     28       NON CYTOPLASMIC.
FT   TRANSMEM     29     54
FT   TOPO_DOM     55     64       CYTOPLASMIC.
FT   TRANSMEM     65     86
FT   TOPO_DOM     87    101       NON CYTOPLASMIC.
FT   TRANSMEM    102    124
FT   TOPO_DOM    125    144       CYTOPLASMIC.
FT   TRANSMEM    145    167
FT   TOPO_DOM    168    199       NON CYTOPLASMIC.
FT   TRANSMEM    200    223
FT   TOPO_DOM    224    244       CYTOPLASMIC.
FT   TRANSMEM    245    267
FT   TOPO_DOM    268    277       NON CYTOPLASMIC.
FT   TRANSMEM    278    298
FT   TOPO_DOM    299    314       CYTOPLASMIC.
//
```

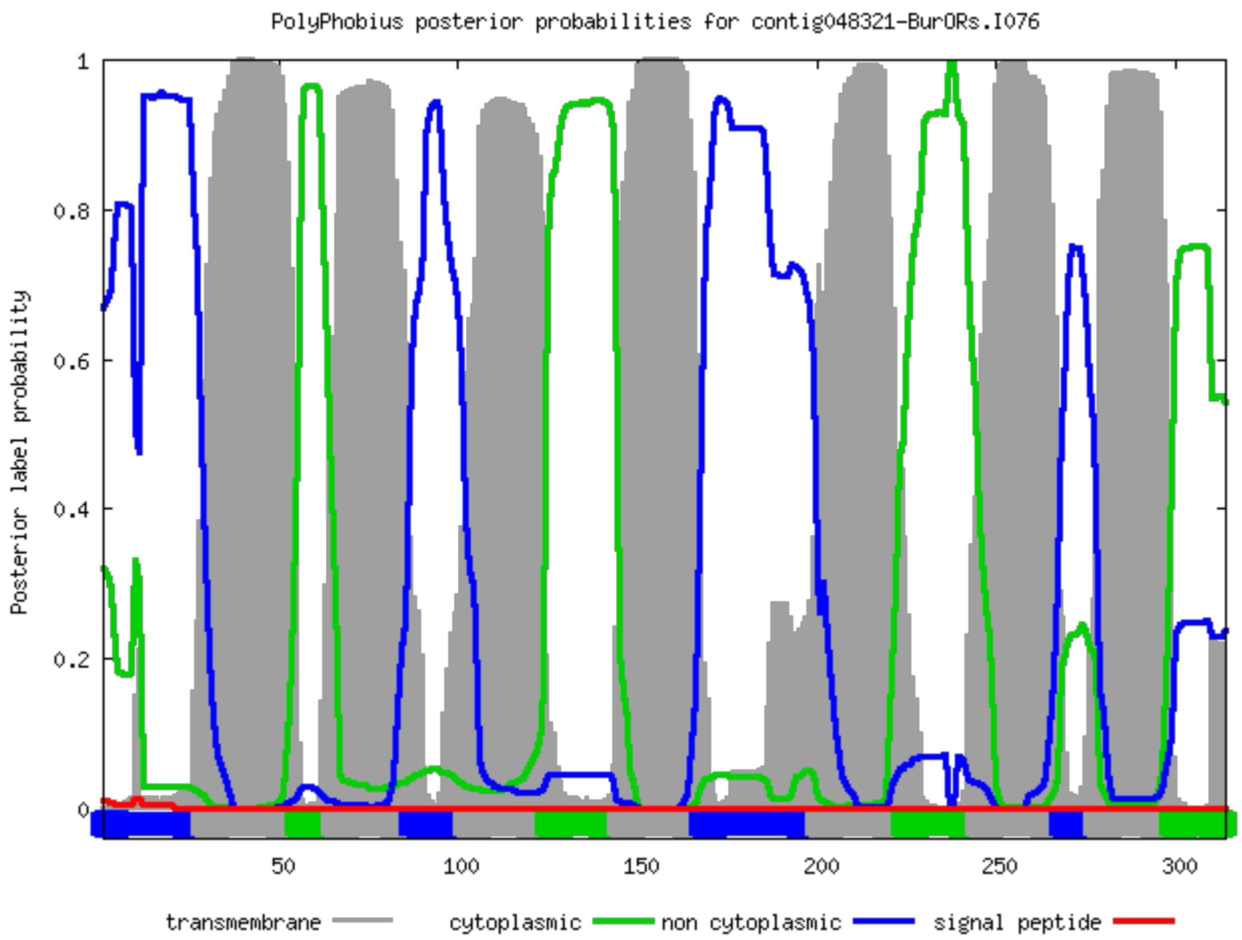

The prediction is based on an alignment. The probability data used in the plot is found here, and the gnuplot script is here.

Prediction of contig034988-NyeORs.A033

```
ID    contig034988-NyeORs.A033
FT    TOPO_DOM      1      22       NON CYTOPLASMIC.
FT    TRANSMEM     23      48
FT    TOPO_DOM     49      56       CYTOPLASMIC.
FT    TRANSMEM     57      77
FT    TOPO_DOM     78      95       NON CYTOPLASMIC.
FT    TRANSMEM     96     118
FT    TOPO_DOM    119     138       CYTOPLASMIC.
FT    TRANSMEM    139     159
FT    TOPO_DOM    160     192       NON CYTOPLASMIC.
FT    TRANSMEM    193     215
FT    TOPO_DOM    216     235       CYTOPLASMIC.
FT    TRANSMEM    236     257
FT    TOPO_DOM    258     268       NON CYTOPLASMIC.
FT    TRANSMEM    269     289
FT    TOPO_DOM    290     304       CYTOPLASMIC.
//
```

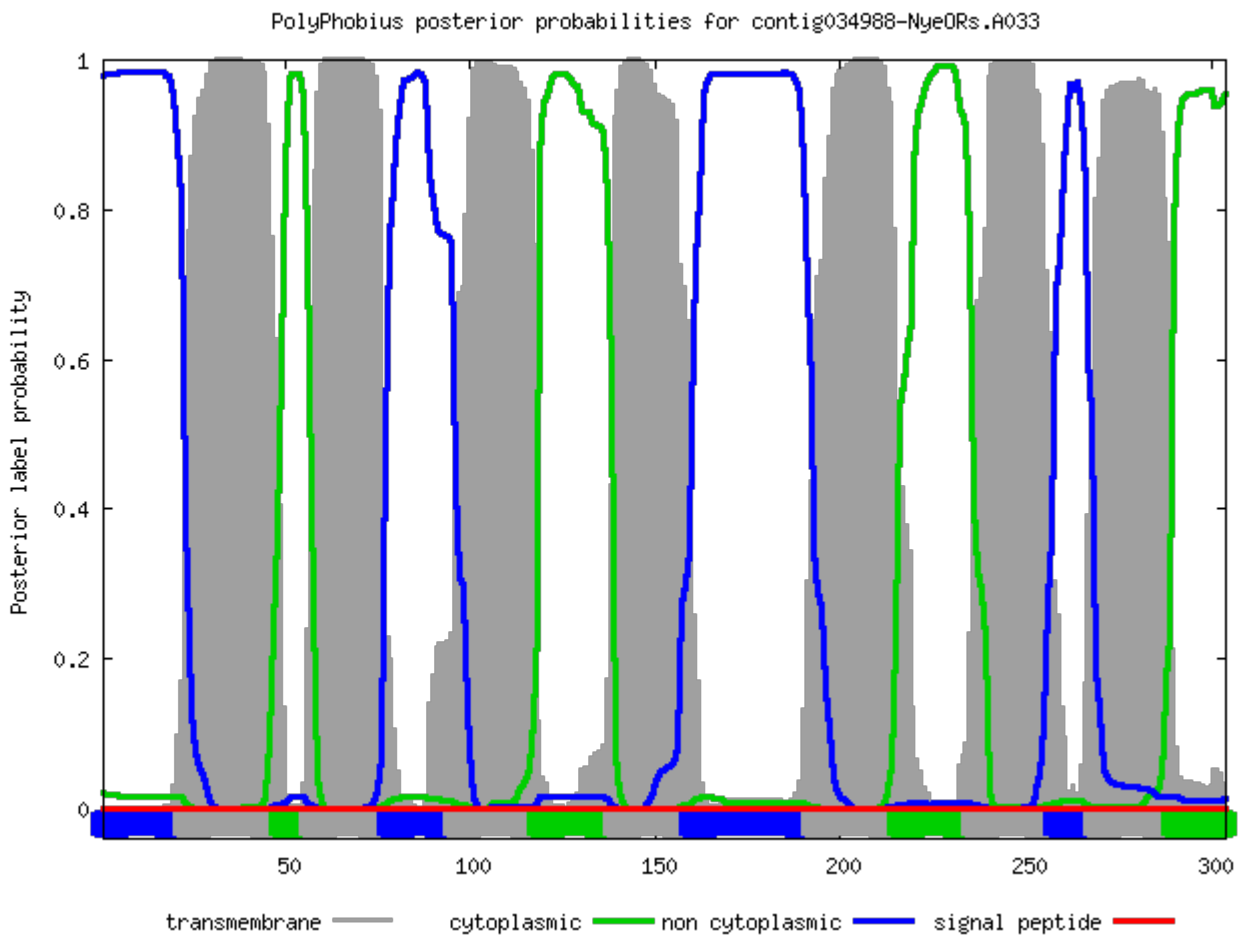

The prediction is based on an alignment. The probability data used in the plot is found here, and the gnuplot script is here.

Prediction of contig090301-BriORs.U109

```
ID   contig090301-BriORs.U109
FT   TOPO_DOM      1      30        NON CYTOPLASMIC.
FT   TRANSMEM     31      52
FT   TOPO_DOM     53      63        CYTOPLASMIC.
FT   TRANSMEM     64      87
FT   TOPO_DOM     88      89        NON CYTOPLASMIC.
FT   TRANSMEM     90     110
FT   TOPO_DOM    111     143        CYTOPLASMIC.
FT   TRANSMEM    144     167
FT   TOPO_DOM    168     197        NON CYTOPLASMIC.
FT   TRANSMEM    198     219
FT   TOPO_DOM    220     237        CYTOPLASMIC.
FT   TRANSMEM    238     255
FT   TOPO_DOM    256     273        NON CYTOPLASMIC.
FT   TRANSMEM    274     294
FT   TOPO_DOM    295     328        CYTOPLASMIC.
//
```

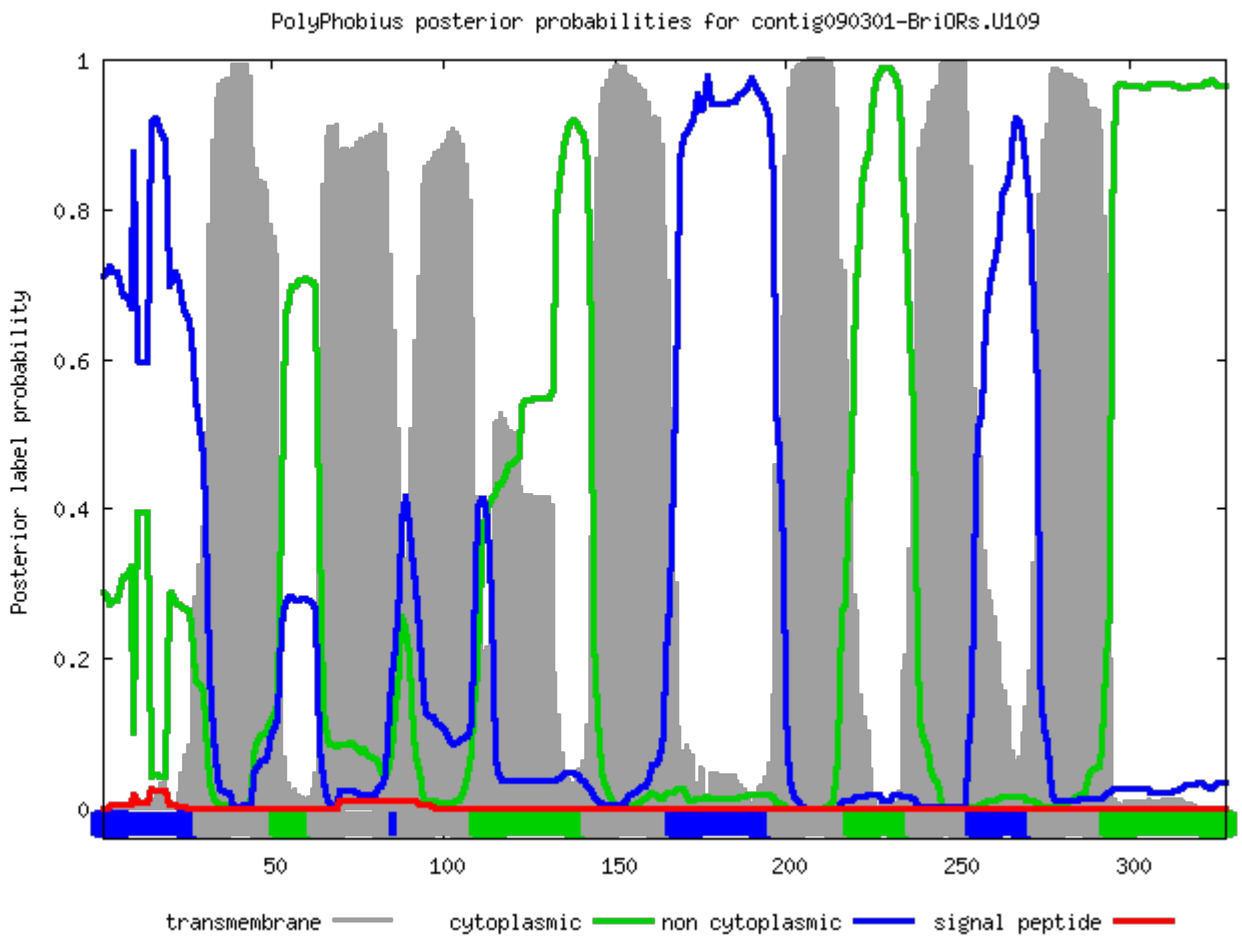

The prediction is based on an alignment. The probability data used in the plot is found here, and the gnuplot script is here.

Prediction of contig046708-TilORs.K143

```
ID   contig046708-TilORs.K143
FT   TOPO_DOM      1     23       NON CYTOPLASMIC.
FT   TRANSMEM     24     49
FT   TOPO_DOM     50     57       CYTOPLASMIC.
FT   TRANSMEM     58     80
FT   TOPO_DOM     81     99       NON CYTOPLASMIC.
FT   TRANSMEM    100    120
FT   TOPO_DOM    121    140       CYTOPLASMIC.
FT   TRANSMEM    141    164
FT   TOPO_DOM    165    195       NON CYTOPLASMIC.
FT   TRANSMEM    196    223
FT   TOPO_DOM    224    242       CYTOPLASMIC.
FT   TRANSMEM    243    263
FT   TOPO_DOM    264    270       NON CYTOPLASMIC.
FT   TRANSMEM    271    291
FT   TOPO_DOM    292    313       CYTOPLASMIC.
//
```

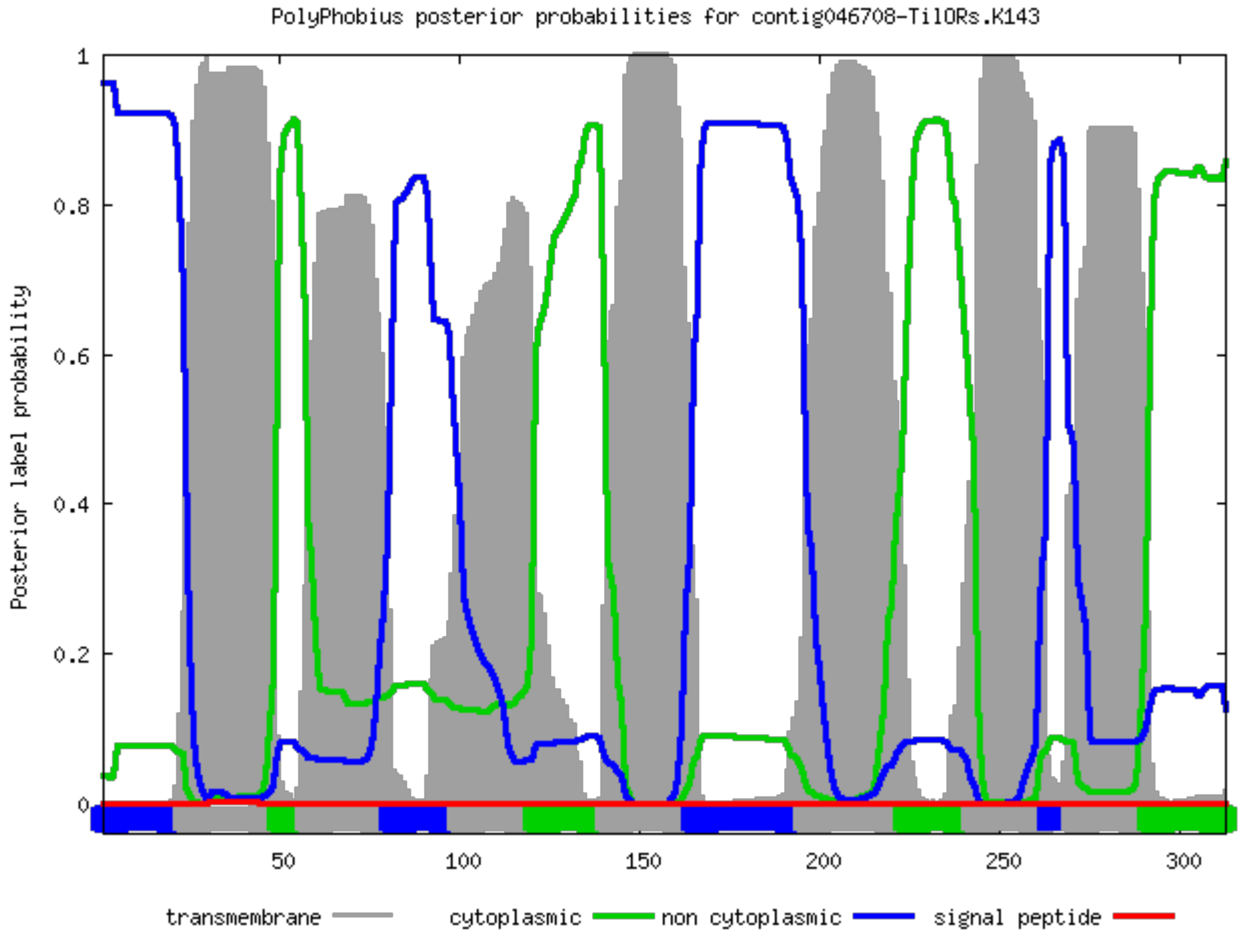

The prediction is based on an alignment. The probability data used in the plot is found here, and the gnuplot script is here.

Prediction of contig041641-BurORs.T129

```
ID    contig041641-BurORs.T129
FT    TOPO_DOM      1      16       NON CYTOPLASMIC.
FT    TRANSMEM     17      38
FT    TOPO_DOM     39      49       CYTOPLASMIC.
FT    TRANSMEM     50      73
FT    TOPO_DOM     74      77       NON CYTOPLASMIC.
FT    TRANSMEM     78      95
FT    TOPO_DOM     96     128       CYTOPLASMIC.
FT    TRANSMEM    129     152
FT    TOPO_DOM    153     181       NON CYTOPLASMIC.
FT    TRANSMEM    182     203
FT    TOPO_DOM    204     223       CYTOPLASMIC.
FT    TRANSMEM    224     243
FT    TOPO_DOM    244     258       NON CYTOPLASMIC.
FT    TRANSMEM    259     280
FT    TOPO_DOM    281     314       CYTOPLASMIC.
//
```

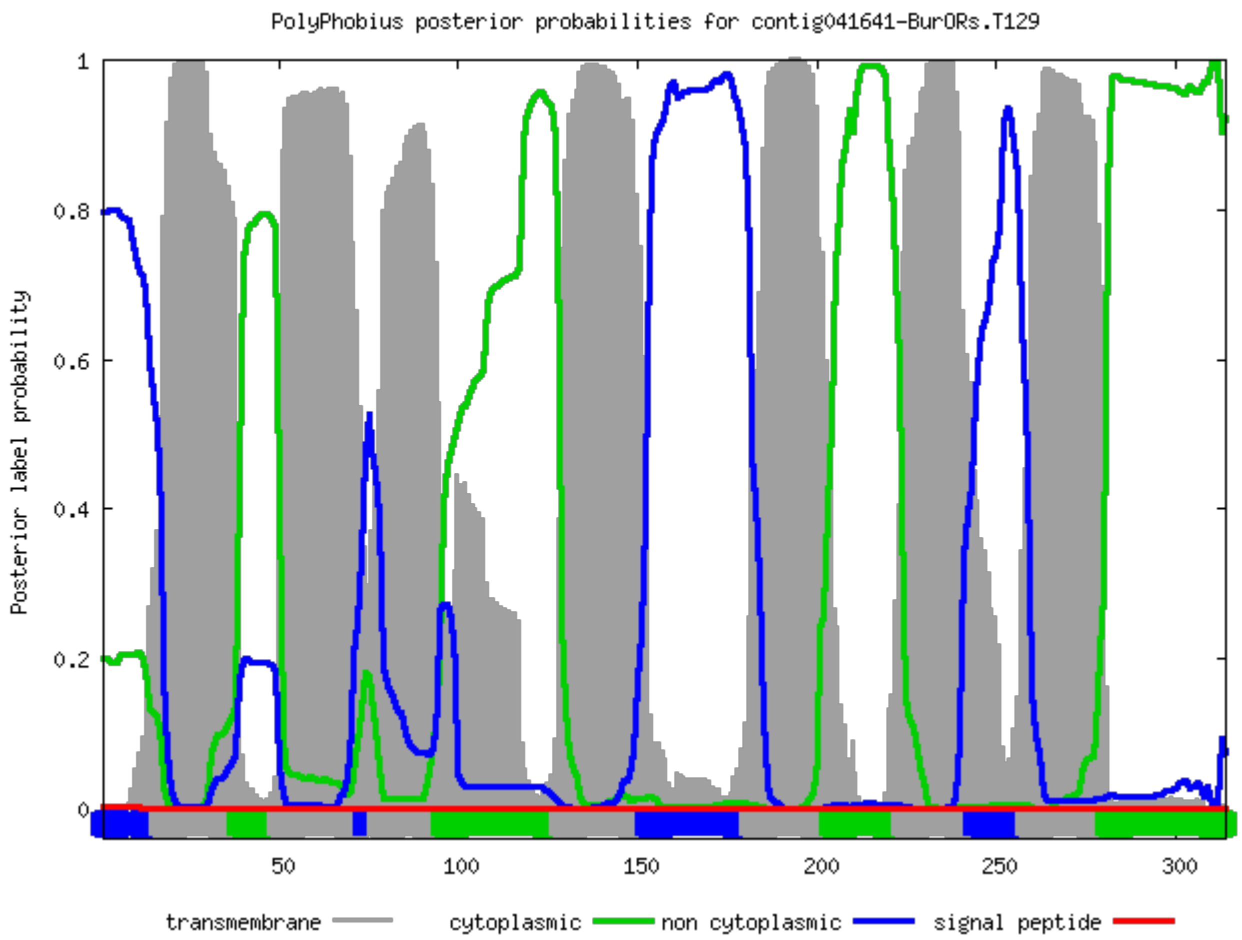

The prediction is based on an alignment. The probability data used in the plot is found here, and the gnuplot script is here.

Prediction of contig027204-TilORs.W239

```
ID   contig027204-TilORs.W239
FT   TOPO_DOM      1     24       NON CYTOPLASMIC.
FT   TRANSMEM     25     46
FT   TOPO_DOM     47     57       CYTOPLASMIC.
FT   TRANSMEM     58     81
FT   TOPO_DOM     82     84       NON CYTOPLASMIC.
FT   TRANSMEM     85    104
FT   TOPO_DOM    105    137       CYTOPLASMIC.
FT   TRANSMEM    138    161
FT   TOPO_DOM    162    191       NON CYTOPLASMIC.
FT   TRANSMEM    192    213
FT   TOPO_DOM    214    228       CYTOPLASMIC.
FT   TRANSMEM    229    247
FT   TOPO_DOM    248    263       NON CYTOPLASMIC.
FT   TRANSMEM    264    285
FT   TOPO_DOM    286    305       CYTOPLASMIC.
//
```

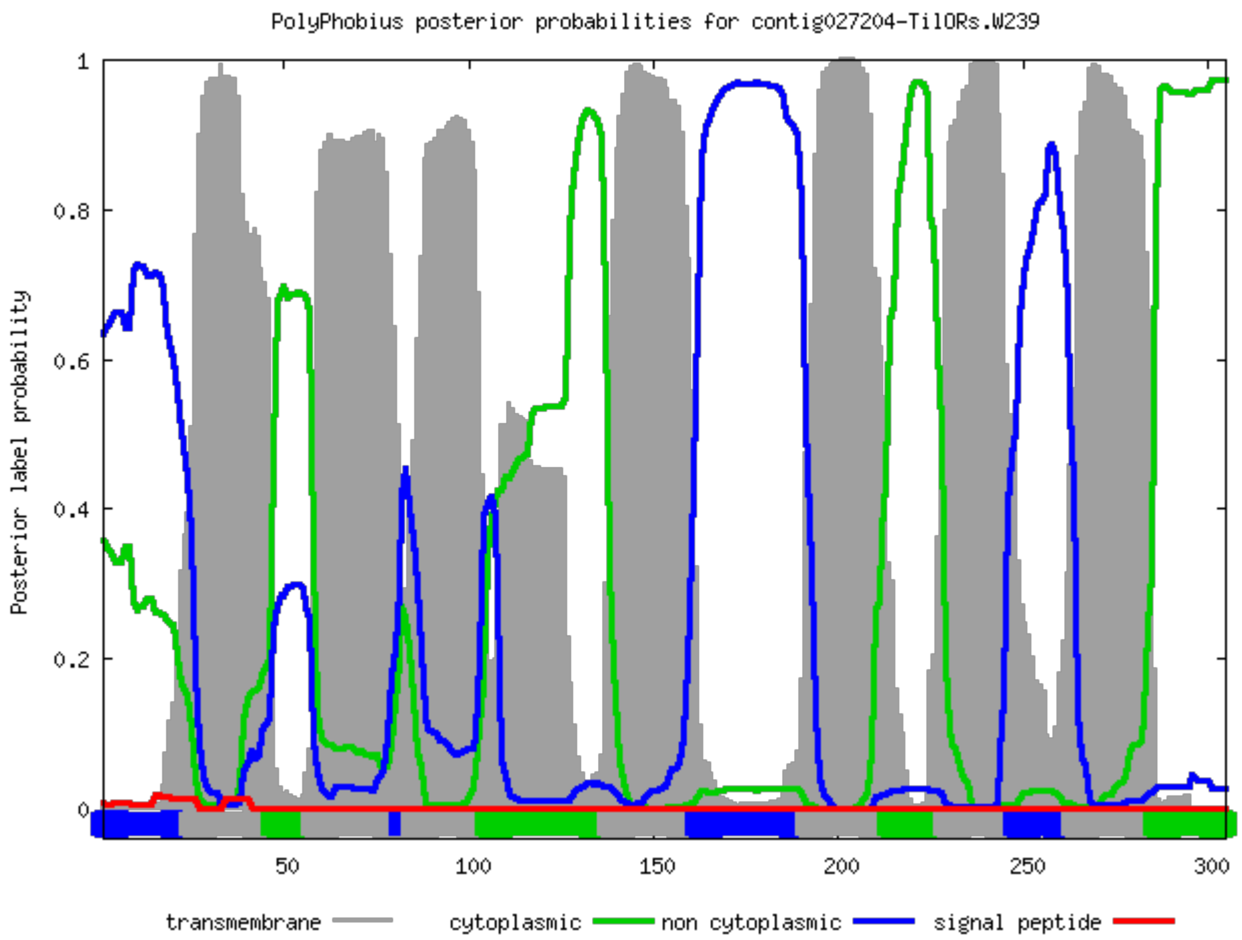

The prediction is based on an alignment. The probability data used in the plot is found here, and the gnuplot script is here.

Prediction of contig090291-BriORs.W114

```
ID   contig090291-BriORs.W114
FT   TOPO_DOM      1     26       NON CYTOPLASMIC.
FT   TRANSMEM     27     48
FT   TOPO_DOM     49     60       CYTOPLASMIC.
FT   TRANSMEM     61     84
FT   TOPO_DOM     85     87       NON CYTOPLASMIC.
FT   TRANSMEM     88    119
FT   TOPO_DOM    120    140       CYTOPLASMIC.
FT   TRANSMEM    141    164
FT   TOPO_DOM    165    194       NON CYTOPLASMIC.
FT   TRANSMEM    195    216
FT   TOPO_DOM    217    231       CYTOPLASMIC.
FT   TRANSMEM    232    251
FT   TOPO_DOM    252    266       NON CYTOPLASMIC.
FT   TRANSMEM    267    288
FT   TOPO_DOM    289    315       CYTOPLASMIC.
//
```

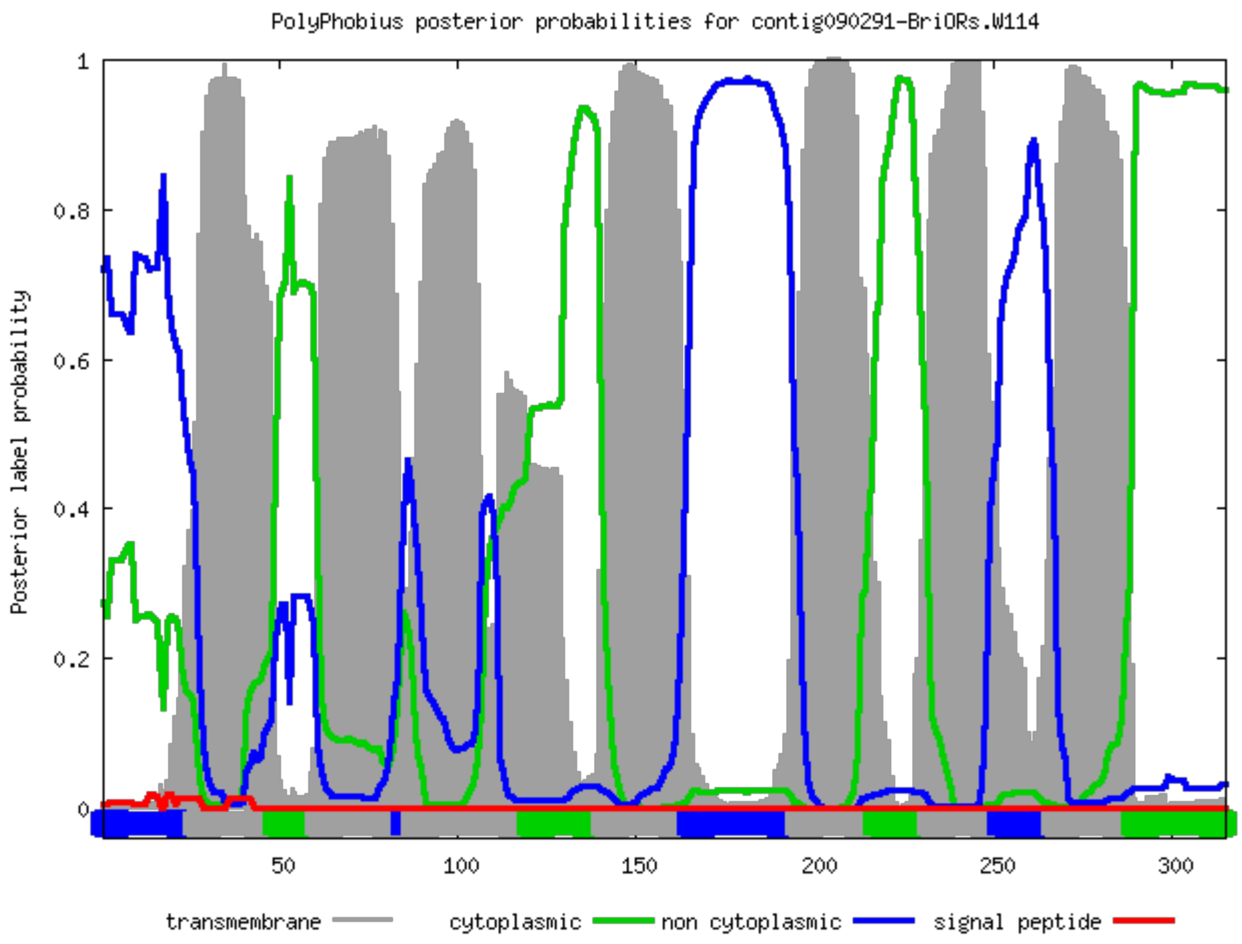

The prediction is based on an alignment. The probability data used in the plot is found here, and the gnuplot script is here.

Prediction of contig026932-ZebORs.I082

```
ID   contig026932-ZebORs.I082
FT   TOPO_DOM      1     28       NON CYTOPLASMIC.
FT   TRANSMEM     29     54
FT   TOPO_DOM     55     64       CYTOPLASMIC.
FT   TRANSMEM     65     86
FT   TOPO_DOM     87    101       NON CYTOPLASMIC.
FT   TRANSMEM    102    124
FT   TOPO_DOM    125    144       CYTOPLASMIC.
FT   TRANSMEM    145    167
FT   TOPO_DOM    168    199       NON CYTOPLASMIC.
FT   TRANSMEM    200    221
FT   TOPO_DOM    222    243       CYTOPLASMIC.
FT   TRANSMEM    244    266
FT   TOPO_DOM    267    276       NON CYTOPLASMIC.
FT   TRANSMEM    277    297
FT   TOPO_DOM    298    313       CYTOPLASMIC.
//
```

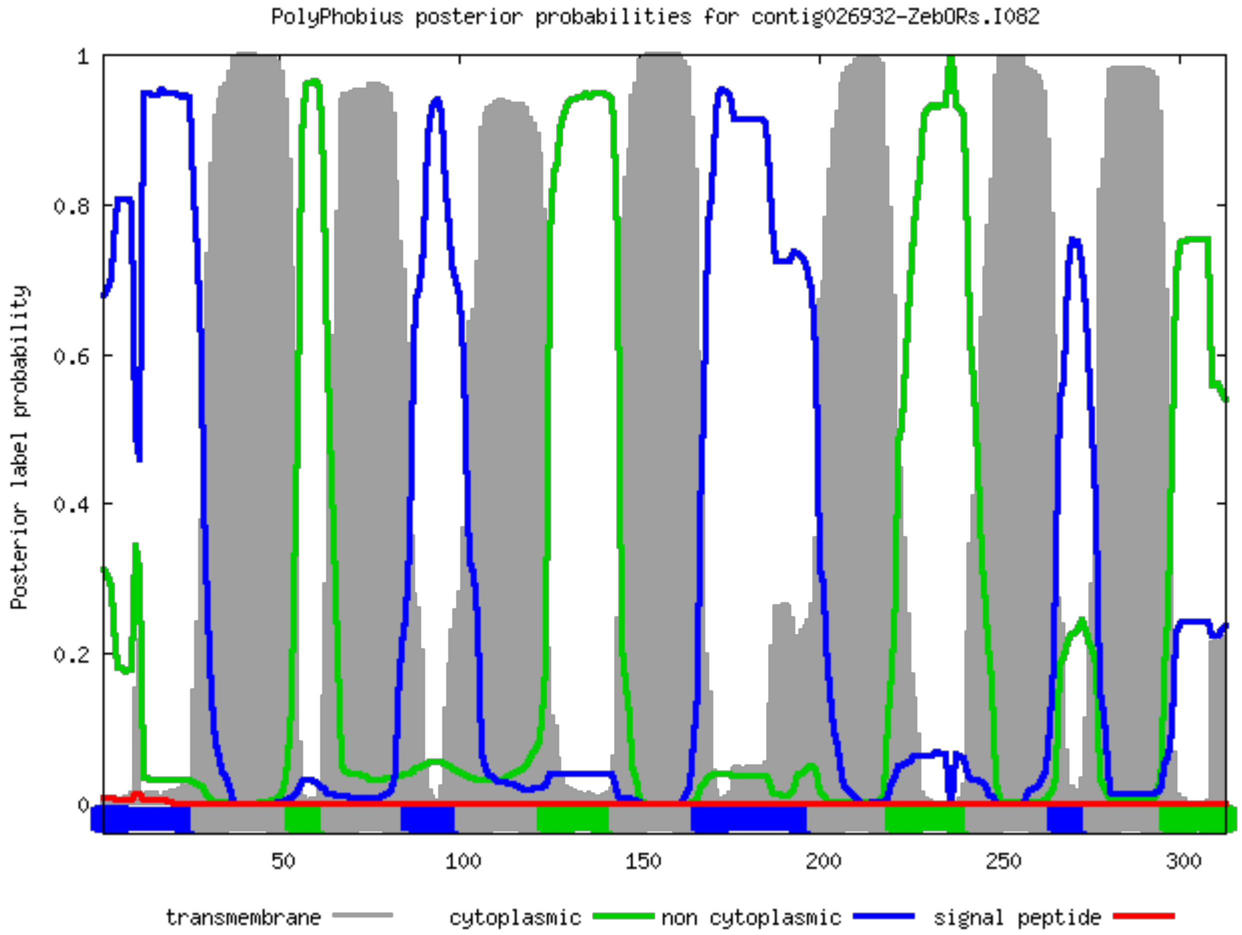

The prediction is based on an alignment. The probability data used in the plot is found here, and the gnuplot script is here.

Prediction of contig025847-ZebORs.V149

```
ID   contig025847-ZebORs.V149
FT   TOPO_DOM      1     37       NON CYTOPLASMIC.
FT   TRANSMEM     38     59
FT   TOPO_DOM     60     70       CYTOPLASMIC.
FT   TRANSMEM     71     94
FT   TOPO_DOM     95     97       NON CYTOPLASMIC.
FT   TRANSMEM     98    130
FT   TOPO_DOM    131    151       CYTOPLASMIC.
FT   TRANSMEM    152    175
FT   TOPO_DOM    176    205       NON CYTOPLASMIC.
FT   TRANSMEM    206    227
FT   TOPO_DOM    228    244       CYTOPLASMIC.
FT   TRANSMEM    245    259
FT   TOPO_DOM    260    273       NON CYTOPLASMIC.
FT   TRANSMEM    274    295
FT   TOPO_DOM    296    339       CYTOPLASMIC.
//
```

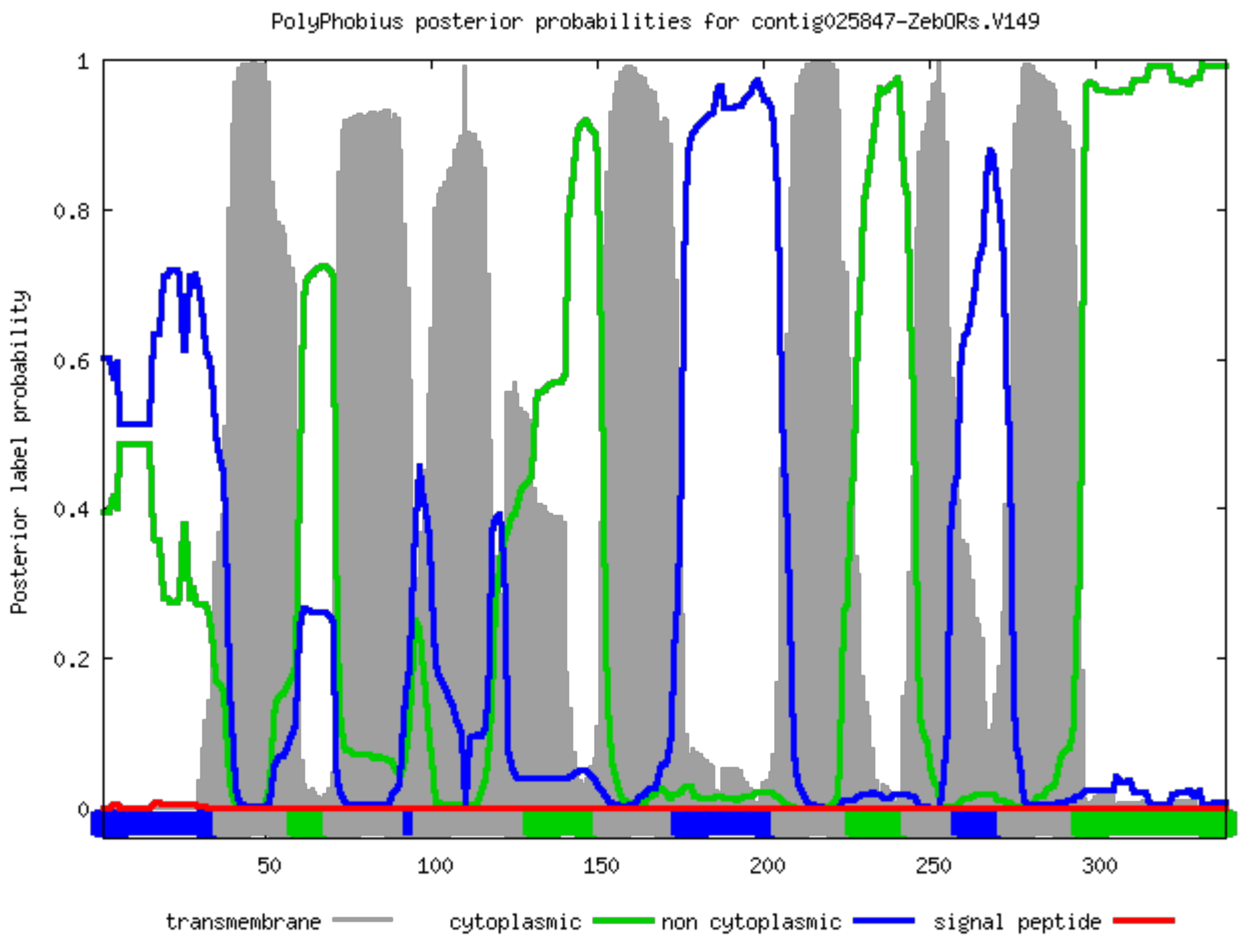

The prediction is based on an alignment. The probability data used in the plot is found here, and the gnuplot script is here.

Prediction of contig027202-TilORs.W243

```
ID   contig027202-TilORs.W243
FT   TOPO_DOM      1     26       NON CYTOPLASMIC.
FT   TRANSMEM     27     48
FT   TOPO_DOM     49     59       CYTOPLASMIC.
FT   TRANSMEM     60     83
FT   TOPO_DOM     84     86       NON CYTOPLASMIC.
FT   TRANSMEM     87    118
FT   TOPO_DOM    119    139       CYTOPLASMIC.
FT   TRANSMEM    140    163
FT   TOPO_DOM    164    193       NON CYTOPLASMIC.
FT   TRANSMEM    194    215
FT   TOPO_DOM    216    233       CYTOPLASMIC.
FT   TRANSMEM    234    253
FT   TOPO_DOM    254    267       NON CYTOPLASMIC.
FT   TRANSMEM    268    290
FT   TOPO_DOM    291    318       CYTOPLASMIC.
//
```

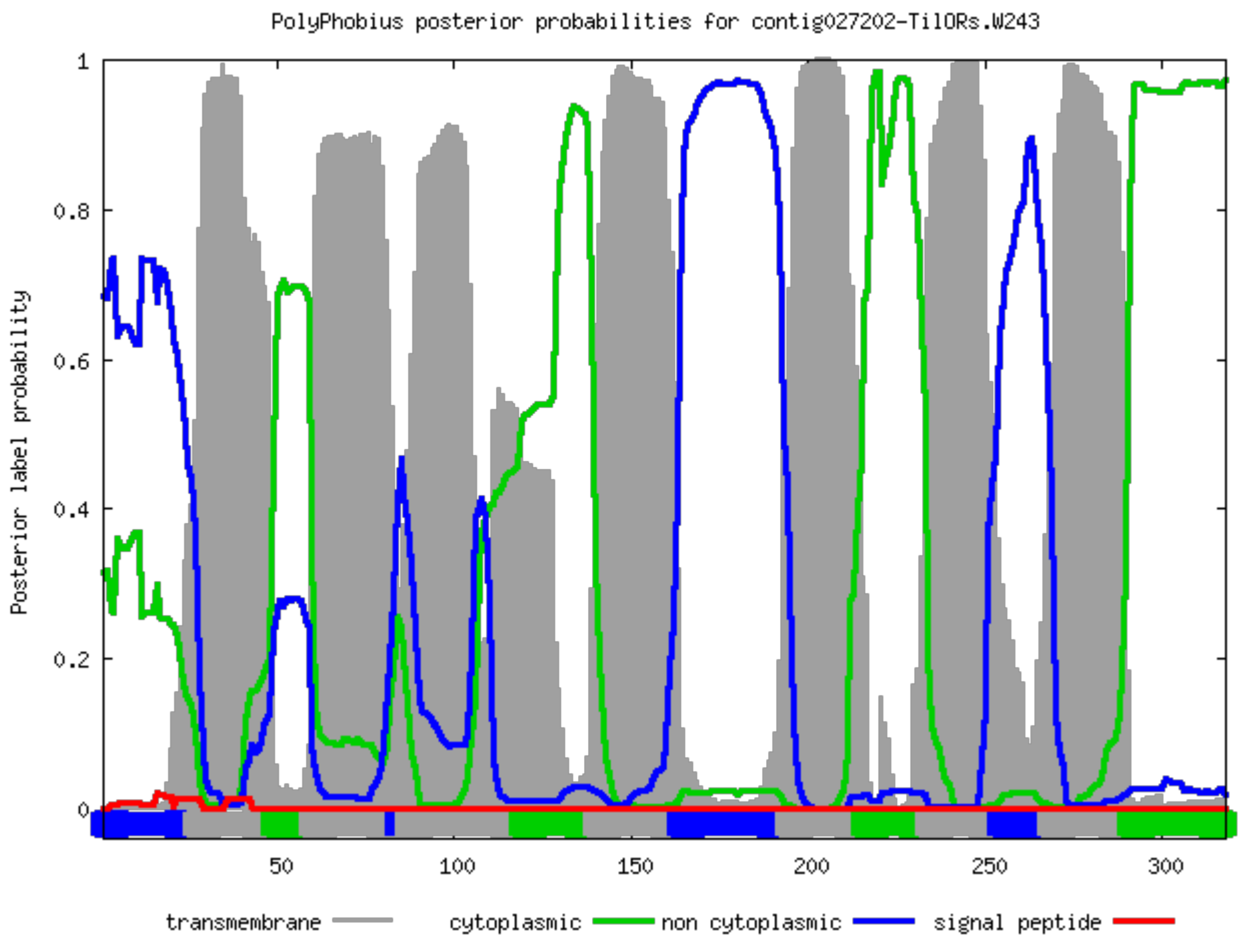

The prediction is based on an alignment. The probability data used in the plot is found here, and the gnuplot script is here.

Prediction of contig025847-ZebORs.U137

```
ID    contig025847-ZebORs.U137
FT    TOPO_DOM      1      28       NON CYTOPLASMIC.
FT    TRANSMEM     29      50
FT    TOPO_DOM     51      65       CYTOPLASMIC.
FT    TRANSMEM     66      89
FT    TOPO_DOM     90      91       NON CYTOPLASMIC.
FT    TRANSMEM     92     112
FT    TOPO_DOM    113     145       CYTOPLASMIC.
FT    TRANSMEM    146     169
FT    TOPO_DOM    170     199       NON CYTOPLASMIC.
FT    TRANSMEM    200     221
FT    TOPO_DOM    222     239       CYTOPLASMIC.
FT    TRANSMEM    240     258
FT    TOPO_DOM    259     275       NON CYTOPLASMIC.
FT    TRANSMEM    276     296
FT    TOPO_DOM    297     330       CYTOPLASMIC.
//
```

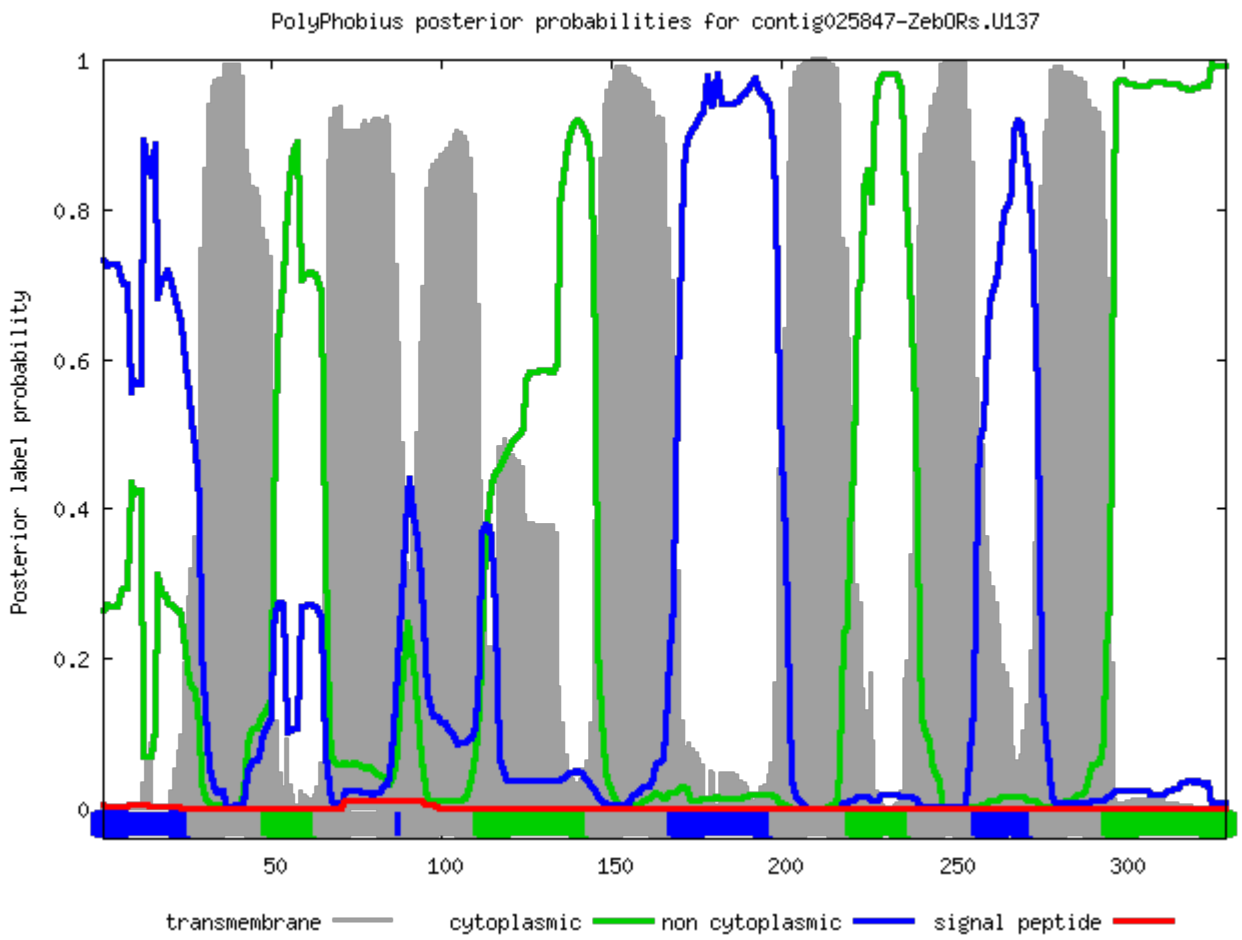

The prediction is based on an alignment. The probability data used in the plot is found here, and the gnuplot script is here.

Prediction of contig025842-ZebORs.W142

```
ID   contig025842-ZebORs.W142
FT   TOPO_DOM      1     26       NON CYTOPLASMIC.
FT   TRANSMEM     27     48
FT   TOPO_DOM     49     59       CYTOPLASMIC.
FT   TRANSMEM     60     83
FT   TOPO_DOM     84     86       NON CYTOPLASMIC.
FT   TRANSMEM     87    118
FT   TOPO_DOM    119    139       CYTOPLASMIC.
FT   TRANSMEM    140    163
FT   TOPO_DOM    164    193       NON CYTOPLASMIC.
FT   TRANSMEM    194    215
FT   TOPO_DOM    216    233       CYTOPLASMIC.
FT   TRANSMEM    234    253
FT   TOPO_DOM    254    268       NON CYTOPLASMIC.
FT   TRANSMEM    269    290
FT   TOPO_DOM    291    318       CYTOPLASMIC.
//
```

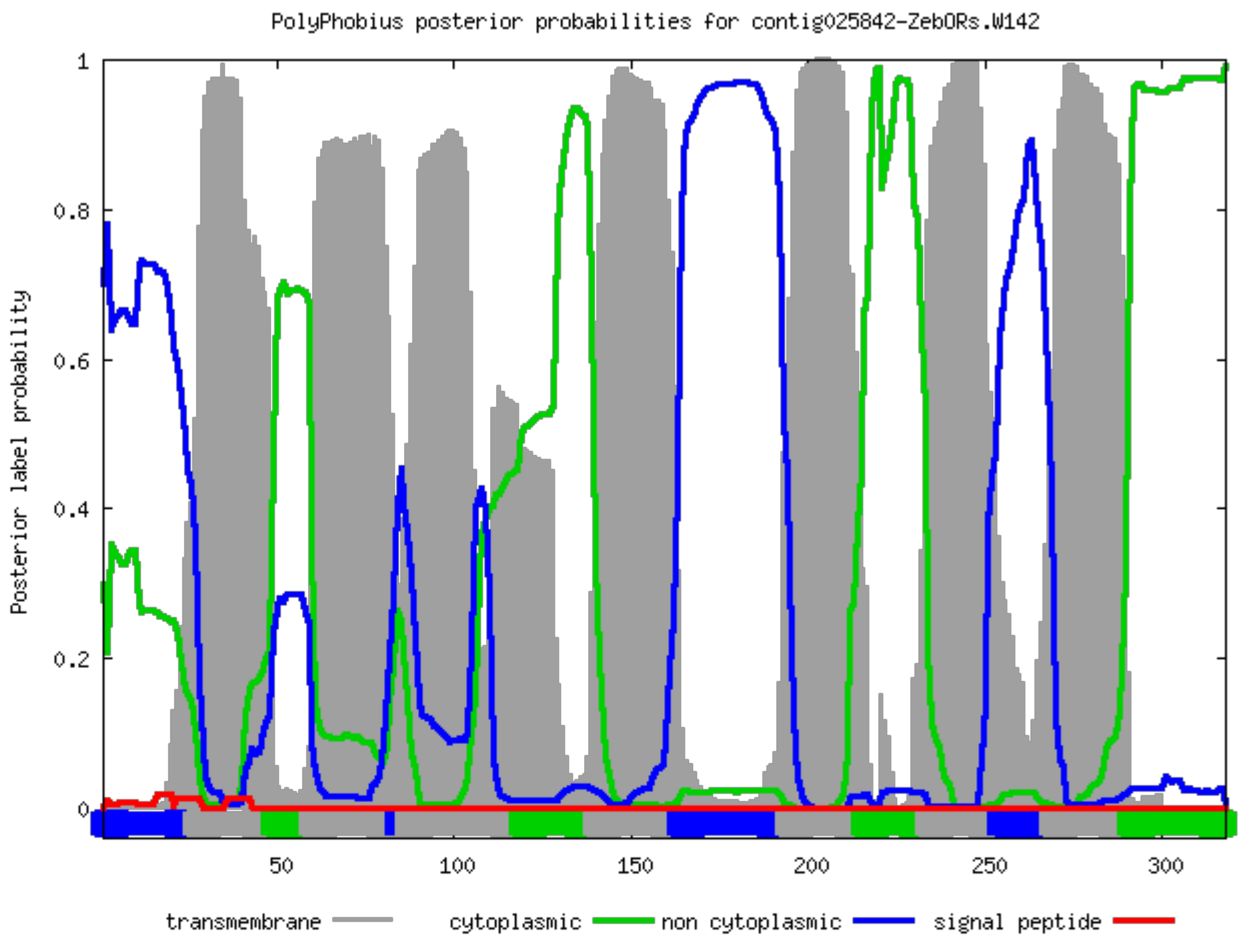

The prediction is based on an alignment. The probability data used in the plot is found here, and the gnuplot script is here.

Prediction of contig027194-TilORs.V262

```
ID   contig027194-TilORs.V262
FT   TOPO_DOM      1     34       NON CYTOPLASMIC.
FT   TRANSMEM     35     56
FT   TOPO_DOM     57     67       CYTOPLASMIC.
FT   TRANSMEM     68     91
FT   TOPO_DOM     92     94       NON CYTOPLASMIC.
FT   TRANSMEM     95    127
FT   TOPO_DOM    128    148       CYTOPLASMIC.
FT   TRANSMEM    149    172
FT   TOPO_DOM    173    202       NON CYTOPLASMIC.
FT   TRANSMEM    203    224
FT   TOPO_DOM    225    242       CYTOPLASMIC.
FT   TRANSMEM    243    262
FT   TOPO_DOM    263    277       NON CYTOPLASMIC.
FT   TRANSMEM    278    299
FT   TOPO_DOM    300    335       CYTOPLASMIC.
//
```

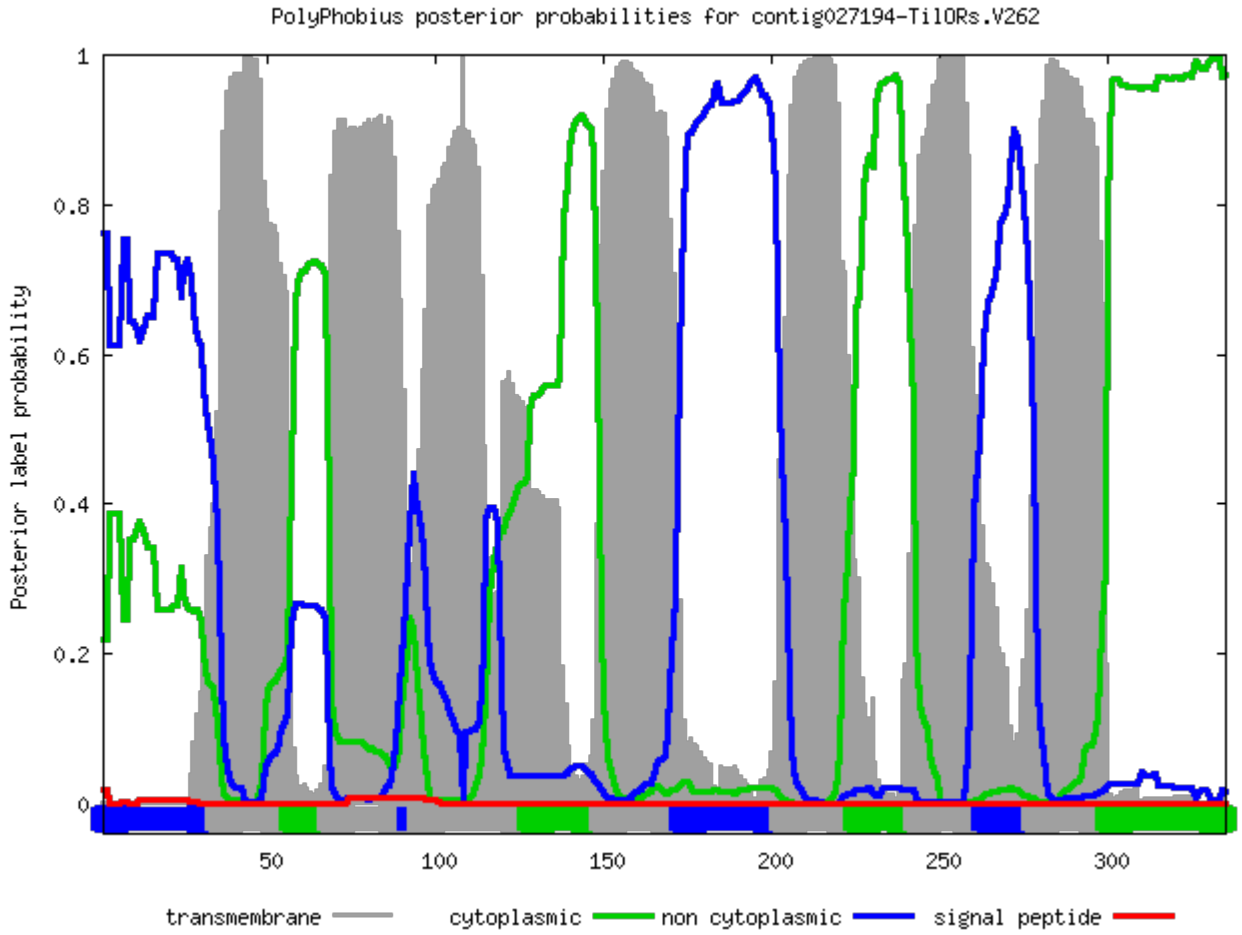

The prediction is based on an alignment. The probability data used in the plot is found here, and the gnuplot script is here.

Prediction of contig045452-BurORs.W133

```
ID   contig045452-BurORs.W133
FT   TOPO_DOM      1      26       NON CYTOPLASMIC.
FT   TRANSMEM     27      48
FT   TOPO_DOM     49      61       CYTOPLASMIC.
FT   TRANSMEM     62      85
FT   TOPO_DOM     86      88       NON CYTOPLASMIC.
FT   TRANSMEM     89     121
FT   TOPO_DOM    122     141       CYTOPLASMIC.
FT   TRANSMEM    142     165
FT   TOPO_DOM    166     195       NON CYTOPLASMIC.
FT   TRANSMEM    196     217
FT   TOPO_DOM    218     235       CYTOPLASMIC.
FT   TRANSMEM    236     255
FT   TOPO_DOM    256     270       NON CYTOPLASMIC.
FT   TRANSMEM    271     292
FT   TOPO_DOM    293     320       CYTOPLASMIC.
//
```

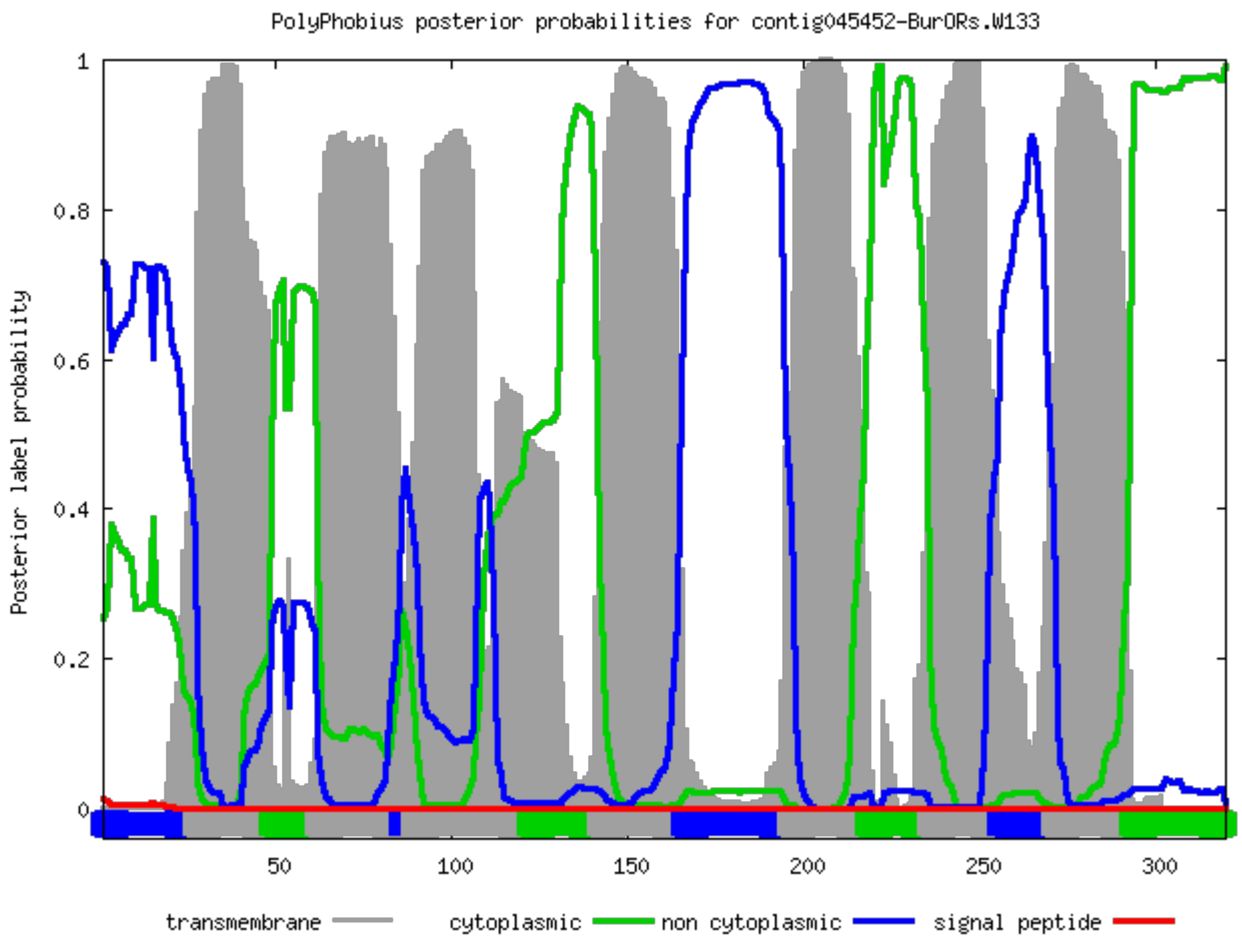

The prediction is based on an alignment. The probability data used in the plot is found here, and the gnuplot script is here.

Prediction of contig090301-BriORs.W116

```
ID   contig090301-BriORs.W116
FT   TOPO_DOM      1     22       NON CYTOPLASMIC.
FT   TRANSMEM     23     45
FT   TOPO_DOM     46     58       CYTOPLASMIC.
FT   TRANSMEM     59     82
FT   TOPO_DOM     83     85       NON CYTOPLASMIC.
FT   TRANSMEM     86    117
FT   TOPO_DOM    118    137       CYTOPLASMIC.
FT   TRANSMEM    138    161
FT   TOPO_DOM    162    191       NON CYTOPLASMIC.
FT   TRANSMEM    192    213
FT   TOPO_DOM    214    228       CYTOPLASMIC.
FT   TRANSMEM    229    248
FT   TOPO_DOM    249    263       NON CYTOPLASMIC.
FT   TRANSMEM    264    285
FT   TOPO_DOM    286    320       CYTOPLASMIC.
//
```

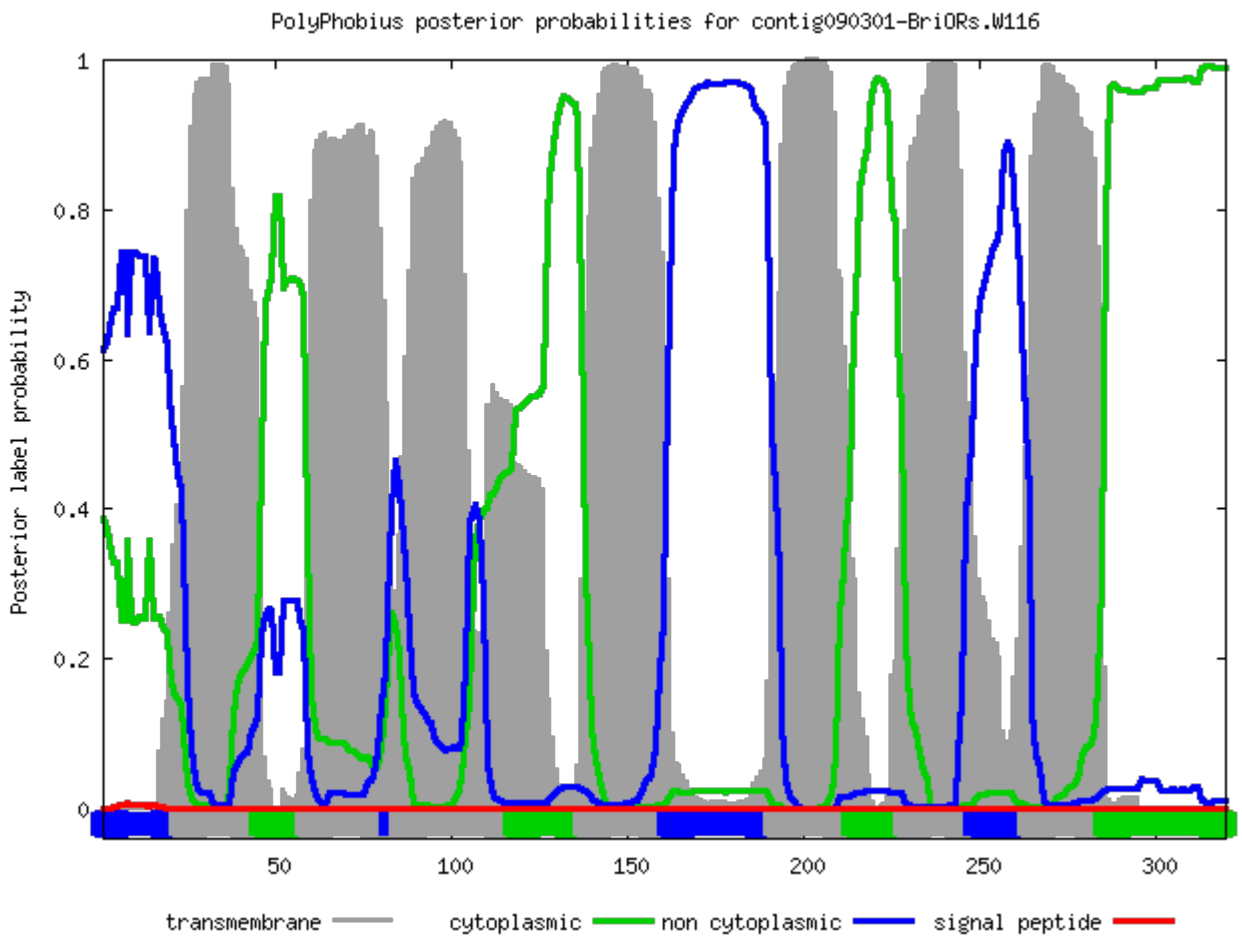

The prediction is based on an alignment. The probability data used in the plot is found here, and the gnuplot script is here.

Prediction of contig090302-BriORs.V122

```
ID   contig090302-BriORs.V122
FT   TOPO_DOM      1     34       NON CYTOPLASMIC.
FT   TRANSMEM     35     56
FT   TOPO_DOM     57     67       CYTOPLASMIC.
FT   TRANSMEM     68     91
FT   TOPO_DOM     92     94       NON CYTOPLASMIC.
FT   TRANSMEM     95    127
FT   TOPO_DOM    128    148       CYTOPLASMIC.
FT   TRANSMEM    149    172
FT   TOPO_DOM    173    202       NON CYTOPLASMIC.
FT   TRANSMEM    203    224
FT   TOPO_DOM    225    241       CYTOPLASMIC.
FT   TRANSMEM    242    256
FT   TOPO_DOM    257    271       NON CYTOPLASMIC.
FT   TRANSMEM    272    292
FT   TOPO_DOM    293    336       CYTOPLASMIC.
//
```

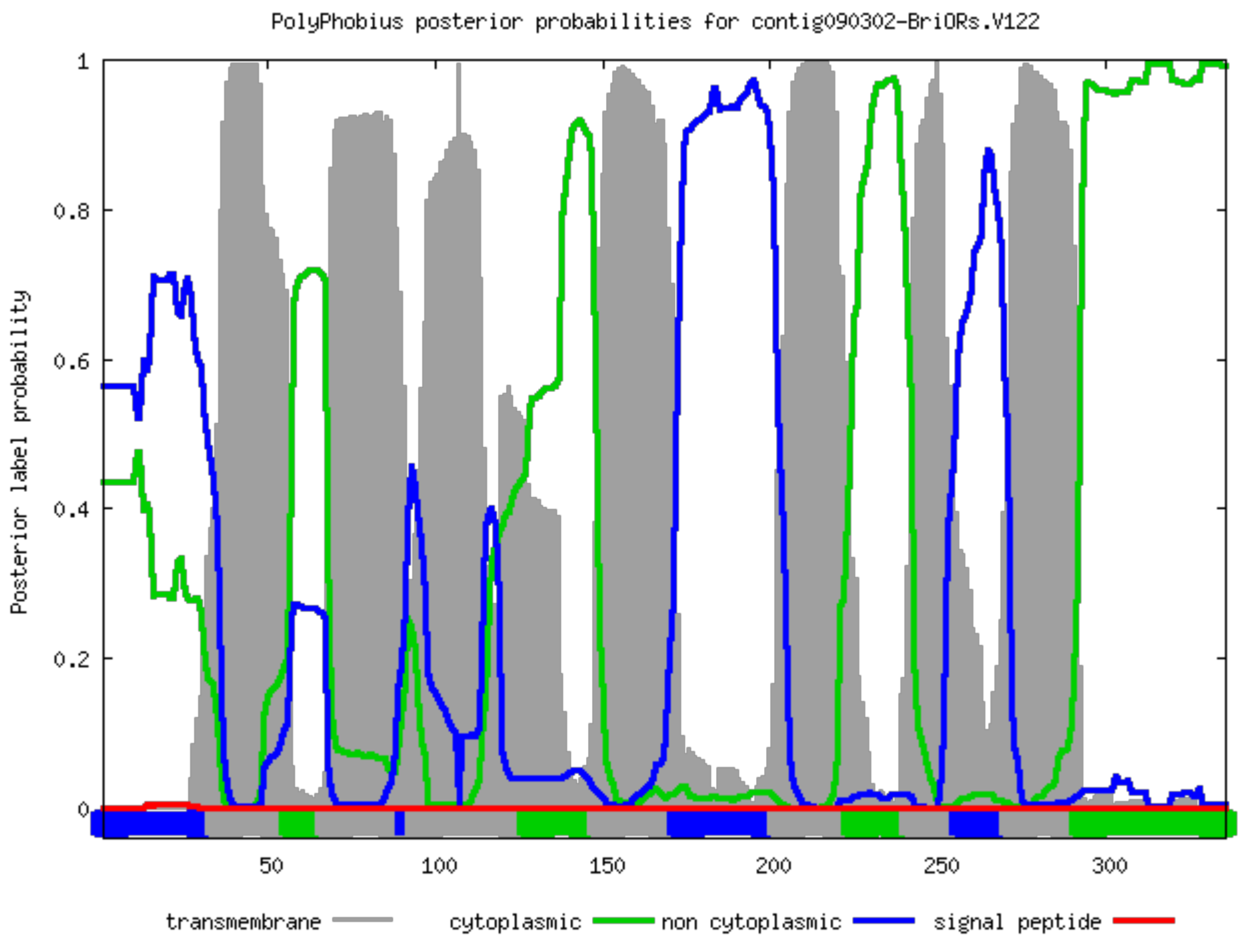

The prediction is based on an alignment. The probability data used in the plot is found here, and the gnuplot script is here.

Prediction of contig090288-BriORs.W113

```
ID   contig090288-BriORs.W113
FT   TOPO_DOM      1     24       NON CYTOPLASMIC.
FT   TRANSMEM     25     46
FT   TOPO_DOM     47     59       CYTOPLASMIC.
FT   TRANSMEM     60     83
FT   TOPO_DOM     84     86       NON CYTOPLASMIC.
FT   TRANSMEM     87    120
FT   TOPO_DOM    121    139       CYTOPLASMIC.
FT   TRANSMEM    140    163
FT   TOPO_DOM    164    193       NON CYTOPLASMIC.
FT   TRANSMEM    194    215
FT   TOPO_DOM    216    230       CYTOPLASMIC.
FT   TRANSMEM    231    249
FT   TOPO_DOM    250    265       NON CYTOPLASMIC.
FT   TRANSMEM    266    287
FT   TOPO_DOM    288    315       CYTOPLASMIC.
//
```

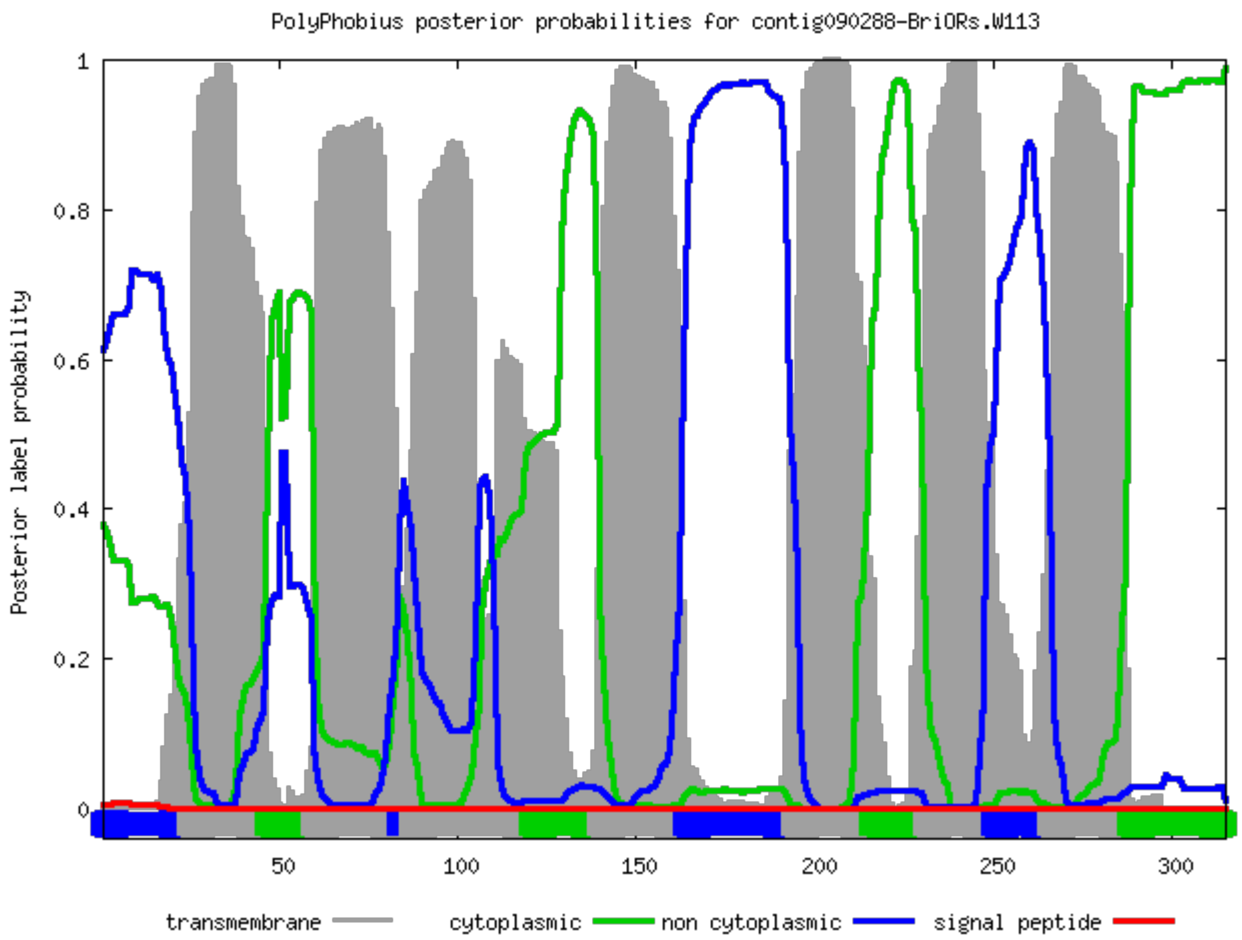

The prediction is based on an alignment. The probability data used in the plot is found here, and the gnuplot script is here.

Prediction of contig046695-TilORs.I130

```
ID   contig046695-TilORs.I130
FT   TOPO_DOM      1     23       NON CYTOPLASMIC.
FT   TRANSMEM     24     49
FT   TOPO_DOM     50     59       CYTOPLASMIC.
FT   TRANSMEM     60     81
FT   TOPO_DOM     82     96       NON CYTOPLASMIC.
FT   TRANSMEM     97    119
FT   TOPO_DOM    120    139       CYTOPLASMIC.
FT   TRANSMEM    140    162
FT   TOPO_DOM    163    194       NON CYTOPLASMIC.
FT   TRANSMEM    195    219
FT   TOPO_DOM    220    240       CYTOPLASMIC.
FT   TRANSMEM    241    262
FT   TOPO_DOM    263    273       NON CYTOPLASMIC.
FT   TRANSMEM    274    294
FT   TOPO_DOM    295    316       CYTOPLASMIC.
//
```

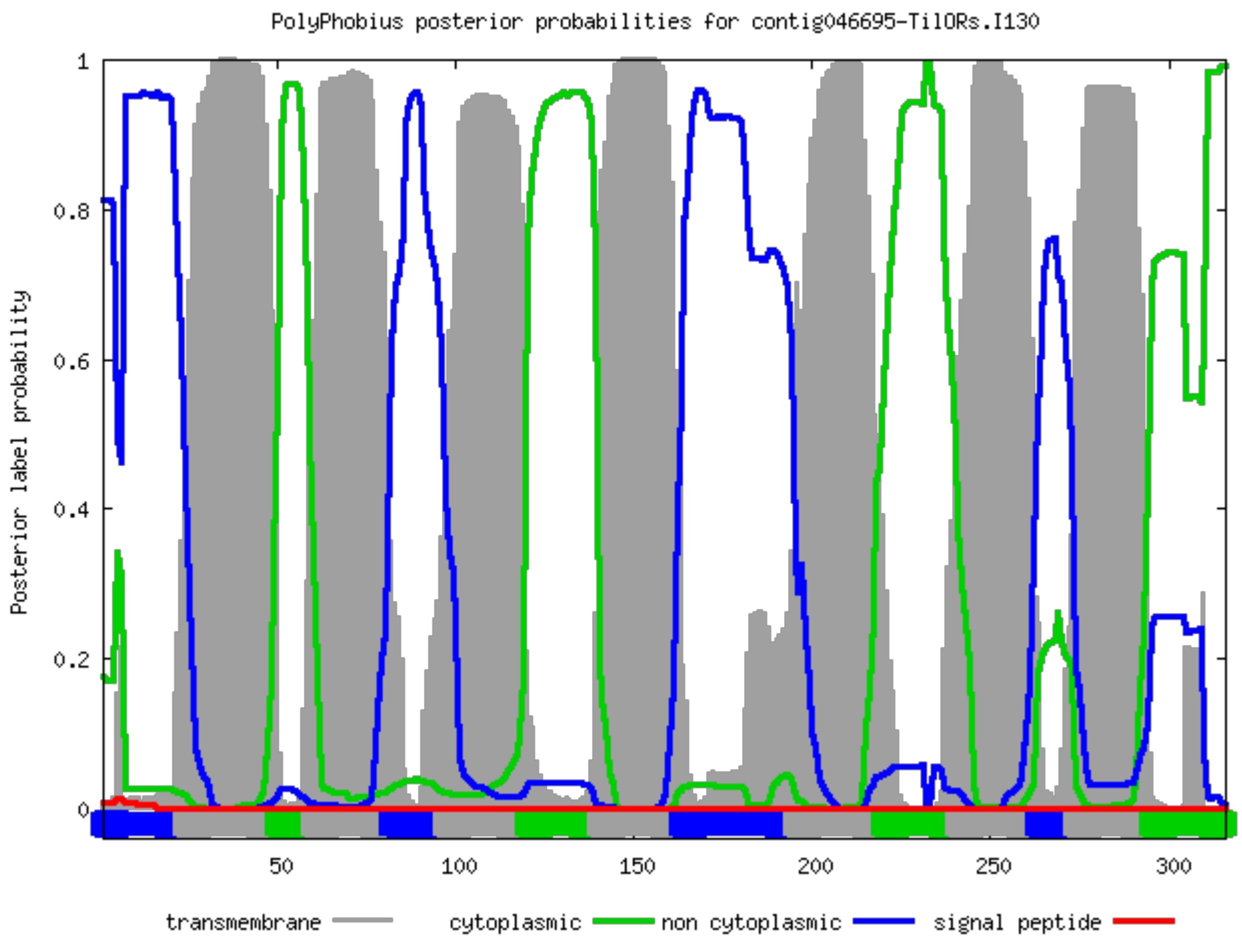

The prediction is based on an alignment. The probability data used in the plot is found here, and the gnuplot script is here.

Prediction of contig051999-NyeORs.U128

```
ID   contig051999-NyeORs.U128
FT   TOPO_DOM      1      30        NON CYTOPLASMIC.
FT   TRANSMEM     31      52
FT   TOPO_DOM     53      63        CYTOPLASMIC.
FT   TRANSMEM     64      87
FT   TOPO_DOM     88      90        NON CYTOPLASMIC.
FT   TRANSMEM     91     110
FT   TOPO_DOM    111     143        CYTOPLASMIC.
FT   TRANSMEM    144     167
FT   TOPO_DOM    168     197        NON CYTOPLASMIC.
FT   TRANSMEM    198     219
FT   TOPO_DOM    220     237        CYTOPLASMIC.
FT   TRANSMEM    238     255
FT   TOPO_DOM    256     273        NON CYTOPLASMIC.
FT   TRANSMEM    274     294
FT   TOPO_DOM    295     328        CYTOPLASMIC.
//
```

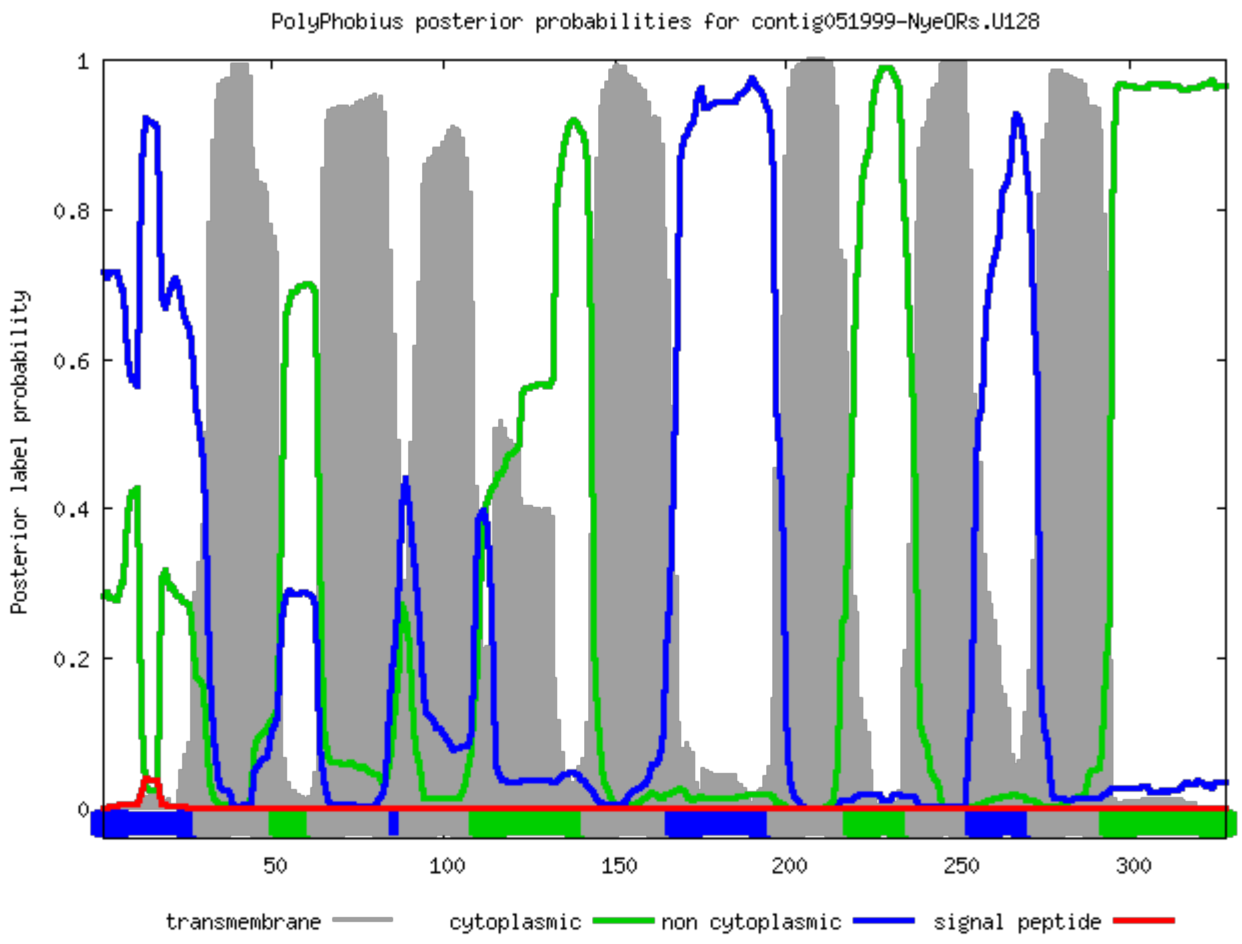

The prediction is based on an alignment. The probability data used in the plot is found here, and the gnuplot script is here.

Prediction of contig046002-ZebORs.K090

```
ID    contig046002-ZebORs.K090
FT    TOPO_DOM      1      24       NON CYTOPLASMIC.
FT    TRANSMEM     25      50
FT    TOPO_DOM     51      58       CYTOPLASMIC.
FT    TRANSMEM     59      81
FT    TOPO_DOM     82     100       NON CYTOPLASMIC.
FT    TRANSMEM    101     121
FT    TOPO_DOM    122     141       CYTOPLASMIC.
FT    TRANSMEM    142     165
FT    TOPO_DOM    166     196       NON CYTOPLASMIC.
FT    TRANSMEM    197     224
FT    TOPO_DOM    225     244       CYTOPLASMIC.
FT    TRANSMEM    245     264
FT    TOPO_DOM    265     271       NON CYTOPLASMIC.
FT    TRANSMEM    272     292
FT    TOPO_DOM    293     307       CYTOPLASMIC.
//
```

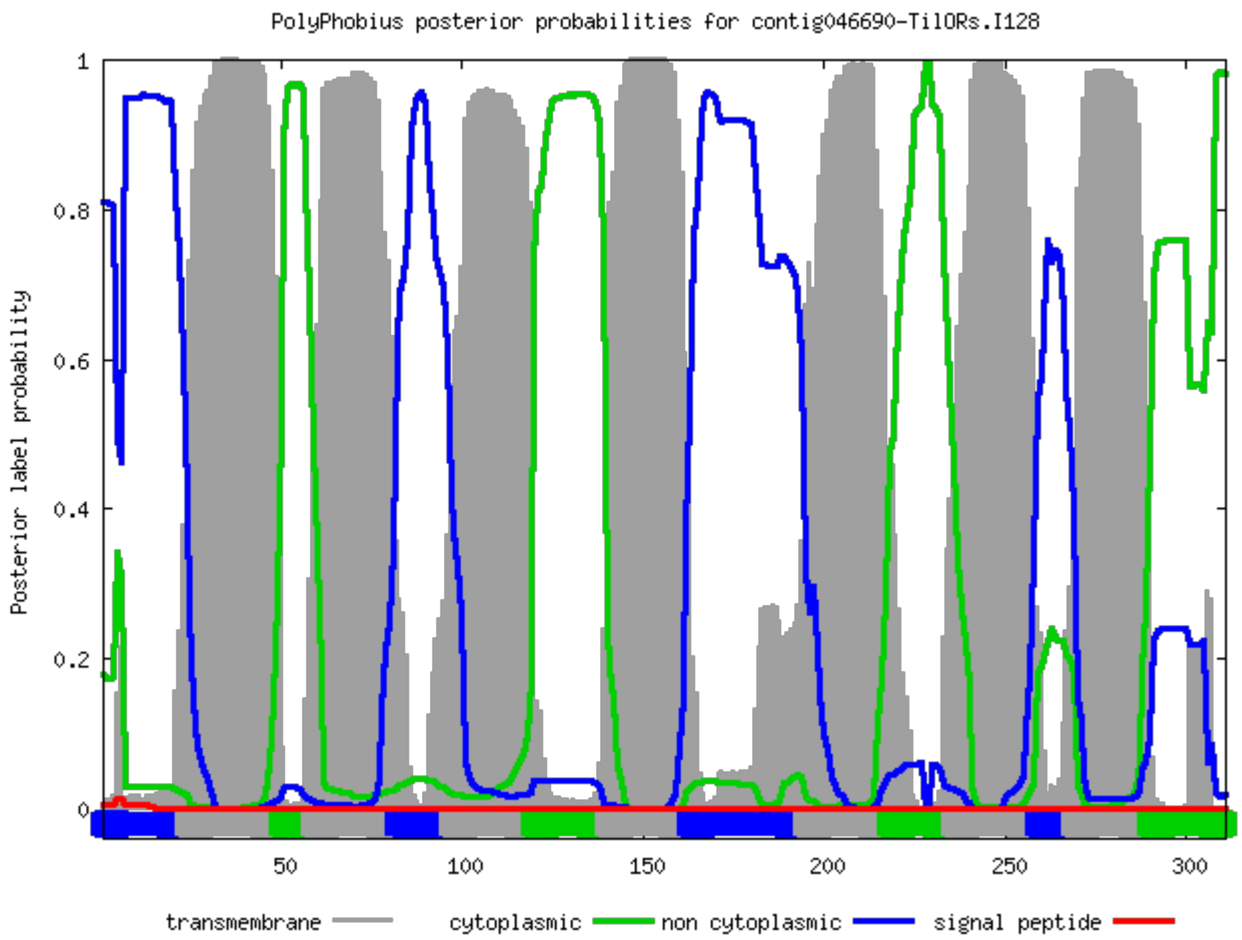

The prediction is based on an alignment. The probability data used in the plot is found here, and the gnuplot script is here.

Prediction of contig027203-TilORs.W238

```
ID   contig027203-TilORs.W238
FT   TOPO_DOM      1     24       NON CYTOPLASMIC.
FT   TRANSMEM     25     46
FT   TOPO_DOM     47     57       CYTOPLASMIC.
FT   TRANSMEM     58     81
FT   TOPO_DOM     82     84       NON CYTOPLASMIC.
FT   TRANSMEM     85    116
FT   TOPO_DOM    117    137       CYTOPLASMIC.
FT   TRANSMEM    138    161
FT   TOPO_DOM    162    191       NON CYTOPLASMIC.
FT   TRANSMEM    192    213
FT   TOPO_DOM    214    228       CYTOPLASMIC.
FT   TRANSMEM    229    247
FT   TOPO_DOM    248    263       NON CYTOPLASMIC.
FT   TRANSMEM    264    285
FT   TOPO_DOM    286    313       CYTOPLASMIC.
//
```

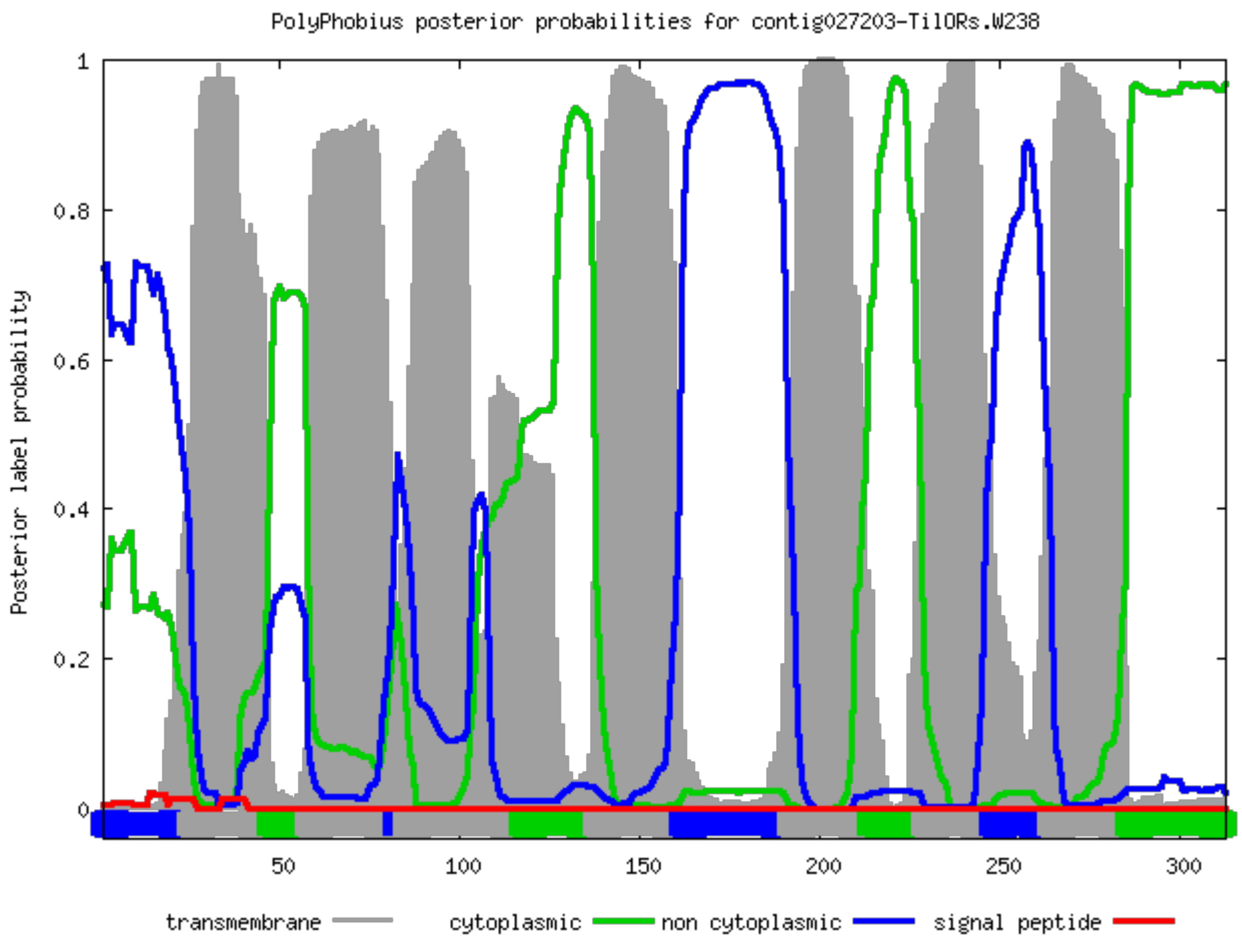

The prediction is based on an alignment. The probability data used in the plot is found here, and the gnuplot script is here.

Prediction of contig045453-BurORs.W132

```
ID   contig045453-BurORs.W132
FT   TOPO_DOM      1     24       NON CYTOPLASMIC.
FT   TRANSMEM     25     46
FT   TOPO_DOM     47     57       CYTOPLASMIC.
FT   TRANSMEM     58     81
FT   TOPO_DOM     82     84       NON CYTOPLASMIC.
FT   TRANSMEM     85    117
FT   TOPO_DOM    118    137       CYTOPLASMIC.
FT   TRANSMEM    138    161
FT   TOPO_DOM    162    191       NON CYTOPLASMIC.
FT   TRANSMEM    192    213
FT   TOPO_DOM    214    228       CYTOPLASMIC.
FT   TRANSMEM    229    247
FT   TOPO_DOM    248    263       NON CYTOPLASMIC.
FT   TRANSMEM    264    285
FT   TOPO_DOM    286    313       CYTOPLASMIC.
//
```

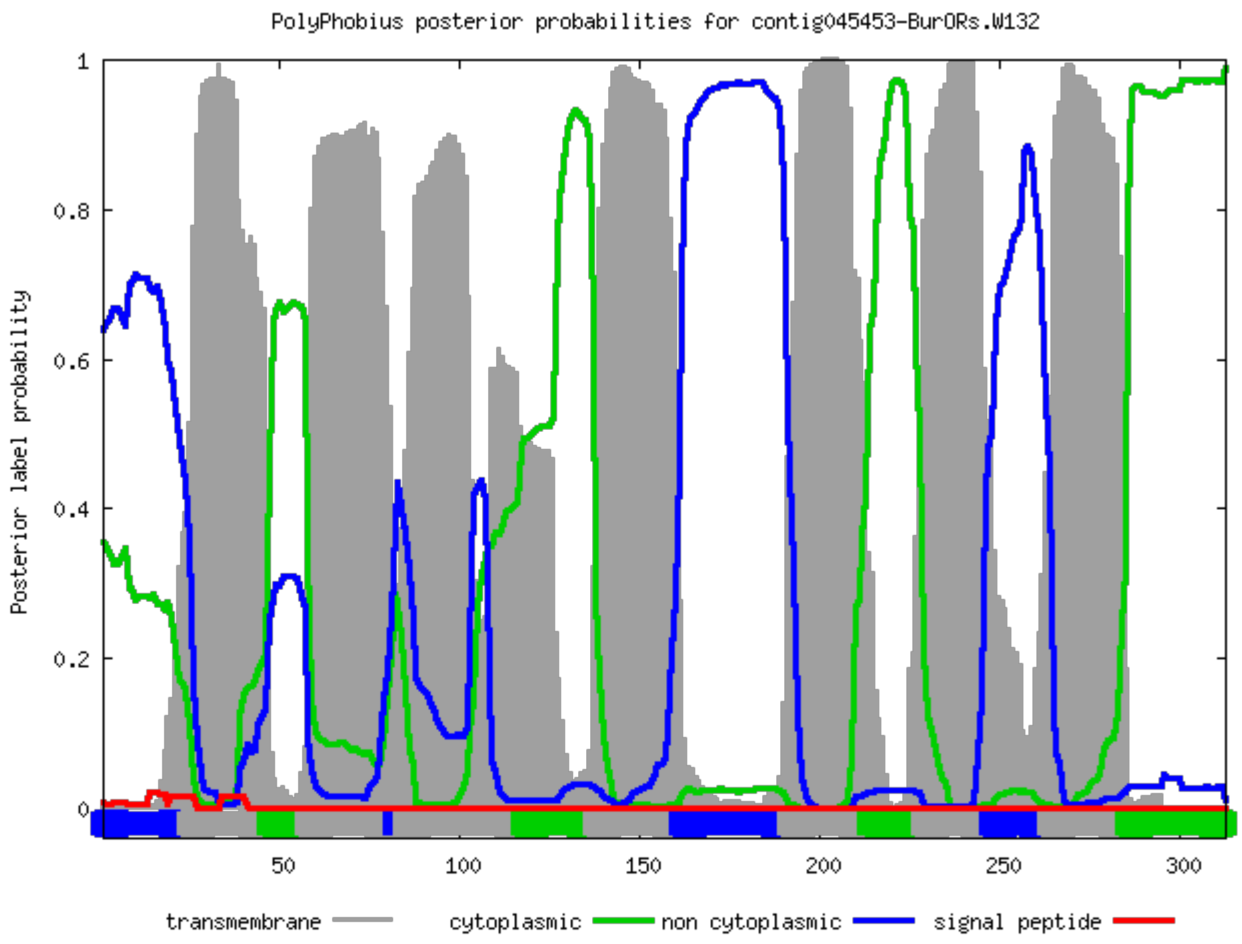

The prediction is based on an alignment. The probability data used in the plot is found here, and the gnuplot script is here.

Prediction of contig027194-TilORs.U236

```
ID    contig027194-TilORs.U236
FT    TOPO_DOM      1     28       NON CYTOPLASMIC.
FT    TRANSMEM     29     50
FT    TOPO_DOM     51     61       CYTOPLASMIC.
FT    TRANSMEM     62     85
FT    TOPO_DOM     86     87       NON CYTOPLASMIC.
FT    TRANSMEM     88    108
FT    TOPO_DOM    109    141       CYTOPLASMIC.
FT    TRANSMEM    142    165
FT    TOPO_DOM    166    195       NON CYTOPLASMIC.
FT    TRANSMEM    196    217
FT    TOPO_DOM    218    235       CYTOPLASMIC.
FT    TRANSMEM    236    253
FT    TOPO_DOM    254    271       NON CYTOPLASMIC.
FT    TRANSMEM    272    292
FT    TOPO_DOM    293    326       CYTOPLASMIC.
//
```

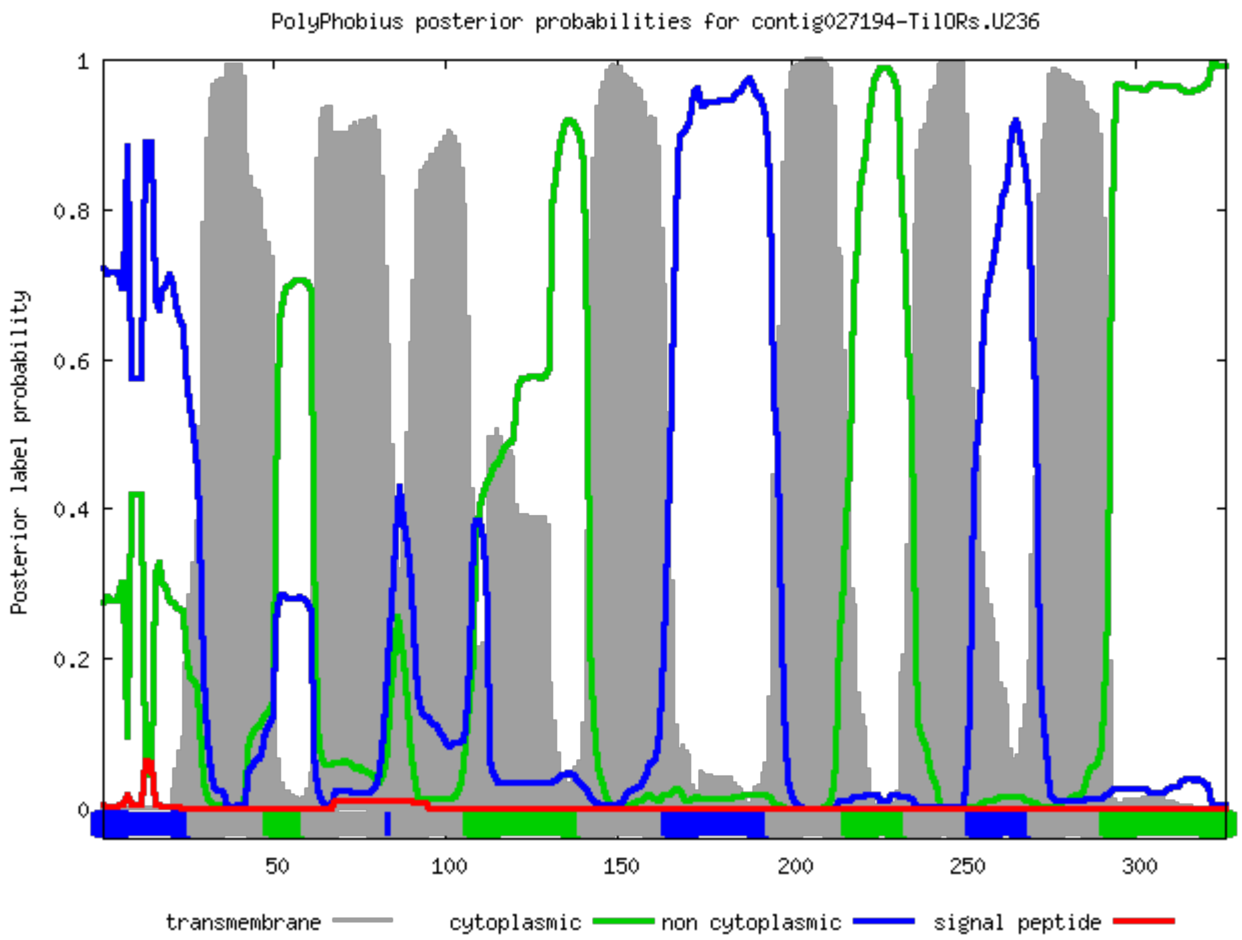

The prediction is based on an alignment. The probability data used in the plot is found here, and the gnuplot script is here.

Prediction of contig041640-BurORs.V144

```
ID   contig041640-BurORs.V144
FT   TOPO_DOM      1     34       NON CYTOPLASMIC.
FT   TRANSMEM     35     56
FT   TOPO_DOM     57     67       CYTOPLASMIC.
FT   TRANSMEM     68     92
FT   TOPO_DOM     93     94       NON CYTOPLASMIC.
FT   TRANSMEM     95    127
FT   TOPO_DOM    128    148       CYTOPLASMIC.
FT   TRANSMEM    149    172
FT   TOPO_DOM    173    202       NON CYTOPLASMIC.
FT   TRANSMEM    203    224
FT   TOPO_DOM    225    242       CYTOPLASMIC.
FT   TRANSMEM    243    262
FT   TOPO_DOM    263    278       NON CYTOPLASMIC.
FT   TRANSMEM    279    299
FT   TOPO_DOM    300    343       CYTOPLASMIC.
//
```

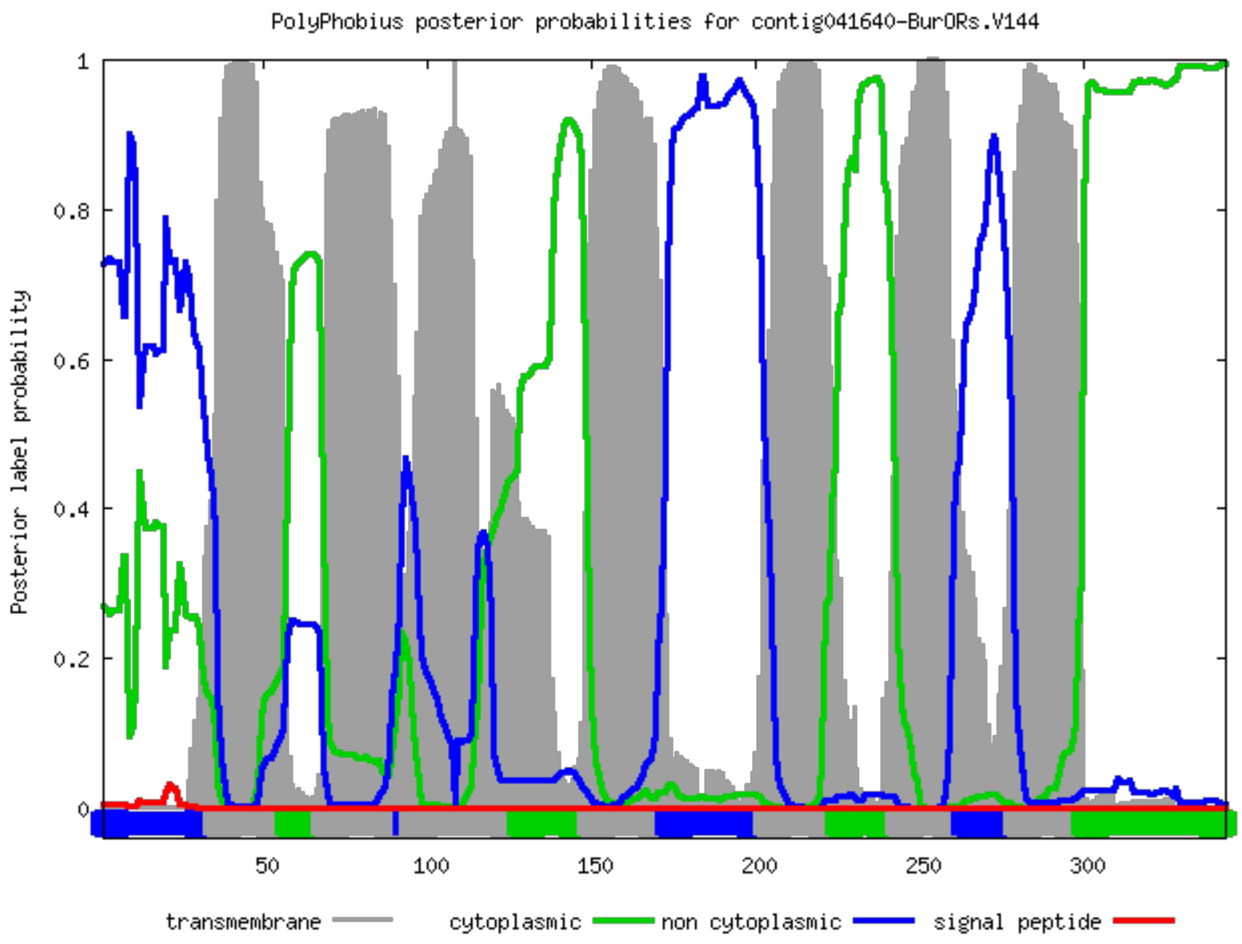

The prediction is based on an alignment. The probability data used in the plot is found here, and the gnuplot script is here.

Prediction of contig066785-BurORs.W148

```
ID   contig066785-BurORs.W148
FT   TOPO_DOM      1     25       NON CYTOPLASMIC.
FT   TRANSMEM     26     59
FT   TOPO_DOM     60     67       CYTOPLASMIC.
FT   TRANSMEM     68     91
FT   TOPO_DOM     92     94       NON CYTOPLASMIC.
FT   TRANSMEM     95    126
FT   TOPO_DOM    127    147       CYTOPLASMIC.
FT   TRANSMEM    148    171
FT   TOPO_DOM    172    200       NON CYTOPLASMIC.
FT   TRANSMEM    201    223
FT   TOPO_DOM    224    238       CYTOPLASMIC.
FT   TRANSMEM    239    258
FT   TOPO_DOM    259    273       NON CYTOPLASMIC.
FT   TRANSMEM    274    295
FT   TOPO_DOM    296    336       CYTOPLASMIC.
//
```

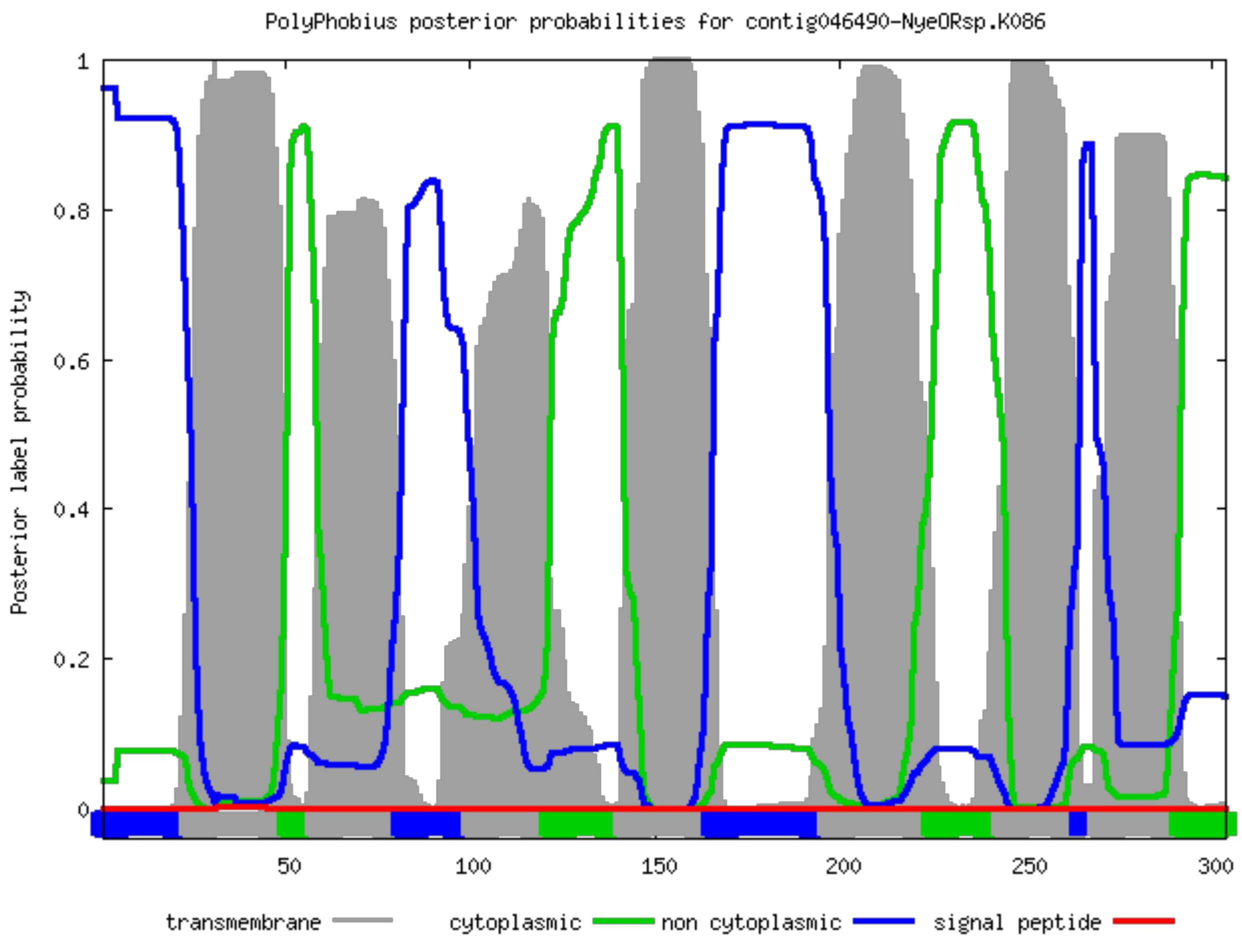

The prediction is based on an alignment. The probability data used in the plot is found here, and the gnuplot script is here.

Prediction of contig027206-TilORs.W240

```
ID   contig027206-TilORs.W240
FT   TOPO_DOM      1     24       NON CYTOPLASMIC.
FT   TRANSMEM     25     46
FT   TOPO_DOM     47     57       CYTOPLASMIC.
FT   TRANSMEM     58     81
FT   TOPO_DOM     82     84       NON CYTOPLASMIC.
FT   TRANSMEM     85    104
FT   TOPO_DOM    105    137       CYTOPLASMIC.
FT   TRANSMEM    138    161
FT   TOPO_DOM    162    191       NON CYTOPLASMIC.
FT   TRANSMEM    192    213
FT   TOPO_DOM    214    228       CYTOPLASMIC.
FT   TRANSMEM    229    247
FT   TOPO_DOM    248    263       NON CYTOPLASMIC.
FT   TRANSMEM    264    285
FT   TOPO_DOM    286    305       CYTOPLASMIC.
//
```

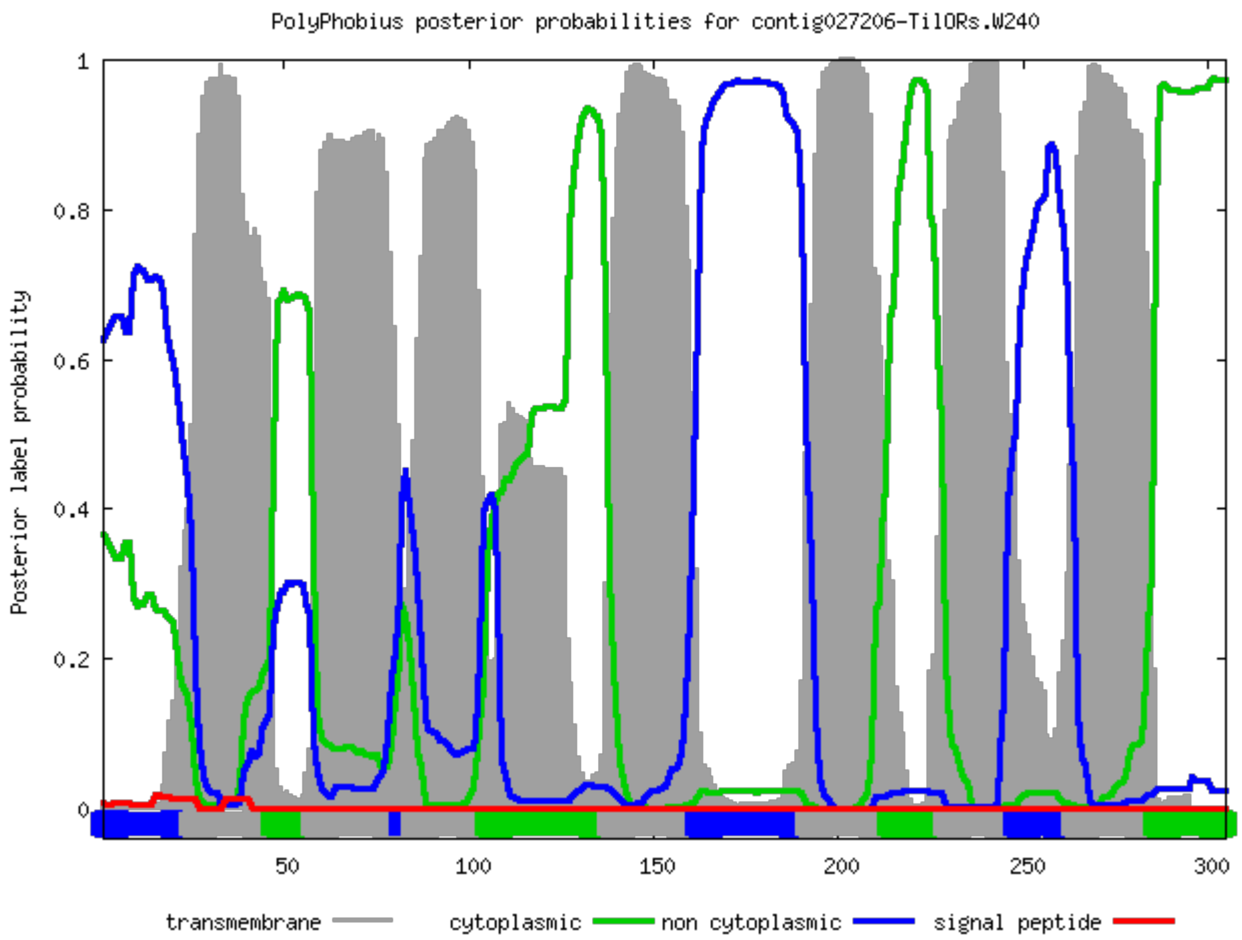

The prediction is based on an alignment. The probability data used in the plot is found here, and the gnuplot script is here.

Prediction of contig050026-NyeORs.W132

```
ID    contig050026-NyeORs.W132
FT    TOPO_DOM      1      26       NON CYTOPLASMIC.
FT    TRANSMEM     27      48
FT    TOPO_DOM     49      59       CYTOPLASMIC.
FT    TRANSMEM     60      83
FT    TOPO_DOM     84      86       NON CYTOPLASMIC.
FT    TRANSMEM     87     118
FT    TOPO_DOM    119     139       CYTOPLASMIC.
FT    TRANSMEM    140     163
FT    TOPO_DOM    164     193       NON CYTOPLASMIC.
FT    TRANSMEM    194     215
FT    TOPO_DOM    216     233       CYTOPLASMIC.
FT    TRANSMEM    234     253
FT    TOPO_DOM    254     268       NON CYTOPLASMIC.
FT    TRANSMEM    269     290
FT    TOPO_DOM    291     318       CYTOPLASMIC.
//
```

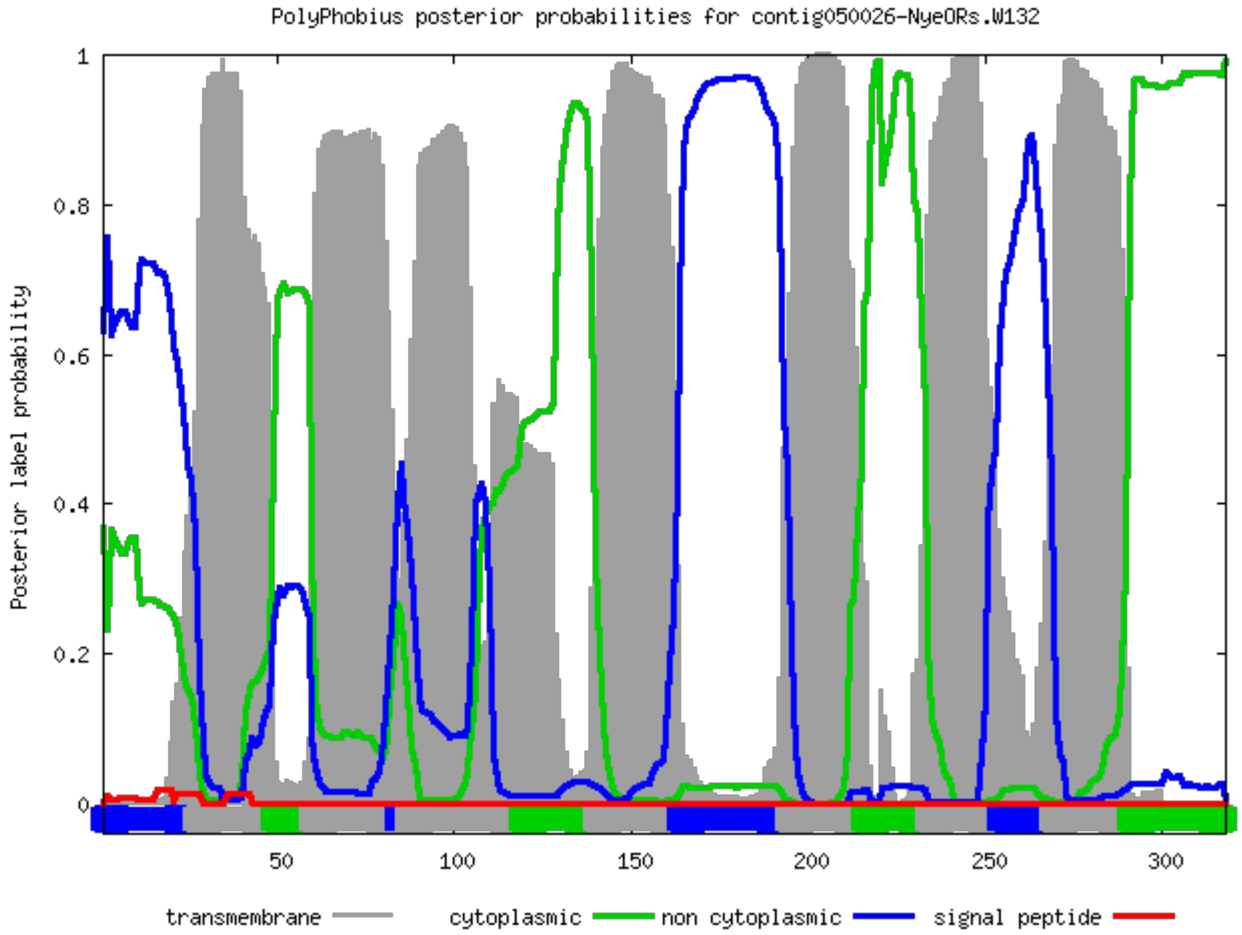

The prediction is based on an alignment. The probability data used in the plot is found here, and the gnuplot script is here.

Prediction of contig067811-ZebORs.W140

```
ID    contig067811-ZebORs.W140
FT    TOPO_DOM      1      24       NON CYTOPLASMIC.
FT    TRANSMEM     25      46
FT    TOPO_DOM     47      57       CYTOPLASMIC.
FT    TRANSMEM     58      81
FT    TOPO_DOM     82      84       NON CYTOPLASMIC.
FT    TRANSMEM     85     104
FT    TOPO_DOM    105     137       CYTOPLASMIC.
FT    TRANSMEM    138     161
FT    TOPO_DOM    162     191       NON CYTOPLASMIC.
FT    TRANSMEM    192     213
FT    TOPO_DOM    214     228       CYTOPLASMIC.
FT    TRANSMEM    229     247
FT    TOPO_DOM    248     263       NON CYTOPLASMIC.
FT    TRANSMEM    264     285
FT    TOPO_DOM    286     305       CYTOPLASMIC.
//
```

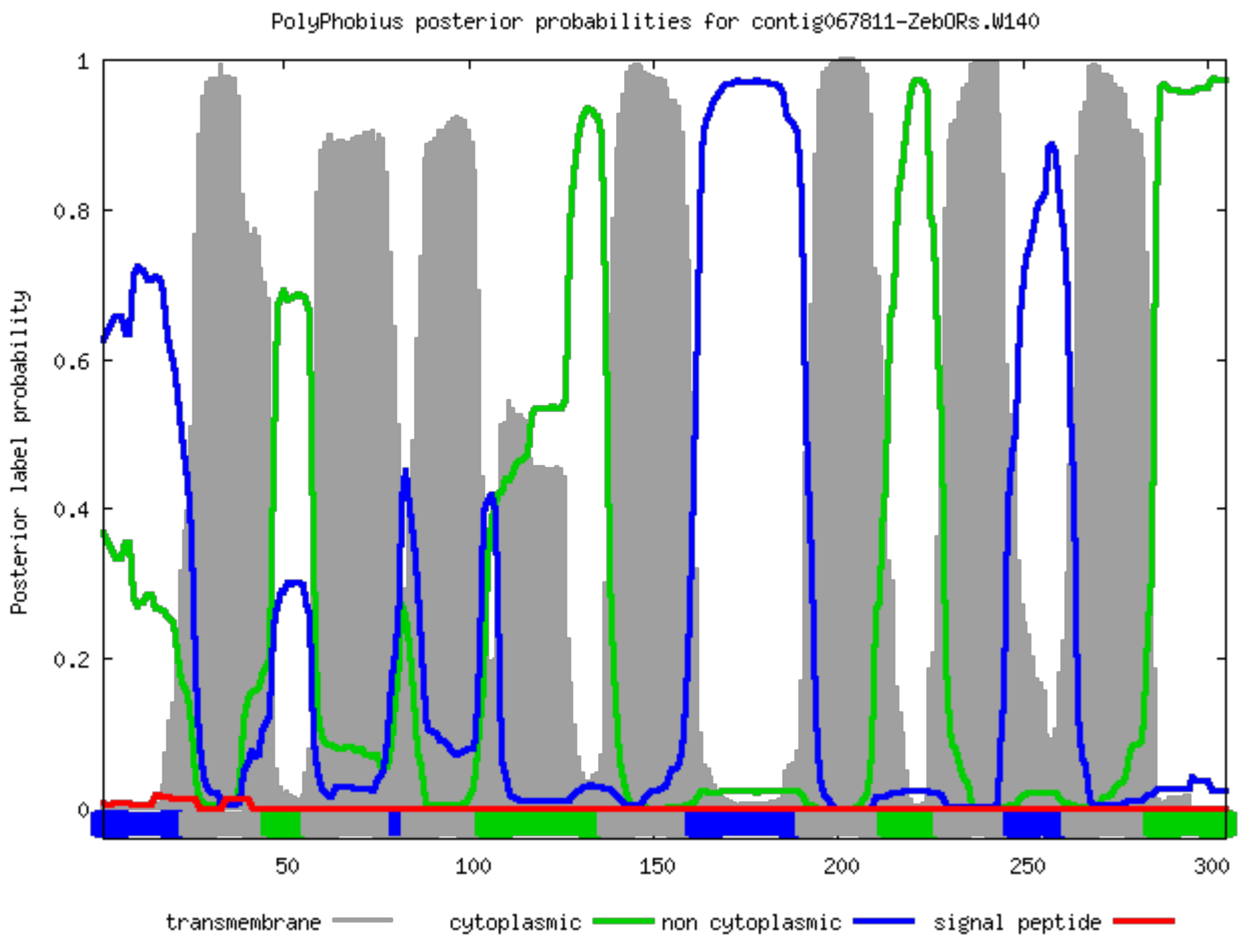

The prediction is based on an alignment. The probability data used in the plot is found here, and the gnuplot script is here.

Prediction of contig041640-BurORs.U130

```
ID    contig041640-BurORs.U130
FT    TOPO_DOM      1     30       NON CYTOPLASMIC.
FT    TRANSMEM     31     52
FT    TOPO_DOM     53     63       CYTOPLASMIC.
FT    TRANSMEM     64     87
FT    TOPO_DOM     88     89       NON CYTOPLASMIC.
FT    TRANSMEM     90    110
FT    TOPO_DOM    111    143       CYTOPLASMIC.
FT    TRANSMEM    144    167
FT    TOPO_DOM    168    197       NON CYTOPLASMIC.
FT    TRANSMEM    198    219
FT    TOPO_DOM    220    237       CYTOPLASMIC.
FT    TRANSMEM    238    255
FT    TOPO_DOM    256    273       NON CYTOPLASMIC.
FT    TRANSMEM    274    294
FT    TOPO_DOM    295    328       CYTOPLASMIC.
//
```

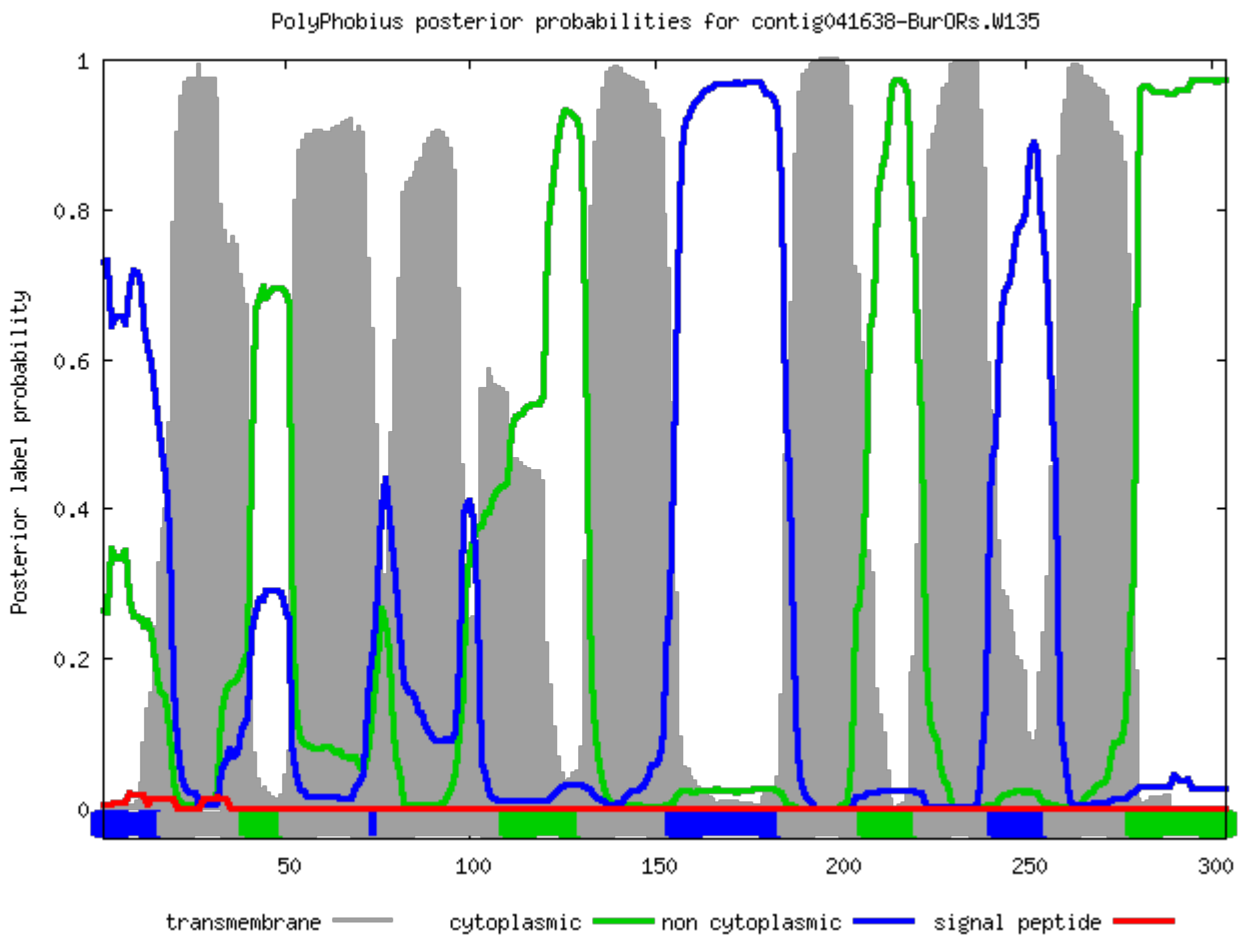

The prediction is based on an alignment. The probability data used in the plot is found here, and the gnuplot script is here.

Prediction of contig050024-NyeORs.W129

```
ID   contig050024-NyeORs.W129
FT   TOPO_DOM      1     24       NON CYTOPLASMIC.
FT   TRANSMEM     25     46
FT   TOPO_DOM     47     58       CYTOPLASMIC.
FT   TRANSMEM     59     82
FT   TOPO_DOM     83     85       NON CYTOPLASMIC.
FT   TRANSMEM     86    105
FT   TOPO_DOM    106    138       CYTOPLASMIC.
FT   TRANSMEM    139    162
FT   TOPO_DOM    163    192       NON CYTOPLASMIC.
FT   TRANSMEM    193    214
FT   TOPO_DOM    215    229       CYTOPLASMIC.
FT   TRANSMEM    230    248
FT   TOPO_DOM    249    264       NON CYTOPLASMIC.
FT   TRANSMEM    265    286
FT   TOPO_DOM    287    306       CYTOPLASMIC.
//
```

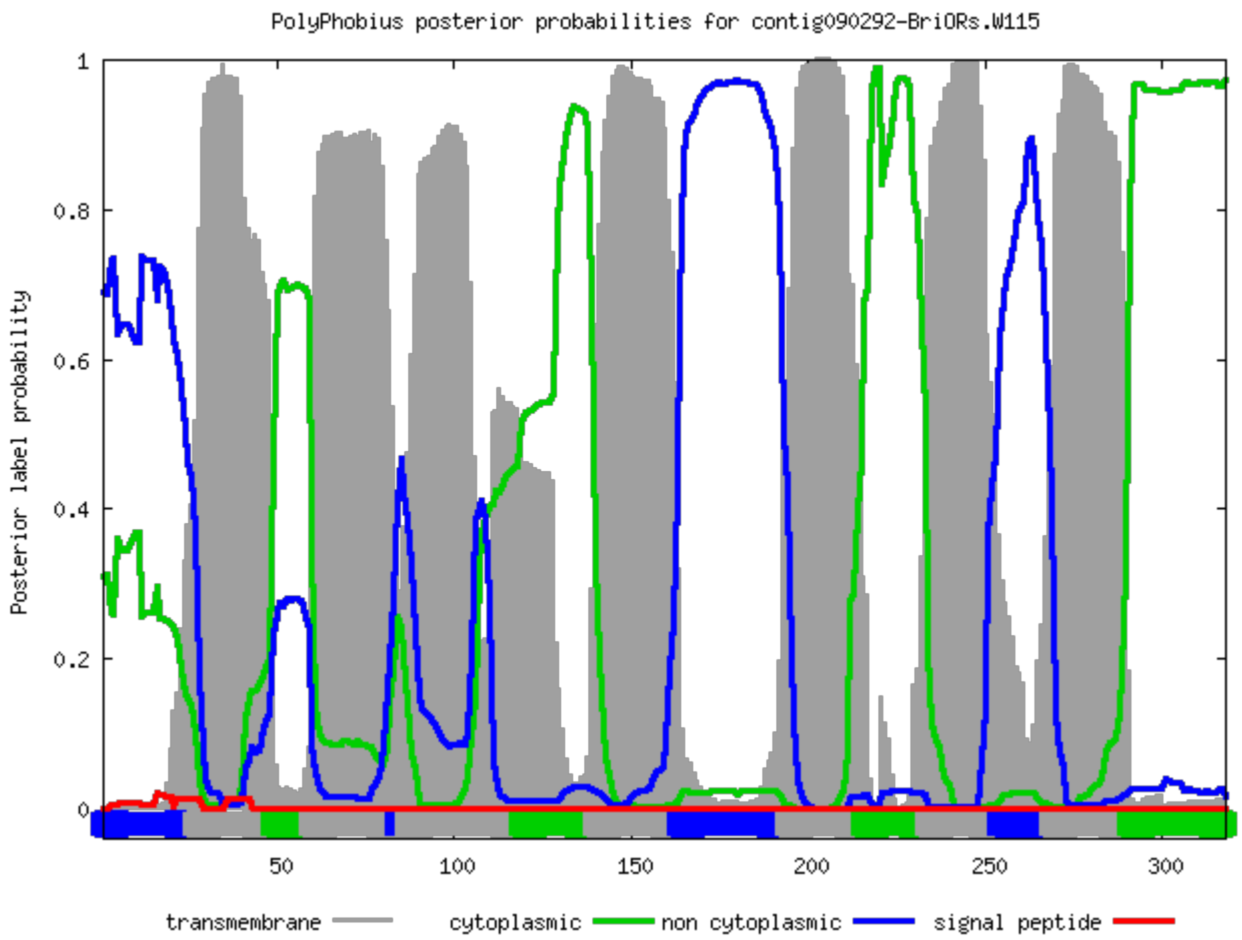

The prediction is based on an alignment. The probability data used in the plot is found here, and the gnuplot script is here.

Prediction of contig009773-BurORs.Q136

```
ID   contig009773-BurORs.Q136
FT   TOPO_DOM      1     17       NON CYTOPLASMIC.
FT   TRANSMEM     18     40
FT   TOPO_DOM     41     51       CYTOPLASMIC.
FT   TRANSMEM     52     76
FT   TOPO_DOM     77     81       NON CYTOPLASMIC.
FT   TRANSMEM     82    110
FT   TOPO_DOM    111    130       CYTOPLASMIC.
FT   TRANSMEM    131    154
FT   TOPO_DOM    155    183       NON CYTOPLASMIC.
FT   TRANSMEM    184    206
FT   TOPO_DOM    207    224       CYTOPLASMIC.
FT   TRANSMEM    225    246
FT   TOPO_DOM    247    259       NON CYTOPLASMIC.
FT   TRANSMEM    260    281
FT   TOPO_DOM    282    310       CYTOPLASMIC.
//
```

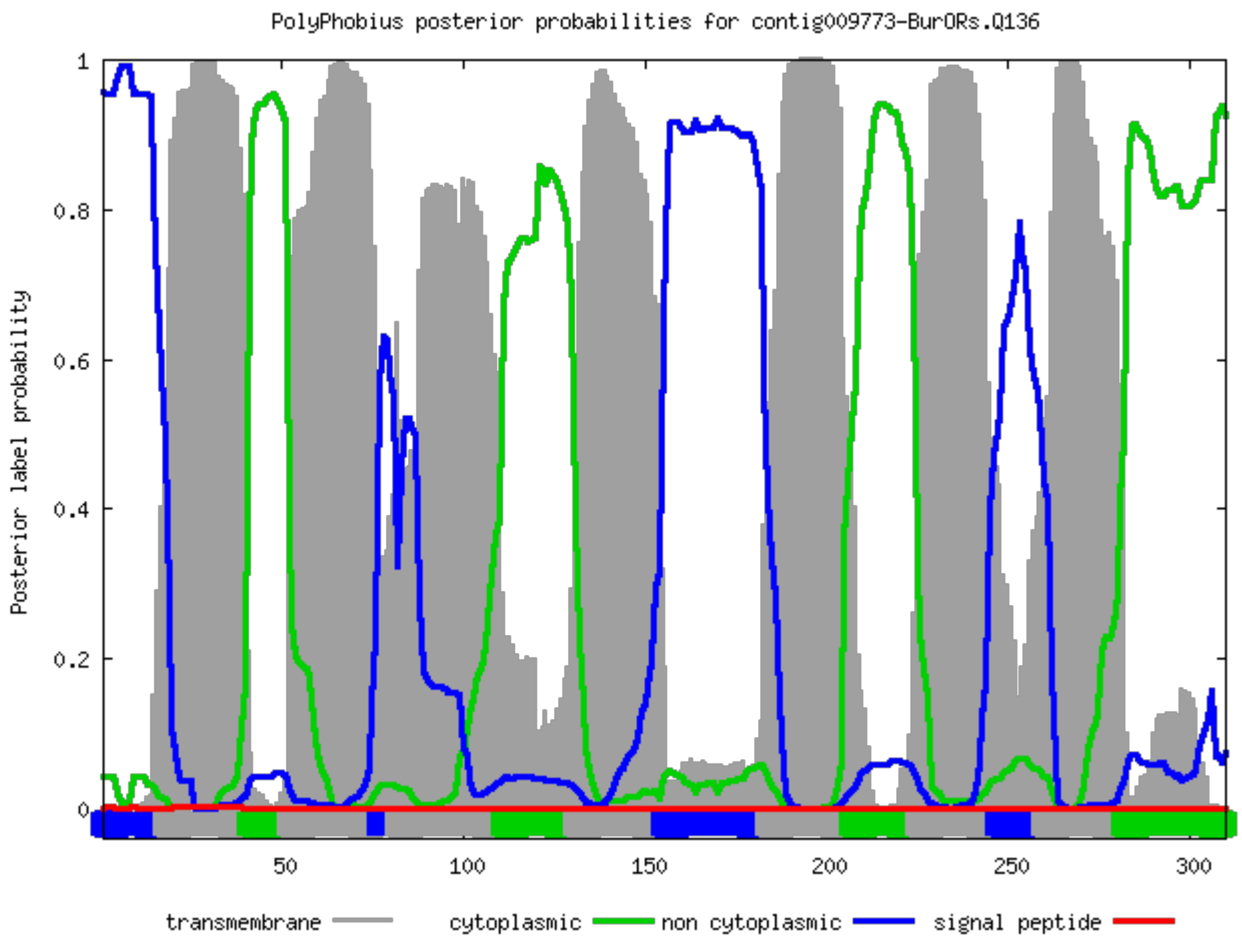

The prediction is based on an alignment. The probability data used in the plot is found here, and the gnuplot script is here.

Prediction of contig049605-BurORs.AB153

```
ID    contig049605-BurORs.AB153
FT    TOPO_DOM      1      48       NON CYTOPLASMIC.
FT    TRANSMEM     49      73
FT    TOPO_DOM     74      94       CYTOPLASMIC.
FT    TRANSMEM     95     114
FT    TOPO_DOM    115     132       NON CYTOPLASMIC.
FT    TRANSMEM    133     155
FT    TOPO_DOM    156     174       CYTOPLASMIC.
FT    TRANSMEM    175     199
FT    TOPO_DOM    200     233       NON CYTOPLASMIC.
FT    TRANSMEM    234     256
FT    TOPO_DOM    257     273       CYTOPLASMIC.
FT    TRANSMEM    274     295
FT    TOPO_DOM    296     306       NON CYTOPLASMIC.
FT    TRANSMEM    307     334
FT    TOPO_DOM    335     345       CYTOPLASMIC.
//
```

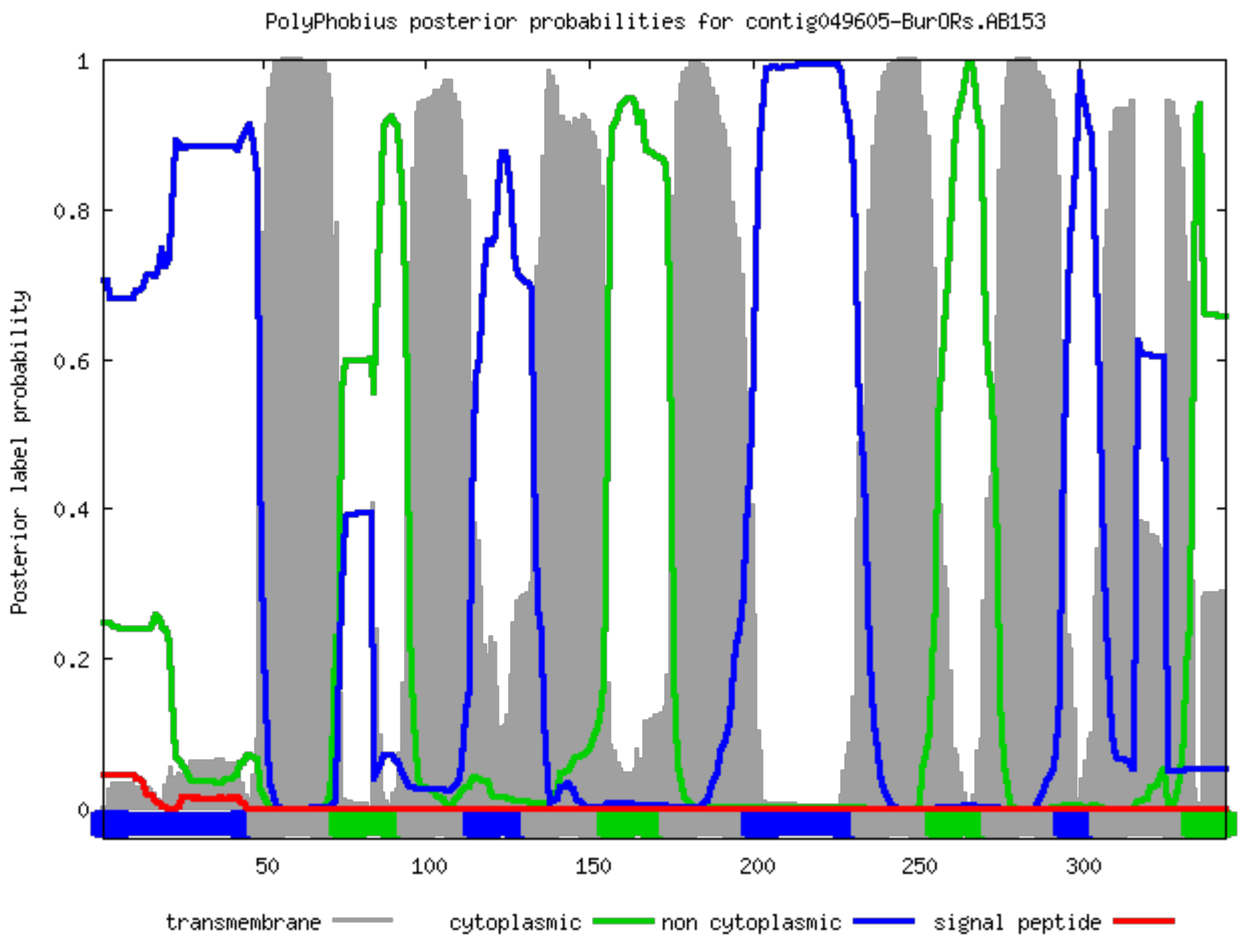

The prediction is based on an alignment. The probability data used in the plot is found here, and the gnuplot script is here.

Prediction of contig046717-TilORs.AB275

```
ID   contig046717-TilORs.AB275
FT   TOPO_DOM      1    48       NON CYTOPLASMIC.
FT   TRANSMEM     49    74
FT   TOPO_DOM     75    75       CYTOPLASMIC.
FT   TRANSMEM     76    92
FT   TOPO_DOM     93    93       NON CYTOPLASMIC.
FT   TRANSMEM     94   124
FT   TOPO_DOM    125   146       CYTOPLASMIC.
FT   TRANSMEM    147   169
FT   TOPO_DOM    170   204       NON CYTOPLASMIC.
FT   TRANSMEM    205   227
FT   TOPO_DOM    228   244       CYTOPLASMIC.
FT   TRANSMEM    245   265
FT   TOPO_DOM    266   276       NON CYTOPLASMIC.
FT   TRANSMEM    277   302
FT   TOPO_DOM    303   317       CYTOPLASMIC.
FT   TRANSMEM    318   332
FT   TOPO_DOM    333   333       NON CYTOPLASMIC.
FT   TRANSMEM    334   348
FT   TOPO_DOM    349   363       CYTOPLASMIC.
//
```

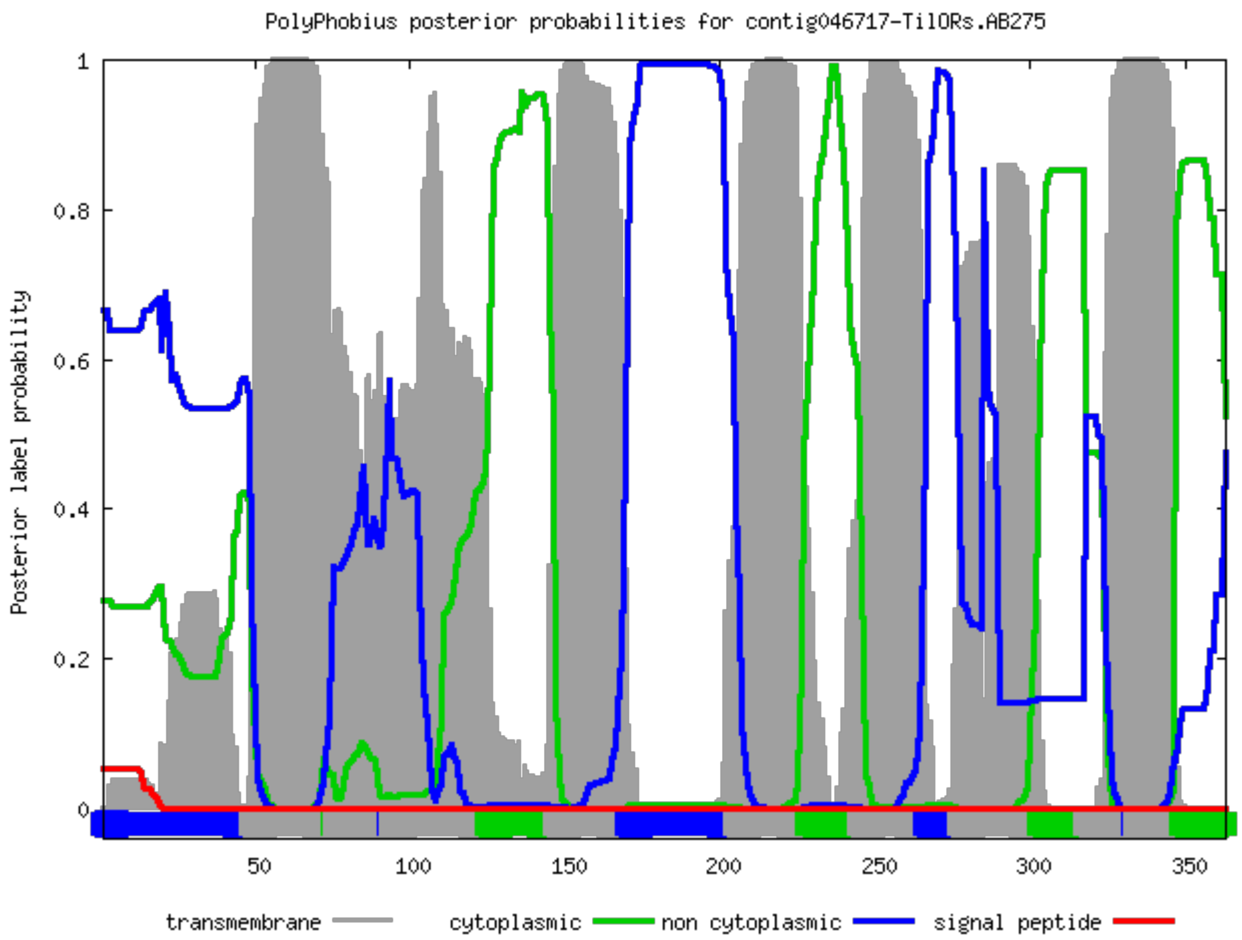

The prediction is based on an alignment. The probability data used in the plot is found here, and the gnuplot script is here.

Prediction of contig050025-NyeORs.W131

```
ID   contig050025-NyeORs.W131
FT   TOPO_DOM      1     26       NON CYTOPLASMIC.
FT   TRANSMEM     27     48
FT   TOPO_DOM     49     58       CYTOPLASMIC.
FT   TRANSMEM     59     82
FT   TOPO_DOM     83     85       NON CYTOPLASMIC.
FT   TRANSMEM     86    117
FT   TOPO_DOM    118    138       CYTOPLASMIC.
FT   TRANSMEM    139    162
FT   TOPO_DOM    163    192       NON CYTOPLASMIC.
FT   TRANSMEM    193    214
FT   TOPO_DOM    215    229       CYTOPLASMIC.
FT   TRANSMEM    230    248
FT   TOPO_DOM    249    264       NON CYTOPLASMIC.
FT   TRANSMEM    265    286
FT   TOPO_DOM    287    313       CYTOPLASMIC.
//
```

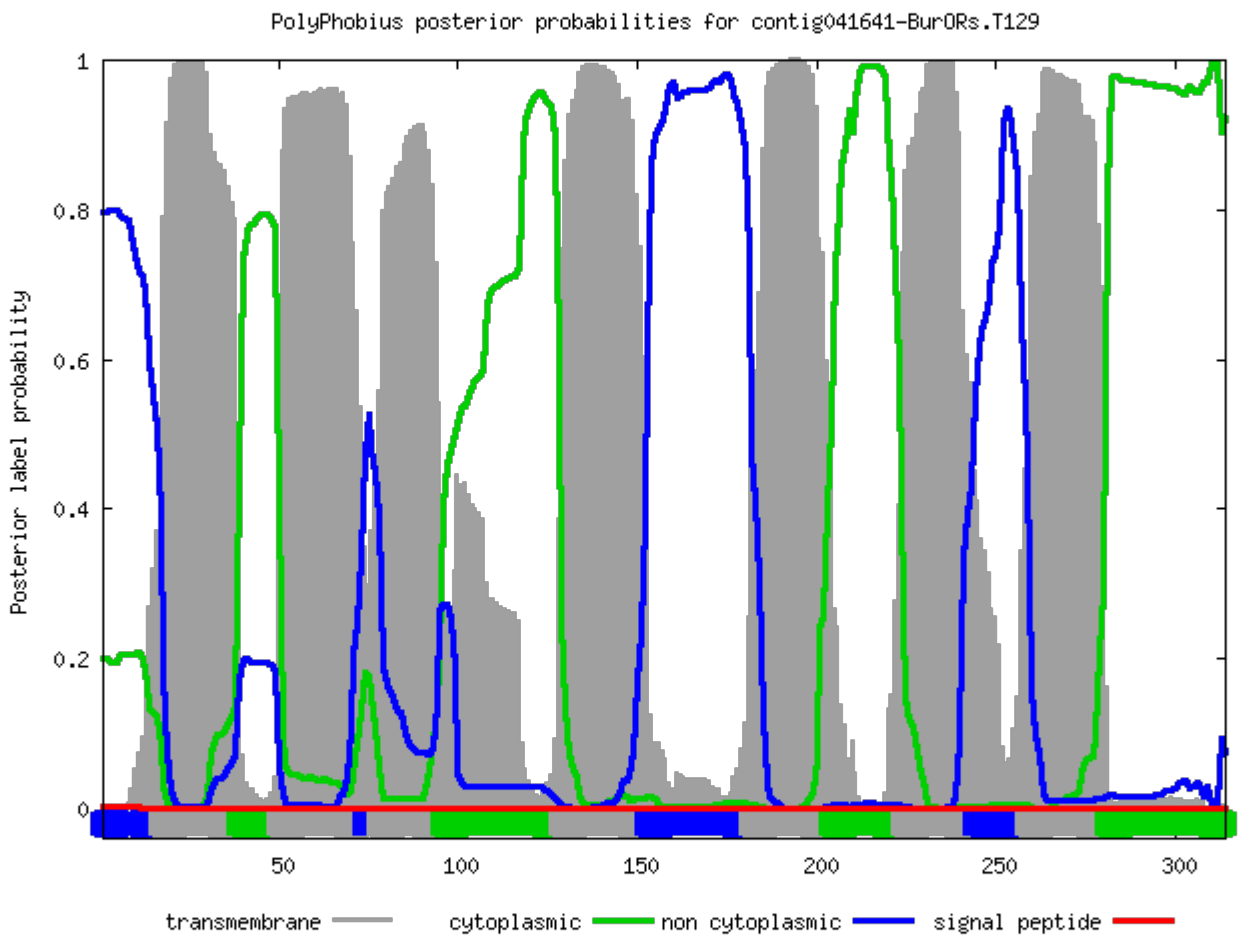

The prediction is based on an alignment. The probability data used in the plot is found here, and the gnuplot script is here.

Prediction of contig045453-BurORs.W134

```
ID   contig045453-BurORs.W134
FT   TOPO_DOM      1     26       NON CYTOPLASMIC.
FT   TRANSMEM     27     48
FT   TOPO_DOM     49     61       CYTOPLASMIC.
FT   TRANSMEM     62     85
FT   TOPO_DOM     86     88       NON CYTOPLASMIC.
FT   TRANSMEM     89    121
FT   TOPO_DOM    122    141       CYTOPLASMIC.
FT   TRANSMEM    142    165
FT   TOPO_DOM    166    195       NON CYTOPLASMIC.
FT   TRANSMEM    196    217
FT   TOPO_DOM    218    232       CYTOPLASMIC.
FT   TRANSMEM    233    251
FT   TOPO_DOM    252    267       NON CYTOPLASMIC.
FT   TRANSMEM    268    289
FT   TOPO_DOM    290    316       CYTOPLASMIC.
//
```

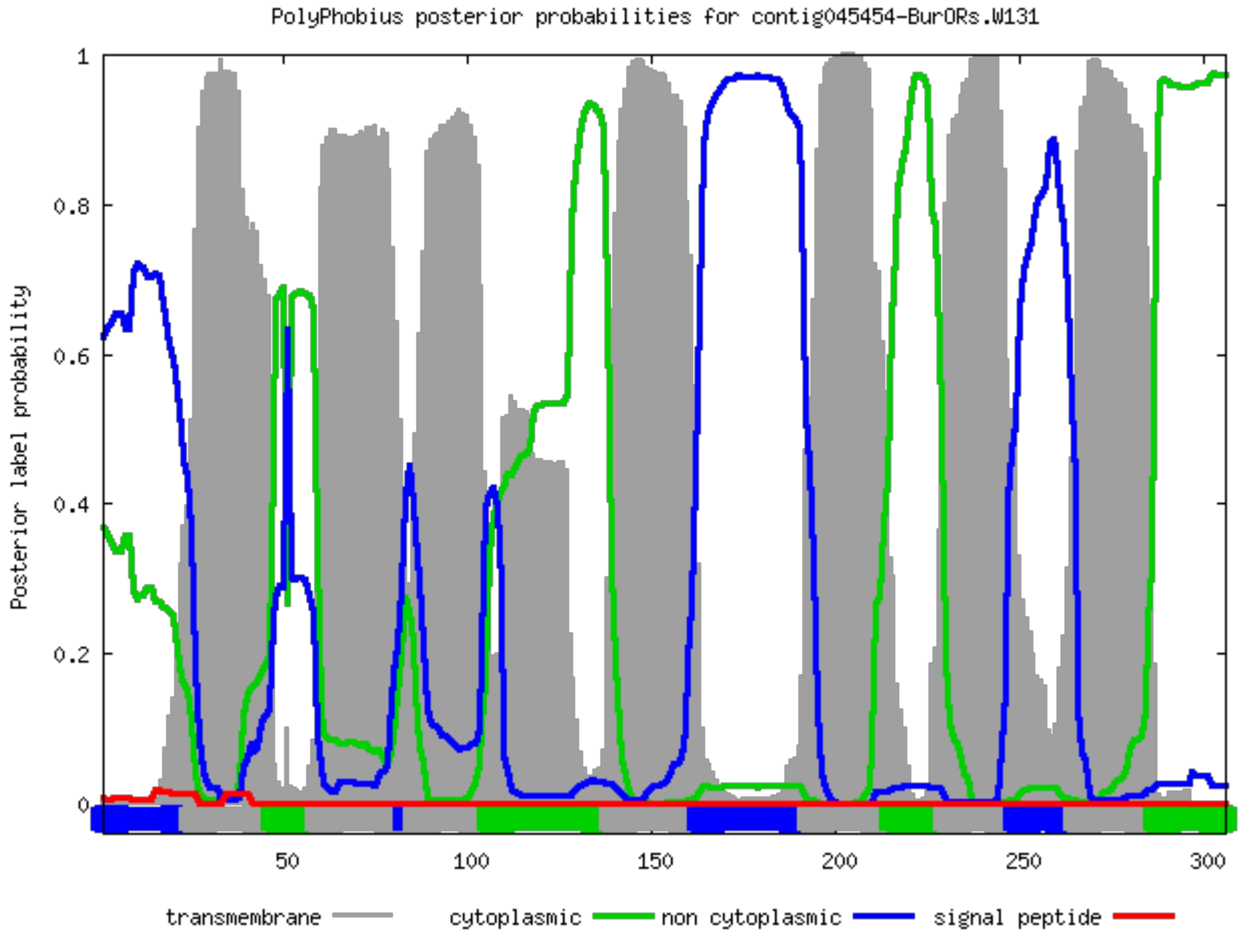

The prediction is based on an alignment. The probability data used in the plot is found here, and the gnuplot script is here.

Prediction of contig057756-NyeOR.A019

```
ID    contig057756-NyeOR.A019
FT    TOPO_DOM      1      22       NON CYTOPLASMIC.
FT    TRANSMEM     23      48
FT    TOPO_DOM     49      56       CYTOPLASMIC.
FT    TRANSMEM     57      77
FT    TOPO_DOM     78      95       NON CYTOPLASMIC.
FT    TRANSMEM     96     118
FT    TOPO_DOM    119     138       CYTOPLASMIC.
FT    TRANSMEM    139     159
FT    TOPO_DOM    160     192       NON CYTOPLASMIC.
FT    TRANSMEM    193     215
FT    TOPO_DOM    216     235       CYTOPLASMIC.
FT    TRANSMEM    236     257
FT    TOPO_DOM    258     268       NON CYTOPLASMIC.
FT    TRANSMEM    269     289
FT    TOPO_DOM    290     307       CYTOPLASMIC.
//
```

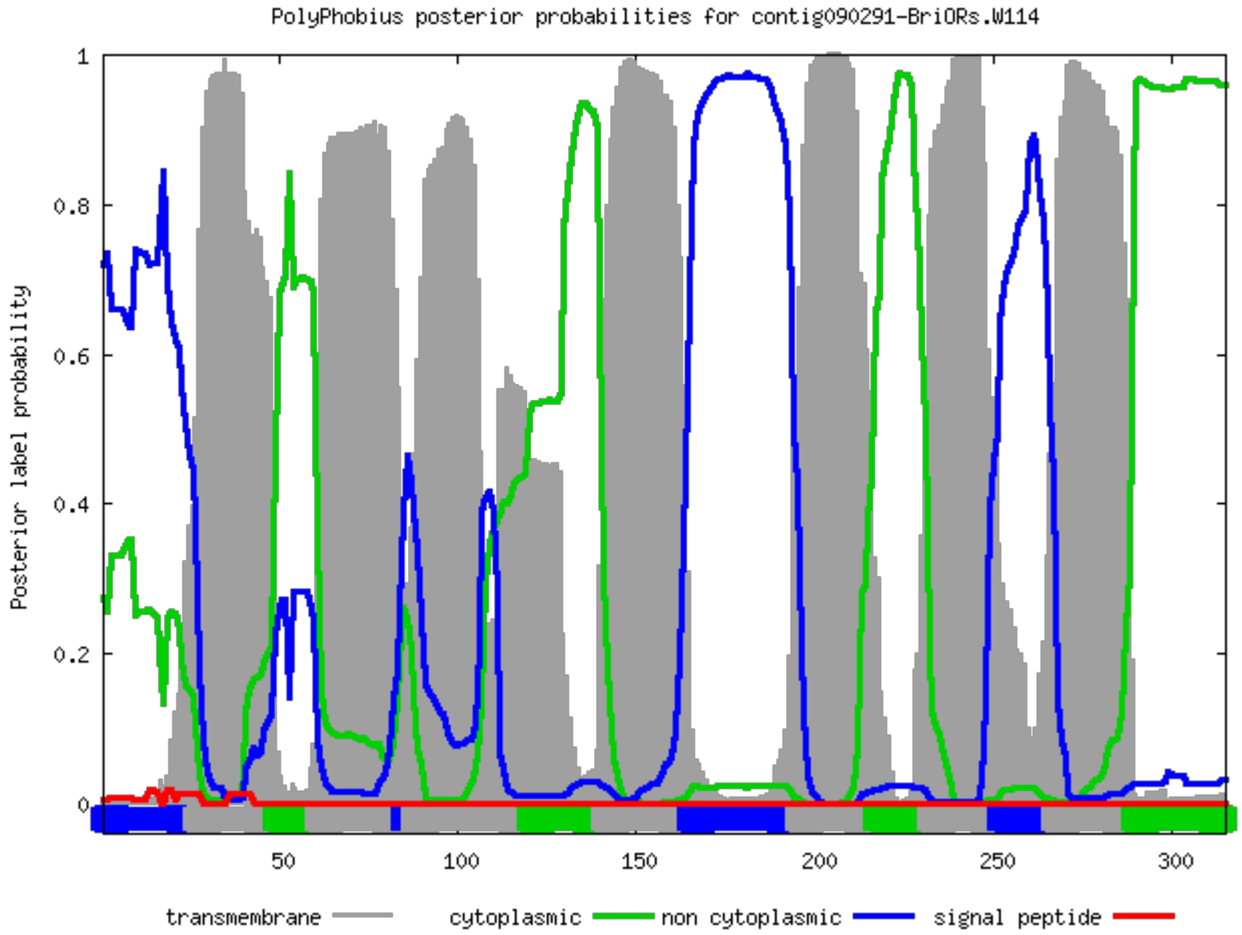

The prediction is based on an alignment. The probability data used in the plot is found here, and the gnuplot script is here.

Prediction of contig042475-BriOR.S102

```
ID   contig042475-BriOR.S102
FT   TOPO_DOM      1     21       NON CYTOPLASMIC.
FT   TRANSMEM     22     43
FT   TOPO_DOM     44     53       CYTOPLASMIC.
FT   TRANSMEM     54     78
FT   TOPO_DOM     79     83       NON CYTOPLASMIC.
FT   TRANSMEM     84    112
FT   TOPO_DOM    113    132       CYTOPLASMIC.
FT   TRANSMEM    133    158
FT   TOPO_DOM    159    186       NON CYTOPLASMIC.
FT   TRANSMEM    187    207
FT   TOPO_DOM    208    227       CYTOPLASMIC.
FT   TRANSMEM    228    253
FT   TOPO_DOM    254    264       NON CYTOPLASMIC.
FT   TRANSMEM    265    285
FT   TOPO_DOM    286    311       CYTOPLASMIC.
//
```

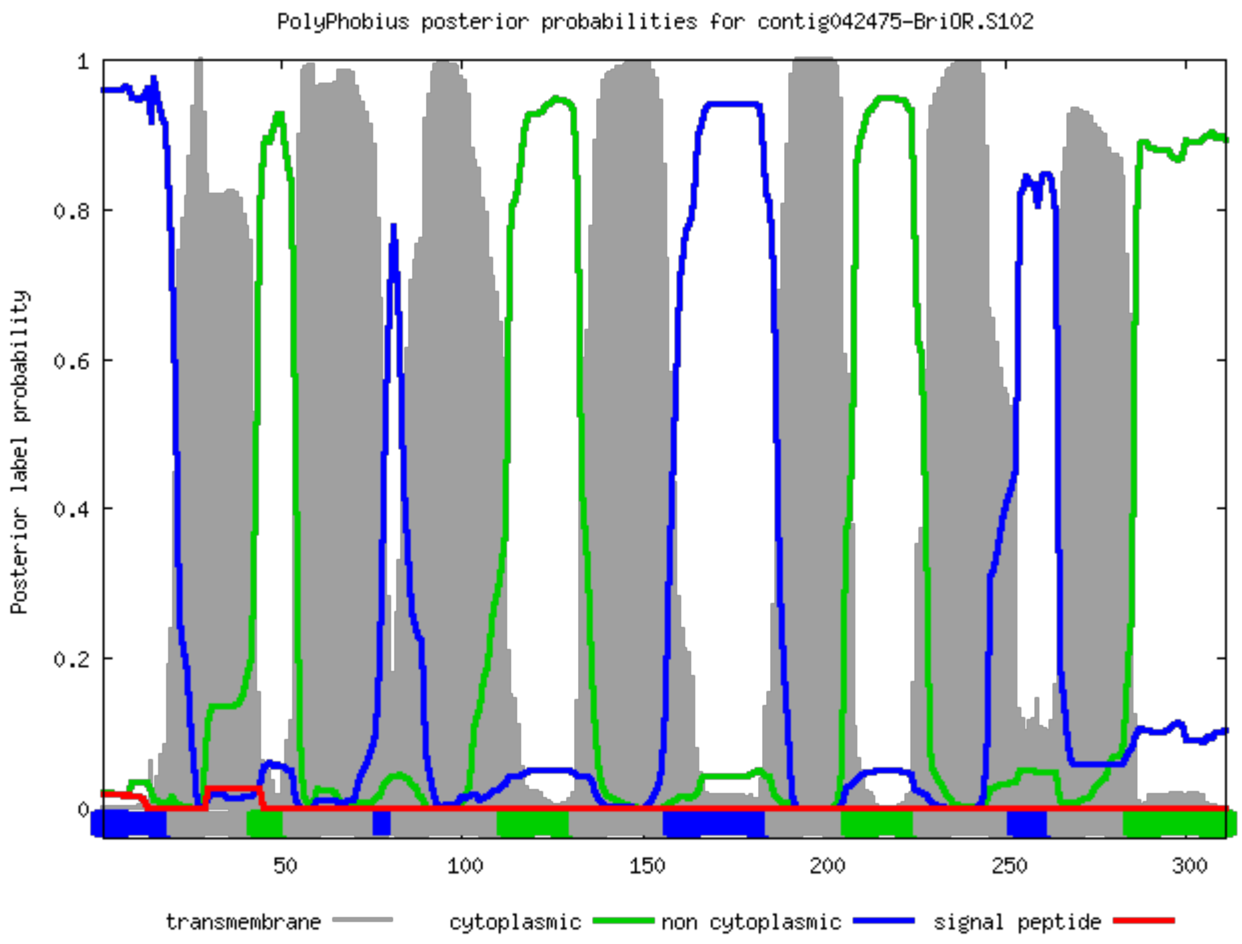

The prediction is based on an alignment. The probability data used in the plot is found here, and the gnuplot script is here.

Prediction of contig049299-BurOR.E046

```
ID   contig049299-BurOR.E046
FT   TOPO_DOM      1     52       NON CYTOPLASMIC.
FT   TRANSMEM     53     78
FT   TOPO_DOM     79     87       CYTOPLASMIC.
FT   TRANSMEM     88    113
FT   TOPO_DOM    114    123       NON CYTOPLASMIC.
FT   TRANSMEM    124    148
FT   TOPO_DOM    149    168       CYTOPLASMIC.
FT   TRANSMEM    169    191
FT   TOPO_DOM    192    223       NON CYTOPLASMIC.
FT   TRANSMEM    224    246
FT   TOPO_DOM    247    266       CYTOPLASMIC.
FT   TRANSMEM    267    286
FT   TOPO_DOM    287    297       NON CYTOPLASMIC.
FT   TRANSMEM    298    321
FT   TOPO_DOM    322    337       CYTOPLASMIC.
//
```

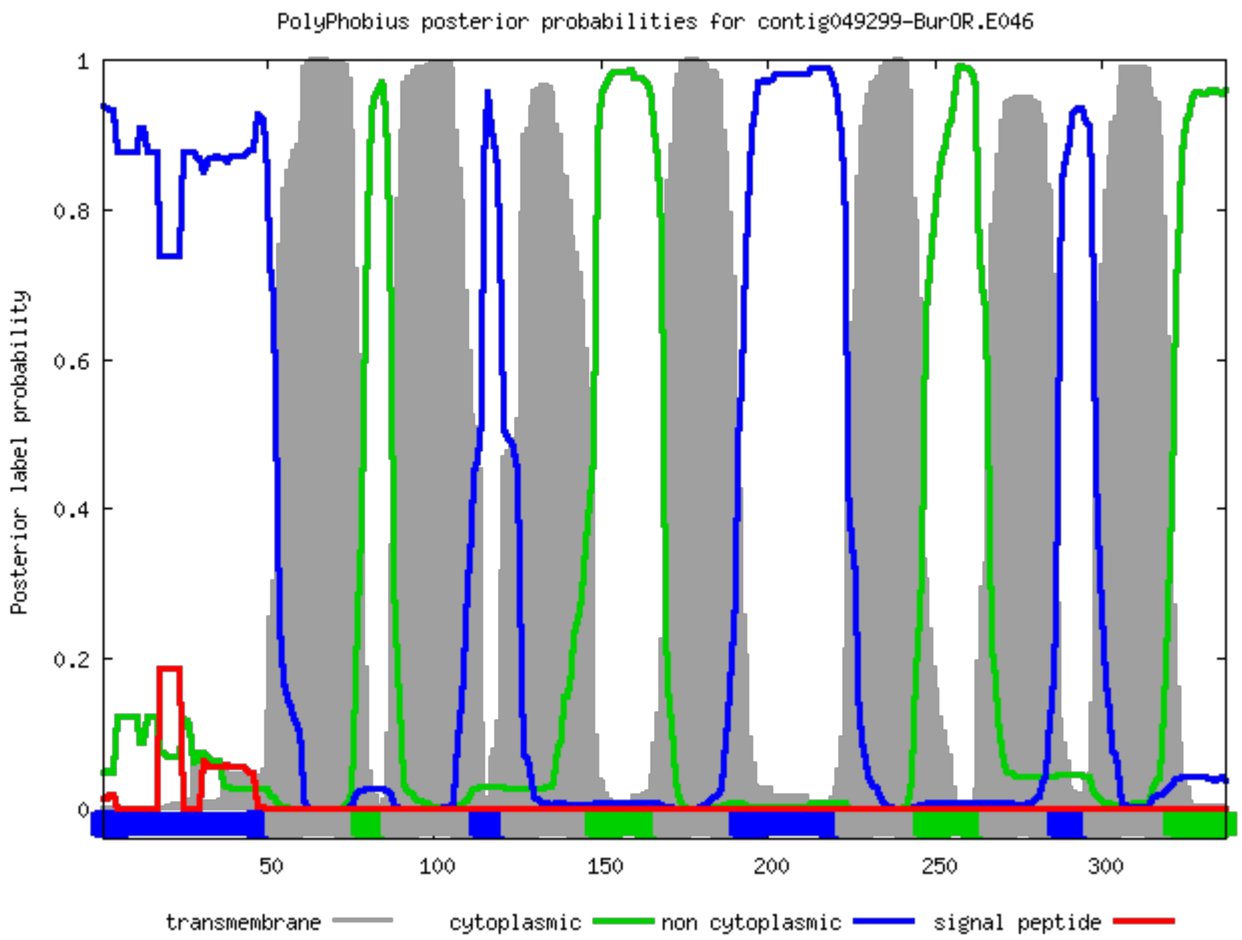

The prediction is based on an alignment. The probability data used in the plot is found here, and the gnuplot script is here.

Prediction of contig054684-NyeOR.A012

```
ID   contig054684-NyeOR.A012
FT   TOPO_DOM      1     25       NON CYTOPLASMIC.
FT   TRANSMEM     26     51
FT   TOPO_DOM     52     59       CYTOPLASMIC.
FT   TRANSMEM     60     80
FT   TOPO_DOM     81     98       NON CYTOPLASMIC.
FT   TRANSMEM     99    121
FT   TOPO_DOM    122    141       CYTOPLASMIC.
FT   TRANSMEM    142    163
FT   TOPO_DOM    164    195       NON CYTOPLASMIC.
FT   TRANSMEM    196    219
FT   TOPO_DOM    220    239       CYTOPLASMIC.
FT   TRANSMEM    240    261
FT   TOPO_DOM    262    272       NON CYTOPLASMIC.
FT   TRANSMEM    273    293
FT   TOPO_DOM    294    319       CYTOPLASMIC.
//
```

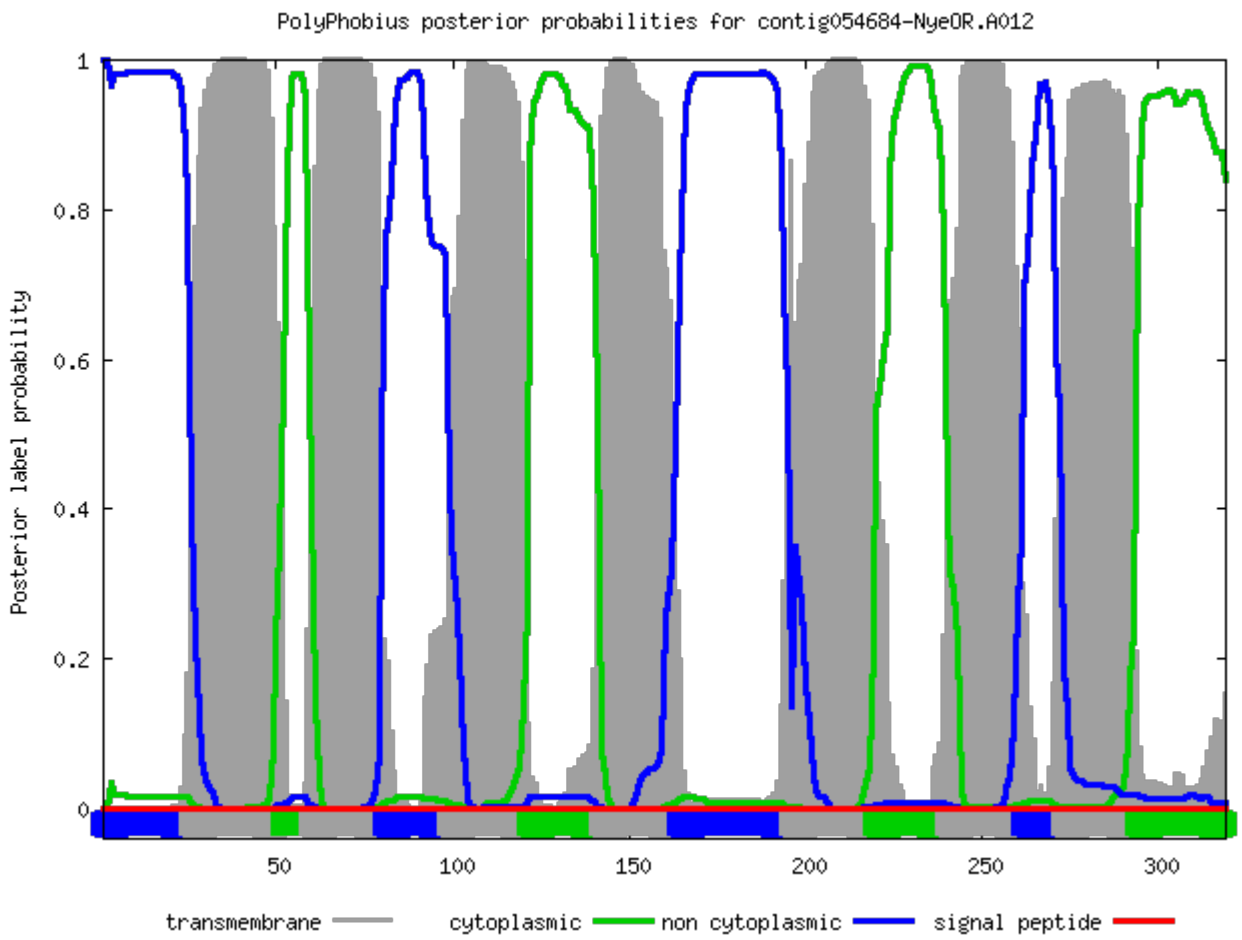

The prediction is based on an alignment. The probability data used in the plot is found here, and the gnuplot script is here.

Prediction of contig106096-BriOR.N089

```
ID   contig106096-BriOR.N089
FT   TOPO_DOM      1     32       NON CYTOPLASMIC.
FT   TRANSMEM     33     58
FT   TOPO_DOM     59     66       CYTOPLASMIC.
FT   TRANSMEM     67     87
FT   TOPO_DOM     88    105       NON CYTOPLASMIC.
FT   TRANSMEM    106    127
FT   TOPO_DOM    128    146       CYTOPLASMIC.
FT   TRANSMEM    147    170
FT   TOPO_DOM    171    207       NON CYTOPLASMIC.
FT   TRANSMEM    208    232
FT   TOPO_DOM    233    249       CYTOPLASMIC.
FT   TRANSMEM    250    271
FT   TOPO_DOM    272    277       NON CYTOPLASMIC.
FT   TRANSMEM    278    298
FT   TOPO_DOM    299    327       CYTOPLASMIC.
//
```

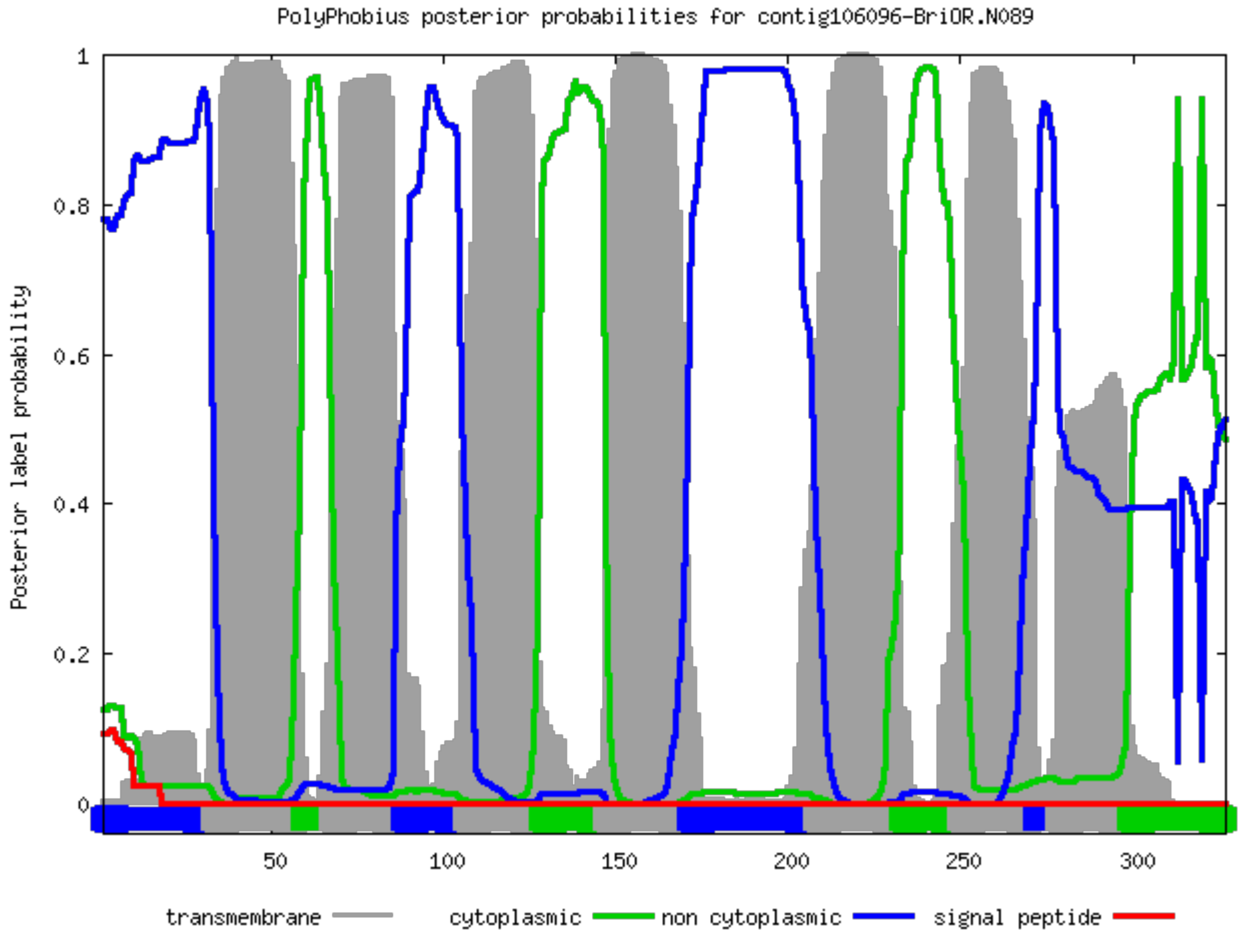

The prediction is based on an alignment. The probability data used in the plot is found here, and the gnuplot script is here.

Prediction of contig017698-BurOR.P120

```
ID   contig017698-BurOR.P120
FT   TOPO_DOM      1     23       NON CYTOPLASMIC.
FT   TRANSMEM     24     47
FT   TOPO_DOM     48     57       CYTOPLASMIC.
FT   TRANSMEM     58     81
FT   TOPO_DOM     82     95       NON CYTOPLASMIC.
FT   TRANSMEM     96    118
FT   TOPO_DOM    119    138       CYTOPLASMIC.
FT   TRANSMEM    139    161
FT   TOPO_DOM    162    197       NON CYTOPLASMIC.
FT   TRANSMEM    198    221
FT   TOPO_DOM    222    235       CYTOPLASMIC.
FT   TRANSMEM    236    258
FT   TOPO_DOM    259    269       NON CYTOPLASMIC.
FT   TRANSMEM    270    290
FT   TOPO_DOM    291    318       CYTOPLASMIC.
//
```

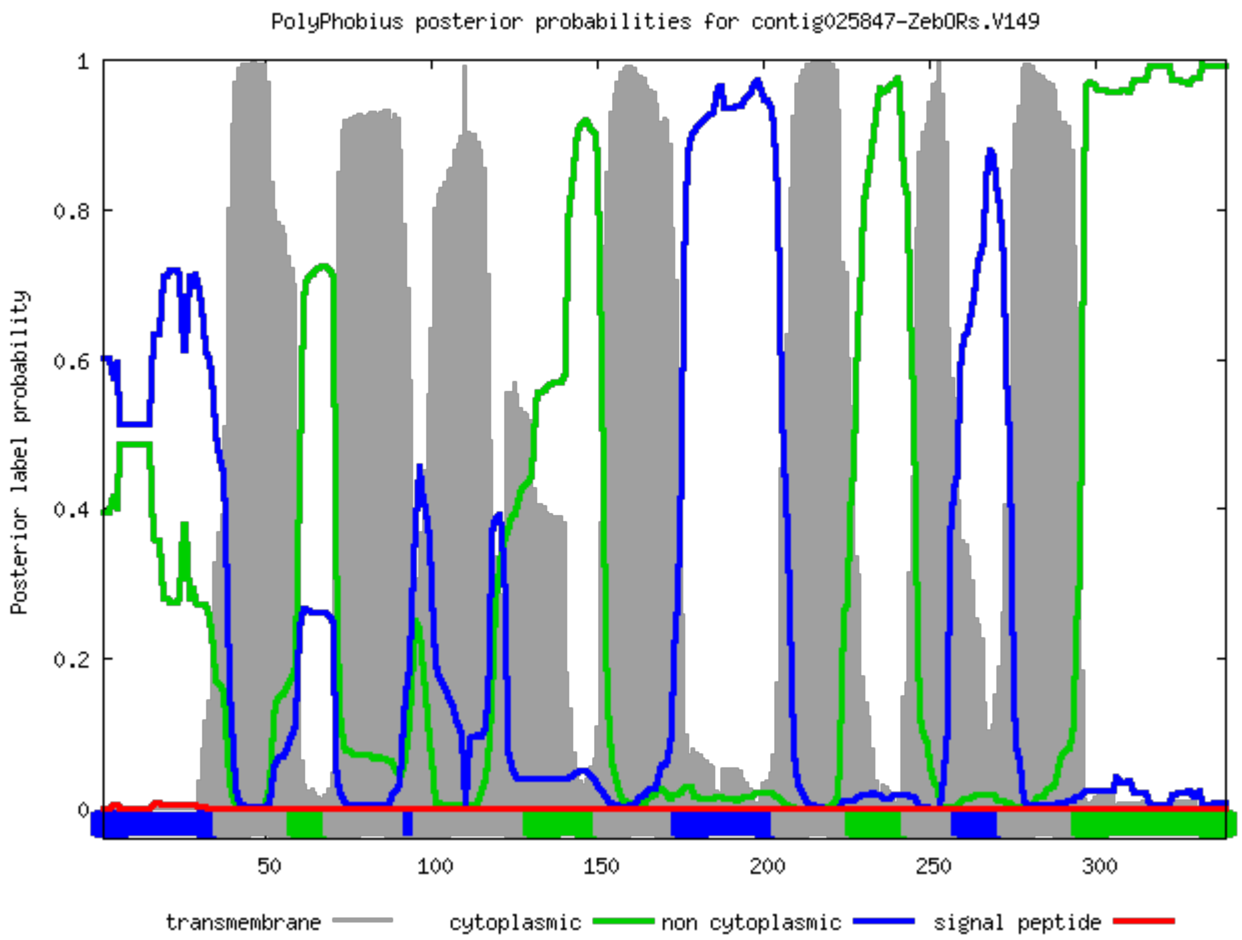

The prediction is based on an alignment. The probability data used in the plot is found here, and the gnuplot script is here.

Prediction of contig022259-TilOR.A015

```
ID   contig022259-TilOR.A015
FT   TOPO_DOM      1      22       NON CYTOPLASMIC.
FT   TRANSMEM     23      48
FT   TOPO_DOM     49      56       CYTOPLASMIC.
FT   TRANSMEM     57      76
FT   TOPO_DOM     77      95       NON CYTOPLASMIC.
FT   TRANSMEM     96     118
FT   TOPO_DOM    119     138       CYTOPLASMIC.
FT   TRANSMEM    139     159
FT   TOPO_DOM    160     192       NON CYTOPLASMIC.
FT   TRANSMEM    193     215
FT   TOPO_DOM    216     235       CYTOPLASMIC.
FT   TRANSMEM    236     257
FT   TOPO_DOM    258     268       NON CYTOPLASMIC.
FT   TRANSMEM    269     289
FT   TOPO_DOM    290     314       CYTOPLASMIC.
//
```

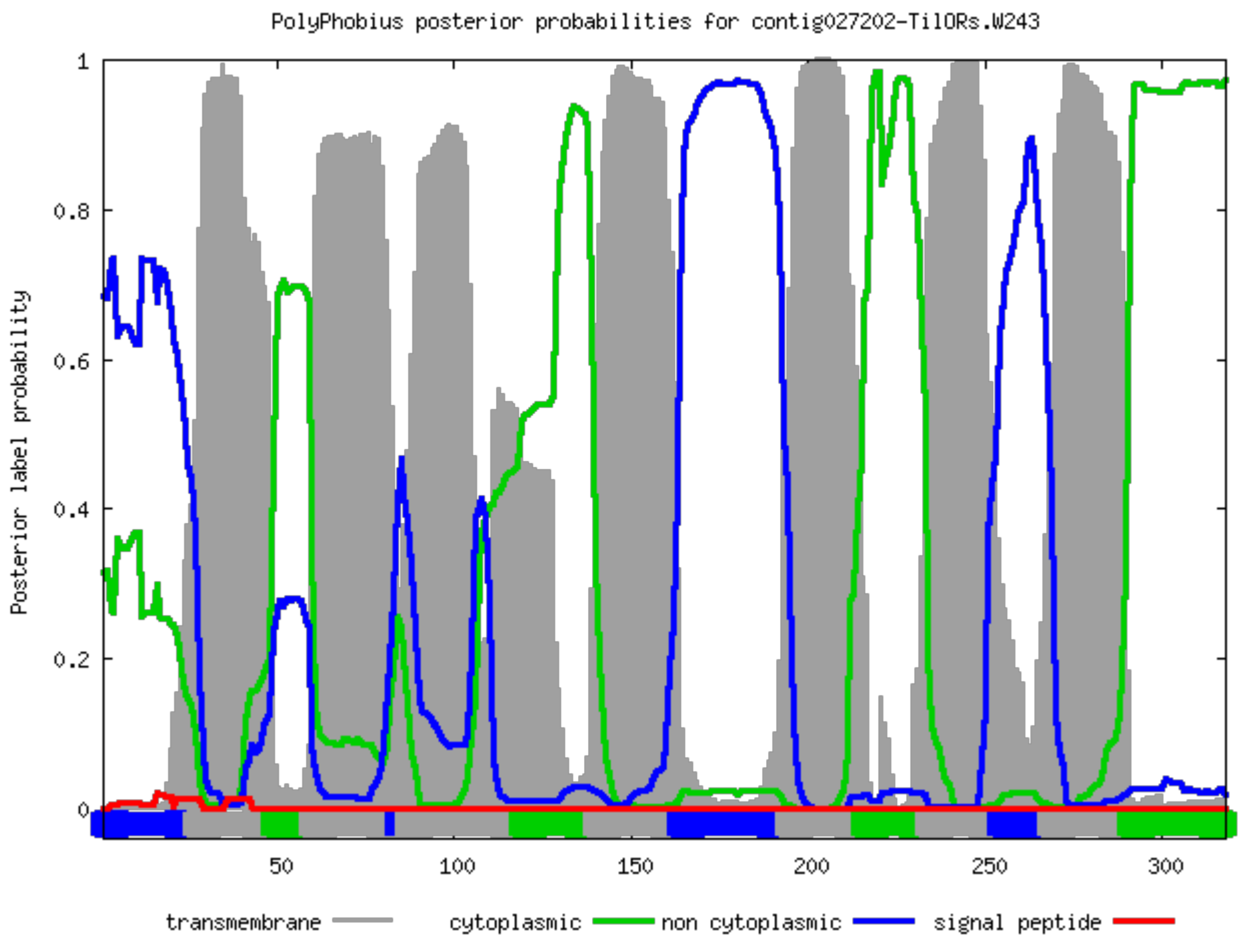

The prediction is based on an alignment. The probability data used in the plot is found here, and the gnuplot script is here.

Prediction of contig039460-TilOR.L145

```
ID    contig039460-TilOR.L145
FT    TOPO_DOM      1     25       NON CYTOPLASMIC.
FT    TRANSMEM     26     50
FT    TOPO_DOM     51     59       CYTOPLASMIC.
FT    TRANSMEM     60     82
FT    TOPO_DOM     83     98       NON CYTOPLASMIC.
FT    TRANSMEM     99    120
FT    TOPO_DOM    121    140       CYTOPLASMIC.
FT    TRANSMEM    141    162
FT    TOPO_DOM    163    198       NON CYTOPLASMIC.
FT    TRANSMEM    199    224
FT    TOPO_DOM    225    235       CYTOPLASMIC.
FT    TRANSMEM    236    259
FT    TOPO_DOM    260    271       NON CYTOPLASMIC.
FT    TRANSMEM    272    292
FT    TOPO_DOM    293    314       CYTOPLASMIC.
//
```

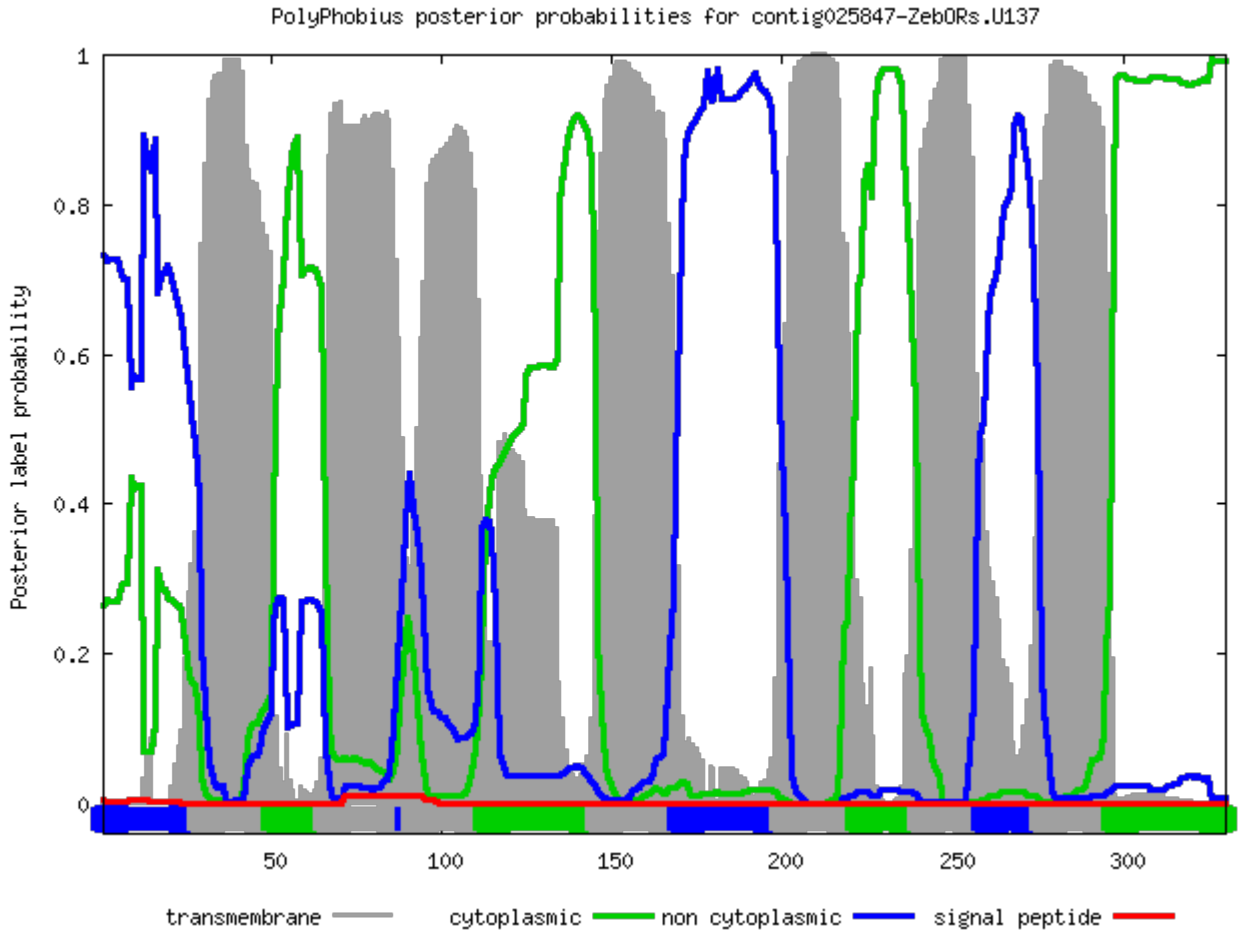

The prediction is based on an alignment. The probability data used in the plot is found here, and the gnuplot script is here.

Prediction of contig020431-ZebOR.O101

```
ID   contig020431-ZebOR.O101
FT   TOPO_DOM      1     24       NON CYTOPLASMIC.
FT   TRANSMEM     25     51
FT   TOPO_DOM     52     60       CYTOPLASMIC.
FT   TRANSMEM     61     83
FT   TOPO_DOM     84     98       NON CYTOPLASMIC.
FT   TRANSMEM     99    121
FT   TOPO_DOM    122    141       CYTOPLASMIC.
FT   TRANSMEM    142    163
FT   TOPO_DOM    164    200       NON CYTOPLASMIC.
FT   TRANSMEM    201    227
FT   TOPO_DOM    228    240       CYTOPLASMIC.
FT   TRANSMEM    241    262
FT   TOPO_DOM    263    273       NON CYTOPLASMIC.
FT   TRANSMEM    274    295
FT   TOPO_DOM    296    331       CYTOPLASMIC.
//
```

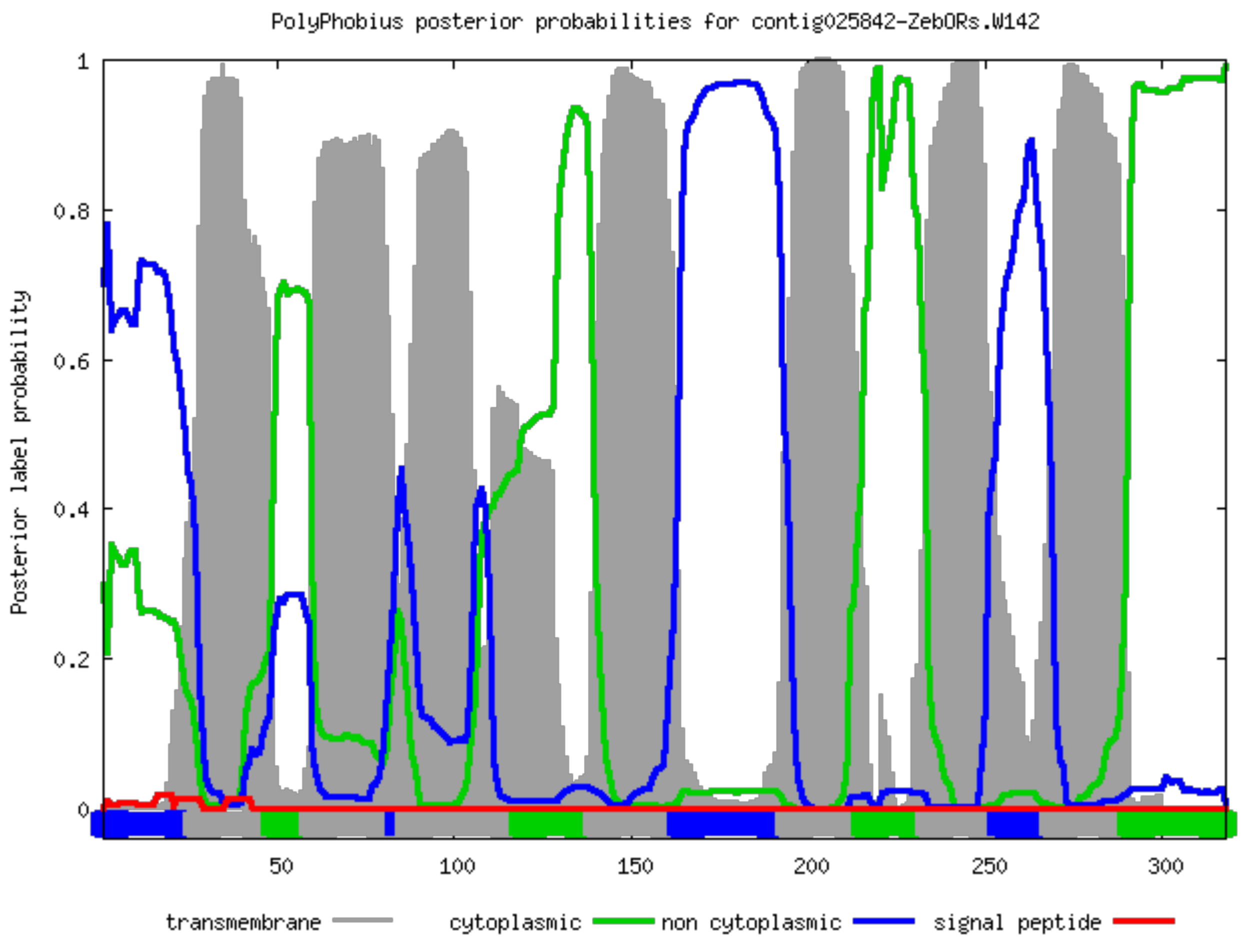

The prediction is based on an alignment. The probability data used in the plot is found here, and the gnuplot script is here.

Prediction of contig028611-TilOR.R246

```
ID   contig028611-TilOR.R246
FT   TOPO_DOM      1     24       NON CYTOPLASMIC.
FT   TRANSMEM     25     48
FT   TOPO_DOM     49     59       CYTOPLASMIC.
FT   TRANSMEM     60     84
FT   TOPO_DOM     85     89       NON CYTOPLASMIC.
FT   TRANSMEM     90    118
FT   TOPO_DOM    119    138       CYTOPLASMIC.
FT   TRANSMEM    139    162
FT   TOPO_DOM    163    194       NON CYTOPLASMIC.
FT   TRANSMEM    195    217
FT   TOPO_DOM    218    235       CYTOPLASMIC.
FT   TRANSMEM    236    259
FT   TOPO_DOM    260    270       NON CYTOPLASMIC.
FT   TRANSMEM    271    293
FT   TOPO_DOM    294    312       CYTOPLASMIC.
//
```

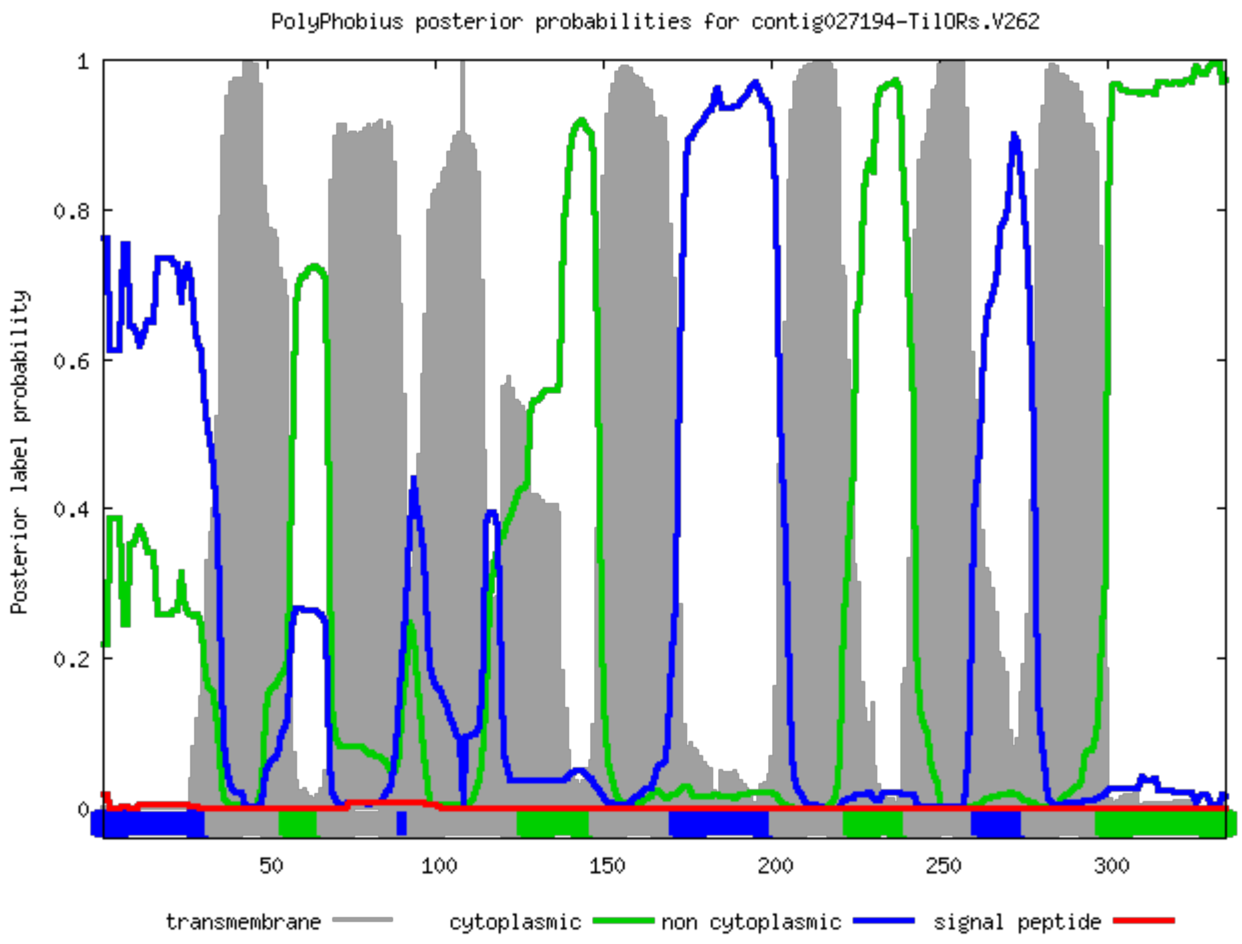

The prediction is based on an alignment. The probability data used in the plot is found here, and the gnuplot script is here.

Prediction of contig060198-BriOR.S104

```
ID   contig060198-BriOR.S104
FT   TOPO_DOM      1      20       NON CYTOPLASMIC.
FT   TRANSMEM     21      42
FT   TOPO_DOM     43      53       CYTOPLASMIC.
FT   TRANSMEM     54      77
FT   TOPO_DOM     78      80       NON CYTOPLASMIC.
FT   TRANSMEM     81     109
FT   TOPO_DOM    110     129       CYTOPLASMIC.
FT   TRANSMEM    130     155
FT   TOPO_DOM    156     183       NON CYTOPLASMIC.
FT   TRANSMEM    184     204
FT   TOPO_DOM    205     224       CYTOPLASMIC.
FT   TRANSMEM    225     250
FT   TOPO_DOM    251     261       NON CYTOPLASMIC.
FT   TRANSMEM    262     282
FT   TOPO_DOM    283     307       CYTOPLASMIC.
//
```

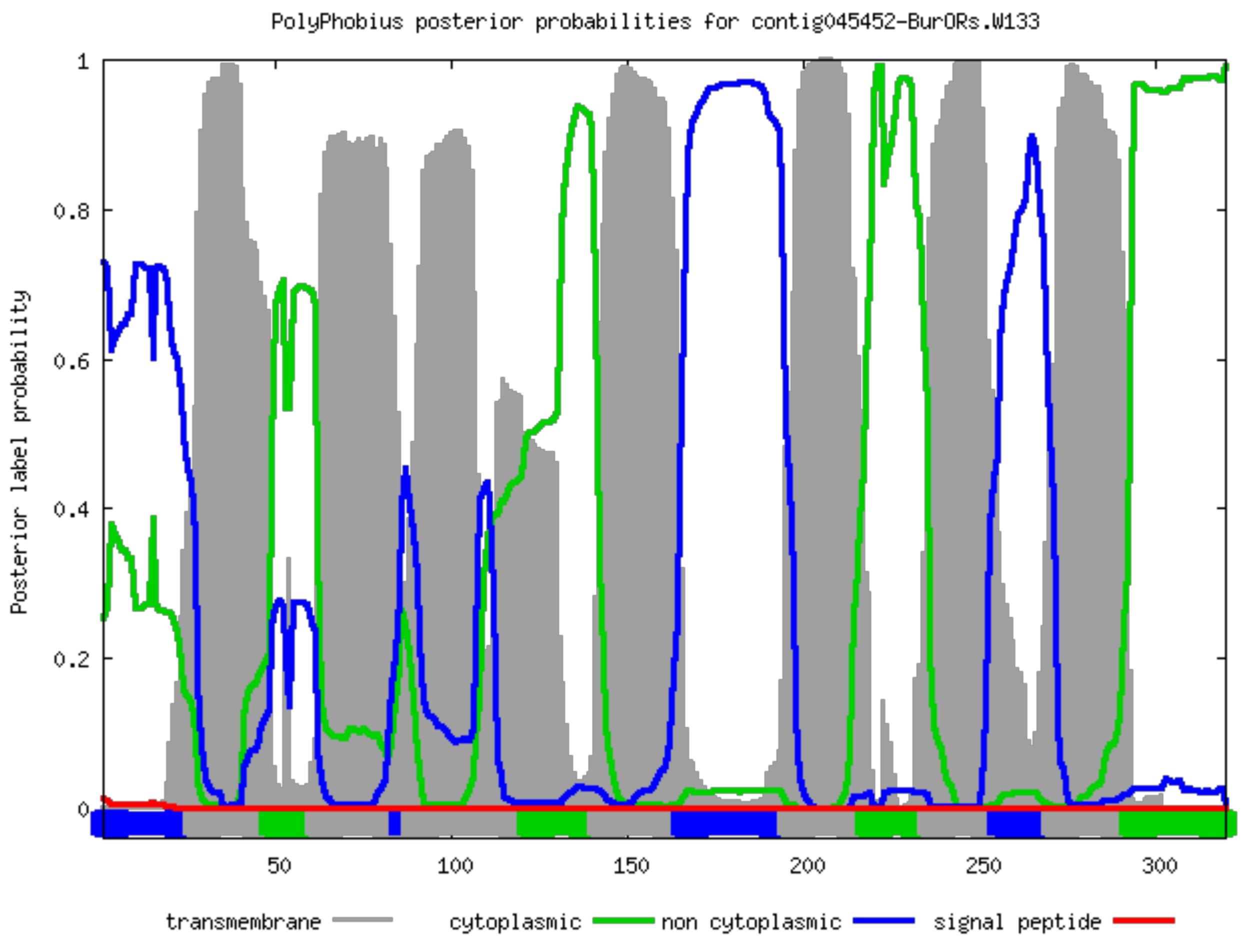

The prediction is based on an alignment. The probability data used in the plot is found here, and the gnuplot script is here.

Prediction of contig017733-ZebOR.S126

```
ID    contig017733-ZebOR.S126
FT    TOPO_DOM      1      20       NON CYTOPLASMIC.
FT    TRANSMEM     21      42
FT    TOPO_DOM     43      53       CYTOPLASMIC.
FT    TRANSMEM     54      77
FT    TOPO_DOM     78      80       NON CYTOPLASMIC.
FT    TRANSMEM     81     109
FT    TOPO_DOM    110     129       CYTOPLASMIC.
FT    TRANSMEM    130     155
FT    TOPO_DOM    156     183       NON CYTOPLASMIC.
FT    TRANSMEM    184     204
FT    TOPO_DOM    205     224       CYTOPLASMIC.
FT    TRANSMEM    225     250
FT    TOPO_DOM    251     261       NON CYTOPLASMIC.
FT    TRANSMEM    262     282
FT    TOPO_DOM    283     307       CYTOPLASMIC.
//
```

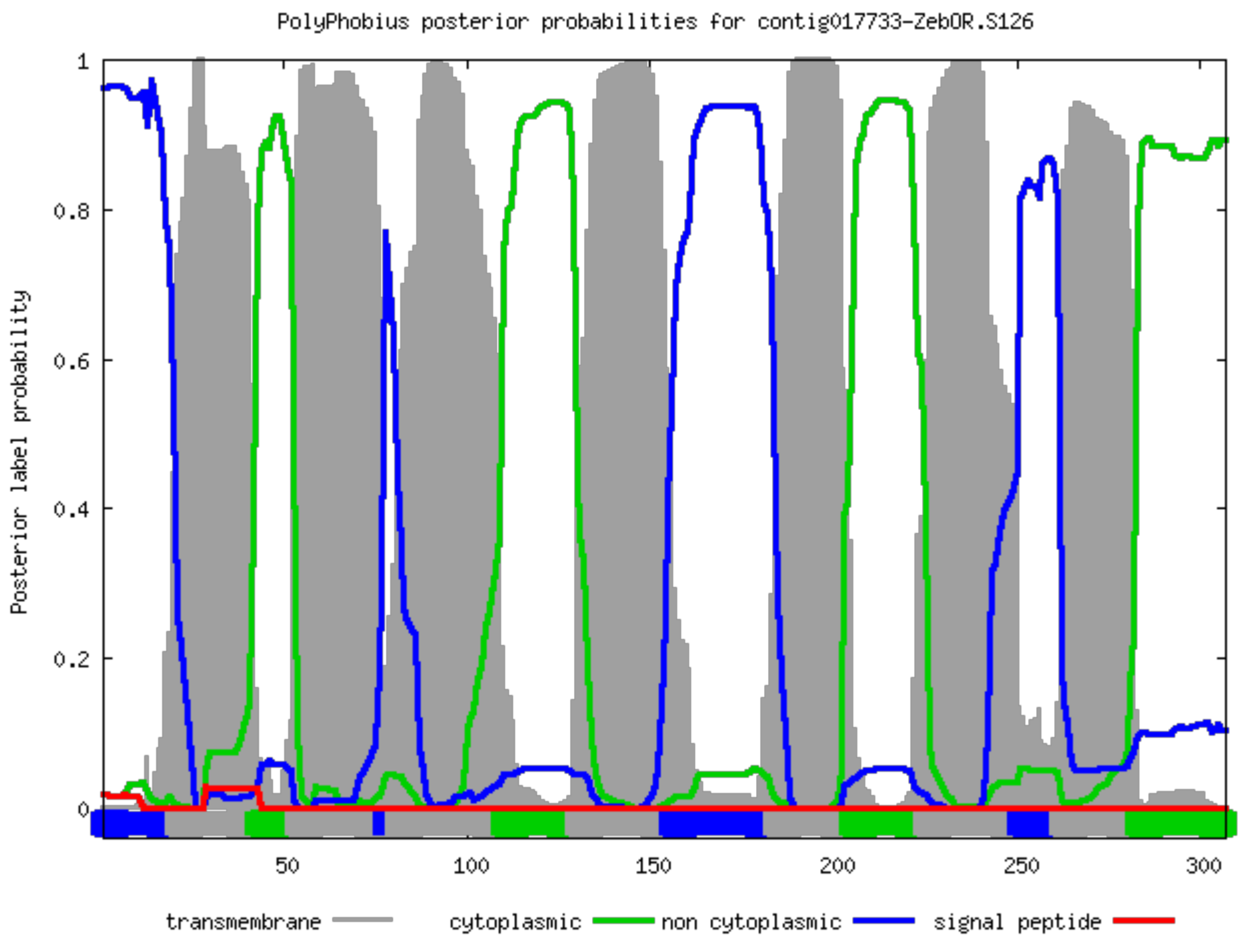

The prediction is based on an alignment. The probability data used in the plot is found here, and the gnuplot script is here.

Prediction of contig048237-ZebOR.G065

```
ID    contig048237-ZebOR.G065
FT    TOPO_DOM      1     22       NON CYTOPLASMIC.
FT    TRANSMEM     23     47
FT    TOPO_DOM     48     55       CYTOPLASMIC.
FT    TRANSMEM     56     76
FT    TOPO_DOM     77     94       NON CYTOPLASMIC.
FT    TRANSMEM     95    117
FT    TOPO_DOM    118    137       CYTOPLASMIC.
FT    TRANSMEM    138    160
FT    TOPO_DOM    161    192       NON CYTOPLASMIC.
FT    TRANSMEM    193    216
FT    TOPO_DOM    217    233       CYTOPLASMIC.
FT    TRANSMEM    234    254
FT    TOPO_DOM    255    268       NON CYTOPLASMIC.
FT    TRANSMEM    269    288
FT    TOPO_DOM    289    312       CYTOPLASMIC.
//
```

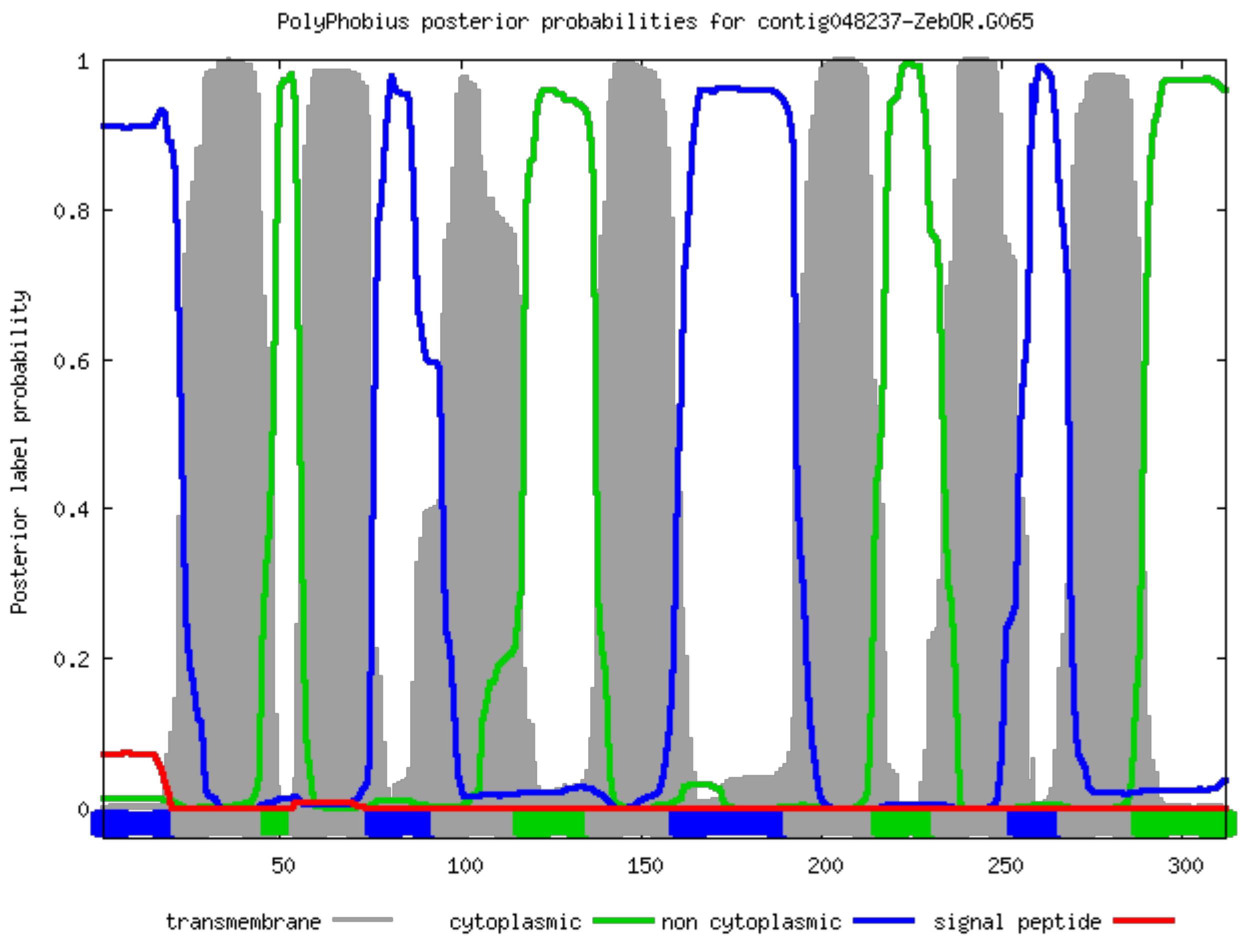

The prediction is based on an alignment. The probability data used in the plot is found here, and the gnuplot script is here.

Prediction of contig042536-BriOR.J063

```
ID   contig042536-BriOR.J063
FT   TOPO_DOM      1      24       NON CYTOPLASMIC.
FT   TRANSMEM     25      50
FT   TOPO_DOM     51      60       CYTOPLASMIC.
FT   TRANSMEM     61      82
FT   TOPO_DOM     83      98       NON CYTOPLASMIC.
FT   TRANSMEM     99     120
FT   TOPO_DOM    121     140       CYTOPLASMIC.
FT   TRANSMEM    141     163
FT   TOPO_DOM    164     195       NON CYTOPLASMIC.
FT   TRANSMEM    196     220
FT   TOPO_DOM    221     238       CYTOPLASMIC.
FT   TRANSMEM    239     261
FT   TOPO_DOM    262     271       NON CYTOPLASMIC.
FT   TRANSMEM    272     292
FT   TOPO_DOM    293     312       CYTOPLASMIC.
//
```

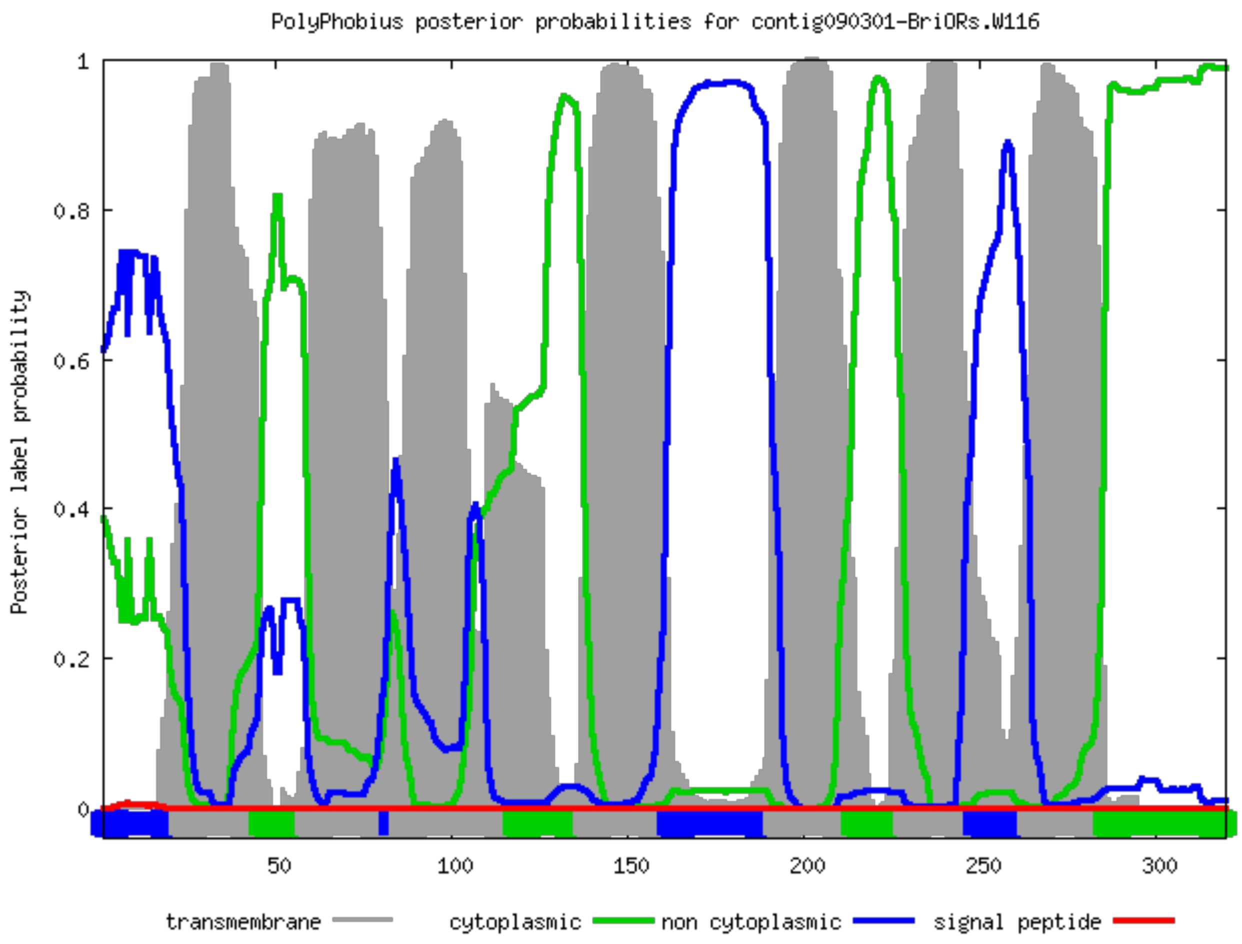

The prediction is based on an alignment. The probability data used in the plot is found here, and the gnuplot script is here.

Prediction of contig014049-ZebOR.D038

```
ID   contig014049-ZebOR.D038
FT   TOPO_DOM      1     22       NON CYTOPLASMIC.
FT   TRANSMEM     23     48
FT   TOPO_DOM     49     57       CYTOPLASMIC.
FT   TRANSMEM     58     81
FT   TOPO_DOM     82     90       NON CYTOPLASMIC.
FT   TRANSMEM     91    118
FT   TOPO_DOM    119    138       CYTOPLASMIC.
FT   TRANSMEM    139    161
FT   TOPO_DOM    162    194       NON CYTOPLASMIC.
FT   TRANSMEM    195    216
FT   TOPO_DOM    217    236       CYTOPLASMIC.
FT   TRANSMEM    237    256
FT   TOPO_DOM    257    267       NON CYTOPLASMIC.
FT   TRANSMEM    268    291
FT   TOPO_DOM    292    309       CYTOPLASMIC.
//
```

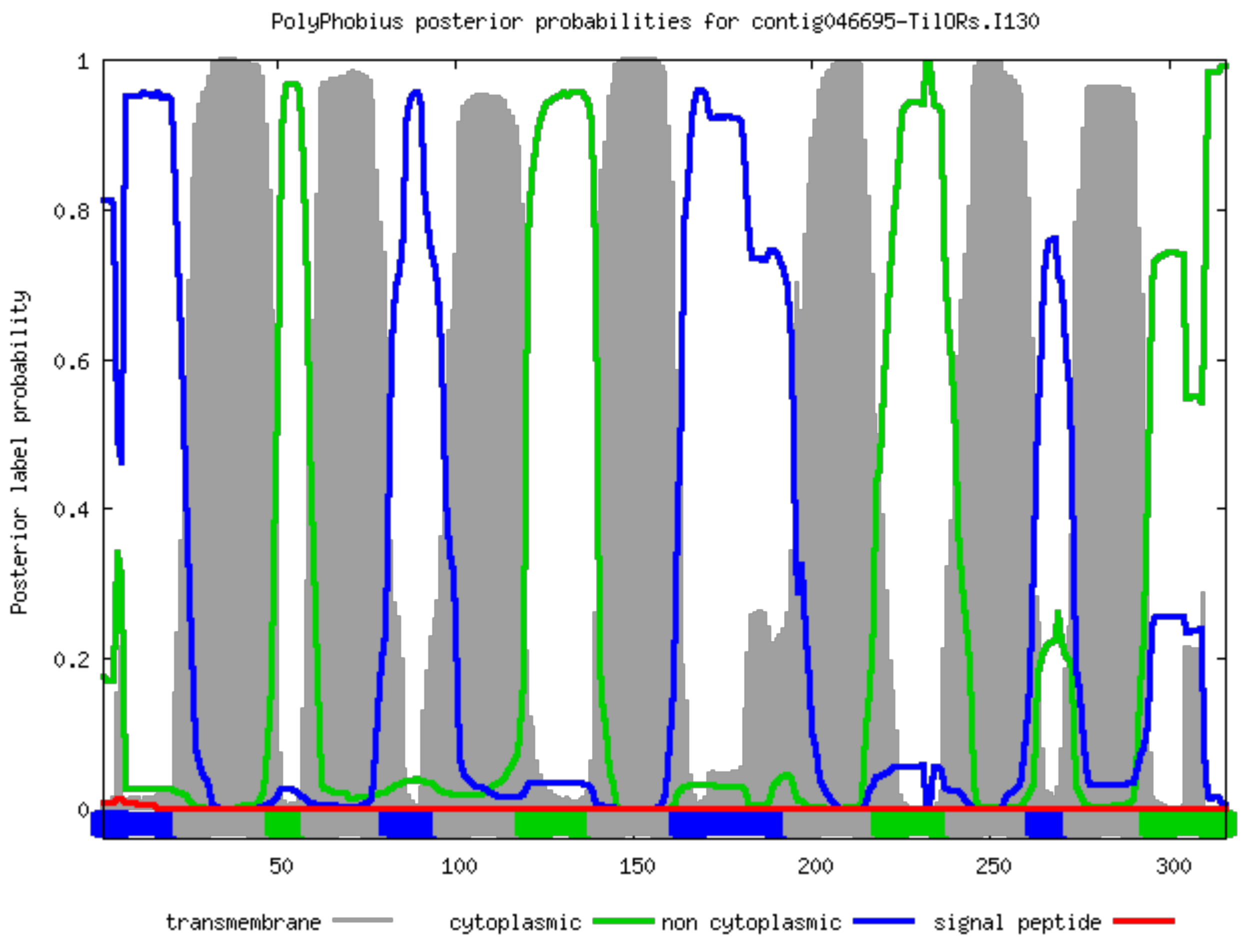

The prediction is based on an alignment. The probability data used in the plot is found here, and the gnuplot script is here.

Prediction of contig065254-TilOR.P213

```
ID   contig065254-TilOR.P213
FT   TOPO_DOM      1     28       NON CYTOPLASMIC.
FT   TRANSMEM     29     52
FT   TOPO_DOM     53     62       CYTOPLASMIC.
FT   TRANSMEM     63     85
FT   TOPO_DOM     86    100       NON CYTOPLASMIC.
FT   TRANSMEM    101    123
FT   TOPO_DOM    124    142       CYTOPLASMIC.
FT   TRANSMEM    143    166
FT   TOPO_DOM    167    200       NON CYTOPLASMIC.
FT   TRANSMEM    201    226
FT   TOPO_DOM    227    241       CYTOPLASMIC.
FT   TRANSMEM    242    265
FT   TOPO_DOM    266    277       NON CYTOPLASMIC.
FT   TRANSMEM    278    298
FT   TOPO_DOM    299    332       CYTOPLASMIC.
//
```

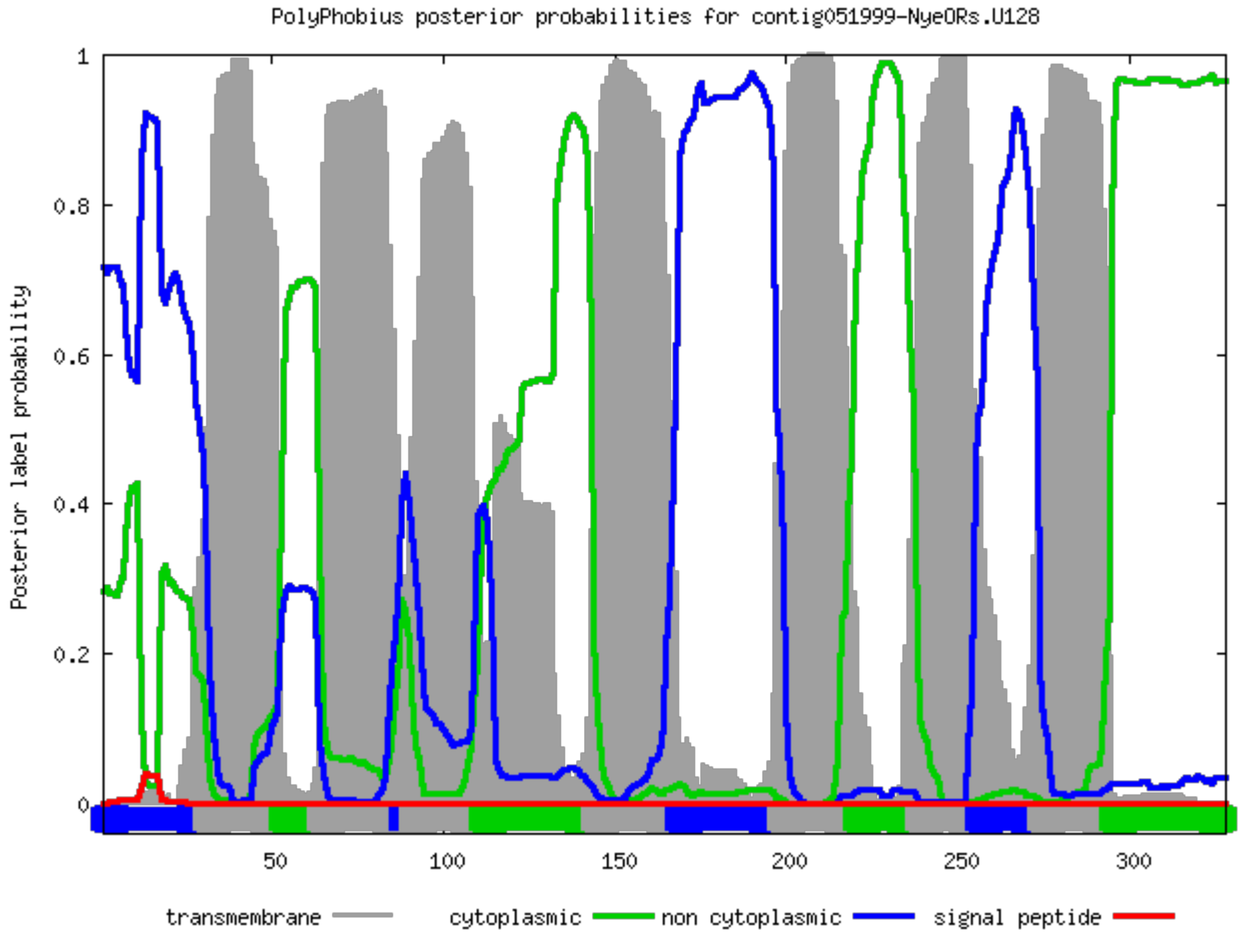

The prediction is based on an alignment. The probability data used in the plot is found here, and the gnuplot script is here.

Prediction of contig049604-BurOR.K081

```
ID   contig049604-BurOR.K081
FT   TOPO_DOM      1     24       NON CYTOPLASMIC.
FT   TRANSMEM     25     50
FT   TOPO_DOM     51     58       CYTOPLASMIC.
FT   TRANSMEM     59     81
FT   TOPO_DOM     82    100       NON CYTOPLASMIC.
FT   TRANSMEM    101    121
FT   TOPO_DOM    122    141       CYTOPLASMIC.
FT   TRANSMEM    142    165
FT   TOPO_DOM    166    196       NON CYTOPLASMIC.
FT   TRANSMEM    197    224
FT   TOPO_DOM    225    244       CYTOPLASMIC.
FT   TRANSMEM    245    264
FT   TOPO_DOM    265    269       NON CYTOPLASMIC.
FT   TRANSMEM    270    292
FT   TOPO_DOM    293    314       CYTOPLASMIC.
//
```

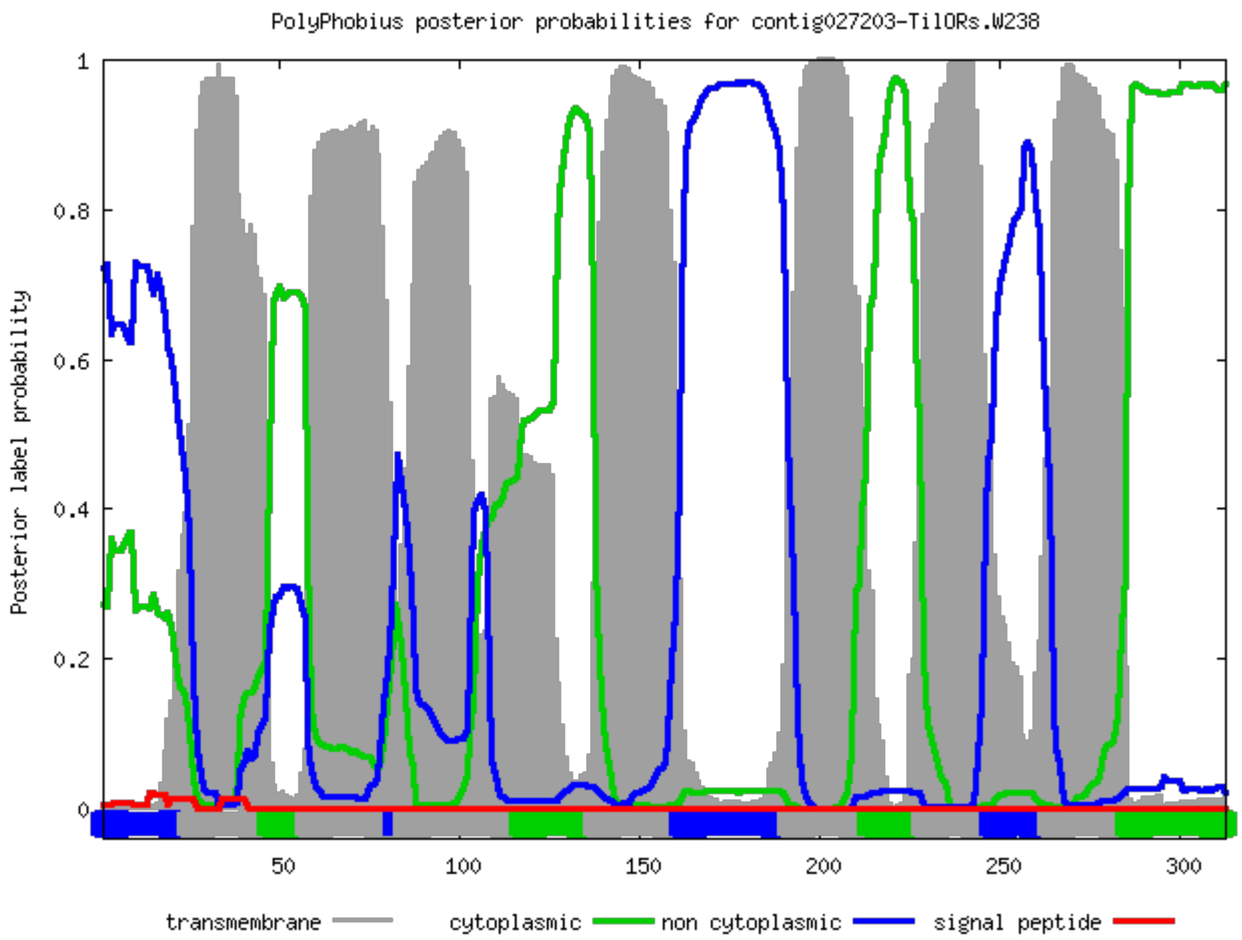

The prediction is based on an alignment. The probability data used in the plot is found here, and the gnuplot script is here.

Prediction of contig032389-BurOR.D033

```
ID    contig032389-BurOR.D033
FT    TOPO_DOM      1    22      NON CYTOPLASMIC.
FT    TRANSMEM     23    48
FT    TOPO_DOM     49    57      CYTOPLASMIC.
FT    TRANSMEM     58    81
FT    TOPO_DOM     82    90      NON CYTOPLASMIC.
FT    TRANSMEM     91   118
FT    TOPO_DOM    119   138      CYTOPLASMIC.
FT    TRANSMEM    139   162
FT    TOPO_DOM    163   193      NON CYTOPLASMIC.
FT    TRANSMEM    194   216
FT    TOPO_DOM    217   236      CYTOPLASMIC.
FT    TRANSMEM    237   256
FT    TOPO_DOM    257   267      NON CYTOPLASMIC.
FT    TRANSMEM    268   291
FT    TOPO_DOM    292   309      CYTOPLASMIC.
//
```

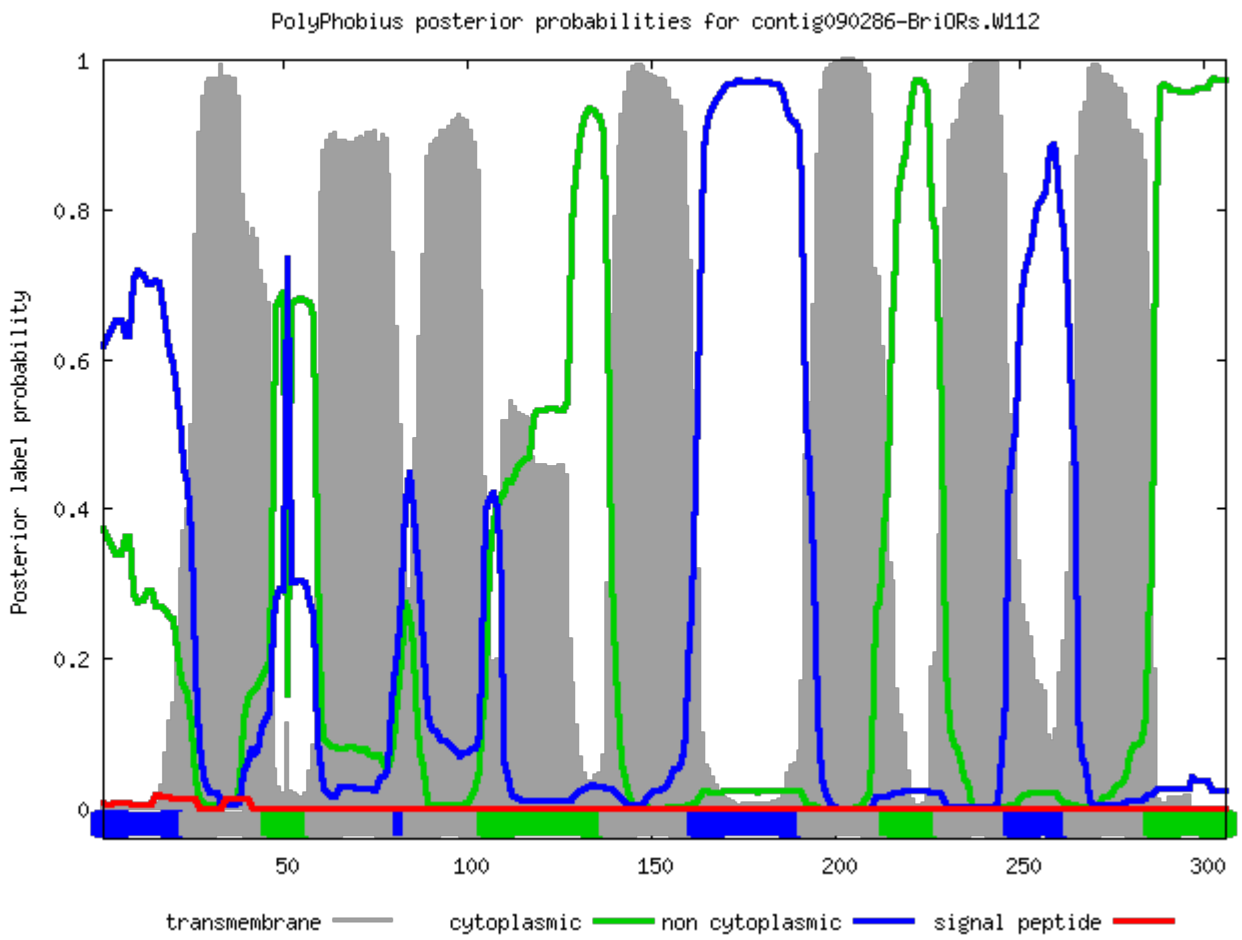

The prediction is based on an alignment. The probability data used in the plot is found here, and the gnuplot script is here.

Prediction of contig048778-NyeOR.M107

```
ID    contig048778-NyeOR.M107
FT    TOPO_DOM      1      31       NON CYTOPLASMIC.
FT    TRANSMEM     32      56
FT    TOPO_DOM     57      66       CYTOPLASMIC.
FT    TRANSMEM     67      88
FT    TOPO_DOM     89     107       NON CYTOPLASMIC.
FT    TRANSMEM    108     126
FT    TOPO_DOM    127     146       CYTOPLASMIC.
FT    TRANSMEM    147     170
FT    TOPO_DOM    171     208       NON CYTOPLASMIC.
FT    TRANSMEM    209     232
FT    TOPO_DOM    233     250       CYTOPLASMIC.
FT    TRANSMEM    251     273
FT    TOPO_DOM    274     279       NON CYTOPLASMIC.
FT    TRANSMEM    280     299
FT    TOPO_DOM    300     321       CYTOPLASMIC.
//
```

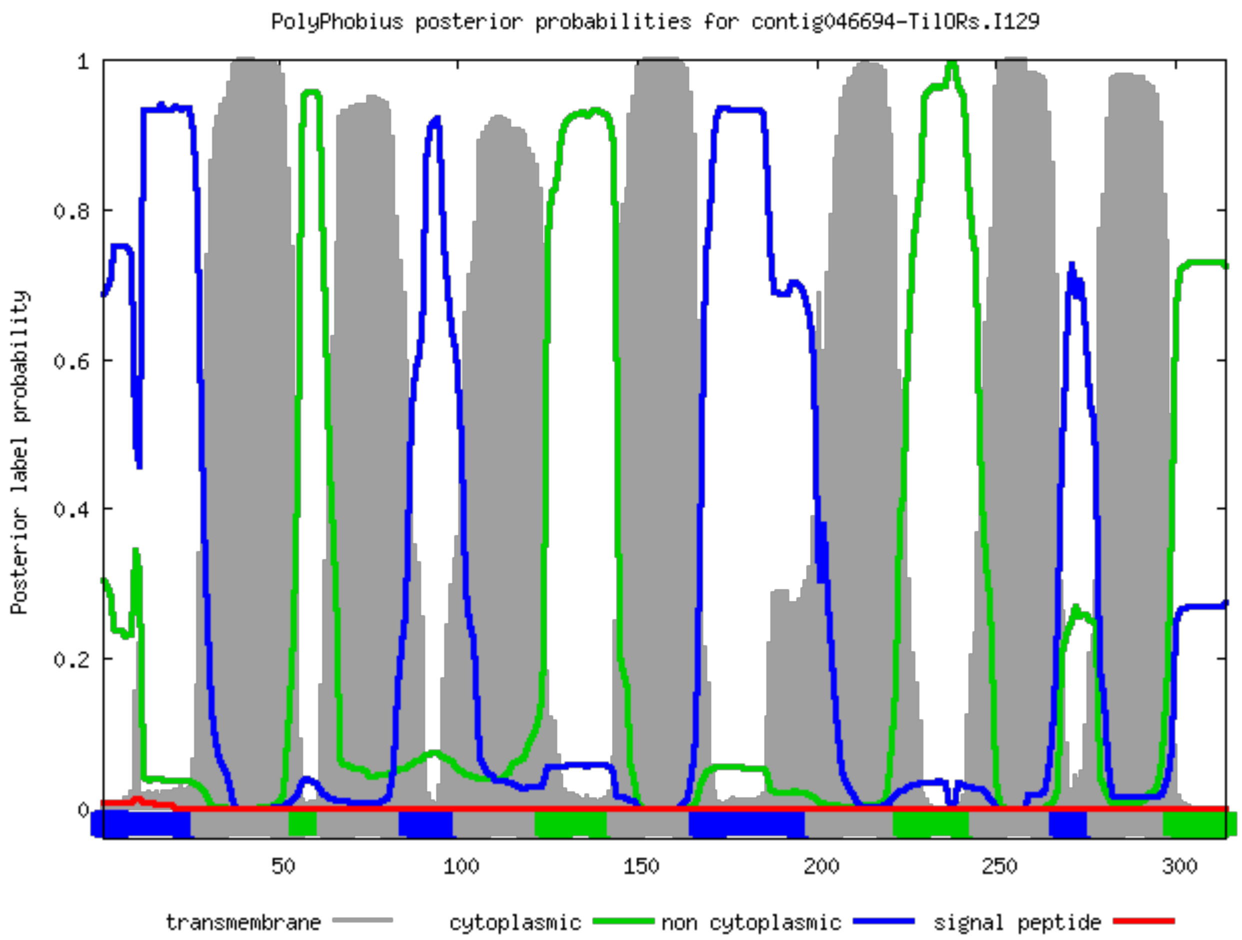

The prediction is based on an alignment. The probability data used in the plot is found here, and the gnuplot script is here.

Prediction of contig017699-BurOR.E042

```
ID   contig017699-BurOR.E042
FT   TOPO_DOM      1     22       NON CYTOPLASMIC.
FT   TRANSMEM     23     48
FT   TOPO_DOM     49     57       CYTOPLASMIC.
FT   TRANSMEM     58     81
FT   TOPO_DOM     82     91       NON CYTOPLASMIC.
FT   TRANSMEM     92    118
FT   TOPO_DOM    119    138       CYTOPLASMIC.
FT   TRANSMEM    139    161
FT   TOPO_DOM    162    193       NON CYTOPLASMIC.
FT   TRANSMEM    194    216
FT   TOPO_DOM    217    236       CYTOPLASMIC.
FT   TRANSMEM    237    256
FT   TOPO_DOM    257    267       NON CYTOPLASMIC.
FT   TRANSMEM    268    291
FT   TOPO_DOM    292    310       CYTOPLASMIC.
//
```

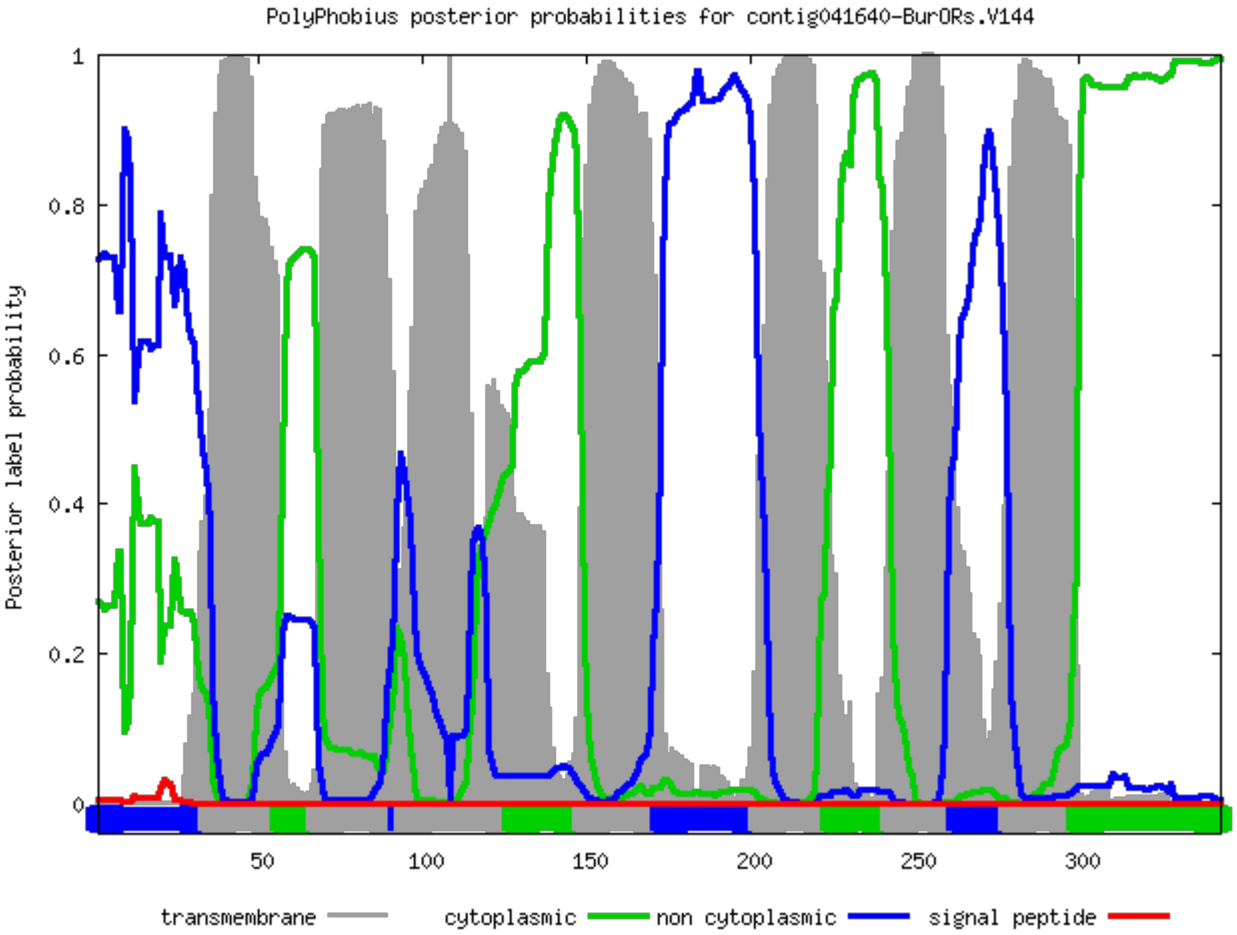

The prediction is based on an alignment. The probability data used in the plot is found here, and the gnuplot script is here.

Prediction of contig032396-BurOR.D034

```
ID   contig032396-BurOR.D034
FT   TOPO_DOM      1     22       NON CYTOPLASMIC.
FT   TRANSMEM     23     48
FT   TOPO_DOM     49     57       CYTOPLASMIC.
FT   TRANSMEM     58     81
FT   TOPO_DOM     82     90       NON CYTOPLASMIC.
FT   TRANSMEM     91    117
FT   TOPO_DOM    118    138       CYTOPLASMIC.
FT   TRANSMEM    139    161
FT   TOPO_DOM    162    193       NON CYTOPLASMIC.
FT   TRANSMEM    194    216
FT   TOPO_DOM    217    236       CYTOPLASMIC.
FT   TRANSMEM    237    256
FT   TOPO_DOM    257    267       NON CYTOPLASMIC.
FT   TRANSMEM    268    291
FT   TOPO_DOM    292    309       CYTOPLASMIC.
//
```

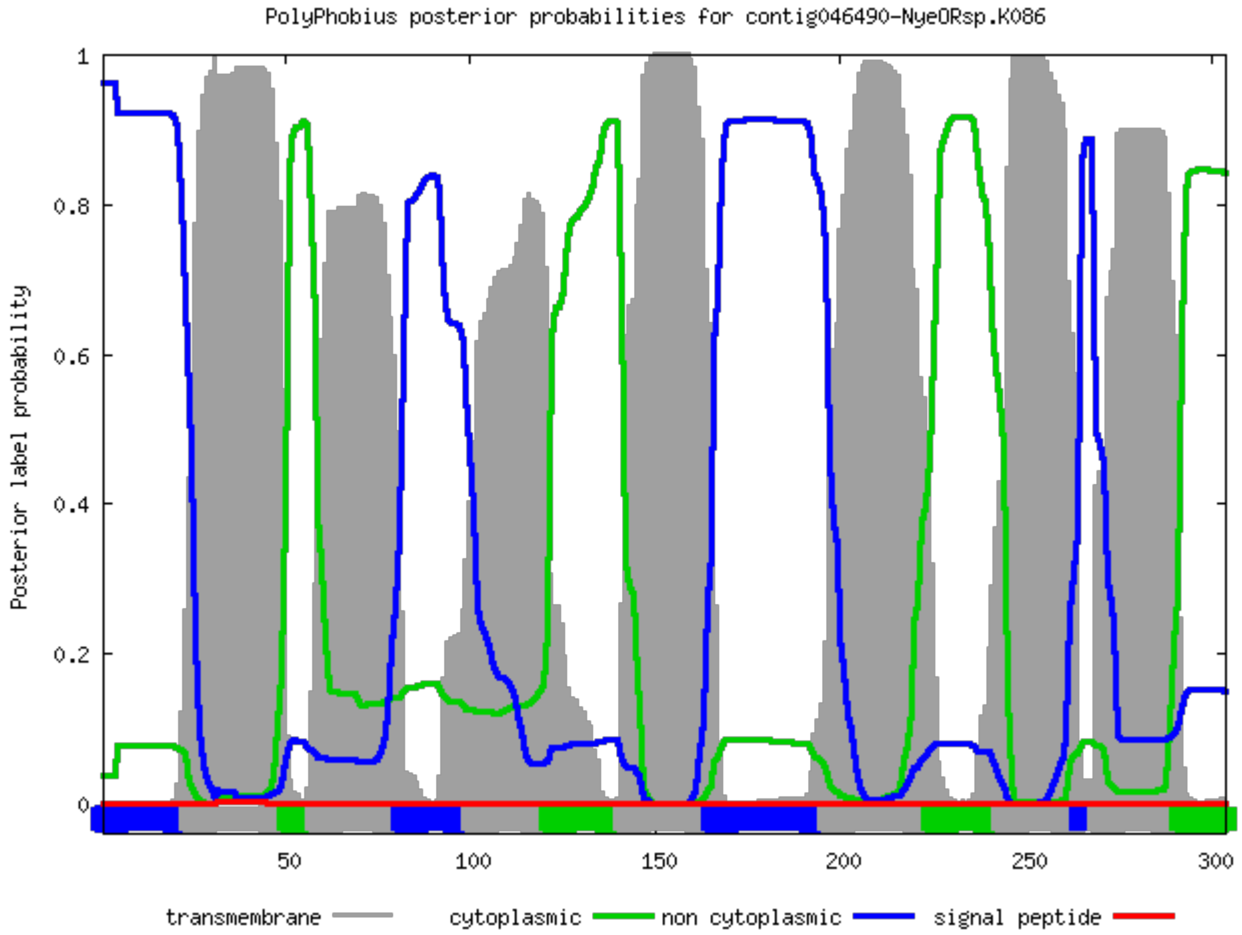

The prediction is based on an alignment. The probability data used in the plot is found here, and the gnuplot script is here.

Prediction of contig057403-ZebOR.H076

```
ID    contig057403-ZebOR.H076
FT    TOPO_DOM      1      23       NON CYTOPLASMIC.
FT    TRANSMEM     24      49
FT    TOPO_DOM     50      56       CYTOPLASMIC.
FT    TRANSMEM     57      76
FT    TOPO_DOM     77      95       NON CYTOPLASMIC.
FT    TRANSMEM     96     118
FT    TOPO_DOM    119     138       CYTOPLASMIC.
FT    TRANSMEM    139     160
FT    TOPO_DOM    161     196       NON CYTOPLASMIC.
FT    TRANSMEM    197     219
FT    TOPO_DOM    220     237       CYTOPLASMIC.
FT    TRANSMEM    238     260
FT    TOPO_DOM    261     271       NON CYTOPLASMIC.
FT    TRANSMEM    272     291
FT    TOPO_DOM    292     310       CYTOPLASMIC.
//
```

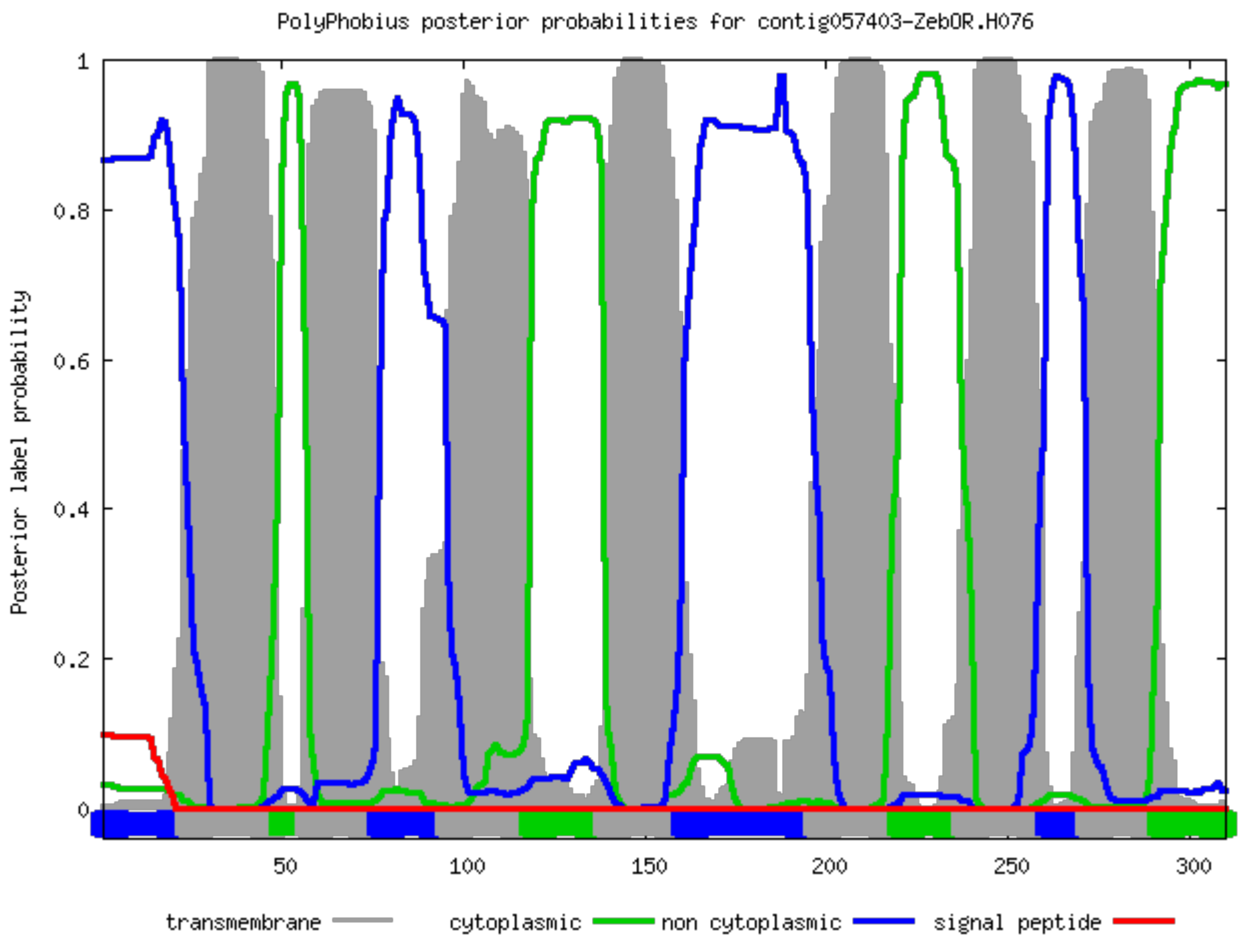

The prediction is based on an alignment. The probability data used in the plot is found here, and the gnuplot script is here.

Prediction of contig028639-TilOR.R251

```
ID   contig028639-TilOR.R251
FT   TOPO_DOM      1     22       NON CYTOPLASMIC.
FT   TRANSMEM     23     46
FT   TOPO_DOM     47     57       CYTOPLASMIC.
FT   TRANSMEM     58     82
FT   TOPO_DOM     83     87       NON CYTOPLASMIC.
FT   TRANSMEM     88    116
FT   TOPO_DOM    117    136       CYTOPLASMIC.
FT   TRANSMEM    137    160
FT   TOPO_DOM    161    191       NON CYTOPLASMIC.
FT   TRANSMEM    192    214
FT   TOPO_DOM    215    232       CYTOPLASMIC.
FT   TRANSMEM    233    256
FT   TOPO_DOM    257    268       NON CYTOPLASMIC.
FT   TRANSMEM    269    290
FT   TOPO_DOM    291    310       CYTOPLASMIC.
//
```

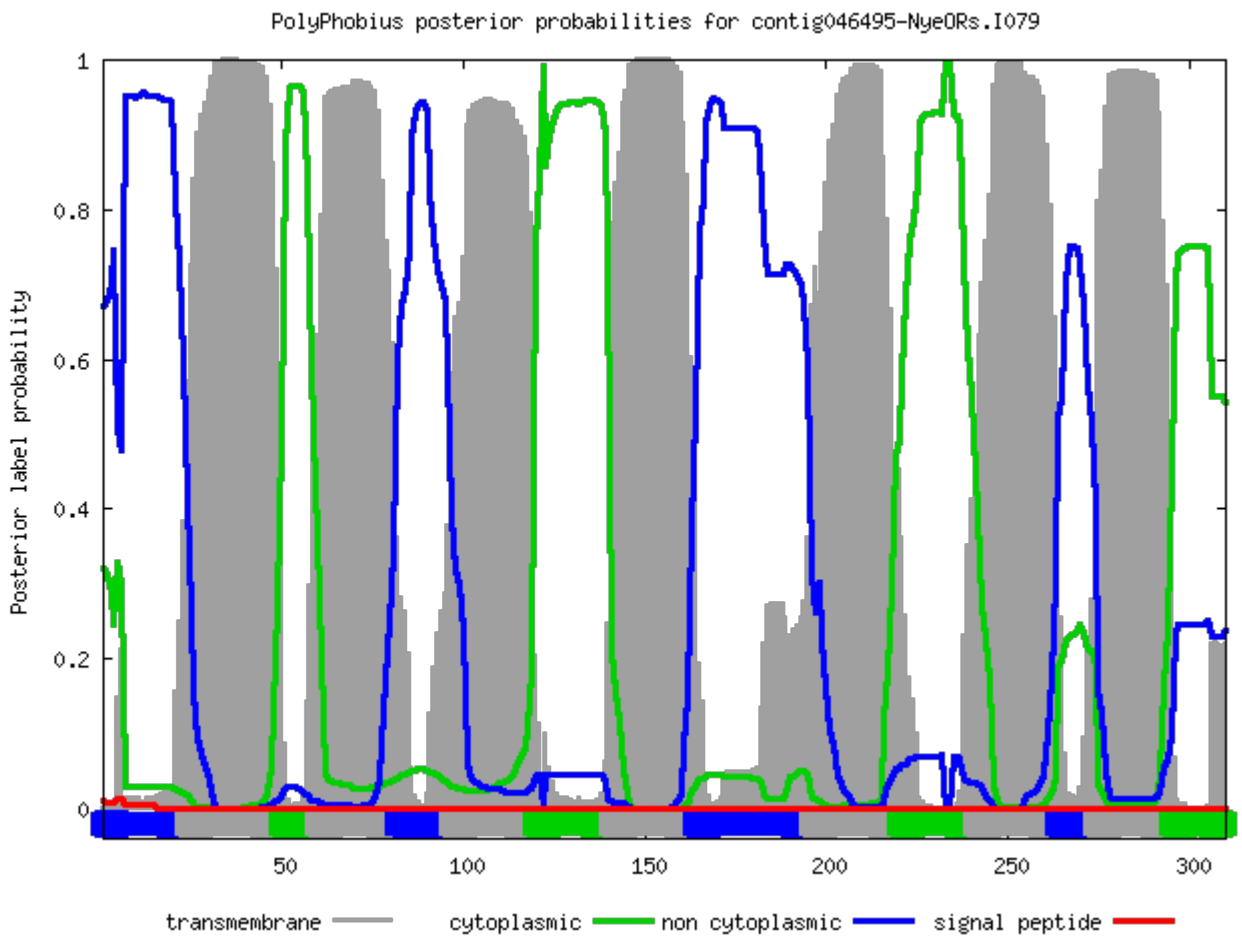

The prediction is based on an alignment. The probability data used in the plot is found here, and the gnuplot script is here.

Prediction of contig064814-BriOR.D029

```
ID   contig064814-BriOR.D029
FT   TOPO_DOM      1     25       NON CYTOPLASMIC.
FT   TRANSMEM     26     51
FT   TOPO_DOM     52     60       CYTOPLASMIC.
FT   TRANSMEM     61     84
FT   TOPO_DOM     85     94       NON CYTOPLASMIC.
FT   TRANSMEM     95    121
FT   TOPO_DOM    122    141       CYTOPLASMIC.
FT   TRANSMEM    142    165
FT   TOPO_DOM    166    196       NON CYTOPLASMIC.
FT   TRANSMEM    197    219
FT   TOPO_DOM    220    239       CYTOPLASMIC.
FT   TRANSMEM    240    259
FT   TOPO_DOM    260    271       NON CYTOPLASMIC.
FT   TRANSMEM    272    294
FT   TOPO_DOM    295    312       CYTOPLASMIC.
//
```

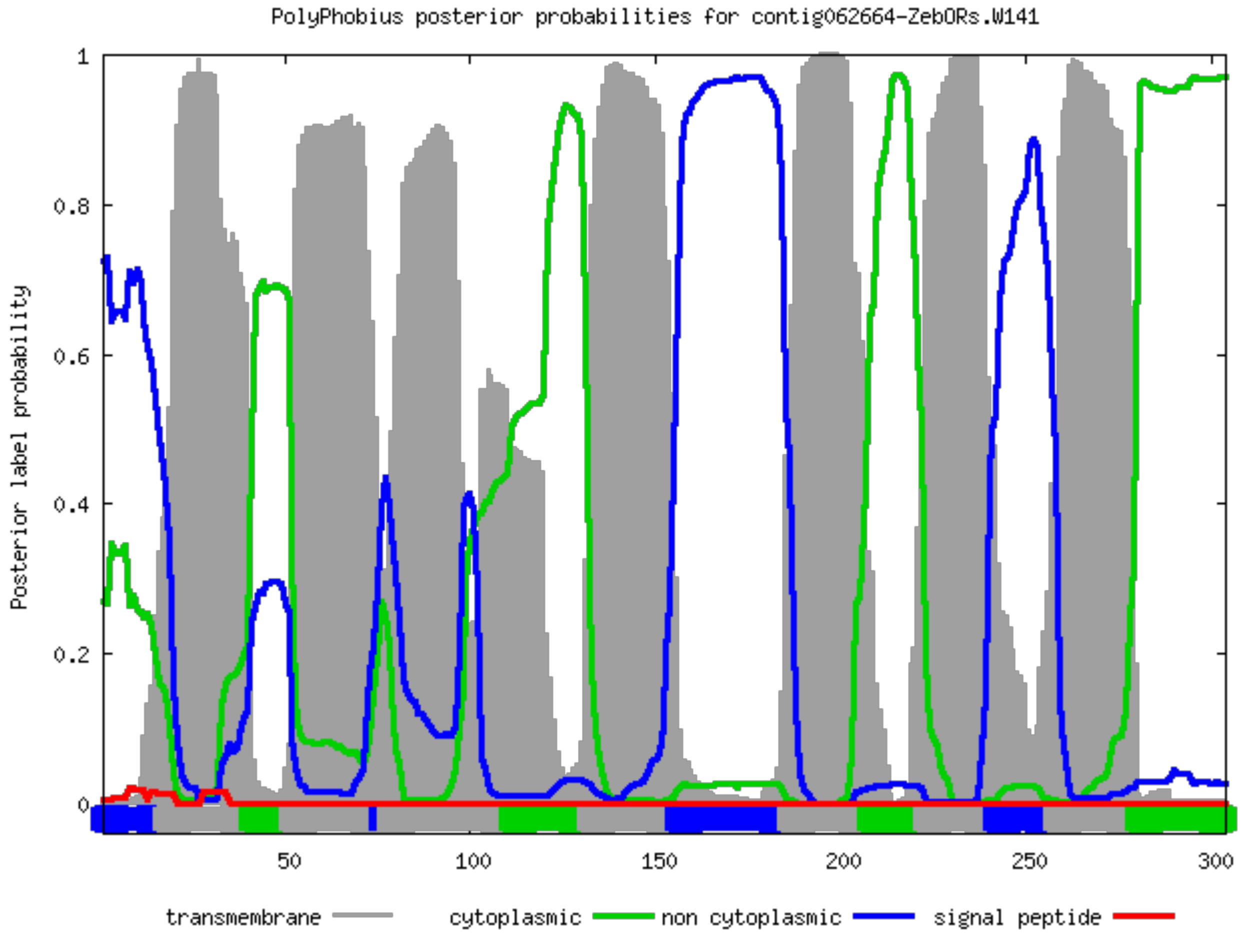

The prediction is based on an alignment. The probability data used in the plot is found here, and the gnuplot script is here.

Prediction of contig042539-BriOR.J064

```
ID    contig042539-BriOR.J064
FT    TOPO_DOM       1      23        NON CYTOPLASMIC.
FT    TRANSMEM      24      49
FT    TOPO_DOM      50      59        CYTOPLASMIC.
FT    TRANSMEM      60      81
FT    TOPO_DOM      82      97        NON CYTOPLASMIC.
FT    TRANSMEM      98     119
FT    TOPO_DOM     120     139        CYTOPLASMIC.
FT    TRANSMEM     140     162
FT    TOPO_DOM     163     194        NON CYTOPLASMIC.
FT    TRANSMEM     195     218
FT    TOPO_DOM     219     236        CYTOPLASMIC.
FT    TRANSMEM     237     260
FT    TOPO_DOM     261     270        NON CYTOPLASMIC.
FT    TRANSMEM     271     291
FT    TOPO_DOM     292     313        CYTOPLASMIC.
//
```

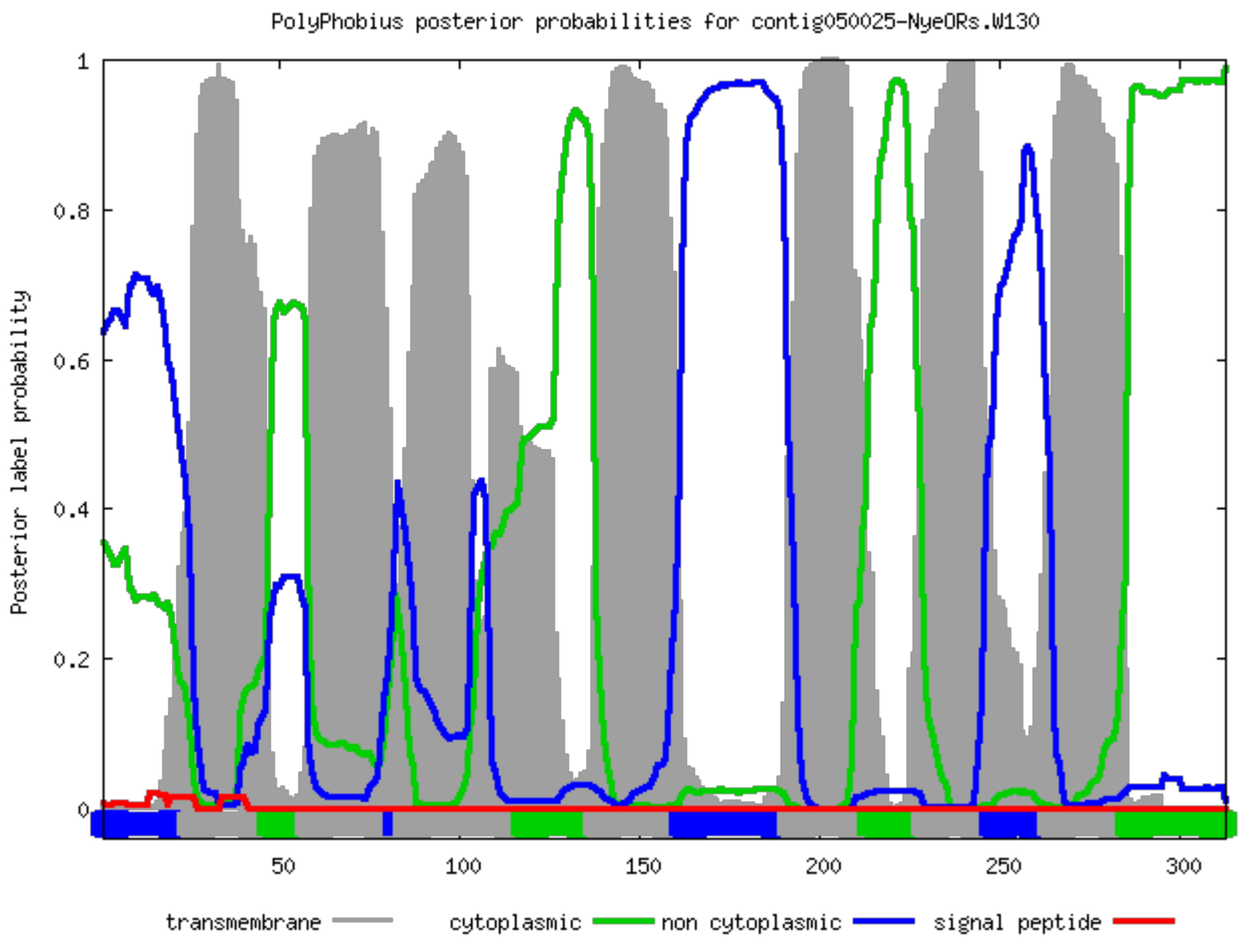

The prediction is based on an alignment. The probability data used in the plot is found here, and the gnuplot script is here.

Prediction of contig082838-BriOR.E040

```
ID    contig082838-BriOR.E040
FT    TOPO_DOM       1      21       NON CYTOPLASMIC.
FT    TRANSMEM      22      47
FT    TOPO_DOM      48      56       CYTOPLASMIC.
FT    TRANSMEM      57      80
FT    TOPO_DOM      81      92       NON CYTOPLASMIC.
FT    TRANSMEM      93     117
FT    TOPO_DOM     118     137       CYTOPLASMIC.
FT    TRANSMEM     138     159
FT    TOPO_DOM     160     192       NON CYTOPLASMIC.
FT    TRANSMEM     193     215
FT    TOPO_DOM     216     235       CYTOPLASMIC.
FT    TRANSMEM     236     255
FT    TOPO_DOM     256     266       NON CYTOPLASMIC.
FT    TRANSMEM     267     290
FT    TOPO_DOM     291     304       CYTOPLASMIC.
//
```

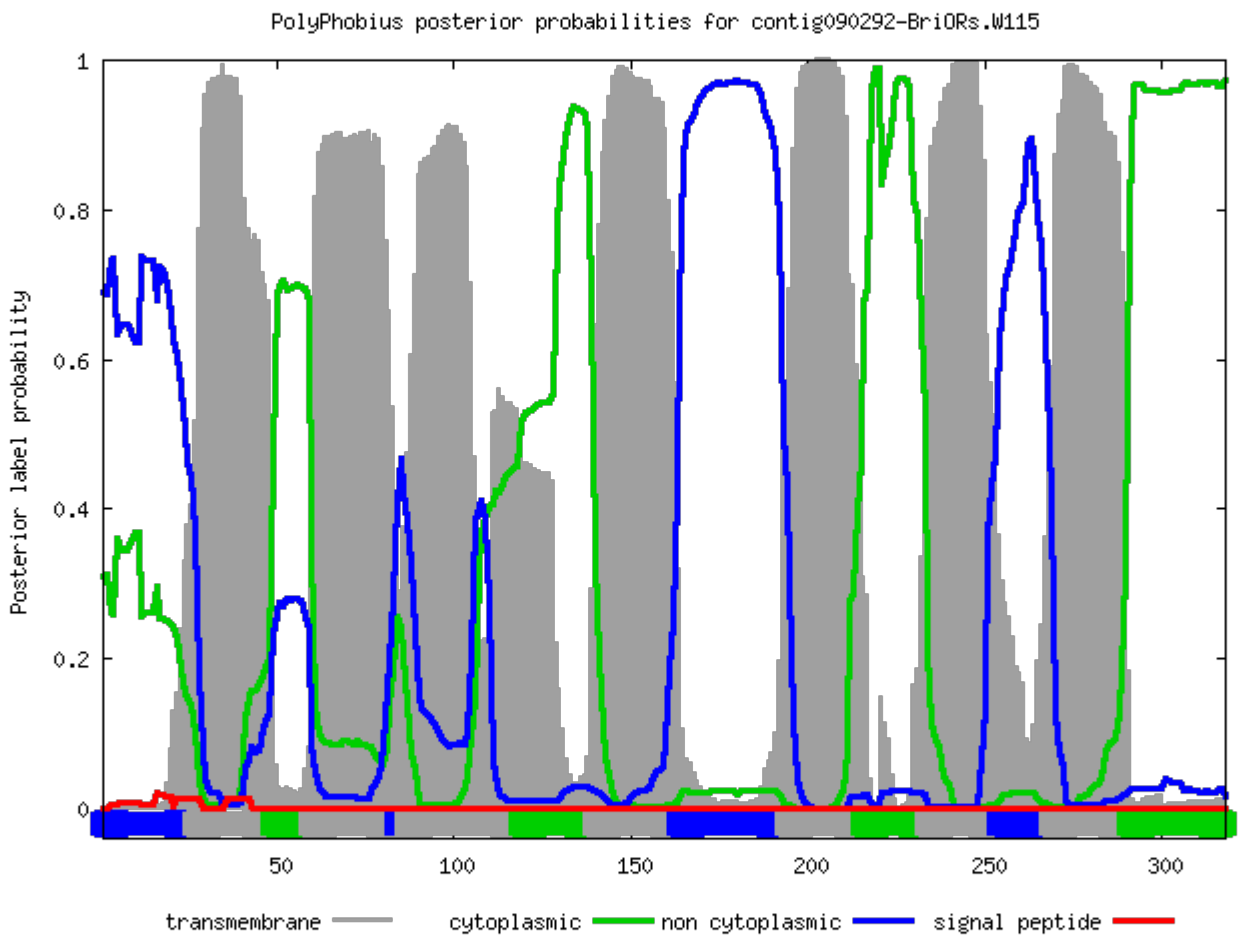

The prediction is based on an alignment. The probability data used in the plot is found here, and the gnuplot script is here.

Prediction of contig066327-ZebOR.F064

```
ID   contig066327-ZebOR.F064
FT   TOPO_DOM      1      22       NON CYTOPLASMIC.
FT   TRANSMEM     23      47
FT   TOPO_DOM     48      56       CYTOPLASMIC.
FT   TRANSMEM     57      77
FT   TOPO_DOM     78      96       NON CYTOPLASMIC.
FT   TRANSMEM     97     118
FT   TOPO_DOM    119     138       CYTOPLASMIC.
FT   TRANSMEM    139     161
FT   TOPO_DOM    162     193       NON CYTOPLASMIC.
FT   TRANSMEM    194     216
FT   TOPO_DOM    217     236       CYTOPLASMIC.
FT   TRANSMEM    237     256
FT   TOPO_DOM    257     267       NON CYTOPLASMIC.
FT   TRANSMEM    268     290
FT   TOPO_DOM    291     310       CYTOPLASMIC.
//
```

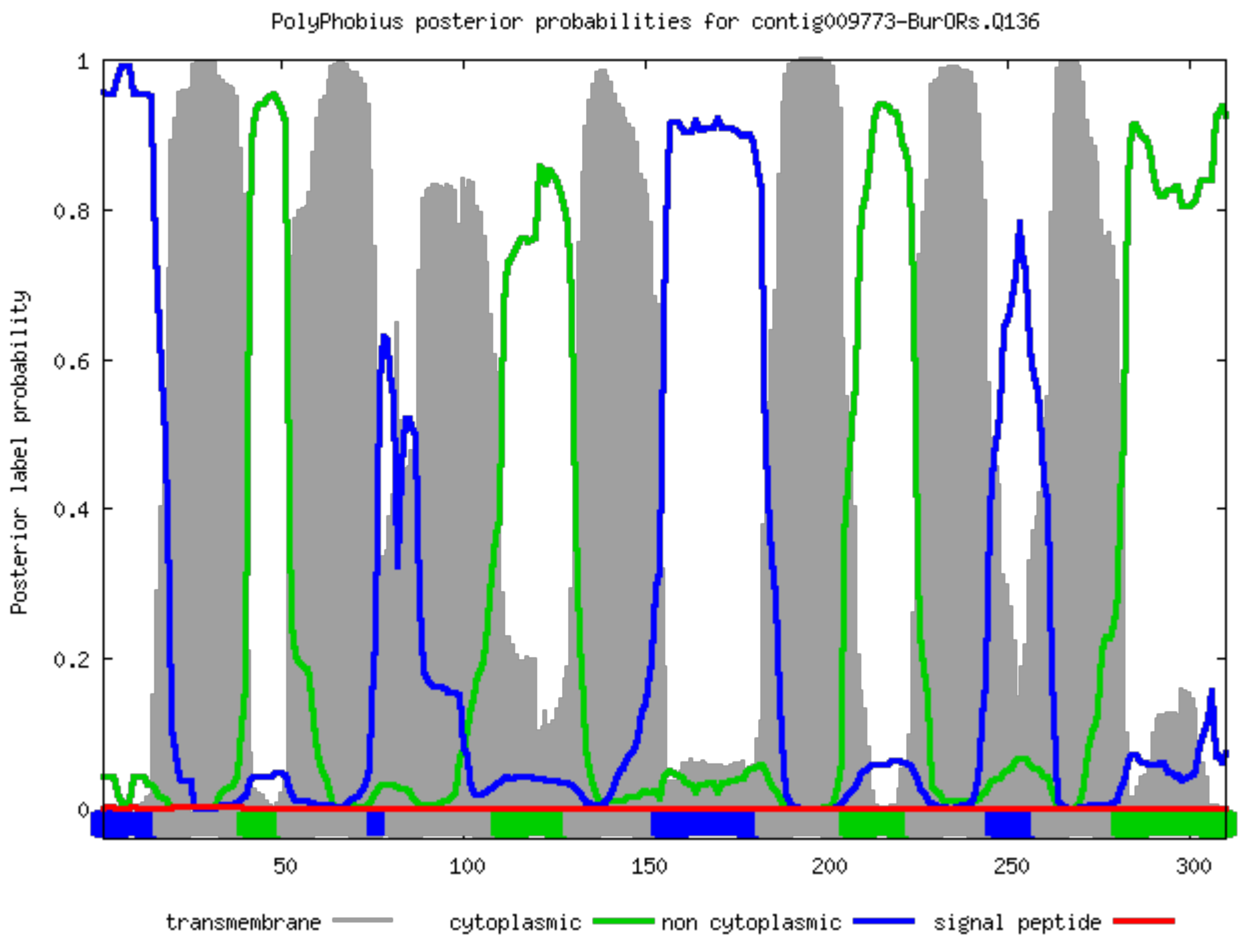

The prediction is based on an alignment. The probability data used in the plot is found here, and the gnuplot script is here.

Prediction of contig064187-BurOR.A016

ID	contig064187-BurOR.A016			
FT	TOPO_DOM	1	18	NON CYTOPLASMIC.
FT	TRANSMEM	19	44	
FT	TOPO_DOM	45	52	CYTOPLASMIC.
FT	TRANSMEM	53	73	
FT	TOPO_DOM	74	91	NON CYTOPLASMIC.
FT	TRANSMEM	92	114	
FT	TOPO_DOM	115	134	CYTOPLASMIC.
FT	TRANSMEM	135	156	
FT	TOPO_DOM	157	188	NON CYTOPLASMIC.
FT	TRANSMEM	189	211	
FT	TOPO_DOM	212	231	CYTOPLASMIC.
FT	TRANSMEM	232	253	
FT	TOPO_DOM	254	264	NON CYTOPLASMIC.
FT	TRANSMEM	265	285	
FT	TOPO_DOM	286	300	CYTOPLASMIC.
//				

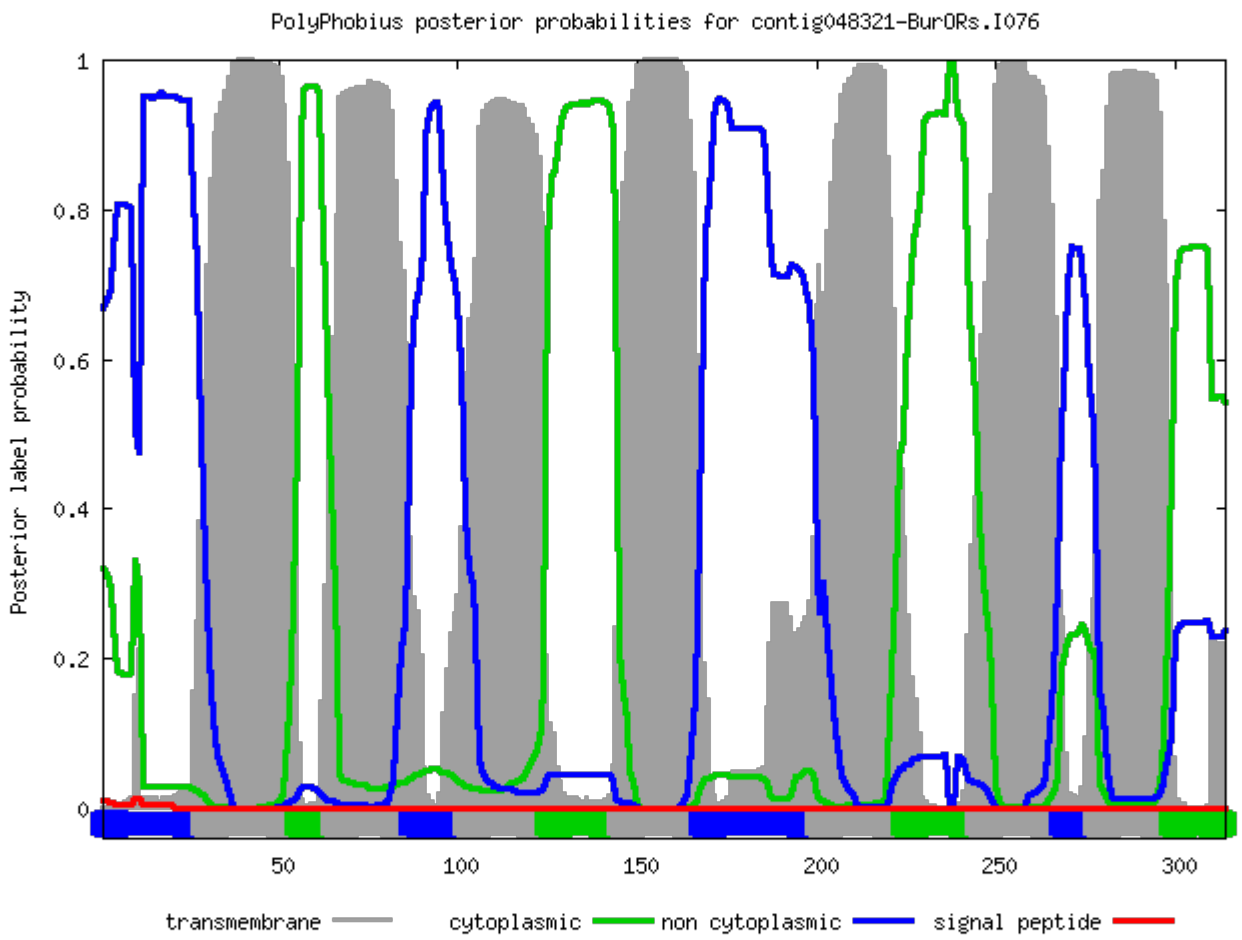

The prediction is based on an alignment. The probability data used in the plot is found here, and the gnuplot script is here.

Prediction of contig039729-NyeOR.F064

```
ID   contig039729-NyeOR.F064
FT   TOPO_DOM      1     22       NON CYTOPLASMIC.
FT   TRANSMEM     23     47
FT   TOPO_DOM     48     57       CYTOPLASMIC.
FT   TRANSMEM     58     78
FT   TOPO_DOM     79     96       NON CYTOPLASMIC.
FT   TRANSMEM     97    118
FT   TOPO_DOM    119    138       CYTOPLASMIC.
FT   TRANSMEM    139    161
FT   TOPO_DOM    162    193       NON CYTOPLASMIC.
FT   TRANSMEM    194    216
FT   TOPO_DOM    217    236       CYTOPLASMIC.
FT   TRANSMEM    237    257
FT   TOPO_DOM    258    267       NON CYTOPLASMIC.
FT   TRANSMEM    268    291
FT   TOPO_DOM    292    305       CYTOPLASMIC.
//
```

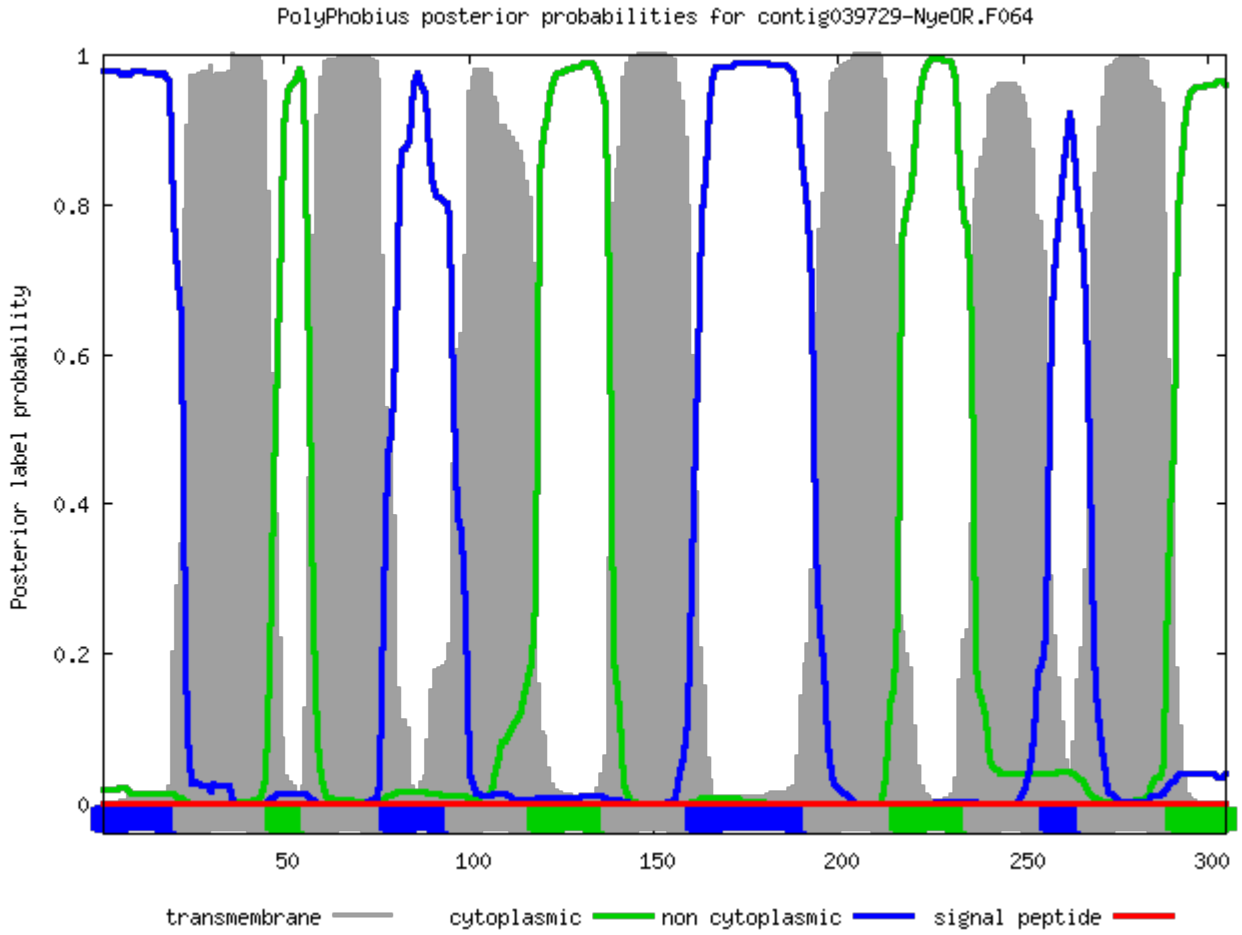

The prediction is based on an alignment. The probability data used in the plot is found here, and the gnuplot script is here.

Prediction of contig064809-BriOR.D028

```
ID    contig064809-BriOR.D028
FT    TOPO_DOM      1      22       NON CYTOPLASMIC.
FT    TRANSMEM     23      48
FT    TOPO_DOM     49      57       CYTOPLASMIC.
FT    TRANSMEM     58      81
FT    TOPO_DOM     82      90       NON CYTOPLASMIC.
FT    TRANSMEM     91     118
FT    TOPO_DOM    119     138       CYTOPLASMIC.
FT    TRANSMEM    139     161
FT    TOPO_DOM    162     193       NON CYTOPLASMIC.
FT    TRANSMEM    194     216
FT    TOPO_DOM    217     236       CYTOPLASMIC.
FT    TRANSMEM    237     256
FT    TOPO_DOM    257     268       NON CYTOPLASMIC.
FT    TRANSMEM    269     291
FT    TOPO_DOM    292     309       CYTOPLASMIC.
//
```

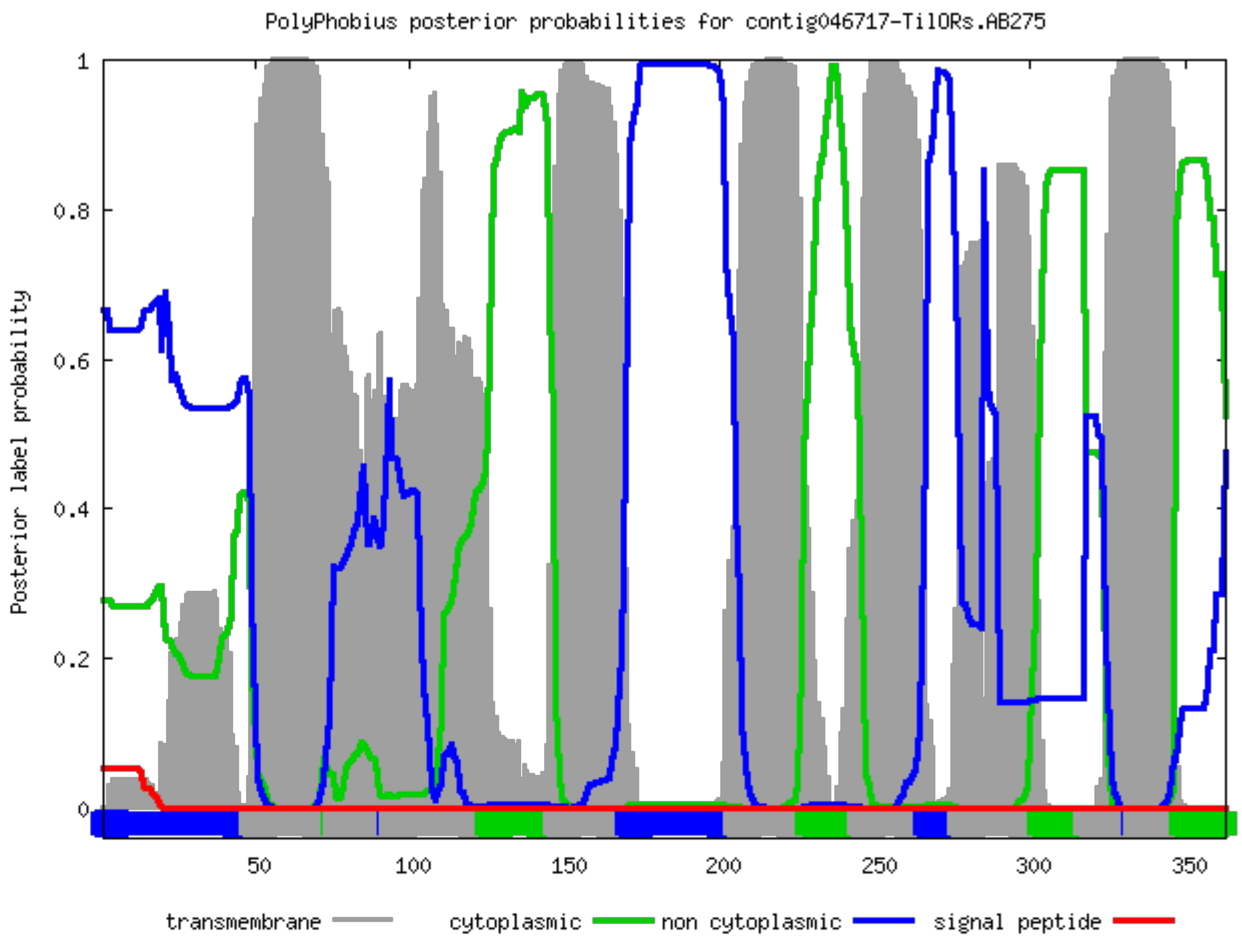

The prediction is based on an alignment. The probability data used in the plot is found here, and the gnuplot script is here.

Prediction of contig013344-TilOR.D058

```
ID   contig013344-TilOR.D058
FT   TOPO_DOM      1      22       NON CYTOPLASMIC.
FT   TRANSMEM     23      48
FT   TOPO_DOM     49      57       CYTOPLASMIC.
FT   TRANSMEM     58      81
FT   TOPO_DOM     82      90       NON CYTOPLASMIC.
FT   TRANSMEM     91     118
FT   TOPO_DOM    119     138       CYTOPLASMIC.
FT   TRANSMEM    139     161
FT   TOPO_DOM    162     193       NON CYTOPLASMIC.
FT   TRANSMEM    194     216
FT   TOPO_DOM    217     236       CYTOPLASMIC.
FT   TRANSMEM    237     256
FT   TOPO_DOM    257     267       NON CYTOPLASMIC.
FT   TRANSMEM    268     291
FT   TOPO_DOM    292     309       CYTOPLASMIC.
//
```

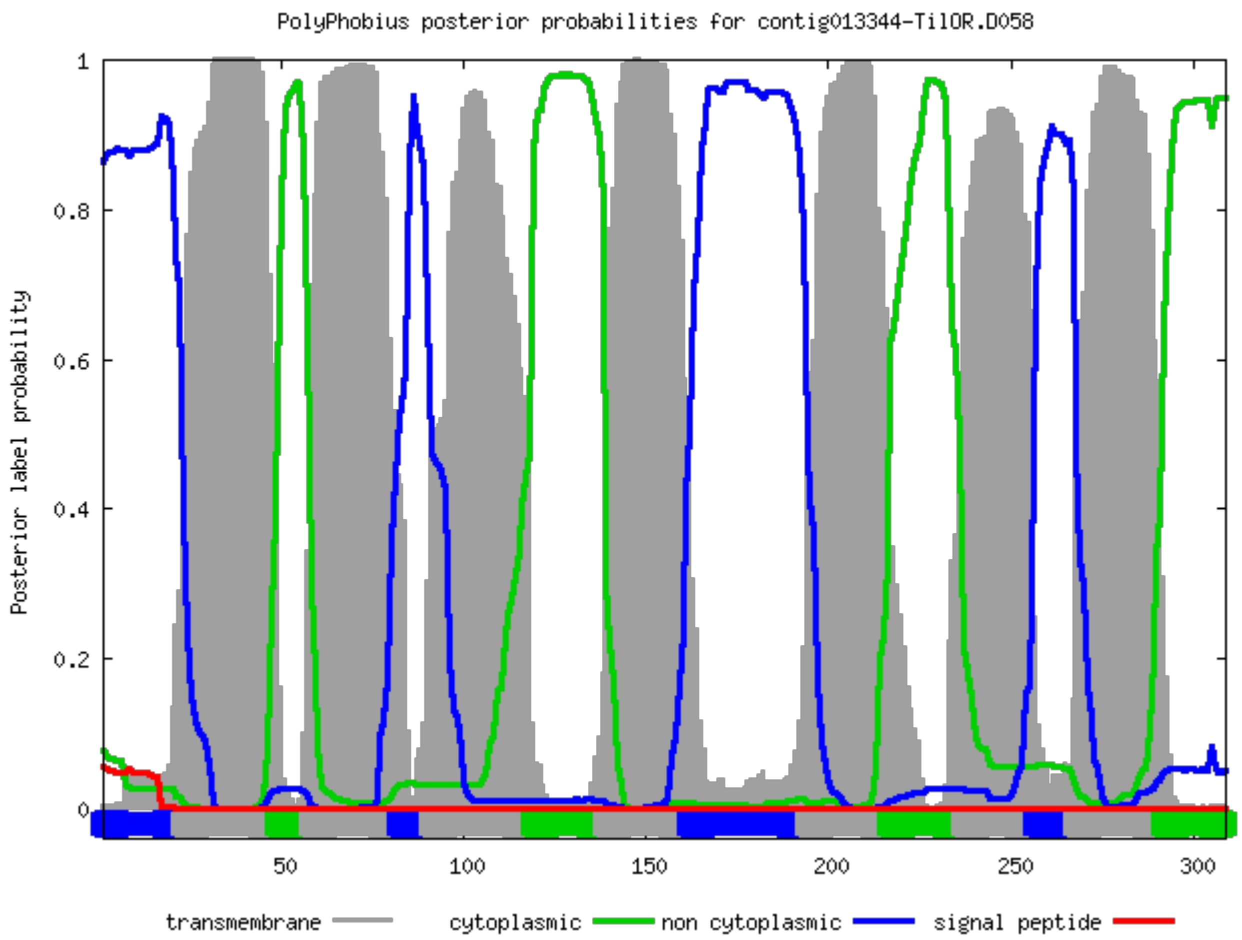

The prediction is based on an alignment. The probability data used in the plot is found here, and the gnuplot script is here.

Prediction of contig039465-TilOR.L150

```
ID   contig039465-TilOR.L150
FT   TOPO_DOM      1      25       NON CYTOPLASMIC.
FT   TRANSMEM     26      50
FT   TOPO_DOM     51      59       CYTOPLASMIC.
FT   TRANSMEM     60      82
FT   TOPO_DOM     83     100       NON CYTOPLASMIC.
FT   TRANSMEM    101     120
FT   TOPO_DOM    121     140       CYTOPLASMIC.
FT   TRANSMEM    141     163
FT   TOPO_DOM    164     199       NON CYTOPLASMIC.
FT   TRANSMEM    200     224
FT   TOPO_DOM    225     237       CYTOPLASMIC.
FT   TRANSMEM    238     260
FT   TOPO_DOM    261     271       NON CYTOPLASMIC.
FT   TRANSMEM    272     292
FT   TOPO_DOM    293     313       CYTOPLASMIC.
//
```

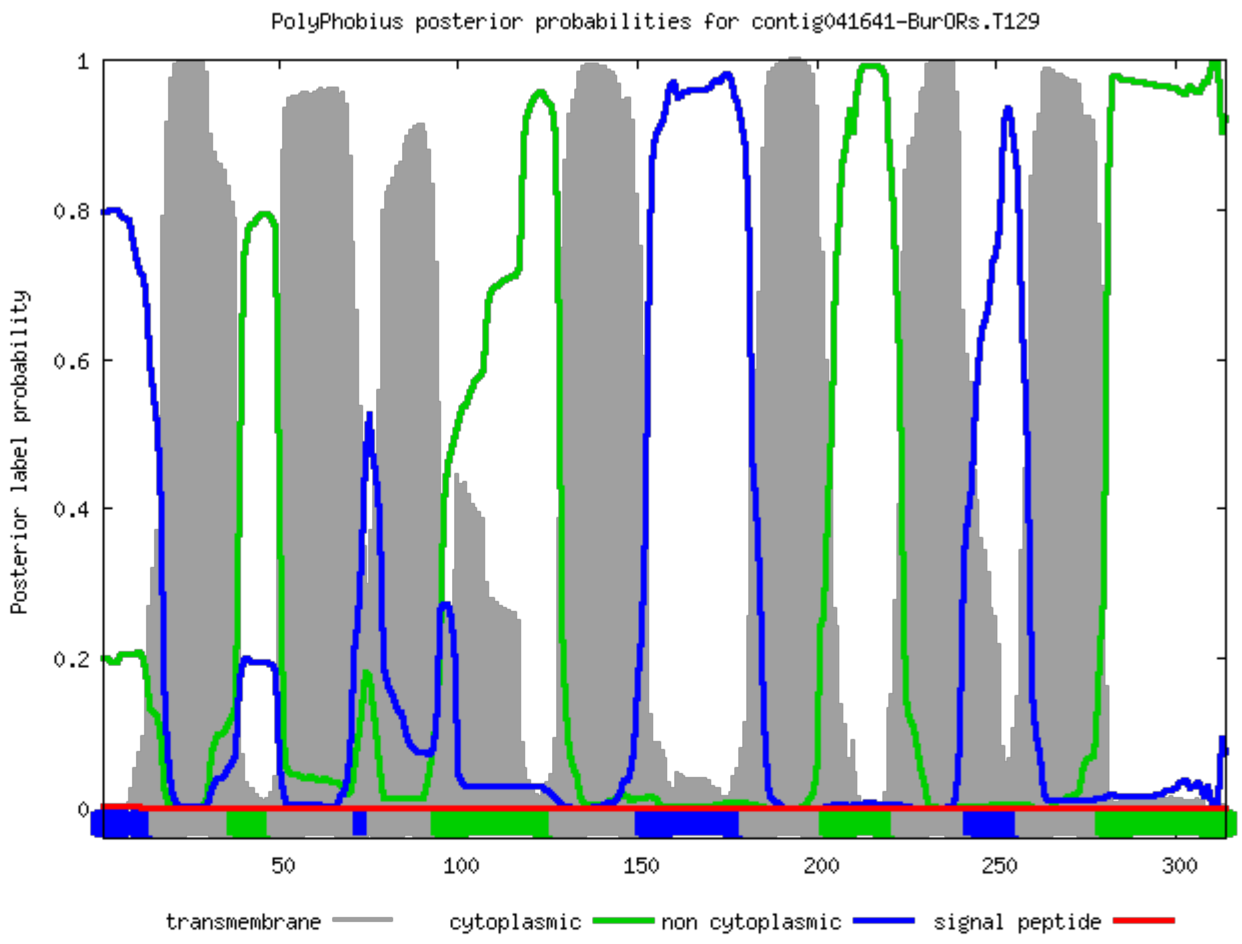

The prediction is based on an alignment. The probability data used in the plot is found here, and the gnuplot script is here.

Prediction of contig054678-NyeOR.A010

```
ID   contig054678-NyeOR.A010
FT   TOPO_DOM      1     25       NON CYTOPLASMIC.
FT   TRANSMEM     26     51
FT   TOPO_DOM     52     59       CYTOPLASMIC.
FT   TRANSMEM     60     80
FT   TOPO_DOM     81     98       NON CYTOPLASMIC.
FT   TRANSMEM     99    121
FT   TOPO_DOM    122    141       CYTOPLASMIC.
FT   TRANSMEM    142    163
FT   TOPO_DOM    164    195       NON CYTOPLASMIC.
FT   TRANSMEM    196    219
FT   TOPO_DOM    220    239       CYTOPLASMIC.
FT   TRANSMEM    240    261
FT   TOPO_DOM    262    272       NON CYTOPLASMIC.
FT   TRANSMEM    273    293
FT   TOPO_DOM    294    337       CYTOPLASMIC.
//
```

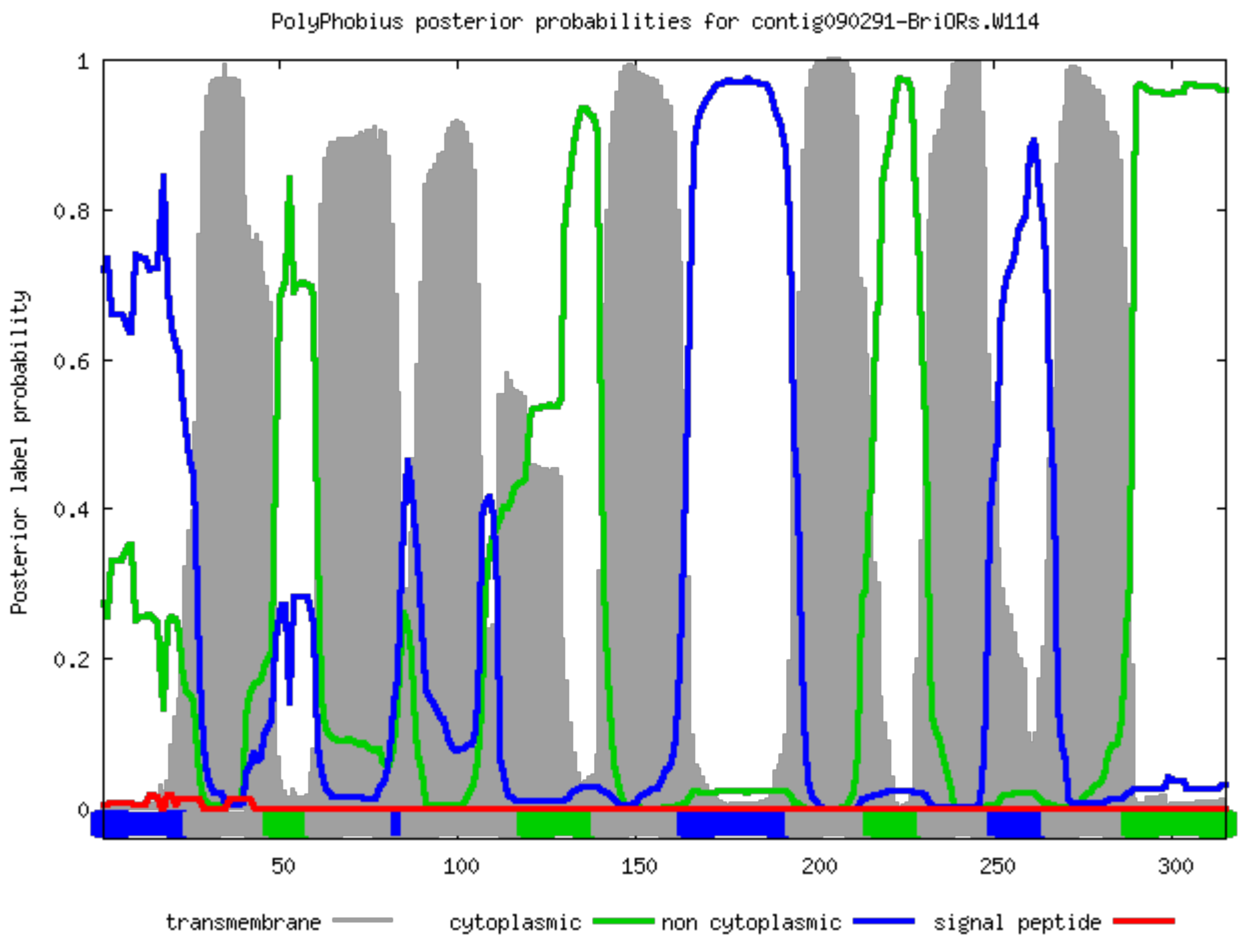

The prediction is based on an alignment. The probability data used in the plot is found here, and the gnuplot script is here.

Prediction of contig057165-NyeOR.A017

```
ID   contig057165-NyeOR.A017
FT   TOPO_DOM      1      22       NON CYTOPLASMIC.
FT   TRANSMEM     23      48
FT   TOPO_DOM     49      56       CYTOPLASMIC.
FT   TRANSMEM     57      77
FT   TOPO_DOM     78      95       NON CYTOPLASMIC.
FT   TRANSMEM     96     118
FT   TOPO_DOM    119     138       CYTOPLASMIC.
FT   TRANSMEM    139     159
FT   TOPO_DOM    160     192       NON CYTOPLASMIC.
FT   TRANSMEM    193     216
FT   TOPO_DOM    217     235       CYTOPLASMIC.
FT   TRANSMEM    236     257
FT   TOPO_DOM    258     268       NON CYTOPLASMIC.
FT   TRANSMEM    269     289
FT   TOPO_DOM    290     309       CYTOPLASMIC.
//
```

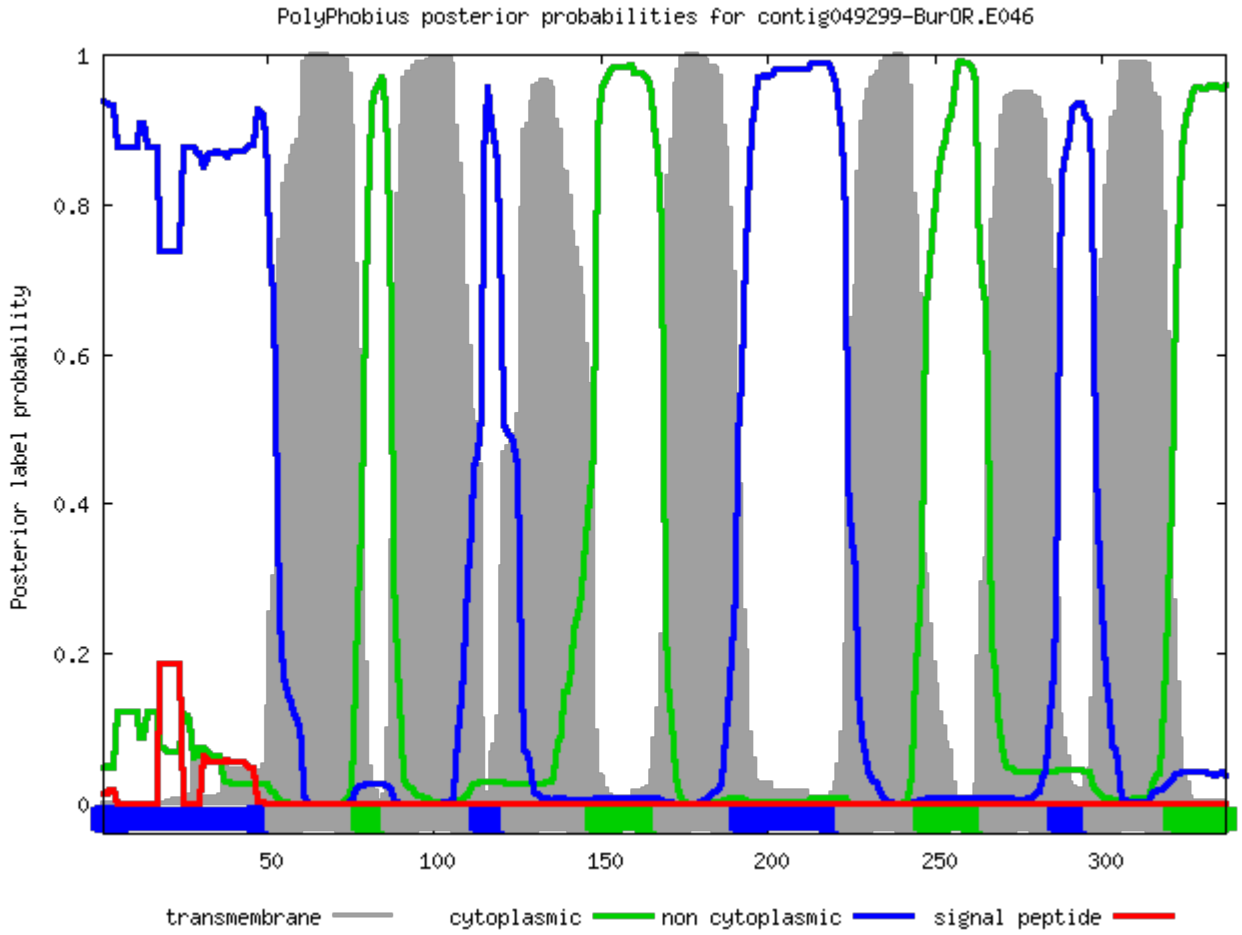

The prediction is based on an alignment. The probability data used in the plot is found here, and the gnuplot script is here.

Prediction of contig025224-ZebOR.C035

```
ID   contig025224-ZebOR.C035
FT   TOPO_DOM      1      21       NON CYTOPLASMIC.
FT   TRANSMEM     22      47
FT   TOPO_DOM     48      56       CYTOPLASMIC.
FT   TRANSMEM     57      79
FT   TOPO_DOM     80      94       NON CYTOPLASMIC.
FT   TRANSMEM     95     117
FT   TOPO_DOM    118     137       CYTOPLASMIC.
FT   TRANSMEM    138     161
FT   TOPO_DOM    162     192       NON CYTOPLASMIC.
FT   TRANSMEM    193     215
FT   TOPO_DOM    216     234       CYTOPLASMIC.
FT   TRANSMEM    235     255
FT   TOPO_DOM    256     266       NON CYTOPLASMIC.
FT   TRANSMEM    267     289
FT   TOPO_DOM    290     321       CYTOPLASMIC.
//
```

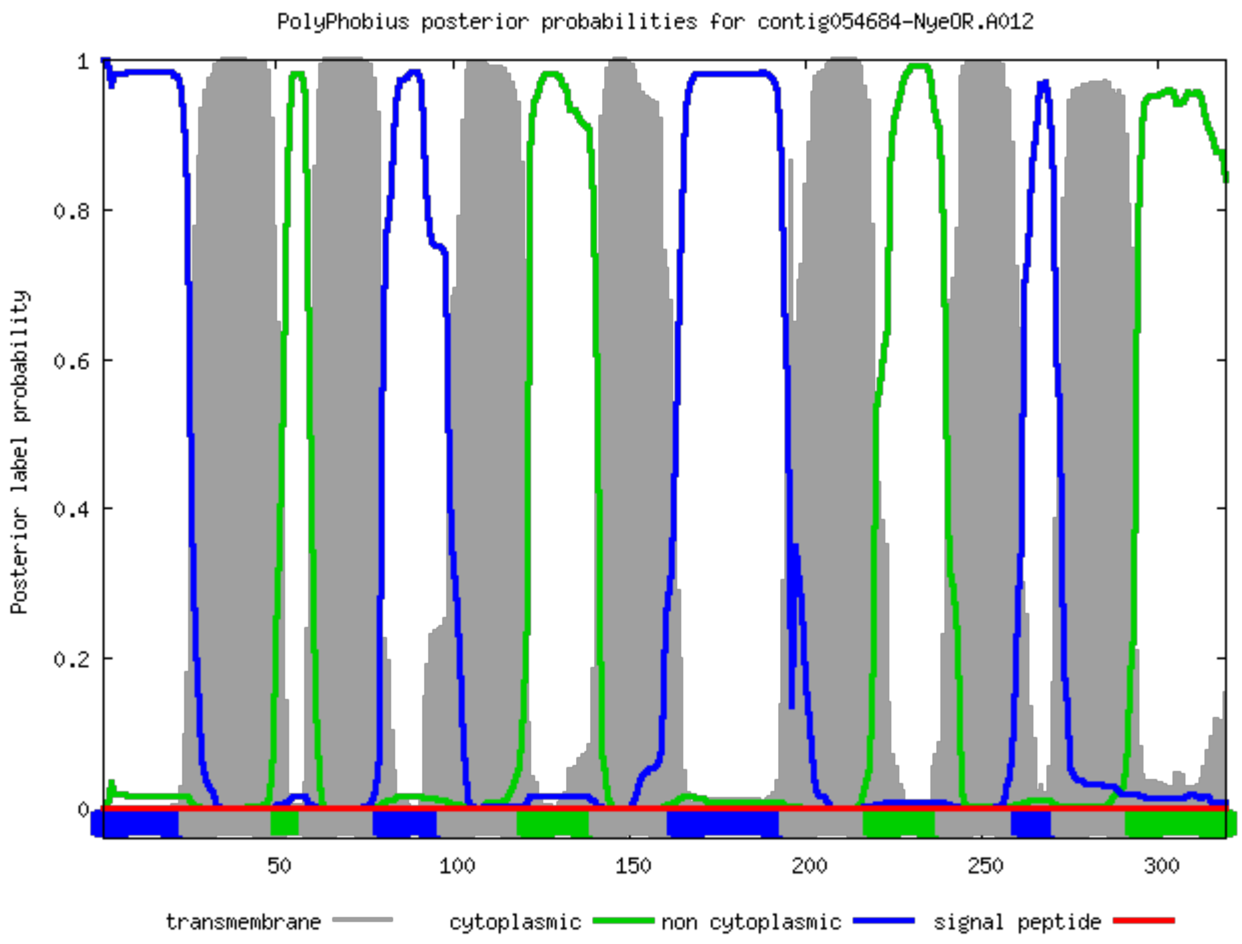

The prediction is based on an alignment. The probability data used in the plot is found here, and the gnuplot script is here.

Prediction of contig039460-TilOR.L146

```
ID   contig039460-TilOR.L146
FT   TOPO_DOM      1     25       NON CYTOPLASMIC.
FT   TRANSMEM     26     50
FT   TOPO_DOM     51     59       CYTOPLASMIC.
FT   TRANSMEM     60     82
FT   TOPO_DOM     83     98       NON CYTOPLASMIC.
FT   TRANSMEM     99    120
FT   TOPO_DOM    121    140       CYTOPLASMIC.
FT   TRANSMEM    141    162
FT   TOPO_DOM    163    198       NON CYTOPLASMIC.
FT   TRANSMEM    199    224
FT   TOPO_DOM    225    235       CYTOPLASMIC.
FT   TRANSMEM    236    260
FT   TOPO_DOM    261    271       NON CYTOPLASMIC.
FT   TRANSMEM    272    292
FT   TOPO_DOM    293    311       CYTOPLASMIC.
//
```

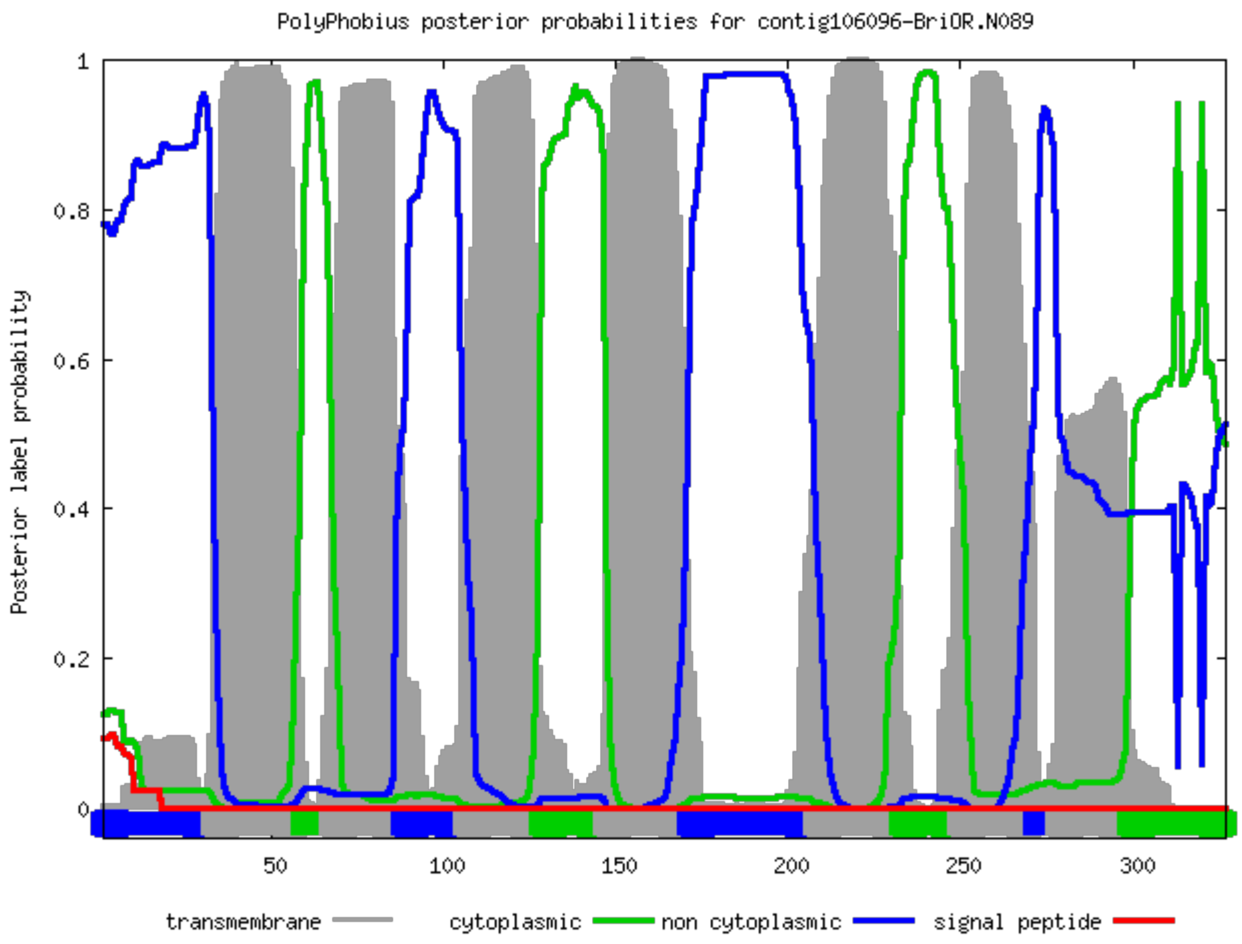

The prediction is based on an alignment. The probability data used in the plot is found here, and the gnuplot script is here.

Prediction of contig013361-TilOR.H108

```
ID   contig013361-TilOR.H108
FT   TOPO_DOM      1     23       NON CYTOPLASMIC.
FT   TRANSMEM     24     49
FT   TOPO_DOM     50     56       CYTOPLASMIC.
FT   TRANSMEM     57     76
FT   TOPO_DOM     77     95       NON CYTOPLASMIC.
FT   TRANSMEM     96    118
FT   TOPO_DOM    119    138       CYTOPLASMIC.
FT   TRANSMEM    139    160
FT   TOPO_DOM    161    196       NON CYTOPLASMIC.
FT   TRANSMEM    197    219
FT   TOPO_DOM    220    237       CYTOPLASMIC.
FT   TRANSMEM    238    259
FT   TOPO_DOM    260    271       NON CYTOPLASMIC.
FT   TRANSMEM    272    291
FT   TOPO_DOM    292    310       CYTOPLASMIC.
//
```

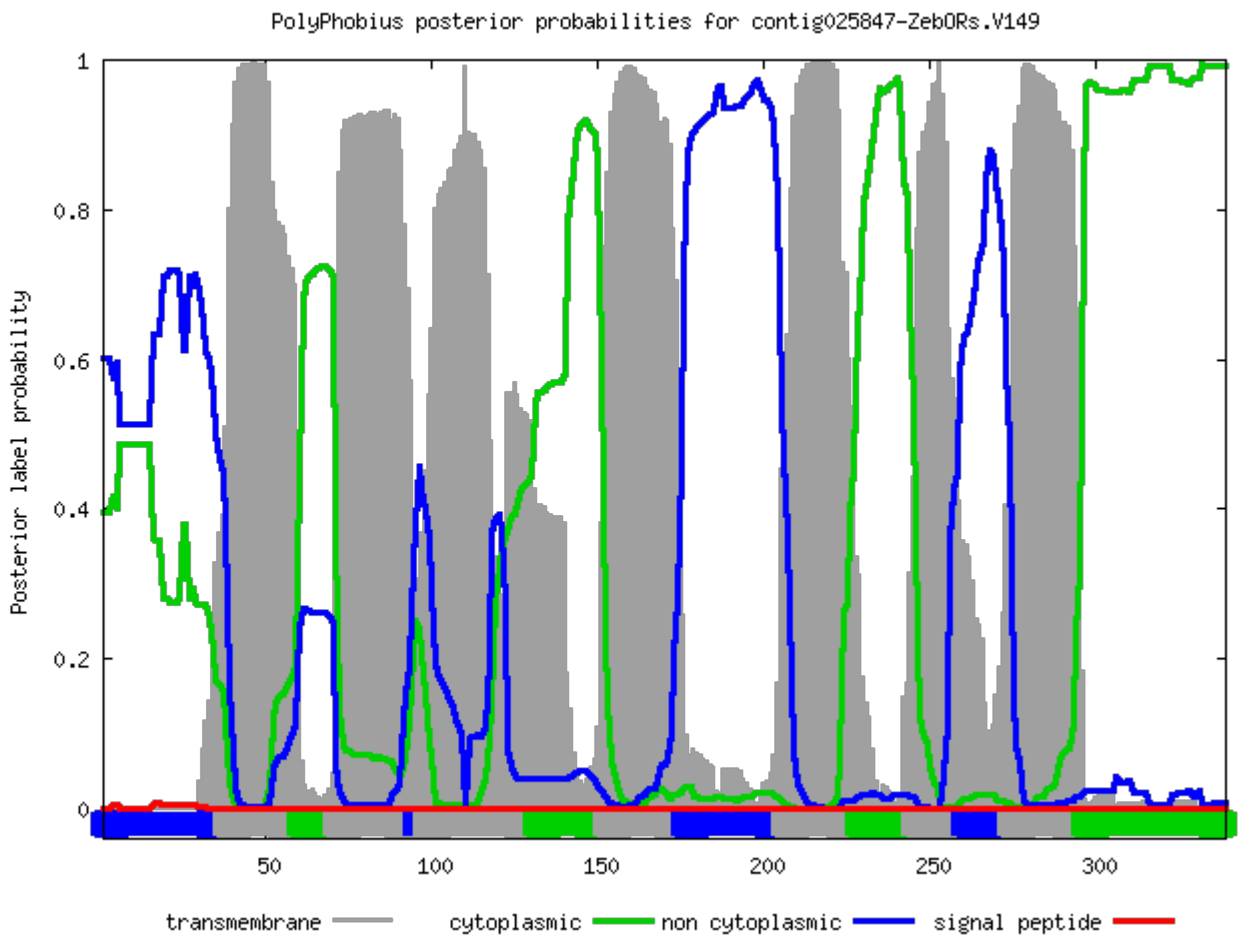

The prediction is based on an alignment. The probability data used in the plot is found here, and the gnuplot script is here.

Prediction of contig038871-NyeOR.S121

```
ID   contig038871-NyeOR.S121
FT   TOPO_DOM      1     21       NON CYTOPLASMIC.
FT   TRANSMEM     22     43
FT   TOPO_DOM     44     53       CYTOPLASMIC.
FT   TRANSMEM     54     78
FT   TOPO_DOM     79     83       NON CYTOPLASMIC.
FT   TRANSMEM     84    112
FT   TOPO_DOM    113    132       CYTOPLASMIC.
FT   TRANSMEM    133    158
FT   TOPO_DOM    159    186       NON CYTOPLASMIC.
FT   TRANSMEM    187    207
FT   TOPO_DOM    208    227       CYTOPLASMIC.
FT   TRANSMEM    228    253
FT   TOPO_DOM    254    264       NON CYTOPLASMIC.
FT   TRANSMEM    265    285
FT   TOPO_DOM    286    313       CYTOPLASMIC.
//
```

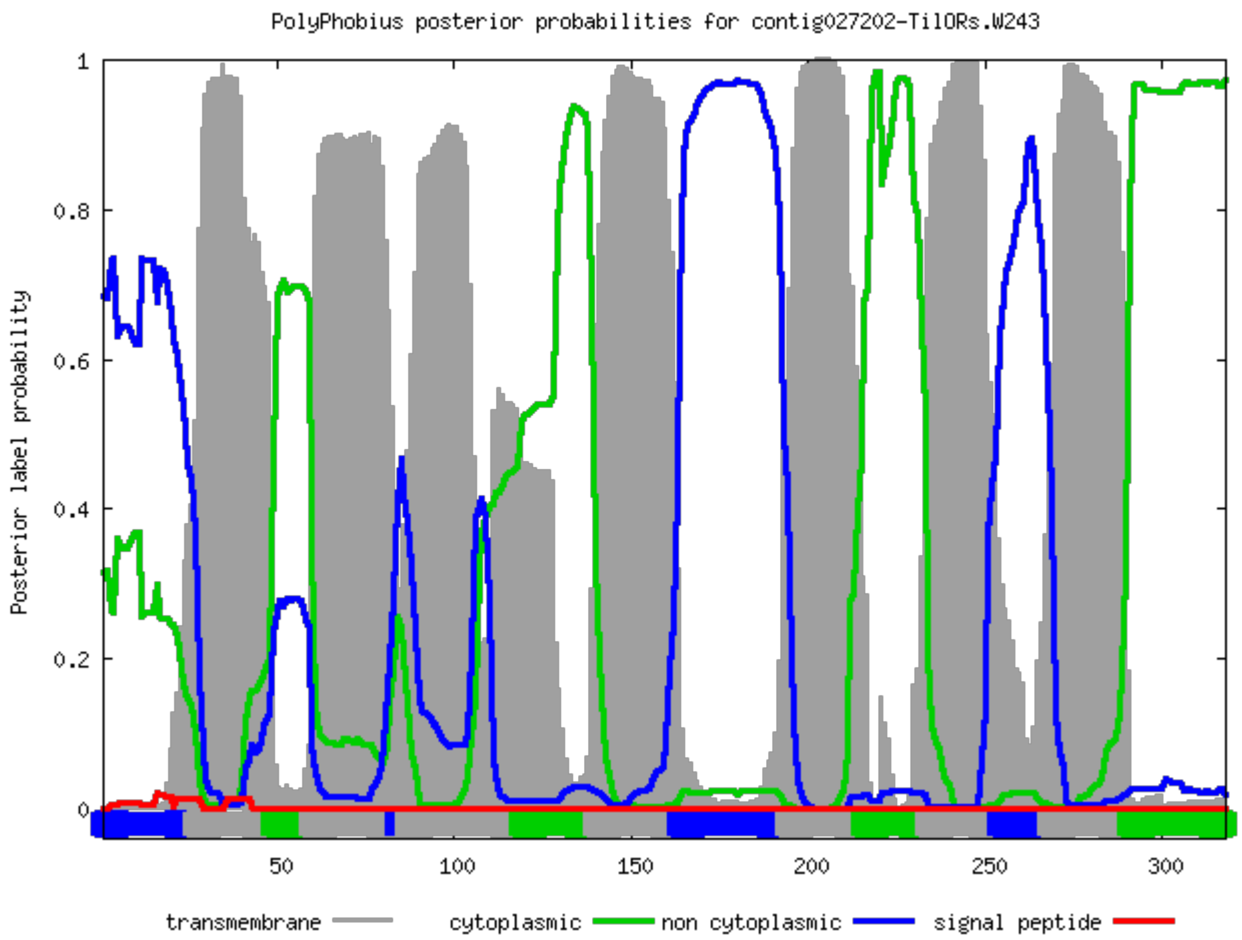

The prediction is based on an alignment. The probability data used in the plot is found here, and the gnuplot script is here.

Prediction of contig006794-BurOR.H061

```
ID    contig006794-BurOR.H061
FT    TOPO_DOM      1     23       NON CYTOPLASMIC.
FT    TRANSMEM     24     49
FT    TOPO_DOM     50     56       CYTOPLASMIC.
FT    TRANSMEM     57     76
FT    TOPO_DOM     77     95       NON CYTOPLASMIC.
FT    TRANSMEM     96    118
FT    TOPO_DOM    119    138       CYTOPLASMIC.
FT    TRANSMEM    139    160
FT    TOPO_DOM    161    196       NON CYTOPLASMIC.
FT    TRANSMEM    197    219
FT    TOPO_DOM    220    237       CYTOPLASMIC.
FT    TRANSMEM    238    260
FT    TOPO_DOM    261    271       NON CYTOPLASMIC.
FT    TRANSMEM    272    291
FT    TOPO_DOM    292    343       CYTOPLASMIC.
//
```

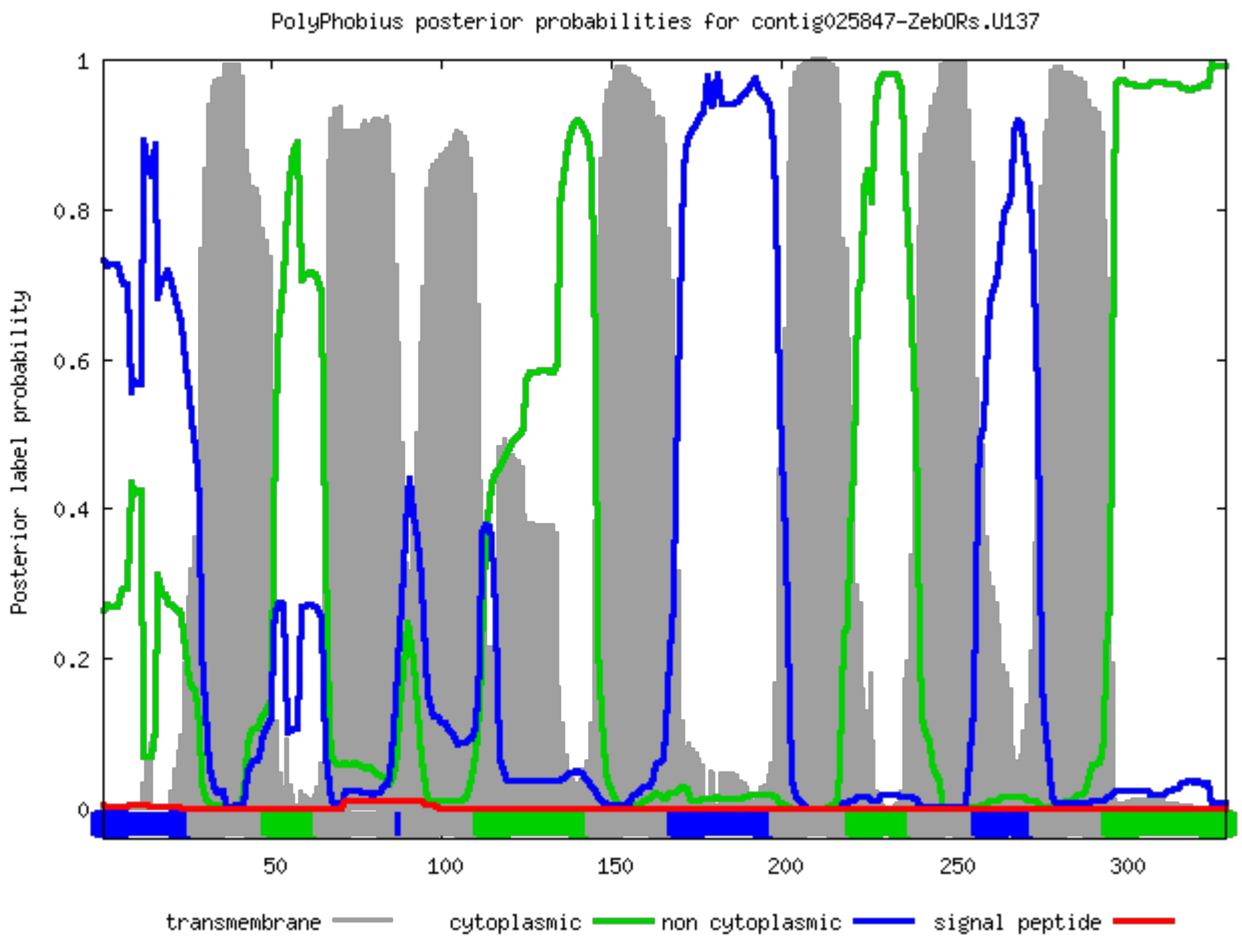

The prediction is based on an alignment. The probability data used in the plot is found here, and the gnuplot script is here.

Prediction of contig047514-ZebOR.A017

```
ID   contig047514-ZebOR.A017
FT   TOPO_DOM      1     22       NON CYTOPLASMIC.
FT   TRANSMEM     23     48
FT   TOPO_DOM     49     56       CYTOPLASMIC.
FT   TRANSMEM     57     76
FT   TOPO_DOM     77     95       NON CYTOPLASMIC.
FT   TRANSMEM     96    118
FT   TOPO_DOM    119    138       CYTOPLASMIC.
FT   TRANSMEM    139    160
FT   TOPO_DOM    161    192       NON CYTOPLASMIC.
FT   TRANSMEM    193    215
FT   TOPO_DOM    216    235       CYTOPLASMIC.
FT   TRANSMEM    236    257
FT   TOPO_DOM    258    268       NON CYTOPLASMIC.
FT   TRANSMEM    269    289
FT   TOPO_DOM    290    306       CYTOPLASMIC.
//
```

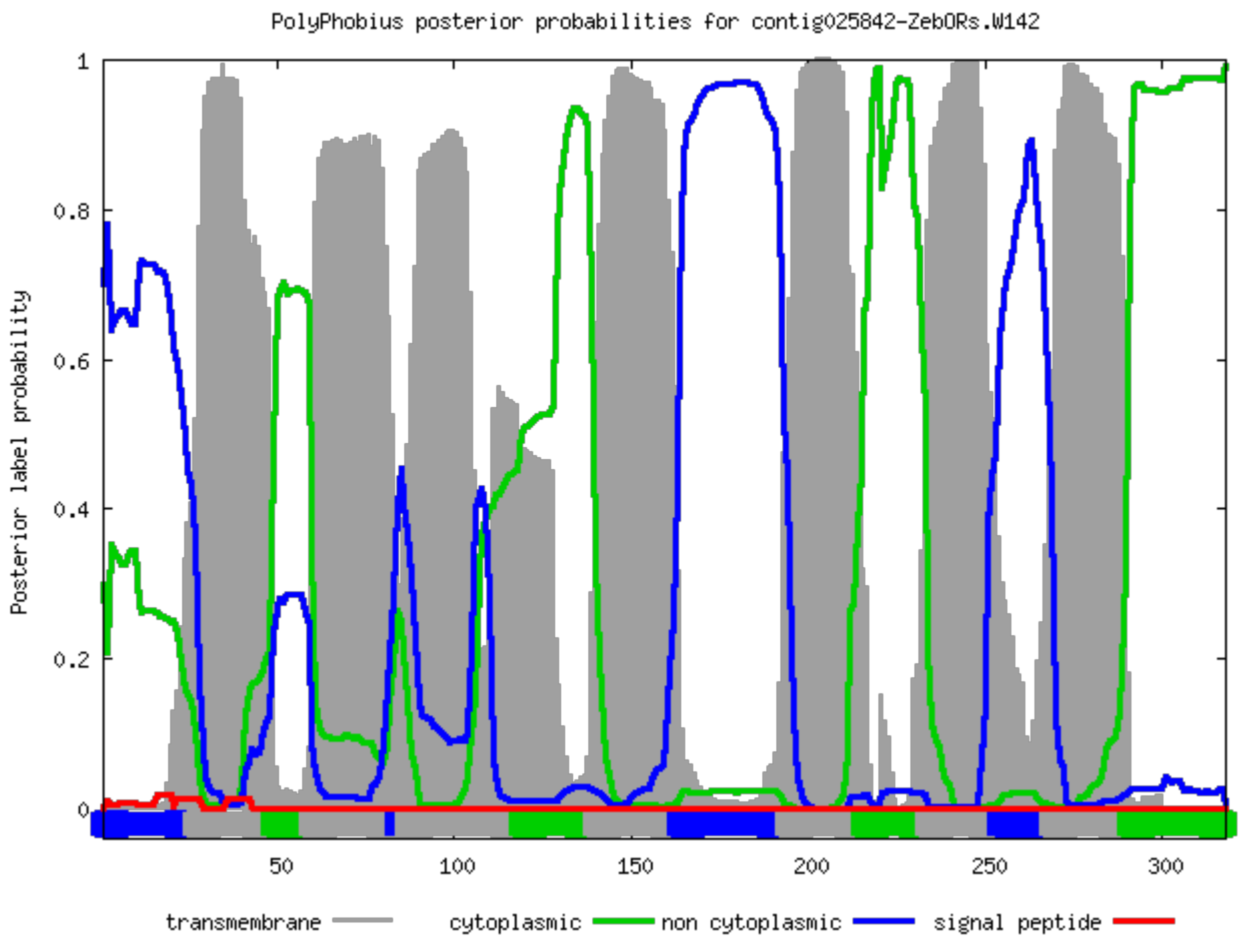

The prediction is based on an alignment. The probability data used in the plot is found here, and the gnuplot script is here.

Prediction of contig030554-ZebOR.A004

```
ID   contig030554-ZebOR.A004
FT   TOPO_DOM      1      24       NON CYTOPLASMIC.
FT   TRANSMEM     25      50
FT   TOPO_DOM     51      58       CYTOPLASMIC.
FT   TRANSMEM     59      79
FT   TOPO_DOM     80      97       NON CYTOPLASMIC.
FT   TRANSMEM     98     120
FT   TOPO_DOM    121     140       CYTOPLASMIC.
FT   TRANSMEM    141     162
FT   TOPO_DOM    163     194       NON CYTOPLASMIC.
FT   TRANSMEM    195     218
FT   TOPO_DOM    219     238       CYTOPLASMIC.
FT   TRANSMEM    239     260
FT   TOPO_DOM    261     271       NON CYTOPLASMIC.
FT   TRANSMEM    272     292
FT   TOPO_DOM    293     328       CYTOPLASMIC.
//
```

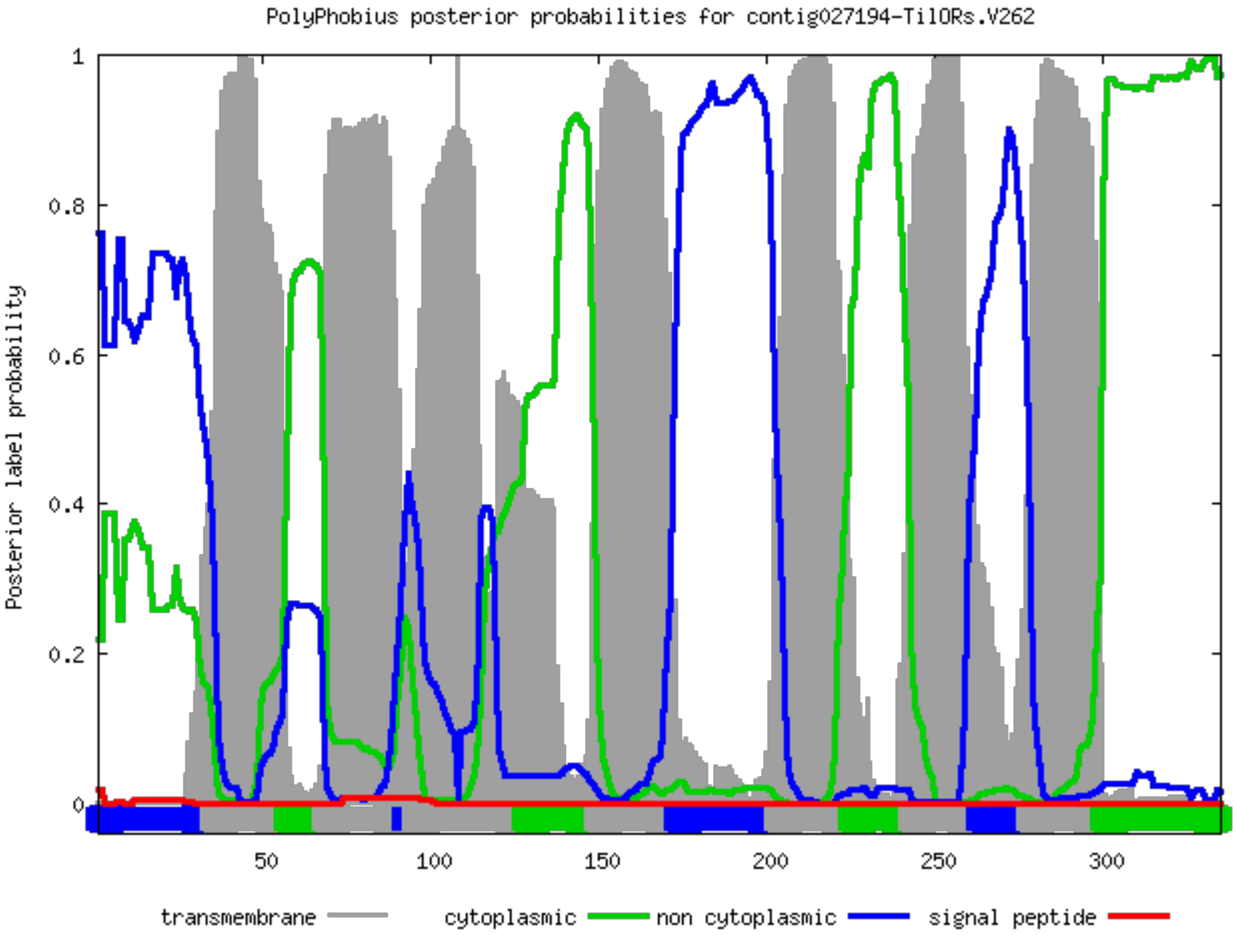

The prediction is based on an alignment. The probability data used in the plot is found here, and the gnuplot script is here.

Prediction of contig062770-NyeOR.E059

```
ID   contig062770-NyeOR.E059
FT   TOPO_DOM      1     23       NON CYTOPLASMIC.
FT   TRANSMEM     24     49
FT   TOPO_DOM     50     58       CYTOPLASMIC.
FT   TRANSMEM     59     82
FT   TOPO_DOM     83     93       NON CYTOPLASMIC.
FT   TRANSMEM     94    119
FT   TOPO_DOM    120    139       CYTOPLASMIC.
FT   TRANSMEM    140    161
FT   TOPO_DOM    162    194       NON CYTOPLASMIC.
FT   TRANSMEM    195    217
FT   TOPO_DOM    218    237       CYTOPLASMIC.
FT   TRANSMEM    238    257
FT   TOPO_DOM    258    268       NON CYTOPLASMIC.
FT   TRANSMEM    269    292
FT   TOPO_DOM    293    310       CYTOPLASMIC.
//
```

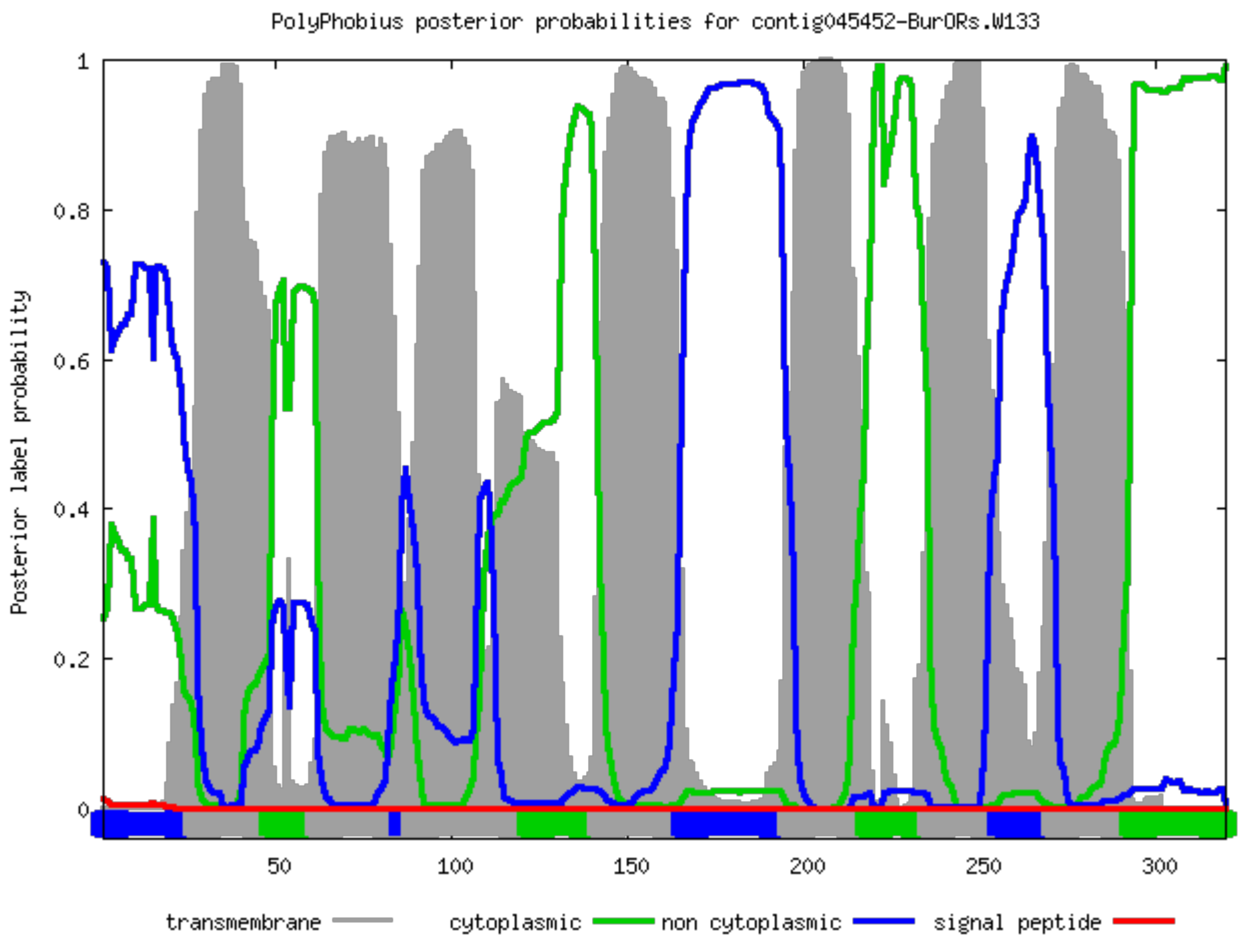

The prediction is based on an alignment. The probability data used in the plot is found here, and the gnuplot script is here.

Prediction of contig055926-NyeOR.N110

```
ID   contig055926-NyeOR.N110
FT   TOPO_DOM      1     32       NON CYTOPLASMIC.
FT   TRANSMEM     33     58
FT   TOPO_DOM     59     66       CYTOPLASMIC.
FT   TRANSMEM     67     86
FT   TOPO_DOM     87    104       NON CYTOPLASMIC.
FT   TRANSMEM    105    127
FT   TOPO_DOM    128    146       CYTOPLASMIC.
FT   TRANSMEM    147    170
FT   TOPO_DOM    171    206       NON CYTOPLASMIC.
FT   TRANSMEM    207    232
FT   TOPO_DOM    233    250       CYTOPLASMIC.
FT   TRANSMEM    251    272
FT   TOPO_DOM    273    277       NON CYTOPLASMIC.
FT   TRANSMEM    278    298
FT   TOPO_DOM    299    323       CYTOPLASMIC.
//
```

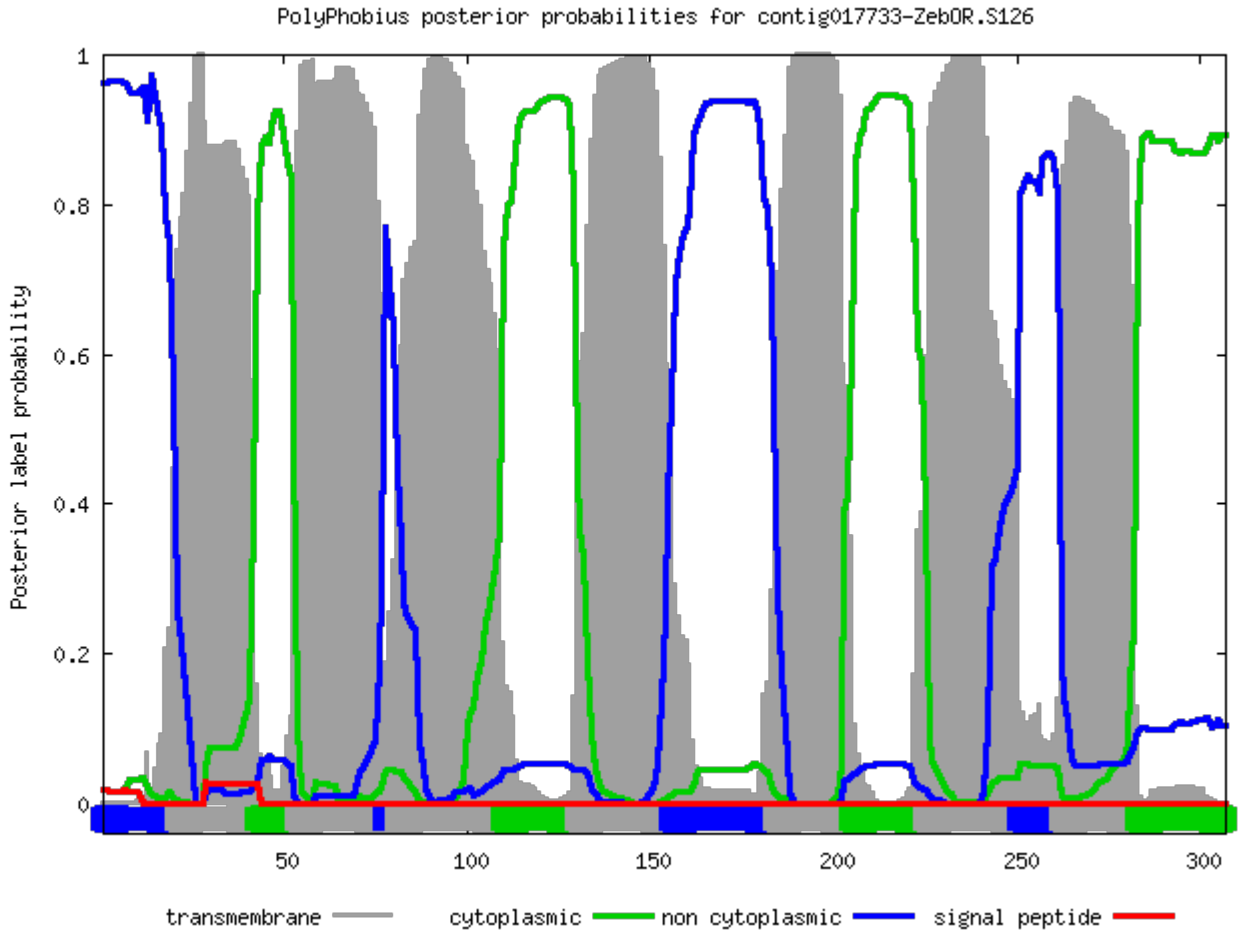

The prediction is based on an alignment. The probability data used in the plot is found here, and the gnuplot script is here.

Prediction of contig084999-BriOR.A002

```
ID    contig084999-BriOR.A002
FT    TOPO_DOM      1      24       NON CYTOPLASMIC.
FT    TRANSMEM     25      50
FT    TOPO_DOM     51      58       CYTOPLASMIC.
FT    TRANSMEM     59      79
FT    TOPO_DOM     80      97       NON CYTOPLASMIC.
FT    TRANSMEM     98     120
FT    TOPO_DOM    121     140       CYTOPLASMIC.
FT    TRANSMEM    141     162
FT    TOPO_DOM    163     194       NON CYTOPLASMIC.
FT    TRANSMEM    195     218
FT    TOPO_DOM    219     238       CYTOPLASMIC.
FT    TRANSMEM    239     260
FT    TOPO_DOM    261     271       NON CYTOPLASMIC.
FT    TRANSMEM    272     292
FT    TOPO_DOM    293     328       CYTOPLASMIC.
//
```

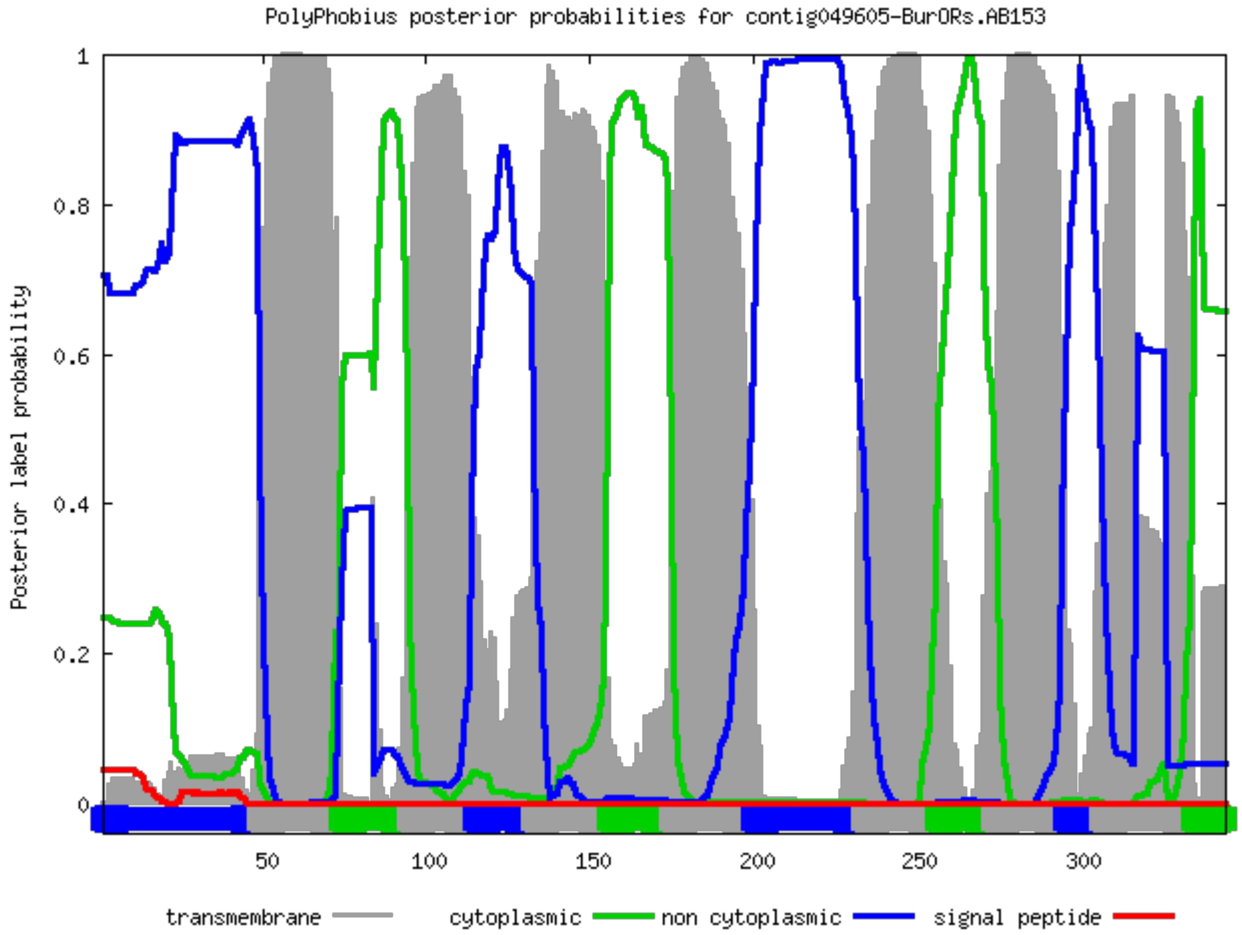

The prediction is based on an alignment. The probability data used in the plot is found here, and the gnuplot script is here.

Prediction of contig010722-ZebOR.N111

```
ID   contig010722-ZebOR.N111
FT   TOPO_DOM      1     32       NON CYTOPLASMIC.
FT   TRANSMEM     33     58
FT   TOPO_DOM     59     66       CYTOPLASMIC.
FT   TRANSMEM     67     87
FT   TOPO_DOM     88    105       NON CYTOPLASMIC.
FT   TRANSMEM    106    127
FT   TOPO_DOM    128    146       CYTOPLASMIC.
FT   TRANSMEM    147    170
FT   TOPO_DOM    171    207       NON CYTOPLASMIC.
FT   TRANSMEM    208    232
FT   TOPO_DOM    233    249       CYTOPLASMIC.
FT   TRANSMEM    250    271
FT   TOPO_DOM    272    277       NON CYTOPLASMIC.
FT   TRANSMEM    278    298
FT   TOPO_DOM    299    324       CYTOPLASMIC.
//
```

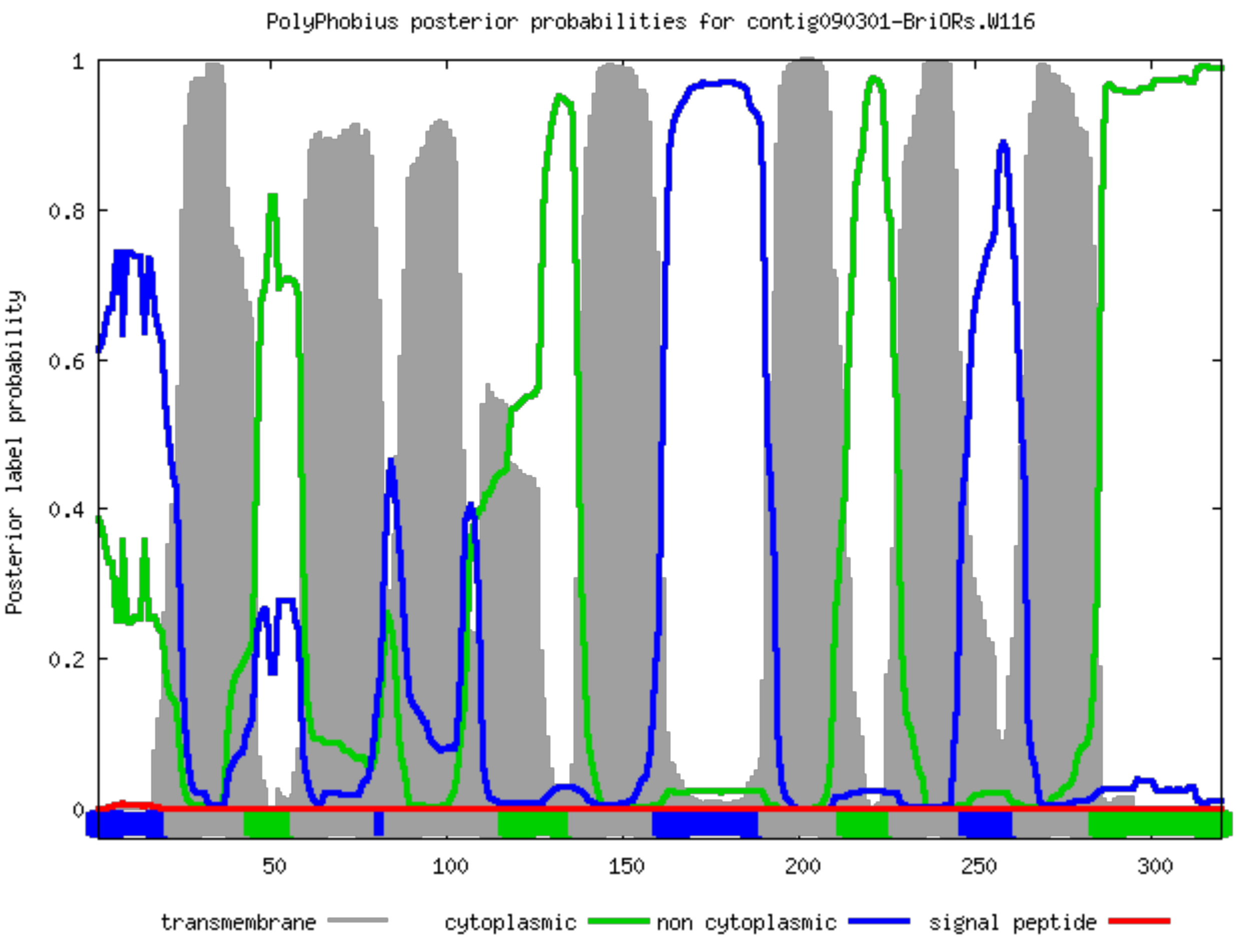

The prediction is based on an alignment. The probability data used in the plot is found here, and the gnuplot script is here.

Prediction of contig030576-ZebOR.A010

```
ID   contig030576-ZebOR.A010
FT   TOPO_DOM      1     22       NON CYTOPLASMIC.
FT   TRANSMEM     23     48
FT   TOPO_DOM     49     56       CYTOPLASMIC.
FT   TRANSMEM     57     76
FT   TOPO_DOM     77     95       NON CYTOPLASMIC.
FT   TRANSMEM     96    118
FT   TOPO_DOM    119    138       CYTOPLASMIC.
FT   TRANSMEM    139    159
FT   TOPO_DOM    160    192       NON CYTOPLASMIC.
FT   TRANSMEM    193    215
FT   TOPO_DOM    216    235       CYTOPLASMIC.
FT   TRANSMEM    236    257
FT   TOPO_DOM    258    268       NON CYTOPLASMIC.
FT   TRANSMEM    269    289
FT   TOPO_DOM    290    316       CYTOPLASMIC.
//
```

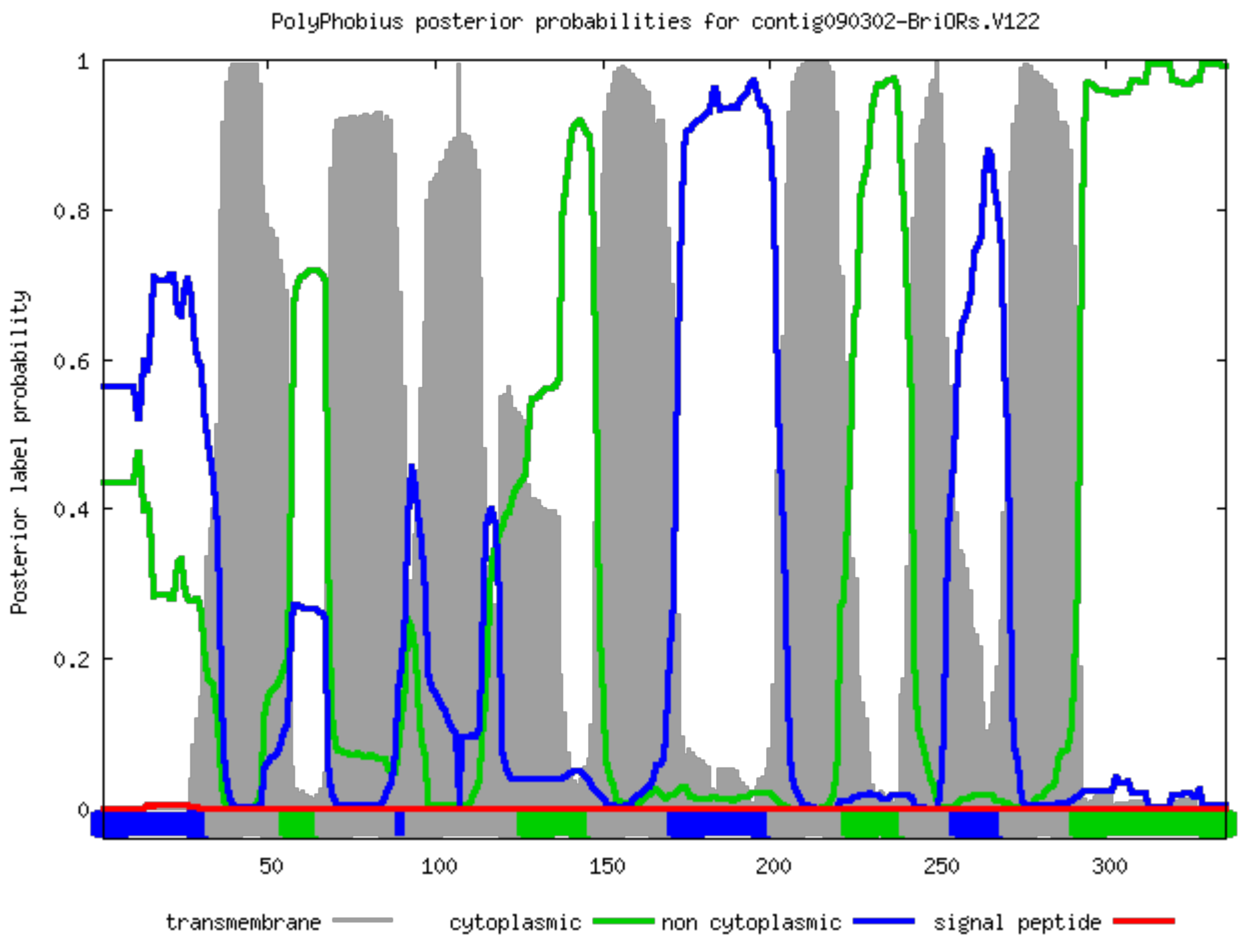

The prediction is based on an alignment. The probability data used in the plot is found here, and the gnuplot script is here.

Prediction of contig053592-NyeOR.E057

```
ID   contig053592-NyeOR.E057
FT   TOPO_DOM      1      21       NON CYTOPLASMIC.
FT   TRANSMEM     22      47
FT   TOPO_DOM     48      56       CYTOPLASMIC.
FT   TRANSMEM     57      81
FT   TOPO_DOM     82      93       NON CYTOPLASMIC.
FT   TRANSMEM     94     117
FT   TOPO_DOM    118     137       CYTOPLASMIC.
FT   TRANSMEM    138     159
FT   TOPO_DOM    160     192       NON CYTOPLASMIC.
FT   TRANSMEM    193     215
FT   TOPO_DOM    216     235       CYTOPLASMIC.
FT   TRANSMEM    236     255
FT   TOPO_DOM    256     266       NON CYTOPLASMIC.
FT   TRANSMEM    267     290
FT   TOPO_DOM    291     306       CYTOPLASMIC.
//
```

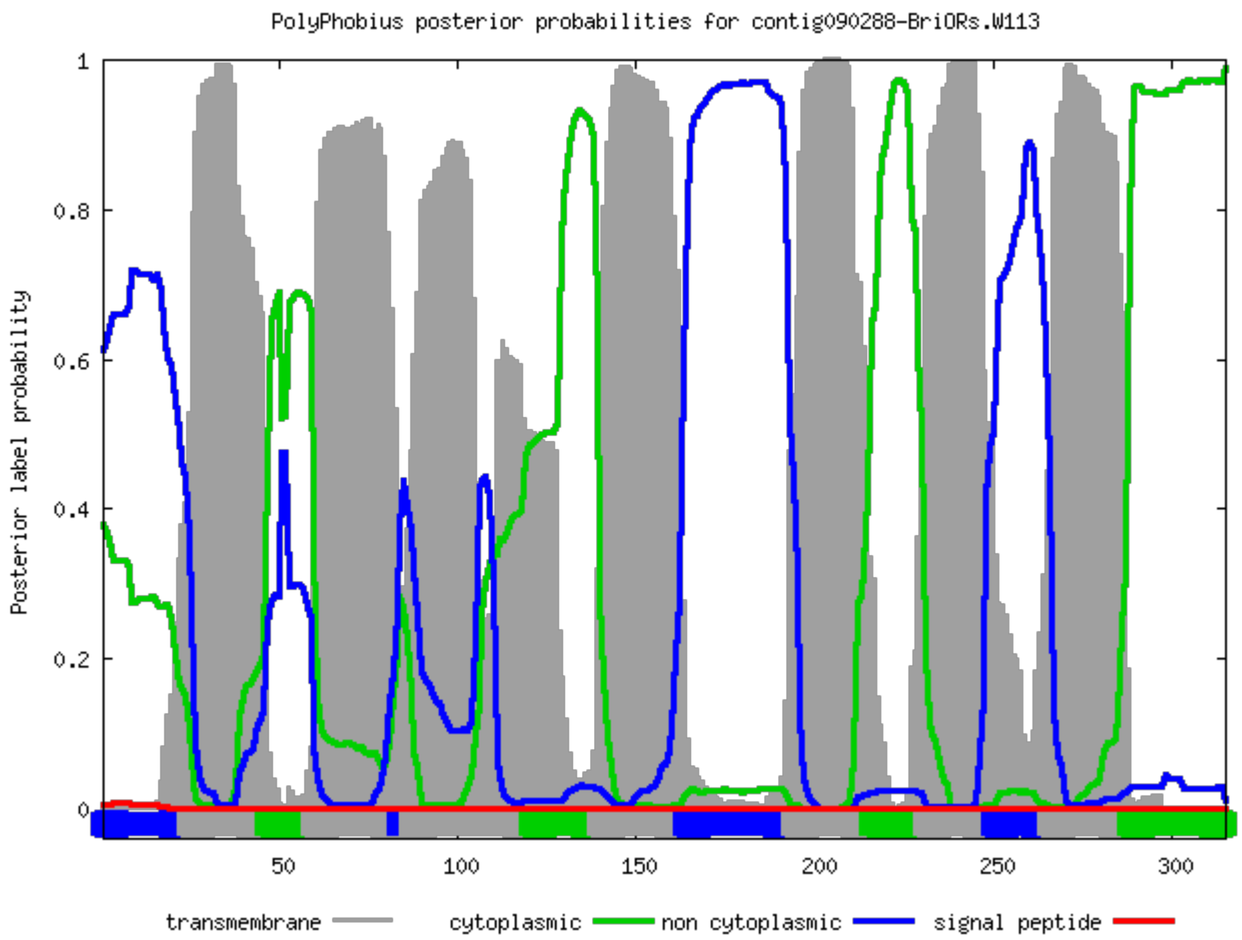

The prediction is based on an alignment. The probability data used in the plot is found here, and the gnuplot script is here.

Prediction of contig068054-ZebOR.S129

```
ID   contig068054-ZebOR.S129
FT   TOPO_DOM      1     20       NON CYTOPLASMIC.
FT   TRANSMEM     21     42
FT   TOPO_DOM     43     52       CYTOPLASMIC.
FT   TRANSMEM     53     77
FT   TOPO_DOM     78     82       NON CYTOPLASMIC.
FT   TRANSMEM     83    111
FT   TOPO_DOM    112    131       CYTOPLASMIC.
FT   TRANSMEM    132    157
FT   TOPO_DOM    158    185       NON CYTOPLASMIC.
FT   TRANSMEM    186    206
FT   TOPO_DOM    207    226       CYTOPLASMIC.
FT   TRANSMEM    227    252
FT   TOPO_DOM    253    263       NON CYTOPLASMIC.
FT   TRANSMEM    264    284
FT   TOPO_DOM    285    307       CYTOPLASMIC.
//
```

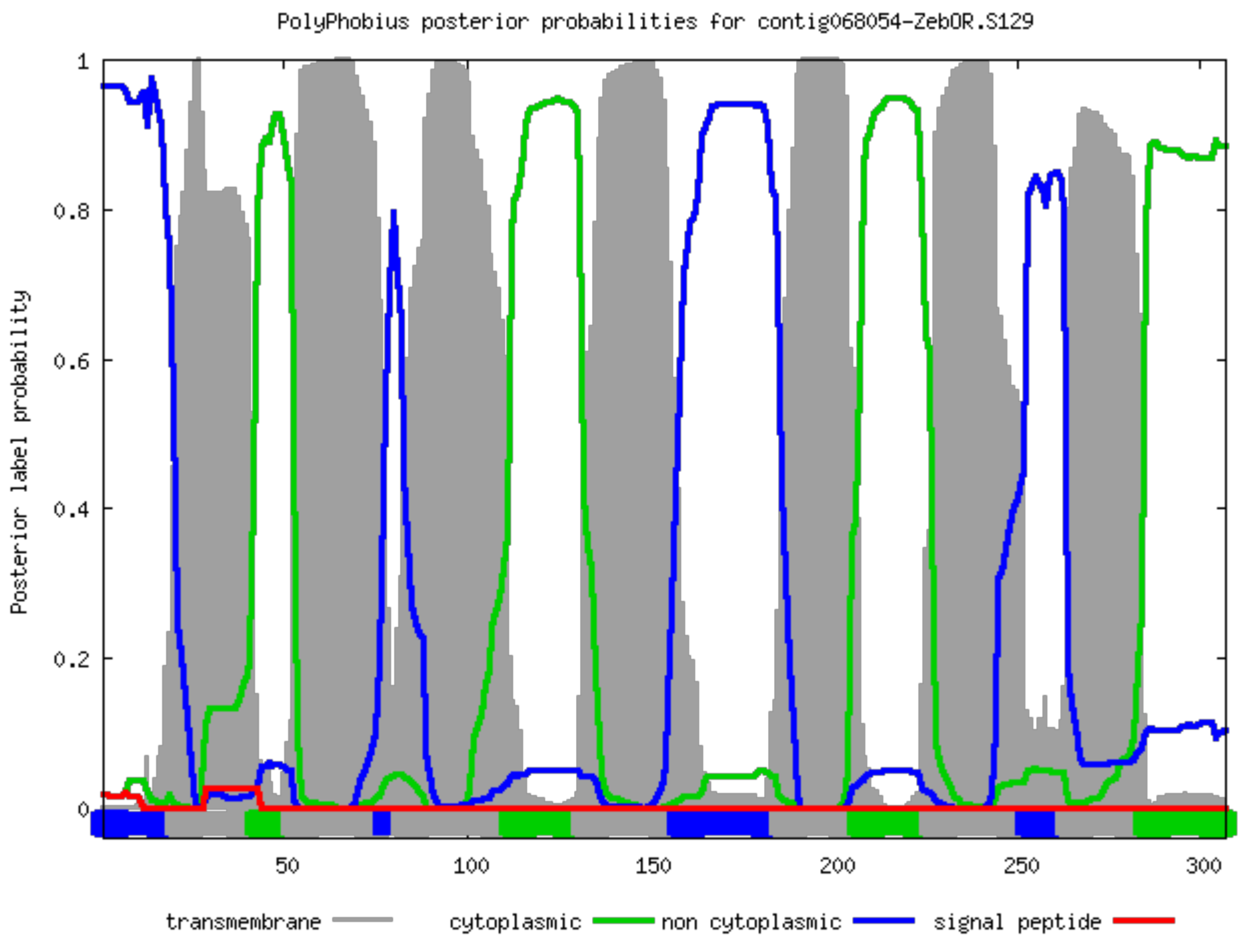

The prediction is based on an alignment. The probability data used in the plot is found here, and the gnuplot script is here.

Prediction of contig036780-BurOR.A001

```
ID   contig036780-BurOR.A001
FT   TOPO_DOM      1     22       NON CYTOPLASMIC.
FT   TRANSMEM     23     48
FT   TOPO_DOM     49     56       CYTOPLASMIC.
FT   TRANSMEM     57     77
FT   TOPO_DOM     78     95       NON CYTOPLASMIC.
FT   TRANSMEM     96    118
FT   TOPO_DOM    119    138       CYTOPLASMIC.
FT   TRANSMEM    139    159
FT   TOPO_DOM    160    192       NON CYTOPLASMIC.
FT   TRANSMEM    193    216
FT   TOPO_DOM    217    235       CYTOPLASMIC.
FT   TRANSMEM    236    257
FT   TOPO_DOM    258    268       NON CYTOPLASMIC.
FT   TRANSMEM    269    289
FT   TOPO_DOM    290    309       CYTOPLASMIC.
//
```

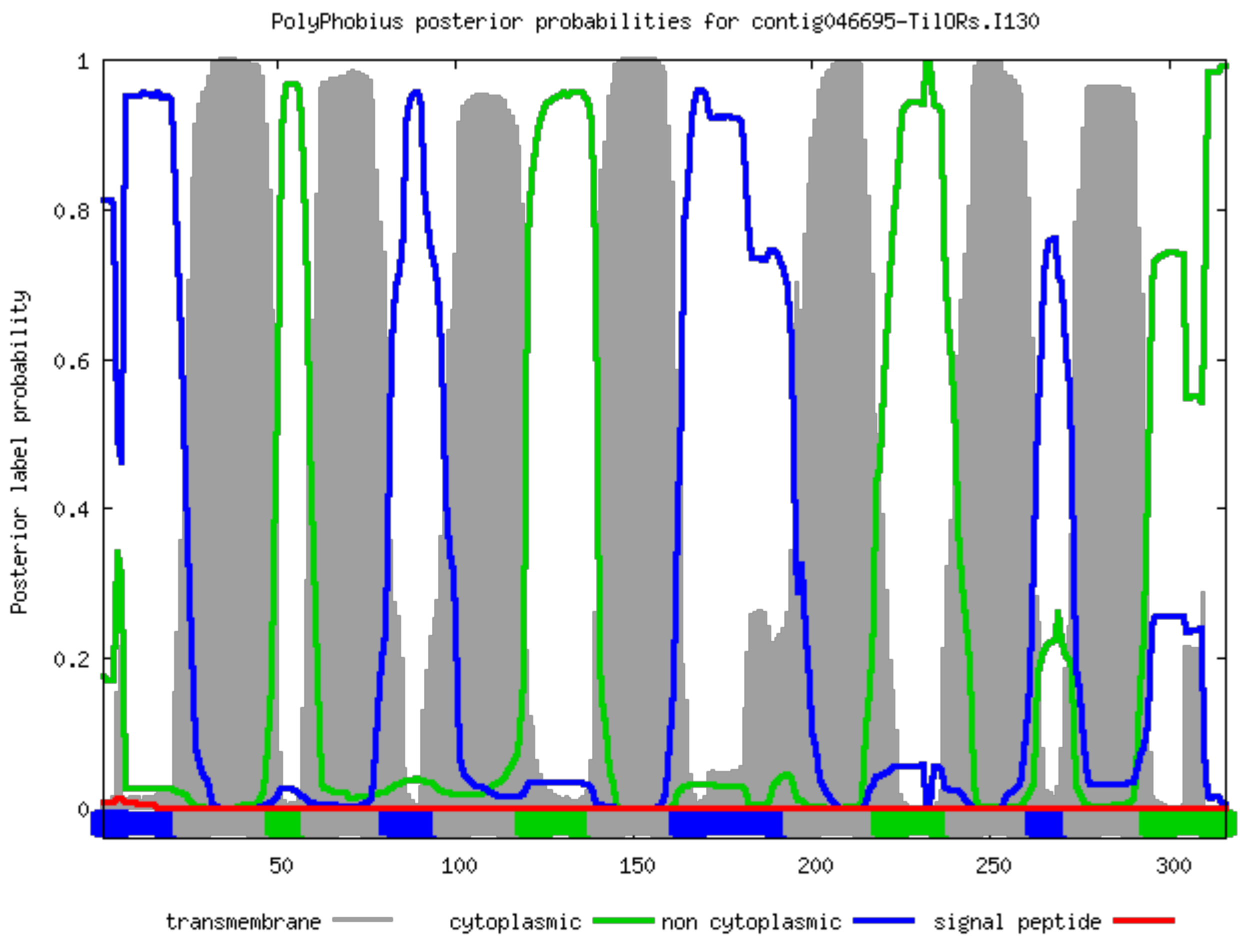

The prediction is based on an alignment. The probability data used in the plot is found here, and the gnuplot script is here.

Prediction of contig025439-ZebOR.E045

```
ID   contig025439-ZebOR.E045
FT   TOPO_DOM      1     22       NON CYTOPLASMIC.
FT   TRANSMEM     23     48
FT   TOPO_DOM     49     57       CYTOPLASMIC.
FT   TRANSMEM     58     82
FT   TOPO_DOM     83     90       NON CYTOPLASMIC.
FT   TRANSMEM     91    118
FT   TOPO_DOM    119    138       CYTOPLASMIC.
FT   TRANSMEM    139    161
FT   TOPO_DOM    162    193       NON CYTOPLASMIC.
FT   TRANSMEM    194    216
FT   TOPO_DOM    217    236       CYTOPLASMIC.
FT   TRANSMEM    237    256
FT   TOPO_DOM    257    267       NON CYTOPLASMIC.
FT   TRANSMEM    268    291
FT   TOPO_DOM    292    309       CYTOPLASMIC.
//
```

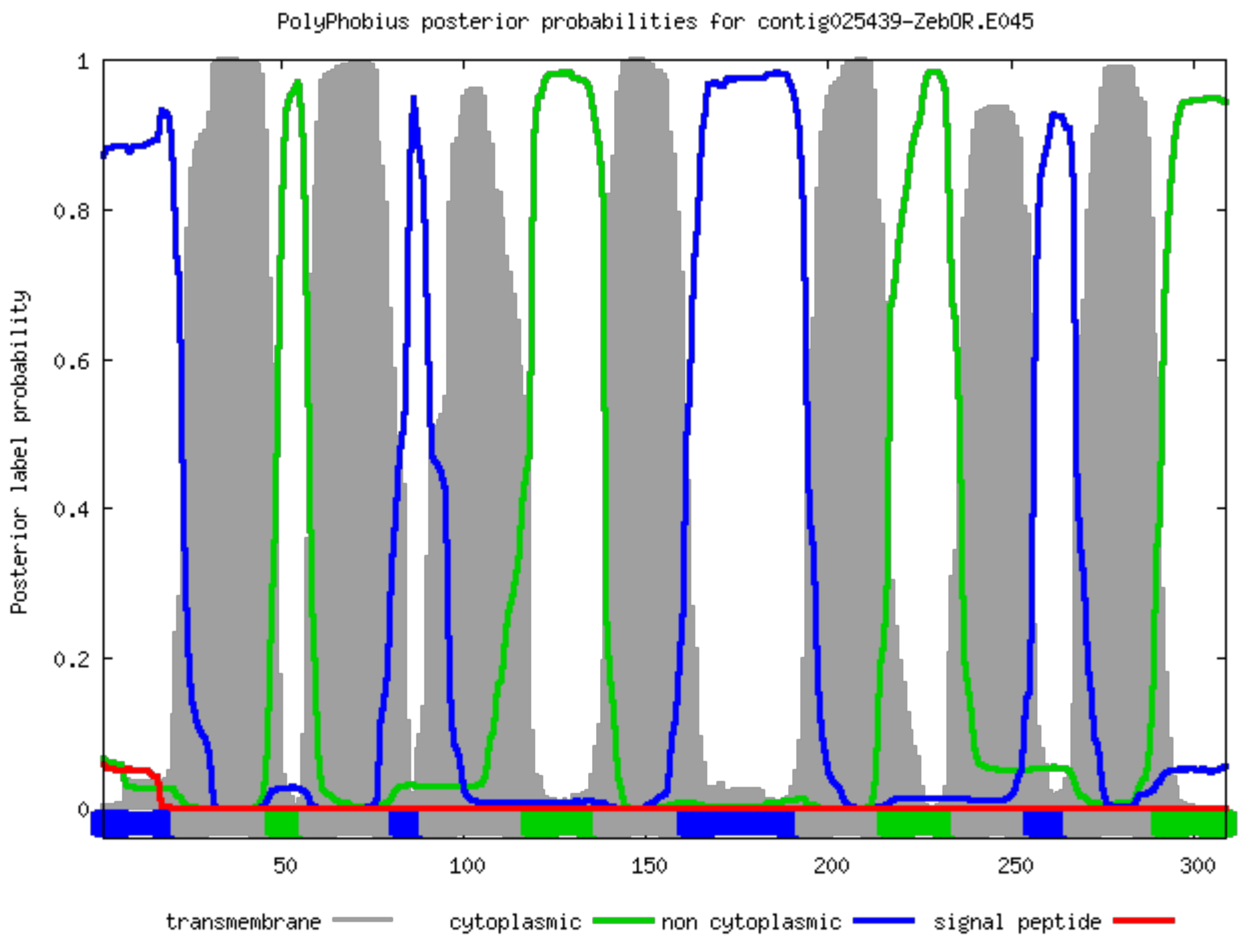

The prediction is based on an alignment. The probability data used in the plot is found here, and the gnuplot script is here.

Prediction of contig049621-BurOR.K082

```
ID   contig049621-BurOR.K082
FT   TOPO_DOM      1     24      NON CYTOPLASMIC.
FT   TRANSMEM     25     48
FT   TOPO_DOM     49     58      CYTOPLASMIC.
FT   TRANSMEM     59     80
FT   TOPO_DOM     81     99      NON CYTOPLASMIC.
FT   TRANSMEM    100    121
FT   TOPO_DOM    122    141      CYTOPLASMIC.
FT   TRANSMEM    142    165
FT   TOPO_DOM    166    198      NON CYTOPLASMIC.
FT   TRANSMEM    199    222
FT   TOPO_DOM    223    242      CYTOPLASMIC.
FT   TRANSMEM    243    262
FT   TOPO_DOM    263    272      NON CYTOPLASMIC.
FT   TRANSMEM    273    292
FT   TOPO_DOM    293    313      CYTOPLASMIC.
//
```

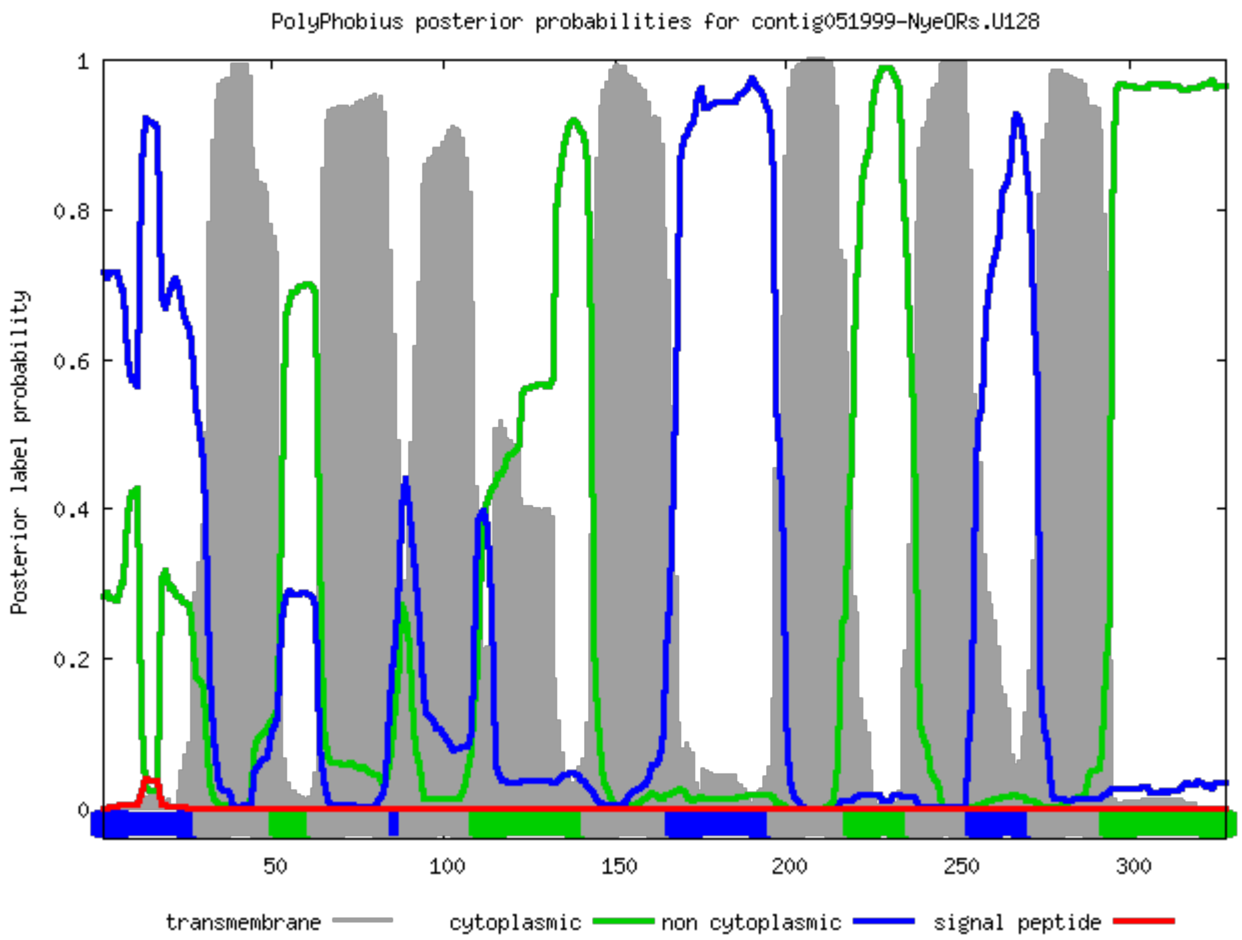

The prediction is based on an alignment. The probability data used in the plot is found here, and the gnuplot script is here.

Prediction of contig043640-BurOR.R137

```
ID   contig043640-BurOR.R137
FT   TOPO_DOM      1     22       NON CYTOPLASMIC.
FT   TRANSMEM     23     46
FT   TOPO_DOM     47     57       CYTOPLASMIC.
FT   TRANSMEM     58     82
FT   TOPO_DOM     83     87       NON CYTOPLASMIC.
FT   TRANSMEM     88    116
FT   TOPO_DOM    117    136       CYTOPLASMIC.
FT   TRANSMEM    137    160
FT   TOPO_DOM    161    191       NON CYTOPLASMIC.
FT   TRANSMEM    192    214
FT   TOPO_DOM    215    232       CYTOPLASMIC.
FT   TRANSMEM    233    256
FT   TOPO_DOM    257    268       NON CYTOPLASMIC.
FT   TRANSMEM    269    290
FT   TOPO_DOM    291    317       CYTOPLASMIC.
//
```

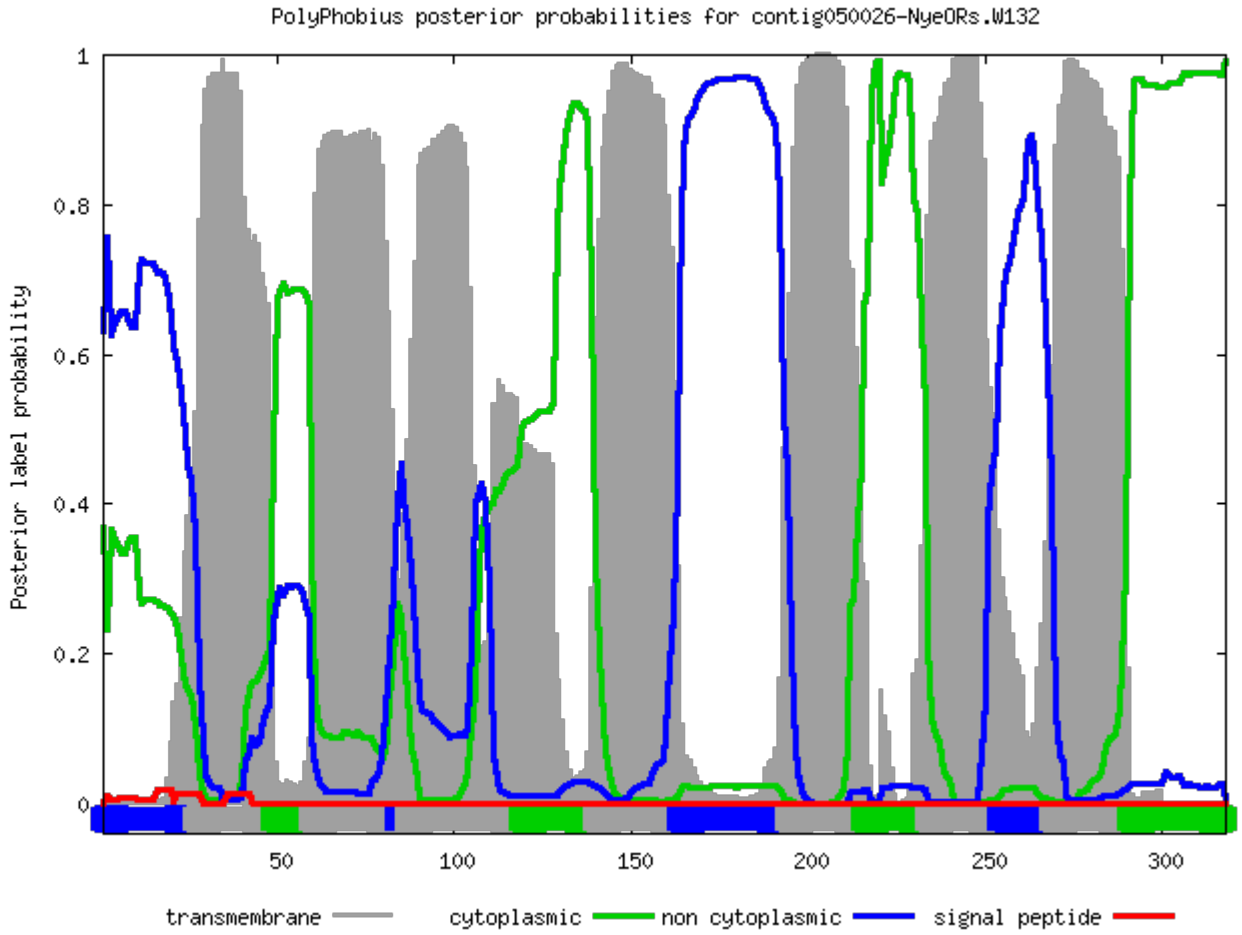

The prediction is based on an alignment. The probability data used in the plot is found here, and the gnuplot script is here.

Prediction of contig041951-TilOR.A022

```
ID   contig041951-TilOR.A022
FT   TOPO_DOM      1     18       NON CYTOPLASMIC.
FT   TRANSMEM     19     44
FT   TOPO_DOM     45     52       CYTOPLASMIC.
FT   TRANSMEM     53     73
FT   TOPO_DOM     74     91       NON CYTOPLASMIC.
FT   TRANSMEM     92    114
FT   TOPO_DOM    115    134       CYTOPLASMIC.
FT   TRANSMEM    135    155
FT   TOPO_DOM    156    188       NON CYTOPLASMIC.
FT   TRANSMEM    189    211
FT   TOPO_DOM    212    231       CYTOPLASMIC.
FT   TRANSMEM    232    253
FT   TOPO_DOM    254    264       NON CYTOPLASMIC.
FT   TRANSMEM    265    285
FT   TOPO_DOM    286    300       CYTOPLASMIC.
//
```

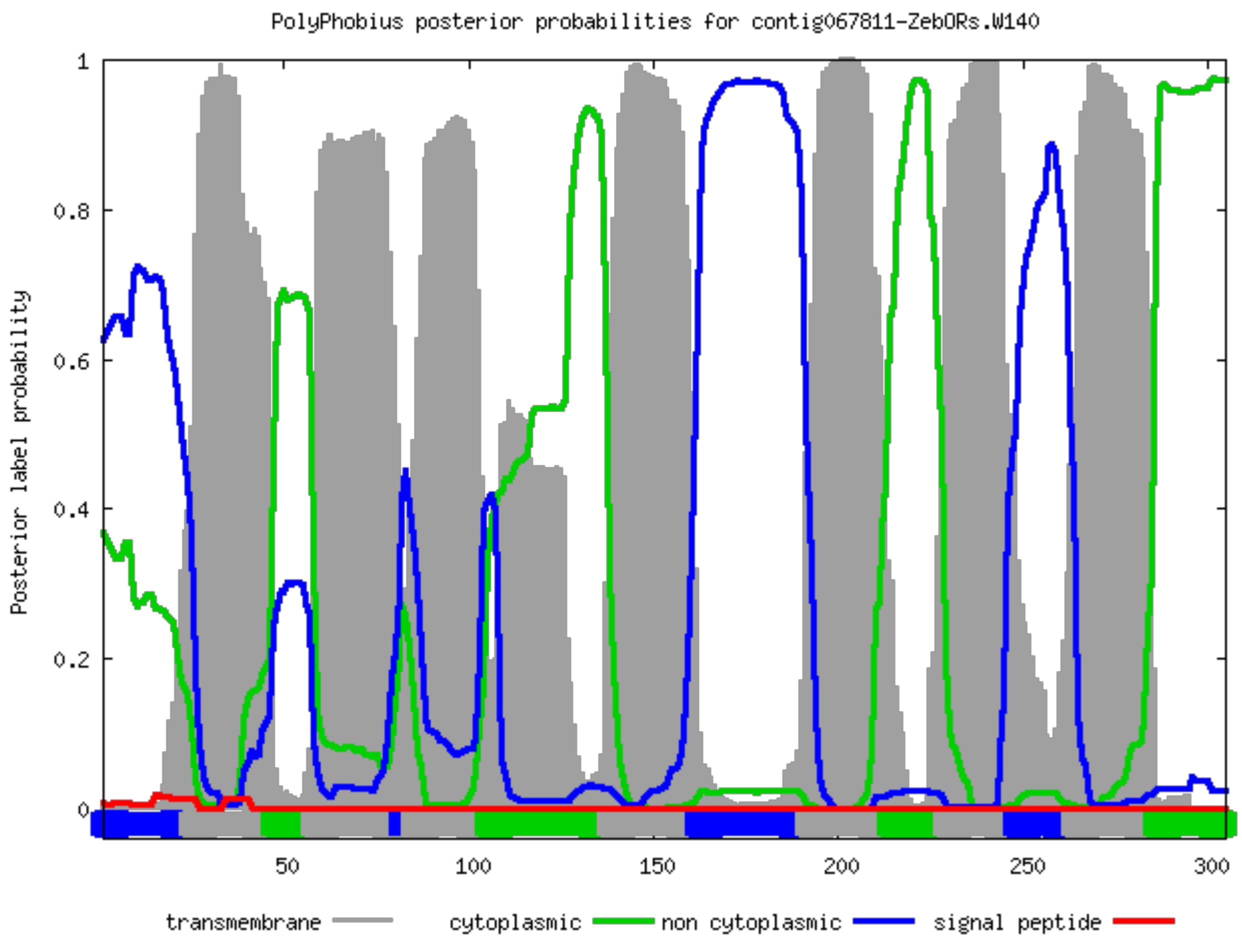

The prediction is based on an alignment. The probability data used in the plot is found here, and the gnuplot script is here.

Prediction of contig014060-ZebOR.H069

```
ID   contig014060-ZebOR.H069
FT   TOPO_DOM      1     22       NON CYTOPLASMIC.
FT   TRANSMEM     23     49
FT   TOPO_DOM     50     56       CYTOPLASMIC.
FT   TRANSMEM     57     77
FT   TOPO_DOM     78     95       NON CYTOPLASMIC.
FT   TRANSMEM     96    118
FT   TOPO_DOM    119    138       CYTOPLASMIC.
FT   TRANSMEM    139    160
FT   TOPO_DOM    161    193       NON CYTOPLASMIC.
FT   TRANSMEM    194    217
FT   TOPO_DOM    218    235       CYTOPLASMIC.
FT   TRANSMEM    236    258
FT   TOPO_DOM    259    269       NON CYTOPLASMIC.
FT   TRANSMEM    270    289
FT   TOPO_DOM    290    314       CYTOPLASMIC.
//
```

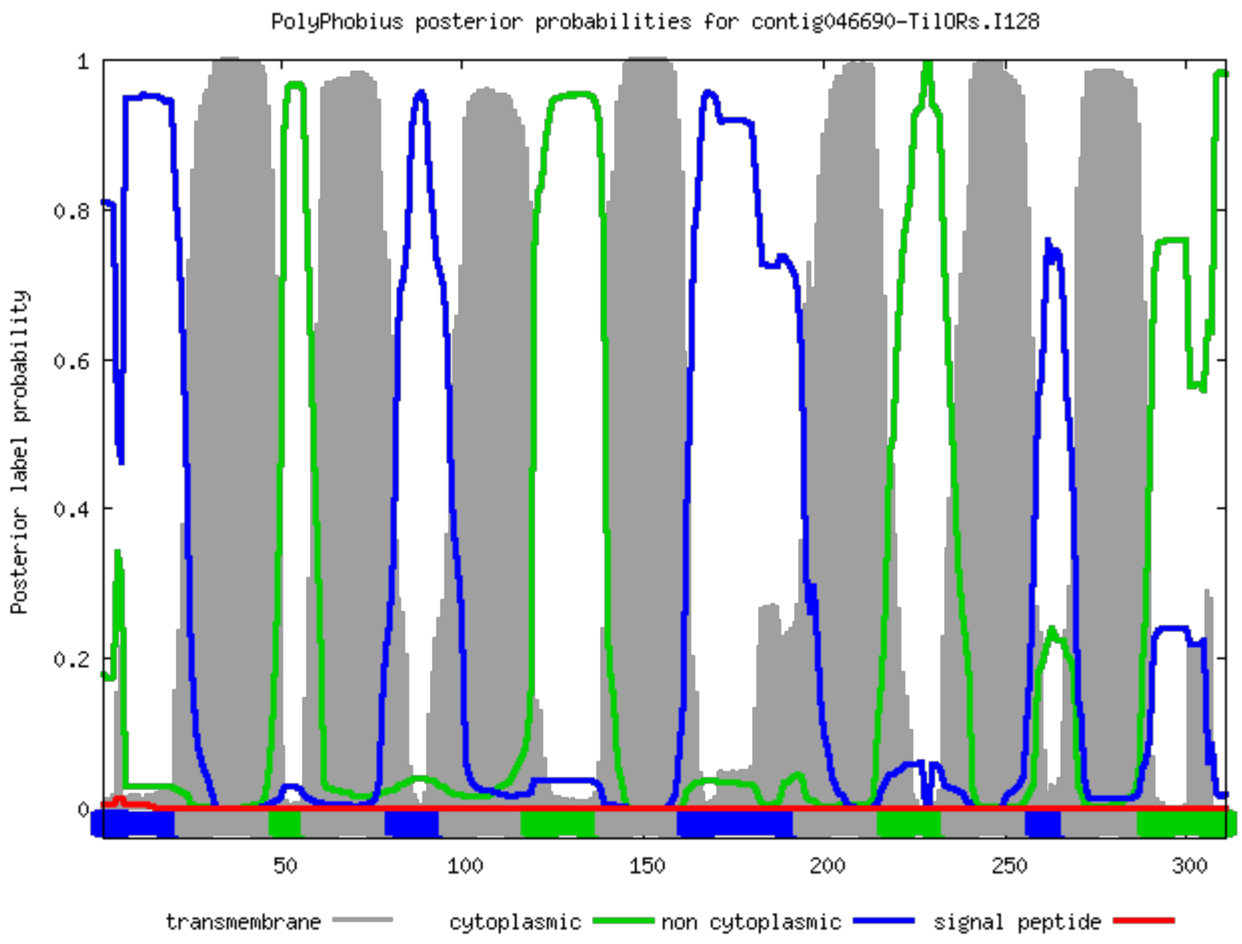

The prediction is based on an alignment. The probability data used in the plot is found here, and the gnuplot script is here.

Prediction of contig074640-TilOR.A002

```
ID   contig074640-TilOR.A002
FT   TOPO_DOM      1     22       NON CYTOPLASMIC.
FT   TRANSMEM     23     48
FT   TOPO_DOM     49     56       CYTOPLASMIC.
FT   TRANSMEM     57     76
FT   TOPO_DOM     77     95       NON CYTOPLASMIC.
FT   TRANSMEM     96    118
FT   TOPO_DOM    119    138       CYTOPLASMIC.
FT   TRANSMEM    139    159
FT   TOPO_DOM    160    192       NON CYTOPLASMIC.
FT   TRANSMEM    193    215
FT   TOPO_DOM    216    235       CYTOPLASMIC.
FT   TRANSMEM    236    257
FT   TOPO_DOM    258    268       NON CYTOPLASMIC.
FT   TRANSMEM    269    289
FT   TOPO_DOM    290    309       CYTOPLASMIC.
//
```

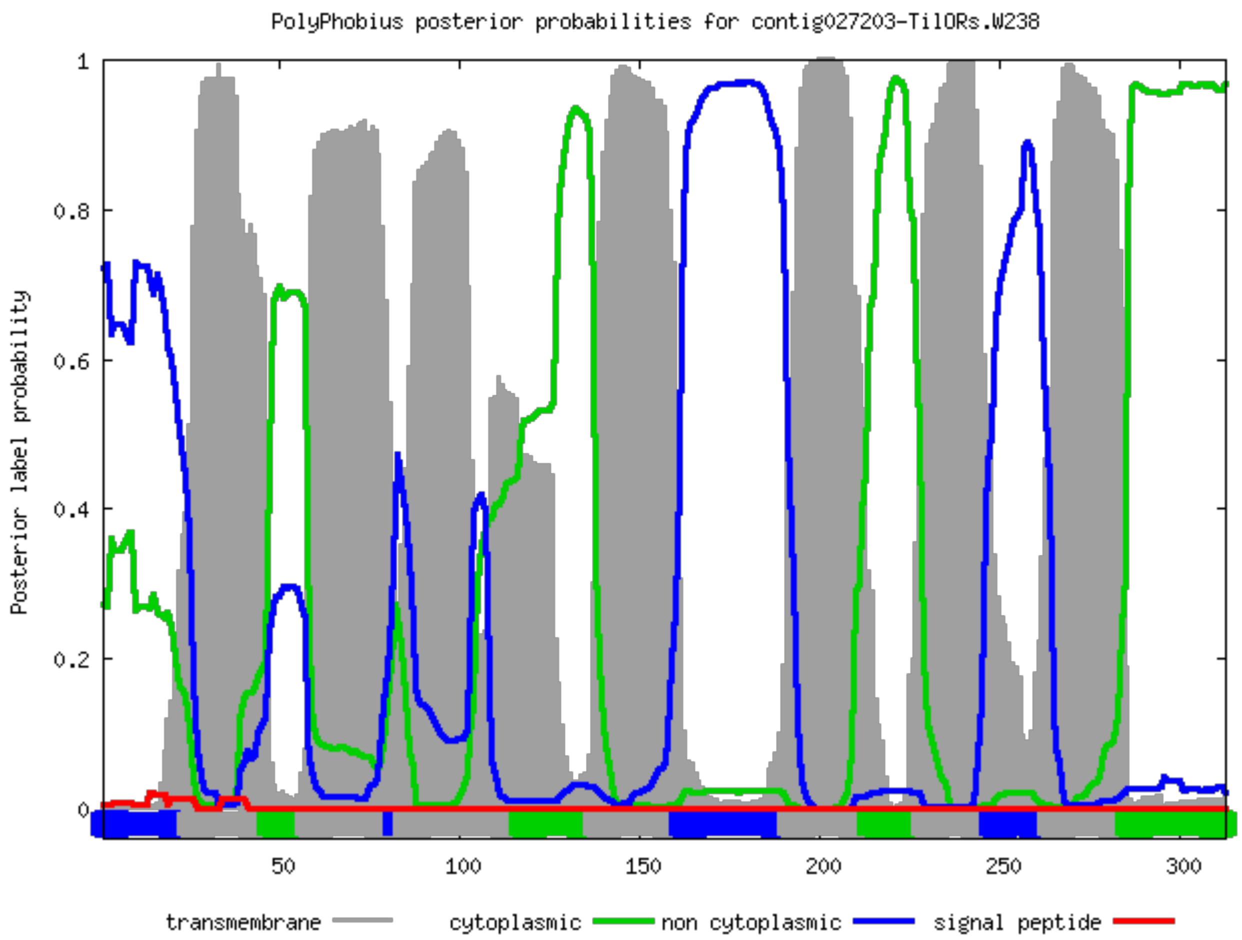

The prediction is based on an alignment. The probability data used in the plot is found here, and the gnuplot script is here.

Prediction of contig021359-NyeOR.O102

```
ID   contig021359-NyeOR.O102
FT   TOPO_DOM      1      24       NON CYTOPLASMIC.
FT   TRANSMEM     25      51
FT   TOPO_DOM     52      60       CYTOPLASMIC.
FT   TRANSMEM     61      83
FT   TOPO_DOM     84      98       NON CYTOPLASMIC.
FT   TRANSMEM     99     121
FT   TOPO_DOM    122     141       CYTOPLASMIC.
FT   TRANSMEM    142     163
FT   TOPO_DOM    164     200       NON CYTOPLASMIC.
FT   TRANSMEM    201     227
FT   TOPO_DOM    228     240       CYTOPLASMIC.
FT   TRANSMEM    241     262
FT   TOPO_DOM    263     273       NON CYTOPLASMIC.
FT   TRANSMEM    274     295
FT   TOPO_DOM    296     331       CYTOPLASMIC.
//
```

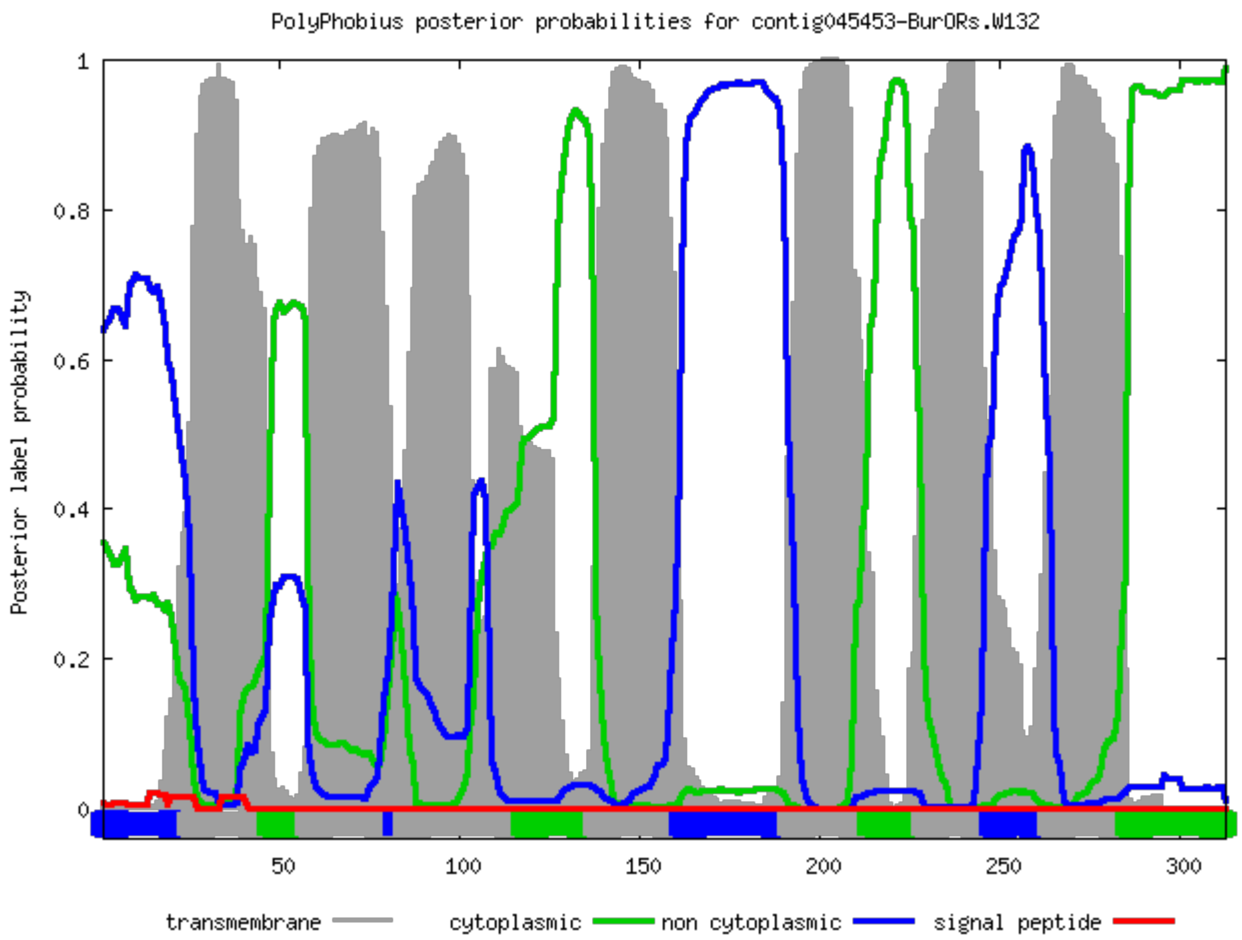

The prediction is based on an alignment. The probability data used in the plot is found here, and the gnuplot script is here.

Prediction of contig053782-BurOR.H066

```
ID   contig053782-BurOR.H066
FT   TOPO_DOM      1     22       NON CYTOPLASMIC.
FT   TRANSMEM     23     49
FT   TOPO_DOM     50     56       CYTOPLASMIC.
FT   TRANSMEM     57     77
FT   TOPO_DOM     78     95       NON CYTOPLASMIC.
FT   TRANSMEM     96    118
FT   TOPO_DOM    119    138       CYTOPLASMIC.
FT   TRANSMEM    139    160
FT   TOPO_DOM    161    193       NON CYTOPLASMIC.
FT   TRANSMEM    194    216
FT   TOPO_DOM    217    235       CYTOPLASMIC.
FT   TRANSMEM    236    258
FT   TOPO_DOM    259    269       NON CYTOPLASMIC.
FT   TRANSMEM    270    289
FT   TOPO_DOM    290    314       CYTOPLASMIC.
//
```

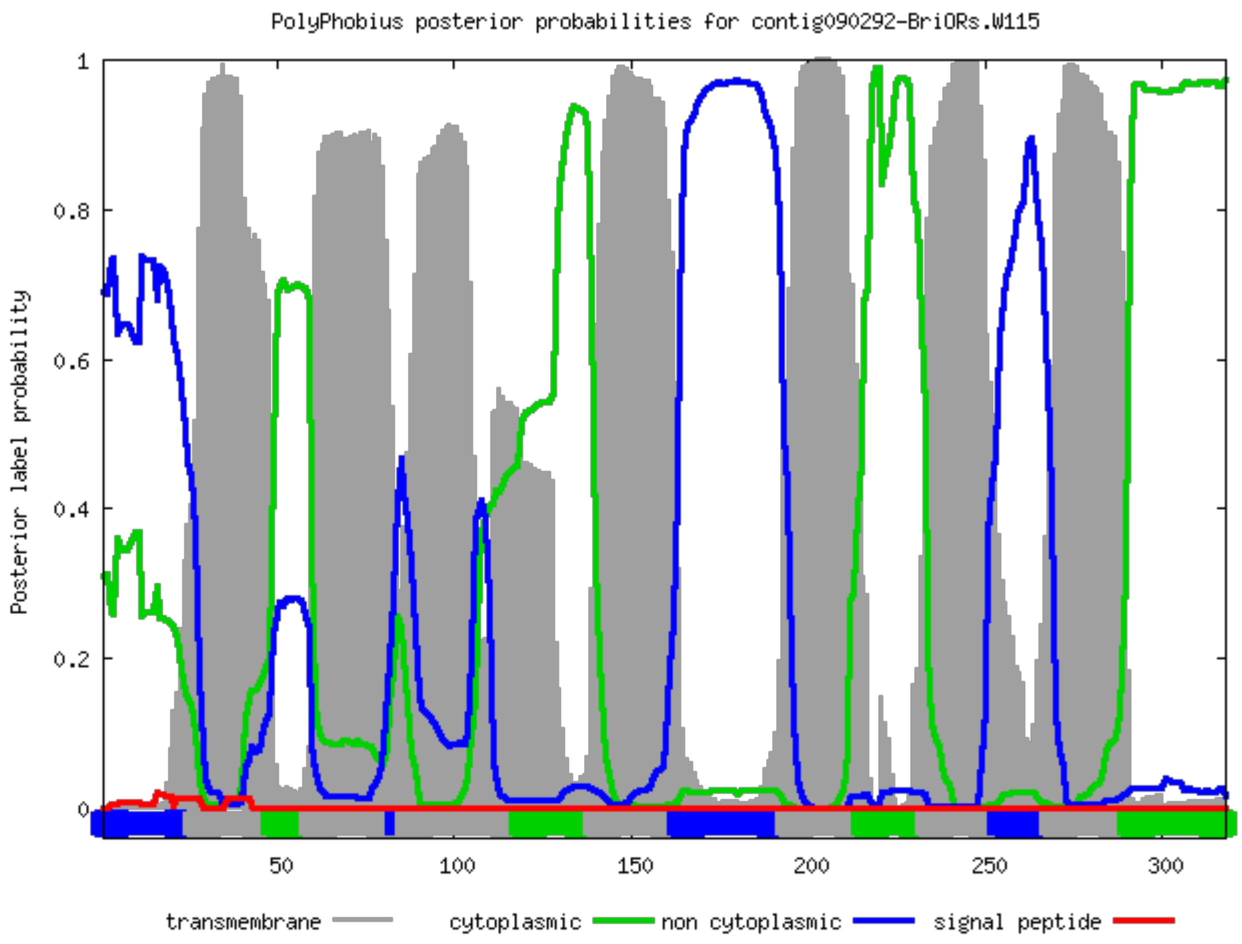

The prediction is based on an alignment. The probability data used in the plot is found here, and the gnuplot script is here.

Prediction of contig059249-BurOR.O098

```
ID   contig059249-BurOR.O098
FT   TOPO_DOM      1     24       NON CYTOPLASMIC.
FT   TRANSMEM     25     51
FT   TOPO_DOM     52     60       CYTOPLASMIC.
FT   TRANSMEM     61     83
FT   TOPO_DOM     84     98       NON CYTOPLASMIC.
FT   TRANSMEM     99    121
FT   TOPO_DOM    122    141       CYTOPLASMIC.
FT   TRANSMEM    142    163
FT   TOPO_DOM    164    200       NON CYTOPLASMIC.
FT   TRANSMEM    201    227
FT   TOPO_DOM    228    240       CYTOPLASMIC.
FT   TRANSMEM    241    262
FT   TOPO_DOM    263    273       NON CYTOPLASMIC.
FT   TRANSMEM    274    295
FT   TOPO_DOM    296    326       CYTOPLASMIC.
//
```

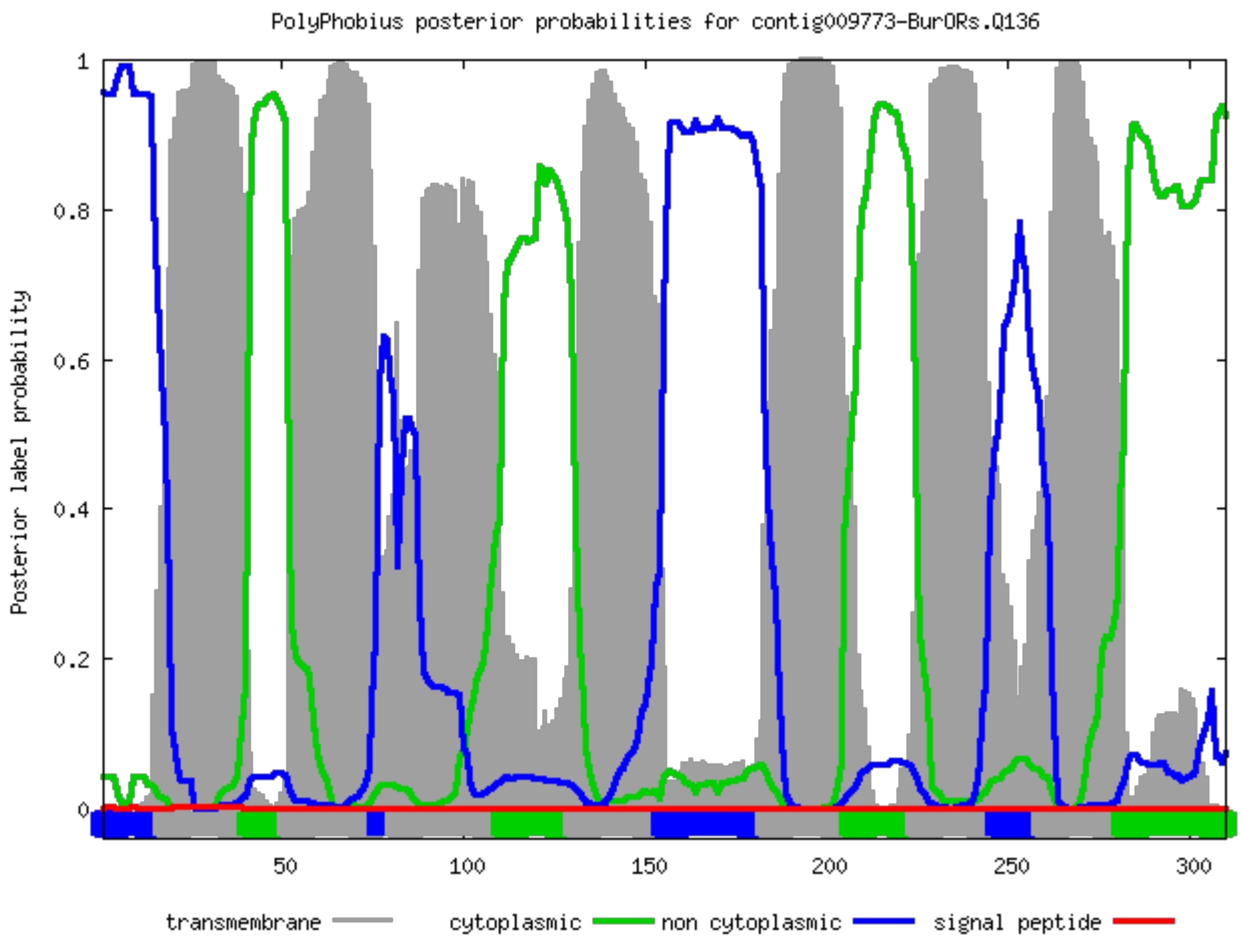

The prediction is based on an alignment. The probability data used in the plot is found here, and the gnuplot script is here.

Prediction of contig013368-TilOR.H112

```
ID   contig013368-TilOR.H112
FT   TOPO_DOM      1     23       NON CYTOPLASMIC.
FT   TRANSMEM     24     49
FT   TOPO_DOM     50     56       CYTOPLASMIC.
FT   TRANSMEM     57     76
FT   TOPO_DOM     77     95       NON CYTOPLASMIC.
FT   TRANSMEM     96    118
FT   TOPO_DOM    119    138       CYTOPLASMIC.
FT   TRANSMEM    139    160
FT   TOPO_DOM    161    196       NON CYTOPLASMIC.
FT   TRANSMEM    197    219
FT   TOPO_DOM    220    237       CYTOPLASMIC.
FT   TRANSMEM    238    260
FT   TOPO_DOM    261    271       NON CYTOPLASMIC.
FT   TRANSMEM    272    291
FT   TOPO_DOM    292    310       CYTOPLASMIC.
//
```

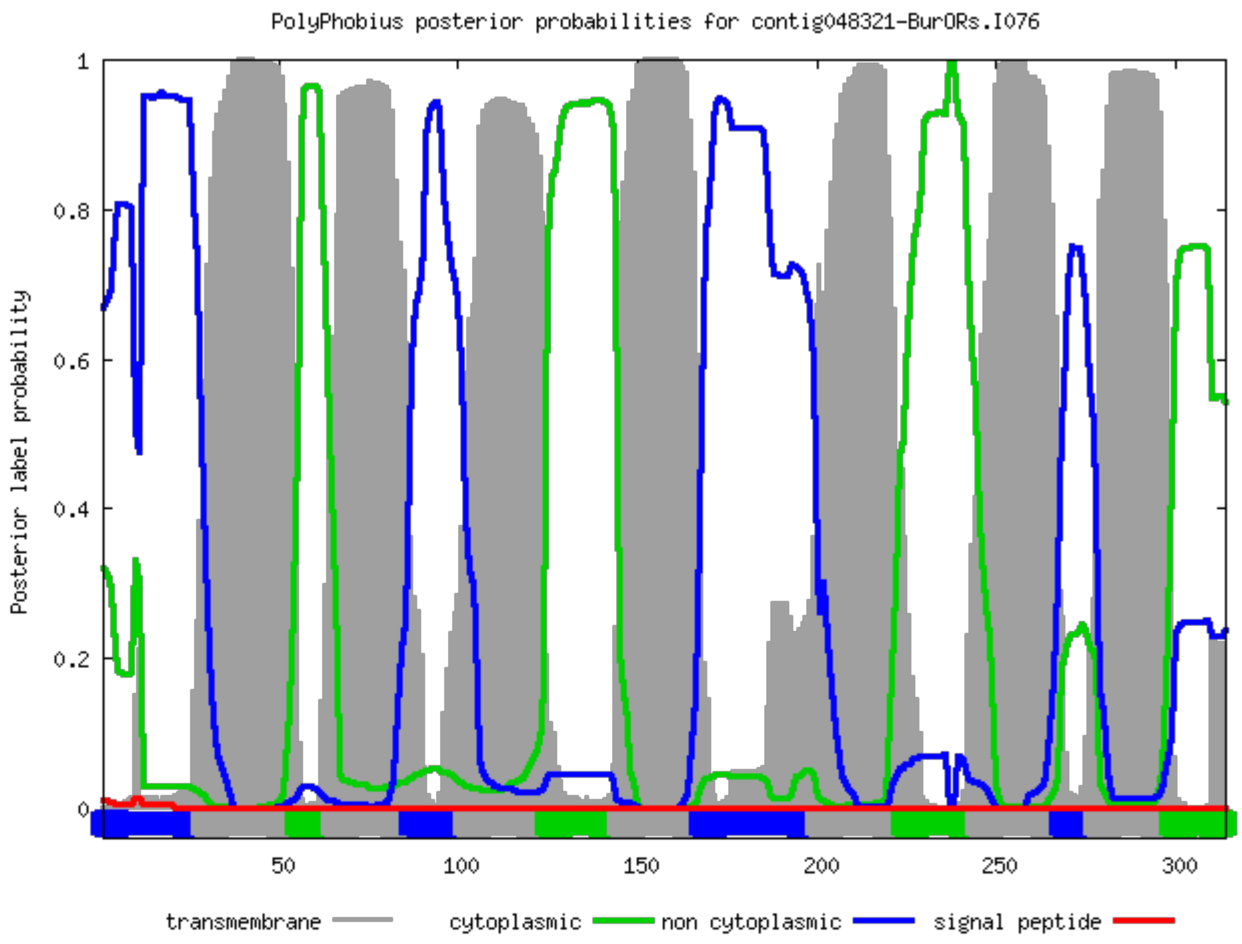

The prediction is based on an alignment. The probability data used in the plot is found here, and the gnuplot script is here.

Prediction of contig065444-TilOR.P214

```
ID   contig065444-TilOR.P214
FT   TOPO_DOM      1     23       NON CYTOPLASMIC.
FT   TRANSMEM     24     47
FT   TOPO_DOM     48     57       CYTOPLASMIC.
FT   TRANSMEM     58     83
FT   TOPO_DOM     84     96       NON CYTOPLASMIC.
FT   TRANSMEM     97    118
FT   TOPO_DOM    119    138       CYTOPLASMIC.
FT   TRANSMEM    139    161
FT   TOPO_DOM    162    196       NON CYTOPLASMIC.
FT   TRANSMEM    197    221
FT   TOPO_DOM    222    234       CYTOPLASMIC.
FT   TRANSMEM    235    258
FT   TOPO_DOM    259    269       NON CYTOPLASMIC.
FT   TRANSMEM    270    290
FT   TOPO_DOM    291    318       CYTOPLASMIC.
//
```

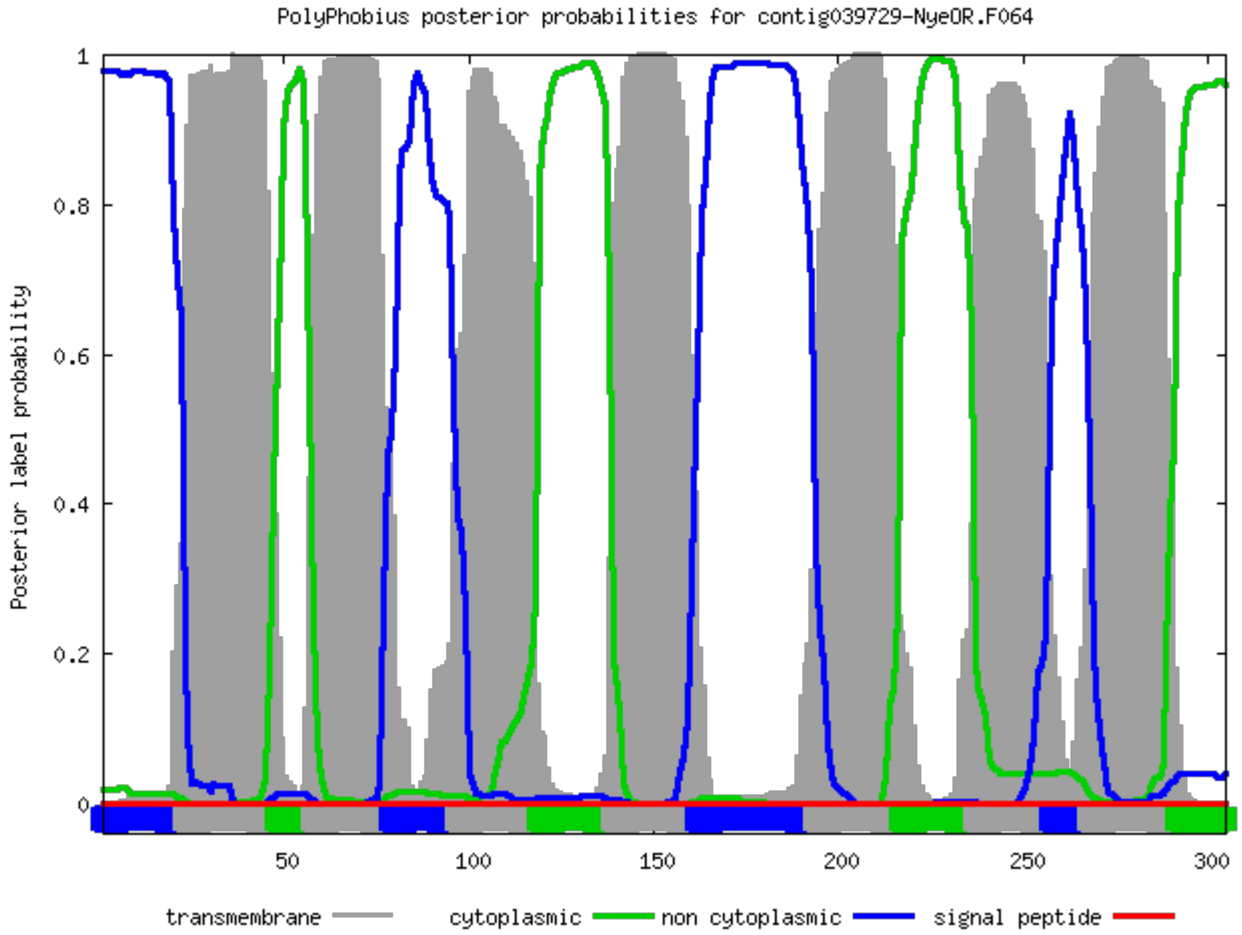

The prediction is based on an alignment. The probability data used in the plot is found here, and the gnuplot script is here.

Prediction of contig023724-TilOR.O176

```
ID    contig023724-TilOR.O176
FT    TOPO_DOM      1      24       NON CYTOPLASMIC.
FT    TRANSMEM     25      51
FT    TOPO_DOM     52      60       CYTOPLASMIC.
FT    TRANSMEM     61      83
FT    TOPO_DOM     84      98       NON CYTOPLASMIC.
FT    TRANSMEM     99     121
FT    TOPO_DOM    122     141       CYTOPLASMIC.
FT    TRANSMEM    142     163
FT    TOPO_DOM    164     200       NON CYTOPLASMIC.
FT    TRANSMEM    201     227
FT    TOPO_DOM    228     240       CYTOPLASMIC.
FT    TRANSMEM    241     262
FT    TOPO_DOM    263     273       NON CYTOPLASMIC.
FT    TRANSMEM    274     295
FT    TOPO_DOM    296     326       CYTOPLASMIC.
//
```

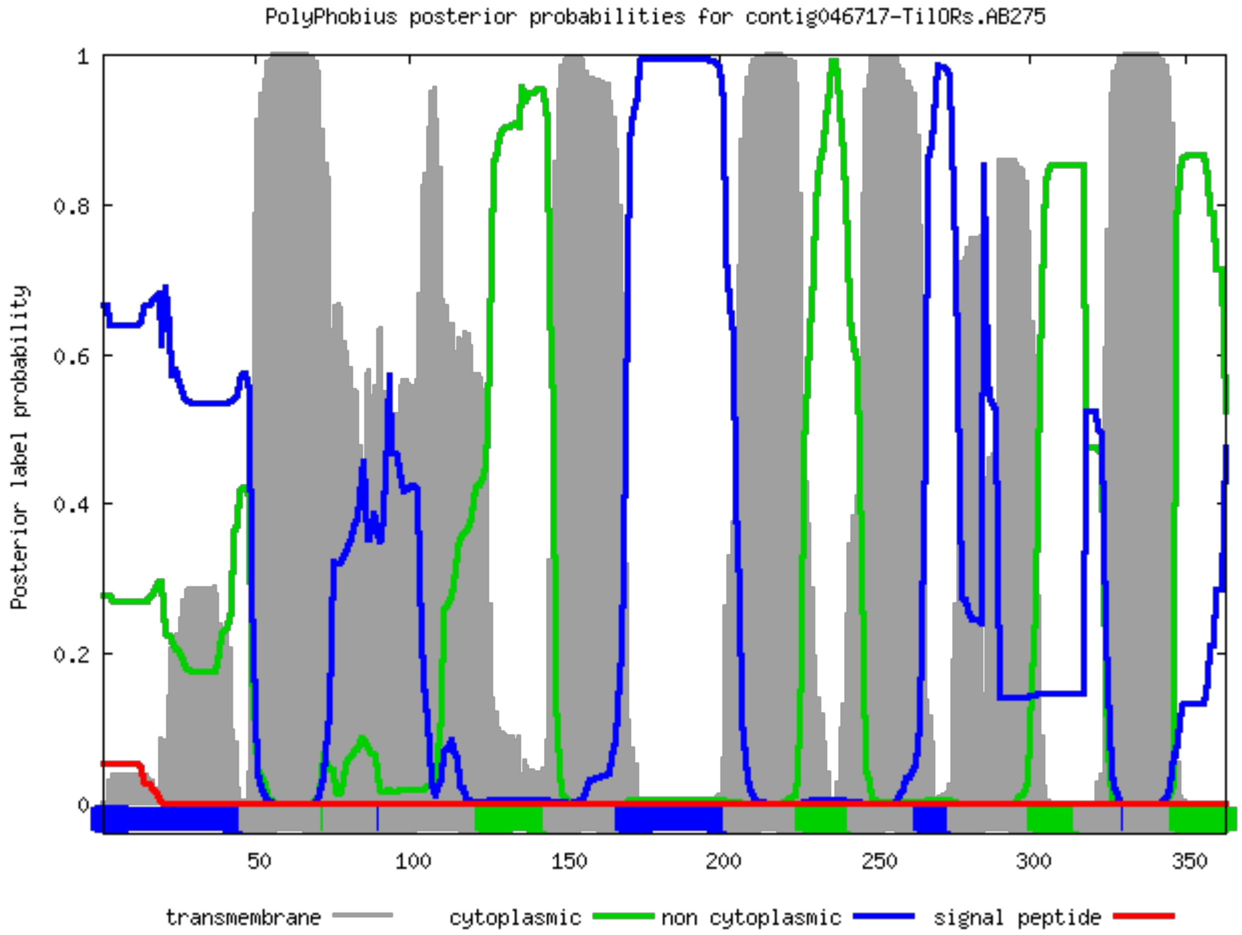

The prediction is based on an alignment. The probability data used in the plot is found here, and the gnuplot script is here.

Prediction of contig013898-BurOR.F058

```
ID   contig013898-BurOR.F058
FT   TOPO_DOM      1     22       NON CYTOPLASMIC.
FT   TRANSMEM     23     47
FT   TOPO_DOM     48     56       CYTOPLASMIC.
FT   TRANSMEM     57     77
FT   TOPO_DOM     78     96       NON CYTOPLASMIC.
FT   TRANSMEM     97    118
FT   TOPO_DOM    119    138       CYTOPLASMIC.
FT   TRANSMEM    139    161
FT   TOPO_DOM    162    193       NON CYTOPLASMIC.
FT   TRANSMEM    194    216
FT   TOPO_DOM    217    236       CYTOPLASMIC.
FT   TRANSMEM    237    256
FT   TOPO_DOM    257    267       NON CYTOPLASMIC.
FT   TRANSMEM    268    290
FT   TOPO_DOM    291    310       CYTOPLASMIC.
//
```

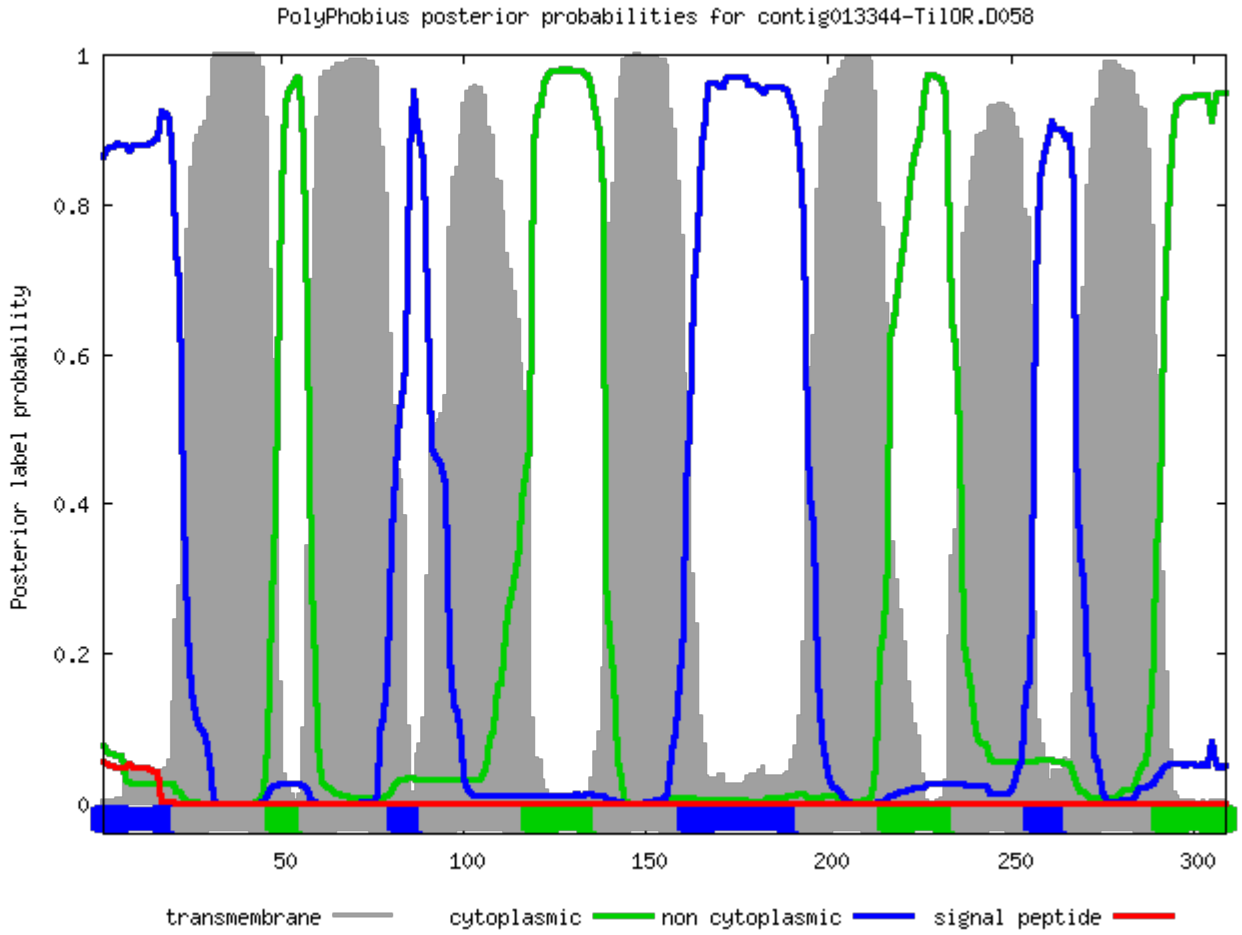

The prediction is based on an alignment. The probability data used in the plot is found here, and the gnuplot script is here.

Prediction of contig042552-BriOR.L071

```
ID    contig042552-BriOR.L071
FT    TOPO_DOM      1     25       NON CYTOPLASMIC.
FT    TRANSMEM     26     50
FT    TOPO_DOM     51     59       CYTOPLASMIC.
FT    TRANSMEM     60     82
FT    TOPO_DOM     83    100       NON CYTOPLASMIC.
FT    TRANSMEM    101    120
FT    TOPO_DOM    121    140       CYTOPLASMIC.
FT    TRANSMEM    141    163
FT    TOPO_DOM    164    199       NON CYTOPLASMIC.
FT    TRANSMEM    200    224
FT    TOPO_DOM    225    237       CYTOPLASMIC.
FT    TRANSMEM    238    260
FT    TOPO_DOM    261    271       NON CYTOPLASMIC.
FT    TRANSMEM    272    292
FT    TOPO_DOM    293    313       CYTOPLASMIC.
//
```

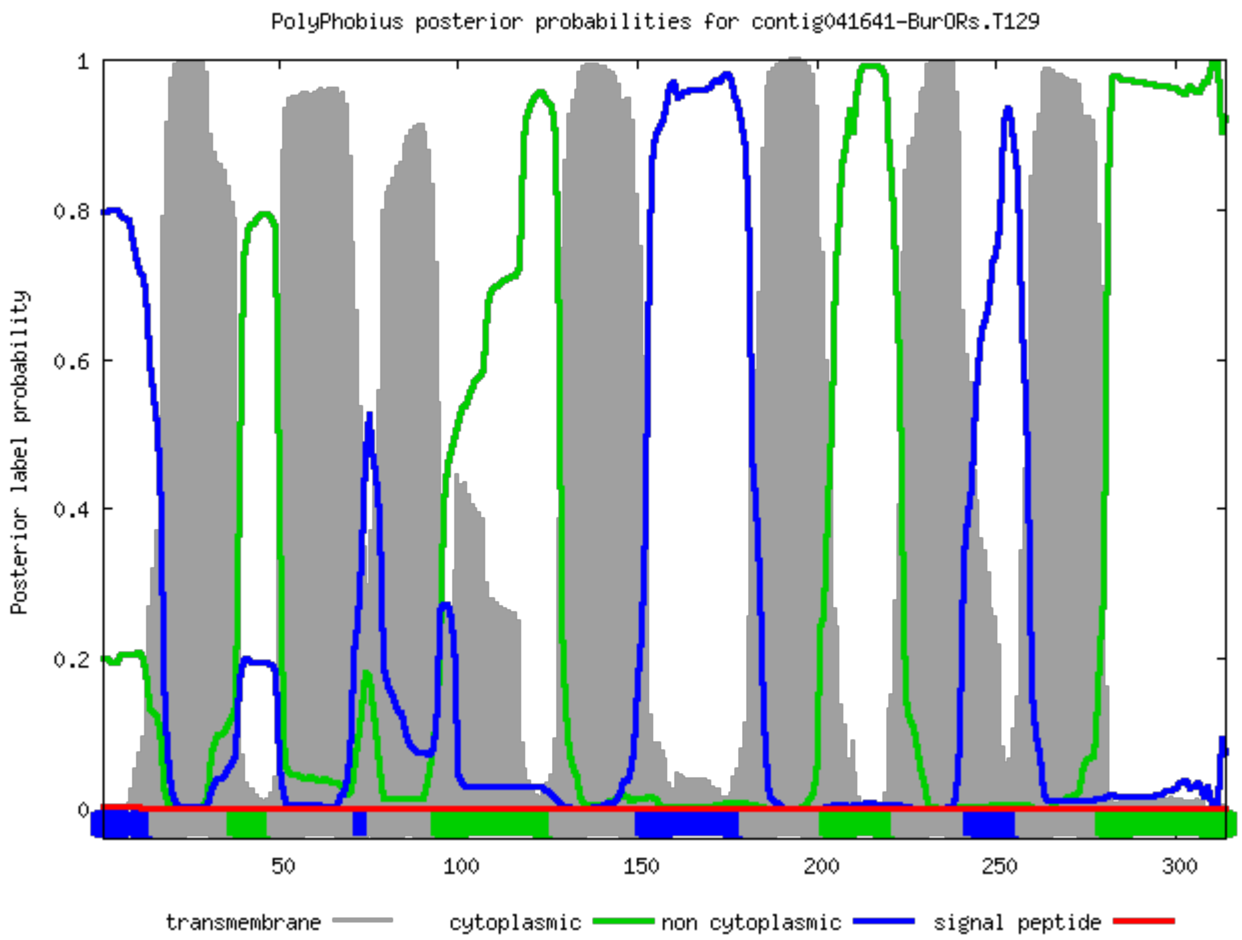

The prediction is based on an alignment. The probability data used in the plot is found here, and the gnuplot script is here.

Prediction of contig055884-BurOR.S125

```
ID   contig055884-BurOR.S125
FT   TOPO_DOM      1      20       NON CYTOPLASMIC.
FT   TRANSMEM     21      42
FT   TOPO_DOM     43      53       CYTOPLASMIC.
FT   TRANSMEM     54      77
FT   TOPO_DOM     78      82       NON CYTOPLASMIC.
FT   TRANSMEM     83     111
FT   TOPO_DOM    112     131       CYTOPLASMIC.
FT   TRANSMEM    132     157
FT   TOPO_DOM    158     185       NON CYTOPLASMIC.
FT   TRANSMEM    186     206
FT   TOPO_DOM    207     226       CYTOPLASMIC.
FT   TRANSMEM    227     252
FT   TOPO_DOM    253     263       NON CYTOPLASMIC.
FT   TRANSMEM    264     284
FT   TOPO_DOM    285     305       CYTOPLASMIC.
//
```

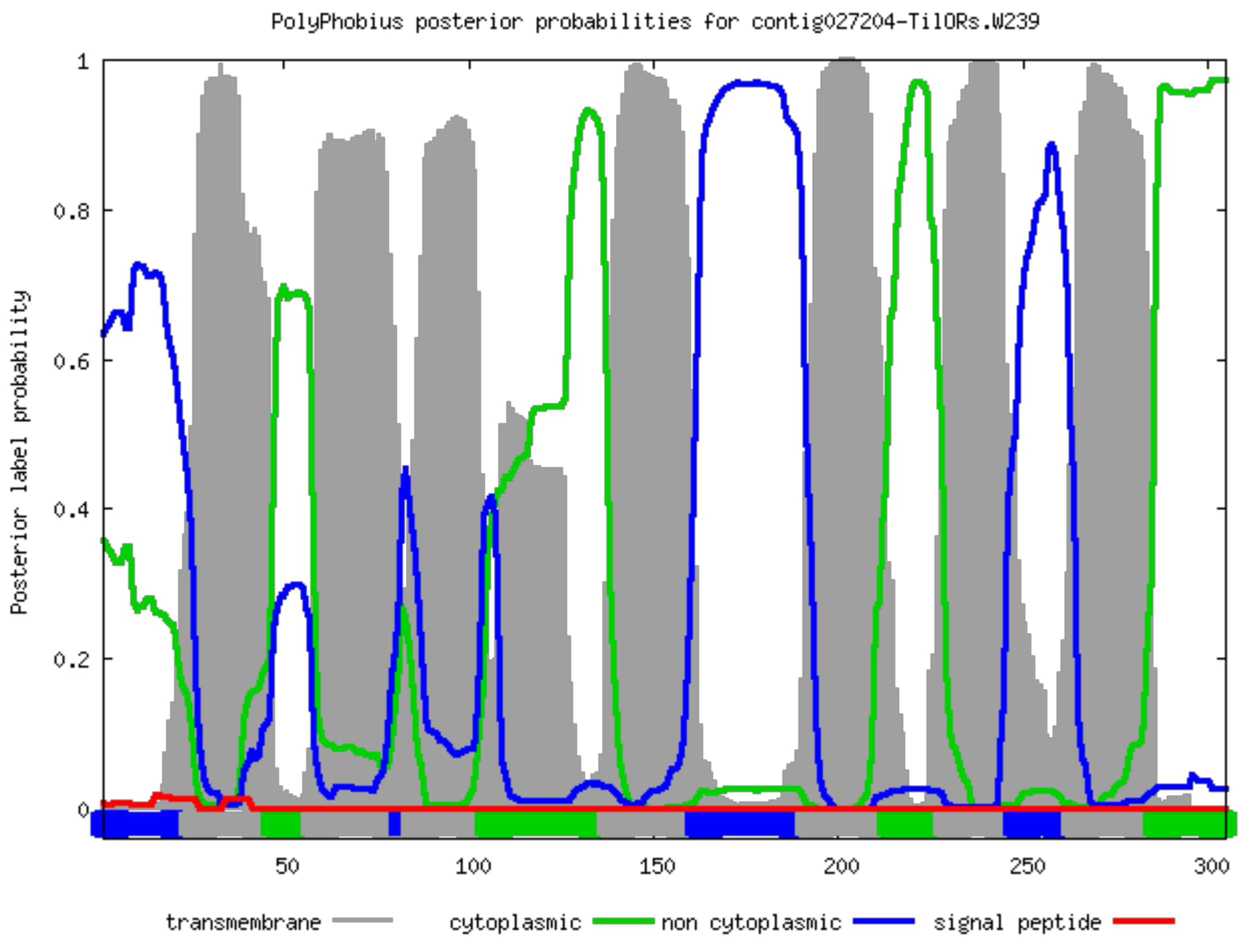

The prediction is based on an alignment. The probability data used in the plot is found here, and the gnuplot script is here.

Prediction of contig116846-BriOR.H055

```
ID   contig116846-BriOR.H055
FT   TOPO_DOM      1      23       NON CYTOPLASMIC.
FT   TRANSMEM     24      49
FT   TOPO_DOM     50      56       CYTOPLASMIC.
FT   TRANSMEM     57      76
FT   TOPO_DOM     77      95       NON CYTOPLASMIC.
FT   TRANSMEM     96     118
FT   TOPO_DOM    119     138       CYTOPLASMIC.
FT   TRANSMEM    139     160
FT   TOPO_DOM    161     196       NON CYTOPLASMIC.
FT   TRANSMEM    197     219
FT   TOPO_DOM    220     237       CYTOPLASMIC.
FT   TRANSMEM    238     259
FT   TOPO_DOM    260     271       NON CYTOPLASMIC.
FT   TRANSMEM    272     291
FT   TOPO_DOM    292     324       CYTOPLASMIC.
//
```

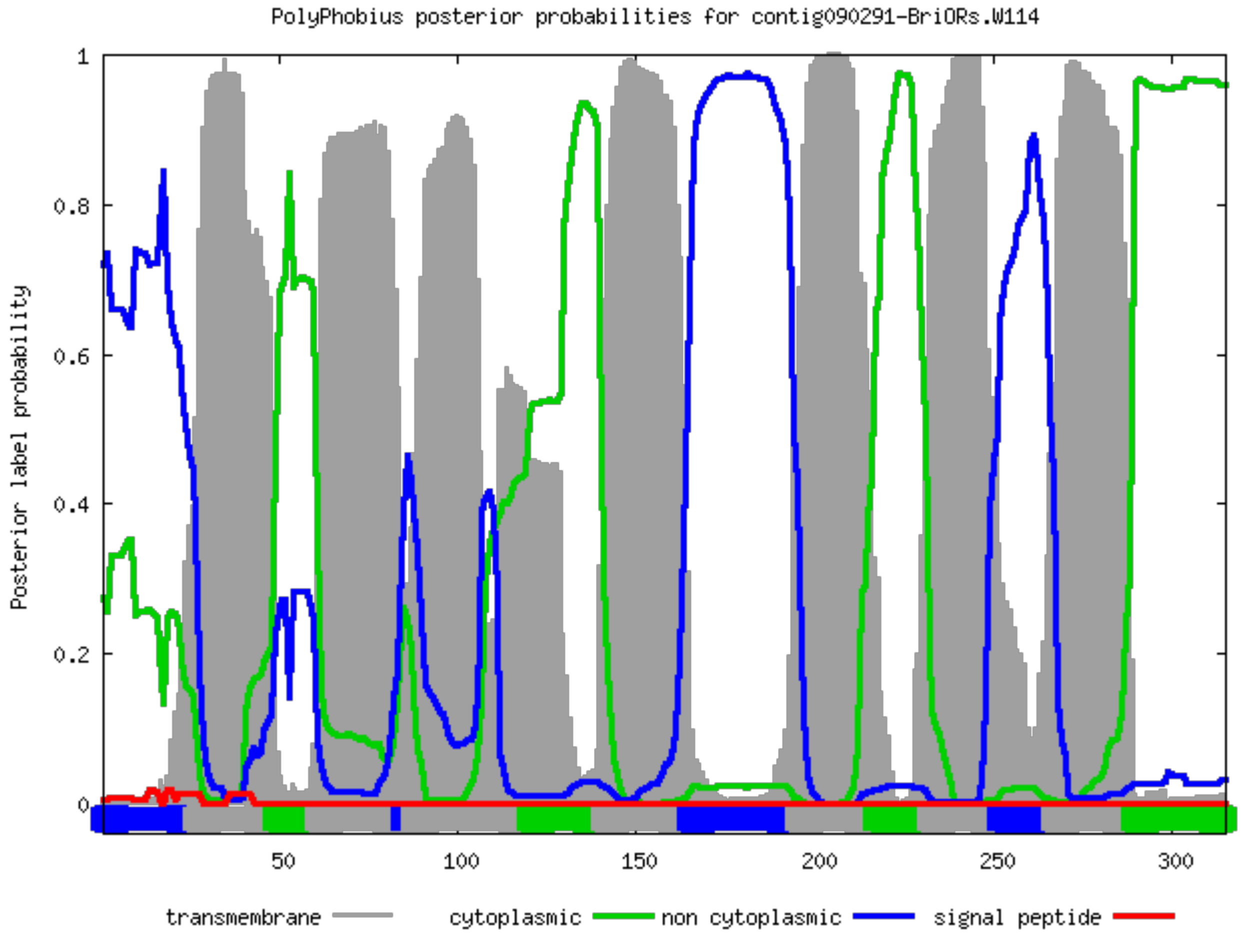

The prediction is based on an alignment. The probability data used in the plot is found here, and the gnuplot script is here.

Prediction of contig028619-TilOR.R249

```
ID   contig028619-TilOR.R249
FT   TOPO_DOM      1     22       NON CYTOPLASMIC.
FT   TRANSMEM     23     46
FT   TOPO_DOM     47     57       CYTOPLASMIC.
FT   TRANSMEM     58     82
FT   TOPO_DOM     83     87       NON CYTOPLASMIC.
FT   TRANSMEM     88    116
FT   TOPO_DOM    117    136       CYTOPLASMIC.
FT   TRANSMEM    137    160
FT   TOPO_DOM    161    191       NON CYTOPLASMIC.
FT   TRANSMEM    192    215
FT   TOPO_DOM    216    232       CYTOPLASMIC.
FT   TRANSMEM    233    256
FT   TOPO_DOM    257    268       NON CYTOPLASMIC.
FT   TRANSMEM    269    290
FT   TOPO_DOM    291    317       CYTOPLASMIC.
//
```

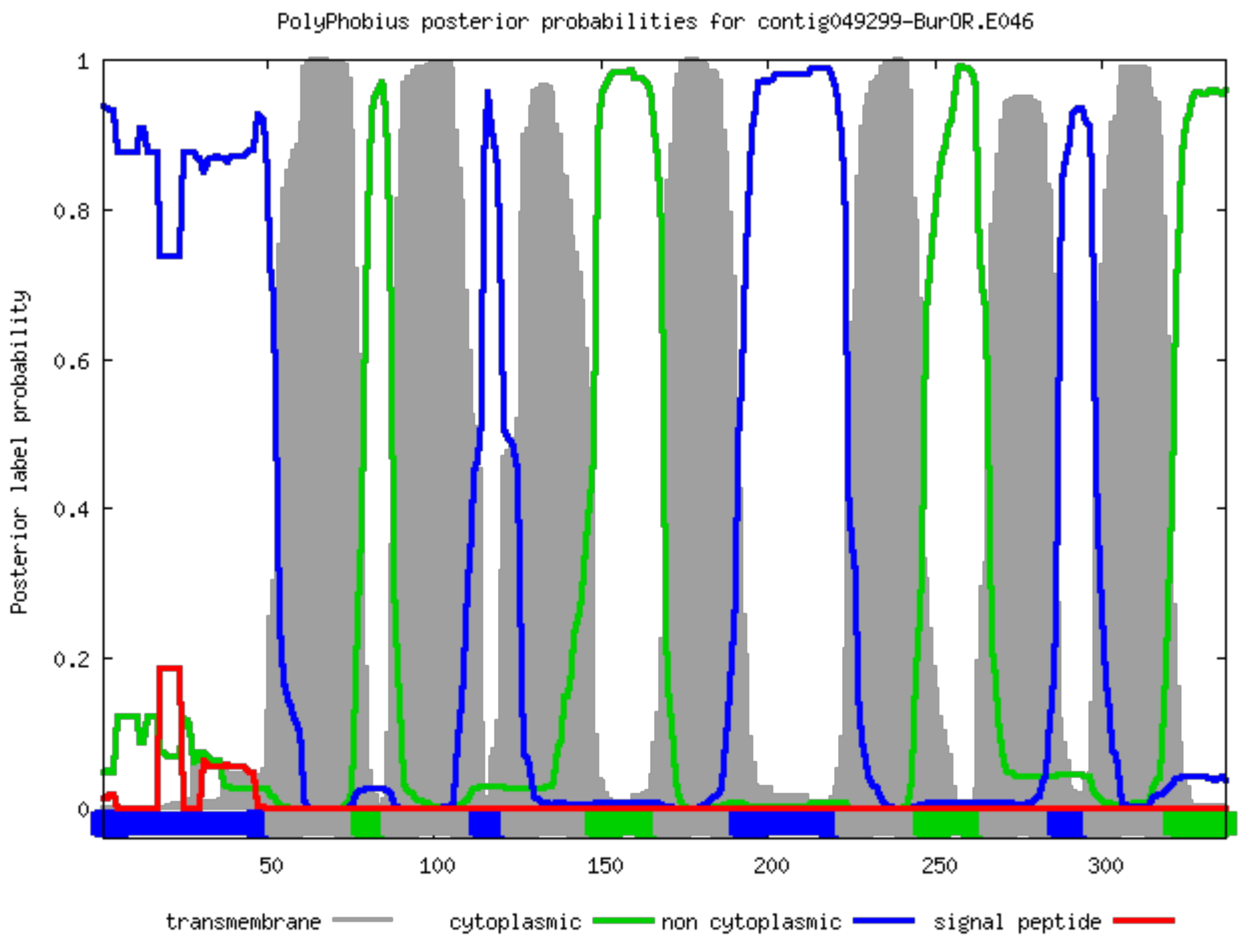

The prediction is based on an alignment. The probability data used in the plot is found here, and the gnuplot script is here.

Prediction of contig035579-NyeOR.H068

```
ID   contig035579-NyeOR.H068
FT   TOPO_DOM      1     22       NON CYTOPLASMIC.
FT   TRANSMEM     23     48
FT   TOPO_DOM     49     56       CYTOPLASMIC.
FT   TRANSMEM     57     77
FT   TOPO_DOM     78     95       NON CYTOPLASMIC.
FT   TRANSMEM     96    118
FT   TOPO_DOM    119    138       CYTOPLASMIC.
FT   TRANSMEM    139    160
FT   TOPO_DOM    161    193       NON CYTOPLASMIC.
FT   TRANSMEM    194    216
FT   TOPO_DOM    217    235       CYTOPLASMIC.
FT   TRANSMEM    236    257
FT   TOPO_DOM    258    269       NON CYTOPLASMIC.
FT   TRANSMEM    270    289
FT   TOPO_DOM    290    314       CYTOPLASMIC.
//
```

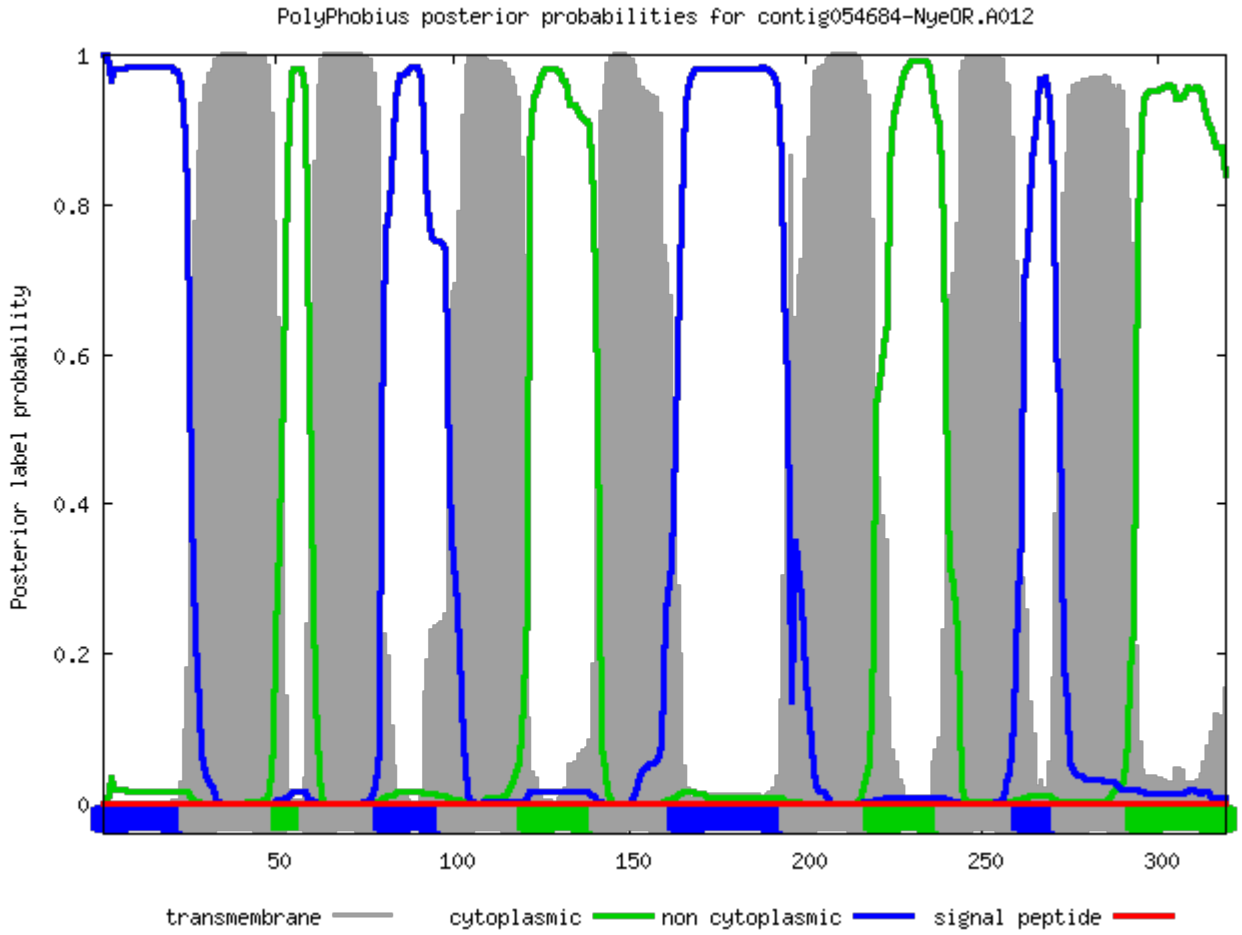

The prediction is based on an alignment. The probability data used in the plot is found here, and the gnuplot script is here.

Prediction of contig054233-BurOR.A012

```
ID   contig054233-BurOR.A012
FT   TOPO_DOM      1     22       NON CYTOPLASMIC.
FT   TRANSMEM     23     48
FT   TOPO_DOM     49     56       CYTOPLASMIC.
FT   TRANSMEM     57     77
FT   TOPO_DOM     78     95       NON CYTOPLASMIC.
FT   TRANSMEM     96    118
FT   TOPO_DOM    119    138       CYTOPLASMIC.
FT   TRANSMEM    139    160
FT   TOPO_DOM    161    192       NON CYTOPLASMIC.
FT   TRANSMEM    193    215
FT   TOPO_DOM    216    235       CYTOPLASMIC.
FT   TRANSMEM    236    257
FT   TOPO_DOM    258    268       NON CYTOPLASMIC.
FT   TRANSMEM    269    289
FT   TOPO_DOM    290    307       CYTOPLASMIC.
//
```

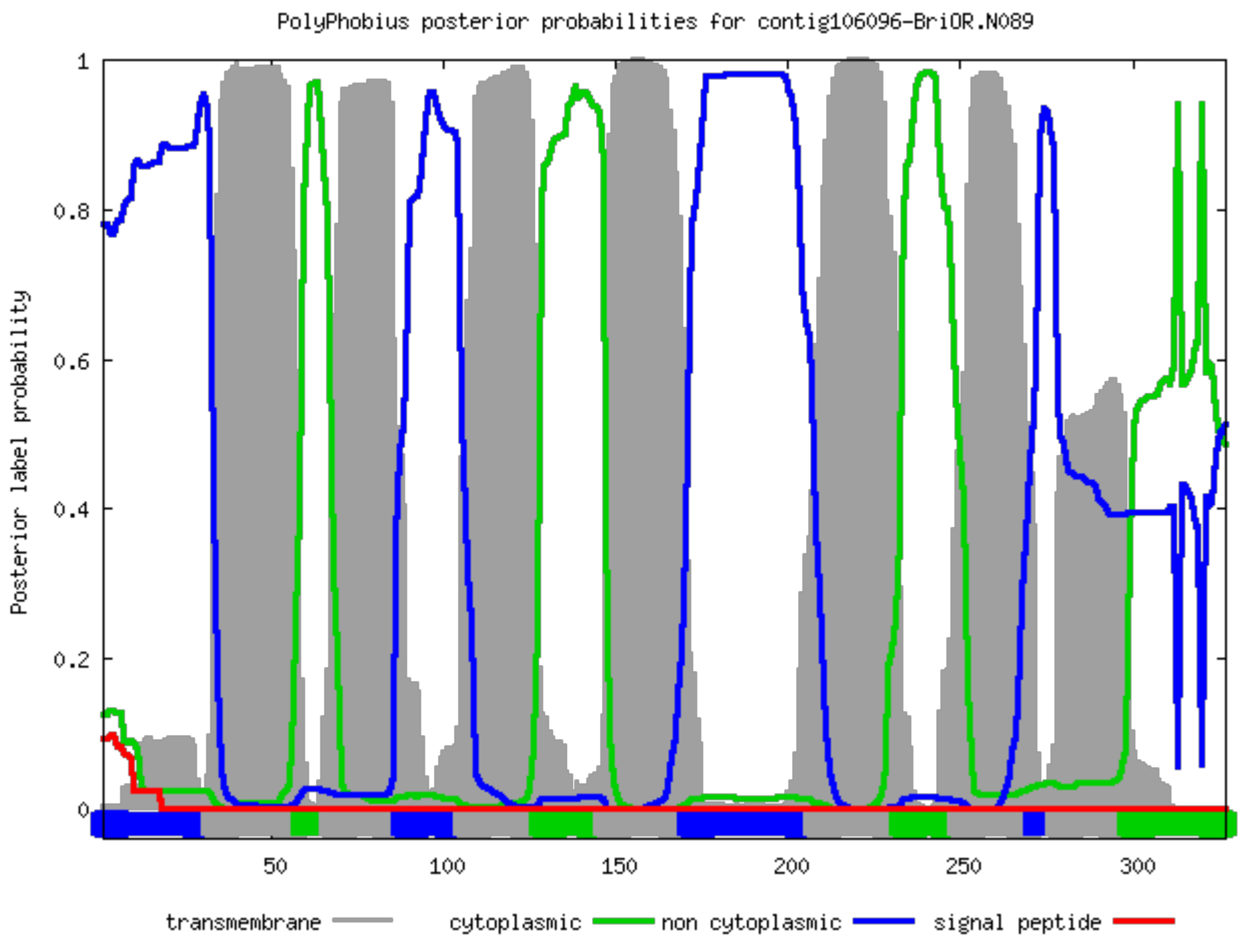

The prediction is based on an alignment. The probability data used in the plot is found here, and the gnuplot script is here.

Prediction of contig039435-TilOR.S229

```
ID    contig039435-TilOR.S229
FT    TOPO_DOM      1      21       NON CYTOPLASMIC.
FT    TRANSMEM     22      43
FT    TOPO_DOM     44      53       CYTOPLASMIC.
FT    TRANSMEM     54      78
FT    TOPO_DOM     79      83       NON CYTOPLASMIC.
FT    TRANSMEM     84     112
FT    TOPO_DOM    113     132       CYTOPLASMIC.
FT    TRANSMEM    133     158
FT    TOPO_DOM    159     186       NON CYTOPLASMIC.
FT    TRANSMEM    187     207
FT    TOPO_DOM    208     227       CYTOPLASMIC.
FT    TRANSMEM    228     253
FT    TOPO_DOM    254     264       NON CYTOPLASMIC.
FT    TRANSMEM    265     285
FT    TOPO_DOM    286     316       CYTOPLASMIC.
//
```

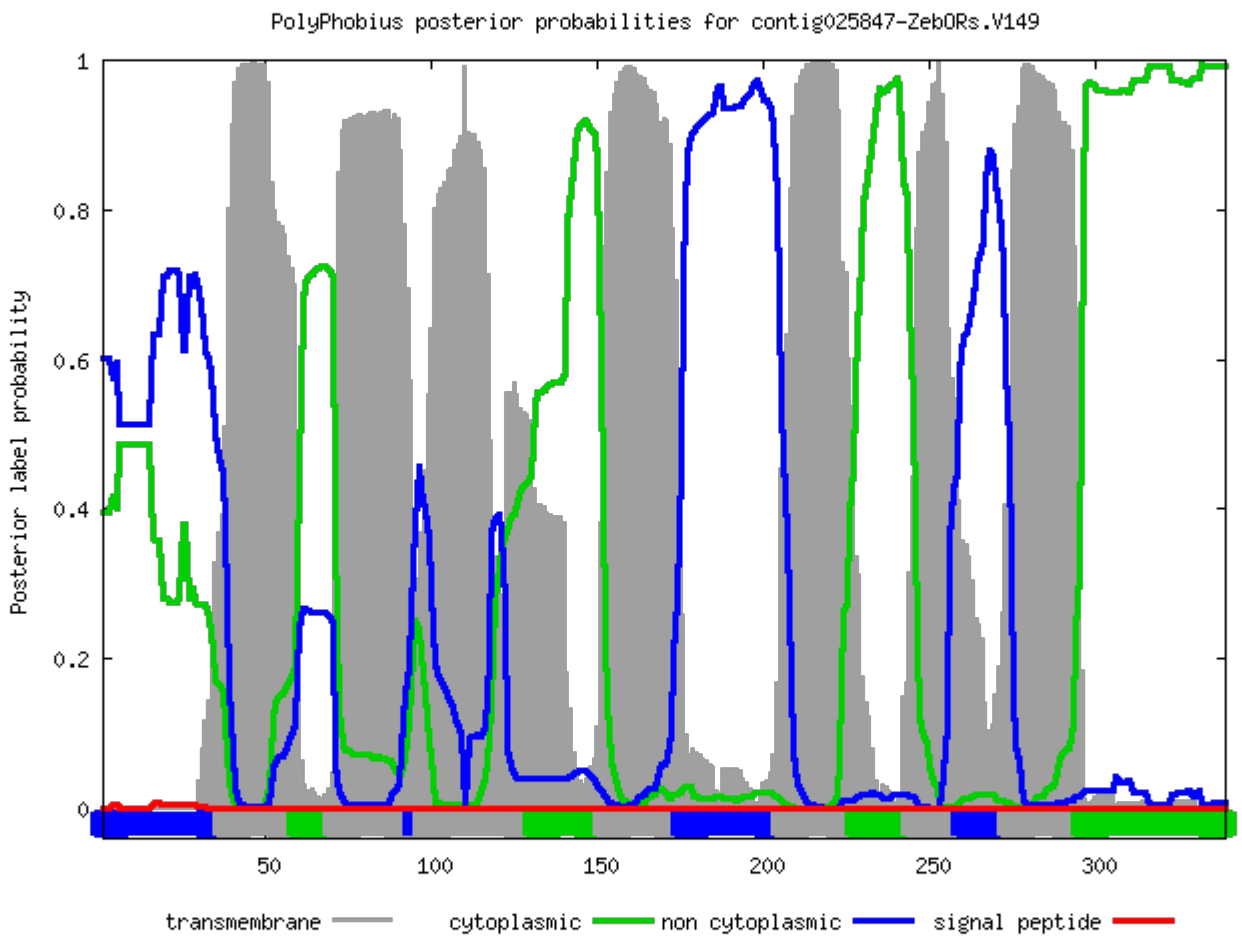

The prediction is based on an alignment. The probability data used in the plot is found here, and the gnuplot script is here.

Prediction of contig022230-TilOR.A007

```
ID   contig022230-TilOR.A007
FT   TOPO_DOM      1     18       NON CYTOPLASMIC.
FT   TRANSMEM     19     44
FT   TOPO_DOM     45     52       CYTOPLASMIC.
FT   TRANSMEM     53     73
FT   TOPO_DOM     74     91       NON CYTOPLASMIC.
FT   TRANSMEM     92    114
FT   TOPO_DOM    115    134       CYTOPLASMIC.
FT   TRANSMEM    135    155
FT   TOPO_DOM    156    188       NON CYTOPLASMIC.
FT   TRANSMEM    189    211
FT   TOPO_DOM    212    231       CYTOPLASMIC.
FT   TRANSMEM    232    253
FT   TOPO_DOM    254    264       NON CYTOPLASMIC.
FT   TRANSMEM    265    285
FT   TOPO_DOM    286    300       CYTOPLASMIC.
//
```

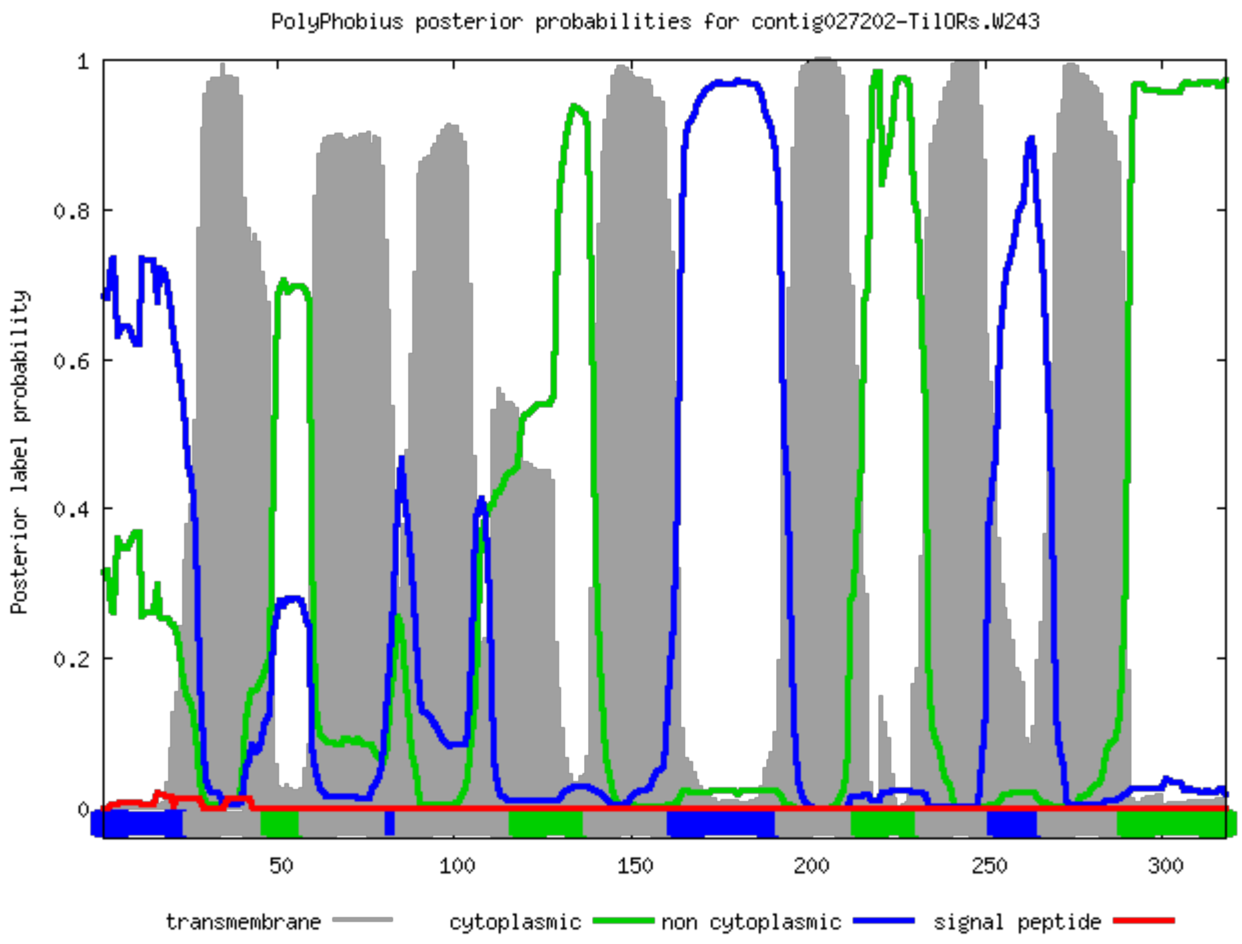

The prediction is based on an alignment. The probability data used in the plot is found here, and the gnuplot script is here.

Prediction of contig047729-TilOR.E076

```
ID   contig047729-TilOR.E076
FT   TOPO_DOM      1     23       NON CYTOPLASMIC.
FT   TRANSMEM     24     49
FT   TOPO_DOM     50     58       CYTOPLASMIC.
FT   TRANSMEM     59     82
FT   TOPO_DOM     83     95       NON CYTOPLASMIC.
FT   TRANSMEM     96    119
FT   TOPO_DOM    120    139       CYTOPLASMIC.
FT   TRANSMEM    140    162
FT   TOPO_DOM    163    194       NON CYTOPLASMIC.
FT   TRANSMEM    195    217
FT   TOPO_DOM    218    237       CYTOPLASMIC.
FT   TRANSMEM    238    257
FT   TOPO_DOM    258    268       NON CYTOPLASMIC.
FT   TRANSMEM    269    292
FT   TOPO_DOM    293    308       CYTOPLASMIC.
//
```

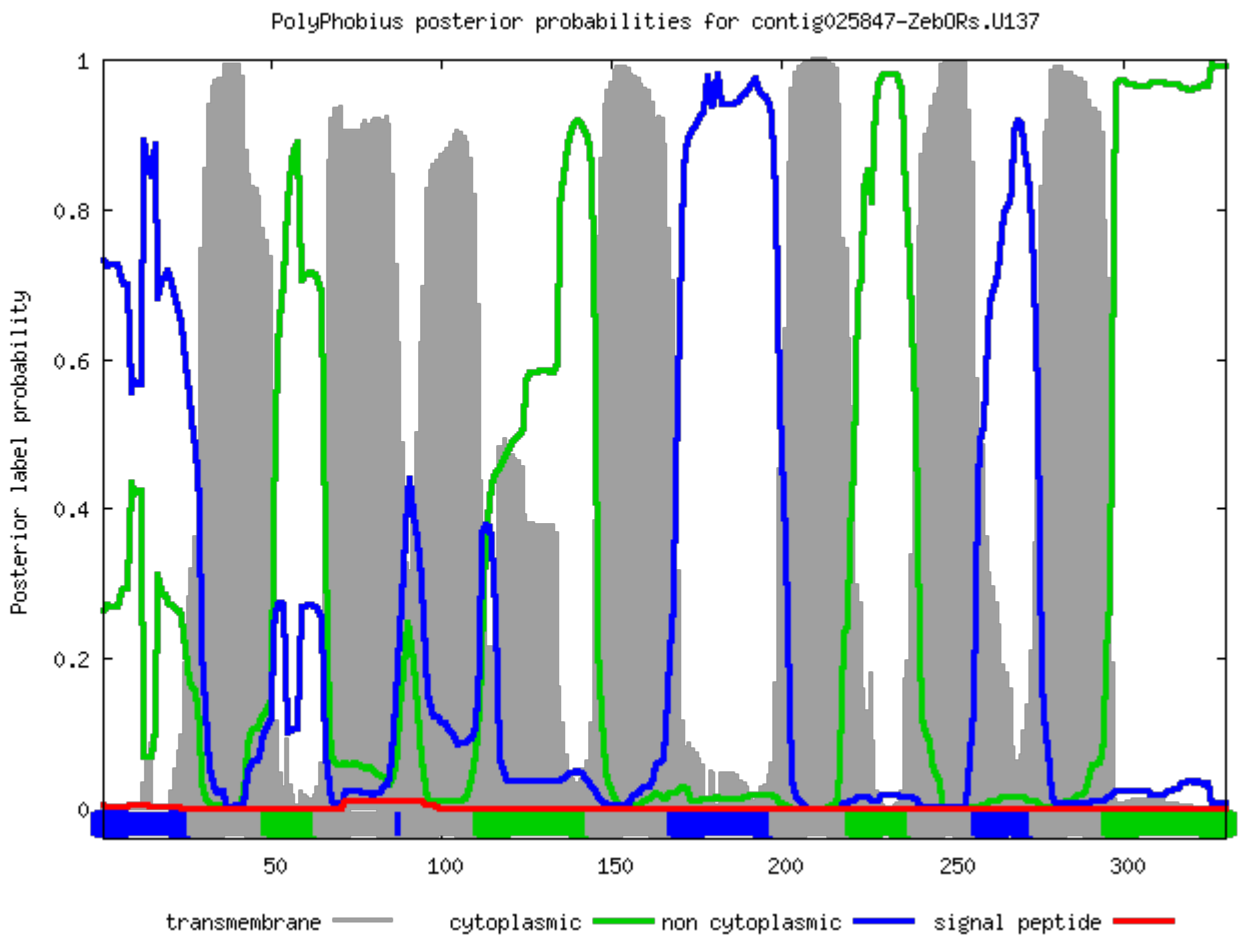

The prediction is based on an alignment. The probability data used in the plot is found here, and the gnuplot script is here.

Prediction of contig034990-NyeOR.A007

```
ID    contig034990-NyeOR.A007
FT    TOPO_DOM      1     22        NON CYTOPLASMIC.
FT    TRANSMEM     23     48
FT    TOPO_DOM     49     56        CYTOPLASMIC.
FT    TRANSMEM     57     77
FT    TOPO_DOM     78     95        NON CYTOPLASMIC.
FT    TRANSMEM     96    118
FT    TOPO_DOM    119    138        CYTOPLASMIC.
FT    TRANSMEM    139    160
FT    TOPO_DOM    161    192        NON CYTOPLASMIC.
FT    TRANSMEM    193    215
FT    TOPO_DOM    216    235        CYTOPLASMIC.
FT    TRANSMEM    236    257
FT    TOPO_DOM    258    268        NON CYTOPLASMIC.
FT    TRANSMEM    269    289
FT    TOPO_DOM    290    320        CYTOPLASMIC.
//
```

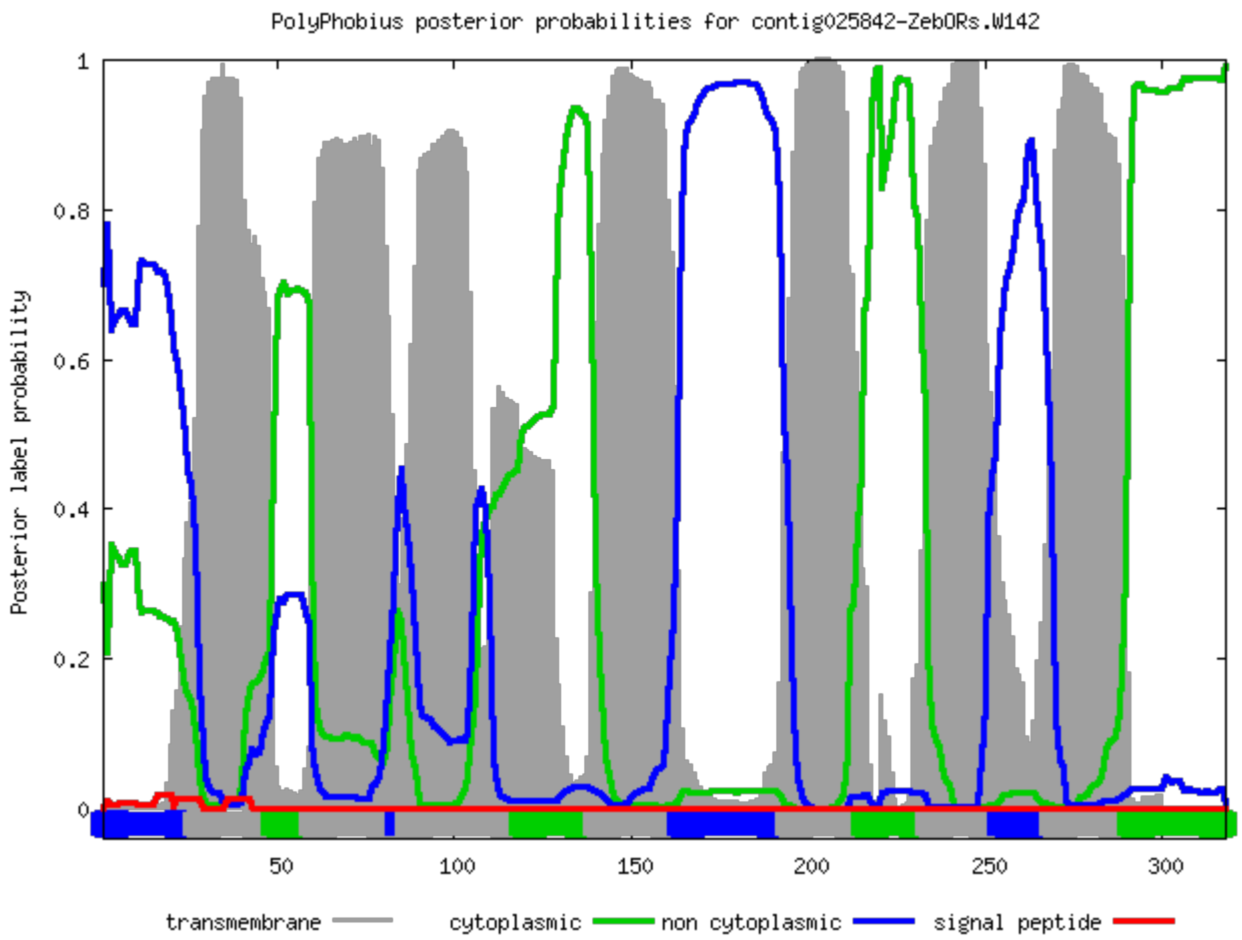

The prediction is based on an alignment. The probability data used in the plot is found here, and the gnuplot script is here.

Prediction of contig013369-TilOR.H114

```
ID   contig013369-TilOR.H114
FT   TOPO_DOM      1     23       NON CYTOPLASMIC.
FT   TRANSMEM     24     49
FT   TOPO_DOM     50     56       CYTOPLASMIC.
FT   TRANSMEM     57     76
FT   TOPO_DOM     77     95       NON CYTOPLASMIC.
FT   TRANSMEM     96    118
FT   TOPO_DOM    119    138       CYTOPLASMIC.
FT   TRANSMEM    139    160
FT   TOPO_DOM    161    196       NON CYTOPLASMIC.
FT   TRANSMEM    197    219
FT   TOPO_DOM    220    237       CYTOPLASMIC.
FT   TRANSMEM    238    260
FT   TOPO_DOM    261    271       NON CYTOPLASMIC.
FT   TRANSMEM    272    291
FT   TOPO_DOM    292    310       CYTOPLASMIC.
//
```

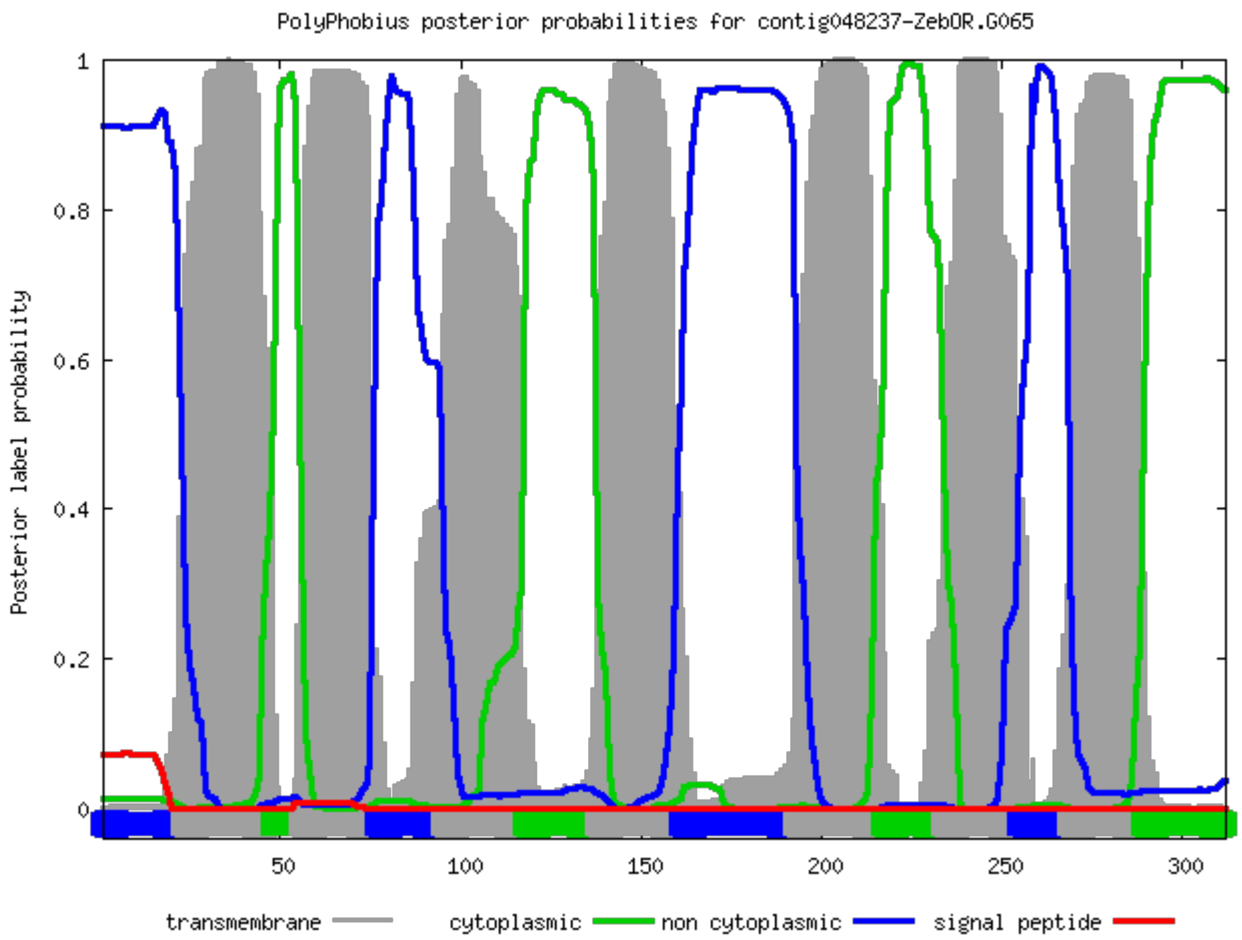

The prediction is based on an alignment. The probability data used in the plot is found here, and the gnuplot script is here.

Prediction of contig063018-ZebOR.E053

```
ID   contig063018-ZebOR.E053
FT   TOPO_DOM      1     21       NON CYTOPLASMIC.
FT   TRANSMEM     22     47
FT   TOPO_DOM     48     56       CYTOPLASMIC.
FT   TRANSMEM     57     82
FT   TOPO_DOM     83     91       NON CYTOPLASMIC.
FT   TRANSMEM     92    117
FT   TOPO_DOM    118    137       CYTOPLASMIC.
FT   TRANSMEM    138    160
FT   TOPO_DOM    161    192       NON CYTOPLASMIC.
FT   TRANSMEM    193    211
FT   TOPO_DOM    212    231       CYTOPLASMIC.
FT   TRANSMEM    232    251
FT   TOPO_DOM    252    262       NON CYTOPLASMIC.
FT   TRANSMEM    263    286
FT   TOPO_DOM    287    306       CYTOPLASMIC.
//
```

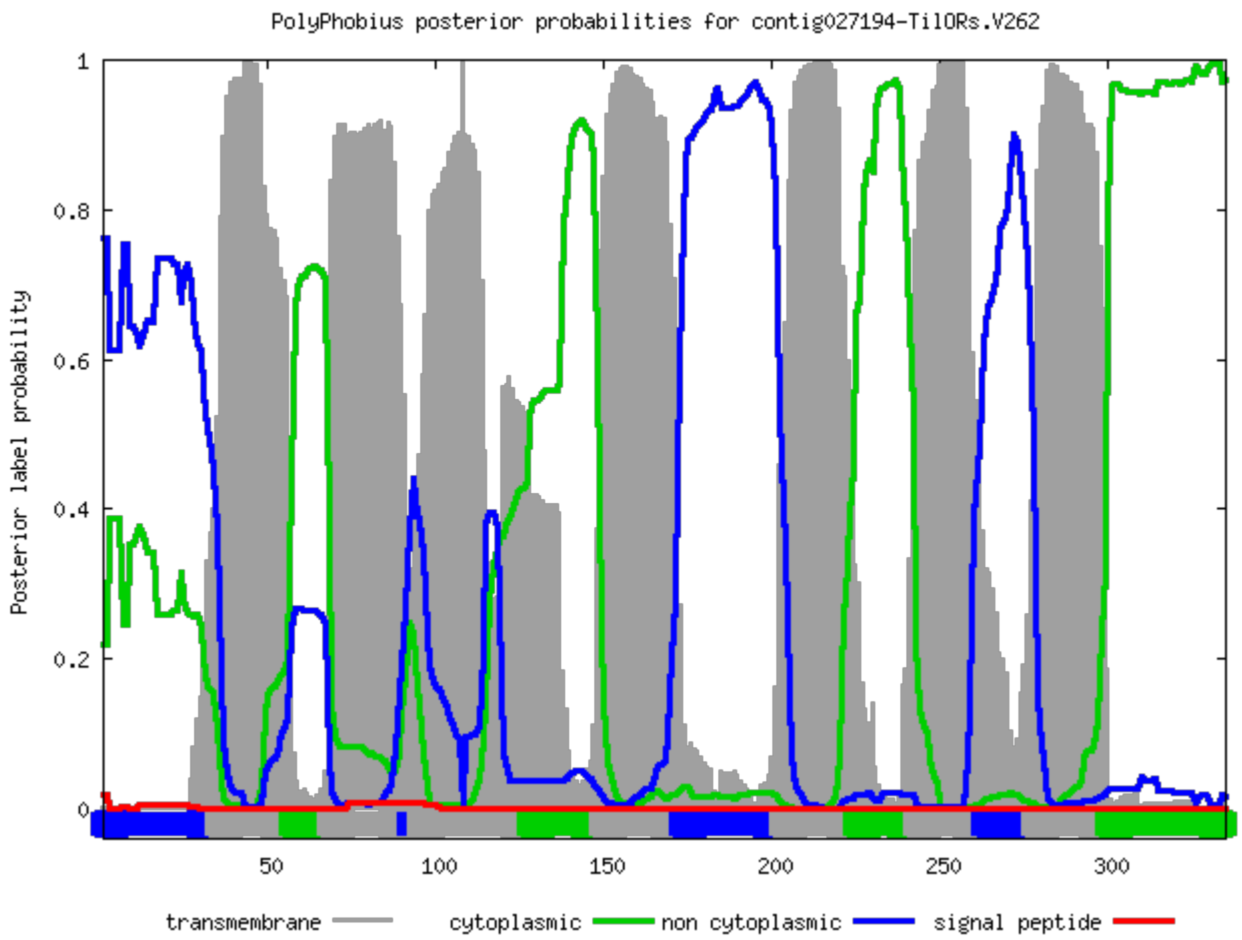

The prediction is based on an alignment. The probability data used in the plot is found here, and the gnuplot script is here.

Prediction of contig028637-TilOR.R250

```
ID   contig028637-TilOR.R250
FT   TOPO_DOM      1     24       NON CYTOPLASMIC.
FT   TRANSMEM     25     48
FT   TOPO_DOM     49     59       CYTOPLASMIC.
FT   TRANSMEM     60     84
FT   TOPO_DOM     85     89       NON CYTOPLASMIC.
FT   TRANSMEM     90    118
FT   TOPO_DOM    119    138       CYTOPLASMIC.
FT   TRANSMEM    139    162
FT   TOPO_DOM    163    194       NON CYTOPLASMIC.
FT   TRANSMEM    195    218
FT   TOPO_DOM    219    235       CYTOPLASMIC.
FT   TRANSMEM    236    259
FT   TOPO_DOM    260    270       NON CYTOPLASMIC.
FT   TRANSMEM    271    293
FT   TOPO_DOM    294    315       CYTOPLASMIC.
//
```

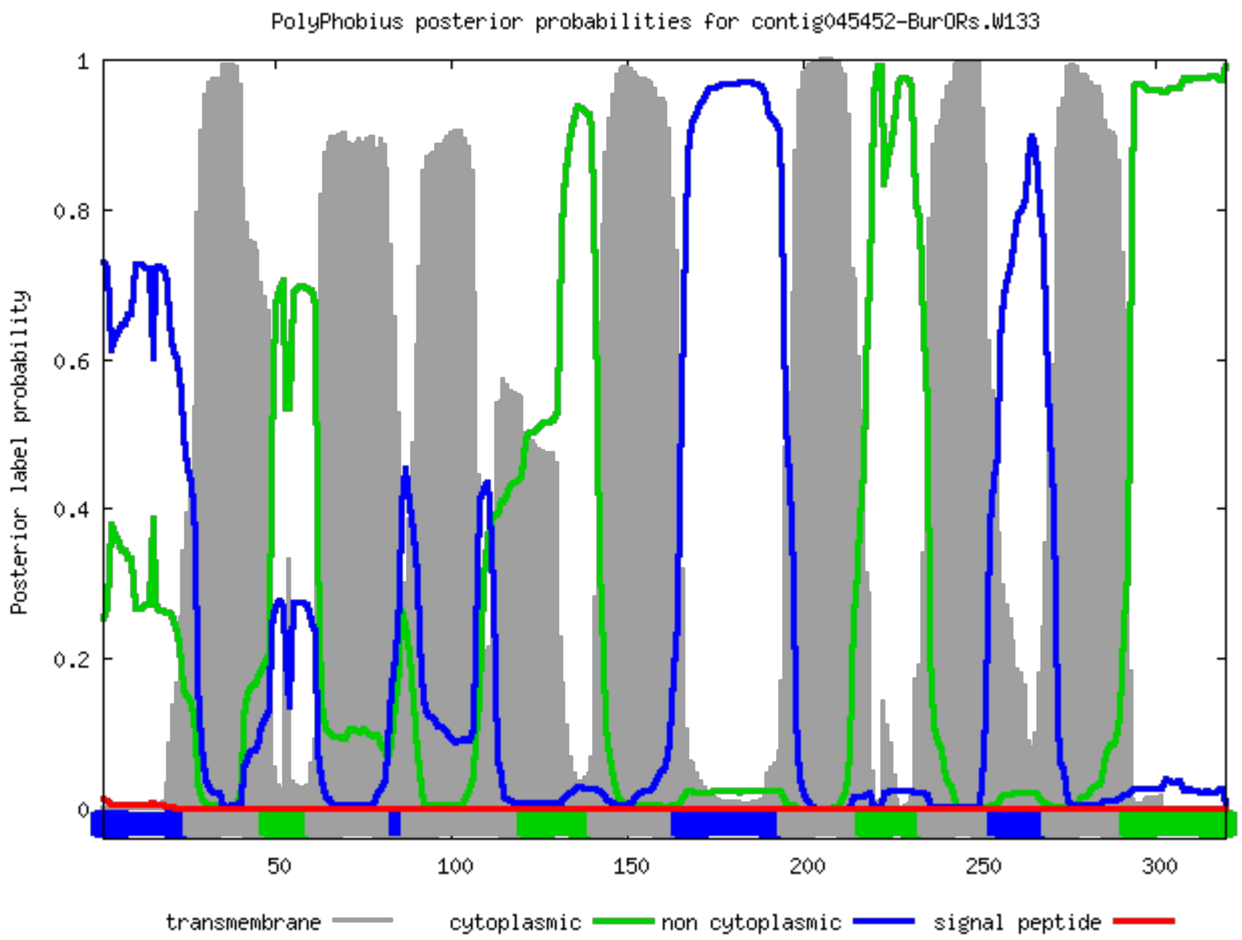

The prediction is based on an alignment. The probability data used in the plot is found here, and the gnuplot script is here.

Prediction of contig009320-TilOR.B045

```
ID   contig009320-TilOR.B045
FT   TOPO_DOM      1     29       NON CYTOPLASMIC.
FT   TRANSMEM     30     55
FT   TOPO_DOM     56     63       CYTOPLASMIC.
FT   TRANSMEM     64     84
FT   TOPO_DOM     85    104       NON CYTOPLASMIC.
FT   TRANSMEM    105    127
FT   TOPO_DOM    128    147       CYTOPLASMIC.
FT   TRANSMEM    148    169
FT   TOPO_DOM    170    204       NON CYTOPLASMIC.
FT   TRANSMEM    205    227
FT   TOPO_DOM    228    247       CYTOPLASMIC.
FT   TRANSMEM    248    269
FT   TOPO_DOM    270    278       NON CYTOPLASMIC.
FT   TRANSMEM    279    301
FT   TOPO_DOM    302    321       CYTOPLASMIC.
//
```

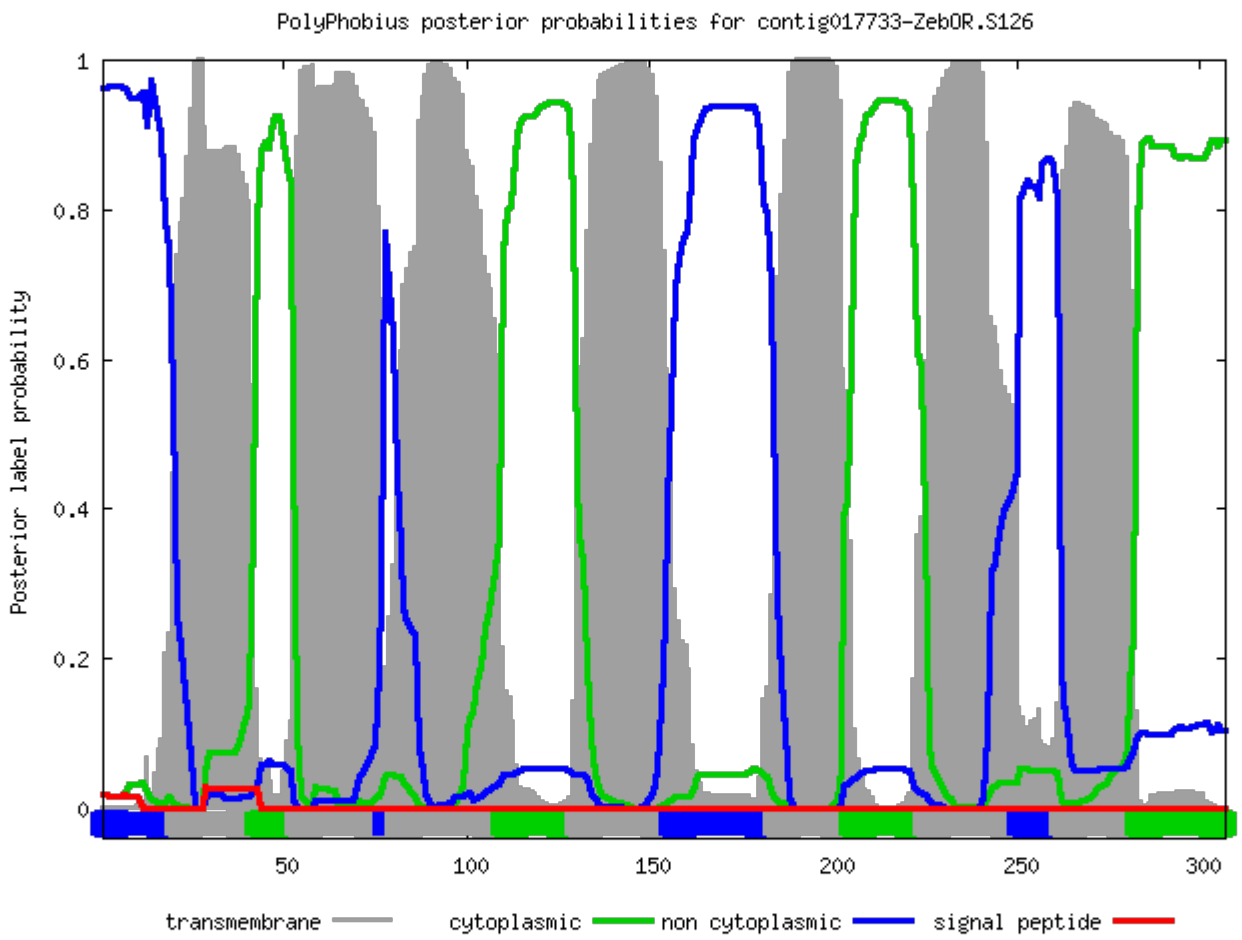

The prediction is based on an alignment. The probability data used in the plot is found here, and the gnuplot script is here.

Prediction of contig042543-BriOR.L069

```
ID   contig042543-BriOR.L069
FT   TOPO_DOM      1     25       NON CYTOPLASMIC.
FT   TRANSMEM     26     50
FT   TOPO_DOM     51     59       CYTOPLASMIC.
FT   TRANSMEM     60     83
FT   TOPO_DOM     84     98       NON CYTOPLASMIC.
FT   TRANSMEM     99    120
FT   TOPO_DOM    121    140       CYTOPLASMIC.
FT   TRANSMEM    141    162
FT   TOPO_DOM    163    198       NON CYTOPLASMIC.
FT   TRANSMEM    199    224
FT   TOPO_DOM    225    235       CYTOPLASMIC.
FT   TRANSMEM    236    259
FT   TOPO_DOM    260    271       NON CYTOPLASMIC.
FT   TRANSMEM    272    292
FT   TOPO_DOM    293    317       CYTOPLASMIC.
//
```

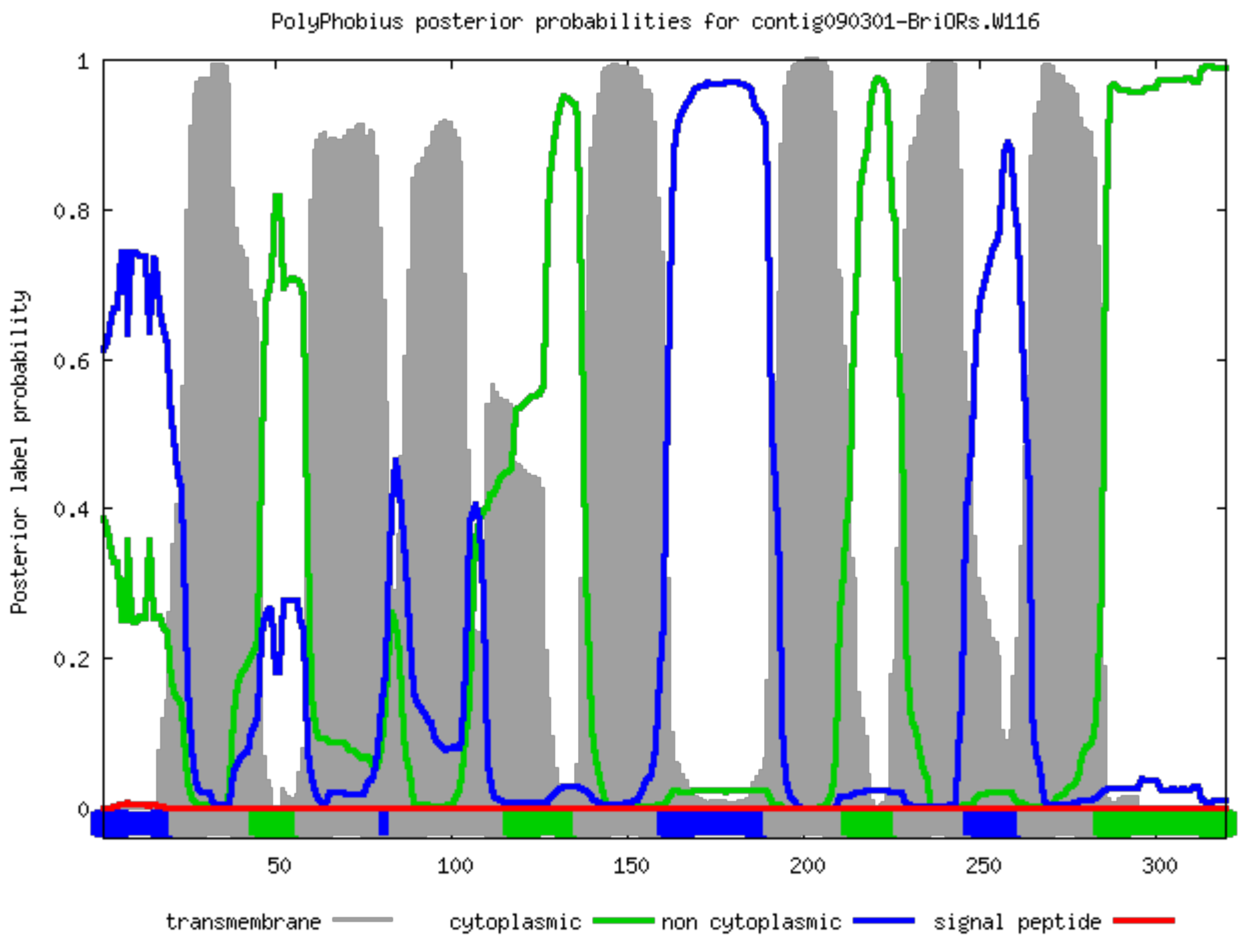

The prediction is based on an alignment. The probability data used in the plot is found here, and the gnuplot script is here.

Prediction of contig052904-BurOR.X128

```
ID   contig052904-BurOR.X128
FT   TOPO_DOM      1     20       NON CYTOPLASMIC.
FT   TRANSMEM     21     44
FT   TOPO_DOM     45     55       CYTOPLASMIC.
FT   TRANSMEM     56     80
FT   TOPO_DOM     81     83       NON CYTOPLASMIC.
FT   TRANSMEM     84    114
FT   TOPO_DOM    115    134       CYTOPLASMIC.
FT   TRANSMEM    135    158
FT   TOPO_DOM    159    188       NON CYTOPLASMIC.
FT   TRANSMEM    189    209
FT   TOPO_DOM    210    229       CYTOPLASMIC.
FT   TRANSMEM    230    248
FT   TOPO_DOM    249    265       NON CYTOPLASMIC.
FT   TRANSMEM    266    286
FT   TOPO_DOM    287    322       CYTOPLASMIC.
//
```

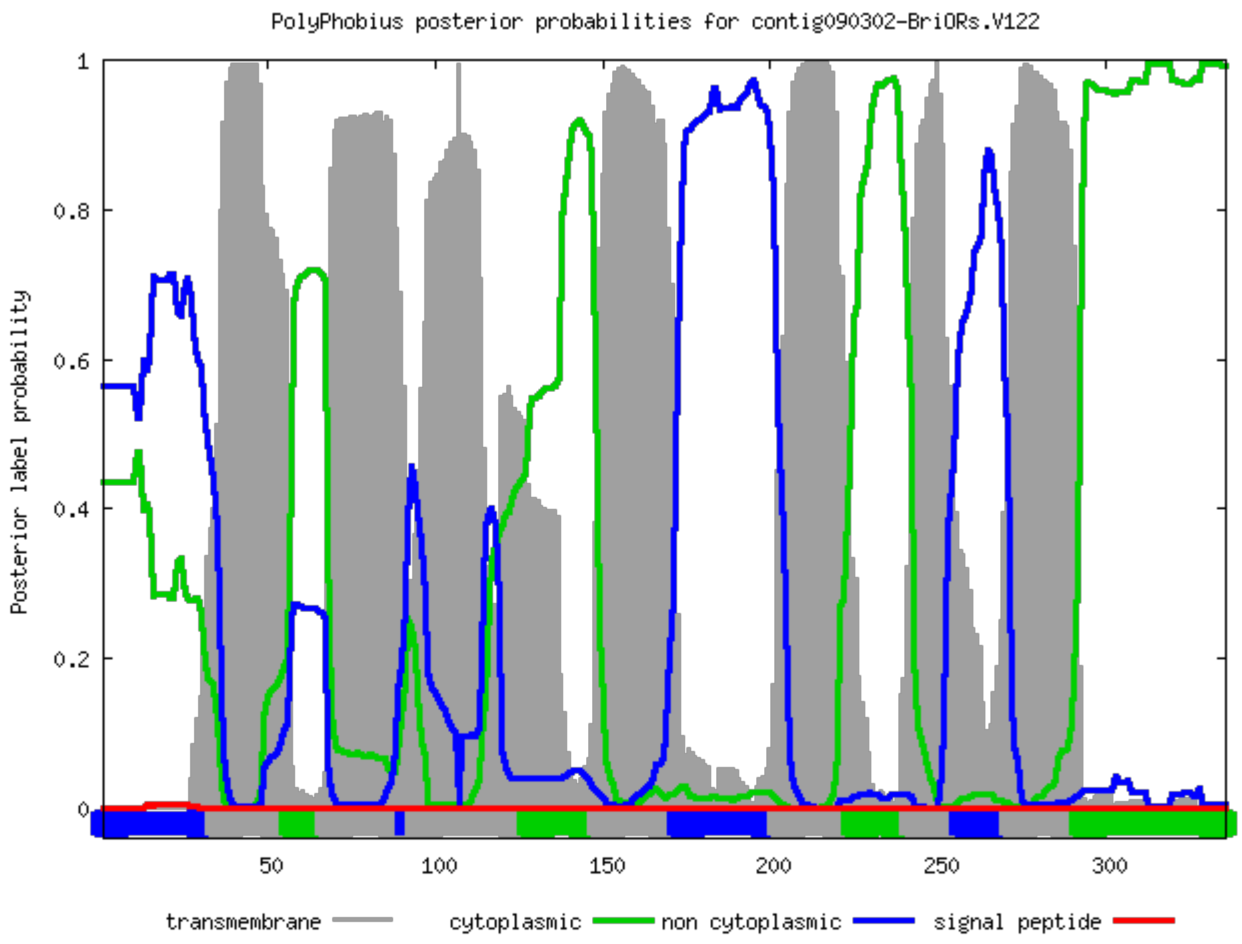

The prediction is based on an alignment. The probability data used in the plot is found here, and the gnuplot script is here.

Prediction of contig039450-TilOR.K133

```
ID   contig039450-TilOR.K133
FT   TOPO_DOM      1     26       NON CYTOPLASMIC.
FT   TRANSMEM     27     50
FT   TOPO_DOM     51     59       CYTOPLASMIC.
FT   TRANSMEM     60     82
FT   TOPO_DOM     83     99       NON CYTOPLASMIC.
FT   TRANSMEM    100    120
FT   TOPO_DOM    121    140       CYTOPLASMIC.
FT   TRANSMEM    141    164
FT   TOPO_DOM    165    195       NON CYTOPLASMIC.
FT   TRANSMEM    196    222
FT   TOPO_DOM    223    241       CYTOPLASMIC.
FT   TRANSMEM    242    261
FT   TOPO_DOM    262    271       NON CYTOPLASMIC.
FT   TRANSMEM    272    291
FT   TOPO_DOM    292    312       CYTOPLASMIC.
//
```

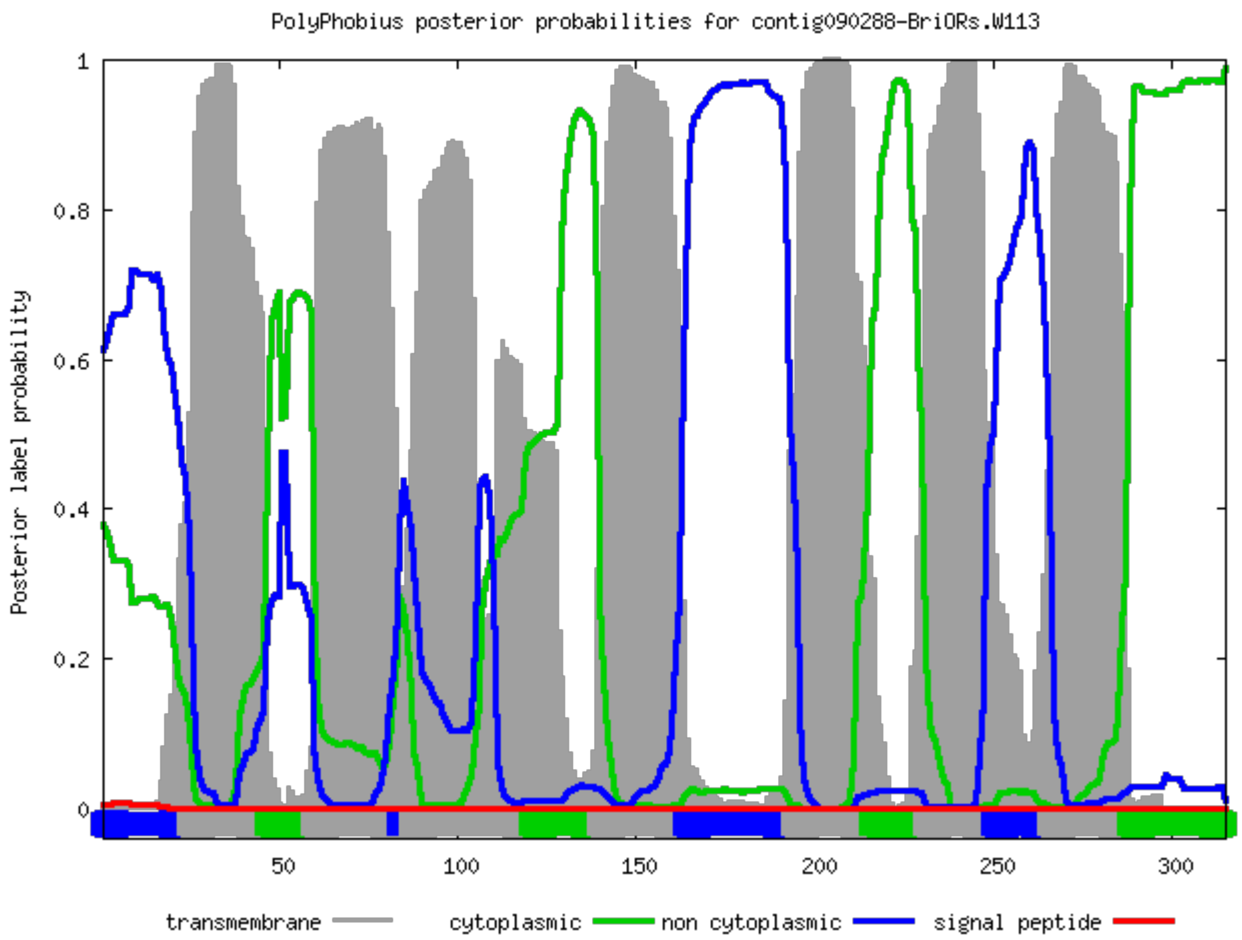

The prediction is based on an alignment. The probability data used in the plot is found here, and the gnuplot script is here.

Prediction of contig014054-ZebOR.D042

```
ID   contig014054-ZebOR.D042
FT   TOPO_DOM      1      22       NON CYTOPLASMIC.
FT   TRANSMEM     23      48
FT   TOPO_DOM     49      57       CYTOPLASMIC.
FT   TRANSMEM     58      81
FT   TOPO_DOM     82      90       NON CYTOPLASMIC.
FT   TRANSMEM     91     118
FT   TOPO_DOM    119     138       CYTOPLASMIC.
FT   TRANSMEM    139     161
FT   TOPO_DOM    162     194       NON CYTOPLASMIC.
FT   TRANSMEM    195     216
FT   TOPO_DOM    217     236       CYTOPLASMIC.
FT   TRANSMEM    237     256
FT   TOPO_DOM    257     267       NON CYTOPLASMIC.
FT   TRANSMEM    268     291
FT   TOPO_DOM    292     319       CYTOPLASMIC.
//
```

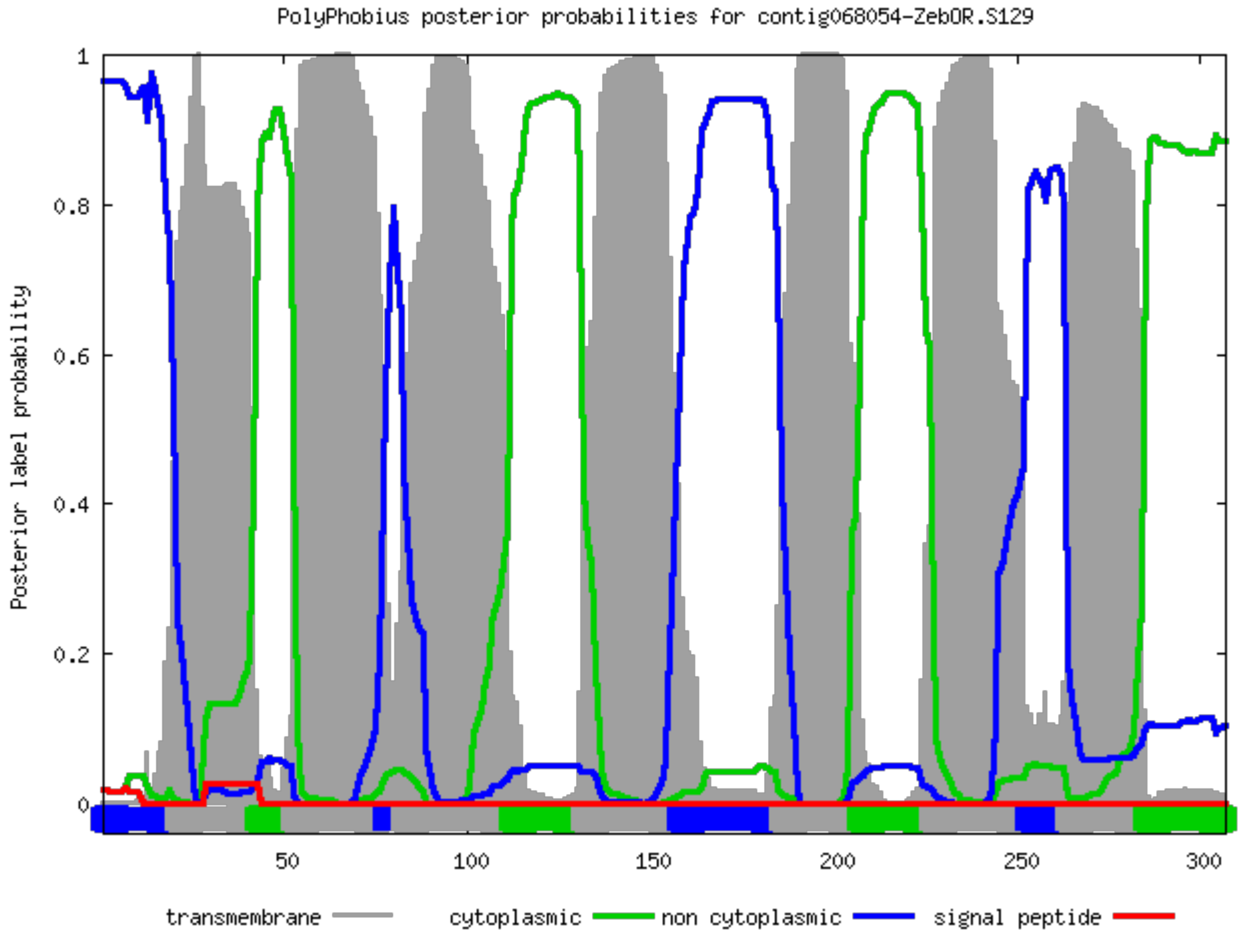

The prediction is based on an alignment. The probability data used in the plot is found here, and the gnuplot script is here.

Prediction of contig046491-NyeOR.K087

```
ID   contig046491-NyeOR.K087
FT   TOPO_DOM      1      24       NON CYTOPLASMIC.
FT   TRANSMEM     25      50
FT   TOPO_DOM     51      58       CYTOPLASMIC.
FT   TRANSMEM     59      81
FT   TOPO_DOM     82     100       NON CYTOPLASMIC.
FT   TRANSMEM    101     121
FT   TOPO_DOM    122     141       CYTOPLASMIC.
FT   TRANSMEM    142     165
FT   TOPO_DOM    166     196       NON CYTOPLASMIC.
FT   TRANSMEM    197     224
FT   TOPO_DOM    225     243       CYTOPLASMIC.
FT   TRANSMEM    244     264
FT   TOPO_DOM    265     272       NON CYTOPLASMIC.
FT   TRANSMEM    273     292
FT   TOPO_DOM    293     314       CYTOPLASMIC.
//
```

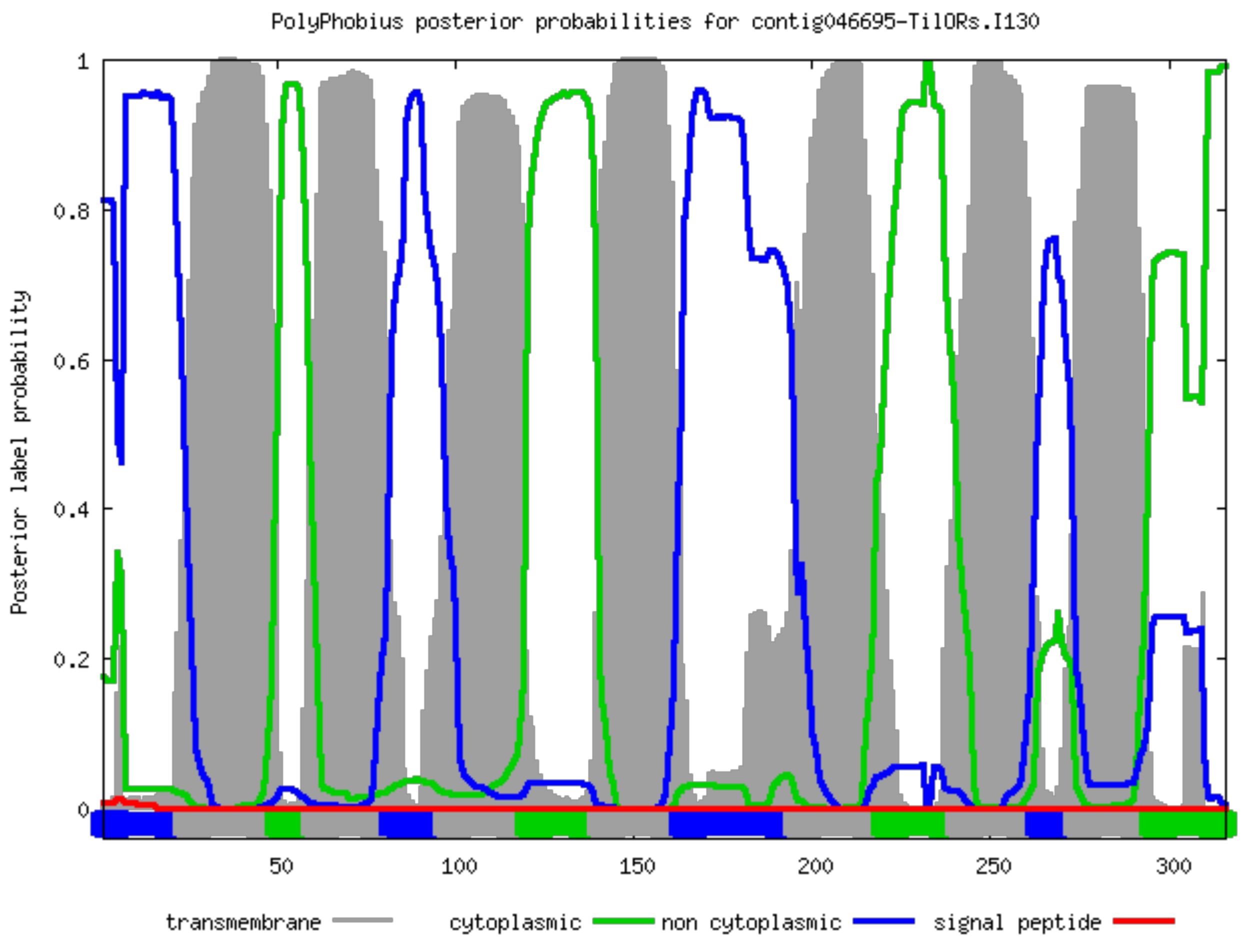

The prediction is based on an alignment. The probability data used in the plot is found here, and the gnuplot script is here.

Prediction of contig017786-ZebOR.L091

```
ID   contig017786-ZebOR.L091
FT   TOPO_DOM      1      25       NON CYTOPLASMIC.
FT   TRANSMEM     26      50
FT   TOPO_DOM     51      59       CYTOPLASMIC.
FT   TRANSMEM     60      82
FT   TOPO_DOM     83      98       NON CYTOPLASMIC.
FT   TRANSMEM     99     120
FT   TOPO_DOM    121     140       CYTOPLASMIC.
FT   TRANSMEM    141     162
FT   TOPO_DOM    163     198       NON CYTOPLASMIC.
FT   TRANSMEM    199     224
FT   TOPO_DOM    225     235       CYTOPLASMIC.
FT   TRANSMEM    236     259
FT   TOPO_DOM    260     271       NON CYTOPLASMIC.
FT   TRANSMEM    272     292
FT   TOPO_DOM    293     314       CYTOPLASMIC.
//
```

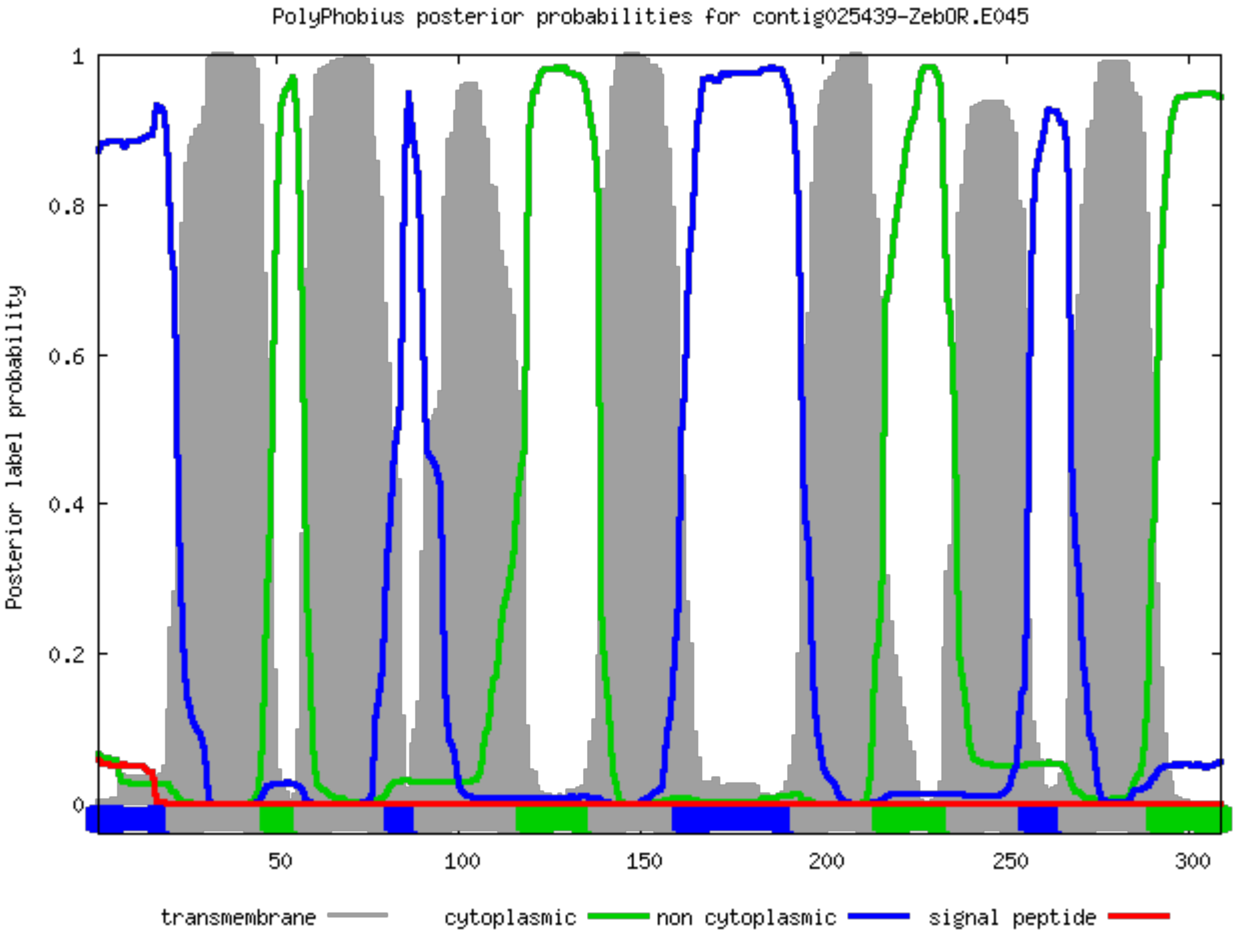

The prediction is based on an alignment. The probability data used in the plot is found here, and the gnuplot script is here.

Prediction of contig004265-BriOR.E035

```
ID   contig004265-BriOR.E035
FT   TOPO_DOM      1     22       NON CYTOPLASMIC.
FT   TRANSMEM     23     48
FT   TOPO_DOM     49     57       CYTOPLASMIC.
FT   TRANSMEM     58     81
FT   TOPO_DOM     82     91       NON CYTOPLASMIC.
FT   TRANSMEM     92    118
FT   TOPO_DOM    119    138       CYTOPLASMIC.
FT   TRANSMEM    139    161
FT   TOPO_DOM    162    193       NON CYTOPLASMIC.
FT   TRANSMEM    194    216
FT   TOPO_DOM    217    236       CYTOPLASMIC.
FT   TRANSMEM    237    256
FT   TOPO_DOM    257    267       NON CYTOPLASMIC.
FT   TRANSMEM    268    291
FT   TOPO_DOM    292    309       CYTOPLASMIC.
//
```

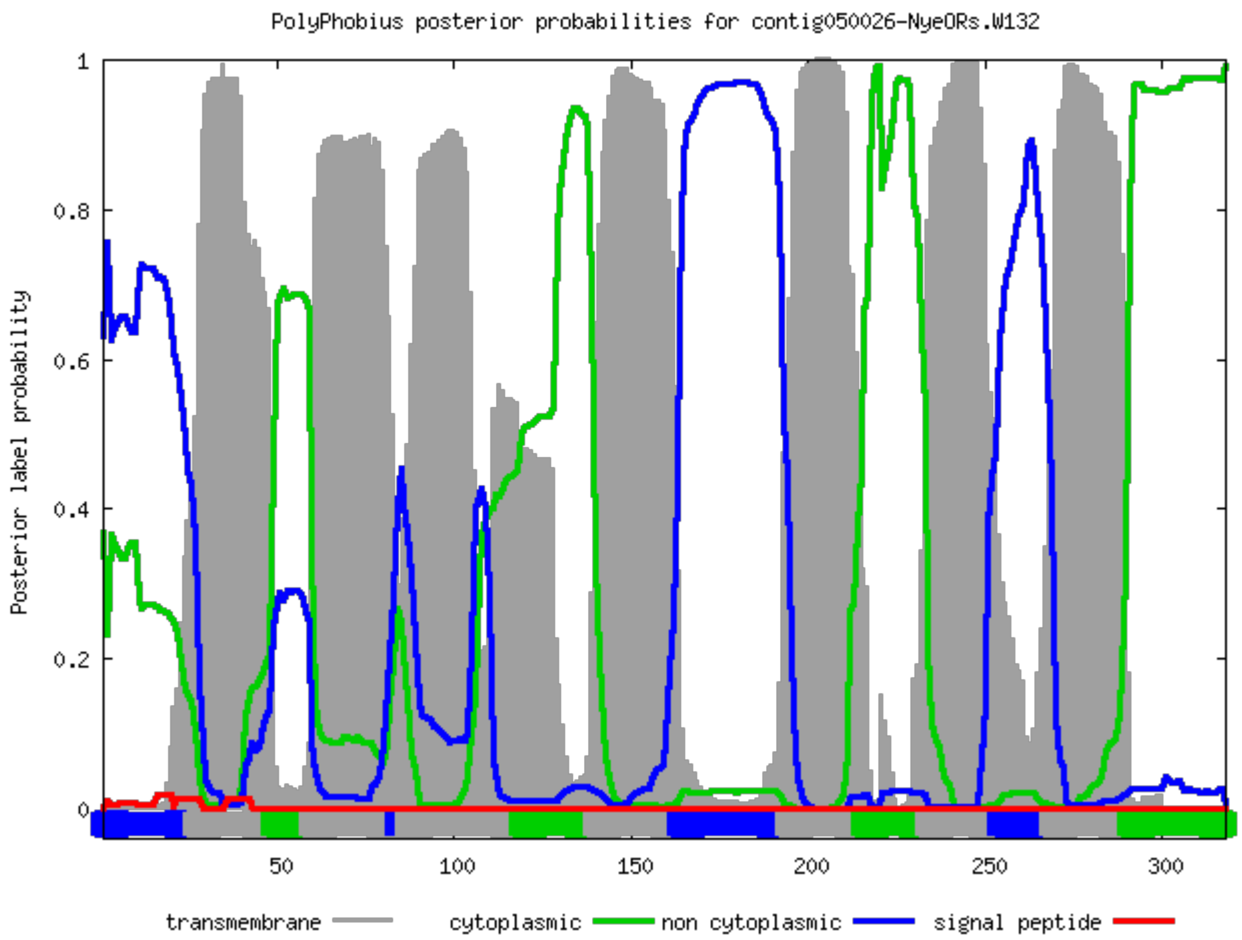

The prediction is based on an alignment. The probability data used in the plot is found here, and the gnuplot script is here.

Prediction of contig046352-TilOR.N193

```
ID   contig046352-TilOR.N193
FT   TOPO_DOM      1     33       NON CYTOPLASMIC.
FT   TRANSMEM     34     59
FT   TOPO_DOM     60     67       CYTOPLASMIC.
FT   TRANSMEM     68     89
FT   TOPO_DOM     90    108       NON CYTOPLASMIC.
FT   TRANSMEM    109    128
FT   TOPO_DOM    129    148       CYTOPLASMIC.
FT   TRANSMEM    149    171
FT   TOPO_DOM    172    207       NON CYTOPLASMIC.
FT   TRANSMEM    208    233
FT   TOPO_DOM    234    252       CYTOPLASMIC.
FT   TRANSMEM    253    275
FT   TOPO_DOM    276    324       NON CYTOPLASMIC.
//
```

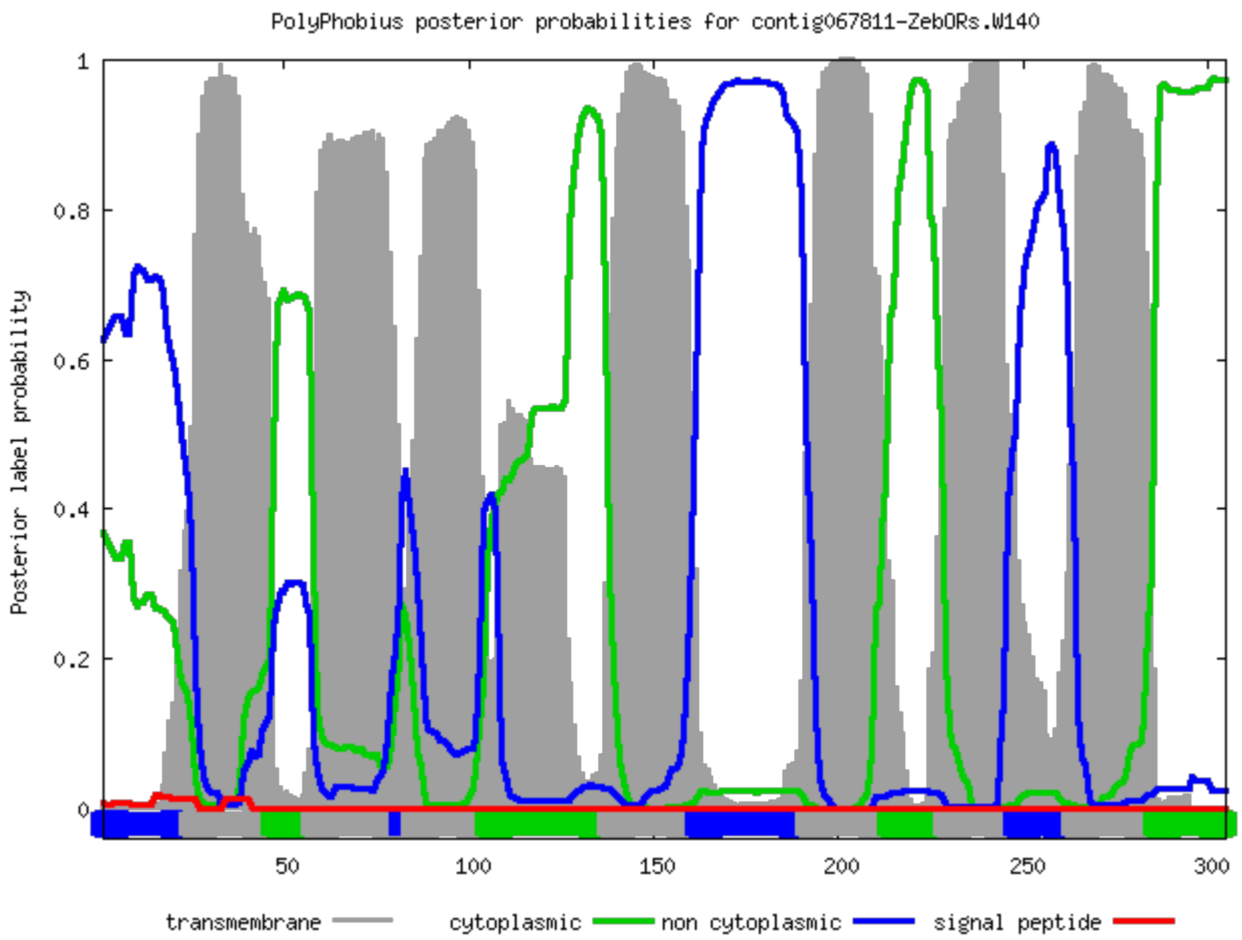

The prediction is based on an alignment. The probability data used in the plot is found here, and the gnuplot script is here.

Prediction of contig014054-ZebOR.D041

```
ID   contig014054-ZebOR.D041
FT   TOPO_DOM      1     25       NON CYTOPLASMIC.
FT   TRANSMEM     26     51
FT   TOPO_DOM     52     60       CYTOPLASMIC.
FT   TRANSMEM     61     84
FT   TOPO_DOM     85     94       NON CYTOPLASMIC.
FT   TRANSMEM     95    121
FT   TOPO_DOM    122    141       CYTOPLASMIC.
FT   TRANSMEM    142    165
FT   TOPO_DOM    166    196       NON CYTOPLASMIC.
FT   TRANSMEM    197    219
FT   TOPO_DOM    220    239       CYTOPLASMIC.
FT   TRANSMEM    240    259
FT   TOPO_DOM    260    271       NON CYTOPLASMIC.
FT   TRANSMEM    272    294
FT   TOPO_DOM    295    312       CYTOPLASMIC.
//
```

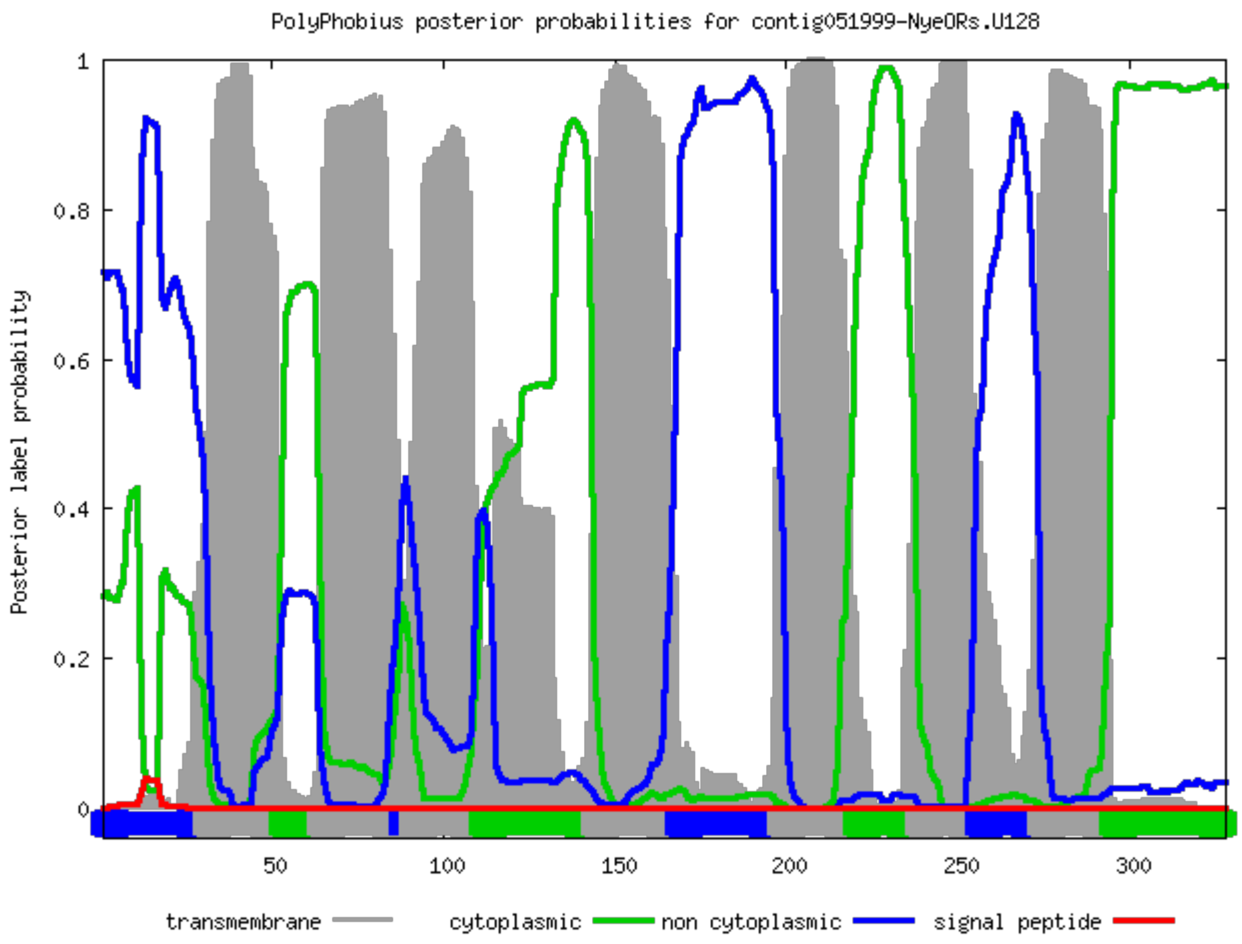

The prediction is based on an alignment. The probability data used in the plot is found here, and the gnuplot script is here.

Prediction of contig020430-ZebOR.O100

```
ID   contig020430-ZebOR.O100
FT   TOPO_DOM      1      25      NON CYTOPLASMIC.
FT   TRANSMEM     26      50
FT   TOPO_DOM     51      59      CYTOPLASMIC.
FT   TRANSMEM     60      82
FT   TOPO_DOM     83      97      NON CYTOPLASMIC.
FT   TRANSMEM     98     120
FT   TOPO_DOM    121     140      CYTOPLASMIC.
FT   TRANSMEM    141     162
FT   TOPO_DOM    163     200      NON CYTOPLASMIC.
FT   TRANSMEM    201     226
FT   TOPO_DOM    227     240      CYTOPLASMIC.
FT   TRANSMEM    241     262
FT   TOPO_DOM    263     274      NON CYTOPLASMIC.
FT   TRANSMEM    275     295
FT   TOPO_DOM    296     322      CYTOPLASMIC.
//
```

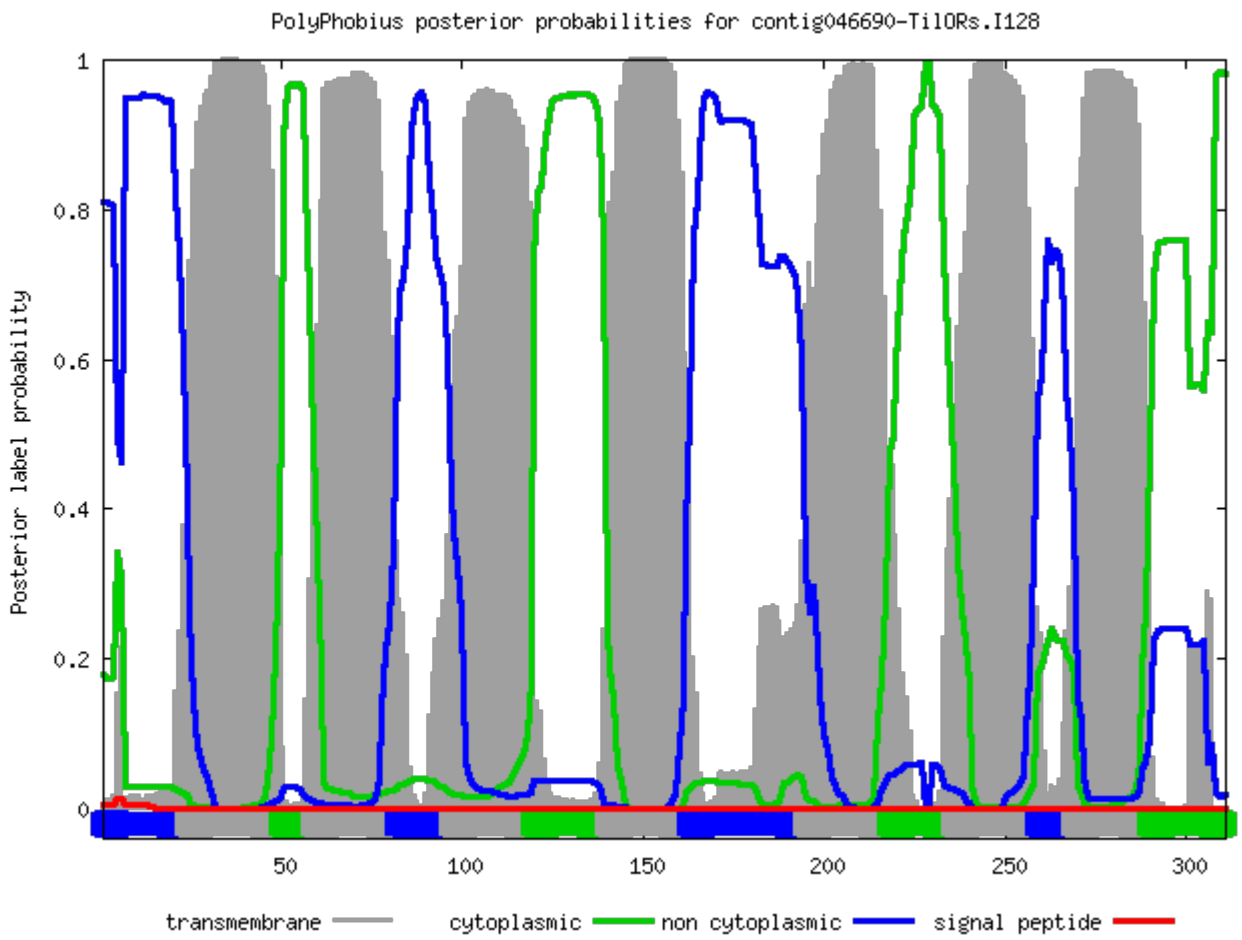

The prediction is based on an alignment. The probability data used in the plot is found here, and the gnuplot script is here.

Prediction of contig028641-TilOR.R253

```
ID   contig028641-TilOR.R253
FT   TOPO_DOM      1     21       NON CYTOPLASMIC.
FT   TRANSMEM     22     45
FT   TOPO_DOM     46     56       CYTOPLASMIC.
FT   TRANSMEM     57     81
FT   TOPO_DOM     82     86       NON CYTOPLASMIC.
FT   TRANSMEM     87    115
FT   TOPO_DOM    116    135       CYTOPLASMIC.
FT   TRANSMEM    136    159
FT   TOPO_DOM    160    191       NON CYTOPLASMIC.
FT   TRANSMEM    192    215
FT   TOPO_DOM    216    232       CYTOPLASMIC.
FT   TRANSMEM    233    255
FT   TOPO_DOM    256    267       NON CYTOPLASMIC.
FT   TRANSMEM    268    290
FT   TOPO_DOM    291    321       CYTOPLASMIC.
//
```

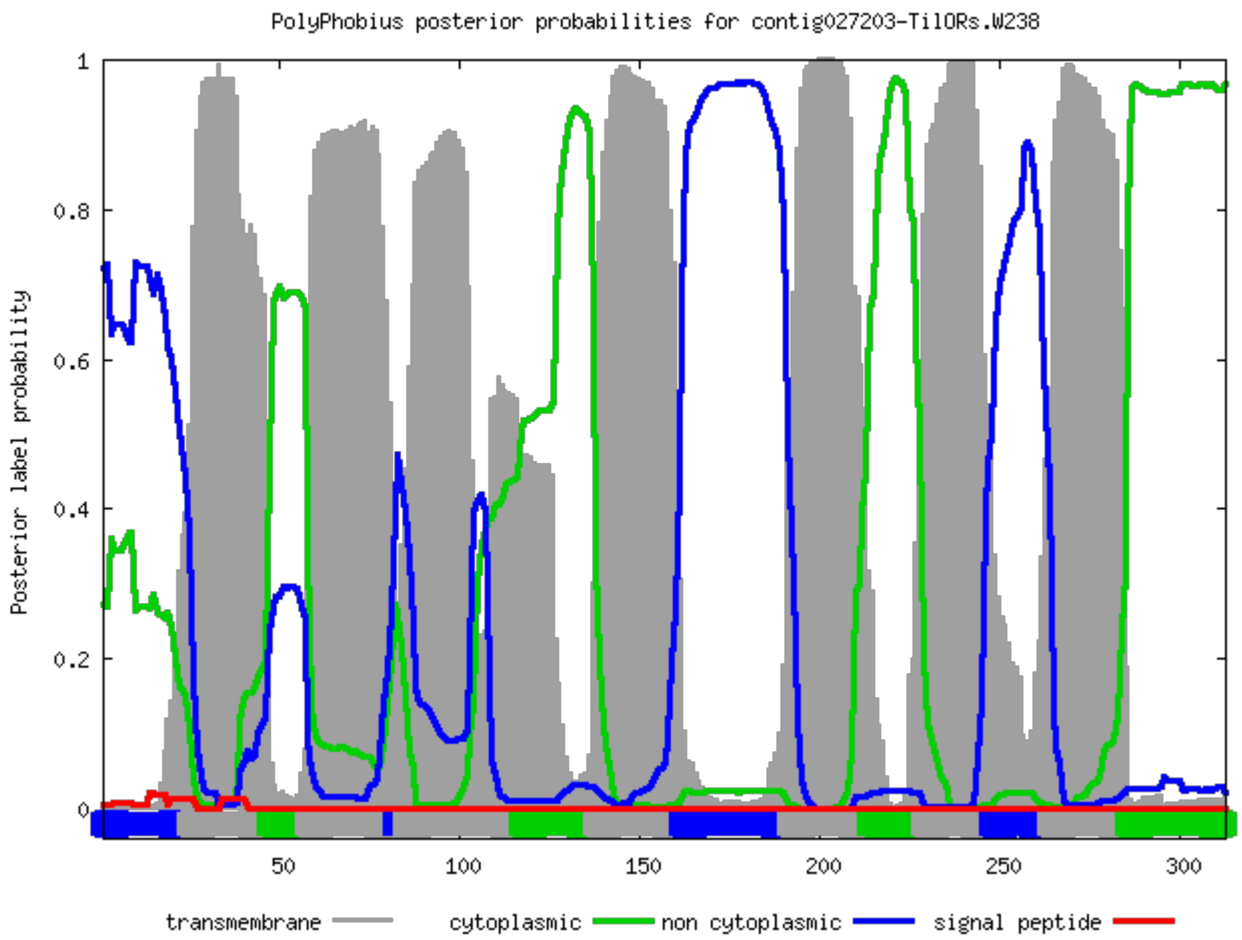

The prediction is based on an alignment. The probability data used in the plot is found here, and the gnuplot script is here.

Prediction of contig013339-TilOR.D056

```
ID   contig013339-TilOR.D056
FT   TOPO_DOM      1     22       NON CYTOPLASMIC.
FT   TRANSMEM     23     48
FT   TOPO_DOM     49     57       CYTOPLASMIC.
FT   TRANSMEM     58     81
FT   TOPO_DOM     82     90       NON CYTOPLASMIC.
FT   TRANSMEM     91    117
FT   TOPO_DOM    118    138       CYTOPLASMIC.
FT   TRANSMEM    139    161
FT   TOPO_DOM    162    194       NON CYTOPLASMIC.
FT   TRANSMEM    195    216
FT   TOPO_DOM    217    236       CYTOPLASMIC.
FT   TRANSMEM    237    256
FT   TOPO_DOM    257    267       NON CYTOPLASMIC.
FT   TRANSMEM    268    291
FT   TOPO_DOM    292    309       CYTOPLASMIC.
//
```

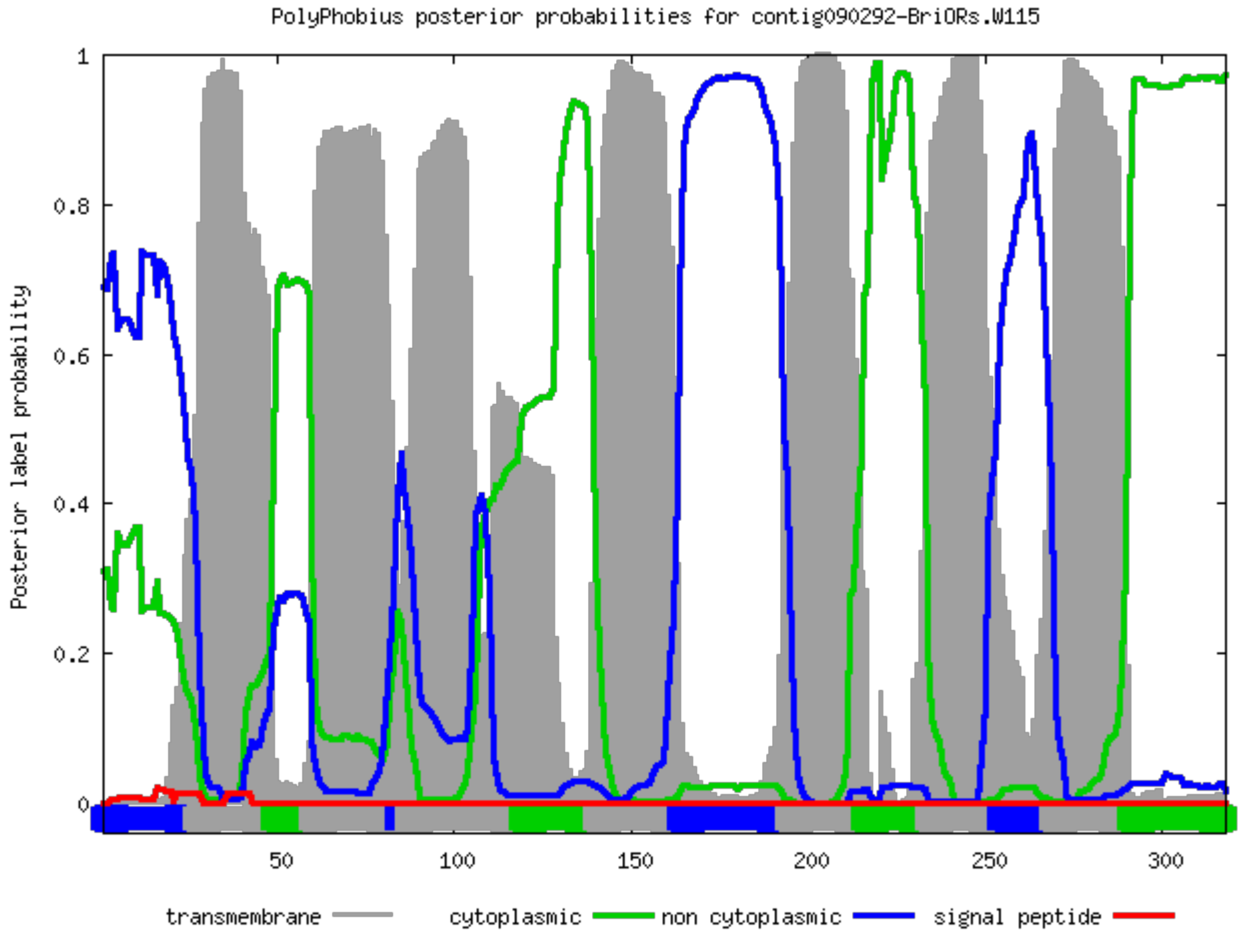

The prediction is based on an alignment. The probability data used in the plot is found here, and the gnuplot script is here.

Prediction of contig021354-NyeOR.O101

```
ID   contig021354-NyeOR.O101
FT   TOPO_DOM      1     26       NON CYTOPLASMIC.
FT   TRANSMEM     27     51
FT   TOPO_DOM     52     60       CYTOPLASMIC.
FT   TRANSMEM     61     83
FT   TOPO_DOM     84     99       NON CYTOPLASMIC.
FT   TRANSMEM    100    121
FT   TOPO_DOM    122    141       CYTOPLASMIC.
FT   TRANSMEM    142    164
FT   TOPO_DOM    165    201       NON CYTOPLASMIC.
FT   TRANSMEM    202    227
FT   TOPO_DOM    228    242       CYTOPLASMIC.
FT   TRANSMEM    243    264
FT   TOPO_DOM    265    275       NON CYTOPLASMIC.
FT   TRANSMEM    276    296
FT   TOPO_DOM    297    324       CYTOPLASMIC.
//
```

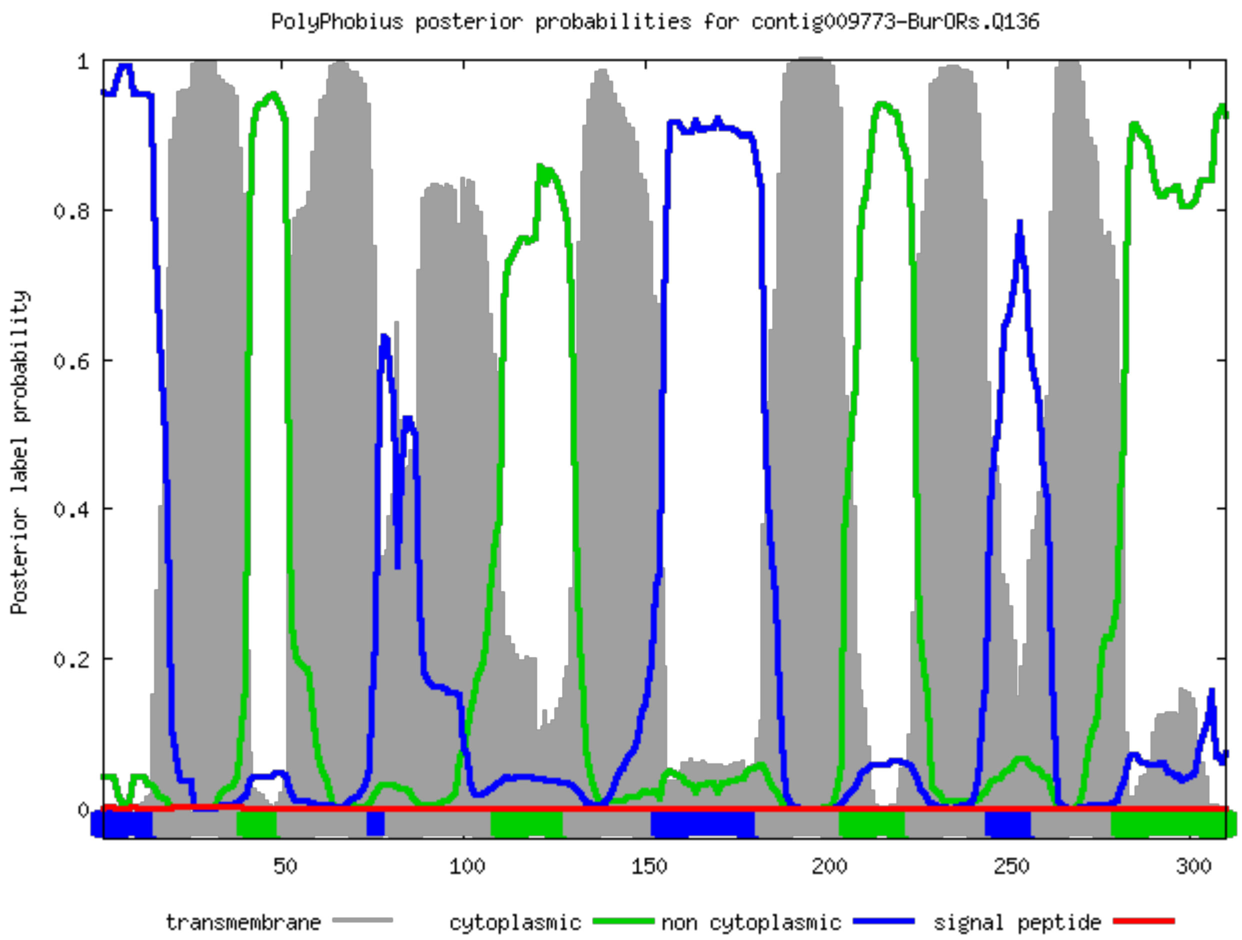

The prediction is based on an alignment. The probability data used in the plot is found here, and the gnuplot script is here.

Prediction of contig065247-TilOR.P211

```
ID   contig065247-TilOR.P211
FT   TOPO_DOM      1     27       NON CYTOPLASMIC.
FT   TRANSMEM     28     51
FT   TOPO_DOM     52     61       CYTOPLASMIC.
FT   TRANSMEM     62     88
FT   TOPO_DOM     89     99       NON CYTOPLASMIC.
FT   TRANSMEM    100    122
FT   TOPO_DOM    123    141       CYTOPLASMIC.
FT   TRANSMEM    142    165
FT   TOPO_DOM    166    199       NON CYTOPLASMIC.
FT   TRANSMEM    200    225
FT   TOPO_DOM    226    237       CYTOPLASMIC.
FT   TRANSMEM    238    261
FT   TOPO_DOM    262    273       NON CYTOPLASMIC.
FT   TRANSMEM    274    294
FT   TOPO_DOM    295    310       CYTOPLASMIC.
//
```

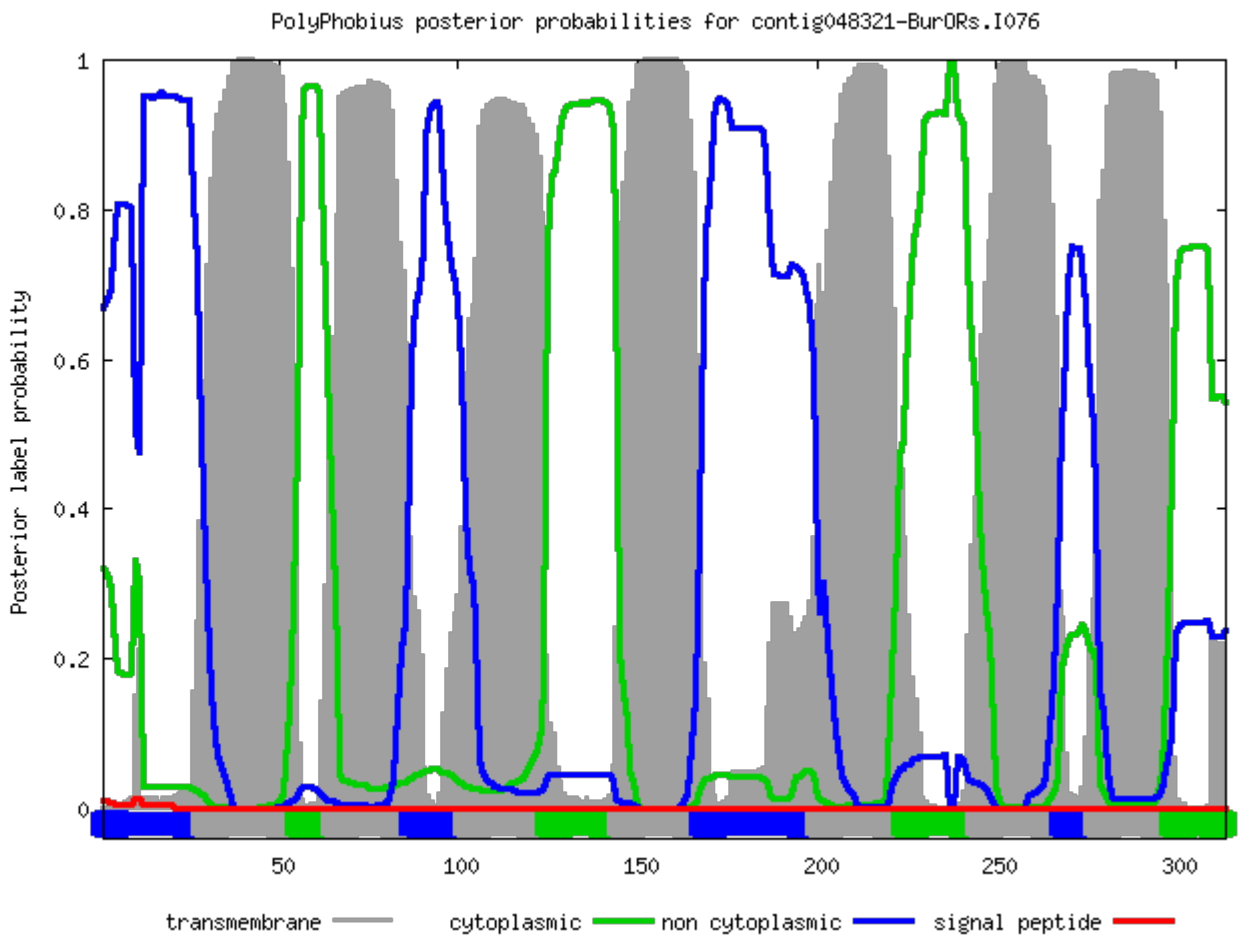

The prediction is based on an alignment. The probability data used in the plot is found here, and the gnuplot script is here.

Prediction of contig051318-BurOR.A005

```
ID   contig051318-BurOR.A005
FT   TOPO_DOM      1      22       NON CYTOPLASMIC.
FT   TRANSMEM     23      48
FT   TOPO_DOM     49      56       CYTOPLASMIC.
FT   TRANSMEM     57      76
FT   TOPO_DOM     77      95       NON CYTOPLASMIC.
FT   TRANSMEM     96     118
FT   TOPO_DOM    119     138       CYTOPLASMIC.
FT   TRANSMEM    139     159
FT   TOPO_DOM    160     192       NON CYTOPLASMIC.
FT   TRANSMEM    193     215
FT   TOPO_DOM    216     235       CYTOPLASMIC.
FT   TRANSMEM    236     257
FT   TOPO_DOM    258     268       NON CYTOPLASMIC.
FT   TRANSMEM    269     289
FT   TOPO_DOM    290     314       CYTOPLASMIC.
//
```

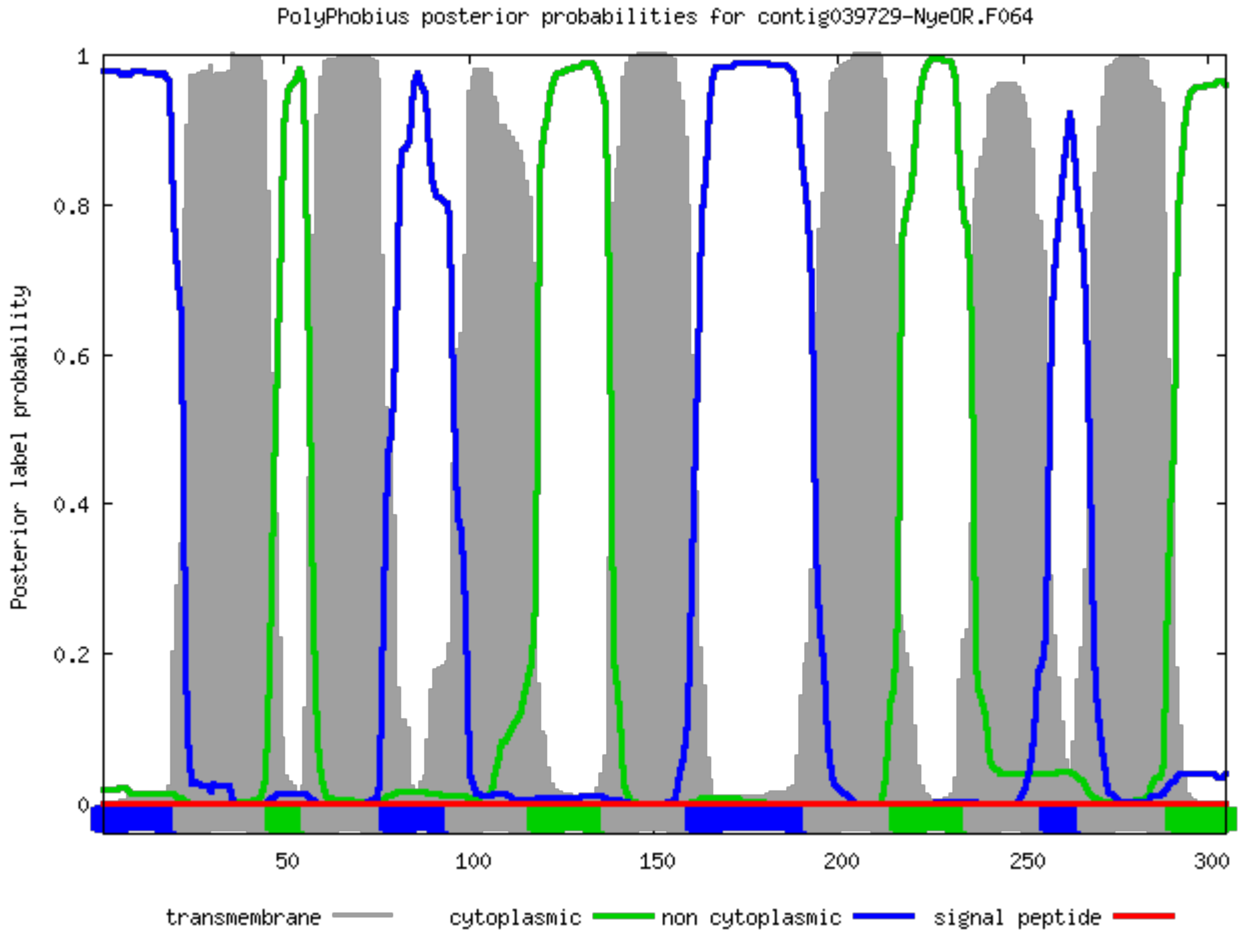

The prediction is based on an alignment. The probability data used in the plot is found here, and the gnuplot script is here.

Prediction of contig046040-ZebOR.R144

```
ID   contig046040-ZebOR.R144
FT   TOPO_DOM      1     24       NON CYTOPLASMIC.
FT   TRANSMEM     25     48
FT   TOPO_DOM     49     59       CYTOPLASMIC.
FT   TRANSMEM     60     84
FT   TOPO_DOM     85     89       NON CYTOPLASMIC.
FT   TRANSMEM     90    118
FT   TOPO_DOM    119    138       CYTOPLASMIC.
FT   TRANSMEM    139    162
FT   TOPO_DOM    163    192       NON CYTOPLASMIC.
FT   TRANSMEM    193    215
FT   TOPO_DOM    216    233       CYTOPLASMIC.
FT   TRANSMEM    234    257
FT   TOPO_DOM    258    268       NON CYTOPLASMIC.
FT   TRANSMEM    269    291
FT   TOPO_DOM    292    313       CYTOPLASMIC.
//
```

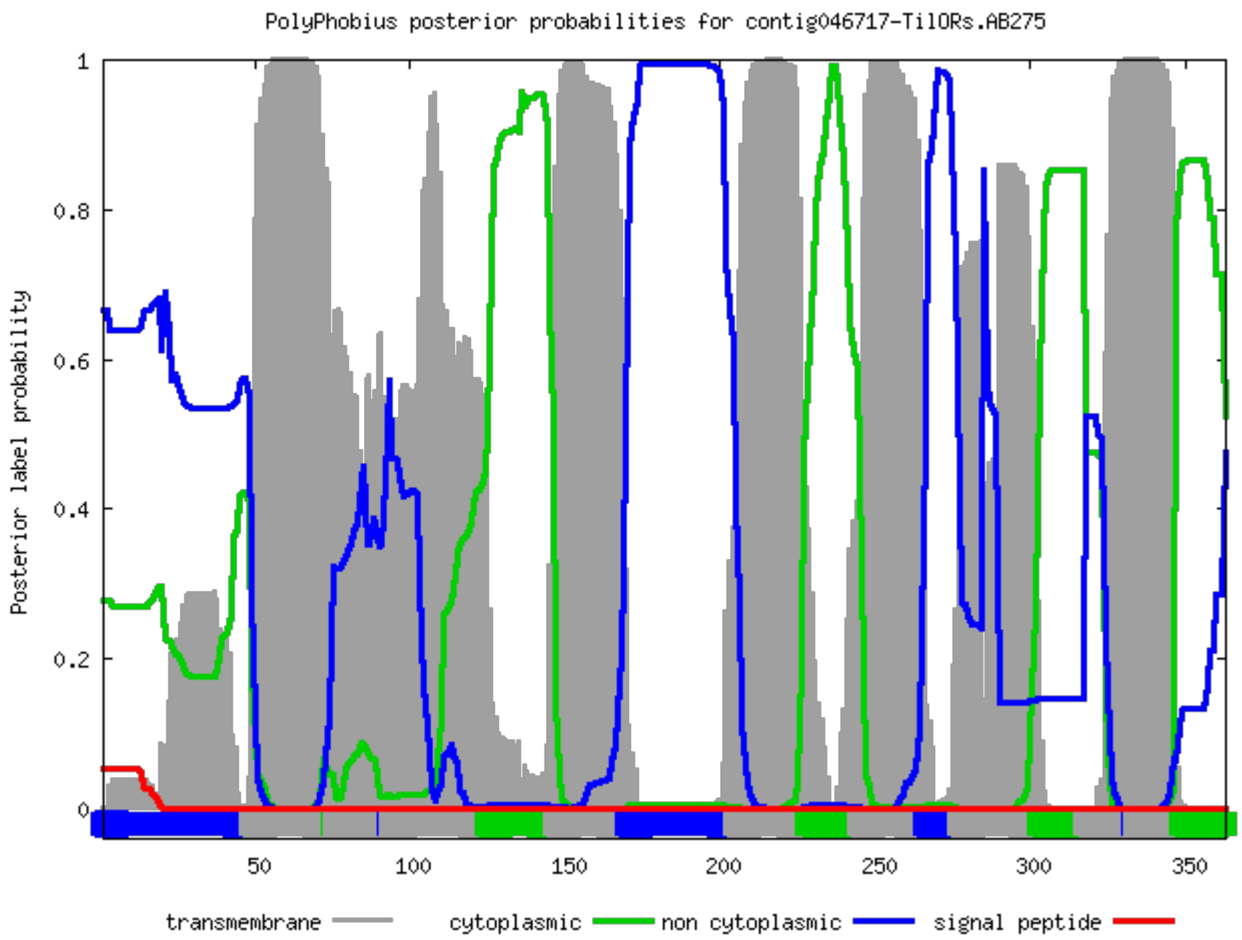

The prediction is based on an alignment. The probability data used in the plot is found here, and the gnuplot script is here.

Prediction of contig017736-ZebOR.S127

```
ID   contig017736-ZebOR.S127
FT   TOPO_DOM      1     20       NON CYTOPLASMIC.
FT   TRANSMEM     21     42
FT   TOPO_DOM     43     53       CYTOPLASMIC.
FT   TRANSMEM     54     77
FT   TOPO_DOM     78     82       NON CYTOPLASMIC.
FT   TRANSMEM     83    111
FT   TOPO_DOM    112    131       CYTOPLASMIC.
FT   TRANSMEM    132    157
FT   TOPO_DOM    158    185       NON CYTOPLASMIC.
FT   TRANSMEM    186    206
FT   TOPO_DOM    207    226       CYTOPLASMIC.
FT   TRANSMEM    227    252
FT   TOPO_DOM    253    263       NON CYTOPLASMIC.
FT   TRANSMEM    264    284
FT   TOPO_DOM    285    305       CYTOPLASMIC.
//
```

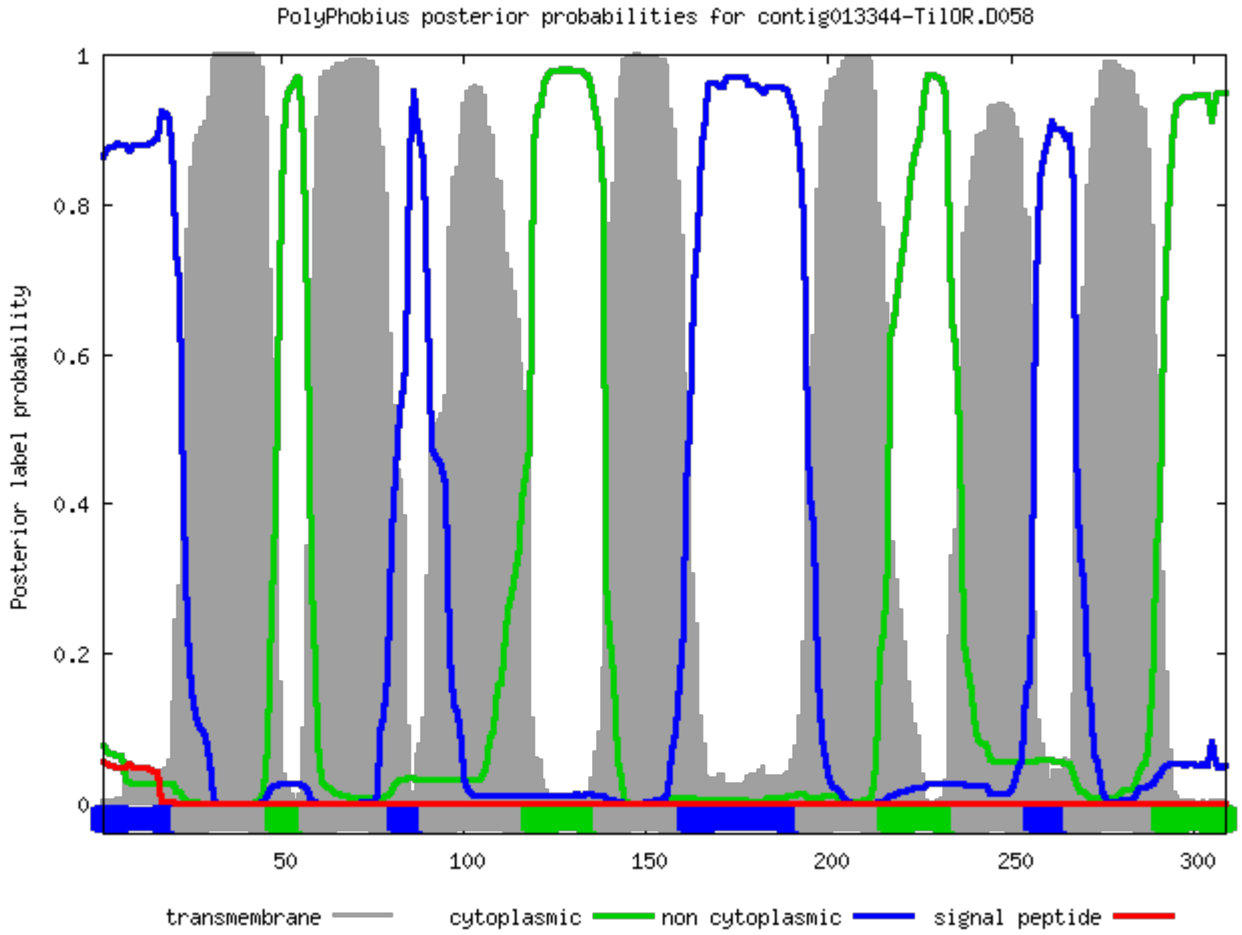

The prediction is based on an alignment. The probability data used in the plot is found here, and the gnuplot script is here.

Prediction of contig042544-BriOR.L070

```
ID    contig042544-BriOR.L070
FT    TOPO_DOM      1     27       NON CYTOPLASMIC.
FT    TRANSMEM     28     51
FT    TOPO_DOM     52     61       CYTOPLASMIC.
FT    TRANSMEM     62     84
FT    TOPO_DOM     85    102       NON CYTOPLASMIC.
FT    TRANSMEM    103    122
FT    TOPO_DOM    123    142       CYTOPLASMIC.
FT    TRANSMEM    143    164
FT    TOPO_DOM    165    200       NON CYTOPLASMIC.
FT    TRANSMEM    201    226
FT    TOPO_DOM    227    239       CYTOPLASMIC.
FT    TRANSMEM    240    262
FT    TOPO_DOM    263    274       NON CYTOPLASMIC.
FT    TRANSMEM    275    294
FT    TOPO_DOM    295    315       CYTOPLASMIC.
//
```

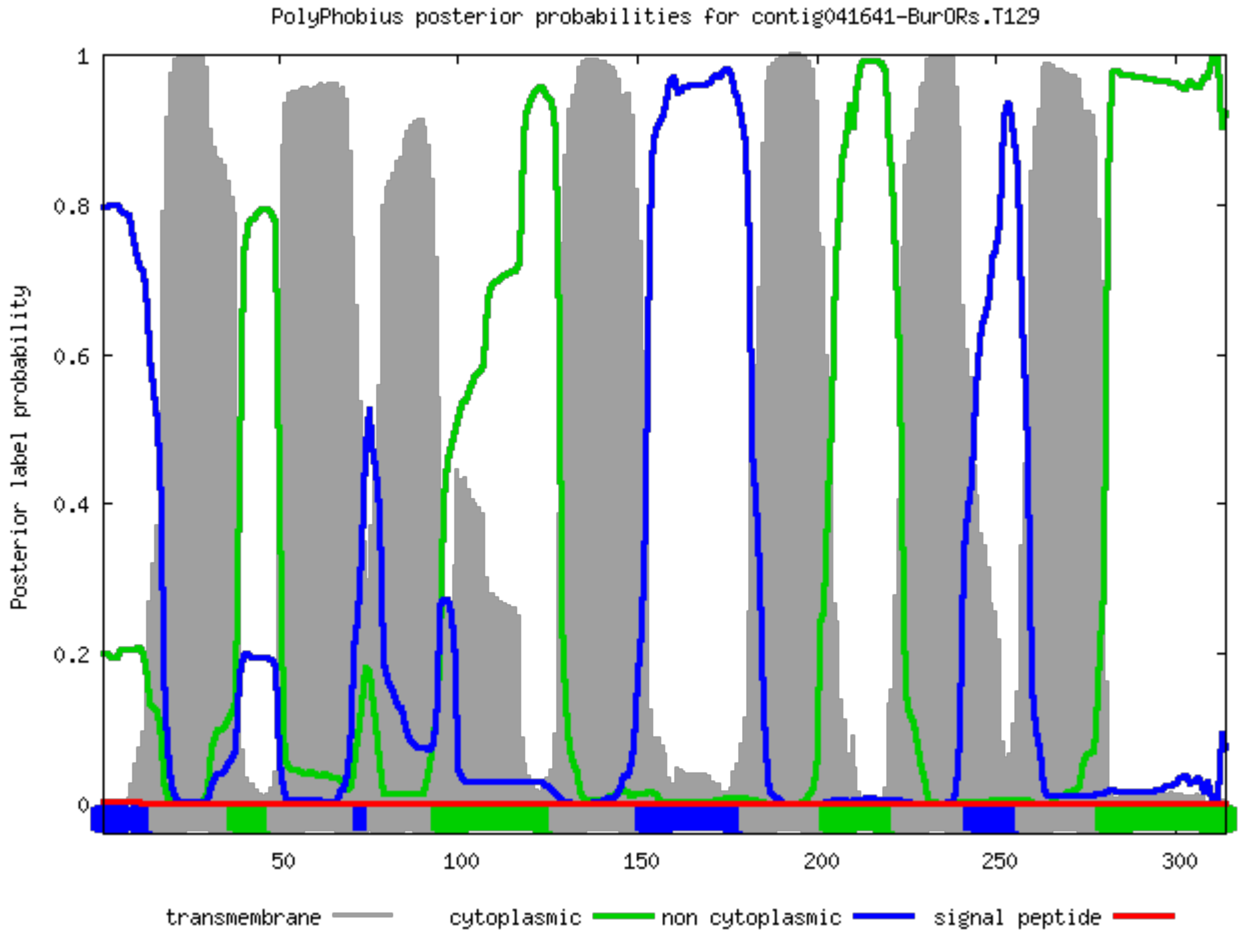

The prediction is based on an alignment. The probability data used in the plot is found here, and the gnuplot script is here.

Prediction of contig046042-ZebOR.R145

```
ID    contig046042-ZebOR.R145
FT    TOPO_DOM       1      22        NON CYTOPLASMIC.
FT    TRANSMEM      23      46
FT    TOPO_DOM      47      57        CYTOPLASMIC.
FT    TRANSMEM      58      82
FT    TOPO_DOM      83      87        NON CYTOPLASMIC.
FT    TRANSMEM      88     116
FT    TOPO_DOM     117     136        CYTOPLASMIC.
FT    TRANSMEM     137     160
FT    TOPO_DOM     161     191        NON CYTOPLASMIC.
FT    TRANSMEM     192     215
FT    TOPO_DOM     216     232        CYTOPLASMIC.
FT    TRANSMEM     233     256
FT    TOPO_DOM     257     268        NON CYTOPLASMIC.
FT    TRANSMEM     269     290
FT    TOPO_DOM     291     317        CYTOPLASMIC.
//
```

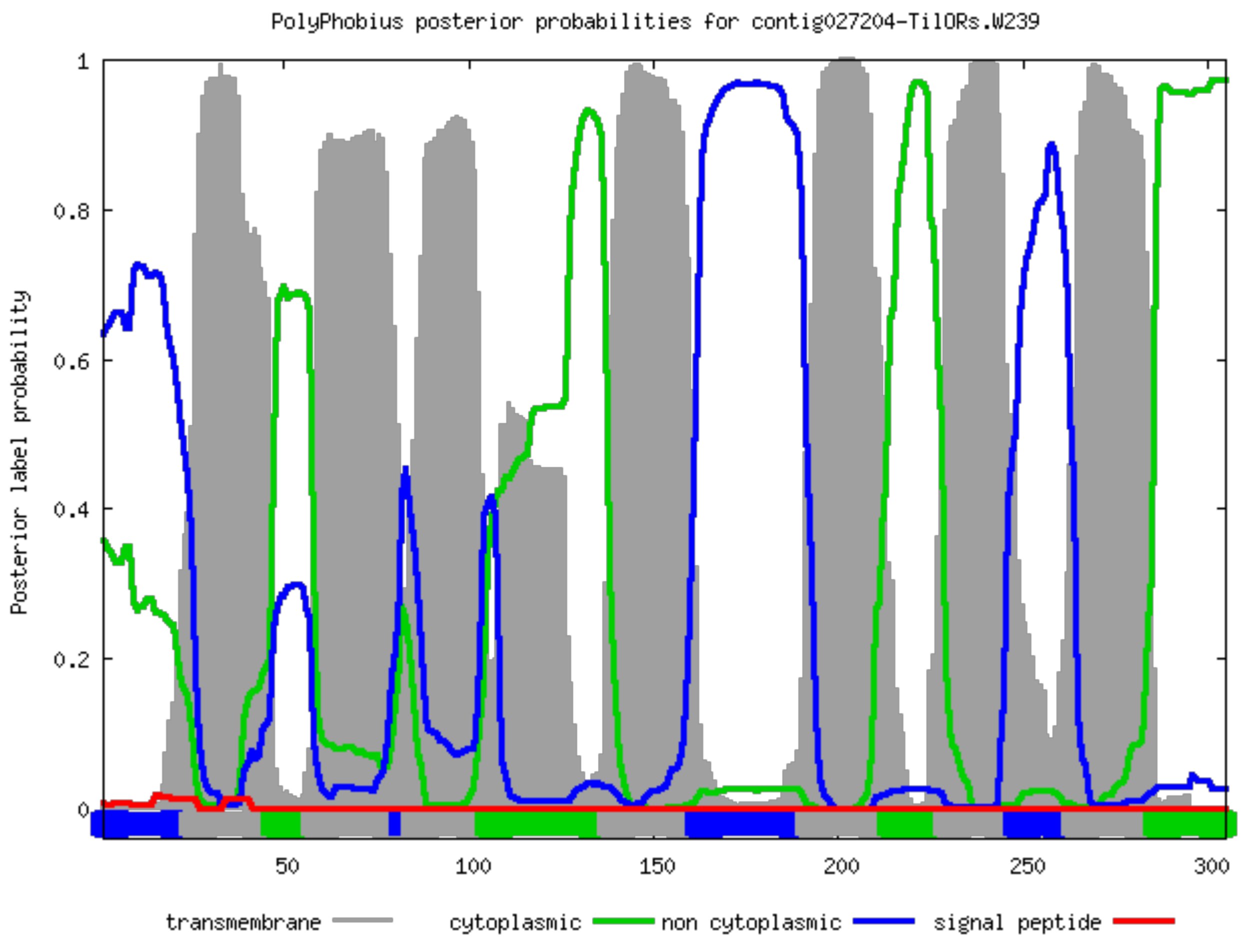

The prediction is based on an alignment. The probability data used in the plot is found here, and the gnuplot script is here.

Prediction of contig039730-NyeOR.D036

```
ID    contig039730-NyeOR.D036
FT    TOPO_DOM      1      22       NON CYTOPLASMIC.
FT    TRANSMEM     23      48
FT    TOPO_DOM     49      57       CYTOPLASMIC.
FT    TRANSMEM     58      81
FT    TOPO_DOM     82      90       NON CYTOPLASMIC.
FT    TRANSMEM     91     118
FT    TOPO_DOM    119     138       CYTOPLASMIC.
FT    TRANSMEM    139     161
FT    TOPO_DOM    162     194       NON CYTOPLASMIC.
FT    TRANSMEM    195     216
FT    TOPO_DOM    217     236       CYTOPLASMIC.
FT    TRANSMEM    237     256
FT    TOPO_DOM    257     268       NON CYTOPLASMIC.
FT    TRANSMEM    269     291
FT    TOPO_DOM    292     319       CYTOPLASMIC.
//
```

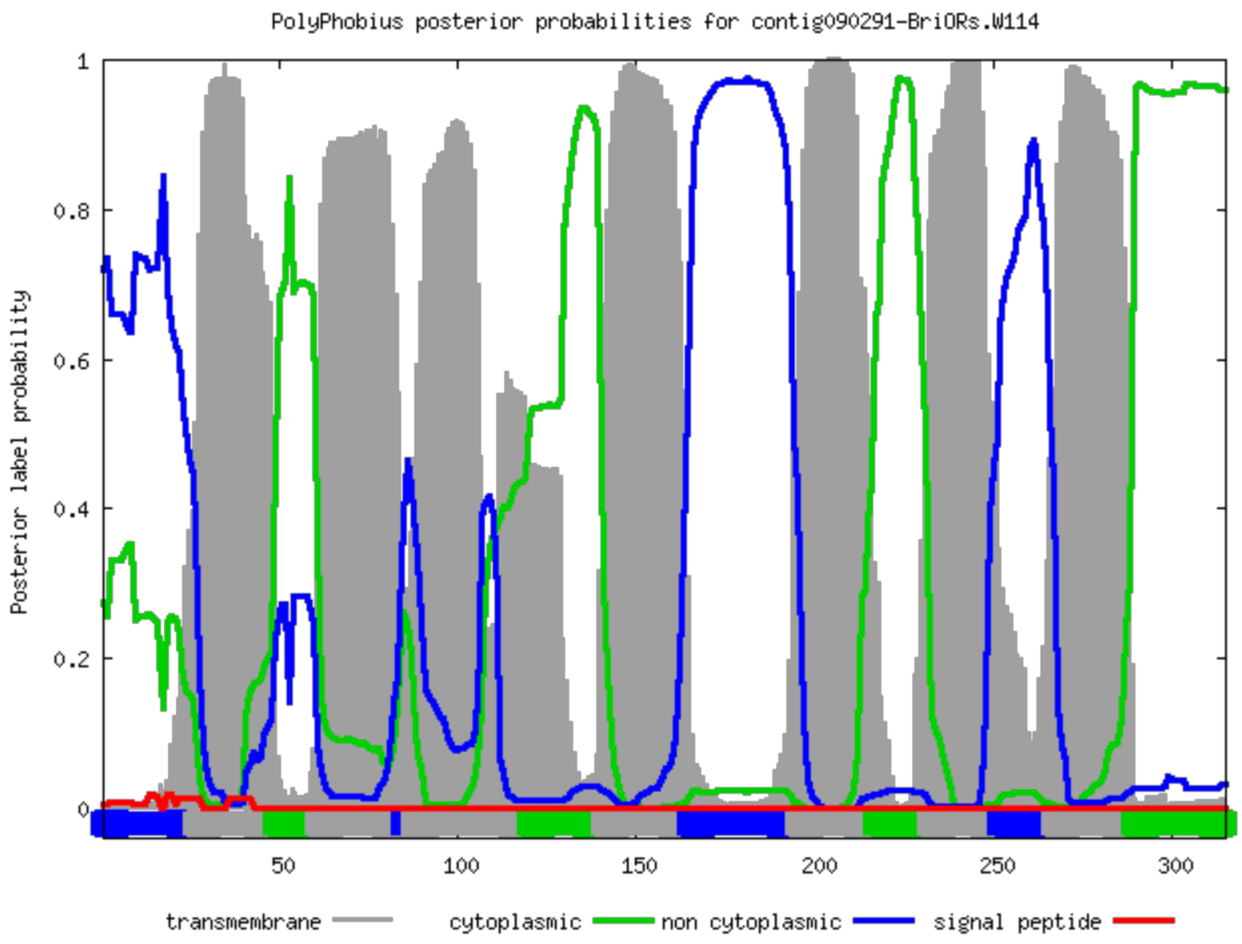

The prediction is based on an alignment. The probability data used in the plot is found here, and the gnuplot script is here.

Prediction of contig057153-BurOR.A014

```
ID    contig057153-BurOR.A014
FT    TOPO_DOM       1      22        NON CYTOPLASMIC.
FT    TRANSMEM      23      48
FT    TOPO_DOM      49      56        CYTOPLASMIC.
FT    TRANSMEM      57      77
FT    TOPO_DOM      78      95        NON CYTOPLASMIC.
FT    TRANSMEM      96     118
FT    TOPO_DOM     119     138        CYTOPLASMIC.
FT    TRANSMEM     139     159
FT    TOPO_DOM     160     192        NON CYTOPLASMIC.
FT    TRANSMEM     193     215
FT    TOPO_DOM     216     235        CYTOPLASMIC.
FT    TRANSMEM     236     257
FT    TOPO_DOM     258     268        NON CYTOPLASMIC.
FT    TRANSMEM     269     289
FT    TOPO_DOM     290     309        CYTOPLASMIC.
//
```

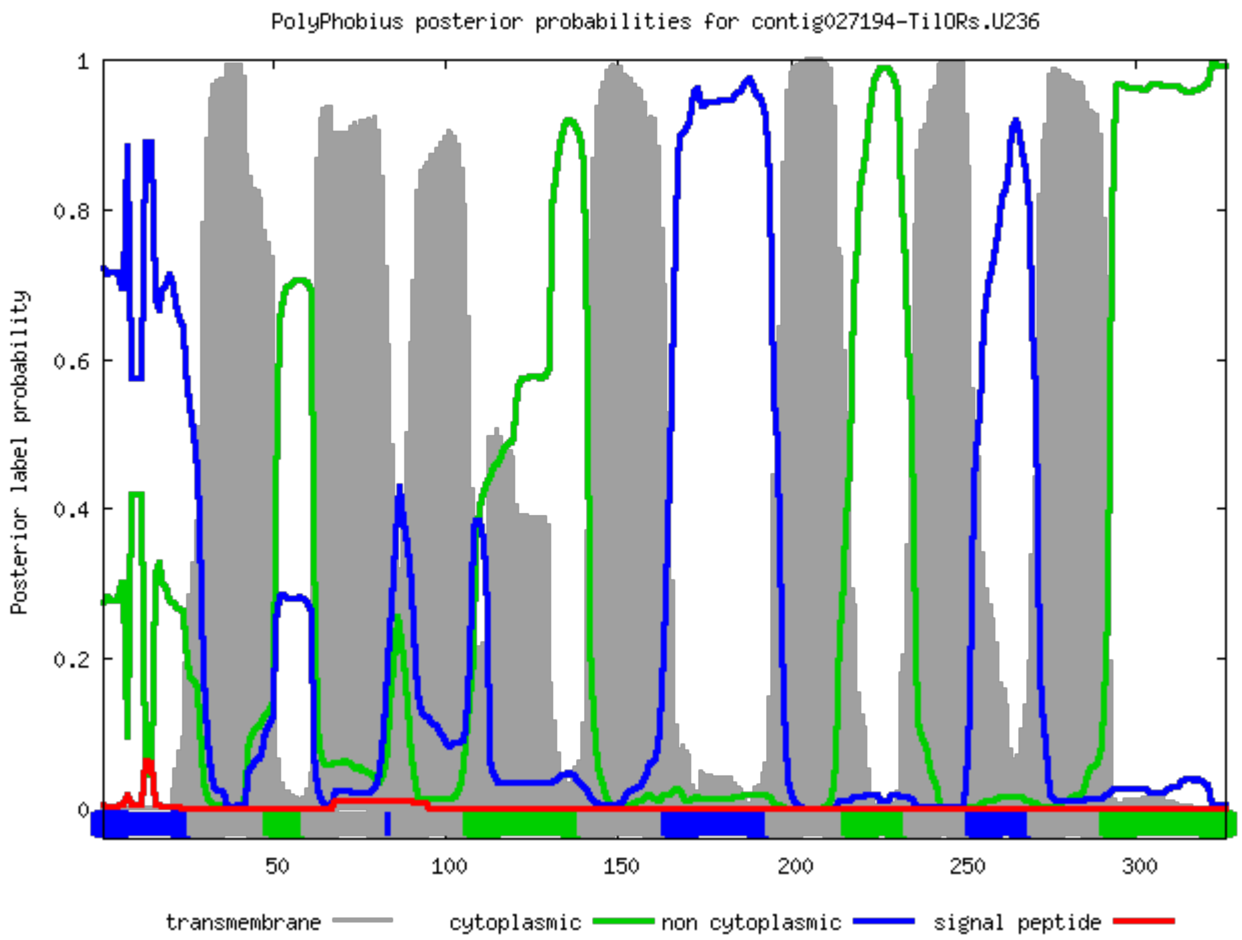

The prediction is based on an alignment. The probability data used in the plot is found here, and the gnuplot script is here.

Prediction of contig047734-TilOR.E077

```
ID   contig047734-TilOR.E077
FT   TOPO_DOM      1     22       NON CYTOPLASMIC.
FT   TRANSMEM     23     48
FT   TOPO_DOM     49     57       CYTOPLASMIC.
FT   TRANSMEM     58     82
FT   TOPO_DOM     83     90       NON CYTOPLASMIC.
FT   TRANSMEM     91    118
FT   TOPO_DOM    119    138       CYTOPLASMIC.
FT   TRANSMEM    139    160
FT   TOPO_DOM    161    193       NON CYTOPLASMIC.
FT   TRANSMEM    194    216
FT   TOPO_DOM    217    236       CYTOPLASMIC.
FT   TRANSMEM    237    256
FT   TOPO_DOM    257    267       NON CYTOPLASMIC.
FT   TRANSMEM    268    291
FT   TOPO_DOM    292    309       CYTOPLASMIC.
//
```

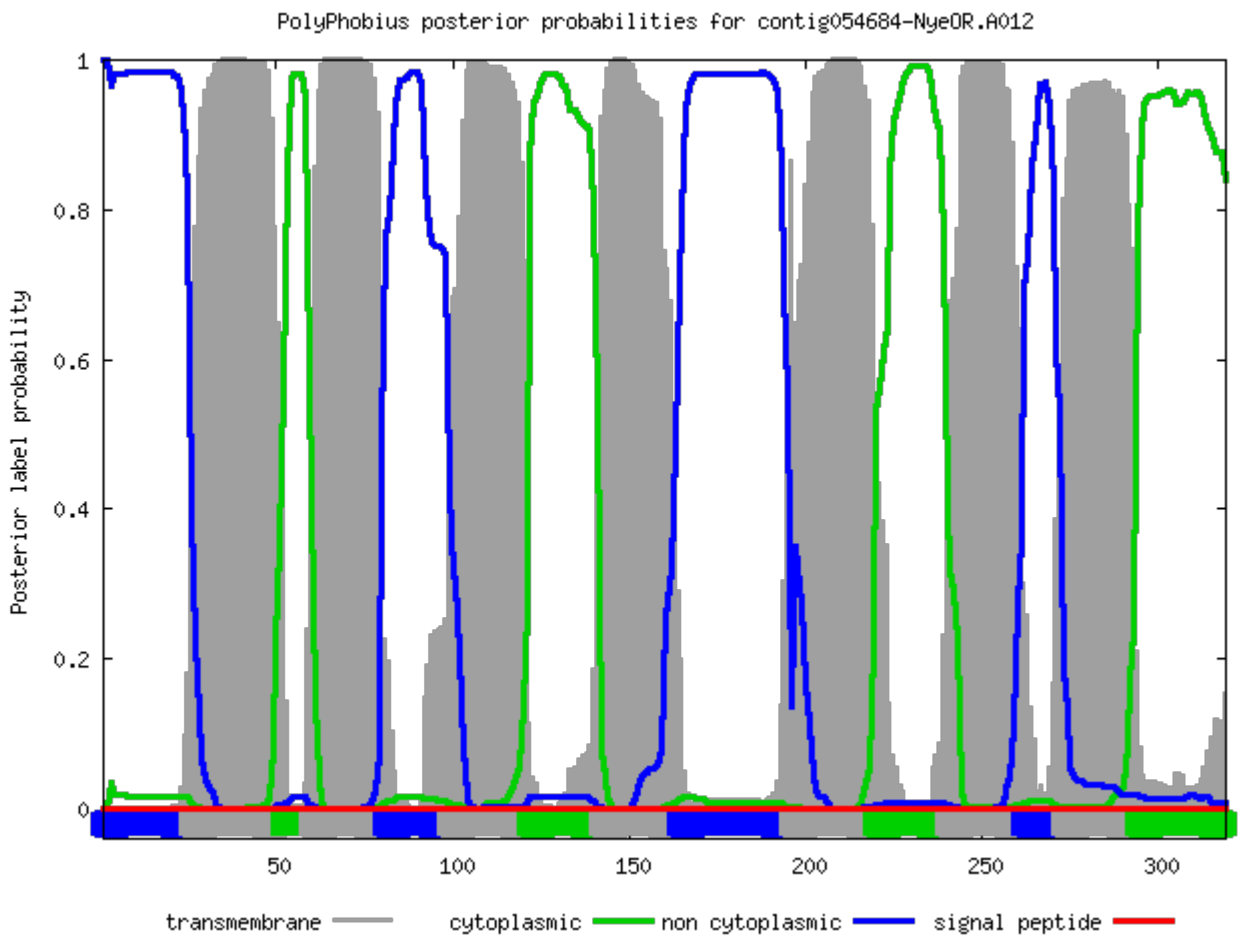

The prediction is based on an alignment. The probability data used in the plot is found here, and the gnuplot script is here.

Prediction of contig047521-ZebOR.A020

```
ID   contig047521-ZebOR.A020
FT   TOPO_DOM      1     22       NON CYTOPLASMIC.
FT   TRANSMEM     23     48
FT   TOPO_DOM     49     56       CYTOPLASMIC.
FT   TRANSMEM     57     76
FT   TOPO_DOM     77     95       NON CYTOPLASMIC.
FT   TRANSMEM     96    118
FT   TOPO_DOM    119    138       CYTOPLASMIC.
FT   TRANSMEM    139    159
FT   TOPO_DOM    160    192       NON CYTOPLASMIC.
FT   TRANSMEM    193    215
FT   TOPO_DOM    216    235       CYTOPLASMIC.
FT   TRANSMEM    236    257
FT   TOPO_DOM    258    268       NON CYTOPLASMIC.
FT   TRANSMEM    269    289
FT   TOPO_DOM    290    316       CYTOPLASMIC.
//
```

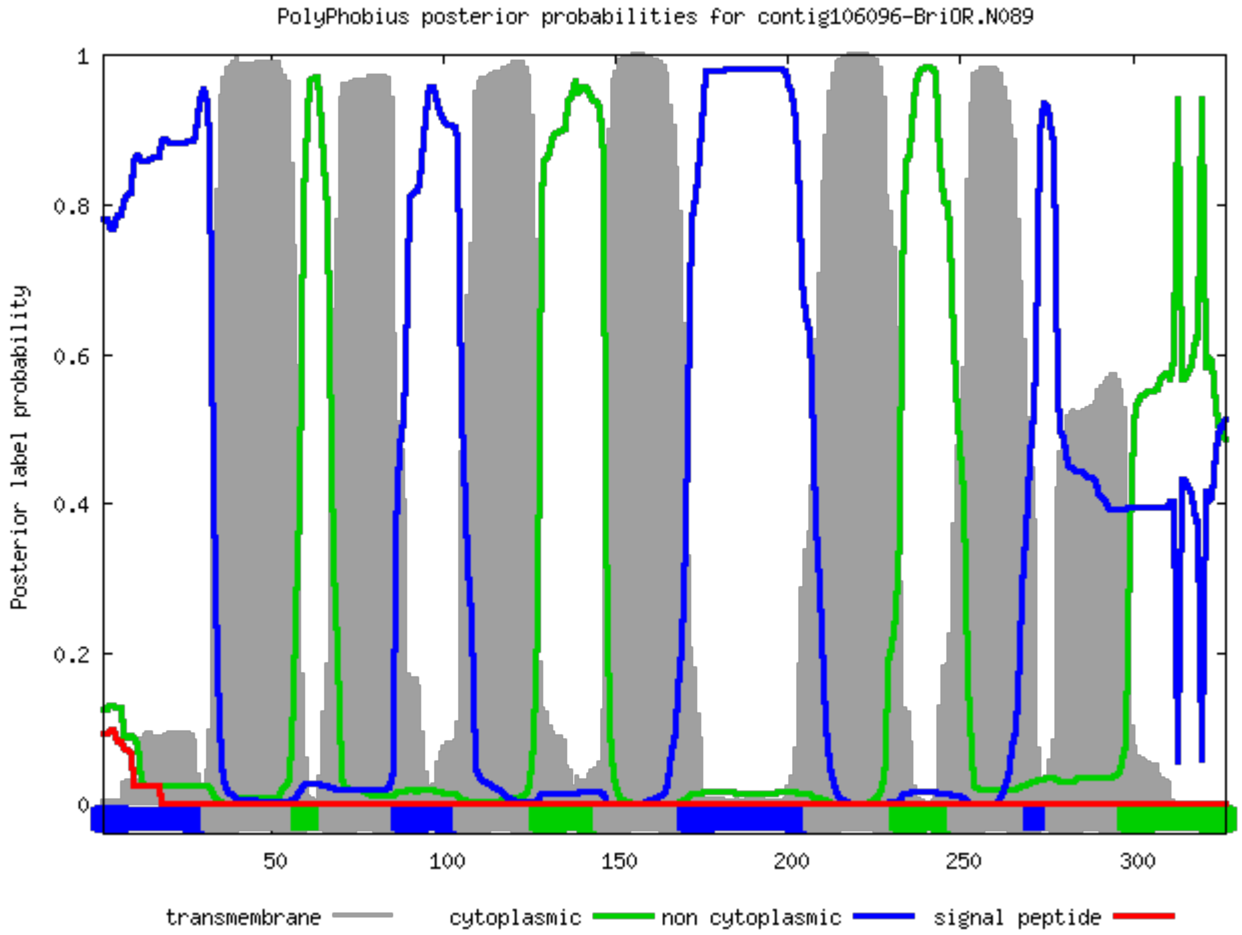

The prediction is based on an alignment. The probability data used in the plot is found here, and the gnuplot script is here.

Prediction of contig096539-BriOR.N087

```
ID   contig096539-BriOR.N087
FT   TOPO_DOM      1     32       NON CYTOPLASMIC.
FT   TRANSMEM     33     58
FT   TOPO_DOM     59     66       CYTOPLASMIC.
FT   TRANSMEM     67     86
FT   TOPO_DOM     87    104       NON CYTOPLASMIC.
FT   TRANSMEM    105    127
FT   TOPO_DOM    128    146       CYTOPLASMIC.
FT   TRANSMEM    147    170
FT   TOPO_DOM    171    206       NON CYTOPLASMIC.
FT   TRANSMEM    207    232
FT   TOPO_DOM    233    249       CYTOPLASMIC.
FT   TRANSMEM    250    271
FT   TOPO_DOM    272    277       NON CYTOPLASMIC.
FT   TRANSMEM    278    298
FT   TOPO_DOM    299    322       CYTOPLASMIC.
//
```

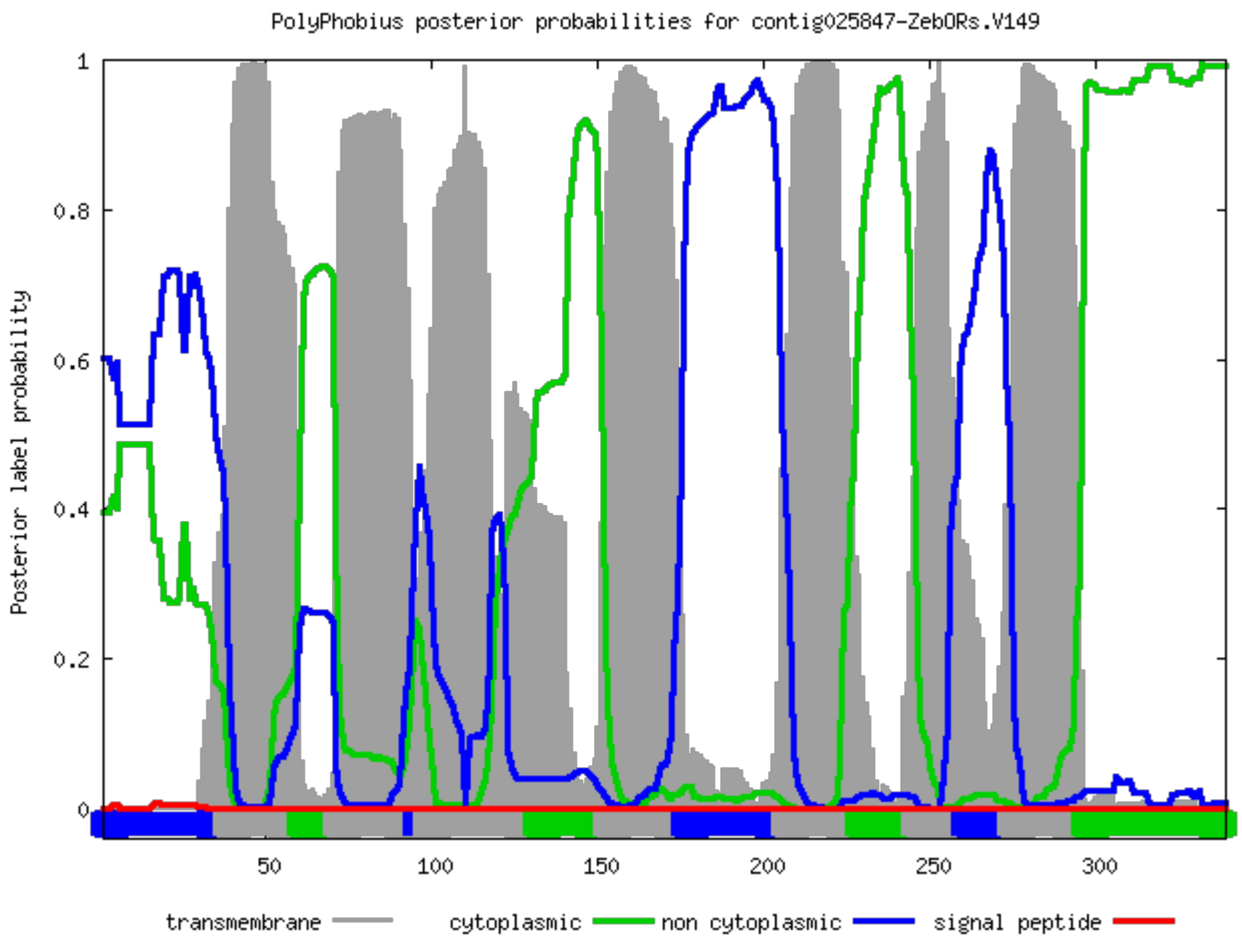

The prediction is based on an alignment. The probability data used in the plot is found here, and the gnuplot script is here.

Prediction of contig065193-ZebOR.R147

```
ID   contig065193-ZebOR.R147
FT   TOPO_DOM      1     24       NON CYTOPLASMIC.
FT   TRANSMEM     25     48
FT   TOPO_DOM     49     59       CYTOPLASMIC.
FT   TRANSMEM     60     84
FT   TOPO_DOM     85     95       NON CYTOPLASMIC.
FT   TRANSMEM     96    118
FT   TOPO_DOM    119    138       CYTOPLASMIC.
FT   TRANSMEM    139    162
FT   TOPO_DOM    163    194       NON CYTOPLASMIC.
FT   TRANSMEM    195    218
FT   TOPO_DOM    219    235       CYTOPLASMIC.
FT   TRANSMEM    236    259
FT   TOPO_DOM    260    270       NON CYTOPLASMIC.
FT   TRANSMEM    271    293
FT   TOPO_DOM    294    328       CYTOPLASMIC.
//
```

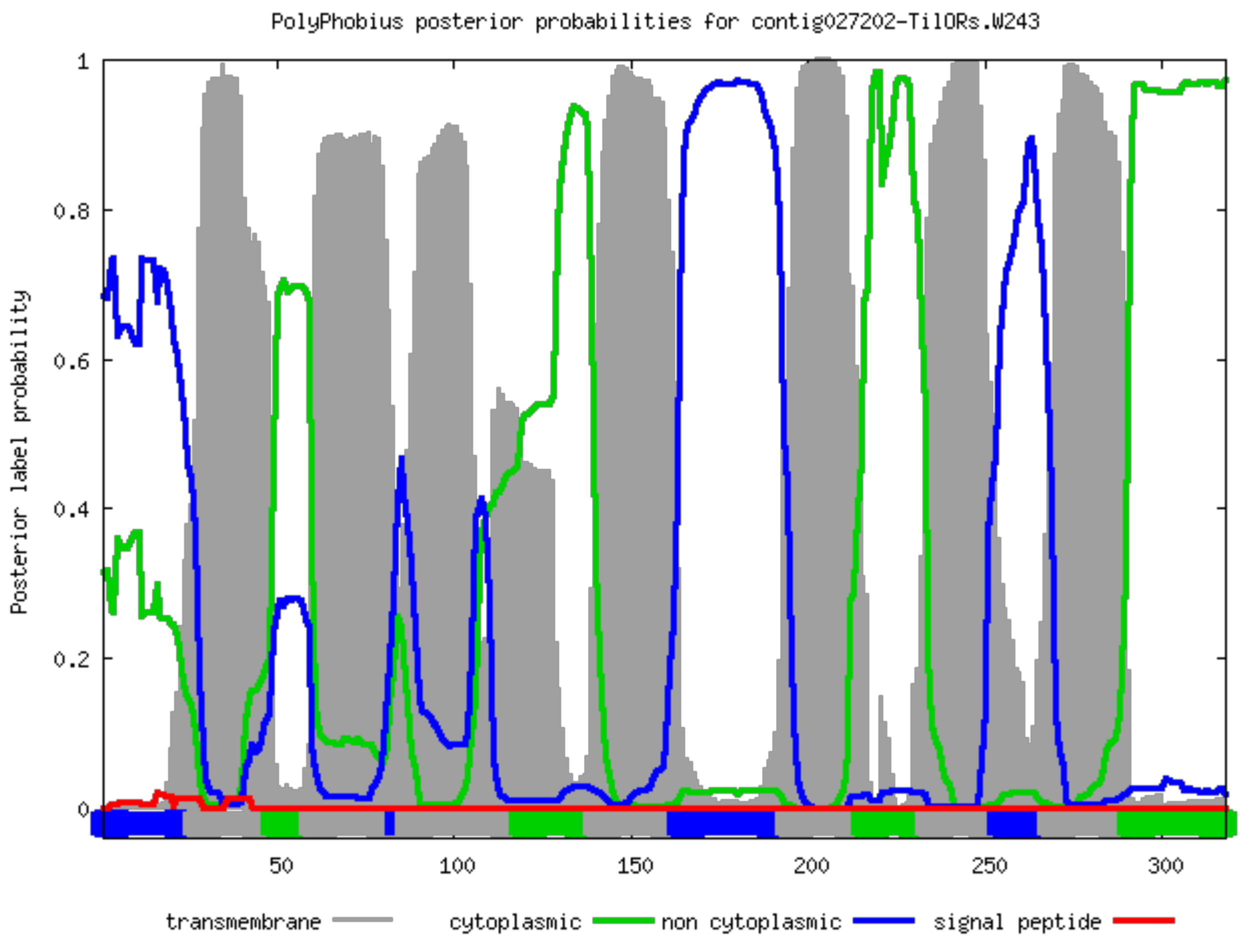

The prediction is based on an alignment. The probability data used in the plot is found here, and the gnuplot script is here.

Prediction of contig055881-BurOR.S123

```
ID   contig055881-BurOR.S123
FT   TOPO_DOM      1     20       NON CYTOPLASMIC.
FT   TRANSMEM     21     42
FT   TOPO_DOM     43     53       CYTOPLASMIC.
FT   TRANSMEM     54     77
FT   TOPO_DOM     78     80       NON CYTOPLASMIC.
FT   TRANSMEM     81    109
FT   TOPO_DOM    110    129       CYTOPLASMIC.
FT   TRANSMEM    130    155
FT   TOPO_DOM    156    183       NON CYTOPLASMIC.
FT   TRANSMEM    184    204
FT   TOPO_DOM    205    224       CYTOPLASMIC.
FT   TRANSMEM    225    250
FT   TOPO_DOM    251    261       NON CYTOPLASMIC.
FT   TRANSMEM    262    282
FT   TOPO_DOM    283    307       CYTOPLASMIC.
//
```

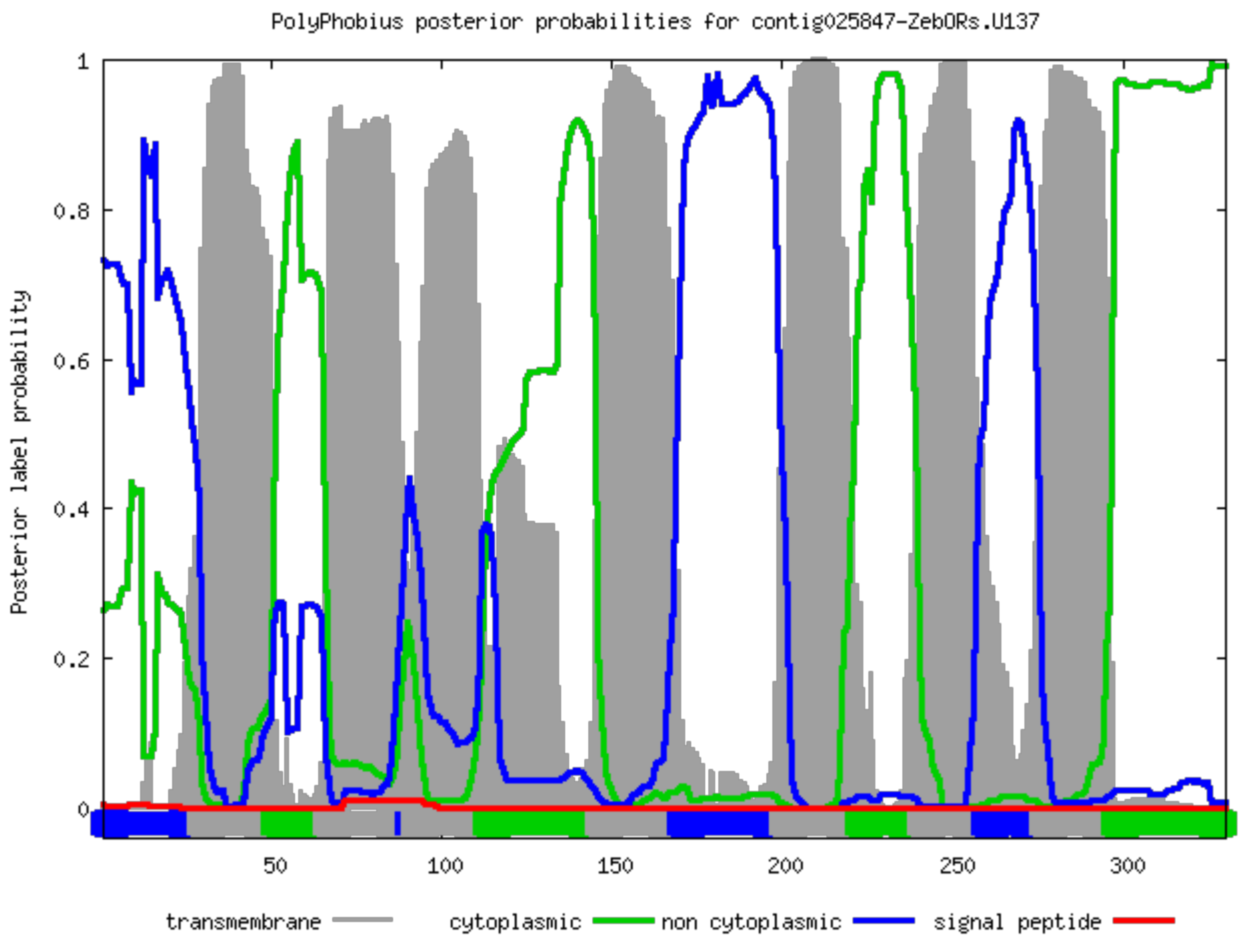

The prediction is based on an alignment. The probability data used in the plot is found here, and the gnuplot script is here.

Prediction of contig066194-BurOR.E054

```
ID   contig066194-BurOR.E054
FT   TOPO_DOM      1     23       NON CYTOPLASMIC.
FT   TRANSMEM     24     49
FT   TOPO_DOM     50     58       CYTOPLASMIC.
FT   TRANSMEM     59     84
FT   TOPO_DOM     85     91       NON CYTOPLASMIC.
FT   TRANSMEM     92    119
FT   TOPO_DOM    120    139       CYTOPLASMIC.
FT   TRANSMEM    140    162
FT   TOPO_DOM    163    194       NON CYTOPLASMIC.
FT   TRANSMEM    195    217
FT   TOPO_DOM    218    237       CYTOPLASMIC.
FT   TRANSMEM    238    257
FT   TOPO_DOM    258    269       NON CYTOPLASMIC.
FT   TRANSMEM    270    292
FT   TOPO_DOM    293    331       CYTOPLASMIC.
//
```

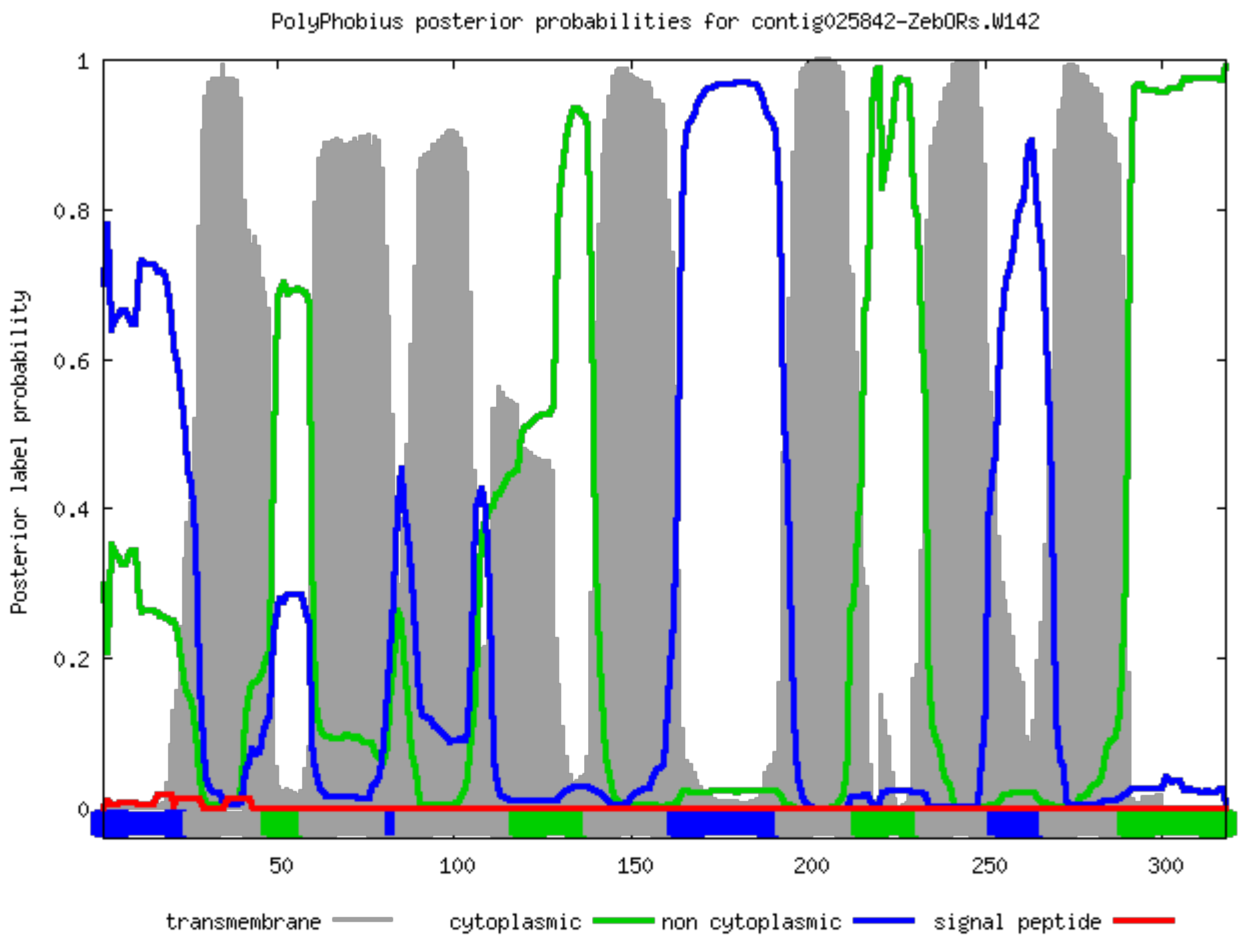

The prediction is based on an alignment. The probability data used in the plot is found here, and the gnuplot script is here.

Prediction of contig062344-NyeOR.A020

```
ID   contig062344-NyeOR.A020
FT   TOPO_DOM      1      22       NON CYTOPLASMIC.
FT   TRANSMEM     23      48
FT   TOPO_DOM     49      56       CYTOPLASMIC.
FT   TRANSMEM     57      76
FT   TOPO_DOM     77      95       NON CYTOPLASMIC.
FT   TRANSMEM     96     118
FT   TOPO_DOM    119     138       CYTOPLASMIC.
FT   TRANSMEM    139     159
FT   TOPO_DOM    160     192       NON CYTOPLASMIC.
FT   TRANSMEM    193     215
FT   TOPO_DOM    216     235       CYTOPLASMIC.
FT   TRANSMEM    236     257
FT   TOPO_DOM    258     268       NON CYTOPLASMIC.
FT   TRANSMEM    269     289
FT   TOPO_DOM    290     312       CYTOPLASMIC.
//
```

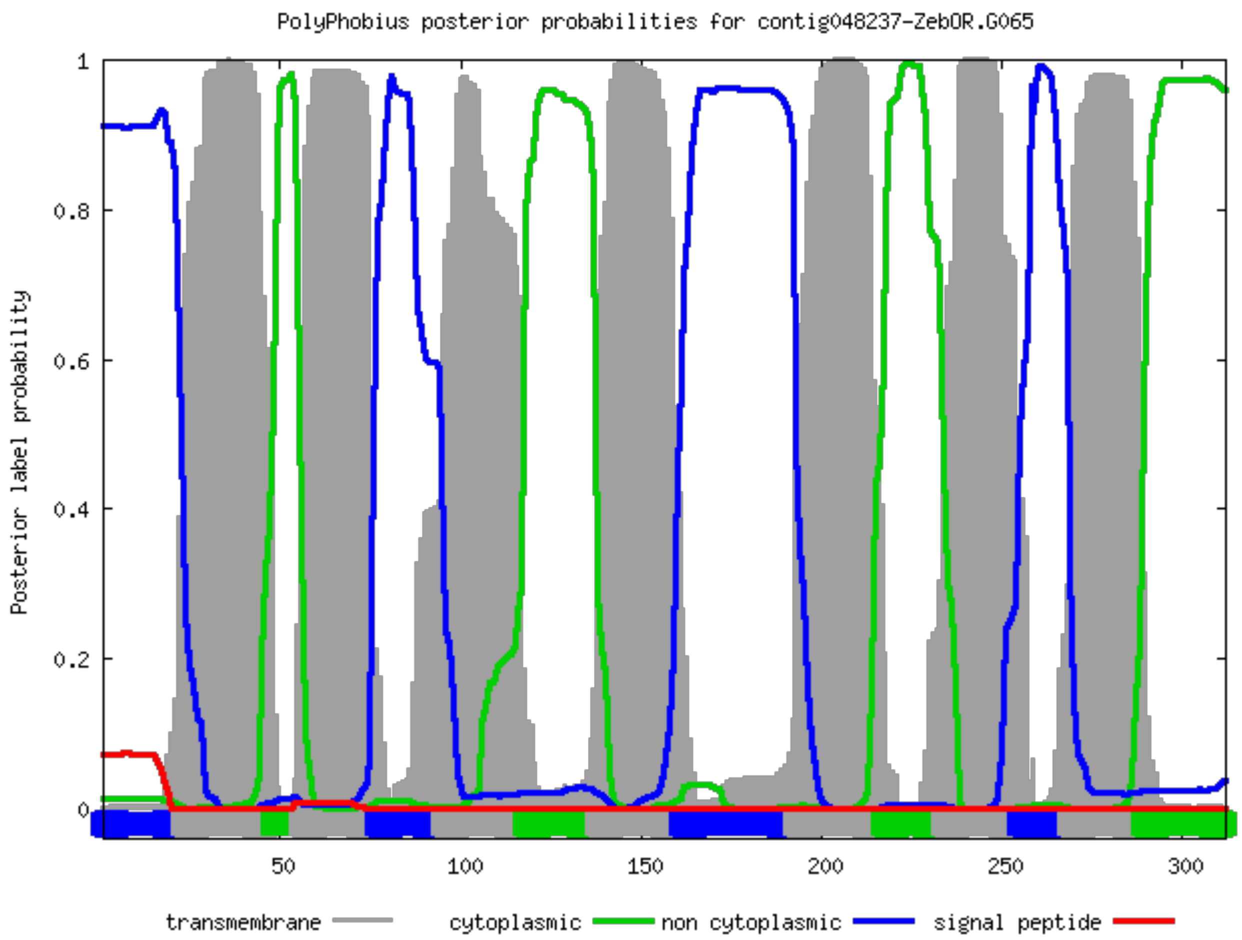

The prediction is based on an alignment. The probability data used in the plot is found here, and the gnuplot script is here.

Prediction of contig093825-BriOR.H053

```
ID    contig093825-BriOR.H053
FT    TOPO_DOM      1      23       NON CYTOPLASMIC.
FT    TRANSMEM     24      49
FT    TOPO_DOM     50      56       CYTOPLASMIC.
FT    TRANSMEM     57      76
FT    TOPO_DOM     77      95       NON CYTOPLASMIC.
FT    TRANSMEM     96     118
FT    TOPO_DOM    119     138       CYTOPLASMIC.
FT    TRANSMEM    139     160
FT    TOPO_DOM    161     196       NON CYTOPLASMIC.
FT    TRANSMEM    197     219
FT    TOPO_DOM    220     237       CYTOPLASMIC.
FT    TRANSMEM    238     260
FT    TOPO_DOM    261     271       NON CYTOPLASMIC.
FT    TRANSMEM    272     291
FT    TOPO_DOM    292     310       CYTOPLASMIC.
//
```

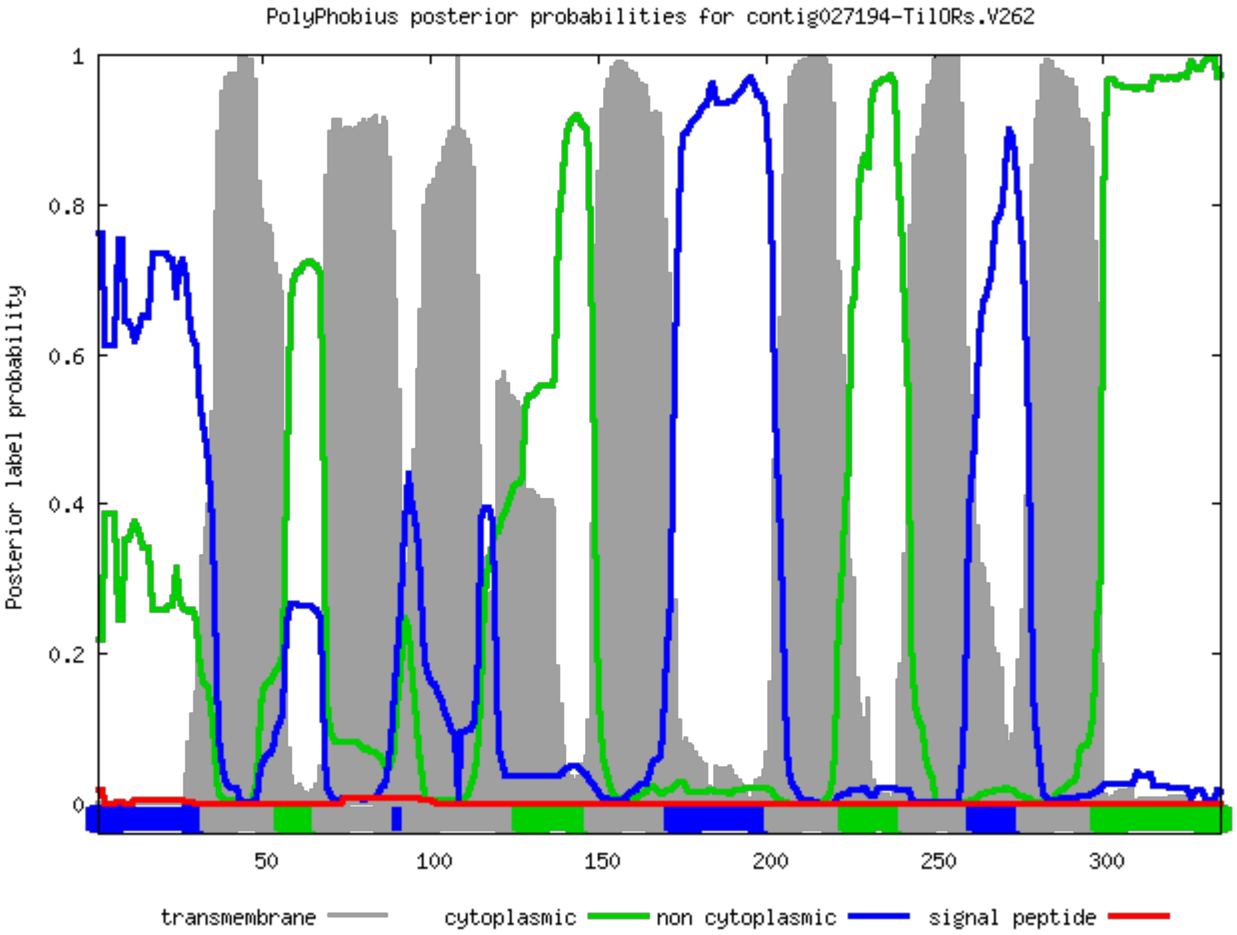

The prediction is based on an alignment. The probability data used in the plot is found here, and the gnuplot script is here.

Prediction of contig085018-BriOR.A006

```
ID   contig085018-BriOR.A006
FT   TOPO_DOM      1      22       NON CYTOPLASMIC.
FT   TRANSMEM     23      48
FT   TOPO_DOM     49      56       CYTOPLASMIC.
FT   TRANSMEM     57      76
FT   TOPO_DOM     77      95       NON CYTOPLASMIC.
FT   TRANSMEM     96     118
FT   TOPO_DOM    119     138       CYTOPLASMIC.
FT   TRANSMEM    139     159
FT   TOPO_DOM    160     192       NON CYTOPLASMIC.
FT   TRANSMEM    193     215
FT   TOPO_DOM    216     235       CYTOPLASMIC.
FT   TRANSMEM    236     257
FT   TOPO_DOM    258     268       NON CYTOPLASMIC.
FT   TRANSMEM    269     289
FT   TOPO_DOM    290     316       CYTOPLASMIC.
//
```

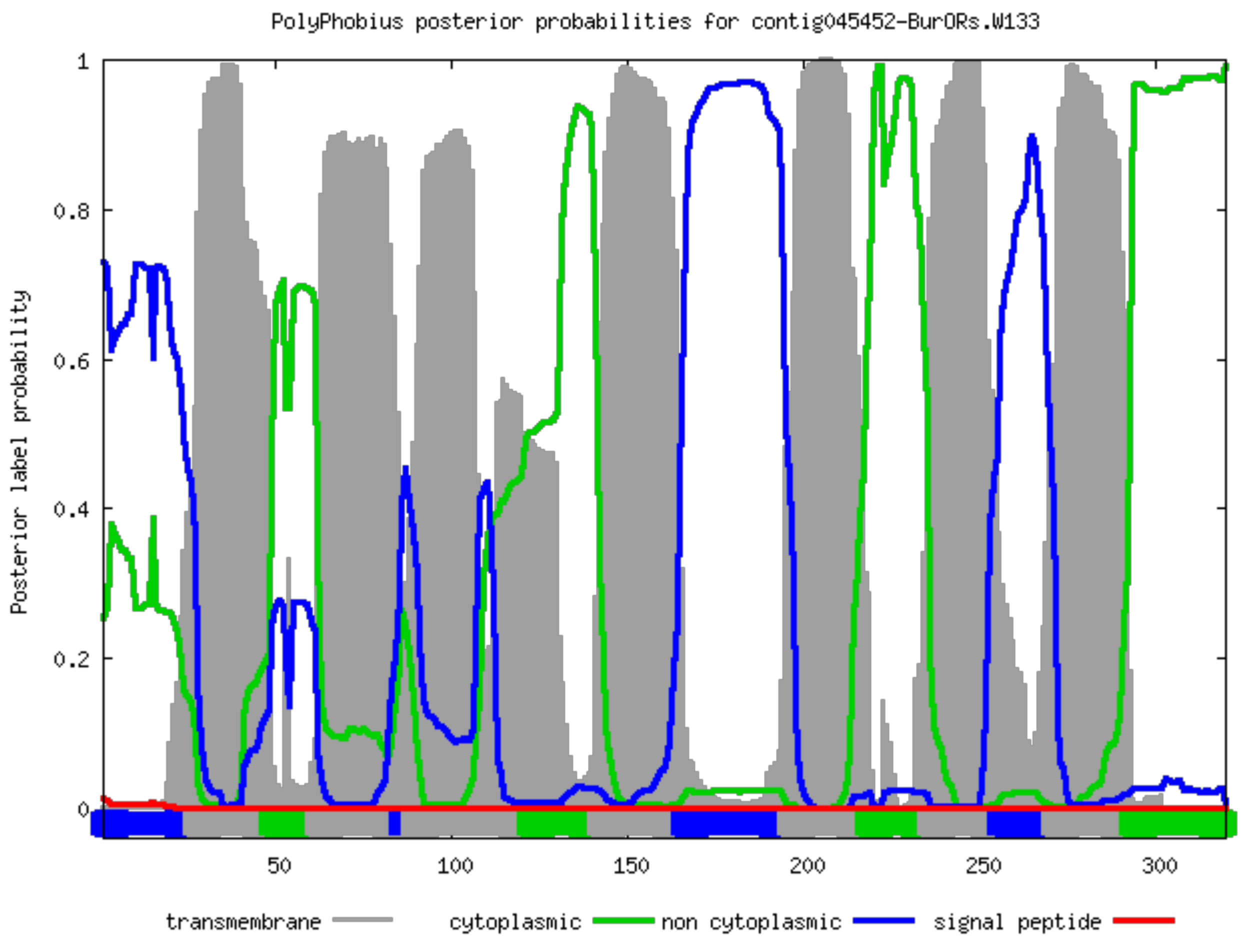

The prediction is based on an alignment. The probability data used in the plot is found here, and the gnuplot script is here.

Prediction of contig028617-TilOR.R248

```
ID   contig028617-TilOR.R248
FT   TOPO_DOM      1     24       NON CYTOPLASMIC.
FT   TRANSMEM     25     48
FT   TOPO_DOM     49     59       CYTOPLASMIC.
FT   TRANSMEM     60     84
FT   TOPO_DOM     85     89       NON CYTOPLASMIC.
FT   TRANSMEM     90    118
FT   TOPO_DOM    119    138       CYTOPLASMIC.
FT   TRANSMEM    139    162
FT   TOPO_DOM    163    194       NON CYTOPLASMIC.
FT   TRANSMEM    195    218
FT   TOPO_DOM    219    235       CYTOPLASMIC.
FT   TRANSMEM    236    259
FT   TOPO_DOM    260    270       NON CYTOPLASMIC.
FT   TRANSMEM    271    293
FT   TOPO_DOM    294    312       CYTOPLASMIC.
//
```

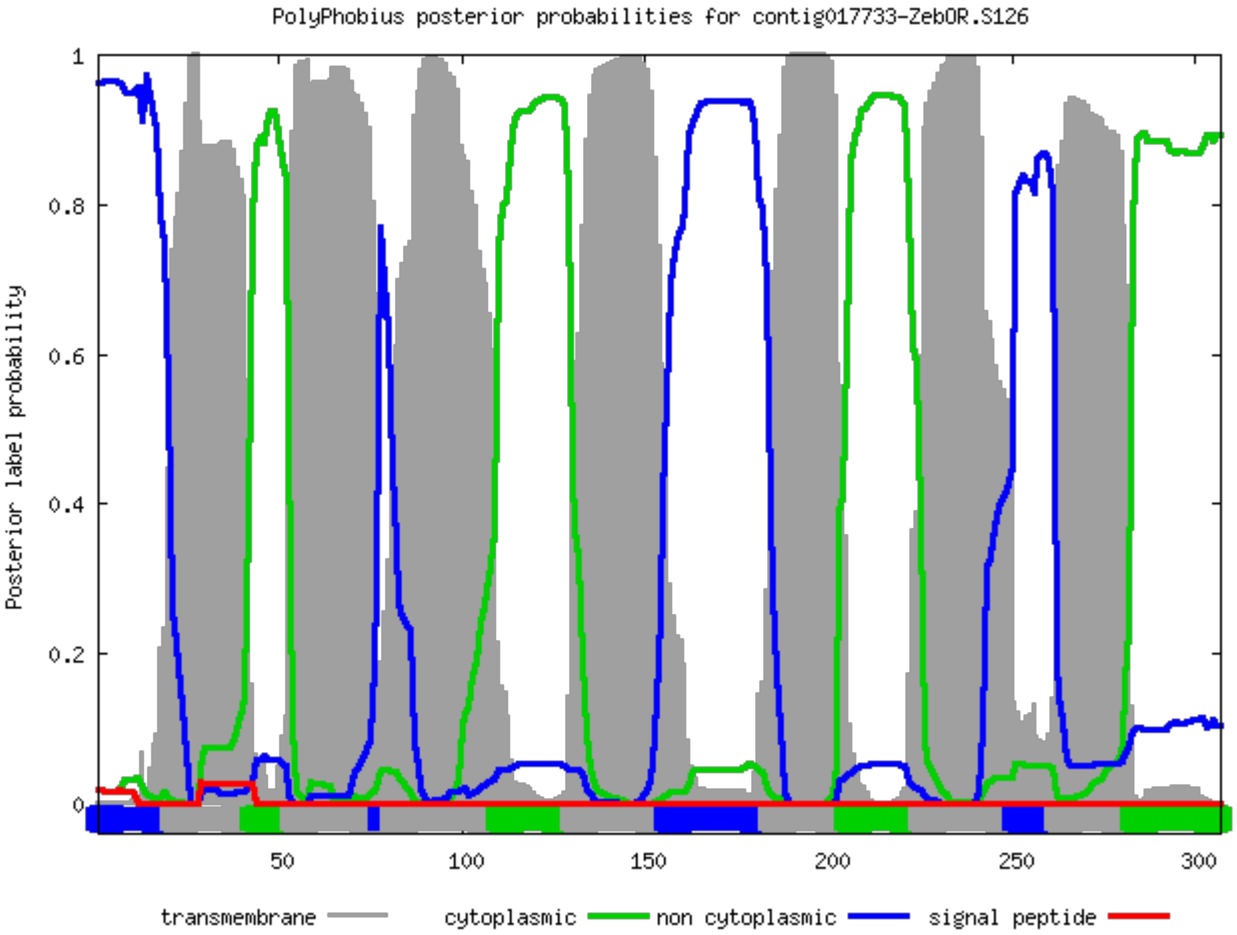

The prediction is based on an alignment. The probability data used in the plot is found here, and the gnuplot script is here.

Prediction of contig034983-NyeOR.A002

```
ID   contig034983-NyeOR.A002
FT   TOPO_DOM      1     18       NON CYTOPLASMIC.
FT   TRANSMEM     19     44
FT   TOPO_DOM     45     52       CYTOPLASMIC.
FT   TRANSMEM     53     73
FT   TOPO_DOM     74     91       NON CYTOPLASMIC.
FT   TRANSMEM     92    114
FT   TOPO_DOM    115    134       CYTOPLASMIC.
FT   TRANSMEM    135    155
FT   TOPO_DOM    156    188       NON CYTOPLASMIC.
FT   TRANSMEM    189    211
FT   TOPO_DOM    212    231       CYTOPLASMIC.
FT   TRANSMEM    232    253
FT   TOPO_DOM    254    264       NON CYTOPLASMIC.
FT   TRANSMEM    265    285
FT   TOPO_DOM    286    300       CYTOPLASMIC.
//
```

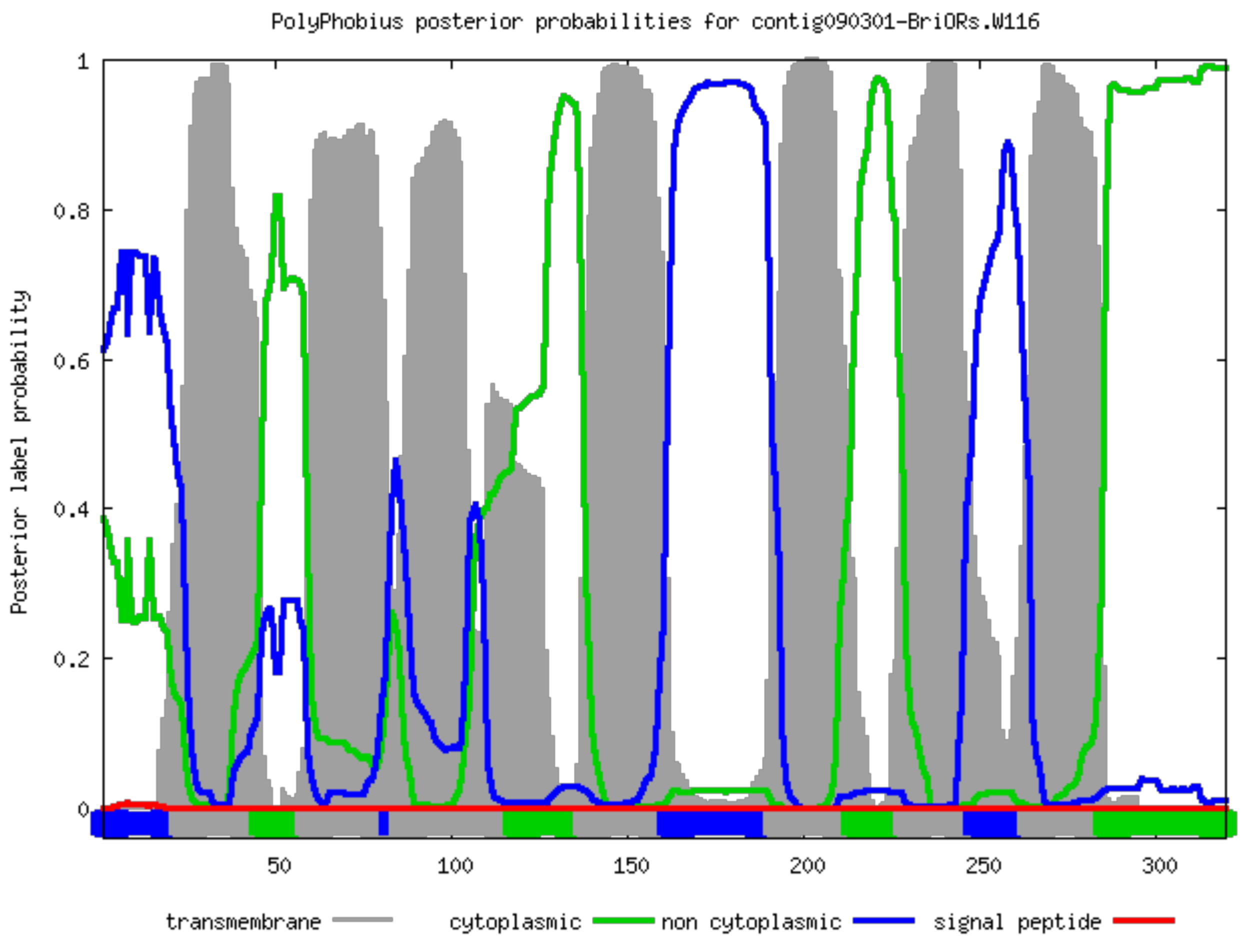

The prediction is based on an alignment. The probability data used in the plot is found here, and the gnuplot script is here.

Prediction of contig014051-ZebOR.D040

```
ID    contig014051-ZebOR.D040
FT    TOPO_DOM      1      22       NON CYTOPLASMIC.
FT    TRANSMEM     23      48
FT    TOPO_DOM     49      57       CYTOPLASMIC.
FT    TRANSMEM     58      81
FT    TOPO_DOM     82      90       NON CYTOPLASMIC.
FT    TRANSMEM     91     118
FT    TOPO_DOM    119     138       CYTOPLASMIC.
FT    TRANSMEM    139     161
FT    TOPO_DOM    162     193       NON CYTOPLASMIC.
FT    TRANSMEM    194     216
FT    TOPO_DOM    217     236       CYTOPLASMIC.
FT    TRANSMEM    237     256
FT    TOPO_DOM    257     268       NON CYTOPLASMIC.
FT    TRANSMEM    269     291
FT    TOPO_DOM    292     309       CYTOPLASMIC.
//
```

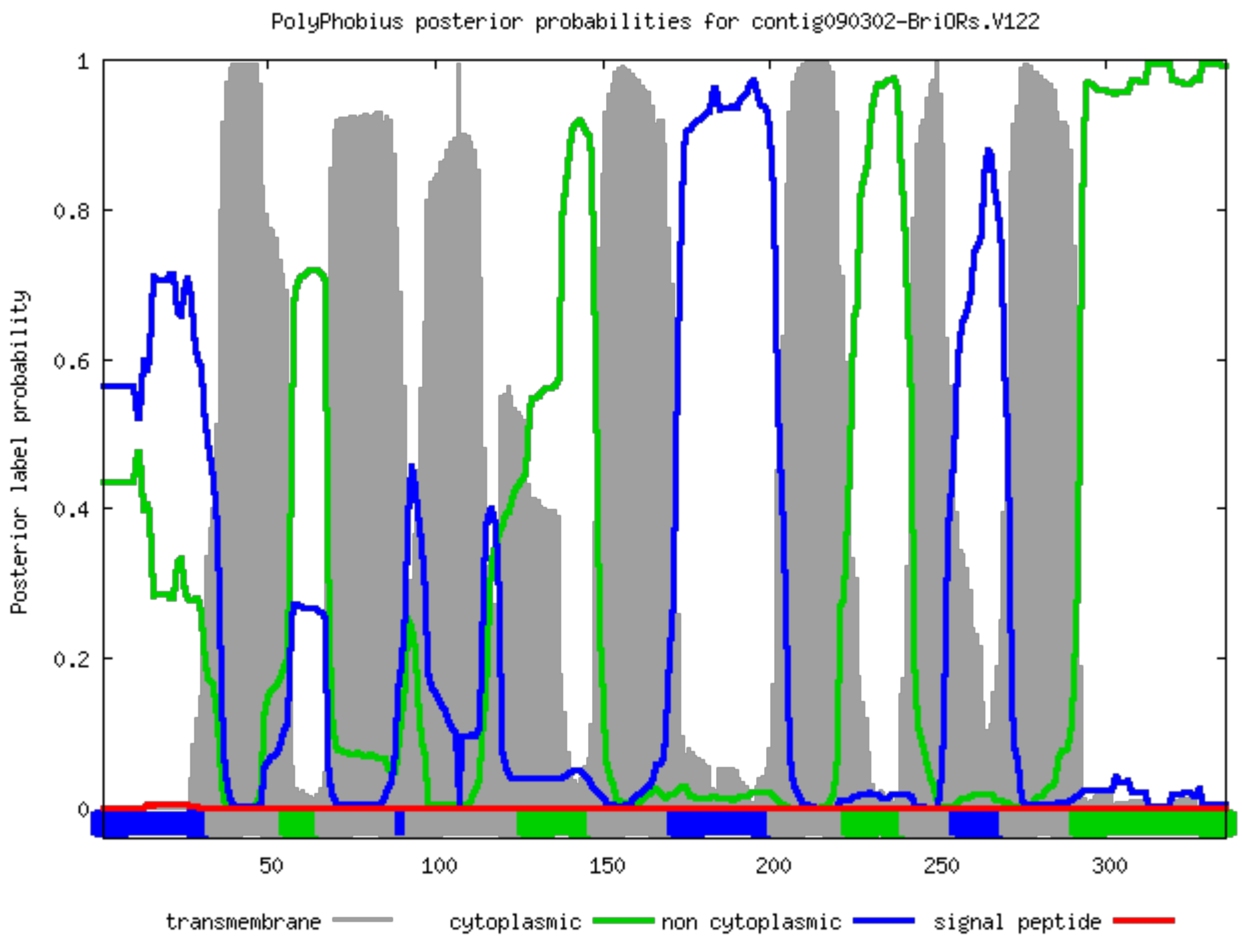

The prediction is based on an alignment. The probability data used in the plot is found here, and the gnuplot script is here.

Prediction of contig053579-NyeOR.E053

```
ID    contig053579-NyeOR.E053
FT    TOPO_DOM      1      23       NON CYTOPLASMIC.
FT    TRANSMEM     24      49
FT    TOPO_DOM     50      58       CYTOPLASMIC.
FT    TRANSMEM     59      83
FT    TOPO_DOM     84      92       NON CYTOPLASMIC.
FT    TRANSMEM     93     119
FT    TOPO_DOM    120     139       CYTOPLASMIC.
FT    TRANSMEM    140     162
FT    TOPO_DOM    163     194       NON CYTOPLASMIC.
FT    TRANSMEM    195     217
FT    TOPO_DOM    218     237       CYTOPLASMIC.
FT    TRANSMEM    238     257
FT    TOPO_DOM    258     268       NON CYTOPLASMIC.
FT    TRANSMEM    269     292
FT    TOPO_DOM    293     308       CYTOPLASMIC.
//
```

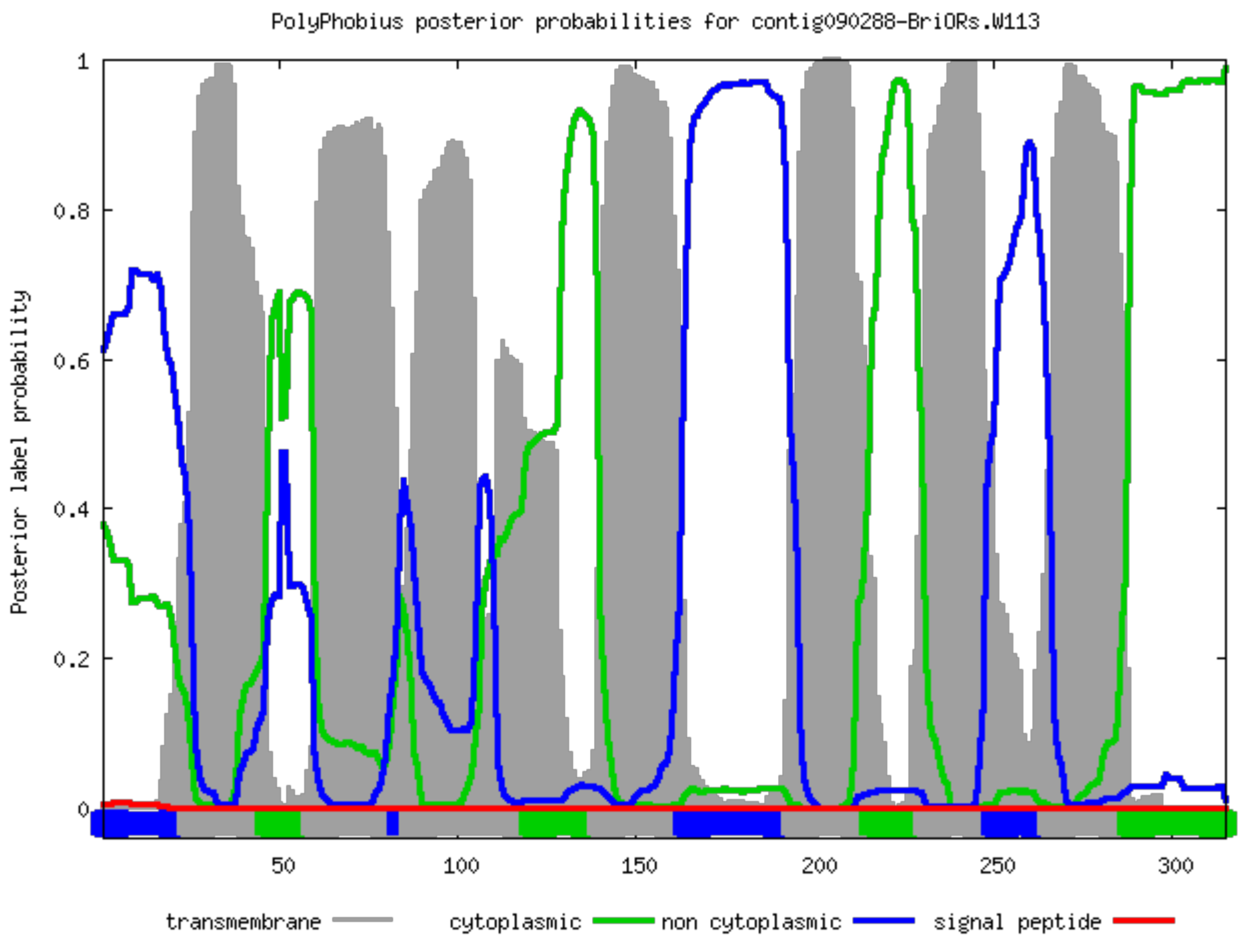

The prediction is based on an alignment. The probability data used in the plot is found here, and the gnuplot script is here.

Prediction of contig035580-NyeOR.H069

```
ID   contig035580-NyeOR.H069
FT   TOPO_DOM      1      23       NON CYTOPLASMIC.
FT   TRANSMEM     24      49
FT   TOPO_DOM     50      56       CYTOPLASMIC.
FT   TRANSMEM     57      76
FT   TOPO_DOM     77      95       NON CYTOPLASMIC.
FT   TRANSMEM     96     118
FT   TOPO_DOM    119     138       CYTOPLASMIC.
FT   TRANSMEM    139     160
FT   TOPO_DOM    161     196       NON CYTOPLASMIC.
FT   TRANSMEM    197     219
FT   TOPO_DOM    220     237       CYTOPLASMIC.
FT   TRANSMEM    238     260
FT   TOPO_DOM    261     271       NON CYTOPLASMIC.
FT   TRANSMEM    272     291
FT   TOPO_DOM    292     310       CYTOPLASMIC.
//
```

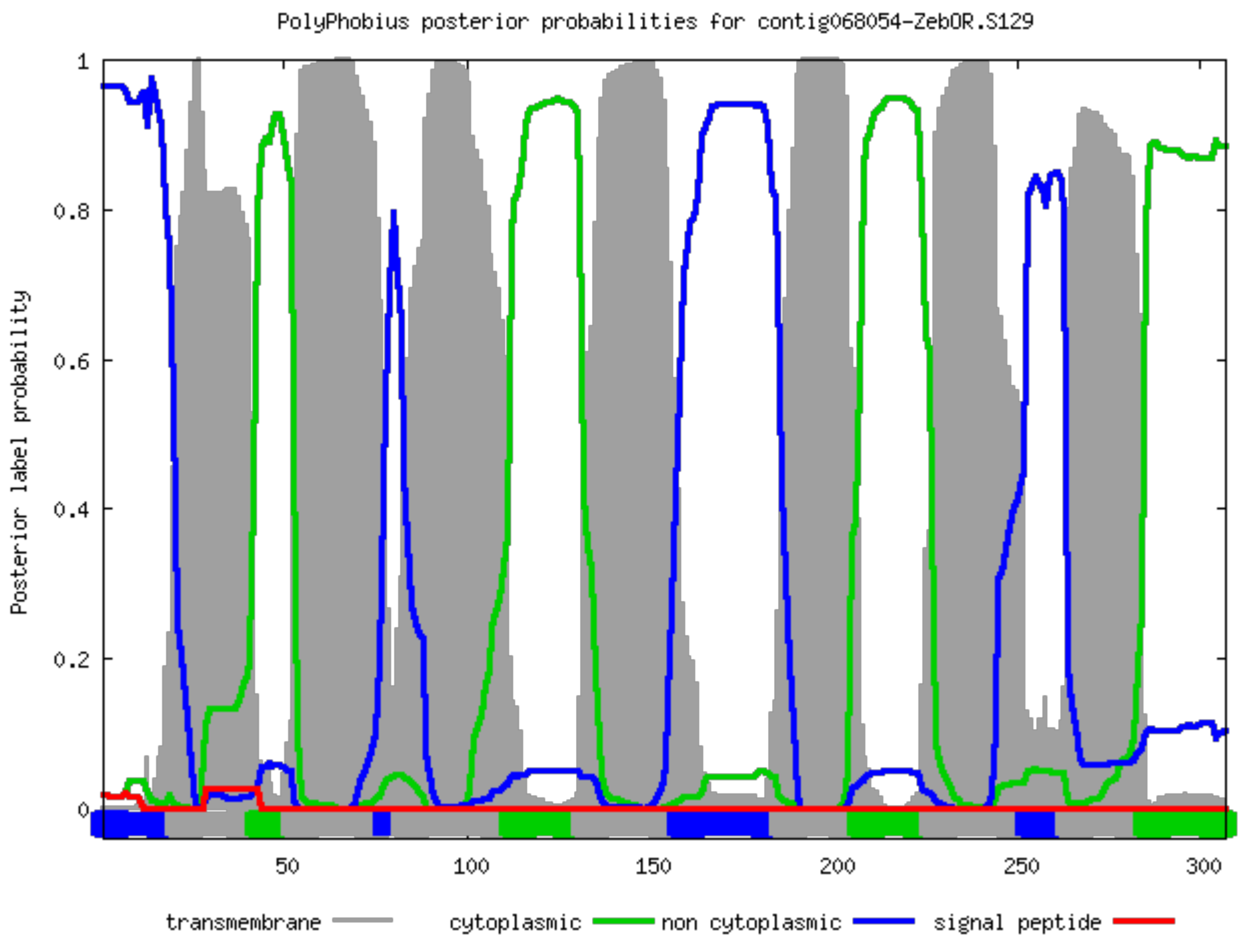

The prediction is based on an alignment. The probability data used in the plot is found here, and the gnuplot script is here.

Prediction of contig064821-BriOR.H052

```
ID   contig064821-BriOR.H052
FT   TOPO_DOM      1     22       NON CYTOPLASMIC.
FT   TRANSMEM     23     49
FT   TOPO_DOM     50     56       CYTOPLASMIC.
FT   TRANSMEM     57     77
FT   TOPO_DOM     78     95       NON CYTOPLASMIC.
FT   TRANSMEM     96    118
FT   TOPO_DOM    119    138       CYTOPLASMIC.
FT   TRANSMEM    139    160
FT   TOPO_DOM    161    193       NON CYTOPLASMIC.
FT   TRANSMEM    194    217
FT   TOPO_DOM    218    235       CYTOPLASMIC.
FT   TRANSMEM    236    258
FT   TOPO_DOM    259    269       NON CYTOPLASMIC.
FT   TRANSMEM    270    289
FT   TOPO_DOM    290    314       CYTOPLASMIC.
//
```

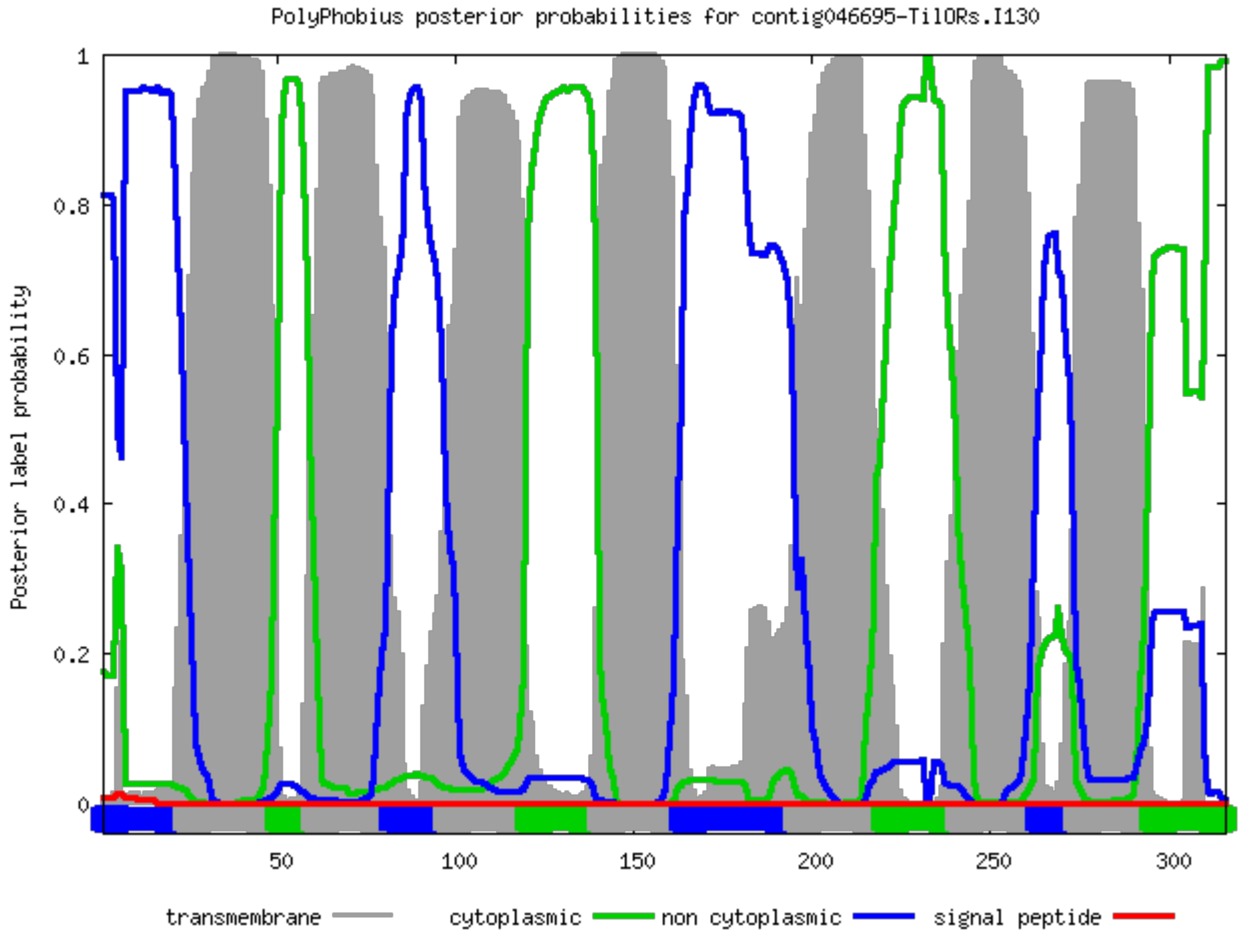

The prediction is based on an alignment. The probability data used in the plot is found here, and the gnuplot script is here.

Prediction of contig033889-BriOR.H049

```
ID   contig033889-BriOR.H049
FT   TOPO_DOM      1      23       NON CYTOPLASMIC.
FT   TRANSMEM     24      49
FT   TOPO_DOM     50      56       CYTOPLASMIC.
FT   TRANSMEM     57      76
FT   TOPO_DOM     77      95       NON CYTOPLASMIC.
FT   TRANSMEM     96     118
FT   TOPO_DOM    119     138       CYTOPLASMIC.
FT   TRANSMEM    139     160
FT   TOPO_DOM    161     196       NON CYTOPLASMIC.
FT   TRANSMEM    197     219
FT   TOPO_DOM    220     237       CYTOPLASMIC.
FT   TRANSMEM    238     259
FT   TOPO_DOM    260     271       NON CYTOPLASMIC.
FT   TRANSMEM    272     291
FT   TOPO_DOM    292     324       CYTOPLASMIC.
//
```

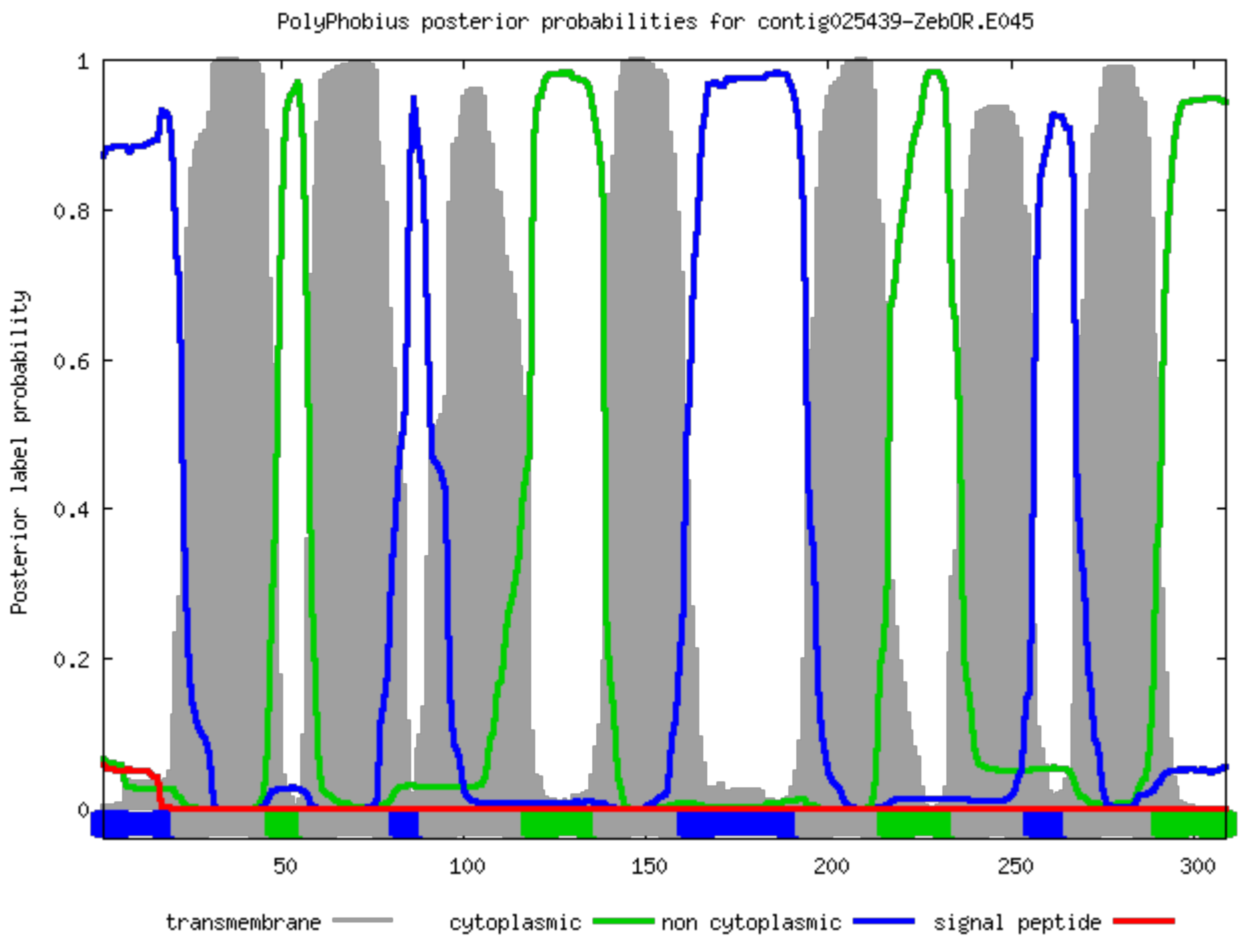

The prediction is based on an alignment. The probability data used in the plot is found here, and the gnuplot script is here.

Prediction of contig047829-TilOR.E081

```
ID   contig047829-TilOR.E081
FT   TOPO_DOM      1    26       NON CYTOPLASMIC.
FT   TRANSMEM     27    52
FT   TOPO_DOM     53    61       CYTOPLASMIC.
FT   TRANSMEM     62    87
FT   TOPO_DOM     88    94       NON CYTOPLASMIC.
FT   TRANSMEM     95   122
FT   TOPO_DOM    123   142       CYTOPLASMIC.
FT   TRANSMEM    143   165
FT   TOPO_DOM    166   197       NON CYTOPLASMIC.
FT   TRANSMEM    198   220
FT   TOPO_DOM    221   239       CYTOPLASMIC.
FT   TRANSMEM    240   260
FT   TOPO_DOM    261   272       NON CYTOPLASMIC.
FT   TRANSMEM    273   295
FT   TOPO_DOM    296   311       CYTOPLASMIC.
//
```

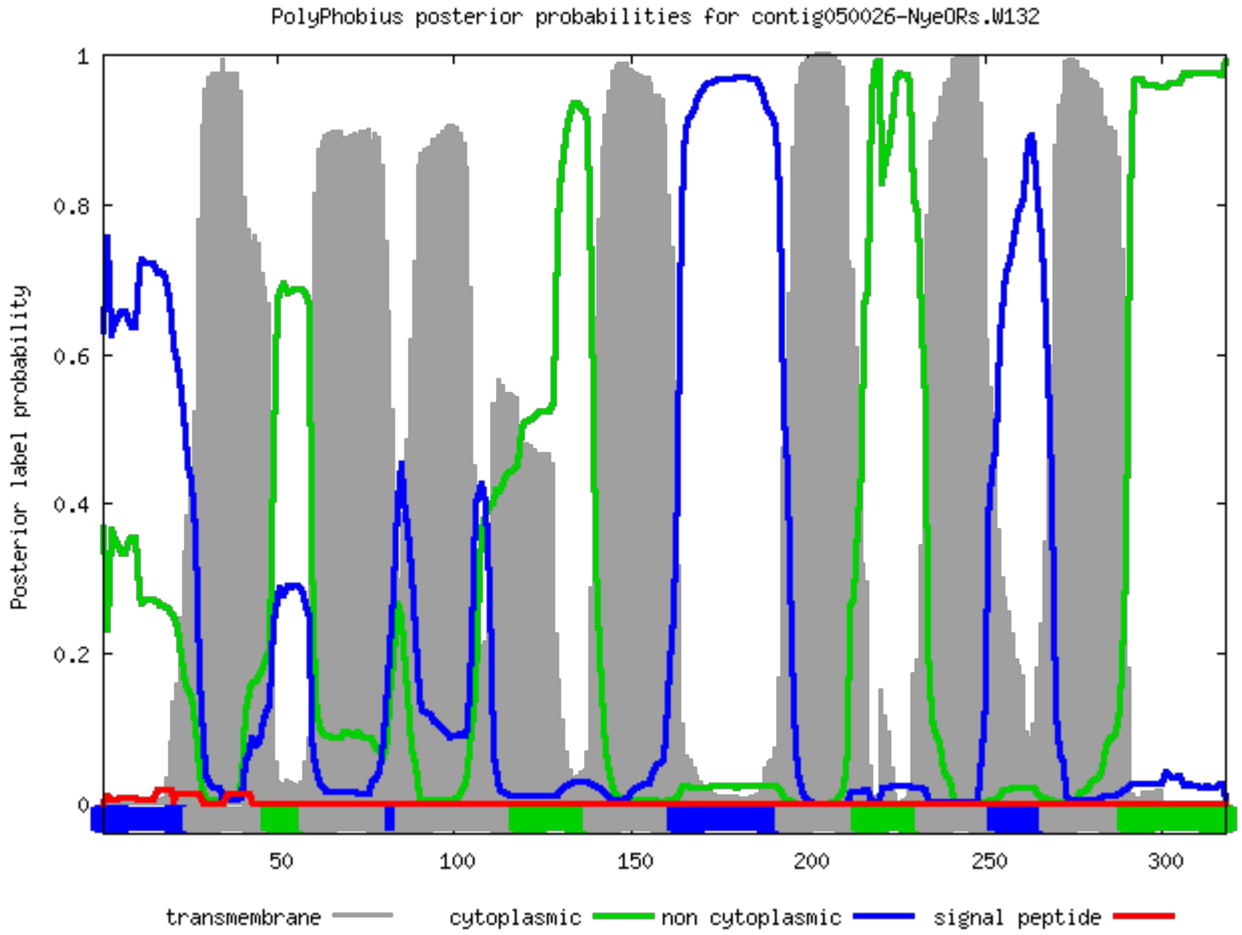

The prediction is based on an alignment. The probability data used in the plot is found here, and the gnuplot script is here.

Prediction of contig047832-TilOR.E082

```
ID   contig047832-TilOR.E082
FT   TOPO_DOM      1     22       NON CYTOPLASMIC.
FT   TRANSMEM     23     48
FT   TOPO_DOM     49     57       CYTOPLASMIC.
FT   TRANSMEM     58     81
FT   TOPO_DOM     82     92       NON CYTOPLASMIC.
FT   TRANSMEM     93    118
FT   TOPO_DOM    119    138       CYTOPLASMIC.
FT   TRANSMEM    139    160
FT   TOPO_DOM    161    193       NON CYTOPLASMIC.
FT   TRANSMEM    194    216
FT   TOPO_DOM    217    236       CYTOPLASMIC.
FT   TRANSMEM    237    256
FT   TOPO_DOM    257    267       NON CYTOPLASMIC.
FT   TRANSMEM    268    291
FT   TOPO_DOM    292    309       CYTOPLASMIC.
//
```

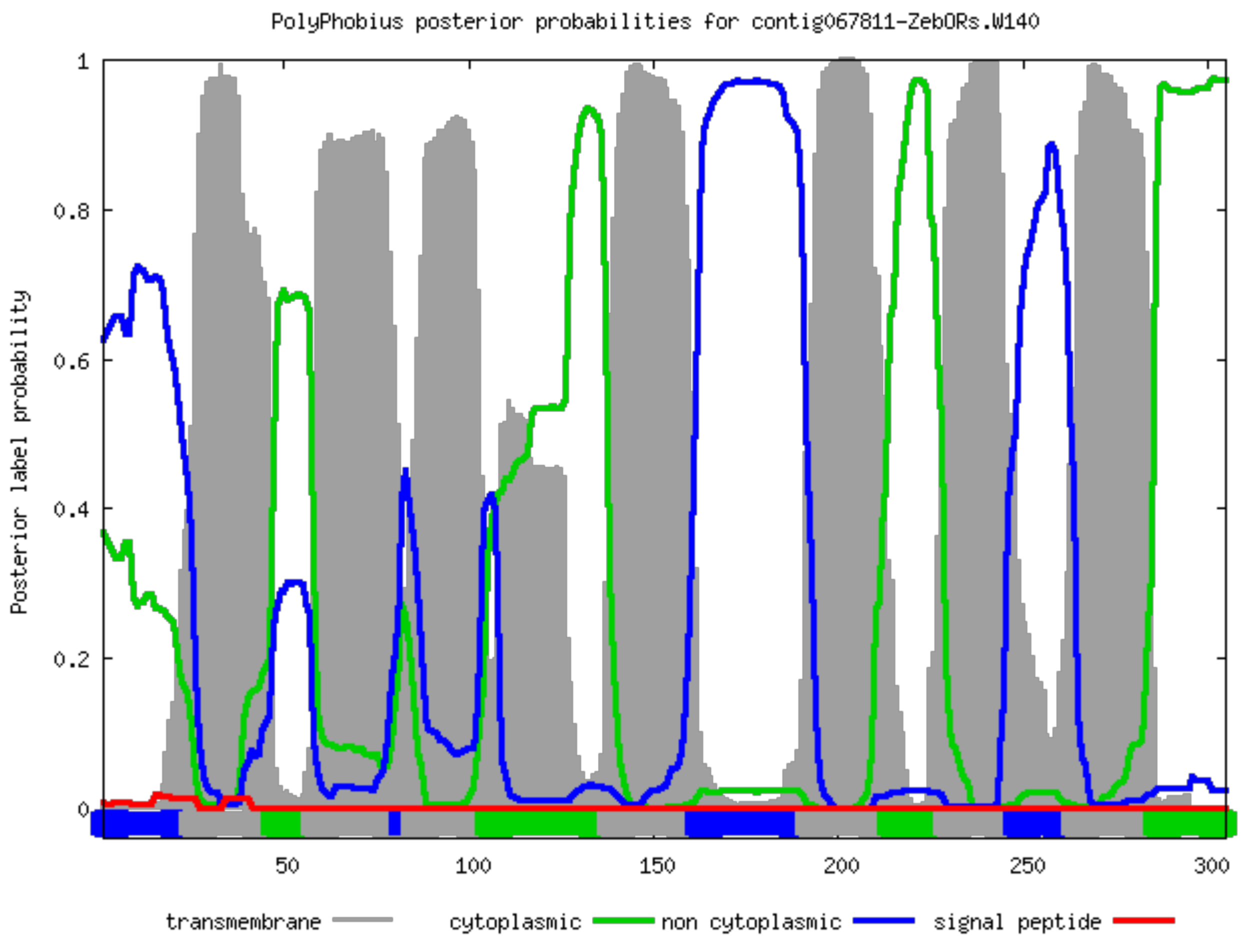

The prediction is based on an alignment. The probability data used in the plot is found here, and the gnuplot script is here.

Prediction of contig018434-ZebOR.H071

```
ID    contig018434-ZebOR.H071
FT    TOPO_DOM      1     23        NON CYTOPLASMIC.
FT    TRANSMEM     24     49
FT    TOPO_DOM     50     56        CYTOPLASMIC.
FT    TRANSMEM     57     76
FT    TOPO_DOM     77     95        NON CYTOPLASMIC.
FT    TRANSMEM     96    118
FT    TOPO_DOM    119    138        CYTOPLASMIC.
FT    TRANSMEM    139    160
FT    TOPO_DOM    161    196        NON CYTOPLASMIC.
FT    TRANSMEM    197    219
FT    TOPO_DOM    220    237        CYTOPLASMIC.
FT    TRANSMEM    238    259
FT    TOPO_DOM    260    271        NON CYTOPLASMIC.
FT    TRANSMEM    272    291
FT    TOPO_DOM    292    310        CYTOPLASMIC.
//
```

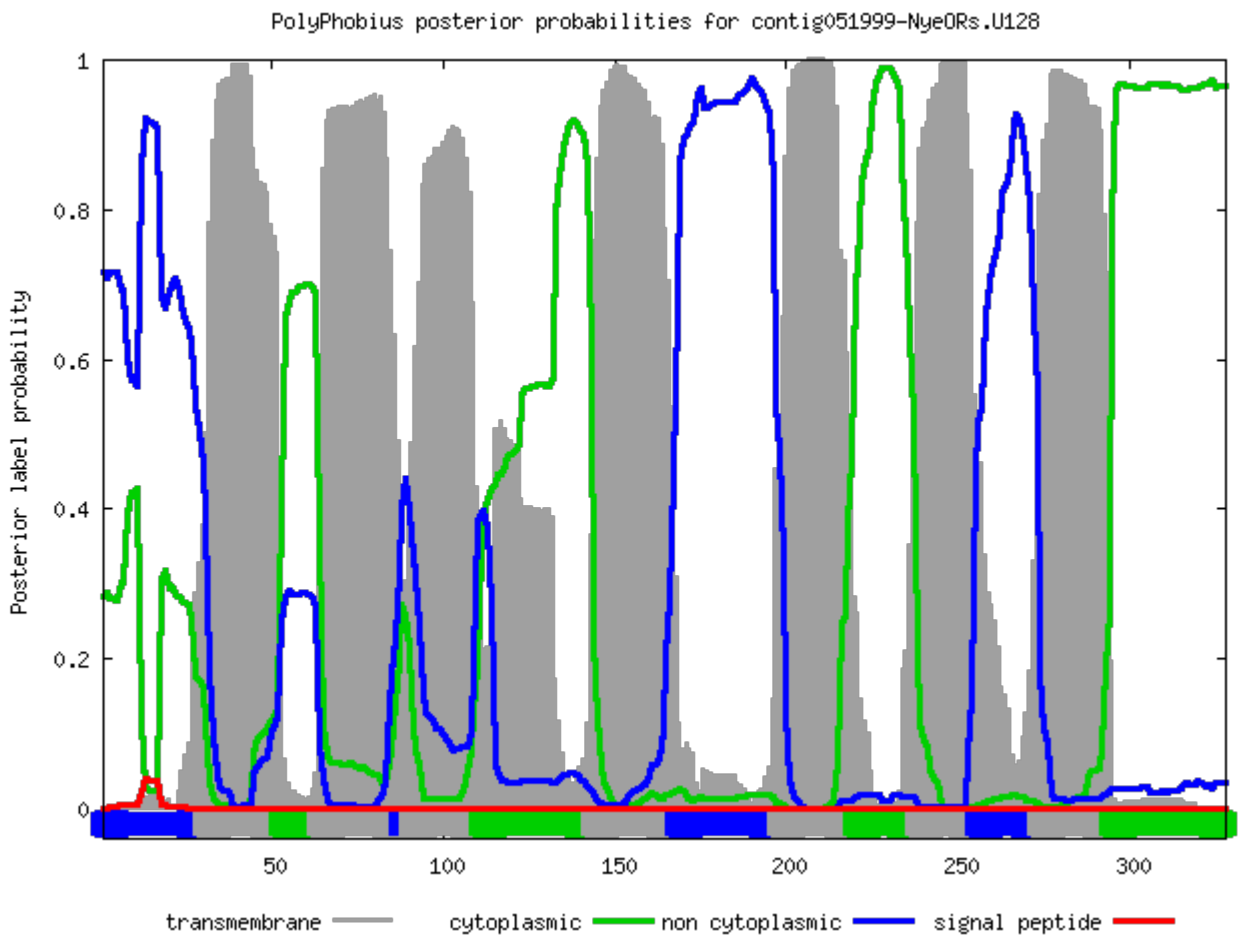

The prediction is based on an alignment. The probability data used in the plot is found here, and the gnuplot script is here.

Prediction of contig046723-TilOR.K138

```
ID   contig046723-TilOR.K138
FT   TOPO_DOM      1     24       NON CYTOPLASMIC.
FT   TRANSMEM     25     48
FT   TOPO_DOM     49     58       CYTOPLASMIC.
FT   TRANSMEM     59     80
FT   TOPO_DOM     81     99       NON CYTOPLASMIC.
FT   TRANSMEM    100    121
FT   TOPO_DOM    122    141       CYTOPLASMIC.
FT   TRANSMEM    142    165
FT   TOPO_DOM    166    198       NON CYTOPLASMIC.
FT   TRANSMEM    199    222
FT   TOPO_DOM    223    242       CYTOPLASMIC.
FT   TRANSMEM    243    262
FT   TOPO_DOM    263    272       NON CYTOPLASMIC.
FT   TRANSMEM    273    292
FT   TOPO_DOM    293    313       CYTOPLASMIC.
//
```

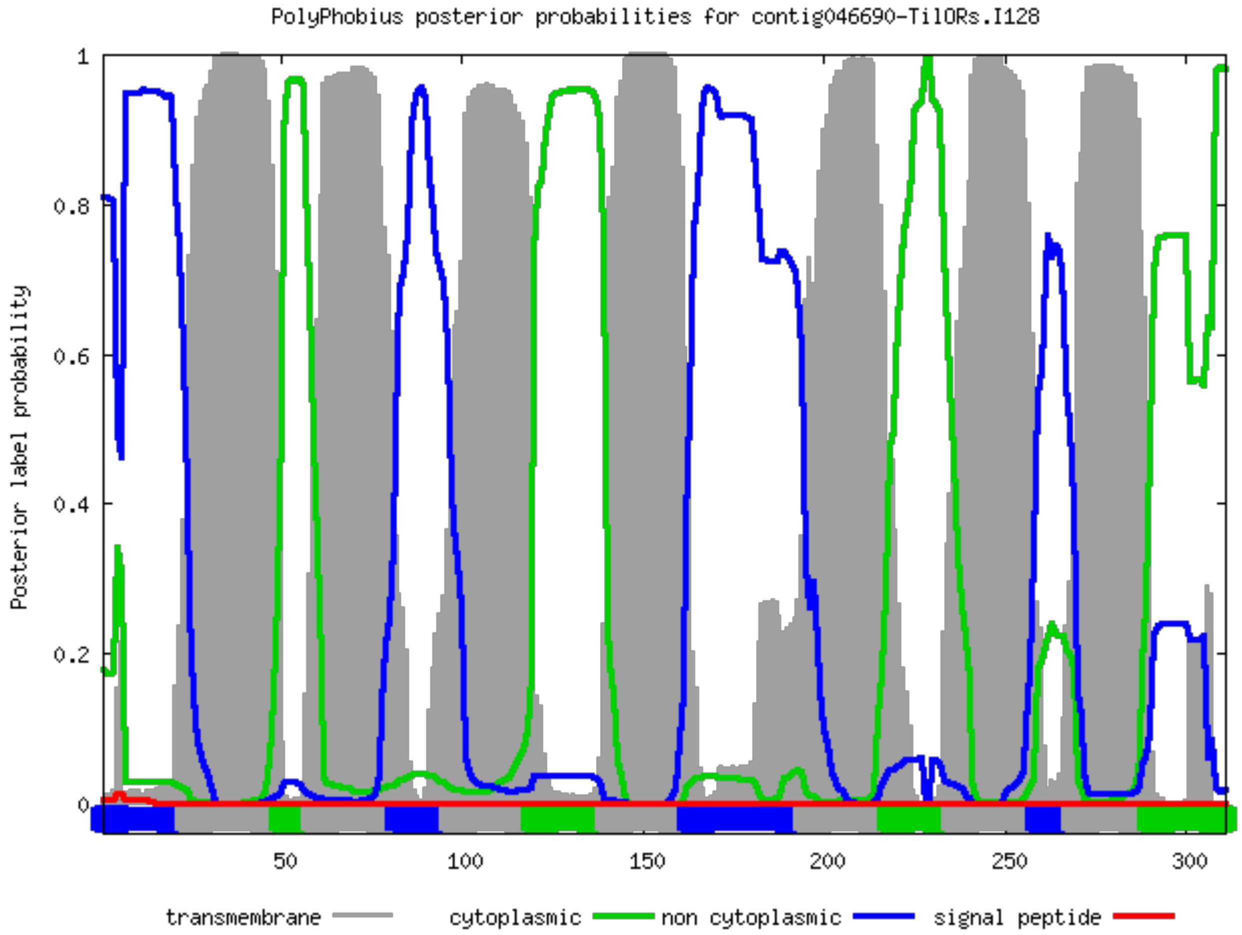

The prediction is based on an alignment. The probability data used in the plot is found here, and the gnuplot script is here.

Prediction of contig065454-TilOR.E088

```
ID   contig065454-TilOR.E088
FT   TOPO_DOM      1      22       NON CYTOPLASMIC.
FT   TRANSMEM     23      48
FT   TOPO_DOM     49      57       CYTOPLASMIC.
FT   TRANSMEM     58      81
FT   TOPO_DOM     82      94       NON CYTOPLASMIC.
FT   TRANSMEM     95     118
FT   TOPO_DOM    119     138       CYTOPLASMIC.
FT   TRANSMEM    139     160
FT   TOPO_DOM    161     193       NON CYTOPLASMIC.
FT   TRANSMEM    194     216
FT   TOPO_DOM    217     236       CYTOPLASMIC.
FT   TRANSMEM    237     256
FT   TOPO_DOM    257     267       NON CYTOPLASMIC.
FT   TRANSMEM    268     291
FT   TOPO_DOM    292     310       CYTOPLASMIC.
//
```

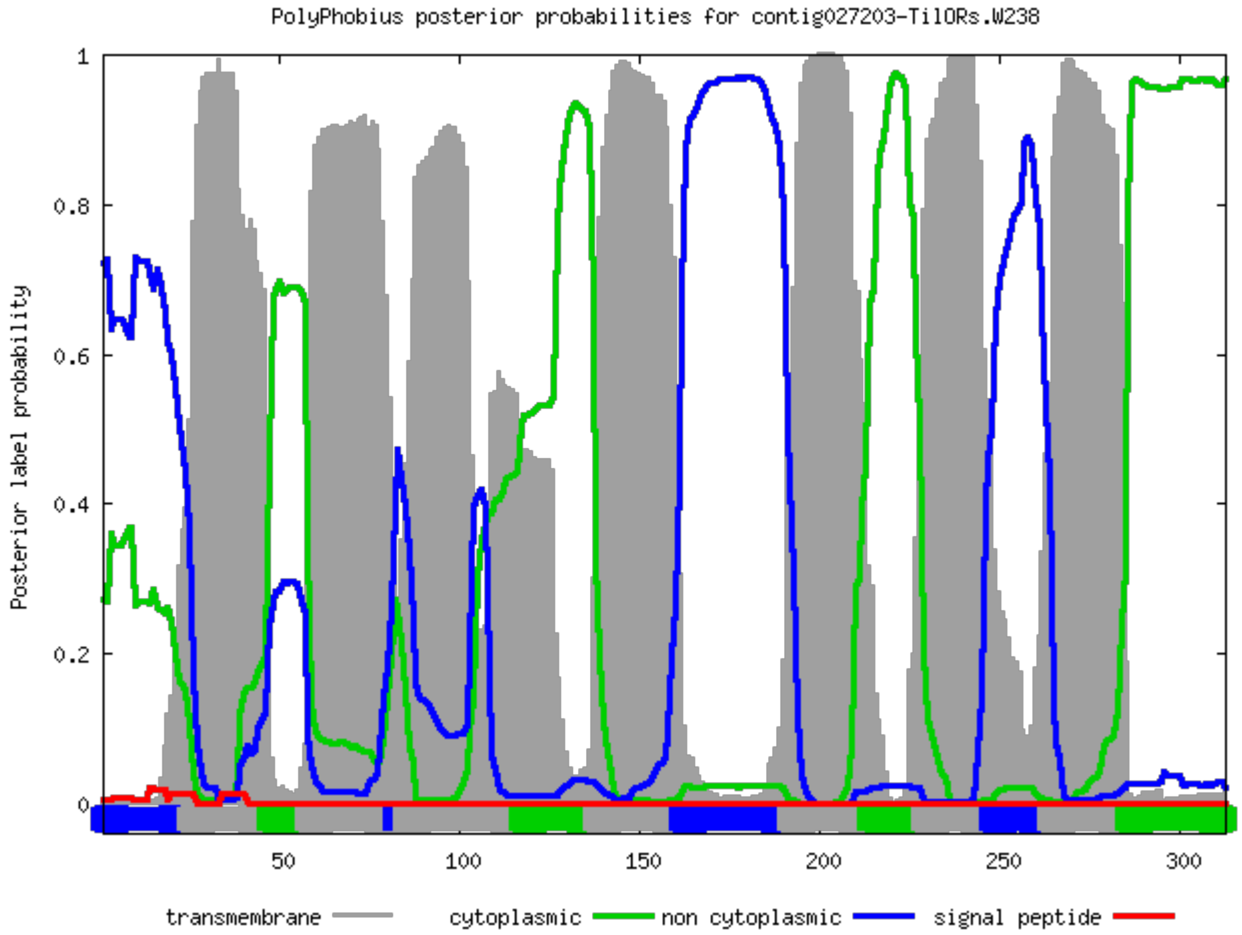

The prediction is based on an alignment. The probability data used in the plot is found here, and the gnuplot script is here.

Prediction of contig004999-TilOR.S217

```
ID   contig004999-TilOR.S217
FT   TOPO_DOM      1     20       NON CYTOPLASMIC.
FT   TRANSMEM     21     42
FT   TOPO_DOM     43     52       CYTOPLASMIC.
FT   TRANSMEM     53     77
FT   TOPO_DOM     78     80       NON CYTOPLASMIC.
FT   TRANSMEM     81    109
FT   TOPO_DOM    110    129       CYTOPLASMIC.
FT   TRANSMEM    130    155
FT   TOPO_DOM    156    183       NON CYTOPLASMIC.
FT   TRANSMEM    184    204
FT   TOPO_DOM    205    224       CYTOPLASMIC.
FT   TRANSMEM    225    250
FT   TOPO_DOM    251    261       NON CYTOPLASMIC.
FT   TRANSMEM    262    282
FT   TOPO_DOM    283    307       CYTOPLASMIC.
//
```

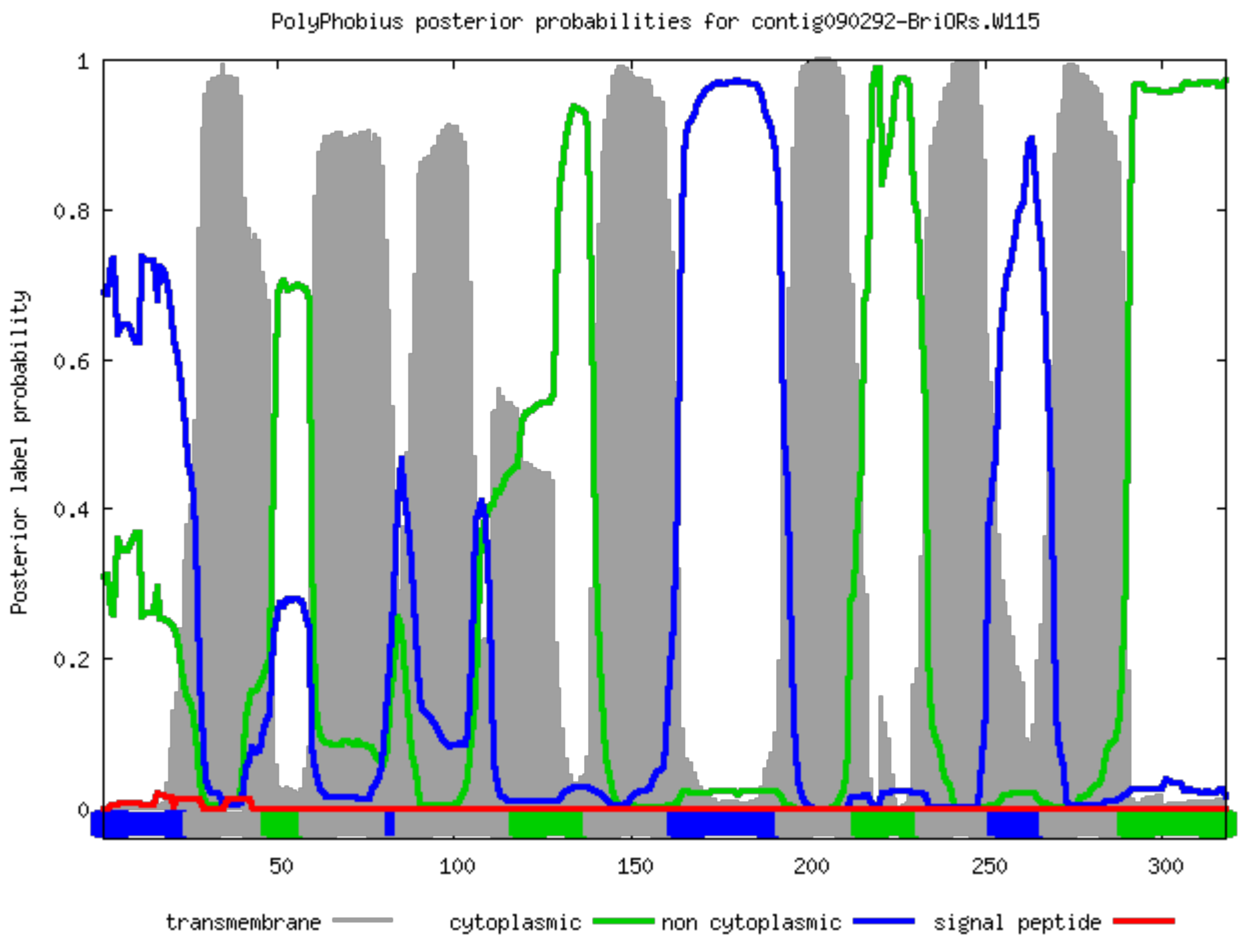

The prediction is based on an alignment. The probability data used in the plot is found here, and the gnuplot script is here.

Prediction of contig090296-BriOR.W110

```
ID    contig090296-BriOR.W110
FT    TOPO_DOM      1      12        NON CYTOPLASMIC.
FT    TRANSMEM     13      34
FT    TOPO_DOM     35      45        CYTOPLASMIC.
FT    TRANSMEM     46      69
FT    TOPO_DOM     70      71        NON CYTOPLASMIC.
FT    TRANSMEM     72     105
FT    TOPO_DOM    106     125        CYTOPLASMIC.
FT    TRANSMEM    126     149
FT    TOPO_DOM    150     179        NON CYTOPLASMIC.
FT    TRANSMEM    180     201
FT    TOPO_DOM    202     216        CYTOPLASMIC.
FT    TRANSMEM    217     236
FT    TOPO_DOM    237     251        NON CYTOPLASMIC.
FT    TRANSMEM    252     273
FT    TOPO_DOM    274     298        CYTOPLASMIC.
//
```

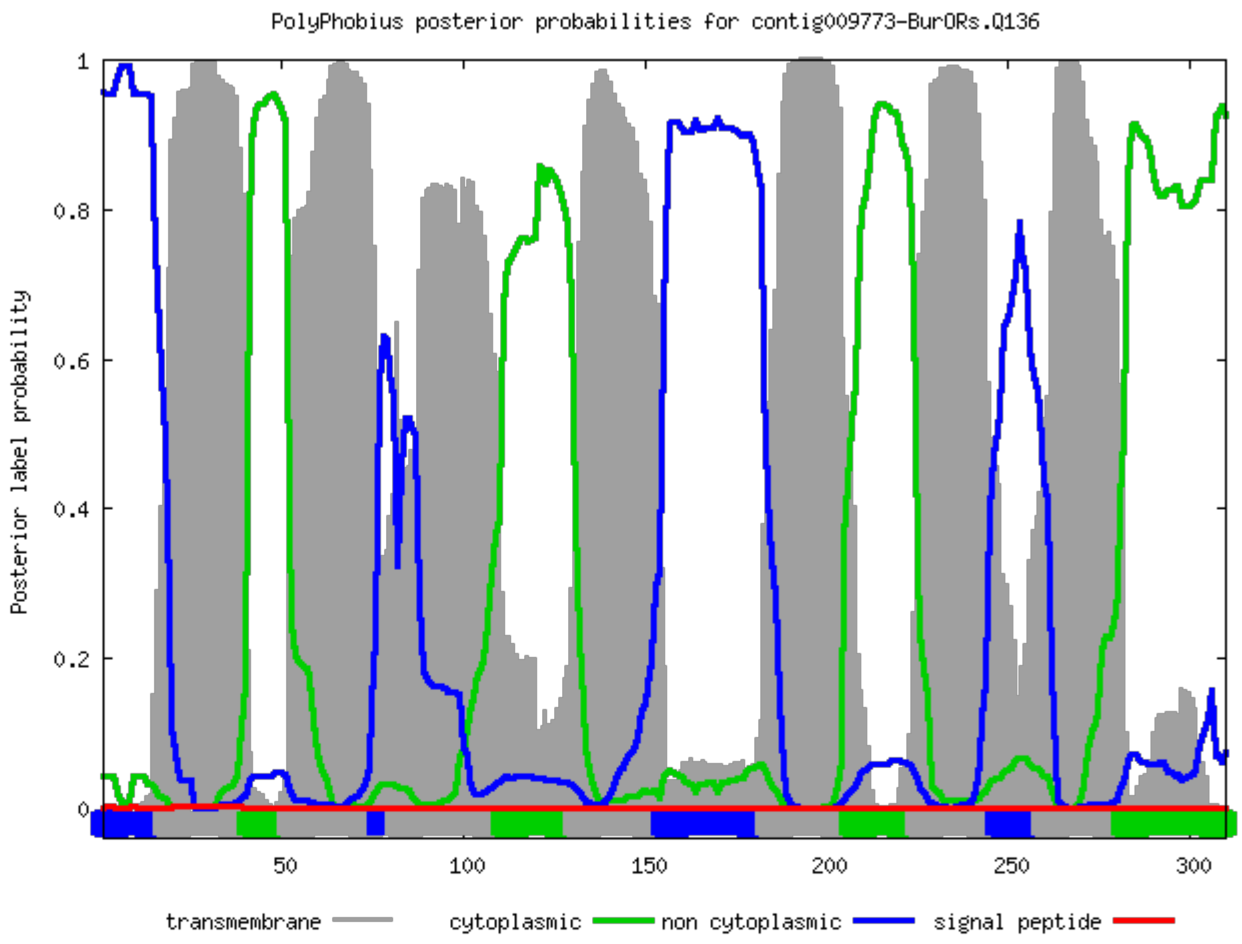

The prediction is based on an alignment. The probability data used in the plot is found here, and the gnuplot script is here.

Prediction of contig030566-ZebOR.A008

```
ID   contig030566-ZebOR.A008
FT   TOPO_DOM      1     22       NON CYTOPLASMIC.
FT   TRANSMEM     23     48
FT   TOPO_DOM     49     56       CYTOPLASMIC.
FT   TRANSMEM     57     77
FT   TOPO_DOM     78     95       NON CYTOPLASMIC.
FT   TRANSMEM     96    118
FT   TOPO_DOM    119    138       CYTOPLASMIC.
FT   TRANSMEM    139    159
FT   TOPO_DOM    160    192       NON CYTOPLASMIC.
FT   TRANSMEM    193    215
FT   TOPO_DOM    216    235       CYTOPLASMIC.
FT   TRANSMEM    236    257
FT   TOPO_DOM    258    268       NON CYTOPLASMIC.
FT   TRANSMEM    269    289
FT   TOPO_DOM    290    309       CYTOPLASMIC.
//
```

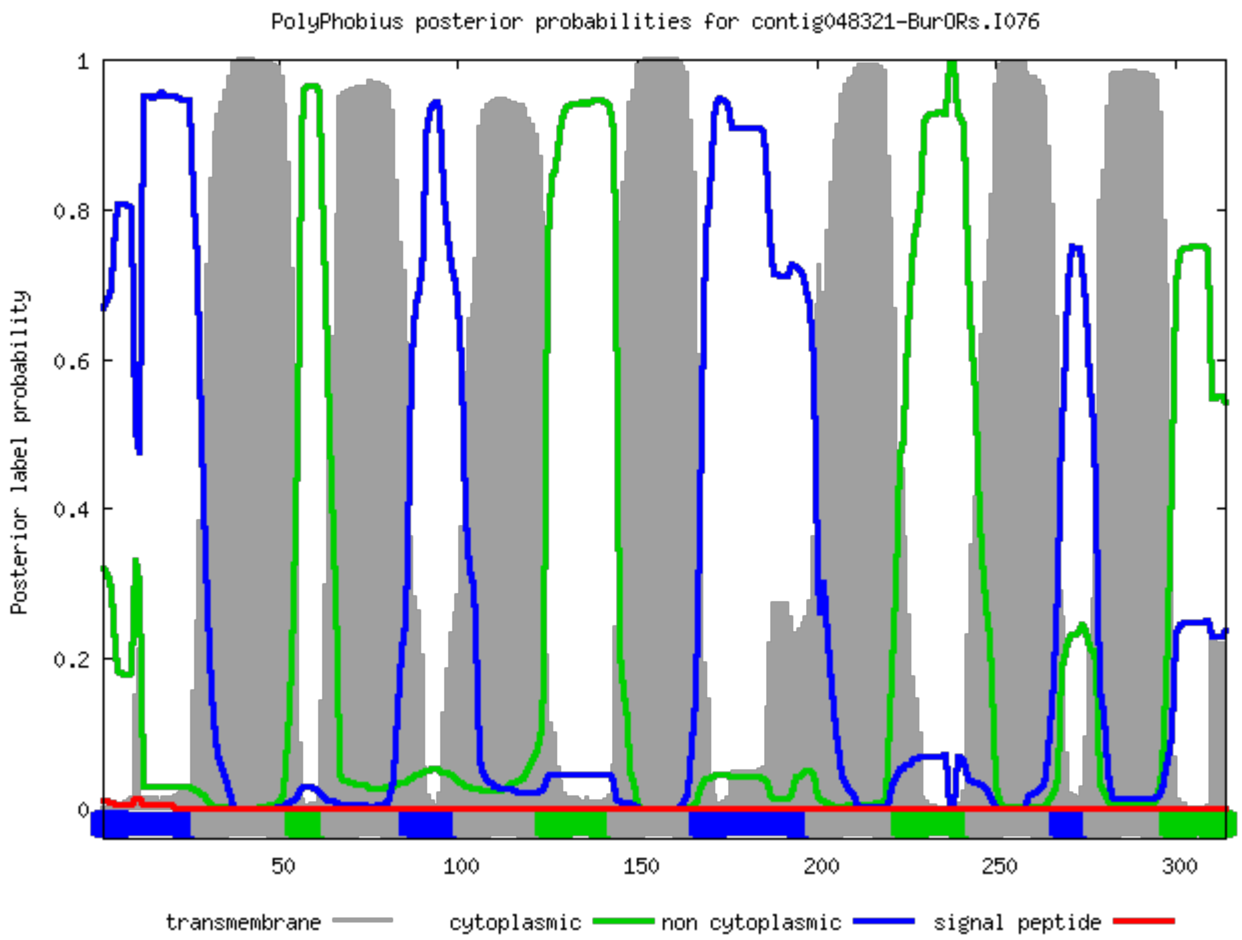

The prediction is based on an alignment. The probability data used in the plot is found here, and the gnuplot script is here.

Prediction of contig036784-BurOR.A003

```
ID   contig036784-BurOR.A003
FT   TOPO_DOM      1     22       NON CYTOPLASMIC.
FT   TRANSMEM     23     48
FT   TOPO_DOM     49     56       CYTOPLASMIC.
FT   TRANSMEM     57     77
FT   TOPO_DOM     78     95       NON CYTOPLASMIC.
FT   TRANSMEM     96    118
FT   TOPO_DOM    119    138       CYTOPLASMIC.
FT   TRANSMEM    139    159
FT   TOPO_DOM    160    192       NON CYTOPLASMIC.
FT   TRANSMEM    193    215
FT   TOPO_DOM    216    235       CYTOPLASMIC.
FT   TRANSMEM    236    257
FT   TOPO_DOM    258    267       NON CYTOPLASMIC.
FT   TRANSMEM    268    289
FT   TOPO_DOM    290    309       CYTOPLASMIC.
//
```

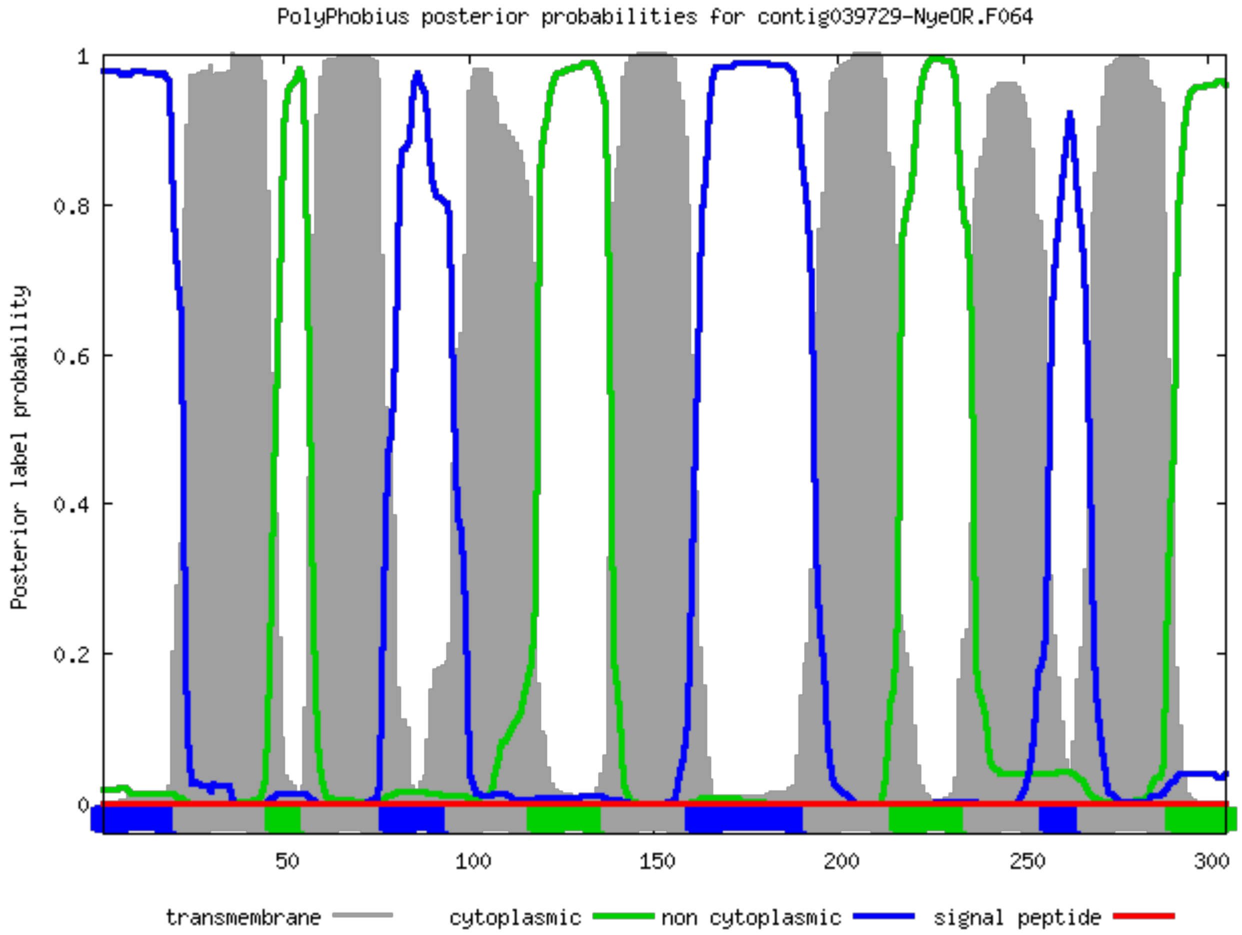

The prediction is based on an alignment. The probability data used in the plot is found here, and the gnuplot script is here.

Prediction of contig039469-TilOR.L152

```
ID   contig039469-TilOR.L152
FT   TOPO_DOM      1     25       NON CYTOPLASMIC.
FT   TRANSMEM     26     50
FT   TOPO_DOM     51     59       CYTOPLASMIC.
FT   TRANSMEM     60     82
FT   TOPO_DOM     83    100       NON CYTOPLASMIC.
FT   TRANSMEM    101    120
FT   TOPO_DOM    121    140       CYTOPLASMIC.
FT   TRANSMEM    141    163
FT   TOPO_DOM    164    199       NON CYTOPLASMIC.
FT   TRANSMEM    200    224
FT   TOPO_DOM    225    237       CYTOPLASMIC.
FT   TRANSMEM    238    260
FT   TOPO_DOM    261    271       NON CYTOPLASMIC.
FT   TRANSMEM    272    292
FT   TOPO_DOM    293    322       CYTOPLASMIC.
//
```

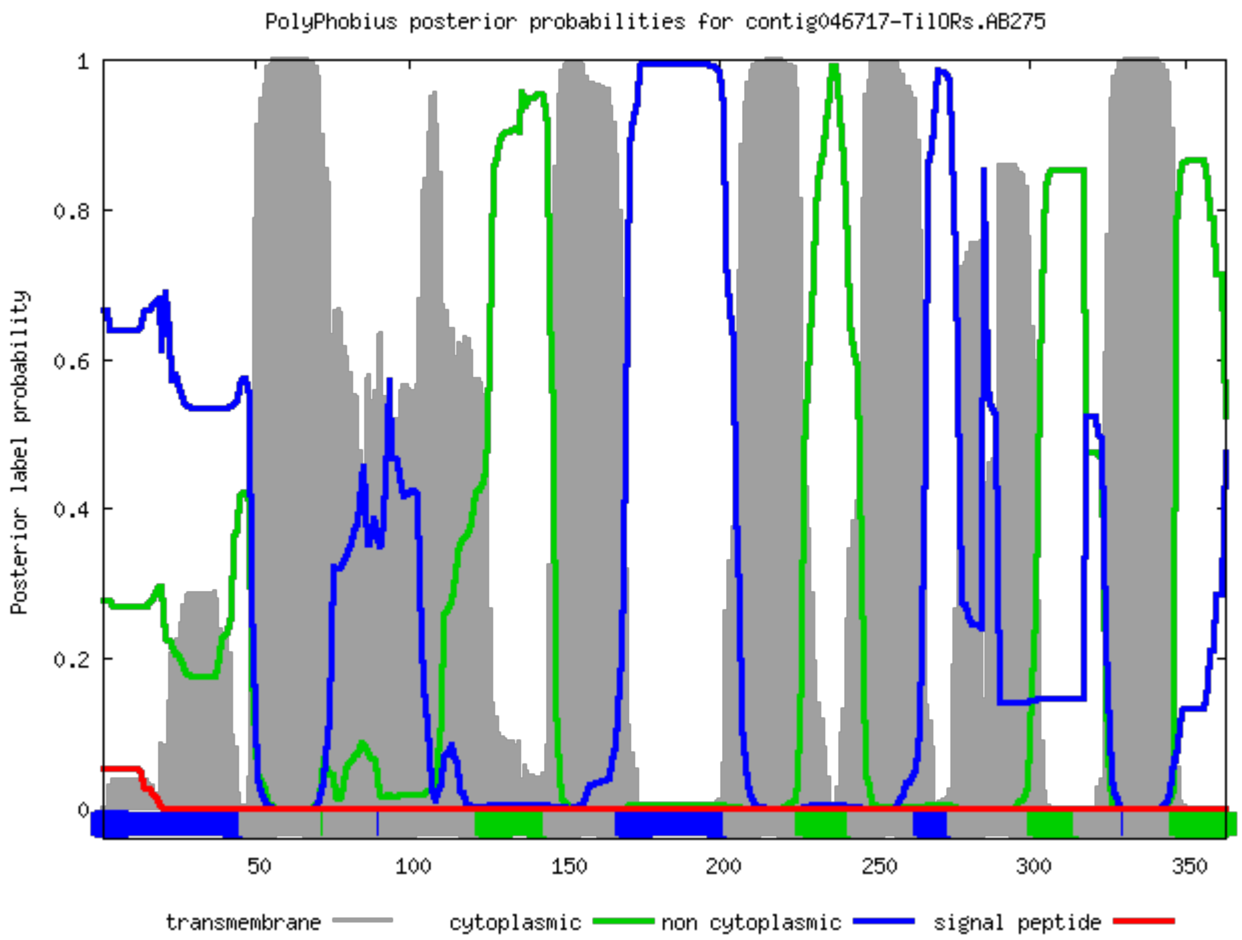

The prediction is based on an alignment. The probability data used in the plot is found here, and the gnuplot script is here.

Prediction of contig039730-NyeOR.H074

```
ID   contig039730-NyeOR.H074
FT   TOPO_DOM      1     22       NON CYTOPLASMIC.
FT   TRANSMEM     23     48
FT   TOPO_DOM     49     55       CYTOPLASMIC.
FT   TRANSMEM     56     76
FT   TOPO_DOM     77     94       NON CYTOPLASMIC.
FT   TRANSMEM     95    117
FT   TOPO_DOM    118    137       CYTOPLASMIC.
FT   TRANSMEM    138    159
FT   TOPO_DOM    160    192       NON CYTOPLASMIC.
FT   TRANSMEM    193    216
FT   TOPO_DOM    217    234       CYTOPLASMIC.
FT   TRANSMEM    235    257
FT   TOPO_DOM    258    268       NON CYTOPLASMIC.
FT   TRANSMEM    269    288
FT   TOPO_DOM    289    314       CYTOPLASMIC.
//
```

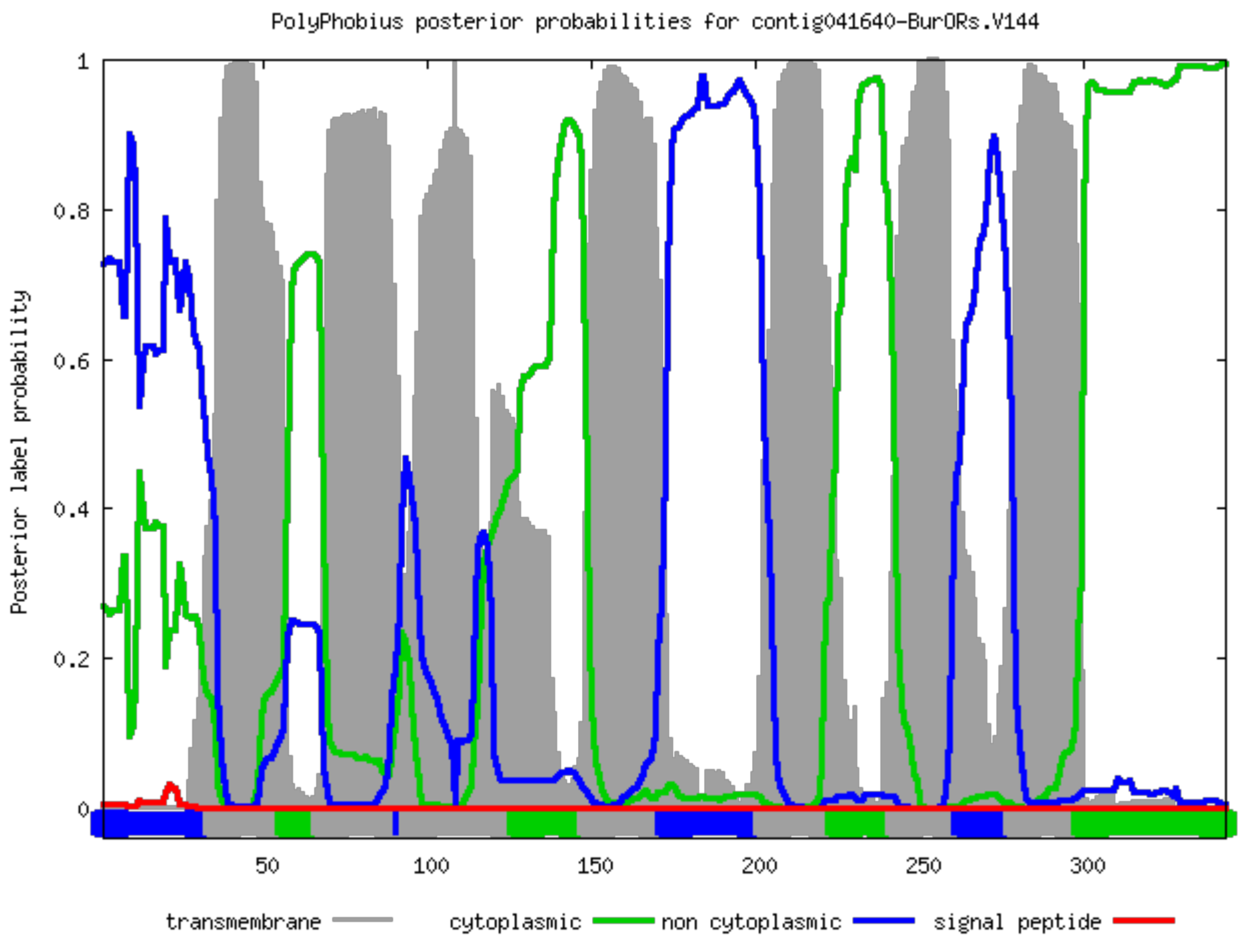

The prediction is based on an alignment. The probability data used in the plot is found here, and the gnuplot script is here.

Prediction of contig034988-NyeOR.A004

```
ID   contig034988-NyeOR.A004
FT   TOPO_DOM      1     22       NON CYTOPLASMIC.
FT   TRANSMEM     23     48
FT   TOPO_DOM     49     56       CYTOPLASMIC.
FT   TRANSMEM     57     77
FT   TOPO_DOM     78     95       NON CYTOPLASMIC.
FT   TRANSMEM     96    118
FT   TOPO_DOM    119    138       CYTOPLASMIC.
FT   TRANSMEM    139    160
FT   TOPO_DOM    161    192       NON CYTOPLASMIC.
FT   TRANSMEM    193    215
FT   TOPO_DOM    216    235       CYTOPLASMIC.
FT   TRANSMEM    236    257
FT   TOPO_DOM    258    268       NON CYTOPLASMIC.
FT   TRANSMEM    269    289
FT   TOPO_DOM    290    306       CYTOPLASMIC.
//
```

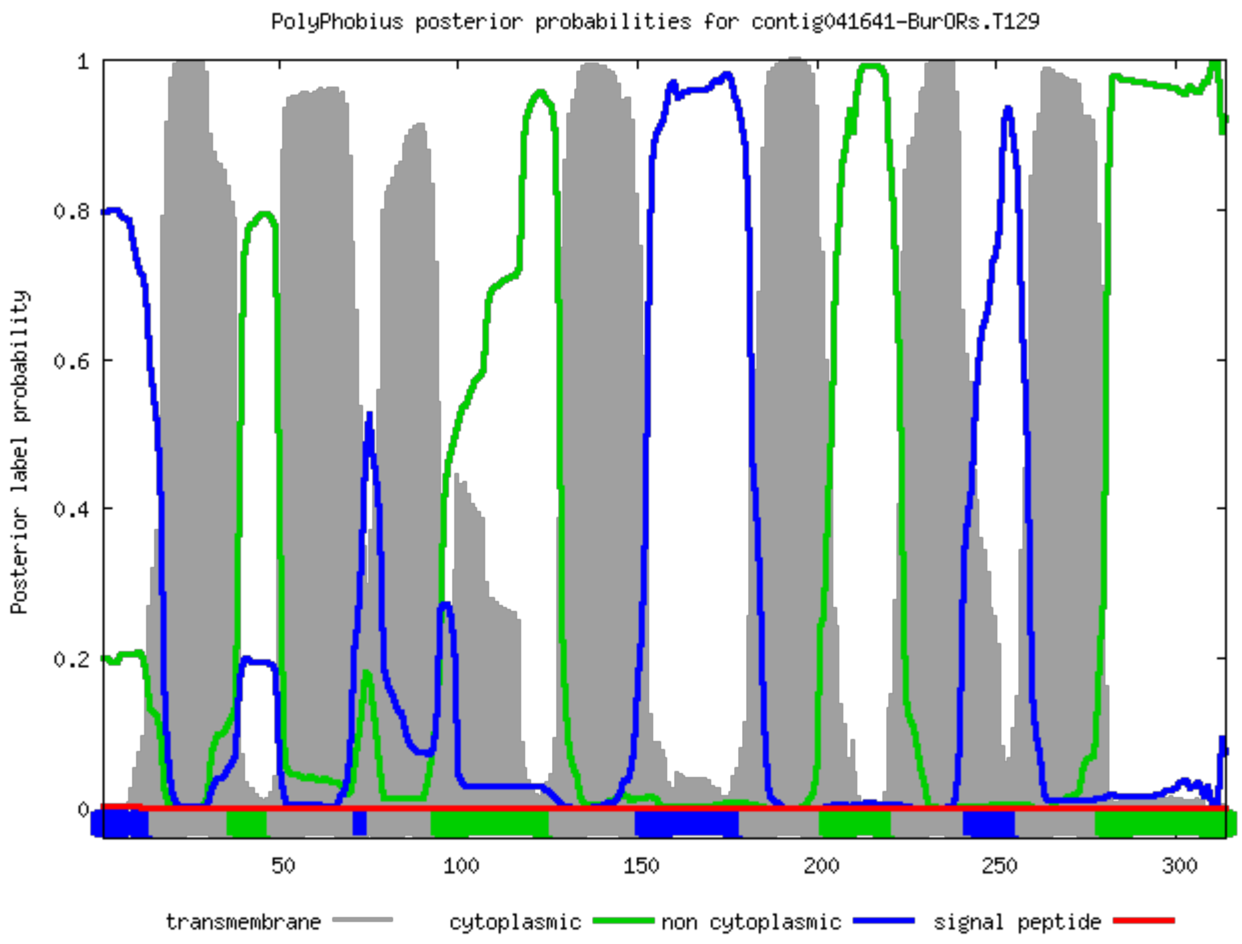

The prediction is based on an alignment. The probability data used in the plot is found here, and the gnuplot script is here.

Prediction of contig047834-TilOR.E084

```
ID   contig047834-TilOR.E084
FT   TOPO_DOM      1     24       NON CYTOPLASMIC.
FT   TRANSMEM     25     50
FT   TOPO_DOM     51     59       CYTOPLASMIC.
FT   TRANSMEM     60     85
FT   TOPO_DOM     86     92       NON CYTOPLASMIC.
FT   TRANSMEM     93    120
FT   TOPO_DOM    121    140       CYTOPLASMIC.
FT   TRANSMEM    141    163
FT   TOPO_DOM    164    195       NON CYTOPLASMIC.
FT   TRANSMEM    196    218
FT   TOPO_DOM    219    238       CYTOPLASMIC.
FT   TRANSMEM    239    258
FT   TOPO_DOM    259    270       NON CYTOPLASMIC.
FT   TRANSMEM    271    293
FT   TOPO_DOM    294    316       CYTOPLASMIC.
//
```

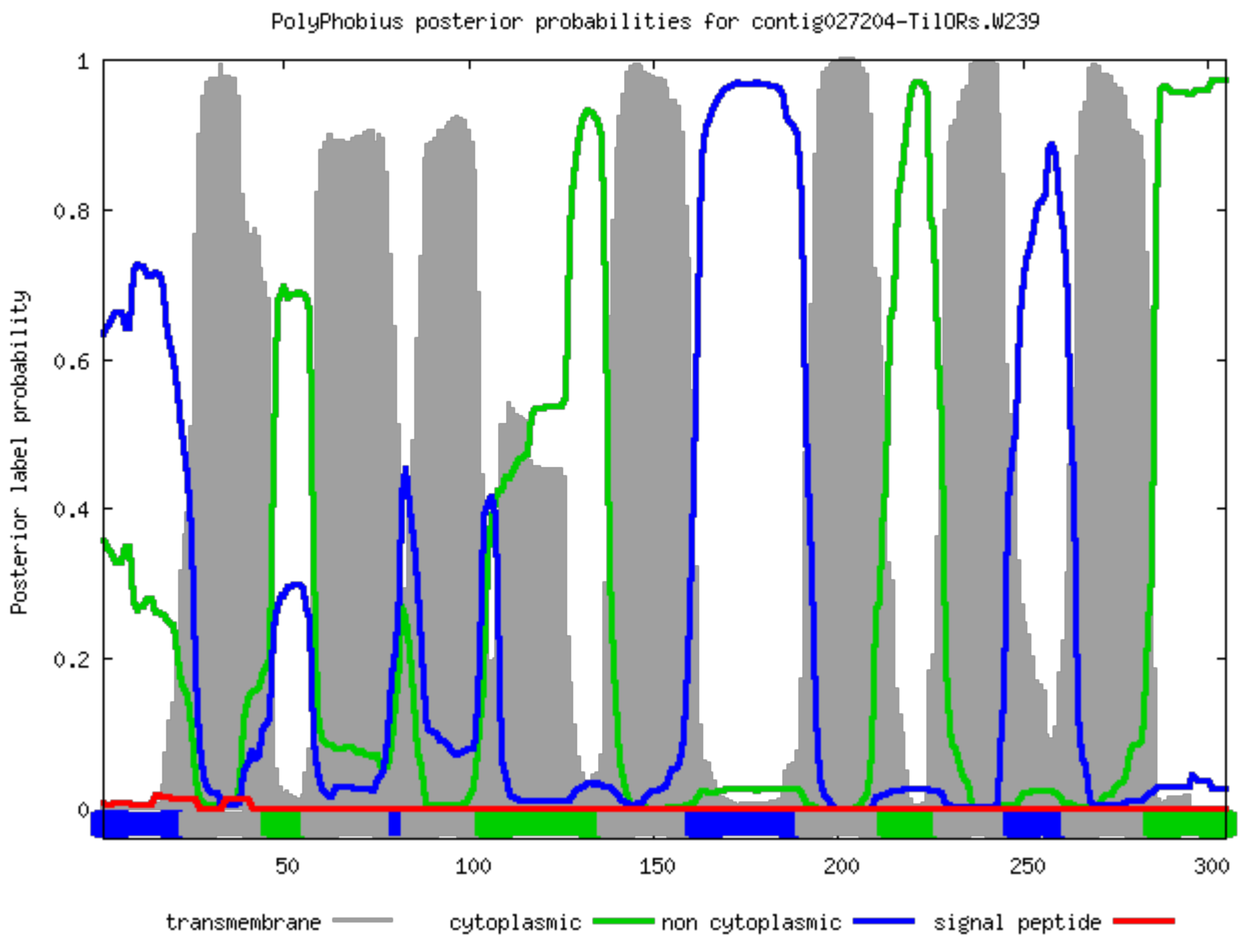

The prediction is based on an alignment. The probability data used in the plot is found here, and the gnuplot script is here.

Prediction of contig013371-TilOR.H117

```
ID    contig013371-TilOR.H117
FT    TOPO_DOM      1     23        NON CYTOPLASMIC.
FT    TRANSMEM     24     49
FT    TOPO_DOM     50     56        CYTOPLASMIC.
FT    TRANSMEM     57     76
FT    TOPO_DOM     77     95        NON CYTOPLASMIC.
FT    TRANSMEM     96    118
FT    TOPO_DOM    119    138        CYTOPLASMIC.
FT    TRANSMEM    139    160
FT    TOPO_DOM    161    196        NON CYTOPLASMIC.
FT    TRANSMEM    197    219
FT    TOPO_DOM    220    237        CYTOPLASMIC.
FT    TRANSMEM    238    260
FT    TOPO_DOM    261    271        NON CYTOPLASMIC.
FT    TRANSMEM    272    291
FT    TOPO_DOM    292    310        CYTOPLASMIC.
//
```

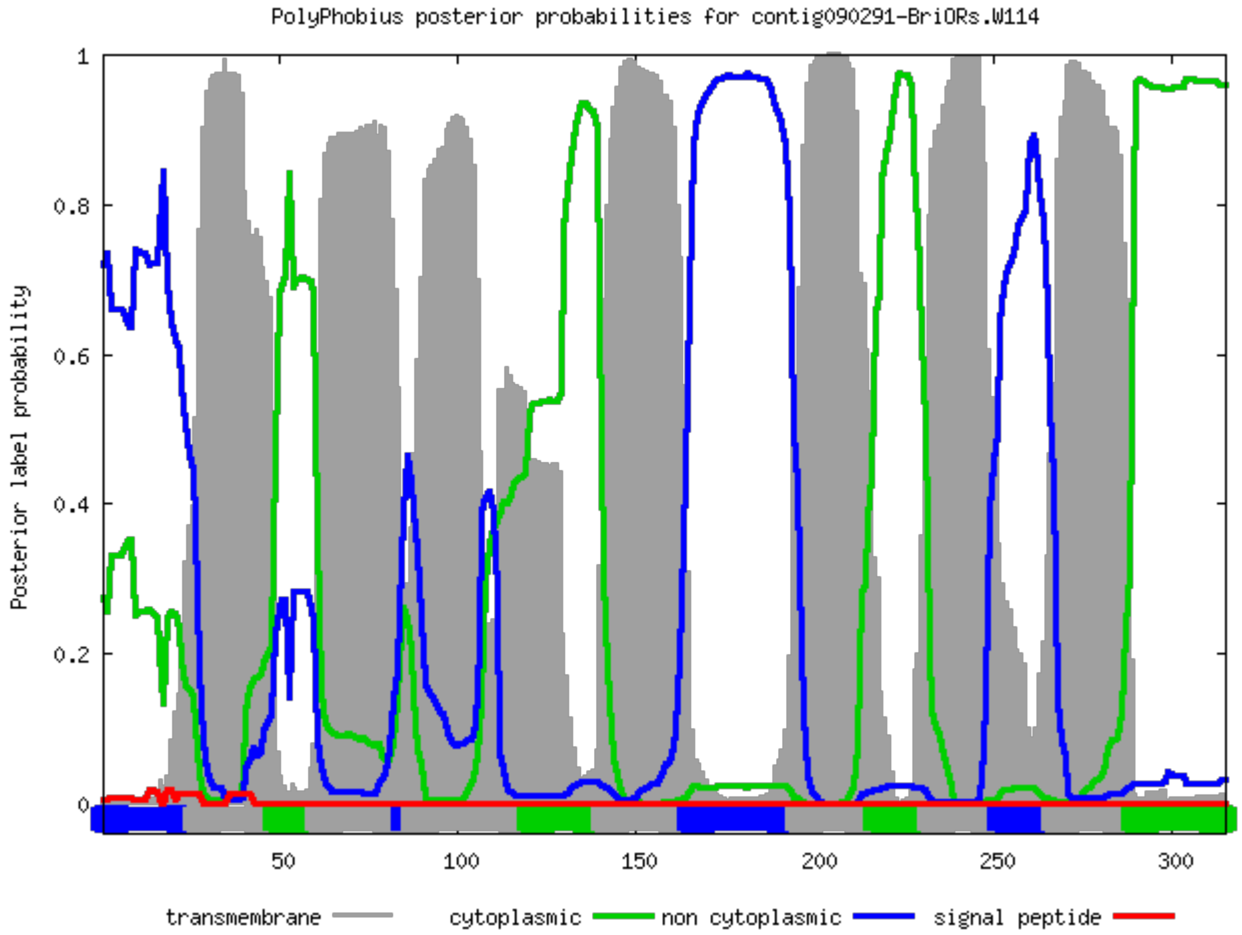

The prediction is based on an alignment. The probability data used in the plot is found here, and the gnuplot script is here.

Prediction of contig039731-NyeOR.D038

```
ID   contig039731-NyeOR.D038
FT   TOPO_DOM      1     25       NON CYTOPLASMIC.
FT   TRANSMEM     26     51
FT   TOPO_DOM     52     60       CYTOPLASMIC.
FT   TRANSMEM     61     84
FT   TOPO_DOM     85     93       NON CYTOPLASMIC.
FT   TRANSMEM     94    121
FT   TOPO_DOM    122    141       CYTOPLASMIC.
FT   TRANSMEM    142    165
FT   TOPO_DOM    166    197       NON CYTOPLASMIC.
FT   TRANSMEM    198    219
FT   TOPO_DOM    220    239       CYTOPLASMIC.
FT   TRANSMEM    240    259
FT   TOPO_DOM    260    271       NON CYTOPLASMIC.
FT   TRANSMEM    272    294
FT   TOPO_DOM    295    312       CYTOPLASMIC.
//
```

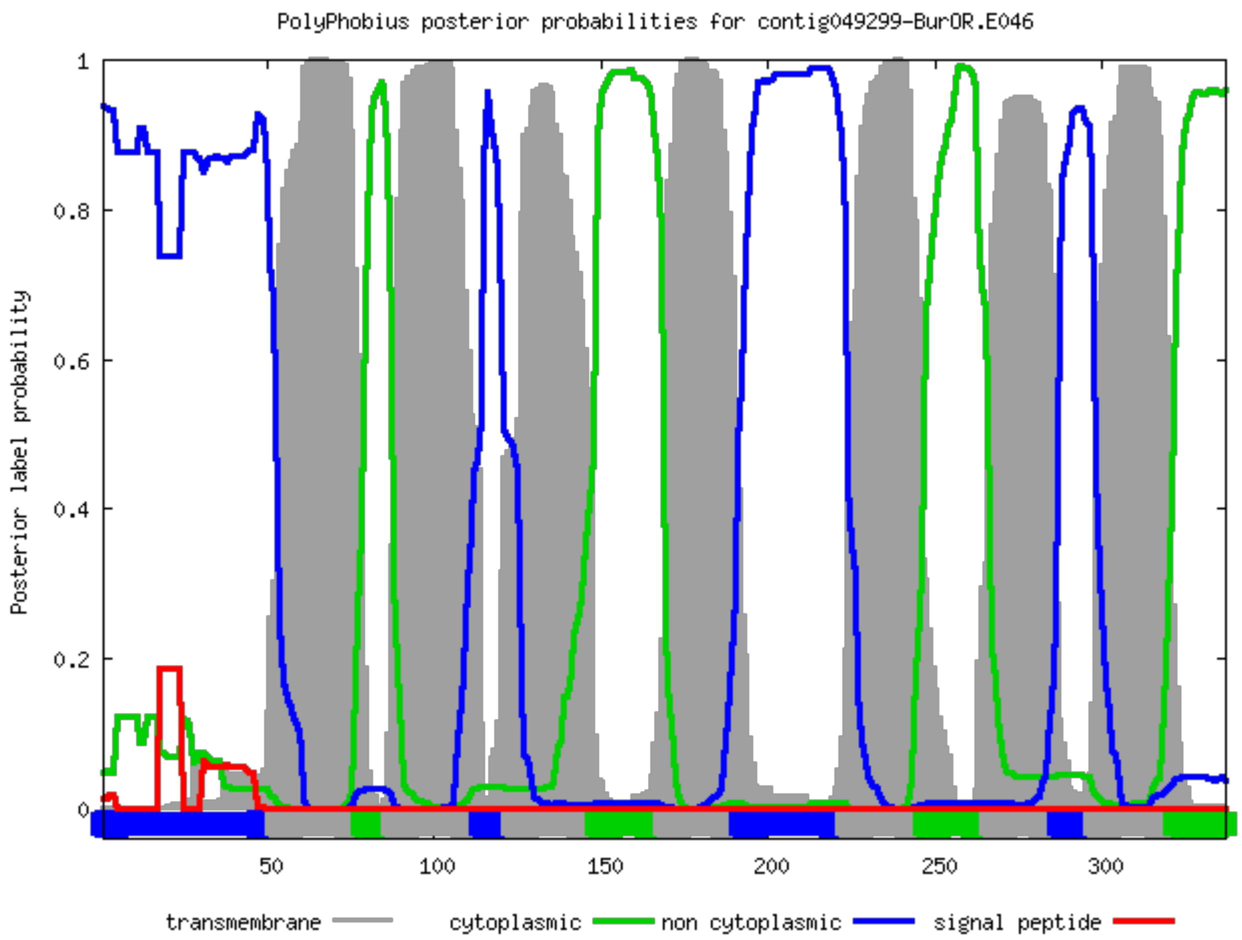

The prediction is based on an alignment. The probability data used in the plot is found here, and the gnuplot script is here.

Prediction of contig052454-BurOR.E050

```
ID   contig052454-BurOR.E050
FT   TOPO_DOM      1      22       NON CYTOPLASMIC.
FT   TRANSMEM     23      48
FT   TOPO_DOM     49      57       CYTOPLASMIC.
FT   TRANSMEM     58      82
FT   TOPO_DOM     83      90       NON CYTOPLASMIC.
FT   TRANSMEM     91     118
FT   TOPO_DOM    119     138       CYTOPLASMIC.
FT   TRANSMEM    139     161
FT   TOPO_DOM    162     193       NON CYTOPLASMIC.
FT   TRANSMEM    194     216
FT   TOPO_DOM    217     236       CYTOPLASMIC.
FT   TRANSMEM    237     256
FT   TOPO_DOM    257     267       NON CYTOPLASMIC.
FT   TRANSMEM    268     291
FT   TOPO_DOM    292     309       CYTOPLASMIC.
//
```

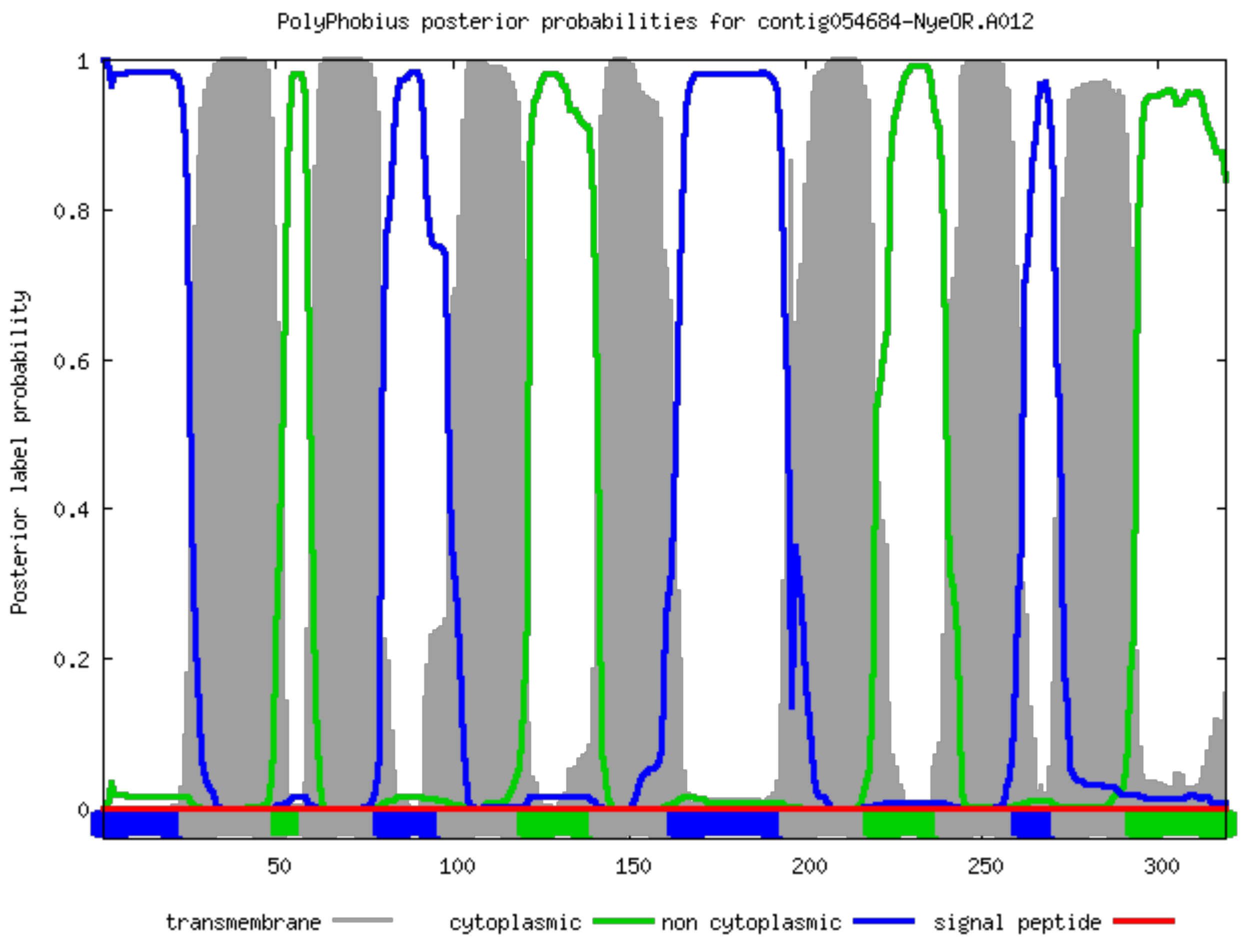

The prediction is based on an alignment. The probability data used in the plot is found here, and the gnuplot script is here.

Prediction of contig054687-NyeOR.A013

```
ID   contig054687-NyeOR.A013
FT   TOPO_DOM      1     22       NON CYTOPLASMIC.
FT   TRANSMEM     23     48
FT   TOPO_DOM     49     56       CYTOPLASMIC.
FT   TRANSMEM     57     77
FT   TOPO_DOM     78     95       NON CYTOPLASMIC.
FT   TRANSMEM     96    118
FT   TOPO_DOM    119    138       CYTOPLASMIC.
FT   TRANSMEM    139    159
FT   TOPO_DOM    160    192       NON CYTOPLASMIC.
FT   TRANSMEM    193    215
FT   TOPO_DOM    216    235       CYTOPLASMIC.
FT   TRANSMEM    236    257
FT   TOPO_DOM    258    268       NON CYTOPLASMIC.
FT   TRANSMEM    269    289
FT   TOPO_DOM    290    309       CYTOPLASMIC.
//
```

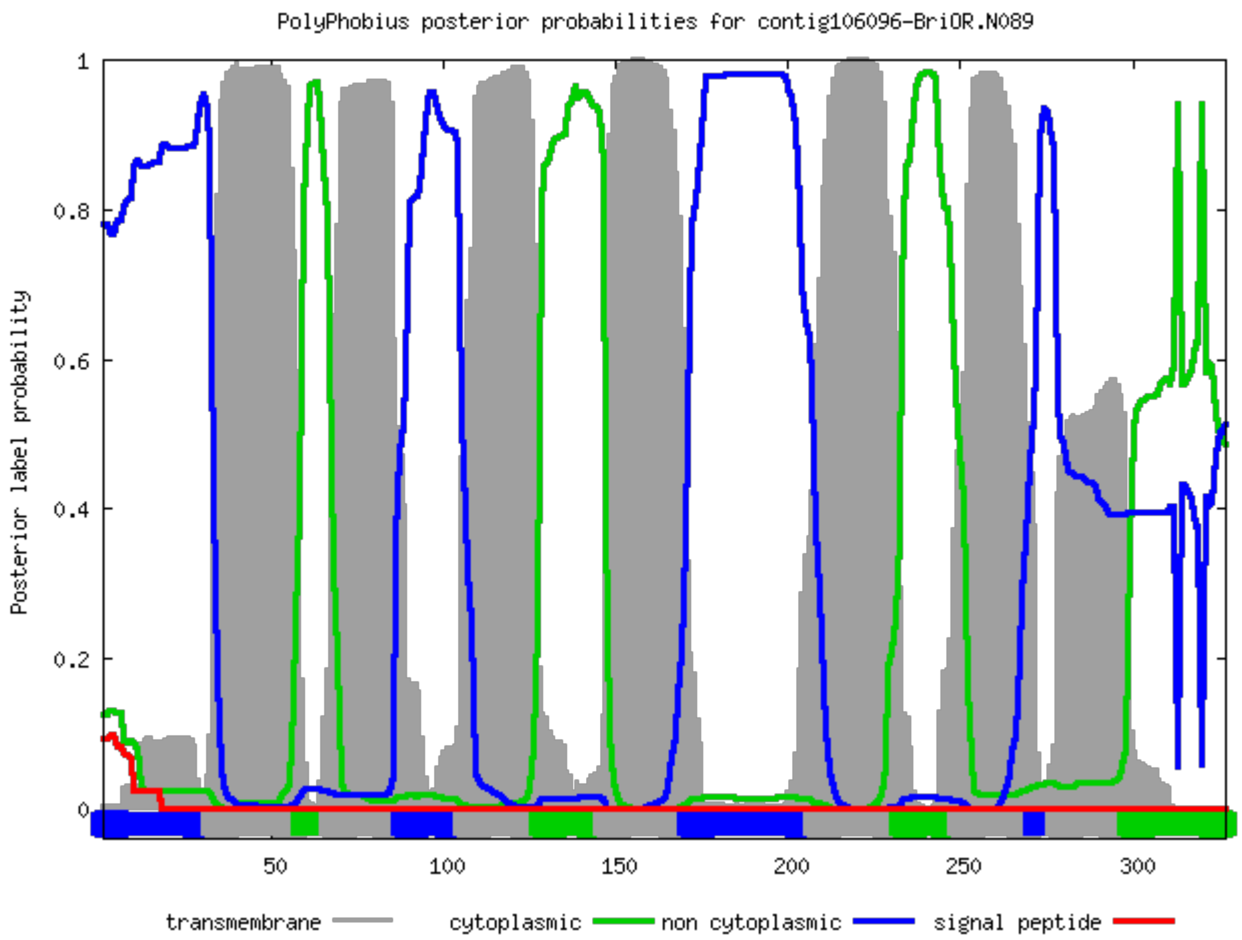

The prediction is based on an alignment. The probability data used in the plot is found here, and the gnuplot script is here.

Prediction of contig048882-BurOR.H064

```
ID   contig048882-BurOR.H064
FT   TOPO_DOM      1      23       NON CYTOPLASMIC.
FT   TRANSMEM     24      49
FT   TOPO_DOM     50      56       CYTOPLASMIC.
FT   TRANSMEM     57      76
FT   TOPO_DOM     77      95       NON CYTOPLASMIC.
FT   TRANSMEM     96     118
FT   TOPO_DOM    119     138       CYTOPLASMIC.
FT   TRANSMEM    139     160
FT   TOPO_DOM    161     196       NON CYTOPLASMIC.
FT   TRANSMEM    197     219
FT   TOPO_DOM    220     237       CYTOPLASMIC.
FT   TRANSMEM    238     259
FT   TOPO_DOM    260     271       NON CYTOPLASMIC.
FT   TRANSMEM    272     291
FT   TOPO_DOM    292     338       CYTOPLASMIC.
//
```

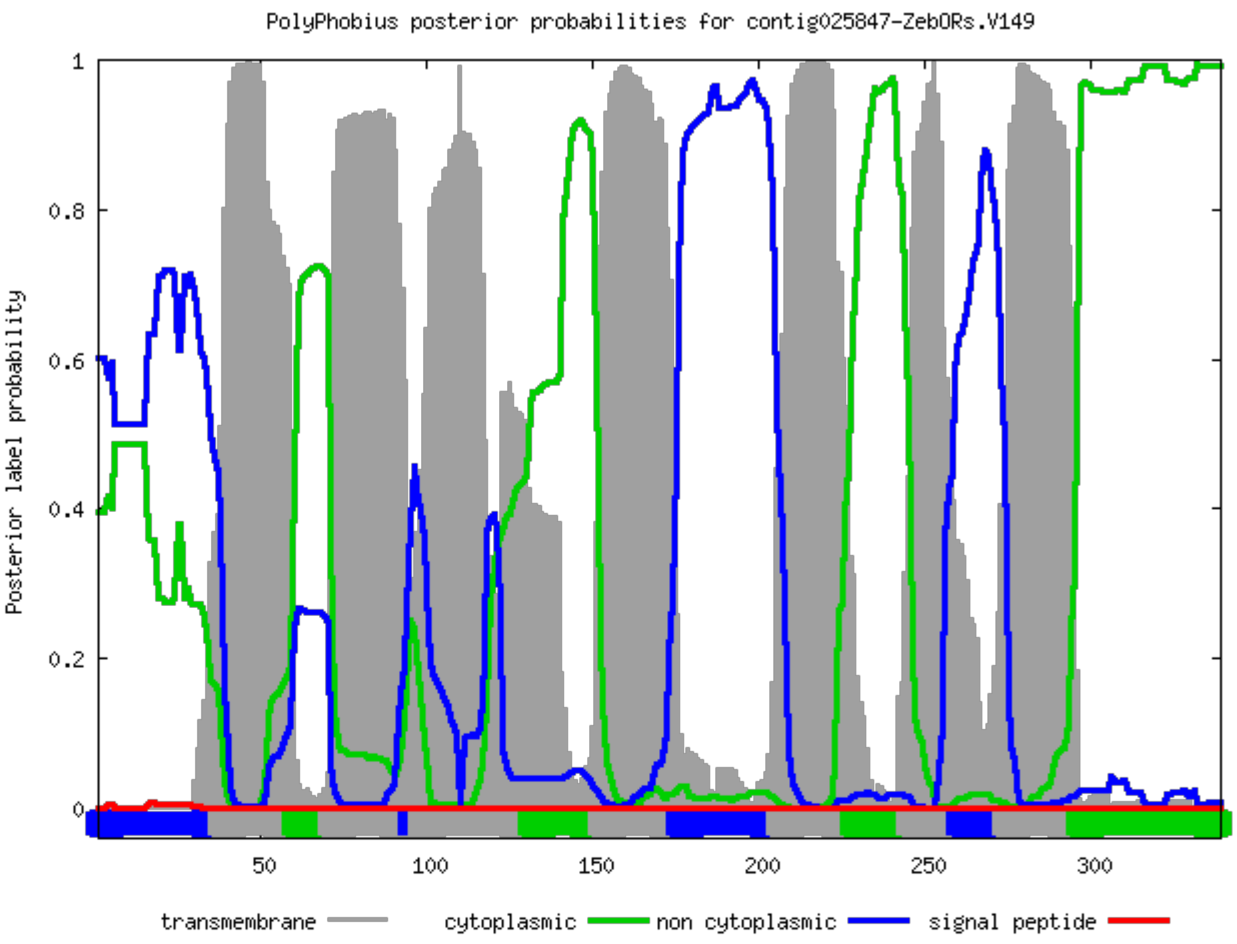

The prediction is based on an alignment. The probability data used in the plot is found here, and the gnuplot script is here.

Prediction of contig053886-ZebOR.B034

```
ID   contig053886-ZebOR.B034
FT   TOPO_DOM      1     28       NON CYTOPLASMIC.
FT   TRANSMEM     29     54
FT   TOPO_DOM     55     62       CYTOPLASMIC.
FT   TRANSMEM     63     83
FT   TOPO_DOM     84    103       NON CYTOPLASMIC.
FT   TRANSMEM    104    126
FT   TOPO_DOM    127    146       CYTOPLASMIC.
FT   TRANSMEM    147    168
FT   TOPO_DOM    169    203       NON CYTOPLASMIC.
FT   TRANSMEM    204    226
FT   TOPO_DOM    227    246       CYTOPLASMIC.
FT   TRANSMEM    247    268
FT   TOPO_DOM    269    277       NON CYTOPLASMIC.
FT   TRANSMEM    278    300
FT   TOPO_DOM    301    320       CYTOPLASMIC.
//
```

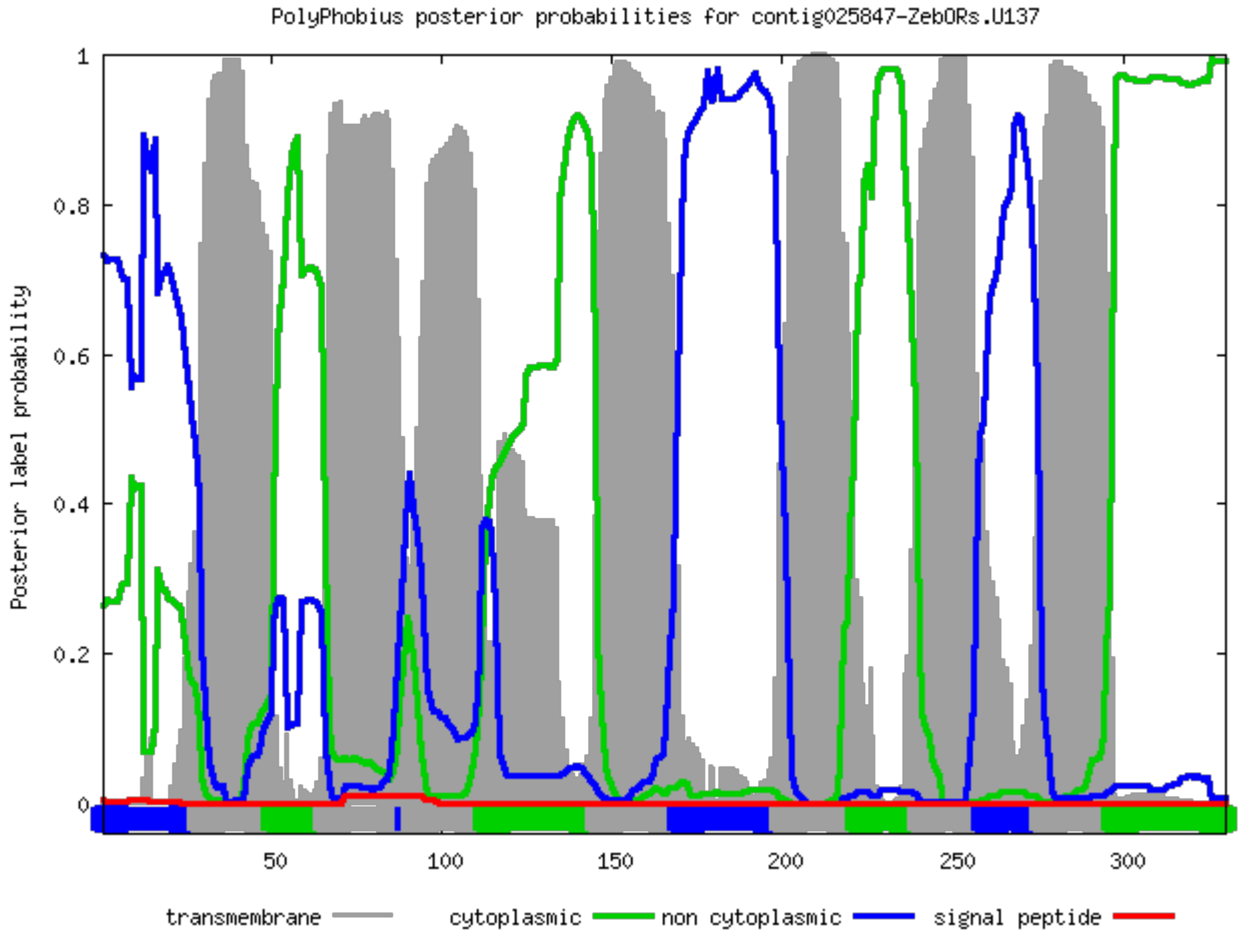

The prediction is based on an alignment. The probability data used in the plot is found here, and the gnuplot script is here.

Prediction of contig040506-NyeOR.J140

```
ID   contig040506-NyeOR.J140
FT   TOPO_DOM      1     23       NON CYTOPLASMIC.
FT   TRANSMEM     24     49
FT   TOPO_DOM     50     59       CYTOPLASMIC.
FT   TRANSMEM     60     81
FT   TOPO_DOM     82     97       NON CYTOPLASMIC.
FT   TRANSMEM     98    119
FT   TOPO_DOM    120    139       CYTOPLASMIC.
FT   TRANSMEM    140    162
FT   TOPO_DOM    163    194       NON CYTOPLASMIC.
FT   TRANSMEM    195    219
FT   TOPO_DOM    220    237       CYTOPLASMIC.
FT   TRANSMEM    238    260
FT   TOPO_DOM    261    270       NON CYTOPLASMIC.
FT   TRANSMEM    271    291
FT   TOPO_DOM    292    312       CYTOPLASMIC.
//
```

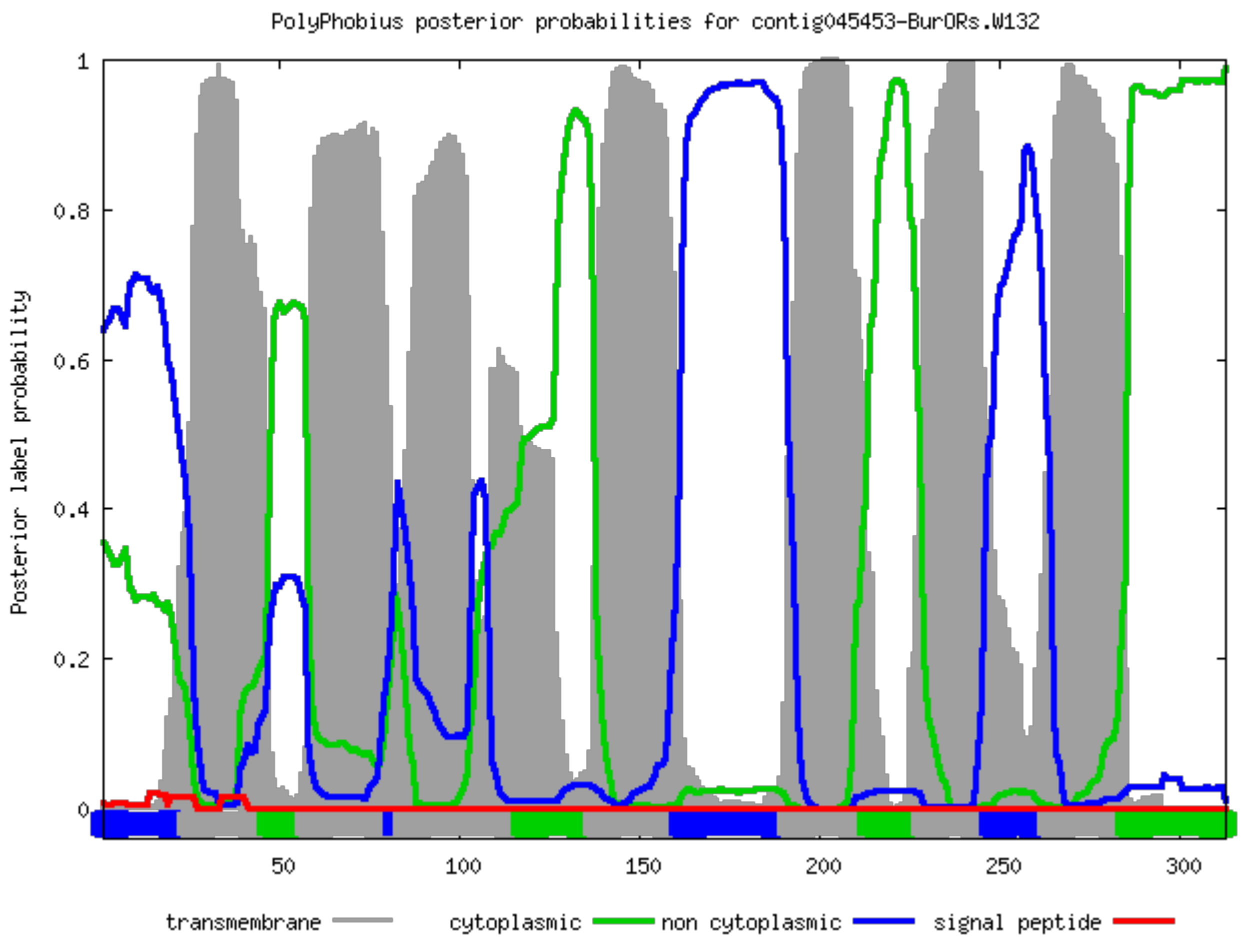

The prediction is based on an alignment. The probability data used in the plot is found here, and the gnuplot script is here.

Prediction of contig039428-TilOR.S227

```
ID   contig039428-TilOR.S227
FT   TOPO_DOM      1     19       NON CYTOPLASMIC.
FT   TRANSMEM     20     41
FT   TOPO_DOM     42     52       CYTOPLASMIC.
FT   TRANSMEM     53     76
FT   TOPO_DOM     77     81       NON CYTOPLASMIC.
FT   TRANSMEM     82    110
FT   TOPO_DOM    111    130       CYTOPLASMIC.
FT   TRANSMEM    131    156
FT   TOPO_DOM    157    184       NON CYTOPLASMIC.
FT   TRANSMEM    185    205
FT   TOPO_DOM    206    225       CYTOPLASMIC.
FT   TRANSMEM    226    251
FT   TOPO_DOM    252    262       NON CYTOPLASMIC.
FT   TRANSMEM    263    283
FT   TOPO_DOM    284    304       CYTOPLASMIC.
//
```

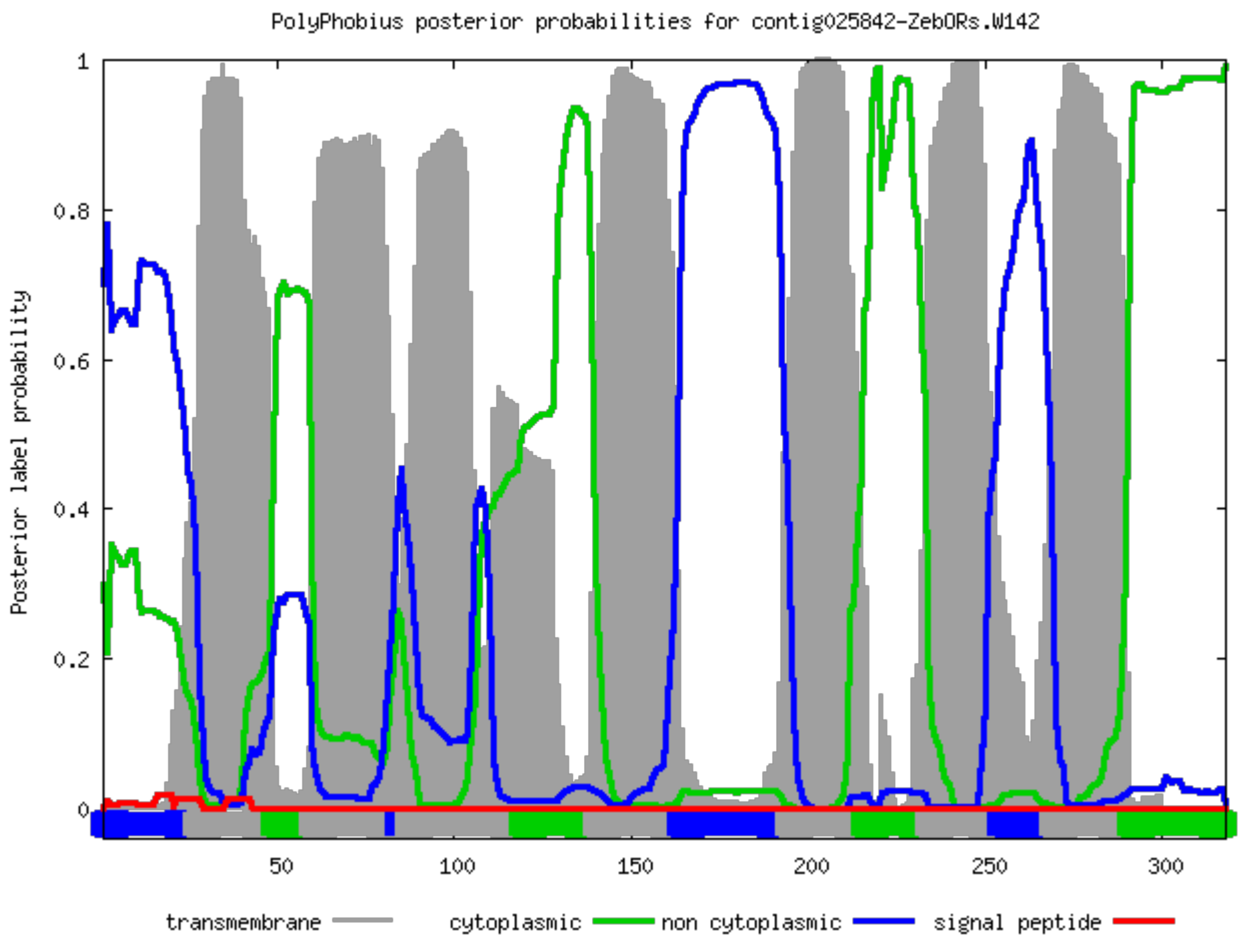

The prediction is based on an alignment. The probability data used in the plot is found here, and the gnuplot script is here.

Prediction of contig039419-TilOR.S224

```
ID   contig039419-TilOR.S224
FT   TOPO_DOM      1     20       NON CYTOPLASMIC.
FT   TRANSMEM     21     42
FT   TOPO_DOM     43     53       CYTOPLASMIC.
FT   TRANSMEM     54     77
FT   TOPO_DOM     78     82       NON CYTOPLASMIC.
FT   TRANSMEM     83    111
FT   TOPO_DOM    112    131       CYTOPLASMIC.
FT   TRANSMEM    132    157
FT   TOPO_DOM    158    185       NON CYTOPLASMIC.
FT   TRANSMEM    186    206
FT   TOPO_DOM    207    226       CYTOPLASMIC.
FT   TRANSMEM    227    252
FT   TOPO_DOM    253    263       NON CYTOPLASMIC.
FT   TRANSMEM    264    284
FT   TOPO_DOM    285    305       CYTOPLASMIC.
//
```

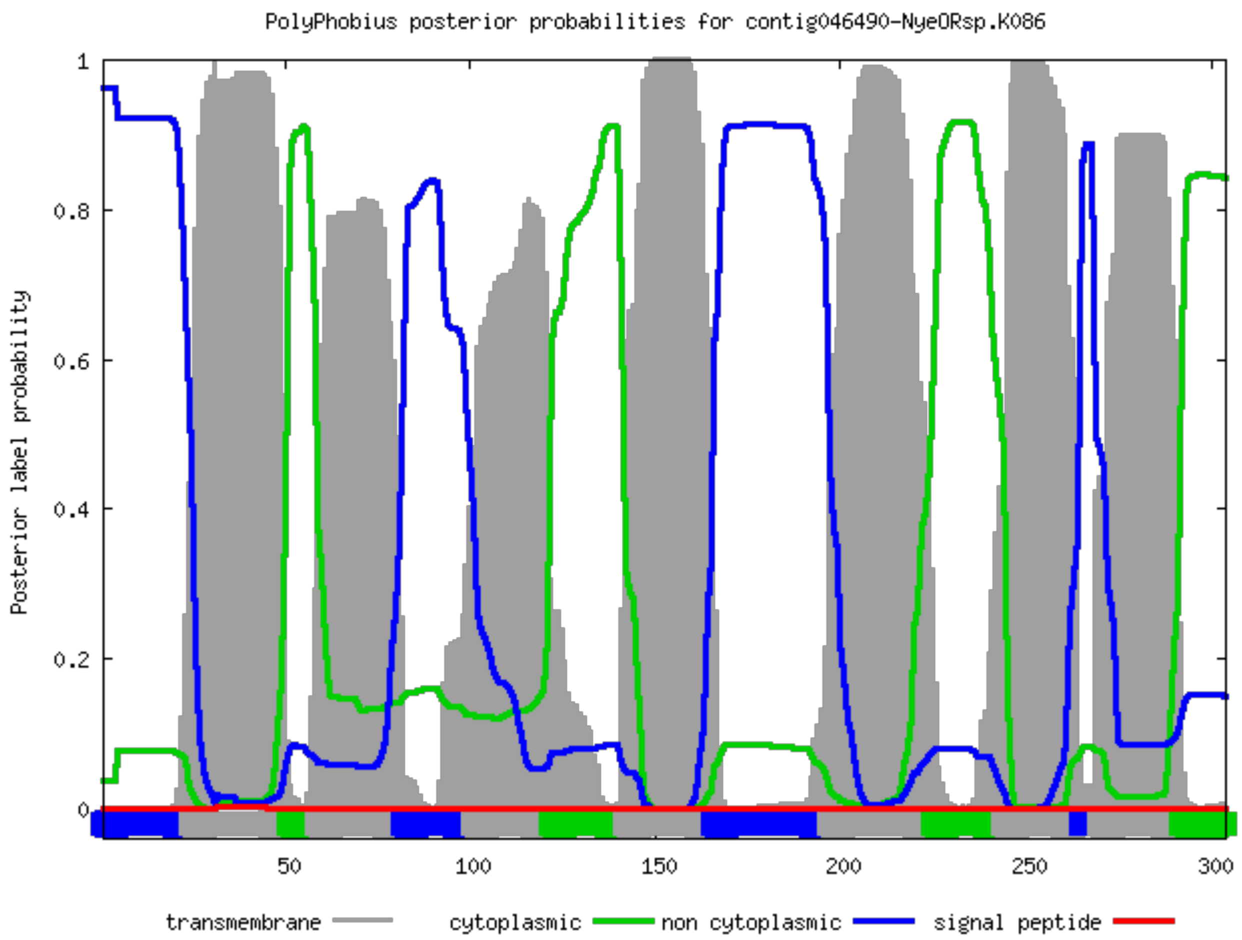

The prediction is based on an alignment. The probability data used in the plot is found here, and the gnuplot script is here.

Prediction of contig004259-BriOR.E038

```
ID   contig004259-BriOR.E038
FT   TOPO_DOM      1     21       NON CYTOPLASMIC.
FT   TRANSMEM     22     47
FT   TOPO_DOM     48     56       CYTOPLASMIC.
FT   TRANSMEM     57     82
FT   TOPO_DOM     83     91       NON CYTOPLASMIC.
FT   TRANSMEM     92    117
FT   TOPO_DOM    118    137       CYTOPLASMIC.
FT   TRANSMEM    138    159
FT   TOPO_DOM    160    192       NON CYTOPLASMIC.
FT   TRANSMEM    193    215
FT   TOPO_DOM    216    235       CYTOPLASMIC.
FT   TRANSMEM    236    255
FT   TOPO_DOM    256    266       NON CYTOPLASMIC.
FT   TRANSMEM    267    290
FT   TOPO_DOM    291    310       CYTOPLASMIC.
//
```

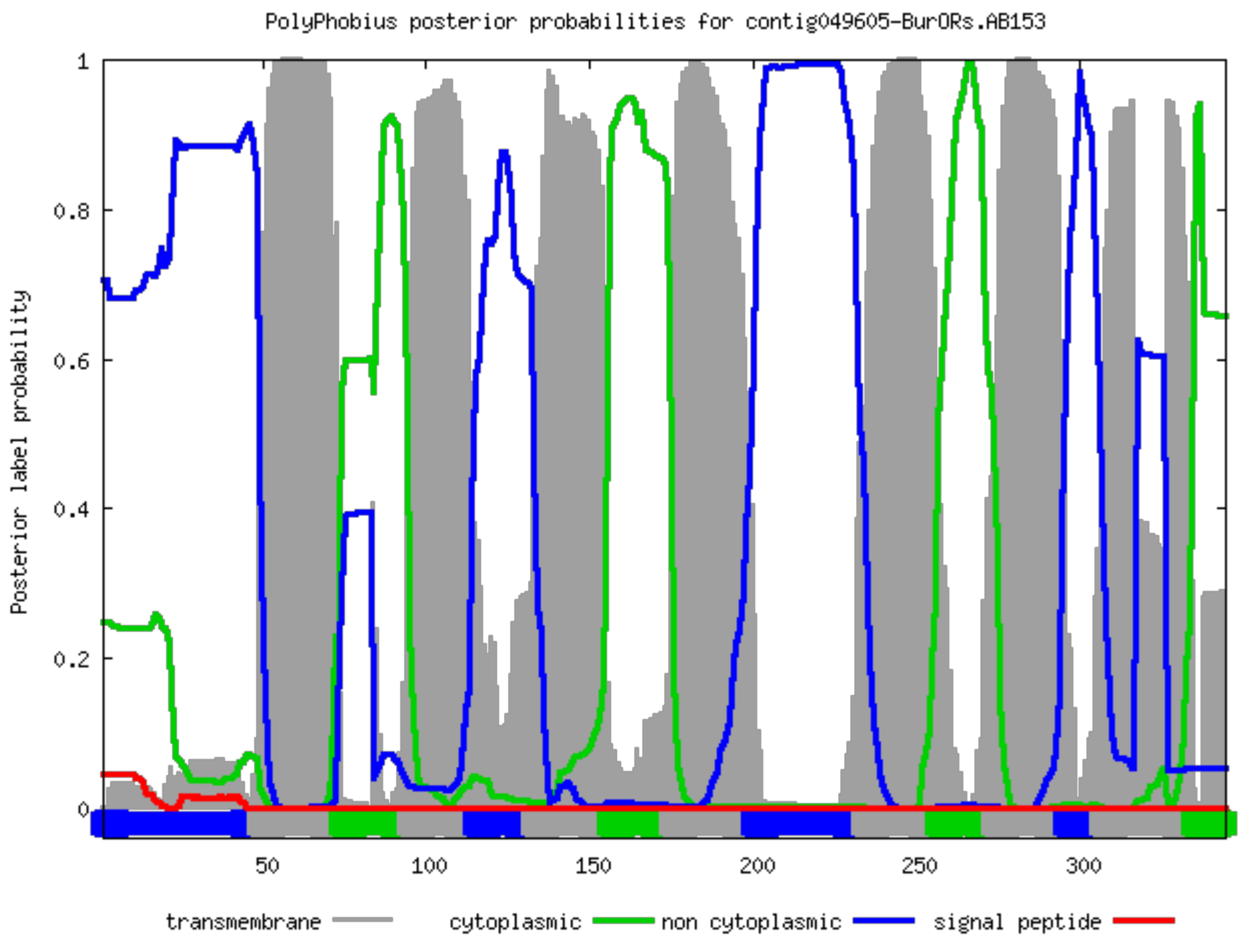

The prediction is based on an alignment. The probability data used in the plot is found here, and the gnuplot script is here.

Prediction of contig021011-NyeOR.Q134

```
ID    contig021011-NyeOR.Q134
FT    TOPO_DOM      1     17       NON CYTOPLASMIC.
FT    TRANSMEM     18     40
FT    TOPO_DOM     41     51       CYTOPLASMIC.
FT    TRANSMEM     52     76
FT    TOPO_DOM     77     81       NON CYTOPLASMIC.
FT    TRANSMEM     82    110
FT    TOPO_DOM    111    130       CYTOPLASMIC.
FT    TRANSMEM    131    154
FT    TOPO_DOM    155    183       NON CYTOPLASMIC.
FT    TRANSMEM    184    206
FT    TOPO_DOM    207    224       CYTOPLASMIC.
FT    TRANSMEM    225    246
FT    TOPO_DOM    247    259       NON CYTOPLASMIC.
FT    TRANSMEM    260    281
FT    TOPO_DOM    282    310       CYTOPLASMIC.
//
```

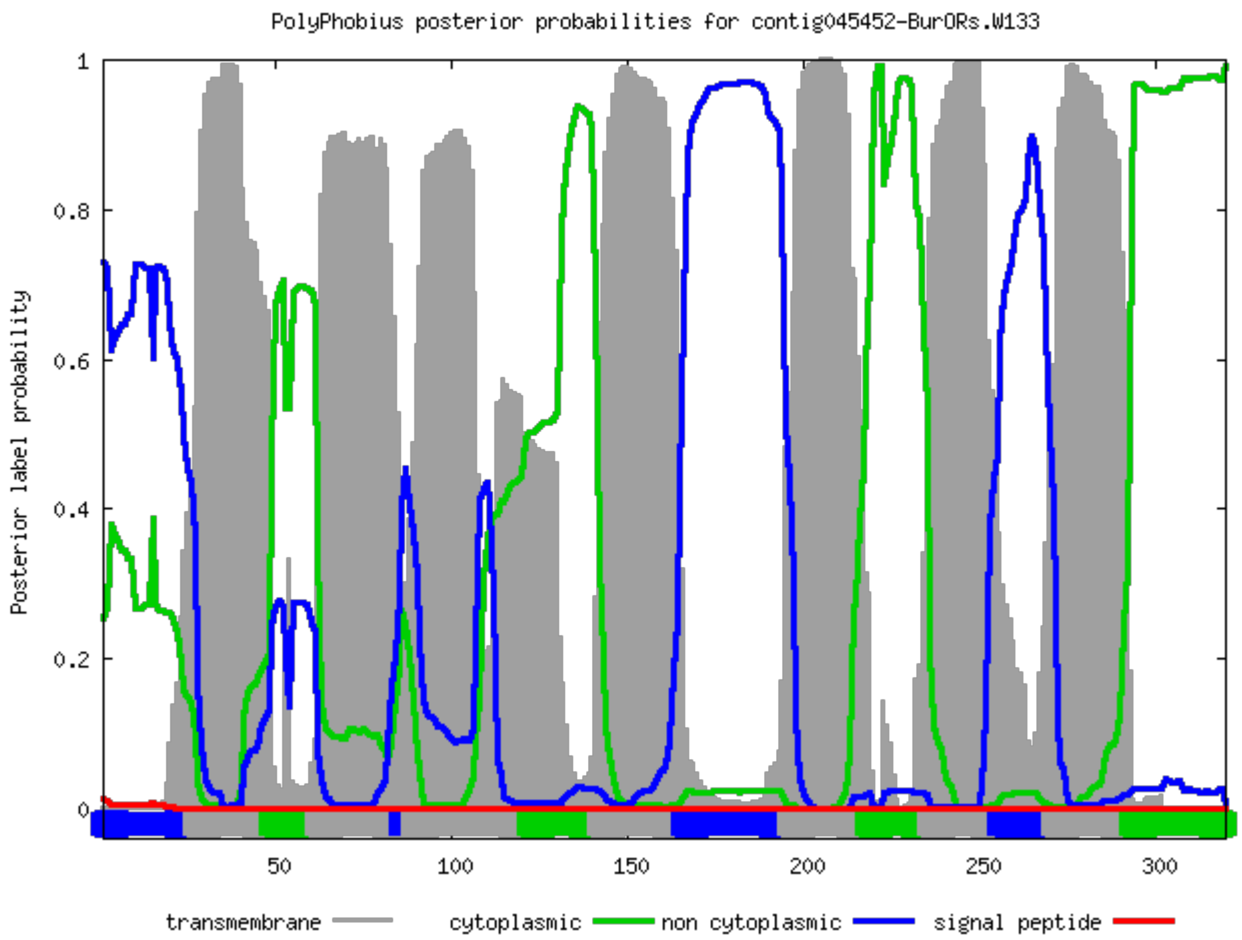

The prediction is based on an alignment. The probability data used in the plot is found here, and the gnuplot script is here.

Prediction of contig049298-BurOR.E045

```
ID    contig049298-BurOR.E045
FT    TOPO_DOM      1      22       NON CYTOPLASMIC.
FT    TRANSMEM     23      48
FT    TOPO_DOM     49      57       CYTOPLASMIC.
FT    TRANSMEM     58      82
FT    TOPO_DOM     83      92       NON CYTOPLASMIC.
FT    TRANSMEM     93     118
FT    TOPO_DOM    119     138       CYTOPLASMIC.
FT    TRANSMEM    139     161
FT    TOPO_DOM    162     193       NON CYTOPLASMIC.
FT    TRANSMEM    194     216
FT    TOPO_DOM    217     236       CYTOPLASMIC.
FT    TRANSMEM    237     256
FT    TOPO_DOM    257     267       NON CYTOPLASMIC.
FT    TRANSMEM    268     291
FT    TOPO_DOM    292     314       CYTOPLASMIC.
//
```

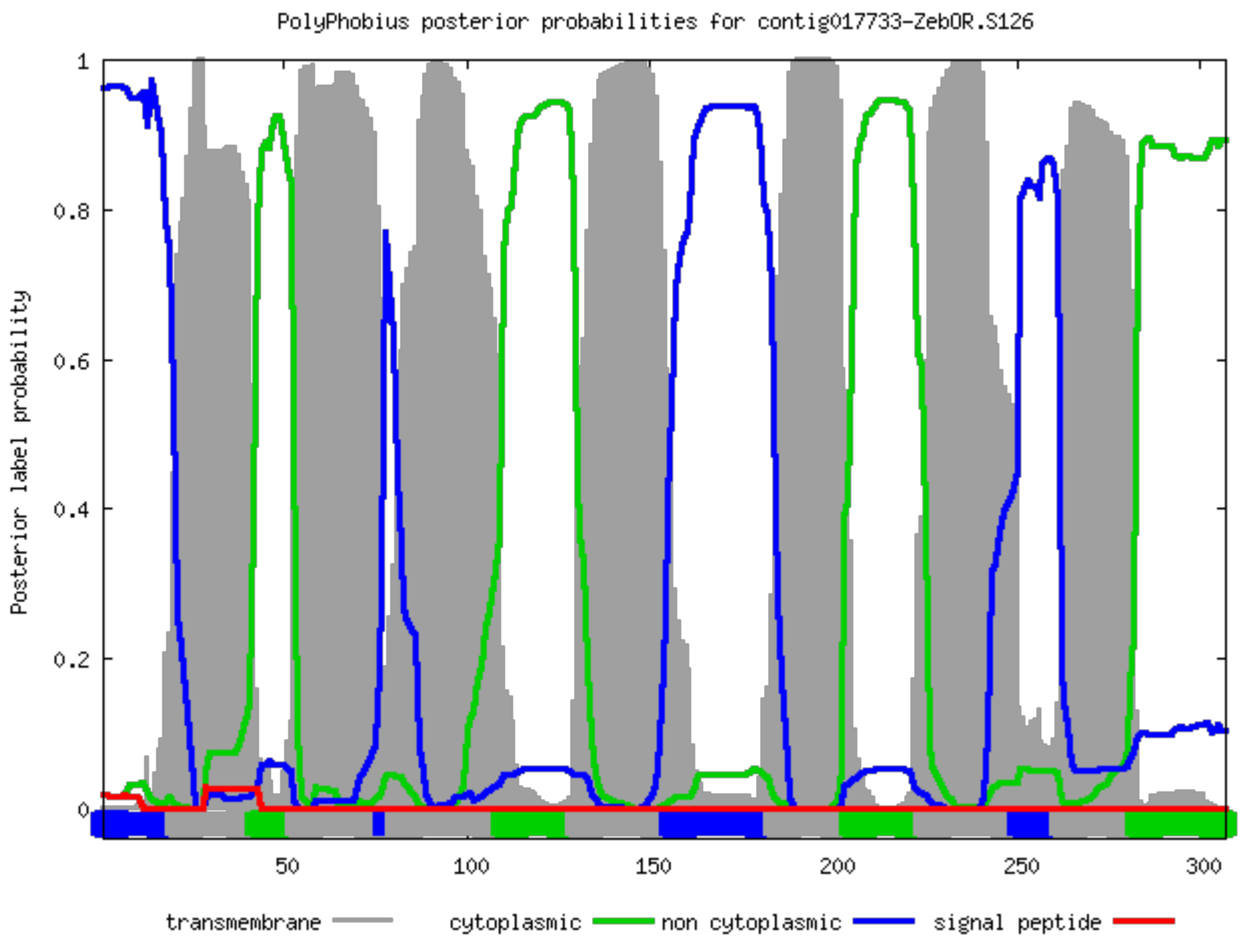

The prediction is based on an alignment. The probability data used in the plot is found here, and the gnuplot script is here.

Prediction of contig055924-NyeOR.N109

```
ID    contig055924-NyeOR.N109
FT    TOPO_DOM      1      32       NON CYTOPLASMIC.
FT    TRANSMEM     33      58
FT    TOPO_DOM     59      66       CYTOPLASMIC.
FT    TRANSMEM     67      86
FT    TOPO_DOM     87     104       NON CYTOPLASMIC.
FT    TRANSMEM    105     127
FT    TOPO_DOM    128     146       CYTOPLASMIC.
FT    TRANSMEM    147     170
FT    TOPO_DOM    171     206       NON CYTOPLASMIC.
FT    TRANSMEM    207     232
FT    TOPO_DOM    233     250       CYTOPLASMIC.
FT    TRANSMEM    251     271
FT    TOPO_DOM    272     277       NON CYTOPLASMIC.
FT    TRANSMEM    278     298
FT    TOPO_DOM    299     324       CYTOPLASMIC.
//
```

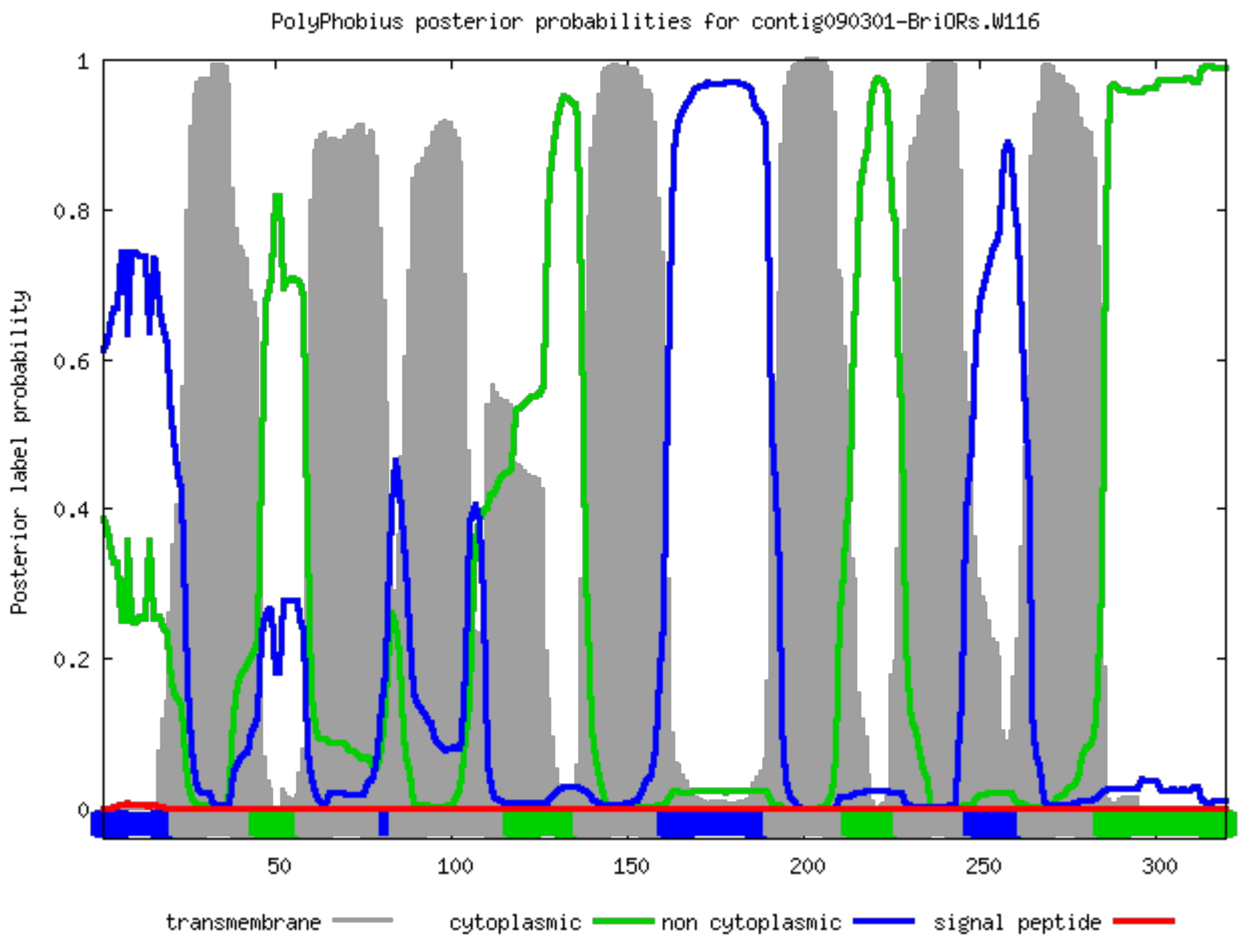

The prediction is based on an alignment. The probability data used in the plot is found here, and the gnuplot script is here.

Prediction of contig010714-ZebOR.N109

```
ID    contig010714-ZebOR.N109
FT    TOPO_DOM      1     32       NON CYTOPLASMIC.
FT    TRANSMEM     33     58
FT    TOPO_DOM     59     66       CYTOPLASMIC.
FT    TRANSMEM     67     86
FT    TOPO_DOM     87    104       NON CYTOPLASMIC.
FT    TRANSMEM    105    127
FT    TOPO_DOM    128    146       CYTOPLASMIC.
FT    TRANSMEM    147    170
FT    TOPO_DOM    171    207       NON CYTOPLASMIC.
FT    TRANSMEM    208    232
FT    TOPO_DOM    233    250       CYTOPLASMIC.
FT    TRANSMEM    251    272
FT    TOPO_DOM    273    277       NON CYTOPLASMIC.
FT    TRANSMEM    278    298
FT    TOPO_DOM    299    324       CYTOPLASMIC.
//
```

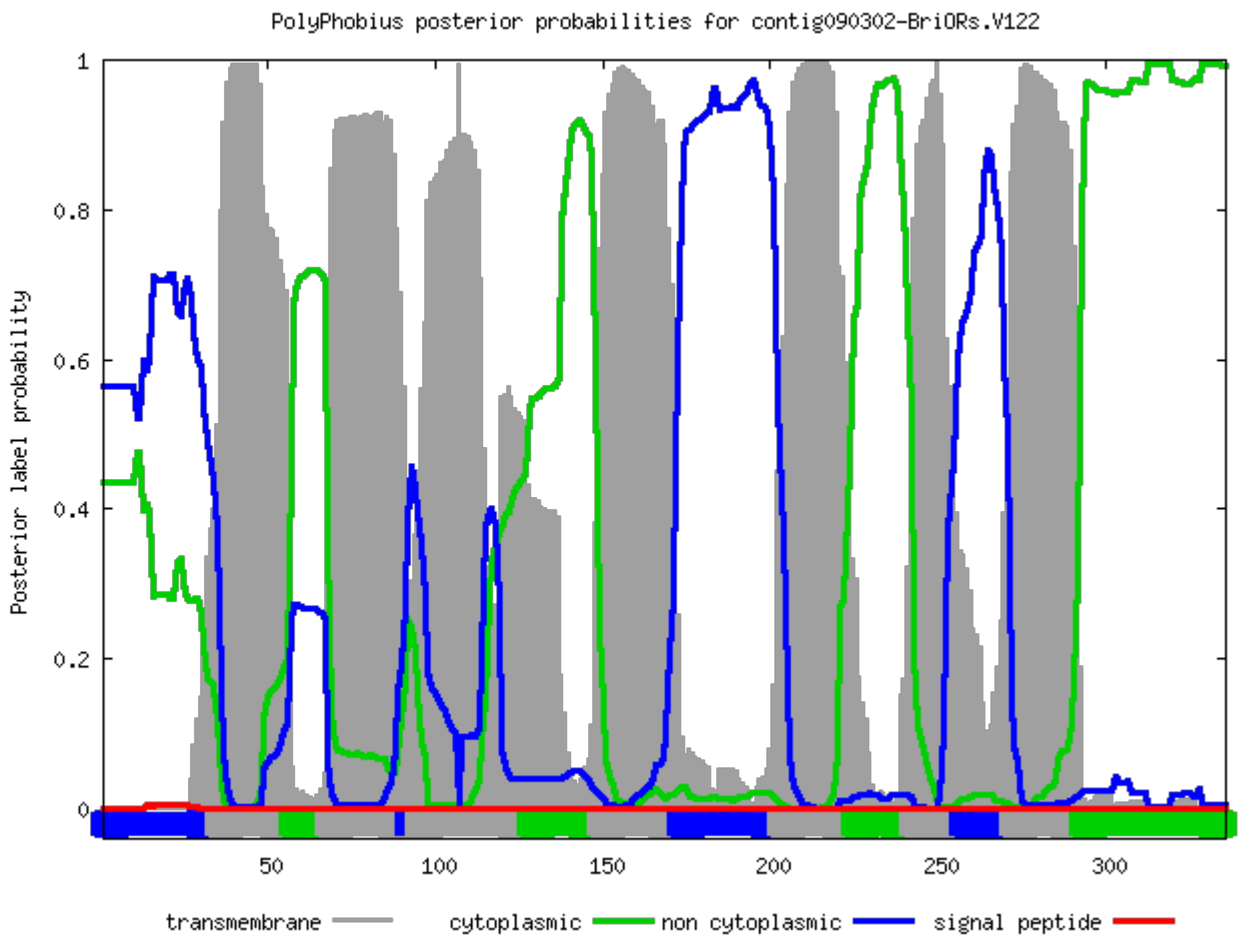

The prediction is based on an alignment. The probability data used in the plot is found here, and the gnuplot script is here.

Prediction of contig064565-BurOR.R139

```
ID   contig064565-BurOR.R139
FT   TOPO_DOM      1     19       NON CYTOPLASMIC.
FT   TRANSMEM     20     43
FT   TOPO_DOM     44     54       CYTOPLASMIC.
FT   TRANSMEM     55     79
FT   TOPO_DOM     80     84       NON CYTOPLASMIC.
FT   TRANSMEM     85    113
FT   TOPO_DOM    114    133       CYTOPLASMIC.
FT   TRANSMEM    134    157
FT   TOPO_DOM    158    191       NON CYTOPLASMIC.
FT   TRANSMEM    192    214
FT   TOPO_DOM    215    231       CYTOPLASMIC.
FT   TRANSMEM    232    254
FT   TOPO_DOM    255    266       NON CYTOPLASMIC.
FT   TRANSMEM    267    289
FT   TOPO_DOM    290    320       CYTOPLASMIC.
//
```

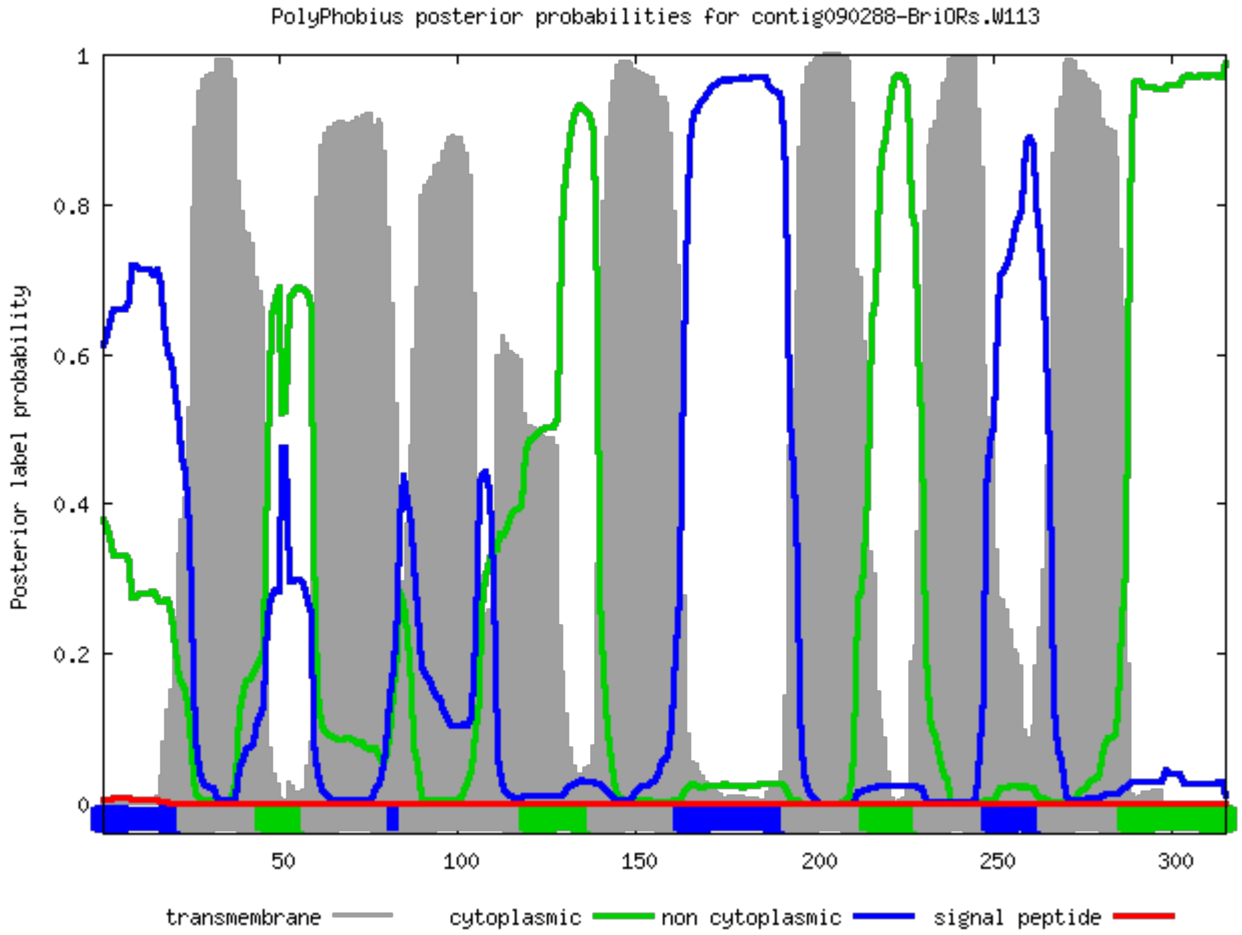

The prediction is based on an alignment. The probability data used in the plot is found here, and the gnuplot script is here.

Prediction of contig022251-TilOR.A013

```
ID   contig022251-TilOR.A013
FT   TOPO_DOM      1     22       NON CYTOPLASMIC.
FT   TRANSMEM     23     48
FT   TOPO_DOM     49     56       CYTOPLASMIC.
FT   TRANSMEM     57     76
FT   TOPO_DOM     77     95       NON CYTOPLASMIC.
FT   TRANSMEM     96    118
FT   TOPO_DOM    119    138       CYTOPLASMIC.
FT   TRANSMEM    139    159
FT   TOPO_DOM    160    192       NON CYTOPLASMIC.
FT   TRANSMEM    193    215
FT   TOPO_DOM    216    235       CYTOPLASMIC.
FT   TRANSMEM    236    257
FT   TOPO_DOM    258    268       NON CYTOPLASMIC.
FT   TRANSMEM    269    289
FT   TOPO_DOM    290    314       CYTOPLASMIC.
//
```

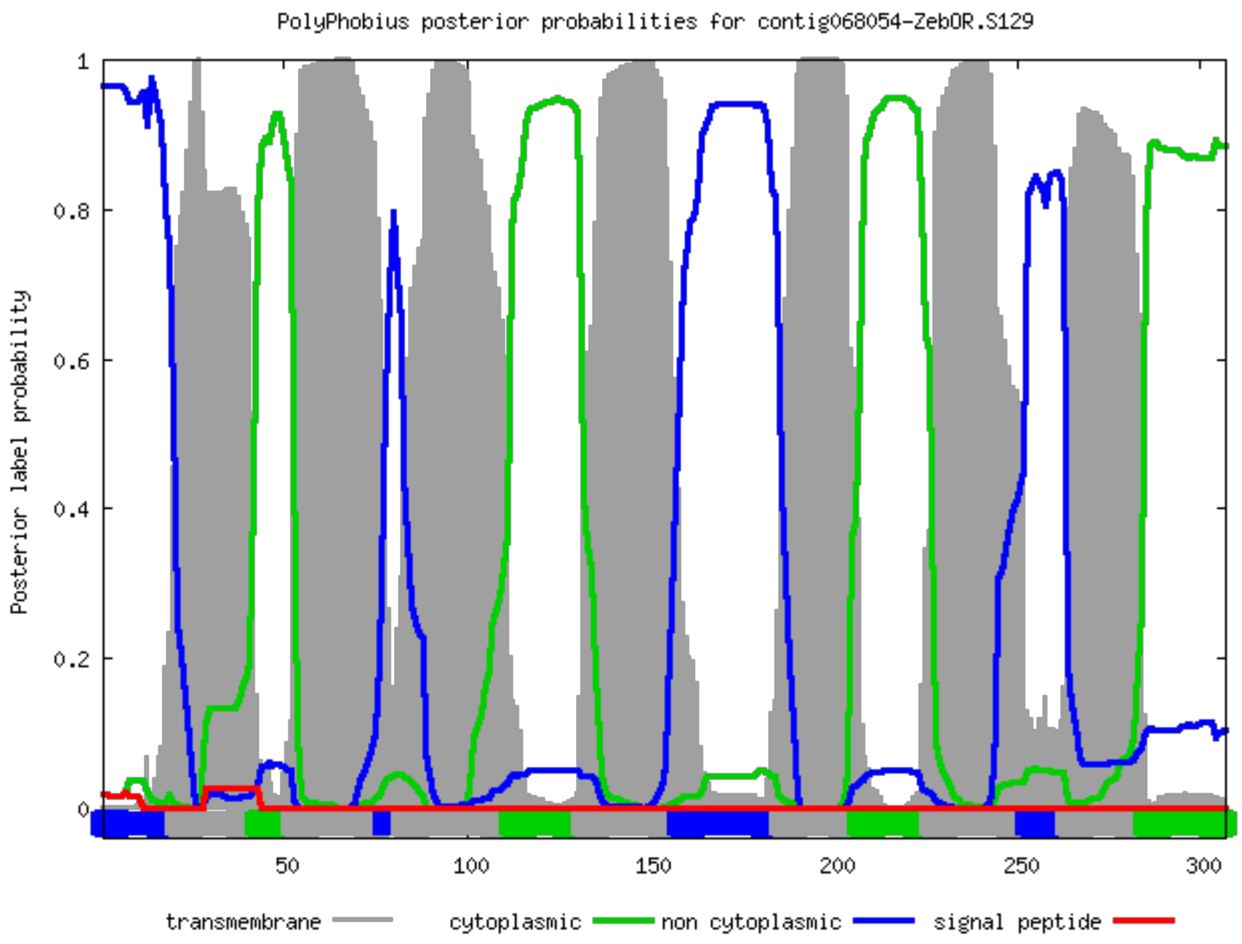

The prediction is based on an alignment. The probability data used in the plot is found here, and the gnuplot script is here.

Prediction of contig014049-ZebOR.D037

```
ID   contig014049-ZebOR.D037
FT   TOPO_DOM      1     22       NON CYTOPLASMIC.
FT   TRANSMEM     23     48
FT   TOPO_DOM     49     57       CYTOPLASMIC.
FT   TRANSMEM     58     81
FT   TOPO_DOM     82     90       NON CYTOPLASMIC.
FT   TRANSMEM     91    118
FT   TOPO_DOM    119    138       CYTOPLASMIC.
FT   TRANSMEM    139    162
FT   TOPO_DOM    163    194       NON CYTOPLASMIC.
FT   TRANSMEM    195    216
FT   TOPO_DOM    217    235       CYTOPLASMIC.
FT   TRANSMEM    236    256
FT   TOPO_DOM    257    267       NON CYTOPLASMIC.
FT   TRANSMEM    268    291
FT   TOPO_DOM    292    309       CYTOPLASMIC.
//
```

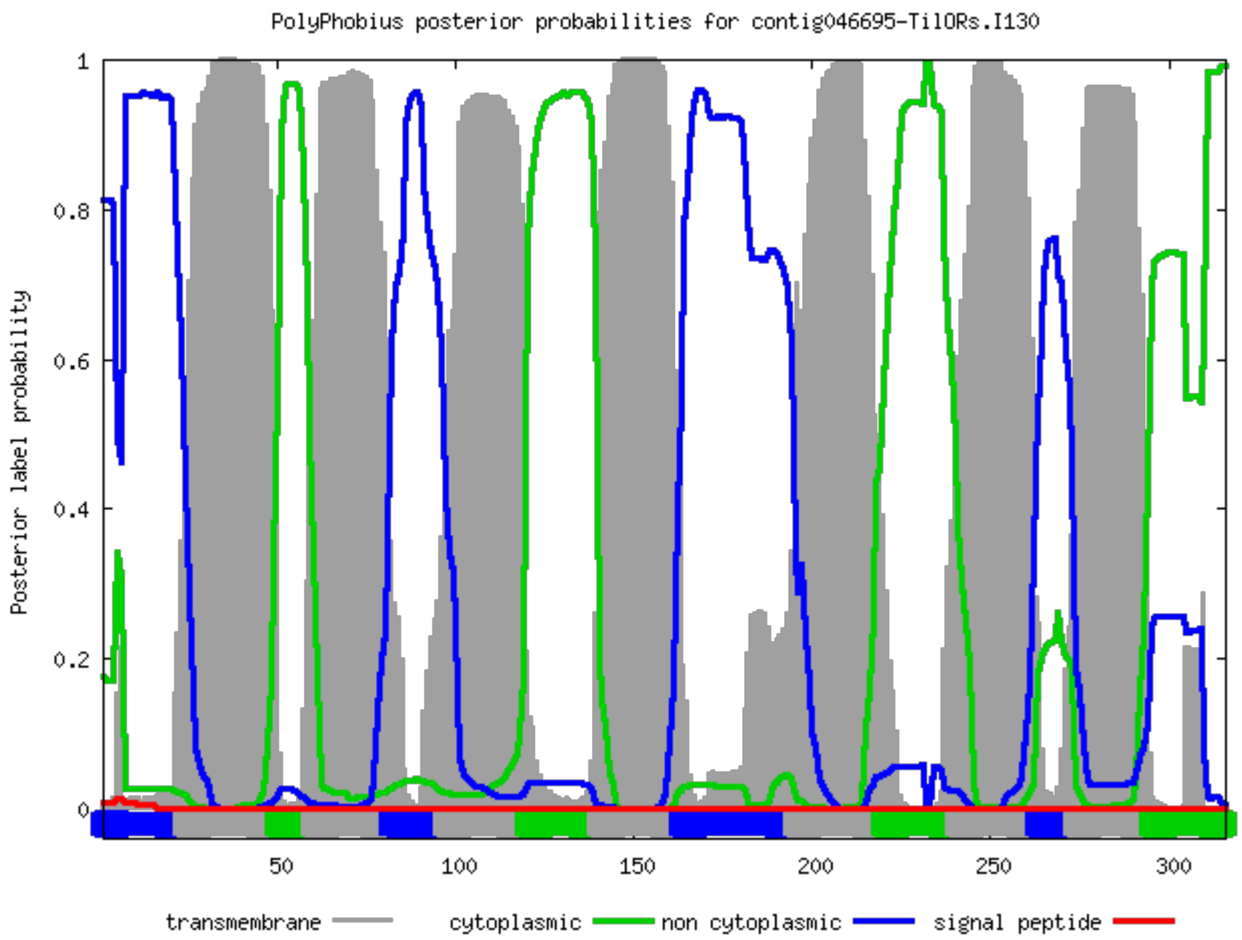

The prediction is based on an alignment. The probability data used in the plot is found here, and the gnuplot script is here.

Prediction of contig064361-NyeOR.L094

```
ID   contig064361-NyeOR.L094
FT   TOPO_DOM      1     25       NON CYTOPLASMIC.
FT   TRANSMEM     26     50
FT   TOPO_DOM     51     59       CYTOPLASMIC.
FT   TRANSMEM     60     85
FT   TOPO_DOM     86     98       NON CYTOPLASMIC.
FT   TRANSMEM     99    120
FT   TOPO_DOM    121    140       CYTOPLASMIC.
FT   TRANSMEM    141    162
FT   TOPO_DOM    163    198       NON CYTOPLASMIC.
FT   TRANSMEM    199    223
FT   TOPO_DOM    224    237       CYTOPLASMIC.
FT   TRANSMEM    238    259
FT   TOPO_DOM    260    271       NON CYTOPLASMIC.
FT   TRANSMEM    272    292
FT   TOPO_DOM    293    313       CYTOPLASMIC.
//
```

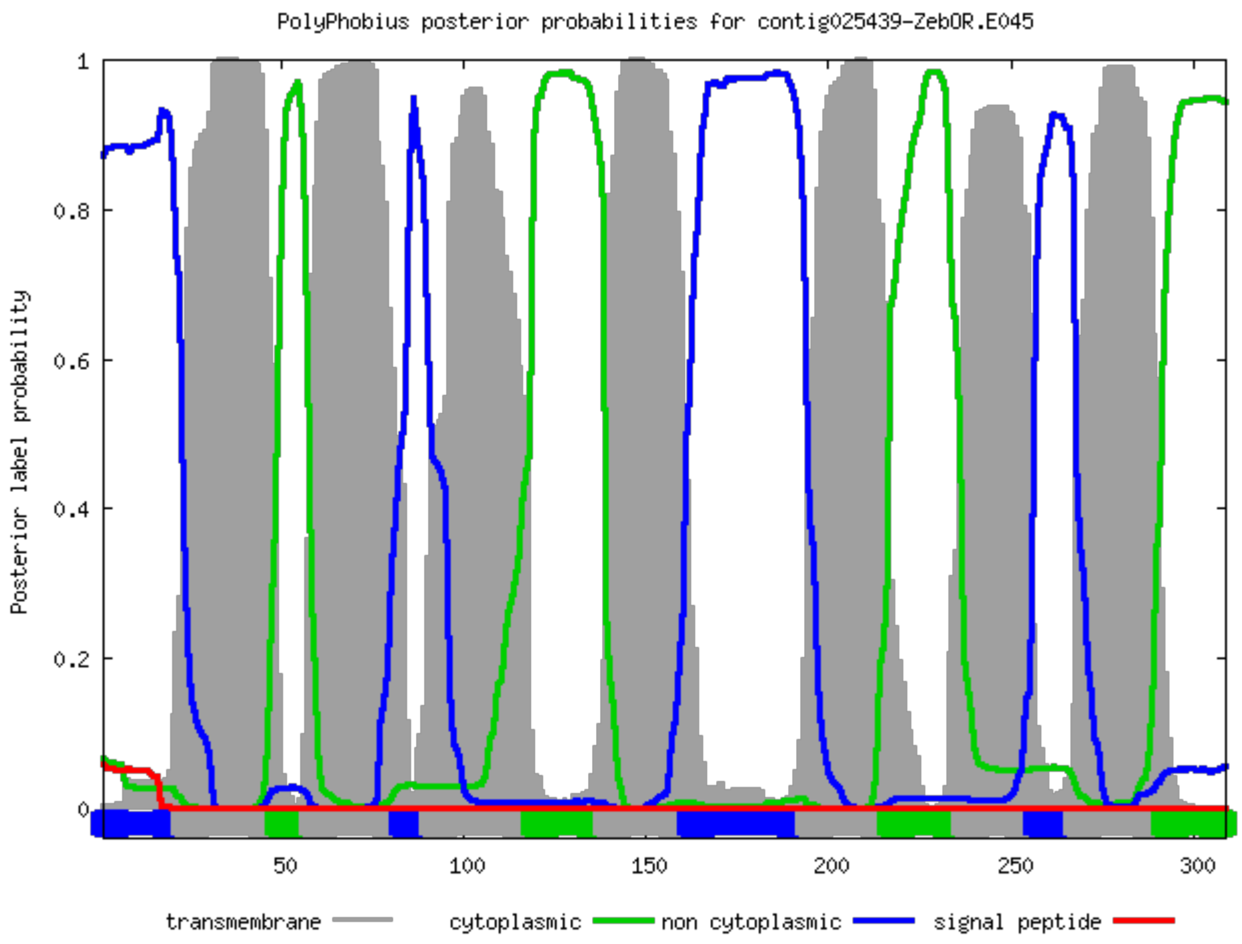

The prediction is based on an alignment. The probability data used in the plot is found here, and the gnuplot script is here.

Prediction of contig054733-BurOR.R138

```
ID    contig054733-BurOR.R138
FT    TOPO_DOM       1      24       NON CYTOPLASMIC.
FT    TRANSMEM      25      48
FT    TOPO_DOM      49      59       CYTOPLASMIC.
FT    TRANSMEM      60      84
FT    TOPO_DOM      85      89       NON CYTOPLASMIC.
FT    TRANSMEM      90     118
FT    TOPO_DOM     119     138       CYTOPLASMIC.
FT    TRANSMEM     139     162
FT    TOPO_DOM     163     192       NON CYTOPLASMIC.
FT    TRANSMEM     193     215
FT    TOPO_DOM     216     233       CYTOPLASMIC.
FT    TRANSMEM     234     255
FT    TOPO_DOM     256     268       NON CYTOPLASMIC.
FT    TRANSMEM     269     291
FT    TOPO_DOM     292     313       CYTOPLASMIC.
//
```

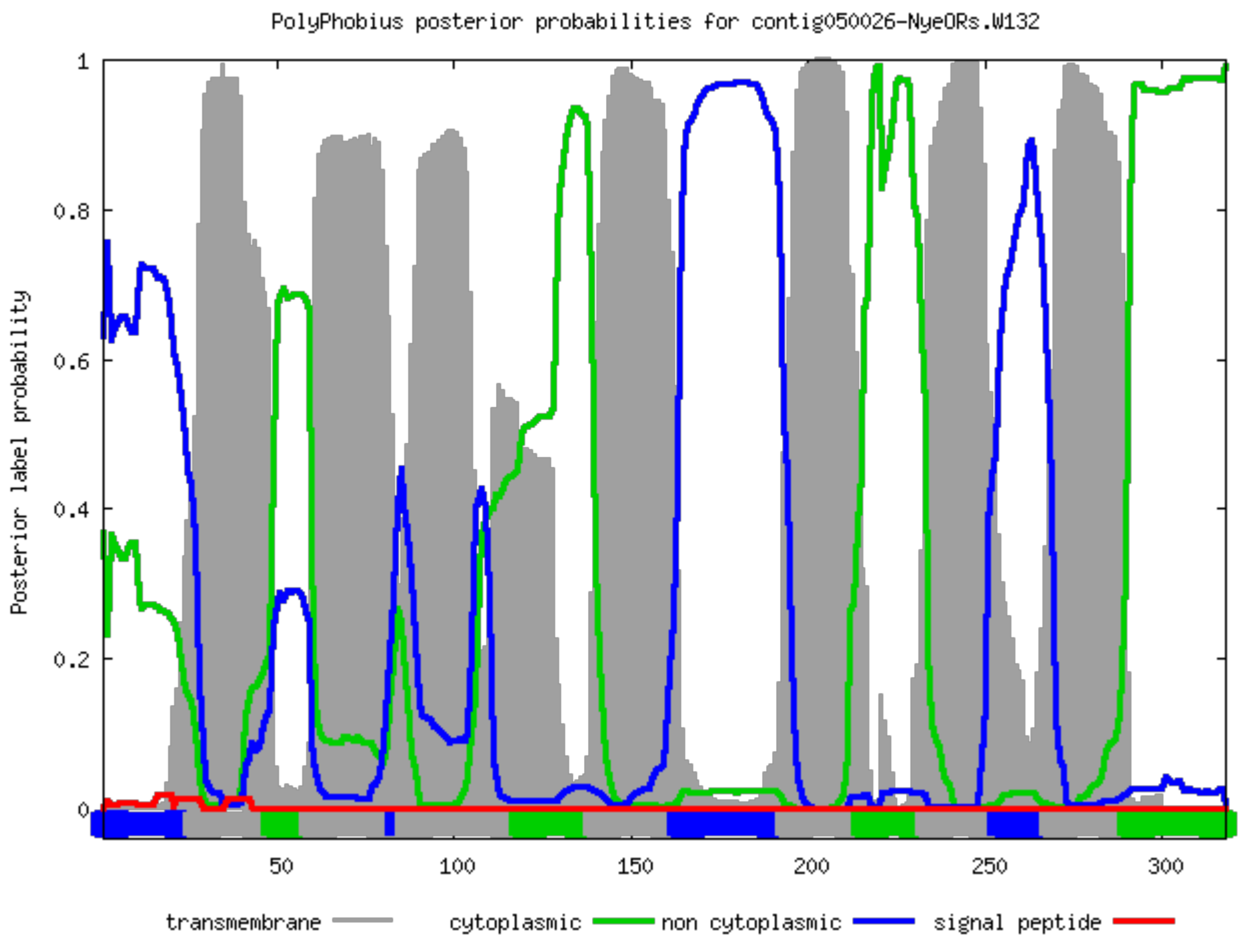

The prediction is based on an alignment. The probability data used in the plot is found here, and the gnuplot script is here.

Prediction of contig048239-ZebOR.E048

```
ID   contig048239-ZebOR.E048
FT   TOPO_DOM      1      23       NON CYTOPLASMIC.
FT   TRANSMEM     24      49
FT   TOPO_DOM     50      58       CYTOPLASMIC.
FT   TRANSMEM     59      82
FT   TOPO_DOM     83      93       NON CYTOPLASMIC.
FT   TRANSMEM     94     119
FT   TOPO_DOM    120     139       CYTOPLASMIC.
FT   TRANSMEM    140     161
FT   TOPO_DOM    162     194       NON CYTOPLASMIC.
FT   TRANSMEM    195     217
FT   TOPO_DOM    218     237       CYTOPLASMIC.
FT   TRANSMEM    238     257
FT   TOPO_DOM    258     268       NON CYTOPLASMIC.
FT   TRANSMEM    269     292
FT   TOPO_DOM    293     310       CYTOPLASMIC.
//
```

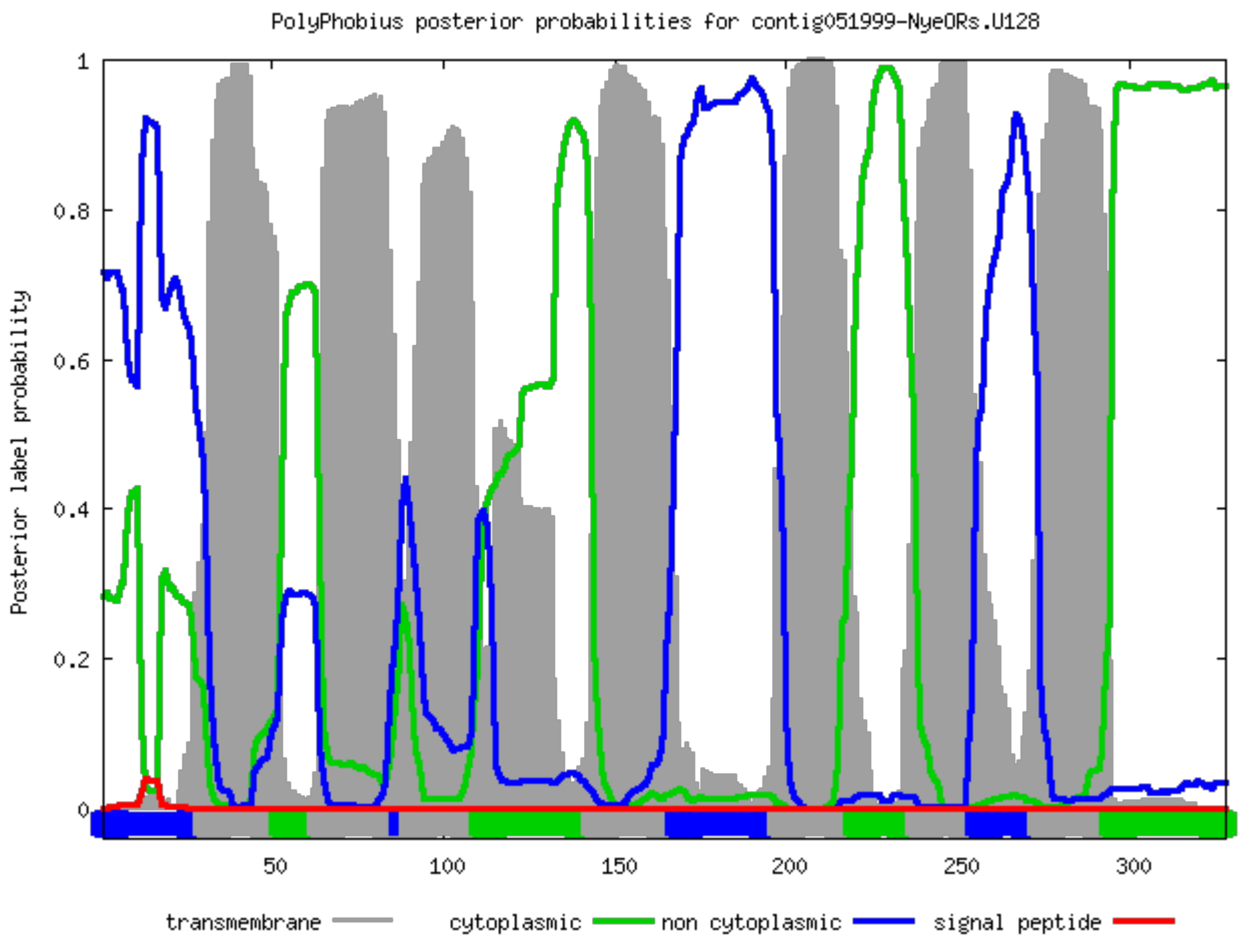

The prediction is based on an alignment. The probability data used in the plot is found here, and the gnuplot script is here.

Prediction of contig054237-BurOR.A013

```
ID   contig054237-BurOR.A013
FT   TOPO_DOM      1     22       NON CYTOPLASMIC.
FT   TRANSMEM     23     48
FT   TOPO_DOM     49     56       CYTOPLASMIC.
FT   TRANSMEM     57     76
FT   TOPO_DOM     77     95       NON CYTOPLASMIC.
FT   TRANSMEM     96    118
FT   TOPO_DOM    119    138       CYTOPLASMIC.
FT   TRANSMEM    139    159
FT   TOPO_DOM    160    192       NON CYTOPLASMIC.
FT   TRANSMEM    193    215
FT   TOPO_DOM    216    235       CYTOPLASMIC.
FT   TRANSMEM    236    257
FT   TOPO_DOM    258    268       NON CYTOPLASMIC.
FT   TRANSMEM    269    289
FT   TOPO_DOM    290    316       CYTOPLASMIC.
//
```

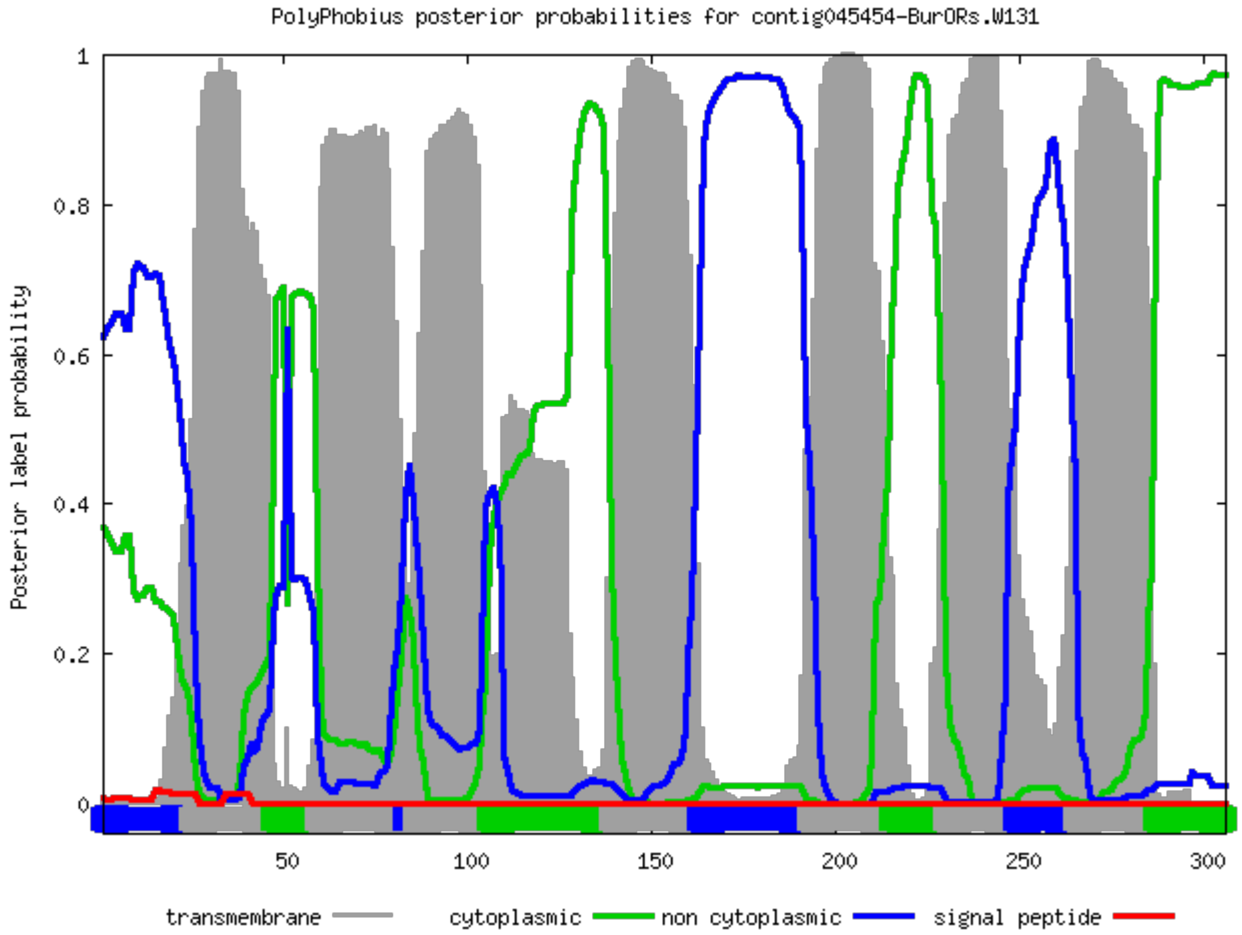

The prediction is based on an alignment. The probability data used in the plot is found here, and the gnuplot script is here.

Prediction of contig013337-TilOR.D055

```
ID    contig013337-TilOR.D055
FT    TOPO_DOM      1      22       NON CYTOPLASMIC.
FT    TRANSMEM     23      48
FT    TOPO_DOM     49      57       CYTOPLASMIC.
FT    TRANSMEM     58      81
FT    TOPO_DOM     82      90       NON CYTOPLASMIC.
FT    TRANSMEM     91     118
FT    TOPO_DOM    119     138       CYTOPLASMIC.
FT    TRANSMEM    139     161
FT    TOPO_DOM    162     193       NON CYTOPLASMIC.
FT    TRANSMEM    194     216
FT    TOPO_DOM    217     236       CYTOPLASMIC.
FT    TRANSMEM    237     256
FT    TOPO_DOM    257     268       NON CYTOPLASMIC.
FT    TRANSMEM    269     291
FT    TOPO_DOM    292     316       CYTOPLASMIC.
//
```

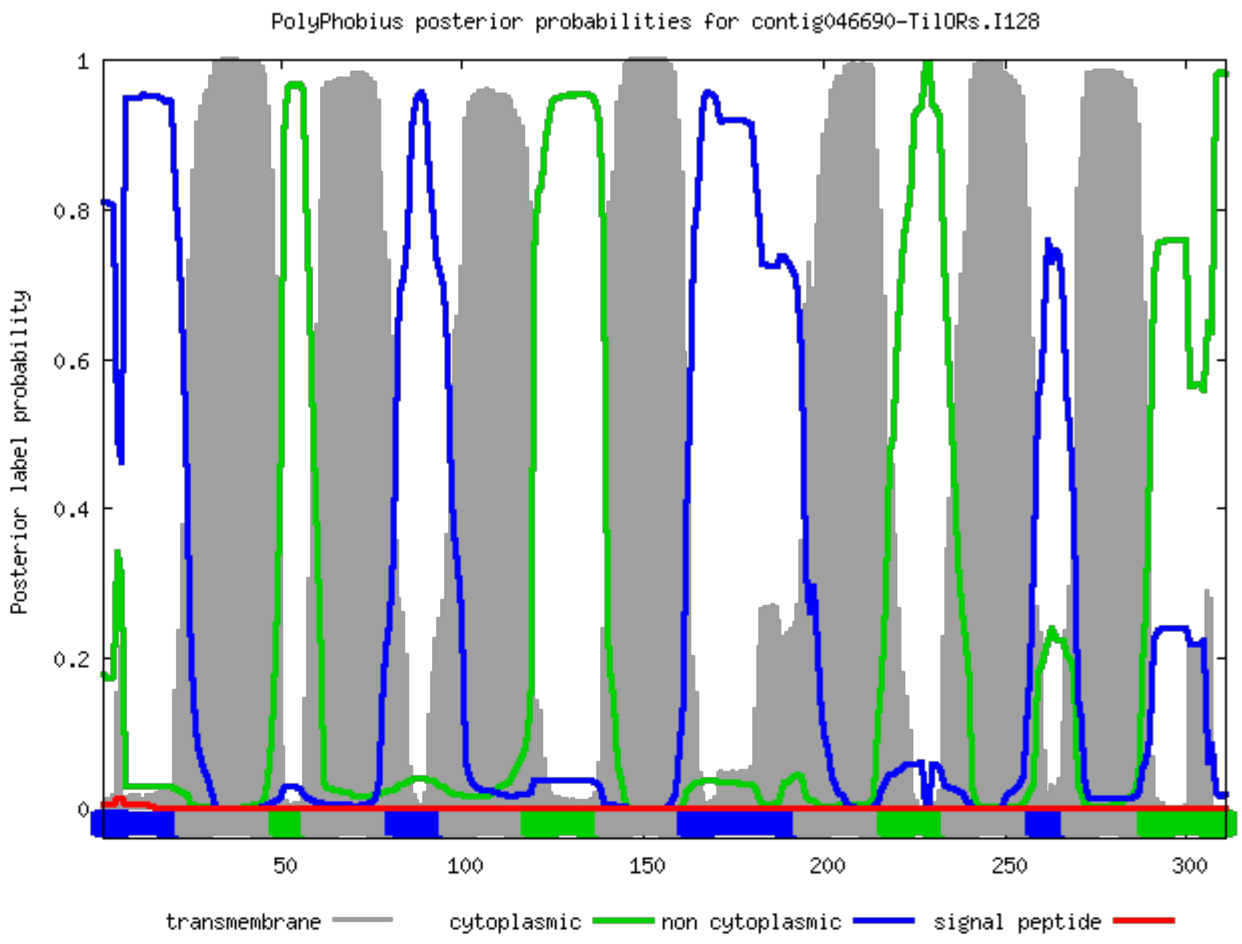

The prediction is based on an alignment. The probability data used in the plot is found here, and the gnuplot script is here.

Prediction of contig065453-TilOR.E087

```
ID   contig065453-TilOR.E087
FT   TOPO_DOM      1     22       NON CYTOPLASMIC.
FT   TRANSMEM     23     48
FT   TOPO_DOM     49     57       CYTOPLASMIC.
FT   TRANSMEM     58     81
FT   TOPO_DOM     82     91       NON CYTOPLASMIC.
FT   TRANSMEM     92    118
FT   TOPO_DOM    119    138       CYTOPLASMIC.
FT   TRANSMEM    139    161
FT   TOPO_DOM    162    193       NON CYTOPLASMIC.
FT   TRANSMEM    194    216
FT   TOPO_DOM    217    236       CYTOPLASMIC.
FT   TRANSMEM    237    256
FT   TOPO_DOM    257    267       NON CYTOPLASMIC.
FT   TRANSMEM    268    291
FT   TOPO_DOM    292    310       CYTOPLASMIC.
//
```

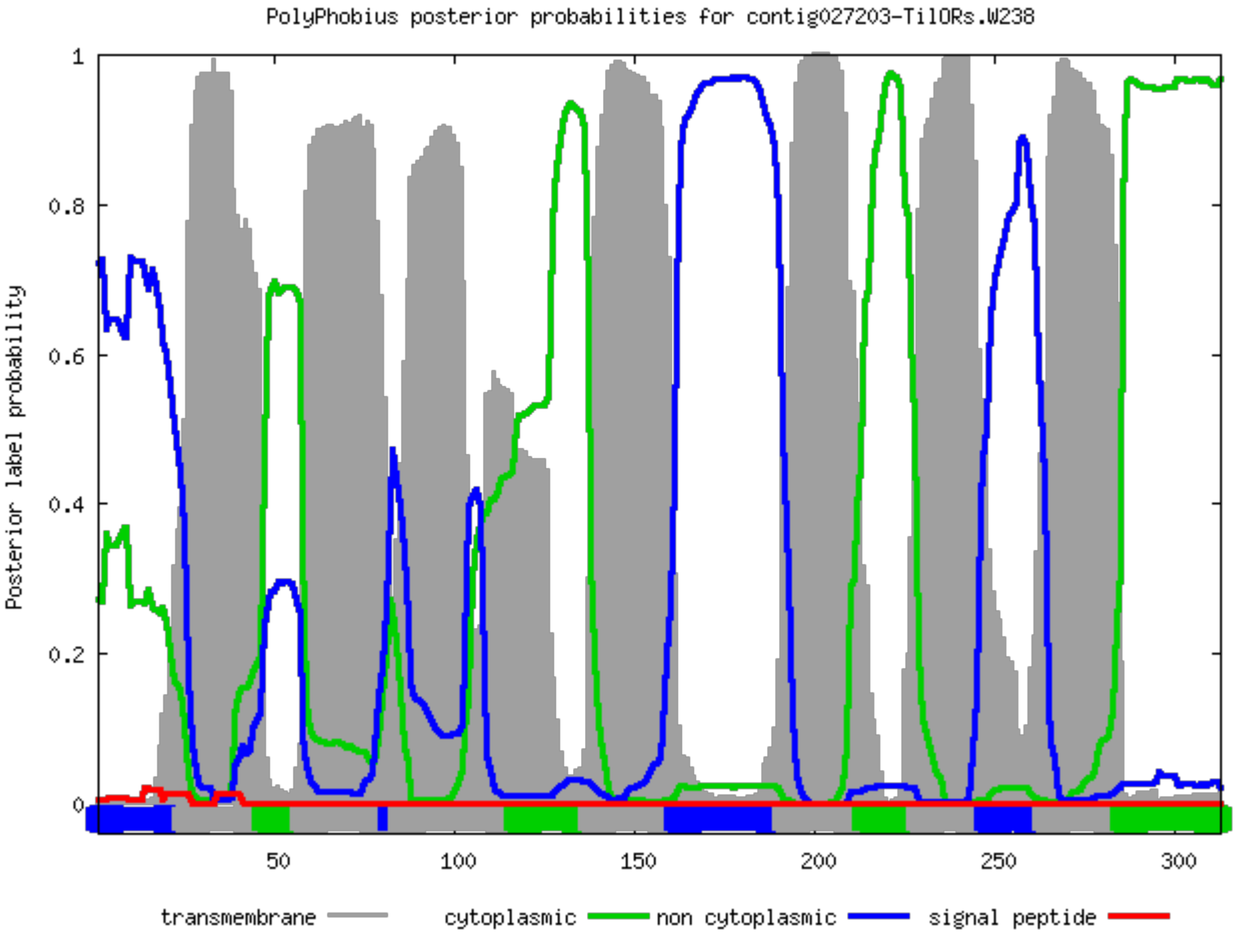

The prediction is based on an alignment. The probability data used in the plot is found here, and the gnuplot script is here.

Prediction of contig042562-BriOR.O079

```
ID    contig042562-BriOR.O079
FT    TOPO_DOM       1      26       NON CYTOPLASMIC.
FT    TRANSMEM      27      51
FT    TOPO_DOM      52      60       CYTOPLASMIC.
FT    TRANSMEM      61      83
FT    TOPO_DOM      84      99       NON CYTOPLASMIC.
FT    TRANSMEM     100     121
FT    TOPO_DOM     122     141       CYTOPLASMIC.
FT    TRANSMEM     142     164
FT    TOPO_DOM     165     201       NON CYTOPLASMIC.
FT    TRANSMEM     202     227
FT    TOPO_DOM     228     242       CYTOPLASMIC.
FT    TRANSMEM     243     263
FT    TOPO_DOM     264     275       NON CYTOPLASMIC.
FT    TRANSMEM     276     296
FT    TOPO_DOM     297     324       CYTOPLASMIC.
//
```

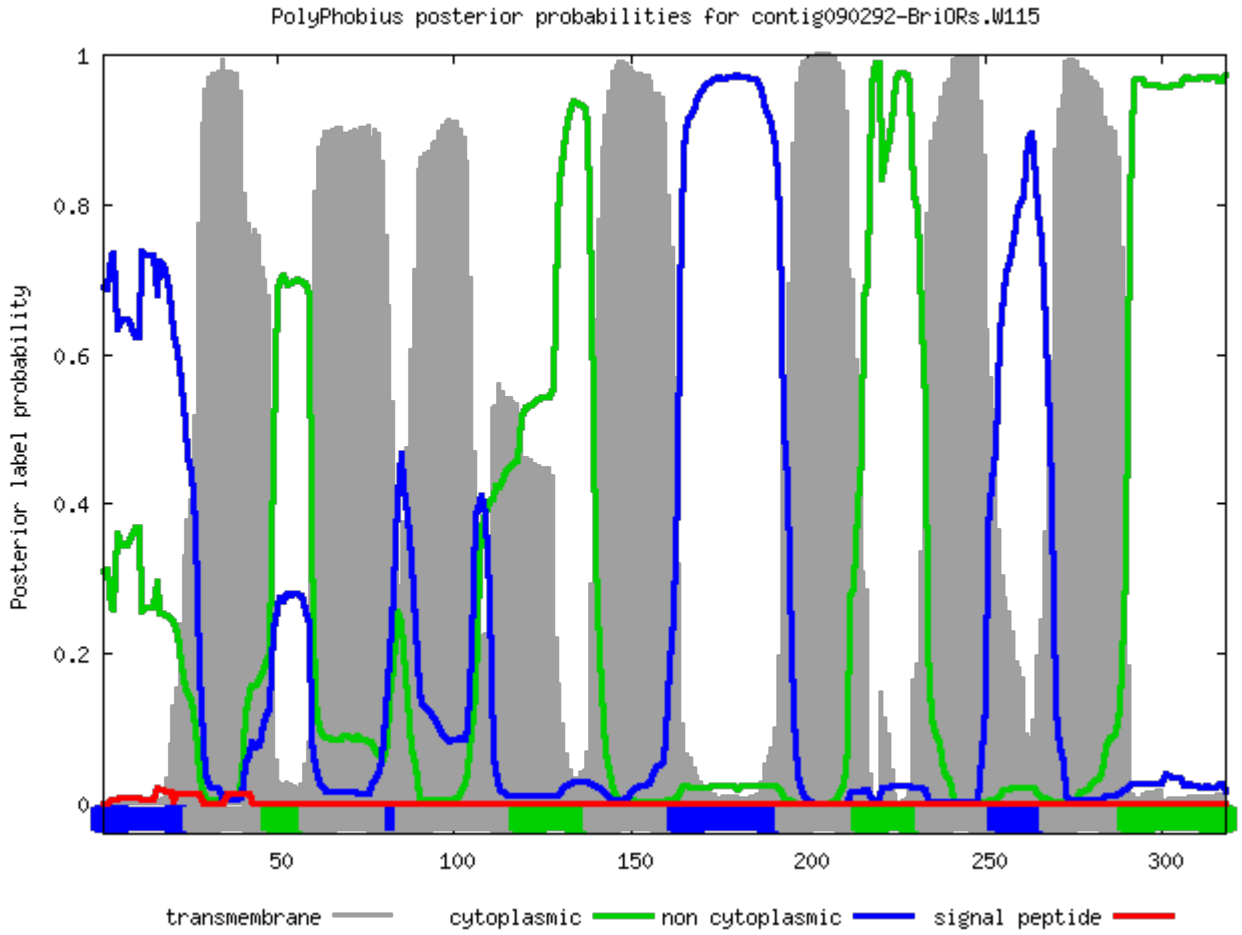

The prediction is based on an alignment. The probability data used in the plot is found here, and the gnuplot script is here.

Prediction of contig017782-ZebOR.J085

```
ID   contig017782-ZebOR.J085
FT   TOPO_DOM      1    23       NON CYTOPLASMIC.
FT   TRANSMEM     24    49
FT   TOPO_DOM     50    59       CYTOPLASMIC.
FT   TRANSMEM     60    81
FT   TOPO_DOM     82    97       NON CYTOPLASMIC.
FT   TRANSMEM     98   119
FT   TOPO_DOM    120   139       CYTOPLASMIC.
FT   TRANSMEM    140   162
FT   TOPO_DOM    163   194       NON CYTOPLASMIC.
FT   TRANSMEM    195   218
FT   TOPO_DOM    219   236       CYTOPLASMIC.
FT   TRANSMEM    237   260
FT   TOPO_DOM    261   270       NON CYTOPLASMIC.
FT   TRANSMEM    271   291
FT   TOPO_DOM    292   313       CYTOPLASMIC.
//
```

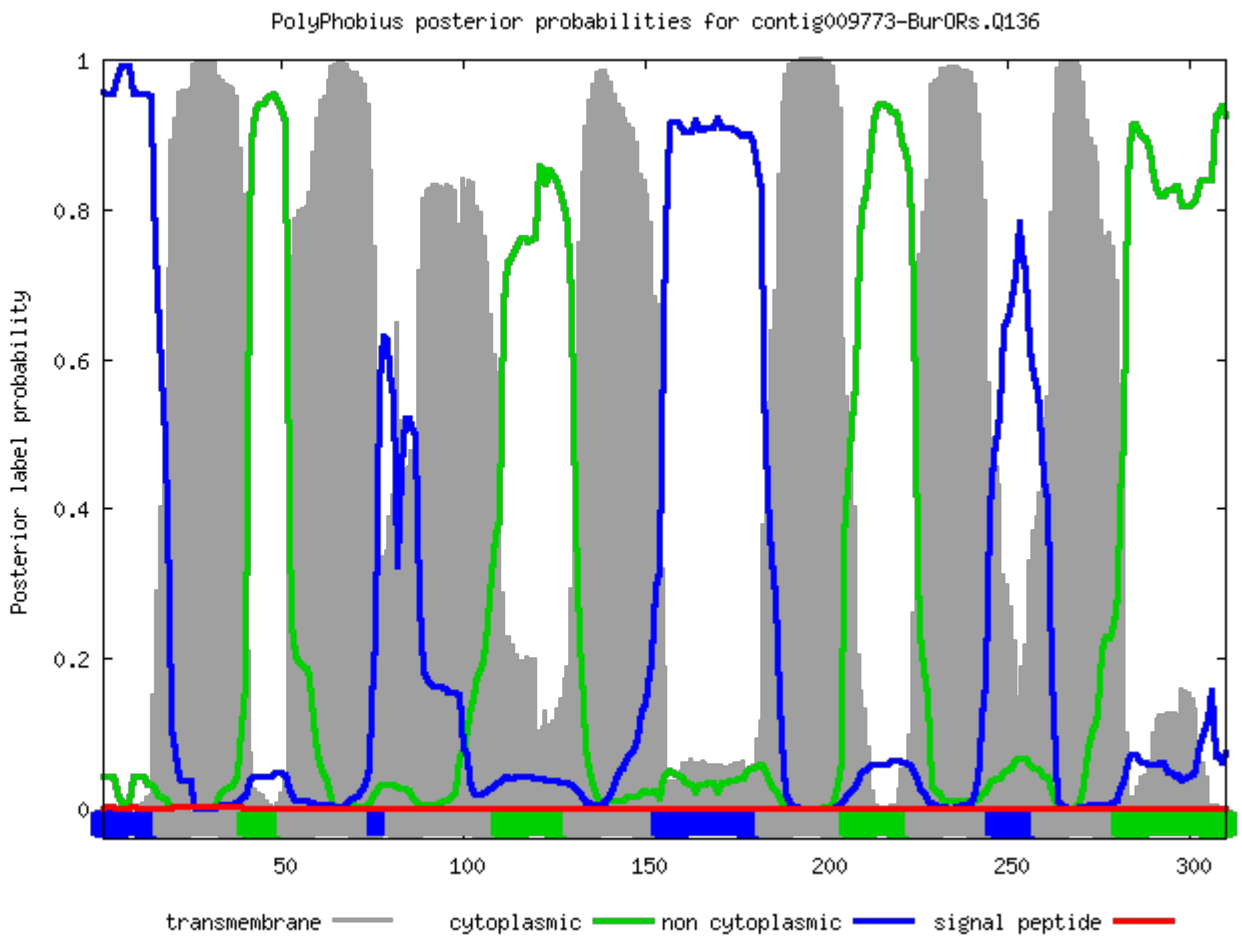

The prediction is based on an alignment. The probability data used in the plot is found here, and the gnuplot script is here.

Prediction of contig061321-BriOR.Y128

```
ID    contig061321-BriOR.Y128
FT    TOPO_DOM      1      17       NON CYTOPLASMIC.
FT    TRANSMEM     18      39
FT    TOPO_DOM     40      50       CYTOPLASMIC.
FT    TRANSMEM     51      74
FT    TOPO_DOM     75      80       NON CYTOPLASMIC.
FT    TRANSMEM     81     109
FT    TOPO_DOM    110     129       CYTOPLASMIC.
FT    TRANSMEM    130     151
FT    TOPO_DOM    152     172       NON CYTOPLASMIC.
FT    TRANSMEM    173     193
FT    TOPO_DOM    194     212       CYTOPLASMIC.
FT    TRANSMEM    213     233
FT    TOPO_DOM    234     248       NON CYTOPLASMIC.
FT    TRANSMEM    249     271
FT    TOPO_DOM    272     312       CYTOPLASMIC.
//
```

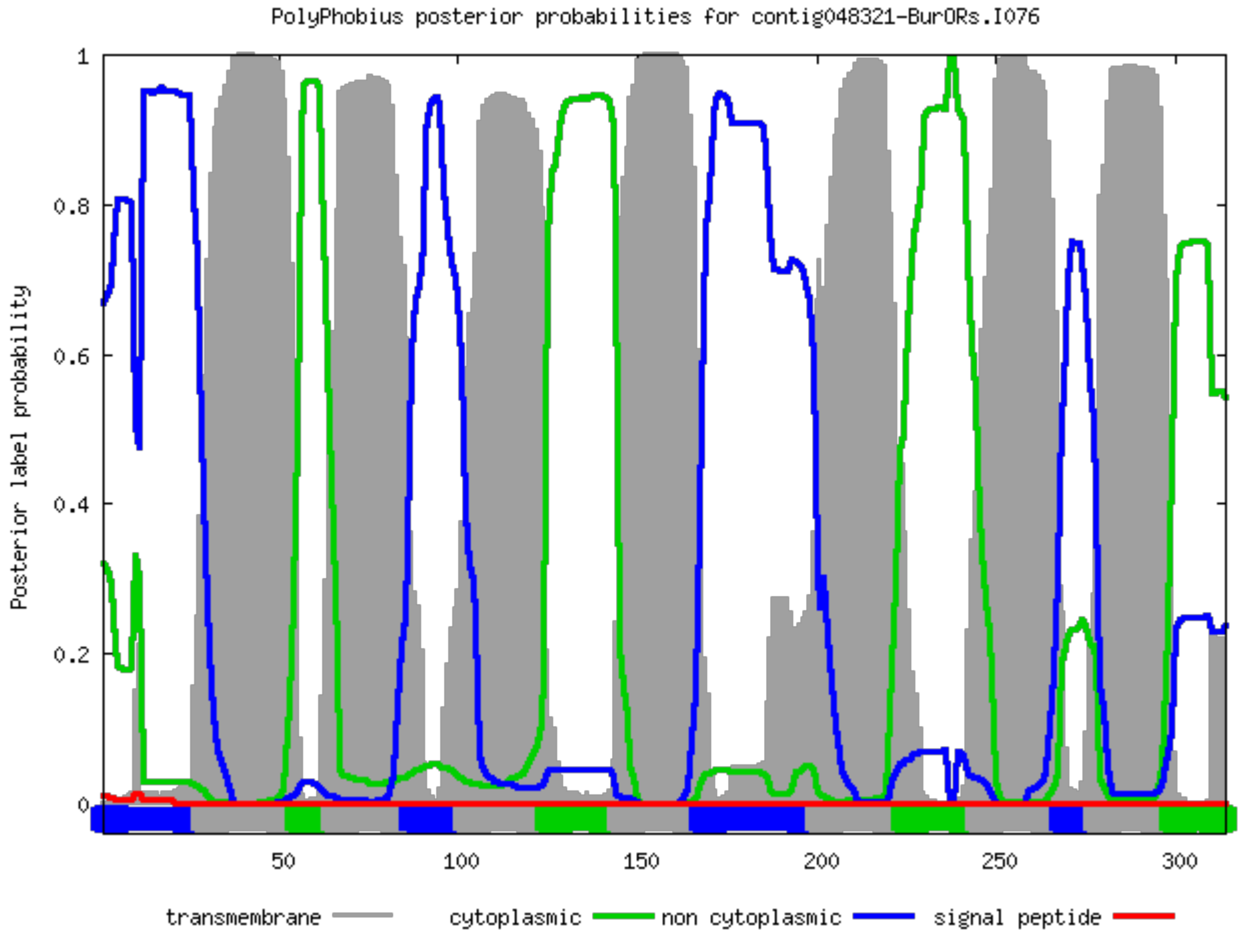

The prediction is based on an alignment. The probability data used in the plot is found here, and the gnuplot script is here.

Prediction of contig064817-BriOR.H051

```
ID    contig064817-BriOR.H051
FT    TOPO_DOM      1      22        NON CYTOPLASMIC.
FT    TRANSMEM     23      49
FT    TOPO_DOM     50      56        CYTOPLASMIC.
FT    TRANSMEM     57      77
FT    TOPO_DOM     78      95        NON CYTOPLASMIC.
FT    TRANSMEM     96     118
FT    TOPO_DOM    119     138        CYTOPLASMIC.
FT    TRANSMEM    139     160
FT    TOPO_DOM    161     193        NON CYTOPLASMIC.
FT    TRANSMEM    194     216
FT    TOPO_DOM    217     235        CYTOPLASMIC.
FT    TRANSMEM    236     258
FT    TOPO_DOM    259     269        NON CYTOPLASMIC.
FT    TRANSMEM    270     289
FT    TOPO_DOM    290     314        CYTOPLASMIC.
//
```

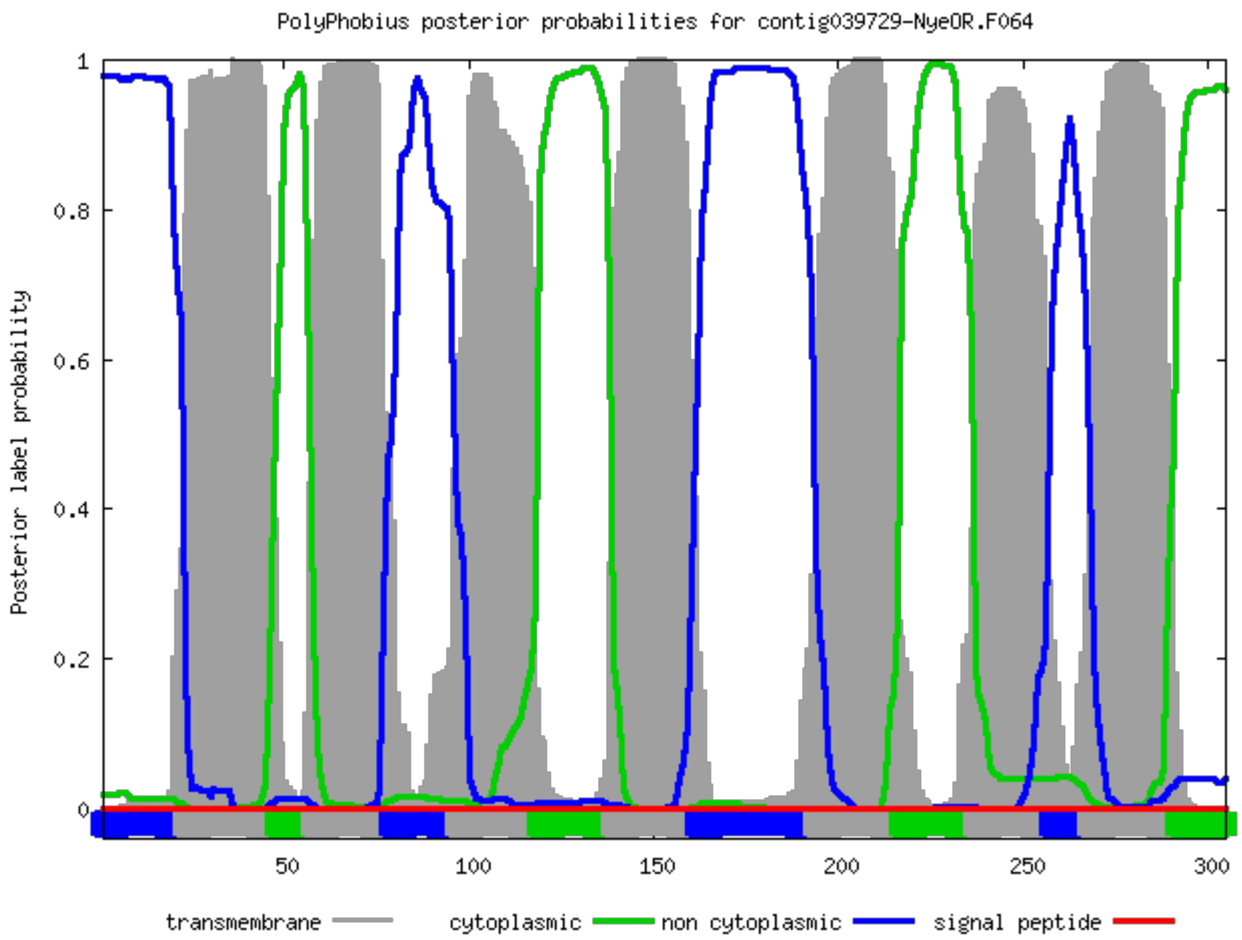

The prediction is based on an alignment. The probability data used in the plot is found here, and the gnuplot script is here.

Prediction of contig096536-BriOR.N085

```
ID   contig096536-BriOR.N085
FT   TOPO_DOM      1     33       NON CYTOPLASMIC.
FT   TRANSMEM     34     59
FT   TOPO_DOM     60     67       CYTOPLASMIC.
FT   TRANSMEM     68     89
FT   TOPO_DOM     90    108       NON CYTOPLASMIC.
FT   TRANSMEM    109    128
FT   TOPO_DOM    129    148       CYTOPLASMIC.
FT   TRANSMEM    149    171
FT   TOPO_DOM    172    207       NON CYTOPLASMIC.
FT   TRANSMEM    208    233
FT   TOPO_DOM    234    252       CYTOPLASMIC.
FT   TRANSMEM    253    275
FT   TOPO_DOM    276    337       NON CYTOPLASMIC.
//
```

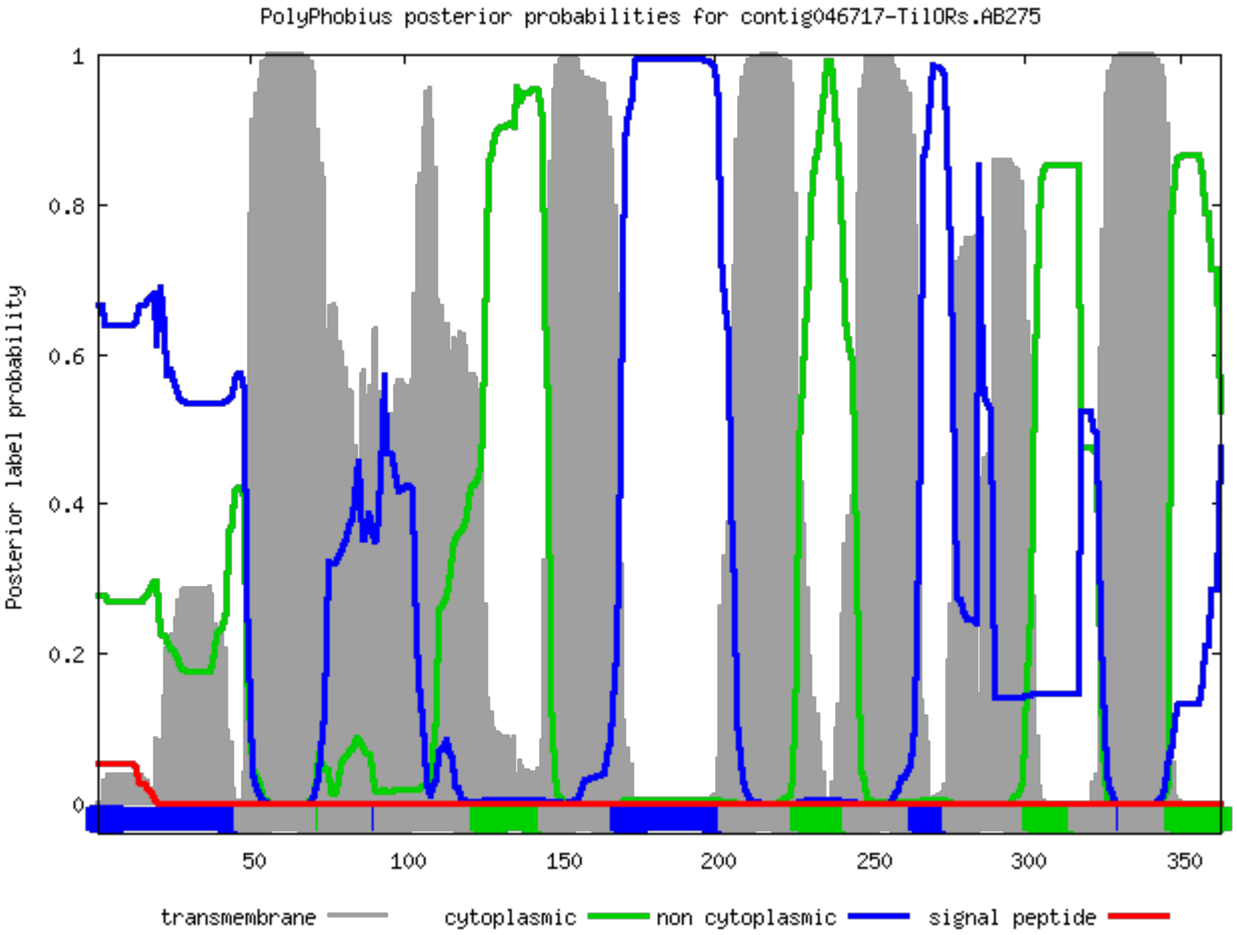

The prediction is based on an alignment. The probability data used in the plot is found here, and the gnuplot script is here.

Prediction of contig010712-ZebOR.N108

```
ID   contig010712-ZebOR.N108
FT   TOPO_DOM      1     32       NON CYTOPLASMIC.
FT   TRANSMEM     33     58
FT   TOPO_DOM     59     66       CYTOPLASMIC.
FT   TRANSMEM     67     86
FT   TOPO_DOM     87    104       NON CYTOPLASMIC.
FT   TRANSMEM    105    127
FT   TOPO_DOM    128    146       CYTOPLASMIC.
FT   TRANSMEM    147    170
FT   TOPO_DOM    171    206       NON CYTOPLASMIC.
FT   TRANSMEM    207    232
FT   TOPO_DOM    233    250       CYTOPLASMIC.
FT   TRANSMEM    251    271
FT   TOPO_DOM    272    277       NON CYTOPLASMIC.
FT   TRANSMEM    278    299
FT   TOPO_DOM    300    324       CYTOPLASMIC.
//
```

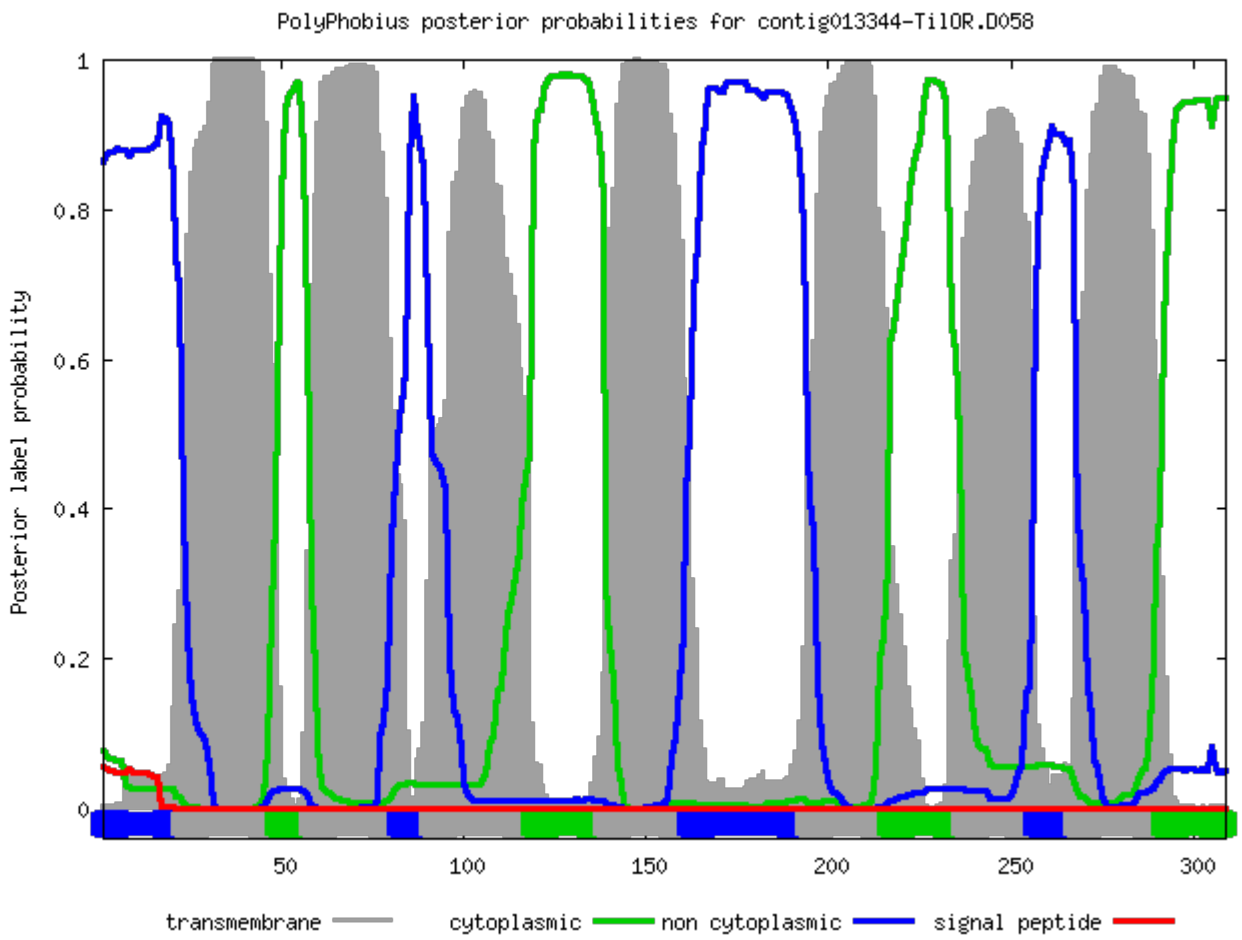

The prediction is based on an alignment. The probability data used in the plot is found here, and the gnuplot script is here.

Prediction of contig053779-BurOR.D035

```
ID   contig053779-BurOR.D035
FT   TOPO_DOM      1     22       NON CYTOPLASMIC.
FT   TRANSMEM     23     48
FT   TOPO_DOM     49     57       CYTOPLASMIC.
FT   TRANSMEM     58     81
FT   TOPO_DOM     82     90       NON CYTOPLASMIC.
FT   TRANSMEM     91    118
FT   TOPO_DOM    119    138       CYTOPLASMIC.
FT   TRANSMEM    139    161
FT   TOPO_DOM    162    193       NON CYTOPLASMIC.
FT   TRANSMEM    194    216
FT   TOPO_DOM    217    236       CYTOPLASMIC.
FT   TRANSMEM    237    256
FT   TOPO_DOM    257    268       NON CYTOPLASMIC.
FT   TRANSMEM    269    291
FT   TOPO_DOM    292    319       CYTOPLASMIC.
//
```

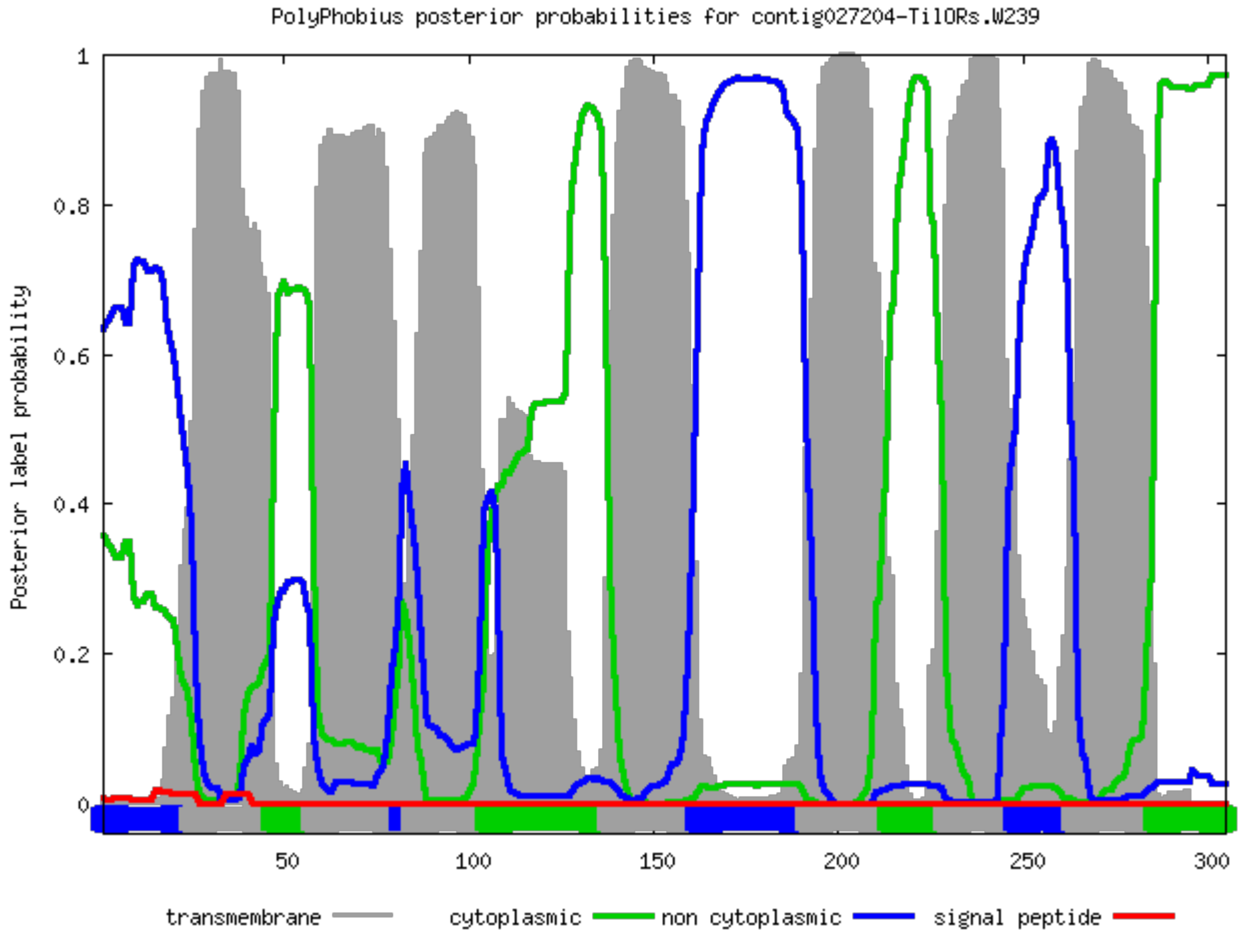

The prediction is based on an alignment. The probability data used in the plot is found here, and the gnuplot script is here.

Prediction of contig046699-TilOR.K134

```
ID   contig046699-TilOR.K134
FT   TOPO_DOM      1     24       NON CYTOPLASMIC.
FT   TRANSMEM     25     50
FT   TOPO_DOM     51     58       CYTOPLASMIC.
FT   TRANSMEM     59     81
FT   TOPO_DOM     82    100       NON CYTOPLASMIC.
FT   TRANSMEM    101    121
FT   TOPO_DOM    122    141       CYTOPLASMIC.
FT   TRANSMEM    142    165
FT   TOPO_DOM    166    196       NON CYTOPLASMIC.
FT   TRANSMEM    197    224
FT   TOPO_DOM    225    244       CYTOPLASMIC.
FT   TRANSMEM    245    264
FT   TOPO_DOM    265    272       NON CYTOPLASMIC.
FT   TRANSMEM    273    292
FT   TOPO_DOM    293    313       CYTOPLASMIC.
//
```

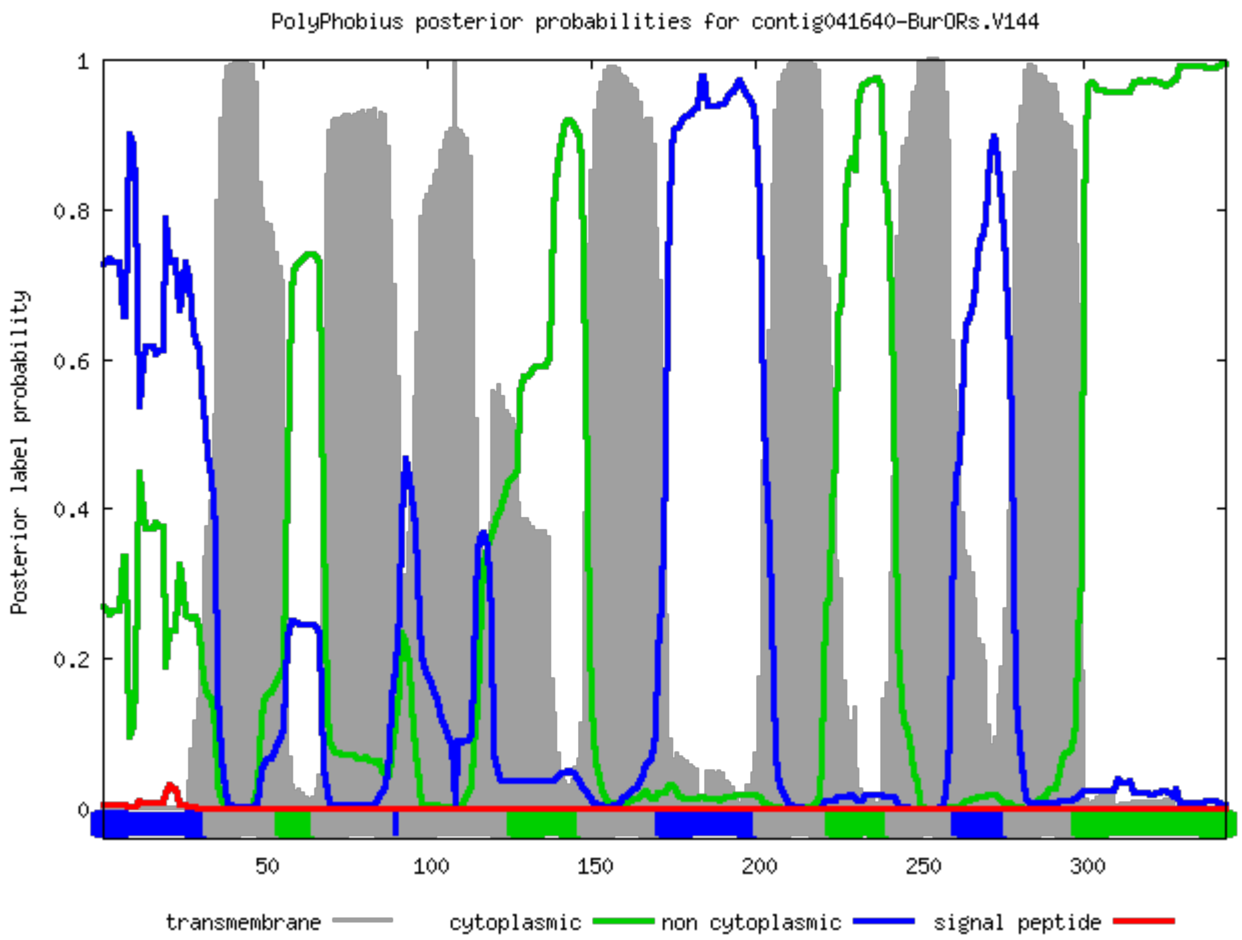

The prediction is based on an alignment. The probability data used in the plot is found here, and the gnuplot script is here.

Prediction of contig035582-NyeOR.H070

```
ID   contig035582-NyeOR.H070
FT   TOPO_DOM      1     23       NON CYTOPLASMIC.
FT   TRANSMEM     24     49
FT   TOPO_DOM     50     56       CYTOPLASMIC.
FT   TRANSMEM     57     76
FT   TOPO_DOM     77     95       NON CYTOPLASMIC.
FT   TRANSMEM     96    118
FT   TOPO_DOM    119    138       CYTOPLASMIC.
FT   TRANSMEM    139    160
FT   TOPO_DOM    161    196       NON CYTOPLASMIC.
FT   TRANSMEM    197    219
FT   TOPO_DOM    220    237       CYTOPLASMIC.
FT   TRANSMEM    238    259
FT   TOPO_DOM    260    271       NON CYTOPLASMIC.
FT   TRANSMEM    272    291
FT   TOPO_DOM    292    310       CYTOPLASMIC.
//
```

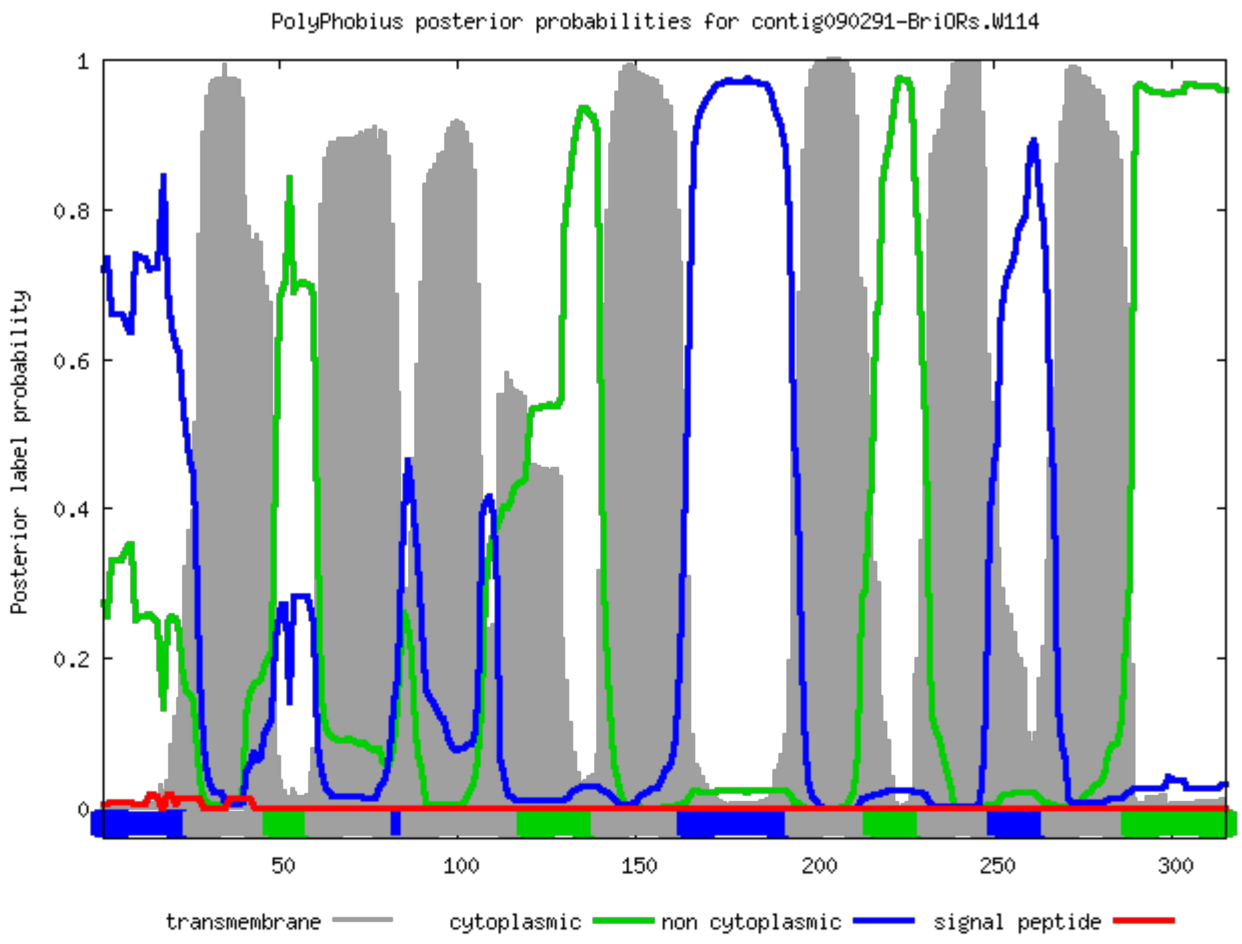

The prediction is based on an alignment. The probability data used in the plot is found here, and the gnuplot script is here.

Prediction of contig085000-BriOR.A003

```
ID   contig085000-BriOR.A003
FT   TOPO_DOM      1     22       NON CYTOPLASMIC.
FT   TRANSMEM     23     48
FT   TOPO_DOM     49     56       CYTOPLASMIC.
FT   TRANSMEM     57     77
FT   TOPO_DOM     78     95       NON CYTOPLASMIC.
FT   TRANSMEM     96    118
FT   TOPO_DOM    119    138       CYTOPLASMIC.
FT   TRANSMEM    139    159
FT   TOPO_DOM    160    192       NON CYTOPLASMIC.
FT   TRANSMEM    193    215
FT   TOPO_DOM    216    235       CYTOPLASMIC.
FT   TRANSMEM    236    257
FT   TOPO_DOM    258    268       NON CYTOPLASMIC.
FT   TRANSMEM    269    289
FT   TOPO_DOM    290    309       CYTOPLASMIC.
//
```

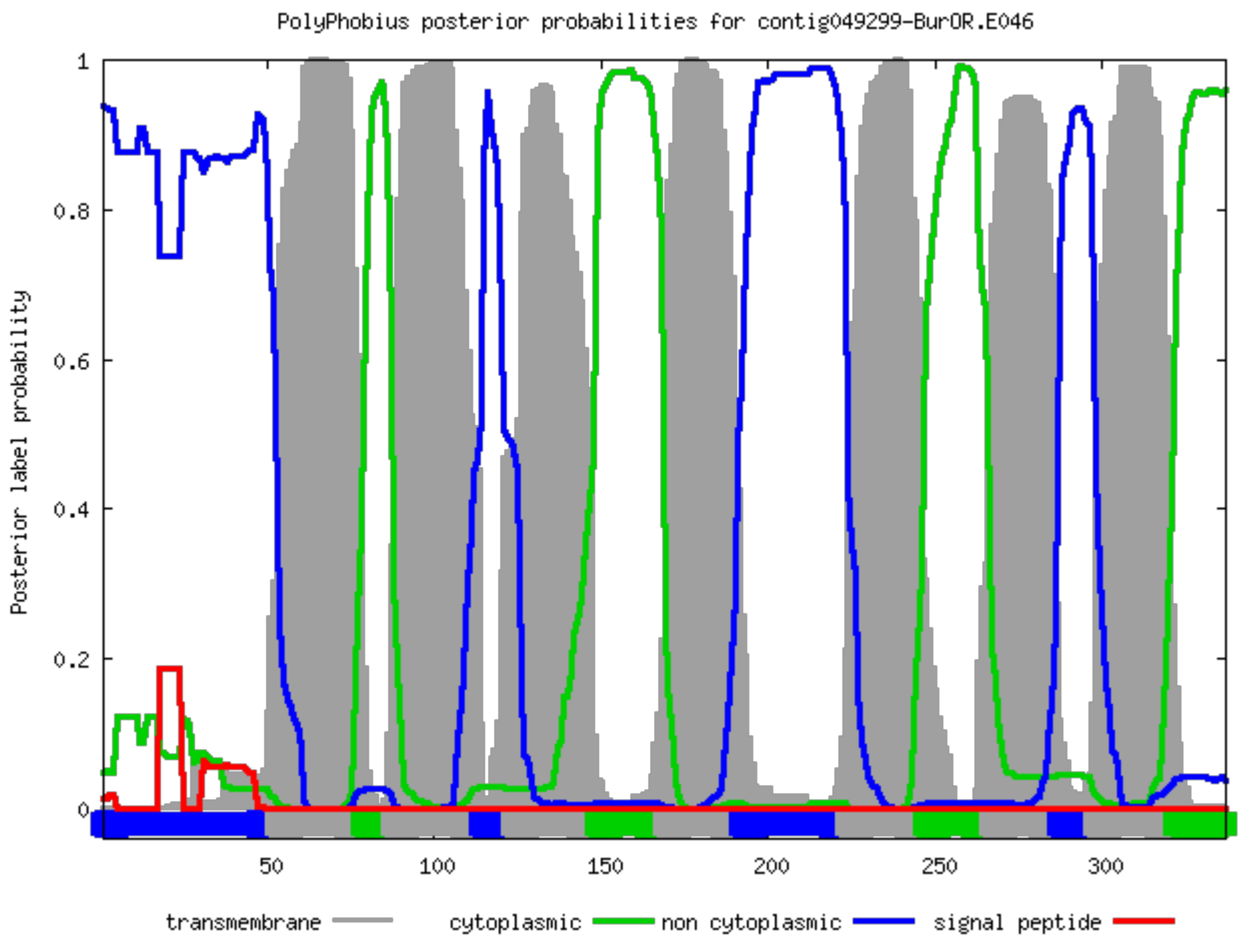

The prediction is based on an alignment. The probability data used in the plot is found here, and the gnuplot script is here.

Prediction of contig014050-ZebOR.D039

```
ID   contig014050-ZebOR.D039
FT   TOPO_DOM      1     22       NON CYTOPLASMIC.
FT   TRANSMEM     23     48
FT   TOPO_DOM     49     57       CYTOPLASMIC.
FT   TRANSMEM     58     81
FT   TOPO_DOM     82     90       NON CYTOPLASMIC.
FT   TRANSMEM     91    117
FT   TOPO_DOM    118    138       CYTOPLASMIC.
FT   TRANSMEM    139    161
FT   TOPO_DOM    162    193       NON CYTOPLASMIC.
FT   TRANSMEM    194    216
FT   TOPO_DOM    217    236       CYTOPLASMIC.
FT   TRANSMEM    237    256
FT   TOPO_DOM    257    267       NON CYTOPLASMIC.
FT   TRANSMEM    268    291
FT   TOPO_DOM    292    309       CYTOPLASMIC.
//
```

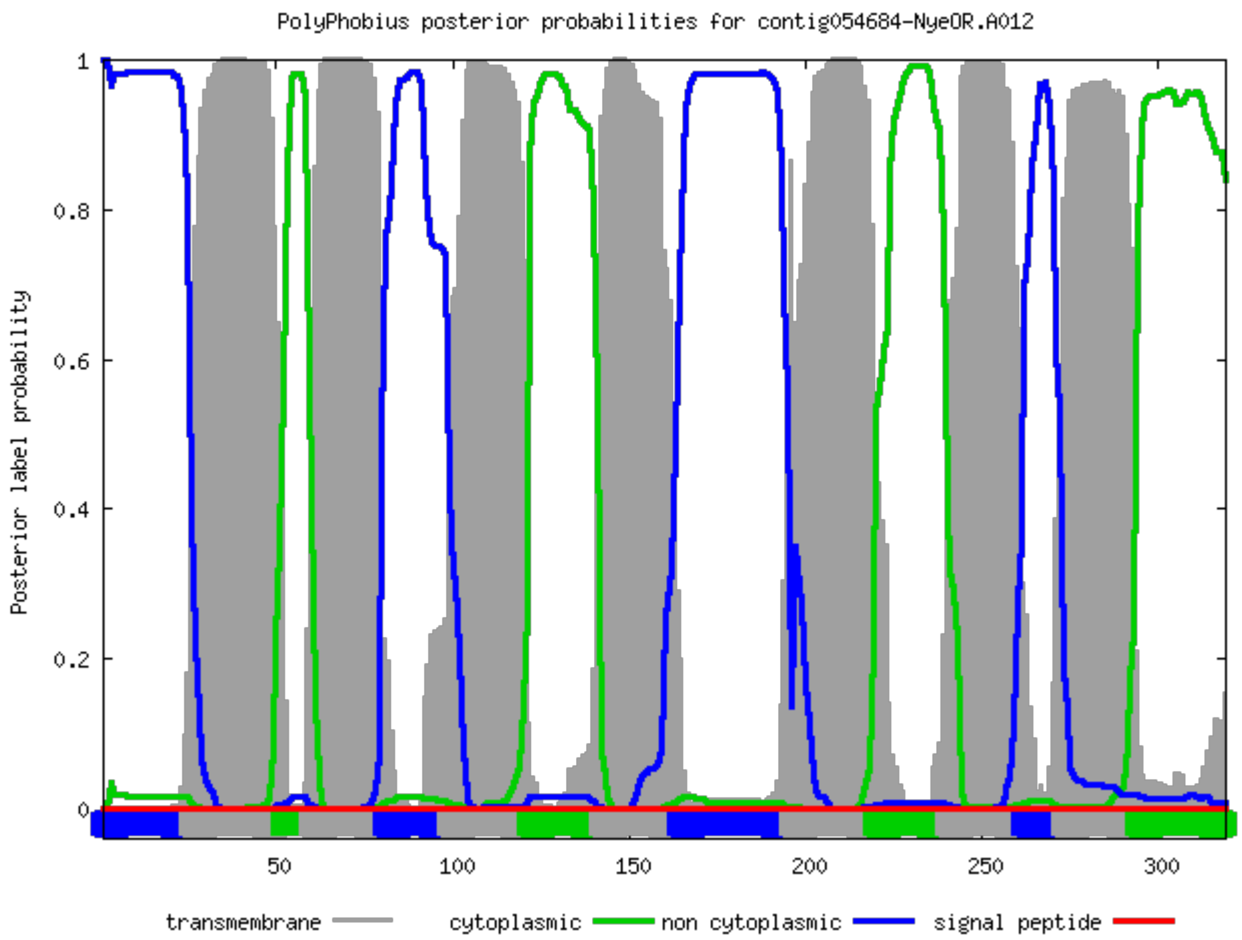

The prediction is based on an alignment. The probability data used in the plot is found here, and the gnuplot script is here.

Prediction of contig065455-TilOR.E085

```
ID   contig065455-TilOR.E085
FT   TOPO_DOM      1     24      NON CYTOPLASMIC.
FT   TRANSMEM     25     50
FT   TOPO_DOM     51     59      CYTOPLASMIC.
FT   TRANSMEM     60     84
FT   TOPO_DOM     85     96      NON CYTOPLASMIC.
FT   TRANSMEM     97    120
FT   TOPO_DOM    121    140      CYTOPLASMIC.
FT   TRANSMEM    141    162
FT   TOPO_DOM    163    195      NON CYTOPLASMIC.
FT   TRANSMEM    196    218
FT   TOPO_DOM    219    238      CYTOPLASMIC.
FT   TRANSMEM    239    258
FT   TOPO_DOM    259    269      NON CYTOPLASMIC.
FT   TRANSMEM    270    293
FT   TOPO_DOM    294    313      CYTOPLASMIC.
//
```

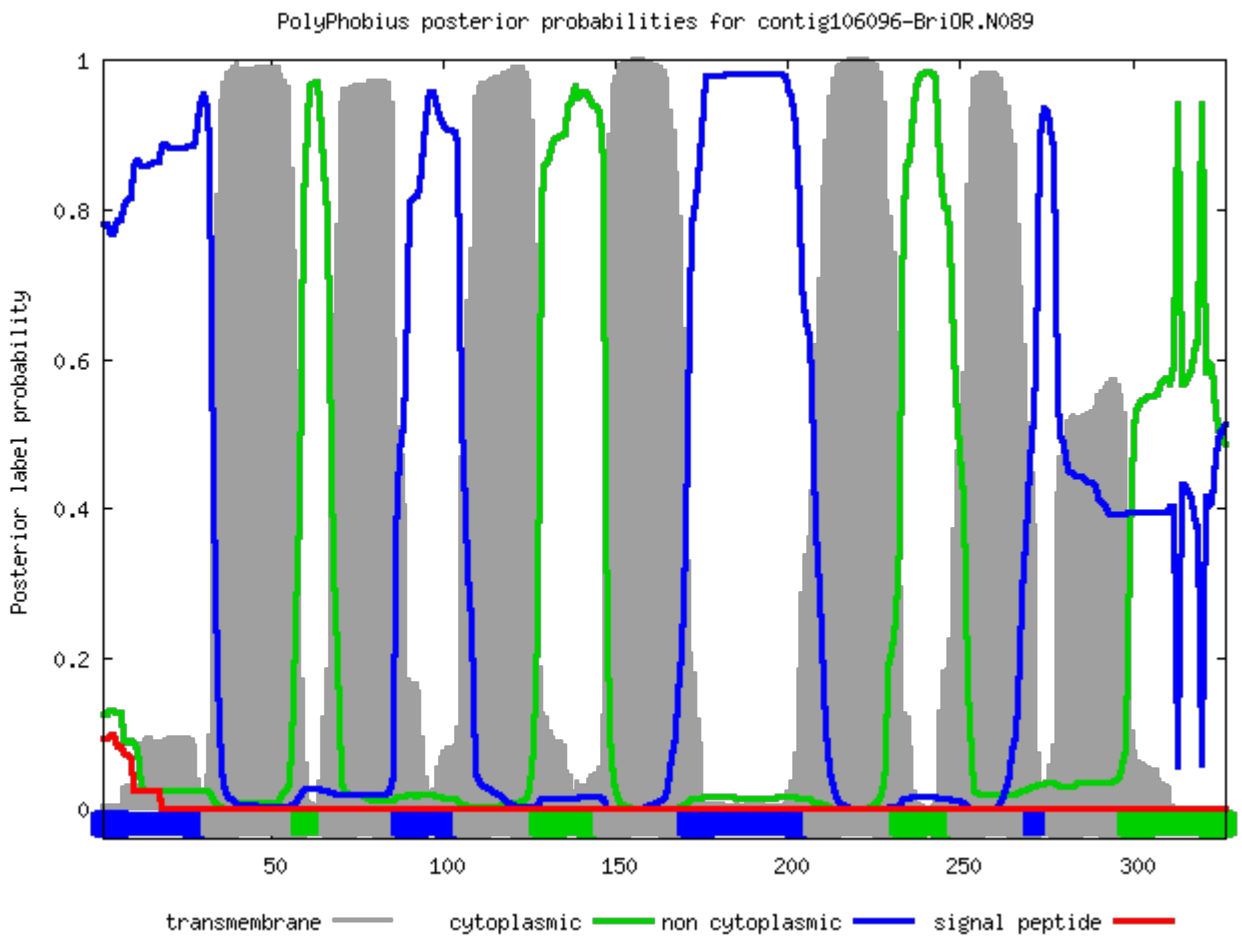

The prediction is based on an alignment. The probability data used in the plot is found here, and the gnuplot script is here.

Prediction of contig110782-BriOR.O080

```
ID   contig110782-BriOR.O080
FT   TOPO_DOM      1      24       NON CYTOPLASMIC.
FT   TRANSMEM     25      51
FT   TOPO_DOM     52      60       CYTOPLASMIC.
FT   TRANSMEM     61      83
FT   TOPO_DOM     84      98       NON CYTOPLASMIC.
FT   TRANSMEM     99     121
FT   TOPO_DOM    122     141       CYTOPLASMIC.
FT   TRANSMEM    142     163
FT   TOPO_DOM    164     200       NON CYTOPLASMIC.
FT   TRANSMEM    201     227
FT   TOPO_DOM    228     240       CYTOPLASMIC.
FT   TRANSMEM    241     262
FT   TOPO_DOM    263     273       NON CYTOPLASMIC.
FT   TRANSMEM    274     295
FT   TOPO_DOM    296     329       CYTOPLASMIC.
//
```

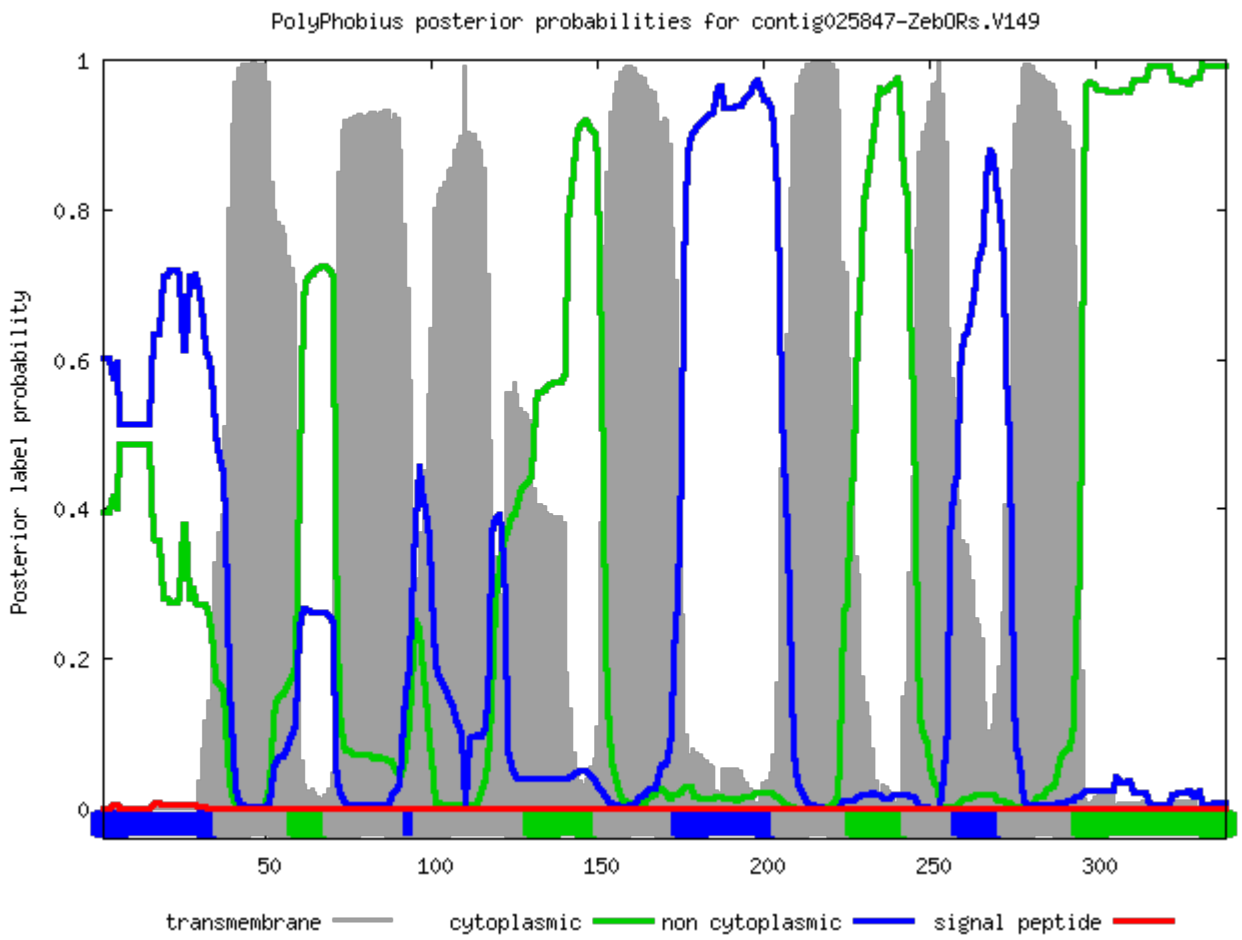

The prediction is based on an alignment. The probability data used in the plot is found here, and the gnuplot script is here.

Prediction of contig013369-TilOR.H116

```
ID   contig013369-TilOR.H116
FT   TOPO_DOM      1      23       NON CYTOPLASMIC.
FT   TRANSMEM     24      49
FT   TOPO_DOM     50      56       CYTOPLASMIC.
FT   TRANSMEM     57      76
FT   TOPO_DOM     77      95       NON CYTOPLASMIC.
FT   TRANSMEM     96     118
FT   TOPO_DOM    119     138       CYTOPLASMIC.
FT   TRANSMEM    139     160
FT   TOPO_DOM    161     196       NON CYTOPLASMIC.
FT   TRANSMEM    197     219
FT   TOPO_DOM    220     237       CYTOPLASMIC.
FT   TRANSMEM    238     259
FT   TOPO_DOM    260     271       NON CYTOPLASMIC.
FT   TRANSMEM    272     291
FT   TOPO_DOM    292     342       CYTOPLASMIC.
//
```

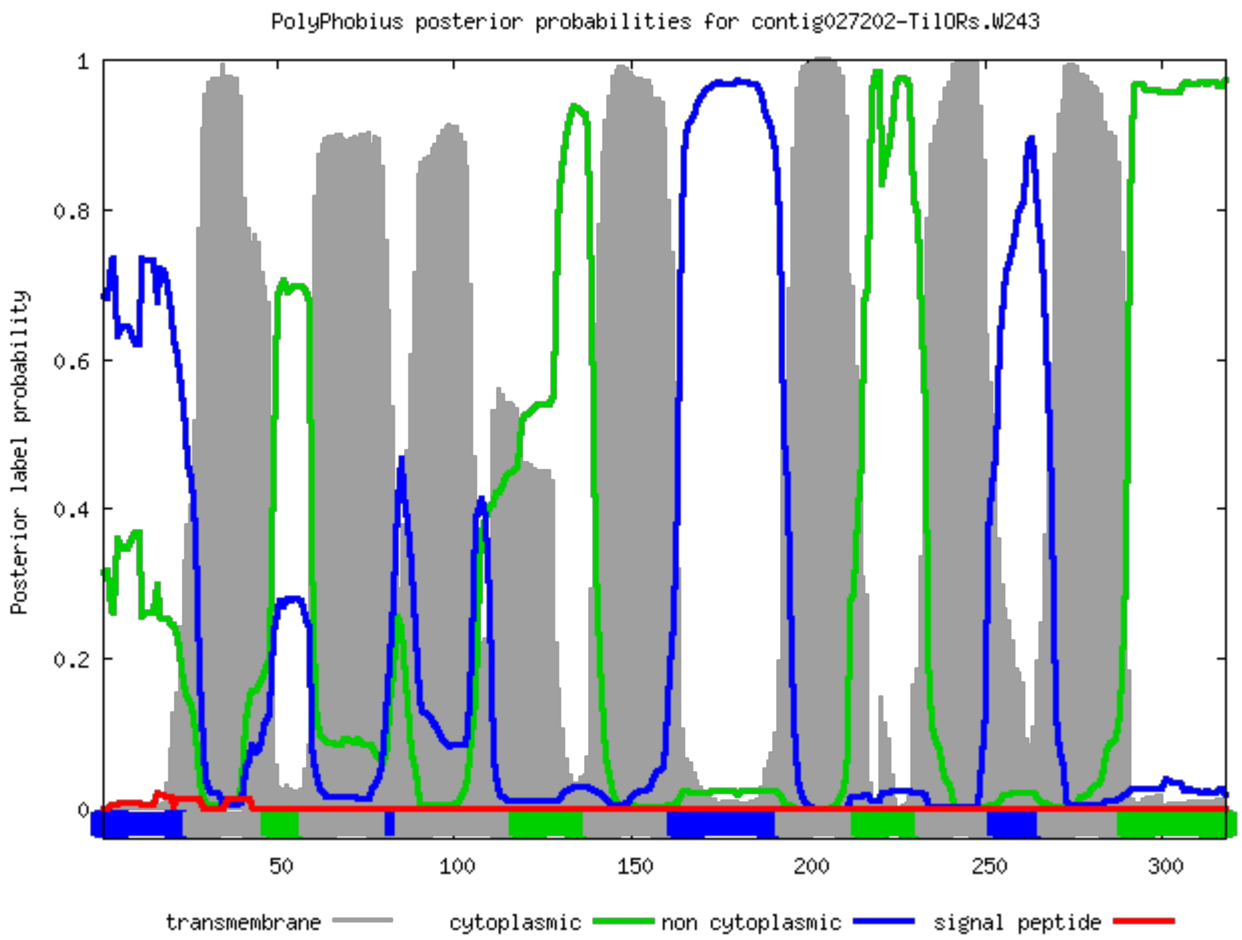

The prediction is based on an alignment. The probability data used in the plot is found here, and the gnuplot script is here.

Prediction of contig073387-BriOR.Z129

```
ID   contig073387-BriOR.Z129
FT   TOPO_DOM      1     49       NON CYTOPLASMIC.
FT   TRANSMEM     50     75
FT   TOPO_DOM     76     87       CYTOPLASMIC.
FT   TRANSMEM     88    109
FT   TOPO_DOM    110    124       NON CYTOPLASMIC.
FT   TRANSMEM    125    147
FT   TOPO_DOM    148    167       CYTOPLASMIC.
FT   TRANSMEM    168    190
FT   TOPO_DOM    191    227       NON CYTOPLASMIC.
FT   TRANSMEM    228    249
FT   TOPO_DOM    250    269       CYTOPLASMIC.
FT   TRANSMEM    270    293
FT   TOPO_DOM    294    325       NON CYTOPLASMIC.
FT   TRANSMEM    326    347
FT   TOPO_DOM    348    391       CYTOPLASMIC.
//
```

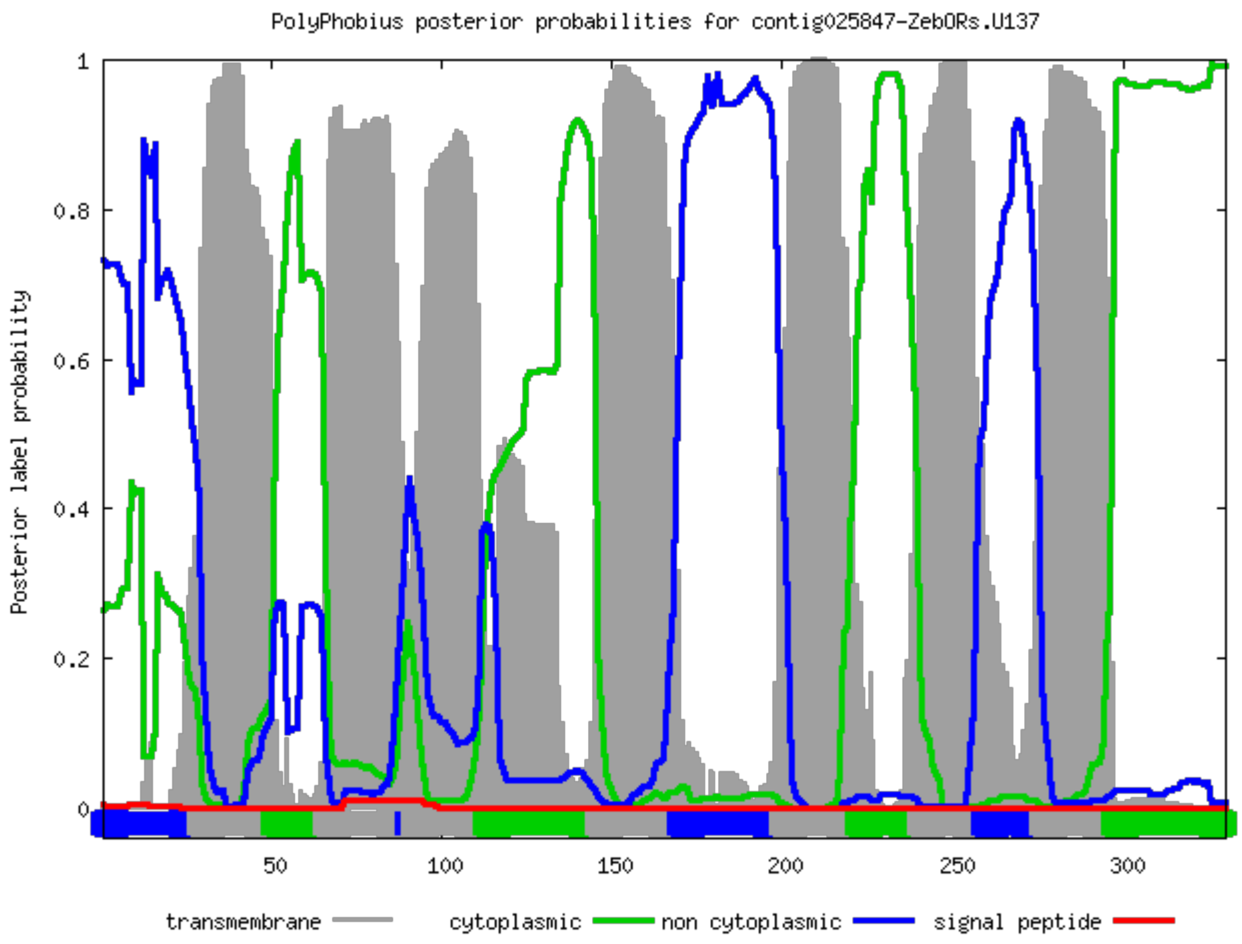

The prediction is based on an alignment. The probability data used in the plot is found here, and the gnuplot script is here.

Prediction of contig034988-NyeOR.A005

```
ID   contig034988-NyeOR.A005
FT   TOPO_DOM      1     22       NON CYTOPLASMIC.
FT   TRANSMEM     23     48
FT   TOPO_DOM     49     56       CYTOPLASMIC.
FT   TRANSMEM     57     76
FT   TOPO_DOM     77     95       NON CYTOPLASMIC.
FT   TRANSMEM     96    118
FT   TOPO_DOM    119    138       CYTOPLASMIC.
FT   TRANSMEM    139    159
FT   TOPO_DOM    160    192       NON CYTOPLASMIC.
FT   TRANSMEM    193    215
FT   TOPO_DOM    216    235       CYTOPLASMIC.
FT   TRANSMEM    236    257
FT   TOPO_DOM    258    268       NON CYTOPLASMIC.
FT   TRANSMEM    269    289
FT   TOPO_DOM    290    311       CYTOPLASMIC.
//
```

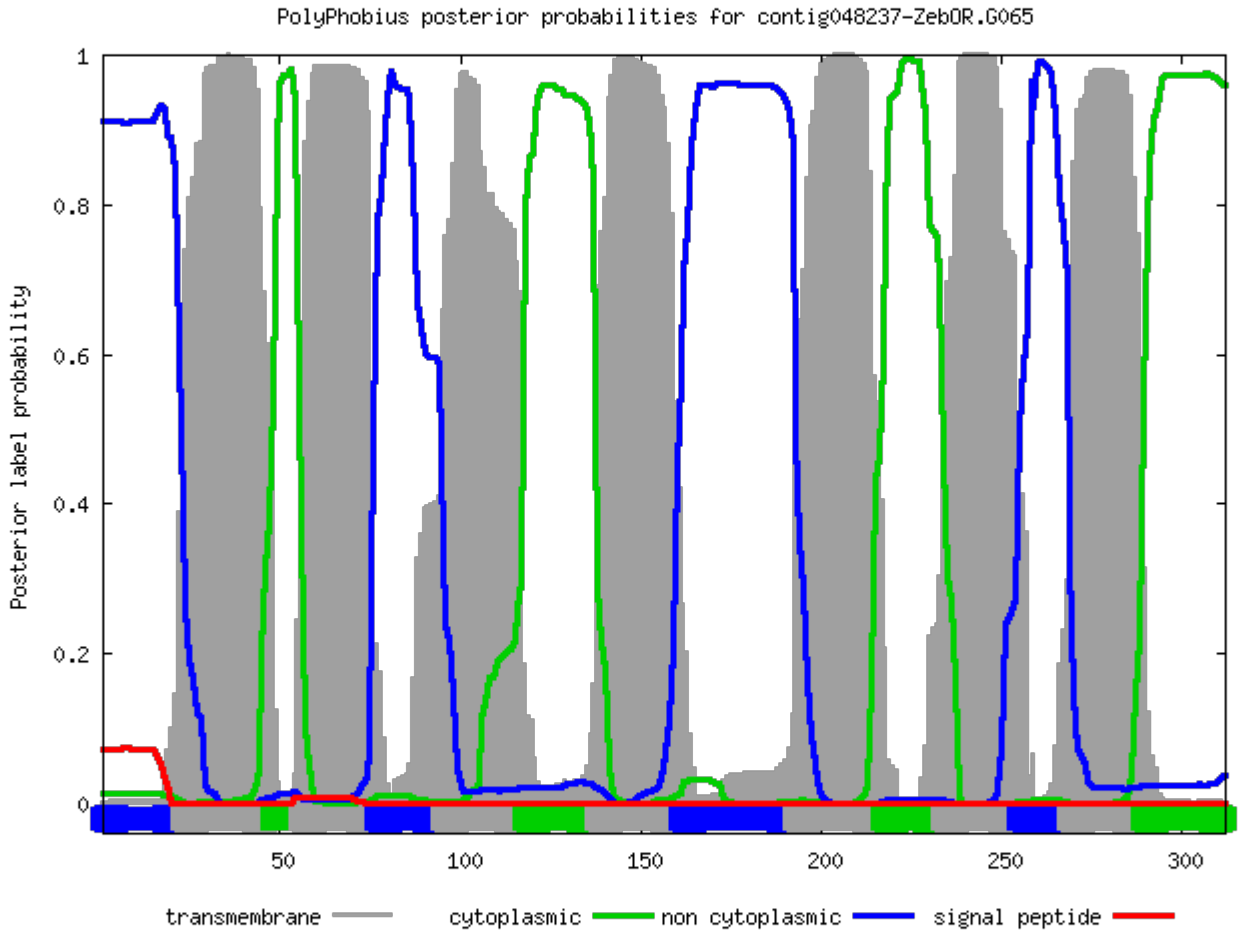

The prediction is based on an alignment. The probability data used in the plot is found here, and the gnuplot script is here.

Prediction of contig039451-TilOR.J266

```
ID   contig039451-TilOR.J266
FT   TOPO_DOM      1     23       NON CYTOPLASMIC.
FT   TRANSMEM     24     49
FT   TOPO_DOM     50     59       CYTOPLASMIC.
FT   TRANSMEM     60     81
FT   TOPO_DOM     82     97       NON CYTOPLASMIC.
FT   TRANSMEM     98    119
FT   TOPO_DOM    120    139       CYTOPLASMIC.
FT   TRANSMEM    140    162
FT   TOPO_DOM    163    194       NON CYTOPLASMIC.
FT   TRANSMEM    195    218
FT   TOPO_DOM    219    236       CYTOPLASMIC.
FT   TRANSMEM    237    260
FT   TOPO_DOM    261    270       NON CYTOPLASMIC.
FT   TRANSMEM    271    291
FT   TOPO_DOM    292    313       CYTOPLASMIC.
//
```

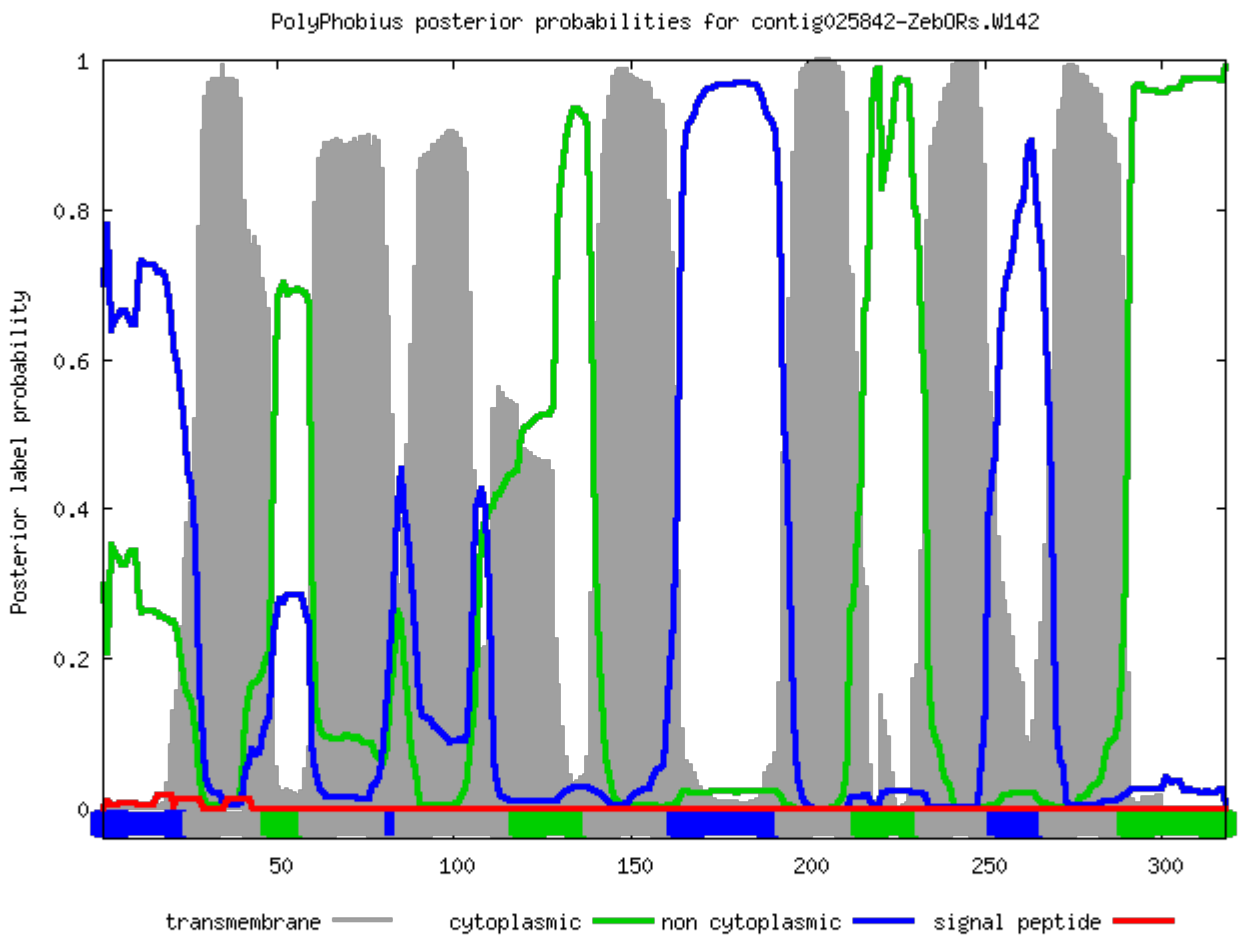

The prediction is based on an alignment. The probability data used in the plot is found here, and the gnuplot script is here.

Prediction of contig048562-BurOR.H062

```
ID    contig048562-BurOR.H062
FT    TOPO_DOM      1      23       NON CYTOPLASMIC.
FT    TRANSMEM     24      49
FT    TOPO_DOM     50      56       CYTOPLASMIC.
FT    TRANSMEM     57      76
FT    TOPO_DOM     77      95       NON CYTOPLASMIC.
FT    TRANSMEM     96     118
FT    TOPO_DOM    119     138       CYTOPLASMIC.
FT    TRANSMEM    139     160
FT    TOPO_DOM    161     196       NON CYTOPLASMIC.
FT    TRANSMEM    197     219
FT    TOPO_DOM    220     237       CYTOPLASMIC.
FT    TRANSMEM    238     260
FT    TOPO_DOM    261     271       NON CYTOPLASMIC.
FT    TRANSMEM    272     291
FT    TOPO_DOM    292     310       CYTOPLASMIC.
//
```

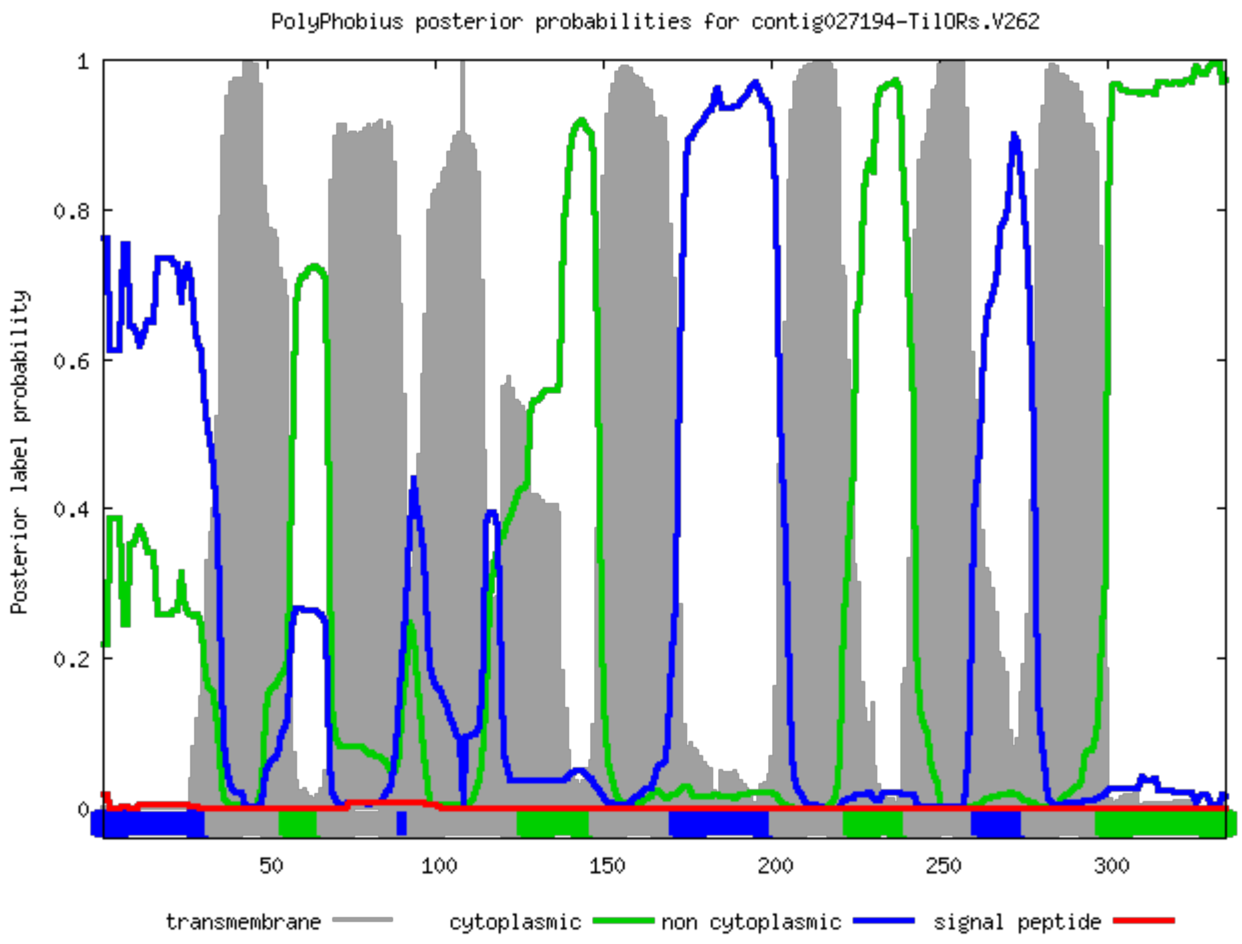

The prediction is based on an alignment. The probability data used in the plot is found here, and the gnuplot script is here.

Prediction of contig042540-BriOR.J123

```
ID   contig042540-BriOR.J123
FT   TOPO_DOM      1     23       NON CYTOPLASMIC.
FT   TRANSMEM     24     49
FT   TOPO_DOM     50     59       CYTOPLASMIC.
FT   TRANSMEM     60     81
FT   TOPO_DOM     82     97       NON CYTOPLASMIC.
FT   TRANSMEM     98    119
FT   TOPO_DOM    120    139       CYTOPLASMIC.
FT   TRANSMEM    140    162
FT   TOPO_DOM    163    194       NON CYTOPLASMIC.
FT   TRANSMEM    195    219
FT   TOPO_DOM    220    237       CYTOPLASMIC.
FT   TRANSMEM    238    260
FT   TOPO_DOM    261    270       NON CYTOPLASMIC.
FT   TRANSMEM    271    291
FT   TOPO_DOM    292    312       CYTOPLASMIC.
//
```

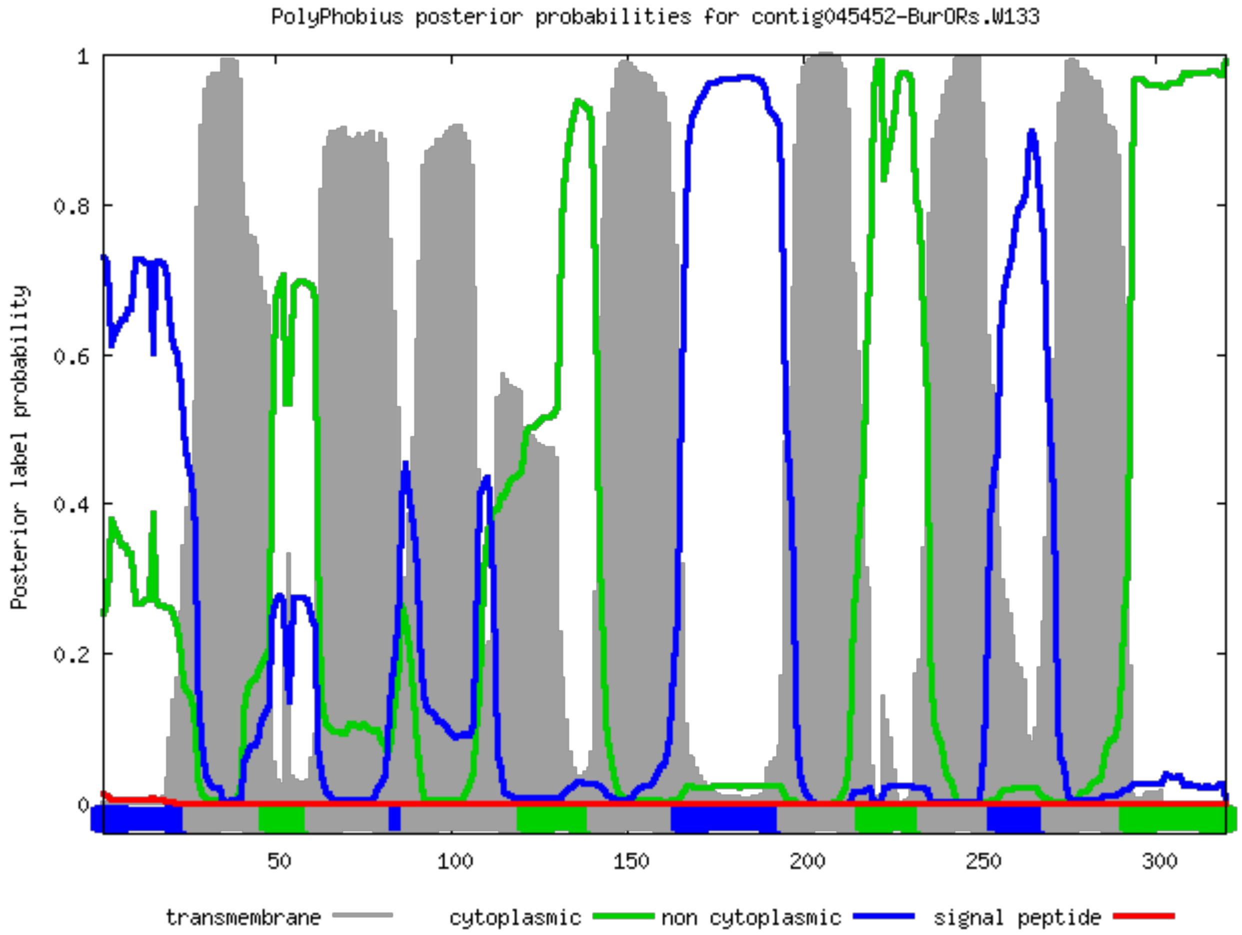

The prediction is based on an alignment. The probability data used in the plot is found here, and the gnuplot script is here.

Prediction of contig017781-ZebOR.J083

```
ID    contig017781-ZebOR.J083
FT    TOPO_DOM      1      25       NON CYTOPLASMIC.
FT    TRANSMEM     26      50
FT    TOPO_DOM     51      60       CYTOPLASMIC.
FT    TRANSMEM     61      82
FT    TOPO_DOM     83      98       NON CYTOPLASMIC.
FT    TRANSMEM     99     120
FT    TOPO_DOM    121     140       CYTOPLASMIC.
FT    TRANSMEM    141     163
FT    TOPO_DOM    164     195       NON CYTOPLASMIC.
FT    TRANSMEM    196     220
FT    TOPO_DOM    221     238       CYTOPLASMIC.
FT    TRANSMEM    239     261
FT    TOPO_DOM    262     271       NON CYTOPLASMIC.
FT    TRANSMEM    272     292
FT    TOPO_DOM    293     312       CYTOPLASMIC.
//
```

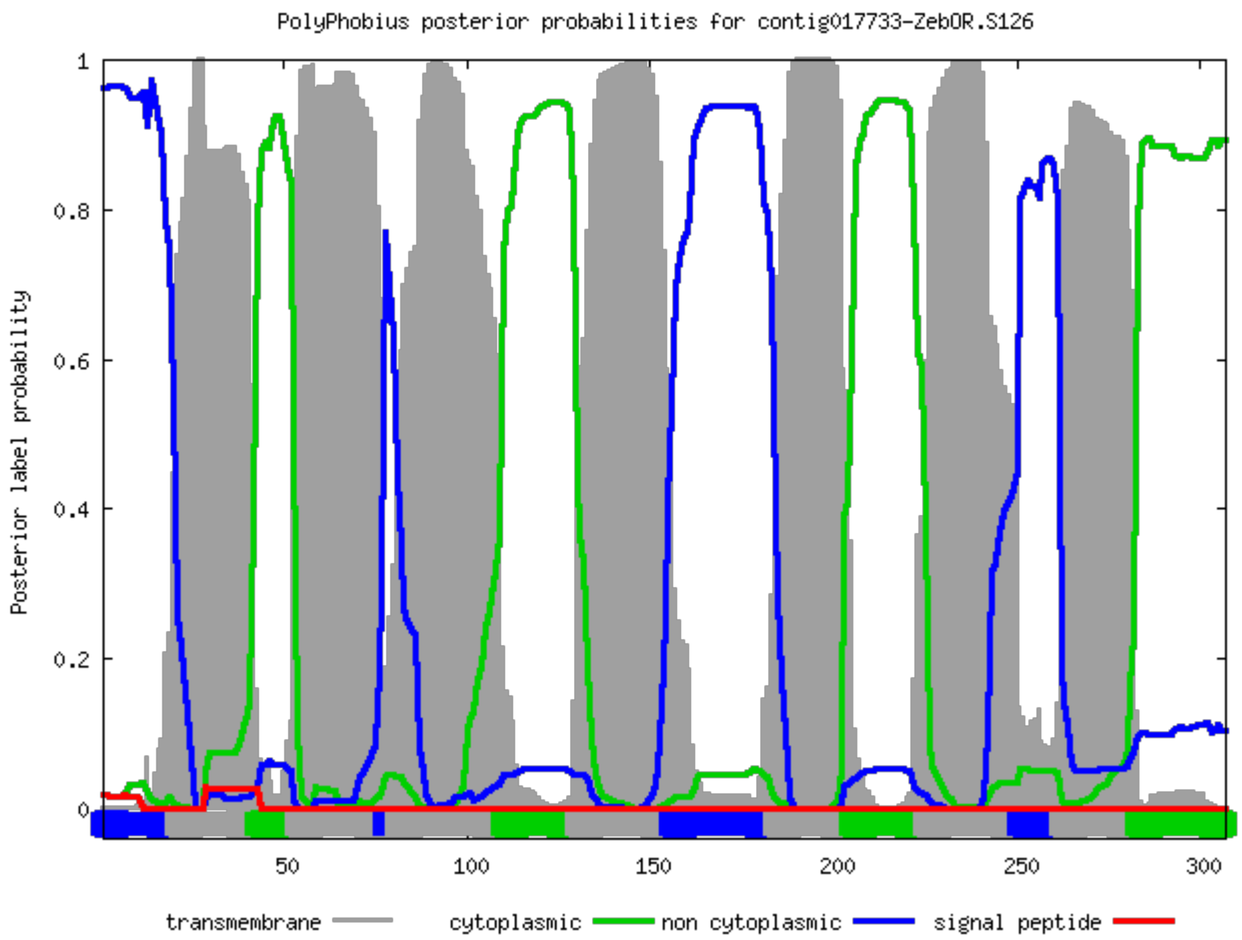

The prediction is based on an alignment. The probability data used in the plot is found here, and the gnuplot script is here.

Prediction of contig004270-BriOR.P100

```
ID    contig004270-BriOR.P100
FT    TOPO_DOM      1     28       NON CYTOPLASMIC.
FT    TRANSMEM     29     52
FT    TOPO_DOM     53     62       CYTOPLASMIC.
FT    TRANSMEM     63     89
FT    TOPO_DOM     90    101       NON CYTOPLASMIC.
FT    TRANSMEM    102    123
FT    TOPO_DOM    124    143       CYTOPLASMIC.
FT    TRANSMEM    144    166
FT    TOPO_DOM    167    201       NON CYTOPLASMIC.
FT    TRANSMEM    202    226
FT    TOPO_DOM    227    240       CYTOPLASMIC.
FT    TRANSMEM    241    263
FT    TOPO_DOM    264    274       NON CYTOPLASMIC.
FT    TRANSMEM    275    295
FT    TOPO_DOM    296    311       CYTOPLASMIC.
//
```

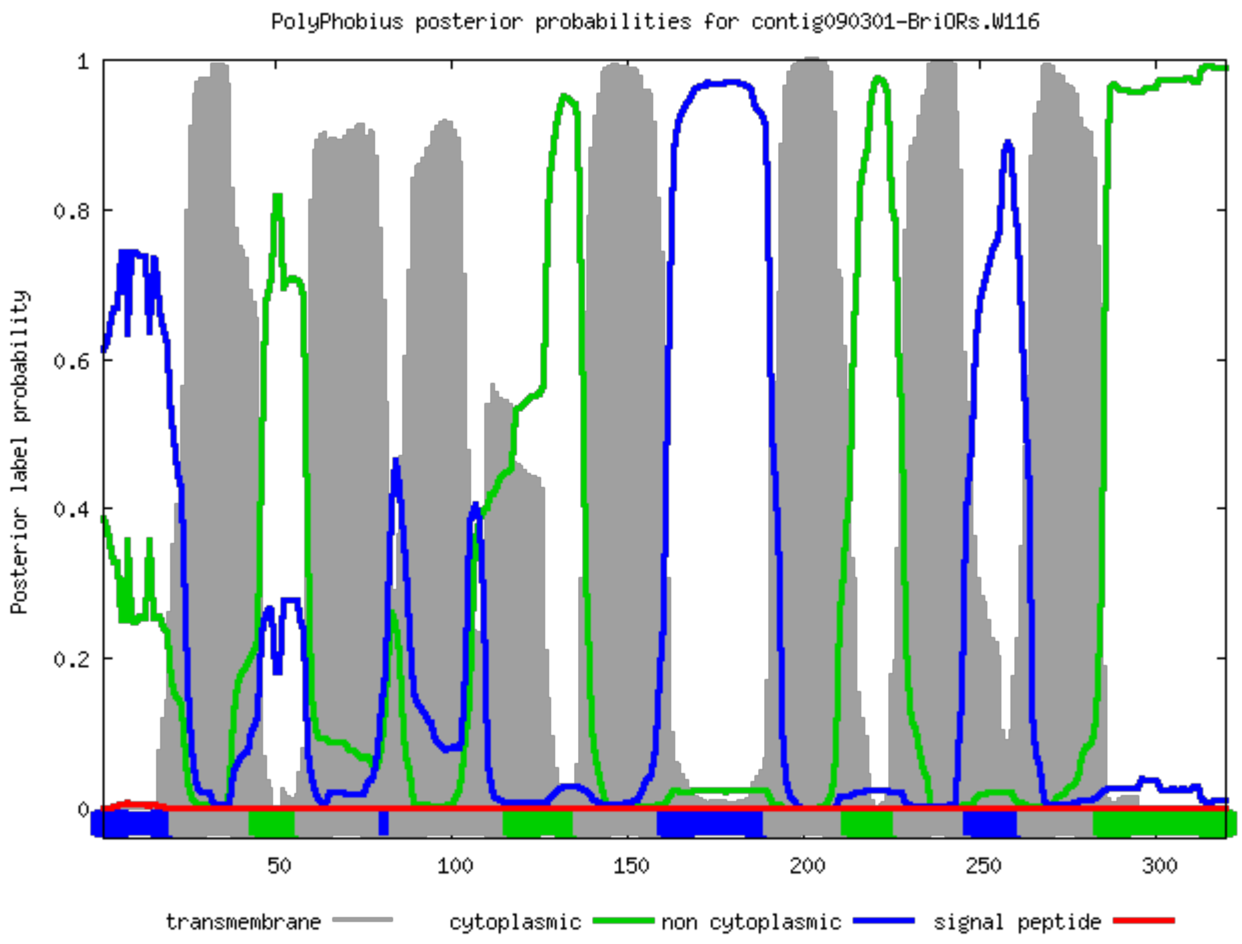

The prediction is based on an alignment. The probability data used in the plot is found here, and the gnuplot script is here.

Prediction of contig042928-BurOR.N108

```
ID   contig042928-BurOR.N108
FT   TOPO_DOM      1     32       NON CYTOPLASMIC.
FT   TRANSMEM     33     58
FT   TOPO_DOM     59     66       CYTOPLASMIC.
FT   TRANSMEM     67     86
FT   TOPO_DOM     87    104       NON CYTOPLASMIC.
FT   TRANSMEM    105    127
FT   TOPO_DOM    128    146       CYTOPLASMIC.
FT   TRANSMEM    147    170
FT   TOPO_DOM    171    207       NON CYTOPLASMIC.
FT   TRANSMEM    208    232
FT   TOPO_DOM    233    250       CYTOPLASMIC.
FT   TRANSMEM    251    272
FT   TOPO_DOM    273    277       NON CYTOPLASMIC.
FT   TRANSMEM    278    298
FT   TOPO_DOM    299    323       CYTOPLASMIC.
//
```

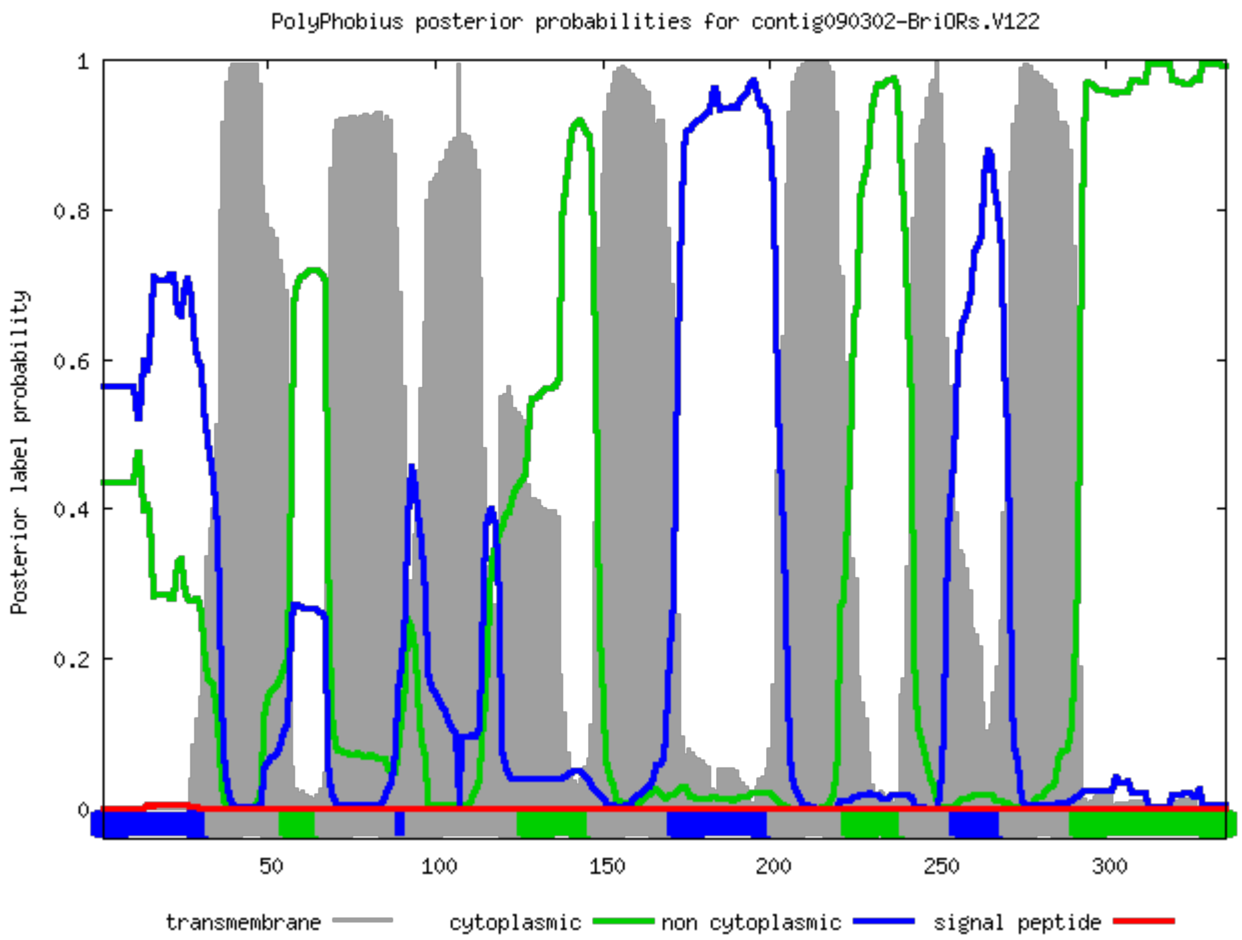

The prediction is based on an alignment. The probability data used in the plot is found here, and the gnuplot script is here.

Prediction of contig046340-TilOR.N188

```
ID   contig046340-TilOR.N188
FT   TOPO_DOM      1     33       NON CYTOPLASMIC.
FT   TRANSMEM     34     59
FT   TOPO_DOM     60     67       CYTOPLASMIC.
FT   TRANSMEM     68     89
FT   TOPO_DOM     90    108       NON CYTOPLASMIC.
FT   TRANSMEM    109    128
FT   TOPO_DOM    129    148       CYTOPLASMIC.
FT   TRANSMEM    149    171
FT   TOPO_DOM    172    207       NON CYTOPLASMIC.
FT   TRANSMEM    208    233
FT   TOPO_DOM    234    252       CYTOPLASMIC.
FT   TRANSMEM    253    274
FT   TOPO_DOM    275    337       NON CYTOPLASMIC.
//
```

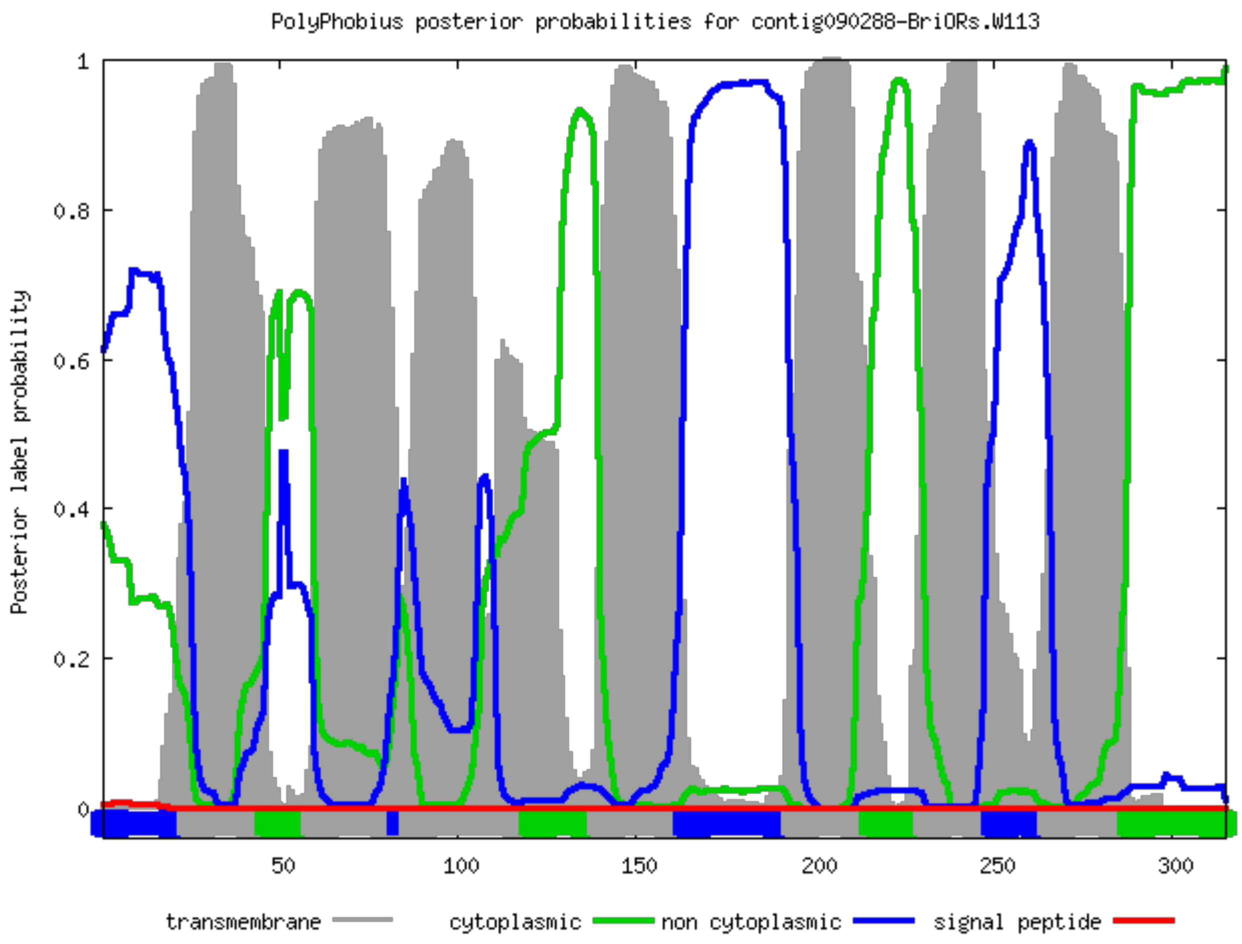

The prediction is based on an alignment. The probability data used in the plot is found here, and the gnuplot script is here.

Prediction of contig040653-BurOR.B030

```
ID   contig040653-BurOR.B030
FT   TOPO_DOM      1      28       NON CYTOPLASMIC.
FT   TRANSMEM     29      54
FT   TOPO_DOM     55      62       CYTOPLASMIC.
FT   TRANSMEM     63      83
FT   TOPO_DOM     84     103       NON CYTOPLASMIC.
FT   TRANSMEM    104     126
FT   TOPO_DOM    127     146       CYTOPLASMIC.
FT   TRANSMEM    147     168
FT   TOPO_DOM    169     203       NON CYTOPLASMIC.
FT   TRANSMEM    204     227
FT   TOPO_DOM    228     246       CYTOPLASMIC.
FT   TRANSMEM    247     268
FT   TOPO_DOM    269     277       NON CYTOPLASMIC.
FT   TRANSMEM    278     300
FT   TOPO_DOM    301     320       CYTOPLASMIC.
//
```

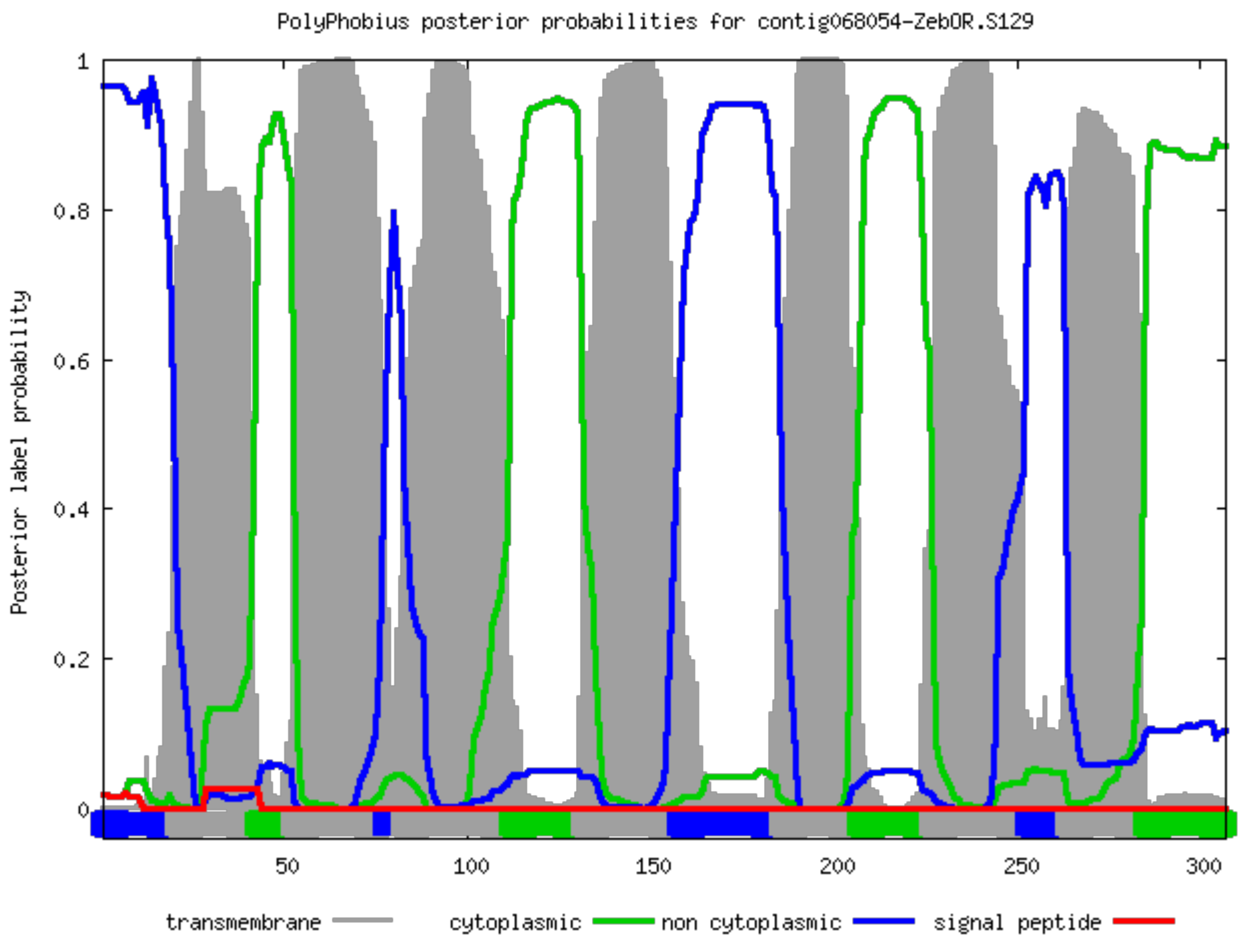

The prediction is based on an alignment. The probability data used in the plot is found here, and the gnuplot script is here.

Prediction of contig052457-BurOR.E051

```
ID    contig052457-BurOR.E051
FT    TOPO_DOM      1      23       NON CYTOPLASMIC.
FT    TRANSMEM     24      49
FT    TOPO_DOM     50      58       CYTOPLASMIC.
FT    TRANSMEM     59      82
FT    TOPO_DOM     83      93       NON CYTOPLASMIC.
FT    TRANSMEM     94     119
FT    TOPO_DOM    120     139       CYTOPLASMIC.
FT    TRANSMEM    140     161
FT    TOPO_DOM    162     194       NON CYTOPLASMIC.
FT    TRANSMEM    195     217
FT    TOPO_DOM    218     237       CYTOPLASMIC.
FT    TRANSMEM    238     257
FT    TOPO_DOM    258     268       NON CYTOPLASMIC.
FT    TRANSMEM    269     292
FT    TOPO_DOM    293     310       CYTOPLASMIC.
//
```

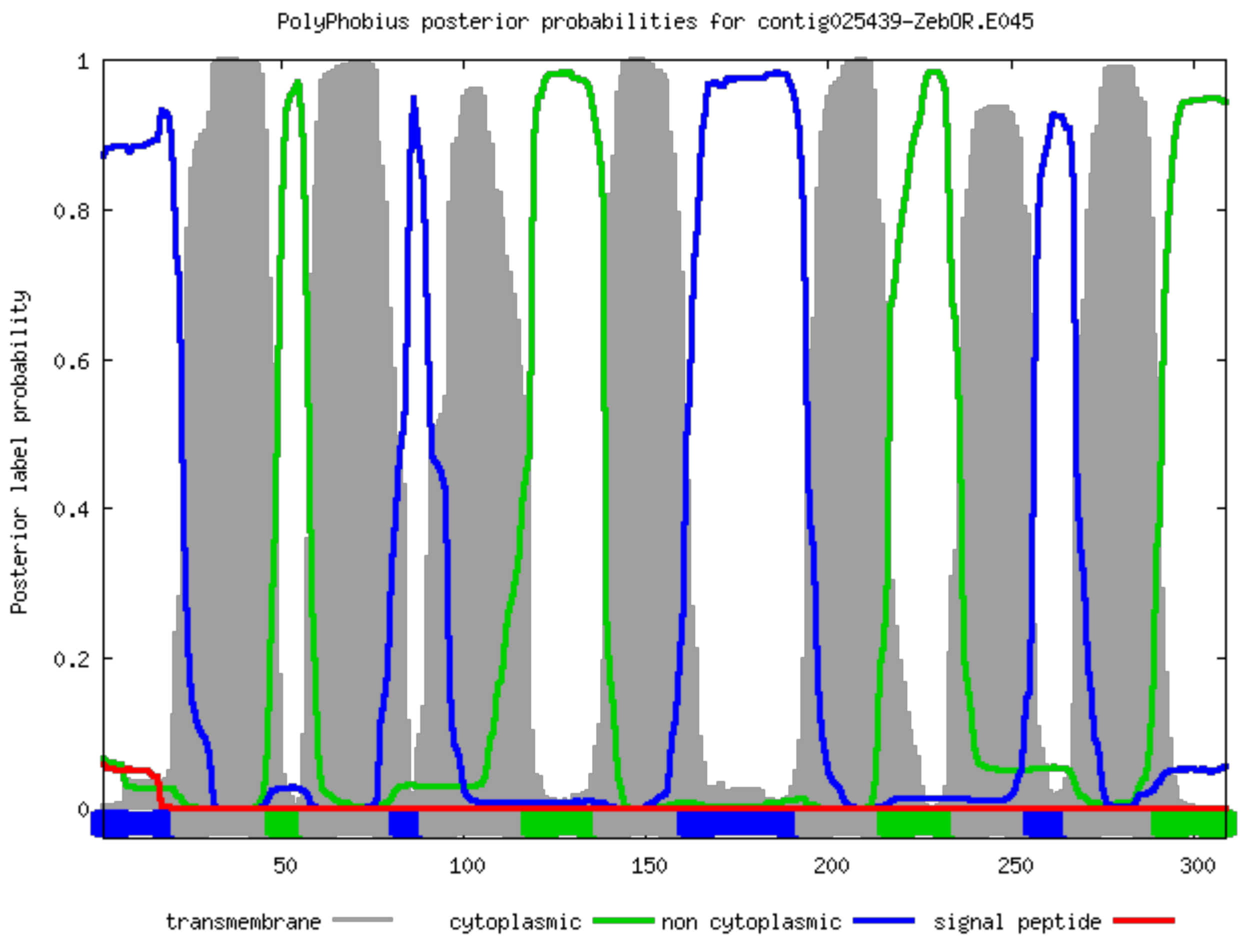

The prediction is based on an alignment. The probability data used in the plot is found here, and the gnuplot script is here.

Prediction of contig004258-BriOR.E037

```
ID   contig004258-BriOR.E037
FT   TOPO_DOM      1     23       NON CYTOPLASMIC.
FT   TRANSMEM     24     49
FT   TOPO_DOM     50     58       CYTOPLASMIC.
FT   TRANSMEM     59     83
FT   TOPO_DOM     84     93       NON CYTOPLASMIC.
FT   TRANSMEM     94    119
FT   TOPO_DOM    120    139       CYTOPLASMIC.
FT   TRANSMEM    140    161
FT   TOPO_DOM    162    194       NON CYTOPLASMIC.
FT   TRANSMEM    195    216
FT   TOPO_DOM    217    236       CYTOPLASMIC.
FT   TRANSMEM    237    256
FT   TOPO_DOM    257    267       NON CYTOPLASMIC.
FT   TRANSMEM    268    291
FT   TOPO_DOM    292    321       CYTOPLASMIC.
//
```

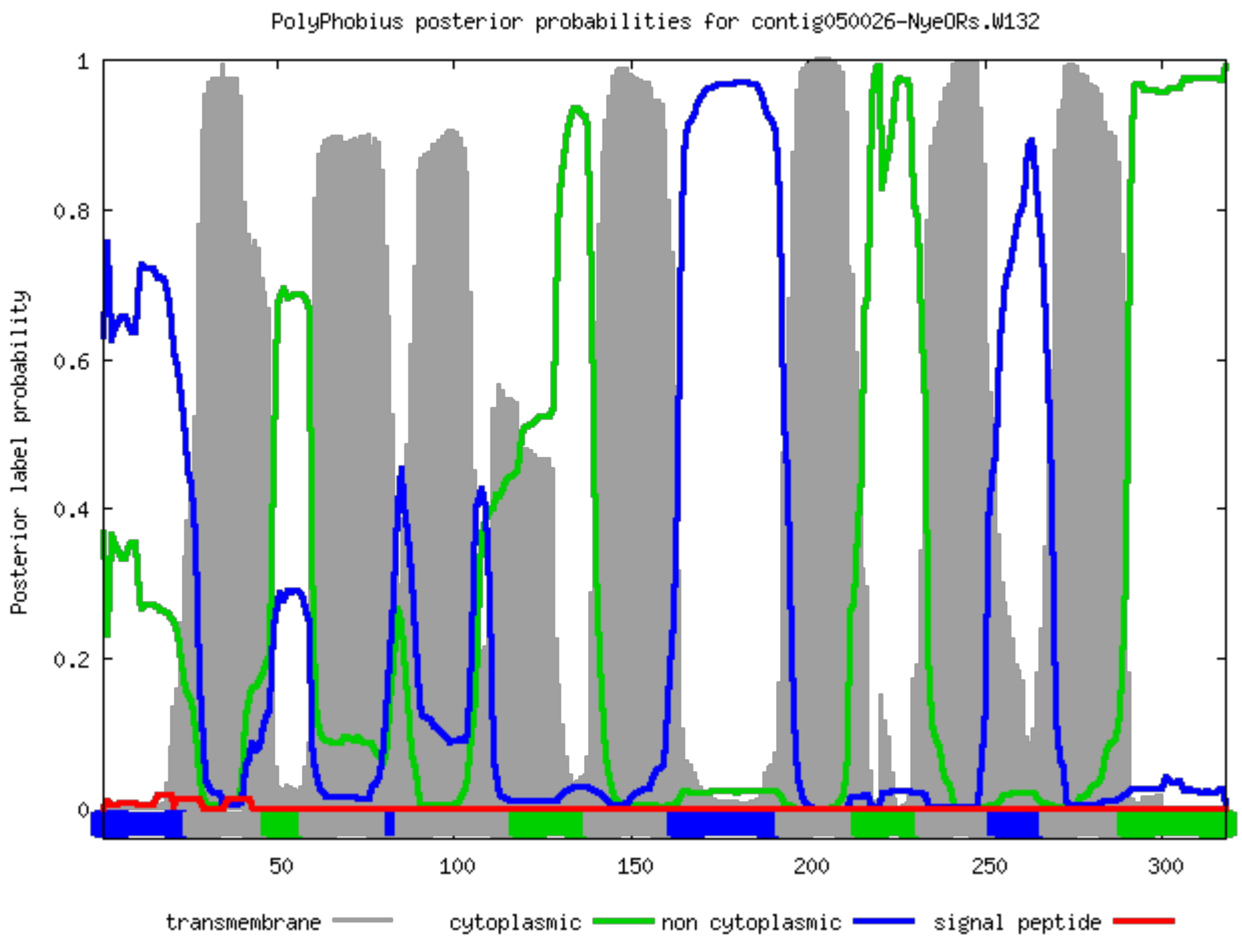

The prediction is based on an alignment. The probability data used in the plot is found here, and the gnuplot script is here.

Prediction of contig085010-BriOR.A005

```
ID    contig085010-BriOR.A005
FT    TOPO_DOM       1      25       NON CYTOPLASMIC.
FT    TRANSMEM      26      51
FT    TOPO_DOM      52      59       CYTOPLASMIC.
FT    TRANSMEM      60      80
FT    TOPO_DOM      81      98       NON CYTOPLASMIC.
FT    TRANSMEM      99     121
FT    TOPO_DOM     122     141       CYTOPLASMIC.
FT    TRANSMEM     142     162
FT    TOPO_DOM     163     195       NON CYTOPLASMIC.
FT    TRANSMEM     196     219
FT    TOPO_DOM     220     239       CYTOPLASMIC.
FT    TRANSMEM     240     261
FT    TOPO_DOM     262     272       NON CYTOPLASMIC.
FT    TRANSMEM     273     293
FT    TOPO_DOM     294     319       CYTOPLASMIC.
//
```

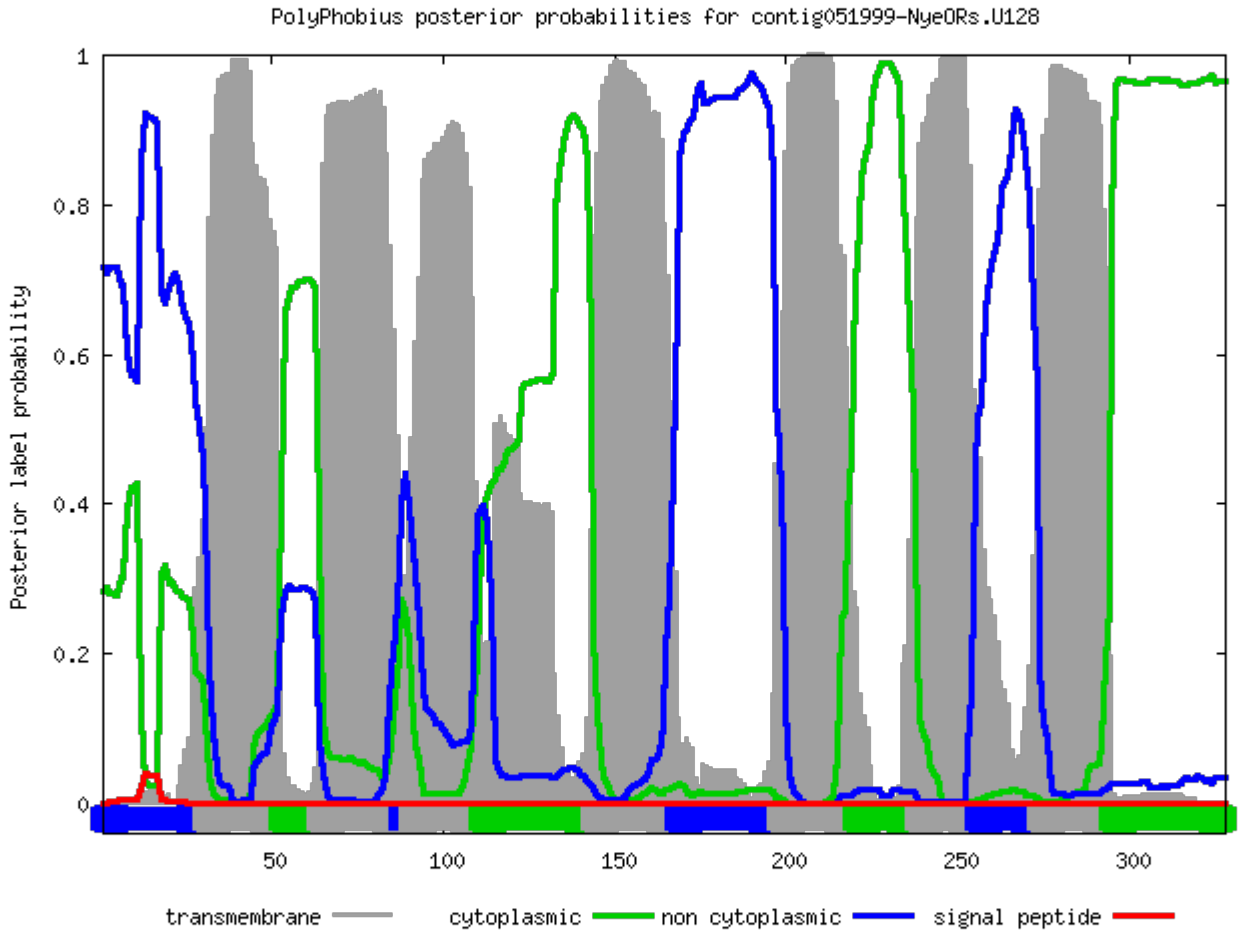

The prediction is based on an alignment. The probability data used in the plot is found here, and the gnuplot script is here.

Prediction of contig014047-ZebOR.D036

```
ID   contig014047-ZebOR.D036
FT   TOPO_DOM      1     22       NON CYTOPLASMIC.
FT   TRANSMEM     23     48
FT   TOPO_DOM     49     57       CYTOPLASMIC.
FT   TRANSMEM     58     81
FT   TOPO_DOM     82     90       NON CYTOPLASMIC.
FT   TRANSMEM     91    118
FT   TOPO_DOM    119    138       CYTOPLASMIC.
FT   TRANSMEM    139    162
FT   TOPO_DOM    163    194       NON CYTOPLASMIC.
FT   TRANSMEM    195    216
FT   TOPO_DOM    217    235       CYTOPLASMIC.
FT   TRANSMEM    236    256
FT   TOPO_DOM    257    266       NON CYTOPLASMIC.
FT   TRANSMEM    267    289
FT   TOPO_DOM    290    308       CYTOPLASMIC.
//
```

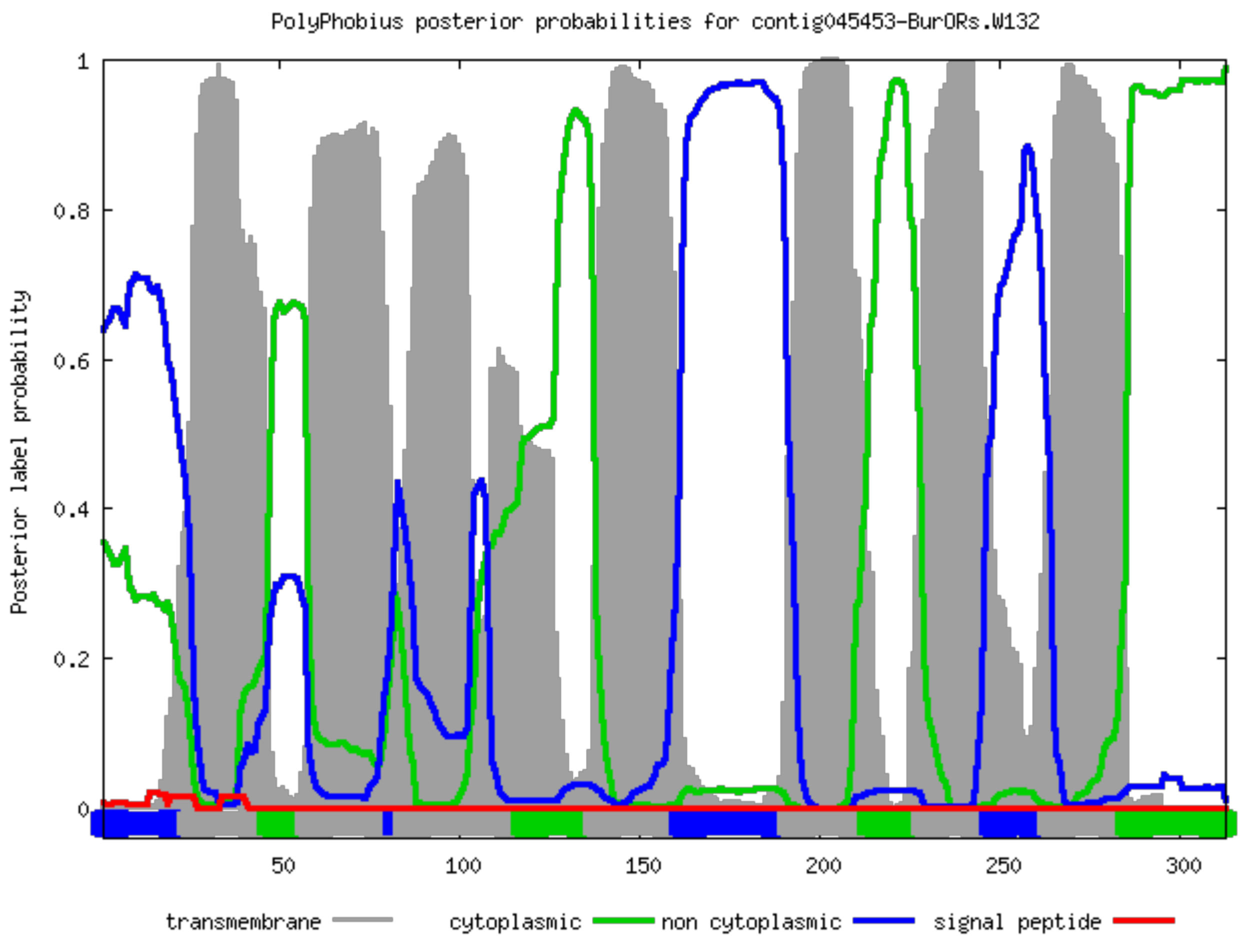

The prediction is based on an alignment. The probability data used in the plot is found here, and the gnuplot script is here.

Prediction of contig047526-ZebOR.A021

```
ID   contig047526-ZebOR.A021
FT   TOPO_DOM      1     22       NON CYTOPLASMIC.
FT   TRANSMEM     23     48
FT   TOPO_DOM     49     56       CYTOPLASMIC.
FT   TRANSMEM     57     77
FT   TOPO_DOM     78     95       NON CYTOPLASMIC.
FT   TRANSMEM     96    118
FT   TOPO_DOM    119    138       CYTOPLASMIC.
FT   TRANSMEM    139    160
FT   TOPO_DOM    161    192       NON CYTOPLASMIC.
FT   TRANSMEM    193    215
FT   TOPO_DOM    216    235       CYTOPLASMIC.
FT   TRANSMEM    236    257
FT   TOPO_DOM    258    268       NON CYTOPLASMIC.
FT   TRANSMEM    269    289
FT   TOPO_DOM    290    306       CYTOPLASMIC.
//
```

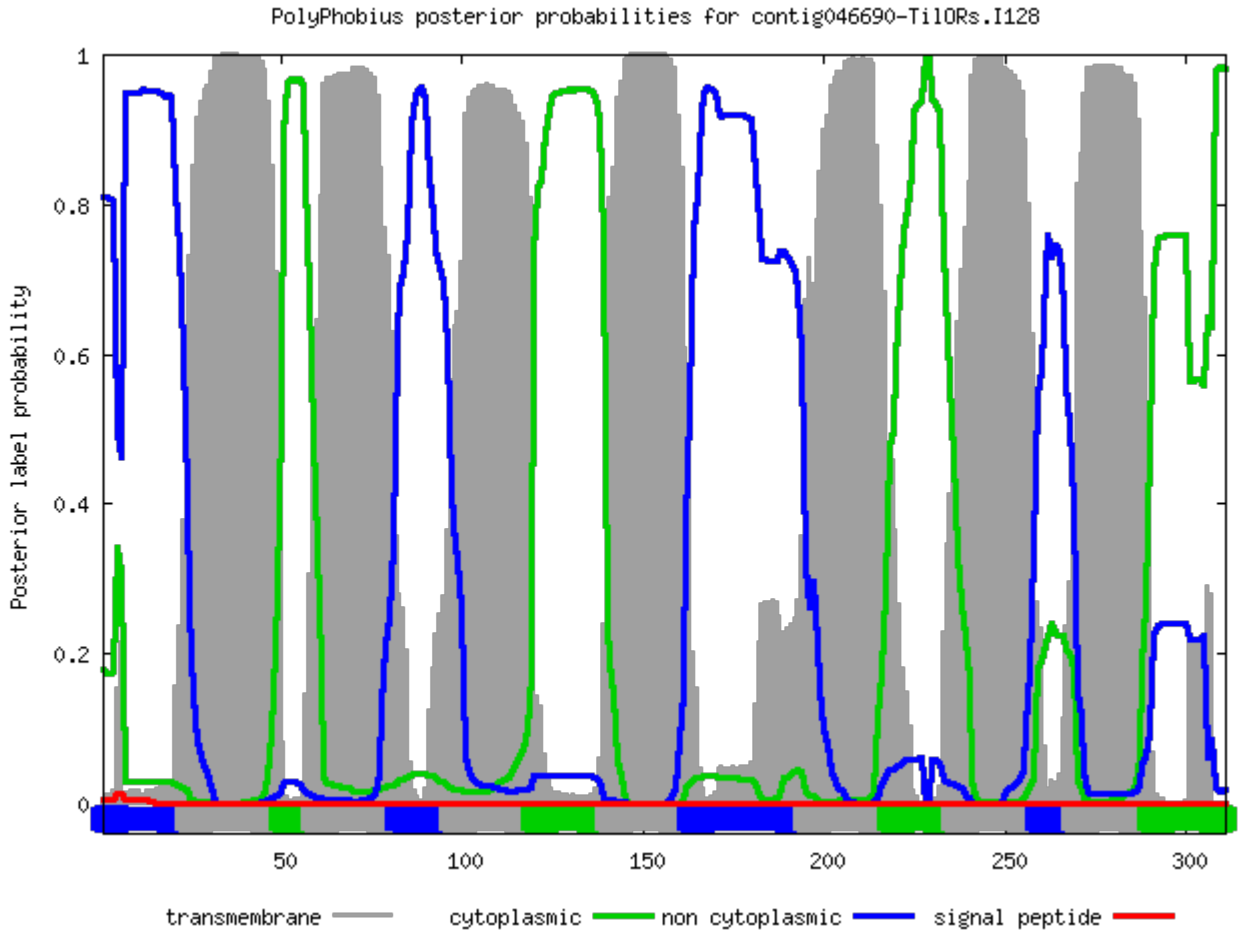

The prediction is based on an alignment. The probability data used in the plot is found here, and the gnuplot script is here.

Prediction of contig065027-BurOR.L090

```
ID    contig065027-BurOR.L090
FT    TOPO_DOM      1      27        NON CYTOPLASMIC.
FT    TRANSMEM     28      52
FT    TOPO_DOM     53      61        CYTOPLASMIC.
FT    TRANSMEM     62      85
FT    TOPO_DOM     86     100        NON CYTOPLASMIC.
FT    TRANSMEM    101     122
FT    TOPO_DOM    123     142        CYTOPLASMIC.
FT    TRANSMEM    143     164
FT    TOPO_DOM    165     200        NON CYTOPLASMIC.
FT    TRANSMEM    201     226
FT    TOPO_DOM    227     240        CYTOPLASMIC.
FT    TRANSMEM    241     262
FT    TOPO_DOM    263     273        NON CYTOPLASMIC.
FT    TRANSMEM    274     294
FT    TOPO_DOM    295     315        CYTOPLASMIC.
//
```

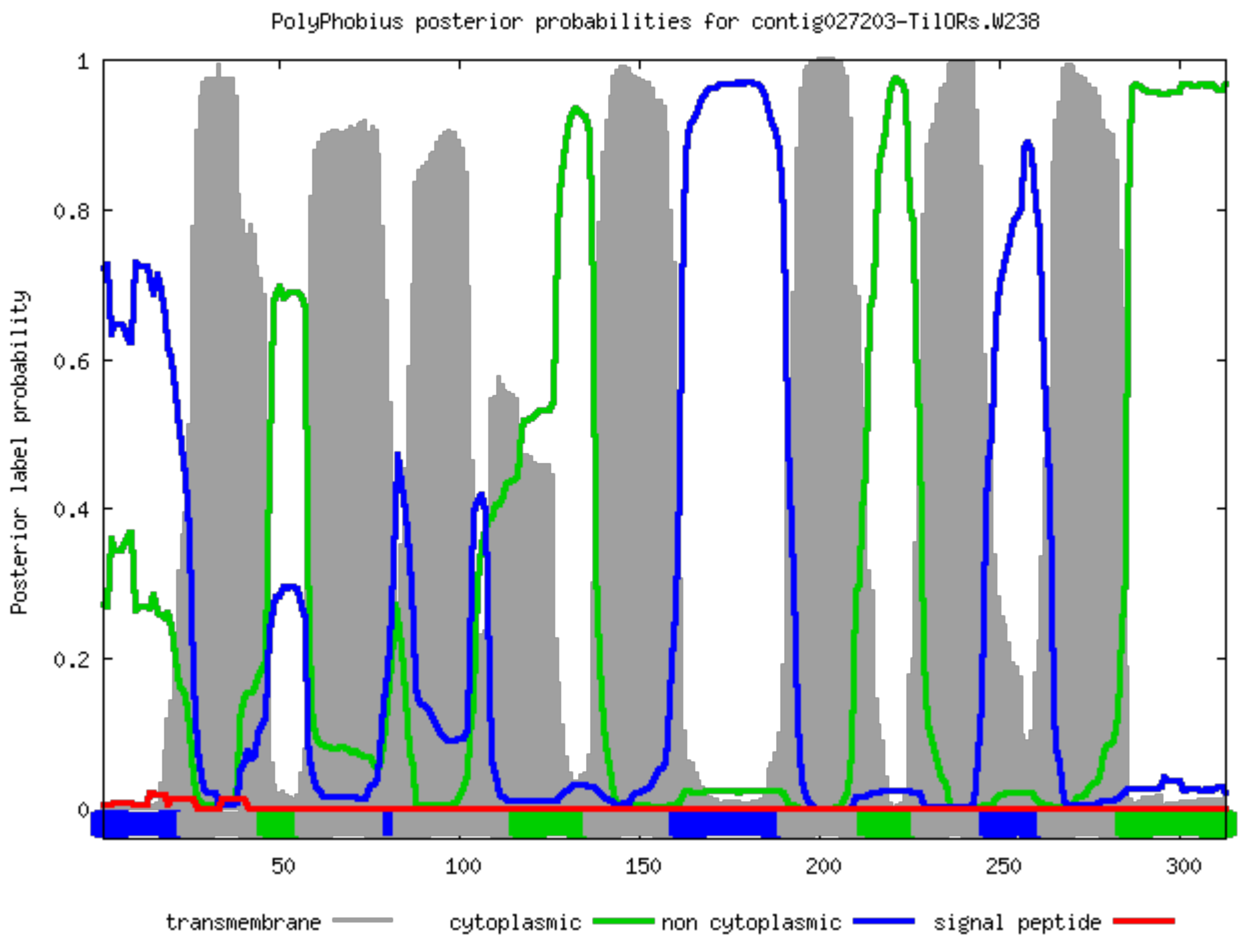

The prediction is based on an alignment. The probability data used in the plot is found here, and the gnuplot script is here.

Prediction of contig010727-ZebOR.N114

```
ID   contig010727-ZebOR.N114
FT   TOPO_DOM      1     33       NON CYTOPLASMIC.
FT   TRANSMEM     34     59
FT   TOPO_DOM     60     67       CYTOPLASMIC.
FT   TRANSMEM     68     89
FT   TOPO_DOM     90    108       NON CYTOPLASMIC.
FT   TRANSMEM    109    128
FT   TOPO_DOM    129    148       CYTOPLASMIC.
FT   TRANSMEM    149    171
FT   TOPO_DOM    172    207       NON CYTOPLASMIC.
FT   TRANSMEM    208    233
FT   TOPO_DOM    234    252       CYTOPLASMIC.
FT   TRANSMEM    253    275
FT   TOPO_DOM    276    327       NON CYTOPLASMIC.
//
```

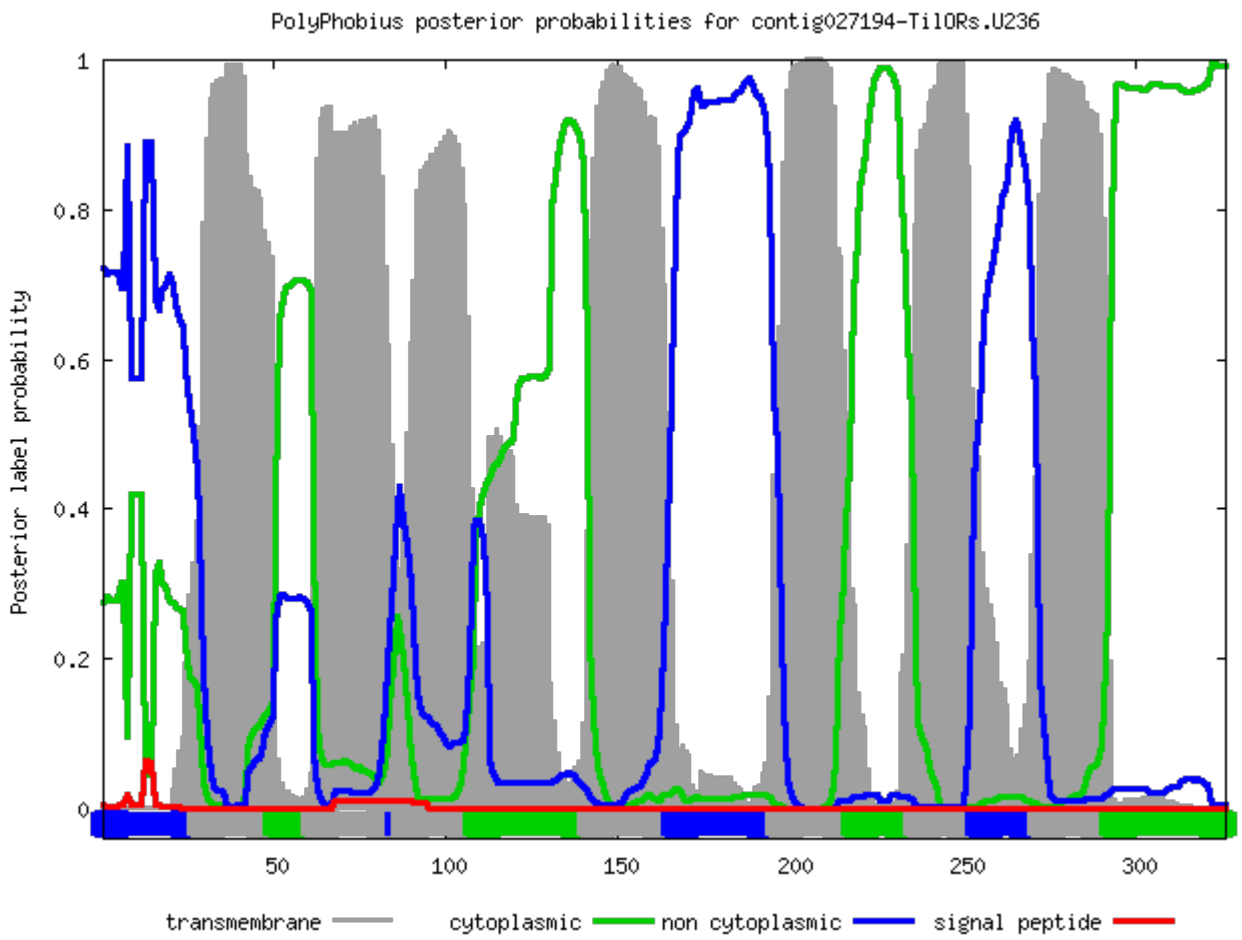

The prediction is based on an alignment. The probability data used in the plot is found here, and the gnuplot script is here.

Prediction of contig062053-NyeOR.O103

```
ID   contig062053-NyeOR.O103
FT   TOPO_DOM      1     24       NON CYTOPLASMIC.
FT   TRANSMEM     25     51
FT   TOPO_DOM     52     59       CYTOPLASMIC.
FT   TRANSMEM     60     83
FT   TOPO_DOM     84     97       NON CYTOPLASMIC.
FT   TRANSMEM     98    121
FT   TOPO_DOM    122    141       CYTOPLASMIC.
FT   TRANSMEM    142    163
FT   TOPO_DOM    164    200       NON CYTOPLASMIC.
FT   TRANSMEM    201    227
FT   TOPO_DOM    228    241       CYTOPLASMIC.
FT   TRANSMEM    242    262
FT   TOPO_DOM    263    273       NON CYTOPLASMIC.
FT   TRANSMEM    274    295
FT   TOPO_DOM    296    326       CYTOPLASMIC.
//
```

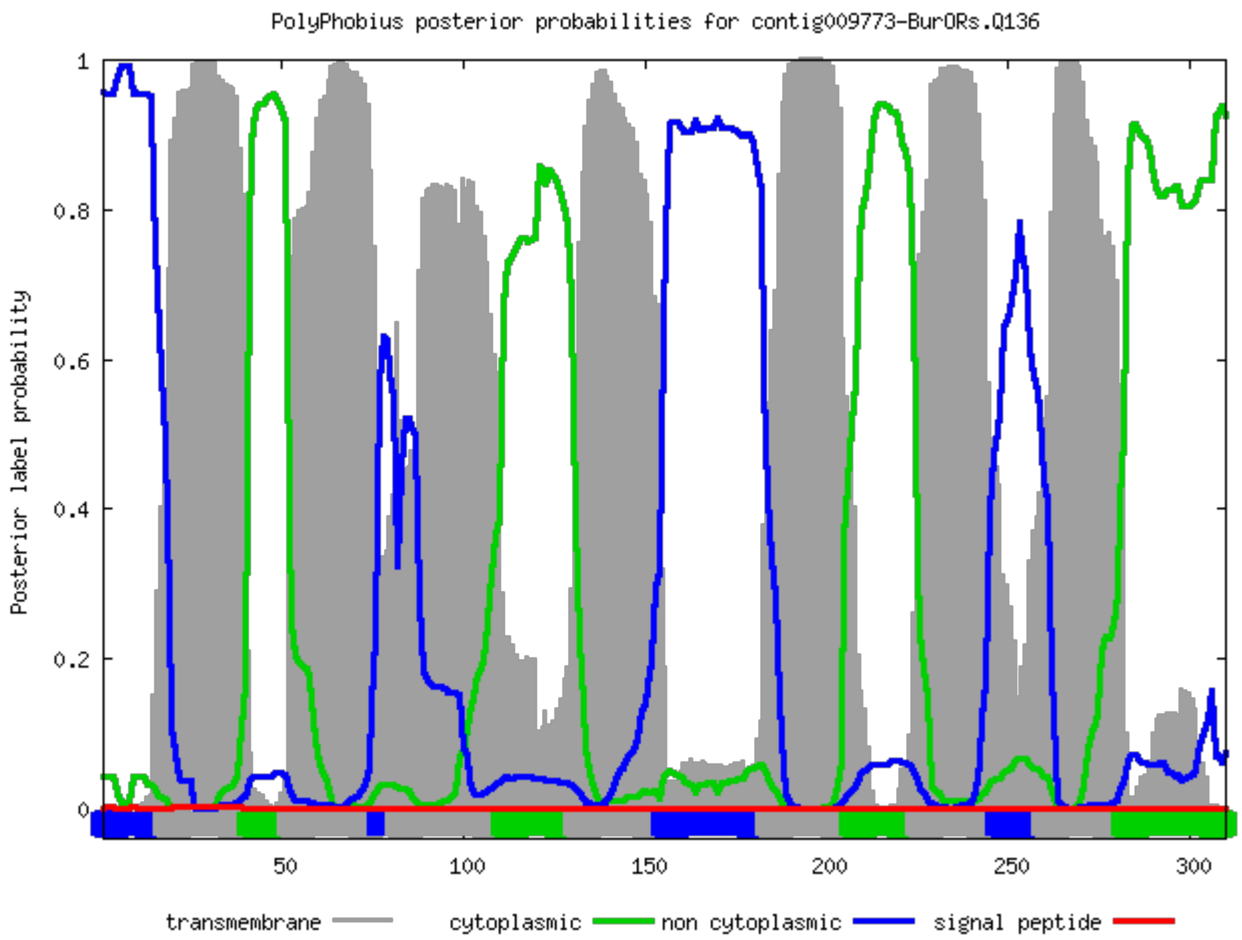

The prediction is based on an alignment. The probability data used in the plot is found here, and the gnuplot script is here.

Prediction of contig039415-TilOR.S221

```
ID    contig039415-TilOR.S221
FT    TOPO_DOM      1      20       NON CYTOPLASMIC.
FT    TRANSMEM     21      42
FT    TOPO_DOM     43      53       CYTOPLASMIC.
FT    TRANSMEM     54      77
FT    TOPO_DOM     78      82       NON CYTOPLASMIC.
FT    TRANSMEM     83     111
FT    TOPO_DOM    112     131       CYTOPLASMIC.
FT    TRANSMEM    132     157
FT    TOPO_DOM    158     185       NON CYTOPLASMIC.
FT    TRANSMEM    186     206
FT    TOPO_DOM    207     226       CYTOPLASMIC.
FT    TRANSMEM    227     252
FT    TOPO_DOM    253     263       NON CYTOPLASMIC.
FT    TRANSMEM    264     284
FT    TOPO_DOM    285     304       CYTOPLASMIC.
//
```

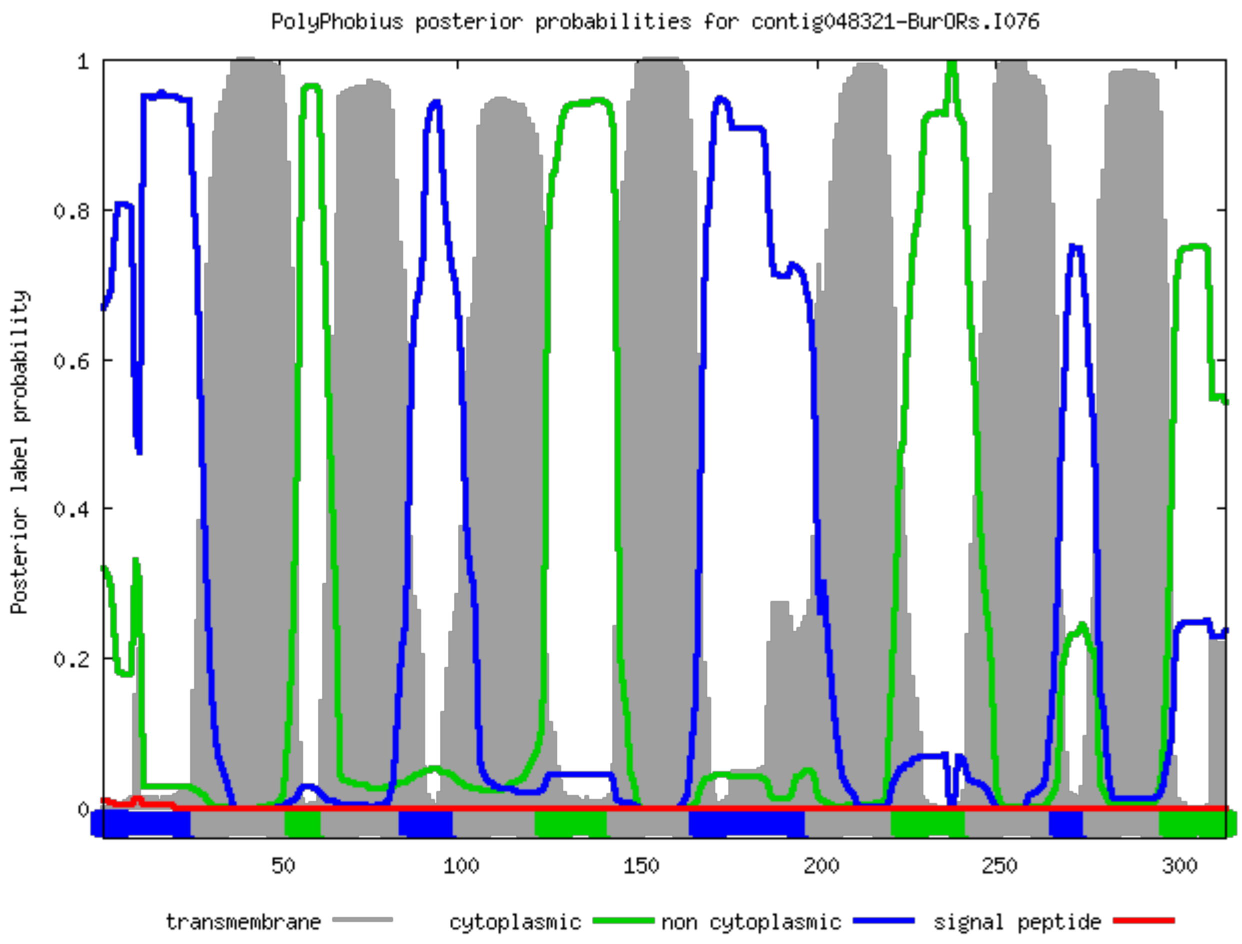

The prediction is based on an alignment. The probability data used in the plot is found here, and the gnuplot script is here.

Prediction of contig028614-TilOR.R247

```
ID   contig028614-TilOR.R247
FT   TOPO_DOM      1      22       NON CYTOPLASMIC.
FT   TRANSMEM     23      46
FT   TOPO_DOM     47      57       CYTOPLASMIC.
FT   TRANSMEM     58      82
FT   TOPO_DOM     83      87       NON CYTOPLASMIC.
FT   TRANSMEM     88     116
FT   TOPO_DOM    117     136       CYTOPLASMIC.
FT   TRANSMEM    137     160
FT   TOPO_DOM    161     191       NON CYTOPLASMIC.
FT   TRANSMEM    192     215
FT   TOPO_DOM    216     232       CYTOPLASMIC.
FT   TRANSMEM    233     256
FT   TOPO_DOM    257     268       NON CYTOPLASMIC.
FT   TRANSMEM    269     290
FT   TOPO_DOM    291     317       CYTOPLASMIC.
//
```

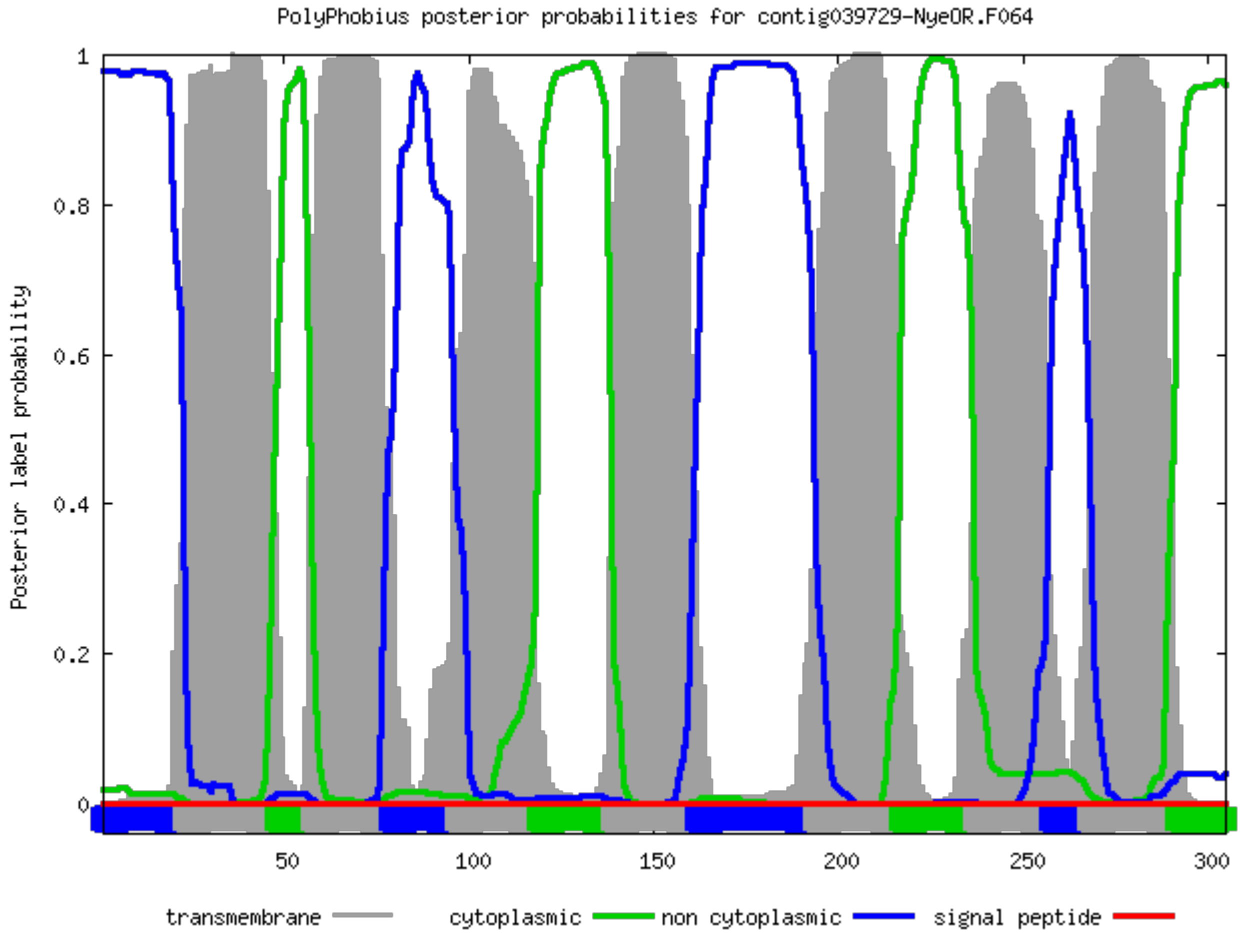

The prediction is based on an alignment. The probability data used in the plot is found here, and the gnuplot script is here.

Prediction of contig049295-BurOR.G060

```
ID   contig049295-BurOR.G060
FT   TOPO_DOM      1     22       NON CYTOPLASMIC.
FT   TRANSMEM     23     47
FT   TOPO_DOM     48     55       CYTOPLASMIC.
FT   TRANSMEM     56     76
FT   TOPO_DOM     77     94       NON CYTOPLASMIC.
FT   TRANSMEM     95    117
FT   TOPO_DOM    118    137       CYTOPLASMIC.
FT   TRANSMEM    138    160
FT   TOPO_DOM    161    192       NON CYTOPLASMIC.
FT   TRANSMEM    193    216
FT   TOPO_DOM    217    233       CYTOPLASMIC.
FT   TRANSMEM    234    255
FT   TOPO_DOM    256    268       NON CYTOPLASMIC.
FT   TRANSMEM    269    288
FT   TOPO_DOM    289    312       CYTOPLASMIC.
//
```

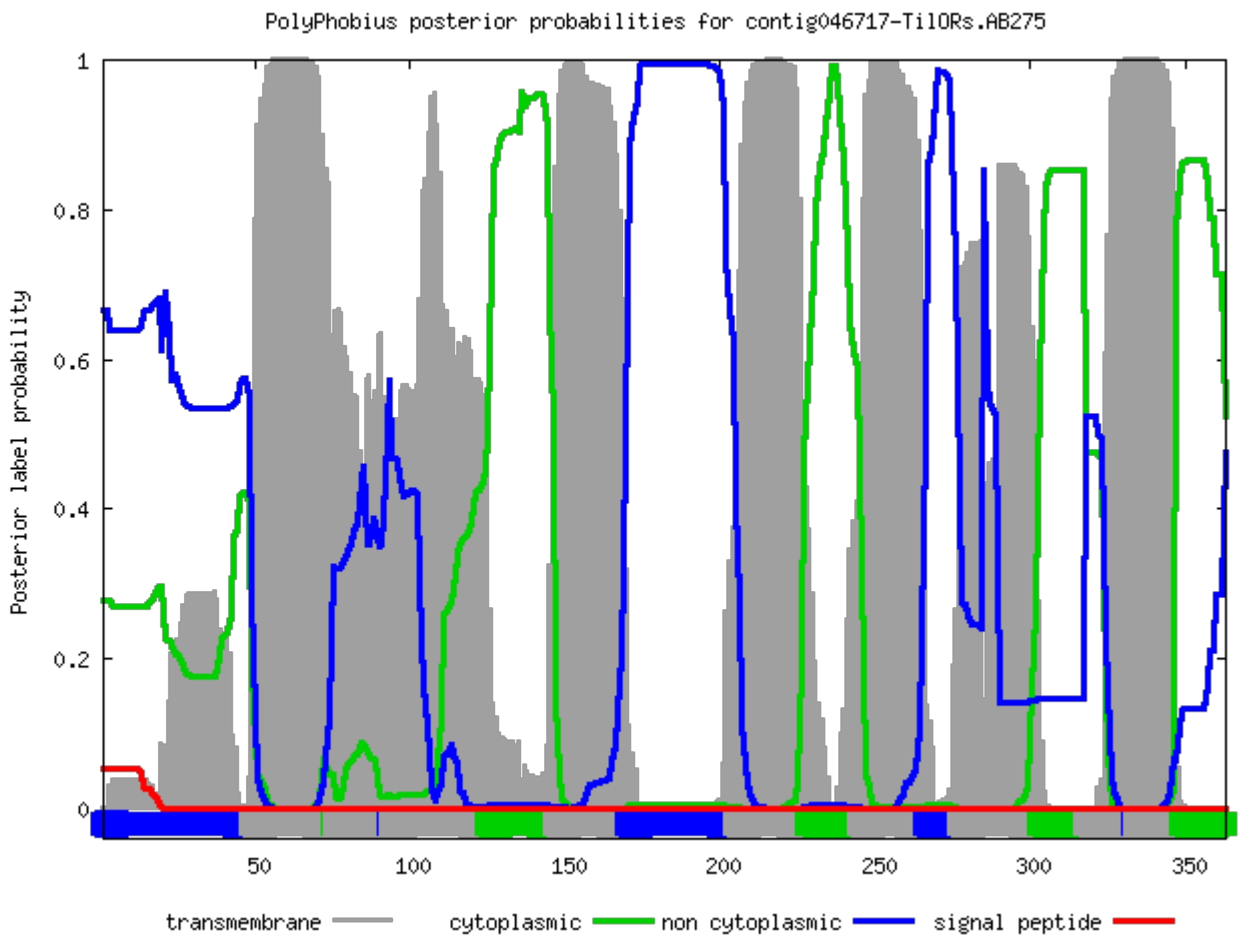

The prediction is based on an alignment. The probability data used in the plot is found here, and the gnuplot script is here.

Prediction of contig064097-ZebOR.N115

```
ID    contig064097-ZebOR.N115
FT    TOPO_DOM      1      32       NON CYTOPLASMIC.
FT    TRANSMEM     33      58
FT    TOPO_DOM     59      66       CYTOPLASMIC.
FT    TRANSMEM     67      86
FT    TOPO_DOM     87     104       NON CYTOPLASMIC.
FT    TRANSMEM    105     127
FT    TOPO_DOM    128     146       CYTOPLASMIC.
FT    TRANSMEM    147     170
FT    TOPO_DOM    171     207       NON CYTOPLASMIC.
FT    TRANSMEM    208     232
FT    TOPO_DOM    233     249       CYTOPLASMIC.
FT    TRANSMEM    250     271
FT    TOPO_DOM    272     277       NON CYTOPLASMIC.
FT    TRANSMEM    278     299
FT    TOPO_DOM    300     322       CYTOPLASMIC.
//
```

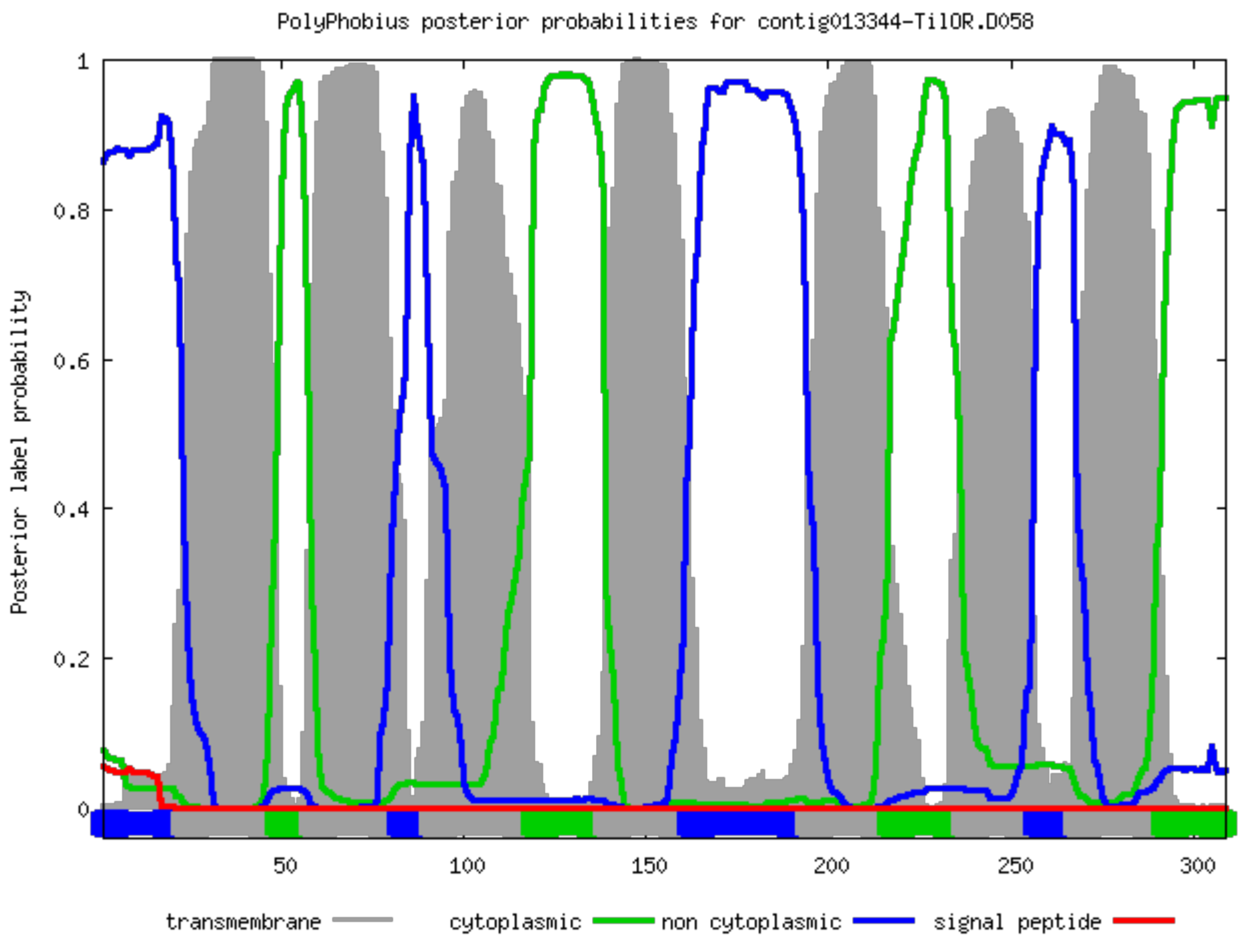

The prediction is based on an alignment. The probability data used in the plot is found here, and the gnuplot script is here.

Prediction of contig104344-BriOR.G048

```
ID   contig104344-BriOR.G048
FT   TOPO_DOM      1     22       NON CYTOPLASMIC.
FT   TRANSMEM     23     47
FT   TOPO_DOM     48     55       CYTOPLASMIC.
FT   TRANSMEM     56     76
FT   TOPO_DOM     77     94       NON CYTOPLASMIC.
FT   TRANSMEM     95    117
FT   TOPO_DOM    118    137       CYTOPLASMIC.
FT   TRANSMEM    138    160
FT   TOPO_DOM    161    192       NON CYTOPLASMIC.
FT   TRANSMEM    193    216
FT   TOPO_DOM    217    233       CYTOPLASMIC.
FT   TRANSMEM    234    255
FT   TOPO_DOM    256    268       NON CYTOPLASMIC.
FT   TRANSMEM    269    288
FT   TOPO_DOM    289    312       CYTOPLASMIC.
//
```

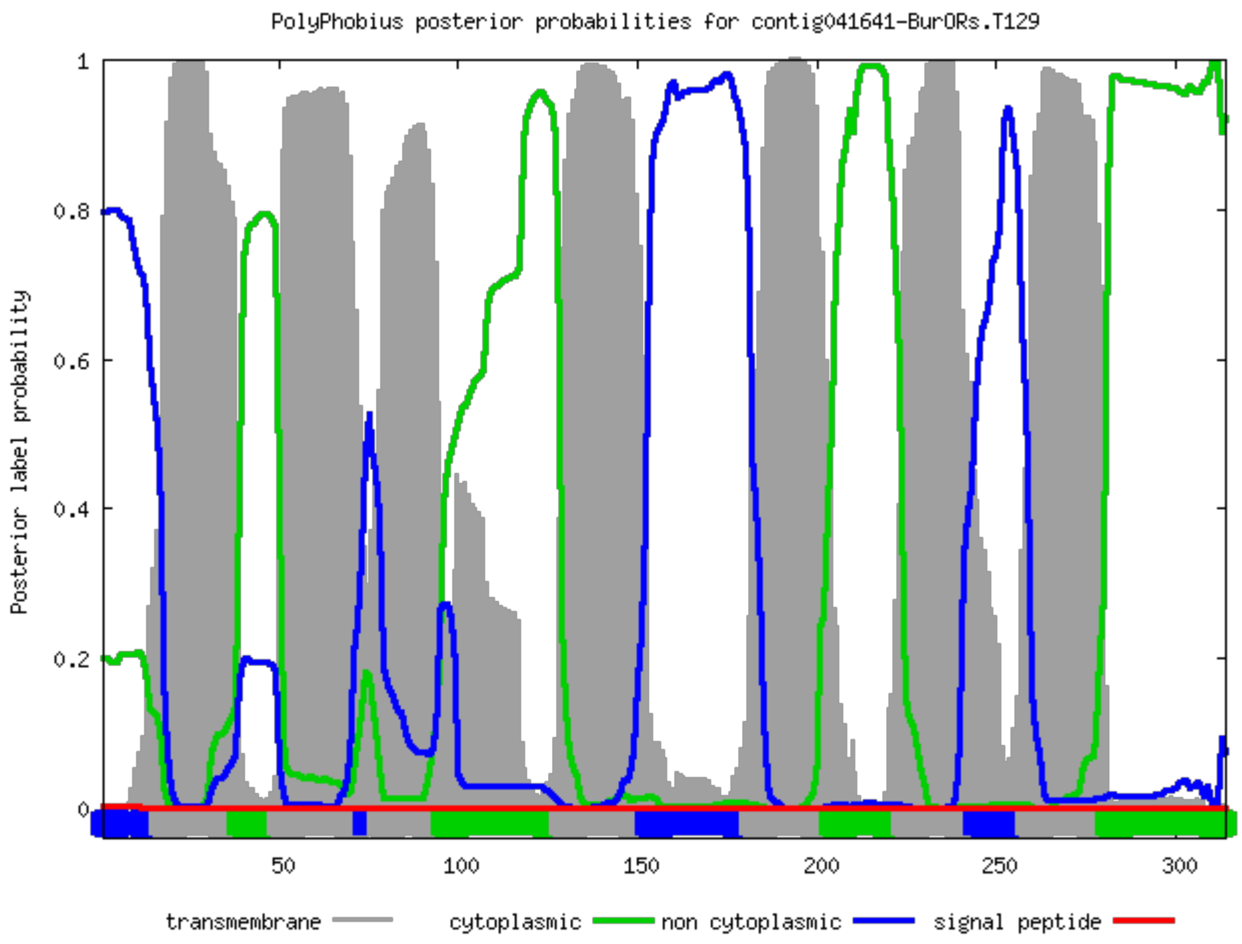

The prediction is based on an alignment. The probability data used in the plot is found here, and the gnuplot script is here.

Prediction of contig093812-BriOR.A010

```
ID   contig093812-BriOR.A010
FT   TOPO_DOM      1     22       NON CYTOPLASMIC.
FT   TRANSMEM     23     48
FT   TOPO_DOM     49     56       CYTOPLASMIC.
FT   TRANSMEM     57     77
FT   TOPO_DOM     78     95       NON CYTOPLASMIC.
FT   TRANSMEM     96    118
FT   TOPO_DOM    119    138       CYTOPLASMIC.
FT   TRANSMEM    139    160
FT   TOPO_DOM    161    192       NON CYTOPLASMIC.
FT   TRANSMEM    193    215
FT   TOPO_DOM    216    235       CYTOPLASMIC.
FT   TRANSMEM    236    257
FT   TOPO_DOM    258    268       NON CYTOPLASMIC.
FT   TRANSMEM    269    289
FT   TOPO_DOM    290    302       CYTOPLASMIC.
FT   TRANSMEM    303    317
FT   TOPO_DOM    318    320       NON CYTOPLASMIC.
//
```

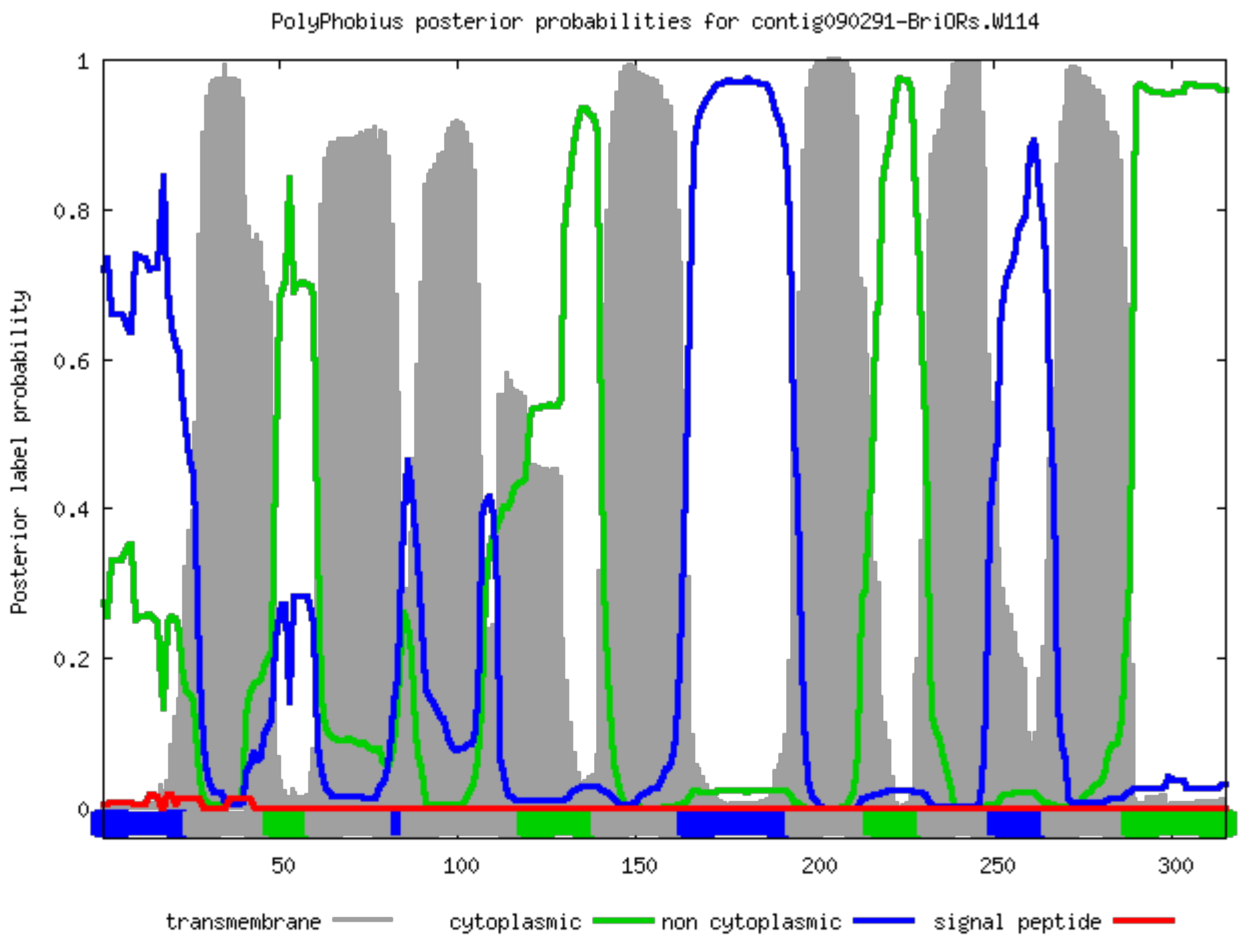

The prediction is based on an alignment. The probability data used in the plot is found here, and the gnuplot script is here.

Prediction of contig047726-TilOR.E075

```
ID   contig047726-TilOR.E075
FT   TOPO_DOM      1     21       NON CYTOPLASMIC.
FT   TRANSMEM     22     47
FT   TOPO_DOM     48     56       CYTOPLASMIC.
FT   TRANSMEM     57     81
FT   TOPO_DOM     82     93       NON CYTOPLASMIC.
FT   TRANSMEM     94    117
FT   TOPO_DOM    118    137       CYTOPLASMIC.
FT   TRANSMEM    138    159
FT   TOPO_DOM    160    192       NON CYTOPLASMIC.
FT   TRANSMEM    193    215
FT   TOPO_DOM    216    235       CYTOPLASMIC.
FT   TRANSMEM    236    255
FT   TOPO_DOM    256    266       NON CYTOPLASMIC.
FT   TRANSMEM    267    290
FT   TOPO_DOM    291    304       CYTOPLASMIC.
//
```

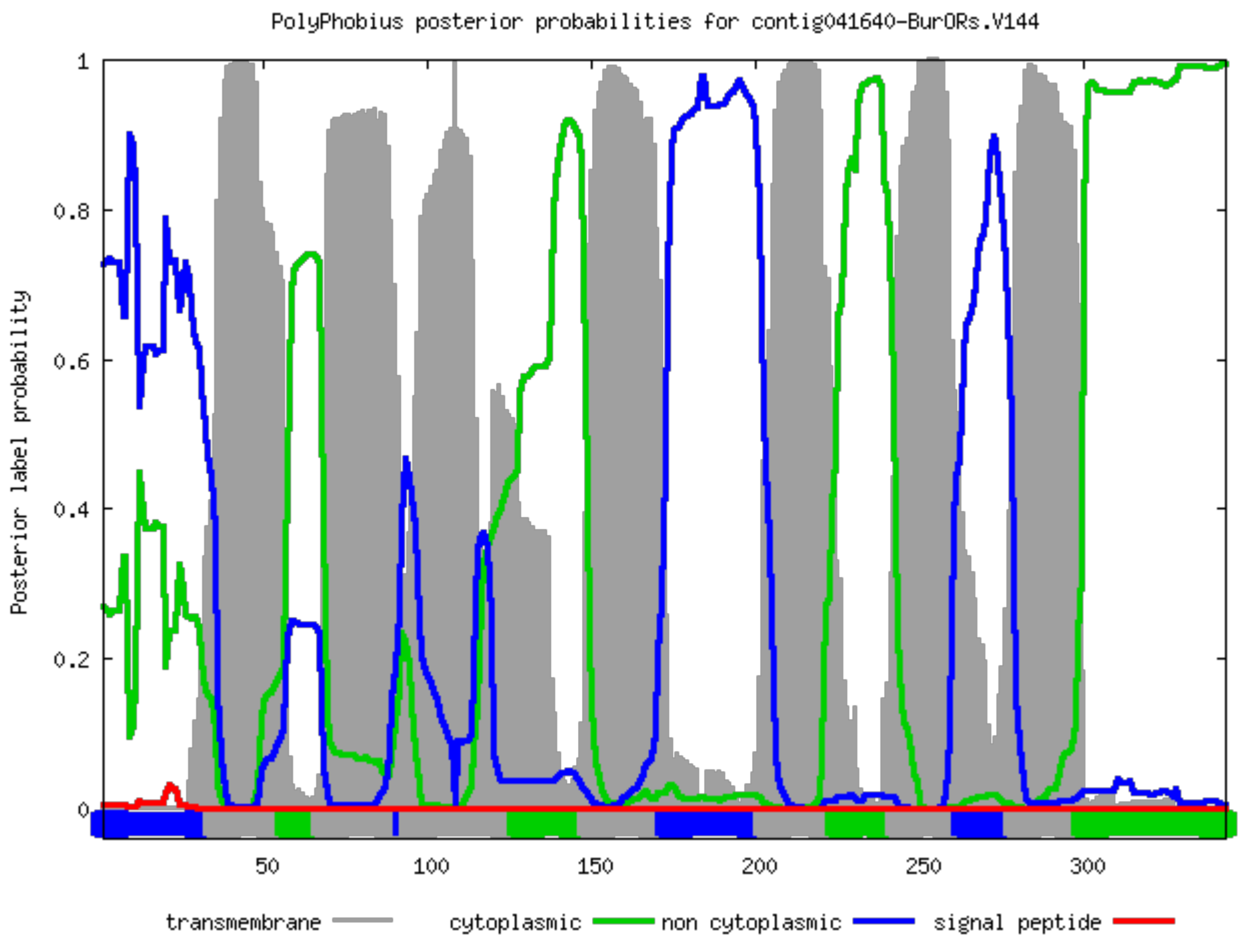

The prediction is based on an alignment. The probability data used in the plot is found here, and the gnuplot script is here.

Prediction of contig046353-TilOR.N195

```
ID   contig046353-TilOR.N195
FT   TOPO_DOM      1     32       NON CYTOPLASMIC.
FT   TRANSMEM     33     58
FT   TOPO_DOM     59     66       CYTOPLASMIC.
FT   TRANSMEM     67     86
FT   TOPO_DOM     87    104       NON CYTOPLASMIC.
FT   TRANSMEM    105    127
FT   TOPO_DOM    128    146       CYTOPLASMIC.
FT   TRANSMEM    147    170
FT   TOPO_DOM    171    207       NON CYTOPLASMIC.
FT   TRANSMEM    208    232
FT   TOPO_DOM    233    250       CYTOPLASMIC.
FT   TRANSMEM    251    271
FT   TOPO_DOM    272    277       NON CYTOPLASMIC.
FT   TRANSMEM    278    298
FT   TOPO_DOM    299    322       CYTOPLASMIC.
//
```

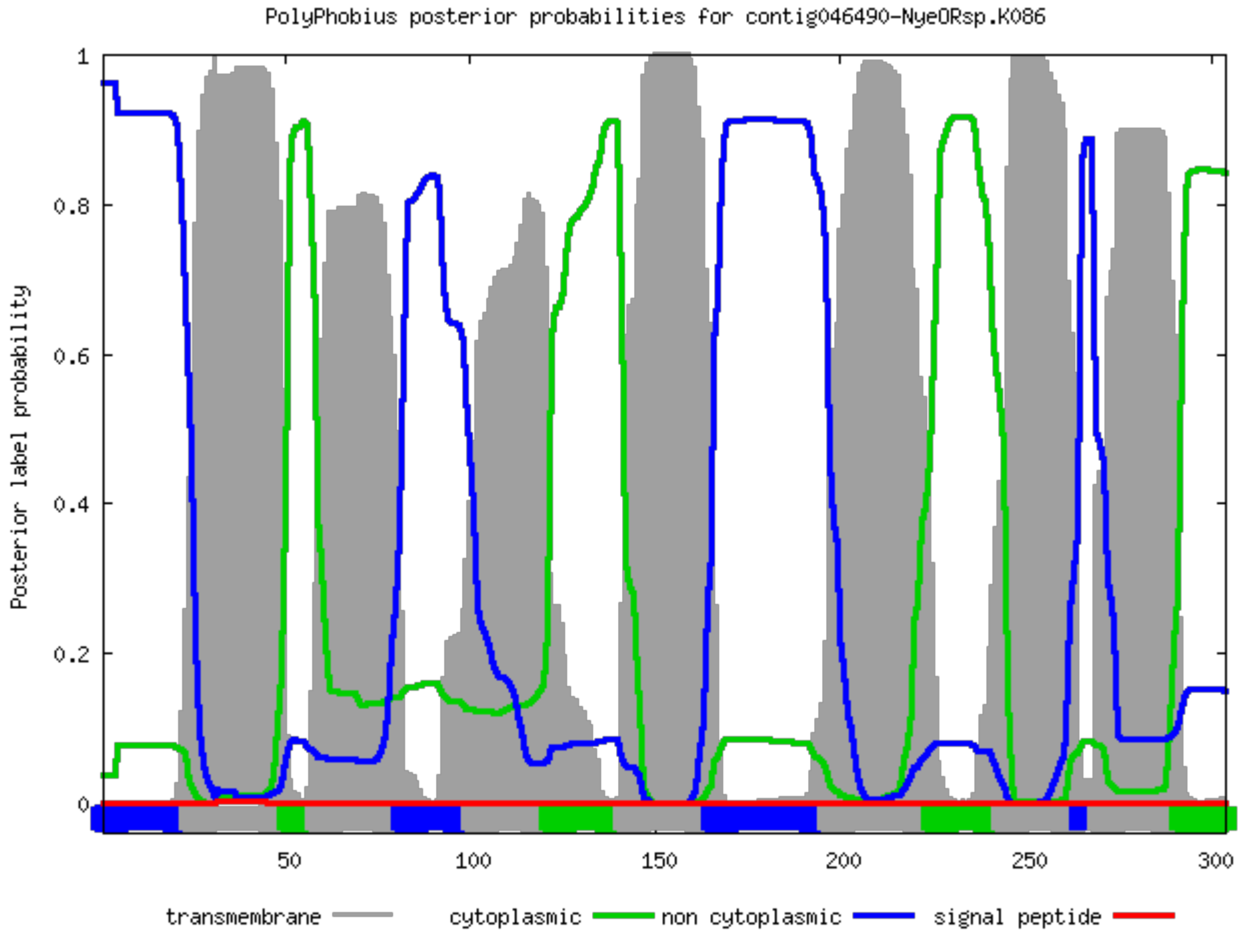

The prediction is based on an alignment. The probability data used in the plot is found here, and the gnuplot script is here.

Prediction of contig013339-TilOR.D057

```
ID   contig013339-TilOR.D057
FT   TOPO_DOM      1     22       NON CYTOPLASMIC.
FT   TRANSMEM     23     48
FT   TOPO_DOM     49     57       CYTOPLASMIC.
FT   TRANSMEM     58     81
FT   TOPO_DOM     82     90       NON CYTOPLASMIC.
FT   TRANSMEM     91    118
FT   TOPO_DOM    119    138       CYTOPLASMIC.
FT   TRANSMEM    139    161
FT   TOPO_DOM    162    193       NON CYTOPLASMIC.
FT   TRANSMEM    194    216
FT   TOPO_DOM    217    236       CYTOPLASMIC.
FT   TRANSMEM    237    256
FT   TOPO_DOM    257    267       NON CYTOPLASMIC.
FT   TRANSMEM    268    291
FT   TOPO_DOM    292    309       CYTOPLASMIC.
//
```

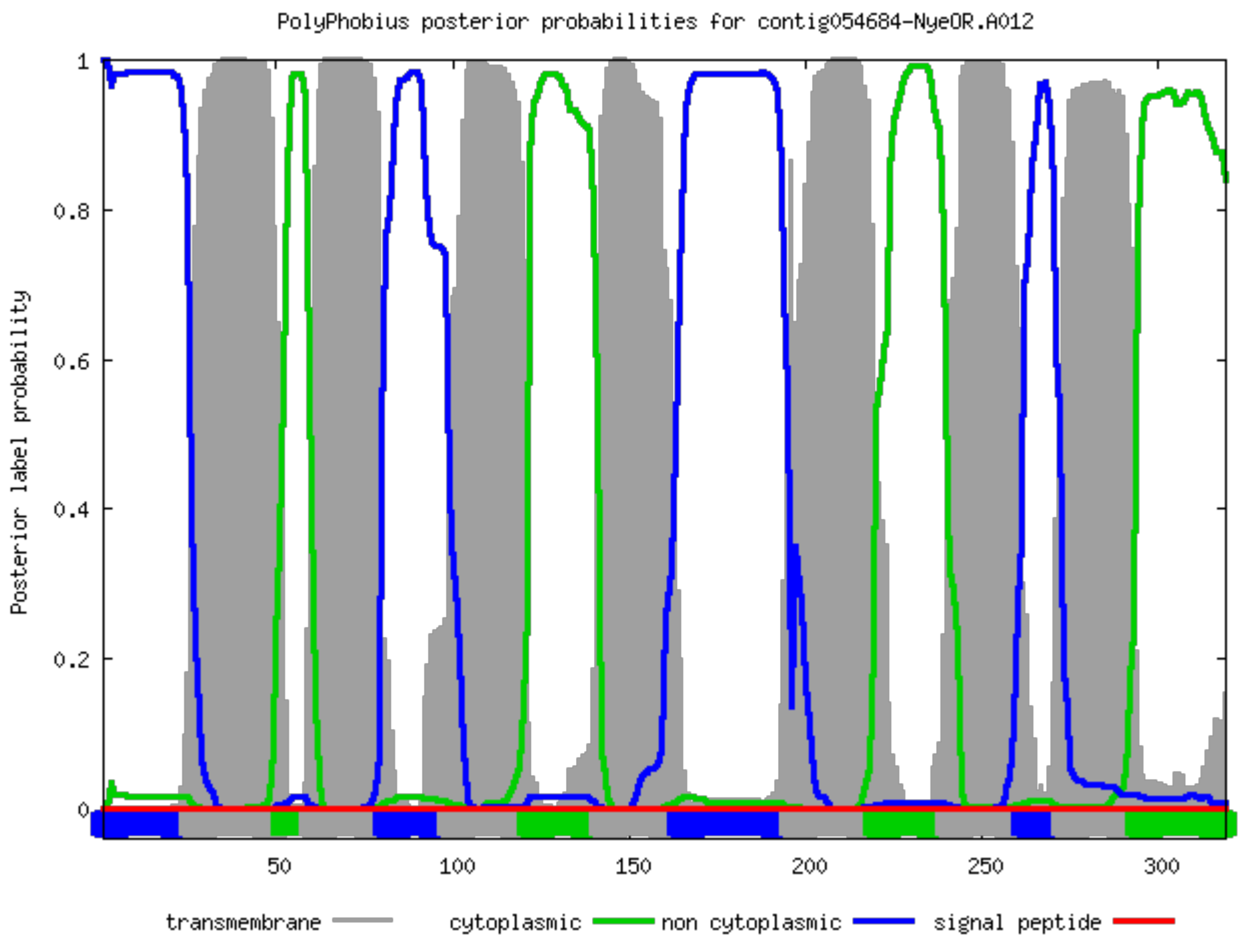

The prediction is based on an alignment. The probability data used in the plot is found here, and the gnuplot script is here.

Prediction of contig039426-TilOR.S226

```
ID   contig039426-TilOR.S226
FT   TOPO_DOM      1     20       NON CYTOPLASMIC.
FT   TRANSMEM     21     42
FT   TOPO_DOM     43     53       CYTOPLASMIC.
FT   TRANSMEM     54     77
FT   TOPO_DOM     78     82       NON CYTOPLASMIC.
FT   TRANSMEM     83    111
FT   TOPO_DOM    112    131       CYTOPLASMIC.
FT   TRANSMEM    132    157
FT   TOPO_DOM    158    185       NON CYTOPLASMIC.
FT   TRANSMEM    186    206
FT   TOPO_DOM    207    226       CYTOPLASMIC.
FT   TRANSMEM    227    252
FT   TOPO_DOM    253    263       NON CYTOPLASMIC.
FT   TRANSMEM    264    284
FT   TOPO_DOM    285    305       CYTOPLASMIC.
//
```

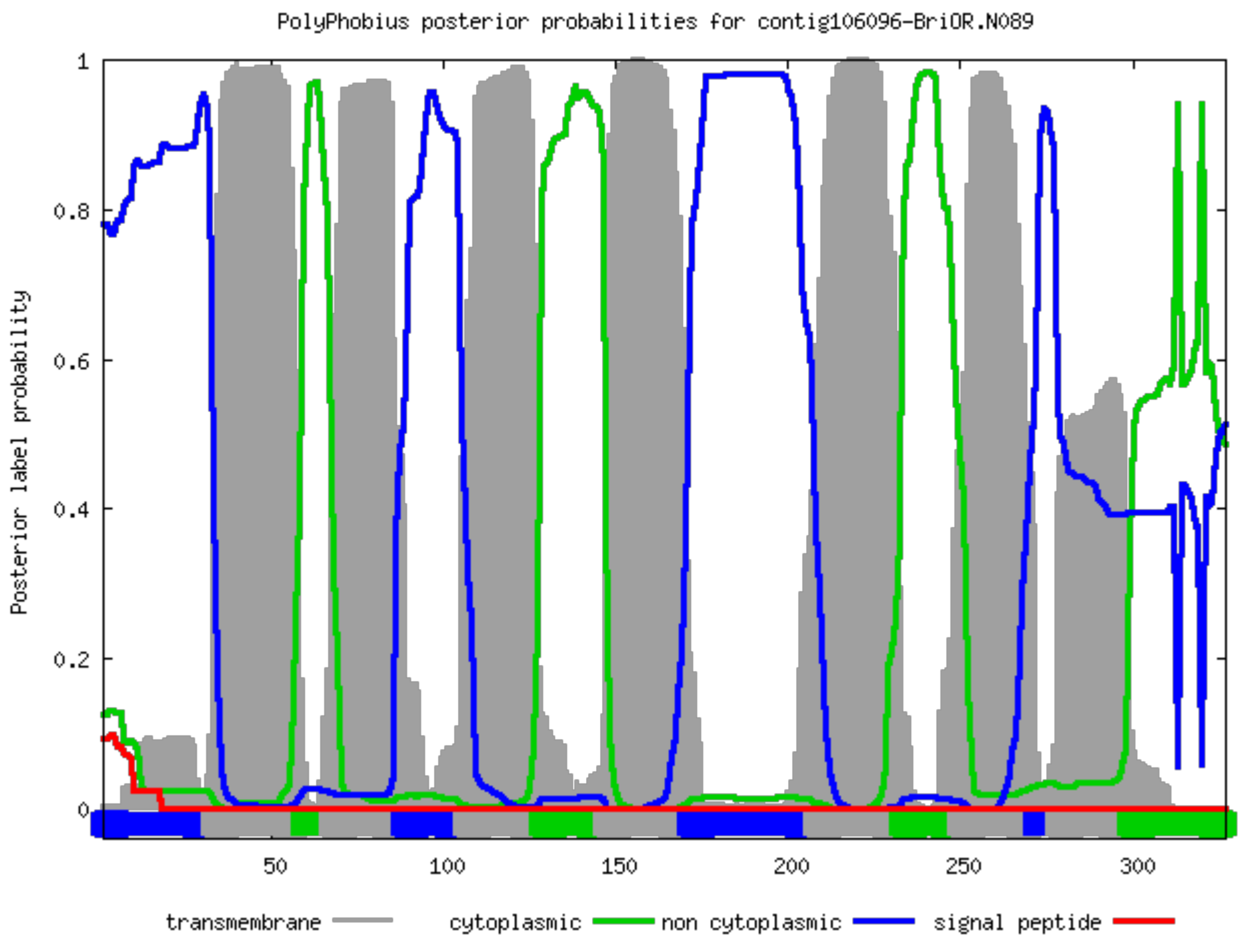

The prediction is based on an alignment. The probability data used in the plot is found here, and the gnuplot script is here.

Prediction of contig028565-BurOR.K080

```
ID    contig028565-BurOR.K080
FT    TOPO_DOM      1      25       NON CYTOPLASMIC.
FT    TRANSMEM     26      50
FT    TOPO_DOM     51      59       CYTOPLASMIC.
FT    TRANSMEM     60      82
FT    TOPO_DOM     83      99       NON CYTOPLASMIC.
FT    TRANSMEM    100     120
FT    TOPO_DOM    121     140       CYTOPLASMIC.
FT    TRANSMEM    141     164
FT    TOPO_DOM    165     196       NON CYTOPLASMIC.
FT    TRANSMEM    197     222
FT    TOPO_DOM    223     241       CYTOPLASMIC.
FT    TRANSMEM    242     261
FT    TOPO_DOM    262     271       NON CYTOPLASMIC.
FT    TRANSMEM    272     291
FT    TOPO_DOM    292     312       CYTOPLASMIC.
//
```

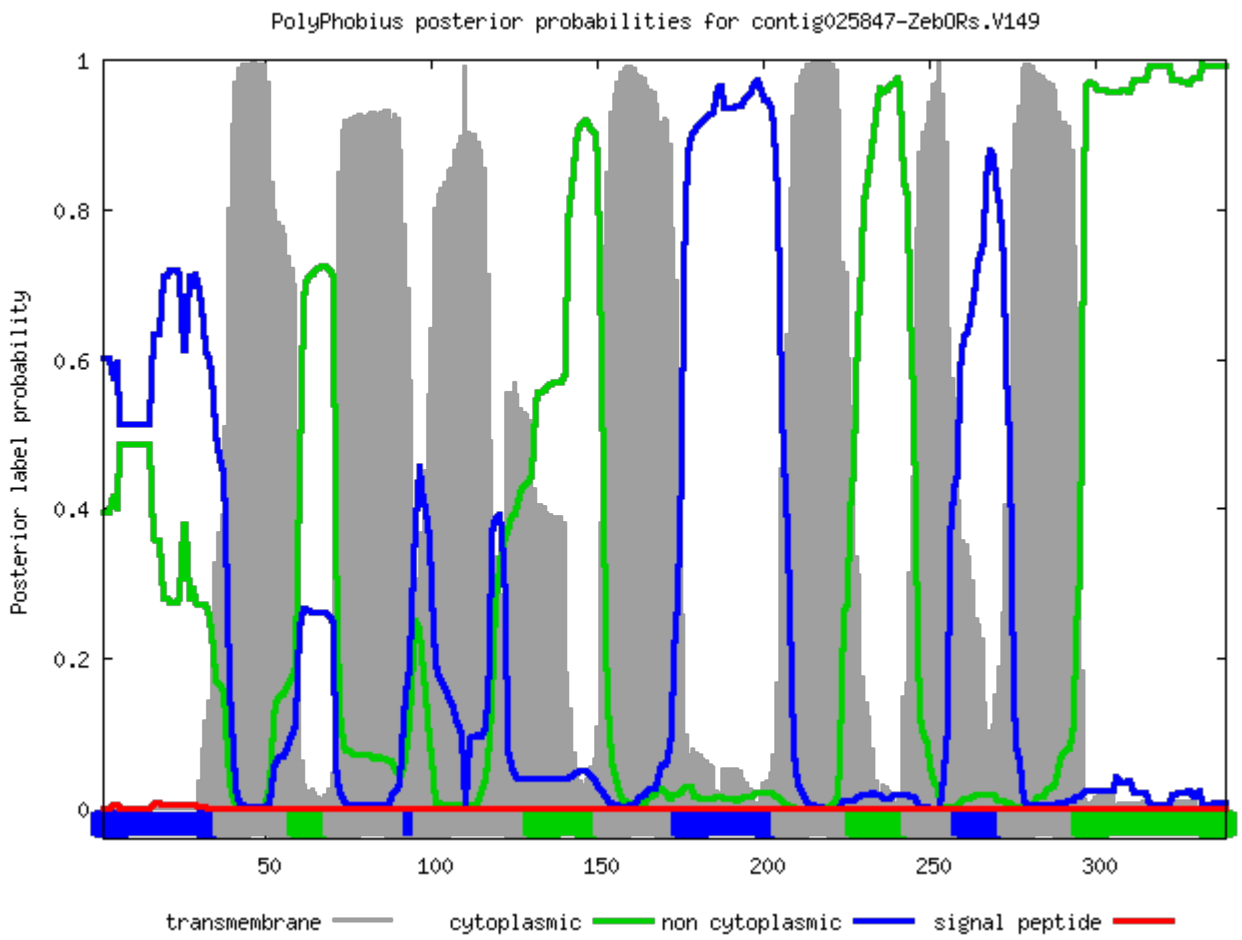

The prediction is based on an alignment. The probability data used in the plot is found here, and the gnuplot script is here.

Prediction of contig032388-BurOR.D032

```
ID   contig032388-BurOR.D032
FT   TOPO_DOM      1     24       NON CYTOPLASMIC.
FT   TRANSMEM     25     50
FT   TOPO_DOM     51     59       CYTOPLASMIC.
FT   TRANSMEM     60     83
FT   TOPO_DOM     84     92       NON CYTOPLASMIC.
FT   TRANSMEM     93    120
FT   TOPO_DOM    121    140       CYTOPLASMIC.
FT   TRANSMEM    141    164
FT   TOPO_DOM    165    195       NON CYTOPLASMIC.
FT   TRANSMEM    196    218
FT   TOPO_DOM    219    238       CYTOPLASMIC.
FT   TRANSMEM    239    258
FT   TOPO_DOM    259    269       NON CYTOPLASMIC.
FT   TRANSMEM    270    293
FT   TOPO_DOM    294    311       CYTOPLASMIC.
//
```

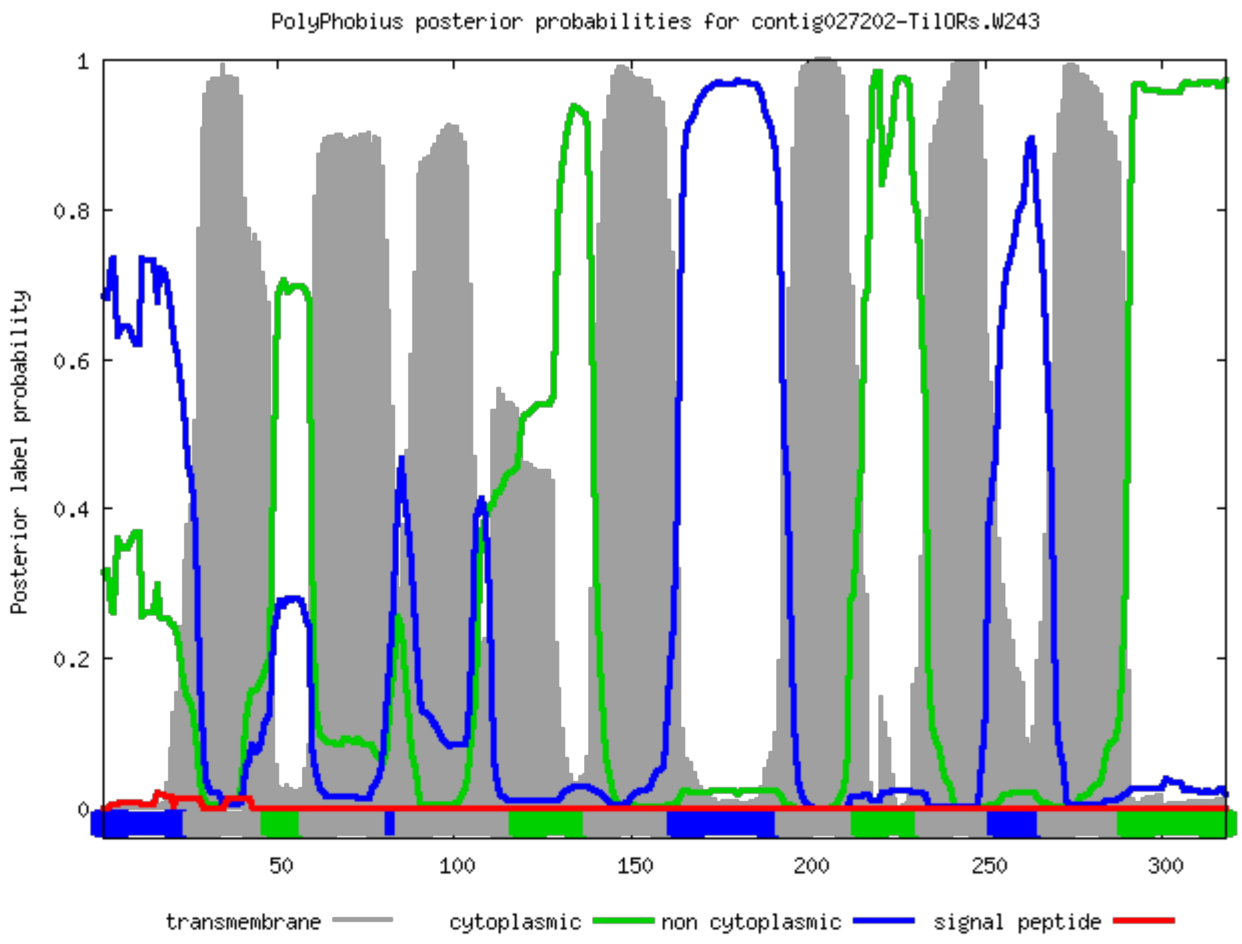

The prediction is based on an alignment. The probability data used in the plot is found here, and the gnuplot script is here.

Prediction of contig085012-BriOR.A006

```
ID    contig085012-BriOR.A006
FT    TOPO_DOM      1      22       NON CYTOPLASMIC.
FT    TRANSMEM     23      48
FT    TOPO_DOM     49      56       CYTOPLASMIC.
FT    TRANSMEM     57      76
FT    TOPO_DOM     77      95       NON CYTOPLASMIC.
FT    TRANSMEM     96     118
FT    TOPO_DOM    119     138       CYTOPLASMIC.
FT    TRANSMEM    139     159
FT    TOPO_DOM    160     192       NON CYTOPLASMIC.
FT    TRANSMEM    193     215
FT    TOPO_DOM    216     235       CYTOPLASMIC.
FT    TRANSMEM    236     257
FT    TOPO_DOM    258     268       NON CYTOPLASMIC.
FT    TRANSMEM    269     289
FT    TOPO_DOM    290     315       CYTOPLASMIC.
//
```

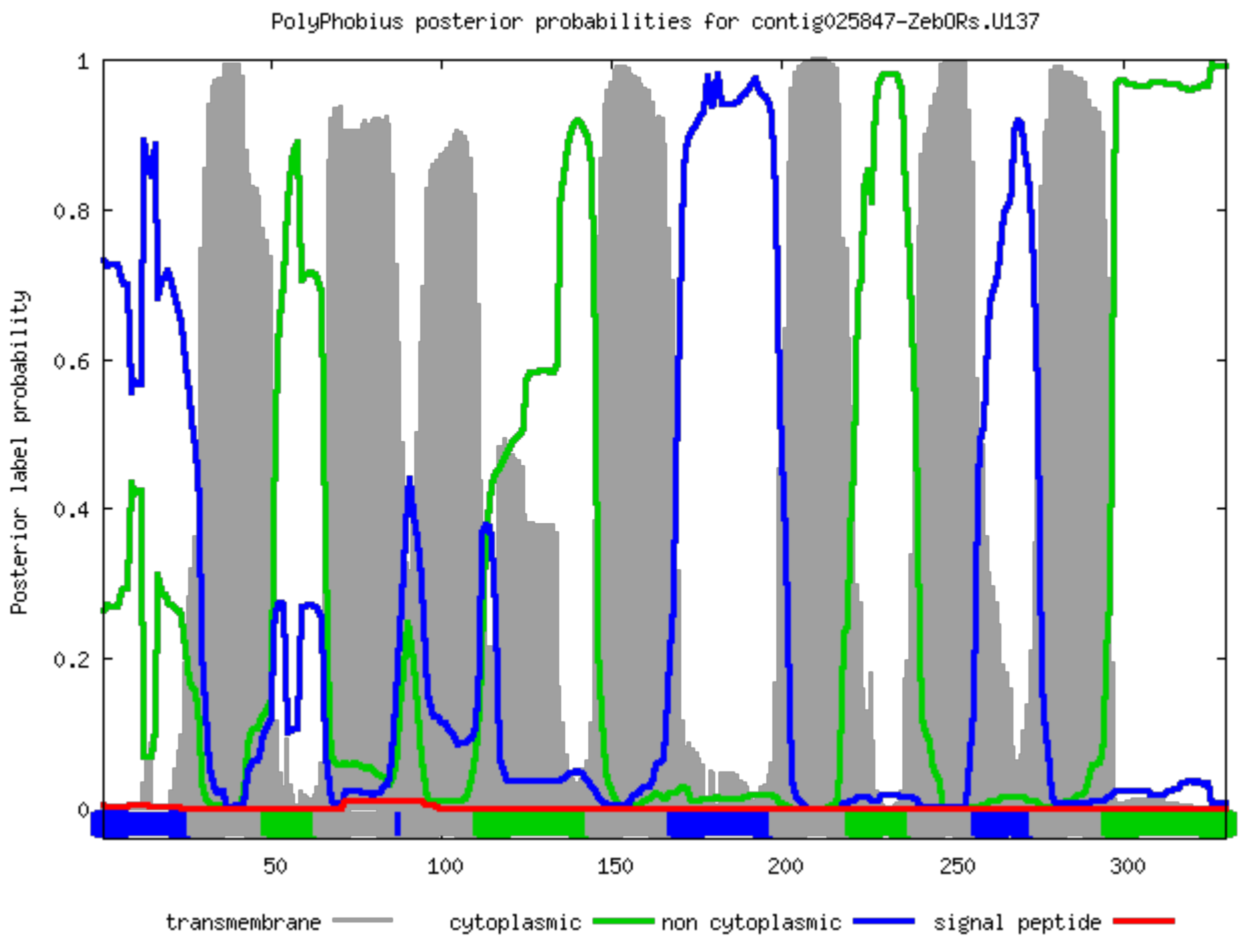

The prediction is based on an alignment. The probability data used in the plot is found here, and the gnuplot script is here.

Prediction of contig039461-TilOR.L148

```
ID   contig039461-TilOR.L148
FT   TOPO_DOM      1     25       NON CYTOPLASMIC.
FT   TRANSMEM     26     50
FT   TOPO_DOM     51     59       CYTOPLASMIC.
FT   TRANSMEM     60     86
FT   TOPO_DOM     87     98       NON CYTOPLASMIC.
FT   TRANSMEM     99    120
FT   TOPO_DOM    121    140       CYTOPLASMIC.
FT   TRANSMEM    141    162
FT   TOPO_DOM    163    198       NON CYTOPLASMIC.
FT   TRANSMEM    199    224
FT   TOPO_DOM    225    237       CYTOPLASMIC.
FT   TRANSMEM    238    260
FT   TOPO_DOM    261    271       NON CYTOPLASMIC.
FT   TRANSMEM    272    292
FT   TOPO_DOM    293    313       CYTOPLASMIC.
//
```

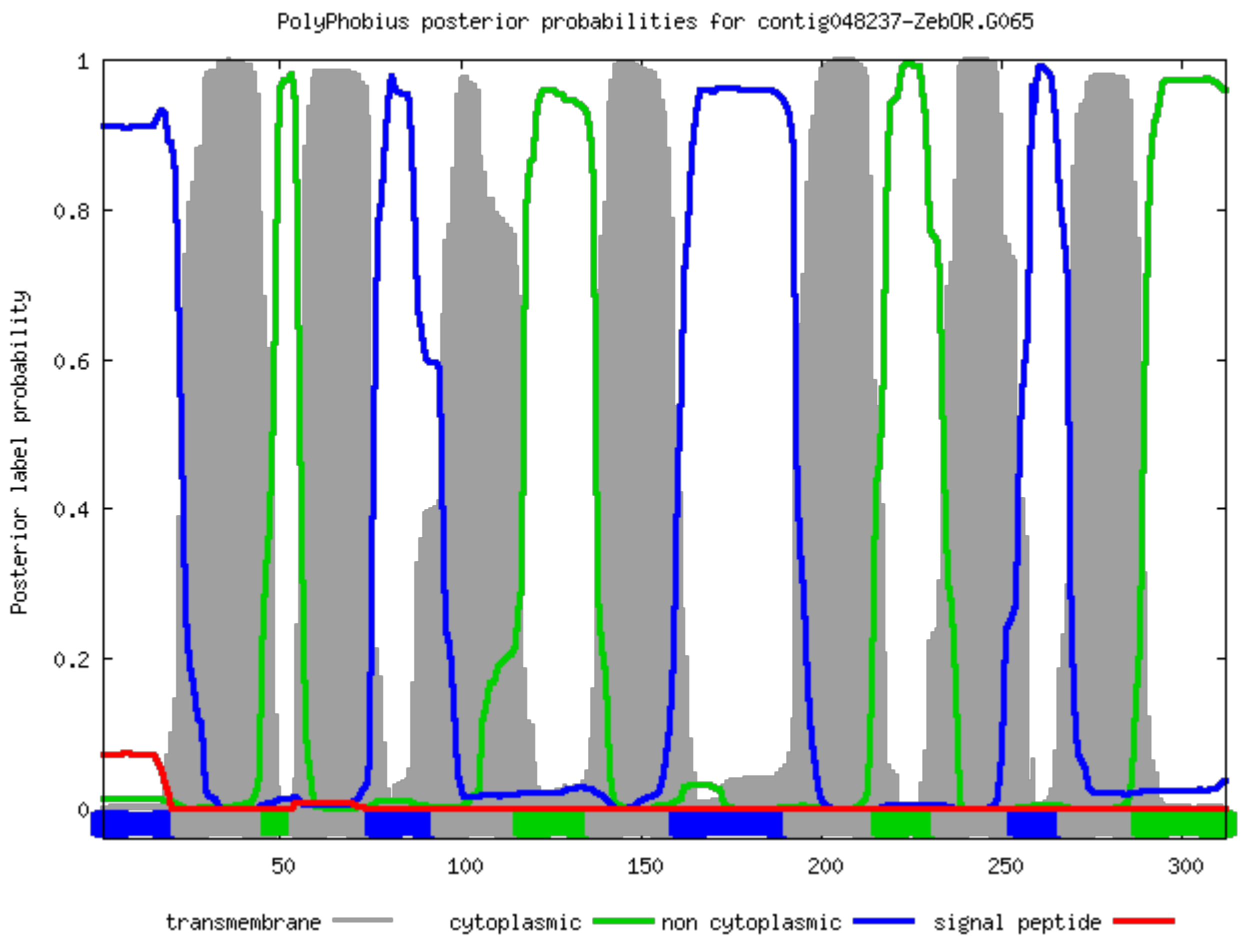

The prediction is based on an alignment. The probability data used in the plot is found here, and the gnuplot script is here.

Prediction of contig039738-NyeOR.D041

```
ID   contig039738-NyeOR.D041
FT   TOPO_DOM      1      24       NON CYTOPLASMIC.
FT   TRANSMEM     25      50
FT   TOPO_DOM     51      59       CYTOPLASMIC.
FT   TRANSMEM     60      83
FT   TOPO_DOM     84      92       NON CYTOPLASMIC.
FT   TRANSMEM     93     120
FT   TOPO_DOM    121     140       CYTOPLASMIC.
FT   TRANSMEM    141     164
FT   TOPO_DOM    165     195       NON CYTOPLASMIC.
FT   TRANSMEM    196     218
FT   TOPO_DOM    219     238       CYTOPLASMIC.
FT   TRANSMEM    239     258
FT   TOPO_DOM    259     269       NON CYTOPLASMIC.
FT   TRANSMEM    270     293
FT   TOPO_DOM    294     311       CYTOPLASMIC.
//
```

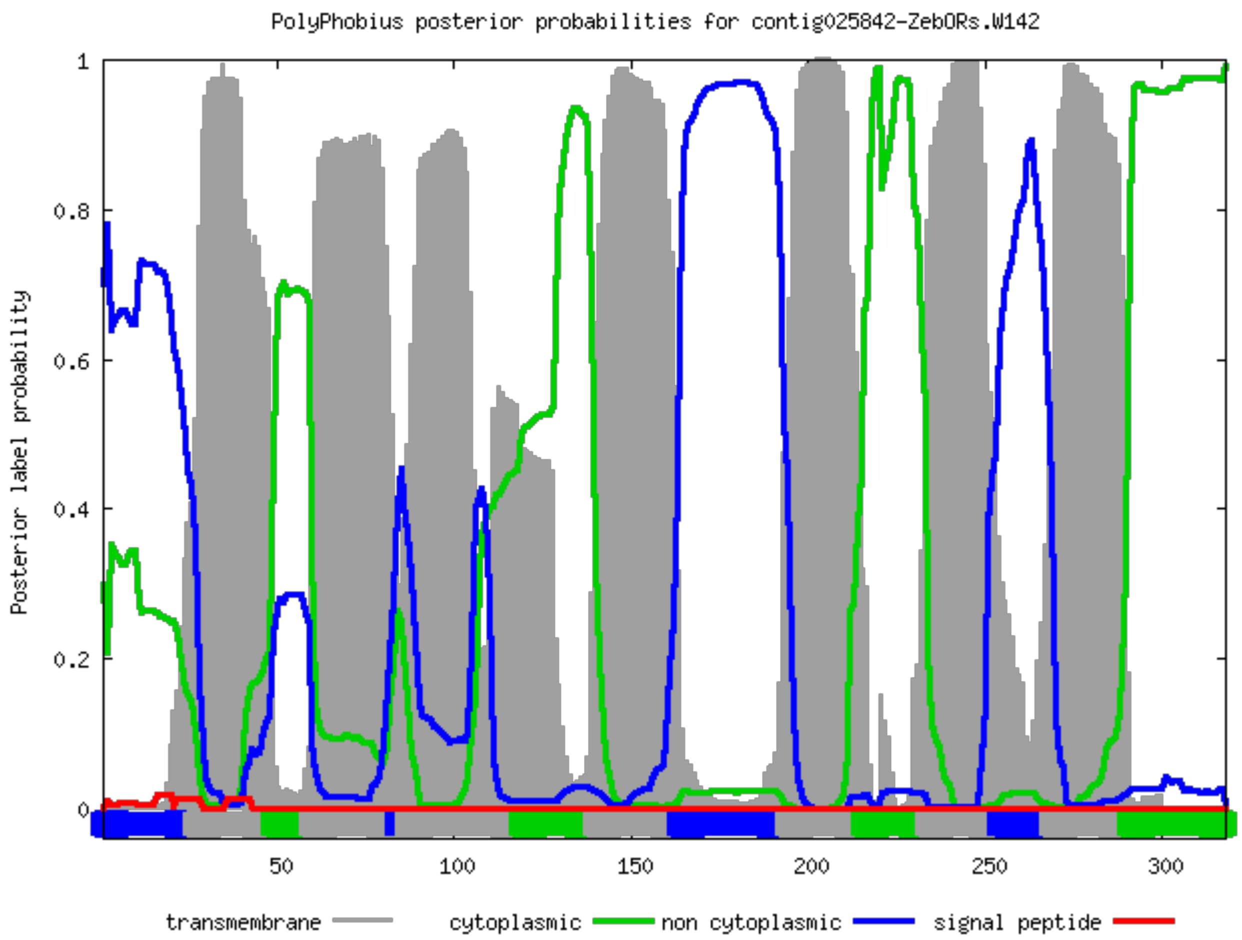

The prediction is based on an alignment. The probability data used in the plot is found here, and the gnuplot script is here.

Prediction of contig065449-TilOR.P215

```
ID   contig065449-TilOR.P215
FT   TOPO_DOM      1     27       NON CYTOPLASMIC.
FT   TRANSMEM     28     51
FT   TOPO_DOM     52     61       CYTOPLASMIC.
FT   TRANSMEM     62     87
FT   TOPO_DOM     88    100       NON CYTOPLASMIC.
FT   TRANSMEM    101    122
FT   TOPO_DOM    123    142       CYTOPLASMIC.
FT   TRANSMEM    143    165
FT   TOPO_DOM    166    199       NON CYTOPLASMIC.
FT   TRANSMEM    200    225
FT   TOPO_DOM    226    238       CYTOPLASMIC.
FT   TRANSMEM    239    261
FT   TOPO_DOM    262    273       NON CYTOPLASMIC.
FT   TRANSMEM    274    294
FT   TOPO_DOM    295    310       CYTOPLASMIC.
//
```

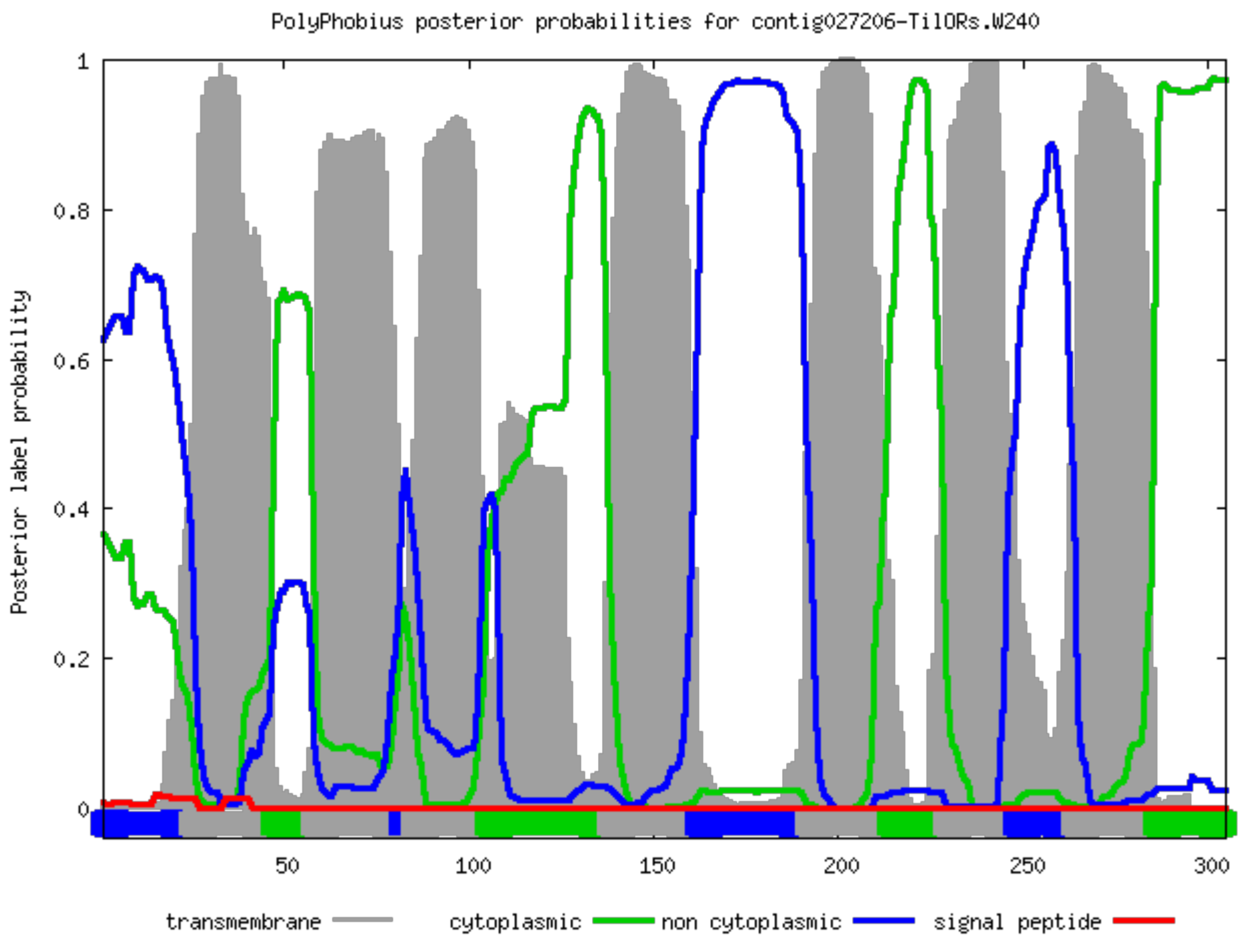

The prediction is based on an alignment. The probability data used in the plot is found here, and the gnuplot script is here.

Prediction of contig065458-TilOR.E086

```
ID   contig065458-TilOR.E086
FT   TOPO_DOM      1     23       NON CYTOPLASMIC.
FT   TRANSMEM     24     49
FT   TOPO_DOM     50     58       CYTOPLASMIC.
FT   TRANSMEM     59     83
FT   TOPO_DOM     84     92       NON CYTOPLASMIC.
FT   TRANSMEM     93    119
FT   TOPO_DOM    120    139       CYTOPLASMIC.
FT   TRANSMEM    140    161
FT   TOPO_DOM    162    194       NON CYTOPLASMIC.
FT   TRANSMEM    195    216
FT   TOPO_DOM    217    236       CYTOPLASMIC.
FT   TRANSMEM    237    256
FT   TOPO_DOM    257    267       NON CYTOPLASMIC.
FT   TRANSMEM    268    291
FT   TOPO_DOM    292    321       CYTOPLASMIC.
//
```

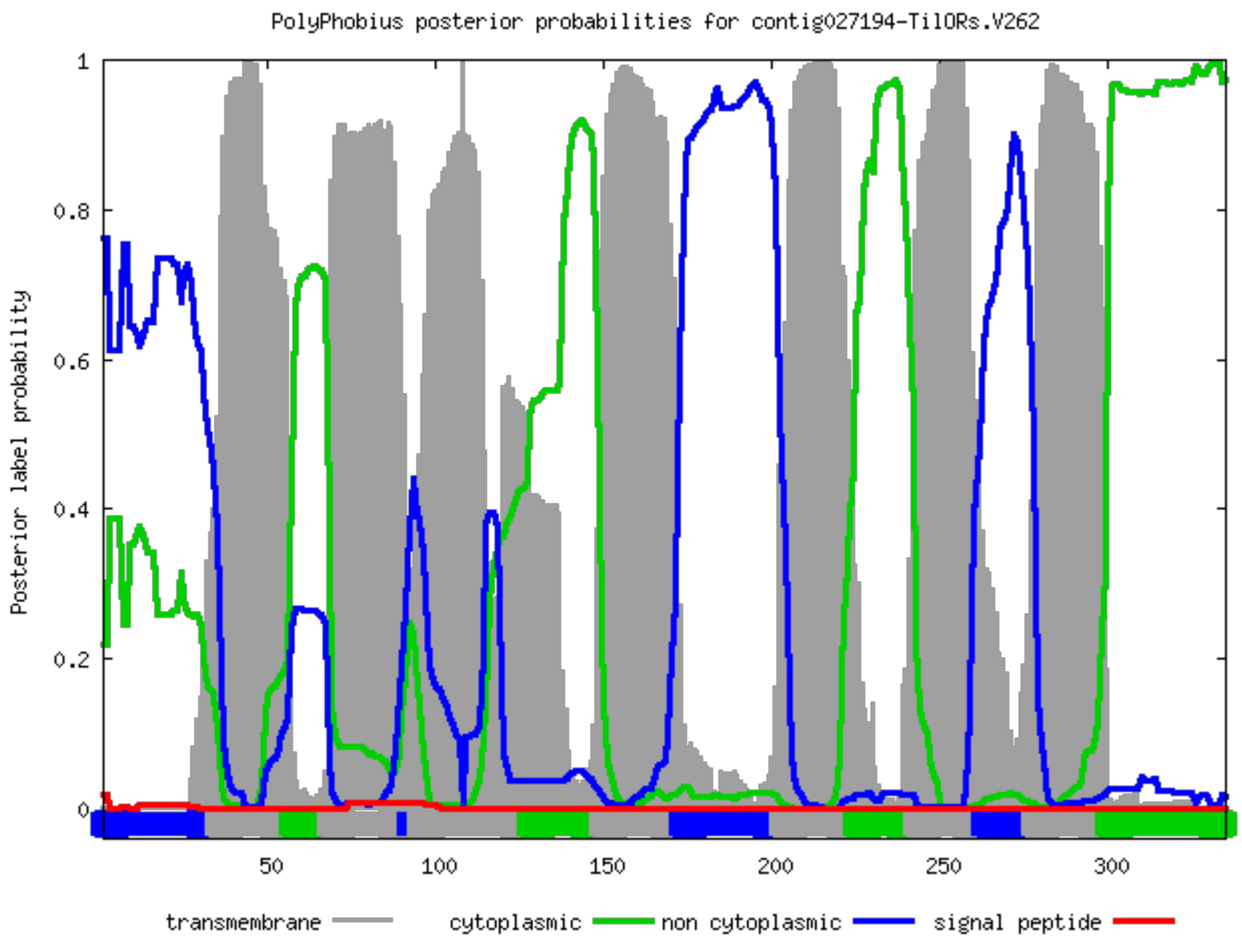

The prediction is based on an alignment. The probability data used in the plot is found here, and the gnuplot script is here.

Prediction of contig056940-NyeOR.L095

```
ID   contig056940-NyeOR.L095
FT   TOPO_DOM      1     25       NON CYTOPLASMIC.
FT   TRANSMEM     26     50
FT   TOPO_DOM     51     59       CYTOPLASMIC.
FT   TRANSMEM     60     86
FT   TOPO_DOM     87     98       NON CYTOPLASMIC.
FT   TRANSMEM     99    120
FT   TOPO_DOM    121    140       CYTOPLASMIC.
FT   TRANSMEM    141    162
FT   TOPO_DOM    163    198       NON CYTOPLASMIC.
FT   TRANSMEM    199    224
FT   TOPO_DOM    225    237       CYTOPLASMIC.
FT   TRANSMEM    238    259
FT   TOPO_DOM    260    271       NON CYTOPLASMIC.
FT   TRANSMEM    272    292
FT   TOPO_DOM    293    313       CYTOPLASMIC.
//
```

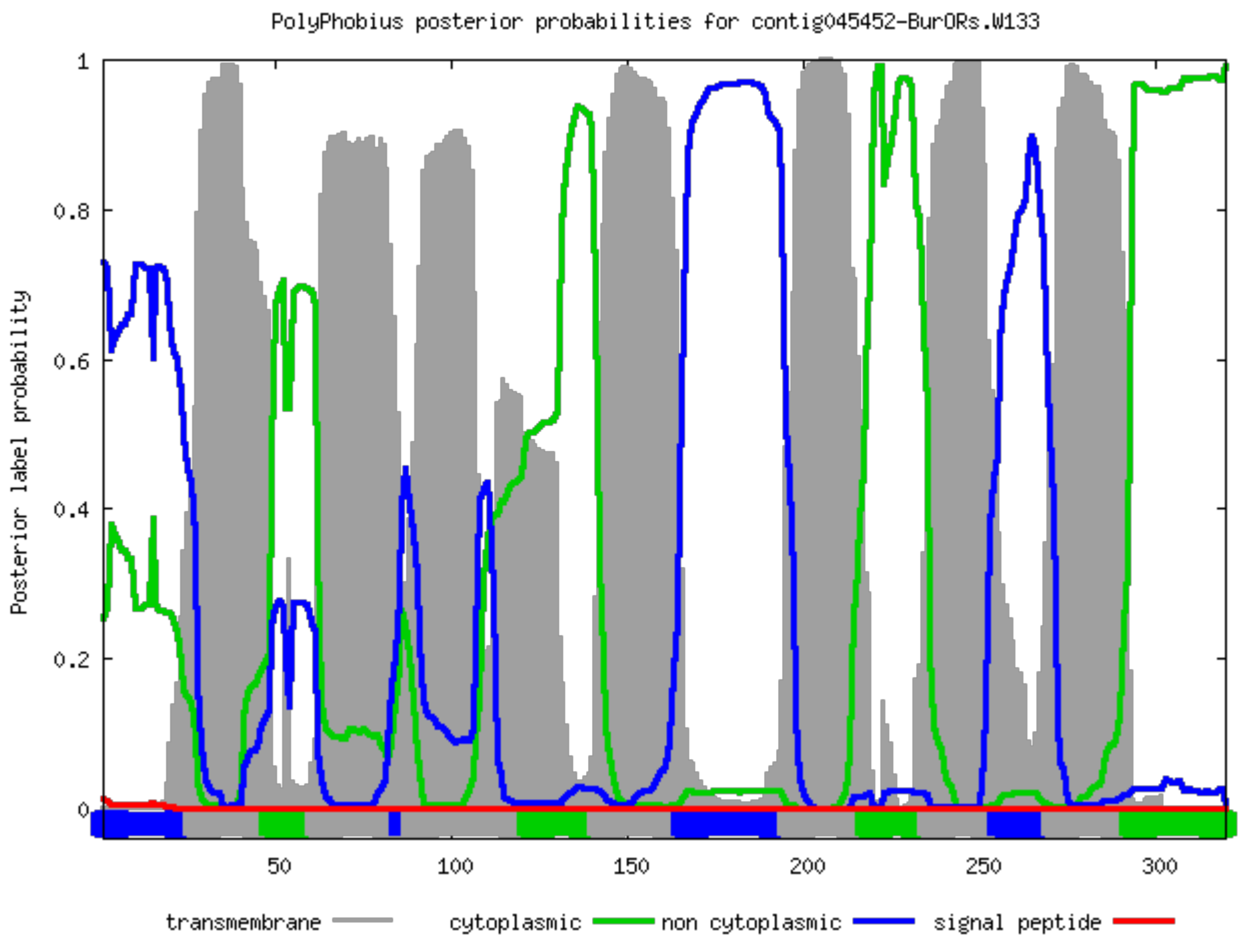

The prediction is based on an alignment. The probability data used in the plot is found here, and the gnuplot script is here.

Prediction of contig022266-TilOR.A018

```
ID    contig022266-TilOR.A018
FT    TOPO_DOM      1     22       NON CYTOPLASMIC.
FT    TRANSMEM     23     48
FT    TOPO_DOM     49     56       CYTOPLASMIC.
FT    TRANSMEM     57     77
FT    TOPO_DOM     78     95       NON CYTOPLASMIC.
FT    TRANSMEM     96    118
FT    TOPO_DOM    119    138       CYTOPLASMIC.
FT    TRANSMEM    139    159
FT    TOPO_DOM    160    192       NON CYTOPLASMIC.
FT    TRANSMEM    193    215
FT    TOPO_DOM    216    235       CYTOPLASMIC.
FT    TRANSMEM    236    257
FT    TOPO_DOM    258    268       NON CYTOPLASMIC.
FT    TRANSMEM    269    289
FT    TOPO_DOM    290    309       CYTOPLASMIC.
//
```

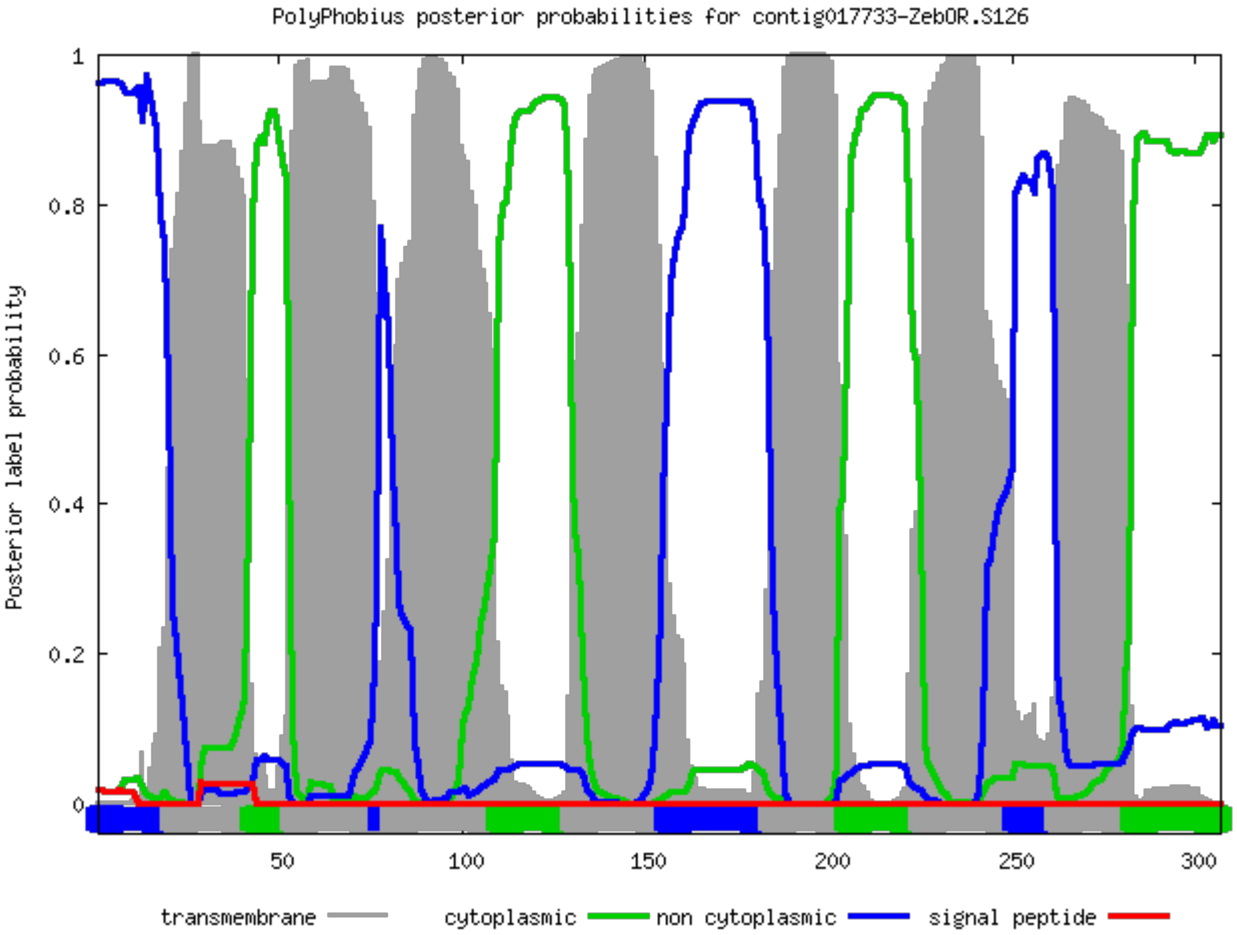

The prediction is based on an alignment. The probability data used in the plot is found here, and the gnuplot script is here.

Prediction of contig047508-ZebOR.A015

```
ID   contig047508-ZebOR.A015
FT   TOPO_DOM      1     22       NON CYTOPLASMIC.
FT   TRANSMEM     23     48
FT   TOPO_DOM     49     56       CYTOPLASMIC.
FT   TRANSMEM     57     76
FT   TOPO_DOM     77     95       NON CYTOPLASMIC.
FT   TRANSMEM     96    118
FT   TOPO_DOM    119    138       CYTOPLASMIC.
FT   TRANSMEM    139    159
FT   TOPO_DOM    160    192       NON CYTOPLASMIC.
FT   TRANSMEM    193    215
FT   TOPO_DOM    216    235       CYTOPLASMIC.
FT   TRANSMEM    236    257
FT   TOPO_DOM    258    268       NON CYTOPLASMIC.
FT   TRANSMEM    269    289
FT   TOPO_DOM    290    311       CYTOPLASMIC.
//
```

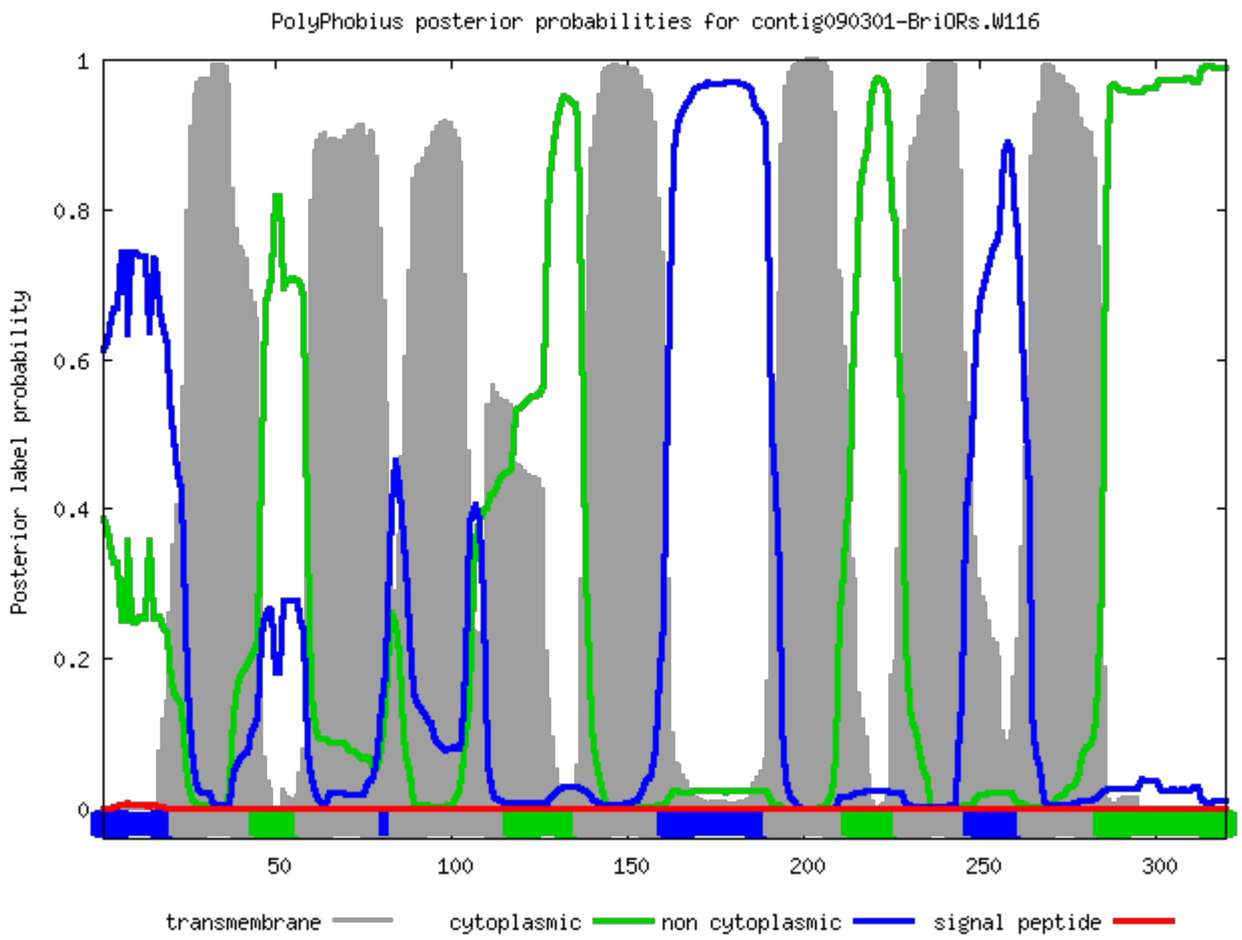

The prediction is based on an alignment. The probability data used in the plot is found here, and the gnuplot script is here.

Prediction of contig055927-NyeOR.N111

```
ID   contig055927-NyeOR.N111
FT   TOPO_DOM      1      32        NON CYTOPLASMIC.
FT   TRANSMEM     33      58
FT   TOPO_DOM     59      66        CYTOPLASMIC.
FT   TRANSMEM     67      86
FT   TOPO_DOM     87     104        NON CYTOPLASMIC.
FT   TRANSMEM    105     127
FT   TOPO_DOM    128     146        CYTOPLASMIC.
FT   TRANSMEM    147     170
FT   TOPO_DOM    171     207        NON CYTOPLASMIC.
FT   TRANSMEM    208     232
FT   TOPO_DOM    233     249        CYTOPLASMIC.
FT   TRANSMEM    250     271
FT   TOPO_DOM    272     277        NON CYTOPLASMIC.
FT   TRANSMEM    278     298
FT   TOPO_DOM    299     322        CYTOPLASMIC.
//
```

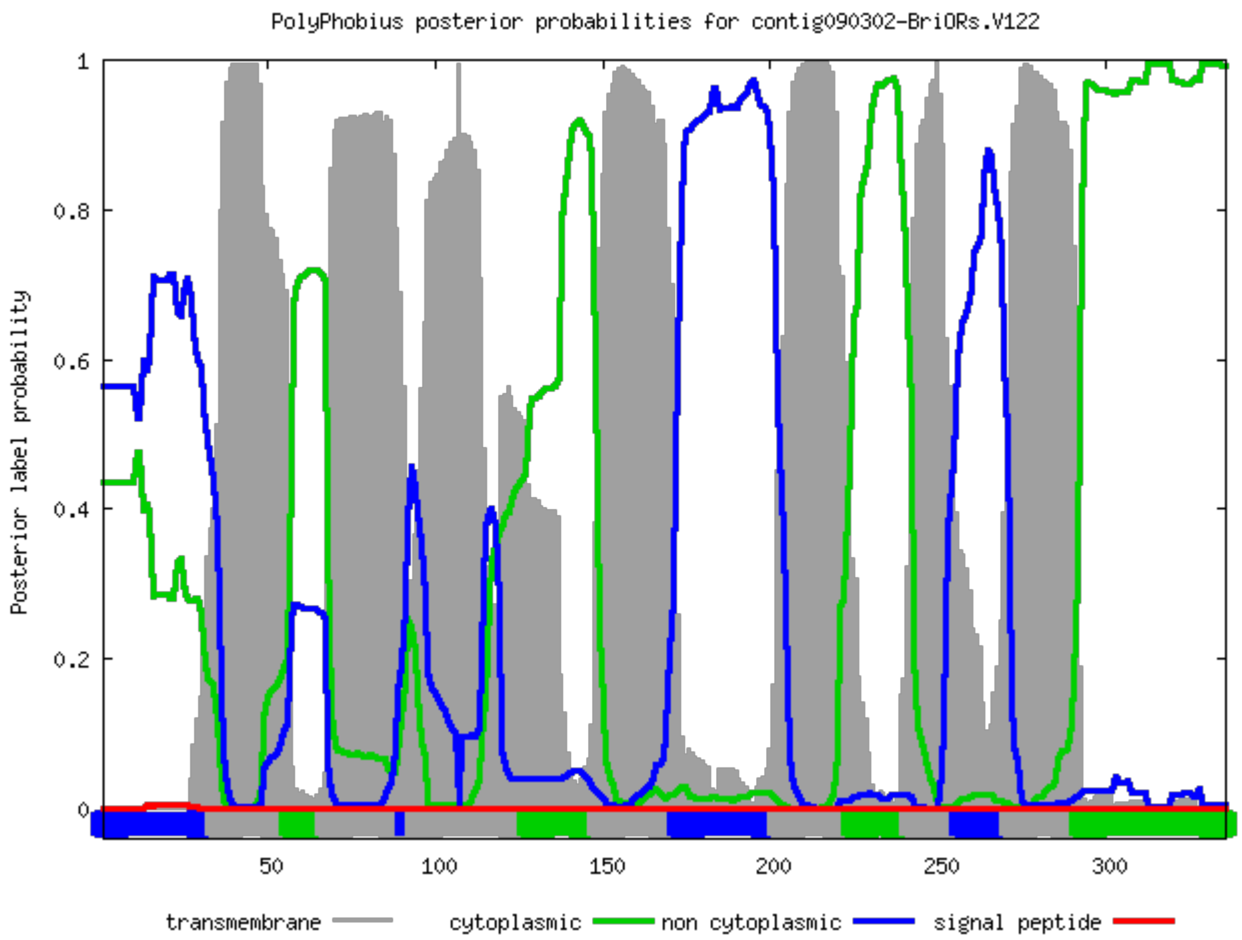

The prediction is based on an alignment. The probability data used in the plot is found here, and the gnuplot script is here.

Prediction of contig023268-NyeOR.P118

```
ID   contig023268-NyeOR.P118
FT   TOPO_DOM      1     28       NON CYTOPLASMIC.
FT   TRANSMEM     29     52
FT   TOPO_DOM     53     62       CYTOPLASMIC.
FT   TRANSMEM     63     86
FT   TOPO_DOM     87    100       NON CYTOPLASMIC.
FT   TRANSMEM    101    123
FT   TOPO_DOM    124    142       CYTOPLASMIC.
FT   TRANSMEM    143    166
FT   TOPO_DOM    167    200       NON CYTOPLASMIC.
FT   TRANSMEM    201    225
FT   TOPO_DOM    226    242       CYTOPLASMIC.
FT   TRANSMEM    243    265
FT   TOPO_DOM    266    277       NON CYTOPLASMIC.
FT   TRANSMEM    278    298
FT   TOPO_DOM    299    332       CYTOPLASMIC.
//
```

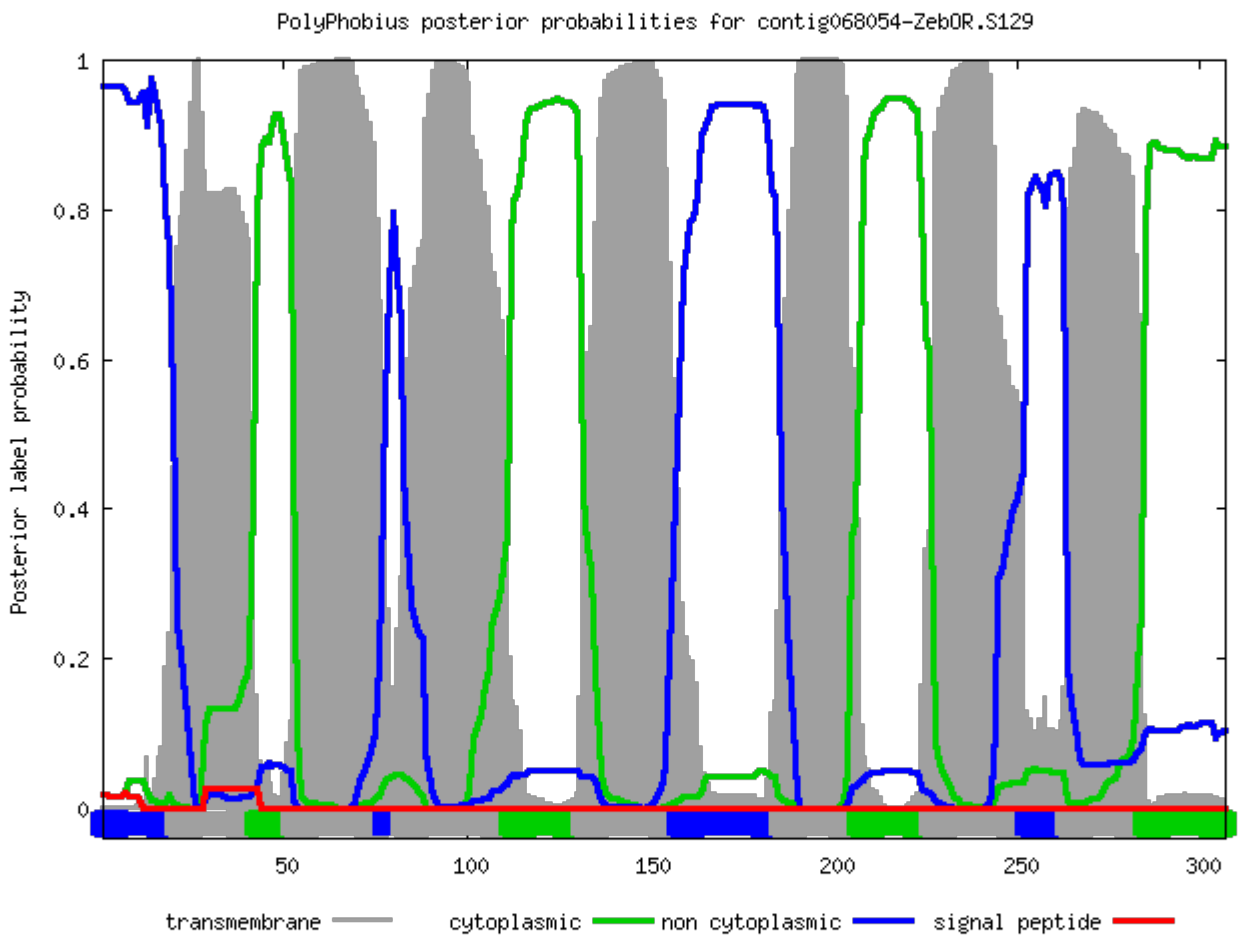

The prediction is based on an alignment. The probability data used in the plot is found here, and the gnuplot script is here.

Prediction of contig056375-NyeOR.A015

```
ID   contig056375-NyeOR.A015
FT   TOPO_DOM      1     22       NON CYTOPLASMIC.
FT   TRANSMEM     23     48
FT   TOPO_DOM     49     56       CYTOPLASMIC.
FT   TRANSMEM     57     76
FT   TOPO_DOM     77     95       NON CYTOPLASMIC.
FT   TRANSMEM     96    118
FT   TOPO_DOM    119    138       CYTOPLASMIC.
FT   TRANSMEM    139    159
FT   TOPO_DOM    160    192       NON CYTOPLASMIC.
FT   TRANSMEM    193    215
FT   TOPO_DOM    216    235       CYTOPLASMIC.
FT   TRANSMEM    236    257
FT   TOPO_DOM    258    268       NON CYTOPLASMIC.
FT   TRANSMEM    269    289
FT   TOPO_DOM    290    304       CYTOPLASMIC.
//
```

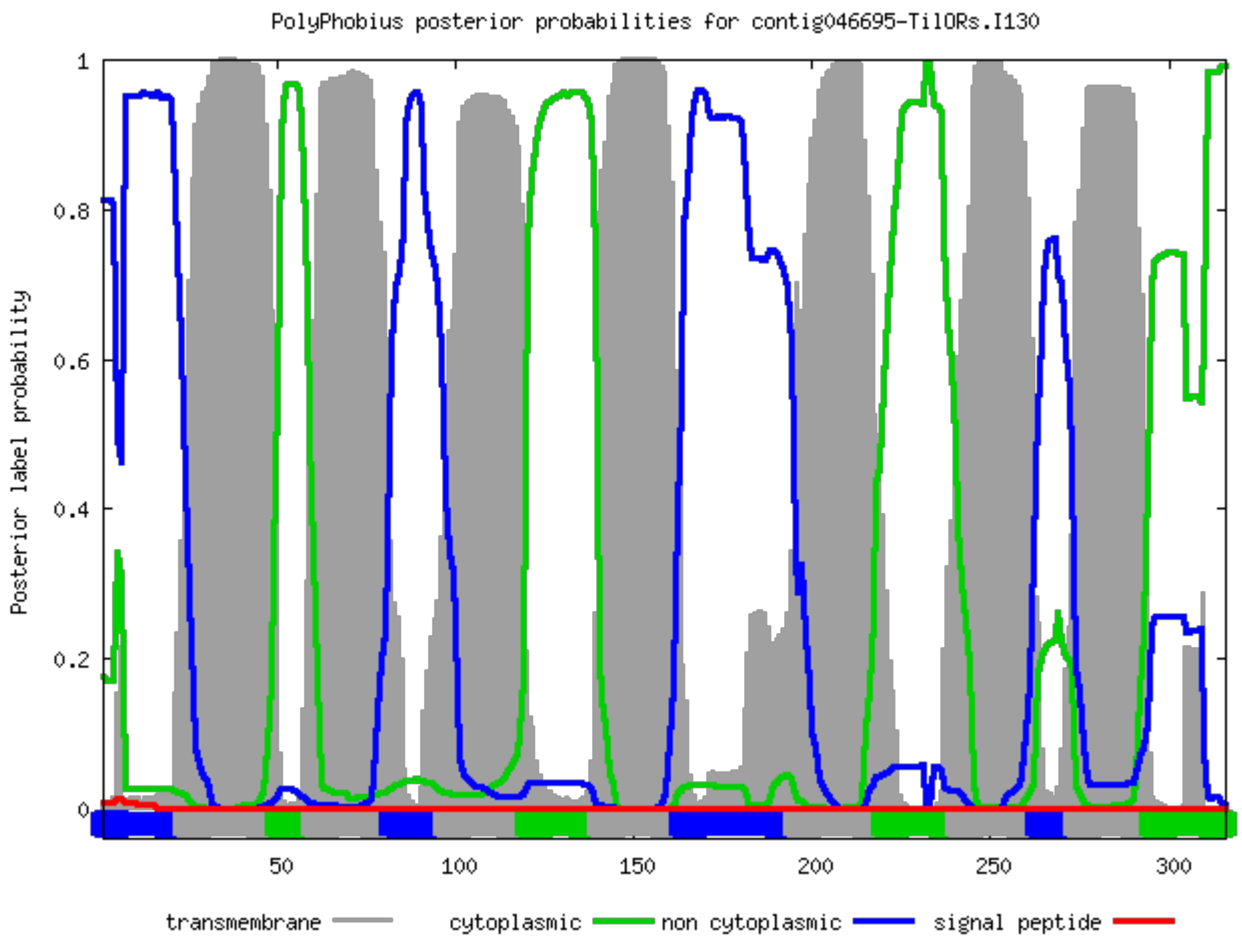

The prediction is based on an alignment. The probability data used in the plot is found here, and the gnuplot script is here.

Prediction of contig051321-BurOR.A006

```
ID   contig051321-BurOR.A006
FT   TOPO_DOM      1     22       NON CYTOPLASMIC.
FT   TRANSMEM     23     48
FT   TOPO_DOM     49     56       CYTOPLASMIC.
FT   TRANSMEM     57     76
FT   TOPO_DOM     77     95       NON CYTOPLASMIC.
FT   TRANSMEM     96    118
FT   TOPO_DOM    119    138       CYTOPLASMIC.
FT   TRANSMEM    139    159
FT   TOPO_DOM    160    192       NON CYTOPLASMIC.
FT   TRANSMEM    193    215
FT   TOPO_DOM    216    235       CYTOPLASMIC.
FT   TRANSMEM    236    257
FT   TOPO_DOM    258    268       NON CYTOPLASMIC.
FT   TRANSMEM    269    289
FT   TOPO_DOM    290    314       CYTOPLASMIC.
//
```

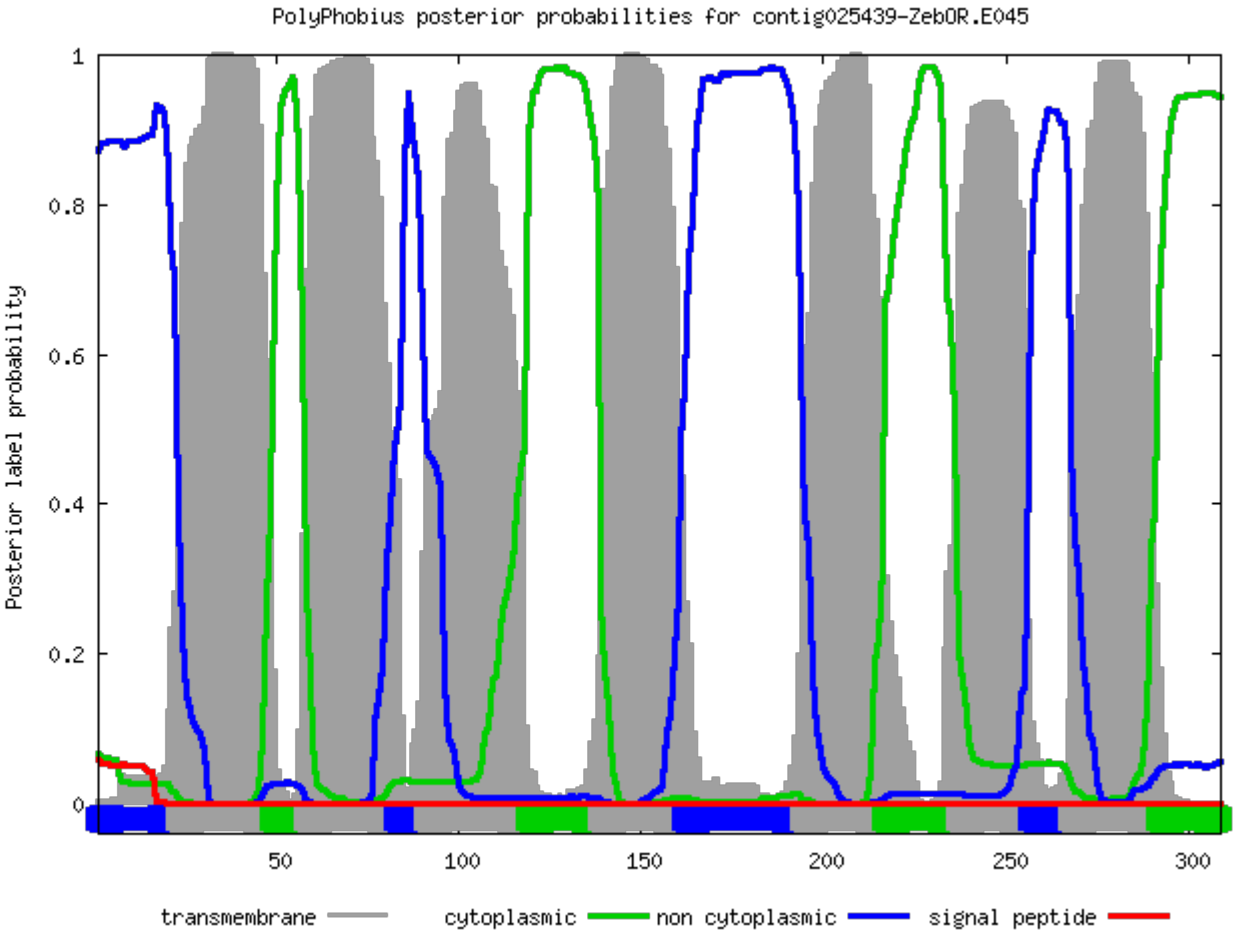

The prediction is based on an alignment. The probability data used in the plot is found here, and the gnuplot script is here.

Prediction of contig042560-BriOR.O078

```
ID   contig042560-BriOR.O078
FT   TOPO_DOM      1      25       NON CYTOPLASMIC.
FT   TRANSMEM     26      50
FT   TOPO_DOM     51      59       CYTOPLASMIC.
FT   TRANSMEM     60      82
FT   TOPO_DOM     83      97       NON CYTOPLASMIC.
FT   TRANSMEM     98     120
FT   TOPO_DOM    121     140       CYTOPLASMIC.
FT   TRANSMEM    141     162
FT   TOPO_DOM    163     200       NON CYTOPLASMIC.
FT   TRANSMEM    201     226
FT   TOPO_DOM    227     239       CYTOPLASMIC.
FT   TRANSMEM    240     262
FT   TOPO_DOM    263     274       NON CYTOPLASMIC.
FT   TRANSMEM    275     295
FT   TOPO_DOM    296     322       CYTOPLASMIC.
//
```

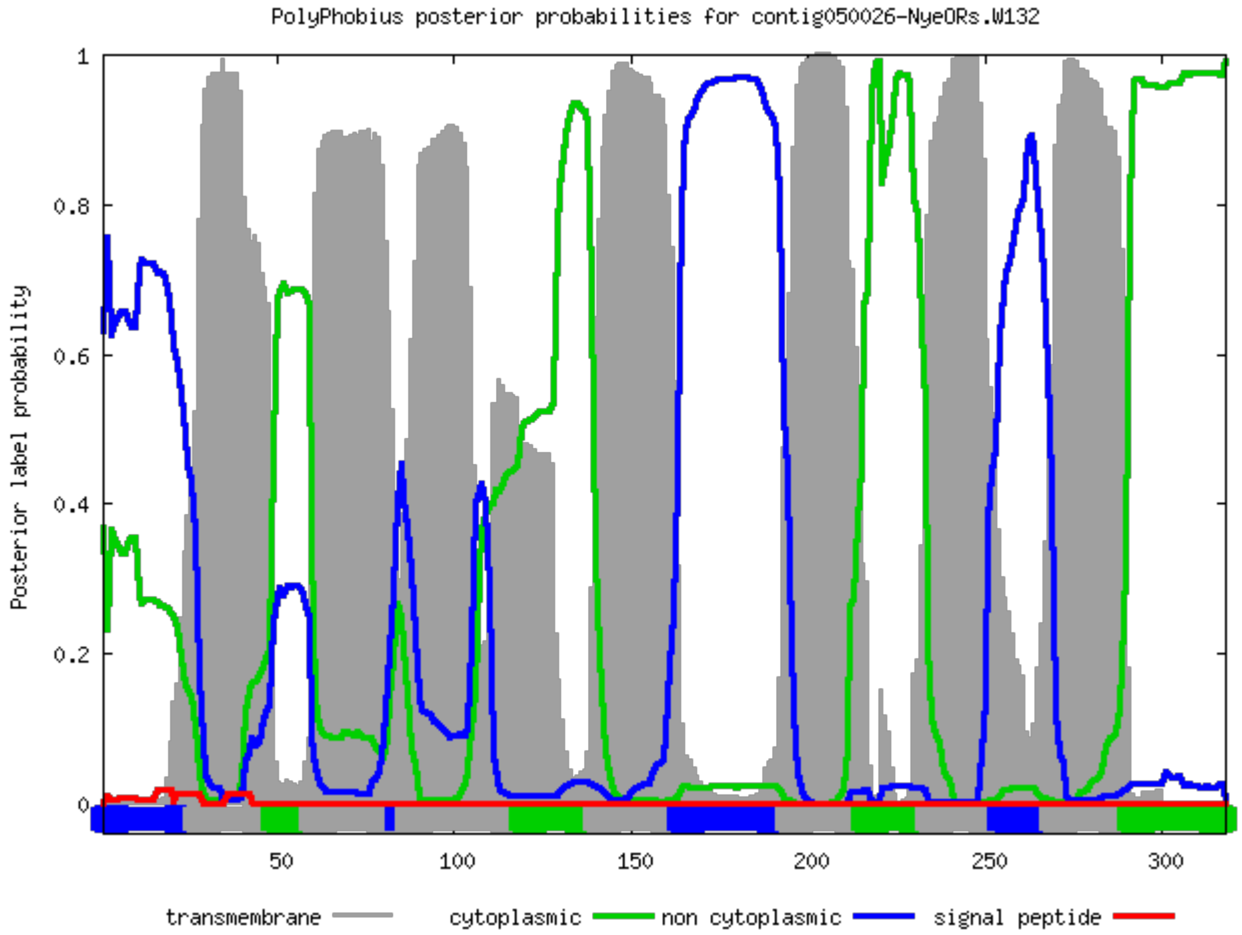

The prediction is based on an alignment. The probability data used in the plot is found here, and the gnuplot script is here.

Prediction of contig014348-BriOR.K066

```
ID   contig014348-BriOR.K066
FT   TOPO_DOM      1     22       NON CYTOPLASMIC.
FT   TRANSMEM     23     46
FT   TOPO_DOM     47     56       CYTOPLASMIC.
FT   TRANSMEM     57     78
FT   TOPO_DOM     79     97       NON CYTOPLASMIC.
FT   TRANSMEM     98    119
FT   TOPO_DOM    120    139       CYTOPLASMIC.
FT   TRANSMEM    140    163
FT   TOPO_DOM    164    195       NON CYTOPLASMIC.
FT   TRANSMEM    196    220
FT   TOPO_DOM    221    240       CYTOPLASMIC.
FT   TRANSMEM    241    260
FT   TOPO_DOM    261    270       NON CYTOPLASMIC.
FT   TRANSMEM    271    290
FT   TOPO_DOM    291    315       CYTOPLASMIC.
//
```

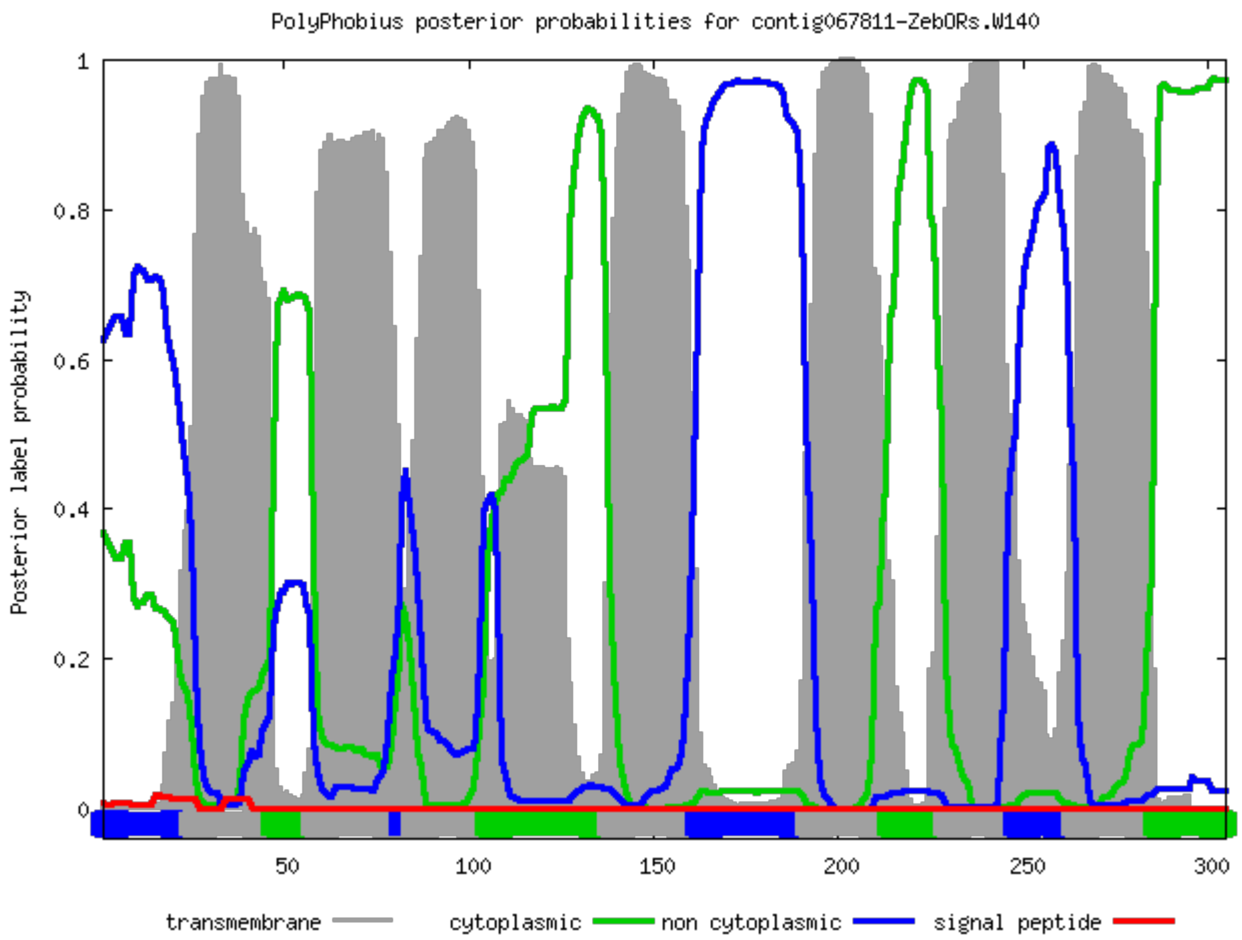

The prediction is based on an alignment. The probability data used in the plot is found here, and the gnuplot script is here.

Prediction of contig022259-TilOR.A014

```
ID   contig022259-TilOR.A014
FT   TOPO_DOM     1    22      NON CYTOPLASMIC.
FT   TRANSMEM    23    48
FT   TOPO_DOM    49    56      CYTOPLASMIC.
FT   TRANSMEM    57    76
FT   TOPO_DOM    77    95      NON CYTOPLASMIC.
FT   TRANSMEM    96   118
FT   TOPO_DOM   119   138      CYTOPLASMIC.
FT   TRANSMEM   139   159
FT   TOPO_DOM   160   192      NON CYTOPLASMIC.
FT   TRANSMEM   193   215
FT   TOPO_DOM   216   235      CYTOPLASMIC.
FT   TRANSMEM   236   257
FT   TOPO_DOM   258   268      NON CYTOPLASMIC.
FT   TRANSMEM   269   289
FT   TOPO_DOM   290   314      CYTOPLASMIC.
//
```

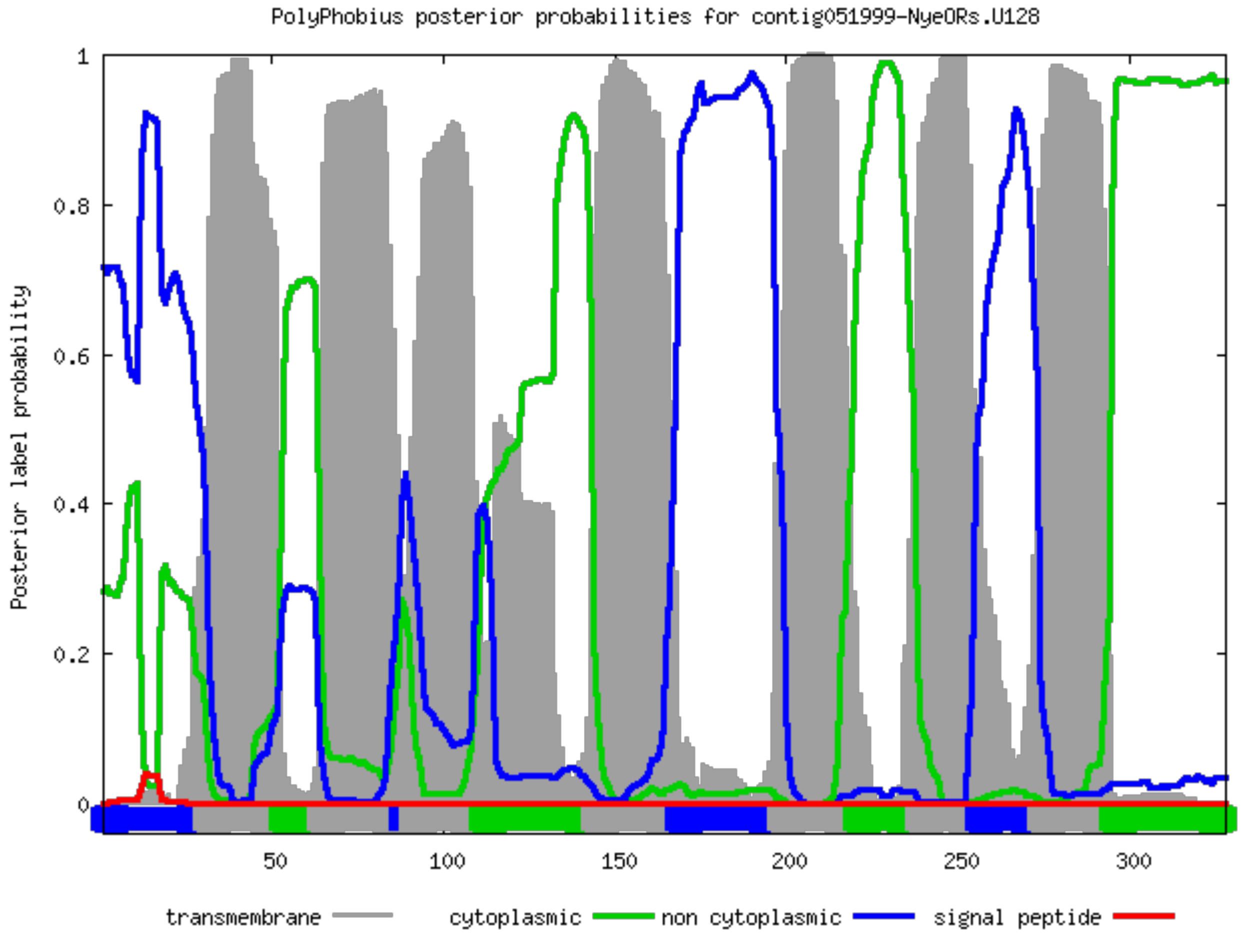

The prediction is based on an alignment. The probability data used in the plot is found here, and the gnuplot script is here.

Prediction of contig042556-BriOR.L073

```
ID    contig042556-BriOR.L073
FT    TOPO_DOM      1      25       NON CYTOPLASMIC.
FT    TRANSMEM     26      50
FT    TOPO_DOM     51      59       CYTOPLASMIC.
FT    TRANSMEM     60      86
FT    TOPO_DOM     87      97       NON CYTOPLASMIC.
FT    TRANSMEM     98     120
FT    TOPO_DOM    121     140       CYTOPLASMIC.
FT    TRANSMEM    141     163
FT    TOPO_DOM    164     198       NON CYTOPLASMIC.
FT    TRANSMEM    199     224
FT    TOPO_DOM    225     237       CYTOPLASMIC.
FT    TRANSMEM    238     259
FT    TOPO_DOM    260     271       NON CYTOPLASMIC.
FT    TRANSMEM    272     292
FT    TOPO_DOM    293     313       CYTOPLASMIC.
//
```

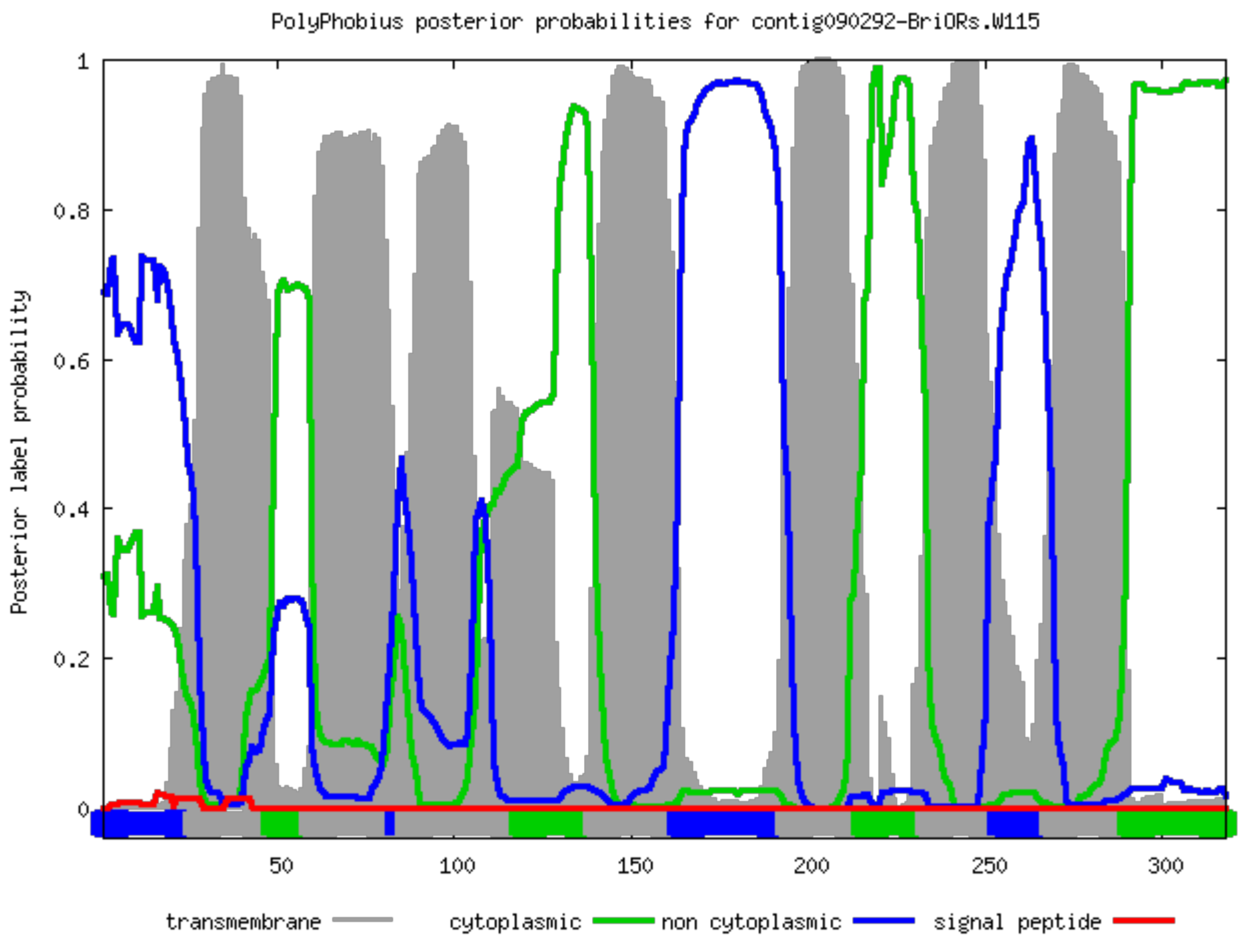

The prediction is based on an alignment. The probability data used in the plot is found here, and the gnuplot script is here.

Prediction of contig039435-TilOR.S228

```
ID   contig039435-TilOR.S228
FT   TOPO_DOM      1     21       NON CYTOPLASMIC.
FT   TRANSMEM     22     43
FT   TOPO_DOM     44     53       CYTOPLASMIC.
FT   TRANSMEM     54     78
FT   TOPO_DOM     79     83       NON CYTOPLASMIC.
FT   TRANSMEM     84    112
FT   TOPO_DOM    113    132       CYTOPLASMIC.
FT   TRANSMEM    133    158
FT   TOPO_DOM    159    186       NON CYTOPLASMIC.
FT   TRANSMEM    187    207
FT   TOPO_DOM    208    227       CYTOPLASMIC.
FT   TRANSMEM    228    253
FT   TOPO_DOM    254    264       NON CYTOPLASMIC.
FT   TRANSMEM    265    285
FT   TOPO_DOM    286    310       CYTOPLASMIC.
//
```

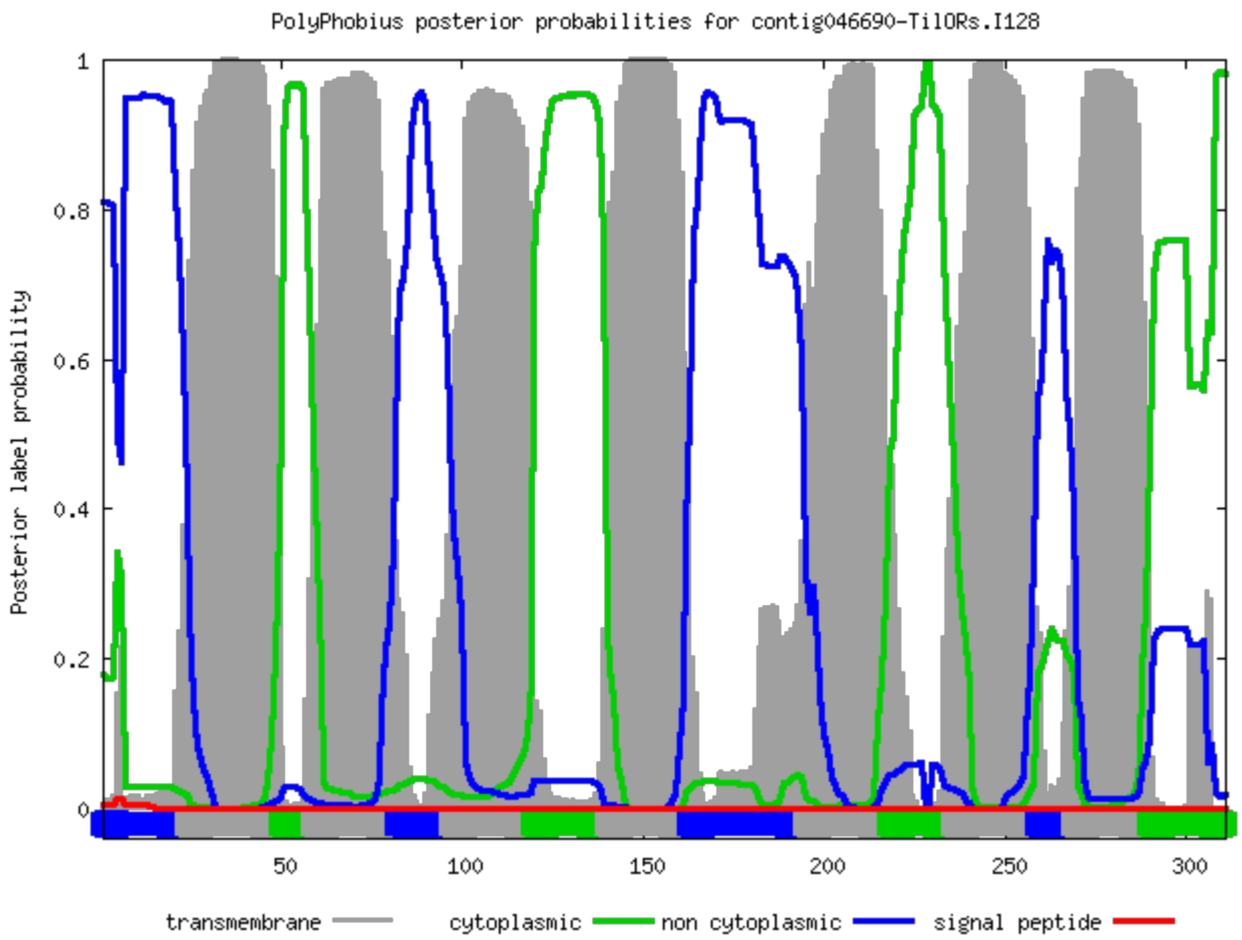

The prediction is based on an alignment. The probability data used in the plot is found here, and the gnuplot script is here.

Prediction of contig039450-TilOR.J131

```
ID    contig039450-TilOR.J131
FT    TOPO_DOM      1     25       NON CYTOPLASMIC.
FT    TRANSMEM     26     50
FT    TOPO_DOM     51     60       CYTOPLASMIC.
FT    TRANSMEM     61     82
FT    TOPO_DOM     83     98       NON CYTOPLASMIC.
FT    TRANSMEM     99    120
FT    TOPO_DOM    121    140       CYTOPLASMIC.
FT    TRANSMEM    141    163
FT    TOPO_DOM    164    195       NON CYTOPLASMIC.
FT    TRANSMEM    196    220
FT    TOPO_DOM    221    238       CYTOPLASMIC.
FT    TRANSMEM    239    261
FT    TOPO_DOM    262    271       NON CYTOPLASMIC.
FT    TRANSMEM    272    292
FT    TOPO_DOM    293    312       CYTOPLASMIC.
//
```

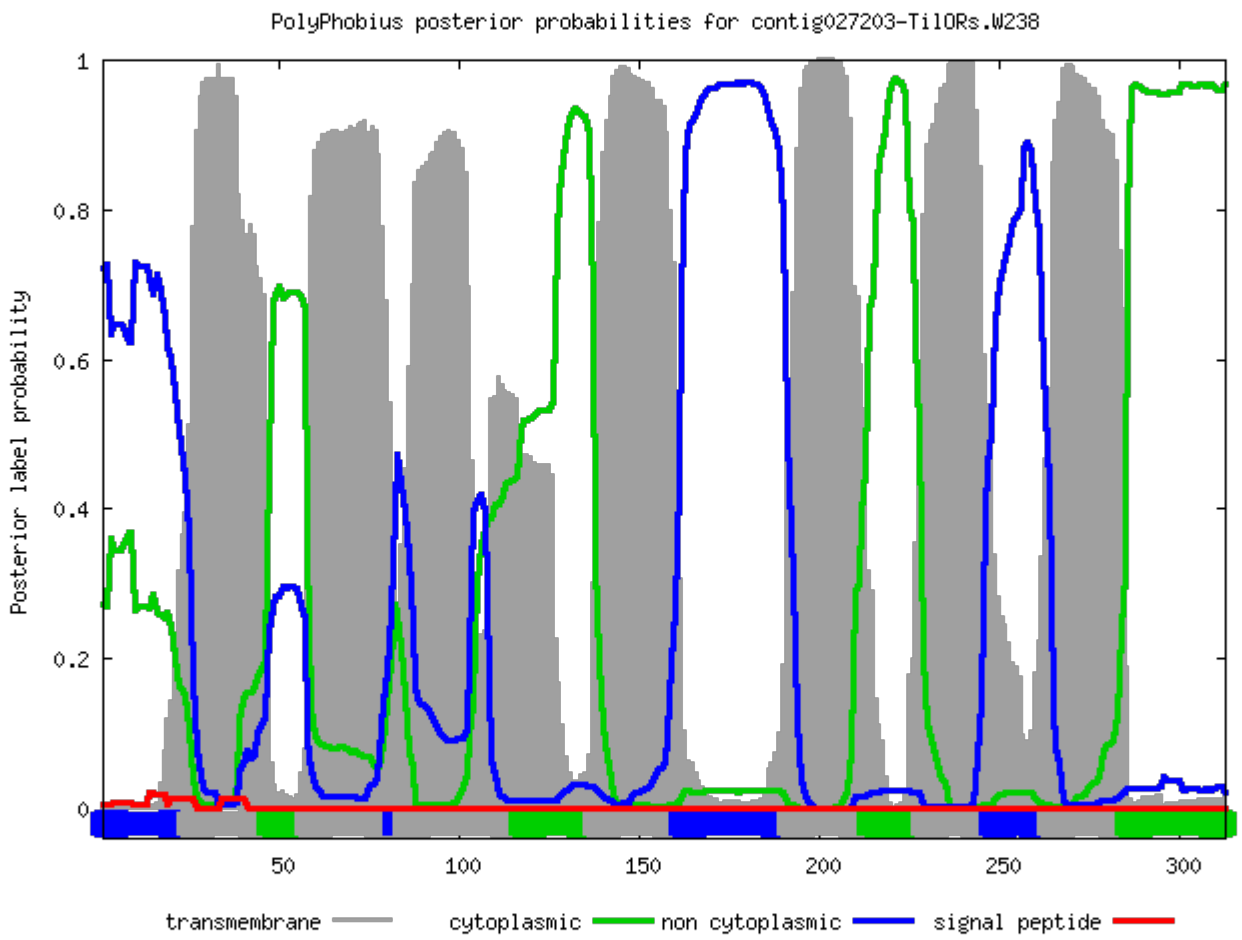

The prediction is based on an alignment. The probability data used in the plot is found here, and the gnuplot script is here.

Prediction of contig022211-TilOR.A003

```
ID   contig022211-TilOR.A003
FT   TOPO_DOM      1     22       NON CYTOPLASMIC.
FT   TRANSMEM     23     48
FT   TOPO_DOM     49     56       CYTOPLASMIC.
FT   TRANSMEM     57     76
FT   TOPO_DOM     77     95       NON CYTOPLASMIC.
FT   TRANSMEM     96    118
FT   TOPO_DOM    119    138       CYTOPLASMIC.
FT   TRANSMEM    139    159
FT   TOPO_DOM    160    192       NON CYTOPLASMIC.
FT   TRANSMEM    193    215
FT   TOPO_DOM    216    235       CYTOPLASMIC.
FT   TRANSMEM    236    257
FT   TOPO_DOM    258    268       NON CYTOPLASMIC.
FT   TRANSMEM    269    289
FT   TOPO_DOM    290    316       CYTOPLASMIC.
//
```

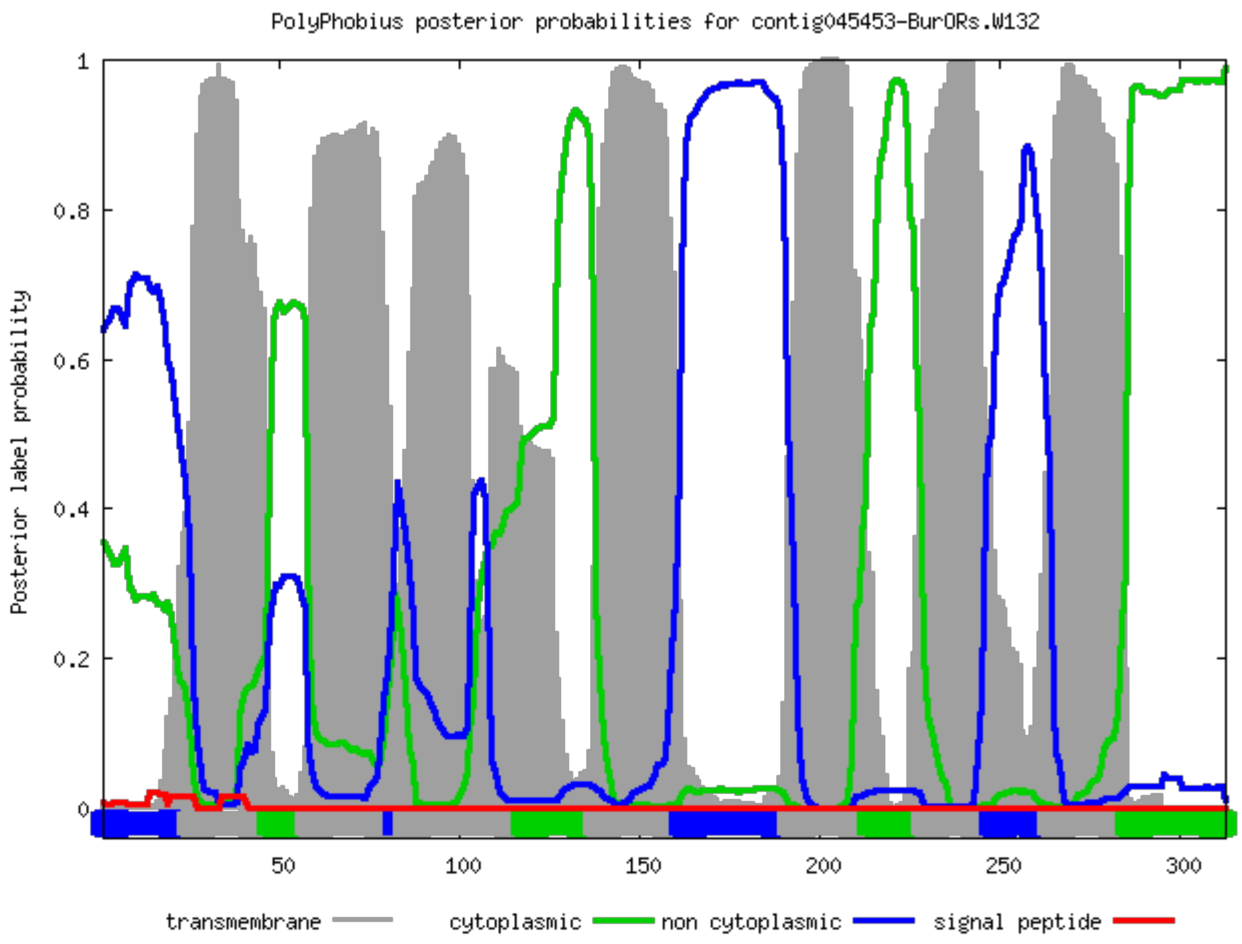

The prediction is based on an alignment. The probability data used in the plot is found here, and the gnuplot script is here.

Prediction of contig042559-BriOR.O077

```
ID   contig042559-BriOR.O077
FT   TOPO_DOM      1     24       NON CYTOPLASMIC.
FT   TRANSMEM     25     51
FT   TOPO_DOM     52     60       CYTOPLASMIC.
FT   TRANSMEM     61     83
FT   TOPO_DOM     84     98       NON CYTOPLASMIC.
FT   TRANSMEM     99    121
FT   TOPO_DOM    122    141       CYTOPLASMIC.
FT   TRANSMEM    142    163
FT   TOPO_DOM    164    200       NON CYTOPLASMIC.
FT   TRANSMEM    201    227
FT   TOPO_DOM    228    240       CYTOPLASMIC.
FT   TRANSMEM    241    262
FT   TOPO_DOM    263    273       NON CYTOPLASMIC.
FT   TRANSMEM    274    295
FT   TOPO_DOM    296    331       CYTOPLASMIC.
//
```

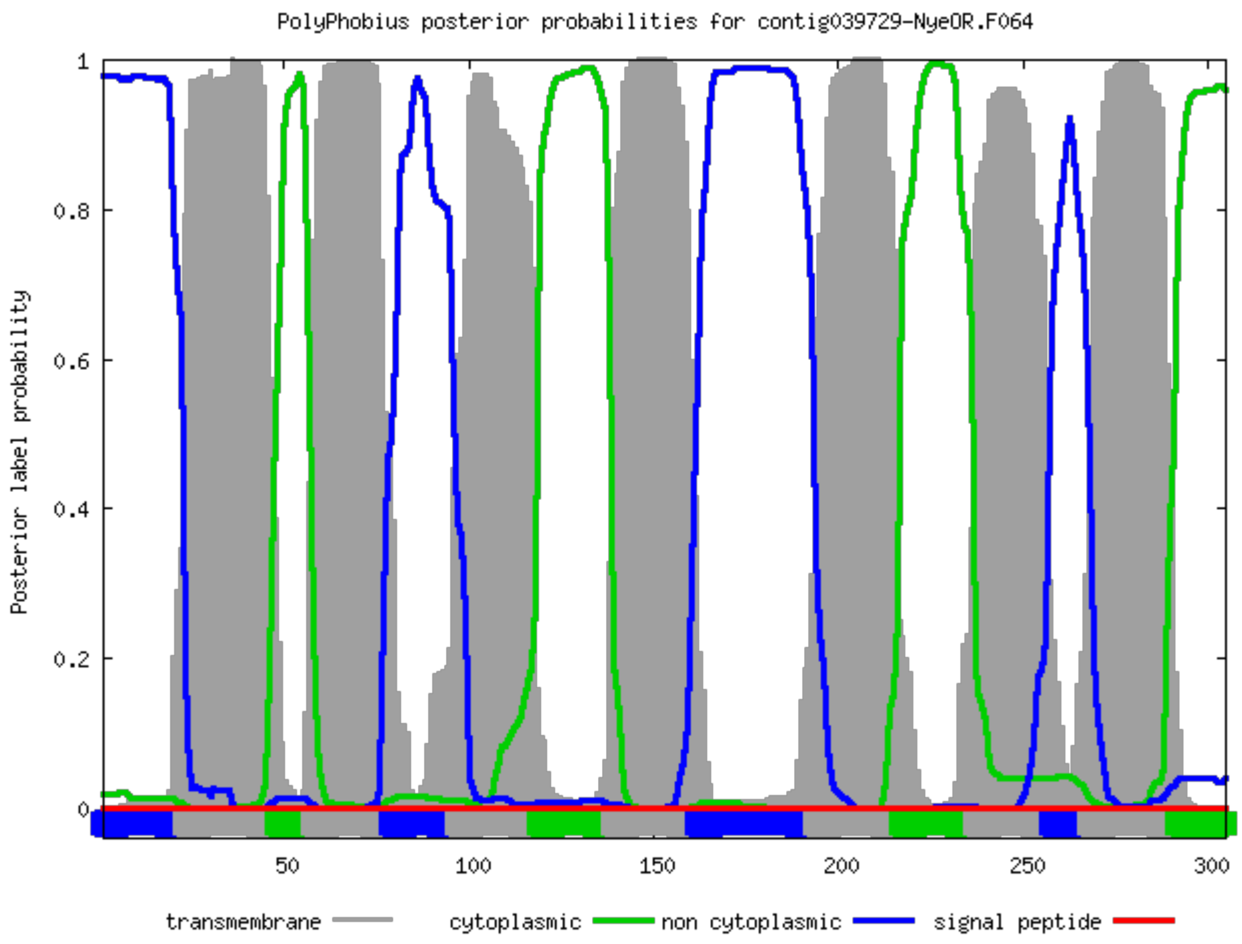

The prediction is based on an alignment. The probability data used in the plot is found here, and the gnuplot script is here.

Prediction of contig020437-ZebOR.L093

```
ID    contig020437-ZebOR.L093
FT    TOPO_DOM      1      25       NON CYTOPLASMIC.
FT    TRANSMEM     26      50
FT    TOPO_DOM     51      59       CYTOPLASMIC.
FT    TRANSMEM     60      86
FT    TOPO_DOM     87      98       NON CYTOPLASMIC.
FT    TRANSMEM     99     120
FT    TOPO_DOM    121     140       CYTOPLASMIC.
FT    TRANSMEM    141     163
FT    TOPO_DOM    164     198       NON CYTOPLASMIC.
FT    TRANSMEM    199     224
FT    TOPO_DOM    225     237       CYTOPLASMIC.
FT    TRANSMEM    238     260
FT    TOPO_DOM    261     271       NON CYTOPLASMIC.
FT    TRANSMEM    272     292
FT    TOPO_DOM    293     313       CYTOPLASMIC.
//
```

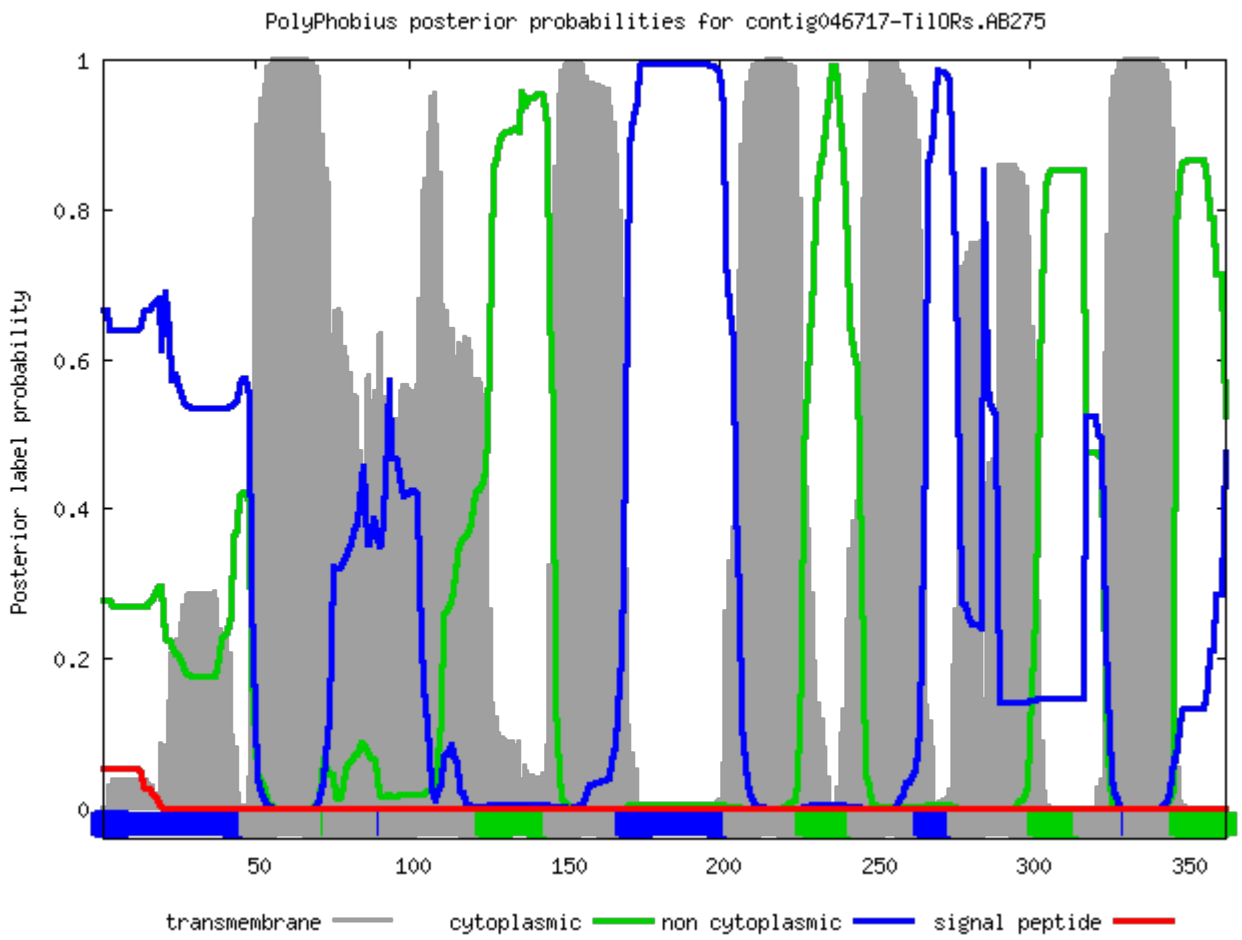

The prediction is based on an alignment. The probability data used in the plot is found here, and the gnuplot script is here.

Prediction of contig013323-TilOR.D050

```
ID   contig013323-TilOR.D050
FT   TOPO_DOM      1     24       NON CYTOPLASMIC.
FT   TRANSMEM     25     50
FT   TOPO_DOM     51     59       CYTOPLASMIC.
FT   TRANSMEM     60     83
FT   TOPO_DOM     84     92       NON CYTOPLASMIC.
FT   TRANSMEM     93    120
FT   TOPO_DOM    121    140       CYTOPLASMIC.
FT   TRANSMEM    141    163
FT   TOPO_DOM    164    196       NON CYTOPLASMIC.
FT   TRANSMEM    197    218
FT   TOPO_DOM    219    238       CYTOPLASMIC.
FT   TRANSMEM    239    258
FT   TOPO_DOM    259    269       NON CYTOPLASMIC.
FT   TRANSMEM    270    293
FT   TOPO_DOM    294    311       CYTOPLASMIC.
//
```

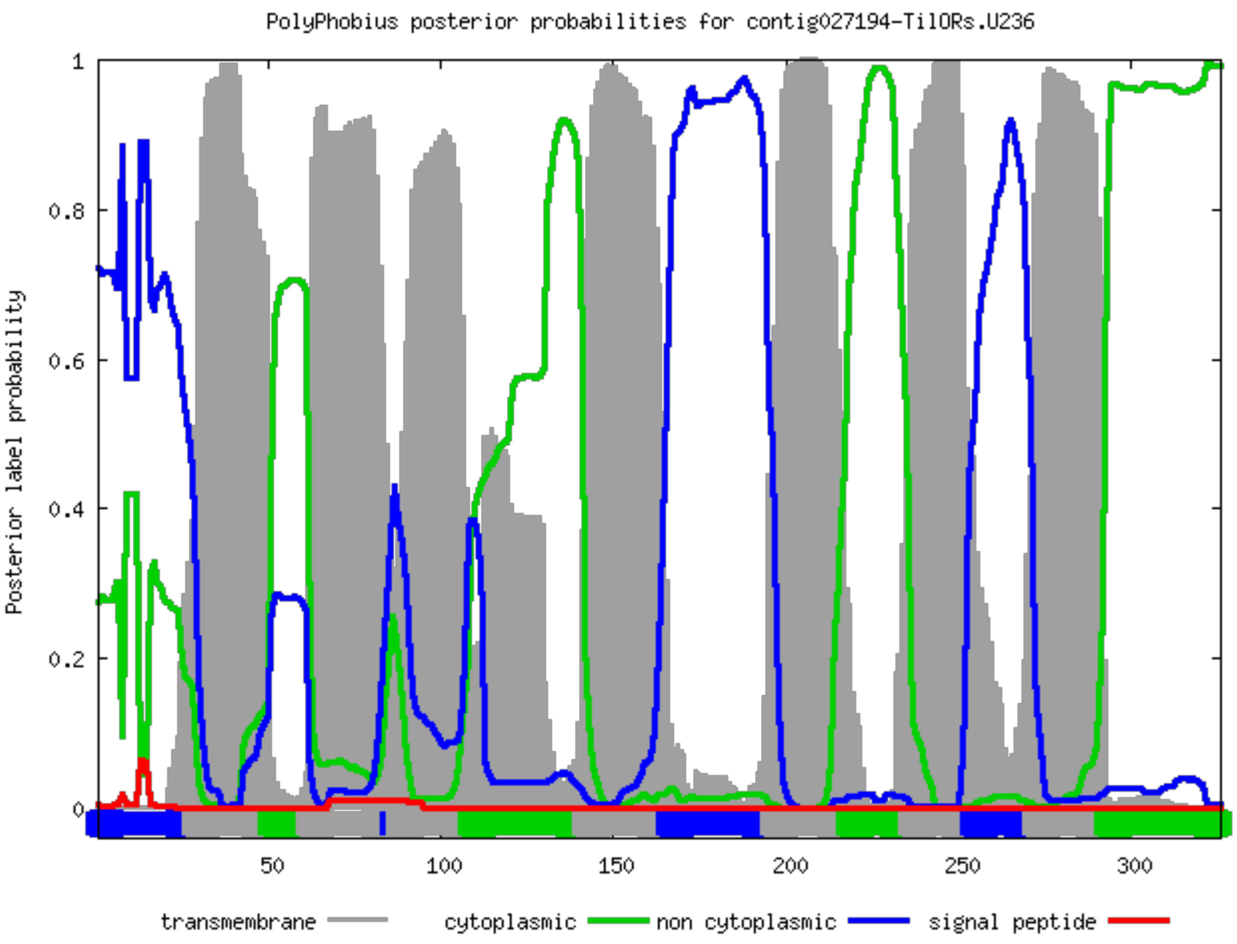

The prediction is based on an alignment. The probability data used in the plot is found here, and the gnuplot script is here.

Prediction of contig035583-NyeOR.H071

```
ID   contig035583-NyeOR.H071
FT   TOPO_DOM      1     23       NON CYTOPLASMIC.
FT   TRANSMEM     24     49
FT   TOPO_DOM     50     56       CYTOPLASMIC.
FT   TRANSMEM     57     76
FT   TOPO_DOM     77     95       NON CYTOPLASMIC.
FT   TRANSMEM     96    118
FT   TOPO_DOM    119    138       CYTOPLASMIC.
FT   TRANSMEM    139    160
FT   TOPO_DOM    161    196       NON CYTOPLASMIC.
FT   TRANSMEM    197    219
FT   TOPO_DOM    220    237       CYTOPLASMIC.
FT   TRANSMEM    238    260
FT   TOPO_DOM    261    271       NON CYTOPLASMIC.
FT   TRANSMEM    272    291
FT   TOPO_DOM    292    343       CYTOPLASMIC.
//
```

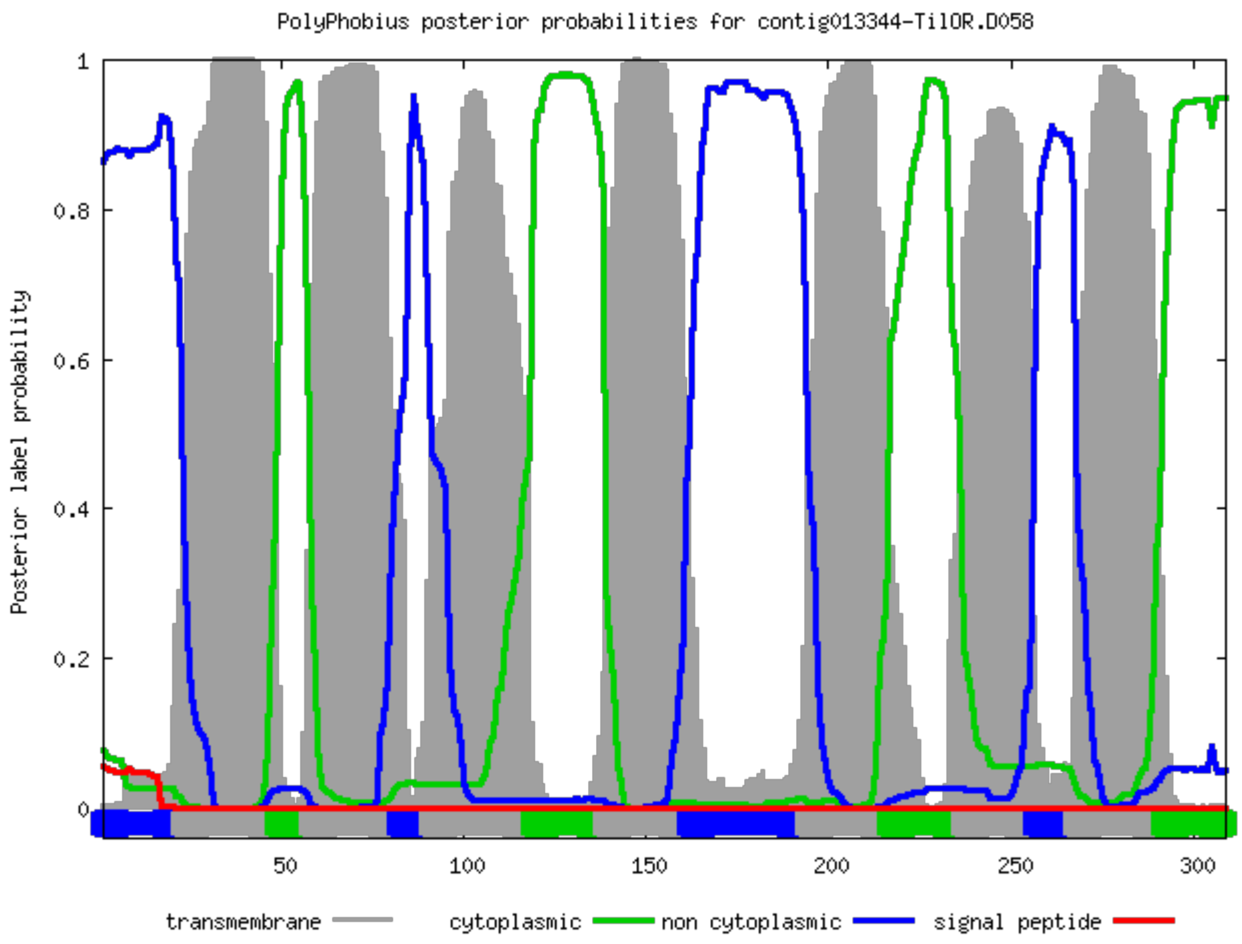

The prediction is based on an alignment. The probability data used in the plot is found here, and the gnuplot script is here.

Prediction of contig017781-ZebOR.J084

```
ID   contig017781-ZebOR.J084
FT   TOPO_DOM      1      23       NON CYTOPLASMIC.
FT   TRANSMEM     24      49
FT   TOPO_DOM     50      59       CYTOPLASMIC.
FT   TRANSMEM     60      81
FT   TOPO_DOM     82      97       NON CYTOPLASMIC.
FT   TRANSMEM     98     119
FT   TOPO_DOM    120     139       CYTOPLASMIC.
FT   TRANSMEM    140     162
FT   TOPO_DOM    163     194       NON CYTOPLASMIC.
FT   TRANSMEM    195     219
FT   TOPO_DOM    220     237       CYTOPLASMIC.
FT   TRANSMEM    238     260
FT   TOPO_DOM    261     270       NON CYTOPLASMIC.
FT   TRANSMEM    271     291
FT   TOPO_DOM    292     312       CYTOPLASMIC.
//
```

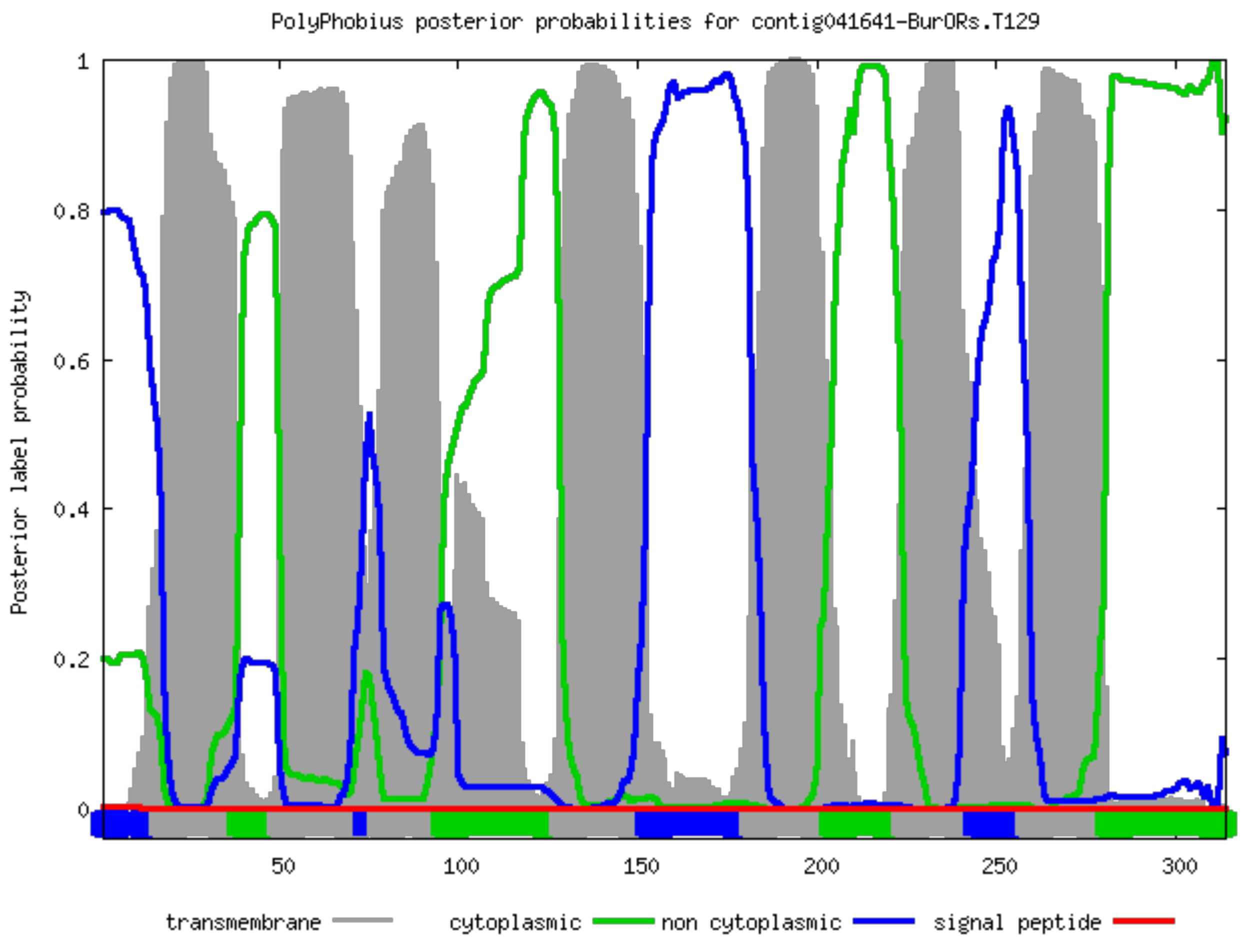

The prediction is based on an alignment. The probability data used in the plot is found here, and the gnuplot script is here.

Prediction of contig041955-TilOR.H119

```
ID   contig041955-TilOR.H119
FT   TOPO_DOM      1     22       NON CYTOPLASMIC.
FT   TRANSMEM     23     49
FT   TOPO_DOM     50     56       CYTOPLASMIC.
FT   TRANSMEM     57     76
FT   TOPO_DOM     77     95       NON CYTOPLASMIC.
FT   TRANSMEM     96    118
FT   TOPO_DOM    119    138       CYTOPLASMIC.
FT   TRANSMEM    139    160
FT   TOPO_DOM    161    196       NON CYTOPLASMIC.
FT   TRANSMEM    197    219
FT   TOPO_DOM    220    237       CYTOPLASMIC.
FT   TRANSMEM    238    260
FT   TOPO_DOM    261    271       NON CYTOPLASMIC.
FT   TRANSMEM    272    291
FT   TOPO_DOM    292    310       CYTOPLASMIC.
//
```

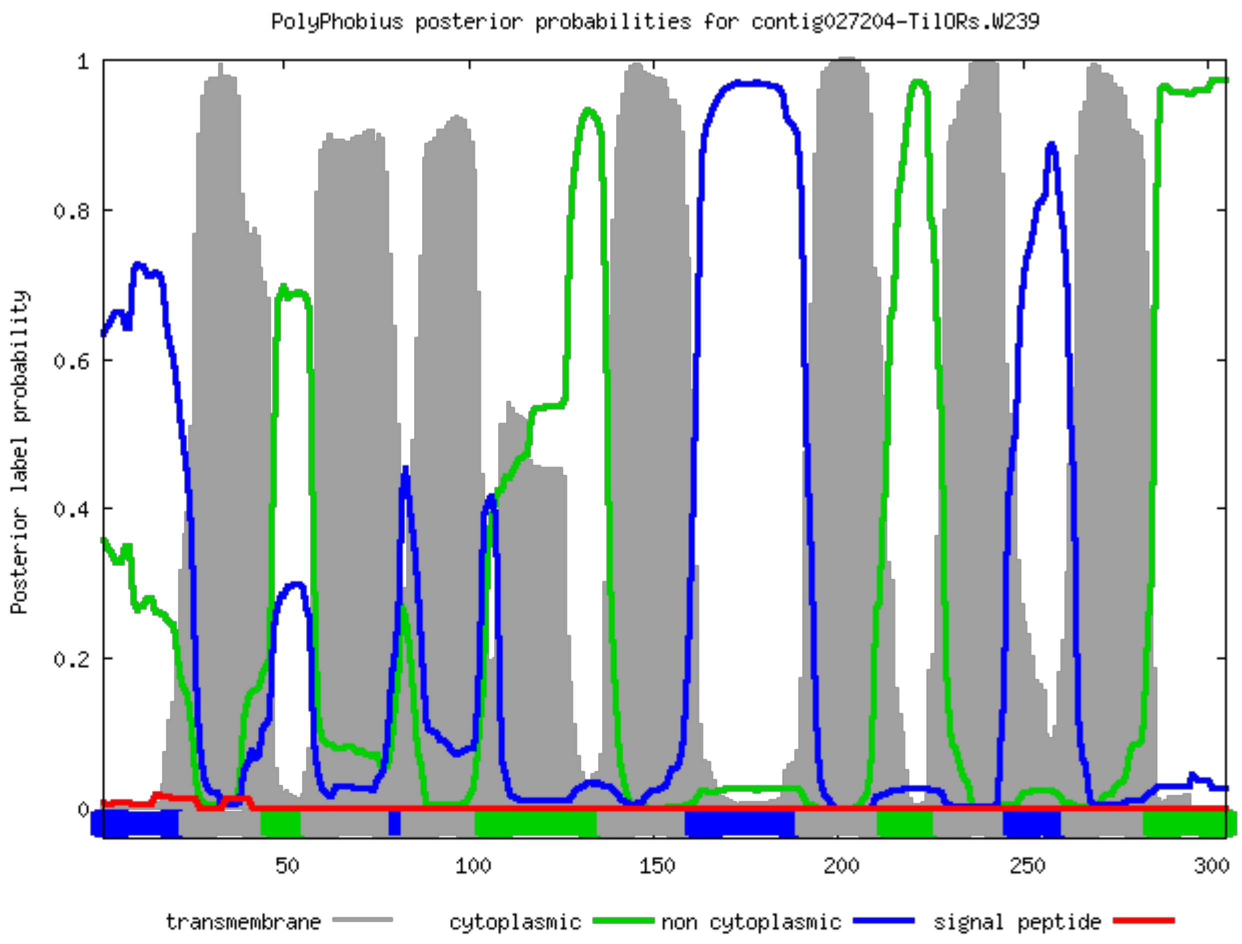

The prediction is based on an alignment. The probability data used in the plot is found here, and the gnuplot script is here.

Prediction of contig034998-NyeOR.H067

```
ID   contig034998-NyeOR.H067
FT   TOPO_DOM      1     23       NON CYTOPLASMIC.
FT   TRANSMEM     24     49
FT   TOPO_DOM     50     56       CYTOPLASMIC.
FT   TRANSMEM     57     76
FT   TOPO_DOM     77     95       NON CYTOPLASMIC.
FT   TRANSMEM     96    118
FT   TOPO_DOM    119    138       CYTOPLASMIC.
FT   TRANSMEM    139    160
FT   TOPO_DOM    161    196       NON CYTOPLASMIC.
FT   TRANSMEM    197    219
FT   TOPO_DOM    220    237       CYTOPLASMIC.
FT   TRANSMEM    238    260
FT   TOPO_DOM    261    271       NON CYTOPLASMIC.
FT   TRANSMEM    272    291
FT   TOPO_DOM    292    310       CYTOPLASMIC.
//
```

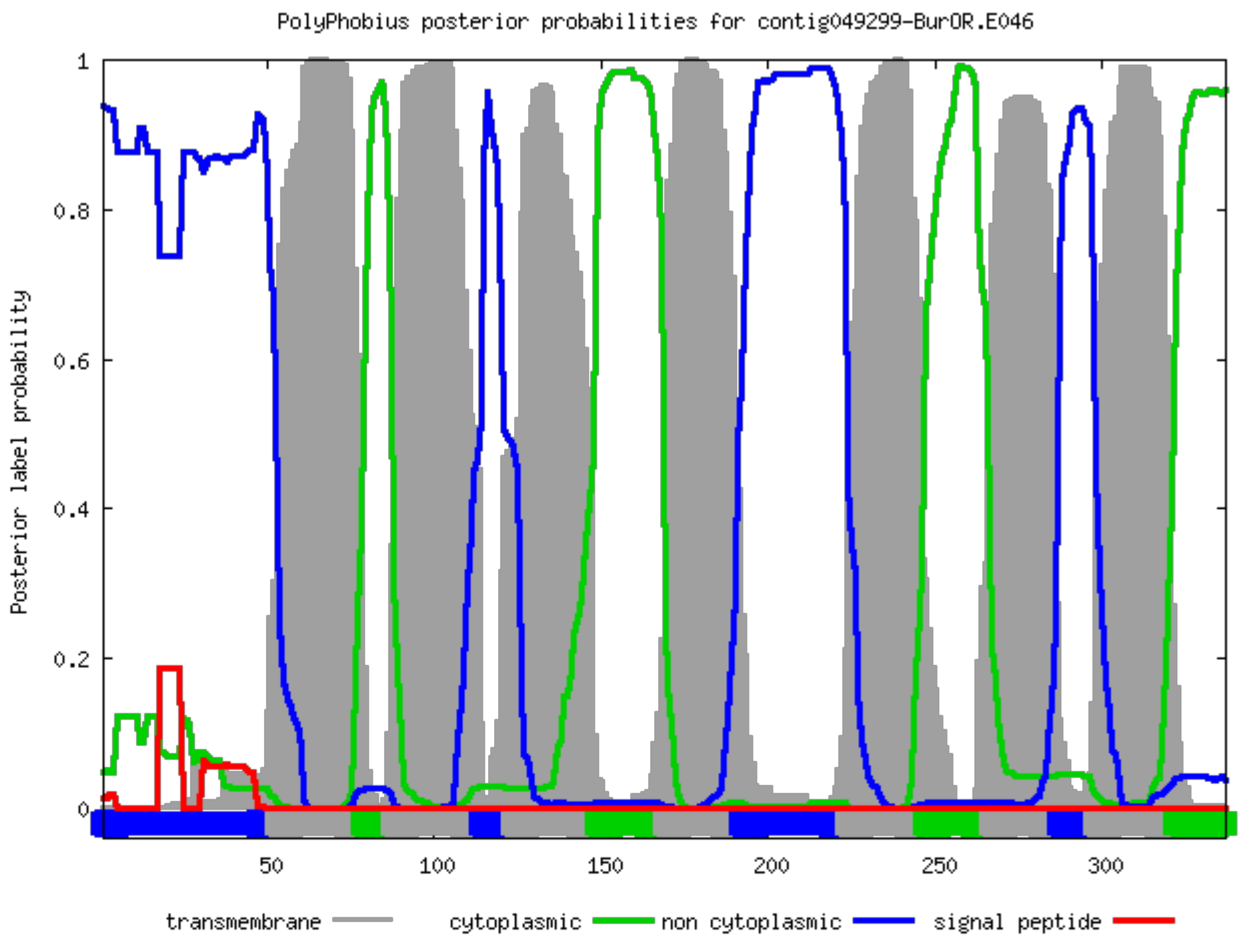

The prediction is based on an alignment. The probability data used in the plot is found here, and the gnuplot script is here.

Prediction of contig013363-TilOR.H110

```
ID   contig013363-TilOR.H110
FT   TOPO_DOM      1     22       NON CYTOPLASMIC.
FT   TRANSMEM     23     48
FT   TOPO_DOM     49     56       CYTOPLASMIC.
FT   TRANSMEM     57     77
FT   TOPO_DOM     78     95       NON CYTOPLASMIC.
FT   TRANSMEM     96    118
FT   TOPO_DOM    119    138       CYTOPLASMIC.
FT   TRANSMEM    139    160
FT   TOPO_DOM    161    193       NON CYTOPLASMIC.
FT   TRANSMEM    194    217
FT   TOPO_DOM    218    235       CYTOPLASMIC.
FT   TRANSMEM    236    258
FT   TOPO_DOM    259    269       NON CYTOPLASMIC.
FT   TRANSMEM    270    289
FT   TOPO_DOM    290    314       CYTOPLASMIC.
//
```

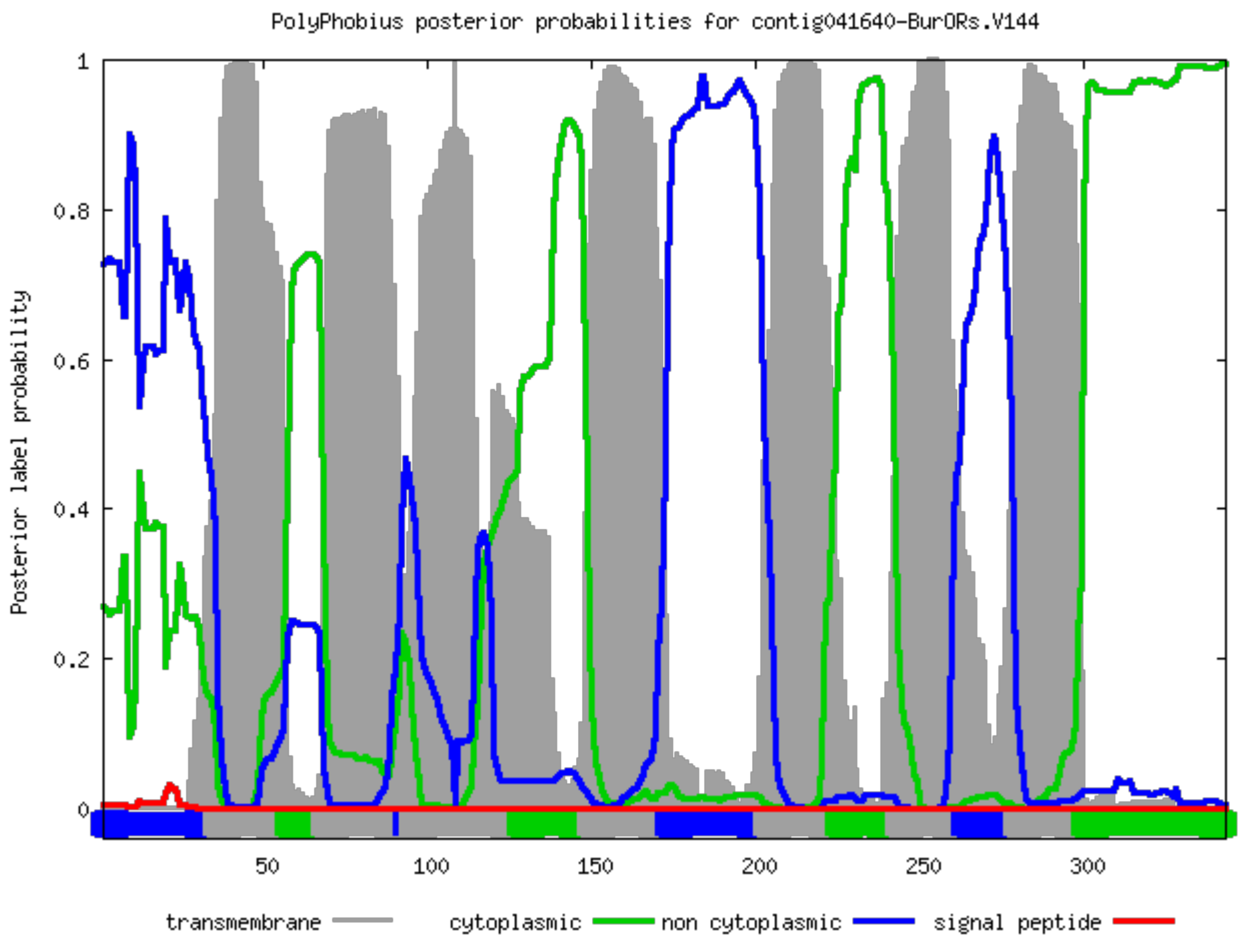

The prediction is based on an alignment. The probability data used in the plot is found here, and the gnuplot script is here.

Prediction of contig005000-TilOR.S218

```
ID   contig005000-TilOR.S218
FT   TOPO_DOM      1      20       NON CYTOPLASMIC.
FT   TRANSMEM     21      42
FT   TOPO_DOM     43      53       CYTOPLASMIC.
FT   TRANSMEM     54      77
FT   TOPO_DOM     78      82       NON CYTOPLASMIC.
FT   TRANSMEM     83     111
FT   TOPO_DOM    112     131       CYTOPLASMIC.
FT   TRANSMEM    132     157
FT   TOPO_DOM    158     185       NON CYTOPLASMIC.
FT   TRANSMEM    186     206
FT   TOPO_DOM    207     226       CYTOPLASMIC.
FT   TRANSMEM    227     252
FT   TOPO_DOM    253     263       NON CYTOPLASMIC.
FT   TRANSMEM    264     284
FT   TOPO_DOM    285     307       CYTOPLASMIC.
//
```

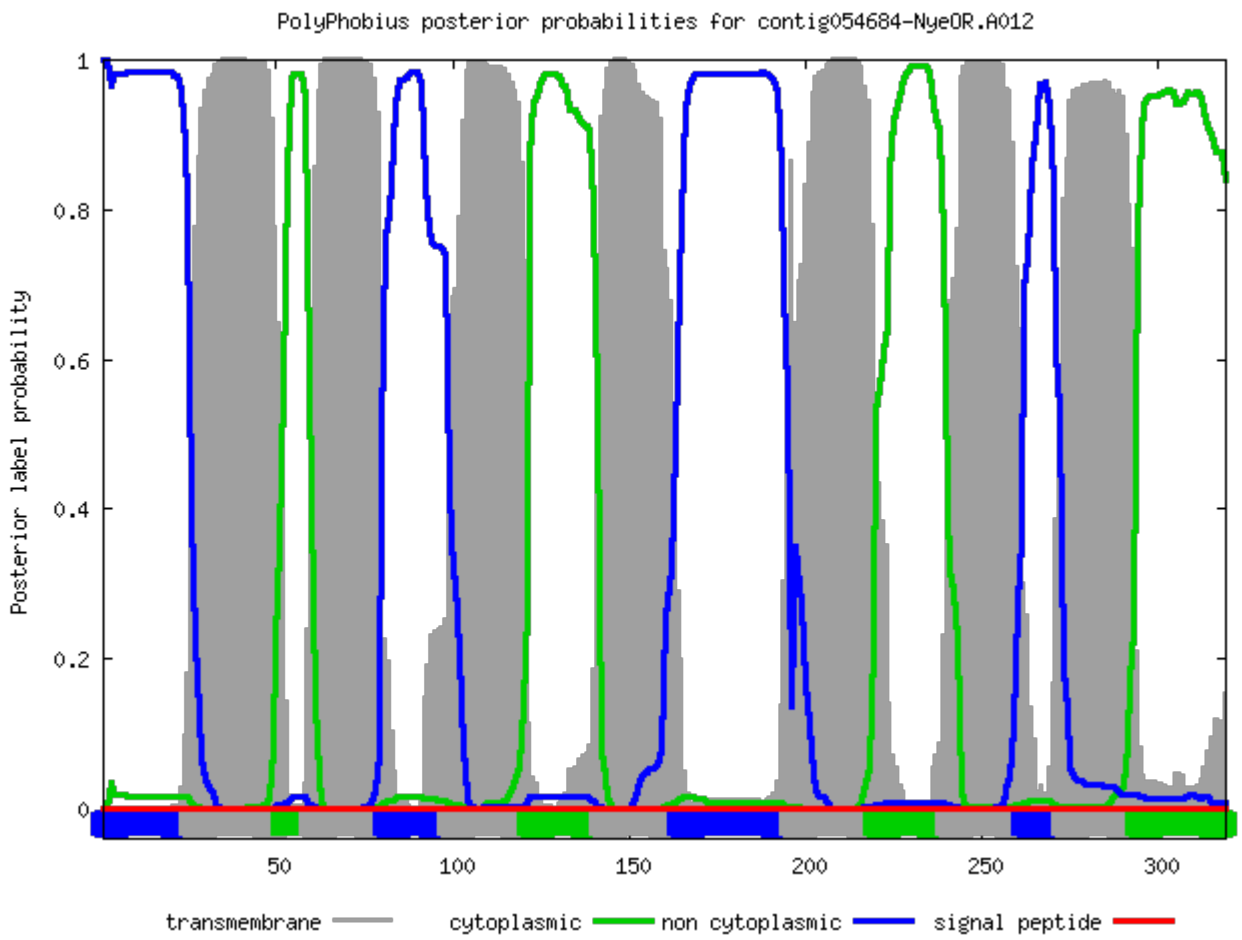

The prediction is based on an alignment. The probability data used in the plot is found here, and the gnuplot script is here.

Prediction of contig039416-TilOR.S222

```
ID   contig039416-TilOR.S222
FT   TOPO_DOM      1     20       NON CYTOPLASMIC.
FT   TRANSMEM     21     42
FT   TOPO_DOM     43     53       CYTOPLASMIC.
FT   TRANSMEM     54     77
FT   TOPO_DOM     78     82       NON CYTOPLASMIC.
FT   TRANSMEM     83    111
FT   TOPO_DOM    112    131       CYTOPLASMIC.
FT   TRANSMEM    132    157
FT   TOPO_DOM    158    185       NON CYTOPLASMIC.
FT   TRANSMEM    186    206
FT   TOPO_DOM    207    226       CYTOPLASMIC.
FT   TRANSMEM    227    252
FT   TOPO_DOM    253    263       NON CYTOPLASMIC.
FT   TRANSMEM    264    284
FT   TOPO_DOM    285    305       CYTOPLASMIC.
//
```

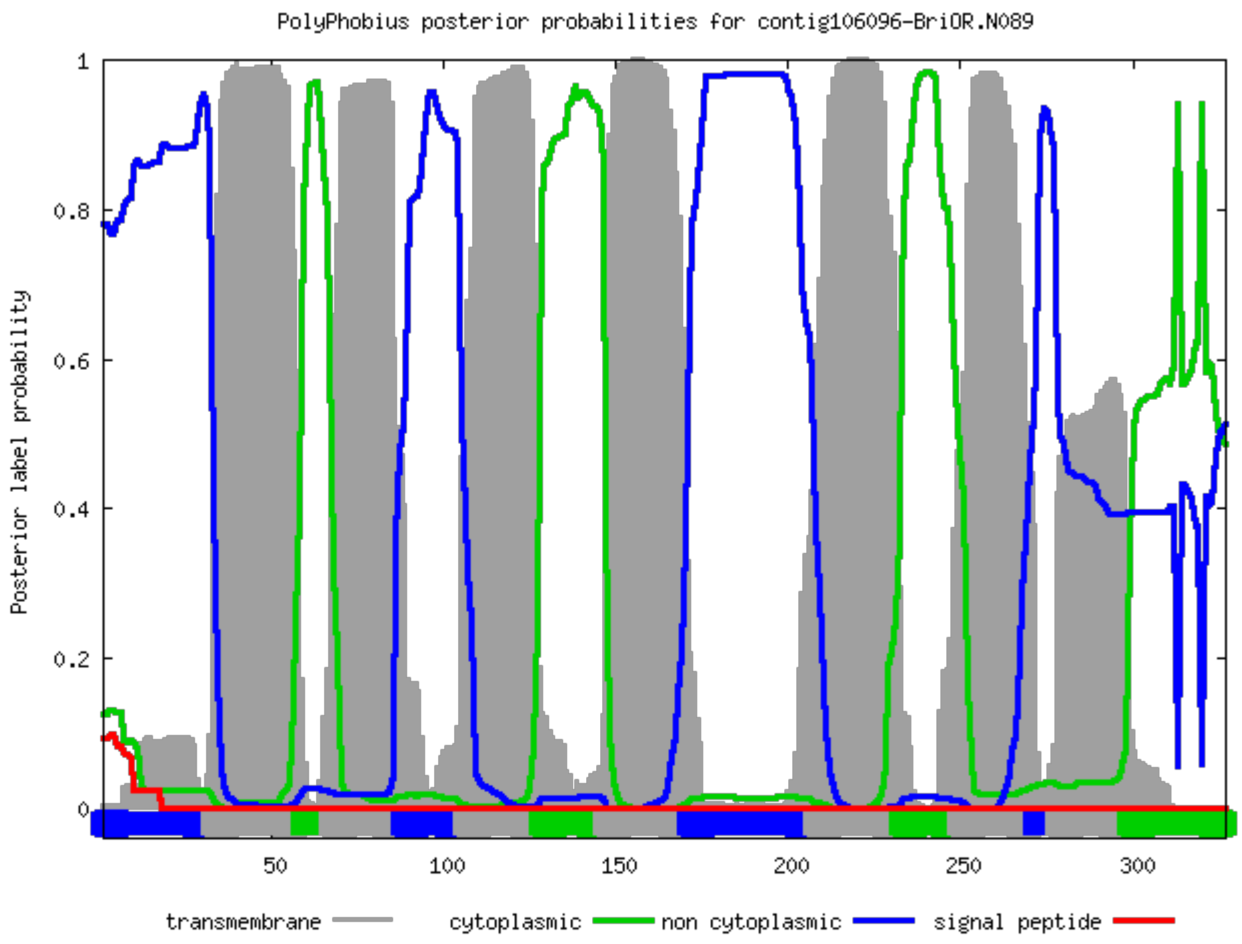

The prediction is based on an alignment. The probability data used in the plot is found here, and the gnuplot script is here.

Prediction of contig073309-TilOR.A026

```
ID   contig073309-TilOR.A026
FT   TOPO_DOM      1     22       NON CYTOPLASMIC.
FT   TRANSMEM     23     48
FT   TOPO_DOM     49     56       CYTOPLASMIC.
FT   TRANSMEM     57     77
FT   TOPO_DOM     78     95       NON CYTOPLASMIC.
FT   TRANSMEM     96    118
FT   TOPO_DOM    119    138       CYTOPLASMIC.
FT   TRANSMEM    139    160
FT   TOPO_DOM    161    192       NON CYTOPLASMIC.
FT   TRANSMEM    193    215
FT   TOPO_DOM    216    235       CYTOPLASMIC.
FT   TRANSMEM    236    257
FT   TOPO_DOM    258    268       NON CYTOPLASMIC.
FT   TRANSMEM    269    289
FT   TOPO_DOM    290    312       CYTOPLASMIC.
//
```

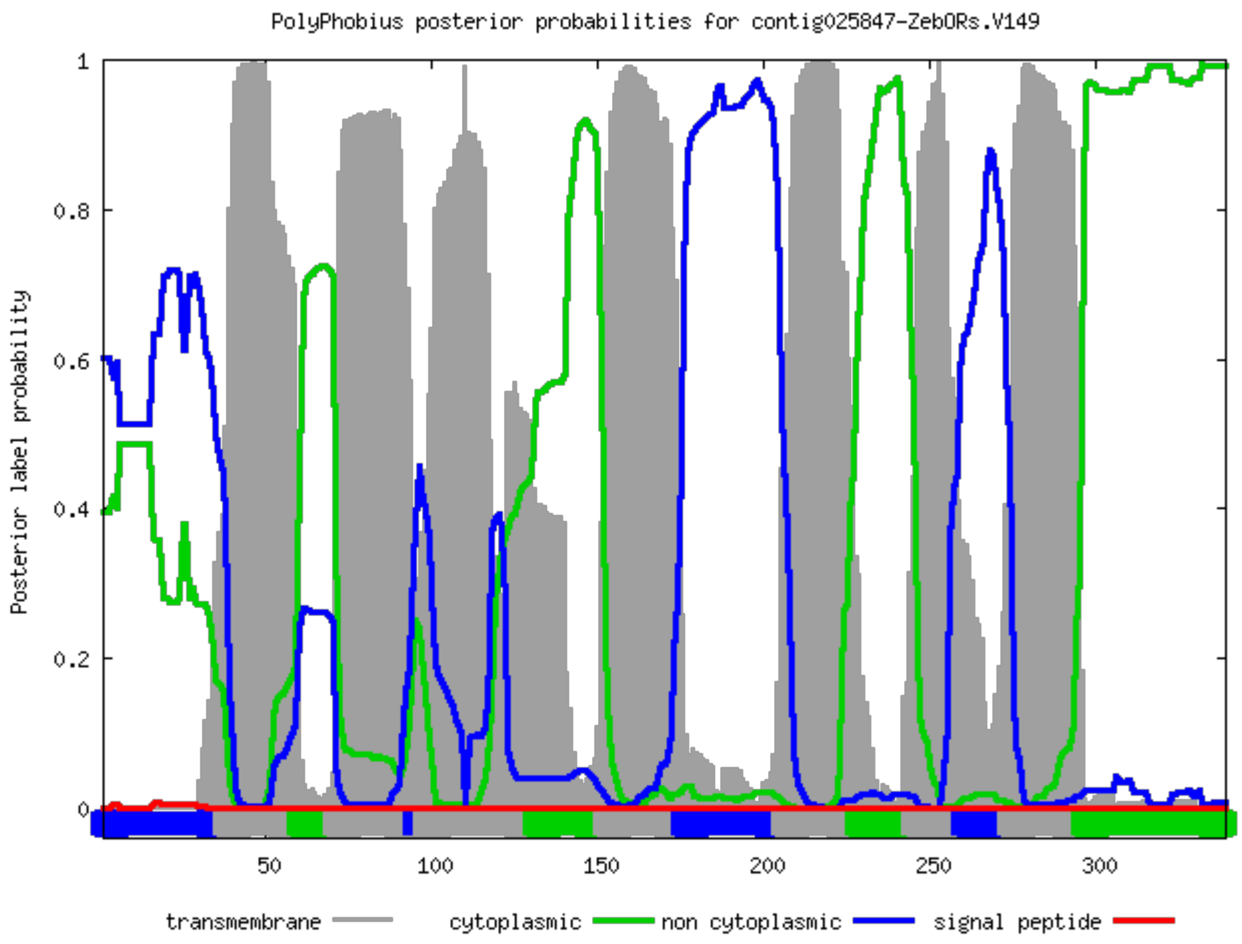

The prediction is based on an alignment. The probability data used in the plot is found here, and the gnuplot script is here.

Prediction of contig063874-BriOR.C026

```
ID   contig063874-BriOR.C026
FT   TOPO_DOM      1     21       NON CYTOPLASMIC.
FT   TRANSMEM     22     47
FT   TOPO_DOM     48     56       CYTOPLASMIC.
FT   TRANSMEM     57     79
FT   TOPO_DOM     80     94       NON CYTOPLASMIC.
FT   TRANSMEM     95    117
FT   TOPO_DOM    118    137       CYTOPLASMIC.
FT   TRANSMEM    138    161
FT   TOPO_DOM    162    192       NON CYTOPLASMIC.
FT   TRANSMEM    193    215
FT   TOPO_DOM    216    234       CYTOPLASMIC.
FT   TRANSMEM    235    255
FT   TOPO_DOM    256    266       NON CYTOPLASMIC.
FT   TRANSMEM    267    289
FT   TOPO_DOM    290    321       CYTOPLASMIC.
//
```

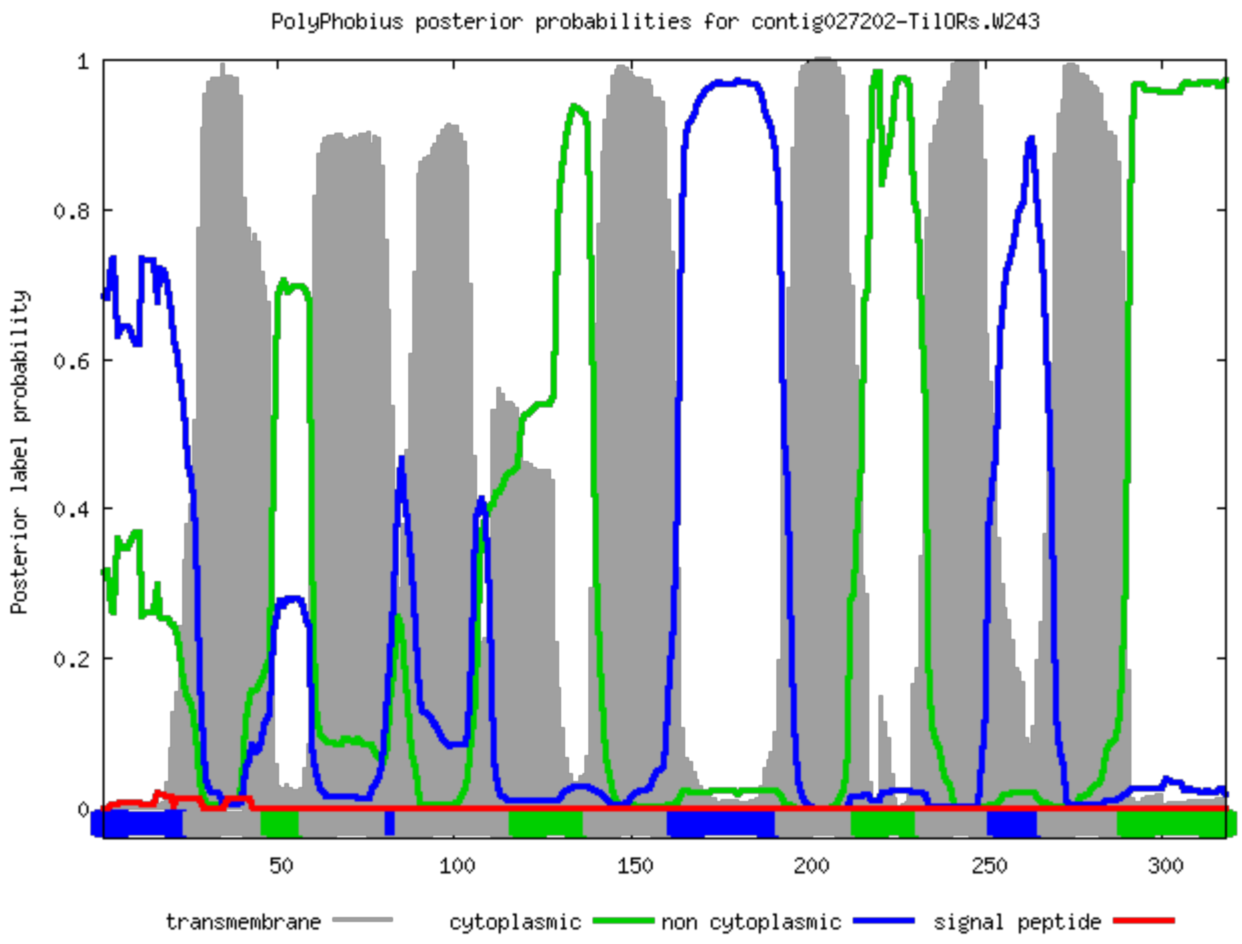

The prediction is based on an alignment. The probability data used in the plot is found here, and the gnuplot script is here.

Prediction of contig064570-BurOR.A017

```
ID   contig064570-BurOR.A017
FT   TOPO_DOM      1     22       NON CYTOPLASMIC.
FT   TRANSMEM     23     48
FT   TOPO_DOM     49     56       CYTOPLASMIC.
FT   TRANSMEM     57     76
FT   TOPO_DOM     77     95       NON CYTOPLASMIC.
FT   TRANSMEM     96    118
FT   TOPO_DOM    119    138       CYTOPLASMIC.
FT   TRANSMEM    139    160
FT   TOPO_DOM    161    192       NON CYTOPLASMIC.
FT   TRANSMEM    193    215
FT   TOPO_DOM    216    235       CYTOPLASMIC.
FT   TRANSMEM    236    257
FT   TOPO_DOM    258    268       NON CYTOPLASMIC.
FT   TRANSMEM    269    289
FT   TOPO_DOM    290    304       CYTOPLASMIC.
//
```

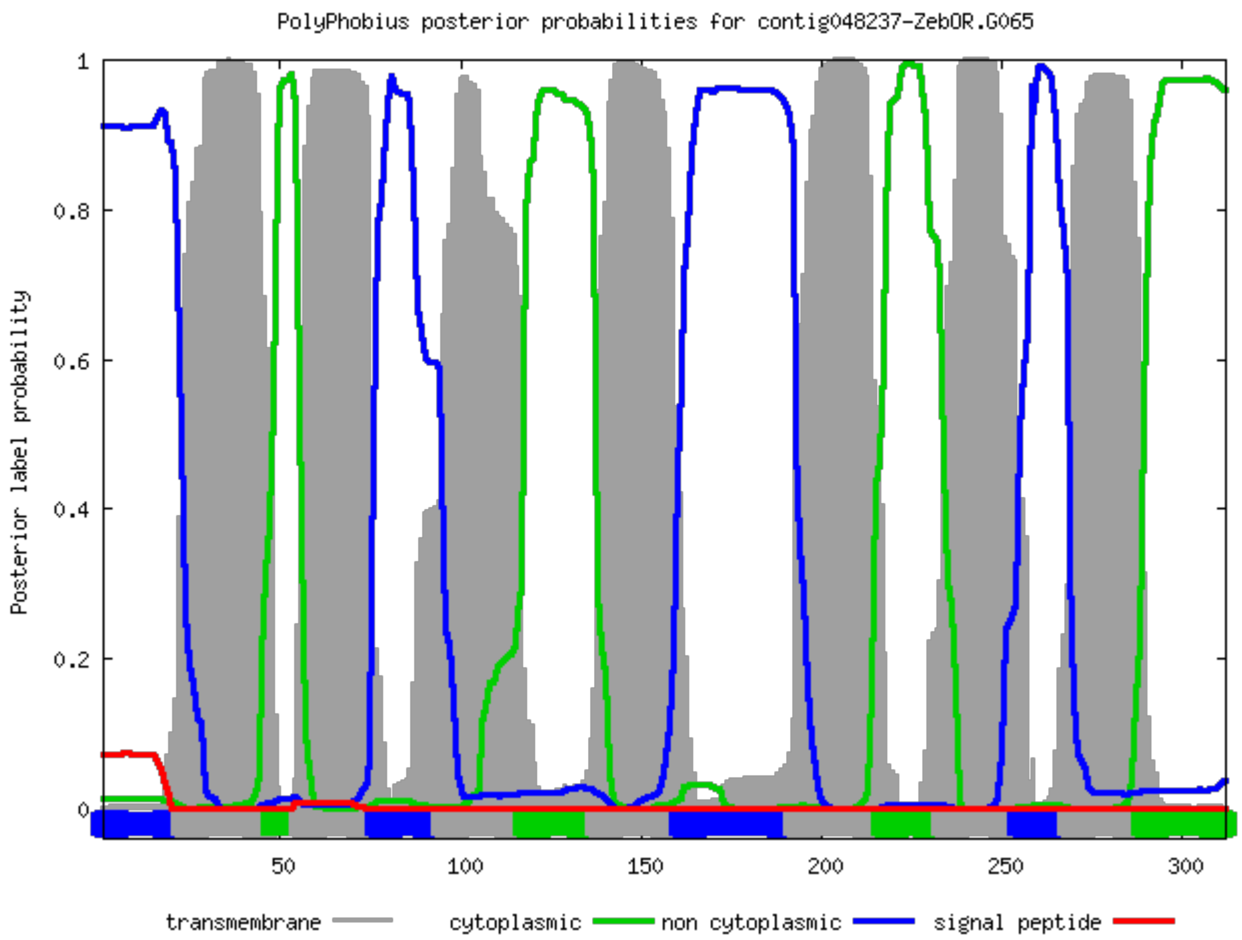

The prediction is based on an alignment. The probability data used in the plot is found here, and the gnuplot script is here.

Prediction of contig057156-BurOR.A015

```
ID   contig057156-BurOR.A015
FT   TOPO_DOM      1     25       NON CYTOPLASMIC.
FT   TRANSMEM     26     51
FT   TOPO_DOM     52     59       CYTOPLASMIC.
FT   TRANSMEM     60     80
FT   TOPO_DOM     81     98       NON CYTOPLASMIC.
FT   TRANSMEM     99    121
FT   TOPO_DOM    122    141       CYTOPLASMIC.
FT   TRANSMEM    142    163
FT   TOPO_DOM    164    195       NON CYTOPLASMIC.
FT   TRANSMEM    196    219
FT   TOPO_DOM    220    239       CYTOPLASMIC.
FT   TRANSMEM    240    261
FT   TOPO_DOM    262    272       NON CYTOPLASMIC.
FT   TRANSMEM    273    293
FT   TOPO_DOM    294    319       CYTOPLASMIC.
//
```

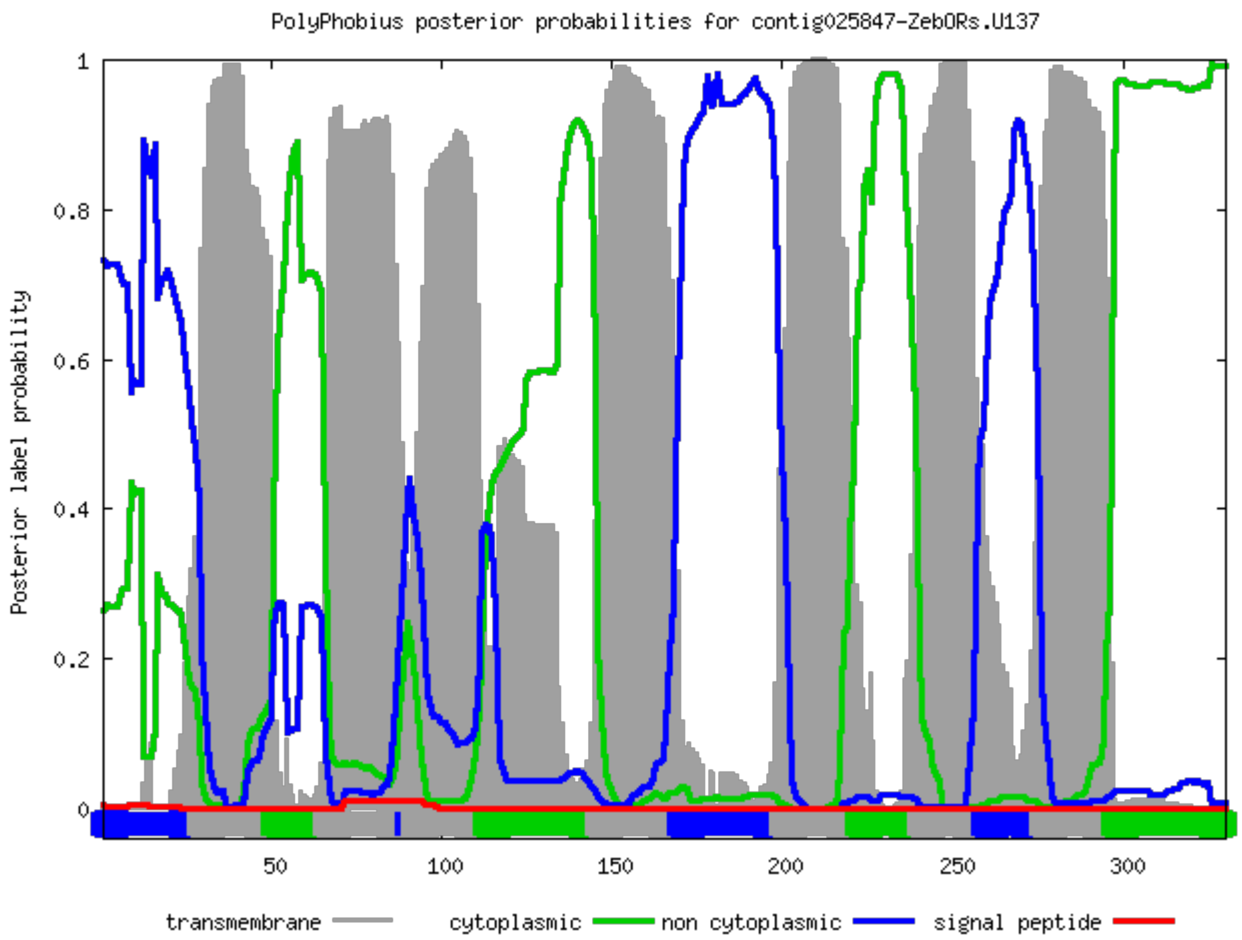

The prediction is based on an alignment. The probability data used in the plot is found here, and the gnuplot script is here.

Prediction of contig054868-NyeOR.A014

```
ID   contig054868-NyeOR.A014
FT   TOPO_DOM      1      22       NON CYTOPLASMIC.
FT   TRANSMEM     23      48
FT   TOPO_DOM     49      56       CYTOPLASMIC.
FT   TRANSMEM     57      77
FT   TOPO_DOM     78      95       NON CYTOPLASMIC.
FT   TRANSMEM     96     118
FT   TOPO_DOM    119     138       CYTOPLASMIC.
FT   TRANSMEM    139     160
FT   TOPO_DOM    161     192       NON CYTOPLASMIC.
FT   TRANSMEM    193     215
FT   TOPO_DOM    216     235       CYTOPLASMIC.
FT   TRANSMEM    236     257
FT   TOPO_DOM    258     268       NON CYTOPLASMIC.
FT   TRANSMEM    269     289
FT   TOPO_DOM    290     307       CYTOPLASMIC.
//
```

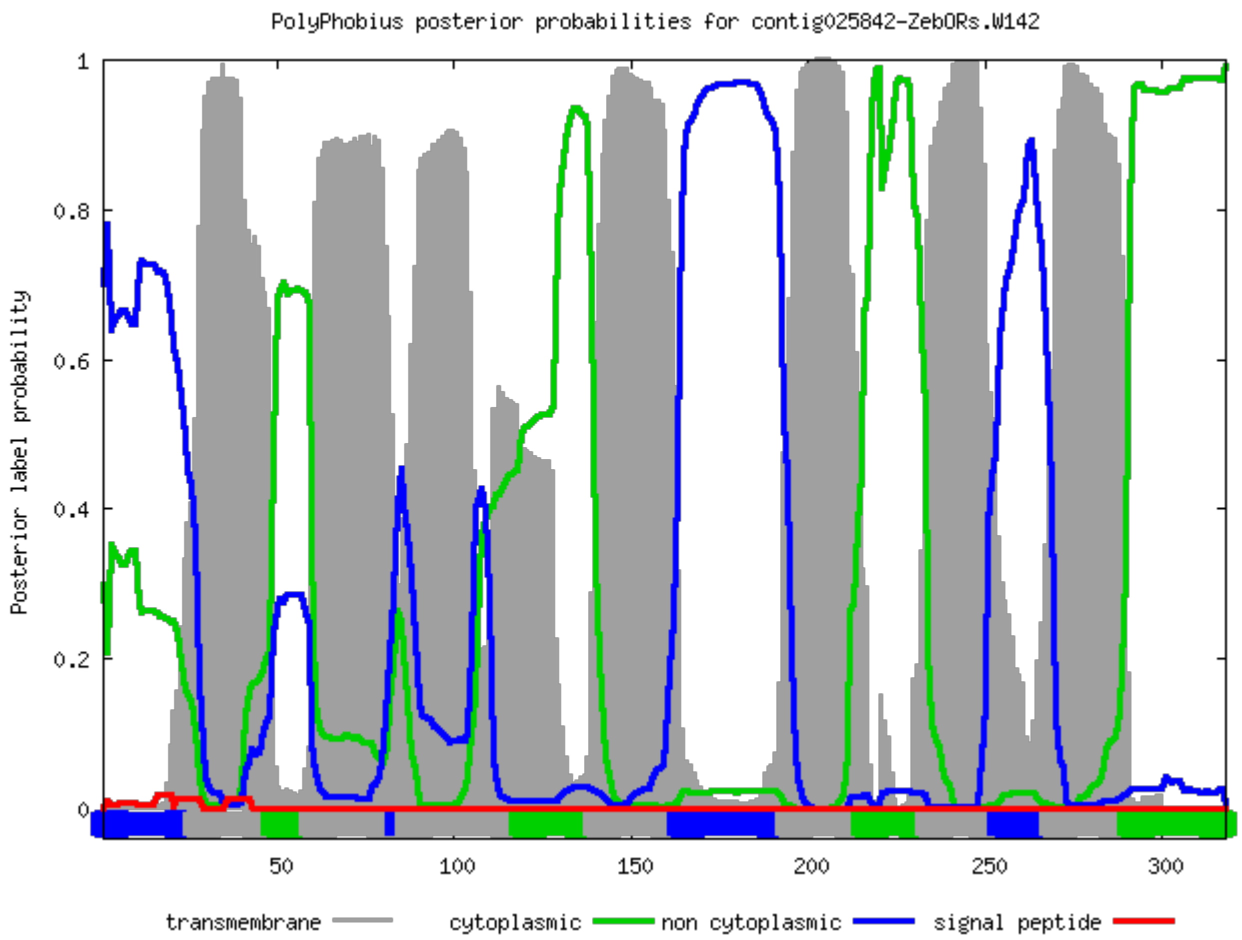

The prediction is based on an alignment. The probability data used in the plot is found here, and the gnuplot script is here.

Prediction of contig039484-TilOR.L154

```
ID   contig039484-TilOR.L154
FT   TOPO_DOM      1    25       NON CYTOPLASMIC.
FT   TRANSMEM     26    50
FT   TOPO_DOM     51    59       CYTOPLASMIC.
FT   TRANSMEM     60    85
FT   TOPO_DOM     86    97       NON CYTOPLASMIC.
FT   TRANSMEM     98   120
FT   TOPO_DOM    121   140       CYTOPLASMIC.
FT   TRANSMEM    141   162
FT   TOPO_DOM    163   198       NON CYTOPLASMIC.
FT   TRANSMEM    199   224
FT   TOPO_DOM    225   237       CYTOPLASMIC.
FT   TRANSMEM    238   259
FT   TOPO_DOM    260   271       NON CYTOPLASMIC.
FT   TRANSMEM    272   292
FT   TOPO_DOM    293   313       CYTOPLASMIC.
//
```

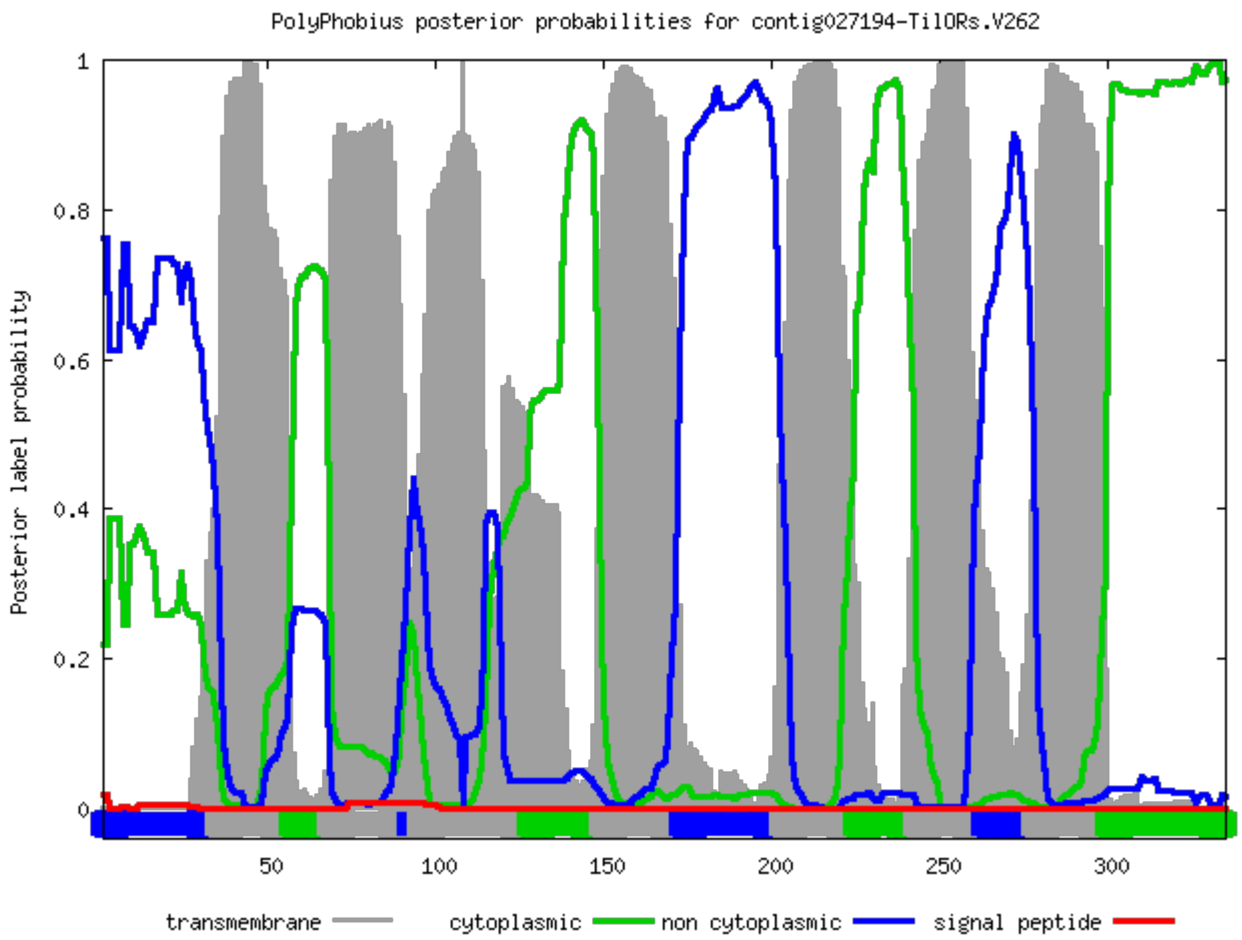

The prediction is based on an alignment. The probability data used in the plot is found here, and the gnuplot script is here.

Prediction of contig013369-TilOR.H115

```
ID   contig013369-TilOR.H115
FT   TOPO_DOM      1      23       NON CYTOPLASMIC.
FT   TRANSMEM     24      49
FT   TOPO_DOM     50      56       CYTOPLASMIC.
FT   TRANSMEM     57      76
FT   TOPO_DOM     77      95       NON CYTOPLASMIC.
FT   TRANSMEM     96     118
FT   TOPO_DOM    119     138       CYTOPLASMIC.
FT   TRANSMEM    139     160
FT   TOPO_DOM    161     196       NON CYTOPLASMIC.
FT   TRANSMEM    197     219
FT   TOPO_DOM    220     237       CYTOPLASMIC.
FT   TRANSMEM    238     260
FT   TOPO_DOM    261     271       NON CYTOPLASMIC.
FT   TRANSMEM    272     291
FT   TOPO_DOM    292     310       CYTOPLASMIC.
//
```

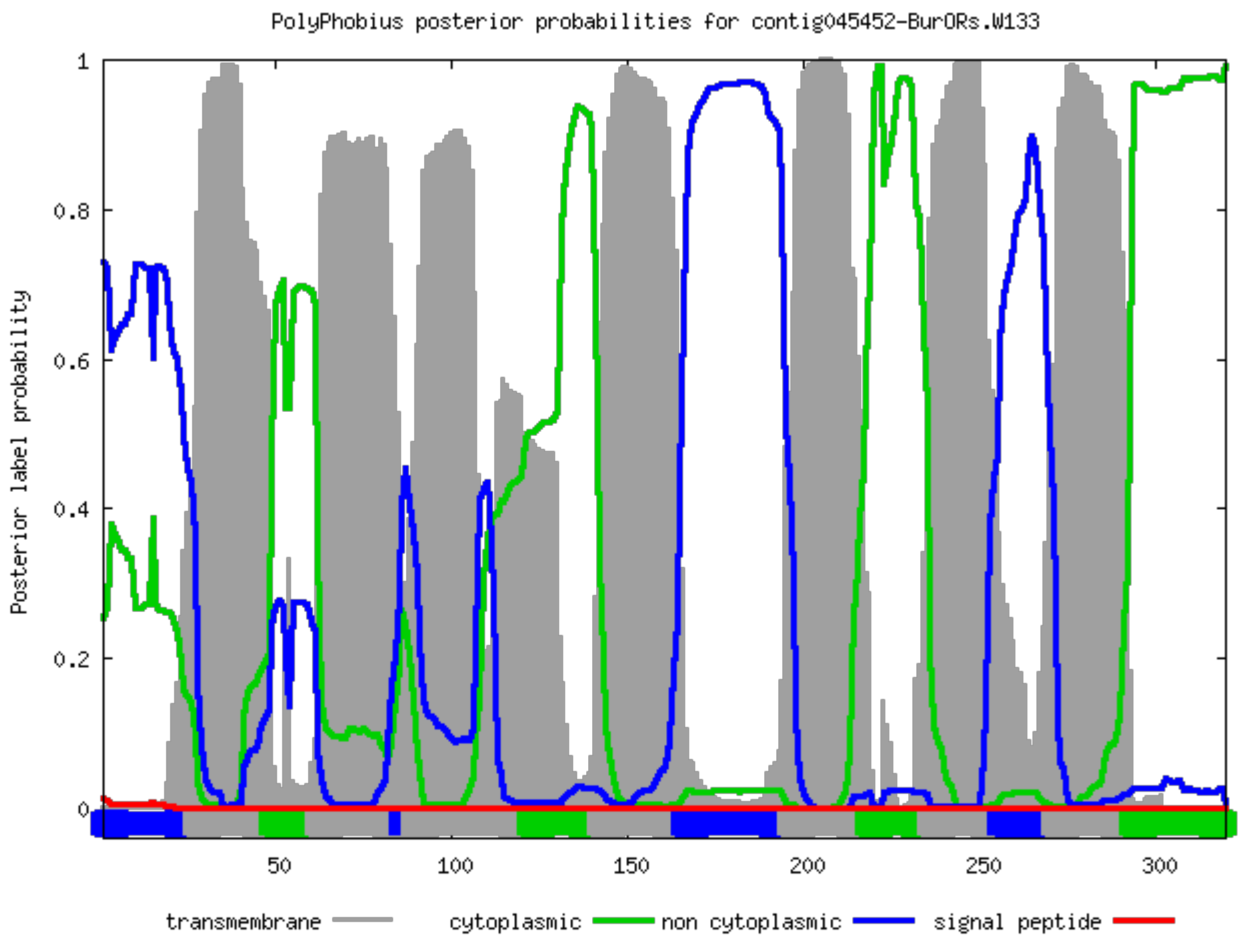

The prediction is based on an alignment. The probability data used in the plot is found here, and the gnuplot script is here.

Prediction of contig053581-NyeOR.G066

```
ID    contig053581-NyeOR.G066
FT    TOPO_DOM      1     22       NON CYTOPLASMIC.
FT    TRANSMEM     23     47
FT    TOPO_DOM     48     55       CYTOPLASMIC.
FT    TRANSMEM     56     76
FT    TOPO_DOM     77     94       NON CYTOPLASMIC.
FT    TRANSMEM     95    117
FT    TOPO_DOM    118    137       CYTOPLASMIC.
FT    TRANSMEM    138    160
FT    TOPO_DOM    161    192       NON CYTOPLASMIC.
FT    TRANSMEM    193    216
FT    TOPO_DOM    217    233       CYTOPLASMIC.
FT    TRANSMEM    234    255
FT    TOPO_DOM    256    268       NON CYTOPLASMIC.
FT    TRANSMEM    269    288
FT    TOPO_DOM    289    312       CYTOPLASMIC.
//
```

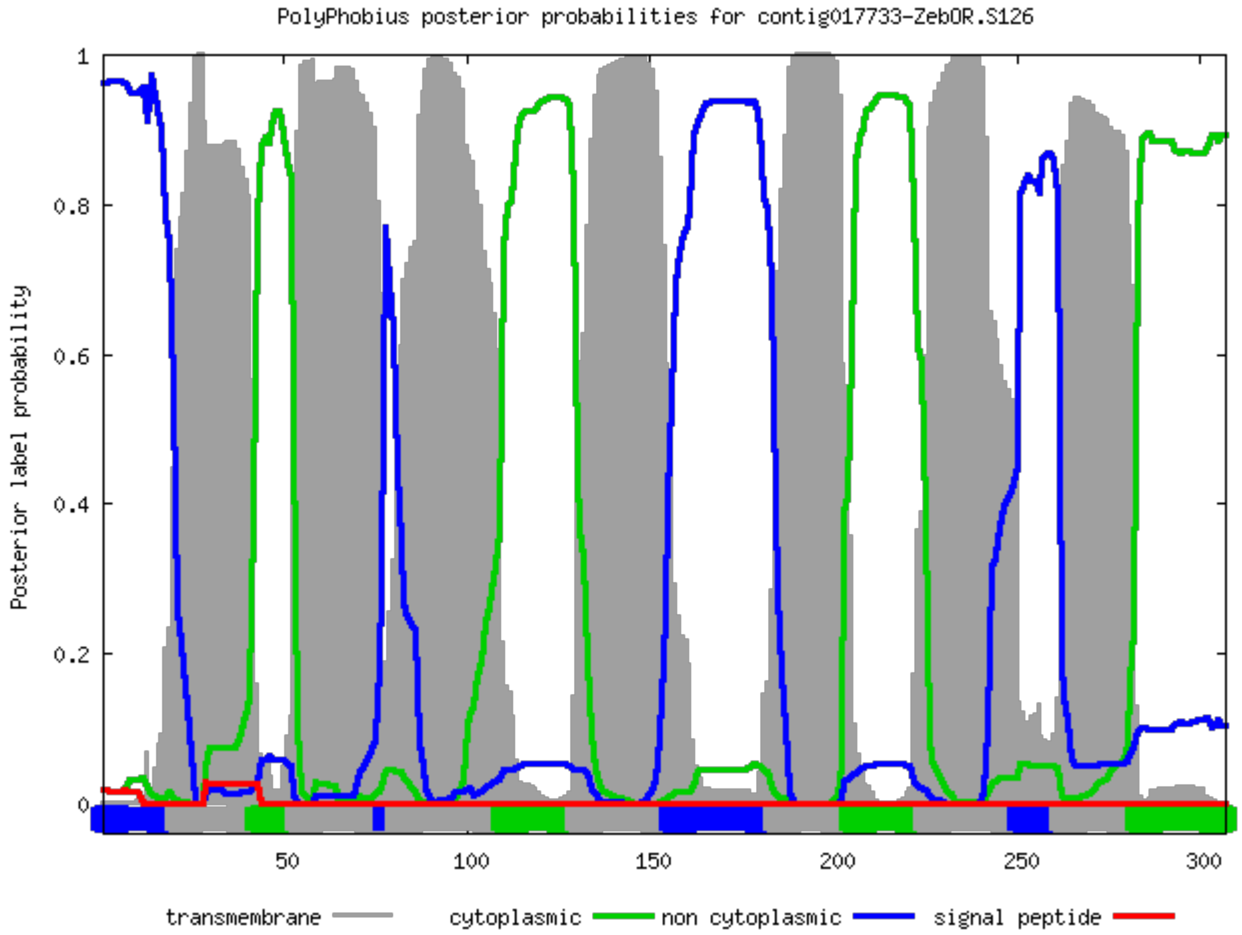

The prediction is based on an alignment. The probability data used in the plot is found here, and the gnuplot script is here.

Prediction of contig056384-BurOR.L087

```
ID   contig056384-BurOR.L087
FT   TOPO_DOM      1     25       NON CYTOPLASMIC.
FT   TRANSMEM     26     50
FT   TOPO_DOM     51     59       CYTOPLASMIC.
FT   TRANSMEM     60     85
FT   TOPO_DOM     86     98       NON CYTOPLASMIC.
FT   TRANSMEM     99    120
FT   TOPO_DOM    121    140       CYTOPLASMIC.
FT   TRANSMEM    141    162
FT   TOPO_DOM    163    198       NON CYTOPLASMIC.
FT   TRANSMEM    199    224
FT   TOPO_DOM    225    236       CYTOPLASMIC.
FT   TRANSMEM    237    259
FT   TOPO_DOM    260    271       NON CYTOPLASMIC.
FT   TRANSMEM    272    292
FT   TOPO_DOM    293    317       CYTOPLASMIC.
//
```

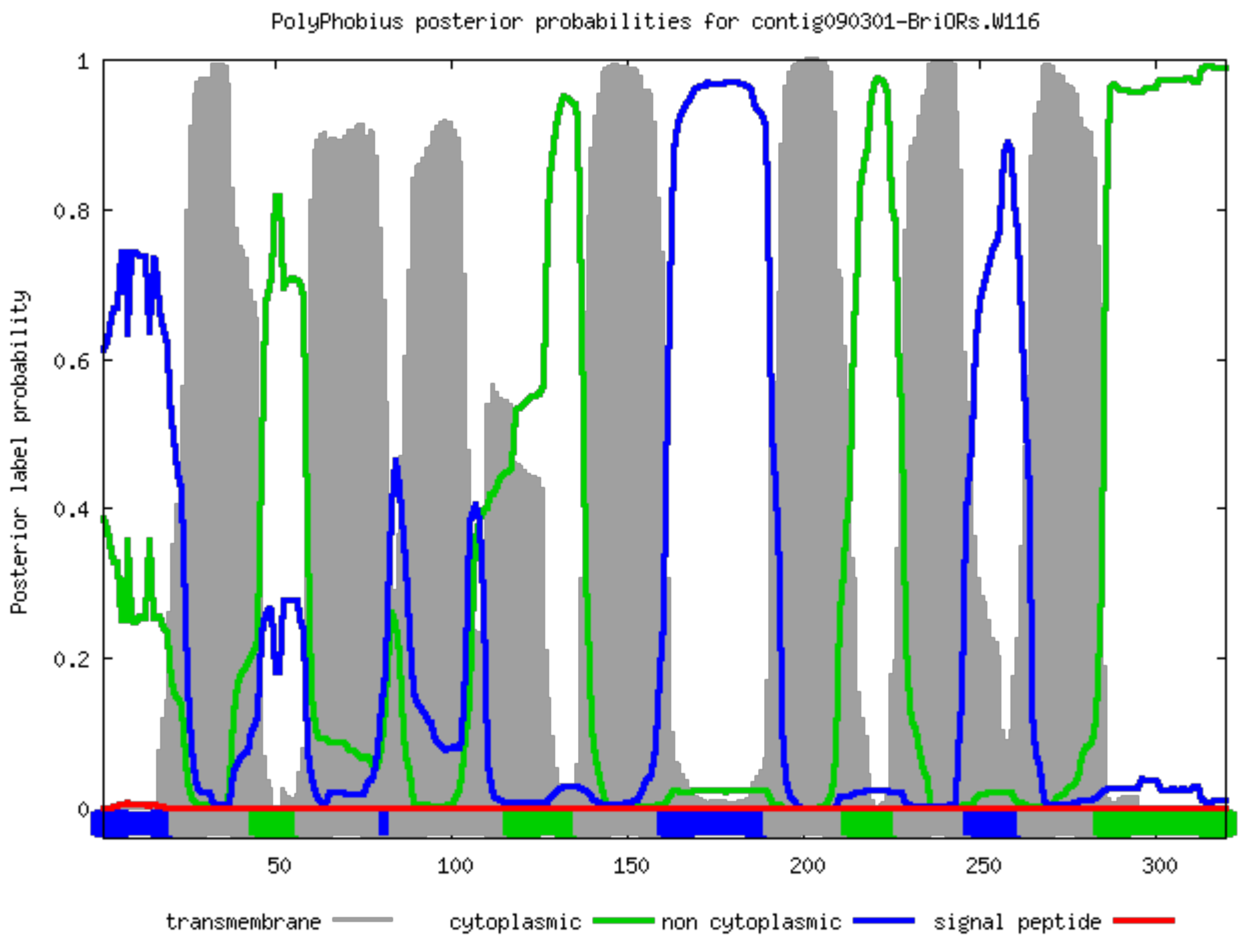

The prediction is based on an alignment. The probability data used in the plot is found here, and the gnuplot script is here.

Prediction of contig053781-BurOR.F059

```
ID   contig053781-BurOR.F059
FT   TOPO_DOM      1     22       NON CYTOPLASMIC.
FT   TRANSMEM     23     47
FT   TOPO_DOM     48     57       CYTOPLASMIC.
FT   TRANSMEM     58     78
FT   TOPO_DOM     79     96       NON CYTOPLASMIC.
FT   TRANSMEM     97    118
FT   TOPO_DOM    119    138       CYTOPLASMIC.
FT   TRANSMEM    139    161
FT   TOPO_DOM    162    193       NON CYTOPLASMIC.
FT   TRANSMEM    194    216
FT   TOPO_DOM    217    236       CYTOPLASMIC.
FT   TRANSMEM    237    257
FT   TOPO_DOM    258    267       NON CYTOPLASMIC.
FT   TRANSMEM    268    291
FT   TOPO_DOM    292    305       CYTOPLASMIC.
//
```

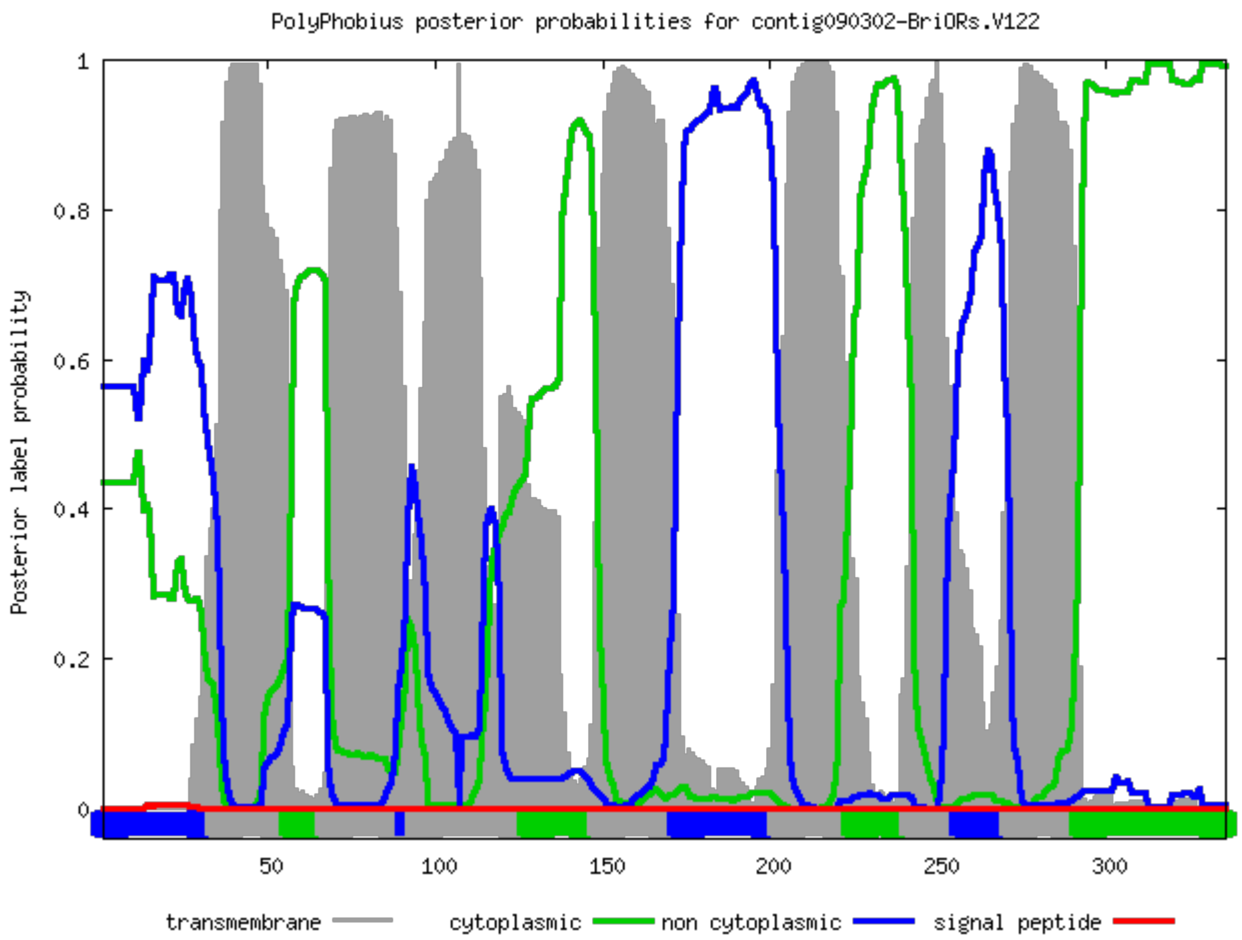

The prediction is based on an alignment. The probability data used in the plot is found here, and the gnuplot script is here.

Prediction of contig013351-TilOR.H104

```
ID   contig013351-TilOR.H104
FT   TOPO_DOM      1     22       NON CYTOPLASMIC.
FT   TRANSMEM     23     49
FT   TOPO_DOM     50     56       CYTOPLASMIC.
FT   TRANSMEM     57     77
FT   TOPO_DOM     78     95       NON CYTOPLASMIC.
FT   TRANSMEM     96    118
FT   TOPO_DOM    119    138       CYTOPLASMIC.
FT   TRANSMEM    139    160
FT   TOPO_DOM    161    193       NON CYTOPLASMIC.
FT   TRANSMEM    194    216
FT   TOPO_DOM    217    235       CYTOPLASMIC.
FT   TRANSMEM    236    258
FT   TOPO_DOM    259    269       NON CYTOPLASMIC.
FT   TRANSMEM    270    289
FT   TOPO_DOM    290    314       CYTOPLASMIC.
//
```

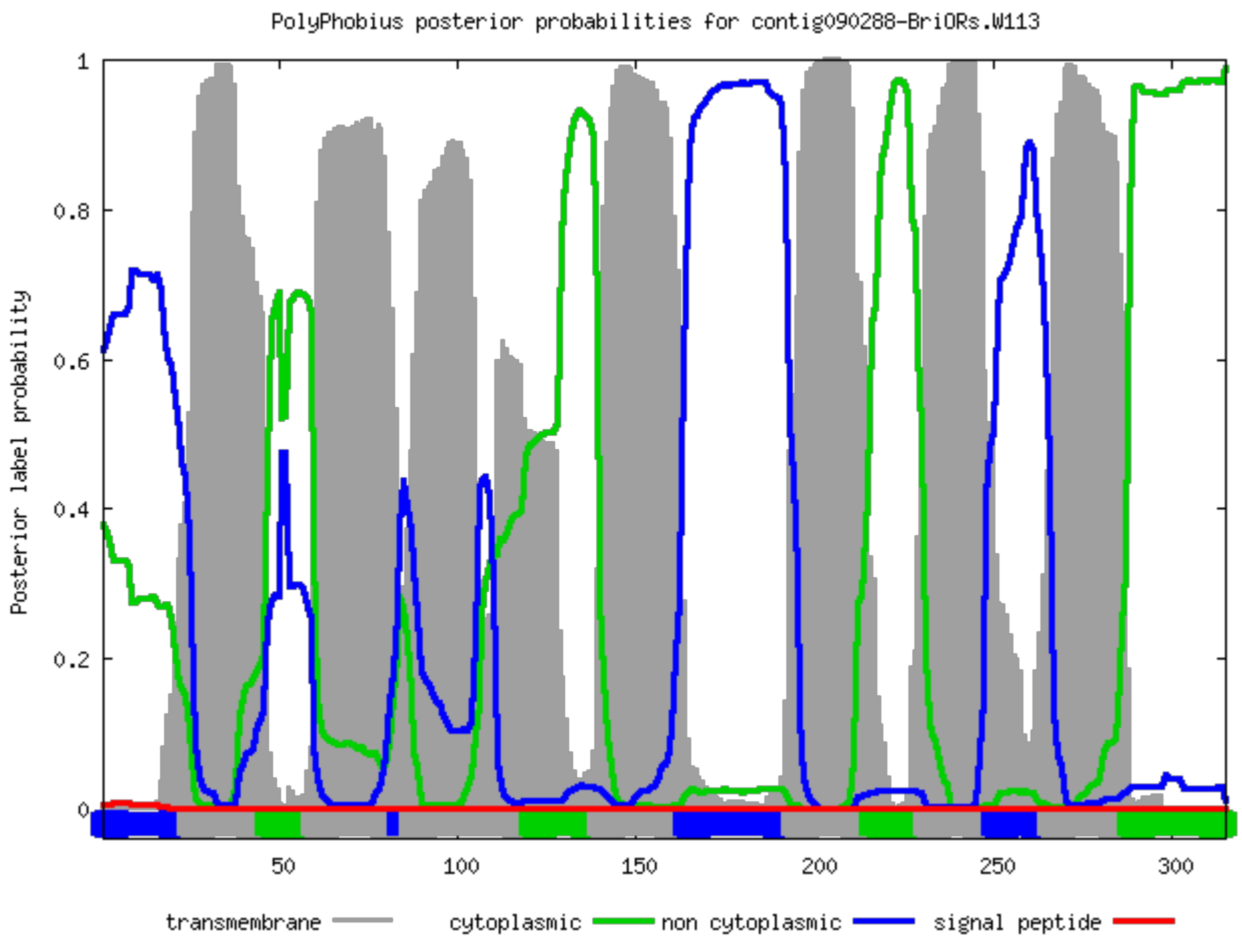

The prediction is based on an alignment. The probability data used in the plot is found here, and the gnuplot script is here.

Prediction of contig093816-BriOR.A011

```
ID   contig093816-BriOR.A011
FT   TOPO_DOM      1      22       NON CYTOPLASMIC.
FT   TRANSMEM     23      48
FT   TOPO_DOM     49      56       CYTOPLASMIC.
FT   TRANSMEM     57      76
FT   TOPO_DOM     77      95       NON CYTOPLASMIC.
FT   TRANSMEM     96     118
FT   TOPO_DOM    119     138       CYTOPLASMIC.
FT   TRANSMEM    139     160
FT   TOPO_DOM    161     192       NON CYTOPLASMIC.
FT   TRANSMEM    193     215
FT   TOPO_DOM    216     235       CYTOPLASMIC.
FT   TRANSMEM    236     257
FT   TOPO_DOM    258     268       NON CYTOPLASMIC.
FT   TRANSMEM    269     289
FT   TOPO_DOM    290     307       CYTOPLASMIC.
//
```

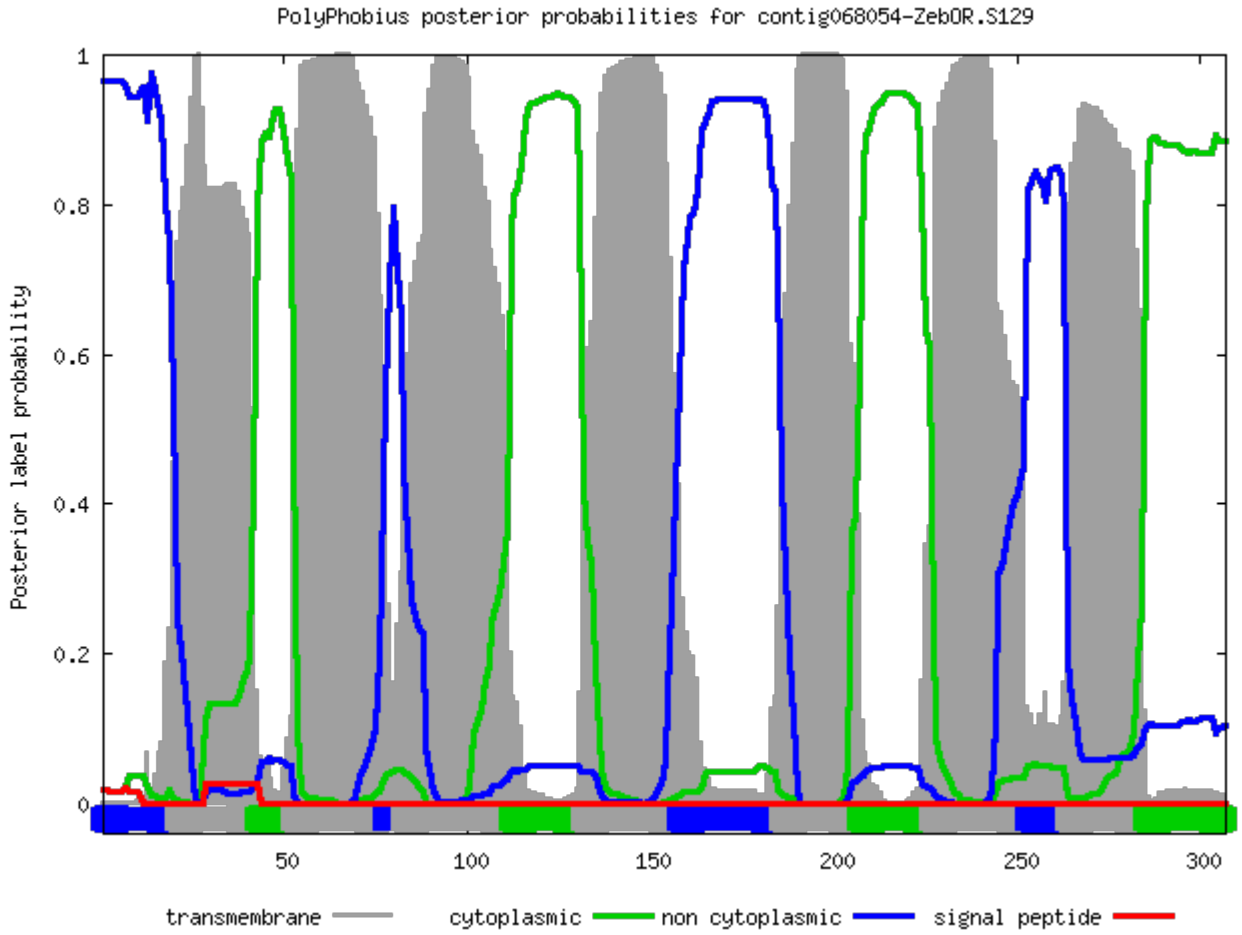

The prediction is based on an alignment. The probability data used in the plot is found here, and the gnuplot script is here.

Prediction of contig040502-NyeOR.L092

```
ID   contig040502-NyeOR.L092
FT   TOPO_DOM      1     25       NON CYTOPLASMIC.
FT   TRANSMEM     26     50
FT   TOPO_DOM     51     59       CYTOPLASMIC.
FT   TRANSMEM     60     82
FT   TOPO_DOM     83     98       NON CYTOPLASMIC.
FT   TRANSMEM     99    120
FT   TOPO_DOM    121    140       CYTOPLASMIC.
FT   TRANSMEM    141    162
FT   TOPO_DOM    163    198       NON CYTOPLASMIC.
FT   TRANSMEM    199    224
FT   TOPO_DOM    225    235       CYTOPLASMIC.
FT   TRANSMEM    236    259
FT   TOPO_DOM    260    271       NON CYTOPLASMIC.
FT   TRANSMEM    272    292
FT   TOPO_DOM    293    314       CYTOPLASMIC.
//
```

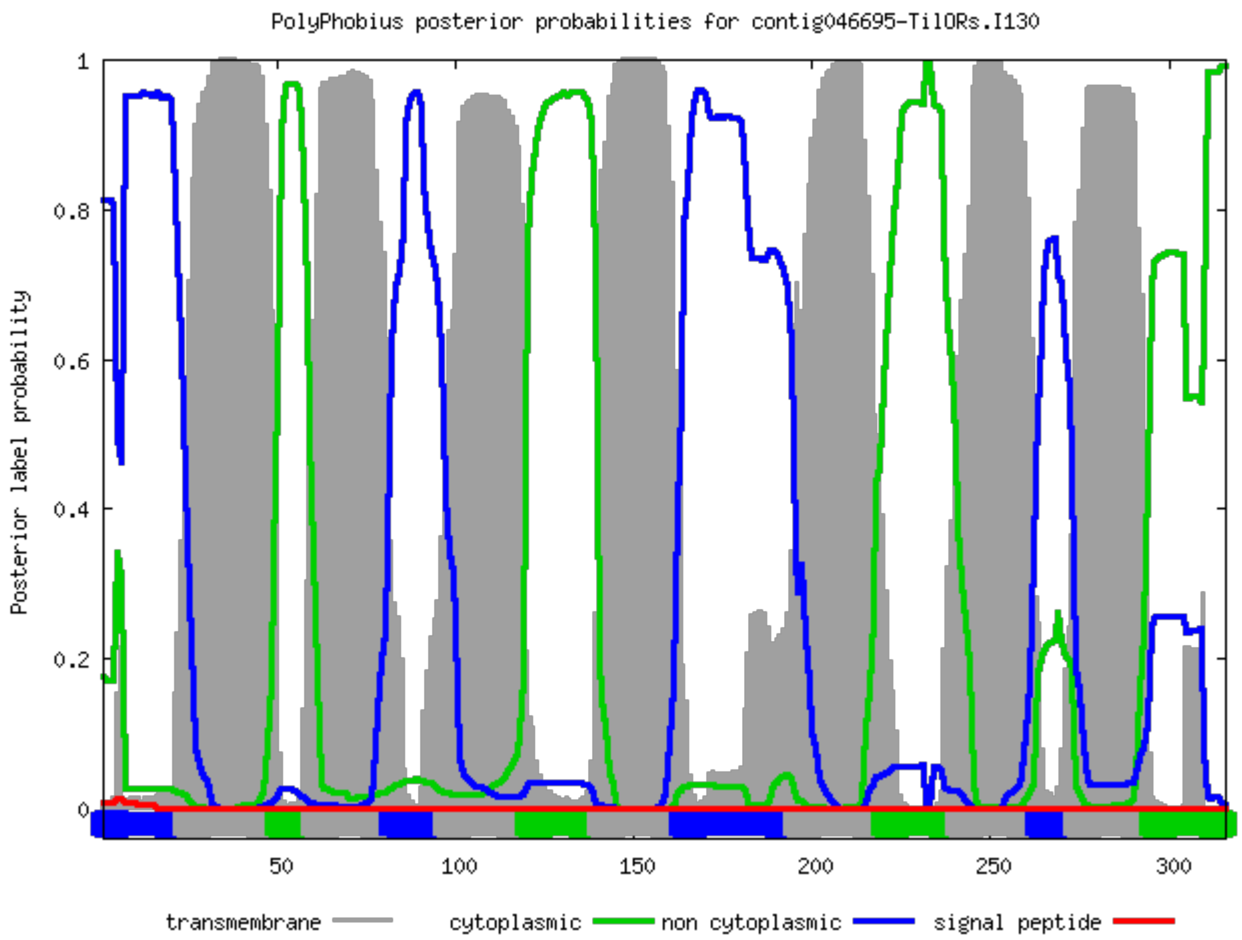

The prediction is based on an alignment. The probability data used in the plot is found here, and the gnuplot script is here.

Prediction of contig025452-ZebOR.P123

```
ID    contig025452-ZebOR.P123
FT    TOPO_DOM      1     23      NON CYTOPLASMIC.
FT    TRANSMEM     24     47
FT    TOPO_DOM     48     57      CYTOPLASMIC.
FT    TRANSMEM     58     83
FT    TOPO_DOM     84     95      NON CYTOPLASMIC.
FT    TRANSMEM     96    118
FT    TOPO_DOM    119    138      CYTOPLASMIC.
FT    TRANSMEM    139    161
FT    TOPO_DOM    162    196      NON CYTOPLASMIC.
FT    TRANSMEM    197    221
FT    TOPO_DOM    222    235      CYTOPLASMIC.
FT    TRANSMEM    236    257
FT    TOPO_DOM    258    269      NON CYTOPLASMIC.
FT    TRANSMEM    270    290
FT    TOPO_DOM    291    318      CYTOPLASMIC.
//
```

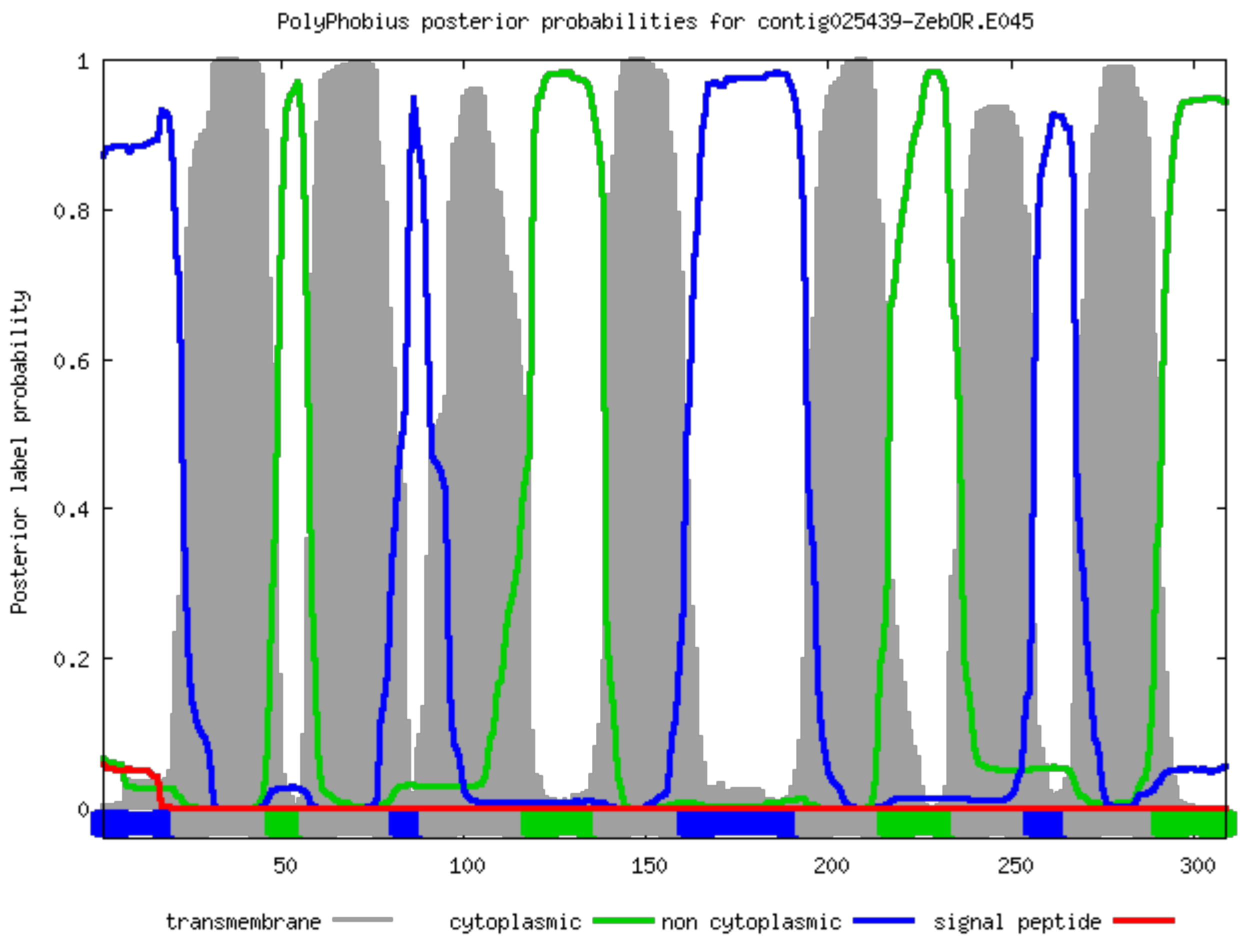

The prediction is based on an alignment. The probability data used in the plot is found here, and the gnuplot script is here.

Prediction of contig053576-NyeOR.E052

```
ID   contig053576-NyeOR.E052
FT   TOPO_DOM      1      25       NON CYTOPLASMIC.
FT   TRANSMEM     26      51
FT   TOPO_DOM     52      60       CYTOPLASMIC.
FT   TRANSMEM     61      86
FT   TOPO_DOM     87      93       NON CYTOPLASMIC.
FT   TRANSMEM     94     121
FT   TOPO_DOM    122     141       CYTOPLASMIC.
FT   TRANSMEM    142     164
FT   TOPO_DOM    165     196       NON CYTOPLASMIC.
FT   TRANSMEM    197     219
FT   TOPO_DOM    220     239       CYTOPLASMIC.
FT   TRANSMEM    240     259
FT   TOPO_DOM    260     270       NON CYTOPLASMIC.
FT   TRANSMEM    271     294
FT   TOPO_DOM    295     326       CYTOPLASMIC.
//
```

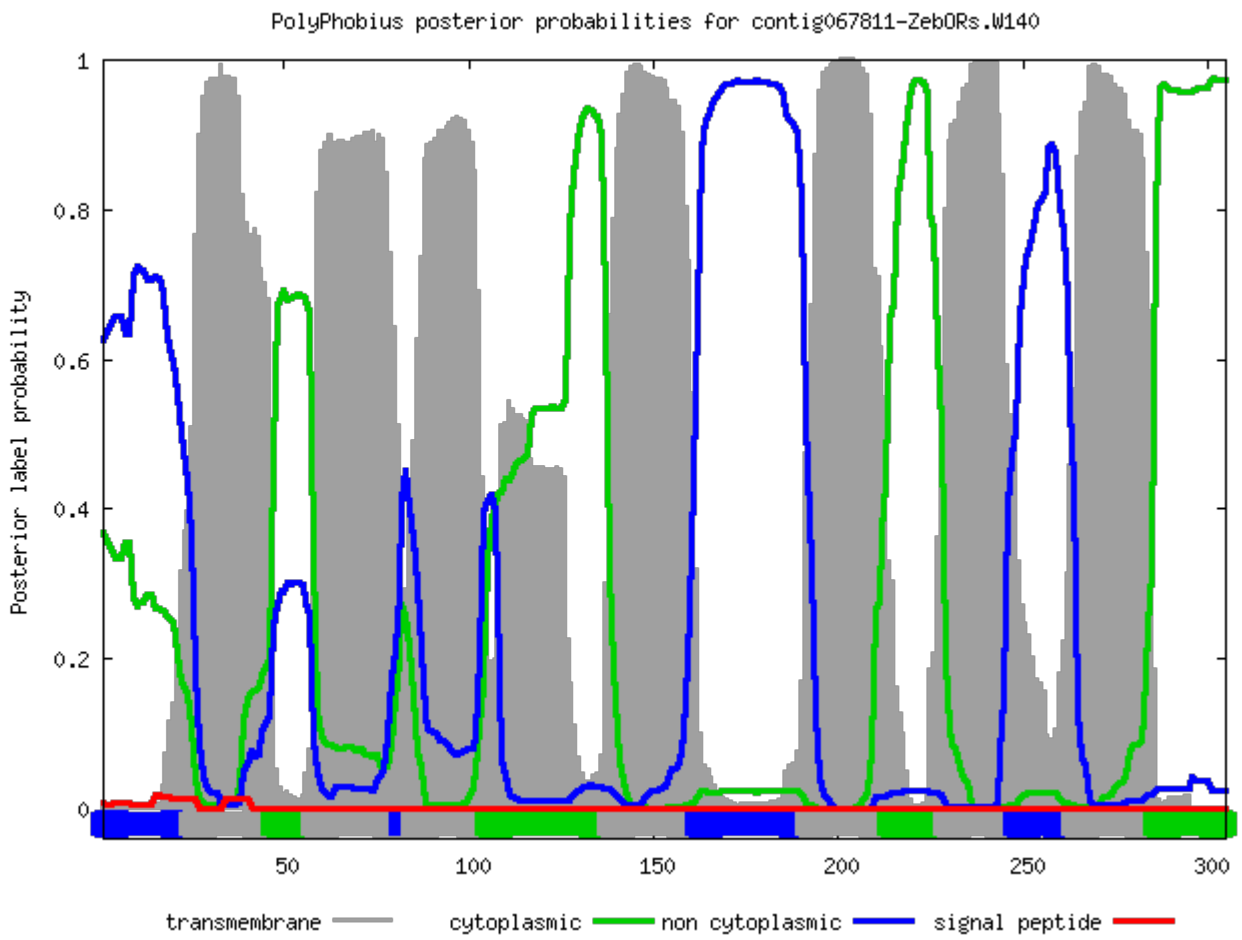

The prediction is based on an alignment. The probability data used in the plot is found here, and the gnuplot script is here.

Prediction of contig057754-NyeOR.A018

```
ID   contig057754-NyeOR.A018
FT   TOPO_DOM     1     22      NON CYTOPLASMIC.
FT   TRANSMEM    23     48
FT   TOPO_DOM    49     56      CYTOPLASMIC.
FT   TRANSMEM    57     77
FT   TOPO_DOM    78     95      NON CYTOPLASMIC.
FT   TRANSMEM    96    118
FT   TOPO_DOM   119    138      CYTOPLASMIC.
FT   TRANSMEM   139    159
FT   TOPO_DOM   160    192      NON CYTOPLASMIC.
FT   TRANSMEM   193    215
FT   TOPO_DOM   216    235      CYTOPLASMIC.
FT   TRANSMEM   236    257
FT   TOPO_DOM   258    267      NON CYTOPLASMIC.
FT   TRANSMEM   268    289
FT   TOPO_DOM   290    309      CYTOPLASMIC.
//
```

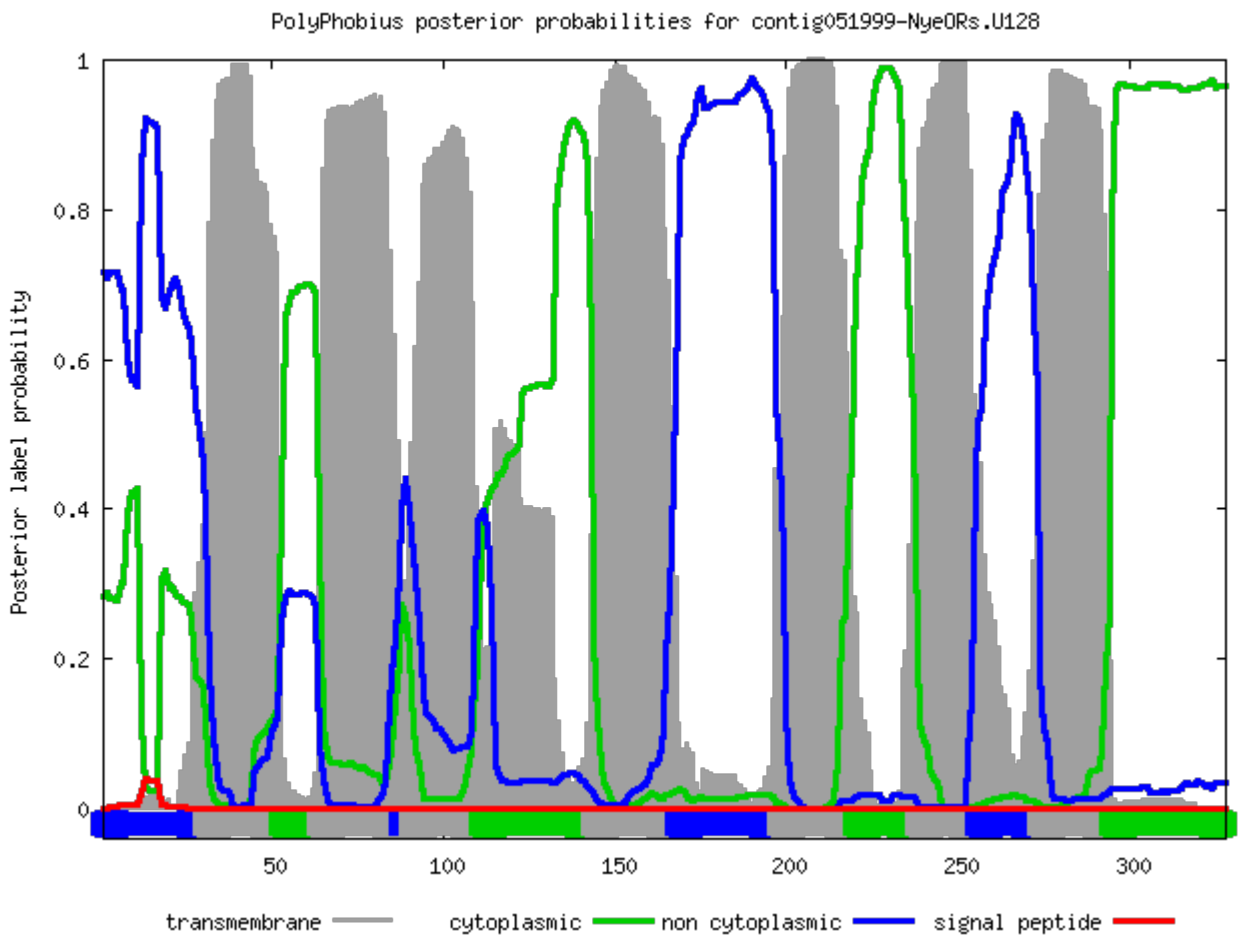

The prediction is based on an alignment. The probability data used in the plot is found here, and the gnuplot script is here.

Prediction of contig053784-BurOR.H067

```
ID   contig053784-BurOR.H067
FT   TOPO_DOM      1     22       NON CYTOPLASMIC.
FT   TRANSMEM     23     48
FT   TOPO_DOM     49     56       CYTOPLASMIC.
FT   TRANSMEM     57     77
FT   TOPO_DOM     78     95       NON CYTOPLASMIC.
FT   TRANSMEM     96    118
FT   TOPO_DOM    119    138       CYTOPLASMIC.
FT   TRANSMEM    139    160
FT   TOPO_DOM    161    193       NON CYTOPLASMIC.
FT   TRANSMEM    194    217
FT   TOPO_DOM    218    235       CYTOPLASMIC.
FT   TRANSMEM    236    258
FT   TOPO_DOM    259    269       NON CYTOPLASMIC.
FT   TRANSMEM    270    289
FT   TOPO_DOM    290    314       CYTOPLASMIC.
//
```

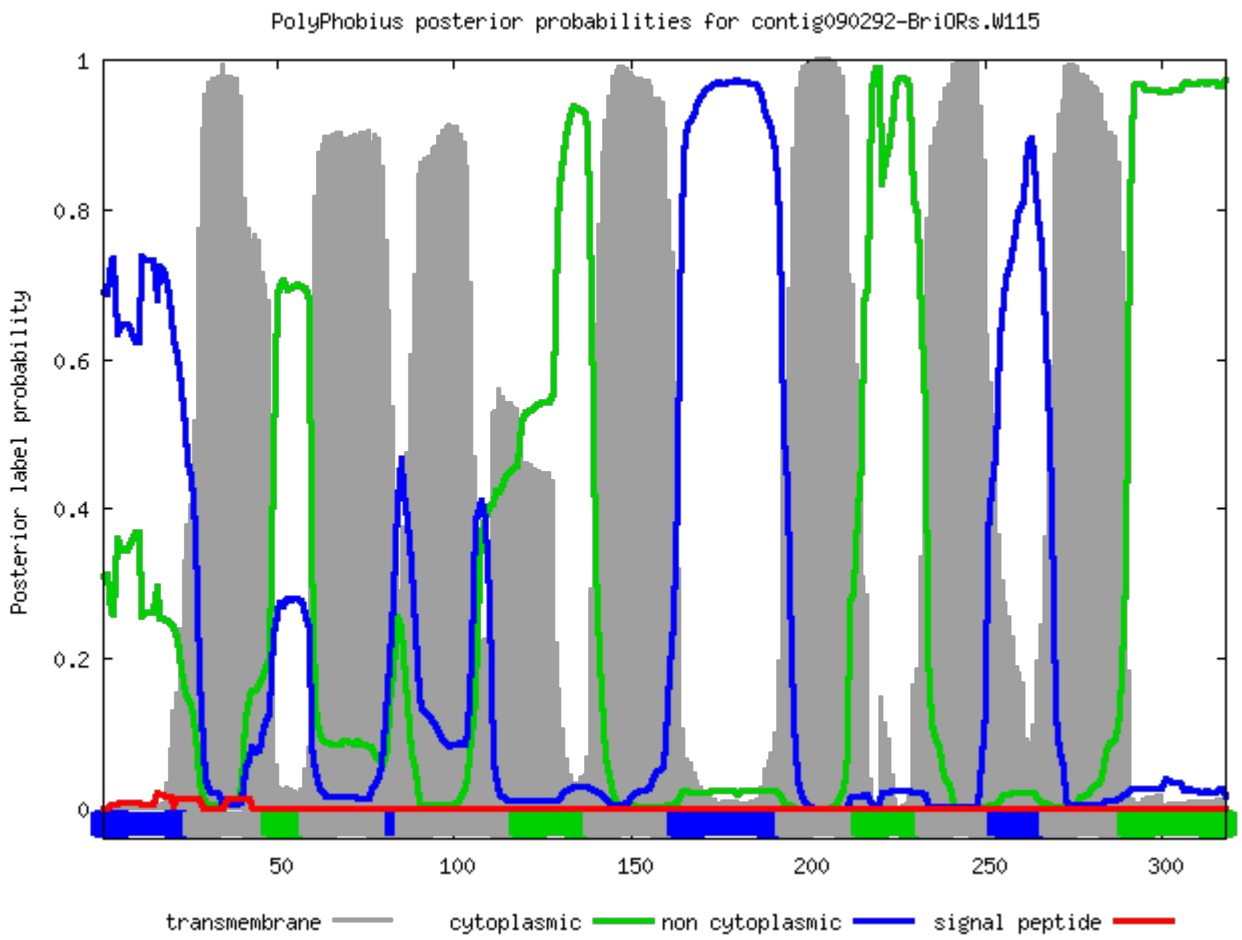

The prediction is based on an alignment. The probability data used in the plot is found here, and the gnuplot script is here.

Prediction of contig051559-BurOR.A008

```
ID   contig051559-BurOR.A008
FT   TOPO_DOM      1     22       NON CYTOPLASMIC.
FT   TRANSMEM     23     48
FT   TOPO_DOM     49     56       CYTOPLASMIC.
FT   TRANSMEM     57     77
FT   TOPO_DOM     78     95       NON CYTOPLASMIC.
FT   TRANSMEM     96    118
FT   TOPO_DOM    119    138       CYTOPLASMIC.
FT   TRANSMEM    139    160
FT   TOPO_DOM    161    192       NON CYTOPLASMIC.
FT   TRANSMEM    193    215
FT   TOPO_DOM    216    235       CYTOPLASMIC.
FT   TRANSMEM    236    257
FT   TOPO_DOM    258    268       NON CYTOPLASMIC.
FT   TRANSMEM    269    289
FT   TOPO_DOM    290    316       CYTOPLASMIC.
//
```

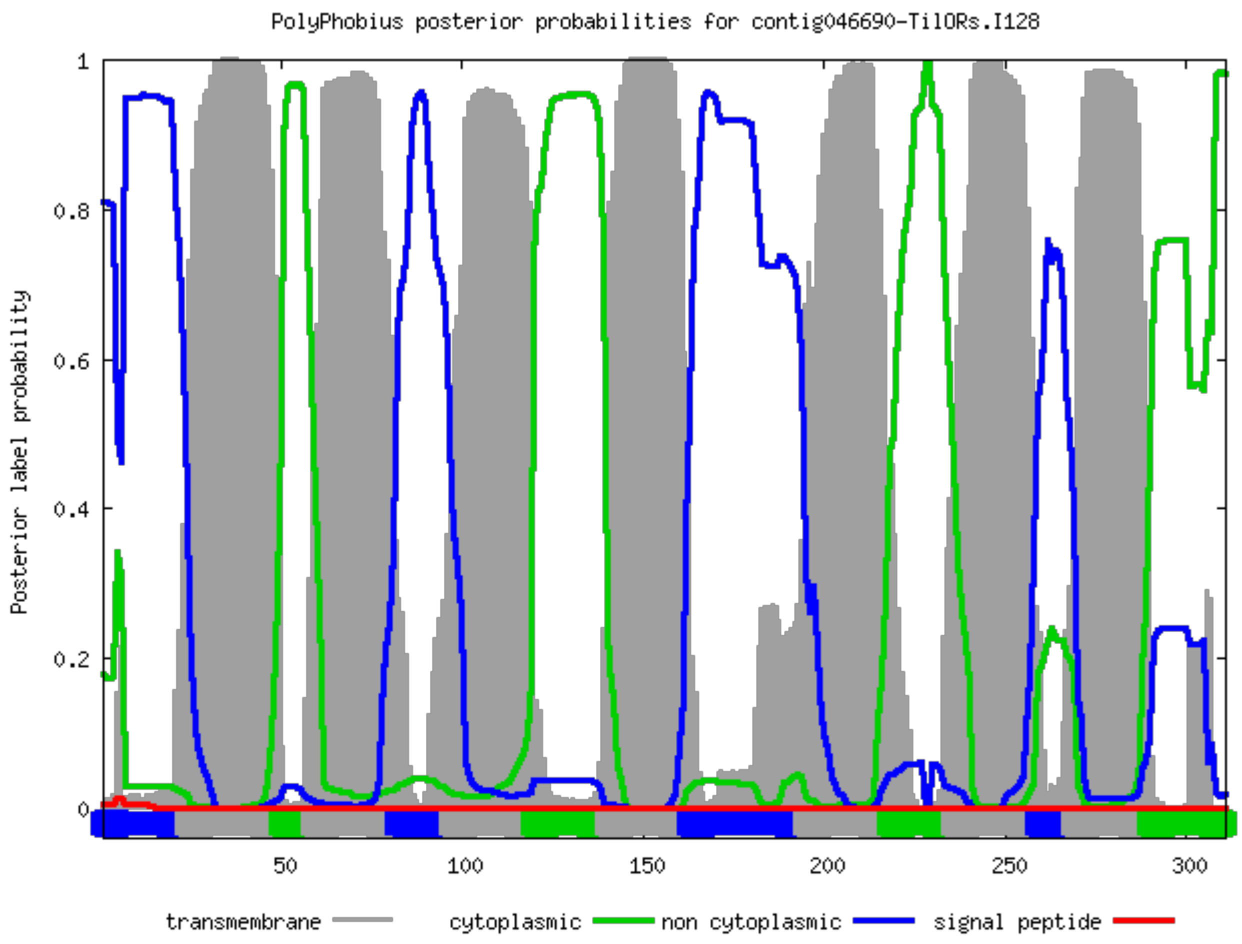

The prediction is based on an alignment. The probability data used in the plot is found here, and the gnuplot script is here.

Prediction of contig065253-TilOR.P212

```
ID   contig065253-TilOR.P212
FT   TOPO_DOM      1     27       NON CYTOPLASMIC.
FT   TRANSMEM     28     51
FT   TOPO_DOM     52     61       CYTOPLASMIC.
FT   TRANSMEM     62     88
FT   TOPO_DOM     89    100       NON CYTOPLASMIC.
FT   TRANSMEM    101    122
FT   TOPO_DOM    123    142       CYTOPLASMIC.
FT   TRANSMEM    143    165
FT   TOPO_DOM    166    199       NON CYTOPLASMIC.
FT   TRANSMEM    200    224
FT   TOPO_DOM    225    238       CYTOPLASMIC.
FT   TRANSMEM    239    261
FT   TOPO_DOM    262    273       NON CYTOPLASMIC.
FT   TRANSMEM    274    294
FT   TOPO_DOM    295    310       CYTOPLASMIC.
//
```

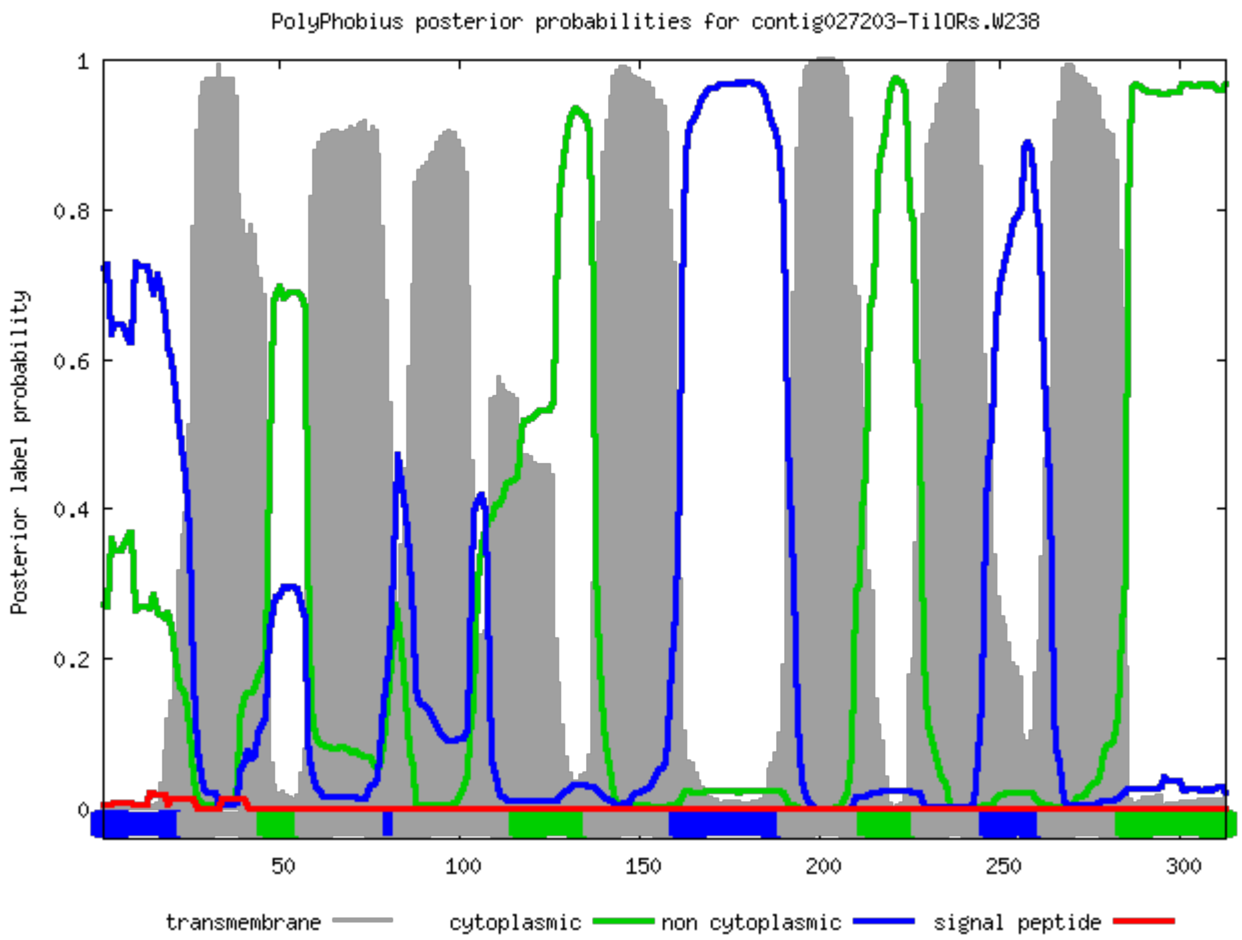

The prediction is based on an alignment. The probability data used in the plot is found here, and the gnuplot script is here.

Prediction of contig013356-TilOR.H105

```
ID    contig013356-TilOR.H105
FT    TOPO_DOM       1      22       NON CYTOPLASMIC.
FT    TRANSMEM      23      49
FT    TOPO_DOM      50      56       CYTOPLASMIC.
FT    TRANSMEM      57      77
FT    TOPO_DOM      78      95       NON CYTOPLASMIC.
FT    TRANSMEM      96     118
FT    TOPO_DOM     119     138       CYTOPLASMIC.
FT    TRANSMEM     139     160
FT    TOPO_DOM     161     193       NON CYTOPLASMIC.
FT    TRANSMEM     194     216
FT    TOPO_DOM     217     235       CYTOPLASMIC.
FT    TRANSMEM     236     258
FT    TOPO_DOM     259     269       NON CYTOPLASMIC.
FT    TRANSMEM     270     289
FT    TOPO_DOM     290     314       CYTOPLASMIC.
//
```

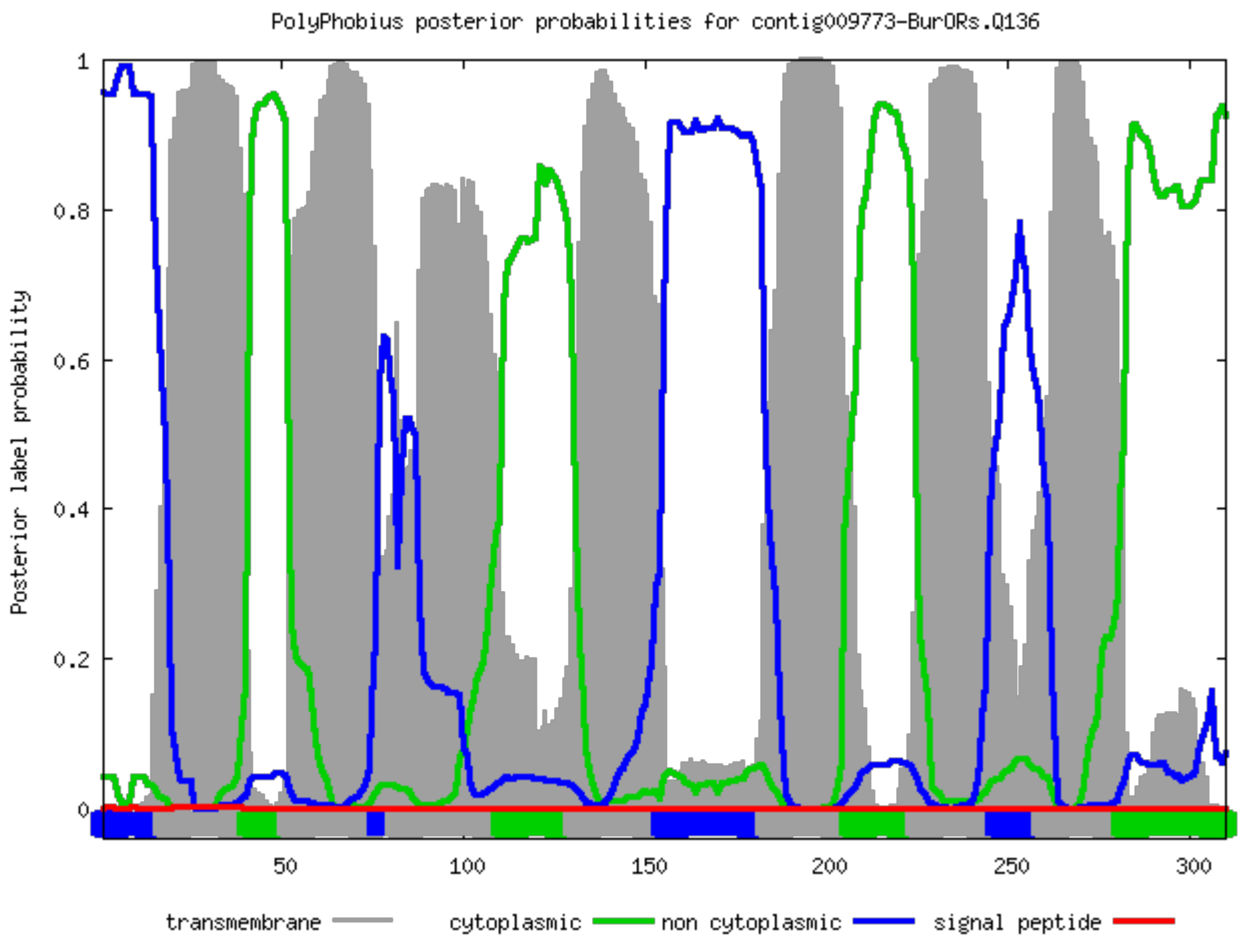

The prediction is based on an alignment. The probability data used in the plot is found here, and the gnuplot script is here.

Prediction of contig064802-BriOR.D027

```
ID    contig064802-BriOR.D027
FT    TOPO_DOM      1     22       NON CYTOPLASMIC.
FT    TRANSMEM     23     48
FT    TOPO_DOM     49     57       CYTOPLASMIC.
FT    TRANSMEM     58     81
FT    TOPO_DOM     82     90       NON CYTOPLASMIC.
FT    TRANSMEM     91    118
FT    TOPO_DOM    119    138       CYTOPLASMIC.
FT    TRANSMEM    139    162
FT    TOPO_DOM    163    194       NON CYTOPLASMIC.
FT    TRANSMEM    195    216
FT    TOPO_DOM    217    236       CYTOPLASMIC.
FT    TRANSMEM    237    256
FT    TOPO_DOM    257    266       NON CYTOPLASMIC.
FT    TRANSMEM    267    289
FT    TOPO_DOM    290    308       CYTOPLASMIC.
//
```

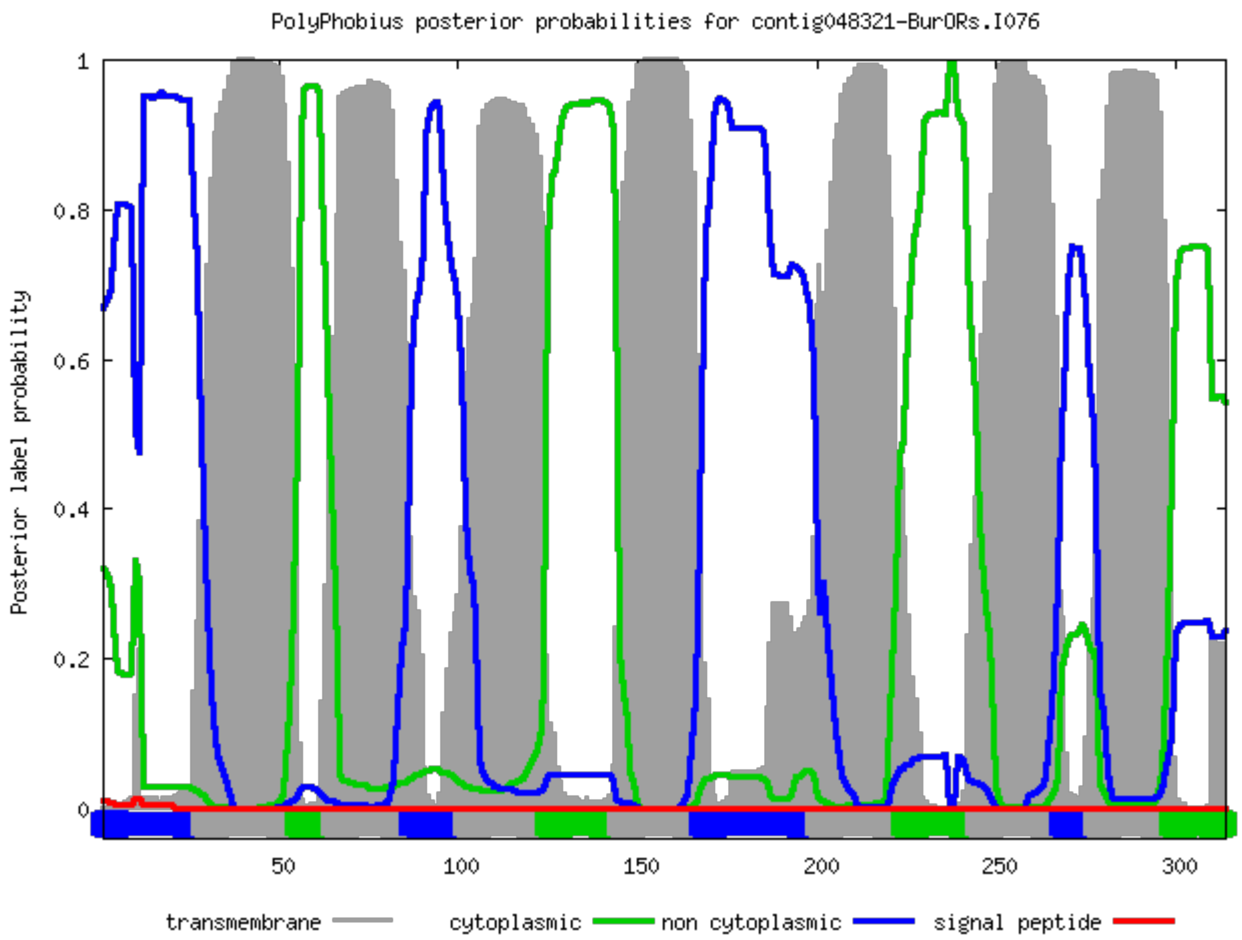

The prediction is based on an alignment. The probability data used in the plot is found here, and the gnuplot script is here.

Prediction of contig046360-TilOR.N197

```
ID    contig046360-TilOR.N197
FT    TOPO_DOM      1      32       NON CYTOPLASMIC.
FT    TRANSMEM     33      58
FT    TOPO_DOM     59      66       CYTOPLASMIC.
FT    TRANSMEM     67      86
FT    TOPO_DOM     87     104       NON CYTOPLASMIC.
FT    TRANSMEM    105     127
FT    TOPO_DOM    128     146       CYTOPLASMIC.
FT    TRANSMEM    147     170
FT    TOPO_DOM    171     206       NON CYTOPLASMIC.
FT    TRANSMEM    207     232
FT    TOPO_DOM    233     250       CYTOPLASMIC.
FT    TRANSMEM    251     272
FT    TOPO_DOM    273     277       NON CYTOPLASMIC.
FT    TRANSMEM    278     298
FT    TOPO_DOM    299     318       CYTOPLASMIC.
//
```

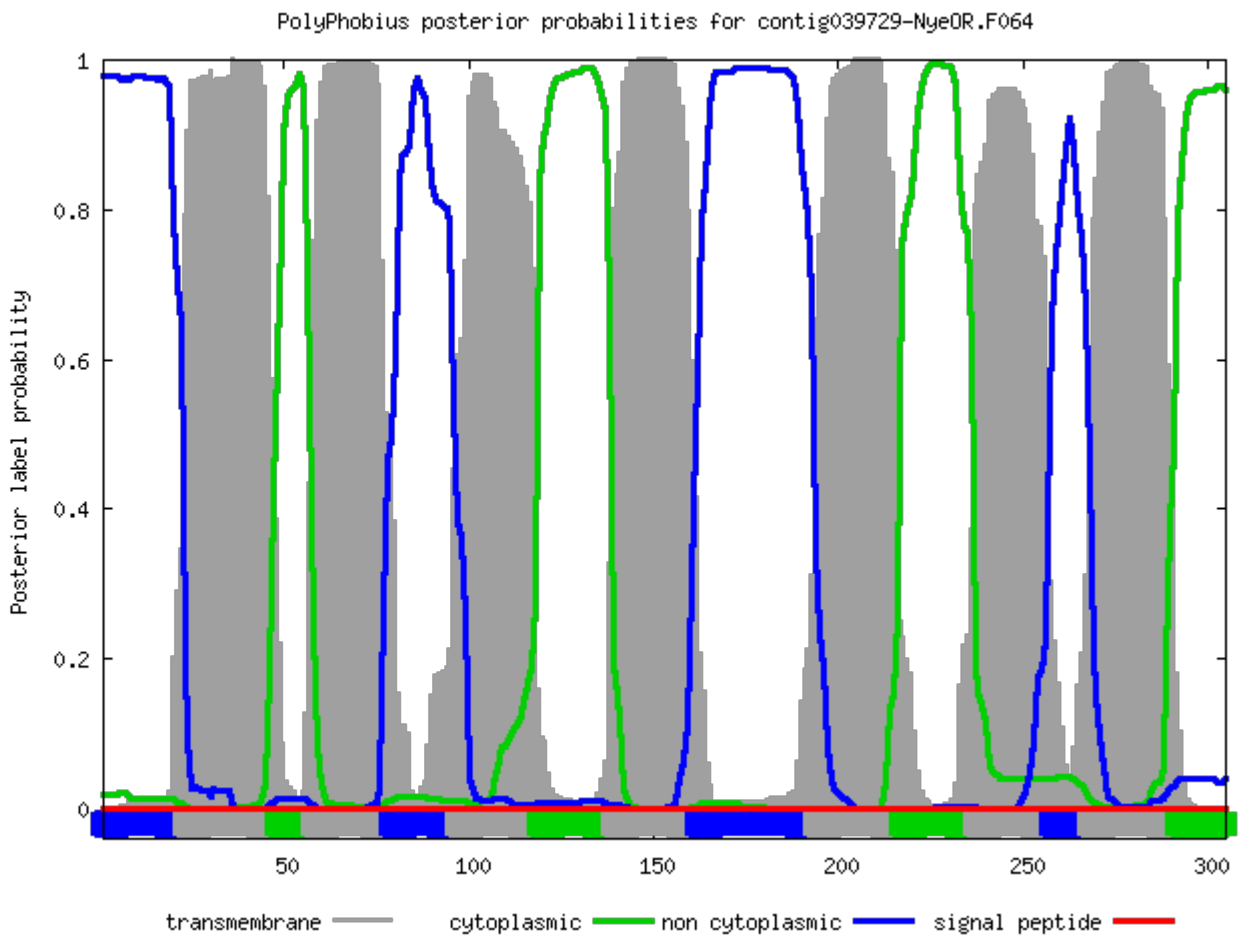

The prediction is based on an alignment. The probability data used in the plot is found here, and the gnuplot script is here.

Prediction of contig096539-BriOR.N088

```
ID   contig096539-BriOR.N088
FT   TOPO_DOM      1     32       NON CYTOPLASMIC.
FT   TRANSMEM     33     58
FT   TOPO_DOM     59     66       CYTOPLASMIC.
FT   TRANSMEM     67     86
FT   TOPO_DOM     87    104       NON CYTOPLASMIC.
FT   TRANSMEM    105    127
FT   TOPO_DOM    128    146       CYTOPLASMIC.
FT   TRANSMEM    147    170
FT   TOPO_DOM    171    207       NON CYTOPLASMIC.
FT   TRANSMEM    208    232
FT   TOPO_DOM    233    250       CYTOPLASMIC.
FT   TRANSMEM    251    271
FT   TOPO_DOM    272    277       NON CYTOPLASMIC.
FT   TRANSMEM    278    298
FT   TOPO_DOM    299    323       CYTOPLASMIC.
//
```

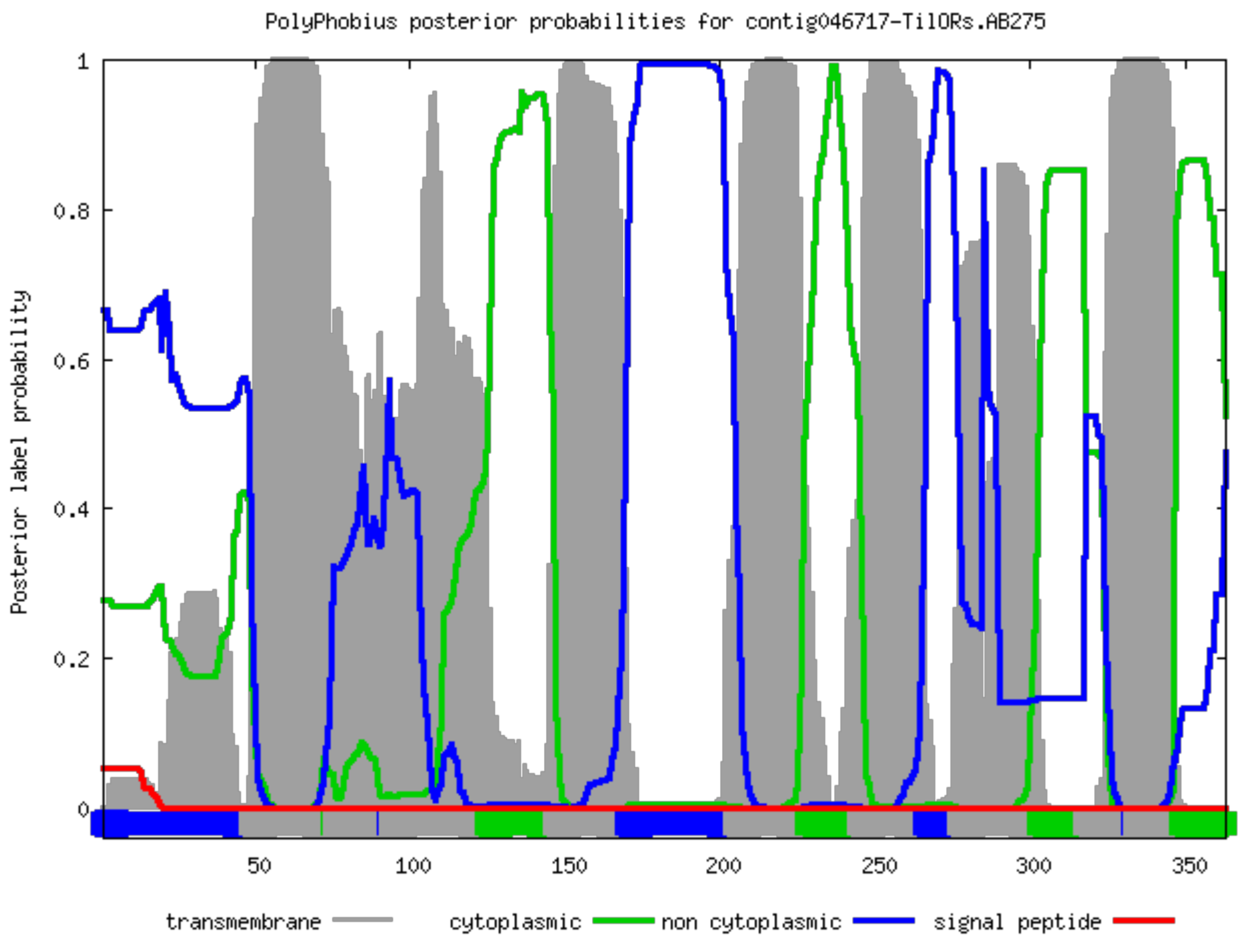

The prediction is based on an alignment. The probability data used in the plot is found here, and the gnuplot script is here.

Prediction of contig030011-ZebOR.H073

```
ID   contig030011-ZebOR.H073
FT   TOPO_DOM      1     23       NON CYTOPLASMIC.
FT   TRANSMEM     24     49
FT   TOPO_DOM     50     56       CYTOPLASMIC.
FT   TRANSMEM     57     76
FT   TOPO_DOM     77     95       NON CYTOPLASMIC.
FT   TRANSMEM     96    118
FT   TOPO_DOM    119    138       CYTOPLASMIC.
FT   TRANSMEM    139    160
FT   TOPO_DOM    161    196       NON CYTOPLASMIC.
FT   TRANSMEM    197    219
FT   TOPO_DOM    220    237       CYTOPLASMIC.
FT   TRANSMEM    238    259
FT   TOPO_DOM    260    271       NON CYTOPLASMIC.
FT   TRANSMEM    272    291
FT   TOPO_DOM    292    324       CYTOPLASMIC.
//
```

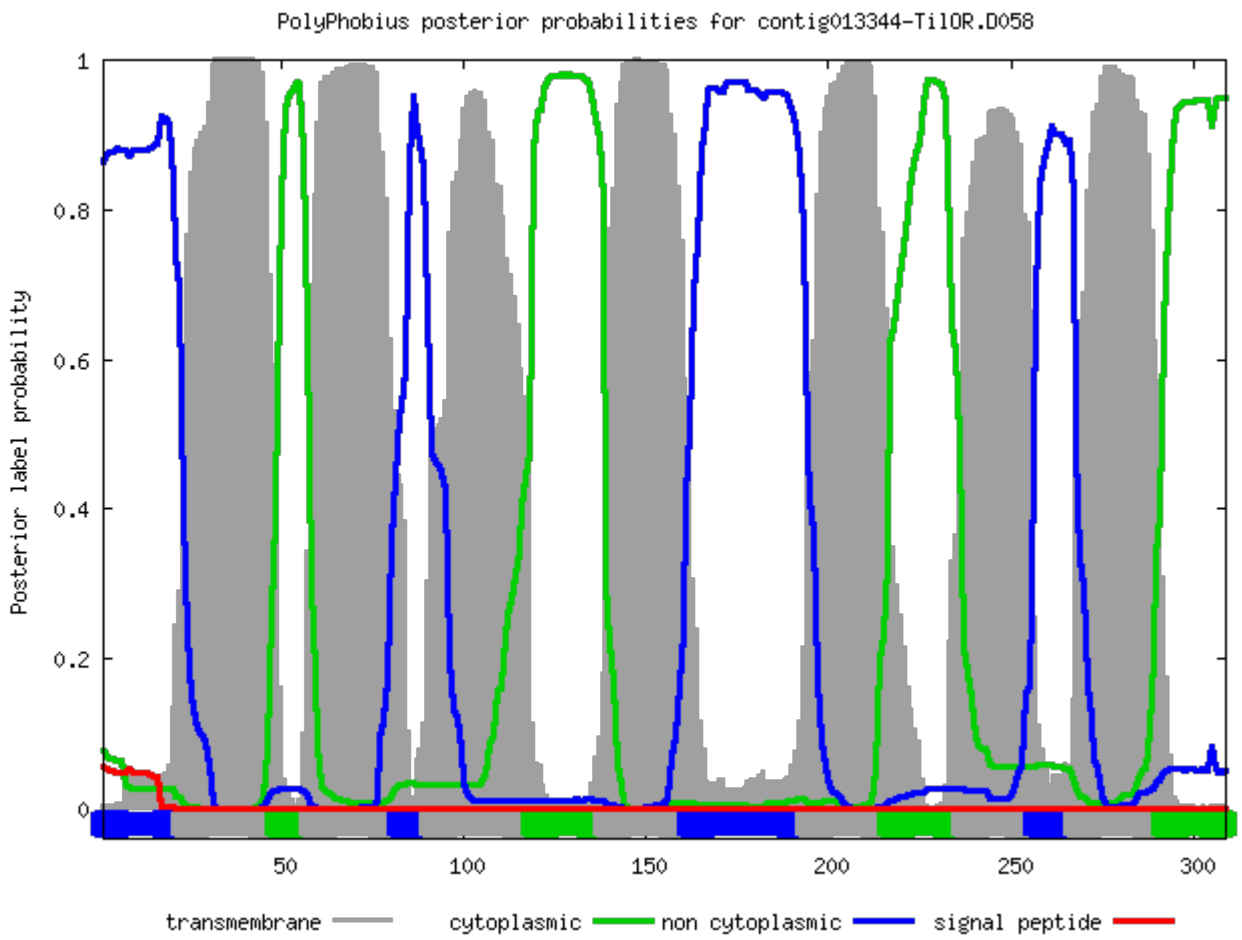

The prediction is based on an alignment. The probability data used in the plot is found here, and the gnuplot script is here.

Prediction of contig047515-ZebOR.A018

```
ID    contig047515-ZebOR.A018
FT    TOPO_DOM      1      22       NON CYTOPLASMIC.
FT    TRANSMEM     23      48
FT    TOPO_DOM     49      56       CYTOPLASMIC.
FT    TRANSMEM     57      77
FT    TOPO_DOM     78      95       NON CYTOPLASMIC.
FT    TRANSMEM     96     118
FT    TOPO_DOM    119     138       CYTOPLASMIC.
FT    TRANSMEM    139     160
FT    TOPO_DOM    161     192       NON CYTOPLASMIC.
FT    TRANSMEM    193     215
FT    TOPO_DOM    216     235       CYTOPLASMIC.
FT    TRANSMEM    236     257
FT    TOPO_DOM    258     268       NON CYTOPLASMIC.
FT    TRANSMEM    269     289
FT    TOPO_DOM    290     316       CYTOPLASMIC.
//
```

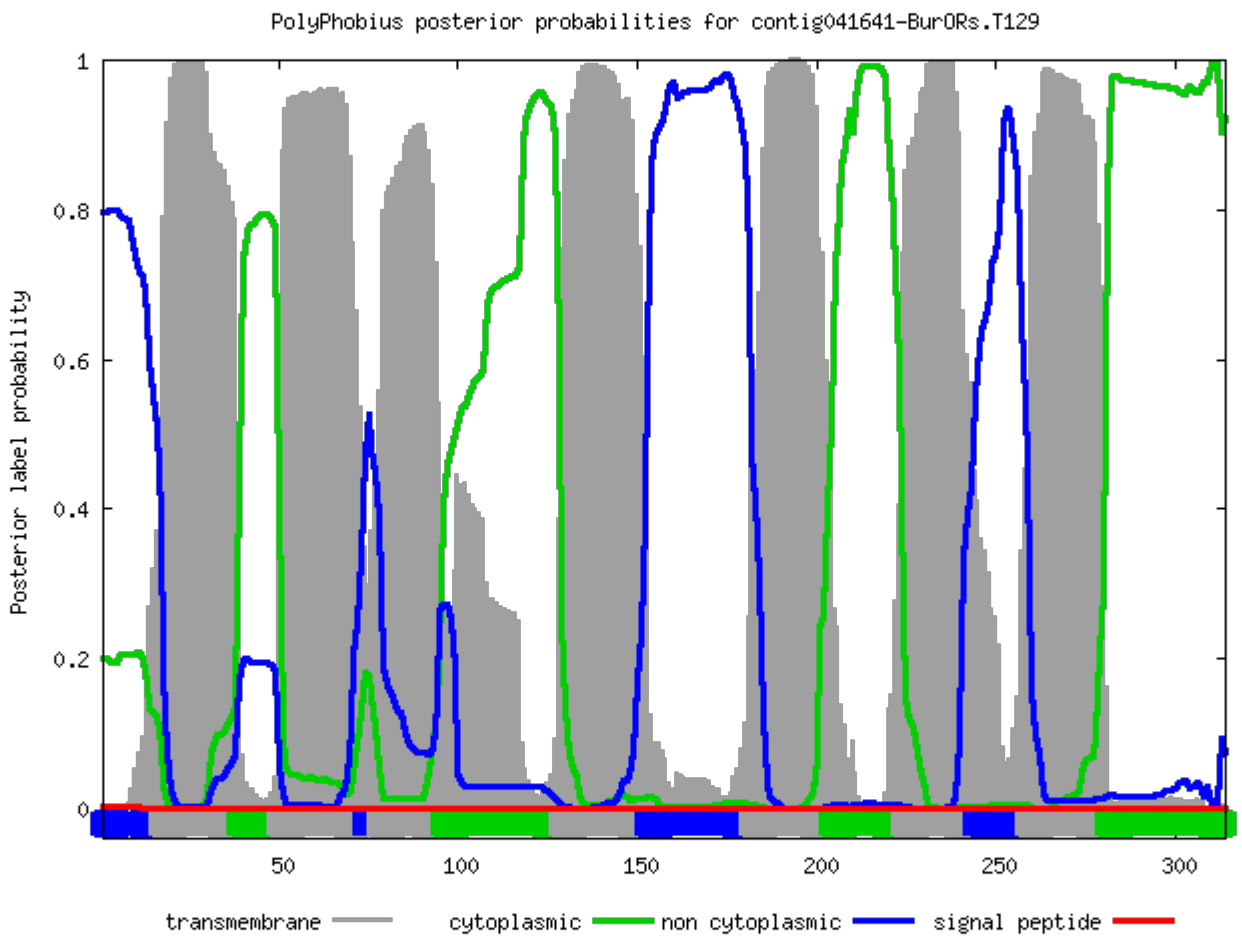

The prediction is based on an alignment. The probability data used in the plot is found here, and the gnuplot script is here.

Prediction of contig068425-TilOR.L155

```
ID   contig068425-TilOR.L155
FT   TOPO_DOM      1     25       NON CYTOPLASMIC.
FT   TRANSMEM     26     50
FT   TOPO_DOM     51     59       CYTOPLASMIC.
FT   TRANSMEM     60     86
FT   TOPO_DOM     87     98       NON CYTOPLASMIC.
FT   TRANSMEM     99    120
FT   TOPO_DOM    121    140       CYTOPLASMIC.
FT   TRANSMEM    141    163
FT   TOPO_DOM    164    199       NON CYTOPLASMIC.
FT   TRANSMEM    200    224
FT   TOPO_DOM    225    237       CYTOPLASMIC.
FT   TRANSMEM    238    260
FT   TOPO_DOM    261    271       NON CYTOPLASMIC.
FT   TRANSMEM    272    292
FT   TOPO_DOM    293    313       CYTOPLASMIC.
//
```

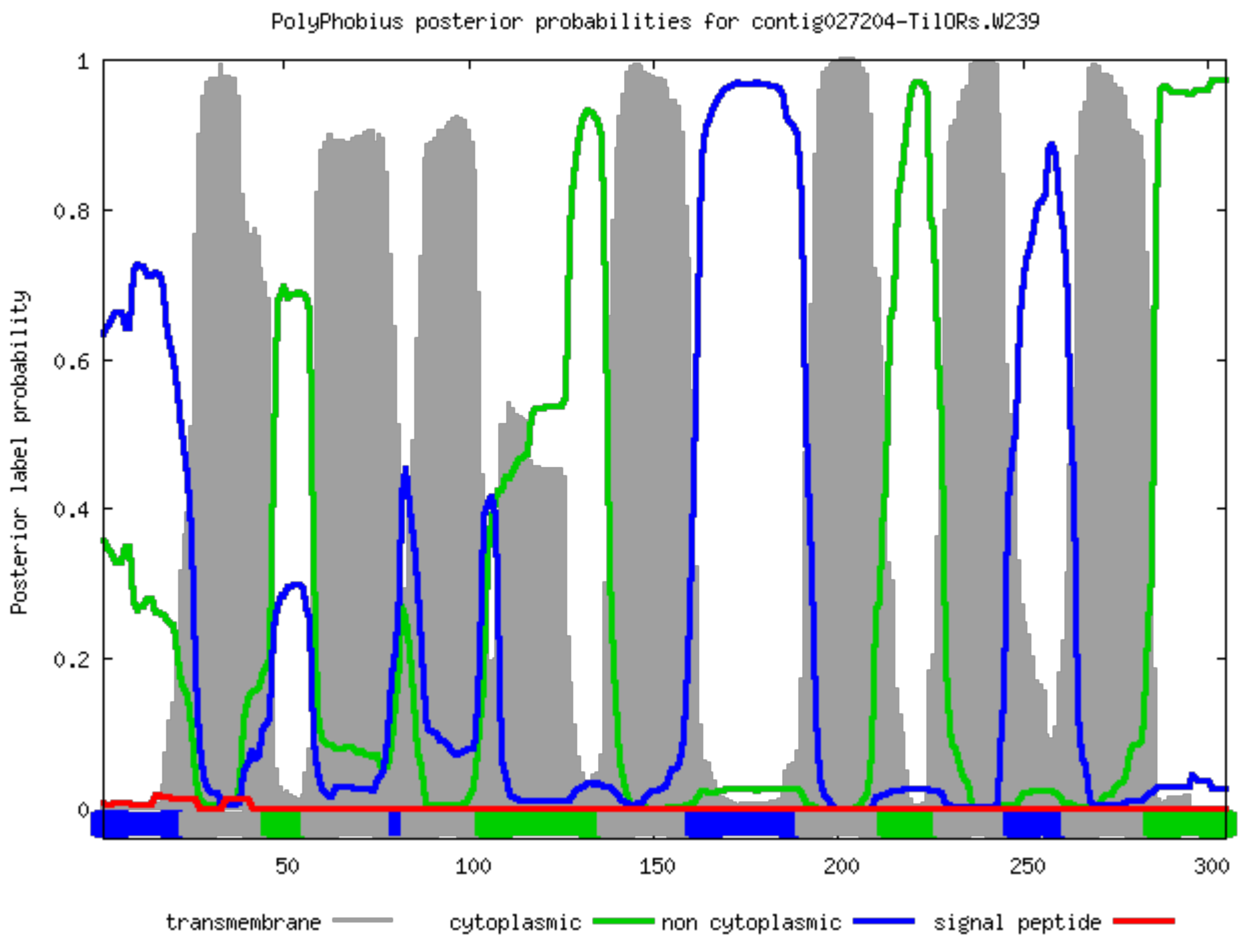

The prediction is based on an alignment. The probability data used in the plot is found here, and the gnuplot script is here.

Prediction of contig046048-ZebOR.R146

```
ID   contig046048-ZebOR.R146
FT   TOPO_DOM      1     24       NON CYTOPLASMIC.
FT   TRANSMEM     25     48
FT   TOPO_DOM     49     59       CYTOPLASMIC.
FT   TRANSMEM     60     84
FT   TOPO_DOM     85     89       NON CYTOPLASMIC.
FT   TRANSMEM     90    118
FT   TOPO_DOM    119    138       CYTOPLASMIC.
FT   TRANSMEM    139    162
FT   TOPO_DOM    163    194       NON CYTOPLASMIC.
FT   TRANSMEM    195    218
FT   TOPO_DOM    219    235       CYTOPLASMIC.
FT   TRANSMEM    236    257
FT   TOPO_DOM    258    270       NON CYTOPLASMIC.
FT   TRANSMEM    271    293
FT   TOPO_DOM    294    317       CYTOPLASMIC.
//
```

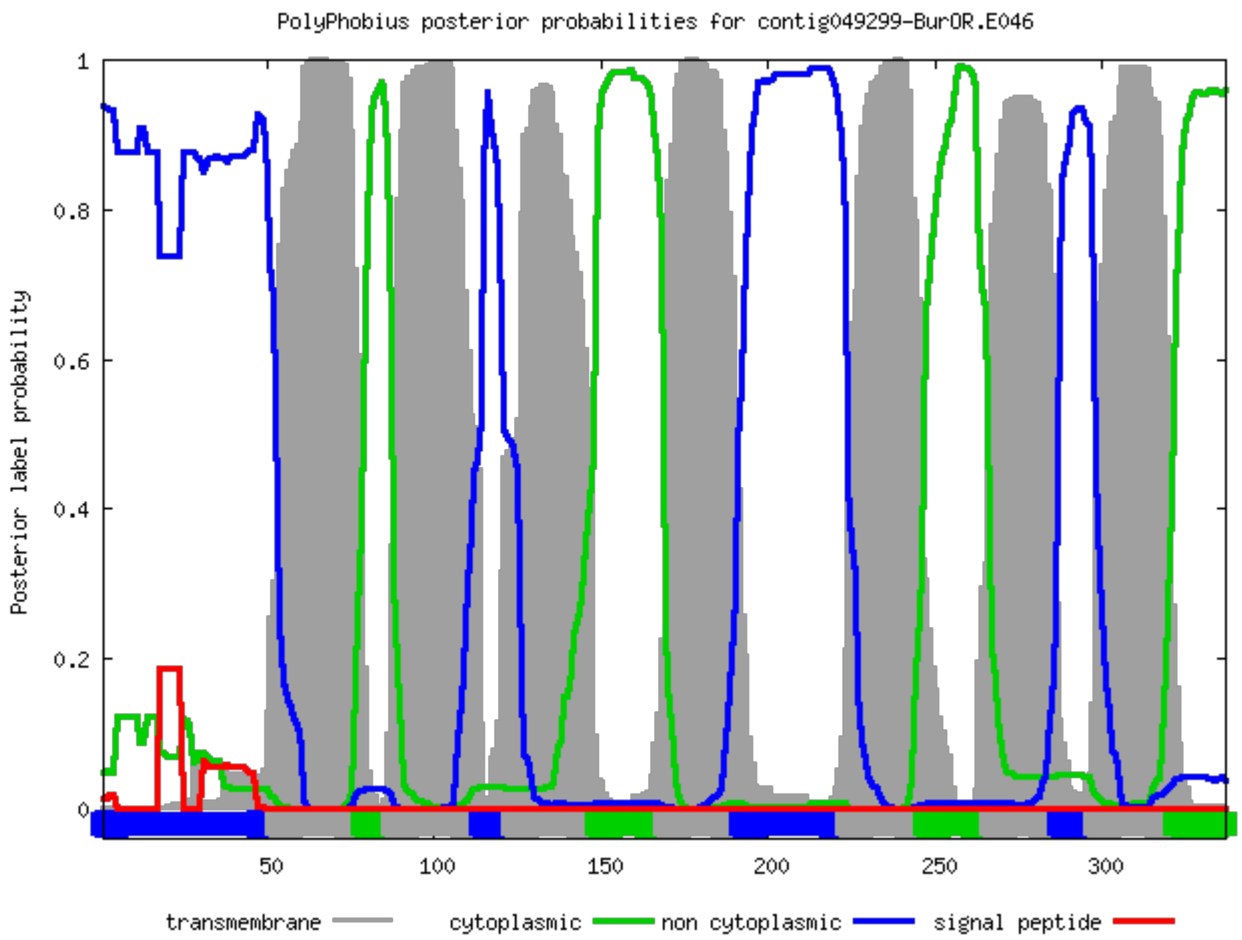

The prediction is based on an alignment. The probability data used in the plot is found here, and the gnuplot script is here.

Prediction of contig062094-ZebOR.A023

```
ID   contig062094-ZebOR.A023
FT   TOPO_DOM      1     22       NON CYTOPLASMIC.
FT   TRANSMEM     23     48
FT   TOPO_DOM     49     56       CYTOPLASMIC.
FT   TRANSMEM     57     76
FT   TOPO_DOM     77     95       NON CYTOPLASMIC.
FT   TRANSMEM     96    118
FT   TOPO_DOM    119    138       CYTOPLASMIC.
FT   TRANSMEM    139    159
FT   TOPO_DOM    160    192       NON CYTOPLASMIC.
FT   TRANSMEM    193    215
FT   TOPO_DOM    216    235       CYTOPLASMIC.
FT   TRANSMEM    236    257
FT   TOPO_DOM    258    268       NON CYTOPLASMIC.
FT   TRANSMEM    269    289
FT   TOPO_DOM    290    314       CYTOPLASMIC.
//
```

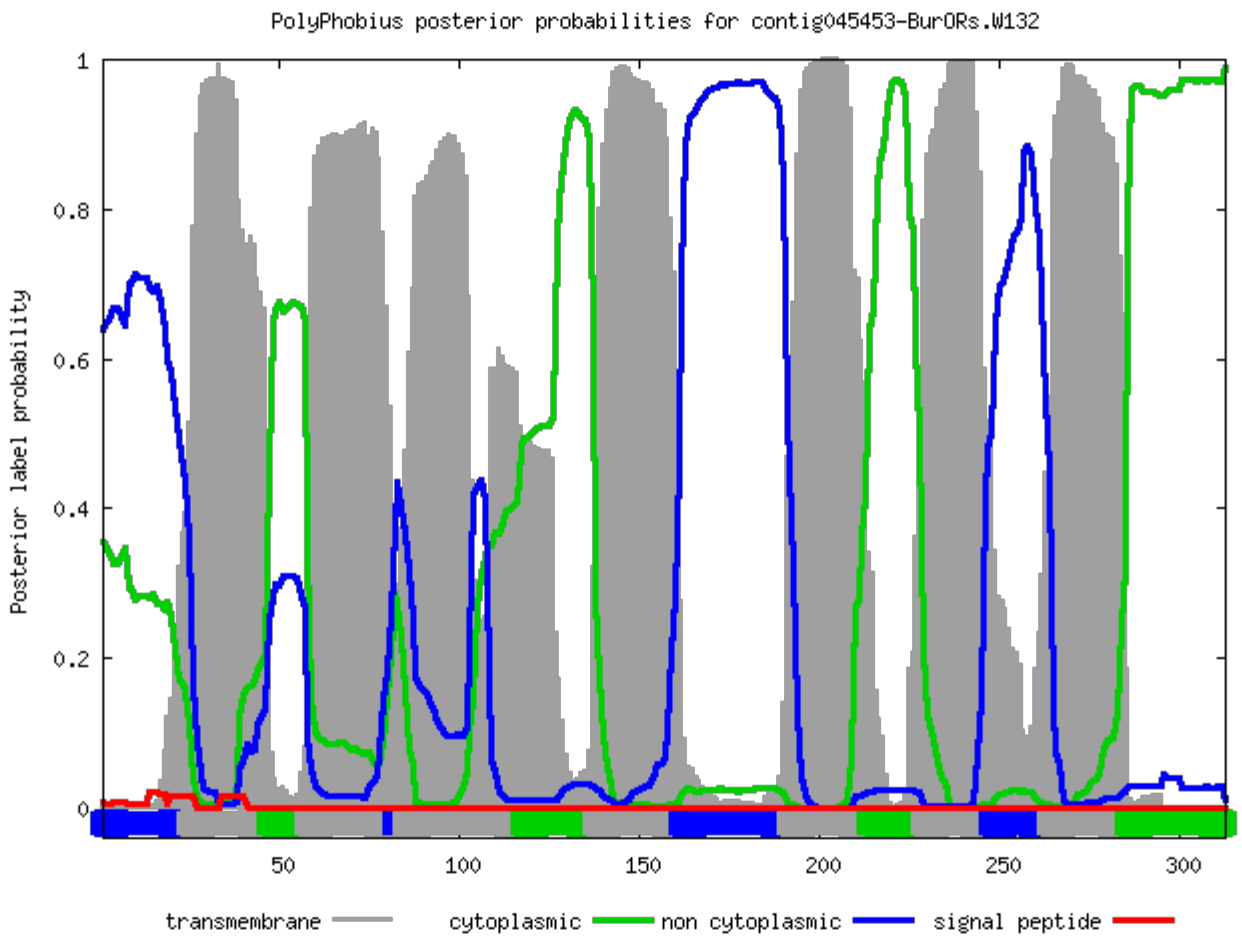

The prediction is based on an alignment. The probability data used in the plot is found here, and the gnuplot script is here.

Prediction of contig041951-TilOR.A021

```
ID   contig041951-TilOR.A021
FT   TOPO_DOM      1     22       NON CYTOPLASMIC.
FT   TRANSMEM     23     48
FT   TOPO_DOM     49     56       CYTOPLASMIC.
FT   TRANSMEM     57     77
FT   TOPO_DOM     78     95       NON CYTOPLASMIC.
FT   TRANSMEM     96    118
FT   TOPO_DOM    119    138       CYTOPLASMIC.
FT   TRANSMEM    139    159
FT   TOPO_DOM    160    192       NON CYTOPLASMIC.
FT   TRANSMEM    193    215
FT   TOPO_DOM    216    235       CYTOPLASMIC.
FT   TRANSMEM    236    257
FT   TOPO_DOM    258    268       NON CYTOPLASMIC.
FT   TRANSMEM    269    289
FT   TOPO_DOM    290    307       CYTOPLASMIC.
//
```

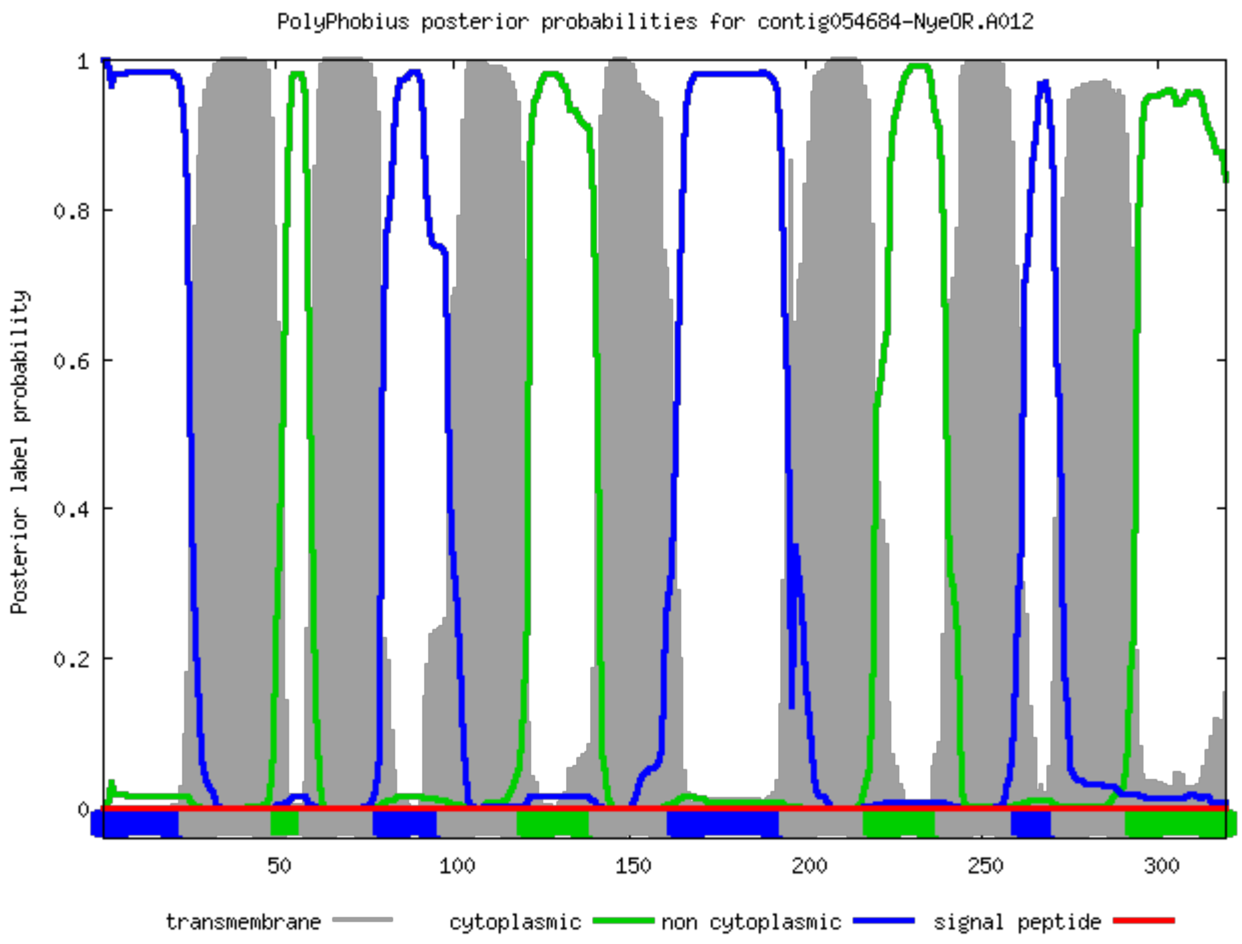

The prediction is based on an alignment. The probability data used in the plot is found here, and the gnuplot script is here.

Prediction of contig004261-BriOR.E034

```
ID   contig004261-BriOR.E034
FT   TOPO_DOM      1     22       NON CYTOPLASMIC.
FT   TRANSMEM     23     48
FT   TOPO_DOM     49     57       CYTOPLASMIC.
FT   TRANSMEM     58     82
FT   TOPO_DOM     83     90       NON CYTOPLASMIC.
FT   TRANSMEM     91    118
FT   TOPO_DOM    119    138       CYTOPLASMIC.
FT   TRANSMEM    139    161
FT   TOPO_DOM    162    193       NON CYTOPLASMIC.
FT   TRANSMEM    194    216
FT   TOPO_DOM    217    235       CYTOPLASMIC.
FT   TRANSMEM    236    256
FT   TOPO_DOM    257    267       NON CYTOPLASMIC.
FT   TRANSMEM    268    291
FT   TOPO_DOM    292    310       CYTOPLASMIC.
//
```

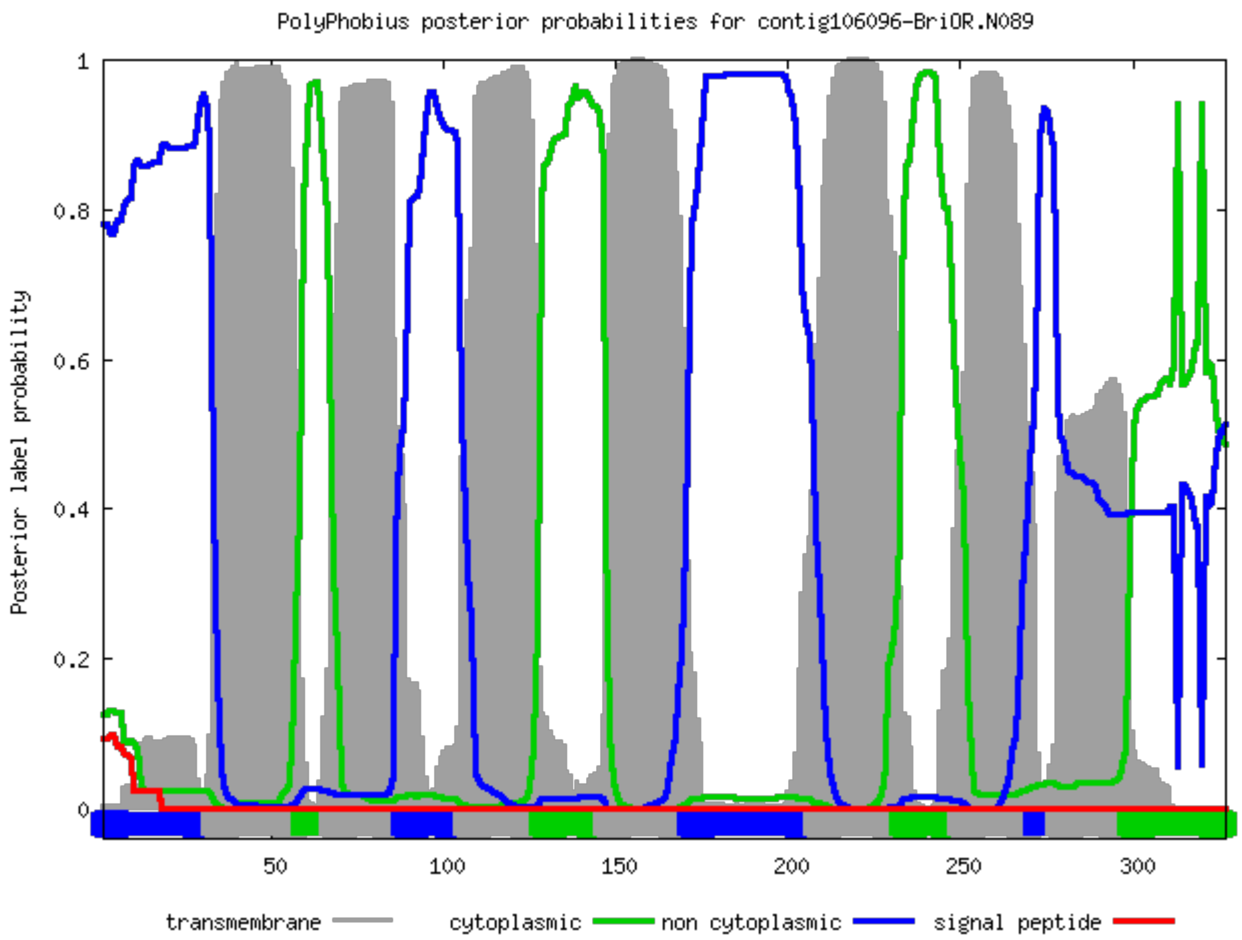

The prediction is based on an alignment. The probability data used in the plot is found here, and the gnuplot script is here.

Prediction of contig013330-TilOR.D053

```
ID   contig013330-TilOR.D053
FT   TOPO_DOM      1     22       NON CYTOPLASMIC.
FT   TRANSMEM     23     48
FT   TOPO_DOM     49     57       CYTOPLASMIC.
FT   TRANSMEM     58     81
FT   TOPO_DOM     82     90       NON CYTOPLASMIC.
FT   TRANSMEM     91    118
FT   TOPO_DOM    119    138       CYTOPLASMIC.
FT   TRANSMEM    139    161
FT   TOPO_DOM    162    193       NON CYTOPLASMIC.
FT   TRANSMEM    194    216
FT   TOPO_DOM    217    236       CYTOPLASMIC.
FT   TRANSMEM    237    256
FT   TOPO_DOM    257    267       NON CYTOPLASMIC.
FT   TRANSMEM    268    291
FT   TOPO_DOM    292    309       CYTOPLASMIC.
//
```

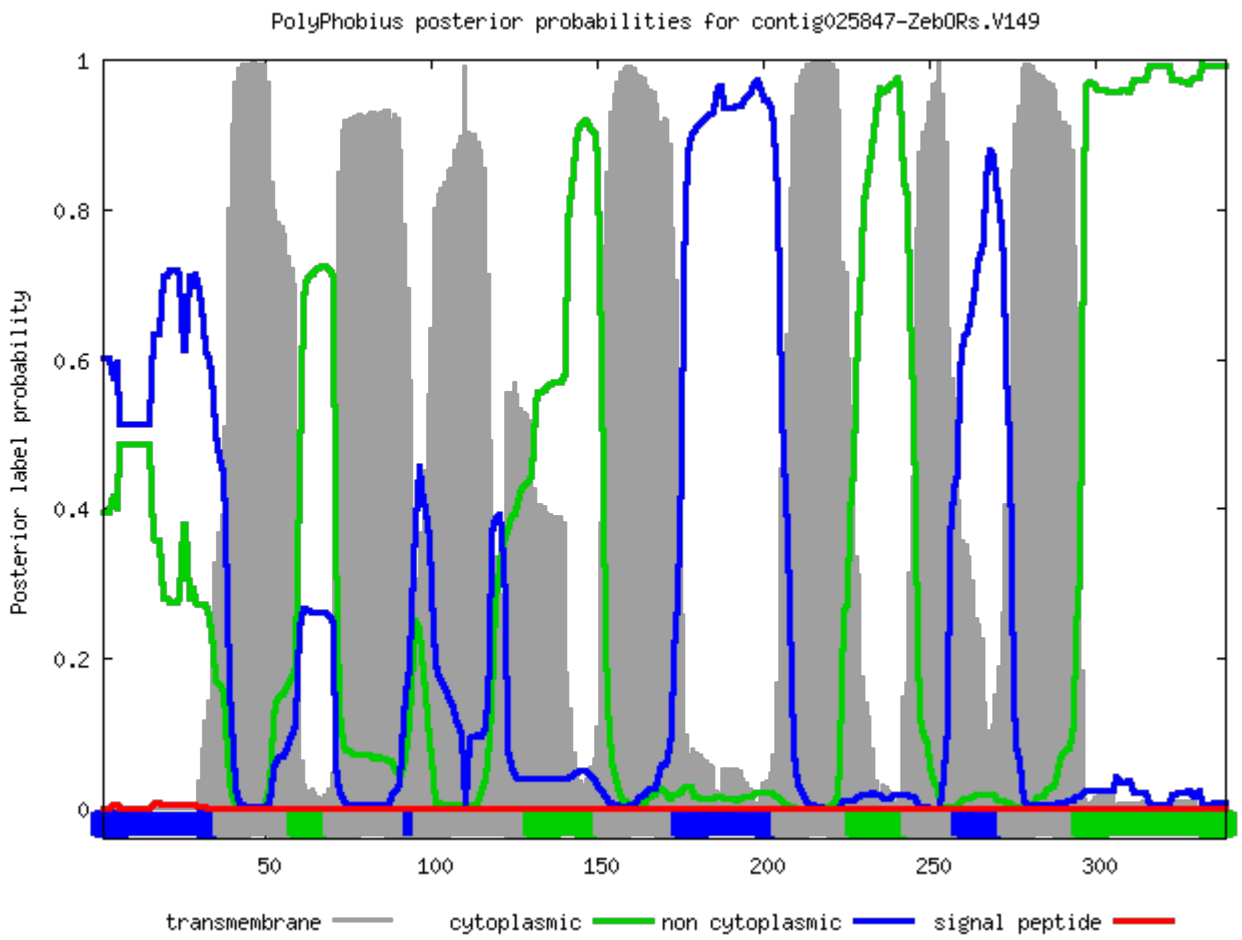

The prediction is based on an alignment. The probability data used in the plot is found here, and the gnuplot script is here.

Prediction of contig009805-BurOR.C031

```
ID   contig009805-BurOR.C031
FT   TOPO_DOM      1     21       NON CYTOPLASMIC.
FT   TRANSMEM     22     47
FT   TOPO_DOM     48     56       CYTOPLASMIC.
FT   TRANSMEM     57     79
FT   TOPO_DOM     80     94       NON CYTOPLASMIC.
FT   TRANSMEM     95    117
FT   TOPO_DOM    118    137       CYTOPLASMIC.
FT   TRANSMEM    138    161
FT   TOPO_DOM    162    192       NON CYTOPLASMIC.
FT   TRANSMEM    193    215
FT   TOPO_DOM    216    235       CYTOPLASMIC.
FT   TRANSMEM    236    255
FT   TOPO_DOM    256    266       NON CYTOPLASMIC.
FT   TRANSMEM    267    289
FT   TOPO_DOM    290    321       CYTOPLASMIC.
//
```

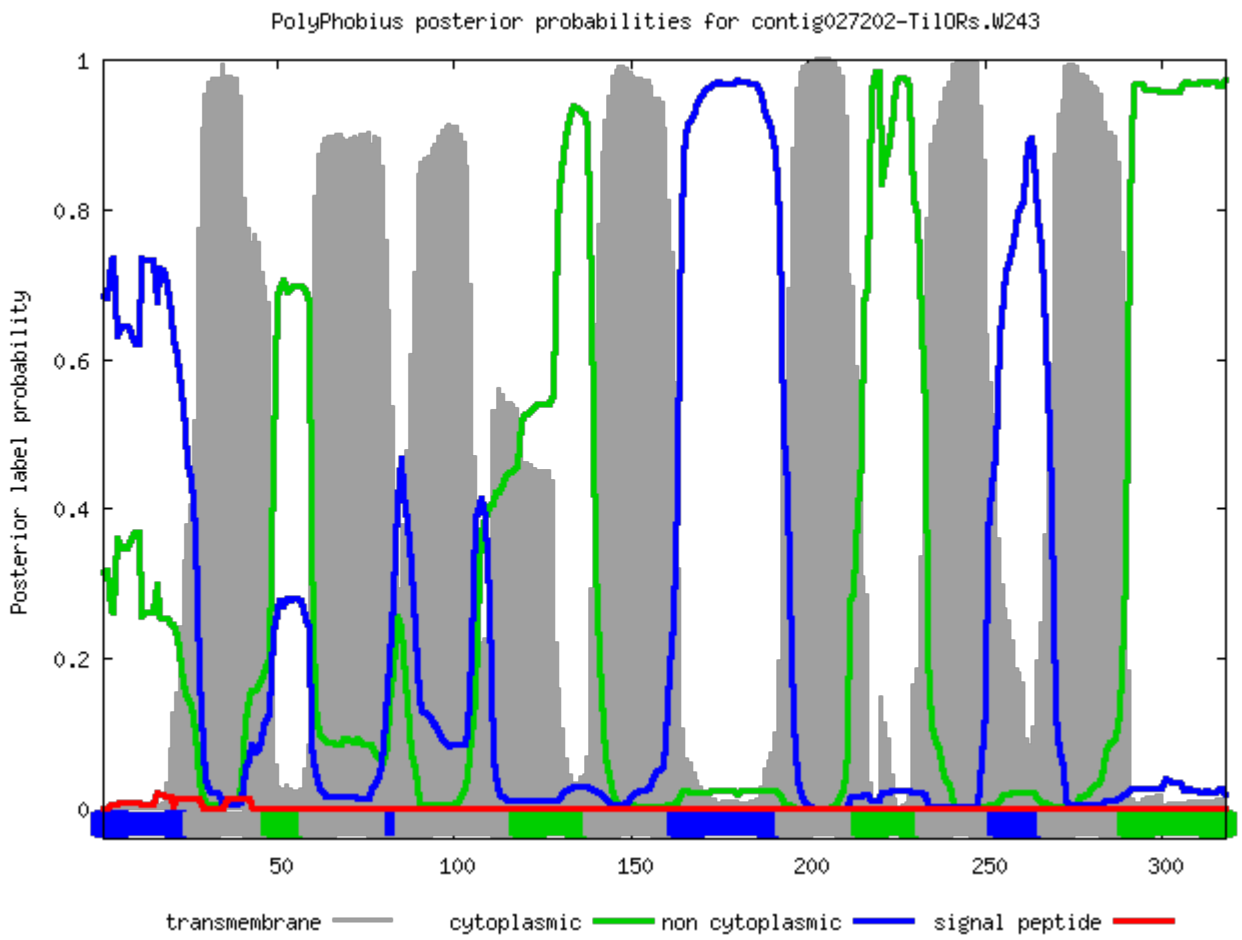

The prediction is based on an alignment. The probability data used in the plot is found here, and the gnuplot script is here.

Prediction of contig022217-TilOR.A004

```
ID    contig022217-TilOR.A004
FT    TOPO_DOM      1     22       NON CYTOPLASMIC.
FT    TRANSMEM     23     48
FT    TOPO_DOM     49     56       CYTOPLASMIC.
FT    TRANSMEM     57     76
FT    TOPO_DOM     77     95       NON CYTOPLASMIC.
FT    TRANSMEM     96    118
FT    TOPO_DOM    119    138       CYTOPLASMIC.
FT    TRANSMEM    139    159
FT    TOPO_DOM    160    192       NON CYTOPLASMIC.
FT    TRANSMEM    193    215
FT    TOPO_DOM    216    235       CYTOPLASMIC.
FT    TRANSMEM    236    257
FT    TOPO_DOM    258    268       NON CYTOPLASMIC.
FT    TRANSMEM    269    289
FT    TOPO_DOM    290    316       CYTOPLASMIC.
//
```

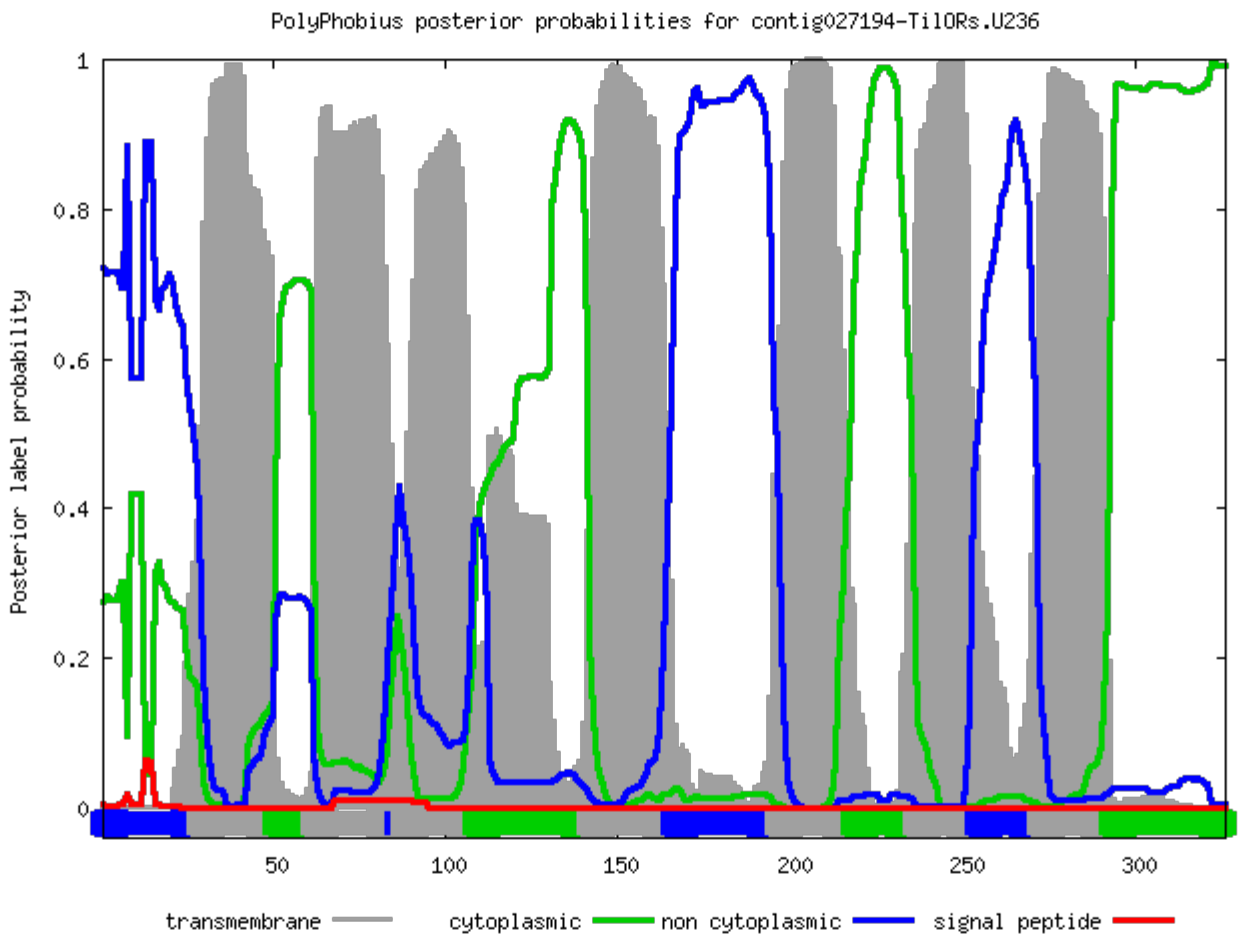

The prediction is based on an alignment. The probability data used in the plot is found here, and the gnuplot script is here.

Prediction of contig068527-TilOR.L157

```
ID   contig068527-TilOR.L157
FT   TOPO_DOM      1     25       NON CYTOPLASMIC.
FT   TRANSMEM     26     50
FT   TOPO_DOM     51     59       CYTOPLASMIC.
FT   TRANSMEM     60     82
FT   TOPO_DOM     83    100       NON CYTOPLASMIC.
FT   TRANSMEM    101    120
FT   TOPO_DOM    121    140       CYTOPLASMIC.
FT   TRANSMEM    141    163
FT   TOPO_DOM    164    199       NON CYTOPLASMIC.
FT   TRANSMEM    200    224
FT   TOPO_DOM    225    238       CYTOPLASMIC.
FT   TRANSMEM    239    261
FT   TOPO_DOM    262    271       NON CYTOPLASMIC.
FT   TRANSMEM    272    292
FT   TOPO_DOM    293    313       CYTOPLASMIC.
//
```

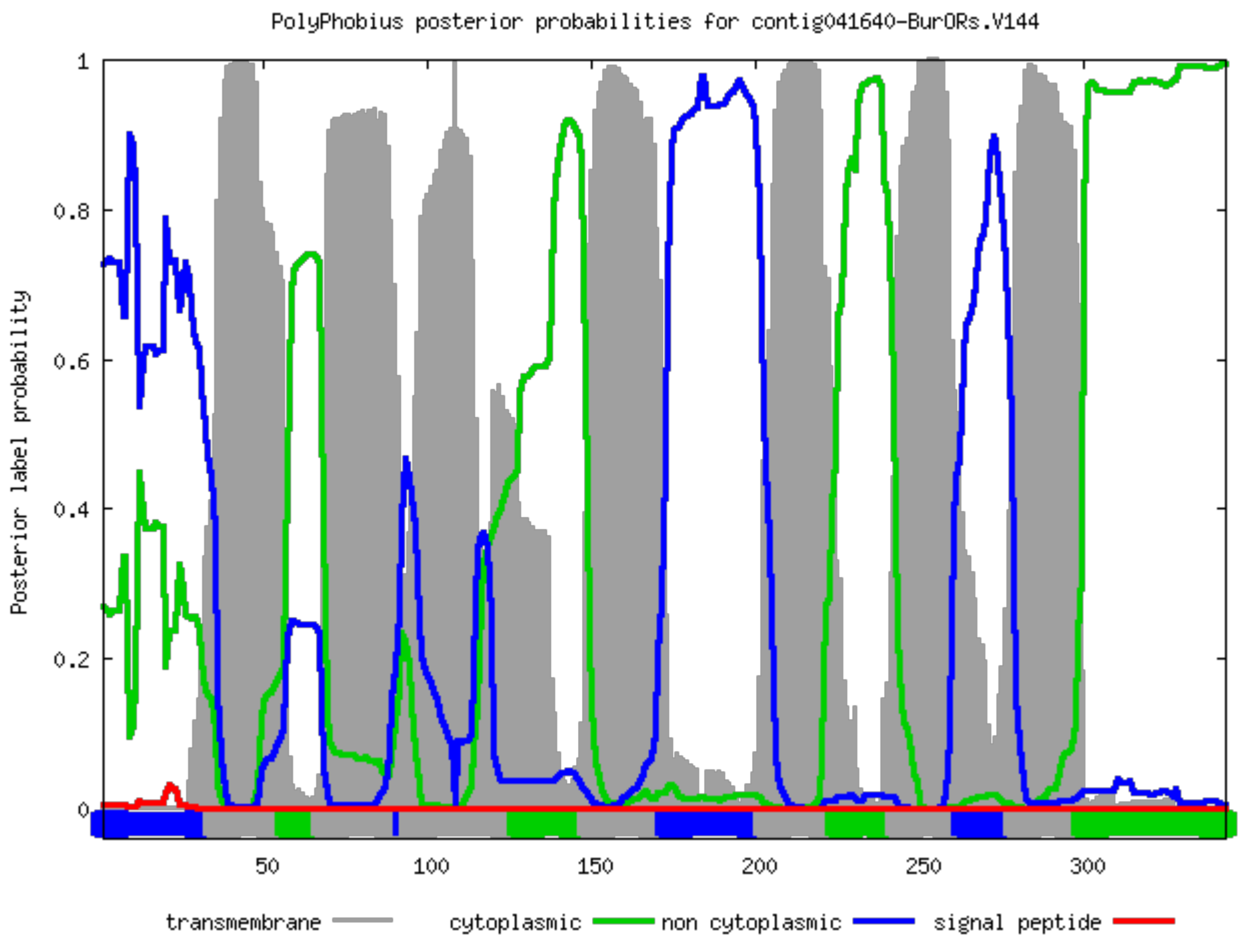

The prediction is based on an alignment. The probability data used in the plot is found here, and the gnuplot script is here.

Prediction of contig053579-NyeOR.E055

```
ID   contig053579-NyeOR.E055
FT   TOPO_DOM      1    22       NON CYTOPLASMIC.
FT   TRANSMEM     23    48
FT   TOPO_DOM     49    57       CYTOPLASMIC.
FT   TRANSMEM     58    82
FT   TOPO_DOM     83    93       NON CYTOPLASMIC.
FT   TRANSMEM     94   118
FT   TOPO_DOM    119   138       CYTOPLASMIC.
FT   TRANSMEM    139   160
FT   TOPO_DOM    161   193       NON CYTOPLASMIC.
FT   TRANSMEM    194   216
FT   TOPO_DOM    217   236       CYTOPLASMIC.
FT   TRANSMEM    237   256
FT   TOPO_DOM    257   267       NON CYTOPLASMIC.
FT   TRANSMEM    268   291
FT   TOPO_DOM    292   312       CYTOPLASMIC.
//
```

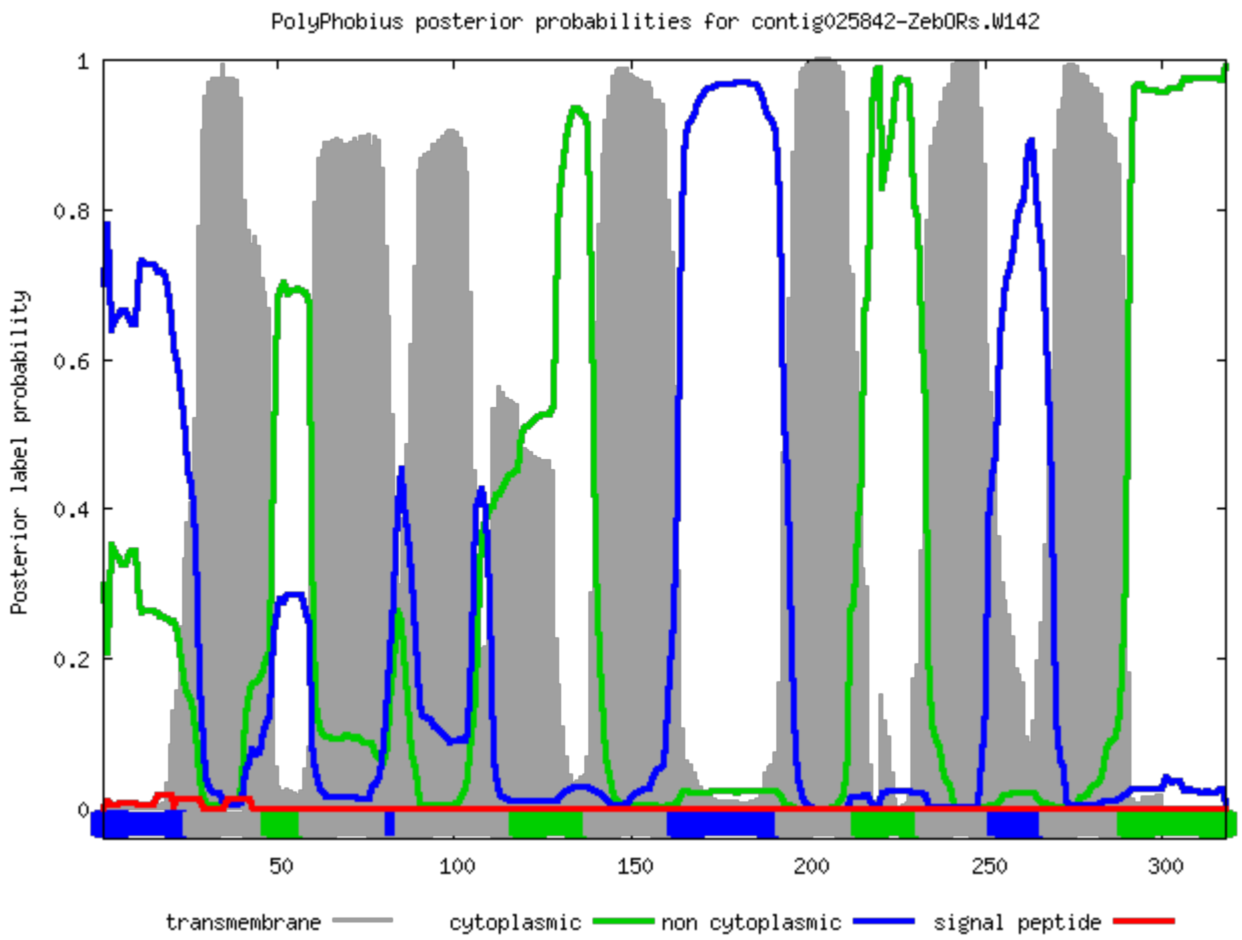

The prediction is based on an alignment. The probability data used in the plot is found here, and the gnuplot script is here.

Prediction of contig046347-TilOR.N190

```
ID   contig046347-TilOR.N190
FT   TOPO_DOM      1     33       NON CYTOPLASMIC.
FT   TRANSMEM     34     59
FT   TOPO_DOM     60     67       CYTOPLASMIC.
FT   TRANSMEM     68     89
FT   TOPO_DOM     90    108       NON CYTOPLASMIC.
FT   TRANSMEM    109    128
FT   TOPO_DOM    129    147       CYTOPLASMIC.
FT   TRANSMEM    148    171
FT   TOPO_DOM    172    207       NON CYTOPLASMIC.
FT   TRANSMEM    208    233
FT   TOPO_DOM    234    252       CYTOPLASMIC.
FT   TRANSMEM    253    275
FT   TOPO_DOM    276    324       NON CYTOPLASMIC.
//
```

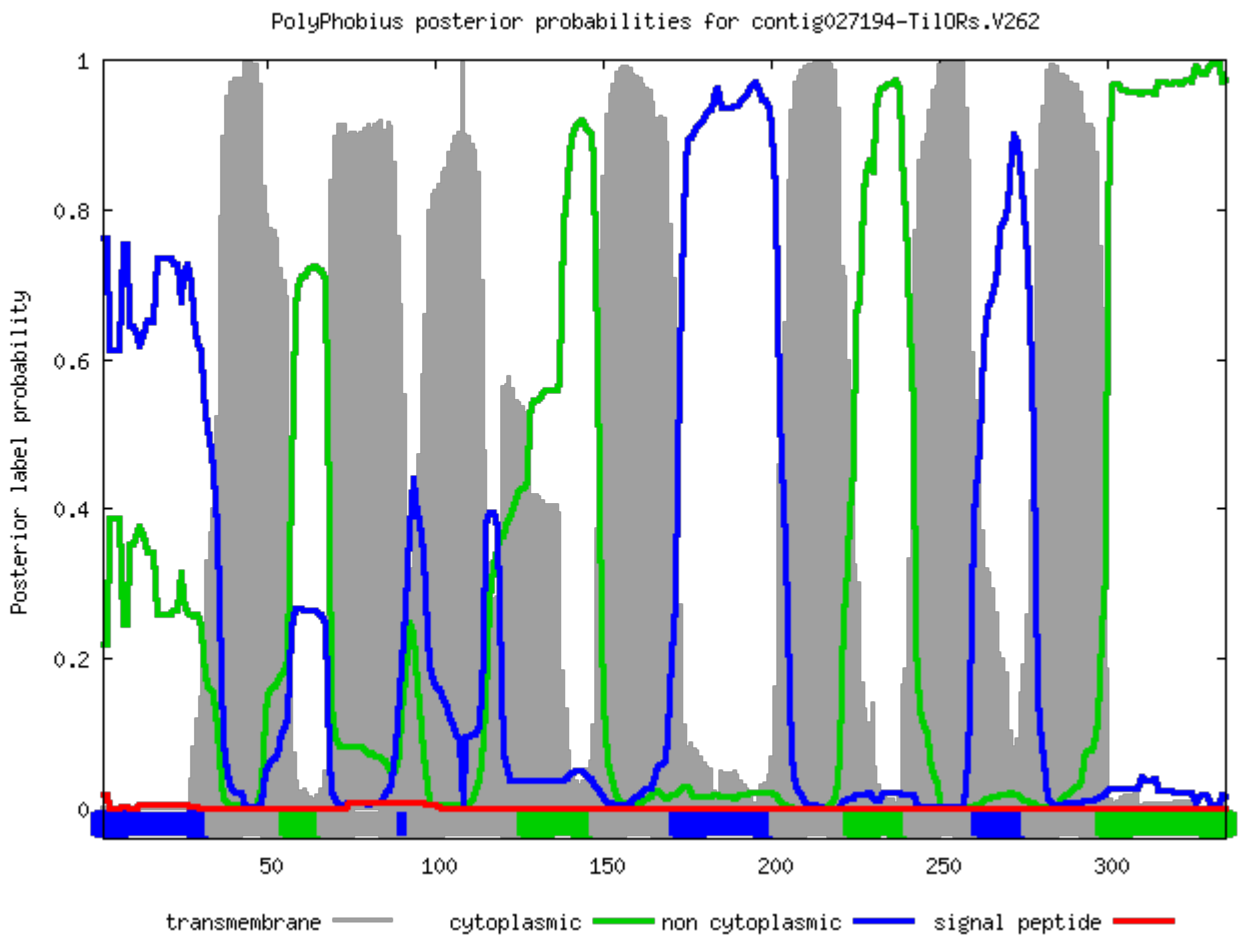

The prediction is based on an alignment. The probability data used in the plot is found here, and the gnuplot script is here.

Prediction of contig039461-TilOR.L147

```
ID   contig039461-TilOR.L147
FT   TOPO_DOM      1      25       NON CYTOPLASMIC.
FT   TRANSMEM     26      50
FT   TOPO_DOM     51      59       CYTOPLASMIC.
FT   TRANSMEM     60      82
FT   TOPO_DOM     83     100       NON CYTOPLASMIC.
FT   TRANSMEM    101     120
FT   TOPO_DOM    121     140       CYTOPLASMIC.
FT   TRANSMEM    141     163
FT   TOPO_DOM    164     199       NON CYTOPLASMIC.
FT   TRANSMEM    200     224
FT   TOPO_DOM    225     237       CYTOPLASMIC.
FT   TRANSMEM    238     260
FT   TOPO_DOM    261     271       NON CYTOPLASMIC.
FT   TRANSMEM    272     291
FT   TOPO_DOM    292     311       CYTOPLASMIC.
//
```

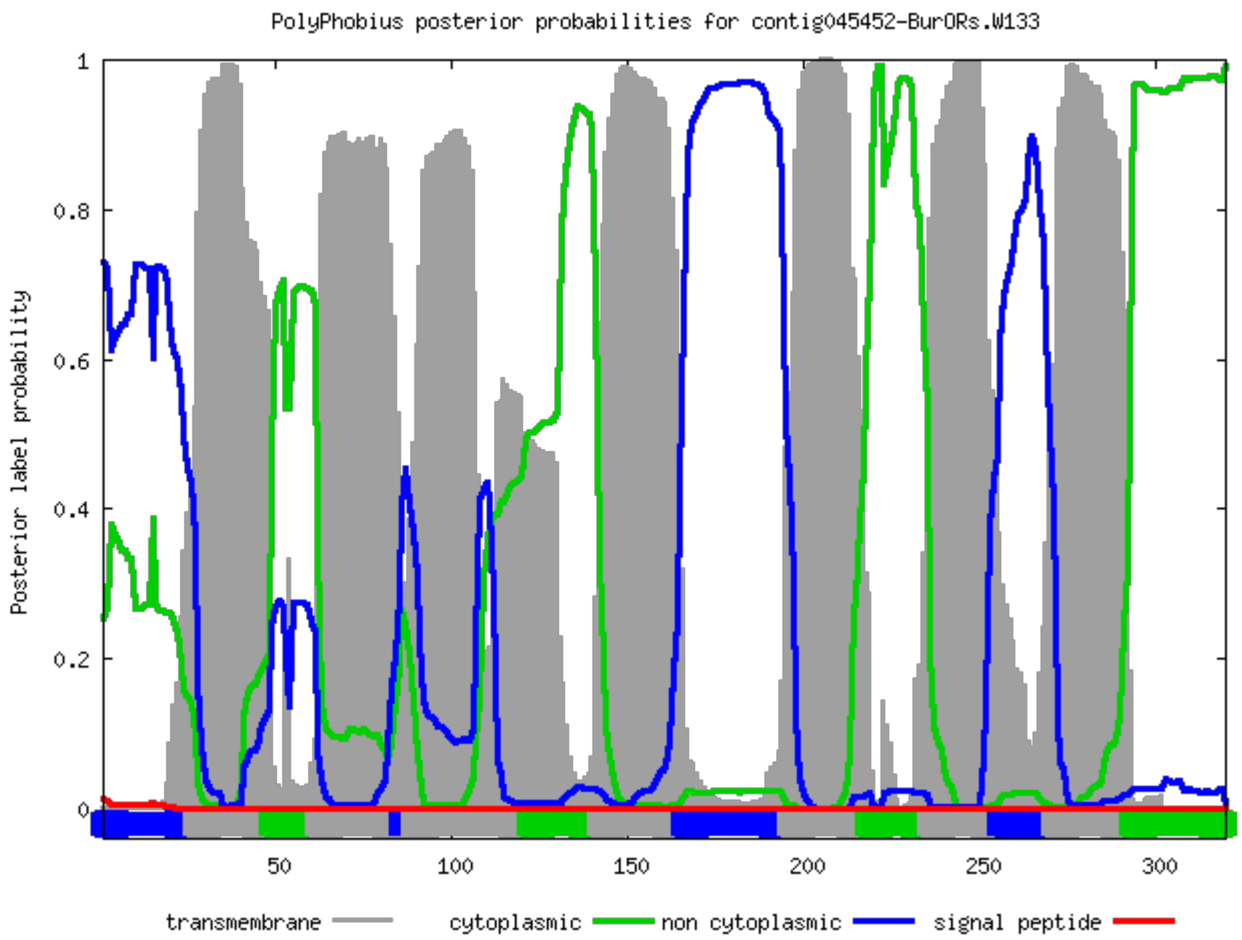

The prediction is based on an alignment. The probability data used in the plot is found here, and the gnuplot script is here.

Prediction of contig055882-BurOR.S124

```
ID   contig055882-BurOR.S124
FT   TOPO_DOM      1     20       NON CYTOPLASMIC.
FT   TRANSMEM     21     42
FT   TOPO_DOM     43     53       CYTOPLASMIC.
FT   TRANSMEM     54     77
FT   TOPO_DOM     78     82       NON CYTOPLASMIC.
FT   TRANSMEM     83    111
FT   TOPO_DOM    112    131       CYTOPLASMIC.
FT   TRANSMEM    132    157
FT   TOPO_DOM    158    185       NON CYTOPLASMIC.
FT   TRANSMEM    186    206
FT   TOPO_DOM    207    226       CYTOPLASMIC.
FT   TRANSMEM    227    252
FT   TOPO_DOM    253    263       NON CYTOPLASMIC.
FT   TRANSMEM    264    284
FT   TOPO_DOM    285    305       CYTOPLASMIC.
//
```

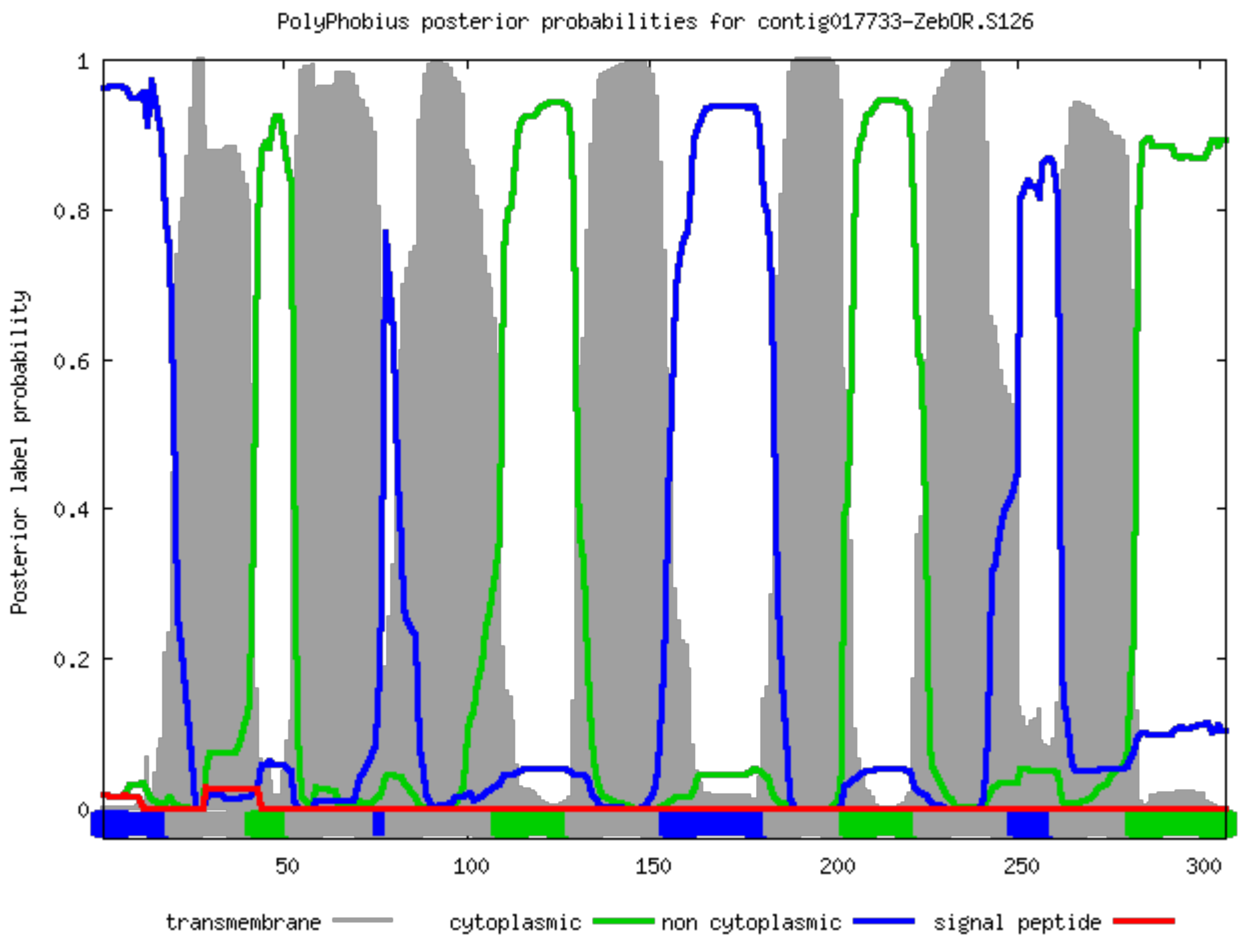

The prediction is based on an alignment. The probability data used in the plot is found here, and the gnuplot script is here.

Prediction of contig013349-TilOR.H103

```
ID   contig013349-TilOR.H103
FT   TOPO_DOM      1     22       NON CYTOPLASMIC.
FT   TRANSMEM     23     48
FT   TOPO_DOM     49     55       CYTOPLASMIC.
FT   TRANSMEM     56     76
FT   TOPO_DOM     77     94       NON CYTOPLASMIC.
FT   TRANSMEM     95    117
FT   TOPO_DOM    118    137       CYTOPLASMIC.
FT   TRANSMEM    138    159
FT   TOPO_DOM    160    192       NON CYTOPLASMIC.
FT   TRANSMEM    193    216
FT   TOPO_DOM    217    234       CYTOPLASMIC.
FT   TRANSMEM    235    256
FT   TOPO_DOM    257    268       NON CYTOPLASMIC.
FT   TRANSMEM    269    288
FT   TOPO_DOM    289    314       CYTOPLASMIC.
//
```

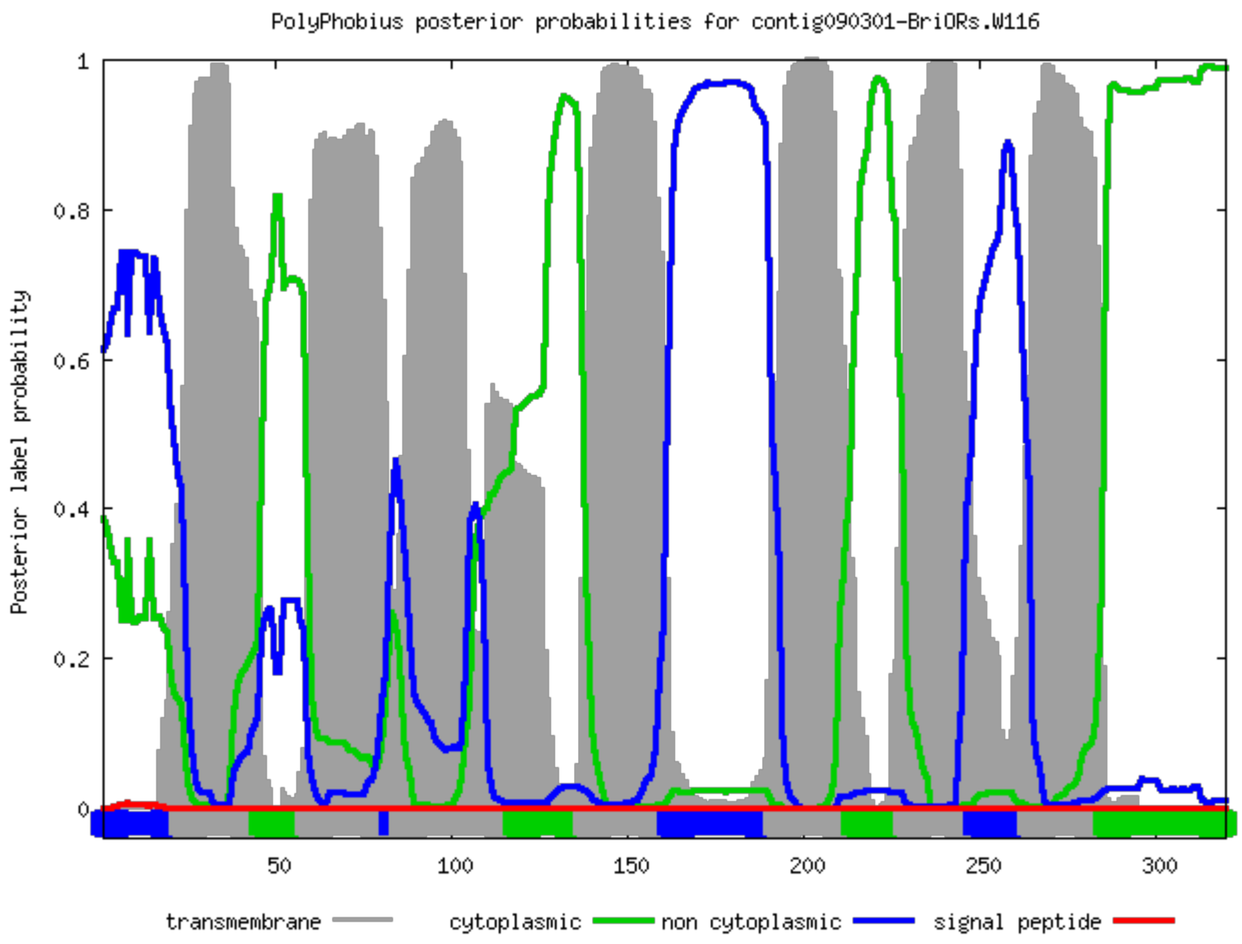

The prediction is based on an alignment. The probability data used in the plot is found here, and the gnuplot script is here.

Prediction of contig046352-TilOR.N194

```
ID   contig046352-TilOR.N194
FT   TOPO_DOM      1     33       NON CYTOPLASMIC.
FT   TRANSMEM     34     59
FT   TOPO_DOM     60     67       CYTOPLASMIC.
FT   TRANSMEM     68     89
FT   TOPO_DOM     90    108       NON CYTOPLASMIC.
FT   TRANSMEM    109    128
FT   TOPO_DOM    129    147       CYTOPLASMIC.
FT   TRANSMEM    148    171
FT   TOPO_DOM    172    208       NON CYTOPLASMIC.
FT   TRANSMEM    209    233
FT   TOPO_DOM    234    252       CYTOPLASMIC.
FT   TRANSMEM    253    275
FT   TOPO_DOM    276    327       NON CYTOPLASMIC.
//
```

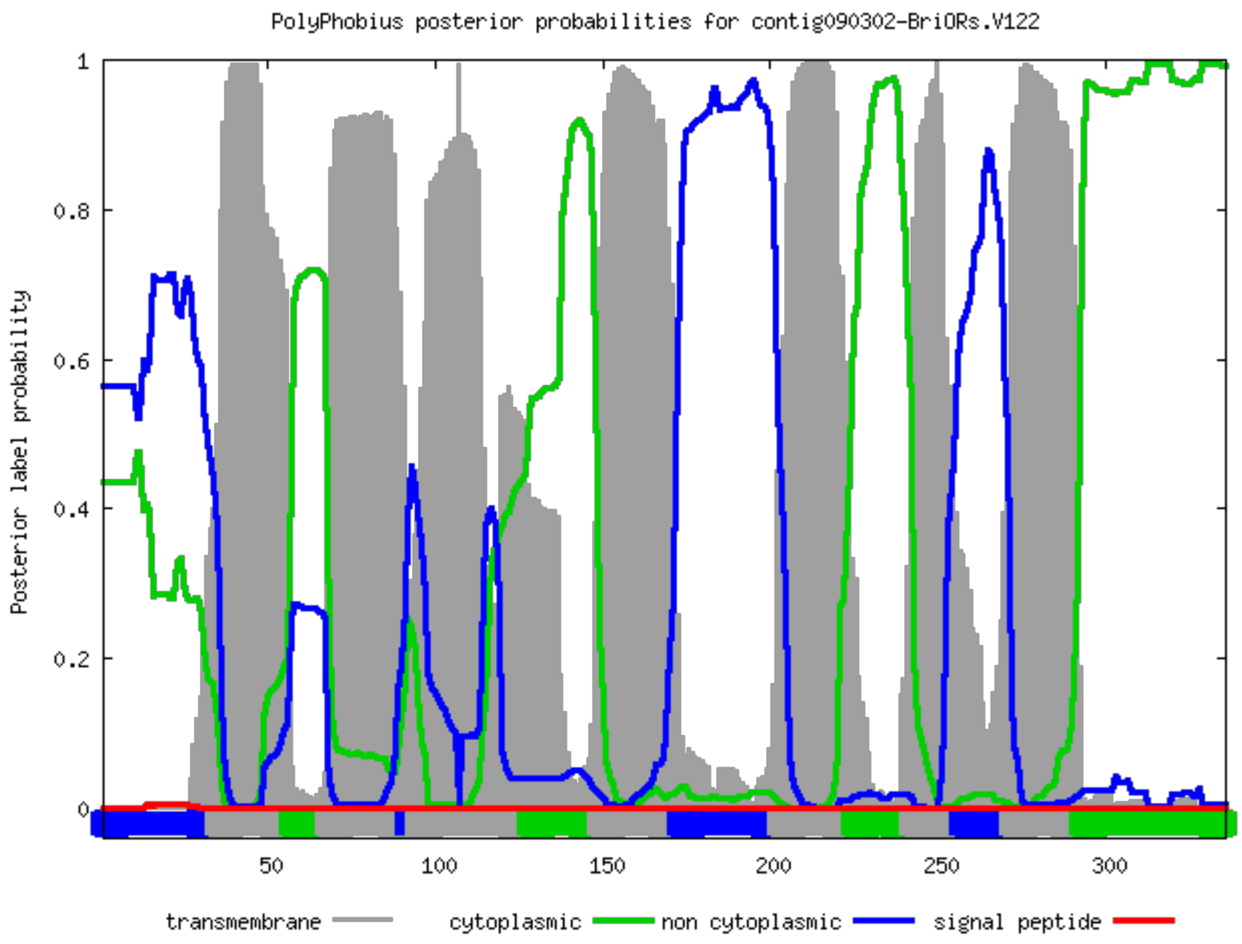

The prediction is based on an alignment. The probability data used in the plot is found here, and the gnuplot script is here.

Prediction of contig022268-TilOR.A019

```
ID   contig022268-TilOR.A019
FT   TOPO_DOM      1      24       NON CYTOPLASMIC.
FT   TRANSMEM     25      50
FT   TOPO_DOM     51      58       CYTOPLASMIC.
FT   TRANSMEM     59      79
FT   TOPO_DOM     80      97       NON CYTOPLASMIC.
FT   TRANSMEM     98     120
FT   TOPO_DOM    121     140       CYTOPLASMIC.
FT   TRANSMEM    141     162
FT   TOPO_DOM    163     194       NON CYTOPLASMIC.
FT   TRANSMEM    195     218
FT   TOPO_DOM    219     238       CYTOPLASMIC.
FT   TRANSMEM    239     260
FT   TOPO_DOM    261     271       NON CYTOPLASMIC.
FT   TRANSMEM    272     292
FT   TOPO_DOM    293     328       CYTOPLASMIC.
//
```

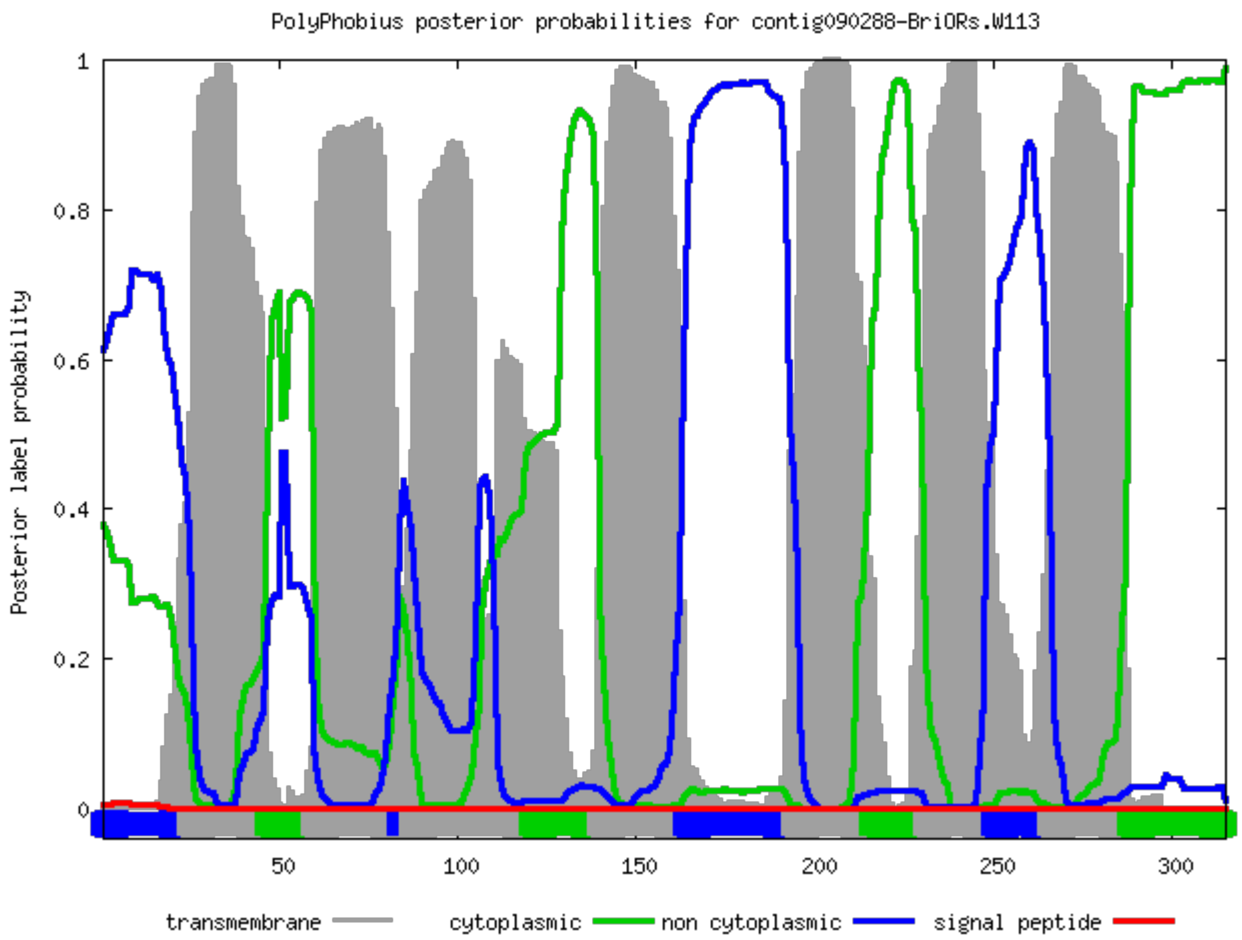

The prediction is based on an alignment. The probability data used in the plot is found here, and the gnuplot script is here.

Prediction of contig047825-TilOR.E079

```
ID   contig047825-TilOR.E079
FT   TOPO_DOM      1     22       NON CYTOPLASMIC.
FT   TRANSMEM     23     48
FT   TOPO_DOM     49     57       CYTOPLASMIC.
FT   TRANSMEM     58     83
FT   TOPO_DOM     84     90       NON CYTOPLASMIC.
FT   TRANSMEM     91    118
FT   TOPO_DOM    119    138       CYTOPLASMIC.
FT   TRANSMEM    139    161
FT   TOPO_DOM    162    193       NON CYTOPLASMIC.
FT   TRANSMEM    194    216
FT   TOPO_DOM    217    236       CYTOPLASMIC.
FT   TRANSMEM    237    256
FT   TOPO_DOM    257    268       NON CYTOPLASMIC.
FT   TRANSMEM    269    291
FT   TOPO_DOM    292    330       CYTOPLASMIC.
//
```

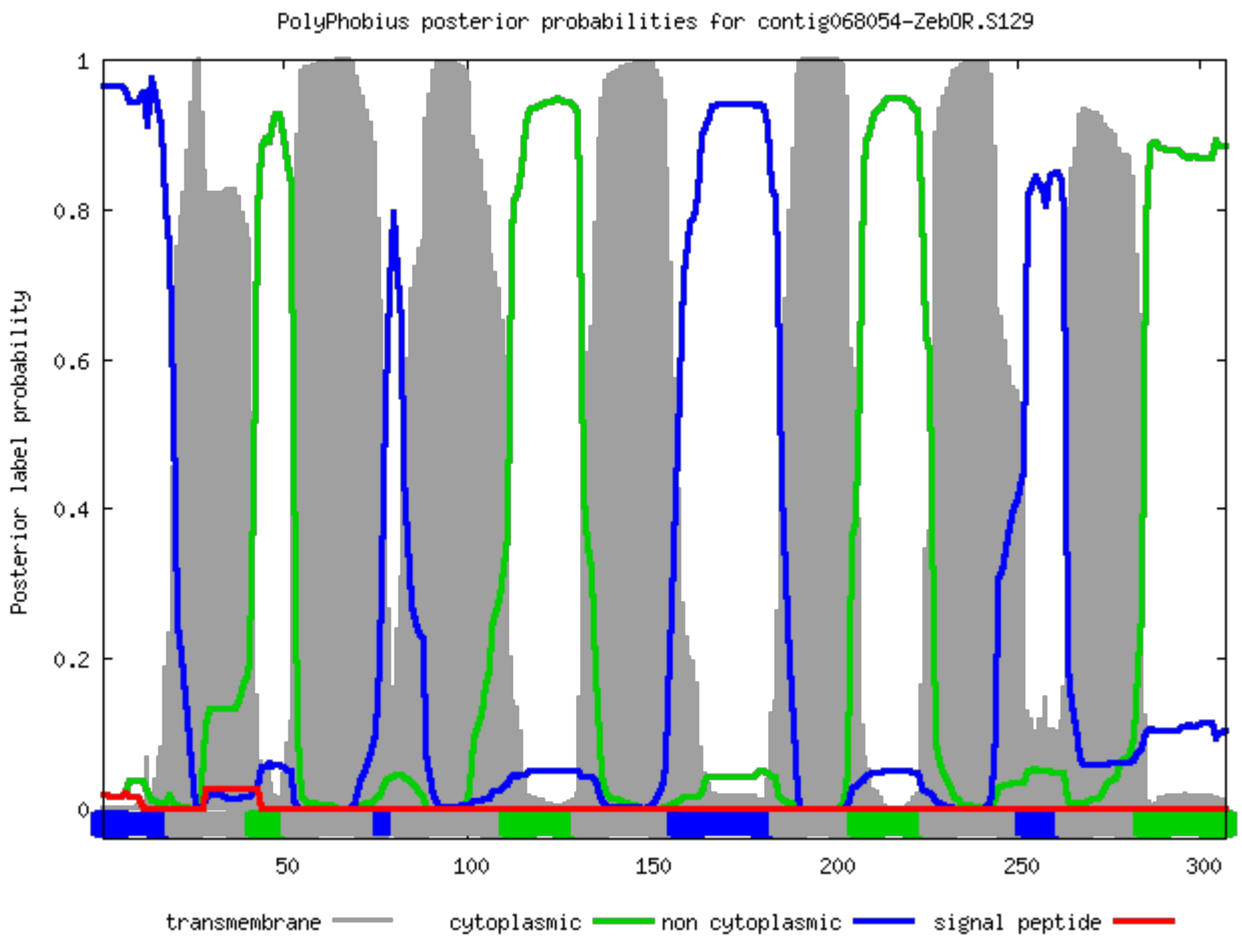

The prediction is based on an alignment. The probability data used in the plot is found here, and the gnuplot script is here.

Prediction of contig013321-TilOR.D048

```
ID   contig013321-TilOR.D048
FT   TOPO_DOM      1     22       NON CYTOPLASMIC.
FT   TRANSMEM     23     48
FT   TOPO_DOM     49     57       CYTOPLASMIC.
FT   TRANSMEM     58     81
FT   TOPO_DOM     82     90       NON CYTOPLASMIC.
FT   TRANSMEM     91    118
FT   TOPO_DOM    119    138       CYTOPLASMIC.
FT   TRANSMEM    139    162
FT   TOPO_DOM    163    193       NON CYTOPLASMIC.
FT   TRANSMEM    194    216
FT   TOPO_DOM    217    235       CYTOPLASMIC.
FT   TRANSMEM    236    256
FT   TOPO_DOM    257    267       NON CYTOPLASMIC.
FT   TRANSMEM    268    291
FT   TOPO_DOM    292    314       CYTOPLASMIC.
//
```

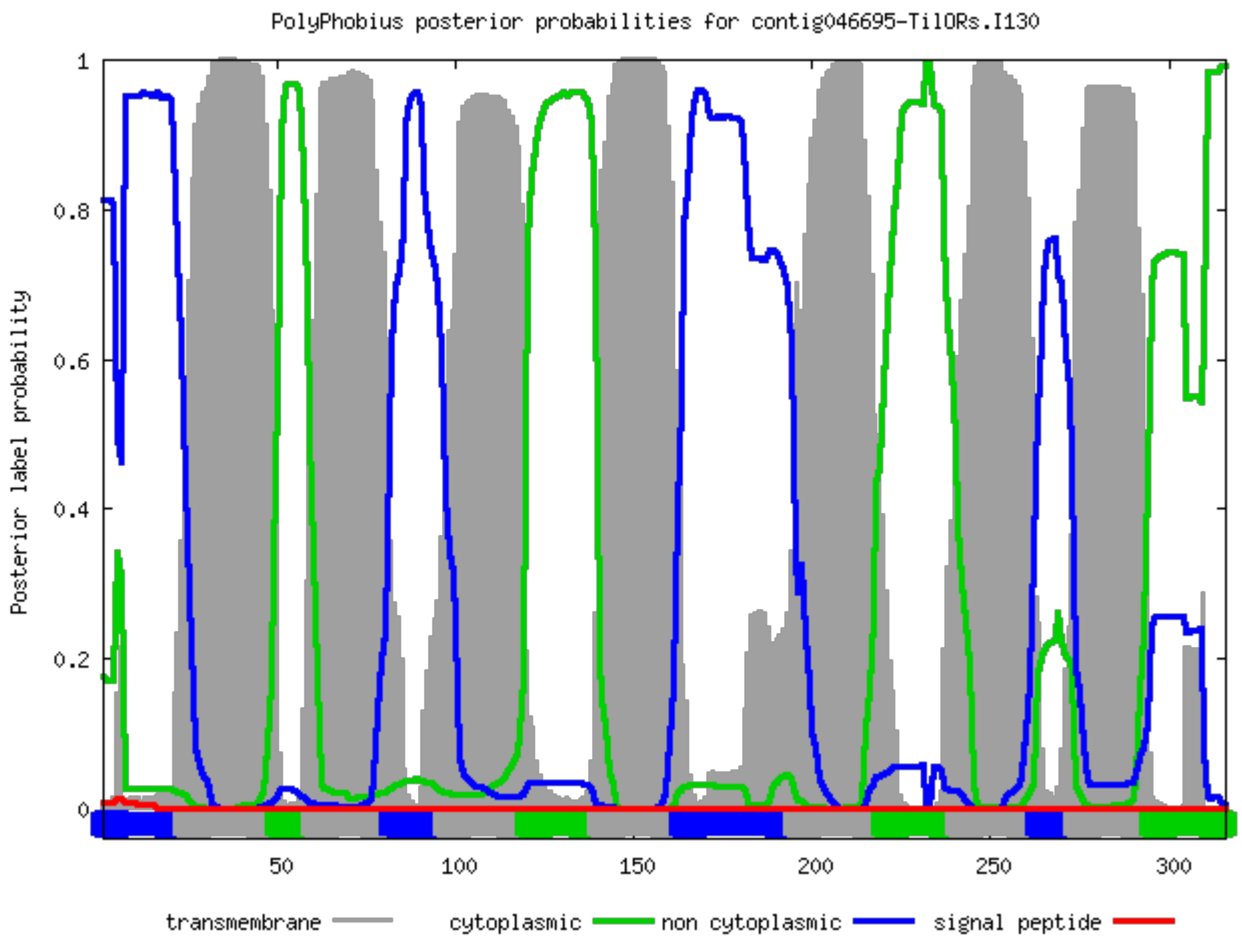

The prediction is based on an alignment. The probability data used in the plot is found here, and the gnuplot script is here.

Prediction of contig028594-BurOR.S122

```
ID   contig028594-BurOR.S122
FT   TOPO_DOM      1     21       NON CYTOPLASMIC.
FT   TRANSMEM     22     43
FT   TOPO_DOM     44     53       CYTOPLASMIC.
FT   TRANSMEM     54     78
FT   TOPO_DOM     79     83       NON CYTOPLASMIC.
FT   TRANSMEM     84    112
FT   TOPO_DOM    113    132       CYTOPLASMIC.
FT   TRANSMEM    133    158
FT   TOPO_DOM    159    186       NON CYTOPLASMIC.
FT   TRANSMEM    187    207
FT   TOPO_DOM    208    227       CYTOPLASMIC.
FT   TRANSMEM    228    253
FT   TOPO_DOM    254    264       NON CYTOPLASMIC.
FT   TRANSMEM    265    285
FT   TOPO_DOM    286    311       CYTOPLASMIC.
//
```

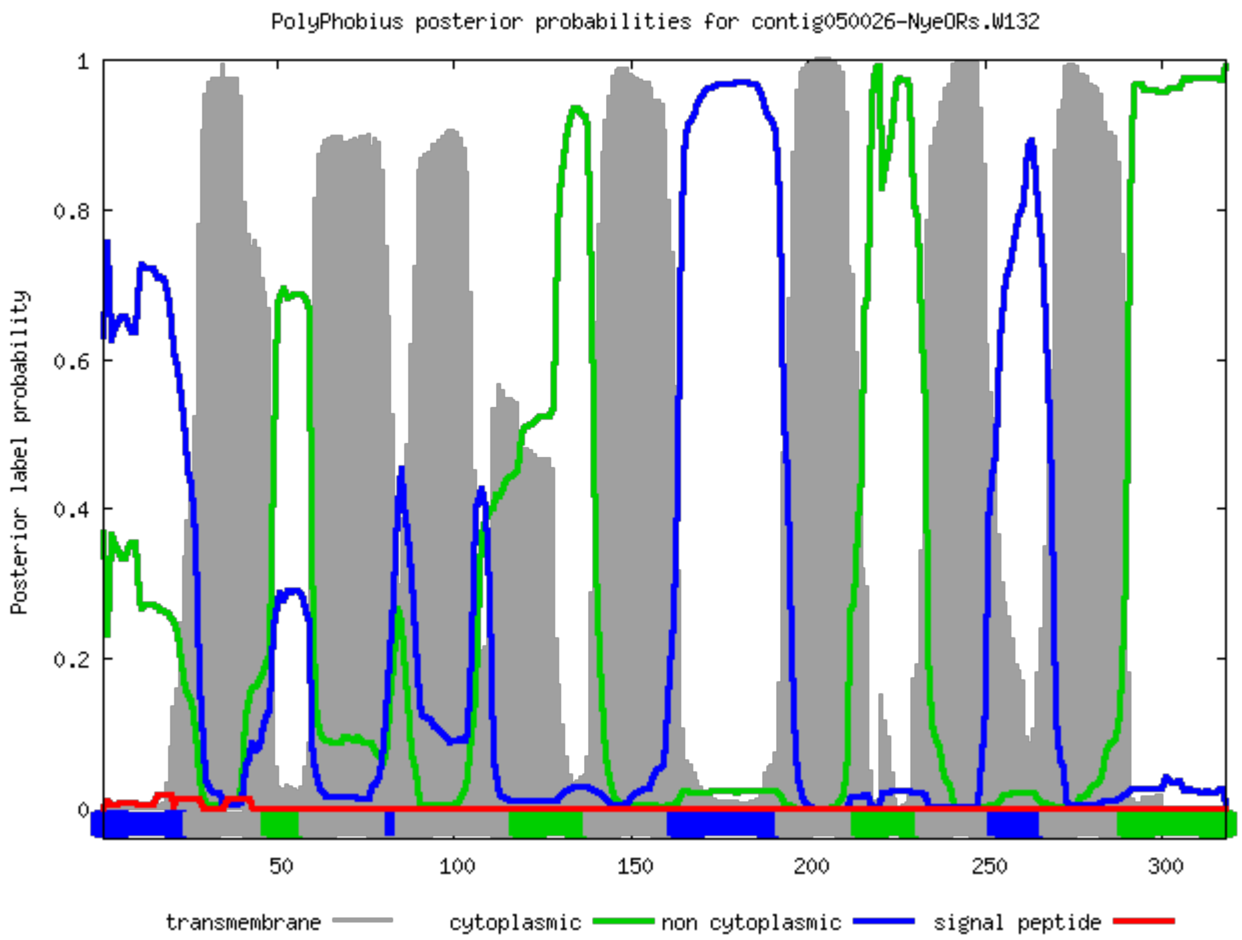

The prediction is based on an alignment. The probability data used in the plot is found here, and the gnuplot script is here.

Prediction of contig009547-TilOR.H101

```
ID   contig009547-TilOR.H101
FT   TOPO_DOM      1     23       NON CYTOPLASMIC.
FT   TRANSMEM     24     49
FT   TOPO_DOM     50     56       CYTOPLASMIC.
FT   TRANSMEM     57     76
FT   TOPO_DOM     77     95       NON CYTOPLASMIC.
FT   TRANSMEM     96    118
FT   TOPO_DOM    119    138       CYTOPLASMIC.
FT   TRANSMEM    139    160
FT   TOPO_DOM    161    196       NON CYTOPLASMIC.
FT   TRANSMEM    197    219
FT   TOPO_DOM    220    237       CYTOPLASMIC.
FT   TRANSMEM    238    260
FT   TOPO_DOM    261    271       NON CYTOPLASMIC.
FT   TRANSMEM    272    291
FT   TOPO_DOM    292    325       CYTOPLASMIC.
//
```

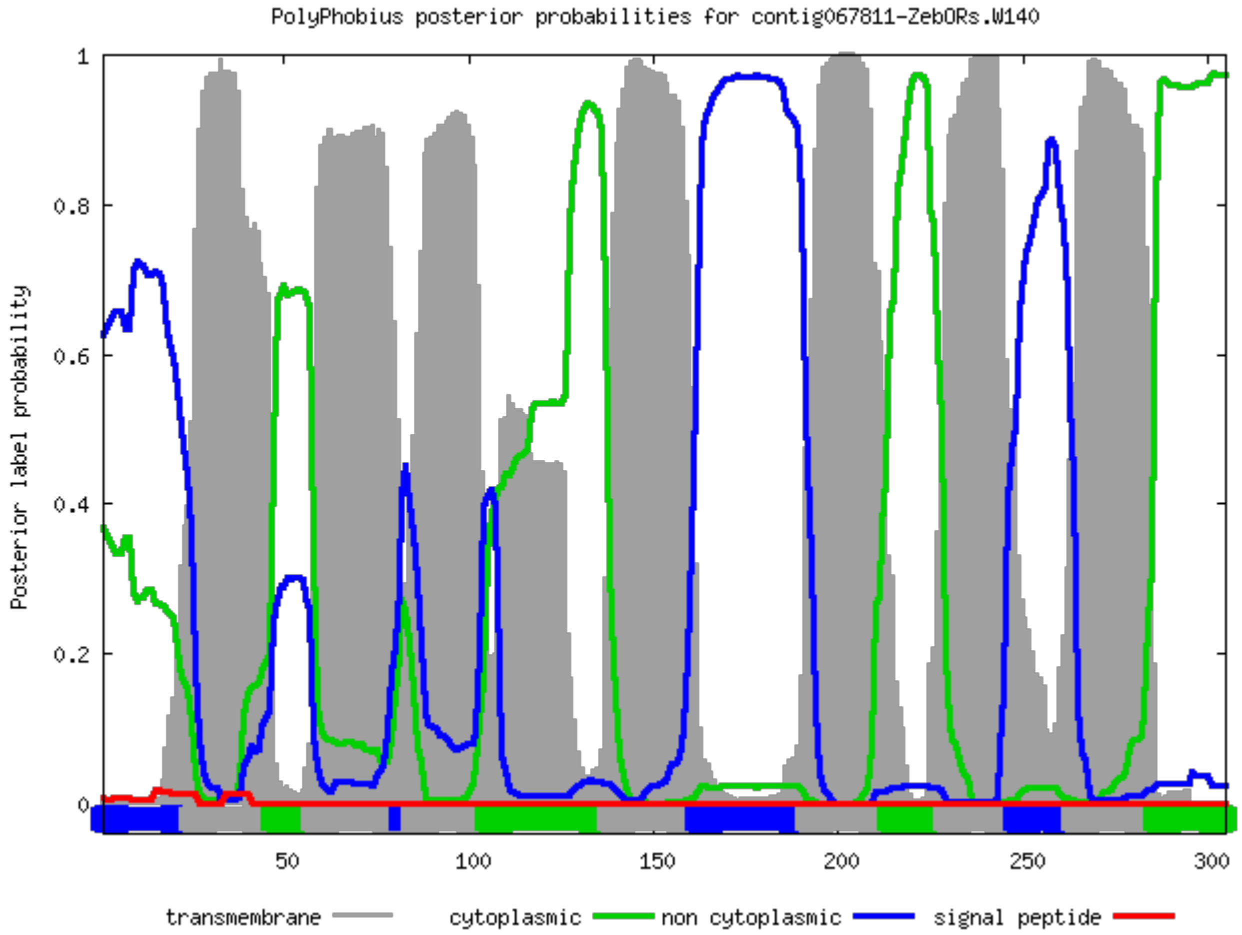

The prediction is based on an alignment. The probability data used in the plot is found here, and the gnuplot script is here.

Prediction of contig013358-TilOR.H106

```
ID   contig013358-TilOR.H106
FT   TOPO_DOM      1     22      NON CYTOPLASMIC.
FT   TRANSMEM     23     48
FT   TOPO_DOM     49     56      CYTOPLASMIC.
FT   TRANSMEM     57     77
FT   TOPO_DOM     78     95      NON CYTOPLASMIC.
FT   TRANSMEM     96    118
FT   TOPO_DOM    119    138      CYTOPLASMIC.
FT   TRANSMEM    139    160
FT   TOPO_DOM    161    193      NON CYTOPLASMIC.
FT   TRANSMEM    194    217
FT   TOPO_DOM    218    235      CYTOPLASMIC.
FT   TRANSMEM    236    258
FT   TOPO_DOM    259    269      NON CYTOPLASMIC.
FT   TRANSMEM    270    289
FT   TOPO_DOM    290    314      CYTOPLASMIC.
//
```

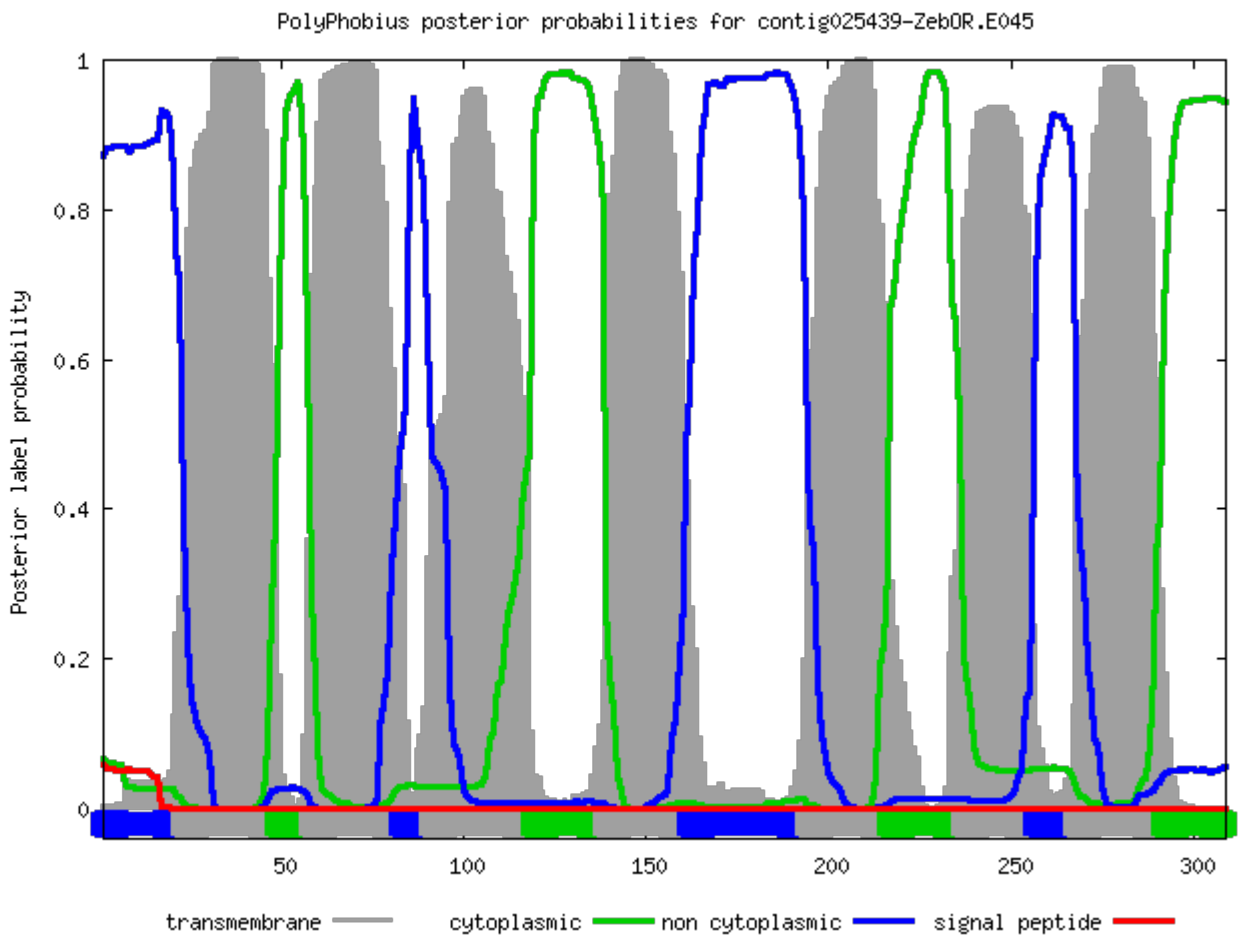

The prediction is based on an alignment. The probability data used in the plot is found here, and the gnuplot script is here.

Prediction of contig014059-ZebOR.H068

```
ID   contig014059-ZebOR.H068
FT   TOPO_DOM      1     22       NON CYTOPLASMIC.
FT   TRANSMEM     23     49
FT   TOPO_DOM     50     56       CYTOPLASMIC.
FT   TRANSMEM     57     77
FT   TOPO_DOM     78     95       NON CYTOPLASMIC.
FT   TRANSMEM     96    118
FT   TOPO_DOM    119    138       CYTOPLASMIC.
FT   TRANSMEM    139    160
FT   TOPO_DOM    161    193       NON CYTOPLASMIC.
FT   TRANSMEM    194    216
FT   TOPO_DOM    217    235       CYTOPLASMIC.
FT   TRANSMEM    236    258
FT   TOPO_DOM    259    269       NON CYTOPLASMIC.
FT   TRANSMEM    270    289
FT   TOPO_DOM    290    314       CYTOPLASMIC.
//
```

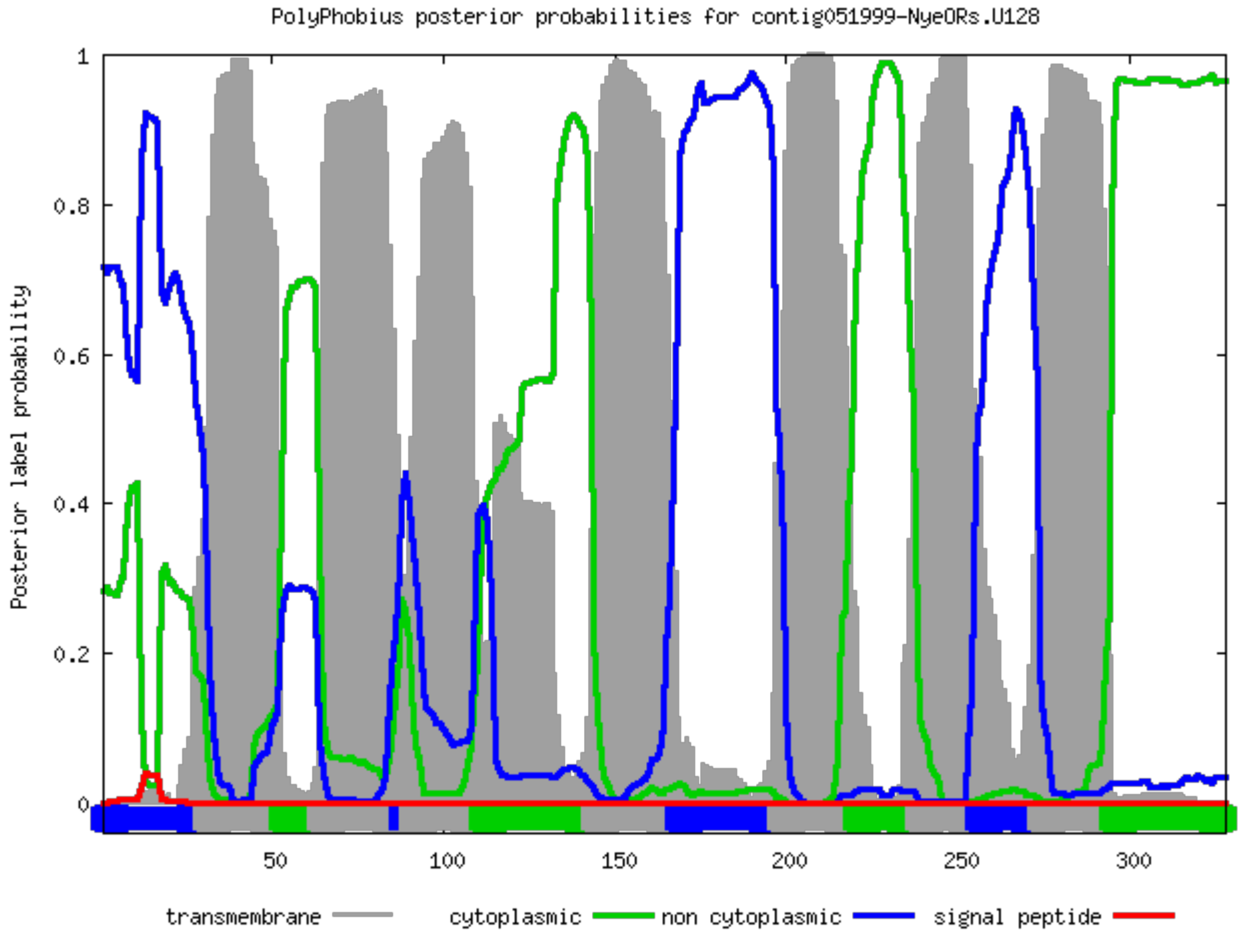

The prediction is based on an alignment. The probability data used in the plot is found here, and the gnuplot script is here.

Prediction of contig053572-NyeOR.E051

```
ID   contig053572-NyeOR.E051
FT   TOPO_DOM      1     22       NON CYTOPLASMIC.
FT   TRANSMEM     23     48
FT   TOPO_DOM     49     57       CYTOPLASMIC.
FT   TRANSMEM     58     83
FT   TOPO_DOM     84     90       NON CYTOPLASMIC.
FT   TRANSMEM     91    118
FT   TOPO_DOM    119    138       CYTOPLASMIC.
FT   TRANSMEM    139    161
FT   TOPO_DOM    162    193       NON CYTOPLASMIC.
FT   TRANSMEM    194    216
FT   TOPO_DOM    217    236       CYTOPLASMIC.
FT   TRANSMEM    237    256
FT   TOPO_DOM    257    268       NON CYTOPLASMIC.
FT   TRANSMEM    269    291
FT   TOPO_DOM    292    330       CYTOPLASMIC.
//
```

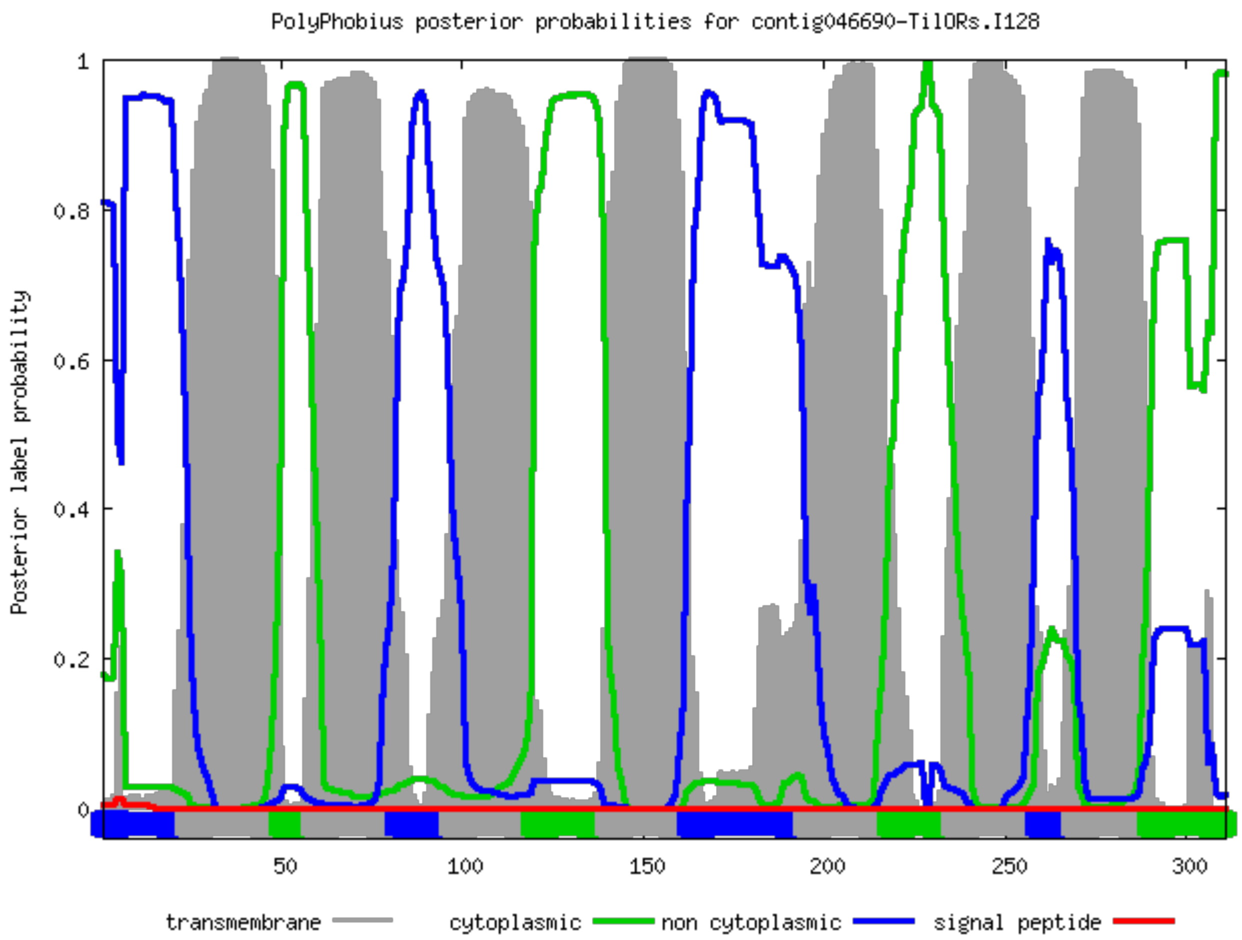

The prediction is based on an alignment. The probability data used in the plot is found here, and the gnuplot script is here.

Prediction of contig005005-TilOR.S219

```
ID   contig005005-TilOR.S219
FT   TOPO_DOM      1     20       NON CYTOPLASMIC.
FT   TRANSMEM     21     42
FT   TOPO_DOM     43     53       CYTOPLASMIC.
FT   TRANSMEM     54     77
FT   TOPO_DOM     78     82       NON CYTOPLASMIC.
FT   TRANSMEM     83    111
FT   TOPO_DOM    112    131       CYTOPLASMIC.
FT   TRANSMEM    132    157
FT   TOPO_DOM    158    185       NON CYTOPLASMIC.
FT   TRANSMEM    186    206
FT   TOPO_DOM    207    226       CYTOPLASMIC.
FT   TRANSMEM    227    252
FT   TOPO_DOM    253    263       NON CYTOPLASMIC.
FT   TRANSMEM    264    284
FT   TOPO_DOM    285    305       CYTOPLASMIC.
//
```

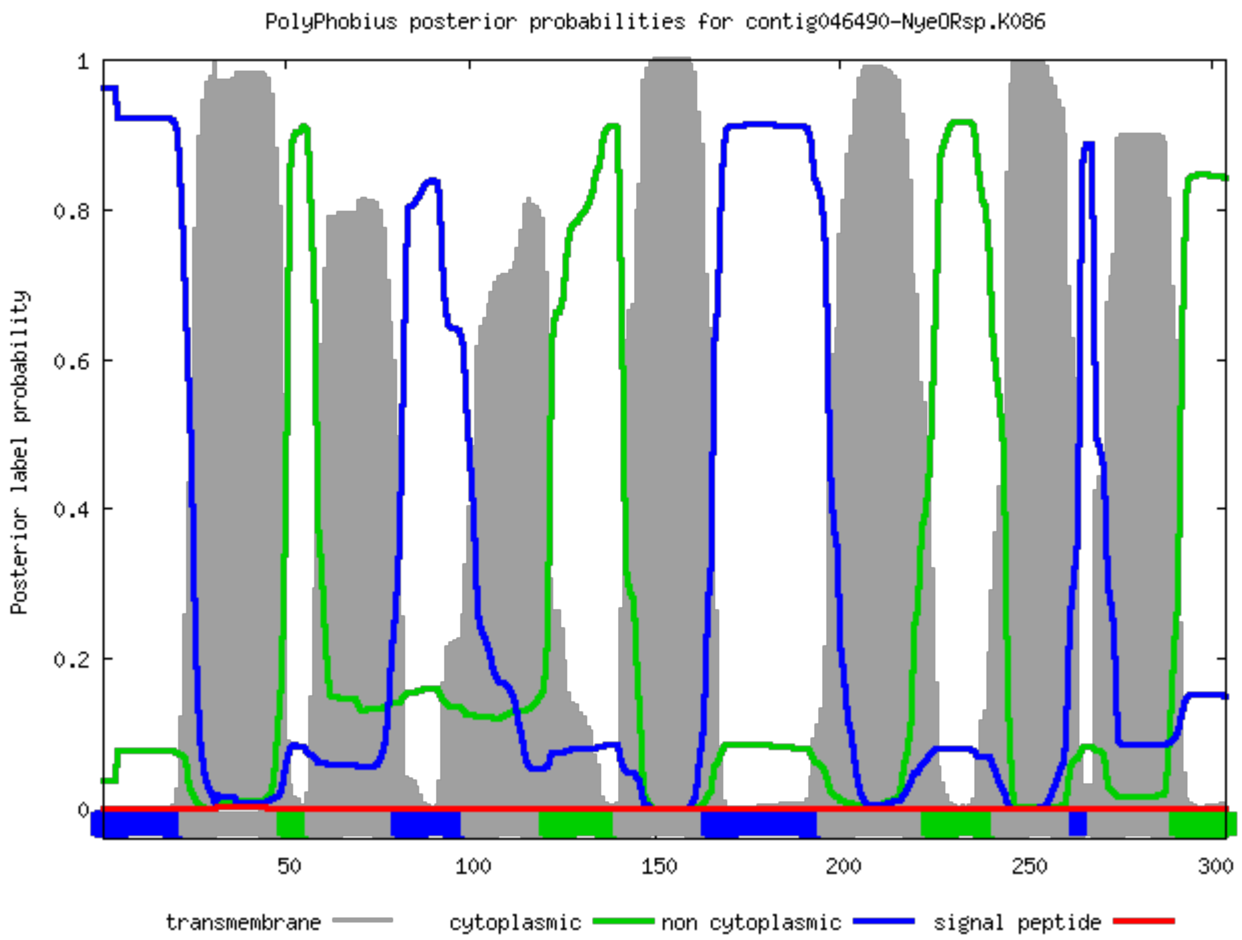

The prediction is based on an alignment. The probability data used in the plot is found here, and the gnuplot script is here.

Prediction of contig056386-BurOR.L088

```
ID   contig056386-BurOR.L088
FT   TOPO_DOM      1     25       NON CYTOPLASMIC.
FT   TRANSMEM     26     50
FT   TOPO_DOM     51     59       CYTOPLASMIC.
FT   TRANSMEM     60     85
FT   TOPO_DOM     86     97       NON CYTOPLASMIC.
FT   TRANSMEM     98    120
FT   TOPO_DOM    121    140       CYTOPLASMIC.
FT   TRANSMEM    141    163
FT   TOPO_DOM    164    198       NON CYTOPLASMIC.
FT   TRANSMEM    199    223
FT   TOPO_DOM    224    238       CYTOPLASMIC.
FT   TRANSMEM    239    260
FT   TOPO_DOM    261    271       NON CYTOPLASMIC.
FT   TRANSMEM    272    292
FT   TOPO_DOM    293    313       CYTOPLASMIC.
//
```

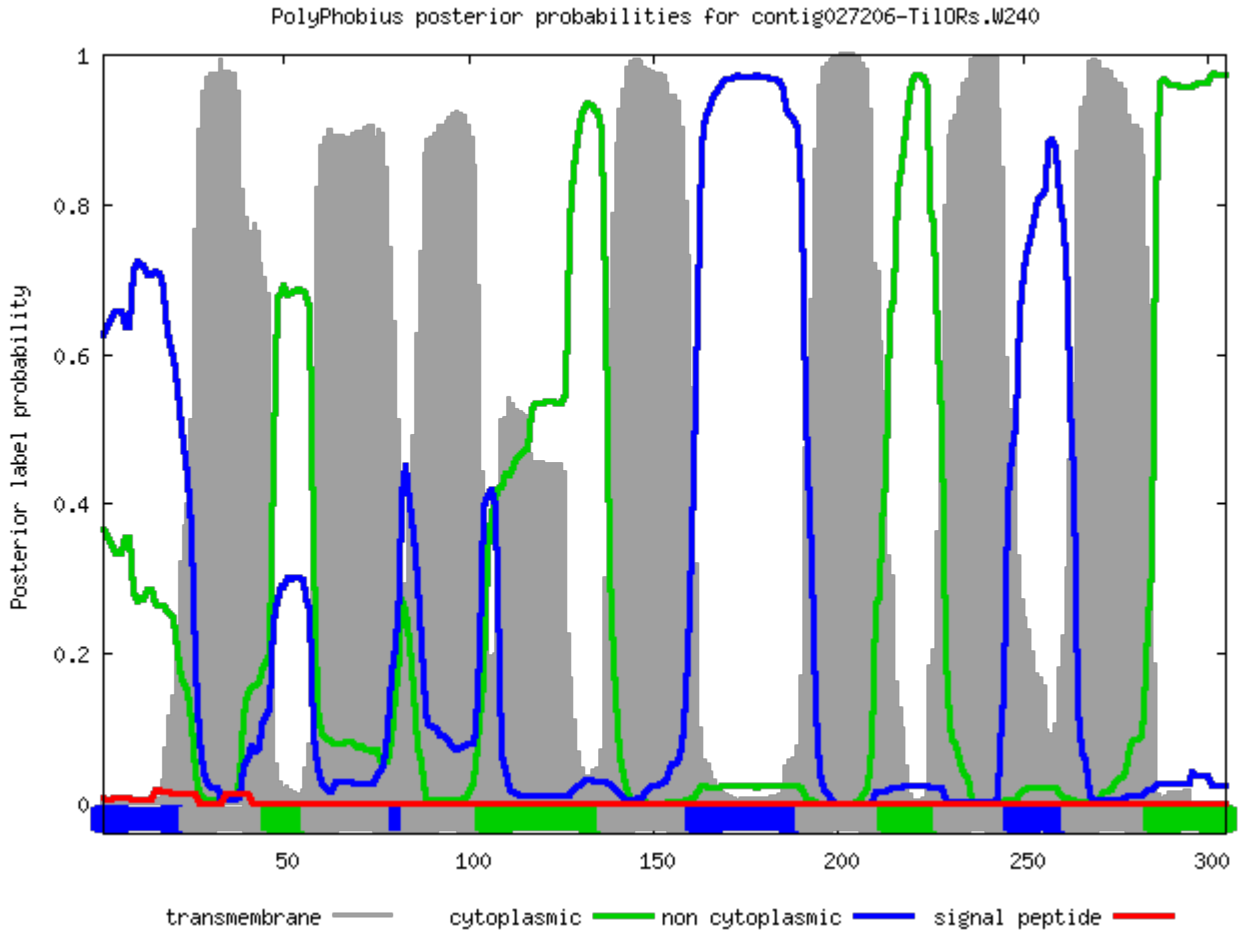

The prediction is based on an alignment. The probability data used in the plot is found here, and the gnuplot script is here.

Prediction of contig009546-TilOR.H100

```
ID    contig009546-TilOR.H100
FT    TOPO_DOM      1     23       NON CYTOPLASMIC.
FT    TRANSMEM     24     49
FT    TOPO_DOM     50     56       CYTOPLASMIC.
FT    TRANSMEM     57     76
FT    TOPO_DOM     77     95       NON CYTOPLASMIC.
FT    TRANSMEM     96    118
FT    TOPO_DOM    119    138       CYTOPLASMIC.
FT    TRANSMEM    139    160
FT    TOPO_DOM    161    196       NON CYTOPLASMIC.
FT    TRANSMEM    197    219
FT    TOPO_DOM    220    237       CYTOPLASMIC.
FT    TRANSMEM    238    259
FT    TOPO_DOM    260    271       NON CYTOPLASMIC.
FT    TRANSMEM    272    291
FT    TOPO_DOM    292    315       CYTOPLASMIC.
//
```

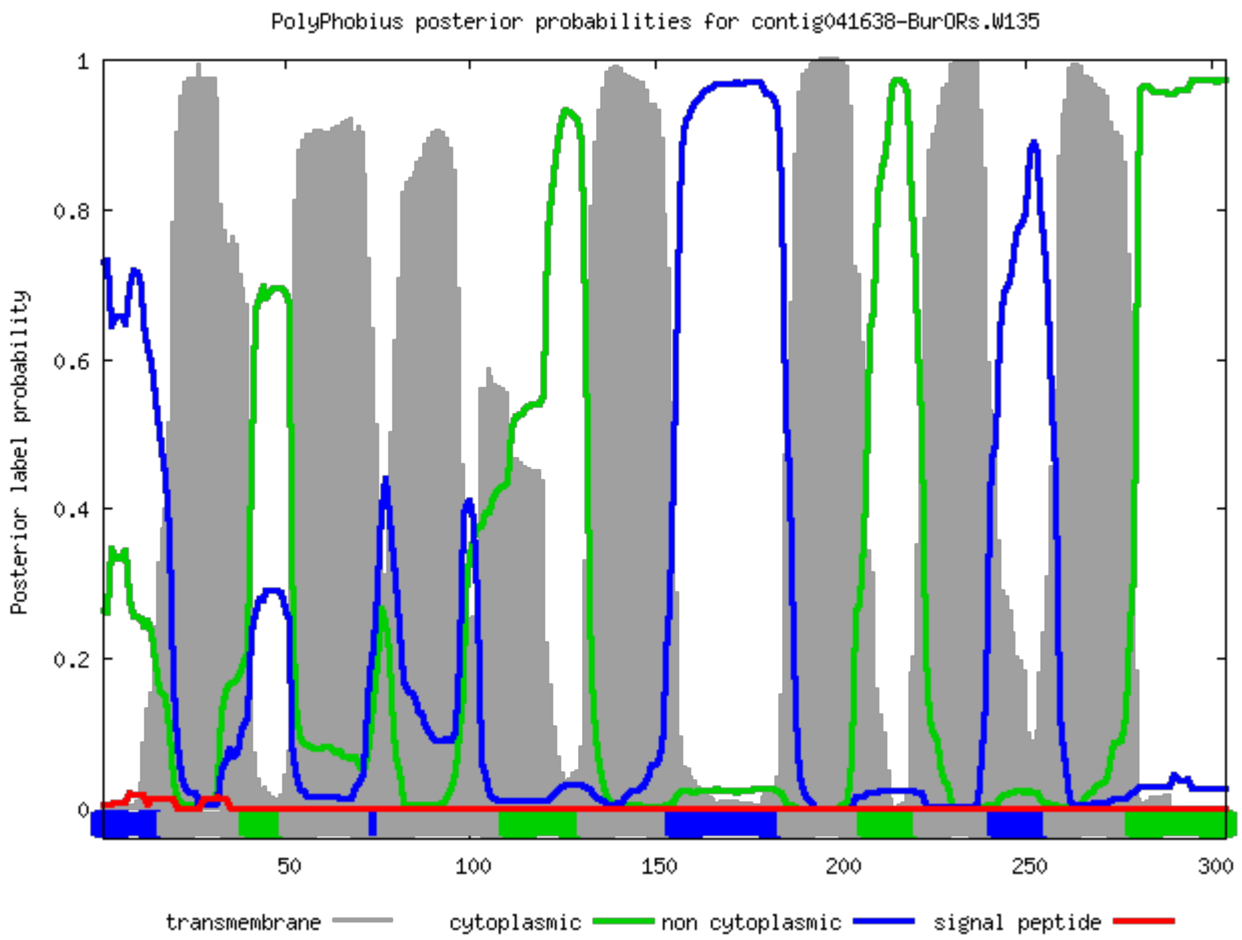

The prediction is based on an alignment. The probability data used in the plot is found here, and the gnuplot script is here.

Prediction of contig034983-NyeOR.A003

```
ID   contig034983-NyeOR.A003
FT   TOPO_DOM      1      22       NON CYTOPLASMIC.
FT   TRANSMEM     23      48
FT   TOPO_DOM     49      56       CYTOPLASMIC.
FT   TRANSMEM     57      76
FT   TOPO_DOM     77      95       NON CYTOPLASMIC.
FT   TRANSMEM     96     118
FT   TOPO_DOM    119     138       CYTOPLASMIC.
FT   TRANSMEM    139     159
FT   TOPO_DOM    160     192       NON CYTOPLASMIC.
FT   TRANSMEM    193     215
FT   TOPO_DOM    216     235       CYTOPLASMIC.
FT   TRANSMEM    236     257
FT   TOPO_DOM    258     268       NON CYTOPLASMIC.
FT   TRANSMEM    269     289
FT   TOPO_DOM    290     316       CYTOPLASMIC.
//
```

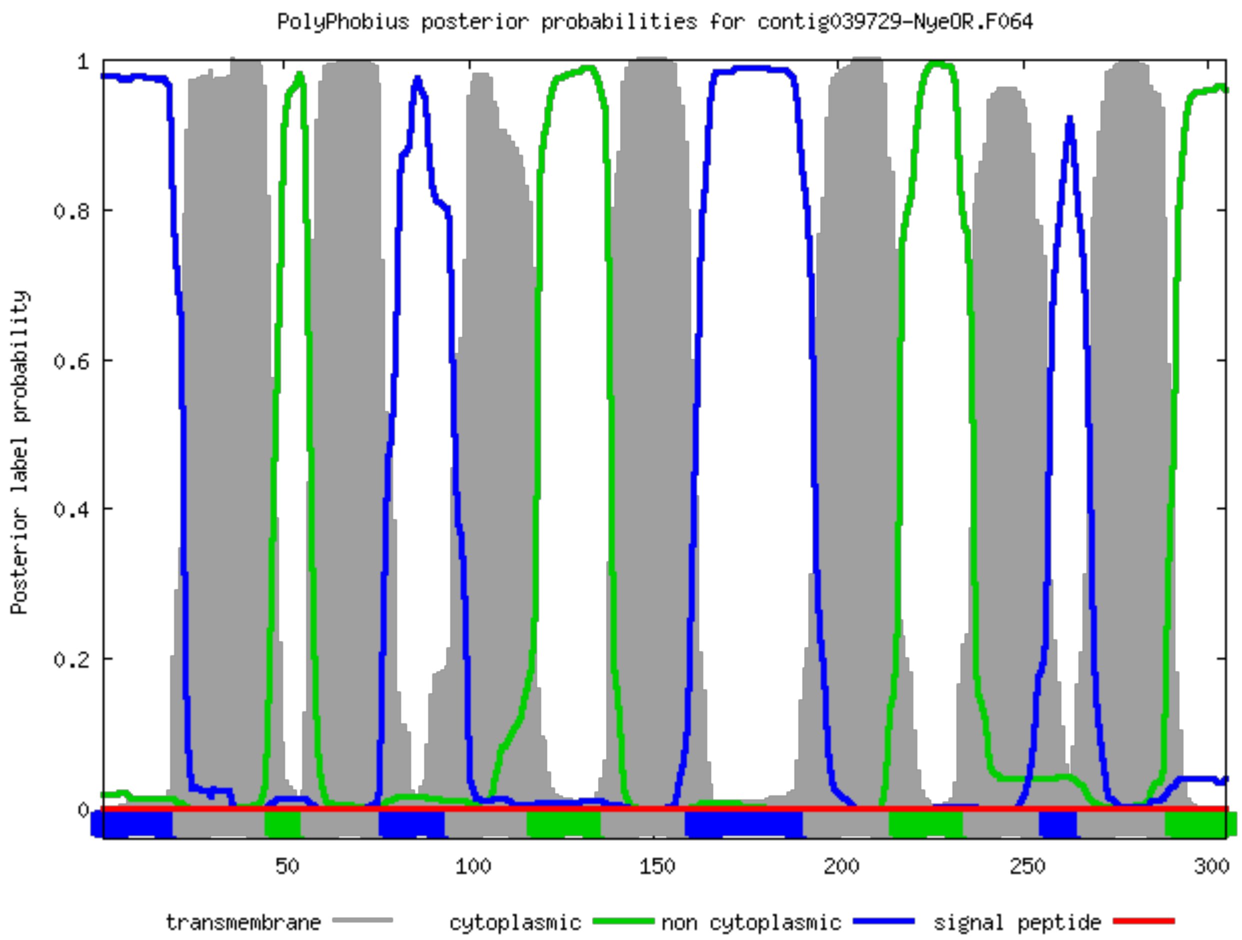

The prediction is based on an alignment. The probability data used in the plot is found here, and the gnuplot script is here.

Prediction of contig017778-ZebOR.K086

```
ID   contig017778-ZebOR.K086
FT   TOPO_DOM      1     25       NON CYTOPLASMIC.
FT   TRANSMEM     26     50
FT   TOPO_DOM     51     59       CYTOPLASMIC.
FT   TRANSMEM     60     82
FT   TOPO_DOM     83     99       NON CYTOPLASMIC.
FT   TRANSMEM    100    120
FT   TOPO_DOM    121    140       CYTOPLASMIC.
FT   TRANSMEM    141    164
FT   TOPO_DOM    165    196       NON CYTOPLASMIC.
FT   TRANSMEM    197    222
FT   TOPO_DOM    223    241       CYTOPLASMIC.
FT   TRANSMEM    242    261
FT   TOPO_DOM    262    271       NON CYTOPLASMIC.
FT   TRANSMEM    272    291
FT   TOPO_DOM    292    312       CYTOPLASMIC.
//
```

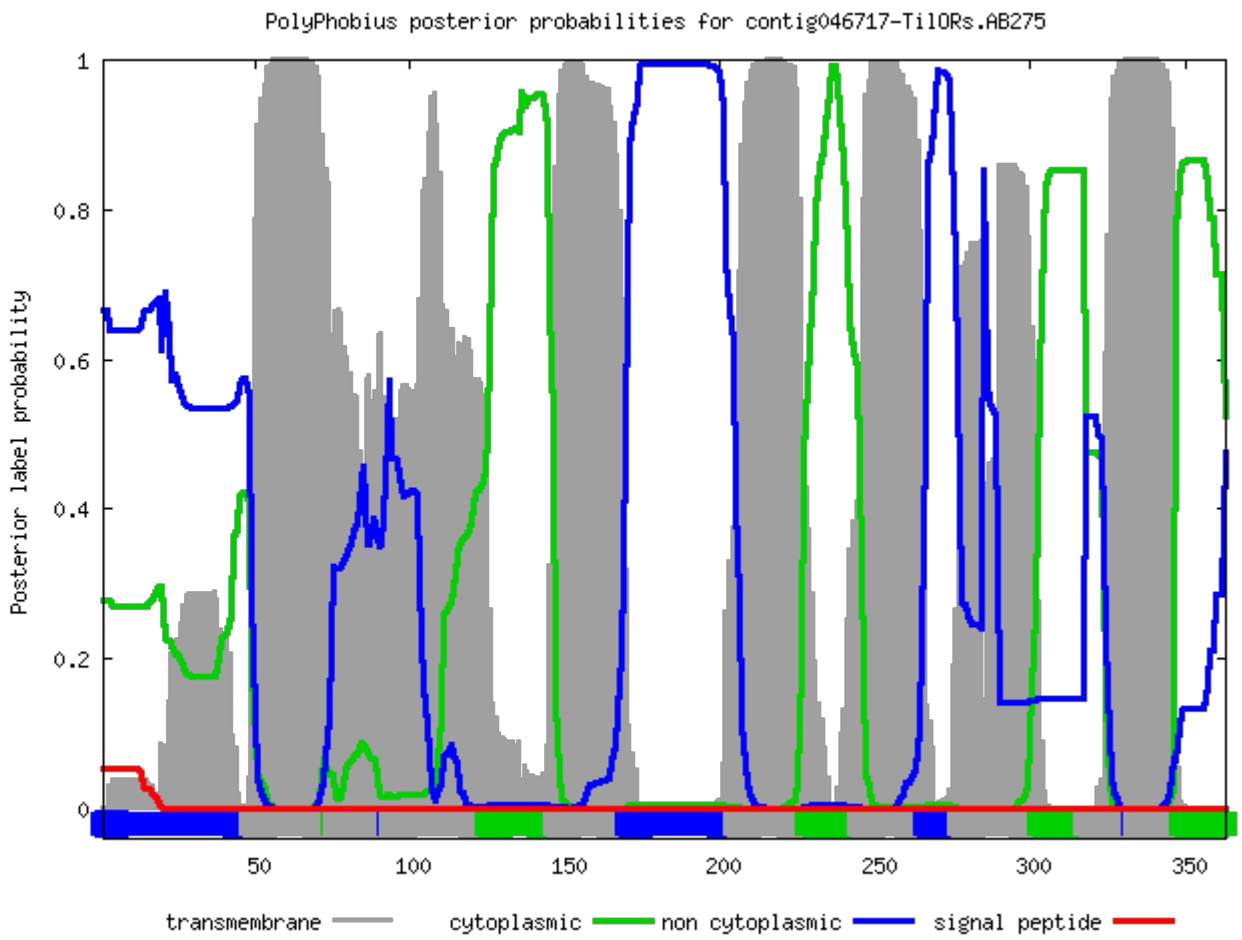

The prediction is based on an alignment. The probability data used in the plot is found here, and the gnuplot script is here.

Prediction of contig052452-BurOR.E048

```
ID    contig052452-BurOR.E048
FT    TOPO_DOM      1     22        NON CYTOPLASMIC.
FT    TRANSMEM     23     48
FT    TOPO_DOM     49     57        CYTOPLASMIC.
FT    TRANSMEM     58     81
FT    TOPO_DOM     82     91        NON CYTOPLASMIC.
FT    TRANSMEM     92    118
FT    TOPO_DOM    119    138        CYTOPLASMIC.
FT    TRANSMEM    139    160
FT    TOPO_DOM    161    193        NON CYTOPLASMIC.
FT    TRANSMEM    194    215
FT    TOPO_DOM    216    235        CYTOPLASMIC.
FT    TRANSMEM    236    255
FT    TOPO_DOM    256    266        NON CYTOPLASMIC.
FT    TRANSMEM    267    290
FT    TOPO_DOM    291    320        CYTOPLASMIC.
//
```

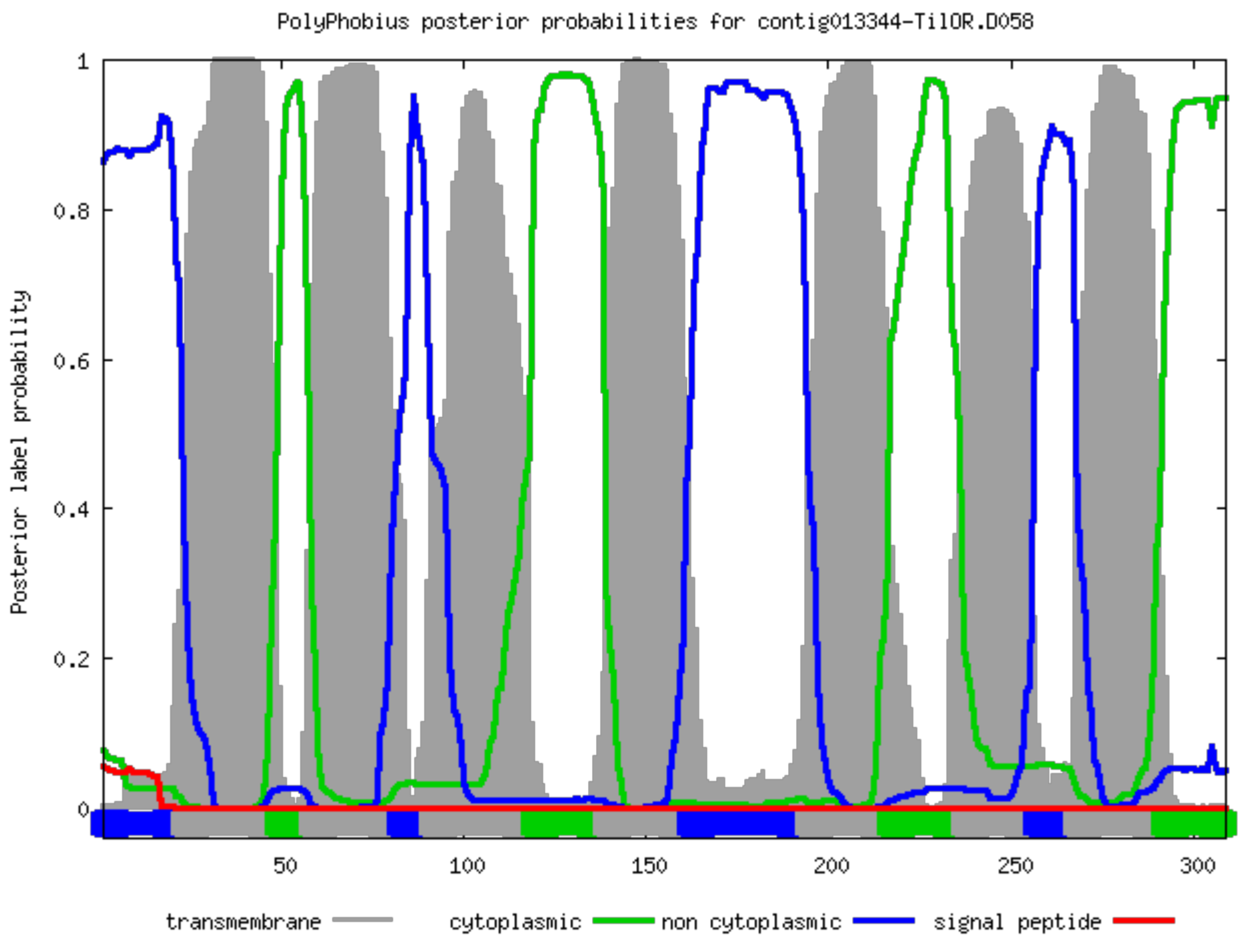

The prediction is based on an alignment. The probability data used in the plot is found here, and the gnuplot script is here.

Prediction of contig022204-TilOR.A001

```
ID   contig022204-TilOR.A001
FT   TOPO_DOM      1      22       NON CYTOPLASMIC.
FT   TRANSMEM     23      48
FT   TOPO_DOM     49      56       CYTOPLASMIC.
FT   TRANSMEM     57      77
FT   TOPO_DOM     78      95       NON CYTOPLASMIC.
FT   TRANSMEM     96     118
FT   TOPO_DOM    119     138       CYTOPLASMIC.
FT   TRANSMEM    139     159
FT   TOPO_DOM    160     192       NON CYTOPLASMIC.
FT   TRANSMEM    193     215
FT   TOPO_DOM    216     235       CYTOPLASMIC.
FT   TRANSMEM    236     257
FT   TOPO_DOM    258     268       NON CYTOPLASMIC.
FT   TRANSMEM    269     289
FT   TOPO_DOM    290     308       CYTOPLASMIC.
//
```

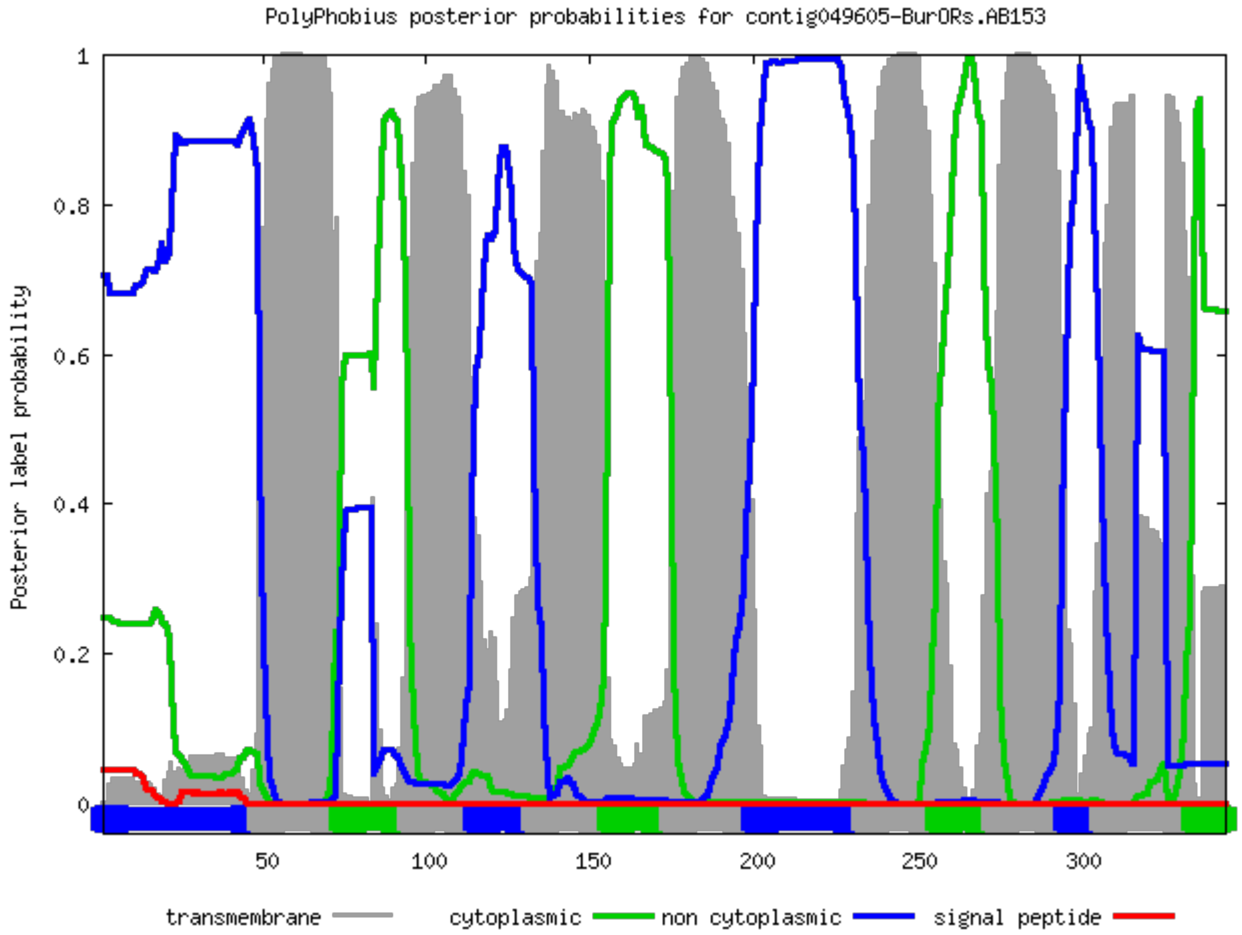

The prediction is based on an alignment. The probability data used in the plot is found here, and the gnuplot script is here.

Prediction of contig039737-NyeOR.D039

```
ID    contig039737-NyeOR.D039
FT    TOPO_DOM       1      22       NON CYTOPLASMIC.
FT    TRANSMEM      23      48
FT    TOPO_DOM      49      57       CYTOPLASMIC.
FT    TRANSMEM      58      81
FT    TOPO_DOM      82      90       NON CYTOPLASMIC.
FT    TRANSMEM      91     118
FT    TOPO_DOM     119     138       CYTOPLASMIC.
FT    TRANSMEM     139     162
FT    TOPO_DOM     163     193       NON CYTOPLASMIC.
FT    TRANSMEM     194     216
FT    TOPO_DOM     217     235       CYTOPLASMIC.
FT    TRANSMEM     236     256
FT    TOPO_DOM     257     267       NON CYTOPLASMIC.
FT    TRANSMEM     268     291
FT    TOPO_DOM     292     309       CYTOPLASMIC.
//
```

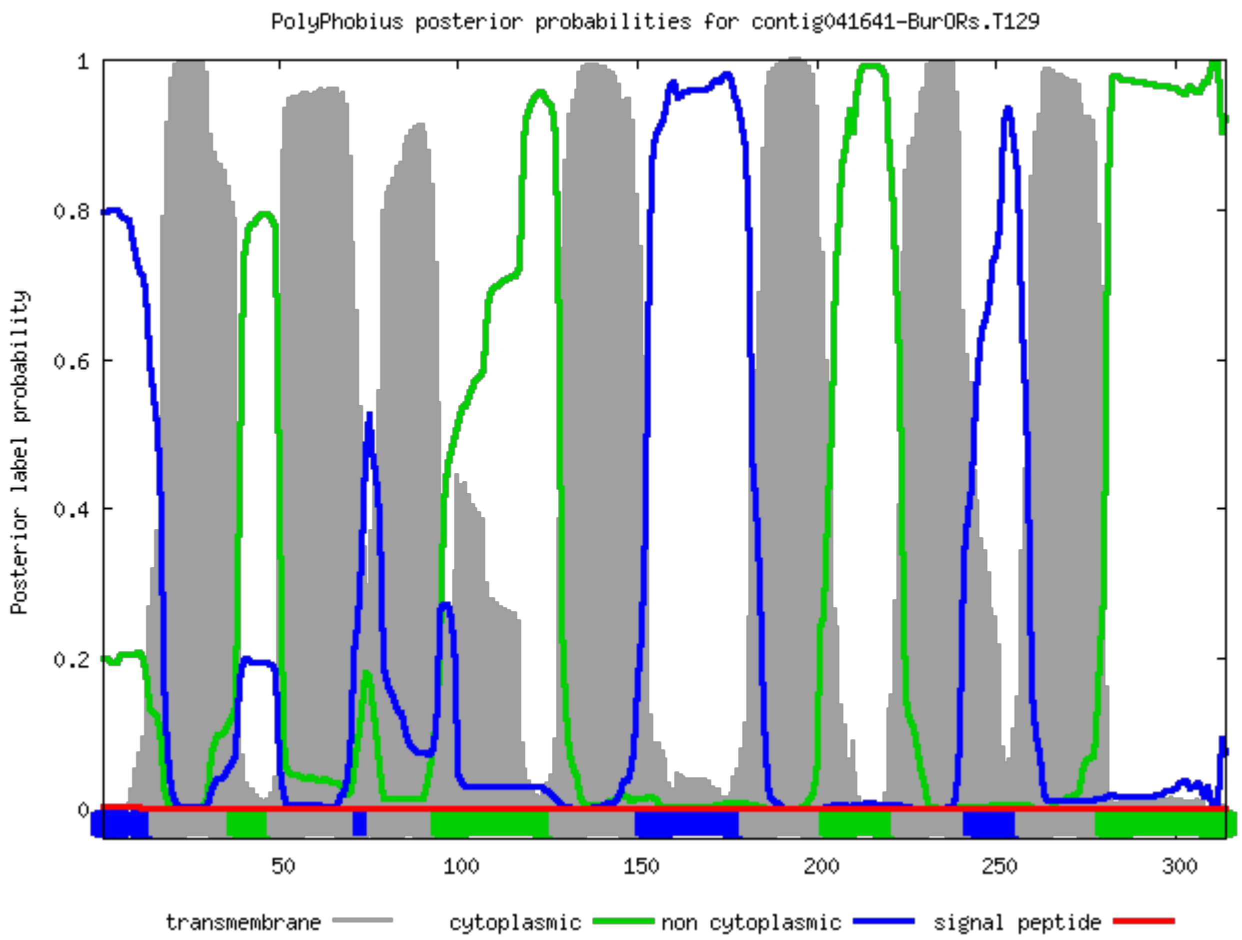

The prediction is based on an alignment. The probability data used in the plot is found here, and the gnuplot script is here.

Prediction of contig042478-BriOR.S103

```
ID   contig042478-BriOR.S103
FT   TOPO_DOM      1     21       NON CYTOPLASMIC.
FT   TRANSMEM     22     43
FT   TOPO_DOM     44     53       CYTOPLASMIC.
FT   TRANSMEM     54     78
FT   TOPO_DOM     79     83       NON CYTOPLASMIC.
FT   TRANSMEM     84    112
FT   TOPO_DOM    113    132       CYTOPLASMIC.
FT   TRANSMEM    133    158
FT   TOPO_DOM    159    186       NON CYTOPLASMIC.
FT   TRANSMEM    187    207
FT   TOPO_DOM    208    227       CYTOPLASMIC.
FT   TRANSMEM    228    253
FT   TOPO_DOM    254    264       NON CYTOPLASMIC.
FT   TRANSMEM    265    285
FT   TOPO_DOM    286    313       CYTOPLASMIC.
//
```

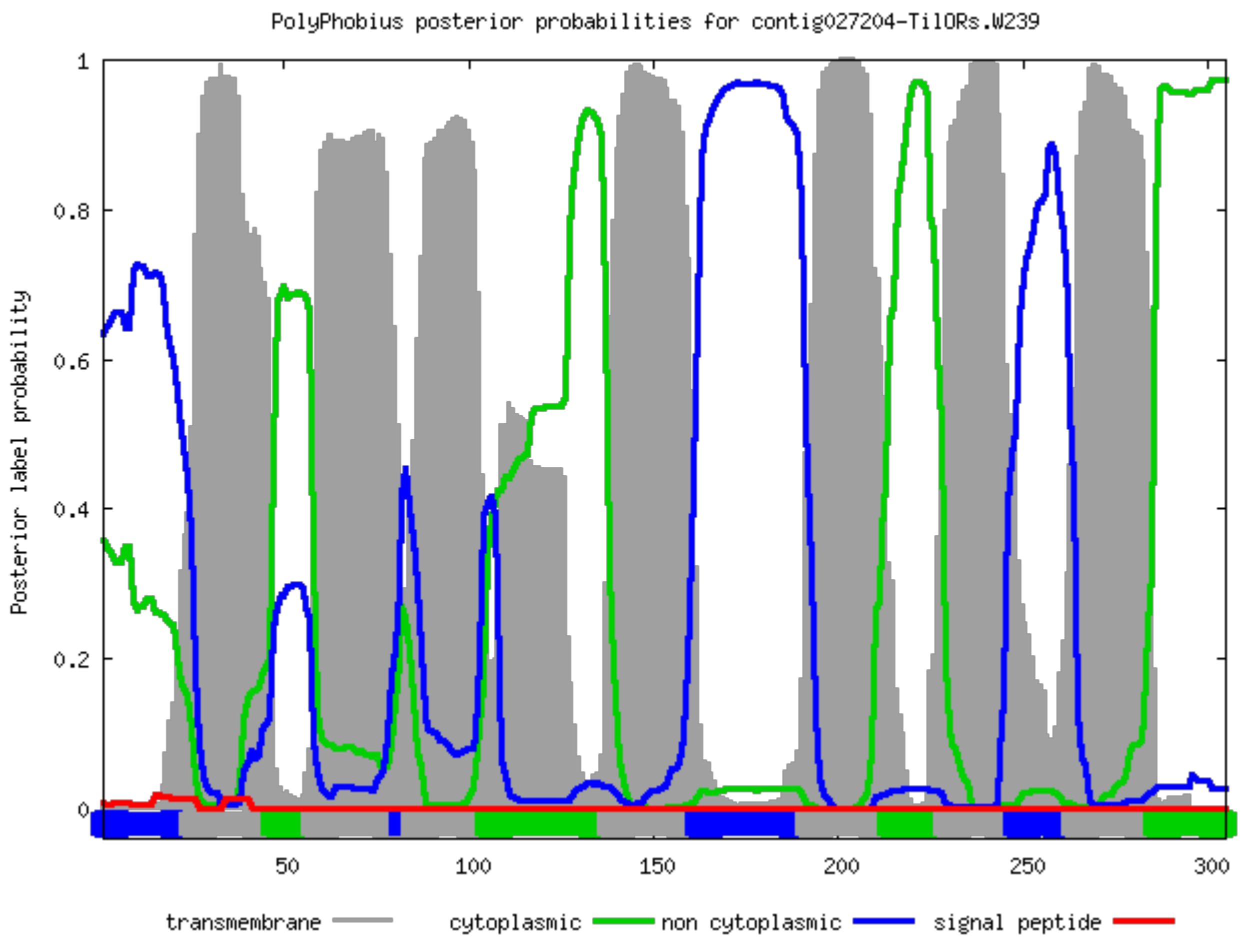

The prediction is based on an alignment. The probability data used in the plot is found here, and the gnuplot script is here.

Prediction of contig005007-TilOR.S220

```
ID    contig005007-TilOR.S220
FT    TOPO_DOM      1     20       NON CYTOPLASMIC.
FT    TRANSMEM     21     42
FT    TOPO_DOM     43     53       CYTOPLASMIC.
FT    TRANSMEM     54     77
FT    TOPO_DOM     78     82       NON CYTOPLASMIC.
FT    TRANSMEM     83    111
FT    TOPO_DOM    112    131       CYTOPLASMIC.
FT    TRANSMEM    132    157
FT    TOPO_DOM    158    185       NON CYTOPLASMIC.
FT    TRANSMEM    186    206
FT    TOPO_DOM    207    226       CYTOPLASMIC.
FT    TRANSMEM    227    252
FT    TOPO_DOM    253    263       NON CYTOPLASMIC.
FT    TRANSMEM    264    284
FT    TOPO_DOM    285    305       CYTOPLASMIC.
//
```

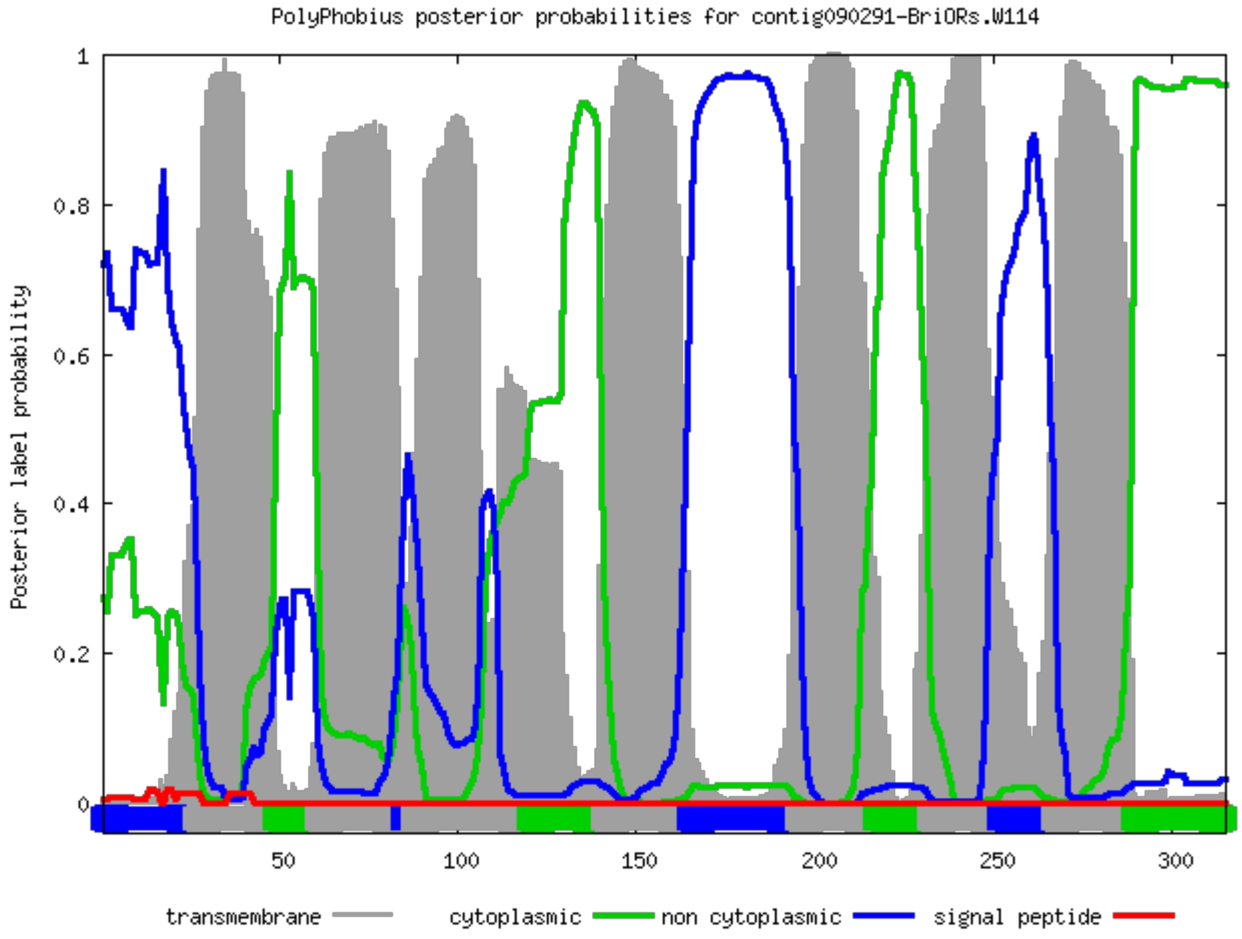

The prediction is based on an alignment. The probability data used in the plot is found here, and the gnuplot script is here.

Prediction of contig040507-NyeOR.J080

```
ID   contig040507-NyeOR.J080
FT   TOPO_DOM      1     25       NON CYTOPLASMIC.
FT   TRANSMEM     26     50
FT   TOPO_DOM     51     60       CYTOPLASMIC.
FT   TRANSMEM     61     82
FT   TOPO_DOM     83     98       NON CYTOPLASMIC.
FT   TRANSMEM     99    120
FT   TOPO_DOM    121    140       CYTOPLASMIC.
FT   TRANSMEM    141    163
FT   TOPO_DOM    164    195       NON CYTOPLASMIC.
FT   TRANSMEM    196    220
FT   TOPO_DOM    221    238       CYTOPLASMIC.
FT   TRANSMEM    239    261
FT   TOPO_DOM    262    271       NON CYTOPLASMIC.
FT   TRANSMEM    272    292
FT   TOPO_DOM    293    312       CYTOPLASMIC.
//
```

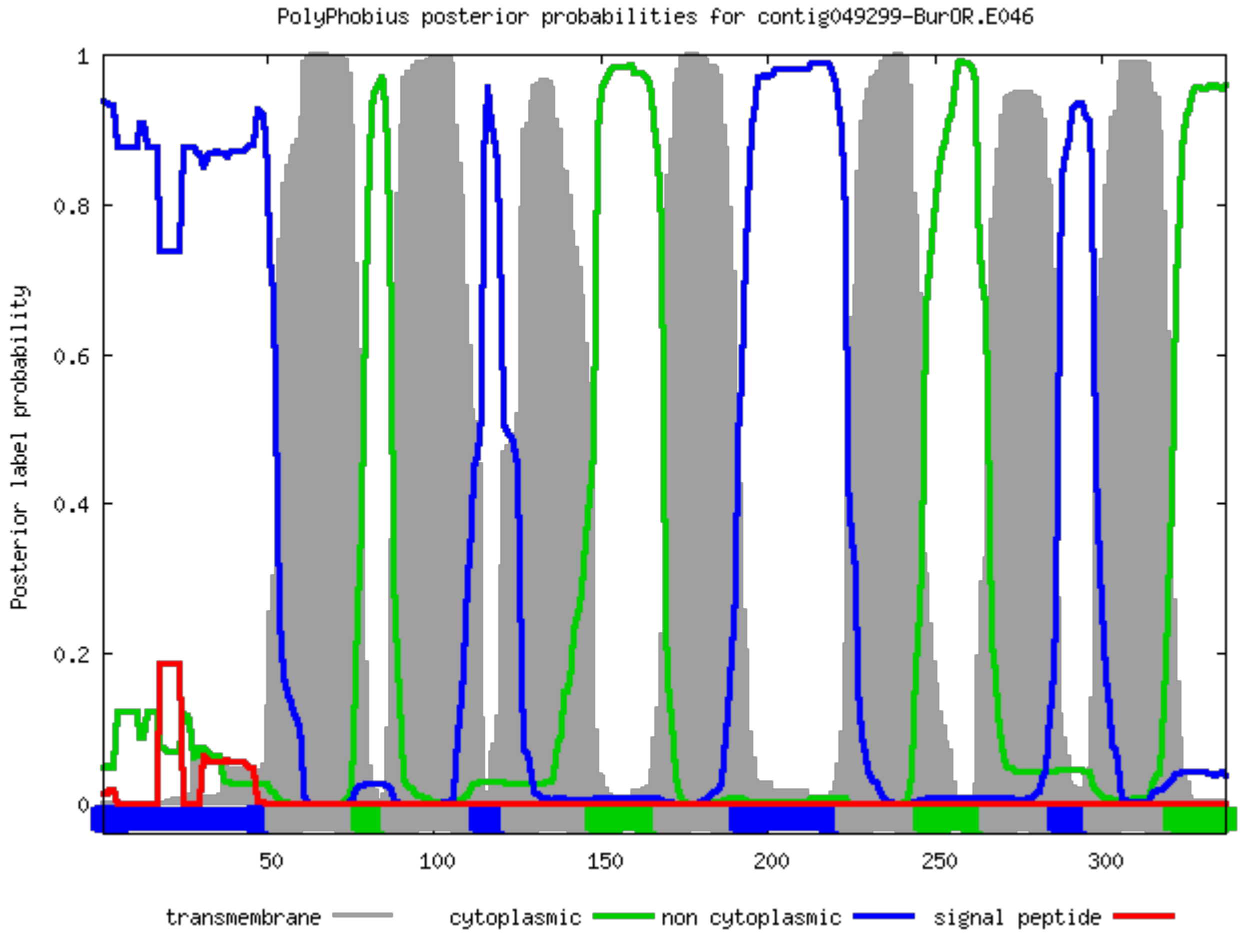

The prediction is based on an alignment. The probability data used in the plot is found here, and the gnuplot script is here.

Prediction of contig068521-TilOR.L156

```
ID    contig068521-TilOR.L156
FT    TOPO_DOM      1      25       NON CYTOPLASMIC.
FT    TRANSMEM     26      49
FT    TOPO_DOM     50      59       CYTOPLASMIC.
FT    TRANSMEM     60      82
FT    TOPO_DOM     83      98       NON CYTOPLASMIC.
FT    TRANSMEM     99     120
FT    TOPO_DOM    121     140       CYTOPLASMIC.
FT    TRANSMEM    141     162
FT    TOPO_DOM    163     198       NON CYTOPLASMIC.
FT    TRANSMEM    199     223
FT    TOPO_DOM    224     235       CYTOPLASMIC.
FT    TRANSMEM    236     259
FT    TOPO_DOM    260     271       NON CYTOPLASMIC.
FT    TRANSMEM    272     292
FT    TOPO_DOM    293     313       CYTOPLASMIC.
//
```

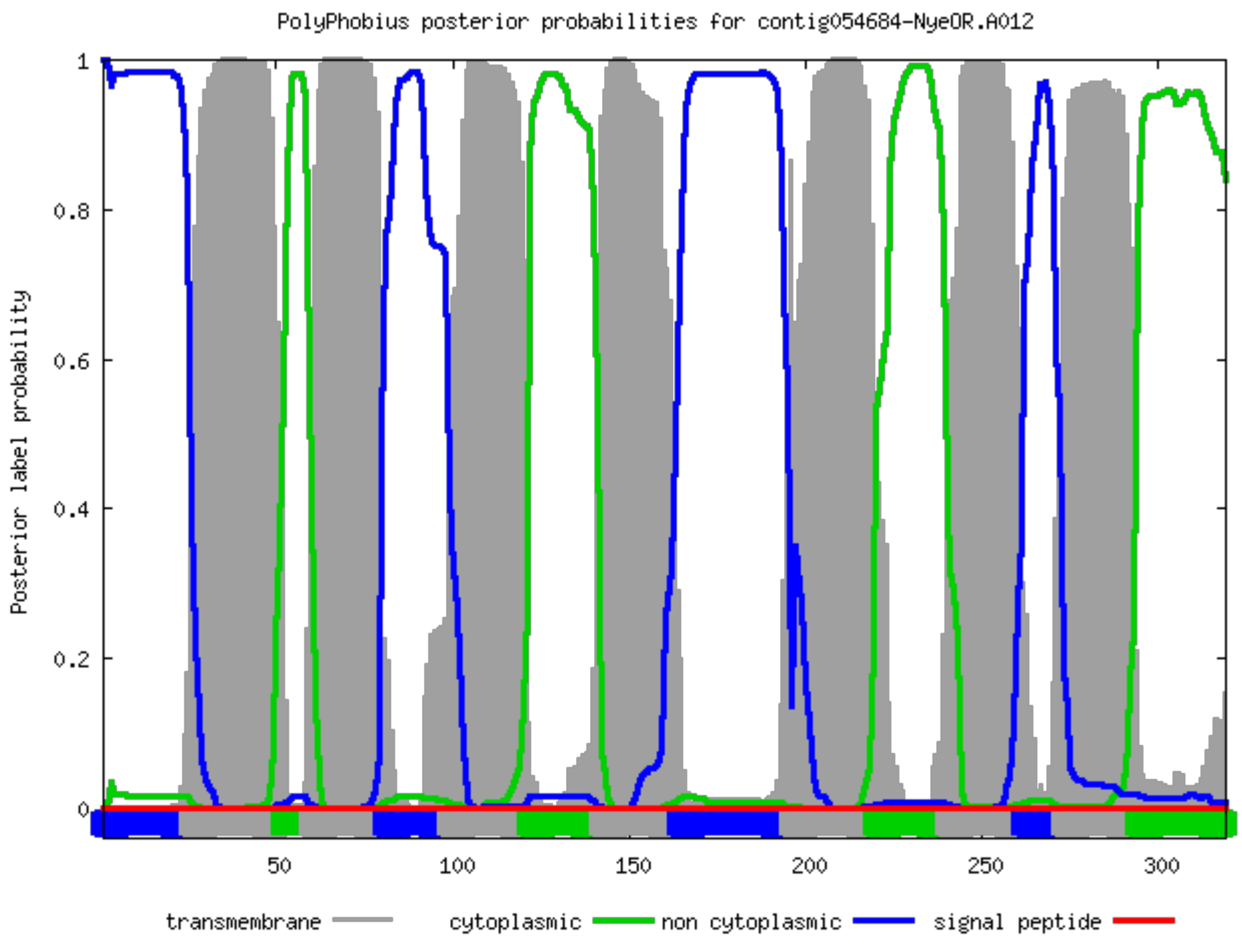

The prediction is based on an alignment. The probability data used in the plot is found here, and the gnuplot script is here.

Prediction of contig047523-ZebOR.A022

```
ID    contig047523-ZebOR.A022
FT    TOPO_DOM      1      22       NON CYTOPLASMIC.
FT    TRANSMEM     23      48
FT    TOPO_DOM     49      56       CYTOPLASMIC.
FT    TRANSMEM     57      77
FT    TOPO_DOM     78      95       NON CYTOPLASMIC.
FT    TRANSMEM     96     118
FT    TOPO_DOM    119     138       CYTOPLASMIC.
FT    TRANSMEM    139     160
FT    TOPO_DOM    161     192       NON CYTOPLASMIC.
FT    TRANSMEM    193     215
FT    TOPO_DOM    216     235       CYTOPLASMIC.
FT    TRANSMEM    236     257
FT    TOPO_DOM    258     268       NON CYTOPLASMIC.
FT    TRANSMEM    269     289
FT    TOPO_DOM    290     307       CYTOPLASMIC.
//
```

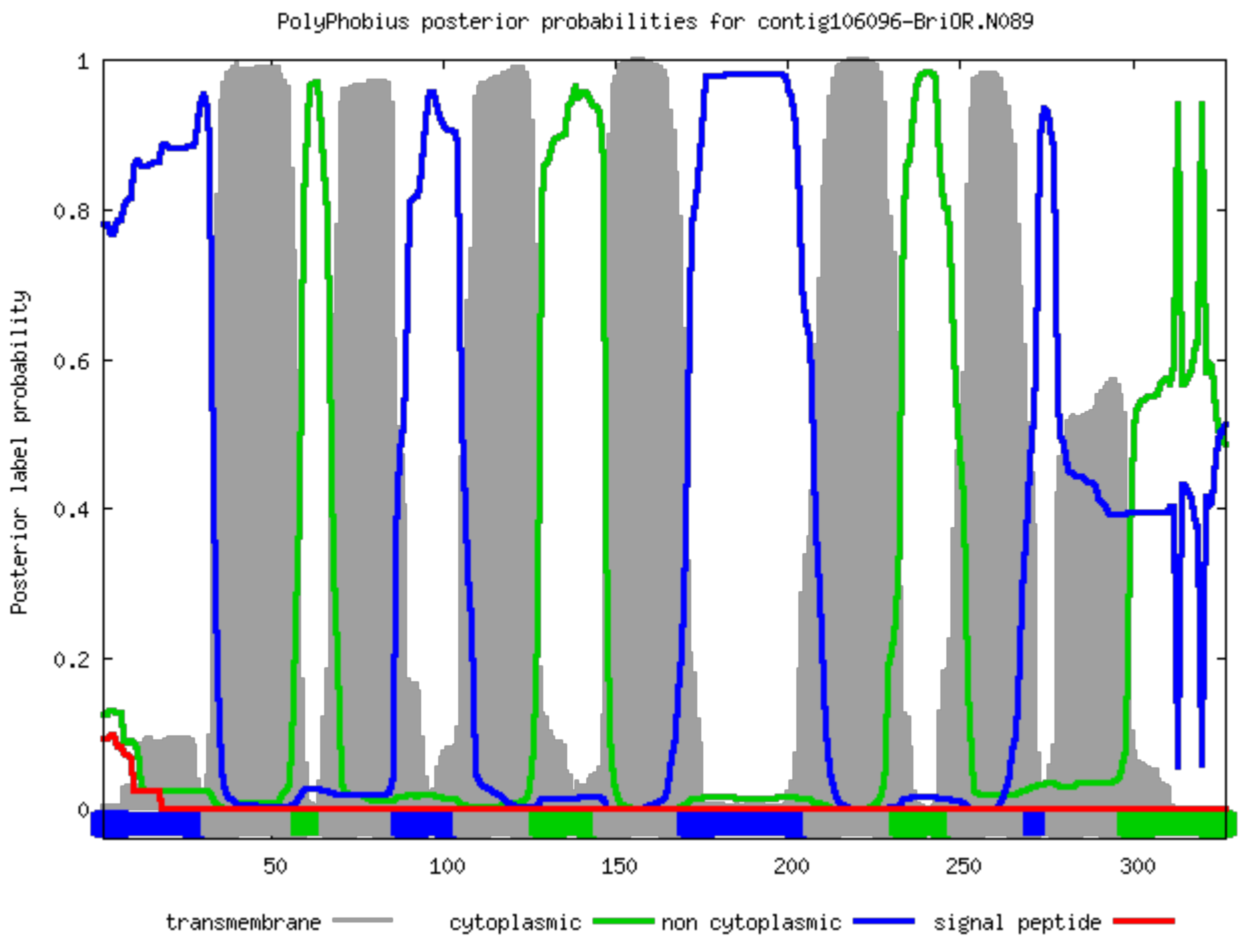

The prediction is based on an alignment. The probability data used in the plot is found here, and the gnuplot script is here.

Prediction of contig051559-BurOR.A007

```
ID    contig051559-BurOR.A007
FT    TOPO_DOM      1      18       NON CYTOPLASMIC.
FT    TRANSMEM     19      44
FT    TOPO_DOM     45      52       CYTOPLASMIC.
FT    TRANSMEM     53      73
FT    TOPO_DOM     74      91       NON CYTOPLASMIC.
FT    TRANSMEM     92     114
FT    TOPO_DOM    115     134       CYTOPLASMIC.
FT    TRANSMEM    135     155
FT    TOPO_DOM    156     188       NON CYTOPLASMIC.
FT    TRANSMEM    189     211
FT    TOPO_DOM    212     231       CYTOPLASMIC.
FT    TRANSMEM    232     253
FT    TOPO_DOM    254     264       NON CYTOPLASMIC.
FT    TRANSMEM    265     285
FT    TOPO_DOM    286     300       CYTOPLASMIC.
//
```

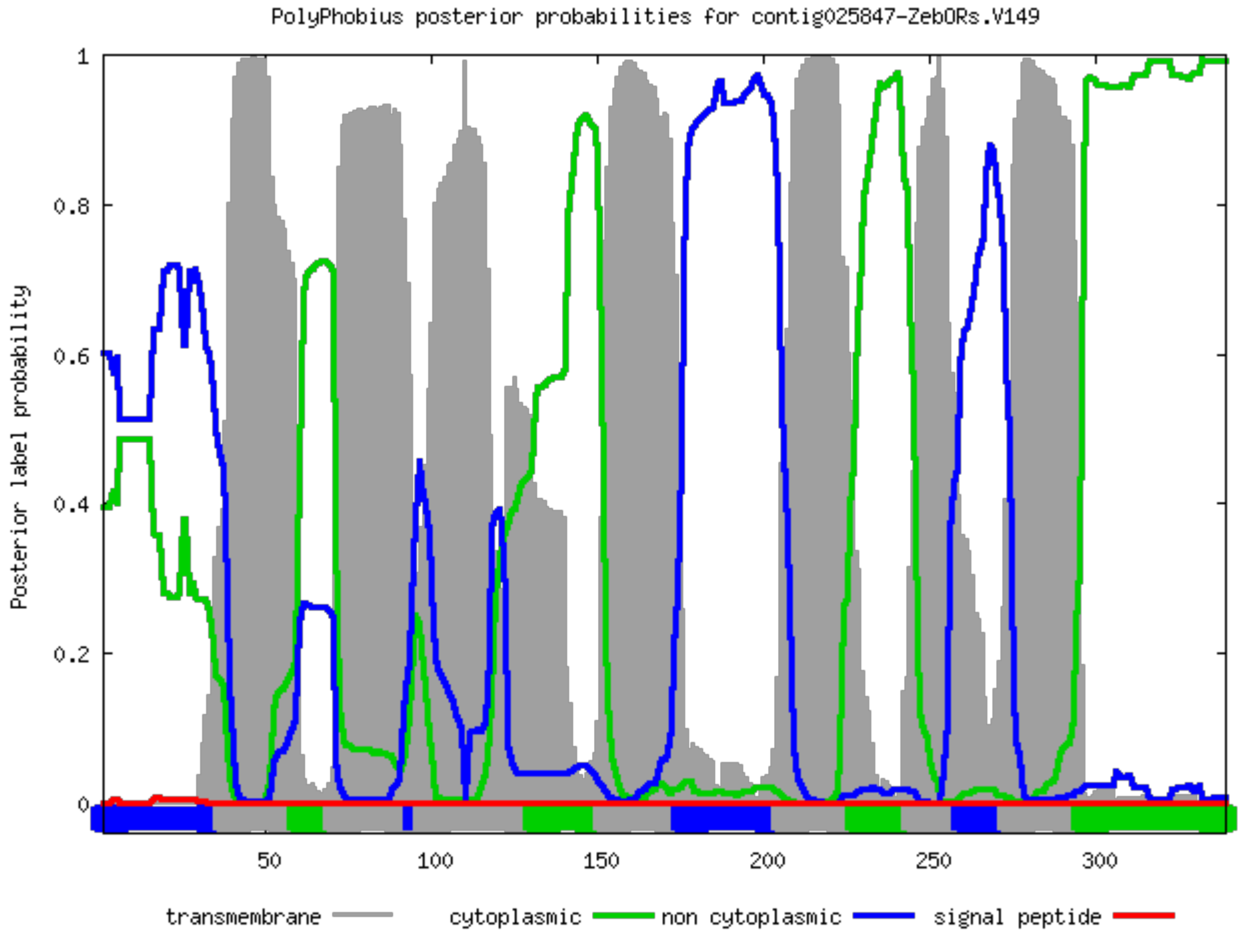

The prediction is based on an alignment. The probability data used in the plot is found here, and the gnuplot script is here.

Prediction of contig034981-NyeOR.A001

```
ID   contig034981-NyeOR.A001
FT   TOPO_DOM      1     22       NON CYTOPLASMIC.
FT   TRANSMEM     23     48
FT   TOPO_DOM     49     56       CYTOPLASMIC.
FT   TRANSMEM     57     76
FT   TOPO_DOM     77     95       NON CYTOPLASMIC.
FT   TRANSMEM     96    118
FT   TOPO_DOM    119    138       CYTOPLASMIC.
FT   TRANSMEM    139    159
FT   TOPO_DOM    160    192       NON CYTOPLASMIC.
FT   TRANSMEM    193    215
FT   TOPO_DOM    216    235       CYTOPLASMIC.
FT   TRANSMEM    236    257
FT   TOPO_DOM    258    268       NON CYTOPLASMIC.
FT   TRANSMEM    269    289
FT   TOPO_DOM    290    313       CYTOPLASMIC.
FT   TRANSMEM    314    328
FT   TOPO_DOM    329    334       NON CYTOPLASMIC.
//
```

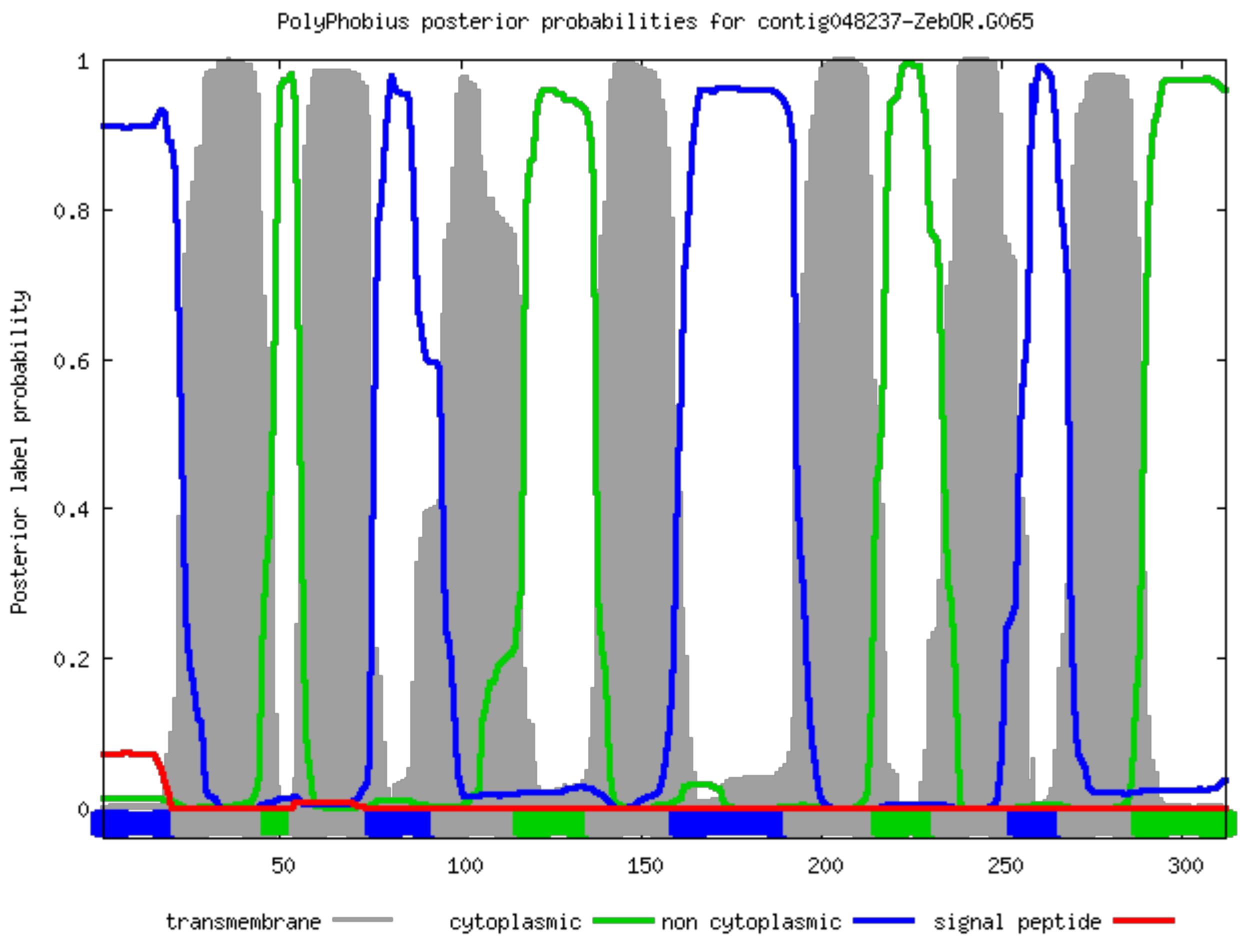

The prediction is based on an alignment. The probability data used in the plot is found here, and the gnuplot script is here.

Prediction of contig018434-ZebOR.H070

```
ID   contig018434-ZebOR.H070
FT   TOPO_DOM      1     23       NON CYTOPLASMIC.
FT   TRANSMEM     24     49
FT   TOPO_DOM     50     56       CYTOPLASMIC.
FT   TRANSMEM     57     76
FT   TOPO_DOM     77     95       NON CYTOPLASMIC.
FT   TRANSMEM     96    118
FT   TOPO_DOM    119    138       CYTOPLASMIC.
FT   TRANSMEM    139    160
FT   TOPO_DOM    161    196       NON CYTOPLASMIC.
FT   TRANSMEM    197    219
FT   TOPO_DOM    220    237       CYTOPLASMIC.
FT   TRANSMEM    238    259
FT   TOPO_DOM    260    271       NON CYTOPLASMIC.
FT   TRANSMEM    272    291
FT   TOPO_DOM    292    309       CYTOPLASMIC.
//
```

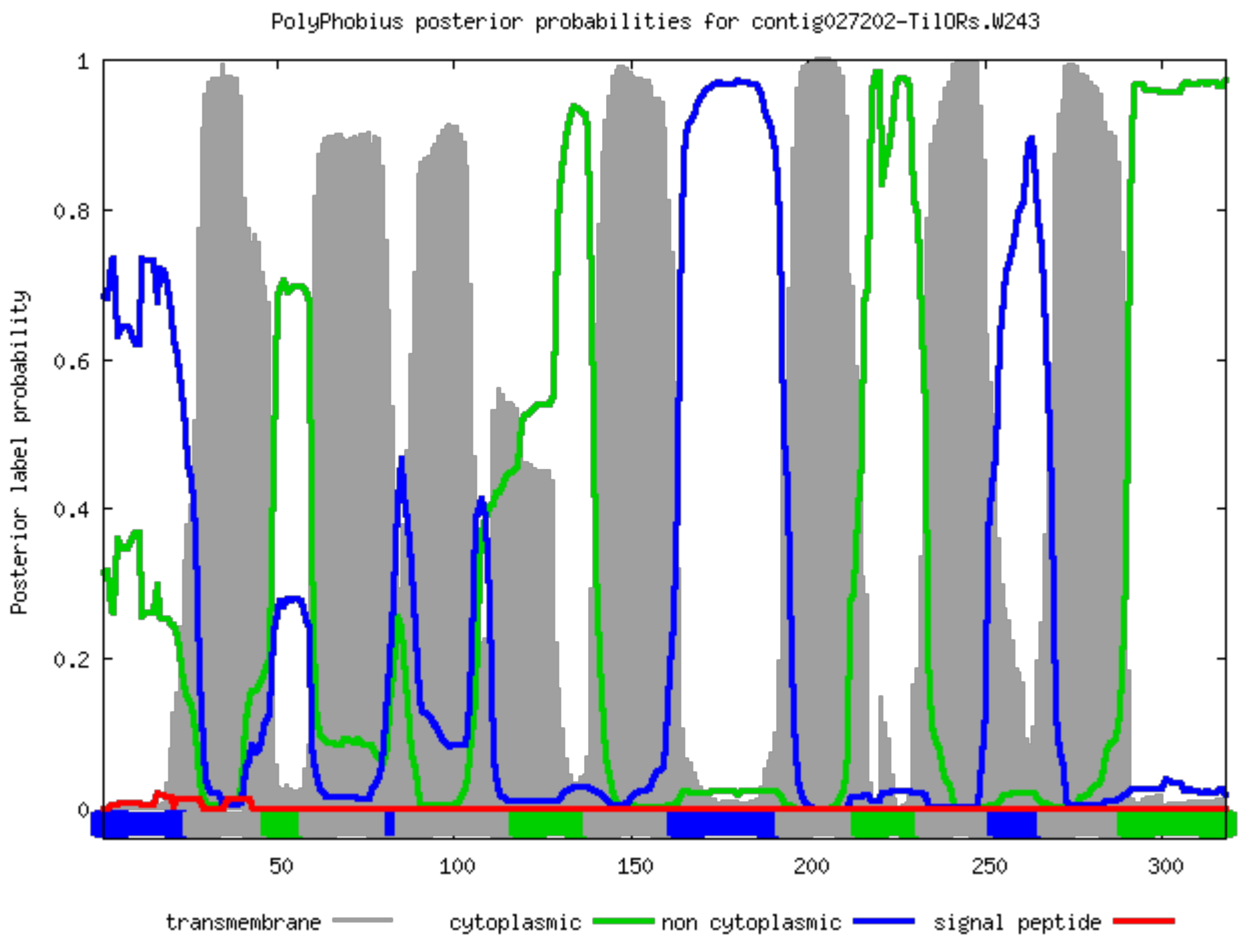

The prediction is based on an alignment. The probability data used in the plot is found here, and the gnuplot script is here.

Prediction of contig062547-NyeOR.J081

```
ID    contig062547-NyeOR.J081
FT    TOPO_DOM      1     23      NON CYTOPLASMIC.
FT    TRANSMEM     24     49
FT    TOPO_DOM     50     59      CYTOPLASMIC.
FT    TRANSMEM     60     81
FT    TOPO_DOM     82     97      NON CYTOPLASMIC.
FT    TRANSMEM     98    119
FT    TOPO_DOM    120    139      CYTOPLASMIC.
FT    TRANSMEM    140    162
FT    TOPO_DOM    163    194      NON CYTOPLASMIC.
FT    TRANSMEM    195    218
FT    TOPO_DOM    219    236      CYTOPLASMIC.
FT    TRANSMEM    237    260
FT    TOPO_DOM    261    270      NON CYTOPLASMIC.
FT    TRANSMEM    271    291
FT    TOPO_DOM    292    313      CYTOPLASMIC.
//
```

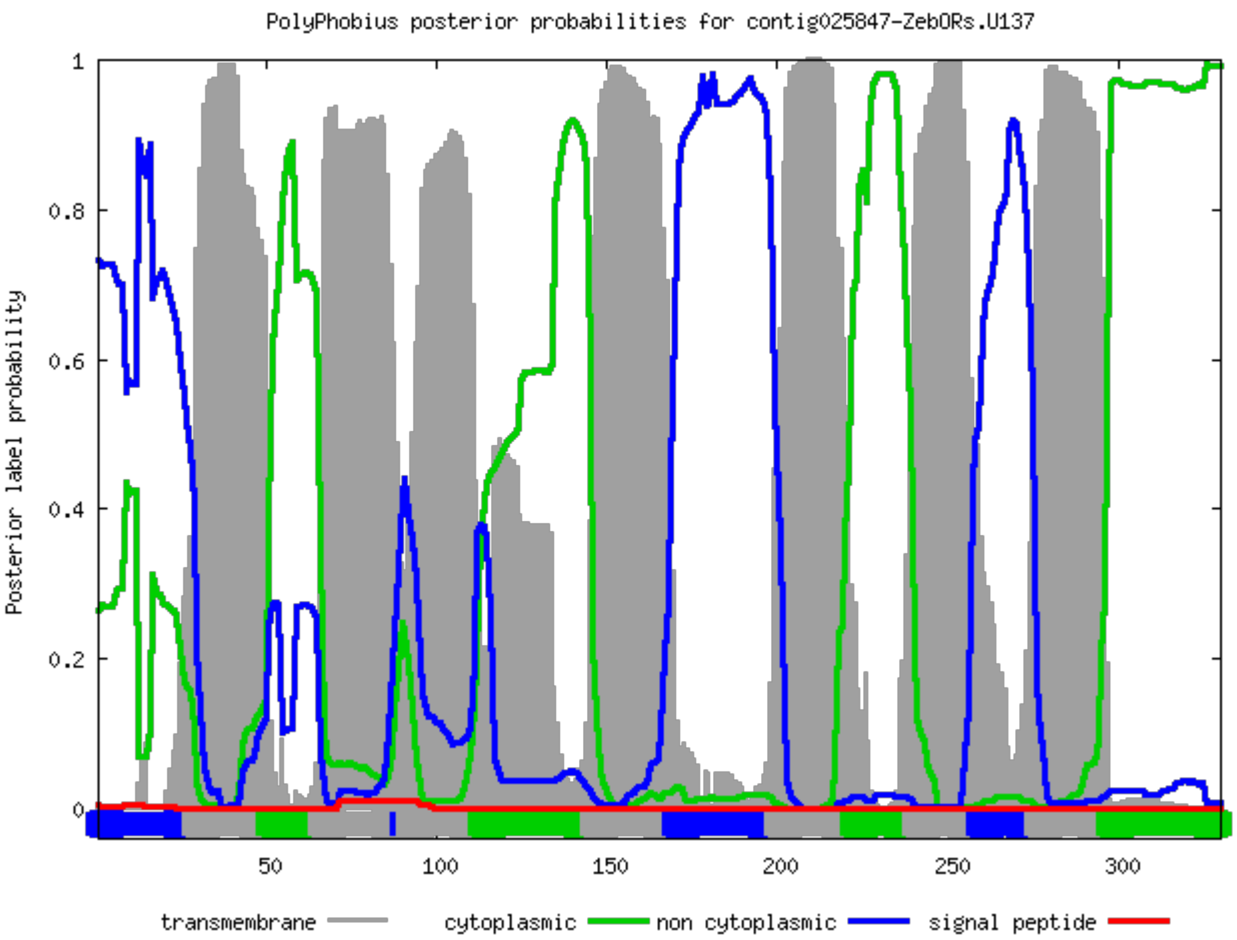

The prediction is based on an alignment. The probability data used in the plot is found here, and the gnuplot script is here.

Prediction of contig013326-TilOR.D051

```
ID   contig013326-TilOR.D051
FT   TOPO_DOM      1     22       NON CYTOPLASMIC.
FT   TRANSMEM     23     48
FT   TOPO_DOM     49     57       CYTOPLASMIC.
FT   TRANSMEM     58     81
FT   TOPO_DOM     82     90       NON CYTOPLASMIC.
FT   TRANSMEM     91    117
FT   TOPO_DOM    118    138       CYTOPLASMIC.
FT   TRANSMEM    139    162
FT   TOPO_DOM    163    194       NON CYTOPLASMIC.
FT   TRANSMEM    195    216
FT   TOPO_DOM    217    236       CYTOPLASMIC.
FT   TRANSMEM    237    256
FT   TOPO_DOM    257    267       NON CYTOPLASMIC.
FT   TRANSMEM    268    291
FT   TOPO_DOM    292    309       CYTOPLASMIC.
//
```

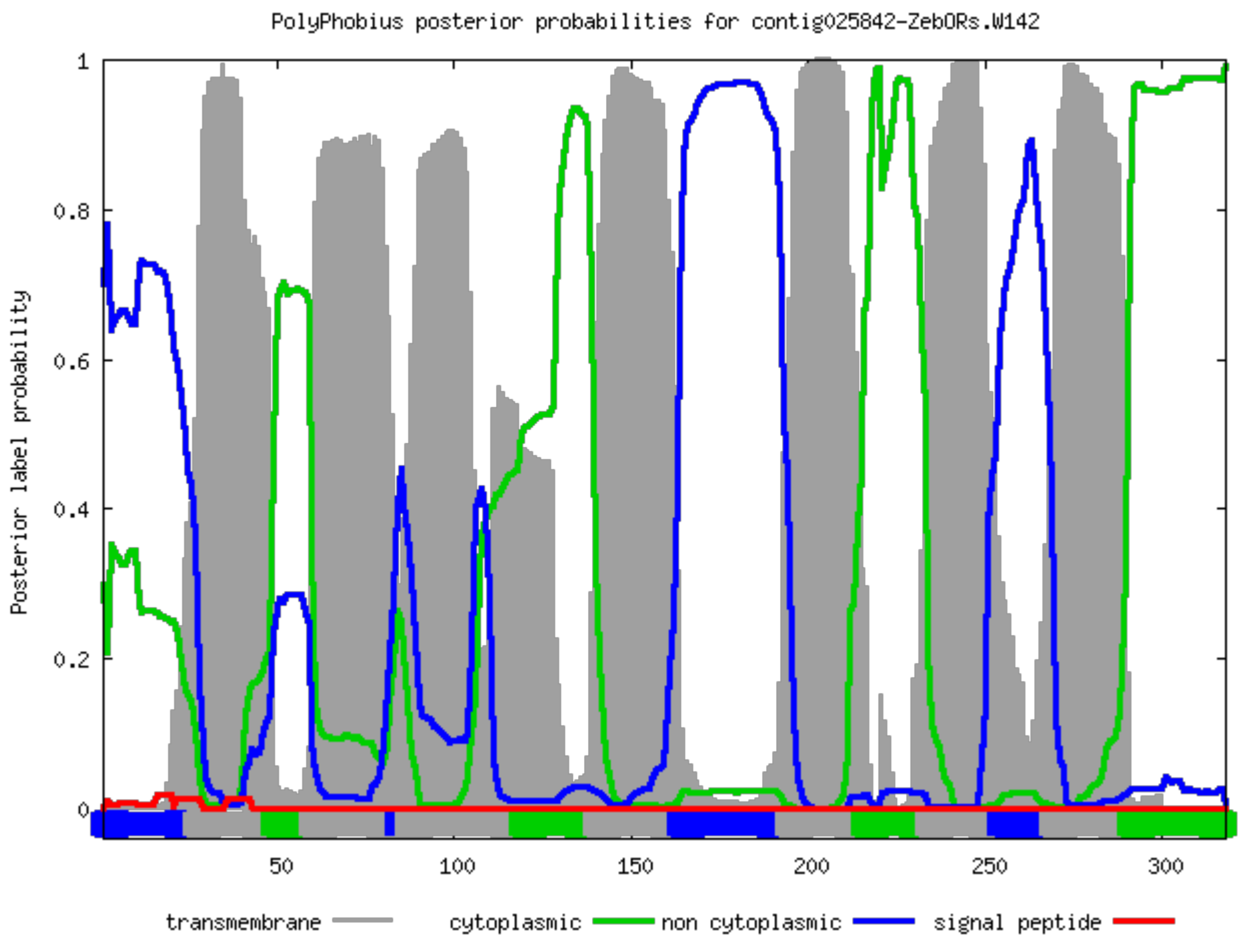

The prediction is based on an alignment. The probability data used in the plot is found here, and the gnuplot script is here.

Prediction of contig039730-NyeOR.D037

```
ID   contig039730-NyeOR.D037
FT   TOPO_DOM      1     25       NON CYTOPLASMIC.
FT   TRANSMEM     26     51
FT   TOPO_DOM     52     60       CYTOPLASMIC.
FT   TRANSMEM     61     85
FT   TOPO_DOM     86     93       NON CYTOPLASMIC.
FT   TRANSMEM     94    121
FT   TOPO_DOM    122    141       CYTOPLASMIC.
FT   TRANSMEM    142    164
FT   TOPO_DOM    165    197       NON CYTOPLASMIC.
FT   TRANSMEM    198    219
FT   TOPO_DOM    220    239       CYTOPLASMIC.
FT   TRANSMEM    240    259
FT   TOPO_DOM    260    271       NON CYTOPLASMIC.
FT   TRANSMEM    272    294
FT   TOPO_DOM    295    322       CYTOPLASMIC.
//
```

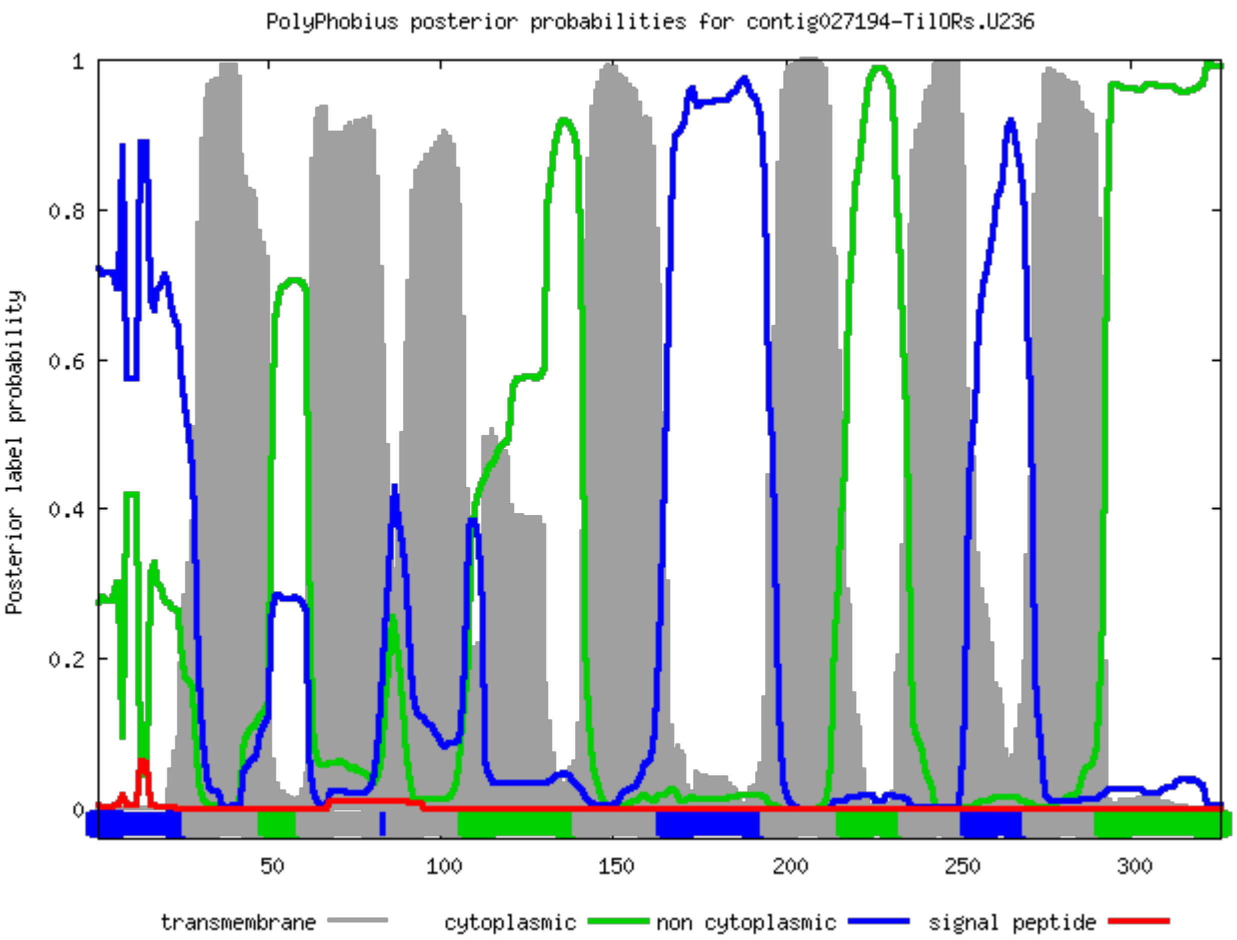

The prediction is based on an alignment. The probability data used in the plot is found here, and the gnuplot script is here.

Prediction of contig053788-BurOR.H069

```
ID   contig053788-BurOR.H069
FT   TOPO_DOM      1      22       NON CYTOPLASMIC.
FT   TRANSMEM     23      49
FT   TOPO_DOM     50      56       CYTOPLASMIC.
FT   TRANSMEM     57      77
FT   TOPO_DOM     78      95       NON CYTOPLASMIC.
FT   TRANSMEM     96     118
FT   TOPO_DOM    119     138       CYTOPLASMIC.
FT   TRANSMEM    139     160
FT   TOPO_DOM    161     193       NON CYTOPLASMIC.
FT   TRANSMEM    194     216
FT   TOPO_DOM    217     235       CYTOPLASMIC.
FT   TRANSMEM    236     258
FT   TOPO_DOM    259     269       NON CYTOPLASMIC.
FT   TRANSMEM    270     289
FT   TOPO_DOM    290     314       CYTOPLASMIC.
//
```

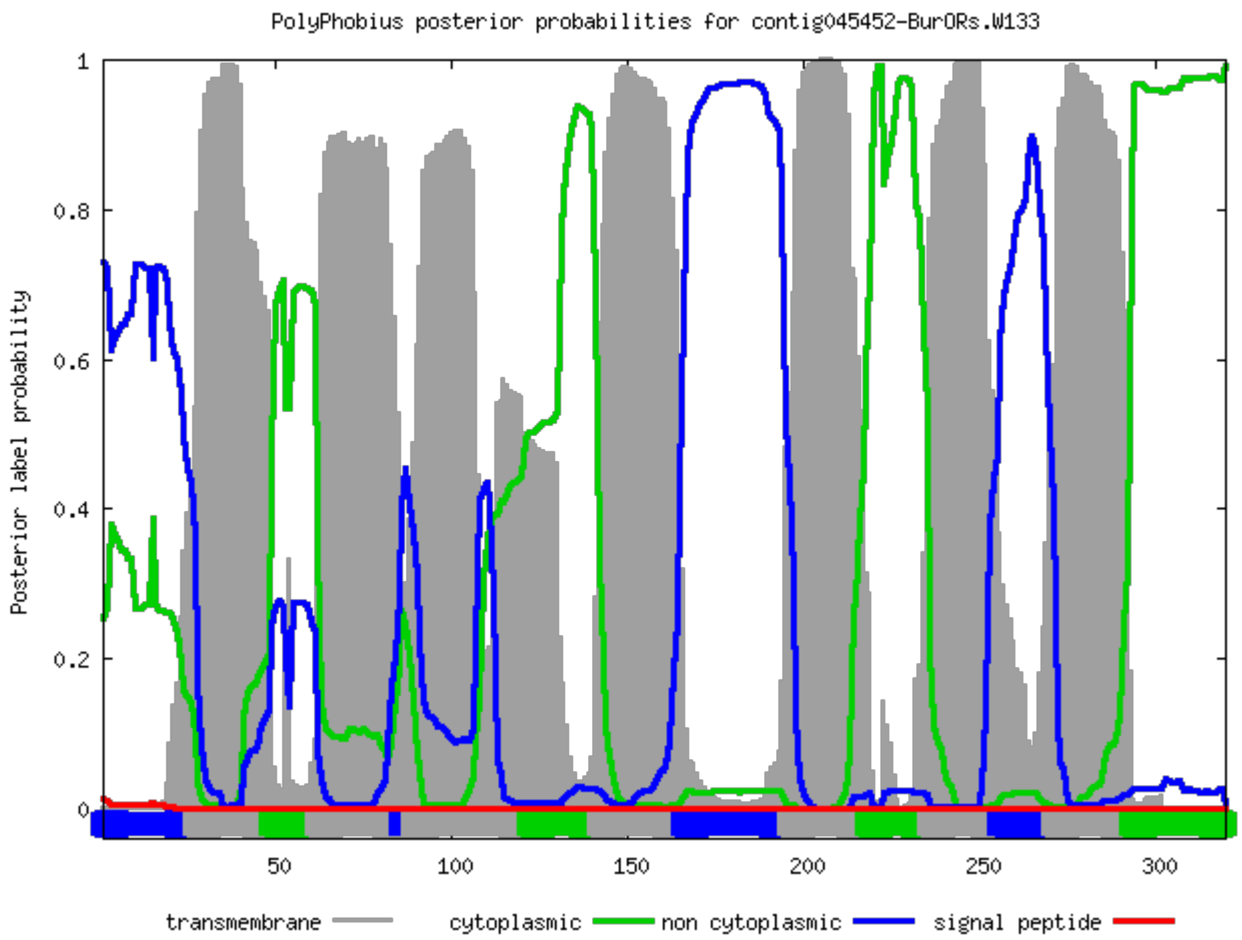

The prediction is based on an alignment. The probability data used in the plot is found here, and the gnuplot script is here.

Prediction of contig030572-ZebOR.A009

```
ID   contig030572-ZebOR.A009
FT   TOPO_DOM      1     22       NON CYTOPLASMIC.
FT   TRANSMEM     23     48
FT   TOPO_DOM     49     56       CYTOPLASMIC.
FT   TRANSMEM     57     76
FT   TOPO_DOM     77     95       NON CYTOPLASMIC.
FT   TRANSMEM     96    118
FT   TOPO_DOM    119    138       CYTOPLASMIC.
FT   TRANSMEM    139    159
FT   TOPO_DOM    160    192       NON CYTOPLASMIC.
FT   TRANSMEM    193    215
FT   TOPO_DOM    216    235       CYTOPLASMIC.
FT   TRANSMEM    236    257
FT   TOPO_DOM    258    268       NON CYTOPLASMIC.
FT   TRANSMEM    269    289
FT   TOPO_DOM    290    314       CYTOPLASMIC.
//
```

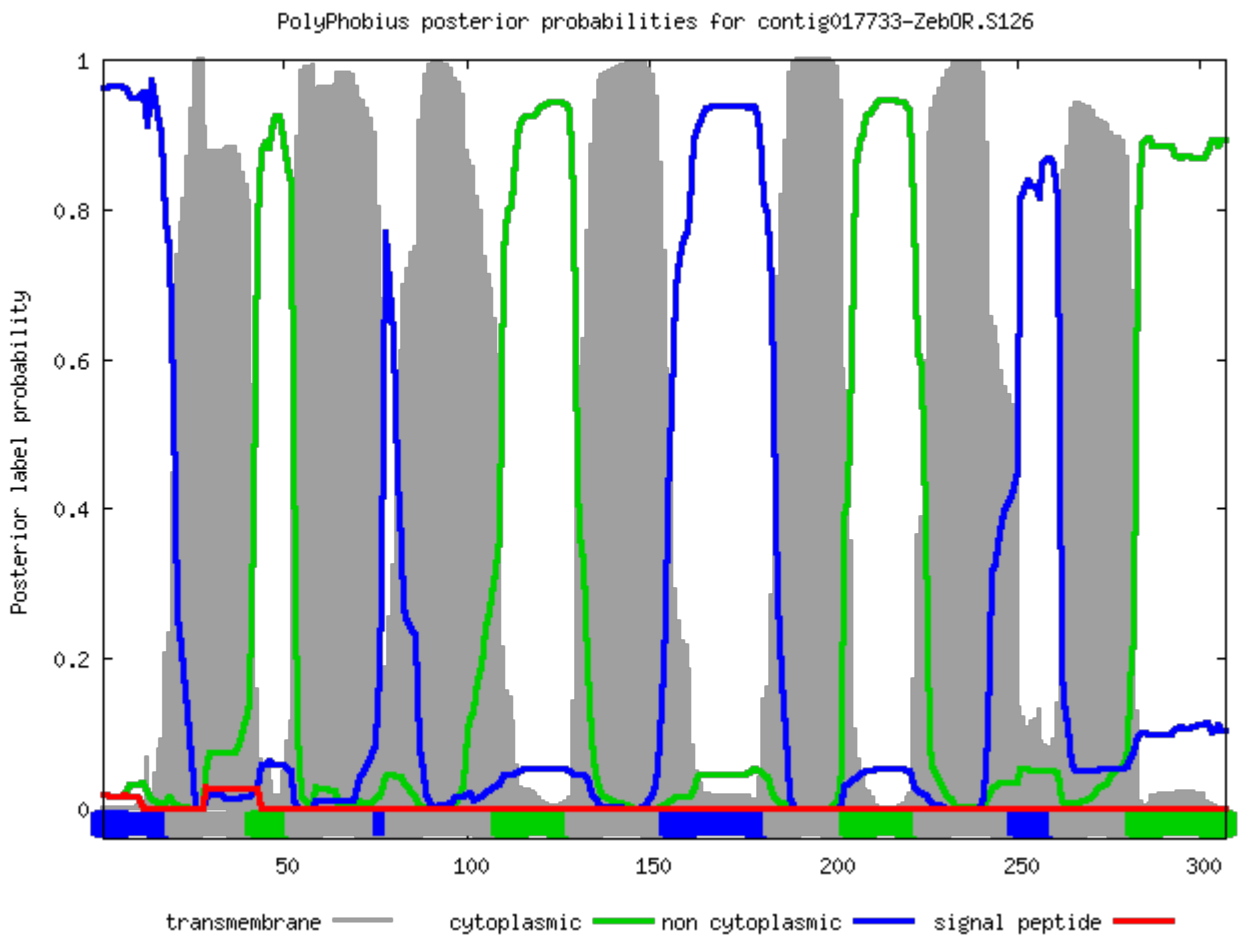

The prediction is based on an alignment. The probability data used in the plot is found here, and the gnuplot script is here.

Prediction of contig096536-BriOR.N086

```
ID    contig096536-BriOR.N086
FT    TOPO_DOM      1      33       NON CYTOPLASMIC.
FT    TRANSMEM     34      59
FT    TOPO_DOM     60      67       CYTOPLASMIC.
FT    TRANSMEM     68      89
FT    TOPO_DOM     90     108       NON CYTOPLASMIC.
FT    TRANSMEM    109     128
FT    TOPO_DOM    129     148       CYTOPLASMIC.
FT    TRANSMEM    149     171
FT    TOPO_DOM    172     208       NON CYTOPLASMIC.
FT    TRANSMEM    209     233
FT    TOPO_DOM    234     252       CYTOPLASMIC.
FT    TRANSMEM    253     275
FT    TOPO_DOM    276     327       NON CYTOPLASMIC.
//
```

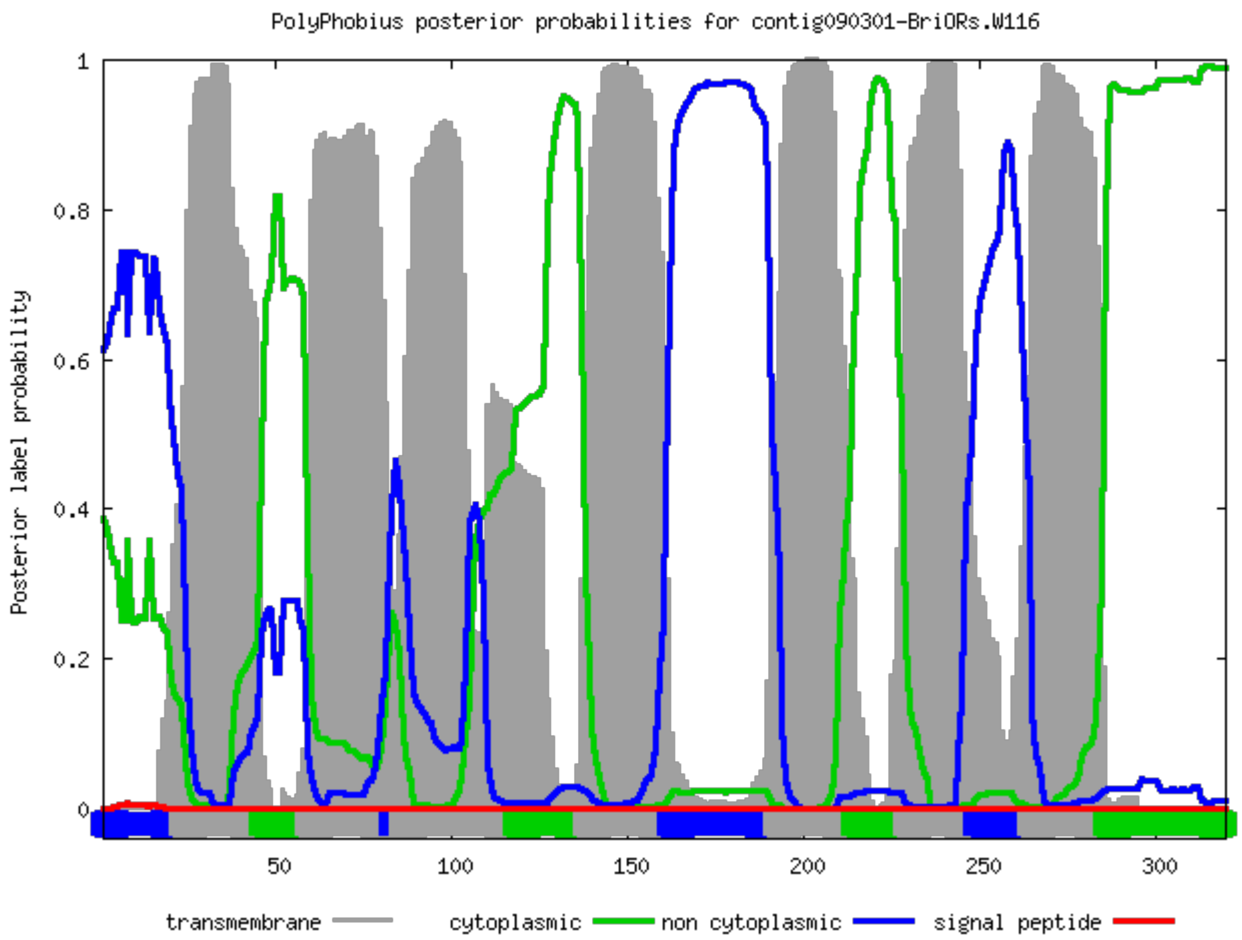

The prediction is based on an alignment. The probability data used in the plot is found here, and the gnuplot script is here.

Prediction of Contig046714+contig046713-TilOR.K136

```
ID   Contig046714+contig046713-TilOR.K136
FT   TOPO_DOM      1     24       NON CYTOPLASMIC.
FT   TRANSMEM     25     50
FT   TOPO_DOM     51     58       CYTOPLASMIC.
FT   TRANSMEM     59     81
FT   TOPO_DOM     82    100       NON CYTOPLASMIC.
FT   TRANSMEM    101    121
FT   TOPO_DOM    122    141       CYTOPLASMIC.
FT   TRANSMEM    142    165
FT   TOPO_DOM    166    196       NON CYTOPLASMIC.
FT   TRANSMEM    197    223
FT   TOPO_DOM    224    243       CYTOPLASMIC.
FT   TRANSMEM    244    264
FT   TOPO_DOM    265    269       NON CYTOPLASMIC.
FT   TRANSMEM    270    292
FT   TOPO_DOM    293    319       CYTOPLASMIC.
//
```

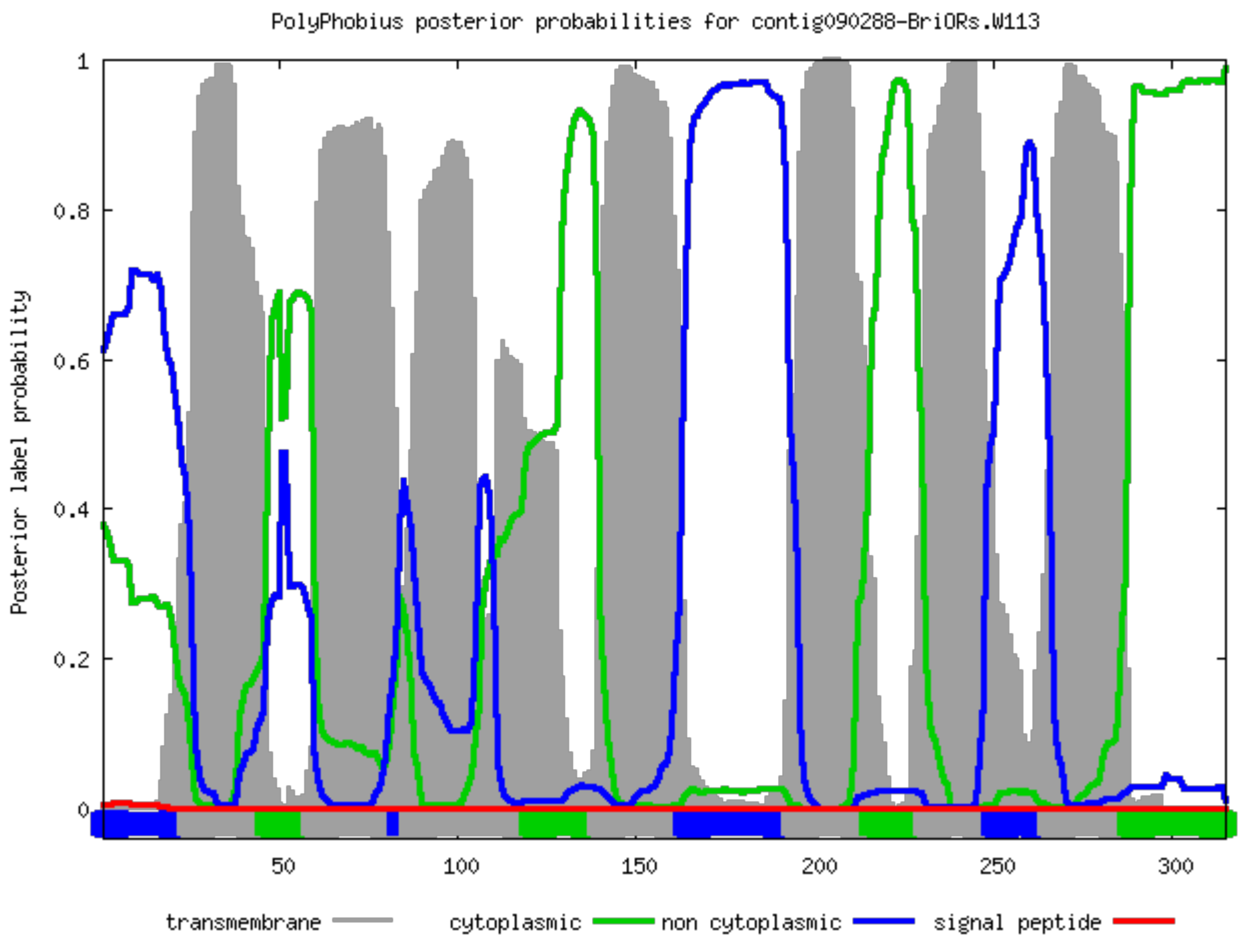

The prediction is based on an alignment. The probability data used in the plot is found here, and the gnuplot script is here.

Prediction of contig047833-TilOR.E083

```
ID   contig047833-TilOR.E083
FT   TOPO_DOM      1    22       NON CYTOPLASMIC.
FT   TRANSMEM     23    48
FT   TOPO_DOM     49    57       CYTOPLASMIC.
FT   TRANSMEM     58    83
FT   TOPO_DOM     84    92       NON CYTOPLASMIC.
FT   TRANSMEM     93   118
FT   TOPO_DOM    119   138       CYTOPLASMIC.
FT   TRANSMEM    139   161
FT   TOPO_DOM    162   193       NON CYTOPLASMIC.
FT   TRANSMEM    194   216
FT   TOPO_DOM    217   236       CYTOPLASMIC.
FT   TRANSMEM    237   256
FT   TOPO_DOM    257   267       NON CYTOPLASMIC.
FT   TRANSMEM    268   291
FT   TOPO_DOM    292   314       CYTOPLASMIC.
//
```

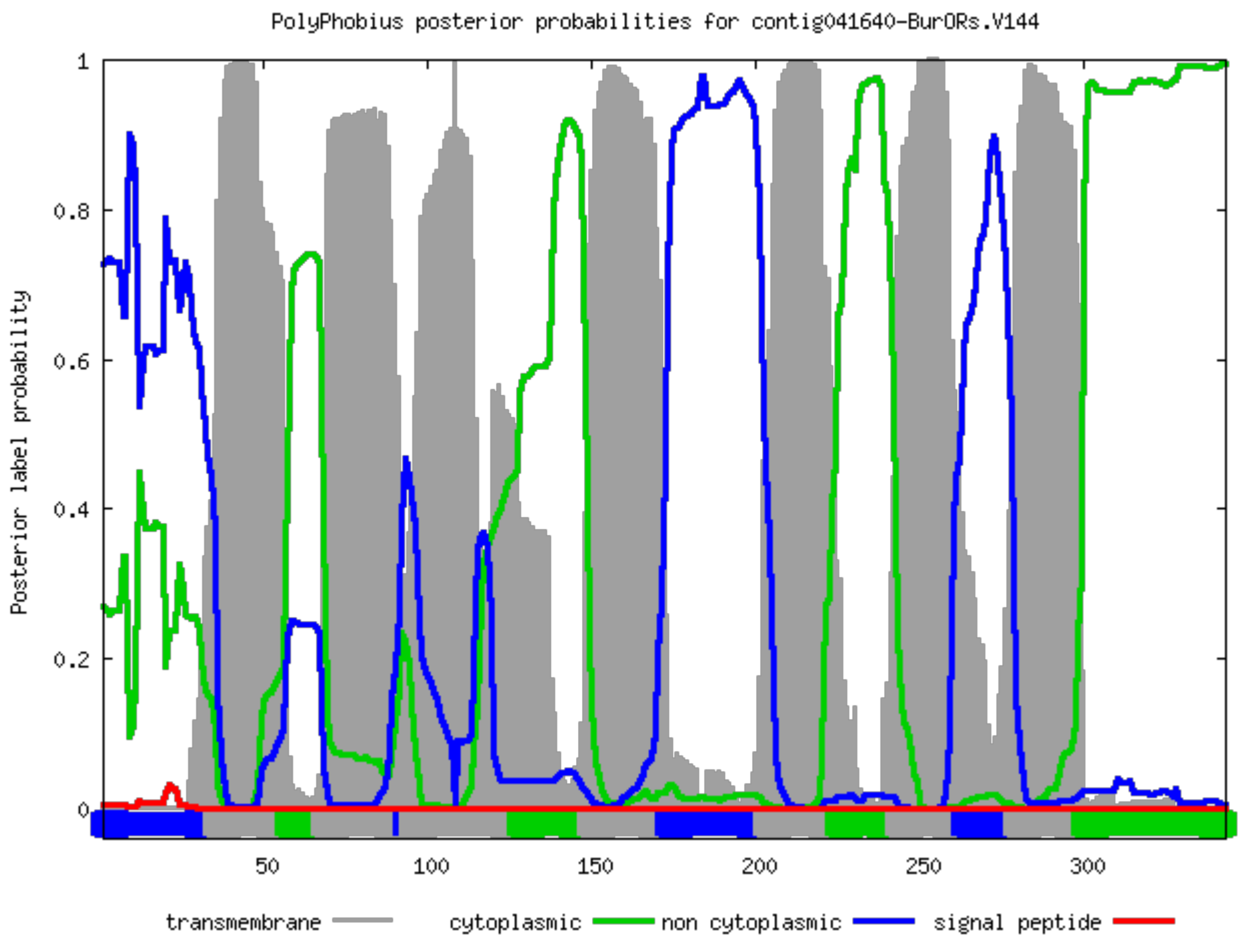

The prediction is based on an alignment. The probability data used in the plot is found here, and the gnuplot script is here.

Prediction of contig022241-TilOR.A011

```
ID   contig022241-TilOR.A011
FT   TOPO_DOM      1     22       NON CYTOPLASMIC.
FT   TRANSMEM     23     48
FT   TOPO_DOM     49     56       CYTOPLASMIC.
FT   TRANSMEM     57     76
FT   TOPO_DOM     77     95       NON CYTOPLASMIC.
FT   TRANSMEM     96    118
FT   TOPO_DOM    119    138       CYTOPLASMIC.
FT   TRANSMEM    139    159
FT   TOPO_DOM    160    192       NON CYTOPLASMIC.
FT   TRANSMEM    193    215
FT   TOPO_DOM    216    235       CYTOPLASMIC.
FT   TRANSMEM    236    257
FT   TOPO_DOM    258    268       NON CYTOPLASMIC.
FT   TRANSMEM    269    289
FT   TOPO_DOM    290    307       CYTOPLASMIC.
//
```

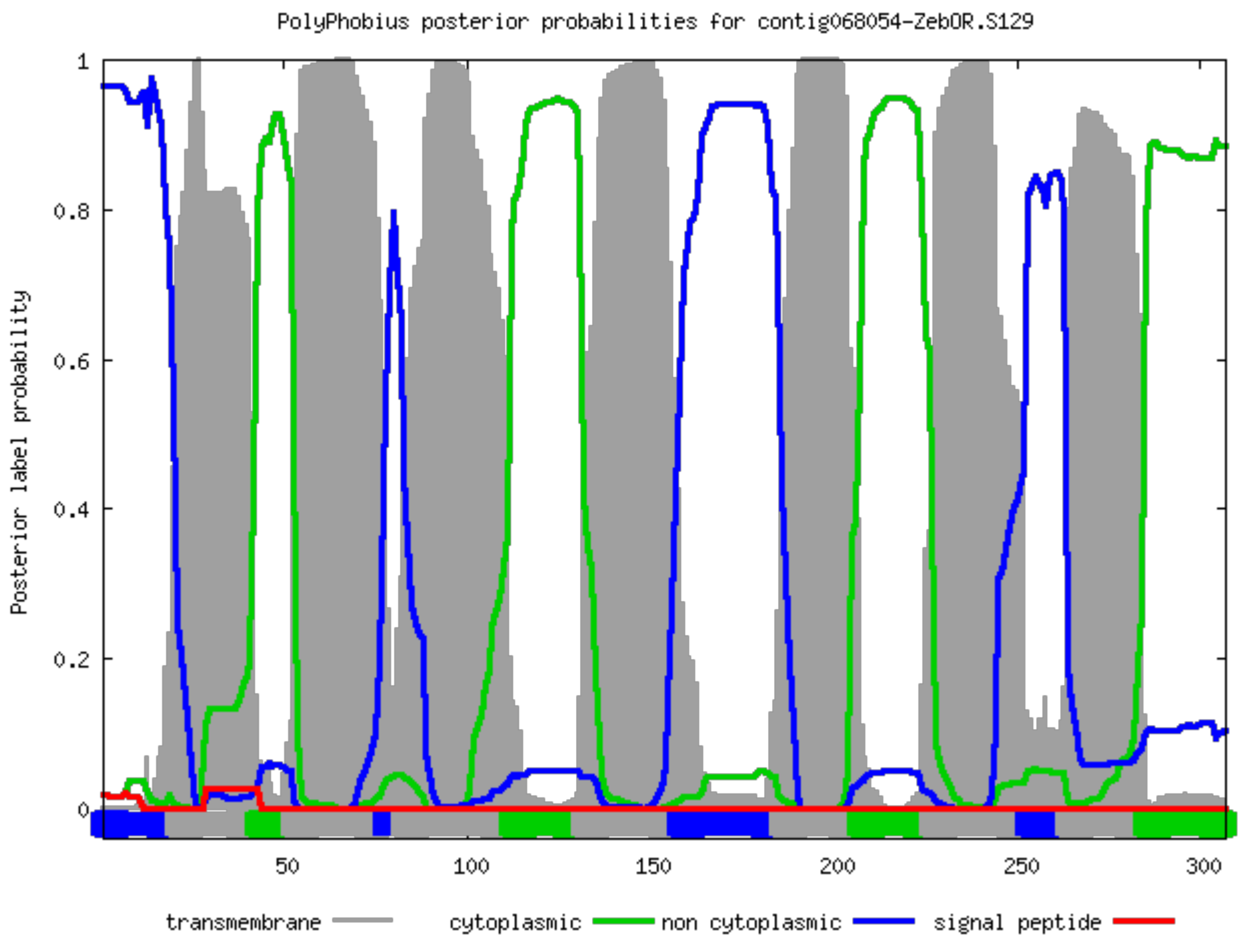

The prediction is based on an alignment. The probability data used in the plot is found here, and the gnuplot script is here.

Prediction of contig042534-BriOR.K067

```
ID   contig042534-BriOR.K067
FT   TOPO_DOM      1     25       NON CYTOPLASMIC.
FT   TRANSMEM     26     50
FT   TOPO_DOM     51     59       CYTOPLASMIC.
FT   TRANSMEM     60     81
FT   TOPO_DOM     82     99       NON CYTOPLASMIC.
FT   TRANSMEM    100    120
FT   TOPO_DOM    121    140       CYTOPLASMIC.
FT   TRANSMEM    141    164
FT   TOPO_DOM    165    196       NON CYTOPLASMIC.
FT   TRANSMEM    197    222
FT   TOPO_DOM    223    241       CYTOPLASMIC.
FT   TRANSMEM    242    261
FT   TOPO_DOM    262    271       NON CYTOPLASMIC.
FT   TRANSMEM    272    291
FT   TOPO_DOM    292    312       CYTOPLASMIC.
//
```

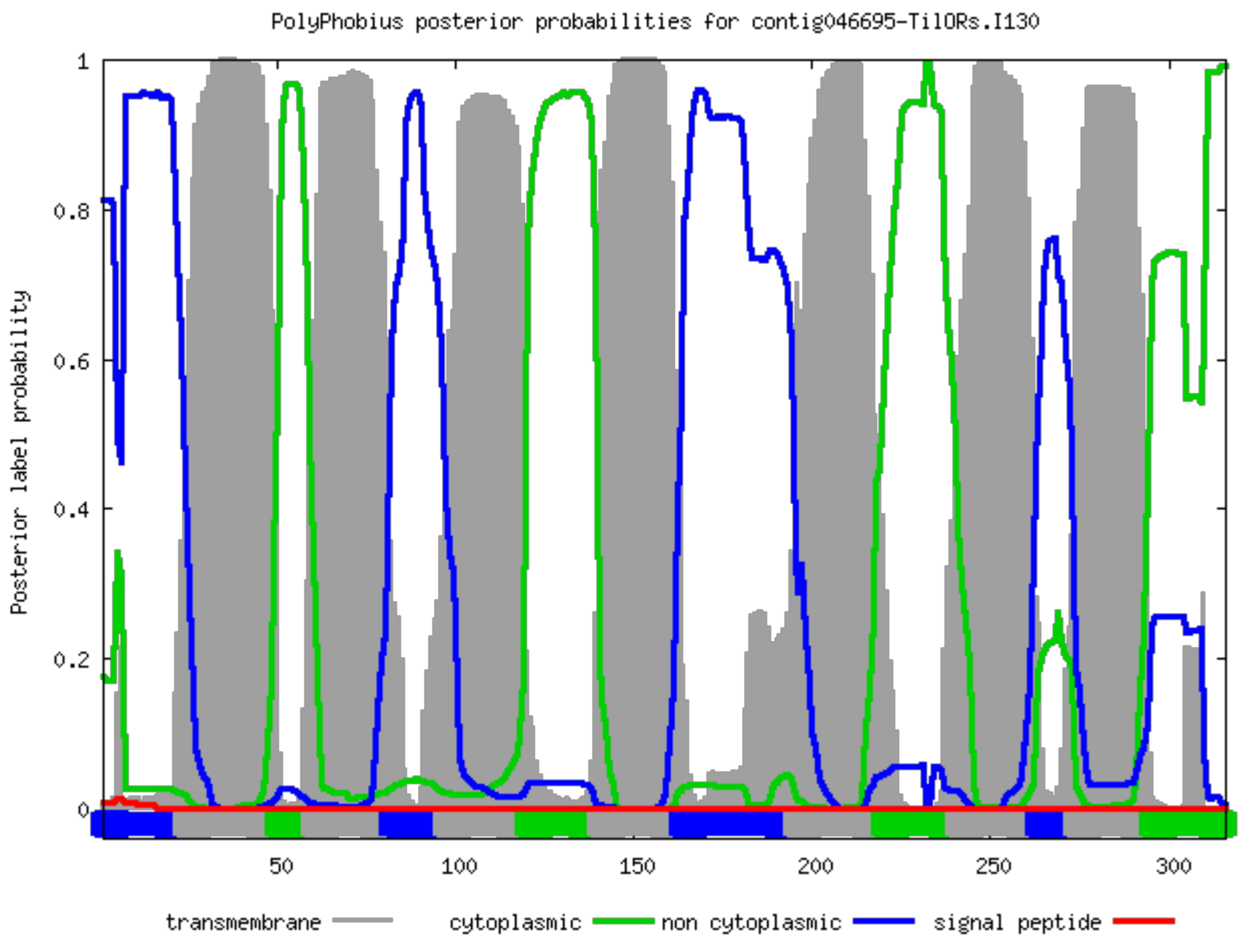

The prediction is based on an alignment. The probability data used in the plot is found here, and the gnuplot script is here.

Prediction of contig017743-ZebOR.S128

```
ID   contig017743-ZebOR.S128
FT   TOPO_DOM      1     19       NON CYTOPLASMIC.
FT   TRANSMEM     20     41
FT   TOPO_DOM     42     51       CYTOPLASMIC.
FT   TRANSMEM     52     76
FT   TOPO_DOM     77     81       NON CYTOPLASMIC.
FT   TRANSMEM     82    110
FT   TOPO_DOM    111    130       CYTOPLASMIC.
FT   TRANSMEM    131    156
FT   TOPO_DOM    157    184       NON CYTOPLASMIC.
FT   TRANSMEM    185    205
FT   TOPO_DOM    206    225       CYTOPLASMIC.
FT   TRANSMEM    226    251
FT   TOPO_DOM    252    262       NON CYTOPLASMIC.
FT   TRANSMEM    263    283
FT   TOPO_DOM    284    304       CYTOPLASMIC.
//
```

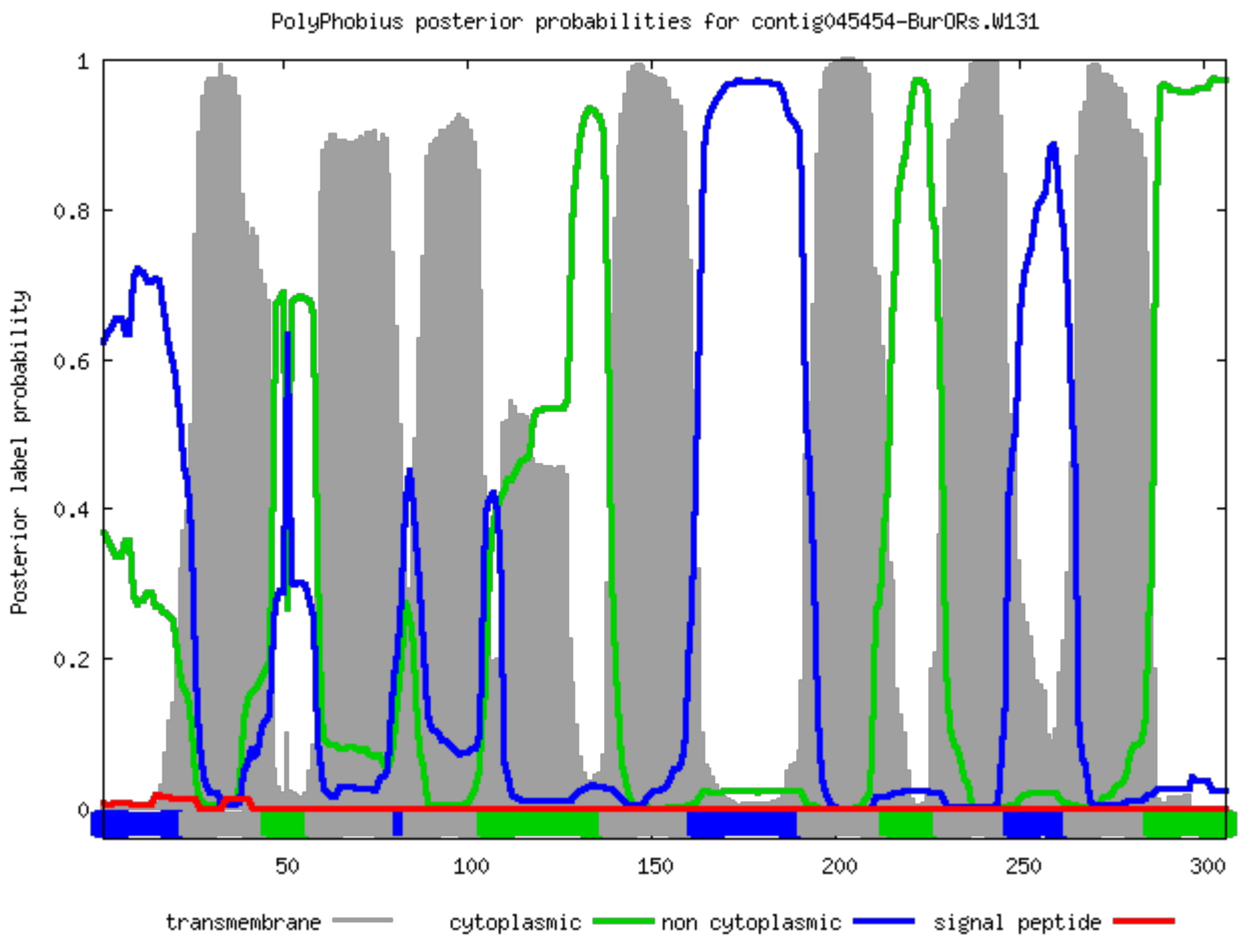

The prediction is based on an alignment. The probability data used in the plot is found here, and the gnuplot script is here.

Prediction of contig040509-NyeOR.K083

```
ID    contig040509-NyeOR.K083
FT    TOPO_DOM      1      25       NON CYTOPLASMIC.
FT    TRANSMEM     26      50
FT    TOPO_DOM     51      59       CYTOPLASMIC.
FT    TRANSMEM     60      81
FT    TOPO_DOM     82      99       NON CYTOPLASMIC.
FT    TRANSMEM    100     120
FT    TOPO_DOM    121     140       CYTOPLASMIC.
FT    TRANSMEM    141     164
FT    TOPO_DOM    165     196       NON CYTOPLASMIC.
FT    TRANSMEM    197     222
FT    TOPO_DOM    223     241       CYTOPLASMIC.
FT    TRANSMEM    242     261
FT    TOPO_DOM    262     271       NON CYTOPLASMIC.
FT    TRANSMEM    272     291
FT    TOPO_DOM    292     312       CYTOPLASMIC.
//
```

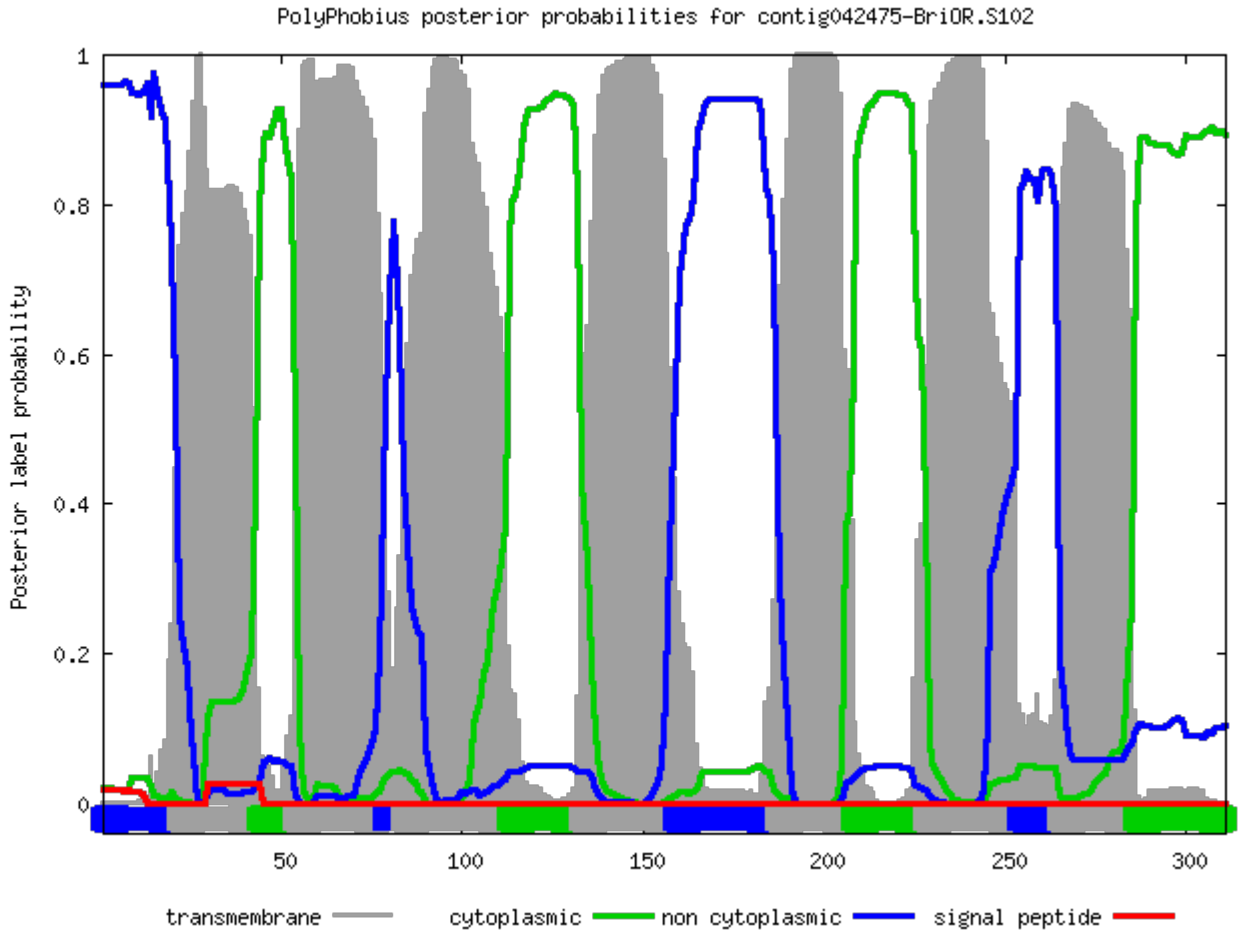

The prediction is based on an alignment. The probability data used in the plot is found here, and the gnuplot script is here.

Prediction of contig013330-TilOR.D054

```
ID   contig013330-TilOR.D054
FT   TOPO_DOM      1     22       NON CYTOPLASMIC.
FT   TRANSMEM     23     48
FT   TOPO_DOM     49     57       CYTOPLASMIC.
FT   TRANSMEM     58     81
FT   TOPO_DOM     82     90       NON CYTOPLASMIC.
FT   TRANSMEM     91    118
FT   TOPO_DOM    119    138       CYTOPLASMIC.
FT   TRANSMEM    139    161
FT   TOPO_DOM    162    193       NON CYTOPLASMIC.
FT   TRANSMEM    194    216
FT   TOPO_DOM    217    236       CYTOPLASMIC.
FT   TRANSMEM    237    256
FT   TOPO_DOM    257    267       NON CYTOPLASMIC.
FT   TRANSMEM    268    291
FT   TOPO_DOM    292    316       CYTOPLASMIC.
//
```

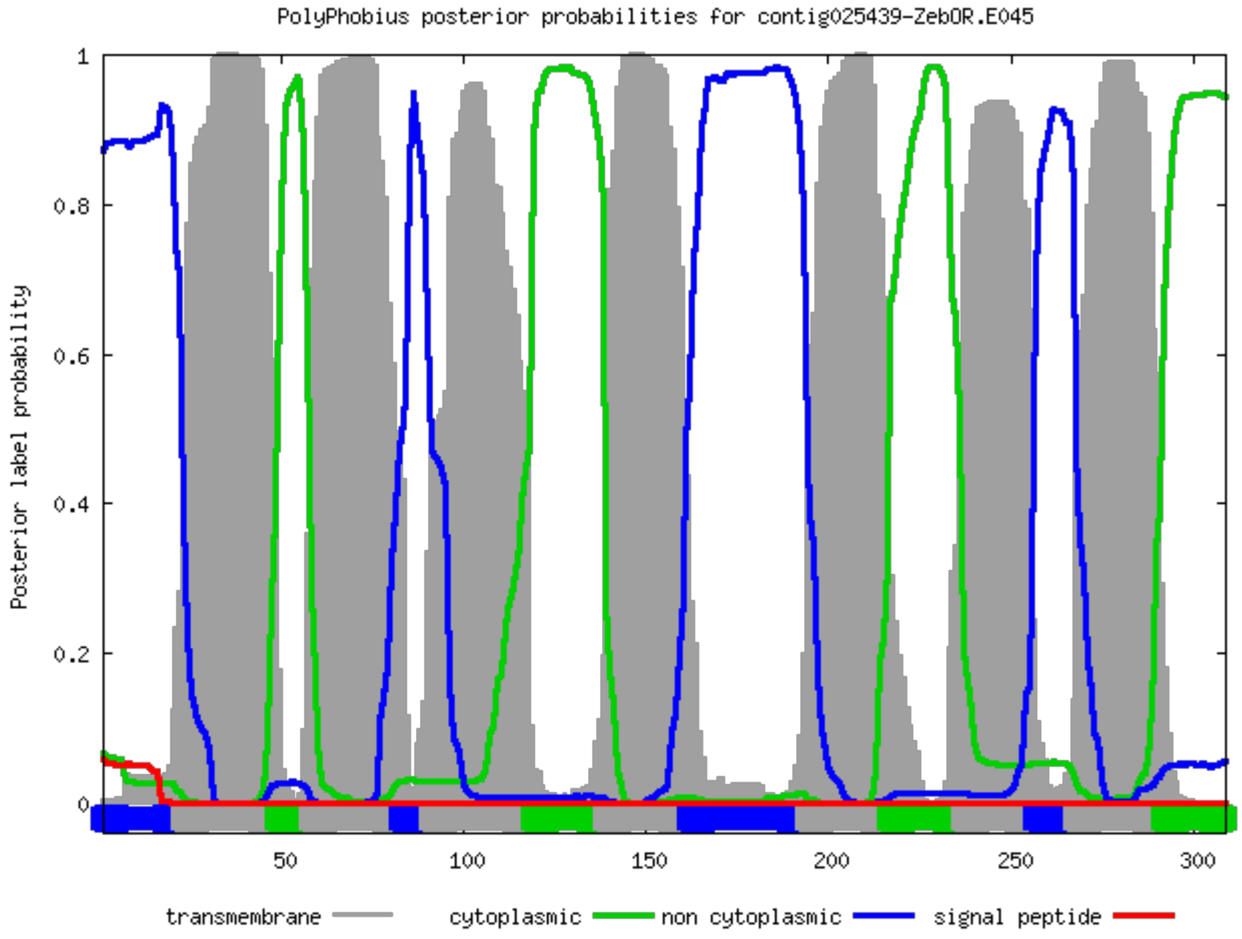

The prediction is based on an alignment. The probability data used in the plot is found here, and the gnuplot script is here.

Prediction of contig030557-ZebOR.A006

```
ID   contig030557-ZebOR.A006
FT   TOPO_DOM      1     22       NON CYTOPLASMIC.
FT   TRANSMEM     23     48
FT   TOPO_DOM     49     56       CYTOPLASMIC.
FT   TRANSMEM     57     77
FT   TOPO_DOM     78     95       NON CYTOPLASMIC.
FT   TRANSMEM     96    118
FT   TOPO_DOM    119    138       CYTOPLASMIC.
FT   TRANSMEM    139    160
FT   TOPO_DOM    161    192       NON CYTOPLASMIC.
FT   TRANSMEM    193    215
FT   TOPO_DOM    216    235       CYTOPLASMIC.
FT   TRANSMEM    236    257
FT   TOPO_DOM    258    268       NON CYTOPLASMIC.
FT   TRANSMEM    269    289
FT   TOPO_DOM    290    309       CYTOPLASMIC.
//
```

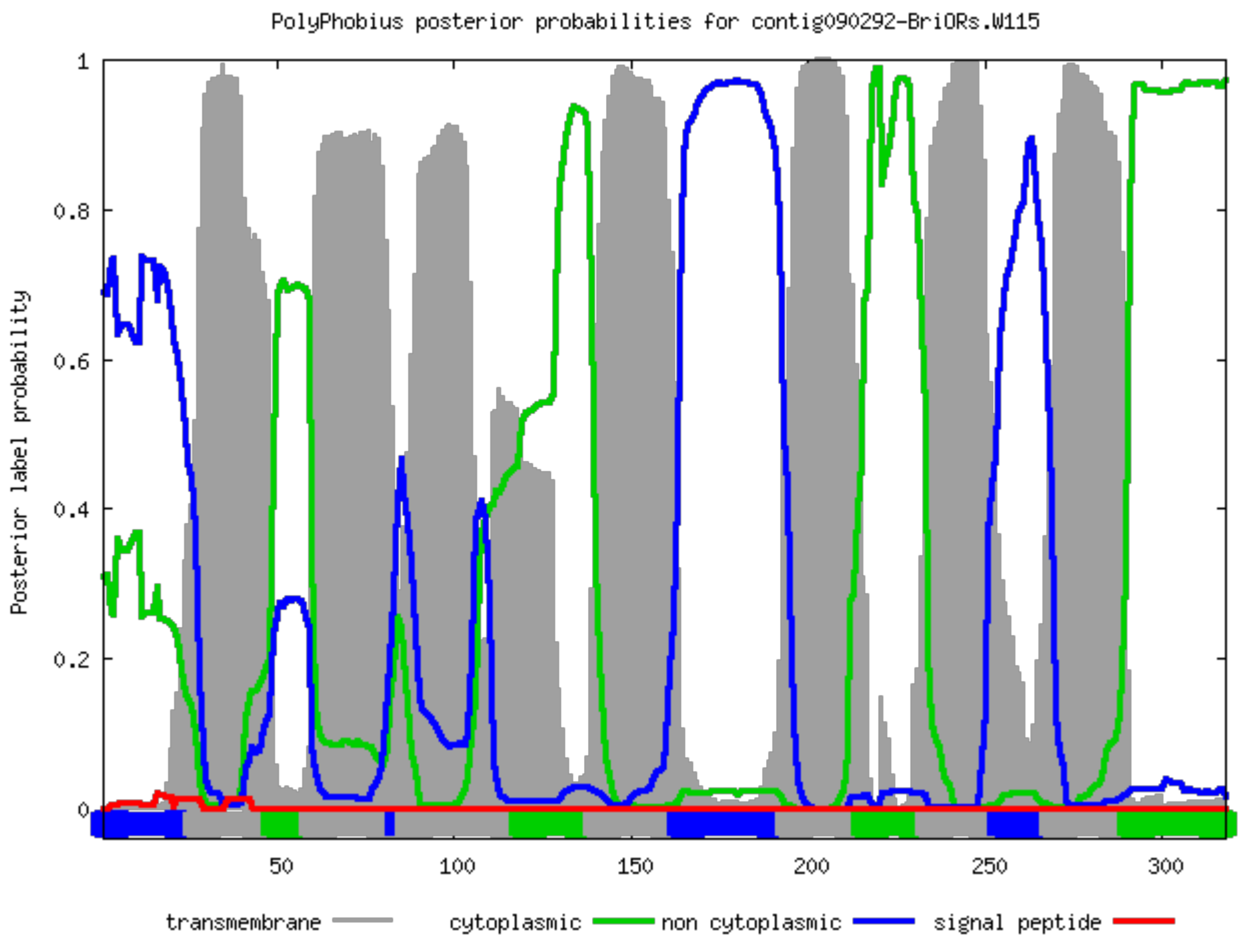

The prediction is based on an alignment. The probability data used in the plot is found here, and the gnuplot script is here.

Prediction of contig004266-BriOR.E039

```
ID   contig004266-BriOR.E039
FT   TOPO_DOM      1     22       NON CYTOPLASMIC.
FT   TRANSMEM     23     48
FT   TOPO_DOM     49     57       CYTOPLASMIC.
FT   TRANSMEM     58     82
FT   TOPO_DOM     83     90       NON CYTOPLASMIC.
FT   TRANSMEM     91    118
FT   TOPO_DOM    119    138       CYTOPLASMIC.
FT   TRANSMEM    139    161
FT   TOPO_DOM    162    193       NON CYTOPLASMIC.
FT   TRANSMEM    194    216
FT   TOPO_DOM    217    236       CYTOPLASMIC.
FT   TRANSMEM    237    256
FT   TOPO_DOM    257    267       NON CYTOPLASMIC.
FT   TRANSMEM    268    291
FT   TOPO_DOM    292    310       CYTOPLASMIC.
//
```

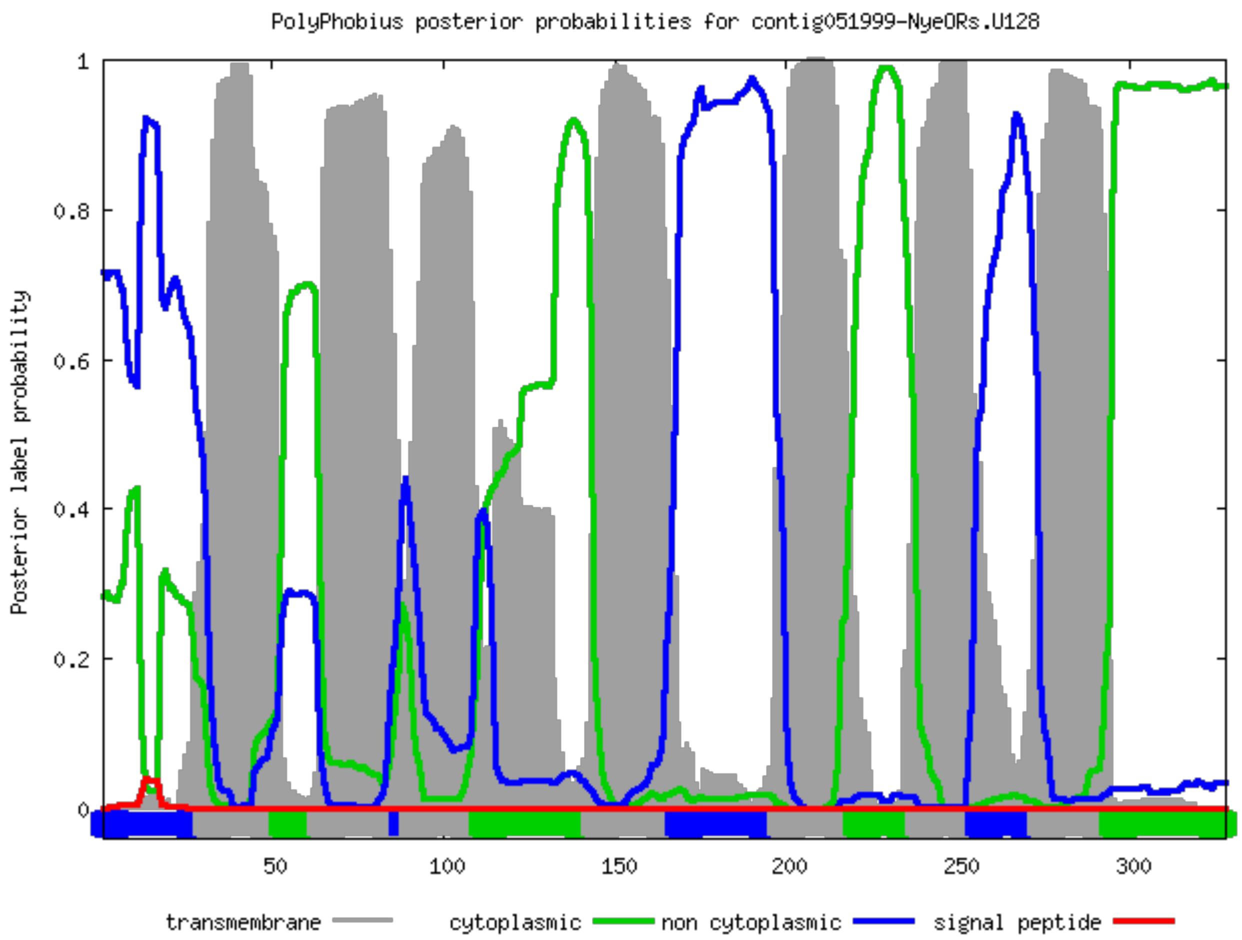

The prediction is based on an alignment. The probability data used in the plot is found here, and the gnuplot script is here.

Prediction of contig039462-TilOR.L149

```
ID   contig039462-TilOR.L149
FT   TOPO_DOM      1     25       NON CYTOPLASMIC.
FT   TRANSMEM     26     50
FT   TOPO_DOM     51     59       CYTOPLASMIC.
FT   TRANSMEM     60     82
FT   TOPO_DOM     83    100       NON CYTOPLASMIC.
FT   TRANSMEM    101    120
FT   TOPO_DOM    121    140       CYTOPLASMIC.
FT   TRANSMEM    141    163
FT   TOPO_DOM    164    199       NON CYTOPLASMIC.
FT   TRANSMEM    200    224
FT   TOPO_DOM    225    238       CYTOPLASMIC.
FT   TRANSMEM    239    261
FT   TOPO_DOM    262    271       NON CYTOPLASMIC.
FT   TRANSMEM    272    292
FT   TOPO_DOM    293    313       CYTOPLASMIC.
//
```

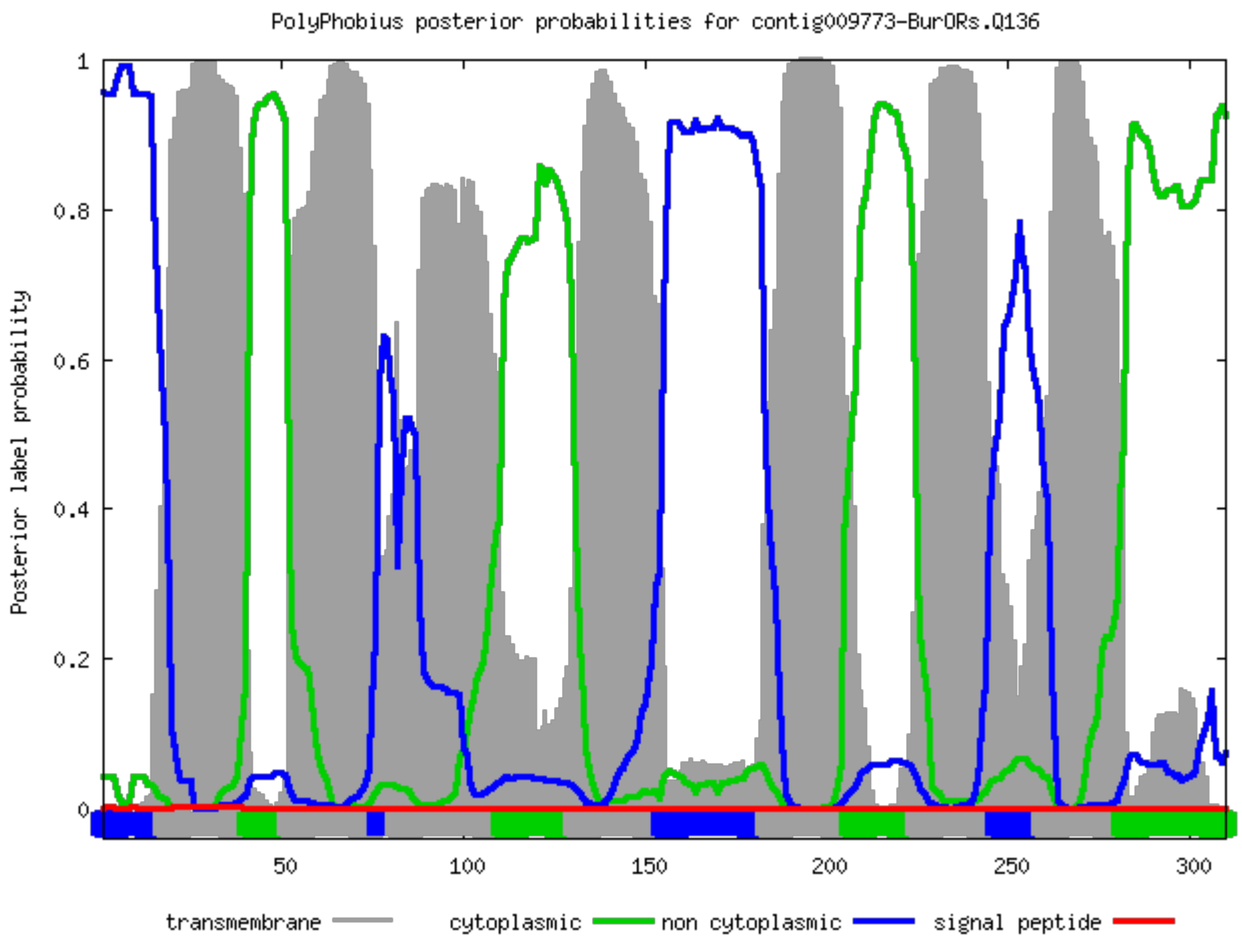

The prediction is based on an alignment. The probability data used in the plot is found here, and the gnuplot script is here.

Prediction of contig084999-BriOR.A001

```
ID   contig084999-BriOR.A001
FT   TOPO_DOM      1     22       NON CYTOPLASMIC.
FT   TRANSMEM     23     48
FT   TOPO_DOM     49     56       CYTOPLASMIC.
FT   TRANSMEM     57     77
FT   TOPO_DOM     78     95       NON CYTOPLASMIC.
FT   TRANSMEM     96    118
FT   TOPO_DOM    119    138       CYTOPLASMIC.
FT   TRANSMEM    139    159
FT   TOPO_DOM    160    192       NON CYTOPLASMIC.
FT   TRANSMEM    193    215
FT   TOPO_DOM    216    235       CYTOPLASMIC.
FT   TRANSMEM    236    257
FT   TOPO_DOM    258    268       NON CYTOPLASMIC.
FT   TRANSMEM    269    289
FT   TOPO_DOM    290    307       CYTOPLASMIC.
//
```

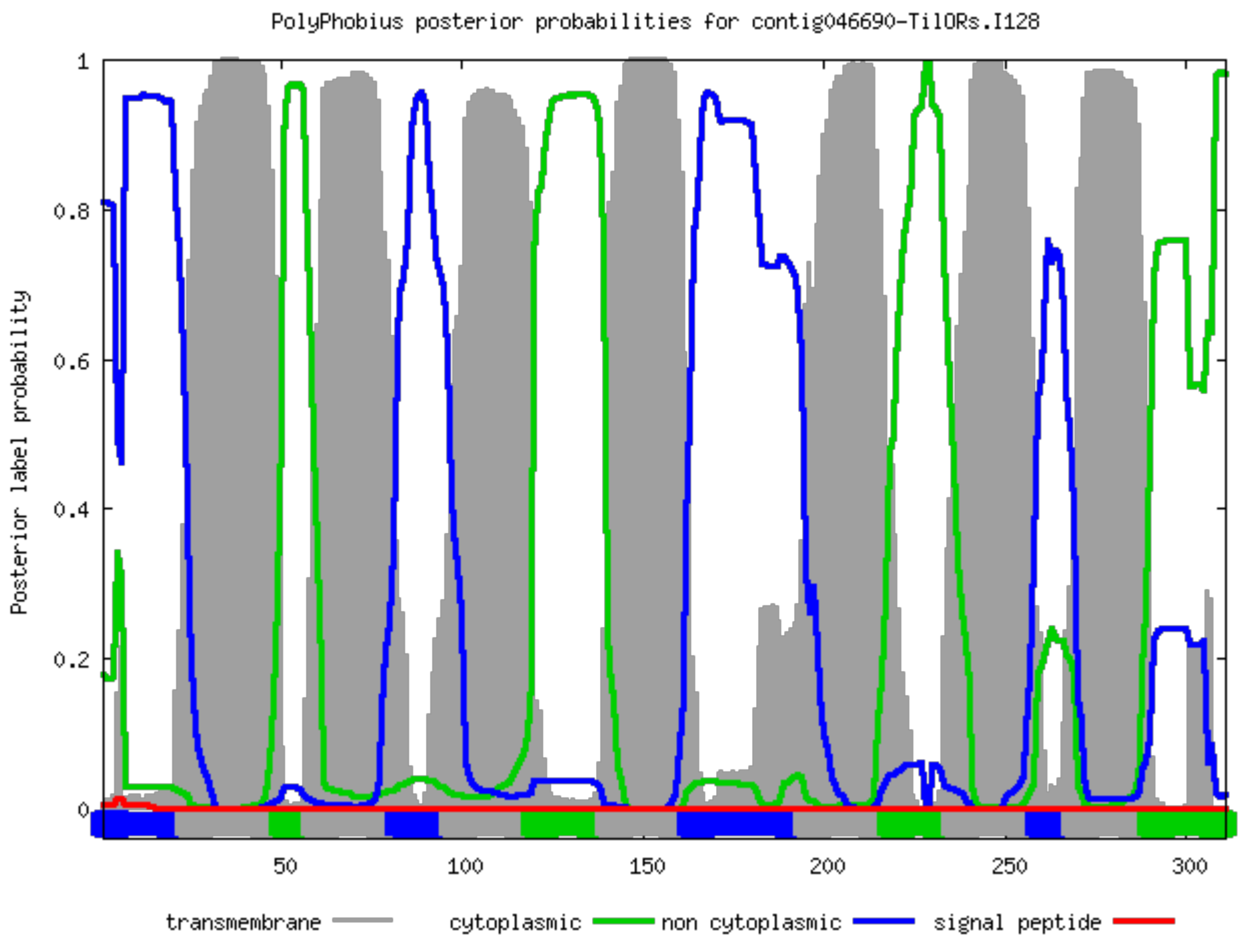

The prediction is based on an alignment. The probability data used in the plot is found here, and the gnuplot script is here.

Prediction of contig059270-NyeOR.S122

```
ID   contig059270-NyeOR.S122
FT   TOPO_DOM      1     20       NON CYTOPLASMIC.
FT   TRANSMEM     21     42
FT   TOPO_DOM     43     53       CYTOPLASMIC.
FT   TRANSMEM     54     77
FT   TOPO_DOM     78     80       NON CYTOPLASMIC.
FT   TRANSMEM     81    109
FT   TOPO_DOM    110    129       CYTOPLASMIC.
FT   TRANSMEM    130    155
FT   TOPO_DOM    156    183       NON CYTOPLASMIC.
FT   TRANSMEM    184    204
FT   TOPO_DOM    205    224       CYTOPLASMIC.
FT   TRANSMEM    225    250
FT   TOPO_DOM    251    261       NON CYTOPLASMIC.
FT   TRANSMEM    262    282
FT   TOPO_DOM    283    307       CYTOPLASMIC.
//
```

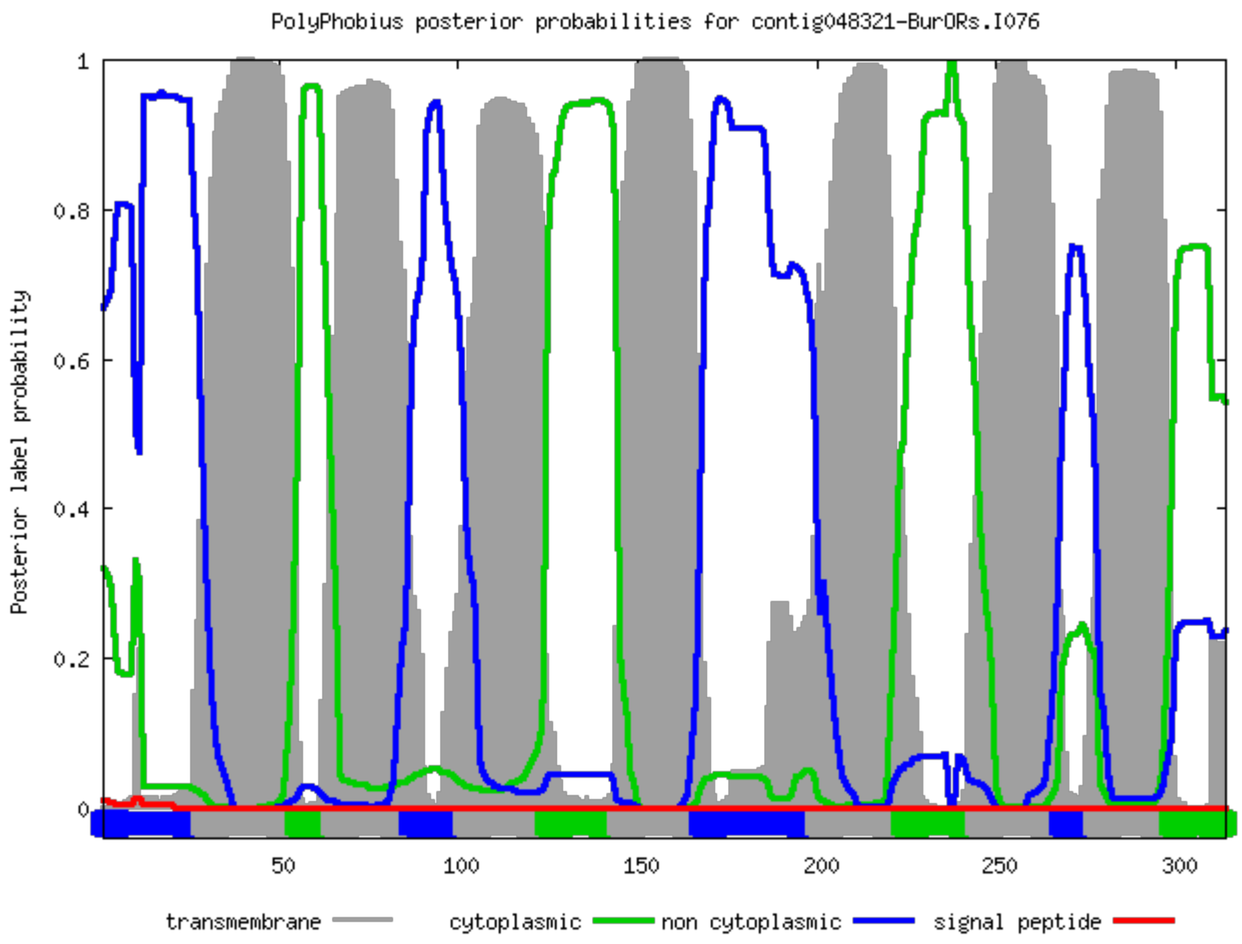

The prediction is based on an alignment. The probability data used in the plot is found here, and the gnuplot script is here.

Prediction of contig047826-TilOR.E080

```
ID    contig047826-TilOR.E080
FT    TOPO_DOM      1      25       NON CYTOPLASMIC.
FT    TRANSMEM     26      51
FT    TOPO_DOM     52      60       CYTOPLASMIC.
FT    TRANSMEM     61      86
FT    TOPO_DOM     87      93       NON CYTOPLASMIC.
FT    TRANSMEM     94     121
FT    TOPO_DOM    122     141       CYTOPLASMIC.
FT    TRANSMEM    142     164
FT    TOPO_DOM    165     196       NON CYTOPLASMIC.
FT    TRANSMEM    197     219
FT    TOPO_DOM    220     239       CYTOPLASMIC.
FT    TRANSMEM    240     259
FT    TOPO_DOM    260     271       NON CYTOPLASMIC.
FT    TRANSMEM    272     294
FT    TOPO_DOM    295     326       CYTOPLASMIC.
//
```

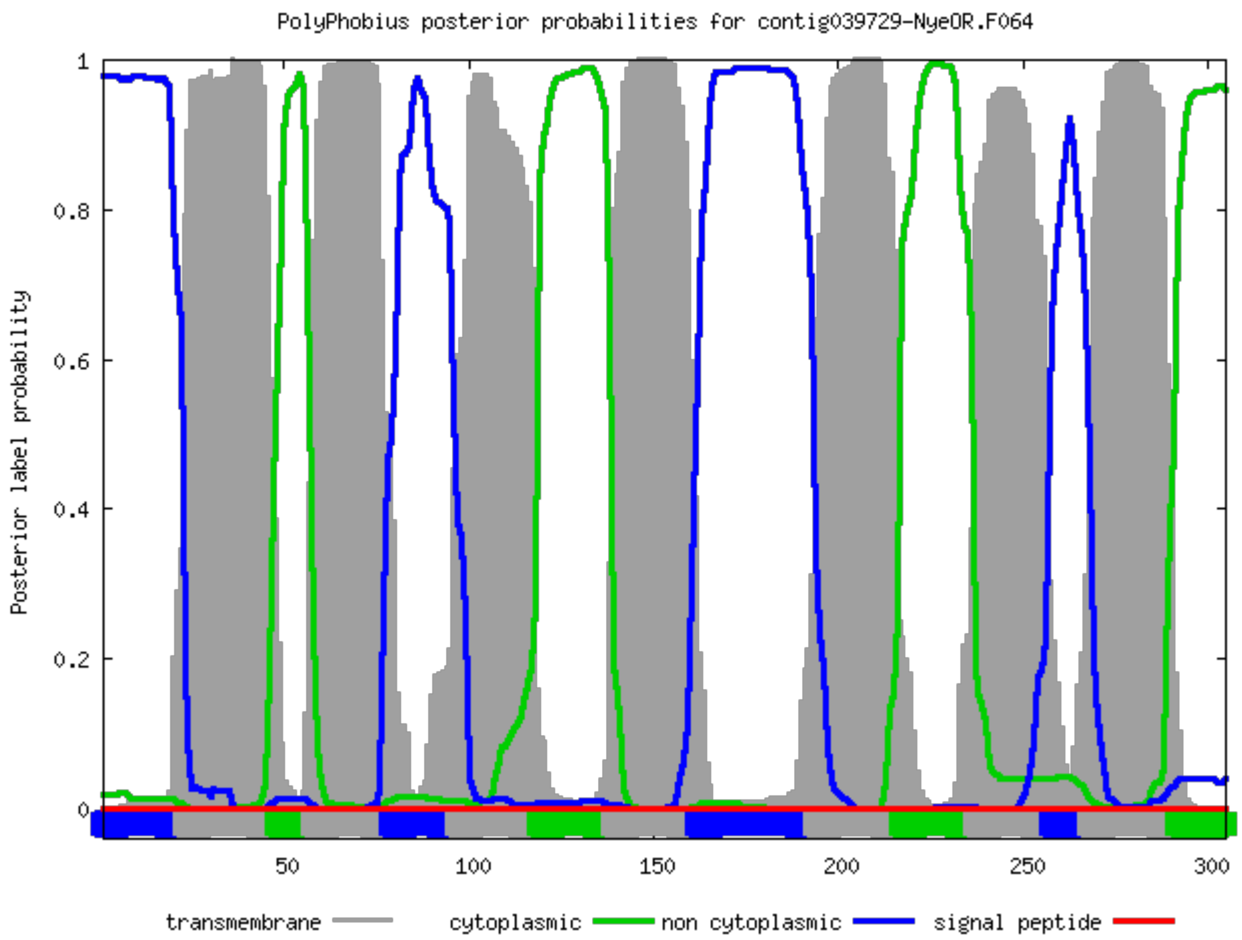

The prediction is based on an alignment. The probability data used in the plot is found here, and the gnuplot script is here.

Prediction of contig054681-NyeOR.A011

```
ID   contig054681-NyeOR.A011
FT   TOPO_DOM      1      22       NON CYTOPLASMIC.
FT   TRANSMEM     23      48
FT   TOPO_DOM     49      56       CYTOPLASMIC.
FT   TRANSMEM     57      77
FT   TOPO_DOM     78      95       NON CYTOPLASMIC.
FT   TRANSMEM     96     118
FT   TOPO_DOM    119     138       CYTOPLASMIC.
FT   TRANSMEM    139     159
FT   TOPO_DOM    160     192       NON CYTOPLASMIC.
FT   TRANSMEM    193     215
FT   TOPO_DOM    216     235       CYTOPLASMIC.
FT   TRANSMEM    236     257
FT   TOPO_DOM    258     268       NON CYTOPLASMIC.
FT   TRANSMEM    269     289
FT   TOPO_DOM    290     309       CYTOPLASMIC.
//
```

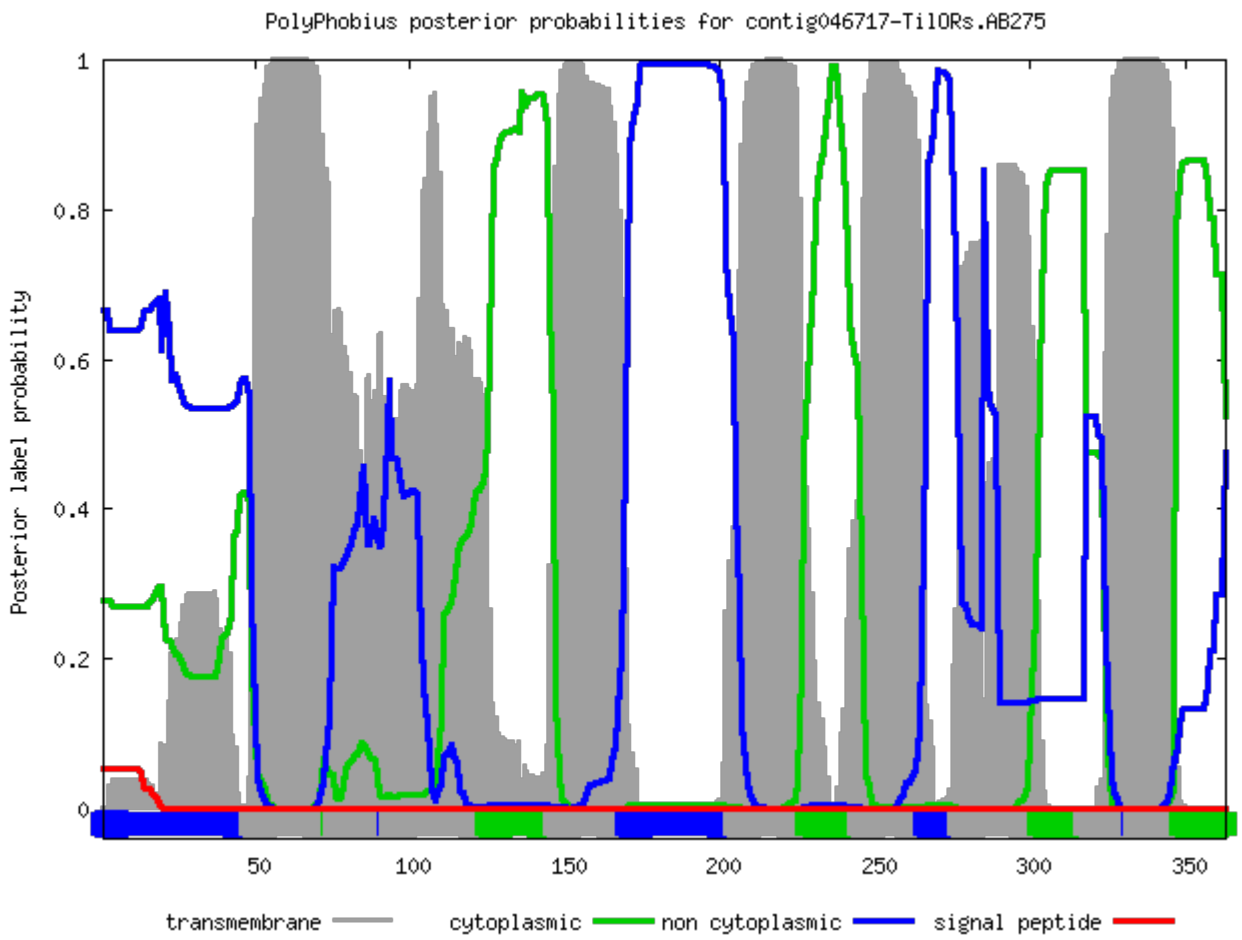

The prediction is based on an alignment. The probability data used in the plot is found here, and the gnuplot script is here.

Prediction of contig014056-ZebOR.F063

```
ID   contig014056-ZebOR.F063
FT   TOPO_DOM      1      22       NON CYTOPLASMIC.
FT   TRANSMEM     23      47
FT   TOPO_DOM     48      57       CYTOPLASMIC.
FT   TRANSMEM     58      78
FT   TOPO_DOM     79      96       NON CYTOPLASMIC.
FT   TRANSMEM     97     118
FT   TOPO_DOM    119     138       CYTOPLASMIC.
FT   TRANSMEM    139     161
FT   TOPO_DOM    162     193       NON CYTOPLASMIC.
FT   TRANSMEM    194     216
FT   TOPO_DOM    217     236       CYTOPLASMIC.
FT   TRANSMEM    237     257
FT   TOPO_DOM    258     267       NON CYTOPLASMIC.
FT   TRANSMEM    268     291
FT   TOPO_DOM    292     305       CYTOPLASMIC.
//
```

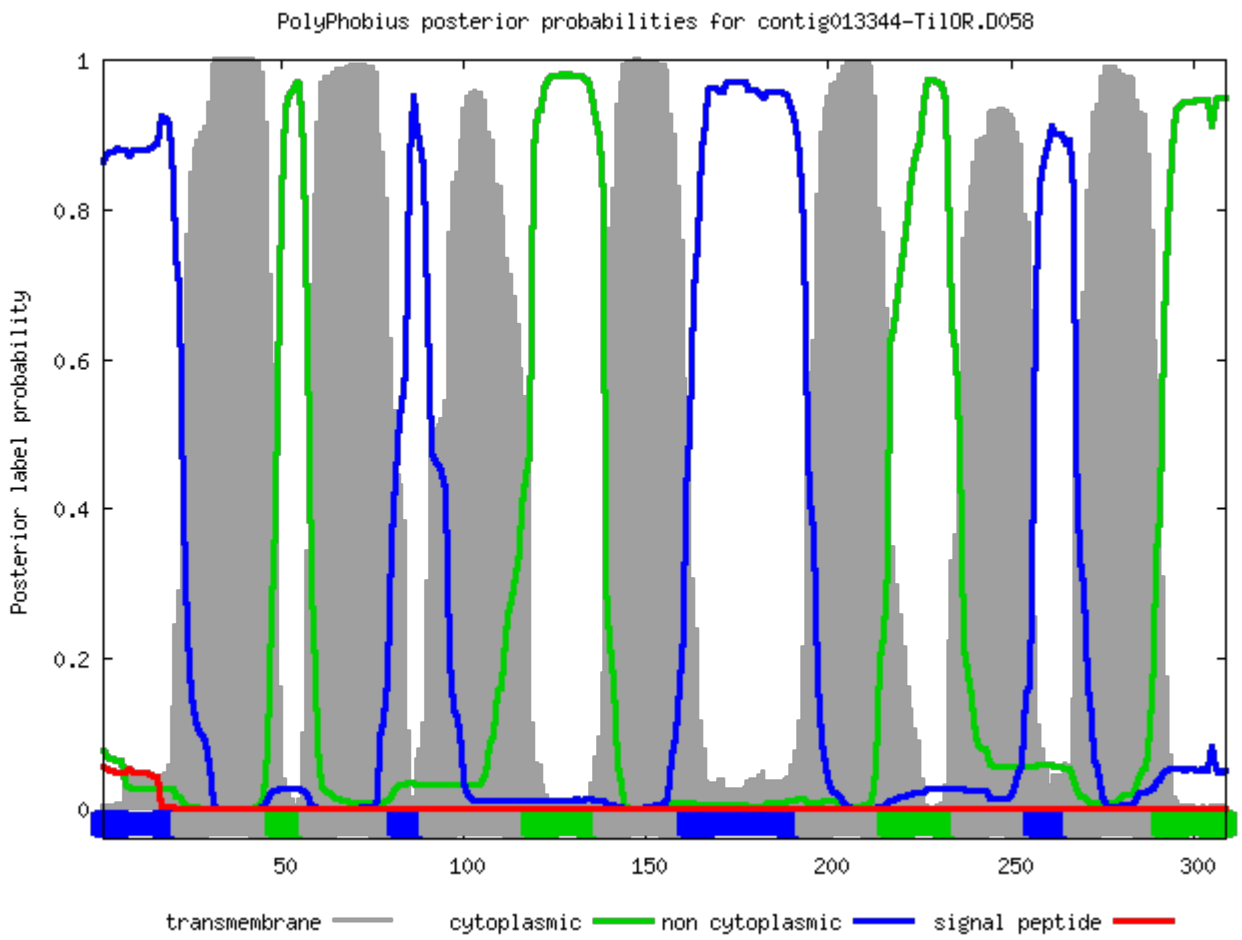

The prediction is based on an alignment. The probability data used in the plot is found here, and the gnuplot script is here.

Prediction of contig028593-BurOR.S121

```
ID   contig028593-BurOR.S121
FT   TOPO_DOM      1     21       NON CYTOPLASMIC.
FT   TRANSMEM     22     43
FT   TOPO_DOM     44     53       CYTOPLASMIC.
FT   TRANSMEM     54     78
FT   TOPO_DOM     79     83       NON CYTOPLASMIC.
FT   TRANSMEM     84    112
FT   TOPO_DOM    113    132       CYTOPLASMIC.
FT   TRANSMEM    133    158
FT   TOPO_DOM    159    186       NON CYTOPLASMIC.
FT   TRANSMEM    187    207
FT   TOPO_DOM    208    227       CYTOPLASMIC.
FT   TRANSMEM    228    253
FT   TOPO_DOM    254    264       NON CYTOPLASMIC.
FT   TRANSMEM    265    285
FT   TOPO_DOM    286    313       CYTOPLASMIC.
//
```

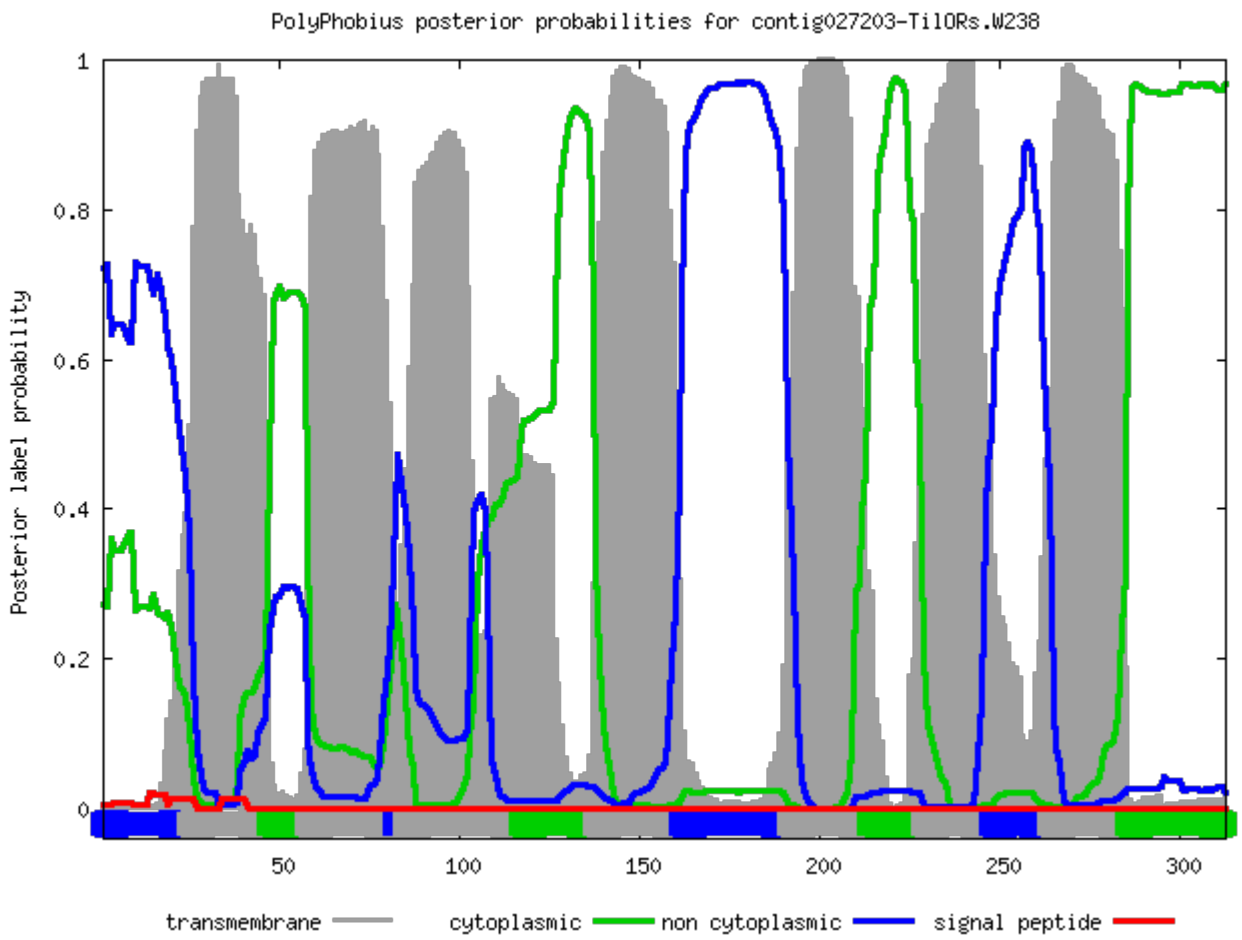

The prediction is based on an alignment. The probability data used in the plot is found here, and the gnuplot script is here.

Prediction of contig064098-ZebOR.N116

```
ID   contig064098-ZebOR.N116
FT   TOPO_DOM      1     32       NON CYTOPLASMIC.
FT   TRANSMEM     33     58
FT   TOPO_DOM     59     66       CYTOPLASMIC.
FT   TRANSMEM     67     86
FT   TOPO_DOM     87    104       NON CYTOPLASMIC.
FT   TRANSMEM    105    127
FT   TOPO_DOM    128    146       CYTOPLASMIC.
FT   TRANSMEM    147    170
FT   TOPO_DOM    171    206       NON CYTOPLASMIC.
FT   TRANSMEM    207    232
FT   TOPO_DOM    233    250       CYTOPLASMIC.
FT   TRANSMEM    251    272
FT   TOPO_DOM    273    277       NON CYTOPLASMIC.
FT   TRANSMEM    278    298
FT   TOPO_DOM    299    323       CYTOPLASMIC.
//
```

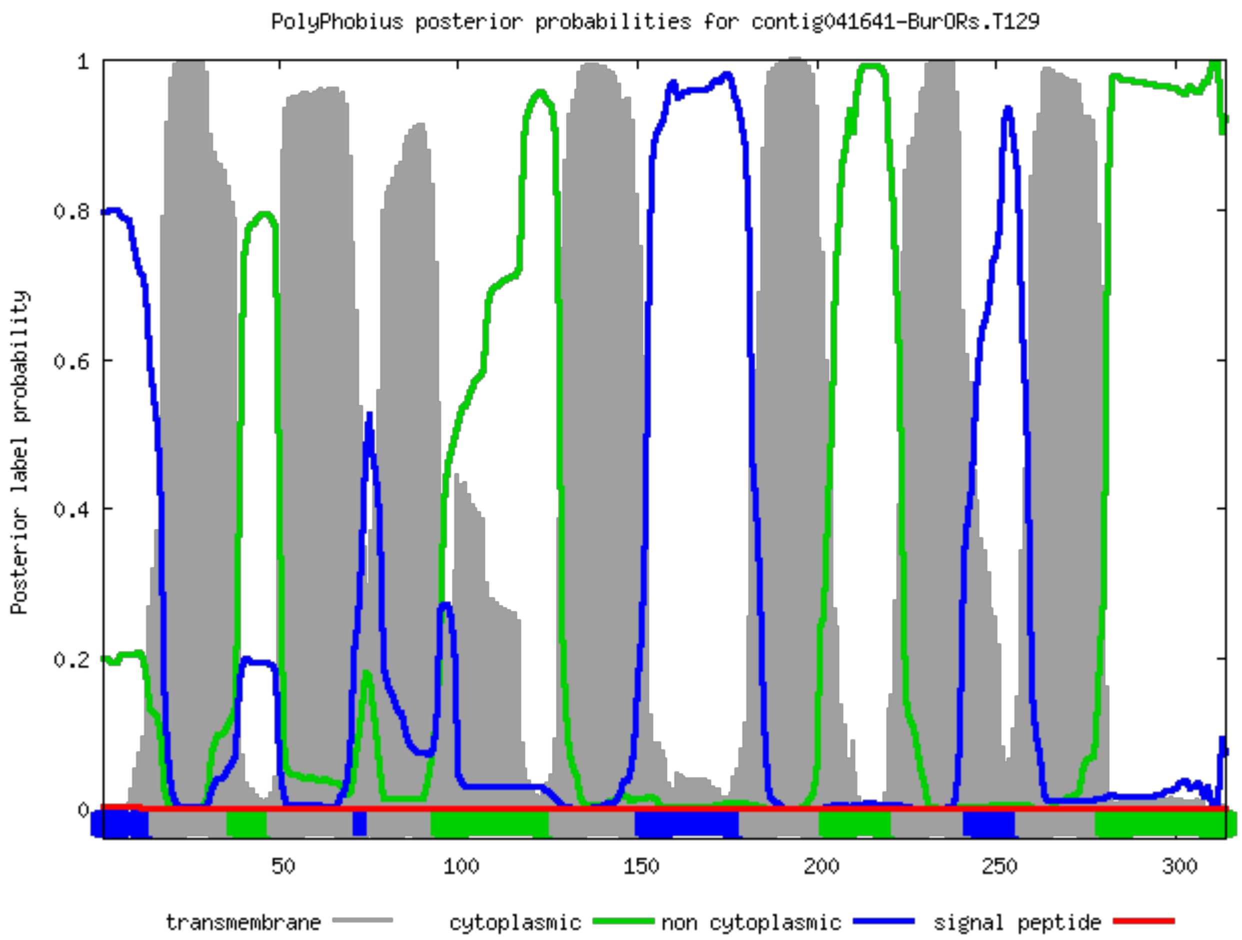

The prediction is based on an alignment. The probability data used in the plot is found here, and the gnuplot script is here.

Prediction of contig036787-BurOR.A004

```
ID   contig036787-BurOR.A004
FT   TOPO_DOM      1     25       NON CYTOPLASMIC.
FT   TRANSMEM     26     51
FT   TOPO_DOM     52     59       CYTOPLASMIC.
FT   TRANSMEM     60     80
FT   TOPO_DOM     81     98       NON CYTOPLASMIC.
FT   TRANSMEM     99    121
FT   TOPO_DOM    122    141       CYTOPLASMIC.
FT   TRANSMEM    142    163
FT   TOPO_DOM    164    195       NON CYTOPLASMIC.
FT   TRANSMEM    196    219
FT   TOPO_DOM    220    239       CYTOPLASMIC.
FT   TRANSMEM    240    261
FT   TOPO_DOM    262    272       NON CYTOPLASMIC.
FT   TRANSMEM    273    293
FT   TOPO_DOM    294    337       CYTOPLASMIC.
//
```

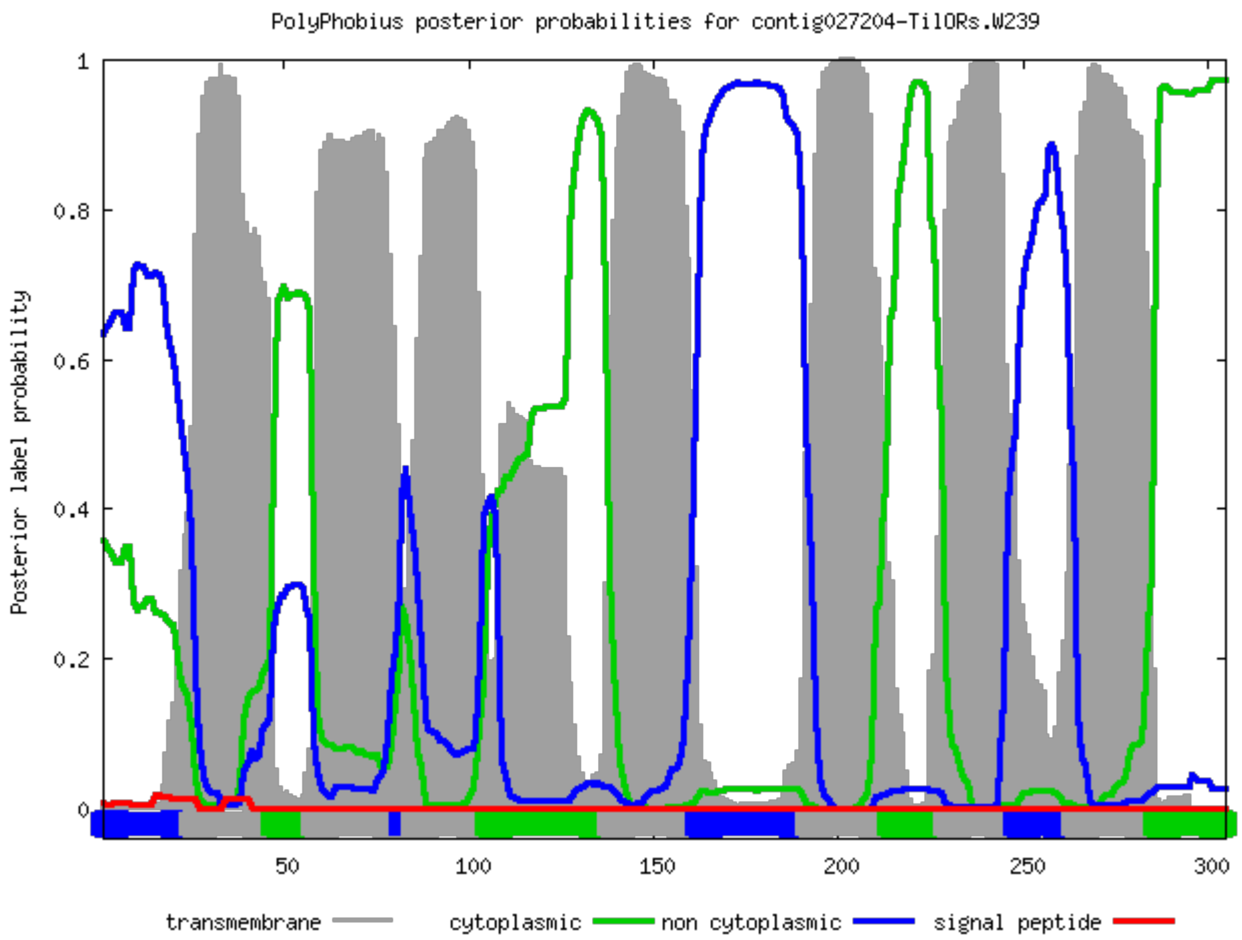

The prediction is based on an alignment. The probability data used in the plot is found here, and the gnuplot script is here.

Prediction of contig046718-TilOR.K137

```
ID    contig046718-TilOR.K137
FT    TOPO_DOM       1      22       NON CYTOPLASMIC.
FT    TRANSMEM      23      46
FT    TOPO_DOM      47      56       CYTOPLASMIC.
FT    TRANSMEM      57      78
FT    TOPO_DOM      79      97       NON CYTOPLASMIC.
FT    TRANSMEM      98     119
FT    TOPO_DOM     120     139       CYTOPLASMIC.
FT    TRANSMEM     140     163
FT    TOPO_DOM     164     196       NON CYTOPLASMIC.
FT    TRANSMEM     197     220
FT    TOPO_DOM     221     240       CYTOPLASMIC.
FT    TRANSMEM     241     260
FT    TOPO_DOM     261     270       NON CYTOPLASMIC.
FT    TRANSMEM     271     290
FT    TOPO_DOM     291     315       CYTOPLASMIC.
//
```

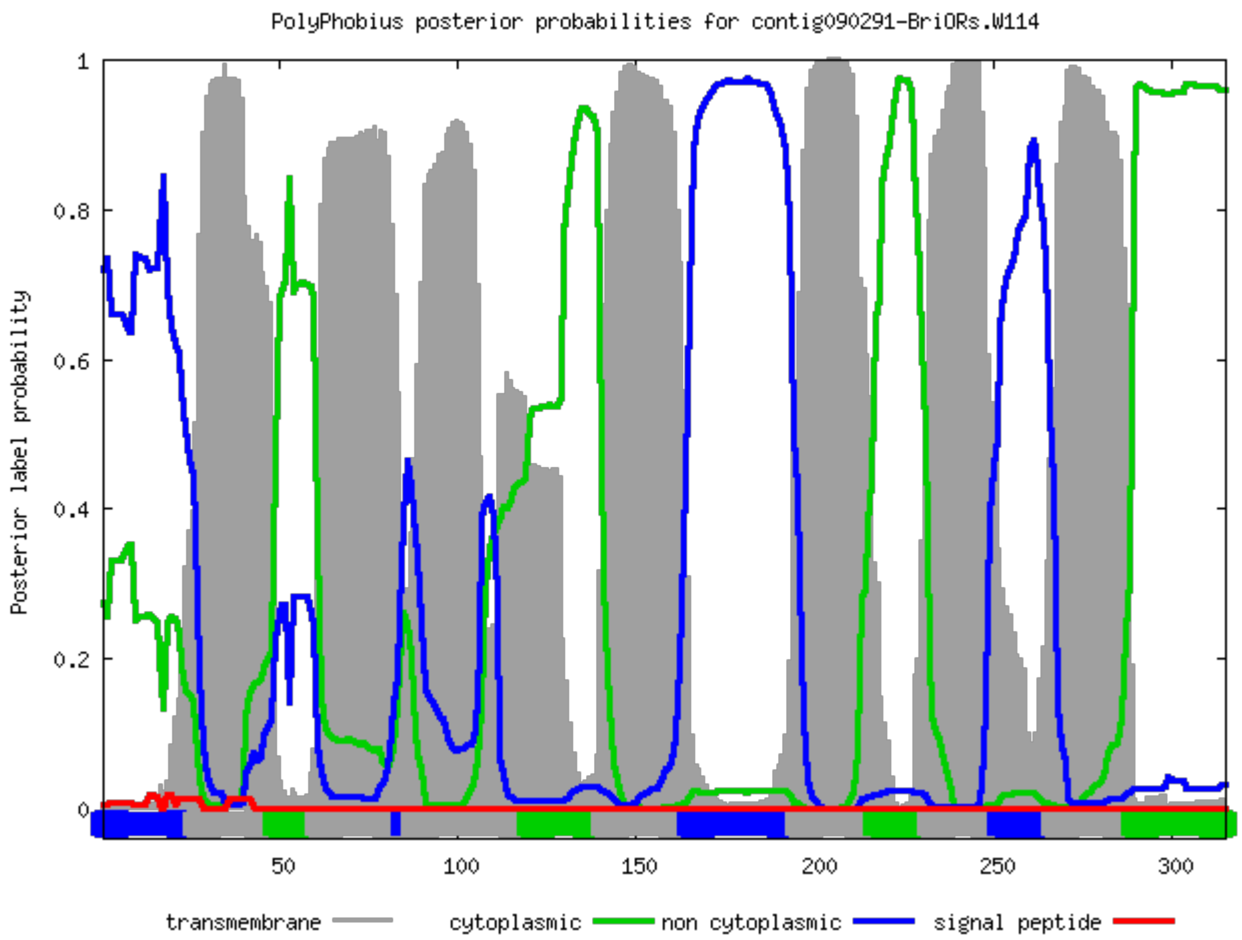

The prediction is based on an alignment. The probability data used in the plot is found here, and the gnuplot script is here.

Prediction of contig010734-ZebOR.M107

```
ID   contig010734-ZebOR.M107
FT   TOPO_DOM      1     31       NON CYTOPLASMIC.
FT   TRANSMEM     32     56
FT   TOPO_DOM     57     66       CYTOPLASMIC.
FT   TRANSMEM     67     88
FT   TOPO_DOM     89    107       NON CYTOPLASMIC.
FT   TRANSMEM    108    126
FT   TOPO_DOM    127    146       CYTOPLASMIC.
FT   TRANSMEM    147    170
FT   TOPO_DOM    171    208       NON CYTOPLASMIC.
FT   TRANSMEM    209    232
FT   TOPO_DOM    233    250       CYTOPLASMIC.
FT   TRANSMEM    251    273
FT   TOPO_DOM    274    279       NON CYTOPLASMIC.
FT   TRANSMEM    280    299
FT   TOPO_DOM    300    327       CYTOPLASMIC.
//
```

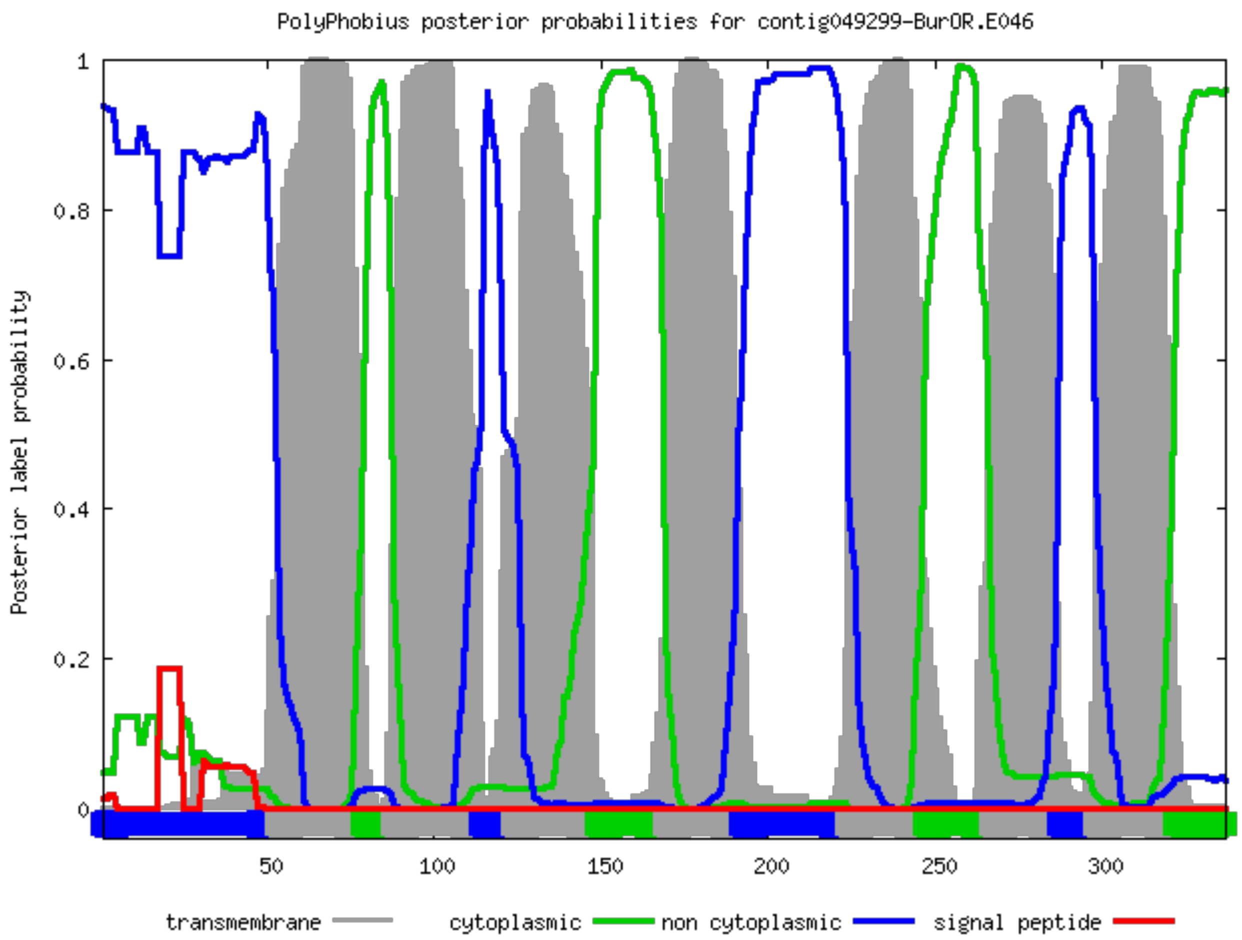

The prediction is based on an alignment. The probability data used in the plot is found here, and the gnuplot script is here.

Prediction of contig047497-ZebOR.A011

```
ID   contig047497-ZebOR.A011
FT   TOPO_DOM      1      22       NON CYTOPLASMIC.
FT   TRANSMEM     23      48
FT   TOPO_DOM     49      56       CYTOPLASMIC.
FT   TRANSMEM     57      77
FT   TOPO_DOM     78      95       NON CYTOPLASMIC.
FT   TRANSMEM     96     118
FT   TOPO_DOM    119     138       CYTOPLASMIC.
FT   TRANSMEM    139     160
FT   TOPO_DOM    161     192       NON CYTOPLASMIC.
FT   TRANSMEM    193     215
FT   TOPO_DOM    216     235       CYTOPLASMIC.
FT   TRANSMEM    236     257
FT   TOPO_DOM    258     268       NON CYTOPLASMIC.
FT   TRANSMEM    269     289
FT   TOPO_DOM    290     320       CYTOPLASMIC.
//
```

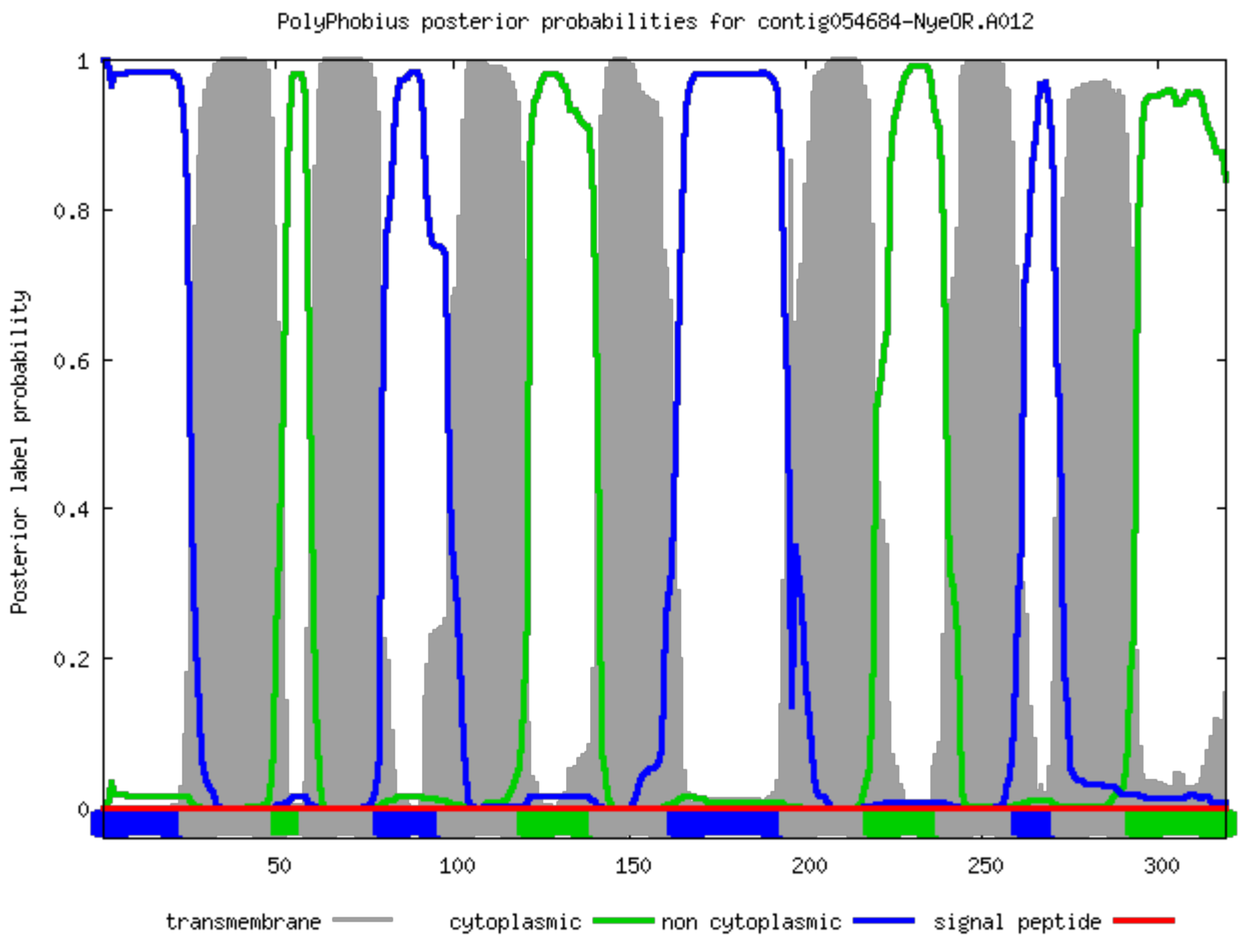

The prediction is based on an alignment. The probability data used in the plot is found here, and the gnuplot script is here.

Prediction of contig065887-BurOR.A018

```
ID   contig065887-BurOR.A018
FT   TOPO_DOM      1     22       NON CYTOPLASMIC.
FT   TRANSMEM     23     48
FT   TOPO_DOM     49     56       CYTOPLASMIC.
FT   TRANSMEM     57     77
FT   TOPO_DOM     78     95       NON CYTOPLASMIC.
FT   TRANSMEM     96    118
FT   TOPO_DOM    119    138       CYTOPLASMIC.
FT   TRANSMEM    139    159
FT   TOPO_DOM    160    192       NON CYTOPLASMIC.
FT   TRANSMEM    193    215
FT   TOPO_DOM    216    235       CYTOPLASMIC.
FT   TRANSMEM    236    257
FT   TOPO_DOM    258    268       NON CYTOPLASMIC.
FT   TRANSMEM    269    289
FT   TOPO_DOM    290    309       CYTOPLASMIC.
//
```

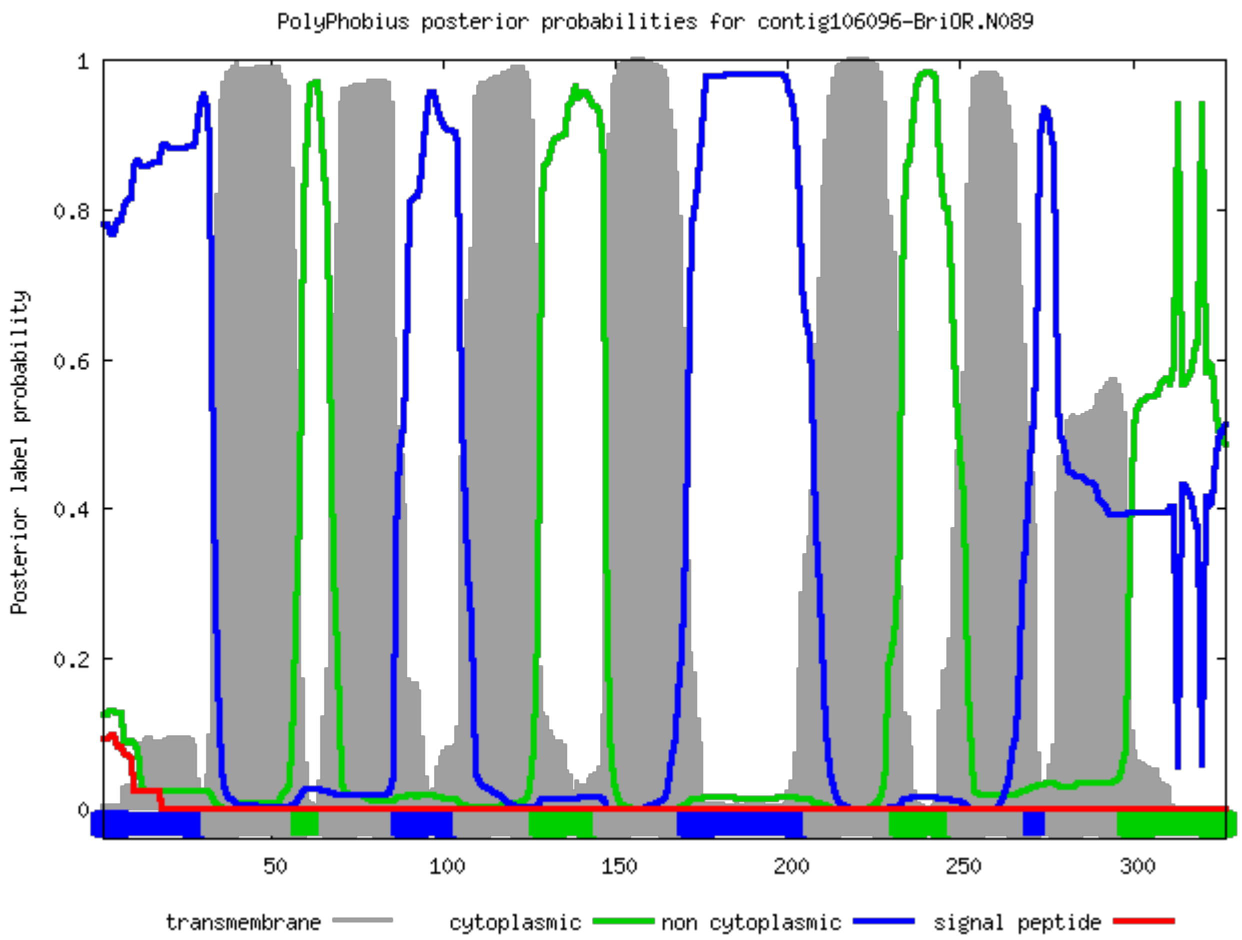

The prediction is based on an alignment. The probability data used in the plot is found here, and the gnuplot script is here.

Prediction of contig053590-NyeOR.E056

```
ID   contig053590-NyeOR.E056
FT   TOPO_DOM      1      24       NON CYTOPLASMIC.
FT   TRANSMEM     25      50
FT   TOPO_DOM     51      59       CYTOPLASMIC.
FT   TRANSMEM     60      84
FT   TOPO_DOM     85      92       NON CYTOPLASMIC.
FT   TRANSMEM     93     120
FT   TOPO_DOM    121     140       CYTOPLASMIC.
FT   TRANSMEM    141     163
FT   TOPO_DOM    164     195       NON CYTOPLASMIC.
FT   TRANSMEM    196     218
FT   TOPO_DOM    219     238       CYTOPLASMIC.
FT   TRANSMEM    239     258
FT   TOPO_DOM    259     269       NON CYTOPLASMIC.
FT   TRANSMEM    270     293
FT   TOPO_DOM    294     322       CYTOPLASMIC.
//
```

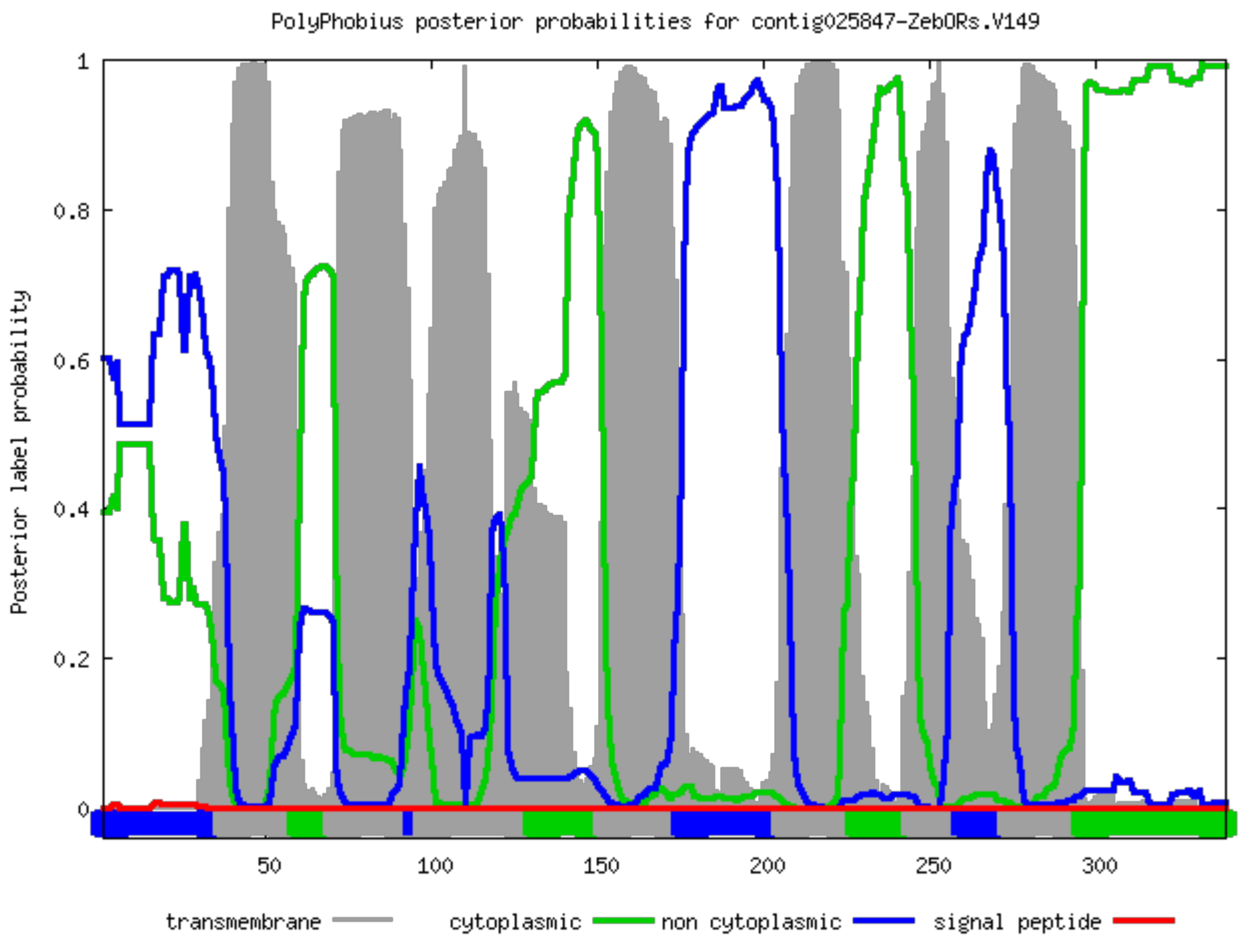

The prediction is based on an alignment. The probability data used in the plot is found here, and the gnuplot script is here.

Prediction of contig030556-ZebOR.A005

```
ID   contig030556-ZebOR.A005
FT   TOPO_DOM      1     25       NON CYTOPLASMIC.
FT   TRANSMEM     26     51
FT   TOPO_DOM     52     59       CYTOPLASMIC.
FT   TRANSMEM     60     80
FT   TOPO_DOM     81     98       NON CYTOPLASMIC.
FT   TRANSMEM     99    121
FT   TOPO_DOM    122    141       CYTOPLASMIC.
FT   TRANSMEM    142    163
FT   TOPO_DOM    164    195       NON CYTOPLASMIC.
FT   TRANSMEM    196    219
FT   TOPO_DOM    220    239       CYTOPLASMIC.
FT   TRANSMEM    240    261
FT   TOPO_DOM    262    272       NON CYTOPLASMIC.
FT   TRANSMEM    273    293
FT   TOPO_DOM    294    337       CYTOPLASMIC.
//
```

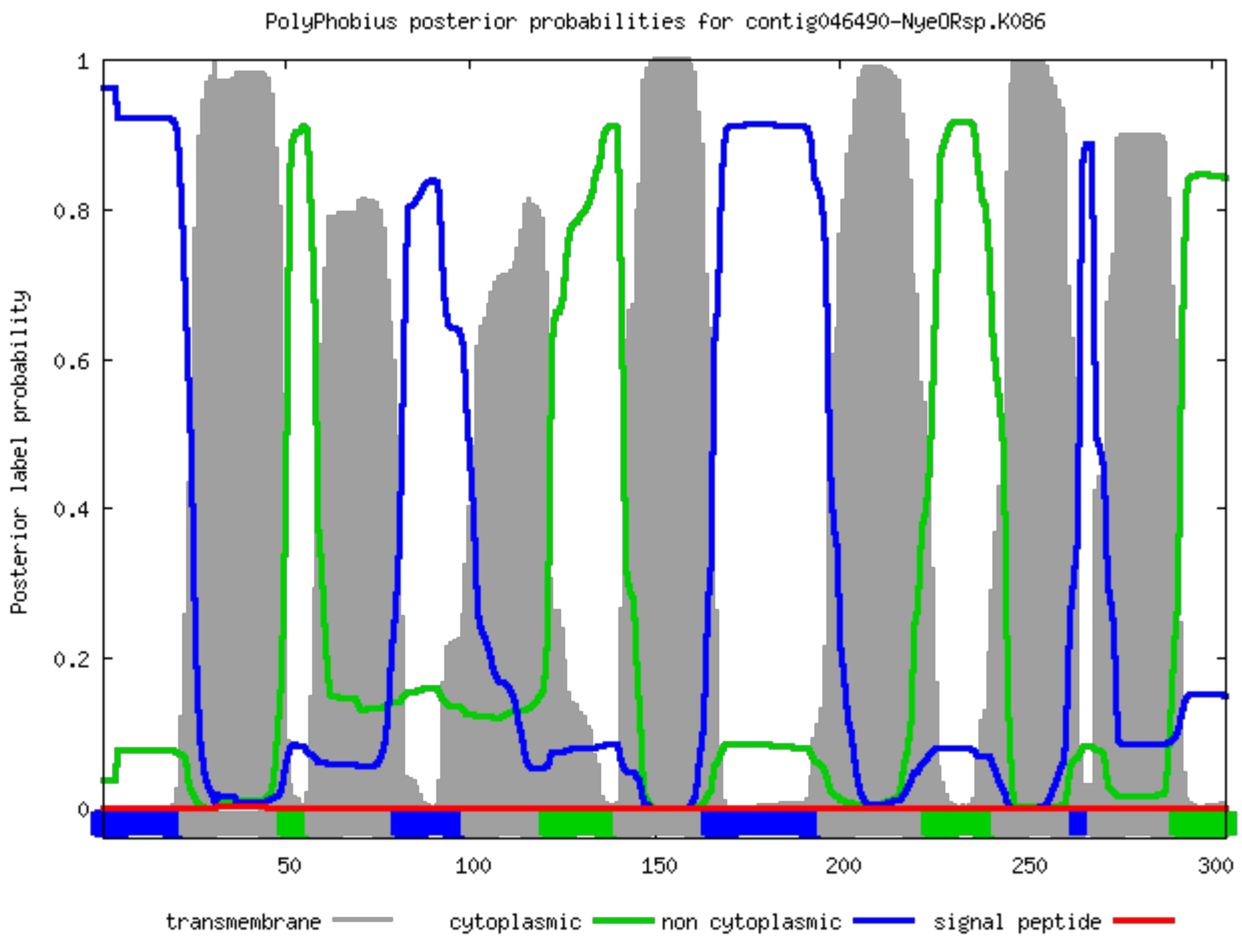

The prediction is based on an alignment. The probability data used in the plot is found here, and the gnuplot script is here.

Prediction of contig017787-ZebOR.L092

```
ID    contig017787-ZebOR.L092
FT    TOPO_DOM      1     25       NON CYTOPLASMIC.
FT    TRANSMEM     26     50
FT    TOPO_DOM     51     59       CYTOPLASMIC.
FT    TRANSMEM     60     83
FT    TOPO_DOM     84     98       NON CYTOPLASMIC.
FT    TRANSMEM     99    120
FT    TOPO_DOM    121    140       CYTOPLASMIC.
FT    TRANSMEM    141    162
FT    TOPO_DOM    163    198       NON CYTOPLASMIC.
FT    TRANSMEM    199    224
FT    TOPO_DOM    225    238       CYTOPLASMIC.
FT    TRANSMEM    239    260
FT    TOPO_DOM    261    271       NON CYTOPLASMIC.
FT    TRANSMEM    272    292
FT    TOPO_DOM    293    313       CYTOPLASMIC.
//
```

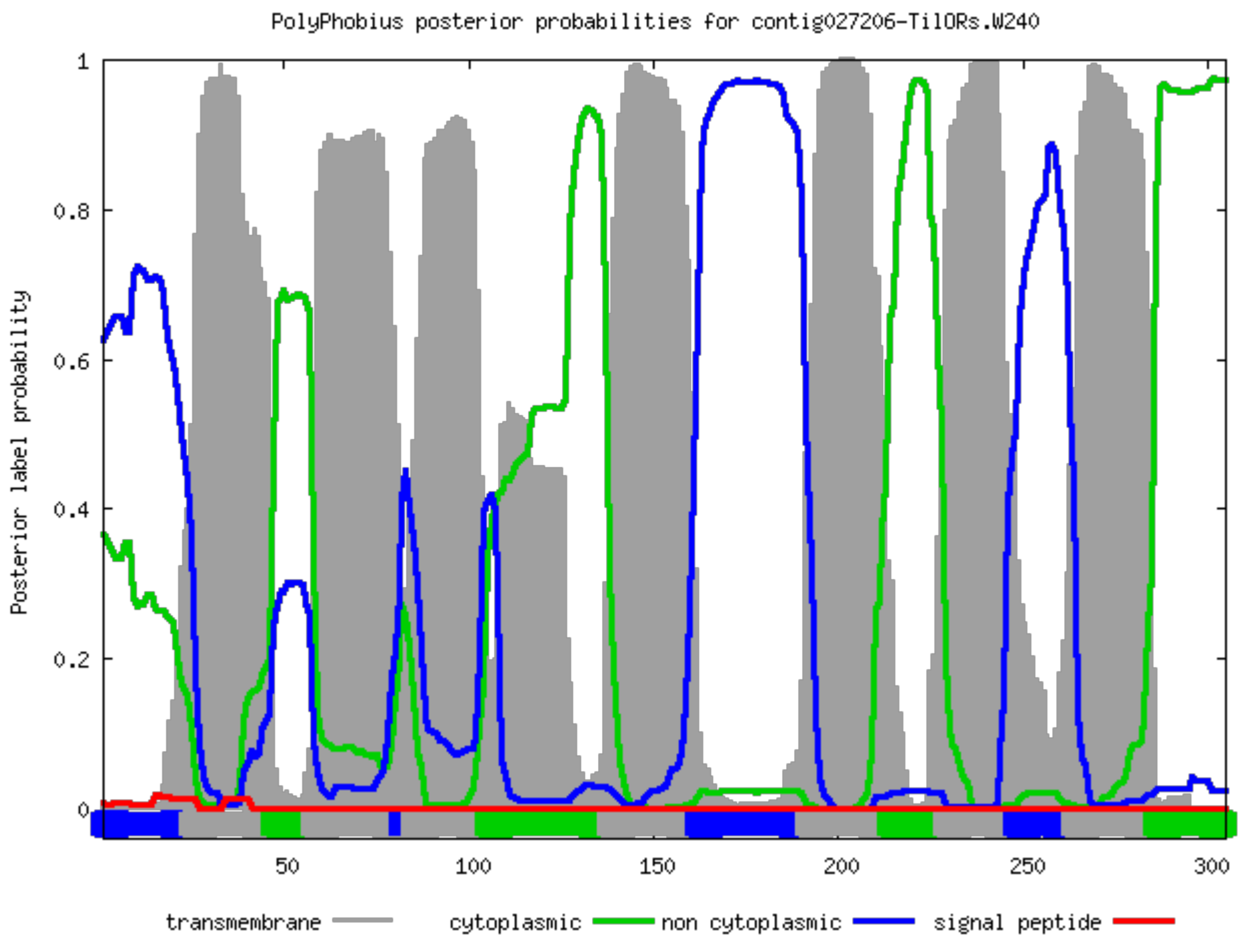

The prediction is based on an alignment. The probability data used in the plot is found here, and the gnuplot script is here.

Prediction of contig013371-TilOR.H118

```
ID    contig013371-TilOR.H118
FT    TOPO_DOM      1     23       NON CYTOPLASMIC.
FT    TRANSMEM     24     49
FT    TOPO_DOM     50     56       CYTOPLASMIC.
FT    TRANSMEM     57     76
FT    TOPO_DOM     77     95       NON CYTOPLASMIC.
FT    TRANSMEM     96    118
FT    TOPO_DOM    119    138       CYTOPLASMIC.
FT    TRANSMEM    139    160
FT    TOPO_DOM    161    196       NON CYTOPLASMIC.
FT    TRANSMEM    197    219
FT    TOPO_DOM    220    237       CYTOPLASMIC.
FT    TRANSMEM    238    260
FT    TOPO_DOM    261    271       NON CYTOPLASMIC.
FT    TRANSMEM    272    291
FT    TOPO_DOM    292    307       CYTOPLASMIC.
//
```

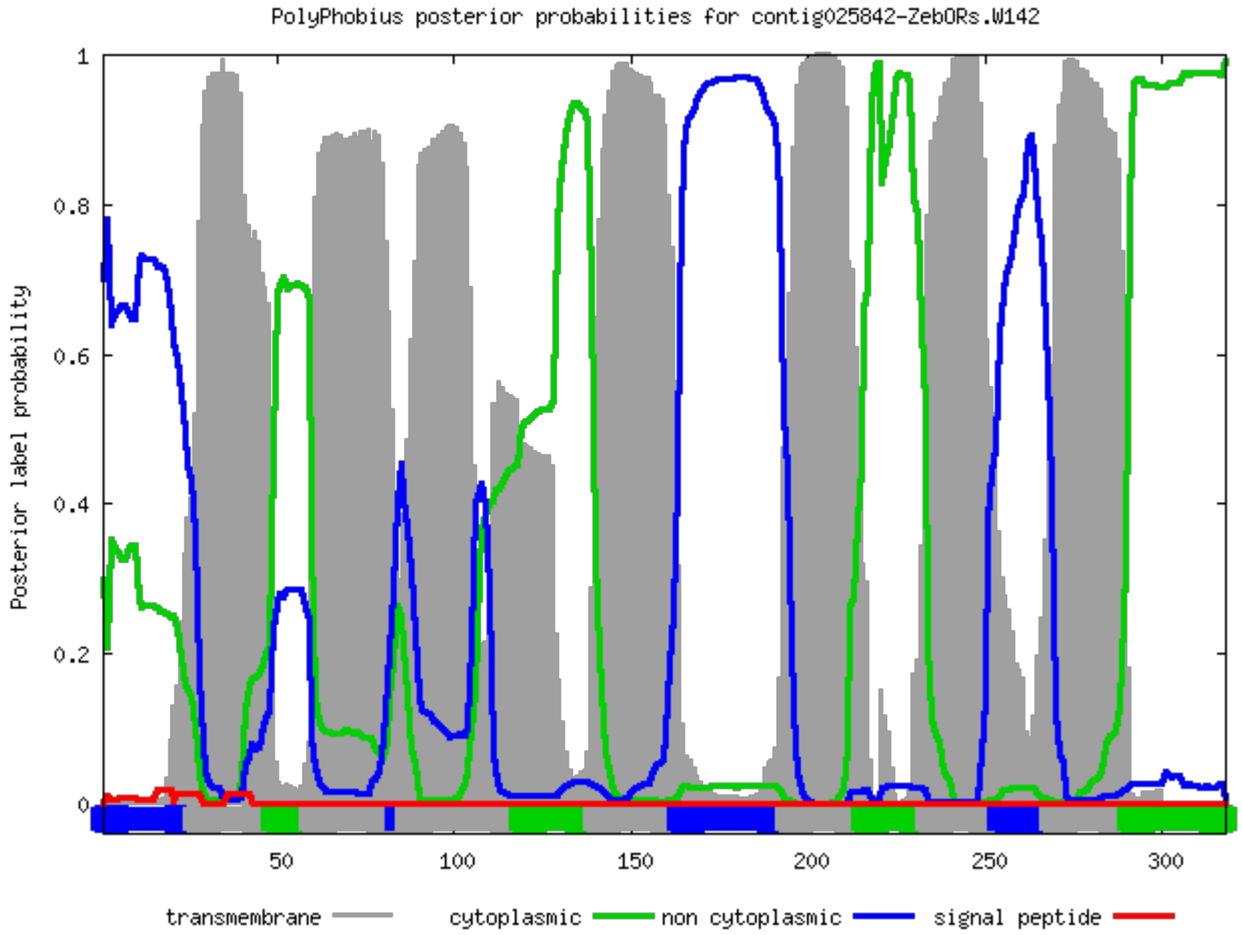

The prediction is based on an alignment. The probability data used in the plot is found here, and the gnuplot script is here.

Prediction of contig041756-NyeOR.H075

```
ID   contig041756-NyeOR.H075
FT   TOPO_DOM      1     22       NON CYTOPLASMIC.
FT   TRANSMEM     23     48
FT   TOPO_DOM     49     56       CYTOPLASMIC.
FT   TRANSMEM     57     77
FT   TOPO_DOM     78     95       NON CYTOPLASMIC.
FT   TRANSMEM     96    118
FT   TOPO_DOM    119    138       CYTOPLASMIC.
FT   TRANSMEM    139    160
FT   TOPO_DOM    161    193       NON CYTOPLASMIC.
FT   TRANSMEM    194    217
FT   TOPO_DOM    218    235       CYTOPLASMIC.
FT   TRANSMEM    236    258
FT   TOPO_DOM    259    269       NON CYTOPLASMIC.
FT   TRANSMEM    270    289
FT   TOPO_DOM    290    314       CYTOPLASMIC.
//
```

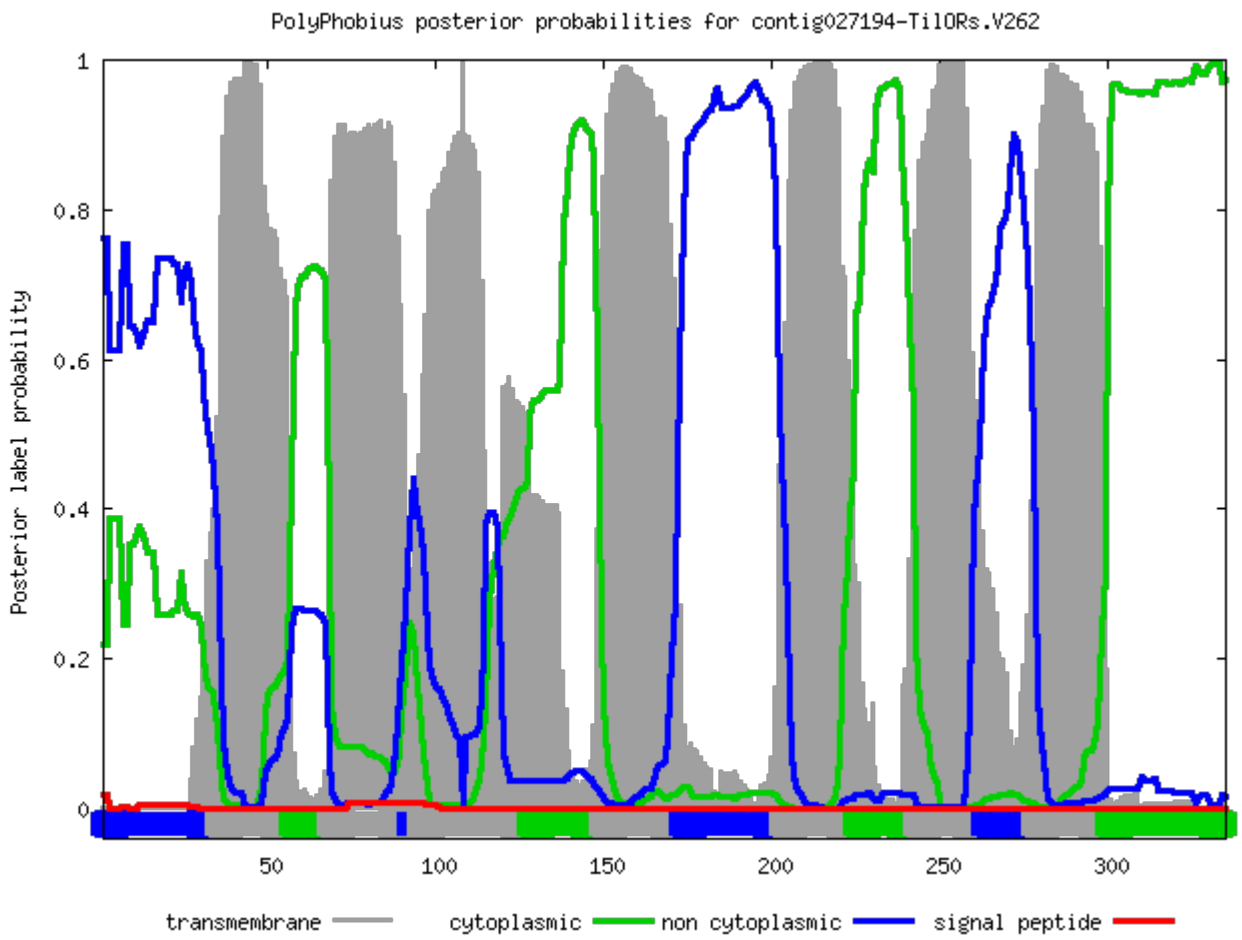

The prediction is based on an alignment. The probability data used in the plot is found here, and the gnuplot script is here.

Prediction of contig046351-TilOR.N192

```
ID   contig046351-TilOR.N192
FT   TOPO_DOM      1      33       NON CYTOPLASMIC.
FT   TRANSMEM     34      59
FT   TOPO_DOM     60      67       CYTOPLASMIC.
FT   TRANSMEM     68      89
FT   TOPO_DOM     90     108       NON CYTOPLASMIC.
FT   TRANSMEM    109     128
FT   TOPO_DOM    129     148       CYTOPLASMIC.
FT   TRANSMEM    149     171
FT   TOPO_DOM    172     207       NON CYTOPLASMIC.
FT   TRANSMEM    208     233
FT   TOPO_DOM    234     252       CYTOPLASMIC.
FT   TRANSMEM    253     275
FT   TOPO_DOM    276     327       NON CYTOPLASMIC.
//
```

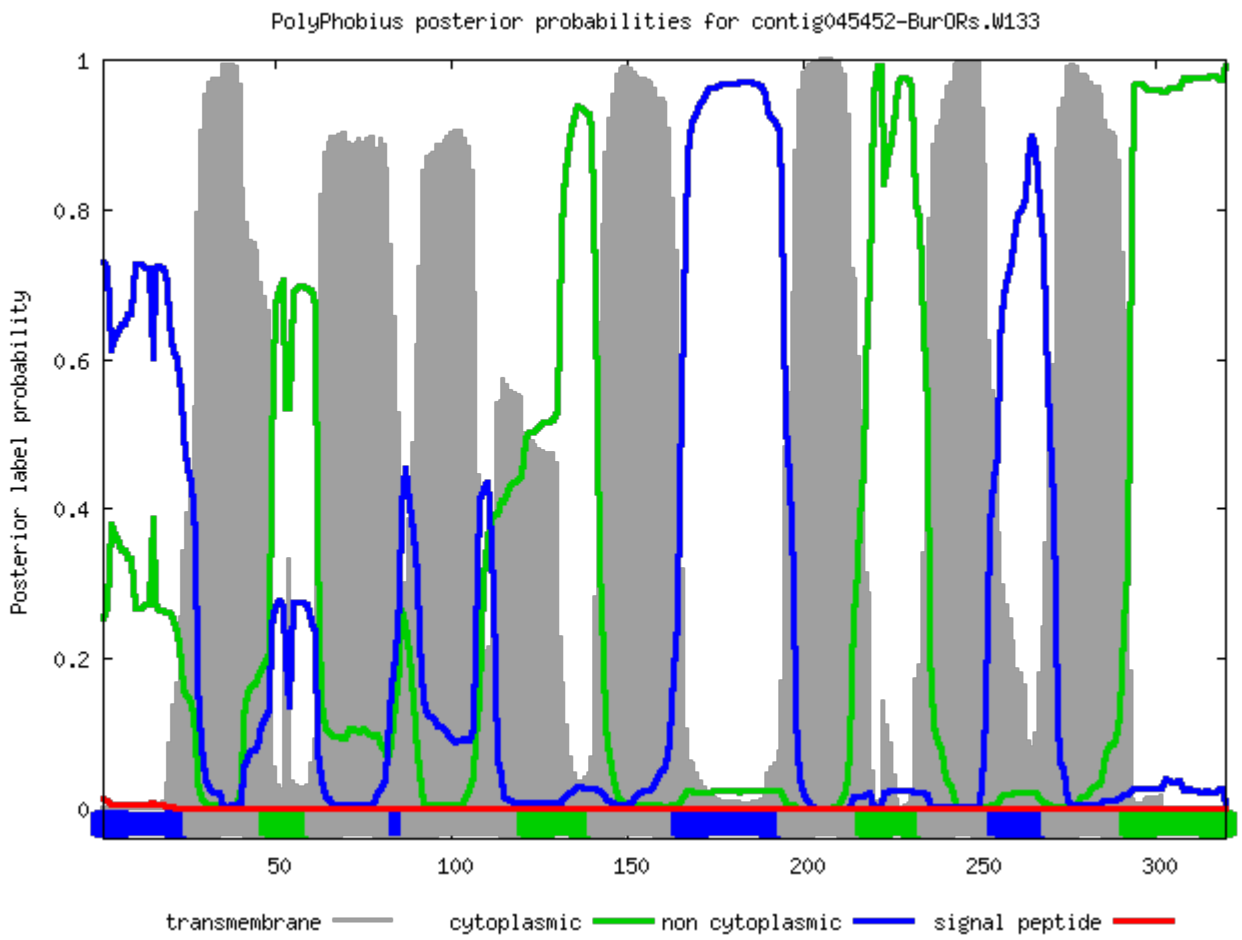

The prediction is based on an alignment. The probability data used in the plot is found here, and the gnuplot script is here.

Prediction of contig014055-ZebOR.H066

```
ID   contig014055-ZebOR.H066
FT   TOPO_DOM      1     22       NON CYTOPLASMIC.
FT   TRANSMEM     23     48
FT   TOPO_DOM     49     55       CYTOPLASMIC.
FT   TRANSMEM     56     76
FT   TOPO_DOM     77     94       NON CYTOPLASMIC.
FT   TRANSMEM     95    117
FT   TOPO_DOM    118    137       CYTOPLASMIC.
FT   TRANSMEM    138    159
FT   TOPO_DOM    160    192       NON CYTOPLASMIC.
FT   TRANSMEM    193    216
FT   TOPO_DOM    217    234       CYTOPLASMIC.
FT   TRANSMEM    235    257
FT   TOPO_DOM    258    268       NON CYTOPLASMIC.
FT   TRANSMEM    269    288
FT   TOPO_DOM    289    314       CYTOPLASMIC.
//
```

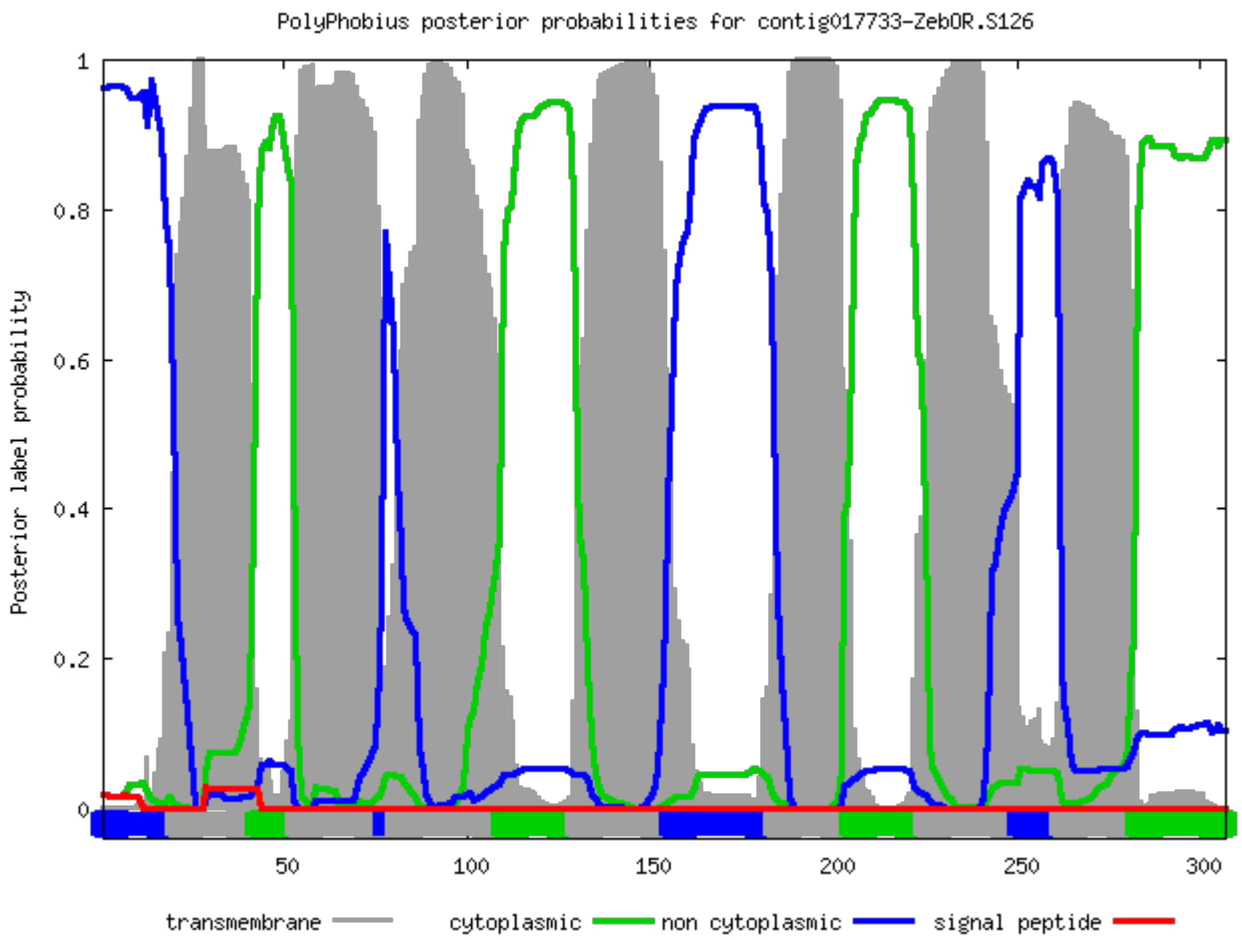

The prediction is based on an alignment. The probability data used in the plot is found here, and the gnuplot script is here.

Prediction of contig065025-BurOR.N111

```
ID   contig065025-BurOR.N111
FT   TOPO_DOM      1     33       NON CYTOPLASMIC.
FT   TRANSMEM     34     59
FT   TOPO_DOM     60     67       CYTOPLASMIC.
FT   TRANSMEM     68     89
FT   TOPO_DOM     90    108       NON CYTOPLASMIC.
FT   TRANSMEM    109    128
FT   TOPO_DOM    129    148       CYTOPLASMIC.
FT   TRANSMEM    149    171
FT   TOPO_DOM    172    207       NON CYTOPLASMIC.
FT   TRANSMEM    208    233
FT   TOPO_DOM    234    252       CYTOPLASMIC.
FT   TRANSMEM    253    274
FT   TOPO_DOM    275    337       NON CYTOPLASMIC.
//
```

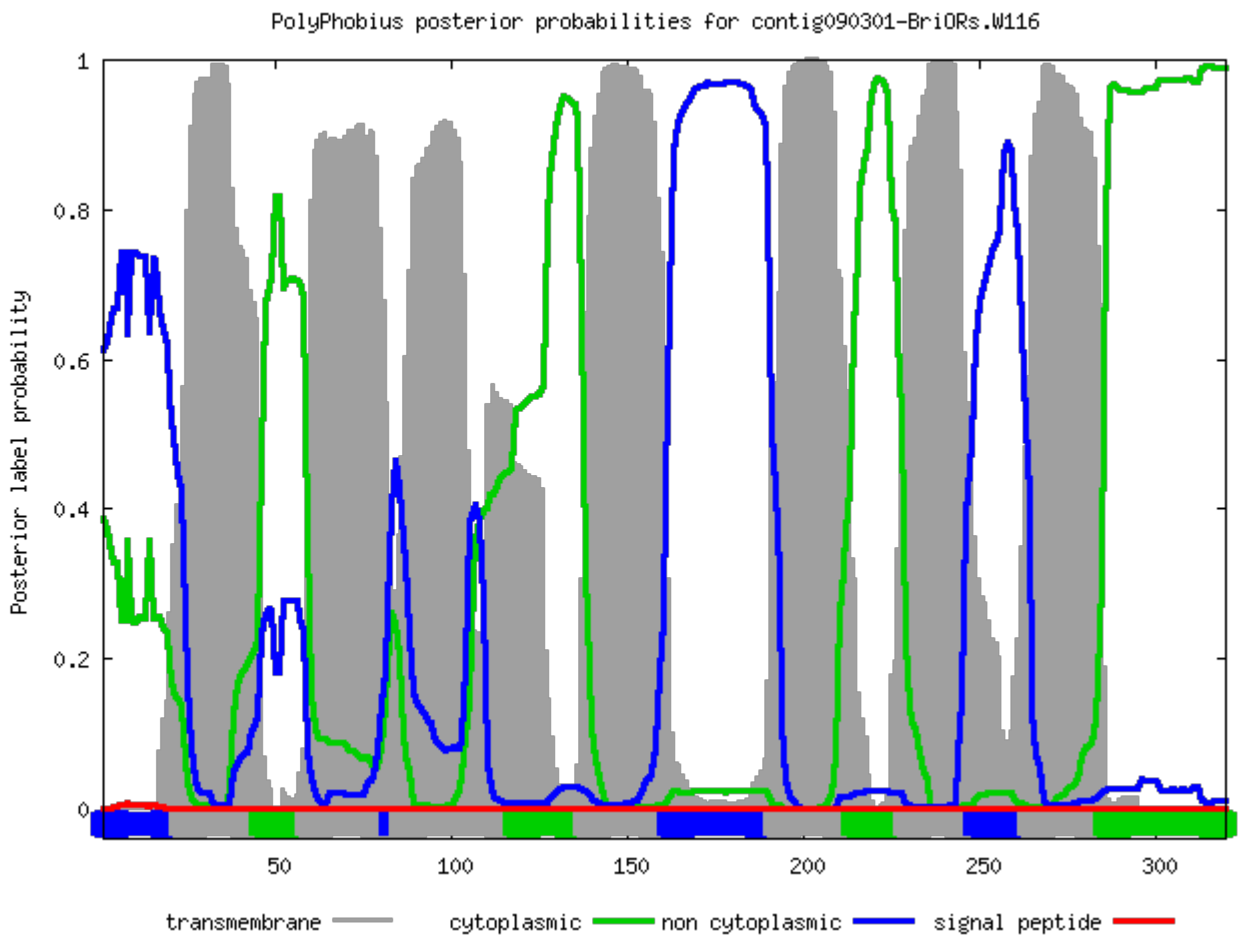

The prediction is based on an alignment. The probability data used in the plot is found here, and the gnuplot script is here.

Prediction of contig046724-TilOR.K139

```
ID   contig046724-TilOR.K139
FT   TOPO_DOM      1     24       NON CYTOPLASMIC.
FT   TRANSMEM     25     50
FT   TOPO_DOM     51     58       CYTOPLASMIC.
FT   TRANSMEM     59     81
FT   TOPO_DOM     82    100       NON CYTOPLASMIC.
FT   TRANSMEM    101    121
FT   TOPO_DOM    122    141       CYTOPLASMIC.
FT   TRANSMEM    142    165
FT   TOPO_DOM    166    196       NON CYTOPLASMIC.
FT   TRANSMEM    197    224
FT   TOPO_DOM    225    243       CYTOPLASMIC.
FT   TRANSMEM    244    264
FT   TOPO_DOM    265    269       NON CYTOPLASMIC.
FT   TRANSMEM    270    292
FT   TOPO_DOM    293    315       CYTOPLASMIC.
//
```

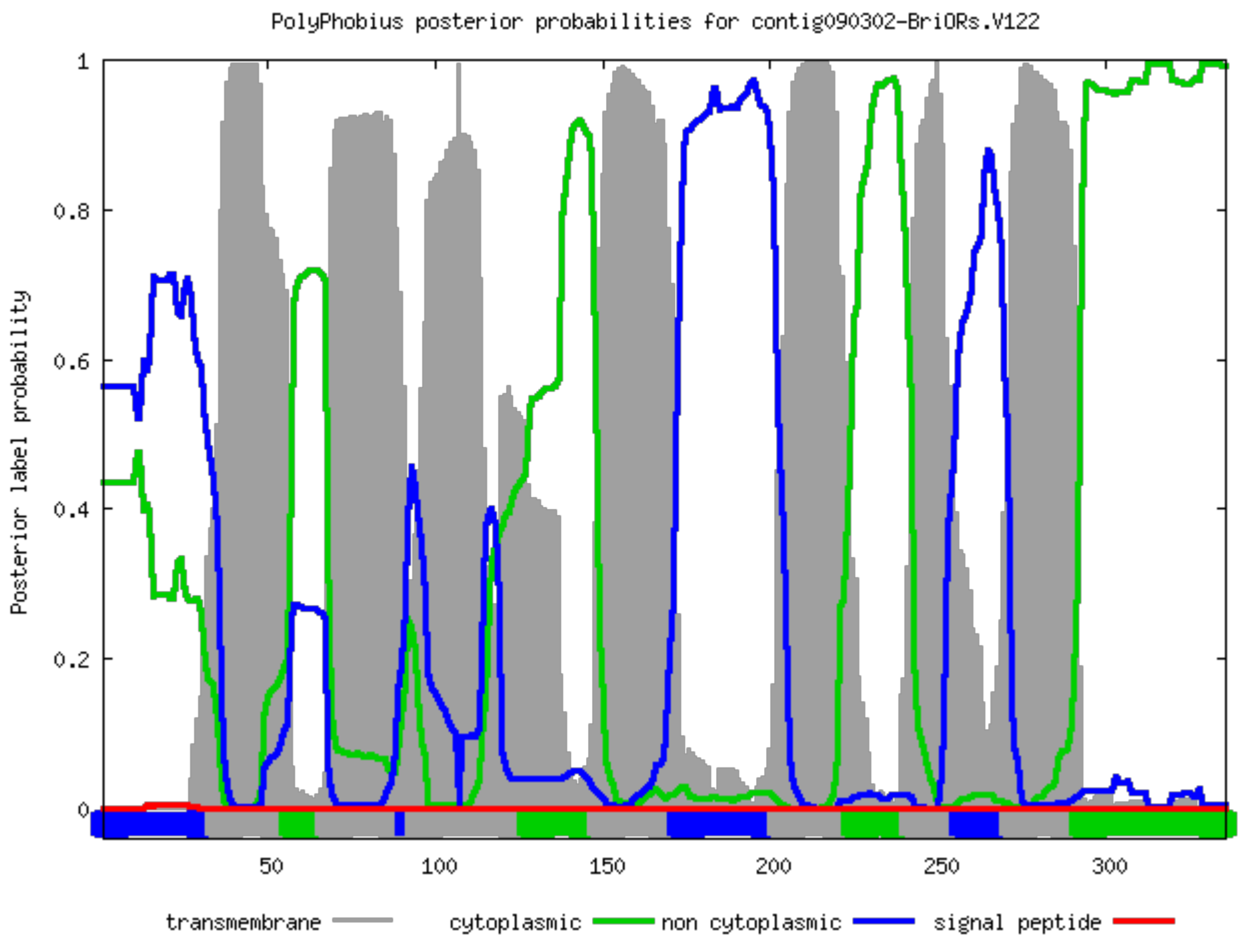

The prediction is based on an alignment. The probability data used in the plot is found here, and the gnuplot script is here.

Prediction of contig094282-BriOR.R118

```
ID    contig094282-BriOR.R118
FT    TOPO_DOM      1      24       NON CYTOPLASMIC.
FT    TRANSMEM     25      48
FT    TOPO_DOM     49      59       CYTOPLASMIC.
FT    TRANSMEM     60      84
FT    TOPO_DOM     85      89       NON CYTOPLASMIC.
FT    TRANSMEM     90     118
FT    TOPO_DOM    119     138       CYTOPLASMIC.
FT    TRANSMEM    139     162
FT    TOPO_DOM    163     194       NON CYTOPLASMIC.
FT    TRANSMEM    195     217
FT    TOPO_DOM    218     235       CYTOPLASMIC.
FT    TRANSMEM    236     256
FT    TOPO_DOM    257     271       NON CYTOPLASMIC.
FT    TRANSMEM    272     293
FT    TOPO_DOM    294     322       CYTOPLASMIC.
//
```

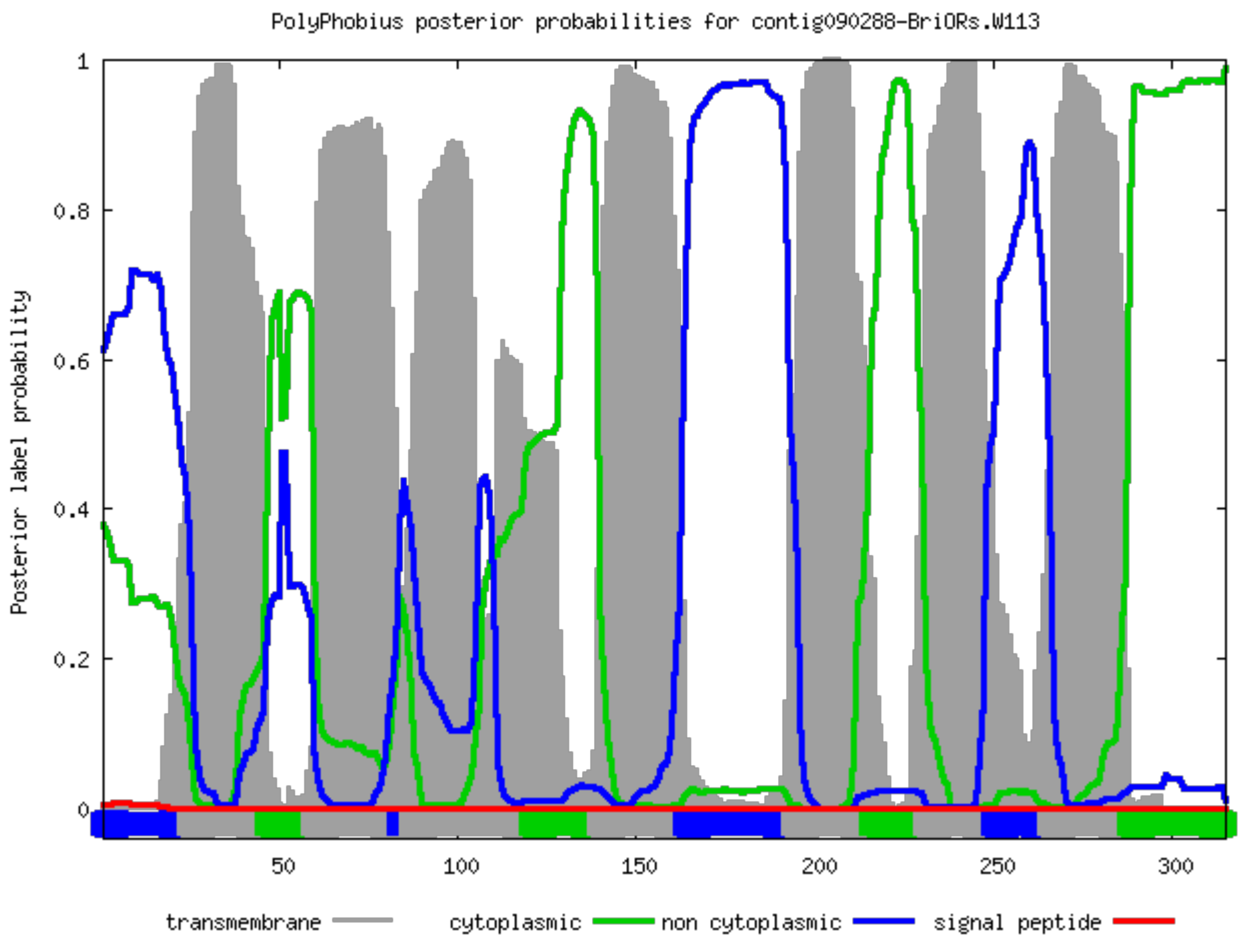

The prediction is based on an alignment. The probability data used in the plot is found here, and the gnuplot script is here.

Prediction of contig034995-NyeOR.A009

```
ID   contig034995-NyeOR.A009
FT   TOPO_DOM       1      22       NON CYTOPLASMIC.
FT   TRANSMEM      23      48
FT   TOPO_DOM      49      56       CYTOPLASMIC.
FT   TRANSMEM      57      77
FT   TOPO_DOM      78      95       NON CYTOPLASMIC.
FT   TRANSMEM      96     118
FT   TOPO_DOM     119     138       CYTOPLASMIC.
FT   TRANSMEM     139     159
FT   TOPO_DOM     160     192       NON CYTOPLASMIC.
FT   TRANSMEM     193     215
FT   TOPO_DOM     216     235       CYTOPLASMIC.
FT   TRANSMEM     236     257
FT   TOPO_DOM     258     268       NON CYTOPLASMIC.
FT   TRANSMEM     269     289
FT   TOPO_DOM     290     320       CYTOPLASMIC.
//
```

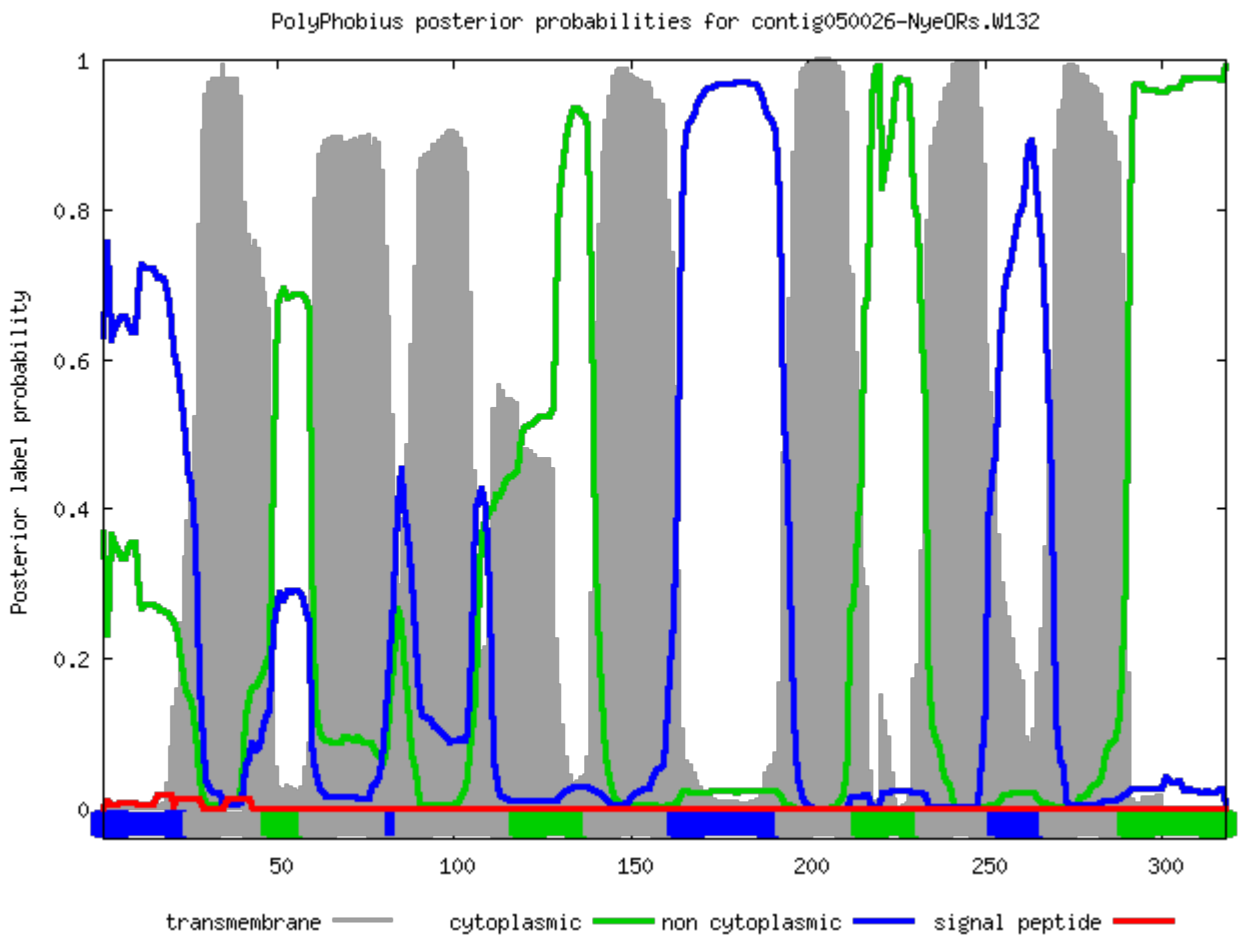

The prediction is based on an alignment. The probability data used in the plot is found here, and the gnuplot script is here.

Prediction of contig059404-NyeOR.E058

```
ID   contig059404-NyeOR.E058
FT   TOPO_DOM      1     22       NON CYTOPLASMIC.
FT   TRANSMEM     23     48
FT   TOPO_DOM     49     57       CYTOPLASMIC.
FT   TRANSMEM     58     83
FT   TOPO_DOM     84     90       NON CYTOPLASMIC.
FT   TRANSMEM     91    118
FT   TOPO_DOM    119    138       CYTOPLASMIC.
FT   TRANSMEM    139    161
FT   TOPO_DOM    162    193       NON CYTOPLASMIC.
FT   TRANSMEM    194    216
FT   TOPO_DOM    217    236       CYTOPLASMIC.
FT   TRANSMEM    237    256
FT   TOPO_DOM    257    267       NON CYTOPLASMIC.
FT   TRANSMEM    268    291
FT   TOPO_DOM    292    326       CYTOPLASMIC.
//
```

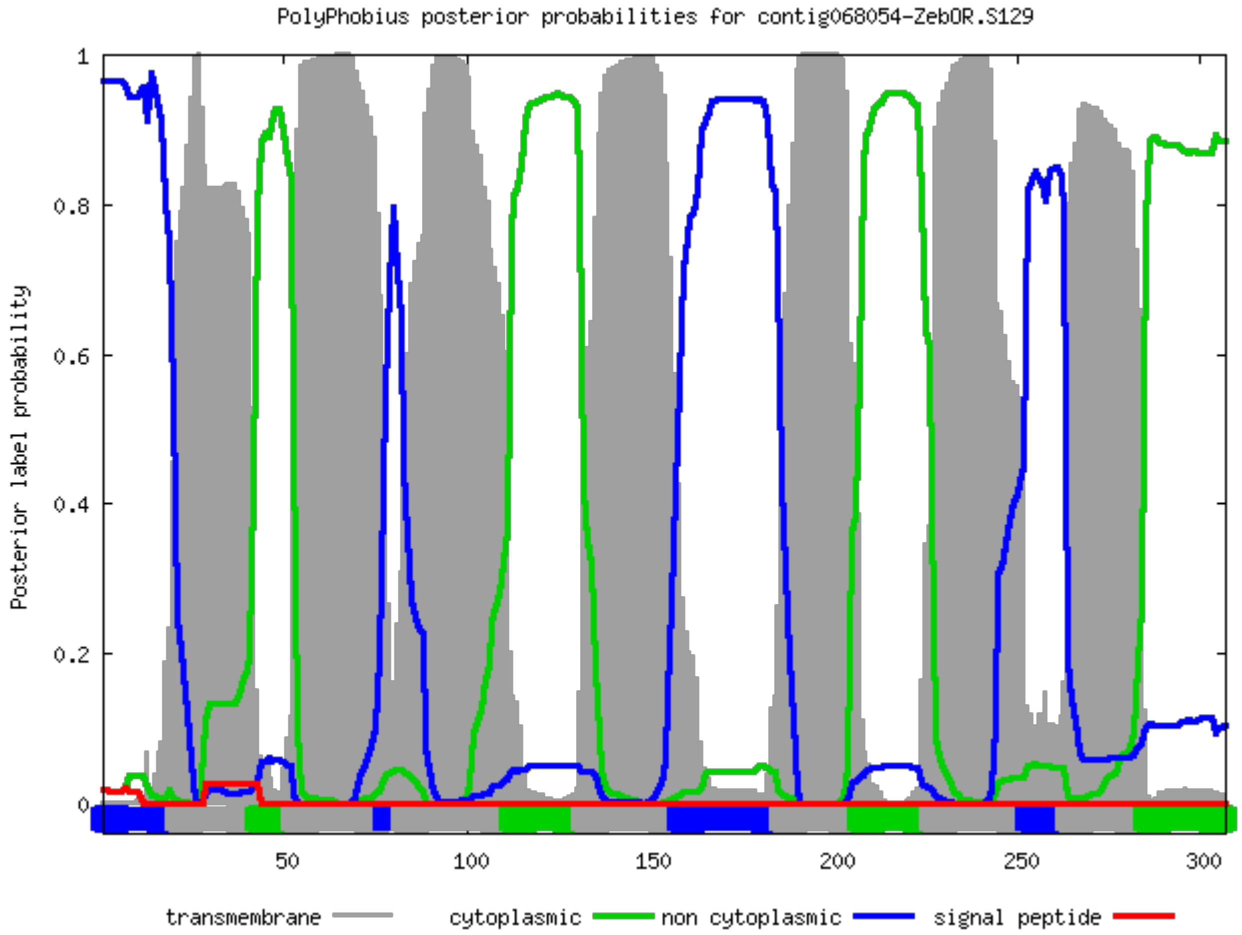

The prediction is based on an alignment. The probability data used in the plot is found here, and the gnuplot script is here.

Prediction of contig051566-BurOR.A009

```
ID   contig051566-BurOR.A009
FT   TOPO_DOM      1     22       NON CYTOPLASMIC.
FT   TRANSMEM     23     48
FT   TOPO_DOM     49     56       CYTOPLASMIC.
FT   TRANSMEM     57     77
FT   TOPO_DOM     78     95       NON CYTOPLASMIC.
FT   TRANSMEM     96    118
FT   TOPO_DOM    119    138       CYTOPLASMIC.
FT   TRANSMEM    139    160
FT   TOPO_DOM    161    192       NON CYTOPLASMIC.
FT   TRANSMEM    193    215
FT   TOPO_DOM    216    235       CYTOPLASMIC.
FT   TRANSMEM    236    257
FT   TOPO_DOM    258    268       NON CYTOPLASMIC.
FT   TRANSMEM    269    289
FT   TOPO_DOM    290    306       CYTOPLASMIC.
//
```

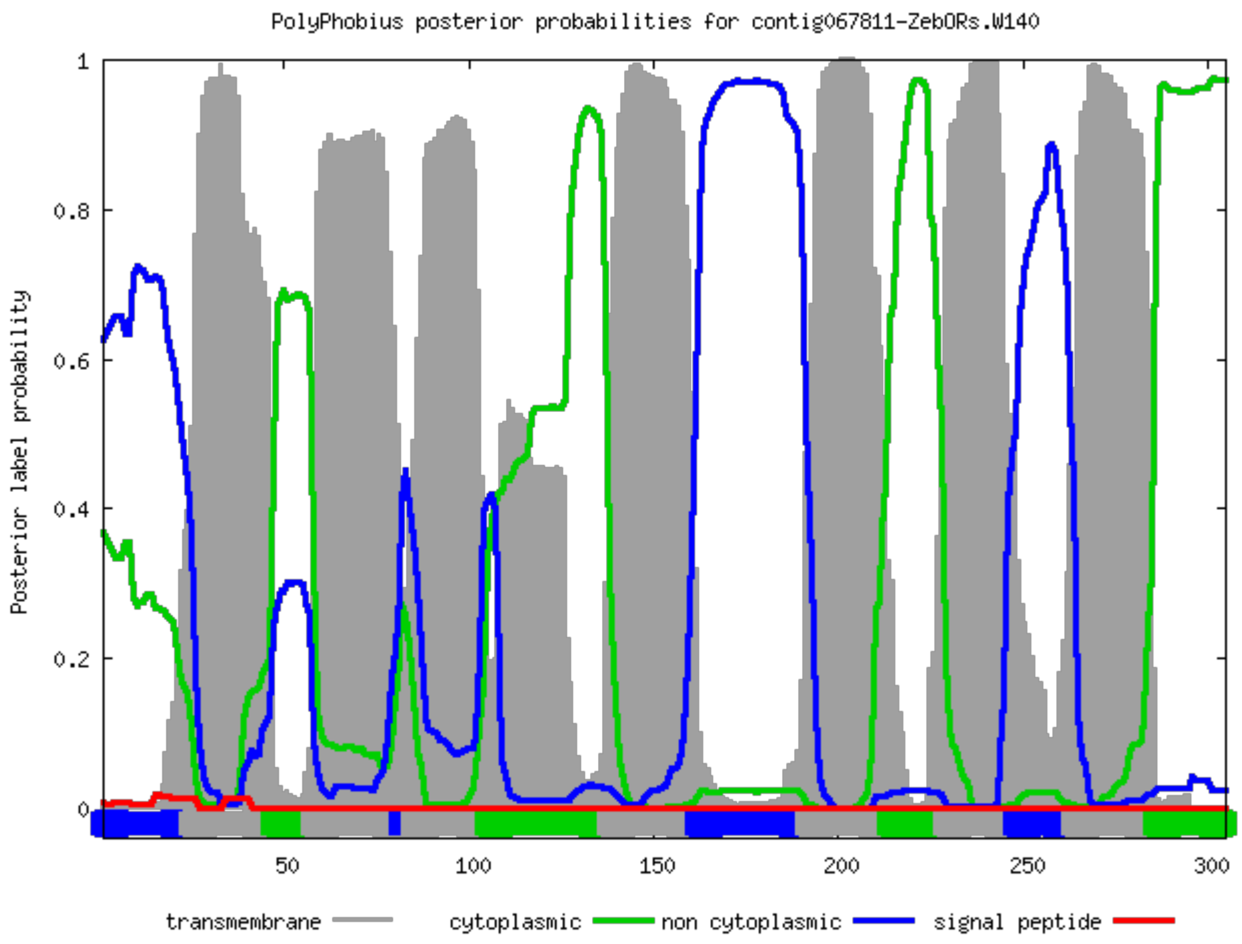

The prediction is based on an alignment. The probability data used in the plot is found here, and the gnuplot script is here.

Prediction of contig020440-ZebOR.L094

```
ID   contig020440-ZebOR.L094
FT   TOPO_DOM      1    25      NON CYTOPLASMIC.
FT   TRANSMEM     26    50
FT   TOPO_DOM     51    59      CYTOPLASMIC.
FT   TRANSMEM     60    86
FT   TOPO_DOM     87    98      NON CYTOPLASMIC.
FT   TRANSMEM     99   120
FT   TOPO_DOM    121   140      CYTOPLASMIC.
FT   TRANSMEM    141   162
FT   TOPO_DOM    163   198      NON CYTOPLASMIC.
FT   TRANSMEM    199   224
FT   TOPO_DOM    225   237      CYTOPLASMIC.
FT   TRANSMEM    238   259
FT   TOPO_DOM    260   271      NON CYTOPLASMIC.
FT   TRANSMEM    272   292
FT   TOPO_DOM    293   313      CYTOPLASMIC.
//
```

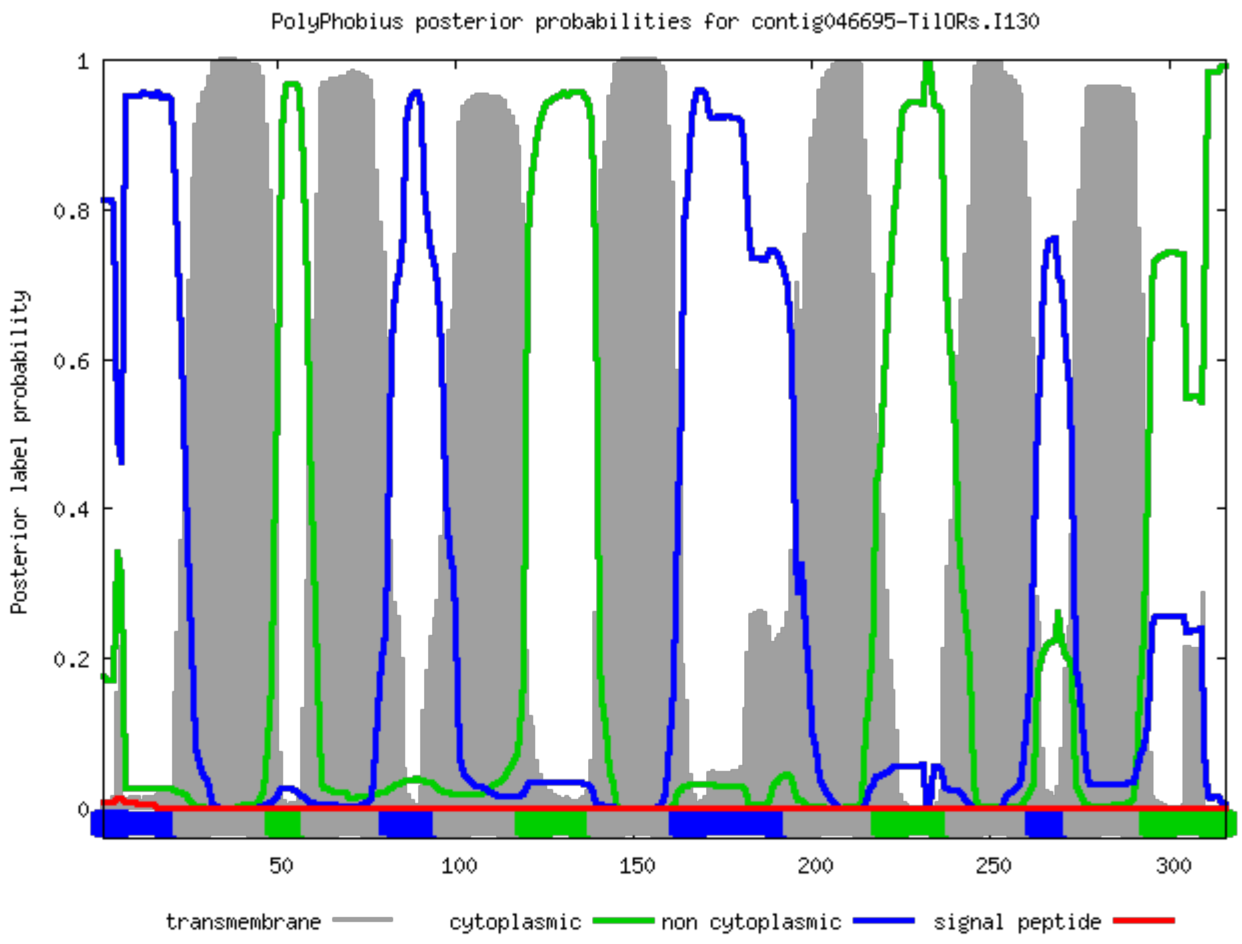

The prediction is based on an alignment. The probability data used in the plot is found here, and the gnuplot script is here.

Prediction of contig046350-TilOR.N191

```
ID   contig046350-TilOR.N191
FT   TOPO_DOM      1     24       NON CYTOPLASMIC.
FT   TRANSMEM     25     50
FT   TOPO_DOM     51     58       CYTOPLASMIC.
FT   TRANSMEM     59     80
FT   TOPO_DOM     81     99       NON CYTOPLASMIC.
FT   TRANSMEM    100    119
FT   TOPO_DOM    120    139       CYTOPLASMIC.
FT   TRANSMEM    140    162
FT   TOPO_DOM    163    198       NON CYTOPLASMIC.
FT   TRANSMEM    199    224
FT   TOPO_DOM    225    243       CYTOPLASMIC.
FT   TRANSMEM    244    266
FT   TOPO_DOM    267    319       NON CYTOPLASMIC.
//
```

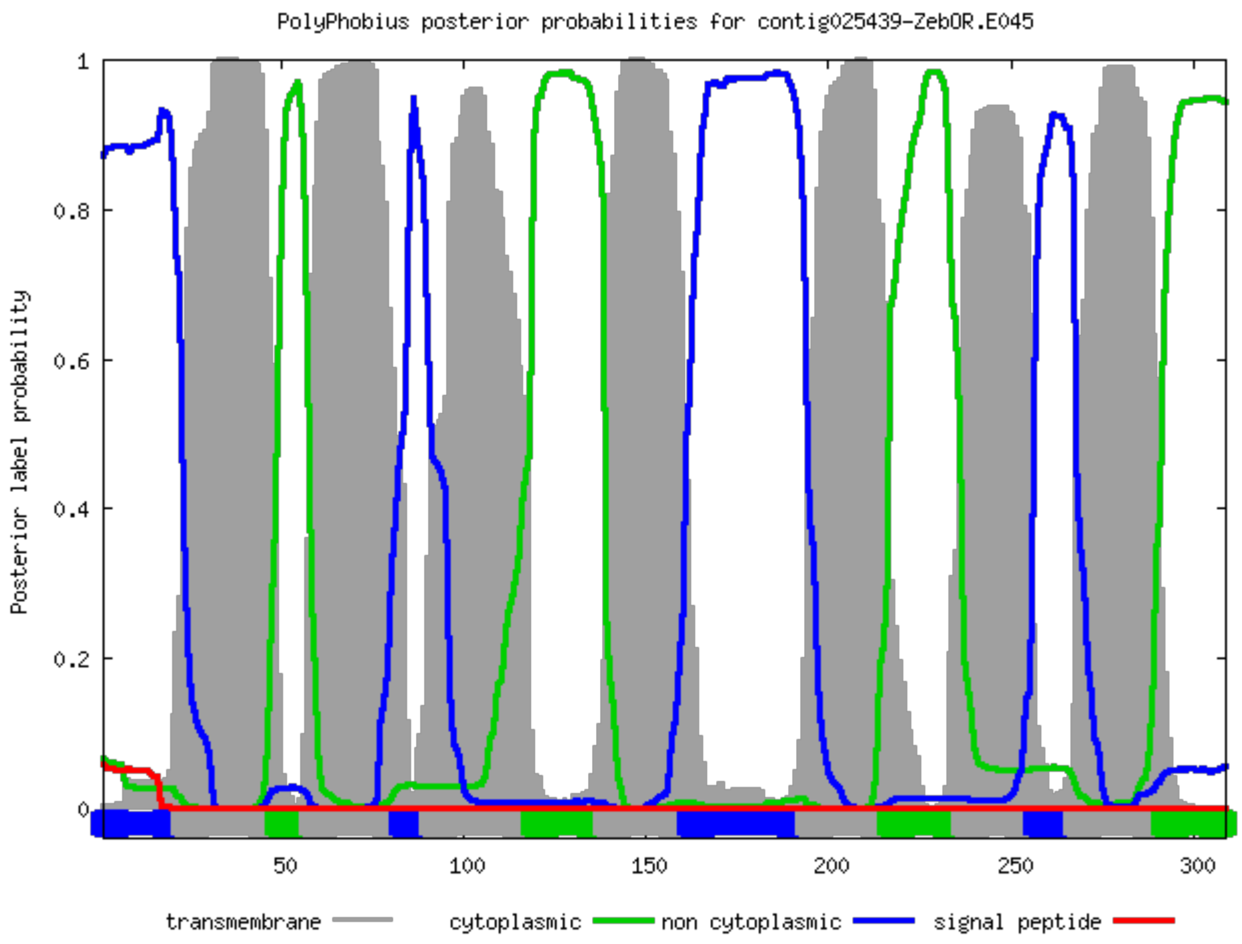

The prediction is based on an alignment. The probability data used in the plot is found here, and the gnuplot script is here.

Prediction of contig039725-NyeOR.H072

```
ID   contig039725-NyeOR.H072
FT   TOPO_DOM      1     22       NON CYTOPLASMIC.
FT   TRANSMEM     23     49
FT   TOPO_DOM     50     56       CYTOPLASMIC.
FT   TRANSMEM     57     77
FT   TOPO_DOM     78     95       NON CYTOPLASMIC.
FT   TRANSMEM     96    118
FT   TOPO_DOM    119    138       CYTOPLASMIC.
FT   TRANSMEM    139    160
FT   TOPO_DOM    161    193       NON CYTOPLASMIC.
FT   TRANSMEM    194    216
FT   TOPO_DOM    217    235       CYTOPLASMIC.
FT   TRANSMEM    236    258
FT   TOPO_DOM    259    269       NON CYTOPLASMIC.
FT   TRANSMEM    270    289
FT   TOPO_DOM    290    314       CYTOPLASMIC.
//
```

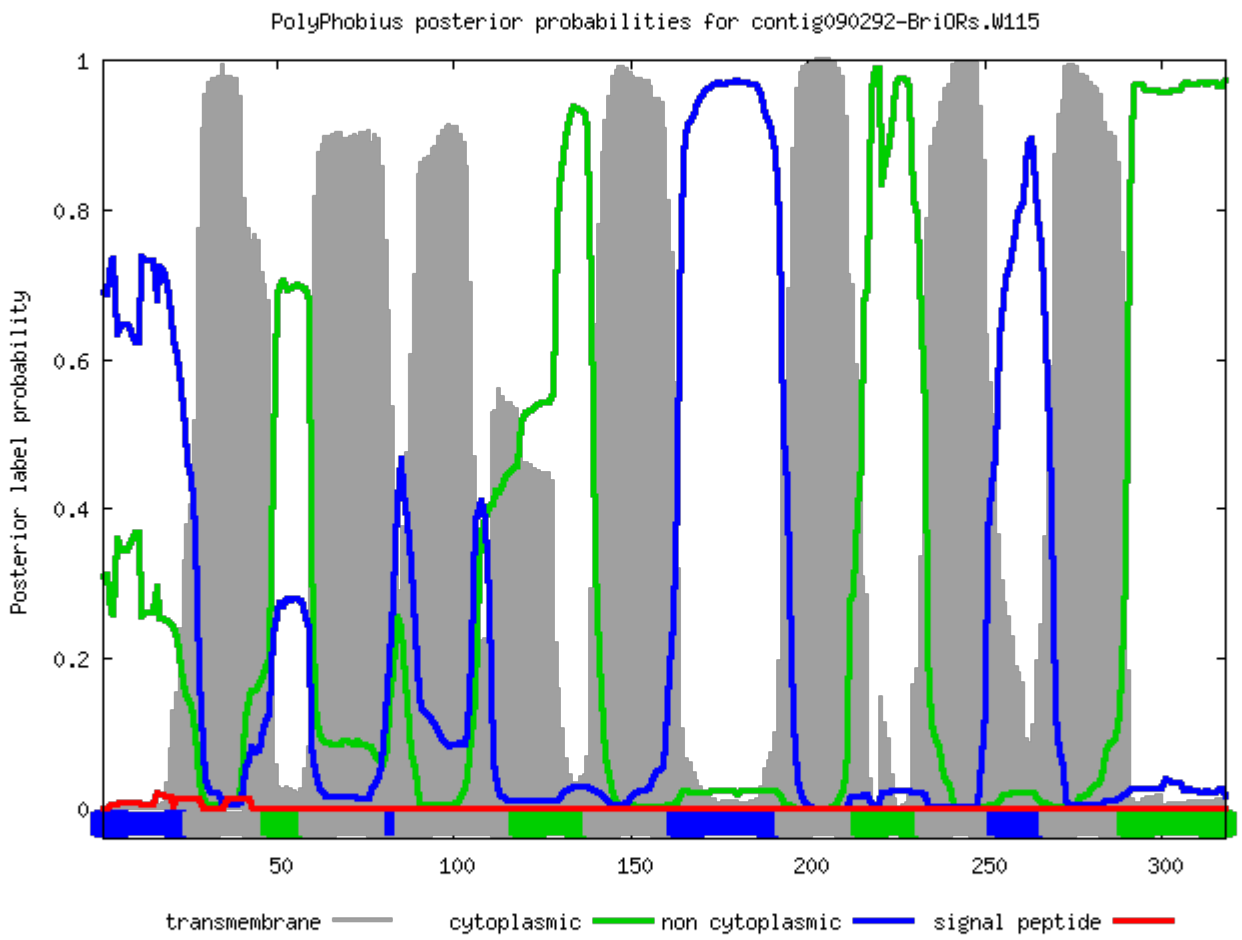

The prediction is based on an alignment. The probability data used in the plot is found here, and the gnuplot script is here.

Prediction of contig042920-BurOR.N107

```
ID   contig042920-BurOR.N107
FT   TOPO_DOM      1     33       NON CYTOPLASMIC.
FT   TRANSMEM     34     59
FT   TOPO_DOM     60     67       CYTOPLASMIC.
FT   TRANSMEM     68     89
FT   TOPO_DOM     90    108       NON CYTOPLASMIC.
FT   TRANSMEM    109    128
FT   TOPO_DOM    129    148       CYTOPLASMIC.
FT   TRANSMEM    149    171
FT   TOPO_DOM    172    207       NON CYTOPLASMIC.
FT   TRANSMEM    208    233
FT   TOPO_DOM    234    252       CYTOPLASMIC.
FT   TRANSMEM    253    275
FT   TOPO_DOM    276    327       NON CYTOPLASMIC.
//
```

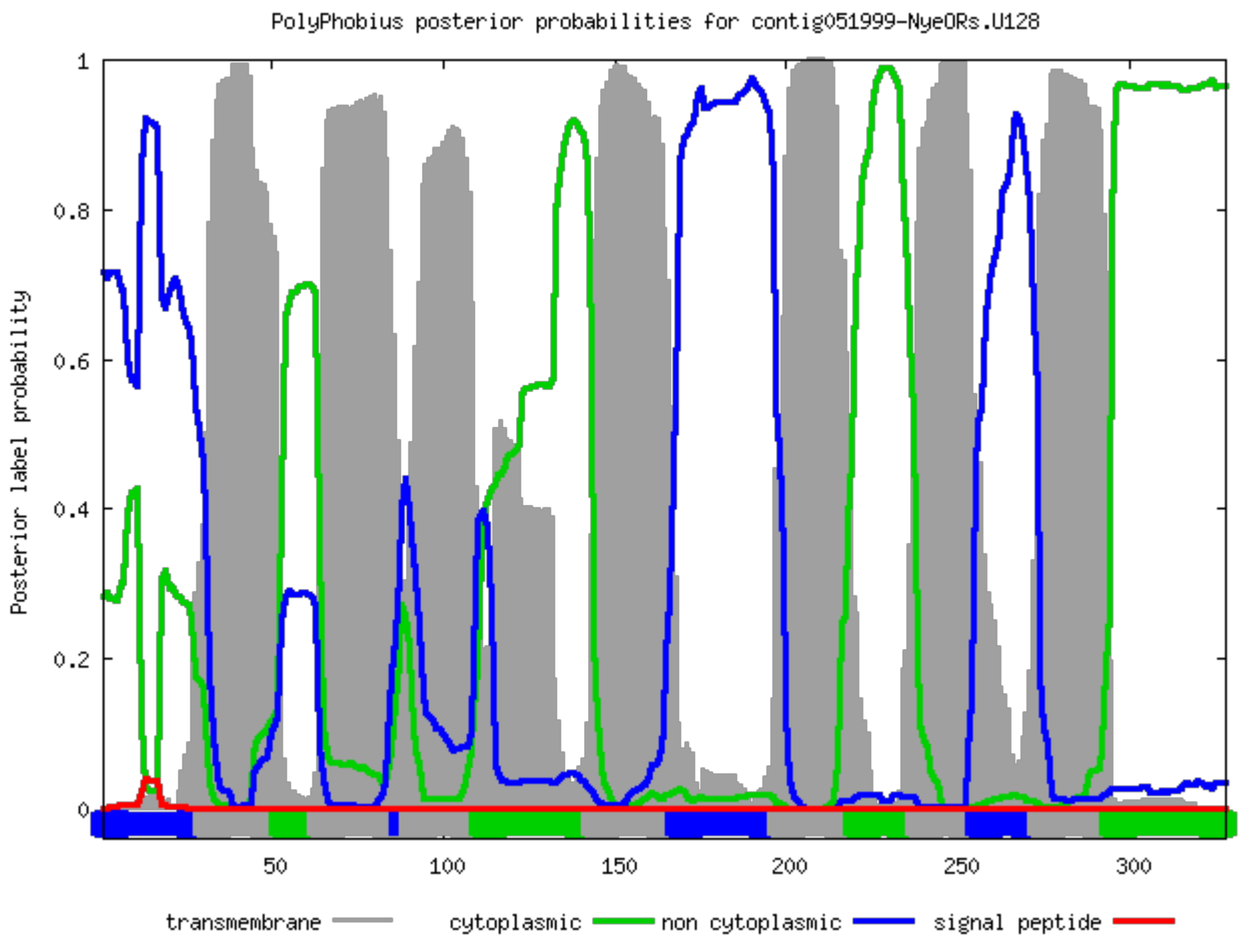

The prediction is based on an alignment. The probability data used in the plot is found here, and the gnuplot script is here.

Prediction of contig062095-ZebOR.A024

```
ID   contig062095-ZebOR.A024
FT   TOPO_DOM      1      22       NON CYTOPLASMIC.
FT   TRANSMEM     23      48
FT   TOPO_DOM     49      56       CYTOPLASMIC.
FT   TRANSMEM     57      76
FT   TOPO_DOM     77      95       NON CYTOPLASMIC.
FT   TRANSMEM     96     118
FT   TOPO_DOM    119     138       CYTOPLASMIC.
FT   TRANSMEM    139     159
FT   TOPO_DOM    160     192       NON CYTOPLASMIC.
FT   TRANSMEM    193     215
FT   TOPO_DOM    216     235       CYTOPLASMIC.
FT   TRANSMEM    236     257
FT   TOPO_DOM    258     268       NON CYTOPLASMIC.
FT   TRANSMEM    269     289
FT   TOPO_DOM    290     304       CYTOPLASMIC.
//
```

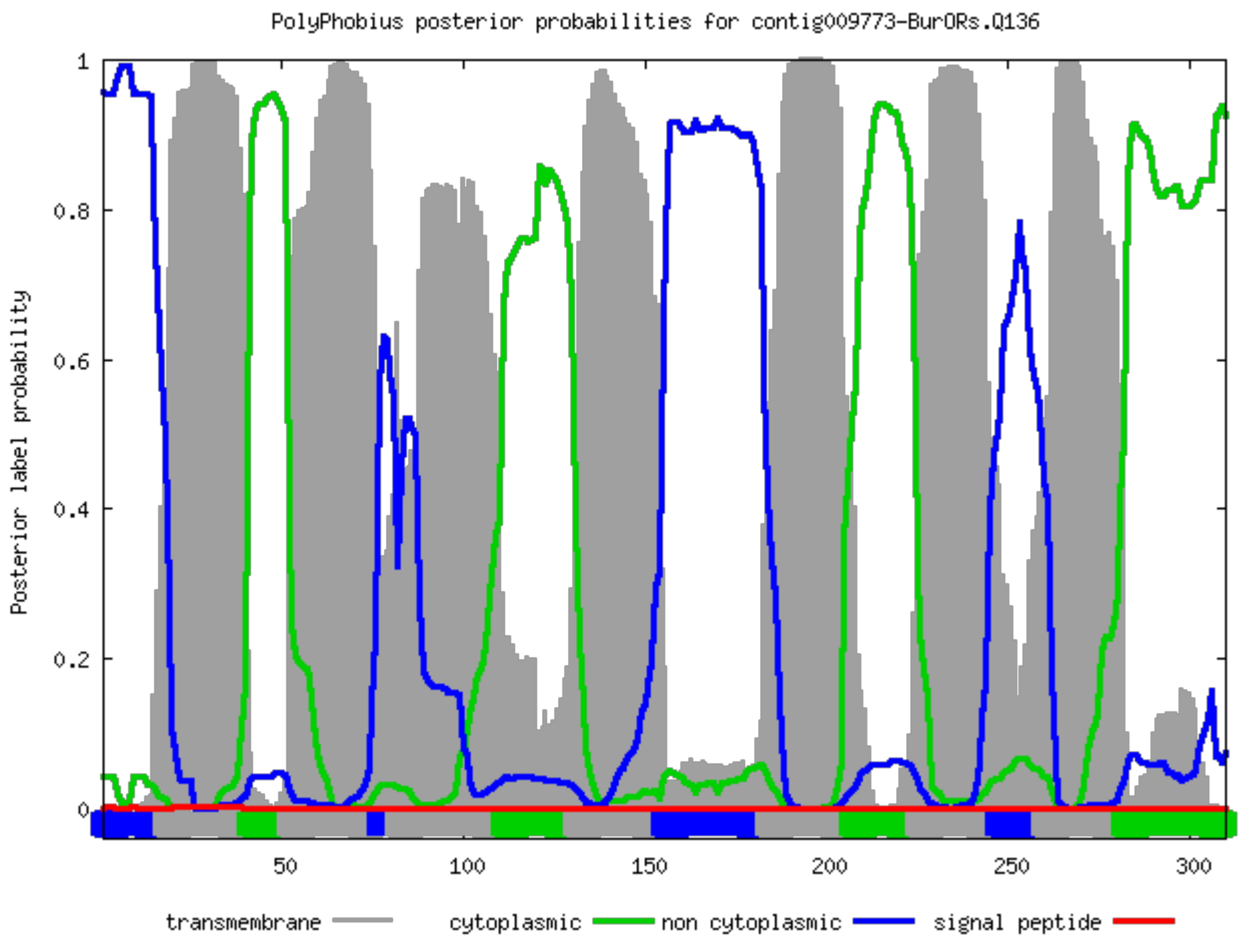

The prediction is based on an alignment. The probability data used in the plot is found here, and the gnuplot script is here.

Prediction of contig047508-ZebOR.A016

```
ID   contig047508-ZebOR.A016
FT   TOPO_DOM      1     22       NON CYTOPLASMIC.
FT   TRANSMEM     23     48
FT   TOPO_DOM     49     56       CYTOPLASMIC.
FT   TRANSMEM     57     77
FT   TOPO_DOM     78     95       NON CYTOPLASMIC.
FT   TRANSMEM     96    118
FT   TOPO_DOM    119    138       CYTOPLASMIC.
FT   TRANSMEM    139    160
FT   TOPO_DOM    161    192       NON CYTOPLASMIC.
FT   TRANSMEM    193    215
FT   TOPO_DOM    216    235       CYTOPLASMIC.
FT   TRANSMEM    236    257
FT   TOPO_DOM    258    268       NON CYTOPLASMIC.
FT   TRANSMEM    269    289
FT   TOPO_DOM    290    306       CYTOPLASMIC.
//
```

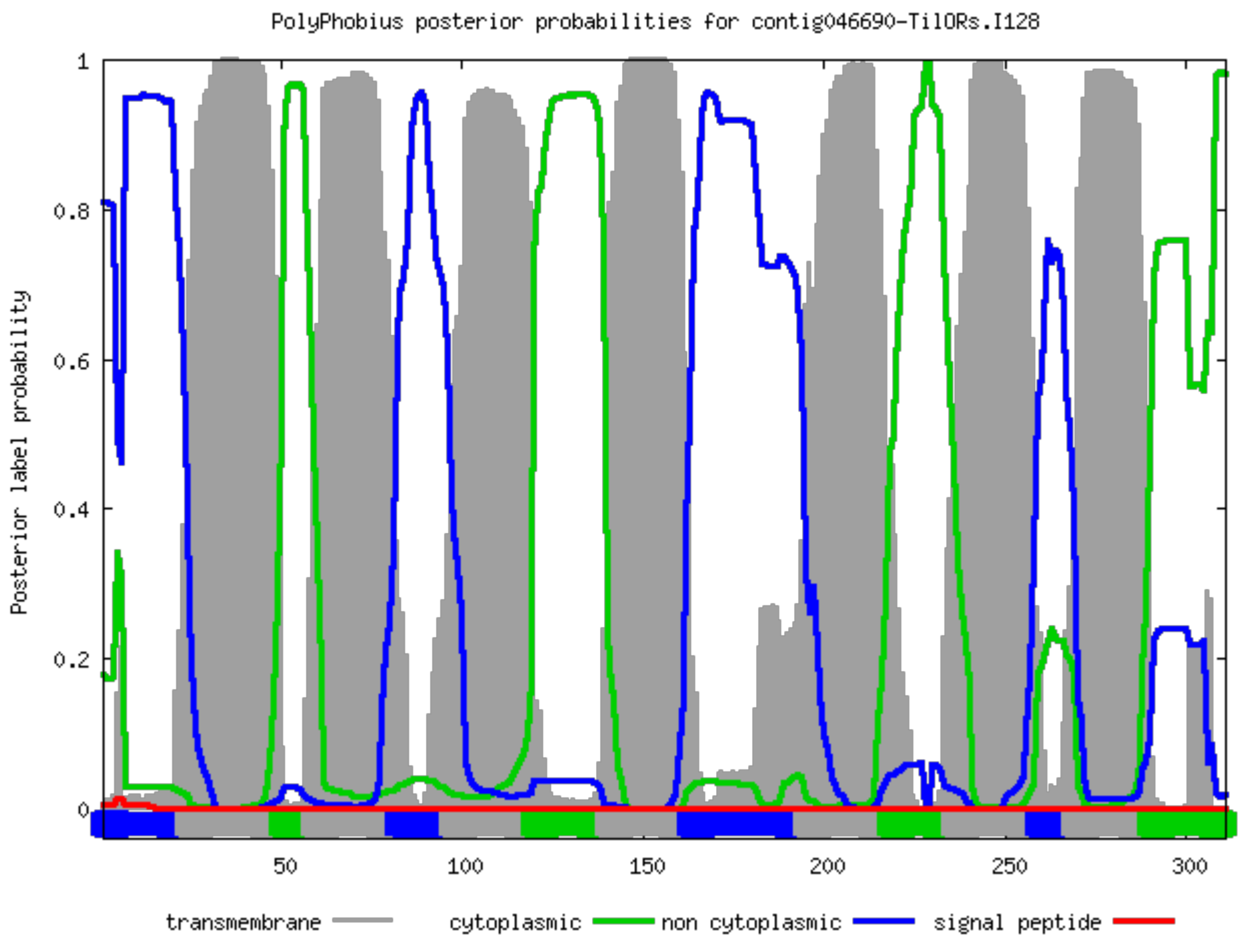

The prediction is based on an alignment. The probability data used in the plot is found here, and the gnuplot script is here.

Prediction of contig049287-BurOR.E043

```
ID   contig049287-BurOR.E043
FT   TOPO_DOM      1     21       NON CYTOPLASMIC.
FT   TRANSMEM     22     47
FT   TOPO_DOM     48     56       CYTOPLASMIC.
FT   TRANSMEM     57     81
FT   TOPO_DOM     82     93       NON CYTOPLASMIC.
FT   TRANSMEM     94    117
FT   TOPO_DOM    118    137       CYTOPLASMIC.
FT   TRANSMEM    138    159
FT   TOPO_DOM    160    192       NON CYTOPLASMIC.
FT   TRANSMEM    193    215
FT   TOPO_DOM    216    235       CYTOPLASMIC.
FT   TRANSMEM    236    255
FT   TOPO_DOM    256    266       NON CYTOPLASMIC.
FT   TRANSMEM    267    290
FT   TOPO_DOM    291    306       CYTOPLASMIC.
//
```

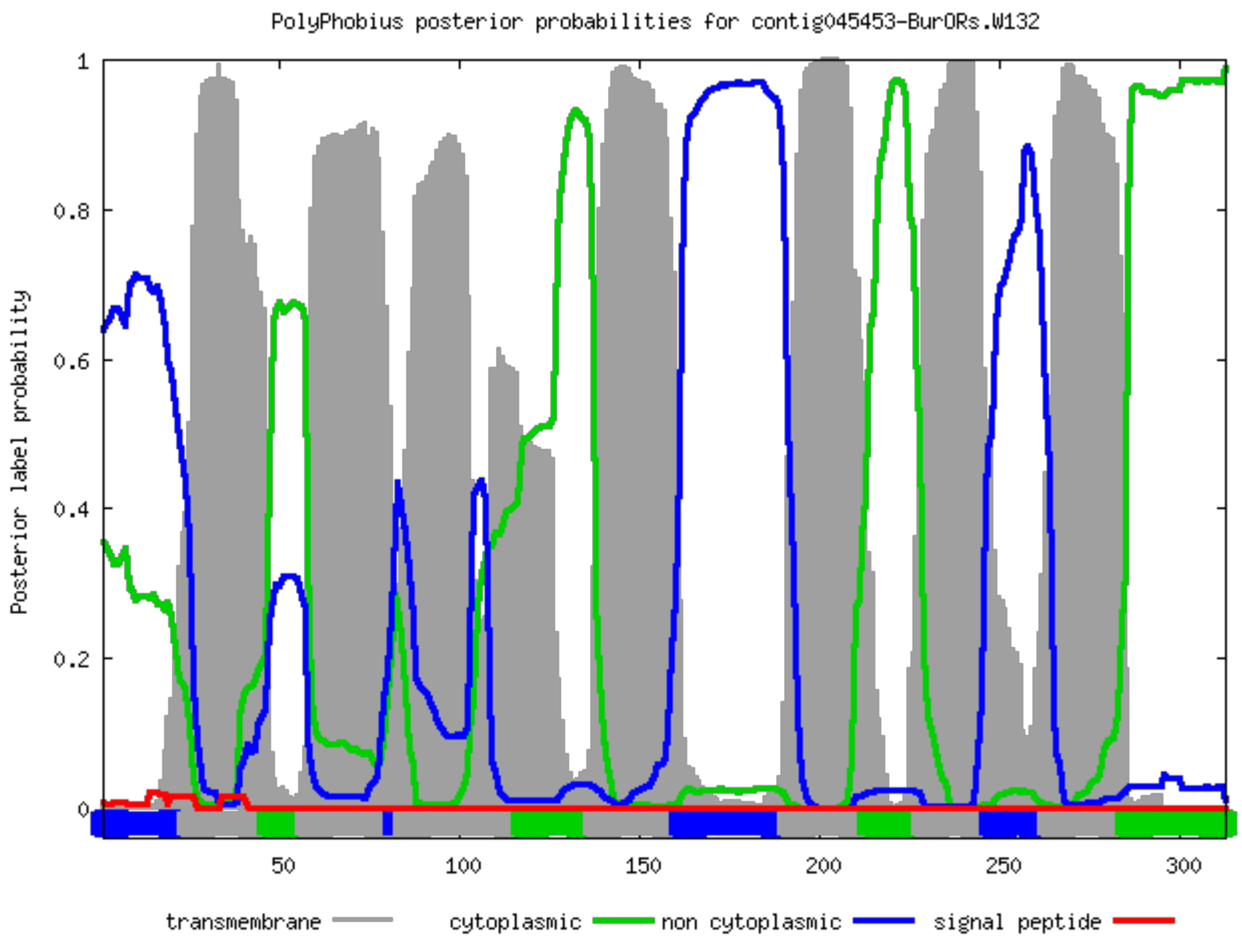

The prediction is based on an alignment. The probability data used in the plot is found here, and the gnuplot script is here.

Prediction of contig058162-BurOR.L089

```
ID   contig058162-BurOR.L089
FT   TOPO_DOM      1     25       NON CYTOPLASMIC.
FT   TRANSMEM     26     50
FT   TOPO_DOM     51     59       CYTOPLASMIC.
FT   TRANSMEM     60     82
FT   TOPO_DOM     83     98       NON CYTOPLASMIC.
FT   TRANSMEM     99    120
FT   TOPO_DOM    121    140       CYTOPLASMIC.
FT   TRANSMEM    141    162
FT   TOPO_DOM    163    198       NON CYTOPLASMIC.
FT   TRANSMEM    199    224
FT   TOPO_DOM    225    235       CYTOPLASMIC.
FT   TRANSMEM    236    259
FT   TOPO_DOM    260    271       NON CYTOPLASMIC.
FT   TRANSMEM    272    292
FT   TOPO_DOM    293    314       CYTOPLASMIC.
//
```

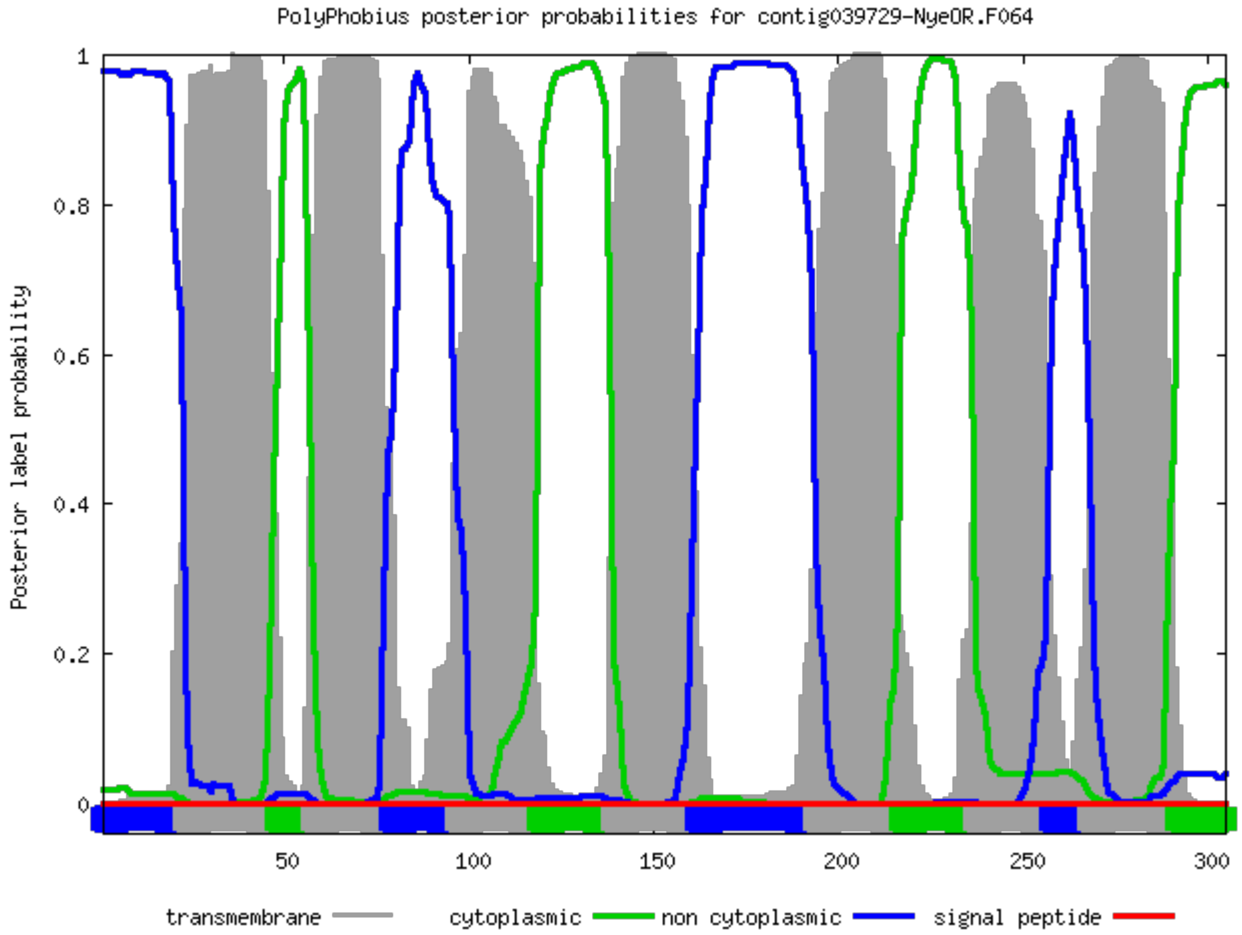

The prediction is based on an alignment. The probability data used in the plot is found here, and the gnuplot script is here.

Prediction of contig010725-ZebOR.N112

```
ID    contig010725-ZebOR.N112
FT    TOPO_DOM      1      32       NON CYTOPLASMIC.
FT    TRANSMEM     33      58
FT    TOPO_DOM     59      66       CYTOPLASMIC.
FT    TRANSMEM     67      86
FT    TOPO_DOM     87     104       NON CYTOPLASMIC.
FT    TRANSMEM    105     127
FT    TOPO_DOM    128     146       CYTOPLASMIC.
FT    TRANSMEM    147     170
FT    TOPO_DOM    171     206       NON CYTOPLASMIC.
FT    TRANSMEM    207     232
FT    TOPO_DOM    233     250       CYTOPLASMIC.
FT    TRANSMEM    251     272
FT    TOPO_DOM    273     277       NON CYTOPLASMIC.
FT    TRANSMEM    278     298
FT    TOPO_DOM    299     324       CYTOPLASMIC.
//
```

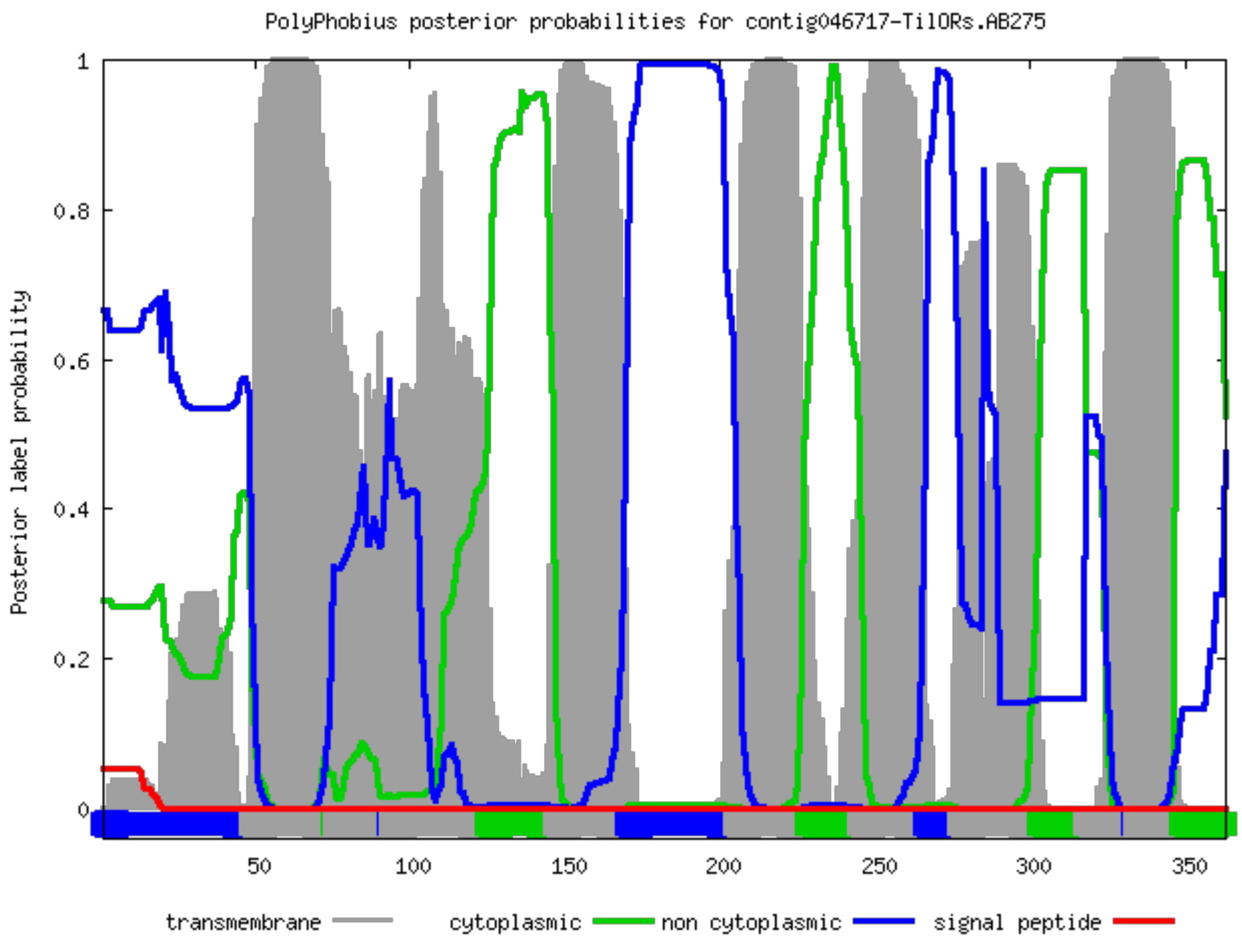

The prediction is based on an alignment. The probability data used in the plot is found here, and the gnuplot script is here.

Prediction of contig046490-NyeOR.K085

```
ID   contig046490-NyeOR.K085
FT   TOPO_DOM      1     24       NON CYTOPLASMIC.
FT   TRANSMEM     25     50
FT   TOPO_DOM     51     58       CYTOPLASMIC.
FT   TRANSMEM     59     81
FT   TOPO_DOM     82    100       NON CYTOPLASMIC.
FT   TRANSMEM    101    121
FT   TOPO_DOM    122    141       CYTOPLASMIC.
FT   TRANSMEM    142    165
FT   TOPO_DOM    166    196       NON CYTOPLASMIC.
FT   TRANSMEM    197    224
FT   TOPO_DOM    225    244       CYTOPLASMIC.
FT   TRANSMEM    245    264
FT   TOPO_DOM    265    269       NON CYTOPLASMIC.
FT   TRANSMEM    270    292
FT   TOPO_DOM    293    314       CYTOPLASMIC.
//
```

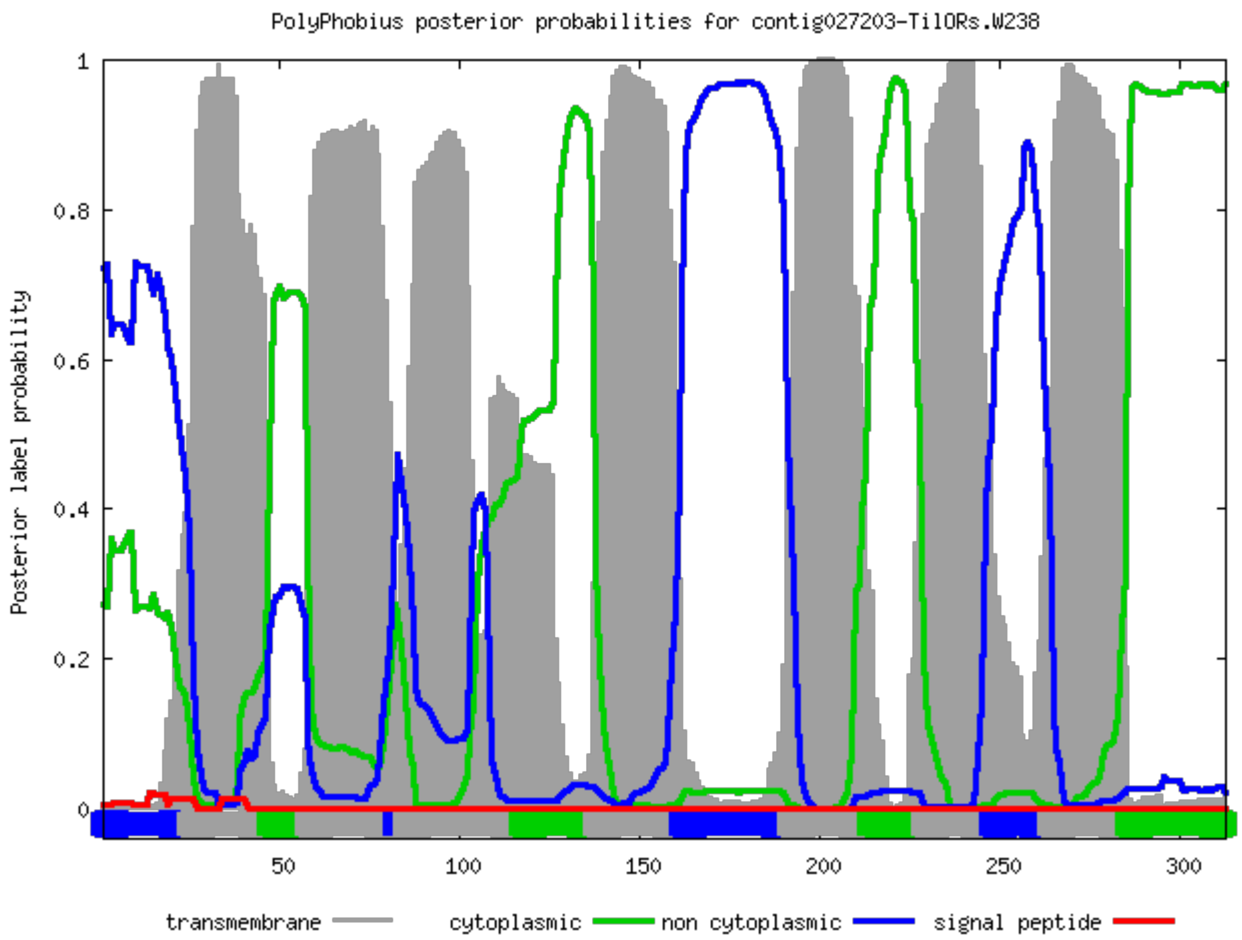

The prediction is based on an alignment. The probability data used in the plot is found here, and the gnuplot script is here.

Prediction of contig061663-NyeOR.N114

```
ID    contig061663-NyeOR.N114
FT    TOPO_DOM      1      32       NON CYTOPLASMIC.
FT    TRANSMEM     33      58
FT    TOPO_DOM     59      66       CYTOPLASMIC.
FT    TRANSMEM     67      87
FT    TOPO_DOM     88     105       NON CYTOPLASMIC.
FT    TRANSMEM    106     127
FT    TOPO_DOM    128     146       CYTOPLASMIC.
FT    TRANSMEM    147     170
FT    TOPO_DOM    171     207       NON CYTOPLASMIC.
FT    TRANSMEM    208     232
FT    TOPO_DOM    233     249       CYTOPLASMIC.
FT    TRANSMEM    250     271
FT    TOPO_DOM    272     277       NON CYTOPLASMIC.
FT    TRANSMEM    278     298
FT    TOPO_DOM    299     327       CYTOPLASMIC.
//
```

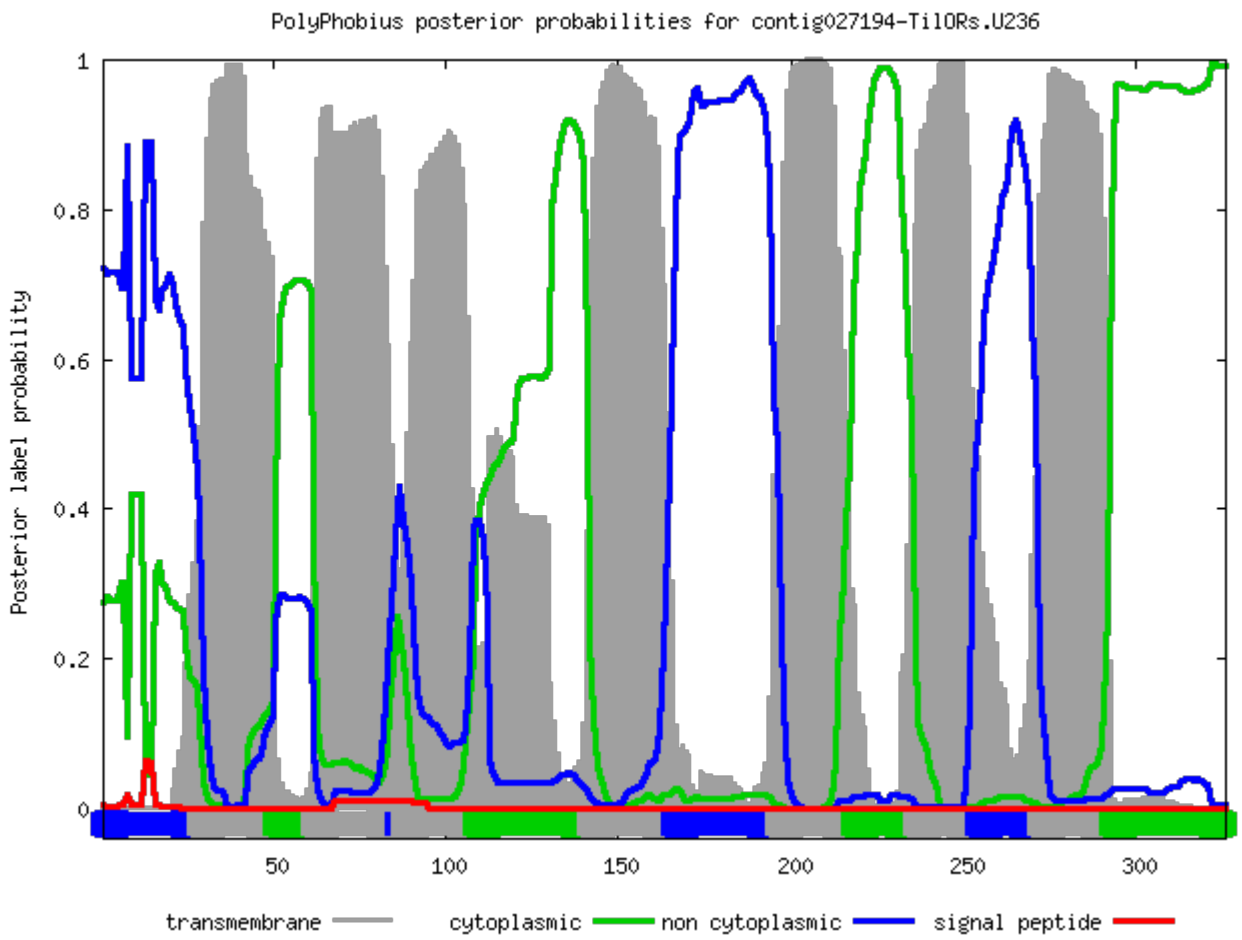

The prediction is based on an alignment. The probability data used in the plot is found here, and the gnuplot script is here.

Prediction of contig013365-TilOR.H111

```
ID   contig013365-TilOR.H111
FT   TOPO_DOM      1     22       NON CYTOPLASMIC.
FT   TRANSMEM     23     49
FT   TOPO_DOM     50     56       CYTOPLASMIC.
FT   TRANSMEM     57     77
FT   TOPO_DOM     78     95       NON CYTOPLASMIC.
FT   TRANSMEM     96    118
FT   TOPO_DOM    119    138       CYTOPLASMIC.
FT   TRANSMEM    139    160
FT   TOPO_DOM    161    193       NON CYTOPLASMIC.
FT   TRANSMEM    194    216
FT   TOPO_DOM    217    235       CYTOPLASMIC.
FT   TRANSMEM    236    257
FT   TOPO_DOM    258    269       NON CYTOPLASMIC.
FT   TRANSMEM    270    289
FT   TOPO_DOM    290    314       CYTOPLASMIC.
//
```

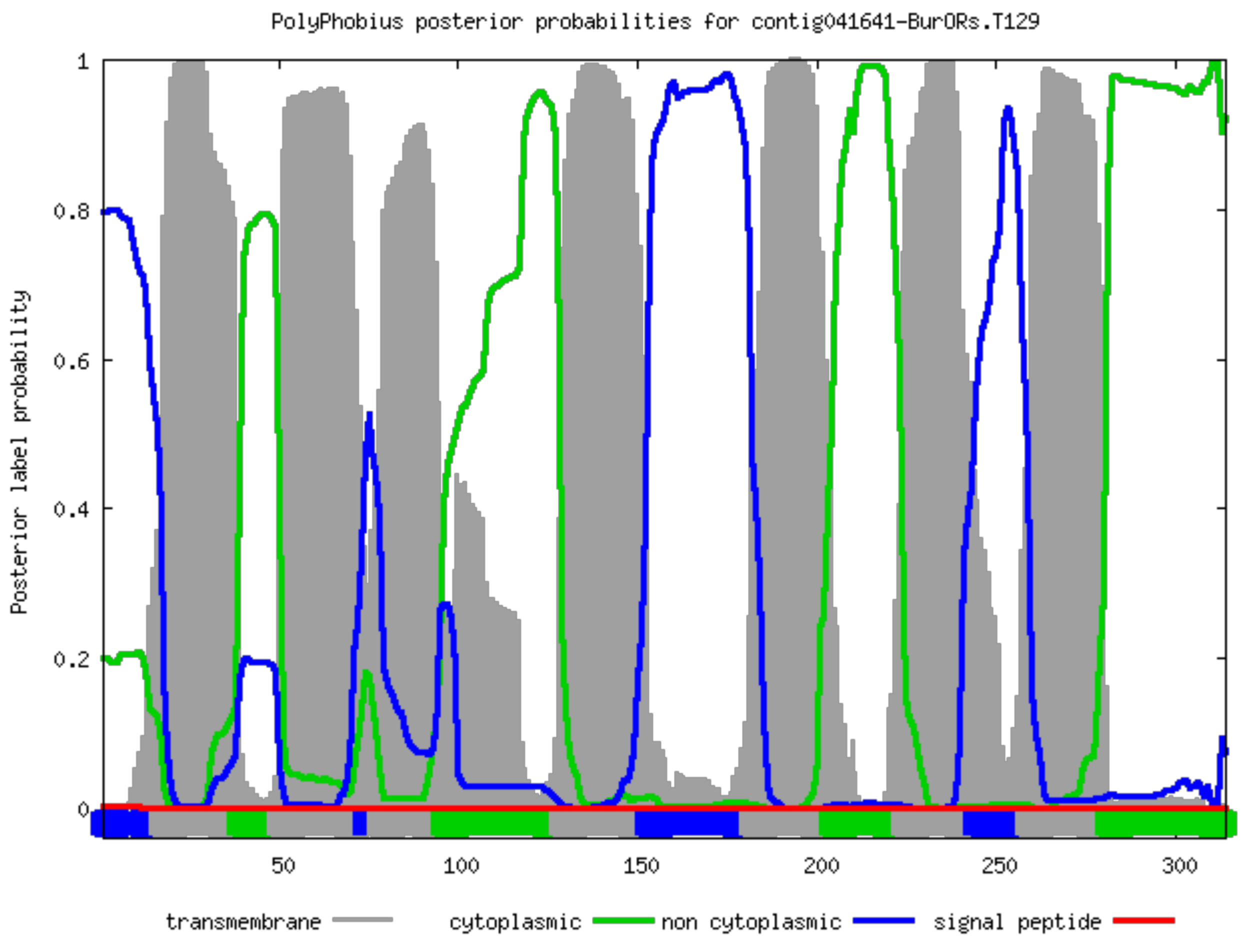

The prediction is based on an alignment. The probability data used in the plot is found here, and the gnuplot script is here.

Prediction of contig057383-BurOR.N109

```
ID   contig057383-BurOR.N109
FT   TOPO_DOM      1     32       NON CYTOPLASMIC.
FT   TRANSMEM     33     58
FT   TOPO_DOM     59     66       CYTOPLASMIC.
FT   TRANSMEM     67     87
FT   TOPO_DOM     88    105       NON CYTOPLASMIC.
FT   TRANSMEM    106    127
FT   TOPO_DOM    128    146       CYTOPLASMIC.
FT   TRANSMEM    147    170
FT   TOPO_DOM    171    207       NON CYTOPLASMIC.
FT   TRANSMEM    208    232
FT   TOPO_DOM    233    249       CYTOPLASMIC.
FT   TRANSMEM    250    271
FT   TOPO_DOM    272    277       NON CYTOPLASMIC.
FT   TRANSMEM    278    298
FT   TOPO_DOM    299    327       CYTOPLASMIC.
//
```

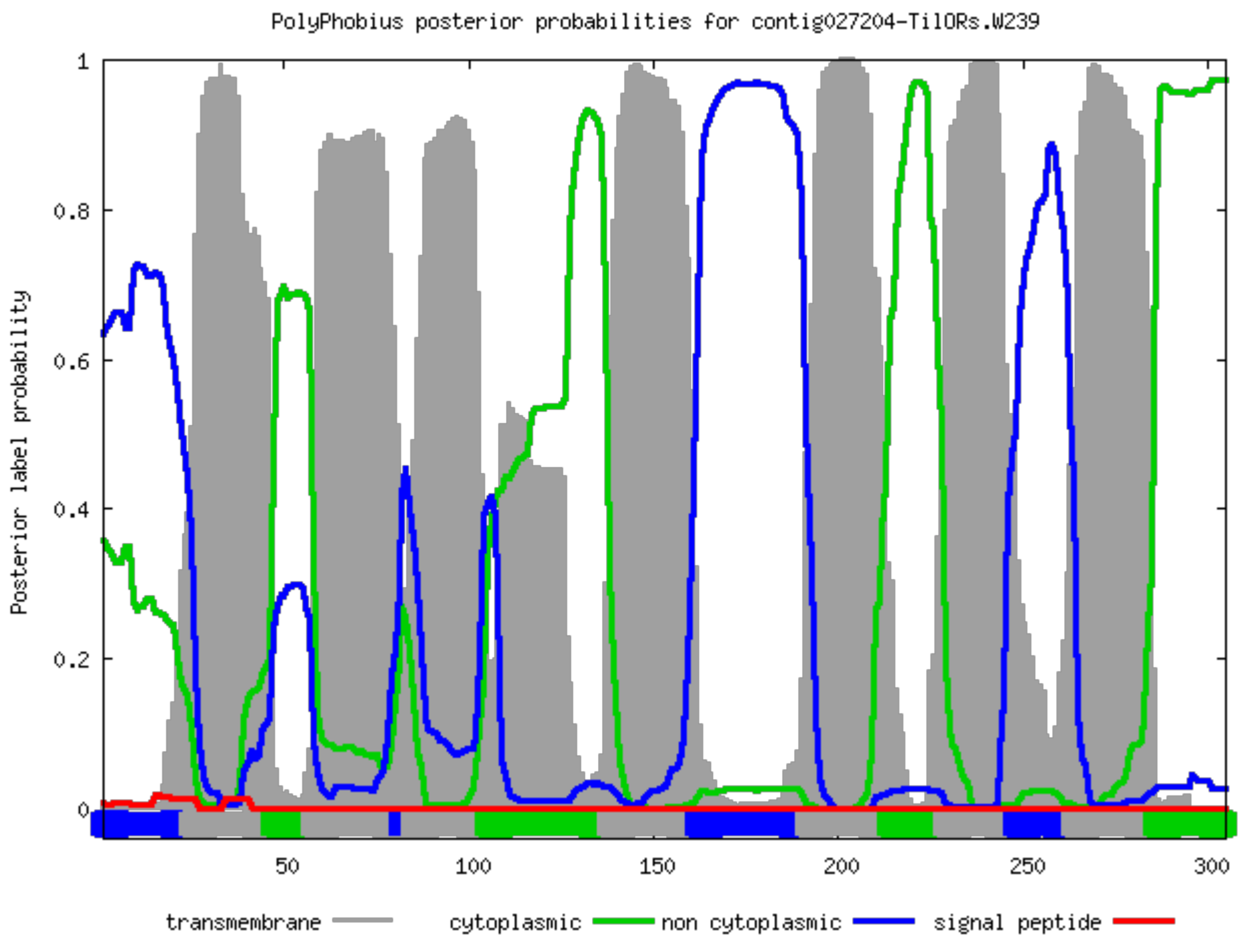

The prediction is based on an alignment. The probability data used in the plot is found here, and the gnuplot script is here.

Prediction of contig017697-BurOR.P119

```
ID   contig017697-BurOR.P119
FT   TOPO_DOM      1     27       NON CYTOPLASMIC.
FT   TRANSMEM     28     51
FT   TOPO_DOM     52     61       CYTOPLASMIC.
FT   TRANSMEM     62     88
FT   TOPO_DOM     89    100       NON CYTOPLASMIC.
FT   TRANSMEM    101    122
FT   TOPO_DOM    123    142       CYTOPLASMIC.
FT   TRANSMEM    143    165
FT   TOPO_DOM    166    200       NON CYTOPLASMIC.
FT   TRANSMEM    201    225
FT   TOPO_DOM    226    239       CYTOPLASMIC.
FT   TRANSMEM    240    262
FT   TOPO_DOM    263    273       NON CYTOPLASMIC.
FT   TRANSMEM    274    294
FT   TOPO_DOM    295    310       CYTOPLASMIC.
//
```

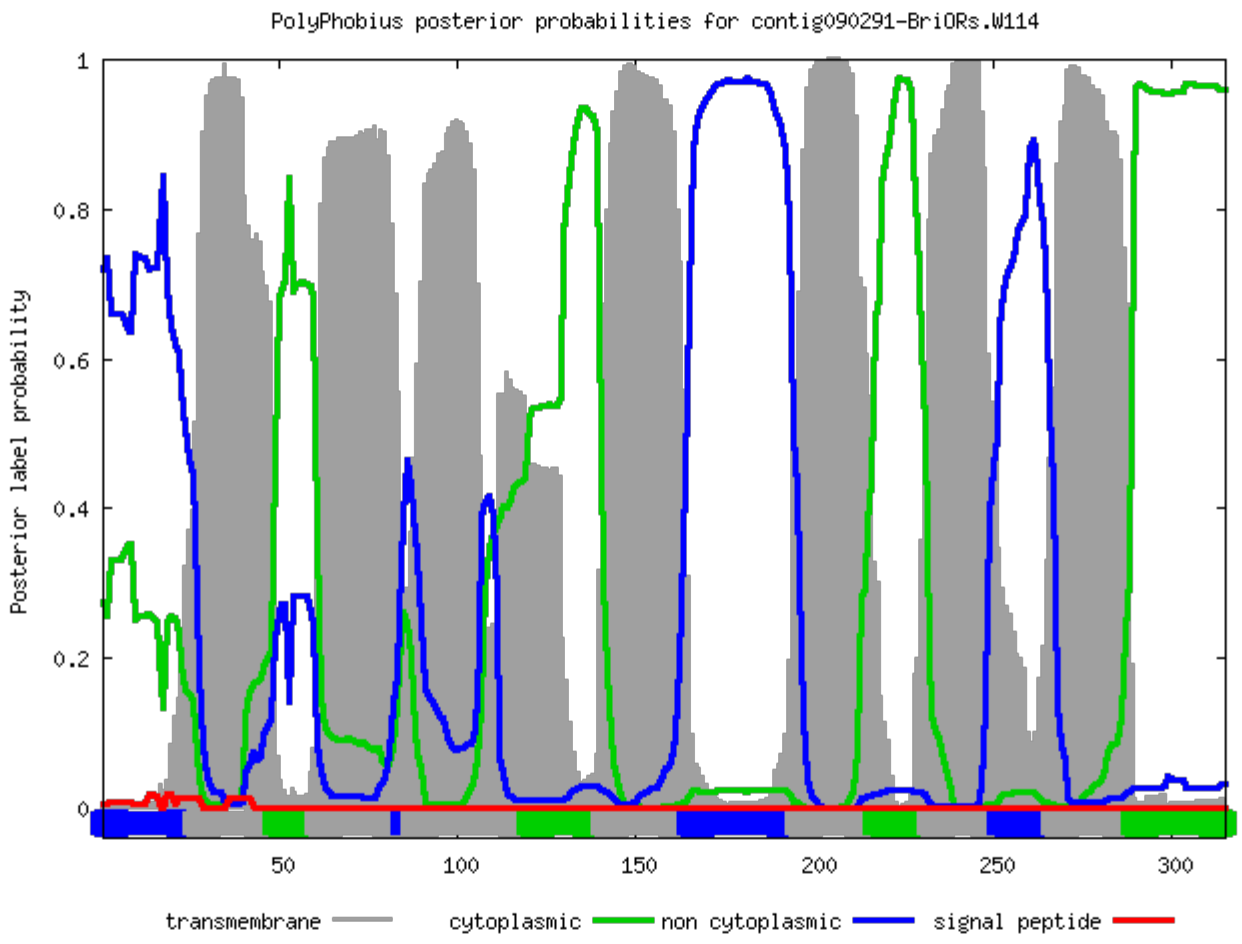

The prediction is based on an alignment. The probability data used in the plot is found here, and the gnuplot script is here.

Prediction of contig047725-TilOR.E074

```
ID    contig047725-TilOR.E074
FT    TOPO_DOM       1      24       NON CYTOPLASMIC.
FT    TRANSMEM      25      50
FT    TOPO_DOM      51      59       CYTOPLASMIC.
FT    TRANSMEM      60      85
FT    TOPO_DOM      86      92       NON CYTOPLASMIC.
FT    TRANSMEM      93     119
FT    TOPO_DOM     120     140       CYTOPLASMIC.
FT    TRANSMEM     141     163
FT    TOPO_DOM     164     195       NON CYTOPLASMIC.
FT    TRANSMEM     196     218
FT    TOPO_DOM     219     238       CYTOPLASMIC.
FT    TRANSMEM     239     258
FT    TOPO_DOM     259     269       NON CYTOPLASMIC.
FT    TRANSMEM     270     293
FT    TOPO_DOM     294     322       CYTOPLASMIC.
//
```

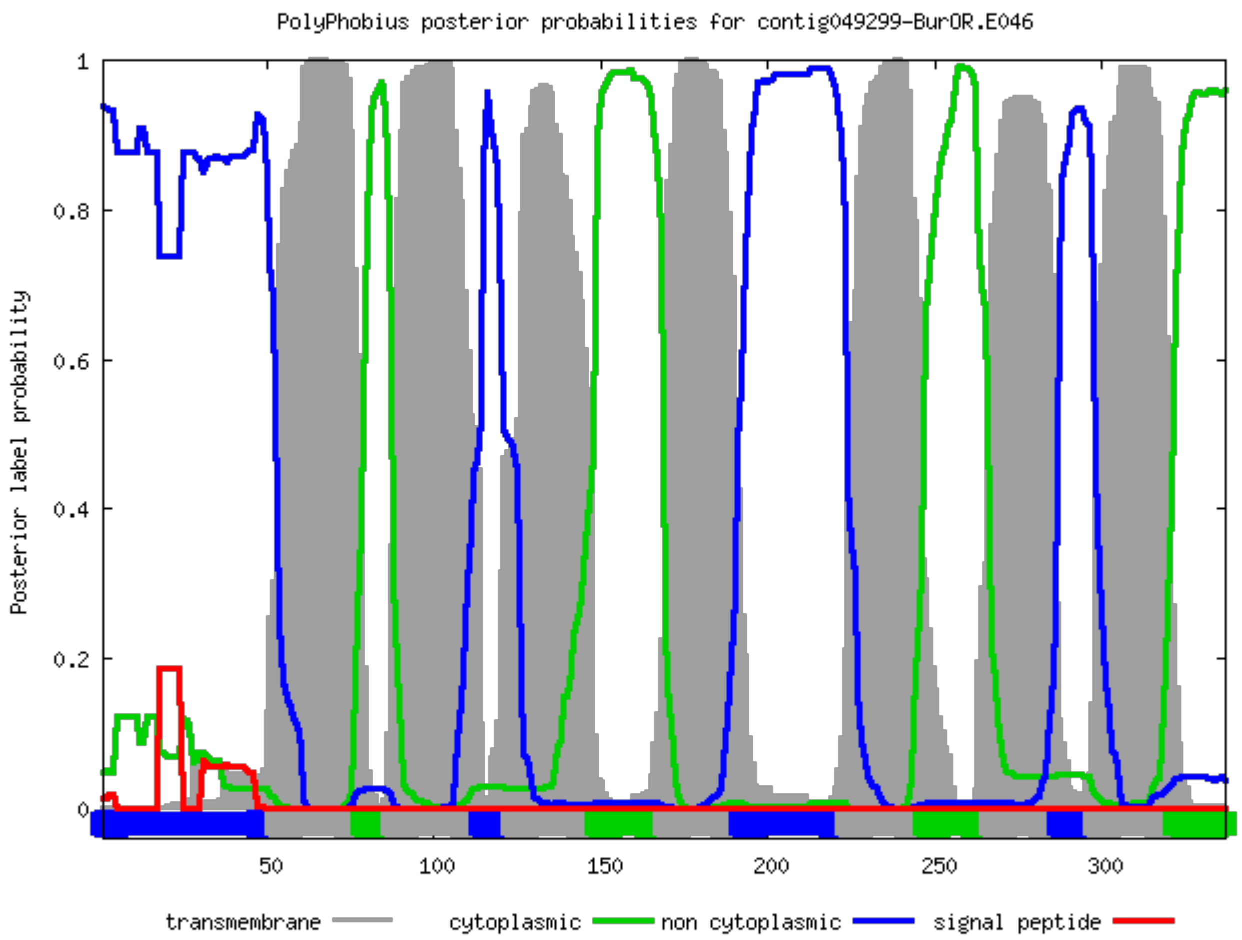

The prediction is based on an alignment. The probability data used in the plot is found here, and the gnuplot script is here.

Prediction of contig065454-TilOR.E089

```
ID    contig065454-TilOR.E089
FT    TOPO_DOM      1     22       NON CYTOPLASMIC.
FT    TRANSMEM     23     48
FT    TOPO_DOM     49     57       CYTOPLASMIC.
FT    TRANSMEM     58     81
FT    TOPO_DOM     82     95       NON CYTOPLASMIC.
FT    TRANSMEM     96    118
FT    TOPO_DOM    119    138       CYTOPLASMIC.
FT    TRANSMEM    139    161
FT    TOPO_DOM    162    193       NON CYTOPLASMIC.
FT    TRANSMEM    194    216
FT    TOPO_DOM    217    236       CYTOPLASMIC.
FT    TRANSMEM    237    256
FT    TOPO_DOM    257    267       NON CYTOPLASMIC.
FT    TRANSMEM    268    291
FT    TOPO_DOM    292    309       CYTOPLASMIC.
//
```

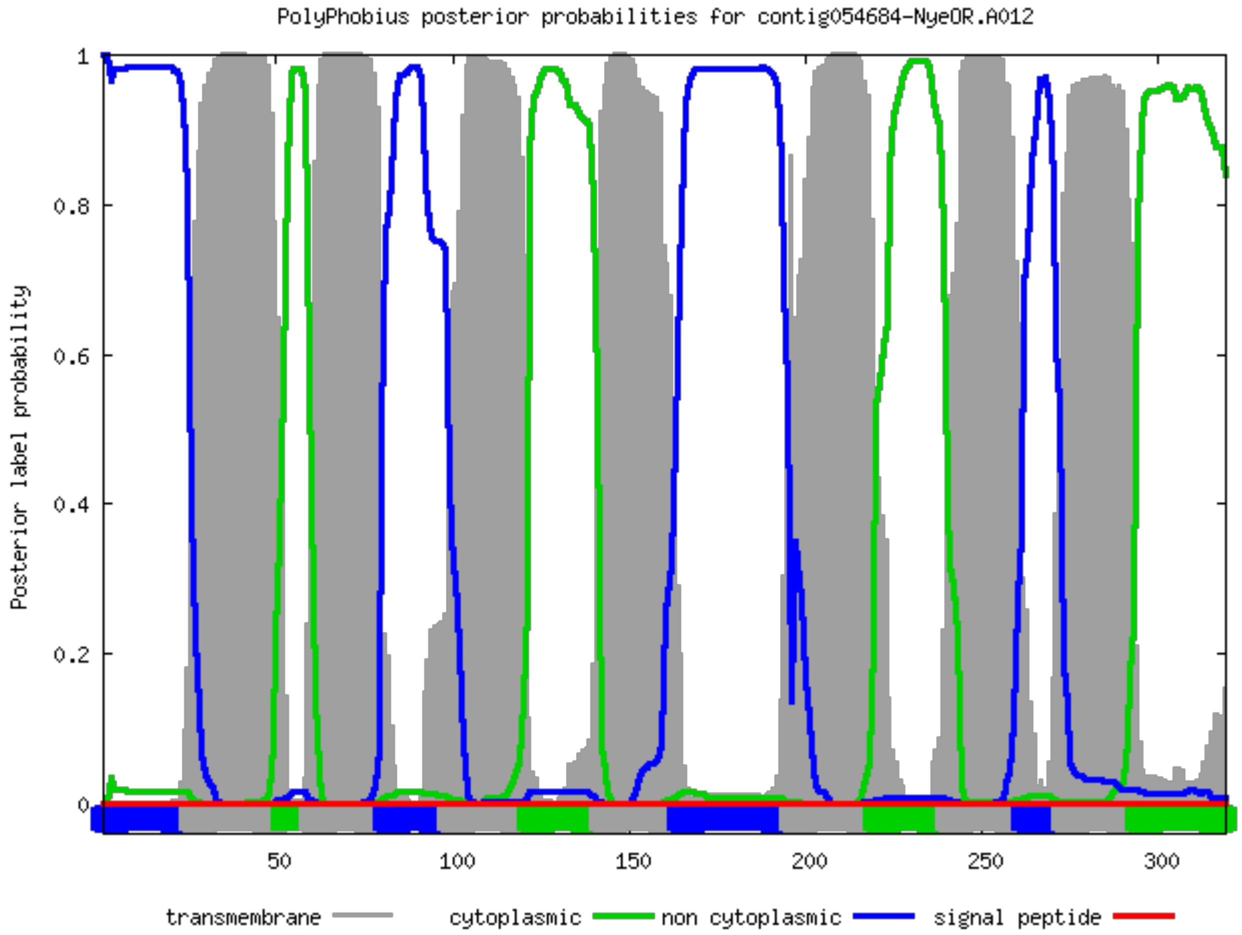

The prediction is based on an alignment. The probability data used in the plot is found here, and the gnuplot script is here.

Prediction of contig025458-ZebOR.P125

```
ID   contig025458-ZebOR.P125
FT   TOPO_DOM      1     28       NON CYTOPLASMIC.
FT   TRANSMEM     29     52
FT   TOPO_DOM     53     62       CYTOPLASMIC.
FT   TRANSMEM     63     86
FT   TOPO_DOM     87    100       NON CYTOPLASMIC.
FT   TRANSMEM    101    123
FT   TOPO_DOM    124    142       CYTOPLASMIC.
FT   TRANSMEM    143    166
FT   TOPO_DOM    167    200       NON CYTOPLASMIC.
FT   TRANSMEM    201    225
FT   TOPO_DOM    226    243       CYTOPLASMIC.
FT   TRANSMEM    244    265
FT   TOPO_DOM    266    277       NON CYTOPLASMIC.
FT   TRANSMEM    278    298
FT   TOPO_DOM    299    332       CYTOPLASMIC.
//
```

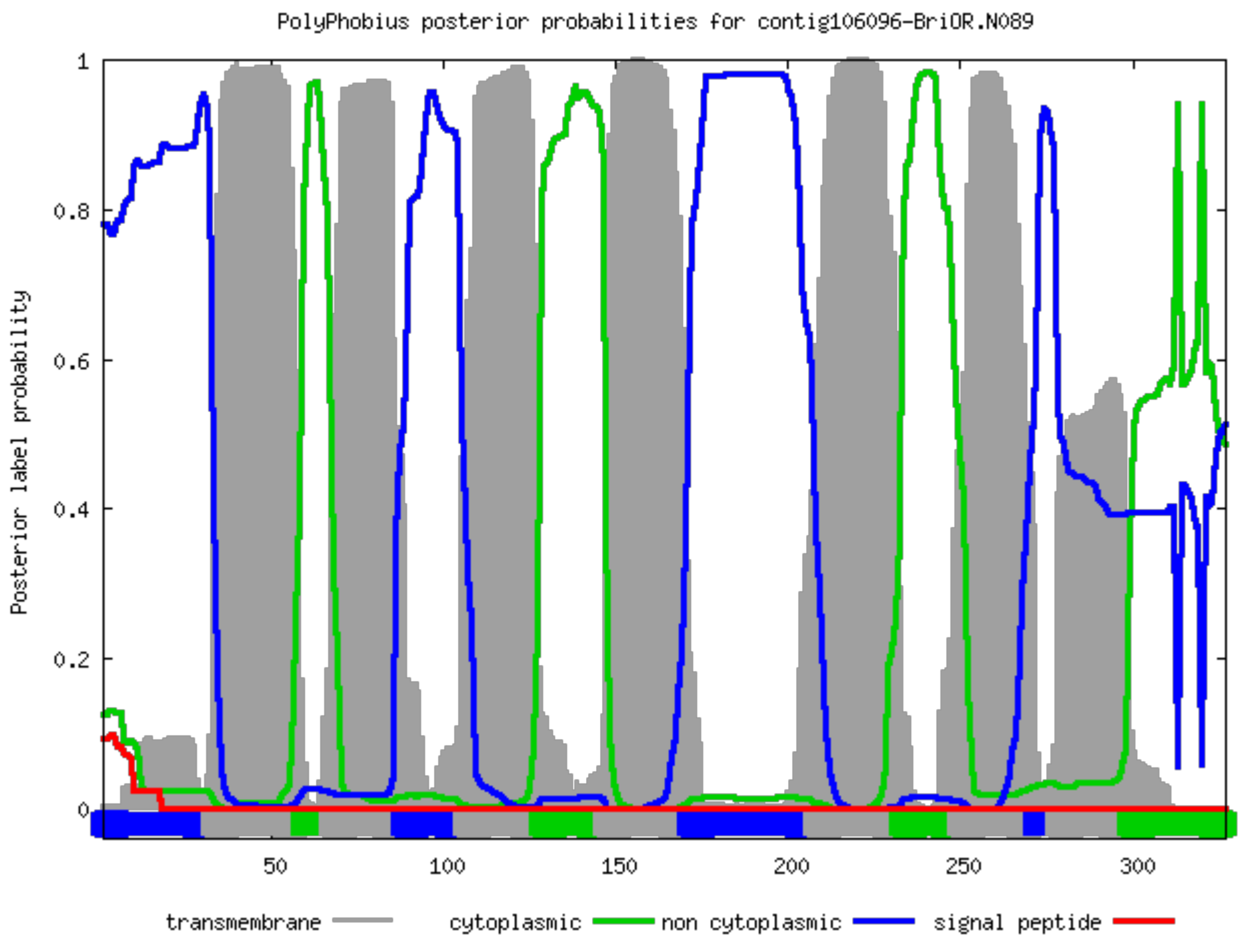

The prediction is based on an alignment. The probability data used in the plot is found here, and the gnuplot script is here.

Prediction of contig053579-NyeOR.E054

```
ID   contig053579-NyeOR.E054
FT   TOPO_DOM      1     22       NON CYTOPLASMIC.
FT   TRANSMEM     23     48
FT   TOPO_DOM     49     57       CYTOPLASMIC.
FT   TRANSMEM     58     82
FT   TOPO_DOM     83     94       NON CYTOPLASMIC.
FT   TRANSMEM     95    118
FT   TOPO_DOM    119    138       CYTOPLASMIC.
FT   TRANSMEM    139    160
FT   TOPO_DOM    161    193       NON CYTOPLASMIC.
FT   TRANSMEM    194    216
FT   TOPO_DOM    217    236       CYTOPLASMIC.
FT   TRANSMEM    237    256
FT   TOPO_DOM    257    267       NON CYTOPLASMIC.
FT   TRANSMEM    268    291
FT   TOPO_DOM    292    314       CYTOPLASMIC.
//
```

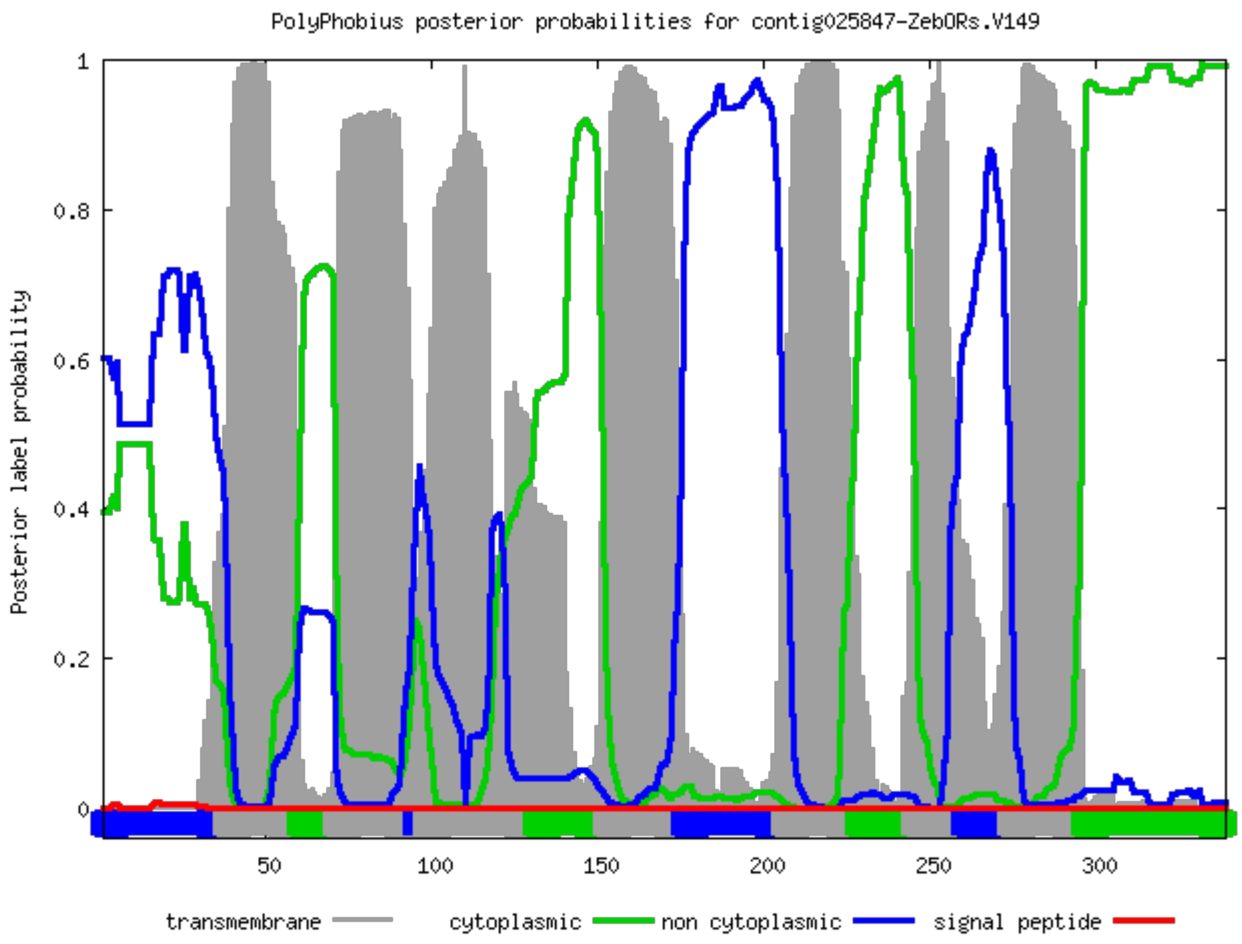

The prediction is based on an alignment. The probability data used in the plot is found here, and the gnuplot script is here.

Prediction of contig057403-ZebOR.H077

```
ID   contig057403-ZebOR.H077
FT   TOPO_DOM      1     23       NON CYTOPLASMIC.
FT   TRANSMEM     24     49
FT   TOPO_DOM     50     56       CYTOPLASMIC.
FT   TRANSMEM     57     76
FT   TOPO_DOM     77     95       NON CYTOPLASMIC.
FT   TRANSMEM     96    118
FT   TOPO_DOM    119    138       CYTOPLASMIC.
FT   TRANSMEM    139    160
FT   TOPO_DOM    161    196       NON CYTOPLASMIC.
FT   TRANSMEM    197    219
FT   TOPO_DOM    220    237       CYTOPLASMIC.
FT   TRANSMEM    238    260
FT   TOPO_DOM    261    271       NON CYTOPLASMIC.
FT   TRANSMEM    272    291
FT   TOPO_DOM    292    343       CYTOPLASMIC.
//
```

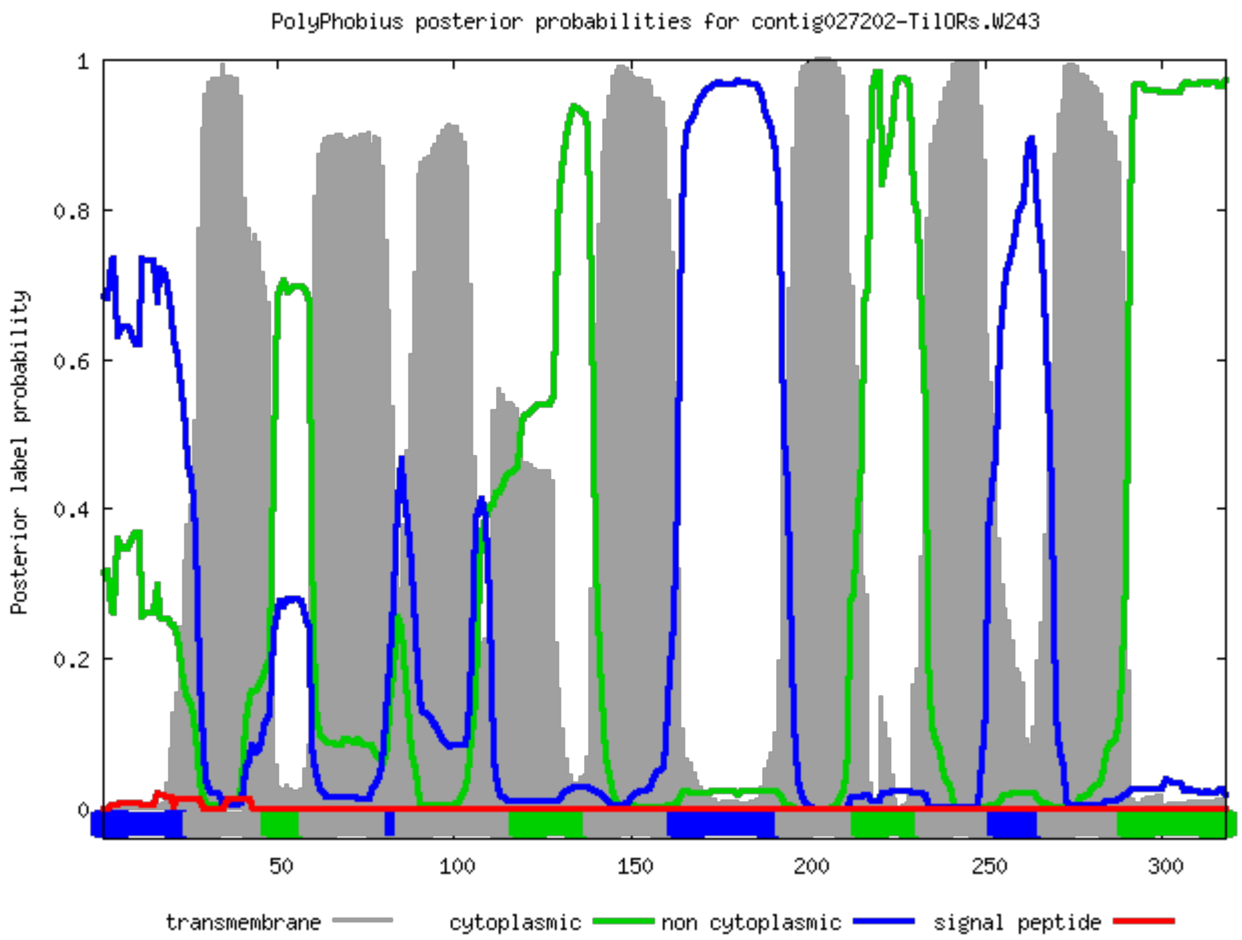

The prediction is based on an alignment. The probability data used in the plot is found here, and the gnuplot script is here.

Prediction of contig010726-ZebOR.N113

```
ID    contig010726-ZebOR.N113
FT    TOPO_DOM      1      32       NON CYTOPLASMIC.
FT    TRANSMEM     33      58
FT    TOPO_DOM     59      66       CYTOPLASMIC.
FT    TRANSMEM     67      86
FT    TOPO_DOM     87     104       NON CYTOPLASMIC.
FT    TRANSMEM    105     127
FT    TOPO_DOM    128     146       CYTOPLASMIC.
FT    TRANSMEM    147     170
FT    TOPO_DOM    171     206       NON CYTOPLASMIC.
FT    TRANSMEM    207     232
FT    TOPO_DOM    233     250       CYTOPLASMIC.
FT    TRANSMEM    251     271
FT    TOPO_DOM    272     277       NON CYTOPLASMIC.
FT    TRANSMEM    278     298
FT    TOPO_DOM    299     324       CYTOPLASMIC.
//
```

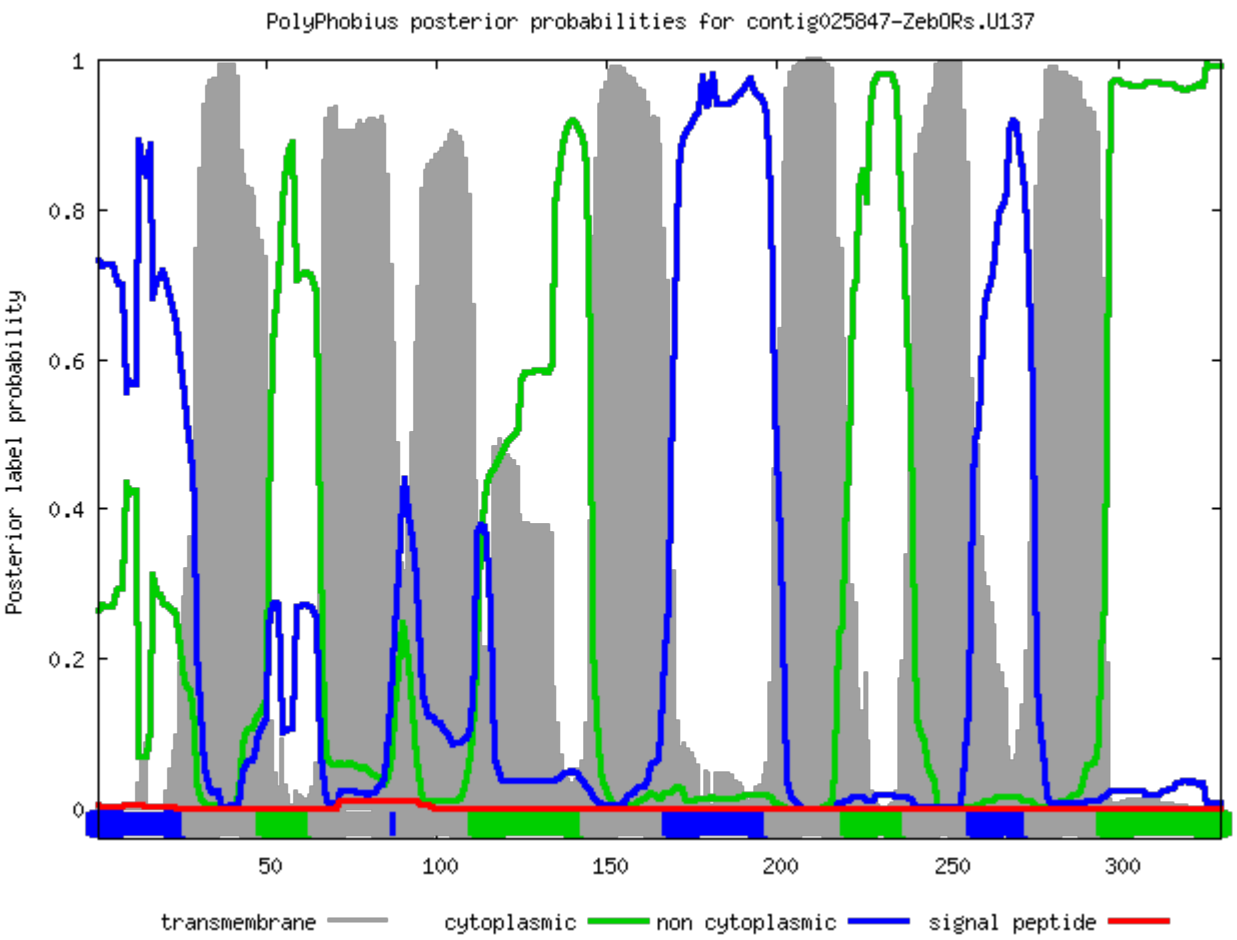

The prediction is based on an alignment. The probability data used in the plot is found here, and the gnuplot script is here.

Prediction of contig048880-BurOR.H063

```
ID   contig048880-BurOR.H063
FT   TOPO_DOM      1     22       NON CYTOPLASMIC.
FT   TRANSMEM     23     48
FT   TOPO_DOM     49     56       CYTOPLASMIC.
FT   TRANSMEM     57     77
FT   TOPO_DOM     78     95       NON CYTOPLASMIC.
FT   TRANSMEM     96    118
FT   TOPO_DOM    119    138       CYTOPLASMIC.
FT   TRANSMEM    139    160
FT   TOPO_DOM    161    193       NON CYTOPLASMIC.
FT   TRANSMEM    194    216
FT   TOPO_DOM    217    235       CYTOPLASMIC.
FT   TRANSMEM    236    258
FT   TOPO_DOM    259    269       NON CYTOPLASMIC.
FT   TRANSMEM    270    289
FT   TOPO_DOM    290    314       CYTOPLASMIC.
//
```

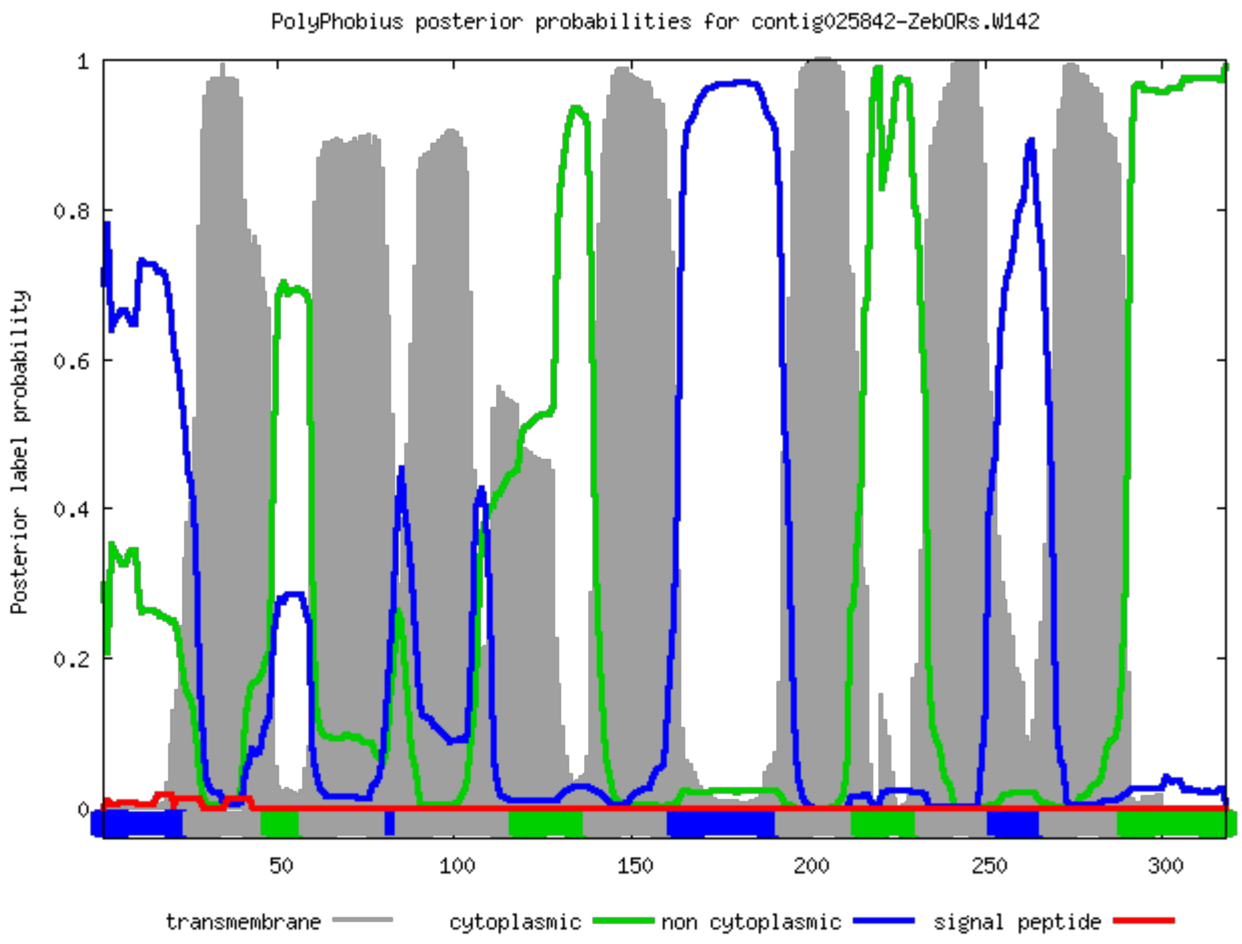

The prediction is based on an alignment. The probability data used in the plot is found here, and the gnuplot script is here.

Prediction of contig048263-ZebOR.E052

```
ID   contig048263-ZebOR.E052
FT   TOPO_DOM      1     25       NON CYTOPLASMIC.
FT   TRANSMEM     26     51
FT   TOPO_DOM     52     60       CYTOPLASMIC.
FT   TRANSMEM     61     86
FT   TOPO_DOM     87     93       NON CYTOPLASMIC.
FT   TRANSMEM     94    121
FT   TOPO_DOM    122    141       CYTOPLASMIC.
FT   TRANSMEM    142    164
FT   TOPO_DOM    165    196       NON CYTOPLASMIC.
FT   TRANSMEM    197    219
FT   TOPO_DOM    220    239       CYTOPLASMIC.
FT   TRANSMEM    240    259
FT   TOPO_DOM    260    270       NON CYTOPLASMIC.
FT   TRANSMEM    271    294
FT   TOPO_DOM    295    326       CYTOPLASMIC.
//
```

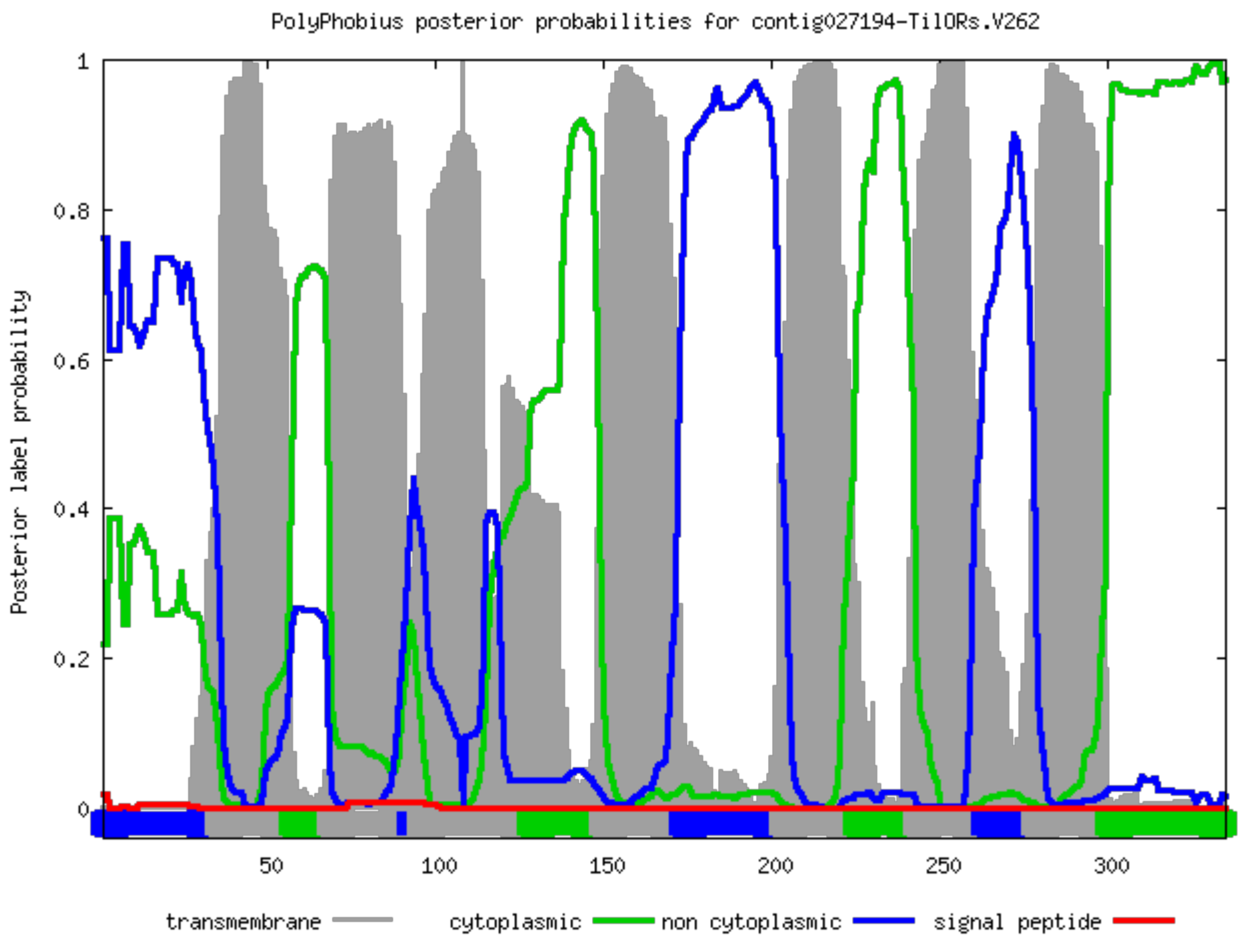

The prediction is based on an alignment. The probability data used in the plot is found here, and the gnuplot script is here.

Prediction of contig023731-TilOR.O177

```
ID   contig023731-TilOR.O177
FT   TOPO_DOM      1     26       NON CYTOPLASMIC.
FT   TRANSMEM     27     51
FT   TOPO_DOM     52     60       CYTOPLASMIC.
FT   TRANSMEM     61     83
FT   TOPO_DOM     84     99       NON CYTOPLASMIC.
FT   TRANSMEM    100    121
FT   TOPO_DOM    122    141       CYTOPLASMIC.
FT   TRANSMEM    142    164
FT   TOPO_DOM    165    201       NON CYTOPLASMIC.
FT   TRANSMEM    202    227
FT   TOPO_DOM    228    242       CYTOPLASMIC.
FT   TRANSMEM    243    263
FT   TOPO_DOM    264    275       NON CYTOPLASMIC.
FT   TRANSMEM    276    296
FT   TOPO_DOM    297    324       CYTOPLASMIC.
//
```

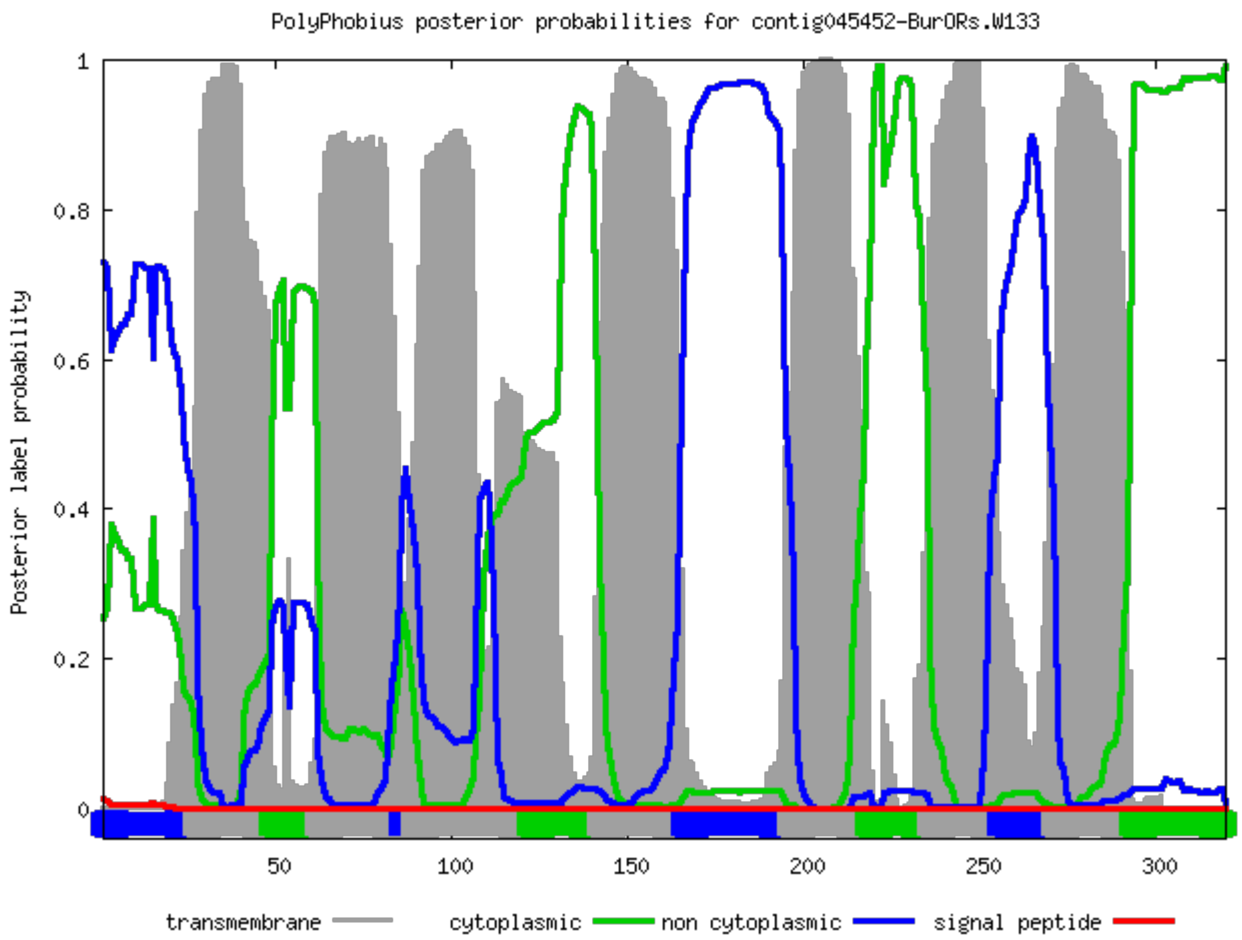

The prediction is based on an alignment. The probability data used in the plot is found here, and the gnuplot script is here.

Prediction of contig068539-TilOR.L158

```
ID   contig068539-TilOR.L158
FT   TOPO_DOM      1     25       NON CYTOPLASMIC.
FT   TRANSMEM     26     50
FT   TOPO_DOM     51     59       CYTOPLASMIC.
FT   TRANSMEM     60     86
FT   TOPO_DOM     87     98       NON CYTOPLASMIC.
FT   TRANSMEM     99    120
FT   TOPO_DOM    121    140       CYTOPLASMIC.
FT   TRANSMEM    141    163
FT   TOPO_DOM    164    199       NON CYTOPLASMIC.
FT   TRANSMEM    200    224
FT   TOPO_DOM    225    237       CYTOPLASMIC.
FT   TRANSMEM    238    260
FT   TOPO_DOM    261    271       NON CYTOPLASMIC.
FT   TRANSMEM    272    292
FT   TOPO_DOM    293    313       CYTOPLASMIC.
//
```

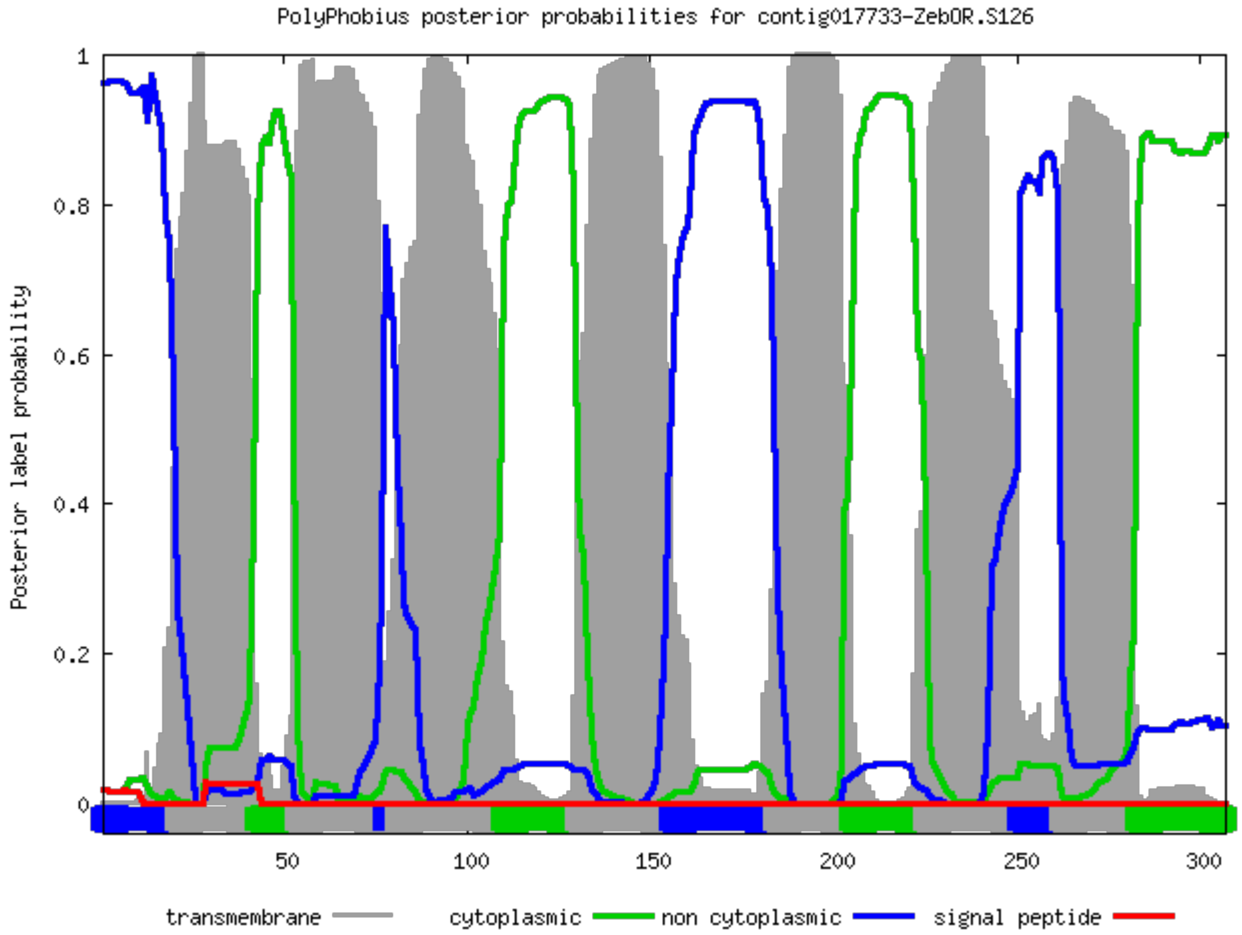

The prediction is based on an alignment. The probability data used in the plot is found here, and the gnuplot script is here.

Prediction of contig047515-ZebOR.A019

```
ID   contig047515-ZebOR.A019
FT   TOPO_DOM      1     18       NON CYTOPLASMIC.
FT   TRANSMEM     19     44
FT   TOPO_DOM     45     52       CYTOPLASMIC.
FT   TRANSMEM     53     73
FT   TOPO_DOM     74     91       NON CYTOPLASMIC.
FT   TRANSMEM     92    114
FT   TOPO_DOM    115    134       CYTOPLASMIC.
FT   TRANSMEM    135    155
FT   TOPO_DOM    156    188       NON CYTOPLASMIC.
FT   TRANSMEM    189    211
FT   TOPO_DOM    212    231       CYTOPLASMIC.
FT   TRANSMEM    232    253
FT   TOPO_DOM    254    264       NON CYTOPLASMIC.
FT   TRANSMEM    265    285
FT   TOPO_DOM    286    300       CYTOPLASMIC.
//
```

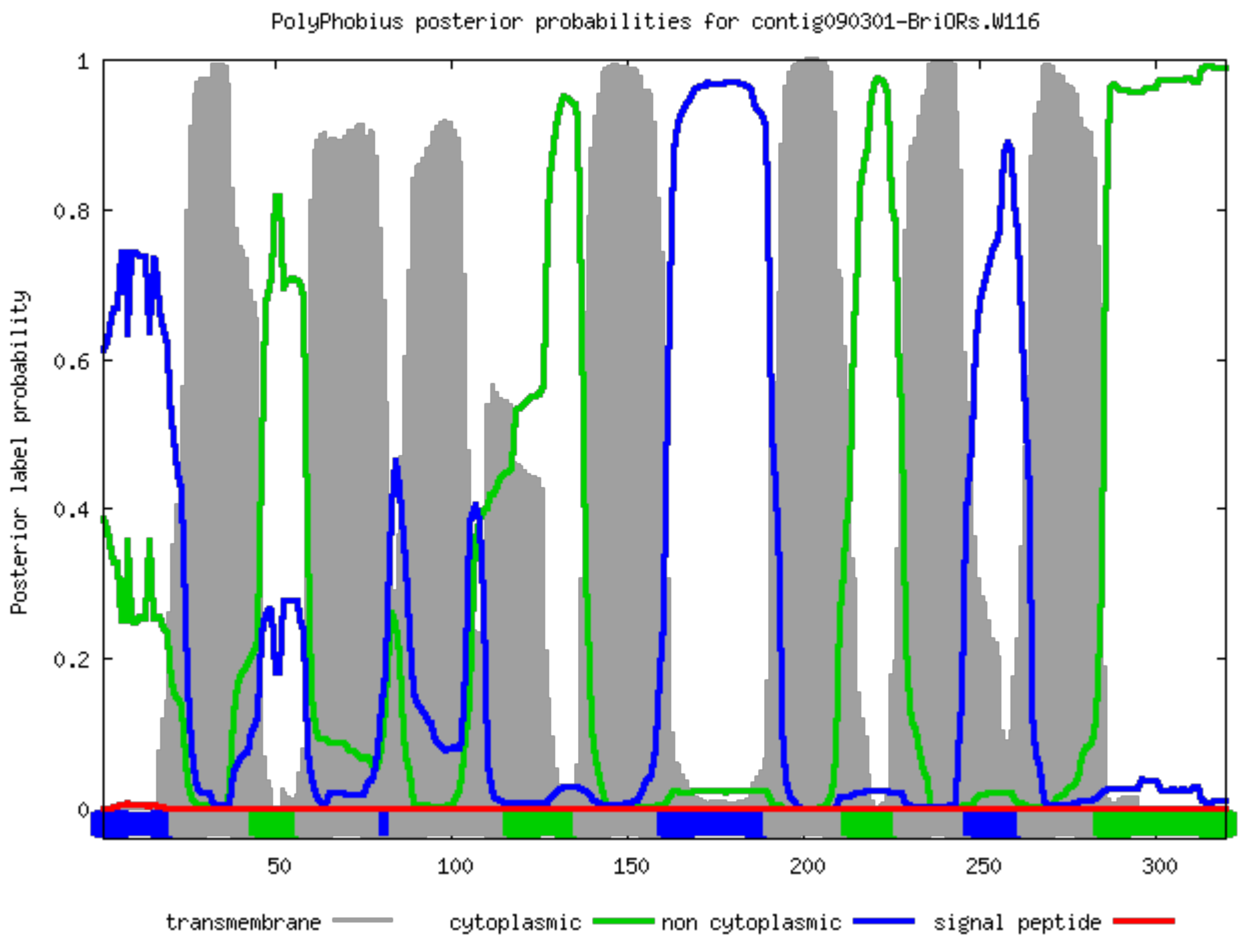

The prediction is based on an alignment. The probability data used in the plot is found here, and the gnuplot script is here.

Prediction of contig048260-ZebOR.E051

```
ID   contig048260-ZebOR.E051
FT   TOPO_DOM      1      22       NON CYTOPLASMIC.
FT   TRANSMEM     23      48
FT   TOPO_DOM     49      57       CYTOPLASMIC.
FT   TRANSMEM     58      83
FT   TOPO_DOM     84      90       NON CYTOPLASMIC.
FT   TRANSMEM     91     118
FT   TOPO_DOM    119     138       CYTOPLASMIC.
FT   TRANSMEM    139     161
FT   TOPO_DOM    162     193       NON CYTOPLASMIC.
FT   TRANSMEM    194     216
FT   TOPO_DOM    217     236       CYTOPLASMIC.
FT   TRANSMEM    237     256
FT   TOPO_DOM    257     267       NON CYTOPLASMIC.
FT   TRANSMEM    268     291
FT   TOPO_DOM    292     314       CYTOPLASMIC.
//
```

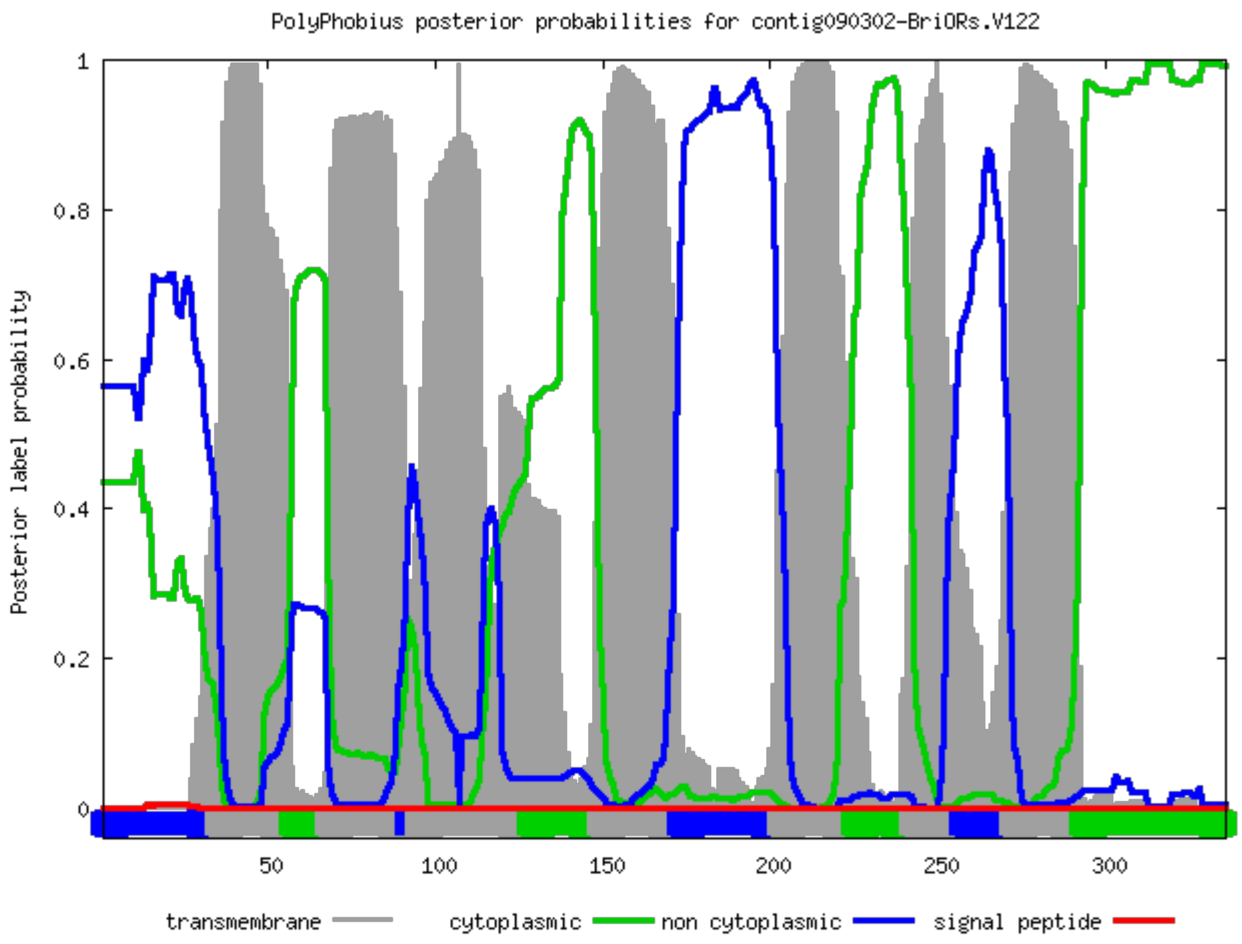

The prediction is based on an alignment. The probability data used in the plot is found here, and the gnuplot script is here.

Prediction of contig020427-ZebOR.O099

```
ID   contig020427-ZebOR.O099
FT   TOPO_DOM      1      26       NON CYTOPLASMIC.
FT   TRANSMEM     27      51
FT   TOPO_DOM     52      60       CYTOPLASMIC.
FT   TRANSMEM     61      83
FT   TOPO_DOM     84      99       NON CYTOPLASMIC.
FT   TRANSMEM    100     121
FT   TOPO_DOM    122     141       CYTOPLASMIC.
FT   TRANSMEM    142     164
FT   TOPO_DOM    165     201       NON CYTOPLASMIC.
FT   TRANSMEM    202     227
FT   TOPO_DOM    228     242       CYTOPLASMIC.
FT   TRANSMEM    243     263
FT   TOPO_DOM    264     275       NON CYTOPLASMIC.
FT   TRANSMEM    276     296
FT   TOPO_DOM    297     324       CYTOPLASMIC.
//
```

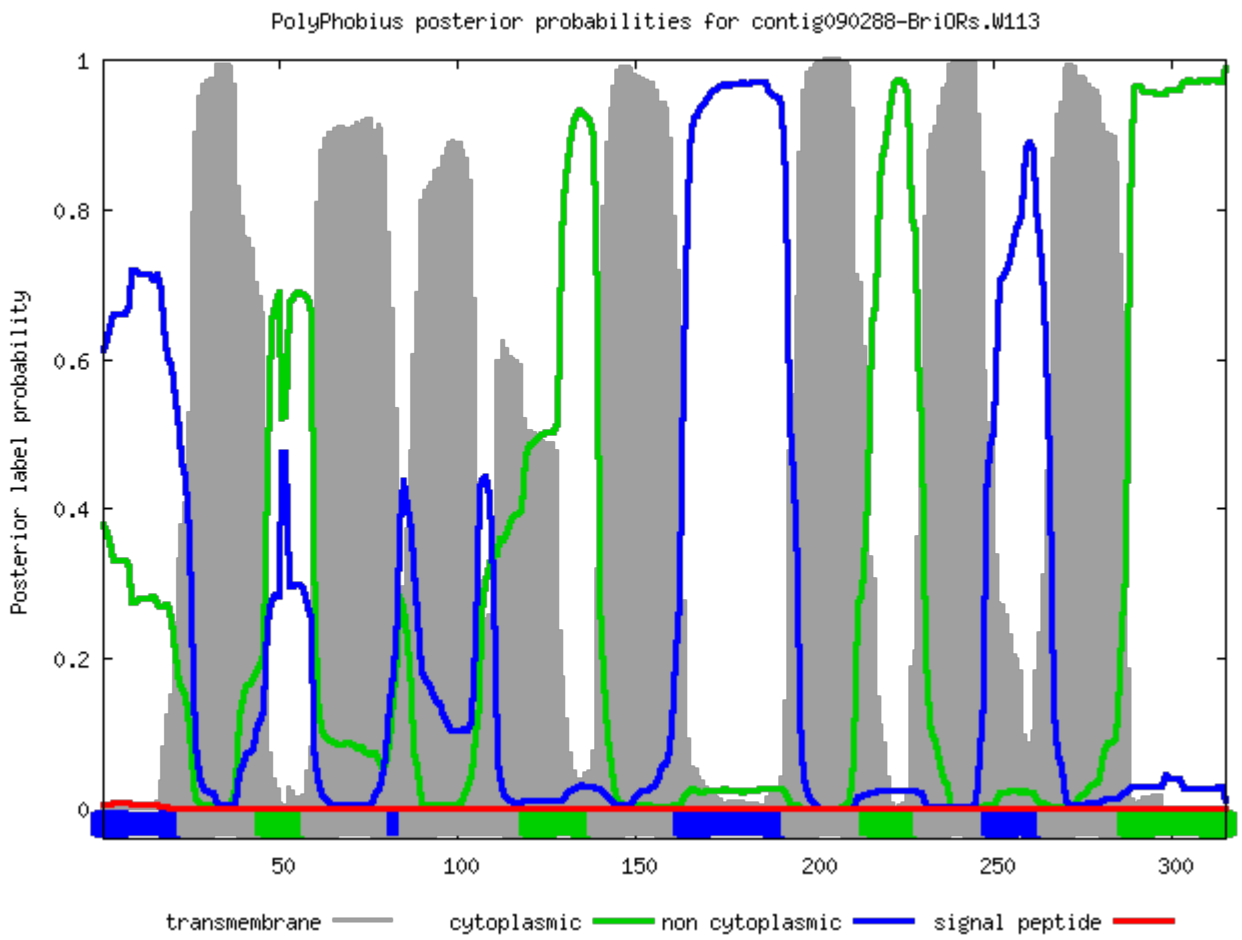

The prediction is based on an alignment. The probability data used in the plot is found here, and the gnuplot script is here.

Prediction of contig055927-NyeOR.N112

```
ID   contig055927-NyeOR.N112
FT   TOPO_DOM      1     33       NON CYTOPLASMIC.
FT   TRANSMEM     34     59
FT   TOPO_DOM     60     67       CYTOPLASMIC.
FT   TRANSMEM     68     89
FT   TOPO_DOM     90    108       NON CYTOPLASMIC.
FT   TRANSMEM    109    128
FT   TOPO_DOM    129    148       CYTOPLASMIC.
FT   TRANSMEM    149    171
FT   TOPO_DOM    172    207       NON CYTOPLASMIC.
FT   TRANSMEM    208    233
FT   TOPO_DOM    234    252       CYTOPLASMIC.
FT   TRANSMEM    253    275
FT   TOPO_DOM    276    327       NON CYTOPLASMIC.
//
```

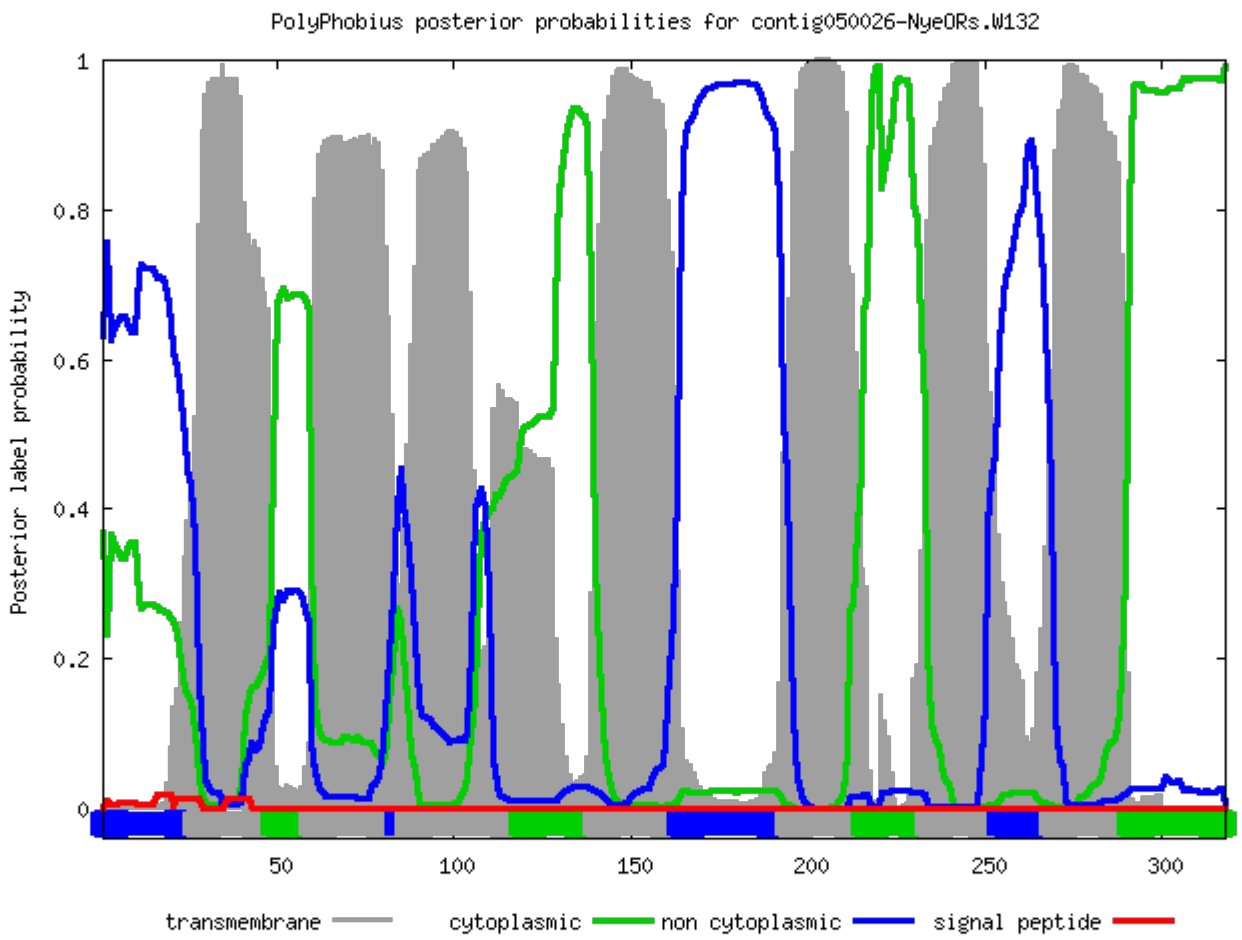

The prediction is based on an alignment. The probability data used in the plot is found here, and the gnuplot script is here.

Prediction of contig047492-ZebOR.H074

```
ID    contig047492-ZebOR.H074
FT    TOPO_DOM      1      23       NON CYTOPLASMIC.
FT    TRANSMEM     24      49
FT    TOPO_DOM     50      56       CYTOPLASMIC.
FT    TRANSMEM     57      76
FT    TOPO_DOM     77      95       NON CYTOPLASMIC.
FT    TRANSMEM     96     118
FT    TOPO_DOM    119     138       CYTOPLASMIC.
FT    TRANSMEM    139     160
FT    TOPO_DOM    161     196       NON CYTOPLASMIC.
FT    TRANSMEM    197     219
FT    TOPO_DOM    220     237       CYTOPLASMIC.
FT    TRANSMEM    238     260
FT    TOPO_DOM    261     271       NON CYTOPLASMIC.
FT    TRANSMEM    272     291
FT    TOPO_DOM    292     310       CYTOPLASMIC.
//
```

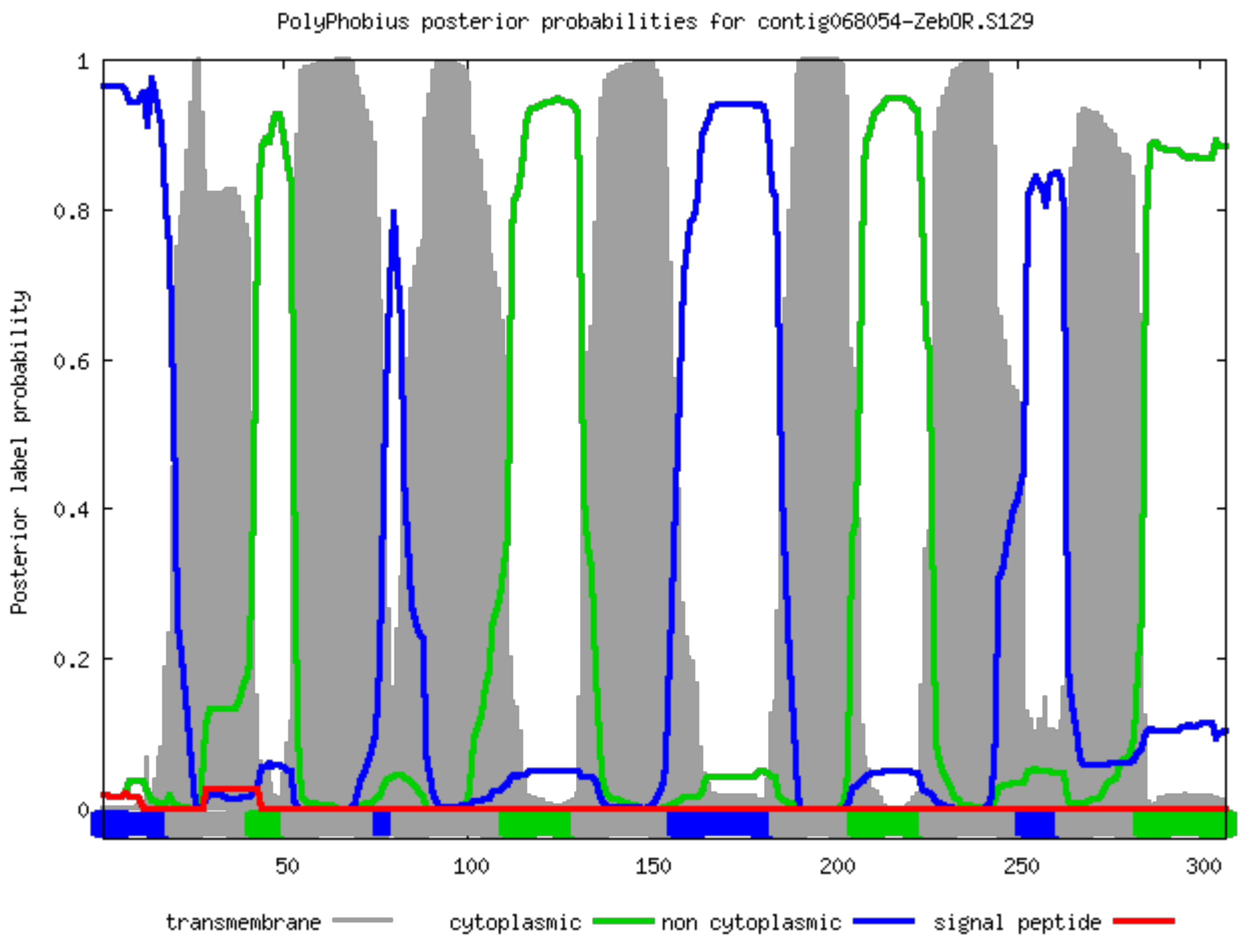

The prediction is based on an alignment. The probability data used in the plot is found here, and the gnuplot script is here.

Prediction of contig010718-ZebOR.N110

```
ID   contig010718-ZebOR.N110
FT   TOPO_DOM      1     32       NON CYTOPLASMIC.
FT   TRANSMEM     33     58
FT   TOPO_DOM     59     66       CYTOPLASMIC.
FT   TRANSMEM     67     86
FT   TOPO_DOM     87    104       NON CYTOPLASMIC.
FT   TRANSMEM    105    127
FT   TOPO_DOM    128    146       CYTOPLASMIC.
FT   TRANSMEM    147    170
FT   TOPO_DOM    171    206       NON CYTOPLASMIC.
FT   TRANSMEM    207    232
FT   TOPO_DOM    233    250       CYTOPLASMIC.
FT   TRANSMEM    251    272
FT   TOPO_DOM    273    277       NON CYTOPLASMIC.
FT   TRANSMEM    278    298
FT   TOPO_DOM    299    324       CYTOPLASMIC.
//
```

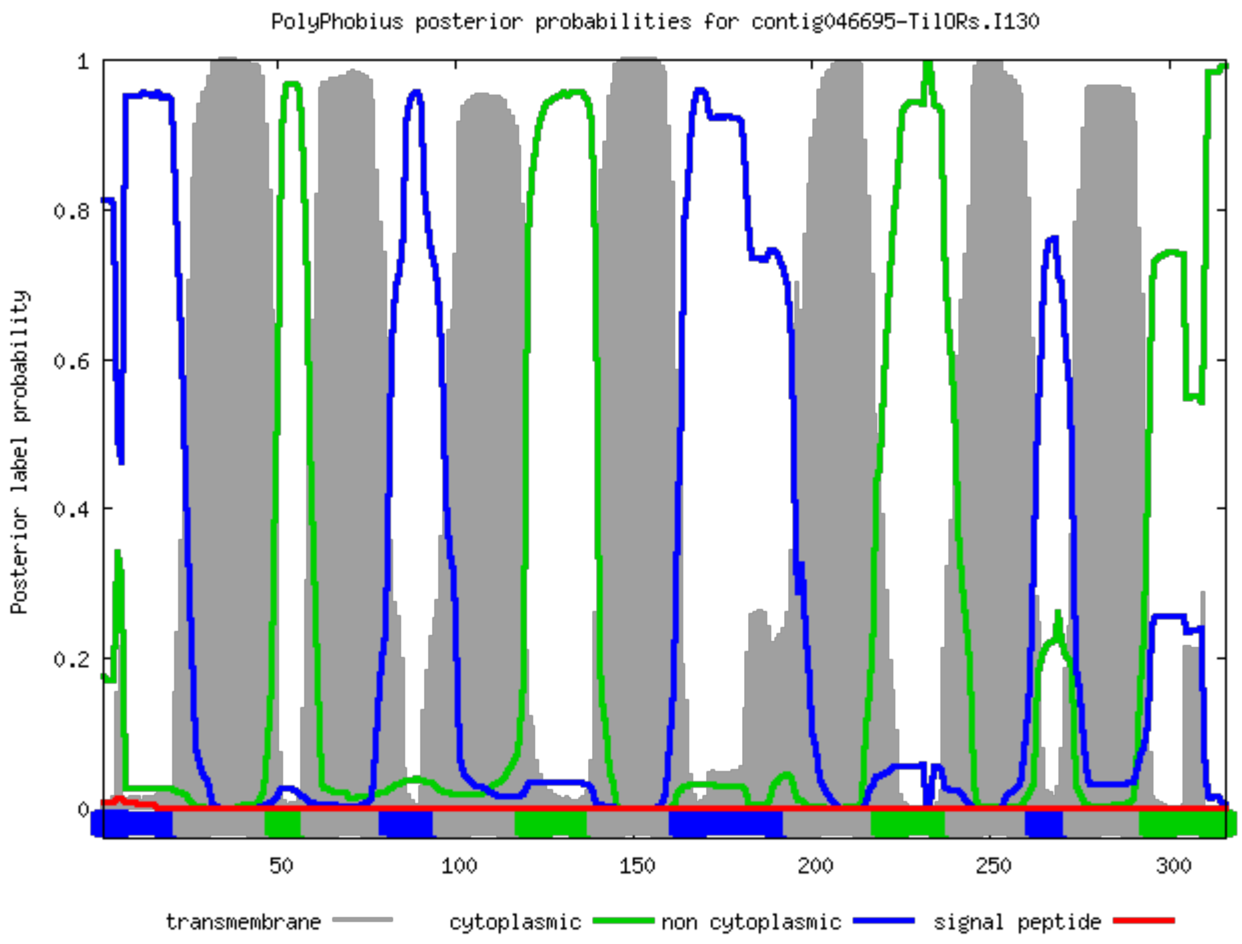

The prediction is based on an alignment. The probability data used in the plot is found here, and the gnuplot script is here.

Prediction of contig047506-ZebOR.A014

```
ID   contig047506-ZebOR.A014
FT   TOPO_DOM      1      18       NON CYTOPLASMIC.
FT   TRANSMEM     19      44
FT   TOPO_DOM     45      52       CYTOPLASMIC.
FT   TRANSMEM     53      73
FT   TOPO_DOM     74      91       NON CYTOPLASMIC.
FT   TRANSMEM     92     114
FT   TOPO_DOM    115     134       CYTOPLASMIC.
FT   TRANSMEM    135     156
FT   TOPO_DOM    157     188       NON CYTOPLASMIC.
FT   TRANSMEM    189     211
FT   TOPO_DOM    212     231       CYTOPLASMIC.
FT   TRANSMEM    232     253
FT   TOPO_DOM    254     264       NON CYTOPLASMIC.
FT   TRANSMEM    265     285
FT   TOPO_DOM    286     300       CYTOPLASMIC.
//
```

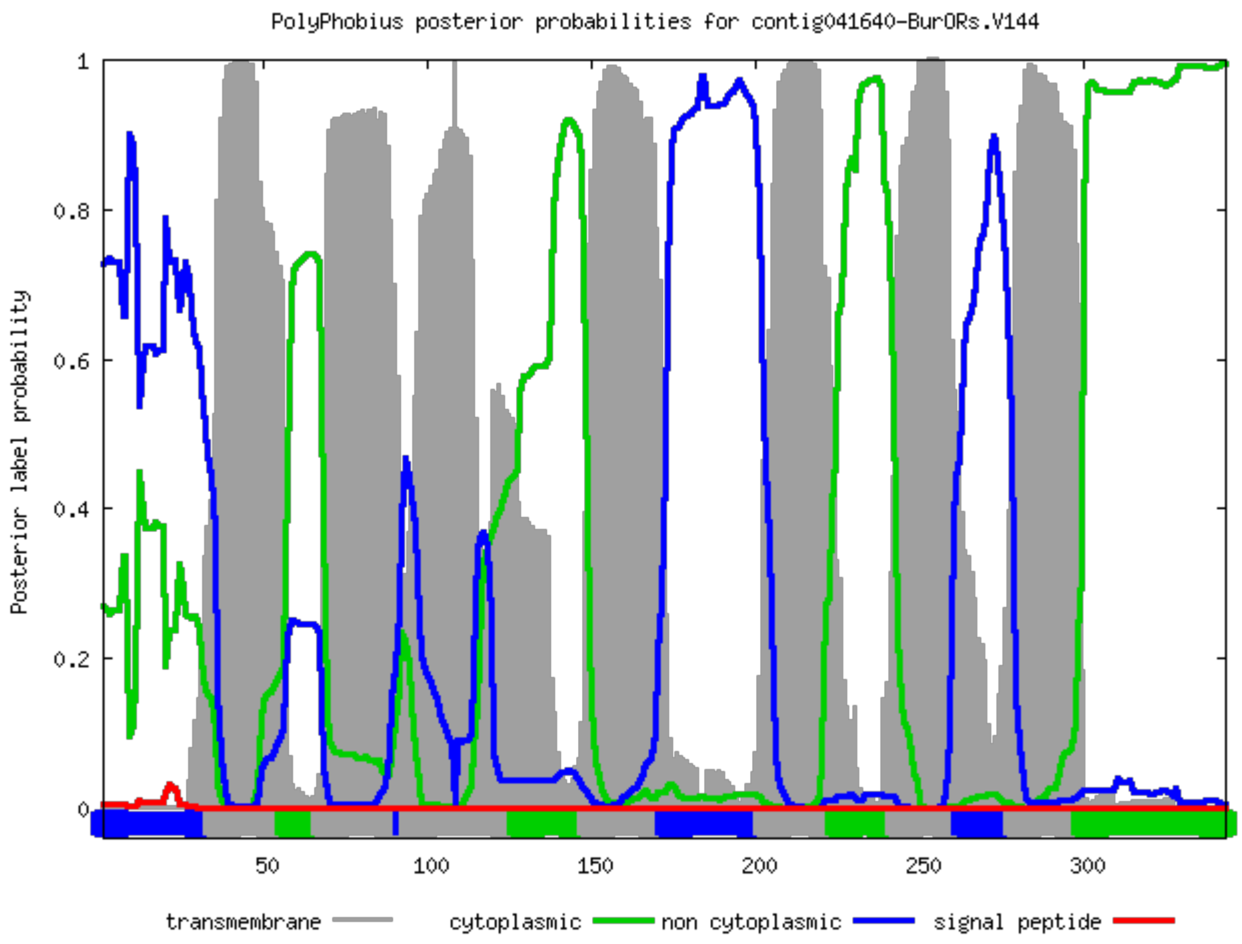

The prediction is based on an alignment. The probability data used in the plot is found here, and the gnuplot script is here.

Prediction of contig034988-NyeOR.A006

```
ID   contig034988-NyeOR.A006
FT   TOPO_DOM      1     30       NON CYTOPLASMIC.
FT   TRANSMEM     31     56
FT   TOPO_DOM     57     64       CYTOPLASMIC.
FT   TRANSMEM     65     85
FT   TOPO_DOM     86    103       NON CYTOPLASMIC.
FT   TRANSMEM    104    126
FT   TOPO_DOM    127    146       CYTOPLASMIC.
FT   TRANSMEM    147    168
FT   TOPO_DOM    169    200       NON CYTOPLASMIC.
FT   TRANSMEM    201    223
FT   TOPO_DOM    224    243       CYTOPLASMIC.
FT   TRANSMEM    244    265
FT   TOPO_DOM    266    276       NON CYTOPLASMIC.
FT   TRANSMEM    277    297
FT   TOPO_DOM    298    312       CYTOPLASMIC.
//
```

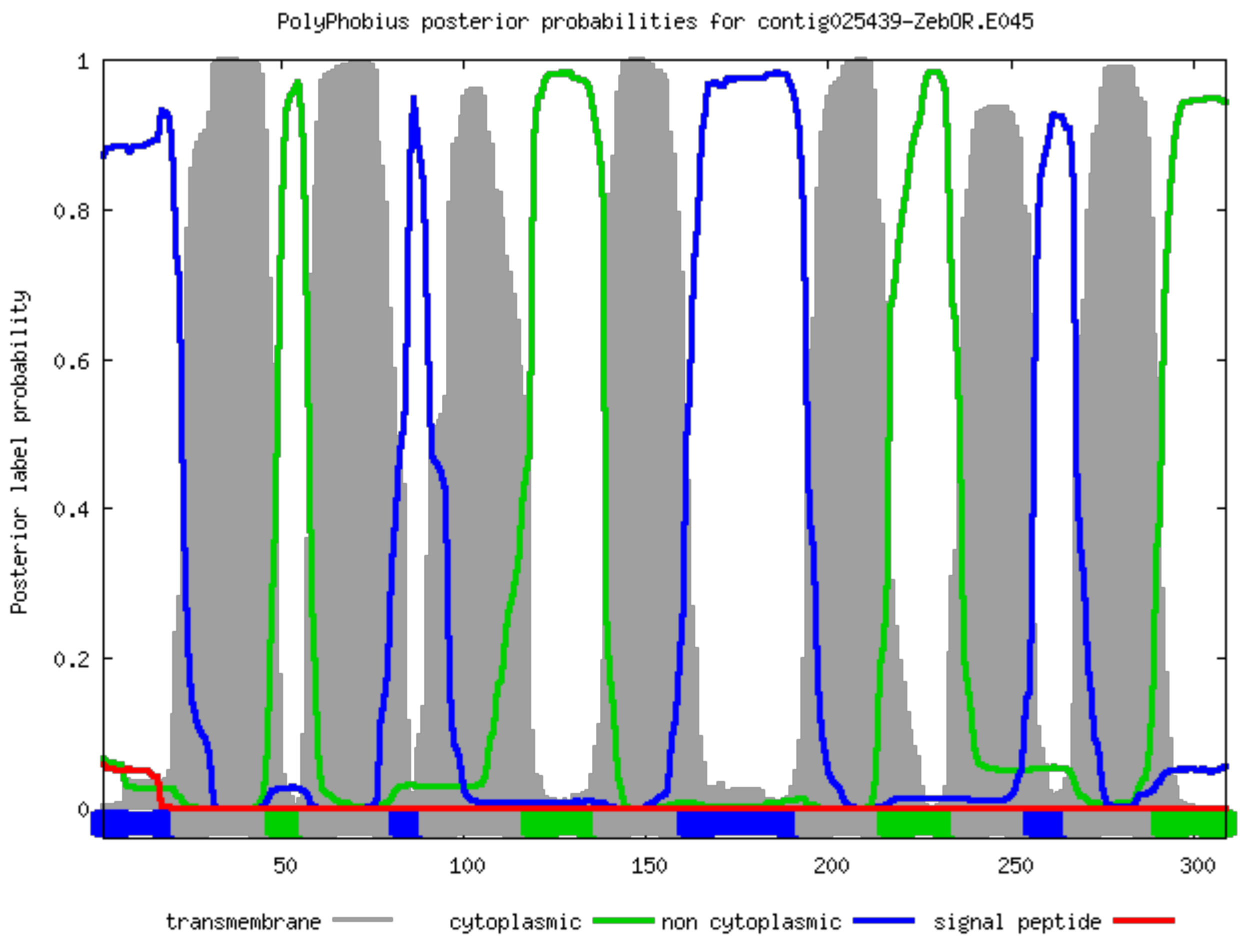

The prediction is based on an alignment. The probability data used in the plot is found here, and the gnuplot script is here.

Prediction of contig060525-NyeOR.K088

```
ID   contig060525-NyeOR.K088
FT   TOPO_DOM      1     24       NON CYTOPLASMIC.
FT   TRANSMEM     25     48
FT   TOPO_DOM     49     58       CYTOPLASMIC.
FT   TRANSMEM     59     80
FT   TOPO_DOM     81     99       NON CYTOPLASMIC.
FT   TRANSMEM    100    121
FT   TOPO_DOM    122    141       CYTOPLASMIC.
FT   TRANSMEM    142    165
FT   TOPO_DOM    166    198       NON CYTOPLASMIC.
FT   TRANSMEM    199    222
FT   TOPO_DOM    223    242       CYTOPLASMIC.
FT   TRANSMEM    243    262
FT   TOPO_DOM    263    272       NON CYTOPLASMIC.
FT   TRANSMEM    273    292
FT   TOPO_DOM    293    313       CYTOPLASMIC.
//
```

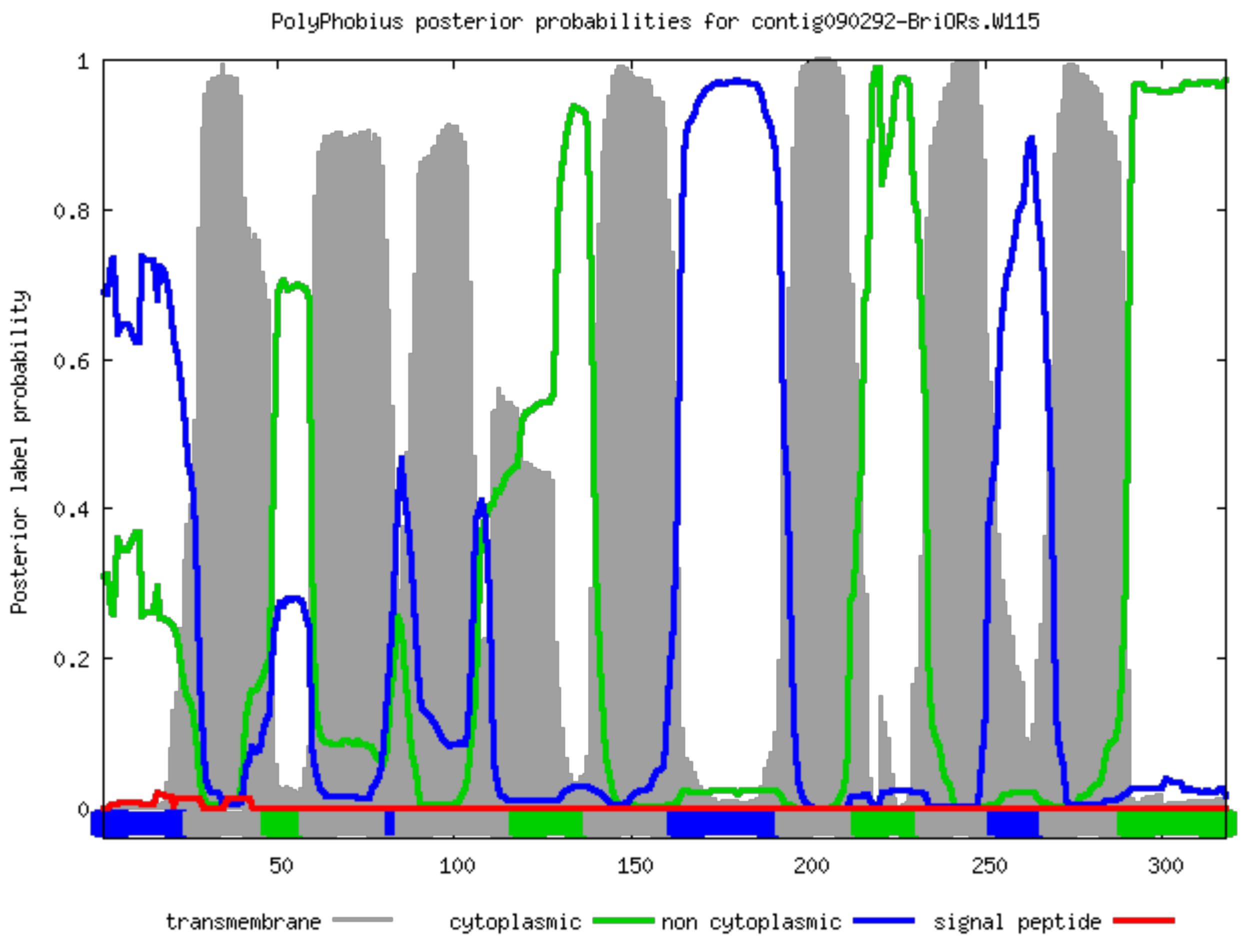

The prediction is based on an alignment. The probability data used in the plot is found here, and the gnuplot script is here.

Prediction of contig056380-NyeOR.A016

```
ID   contig056380-NyeOR.A016
FT   TOPO_DOM      1      22       NON CYTOPLASMIC.
FT   TRANSMEM     23      48
FT   TOPO_DOM     49      56       CYTOPLASMIC.
FT   TRANSMEM     57      76
FT   TOPO_DOM     77      95       NON CYTOPLASMIC.
FT   TRANSMEM     96     118
FT   TOPO_DOM    119     138       CYTOPLASMIC.
FT   TRANSMEM    139     159
FT   TOPO_DOM    160     192       NON CYTOPLASMIC.
FT   TRANSMEM    193     215
FT   TOPO_DOM    216     235       CYTOPLASMIC.
FT   TRANSMEM    236     257
FT   TOPO_DOM    258     268       NON CYTOPLASMIC.
FT   TRANSMEM    269     289
FT   TOPO_DOM    290     314       CYTOPLASMIC.
//
```

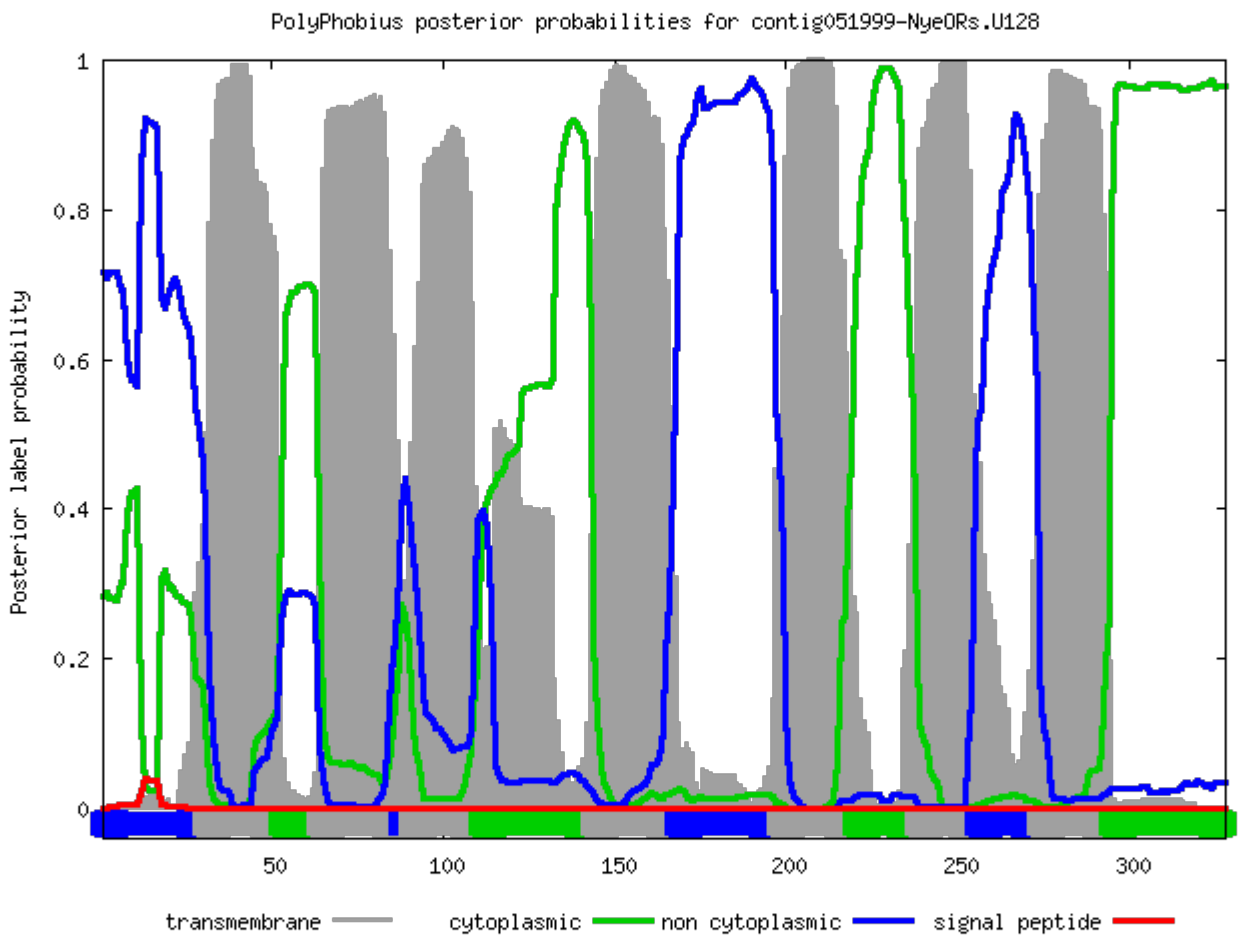

The prediction is based on an alignment. The probability data used in the plot is found here, and the gnuplot script is here.

Prediction of contig047820-TilOR.E078

```
ID   contig047820-TilOR.E078
FT   TOPO_DOM      1     21       NON CYTOPLASMIC.
FT   TRANSMEM     22     47
FT   TOPO_DOM     48     56       CYTOPLASMIC.
FT   TRANSMEM     57     81
FT   TOPO_DOM     82     91       NON CYTOPLASMIC.
FT   TRANSMEM     92    117
FT   TOPO_DOM    118    137       CYTOPLASMIC.
FT   TRANSMEM    138    160
FT   TOPO_DOM    161    193       NON CYTOPLASMIC.
FT   TRANSMEM    194    216
FT   TOPO_DOM    217    236       CYTOPLASMIC.
FT   TRANSMEM    237    256
FT   TOPO_DOM    257    267       NON CYTOPLASMIC.
FT   TRANSMEM    268    291
FT   TOPO_DOM    292    309       CYTOPLASMIC.
//
```

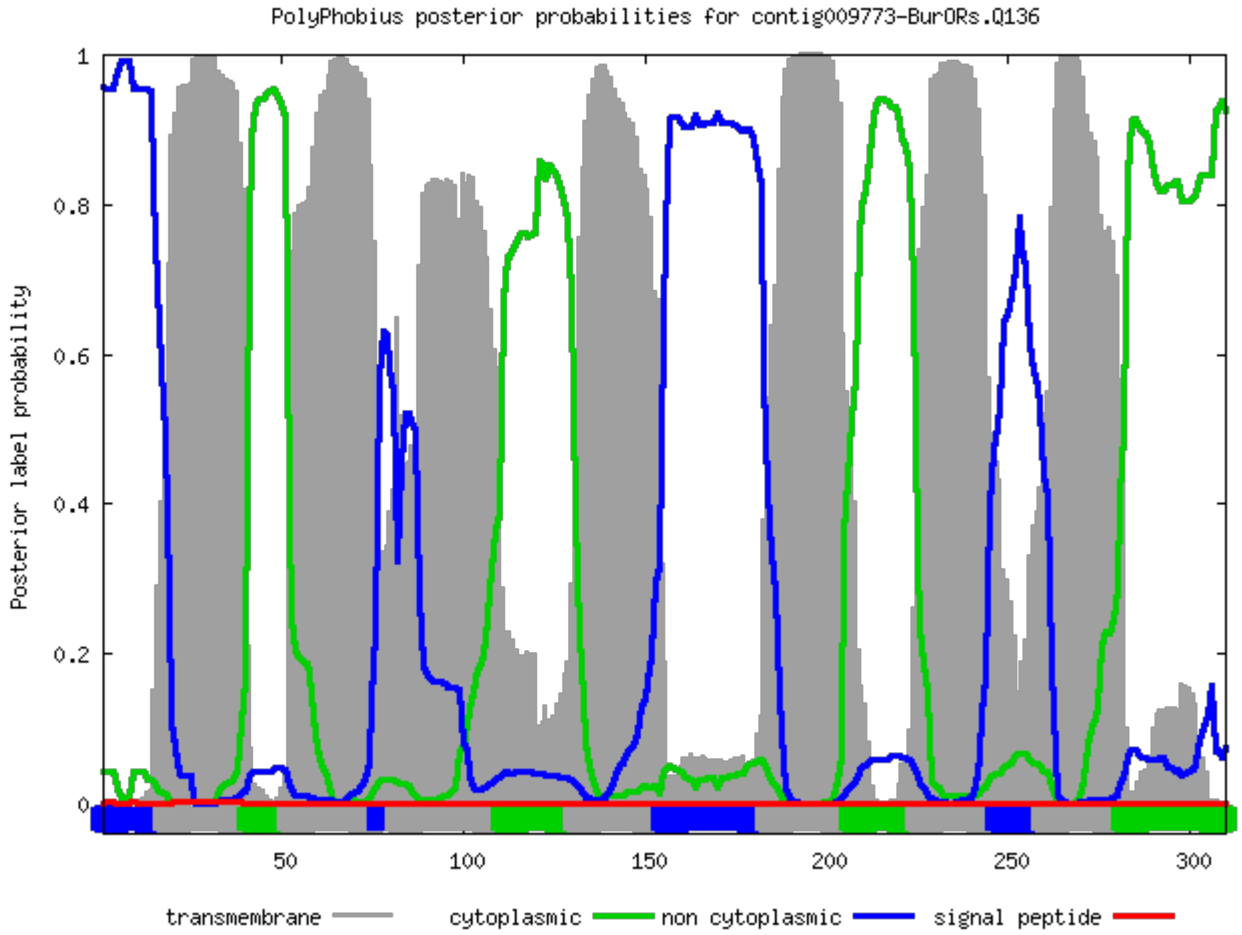

The prediction is based on an alignment. The probability data used in the plot is found here, and the gnuplot script is here.

Prediction of contig028607-TilOR.R245

```
ID   contig028607-TilOR.R245
FT   TOPO_DOM      1     24       NON CYTOPLASMIC.
FT   TRANSMEM     25     48
FT   TOPO_DOM     49     59       CYTOPLASMIC.
FT   TRANSMEM     60     84
FT   TOPO_DOM     85     89       NON CYTOPLASMIC.
FT   TRANSMEM     90    118
FT   TOPO_DOM    119    138       CYTOPLASMIC.
FT   TRANSMEM    139    162
FT   TOPO_DOM    163    194       NON CYTOPLASMIC.
FT   TRANSMEM    195    218
FT   TOPO_DOM    219    235       CYTOPLASMIC.
FT   TRANSMEM    236    256
FT   TOPO_DOM    257    270       NON CYTOPLASMIC.
FT   TRANSMEM    271    293
FT   TOPO_DOM    294    326       CYTOPLASMIC.
//
```

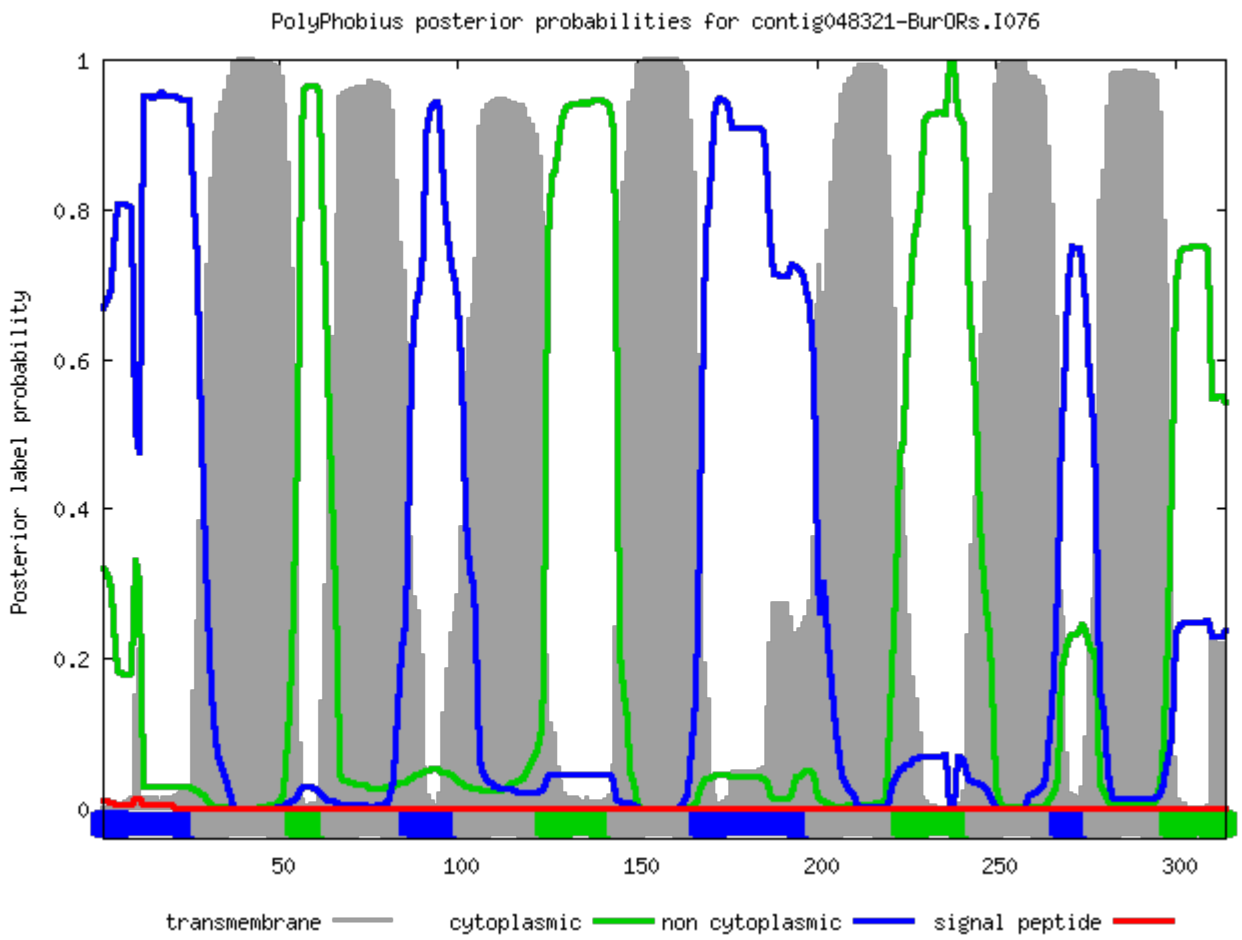

The prediction is based on an alignment. The probability data used in the plot is found here, and the gnuplot script is here.

Prediction of contig057400-ZebOR.H075

```
ID   contig057400-ZebOR.H075
FT   TOPO_DOM      1     23       NON CYTOPLASMIC.
FT   TRANSMEM     24     49
FT   TOPO_DOM     50     56       CYTOPLASMIC.
FT   TRANSMEM     57     76
FT   TOPO_DOM     77     95       NON CYTOPLASMIC.
FT   TRANSMEM     96    118
FT   TOPO_DOM    119    138       CYTOPLASMIC.
FT   TRANSMEM    139    160
FT   TOPO_DOM    161    196       NON CYTOPLASMIC.
FT   TRANSMEM    197    219
FT   TOPO_DOM    220    237       CYTOPLASMIC.
FT   TRANSMEM    238    260
FT   TOPO_DOM    261    271       NON CYTOPLASMIC.
FT   TRANSMEM    272    291
FT   TOPO_DOM    292    310       CYTOPLASMIC.
//
```

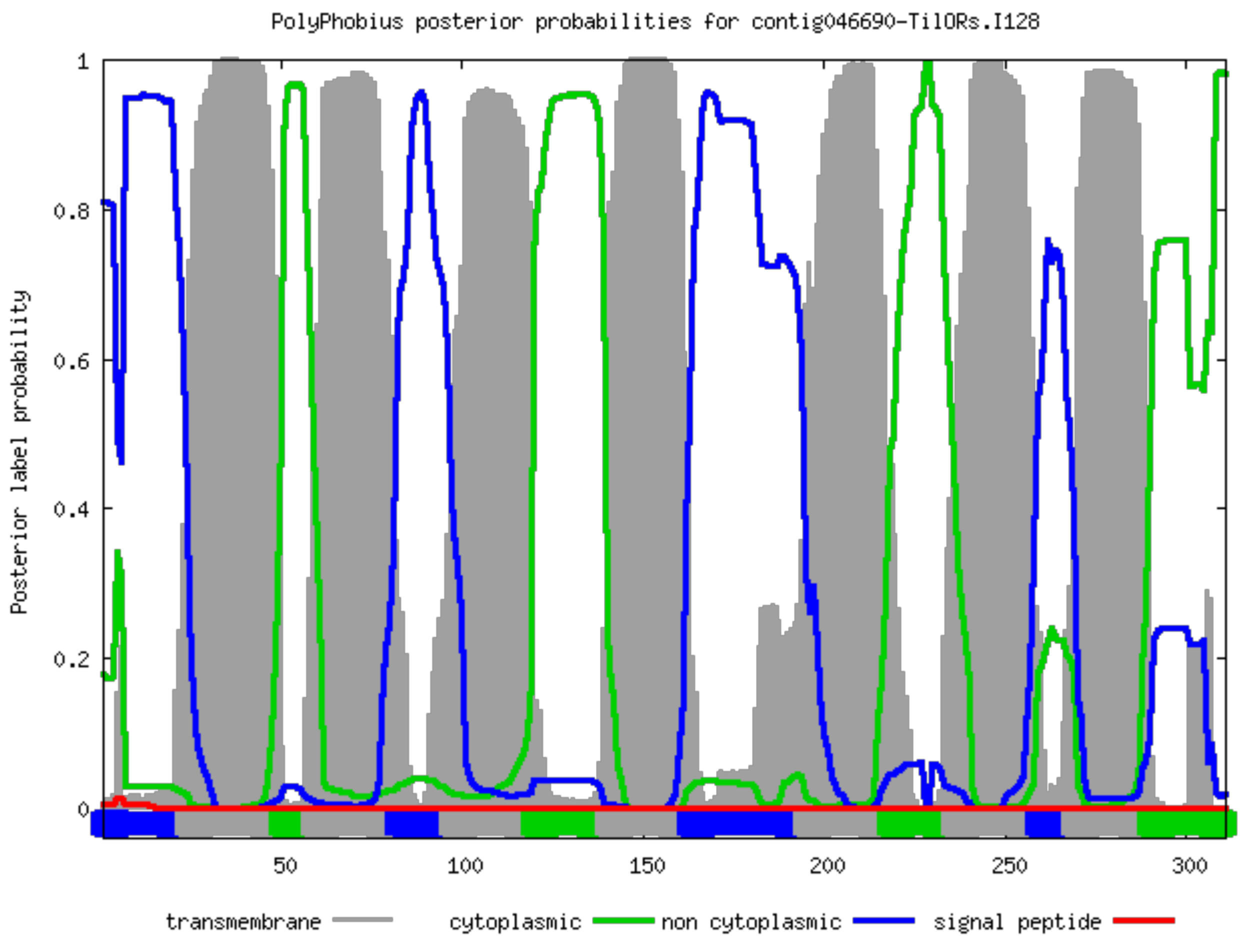

The prediction is based on an alignment. The probability data used in the plot is found here, and the gnuplot script is here.

Prediction of contig052451-BurOR.E047

```
ID   contig052451-BurOR.E047
FT   TOPO_DOM      1     21       NON CYTOPLASMIC.
FT   TRANSMEM     22     47
FT   TOPO_DOM     48     56       CYTOPLASMIC.
FT   TRANSMEM     57     82
FT   TOPO_DOM     83     91       NON CYTOPLASMIC.
FT   TRANSMEM     92    117
FT   TOPO_DOM    118    137       CYTOPLASMIC.
FT   TRANSMEM    138    159
FT   TOPO_DOM    160    192       NON CYTOPLASMIC.
FT   TRANSMEM    193    215
FT   TOPO_DOM    216    235       CYTOPLASMIC.
FT   TRANSMEM    236    255
FT   TOPO_DOM    256    266       NON CYTOPLASMIC.
FT   TRANSMEM    267    290
FT   TOPO_DOM    291    310       CYTOPLASMIC.
//
```

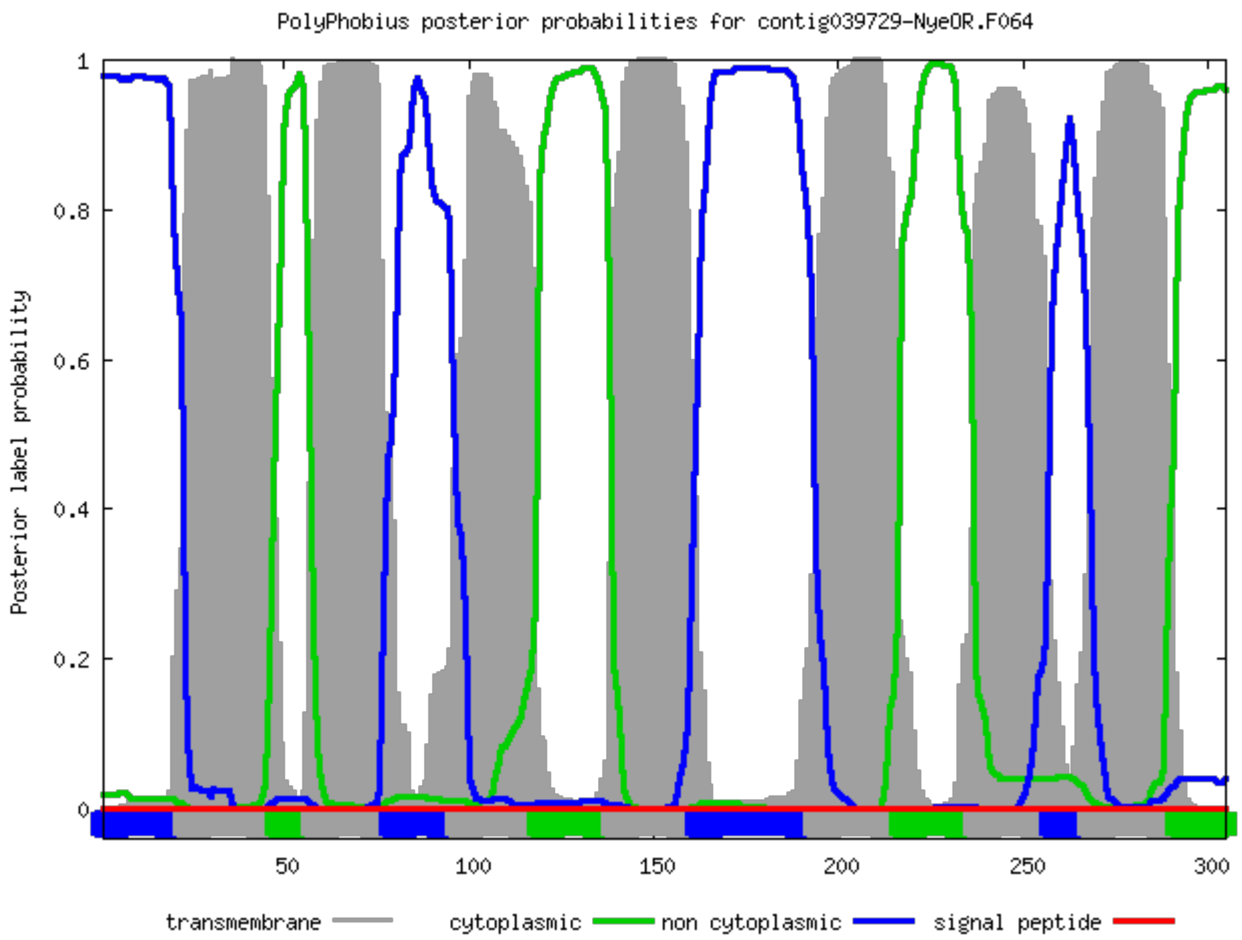

The prediction is based on an alignment. The probability data used in the plot is found here, and the gnuplot script is here.

Prediction of contig064724-BurOR.E052

```
ID   contig064724-BurOR.E052
FT   TOPO_DOM      1     25       NON CYTOPLASMIC.
FT   TRANSMEM     26     51
FT   TOPO_DOM     52     60       CYTOPLASMIC.
FT   TRANSMEM     61     86
FT   TOPO_DOM     87     93       NON CYTOPLASMIC.
FT   TRANSMEM     94    121
FT   TOPO_DOM    122    141       CYTOPLASMIC.
FT   TRANSMEM    142    164
FT   TOPO_DOM    165    196       NON CYTOPLASMIC.
FT   TRANSMEM    197    219
FT   TOPO_DOM    220    239       CYTOPLASMIC.
FT   TRANSMEM    240    259
FT   TOPO_DOM    260    270       NON CYTOPLASMIC.
FT   TRANSMEM    271    294
FT   TOPO_DOM    295    326       CYTOPLASMIC.
//
```

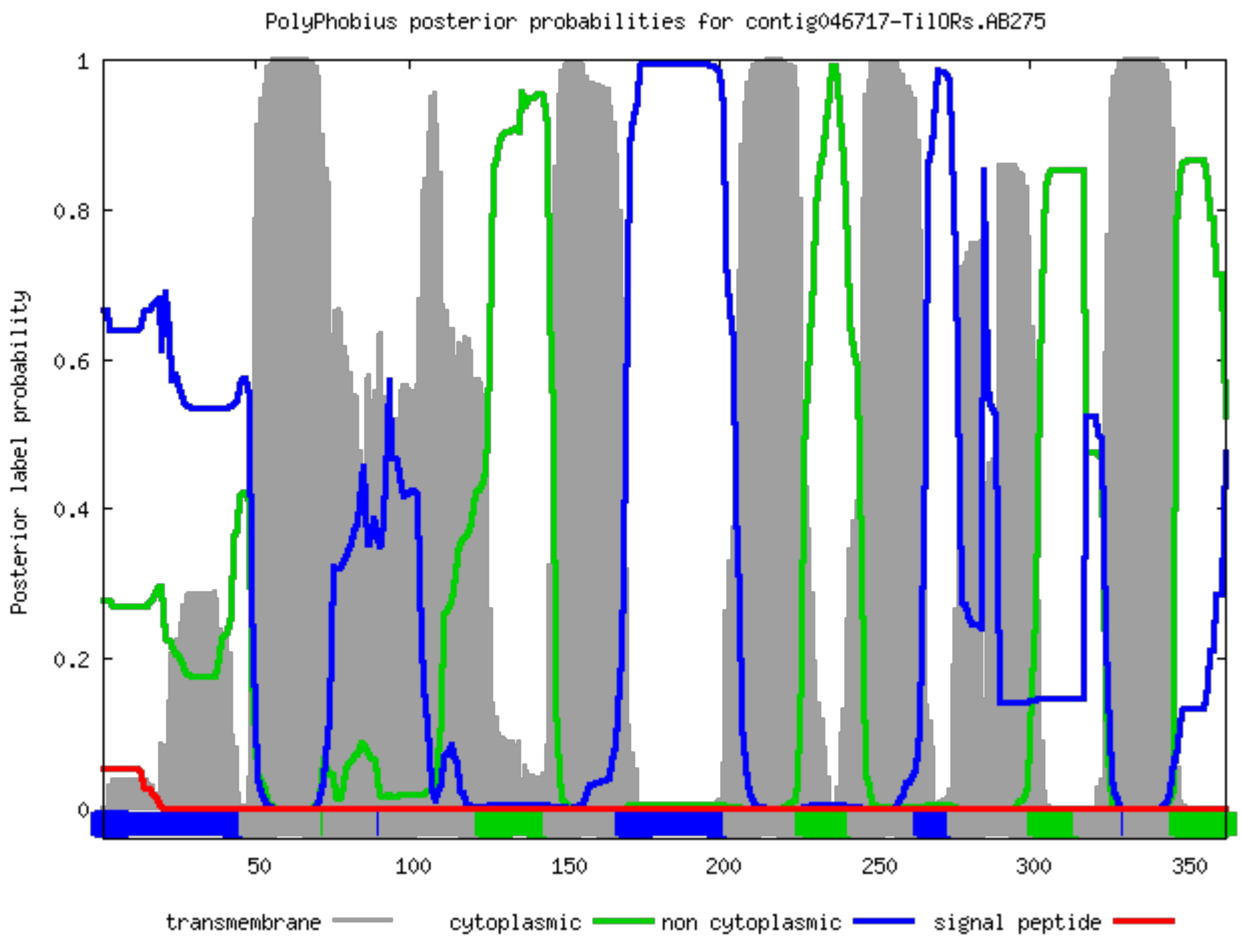

The prediction is based on an alignment. The probability data used in the plot is found here, and the gnuplot script is here.

Prediction of contig034994-NyeOR.A008

```
ID   contig034994-NyeOR.A008
FT   TOPO_DOM      1     22       NON CYTOPLASMIC.
FT   TRANSMEM     23     48
FT   TOPO_DOM     49     56       CYTOPLASMIC.
FT   TRANSMEM     57     77
FT   TOPO_DOM     78     95       NON CYTOPLASMIC.
FT   TRANSMEM     96    118
FT   TOPO_DOM    119    138       CYTOPLASMIC.
FT   TRANSMEM    139    160
FT   TOPO_DOM    161    192       NON CYTOPLASMIC.
FT   TRANSMEM    193    215
FT   TOPO_DOM    216    235       CYTOPLASMIC.
FT   TRANSMEM    236    257
FT   TOPO_DOM    258    268       NON CYTOPLASMIC.
FT   TRANSMEM    269    289
FT   TOPO_DOM    290    320       CYTOPLASMIC.
//
```

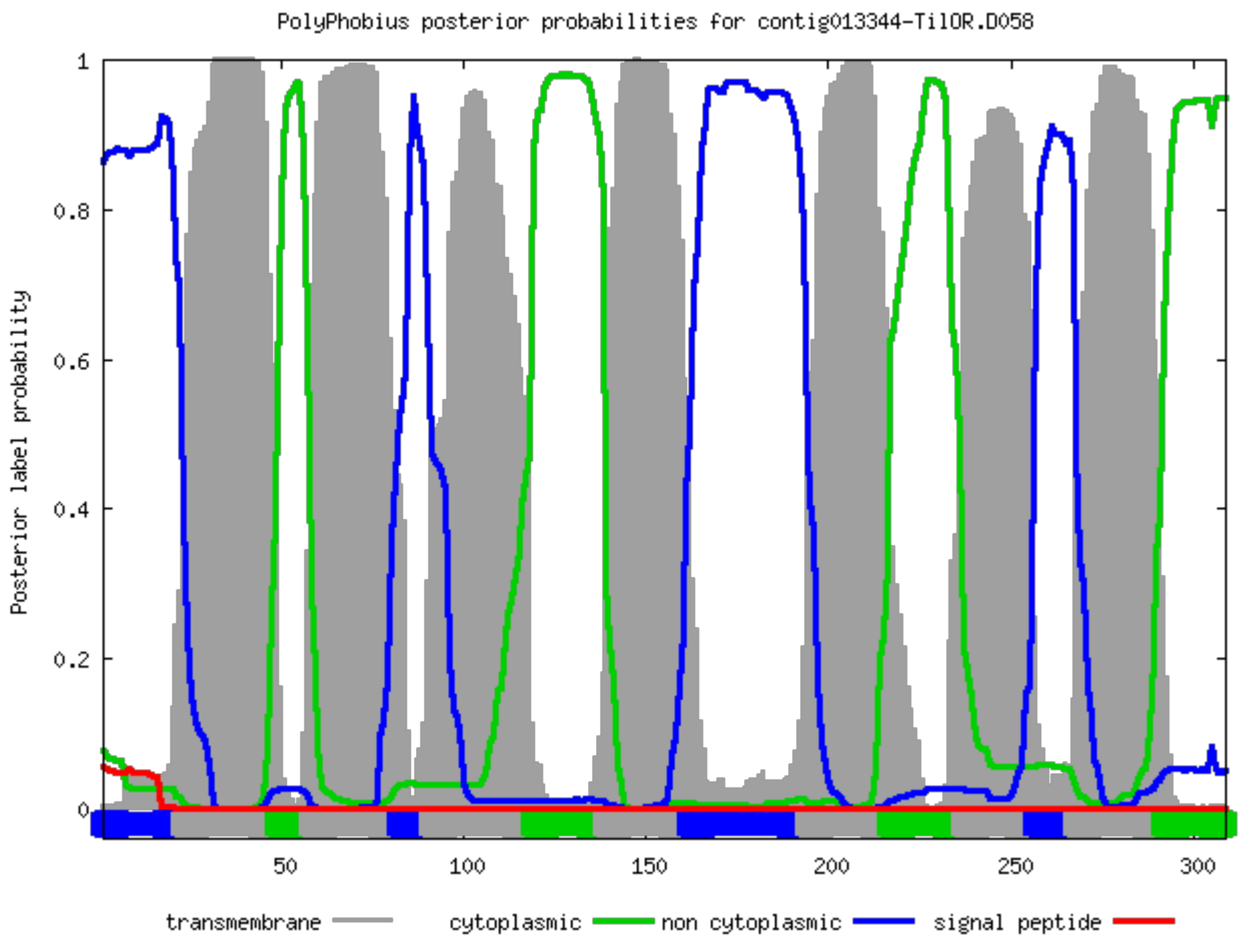

The prediction is based on an alignment. The probability data used in the plot is found here, and the gnuplot script is here.

Prediction of contig033883-BriOR.F045

```
ID   contig033883-BriOR.F045
FT   TOPO_DOM      1     22       NON CYTOPLASMIC.
FT   TRANSMEM     23     47
FT   TOPO_DOM     48     56       CYTOPLASMIC.
FT   TRANSMEM     57     77
FT   TOPO_DOM     78     96       NON CYTOPLASMIC.
FT   TRANSMEM     97    118
FT   TOPO_DOM    119    138       CYTOPLASMIC.
FT   TRANSMEM    139    161
FT   TOPO_DOM    162    193       NON CYTOPLASMIC.
FT   TRANSMEM    194    216
FT   TOPO_DOM    217    236       CYTOPLASMIC.
FT   TRANSMEM    237    256
FT   TOPO_DOM    257    267       NON CYTOPLASMIC.
FT   TRANSMEM    268    290
FT   TOPO_DOM    291    310       CYTOPLASMIC.
//
```

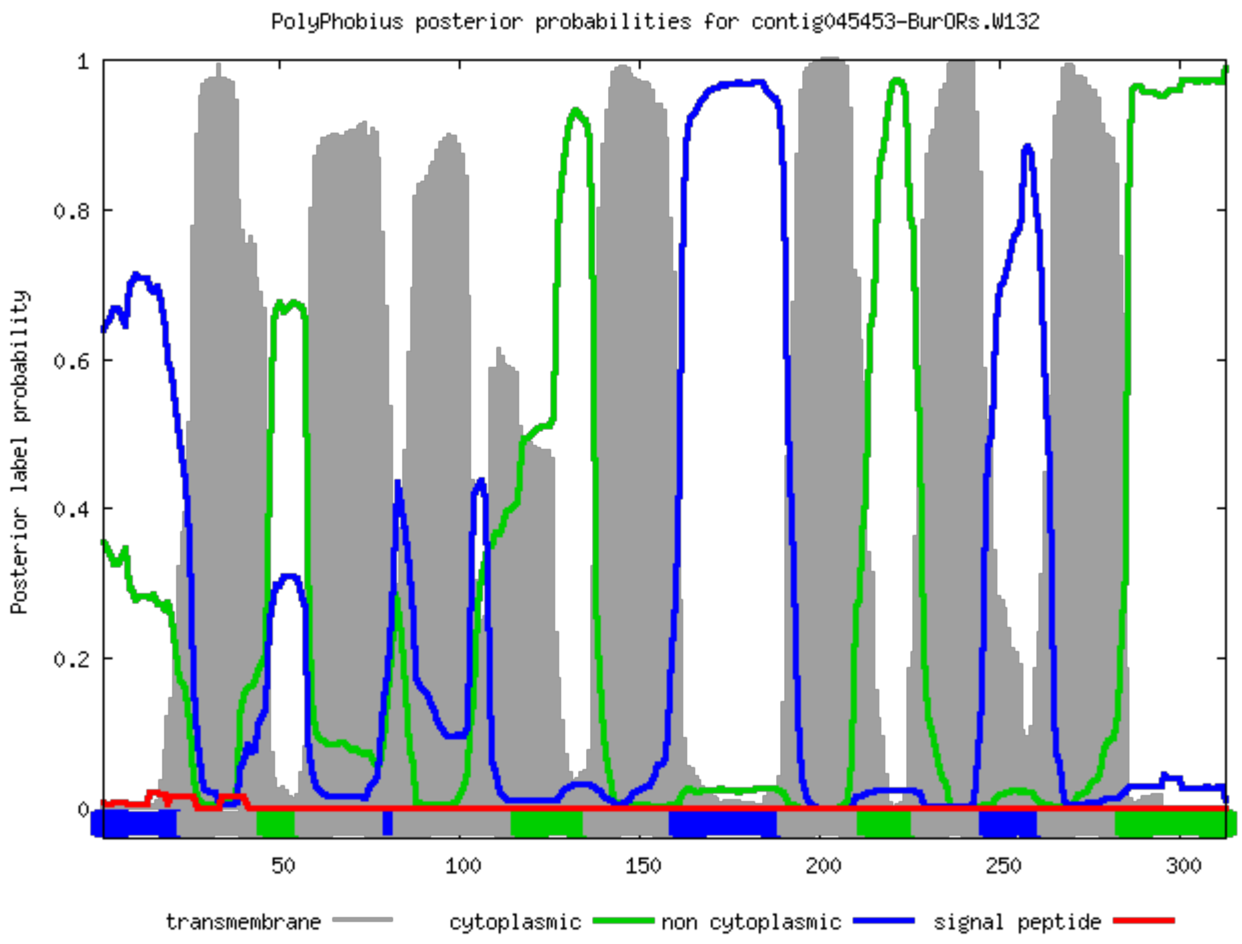

The prediction is based on an alignment. The probability data used in the plot is found here, and the gnuplot script is here.

Prediction of contig013368-TilOR.H113

```
ID   contig013368-TilOR.H113
FT   TOPO_DOM      1     23       NON CYTOPLASMIC.
FT   TRANSMEM     24     49
FT   TOPO_DOM     50     56       CYTOPLASMIC.
FT   TRANSMEM     57     76
FT   TOPO_DOM     77     95       NON CYTOPLASMIC.
FT   TRANSMEM     96    118
FT   TOPO_DOM    119    138       CYTOPLASMIC.
FT   TRANSMEM    139    160
FT   TOPO_DOM    161    196       NON CYTOPLASMIC.
FT   TRANSMEM    197    219
FT   TOPO_DOM    220    237       CYTOPLASMIC.
FT   TRANSMEM    238    260
FT   TOPO_DOM    261    271       NON CYTOPLASMIC.
FT   TRANSMEM    272    291
FT   TOPO_DOM    292    319       CYTOPLASMIC.
//
```

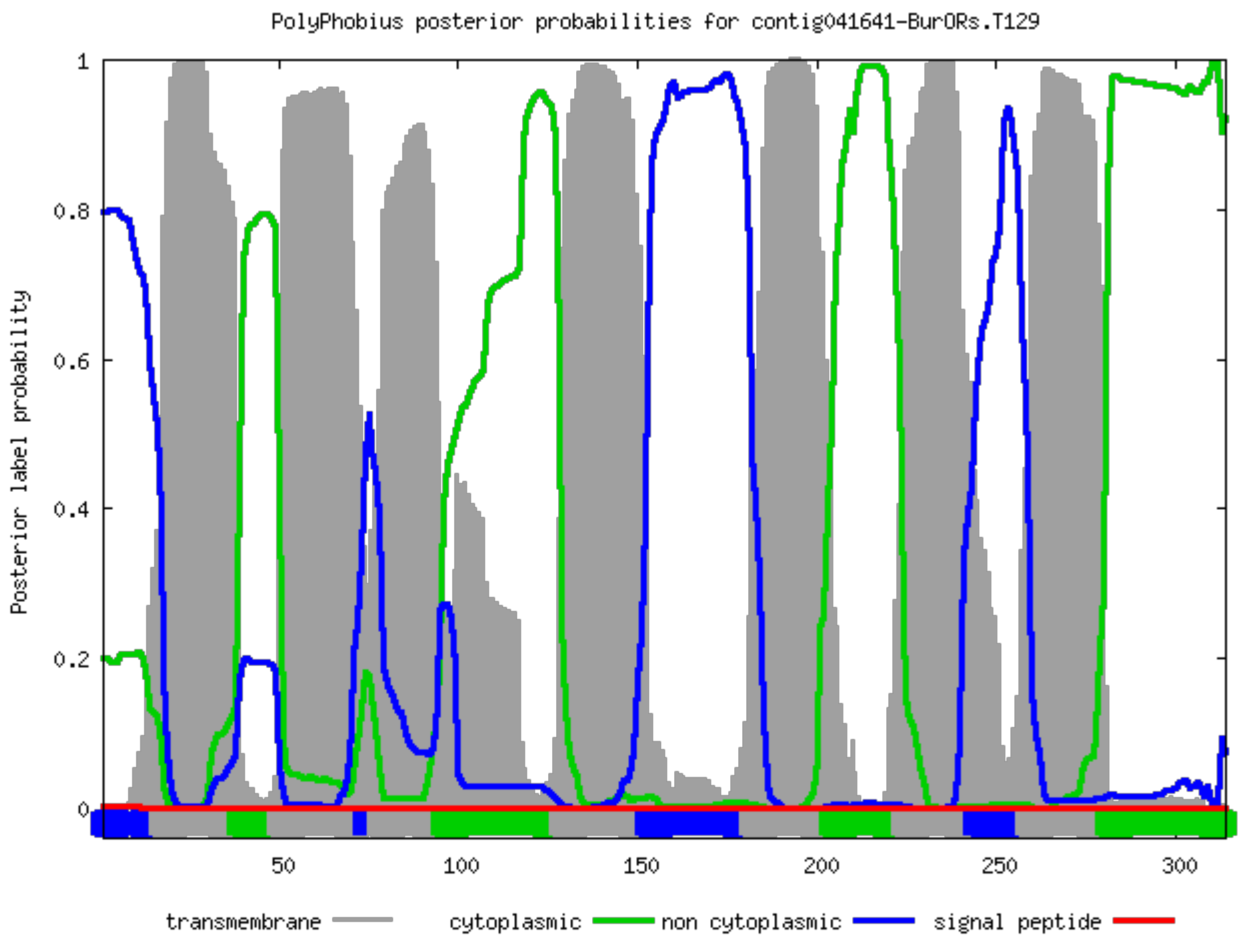

The prediction is based on an alignment. The probability data used in the plot is found here, and the gnuplot script is here.

Prediction of contig039437-TilOR.S231

```
ID    contig039437-TilOR.S231
FT    TOPO_DOM      1      21       NON CYTOPLASMIC.
FT    TRANSMEM     22      43
FT    TOPO_DOM     44      53       CYTOPLASMIC.
FT    TRANSMEM     54      78
FT    TOPO_DOM     79      83       NON CYTOPLASMIC.
FT    TRANSMEM     84     112
FT    TOPO_DOM    113     132       CYTOPLASMIC.
FT    TRANSMEM    133     158
FT    TOPO_DOM    159     186       NON CYTOPLASMIC.
FT    TRANSMEM    187     207
FT    TOPO_DOM    208     227       CYTOPLASMIC.
FT    TRANSMEM    228     253
FT    TOPO_DOM    254     264       NON CYTOPLASMIC.
FT    TRANSMEM    265     285
FT    TOPO_DOM    286     313       CYTOPLASMIC.
//
```

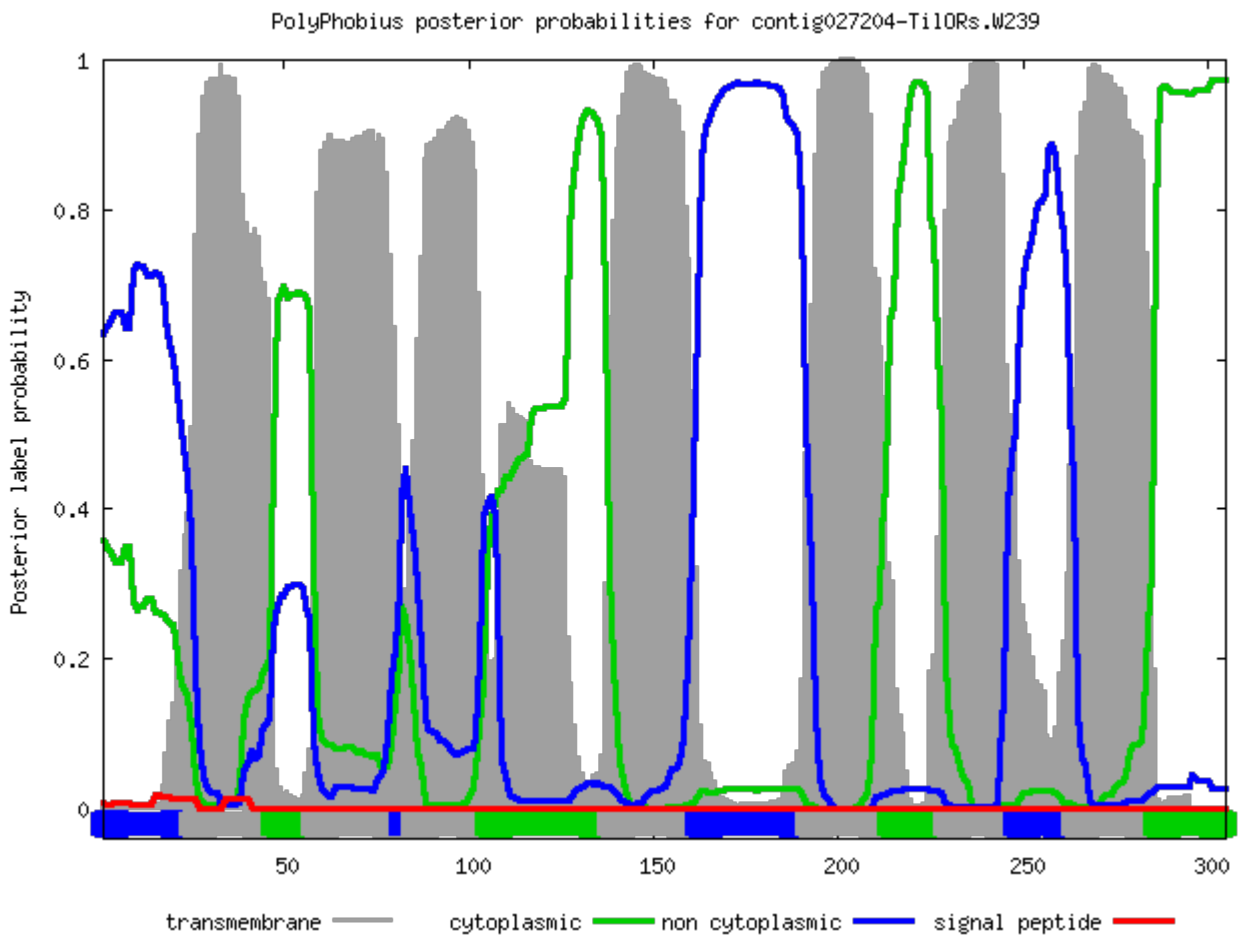

The prediction is based on an alignment. The probability data used in the plot is found here, and the gnuplot script is here.

Prediction of contig042554-BriOR.L072

```
ID   contig042554-BriOR.L072
FT   TOPO_DOM      1     25       NON CYTOPLASMIC.
FT   TRANSMEM     26     50
FT   TOPO_DOM     51     59       CYTOPLASMIC.
FT   TRANSMEM     60     86
FT   TOPO_DOM     87     97       NON CYTOPLASMIC.
FT   TRANSMEM     98    120
FT   TOPO_DOM    121    140       CYTOPLASMIC.
FT   TRANSMEM    141    163
FT   TOPO_DOM    164    198       NON CYTOPLASMIC.
FT   TRANSMEM    199    223
FT   TOPO_DOM    224    238       CYTOPLASMIC.
FT   TRANSMEM    239    260
FT   TOPO_DOM    261    271       NON CYTOPLASMIC.
FT   TRANSMEM    272    292
FT   TOPO_DOM    293    313       CYTOPLASMIC.
//
```

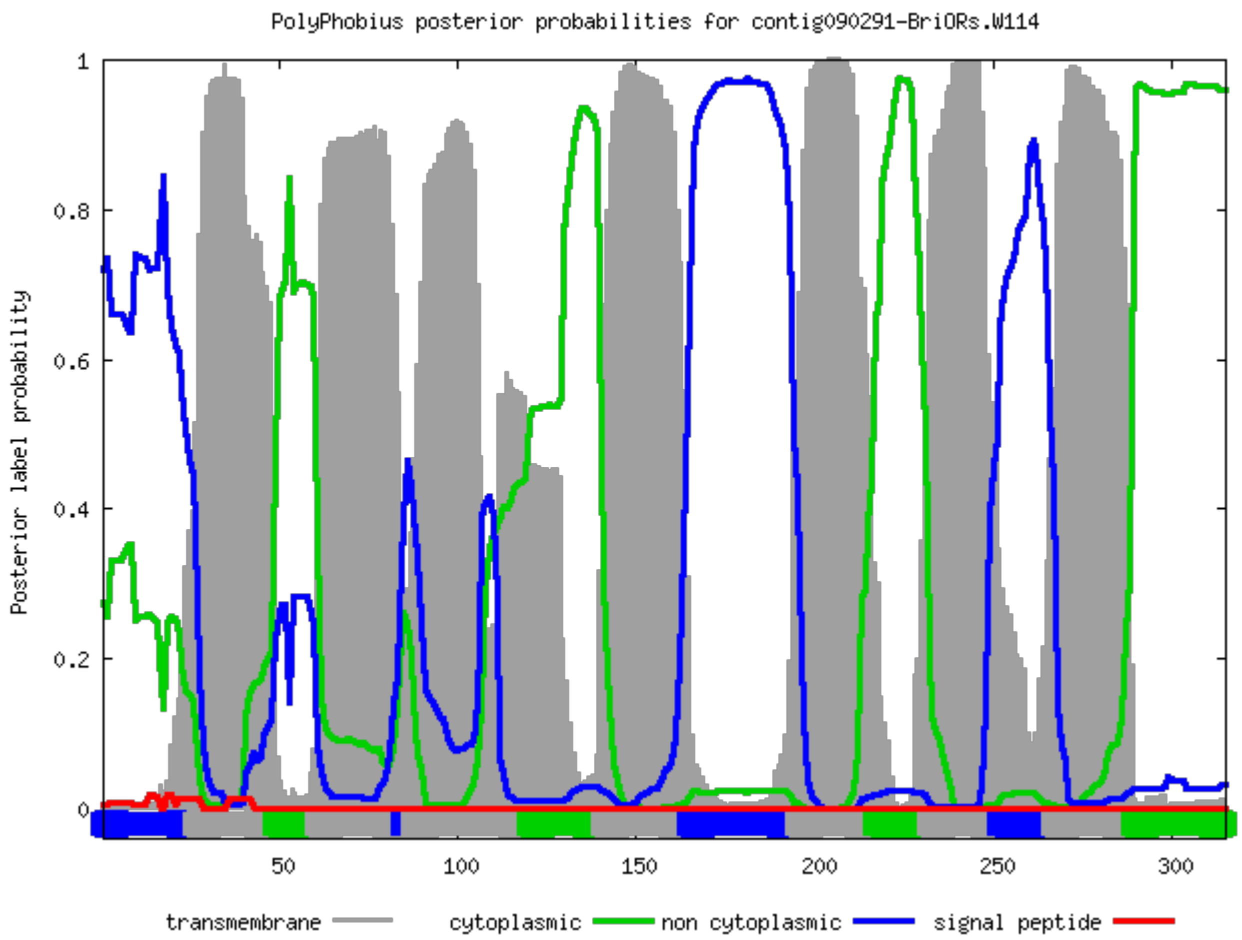

The prediction is based on an alignment. The probability data used in the plot is found here, and the gnuplot script is here.

Prediction of contig049289-BurOR.E044

```
ID   contig049289-BurOR.E044
FT   TOPO_DOM      1     24       NON CYTOPLASMIC.
FT   TRANSMEM     25     50
FT   TOPO_DOM     51     59       CYTOPLASMIC.
FT   TRANSMEM     60     85
FT   TOPO_DOM     86     92       NON CYTOPLASMIC.
FT   TRANSMEM     93    120
FT   TOPO_DOM    121    140       CYTOPLASMIC.
FT   TRANSMEM    141    163
FT   TOPO_DOM    164    195       NON CYTOPLASMIC.
FT   TRANSMEM    196    218
FT   TOPO_DOM    219    238       CYTOPLASMIC.
FT   TRANSMEM    239    258
FT   TOPO_DOM    259    269       NON CYTOPLASMIC.
FT   TRANSMEM    270    293
FT   TOPO_DOM    294    322       CYTOPLASMIC.
//
```

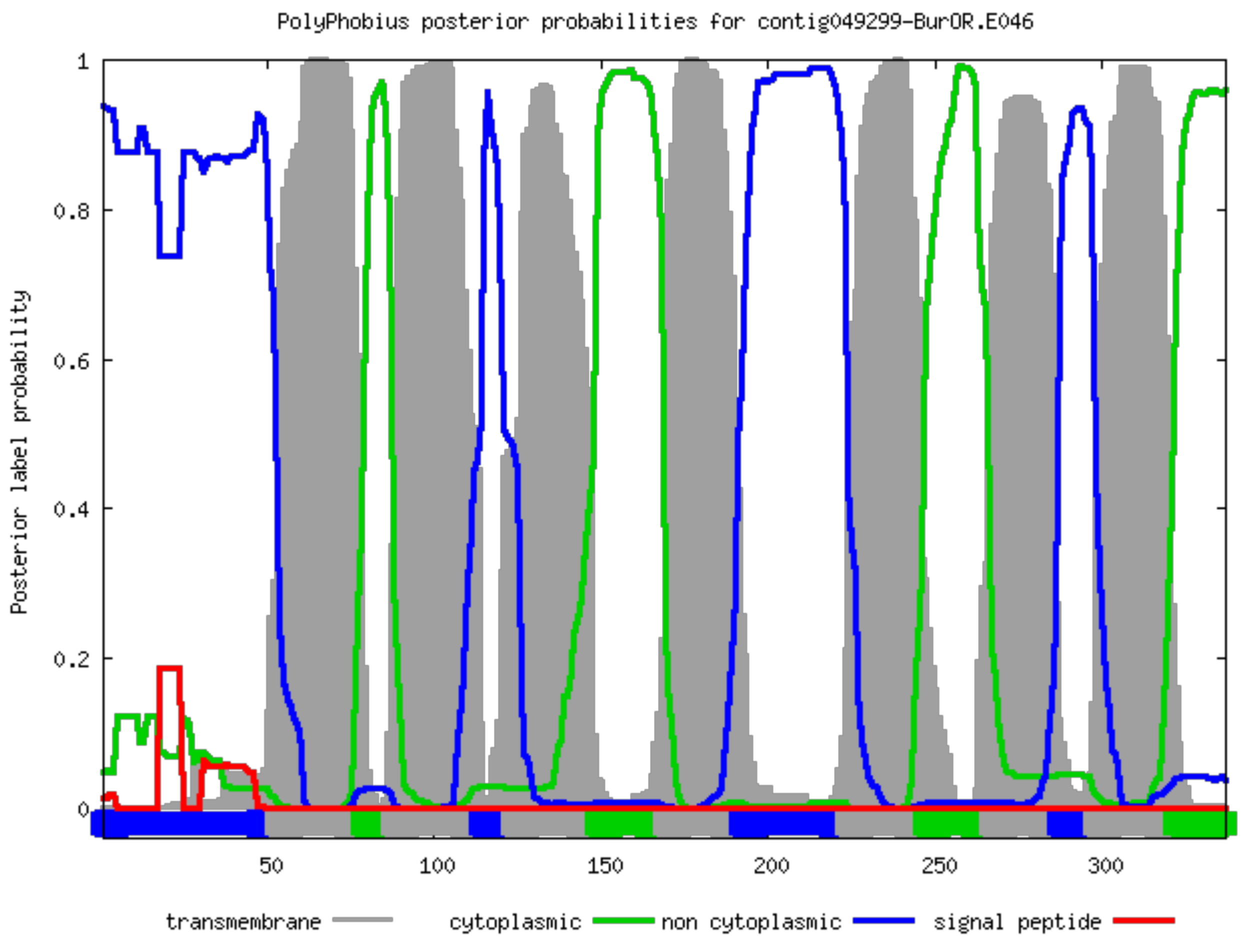

The prediction is based on an alignment. The probability data used in the plot is found here, and the gnuplot script is here.

Prediction of contig046488-NyeOR.K084

```
ID   contig046488-NyeOR.K084
FT   TOPO_DOM      1     22       NON CYTOPLASMIC.
FT   TRANSMEM     23     46
FT   TOPO_DOM     47     56       CYTOPLASMIC.
FT   TRANSMEM     57     78
FT   TOPO_DOM     79     97       NON CYTOPLASMIC.
FT   TRANSMEM     98    119
FT   TOPO_DOM    120    139       CYTOPLASMIC.
FT   TRANSMEM    140    163
FT   TOPO_DOM    164    196       NON CYTOPLASMIC.
FT   TRANSMEM    197    221
FT   TOPO_DOM    222    240       CYTOPLASMIC.
FT   TRANSMEM    241    260
FT   TOPO_DOM    261    270       NON CYTOPLASMIC.
FT   TRANSMEM    271    290
FT   TOPO_DOM    291    315       CYTOPLASMIC.
//
```

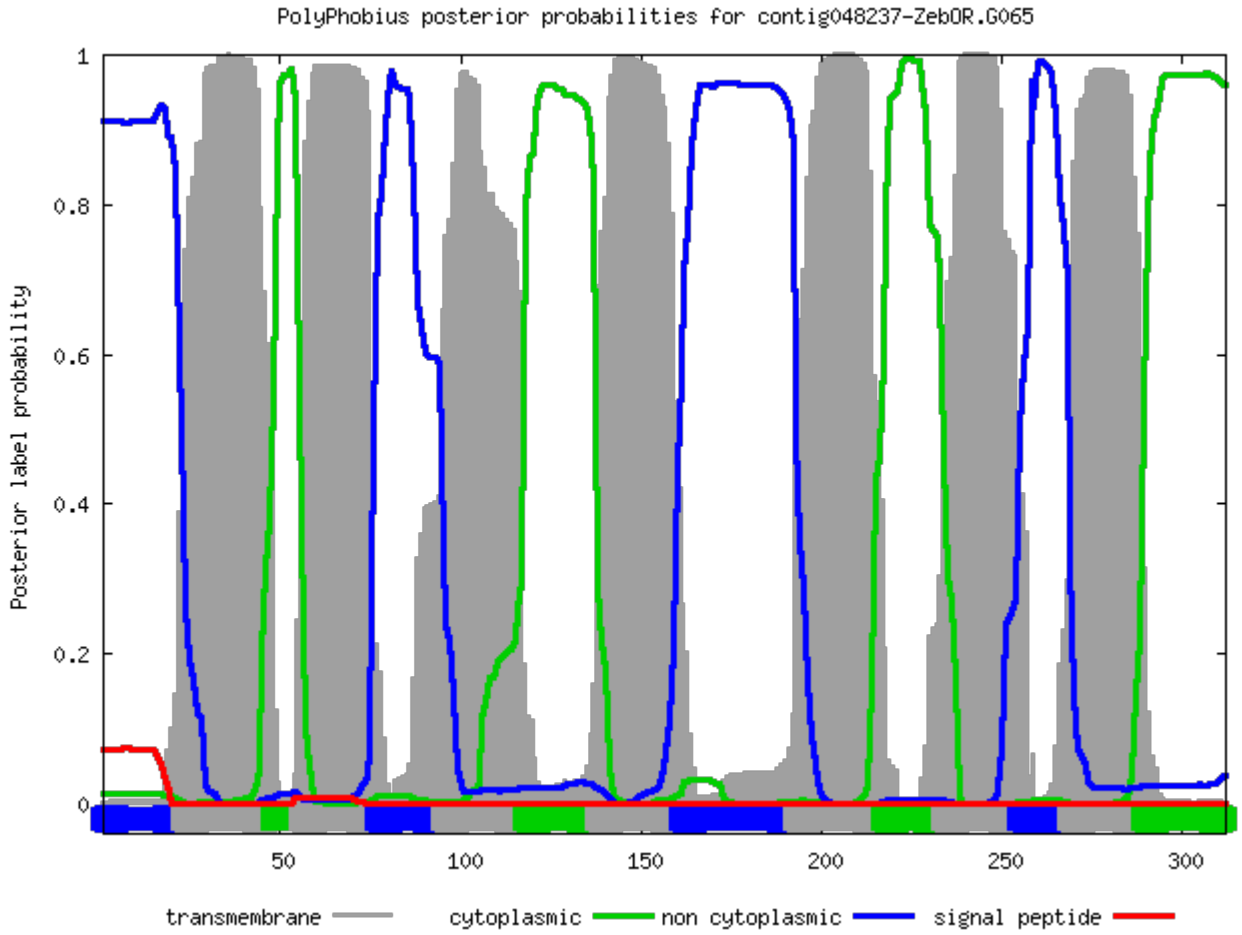

The prediction is based on an alignment. The probability data used in the plot is found here, and the gnuplot script is here.

Prediction of contig039416-TilOR.S223

```
ID    contig039416-TilOR.S223
FT    TOPO_DOM      1      20       NON CYTOPLASMIC.
FT    TRANSMEM     21      42
FT    TOPO_DOM     43      53       CYTOPLASMIC.
FT    TRANSMEM     54      77
FT    TOPO_DOM     78      82       NON CYTOPLASMIC.
FT    TRANSMEM     83     111
FT    TOPO_DOM    112     131       CYTOPLASMIC.
FT    TRANSMEM    132     157
FT    TOPO_DOM    158     185       NON CYTOPLASMIC.
FT    TRANSMEM    186     206
FT    TOPO_DOM    207     226       CYTOPLASMIC.
FT    TRANSMEM    227     252
FT    TOPO_DOM    253     263       NON CYTOPLASMIC.
FT    TRANSMEM    264     284
FT    TOPO_DOM    285     305       CYTOPLASMIC.
//
```

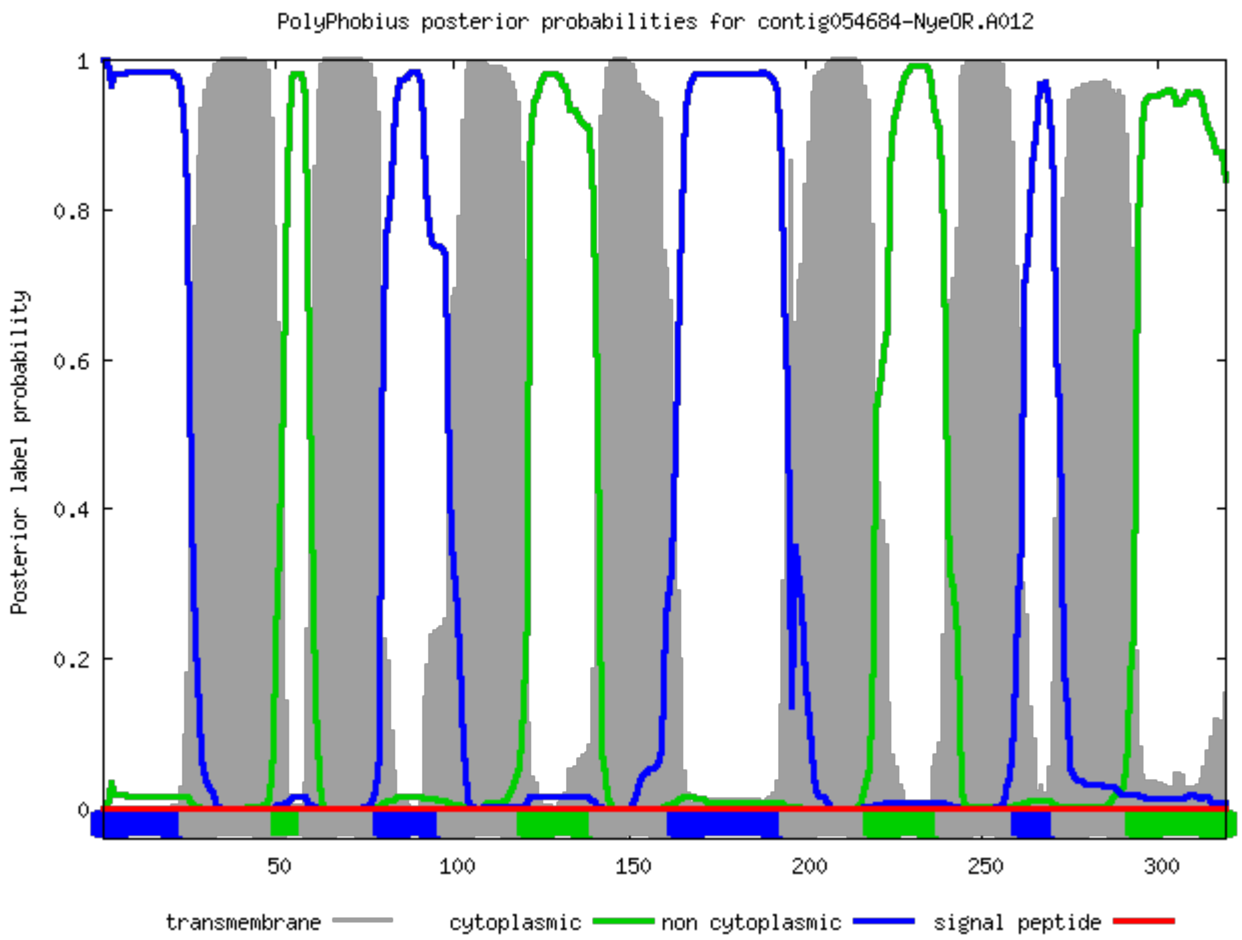

The prediction is based on an alignment. The probability data used in the plot is found here, and the gnuplot script is here.

Prediction of contig004267-BriOR.P099

```
ID    contig004267-BriOR.P099
FT    TOPO_DOM      1     23       NON CYTOPLASMIC.
FT    TRANSMEM     24     47
FT    TOPO_DOM     48     57       CYTOPLASMIC.
FT    TRANSMEM     58     83
FT    TOPO_DOM     84     96       NON CYTOPLASMIC.
FT    TRANSMEM     97    118
FT    TOPO_DOM    119    138       CYTOPLASMIC.
FT    TRANSMEM    139    161
FT    TOPO_DOM    162    196       NON CYTOPLASMIC.
FT    TRANSMEM    197    221
FT    TOPO_DOM    222    235       CYTOPLASMIC.
FT    TRANSMEM    236    258
FT    TOPO_DOM    259    269       NON CYTOPLASMIC.
FT    TRANSMEM    270    290
FT    TOPO_DOM    291    318       CYTOPLASMIC.
//
```

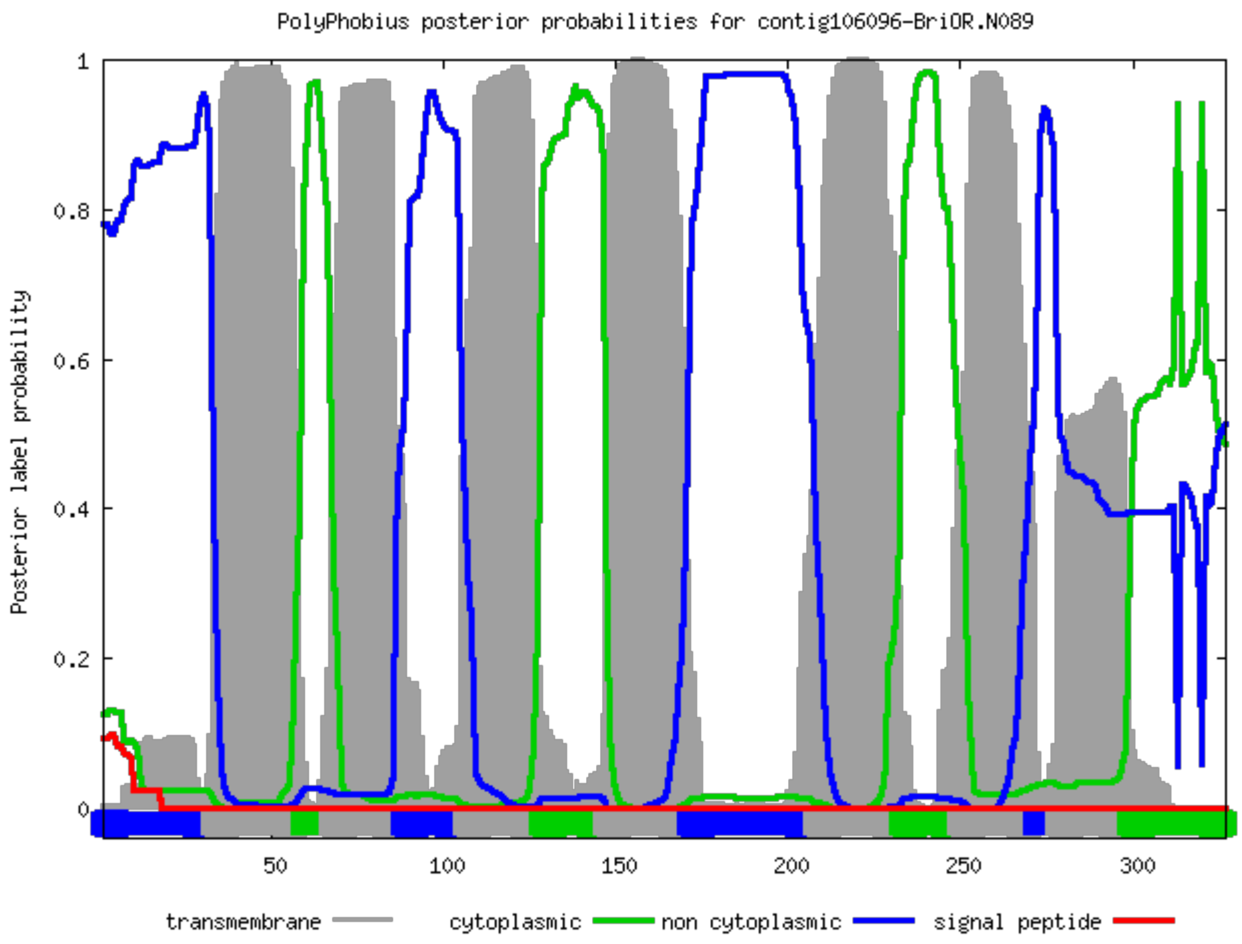

The prediction is based on an alignment. The probability data used in the plot is found here, and the gnuplot script is here.

Prediction of contig093807-BriOR.A009

```
ID   contig093807-BriOR.A009
FT   TOPO_DOM      1     22       NON CYTOPLASMIC.
FT   TRANSMEM     23     48
FT   TOPO_DOM     49     56       CYTOPLASMIC.
FT   TRANSMEM     57     76
FT   TOPO_DOM     77     95       NON CYTOPLASMIC.
FT   TRANSMEM     96    118
FT   TOPO_DOM    119    138       CYTOPLASMIC.
FT   TRANSMEM    139    160
FT   TOPO_DOM    161    192       NON CYTOPLASMIC.
FT   TRANSMEM    193    215
FT   TOPO_DOM    216    235       CYTOPLASMIC.
FT   TRANSMEM    236    257
FT   TOPO_DOM    258    268       NON CYTOPLASMIC.
FT   TRANSMEM    269    289
FT   TOPO_DOM    290    306       CYTOPLASMIC.
//
```

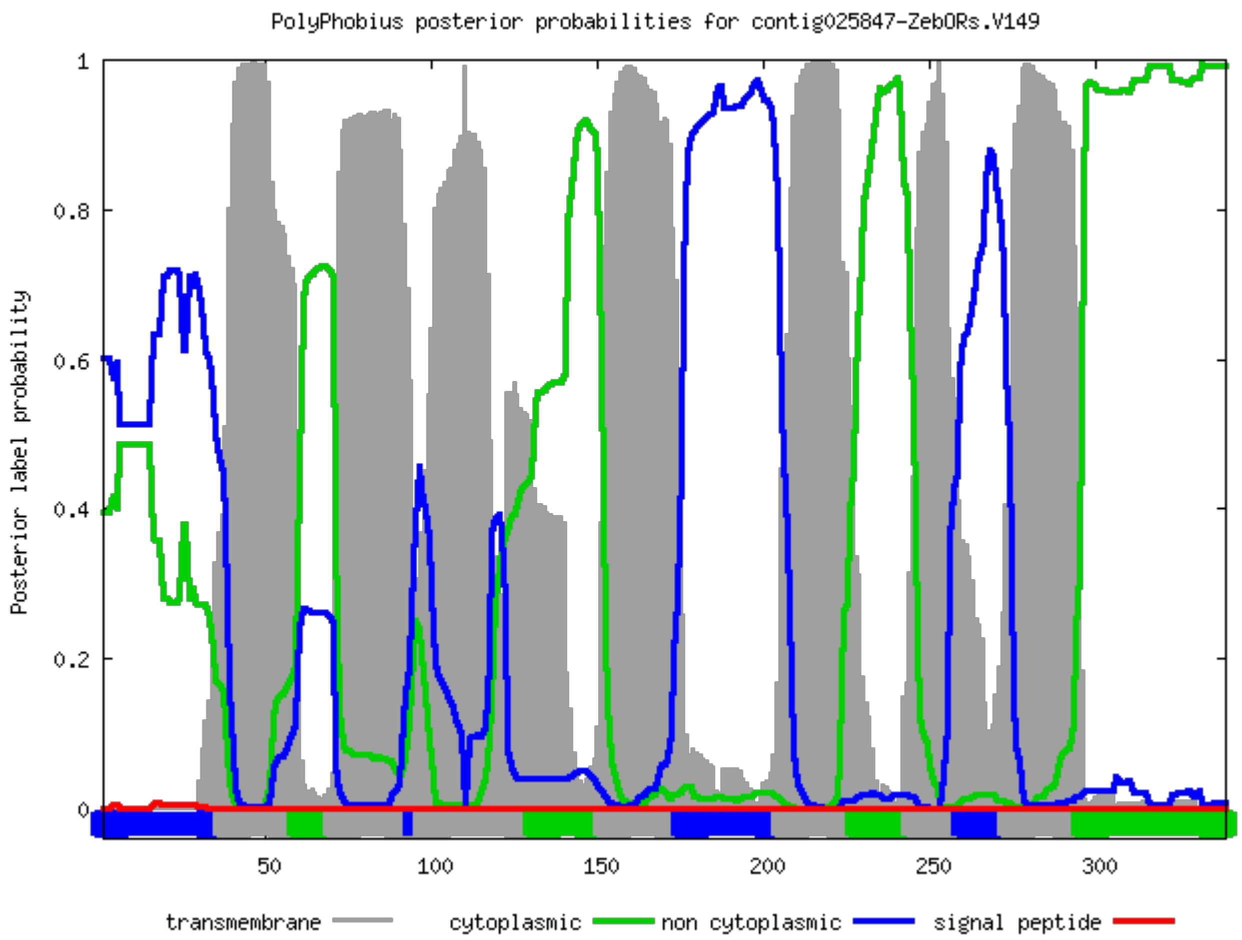

The prediction is based on an alignment. The probability data used in the plot is found here, and the gnuplot script is here.

Prediction of contig022238-TilOR.A010

```
ID   contig022238-TilOR.A010
FT   TOPO_DOM      1    22      NON CYTOPLASMIC.
FT   TRANSMEM     23    48
FT   TOPO_DOM     49    56      CYTOPLASMIC.
FT   TRANSMEM     57    77
FT   TOPO_DOM     78    95      NON CYTOPLASMIC.
FT   TRANSMEM     96   118
FT   TOPO_DOM    119   138      CYTOPLASMIC.
FT   TRANSMEM    139   160
FT   TOPO_DOM    161   192      NON CYTOPLASMIC.
FT   TRANSMEM    193   215
FT   TOPO_DOM    216   235      CYTOPLASMIC.
FT   TRANSMEM    236   257
FT   TOPO_DOM    258   268      NON CYTOPLASMIC.
FT   TRANSMEM    269   289
FT   TOPO_DOM    290   306      CYTOPLASMIC.
//
```

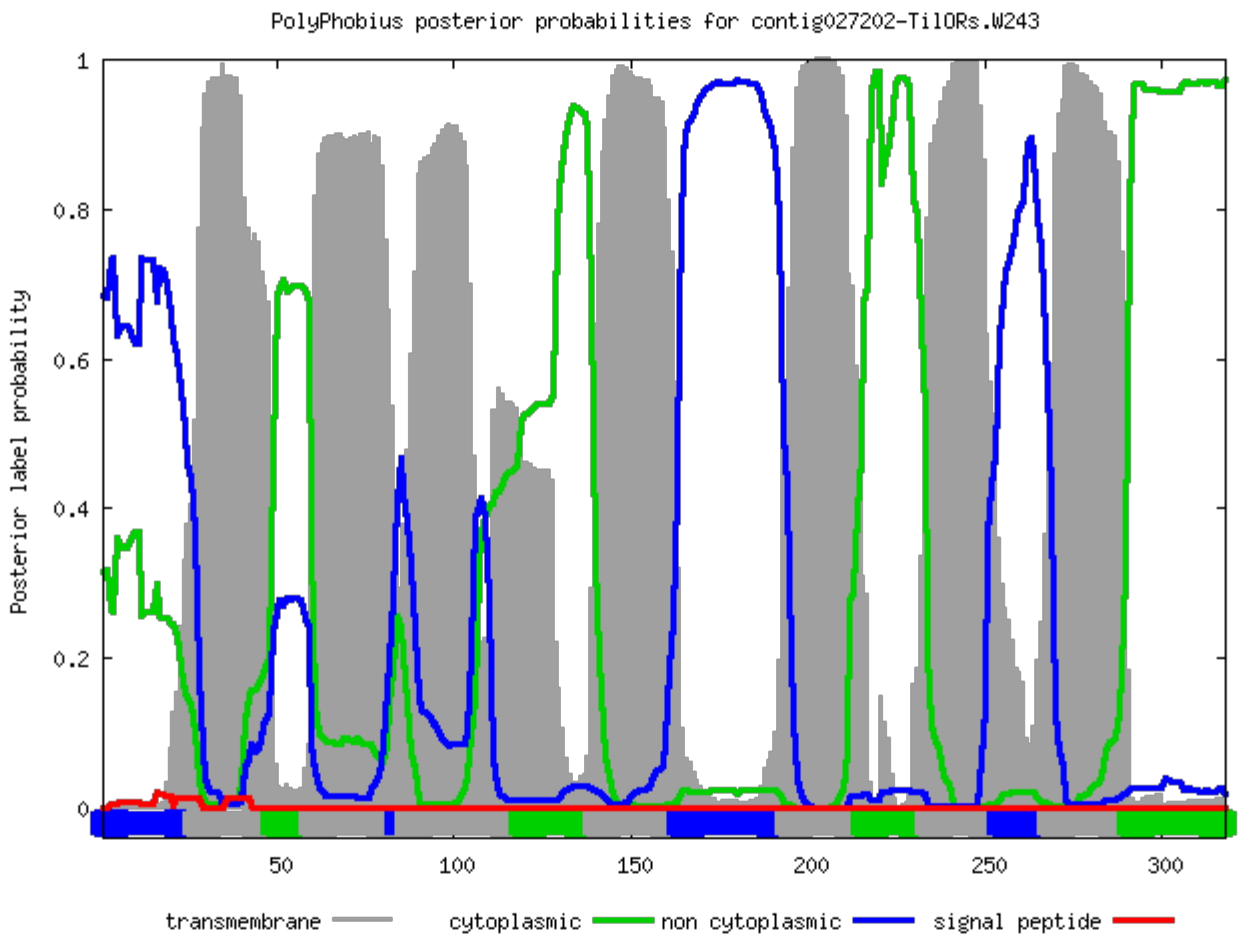

The prediction is based on an alignment. The probability data used in the plot is found here, and the gnuplot script is here.

Prediction of contig013327-TilOR.D052

```
ID   contig013327-TilOR.D052
FT   TOPO_DOM      1     22       NON CYTOPLASMIC.
FT   TRANSMEM     23     48
FT   TOPO_DOM     49     57       CYTOPLASMIC.
FT   TRANSMEM     58     81
FT   TOPO_DOM     82     90       NON CYTOPLASMIC.
FT   TRANSMEM     91    118
FT   TOPO_DOM    119    138       CYTOPLASMIC.
FT   TRANSMEM    139    161
FT   TOPO_DOM    162    193       NON CYTOPLASMIC.
FT   TRANSMEM    194    216
FT   TOPO_DOM    217    236       CYTOPLASMIC.
FT   TRANSMEM    237    256
FT   TOPO_DOM    257    267       NON CYTOPLASMIC.
FT   TRANSMEM    268    291
FT   TOPO_DOM    292    309       CYTOPLASMIC.
//
```

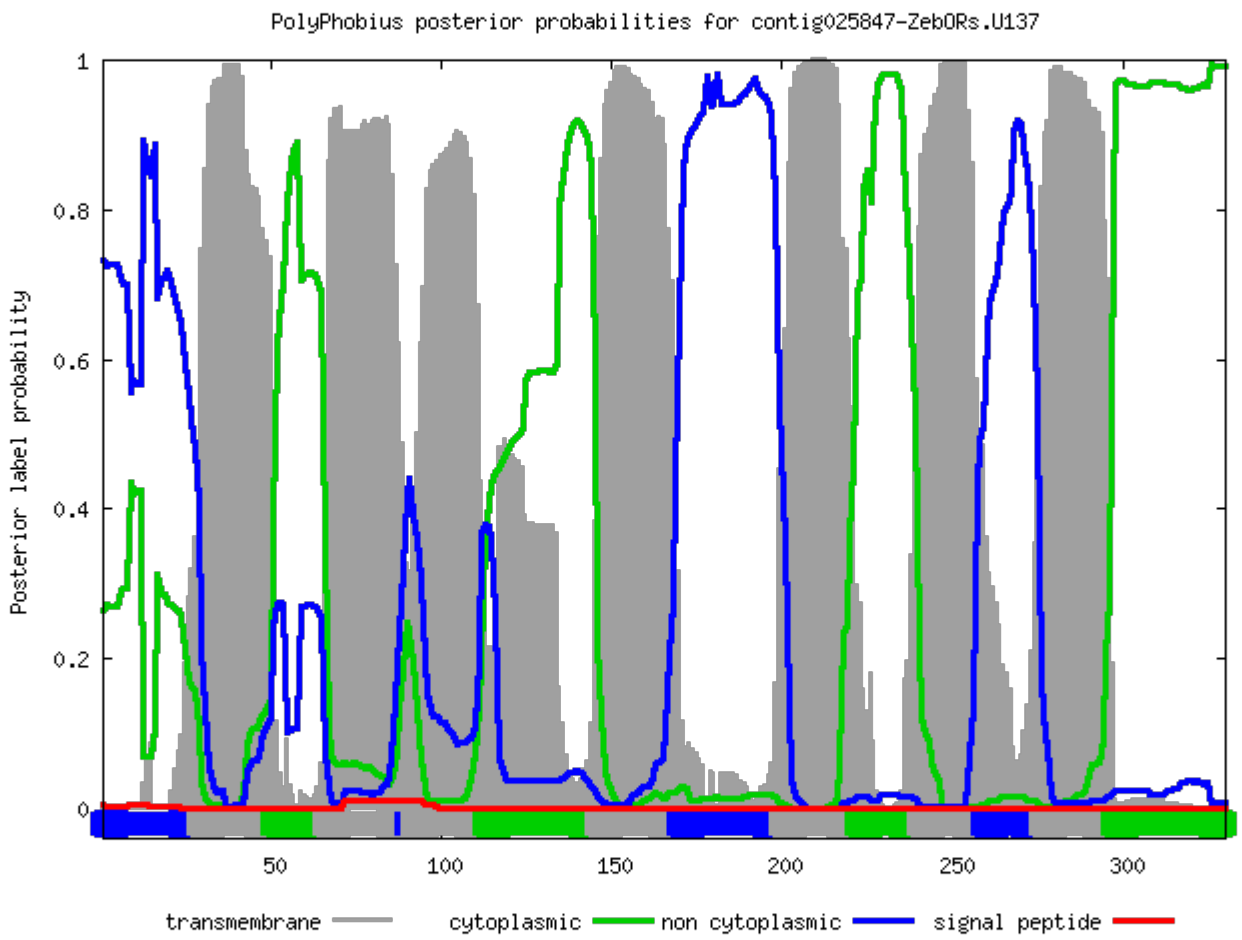

The prediction is based on an alignment. The probability data used in the plot is found here, and the gnuplot script is here.

Prediction of contig013359-TilOR.H107

```
ID   contig013359-TilOR.H107
FT   TOPO_DOM      1     22       NON CYTOPLASMIC.
FT   TRANSMEM     23     49
FT   TOPO_DOM     50     56       CYTOPLASMIC.
FT   TRANSMEM     57     77
FT   TOPO_DOM     78     95       NON CYTOPLASMIC.
FT   TRANSMEM     96    118
FT   TOPO_DOM    119    138       CYTOPLASMIC.
FT   TRANSMEM    139    160
FT   TOPO_DOM    161    193       NON CYTOPLASMIC.
FT   TRANSMEM    194    216
FT   TOPO_DOM    217    235       CYTOPLASMIC.
FT   TRANSMEM    236    257
FT   TOPO_DOM    258    269       NON CYTOPLASMIC.
FT   TRANSMEM    270    289
FT   TOPO_DOM    290    314       CYTOPLASMIC.
//
```

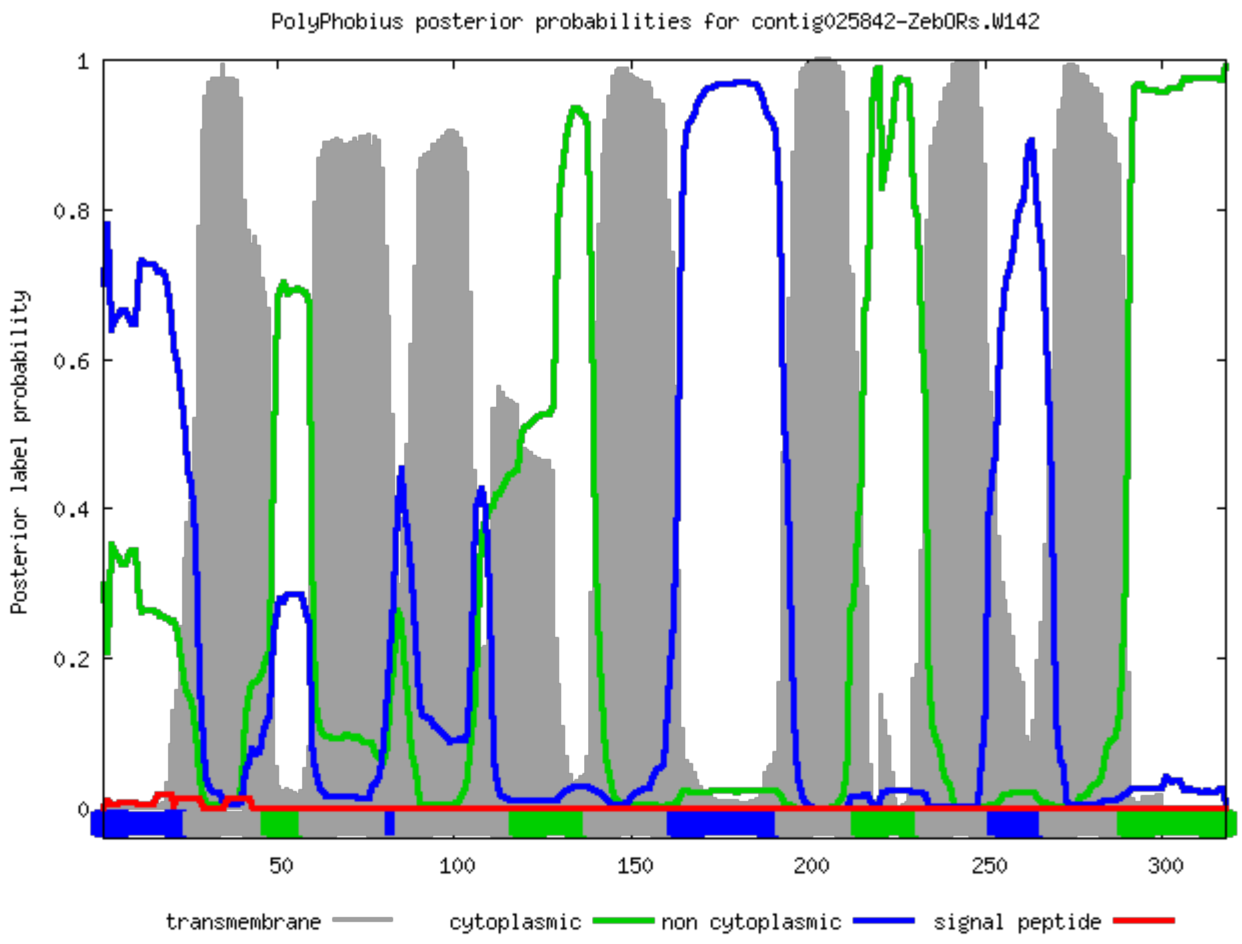

The prediction is based on an alignment. The probability data used in the plot is found here, and the gnuplot script is here.

Prediction of contig039469-TilOR.L151

```
ID   contig039469-TilOR.L151
FT   TOPO_DOM      1     25       NON CYTOPLASMIC.
FT   TRANSMEM     26     50
FT   TOPO_DOM     51     59       CYTOPLASMIC.
FT   TRANSMEM     60     82
FT   TOPO_DOM     83     98       NON CYTOPLASMIC.
FT   TRANSMEM     99    120
FT   TOPO_DOM    121    140       CYTOPLASMIC.
FT   TRANSMEM    141    163
FT   TOPO_DOM    164    198       NON CYTOPLASMIC.
FT   TRANSMEM    199    223
FT   TOPO_DOM    224    235       CYTOPLASMIC.
FT   TRANSMEM    236    259
FT   TOPO_DOM    260    271       NON CYTOPLASMIC.
FT   TRANSMEM    272    292
FT   TOPO_DOM    293    313       CYTOPLASMIC.
//
```

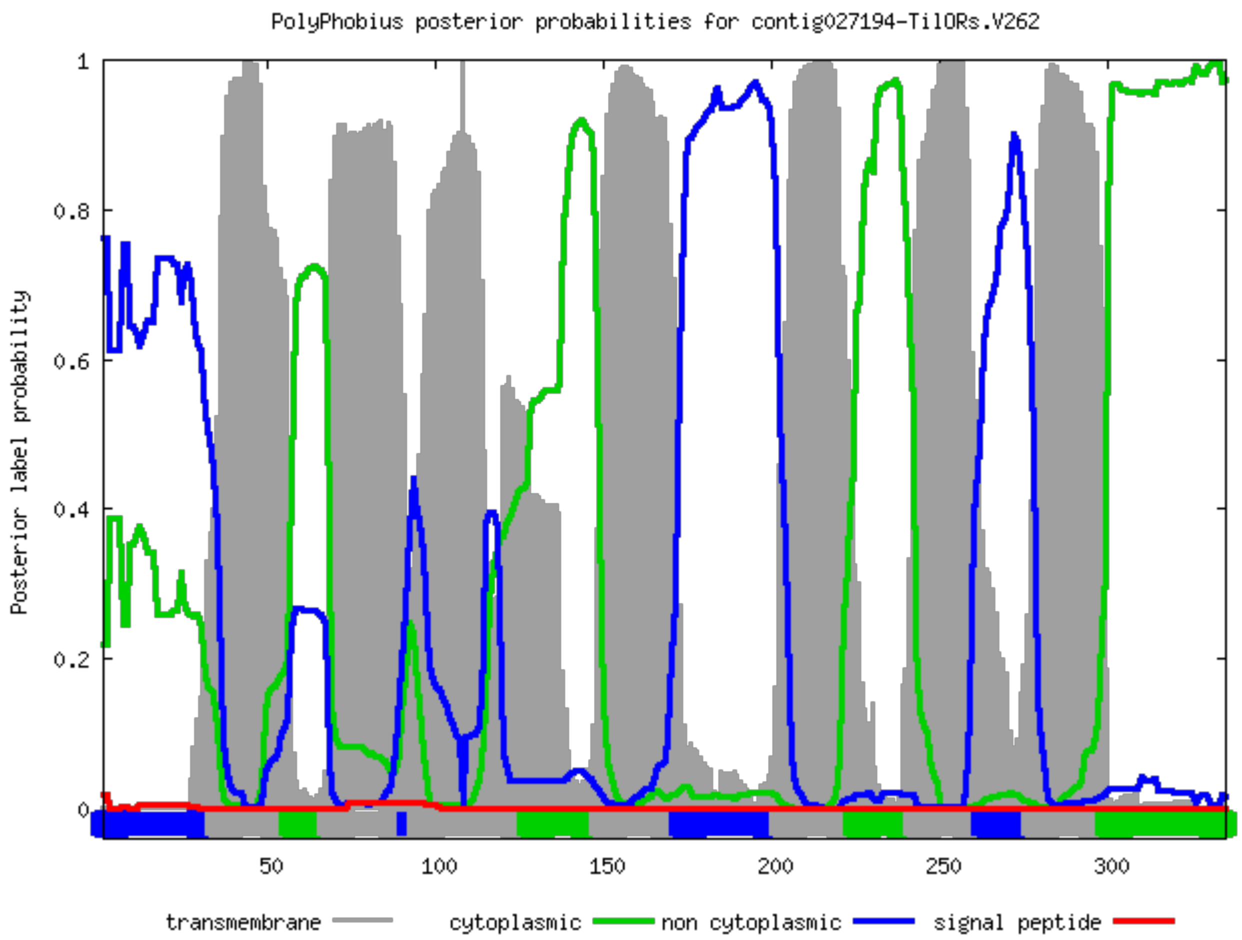

The prediction is based on an alignment. The probability data used in the plot is found here, and the gnuplot script is here.

Prediction of contig028564-BurOR.J077

```
ID   contig028564-BurOR.J077
FT   TOPO_DOM      1     24       NON CYTOPLASMIC.
FT   TRANSMEM     25     50
FT   TOPO_DOM     51     60       CYTOPLASMIC.
FT   TRANSMEM     61     82
FT   TOPO_DOM     83     98       NON CYTOPLASMIC.
FT   TRANSMEM     99    120
FT   TOPO_DOM    121    140       CYTOPLASMIC.
FT   TRANSMEM    141    163
FT   TOPO_DOM    164    195       NON CYTOPLASMIC.
FT   TRANSMEM    196    220
FT   TOPO_DOM    221    238       CYTOPLASMIC.
FT   TRANSMEM    239    261
FT   TOPO_DOM    262    271       NON CYTOPLASMIC.
FT   TRANSMEM    272    292
FT   TOPO_DOM    293    312       CYTOPLASMIC.
//
```

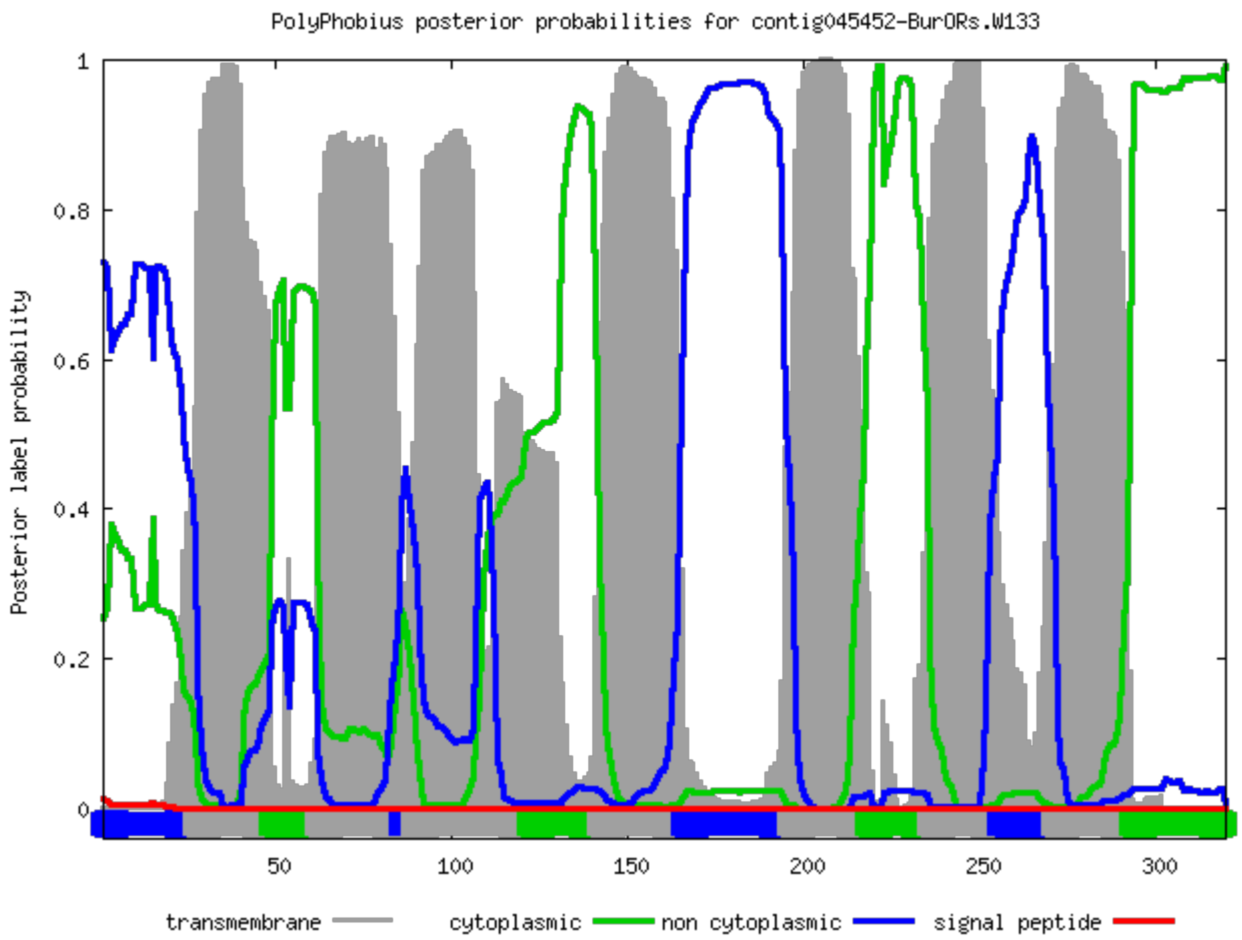

The prediction is based on an alignment. The probability data used in the plot is found here, and the gnuplot script is here.

Prediction of contig048778-NyeOR.M108

```
ID   contig048778-NyeOR.M108
FT   TOPO_DOM      1     31       NON CYTOPLASMIC.
FT   TRANSMEM     32     56
FT   TOPO_DOM     57     66       CYTOPLASMIC.
FT   TRANSMEM     67     88
FT   TOPO_DOM     89    107       NON CYTOPLASMIC.
FT   TRANSMEM    108    126
FT   TOPO_DOM    127    146       CYTOPLASMIC.
FT   TRANSMEM    147    170
FT   TOPO_DOM    171    208       NON CYTOPLASMIC.
FT   TRANSMEM    209    232
FT   TOPO_DOM    233    250       CYTOPLASMIC.
FT   TRANSMEM    251    273
FT   TOPO_DOM    274    279       NON CYTOPLASMIC.
FT   TRANSMEM    280    299
FT   TOPO_DOM    300    327       CYTOPLASMIC.
//
```

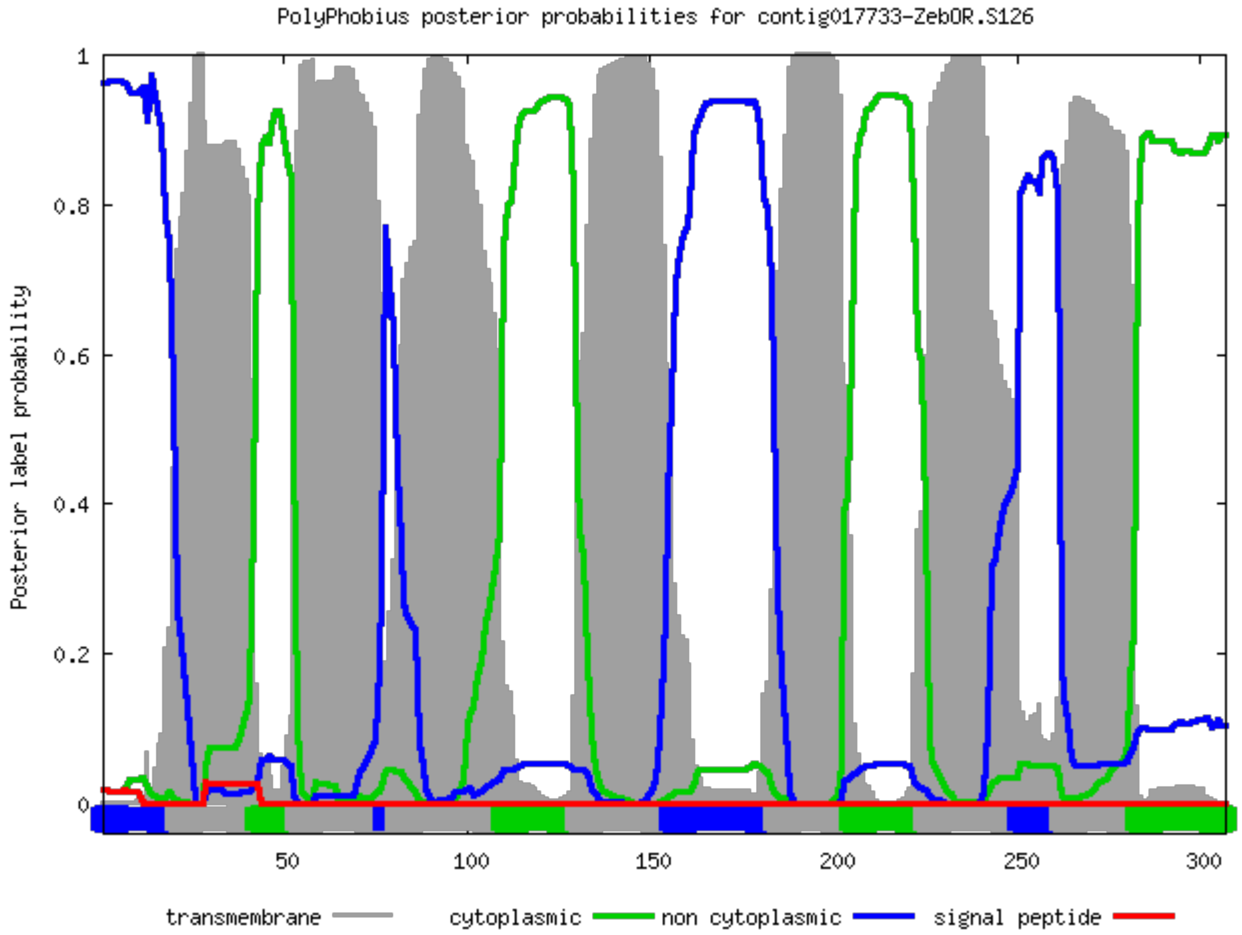

The prediction is based on an alignment. The probability data used in the plot is found here, and the gnuplot script is here.

Prediction of contig020980-NyeOR.C035

```
ID   contig020980-NyeOR.C035
FT   TOPO_DOM      1      21       NON CYTOPLASMIC.
FT   TRANSMEM     22      47
FT   TOPO_DOM     48      56       CYTOPLASMIC.
FT   TRANSMEM     57      79
FT   TOPO_DOM     80      94       NON CYTOPLASMIC.
FT   TRANSMEM     95     117
FT   TOPO_DOM    118     137       CYTOPLASMIC.
FT   TRANSMEM    138     161
FT   TOPO_DOM    162     192       NON CYTOPLASMIC.
FT   TRANSMEM    193     215
FT   TOPO_DOM    216     234       CYTOPLASMIC.
FT   TRANSMEM    235     255
FT   TOPO_DOM    256     266       NON CYTOPLASMIC.
FT   TRANSMEM    267     289
FT   TOPO_DOM    290     321       CYTOPLASMIC.
//
```

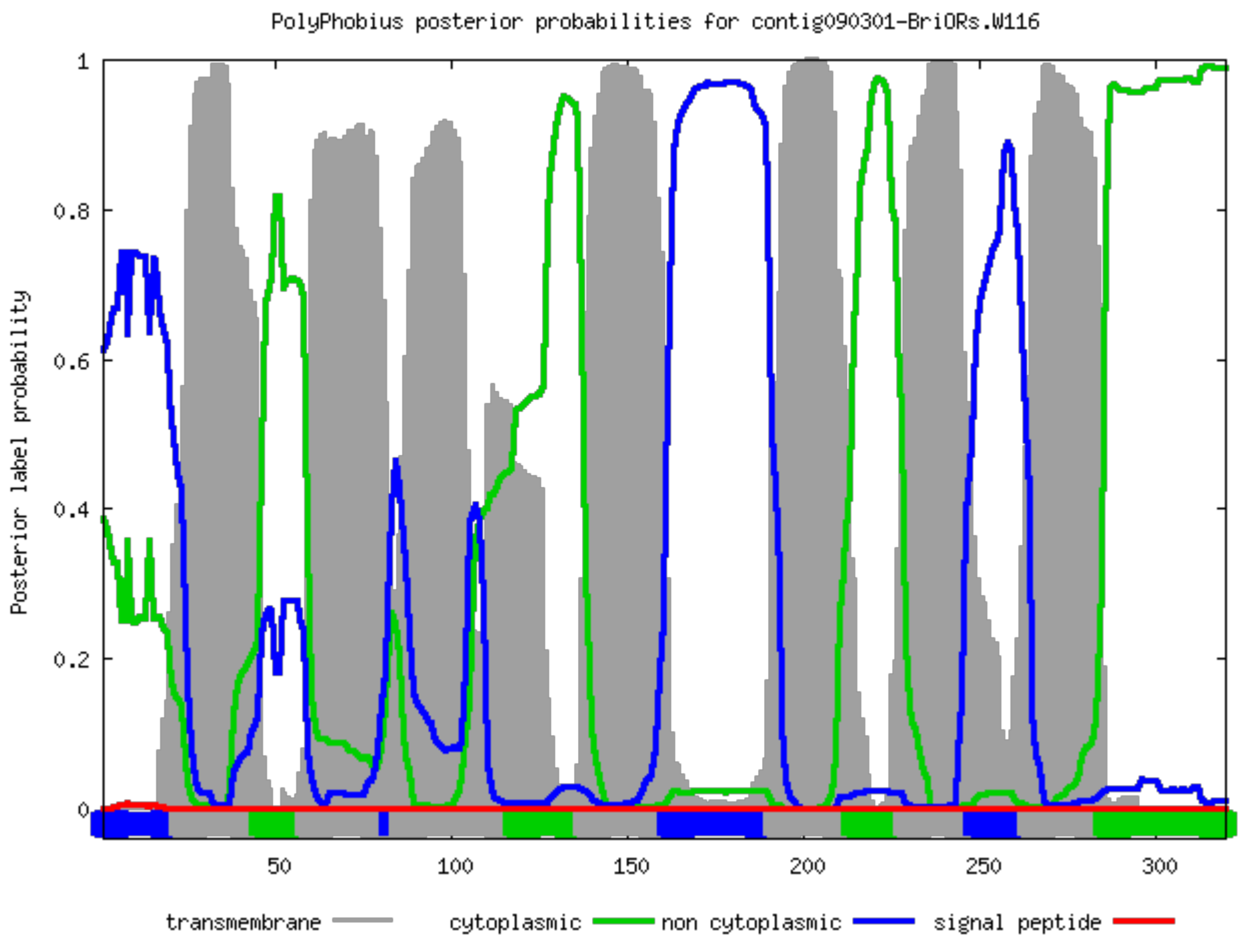

The prediction is based on an alignment. The probability data used in the plot is found here, and the gnuplot script is here.

Prediction of contig048242-ZebOR.E049

```
ID   contig048242-ZebOR.E049
FT   TOPO_DOM      1     21       NON CYTOPLASMIC.
FT   TRANSMEM     22     47
FT   TOPO_DOM     48     56       CYTOPLASMIC.
FT   TRANSMEM     57     82
FT   TOPO_DOM     83     91       NON CYTOPLASMIC.
FT   TRANSMEM     92    117
FT   TOPO_DOM    118    137       CYTOPLASMIC.
FT   TRANSMEM    138    160
FT   TOPO_DOM    161    192       NON CYTOPLASMIC.
FT   TRANSMEM    193    215
FT   TOPO_DOM    216    235       CYTOPLASMIC.
FT   TRANSMEM    236    255
FT   TOPO_DOM    256    266       NON CYTOPLASMIC.
FT   TRANSMEM    267    290
FT   TOPO_DOM    291    306       CYTOPLASMIC.
//
```

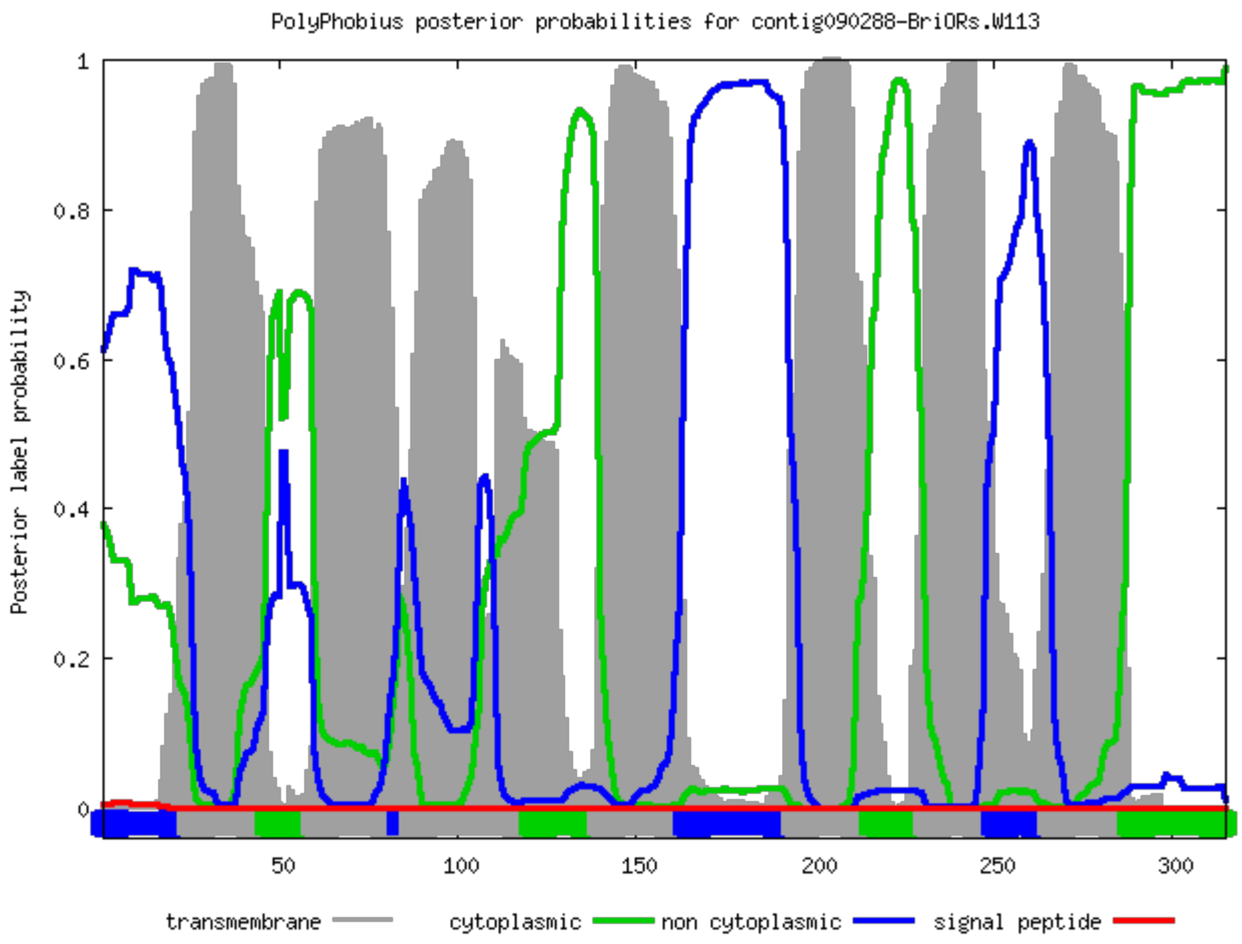

The prediction is based on an alignment. The probability data used in the plot is found here, and the gnuplot script is here.

Prediction of contig022265-TilOR.A017

```
ID   contig022265-TilOR.A017
FT   TOPO_DOM      1     25       NON CYTOPLASMIC.
FT   TRANSMEM     26     51
FT   TOPO_DOM     52     59       CYTOPLASMIC.
FT   TRANSMEM     60     80
FT   TOPO_DOM     81     98       NON CYTOPLASMIC.
FT   TRANSMEM     99    121
FT   TOPO_DOM    122    141       CYTOPLASMIC.
FT   TRANSMEM    142    163
FT   TOPO_DOM    164    195       NON CYTOPLASMIC.
FT   TRANSMEM    196    219
FT   TOPO_DOM    220    239       CYTOPLASMIC.
FT   TRANSMEM    240    261
FT   TOPO_DOM    262    272       NON CYTOPLASMIC.
FT   TRANSMEM    273    293
FT   TOPO_DOM    294    319       CYTOPLASMIC.
//
```

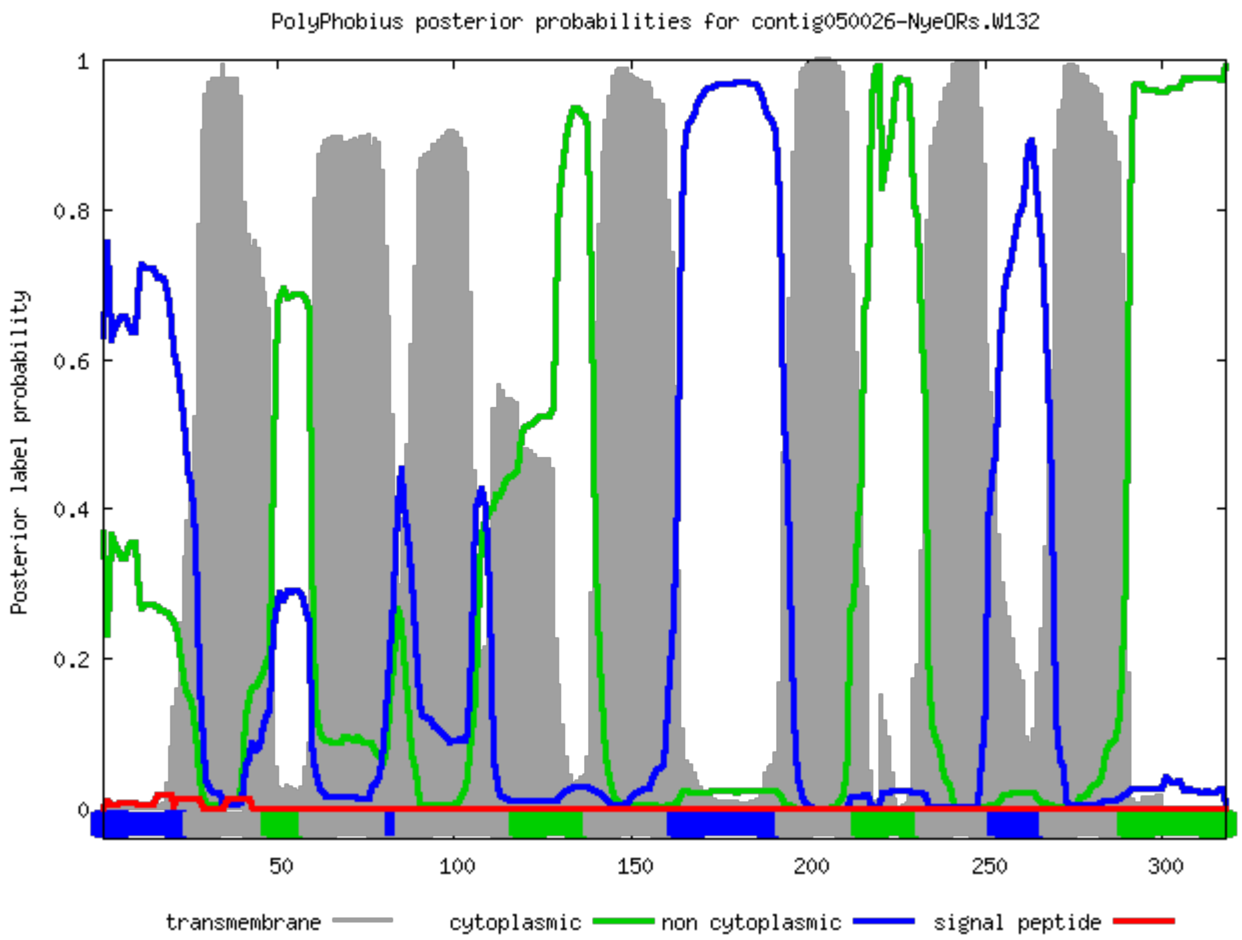

The prediction is based on an alignment. The probability data used in the plot is found here, and the gnuplot script is here.

Prediction of contig044492-NyeOR.B034

```
ID   contig044492-NyeOR.B034
FT   TOPO_DOM      1     29       NON CYTOPLASMIC.
FT   TRANSMEM     30     55
FT   TOPO_DOM     56     63       CYTOPLASMIC.
FT   TRANSMEM     64     84
FT   TOPO_DOM     85    104       NON CYTOPLASMIC.
FT   TRANSMEM    105    127
FT   TOPO_DOM    128    147       CYTOPLASMIC.
FT   TRANSMEM    148    169
FT   TOPO_DOM    170    204       NON CYTOPLASMIC.
FT   TRANSMEM    205    227
FT   TOPO_DOM    228    247       CYTOPLASMIC.
FT   TRANSMEM    248    269
FT   TOPO_DOM    270    278       NON CYTOPLASMIC.
FT   TRANSMEM    279    301
FT   TOPO_DOM    302    321       CYTOPLASMIC.
//
```

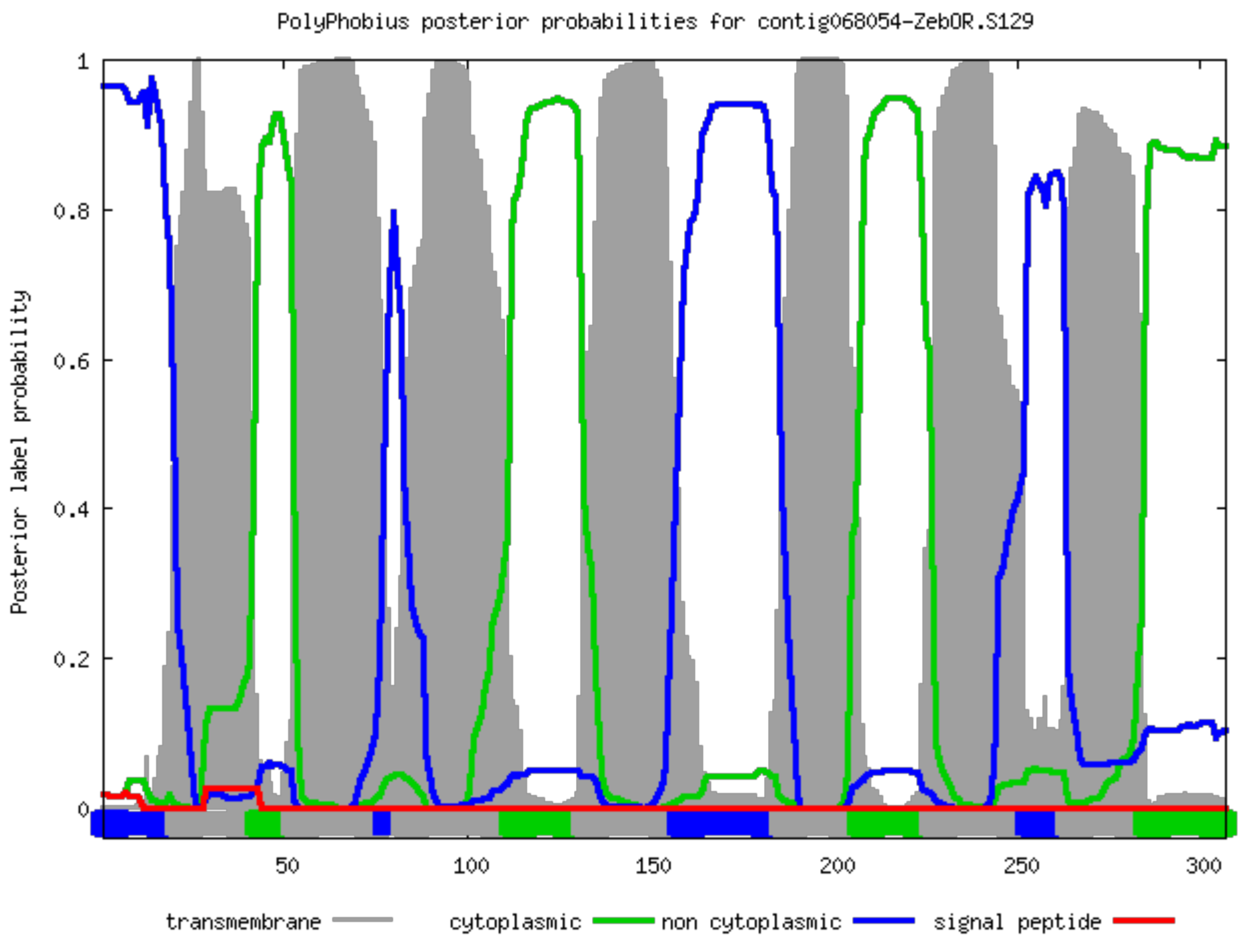

The prediction is based on an alignment. The probability data used in the plot is found here, and the gnuplot script is here.

Prediction of contig046010-ZebOR.K088

```
ID    contig046010-ZebOR.K088
FT    TOPO_DOM      1      24       NON CYTOPLASMIC.
FT    TRANSMEM     25      48
FT    TOPO_DOM     49      58       CYTOPLASMIC.
FT    TRANSMEM     59      80
FT    TOPO_DOM     81      99       NON CYTOPLASMIC.
FT    TRANSMEM    100     121
FT    TOPO_DOM    122     141       CYTOPLASMIC.
FT    TRANSMEM    142     165
FT    TOPO_DOM    166     198       NON CYTOPLASMIC.
FT    TRANSMEM    199     222
FT    TOPO_DOM    223     242       CYTOPLASMIC.
FT    TRANSMEM    243     262
FT    TOPO_DOM    263     272       NON CYTOPLASMIC.
FT    TRANSMEM    273     292
FT    TOPO_DOM    293     313       CYTOPLASMIC.
//
```

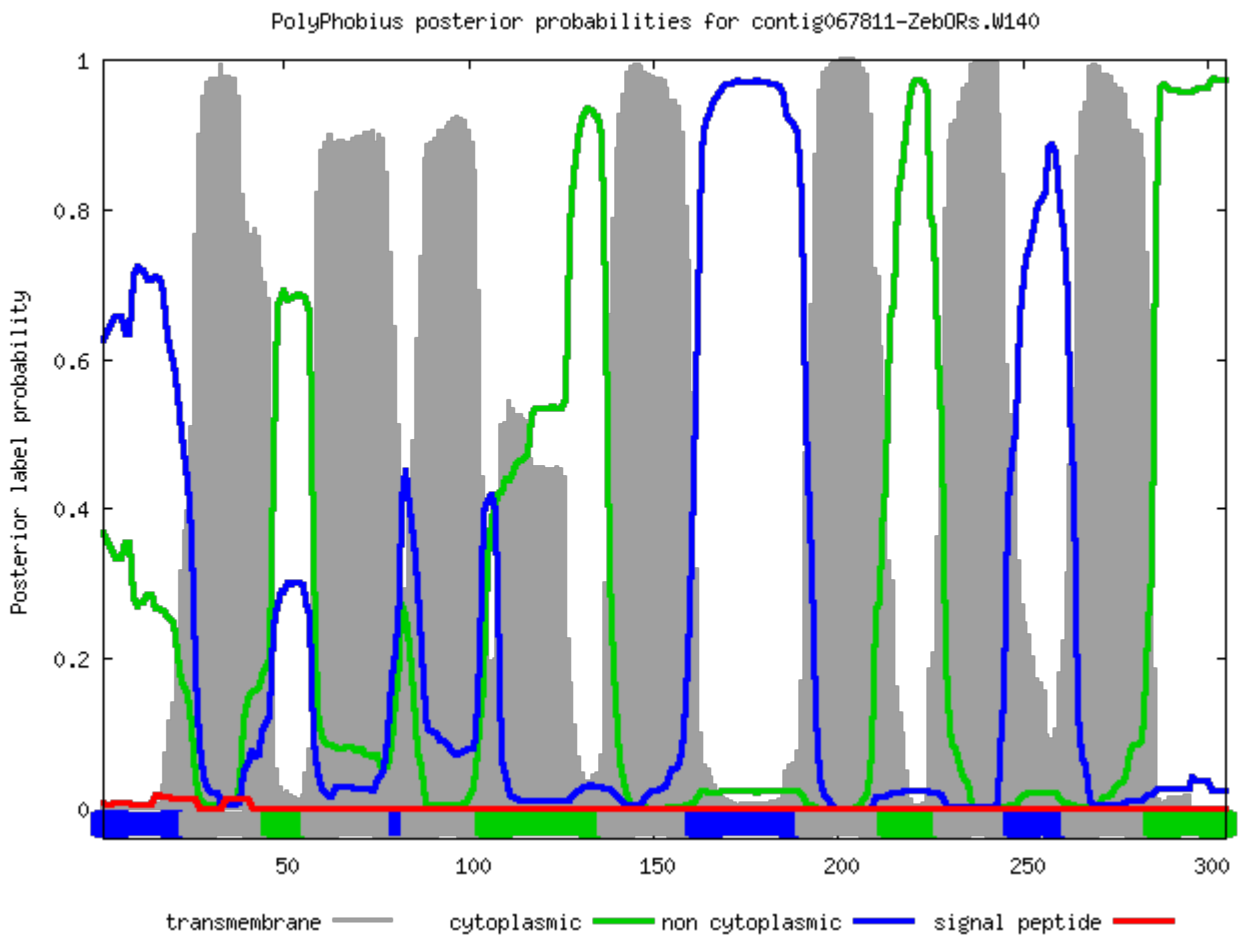

The prediction is based on an alignment. The probability data used in the plot is found here, and the gnuplot script is here.

Prediction of contig050080-TilOR.N198

```
ID   contig050080-TilOR.N198
FT   TOPO_DOM      1     31       NON CYTOPLASMIC.
FT   TRANSMEM     32     57
FT   TOPO_DOM     58     65       CYTOPLASMIC.
FT   TRANSMEM     66     85
FT   TOPO_DOM     86    103       NON CYTOPLASMIC.
FT   TRANSMEM    104    126
FT   TOPO_DOM    127    145       CYTOPLASMIC.
FT   TRANSMEM    146    169
FT   TOPO_DOM    170    205       NON CYTOPLASMIC.
FT   TRANSMEM    206    231
FT   TOPO_DOM    232    249       CYTOPLASMIC.
FT   TRANSMEM    250    271
FT   TOPO_DOM    272    276       NON CYTOPLASMIC.
FT   TRANSMEM    277    297
FT   TOPO_DOM    298    323       CYTOPLASMIC.
//
```

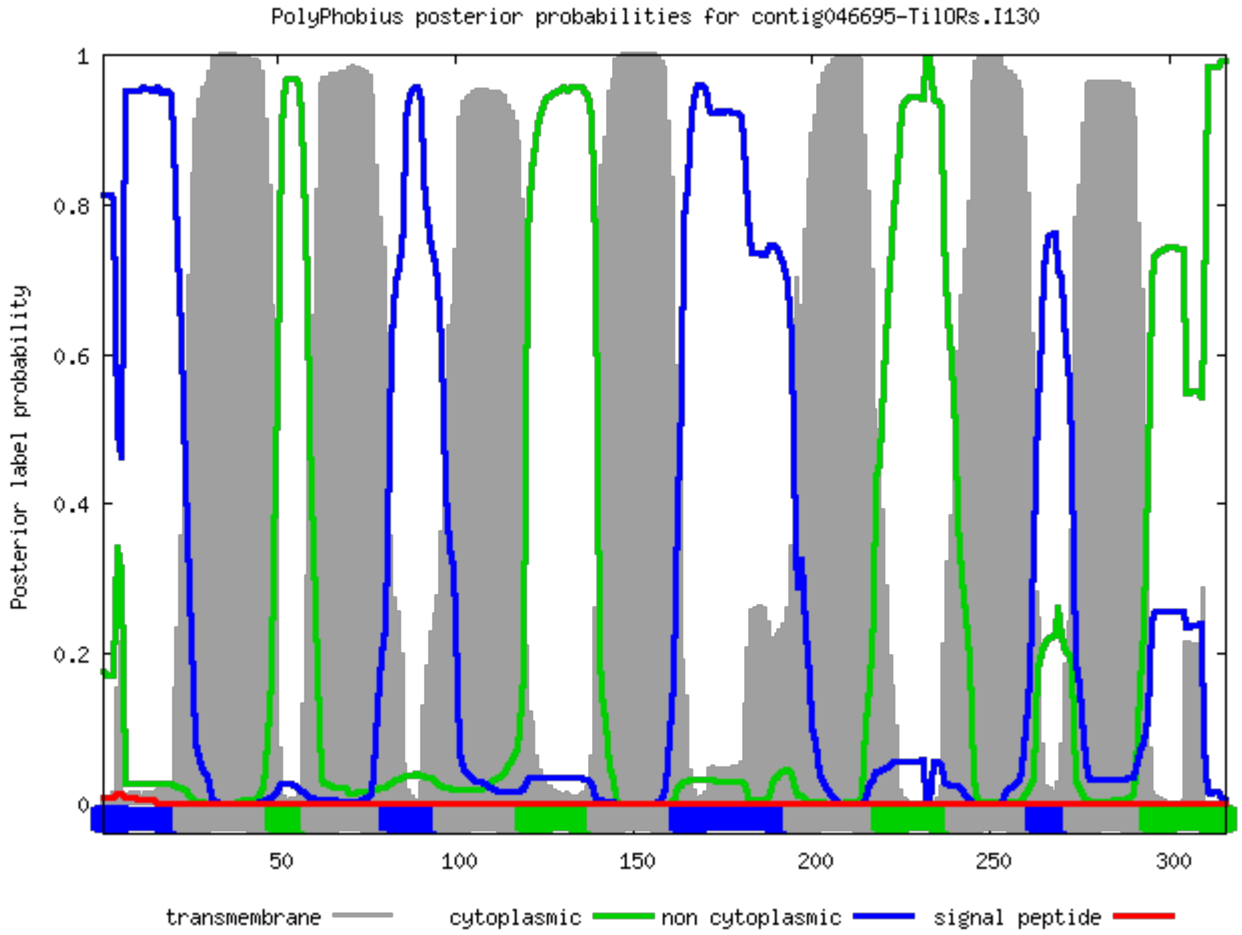

The prediction is based on an alignment. The probability data used in the plot is found here, and the gnuplot script is here.

Prediction of contig013322-TilOR.D049

```
ID    contig013322-TilOR.D049
FT    TOPO_DOM      1      22       NON CYTOPLASMIC.
FT    TRANSMEM     23      48
FT    TOPO_DOM     49      57       CYTOPLASMIC.
FT    TRANSMEM     58      81
FT    TOPO_DOM     82      90       NON CYTOPLASMIC.
FT    TRANSMEM     91     118
FT    TOPO_DOM    119     138       CYTOPLASMIC.
FT    TRANSMEM    139     162
FT    TOPO_DOM    163     193       NON CYTOPLASMIC.
FT    TRANSMEM    194     216
FT    TOPO_DOM    217     236       CYTOPLASMIC.
FT    TRANSMEM    237     256
FT    TOPO_DOM    257     267       NON CYTOPLASMIC.
FT    TRANSMEM    268     291
FT    TOPO_DOM    292     309       CYTOPLASMIC.
//
```

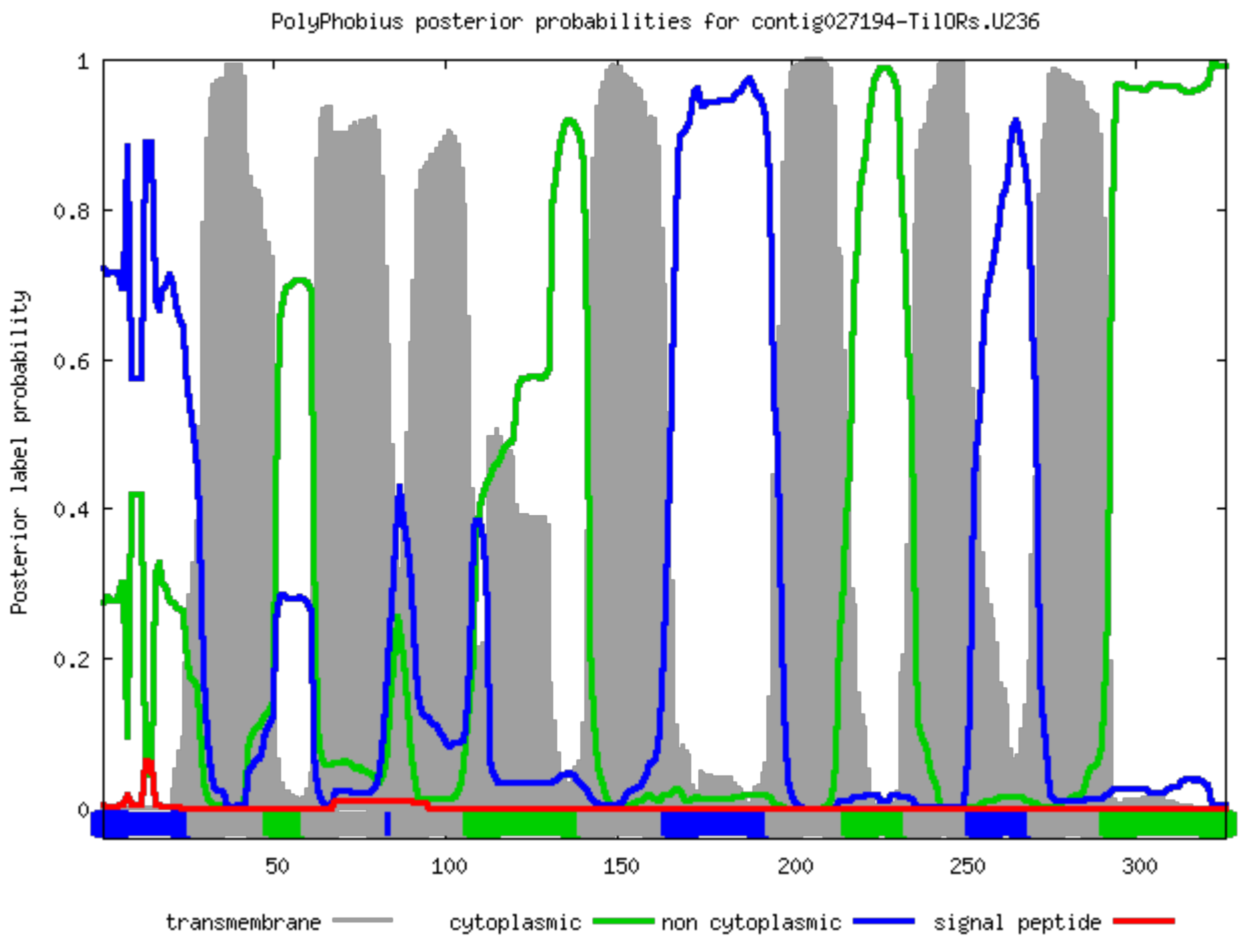

The prediction is based on an alignment. The probability data used in the plot is found here, and the gnuplot script is here.

Prediction of contig096535-BriOR.N084

```
ID   contig096535-BriOR.N084
FT   TOPO_DOM      1     33       NON CYTOPLASMIC.
FT   TRANSMEM     34     59
FT   TOPO_DOM     60     67       CYTOPLASMIC.
FT   TRANSMEM     68     89
FT   TOPO_DOM     90    108       NON CYTOPLASMIC.
FT   TRANSMEM    109    128
FT   TOPO_DOM    129    148       CYTOPLASMIC.
FT   TRANSMEM    149    171
FT   TOPO_DOM    172    207       NON CYTOPLASMIC.
FT   TRANSMEM    208    233
FT   TOPO_DOM    234    252       CYTOPLASMIC.
FT   TRANSMEM    253    275
FT   TOPO_DOM    276    337       NON CYTOPLASMIC.
//
```

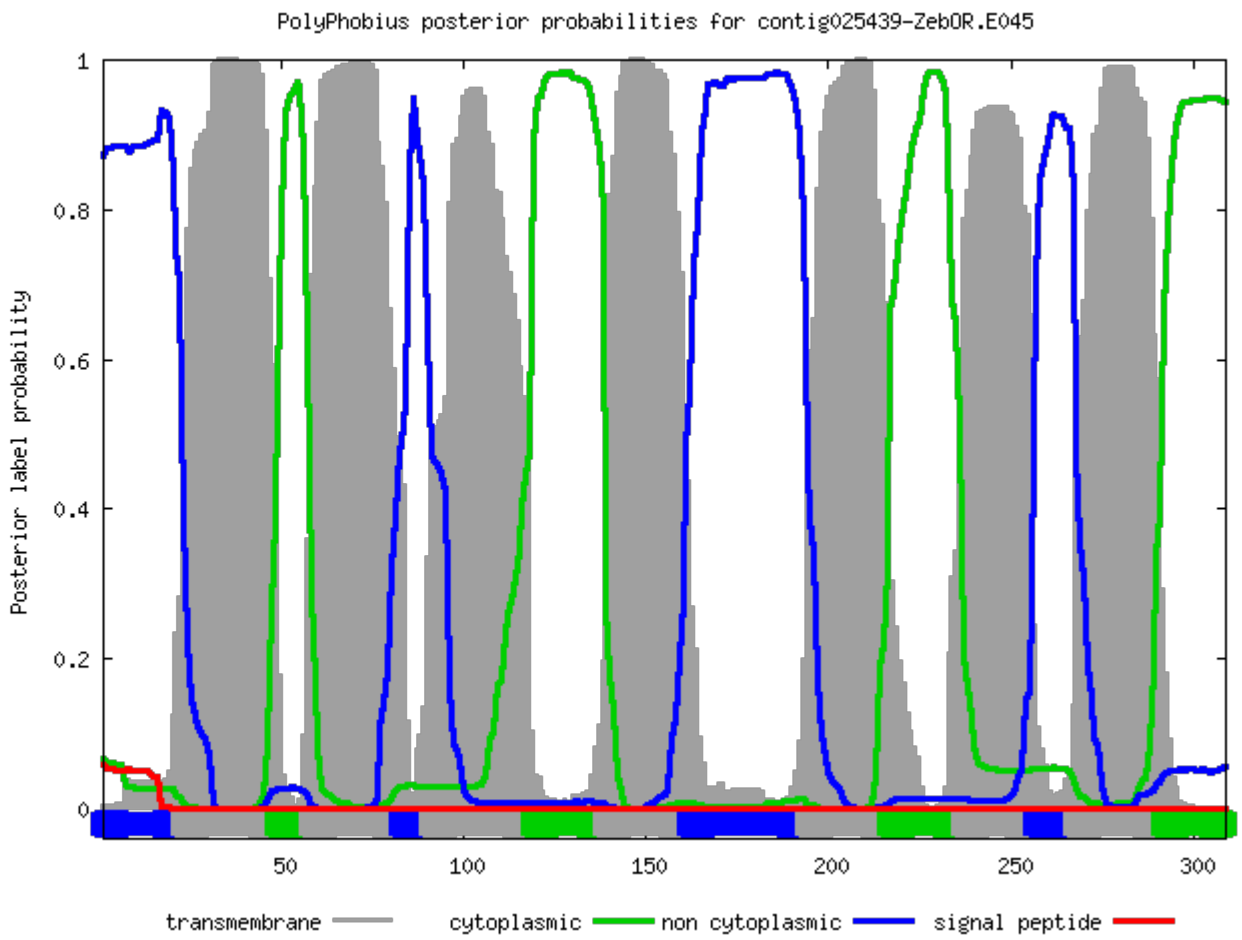

The prediction is based on an alignment. The probability data used in the plot is found here, and the gnuplot script is here.

Prediction of contig023716-TilOR.L144

```
ID   contig023716-TilOR.L144
FT   TOPO_DOM      1     25       NON CYTOPLASMIC.
FT   TRANSMEM     26     50
FT   TOPO_DOM     51     59       CYTOPLASMIC.
FT   TRANSMEM     60     82
FT   TOPO_DOM     83    100       NON CYTOPLASMIC.
FT   TRANSMEM    101    120
FT   TOPO_DOM    121    140       CYTOPLASMIC.
FT   TRANSMEM    141    162
FT   TOPO_DOM    163    200       NON CYTOPLASMIC.
FT   TRANSMEM    201    224
FT   TOPO_DOM    225    235       CYTOPLASMIC.
FT   TRANSMEM    236    259
FT   TOPO_DOM    260    272       NON CYTOPLASMIC.
FT   TRANSMEM    273    292
FT   TOPO_DOM    293    313       CYTOPLASMIC.
//
```

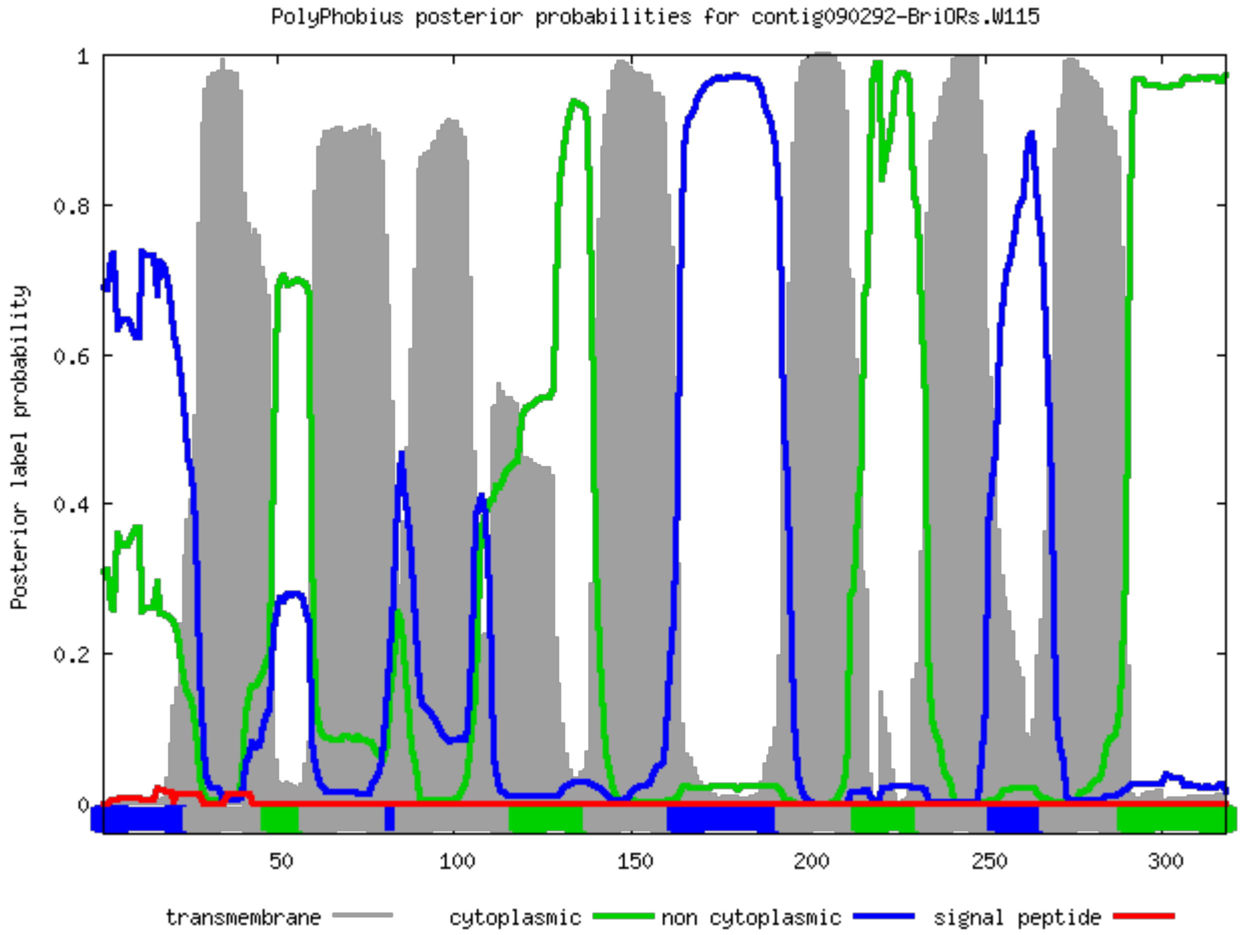

The prediction is based on an alignment. The probability data used in the plot is found here, and the gnuplot script is here.

Prediction of contig017696-BurOR.P118

```
ID   contig017696-BurOR.P118
FT   TOPO_DOM      1      28       NON CYTOPLASMIC.
FT   TRANSMEM     29      52
FT   TOPO_DOM     53      62       CYTOPLASMIC.
FT   TRANSMEM     63      86
FT   TOPO_DOM     87     100       NON CYTOPLASMIC.
FT   TRANSMEM    101     123
FT   TOPO_DOM    124     142       CYTOPLASMIC.
FT   TRANSMEM    143     166
FT   TOPO_DOM    167     200       NON CYTOPLASMIC.
FT   TRANSMEM    201     225
FT   TOPO_DOM    226     242       CYTOPLASMIC.
FT   TRANSMEM    243     265
FT   TOPO_DOM    266     277       NON CYTOPLASMIC.
FT   TRANSMEM    278     298
FT   TOPO_DOM    299     332       CYTOPLASMIC.
//
```

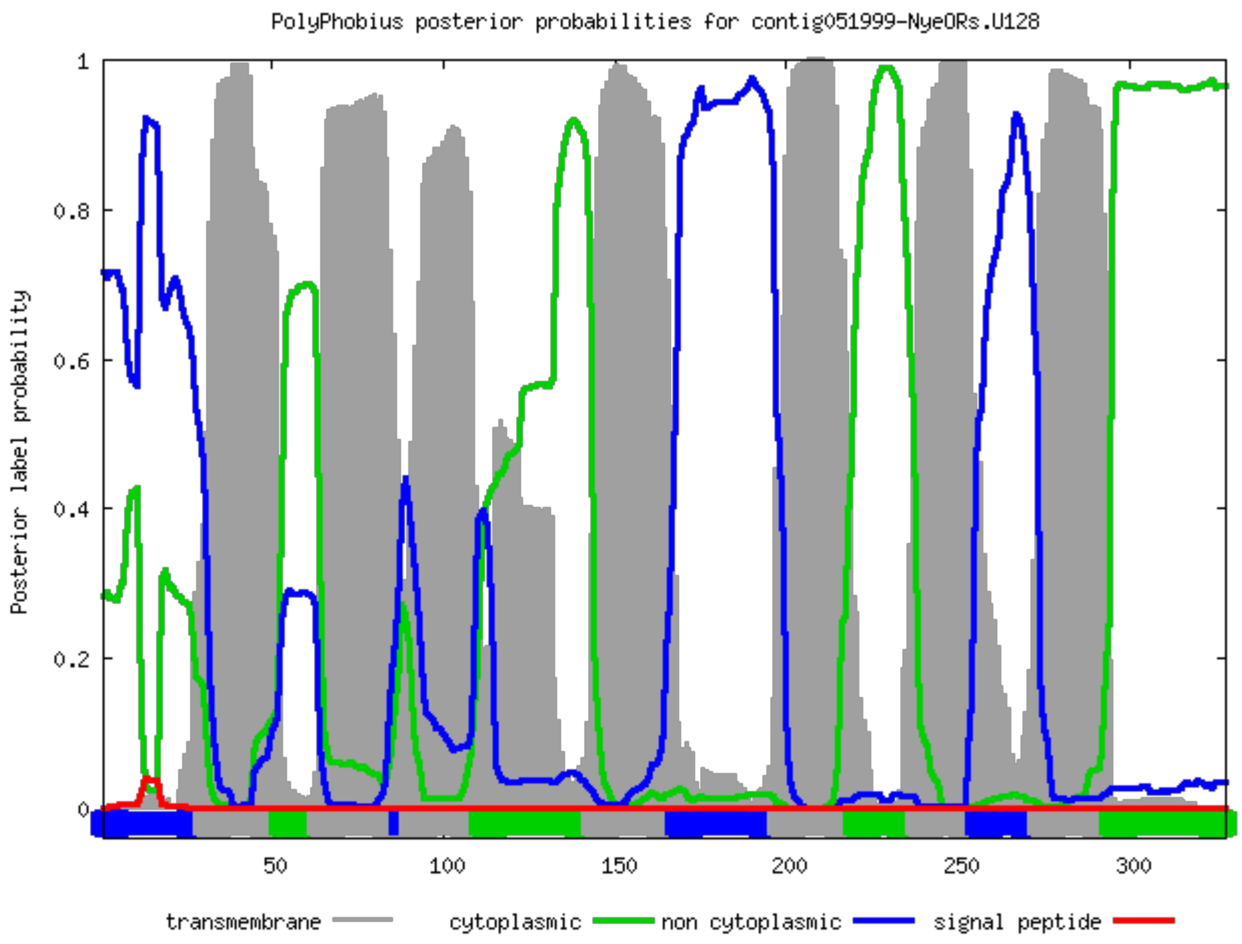

The prediction is based on an alignment. The probability data used in the plot is found here, and the gnuplot script is here.

Prediction of contig025453-ZebOR.P124

```
ID   contig025453-ZebOR.P124
FT   TOPO_DOM      1     27       NON CYTOPLASMIC.
FT   TRANSMEM     28     51
FT   TOPO_DOM     52     61       CYTOPLASMIC.
FT   TRANSMEM     62     87
FT   TOPO_DOM     88    100       NON CYTOPLASMIC.
FT   TRANSMEM    101    122
FT   TOPO_DOM    123    142       CYTOPLASMIC.
FT   TRANSMEM    143    165
FT   TOPO_DOM    166    200       NON CYTOPLASMIC.
FT   TRANSMEM    201    225
FT   TOPO_DOM    226    239       CYTOPLASMIC.
FT   TRANSMEM    240    262
FT   TOPO_DOM    263    273       NON CYTOPLASMIC.
FT   TRANSMEM    274    294
FT   TOPO_DOM    295    310       CYTOPLASMIC.
//
```

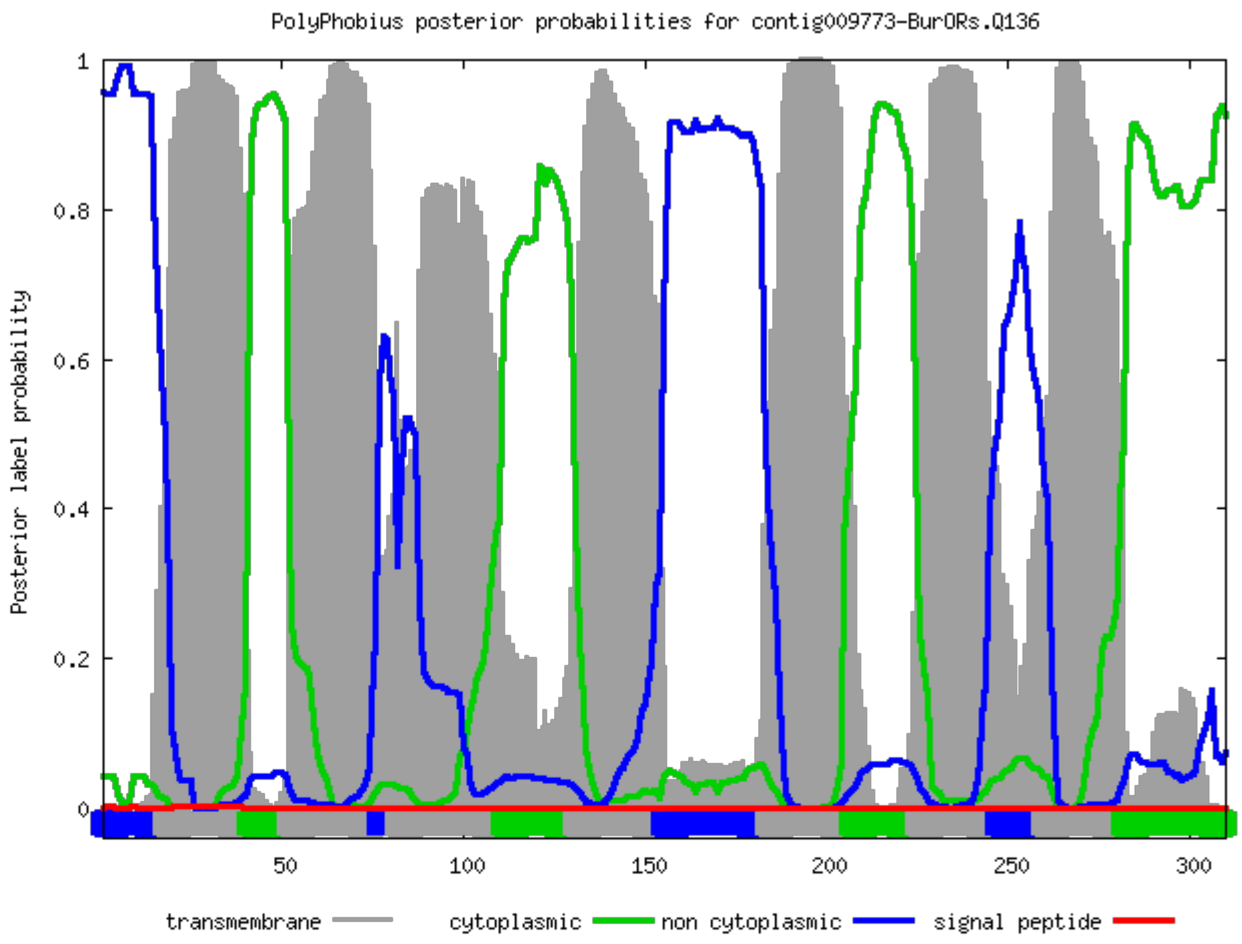

The prediction is based on an alignment. The probability data used in the plot is found here, and the gnuplot script is here.

Prediction of contig022268-TilOR.A020

```
ID    contig022268-TilOR.A020
FT    TOPO_DOM      1      22       NON CYTOPLASMIC.
FT    TRANSMEM     23      48
FT    TOPO_DOM     49      56       CYTOPLASMIC.
FT    TRANSMEM     57      77
FT    TOPO_DOM     78      95       NON CYTOPLASMIC.
FT    TRANSMEM     96     118
FT    TOPO_DOM    119     138       CYTOPLASMIC.
FT    TRANSMEM    139     159
FT    TOPO_DOM    160     192       NON CYTOPLASMIC.
FT    TRANSMEM    193     215
FT    TOPO_DOM    216     235       CYTOPLASMIC.
FT    TRANSMEM    236     257
FT    TOPO_DOM    258     268       NON CYTOPLASMIC.
FT    TRANSMEM    269     289
FT    TOPO_DOM    290     307       CYTOPLASMIC.
//
```

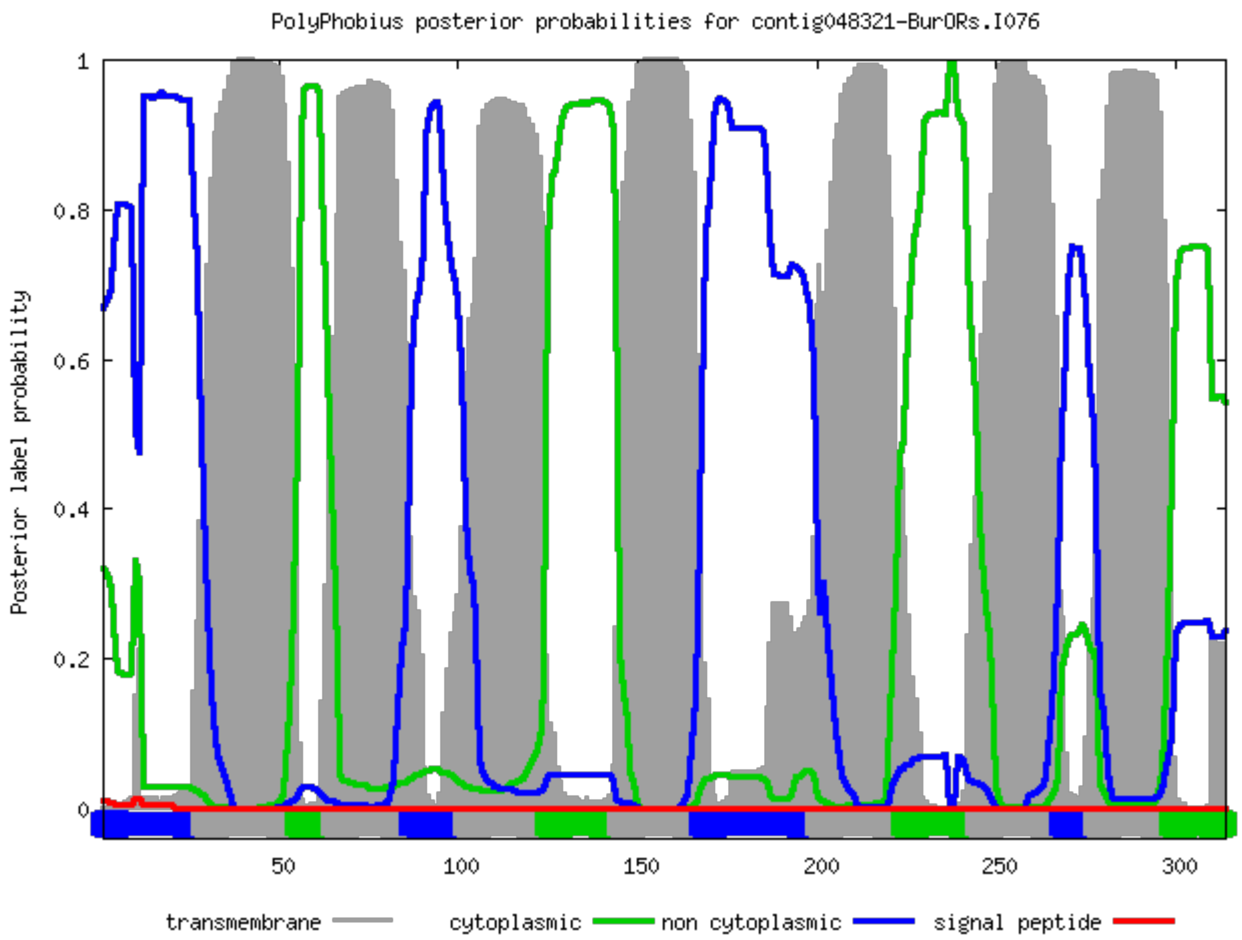

The prediction is based on an alignment. The probability data used in the plot is found here, and the gnuplot script is here.

Prediction of contig041757-NyeOR.H076

```
ID    contig041757-NyeOR.H076
FT    TOPO_DOM      1      22       NON CYTOPLASMIC.
FT    TRANSMEM     23      49
FT    TOPO_DOM     50      56       CYTOPLASMIC.
FT    TRANSMEM     57      77
FT    TOPO_DOM     78      95       NON CYTOPLASMIC.
FT    TRANSMEM     96     118
FT    TOPO_DOM    119     138       CYTOPLASMIC.
FT    TRANSMEM    139     160
FT    TOPO_DOM    161     193       NON CYTOPLASMIC.
FT    TRANSMEM    194     216
FT    TOPO_DOM    217     235       CYTOPLASMIC.
FT    TRANSMEM    236     258
FT    TOPO_DOM    259     269       NON CYTOPLASMIC.
FT    TRANSMEM    270     289
FT    TOPO_DOM    290     314       CYTOPLASMIC.
//
```

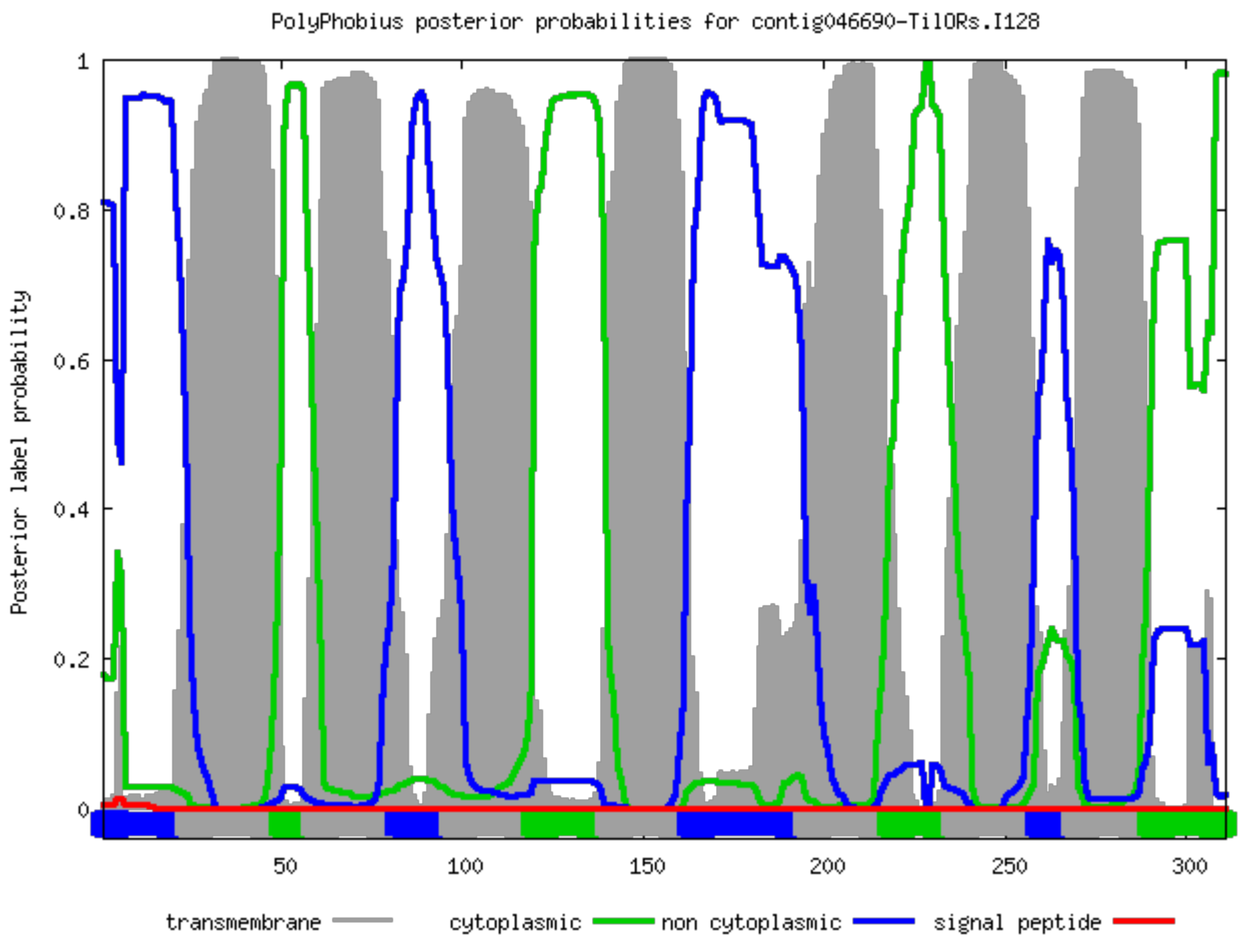

The prediction is based on an alignment. The probability data used in the plot is found here, and the gnuplot script is here.

Prediction of contig067265-BurOR.R141

```
ID   contig067265-BurOR.R141
FT   TOPO_DOM      1     24       NON CYTOPLASMIC.
FT   TRANSMEM     25     48
FT   TOPO_DOM     49     59       CYTOPLASMIC.
FT   TRANSMEM     60     84
FT   TOPO_DOM     85     89       NON CYTOPLASMIC.
FT   TRANSMEM     90    118
FT   TOPO_DOM    119    138       CYTOPLASMIC.
FT   TRANSMEM    139    162
FT   TOPO_DOM    163    194       NON CYTOPLASMIC.
FT   TRANSMEM    195    218
FT   TOPO_DOM    219    235       CYTOPLASMIC.
FT   TRANSMEM    236    258
FT   TOPO_DOM    259    270       NON CYTOPLASMIC.
FT   TRANSMEM    271    293
FT   TOPO_DOM    294    317       CYTOPLASMIC.
//
```

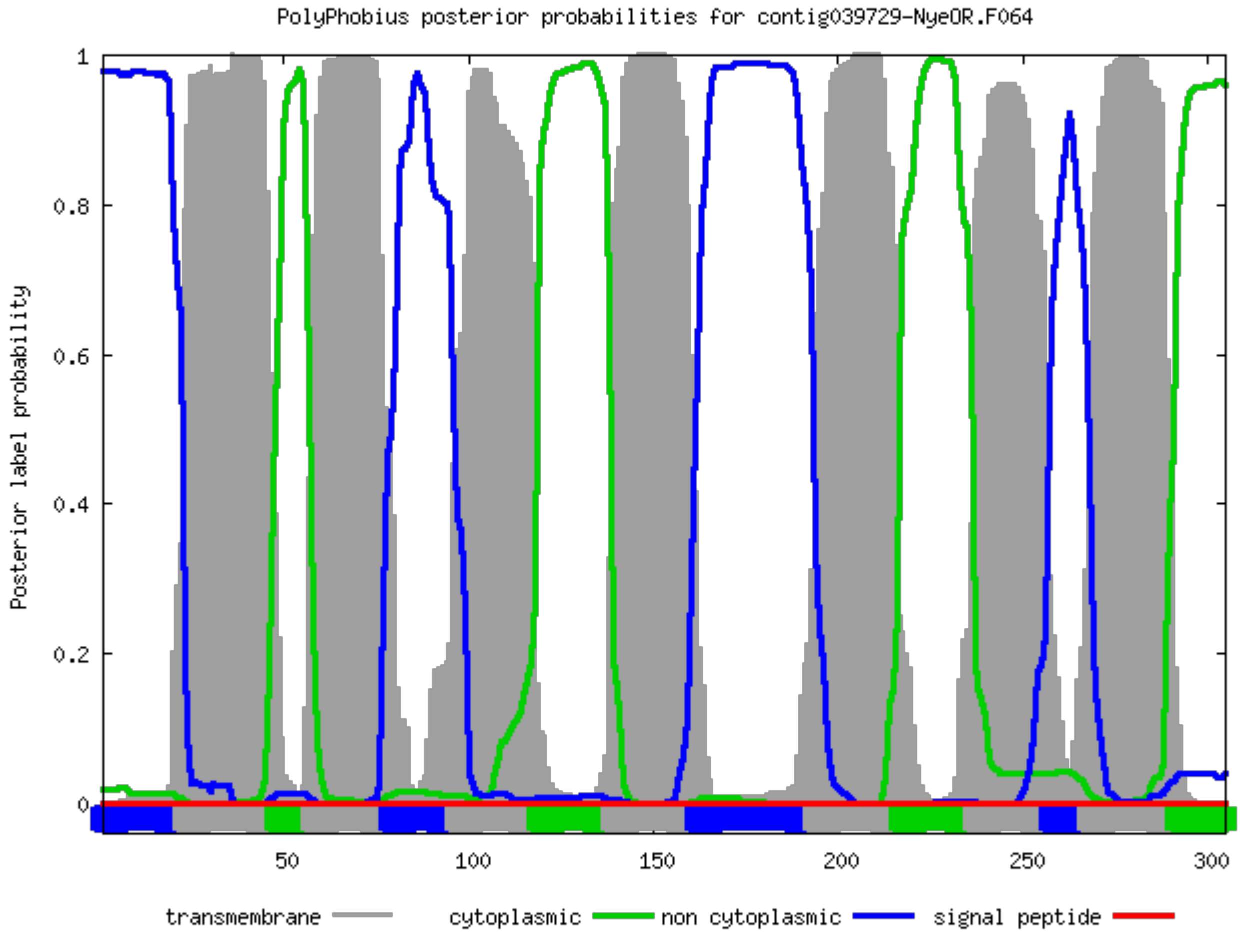

The prediction is based on an alignment. The probability data used in the plot is found here, and the gnuplot script is here.

Prediction of contig040502-NyeOR.L091

```
ID   contig040502-NyeOR.L091
FT   TOPO_DOM      1      25       NON CYTOPLASMIC.
FT   TRANSMEM     26      50
FT   TOPO_DOM     51      59       CYTOPLASMIC.
FT   TRANSMEM     60      83
FT   TOPO_DOM     84      98       NON CYTOPLASMIC.
FT   TRANSMEM     99     120
FT   TOPO_DOM    121     140       CYTOPLASMIC.
FT   TRANSMEM    141     162
FT   TOPO_DOM    163     198       NON CYTOPLASMIC.
FT   TRANSMEM    199     224
FT   TOPO_DOM    225     238       CYTOPLASMIC.
FT   TRANSMEM    239     262
FT   TOPO_DOM    263     271       NON CYTOPLASMIC.
FT   TRANSMEM    272     292
FT   TOPO_DOM    293     313       CYTOPLASMIC.
//
```

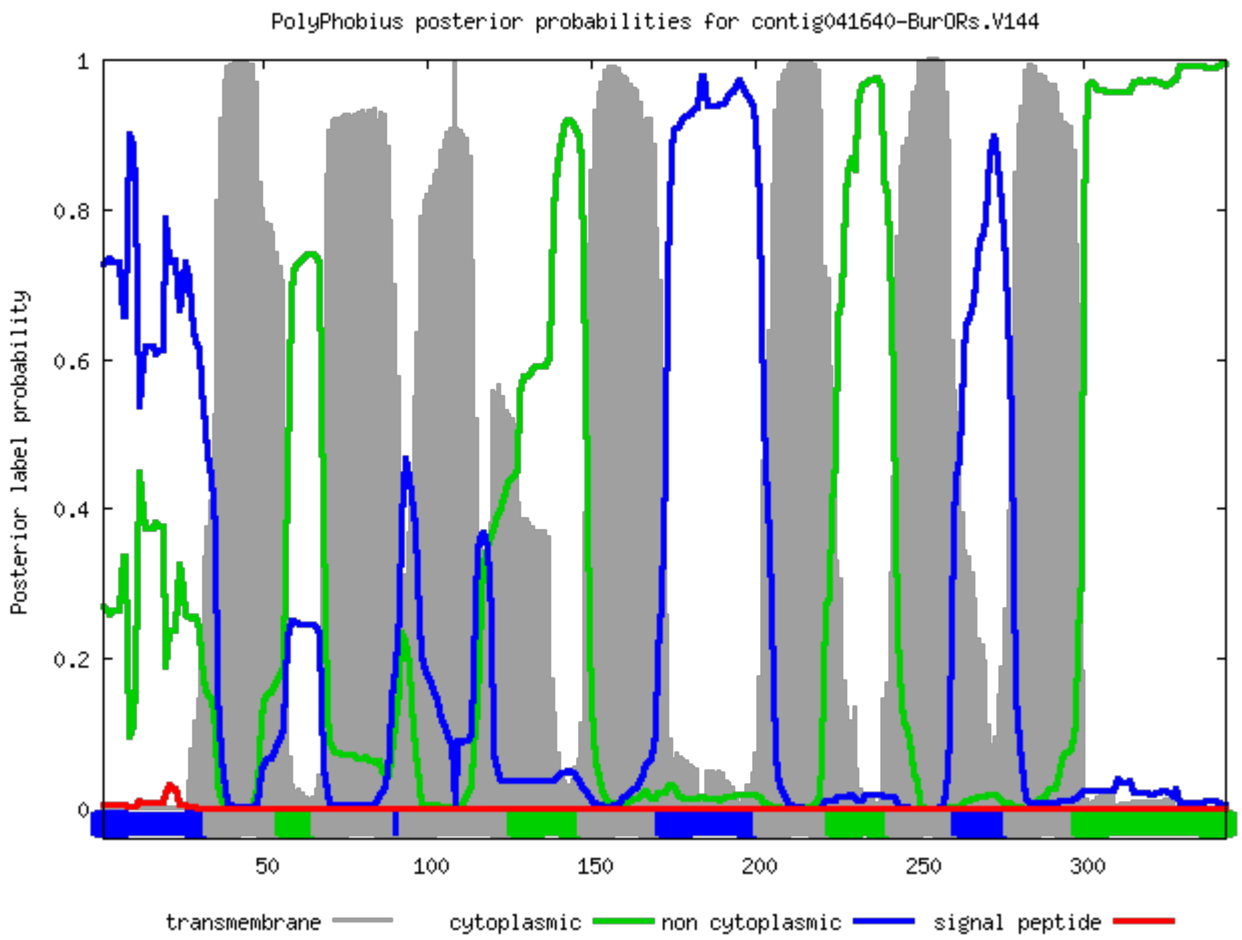

The prediction is based on an alignment. The probability data used in the plot is found here, and the gnuplot script is here.

Prediction of contig025443-ZebOR.E046

```
ID   contig025443-ZebOR.E046
FT   TOPO_DOM      1     22       NON CYTOPLASMIC.
FT   TRANSMEM     23     48
FT   TOPO_DOM     49     57       CYTOPLASMIC.
FT   TRANSMEM     58     82
FT   TOPO_DOM     83     92       NON CYTOPLASMIC.
FT   TRANSMEM     93    118
FT   TOPO_DOM    119    138       CYTOPLASMIC.
FT   TRANSMEM    139    160
FT   TOPO_DOM    161    193       NON CYTOPLASMIC.
FT   TRANSMEM    194    215
FT   TOPO_DOM    216    235       CYTOPLASMIC.
FT   TRANSMEM    236    255
FT   TOPO_DOM    256    266       NON CYTOPLASMIC.
FT   TRANSMEM    267    290
FT   TOPO_DOM    291    320       CYTOPLASMIC.
//
```

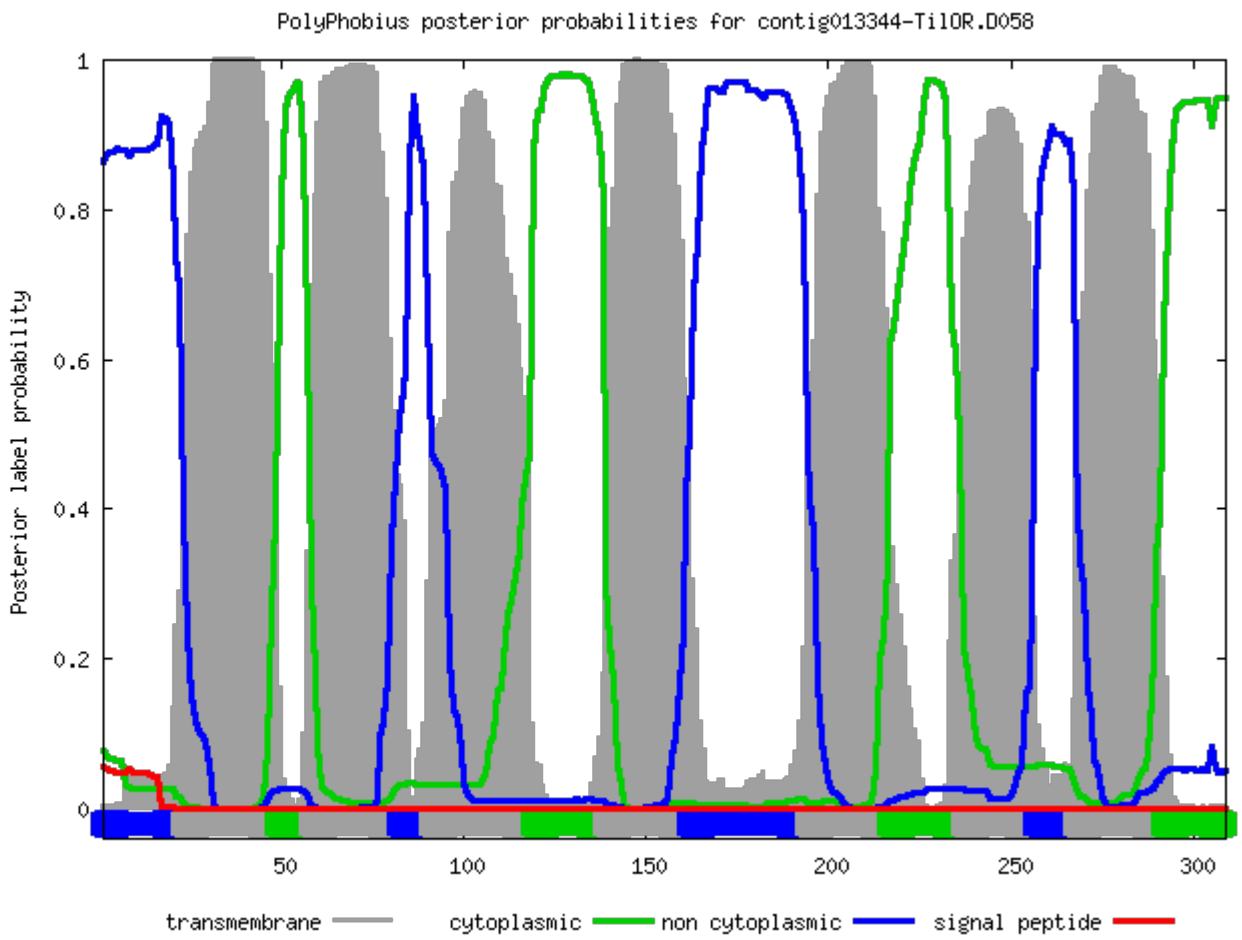

The prediction is based on an alignment. The probability data used in the plot is found here, and the gnuplot script is here.

Prediction of contig014057-ZebOR.H067

```
ID    contig014057-ZebOR.H067
FT    TOPO_DOM       1      22       NON CYTOPLASMIC.
FT    TRANSMEM      23      49
FT    TOPO_DOM      50      56       CYTOPLASMIC.
FT    TRANSMEM      57      77
FT    TOPO_DOM      78      95       NON CYTOPLASMIC.
FT    TRANSMEM      96     118
FT    TOPO_DOM     119     138       CYTOPLASMIC.
FT    TRANSMEM     139     160
FT    TOPO_DOM     161     193       NON CYTOPLASMIC.
FT    TRANSMEM     194     216
FT    TOPO_DOM     217     235       CYTOPLASMIC.
FT    TRANSMEM     236     258
FT    TOPO_DOM     259     269       NON CYTOPLASMIC.
FT    TRANSMEM     270     289
FT    TOPO_DOM     290     314       CYTOPLASMIC.
//
```

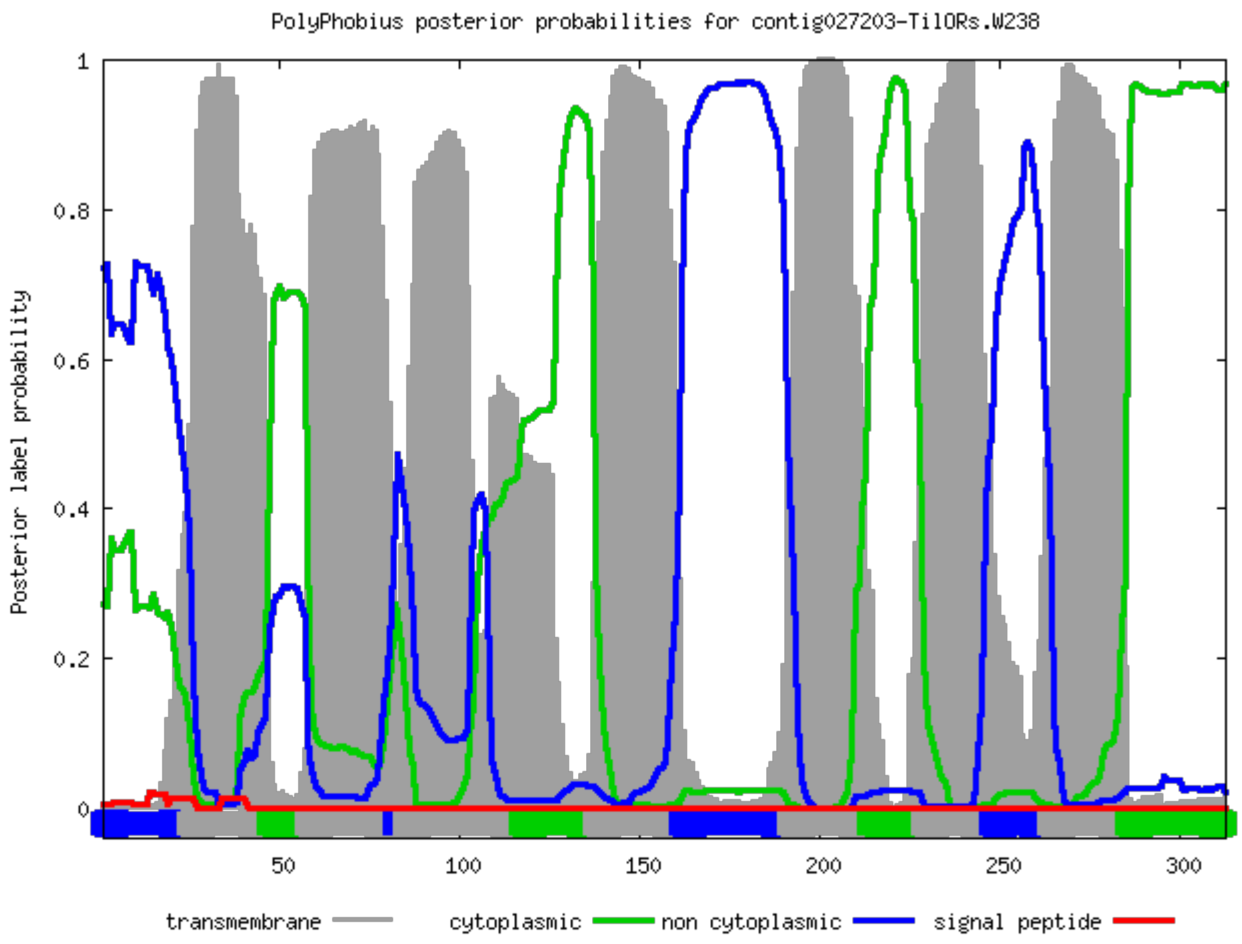

The prediction is based on an alignment. The probability data used in the plot is found here, and the gnuplot script is here.

Prediction of contig051570-BurOR.A010

```
ID   contig051570-BurOR.A010
FT   TOPO_DOM      1     22       NON CYTOPLASMIC.
FT   TRANSMEM     23     48
FT   TOPO_DOM     49     56       CYTOPLASMIC.
FT   TRANSMEM     57     76
FT   TOPO_DOM     77     95       NON CYTOPLASMIC.
FT   TRANSMEM     96    118
FT   TOPO_DOM    119    138       CYTOPLASMIC.
FT   TRANSMEM    139    159
FT   TOPO_DOM    160    192       NON CYTOPLASMIC.
FT   TRANSMEM    193    215
FT   TOPO_DOM    216    235       CYTOPLASMIC.
FT   TRANSMEM    236    257
FT   TOPO_DOM    258    268       NON CYTOPLASMIC.
FT   TRANSMEM    269    289
FT   TOPO_DOM    290    311       CYTOPLASMIC.
//
```

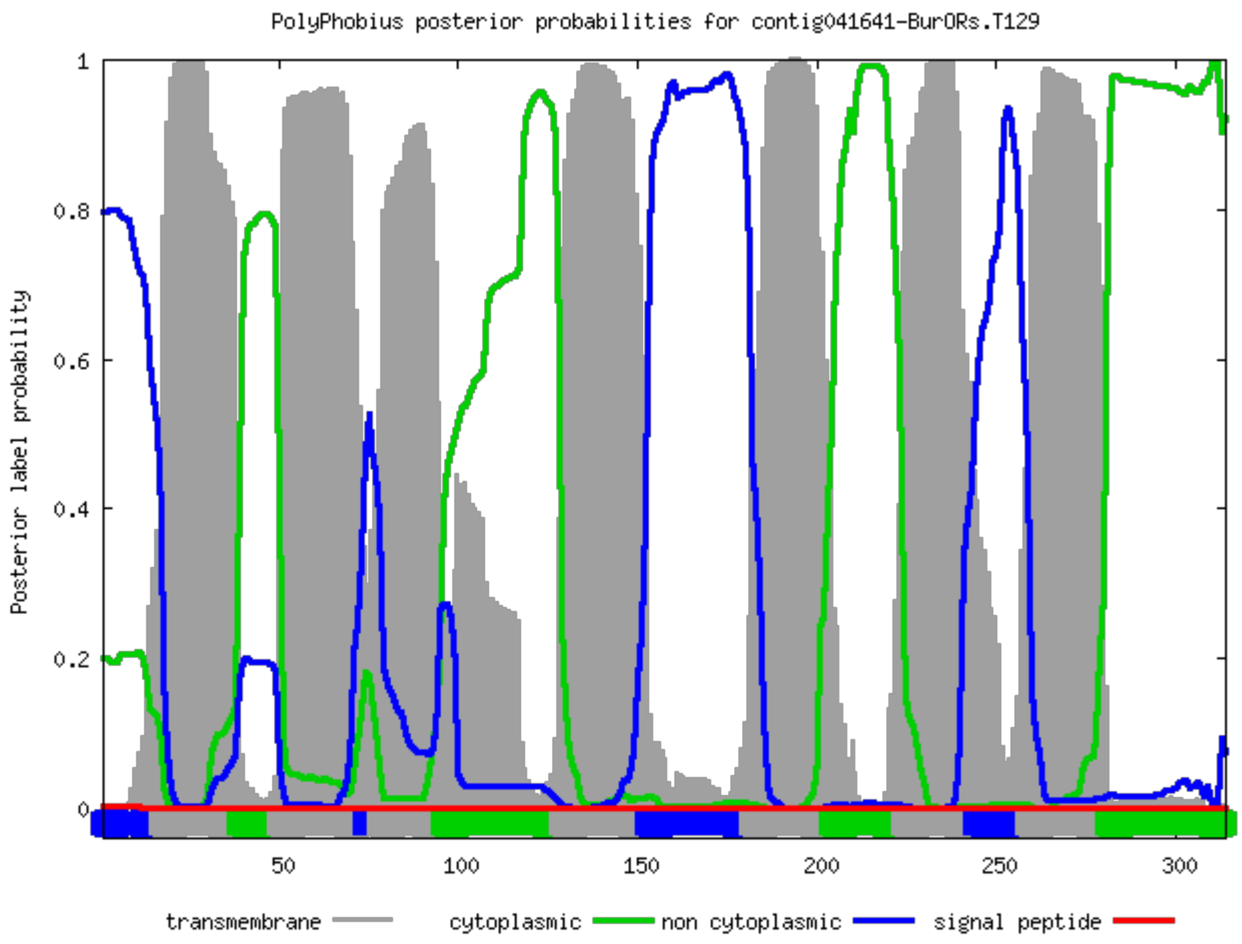

The prediction is based on an alignment. The probability data used in the plot is found here, and the gnuplot script is here.

Prediction of contig050422-ZebOR.Q143

```
ID   contig050422-ZebOR.Q143
FT   TOPO_DOM      1     17       NON CYTOPLASMIC.
FT   TRANSMEM     18     40
FT   TOPO_DOM     41     51       CYTOPLASMIC.
FT   TRANSMEM     52     76
FT   TOPO_DOM     77     81       NON CYTOPLASMIC.
FT   TRANSMEM     82    110
FT   TOPO_DOM    111    130       CYTOPLASMIC.
FT   TRANSMEM    131    154
FT   TOPO_DOM    155    183       NON CYTOPLASMIC.
FT   TRANSMEM    184    206
FT   TOPO_DOM    207    224       CYTOPLASMIC.
FT   TRANSMEM    225    246
FT   TOPO_DOM    247    259       NON CYTOPLASMIC.
FT   TRANSMEM    260    281
FT   TOPO_DOM    282    310       CYTOPLASMIC.
//
```

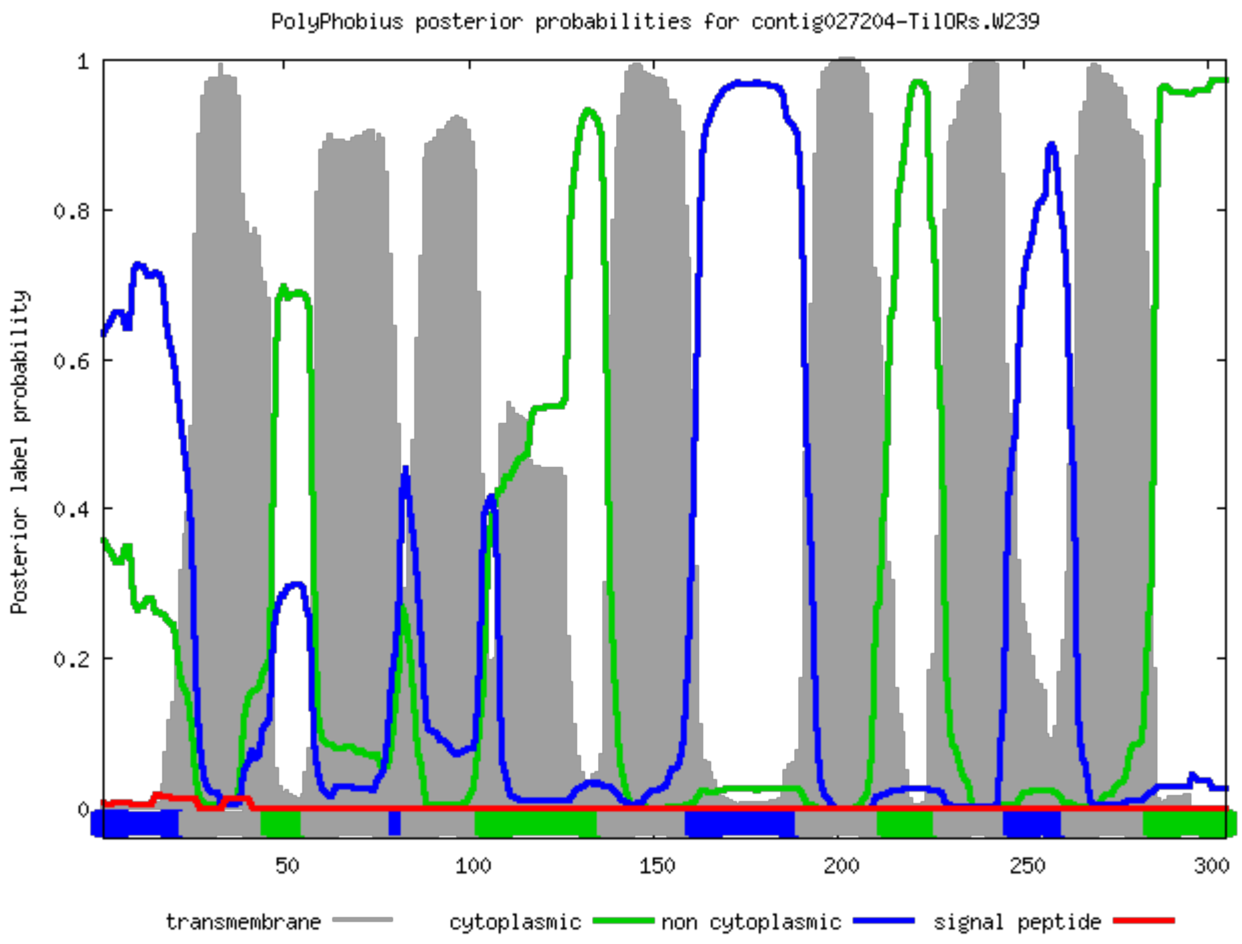

The prediction is based on an alignment. The probability data used in the plot is found here, and the gnuplot script is here.

Prediction of contig063829-BriOR.Q117

```
ID   contig063829-BriOR.Q117
FT   TOPO_DOM      1     17       NON CYTOPLASMIC.
FT   TRANSMEM     18     40
FT   TOPO_DOM     41     51       CYTOPLASMIC.
FT   TRANSMEM     52     76
FT   TOPO_DOM     77     81       NON CYTOPLASMIC.
FT   TRANSMEM     82    110
FT   TOPO_DOM    111    130       CYTOPLASMIC.
FT   TRANSMEM    131    154
FT   TOPO_DOM    155    183       NON CYTOPLASMIC.
FT   TRANSMEM    184    206
FT   TOPO_DOM    207    224       CYTOPLASMIC.
FT   TRANSMEM    225    246
FT   TOPO_DOM    247    259       NON CYTOPLASMIC.
FT   TRANSMEM    260    281
FT   TOPO_DOM    282    310       CYTOPLASMIC.
//
```

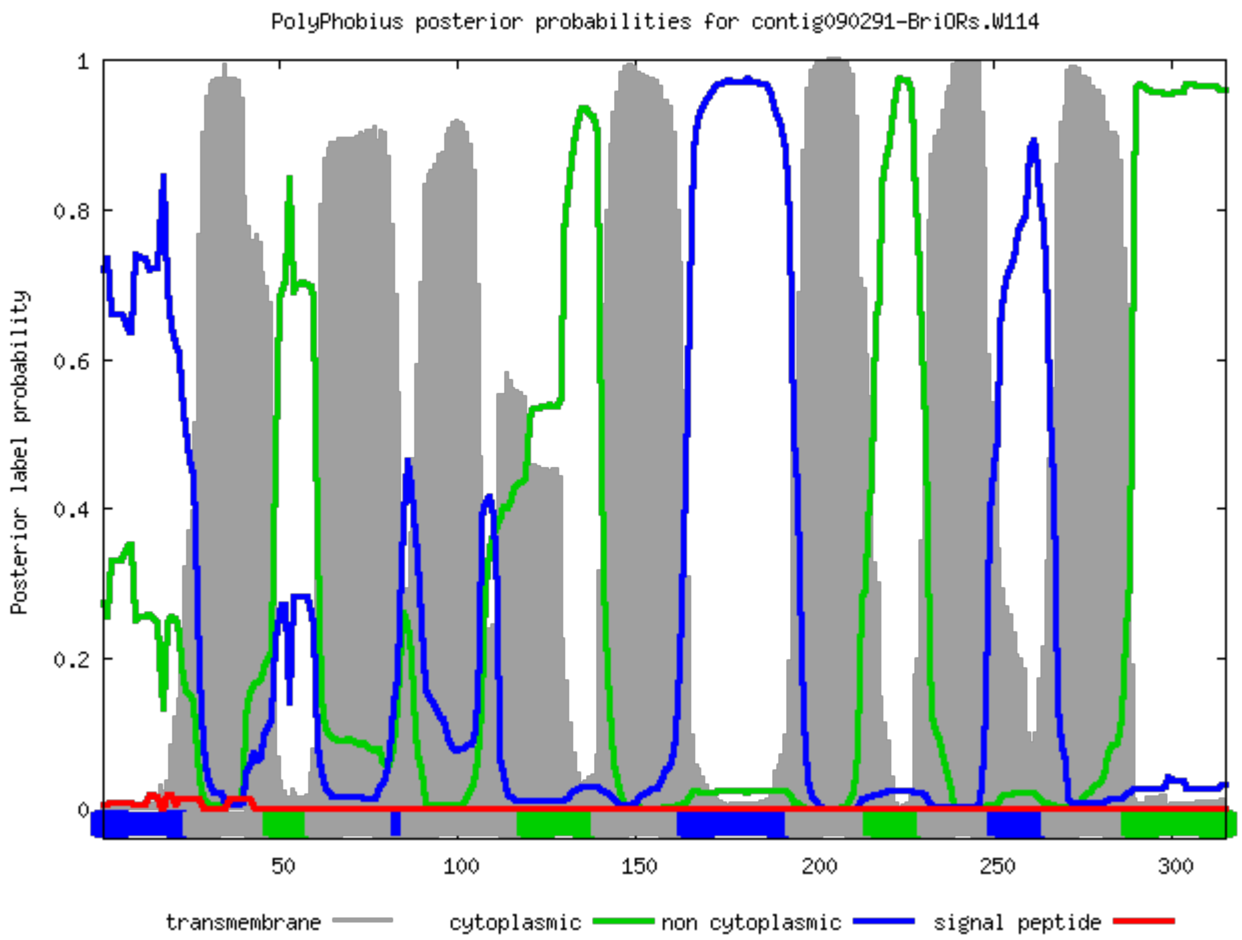

The prediction is based on an alignment. The probability data used in the plot is found here, and the gnuplot script is here.

Prediction of contig030553-ZebOR.A002

```
ID   contig030553-ZebOR.A002
FT   TOPO_DOM      1      22       NON CYTOPLASMIC.
FT   TRANSMEM     23      48
FT   TOPO_DOM     49      56       CYTOPLASMIC.
FT   TRANSMEM     57      77
FT   TOPO_DOM     78      95       NON CYTOPLASMIC.
FT   TRANSMEM     96     118
FT   TOPO_DOM    119     138       CYTOPLASMIC.
FT   TRANSMEM    139     159
FT   TOPO_DOM    160     192       NON CYTOPLASMIC.
FT   TRANSMEM    193     215
FT   TOPO_DOM    216     235       CYTOPLASMIC.
FT   TRANSMEM    236     257
FT   TOPO_DOM    258     268       NON CYTOPLASMIC.
FT   TRANSMEM    269     289
FT   TOPO_DOM    290     307       CYTOPLASMIC.
//
```

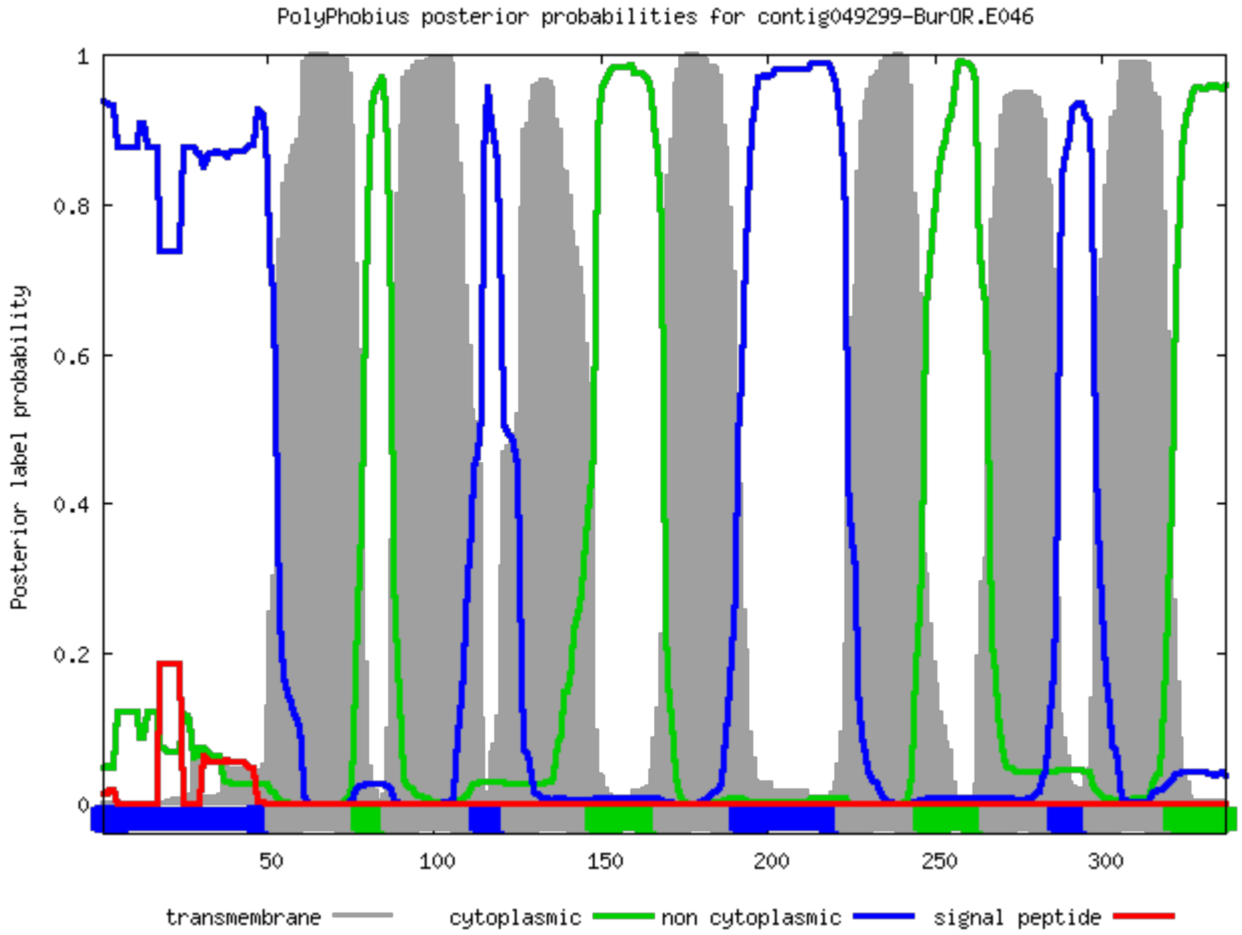

The prediction is based on an alignment. The probability data used in the plot is found here, and the gnuplot script is here.

Prediction of contig022225-TilOR.A005

```
ID   contig022225-TilOR.A005
FT   TOPO_DOM      1      22       NON CYTOPLASMIC.
FT   TRANSMEM     23      48
FT   TOPO_DOM     49      56       CYTOPLASMIC.
FT   TRANSMEM     57      77
FT   TOPO_DOM     78      95       NON CYTOPLASMIC.
FT   TRANSMEM     96     118
FT   TOPO_DOM    119     138       CYTOPLASMIC.
FT   TRANSMEM    139     159
FT   TOPO_DOM    160     192       NON CYTOPLASMIC.
FT   TRANSMEM    193     215
FT   TOPO_DOM    216     235       CYTOPLASMIC.
FT   TRANSMEM    236     257
FT   TOPO_DOM    258     268       NON CYTOPLASMIC.
FT   TRANSMEM    269     289
FT   TOPO_DOM    290     303       CYTOPLASMIC.
//
```

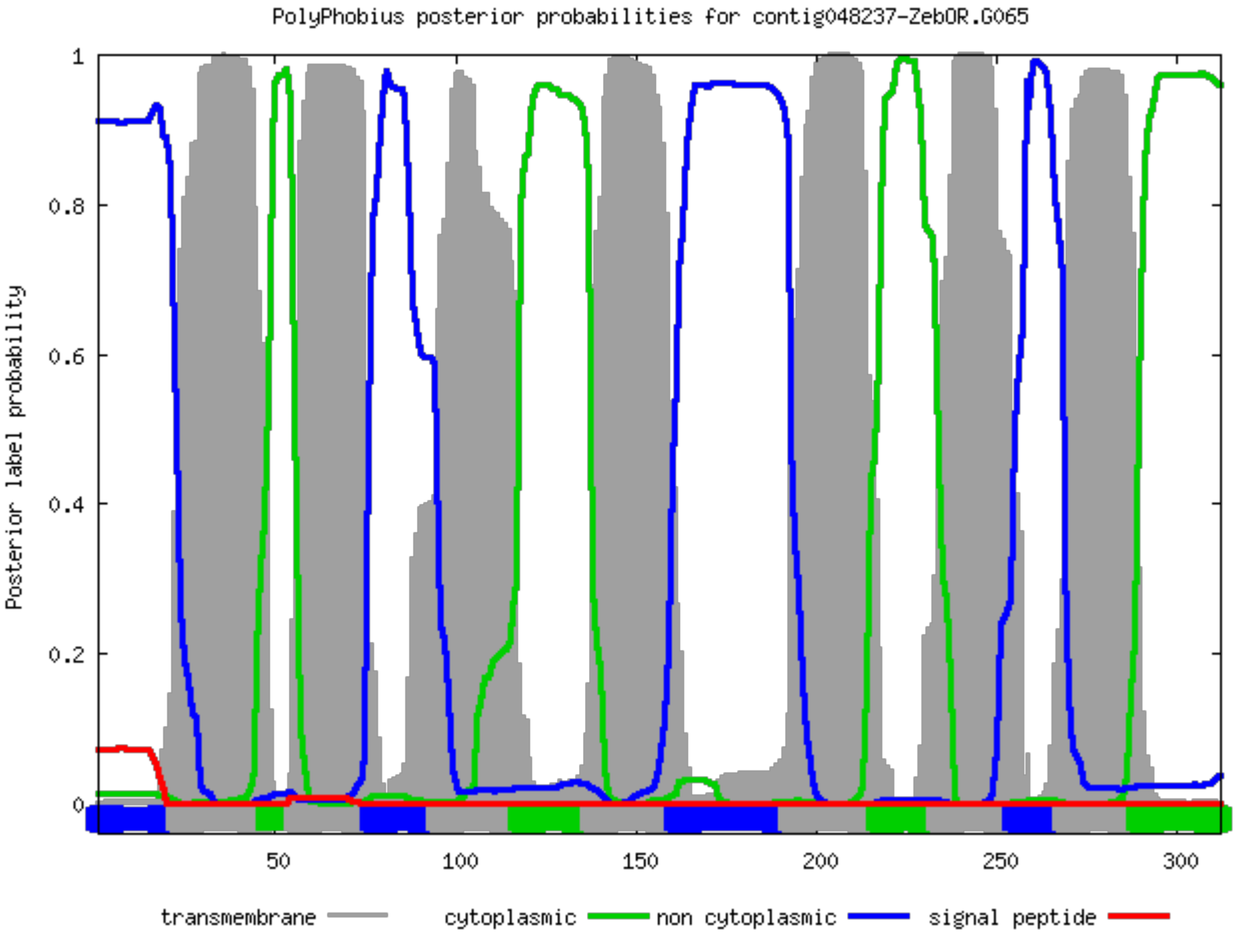

The prediction is based on an alignment. The probability data used in the plot is found here, and the gnuplot script is here.

Prediction of contig022245-TilOR.A012

```
ID   contig022245-TilOR.A012
FT   TOPO_DOM      1      22       NON CYTOPLASMIC.
FT   TRANSMEM     23      48
FT   TOPO_DOM     49      56       CYTOPLASMIC.
FT   TRANSMEM     57      76
FT   TOPO_DOM     77      95       NON CYTOPLASMIC.
FT   TRANSMEM     96     118
FT   TOPO_DOM    119     138       CYTOPLASMIC.
FT   TRANSMEM    139     159
FT   TOPO_DOM    160     192       NON CYTOPLASMIC.
FT   TRANSMEM    193     215
FT   TOPO_DOM    216     235       CYTOPLASMIC.
FT   TRANSMEM    236     257
FT   TOPO_DOM    258     268       NON CYTOPLASMIC.
FT   TRANSMEM    269     289
FT   TOPO_DOM    290     316       CYTOPLASMIC.
//
```

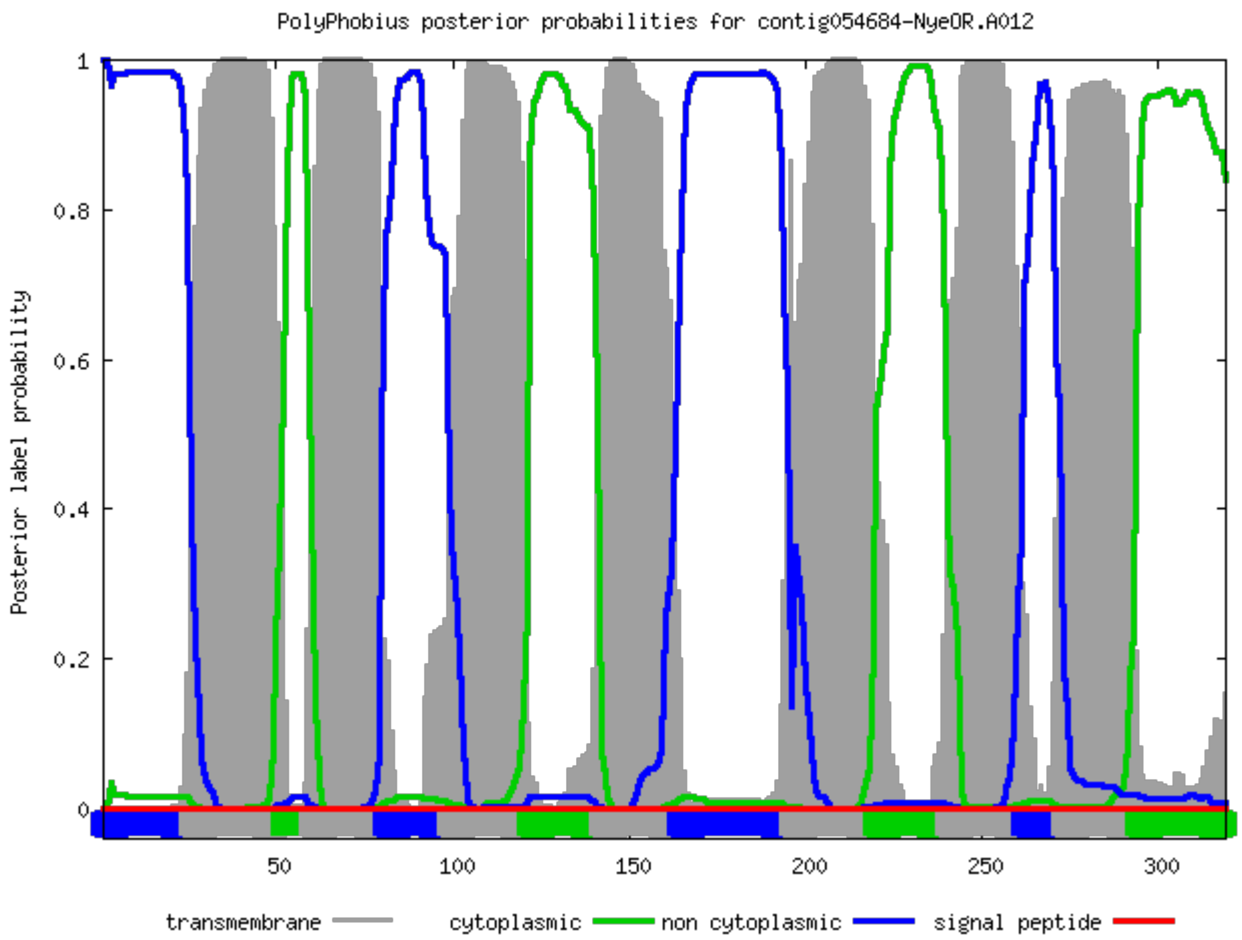

The prediction is based on an alignment. The probability data used in the plot is found here, and the gnuplot script is here.

Prediction of contig039425-TilOR.S225

```
ID   contig039425-TilOR.S225
FT   TOPO_DOM      1     20       NON CYTOPLASMIC.
FT   TRANSMEM     21     42
FT   TOPO_DOM     43     53       CYTOPLASMIC.
FT   TRANSMEM     54     77
FT   TOPO_DOM     78     82       NON CYTOPLASMIC.
FT   TRANSMEM     83    111
FT   TOPO_DOM    112    131       CYTOPLASMIC.
FT   TRANSMEM    132    157
FT   TOPO_DOM    158    185       NON CYTOPLASMIC.
FT   TRANSMEM    186    206
FT   TOPO_DOM    207    226       CYTOPLASMIC.
FT   TRANSMEM    227    252
FT   TOPO_DOM    253    263       NON CYTOPLASMIC.
FT   TRANSMEM    264    284
FT   TOPO_DOM    285    305       CYTOPLASMIC.
//
```

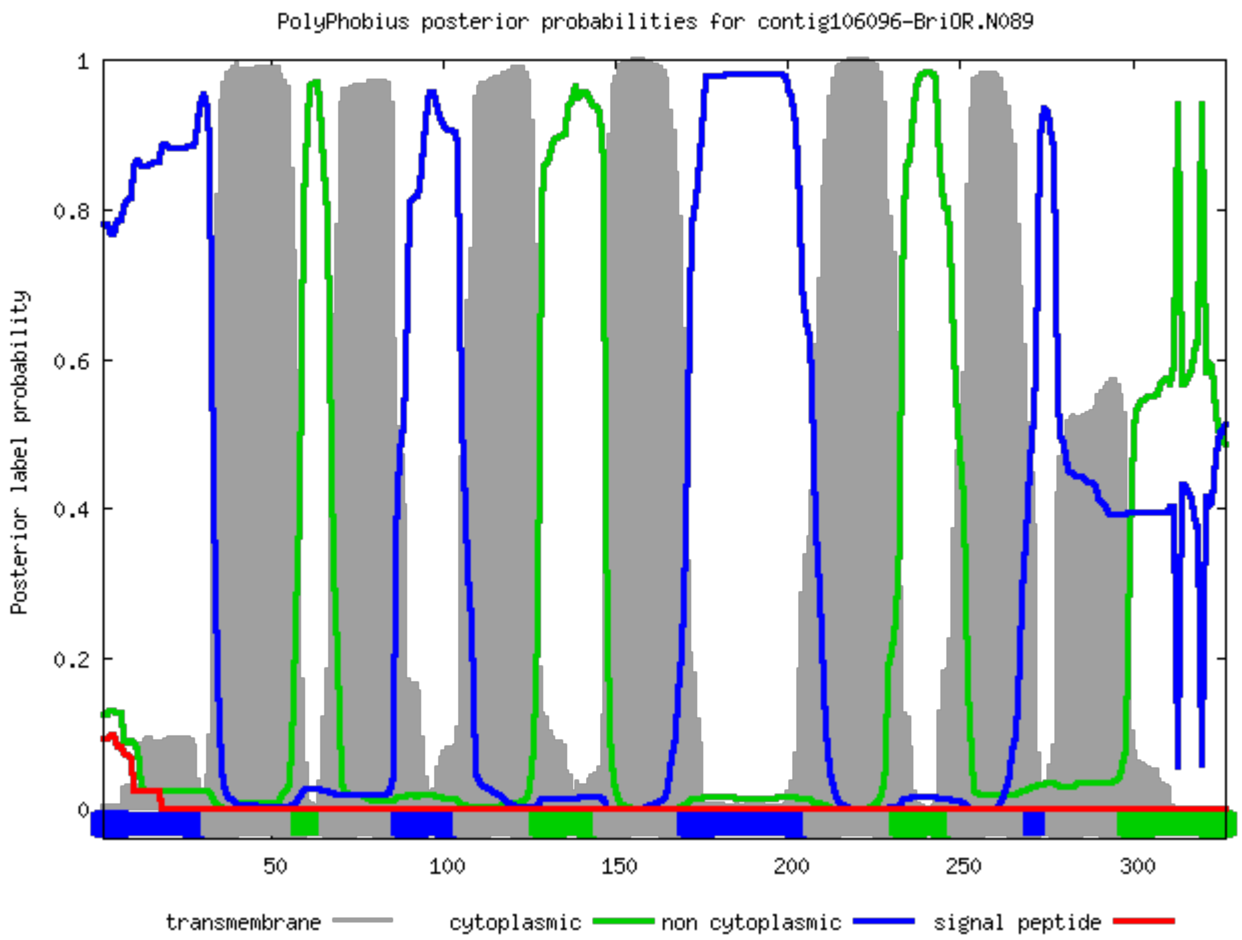

The prediction is based on an alignment. The probability data used in the plot is found here, and the gnuplot script is here.

Prediction of contig018437-ZebOR.H072

```
ID    contig018437-ZebOR.H072
FT    TOPO_DOM      1      23       NON CYTOPLASMIC.
FT    TRANSMEM     24      49
FT    TOPO_DOM     50      56       CYTOPLASMIC.
FT    TRANSMEM     57      76
FT    TOPO_DOM     77      95       NON CYTOPLASMIC.
FT    TRANSMEM     96     118
FT    TOPO_DOM    119     138       CYTOPLASMIC.
FT    TRANSMEM    139     160
FT    TOPO_DOM    161     196       NON CYTOPLASMIC.
FT    TRANSMEM    197     219
FT    TOPO_DOM    220     237       CYTOPLASMIC.
FT    TRANSMEM    238     259
FT    TOPO_DOM    260     271       NON CYTOPLASMIC.
FT    TRANSMEM    272     291
FT    TOPO_DOM    292     324       CYTOPLASMIC.
//
```

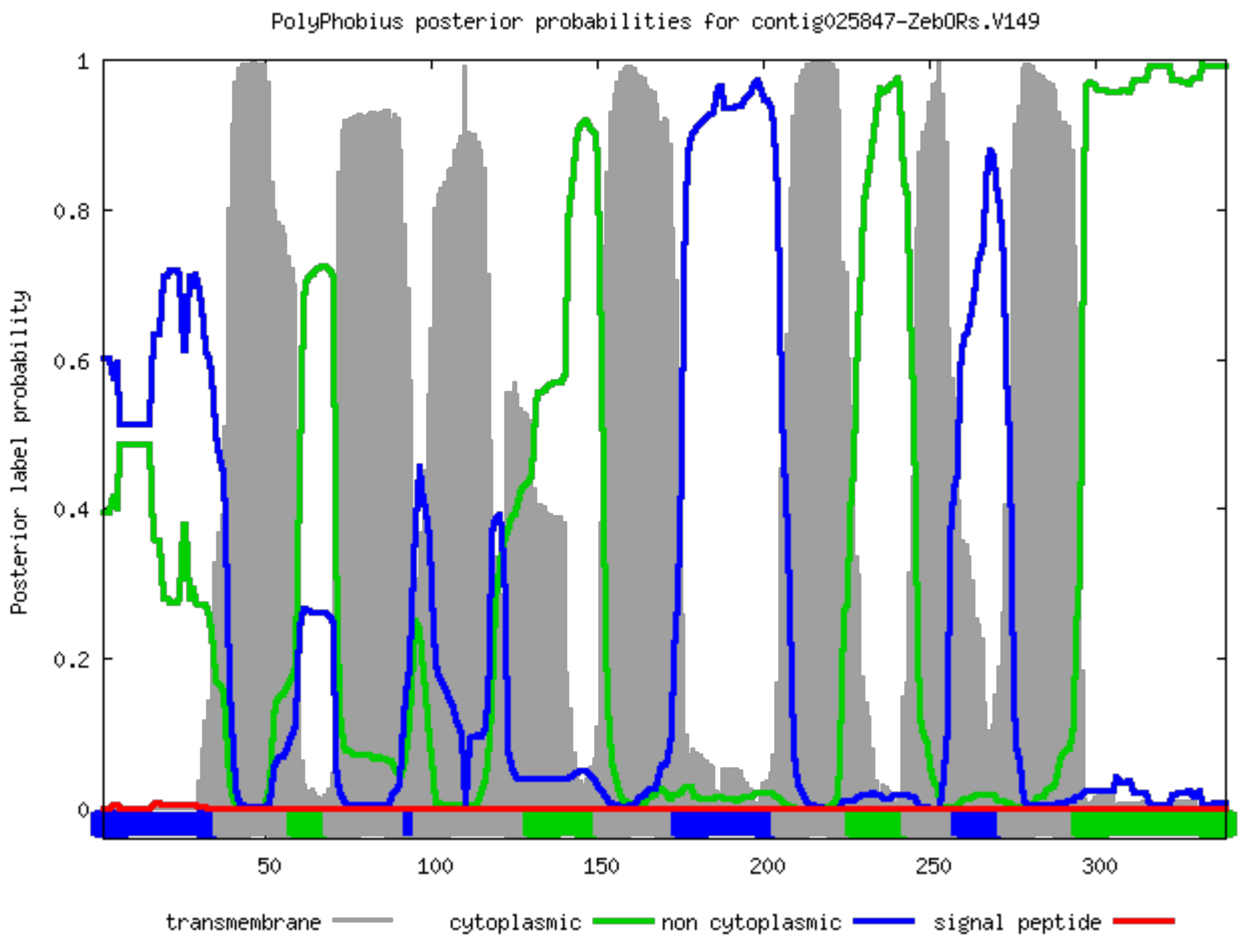

The prediction is based on an alignment. The probability data used in the plot is found here, and the gnuplot script is here.

Prediction of contig020445-ZebOR.L096

```
ID   contig020445-ZebOR.L096
FT   TOPO_DOM      1     25       NON CYTOPLASMIC.
FT   TRANSMEM     26     50
FT   TOPO_DOM     51     59       CYTOPLASMIC.
FT   TRANSMEM     60     86
FT   TOPO_DOM     87    100       NON CYTOPLASMIC.
FT   TRANSMEM    101    120
FT   TOPO_DOM    121    140       CYTOPLASMIC.
FT   TRANSMEM    141    163
FT   TOPO_DOM    164    199       NON CYTOPLASMIC.
FT   TRANSMEM    200    224
FT   TOPO_DOM    225    238       CYTOPLASMIC.
FT   TRANSMEM    239    260
FT   TOPO_DOM    261    271       NON CYTOPLASMIC.
FT   TRANSMEM    272    292
FT   TOPO_DOM    293    313       CYTOPLASMIC.
//
```

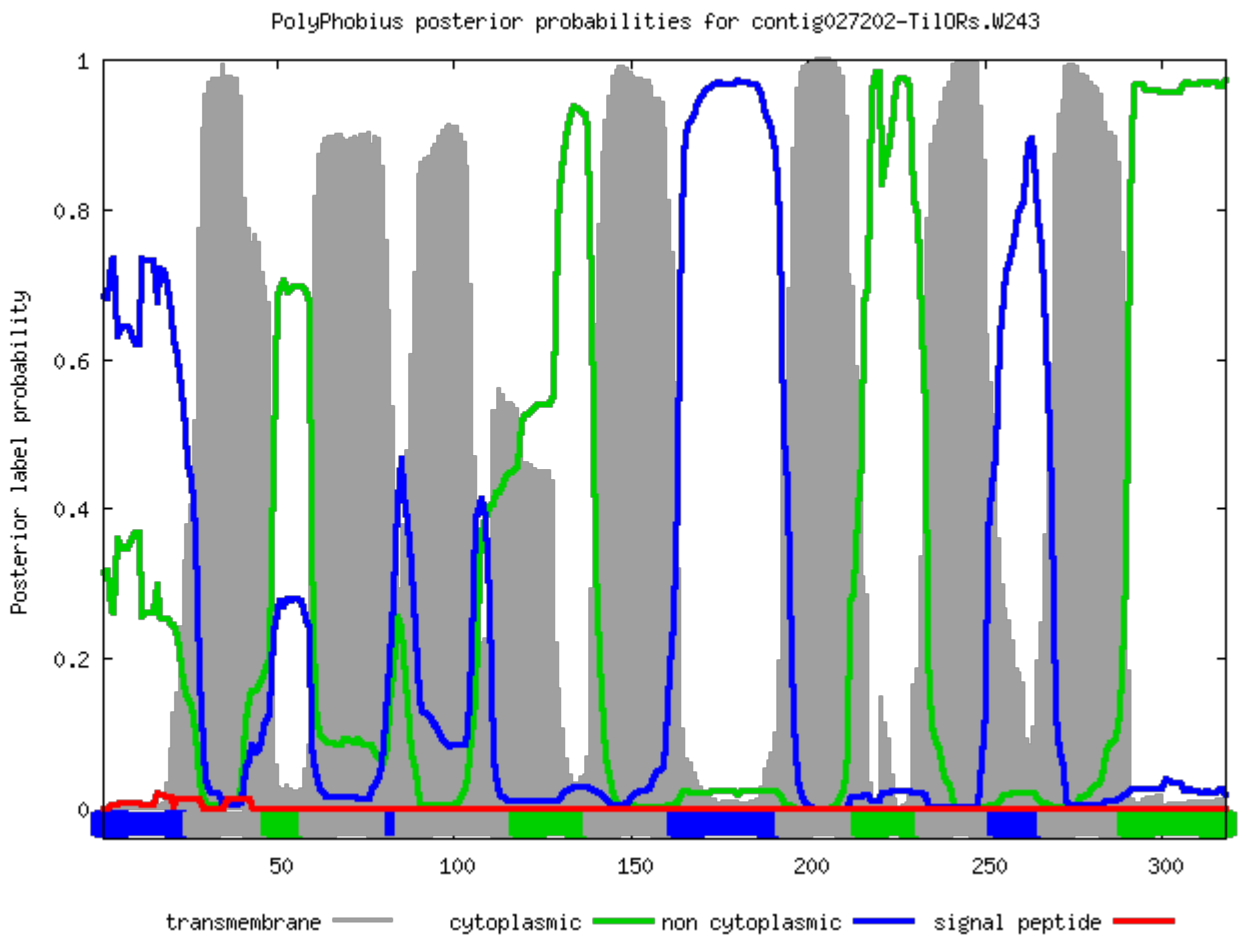

The prediction is based on an alignment. The probability data used in the plot is found here, and the gnuplot script is here.

Prediction of contig009548-TilOR.H102

```
ID    contig009548-TilOR.H102
FT    TOPO_DOM      1      22       NON CYTOPLASMIC.
FT    TRANSMEM     23      49
FT    TOPO_DOM     50      56       CYTOPLASMIC.
FT    TRANSMEM     57      76
FT    TOPO_DOM     77      95       NON CYTOPLASMIC.
FT    TRANSMEM     96     118
FT    TOPO_DOM    119     138       CYTOPLASMIC.
FT    TRANSMEM    139     160
FT    TOPO_DOM    161     196       NON CYTOPLASMIC.
FT    TRANSMEM    197     219
FT    TOPO_DOM    220     237       CYTOPLASMIC.
FT    TRANSMEM    238     260
FT    TOPO_DOM    261     271       NON CYTOPLASMIC.
FT    TRANSMEM    272     291
FT    TOPO_DOM    292     310       CYTOPLASMIC.
//
```

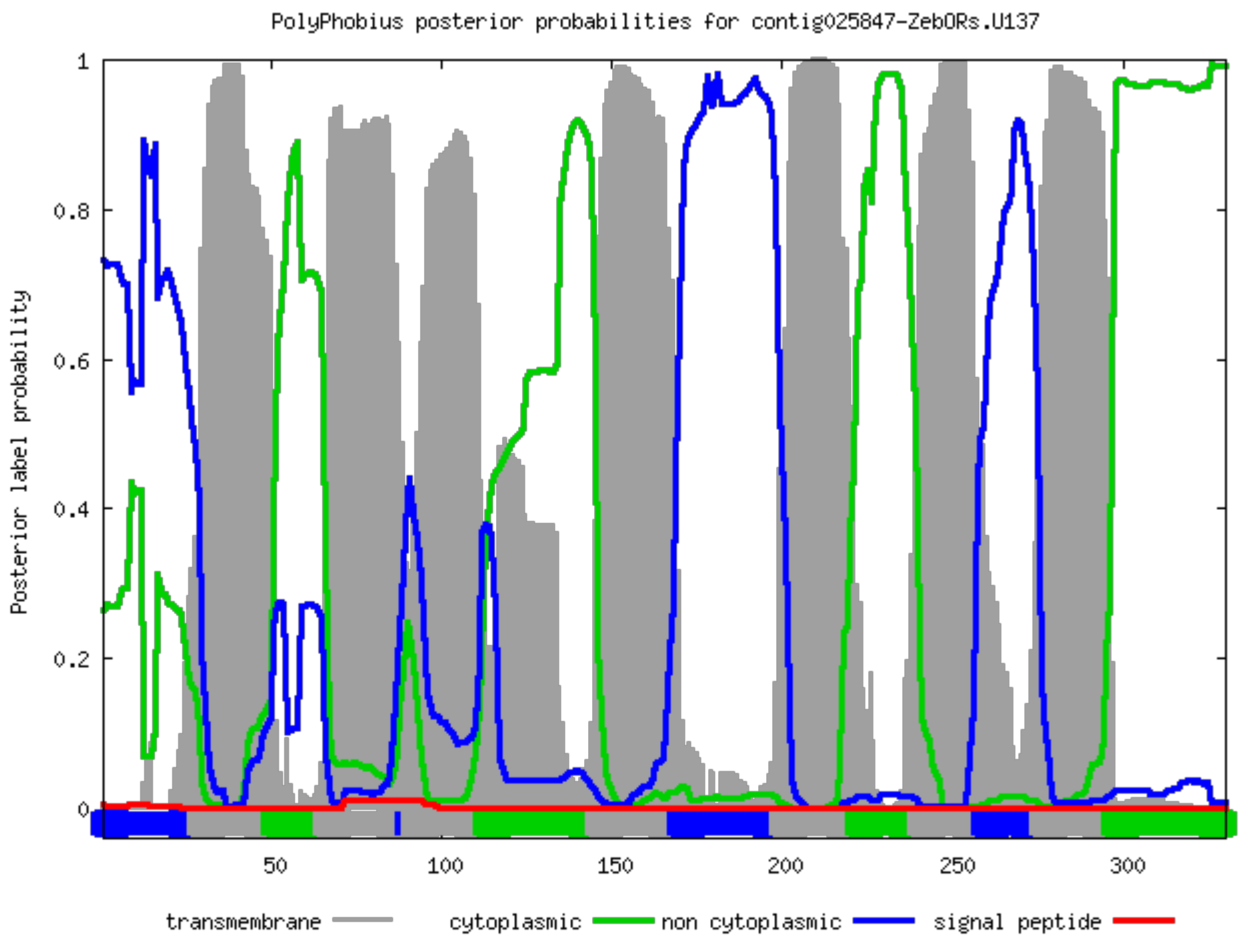

The prediction is based on an alignment. The probability data used in the plot is found here, and the gnuplot script is here.

Prediction of contig030553-ZebOR.A003

```
ID   contig030553-ZebOR.A003
FT   TOPO_DOM      1     22       NON CYTOPLASMIC.
FT   TRANSMEM     23     48
FT   TOPO_DOM     49     56       CYTOPLASMIC.
FT   TRANSMEM     57     77
FT   TOPO_DOM     78     95       NON CYTOPLASMIC.
FT   TRANSMEM     96    118
FT   TOPO_DOM    119    138       CYTOPLASMIC.
FT   TRANSMEM    139    159
FT   TOPO_DOM    160    192       NON CYTOPLASMIC.
FT   TRANSMEM    193    215
FT   TOPO_DOM    216    235       CYTOPLASMIC.
FT   TRANSMEM    236    257
FT   TOPO_DOM    258    267       NON CYTOPLASMIC.
FT   TRANSMEM    268    289
FT   TOPO_DOM    290    309       CYTOPLASMIC.
//
```

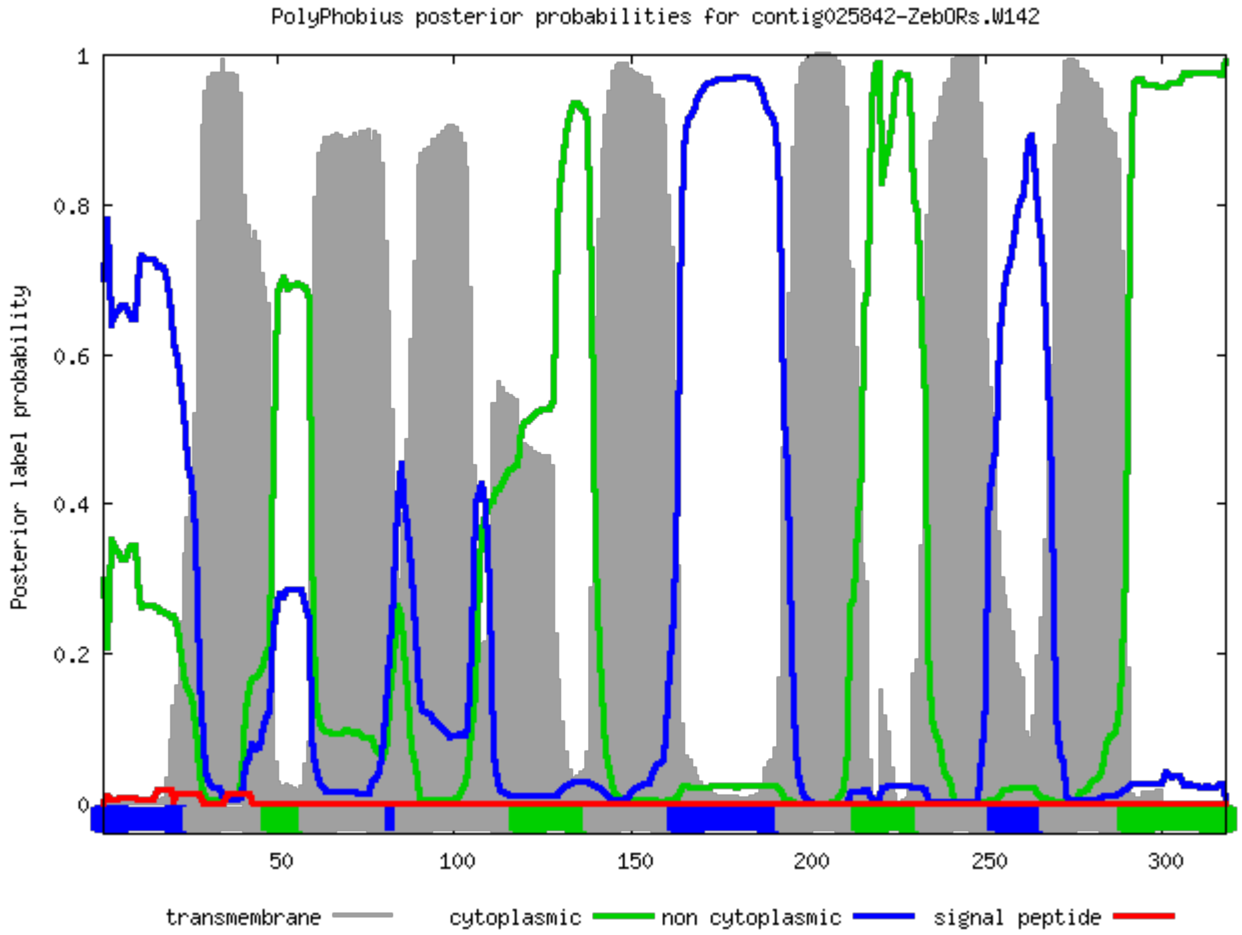

The prediction is based on an alignment. The probability data used in the plot is found here, and the gnuplot script is here.

Prediction of contig053780-BurOR.H065

```
ID   contig053780-BurOR.H065
FT   TOPO_DOM      1     22       NON CYTOPLASMIC.
FT   TRANSMEM     23     48
FT   TOPO_DOM     49     55       CYTOPLASMIC.
FT   TRANSMEM     56     76
FT   TOPO_DOM     77     94       NON CYTOPLASMIC.
FT   TRANSMEM     95    117
FT   TOPO_DOM    118    137       CYTOPLASMIC.
FT   TRANSMEM    138    159
FT   TOPO_DOM    160    192       NON CYTOPLASMIC.
FT   TRANSMEM    193    216
FT   TOPO_DOM    217    234       CYTOPLASMIC.
FT   TRANSMEM    235    257
FT   TOPO_DOM    258    268       NON CYTOPLASMIC.
FT   TRANSMEM    269    288
FT   TOPO_DOM    289    314       CYTOPLASMIC.
//
```

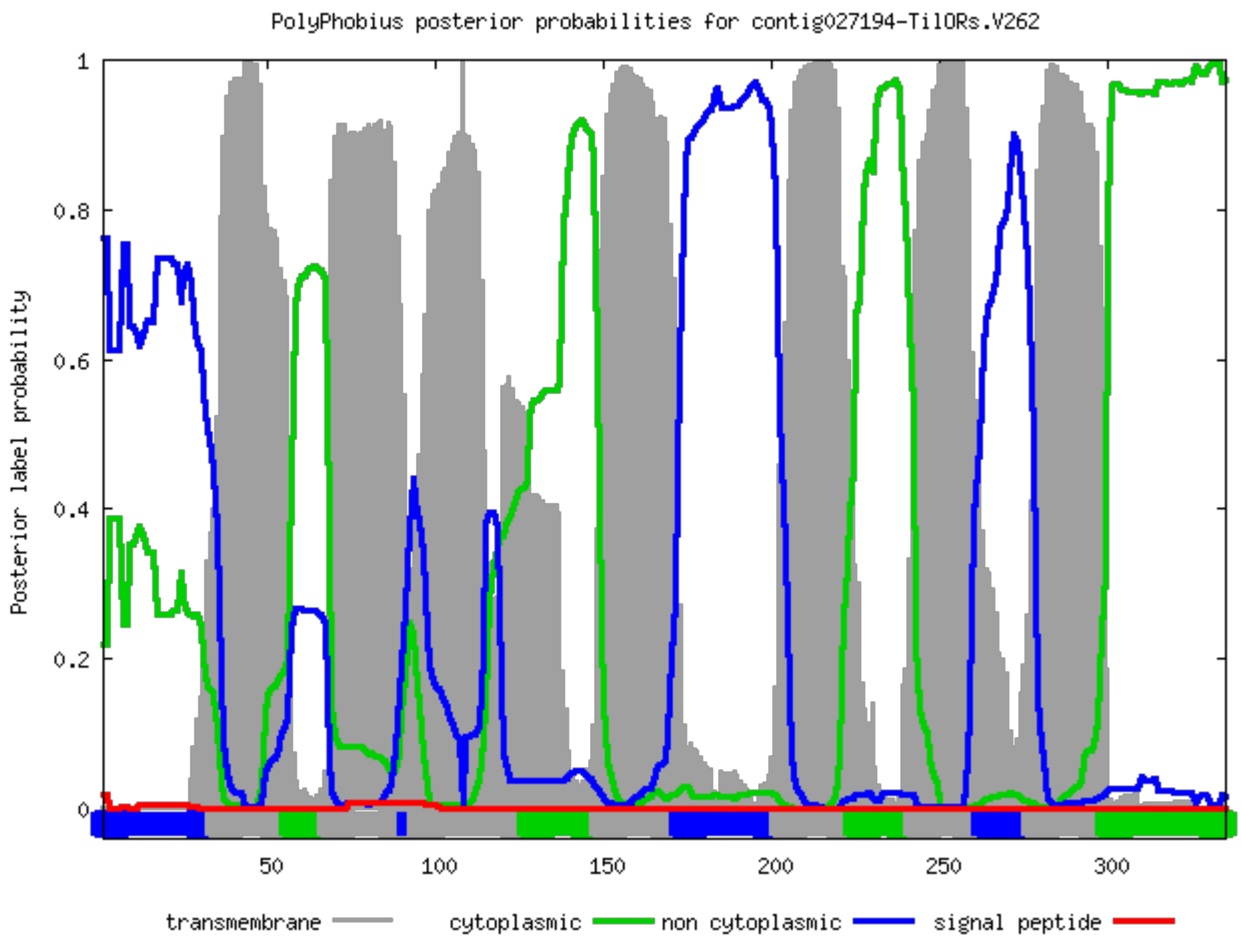

The prediction is based on an alignment. The probability data used in the plot is found here, and the gnuplot script is here.

Prediction of contig025447-ZebOR.E047

ID	contig025447-ZebOR.E047			
FT	TOPO_DOM	1	22	NON CYTOPLASMIC.
FT	TRANSMEM	23	48	
FT	TOPO_DOM	49	57	CYTOPLASMIC.
FT	TRANSMEM	58	83	
FT	TOPO_DOM	84	90	NON CYTOPLASMIC.
FT	TRANSMEM	91	118	
FT	TOPO_DOM	119	138	CYTOPLASMIC.
FT	TRANSMEM	139	161	
FT	TOPO_DOM	162	193	NON CYTOPLASMIC.
FT	TRANSMEM	194	216	
FT	TOPO_DOM	217	236	CYTOPLASMIC.
FT	TRANSMEM	237	256	
FT	TOPO_DOM	257	268	NON CYTOPLASMIC.
FT	TRANSMEM	269	291	
FT	TOPO_DOM	292	310	CYTOPLASMIC.
//				

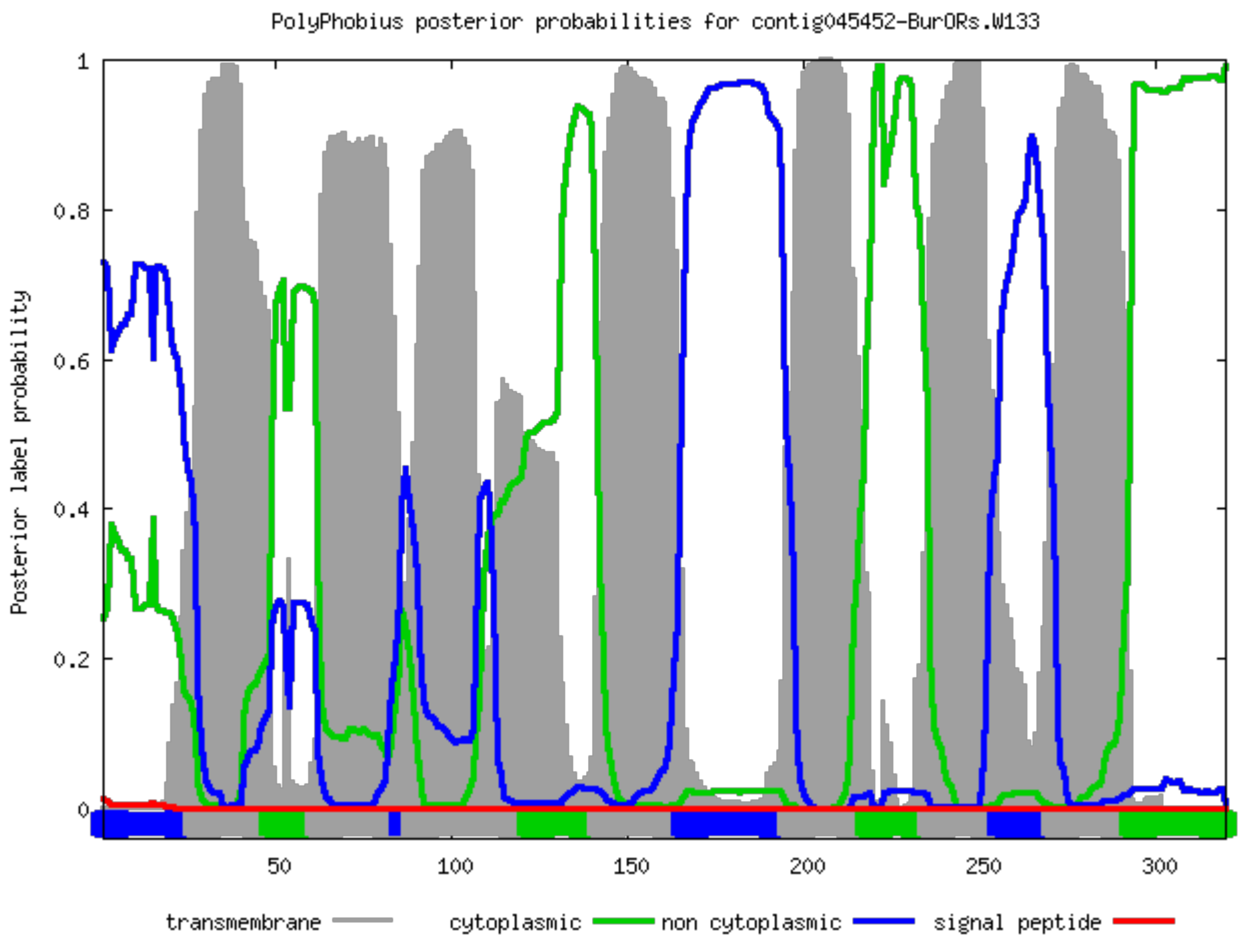

The prediction is based on an alignment. The probability data used in the plot is found here, and the gnuplot script is here.

Prediction of contig047499-ZebOR.A012

```
ID   contig047499-ZebOR.A012
FT   TOPO_DOM      1     22       NON CYTOPLASMIC.
FT   TRANSMEM     23     48
FT   TOPO_DOM     49     56       CYTOPLASMIC.
FT   TRANSMEM     57     76
FT   TOPO_DOM     77     95       NON CYTOPLASMIC.
FT   TRANSMEM     96    118
FT   TOPO_DOM    119    138       CYTOPLASMIC.
FT   TRANSMEM    139    160
FT   TOPO_DOM    161    192       NON CYTOPLASMIC.
FT   TRANSMEM    193    215
FT   TOPO_DOM    216    235       CYTOPLASMIC.
FT   TRANSMEM    236    257
FT   TOPO_DOM    258    268       NON CYTOPLASMIC.
FT   TRANSMEM    269    289
FT   TOPO_DOM    290    302       CYTOPLASMIC.
FT   TRANSMEM    303    317
FT   TOPO_DOM    318    320       NON CYTOPLASMIC.
//
```

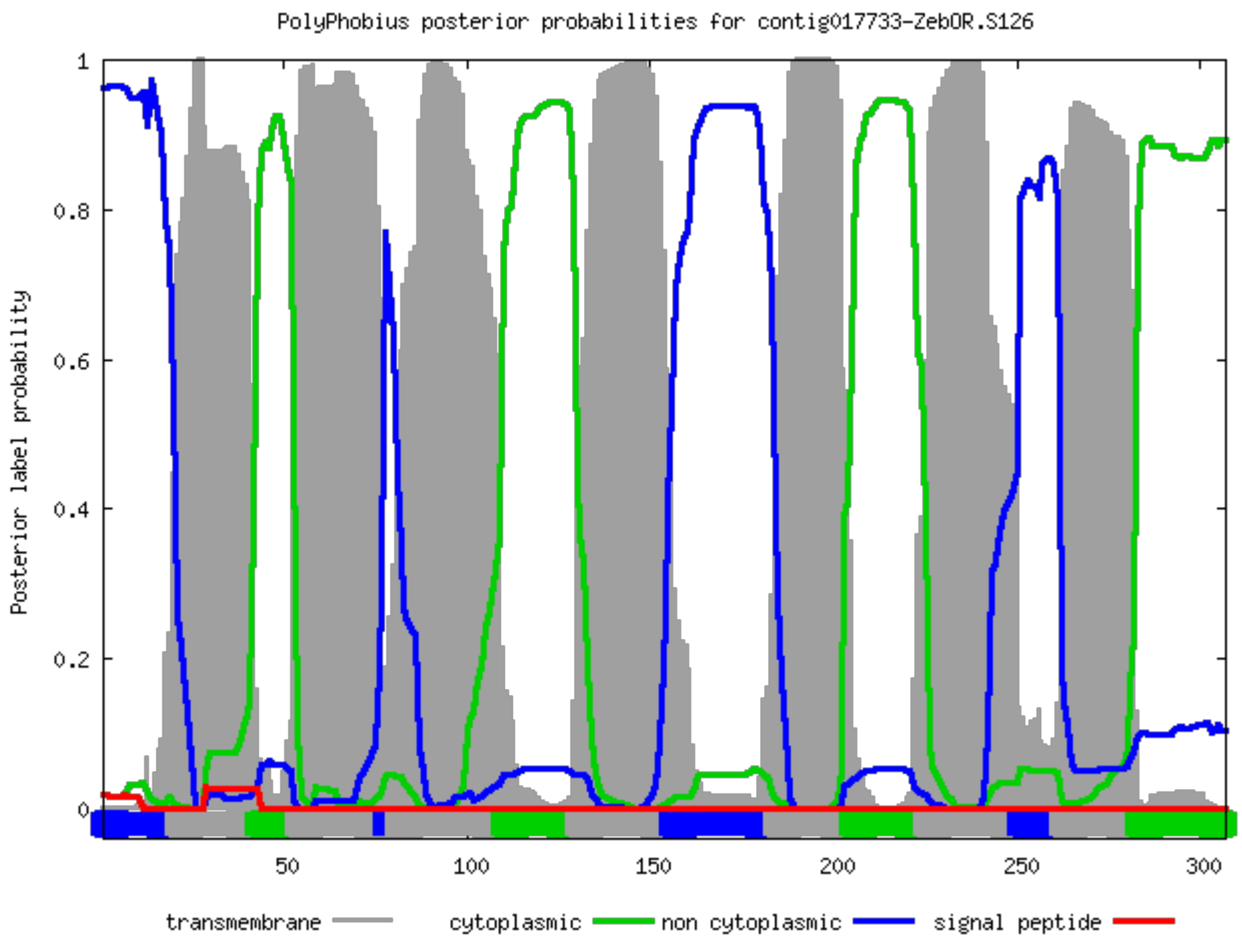

The prediction is based on an alignment. The probability data used in the plot is found here, and the gnuplot script is here.

Prediction of contig039436-TilOR.S230

```
ID   contig039436-TilOR.S230
FT   TOPO_DOM      1     21       NON CYTOPLASMIC.
FT   TRANSMEM     22     43
FT   TOPO_DOM     44     53       CYTOPLASMIC.
FT   TRANSMEM     54     78
FT   TOPO_DOM     79     83       NON CYTOPLASMIC.
FT   TRANSMEM     84    112
FT   TOPO_DOM    113    132       CYTOPLASMIC.
FT   TRANSMEM    133    158
FT   TOPO_DOM    159    186       NON CYTOPLASMIC.
FT   TRANSMEM    187    207
FT   TOPO_DOM    208    227       CYTOPLASMIC.
FT   TRANSMEM    228    253
FT   TOPO_DOM    254    264       NON CYTOPLASMIC.
FT   TRANSMEM    265    285
FT   TOPO_DOM    286    311       CYTOPLASMIC.
//
```

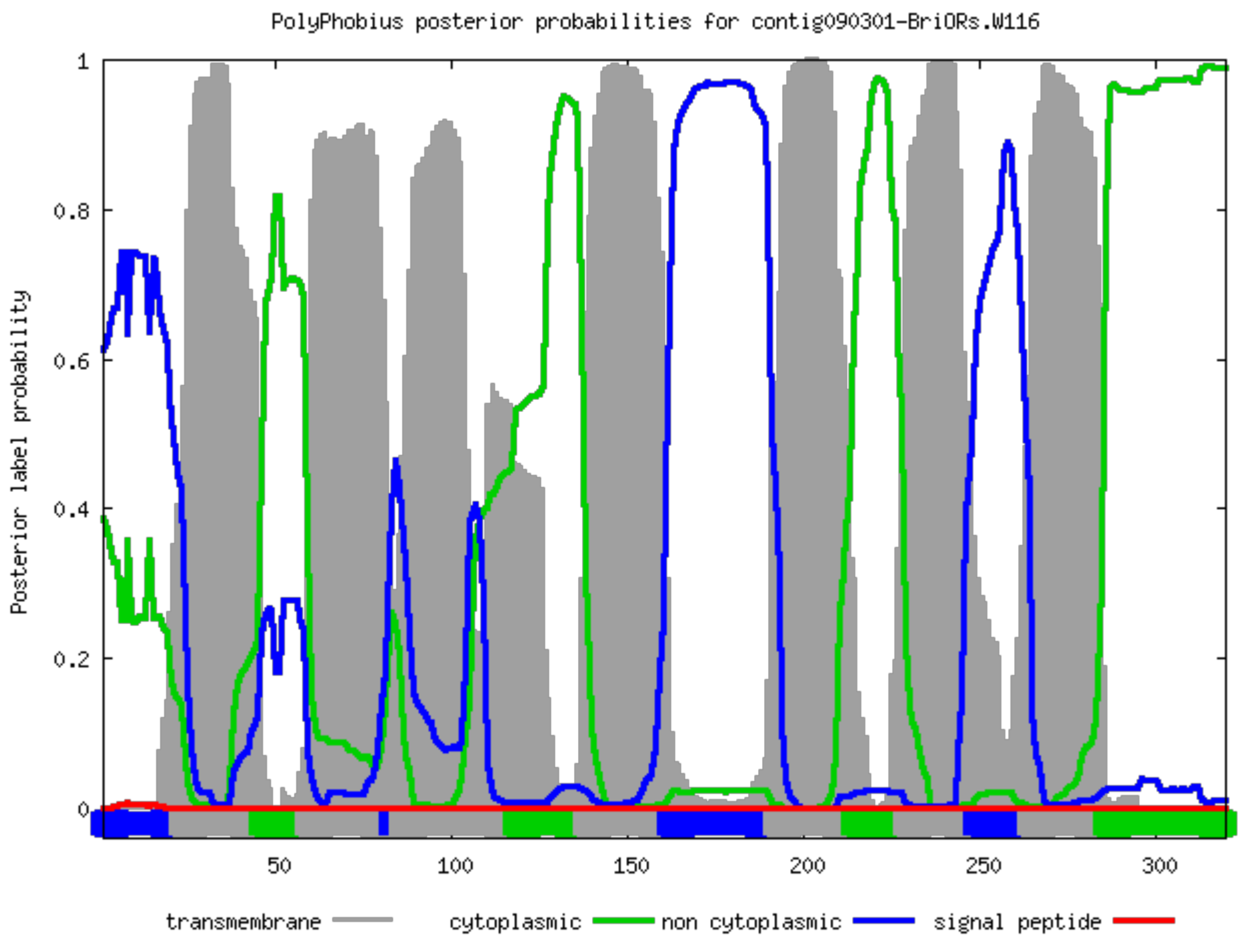

The prediction is based on an alignment. The probability data used in the plot is found here, and the gnuplot script is here.

Prediction of contig075822-TilOR.F098

```
ID    contig075822-TilOR.F098
FT    TOPO_DOM      1      22       NON CYTOPLASMIC.
FT    TRANSMEM     23      47
FT    TOPO_DOM     48      57       CYTOPLASMIC.
FT    TRANSMEM     58      77
FT    TOPO_DOM     78      96       NON CYTOPLASMIC.
FT    TRANSMEM     97     118
FT    TOPO_DOM    119     138       CYTOPLASMIC.
FT    TRANSMEM    139     161
FT    TOPO_DOM    162     193       NON CYTOPLASMIC.
FT    TRANSMEM    194     216
FT    TOPO_DOM    217     236       CYTOPLASMIC.
FT    TRANSMEM    237     257
FT    TOPO_DOM    258     267       NON CYTOPLASMIC.
FT    TRANSMEM    268     291
FT    TOPO_DOM    292     305       CYTOPLASMIC.
//
```

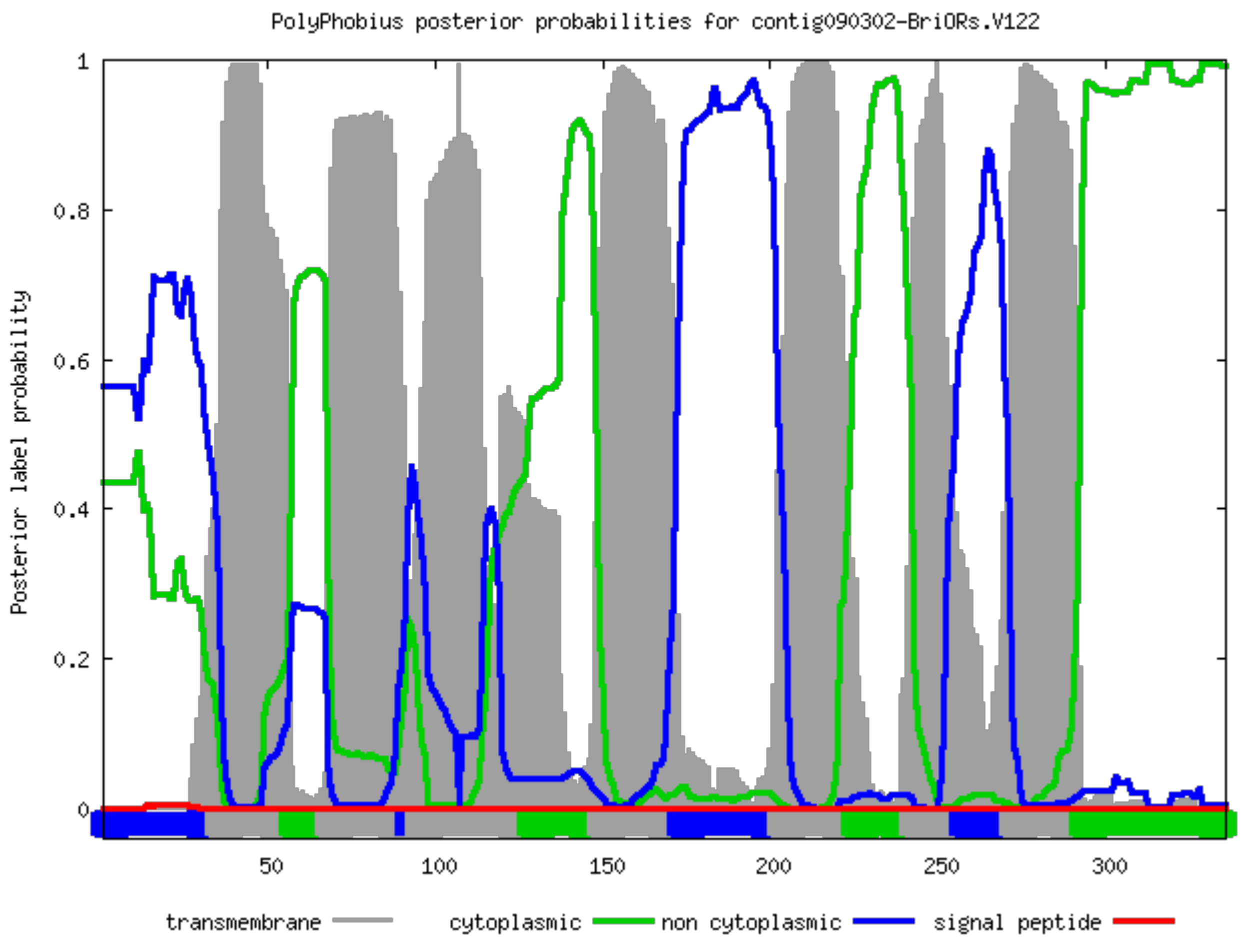

The prediction is based on an alignment. The probability data used in the plot is found here, and the gnuplot script is here.

Prediction of contig051573-BurOR.A011

```
ID   contig051573-BurOR.A011
FT   TOPO_DOM      1     22       NON CYTOPLASMIC.
FT   TRANSMEM     23     48
FT   TOPO_DOM     49     56       CYTOPLASMIC.
FT   TRANSMEM     57     76
FT   TOPO_DOM     77     95       NON CYTOPLASMIC.
FT   TRANSMEM     96    118
FT   TOPO_DOM    119    138       CYTOPLASMIC.
FT   TRANSMEM    139    160
FT   TOPO_DOM    161    192       NON CYTOPLASMIC.
FT   TRANSMEM    193    215
FT   TOPO_DOM    216    235       CYTOPLASMIC.
FT   TRANSMEM    236    257
FT   TOPO_DOM    258    268       NON CYTOPLASMIC.
FT   TRANSMEM    269    289
FT   TOPO_DOM    290    307       CYTOPLASMIC.
//
```

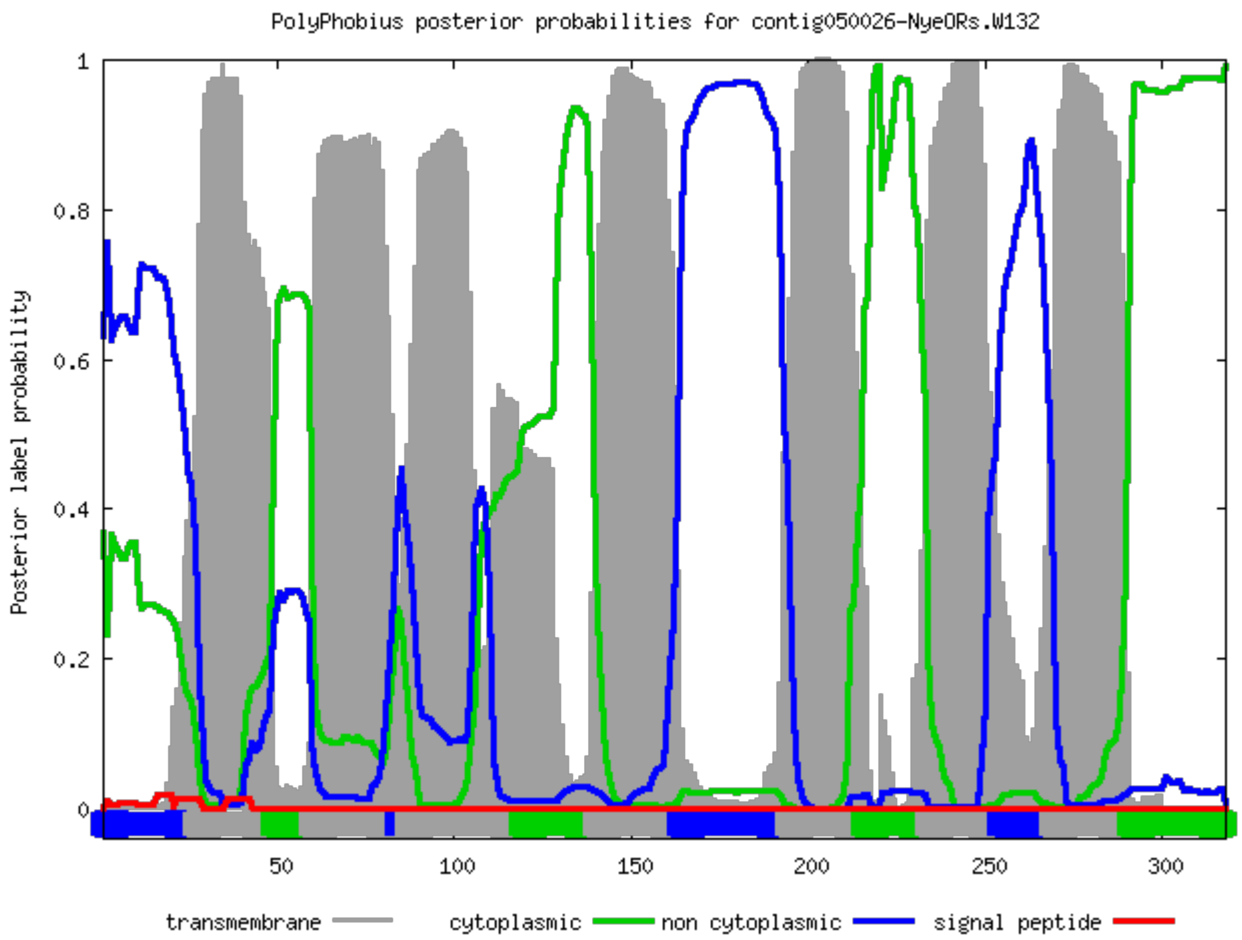

The prediction is based on an alignment. The probability data used in the plot is found here, and the gnuplot script is here.

Prediction of contig067209-BurOR.R140

```
ID   contig067209-BurOR.R140
FT   TOPO_DOM      1     24       NON CYTOPLASMIC.
FT   TRANSMEM     25     48
FT   TOPO_DOM     49     59       CYTOPLASMIC.
FT   TRANSMEM     60     84
FT   TOPO_DOM     85     95       NON CYTOPLASMIC.
FT   TRANSMEM     96    118
FT   TOPO_DOM    119    138       CYTOPLASMIC.
FT   TRANSMEM    139    162
FT   TOPO_DOM    163    194       NON CYTOPLASMIC.
FT   TRANSMEM    195    218
FT   TOPO_DOM    219    235       CYTOPLASMIC.
FT   TRANSMEM    236    259
FT   TOPO_DOM    260    271       NON CYTOPLASMIC.
FT   TRANSMEM    272    293
FT   TOPO_DOM    294    328       CYTOPLASMIC.
//
```

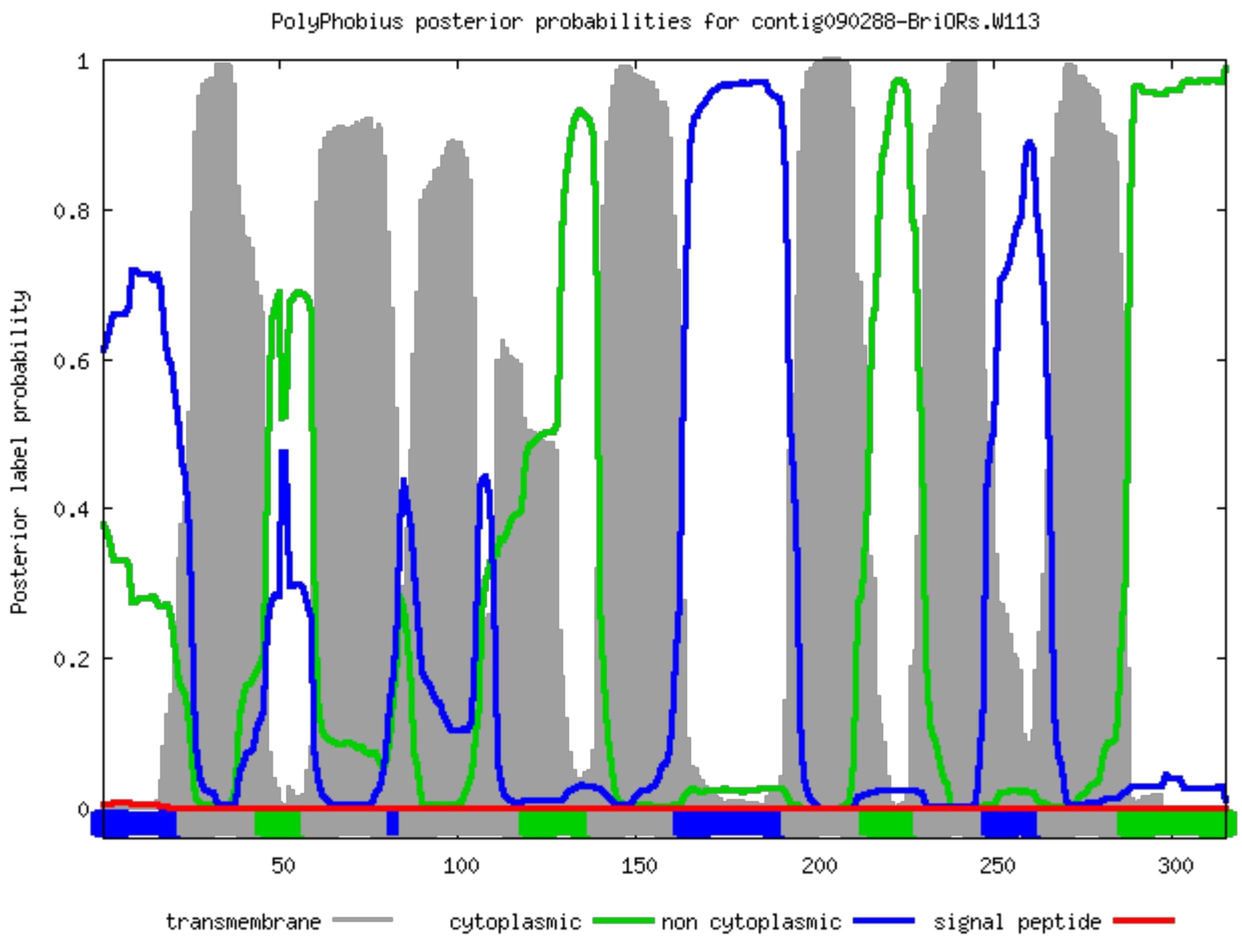

The prediction is based on an alignment. The probability data used in the plot is found here, and the gnuplot script is here.

Prediction of contig056942-NyeOR.L093

```
ID   contig056942-NyeOR.L093
FT   TOPO_DOM      1     25       NON CYTOPLASMIC.
FT   TRANSMEM     26     50
FT   TOPO_DOM     51     59       CYTOPLASMIC.
FT   TRANSMEM     60     83
FT   TOPO_DOM     84    100       NON CYTOPLASMIC.
FT   TRANSMEM    101    120
FT   TOPO_DOM    121    140       CYTOPLASMIC.
FT   TRANSMEM    141    162
FT   TOPO_DOM    163    199       NON CYTOPLASMIC.
FT   TRANSMEM    200    224
FT   TOPO_DOM    225    238       CYTOPLASMIC.
FT   TRANSMEM    239    260
FT   TOPO_DOM    261    271       NON CYTOPLASMIC.
FT   TRANSMEM    272    292
FT   TOPO_DOM    293    313       CYTOPLASMIC.
//
```

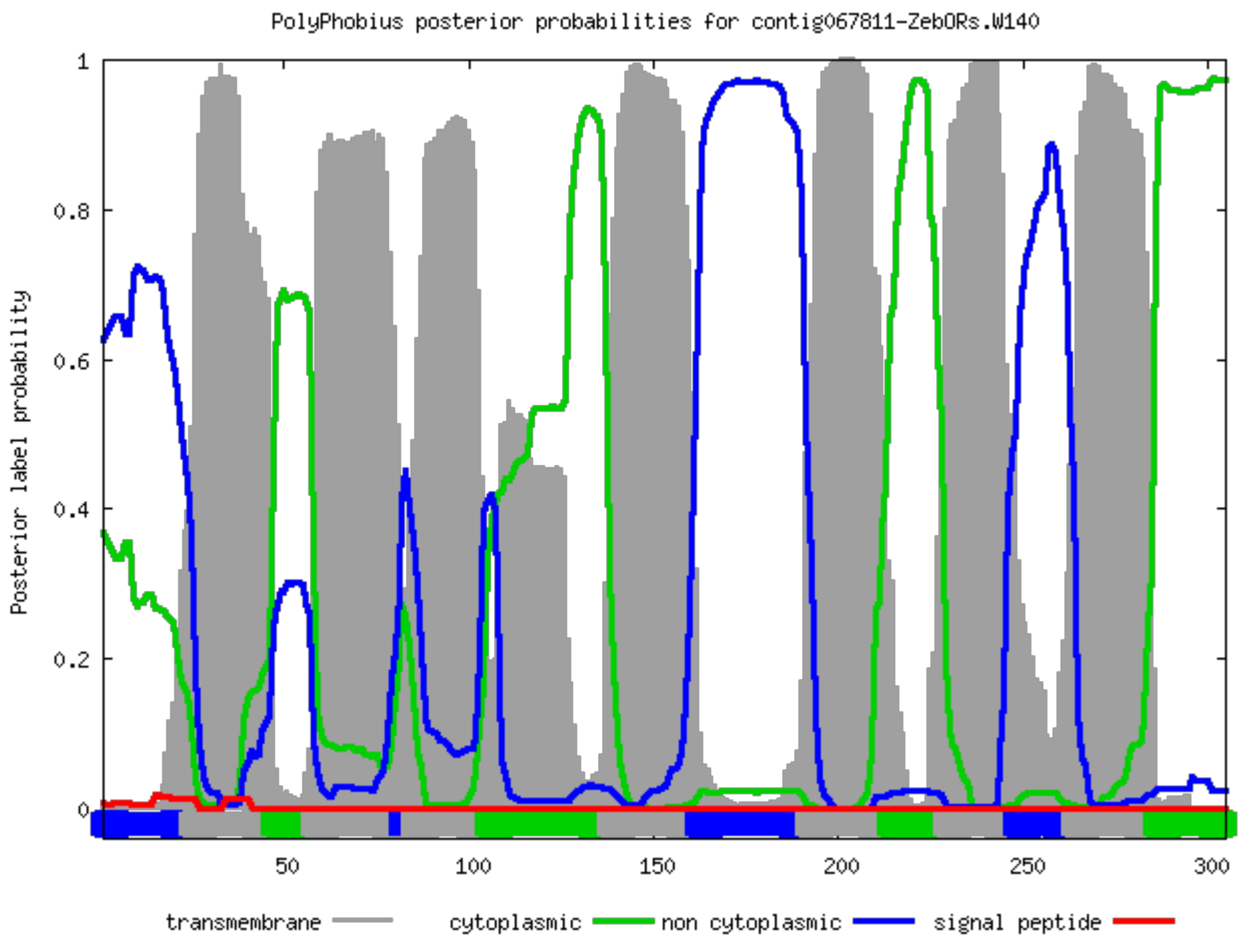

The prediction is based on an alignment. The probability data used in the plot is found here, and the gnuplot script is here.

Prediction of contig107626-BriOR.H054

```
ID   contig107626-BriOR.H054
FT   TOPO_DOM      1     22       NON CYTOPLASMIC.
FT   TRANSMEM     23     48
FT   TOPO_DOM     49     55       CYTOPLASMIC.
FT   TRANSMEM     56     76
FT   TOPO_DOM     77     94       NON CYTOPLASMIC.
FT   TRANSMEM     95    117
FT   TOPO_DOM    118    137       CYTOPLASMIC.
FT   TRANSMEM    138    159
FT   TOPO_DOM    160    192       NON CYTOPLASMIC.
FT   TRANSMEM    193    216
FT   TOPO_DOM    217    234       CYTOPLASMIC.
FT   TRANSMEM    235    257
FT   TOPO_DOM    258    268       NON CYTOPLASMIC.
FT   TRANSMEM    269    288
FT   TOPO_DOM    289    314       CYTOPLASMIC.
//
```

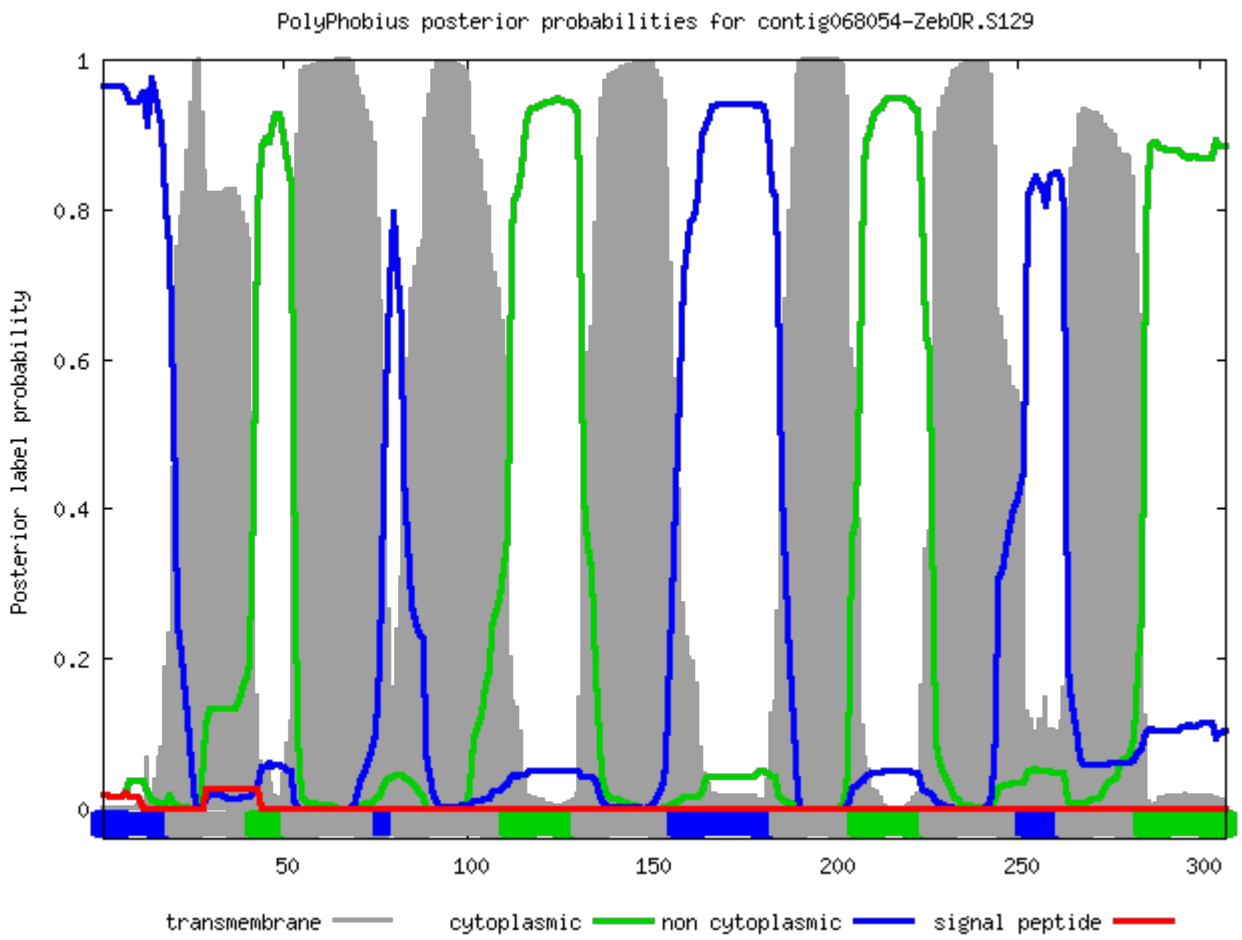

The prediction is based on an alignment. The probability data used in the plot is found here, and the gnuplot script is here.

Prediction of contig053787-BurOR.H068

```
ID   contig053787-BurOR.H068
FT   TOPO_DOM      1     22       NON CYTOPLASMIC.
FT   TRANSMEM     23     49
FT   TOPO_DOM     50     56       CYTOPLASMIC.
FT   TRANSMEM     57     77
FT   TOPO_DOM     78     95       NON CYTOPLASMIC.
FT   TRANSMEM     96    118
FT   TOPO_DOM    119    138       CYTOPLASMIC.
FT   TRANSMEM    139    160
FT   TOPO_DOM    161    193       NON CYTOPLASMIC.
FT   TRANSMEM    194    216
FT   TOPO_DOM    217    235       CYTOPLASMIC.
FT   TRANSMEM    236    258
FT   TOPO_DOM    259    269       NON CYTOPLASMIC.
FT   TRANSMEM    270    289
FT   TOPO_DOM    290    314       CYTOPLASMIC.
//
```

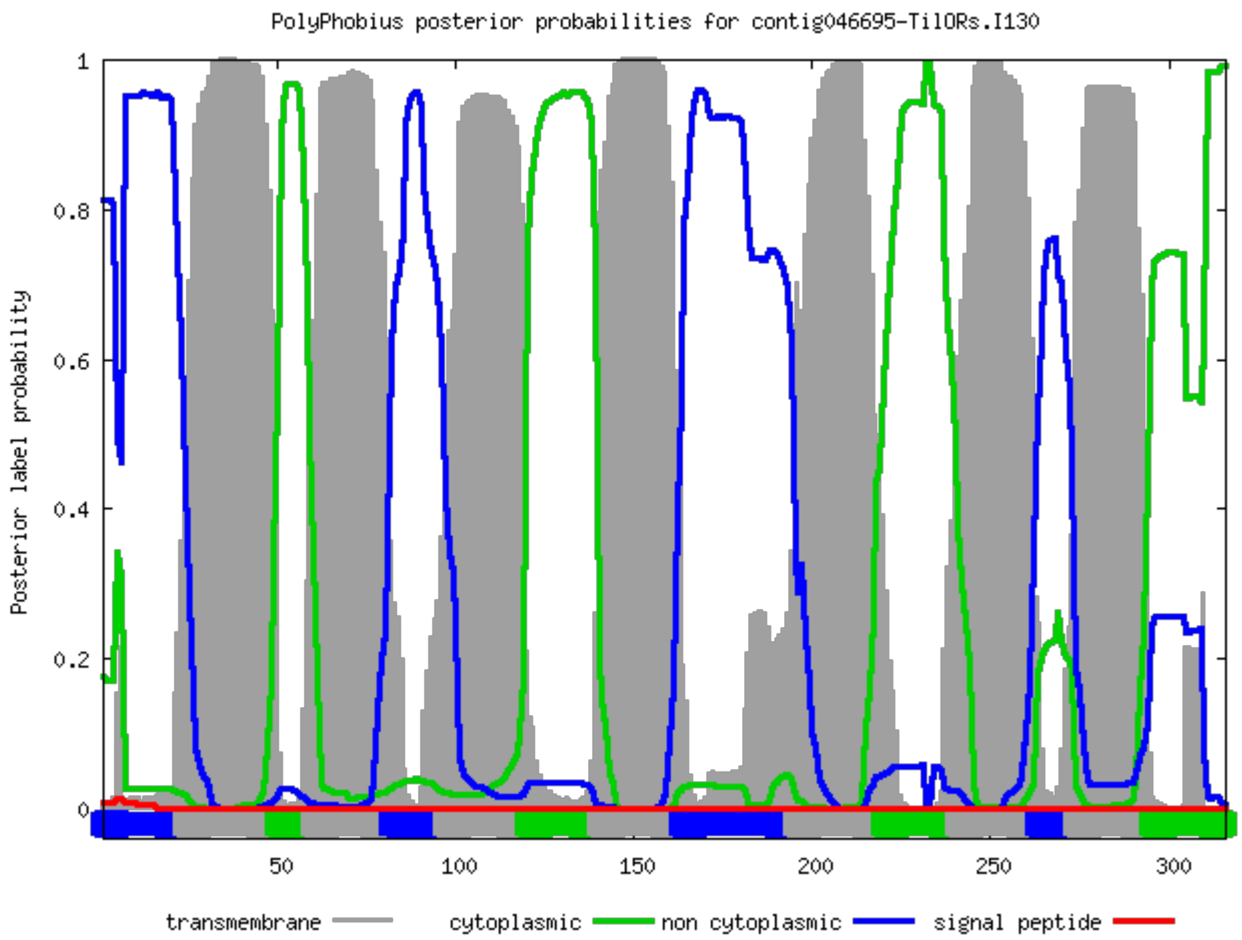

The prediction is based on an alignment. The probability data used in the plot is found here, and the gnuplot script is here.

Prediction of contig085002-BriOR.A004

```
ID   contig085002-BriOR.A004
FT   TOPO_DOM      1     25       NON CYTOPLASMIC.
FT   TRANSMEM     26     51
FT   TOPO_DOM     52     59       CYTOPLASMIC.
FT   TRANSMEM     60     80
FT   TOPO_DOM     81     98       NON CYTOPLASMIC.
FT   TRANSMEM     99    121
FT   TOPO_DOM    122    141       CYTOPLASMIC.
FT   TRANSMEM    142    163
FT   TOPO_DOM    164    195       NON CYTOPLASMIC.
FT   TRANSMEM    196    219
FT   TOPO_DOM    220    239       CYTOPLASMIC.
FT   TRANSMEM    240    261
FT   TOPO_DOM    262    272       NON CYTOPLASMIC.
FT   TRANSMEM    273    293
FT   TOPO_DOM    294    337       CYTOPLASMIC.
//
```

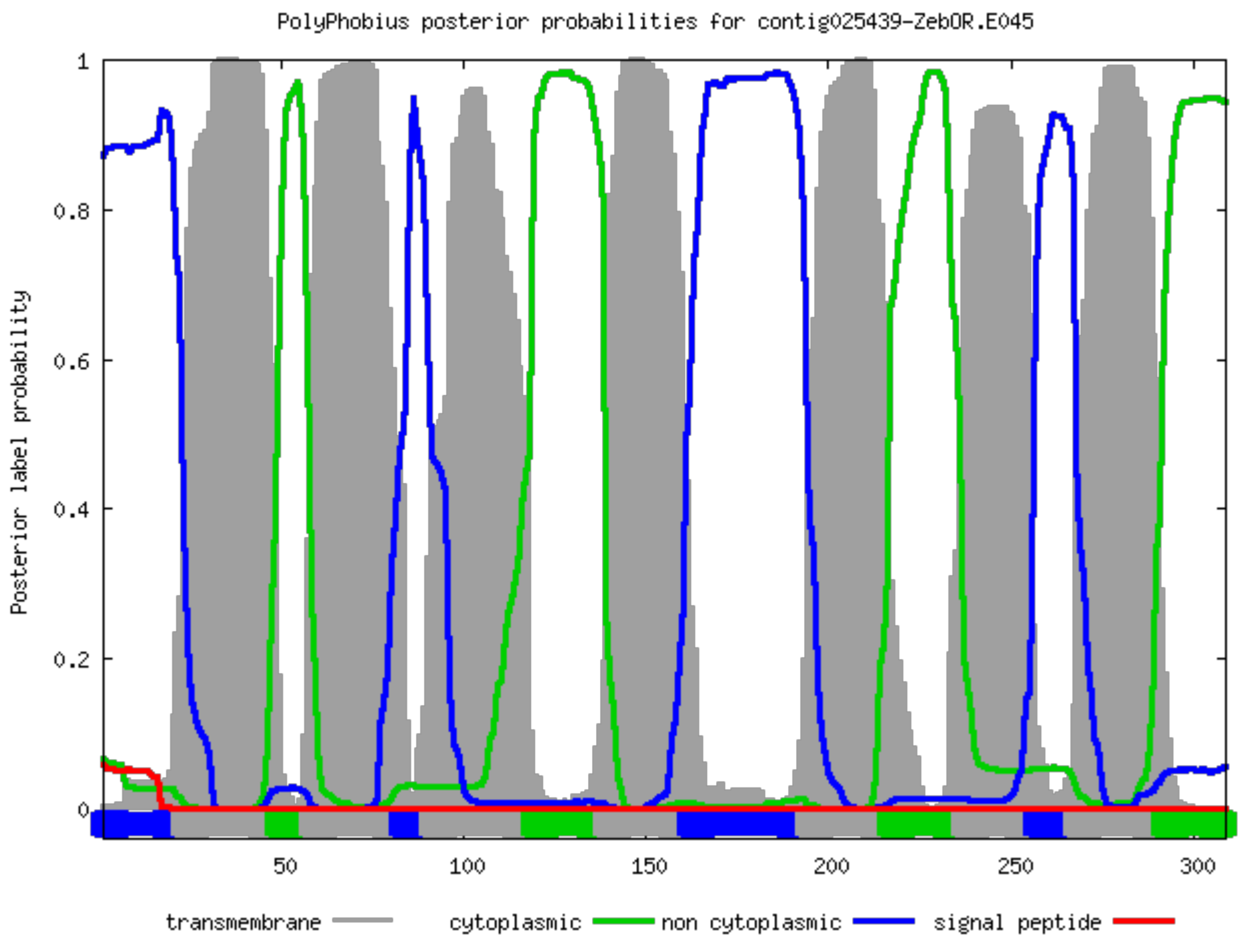

The prediction is based on an alignment. The probability data used in the plot is found here, and the gnuplot script is here.

Prediction of contig047503-ZebOR.A013

```
ID    contig047503-ZebOR.A013
FT    TOPO_DOM      1      22        NON CYTOPLASMIC.
FT    TRANSMEM     23      48
FT    TOPO_DOM     49      56        CYTOPLASMIC.
FT    TRANSMEM     57      77
FT    TOPO_DOM     78      95        NON CYTOPLASMIC.
FT    TRANSMEM     96     118
FT    TOPO_DOM    119     138        CYTOPLASMIC.
FT    TRANSMEM    139     159
FT    TOPO_DOM    160     192        NON CYTOPLASMIC.
FT    TRANSMEM    193     215
FT    TOPO_DOM    216     235        CYTOPLASMIC.
FT    TRANSMEM    236     257
FT    TOPO_DOM    258     268        NON CYTOPLASMIC.
FT    TRANSMEM    269     289
FT    TOPO_DOM    290     317        CYTOPLASMIC.
//
```

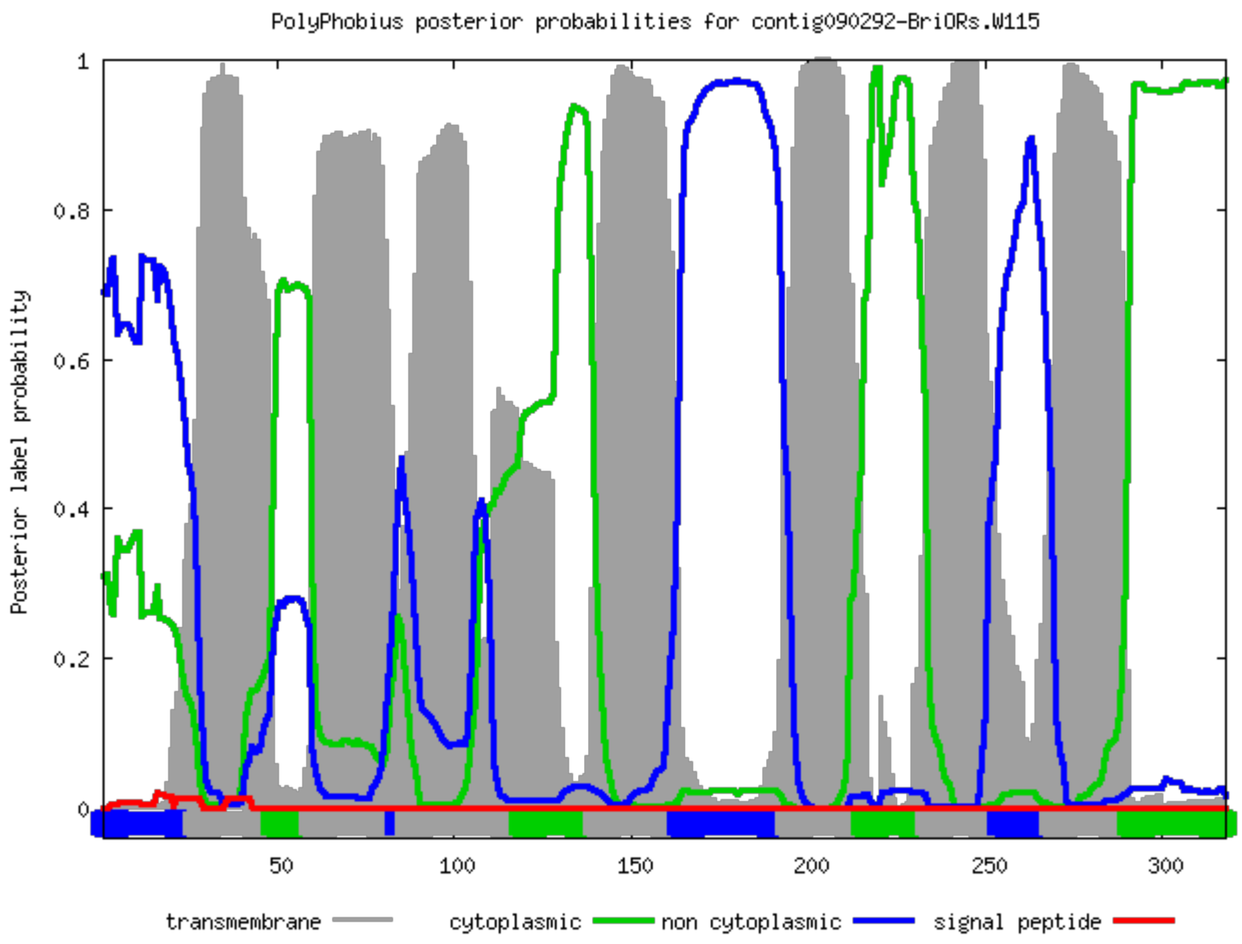

The prediction is based on an alignment. The probability data used in the plot is found here, and the gnuplot script is here.

Prediction of contig046002-ZebOR.K087

```
ID    contig046002-ZebOR.K087
FT    TOPO_DOM      1     24      NON CYTOPLASMIC.
FT    TRANSMEM     25     50
FT    TOPO_DOM     51     58      CYTOPLASMIC.
FT    TRANSMEM     59     81
FT    TOPO_DOM     82    100      NON CYTOPLASMIC.
FT    TRANSMEM    101    121
FT    TOPO_DOM    122    141      CYTOPLASMIC.
FT    TRANSMEM    142    165
FT    TOPO_DOM    166    196      NON CYTOPLASMIC.
FT    TRANSMEM    197    224
FT    TOPO_DOM    225    244      CYTOPLASMIC.
FT    TRANSMEM    245    264
FT    TOPO_DOM    265    269      NON CYTOPLASMIC.
FT    TRANSMEM    270    292
FT    TOPO_DOM    293    314      CYTOPLASMIC.
//
```

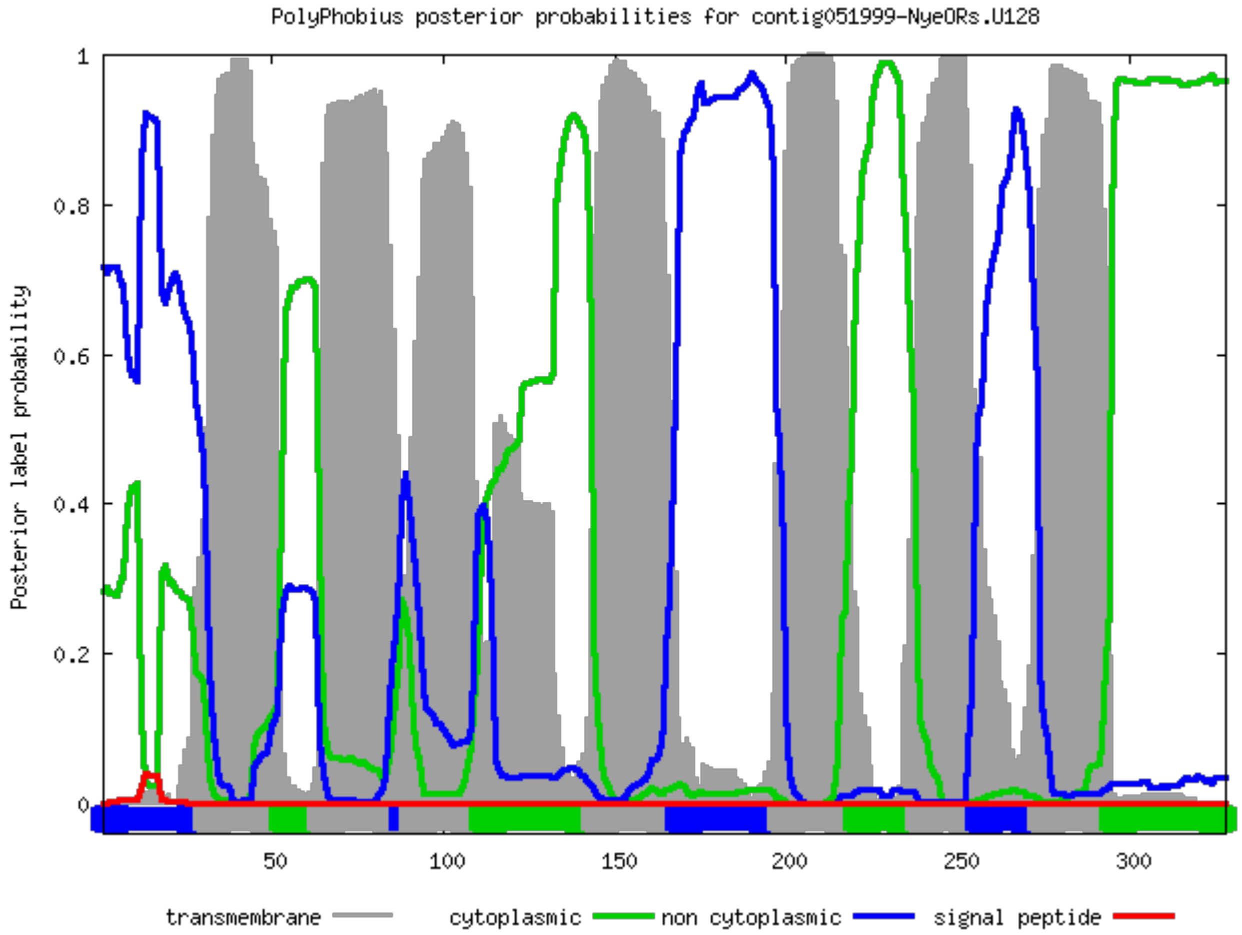

The prediction is based on an alignment. The probability data used in the plot is found here, and the gnuplot script is here.

Prediction of contig022234-TilOR.A009

```
ID   contig022234-TilOR.A009
FT   TOPO_DOM      1     22       NON CYTOPLASMIC.
FT   TRANSMEM     23     48
FT   TOPO_DOM     49     56       CYTOPLASMIC.
FT   TRANSMEM     57     77
FT   TOPO_DOM     78     95       NON CYTOPLASMIC.
FT   TRANSMEM     96    118
FT   TOPO_DOM    119    138       CYTOPLASMIC.
FT   TRANSMEM    139    160
FT   TOPO_DOM    161    192       NON CYTOPLASMIC.
FT   TRANSMEM    193    215
FT   TOPO_DOM    216    235       CYTOPLASMIC.
FT   TRANSMEM    236    257
FT   TOPO_DOM    258    268       NON CYTOPLASMIC.
FT   TRANSMEM    269    289
FT   TOPO_DOM    290    320       CYTOPLASMIC.
//
```

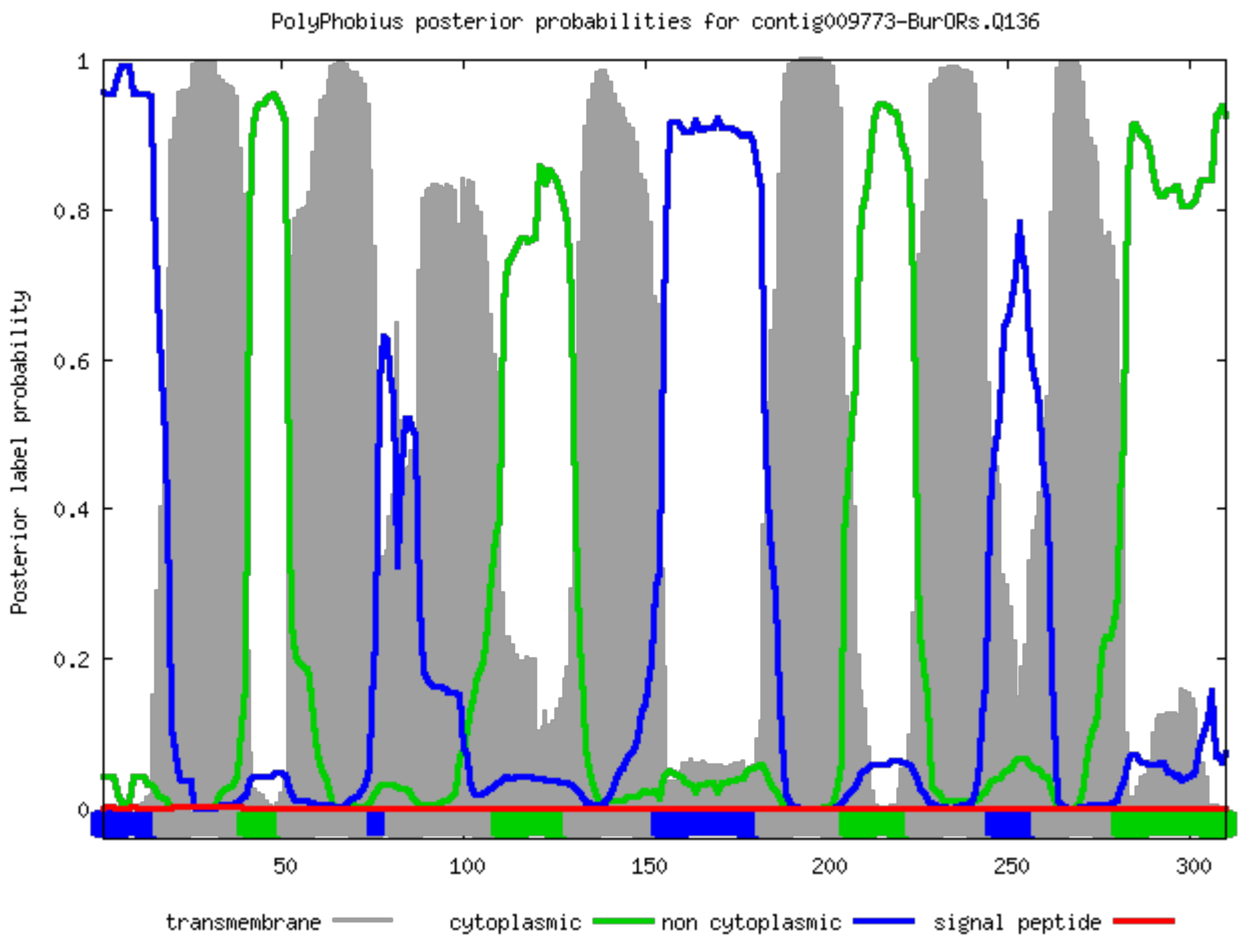

The prediction is based on an alignment. The probability data used in the plot is found here, and the gnuplot script is here.

Prediction of contig039737-NyeOR.D040

```
ID   contig039737-NyeOR.D040
FT   TOPO_DOM      1     22       NON CYTOPLASMIC.
FT   TRANSMEM     23     48
FT   TOPO_DOM     49     57       CYTOPLASMIC.
FT   TRANSMEM     58     81
FT   TOPO_DOM     82     90       NON CYTOPLASMIC.
FT   TRANSMEM     91    117
FT   TOPO_DOM    118    138       CYTOPLASMIC.
FT   TRANSMEM    139    161
FT   TOPO_DOM    162    194       NON CYTOPLASMIC.
FT   TRANSMEM    195    216
FT   TOPO_DOM    217    236       CYTOPLASMIC.
FT   TRANSMEM    237    256
FT   TOPO_DOM    257    267       NON CYTOPLASMIC.
FT   TRANSMEM    268    291
FT   TOPO_DOM    292    309       CYTOPLASMIC.
//
```

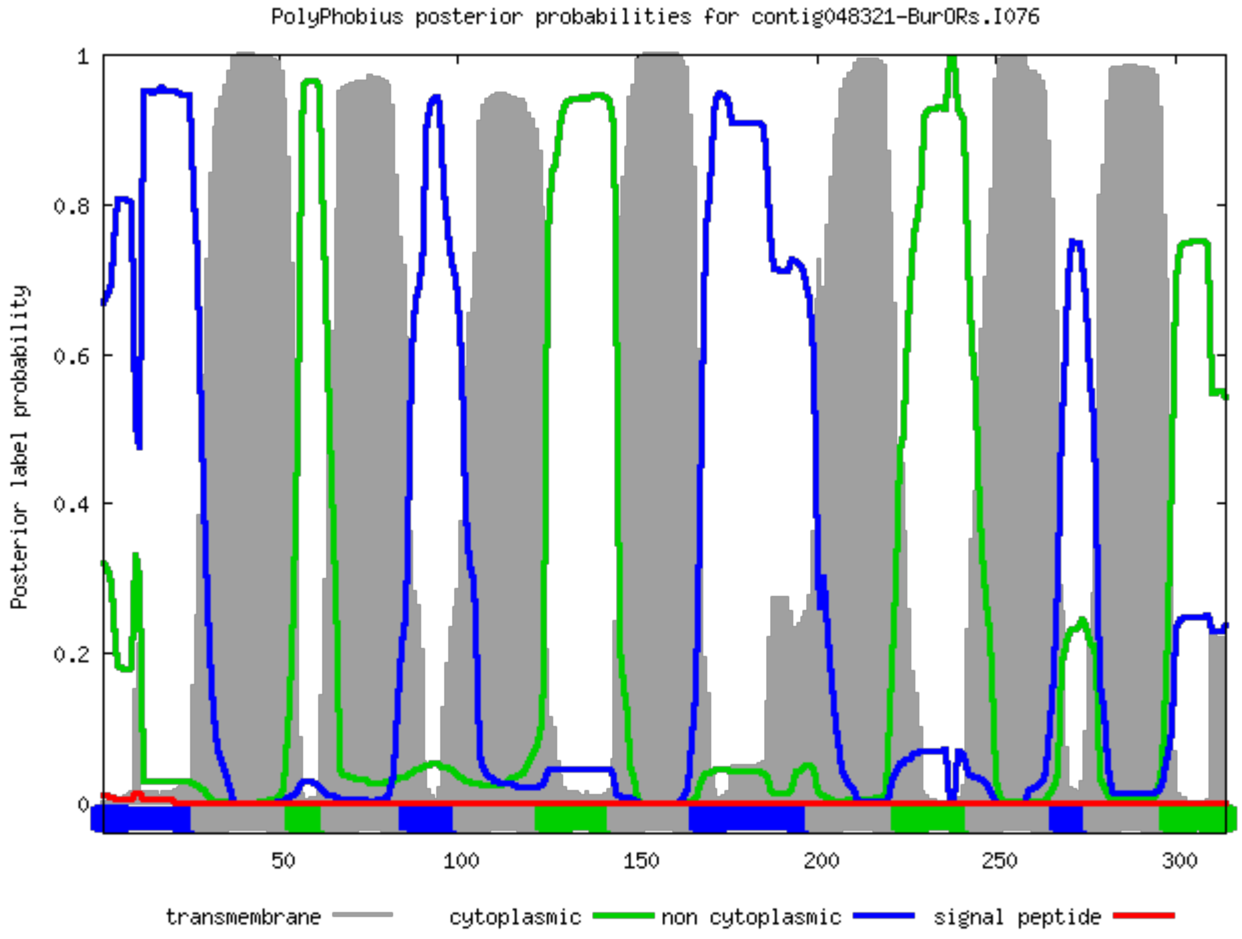

The prediction is based on an alignment. The probability data used in the plot is found here, and the gnuplot script is here.

Prediction of contig039481-TilOR.L153

```
ID   contig039481-TilOR.L153
FT   TOPO_DOM      1     25       NON CYTOPLASMIC.
FT   TRANSMEM     26     50
FT   TOPO_DOM     51     59       CYTOPLASMIC.
FT   TRANSMEM     60     86
FT   TOPO_DOM     87     97       NON CYTOPLASMIC.
FT   TRANSMEM     98    120
FT   TOPO_DOM    121    140       CYTOPLASMIC.
FT   TRANSMEM    141    163
FT   TOPO_DOM    164    198       NON CYTOPLASMIC.
FT   TRANSMEM    199    223
FT   TOPO_DOM    224    238       CYTOPLASMIC.
FT   TRANSMEM    239    259
FT   TOPO_DOM    260    271       NON CYTOPLASMIC.
FT   TRANSMEM    272    292
FT   TOPO_DOM    293    313       CYTOPLASMIC.
//
```

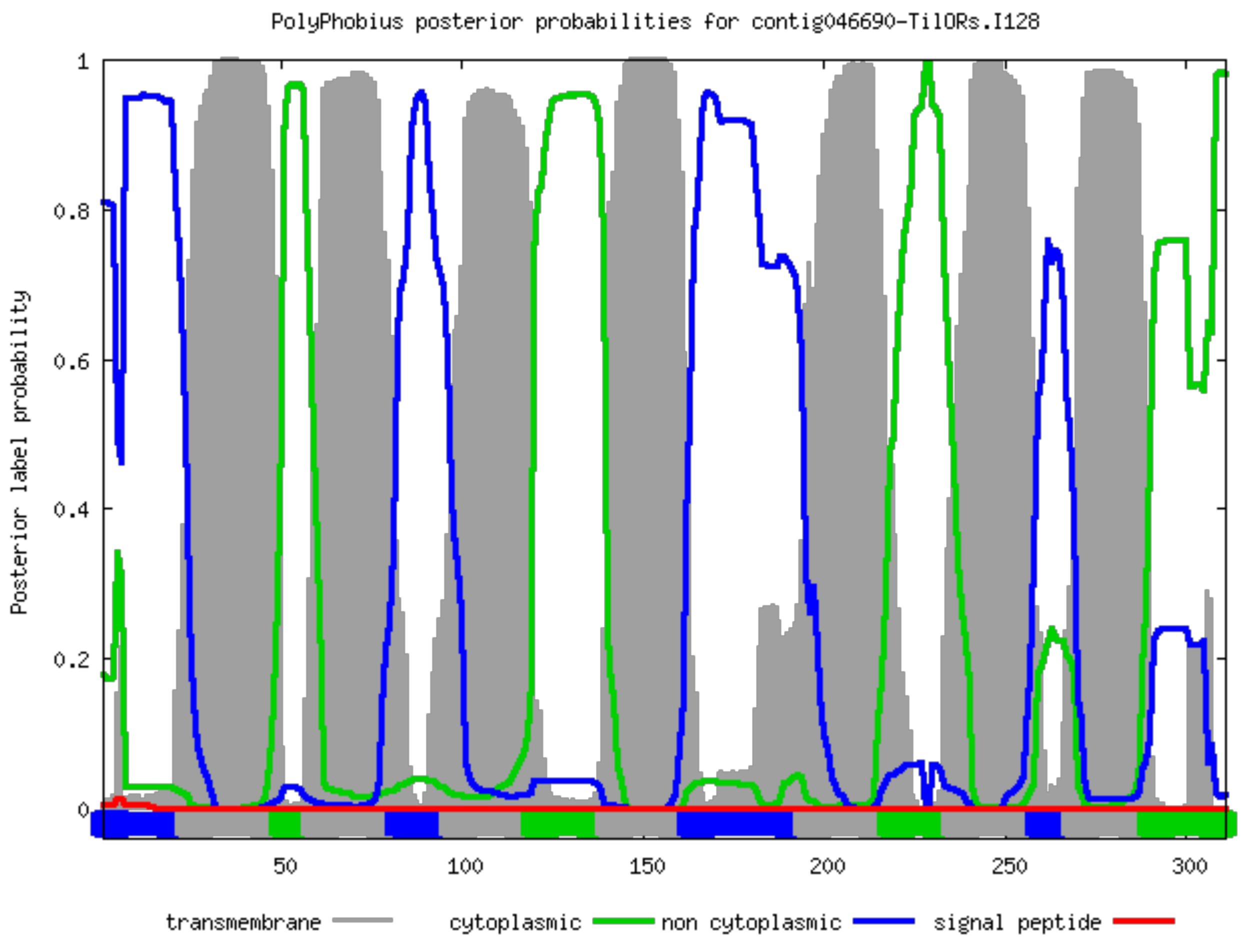

The prediction is based on an alignment. The probability data used in the plot is found here, and the gnuplot script is here.

Prediction of contig013362-TilOR.H109

```
ID    contig013362-TilOR.H109
FT    TOPO_DOM      1      23       NON CYTOPLASMIC.
FT    TRANSMEM     24      49
FT    TOPO_DOM     50      56       CYTOPLASMIC.
FT    TRANSMEM     57      76
FT    TOPO_DOM     77      95       NON CYTOPLASMIC.
FT    TRANSMEM     96     118
FT    TOPO_DOM    119     138       CYTOPLASMIC.
FT    TRANSMEM    139     160
FT    TOPO_DOM    161     196       NON CYTOPLASMIC.
FT    TRANSMEM    197     219
FT    TOPO_DOM    220     237       CYTOPLASMIC.
FT    TRANSMEM    238     259
FT    TOPO_DOM    260     271       NON CYTOPLASMIC.
FT    TRANSMEM    272     291
FT    TOPO_DOM    292     310       CYTOPLASMIC.
//
```

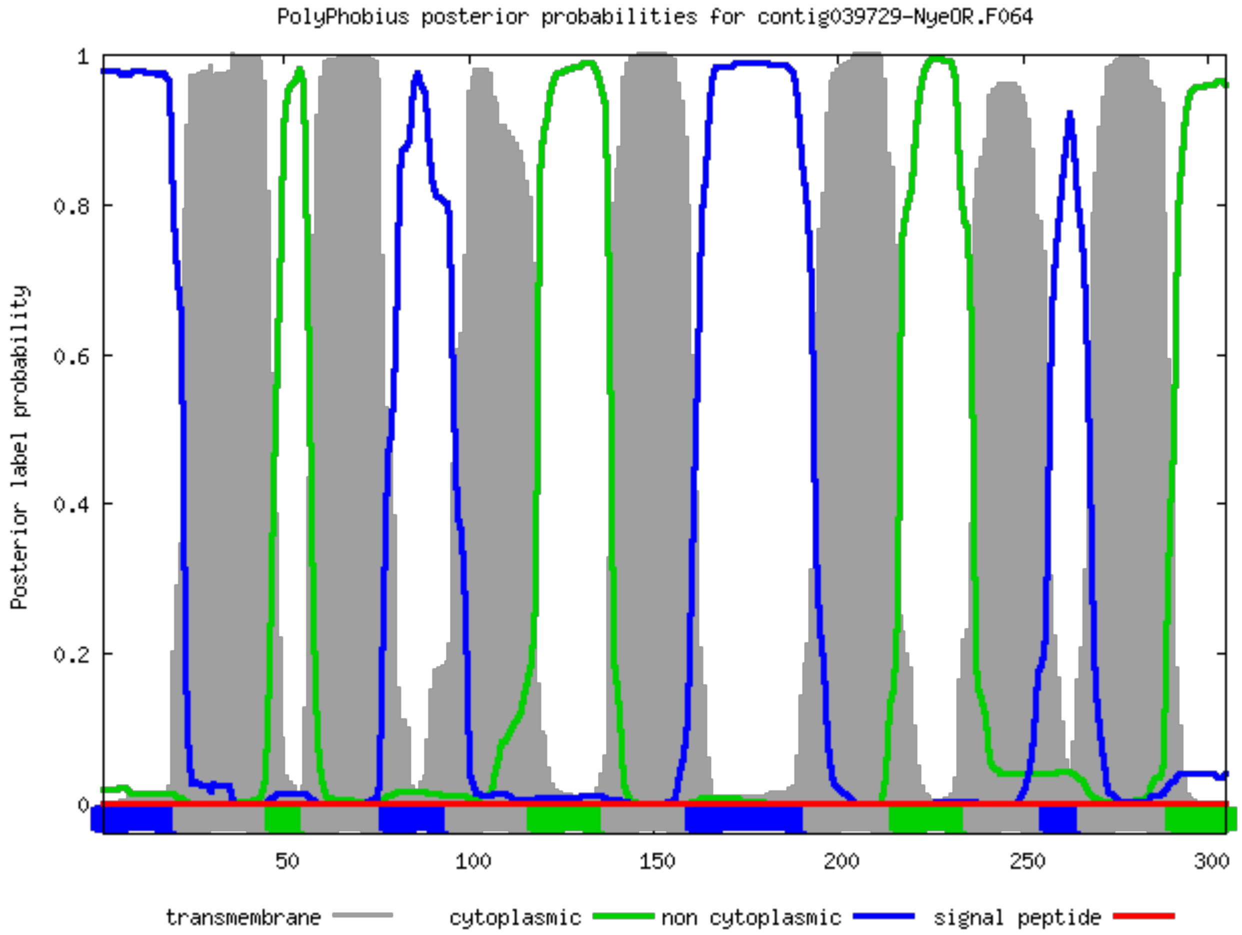

The prediction is based on an alignment. The probability data used in the plot is found here, and the gnuplot script is here.

Prediction of contig041756-NyeOR.H139

```
ID    contig041756-NyeOR.H139
FT    TOPO_DOM       1      22       NON CYTOPLASMIC.
FT    TRANSMEM      23      49
FT    TOPO_DOM      50      56       CYTOPLASMIC.
FT    TRANSMEM      57      77
FT    TOPO_DOM      78      95       NON CYTOPLASMIC.
FT    TRANSMEM      96     118
FT    TOPO_DOM     119     138       CYTOPLASMIC.
FT    TRANSMEM     139     160
FT    TOPO_DOM     161     193       NON CYTOPLASMIC.
FT    TRANSMEM     194     217
FT    TOPO_DOM     218     235       CYTOPLASMIC.
FT    TRANSMEM     236     258
FT    TOPO_DOM     259     269       NON CYTOPLASMIC.
FT    TRANSMEM     270     289
FT    TOPO_DOM     290     314       CYTOPLASMIC.
//
```

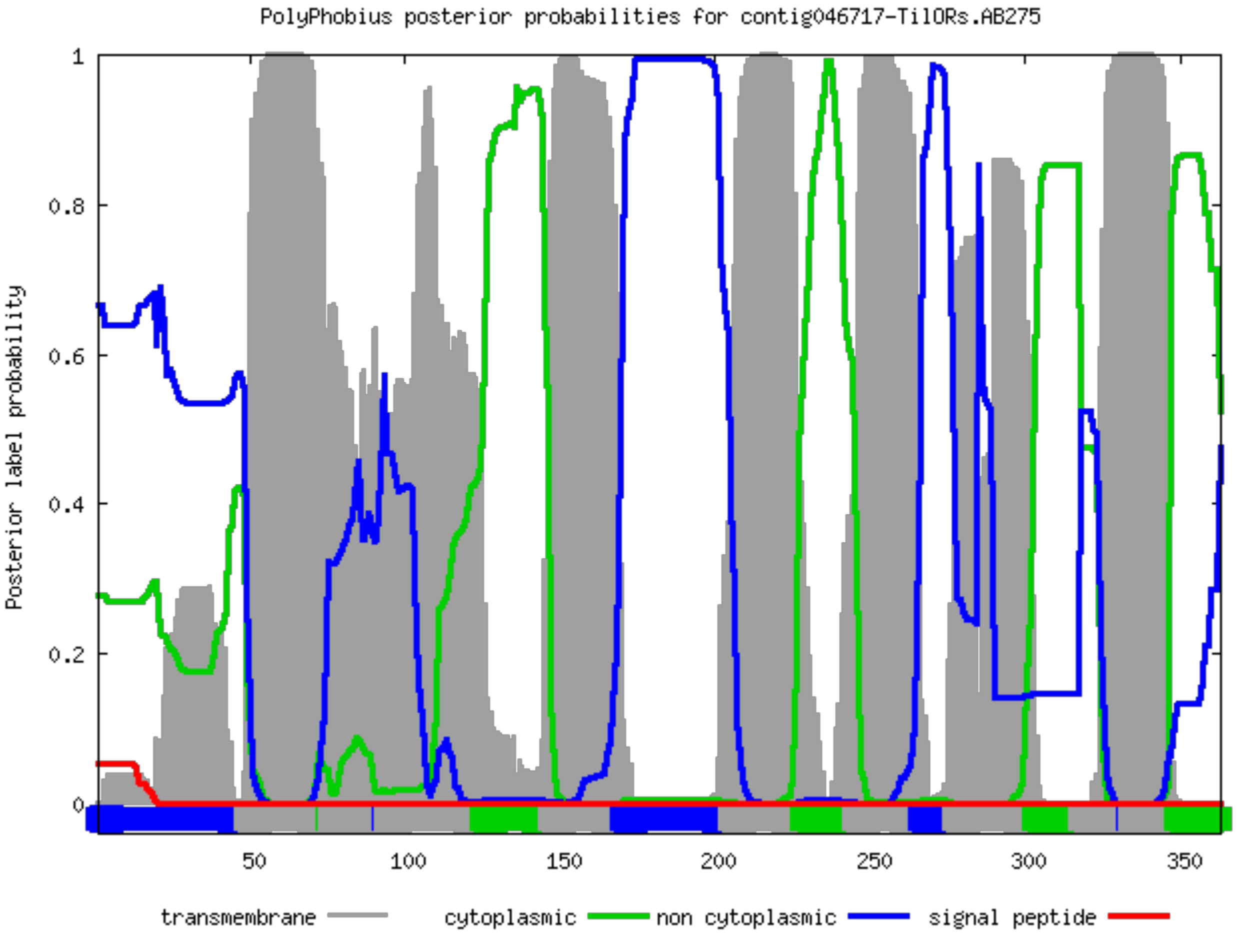

The prediction is based on an alignment. The probability data used in the plot is found here, and the gnuplot script is here.

Prediction of contig009545-TilOR.F097

ID	contig009545-TilOR.F097			
FT	TOPO_DOM	1	22	NON CYTOPLASMIC.
FT	TRANSMEM	23	47	
FT	TOPO_DOM	48	57	CYTOPLASMIC.
FT	TRANSMEM	58	77	
FT	TOPO_DOM	78	96	NON CYTOPLASMIC.
FT	TRANSMEM	97	118	
FT	TOPO_DOM	119	138	CYTOPLASMIC.
FT	TRANSMEM	139	161	
FT	TOPO_DOM	162	193	NON CYTOPLASMIC.
FT	TRANSMEM	194	216	
FT	TOPO_DOM	217	236	CYTOPLASMIC.
FT	TRANSMEM	237	256	
FT	TOPO_DOM	257	267	NON CYTOPLASMIC.
FT	TRANSMEM	268	290	
FT	TOPO_DOM	291	321	CYTOPLASMIC.
//				

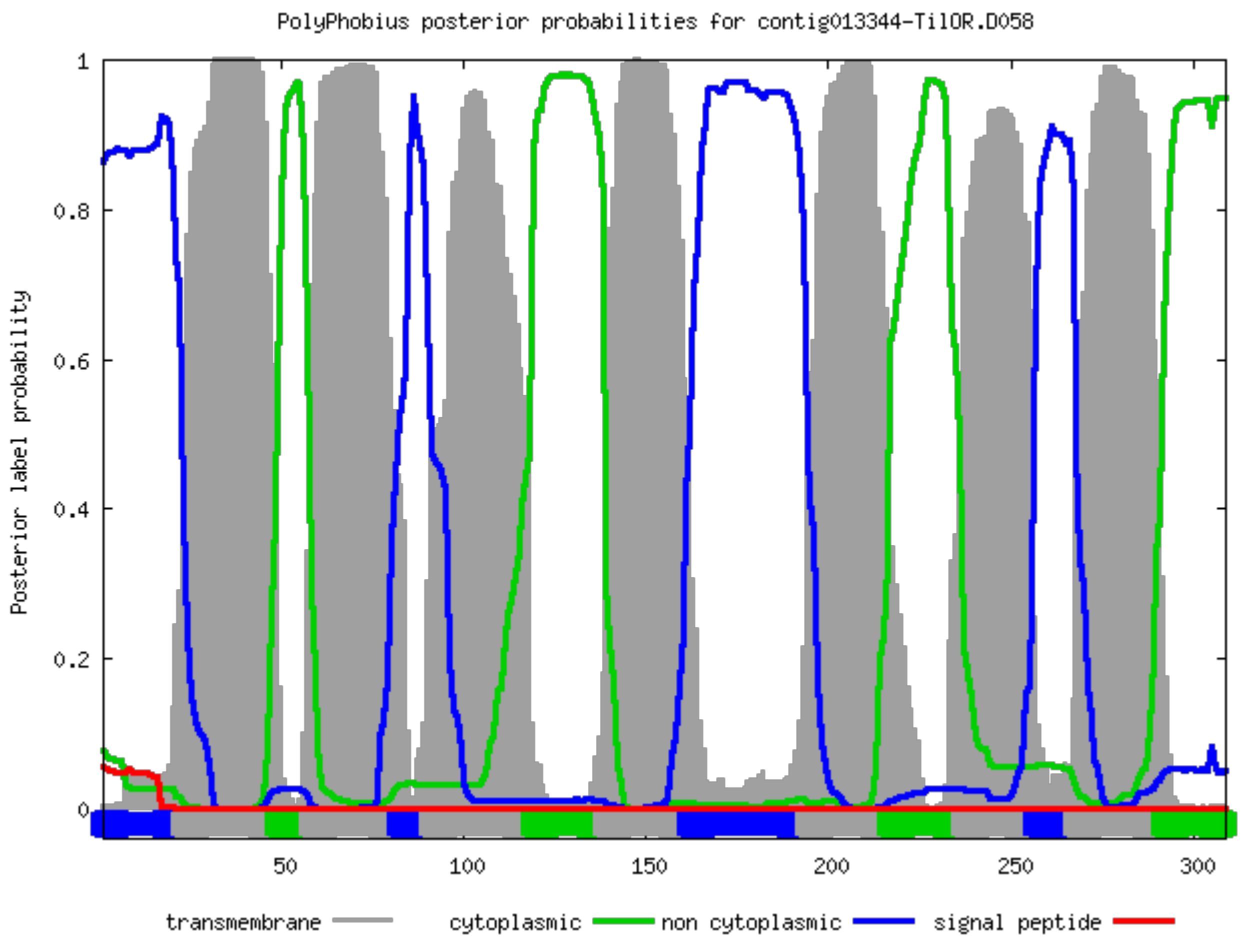

The prediction is based on an alignment. The probability data used in the plot is found here, and the gnuplot script is here.

Prediction of contig041952-TilOR.A023

```
ID    contig041952-TilOR.A023
FT    TOPO_DOM      1      22       NON CYTOPLASMIC.
FT    TRANSMEM     23      48
FT    TOPO_DOM     49      56       CYTOPLASMIC.
FT    TRANSMEM     57      77
FT    TOPO_DOM     78      95       NON CYTOPLASMIC.
FT    TRANSMEM     96     118
FT    TOPO_DOM    119     138       CYTOPLASMIC.
FT    TRANSMEM    139     159
FT    TOPO_DOM    160     192       NON CYTOPLASMIC.
FT    TRANSMEM    193     215
FT    TOPO_DOM    216     235       CYTOPLASMIC.
FT    TRANSMEM    236     257
FT    TOPO_DOM    258     268       NON CYTOPLASMIC.
FT    TRANSMEM    269     289
FT    TOPO_DOM    290     327       CYTOPLASMIC.
//
```

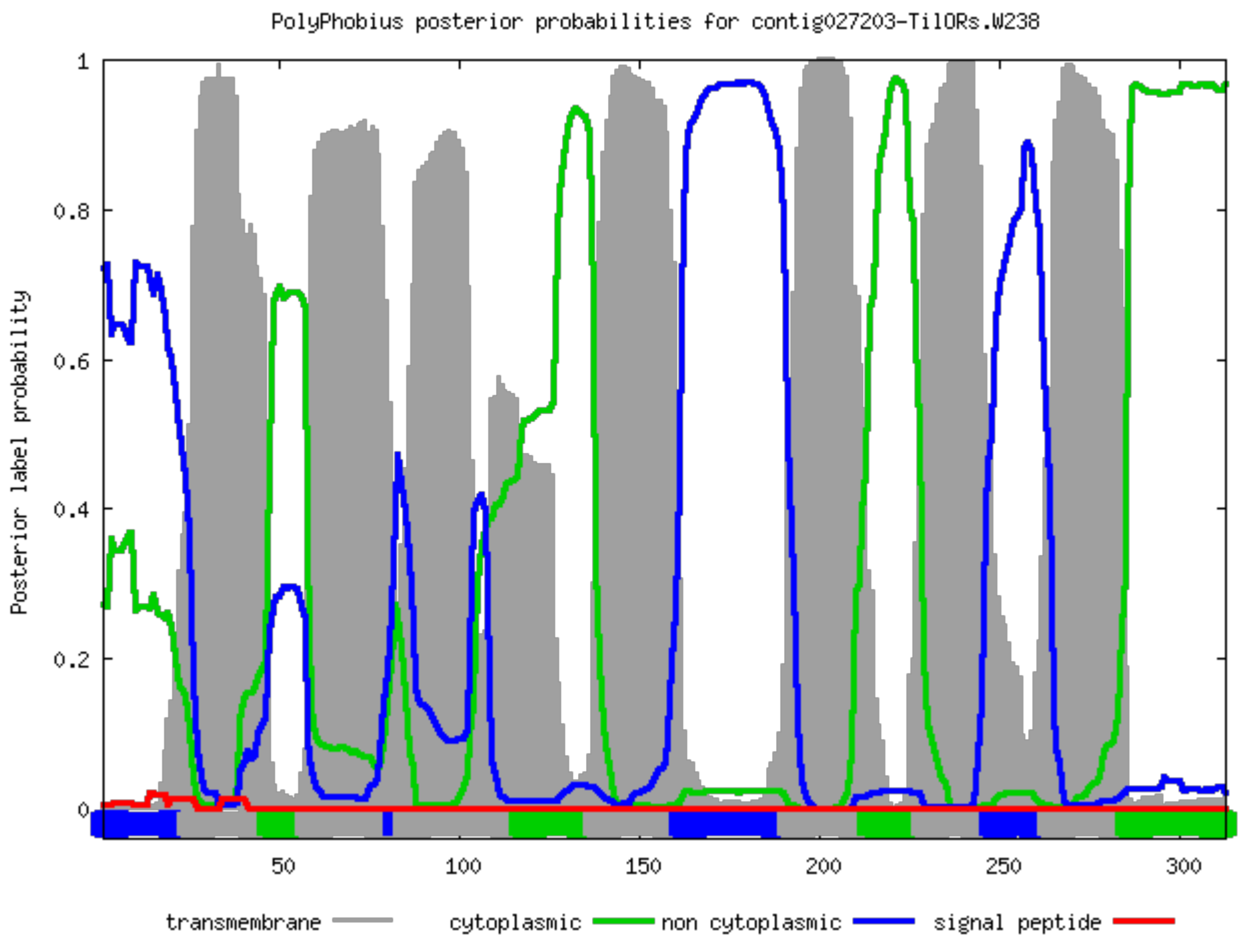

The prediction is based on an alignment. The probability data used in the plot is found here, and the gnuplot script is here.

Prediction of contig004255-BriOR.E036

```
ID    contig004255-BriOR.E036
FT    TOPO_DOM      1     22       NON CYTOPLASMIC.
FT    TRANSMEM     23     48
FT    TOPO_DOM     49     57       CYTOPLASMIC.
FT    TRANSMEM     58     83
FT    TOPO_DOM     84     90       NON CYTOPLASMIC.
FT    TRANSMEM     91    118
FT    TOPO_DOM    119    138       CYTOPLASMIC.
FT    TRANSMEM    139    161
FT    TOPO_DOM    162    193       NON CYTOPLASMIC.
FT    TRANSMEM    194    216
FT    TOPO_DOM    217    236       CYTOPLASMIC.
FT    TRANSMEM    237    256
FT    TOPO_DOM    257    268       NON CYTOPLASMIC.
FT    TRANSMEM    269    291
FT    TOPO_DOM    292    309       CYTOPLASMIC.
//
```

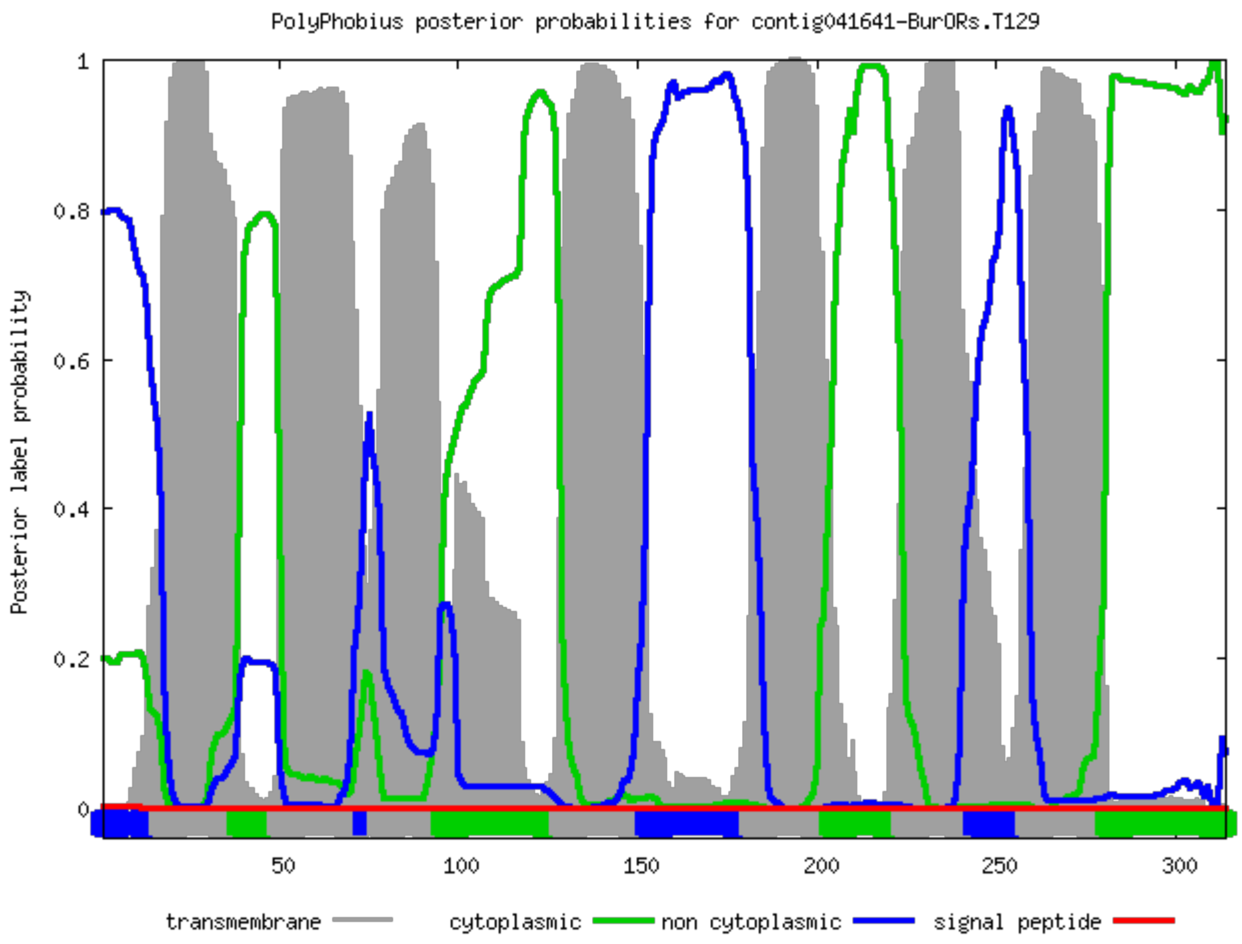

The prediction is based on an alignment. The probability data used in the plot is found here, and the gnuplot script is here.

Prediction of contig072645-TilOR.L159

```
ID   contig072645-TilOR.L159
FT   TOPO_DOM      1     26       NON CYTOPLASMIC.
FT   TRANSMEM     27     50
FT   TOPO_DOM     51     59       CYTOPLASMIC.
FT   TRANSMEM     60     86
FT   TOPO_DOM     87     97       NON CYTOPLASMIC.
FT   TRANSMEM     98    120
FT   TOPO_DOM    121    140       CYTOPLASMIC.
FT   TRANSMEM    141    163
FT   TOPO_DOM    164    198       NON CYTOPLASMIC.
FT   TRANSMEM    199    223
FT   TOPO_DOM    224    238       CYTOPLASMIC.
FT   TRANSMEM    239    259
FT   TOPO_DOM    260    271       NON CYTOPLASMIC.
FT   TRANSMEM    272    292
FT   TOPO_DOM    293    313       CYTOPLASMIC.
//
```

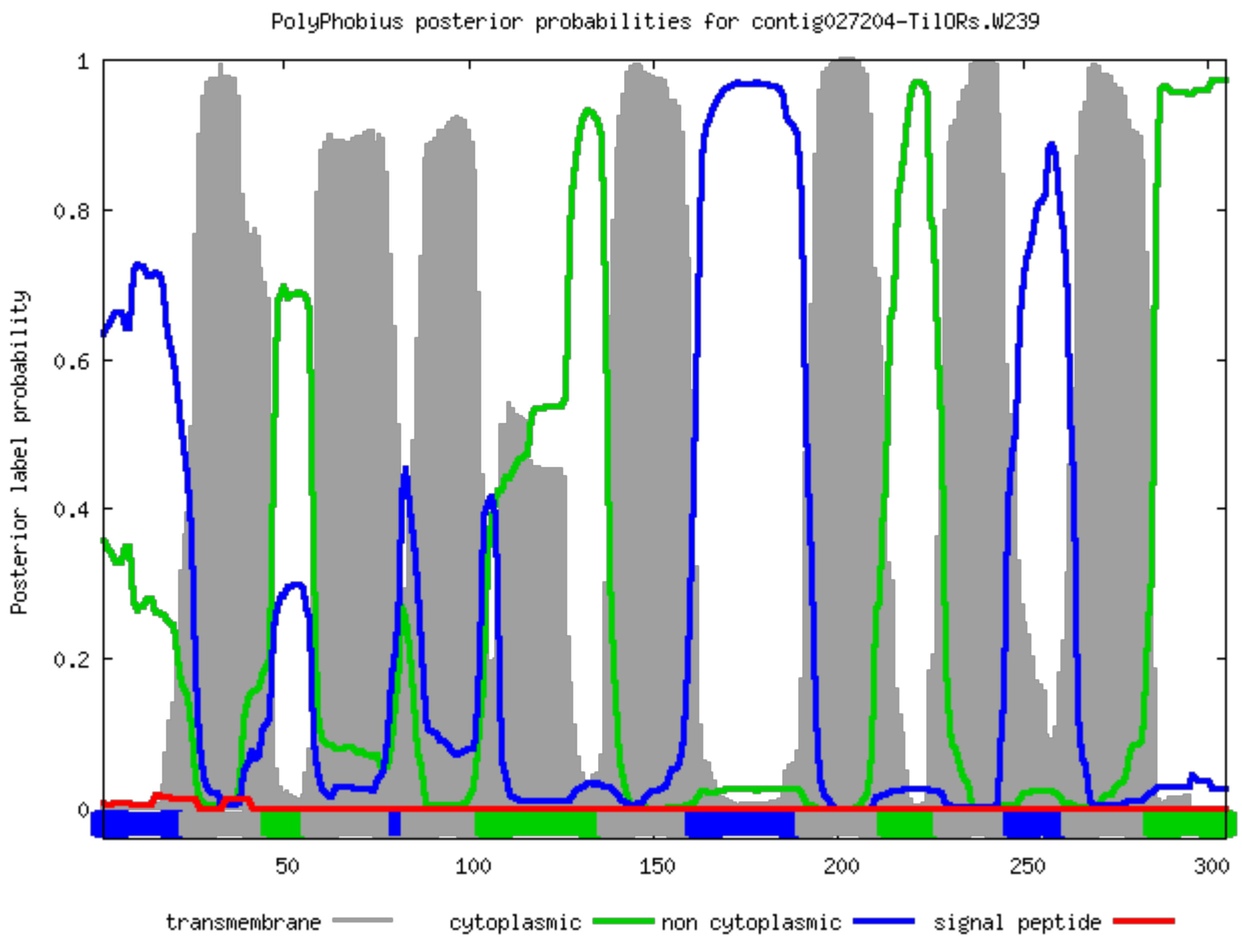

The prediction is based on an alignment. The probability data used in the plot is found here, and the gnuplot script is here.

Prediction of contig020442-ZebOR.L095

```
ID   contig020442-ZebOR.L095
FT   TOPO_DOM      1      25       NON CYTOPLASMIC.
FT   TRANSMEM     26      50
FT   TOPO_DOM     51      59       CYTOPLASMIC.
FT   TRANSMEM     60      83
FT   TOPO_DOM     84      98       NON CYTOPLASMIC.
FT   TRANSMEM     99     120
FT   TOPO_DOM    121     140       CYTOPLASMIC.
FT   TRANSMEM    141     162
FT   TOPO_DOM    163     198       NON CYTOPLASMIC.
FT   TRANSMEM    199     224
FT   TOPO_DOM    225     237       CYTOPLASMIC.
FT   TRANSMEM    238     259
FT   TOPO_DOM    260     271       NON CYTOPLASMIC.
FT   TRANSMEM    272     292
FT   TOPO_DOM    293     313       CYTOPLASMIC.
//
```

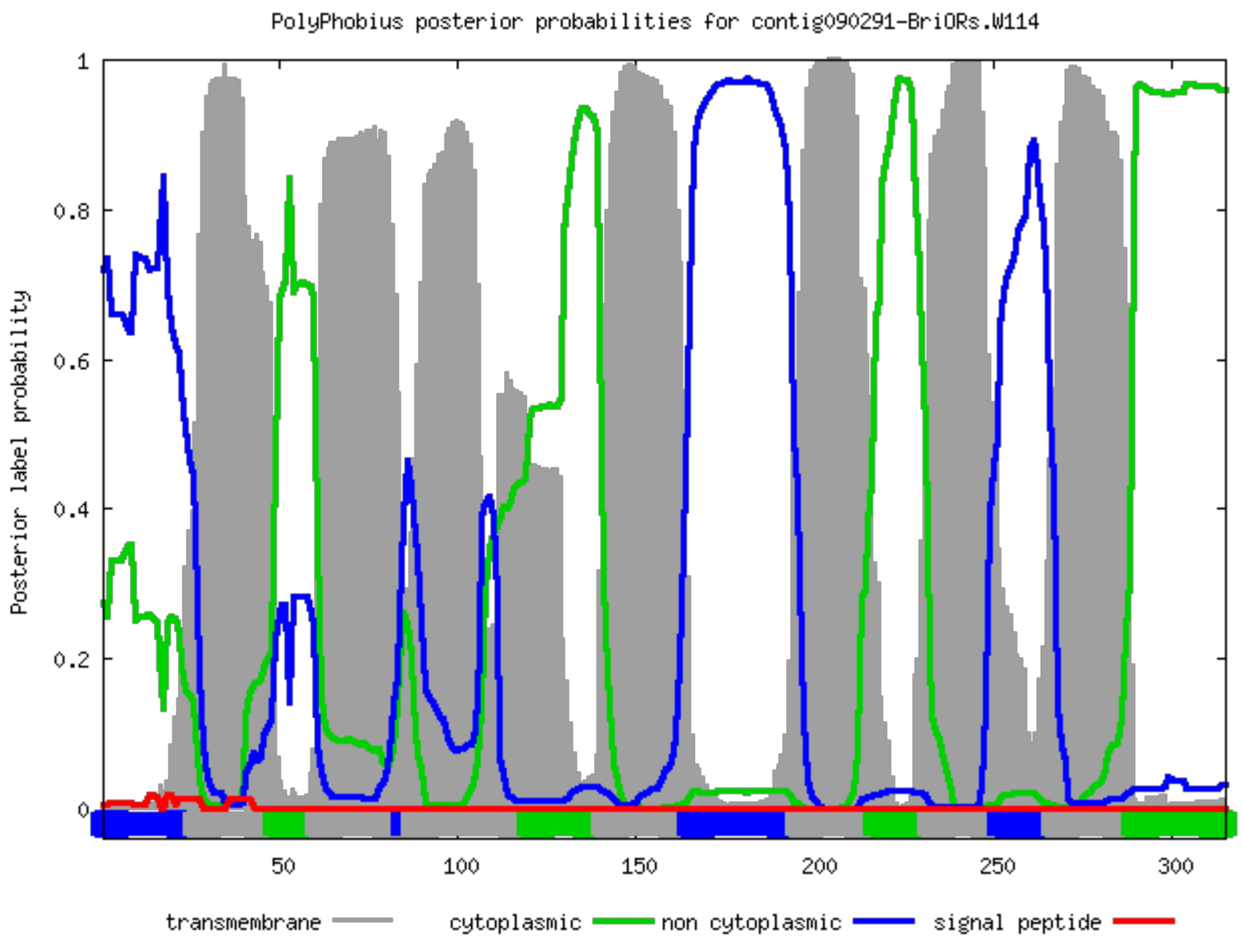

The prediction is based on an alignment. The probability data used in the plot is found here, and the gnuplot script is here.

Prediction of contig047714-TilOR.G099

```
ID   contig047714-TilOR.G099
FT   TOPO_DOM      1     22       NON CYTOPLASMIC.
FT   TRANSMEM     23     47
FT   TOPO_DOM     48     55       CYTOPLASMIC.
FT   TRANSMEM     56     76
FT   TOPO_DOM     77     94       NON CYTOPLASMIC.
FT   TRANSMEM     95    117
FT   TOPO_DOM    118    137       CYTOPLASMIC.
FT   TRANSMEM    138    160
FT   TOPO_DOM    161    192       NON CYTOPLASMIC.
FT   TRANSMEM    193    216
FT   TOPO_DOM    217    233       CYTOPLASMIC.
FT   TRANSMEM    234    254
FT   TOPO_DOM    255    268       NON CYTOPLASMIC.
FT   TRANSMEM    269    288
FT   TOPO_DOM    289    312       CYTOPLASMIC.
//
```

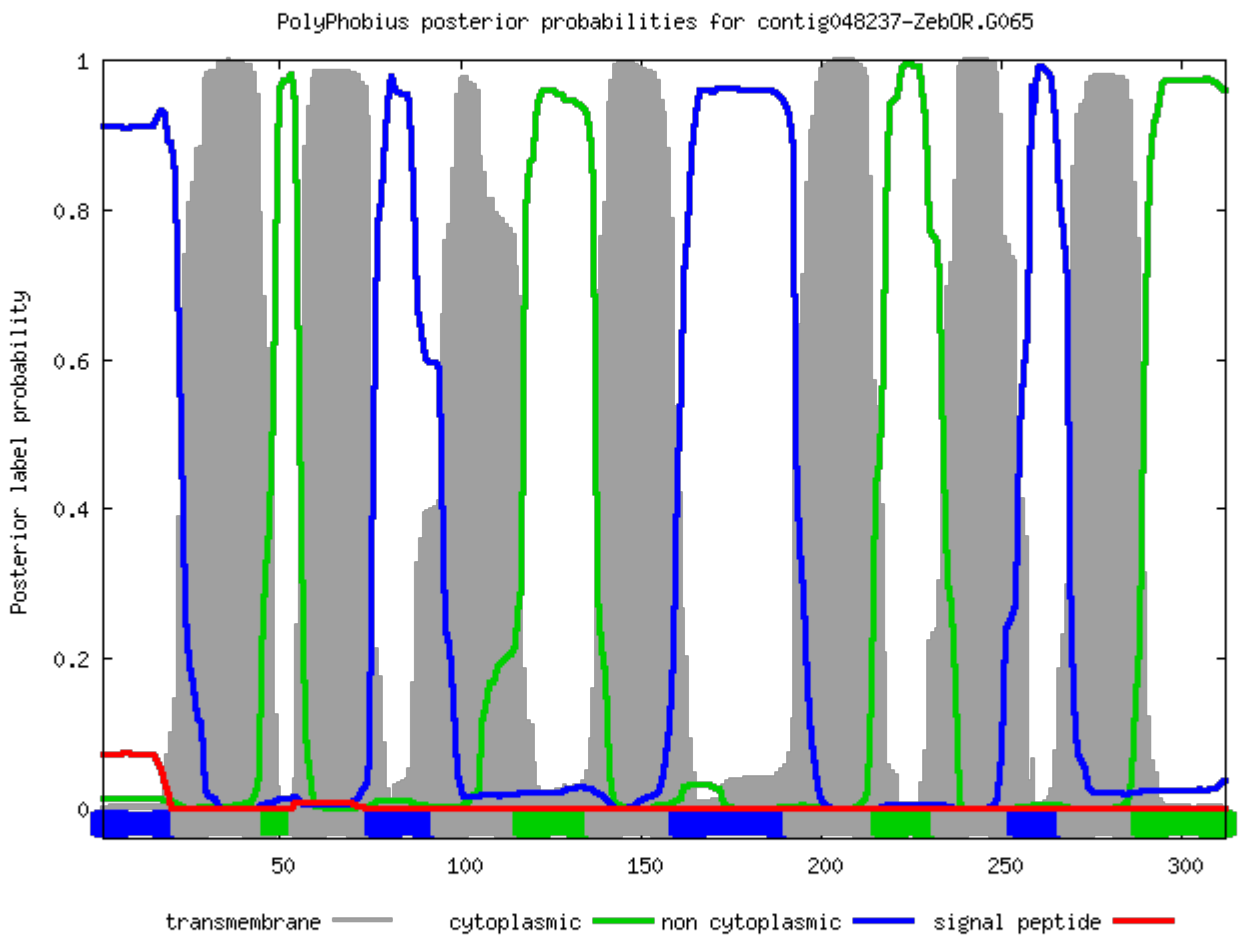

The prediction is based on an alignment. The probability data used in the plot is found here, and the gnuplot script is here.

Prediction of contig070886-TilOR.A025

```
ID   contig070886-TilOR.A025
FT   TOPO_DOM      1      22       NON CYTOPLASMIC.
FT   TRANSMEM     23      48
FT   TOPO_DOM     49      56       CYTOPLASMIC.
FT   TRANSMEM     57      77
FT   TOPO_DOM     78      95       NON CYTOPLASMIC.
FT   TRANSMEM     96     118
FT   TOPO_DOM    119     138       CYTOPLASMIC.
FT   TRANSMEM    139     160
FT   TOPO_DOM    161     192       NON CYTOPLASMIC.
FT   TRANSMEM    193     215
FT   TOPO_DOM    216     235       CYTOPLASMIC.
FT   TRANSMEM    236     257
FT   TOPO_DOM    258     268       NON CYTOPLASMIC.
FT   TRANSMEM    269     289
FT   TOPO_DOM    290     306       CYTOPLASMIC.
//
```

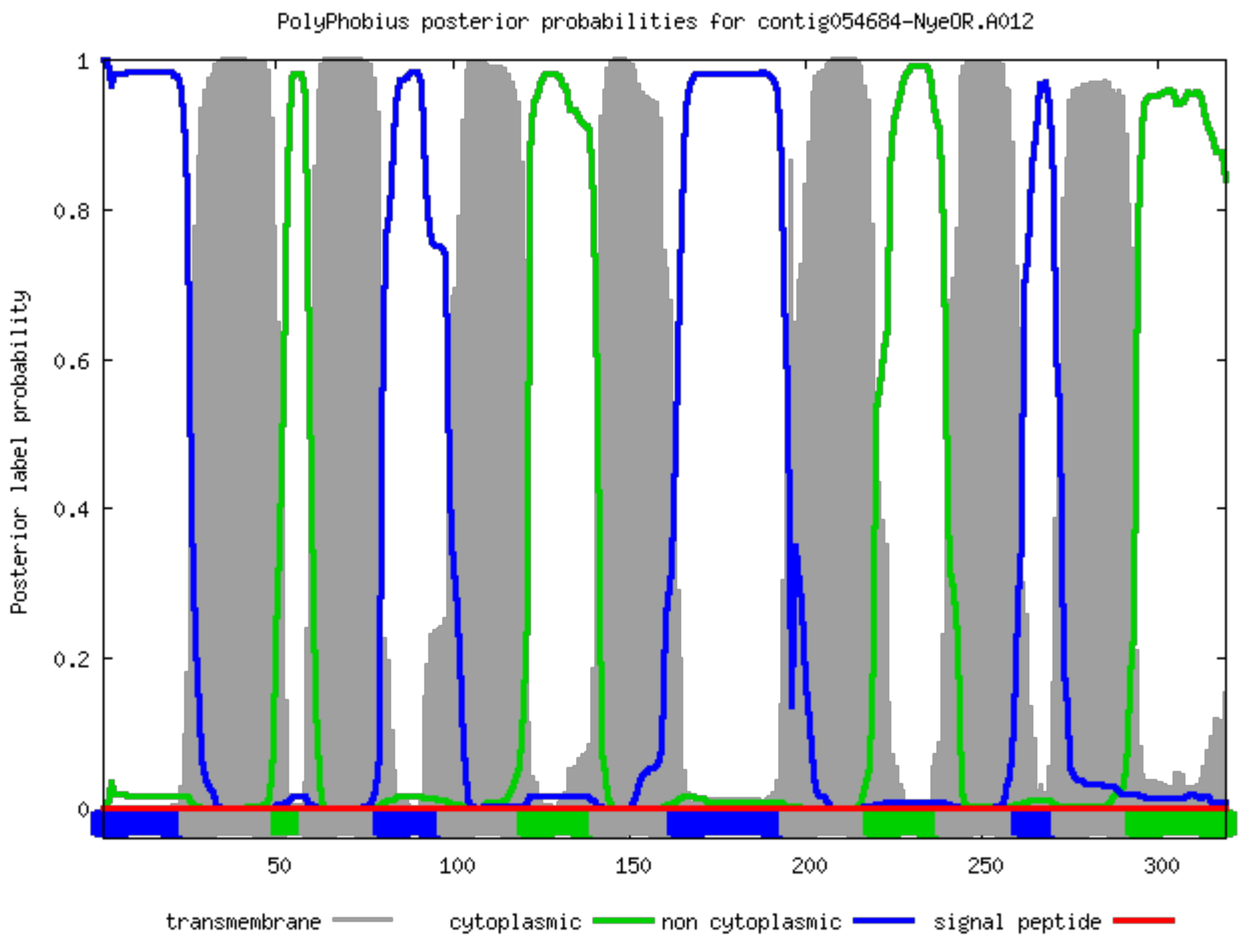

The prediction is based on an alignment. The probability data used in the plot is found here, and the gnuplot script is here.

Prediction of contig085026-BriOR.A008

```
ID   contig085026-BriOR.A008
FT   TOPO_DOM      1     22       NON CYTOPLASMIC.
FT   TRANSMEM     23     48
FT   TOPO_DOM     49     56       CYTOPLASMIC.
FT   TRANSMEM     57     76
FT   TOPO_DOM     77     95       NON CYTOPLASMIC.
FT   TRANSMEM     96    118
FT   TOPO_DOM    119    138       CYTOPLASMIC.
FT   TRANSMEM    139    159
FT   TOPO_DOM    160    192       NON CYTOPLASMIC.
FT   TRANSMEM    193    215
FT   TOPO_DOM    216    235       CYTOPLASMIC.
FT   TRANSMEM    236    257
FT   TOPO_DOM    258    268       NON CYTOPLASMIC.
FT   TRANSMEM    269    289
FT   TOPO_DOM    290    316       CYTOPLASMIC.
//
```

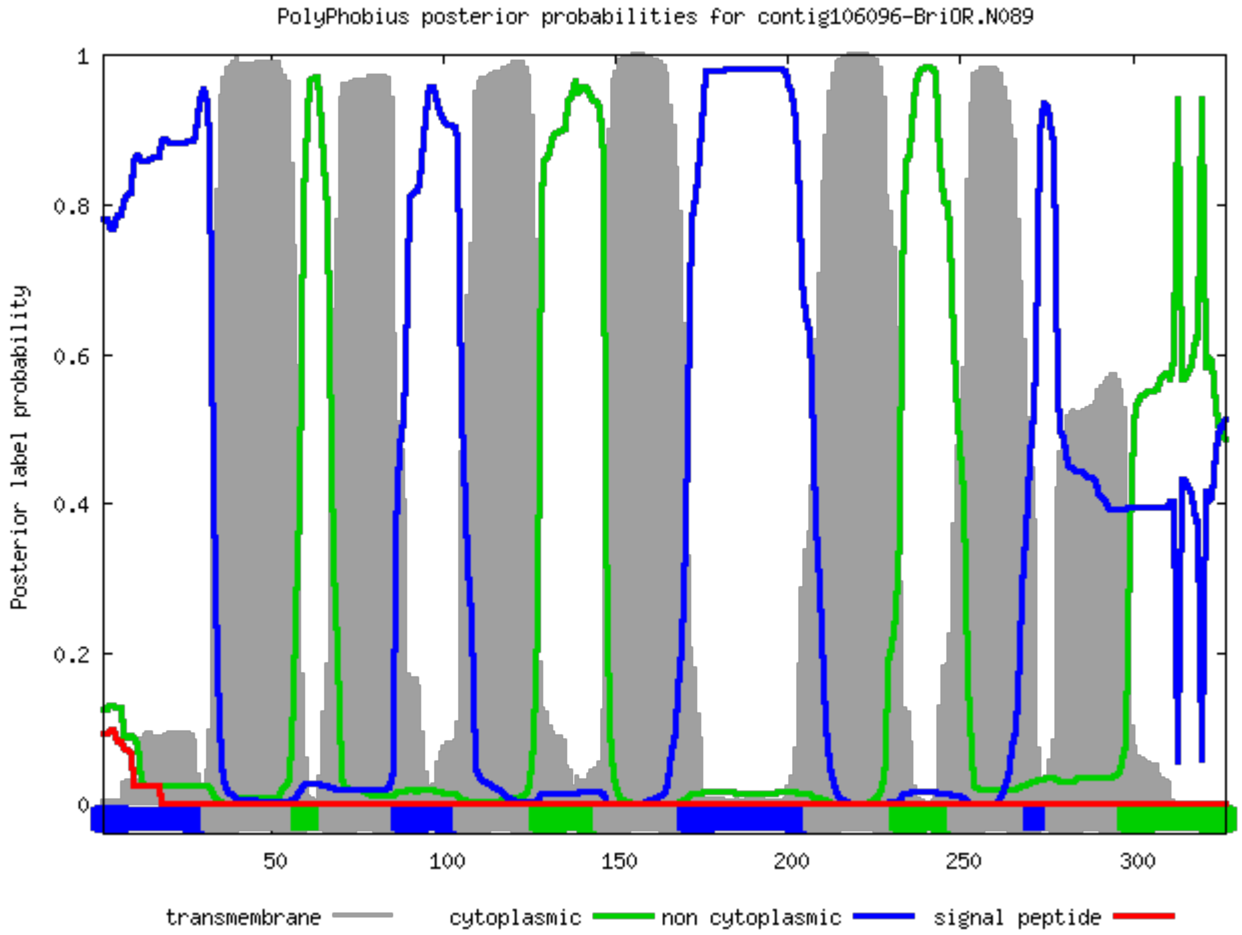

The prediction is based on an alignment. The probability data used in the plot is found here, and the gnuplot script is here.

Prediction of contig044295-NyeOR.R135

```
ID    contig044295-NyeOR.R135
FT    TOPO_DOM      1      24       NON CYTOPLASMIC.
FT    TRANSMEM     25      48
FT    TOPO_DOM     49      59       CYTOPLASMIC.
FT    TRANSMEM     60      84
FT    TOPO_DOM     85      89       NON CYTOPLASMIC.
FT    TRANSMEM     90     118
FT    TOPO_DOM    119     138       CYTOPLASMIC.
FT    TRANSMEM    139     162
FT    TOPO_DOM    163     194       NON CYTOPLASMIC.
FT    TRANSMEM    195     218
FT    TOPO_DOM    219     235       CYTOPLASMIC.
FT    TRANSMEM    236     257
FT    TOPO_DOM    258     270       NON CYTOPLASMIC.
FT    TRANSMEM    271     293
FT    TOPO_DOM    294     317       CYTOPLASMIC.
//
```

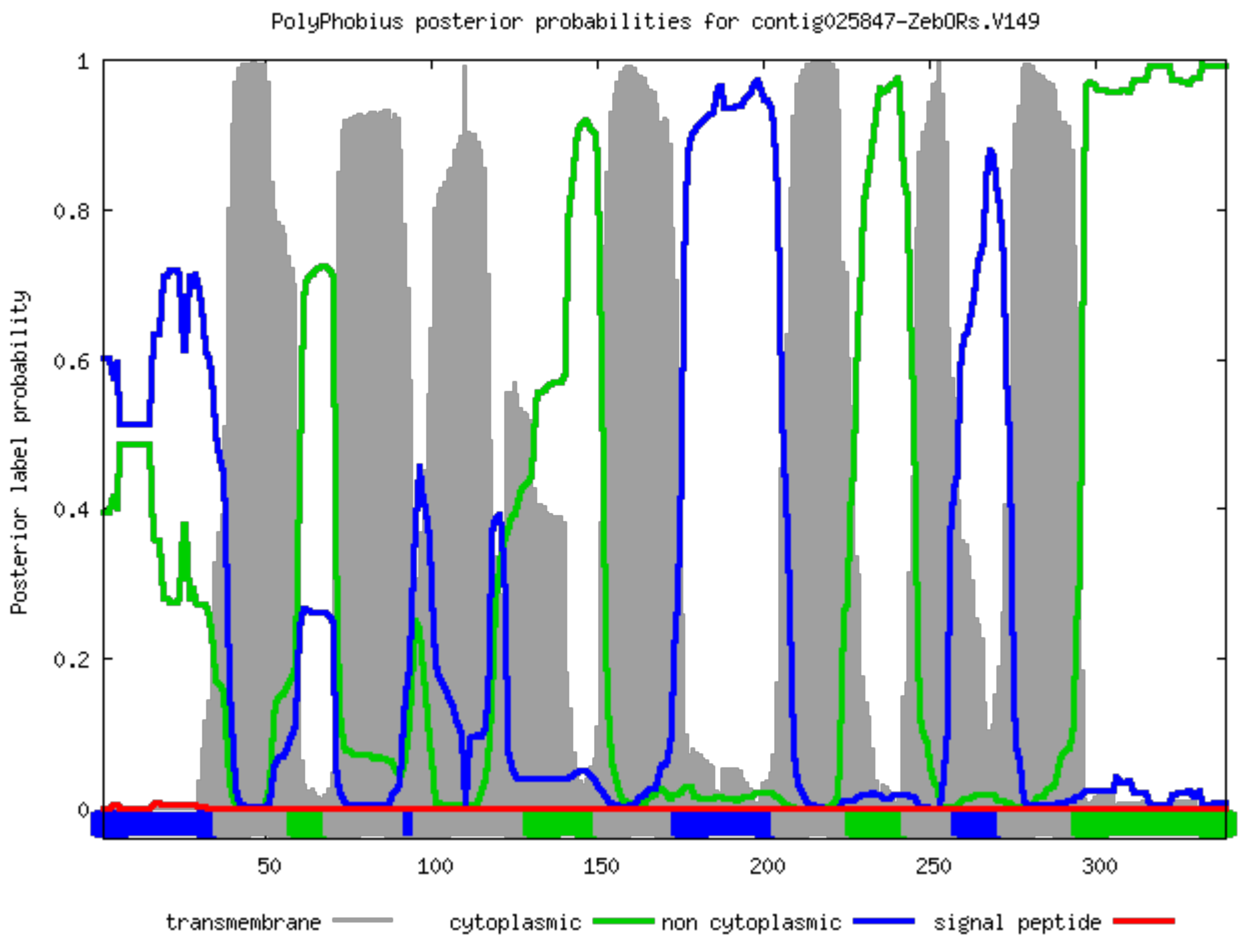

The prediction is based on an alignment. The probability data used in the plot is found here, and the gnuplot script is here.

Prediction of contig046706-TilOR.K135

```
ID   contig046706-TilOR.K135
FT   TOPO_DOM      1     25       NON CYTOPLASMIC.
FT   TRANSMEM     26     51
FT   TOPO_DOM     52     59       CYTOPLASMIC.
FT   TRANSMEM     60     82
FT   TOPO_DOM     83    101       NON CYTOPLASMIC.
FT   TRANSMEM    102    122
FT   TOPO_DOM    123    142       CYTOPLASMIC.
FT   TRANSMEM    143    166
FT   TOPO_DOM    167    197       NON CYTOPLASMIC.
FT   TRANSMEM    198    225
FT   TOPO_DOM    226    244       CYTOPLASMIC.
FT   TRANSMEM    245    265
FT   TOPO_DOM    266    273       NON CYTOPLASMIC.
FT   TRANSMEM    274    293
FT   TOPO_DOM    294    315       CYTOPLASMIC.
//
```

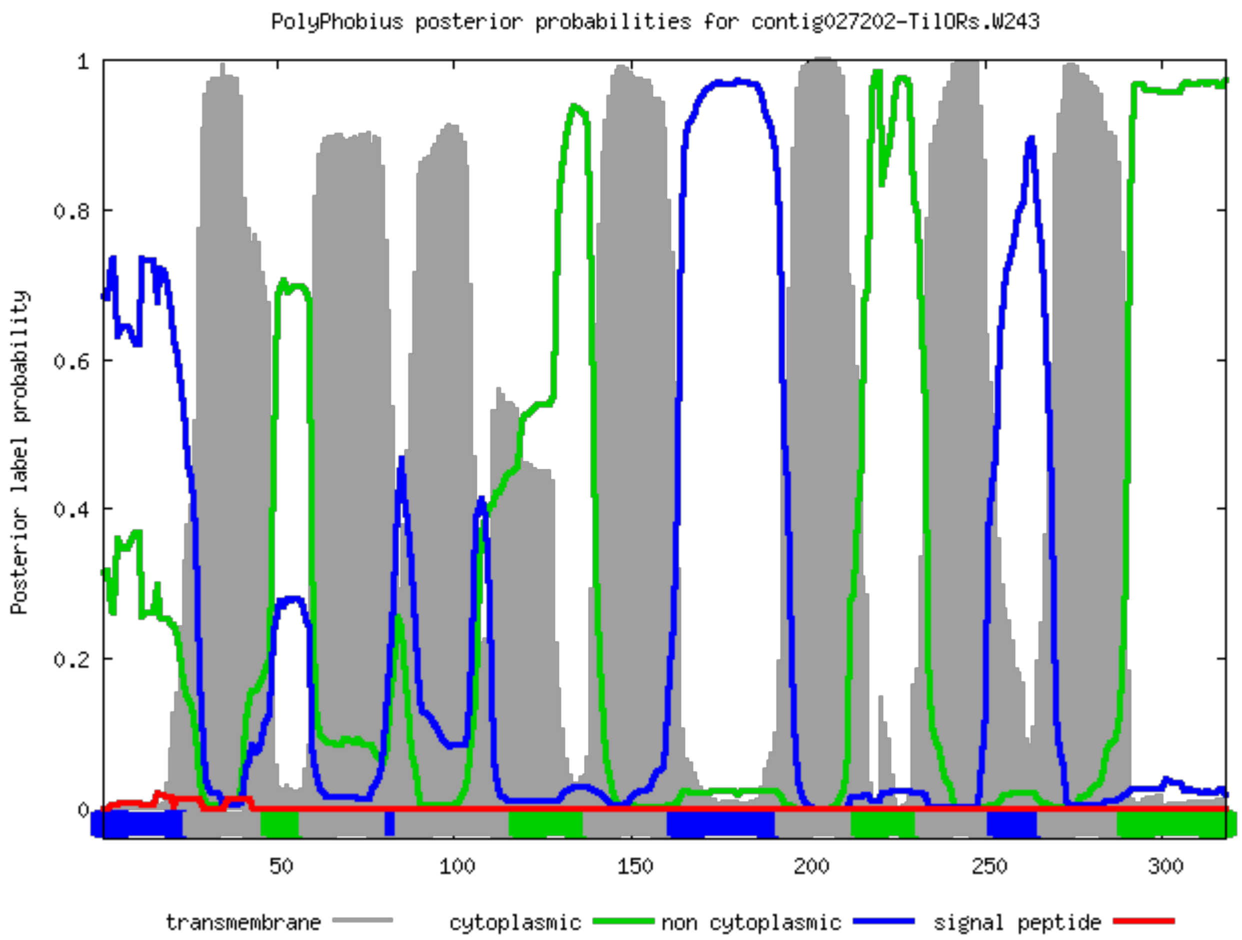

The prediction is based on an alignment. The probability data used in the plot is found here, and the gnuplot script is here.

Prediction of contig046356-TilOR.N196

```
ID    contig046356-TilOR.N196
FT    TOPO_DOM      1      32       NON CYTOPLASMIC.
FT    TRANSMEM     33      58
FT    TOPO_DOM     59      66       CYTOPLASMIC.
FT    TRANSMEM     67      86
FT    TOPO_DOM     87     104       NON CYTOPLASMIC.
FT    TRANSMEM    105     127
FT    TOPO_DOM    128     146       CYTOPLASMIC.
FT    TRANSMEM    147     170
FT    TOPO_DOM    171     207       NON CYTOPLASMIC.
FT    TRANSMEM    208     232
FT    TOPO_DOM    233     250       CYTOPLASMIC.
FT    TRANSMEM    251     271
FT    TOPO_DOM    272     277       NON CYTOPLASMIC.
FT    TRANSMEM    278     298
FT    TOPO_DOM    299     330       CYTOPLASMIC.
//
```

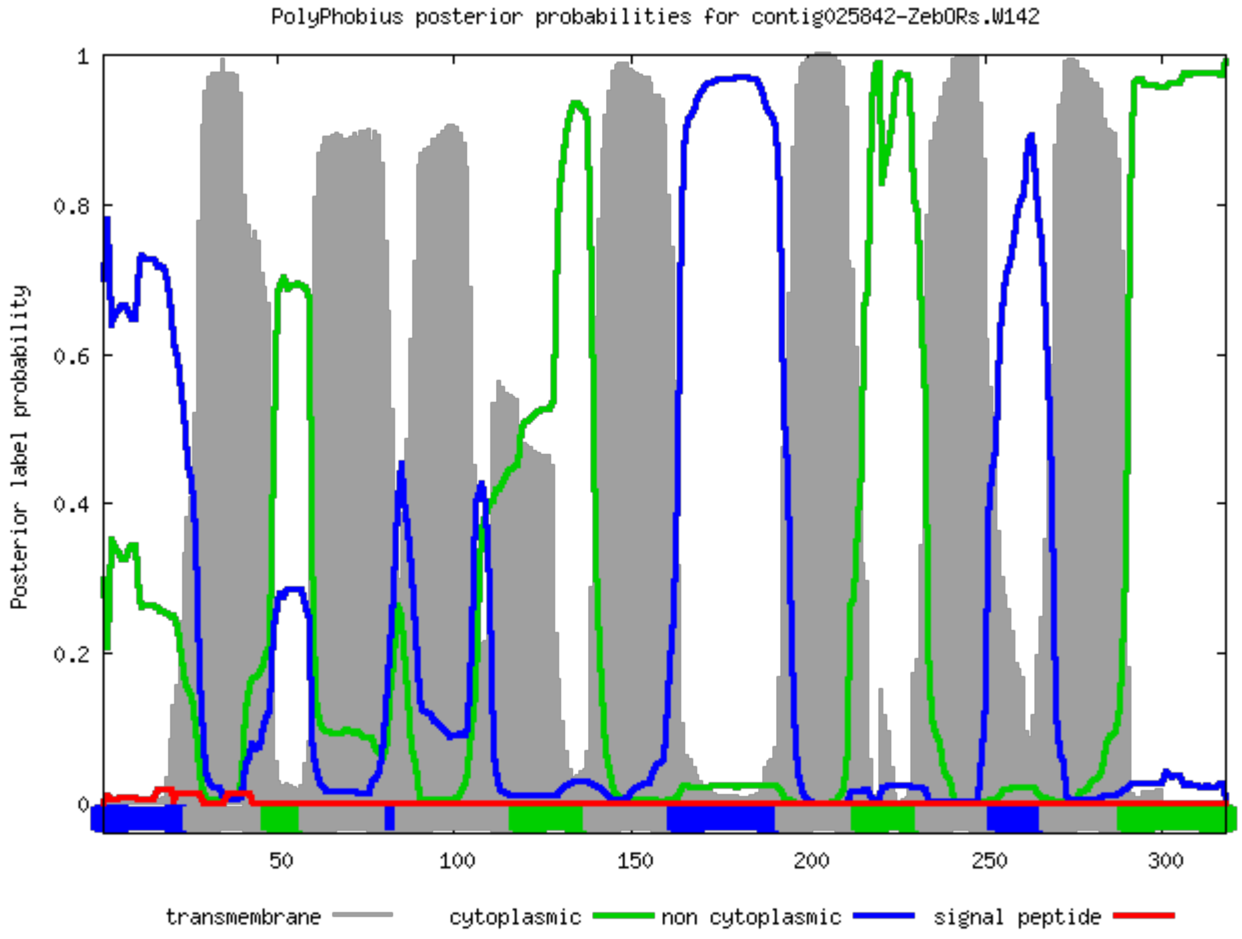

The prediction is based on an alignment. The probability data used in the plot is found here, and the gnuplot script is here.

Prediction of contig052450-BurOR.E055

```
ID   contig052450-BurOR.E055
FT   TOPO_DOM      1     22       NON CYTOPLASMIC.
FT   TRANSMEM     23     48
FT   TOPO_DOM     49     57       CYTOPLASMIC.
FT   TRANSMEM     58     81
FT   TOPO_DOM     82     90       NON CYTOPLASMIC.
FT   TRANSMEM     91    118
FT   TOPO_DOM    119    138       CYTOPLASMIC.
FT   TRANSMEM    139    161
FT   TOPO_DOM    162    193       NON CYTOPLASMIC.
FT   TRANSMEM    194    216
FT   TOPO_DOM    217    236       CYTOPLASMIC.
FT   TRANSMEM    237    256
FT   TOPO_DOM    257    267       NON CYTOPLASMIC.
FT   TRANSMEM    268    291
FT   TOPO_DOM    292    310       CYTOPLASMIC.
//
```

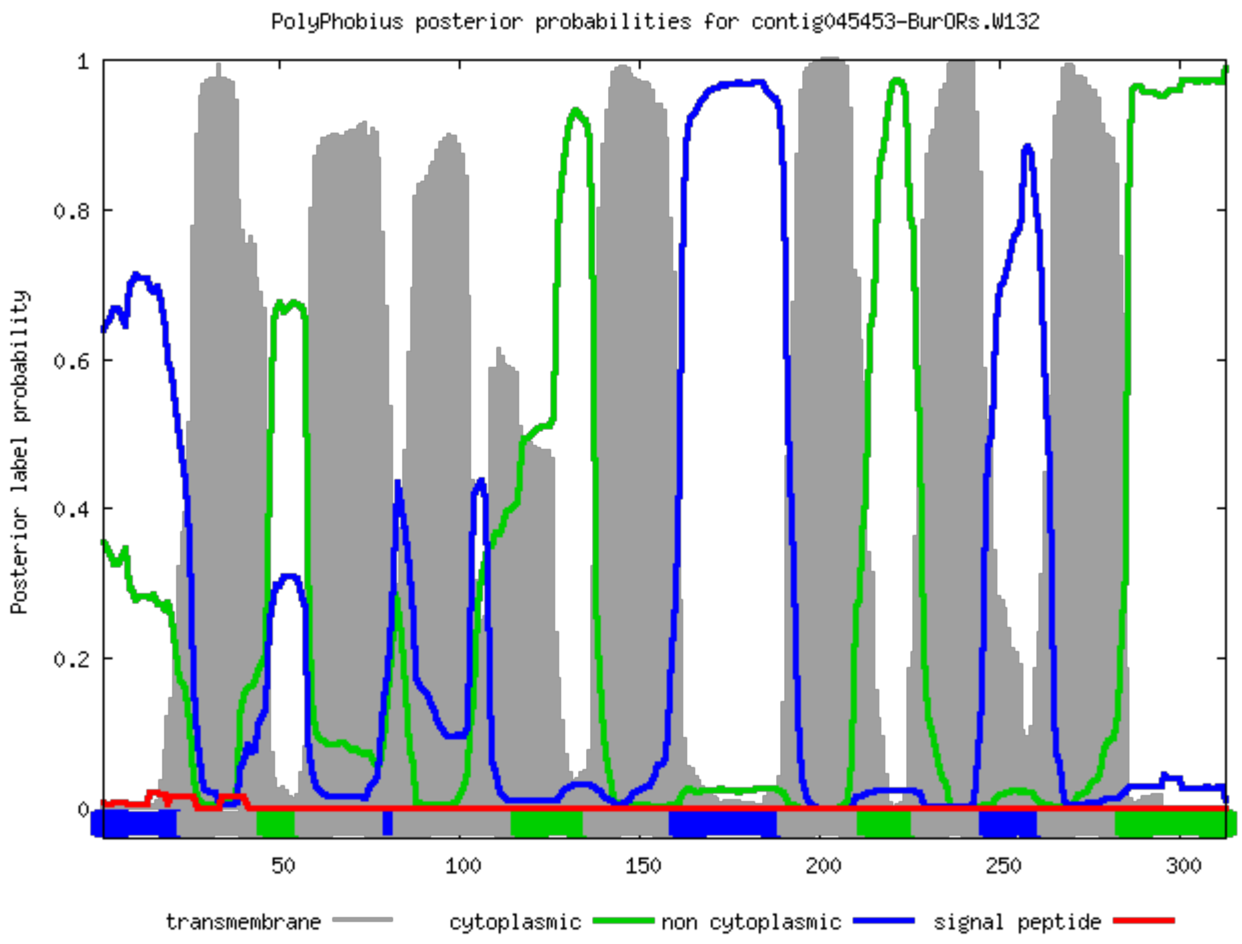

The prediction is based on an alignment. The probability data used in the plot is found here, and the gnuplot script is here.

Prediction of contig070885-TilOR.A024

```
ID   contig070885-TilOR.A024
FT   TOPO_DOM      1     22       NON CYTOPLASMIC.
FT   TRANSMEM     23     48
FT   TOPO_DOM     49     56       CYTOPLASMIC.
FT   TRANSMEM     57     77
FT   TOPO_DOM     78     95       NON CYTOPLASMIC.
FT   TRANSMEM     96    118
FT   TOPO_DOM    119    138       CYTOPLASMIC.
FT   TRANSMEM    139    160
FT   TOPO_DOM    161    192       NON CYTOPLASMIC.
FT   TRANSMEM    193    215
FT   TOPO_DOM    216    235       CYTOPLASMIC.
FT   TRANSMEM    236    257
FT   TOPO_DOM    258    268       NON CYTOPLASMIC.
FT   TRANSMEM    269    289
FT   TOPO_DOM    290    309       CYTOPLASMIC.
//
```

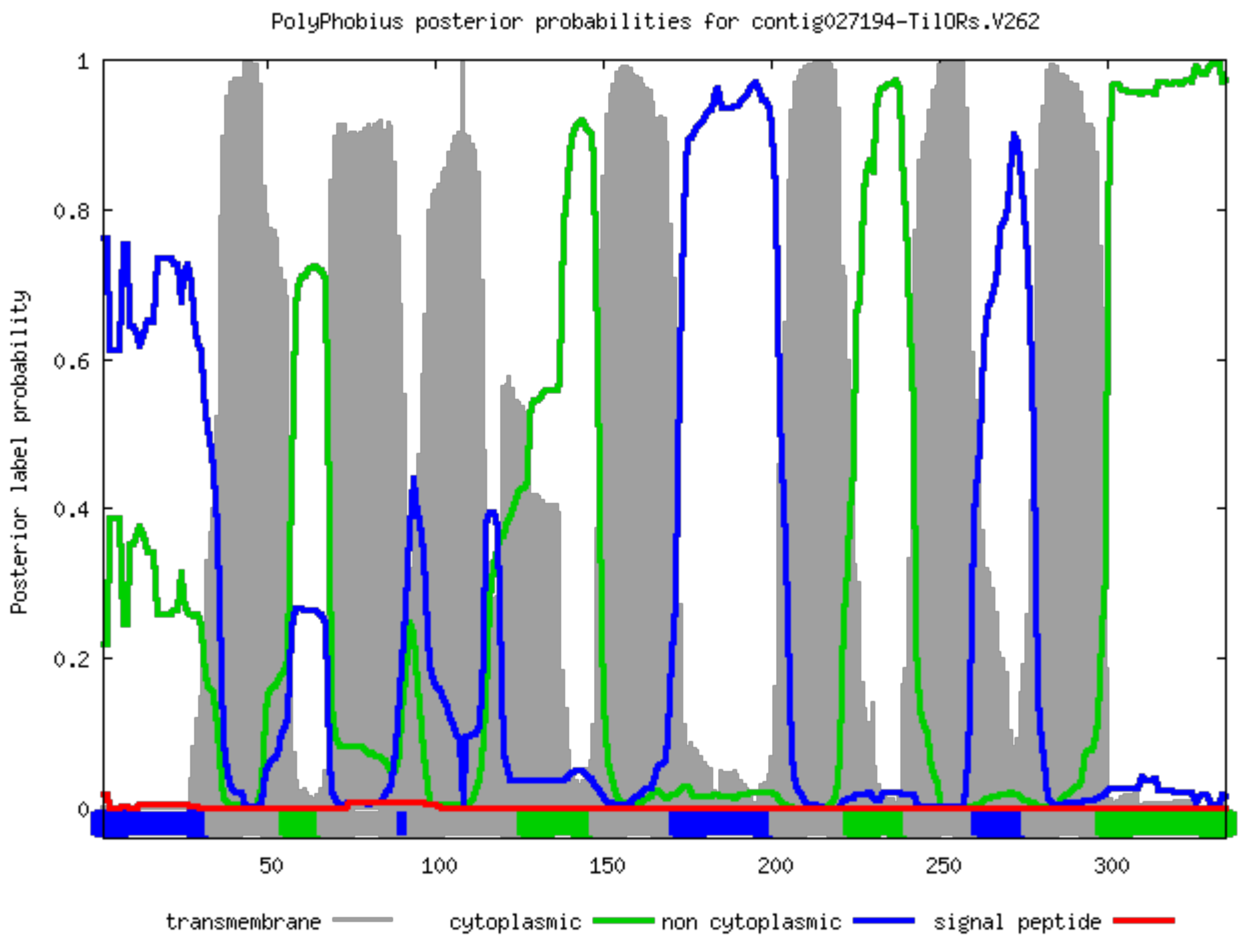

The prediction is based on an alignment. The probability data used in the plot is found here, and the gnuplot script is here.

Prediction of contig052453-BurOR.E049

```
ID   contig052453-BurOR.E049
FT   TOPO_DOM      1     22       NON CYTOPLASMIC.
FT   TRANSMEM     23     48
FT   TOPO_DOM     49     57       CYTOPLASMIC.
FT   TRANSMEM     58     83
FT   TOPO_DOM     84     90       NON CYTOPLASMIC.
FT   TRANSMEM     91    118
FT   TOPO_DOM    119    138       CYTOPLASMIC.
FT   TRANSMEM    139    161
FT   TOPO_DOM    162    193       NON CYTOPLASMIC.
FT   TRANSMEM    194    216
FT   TOPO_DOM    217    236       CYTOPLASMIC.
FT   TRANSMEM    237    256
FT   TOPO_DOM    257    267       NON CYTOPLASMIC.
FT   TRANSMEM    268    291
FT   TOPO_DOM    292    326       CYTOPLASMIC.
//
```

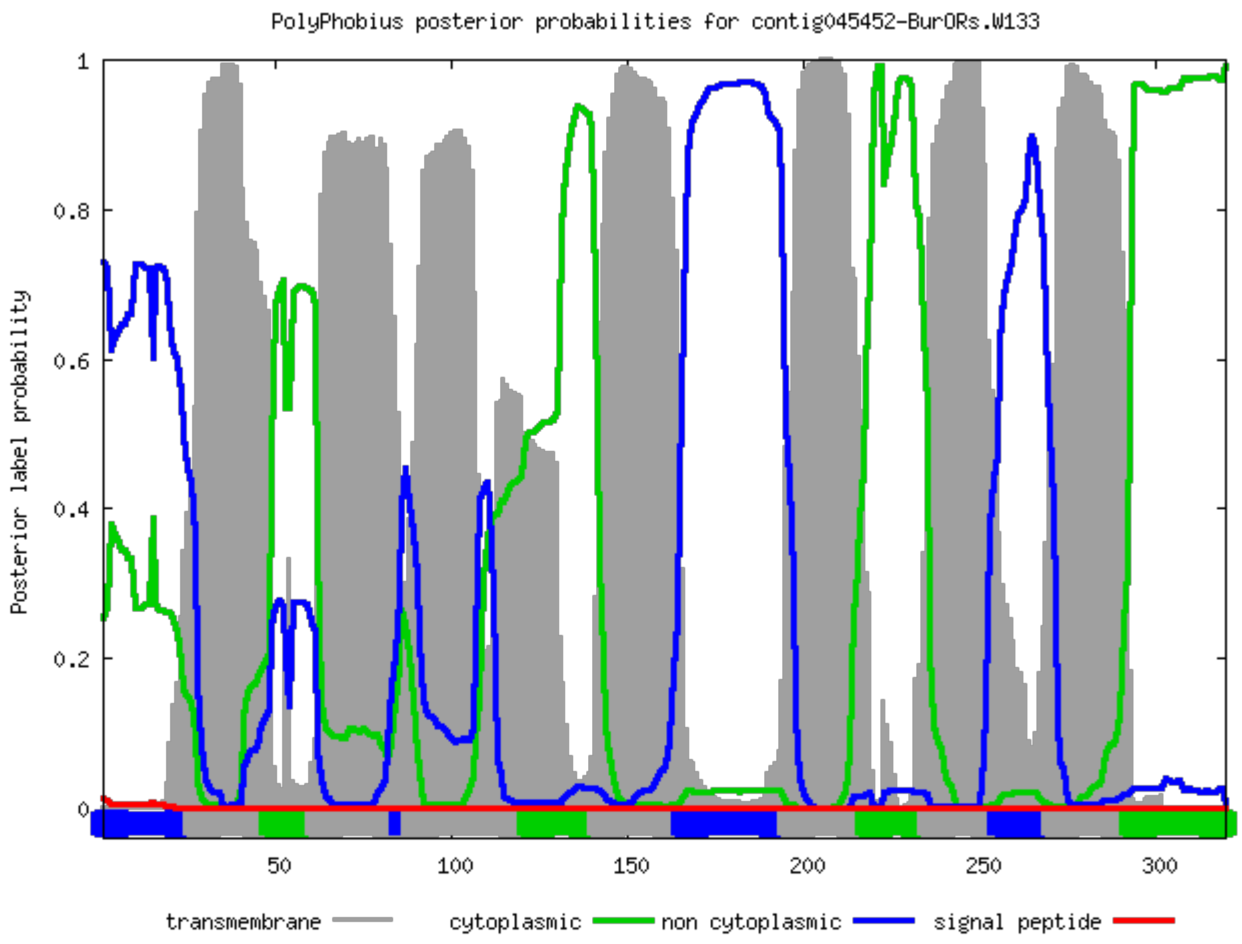

The prediction is based on an alignment. The probability data used in the plot is found here, and the gnuplot script is here.

Prediction of contig060631-BurOR.N110

```
ID   contig060631-BurOR.N110
//
```

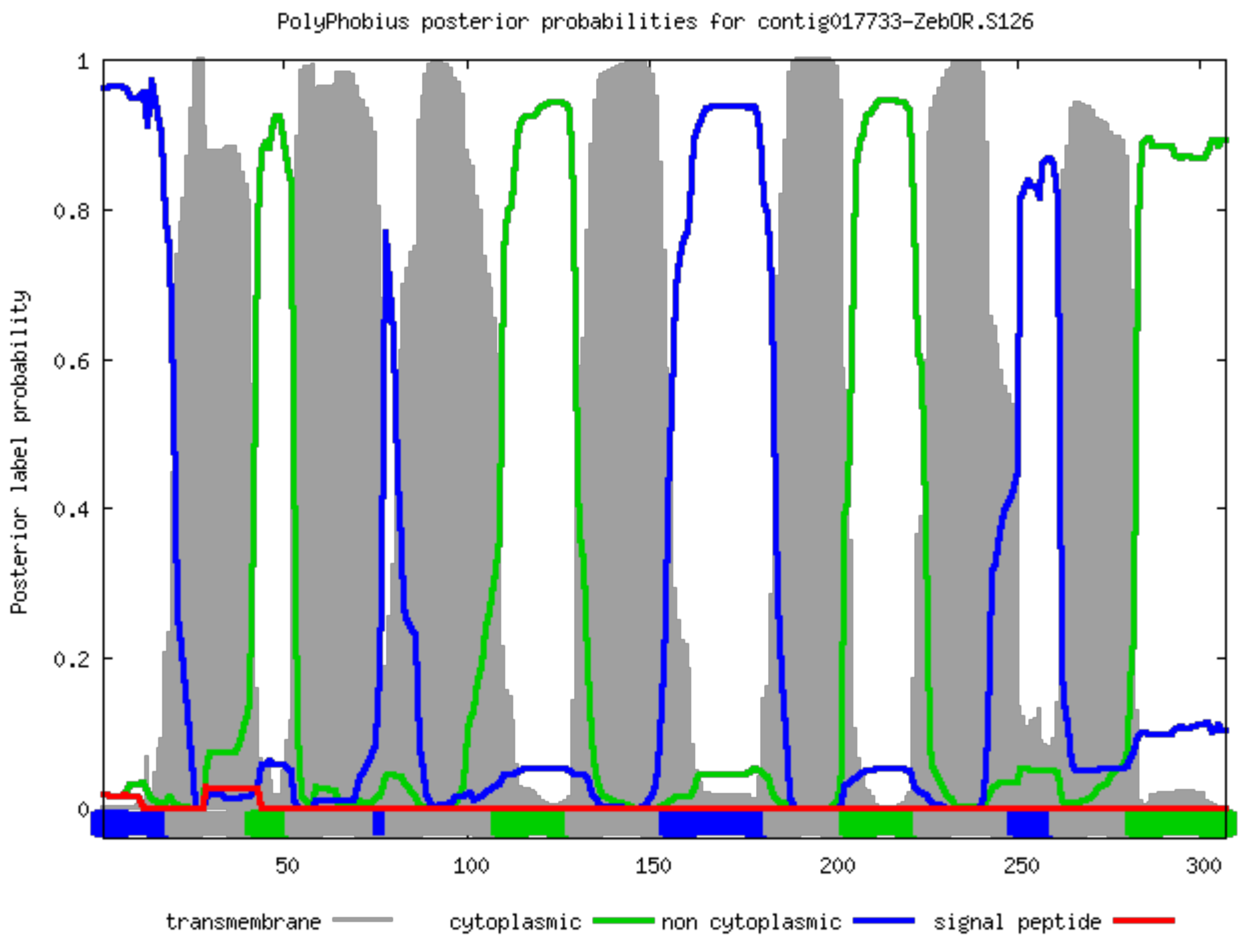

The prediction is based on an alignment. The probability data used in the plot is found here, and the gnuplot script is here.

Prediction of contig022232-TilOR.A008

```
ID   contig022232-TilOR.A008
FT   TOPO_DOM      1     22       NON CYTOPLASMIC.
FT   TRANSMEM     23     48
FT   TOPO_DOM     49     56       CYTOPLASMIC.
FT   TRANSMEM     57     76
FT   TOPO_DOM     77     95       NON CYTOPLASMIC.
FT   TRANSMEM     96    118
FT   TOPO_DOM    119    138       CYTOPLASMIC.
FT   TRANSMEM    139    159
FT   TOPO_DOM    160    192       NON CYTOPLASMIC.
FT   TRANSMEM    193    215
FT   TOPO_DOM    216    235       CYTOPLASMIC.
FT   TRANSMEM    236    257
FT   TOPO_DOM    258    268       NON CYTOPLASMIC.
FT   TRANSMEM    269    289
FT   TOPO_DOM    290    306       CYTOPLASMIC.
//
```

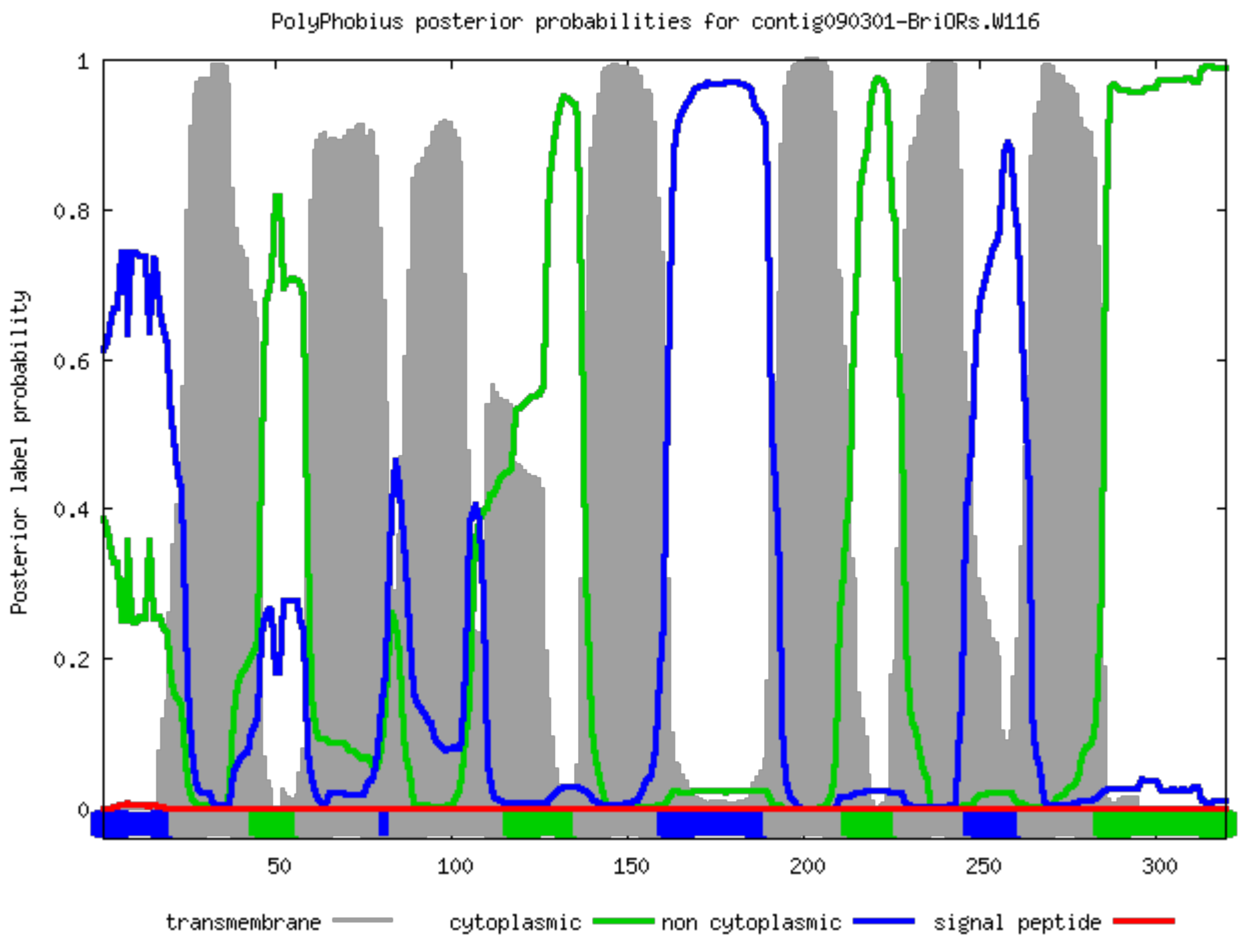

The prediction is based on an alignment. The probability data used in the plot is found here, and the gnuplot script is here.

Prediction of contig039738-NyeOR.D042

```
ID   contig039738-NyeOR.D042
FT   TOPO_DOM      1     22       NON CYTOPLASMIC.
FT   TRANSMEM     23     48
FT   TOPO_DOM     49     57       CYTOPLASMIC.
FT   TRANSMEM     58     81
FT   TOPO_DOM     82     90       NON CYTOPLASMIC.
FT   TRANSMEM     91    118
FT   TOPO_DOM    119    138       CYTOPLASMIC.
FT   TRANSMEM    139    162
FT   TOPO_DOM    163    194       NON CYTOPLASMIC.
FT   TRANSMEM    195    216
FT   TOPO_DOM    217    236       CYTOPLASMIC.
FT   TRANSMEM    237    256
FT   TOPO_DOM    257    267       NON CYTOPLASMIC.
FT   TRANSMEM    268    291
FT   TOPO_DOM    292    309       CYTOPLASMIC.
//
```

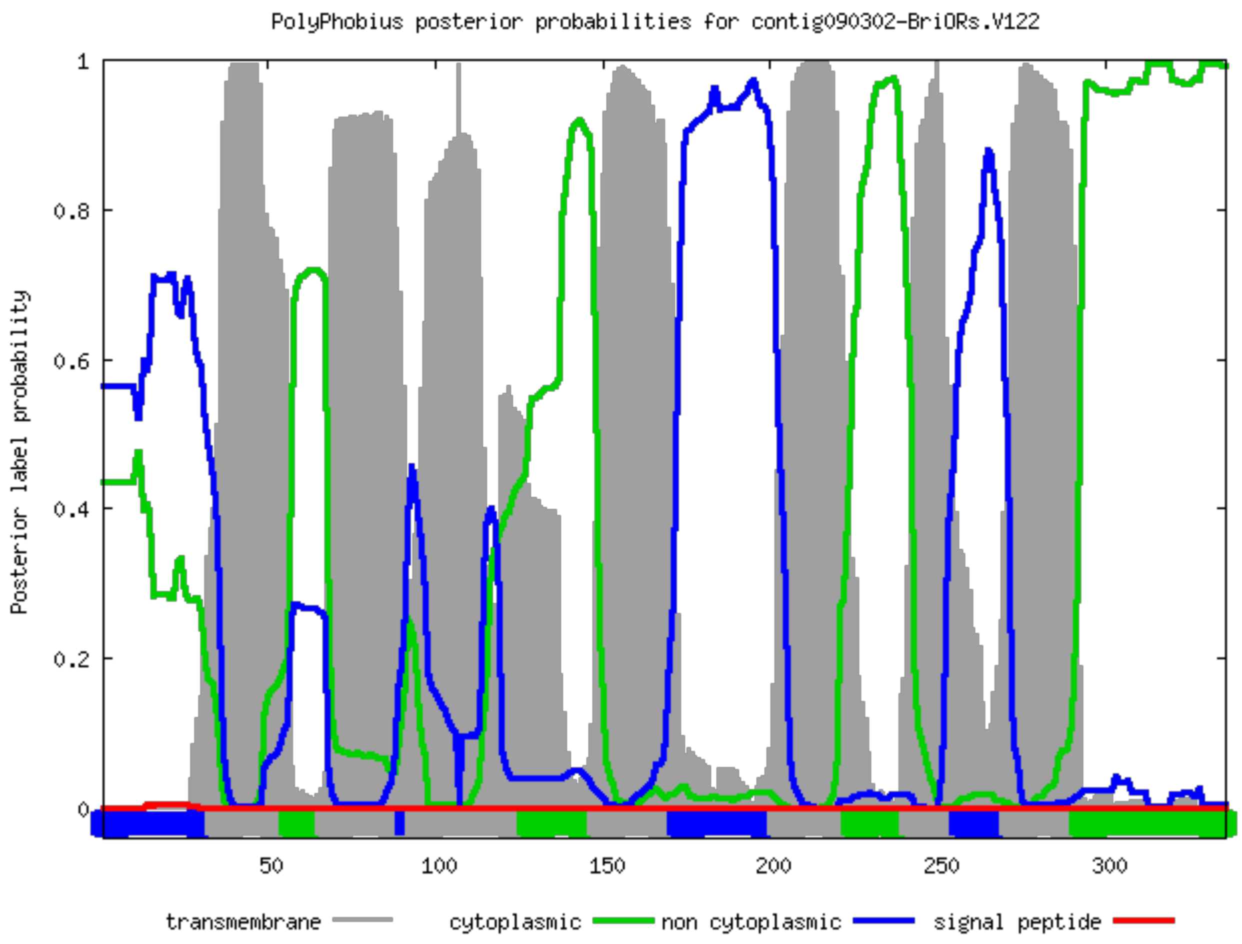

The prediction is based on an alignment. The probability data used in the plot is found here, and the gnuplot script is here.

Prediction of contig023280-NyeOR.E050

```
ID   contig023280-NyeOR.E050
FT   TOPO_DOM      1     22       NON CYTOPLASMIC.
FT   TRANSMEM     23     48
FT   TOPO_DOM     49     57       CYTOPLASMIC.
FT   TRANSMEM     58     81
FT   TOPO_DOM     82     91       NON CYTOPLASMIC.
FT   TRANSMEM     92    118
FT   TOPO_DOM    119    138       CYTOPLASMIC.
FT   TRANSMEM    139    161
FT   TOPO_DOM    162    193       NON CYTOPLASMIC.
FT   TRANSMEM    194    216
FT   TOPO_DOM    217    236       CYTOPLASMIC.
FT   TRANSMEM    237    256
FT   TOPO_DOM    257    267       NON CYTOPLASMIC.
FT   TRANSMEM    268    291
FT   TOPO_DOM    292    310       CYTOPLASMIC.
//
```

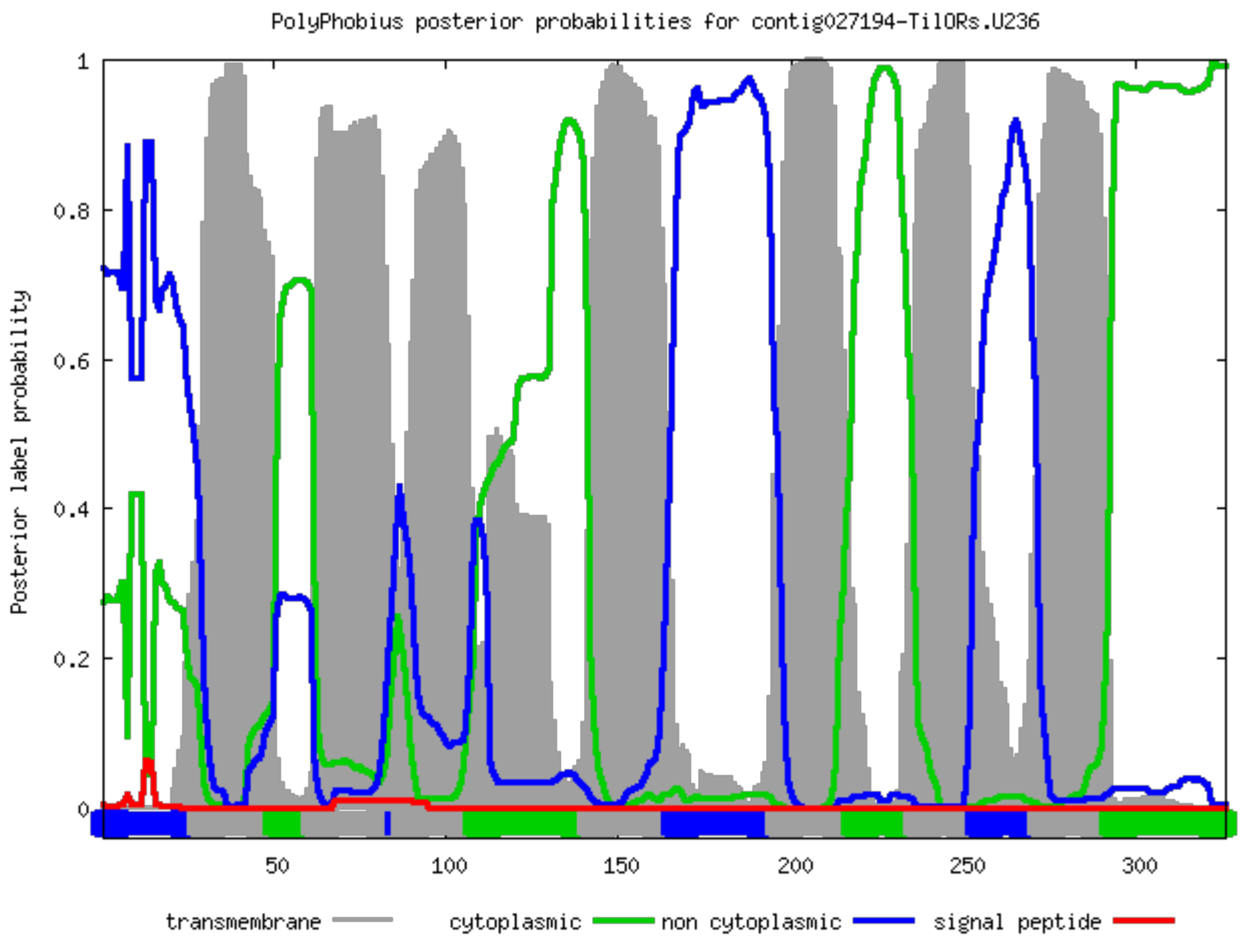

The prediction is based on an alignment. The probability data used in the plot is found here, and the gnuplot script is here.

Prediction of contig009565-TilOR.H126

```
ID   contig009565-TilOR.H126
FT   TOPO_DOM      1      1       NON CYTOPLASMIC.
FT   TRANSMEM      2     20
FT   TOPO_DOM     21     27       CYTOPLASMIC.
FT   TRANSMEM     28     47
FT   TOPO_DOM     48     66       NON CYTOPLASMIC.
FT   TRANSMEM     67     89
FT   TOPO_DOM     90    109       CYTOPLASMIC.
FT   TRANSMEM    110    131
FT   TOPO_DOM    132    167       NON CYTOPLASMIC.
FT   TRANSMEM    168    190
FT   TOPO_DOM    191    208       CYTOPLASMIC.
FT   TRANSMEM    209    230
FT   TOPO_DOM    231    242       NON CYTOPLASMIC.
FT   TRANSMEM    243    262
FT   TOPO_DOM    263    280       CYTOPLASMIC.
//
```

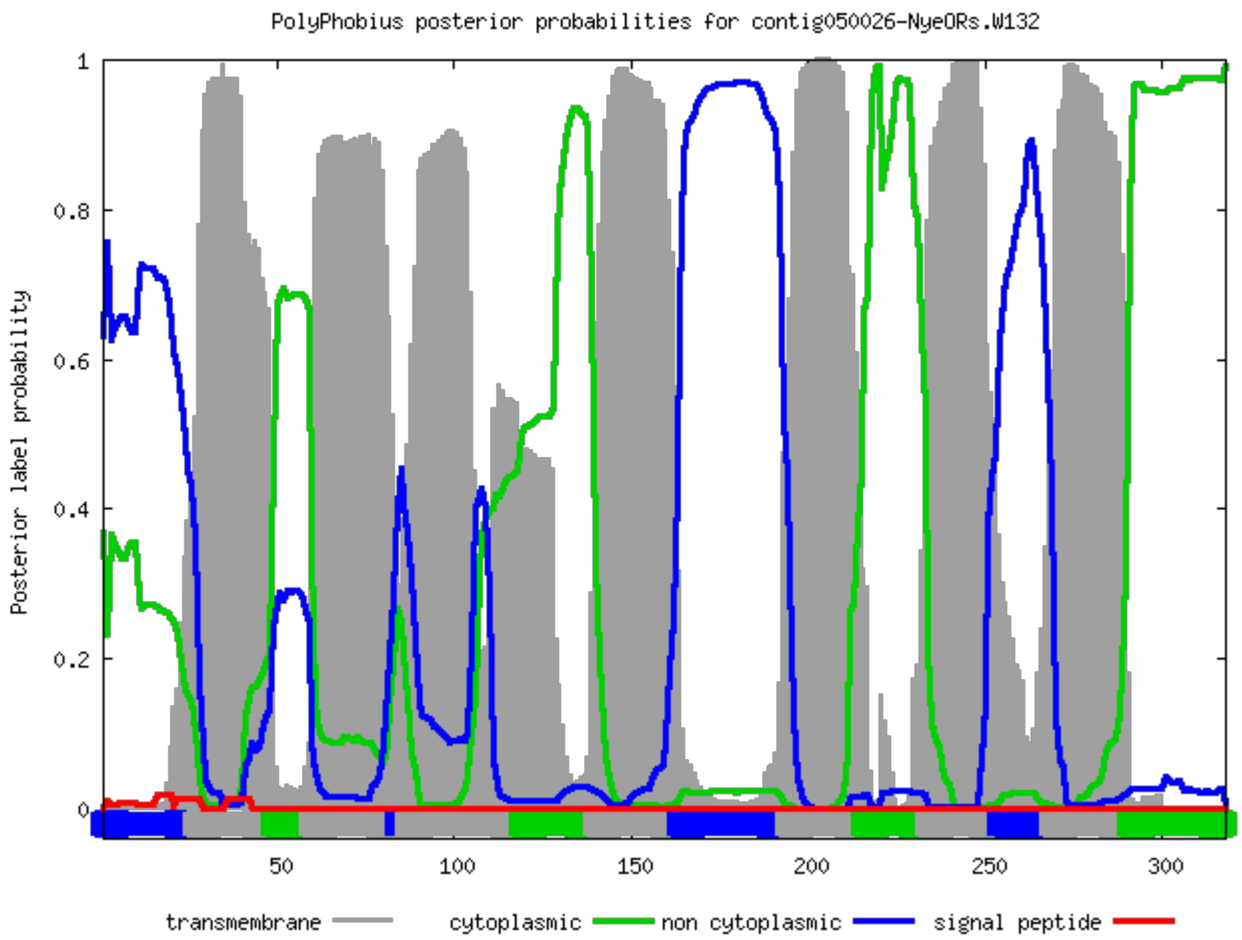

The prediction is based on an alignment. The probability data used in the plot is found here, and the gnuplot script is here.

Prediction of contig023717-TilOR.O175

```
ID   contig023717-TilOR.O175
FT   TOPO_DOM      1     24       NON CYTOPLASMIC.
FT   TRANSMEM     25     51
FT   TOPO_DOM     52     60       CYTOPLASMIC.
FT   TRANSMEM     61     83
FT   TOPO_DOM     84     98       NON CYTOPLASMIC.
FT   TRANSMEM     99    121
FT   TOPO_DOM    122    141       CYTOPLASMIC.
FT   TRANSMEM    142    163
FT   TOPO_DOM    164    200       NON CYTOPLASMIC.
FT   TRANSMEM    201    227
FT   TOPO_DOM    228    240       CYTOPLASMIC.
FT   TRANSMEM    241    262
FT   TOPO_DOM    263    273       NON CYTOPLASMIC.
FT   TRANSMEM    274    295
FT   TOPO_DOM    296    326       CYTOPLASMIC.
//
```

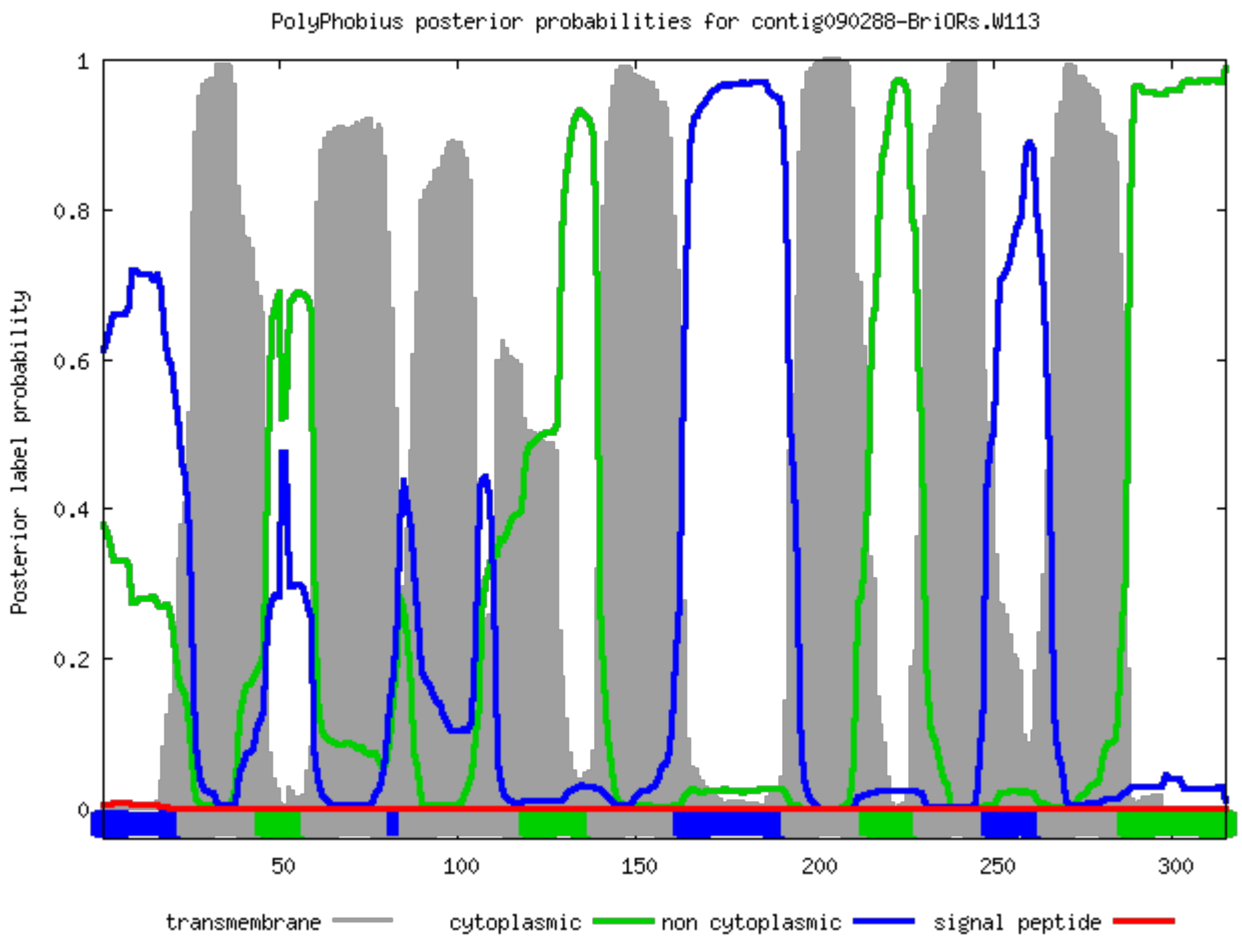

The prediction is based on an alignment. The probability data used in the plot is found here, and the gnuplot script is here.

Prediction of contig049873-BriOR.H050

```
ID   contig049873-BriOR.H050
FT   TOPO_DOM      1      23       NON CYTOPLASMIC.
FT   TRANSMEM     24      49
FT   TOPO_DOM     50      56       CYTOPLASMIC.
FT   TRANSMEM     57      76
FT   TOPO_DOM     77      95       NON CYTOPLASMIC.
FT   TRANSMEM     96     118
FT   TOPO_DOM    119     138       CYTOPLASMIC.
FT   TRANSMEM    139     160
FT   TOPO_DOM    161     196       NON CYTOPLASMIC.
FT   TRANSMEM    197     219
FT   TOPO_DOM    220     237       CYTOPLASMIC.
FT   TRANSMEM    238     260
FT   TOPO_DOM    261     271       NON CYTOPLASMIC.
FT   TRANSMEM    272     291
FT   TOPO_DOM    292     329       CYTOPLASMIC.
//
```

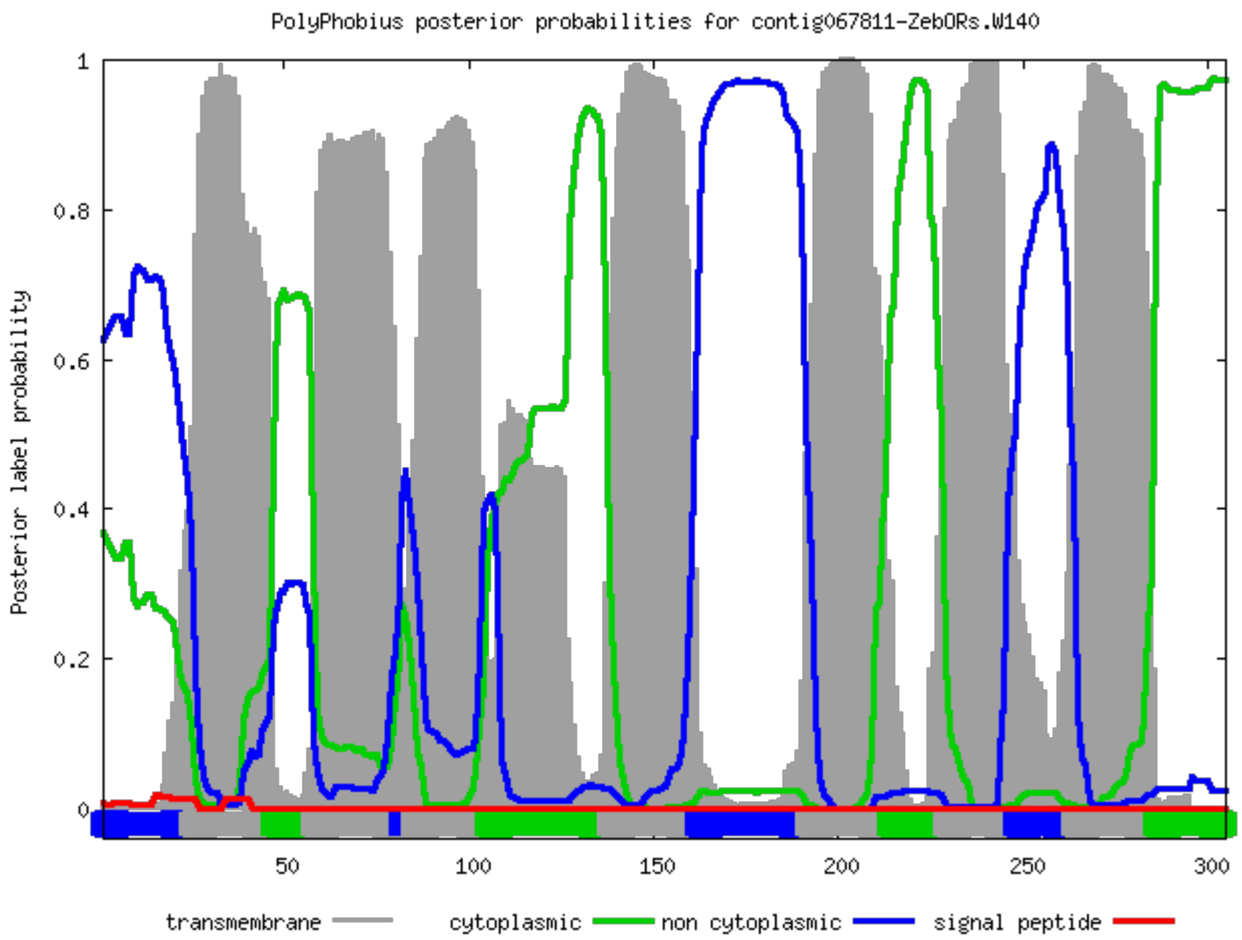

The prediction is based on an alignment. The probability data used in the plot is found here, and the gnuplot script is here.

Prediction of contig055933-NyeOR.N113

```
ID   contig055933-NyeOR.N113
FT   TOPO_DOM      1     33       NON CYTOPLASMIC.
FT   TRANSMEM     34     59
FT   TOPO_DOM     60     67       CYTOPLASMIC.
FT   TRANSMEM     68     89
FT   TOPO_DOM     90    108       NON CYTOPLASMIC.
FT   TRANSMEM    109    128
FT   TOPO_DOM    129    148       CYTOPLASMIC.
FT   TRANSMEM    149    171
FT   TOPO_DOM    172    207       NON CYTOPLASMIC.
FT   TRANSMEM    208    233
FT   TOPO_DOM    234    252       CYTOPLASMIC.
FT   TRANSMEM    253    275
FT   TOPO_DOM    276    337       NON CYTOPLASMIC.
//
```

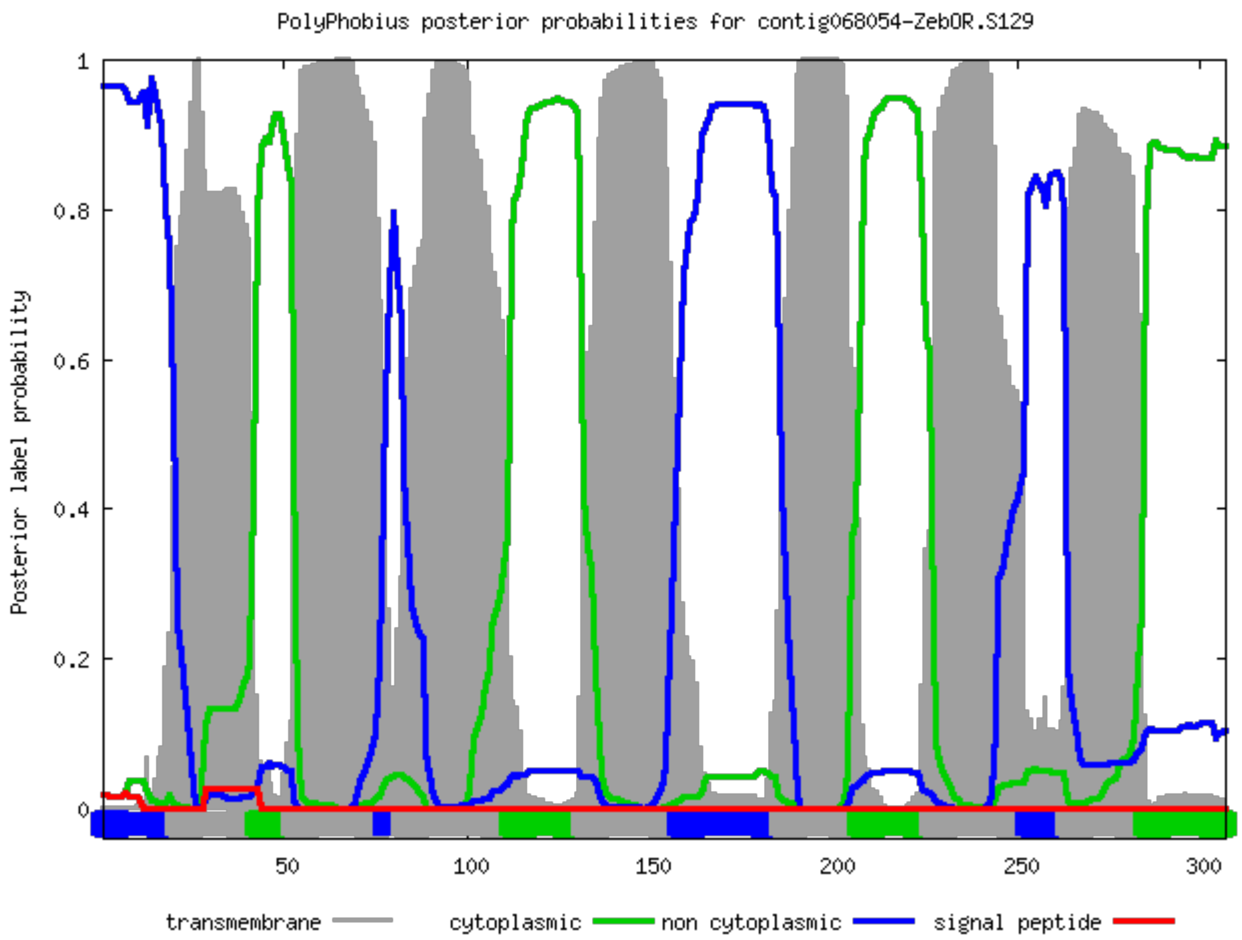

The prediction is based on an alignment. The probability data used in the plot is found here, and the gnuplot script is here.

Prediction of contig046344-TilOR.N189

```
ID   contig046344-TilOR.N189
FT   TOPO_DOM      1     33       NON CYTOPLASMIC.
FT   TRANSMEM     34     59
FT   TOPO_DOM     60     67       CYTOPLASMIC.
FT   TRANSMEM     68     89
FT   TOPO_DOM     90    108       NON CYTOPLASMIC.
FT   TRANSMEM    109    128
FT   TOPO_DOM    129    148       CYTOPLASMIC.
FT   TRANSMEM    149    170
FT   TOPO_DOM    171    207       NON CYTOPLASMIC.
FT   TRANSMEM    208    233
FT   TOPO_DOM    234    252       CYTOPLASMIC.
FT   TRANSMEM    253    275
FT   TOPO_DOM    276    328       NON CYTOPLASMIC.
//
```

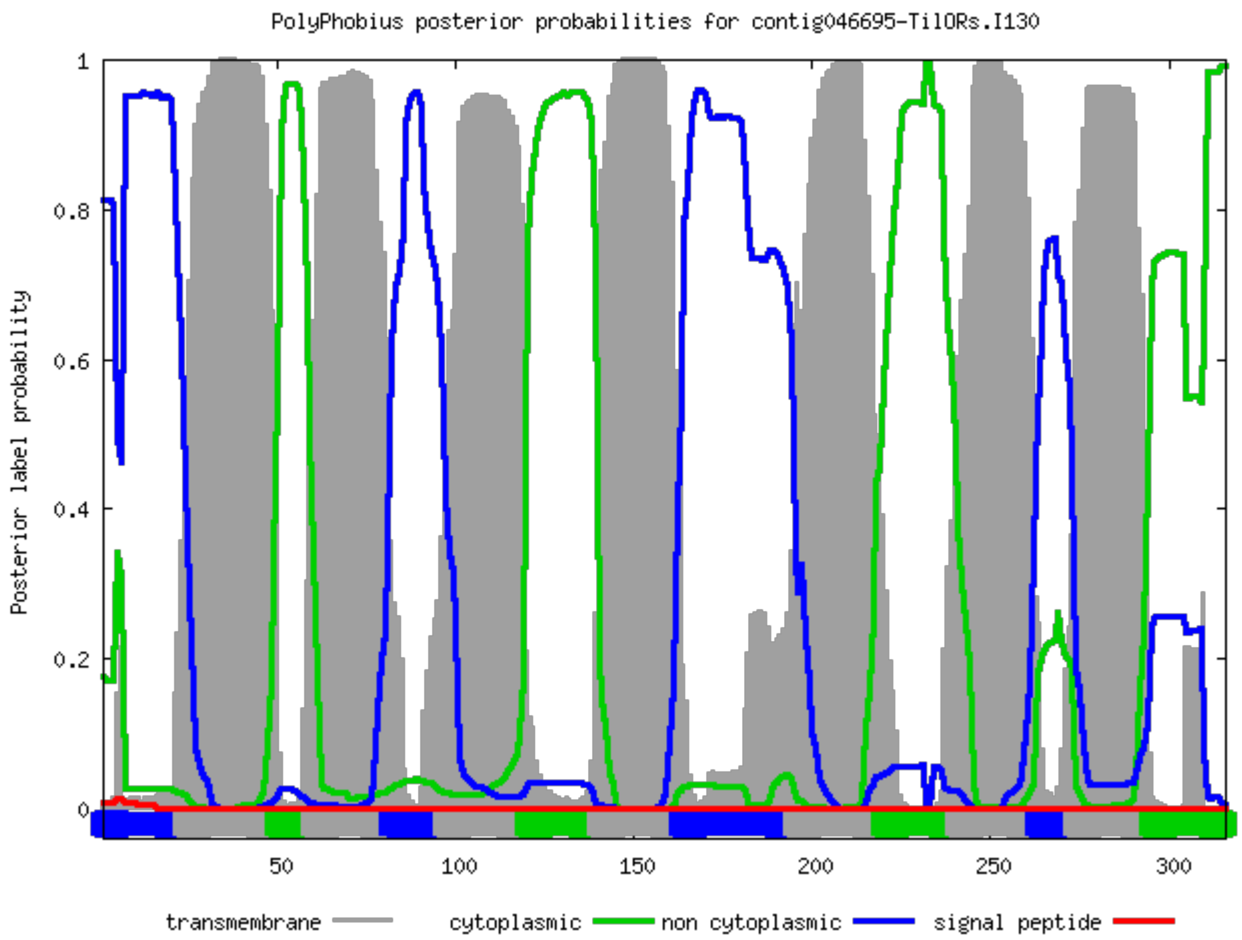

The prediction is based on an alignment. The probability data used in the plot is found here, and the gnuplot script is here.

Prediction of contig030560-ZebOR.A007

```
ID   contig030560-ZebOR.A007
FT   TOPO_DOM      1     25       NON CYTOPLASMIC.
FT   TRANSMEM     26     51
FT   TOPO_DOM     52     59       CYTOPLASMIC.
FT   TRANSMEM     60     80
FT   TOPO_DOM     81     98       NON CYTOPLASMIC.
FT   TRANSMEM     99    121
FT   TOPO_DOM    122    141       CYTOPLASMIC.
FT   TRANSMEM    142    163
FT   TOPO_DOM    164    195       NON CYTOPLASMIC.
FT   TRANSMEM    196    219
FT   TOPO_DOM    220    239       CYTOPLASMIC.
FT   TRANSMEM    240    261
FT   TOPO_DOM    262    272       NON CYTOPLASMIC.
FT   TRANSMEM    273    293
FT   TOPO_DOM    294    319       CYTOPLASMIC.
//
```

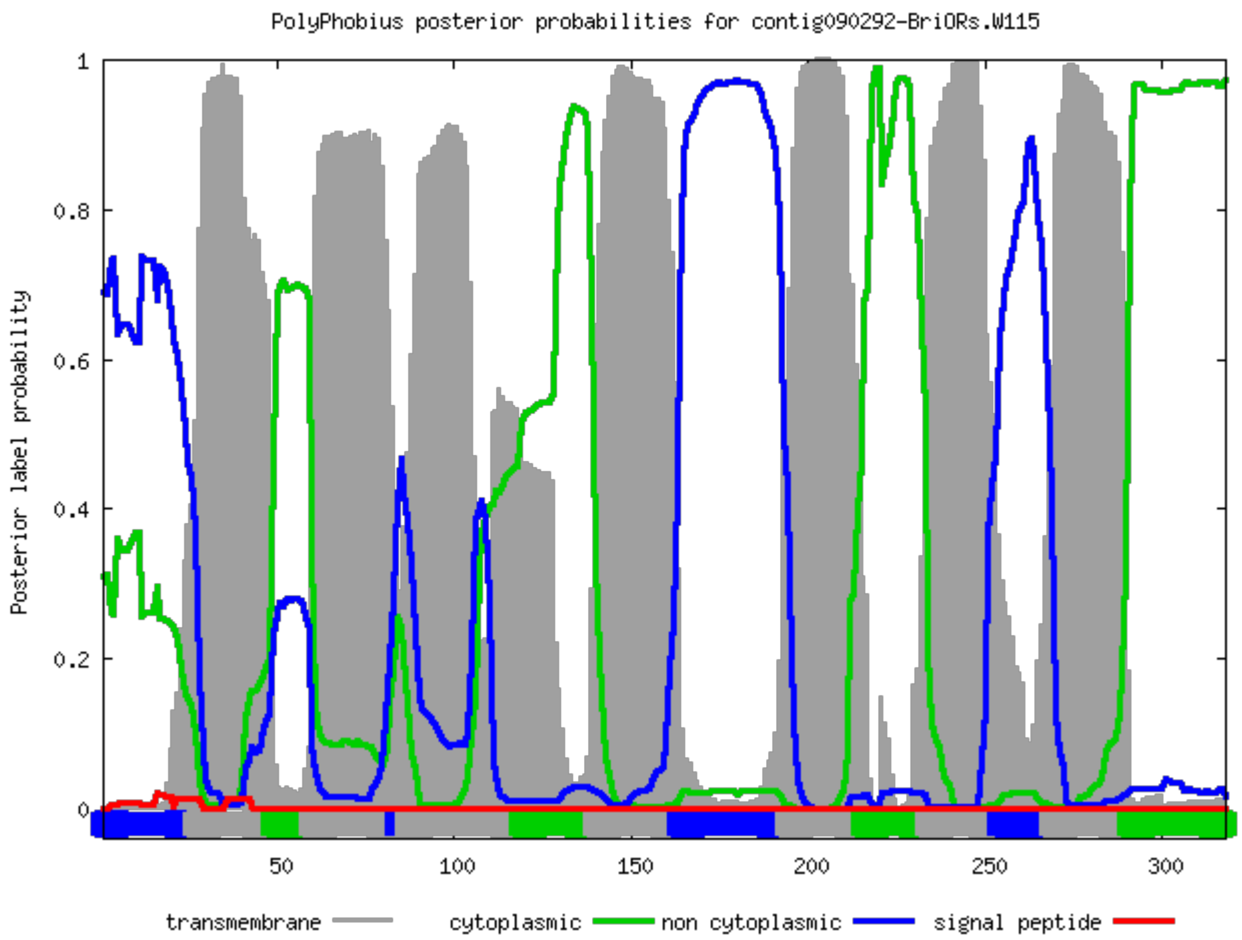

The prediction is based on an alignment. The probability data used in the plot is found here, and the gnuplot script is here.

Prediction of contig048243-ZebOR.E050

```
ID   contig048243-ZebOR.E050
FT   TOPO_DOM      1    24       NON CYTOPLASMIC.
FT   TRANSMEM     25    50
FT   TOPO_DOM     51    59       CYTOPLASMIC.
FT   TRANSMEM     60    85
FT   TOPO_DOM     86    92       NON CYTOPLASMIC.
FT   TRANSMEM     93   120
FT   TOPO_DOM    121   140       CYTOPLASMIC.
FT   TRANSMEM    141   163
FT   TOPO_DOM    164   195       NON CYTOPLASMIC.
FT   TRANSMEM    196   218
FT   TOPO_DOM    219   238       CYTOPLASMIC.
FT   TRANSMEM    239   258
FT   TOPO_DOM    259   269       NON CYTOPLASMIC.
FT   TRANSMEM    270   293
FT   TOPO_DOM    294   322       CYTOPLASMIC.
//
```

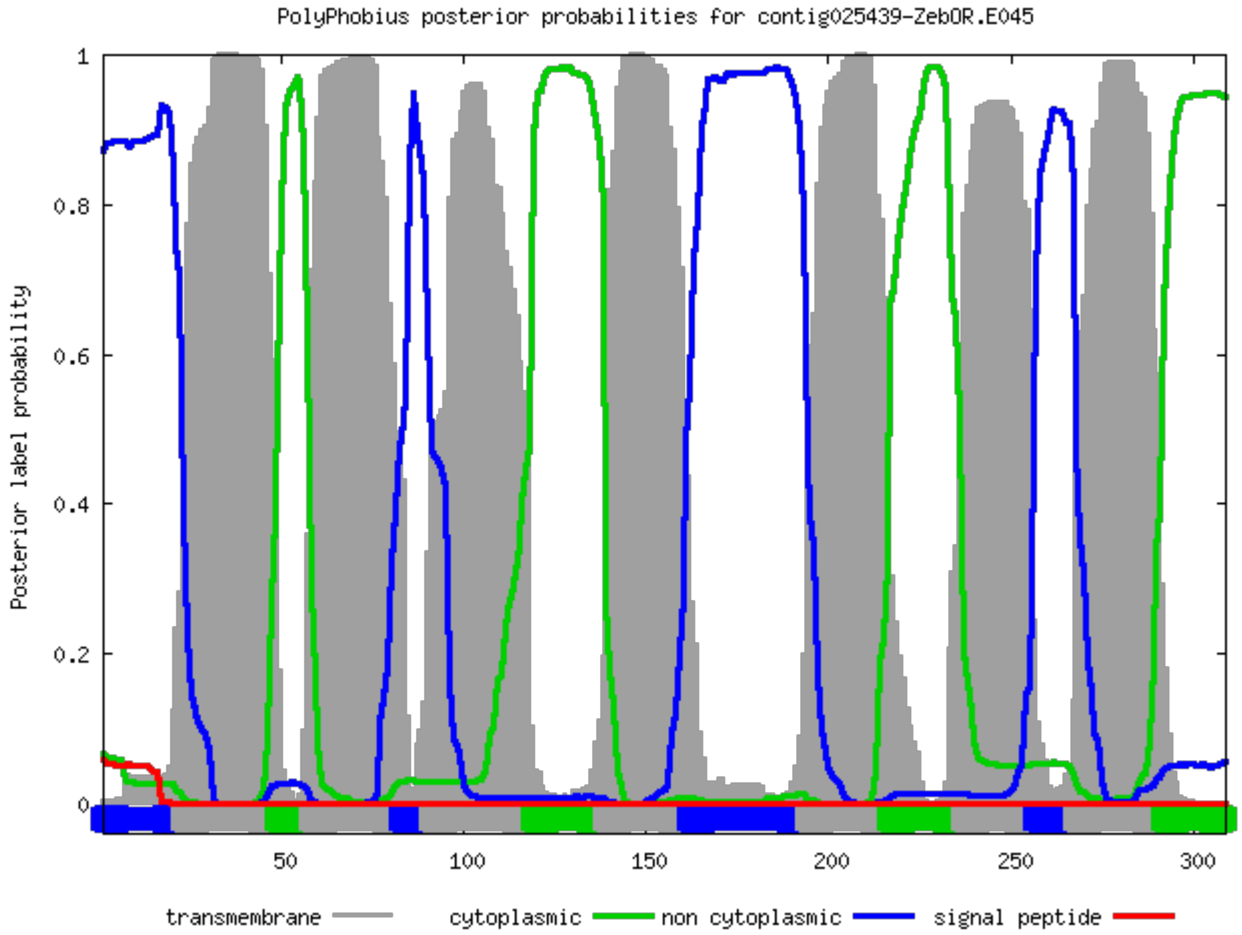

The prediction is based on an alignment. The probability data used in the plot is found here, and the gnuplot script is here.

Prediction of contig022264-TilOR.A016

```
ID    contig022264-TilOR.A016
FT    TOPO_DOM      1      22       NON CYTOPLASMIC.
FT    TRANSMEM     23      48
FT    TOPO_DOM     49      56       CYTOPLASMIC.
FT    TRANSMEM     57      77
FT    TOPO_DOM     78      95       NON CYTOPLASMIC.
FT    TRANSMEM     96     118
FT    TOPO_DOM    119     138       CYTOPLASMIC.
FT    TRANSMEM    139     159
FT    TOPO_DOM    160     192       NON CYTOPLASMIC.
FT    TRANSMEM    193     215
FT    TOPO_DOM    216     235       CYTOPLASMIC.
FT    TRANSMEM    236     257
FT    TOPO_DOM    258     268       NON CYTOPLASMIC.
FT    TRANSMEM    269     289
FT    TOPO_DOM    290     309       CYTOPLASMIC.
//
```

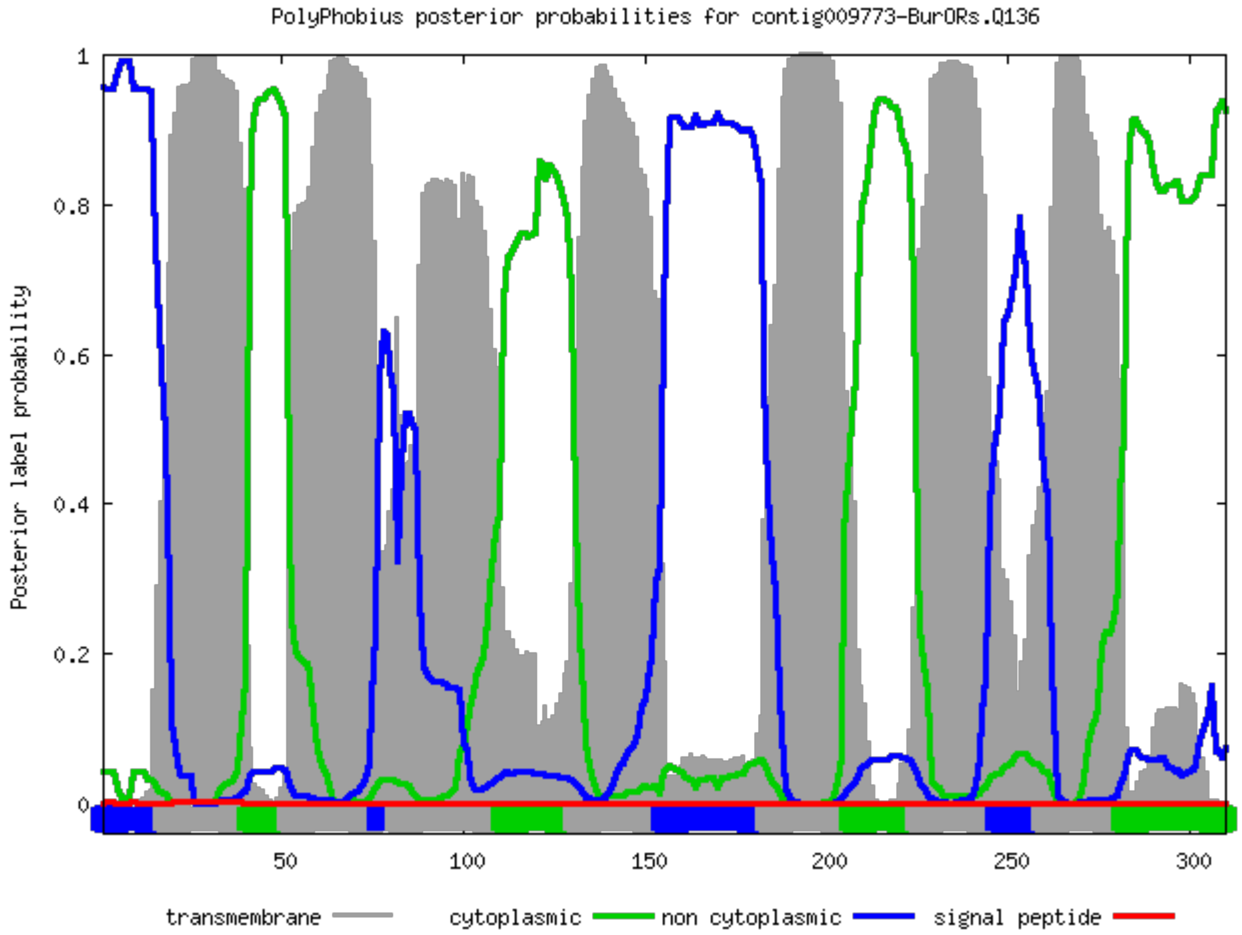

The prediction is based on an alignment. The probability data used in the plot is found here, and the gnuplot script is here.

Prediction of contig022227-TilOR.A006

```
ID   contig022227-TilOR.A006
FT   TOPO_DOM      1     22       NON CYTOPLASMIC.
FT   TRANSMEM     23     48
FT   TOPO_DOM     49     56       CYTOPLASMIC.
FT   TRANSMEM     57     77
FT   TOPO_DOM     78     95       NON CYTOPLASMIC.
FT   TRANSMEM     96    118
FT   TOPO_DOM    119    138       CYTOPLASMIC.
FT   TRANSMEM    139    159
FT   TOPO_DOM    160    192       NON CYTOPLASMIC.
FT   TRANSMEM    193    215
FT   TOPO_DOM    216    235       CYTOPLASMIC.
FT   TRANSMEM    236    257
FT   TOPO_DOM    258    268       NON CYTOPLASMIC.
FT   TRANSMEM    269    289
FT   TOPO_DOM    290    313       CYTOPLASMIC.
//
```

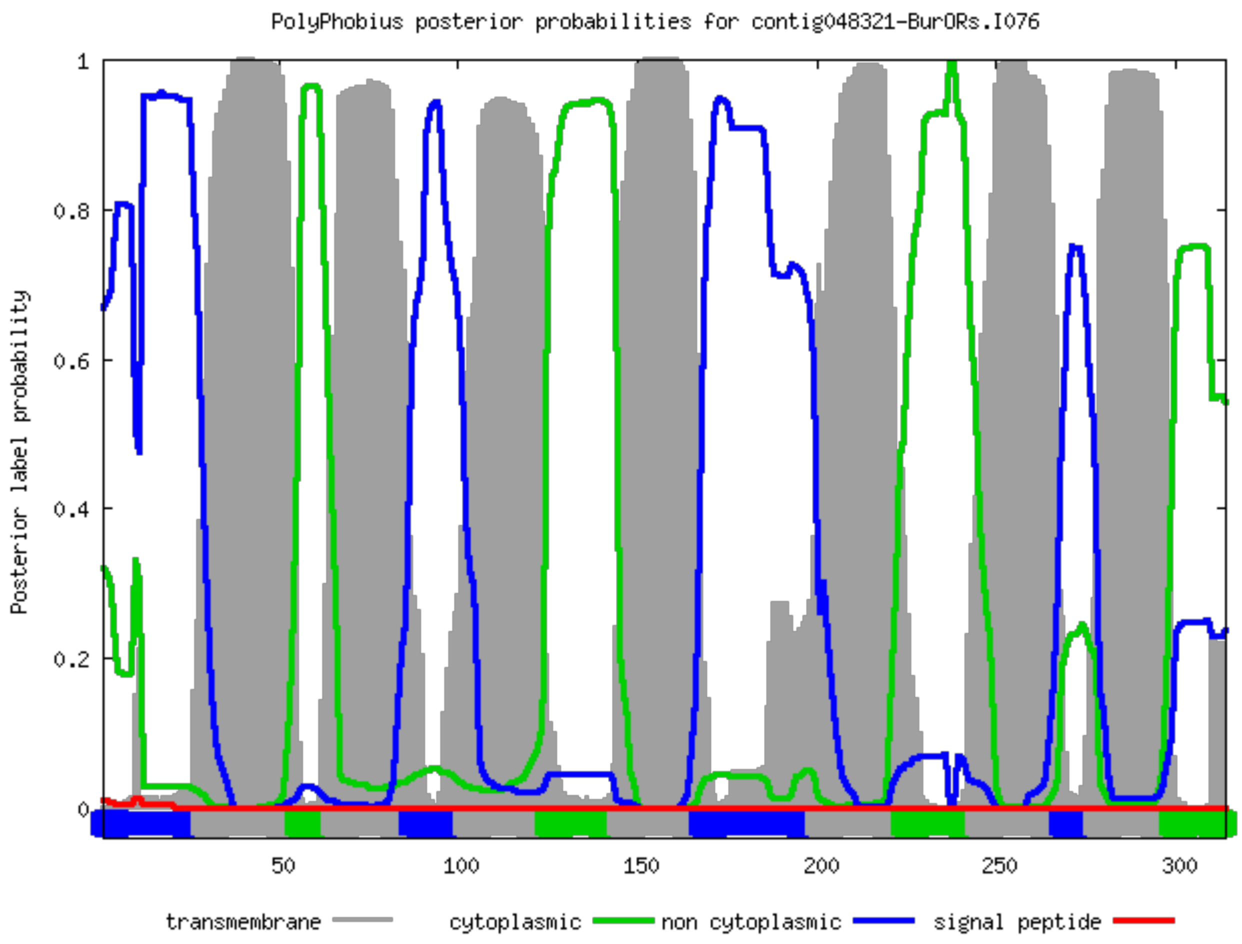

The prediction is based on an alignment. The probability data used in the plot is found here, and the gnuplot script is here.

Prediction of contig064938-BurOR.E053

```
ID    contig064938-BurOR.E053
FT    TOPO_DOM      1      22       NON CYTOPLASMIC.
FT    TRANSMEM     23      48
FT    TOPO_DOM     49      57       CYTOPLASMIC.
FT    TRANSMEM     58      82
FT    TOPO_DOM     83      93       NON CYTOPLASMIC.
FT    TRANSMEM     94     118
FT    TOPO_DOM    119     138       CYTOPLASMIC.
FT    TRANSMEM    139     161
FT    TOPO_DOM    162     193       NON CYTOPLASMIC.
FT    TRANSMEM    194     216
FT    TOPO_DOM    217     236       CYTOPLASMIC.
FT    TRANSMEM    237     256
FT    TOPO_DOM    257     267       NON CYTOPLASMIC.
FT    TRANSMEM    268     291
FT    TOPO_DOM    292     309       CYTOPLASMIC.
//
```

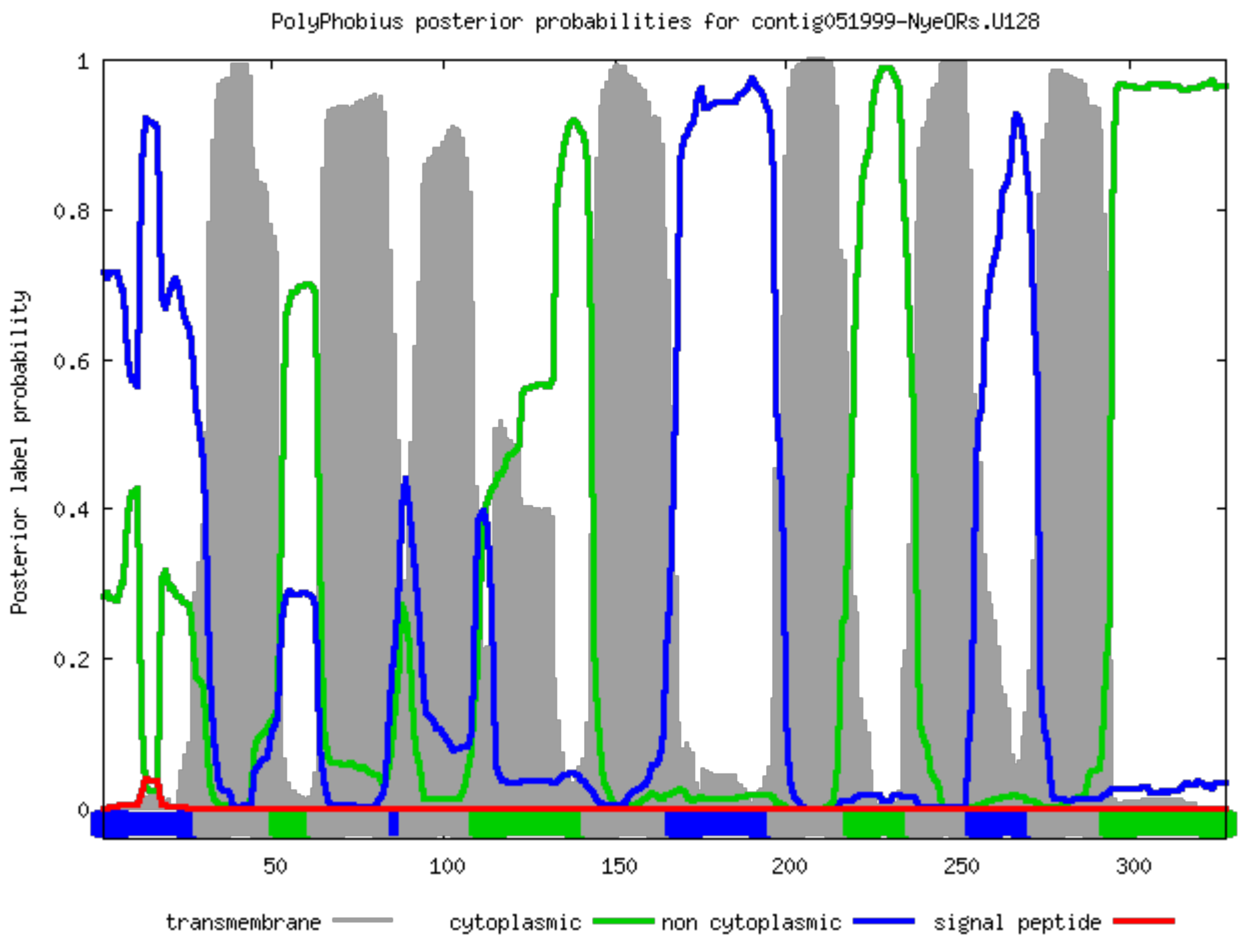

The prediction is based on an alignment. The probability data used in the plot is found here, and the gnuplot script is here.

Prediction of contig004275-BriOR.P101

```
ID   contig004275-BriOR.P101
FT   TOPO_DOM      1     28       NON CYTOPLASMIC.
FT   TRANSMEM     29     52
FT   TOPO_DOM     53     62       CYTOPLASMIC.
FT   TRANSMEM     63     86
FT   TOPO_DOM     87    101       NON CYTOPLASMIC.
FT   TRANSMEM    102    123
FT   TOPO_DOM    124    142       CYTOPLASMIC.
FT   TRANSMEM    143    166
FT   TOPO_DOM    167    200       NON CYTOPLASMIC.
FT   TRANSMEM    201    225
FT   TOPO_DOM    226    242       CYTOPLASMIC.
FT   TRANSMEM    243    265
FT   TOPO_DOM    266    277       NON CYTOPLASMIC.
FT   TRANSMEM    278    298
FT   TOPO_DOM    299    332       CYTOPLASMIC.
//
```

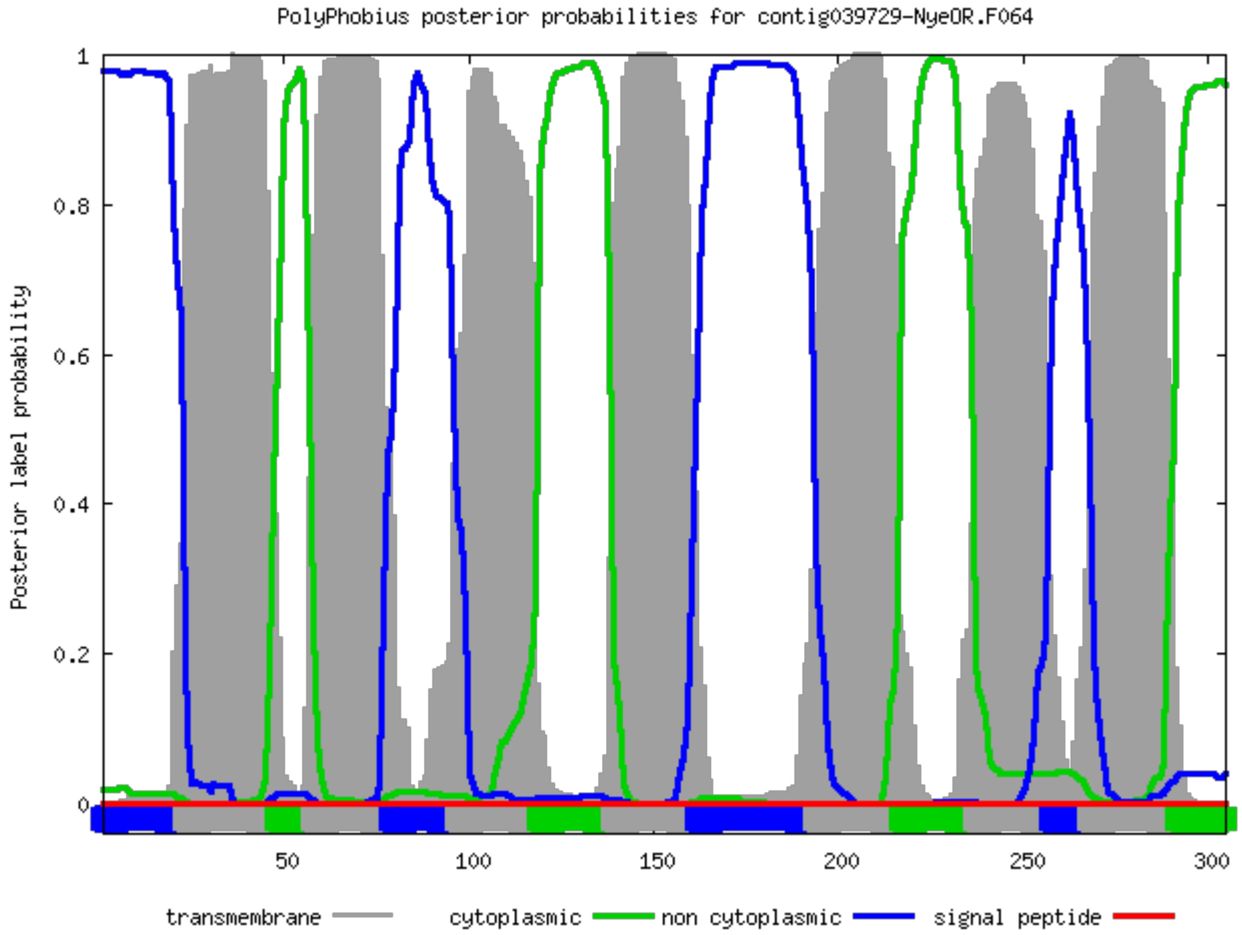

The prediction is based on an alignment. The probability data used in the plot is found here, and the gnuplot script is here.

Prediction of contig036782-BurOR.A002

```
ID   contig036782-BurOR.A002
FT   TOPO_DOM      1      22       NON CYTOPLASMIC.
FT   TRANSMEM     23      48
FT   TOPO_DOM     49      56       CYTOPLASMIC.
FT   TRANSMEM     57      77
FT   TOPO_DOM     78      95       NON CYTOPLASMIC.
FT   TRANSMEM     96     118
FT   TOPO_DOM    119     138       CYTOPLASMIC.
FT   TRANSMEM    139     159
FT   TOPO_DOM    160     192       NON CYTOPLASMIC.
FT   TRANSMEM    193     215
FT   TOPO_DOM    216     235       CYTOPLASMIC.
FT   TRANSMEM    236     257
FT   TOPO_DOM    258     268       NON CYTOPLASMIC.
FT   TRANSMEM    269     289
FT   TOPO_DOM    290     307       CYTOPLASMIC.
//
```

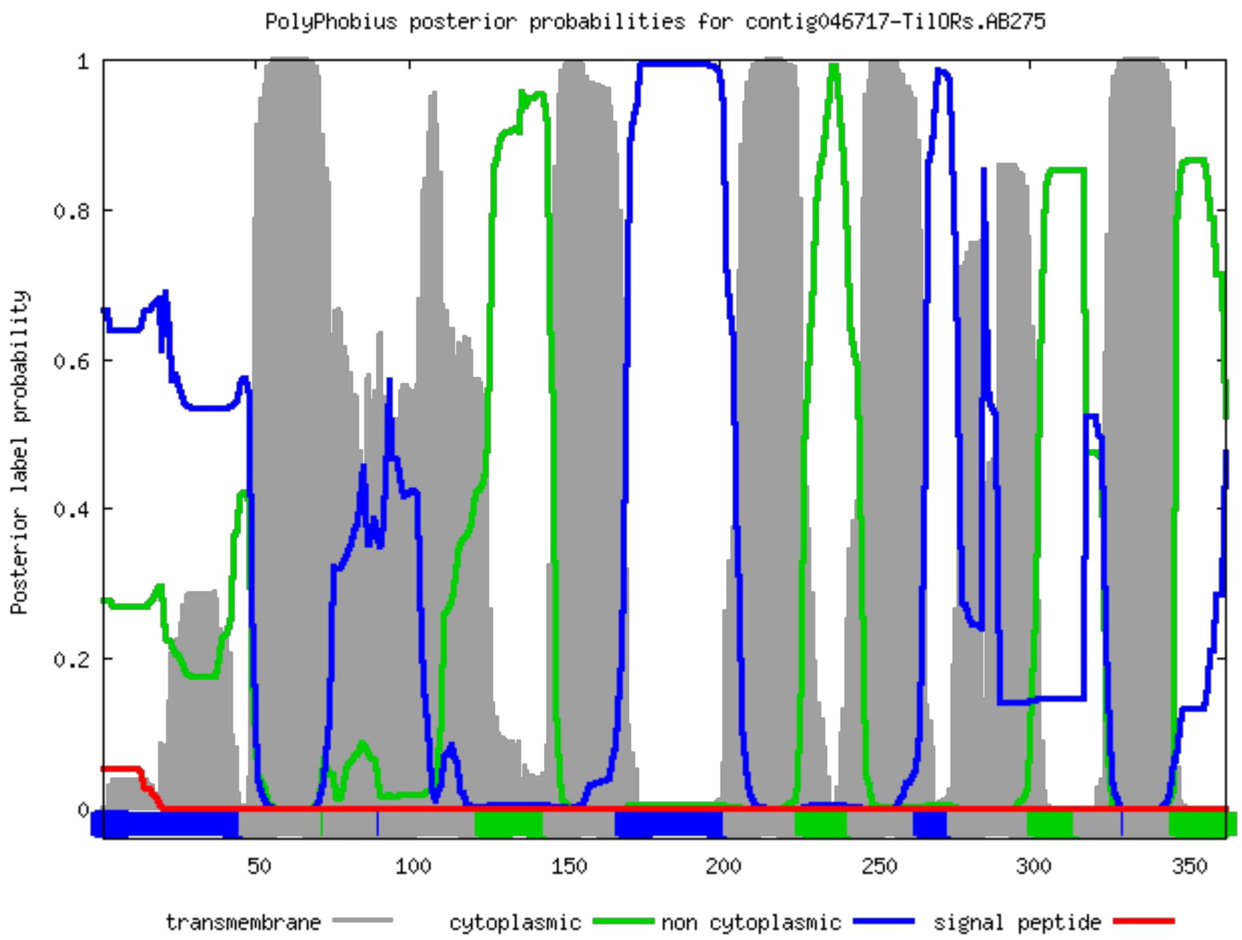

The prediction is based on an alignment. The probability data used in the plot is found here, and the gnuplot script is here.

Prediction of contig039729-NyeOR.H073

```
ID   contig039729-NyeOR.H073
FT   TOPO_DOM      1     22       NON CYTOPLASMIC.
FT   TRANSMEM     23     49
FT   TOPO_DOM     50     56       CYTOPLASMIC.
FT   TRANSMEM     57     77
FT   TOPO_DOM     78     95       NON CYTOPLASMIC.
FT   TRANSMEM     96    118
FT   TOPO_DOM    119    138       CYTOPLASMIC.
FT   TRANSMEM    139    160
FT   TOPO_DOM    161    193       NON CYTOPLASMIC.
FT   TRANSMEM    194    216
FT   TOPO_DOM    217    235       CYTOPLASMIC.
FT   TRANSMEM    236    258
FT   TOPO_DOM    259    269       NON CYTOPLASMIC.
FT   TRANSMEM    270    289
FT   TOPO_DOM    290    314       CYTOPLASMIC.
//
```

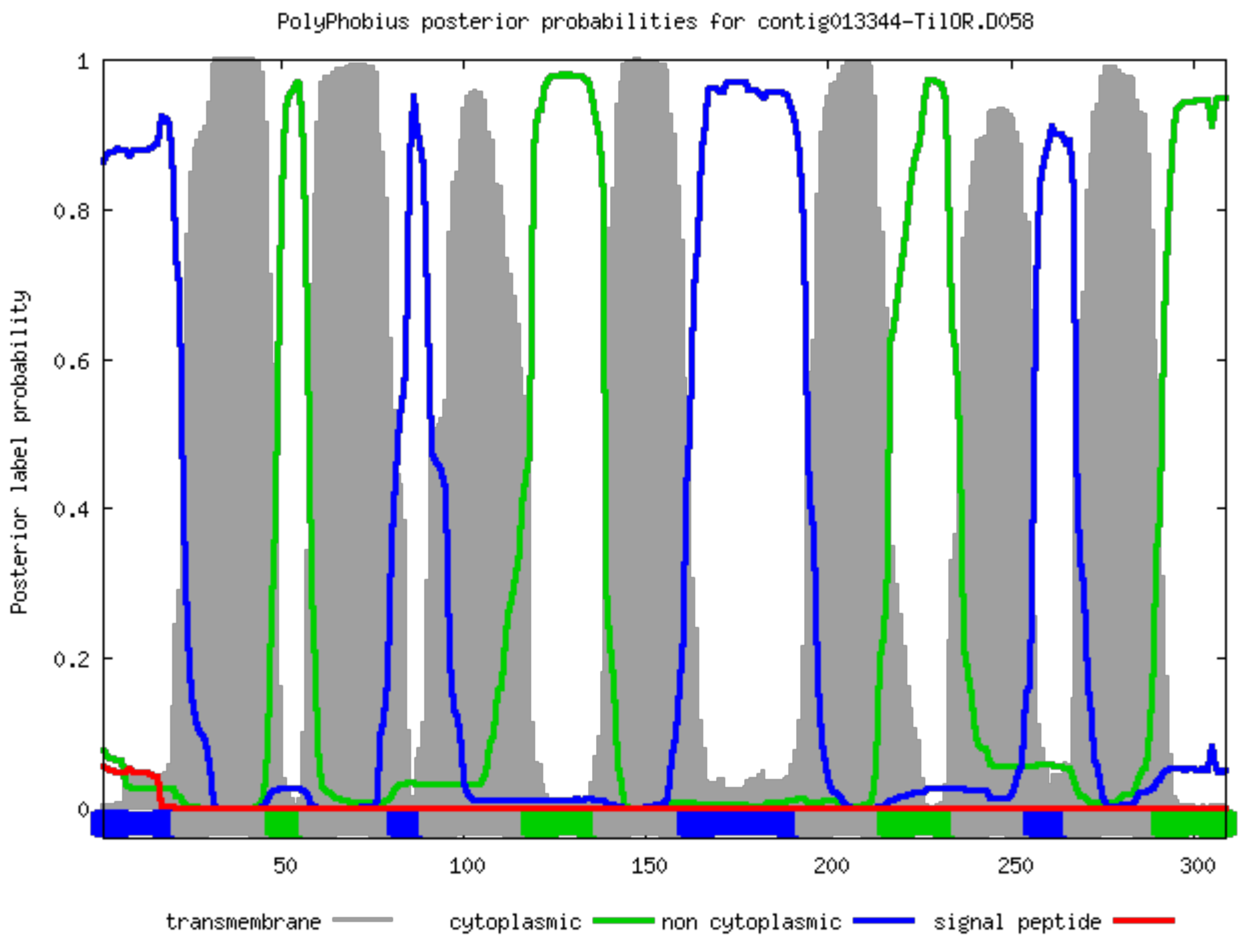

The prediction is based on an alignment. The probability data used in the plot is found here, and the gnuplot script is here.

Prediction of contig028644-TilOR.R252

```
ID   contig028644-TilOR.R252
FT   TOPO_DOM      1     21       NON CYTOPLASMIC.
FT   TRANSMEM     22     45
FT   TOPO_DOM     46     56       CYTOPLASMIC.
FT   TRANSMEM     57     81
FT   TOPO_DOM     82     86       NON CYTOPLASMIC.
FT   TRANSMEM     87    115
FT   TOPO_DOM    116    135       CYTOPLASMIC.
FT   TRANSMEM    136    159
FT   TOPO_DOM    160    191       NON CYTOPLASMIC.
FT   TRANSMEM    192    215
FT   TOPO_DOM    216    232       CYTOPLASMIC.
FT   TRANSMEM    233    255
FT   TOPO_DOM    256    267       NON CYTOPLASMIC.
FT   TRANSMEM    268    290
FT   TOPO_DOM    291    321       CYTOPLASMIC.
//
```

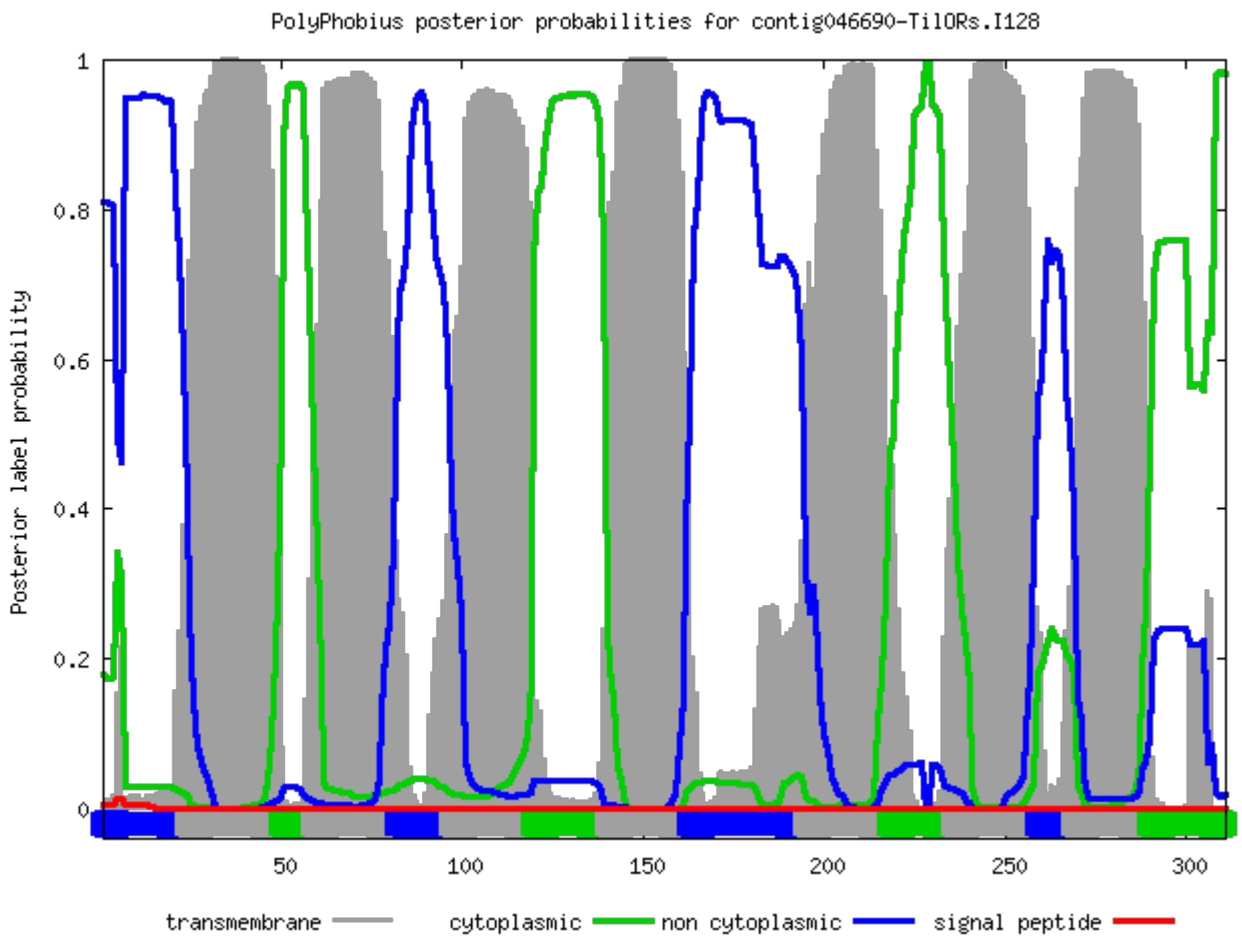

The prediction is based on an alignment. The probability data used in the plot is found here, and the gnuplot script is here.

Prediction of contig030552-ZebOR.A001

```
ID   contig030552-ZebOR.A001
FT   TOPO_DOM      1     22       NON CYTOPLASMIC.
FT   TRANSMEM     23     48
FT   TOPO_DOM     49     56       CYTOPLASMIC.
FT   TRANSMEM     57     77
FT   TOPO_DOM     78     95       NON CYTOPLASMIC.
FT   TRANSMEM     96    118
FT   TOPO_DOM    119    138       CYTOPLASMIC.
FT   TRANSMEM    139    159
FT   TOPO_DOM    160    192       NON CYTOPLASMIC.
FT   TRANSMEM    193    216
FT   TOPO_DOM    217    235       CYTOPLASMIC.
FT   TRANSMEM    236    257
FT   TOPO_DOM    258    268       NON CYTOPLASMIC.
FT   TRANSMEM    269    289
FT   TOPO_DOM    290    309       CYTOPLASMIC.
//
```

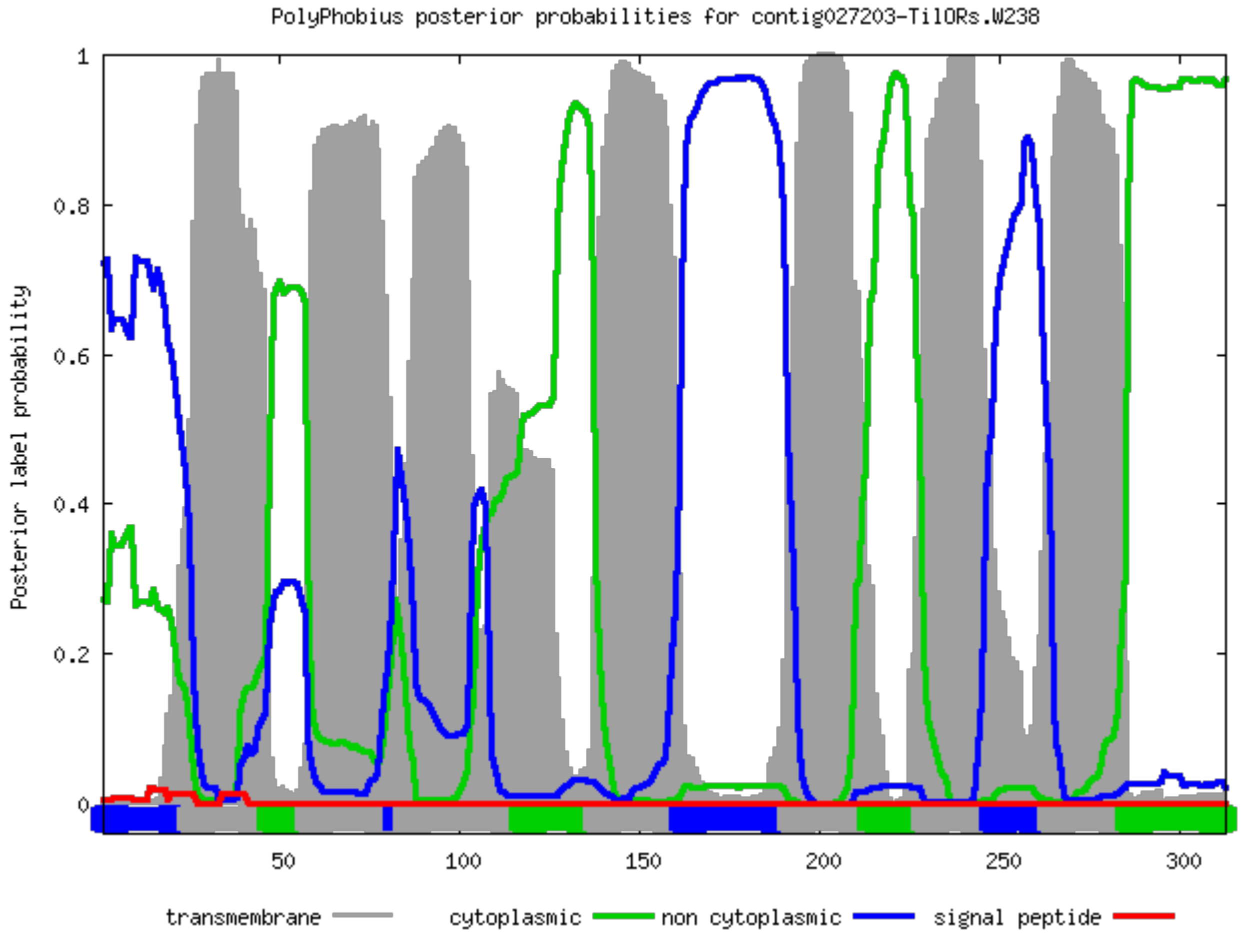

The prediction is based on an alignment. The probability data used in the plot is found here, and the gnuplot script is here.
