# Supplementary material for: Inventory of the cichlid olfactory receptor gene repertoires: identification of olfactory genes with more than one coding exon
Source: BMC Genomics. 2014 Jul 11;15(1):586. doi: 10.1186/1471-2164-15-586 (PMC4122780; doi:10.1186/1471-2164-15-586)
Supplement: Supplementary file 5 — Additional file 5: List of pairs, triplets and quadruplets of genes with 99% of identity or more. (PDF 328 KB) [file 12864_2014_6314_MOESM5_ESM.pdf]

Examples of model fish OR genes identified in ENSEMBL and presenting more than 1 coding exon. Bold letters correspond to exons

>M'Buna MZ_S2_DNA S2 LOC101472154

ggataagaagataaaaaagtataattcaagcttttcgagtttttgatcattagacattgtctgtcattctcttctttgattccagatcttgtggtgtattaattcaaagctttttaaaaaa
tctttttattacattttactaaaacatgtatctatgaatctaaaagctttgtctggtttgtgttacctgtttcttgggttttgatgtgttttgcagaaacgagctgctgtggatggagaacc
taaca**atggcgactcctctcaaacagccgattgtgtttgaacttgaaggcttctatatacctccagggtttggccctttactgttcttcctggctctg**
tttacctacatgttggtgttgctgggcaatggtgtcatagtatccgtgattgttattgacaagaacctgcacagacccatgtttgtaatggtgtgcc
acctgctagtctgtgatcttttggggtccacagctgtgctgcctggtctcatgatgcacttcttgatggggcagaagaggattgcatacatcccagc
cattgcccaggccttttctgtgcacacatatgggctagcagtgcaggctattctgggtgtgatggcctacgacaggtaataaaactatcagctcaacgtaaaa
atcaaagaaaaaagaaaatcgaactttcctttttgatgctgctgtttgaattgataatggattatgtctccaacctttagctgtatgtcagcattgtgttcttttcgtctgttcttcttct
gtttttatcttctttttgtttgtagaattgtaagaaaaagtttttttttccaaagaaatgagaaagcacaggatttgaaaagacttaaattttttttttcattaacaaaacttctaaat
taaaacctactacatatctttagtataaatgccccttctatttctctgtg**caggtacattgcagtctgtgagcctctcaggtatcacgctatcatgacctcggctt**
ggctgcactcctgctgcgccctggcctggttgcttgctctgctgcctattgctgtgctcttcagcttccacatgaatgtcccattgtgtggcagagt
cattctgcatgtttactgcagcaaccggggtatcctaggcctggcctgcattcccacacctgccagtgacatctatggtcagtcctaagtgtatgcagtaga
tggttgaacattttatcagcttcaaatcagcttgattcatagccttagtttcacagtttaatttttttttctctccctcaatagttaaaatgatgagtgcagtattgcaaaccattgtctcc
actttatactgttgtacaagcaacttctgtgttttgttggatttttagagaaaggaattaaactgattgtgttgtcatggtttttttttttaaagaaaagcttgtccccacagtgagatct
caagcatttattagctgtttttccagatataaacagattttaactgagggaacaattaaaccacatgggtatggggacatgtaatacttgattgaaaggacagttgtctctttttattcg
gta**ggtctggccatgacctggactgtgagtacagggatcttccttatcattgctttttcctacatcaggatcctgcaagtatctctaaaacacagca**
gaatcgacaccagcatccgcagcaaggcctttcaaacctgtgcttctcacctcgtcgtgtacgtgctctaccaaatagcctcagtgatcataattgt
gagttacaggtttccttcagtgtctgaaaacctaaagaaattctttagcattctgttcatcatcgttccaccagccatcaaccccattatctacgga
ctggtcagtaaagagttacgcagcagcatcattaaacacttcacaatctagtgcagtgtaaatgttccaaaaatactggatactcatacaagaggcagaacaaagccc
aacaaagtttttaatattattgatattattaattaatatttatttattaattgttgttgtgatttatatataaatgataccaaattgtattaatgtcaagatgaatggacccaaactcct
gtaatttcagaatgaatgacctgatttgtcttcctttctacatctatgaaaaaaagtgaatgtctta

>Takifugu TR_S3_DNA LOC101079185

gatgttgaggaggatgatgattgtgggcagctaatgatgctgctgatggtccaaaccctccagtcagtgattaaaagccaacacagtcttgctgctgagtcagagcatcagcgctctggag
acaggacaccttgtgatcttcaacttttacagtgttgtgagcctcaaacattctctttttagttttatgtgtttgataatacattgaattttgaagtttcctgttttctttgaaggtatga
tggtttactgcgttcatgtgcagtcacgacctgtttgctgcctgactacttcttaatatcctttcccccagaacttttgatggagaacttaacg**atgctgactcctctcaaac**
agccggtggtgtttgagttggtgggcttctatgtccctccaggctctggccctttgttcttctgcgtgactctgattgcctacctgctgacactgc
tggccaacggtgtggtggccggtgtcattcttatagacaagagtctgcacagacccatgttcatcatgatttgtcatctggtggtctgtgatctgct
gggggcaacagcgatgctgcctcgcctcatggtgcactttctcacagggcataaaggatcccctatgtggtggccatcatccaggccctctgtgta
cacacatatggtgccgggattcagactattctggctgtgatggcctacgacaggtagctatggaaacacagcagtctggtaatgaaatggattgtgatgttataaacca
tgggaacagctgagggaagcagatgtaagagatggttctcaagttgagcttgtctctgatgataatggaggttctgttgacttgttgtttctatttatctcttctaatgagggaaaagttc
aggcaacaagcttttggattgaaatgattgtcttcagaggcttccgatcttttcagaacagtcagtgtaccgacccaagggtccaaatgtctgggaacctcctaacagcagaactgtacaa
taacagtaggaattatagatgcacctttcctgatcaggatactcagggtccagatttgagagcaaagctaagacctataaataaataaataaataaggaataatcttttatagtgatgttt
ttagttcatggtagcaaattgatttgacatattaaagcagcaatgagttaaagcacatgtttatgcatattttgcagtagatgaaacatttctaagataaaagcatcagactgtgggtt
tattctgggttgtagccctgaggcagtaatgtgtcttttctctct**caggtatgtggctgtgtgtgagccgctcaggtacgccaccatcatgacgccagctcgcctg**
cacacctgctgctcgctggcctggcttgctgctgtgctgtctattggcgtgctctttgcttttcacgcagatgtccagctgtgtggaaacatcatcc
aacatgtctacatcagcaaccgtgggatctggatctggcctgcagccccacccccataaacaacatctatggtcagtgcatcattttagattaatctaaaaatcagaattacacca
agagcataataatcacatagacctgaaatggtaataatgtcataaaaagagtgcaggaaaaaaacacagtaatttttgacactgaaattgaggcaatttgtcttatttttagcacatttct
gaatacagaaaattaaattttatatatgtgtgaaaatactatggaatcaatacctgatgattaatctgttgcaatatttaaaagaatgaattacttagaatgaaaagatatatgcttcaac
ctcagccatggttaaaaattaagttaaaaagttaaaaaagaataaatgacatgaataacaaagagaaaaggtggttgcagactctatatacaaccattataactgaaacgctgaattgaaa
gaccttatagtgatgctactgaaaggttgaatagctgaaatcatcaggcactaacaagcataaaaggttttatttgtagcatagaaaaagaaaaagaatgataacggtgcctgtgaagaca
ctgtaggtttcctgcactccaggaaactcccgagagatcttcatatgacacctaaagcaacattacacaagtggaaatacctaaatgtggaatgctactgttgaaaaaatgggtcgggga
tgttggttgaaggggattgtgtcattttcaataattcaggaataaatagataattgtgacatgttttgtag**gcctgtccatgacctggactttgagtacaagtgtcttcct**
gatcatcgcgttgtcctacttcagaatcctgaatgccggagtcaaacataatcggacaggcatcaacagcaaagccctgcgcacgtgtgccacacac
ctggttgtttatgtggtctacgaagtagccacactggtcatcattgtcagtcttcggttcccctcactgtctcccaacgtcaagaaattctgcagca
tcctgttcatcgtagtcccccagccgtcaaccctgtgatctacggactggtcagtaaagagctacgtgctagcatcatcaagcagctcagcactaa
agcacacaaatgaactctagtgcctctccgtctacataagagctaatatagttaccgtacatatatatctcatcaatgttagacagagcttctcgctgaacagttattaatgacagcg
cataaatgagaggtgt

>MEDAKA LOC101159375

tctgtttctttaaatcagaggtcagtgtttgtgaacgagacatgggagcttggtaccccc**atgttcttcatcatccagggacttgccagcattggcgagaagagggtgg**
tcctttttgtcatcctactgctgggatacctggttatcctgggggaaacagcatgatcatctttgtggtacgaggcagatgttaactcgtgtttagcctgaag
cacaggtcttgattctgtcgtcttcctctgcag**acgctctctgaccccaggctcacctcccccatgtacttctttctccaaaacctgtcttttgtggacctgg**
tctataccacaaccaccatccccaacatgctggctggtttcctcatagaccaactgaccatctccatcccggctgcttcctacagatgtacttctt
cactcaacttgctgtgacggacgtgccatcctcaccgtcatggcttatgaccgctacttggccgtctgcaacccctgcgctacaccgccatcatg
acccggtcagtgcggatgctgctggtcacaggagcctggtgcttcggcttgctctgcacactgccgcctacagtactggccttcctgcggccttact
gcggccccaacatggtccggcacagctggtgtgacctgtcgtctgtcagaaccctggcgtgcgccgacacctccattgacaatgtcctgtccctgtc
ctttgccatggttgcgctgctcaccacaggggtcctcattctcacctcgtacttcctaatcgccacctcaatgtaccagatgggcgtccccgagagg
ctgaaggccttcagcacatgtgccgcacacctgactgtggtctccatctcgtacagcgccgcctcctttgtctacatttcatatcgagtgggaaact
tttcatcagaggtatgacatgcagctcacttgaaaaatacagagcaatctaaccactgcaccggggtccacatattaaaacgcgacaaaggcataaattaatataatataattccttt
gtgattaaatttacatttggagcgctgaacacaaaaatgttaggcaaaaagaaaagactgcatacagtttagggctgcaaccagtcgctgtctgaatattatttcaaacttcattttct
atgatcttttgtaaacttcaaacgagtaagattgcctttaaagacctacaccaacaaaaatggtgttttctgtgcttttaacatgttcttgtagcatttttctgatgacgtaagaaatata
tagaagaatttaacattagaactgtgtttctgaggatttctttagtcaaatcgtaatgagtcaggagcagataaaaacctgcagtttgaaaaagatcaaagttgtgatgcaacaactgaaa
tggtcggaccaactctctgtgttccgctccattctgattcaaccacttgcagacaaatagatccatgtacgtcttttttttttcctcgtctgagctggagtctggatcaaaactgaacagc
tggatagattcaatatttttcaccattttgttgcattgccaatgttagcttggggttgtgaaactagcgagagagagattatagcaggaatgatgggaaatcagtggatggttgcttcca
tgccaacagacccgcccacaactcagagtcagatttctaattaactcctcccactccacagaaagtgtatccaataaaacgacacaggtttttagattgtggcaaaaacagtttaatcata
attaccagacatctgggaacactttgaaaacaaatcaaaaggtgatcagagtgggtctttaaaaaaaaagggaagttgacttactttccacactatgtcacacttaaaagcctttttttatt
tttcaaaaaaaatgcaacagcaggcctatgtcttacatattgataaattctggtcaaaatgtttggttaggggggttattgtgaatacgctaacaaggctgggtttactgggctaacaaggct
gggagtcagagtagtcacaagtaatgattgttttagcggatgctttgttgtgttgcaaacaaggttgagagttactgtgagcacagttaatgaagtttactgaaagtcaaacttatctctg
actctttaaactcactcaatgtgccttatggttaagtgcaccatttgacacacagtaatttcaaaaatagaccattcattgatgttgtgccttaaaataacccttaatgccgaaaataag
taattttaactttgccatcaatagttactccacatctattgttacccaaaaaaaattattttacacaatctattcgttttgcagctgaagctggttttcaagataacaaactcaagttttt
ttacacaaaaaagaaaaagtgataacctttccaagttgatcagtaagccttgatcttttcactagcgagtctgtcctttggggagaaaacagtcagtacattaaagaaggtttctttatta
ccacagaggaaaacatggtttcacagttgctgtgggtcatatttatagatggctgcagctctgagtctttggtggatacttcaccccatccctgaatctctgcatcctcacagcggtgag
atggaagaacggtcagatgacagctgctcttacttttcag**gtgcgcatcattgtgtctgtgctgtactccgcgctgaccccattcctcaaccccatgatctaca**
gtctgaggaacaaggagctgaggatagccatcaggaagactctggcctccttcagacctgctgccacgtctactgtaaaggccatcagtacataagc
catcagtacataacaaccatcaaccatccaaccagaactatcaagagttcagatc

gatggaggaaaagggggatgttctttgactccctctggggctcggctgcactcctgcctggcgctggcctggtccgtcgcggtgctgctcatcgctc**caggtacattgccgtgtgcg**
aacctctccggtatcacgccatcatgacggggctcggctgcactcctgcctggcgctggcctggtccgtcgcggtgctgctcatcgctctgctctt
catcttccacatgaactccccgctgtgtggcaacatcatccagcacgtctactgcagcaaccggggcatcctgaacctggcatgcggccccaccccc
ttaaacaacatctatggtcagtgcatcgcagcaaacgcgtctcctgctgttttcacgattctgaaccagcagaataacgccgtgtgctctgtatgtttctgca**ggtctctccat**
gagctggtctttgaataccagcatcttcctcatcatttctttctcctacatcagaatccttcatgcttgtgtgaaacagggcgcggccgacagcggg
atgcgcagtaagaccttccagacgtgcgctccccacctcgtggtttatgttctctaccagatagcttcagtggtgataattgtaagttaccggttcc
cctcgctctcacagaacatcaagaaattcttaagcatactgtttatcattattccacctctcataaccctctgatatatggactggtcagtaaaga
gatccgtgcaggcatcatcaagcatttgaccttttttacatccatccaagagatgagaggcttcacccctttttcctcatcattgtttgtaacccatgttagtgacttt
taaacttttaatgtcttt

> **Stickelback** ENSGACG00000005783

tctagcttggttaacggcacccgggggcctgatactgtcactttcttcatcattgagggccttgccggccttggcaaagaaaag**atggtgctttttgtcatcctcctcctgggt**
tacatgattatcctgggaggaaacagtatgattatctttgtggtaaaaactctcttctttctcactctttctagtcatgccaatttaaagggtaatccctctttctgaca
aagatgtattctctatcaatattgtatttgctgaagacgttttcttctcacgca**ggcgttaactgattccaagctcaactctcccatgtatttttcctctacaacct**
ctcctttgtggacatcatctacaccaccattaccatccccagcatgctgtctggcctcctggttggcatgaaaactatttctgtgccgggctgcttc
ctccagatgtttgttttatccagctggctgtaactggtcggccatcctgactgtcatggcgtatgaccgctatgtggcaatctgcaaccctctgc
gctacgcatccatcatgaccatgcccgcccgggtgctccttgtcgcaggagcctggagcttcggtaccctctgcacactgccggccacctccatagc
cttccggcaattttactgcggccctaatgtggttaaacatggctggtgcgacccatcatctgttaggcggctagtgtgcggggacgttacagtagat
aacattatttcactttcctttgccatggtggcacttttgacaacaggagtcttcatcctcacttcctatatcctgattggggtttccatattaagga
tgggagtagttcagaggctgaaagctttcgggacttgcgccgcccacctgactgtggtgtccatctcttacagctcggcctcctgtgtatacatctc
ctaccgggtgggaaacattccaccagaggtacgtcaatcgtcctacatatcctgtttagtttttcctttttgagtacatatctcatatttgttcaacgaataggacatttaaaat
catatgtttagtactagaattttaaacaaggcaggcaaatgataattgtttgggagtcggaaaatcagctggagatataatctttcatgtttcctgcctatcgcgtccctgagttaacttc
acttcctgccctagtgttttctcctctaggcgattgtctgtttcccaatgtttttaaaccatgtgtgttccttctctcggcggttcattgagtttcccatgtcggtgttgagattcgtgtt
tatgtcggtgaggttttgtttacaccccaaggattttcgatttctgcattaatatagtagttttgttcgtggaatctgcatttgagtcctcctgtcccctgtcctggacttgaatgaatga
gtggtcatggaggttgcatgttcttatcaagtgacccaagtttgcagtttctgttacattaaaacacagaattgaaggactctgtaaacaaagaaaacagacccactcagatggaaaatat
aaaatagattgaaaaaatctaaaatgtccattggttcagtgatttgtcctgactgaaatatctactatctatatcttttgacagattgcagcaaaatttggtcacaccttttttggcttg
acagacttttttaagtcacggtgattgactgtgcatatgtggggacctgacgtgattttttccaatggcaggtaatccagtggcactgccttggccatggaccttccgggcacccctgc
ttcaatagcatcattagtacatttgtttttatatggcatgtgtcgggatctccgatccccccgagttggttgatgagtcaaaaagtcaaaaagttaacatttatttagaagagaagaata
ttaccgcagatatctggctctaactattgcttataagcaggaggtcaaacacacaaccacgtatttcagcgctcggtgtaatggcgtctccgagcagtaaaaacgctgagtttttatactc
ataaagaccatcctcagtgccagttacgagaagctacaaaagttgagataagaacccaggtgaagatgagggtaacacacactgctcctttgttcgagcttctgtgagagcaagaactccc
ttcttcagacacagctgcaaaccttaaatctttctgtctttgcacataacggtcaaatacagacacttgagacccgggtttagcacaagacaaatgttacaacatgttttgccattacatg
catctcacacatgatattaaaaaataaagttgaagcgttcaaaaccctgaaataagatggtggacatggtaaacattatacctgccaagtgctgccattgtgagtatttttgactgaccaac
ctcgtcttccatcggaactgaagatagaaataaatacatggtgcgttctctaaattatctgttataattctgttat**aggttcgtatcatcgtgtcagtgatgtacgctgcac**
tcactcctttcctaaacccaatgatctacagtctgaagaacaaggagctacgagagtccatgggagaactctgatgaggttcagacctgctgctgtg
ttaccaaccaaagacctcaacaccgtgaccacatcgtactgactgggaaagaaacccagtaattaggactaatcactgcatactgccacacctgtcagatgtgaataaaga
atcaagcacaaaaactaa

>Zebrafish **or112-1** ENSDARG00000077211

agtccaggctatgtttctcaaagcaatctattatcattttgaagcttgcagcatctaacagctttttaggtactagtaaatttagttatgtctgtaaatgtaacagcctacttgtagca
tgatttcaatgtaatgttaaagttccaattcattaacatttaaatcagaacacgtactaattgtggtaacatgcacatttaacatagtctattccctccaatactcaagggaatgcttgtt
ttatttcattttcaggaagaattcattttaaaatgattttttttttacatttacattgccttgtaatgttgccttactgcacacttaaagcaaaataaaaaaaaaactaataactttctg
cttactccttttacaaaattctagctaattattctgttccacttgaactaactatgctatcgcaattaaaaacaagcataactgccttcagcagaactaattaacccagtcaaaaatgatc
aagtaattgcttcgttcttcctggaacaataccttcatcagcttgtctgagtggatggagtgtgaggtcattagaacgtagaagaggtcaatggctgtacagtttgttgtgttcct**atgg**
gagcagagatcaaatgtataaatgctcccattccaatggcttcaaatgttaatggggtccagctctattgacgaaagaccaaacatcagacactct
ggcaggtatgtttcgccctgcagcaattgtatacatttttatgtaattgtaaaaactgacaaataataatatgcaaaattaataaaatgaaaatgacttctctttgtttttgaaagttgt
cgatattgtggattattgattgactggagaatgttgattgtccaatattaatcttcaatattaagttgttgttgttttacaaatgcattggttttactaaccattacttttgtacaaacaa
aactatttcgaataattgtcaaacatttgacctatttttaaatagactgtactgtatctaagaggcagtttgttcttaaaatgacaggagaatttagttgttggaactgttggagaagcca
gagatgatatttttctgtgtaagaaattctgtgtaagttacaaaatttcgtctacactctacaaaatggtggattgtttcaacccaaagttgggtaatgtatggacaaacccaatggttgg
ctgctgtattacaatctagatttcgttttcaatcatatgaacgatctaataattttgctcatgcatttacttacattttgtaatttaaaatag**gttgcgccgaccacattcaatg**
tgccatgactgaggaactccaaggagcaaacttctcgcacacaaagttttattaacgggatttccagagatctacaaatacagacgttttctttc
ctgccattcttcctaacttacctttgcttctggtcgggaattctttgctgctttttgtgatcaaaagtacagagagtcttcacagtcccatgtaca
tttttgtgtctgctttggcagttgtggatatcattgtttccactgccattatcccggcaatgcttcttggttttctgtttgacttgaatgagataac
tttatccggatgtttgacgcaaatgtttgtcactcatttcttttcttctgttgagtccaccatacttttagccatggctttggatcgctttgtggcc
atttgcaagccgttgcattacaatgaaatcatgaactcgtccatgttttgaagctgtttcttttcacactgatcaggagtggcacaataatgtcca
cactggtcgctcttgcttcacctttgtctttctgtgggtctaatgtaatctatcactgttactgcgaccacatggctcttgtcagcttggcctgtga
ttcaatagctcaaaatcaaacaatggggttaattgtgattatctgttttgtgggaatcgatacatctgttatattcttctcctatgtgaaaatcctg
catgttgtgttgggaactgcagctggagaggatcgctggaaagcttttcatacctgtggtacacacttaatggtcatgatatgtttttactttgtcg
gtagtgtaacctttctgtcaagaaacctgaacattccaatcccgattgatgttaatacgtttctaggtgttatgtatatagtatttcctgcaagtgt
caatccaatcatctatggtgtgcggacaaaagaaataaggactgctttgtttaagatttttaaaaataaaatcaacaaagtttccattgtaaaggtt
tcaactataaggaattgattgcccgagaaatctttcaatttgacaatttttctattaatctaagctttctaagcaatgtatgaaggtgtattttaatgttcgattataaacaaataa
acgatgctgttctgttatctactcacccagtggaacttaagtctcactatgagtatcaatcttggtaaaaaaaaaggaaaaattttgttaatcgtggttttaatttacaaactaagtgcag
ctctgtttgctctgttttgaggagaaactgaattgtctgtaagaaattgattttttttatgacatttctatgaaaataaaatatttcaaagaattacaaatcaatcatgctaaaaattaa
gttattatttt
