# Supplementary material for: Inventory of the cichlid olfactory receptor gene repertoires: identification of olfactory genes with more than one coding exon
Source: BMC Genomics. 2014 Jul 11;15(1):586. doi: 10.1186/1471-2164-15-586 (PMC4122780; doi:10.1186/1471-2164-15-586)
Supplement: Supplementary file 6 — Additional file 6: Details of dN/dS ratios for families A, D, E, G, H, I, K, L, N, O, P, R, S, and W. (PDF 5 MB) [file 12864_2014_6314_MOESM6_ESM.pdf]

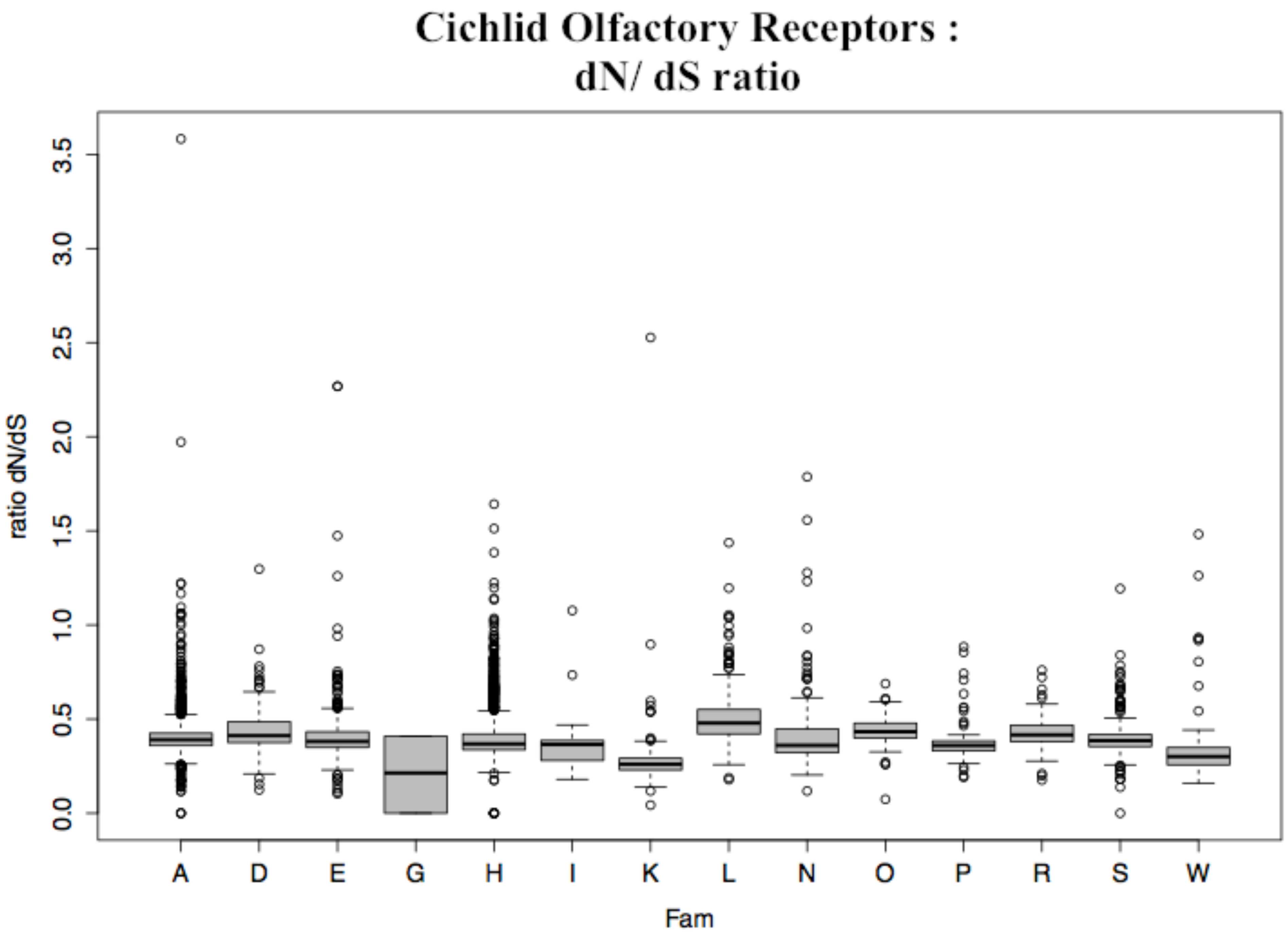
Cichlid Olfactory Receptors :
dN/ dS ratio
ratio dN/dS
0.0
0.5
1.0
1.5
2.0
2.5
3.0
3.5
A
D
E
G
H
I
K
L
N
O
P
R
S
W
Fam

Cichlid Olfactory Receptors :
dN/dS ratio

Fam A

OR pairs		dN	dS	dN/dS
contig034983-NyeOR.A002	contig047515-ZebOR.A019	0.000	0.000	0.000
contig054233-BurOR.A012	contig047523-ZebOR.A022	0.000	0.000	0.000
contig065887-BurOR.A018	contig030566-ZebOR.A008	0.000	0.004	0.000
contig051570-BurOR.A010	contig047508-ZebOR.A015	0.000	0.012	0.000
contig051318-BurOR.A005	contig030572-ZebOR.A009	0.001	0.000	>10
contig057165-NyeOR.A017	contig030552-ZebOR.A001	0.001	0.004	0.348
contig034981-NyeOR.A001	contig047521-ZebOR.A020	0.001	0.004	0.349
contig034983-NyeOR.A003	contig047515-ZebOR.A018	0.001	0.004	0.352
contig054687-NyeOR.A013	contig030566-ZebOR.A008	0.001	0.004	0.355
contig036780-BurOR.A001	contig030552-ZebOR.A001	0.001	0.008	0.173
contig054684-NyeOR.A012	contig030560-ZebOR.A007	0.001	0.008	0.174
contig065887-BurOR.A018	contig054687-NyeOR.A013	0.001	0.008	0.177
contig034988-NyeOR.A004	contig047508-ZebOR.A016	0.001	0.008	0.179
contig057156-BurOR.A015	contig054684-NyeOR.A012	0.001	0.012	0.116
contig057754-NyeOR.A018	contig030553-ZebOR.A003	0.001	0.013	0.117
contig057153-BurOR.A014	contig030557-ZebOR.A006	0.002	0.000	>10
contig064187-BurOR.A016	contig047506-ZebOR.A014	0.002	0.009	0.175
contig036780-BurOR.A001	contig057165-NyeOR.A017	0.003	0.004	0.697
contig057153-BurOR.A014	contig054681-NyeOR.A011	0.003	0.004	0.703
contig064187-BurOR.A016	contig034988-NyeOR.A006	0.003	0.004	0.704
contig054681-NyeOR.A011	contig030557-ZebOR.A006	0.003	0.004	0.704
contig051559-BurOR.A008	contig034983-NyeOR.A003	0.003	0.004	0.707
contig036784-BurOR.A003	contig057754-NyeOR.A018	0.003	0.008	0.352
contig054233-BurOR.A012	contig054868-NyeOR.A014	0.003	0.008	0.366
contig054868-NyeOR.A014	contig047523-ZebOR.A022	0.003	0.008	0.366
contig051559-BurOR.A007	contig034983-NyeOR.A002	0.003	0.009	0.346
contig051559-BurOR.A007	contig047515-ZebOR.A019	0.003	0.009	0.346
contig057156-BurOR.A015	contig030560-ZebOR.A007	0.003	0.012	0.232
contig036787-BurOR.A004	contig030556-ZebOR.A005	0.003	0.012	0.233
contig036782-BurOR.A002	contig030553-ZebOR.A002	0.003	0.013	0.237
contig036784-BurOR.A003	contig030553-ZebOR.A003	0.004	0.004	1.061
contig051570-BurOR.A010	contig034988-NyeOR.A005	0.004	0.004	1.062
contig036787-BurOR.A004	contig054678-NyeOR.A010	0.004	0.008	0.525
contig051559-BurOR.A008	contig047515-ZebOR.A018	0.004	0.008	0.529
contig054678-NyeOR.A010	contig030556-ZebOR.A005	0.004	0.012	0.349
contig034988-NyeOR.A005	contig047508-ZebOR.A015	0.004	0.017	0.263
contig051321-BurOR.A006	contig056380-NyeOR.A016	0.004	0.021	0.211
contig057756-NyeOR.A019	contig030553-ZebOR.A002	0.004	0.021	0.213
contig034988-NyeOR.A006	contig047506-ZebOR.A014	0.005	0.004	1.054
contig054237-BurOR.A013	contig047521-ZebOR.A020	0.006	0.012	0.463
contig051566-BurOR.A009	contig047508-ZebOR.A016	0.006	0.012	0.481
contig056380-NyeOR.A016	contig062094-ZebOR.A023	0.006	0.021	0.280

contig051566-BurOR.A009	contig034988-NyeOR.A004	0.007	0.012	0.600
contig054237-BurOR.A013	contig034981-NyeOR.A001	0.007	0.017	0.434
contig036782-BurOR.A002	contig057756-NyeOR.A019	0.007	0.025	0.296
contig051321-BurOR.A006	contig062094-ZebOR.A023	0.008	0.014	0.552
contig034990-NyeOR.A007	contig047499-ZebOR.A012	0.008	0.018	0.449
contig064570-BurOR.A017	contig062095-ZebOR.A024	0.008	0.023	0.357
contig085010-BriOR.A005	contig054684-NyeOR.A012	0.009	0.008	1.045
contig085010-BriOR.A005	contig030560-ZebOR.A007	0.010	0.008	1.222
contig085010-BriOR.A005	contig057156-BurOR.A015	0.010	0.012	0.812
contig085026-BriOR.A008	contig047521-ZebOR.A020	0.010	0.017	0.610
contig085026-BriOR.A008	contig034981-NyeOR.A001	0.012	0.021	0.557
contig085000-BriOR.A003	contig057754-NyeOR.A018	0.013	0.017	0.798
contig047508-ZebOR.A016	contig047526-ZebOR.A021	0.013	0.027	0.470
contig084999-BriOR.A002	contig030554-ZebOR.A004	0.013	0.043	0.309
contig085026-BriOR.A008	contig054237-BurOR.A013	0.014	0.012	1.167
contig034994-NyeOR.A008	contig034995-NyeOR.A009	0.014	0.020	0.725
contig034988-NyeOR.A004	contig047526-ZebOR.A021	0.014	0.027	0.524
contig064187-BurOR.A016	contig041951-TilOR.A022	0.014	0.096	0.143
contig085000-BriOR.A003	contig030553-ZebOR.A003	0.015	0.004	3.582
contig056375-NyeOR.A015	contig062095-ZebOR.A024	0.015	0.047	0.321
contig041951-TilOR.A022	contig047506-ZebOR.A014	0.015	0.086	0.177
contig051570-BurOR.A010	contig070885-TilOR.A024	0.015	0.107	0.138
contig085000-BriOR.A003	contig036784-BurOR.A003	0.016	0.008	1.972
contig070885-TilOR.A024	contig047508-ZebOR.A015	0.016	0.100	0.155
contig034994-NyeOR.A008	contig047497-ZebOR.A011	0.017	0.016	1.095
contig062344-NyeOR.A020	contig030576-ZebOR.A010	0.017	0.019	0.902
contig093812-BriOR.A010	contig047499-ZebOR.A012	0.017	0.048	0.361
contig034988-NyeOR.A006	contig041951-TilOR.A022	0.017	0.091	0.185
contig093816-BriOR.A011	contig051573-BurOR.A011	0.018	0.038	0.473
contig093807-BriOR.A009	contig047514-ZebOR.A017	0.018	0.038	0.477
contig093812-BriOR.A010	contig034990-NyeOR.A007	0.018	0.042	0.429
contig022238-TilOR.A010	contig047514-ZebOR.A017	0.018	0.069	0.264
contig022230-TilOR.A007	contig047515-ZebOR.A019	0.018	0.077	0.237
contig034983-NyeOR.A002	contig022230-TilOR.A007	0.018	0.077	0.237
contig051559-BurOR.A007	contig022230-TilOR.A007	0.018	0.077	0.237
contig034995-NyeOR.A009	contig047497-ZebOR.A011	0.019	0.020	0.952
contig051566-BurOR.A009	contig047526-ZebOR.A021	0.019	0.031	0.600
contig085026-BriOR.A008	contig022211-TilOR.A003	0.019	0.068	0.276
contig084999-BriOR.A001	contig030553-ZebOR.A002	0.019	0.078	0.249
contig034988-NyeOR.A005	contig070885-TilOR.A024	0.019	0.112	0.172
contig054684-NyeOR.A012	contig022265-TilOR.A017	0.019	0.133	0.140
contig064570-BurOR.A017	contig056375-NyeOR.A015	0.020	0.065	0.304
contig022211-TilOR.A003	contig047521-ZebOR.A020	0.020	0.068	0.297

contig022232-TilOR.A008	contig047515-ZebOR.A018	0.020	0.077	0.262
contig057156-BurOR.A015	contig022265-TilOR.A017	0.020	0.119	0.169
contig022265-TilOR.A017	contig030560-ZebOR.A007	0.020	0.133	0.151
contig051566-BurOR.A009	contig070886-TilOR.A025	0.021	0.082	0.257
contig085002-BriOR.A004	contig036787-BurOR.A004	0.022	0.047	0.461
contig085002-BriOR.A004	contig030556-ZebOR.A005	0.022	0.051	0.424
contig093807-BriOR.A009	contig022238-TilOR.A010	0.022	0.053	0.413
contig034981-NyeOR.A001	contig022211-TilOR.A003	0.022	0.073	0.299
contig034983-NyeOR.A003	contig022232-TilOR.A008	0.022	0.073	0.300
contig085018-BriOR.A006	contig030576-ZebOR.A010	0.023	0.033	0.701
contig085002-BriOR.A004	contig054678-NyeOR.A010	0.023	0.047	0.490
contig084999-BriOR.A001	contig036782-BurOR.A002	0.023	0.083	0.273
contig085012-BriOR.A130	contig030572-ZebOR.A009	0.024	0.026	0.940
contig070886-TilOR.A025	contig047508-ZebOR.A016	0.024	0.078	0.311
contig084999-BriOR.A001	contig057756-NyeOR.A019	0.024	0.087	0.276
contig085010-BriOR.A005	contig022265-TilOR.A017	0.024	0.124	0.196
contig034990-NyeOR.A007	contig047497-ZebOR.A011	0.025	0.069	0.357
contig051559-BurOR.A008	contig022232-TilOR.A008	0.025	0.077	0.322
contig022266-TilOR.A018	contig030557-ZebOR.A006	0.025	0.088	0.289
contig022259-TilOR.A015	contig062094-ZebOR.A023	0.025	0.102	0.242
contig085012-BriOR.A130	contig051318-BurOR.A005	0.026	0.026	0.998
contig054237-BurOR.A013	contig022211-TilOR.A003	0.026	0.064	0.410
contig034988-NyeOR.A004	contig070886-TilOR.A025	0.026	0.078	0.330
contig070886-TilOR.A025	contig047526-ZebOR.A021	0.026	0.078	0.332
contig051321-BurOR.A006	contig022259-TilOR.A015	0.027	0.109	0.247
contig054681-NyeOR.A011	contig022266-TilOR.A018	0.028	0.083	0.342
contig057153-BurOR.A014	contig022266-TilOR.A018	0.028	0.088	0.323
contig084999-BriOR.A002	contig022268-TilOR.A019	0.028	0.102	0.275
contig056380-NyeOR.A016	contig022259-TilOR.A015	0.028	0.116	0.239
contig054687-NyeOR.A013	contig022264-TilOR.A016	0.029	0.076	0.386
contig034990-NyeOR.A007	contig034995-NyeOR.A009	0.029	0.082	0.354
contig054233-BurOR.A012	contig073309-TilOR.A026	0.030	0.077	0.393
contig073309-TilOR.A026	contig047523-ZebOR.A022	0.030	0.077	0.393
contig054868-NyeOR.A014	contig073309-TilOR.A026	0.030	0.077	0.394
contig034995-NyeOR.A009	contig047499-ZebOR.A012	0.030	0.084	0.356
contig022268-TilOR.A019	contig030554-ZebOR.A004	0.030	0.086	0.352
contig065887-BurOR.A018	contig022264-TilOR.A016	0.031	0.067	0.461
contig022264-TilOR.A016	contig030566-ZebOR.A008	0.031	0.071	0.432
contig034990-NyeOR.A007	contig034994-NyeOR.A008	0.031	0.076	0.412
contig047497-ZebOR.A011	contig047499-ZebOR.A012	0.031	0.088	0.355
contig022238-TilOR.A010	contig070886-TilOR.A025	0.031	0.108	0.290
contig070885-TilOR.A024	contig047503-ZebOR.A013	0.031	0.154	0.199
contig022204-TilOR.A001	contig022225-TilOR.A005	0.032	0.026	1.218

contig034994-NyeOR.A008	contig047499-ZebOR.A012	0.032	0.078	0.412
contig051570-BurOR.A010	contig047503-ZebOR.A013	0.033	0.087	0.375
contig047503-ZebOR.A013	contig047508-ZebOR.A015	0.033	0.101	0.323
contig085018-BriOR.A006	contig062344-NyeOR.A020	0.034	0.038	0.898
contig034988-NyeOR.A005	contig047503-ZebOR.A013	0.034	0.092	0.372
contig093812-BriOR.A010	contig034995-NyeOR.A009	0.034	0.093	0.367
contig022251-TilOR.A013	contig030572-ZebOR.A009	0.034	0.099	0.342
contig051318-BurOR.A005	contig022251-TilOR.A013	0.036	0.099	0.357
contig064570-BurOR.A017	contig022259-TilOR.A014	0.036	0.155	0.231
contig093812-BriOR.A010	contig047497-ZebOR.A011	0.037	0.089	0.420
contig093812-BriOR.A010	contig034994-NyeOR.A008	0.037	0.096	0.380
contig022251-TilOR.A013	contig062095-ZebOR.A024	0.037	0.127	0.290
contig034988-NyeORs.A033	contig041951-TilOR.A021	0.038	0.038	1.012
contig022259-TilOR.A014	contig062095-ZebOR.A024	0.038	0.153	0.250
contig056375-NyeOR.A015	contig022259-TilOR.A014	0.039	0.130	0.300
contig064570-BurOR.A017	contig022251-TilOR.A013	0.040	0.127	0.315
contig056375-NyeOR.A015	contig022251-TilOR.A013	0.041	0.100	0.406
contig070886-TilOR.A025	contig047514-ZebOR.A017	0.041	0.132	0.308
contig064570-BurOR.A017	contig062094-ZebOR.A023	0.041	0.138	0.298
contig064570-BurOR.A017	contig022259-TilOR.A015	0.041	0.138	0.300
contig064570-BurOR.A017	contig056380-NyeOR.A016	0.041	0.163	0.253
contig041952-TilOR.A023	contig070886-TilOR.A025	0.042	0.131	0.321
contig051321-BurOR.A006	contig064570-BurOR.A017	0.042	0.156	0.270
contig022259-TilOR.A014	contig022259-TilOR.A015	0.043	0.145	0.296
contig051321-BurOR.A006	contig062095-ZebOR.A024	0.043	0.163	0.262
contig056380-NyeOR.A016	contig062095-ZebOR.A024	0.043	0.174	0.246
contig022259-TilOR.A015	contig062095-ZebOR.A024	0.044	0.136	0.323
contig022259-TilOR.A014	contig062094-ZebOR.A023	0.044	0.147	0.298
contig051566-BurOR.A009	contig022238-TilOR.A010	0.045	0.142	0.320
contig022238-TilOR.A010	contig047526-ZebOR.A021	0.045	0.144	0.310
contig051321-BurOR.A006	contig022259-TilOR.A014	0.046	0.164	0.281
contig085012-BriOR.A130	contig022251-TilOR.A013	0.047	0.106	0.446
contig062094-ZebOR.A023	contig062095-ZebOR.A024	0.047	0.146	0.320
contig056380-NyeOR.A016	contig022259-TilOR.A014	0.047	0.171	0.273
contig022241-TilOR.A011	contig022245-TilOR.A012	0.048	0.054	0.890
contig093807-BriOR.A009	contig070886-TilOR.A025	0.048	0.125	0.382
contig022238-TilOR.A010	contig047508-ZebOR.A016	0.048	0.137	0.354
contig051321-BurOR.A006	contig056375-NyeOR.A015	0.048	0.153	0.310
contig056375-NyeOR.A015	contig056380-NyeOR.A016	0.048	0.158	0.301
contig022241-TilOR.A011	contig030576-ZebOR.A010	0.049	0.082	0.596
contig062344-NyeOR.A020	contig022241-TilOR.A011	0.049	0.082	0.597
contig056375-NyeOR.A015	contig022259-TilOR.A015	0.049	0.118	0.417
contig051318-BurOR.A005	contig064570-BurOR.A017	0.049	0.129	0.378

contig064570-BurOR.A017	contig030572-ZebOR.A009	0.049	0.129	0.378
contig030572-ZebOR.A009	contig062095-ZebOR.A024	0.049	0.136	0.363
contig051318-BurOR.A005	contig062095-ZebOR.A024	0.049	0.136	0.363
contig051566-BurOR.A009	contig047514-ZebOR.A017	0.049	0.145	0.337
contig034988-NyeOR.A004	contig022238-TilOR.A010	0.050	0.137	0.365
contig022238-TilOR.A010	contig041952-TilOR.A023	0.050	0.156	0.322
contig085018-BriOR.A006	contig022241-TilOR.A011	0.051	0.064	0.789
contig022217-TilOR.A004	contig030576-ZebOR.A010	0.051	0.074	0.690
contig056375-NyeOR.A015	contig062094-ZebOR.A023	0.051	0.133	0.380
contig047514-ZebOR.A017	contig047526-ZebOR.A021	0.051	0.140	0.361
contig022251-TilOR.A013	contig022259-TilOR.A014	0.051	0.144	0.353
contig051573-BurOR.A011	contig034994-NyeOR.A008	0.051	0.148	0.345
contig022217-TilOR.A004	contig022241-TilOR.A011	0.052	0.096	0.545
contig022251-TilOR.A013	contig022259-TilOR.A015	0.052	0.101	0.519
contig093807-BriOR.A009	contig047526-ZebOR.A021	0.052	0.122	0.422
contig047508-ZebOR.A016	contig047514-ZebOR.A017	0.052	0.140	0.372
contig051573-BurOR.A011	contig034995-NyeOR.A009	0.052	0.160	0.325
contig085018-BriOR.A006	contig022217-TilOR.A004	0.053	0.074	0.707
contig051318-BurOR.A005	contig051321-BurOR.A006	0.053	0.097	0.546
contig093807-BriOR.A009	contig047508-ZebOR.A016	0.053	0.132	0.402
contig051573-BurOR.A011	contig047497-ZebOR.A011	0.054	0.137	0.396
contig034988-NyeOR.A004	contig047514-ZebOR.A017	0.054	0.141	0.382
contig051318-BurOR.A005	contig062094-ZebOR.A023	0.055	0.086	0.644
contig051321-BurOR.A006	contig030572-ZebOR.A009	0.055	0.097	0.562
contig051318-BurOR.A005	contig056380-NyeOR.A016	0.055	0.111	0.492
contig093807-BriOR.A009	contig034988-NyeOR.A004	0.055	0.133	0.412
contig056380-NyeOR.A016	contig030572-ZebOR.A009	0.056	0.111	0.506
contig093807-BriOR.A009	contig051566-BurOR.A009	0.056	0.137	0.411
contig085012-BriOR.A130	contig064570-BurOR.A017	0.056	0.146	0.382
contig062344-NyeOR.A020	contig022217-TilOR.A004	0.057	0.082	0.698
contig030572-ZebOR.A009	contig062094-ZebOR.A023	0.057	0.086	0.661
contig051321-BurOR.A006	contig022251-TilOR.A013	0.057	0.105	0.545
contig057754-NyeOR.A018	contig022264-TilOR.A016	0.057	0.130	0.437
contig085012-BriOR.A130	contig062095-ZebOR.A024	0.057	0.154	0.367
contig093812-BriOR.A010	contig070886-TilOR.A025	0.057	0.160	0.354
contig051559-BurOR.A007	contig064187-BurOR.A016	0.057	0.187	0.305
contig022264-TilOR.A016	contig030553-ZebOR.A003	0.058	0.115	0.506
contig051318-BurOR.A005	contig022259-TilOR.A015	0.058	0.126	0.456
contig041952-TilOR.A023	contig047514-ZebOR.A017	0.058	0.132	0.442
contig085002-BriOR.A004	contig022268-TilOR.A019	0.058	0.132	0.443
contig054687-NyeOR.A013	contig057754-NyeOR.A018	0.058	0.150	0.388
contig093816-BriOR.A011	contig034995-NyeOR.A009	0.058	0.162	0.359
contig022251-TilOR.A013	contig062094-ZebOR.A023	0.059	0.103	0.578

contig056380-NyeOR.A016	contig022251-TilOR.A013	0.059	0.119	0.493
contig022259-TilOR.A015	contig030572-ZebOR.A009	0.059	0.126	0.468
contig051318-BurOR.A005	contig056375-NyeOR.A015	0.059	0.146	0.404
contig056375-NyeOR.A015	contig030572-ZebOR.A009	0.059	0.146	0.404
contig051559-BurOR.A007	contig047506-ZebOR.A014	0.059	0.176	0.333
contig051559-BurOR.A007	contig041951-TilOR.A022	0.059	0.176	0.334
contig064187-BurOR.A016	contig034983-NyeOR.A002	0.059	0.187	0.313
contig064187-BurOR.A016	contig047515-ZebOR.A019	0.059	0.187	0.313
contig036784-BurOR.A003	contig022264-TilOR.A016	0.060	0.120	0.500
contig054687-NyeOR.A013	contig030553-ZebOR.A003	0.060	0.135	0.443
contig085000-BriOR.A003	contig054687-NyeOR.A013	0.060	0.139	0.431
contig065887-BurOR.A018	contig057754-NyeOR.A018	0.060	0.140	0.427
contig085012-BriOR.A130	contig056375-NyeOR.A015	0.060	0.144	0.416
contig030554-ZebOR.A004	contig030556-ZebOR.A005	0.060	0.145	0.412
contig057754-NyeOR.A018	contig030566-ZebOR.A008	0.060	0.145	0.413
contig093816-BriOR.A011	contig034994-NyeOR.A008	0.060	0.147	0.405
contig036787-BurOR.A004	contig030554-ZebOR.A004	0.060	0.155	0.387
contig070886-TilOR.A025	contig073309-TilOR.A026	0.060	0.160	0.378
contig034983-NyeOR.A002	contig041951-TilOR.A022	0.060	0.176	0.342
contig041951-TilOR.A022	contig047515-ZebOR.A019	0.060	0.176	0.342
contig034983-NyeOR.A002	contig047506-ZebOR.A014	0.060	0.177	0.341
contig047506-ZebOR.A014	contig047515-ZebOR.A019	0.060	0.177	0.341
contig051559-BurOR.A007	contig034988-NyeOR.A006	0.060	0.182	0.332
contig085000-BriOR.A003	contig022264-TilOR.A016	0.061	0.119	0.516
contig065887-BurOR.A018	contig030553-ZebOR.A003	0.061	0.125	0.490
contig085000-BriOR.A003	contig065887-BurOR.A018	0.061	0.129	0.476
contig030553-ZebOR.A003	contig030566-ZebOR.A008	0.061	0.130	0.472
contig085000-BriOR.A003	contig030566-ZebOR.A008	0.061	0.134	0.459
contig022232-TilOR.A008	contig022234-TilOR.A009	0.061	0.136	0.446
contig036784-BurOR.A003	contig054687-NyeOR.A013	0.061	0.140	0.440
contig034990-NyeOR.A007	contig070886-TilOR.A025	0.061	0.167	0.365
contig041952-TilOR.A023	contig047526-ZebOR.A021	0.061	0.177	0.347
contig051566-BurOR.A009	contig041952-TilOR.A023	0.061	0.185	0.328
contig070886-TilOR.A025	contig047499-ZebOR.A012	0.062	0.164	0.378
contig093812-BriOR.A010	contig047523-ZebOR.A022	0.062	0.173	0.359
contig093812-BriOR.A010	contig054233-BurOR.A012	0.062	0.173	0.359
contig034983-NyeOR.A002	contig034988-NyeOR.A006	0.062	0.182	0.340
contig034988-NyeOR.A006	contig047515-ZebOR.A019	0.062	0.182	0.340
contig064187-BurOR.A016	contig022230-TilOR.A007	0.062	0.186	0.333
contig070886-TilOR.A025	contig047497-ZebOR.A011	0.062	0.205	0.304
contig022268-TilOR.A019	contig030556-ZebOR.A005	0.063	0.127	0.494
contig036784-BurOR.A003	contig065887-BurOR.A018	0.063	0.130	0.485
contig036784-BurOR.A003	contig030566-ZebOR.A008	0.063	0.134	0.468

contig036787-BurOR.A004	contig022268-TilOR.A019	0.063	0.136	0.461
contig051318-BurOR.A005	contig022259-TilOR.A014	0.063	0.138	0.457
contig093816-BriOR.A011	contig047497-ZebOR.A011	0.063	0.141	0.445
contig034994-NyeOR.A008	contig070886-TilOR.A025	0.063	0.220	0.287
contig022245-TilOR.A012	contig030576-ZebOR.A010	0.064	0.087	0.729
contig022217-TilOR.A004	contig022245-TilOR.A012	0.064	0.092	0.695
contig054678-NyeOR.A010	contig022268-TilOR.A019	0.064	0.141	0.455
contig084999-BriOR.A002	contig030556-ZebOR.A005	0.064	0.156	0.413
contig054678-NyeOR.A010	contig030554-ZebOR.A004	0.064	0.160	0.403
contig022230-TilOR.A007	contig047506-ZebOR.A014	0.064	0.186	0.342
contig041952-TilOR.A023	contig047508-ZebOR.A016	0.064	0.190	0.335
contig034990-NyeOR.A007	contig047523-ZebOR.A022	0.064	0.194	0.328
contig054233-BurOR.A012	contig034990-NyeOR.A007	0.064	0.194	0.328
contig034995-NyeOR.A009	contig070886-TilOR.A025	0.064	0.232	0.277
contig051573-BurOR.A011	contig047523-ZebOR.A022	0.064	0.252	0.255
contig051573-BurOR.A011	contig054233-BurOR.A012	0.064	0.252	0.255
contig022259-TilOR.A014	contig030572-ZebOR.A009	0.065	0.138	0.468
contig093807-BriOR.A009	contig041952-TilOR.A023	0.065	0.154	0.424
contig022230-TilOR.A007	contig041951-TilOR.A022	0.065	0.159	0.411
contig093812-BriOR.A010	contig054868-NyeOR.A014	0.065	0.178	0.367
contig034988-NyeOR.A006	contig022230-TilOR.A007	0.065	0.181	0.361
contig034988-NyeOR.A004	contig041952-TilOR.A023	0.065	0.191	0.343
contig051573-BurOR.A011	contig034990-NyeOR.A007	0.065	0.218	0.298
contig084999-BriOR.A002	contig085002-BriOR.A004	0.066	0.136	0.483
contig093812-BriOR.A010	contig051566-BurOR.A009	0.066	0.161	0.414
contig054233-BurOR.A012	contig070886-TilOR.A025	0.066	0.166	0.398
contig070886-TilOR.A025	contig047523-ZebOR.A022	0.066	0.166	0.398
contig054868-NyeOR.A014	contig070886-TilOR.A025	0.066	0.166	0.398
contig093812-BriOR.A010	contig022238-TilOR.A010	0.066	0.173	0.380
contig093812-BriOR.A010	contig047514-ZebOR.A017	0.066	0.189	0.348
contig093812-BriOR.A010	contig051573-BurOR.A011	0.066	0.208	0.316
contig051573-BurOR.A011	contig047499-ZebOR.A012	0.066	0.219	0.300
contig085002-BriOR.A004	contig030554-ZebOR.A004	0.067	0.145	0.464
contig084999-BriOR.A002	contig036787-BurOR.A004	0.067	0.155	0.434
contig034990-NyeOR.A007	contig054868-NyeOR.A014	0.067	0.199	0.336
contig047497-ZebOR.A011	contig047523-ZebOR.A022	0.067	0.200	0.333
contig054233-BurOR.A012	contig047497-ZebOR.A011	0.067	0.200	0.333
contig047499-ZebOR.A012	contig047523-ZebOR.A022	0.067	0.204	0.329
contig054233-BurOR.A012	contig047499-ZebOR.A012	0.067	0.204	0.329
contig034994-NyeOR.A008	contig047523-ZebOR.A022	0.067	0.215	0.313
contig054233-BurOR.A012	contig034994-NyeOR.A008	0.067	0.215	0.313
contig085018-BriOR.A006	contig022245-TilOR.A012	0.068	0.088	0.779
contig022238-TilOR.A010	contig073309-TilOR.A026	0.068	0.170	0.402

contig093812-BriOR.A010	contig047526-ZebOR.A021	0.068	0.176	0.387
contig034995-NyeOR.A009	contig047523-ZebOR.A022	0.068	0.216	0.317
contig054233-BurOR.A012	contig034995-NyeOR.A009	0.068	0.216	0.317
contig051573-BurOR.A011	contig054868-NyeOR.A014	0.068	0.258	0.262
contig084999-BriOR.A002	contig054678-NyeOR.A010	0.069	0.160	0.429
contig073309-TilOR.A026	contig047514-ZebOR.A017	0.069	0.168	0.412
contig022238-TilOR.A010	contig047499-ZebOR.A012	0.069	0.187	0.371
contig051573-BurOR.A011	contig041952-TilOR.A023	0.069	0.215	0.319
contig041952-TilOR.A023	contig047523-ZebOR.A022	0.070	0.158	0.441
contig054233-BurOR.A012	contig041952-TilOR.A023	0.070	0.158	0.441
contig093812-BriOR.A010	contig047508-ZebOR.A016	0.070	0.166	0.420
contig054868-NyeOR.A014	contig047497-ZebOR.A011	0.070	0.205	0.340
contig034994-NyeOR.A008	contig047526-ZebOR.A021	0.070	0.209	0.333
contig054868-NyeOR.A014	contig047499-ZebOR.A012	0.070	0.209	0.336
contig093812-BriOR.A010	contig093816-BriOR.A011	0.070	0.212	0.330
contig093816-BriOR.A011	contig041952-TilOR.A023	0.070	0.222	0.317
contig051573-BurOR.A011	contig073309-TilOR.A026	0.070	0.251	0.279
contig093812-BriOR.A010	contig034988-NyeOR.A004	0.071	0.166	0.428
contig047526-ZebOR.A021	contig047523-ZebOR.A022	0.071	0.168	0.419
contig054233-BurOR.A012	contig047526-ZebOR.A021	0.071	0.168	0.419
contig054868-NyeOR.A014	contig047526-ZebOR.A021	0.071	0.168	0.419
contig047499-ZebOR.A012	contig047514-ZebOR.A017	0.071	0.193	0.368
contig051566-BurOR.A009	contig034994-NyeOR.A008	0.071	0.193	0.369
contig034994-NyeOR.A008	contig054868-NyeOR.A014	0.071	0.220	0.321
contig093816-BriOR.A011	contig034990-NyeOR.A007	0.071	0.225	0.316
contig093816-BriOR.A011	contig047523-ZebOR.A022	0.071	0.253	0.279
contig093816-BriOR.A011	contig054233-BurOR.A012	0.071	0.253	0.279
contig062344-NyeOR.A020	contig022245-TilOR.A012	0.072	0.096	0.750
contig085012-BriOR.A130	contig022259-TilOR.A015	0.072	0.145	0.496
contig022259-TilOR.A014	contig030576-ZebOR.A010	0.072	0.167	0.430
contig034990-NyeOR.A007	contig022238-TilOR.A010	0.072	0.175	0.410
contig084999-BriOR.A001	contig022268-TilOR.A020	0.072	0.179	0.404
contig034995-NyeOR.A009	contig054868-NyeOR.A014	0.072	0.221	0.324
contig093816-BriOR.A011	contig047499-ZebOR.A012	0.072	0.227	0.317
contig051566-BurOR.A009	contig047499-ZebOR.A012	0.073	0.154	0.476
contig022217-TilOR.A004	contig022259-TilOR.A014	0.073	0.161	0.456
contig054868-NyeOR.A014	contig041952-TilOR.A023	0.073	0.163	0.448
contig093807-BriOR.A009	contig093812-BriOR.A010	0.073	0.181	0.402
contig022238-TilOR.A010	contig047497-ZebOR.A011	0.073	0.213	0.343
contig051573-BurOR.A011	contig070886-TilOR.A025	0.073	0.282	0.258
contig051566-BurOR.A009	contig034990-NyeOR.A007	0.074	0.157	0.471
contig022238-TilOR.A010	contig047523-ZebOR.A022	0.074	0.167	0.446
contig054233-BurOR.A012	contig022238-TilOR.A010	0.074	0.167	0.446

contig054868-NyeOR.A014	contig022238-TilOR.A010	0.074	0.167	0.446
contig093812-BriOR.A010	contig041952-TilOR.A023	0.074	0.193	0.383
contig047497-ZebOR.A011	contig047526-ZebOR.A021	0.074	0.195	0.380
contig034994-NyeOR.A008	contig047508-ZebOR.A016	0.074	0.199	0.375
contig034995-NyeOR.A009	contig047526-ZebOR.A021	0.074	0.221	0.335
contig034994-NyeOR.A008	contig022238-TilOR.A010	0.074	0.228	0.324
contig093816-BriOR.A011	contig054868-NyeOR.A014	0.074	0.259	0.286
contig022225-TilOR.A005	contig022227-TilOR.A006	0.075	0.086	0.872
contig085012-BriOR.A130	contig051321-BurOR.A006	0.075	0.104	0.720
contig047508-ZebOR.A016	contig047523-ZebOR.A022	0.075	0.158	0.476
contig054233-BurOR.A012	contig047508-ZebOR.A016	0.075	0.158	0.476
contig054868-NyeOR.A014	contig047508-ZebOR.A016	0.075	0.158	0.476
contig047499-ZebOR.A012	contig047526-ZebOR.A021	0.075	0.169	0.443
contig051566-BurOR.A009	contig047497-ZebOR.A011	0.075	0.179	0.422
contig034990-NyeOR.A007	contig047514-ZebOR.A017	0.075	0.180	0.416
contig034994-NyeOR.A008	contig047514-ZebOR.A017	0.075	0.234	0.322
contig034995-NyeOR.A009	contig022238-TilOR.A010	0.075	0.240	0.312
contig093816-BriOR.A011	contig070886-TilOR.A025	0.075	0.277	0.269
contig085012-BriOR.A130	contig056380-NyeOR.A016	0.076	0.118	0.648
contig051566-BurOR.A009	contig073309-TilOR.A026	0.076	0.152	0.502
contig085018-BriOR.A006	contig022259-TilOR.A014	0.076	0.157	0.487
contig047514-ZebOR.A017	contig047523-ZebOR.A022	0.076	0.172	0.441
contig054233-BurOR.A012	contig047514-ZebOR.A017	0.076	0.172	0.441
contig054868-NyeOR.A014	contig047514-ZebOR.A017	0.076	0.172	0.441
contig034990-NyeOR.A007	contig047526-ZebOR.A021	0.076	0.173	0.439
contig034988-NyeOR.A004	contig034994-NyeOR.A008	0.076	0.199	0.382
contig051566-BurOR.A009	contig034995-NyeOR.A009	0.076	0.205	0.370
contig047497-ZebOR.A011	contig047514-ZebOR.A017	0.076	0.219	0.349
contig093812-BriOR.A010	contig073309-TilOR.A026	0.076	0.221	0.343
contig093816-BriOR.A011	contig073309-TilOR.A026	0.076	0.252	0.304
contig051570-BurOR.A010	contig022232-TilOR.A008	0.076	0.258	0.295
contig085012-BriOR.A130	contig062094-ZebOR.A023	0.077	0.092	0.833
contig022245-TilOR.A012	contig022259-TilOR.A014	0.077	0.128	0.604
contig056375-NyeOR.A015	contig022245-TilOR.A012	0.077	0.151	0.511
contig041952-TilOR.A023	contig073309-TilOR.A026	0.077	0.154	0.501
contig047499-ZebOR.A012	contig047508-ZebOR.A016	0.077	0.159	0.481
contig034988-NyeOR.A004	contig047523-ZebOR.A022	0.077	0.159	0.485
contig054233-BurOR.A012	contig034988-NyeOR.A004	0.077	0.159	0.485
contig034988-NyeOR.A004	contig054868-NyeOR.A014	0.077	0.159	0.485
contig022245-TilOR.A012	contig062095-ZebOR.A024	0.077	0.161	0.479
contig034990-NyeOR.A007	contig047508-ZebOR.A016	0.077	0.163	0.476
contig093807-BriOR.A009	contig073309-TilOR.A026	0.077	0.168	0.459
contig093807-BriOR.A009	contig047523-ZebOR.A022	0.077	0.175	0.438

contig093807-BriOR.A009	contig054233-BurOR.A012	0.077	0.175	0.438
contig093807-BriOR.A009	contig054868-NyeOR.A014	0.077	0.175	0.438
contig022268-TilOR.A020	contig030553-ZebOR.A002	0.077	0.217	0.355
contig022232-TilOR.A008	contig047508-ZebOR.A015	0.077	0.243	0.316
contig034995-NyeOR.A009	contig047514-ZebOR.A017	0.077	0.246	0.311
contig022232-TilOR.A008	contig070885-TilOR.A024	0.077	0.262	0.294
contig085012-BriOR.A130	contig022259-TilOR.A014	0.078	0.155	0.506
contig073309-TilOR.A026	contig047526-ZebOR.A021	0.078	0.156	0.497
contig034988-NyeOR.A004	contig047499-ZebOR.A012	0.078	0.160	0.490
contig093807-BriOR.A009	contig047499-ZebOR.A012	0.078	0.175	0.448
contig034990-NyeOR.A007	contig041952-TilOR.A023	0.078	0.196	0.396
contig041952-TilOR.A023	contig047499-ZebOR.A012	0.078	0.211	0.370
contig070885-TilOR.A024	contig047515-ZebOR.A018	0.078	0.264	0.295
contig022204-TilOR.A001	contig022227-TilOR.A006	0.079	0.099	0.794
contig051566-BurOR.A009	contig047523-ZebOR.A022	0.079	0.153	0.514
contig051566-BurOR.A009	contig054233-BurOR.A012	0.079	0.153	0.514
contig051566-BurOR.A009	contig054868-NyeOR.A014	0.079	0.153	0.514
contig073309-TilOR.A026	contig047508-ZebOR.A016	0.079	0.157	0.506
contig034988-NyeOR.A004	contig034990-NyeOR.A007	0.079	0.163	0.484
contig047497-ZebOR.A011	contig047508-ZebOR.A016	0.079	0.184	0.427
contig034995-NyeOR.A009	contig047508-ZebOR.A016	0.079	0.210	0.375
contig034983-NyeOR.A003	contig070885-TilOR.A024	0.079	0.263	0.302
contig022245-TilOR.A012	contig022251-TilOR.A013	0.080	0.106	0.753
contig064570-BurOR.A017	contig022245-TilOR.A012	0.080	0.153	0.520
contig034988-NyeOR.A004	contig047497-ZebOR.A011	0.080	0.185	0.435
contig036782-BurOR.A002	contig022268-TilOR.A020	0.080	0.211	0.380
contig034988-NyeOR.A005	contig022232-TilOR.A008	0.080	0.249	0.322
contig034988-NyeOR.A004	contig073309-TilOR.A026	0.081	0.157	0.515
contig034988-NyeOR.A004	contig034995-NyeOR.A009	0.081	0.211	0.382
contig022234-TilOR.A009	contig070885-TilOR.A024	0.081	0.230	0.353
contig051570-BurOR.A010	contig047515-ZebOR.A018	0.081	0.252	0.324
contig022241-TilOR.A011	contig022259-TilOR.A014	0.082	0.143	0.578
contig022251-TilOR.A013	contig030576-ZebOR.A010	0.082	0.168	0.489
contig093807-BriOR.A009	contig034990-NyeOR.A007	0.082	0.173	0.476
contig062344-NyeOR.A020	contig022259-TilOR.A014	0.082	0.178	0.459
contig030576-ZebOR.A010	contig062095-ZebOR.A024	0.082	0.217	0.376
contig062344-NyeOR.A020	contig062095-ZebOR.A024	0.082	0.217	0.376
contig057756-NyeOR.A019	contig022268-TilOR.A020	0.082	0.233	0.352
contig034990-NyeOR.A007	contig073309-TilOR.A026	0.082	0.234	0.348
contig047508-ZebOR.A015	contig047515-ZebOR.A018	0.082	0.249	0.330
contig051573-BurOR.A011	contig047526-ZebOR.A021	0.082	0.284	0.289
contig051318-BurOR.A005	contig022245-TilOR.A012	0.083	0.118	0.702
contig056375-NyeOR.A015	contig062344-NyeOR.A020	0.083	0.217	0.384

contig056375-NyeOR.A015	contig030576-ZebOR.A010	0.083	0.218	0.383
contig093807-BriOR.A009	contig034994-NyeOR.A008	0.083	0.226	0.366
contig051570-BurOR.A010	contig034983-NyeOR.A003	0.083	0.251	0.330
contig034988-NyeOR.A005	contig047515-ZebOR.A018	0.083	0.252	0.329
contig051559-BurOR.A008	contig070885-TilOR.A024	0.083	0.269	0.308
contig022245-TilOR.A012	contig030572-ZebOR.A009	0.084	0.118	0.715
contig022245-TilOR.A012	contig022259-TilOR.A015	0.084	0.148	0.566
contig085018-BriOR.A006	contig022251-TilOR.A013	0.084	0.169	0.497
contig064570-BurOR.A017	contig030576-ZebOR.A010	0.084	0.209	0.403
contig064570-BurOR.A017	contig062344-NyeOR.A020	0.084	0.209	0.404
contig093807-BriOR.A009	contig047497-ZebOR.A011	0.084	0.211	0.396
contig041952-TilOR.A023	contig047497-ZebOR.A011	0.084	0.217	0.388
contig093807-BriOR.A009	contig034995-NyeOR.A009	0.084	0.238	0.352
contig073309-TilOR.A026	contig047497-ZebOR.A011	0.084	0.241	0.351
contig034983-NyeOR.A003	contig047508-ZebOR.A015	0.084	0.249	0.337
contig051566-BurOR.A009	contig051573-BurOR.A011	0.084	0.267	0.314
contig034994-NyeOR.A008	contig041952-TilOR.A023	0.085	0.231	0.367
contig073309-TilOR.A026	contig047499-ZebOR.A012	0.085	0.233	0.364
contig034983-NyeOR.A003	contig034988-NyeOR.A005	0.085	0.252	0.336
contig034994-NyeOR.A008	contig073309-TilOR.A026	0.085	0.256	0.333
contig051321-BurOR.A006	contig022245-TilOR.A012	0.086	0.128	0.671
contig022241-TilOR.A011	contig022251-TilOR.A013	0.086	0.130	0.661
contig022241-TilOR.A011	contig062095-ZebOR.A024	0.086	0.175	0.491
contig022234-TilOR.A009	contig047515-ZebOR.A018	0.086	0.199	0.434
contig034995-NyeOR.A009	contig041952-TilOR.A023	0.086	0.238	0.359
contig051559-BurOR.A008	contig051570-BurOR.A010	0.086	0.257	0.336
contig034995-NyeOR.A009	contig073309-TilOR.A026	0.086	0.257	0.336
contig056380-NyeOR.A016	contig022245-TilOR.A012	0.087	0.142	0.614
contig022217-TilOR.A004	contig062095-ZebOR.A024	0.087	0.200	0.434
contig085018-BriOR.A006	contig062095-ZebOR.A024	0.087	0.212	0.408
contig034981-NyeOR.A001	contig062344-NyeOR.A020	0.087	0.235	0.371
contig051559-BurOR.A008	contig047508-ZebOR.A015	0.087	0.254	0.343
contig093816-BriOR.A011	contig047526-ZebOR.A021	0.087	0.268	0.326
contig051573-BurOR.A011	contig047508-ZebOR.A016	0.087	0.273	0.319
contig022245-TilOR.A012	contig062094-ZebOR.A023	0.088	0.116	0.758
contig022217-TilOR.A004	contig022251-TilOR.A013	0.088	0.155	0.568
contig056375-NyeOR.A015	contig022241-TilOR.A011	0.088	0.176	0.501
contig034983-NyeOR.A003	contig022234-TilOR.A009	0.088	0.194	0.455
contig085018-BriOR.A006	contig056375-NyeOR.A015	0.088	0.224	0.395
contig051559-BurOR.A008	contig034988-NyeOR.A005	0.088	0.257	0.342
contig064570-BurOR.A017	contig022241-TilOR.A011	0.089	0.167	0.530
contig085018-BriOR.A006	contig056380-NyeOR.A016	0.089	0.181	0.493
contig056380-NyeOR.A016	contig030576-ZebOR.A010	0.089	0.181	0.494

contig064570-BurOR.A017	contig022217-TilOR.A004	0.089	0.189	0.469
contig056375-NyeOR.A015	contig022217-TilOR.A004	0.089	0.195	0.455
contig085018-BriOR.A006	contig064570-BurOR.A017	0.089	0.204	0.437
contig051570-BurOR.A010	contig022234-TilOR.A009	0.089	0.214	0.419
contig093816-BriOR.A011	contig051566-BurOR.A009	0.089	0.262	0.339
contig051573-BurOR.A011	contig034988-NyeOR.A004	0.089	0.274	0.324
contig030576-ZebOR.A010	contig062094-ZebOR.A023	0.090	0.164	0.551
contig022217-TilOR.A004	contig022259-TilOR.A015	0.090	0.175	0.514
contig022234-TilOR.A009	contig047508-ZebOR.A015	0.090	0.200	0.451
contig051573-BurOR.A011	contig022238-TilOR.A010	0.090	0.276	0.325
contig051321-BurOR.A006	contig030576-ZebOR.A010	0.091	0.156	0.585
contig022259-TilOR.A015	contig030576-ZebOR.A010	0.091	0.191	0.478
contig051559-BurOR.A008	contig022234-TilOR.A009	0.091	0.199	0.459
contig034988-NyeOR.A005	contig022234-TilOR.A009	0.091	0.203	0.449
contig054237-BurOR.A013	contig062344-NyeOR.A020	0.091	0.217	0.419
contig062344-NyeOR.A020	contig047521-ZebOR.A020	0.091	0.223	0.409
contig051573-BurOR.A011	contig047514-ZebOR.A017	0.091	0.265	0.344
contig022232-TilOR.A008	contig047503-ZebOR.A013	0.091	0.289	0.315
contig051321-BurOR.A006	contig022217-TilOR.A004	0.092	0.148	0.623
contig093816-BriOR.A011	contig022238-TilOR.A010	0.092	0.257	0.359
contig093816-BriOR.A011	contig047508-ZebOR.A016	0.092	0.257	0.359
contig085018-BriOR.A006	contig062094-ZebOR.A023	0.093	0.164	0.569
contig062344-NyeOR.A020	contig022251-TilOR.A013	0.093	0.180	0.515
contig022217-TilOR.A004	contig062094-ZebOR.A023	0.094	0.138	0.676
contig056380-NyeOR.A016	contig022217-TilOR.A004	0.094	0.153	0.614
contig085018-BriOR.A006	contig051321-BurOR.A006	0.094	0.156	0.604
contig051318-BurOR.A005	contig022217-TilOR.A004	0.094	0.162	0.579
contig093816-BriOR.A011	contig034988-NyeOR.A004	0.094	0.257	0.364
contig022217-TilOR.A004	contig030572-ZebOR.A009	0.095	0.162	0.589
contig085018-BriOR.A006	contig022259-TilOR.A015	0.095	0.192	0.493
contig085026-BriOR.A008	contig062344-NyeOR.A020	0.096	0.222	0.435
contig085012-BriOR.A130	contig022245-TilOR.A012	0.097	0.122	0.793
contig056380-NyeOR.A016	contig022241-TilOR.A011	0.097	0.144	0.669
contig093816-BriOR.A011	contig047514-ZebOR.A017	0.097	0.251	0.386
contig085018-BriOR.A006	contig034981-NyeOR.A001	0.098	0.238	0.410
contig022241-TilOR.A011	contig030572-ZebOR.A009	0.099	0.148	0.670
contig051318-BurOR.A005	contig022241-TilOR.A011	0.099	0.148	0.670
contig030572-ZebOR.A009	contig030576-ZebOR.A010	0.099	0.170	0.584
contig051318-BurOR.A005	contig030576-ZebOR.A010	0.099	0.170	0.585
contig047503-ZebOR.A013	contig047515-ZebOR.A018	0.099	0.280	0.352
contig093807-BriOR.A009	contig051573-BurOR.A011	0.099	0.280	0.352
contig051321-BurOR.A006	contig022241-TilOR.A011	0.100	0.130	0.772
contig051321-BurOR.A006	contig062344-NyeOR.A020	0.100	0.152	0.659

contig022241-TilOR.A011	contig022259-TilOR.A015	0.100	0.175	0.573
contig056380-NyeOR.A016	contig062344-NyeOR.A020	0.100	0.177	0.564
contig034983-NyeOR.A003	contig047503-ZebOR.A013	0.100	0.279	0.359
contig022241-TilOR.A011	contig062094-ZebOR.A023	0.101	0.118	0.856
contig062344-NyeOR.A020	contig062094-ZebOR.A023	0.101	0.160	0.630
contig085018-BriOR.A006	contig051318-BurOR.A005	0.101	0.170	0.593
contig085018-BriOR.A006	contig030572-ZebOR.A009	0.101	0.171	0.592
contig062344-NyeOR.A020	contig022211-TilOR.A003	0.101	0.202	0.497
contig047521-ZebOR.A020	contig062095-ZebOR.A024	0.101	0.263	0.384
contig062344-NyeOR.A020	contig022259-TilOR.A015	0.102	0.203	0.501
contig022241-TilOR.A011	contig047521-ZebOR.A020	0.102	0.237	0.428
contig034981-NyeOR.A001	contig022241-TilOR.A011	0.102	0.245	0.417
contig034981-NyeOR.A001	contig062095-ZebOR.A024	0.102	0.259	0.392
contig085012-BriOR.A130	contig022241-TilOR.A011	0.103	0.140	0.735
contig054237-BurOR.A013	contig022241-TilOR.A011	0.103	0.231	0.446
contig054237-BurOR.A013	contig030576-ZebOR.A010	0.103	0.236	0.435
contig064570-BurOR.A017	contig047521-ZebOR.A020	0.103	0.259	0.399
contig022259-TilOR.A014	contig047521-ZebOR.A020	0.103	0.263	0.393
contig064570-BurOR.A017	contig034981-NyeOR.A001	0.104	0.256	0.408
contig034981-NyeOR.A001	contig022259-TilOR.A014	0.104	0.271	0.384
contig056375-NyeOR.A015	contig047521-ZebOR.A020	0.104	0.275	0.380
contig051559-BurOR.A008	contig047503-ZebOR.A013	0.104	0.285	0.363
contig030576-ZebOR.A010	contig047521-ZebOR.A020	0.105	0.240	0.436
contig093807-BriOR.A009	contig093816-BriOR.A011	0.105	0.261	0.401
contig034981-NyeOR.A001	contig056375-NyeOR.A015	0.105	0.271	0.388
contig085026-BriOR.A008	contig062095-ZebOR.A024	0.105	0.285	0.367
contig034981-NyeOR.A001	contig030576-ZebOR.A010	0.106	0.249	0.425
contig054237-BurOR.A013	contig062095-ZebOR.A024	0.106	0.281	0.377
contig085026-BriOR.A008	contig064570-BurOR.A017	0.107	0.282	0.381
contig054237-BurOR.A013	contig022259-TilOR.A014	0.108	0.270	0.399
contig085026-BriOR.A008	contig022259-TilOR.A014	0.108	0.271	0.397
contig085026-BriOR.A008	contig056375-NyeOR.A015	0.108	0.285	0.379
contig085026-BriOR.A008	contig022241-TilOR.A011	0.109	0.224	0.485
contig022217-TilOR.A004	contig047521-ZebOR.A020	0.109	0.225	0.486
contig085026-BriOR.A008	contig030576-ZebOR.A010	0.109	0.237	0.461
contig022234-TilOR.A009	contig047503-ZebOR.A013	0.109	0.248	0.438
contig054237-BurOR.A013	contig064570-BurOR.A017	0.109	0.277	0.391
contig054237-BurOR.A013	contig056375-NyeOR.A015	0.109	0.281	0.389
contig085012-BriOR.A130	contig022217-TilOR.A004	0.110	0.177	0.621
contig062344-NyeOR.A020	contig030572-ZebOR.A009	0.110	0.182	0.606
contig051318-BurOR.A005	contig062344-NyeOR.A020	0.110	0.182	0.607
contig034981-NyeOR.A001	contig022217-TilOR.A004	0.110	0.233	0.473
contig054237-BurOR.A013	contig022217-TilOR.A004	0.111	0.221	0.500

contig085018-BriOR.A006	contig047521-ZebOR.A020	0.111	0.239	0.463
contig085018-BriOR.A006	contig054237-BurOR.A013	0.112	0.234	0.477
contig085012-BriOR.A130	contig062344-NyeOR.A020	0.113	0.188	0.602
contig022211-TilOR.A003	contig022241-TilOR.A011	0.113	0.210	0.537
contig022211-TilOR.A003	contig062095-ZebOR.A024	0.113	0.274	0.413
contig022211-TilOR.A003	contig030576-ZebOR.A010	0.114	0.198	0.577
contig085018-BriOR.A006	contig085026-BriOR.A008	0.115	0.235	0.489
contig056375-NyeOR.A015	contig022211-TilOR.A003	0.115	0.268	0.429
contig022259-TilOR.A014	contig070885-TilOR.A024	0.115	0.383	0.300
contig022211-TilOR.A003	contig022259-TilOR.A014	0.116	0.264	0.440
contig064570-BurOR.A017	contig022211-TilOR.A003	0.116	0.270	0.428
contig085012-BriOR.A130	contig030576-ZebOR.A010	0.117	0.187	0.625
contig085026-BriOR.A008	contig022217-TilOR.A004	0.117	0.217	0.538
contig070885-TilOR.A024	contig062095-ZebOR.A024	0.117	0.390	0.300
contig085018-BriOR.A006	contig085012-BriOR.A130	0.118	0.187	0.632
contig022251-TilOR.A013	contig047521-ZebOR.A020	0.118	0.255	0.464
contig062344-NyeOR.A020	contig070885-TilOR.A024	0.118	0.332	0.355
contig022245-TilOR.A012	contig047521-ZebOR.A020	0.119	0.209	0.571
contig034981-NyeOR.A001	contig022251-TilOR.A013	0.119	0.252	0.474
contig070885-TilOR.A024	contig030576-ZebOR.A010	0.119	0.328	0.361
contig022232-TilOR.A008	contig022251-TilOR.A013	0.119	0.368	0.324
contig054684-NyeOR.A012	contig030554-ZebOR.A004	0.119	0.449	0.264
contig022211-TilOR.A003	contig022217-TilOR.A004	0.120	0.200	0.600
contig054237-BurOR.A013	contig022245-TilOR.A012	0.120	0.205	0.588
contig085018-BriOR.A006	contig022211-TilOR.A003	0.120	0.207	0.580
contig034981-NyeOR.A001	contig022245-TilOR.A012	0.120	0.217	0.555
contig022251-TilOR.A013	contig047515-ZebOR.A018	0.120	0.358	0.336
contig064570-BurOR.A017	contig070885-TilOR.A024	0.120	0.375	0.321
contig056375-NyeOR.A015	contig070885-TilOR.A024	0.120	0.390	0.309
contig057156-BurOR.A015	contig030554-ZebOR.A004	0.120	0.423	0.284
contig022259-TilOR.A015	contig047521-ZebOR.A020	0.121	0.290	0.416
contig022259-TilOR.A014	contig047503-ZebOR.A013	0.121	0.301	0.401
contig030554-ZebOR.A004	contig030560-ZebOR.A007	0.121	0.449	0.268
contig034981-NyeOR.A001	contig022259-TilOR.A015	0.122	0.287	0.424
contig085018-BriOR.A006	contig070885-TilOR.A024	0.122	0.323	0.378
contig022234-TilOR.A009	contig022259-TilOR.A014	0.122	0.353	0.345
contig034983-NyeOR.A003	contig022251-TilOR.A013	0.122	0.365	0.334
contig056375-NyeOR.A015	contig047515-ZebOR.A018	0.122	0.424	0.288
contig084999-BriOR.A002	contig054684-NyeOR.A012	0.122	0.452	0.270
contig054684-NyeOR.A012	contig030556-ZebOR.A005	0.122	0.510	0.239
contig085026-BriOR.A008	contig022251-TilOR.A013	0.123	0.251	0.489
contig054237-BurOR.A013	contig022251-TilOR.A013	0.123	0.251	0.491
contig022245-TilOR.A012	contig070885-TilOR.A024	0.123	0.297	0.415

contig047503-ZebOR.A013	contig062095-ZebOR.A024	0.123	0.300	0.410
contig022251-TilOR.A013	contig070885-TilOR.A024	0.123	0.331	0.372
contig056375-NyeOR.A015	contig022232-TilOR.A008	0.123	0.406	0.303
contig084999-BriOR.A002	contig057156-BurOR.A015	0.123	0.425	0.290
contig057156-BurOR.A015	contig030556-ZebOR.A005	0.123	0.487	0.254
contig030556-ZebOR.A005	contig030560-ZebOR.A007	0.123	0.510	0.242
contig034988-NyeOR.A005	contig022259-TilOR.A014	0.124	0.369	0.336
contig022259-TilOR.A014	contig047515-ZebOR.A018	0.124	0.391	0.317
contig047515-ZebOR.A018	contig062095-ZebOR.A024	0.124	0.416	0.298
contig034983-NyeOR.A003	contig056375-NyeOR.A015	0.124	0.431	0.288
contig084999-BriOR.A002	contig030560-ZebOR.A007	0.124	0.452	0.274
contig051321-BurOR.A006	contig047521-ZebOR.A020	0.125	0.238	0.525
contig085026-BriOR.A008	contig022259-TilOR.A015	0.125	0.286	0.437
contig054237-BurOR.A013	contig022259-TilOR.A015	0.125	0.286	0.438
contig062344-NyeOR.A020	contig022234-TilOR.A009	0.125	0.311	0.401
contig034988-NyeOR.A005	contig062344-NyeOR.A020	0.125	0.325	0.385
contig051559-BurOR.A008	contig022251-TilOR.A013	0.125	0.364	0.344
contig034988-NyeOR.A005	contig062095-ZebOR.A024	0.125	0.371	0.336
contig022232-TilOR.A008	contig022259-TilOR.A014	0.125	0.402	0.311
contig064570-BurOR.A017	contig047515-ZebOR.A018	0.125	0.412	0.303
contig036787-BurOR.A004	contig054684-NyeOR.A012	0.125	0.501	0.250
contig051321-BurOR.A006	contig034981-NyeOR.A001	0.126	0.247	0.511
contig064570-BurOR.A017	contig047503-ZebOR.A013	0.126	0.284	0.443
contig056375-NyeOR.A015	contig047503-ZebOR.A013	0.126	0.306	0.413
contig022259-TilOR.A014	contig047508-ZebOR.A015	0.126	0.339	0.373
contig047508-ZebOR.A015	contig062095-ZebOR.A024	0.126	0.346	0.364
contig051570-BurOR.A010	contig022259-TilOR.A014	0.126	0.355	0.354
contig051570-BurOR.A010	contig062095-ZebOR.A024	0.126	0.361	0.348
contig022232-TilOR.A008	contig062095-ZebOR.A024	0.126	0.393	0.322
contig034983-NyeOR.A003	contig022259-TilOR.A014	0.126	0.398	0.317
contig034983-NyeOR.A003	contig062095-ZebOR.A024	0.126	0.423	0.297
contig022265-TilOR.A017	contig030554-ZebOR.A004	0.126	0.428	0.295
contig085026-BriOR.A008	contig022245-TilOR.A012	0.127	0.200	0.635
contig022241-TilOR.A011	contig047503-ZebOR.A013	0.127	0.231	0.551
contig056380-NyeOR.A016	contig047521-ZebOR.A020	0.127	0.249	0.512
contig022251-TilOR.A013	contig047503-ZebOR.A013	0.127	0.284	0.448
contig062344-NyeOR.A020	contig047508-ZebOR.A015	0.127	0.296	0.430
contig051570-BurOR.A010	contig062344-NyeOR.A020	0.127	0.312	0.406
contig056380-NyeOR.A016	contig070885-TilOR.A024	0.127	0.331	0.382
contig064570-BurOR.A017	contig034988-NyeOR.A005	0.127	0.354	0.360
contig064570-BurOR.A017	contig022232-TilOR.A008	0.127	0.389	0.327
contig022265-TilOR.A017	contig022268-TilOR.A019	0.127	0.406	0.312
contig057156-BurOR.A015	contig022268-TilOR.A019	0.127	0.418	0.303

contig064570-BurOR.A017	contig034983-NyeOR.A003	0.127	0.419	0.302
contig054684-NyeOR.A012	contig022268-TilOR.A019	0.127	0.452	0.281
contig085010-BriOR.A005	contig030554-ZebOR.A004	0.127	0.452	0.281
contig036787-BurOR.A004	contig057156-BurOR.A015	0.127	0.478	0.265
contig036787-BurOR.A004	contig030560-ZebOR.A007	0.127	0.501	0.253
contig054678-NyeOR.A010	contig054684-NyeOR.A012	0.127	0.502	0.252
contig047521-ZebOR.A020	contig062094-ZebOR.A023	0.128	0.234	0.550
contig034981-NyeOR.A001	contig056380-NyeOR.A016	0.128	0.257	0.499
contig062344-NyeOR.A020	contig047503-ZebOR.A013	0.128	0.263	0.486
contig030576-ZebOR.A010	contig047503-ZebOR.A013	0.128	0.263	0.488
contig051570-BurOR.A010	contig064570-BurOR.A017	0.128	0.344	0.374
contig034988-NyeOR.A005	contig056375-NyeOR.A015	0.128	0.378	0.339
contig022259-TilOR.A015	contig070885-TilOR.A024	0.128	0.381	0.336
contig051559-BurOR.A008	contig056375-NyeOR.A015	0.128	0.430	0.297
contig085010-BriOR.A005	contig022268-TilOR.A019	0.128	0.455	0.282
contig057156-BurOR.A015	contig054678-NyeOR.A010	0.128	0.479	0.268
contig054678-NyeOR.A010	contig030560-ZebOR.A007	0.128	0.502	0.256
contig034981-NyeOR.A001	contig062094-ZebOR.A023	0.129	0.242	0.535
contig064570-BurOR.A017	contig047508-ZebOR.A015	0.129	0.330	0.390
contig034988-NyeOR.A005	contig030576-ZebOR.A010	0.129	0.343	0.375
contig051570-BurOR.A010	contig056375-NyeOR.A015	0.129	0.368	0.351
contig051559-BurOR.A008	contig022259-TilOR.A014	0.129	0.404	0.320
contig051559-BurOR.A008	contig062095-ZebOR.A024	0.129	0.423	0.306
contig084999-BriOR.A002	contig022265-TilOR.A017	0.129	0.445	0.290
contig022268-TilOR.A019	contig030560-ZebOR.A007	0.129	0.452	0.285
contig051321-BurOR.A006	contig054237-BurOR.A013	0.130	0.234	0.554
contig085026-BriOR.A008	contig051321-BurOR.A006	0.130	0.235	0.552
contig085018-BriOR.A006	contig047503-ZebOR.A013	0.130	0.262	0.498
contig022241-TilOR.A011	contig070885-TilOR.A024	0.130	0.291	0.449
contig051570-BurOR.A010	contig030576-ZebOR.A010	0.130	0.330	0.395
contig022234-TilOR.A009	contig022251-TilOR.A013	0.130	0.347	0.374
contig056375-NyeOR.A015	contig047508-ZebOR.A015	0.130	0.351	0.371
contig056375-NyeOR.A015	contig022234-TilOR.A009	0.130	0.371	0.351
contig051559-BurOR.A008	contig064570-BurOR.A017	0.130	0.418	0.311
contig084999-BriOR.A002	contig085010-BriOR.A005	0.130	0.454	0.287
contig085010-BriOR.A005	contig030556-ZebOR.A005	0.130	0.513	0.253
contig022211-TilOR.A003	contig022251-TilOR.A013	0.131	0.252	0.522
contig085018-BriOR.A006	contig022234-TilOR.A009	0.131	0.318	0.411
contig030576-ZebOR.A010	contig047508-ZebOR.A015	0.131	0.320	0.410
contig070885-TilOR.A024	contig062094-ZebOR.A023	0.131	0.323	0.405
contig022234-TilOR.A009	contig030576-ZebOR.A010	0.131	0.342	0.383
contig022265-TilOR.A017	contig030556-ZebOR.A005	0.131	0.431	0.305
contig085002-BriOR.A004	contig054684-NyeOR.A012	0.131	0.469	0.279

contig022211-TilOR.A003	contig022245-TilOR.A012	0.132	0.194	0.681
contig054237-BurOR.A013	contig056380-NyeOR.A016	0.132	0.244	0.539
contig085026-BriOR.A008	contig056380-NyeOR.A016	0.132	0.245	0.538
contig022211-TilOR.A003	contig022259-TilOR.A015	0.132	0.273	0.483
contig085018-BriOR.A006	contig034988-NyeOR.A005	0.132	0.319	0.412
contig051321-BurOR.A006	contig070885-TilOR.A024	0.132	0.325	0.406
contig070885-TilOR.A024	contig047521-ZebOR.A020	0.132	0.339	0.388
contig036787-BurOR.A004	contig022265-TilOR.A017	0.132	0.423	0.311
contig085002-BriOR.A004	contig057156-BurOR.A015	0.132	0.461	0.287
contig085002-BriOR.A004	contig030560-ZebOR.A007	0.132	0.468	0.282
contig085026-BriOR.A008	contig062094-ZebOR.A023	0.133	0.230	0.578
contig054237-BurOR.A013	contig062094-ZebOR.A023	0.133	0.230	0.580
contig085018-BriOR.A006	contig051570-BurOR.A010	0.133	0.306	0.436
contig034981-NyeOR.A001	contig070885-TilOR.A024	0.133	0.349	0.380
contig085018-BriOR.A006	contig047515-ZebOR.A018	0.133	0.350	0.380
contig062344-NyeOR.A020	contig022232-TilOR.A008	0.133	0.368	0.363
contig022232-TilOR.A008	contig030576-ZebOR.A010	0.133	0.382	0.349
contig022232-TilOR.A008	contig022259-TilOR.A015	0.133	0.386	0.344
contig030576-ZebOR.A010	contig047515-ZebOR.A018	0.133	0.389	0.343
contig054678-NyeOR.A010	contig022265-TilOR.A017	0.133	0.417	0.319
contig085010-BriOR.A005	contig036787-BurOR.A004	0.133	0.504	0.265
contig051318-BurOR.A005	contig047521-ZebOR.A020	0.134	0.237	0.565
contig085018-BriOR.A006	contig047508-ZebOR.A015	0.134	0.291	0.461
contig022217-TilOR.A004	contig070885-TilOR.A024	0.134	0.314	0.428
contig022234-TilOR.A009	contig062095-ZebOR.A024	0.134	0.332	0.402
contig034988-NyeOR.A005	contig022251-TilOR.A013	0.134	0.353	0.381
contig051318-BurOR.A005	contig054237-BurOR.A013	0.135	0.244	0.553
contig051318-BurOR.A005	contig034981-NyeOR.A001	0.135	0.245	0.550
contig056380-NyeOR.A016	contig047503-ZebOR.A013	0.135	0.263	0.511
contig022259-TilOR.A015	contig047503-ZebOR.A013	0.135	0.298	0.453
contig085018-BriOR.A006	contig034983-NyeOR.A003	0.135	0.343	0.392
contig034983-NyeOR.A003	contig030576-ZebOR.A010	0.135	0.381	0.354
contig022259-TilOR.A015	contig047515-ZebOR.A018	0.135	0.389	0.348
contig056380-NyeOR.A016	contig047515-ZebOR.A018	0.135	0.409	0.329
contig034983-NyeOR.A003	contig056380-NyeOR.A016	0.135	0.416	0.324
contig085010-BriOR.A005	contig054678-NyeOR.A010	0.135	0.497	0.271
contig030572-ZebOR.A009	contig047521-ZebOR.A020	0.136	0.237	0.572
contig051570-BurOR.A010	contig022245-TilOR.A012	0.136	0.308	0.442
contig064570-BurOR.A017	contig022234-TilOR.A009	0.136	0.316	0.432
contig051570-BurOR.A010	contig022251-TilOR.A013	0.136	0.339	0.402
contig022211-TilOR.A003	contig070885-TilOR.A024	0.136	0.382	0.356
contig056380-NyeOR.A016	contig022232-TilOR.A008	0.136	0.414	0.328
contig034981-NyeOR.A001	contig030572-ZebOR.A009	0.137	0.245	0.556

contig054237-BurOR.A013	contig030572-ZebOR.A009	0.137	0.245	0.560
contig022245-TilOR.A012	contig047508-ZebOR.A015	0.137	0.293	0.467
contig051570-BurOR.A010	contig047521-ZebOR.A020	0.137	0.313	0.436
contig022251-TilOR.A013	contig047508-ZebOR.A015	0.137	0.323	0.424
contig085018-BriOR.A006	contig022232-TilOR.A008	0.137	0.342	0.400
contig054237-BurOR.A013	contig070885-TilOR.A024	0.137	0.346	0.396
contig051318-BurOR.A005	contig070885-TilOR.A024	0.137	0.350	0.390
contig022234-TilOR.A009	contig022259-TilOR.A015	0.137	0.374	0.366
contig034983-NyeOR.A003	contig022259-TilOR.A015	0.137	0.395	0.346
contig085002-BriOR.A004	contig022265-TilOR.A017	0.137	0.436	0.314
contig051321-BurOR.A006	contig022211-TilOR.A003	0.138	0.223	0.621
contig022245-TilOR.A012	contig047503-ZebOR.A013	0.138	0.242	0.569
contig085012-BriOR.A130	contig047521-ZebOR.A020	0.138	0.245	0.562
contig085026-BriOR.A008	contig051318-BurOR.A005	0.138	0.245	0.566
contig047503-ZebOR.A013	contig062094-ZebOR.A023	0.138	0.247	0.558
contig047508-ZebOR.A015	contig047521-ZebOR.A020	0.138	0.298	0.462
contig034988-NyeOR.A005	contig022241-TilOR.A011	0.138	0.303	0.455
contig034988-NyeOR.A005	contig056380-NyeOR.A016	0.138	0.313	0.441
contig034988-NyeOR.A005	contig047521-ZebOR.A020	0.138	0.320	0.432
contig034988-NyeOR.A005	contig022245-TilOR.A012	0.138	0.321	0.429
contig051570-BurOR.A010	contig034981-NyeOR.A001	0.138	0.322	0.427
contig051570-BurOR.A010	contig022211-TilOR.A003	0.138	0.341	0.403
contig085018-BriOR.A006	contig051559-BurOR.A008	0.138	0.349	0.395
contig070885-TilOR.A024	contig030572-ZebOR.A009	0.138	0.351	0.395
contig022217-TilOR.A004	contig047515-ZebOR.A018	0.138	0.355	0.389
contig034988-NyeOR.A005	contig022259-TilOR.A015	0.138	0.380	0.364
contig047515-ZebOR.A018	contig062094-ZebOR.A023	0.138	0.384	0.360
contig051559-BurOR.A008	contig030576-ZebOR.A010	0.138	0.388	0.357
contig034983-NyeOR.A003	contig062094-ZebOR.A023	0.138	0.390	0.354
contig051321-BurOR.A006	contig047515-ZebOR.A018	0.138	0.396	0.349
contig051321-BurOR.A006	contig022232-TilOR.A008	0.138	0.400	0.343
contig051321-BurOR.A006	contig034983-NyeOR.A003	0.138	0.402	0.343
contig051559-BurOR.A008	contig056380-NyeOR.A016	0.138	0.416	0.333
contig085012-BriOR.A130	contig034981-NyeOR.A001	0.139	0.253	0.548
contig051570-BurOR.A010	contig022241-TilOR.A011	0.139	0.290	0.481
contig034981-NyeOR.A001	contig047508-ZebOR.A015	0.139	0.307	0.451
contig034981-NyeOR.A001	contig034988-NyeOR.A005	0.139	0.329	0.423
contig022211-TilOR.A003	contig047508-ZebOR.A015	0.139	0.345	0.402
contig034988-NyeOR.A005	contig022211-TilOR.A003	0.139	0.348	0.400
contig085026-BriOR.A008	contig070885-TilOR.A024	0.139	0.352	0.395
contig062344-NyeOR.A020	contig047515-ZebOR.A018	0.139	0.378	0.369
contig022232-TilOR.A008	contig062094-ZebOR.A023	0.139	0.388	0.359
contig085002-BriOR.A004	contig085010-BriOR.A005	0.139	0.471	0.295

contig085026-BriOR.A008	contig030572-ZebOR.A009	0.140	0.245	0.572
contig085012-BriOR.A130	contig054237-BurOR.A013	0.140	0.251	0.556
contig085026-BriOR.A008	contig085012-BriOR.A130	0.140	0.255	0.548
contig051321-BurOR.A006	contig047503-ZebOR.A013	0.140	0.258	0.543
contig022234-TilOR.A009	contig022241-TilOR.A011	0.140	0.275	0.510
contig022241-TilOR.A011	contig047508-ZebOR.A015	0.140	0.275	0.510
contig056380-NyeOR.A016	contig047508-ZebOR.A015	0.140	0.288	0.488
contig051570-BurOR.A010	contig056380-NyeOR.A016	0.140	0.301	0.466
contig051321-BurOR.A006	contig022234-TilOR.A009	0.140	0.334	0.419
contig056380-NyeOR.A016	contig022234-TilOR.A009	0.140	0.340	0.411
contig022217-TilOR.A004	contig022232-TilOR.A008	0.140	0.347	0.404
contig034983-NyeOR.A003	contig022217-TilOR.A004	0.140	0.349	0.401
contig051570-BurOR.A010	contig022259-TilOR.A015	0.140	0.366	0.383
contig051559-BurOR.A008	contig022259-TilOR.A015	0.140	0.395	0.356
contig056380-NyeOR.A016	contig022211-TilOR.A003	0.141	0.244	0.575
contig085026-BriOR.A008	contig047508-ZebOR.A015	0.141	0.309	0.458
contig085026-BriOR.A008	contig051570-BurOR.A010	0.141	0.325	0.433
contig022259-TilOR.A015	contig047508-ZebOR.A015	0.141	0.349	0.404
contig034983-NyeOR.A003	contig062344-NyeOR.A020	0.141	0.371	0.381
contig022232-TilOR.A008	contig047521-ZebOR.A020	0.141	0.404	0.348
contig022211-TilOR.A003	contig062094-ZebOR.A023	0.142	0.229	0.618
contig034988-NyeOR.A005	contig062094-ZebOR.A023	0.142	0.309	0.459
contig022241-TilOR.A011	contig047515-ZebOR.A018	0.142	0.327	0.433
contig085026-BriOR.A008	contig034988-NyeOR.A005	0.142	0.331	0.429
contig085012-BriOR.A130	contig070885-TilOR.A024	0.142	0.345	0.412
contig051559-BurOR.A008	contig062094-ZebOR.A023	0.142	0.390	0.363
contig051321-BurOR.A006	contig051559-BurOR.A008	0.142	0.402	0.353
contig022211-TilOR.A003	contig022232-TilOR.A008	0.142	0.404	0.353
contig034981-NyeOR.A001	contig022232-TilOR.A008	0.142	0.414	0.342
contig051570-BurOR.A010	contig062094-ZebOR.A023	0.143	0.296	0.484
contig022232-TilOR.A008	contig022241-TilOR.A011	0.143	0.318	0.451
contig034983-NyeOR.A003	contig022241-TilOR.A011	0.143	0.320	0.448
contig051321-BurOR.A006	contig034988-NyeOR.A005	0.143	0.320	0.448
contig022234-TilOR.A009	contig062094-ZebOR.A023	0.143	0.323	0.444
contig022232-TilOR.A008	contig022245-TilOR.A012	0.143	0.350	0.409
contig051559-BurOR.A008	contig022217-TilOR.A004	0.143	0.355	0.404
contig022245-TilOR.A012	contig047515-ZebOR.A018	0.143	0.356	0.402
contig085026-BriOR.A008	contig022232-TilOR.A008	0.143	0.403	0.354
contig022217-TilOR.A004	contig047503-ZebOR.A013	0.144	0.240	0.598
contig047508-ZebOR.A015	contig062094-ZebOR.A023	0.144	0.283	0.508
contig051318-BurOR.A005	contig047503-ZebOR.A013	0.144	0.308	0.467
contig034988-NyeOR.A005	contig022217-TilOR.A004	0.144	0.316	0.457
contig022225-TilOR.A005	contig070885-TilOR.A024	0.144	0.375	0.384

contig051321-BurOR.A006	contig051570-BurOR.A010	0.145	0.307	0.473
contig034983-NyeOR.A003	contig022245-TilOR.A012	0.145	0.362	0.400
contig051559-BurOR.A008	contig062344-NyeOR.A020	0.145	0.377	0.384
contig022204-TilOR.A001	contig022234-TilOR.A009	0.146	0.280	0.520
contig051321-BurOR.A006	contig047508-ZebOR.A015	0.146	0.294	0.495
contig051570-BurOR.A010	contig022217-TilOR.A004	0.146	0.303	0.483
contig030572-ZebOR.A009	contig047503-ZebOR.A013	0.146	0.308	0.472
contig051570-BurOR.A010	contig054237-BurOR.A013	0.146	0.324	0.452
contig022232-TilOR.A008	contig030572-ZebOR.A009	0.146	0.386	0.379
contig051318-BurOR.A005	contig022232-TilOR.A008	0.146	0.386	0.379
contig054237-BurOR.A013	contig022232-TilOR.A008	0.146	0.397	0.368
contig051318-BurOR.A005	contig022211-TilOR.A003	0.147	0.239	0.614
contig022217-TilOR.A004	contig047508-ZebOR.A015	0.147	0.288	0.511
contig022225-TilOR.A005	contig022234-TilOR.A009	0.147	0.298	0.492
contig054237-BurOR.A013	contig047508-ZebOR.A015	0.147	0.308	0.477
contig051559-BurOR.A008	contig022241-TilOR.A011	0.147	0.326	0.451
contig054237-BurOR.A013	contig034988-NyeOR.A005	0.148	0.330	0.448
contig051559-BurOR.A008	contig022245-TilOR.A012	0.148	0.368	0.403
contig051318-BurOR.A005	contig047515-ZebOR.A018	0.148	0.368	0.403
contig022211-TilOR.A003	contig030572-ZebOR.A009	0.149	0.240	0.621
contig022217-TilOR.A004	contig022234-TilOR.A009	0.149	0.288	0.516
contig047503-ZebOR.A013	contig047521-ZebOR.A020	0.149	0.317	0.471
contig051318-BurOR.A005	contig047508-ZebOR.A015	0.150	0.320	0.470
contig085026-BriOR.A008	contig047503-ZebOR.A013	0.150	0.326	0.460
contig051318-BurOR.A005	contig051570-BurOR.A010	0.150	0.333	0.451
contig030572-ZebOR.A009	contig047515-ZebOR.A018	0.150	0.369	0.407
contig051318-BurOR.A005	contig034983-NyeOR.A003	0.150	0.375	0.401
contig022227-TilOR.A006	contig070885-TilOR.A024	0.150	0.398	0.376
contig085012-BriOR.A130	contig022211-TilOR.A003	0.151	0.242	0.625
contig022204-TilOR.A001	contig047521-ZebOR.A020	0.151	0.310	0.489
contig022204-TilOR.A001	contig070885-TilOR.A024	0.151	0.363	0.416
contig034981-NyeOR.A001	contig022204-TilOR.A001	0.152	0.306	0.498
contig022225-TilOR.A005	contig022259-TilOR.A014	0.152	0.318	0.479
contig022211-TilOR.A003	contig047503-ZebOR.A013	0.152	0.319	0.476
contig030572-ZebOR.A009	contig047508-ZebOR.A015	0.152	0.320	0.476
contig034981-NyeOR.A001	contig047503-ZebOR.A013	0.152	0.328	0.464
contig022234-TilOR.A009	contig030572-ZebOR.A009	0.152	0.329	0.460
contig051570-BurOR.A010	contig030572-ZebOR.A009	0.152	0.333	0.456
contig051318-BurOR.A005	contig034988-NyeOR.A005	0.152	0.346	0.438
contig034983-NyeOR.A003	contig030572-ZebOR.A009	0.152	0.375	0.405
contig085012-BriOR.A130	contig022232-TilOR.A008	0.152	0.385	0.394
contig022268-TilOR.A020	contig030557-ZebOR.A006	0.152	0.541	0.281
contig085012-BriOR.A130	contig047503-ZebOR.A013	0.153	0.317	0.483

contig074640-TilOR.A002	contig022227-TilOR.A006	0.153	0.338	0.451
contig022204-TilOR.A001	contig022232-TilOR.A008	0.153	0.369	0.415
contig022234-TilOR.A009	contig047521-ZebOR.A020	0.154	0.324	0.476
contig085026-BriOR.A008	contig022234-TilOR.A009	0.154	0.324	0.476
contig054237-BurOR.A013	contig047503-ZebOR.A013	0.154	0.325	0.474
contig034988-NyeOR.A005	contig030572-ZebOR.A009	0.154	0.347	0.443
contig051318-BurOR.A005	contig051559-BurOR.A008	0.154	0.374	0.411
contig022225-TilOR.A005	contig047521-ZebOR.A020	0.155	0.316	0.490
contig022204-TilOR.A001	contig047515-ZebOR.A018	0.155	0.395	0.393
contig030553-ZebOR.A002	contig030557-ZebOR.A006	0.155	0.513	0.302
contig022204-TilOR.A001	contig074640-TilOR.A002	0.156	0.309	0.507
contig034981-NyeOR.A001	contig022225-TilOR.A005	0.156	0.312	0.498
contig051318-BurOR.A005	contig022234-TilOR.A009	0.156	0.320	0.487
contig022204-TilOR.A001	contig022211-TilOR.A003	0.156	0.338	0.462
contig051559-BurOR.A008	contig030572-ZebOR.A009	0.156	0.374	0.415
contig051570-BurOR.A010	contig022225-TilOR.A005	0.156	0.377	0.413
contig057153-BurOR.A014	contig022268-TilOR.A020	0.156	0.538	0.291
contig054681-NyeOR.A011	contig022268-TilOR.A020	0.156	0.544	0.287
contig022204-TilOR.A001	contig022259-TilOR.A014	0.157	0.300	0.524
contig054237-BurOR.A013	contig022204-TilOR.A001	0.157	0.329	0.476
contig085012-BriOR.A130	contig051570-BurOR.A010	0.157	0.340	0.462
contig022211-TilOR.A003	contig022225-TilOR.A005	0.157	0.359	0.438
contig022225-TilOR.A005	contig047508-ZebOR.A015	0.157	0.360	0.435
contig034983-NyeOR.A003	contig022204-TilOR.A001	0.157	0.387	0.405
contig057153-BurOR.A014	contig030553-ZebOR.A002	0.157	0.501	0.314
contig022266-TilOR.A018	contig022268-TilOR.A020	0.157	0.559	0.280
contig054237-BurOR.A013	contig022234-TilOR.A009	0.158	0.318	0.496
contig085012-BriOR.A130	contig047508-ZebOR.A015	0.158	0.325	0.487
contig085026-BriOR.A008	contig022204-TilOR.A001	0.158	0.338	0.467
contig022225-TilOR.A005	contig047503-ZebOR.A013	0.158	0.356	0.444
contig051570-BurOR.A010	contig022204-TilOR.A001	0.158	0.368	0.430
contig034988-NyeOR.A005	contig022225-TilOR.A005	0.158	0.392	0.402
contig036782-BurOR.A002	contig030557-ZebOR.A006	0.158	0.528	0.300
contig085026-BriOR.A008	contig022225-TilOR.A005	0.159	0.345	0.462
contig022204-TilOR.A001	contig047508-ZebOR.A015	0.159	0.352	0.452
contig085012-BriOR.A130	contig034988-NyeOR.A005	0.159	0.354	0.449
contig022211-TilOR.A003	contig022234-TilOR.A009	0.159	0.366	0.434
contig022225-TilOR.A005	contig022232-TilOR.A008	0.159	0.400	0.398
contig047515-ZebOR.A018	contig047521-ZebOR.A020	0.159	0.413	0.385
contig054681-NyeOR.A011	contig030553-ZebOR.A002	0.159	0.516	0.309
contig084999-BriOR.A001	contig030557-ZebOR.A006	0.159	0.523	0.305
contig054237-BurOR.A013	contig022225-TilOR.A005	0.160	0.336	0.476
contig034988-NyeOR.A005	contig022204-TilOR.A001	0.160	0.383	0.418

contig022211-TilOR.A003	contig047515-ZebOR.A018	0.160	0.422	0.378
contig034981-NyeOR.A001	contig047515-ZebOR.A018	0.160	0.423	0.378
contig022227-TilOR.A006	contig022259-TilOR.A014	0.160	0.426	0.376
contig022266-TilOR.A018	contig030553-ZebOR.A002	0.160	0.522	0.306
contig022234-TilOR.A009	contig022245-TilOR.A012	0.161	0.288	0.559
contig034981-NyeOR.A001	contig022234-TilOR.A009	0.161	0.350	0.459
contig051559-BurOR.A008	contig022204-TilOR.A001	0.161	0.394	0.408
contig085026-BriOR.A008	contig047515-ZebOR.A018	0.161	0.409	0.394
contig034983-NyeOR.A003	contig047521-ZebOR.A020	0.161	0.419	0.383
contig057756-NyeOR.A019	contig030557-ZebOR.A006	0.161	0.515	0.313
contig036782-BurOR.A002	contig057153-BurOR.A014	0.161	0.517	0.312
contig074640-TilOR.A002	contig022225-TilOR.A005	0.162	0.315	0.513
contig022225-TilOR.A005	contig022259-TilOR.A015	0.162	0.382	0.425
contig034983-NyeOR.A003	contig022211-TilOR.A003	0.162	0.415	0.390
contig022225-TilOR.A005	contig047515-ZebOR.A018	0.162	0.424	0.382
contig034981-NyeOR.A001	contig034983-NyeOR.A003	0.162	0.429	0.376
contig084999-BriOR.A001	contig057153-BurOR.A014	0.162	0.511	0.317
contig022227-TilOR.A006	contig022234-TilOR.A009	0.163	0.386	0.423
contig085026-BriOR.A008	contig034983-NyeOR.A003	0.163	0.416	0.392
contig036782-BurOR.A002	contig054681-NyeOR.A011	0.163	0.532	0.307
contig084999-BriOR.A001	contig022266-TilOR.A018	0.163	0.536	0.305
contig036782-BurOR.A002	contig022266-TilOR.A018	0.163	0.546	0.299
contig022204-TilOR.A001	contig047503-ZebOR.A013	0.164	0.348	0.472
contig054237-BurOR.A013	contig047515-ZebOR.A018	0.164	0.408	0.401
contig034983-NyeOR.A003	contig022225-TilOR.A005	0.164	0.417	0.394
contig051559-BurOR.A008	contig047521-ZebOR.A020	0.164	0.426	0.386
contig022227-TilOR.A006	contig047503-ZebOR.A013	0.164	0.434	0.377
contig057153-BurOR.A014	contig057756-NyeOR.A019	0.164	0.503	0.326
contig084999-BriOR.A001	contig054681-NyeOR.A011	0.164	0.526	0.312
contig064570-BurOR.A017	contig022204-TilOR.A001	0.165	0.285	0.580
contig085018-BriOR.A006	contig022204-TilOR.A001	0.165	0.312	0.529
contig022225-TilOR.A005	contig062095-ZebOR.A024	0.165	0.330	0.499
contig022225-TilOR.A005	contig022251-TilOR.A013	0.165	0.356	0.463
contig051559-BurOR.A008	contig022211-TilOR.A003	0.165	0.407	0.406
contig054237-BurOR.A013	contig034983-NyeOR.A003	0.165	0.414	0.399
contig074640-TilOR.A002	contig022234-TilOR.A009	0.165	0.421	0.392
contig051559-BurOR.A008	contig034981-NyeOR.A001	0.165	0.436	0.379
contig051570-BurOR.A010	contig022227-TilOR.A006	0.165	0.444	0.372
contig064570-BurOR.A017	contig022225-TilOR.A005	0.166	0.333	0.497
contig022227-TilOR.A006	contig047508-ZebOR.A015	0.166	0.426	0.390
contig054681-NyeOR.A011	contig057756-NyeOR.A019	0.166	0.518	0.320
contig057756-NyeOR.A019	contig022266-TilOR.A018	0.166	0.541	0.307
contig085018-BriOR.A006	contig022225-TilOR.A005	0.167	0.328	0.511

contig064570-BurOR.A017	contig022227-TilOR.A006	0.167	0.406	0.410
contig022227-TilOR.A006	contig062095-ZebOR.A024	0.167	0.407	0.410
contig085026-BriOR.A008	contig051559-BurOR.A008	0.167	0.422	0.395
contig022227-TilOR.A006	contig022232-TilOR.A008	0.167	0.452	0.369
contig034988-NyeOR.A005	contig022227-TilOR.A006	0.167	0.459	0.363
contig022204-TilOR.A001	contig062095-ZebOR.A024	0.168	0.282	0.595
contig085012-BriOR.A130	contig022234-TilOR.A009	0.168	0.353	0.477
contig085012-BriOR.A130	contig047515-ZebOR.A018	0.168	0.380	0.440
contig051559-BurOR.A008	contig022225-TilOR.A005	0.168	0.423	0.397
contig022227-TilOR.A006	contig047515-ZebOR.A018	0.168	0.507	0.332
contig074640-TilOR.A002	contig047503-ZebOR.A013	0.169	0.383	0.442
contig085012-BriOR.A130	contig034983-NyeOR.A003	0.169	0.387	0.438
contig051559-BurOR.A008	contig054237-BurOR.A013	0.169	0.421	0.402
contig074640-TilOR.A002	contig070885-TilOR.A024	0.170	0.460	0.370
contig034983-NyeOR.A003	contig022227-TilOR.A006	0.170	0.498	0.341
contig056375-NyeOR.A015	contig022204-TilOR.A001	0.171	0.304	0.562
contig022204-TilOR.A001	contig030576-ZebOR.A010	0.171	0.341	0.502
contig056375-NyeOR.A015	contig022225-TilOR.A005	0.171	0.347	0.493
contig022204-TilOR.A001	contig022259-TilOR.A015	0.171	0.348	0.492
contig085012-BriOR.A130	contig051559-BurOR.A008	0.171	0.386	0.443
contig056375-NyeOR.A015	contig022227-TilOR.A006	0.171	0.425	0.403
contig022204-TilOR.A001	contig022251-TilOR.A013	0.172	0.325	0.529
contig085018-BriOR.A006	contig022227-TilOR.A006	0.172	0.408	0.423
contig022227-TilOR.A006	contig022259-TilOR.A015	0.172	0.469	0.367
contig051559-BurOR.A008	contig022227-TilOR.A006	0.172	0.506	0.340
contig022225-TilOR.A005	contig062094-ZebOR.A023	0.173	0.376	0.459
contig056380-NyeOR.A016	contig022225-TilOR.A005	0.173	0.382	0.454
contig034988-NyeOR.A005	contig074640-TilOR.A002	0.173	0.440	0.393
contig022227-TilOR.A006	contig022251-TilOR.A013	0.173	0.452	0.383
contig062344-NyeOR.A020	contig022225-TilOR.A005	0.174	0.345	0.505
contig022225-TilOR.A005	contig030576-ZebOR.A010	0.174	0.358	0.485
contig051570-BurOR.A010	contig074640-TilOR.A002	0.174	0.428	0.406
contig062344-NyeOR.A020	contig022204-TilOR.A001	0.175	0.335	0.523
contig074640-TilOR.A002	contig022259-TilOR.A014	0.175	0.354	0.495
contig074640-TilOR.A002	contig047508-ZebOR.A015	0.175	0.425	0.412
contig022230-TilOR.A007	contig022268-TilOR.A020	0.175	0.563	0.312
contig022225-TilOR.A005	contig030572-ZebOR.A009	0.176	0.344	0.511
contig051318-BurOR.A005	contig022225-TilOR.A005	0.176	0.344	0.511
contig022227-TilOR.A006	contig047521-ZebOR.A020	0.176	0.400	0.441
contig056380-NyeOR.A016	contig022227-TilOR.A006	0.176	0.472	0.373
contig022217-TilOR.A004	contig022225-TilOR.A005	0.177	0.333	0.531
contig022204-TilOR.A001	contig062094-ZebOR.A023	0.177	0.342	0.517
contig056380-NyeOR.A016	contig022204-TilOR.A001	0.177	0.347	0.510

contig034981-NyeOR.A001	contig022227-TilOR.A006	0.177	0.396	0.448
contig022227-TilOR.A006	contig062094-ZebOR.A023	0.177	0.462	0.382
contig022230-TilOR.A007	contig030553-ZebOR.A002	0.177	0.547	0.324
contig022204-TilOR.A001	contig022245-TilOR.A012	0.178	0.298	0.596
contig022204-TilOR.A001	contig022241-TilOR.A011	0.178	0.302	0.589
contig022225-TilOR.A005	contig022245-TilOR.A012	0.178	0.334	0.535
contig051321-BurOR.A006	contig022225-TilOR.A005	0.178	0.372	0.478
contig051318-BurOR.A005	contig022227-TilOR.A006	0.178	0.457	0.389
contig022204-TilOR.A001	contig022217-TilOR.A004	0.179	0.320	0.560
contig022227-TilOR.A006	contig030576-ZebOR.A010	0.179	0.446	0.400
contig022227-TilOR.A006	contig030572-ZebOR.A009	0.179	0.457	0.393
contig074640-TilOR.A002	contig022232-TilOR.A008	0.179	0.467	0.384
contig022268-TilOR.A020	contig047506-ZebOR.A014	0.179	0.613	0.292
contig022225-TilOR.A005	contig022241-TilOR.A011	0.180	0.336	0.535
contig051559-BurOR.A007	contig030553-ZebOR.A002	0.180	0.527	0.342
contig051559-BurOR.A007	contig022268-TilOR.A020	0.180	0.569	0.317
contig085018-BriOR.A006	contig074640-TilOR.A002	0.181	0.338	0.537
contig085012-BriOR.A130	contig022225-TilOR.A005	0.181	0.358	0.507
contig054237-BurOR.A013	contig022227-TilOR.A006	0.181	0.423	0.428
contig051321-BurOR.A006	contig022227-TilOR.A006	0.181	0.476	0.380
contig036782-BurOR.A002	contig022230-TilOR.A007	0.181	0.573	0.316
contig064187-BurOR.A016	contig022268-TilOR.A020	0.181	0.611	0.296
contig084999-BriOR.A001	contig022230-TilOR.A007	0.182	0.515	0.354
contig034988-NyeOR.A006	contig022268-TilOR.A020	0.182	0.598	0.305
contig022230-TilOR.A007	contig030557-ZebOR.A006	0.183	0.524	0.349
contig064187-BurOR.A016	contig030557-ZebOR.A006	0.183	0.548	0.335
contig057756-NyeOR.A019	contig022230-TilOR.A007	0.183	0.565	0.324
contig022204-TilOR.A001	contig030572-ZebOR.A009	0.184	0.308	0.598
contig022211-TilOR.A003	contig022227-TilOR.A006	0.184	0.442	0.416
contig022230-TilOR.A007	contig022266-TilOR.A018	0.184	0.508	0.363
contig036782-BurOR.A002	contig051559-BurOR.A007	0.184	0.552	0.333
contig022268-TilOR.A020	contig047515-ZebOR.A019	0.184	0.580	0.317
contig034983-NyeOR.A002	contig022268-TilOR.A020	0.184	0.580	0.317
contig051321-BurOR.A006	contig022204-TilOR.A001	0.185	0.353	0.523
contig056375-NyeOR.A015	contig074640-TilOR.A002	0.185	0.385	0.480
contig074640-TilOR.A002	contig047521-ZebOR.A020	0.185	0.417	0.443
contig085026-BriOR.A008	contig022227-TilOR.A006	0.185	0.421	0.440
contig074640-TilOR.A002	contig047515-ZebOR.A018	0.185	0.474	0.390
contig084999-BriOR.A001	contig051559-BurOR.A007	0.185	0.479	0.387
contig030553-ZebOR.A002	contig047515-ZebOR.A019	0.185	0.533	0.347
contig034983-NyeOR.A002	contig030553-ZebOR.A002	0.185	0.533	0.347
contig057153-BurOR.A014	contig064187-BurOR.A016	0.185	0.540	0.343
contig030557-ZebOR.A006	contig047506-ZebOR.A014	0.185	0.549	0.337

contig051318-BurOR.A005	contig022204-TilOR.A001	0.186	0.308	0.606
contig022227-TilOR.A006	contig022245-TilOR.A012	0.186	0.396	0.470
contig034981-NyeOR.A001	contig074640-TilOR.A002	0.186	0.427	0.435
contig051559-BurOR.A007	contig057756-NyeOR.A019	0.186	0.544	0.341
contig074640-TilOR.A002	contig030576-ZebOR.A010	0.187	0.363	0.515
contig062344-NyeOR.A020	contig022227-TilOR.A006	0.187	0.418	0.447
contig034983-NyeOR.A003	contig074640-TilOR.A002	0.187	0.474	0.395
contig051559-BurOR.A007	contig030557-ZebOR.A006	0.187	0.509	0.367
contig057153-BurOR.A014	contig047506-ZebOR.A014	0.187	0.541	0.346
contig064187-BurOR.A016	contig054681-NyeOR.A011	0.187	0.556	0.337
contig085012-BriOR.A130	contig022204-TilOR.A001	0.188	0.328	0.574
contig074640-TilOR.A002	contig062095-ZebOR.A024	0.188	0.380	0.496
contig051559-BurOR.A007	contig022266-TilOR.A018	0.188	0.501	0.375
contig057153-BurOR.A014	contig022230-TilOR.A007	0.188	0.521	0.360
contig054681-NyeOR.A011	contig022230-TilOR.A007	0.188	0.528	0.356
contig064187-BurOR.A016	contig022266-TilOR.A018	0.188	0.562	0.334
contig022227-TilOR.A006	contig022241-TilOR.A011	0.189	0.397	0.476
contig022217-TilOR.A004	contig022227-TilOR.A006	0.189	0.412	0.460
contig084999-BriOR.A001	contig064187-BurOR.A016	0.189	0.545	0.347
contig054681-NyeOR.A011	contig047506-ZebOR.A014	0.189	0.557	0.340
contig036782-BurOR.A002	contig034983-NyeOR.A002	0.189	0.558	0.338
contig036782-BurOR.A002	contig047515-ZebOR.A019	0.189	0.558	0.338
contig064187-BurOR.A016	contig030553-ZebOR.A002	0.189	0.612	0.308
contig064570-BurOR.A017	contig074640-TilOR.A002	0.190	0.393	0.484
contig054237-BurOR.A013	contig074640-TilOR.A002	0.190	0.425	0.449
contig084999-BriOR.A001	contig034983-NyeOR.A002	0.190	0.485	0.392
contig084999-BriOR.A001	contig047515-ZebOR.A019	0.190	0.485	0.392
contig034983-NyeOR.A002	contig057756-NyeOR.A019	0.190	0.550	0.346
contig057756-NyeOR.A019	contig047515-ZebOR.A019	0.190	0.550	0.346
contig022266-TilOR.A018	contig047506-ZebOR.A014	0.190	0.563	0.336
contig030553-ZebOR.A002	contig047506-ZebOR.A014	0.190	0.604	0.315
contig074640-TilOR.A002	contig022251-TilOR.A013	0.191	0.413	0.462
contig051559-BurOR.A008	contig074640-TilOR.A002	0.191	0.481	0.397
contig084999-BriOR.A001	contig047506-ZebOR.A014	0.191	0.529	0.361
contig034988-NyeOR.A006	contig030557-ZebOR.A006	0.191	0.544	0.351
contig022268-TilOR.A020	contig041951-TilOR.A022	0.191	0.617	0.310
contig074640-TilOR.A002	contig022217-TilOR.A004	0.192	0.335	0.573
contig074640-TilOR.A002	contig022259-TilOR.A015	0.192	0.405	0.473
contig030557-ZebOR.A006	contig047515-ZebOR.A019	0.192	0.498	0.385
contig034983-NyeOR.A002	contig030557-ZebOR.A006	0.192	0.498	0.385
contig051559-BurOR.A007	contig057153-BurOR.A014	0.192	0.505	0.379
contig041951-TilOR.A022	contig030557-ZebOR.A006	0.192	0.510	0.376
contig051559-BurOR.A007	contig054681-NyeOR.A011	0.192	0.512	0.374

contig022266-TilOR.A018	contig047515-ZebOR.A019	0.193	0.490	0.393
contig034983-NyeOR.A002	contig022266-TilOR.A018	0.193	0.490	0.393
contig057153-BurOR.A014	contig034988-NyeOR.A006	0.193	0.537	0.359
contig036782-BurOR.A002	contig064187-BurOR.A016	0.193	0.620	0.311
contig085026-BriOR.A008	contig074640-TilOR.A002	0.194	0.423	0.460
contig057153-BurOR.A014	contig041951-TilOR.A022	0.194	0.503	0.386
contig084999-BriOR.A001	contig034988-NyeOR.A006	0.194	0.534	0.364
contig034988-NyeOR.A006	contig030553-ZebOR.A002	0.194	0.599	0.324
contig036782-BurOR.A002	contig047506-ZebOR.A014	0.194	0.612	0.318
contig062344-NyeOR.A020	contig074640-TilOR.A002	0.195	0.350	0.558
contig085012-BriOR.A130	contig022227-TilOR.A006	0.195	0.469	0.416
contig084999-BriOR.A001	contig041951-TilOR.A022	0.195	0.524	0.373
contig034988-NyeOR.A006	contig022266-TilOR.A018	0.195	0.550	0.355
contig034988-NyeOR.A006	contig054681-NyeOR.A011	0.195	0.552	0.353
contig041951-TilOR.A022	contig030553-ZebOR.A002	0.195	0.608	0.321
contig074640-TilOR.A002	contig022211-TilOR.A003	0.196	0.395	0.497
contig057153-BurOR.A014	contig034983-NyeOR.A002	0.196	0.494	0.397
contig057153-BurOR.A014	contig047515-ZebOR.A019	0.196	0.494	0.397
contig022266-TilOR.A018	contig041951-TilOR.A022	0.196	0.507	0.387
contig054681-NyeOR.A011	contig041951-TilOR.A022	0.196	0.518	0.378
contig064187-BurOR.A016	contig057756-NyeOR.A019	0.196	0.604	0.324
contig034983-NyeOR.A002	contig054681-NyeOR.A011	0.197	0.501	0.392
contig054681-NyeOR.A011	contig047515-ZebOR.A019	0.197	0.501	0.392
contig057756-NyeOR.A019	contig047506-ZebOR.A014	0.198	0.596	0.331
contig036782-BurOR.A002	contig034988-NyeOR.A006	0.198	0.607	0.326
contig036782-BurOR.A002	contig041951-TilOR.A022	0.199	0.616	0.323
contig074640-TilOR.A002	contig022245-TilOR.A012	0.200	0.364	0.550
contig051321-BurOR.A006	contig074640-TilOR.A002	0.200	0.403	0.495
contig056380-NyeOR.A016	contig074640-TilOR.A002	0.200	0.424	0.471
contig034988-NyeOR.A006	contig057756-NyeOR.A019	0.201	0.591	0.340
contig057756-NyeOR.A019	contig041951-TilOR.A022	0.202	0.610	0.332
contig074640-TilOR.A002	contig022241-TilOR.A011	0.203	0.356	0.571
contig074640-TilOR.A002	contig062094-ZebOR.A023	0.203	0.397	0.511
contig074640-TilOR.A002	contig030572-ZebOR.A009	0.205	0.430	0.477
contig085012-BriOR.A130	contig074640-TilOR.A002	0.205	0.448	0.457
contig051318-BurOR.A005	contig074640-TilOR.A002	0.206	0.427	0.482
contig057153-BurOR.A014	contig030554-ZebOR.A004	0.239	0.762	0.314
contig030554-ZebOR.A004	contig030557-ZebOR.A006	0.239	0.767	0.311
contig057153-BurOR.A014	contig054684-NyeOR.A012	0.240	0.667	0.360
contig054684-NyeOR.A012	contig030557-ZebOR.A006	0.240	0.671	0.357
contig034988-NyeORs.A033	contig022268-TilOR.A020	0.240	0.678	0.354
contig054681-NyeOR.A011	contig030554-ZebOR.A004	0.240	0.771	0.311
contig057156-BurOR.A015	contig030557-ZebOR.A006	0.241	0.643	0.375

contig030557-ZebOR.A006	contig030560-ZebOR.A007	0.241	0.656	0.367
contig054681-NyeOR.A011	contig054684-NyeOR.A012	0.241	0.675	0.356
contig057153-BurOR.A014	contig057156-BurOR.A015	0.242	0.639	0.379
contig057156-BurOR.A015	contig054681-NyeOR.A011	0.242	0.647	0.375
contig057153-BurOR.A014	contig030560-ZebOR.A007	0.242	0.652	0.371
contig054681-NyeOR.A011	contig030560-ZebOR.A007	0.242	0.659	0.367
contig085010-BriOR.A005	contig030557-ZebOR.A006	0.242	0.681	0.356
contig022265-TilOR.A017	contig030557-ZebOR.A006	0.242	0.703	0.344
contig085010-BriOR.A005	contig057153-BurOR.A014	0.243	0.677	0.359
contig085010-BriOR.A005	contig054681-NyeOR.A011	0.243	0.685	0.355
contig057153-BurOR.A014	contig022265-TilOR.A017	0.243	0.699	0.348
contig054681-NyeOR.A011	contig022265-TilOR.A017	0.243	0.707	0.344
contig022268-TilOR.A020	contig041951-TilOR.A021	0.244	0.687	0.355
contig054868-NyeOR.A014	contig041951-TilOR.A021	0.245	0.740	0.331
contig084999-BriOR.A002	contig030557-ZebOR.A006	0.245	0.755	0.325
contig041951-TilOR.A021	contig030557-ZebOR.A006	0.246	0.591	0.417
contig041951-TilOR.A021	contig047523-ZebOR.A022	0.246	0.735	0.334
contig054233-BurOR.A012	contig041951-TilOR.A021	0.246	0.735	0.334
contig022266-TilOR.A018	contig030554-ZebOR.A004	0.246	0.744	0.330
contig084999-BriOR.A002	contig057153-BurOR.A014	0.246	0.750	0.328
contig084999-BriOR.A002	contig054681-NyeOR.A011	0.246	0.759	0.324
contig036787-BurOR.A004	contig057153-BurOR.A014	0.246	0.789	0.312
contig036787-BurOR.A004	contig030557-ZebOR.A006	0.246	0.794	0.309
contig036787-BurOR.A004	contig054681-NyeOR.A011	0.247	0.798	0.309
contig030556-ZebOR.A005	contig030557-ZebOR.A006	0.247	0.808	0.306
contig054684-NyeOR.A012	contig022266-TilOR.A018	0.248	0.629	0.393
contig064187-BurOR.A016	contig030552-ZebOR.A001	0.248	0.788	0.315
contig064187-BurOR.A016	contig057165-NyeOR.A017	0.248	0.799	0.311
contig057153-BurOR.A014	contig030556-ZebOR.A005	0.248	0.804	0.309
contig054678-NyeOR.A010	contig030557-ZebOR.A006	0.248	0.827	0.300
contig057153-BurOR.A014	contig041951-TilOR.A021	0.249	0.579	0.431
contig057156-BurOR.A015	contig022266-TilOR.A018	0.249	0.603	0.413
contig022266-TilOR.A018	contig041951-TilOR.A021	0.249	0.607	0.409
contig022266-TilOR.A018	contig030560-ZebOR.A007	0.249	0.615	0.404
contig041951-TilOR.A021	contig073309-TilOR.A026	0.249	0.708	0.352
contig084999-BriOR.A002	contig022266-TilOR.A018	0.249	0.750	0.333
contig036780-BurOR.A001	contig064187-BurOR.A016	0.249	0.807	0.309
contig054681-NyeOR.A011	contig030556-ZebOR.A005	0.249	0.813	0.306
contig036787-BurOR.A004	contig022266-TilOR.A018	0.249	0.820	0.303
contig057153-BurOR.A014	contig054678-NyeOR.A010	0.249	0.822	0.303
contig085010-BriOR.A005	contig022266-TilOR.A018	0.250	0.638	0.392
contig022265-TilOR.A017	contig022266-TilOR.A018	0.250	0.649	0.385
contig085002-BriOR.A004	contig030557-ZebOR.A006	0.250	0.747	0.335

contig030552-ZebOR.A001	contig047506-ZebOR.A014	0.250	0.778	0.321
contig057165-NyeOR.A017	contig047506-ZebOR.A014	0.250	0.788	0.318
contig054678-NyeOR.A010	contig054681-NyeOR.A011	0.250	0.832	0.300
contig022266-TilOR.A018	contig030556-ZebOR.A005	0.250	0.835	0.300
contig085002-BriOR.A004	contig057153-BurOR.A014	0.251	0.742	0.338
contig085002-BriOR.A004	contig054681-NyeOR.A011	0.251	0.751	0.335
contig022268-TilOR.A019	contig030557-ZebOR.A006	0.251	0.755	0.333
contig036780-BurOR.A001	contig047506-ZebOR.A014	0.251	0.797	0.315
contig054678-NyeOR.A010	contig022266-TilOR.A018	0.251	0.854	0.294
contig054681-NyeOR.A011	contig041951-TilOR.A021	0.252	0.595	0.423
contig022230-TilOR.A007	contig022268-TilOR.A019	0.252	0.689	0.365
contig022230-TilOR.A007	contig022234-TilOR.A009	0.252	0.751	0.336
contig057153-BurOR.A014	contig022268-TilOR.A019	0.252	0.751	0.336
contig085002-BriOR.A004	contig022266-TilOR.A018	0.252	0.776	0.325
contig022230-TilOR.A007	contig047503-ZebOR.A013	0.252	0.799	0.316
contig051573-BurOR.A011	contig041951-TilOR.A021	0.253	0.702	0.361
contig036787-BurOR.A004	contig022230-TilOR.A007	0.253	0.724	0.349
contig084999-BriOR.A002	contig041951-TilOR.A021	0.253	0.742	0.342
contig054681-NyeOR.A011	contig022268-TilOR.A019	0.253	0.759	0.333
contig034988-NyeOR.A005	contig022230-TilOR.A007	0.253	0.846	0.299
contig022230-TilOR.A007	contig070885-TilOR.A024	0.253	0.872	0.290
contig034988-NyeORs.A033	contig030557-ZebOR.A006	0.254	0.589	0.431
contig034988-NyeORs.A033	contig022266-TilOR.A018	0.254	0.624	0.407
contig022230-TilOR.A007	contig030552-ZebOR.A001	0.254	0.645	0.394
contig057165-NyeOR.A017	contig022230-TilOR.A007	0.254	0.655	0.388
contig084999-BriOR.A002	contig022230-TilOR.A007	0.254	0.655	0.388
contig034988-NyeORs.A033	contig073309-TilOR.A026	0.254	0.714	0.355
contig041951-TilOR.A021	contig070886-TilOR.A025	0.254	0.724	0.351
contig054868-NyeOR.A014	contig034988-NyeORs.A033	0.254	0.740	0.343
contig054678-NyeOR.A010	contig022230-TilOR.A007	0.254	0.755	0.336
contig022230-TilOR.A007	contig030554-ZebOR.A004	0.255	0.641	0.398
contig036780-BurOR.A001	contig022230-TilOR.A007	0.255	0.650	0.393
contig051559-BurOR.A007	contig047503-ZebOR.A013	0.255	0.723	0.353
contig034988-NyeORs.A033	contig047523-ZebOR.A022	0.255	0.735	0.347
contig054233-BurOR.A012	contig034988-NyeORs.A033	0.255	0.735	0.347
contig022266-TilOR.A018	contig022268-TilOR.A019	0.255	0.740	0.345
contig041951-TilOR.A021	contig047521-ZebOR.A020	0.255	0.742	0.344
contig093816-BriOR.A011	contig041951-TilOR.A021	0.255	0.758	0.336
contig022230-TilOR.A007	contig022232-TilOR.A008	0.255	0.805	0.318
contig022225-TilOR.A005	contig022266-TilOR.A018	0.256	0.633	0.404
contig022225-TilOR.A005	contig022268-TilOR.A020	0.256	0.639	0.401
contig022225-TilOR.A005	contig030557-ZebOR.A006	0.256	0.663	0.387
contig041951-TilOR.A022	contig030552-ZebOR.A001	0.256	0.712	0.359

contig057165-NyeOR.A017	contig041951-TilOR.A022	0.256	0.721	0.355
contig051559-BurOR.A007	contig022234-TilOR.A009	0.256	0.746	0.343
contig034981-NyeOR.A001	contig041951-TilOR.A021	0.256	0.757	0.339
contig036787-BurOR.A004	contig051559-BurOR.A007	0.256	0.781	0.328
contig051570-BurOR.A010	contig022230-TilOR.A007	0.256	0.825	0.310
contig057153-BurOR.A014	contig034988-NyeORs.A033	0.257	0.577	0.446
contig051559-BurOR.A007	contig030552-ZebOR.A001	0.257	0.634	0.405
contig051559-BurOR.A007	contig057165-NyeOR.A017	0.257	0.644	0.399
contig051559-BurOR.A007	contig030554-ZebOR.A004	0.257	0.684	0.375
contig084999-BriOR.A002	contig051559-BurOR.A007	0.257	0.705	0.365
contig041951-TilOR.A021	contig030554-ZebOR.A004	0.257	0.720	0.357
contig036780-BurOR.A001	contig041951-TilOR.A022	0.257	0.729	0.352
contig051559-BurOR.A007	contig022268-TilOR.A019	0.257	0.732	0.351
contig022230-TilOR.A007	contig030556-ZebOR.A005	0.257	0.737	0.348
contig051559-BurOR.A007	contig034988-NyeOR.A005	0.257	0.772	0.333
contig022230-TilOR.A007	contig047515-ZebOR.A018	0.257	0.794	0.323
contig051559-BurOR.A007	contig054678-NyeOR.A010	0.257	0.814	0.315
contig022230-TilOR.A007	contig047508-ZebOR.A015	0.257	0.819	0.314
contig022232-TilOR.A008	contig041951-TilOR.A022	0.257	0.830	0.310
contig036780-BurOR.A001	contig051559-BurOR.A007	0.258	0.639	0.404
contig064187-BurOR.A016	contig041951-TilOR.A021	0.258	0.657	0.393
contig057165-NyeOR.A017	contig022268-TilOR.A020	0.258	0.684	0.377
contig022268-TilOR.A020	contig030552-ZebOR.A001	0.258	0.695	0.372
contig034983-NyeOR.A002	contig047503-ZebOR.A013	0.258	0.720	0.359
contig047503-ZebOR.A013	contig047515-ZebOR.A019	0.258	0.720	0.359
contig041951-TilOR.A021	contig041952-TilOR.A023	0.258	0.734	0.352
contig022234-TilOR.A009	contig047515-ZebOR.A019	0.258	0.737	0.349
contig034983-NyeOR.A002	contig022234-TilOR.A009	0.258	0.737	0.349
contig034988-NyeOR.A006	contig030552-ZebOR.A001	0.258	0.776	0.332
contig034988-NyeOR.A006	contig057165-NyeOR.A017	0.258	0.786	0.329
contig041951-TilOR.A022	contig047515-ZebOR.A018	0.258	0.834	0.310
contig064187-BurOR.A016	contig022232-TilOR.A008	0.258	0.851	0.304
contig054681-NyeOR.A011	contig034988-NyeORs.A033	0.259	0.593	0.437
contig057153-BurOR.A014	contig022225-TilOR.A005	0.259	0.659	0.394
contig041951-TilOR.A021	contig047515-ZebOR.A018	0.259	0.709	0.365
contig022232-TilOR.A008	contig041951-TilOR.A021	0.259	0.727	0.357
contig051559-BurOR.A007	contig022232-TilOR.A008	0.259	0.729	0.355
contig036787-BurOR.A004	contig034983-NyeOR.A002	0.259	0.790	0.328
contig036787-BurOR.A004	contig047515-ZebOR.A019	0.259	0.790	0.328
contig036780-BurOR.A001	contig034988-NyeOR.A006	0.259	0.794	0.326
contig022268-TilOR.A020	contig047521-ZebOR.A020	0.259	0.806	0.321
contig034983-NyeOR.A003	contig022230-TilOR.A007	0.259	0.806	0.322
contig051559-BurOR.A007	contig070885-TilOR.A024	0.259	0.814	0.317

contig034983-NyeOR.A002	contig054678-NyeOR.A010	0.259	0.823	0.315
contig054678-NyeOR.A010	contig047515-ZebOR.A019	0.259	0.823	0.315
contig064187-BurOR.A016	contig047515-ZebOR.A018	0.259	0.829	0.313
contig030552-ZebOR.A001	contig030557-ZebOR.A006	0.260	0.577	0.452
contig057165-NyeOR.A017	contig030557-ZebOR.A006	0.260	0.586	0.445
contig030552-ZebOR.A001	contig047515-ZebOR.A019	0.260	0.631	0.412
contig034983-NyeOR.A002	contig030552-ZebOR.A001	0.260	0.631	0.412
contig034983-NyeOR.A002	contig057165-NyeOR.A017	0.260	0.641	0.405
contig057165-NyeOR.A017	contig047515-ZebOR.A019	0.260	0.641	0.405
contig041951-TilOR.A021	contig047506-ZebOR.A014	0.260	0.648	0.401
contig022204-TilOR.A001	contig022230-TilOR.A007	0.260	0.655	0.398
contig036780-BurOR.A001	contig022268-TilOR.A020	0.260	0.684	0.381
contig064187-BurOR.A016	contig034988-NyeORs.A033	0.260	0.691	0.376
contig030554-ZebOR.A004	contig047515-ZebOR.A019	0.260	0.692	0.375
contig034983-NyeOR.A002	contig030554-ZebOR.A004	0.260	0.692	0.375
contig022234-TilOR.A009	contig041951-TilOR.A021	0.260	0.707	0.367
contig084999-BriOR.A002	contig034983-NyeOR.A002	0.260	0.714	0.365
contig084999-BriOR.A002	contig047515-ZebOR.A019	0.260	0.714	0.365
contig051559-BurOR.A007	contig047515-ZebOR.A018	0.260	0.719	0.362
contig022268-TilOR.A019	contig041951-TilOR.A022	0.260	0.721	0.361
contig022268-TilOR.A019	contig047515-ZebOR.A019	0.260	0.740	0.351
contig034983-NyeOR.A002	contig022268-TilOR.A019	0.260	0.740	0.351
contig064187-BurOR.A016	contig022268-TilOR.A019	0.260	0.756	0.344
contig034983-NyeOR.A002	contig034988-NyeOR.A005	0.260	0.769	0.338
contig034988-NyeOR.A005	contig047515-ZebOR.A019	0.260	0.769	0.338
contig051559-BurOR.A007	contig030556-ZebOR.A005	0.260	0.783	0.332
contig034981-NyeOR.A001	contig022268-TilOR.A020	0.260	0.798	0.326
contig064187-BurOR.A016	contig054678-NyeOR.A010	0.260	0.812	0.320
contig022232-TilOR.A008	contig047506-ZebOR.A014	0.260	0.840	0.310
contig022225-TilOR.A005	contig030552-ZebOR.A001	0.261	0.621	0.421
contig036780-BurOR.A001	contig034983-NyeOR.A002	0.261	0.636	0.410
contig036780-BurOR.A001	contig047515-ZebOR.A019	0.261	0.636	0.410
contig022225-TilOR.A005	contig022230-TilOR.A007	0.261	0.675	0.387
contig054868-NyeOR.A014	contig022230-TilOR.A007	0.261	0.682	0.383
contig034988-NyeORs.A033	contig047521-ZebOR.A020	0.261	0.685	0.382
contig022230-TilOR.A007	contig073309-TilOR.A026	0.261	0.699	0.373
contig041951-TilOR.A021	contig070885-TilOR.A024	0.261	0.712	0.366
contig034983-NyeOR.A003	contig041951-TilOR.A021	0.261	0.719	0.363
contig084999-BriOR.A002	contig034988-NyeORs.A033	0.261	0.741	0.352
contig051559-BurOR.A007	contig051570-BurOR.A010	0.261	0.747	0.350
contig054237-BurOR.A013	contig041951-TilOR.A021	0.261	0.754	0.347
contig047506-ZebOR.A014	contig047515-ZebOR.A018	0.261	0.818	0.319
contig034983-NyeOR.A002	contig070885-TilOR.A024	0.261	0.824	0.317

contig070885-TilOR.A024	contig047515-ZebOR.A019	0.261	0.824	0.317
contig034983-NyeOR.A003	contig041951-TilOR.A022	0.261	0.846	0.308
contig022268-TilOR.A020	contig030553-ZebOR.A003	0.261	0.916	0.285
contig054681-NyeOR.A011	contig030552-ZebOR.A001	0.262	0.580	0.451
contig054681-NyeOR.A011	contig057165-NyeOR.A017	0.262	0.589	0.444
contig054681-NyeOR.A011	contig022225-TilOR.A005	0.262	0.646	0.405
contig034988-NyeORs.A033	contig047506-ZebOR.A014	0.262	0.683	0.383
contig036787-BurOR.A004	contig041951-TilOR.A022	0.262	0.732	0.358
contig022232-TilOR.A008	contig047515-ZebOR.A019	0.262	0.737	0.355
contig034983-NyeOR.A002	contig022232-TilOR.A008	0.262	0.737	0.355
contig051559-BurOR.A007	contig047508-ZebOR.A015	0.262	0.742	0.354
contig022268-TilOR.A019	contig047506-ZebOR.A014	0.262	0.746	0.352
contig036787-BurOR.A004	contig064187-BurOR.A016	0.262	0.777	0.337
contig054237-BurOR.A013	contig022268-TilOR.A020	0.262	0.800	0.327
contig054678-NyeOR.A010	contig047506-ZebOR.A014	0.262	0.802	0.327
contig022268-TilOR.A020	contig030556-ZebOR.A005	0.262	0.809	0.324
contig064187-BurOR.A016	contig034983-NyeOR.A003	0.262	0.841	0.311
contig057754-NyeOR.A018	contig022268-TilOR.A020	0.262	0.952	0.275
contig036780-BurOR.A001	contig030557-ZebOR.A006	0.263	0.586	0.448
contig036780-BurOR.A001	contig022225-TilOR.A005	0.263	0.612	0.431
contig057165-NyeOR.A017	contig022225-TilOR.A005	0.263	0.612	0.431
contig022204-TilOR.A001	contig022266-TilOR.A018	0.263	0.623	0.423
contig057156-BurOR.A015	contig022268-TilOR.A020	0.263	0.689	0.382
contig054237-BurOR.A013	contig034988-NyeORs.A033	0.263	0.696	0.379
contig034981-NyeOR.A001	contig034988-NyeORs.A033	0.263	0.699	0.375
contig085002-BriOR.A004	contig022230-TilOR.A007	0.263	0.704	0.374
contig073309-TilOR.A026	contig047506-ZebOR.A014	0.263	0.708	0.371
contig054684-NyeOR.A012	contig022268-TilOR.A020	0.263	0.710	0.371
contig022268-TilOR.A020	contig073309-TilOR.A026	0.263	0.715	0.368
contig034983-NyeOR.A002	contig047515-ZebOR.A018	0.263	0.728	0.362
contig047515-ZebOR.A018	contig047515-ZebOR.A019	0.263	0.728	0.362
contig064187-BurOR.A016	contig073309-TilOR.A026	0.263	0.729	0.361
contig051559-BurOR.A007	contig034983-NyeOR.A003	0.263	0.730	0.360
contig054678-NyeOR.A010	contig041951-TilOR.A022	0.263	0.763	0.344
contig030556-ZebOR.A005	contig047515-ZebOR.A019	0.263	0.792	0.332
contig034983-NyeOR.A002	contig030556-ZebOR.A005	0.263	0.792	0.332
contig054868-NyeOR.A014	contig022234-TilOR.A009	0.263	0.800	0.329
contig064187-BurOR.A016	contig022234-TilOR.A009	0.263	0.812	0.324
contig051559-BurOR.A008	contig022230-TilOR.A007	0.263	0.817	0.323
contig041951-TilOR.A021	contig030553-ZebOR.A003	0.263	0.824	0.319
contig064187-BurOR.A016	contig030553-ZebOR.A003	0.263	0.847	0.310
contig054868-NyeOR.A014	contig070885-TilOR.A024	0.263	0.874	0.301
contig054233-BurOR.A012	contig070885-TilOR.A024	0.263	0.888	0.296

contig070885-TilOR.A024	contig047523-ZebOR.A022	0.263	0.888	0.296
contig036780-BurOR.A001	contig054681-NyeOR.A011	0.264	0.589	0.447
contig022204-TilOR.A001	contig030557-ZebOR.A006	0.264	0.652	0.405
contig041951-TilOR.A022	contig030554-ZebOR.A004	0.264	0.667	0.396
contig041951-TilOR.A022	contig073309-TilOR.A026	0.264	0.702	0.376
contig064187-BurOR.A016	contig030554-ZebOR.A004	0.264	0.708	0.373
contig051570-BurOR.A010	contig034983-NyeOR.A002	0.264	0.744	0.355
contig051570-BurOR.A010	contig047515-ZebOR.A019	0.264	0.744	0.355
contig036787-BurOR.A004	contig047506-ZebOR.A014	0.264	0.767	0.344
contig036787-BurOR.A004	contig022268-TilOR.A020	0.264	0.782	0.338
contig022234-TilOR.A009	contig041951-TilOR.A022	0.264	0.792	0.333
contig064187-BurOR.A016	contig030556-ZebOR.A005	0.264	0.794	0.332
contig085026-BriOR.A008	contig022268-TilOR.A020	0.264	0.801	0.329
contig054868-NyeOR.A014	contig047515-ZebOR.A018	0.264	0.801	0.330
contig034983-NyeOR.A003	contig047506-ZebOR.A014	0.264	0.830	0.318
contig041951-TilOR.A022	contig030553-ZebOR.A003	0.264	0.836	0.316
contig034988-NyeOR.A006	contig022232-TilOR.A008	0.264	0.837	0.316
contig030553-ZebOR.A003	contig047506-ZebOR.A014	0.264	0.842	0.313
contig057754-NyeOR.A018	contig041951-TilOR.A021	0.264	0.855	0.309
contig064187-BurOR.A016	contig057754-NyeOR.A018	0.264	0.867	0.304
contig034988-NyeOR.A006	contig041951-TilOR.A021	0.265	0.654	0.405
contig022230-TilOR.A007	contig041951-TilOR.A021	0.265	0.677	0.391
contig022230-TilOR.A007	contig047523-ZebOR.A022	0.265	0.677	0.391
contig054233-BurOR.A012	contig022230-TilOR.A007	0.265	0.677	0.391
contig041951-TilOR.A021	contig041951-TilOR.A022	0.265	0.684	0.388
contig022268-TilOR.A020	contig030560-ZebOR.A007	0.265	0.688	0.385
contig022268-TilOR.A019	contig041951-TilOR.A021	0.265	0.692	0.383
contig051559-BurOR.A008	contig041951-TilOR.A021	0.265	0.707	0.375
contig034988-NyeORs.A033	contig030554-ZebOR.A004	0.265	0.719	0.368
contig054868-NyeOR.A014	contig047506-ZebOR.A014	0.265	0.737	0.360
contig034983-NyeOR.A002	contig047508-ZebOR.A015	0.265	0.739	0.359
contig047508-ZebOR.A015	contig047515-ZebOR.A019	0.265	0.739	0.359
contig034988-NyeORs.A033	contig070886-TilOR.A025	0.265	0.753	0.352
contig085026-BriOR.A008	contig041951-TilOR.A021	0.265	0.756	0.350
contig022234-TilOR.A009	contig047506-ZebOR.A014	0.265	0.801	0.330
contig022234-TilOR.A009	contig047523-ZebOR.A022	0.265	0.813	0.326
contig054233-BurOR.A012	contig022234-TilOR.A009	0.265	0.813	0.326
contig034988-NyeOR.A006	contig047515-ZebOR.A018	0.265	0.828	0.320
contig085000-BriOR.A003	contig047506-ZebOR.A014	0.265	0.839	0.316
contig085000-BriOR.A003	contig064187-BurOR.A016	0.265	0.843	0.314
contig034988-NyeOR.A005	contig041951-TilOR.A022	0.265	0.845	0.314
contig051559-BurOR.A008	contig041951-TilOR.A022	0.265	0.858	0.309
contig057754-NyeOR.A018	contig047506-ZebOR.A014	0.265	0.862	0.307

contig022232-TilOR.A008	contig022268-TilOR.A020	0.265	0.878	0.302
contig022225-TilOR.A005	contig030553-ZebOR.A002	0.266	0.560	0.474
contig057153-BurOR.A014	contig030552-ZebOR.A001	0.266	0.573	0.463
contig057153-BurOR.A014	contig057165-NyeOR.A017	0.266	0.582	0.456
contig041951-TilOR.A021	contig030552-ZebOR.A001	0.266	0.590	0.450
contig054868-NyeOR.A014	contig041951-TilOR.A022	0.266	0.687	0.388
contig030554-ZebOR.A004	contig047506-ZebOR.A014	0.266	0.699	0.381
contig057156-BurOR.A015	contig054868-NyeOR.A014	0.266	0.703	0.378
contig084999-BriOR.A002	contig064187-BurOR.A016	0.266	0.704	0.377
contig034988-NyeOR.A006	contig073309-TilOR.A026	0.266	0.713	0.373
contig054684-NyeOR.A012	contig054868-NyeOR.A014	0.266	0.724	0.367
contig034983-NyeOR.A002	contig034983-NyeOR.A003	0.266	0.738	0.360
contig034983-NyeOR.A003	contig047515-ZebOR.A019	0.266	0.738	0.360
contig041951-TilOR.A022	contig030556-ZebOR.A005	0.266	0.745	0.357
contig064187-BurOR.A016	contig054868-NyeOR.A014	0.266	0.758	0.351
contig030556-ZebOR.A005	contig047506-ZebOR.A014	0.266	0.783	0.339
contig022211-TilOR.A003	contig022268-TilOR.A020	0.266	0.801	0.332
contig034983-NyeOR.A003	contig054868-NyeOR.A014	0.266	0.812	0.328
contig047515-ZebOR.A018	contig047523-ZebOR.A022	0.266	0.814	0.327
contig054233-BurOR.A012	contig047515-ZebOR.A018	0.266	0.814	0.327
contig085000-BriOR.A003	contig041951-TilOR.A022	0.266	0.832	0.320
contig051559-BurOR.A008	contig064187-BurOR.A016	0.266	0.852	0.312
contig064187-BurOR.A016	contig034988-NyeOR.A005	0.266	0.852	0.312
contig057754-NyeOR.A018	contig041951-TilOR.A022	0.266	0.855	0.310
contig036784-BurOR.A003	contig022268-TilOR.A020	0.266	0.926	0.287
contig022204-TilOR.A001	contig022268-TilOR.A020	0.267	0.631	0.423
contig057153-BurOR.A014	contig022204-TilOR.A001	0.267	0.648	0.412
contig054868-NyeOR.A014	contig030552-ZebOR.A001	0.267	0.657	0.407
contig054868-NyeOR.A014	contig057165-NyeOR.A017	0.267	0.657	0.407
contig085010-BriOR.A005	contig022230-TilOR.A007	0.267	0.675	0.395
contig034988-NyeOR.A006	contig034988-NyeORs.A033	0.267	0.688	0.388
contig054233-BurOR.A012	contig057156-BurOR.A015	0.267	0.698	0.382
contig057156-BurOR.A015	contig047523-ZebOR.A022	0.267	0.698	0.382
contig051573-BurOR.A011	contig057156-BurOR.A015	0.267	0.698	0.383
contig085010-BriOR.A005	contig030552-ZebOR.A001	0.267	0.712	0.376
contig085010-BriOR.A005	contig036780-BurOR.A001	0.267	0.712	0.376
contig085010-BriOR.A005	contig057165-NyeOR.A017	0.267	0.712	0.376
contig085010-BriOR.A005	contig022268-TilOR.A020	0.267	0.714	0.373
contig036780-BurOR.A001	contig047521-ZebOR.A020	0.267	0.715	0.374
contig034988-NyeORs.A033	contig041951-TilOR.A022	0.267	0.718	0.373
contig054233-BurOR.A012	contig054684-NyeOR.A012	0.267	0.719	0.371
contig054684-NyeOR.A012	contig047523-ZebOR.A022	0.267	0.719	0.371
contig051573-BurOR.A011	contig054684-NyeOR.A012	0.267	0.719	0.372

contig051573-BurOR.A011	contig034988-NyeORs.A033	0.267	0.722	0.370
contig030552-ZebOR.A001	contig047521-ZebOR.A020	0.267	0.725	0.368
contig051559-BurOR.A007	contig051559-BurOR.A008	0.267	0.740	0.361
contig022211-TilOR.A003	contig041951-TilOR.A021	0.267	0.745	0.358
contig085002-BriOR.A004	contig051559-BurOR.A007	0.267	0.753	0.355
contig041951-TilOR.A021	contig030576-ZebOR.A010	0.267	0.763	0.351
contig022230-TilOR.A007	contig047521-ZebOR.A020	0.267	0.767	0.348
contig041951-TilOR.A022	contig047503-ZebOR.A013	0.267	0.785	0.340
contig022259-TilOR.A014	contig022268-TilOR.A020	0.267	0.806	0.331
contig054678-NyeOR.A010	contig022268-TilOR.A020	0.267	0.815	0.328
contig085000-BriOR.A003	contig022268-TilOR.A020	0.267	0.929	0.287
contig036780-BurOR.A001	contig057153-BurOR.A014	0.268	0.582	0.460
contig084999-BriOR.A001	contig022225-TilOR.A005	0.268	0.586	0.457
contig057165-NyeOR.A017	contig041951-TilOR.A021	0.268	0.598	0.448
contig036780-BurOR.A001	contig041951-TilOR.A021	0.268	0.600	0.446
contig041951-TilOR.A021	contig047508-ZebOR.A015	0.268	0.616	0.435
contig051570-BurOR.A010	contig041951-TilOR.A021	0.268	0.617	0.433
contig034988-NyeOR.A005	contig041951-TilOR.A021	0.268	0.642	0.418
contig022227-TilOR.A006	contig022230-TilOR.A007	0.268	0.651	0.411
contig041951-TilOR.A021	contig047503-ZebOR.A013	0.268	0.652	0.412
contig084999-BriOR.A002	contig041951-TilOR.A022	0.268	0.661	0.405
contig022204-TilOR.A001	contig041951-TilOR.A021	0.268	0.666	0.402
contig054868-NyeOR.A014	contig030557-ZebOR.A006	0.268	0.674	0.397
contig054868-NyeOR.A014	contig030556-ZebOR.A005	0.268	0.674	0.397
contig054681-NyeOR.A011	contig054868-NyeOR.A014	0.268	0.683	0.392
contig041951-TilOR.A021	contig047526-ZebOR.A021	0.268	0.686	0.390
contig084999-BriOR.A002	contig047506-ZebOR.A014	0.268	0.695	0.385
contig041951-TilOR.A021	contig047514-ZebOR.A017	0.268	0.699	0.383
contig054868-NyeOR.A014	contig030560-ZebOR.A007	0.268	0.702	0.382
contig034988-NyeORs.A033	contig070885-TilOR.A024	0.268	0.725	0.369
contig034983-NyeOR.A002	contig047521-ZebOR.A020	0.268	0.730	0.367
contig047515-ZebOR.A019	contig047521-ZebOR.A020	0.268	0.730	0.367
contig036780-BurOR.A001	contig034981-NyeOR.A001	0.268	0.730	0.368
contig034994-NyeOR.A008	contig041951-TilOR.A021	0.268	0.733	0.366
contig062344-NyeOR.A020	contig041951-TilOR.A021	0.268	0.739	0.362
contig034981-NyeOR.A001	contig030552-ZebOR.A001	0.268	0.741	0.362
contig034988-NyeOR.A006	contig054868-NyeOR.A014	0.268	0.741	0.362
contig085010-BriOR.A005	contig064187-BurOR.A016	0.268	0.743	0.361
contig034981-NyeOR.A001	contig022230-TilOR.A007	0.268	0.759	0.353
contig064187-BurOR.A016	contig022265-TilOR.A017	0.268	0.770	0.348
contig070885-TilOR.A024	contig030557-ZebOR.A006	0.268	0.774	0.346
contig036780-BurOR.A001	contig022211-TilOR.A003	0.268	0.789	0.340
contig034988-NyeORs.A033	contig030553-ZebOR.A003	0.268	0.791	0.339

contig064187-BurOR.A016	contig047503-ZebOR.A013	0.268	0.792	0.338
contig022211-TilOR.A003	contig030552-ZebOR.A001	0.268	0.801	0.335
contig034983-NyeOR.A003	contig047523-ZebOR.A022	0.268	0.825	0.325
contig054233-BurOR.A012	contig034983-NyeOR.A003	0.268	0.825	0.325
contig036784-BurOR.A003	contig041951-TilOR.A021	0.268	0.827	0.325
contig034988-NyeOR.A005	contig054868-NyeOR.A014	0.268	0.839	0.320
contig034983-NyeOR.A003	contig034988-NyeOR.A006	0.268	0.840	0.318
contig051559-BurOR.A008	contig047506-ZebOR.A014	0.268	0.841	0.319
contig034988-NyeOR.A005	contig047506-ZebOR.A014	0.268	0.841	0.319
contig036784-BurOR.A003	contig064187-BurOR.A016	0.268	0.850	0.316
contig034988-NyeOR.A005	contig047523-ZebOR.A022	0.268	0.853	0.315
contig054233-BurOR.A012	contig034988-NyeOR.A005	0.268	0.853	0.315
contig036782-BurOR.A002	contig022225-TilOR.A005	0.269	0.591	0.456
contig054681-NyeOR.A011	contig022204-TilOR.A001	0.269	0.636	0.423
contig057156-BurOR.A015	contig022230-TilOR.A007	0.269	0.650	0.415
contig084999-BriOR.A001	contig041951-TilOR.A021	0.269	0.655	0.411
contig054684-NyeOR.A012	contig022230-TilOR.A007	0.269	0.681	0.396
contig051566-BurOR.A009	contig041951-TilOR.A021	0.269	0.684	0.393
contig085002-BriOR.A004	contig041951-TilOR.A021	0.269	0.693	0.387
contig030560-ZebOR.A007	contig047523-ZebOR.A022	0.269	0.697	0.386
contig054233-BurOR.A012	contig030560-ZebOR.A007	0.269	0.697	0.386
contig034988-NyeORs.A033	contig022230-TilOR.A007	0.269	0.702	0.383
contig085002-BriOR.A004	contig034988-NyeORs.A033	0.269	0.709	0.379
contig034988-NyeORs.A033	contig022232-TilOR.A008	0.269	0.709	0.379
contig036780-BurOR.A001	contig054684-NyeOR.A012	0.269	0.710	0.379
contig054684-NyeOR.A012	contig030552-ZebOR.A001	0.269	0.710	0.379
contig054684-NyeOR.A012	contig057165-NyeOR.A017	0.269	0.710	0.379
contig057165-NyeOR.A017	contig047521-ZebOR.A020	0.269	0.715	0.377
contig022268-TilOR.A019	contig022268-TilOR.A020	0.269	0.717	0.375
contig085002-BriOR.A004	contig064187-BurOR.A016	0.269	0.718	0.375
contig036780-BurOR.A001	contig057156-BurOR.A015	0.269	0.721	0.373
contig057156-BurOR.A015	contig030552-ZebOR.A001	0.269	0.721	0.373
contig057156-BurOR.A015	contig057165-NyeOR.A017	0.269	0.721	0.373
contig051559-BurOR.A007	contig054868-NyeOR.A014	0.269	0.722	0.372
contig034981-NyeOR.A001	contig034983-NyeOR.A002	0.269	0.723	0.372
contig034981-NyeOR.A001	contig047515-ZebOR.A019	0.269	0.723	0.372
contig022268-TilOR.A020	contig030554-ZebOR.A004	0.269	0.726	0.370
contig036780-BurOR.A001	contig054237-BurOR.A013	0.269	0.726	0.371
contig047506-ZebOR.A014	contig047523-ZebOR.A022	0.269	0.732	0.367
contig054233-BurOR.A012	contig047506-ZebOR.A014	0.269	0.732	0.367
contig051559-BurOR.A007	contig047521-ZebOR.A020	0.269	0.733	0.367
contig054237-BurOR.A013	contig030552-ZebOR.A001	0.269	0.737	0.365
contig085002-BriOR.A004	contig022268-TilOR.A020	0.269	0.745	0.361

contig085000-BriOR.A003	contig085002-BriOR.A004	0.269	0.750	0.359
contig054233-BurOR.A012	contig064187-BurOR.A016	0.269	0.752	0.357
contig064187-BurOR.A016	contig047523-ZebOR.A022	0.269	0.752	0.357
contig054681-NyeOR.A011	contig070885-TilOR.A024	0.269	0.767	0.351
contig034988-NyeORs.A033	contig041952-TilOR.A023	0.269	0.777	0.346
contig054237-BurOR.A013	contig022230-TilOR.A007	0.269	0.804	0.334
contig034988-NyeOR.A006	contig022234-TilOR.A009	0.269	0.811	0.331
contig057754-NyeOR.A018	contig034988-NyeORs.A033	0.269	0.823	0.326
contig051570-BurOR.A010	contig041951-TilOR.A022	0.269	0.824	0.326
contig051570-BurOR.A010	contig047523-ZebOR.A022	0.269	0.845	0.319
contig051570-BurOR.A010	contig054233-BurOR.A012	0.269	0.845	0.319
contig036784-BurOR.A003	contig047506-ZebOR.A014	0.269	0.846	0.319
contig057156-BurOR.A015	contig070885-TilOR.A024	0.269	0.878	0.306
contig022230-TilOR.A007	contig022265-TilOR.A017	0.270	0.622	0.434
contig041951-TilOR.A021	contig030553-ZebOR.A002	0.270	0.622	0.435
contig022204-TilOR.A001	contig047515-ZebOR.A019	0.270	0.643	0.420
contig034983-NyeOR.A002	contig022204-TilOR.A001	0.270	0.643	0.420
contig036780-BurOR.A001	contig054868-NyeOR.A014	0.270	0.657	0.411
contig084999-BriOR.A001	contig034988-NyeORs.A033	0.270	0.657	0.411
contig051573-BurOR.A011	contig030557-ZebOR.A006	0.270	0.674	0.401
contig041951-TilOR.A022	contig047523-ZebOR.A022	0.270	0.682	0.395
contig054233-BurOR.A012	contig041951-TilOR.A022	0.270	0.682	0.395
contig036780-BurOR.A001	contig034988-NyeOR.A005	0.270	0.686	0.394
contig034988-NyeOR.A005	contig022266-TilOR.A018	0.270	0.691	0.391
contig034988-NyeOR.A005	contig030552-ZebOR.A001	0.270	0.697	0.388
contig085000-BriOR.A003	contig030556-ZebOR.A005	0.270	0.700	0.386
contig085026-BriOR.A008	contig034988-NyeORs.A033	0.270	0.703	0.384
contig093816-BriOR.A011	contig057156-BurOR.A015	0.270	0.707	0.382
contig085010-BriOR.A005	contig054868-NyeOR.A014	0.270	0.713	0.380
contig051573-BurOR.A011	contig030560-ZebOR.A007	0.270	0.718	0.375
contig022238-TilOR.A010	contig041951-TilOR.A021	0.270	0.719	0.375
contig030552-ZebOR.A001	contig030560-ZebOR.A007	0.270	0.726	0.372
contig036780-BurOR.A001	contig030560-ZebOR.A007	0.270	0.726	0.372
contig057165-NyeOR.A017	contig030560-ZebOR.A007	0.270	0.726	0.372
contig051559-BurOR.A007	contig034981-NyeOR.A001	0.270	0.726	0.373
contig093816-BriOR.A011	contig054684-NyeOR.A012	0.270	0.729	0.371
contig034981-NyeOR.A001	contig057165-NyeOR.A017	0.270	0.730	0.371
contig036780-BurOR.A001	contig022234-TilOR.A009	0.270	0.731	0.370
contig054868-NyeOR.A014	contig022268-TilOR.A020	0.270	0.733	0.368
contig085010-BriOR.A005	contig047506-ZebOR.A014	0.270	0.733	0.369
contig022234-TilOR.A009	contig030552-ZebOR.A001	0.270	0.742	0.364
contig051559-BurOR.A008	contig034983-NyeOR.A002	0.270	0.748	0.361
contig051559-BurOR.A008	contig047515-ZebOR.A019	0.270	0.748	0.361

contig034988-NyeOR.A006	contig022268-TilOR.A019	0.270	0.751	0.359
contig022265-TilOR.A017	contig047506-ZebOR.A014	0.270	0.760	0.355
contig085002-BriOR.A004	contig034983-NyeOR.A002	0.270	0.762	0.355
contig085002-BriOR.A004	contig047515-ZebOR.A019	0.270	0.762	0.355
contig054237-BurOR.A013	contig034983-NyeOR.A002	0.270	0.766	0.353
contig054237-BurOR.A013	contig047515-ZebOR.A019	0.270	0.766	0.353
contig054868-NyeOR.A014	contig022259-TilOR.A014	0.270	0.770	0.351
contig047503-ZebOR.A013	contig047506-ZebOR.A014	0.270	0.782	0.345
contig057165-NyeOR.A017	contig022211-TilOR.A003	0.270	0.789	0.343
contig041951-TilOR.A022	contig047508-ZebOR.A015	0.270	0.818	0.330
contig047508-ZebOR.A015	contig047523-ZebOR.A022	0.270	0.827	0.327
contig054233-BurOR.A012	contig047508-ZebOR.A015	0.270	0.827	0.327
contig034988-NyeOR.A006	contig030553-ZebOR.A003	0.270	0.828	0.326
contig051570-BurOR.A010	contig054868-NyeOR.A014	0.270	0.831	0.324
contig051570-BurOR.A010	contig064187-BurOR.A016	0.270	0.831	0.324
contig036784-BurOR.A003	contig041951-TilOR.A022	0.270	0.839	0.322
contig034988-NyeOR.A005	contig034988-NyeOR.A006	0.270	0.850	0.318
contig085010-BriOR.A005	contig070885-TilOR.A024	0.270	0.872	0.310
contig041951-TilOR.A022	contig070885-TilOR.A024	0.270	0.884	0.305
contig022265-TilOR.A017	contig041951-TilOR.A021	0.271	0.653	0.415
contig051559-BurOR.A007	contig041951-TilOR.A021	0.271	0.657	0.413
contig022225-TilOR.A005	contig047515-ZebOR.A019	0.271	0.662	0.409
contig034983-NyeOR.A002	contig022225-TilOR.A005	0.271	0.662	0.409
contig034988-NyeORs.A033	contig047515-ZebOR.A018	0.271	0.667	0.406
contig057153-BurOR.A014	contig054868-NyeOR.A014	0.271	0.670	0.404
contig030557-ZebOR.A006	contig047523-ZebOR.A022	0.271	0.670	0.404
contig054233-BurOR.A012	contig030557-ZebOR.A006	0.271	0.670	0.404
contig030556-ZebOR.A005	contig047523-ZebOR.A022	0.271	0.670	0.405
contig054233-BurOR.A012	contig030556-ZebOR.A005	0.271	0.670	0.405
contig085002-BriOR.A004	contig041951-TilOR.A022	0.271	0.674	0.402
contig054233-BurOR.A012	contig054681-NyeOR.A011	0.271	0.679	0.399
contig054681-NyeOR.A011	contig047523-ZebOR.A022	0.271	0.679	0.399
contig085000-BriOR.A003	contig036787-BurOR.A004	0.271	0.688	0.394
contig051559-BurOR.A007	contig073309-TilOR.A026	0.271	0.705	0.384
contig036787-BurOR.A004	contig034988-NyeORs.A033	0.271	0.707	0.383
contig085002-BriOR.A004	contig047506-ZebOR.A014	0.271	0.708	0.382
contig085010-BriOR.A005	contig047523-ZebOR.A022	0.271	0.708	0.383
contig085010-BriOR.A005	contig054233-BurOR.A012	0.271	0.708	0.383
contig036787-BurOR.A004	contig041951-TilOR.A021	0.271	0.714	0.379
contig057156-BurOR.A015	contig064187-BurOR.A016	0.271	0.715	0.379
contig022230-TilOR.A007	contig022259-TilOR.A014	0.271	0.717	0.378
contig054237-BurOR.A013	contig057165-NyeOR.A017	0.271	0.726	0.374
contig022266-TilOR.A018	contig070885-TilOR.A024	0.271	0.733	0.369

contig085026-BriOR.A008	contig036780-BurOR.A001	0.271	0.735	0.369
contig093812-BriOR.A010	contig041951-TilOR.A021	0.271	0.738	0.367
contig085026-BriOR.A008	contig030552-ZebOR.A001	0.271	0.747	0.364
contig064187-BurOR.A016	contig054684-NyeOR.A012	0.271	0.749	0.362
contig064187-BurOR.A016	contig022204-TilOR.A001	0.271	0.766	0.353
contig057153-BurOR.A014	contig070885-TilOR.A024	0.271	0.769	0.352
contig051559-BurOR.A007	contig054237-BurOR.A013	0.271	0.769	0.353
contig093816-BriOR.A011	contig034988-NyeORs.A033	0.271	0.771	0.352
contig034988-NyeOR.A006	contig047503-ZebOR.A013	0.271	0.794	0.342
contig051559-BurOR.A008	contig054868-NyeOR.A014	0.271	0.798	0.339
contig085000-BriOR.A003	contig041951-TilOR.A021	0.271	0.810	0.334
contig054868-NyeOR.A014	contig047508-ZebOR.A015	0.271	0.813	0.333
contig085000-BriOR.A003	contig034988-NyeOR.A006	0.271	0.825	0.329
contig064187-BurOR.A016	contig047508-ZebOR.A015	0.271	0.826	0.328
contig034988-NyeOR.A006	contig057754-NyeOR.A018	0.271	0.848	0.319
contig054684-NyeOR.A012	contig070885-TilOR.A024	0.271	0.891	0.304
contig054868-NyeOR.A014	contig030576-ZebOR.A010	0.271	0.899	0.302
contig064187-BurOR.A016	contig070885-TilOR.A024	0.271	0.907	0.299
contig051573-BurOR.A011	contig054681-NyeOR.A011	0.272	0.658	0.413
contig022230-TilOR.A007	contig030560-ZebOR.A007	0.272	0.670	0.406
contig051573-BurOR.A011	contig022230-TilOR.A007	0.272	0.678	0.401
contig084999-BriOR.A001	contig085010-BriOR.A005	0.272	0.679	0.400
contig036787-BurOR.A004	contig054868-NyeOR.A014	0.272	0.683	0.399
contig034988-NyeOR.A005	contig057165-NyeOR.A017	0.272	0.686	0.397
contig084999-BriOR.A001	contig054868-NyeOR.A014	0.272	0.688	0.396
contig085000-BriOR.A003	contig054678-NyeOR.A010	0.272	0.695	0.391
contig034988-NyeORs.A033	contig022211-TilOR.A003	0.272	0.706	0.385
contig085010-BriOR.A005	contig051573-BurOR.A011	0.272	0.708	0.385
contig051559-BurOR.A007	contig047523-ZebOR.A022	0.272	0.717	0.379
contig051559-BurOR.A007	contig054233-BurOR.A012	0.272	0.717	0.379
contig034988-NyeOR.A005	contig030557-ZebOR.A006	0.272	0.719	0.378
contig093816-BriOR.A011	contig030556-ZebOR.A005	0.272	0.721	0.378
contig093816-BriOR.A011	contig030560-ZebOR.A007	0.272	0.728	0.374
contig057165-NyeOR.A017	contig022234-TilOR.A009	0.272	0.731	0.373
contig030553-ZebOR.A002	contig047521-ZebOR.A020	0.272	0.734	0.371
contig085010-BriOR.A005	contig041951-TilOR.A022	0.272	0.736	0.369
contig034988-NyeOR.A006	contig047523-ZebOR.A022	0.272	0.736	0.369
contig054233-BurOR.A012	contig034988-NyeOR.A006	0.272	0.736	0.369
contig022265-TilOR.A017	contig030552-ZebOR.A001	0.272	0.742	0.367
contig036780-BurOR.A001	contig022265-TilOR.A017	0.272	0.742	0.367
contig057165-NyeOR.A017	contig022265-TilOR.A017	0.272	0.742	0.367
contig036780-BurOR.A001	contig022232-TilOR.A008	0.272	0.748	0.364
contig022265-TilOR.A017	contig041951-TilOR.A022	0.272	0.757	0.360

contig022232-TilOR.A008	contig030552-ZebOR.A001	0.272	0.759	0.358
contig054868-NyeOR.A014	contig022232-TilOR.A008	0.272	0.772	0.352
contig051570-BurOR.A010	contig047506-ZebOR.A014	0.272	0.821	0.331
contig022265-TilOR.A017	contig070885-TilOR.A024	0.272	0.839	0.324
contig051559-BurOR.A008	contig034988-NyeOR.A006	0.272	0.851	0.319
contig054868-NyeOR.A014	contig062344-NyeOR.A020	0.272	0.867	0.314
contig070885-TilOR.A024	contig030560-ZebOR.A007	0.272	0.870	0.312
contig022268-TilOR.A020	contig047515-ZebOR.A018	0.272	0.886	0.307
contig084999-BriOR.A001	contig057156-BurOR.A015	0.273	0.642	0.425
contig030552-ZebOR.A001	contig047523-ZebOR.A022	0.273	0.652	0.418
contig054233-BurOR.A012	contig030552-ZebOR.A001	0.273	0.652	0.418
contig054233-BurOR.A012	contig057165-NyeOR.A017	0.273	0.652	0.418
contig057165-NyeOR.A017	contig047523-ZebOR.A022	0.273	0.652	0.418
contig093816-BriOR.A011	contig030557-ZebOR.A006	0.273	0.655	0.417
contig022227-TilOR.A006	contig030552-ZebOR.A001	0.273	0.662	0.413
contig084999-BriOR.A001	contig054684-NyeOR.A012	0.273	0.671	0.407
contig084999-BriOR.A002	contig022268-TilOR.A020	0.273	0.673	0.407
contig034983-NyeOR.A003	contig034988-NyeORs.A033	0.273	0.676	0.403
contig041951-TilOR.A021	contig030560-ZebOR.A007	0.273	0.681	0.400
contig051559-BurOR.A007	contig034988-NyeORs.A033	0.273	0.682	0.401
contig041951-TilOR.A021	contig047508-ZebOR.A016	0.273	0.685	0.398
contig022230-TilOR.A007	contig047499-ZebOR.A012	0.273	0.686	0.398
contig034988-NyeOR.A004	contig041951-TilOR.A021	0.273	0.688	0.396
contig034988-NyeORs.A033	contig022268-TilOR.A019	0.273	0.690	0.395
contig054678-NyeOR.A010	contig054868-NyeOR.A014	0.273	0.700	0.390
contig057156-BurOR.A015	contig047506-ZebOR.A014	0.273	0.706	0.387
contig034988-NyeOR.A005	contig054681-NyeOR.A011	0.273	0.712	0.383
contig034988-NyeORs.A033	contig030556-ZebOR.A005	0.273	0.720	0.379
contig041951-TilOR.A021	contig030556-ZebOR.A005	0.273	0.726	0.375
contig022268-TilOR.A020	contig047523-ZebOR.A022	0.273	0.728	0.375
contig054233-BurOR.A012	contig022268-TilOR.A020	0.273	0.728	0.375
contig064187-BurOR.A016	contig030560-ZebOR.A007	0.273	0.737	0.371
contig054684-NyeOR.A012	contig047506-ZebOR.A014	0.273	0.739	0.369
contig022204-TilOR.A001	contig047506-ZebOR.A014	0.273	0.756	0.361
contig034988-NyeOR.A006	contig022204-TilOR.A001	0.273	0.762	0.359
contig022259-TilOR.A014	contig047523-ZebOR.A022	0.273	0.765	0.357
contig054233-BurOR.A012	contig022259-TilOR.A014	0.273	0.765	0.357
contig036780-BurOR.A001	contig034983-NyeOR.A003	0.273	0.768	0.355
contig022211-TilOR.A003	contig022230-TilOR.A007	0.273	0.779	0.351
contig034983-NyeOR.A003	contig030552-ZebOR.A001	0.273	0.780	0.350
contig085010-BriOR.A005	contig034988-NyeOR.A005	0.273	0.794	0.344
contig036784-BurOR.A003	contig034988-NyeORs.A033	0.273	0.796	0.343
contig085026-BriOR.A008	contig022230-TilOR.A007	0.273	0.800	0.342

contig051559-BurOR.A008	contig047523-ZebOR.A022	0.273	0.811	0.336
contig051559-BurOR.A008	contig054233-BurOR.A012	0.273	0.811	0.336
contig022230-TilOR.A007	contig030553-ZebOR.A003	0.273	0.814	0.335
contig047506-ZebOR.A014	contig047508-ZebOR.A015	0.273	0.815	0.335
contig030553-ZebOR.A003	contig047521-ZebOR.A020	0.273	0.841	0.325
contig030553-ZebOR.A003	contig030557-ZebOR.A006	0.273	0.843	0.323
contig085000-BriOR.A003	contig030557-ZebOR.A006	0.273	0.849	0.321
contig070885-TilOR.A024	contig047506-ZebOR.A014	0.273	0.895	0.305
contig034988-NyeORs.A033	contig030552-ZebOR.A001	0.274	0.550	0.499
contig093816-BriOR.A011	contig054681-NyeOR.A011	0.274	0.639	0.429
contig051559-BurOR.A007	contig022204-TilOR.A001	0.274	0.645	0.424
contig034988-NyeORs.A033	contig022265-TilOR.A017	0.274	0.652	0.421
contig034983-NyeOR.A002	contig041951-TilOR.A021	0.274	0.655	0.419
contig041951-TilOR.A021	contig047515-ZebOR.A019	0.274	0.655	0.419
contig034990-NyeOR.A007	contig022230-TilOR.A007	0.274	0.656	0.417
contig084999-BriOR.A001	contig030560-ZebOR.A007	0.274	0.656	0.418
contig051559-BurOR.A007	contig022225-TilOR.A005	0.274	0.665	0.412
contig054233-BurOR.A012	contig057153-BurOR.A014	0.274	0.666	0.411
contig057153-BurOR.A014	contig047523-ZebOR.A022	0.274	0.666	0.411
contig084999-BriOR.A001	contig073309-TilOR.A026	0.274	0.671	0.409
contig022227-TilOR.A006	contig041951-TilOR.A021	0.274	0.679	0.403
contig036780-BurOR.A001	contig051570-BurOR.A010	0.274	0.685	0.401
contig054868-NyeOR.A014	contig022265-TilOR.A017	0.274	0.686	0.399
contig051570-BurOR.A010	contig030552-ZebOR.A001	0.274	0.695	0.395
contig030557-ZebOR.A006	contig047508-ZebOR.A015	0.274	0.697	0.393
contig051573-BurOR.A011	contig030556-ZebOR.A005	0.274	0.697	0.394
contig057156-BurOR.A015	contig041951-TilOR.A022	0.274	0.709	0.387
contig051570-BurOR.A010	contig030557-ZebOR.A006	0.274	0.717	0.382
contig085002-BriOR.A004	contig036784-BurOR.A003	0.274	0.730	0.375
contig041951-TilOR.A021	contig047499-ZebOR.A012	0.274	0.730	0.375
contig034983-NyeOR.A002	contig054868-NyeOR.A014	0.274	0.730	0.375
contig054868-NyeOR.A014	contig047515-ZebOR.A019	0.274	0.730	0.375
contig051573-BurOR.A011	contig022265-TilOR.A017	0.274	0.731	0.375
contig085026-BriOR.A008	contig057165-NyeOR.A017	0.274	0.735	0.372
contig054678-NyeOR.A010	contig041951-TilOR.A021	0.274	0.739	0.371
contig054684-NyeOR.A012	contig041951-TilOR.A022	0.274	0.742	0.369
contig034981-NyeOR.A001	contig030553-ZebOR.A002	0.274	0.749	0.365
contig022211-TilOR.A003	contig047515-ZebOR.A019	0.274	0.753	0.364
contig034983-NyeOR.A002	contig022211-TilOR.A003	0.274	0.753	0.364
contig034988-NyeOR.A005	contig022265-TilOR.A017	0.274	0.777	0.353
contig022232-TilOR.A008	contig047523-ZebOR.A022	0.274	0.784	0.349
contig054233-BurOR.A012	contig022232-TilOR.A008	0.274	0.784	0.349
contig057156-BurOR.A015	contig034988-NyeOR.A005	0.274	0.789	0.347

contig051570-BurOR.A010	contig034988-NyeOR.A006	0.274	0.829	0.330
contig054868-NyeOR.A014	contig047503-ZebOR.A013	0.274	0.833	0.329
contig047503-ZebOR.A013	contig047523-ZebOR.A022	0.274	0.834	0.328
contig054233-BurOR.A012	contig047503-ZebOR.A013	0.274	0.834	0.328
contig057754-NyeOR.A018	contig022230-TilOR.A007	0.274	0.834	0.328
contig036784-BurOR.A003	contig047521-ZebOR.A020	0.274	0.859	0.319
contig034983-NyeOR.A003	contig022268-TilOR.A020	0.274	0.871	0.315
contig057754-NyeOR.A018	contig030557-ZebOR.A006	0.274	0.876	0.313
contig057754-NyeOR.A018	contig030553-ZebOR.A002	0.274	0.889	0.309
contig030576-ZebOR.A010	contig047523-ZebOR.A022	0.274	0.893	0.307
contig054233-BurOR.A012	contig030576-ZebOR.A010	0.274	0.893	0.307
contig057756-NyeOR.A019	contig022225-TilOR.A005	0.275	0.594	0.462
contig034988-NyeORs.A033	contig047508-ZebOR.A015	0.275	0.627	0.439
contig051570-BurOR.A010	contig034988-NyeORs.A033	0.275	0.628	0.438
contig036782-BurOR.A002	contig041951-TilOR.A021	0.275	0.649	0.423
contig036780-BurOR.A001	contig047523-ZebOR.A022	0.275	0.652	0.421
contig036780-BurOR.A001	contig054233-BurOR.A012	0.275	0.652	0.421
contig036780-BurOR.A001	contig022227-TilOR.A006	0.275	0.652	0.422
contig057165-NyeOR.A017	contig022227-TilOR.A006	0.275	0.652	0.422
contig051573-BurOR.A011	contig057153-BurOR.A014	0.275	0.665	0.413
contig036780-BurOR.A001	contig047508-ZebOR.A015	0.275	0.670	0.411
contig057156-BurOR.A015	contig041951-TilOR.A021	0.275	0.672	0.409
contig051573-BurOR.A011	contig057165-NyeOR.A017	0.275	0.673	0.409
contig036787-BurOR.A004	contig047523-ZebOR.A022	0.275	0.678	0.406
contig036787-BurOR.A004	contig054233-BurOR.A012	0.275	0.678	0.406
contig030552-ZebOR.A001	contig047508-ZebOR.A015	0.275	0.680	0.405
contig054684-NyeOR.A012	contig041951-TilOR.A021	0.275	0.682	0.403
contig022265-TilOR.A017	contig047523-ZebOR.A022	0.275	0.682	0.404
contig054233-BurOR.A012	contig022265-TilOR.A017	0.275	0.682	0.404
contig054681-NyeOR.A011	contig047508-ZebOR.A015	0.275	0.690	0.399
contig085010-BriOR.A005	contig051559-BurOR.A007	0.275	0.691	0.397
contig034988-NyeOR.A006	contig030554-ZebOR.A004	0.275	0.699	0.393
contig093812-BriOR.A010	contig022230-TilOR.A007	0.275	0.703	0.390
contig051570-BurOR.A010	contig054681-NyeOR.A011	0.275	0.711	0.387
contig085002-BriOR.A004	contig054868-NyeOR.A014	0.275	0.713	0.386
contig057153-BurOR.A014	contig034988-NyeOR.A005	0.275	0.714	0.385
contig085010-BriOR.A005	contig093816-BriOR.A011	0.275	0.717	0.383
contig030560-ZebOR.A007	contig047506-ZebOR.A014	0.275	0.727	0.378
contig054678-NyeOR.A010	contig034988-NyeORs.A033	0.275	0.733	0.375
contig057165-NyeOR.A017	contig022232-TilOR.A008	0.275	0.746	0.368
contig022265-TilOR.A017	contig022268-TilOR.A020	0.275	0.749	0.367
contig036780-BurOR.A001	contig047515-ZebOR.A018	0.275	0.758	0.363
contig085010-BriOR.A005	contig047508-ZebOR.A015	0.275	0.759	0.363

contig034994-NyeOR.A008	contig034988-NyeORs.A033	0.275	0.761	0.361
contig085026-BriOR.A008	contig034983-NyeOR.A002	0.275	0.762	0.360
contig085026-BriOR.A008	contig047515-ZebOR.A019	0.275	0.762	0.360
contig030552-ZebOR.A001	contig047515-ZebOR.A018	0.275	0.769	0.358
contig085010-BriOR.A005	contig051570-BurOR.A010	0.275	0.781	0.352
contig034988-NyeOR.A006	contig054678-NyeOR.A010	0.275	0.792	0.347
contig034988-NyeOR.A005	contig022268-TilOR.A019	0.275	0.799	0.344
contig041952-TilOR.A023	contig070885-TilOR.A024	0.275	0.805	0.342
contig085000-BriOR.A003	contig022230-TilOR.A007	0.275	0.811	0.338
contig034988-NyeOR.A006	contig047508-ZebOR.A015	0.275	0.823	0.334
contig036784-BurOR.A003	contig034988-NyeOR.A006	0.275	0.831	0.331
contig030553-ZebOR.A002	contig030553-ZebOR.A003	0.275	0.856	0.322
contig034981-NyeOR.A001	contig030553-ZebOR.A003	0.275	0.859	0.320
contig054233-BurOR.A012	contig062344-NyeOR.A020	0.275	0.861	0.319
contig062344-NyeOR.A020	contig047523-ZebOR.A022	0.275	0.861	0.319
contig034988-NyeOR.A006	contig070885-TilOR.A024	0.275	0.907	0.303
contig036780-BurOR.A001	contig034988-NyeORs.A033	0.276	0.559	0.495
contig034988-NyeORs.A033	contig030553-ZebOR.A002	0.276	0.619	0.447
contig022204-TilOR.A001	contig030552-ZebOR.A001	0.276	0.638	0.432
contig084999-BriOR.A002	contig054868-NyeOR.A014	0.276	0.640	0.431
contig034988-NyeORs.A033	contig047503-ZebOR.A013	0.276	0.654	0.422
contig034988-NyeOR.A005	contig034988-NyeORs.A033	0.276	0.654	0.422
contig054868-NyeOR.A014	contig022266-TilOR.A018	0.276	0.664	0.415
contig084999-BriOR.A002	contig093816-BriOR.A011	0.276	0.665	0.415
contig073309-TilOR.A026	contig030557-ZebOR.A006	0.276	0.671	0.411
contig022225-TilOR.A005	contig041951-TilOR.A021	0.276	0.673	0.410
contig051559-BurOR.A007	contig022265-TilOR.A017	0.276	0.673	0.411
contig034988-NyeORs.A033	contig022204-TilOR.A001	0.276	0.675	0.409
contig022259-TilOR.A014	contig047515-ZebOR.A019	0.276	0.677	0.407
contig034983-NyeOR.A002	contig022259-TilOR.A014	0.276	0.677	0.407
contig034983-NyeOR.A002	contig034988-NyeORs.A033	0.276	0.679	0.407
contig034988-NyeORs.A033	contig047515-ZebOR.A019	0.276	0.679	0.407
contig034988-NyeORs.A033	contig030560-ZebOR.A007	0.276	0.681	0.405
contig073309-TilOR.A026	contig030553-ZebOR.A002	0.276	0.685	0.403
contig054233-BurOR.A012	contig054678-NyeOR.A010	0.276	0.695	0.397
contig054678-NyeOR.A010	contig047523-ZebOR.A022	0.276	0.695	0.397
contig084999-BriOR.A002	contig034988-NyeOR.A006	0.276	0.695	0.398
contig054868-NyeOR.A014	contig030553-ZebOR.A002	0.276	0.702	0.393
contig022204-TilOR.A001	contig041951-TilOR.A022	0.276	0.705	0.392
contig036780-BurOR.A001	contig070885-TilOR.A024	0.276	0.709	0.390
contig034983-NyeOR.A002	contig073309-TilOR.A026	0.276	0.713	0.387
contig073309-TilOR.A026	contig047515-ZebOR.A019	0.276	0.713	0.387
contig070885-TilOR.A024	contig030552-ZebOR.A001	0.276	0.720	0.384

contig085000-BriOR.A003	contig054684-NyeOR.A012	0.276	0.735	0.375
contig085000-BriOR.A003	contig057156-BurOR.A015	0.276	0.735	0.375
contig057156-BurOR.A015	contig047508-ZebOR.A015	0.276	0.753	0.366
contig051559-BurOR.A007	contig022211-TilOR.A003	0.276	0.756	0.364
contig085002-BriOR.A004	contig057754-NyeOR.A018	0.276	0.763	0.362
contig085026-BriOR.A008	contig051559-BurOR.A007	0.276	0.765	0.361
contig034983-NyeOR.A003	contig057165-NyeOR.A017	0.276	0.766	0.360
contig085000-BriOR.A003	contig022265-TilOR.A017	0.276	0.772	0.358
contig051570-BurOR.A010	contig057156-BurOR.A015	0.276	0.775	0.356
contig085000-BriOR.A003	contig034988-NyeORs.A033	0.276	0.780	0.353
contig034988-NyeOR.A005	contig054684-NyeOR.A012	0.276	0.800	0.344
contig022268-TilOR.A019	contig047508-ZebOR.A015	0.276	0.804	0.343
contig057153-BurOR.A014	contig030553-ZebOR.A003	0.276	0.834	0.331
contig034988-NyeOR.A005	contig022268-TilOR.A020	0.276	0.837	0.330
contig085018-BriOR.A006	contig054868-NyeOR.A014	0.276	0.861	0.320
contig036784-BurOR.A003	contig034981-NyeOR.A001	0.276	0.878	0.314
contig054868-NyeOR.A014	contig062095-ZebOR.A024	0.276	0.898	0.308
contig064570-BurOR.A017	contig054868-NyeOR.A014	0.276	0.910	0.304
contig084999-BriOR.A001	contig057754-NyeOR.A018	0.276	0.947	0.291
contig057165-NyeOR.A017	contig034988-NyeORs.A033	0.277	0.557	0.497
contig022266-TilOR.A018	contig030552-ZebOR.A001	0.277	0.567	0.488
contig057165-NyeOR.A017	contig022266-TilOR.A018	0.277	0.576	0.480
contig093816-BriOR.A011	contig057153-BurOR.A014	0.277	0.646	0.429
contig051573-BurOR.A011	contig064187-BurOR.A016	0.277	0.662	0.419
contig051573-BurOR.A011	contig030552-ZebOR.A001	0.277	0.662	0.419
contig051559-BurOR.A008	contig034988-NyeORs.A033	0.277	0.665	0.417
contig051559-BurOR.A007	contig057156-BurOR.A015	0.277	0.666	0.417
contig036784-BurOR.A003	contig036787-BurOR.A004	0.277	0.669	0.414
contig022227-TilOR.A006	contig047515-ZebOR.A019	0.277	0.669	0.414
contig034983-NyeOR.A002	contig022227-TilOR.A006	0.277	0.669	0.414
contig022266-TilOR.A018	contig047508-ZebOR.A015	0.277	0.670	0.413
contig054681-NyeOR.A011	contig073309-TilOR.A026	0.277	0.675	0.410
contig051559-BurOR.A007	contig022259-TilOR.A014	0.277	0.680	0.407
contig054684-NyeOR.A012	contig022204-TilOR.A001	0.277	0.680	0.407
contig034988-NyeORs.A033	contig022234-TilOR.A009	0.277	0.681	0.406
contig051573-BurOR.A011	contig022268-TilOR.A020	0.277	0.681	0.406
contig036784-BurOR.A003	contig030556-ZebOR.A005	0.277	0.681	0.406
contig051570-BurOR.A010	contig057165-NyeOR.A017	0.277	0.685	0.404
contig051559-BurOR.A007	contig030560-ZebOR.A007	0.277	0.686	0.404
contig051570-BurOR.A010	contig022266-TilOR.A018	0.277	0.690	0.401
contig085010-BriOR.A005	contig034983-NyeOR.A002	0.277	0.690	0.402
contig085010-BriOR.A005	contig047515-ZebOR.A019	0.277	0.690	0.402
contig057153-BurOR.A014	contig047508-ZebOR.A015	0.277	0.692	0.400

contig093807-BriOR.A009	contig041951-TilOR.A021	0.277	0.697	0.397
contig051559-BurOR.A007	contig054684-NyeOR.A012	0.277	0.697	0.398
contig062344-NyeOR.A020	contig034988-NyeORs.A033	0.277	0.698	0.397
contig093816-BriOR.A011	contig036787-BurOR.A004	0.277	0.708	0.391
contig051570-BurOR.A010	contig057153-BurOR.A014	0.277	0.713	0.388
contig034988-NyeORs.A033	contig030576-ZebOR.A010	0.277	0.720	0.385
contig034983-NyeOR.A002	contig047523-ZebOR.A022	0.277	0.725	0.382
contig047515-ZebOR.A019	contig047523-ZebOR.A022	0.277	0.725	0.382
contig054233-BurOR.A012	contig034983-NyeOR.A002	0.277	0.725	0.382
contig054233-BurOR.A012	contig047515-ZebOR.A019	0.277	0.725	0.382
contig041951-TilOR.A021	contig047497-ZebOR.A011	0.277	0.727	0.381
contig041951-TilOR.A022	contig030560-ZebOR.A007	0.277	0.730	0.379
contig093816-BriOR.A011	contig022265-TilOR.A017	0.277	0.741	0.374
contig022265-TilOR.A017	contig047508-ZebOR.A015	0.277	0.742	0.373
contig034990-NyeOR.A007	contig041951-TilOR.A021	0.277	0.748	0.371
contig054237-BurOR.A013	contig030553-ZebOR.A002	0.277	0.757	0.365
contig036787-BurOR.A004	contig034988-NyeOR.A006	0.277	0.758	0.365
contig022232-TilOR.A008	contig073309-TilOR.A026	0.277	0.758	0.365
contig051570-BurOR.A010	contig022265-TilOR.A017	0.277	0.764	0.362
contig036782-BurOR.A002	contig047521-ZebOR.A020	0.277	0.766	0.361
contig084999-BriOR.A001	contig047521-ZebOR.A020	0.277	0.777	0.356
contig036780-BurOR.A001	contig051559-BurOR.A008	0.277	0.779	0.356
contig034988-NyeOR.A005	contig030560-ZebOR.A007	0.277	0.782	0.354
contig064187-BurOR.A016	contig022225-TilOR.A005	0.277	0.783	0.354
contig051570-BurOR.A010	contig022268-TilOR.A019	0.277	0.785	0.353
contig051559-BurOR.A008	contig030552-ZebOR.A001	0.277	0.790	0.351
contig084999-BriOR.A002	contig022204-TilOR.A001	0.277	0.807	0.344
contig022259-TilOR.A015	contig022268-TilOR.A020	0.277	0.822	0.337
contig022268-TilOR.A019	contig070885-TilOR.A024	0.277	0.826	0.335
contig085000-BriOR.A003	contig057153-BurOR.A014	0.277	0.840	0.329
contig070885-TilOR.A024	contig073309-TilOR.A026	0.277	0.843	0.329
contig084999-BriOR.A001	contig070885-TilOR.A024	0.277	0.858	0.323
contig085000-BriOR.A003	contig047521-ZebOR.A020	0.277	0.862	0.322
contig054868-NyeOR.A014	contig056375-NyeOR.A015	0.277	0.865	0.321
contig057153-BurOR.A014	contig057754-NyeOR.A018	0.277	0.866	0.320
contig084999-BriOR.A001	contig034988-NyeOR.A005	0.277	0.869	0.319
contig084999-BriOR.A001	contig030553-ZebOR.A003	0.277	0.897	0.309
contig022211-TilOR.A003	contig030553-ZebOR.A003	0.277	0.955	0.290
contig084999-BriOR.A002	contig051573-BurOR.A011	0.278	0.624	0.446
contig036780-BurOR.A001	contig022204-TilOR.A001	0.278	0.628	0.442
contig057165-NyeOR.A017	contig022204-TilOR.A001	0.278	0.628	0.442
contig022259-TilOR.A014	contig030553-ZebOR.A002	0.278	0.650	0.428
contig057756-NyeOR.A019	contig041951-TilOR.A021	0.278	0.662	0.420

contig057165-NyeOR.A017	contig047508-ZebOR.A015	0.278	0.670	0.414
contig057156-BurOR.A015	contig034988-NyeORs.A033	0.278	0.671	0.414
contig036784-BurOR.A003	contig054678-NyeOR.A010	0.278	0.676	0.411
contig054684-NyeOR.A012	contig034988-NyeORs.A033	0.278	0.682	0.408
contig093816-BriOR.A011	contig022230-TilOR.A007	0.278	0.683	0.407
contig084999-BriOR.A001	contig047523-ZebOR.A022	0.278	0.684	0.406
contig084999-BriOR.A001	contig054233-BurOR.A012	0.278	0.684	0.406
contig085010-BriOR.A005	contig036782-BurOR.A002	0.278	0.690	0.403
contig093816-BriOR.A011	contig030554-ZebOR.A004	0.278	0.691	0.402
contig022232-TilOR.A008	contig030557-ZebOR.A006	0.278	0.695	0.399
contig085002-BriOR.A004	contig093816-BriOR.A011	0.278	0.701	0.397
contig057156-BurOR.A015	contig062095-ZebOR.A024	0.278	0.703	0.396
contig085002-BriOR.A004	contig047523-ZebOR.A022	0.278	0.708	0.393
contig085002-BriOR.A004	contig054233-BurOR.A012	0.278	0.708	0.393
contig057165-NyeOR.A017	contig070885-TilOR.A024	0.278	0.709	0.393
contig054684-NyeOR.A012	contig062095-ZebOR.A024	0.278	0.713	0.390
contig036782-BurOR.A002	contig073309-TilOR.A026	0.278	0.715	0.389
contig064187-BurOR.A016	contig034990-NyeOR.A007	0.278	0.719	0.386
contig022241-TilOR.A011	contig041951-TilOR.A021	0.278	0.720	0.386
contig093816-BriOR.A011	contig054678-NyeOR.A010	0.278	0.726	0.383
contig064187-BurOR.A016	contig047499-ZebOR.A012	0.278	0.729	0.381
contig085002-BriOR.A004	contig030553-ZebOR.A003	0.278	0.730	0.381
contig036782-BurOR.A002	contig054868-NyeOR.A014	0.278	0.732	0.380
contig074640-TilOR.A002	contig022230-TilOR.A007	0.278	0.733	0.380
contig085000-BriOR.A003	contig030560-ZebOR.A007	0.278	0.734	0.379
contig022268-TilOR.A020	contig062095-ZebOR.A024	0.278	0.744	0.374
contig057165-NyeOR.A017	contig047515-ZebOR.A018	0.278	0.756	0.367
contig070885-TilOR.A024	contig030553-ZebOR.A002	0.278	0.756	0.368
contig034988-NyeOR.A005	contig030553-ZebOR.A002	0.278	0.759	0.366
contig022230-TilOR.A007	contig030576-ZebOR.A010	0.278	0.761	0.365
contig054684-NyeOR.A012	contig047508-ZebOR.A015	0.278	0.765	0.363
contig084999-BriOR.A001	contig034981-NyeOR.A001	0.278	0.770	0.361
contig034988-NyeOR.A006	contig030556-ZebOR.A005	0.278	0.774	0.360
contig022268-TilOR.A020	contig041952-TilOR.A023	0.278	0.779	0.357
contig036782-BurOR.A002	contig034981-NyeOR.A001	0.278	0.782	0.355
contig051570-BurOR.A010	contig054684-NyeOR.A012	0.278	0.787	0.353
contig036784-BurOR.A003	contig022230-TilOR.A007	0.278	0.818	0.340
contig036784-BurOR.A003	contig030557-ZebOR.A006	0.278	0.846	0.329
contig054681-NyeOR.A011	contig030553-ZebOR.A003	0.278	0.848	0.328
contig085000-BriOR.A003	contig054681-NyeOR.A011	0.278	0.854	0.326
contig054237-BurOR.A013	contig030553-ZebOR.A003	0.278	0.855	0.325
contig057754-NyeOR.A018	contig047521-ZebOR.A020	0.278	0.863	0.323
contig036784-BurOR.A003	contig030553-ZebOR.A002	0.278	0.866	0.321

contig036784-BurOR.A003	contig054237-BurOR.A013	0.278	0.873	0.319
contig036784-BurOR.A003	contig022211-TilOR.A003	0.278	0.976	0.285
contig036780-BurOR.A001	contig022266-TilOR.A018	0.279	0.576	0.484
contig084999-BriOR.A002	contig047523-ZebOR.A022	0.279	0.635	0.439
contig084999-BriOR.A002	contig054233-BurOR.A012	0.279	0.635	0.439
contig085002-BriOR.A004	contig030552-ZebOR.A001	0.279	0.636	0.438
contig085002-BriOR.A004	contig036780-BurOR.A001	0.279	0.636	0.438
contig036782-BurOR.A002	contig057156-BurOR.A015	0.279	0.652	0.429
contig051573-BurOR.A011	contig047506-ZebOR.A014	0.279	0.653	0.427
contig054868-NyeOR.A014	contig030554-ZebOR.A004	0.279	0.654	0.426
contig022204-TilOR.A001	contig030560-ZebOR.A007	0.279	0.659	0.424
contig022266-TilOR.A018	contig047523-ZebOR.A022	0.279	0.660	0.422
contig054233-BurOR.A012	contig022266-TilOR.A018	0.279	0.660	0.422
contig085010-BriOR.A005	contig022204-TilOR.A001	0.279	0.674	0.413
contig036782-BurOR.A002	contig054684-NyeOR.A012	0.279	0.682	0.410
contig036787-BurOR.A004	contig051573-BurOR.A011	0.279	0.685	0.407
contig057156-BurOR.A015	contig022225-TilOR.A005	0.279	0.686	0.407
contig051559-BurOR.A007	contig051573-BurOR.A011	0.279	0.698	0.399
contig030553-ZebOR.A002	contig047523-ZebOR.A022	0.279	0.698	0.400
contig054233-BurOR.A012	contig030553-ZebOR.A002	0.279	0.698	0.400
contig022230-TilOR.A007	contig022241-TilOR.A011	0.279	0.699	0.399
contig036787-BurOR.A004	contig057754-NyeOR.A018	0.279	0.700	0.399
contig057754-NyeOR.A018	contig030556-ZebOR.A005	0.279	0.712	0.392
contig054684-NyeOR.A012	contig022225-TilOR.A005	0.279	0.719	0.388
contig022230-TilOR.A007	contig022259-TilOR.A015	0.279	0.722	0.387
contig084999-BriOR.A002	contig034988-NyeOR.A005	0.279	0.742	0.376
contig030560-ZebOR.A007	contig047508-ZebOR.A015	0.279	0.747	0.373
contig051570-BurOR.A010	contig030560-ZebOR.A007	0.279	0.769	0.363
contig022225-TilOR.A005	contig047506-ZebOR.A014	0.279	0.773	0.361
contig084999-BriOR.A002	contig022225-TilOR.A005	0.279	0.806	0.347
contig054868-NyeOR.A014	contig022241-TilOR.A011	0.279	0.837	0.334
contig085018-BriOR.A006	contig047523-ZebOR.A022	0.279	0.855	0.326
contig085018-BriOR.A006	contig054233-BurOR.A012	0.279	0.855	0.326
contig085000-BriOR.A003	contig034981-NyeOR.A001	0.279	0.881	0.316
contig054681-NyeOR.A011	contig057754-NyeOR.A018	0.279	0.881	0.317
contig034981-NyeOR.A001	contig057754-NyeOR.A018	0.279	0.881	0.317
contig051559-BurOR.A008	contig022268-TilOR.A020	0.279	0.883	0.316
contig047523-ZebOR.A022	contig062095-ZebOR.A024	0.279	0.892	0.313
contig054233-BurOR.A012	contig062095-ZebOR.A024	0.279	0.892	0.313
contig036782-BurOR.A002	contig057754-NyeOR.A018	0.279	0.914	0.305
contig084999-BriOR.A001	contig036784-BurOR.A003	0.279	0.921	0.303
contig022204-TilOR.A001	contig030553-ZebOR.A002	0.280	0.561	0.498
contig051573-BurOR.A011	contig034988-NyeOR.A006	0.280	0.647	0.432

contig051573-BurOR.A011	contig030554-ZebOR.A004	0.280	0.649	0.432
contig057153-BurOR.A014	contig073309-TilOR.A026	0.280	0.662	0.423
contig057156-BurOR.A015	contig034983-NyeOR.A002	0.280	0.664	0.421
contig057156-BurOR.A015	contig047515-ZebOR.A019	0.280	0.664	0.421
contig036780-BurOR.A001	contig051573-BurOR.A011	0.280	0.673	0.416
contig022265-TilOR.A017	contig047515-ZebOR.A019	0.280	0.679	0.412
contig034983-NyeOR.A002	contig022265-TilOR.A017	0.280	0.679	0.412
contig030560-ZebOR.A007	contig047515-ZebOR.A019	0.280	0.685	0.409
contig034983-NyeOR.A002	contig030560-ZebOR.A007	0.280	0.685	0.409
contig084999-BriOR.A001	contig030554-ZebOR.A004	0.280	0.689	0.407
contig034983-NyeOR.A002	contig054684-NyeOR.A012	0.280	0.696	0.402
contig054684-NyeOR.A012	contig047515-ZebOR.A019	0.280	0.696	0.402
contig054678-NyeOR.A010	contig057754-NyeOR.A018	0.280	0.696	0.402
contig030560-ZebOR.A007	contig062095-ZebOR.A024	0.280	0.697	0.401
contig085010-BriOR.A005	contig030553-ZebOR.A002	0.280	0.702	0.399
contig022225-TilOR.A005	contig030560-ZebOR.A007	0.280	0.702	0.399
contig051573-BurOR.A011	contig054678-NyeOR.A010	0.280	0.703	0.398
contig034988-NyeOR.A006	contig034990-NyeOR.A007	0.280	0.704	0.399
contig030557-ZebOR.A006	contig047515-ZebOR.A018	0.280	0.708	0.396
contig034990-NyeOR.A007	contig047506-ZebOR.A014	0.280	0.710	0.394
contig022232-TilOR.A008	contig022266-TilOR.A018	0.280	0.711	0.394
contig030553-ZebOR.A002	contig047515-ZebOR.A018	0.280	0.712	0.393
contig034988-NyeOR.A006	contig047499-ZebOR.A012	0.280	0.713	0.393
contig022266-TilOR.A018	contig047515-ZebOR.A018	0.280	0.717	0.390
contig047499-ZebOR.A012	contig047506-ZebOR.A014	0.280	0.720	0.388
contig085018-BriOR.A006	contig041951-TilOR.A021	0.280	0.743	0.377
contig034995-NyeOR.A009	contig041951-TilOR.A021	0.280	0.760	0.368
contig093812-BriOR.A010	contig034988-NyeORs.A033	0.280	0.763	0.368
contig051559-BurOR.A008	contig057165-NyeOR.A017	0.280	0.777	0.360
contig064570-BurOR.A017	contig022268-TilOR.A020	0.280	0.777	0.361
contig085026-BriOR.A008	contig030553-ZebOR.A002	0.280	0.783	0.358
contig034988-NyeOR.A006	contig022225-TilOR.A005	0.280	0.784	0.358
contig022234-TilOR.A009	contig022268-TilOR.A020	0.280	0.817	0.342
contig022268-TilOR.A020	contig070885-TilOR.A024	0.280	0.827	0.339
contig022268-TilOR.A020	contig062094-ZebOR.A023	0.280	0.837	0.335
contig054868-NyeOR.A014	contig056380-NyeOR.A016	0.280	0.859	0.326
contig085000-BriOR.A003	contig030553-ZebOR.A002	0.280	0.862	0.325
contig054868-NyeOR.A014	contig022217-TilOR.A004	0.280	0.866	0.324
contig036782-BurOR.A002	contig030553-ZebOR.A003	0.280	0.880	0.318
contig054233-BurOR.A012	contig064570-BurOR.A017	0.280	0.904	0.309
contig064570-BurOR.A017	contig047523-ZebOR.A022	0.280	0.904	0.309
contig085002-BriOR.A004	contig057165-NyeOR.A017	0.281	0.636	0.441
contig054868-NyeOR.A014	contig022268-TilOR.A019	0.281	0.636	0.441

contig036782-BurOR.A002	contig034988-NyeORs.A033	0.281	0.646	0.435
contig036780-BurOR.A001	contig036787-BurOR.A004	0.281	0.655	0.429
contig036787-BurOR.A004	contig030552-ZebOR.A001	0.281	0.655	0.429
contig022227-TilOR.A006	contig022268-TilOR.A020	0.281	0.657	0.427
contig057156-BurOR.A015	contig022204-TilOR.A001	0.281	0.662	0.424
contig057156-BurOR.A015	contig030553-ZebOR.A002	0.281	0.663	0.424
contig022259-TilOR.A014	contig041951-TilOR.A021	0.281	0.666	0.421
contig036782-BurOR.A002	contig030560-ZebOR.A007	0.281	0.666	0.421
contig051559-BurOR.A007	contig022227-TilOR.A006	0.281	0.672	0.418
contig085002-BriOR.A004	contig051573-BurOR.A011	0.281	0.677	0.415
contig093816-BriOR.A011	contig022268-TilOR.A019	0.281	0.678	0.415
contig093816-BriOR.A011	contig057165-NyeOR.A017	0.281	0.680	0.413
contig030553-ZebOR.A003	contig030556-ZebOR.A005	0.281	0.681	0.413
contig057153-BurOR.A014	contig022232-TilOR.A008	0.281	0.691	0.406
contig054684-NyeOR.A012	contig030553-ZebOR.A002	0.281	0.694	0.406
contig084999-BriOR.A001	contig041952-TilOR.A023	0.281	0.696	0.404
contig054681-NyeOR.A011	contig022232-TilOR.A008	0.281	0.699	0.402
contig051559-BurOR.A007	contig034990-NyeOR.A007	0.281	0.707	0.397
contig022259-TilOR.A014	contig030557-ZebOR.A006	0.281	0.707	0.397
contig022217-TilOR.A004	contig022230-TilOR.A007	0.281	0.718	0.392
contig022225-TilOR.A005	contig041951-TilOR.A022	0.281	0.725	0.388
contig084999-BriOR.A002	contig051570-BurOR.A010	0.281	0.730	0.385
contig062344-NyeOR.A020	contig022230-TilOR.A007	0.281	0.733	0.383
contig022217-TilOR.A004	contig041951-TilOR.A021	0.281	0.735	0.382
contig051559-BurOR.A007	contig047499-ZebOR.A012	0.281	0.740	0.379
contig084999-BriOR.A002	contig047508-ZebOR.A015	0.281	0.741	0.379
contig084999-BriOR.A002	contig070885-TilOR.A024	0.281	0.744	0.377
contig022266-TilOR.A018	contig047521-ZebOR.A020	0.281	0.757	0.372
contig093812-BriOR.A010	contig064187-BurOR.A016	0.281	0.771	0.364
contig022265-TilOR.A017	contig030553-ZebOR.A003	0.281	0.772	0.364
contig057756-NyeOR.A019	contig047521-ZebOR.A020	0.281	0.776	0.363
contig062344-NyeOR.A020	contig022268-TilOR.A019	0.281	0.777	0.362
contig064187-BurOR.A016	contig047521-ZebOR.A020	0.281	0.781	0.360
contig062344-NyeOR.A020	contig022268-TilOR.A020	0.281	0.783	0.359
contig022268-TilOR.A020	contig030576-ZebOR.A010	0.281	0.784	0.359
contig036782-BurOR.A002	contig054237-BurOR.A013	0.281	0.790	0.356
contig084999-BriOR.A001	contig054237-BurOR.A013	0.281	0.802	0.350
contig022266-TilOR.A018	contig030553-ZebOR.A003	0.281	0.804	0.349
contig022217-TilOR.A004	contig022268-TilOR.A020	0.281	0.804	0.350
contig085000-BriOR.A003	contig022266-TilOR.A018	0.281	0.810	0.347
contig054868-NyeOR.A014	contig062094-ZebOR.A023	0.281	0.851	0.330
contig022264-TilOR.A016	contig041951-TilOR.A022	0.281	0.877	0.320
contig084999-BriOR.A001	contig085000-BriOR.A003	0.281	0.917	0.307

contig084999-BriOR.A001	contig051573-BurOR.A011	0.282	0.636	0.444
contig051573-BurOR.A011	contig022266-TilOR.A018	0.282	0.648	0.435
contig030554-ZebOR.A004	contig047523-ZebOR.A022	0.282	0.649	0.434
contig054233-BurOR.A012	contig030554-ZebOR.A004	0.282	0.649	0.434
contig036787-BurOR.A004	contig030553-ZebOR.A003	0.282	0.669	0.421
contig084999-BriOR.A001	contig022268-TilOR.A019	0.282	0.675	0.418
contig054678-NyeOR.A010	contig030553-ZebOR.A003	0.282	0.676	0.418
contig057756-NyeOR.A019	contig073309-TilOR.A026	0.282	0.685	0.412
contig022266-TilOR.A018	contig073309-TilOR.A026	0.282	0.691	0.408
contig085010-BriOR.A005	contig041951-TilOR.A021	0.282	0.692	0.407
contig022268-TilOR.A020	contig047514-ZebOR.A017	0.282	0.694	0.406
contig022245-TilOR.A012	contig041951-TilOR.A021	0.282	0.695	0.406
contig034988-NyeORs.A033	contig022241-TilOR.A011	0.282	0.695	0.406
contig054868-NyeOR.A014	contig057756-NyeOR.A019	0.282	0.702	0.402
contig036780-BurOR.A001	contig022259-TilOR.A014	0.282	0.702	0.402
contig085010-BriOR.A005	contig022225-TilOR.A005	0.282	0.707	0.398
contig085010-BriOR.A005	contig062095-ZebOR.A024	0.282	0.708	0.399
contig054681-NyeOR.A011	contig022259-TilOR.A014	0.282	0.711	0.397
contig022259-TilOR.A014	contig030552-ZebOR.A001	0.282	0.712	0.396
contig036780-BurOR.A001	contig056375-NyeOR.A015	0.282	0.713	0.395
contig022259-TilOR.A014	contig022266-TilOR.A018	0.282	0.716	0.394
contig034983-NyeOR.A003	contig030557-ZebOR.A006	0.282	0.718	0.393
contig022265-TilOR.A017	contig062095-ZebOR.A024	0.282	0.722	0.391
contig034983-NyeOR.A003	contig030553-ZebOR.A002	0.282	0.722	0.391
contig056375-NyeOR.A015	contig030552-ZebOR.A001	0.282	0.724	0.389
contig034983-NyeOR.A003	contig022266-TilOR.A018	0.282	0.727	0.388
contig036782-BurOR.A002	contig047523-ZebOR.A022	0.282	0.728	0.387
contig036782-BurOR.A002	contig054233-BurOR.A012	0.282	0.728	0.387
contig085000-BriOR.A003	contig085010-BriOR.A005	0.282	0.740	0.381
contig084999-BriOR.A002	contig062344-NyeOR.A020	0.282	0.747	0.378
contig034988-NyeORs.A033	contig047497-ZebOR.A011	0.282	0.755	0.374
contig093812-BriOR.A010	contig051559-BurOR.A007	0.282	0.759	0.372
contig054237-BurOR.A013	contig022266-TilOR.A018	0.282	0.763	0.370
contig034981-NyeOR.A001	contig022266-TilOR.A018	0.282	0.772	0.366
contig036784-BurOR.A003	contig022265-TilOR.A017	0.282	0.786	0.359
contig022268-TilOR.A020	contig047503-ZebOR.A013	0.282	0.790	0.357
contig036782-BurOR.A002	contig034988-NyeOR.A005	0.282	0.792	0.356
contig034981-NyeOR.A001	contig057756-NyeOR.A019	0.282	0.792	0.357
contig054868-NyeOR.A014	contig022245-TilOR.A012	0.282	0.802	0.352
contig051570-BurOR.A010	contig022268-TilOR.A020	0.282	0.811	0.348
contig057754-NyeOR.A018	contig022266-TilOR.A018	0.282	0.822	0.343
contig022241-TilOR.A011	contig047523-ZebOR.A022	0.282	0.831	0.340
contig054233-BurOR.A012	contig022241-TilOR.A011	0.282	0.831	0.340

contig036784-BurOR.A003	contig057153-BurOR.A014	0.282	0.837	0.337
contig085026-BriOR.A008	contig030553-ZebOR.A003	0.282	0.838	0.337
contig051559-BurOR.A007	contig030553-ZebOR.A003	0.282	0.853	0.331
contig022217-TilOR.A004	contig047523-ZebOR.A022	0.282	0.854	0.331
contig054233-BurOR.A012	contig022217-TilOR.A004	0.282	0.854	0.331
contig085000-BriOR.A003	contig054237-BurOR.A013	0.282	0.877	0.321
contig054237-BurOR.A013	contig057754-NyeOR.A018	0.282	0.877	0.322
contig036782-BurOR.A002	contig036784-BurOR.A003	0.282	0.890	0.317
contig054868-NyeOR.A014	contig022259-TilOR.A015	0.282	0.918	0.307
contig085000-BriOR.A003	contig022211-TilOR.A003	0.282	0.971	0.290
contig057754-NyeOR.A018	contig022211-TilOR.A003	0.282	0.980	0.287
contig022268-TilOR.A019	contig030552-ZebOR.A001	0.283	0.562	0.504
contig036780-BurOR.A001	contig022268-TilOR.A019	0.283	0.562	0.504
contig084999-BriOR.A001	contig022204-TilOR.A001	0.283	0.591	0.479
contig036782-BurOR.A002	contig022204-TilOR.A001	0.283	0.591	0.479
contig030552-ZebOR.A001	contig030556-ZebOR.A005	0.283	0.637	0.444
contig036780-BurOR.A001	contig030556-ZebOR.A005	0.283	0.637	0.444
contig022204-TilOR.A001	contig022265-TilOR.A017	0.283	0.654	0.433
contig036787-BurOR.A004	contig057165-NyeOR.A017	0.283	0.655	0.432
contig036782-BurOR.A002	contig022259-TilOR.A014	0.283	0.669	0.423
contig093816-BriOR.A011	contig030552-ZebOR.A001	0.283	0.670	0.422
contig030553-ZebOR.A002	contig030560-ZebOR.A007	0.283	0.678	0.417
contig073309-TilOR.A026	contig030552-ZebOR.A001	0.283	0.679	0.417
contig022266-TilOR.A018	contig047503-ZebOR.A013	0.283	0.680	0.417
contig041952-TilOR.A023	contig030553-ZebOR.A002	0.283	0.681	0.415
contig041951-TilOR.A021	contig062095-ZebOR.A024	0.283	0.686	0.413
contig093816-BriOR.A011	contig064187-BurOR.A016	0.283	0.687	0.412
contig084999-BriOR.A001	contig022259-TilOR.A014	0.283	0.689	0.410
contig057165-NyeOR.A017	contig073309-TilOR.A026	0.283	0.689	0.411
contig093812-BriOR.A010	contig041951-TilOR.A022	0.283	0.703	0.403
contig085002-BriOR.A004	contig034988-NyeOR.A006	0.283	0.704	0.402
contig057153-BurOR.A014	contig047515-ZebOR.A018	0.283	0.704	0.402
contig036780-BurOR.A001	contig062344-NyeOR.A020	0.283	0.707	0.400
contig057156-BurOR.A015	contig070886-TilOR.A025	0.283	0.711	0.398
contig054681-NyeOR.A011	contig047515-ZebOR.A018	0.283	0.712	0.398
contig022234-TilOR.A009	contig022266-TilOR.A018	0.283	0.713	0.397
contig062344-NyeOR.A020	contig030552-ZebOR.A001	0.283	0.716	0.395
contig085010-BriOR.A005	contig034988-NyeOR.A006	0.283	0.723	0.392
contig054684-NyeOR.A012	contig070886-TilOR.A025	0.283	0.733	0.386
contig084999-BriOR.A001	contig030556-ZebOR.A005	0.283	0.743	0.380
contig085000-BriOR.A003	contig022268-TilOR.A019	0.283	0.750	0.377
contig022204-TilOR.A001	contig030556-ZebOR.A005	0.283	0.750	0.377
contig093812-BriOR.A010	contig047506-ZebOR.A014	0.283	0.761	0.372

contig034988-NyeORs.A033	contig047499-ZebOR.A012	0.283	0.769	0.368
contig047506-ZebOR.A014	contig047521-ZebOR.A020	0.283	0.771	0.368
contig064187-BurOR.A016	contig034981-NyeOR.A001	0.283	0.773	0.365
contig084999-BriOR.A001	contig047515-ZebOR.A018	0.283	0.783	0.362
contig036782-BurOR.A002	contig070885-TilOR.A024	0.283	0.788	0.359
contig064187-BurOR.A016	contig022211-TilOR.A003	0.283	0.794	0.357
contig022268-TilOR.A020	contig047508-ZebOR.A015	0.283	0.794	0.357
contig034988-NyeOR.A005	contig057756-NyeOR.A019	0.283	0.799	0.354
contig085010-BriOR.A005	contig022232-TilOR.A008	0.283	0.804	0.352
contig030557-ZebOR.A006	contig047521-ZebOR.A020	0.283	0.808	0.351
contig022232-TilOR.A008	contig022268-TilOR.A019	0.283	0.820	0.345
contig051570-BurOR.A010	contig073309-TilOR.A026	0.283	0.835	0.339
contig062344-NyeOR.A020	contig030553-ZebOR.A003	0.283	0.841	0.337
contig084999-BriOR.A001	contig051570-BurOR.A010	0.283	0.848	0.333
contig054233-BurOR.A012	contig056380-NyeOR.A016	0.283	0.853	0.332
contig056380-NyeOR.A016	contig047523-ZebOR.A022	0.283	0.853	0.332
contig085000-BriOR.A003	contig051559-BurOR.A007	0.283	0.856	0.330
contig085026-BriOR.A008	contig036784-BurOR.A003	0.283	0.856	0.331
contig054233-BurOR.A012	contig056375-NyeOR.A015	0.283	0.859	0.329
contig056375-NyeOR.A015	contig047523-ZebOR.A022	0.283	0.859	0.329
contig051559-BurOR.A007	contig057754-NyeOR.A018	0.283	0.873	0.324
contig064570-BurOR.A017	contig073309-TilOR.A026	0.283	0.885	0.320
contig073309-TilOR.A026	contig062095-ZebOR.A024	0.283	0.900	0.314
contig022268-TilOR.A019	contig047523-ZebOR.A022	0.284	0.632	0.449
contig054233-BurOR.A012	contig022268-TilOR.A019	0.284	0.632	0.449
contig084999-BriOR.A001	contig057165-NyeOR.A017	0.284	0.643	0.442
contig051573-BurOR.A011	contig022268-TilOR.A019	0.284	0.646	0.439
contig022230-TilOR.A007	contig041952-TilOR.A023	0.284	0.650	0.436
contig084999-BriOR.A001	contig030552-ZebOR.A001	0.284	0.653	0.435
contig057756-NyeOR.A019	contig034988-NyeORs.A033	0.284	0.659	0.431
contig036780-BurOR.A001	contig054678-NyeOR.A010	0.284	0.662	0.429
contig054678-NyeOR.A010	contig030552-ZebOR.A001	0.284	0.662	0.429
contig057756-NyeOR.A019	contig022259-TilOR.A014	0.284	0.672	0.423
contig084999-BriOR.A001	contig047514-ZebOR.A017	0.284	0.684	0.415
contig041951-TilOR.A022	contig047499-ZebOR.A012	0.284	0.685	0.415
contig084999-BriOR.A002	contig085000-BriOR.A003	0.284	0.687	0.414
contig051566-BurOR.A009	contig034988-NyeORs.A033	0.284	0.700	0.406
contig022230-TilOR.A007	contig022245-TilOR.A012	0.284	0.701	0.406
contig057153-BurOR.A014	contig022259-TilOR.A014	0.284	0.702	0.404
contig030557-ZebOR.A006	contig047503-ZebOR.A013	0.284	0.702	0.404
contig057165-NyeOR.A017	contig022259-TilOR.A014	0.284	0.702	0.405
contig034988-NyeORs.A033	contig047526-ZebOR.A021	0.284	0.703	0.404
contig051573-BurOR.A011	contig034983-NyeOR.A002	0.284	0.706	0.402

contig051573-BurOR.A011	contig047515-ZebOR.A019	0.284	0.706	0.402
contig056375-NyeOR.A015	contig057165-NyeOR.A017	0.284	0.713	0.398
contig034988-NyeOR.A005	contig030554-ZebOR.A004	0.284	0.715	0.397
contig034988-NyeORs.A033	contig022238-TilOR.A010	0.284	0.735	0.386
contig051570-BurOR.A010	contig030553-ZebOR.A002	0.284	0.735	0.386
contig054687-NyeOR.A013	contig022230-TilOR.A007	0.284	0.740	0.383
contig085018-BriOR.A006	contig036780-BurOR.A001	0.284	0.745	0.382
contig034995-NyeOR.A009	contig022230-TilOR.A007	0.284	0.746	0.381
contig034988-NyeOR.A006	contig022265-TilOR.A017	0.284	0.747	0.380
contig093812-BriOR.A010	contig034988-NyeOR.A006	0.284	0.754	0.376
contig085018-BriOR.A006	contig030552-ZebOR.A001	0.284	0.756	0.376
contig022268-TilOR.A019	contig030576-ZebOR.A010	0.284	0.775	0.367
contig057754-NyeOR.A018	contig022265-TilOR.A017	0.284	0.777	0.366
contig034990-NyeOR.A007	contig034988-NyeORs.A033	0.284	0.788	0.361
contig054678-NyeOR.A010	contig022204-TilOR.A001	0.284	0.791	0.359
contig057756-NyeOR.A019	contig070885-TilOR.A024	0.284	0.795	0.357
contig022211-TilOR.A003	contig030553-ZebOR.A002	0.284	0.807	0.352
contig084999-BriOR.A001	contig047508-ZebOR.A015	0.284	0.817	0.347
contig073309-TilOR.A026	contig047508-ZebOR.A015	0.284	0.817	0.347
contig084999-BriOR.A001	contig085026-BriOR.A008	0.284	0.829	0.343
contig022217-TilOR.A004	contig030553-ZebOR.A003	0.284	0.833	0.341
contig085000-BriOR.A003	contig070885-TilOR.A024	0.284	0.836	0.339
contig051321-BurOR.A006	contig022268-TilOR.A020	0.284	0.837	0.340
contig034988-NyeOR.A005	contig073309-TilOR.A026	0.284	0.843	0.337
contig036784-BurOR.A003	contig054681-NyeOR.A011	0.284	0.851	0.333
contig030553-ZebOR.A003	contig030576-ZebOR.A010	0.284	0.862	0.330
contig085010-BriOR.A005	contig062344-NyeOR.A020	0.284	0.868	0.327
contig056380-NyeOR.A016	contig022268-TilOR.A020	0.284	0.874	0.325
contig057754-NyeOR.A018	contig057756-NyeOR.A019	0.284	0.879	0.323
contig085000-BriOR.A003	contig036782-BurOR.A002	0.284	0.886	0.321
contig064187-BurOR.A016	contig022264-TilOR.A016	0.284	0.889	0.319
contig057165-NyeOR.A017	contig022268-TilOR.A019	0.285	0.562	0.508
contig093816-BriOR.A011	contig022266-TilOR.A018	0.285	0.629	0.452
contig057165-NyeOR.A017	contig030556-ZebOR.A005	0.285	0.637	0.448
contig084999-BriOR.A001	contig084999-BriOR.A002	0.285	0.648	0.440
contig034990-NyeOR.A007	contig041951-TilOR.A022	0.285	0.655	0.434
contig051573-BurOR.A011	contig041951-TilOR.A022	0.285	0.656	0.434
contig030553-ZebOR.A002	contig047514-ZebOR.A017	0.285	0.672	0.424
contig093816-BriOR.A011	contig047506-ZebOR.A014	0.285	0.678	0.420
contig093816-BriOR.A011	contig036780-BurOR.A001	0.285	0.680	0.419
contig036780-BurOR.A001	contig073309-TilOR.A026	0.285	0.689	0.414
contig022225-TilOR.A005	contig022265-TilOR.A017	0.285	0.692	0.412
contig054681-NyeOR.A011	contig047503-ZebOR.A013	0.285	0.695	0.410

contig093816-BriOR.A011	contig022268-TilOR.A020	0.285	0.695	0.410
contig054233-BurOR.A012	contig057756-NyeOR.A019	0.285	0.698	0.409
contig057756-NyeOR.A019	contig047523-ZebOR.A022	0.285	0.698	0.409
contig093816-BriOR.A011	contig051559-BurOR.A007	0.285	0.703	0.405
contig057165-NyeOR.A017	contig062344-NyeOR.A020	0.285	0.705	0.405
contig030553-ZebOR.A002	contig047508-ZebOR.A015	0.285	0.709	0.402
contig084999-BriOR.A001	contig022265-TilOR.A017	0.285	0.709	0.403
contig036782-BurOR.A002	contig041952-TilOR.A023	0.285	0.711	0.401
contig070886-TilOR.A025	contig030560-ZebOR.A007	0.285	0.711	0.402
contig022234-TilOR.A009	contig030553-ZebOR.A002	0.285	0.713	0.400
contig057153-BurOR.A014	contig034983-NyeOR.A003	0.285	0.713	0.400
contig070885-TilOR.A024	contig030554-ZebOR.A004	0.285	0.716	0.398
contig036780-BurOR.A001	contig030576-ZebOR.A010	0.285	0.717	0.397
contig054684-NyeOR.A012	contig030553-ZebOR.A003	0.285	0.723	0.394
contig057156-BurOR.A015	contig030553-ZebOR.A003	0.285	0.723	0.394
contig030557-ZebOR.A006	contig062095-ZebOR.A024	0.285	0.725	0.393
contig030576-ZebOR.A010	contig047515-ZebOR.A019	0.285	0.725	0.394
contig034983-NyeOR.A002	contig030576-ZebOR.A010	0.285	0.725	0.394
contig030552-ZebOR.A001	contig030576-ZebOR.A010	0.285	0.728	0.391
contig084999-BriOR.A001	contig036787-BurOR.A004	0.285	0.730	0.390
contig022259-TilOR.A015	contig047515-ZebOR.A019	0.285	0.732	0.389
contig034983-NyeOR.A002	contig022259-TilOR.A015	0.285	0.732	0.389
contig057156-BurOR.A015	contig064570-BurOR.A017	0.285	0.734	0.388
contig062344-NyeOR.A020	contig030554-ZebOR.A004	0.285	0.742	0.384
contig036782-BurOR.A002	contig047515-ZebOR.A018	0.285	0.743	0.383
contig064570-BurOR.A017	contig054684-NyeOR.A012	0.285	0.745	0.383
contig084999-BriOR.A002	contig030576-ZebOR.A010	0.285	0.746	0.383
contig022232-TilOR.A008	contig041952-TilOR.A023	0.285	0.748	0.381
contig034981-NyeOR.A001	contig047506-ZebOR.A014	0.285	0.763	0.373
contig022268-TilOR.A020	contig070886-TilOR.A025	0.285	0.766	0.372
contig036787-BurOR.A004	contig022204-TilOR.A001	0.285	0.771	0.370
contig034995-NyeOR.A009	contig034988-NyeORs.A033	0.285	0.771	0.370
contig022211-TilOR.A003	contig047506-ZebOR.A014	0.285	0.784	0.364
contig054237-BurOR.A013	contig057756-NyeOR.A019	0.285	0.800	0.357
contig073309-TilOR.A026	contig047515-ZebOR.A018	0.285	0.801	0.356
contig054681-NyeOR.A011	contig047521-ZebOR.A020	0.285	0.813	0.350
contig022232-TilOR.A008	contig030560-ZebOR.A007	0.285	0.815	0.349
contig085026-BriOR.A008	contig036782-BurOR.A002	0.285	0.817	0.348
contig034981-NyeOR.A001	contig030557-ZebOR.A006	0.285	0.825	0.345
contig062344-NyeOR.A020	contig022265-TilOR.A017	0.285	0.840	0.339
contig047523-ZebOR.A022	contig062094-ZebOR.A023	0.285	0.846	0.336
contig054233-BurOR.A012	contig062094-ZebOR.A023	0.285	0.846	0.336
contig057756-NyeOR.A019	contig030553-ZebOR.A003	0.285	0.847	0.337

contig036784-BurOR.A003	contig022217-TilOR.A004	0.285	0.851	0.335
contig036784-BurOR.A003	contig062344-NyeOR.A020	0.285	0.857	0.332
contig064187-BurOR.A016	contig054687-NyeOR.A013	0.285	0.870	0.327
contig036784-BurOR.A003	contig030576-ZebOR.A010	0.285	0.878	0.325
contig022264-TilOR.A016	contig047506-ZebOR.A014	0.285	0.884	0.322
contig054684-NyeOR.A012	contig062344-NyeOR.A020	0.285	0.888	0.321
contig022259-TilOR.A015	contig047523-ZebOR.A022	0.285	0.912	0.313
contig054233-BurOR.A012	contig022259-TilOR.A015	0.285	0.912	0.313
contig036782-BurOR.A002	contig057165-NyeOR.A017	0.286	0.614	0.466
contig036782-BurOR.A002	contig030552-ZebOR.A001	0.286	0.624	0.459
contig051573-BurOR.A011	contig030553-ZebOR.A002	0.286	0.640	0.446
contig084999-BriOR.A001	contig036780-BurOR.A001	0.286	0.643	0.445
contig054678-NyeOR.A010	contig057165-NyeOR.A017	0.286	0.662	0.432
contig093816-BriOR.A011	contig034988-NyeOR.A006	0.286	0.673	0.425
contig085010-BriOR.A005	contig034988-NyeORs.A033	0.286	0.686	0.417
contig022266-TilOR.A018	contig062095-ZebOR.A024	0.286	0.688	0.415
contig030557-ZebOR.A006	contig047514-ZebOR.A017	0.286	0.692	0.412
contig057156-BurOR.A015	contig034988-NyeOR.A006	0.286	0.697	0.410
contig034988-NyeORs.A033	contig022227-TilOR.A006	0.286	0.697	0.411
contig022238-TilOR.A010	contig030557-ZebOR.A006	0.286	0.699	0.409
contig051570-BurOR.A010	contig030554-ZebOR.A004	0.286	0.703	0.406
contig022234-TilOR.A009	contig030557-ZebOR.A006	0.286	0.703	0.406
contig034988-NyeOR.A004	contig034988-NyeORs.A033	0.286	0.704	0.406
contig022238-TilOR.A010	contig022268-TilOR.A020	0.286	0.706	0.405
contig054681-NyeOR.A011	contig062095-ZebOR.A024	0.286	0.708	0.404
contig064187-BurOR.A016	contig022259-TilOR.A014	0.286	0.711	0.402
contig034988-NyeORs.A033	contig047514-ZebOR.A017	0.286	0.714	0.401
contig034983-NyeOR.A002	contig034990-NyeOR.A007	0.286	0.715	0.400
contig034990-NyeOR.A007	contig047515-ZebOR.A019	0.286	0.715	0.400
contig030554-ZebOR.A004	contig047508-ZebOR.A015	0.286	0.715	0.400
contig051559-BurOR.A008	contig022266-TilOR.A018	0.286	0.715	0.400
contig034983-NyeOR.A003	contig054681-NyeOR.A011	0.286	0.722	0.396
contig064570-BurOR.A017	contig030560-ZebOR.A007	0.286	0.728	0.393
contig065887-BurOR.A018	contig022230-TilOR.A007	0.286	0.729	0.392
contig034988-NyeOR.A006	contig054684-NyeOR.A012	0.286	0.729	0.392
contig070886-TilOR.A025	contig030557-ZebOR.A006	0.286	0.732	0.391
contig022230-TilOR.A007	contig030566-ZebOR.A008	0.286	0.739	0.387
contig064570-BurOR.A017	contig022265-TilOR.A017	0.286	0.743	0.386
contig085018-BriOR.A006	contig057165-NyeOR.A017	0.286	0.745	0.384
contig034983-NyeOR.A002	contig047499-ZebOR.A012	0.286	0.748	0.382
contig047499-ZebOR.A012	contig047515-ZebOR.A019	0.286	0.748	0.382
contig056375-NyeOR.A015	contig022268-TilOR.A020	0.286	0.750	0.381
contig054684-NyeOR.A012	contig073309-TilOR.A026	0.286	0.750	0.382

contig064187-BurOR.A016	contig022227-TilOR.A006	0.286	0.756	0.378
contig054684-NyeOR.A012	contig057754-NyeOR.A018	0.286	0.762	0.375
contig057156-BurOR.A015	contig057754-NyeOR.A018	0.286	0.762	0.375
contig022241-TilOR.A011	contig022268-TilOR.A020	0.286	0.781	0.365
contig084999-BriOR.A001	contig034983-NyeOR.A003	0.286	0.794	0.360
contig036784-BurOR.A003	contig022266-TilOR.A018	0.286	0.795	0.360
contig057156-BurOR.A015	contig022232-TilOR.A008	0.286	0.810	0.353
contig085026-BriOR.A008	contig064187-BurOR.A016	0.286	0.816	0.350
contig084999-BriOR.A002	contig022232-TilOR.A008	0.286	0.817	0.350
contig054237-BurOR.A013	contig064187-BurOR.A016	0.286	0.819	0.349
contig034981-NyeOR.A001	contig054681-NyeOR.A011	0.286	0.830	0.344
contig073309-TilOR.A026	contig047503-ZebOR.A013	0.286	0.831	0.345
contig054684-NyeOR.A012	contig022232-TilOR.A008	0.286	0.835	0.342
contig030553-ZebOR.A003	contig047515-ZebOR.A019	0.286	0.843	0.340
contig034983-NyeOR.A002	contig030553-ZebOR.A003	0.286	0.843	0.340
contig085000-BriOR.A003	contig085026-BriOR.A008	0.286	0.859	0.333
contig054687-NyeOR.A013	contig047506-ZebOR.A014	0.286	0.866	0.330
contig062344-NyeOR.A020	contig030560-ZebOR.A007	0.286	0.867	0.330
contig056375-NyeOR.A015	contig073309-TilOR.A026	0.286	0.867	0.330
contig051321-BurOR.A006	contig054868-NyeOR.A014	0.286	0.887	0.323
contig093816-BriOR.A011	contig041951-TilOR.A022	0.287	0.640	0.447
contig057156-BurOR.A015	contig056375-NyeOR.A015	0.287	0.663	0.432
contig085000-BriOR.A003	contig030554-ZebOR.A004	0.287	0.663	0.433
contig054684-NyeOR.A012	contig056375-NyeOR.A015	0.287	0.673	0.426
contig054681-NyeOR.A011	contig047514-ZebOR.A017	0.287	0.676	0.424
contig054681-NyeOR.A011	contig022238-TilOR.A010	0.287	0.682	0.421
contig036780-BurOR.A001	contig047503-ZebOR.A013	0.287	0.684	0.420
contig030552-ZebOR.A001	contig047503-ZebOR.A013	0.287	0.695	0.414
contig057153-BurOR.A014	contig047503-ZebOR.A013	0.287	0.697	0.411
contig054681-NyeOR.A011	contig022234-TilOR.A009	0.287	0.697	0.412
contig022232-TilOR.A008	contig030553-ZebOR.A002	0.287	0.701	0.409
contig036782-BurOR.A002	contig047514-ZebOR.A017	0.287	0.701	0.409
contig022225-TilOR.A005	contig041952-TilOR.A023	0.287	0.705	0.407
contig064187-BurOR.A016	contig041952-TilOR.A023	0.287	0.705	0.408
contig022204-TilOR.A001	contig041952-TilOR.A023	0.287	0.708	0.405
contig054681-NyeOR.A011	contig070886-TilOR.A025	0.287	0.715	0.402
contig057165-NyeOR.A017	contig030576-ZebOR.A010	0.287	0.717	0.400
contig085000-BriOR.A003	contig022225-TilOR.A005	0.287	0.718	0.401
contig030553-ZebOR.A003	contig030560-ZebOR.A007	0.287	0.723	0.398
contig057156-BurOR.A015	contig041952-TilOR.A023	0.287	0.725	0.396
contig051559-BurOR.A008	contig030557-ZebOR.A006	0.287	0.727	0.394
contig051559-BurOR.A007	contig030576-ZebOR.A010	0.287	0.728	0.394
contig051559-BurOR.A007	contig022259-TilOR.A015	0.287	0.729	0.394

contig051559-BurOR.A008	contig030553-ZebOR.A002	0.287	0.732	0.392
contig084999-BriOR.A001	contig070886-TilOR.A025	0.287	0.734	0.391
contig036784-BurOR.A003	contig054684-NyeOR.A012	0.287	0.737	0.389
contig036784-BurOR.A003	contig057156-BurOR.A015	0.287	0.737	0.389
contig085010-BriOR.A005	contig064570-BurOR.A017	0.287	0.739	0.388
contig036784-BurOR.A003	contig022268-TilOR.A019	0.287	0.741	0.387
contig054684-NyeOR.A012	contig041952-TilOR.A023	0.287	0.747	0.385
contig022265-TilOR.A017	contig070886-TilOR.A025	0.287	0.751	0.382
contig036782-BurOR.A002	contig034983-NyeOR.A003	0.287	0.753	0.381
contig084999-BriOR.A001	contig022234-TilOR.A009	0.287	0.757	0.379
contig022204-TilOR.A001	contig030554-ZebOR.A004	0.287	0.763	0.376
contig093812-BriOR.A010	contig034983-NyeOR.A002	0.287	0.767	0.374
contig093812-BriOR.A010	contig047515-ZebOR.A019	0.287	0.767	0.374
contig085010-BriOR.A005	contig022234-TilOR.A009	0.287	0.797	0.361
contig057153-BurOR.A014	contig047521-ZebOR.A020	0.287	0.803	0.357
contig034983-NyeOR.A003	contig073309-TilOR.A026	0.287	0.812	0.353
contig054237-BurOR.A013	contig030557-ZebOR.A006	0.287	0.815	0.352
contig085018-BriOR.A006	contig022268-TilOR.A020	0.287	0.823	0.349
contig051570-BurOR.A010	contig041952-TilOR.A023	0.287	0.824	0.348
contig022259-TilOR.A014	contig070886-TilOR.A025	0.287	0.831	0.346
contig034988-NyeOR.A005	contig041952-TilOR.A023	0.287	0.834	0.344
contig084999-BriOR.A002	contig062095-ZebOR.A024	0.287	0.839	0.342
contig085000-BriOR.A003	contig034983-NyeOR.A002	0.287	0.846	0.339
contig085000-BriOR.A003	contig047515-ZebOR.A019	0.287	0.846	0.339
contig085000-BriOR.A003	contig022217-TilOR.A004	0.287	0.847	0.339
contig085000-BriOR.A003	contig062344-NyeOR.A020	0.287	0.854	0.336
contig064187-BurOR.A016	contig065887-BurOR.A018	0.287	0.858	0.334
contig085026-BriOR.A008	contig057754-NyeOR.A018	0.287	0.859	0.334
contig034983-NyeOR.A002	contig057754-NyeOR.A018	0.287	0.863	0.333
contig057754-NyeOR.A018	contig047515-ZebOR.A019	0.287	0.863	0.333
contig057754-NyeOR.A018	contig022217-TilOR.A004	0.287	0.867	0.331
contig064187-BurOR.A016	contig030566-ZebOR.A008	0.287	0.869	0.330
contig057156-BurOR.A015	contig062344-NyeOR.A020	0.287	0.874	0.328
contig022230-TilOR.A007	contig022238-TilOR.A010	0.288	0.583	0.494
contig057165-NyeOR.A017	contig030553-ZebOR.A002	0.288	0.606	0.476
contig036780-BurOR.A001	contig036782-BurOR.A002	0.288	0.614	0.470
contig030552-ZebOR.A001	contig030553-ZebOR.A002	0.288	0.616	0.468
contig036782-BurOR.A002	contig051573-BurOR.A011	0.288	0.629	0.458
contig084999-BriOR.A001	contig093816-BriOR.A011	0.288	0.629	0.458
contig022238-TilOR.A010	contig041951-TilOR.A022	0.288	0.631	0.456
contig056375-NyeOR.A015	contig030560-ZebOR.A007	0.288	0.658	0.438
contig022227-TilOR.A006	contig022266-TilOR.A018	0.288	0.660	0.436
contig056375-NyeOR.A015	contig022265-TilOR.A017	0.288	0.660	0.436

contig041951-TilOR.A022	contig070886-TilOR.A025	0.288	0.661	0.436
contig085010-BriOR.A005	contig057756-NyeOR.A019	0.288	0.673	0.428
contig070886-TilOR.A025	contig030553-ZebOR.A002	0.288	0.676	0.426
contig085010-BriOR.A005	contig056375-NyeOR.A015	0.288	0.678	0.425
contig084999-BriOR.A001	contig022238-TilOR.A010	0.288	0.697	0.412
contig022259-TilOR.A014	contig047506-ZebOR.A014	0.288	0.702	0.410
contig034988-NyeOR.A006	contig030560-ZebOR.A007	0.288	0.718	0.402
contig057153-BurOR.A014	contig062095-ZebOR.A024	0.288	0.721	0.400
contig022230-TilOR.A007	contig062095-ZebOR.A024	0.288	0.726	0.397
contig073309-TilOR.A026	contig030560-ZebOR.A007	0.288	0.727	0.396
contig030554-ZebOR.A004	contig030576-ZebOR.A010	0.288	0.741	0.389
contig022227-TilOR.A006	contig047506-ZebOR.A014	0.288	0.747	0.385
contig034988-NyeOR.A006	contig022227-TilOR.A006	0.288	0.752	0.383
contig022251-TilOR.A013	contig041951-TilOR.A021	0.288	0.755	0.381
contig057754-NyeOR.A018	contig030560-ZebOR.A007	0.288	0.762	0.379
contig022225-TilOR.A005	contig030556-ZebOR.A005	0.288	0.766	0.376
contig085002-BriOR.A004	contig070885-TilOR.A024	0.288	0.778	0.371
contig034988-NyeOR.A006	contig047521-ZebOR.A020	0.288	0.779	0.369
contig022204-TilOR.A001	contig022268-TilOR.A019	0.288	0.793	0.363
contig022245-TilOR.A012	contig047523-ZebOR.A022	0.288	0.797	0.361
contig054233-BurOR.A012	contig022245-TilOR.A012	0.288	0.797	0.361
contig022234-TilOR.A009	contig073309-TilOR.A026	0.288	0.801	0.360
contig085026-BriOR.A008	contig047506-ZebOR.A014	0.288	0.805	0.357
contig084999-BriOR.A001	contig047503-ZebOR.A013	0.288	0.806	0.357
contig041952-TilOR.A023	contig047508-ZebOR.A015	0.288	0.807	0.356
contig054237-BurOR.A013	contig047506-ZebOR.A014	0.288	0.808	0.356
contig057153-BurOR.A014	contig034981-NyeOR.A001	0.288	0.820	0.351
contig054237-BurOR.A013	contig054681-NyeOR.A011	0.288	0.820	0.351
contig065887-BurOR.A018	contig047506-ZebOR.A014	0.288	0.853	0.337
contig036784-BurOR.A003	contig057756-NyeOR.A019	0.288	0.856	0.336
contig036784-BurOR.A003	contig051559-BurOR.A007	0.288	0.856	0.336
contig030566-ZebOR.A008	contig047506-ZebOR.A014	0.288	0.865	0.333
contig085000-BriOR.A003	contig030576-ZebOR.A010	0.288	0.875	0.329
contig051318-BurOR.A005	contig054868-NyeOR.A014	0.288	0.885	0.325
contig054868-NyeOR.A014	contig030572-ZebOR.A009	0.288	0.885	0.325
contig085012-BriOR.A130	contig054868-NyeOR.A014	0.288	0.899	0.320
contig030552-ZebOR.A001	contig030554-ZebOR.A004	0.289	0.564	0.512
contig036780-BurOR.A001	contig030554-ZebOR.A004	0.289	0.564	0.512
contig057756-NyeOR.A019	contig022204-TilOR.A001	0.289	0.594	0.486
contig022227-TilOR.A006	contig030553-ZebOR.A002	0.289	0.600	0.482
contig057156-BurOR.A015	contig057756-NyeOR.A019	0.289	0.635	0.456
contig022230-TilOR.A007	contig070886-TilOR.A025	0.289	0.638	0.453
contig084999-BriOR.A001	contig047499-ZebOR.A012	0.289	0.659	0.439

contig054684-NyeOR.A012	contig057756-NyeOR.A019	0.289	0.665	0.435
contig022227-TilOR.A006	contig030557-ZebOR.A006	0.289	0.676	0.427
contig022268-TilOR.A020	contig047499-ZebOR.A012	0.289	0.678	0.427
contig064187-BurOR.A016	contig022238-TilOR.A010	0.289	0.686	0.421
contig064187-BurOR.A016	contig070886-TilOR.A025	0.289	0.687	0.420
contig064570-BurOR.A017	contig041951-TilOR.A021	0.289	0.690	0.418
contig084999-BriOR.A001	contig093812-BriOR.A010	0.289	0.691	0.418
contig041952-TilOR.A023	contig047506-ZebOR.A014	0.289	0.696	0.416
contig022230-TilOR.A007	contig047497-ZebOR.A011	0.289	0.697	0.415
contig034983-NyeOR.A002	contig062344-NyeOR.A020	0.289	0.698	0.413
contig062344-NyeOR.A020	contig047515-ZebOR.A019	0.289	0.698	0.413
contig057153-BurOR.A014	contig022234-TilOR.A009	0.289	0.699	0.413
contig030553-ZebOR.A002	contig047503-ZebOR.A013	0.289	0.701	0.413
contig034988-NyeORs.A033	contig047508-ZebOR.A016	0.289	0.702	0.411
contig022238-TilOR.A010	contig030553-ZebOR.A002	0.289	0.703	0.411
contig056375-NyeOR.A015	contig030557-ZebOR.A006	0.289	0.704	0.411
contig034988-NyeOR.A006	contig022259-TilOR.A014	0.289	0.713	0.406
contig093812-BriOR.A010	contig022268-TilOR.A020	0.289	0.714	0.404
contig022217-TilOR.A004	contig047515-ZebOR.A019	0.289	0.716	0.404
contig034983-NyeOR.A002	contig022217-TilOR.A004	0.289	0.716	0.404
contig041952-TilOR.A023	contig030560-ZebOR.A007	0.289	0.725	0.399
contig036780-BurOR.A001	contig062095-ZebOR.A024	0.289	0.731	0.395
contig036784-BurOR.A003	contig030560-ZebOR.A007	0.289	0.737	0.392
contig022232-TilOR.A008	contig022265-TilOR.A017	0.289	0.739	0.391
contig057165-NyeOR.A017	contig041952-TilOR.A023	0.289	0.741	0.390
contig030552-ZebOR.A001	contig062095-ZebOR.A024	0.289	0.742	0.389
contig034994-NyeOR.A008	contig022230-TilOR.A007	0.289	0.743	0.390
contig036782-BurOR.A002	contig022234-TilOR.A009	0.289	0.744	0.389
contig022241-TilOR.A011	contig022268-TilOR.A019	0.289	0.751	0.385
contig022259-TilOR.A015	contig041951-TilOR.A021	0.289	0.753	0.384
contig084999-BriOR.A001	contig054678-NyeOR.A010	0.289	0.754	0.383
contig062344-NyeOR.A020	contig030553-ZebOR.A002	0.289	0.754	0.384
contig022259-TilOR.A015	contig022266-TilOR.A018	0.289	0.763	0.378
contig057156-BurOR.A015	contig047503-ZebOR.A013	0.289	0.766	0.378
contig036782-BurOR.A002	contig051570-BurOR.A010	0.289	0.767	0.376
contig034981-NyeOR.A001	contig034988-NyeOR.A006	0.289	0.772	0.374
contig022225-TilOR.A005	contig030554-ZebOR.A004	0.289	0.773	0.373
contig085002-BriOR.A004	contig034988-NyeOR.A005	0.289	0.773	0.375
contig051570-BurOR.A010	contig057756-NyeOR.A019	0.289	0.774	0.374
contig057754-NyeOR.A018	contig022268-TilOR.A019	0.289	0.776	0.373
contig022234-TilOR.A009	contig030560-ZebOR.A007	0.289	0.783	0.370
contig085010-BriOR.A005	contig047503-ZebOR.A013	0.289	0.794	0.364
contig054678-NyeOR.A010	contig022225-TilOR.A005	0.289	0.808	0.358

contig036784-BurOR.A003	contig070885-TilOR.A024	0.289	0.818	0.354
contig085026-BriOR.A008	contig057756-NyeOR.A019	0.289	0.827	0.349
contig093816-BriOR.A011	contig070885-TilOR.A024	0.289	0.830	0.348
contig036782-BurOR.A002	contig022211-TilOR.A003	0.289	0.830	0.348
contig054687-NyeOR.A013	contig022268-TilOR.A020	0.289	0.833	0.347
contig073309-TilOR.A026	contig030576-ZebOR.A010	0.289	0.848	0.341
contig084999-BriOR.A001	contig022211-TilOR.A003	0.289	0.855	0.338
contig054687-NyeOR.A013	contig041951-TilOR.A022	0.289	0.859	0.336
contig057754-NyeOR.A018	contig062344-NyeOR.A020	0.289	0.860	0.336
contig051321-BurOR.A006	contig047523-ZebOR.A022	0.289	0.881	0.328
contig051321-BurOR.A006	contig054233-BurOR.A012	0.289	0.881	0.328
contig057754-NyeOR.A018	contig030576-ZebOR.A010	0.289	0.882	0.328
contig085000-BriOR.A003	contig034988-NyeOR.A005	0.289	0.910	0.317
contig036780-BurOR.A001	contig030553-ZebOR.A002	0.290	0.606	0.479
contig022266-TilOR.A018	contig047499-ZebOR.A012	0.290	0.612	0.473
contig093816-BriOR.A011	contig030553-ZebOR.A002	0.290	0.634	0.457
contig051573-BurOR.A011	contig057756-NyeOR.A019	0.290	0.649	0.447
contig034990-NyeOR.A007	contig022268-TilOR.A020	0.290	0.659	0.440
contig054681-NyeOR.A011	contig022227-TilOR.A006	0.290	0.660	0.440
contig051566-BurOR.A009	contig030557-ZebOR.A006	0.290	0.662	0.437
contig030553-ZebOR.A002	contig030554-ZebOR.A004	0.290	0.667	0.434
contig030557-ZebOR.A006	contig047526-ZebOR.A021	0.290	0.673	0.430
contig057153-BurOR.A014	contig047514-ZebOR.A017	0.290	0.683	0.424
contig057165-NyeOR.A017	contig047503-ZebOR.A013	0.290	0.684	0.423
contig034988-NyeOR.A006	contig041952-TilOR.A023	0.290	0.689	0.420
contig057153-BurOR.A014	contig022238-TilOR.A010	0.290	0.689	0.421
contig051559-BurOR.A007	contig062344-NyeOR.A020	0.290	0.701	0.414
contig084999-BriOR.A001	contig085002-BriOR.A004	0.290	0.704	0.413
contig057756-NyeOR.A019	contig041952-TilOR.A023	0.290	0.707	0.410
contig093816-BriOR.A011	contig034983-NyeOR.A002	0.290	0.711	0.408
contig093816-BriOR.A011	contig047515-ZebOR.A019	0.290	0.711	0.408
contig085010-BriOR.A005	contig070886-TilOR.A025	0.290	0.714	0.405
contig057156-BurOR.A015	contig073309-TilOR.A026	0.290	0.717	0.405
contig051559-BurOR.A007	contig022217-TilOR.A004	0.290	0.719	0.404
contig051559-BurOR.A008	contig057153-BurOR.A014	0.290	0.723	0.401
contig085018-BriOR.A006	contig034988-NyeORs.A033	0.290	0.723	0.401
contig057153-BurOR.A014	contig070886-TilOR.A025	0.290	0.723	0.402
contig051559-BurOR.A008	contig054681-NyeOR.A011	0.290	0.731	0.397
contig022227-TilOR.A006	contig041952-TilOR.A023	0.290	0.735	0.394
contig036782-BurOR.A002	contig047508-ZebOR.A015	0.290	0.740	0.392
contig057756-NyeOR.A019	contig047508-ZebOR.A015	0.290	0.746	0.389
contig057756-NyeOR.A019	contig047515-ZebOR.A018	0.290	0.749	0.387
contig022259-TilOR.A014	contig073309-TilOR.A026	0.290	0.751	0.387

contig085002-BriOR.A004	contig062095-ZebOR.A024	0.290	0.756	0.384
contig085002-BriOR.A004	contig022204-TilOR.A001	0.290	0.770	0.376
contig084999-BriOR.A001	contig022232-TilOR.A008	0.290	0.773	0.375
contig057156-BurOR.A015	contig022234-TilOR.A009	0.290	0.777	0.373
contig085002-BriOR.A004	contig022232-TilOR.A008	0.290	0.790	0.368
contig036787-BurOR.A004	contig022232-TilOR.A008	0.290	0.791	0.367
contig034988-NyeOR.A006	contig022211-TilOR.A003	0.290	0.792	0.365
contig022225-TilOR.A005	contig022268-TilOR.A019	0.290	0.792	0.367
contig054684-NyeOR.A012	contig022234-TilOR.A009	0.290	0.801	0.362
contig084999-BriOR.A001	contig051559-BurOR.A008	0.290	0.804	0.361
contig022232-TilOR.A008	contig030556-ZebOR.A005	0.290	0.805	0.360
contig085026-BriOR.A008	contig057156-BurOR.A015	0.290	0.808	0.359
contig054237-BurOR.A013	contig057153-BurOR.A014	0.290	0.810	0.358
contig070885-TilOR.A024	contig030553-ZebOR.A003	0.290	0.814	0.356
contig022241-TilOR.A011	contig073309-TilOR.A026	0.290	0.818	0.354
contig093816-BriOR.A011	contig034988-NyeOR.A005	0.290	0.836	0.346
contig036784-BurOR.A003	contig034983-NyeOR.A002	0.290	0.846	0.342
contig036784-BurOR.A003	contig047515-ZebOR.A019	0.290	0.846	0.342
contig085000-BriOR.A003	contig057756-NyeOR.A019	0.290	0.853	0.340
contig034988-NyeOR.A006	contig022264-TilOR.A016	0.290	0.869	0.334
contig085000-BriOR.A003	contig051570-BurOR.A010	0.290	0.887	0.327
contig036784-BurOR.A003	contig034988-NyeOR.A005	0.290	0.889	0.326
contig085000-BriOR.A003	contig022232-TilOR.A008	0.290	0.929	0.312
contig084999-BriOR.A002	contig030552-ZebOR.A001	0.291	0.550	0.528
contig084999-BriOR.A002	contig036780-BurOR.A001	0.291	0.550	0.528
contig057165-NyeOR.A017	contig030554-ZebOR.A004	0.291	0.564	0.516
contig030552-ZebOR.A001	contig047526-ZebOR.A021	0.291	0.629	0.462
contig084999-BriOR.A001	contig034990-NyeOR.A007	0.291	0.636	0.457
contig034988-NyeORs.A033	contig022259-TilOR.A014	0.291	0.637	0.456
contig057165-NyeOR.A017	contig047526-ZebOR.A021	0.291	0.639	0.455
contig041951-TilOR.A022	contig041952-TilOR.A023	0.291	0.647	0.449
contig057756-NyeOR.A019	contig030560-ZebOR.A007	0.291	0.649	0.447
contig030553-ZebOR.A002	contig047499-ZebOR.A012	0.291	0.653	0.446
contig022266-TilOR.A018	contig070886-TilOR.A025	0.291	0.662	0.440
contig093812-BriOR.A010	contig030553-ZebOR.A002	0.291	0.664	0.438
contig051566-BurOR.A009	contig054681-NyeOR.A011	0.291	0.666	0.437
contig034988-NyeOR.A006	contig070886-TilOR.A025	0.291	0.672	0.433
contig022268-TilOR.A019	contig030553-ZebOR.A002	0.291	0.673	0.433
contig054681-NyeOR.A011	contig047526-ZebOR.A021	0.291	0.677	0.429
contig022238-TilOR.A010	contig047506-ZebOR.A014	0.291	0.677	0.430
contig064570-BurOR.A017	contig022266-TilOR.A018	0.291	0.682	0.427
contig054681-NyeOR.A011	contig056375-NyeOR.A015	0.291	0.687	0.423
contig084999-BriOR.A002	contig036784-BurOR.A003	0.291	0.687	0.424

contig085002-BriOR.A004	contig022259-TilOR.A015	0.291	0.704	0.413
contig036782-BurOR.A002	contig070886-TilOR.A025	0.291	0.706	0.412
contig084999-BriOR.A002	contig057754-NyeOR.A018	0.291	0.710	0.410
contig056375-NyeOR.A015	contig022230-TilOR.A007	0.291	0.711	0.410
contig085002-BriOR.A004	contig073309-TilOR.A026	0.291	0.714	0.408
contig057156-BurOR.A015	contig047514-ZebOR.A017	0.291	0.714	0.408
contig022204-TilOR.A001	contig030553-ZebOR.A003	0.291	0.715	0.408
contig057156-BurOR.A015	contig022259-TilOR.A015	0.291	0.726	0.401
contig085010-BriOR.A005	contig030553-ZebOR.A003	0.291	0.728	0.400
contig041952-TilOR.A023	contig030552-ZebOR.A001	0.291	0.730	0.399
contig057165-NyeOR.A017	contig062095-ZebOR.A024	0.291	0.731	0.398
contig036782-BurOR.A002	contig022232-TilOR.A008	0.291	0.732	0.398
contig036782-BurOR.A002	contig022238-TilOR.A010	0.291	0.733	0.397
contig054678-NyeOR.A010	contig022259-TilOR.A015	0.291	0.735	0.396
contig054684-NyeOR.A012	contig047514-ZebOR.A017	0.291	0.736	0.396
contig085010-BriOR.A005	contig073309-TilOR.A026	0.291	0.738	0.394
contig064570-BurOR.A017	contig030557-ZebOR.A006	0.291	0.741	0.392
contig036782-BurOR.A002	contig051559-BurOR.A008	0.291	0.763	0.382
contig036780-BurOR.A001	contig022217-TilOR.A004	0.291	0.775	0.375
contig036787-BurOR.A004	contig070885-TilOR.A024	0.291	0.775	0.375
contig041951-TilOR.A022	contig047521-ZebOR.A020	0.291	0.777	0.374
contig030553-ZebOR.A002	contig030576-ZebOR.A010	0.291	0.780	0.373
contig036787-BurOR.A004	contig022225-TilOR.A005	0.291	0.787	0.369
contig022217-TilOR.A004	contig030552-ZebOR.A001	0.291	0.787	0.369
contig070885-TilOR.A024	contig030556-ZebOR.A005	0.291	0.789	0.368
contig051559-BurOR.A008	contig073309-TilOR.A026	0.291	0.799	0.365
contig051559-BurOR.A007	contig034995-NyeOR.A009	0.291	0.805	0.362
contig085000-BriOR.A003	contig022234-TilOR.A009	0.291	0.809	0.360
contig054678-NyeOR.A010	contig022232-TilOR.A008	0.291	0.811	0.358
contig070886-TilOR.A025	contig047515-ZebOR.A018	0.291	0.818	0.356
contig065887-BurOR.A018	contig041951-TilOR.A022	0.291	0.834	0.349
contig093816-BriOR.A011	contig051570-BurOR.A010	0.291	0.839	0.347
contig041951-TilOR.A022	contig030566-ZebOR.A008	0.291	0.845	0.344
contig022268-TilOR.A020	contig030566-ZebOR.A008	0.291	0.845	0.345
contig034988-NyeOR.A006	contig054687-NyeOR.A013	0.291	0.851	0.342
contig093812-BriOR.A010	contig070885-TilOR.A024	0.291	0.859	0.339
contig065887-BurOR.A018	contig022268-TilOR.A020	0.291	0.860	0.339
contig085000-BriOR.A003	contig047508-ZebOR.A015	0.291	0.868	0.335
contig036784-BurOR.A003	contig051570-BurOR.A010	0.291	0.868	0.336
contig022264-TilOR.A016	contig047521-ZebOR.A020	0.291	0.873	0.333
contig030572-ZebOR.A009	contig047523-ZebOR.A022	0.291	0.880	0.331
contig051318-BurOR.A005	contig047523-ZebOR.A022	0.291	0.880	0.331
contig051318-BurOR.A005	contig054233-BurOR.A012	0.291	0.880	0.331

contig054233-BurOR.A012	contig030572-ZebOR.A009	0.291	0.880	0.331
contig034988-NyeOR.A005	contig030553-ZebOR.A003	0.291	0.886	0.328
contig085000-BriOR.A003	contig047503-ZebOR.A013	0.291	0.895	0.325
contig036784-BurOR.A003	contig022232-TilOR.A008	0.291	0.908	0.321
contig051559-BurOR.A007	contig022238-TilOR.A010	0.292	0.605	0.483
contig022266-TilOR.A018	contig047514-ZebOR.A017	0.292	0.612	0.477
contig093816-BriOR.A011	contig036782-BurOR.A002	0.292	0.623	0.469
contig022238-TilOR.A010	contig022266-TilOR.A018	0.292	0.627	0.466
contig034990-NyeOR.A007	contig030553-ZebOR.A002	0.292	0.629	0.465
contig034988-NyeORs.A033	contig022245-TilOR.A012	0.292	0.646	0.452
contig034988-NyeORs.A033	contig062095-ZebOR.A024	0.292	0.654	0.447
contig036782-BurOR.A002	contig030554-ZebOR.A004	0.292	0.655	0.446
contig084999-BriOR.A002	contig070886-TilOR.A025	0.292	0.663	0.440
contig057156-BurOR.A015	contig047526-ZebOR.A021	0.292	0.671	0.435
contig057153-BurOR.A014	contig022227-TilOR.A006	0.292	0.672	0.435
contig070886-TilOR.A025	contig047506-ZebOR.A014	0.292	0.675	0.432
contig073309-TilOR.A026	contig030556-ZebOR.A005	0.292	0.679	0.430
contig034983-NyeOR.A002	contig074640-TilOR.A002	0.292	0.681	0.428
contig074640-TilOR.A002	contig047515-ZebOR.A019	0.292	0.681	0.428
contig051559-BurOR.A007	contig041952-TilOR.A023	0.292	0.687	0.425
contig054684-NyeOR.A012	contig047526-ZebOR.A021	0.292	0.691	0.422
contig034988-NyeORs.A033	contig022225-TilOR.A005	0.292	0.692	0.422
contig070886-TilOR.A025	contig030552-ZebOR.A001	0.292	0.693	0.422
contig041952-TilOR.A023	contig030557-ZebOR.A006	0.292	0.693	0.422
contig034994-NyeOR.A008	contig022266-TilOR.A018	0.292	0.695	0.420
contig057165-NyeOR.A017	contig070886-TilOR.A025	0.292	0.703	0.415
contig064570-BurOR.A017	contig022230-TilOR.A007	0.292	0.708	0.412
contig057756-NyeOR.A019	contig047514-ZebOR.A017	0.292	0.708	0.412
contig093816-BriOR.A011	contig022232-TilOR.A008	0.292	0.709	0.411
contig085002-BriOR.A004	contig022241-TilOR.A011	0.292	0.709	0.412
contig030553-ZebOR.A002	contig030556-ZebOR.A005	0.292	0.719	0.406
contig036784-BurOR.A003	contig022204-TilOR.A001	0.292	0.719	0.406
contig064570-BurOR.A017	contig054681-NyeOR.A011	0.292	0.723	0.404
contig034995-NyeOR.A009	contig022266-TilOR.A018	0.292	0.723	0.404
contig051566-BurOR.A009	contig022265-TilOR.A017	0.292	0.725	0.403
contig085000-BriOR.A003	contig022204-TilOR.A001	0.292	0.728	0.402
contig022245-TilOR.A012	contig022268-TilOR.A020	0.292	0.730	0.401
contig036780-BurOR.A001	contig022245-TilOR.A012	0.292	0.732	0.399
contig084999-BriOR.A002	contig022241-TilOR.A011	0.292	0.735	0.397
contig022268-TilOR.A019	contig030553-ZebOR.A003	0.292	0.741	0.393
contig085010-BriOR.A005	contig036784-BurOR.A003	0.292	0.742	0.394
contig022245-TilOR.A012	contig030552-ZebOR.A001	0.292	0.743	0.393
contig085010-BriOR.A005	contig057754-NyeOR.A018	0.292	0.745	0.392

contig022234-TilOR.A009	contig070886-TilOR.A025	0.292	0.748	0.390
contig085002-BriOR.A004	contig047508-ZebOR.A015	0.292	0.749	0.389
contig085002-BriOR.A004	contig051570-BurOR.A010	0.292	0.760	0.384
contig034983-NyeOR.A003	contig057756-NyeOR.A019	0.292	0.760	0.384
contig034981-NyeOR.A001	contig041951-TilOR.A022	0.292	0.769	0.380
contig036780-BurOR.A001	contig064570-BurOR.A017	0.292	0.769	0.380
contig084999-BriOR.A002	contig056375-NyeOR.A015	0.292	0.774	0.377
contig051559-BurOR.A007	contig054687-NyeOR.A013	0.292	0.780	0.374
contig022232-TilOR.A008	contig030554-ZebOR.A004	0.292	0.781	0.374
contig064570-BurOR.A017	contig030552-ZebOR.A001	0.292	0.781	0.374
contig022234-TilOR.A009	contig022265-TilOR.A017	0.292	0.787	0.370
contig085018-BriOR.A006	contig022268-TilOR.A019	0.292	0.790	0.370
contig054684-NyeOR.A012	contig047503-ZebOR.A013	0.292	0.800	0.364
contig022230-TilOR.A007	contig022264-TilOR.A016	0.292	0.804	0.363
contig054678-NyeOR.A010	contig070885-TilOR.A024	0.292	0.807	0.361
contig085000-BriOR.A003	contig030552-ZebOR.A001	0.292	0.813	0.359
contig085000-BriOR.A003	contig057165-NyeOR.A017	0.292	0.813	0.359
contig085026-BriOR.A008	contig034988-NyeOR.A006	0.292	0.814	0.359
contig070885-TilOR.A024	contig047499-ZebOR.A012	0.292	0.815	0.359
contig054237-BurOR.A013	contig034988-NyeOR.A006	0.292	0.817	0.357
contig093816-BriOR.A011	contig047508-ZebOR.A015	0.292	0.821	0.356
contig062344-NyeOR.A020	contig041952-TilOR.A023	0.292	0.823	0.354
contig036784-BurOR.A003	contig047508-ZebOR.A015	0.292	0.849	0.344
contig054687-NyeOR.A013	contig030557-ZebOR.A006	0.292	0.851	0.344
contig070885-TilOR.A024	contig070886-TilOR.A025	0.292	0.856	0.341
contig041952-TilOR.A023	contig047515-ZebOR.A018	0.292	0.863	0.338
contig051570-BurOR.A010	contig030553-ZebOR.A003	0.292	0.864	0.338
contig085010-BriOR.A005	contig030576-ZebOR.A010	0.292	0.912	0.321
contig054868-NyeOR.A014	contig022251-TilOR.A013	0.292	0.930	0.314
contig085000-BriOR.A003	contig047515-ZebOR.A018	0.292	0.957	0.305
contig070886-TilOR.A025	contig030576-ZebOR.A010	0.292	0.963	0.303
contig084999-BriOR.A002	contig057165-NyeOR.A017	0.293	0.550	0.532
contig022230-TilOR.A007	contig047514-ZebOR.A017	0.293	0.602	0.487
contig084999-BriOR.A001	contig022227-TilOR.A006	0.293	0.602	0.487
contig034990-NyeOR.A007	contig022266-TilOR.A018	0.293	0.608	0.482
contig074640-TilOR.A002	contig041951-TilOR.A021	0.293	0.625	0.469
contig022268-TilOR.A020	contig047497-ZebOR.A011	0.293	0.634	0.463
contig036780-BurOR.A001	contig047526-ZebOR.A021	0.293	0.639	0.458
contig084999-BriOR.A001	contig093807-BriOR.A009	0.293	0.662	0.443
contig051566-BurOR.A009	contig057156-BurOR.A015	0.293	0.677	0.433
contig054681-NyeOR.A011	contig041952-TilOR.A023	0.293	0.677	0.434
contig084999-BriOR.A002	contig030553-ZebOR.A003	0.293	0.679	0.432
contig036782-BurOR.A002	contig047499-ZebOR.A012	0.293	0.681	0.431

contig034988-NyeORs.A033	contig022217-TilOR.A004	0.293	0.683	0.429
contig057156-BurOR.A015	contig034988-NyeOR.A004	0.293	0.685	0.428
contig093812-BriOR.A010	contig036782-BurOR.A002	0.293	0.693	0.423
contig051566-BurOR.A009	contig054684-NyeOR.A012	0.293	0.698	0.420
contig057153-BurOR.A014	contig056375-NyeOR.A015	0.293	0.700	0.418
contig034988-NyeOR.A004	contig054684-NyeOR.A012	0.293	0.706	0.415
contig093812-BriOR.A010	contig057165-NyeOR.A017	0.293	0.714	0.410
contig085002-BriOR.A004	contig070886-TilOR.A025	0.293	0.729	0.402
contig085010-BriOR.A005	contig041952-TilOR.A023	0.293	0.730	0.401
contig034988-NyeOR.A004	contig022265-TilOR.A017	0.293	0.735	0.399
contig054684-NyeOR.A012	contig022259-TilOR.A015	0.293	0.737	0.398
contig085026-BriOR.A008	contig022266-TilOR.A018	0.293	0.738	0.397
contig036780-BurOR.A001	contig022241-TilOR.A011	0.293	0.739	0.396
contig057165-NyeOR.A017	contig022241-TilOR.A011	0.293	0.739	0.396
contig085002-BriOR.A004	contig062344-NyeOR.A020	0.293	0.742	0.395
contig022241-TilOR.A011	contig030552-ZebOR.A001	0.293	0.750	0.390
contig085018-BriOR.A006	contig022230-TilOR.A007	0.293	0.752	0.390
contig064187-BurOR.A016	contig034995-NyeOR.A009	0.293	0.756	0.388
contig084999-BriOR.A002	contig085018-BriOR.A006	0.293	0.761	0.386
contig051573-BurOR.A011	contig022259-TilOR.A014	0.293	0.768	0.381
contig057156-BurOR.A015	contig034981-NyeOR.A001	0.293	0.770	0.381
contig057165-NyeOR.A017	contig022217-TilOR.A004	0.293	0.775	0.378
contig022251-TilOR.A013	contig022268-TilOR.A020	0.293	0.777	0.377
contig056380-NyeOR.A016	contig030557-ZebOR.A006	0.293	0.784	0.374
contig022211-TilOR.A003	contig041951-TilOR.A022	0.293	0.799	0.367
contig022259-TilOR.A015	contig030557-ZebOR.A006	0.293	0.800	0.366
contig051573-BurOR.A011	contig070885-TilOR.A024	0.293	0.813	0.361
contig084999-BriOR.A002	contig064570-BurOR.A017	0.293	0.825	0.356
contig022266-TilOR.A018	contig030576-ZebOR.A010	0.293	0.831	0.352
contig065887-BurOR.A018	contig034988-NyeOR.A006	0.293	0.839	0.350
contig030553-ZebOR.A003	contig047508-ZebOR.A015	0.293	0.846	0.347
contig057756-NyeOR.A019	contig022211-TilOR.A003	0.293	0.853	0.344
contig064570-BurOR.A017	contig070886-TilOR.A025	0.293	0.865	0.339
contig022265-TilOR.A017	contig030576-ZebOR.A010	0.293	0.883	0.332
contig034981-NyeOR.A001	contig022264-TilOR.A016	0.293	0.884	0.332
contig036784-BurOR.A003	contig062095-ZebOR.A024	0.293	0.885	0.332
contig070886-TilOR.A025	contig062095-ZebOR.A024	0.293	0.893	0.328
contig085012-BriOR.A130	contig047523-ZebOR.A022	0.293	0.893	0.329
contig085012-BriOR.A130	contig054233-BurOR.A012	0.293	0.893	0.329
contig022264-TilOR.A016	contig022268-TilOR.A020	0.293	0.900	0.326
contig022232-TilOR.A008	contig030553-ZebOR.A003	0.293	0.902	0.324
contig062344-NyeOR.A020	contig070886-TilOR.A025	0.293	0.927	0.316
contig054684-NyeOR.A012	contig030576-ZebOR.A010	0.293	0.932	0.314

contig022211-TilOR.A003	contig022264-TilOR.A016	0.293	0.999	0.294
contig036782-BurOR.A002	contig022227-TilOR.A006	0.294	0.627	0.469
contig022259-TilOR.A014	contig041951-TilOR.A022	0.294	0.640	0.459
contig051566-BurOR.A009	contig057153-BurOR.A014	0.294	0.654	0.450
contig036782-BurOR.A002	contig022268-TilOR.A019	0.294	0.661	0.444
contig036784-BurOR.A003	contig030554-ZebOR.A004	0.294	0.662	0.444
contig057153-BurOR.A014	contig047526-ZebOR.A021	0.294	0.664	0.442
contig056375-NyeOR.A015	contig041951-TilOR.A021	0.294	0.666	0.442
contig051559-BurOR.A007	contig022241-TilOR.A011	0.294	0.668	0.441
contig030560-ZebOR.A007	contig047526-ZebOR.A021	0.294	0.670	0.439
contig034988-NyeOR.A006	contig022238-TilOR.A010	0.294	0.671	0.438
contig093807-BriOR.A009	contig030553-ZebOR.A002	0.294	0.671	0.438
contig034994-NyeOR.A008	contig057165-NyeOR.A017	0.294	0.678	0.434
contig022241-TilOR.A011	contig047515-ZebOR.A019	0.294	0.681	0.432
contig034983-NyeOR.A002	contig022241-TilOR.A011	0.294	0.681	0.432
contig057754-NyeOR.A018	contig030554-ZebOR.A004	0.294	0.685	0.429
contig022266-TilOR.A018	contig047497-ZebOR.A011	0.294	0.690	0.427
contig093807-BriOR.A009	contig030557-ZebOR.A006	0.294	0.696	0.422
contig022225-TilOR.A005	contig030553-ZebOR.A003	0.294	0.700	0.420
contig051559-BurOR.A007	contig074640-TilOR.A002	0.294	0.700	0.420
contig022230-TilOR.A007	contig062094-ZebOR.A023	0.294	0.701	0.420
contig036780-BurOR.A001	contig070886-TilOR.A025	0.294	0.703	0.418
contig051573-BurOR.A011	contig022232-TilOR.A008	0.294	0.705	0.417
contig064187-BurOR.A016	contig047497-ZebOR.A011	0.294	0.706	0.417
contig036787-BurOR.A004	contig030553-ZebOR.A002	0.294	0.707	0.416
contig022265-TilOR.A017	contig030553-ZebOR.A002	0.294	0.711	0.414
contig030560-ZebOR.A007	contig047514-ZebOR.A017	0.294	0.714	0.412
contig022259-TilOR.A015	contig030560-ZebOR.A007	0.294	0.720	0.409
contig057754-NyeOR.A018	contig022204-TilOR.A001	0.294	0.722	0.408
contig084999-BriOR.A002	contig047521-ZebOR.A020	0.294	0.724	0.407
contig036782-BurOR.A002	contig030556-ZebOR.A005	0.294	0.728	0.404
contig036782-BurOR.A002	contig047503-ZebOR.A013	0.294	0.731	0.402
contig057165-NyeOR.A017	contig022245-TilOR.A012	0.294	0.732	0.402
contig057153-BurOR.A014	contig064570-BurOR.A017	0.294	0.736	0.399
contig022265-TilOR.A017	contig047508-ZebOR.A016	0.294	0.738	0.399
contig036780-BurOR.A001	contig041952-TilOR.A023	0.294	0.741	0.396
contig057756-NyeOR.A019	contig022234-TilOR.A009	0.294	0.753	0.390
contig036787-BurOR.A004	contig034988-NyeOR.A005	0.294	0.758	0.388
contig051559-BurOR.A007	contig065887-BurOR.A018	0.294	0.769	0.383
contig064570-BurOR.A017	contig057165-NyeOR.A017	0.294	0.769	0.383
contig084999-BriOR.A001	contig062344-NyeOR.A020	0.294	0.772	0.381
contig034988-NyeOR.A005	contig030556-ZebOR.A005	0.294	0.772	0.381
contig030560-ZebOR.A007	contig047503-ZebOR.A013	0.294	0.776	0.378

contig056380-NyeOR.A016	contig022266-TilOR.A018	0.294	0.777	0.378
contig051559-BurOR.A007	contig030566-ZebOR.A008	0.294	0.779	0.378
contig022234-TilOR.A009	contig030553-ZebOR.A003	0.294	0.785	0.374
contig036782-BurOR.A002	contig062344-NyeOR.A020	0.294	0.787	0.373
contig057156-BurOR.A015	contig047521-ZebOR.A020	0.294	0.787	0.374
contig054681-NyeOR.A011	contig056380-NyeOR.A016	0.294	0.789	0.373
contig085002-BriOR.A004	contig022225-TilOR.A005	0.294	0.792	0.372
contig085026-BriOR.A008	contig030557-ZebOR.A006	0.294	0.793	0.371
contig030554-ZebOR.A004	contig062095-ZebOR.A024	0.294	0.796	0.369
contig022259-TilOR.A014	contig041952-TilOR.A023	0.294	0.800	0.368
contig051573-BurOR.A011	contig034988-NyeOR.A005	0.294	0.806	0.365
contig085000-BriOR.A003	contig036780-BurOR.A001	0.294	0.813	0.362
contig022268-TilOR.A019	contig062095-ZebOR.A024	0.294	0.821	0.358
contig065887-BurOR.A018	contig030557-ZebOR.A006	0.294	0.826	0.356
contig034983-NyeOR.A003	contig070886-TilOR.A025	0.294	0.829	0.354
contig030557-ZebOR.A006	contig030566-ZebOR.A008	0.294	0.837	0.352
contig054868-NyeOR.A014	contig030553-ZebOR.A003	0.294	0.837	0.352
contig034988-NyeOR.A006	contig030566-ZebOR.A008	0.294	0.850	0.345
contig064570-BurOR.A017	contig041952-TilOR.A023	0.294	0.869	0.339
contig034983-NyeOR.A003	contig041952-TilOR.A023	0.294	0.874	0.336
contig041952-TilOR.A023	contig062095-ZebOR.A024	0.294	0.897	0.328
contig034988-NyeOR.A005	contig057754-NyeOR.A018	0.294	0.907	0.325
contig030560-ZebOR.A007	contig030576-ZebOR.A010	0.294	0.911	0.323
contig036784-BurOR.A003	contig047515-ZebOR.A018	0.294	0.936	0.314
contig085000-BriOR.A003	contig034983-NyeOR.A003	0.294	0.941	0.313
contig057165-NyeOR.A017	contig057756-NyeOR.A019	0.295	0.603	0.489
contig057756-NyeOR.A019	contig030552-ZebOR.A001	0.295	0.613	0.482
contig084999-BriOR.A001	contig047497-ZebOR.A011	0.295	0.628	0.469
contig084999-BriOR.A002	contig030553-ZebOR.A002	0.295	0.646	0.456
contig051566-BurOR.A009	contig022266-TilOR.A018	0.295	0.646	0.457
contig074640-TilOR.A002	contig030552-ZebOR.A001	0.295	0.647	0.456
contig093807-BriOR.A009	contig041951-TilOR.A022	0.295	0.648	0.456
contig022268-TilOR.A019	contig070886-TilOR.A025	0.295	0.650	0.454
contig022238-TilOR.A010	contig030552-ZebOR.A001	0.295	0.656	0.450
contig036782-BurOR.A002	contig034990-NyeOR.A007	0.295	0.657	0.449
contig022266-TilOR.A018	contig047526-ZebOR.A021	0.295	0.657	0.449
contig057165-NyeOR.A017	contig022238-TilOR.A010	0.295	0.666	0.444
contig030552-ZebOR.A001	contig047514-ZebOR.A017	0.295	0.669	0.441
contig085002-BriOR.A004	contig022259-TilOR.A014	0.295	0.671	0.440
contig093807-BriOR.A009	contig022268-TilOR.A020	0.295	0.674	0.438
contig057156-BurOR.A015	contig022238-TilOR.A010	0.295	0.674	0.438
contig051566-BurOR.A009	contig030560-ZebOR.A007	0.295	0.676	0.436
contig070886-TilOR.A025	contig030554-ZebOR.A004	0.295	0.679	0.434

contig093807-BriOR.A009	contig054681-NyeOR.A011	0.295	0.679	0.434
contig057165-NyeOR.A017	contig047514-ZebOR.A017	0.295	0.679	0.434
contig056375-NyeOR.A015	contig022266-TilOR.A018	0.295	0.683	0.433
contig057156-BurOR.A015	contig047508-ZebOR.A016	0.295	0.688	0.428
contig057156-BurOR.A015	contig022259-TilOR.A014	0.295	0.692	0.427
contig057165-NyeOR.A017	contig047499-ZebOR.A012	0.295	0.693	0.425
contig054684-NyeOR.A012	contig022238-TilOR.A010	0.295	0.694	0.425
contig070886-TilOR.A025	contig030556-ZebOR.A005	0.295	0.698	0.423
contig093812-BriOR.A010	contig030552-ZebOR.A001	0.295	0.703	0.419
contig036784-BurOR.A003	contig022225-TilOR.A005	0.295	0.704	0.419
contig022227-TilOR.A006	contig041951-TilOR.A022	0.295	0.709	0.416
contig054684-NyeOR.A012	contig047508-ZebOR.A016	0.295	0.709	0.416
contig093807-BriOR.A009	contig034988-NyeORs.A033	0.295	0.712	0.414
contig057756-NyeOR.A019	contig070886-TilOR.A025	0.295	0.713	0.415
contig034981-NyeOR.A001	contig030560-ZebOR.A007	0.295	0.718	0.410
contig064187-BurOR.A016	contig062344-NyeOR.A020	0.295	0.718	0.411
contig022265-TilOR.A017	contig073309-TilOR.A026	0.295	0.718	0.411
contig022265-TilOR.A017	contig041952-TilOR.A023	0.295	0.721	0.410
contig093816-BriOR.A011	contig047515-ZebOR.A018	0.295	0.725	0.406
contig085010-BriOR.A005	contig034981-NyeOR.A001	0.295	0.729	0.405
contig085010-BriOR.A005	contig022259-TilOR.A015	0.295	0.731	0.403
contig064187-BurOR.A016	contig034994-NyeOR.A008	0.295	0.735	0.401
contig022241-TilOR.A011	contig022266-TilOR.A018	0.295	0.736	0.400
contig034988-NyeOR.A006	contig034995-NyeOR.A009	0.295	0.739	0.400
contig034981-NyeOR.A001	contig054684-NyeOR.A012	0.295	0.740	0.398
contig034995-NyeOR.A009	contig047506-ZebOR.A014	0.295	0.746	0.396
contig093816-BriOR.A011	contig022234-TilOR.A009	0.295	0.749	0.393
contig022230-TilOR.A007	contig022251-TilOR.A013	0.295	0.749	0.394
contig093816-BriOR.A011	contig022259-TilOR.A014	0.295	0.750	0.393
contig054868-NyeOR.A014	contig022225-TilOR.A005	0.295	0.750	0.394
contig085002-BriOR.A004	contig085018-BriOR.A006	0.295	0.759	0.389
contig022265-TilOR.A017	contig047503-ZebOR.A013	0.295	0.765	0.385
contig054681-NyeOR.A011	contig022259-TilOR.A015	0.295	0.781	0.377
contig022259-TilOR.A014	contig047526-ZebOR.A021	0.295	0.787	0.375
contig051566-BurOR.A009	contig022259-TilOR.A014	0.295	0.788	0.374
contig034988-NyeOR.A005	contig054678-NyeOR.A010	0.295	0.789	0.374
contig036784-BurOR.A003	contig022234-TilOR.A009	0.295	0.790	0.373
contig051573-BurOR.A011	contig062095-ZebOR.A024	0.295	0.793	0.372
contig062344-NyeOR.A020	contig022266-TilOR.A018	0.295	0.793	0.372
contig057754-NyeOR.A018	contig022234-TilOR.A009	0.295	0.799	0.370
contig030556-ZebOR.A005	contig062095-ZebOR.A024	0.295	0.799	0.370
contig036782-BurOR.A002	contig030576-ZebOR.A010	0.295	0.801	0.369
contig054687-NyeOR.A013	contig030553-ZebOR.A002	0.295	0.801	0.369

contig054237-BurOR.A013	contig041951-TilOR.A022	0.295	0.815	0.362
contig085026-BriOR.A008	contig054868-NyeOR.A014	0.295	0.847	0.349
contig054868-NyeOR.A014	contig022211-TilOR.A003	0.295	0.850	0.347
contig022211-TilOR.A003	contig030557-ZebOR.A006	0.295	0.866	0.341
contig030553-ZebOR.A003	contig062095-ZebOR.A024	0.295	0.879	0.335
contig051570-BurOR.A010	contig057754-NyeOR.A018	0.295	0.884	0.334
contig054237-BurOR.A013	contig022264-TilOR.A016	0.295	0.887	0.333
contig057156-BurOR.A015	contig030576-ZebOR.A010	0.295	0.918	0.321
contig022251-TilOR.A013	contig047523-ZebOR.A022	0.295	0.924	0.319
contig054233-BurOR.A012	contig022251-TilOR.A013	0.295	0.924	0.319
contig030553-ZebOR.A003	contig047515-ZebOR.A018	0.295	0.930	0.317
contig034988-NyeORs.A033	contig074640-TilOR.A002	0.296	0.594	0.498
contig093807-BriOR.A009	contig022230-TilOR.A007	0.296	0.618	0.479
contig041951-TilOR.A022	contig047514-ZebOR.A017	0.296	0.623	0.475
contig030553-ZebOR.A002	contig047497-ZebOR.A011	0.296	0.624	0.474
contig030553-ZebOR.A003	contig030554-ZebOR.A004	0.296	0.654	0.452
contig034988-NyeOR.A004	contig030557-ZebOR.A006	0.296	0.656	0.451
contig034994-NyeOR.A008	contig022268-TilOR.A020	0.296	0.665	0.445
contig034994-NyeOR.A008	contig030552-ZebOR.A001	0.296	0.668	0.444
contig034994-NyeOR.A008	contig030557-ZebOR.A006	0.296	0.677	0.438
contig064187-BurOR.A016	contig022241-TilOR.A011	0.296	0.679	0.436
contig034995-NyeOR.A009	contig022268-TilOR.A020	0.296	0.683	0.433
contig057153-BurOR.A014	contig041952-TilOR.A023	0.296	0.684	0.433
contig034988-NyeOR.A004	contig030560-ZebOR.A007	0.296	0.685	0.432
contig036787-BurOR.A004	contig073309-TilOR.A026	0.296	0.688	0.431
contig034995-NyeOR.A009	contig057165-NyeOR.A017	0.296	0.690	0.429
contig034988-NyeOR.A006	contig047497-ZebOR.A011	0.296	0.690	0.429
contig022259-TilOR.A015	contig022265-TilOR.A017	0.296	0.692	0.429
contig022259-TilOR.A014	contig022265-TilOR.A017	0.296	0.695	0.426
contig022265-TilOR.A017	contig047526-ZebOR.A021	0.296	0.697	0.424
contig047497-ZebOR.A011	contig047506-ZebOR.A014	0.296	0.697	0.425
contig093807-BriOR.A009	contig036782-BurOR.A002	0.296	0.700	0.423
contig084999-BriOR.A002	contig054237-BurOR.A013	0.296	0.705	0.420
contig051559-BurOR.A007	contig062095-ZebOR.A024	0.296	0.710	0.417
contig034983-NyeOR.A002	contig062095-ZebOR.A024	0.296	0.713	0.415
contig047515-ZebOR.A019	contig062095-ZebOR.A024	0.296	0.713	0.415
contig022241-TilOR.A011	contig030554-ZebOR.A004	0.296	0.714	0.414
contig036782-BurOR.A002	contig022265-TilOR.A017	0.296	0.720	0.412
contig034988-NyeORs.A033	contig022251-TilOR.A013	0.296	0.724	0.409
contig051321-BurOR.A006	contig041951-TilOR.A021	0.296	0.724	0.409
contig036787-BurOR.A004	contig022259-TilOR.A015	0.296	0.728	0.407
contig022234-TilOR.A009	contig041952-TilOR.A023	0.296	0.734	0.403
contig056380-NyeOR.A016	contig041951-TilOR.A021	0.296	0.734	0.404

contig051321-BurOR.A006	contig022230-TilOR.A007	0.296	0.735	0.403
contig057756-NyeOR.A019	contig022232-TilOR.A008	0.296	0.738	0.401
contig057756-NyeOR.A019	contig022238-TilOR.A010	0.296	0.740	0.400
contig022259-TilOR.A015	contig030556-ZebOR.A005	0.296	0.741	0.400
contig056380-NyeOR.A016	contig022230-TilOR.A007	0.296	0.745	0.397
contig064187-BurOR.A016	contig030576-ZebOR.A010	0.296	0.745	0.398
contig036787-BurOR.A004	contig051570-BurOR.A010	0.296	0.746	0.397
contig084999-BriOR.A002	contig022259-TilOR.A015	0.296	0.747	0.396
contig036787-BurOR.A004	contig047508-ZebOR.A015	0.296	0.747	0.397
contig022268-TilOR.A019	contig047521-ZebOR.A020	0.296	0.749	0.395
contig085018-BriOR.A006	contig030554-ZebOR.A004	0.296	0.756	0.392
contig051570-BurOR.A010	contig030556-ZebOR.A005	0.296	0.759	0.390
contig034983-NyeOR.A002	contig054687-NyeOR.A013	0.296	0.759	0.390
contig054687-NyeOR.A013	contig047515-ZebOR.A019	0.296	0.759	0.390
contig030556-ZebOR.A005	contig047508-ZebOR.A015	0.296	0.760	0.390
contig064187-BurOR.A016	contig022259-TilOR.A015	0.296	0.762	0.389
contig022241-TilOR.A011	contig030557-ZebOR.A006	0.296	0.765	0.388
contig022259-TilOR.A015	contig030553-ZebOR.A002	0.296	0.766	0.387
contig051559-BurOR.A008	contig057756-NyeOR.A019	0.296	0.770	0.385
contig057153-BurOR.A014	contig056380-NyeOR.A016	0.296	0.780	0.380
contig036787-BurOR.A004	contig062095-ZebOR.A024	0.296	0.785	0.377
contig057156-BurOR.A015	contig022241-TilOR.A011	0.296	0.787	0.376
contig057153-BurOR.A014	contig022259-TilOR.A015	0.296	0.796	0.373
contig085026-BriOR.A008	contig054681-NyeOR.A011	0.296	0.797	0.371
contig084999-BriOR.A001	contig030576-ZebOR.A010	0.296	0.797	0.371
contig051570-BurOR.A010	contig051573-BurOR.A011	0.296	0.809	0.366
contig085026-BriOR.A008	contig041951-TilOR.A022	0.296	0.809	0.366
contig054237-BurOR.A013	contig057156-BurOR.A015	0.296	0.812	0.365
contig057754-NyeOR.A018	contig070885-TilOR.A024	0.296	0.821	0.360
contig022264-TilOR.A016	contig030553-ZebOR.A002	0.296	0.830	0.357
contig034988-NyeOR.A005	contig022264-TilOR.A016	0.296	0.837	0.354
contig057153-BurOR.A014	contig054687-NyeOR.A013	0.296	0.842	0.352
contig022259-TilOR.A014	contig047514-ZebOR.A017	0.296	0.847	0.349
contig093812-BriOR.A010	contig062095-ZebOR.A024	0.296	0.859	0.345
contig022264-TilOR.A016	contig047503-ZebOR.A013	0.296	0.863	0.343
contig057754-NyeOR.A018	contig047508-ZebOR.A015	0.296	0.865	0.343
contig022241-TilOR.A011	contig070886-TilOR.A025	0.296	0.869	0.340
contig054681-NyeOR.A011	contig022211-TilOR.A003	0.296	0.870	0.341
contig057754-NyeOR.A018	contig022232-TilOR.A008	0.296	0.898	0.329
contig036784-BurOR.A003	contig034983-NyeOR.A003	0.296	0.920	0.322
contig036780-BurOR.A001	contig057756-NyeOR.A019	0.297	0.603	0.493
contig022238-TilOR.A010	contig047515-ZebOR.A019	0.297	0.612	0.486
contig034983-NyeOR.A002	contig022238-TilOR.A010	0.297	0.612	0.486

contig057756-NyeOR.A019	contig022227-TilOR.A006	0.297	0.624	0.476
contig084999-BriOR.A002	contig036782-BurOR.A002	0.297	0.635	0.468
contig036780-BurOR.A001	contig074640-TilOR.A002	0.297	0.637	0.466
contig057165-NyeOR.A017	contig074640-TilOR.A002	0.297	0.637	0.466
contig030557-ZebOR.A006	contig047499-ZebOR.A012	0.297	0.639	0.465
contig093812-BriOR.A010	contig030557-ZebOR.A006	0.297	0.643	0.462
contig034988-NyeOR.A004	contig054681-NyeOR.A011	0.297	0.660	0.451
contig093807-BriOR.A009	contig064187-BurOR.A016	0.297	0.663	0.448
contig084999-BriOR.A001	contig051566-BurOR.A009	0.297	0.663	0.449
contig093807-BriOR.A009	contig030552-ZebOR.A001	0.297	0.671	0.443
contig030553-ZebOR.A002	contig062095-ZebOR.A024	0.297	0.674	0.441
contig036780-BurOR.A001	contig047514-ZebOR.A017	0.297	0.679	0.438
contig093807-BriOR.A009	contig057165-NyeOR.A017	0.297	0.681	0.436
contig030552-ZebOR.A001	contig047499-ZebOR.A012	0.297	0.682	0.435
contig084999-BriOR.A001	contig062095-ZebOR.A024	0.297	0.682	0.436
contig030560-ZebOR.A007	contig047508-ZebOR.A016	0.297	0.688	0.432
contig034983-NyeOR.A002	contig041952-TilOR.A023	0.297	0.694	0.428
contig041952-TilOR.A023	contig047515-ZebOR.A019	0.297	0.694	0.428
contig054678-NyeOR.A010	contig073309-TilOR.A026	0.297	0.705	0.422
contig057754-NyeOR.A018	contig022225-TilOR.A005	0.297	0.707	0.420
contig062344-NyeOR.A020	contig047506-ZebOR.A014	0.297	0.708	0.419
contig093812-BriOR.A010	contig036780-BurOR.A001	0.297	0.714	0.416
contig036782-BurOR.A002	contig036787-BurOR.A004	0.297	0.716	0.415
contig084999-BriOR.A002	contig085026-BriOR.A008	0.297	0.719	0.414
contig034994-NyeOR.A008	contig047506-ZebOR.A014	0.297	0.726	0.409
contig054868-NyeOR.A014	contig022227-TilOR.A006	0.297	0.731	0.407
contig057156-BurOR.A015	contig022227-TilOR.A006	0.297	0.735	0.404
contig093816-BriOR.A011	contig034983-NyeOR.A003	0.297	0.736	0.404
contig054678-NyeOR.A010	contig030553-ZebOR.A002	0.297	0.736	0.404
contig085002-BriOR.A004	contig064570-BurOR.A017	0.297	0.743	0.400
contig051559-BurOR.A007	contig047497-ZebOR.A011	0.297	0.751	0.395
contig093812-BriOR.A010	contig022232-TilOR.A008	0.297	0.756	0.393
contig057156-BurOR.A015	contig022251-TilOR.A013	0.297	0.757	0.392
contig022259-TilOR.A014	contig030553-ZebOR.A003	0.297	0.768	0.386
contig051570-BurOR.A010	contig054678-NyeOR.A010	0.297	0.776	0.383
contig054678-NyeOR.A010	contig047508-ZebOR.A015	0.297	0.777	0.382
contig036780-BurOR.A001	contig022259-TilOR.A015	0.297	0.783	0.379
contig051559-BurOR.A007	contig034994-NyeOR.A008	0.297	0.789	0.376
contig051573-BurOR.A011	contig047508-ZebOR.A015	0.297	0.792	0.375
contig022259-TilOR.A015	contig030552-ZebOR.A001	0.297	0.795	0.373
contig057754-NyeOR.A018	contig022259-TilOR.A014	0.297	0.800	0.372
contig030552-ZebOR.A001	contig030553-ZebOR.A003	0.297	0.805	0.368
contig057165-NyeOR.A017	contig030553-ZebOR.A003	0.297	0.805	0.368

contig051573-BurOR.A011	contig062344-NyeOR.A020	0.297	0.805	0.369
contig054684-NyeOR.A012	contig074640-TilOR.A002	0.297	0.808	0.367
contig022241-TilOR.A011	contig041952-TilOR.A023	0.297	0.811	0.366
contig057165-NyeOR.A017	contig057754-NyeOR.A018	0.297	0.813	0.366
contig057754-NyeOR.A018	contig030552-ZebOR.A001	0.297	0.813	0.366
contig034983-NyeOR.A002	contig034995-NyeOR.A009	0.297	0.814	0.364
contig034995-NyeOR.A009	contig047515-ZebOR.A019	0.297	0.814	0.364
contig030553-ZebOR.A002	contig030566-ZebOR.A008	0.297	0.814	0.365
contig051573-BurOR.A011	contig030576-ZebOR.A010	0.297	0.814	0.365
contig034990-NyeOR.A007	contig070885-TilOR.A024	0.297	0.816	0.364
contig065887-BurOR.A018	contig030553-ZebOR.A002	0.297	0.816	0.364
contig051573-BurOR.A011	contig047521-ZebOR.A020	0.297	0.816	0.365
contig054678-NyeOR.A010	contig062095-ZebOR.A024	0.297	0.818	0.363
contig062344-NyeOR.A020	contig030557-ZebOR.A006	0.297	0.826	0.359
contig054868-NyeOR.A014	contig047521-ZebOR.A020	0.297	0.828	0.358
contig034994-NyeOR.A008	contig062095-ZebOR.A024	0.297	0.834	0.356
contig036784-BurOR.A003	contig064570-BurOR.A017	0.297	0.837	0.355
contig041952-TilOR.A023	contig047503-ZebOR.A013	0.297	0.845	0.351
contig051570-BurOR.A010	contig070886-TilOR.A025	0.297	0.848	0.351
contig054687-NyeOR.A013	contig047521-ZebOR.A020	0.297	0.848	0.351
contig022238-TilOR.A010	contig070885-TilOR.A024	0.297	0.862	0.345
contig056375-NyeOR.A015	contig041952-TilOR.A023	0.297	0.864	0.344
contig030557-ZebOR.A006	contig030576-ZebOR.A010	0.297	0.866	0.342
contig056375-NyeOR.A015	contig070886-TilOR.A025	0.297	0.867	0.343
contig036784-BurOR.A003	contig047503-ZebOR.A013	0.297	0.875	0.339
contig085000-BriOR.A003	contig062095-ZebOR.A024	0.297	0.888	0.334
contig034983-NyeOR.A003	contig030553-ZebOR.A003	0.297	0.914	0.325
contig054681-NyeOR.A011	contig047499-ZebOR.A012	0.298	0.624	0.478
contig093812-BriOR.A010	contig054681-NyeOR.A011	0.298	0.627	0.475
contig036782-BurOR.A002	contig047497-ZebOR.A011	0.298	0.632	0.472
contig093816-BriOR.A011	contig057756-NyeOR.A019	0.298	0.642	0.465
contig030557-ZebOR.A006	contig047508-ZebOR.A016	0.298	0.654	0.457
contig084999-BriOR.A002	contig047526-ZebOR.A021	0.298	0.657	0.453
contig064570-BurOR.A017	contig034988-NyeORs.A033	0.298	0.658	0.453
contig084999-BriOR.A001	contig047526-ZebOR.A021	0.298	0.660	0.451
contig034994-NyeOR.A008	contig054681-NyeOR.A011	0.298	0.661	0.450
contig036780-BurOR.A001	contig022238-TilOR.A010	0.298	0.666	0.447
contig022241-TilOR.A011	contig047506-ZebOR.A014	0.298	0.670	0.445
contig022238-TilOR.A010	contig030560-ZebOR.A007	0.298	0.673	0.442
contig064187-BurOR.A016	contig047514-ZebOR.A017	0.298	0.678	0.439
contig057756-NyeOR.A019	contig030554-ZebOR.A004	0.298	0.679	0.438
contig034995-NyeOR.A009	contig030552-ZebOR.A001	0.298	0.679	0.439
contig051559-BurOR.A007	contig070886-TilOR.A025	0.298	0.679	0.439

contig093807-BriOR.A009	contig057153-BurOR.A014	0.298	0.687	0.434
contig057756-NyeOR.A019	contig022268-TilOR.A019	0.298	0.690	0.432
contig051566-BurOR.A009	contig022268-TilOR.A020	0.298	0.692	0.431
contig084999-BriOR.A002	contig047514-ZebOR.A017	0.298	0.700	0.425
contig054678-NyeOR.A010	contig022259-TilOR.A014	0.298	0.700	0.425
contig054684-NyeOR.A012	contig022259-TilOR.A014	0.298	0.702	0.424
contig057756-NyeOR.A019	contig022265-TilOR.A017	0.298	0.702	0.424
contig084999-BriOR.A001	contig056375-NyeOR.A015	0.298	0.708	0.421
contig022268-TilOR.A020	contig047526-ZebOR.A021	0.298	0.710	0.420
contig084999-BriOR.A002	contig022259-TilOR.A014	0.298	0.714	0.417
contig084999-BriOR.A002	contig074640-TilOR.A002	0.298	0.715	0.416
contig022234-TilOR.A009	contig047514-ZebOR.A017	0.298	0.718	0.415
contig034988-NyeOR.A006	contig034994-NyeOR.A008	0.298	0.719	0.414
contig022234-TilOR.A009	contig047526-ZebOR.A021	0.298	0.731	0.407
contig022266-TilOR.A018	contig062094-ZebOR.A023	0.298	0.732	0.408
contig051573-BurOR.A011	contig022234-TilOR.A009	0.298	0.734	0.405
contig030576-ZebOR.A010	contig047506-ZebOR.A014	0.298	0.735	0.406
contig022251-TilOR.A013	contig047515-ZebOR.A019	0.298	0.736	0.405
contig034983-NyeOR.A002	contig022251-TilOR.A013	0.298	0.736	0.405
contig030557-ZebOR.A006	contig062094-ZebOR.A023	0.298	0.739	0.403
contig022245-TilOR.A012	contig030557-ZebOR.A006	0.298	0.744	0.400
contig065887-BurOR.A018	contig034983-NyeOR.A002	0.298	0.748	0.399
contig065887-BurOR.A018	contig047515-ZebOR.A019	0.298	0.748	0.399
contig022251-TilOR.A013	contig030560-ZebOR.A007	0.298	0.750	0.397
contig022259-TilOR.A015	contig047506-ZebOR.A014	0.298	0.752	0.396
contig085010-BriOR.A005	contig022245-TilOR.A012	0.298	0.753	0.396
contig085010-BriOR.A005	contig022227-TilOR.A006	0.298	0.757	0.393
contig022234-TilOR.A009	contig022268-TilOR.A019	0.298	0.765	0.390
contig054681-NyeOR.A011	contig022241-TilOR.A011	0.298	0.769	0.387
contig054684-NyeOR.A012	contig022245-TilOR.A012	0.298	0.770	0.388
contig022217-TilOR.A004	contig030553-ZebOR.A002	0.298	0.772	0.386
contig073309-TilOR.A026	contig030553-ZebOR.A003	0.298	0.776	0.384
contig057756-NyeOR.A019	contig062344-NyeOR.A020	0.298	0.784	0.380
contig036784-BurOR.A003	contig022259-TilOR.A014	0.298	0.785	0.379
contig085026-BriOR.A008	contig057153-BurOR.A014	0.298	0.788	0.377
contig022211-TilOR.A003	contig022266-TilOR.A018	0.298	0.791	0.378
contig085010-BriOR.A005	contig022241-TilOR.A011	0.298	0.793	0.376
contig054237-BurOR.A013	contig054684-NyeOR.A012	0.298	0.800	0.373
contig054684-NyeOR.A012	contig022241-TilOR.A011	0.298	0.811	0.368
contig051559-BurOR.A008	contig070886-TilOR.A025	0.298	0.815	0.366
contig057153-BurOR.A014	contig065887-BurOR.A018	0.298	0.817	0.365
contig057153-BurOR.A014	contig030566-ZebOR.A008	0.298	0.828	0.360
contig054681-NyeOR.A011	contig062344-NyeOR.A020	0.298	0.830	0.359

contig070886-TilOR.A025	contig047508-ZebOR.A015	0.298	0.830	0.360
contig030553-ZebOR.A003	contig047523-ZebOR.A022	0.298	0.831	0.358
contig054233-BurOR.A012	contig030553-ZebOR.A003	0.298	0.831	0.358
contig054868-NyeOR.A014	contig057754-NyeOR.A018	0.298	0.831	0.358
contig064570-BurOR.A017	contig030553-ZebOR.A003	0.298	0.832	0.358
contig070885-TilOR.A024	contig047514-ZebOR.A017	0.298	0.832	0.359
contig093816-BriOR.A011	contig062344-NyeOR.A020	0.298	0.834	0.358
contig070885-TilOR.A024	contig047526-ZebOR.A021	0.298	0.836	0.356
contig085026-BriOR.A008	contig051573-BurOR.A011	0.298	0.840	0.354
contig034981-NyeOR.A001	contig054868-NyeOR.A014	0.298	0.845	0.353
contig022211-TilOR.A003	contig047523-ZebOR.A022	0.298	0.845	0.353
contig054233-BurOR.A012	contig022211-TilOR.A003	0.298	0.845	0.353
contig070886-TilOR.A025	contig062094-ZebOR.A023	0.298	0.846	0.352
contig054681-NyeOR.A011	contig054687-NyeOR.A013	0.298	0.855	0.348
contig030553-ZebOR.A003	contig047503-ZebOR.A013	0.298	0.856	0.348
contig084999-BriOR.A001	contig022264-TilOR.A016	0.298	0.857	0.348
contig034988-NyeOR.A005	contig070886-TilOR.A025	0.298	0.858	0.347
contig057153-BurOR.A014	contig022211-TilOR.A003	0.298	0.861	0.347
contig041952-TilOR.A023	contig030576-ZebOR.A010	0.298	0.865	0.344
contig054681-NyeOR.A011	contig030576-ZebOR.A010	0.298	0.871	0.342
contig022259-TilOR.A015	contig070886-TilOR.A025	0.298	0.899	0.332
contig057754-NyeOR.A018	contig047515-ZebOR.A018	0.298	0.925	0.322
contig030553-ZebOR.A002	contig047526-ZebOR.A021	0.299	0.626	0.478
contig051566-BurOR.A009	contig030553-ZebOR.A002	0.299	0.629	0.475
contig051559-BurOR.A007	contig047514-ZebOR.A017	0.299	0.644	0.464
contig054687-NyeOR.A013	contig022204-TilOR.A001	0.299	0.647	0.462
contig034994-NyeOR.A008	contig030553-ZebOR.A002	0.299	0.649	0.461
contig084999-BriOR.A001	contig034994-NyeOR.A008	0.299	0.651	0.459
contig093807-BriOR.A009	contig047506-ZebOR.A014	0.299	0.654	0.457
contig030557-ZebOR.A006	contig047497-ZebOR.A011	0.299	0.672	0.445
contig036787-BurOR.A004	contig022259-TilOR.A014	0.299	0.673	0.444
contig034990-NyeOR.A007	contig057165-NyeOR.A017	0.299	0.673	0.444
contig036780-BurOR.A001	contig034994-NyeOR.A008	0.299	0.678	0.440
contig084999-BriOR.A001	contig022245-TilOR.A012	0.299	0.679	0.440
contig093807-BriOR.A009	contig036780-BurOR.A001	0.299	0.681	0.439
contig041951-TilOR.A021	contig062094-ZebOR.A023	0.299	0.682	0.438
contig034994-NyeOR.A008	contig022232-TilOR.A008	0.299	0.683	0.438
contig064187-BurOR.A016	contig022245-TilOR.A012	0.299	0.684	0.437
contig022259-TilOR.A014	contig030556-ZebOR.A005	0.299	0.685	0.436
contig022259-TilOR.A014	contig030560-ZebOR.A007	0.299	0.686	0.435
contig036780-BurOR.A001	contig047499-ZebOR.A012	0.299	0.693	0.432
contig084999-BriOR.A002	contig022238-TilOR.A010	0.299	0.694	0.430
contig022245-TilOR.A012	contig022266-TilOR.A018	0.299	0.695	0.430

contig064570-BurOR.A017	contig034983-NyeOR.A002	0.299	0.695	0.431
contig064570-BurOR.A017	contig047515-ZebOR.A019	0.299	0.695	0.431
contig093812-BriOR.A010	contig057756-NyeOR.A019	0.299	0.695	0.431
contig051559-BurOR.A007	contig056375-NyeOR.A015	0.299	0.696	0.431
contig085010-BriOR.A005	contig022259-TilOR.A014	0.299	0.697	0.429
contig034988-NyeORs.A033	contig022259-TilOR.A015	0.299	0.699	0.428
contig034995-NyeOR.A009	contig030557-ZebOR.A006	0.299	0.704	0.424
contig034983-NyeOR.A002	contig056375-NyeOR.A015	0.299	0.709	0.422
contig056375-NyeOR.A015	contig047515-ZebOR.A019	0.299	0.709	0.422
contig030553-ZebOR.A002	contig062094-ZebOR.A023	0.299	0.718	0.416
contig051559-BurOR.A007	contig022251-TilOR.A013	0.299	0.722	0.414
contig054237-BurOR.A013	contig022268-TilOR.A019	0.299	0.724	0.412
contig054681-NyeOR.A011	contig022245-TilOR.A012	0.299	0.727	0.412
contig084999-BriOR.A002	contig022227-TilOR.A006	0.299	0.728	0.411
contig054687-NyeOR.A013	contig041951-TilOR.A021	0.299	0.729	0.410
contig022241-TilOR.A011	contig022265-TilOR.A017	0.299	0.731	0.409
contig056375-NyeOR.A015	contig030554-ZebOR.A004	0.299	0.734	0.407
contig057756-NyeOR.A019	contig047503-ZebOR.A013	0.299	0.737	0.405
contig054681-NyeOR.A011	contig062094-ZebOR.A023	0.299	0.743	0.402
contig085026-BriOR.A008	contig022268-TilOR.A019	0.299	0.744	0.402
contig022225-TilOR.A005	contig047523-ZebOR.A022	0.299	0.745	0.401
contig054233-BurOR.A012	contig022225-TilOR.A005	0.299	0.745	0.401
contig030560-ZebOR.A007	contig047521-ZebOR.A020	0.299	0.750	0.398
contig022245-TilOR.A012	contig030560-ZebOR.A007	0.299	0.752	0.398
contig034981-NyeOR.A001	contig022265-TilOR.A017	0.299	0.755	0.396
contig030566-ZebOR.A008	contig047515-ZebOR.A019	0.299	0.759	0.394
contig034983-NyeOR.A002	contig030566-ZebOR.A008	0.299	0.759	0.394
contig022251-TilOR.A013	contig022266-TilOR.A018	0.299	0.761	0.393
contig085010-BriOR.A005	contig047521-ZebOR.A020	0.299	0.762	0.393
contig034988-NyeOR.A006	contig022259-TilOR.A015	0.299	0.763	0.392
contig054684-NyeOR.A012	contig022251-TilOR.A013	0.299	0.768	0.389
contig054684-NyeOR.A012	contig022227-TilOR.A006	0.299	0.769	0.389
contig051573-BurOR.A011	contig047515-ZebOR.A018	0.299	0.770	0.388
contig054684-NyeOR.A012	contig047521-ZebOR.A020	0.299	0.774	0.386
contig054237-BurOR.A013	contig030560-ZebOR.A007	0.299	0.776	0.385
contig022268-TilOR.A019	contig047515-ZebOR.A018	0.299	0.776	0.385
contig022234-TilOR.A009	contig022238-TilOR.A010	0.299	0.779	0.384
contig036787-BurOR.A004	contig062344-NyeOR.A020	0.299	0.780	0.383
contig022232-TilOR.A008	contig070886-TilOR.A025	0.299	0.781	0.382
contig057165-NyeOR.A017	contig022259-TilOR.A015	0.299	0.783	0.382
contig074640-TilOR.A002	contig030560-ZebOR.A007	0.299	0.784	0.381
contig085000-BriOR.A003	contig022259-TilOR.A014	0.299	0.788	0.379
contig085010-BriOR.A005	contig054237-BurOR.A013	0.299	0.788	0.379

contig022241-TilOR.A011	contig030560-ZebOR.A007	0.299	0.792	0.378
contig034988-NyeOR.A004	contig022259-TilOR.A014	0.299	0.793	0.377
contig062344-NyeOR.A020	contig030556-ZebOR.A005	0.299	0.794	0.376
contig034994-NyeOR.A008	contig070885-TilOR.A024	0.299	0.796	0.376
contig022251-TilOR.A013	contig030557-ZebOR.A006	0.299	0.801	0.373
contig036780-BurOR.A001	contig030553-ZebOR.A003	0.299	0.805	0.371
contig093816-BriOR.A011	contig047521-ZebOR.A020	0.299	0.808	0.370
contig036780-BurOR.A001	contig057754-NyeOR.A018	0.299	0.813	0.368
contig085010-BriOR.A005	contig074640-TilOR.A002	0.299	0.820	0.365
contig085018-BriOR.A006	contig073309-TilOR.A026	0.299	0.823	0.363
contig022264-TilOR.A016	contig070885-TilOR.A024	0.299	0.824	0.363
contig022238-TilOR.A010	contig022259-TilOR.A014	0.299	0.827	0.361
contig022245-TilOR.A012	contig070886-TilOR.A025	0.299	0.829	0.361
contig085026-BriOR.A008	contig093816-BriOR.A011	0.299	0.832	0.360
contig051573-BurOR.A011	contig034981-NyeOR.A001	0.299	0.833	0.359
contig085026-BriOR.A008	contig047523-ZebOR.A022	0.299	0.841	0.355
contig085026-BriOR.A008	contig054233-BurOR.A012	0.299	0.841	0.355
contig093816-BriOR.A011	contig047503-ZebOR.A013	0.299	0.843	0.355
contig093816-BriOR.A011	contig030576-ZebOR.A010	0.299	0.844	0.354
contig051559-BurOR.A008	contig041952-TilOR.A023	0.299	0.860	0.347
contig085018-BriOR.A006	contig030553-ZebOR.A003	0.299	0.865	0.345
contig093812-BriOR.A010	contig034988-NyeOR.A005	0.299	0.865	0.346
contig093812-BriOR.A010	contig064570-BurOR.A017	0.299	0.871	0.343
contig085026-BriOR.A008	contig022264-TilOR.A016	0.299	0.889	0.336
contig085000-BriOR.A003	contig051559-BurOR.A008	0.299	0.954	0.313
contig093807-BriOR.A009	contig022266-TilOR.A018	0.300	0.634	0.473
contig022266-TilOR.A018	contig041952-TilOR.A023	0.300	0.636	0.471
contig084999-BriOR.A002	contig073309-TilOR.A026	0.300	0.645	0.465
contig057153-BurOR.A014	contig034988-NyeOR.A004	0.300	0.647	0.464
contig022230-TilOR.A007	contig047526-ZebOR.A021	0.300	0.648	0.463
contig054681-NyeOR.A011	contig047497-ZebOR.A011	0.300	0.656	0.458
contig054681-NyeOR.A011	contig047508-ZebOR.A016	0.300	0.657	0.456
contig084999-BriOR.A001	contig034988-NyeOR.A004	0.300	0.661	0.454
contig047506-ZebOR.A014	contig047514-ZebOR.A017	0.300	0.669	0.448
contig051559-BurOR.A007	contig022245-TilOR.A012	0.300	0.670	0.448
contig022245-TilOR.A012	contig047515-ZebOR.A019	0.300	0.682	0.439
contig034983-NyeOR.A002	contig022245-TilOR.A012	0.300	0.682	0.439
contig057756-NyeOR.A019	contig047499-ZebOR.A012	0.300	0.683	0.439
contig064570-BurOR.A017	contig030553-ZebOR.A002	0.300	0.683	0.439
contig085002-BriOR.A004	contig047521-ZebOR.A020	0.300	0.684	0.438
contig034995-NyeOR.A009	contig054681-NyeOR.A011	0.300	0.687	0.437
contig036780-BurOR.A001	contig034995-NyeOR.A009	0.300	0.690	0.435
contig051559-BurOR.A007	contig064570-BurOR.A017	0.300	0.693	0.433

contig085002-BriOR.A004	contig030553-ZebOR.A002	0.300	0.702	0.427
contig036787-BurOR.A004	contig070886-TilOR.A025	0.300	0.707	0.424
contig085002-BriOR.A004	contig047526-ZebOR.A021	0.300	0.707	0.425
contig034983-NyeOR.A002	contig062094-ZebOR.A023	0.300	0.710	0.422
contig047515-ZebOR.A019	contig062094-ZebOR.A023	0.300	0.710	0.422
contig084999-BriOR.A001	contig064570-BurOR.A017	0.300	0.712	0.421
contig085012-BriOR.A130	contig041951-TilOR.A021	0.300	0.717	0.418
contig034988-NyeOR.A006	contig062344-NyeOR.A020	0.300	0.719	0.418
contig022227-TilOR.A006	contig047523-ZebOR.A022	0.300	0.726	0.414
contig054233-BurOR.A012	contig022227-TilOR.A006	0.300	0.726	0.414
contig030554-ZebOR.A004	contig047514-ZebOR.A017	0.300	0.728	0.412
contig057756-NyeOR.A019	contig030556-ZebOR.A005	0.300	0.732	0.410
contig022217-TilOR.A004	contig022268-TilOR.A019	0.300	0.743	0.404
contig036782-BurOR.A002	contig054678-NyeOR.A010	0.300	0.745	0.402
contig085026-BriOR.A008	contig030560-ZebOR.A007	0.300	0.754	0.398
contig074640-TilOR.A002	contig022265-TilOR.A017	0.300	0.757	0.397
contig057156-BurOR.A015	contig022245-TilOR.A012	0.300	0.758	0.396
contig057153-BurOR.A014	contig022241-TilOR.A011	0.300	0.760	0.394
contig051321-BurOR.A006	contig022266-TilOR.A018	0.300	0.766	0.392
contig051321-BurOR.A006	contig030557-ZebOR.A006	0.300	0.773	0.388
contig085026-BriOR.A008	contig054684-NyeOR.A012	0.300	0.778	0.386
contig054681-NyeOR.A011	contig022251-TilOR.A013	0.300	0.782	0.383
contig057156-BurOR.A015	contig074640-TilOR.A002	0.300	0.789	0.380
contig051573-BurOR.A011	contig064570-BurOR.A017	0.300	0.804	0.373
contig084999-BriOR.A001	contig022259-TilOR.A015	0.300	0.805	0.373
contig022264-TilOR.A016	contig041951-TilOR.A021	0.300	0.805	0.373
contig057756-NyeOR.A019	contig030576-ZebOR.A010	0.300	0.811	0.370
contig070886-TilOR.A025	contig047521-ZebOR.A020	0.300	0.812	0.369
contig054678-NyeOR.A010	contig062344-NyeOR.A020	0.300	0.812	0.369
contig051570-BurOR.A010	contig022264-TilOR.A016	0.300	0.817	0.367
contig057153-BurOR.A014	contig062344-NyeOR.A020	0.300	0.821	0.365
contig036782-BurOR.A002	contig054687-NyeOR.A013	0.300	0.823	0.364
contig047521-ZebOR.A020	contig047523-ZebOR.A022	0.300	0.823	0.365
contig054233-BurOR.A012	contig047521-ZebOR.A020	0.300	0.823	0.365
contig093816-BriOR.A011	contig034981-NyeOR.A001	0.300	0.826	0.364
contig065887-BurOR.A018	contig054681-NyeOR.A011	0.300	0.831	0.361
contig085000-BriOR.A003	contig054868-NyeOR.A014	0.300	0.837	0.359
contig085000-BriOR.A003	contig064570-BurOR.A017	0.300	0.840	0.357
contig036784-BurOR.A003	contig054868-NyeOR.A014	0.300	0.840	0.357
contig054681-NyeOR.A011	contig030566-ZebOR.A008	0.300	0.842	0.356
contig051559-BurOR.A007	contig022264-TilOR.A016	0.300	0.848	0.354
contig093812-BriOR.A010	contig051570-BurOR.A010	0.300	0.857	0.350
contig034981-NyeOR.A001	contig054687-NyeOR.A013	0.300	0.859	0.349

contig030566-ZebOR.A008	contig047521-ZebOR.A020	0.300	0.860	0.349
contig057153-BurOR.A014	contig030576-ZebOR.A010	0.300	0.861	0.348
contig065887-BurOR.A018	contig047521-ZebOR.A020	0.300	0.862	0.348
contig093807-BriOR.A009	contig022259-TilOR.A014	0.300	0.863	0.348
contig085018-BriOR.A006	contig036784-BurOR.A003	0.300	0.869	0.346
contig034981-NyeOR.A001	contig073309-TilOR.A026	0.300	0.878	0.342
contig056380-NyeOR.A016	contig070886-TilOR.A025	0.300	0.884	0.339
contig057754-NyeOR.A018	contig062095-ZebOR.A024	0.300	0.888	0.337
contig022259-TilOR.A015	contig073309-TilOR.A026	0.300	0.892	0.337
contig034995-NyeOR.A009	contig062095-ZebOR.A024	0.300	0.894	0.335
contig034983-NyeOR.A003	contig057754-NyeOR.A018	0.300	0.910	0.330
contig036784-BurOR.A003	contig051559-BurOR.A008	0.300	0.932	0.322
contig054687-NyeOR.A013	contig022211-TilOR.A003	0.300	0.955	0.314
contig034990-NyeOR.A007	contig030557-ZebOR.A006	0.301	0.626	0.480
contig057153-BurOR.A014	contig047499-ZebOR.A012	0.301	0.631	0.478
contig093812-BriOR.A010	contig057153-BurOR.A014	0.301	0.634	0.475
contig034988-NyeOR.A004	contig022266-TilOR.A018	0.301	0.640	0.471
contig093807-BriOR.A009	contig034988-NyeOR.A006	0.301	0.648	0.465
contig022204-TilOR.A001	contig030566-ZebOR.A008	0.301	0.656	0.459
contig065887-BurOR.A018	contig022204-TilOR.A001	0.301	0.657	0.458
contig036782-BurOR.A002	contig051566-BurOR.A009	0.301	0.657	0.459
contig034990-NyeOR.A007	contig057756-NyeOR.A019	0.301	0.658	0.457
contig084999-BriOR.A001	contig034995-NyeOR.A009	0.301	0.659	0.457
contig084999-BriOR.A002	contig051566-BurOR.A009	0.301	0.662	0.456
contig030554-ZebOR.A004	contig047526-ZebOR.A021	0.301	0.663	0.453
contig034990-NyeOR.A007	contig030552-ZebOR.A001	0.301	0.663	0.455
contig093807-BriOR.A009	contig051559-BurOR.A007	0.301	0.665	0.453
contig073309-TilOR.A026	contig030554-ZebOR.A004	0.301	0.666	0.453
contig057153-BurOR.A014	contig034994-NyeOR.A008	0.301	0.668	0.450
contig041951-TilOR.A022	contig047497-ZebOR.A011	0.301	0.672	0.448
contig022245-TilOR.A012	contig047506-ZebOR.A014	0.301	0.675	0.446
contig030556-ZebOR.A005	contig047526-ZebOR.A021	0.301	0.682	0.442
contig034983-NyeOR.A002	contig070886-TilOR.A025	0.301	0.687	0.438
contig070886-TilOR.A025	contig047515-ZebOR.A019	0.301	0.687	0.438
contig022265-TilOR.A017	contig047514-ZebOR.A017	0.301	0.694	0.433
contig034994-NyeOR.A008	contig041951-TilOR.A022	0.301	0.694	0.434
contig064187-BurOR.A016	contig062095-ZebOR.A024	0.301	0.698	0.431
contig056375-NyeOR.A015	contig030553-ZebOR.A002	0.301	0.700	0.429
contig034988-NyeOR.A004	contig022268-TilOR.A020	0.301	0.701	0.429
contig034995-NyeOR.A009	contig041951-TilOR.A022	0.301	0.705	0.427
contig093807-BriOR.A009	contig057756-NyeOR.A019	0.301	0.707	0.426
contig030554-ZebOR.A004	contig047521-ZebOR.A020	0.301	0.709	0.425
contig085010-BriOR.A005	contig047514-ZebOR.A017	0.301	0.712	0.423

contig022268-TilOR.A019	contig047514-ZebOR.A017	0.301	0.715	0.421
contig085018-BriOR.A006	contig034983-NyeOR.A002	0.301	0.716	0.420
contig085018-BriOR.A006	contig047515-ZebOR.A019	0.301	0.716	0.420
contig022227-TilOR.A006	contig022265-TilOR.A017	0.301	0.722	0.416
contig022259-TilOR.A015	contig041951-TilOR.A022	0.301	0.724	0.415
contig054678-NyeOR.A010	contig070886-TilOR.A025	0.301	0.725	0.415
contig036787-BurOR.A004	contig022241-TilOR.A011	0.301	0.728	0.413
contig057153-BurOR.A014	contig062094-ZebOR.A023	0.301	0.735	0.409
contig057153-BurOR.A014	contig022245-TilOR.A012	0.301	0.740	0.407
contig022241-TilOR.A011	contig030556-ZebOR.A005	0.301	0.742	0.406
contig022259-TilOR.A015	contig022268-TilOR.A019	0.301	0.742	0.406
contig022238-TilOR.A010	contig030554-ZebOR.A004	0.301	0.743	0.406
contig084999-BriOR.A002	contig022211-TilOR.A003	0.301	0.745	0.403
contig085010-BriOR.A005	contig085026-BriOR.A008	0.301	0.766	0.392
contig057754-NyeOR.A018	contig073309-TilOR.A026	0.301	0.770	0.391
contig051321-BurOR.A006	contig054681-NyeOR.A011	0.301	0.778	0.387
contig064570-BurOR.A017	contig054678-NyeOR.A010	0.301	0.780	0.386
contig051573-BurOR.A011	contig034983-NyeOR.A003	0.301	0.781	0.385
contig064570-BurOR.A017	contig030554-ZebOR.A004	0.301	0.782	0.384
contig036782-BurOR.A002	contig022259-TilOR.A015	0.301	0.787	0.382
contig034983-NyeOR.A003	contig022268-TilOR.A019	0.301	0.787	0.382
contig085018-BriOR.A006	contig022266-TilOR.A018	0.301	0.798	0.378
contig022264-TilOR.A016	contig047508-ZebOR.A015	0.301	0.800	0.376
contig051573-BurOR.A011	contig056375-NyeOR.A015	0.301	0.800	0.376
contig036780-BurOR.A001	contig056380-NyeOR.A016	0.301	0.801	0.376
contig084999-BriOR.A001	contig022217-TilOR.A004	0.301	0.805	0.373
contig064570-BurOR.A017	contig022268-TilOR.A019	0.301	0.807	0.373
contig074640-TilOR.A002	contig041951-TilOR.A022	0.301	0.811	0.371
contig093816-BriOR.A011	contig062095-ZebOR.A024	0.301	0.812	0.371
contig056380-NyeOR.A016	contig030552-ZebOR.A001	0.301	0.813	0.371
contig085010-BriOR.A005	contig047515-ZebOR.A018	0.301	0.820	0.367
contig093816-BriOR.A011	contig064570-BurOR.A017	0.301	0.823	0.366
contig054233-BurOR.A012	contig057754-NyeOR.A018	0.301	0.825	0.365
contig057754-NyeOR.A018	contig047523-ZebOR.A022	0.301	0.825	0.365
contig034981-NyeOR.A001	contig070886-TilOR.A025	0.301	0.829	0.363
contig085018-BriOR.A006	contig030557-ZebOR.A006	0.301	0.830	0.362
contig085026-BriOR.A008	contig070886-TilOR.A025	0.301	0.830	0.363
contig062344-NyeOR.A020	contig073309-TilOR.A026	0.301	0.832	0.362
contig022264-TilOR.A016	contig030557-ZebOR.A006	0.301	0.837	0.359
contig093812-BriOR.A010	contig047508-ZebOR.A015	0.301	0.839	0.359
contig084999-BriOR.A001	contig054687-NyeOR.A013	0.301	0.839	0.359
contig064570-BurOR.A017	contig057754-NyeOR.A018	0.301	0.840	0.358
contig034981-NyeOR.A001	contig047523-ZebOR.A022	0.301	0.840	0.359

contig054233-BurOR.A012	contig034981-NyeOR.A001	0.301	0.840	0.359
contig054237-BurOR.A013	contig054868-NyeOR.A014	0.301	0.841	0.358
contig022217-TilOR.A004	contig073309-TilOR.A026	0.301	0.843	0.357
contig047526-ZebOR.A021	contig062095-ZebOR.A024	0.301	0.846	0.356
contig051566-BurOR.A009	contig062095-ZebOR.A024	0.301	0.848	0.355
contig036782-BurOR.A002	contig022264-TilOR.A016	0.301	0.853	0.353
contig051566-BurOR.A009	contig070885-TilOR.A024	0.301	0.857	0.351
contig062344-NyeOR.A020	contig022264-TilOR.A016	0.301	0.857	0.352
contig051566-BurOR.A009	contig064570-BurOR.A017	0.301	0.859	0.350
contig034988-NyeOR.A005	contig054687-NyeOR.A013	0.301	0.860	0.351
contig022211-TilOR.A003	contig070886-TilOR.A025	0.301	0.882	0.341
contig057754-NyeOR.A018	contig047503-ZebOR.A013	0.301	0.892	0.337
contig022232-TilOR.A008	contig022264-TilOR.A016	0.301	0.915	0.329
contig030576-ZebOR.A010	contig047514-ZebOR.A017	0.301	0.980	0.307
contig034990-NyeOR.A007	contig054681-NyeOR.A011	0.302	0.611	0.494
contig034988-NyeOR.A004	contig030553-ZebOR.A002	0.302	0.627	0.481
contig022241-TilOR.A011	contig041951-TilOR.A022	0.302	0.652	0.463
contig036782-BurOR.A002	contig047526-ZebOR.A021	0.302	0.654	0.462
contig034995-NyeOR.A009	contig030553-ZebOR.A002	0.302	0.654	0.462
contig036782-BurOR.A002	contig034994-NyeOR.A008	0.302	0.658	0.459
contig057165-NyeOR.A017	contig047497-ZebOR.A011	0.302	0.658	0.460
contig051566-BurOR.A009	contig057756-NyeOR.A019	0.302	0.663	0.455
contig034988-NyeOR.A006	contig047514-ZebOR.A017	0.302	0.663	0.456
contig064187-BurOR.A016	contig047526-ZebOR.A021	0.302	0.669	0.451
contig036782-BurOR.A002	contig062095-ZebOR.A024	0.302	0.672	0.449
contig034988-NyeOR.A006	contig022241-TilOR.A011	0.302	0.681	0.443
contig022238-TilOR.A010	contig022268-TilOR.A019	0.302	0.690	0.437
contig085002-BriOR.A004	contig036782-BurOR.A002	0.302	0.690	0.438
contig084999-BriOR.A002	contig034988-NyeOR.A004	0.302	0.691	0.437
contig062344-NyeOR.A020	contig041951-TilOR.A022	0.302	0.693	0.436
contig085002-BriOR.A004	contig056375-NyeOR.A015	0.302	0.697	0.433
contig051559-BurOR.A007	contig062094-ZebOR.A023	0.302	0.708	0.427
contig093812-BriOR.A010	contig022225-TilOR.A005	0.302	0.711	0.424
contig085018-BriOR.A006	contig051559-BurOR.A007	0.302	0.719	0.420
contig036787-BurOR.A004	contig057756-NyeOR.A019	0.302	0.719	0.420
contig093816-BriOR.A011	contig051559-BurOR.A008	0.302	0.723	0.417
contig041951-TilOR.A021	contig030566-ZebOR.A008	0.302	0.740	0.408
contig065887-BurOR.A018	contig041951-TilOR.A021	0.302	0.741	0.407
contig051321-BurOR.A006	contig034983-NyeOR.A002	0.302	0.745	0.405
contig051321-BurOR.A006	contig047515-ZebOR.A019	0.302	0.745	0.405
contig034988-NyeOR.A006	contig030576-ZebOR.A010	0.302	0.746	0.404
contig036787-BurOR.A004	contig064570-BurOR.A017	0.302	0.749	0.404
contig034983-NyeOR.A002	contig056380-NyeOR.A016	0.302	0.755	0.400

contig056380-NyeOR.A016	contig047515-ZebOR.A019	0.302	0.755	0.400
contig056375-NyeOR.A015	contig022268-TilOR.A019	0.302	0.757	0.398
contig022217-TilOR.A004	contig022266-TilOR.A018	0.302	0.757	0.399
contig084999-BriOR.A002	contig022217-TilOR.A004	0.302	0.759	0.397
contig054678-NyeOR.A010	contig022241-TilOR.A011	0.302	0.759	0.398
contig034983-NyeOR.A002	contig047497-ZebOR.A011	0.302	0.760	0.397
contig047497-ZebOR.A011	contig047515-ZebOR.A019	0.302	0.760	0.397
contig064570-BurOR.A017	contig030556-ZebOR.A005	0.302	0.762	0.397
contig030556-ZebOR.A005	contig047514-ZebOR.A017	0.302	0.765	0.394
contig022227-TilOR.A006	contig030560-ZebOR.A007	0.302	0.768	0.393
contig047514-ZebOR.A017	contig047515-ZebOR.A018	0.302	0.773	0.391
contig093807-BriOR.A009	contig022234-TilOR.A009	0.302	0.777	0.389
contig084999-BriOR.A001	contig062094-ZebOR.A023	0.302	0.778	0.388
contig085018-BriOR.A006	contig030553-ZebOR.A002	0.302	0.783	0.385
contig022259-TilOR.A014	contig047508-ZebOR.A016	0.302	0.790	0.382
contig057153-BurOR.A014	contig022251-TilOR.A013	0.302	0.797	0.379
contig047515-ZebOR.A018	contig047526-ZebOR.A021	0.302	0.799	0.378
contig034983-NyeOR.A002	contig034994-NyeOR.A008	0.302	0.799	0.378
contig034994-NyeOR.A008	contig047515-ZebOR.A019	0.302	0.799	0.378
contig036784-BurOR.A003	contig030552-ZebOR.A001	0.302	0.810	0.373
contig036784-BurOR.A003	contig057165-NyeOR.A017	0.302	0.810	0.373
contig057156-BurOR.A015	contig047515-ZebOR.A018	0.302	0.812	0.372
contig047497-ZebOR.A011	contig062095-ZebOR.A024	0.302	0.813	0.372
contig036784-BurOR.A003	contig022245-TilOR.A012	0.302	0.815	0.370
contig073309-TilOR.A026	contig062094-ZebOR.A023	0.302	0.816	0.370
contig034994-NyeOR.A008	contig056375-NyeOR.A015	0.302	0.816	0.371
contig022268-TilOR.A020	contig030572-ZebOR.A009	0.302	0.823	0.366
contig051318-BurOR.A005	contig022268-TilOR.A020	0.302	0.823	0.366
contig085018-BriOR.A006	contig054681-NyeOR.A011	0.302	0.835	0.361
contig036782-BurOR.A002	contig030566-ZebOR.A008	0.302	0.837	0.361
contig036782-BurOR.A002	contig065887-BurOR.A018	0.302	0.838	0.360
contig034988-NyeOR.A005	contig047499-ZebOR.A012	0.302	0.845	0.357
contig064187-BurOR.A016	contig074640-TilOR.A002	0.302	0.845	0.357
contig034988-NyeOR.A005	contig047514-ZebOR.A017	0.302	0.849	0.356
contig070886-TilOR.A025	contig047503-ZebOR.A013	0.302	0.850	0.355
contig054237-BurOR.A013	contig054687-NyeOR.A013	0.302	0.862	0.350
contig085000-BriOR.A003	contig085018-BriOR.A006	0.302	0.865	0.350
contig034981-NyeOR.A001	contig030566-ZebOR.A008	0.302	0.871	0.347
contig065887-BurOR.A018	contig034981-NyeOR.A001	0.302	0.873	0.346
contig051321-BurOR.A006	contig070886-TilOR.A025	0.302	0.886	0.341
contig022217-TilOR.A004	contig070886-TilOR.A025	0.302	0.898	0.336
contig034988-NyeOR.A005	contig022238-TilOR.A010	0.302	0.898	0.337
contig051559-BurOR.A008	contig030553-ZebOR.A003	0.302	0.927	0.326

contig062344-NyeOR.A020	contig047514-ZebOR.A017	0.302	0.943	0.320
contig022211-TilOR.A003	contig030566-ZebOR.A008	0.302	0.969	0.312
contig065887-BurOR.A018	contig022211-TilOR.A003	0.302	0.971	0.311
contig057153-BurOR.A014	contig047508-ZebOR.A016	0.303	0.645	0.469
contig084999-BriOR.A002	contig057756-NyeOR.A019	0.303	0.658	0.460
contig084999-BriOR.A001	contig047508-ZebOR.A016	0.303	0.659	0.459
contig057153-BurOR.A014	contig047497-ZebOR.A011	0.303	0.663	0.457
contig051566-BurOR.A009	contig030552-ZebOR.A001	0.303	0.672	0.451
contig085010-BriOR.A005	contig047526-ZebOR.A021	0.303	0.673	0.449
contig022245-TilOR.A012	contig041951-TilOR.A022	0.303	0.676	0.448
contig093807-BriOR.A009	contig057156-BurOR.A015	0.303	0.680	0.446
contig051566-BurOR.A009	contig057165-NyeOR.A017	0.303	0.683	0.444
contig047506-ZebOR.A014	contig062095-ZebOR.A024	0.303	0.689	0.439
contig022232-TilOR.A008	contig047497-ZebOR.A011	0.303	0.695	0.435
contig057153-BurOR.A014	contig034995-NyeOR.A009	0.303	0.695	0.436
contig022268-TilOR.A020	contig047508-ZebOR.A016	0.303	0.698	0.434
contig074640-TilOR.A002	contig030554-ZebOR.A004	0.303	0.700	0.433
contig093807-BriOR.A009	contig054684-NyeOR.A012	0.303	0.700	0.433
contig041951-TilOR.A022	contig030576-ZebOR.A010	0.303	0.719	0.422
contig022259-TilOR.A015	contig030554-ZebOR.A004	0.303	0.731	0.414
contig022211-TilOR.A003	contig022268-TilOR.A019	0.303	0.736	0.411
contig051321-BurOR.A006	contig030553-ZebOR.A002	0.303	0.741	0.409
contig084999-BriOR.A002	contig022234-TilOR.A009	0.303	0.742	0.408
contig064187-BurOR.A016	contig022251-TilOR.A013	0.303	0.743	0.408
contig054868-NyeOR.A014	contig022204-TilOR.A001	0.303	0.746	0.406
contig036782-BurOR.A002	contig062094-ZebOR.A023	0.303	0.749	0.405
contig085010-BriOR.A005	contig022251-TilOR.A013	0.303	0.751	0.403
contig084999-BriOR.A001	contig030572-ZebOR.A009	0.303	0.766	0.396
contig084999-BriOR.A001	contig051318-BurOR.A005	0.303	0.766	0.396
contig051321-BurOR.A006	contig057153-BurOR.A014	0.303	0.769	0.394
contig054237-BurOR.A013	contig022265-TilOR.A017	0.303	0.769	0.394
contig036782-BurOR.A002	contig022217-TilOR.A004	0.303	0.770	0.393
contig056380-NyeOR.A016	contig030553-ZebOR.A002	0.303	0.773	0.392
contig022217-TilOR.A004	contig030557-ZebOR.A006	0.303	0.776	0.391
contig022238-TilOR.A010	contig047515-ZebOR.A018	0.303	0.778	0.389
contig022265-TilOR.A017	contig047521-ZebOR.A020	0.303	0.789	0.384
contig022245-TilOR.A012	contig030553-ZebOR.A003	0.303	0.797	0.380
contig034988-NyeOR.A005	contig047526-ZebOR.A021	0.303	0.807	0.375
contig093812-BriOR.A010	contig056375-NyeOR.A015	0.303	0.809	0.374
contig070885-TilOR.A024	contig047497-ZebOR.A011	0.303	0.817	0.371
contig051573-BurOR.A011	contig022217-TilOR.A004	0.303	0.826	0.367
contig084999-BriOR.A001	contig030566-ZebOR.A008	0.303	0.828	0.366
contig084999-BriOR.A001	contig065887-BurOR.A018	0.303	0.829	0.365

contig047503-ZebOR.A013	contig047514-ZebOR.A017	0.303	0.830	0.365
contig030560-ZebOR.A007	contig047515-ZebOR.A018	0.303	0.830	0.366
contig051570-BurOR.A010	contig047499-ZebOR.A012	0.303	0.837	0.362
contig022238-TilOR.A010	contig047503-ZebOR.A013	0.303	0.839	0.362
contig051573-BurOR.A011	contig022211-TilOR.A003	0.303	0.848	0.357
contig034988-NyeOR.A004	contig070885-TilOR.A024	0.303	0.875	0.346
contig047499-ZebOR.A012	contig062095-ZebOR.A024	0.303	0.883	0.343
contig051573-BurOR.A011	contig022259-TilOR.A015	0.303	0.887	0.342
contig030576-ZebOR.A010	contig047526-ZebOR.A021	0.303	0.895	0.338
contig022264-TilOR.A016	contig030576-ZebOR.A010	0.303	0.903	0.335
contig085018-BriOR.A006	contig070886-TilOR.A025	0.303	0.921	0.329
contig030553-ZebOR.A002	contig047508-ZebOR.A016	0.304	0.625	0.487
contig054687-NyeOR.A013	contig022225-TilOR.A005	0.304	0.631	0.481
contig056375-NyeOR.A015	contig034988-NyeORs.A033	0.304	0.634	0.479
contig022266-TilOR.A018	contig047508-ZebOR.A016	0.304	0.637	0.477
contig034983-NyeOR.A002	contig047514-ZebOR.A017	0.304	0.651	0.467
contig047514-ZebOR.A017	contig047515-ZebOR.A019	0.304	0.651	0.467
contig036782-BurOR.A002	contig034988-NyeOR.A004	0.304	0.655	0.465
contig034988-NyeOR.A006	contig047526-ZebOR.A021	0.304	0.655	0.465
contig036780-BurOR.A001	contig034990-NyeOR.A007	0.304	0.673	0.451
contig085010-BriOR.A005	contig022238-TilOR.A010	0.304	0.677	0.449
contig093807-BriOR.A009	contig022265-TilOR.A017	0.304	0.680	0.447
contig085010-BriOR.A005	contig051566-BurOR.A009	0.304	0.680	0.447
contig054237-BurOR.A013	contig030554-ZebOR.A004	0.304	0.685	0.444
contig034988-NyeOR.A006	contig022245-TilOR.A012	0.304	0.686	0.444
contig041951-TilOR.A022	contig062094-ZebOR.A023	0.304	0.687	0.442
contig085010-BriOR.A005	contig034988-NyeOR.A004	0.304	0.688	0.442
contig034988-NyeOR.A006	contig062095-ZebOR.A024	0.304	0.689	0.441
contig022238-TilOR.A010	contig022265-TilOR.A017	0.304	0.690	0.440
contig057756-NyeOR.A019	contig062095-ZebOR.A024	0.304	0.691	0.441
contig084999-BriOR.A002	contig047508-ZebOR.A016	0.304	0.694	0.437
contig064187-BurOR.A016	contig064570-BurOR.A017	0.304	0.702	0.433
contig036782-BurOR.A002	contig064570-BurOR.A017	0.304	0.702	0.434
contig054687-NyeOR.A013	contig034988-NyeORs.A033	0.304	0.704	0.432
contig085026-BriOR.A008	contig030554-ZebOR.A004	0.304	0.704	0.432
contig022230-TilOR.A007	contig030572-ZebOR.A009	0.304	0.706	0.430
contig051318-BurOR.A005	contig022230-TilOR.A007	0.304	0.706	0.430
contig051566-BurOR.A009	contig030554-ZebOR.A004	0.304	0.709	0.429
contig022245-TilOR.A012	contig022265-TilOR.A017	0.304	0.723	0.420
contig022232-TilOR.A008	contig047499-ZebOR.A012	0.304	0.726	0.419
contig041951-TilOR.A021	contig030572-ZebOR.A009	0.304	0.728	0.418
contig051318-BurOR.A005	contig041951-TilOR.A021	0.304	0.728	0.418
contig022217-TilOR.A004	contig030554-ZebOR.A004	0.304	0.733	0.415

contig051321-BurOR.A006	contig051559-BurOR.A007	0.304	0.742	0.410
contig034995-NyeOR.A009	contig022232-TilOR.A008	0.304	0.744	0.409
contig064187-BurOR.A016	contig022217-TilOR.A004	0.304	0.747	0.407
contig051559-BurOR.A007	contig056380-NyeOR.A016	0.304	0.752	0.405
contig022251-TilOR.A013	contig030553-ZebOR.A002	0.304	0.755	0.403
contig022225-TilOR.A005	contig073309-TilOR.A026	0.304	0.756	0.402
contig084999-BriOR.A001	contig022251-TilOR.A013	0.304	0.764	0.398
contig085002-BriOR.A004	contig022264-TilOR.A016	0.304	0.772	0.394
contig034988-NyeORs.A033	contig022264-TilOR.A016	0.304	0.773	0.393
contig085000-BriOR.A003	contig073309-TilOR.A026	0.304	0.776	0.391
contig085002-BriOR.A004	contig047514-ZebOR.A017	0.304	0.778	0.391
contig036784-BurOR.A003	contig073309-TilOR.A026	0.304	0.779	0.390
contig054681-NyeOR.A011	contig022217-TilOR.A004	0.304	0.780	0.390
contig034983-NyeOR.A003	contig047514-ZebOR.A017	0.304	0.784	0.388
contig084999-BriOR.A002	contig047515-ZebOR.A018	0.304	0.785	0.386
contig051570-BurOR.A010	contig047526-ZebOR.A021	0.304	0.797	0.382
contig056380-NyeOR.A016	contig057165-NyeOR.A017	0.304	0.801	0.379
contig057156-BurOR.A015	contig056380-NyeOR.A016	0.304	0.809	0.376
contig036780-BurOR.A001	contig036784-BurOR.A003	0.304	0.810	0.375
contig034983-NyeOR.A003	contig047526-ZebOR.A021	0.304	0.810	0.375
contig047499-ZebOR.A012	contig047508-ZebOR.A015	0.304	0.820	0.371
contig036784-BurOR.A003	contig056375-NyeOR.A015	0.304	0.820	0.371
contig057156-BurOR.A015	contig034983-NyeOR.A003	0.304	0.823	0.370
contig022264-TilOR.A016	contig047515-ZebOR.A019	0.304	0.825	0.369
contig034983-NyeOR.A002	contig022264-TilOR.A016	0.304	0.825	0.369
contig085018-BriOR.A006	contig057153-BurOR.A014	0.304	0.826	0.368
contig056380-NyeOR.A016	contig073309-TilOR.A026	0.304	0.827	0.367
contig056375-NyeOR.A015	contig047526-ZebOR.A021	0.304	0.827	0.368
contig054687-NyeOR.A013	contig022266-TilOR.A018	0.304	0.829	0.366
contig051573-BurOR.A011	contig054237-BurOR.A013	0.304	0.829	0.367
contig051566-BurOR.A009	contig056375-NyeOR.A015	0.304	0.829	0.367
contig085000-BriOR.A003	contig047523-ZebOR.A022	0.304	0.831	0.365
contig085000-BriOR.A003	contig054233-BurOR.A012	0.304	0.831	0.365
contig085010-BriOR.A005	contig034983-NyeOR.A003	0.304	0.831	0.365
contig051318-BurOR.A005	contig054678-NyeOR.A010	0.304	0.831	0.366
contig074640-TilOR.A002	contig047506-ZebOR.A014	0.304	0.834	0.364
contig036784-BurOR.A003	contig047523-ZebOR.A022	0.304	0.835	0.364
contig036784-BurOR.A003	contig054233-BurOR.A012	0.304	0.835	0.364
contig054233-BurOR.A012	contig054237-BurOR.A013	0.304	0.836	0.364
contig054237-BurOR.A013	contig047523-ZebOR.A022	0.304	0.836	0.364
contig051573-BurOR.A011	contig047503-ZebOR.A013	0.304	0.837	0.363
contig047514-ZebOR.A017	contig062095-ZebOR.A024	0.304	0.840	0.362
contig093816-BriOR.A011	contig022211-TilOR.A003	0.304	0.841	0.362

contig051318-BurOR.A005	contig070886-TilOR.A025	0.304	0.843	0.360
contig070886-TilOR.A025	contig030572-ZebOR.A009	0.304	0.843	0.360
contig064570-BurOR.A017	contig034994-NyeOR.A008	0.304	0.845	0.359
contig054684-NyeOR.A012	contig047515-ZebOR.A018	0.304	0.849	0.358
contig034995-NyeOR.A009	contig070885-TilOR.A024	0.304	0.850	0.358
contig064570-BurOR.A017	contig047514-ZebOR.A017	0.304	0.851	0.358
contig034990-NyeOR.A007	contig062095-ZebOR.A024	0.304	0.852	0.357
contig085026-BriOR.A008	contig054687-NyeOR.A013	0.304	0.857	0.355
contig062344-NyeOR.A020	contig047526-ZebOR.A021	0.304	0.863	0.352
contig034988-NyeOR.A005	contig030566-ZebOR.A008	0.304	0.872	0.348
contig085018-BriOR.A006	contig057754-NyeOR.A018	0.304	0.872	0.349
contig054237-BurOR.A013	contig030566-ZebOR.A008	0.304	0.874	0.348
contig054237-BurOR.A013	contig065887-BurOR.A018	0.304	0.876	0.347
contig085010-BriOR.A005	contig085018-BriOR.A006	0.304	0.882	0.345
contig022241-TilOR.A011	contig047514-ZebOR.A017	0.304	0.883	0.345
contig051570-BurOR.A010	contig022238-TilOR.A010	0.304	0.887	0.343
contig085018-BriOR.A006	contig054684-NyeOR.A012	0.304	0.901	0.338
contig022259-TilOR.A015	contig030553-ZebOR.A003	0.304	0.933	0.326
contig022238-TilOR.A010	contig030576-ZebOR.A010	0.304	0.971	0.313
contig057153-BurOR.A014	contig034990-NyeOR.A007	0.305	0.617	0.494
contig093812-BriOR.A010	contig022266-TilOR.A018	0.305	0.625	0.488
contig022268-TilOR.A019	contig047526-ZebOR.A021	0.305	0.634	0.482
contig057756-NyeOR.A019	contig047497-ZebOR.A011	0.305	0.641	0.475
contig051566-BurOR.A009	contig022230-TilOR.A007	0.305	0.644	0.474
contig030552-ZebOR.A001	contig047497-ZebOR.A011	0.305	0.648	0.470
contig022268-TilOR.A019	contig073309-TilOR.A026	0.305	0.653	0.466
contig047506-ZebOR.A014	contig047526-ZebOR.A021	0.305	0.657	0.464
contig036782-BurOR.A002	contig034995-NyeOR.A009	0.305	0.662	0.460
contig034988-NyeOR.A004	contig057756-NyeOR.A019	0.305	0.662	0.461
contig041951-TilOR.A022	contig047526-ZebOR.A021	0.305	0.663	0.461
contig036780-BurOR.A001	contig051566-BurOR.A009	0.305	0.683	0.447
contig064570-BurOR.A017	contig041951-TilOR.A022	0.305	0.689	0.442
contig036782-BurOR.A002	contig056375-NyeOR.A015	0.305	0.698	0.437
contig093807-BriOR.A009	contig022268-TilOR.A019	0.305	0.707	0.432
contig022259-TilOR.A014	contig030554-ZebOR.A004	0.305	0.710	0.429
contig085002-BriOR.A004	contig051566-BurOR.A009	0.305	0.717	0.425
contig034988-NyeOR.A004	contig030554-ZebOR.A004	0.305	0.718	0.424
contig022259-TilOR.A014	contig022268-TilOR.A019	0.305	0.731	0.418
contig022251-TilOR.A013	contig047506-ZebOR.A014	0.305	0.733	0.416
contig036787-BurOR.A004	contig022234-TilOR.A009	0.305	0.734	0.416
contig085002-BriOR.A004	contig051321-BurOR.A006	0.305	0.736	0.415
contig085026-BriOR.A008	contig022265-TilOR.A017	0.305	0.747	0.408
contig022234-TilOR.A009	contig030556-ZebOR.A005	0.305	0.747	0.409

contig085002-BriOR.A004	contig022238-TilOR.A010	0.305	0.757	0.403
contig051559-BurOR.A008	contig051573-BurOR.A011	0.305	0.768	0.398
contig085002-BriOR.A004	contig054687-NyeOR.A013	0.305	0.770	0.396
contig051566-BurOR.A009	contig022234-TilOR.A009	0.305	0.771	0.396
contig051559-BurOR.A008	contig022268-TilOR.A019	0.305	0.774	0.395
contig047508-ZebOR.A015	contig047526-ZebOR.A021	0.305	0.780	0.391
contig022245-TilOR.A012	contig073309-TilOR.A026	0.305	0.782	0.390
contig051318-BurOR.A005	contig030557-ZebOR.A006	0.305	0.787	0.388
contig030557-ZebOR.A006	contig030572-ZebOR.A009	0.305	0.788	0.387
contig034983-NyeOR.A003	contig022238-TilOR.A010	0.305	0.789	0.387
contig051321-BurOR.A006	contig054678-NyeOR.A010	0.305	0.792	0.386
contig022234-TilOR.A009	contig022264-TilOR.A016	0.305	0.801	0.381
contig057156-BurOR.A015	contig022211-TilOR.A003	0.305	0.808	0.378
contig064570-BurOR.A017	contig022238-TilOR.A010	0.305	0.811	0.376
contig036780-BurOR.A001	contig022251-TilOR.A013	0.305	0.816	0.374
contig054687-NyeOR.A013	contig057756-NyeOR.A019	0.305	0.817	0.374
contig093816-BriOR.A011	contig056375-NyeOR.A015	0.305	0.819	0.372
contig022238-TilOR.A010	contig062095-ZebOR.A024	0.305	0.824	0.369
contig057153-BurOR.A014	contig022264-TilOR.A016	0.305	0.828	0.368
contig022251-TilOR.A013	contig030552-ZebOR.A001	0.305	0.829	0.368
contig093816-BriOR.A011	contig022217-TilOR.A004	0.305	0.829	0.368
contig051570-BurOR.A010	contig054687-NyeOR.A013	0.305	0.839	0.363
contig073309-TilOR.A026	contig047521-ZebOR.A020	0.305	0.847	0.361
contig034988-NyeOR.A004	contig062095-ZebOR.A024	0.305	0.853	0.358
contig085018-BriOR.A006	contig022265-TilOR.A017	0.305	0.854	0.357
contig084999-BriOR.A002	contig051318-BurOR.A005	0.305	0.855	0.357
contig051570-BurOR.A010	contig047514-ZebOR.A017	0.305	0.856	0.356
contig064570-BurOR.A017	contig034988-NyeOR.A004	0.305	0.864	0.353
contig022238-TilOR.A010	contig047508-ZebOR.A015	0.305	0.868	0.351
contig070885-TilOR.A024	contig047508-ZebOR.A016	0.305	0.872	0.350
contig093807-BriOR.A009	contig070885-TilOR.A024	0.305	0.874	0.349
contig034995-NyeOR.A009	contig056375-NyeOR.A015	0.305	0.874	0.349
contig036784-BurOR.A003	contig022251-TilOR.A013	0.305	0.876	0.348
contig022264-TilOR.A016	contig062095-ZebOR.A024	0.305	0.878	0.348
contig051318-BurOR.A005	contig073309-TilOR.A026	0.305	0.880	0.346
contig073309-TilOR.A026	contig030572-ZebOR.A009	0.305	0.881	0.346
contig064570-BurOR.A017	contig047499-ZebOR.A012	0.305	0.895	0.341
contig051559-BurOR.A008	contig057754-NyeOR.A018	0.305	0.922	0.330
contig022264-TilOR.A016	contig047515-ZebOR.A018	0.305	0.928	0.329
contig062344-NyeOR.A020	contig022238-TilOR.A010	0.305	0.935	0.326
contig036784-BurOR.A003	contig022259-TilOR.A015	0.305	0.954	0.320
contig022225-TilOR.A005	contig030566-ZebOR.A008	0.306	0.641	0.478
contig065887-BurOR.A018	contig022225-TilOR.A005	0.306	0.642	0.477

contig030552-ZebOR.A001	contig047508-ZebOR.A016	0.306	0.661	0.463
contig022245-TilOR.A012	contig030553-ZebOR.A002	0.306	0.670	0.456
contig057165-NyeOR.A017	contig047508-ZebOR.A016	0.306	0.671	0.456
contig036787-BurOR.A004	contig054687-NyeOR.A013	0.306	0.675	0.454
contig093807-BriOR.A009	contig030560-ZebOR.A007	0.306	0.679	0.450
contig041951-TilOR.A022	contig062095-ZebOR.A024	0.306	0.685	0.446
contig084999-BriOR.A002	contig047503-ZebOR.A013	0.306	0.686	0.446
contig054687-NyeOR.A013	contig030556-ZebOR.A005	0.306	0.687	0.445
contig036787-BurOR.A004	contig047526-ZebOR.A021	0.306	0.690	0.443
contig085010-BriOR.A005	contig047508-ZebOR.A016	0.306	0.691	0.442
contig084999-BriOR.A002	contig022245-TilOR.A012	0.306	0.693	0.442
contig064570-BurOR.A017	contig047506-ZebOR.A014	0.306	0.693	0.442
contig022245-TilOR.A012	contig022268-TilOR.A019	0.306	0.703	0.435
contig064187-BurOR.A016	contig056375-NyeOR.A015	0.306	0.705	0.434
contig022268-TilOR.A019	contig047503-ZebOR.A013	0.306	0.706	0.434
contig034988-NyeOR.A004	contig030556-ZebOR.A005	0.306	0.711	0.431
contig051566-BurOR.A009	contig030556-ZebOR.A005	0.306	0.713	0.429
contig065887-BurOR.A018	contig034988-NyeORs.A033	0.306	0.715	0.428
contig030554-ZebOR.A004	contig047508-ZebOR.A016	0.306	0.721	0.425
contig022227-TilOR.A006	contig030556-ZebOR.A005	0.306	0.724	0.423
contig022251-TilOR.A013	contig022265-TilOR.A017	0.306	0.727	0.421
contig022238-TilOR.A010	contig030556-ZebOR.A005	0.306	0.730	0.420
contig036780-BurOR.A001	contig062094-ZebOR.A023	0.306	0.732	0.418
contig085002-BriOR.A004	contig034988-NyeOR.A004	0.306	0.737	0.414
contig022217-TilOR.A004	contig047506-ZebOR.A014	0.306	0.737	0.416
contig022204-TilOR.A001	contig047523-ZebOR.A022	0.306	0.741	0.413
contig054233-BurOR.A012	contig022204-TilOR.A001	0.306	0.741	0.413
contig030552-ZebOR.A001	contig062094-ZebOR.A023	0.306	0.743	0.412
contig034988-NyeOR.A006	contig022251-TilOR.A013	0.306	0.744	0.411
contig054678-NyeOR.A010	contig057756-NyeOR.A019	0.306	0.749	0.408
contig051573-BurOR.A011	contig022245-TilOR.A012	0.306	0.750	0.408
contig022264-TilOR.A016	contig022268-TilOR.A019	0.306	0.753	0.406
contig074640-TilOR.A002	contig030553-ZebOR.A003	0.306	0.758	0.404
contig054678-NyeOR.A010	contig022234-TilOR.A009	0.306	0.763	0.401
contig054681-NyeOR.A011	contig030572-ZebOR.A009	0.306	0.769	0.399
contig057153-BurOR.A014	contig022217-TilOR.A004	0.306	0.771	0.397
contig036787-BurOR.A004	contig047514-ZebOR.A017	0.306	0.775	0.395
contig084999-BriOR.A002	contig022251-TilOR.A013	0.306	0.779	0.393
contig022217-TilOR.A004	contig022265-TilOR.A017	0.306	0.780	0.393
contig057156-BurOR.A015	contig022217-TilOR.A004	0.306	0.788	0.388
contig085010-BriOR.A005	contig022217-TilOR.A004	0.306	0.794	0.385
contig084999-BriOR.A002	contig034983-NyeOR.A003	0.306	0.796	0.384
contig084999-BriOR.A001	contig051321-BurOR.A006	0.306	0.802	0.382

contig056375-NyeOR.A015	contig030553-ZebOR.A003	0.306	0.803	0.381
contig085018-BriOR.A006	contig036782-BurOR.A002	0.306	0.804	0.381
contig065887-BurOR.A018	contig022266-TilOR.A018	0.306	0.806	0.380
contig057756-NyeOR.A019	contig022259-TilOR.A015	0.306	0.809	0.378
contig085000-BriOR.A003	contig022245-TilOR.A012	0.306	0.812	0.377
contig056375-NyeOR.A015	contig047514-ZebOR.A017	0.306	0.815	0.376
contig022266-TilOR.A018	contig030566-ZebOR.A008	0.306	0.816	0.375
contig054687-NyeOR.A013	contig047508-ZebOR.A015	0.306	0.821	0.373
contig054684-NyeOR.A012	contig056380-NyeOR.A016	0.306	0.821	0.373
contig093816-BriOR.A011	contig054237-BurOR.A013	0.306	0.822	0.372
contig051321-BurOR.A006	contig057156-BurOR.A015	0.306	0.822	0.373
contig057756-NyeOR.A019	contig022264-TilOR.A016	0.306	0.824	0.371
contig022241-TilOR.A011	contig047526-ZebOR.A021	0.306	0.825	0.370
contig054237-BurOR.A013	contig070886-TilOR.A025	0.306	0.825	0.371
contig051566-BurOR.A009	contig034988-NyeOR.A005	0.306	0.827	0.370
contig057754-NyeOR.A018	contig022245-TilOR.A012	0.306	0.830	0.368
contig093807-BriOR.A009	contig062095-ZebOR.A024	0.306	0.830	0.369
contig054687-NyeOR.A013	contig070885-TilOR.A024	0.306	0.833	0.367
contig047508-ZebOR.A015	contig047514-ZebOR.A017	0.306	0.837	0.365
contig084999-BriOR.A001	contig056380-NyeOR.A016	0.306	0.837	0.366
contig022259-TilOR.A015	contig047526-ZebOR.A021	0.306	0.838	0.366
contig034983-NyeOR.A003	contig030560-ZebOR.A007	0.306	0.841	0.363
contig054681-NyeOR.A011	contig022264-TilOR.A016	0.306	0.841	0.364
contig093807-BriOR.A009	contig064570-BurOR.A017	0.306	0.841	0.364
contig084999-BriOR.A002	contig030572-ZebOR.A009	0.306	0.849	0.361
contig036784-BurOR.A003	contig022241-TilOR.A011	0.306	0.854	0.359
contig064570-BurOR.A017	contig047526-ZebOR.A021	0.306	0.857	0.356
contig022251-TilOR.A013	contig030553-ZebOR.A003	0.306	0.857	0.357
contig065887-BurOR.A018	contig034988-NyeOR.A005	0.306	0.861	0.355
contig085012-BriOR.A130	contig070886-TilOR.A025	0.306	0.869	0.353
contig022217-TilOR.A004	contig022264-TilOR.A016	0.306	0.881	0.347
contig085018-BriOR.A006	contig030560-ZebOR.A007	0.306	0.881	0.347
contig085026-BriOR.A008	contig065887-BurOR.A018	0.306	0.885	0.346
contig022251-TilOR.A013	contig041952-TilOR.A023	0.306	0.890	0.344
contig054687-NyeOR.A013	contig047503-ZebOR.A013	0.306	0.899	0.340
contig064570-BurOR.A017	contig034995-NyeOR.A009	0.306	0.906	0.338
contig085000-BriOR.A003	contig022259-TilOR.A015	0.306	0.957	0.320
contig036782-BurOR.A002	contig047508-ZebOR.A016	0.307	0.653	0.470
contig051566-BurOR.A009	contig041951-TilOR.A022	0.307	0.656	0.469
contig036780-BurOR.A001	contig047497-ZebOR.A011	0.307	0.658	0.466
contig034988-NyeOR.A004	contig022230-TilOR.A007	0.307	0.658	0.467
contig057756-NyeOR.A019	contig047508-ZebOR.A016	0.307	0.659	0.466
contig057756-NyeOR.A019	contig047526-ZebOR.A021	0.307	0.660	0.465

contig085002-BriOR.A004	contig054237-BurOR.A013	0.307	0.660	0.466
contig085002-BriOR.A004	contig022245-TilOR.A012	0.307	0.665	0.462
contig022204-TilOR.A001	contig022264-TilOR.A016	0.307	0.671	0.457
contig093807-BriOR.A009	contig034983-NyeOR.A002	0.307	0.672	0.456
contig093807-BriOR.A009	contig047515-ZebOR.A019	0.307	0.672	0.456
contig051321-BurOR.A006	contig034988-NyeORs.A033	0.307	0.673	0.455
contig036787-BurOR.A004	contig022264-TilOR.A016	0.307	0.678	0.453
contig056380-NyeOR.A016	contig034988-NyeORs.A033	0.307	0.682	0.449
contig054678-NyeOR.A010	contig054687-NyeOR.A013	0.307	0.682	0.450
contig085002-BriOR.A004	contig022211-TilOR.A003	0.307	0.689	0.446
contig022264-TilOR.A016	contig030556-ZebOR.A005	0.307	0.690	0.445
contig064570-BurOR.A017	contig034988-NyeOR.A006	0.307	0.693	0.443
contig022241-TilOR.A011	contig030553-ZebOR.A002	0.307	0.699	0.439
contig054678-NyeOR.A010	contig047526-ZebOR.A021	0.307	0.708	0.433
contig030553-ZebOR.A002	contig030572-ZebOR.A009	0.307	0.708	0.433
contig051318-BurOR.A005	contig030553-ZebOR.A002	0.307	0.708	0.433
contig034988-NyeORs.A033	contig030566-ZebOR.A008	0.307	0.714	0.429
contig064570-BurOR.A017	contig057756-NyeOR.A019	0.307	0.721	0.425
contig074640-TilOR.A002	contig030556-ZebOR.A005	0.307	0.723	0.425
contig056375-NyeOR.A015	contig030556-ZebOR.A005	0.307	0.726	0.423
contig022227-TilOR.A006	contig022268-TilOR.A019	0.307	0.733	0.419
contig085018-BriOR.A006	contig064187-BurOR.A016	0.307	0.736	0.417
contig085002-BriOR.A004	contig047508-ZebOR.A016	0.307	0.740	0.415
contig022265-TilOR.A017	contig047515-ZebOR.A018	0.307	0.743	0.414
contig085002-BriOR.A004	contig065887-BurOR.A018	0.307	0.747	0.410
contig085002-BriOR.A004	contig030566-ZebOR.A008	0.307	0.757	0.405
contig036784-BurOR.A003	contig074640-TilOR.A002	0.307	0.762	0.403
contig034983-NyeOR.A003	contig030554-ZebOR.A004	0.307	0.762	0.403
contig054678-NyeOR.A010	contig022227-TilOR.A006	0.307	0.763	0.403
contig036787-BurOR.A004	contig034983-NyeOR.A003	0.307	0.764	0.401
contig051318-BurOR.A005	contig054681-NyeOR.A011	0.307	0.768	0.399
contig085000-BriOR.A003	contig074640-TilOR.A002	0.307	0.771	0.398
contig036782-BurOR.A002	contig051321-BurOR.A006	0.307	0.773	0.398
contig034983-NyeOR.A003	contig030556-ZebOR.A005	0.307	0.778	0.394
contig034988-NyeOR.A004	contig022234-TilOR.A009	0.307	0.788	0.390
contig054678-NyeOR.A010	contig047514-ZebOR.A017	0.307	0.795	0.387
contig085018-BriOR.A006	contig051573-BurOR.A011	0.307	0.796	0.386
contig084999-BriOR.A001	contig085018-BriOR.A006	0.307	0.800	0.384
contig051318-BurOR.A005	contig057156-BurOR.A015	0.307	0.804	0.382
contig057156-BurOR.A015	contig030572-ZebOR.A009	0.307	0.805	0.381
contig051318-BurOR.A005	contig030556-ZebOR.A005	0.307	0.813	0.378
contig057165-NyeOR.A017	contig022251-TilOR.A013	0.307	0.816	0.377
contig051566-BurOR.A009	contig051570-BurOR.A010	0.307	0.817	0.376

contig057756-NyeOR.A019	contig030566-ZebOR.A008	0.307	0.818	0.376
contig065887-BurOR.A018	contig057756-NyeOR.A019	0.307	0.819	0.375
contig085012-BriOR.A130	contig022268-TilOR.A020	0.307	0.829	0.371
contig041952-TilOR.A023	contig030572-ZebOR.A009	0.307	0.832	0.369
contig093807-BriOR.A009	contig047503-ZebOR.A013	0.307	0.833	0.369
contig047508-ZebOR.A016	contig062095-ZebOR.A024	0.307	0.849	0.362
contig022241-TilOR.A011	contig030553-ZebOR.A003	0.307	0.849	0.362
contig051570-BurOR.A010	contig030566-ZebOR.A008	0.307	0.851	0.361
contig085018-BriOR.A006	contig047526-ZebOR.A021	0.307	0.856	0.359
contig034983-NyeOR.A003	contig054684-NyeOR.A012	0.307	0.861	0.356
contig064570-BurOR.A017	contig034990-NyeOR.A007	0.307	0.863	0.355
contig093816-BriOR.A011	contig022259-TilOR.A015	0.307	0.866	0.355
contig085026-BriOR.A008	contig030566-ZebOR.A008	0.307	0.870	0.353
contig085018-BriOR.A006	contig057156-BurOR.A015	0.307	0.888	0.345
contig051566-BurOR.A009	contig030576-ZebOR.A010	0.307	0.897	0.342
contig057754-NyeOR.A018	contig022259-TilOR.A015	0.307	0.958	0.320
contig093807-BriOR.A009	contig030576-ZebOR.A010	0.307	0.999	0.308
contig036787-BurOR.A004	contig065887-BurOR.A018	0.308	0.655	0.470
contig036787-BurOR.A004	contig030566-ZebOR.A008	0.308	0.664	0.464
contig065887-BurOR.A018	contig030556-ZebOR.A005	0.308	0.667	0.462
contig034994-NyeOR.A008	contig057756-NyeOR.A019	0.308	0.667	0.462
contig036780-BurOR.A001	contig047508-ZebOR.A016	0.308	0.671	0.459
contig057156-BurOR.A015	contig054687-NyeOR.A013	0.308	0.675	0.456
contig030556-ZebOR.A005	contig030566-ZebOR.A008	0.308	0.676	0.456
contig051566-BurOR.A009	contig064187-BurOR.A016	0.308	0.682	0.452
contig054678-NyeOR.A010	contig022264-TilOR.A016	0.308	0.685	0.450
contig054684-NyeOR.A012	contig054687-NyeOR.A013	0.308	0.696	0.443
contig056375-NyeOR.A015	contig047506-ZebOR.A014	0.308	0.696	0.443
contig051559-BurOR.A007	contig047526-ZebOR.A021	0.308	0.698	0.441
contig054687-NyeOR.A013	contig022265-TilOR.A017	0.308	0.698	0.441
contig056375-NyeOR.A015	contig057756-NyeOR.A019	0.308	0.707	0.435
contig056380-NyeOR.A016	contig041951-TilOR.A022	0.308	0.708	0.435
contig036787-BurOR.A004	contig056375-NyeOR.A015	0.308	0.713	0.431
contig030556-ZebOR.A005	contig047508-ZebOR.A016	0.308	0.714	0.431
contig085002-BriOR.A004	contig057756-NyeOR.A019	0.308	0.715	0.431
contig022211-TilOR.A003	contig030554-ZebOR.A004	0.308	0.717	0.430
contig051321-BurOR.A006	contig041951-TilOR.A022	0.308	0.720	0.427
contig064187-BurOR.A016	contig062094-ZebOR.A023	0.308	0.722	0.426
contig022225-TilOR.A005	contig047499-ZebOR.A012	0.308	0.722	0.426
contig051318-BurOR.A005	contig022266-TilOR.A018	0.308	0.725	0.424
contig022266-TilOR.A018	contig030572-ZebOR.A009	0.308	0.726	0.424
contig057165-NyeOR.A017	contig062094-ZebOR.A023	0.308	0.732	0.421
contig057156-BurOR.A015	contig034990-NyeOR.A007	0.308	0.734	0.420

contig054678-NyeOR.A010	contig056375-NyeOR.A015	0.308	0.743	0.415
contig034990-NyeOR.A007	contig054684-NyeOR.A012	0.308	0.755	0.408
contig057756-NyeOR.A019	contig062094-ZebOR.A023	0.308	0.759	0.406
contig036780-BurOR.A001	contig051321-BurOR.A006	0.308	0.778	0.396
contig084999-BriOR.A002	contig056380-NyeOR.A016	0.308	0.779	0.396
contig051318-BurOR.A005	contig057153-BurOR.A014	0.308	0.782	0.394
contig057153-BurOR.A014	contig030572-ZebOR.A009	0.308	0.783	0.394
contig034983-NyeOR.A003	contig054678-NyeOR.A010	0.308	0.784	0.393
contig051321-BurOR.A006	contig030552-ZebOR.A001	0.308	0.789	0.390
contig056375-NyeOR.A015	contig047497-ZebOR.A011	0.308	0.795	0.387
contig036787-BurOR.A004	contig051318-BurOR.A005	0.308	0.799	0.385
contig051566-BurOR.A009	contig047508-ZebOR.A015	0.308	0.799	0.386
contig054684-NyeOR.A012	contig022217-TilOR.A004	0.308	0.800	0.385
contig057756-NyeOR.A019	contig022217-TilOR.A004	0.308	0.803	0.383
contig056380-NyeOR.A016	contig030560-ZebOR.A007	0.308	0.803	0.383
contig051573-BurOR.A011	contig030553-ZebOR.A003	0.308	0.804	0.383
contig036782-BurOR.A002	contig056380-NyeOR.A016	0.308	0.807	0.381
contig085010-BriOR.A005	contig056380-NyeOR.A016	0.308	0.815	0.378
contig054684-NyeOR.A012	contig022211-TilOR.A003	0.308	0.815	0.378
contig034988-NyeOR.A005	contig034990-NyeOR.A007	0.308	0.816	0.377
contig022264-TilOR.A016	contig022266-TilOR.A018	0.308	0.816	0.378
contig085010-BriOR.A005	contig051559-BurOR.A008	0.308	0.818	0.377
contig085000-BriOR.A003	contig056375-NyeOR.A015	0.308	0.823	0.374
contig093807-BriOR.A009	contig047515-ZebOR.A018	0.308	0.824	0.374
contig047503-ZebOR.A013	contig047526-ZebOR.A021	0.308	0.826	0.373
contig051321-BurOR.A006	contig073309-TilOR.A026	0.308	0.828	0.372
contig034988-NyeOR.A006	contig074640-TilOR.A002	0.308	0.833	0.369
contig030566-ZebOR.A008	contig047508-ZebOR.A015	0.308	0.833	0.370
contig034988-NyeOR.A004	contig056375-NyeOR.A015	0.308	0.834	0.370
contig034988-NyeOR.A004	contig034988-NyeOR.A005	0.308	0.844	0.364
contig070885-TilOR.A024	contig030566-ZebOR.A008	0.308	0.845	0.365
contig065887-BurOR.A018	contig070885-TilOR.A024	0.308	0.847	0.364
contig064570-BurOR.A017	contig047508-ZebOR.A016	0.308	0.861	0.357
contig051566-BurOR.A009	contig062344-NyeOR.A020	0.308	0.864	0.356
contig085000-BriOR.A003	contig022251-TilOR.A013	0.308	0.879	0.351
contig093807-BriOR.A009	contig034988-NyeOR.A005	0.308	0.892	0.346
contig030566-ZebOR.A008	contig047503-ZebOR.A013	0.308	0.912	0.338
contig034983-NyeOR.A003	contig022264-TilOR.A016	0.308	0.913	0.337
contig034988-NyeORs.A033	contig062094-ZebOR.A023	0.309	0.633	0.489
contig034988-NyeOR.A004	contig030552-ZebOR.A001	0.309	0.658	0.469
contig041951-TilOR.A022	contig047508-ZebOR.A016	0.309	0.660	0.467
contig065887-BurOR.A018	contig054678-NyeOR.A010	0.309	0.662	0.467
contig034988-NyeOR.A004	contig057165-NyeOR.A017	0.309	0.668	0.462

contig051566-BurOR.A009	contig022268-TilOR.A019	0.309	0.668	0.462
contig034988-NyeOR.A004	contig041951-TilOR.A022	0.309	0.668	0.464
contig054678-NyeOR.A010	contig030566-ZebOR.A008	0.309	0.671	0.461
contig085010-BriOR.A005	contig093807-BriOR.A009	0.309	0.677	0.456
contig064187-BurOR.A016	contig034988-NyeOR.A004	0.309	0.700	0.442
contig022227-TilOR.A006	contig030554-ZebOR.A004	0.309	0.701	0.441
contig085018-BriOR.A006	contig047506-ZebOR.A014	0.309	0.727	0.426
contig036787-BurOR.A004	contig074640-TilOR.A002	0.309	0.732	0.423
contig022232-TilOR.A008	contig047526-ZebOR.A021	0.309	0.740	0.417
contig036787-BurOR.A004	contig022227-TilOR.A006	0.309	0.744	0.415
contig056380-NyeOR.A016	contig022265-TilOR.A017	0.309	0.744	0.415
contig084999-BriOR.A002	contig034981-NyeOR.A001	0.309	0.744	0.415
contig030554-ZebOR.A004	contig047515-ZebOR.A018	0.309	0.752	0.412
contig036782-BurOR.A002	contig022251-TilOR.A013	0.309	0.753	0.410
contig036787-BurOR.A004	contig047515-ZebOR.A018	0.309	0.754	0.410
contig036787-BurOR.A004	contig051321-BurOR.A006	0.309	0.761	0.406
contig034981-NyeOR.A001	contig022268-TilOR.A019	0.309	0.766	0.403
contig030556-ZebOR.A005	contig047515-ZebOR.A018	0.309	0.767	0.402
contig051559-BurOR.A008	contig047514-ZebOR.A017	0.309	0.771	0.401
contig051321-BurOR.A006	contig030556-ZebOR.A005	0.309	0.774	0.399
contig057754-NyeOR.A018	contig074640-TilOR.A002	0.309	0.777	0.398
contig022217-TilOR.A004	contig030560-ZebOR.A007	0.309	0.782	0.396
contig057156-BurOR.A015	contig062094-ZebOR.A023	0.309	0.787	0.393
contig022211-TilOR.A003	contig030560-ZebOR.A007	0.309	0.791	0.390
contig051559-BurOR.A008	contig047526-ZebOR.A021	0.309	0.796	0.388
contig056375-NyeOR.A015	contig022238-TilOR.A010	0.309	0.800	0.386
contig085010-BriOR.A005	contig022211-TilOR.A003	0.309	0.803	0.385
contig051570-BurOR.A010	contig034990-NyeOR.A007	0.309	0.808	0.382
contig051559-BurOR.A008	contig057156-BurOR.A015	0.309	0.810	0.382
contig054684-NyeOR.A012	contig030572-ZebOR.A009	0.309	0.815	0.380
contig051566-BurOR.A009	contig047515-ZebOR.A018	0.309	0.818	0.378
contig034988-NyeOR.A005	contig034994-NyeOR.A008	0.309	0.822	0.376
contig064570-BurOR.A017	contig047497-ZebOR.A011	0.309	0.823	0.376
contig064570-BurOR.A017	contig022264-TilOR.A016	0.309	0.830	0.372
contig051318-BurOR.A005	contig041952-TilOR.A023	0.309	0.832	0.372
contig051321-BurOR.A006	contig054684-NyeOR.A012	0.309	0.834	0.370
contig051570-BurOR.A010	contig034988-NyeOR.A004	0.309	0.834	0.371
contig056375-NyeOR.A015	contig057754-NyeOR.A018	0.309	0.836	0.369
contig051570-BurOR.A010	contig065887-BurOR.A018	0.309	0.840	0.368
contig022211-TilOR.A003	contig073309-TilOR.A026	0.309	0.841	0.367
contig051566-BurOR.A009	contig022259-TilOR.A015	0.309	0.892	0.346
contig085012-BriOR.A130	contig073309-TilOR.A026	0.309	0.894	0.346
contig022251-TilOR.A013	contig073309-TilOR.A026	0.309	0.911	0.339

contig034988-NyeOR.A004	contig030576-ZebOR.A010	0.309	0.916	0.337
contig022230-TilOR.A007	contig047508-ZebOR.A016	0.310	0.636	0.487
contig057156-BurOR.A015	contig022264-TilOR.A016	0.310	0.654	0.474
contig057156-BurOR.A015	contig030566-ZebOR.A008	0.310	0.664	0.467
contig057156-BurOR.A015	contig065887-BurOR.A018	0.310	0.665	0.466
contig034988-NyeOR.A004	contig022268-TilOR.A019	0.310	0.667	0.465
contig054684-NyeOR.A012	contig022264-TilOR.A016	0.310	0.674	0.460
contig054684-NyeOR.A012	contig030566-ZebOR.A008	0.310	0.685	0.453
contig065887-BurOR.A018	contig054684-NyeOR.A012	0.310	0.686	0.452
contig022265-TilOR.A017	contig030566-ZebOR.A008	0.310	0.687	0.452
contig065887-BurOR.A018	contig022265-TilOR.A017	0.310	0.688	0.451
contig036782-BurOR.A002	contig022245-TilOR.A012	0.310	0.689	0.450
contig022245-TilOR.A012	contig030554-ZebOR.A004	0.310	0.693	0.447
contig054687-NyeOR.A013	contig030560-ZebOR.A007	0.310	0.695	0.446
contig022251-TilOR.A013	contig041951-TilOR.A022	0.310	0.696	0.446
contig085012-BriOR.A130	contig022230-TilOR.A007	0.310	0.697	0.445
contig034990-NyeOR.A007	contig022232-TilOR.A008	0.310	0.700	0.443
contig036787-BurOR.A004	contig051566-BurOR.A009	0.310	0.700	0.443
contig085002-BriOR.A004	contig085026-BriOR.A008	0.310	0.708	0.438
contig047506-ZebOR.A014	contig062094-ZebOR.A023	0.310	0.713	0.434
contig084999-BriOR.A002	contig093807-BriOR.A009	0.310	0.716	0.433
contig022264-TilOR.A016	contig022265-TilOR.A017	0.310	0.719	0.432
contig022232-TilOR.A008	contig022238-TilOR.A010	0.310	0.734	0.422
contig085002-BriOR.A004	contig022234-TilOR.A009	0.310	0.735	0.421
contig034988-NyeOR.A006	contig022217-TilOR.A004	0.310	0.737	0.420
contig022232-TilOR.A008	contig047514-ZebOR.A017	0.310	0.737	0.420
contig085012-BriOR.A130	contig036780-BurOR.A001	0.310	0.742	0.418
contig084999-BriOR.A001	contig022241-TilOR.A011	0.310	0.746	0.416
contig034983-NyeOR.A003	contig022265-TilOR.A017	0.310	0.753	0.411
contig085012-BriOR.A130	contig030552-ZebOR.A001	0.310	0.753	0.412
contig085002-BriOR.A004	contig022251-TilOR.A013	0.310	0.754	0.412
contig093816-BriOR.A011	contig022245-TilOR.A012	0.310	0.755	0.411
contig084999-BriOR.A001	contig085012-BriOR.A130	0.310	0.756	0.410
contig054678-NyeOR.A010	contig047515-ZebOR.A018	0.310	0.773	0.401
contig051559-BurOR.A008	contig022238-TilOR.A010	0.310	0.776	0.399
contig051321-BurOR.A006	contig057165-NyeOR.A017	0.310	0.778	0.399
contig022234-TilOR.A009	contig047508-ZebOR.A016	0.310	0.785	0.395
contig034990-NyeOR.A007	contig047508-ZebOR.A015	0.310	0.791	0.392
contig047514-ZebOR.A017	contig047521-ZebOR.A020	0.310	0.793	0.391
contig085002-BriOR.A004	contig030572-ZebOR.A009	0.310	0.800	0.387
contig084999-BriOR.A002	contig051559-BurOR.A008	0.310	0.807	0.385
contig093816-BriOR.A011	contig030553-ZebOR.A003	0.310	0.809	0.383
contig022245-TilOR.A012	contig047526-ZebOR.A021	0.310	0.810	0.383

contig056380-NyeOR.A016	contig047526-ZebOR.A021	0.310	0.812	0.382
contig051318-BurOR.A005	contig054684-NyeOR.A012	0.310	0.814	0.380
contig051321-BurOR.A006	contig030560-ZebOR.A007	0.310	0.815	0.380
contig034988-NyeOR.A004	contig047508-ZebOR.A015	0.310	0.816	0.380
contig065887-BurOR.A018	contig047508-ZebOR.A015	0.310	0.822	0.378
contig051559-BurOR.A008	contig030560-ZebOR.A007	0.310	0.827	0.375
contig093816-BriOR.A011	contig062094-ZebOR.A023	0.310	0.834	0.372
contig051566-BurOR.A009	contig062094-ZebOR.A023	0.310	0.840	0.369
contig034988-NyeOR.A005	contig047508-ZebOR.A016	0.310	0.841	0.369
contig051573-BurOR.A011	contig062094-ZebOR.A023	0.310	0.841	0.369
contig056375-NyeOR.A015	contig047499-ZebOR.A012	0.310	0.844	0.367
contig054237-BurOR.A013	contig073309-TilOR.A026	0.310	0.861	0.360
contig093816-BriOR.A011	contig056380-NyeOR.A016	0.310	0.871	0.355
contig034988-NyeOR.A004	contig062344-NyeOR.A020	0.310	0.883	0.351
contig065887-BurOR.A018	contig047503-ZebOR.A013	0.310	0.900	0.345
contig022259-TilOR.A015	contig041952-TilOR.A023	0.310	0.911	0.340
contig093807-BriOR.A009	contig062344-NyeOR.A020	0.310	0.962	0.323
contig030554-ZebOR.A004	contig047503-ZebOR.A013	0.311	0.662	0.469
contig051566-BurOR.A009	contig034988-NyeOR.A006	0.311	0.667	0.465
contig036780-BurOR.A001	contig034988-NyeOR.A004	0.311	0.668	0.466
contig022268-TilOR.A019	contig047508-ZebOR.A016	0.311	0.669	0.465
contig056375-NyeOR.A015	contig041951-TilOR.A022	0.311	0.670	0.464
contig051566-BurOR.A009	contig047506-ZebOR.A014	0.311	0.670	0.465
contig034995-NyeOR.A009	contig057756-NyeOR.A019	0.311	0.672	0.463
contig093816-BriOR.A011	contig022227-TilOR.A006	0.311	0.702	0.443
contig074640-TilOR.A002	contig022268-TilOR.A019	0.311	0.704	0.443
contig034983-NyeOR.A002	contig047526-ZebOR.A021	0.311	0.705	0.441
contig047515-ZebOR.A019	contig047526-ZebOR.A021	0.311	0.705	0.441
contig022234-TilOR.A009	contig030554-ZebOR.A004	0.311	0.710	0.437
contig036782-BurOR.A002	contig022241-TilOR.A011	0.311	0.718	0.433
contig051566-BurOR.A009	contig054678-NyeOR.A010	0.311	0.718	0.434
contig036787-BurOR.A004	contig034988-NyeOR.A004	0.311	0.720	0.432
contig085002-BriOR.A004	contig030576-ZebOR.A010	0.311	0.721	0.432
contig034988-NyeOR.A006	contig062094-ZebOR.A023	0.311	0.724	0.429
contig036782-BurOR.A002	contig030572-ZebOR.A009	0.311	0.727	0.428
contig036782-BurOR.A002	contig051318-BurOR.A005	0.311	0.727	0.428
contig034990-NyeOR.A007	contig030560-ZebOR.A007	0.311	0.733	0.424
contig022217-TilOR.A004	contig041951-TilOR.A022	0.311	0.739	0.420
contig036787-BurOR.A004	contig022238-TilOR.A010	0.311	0.739	0.421
contig054687-NyeOR.A013	contig022268-TilOR.A019	0.311	0.740	0.420
contig093812-BriOR.A010	contig057156-BurOR.A015	0.311	0.742	0.419
contig085002-BriOR.A004	contig074640-TilOR.A002	0.311	0.745	0.418
contig054678-NyeOR.A010	contig074640-TilOR.A002	0.311	0.756	0.412

contig022227-TilOR.A006	contig030553-ZebOR.A003	0.311	0.760	0.410
contig093812-BriOR.A010	contig054684-NyeOR.A012	0.311	0.764	0.407
contig057156-BurOR.A015	contig034994-NyeOR.A008	0.311	0.770	0.403
contig036787-BurOR.A004	contig051559-BurOR.A008	0.311	0.774	0.402
contig085002-BriOR.A004	contig047515-ZebOR.A018	0.311	0.776	0.401
contig084999-BriOR.A002	contig062094-ZebOR.A023	0.311	0.780	0.398
contig051559-BurOR.A008	contig030556-ZebOR.A005	0.311	0.788	0.395
contig084999-BriOR.A002	contig051321-BurOR.A006	0.311	0.792	0.392
contig034994-NyeOR.A008	contig054684-NyeOR.A012	0.311	0.793	0.392
contig030560-ZebOR.A007	contig030572-ZebOR.A009	0.311	0.796	0.390
contig051318-BurOR.A005	contig030560-ZebOR.A007	0.311	0.796	0.390
contig051573-BurOR.A011	contig057754-NyeOR.A018	0.311	0.798	0.390
contig054684-NyeOR.A012	contig062094-ZebOR.A023	0.311	0.799	0.390
contig085018-BriOR.A006	contig093816-BriOR.A011	0.311	0.801	0.388
contig093807-BriOR.A009	contig056375-NyeOR.A015	0.311	0.806	0.386
contig085002-BriOR.A004	contig051318-BurOR.A005	0.311	0.806	0.386
contig034981-NyeOR.A001	contig047514-ZebOR.A017	0.311	0.810	0.384
contig051570-BurOR.A010	contig034994-NyeOR.A008	0.311	0.812	0.383
contig085018-BriOR.A006	contig057756-NyeOR.A019	0.311	0.814	0.382
contig034990-NyeOR.A007	contig056375-NyeOR.A015	0.311	0.815	0.382
contig034988-NyeOR.A004	contig047515-ZebOR.A018	0.311	0.823	0.378
contig034994-NyeOR.A008	contig022259-TilOR.A014	0.311	0.827	0.376
contig056375-NyeOR.A015	contig047508-ZebOR.A016	0.311	0.830	0.374
contig093807-BriOR.A009	contig034983-NyeOR.A003	0.311	0.835	0.372
contig051318-BurOR.A005	contig022268-TilOR.A019	0.311	0.837	0.371
contig093812-BriOR.A010	contig022259-TilOR.A014	0.311	0.840	0.370
contig051559-BurOR.A008	contig054684-NyeOR.A012	0.311	0.846	0.368
contig022259-TilOR.A014	contig047499-ZebOR.A012	0.311	0.847	0.367
contig085000-BriOR.A003	contig022241-TilOR.A011	0.311	0.851	0.365
contig074640-TilOR.A002	contig022268-TilOR.A020	0.311	0.871	0.357
contig057754-NyeOR.A018	contig022251-TilOR.A013	0.311	0.879	0.354
contig070886-TilOR.A025	contig030553-ZebOR.A003	0.311	0.886	0.351
contig093807-BriOR.A009	contig022241-TilOR.A011	0.311	0.898	0.347
contig093807-BriOR.A009	contig051570-BurOR.A010	0.311	0.899	0.346
contig030576-ZebOR.A010	contig047508-ZebOR.A016	0.311	0.912	0.341
contig054687-NyeOR.A013	contig022232-TilOR.A008	0.311	0.926	0.336
contig022217-TilOR.A004	contig047514-ZebOR.A017	0.311	0.937	0.332
contig022264-TilOR.A016	contig030560-ZebOR.A007	0.312	0.674	0.464
contig064187-BurOR.A016	contig047508-ZebOR.A016	0.312	0.676	0.461
contig034988-NyeOR.A004	contig034988-NyeOR.A006	0.312	0.685	0.455
contig065887-BurOR.A018	contig030560-ZebOR.A007	0.312	0.685	0.456
contig034988-NyeOR.A004	contig047506-ZebOR.A014	0.312	0.687	0.454
contig034988-NyeORs.A033	contig030572-ZebOR.A009	0.312	0.687	0.454

contig051318-BurOR.A005	contig034988-NyeORs.A033	0.312	0.687	0.454
contig034990-NyeOR.A007	contig022225-TilOR.A005	0.312	0.695	0.449
contig084999-BriOR.A002	contig022264-TilOR.A016	0.312	0.709	0.441
contig030556-ZebOR.A005	contig047521-ZebOR.A020	0.312	0.721	0.432
contig036787-BurOR.A004	contig047521-ZebOR.A020	0.312	0.730	0.427
contig022227-TilOR.A006	contig070886-TilOR.A025	0.312	0.734	0.425
contig085002-BriOR.A004	contig022227-TilOR.A006	0.312	0.737	0.424
contig034988-NyeOR.A004	contig054678-NyeOR.A010	0.312	0.738	0.423
contig085012-BriOR.A130	contig057165-NyeOR.A017	0.312	0.742	0.421
contig093816-BriOR.A011	contig022225-TilOR.A005	0.312	0.744	0.419
contig064187-BurOR.A016	contig056380-NyeOR.A016	0.312	0.744	0.419
contig051559-BurOR.A008	contig030554-ZebOR.A004	0.312	0.750	0.416
contig051573-BurOR.A011	contig022225-TilOR.A005	0.312	0.750	0.416
contig051321-BurOR.A006	contig064187-BurOR.A016	0.312	0.757	0.412
contig054678-NyeOR.A010	contig022238-TilOR.A010	0.312	0.758	0.411
contig036784-BurOR.A003	contig022227-TilOR.A006	0.312	0.765	0.408
contig093807-BriOR.A009	contig022232-TilOR.A008	0.312	0.773	0.403
contig056380-NyeOR.A016	contig022268-TilOR.A019	0.312	0.774	0.403
contig022268-TilOR.A019	contig062094-ZebOR.A023	0.312	0.775	0.402
contig030560-ZebOR.A007	contig062094-ZebOR.A023	0.312	0.780	0.400
contig051321-BurOR.A006	contig057756-NyeOR.A019	0.312	0.782	0.399
contig085002-BriOR.A004	contig093807-BriOR.A009	0.312	0.783	0.398
contig051559-BurOR.A008	contig054678-NyeOR.A010	0.312	0.794	0.393
contig034994-NyeOR.A008	contig047508-ZebOR.A015	0.312	0.795	0.392
contig051573-BurOR.A011	contig022251-TilOR.A013	0.312	0.804	0.388
contig085010-BriOR.A005	contig051321-BurOR.A006	0.312	0.816	0.383
contig056380-NyeOR.A016	contig057756-NyeOR.A019	0.312	0.817	0.382
contig051566-BurOR.A009	contig034983-NyeOR.A003	0.312	0.830	0.376
contig022268-TilOR.A019	contig030572-ZebOR.A009	0.312	0.831	0.375
contig051570-BurOR.A010	contig047508-ZebOR.A016	0.312	0.831	0.375
contig054687-NyeOR.A013	contig062344-NyeOR.A020	0.312	0.839	0.371
contig022217-TilOR.A004	contig047526-ZebOR.A021	0.312	0.849	0.368
contig051566-BurOR.A009	contig056380-NyeOR.A016	0.312	0.852	0.367
contig022211-TilOR.A003	contig047514-ZebOR.A017	0.312	0.856	0.365
contig085018-BriOR.A006	contig051566-BurOR.A009	0.312	0.858	0.363
contig093816-BriOR.A011	contig051321-BurOR.A006	0.312	0.873	0.357
contig022251-TilOR.A013	contig070886-TilOR.A025	0.312	0.877	0.356
contig062344-NyeOR.A020	contig047508-ZebOR.A016	0.312	0.879	0.355
contig093807-BriOR.A009	contig047508-ZebOR.A015	0.312	0.879	0.355
contig051573-BurOR.A011	contig056380-NyeOR.A016	0.312	0.879	0.355
contig054687-NyeOR.A013	contig030576-ZebOR.A010	0.312	0.886	0.352
contig051559-BurOR.A008	contig022264-TilOR.A016	0.312	0.925	0.337
contig085018-BriOR.A006	contig047514-ZebOR.A017	0.312	0.939	0.332

contig022225-TilOR.A005	contig022264-TilOR.A016	0.313	0.642	0.487
contig030560-ZebOR.A007	contig030566-ZebOR.A008	0.313	0.684	0.457
contig085010-BriOR.A005	contig054687-NyeOR.A013	0.313	0.706	0.443
contig022265-TilOR.A017	contig062094-ZebOR.A023	0.313	0.712	0.440
contig030556-ZebOR.A005	contig047503-ZebOR.A013	0.313	0.715	0.437
contig065887-BurOR.A018	contig022268-TilOR.A019	0.313	0.719	0.436
contig036787-BurOR.A004	contig047508-ZebOR.A016	0.313	0.723	0.432
contig036787-BurOR.A004	contig047503-ZebOR.A013	0.313	0.723	0.433
contig054868-NyeOR.A014	contig074640-TilOR.A002	0.313	0.725	0.432
contig022268-TilOR.A019	contig030566-ZebOR.A008	0.313	0.728	0.430
contig085018-BriOR.A006	contig034988-NyeOR.A006	0.313	0.738	0.424
contig056380-NyeOR.A016	contig030554-ZebOR.A004	0.313	0.740	0.423
contig022251-TilOR.A013	contig030554-ZebOR.A004	0.313	0.740	0.424
contig054678-NyeOR.A010	contig047508-ZebOR.A016	0.313	0.741	0.423
contig093807-BriOR.A009	contig030554-ZebOR.A004	0.313	0.744	0.420
contig051321-BurOR.A006	contig022265-TilOR.A017	0.313	0.744	0.421
contig054678-NyeOR.A010	contig047521-ZebOR.A020	0.313	0.759	0.412
contig022211-TilOR.A003	contig022265-TilOR.A017	0.313	0.760	0.412
contig047521-ZebOR.A020	contig047526-ZebOR.A021	0.313	0.764	0.410
contig034994-NyeOR.A008	contig030560-ZebOR.A007	0.313	0.769	0.407
contig054687-NyeOR.A013	contig022234-TilOR.A009	0.313	0.772	0.406
contig057756-NyeOR.A019	contig022251-TilOR.A013	0.313	0.774	0.405
contig051573-BurOR.A011	contig022241-TilOR.A011	0.313	0.775	0.404
contig085010-BriOR.A005	contig062094-ZebOR.A023	0.313	0.781	0.400
contig022264-TilOR.A016	contig030552-ZebOR.A001	0.313	0.784	0.399
contig036780-BurOR.A001	contig022264-TilOR.A016	0.313	0.784	0.399
contig057165-NyeOR.A017	contig022264-TilOR.A016	0.313	0.784	0.399
contig085012-BriOR.A130	contig030557-ZebOR.A006	0.313	0.793	0.395
contig047526-ZebOR.A021	contig062094-ZebOR.A023	0.313	0.801	0.390
contig093816-BriOR.A011	contig057754-NyeOR.A018	0.313	0.803	0.390
contig041952-TilOR.A023	contig062094-ZebOR.A023	0.313	0.804	0.389
contig051318-BurOR.A005	contig030554-ZebOR.A004	0.313	0.806	0.389
contig085010-BriOR.A005	contig051318-BurOR.A005	0.313	0.808	0.388
contig085010-BriOR.A005	contig030572-ZebOR.A009	0.313	0.809	0.387
contig047508-ZebOR.A015	contig047508-ZebOR.A016	0.313	0.813	0.385
contig051566-BurOR.A009	contig022241-TilOR.A011	0.313	0.820	0.382
contig085018-BriOR.A006	contig041952-TilOR.A023	0.313	0.828	0.378
contig041952-TilOR.A023	contig047521-ZebOR.A020	0.313	0.833	0.375
contig054687-NyeOR.A013	contig022217-TilOR.A004	0.313	0.841	0.372
contig057754-NyeOR.A018	contig022241-TilOR.A011	0.313	0.857	0.365
contig051318-BurOR.A005	contig051566-BurOR.A009	0.313	0.857	0.365
contig051566-BurOR.A009	contig030572-ZebOR.A009	0.313	0.857	0.365
contig054678-NyeOR.A010	contig030572-ZebOR.A009	0.313	0.869	0.360

contig093812-BriOR.A010	contig022241-TilOR.A011	0.313	0.870	0.360
contig022238-TilOR.A010	contig022241-TilOR.A011	0.313	0.874	0.358
contig085018-BriOR.A006	contig034988-NyeOR.A004	0.313	0.876	0.358
contig034988-NyeOR.A004	contig022259-TilOR.A015	0.313	0.884	0.354
contig054687-NyeOR.A013	contig062095-ZebOR.A024	0.313	0.886	0.353
contig022251-TilOR.A013	contig022264-TilOR.A016	0.313	0.935	0.334
contig022232-TilOR.A008	contig030566-ZebOR.A008	0.313	0.939	0.333
contig034988-NyeOR.A006	contig047508-ZebOR.A016	0.314	0.662	0.475
contig034988-NyeOR.A006	contig056375-NyeOR.A015	0.314	0.695	0.452
contig051559-BurOR.A007	contig051566-BurOR.A009	0.314	0.701	0.448
contig051318-BurOR.A005	contig051559-BurOR.A007	0.314	0.702	0.448
contig051559-BurOR.A007	contig030572-ZebOR.A009	0.314	0.702	0.448
contig030572-ZebOR.A009	contig047515-ZebOR.A019	0.314	0.704	0.446
contig034983-NyeOR.A002	contig030572-ZebOR.A009	0.314	0.704	0.446
contig051318-BurOR.A005	contig034983-NyeOR.A002	0.314	0.704	0.446
contig051318-BurOR.A005	contig047515-ZebOR.A019	0.314	0.704	0.446
contig056380-NyeOR.A016	contig047506-ZebOR.A014	0.314	0.735	0.428
contig057156-BurOR.A015	contig047499-ZebOR.A012	0.314	0.738	0.425
contig051559-BurOR.A008	contig022265-TilOR.A017	0.314	0.740	0.424
contig093812-BriOR.A010	contig030560-ZebOR.A007	0.314	0.742	0.423
contig051321-BurOR.A006	contig047506-ZebOR.A014	0.314	0.747	0.420
contig085018-BriOR.A006	contig036787-BurOR.A004	0.314	0.748	0.420
contig093807-BriOR.A009	contig030556-ZebOR.A005	0.314	0.748	0.420
contig036787-BurOR.A004	contig030576-ZebOR.A010	0.314	0.750	0.418
contig054678-NyeOR.A010	contig047503-ZebOR.A013	0.314	0.752	0.417
contig054684-NyeOR.A012	contig047499-ZebOR.A012	0.314	0.760	0.413
contig085018-BriOR.A006	contig030556-ZebOR.A005	0.314	0.761	0.413
contig085010-BriOR.A005	contig034990-NyeOR.A007	0.314	0.761	0.413
contig030556-ZebOR.A005	contig030576-ZebOR.A010	0.314	0.763	0.411
contig057754-NyeOR.A018	contig022227-TilOR.A006	0.314	0.767	0.410
contig022251-TilOR.A013	contig022268-TilOR.A019	0.314	0.773	0.406
contig085012-BriOR.A130	contig054681-NyeOR.A011	0.314	0.774	0.406
contig085000-BriOR.A003	contig051573-BurOR.A011	0.314	0.781	0.402
contig054678-NyeOR.A010	contig062094-ZebOR.A023	0.314	0.785	0.400
contig051321-BurOR.A006	contig022268-TilOR.A019	0.314	0.786	0.399
contig085002-BriOR.A004	contig034983-NyeOR.A003	0.314	0.787	0.399
contig085000-BriOR.A003	contig093816-BriOR.A011	0.314	0.791	0.397
contig030554-ZebOR.A004	contig030572-ZebOR.A009	0.314	0.801	0.393
contig036784-BurOR.A003	contig051573-BurOR.A011	0.314	0.807	0.389
contig085026-BriOR.A008	contig047514-ZebOR.A017	0.314	0.809	0.388
contig051566-BurOR.A009	contig022245-TilOR.A012	0.314	0.811	0.387
contig047508-ZebOR.A016	contig047515-ZebOR.A018	0.314	0.820	0.383
contig062344-NyeOR.A020	contig030566-ZebOR.A008	0.314	0.826	0.380

contig022259-TilOR.A014	contig022264-TilOR.A016	0.314	0.826	0.380
contig065887-BurOR.A018	contig062344-NyeOR.A020	0.314	0.827	0.379
contig034983-NyeOR.A003	contig034988-NyeOR.A004	0.314	0.834	0.376
contig034988-NyeOR.A004	contig062094-ZebOR.A023	0.314	0.845	0.372
contig051321-BurOR.A006	contig051566-BurOR.A009	0.314	0.866	0.363
contig065887-BurOR.A018	contig030576-ZebOR.A010	0.314	0.874	0.360
contig057754-NyeOR.A018	contig070886-TilOR.A025	0.314	0.880	0.357
contig051321-BurOR.A006	contig051573-BurOR.A011	0.314	0.881	0.357
contig034988-NyeOR.A005	contig034995-NyeOR.A009	0.314	0.884	0.355
contig022259-TilOR.A015	contig047514-ZebOR.A017	0.314	0.889	0.353
contig054687-NyeOR.A013	contig022251-TilOR.A013	0.314	0.890	0.352
contig034995-NyeOR.A009	contig022259-TilOR.A014	0.314	0.896	0.351
contig022217-TilOR.A004	contig022238-TilOR.A010	0.314	0.929	0.339
contig047506-ZebOR.A014	contig047508-ZebOR.A016	0.315	0.664	0.474
contig022264-TilOR.A016	contig030554-ZebOR.A004	0.315	0.683	0.461
contig085010-BriOR.A005	contig022264-TilOR.A016	0.315	0.684	0.461
contig085010-BriOR.A005	contig030566-ZebOR.A008	0.315	0.694	0.454
contig085010-BriOR.A005	contig065887-BurOR.A018	0.315	0.696	0.453
contig057756-NyeOR.A019	contig022245-TilOR.A012	0.315	0.698	0.451
contig051559-BurOR.A007	contig034988-NyeOR.A004	0.315	0.708	0.445
contig085018-BriOR.A006	contig041951-TilOR.A022	0.315	0.711	0.442
contig051318-BurOR.A005	contig057756-NyeOR.A019	0.315	0.731	0.431
contig057756-NyeOR.A019	contig030572-ZebOR.A009	0.315	0.731	0.431
contig057756-NyeOR.A019	contig022241-TilOR.A011	0.315	0.733	0.430
contig034988-NyeOR.A006	contig056380-NyeOR.A016	0.315	0.746	0.423
contig051321-BurOR.A006	contig030554-ZebOR.A004	0.315	0.752	0.419
contig093807-BriOR.A009	contig036787-BurOR.A004	0.315	0.757	0.415
contig051321-BurOR.A006	contig034988-NyeOR.A006	0.315	0.759	0.415
contig036787-BurOR.A004	contig022251-TilOR.A013	0.315	0.765	0.412
contig034995-NyeOR.A009	contig022225-TilOR.A005	0.315	0.767	0.411
contig093807-BriOR.A009	contig054678-NyeOR.A010	0.315	0.777	0.406
contig022251-TilOR.A013	contig030556-ZebOR.A005	0.315	0.778	0.404
contig085018-BriOR.A006	contig054678-NyeOR.A010	0.315	0.778	0.405
contig093816-BriOR.A011	contig022241-TilOR.A011	0.315	0.779	0.404
contig085026-BriOR.A008	contig047526-ZebOR.A021	0.315	0.780	0.403
contig054678-NyeOR.A010	contig030576-ZebOR.A010	0.315	0.780	0.403
contig034981-NyeOR.A001	contig047526-ZebOR.A021	0.315	0.780	0.404
contig054678-NyeOR.A010	contig056380-NyeOR.A016	0.315	0.790	0.398
contig093816-BriOR.A011	contig030572-ZebOR.A009	0.315	0.799	0.395
contig093816-BriOR.A011	contig051318-BurOR.A005	0.315	0.799	0.395
contig054237-BurOR.A013	contig047514-ZebOR.A017	0.315	0.806	0.390
contig093816-BriOR.A011	contig036784-BurOR.A003	0.315	0.812	0.388
contig034988-NyeOR.A005	contig047497-ZebOR.A011	0.315	0.813	0.387

contig057156-BurOR.A015	contig034995-NyeOR.A009	0.315	0.813	0.387
contig022211-TilOR.A003	contig047526-ZebOR.A021	0.315	0.816	0.385
contig093807-BriOR.A009	contig051559-BurOR.A008	0.315	0.821	0.384
contig022217-TilOR.A004	contig030566-ZebOR.A008	0.315	0.828	0.381
contig065887-BurOR.A018	contig022217-TilOR.A004	0.315	0.830	0.380
contig034995-NyeOR.A009	contig054684-NyeOR.A012	0.315	0.837	0.376
contig034988-NyeOR.A004	contig022241-TilOR.A011	0.315	0.838	0.376
contig056380-NyeOR.A016	contig041952-TilOR.A023	0.315	0.839	0.375
contig022245-TilOR.A012	contig047514-ZebOR.A017	0.315	0.856	0.368
contig054687-NyeOR.A013	contig054868-NyeOR.A014	0.315	0.859	0.366
contig022241-TilOR.A011	contig047499-ZebOR.A012	0.315	0.869	0.363
contig030566-ZebOR.A008	contig030576-ZebOR.A010	0.315	0.872	0.361
contig051570-BurOR.A010	contig034995-NyeOR.A009	0.315	0.874	0.361
contig022259-TilOR.A015	contig047508-ZebOR.A016	0.315	0.881	0.358
contig030566-ZebOR.A008	contig062095-ZebOR.A024	0.315	0.899	0.351
contig065887-BurOR.A018	contig062095-ZebOR.A024	0.315	0.901	0.350
contig056380-NyeOR.A016	contig030553-ZebOR.A003	0.315	0.925	0.341
contig051321-BurOR.A006	contig030553-ZebOR.A003	0.315	0.926	0.341
contig065887-BurOR.A018	contig022232-TilOR.A008	0.315	0.927	0.340
contig085012-BriOR.A130	contig034988-NyeORs.A033	0.316	0.671	0.470
contig084999-BriOR.A002	contig054687-NyeOR.A013	0.316	0.694	0.455
contig051573-BurOR.A011	contig022227-TilOR.A006	0.316	0.709	0.446
contig054233-BurOR.A012	contig074640-TilOR.A002	0.316	0.720	0.440
contig074640-TilOR.A002	contig047523-ZebOR.A022	0.316	0.720	0.440
contig036787-BurOR.A004	contig022211-TilOR.A003	0.316	0.727	0.435
contig085012-BriOR.A130	contig022266-TilOR.A018	0.316	0.731	0.432
contig034994-NyeOR.A008	contig022225-TilOR.A005	0.316	0.733	0.431
contig030560-ZebOR.A007	contig047499-ZebOR.A012	0.316	0.738	0.428
contig022211-TilOR.A003	contig030556-ZebOR.A005	0.316	0.740	0.427
contig030554-ZebOR.A004	contig062094-ZebOR.A023	0.316	0.741	0.426
contig022265-TilOR.A017	contig030572-ZebOR.A009	0.316	0.760	0.416
contig051318-BurOR.A005	contig022265-TilOR.A017	0.316	0.760	0.416
contig057156-BurOR.A015	contig047497-ZebOR.A011	0.316	0.762	0.415
contig022234-TilOR.A009	contig030566-ZebOR.A008	0.316	0.783	0.403
contig054684-NyeOR.A012	contig047497-ZebOR.A011	0.316	0.784	0.403
contig085012-BriOR.A130	contig057153-BurOR.A014	0.316	0.789	0.401
contig054678-NyeOR.A010	contig022251-TilOR.A013	0.316	0.796	0.397
contig051570-BurOR.A010	contig047497-ZebOR.A011	0.316	0.804	0.394
contig051318-BurOR.A005	contig051573-BurOR.A011	0.316	0.806	0.392
contig051573-BurOR.A011	contig030572-ZebOR.A009	0.316	0.806	0.392
contig054237-BurOR.A013	contig041952-TilOR.A023	0.316	0.813	0.389
contig051559-BurOR.A008	contig051566-BurOR.A009	0.316	0.816	0.388
contig034988-NyeOR.A004	contig022245-TilOR.A012	0.316	0.828	0.382

contig034983-NyeOR.A003	contig047508-ZebOR.A016	0.316	0.831	0.380
contig022217-TilOR.A004	contig041952-TilOR.A023	0.316	0.844	0.374
contig051566-BurOR.A009	contig047503-ZebOR.A013	0.316	0.846	0.373
contig030556-ZebOR.A005	contig030572-ZebOR.A009	0.316	0.850	0.372
contig034995-NyeOR.A009	contig047508-ZebOR.A015	0.316	0.855	0.370
contig047514-ZebOR.A017	contig062094-ZebOR.A023	0.316	0.856	0.370
contig034988-NyeOR.A004	contig056380-NyeOR.A016	0.316	0.857	0.369
contig022211-TilOR.A003	contig022238-TilOR.A010	0.316	0.858	0.368
contig085012-BriOR.A130	contig041952-TilOR.A023	0.316	0.864	0.366
contig085018-BriOR.A006	contig047508-ZebOR.A016	0.316	0.873	0.362
contig030553-ZebOR.A003	contig062094-ZebOR.A023	0.316	0.885	0.357
contig022251-TilOR.A013	contig047514-ZebOR.A017	0.316	0.894	0.354
contig022251-TilOR.A013	contig030566-ZebOR.A008	0.316	0.903	0.350
contig065887-BurOR.A018	contig022251-TilOR.A013	0.316	0.905	0.349
contig054687-NyeOR.A013	contig047515-ZebOR.A018	0.316	0.918	0.344
contig036784-BurOR.A003	contig056380-NyeOR.A016	0.316	0.945	0.335
contig036784-BurOR.A003	contig051321-BurOR.A006	0.316	0.946	0.334
contig093812-BriOR.A010	contig022204-TilOR.A001	0.317	0.703	0.451
contig085012-BriOR.A130	contig030553-ZebOR.A002	0.317	0.704	0.450
contig051566-BurOR.A009	contig034983-NyeOR.A002	0.317	0.708	0.448
contig051566-BurOR.A009	contig047515-ZebOR.A019	0.317	0.708	0.448
contig022204-TilOR.A001	contig070886-TilOR.A025	0.317	0.711	0.446
contig022225-TilOR.A005	contig070886-TilOR.A025	0.317	0.715	0.443
contig085000-BriOR.A003	contig022227-TilOR.A006	0.317	0.751	0.422
contig054678-NyeOR.A010	contig022211-TilOR.A003	0.317	0.757	0.419
contig084999-BriOR.A001	contig074640-TilOR.A002	0.317	0.765	0.414
contig022204-TilOR.A001	contig073309-TilOR.A026	0.317	0.769	0.412
contig051566-BurOR.A009	contig022232-TilOR.A008	0.317	0.770	0.411
contig085010-BriOR.A005	contig093812-BriOR.A010	0.317	0.770	0.412
contig034990-NyeOR.A007	contig022265-TilOR.A017	0.317	0.774	0.409
contig054687-NyeOR.A013	contig030552-ZebOR.A001	0.317	0.775	0.409
contig054687-NyeOR.A013	contig057165-NyeOR.A017	0.317	0.775	0.409
contig093816-BriOR.A011	contig022251-TilOR.A013	0.317	0.791	0.401
contig085010-BriOR.A005	contig034994-NyeOR.A008	0.317	0.798	0.397
contig022238-TilOR.A010	contig022245-TilOR.A012	0.317	0.807	0.392
contig034995-NyeOR.A009	contig030560-ZebOR.A007	0.317	0.813	0.391
contig022259-TilOR.A014	contig047497-ZebOR.A011	0.317	0.818	0.387
contig034990-NyeOR.A007	contig022259-TilOR.A014	0.317	0.818	0.387
contig051321-BurOR.A006	contig047526-ZebOR.A021	0.317	0.826	0.383
contig036787-BurOR.A004	contig030572-ZebOR.A009	0.317	0.835	0.379
contig065887-BurOR.A018	contig054868-NyeOR.A014	0.317	0.835	0.379
contig022238-TilOR.A010	contig062094-ZebOR.A023	0.317	0.836	0.380
contig064570-BurOR.A017	contig054687-NyeOR.A013	0.317	0.838	0.378

contig056375-NyeOR.A015	contig022264-TilOR.A016	0.317	0.839	0.378
contig047508-ZebOR.A016	contig062094-ZebOR.A023	0.317	0.842	0.376
contig022238-TilOR.A010	contig022259-TilOR.A015	0.317	0.842	0.376
contig054868-NyeOR.A014	contig030566-ZebOR.A008	0.317	0.846	0.375
contig034994-NyeOR.A008	contig022265-TilOR.A017	0.317	0.849	0.373
contig051566-BurOR.A009	contig022217-TilOR.A004	0.317	0.850	0.372
contig034988-NyeOR.A004	contig030572-ZebOR.A009	0.317	0.862	0.368
contig051318-BurOR.A005	contig034988-NyeOR.A004	0.317	0.862	0.368
contig085012-BriOR.A130	contig051566-BurOR.A009	0.317	0.876	0.362
contig030553-ZebOR.A003	contig047526-ZebOR.A021	0.317	0.876	0.362
contig051566-BurOR.A009	contig022251-TilOR.A013	0.317	0.877	0.361
contig085000-BriOR.A003	contig070886-TilOR.A025	0.317	0.886	0.357
contig036784-BurOR.A003	contig070886-TilOR.A025	0.317	0.890	0.356
contig036784-BurOR.A003	contig062094-ZebOR.A023	0.317	0.904	0.350
contig085018-BriOR.A006	contig022238-TilOR.A010	0.317	0.931	0.341
contig085000-BriOR.A003	contig056380-NyeOR.A016	0.317	0.948	0.335
contig085000-BriOR.A003	contig051321-BurOR.A006	0.317	0.949	0.334
contig084999-BriOR.A002	contig065887-BurOR.A018	0.318	0.674	0.472
contig084999-BriOR.A002	contig030566-ZebOR.A008	0.318	0.683	0.466
contig051559-BurOR.A007	contig047508-ZebOR.A016	0.318	0.684	0.465
contig041951-TilOR.A022	contig030572-ZebOR.A009	0.318	0.689	0.462
contig051318-BurOR.A005	contig041951-TilOR.A022	0.318	0.689	0.462
contig054237-BurOR.A013	contig030556-ZebOR.A005	0.318	0.691	0.460
contig036787-BurOR.A004	contig054237-BurOR.A013	0.318	0.700	0.454
contig085012-BriOR.A130	contig034983-NyeOR.A002	0.318	0.706	0.451
contig085012-BriOR.A130	contig047515-ZebOR.A019	0.318	0.706	0.451
contig085002-BriOR.A004	contig047503-ZebOR.A013	0.318	0.707	0.450
contig034983-NyeOR.A002	contig034988-NyeOR.A004	0.318	0.716	0.445
contig034988-NyeOR.A004	contig047515-ZebOR.A019	0.318	0.716	0.445
contig034981-NyeOR.A001	contig030554-ZebOR.A004	0.318	0.729	0.436
contig085026-BriOR.A008	contig030556-ZebOR.A005	0.318	0.731	0.435
contig085026-BriOR.A008	contig036787-BurOR.A004	0.318	0.740	0.430
contig036787-BurOR.A004	contig056380-NyeOR.A016	0.318	0.759	0.419
contig085002-BriOR.A004	contig085012-BriOR.A130	0.318	0.760	0.419
contig030560-ZebOR.A007	contig047497-ZebOR.A011	0.318	0.761	0.418
contig051566-BurOR.A009	contig047521-ZebOR.A020	0.318	0.765	0.415
contig036780-BurOR.A001	contig051318-BurOR.A005	0.318	0.768	0.414
contig036780-BurOR.A001	contig030572-ZebOR.A009	0.318	0.769	0.414
contig065887-BurOR.A018	contig022234-TilOR.A009	0.318	0.773	0.411
contig056380-NyeOR.A016	contig030556-ZebOR.A005	0.318	0.773	0.412
contig030552-ZebOR.A001	contig030572-ZebOR.A009	0.318	0.780	0.408
contig051318-BurOR.A005	contig030552-ZebOR.A001	0.318	0.780	0.408
contig047497-ZebOR.A011	contig047508-ZebOR.A015	0.318	0.787	0.404

contig093812-BriOR.A010	contig022234-TilOR.A009	0.318	0.794	0.401
contig085002-BriOR.A004	contig051559-BurOR.A008	0.318	0.797	0.399
contig085012-BriOR.A130	contig057156-BurOR.A015	0.318	0.805	0.395
contig051559-BurOR.A008	contig034988-NyeOR.A004	0.318	0.820	0.388
contig022238-TilOR.A010	contig047521-ZebOR.A020	0.318	0.823	0.386
contig093816-BriOR.A011	contig085012-BriOR.A130	0.318	0.823	0.386
contig085012-BriOR.A130	contig051573-BurOR.A011	0.318	0.830	0.384
contig051321-BurOR.A006	contig041952-TilOR.A023	0.318	0.833	0.381
contig022241-TilOR.A011	contig047508-ZebOR.A016	0.318	0.834	0.381
contig034994-NyeOR.A008	contig022241-TilOR.A011	0.318	0.837	0.379
contig054233-BurOR.A012	contig054687-NyeOR.A013	0.318	0.854	0.372
contig054687-NyeOR.A013	contig047523-ZebOR.A022	0.318	0.854	0.372
contig085026-BriOR.A008	contig073309-TilOR.A026	0.318	0.862	0.369
contig034988-NyeOR.A004	contig047503-ZebOR.A013	0.318	0.864	0.368
contig034988-NyeOR.A004	contig022217-TilOR.A004	0.318	0.868	0.367
contig051321-BurOR.A006	contig034988-NyeOR.A004	0.318	0.871	0.365
contig093807-BriOR.A009	contig062094-ZebOR.A023	0.318	0.872	0.365
contig093807-BriOR.A009	contig022259-TilOR.A015	0.318	0.878	0.362
contig085018-BriOR.A006	contig022264-TilOR.A016	0.318	0.891	0.357
contig056380-NyeOR.A016	contig047514-ZebOR.A017	0.318	0.894	0.356
contig034983-NyeOR.A003	contig054687-NyeOR.A013	0.318	0.903	0.353
contig085000-BriOR.A003	contig062094-ZebOR.A023	0.318	0.907	0.350
contig057754-NyeOR.A018	contig062094-ZebOR.A023	0.318	0.908	0.351
contig030566-ZebOR.A008	contig047515-ZebOR.A018	0.318	0.931	0.342
contig093807-BriOR.A009	contig022217-TilOR.A004	0.318	0.955	0.333
contig085018-BriOR.A006	contig093807-BriOR.A009	0.318	0.957	0.332
contig056380-NyeOR.A016	contig057754-NyeOR.A018	0.318	0.964	0.330
contig054687-NyeOR.A013	contig030554-ZebOR.A004	0.319	0.669	0.476
contig051566-BurOR.A009	contig022225-TilOR.A005	0.319	0.672	0.475
contig085012-BriOR.A130	contig051559-BurOR.A007	0.319	0.692	0.460
contig054237-BurOR.A013	contig054678-NyeOR.A010	0.319	0.728	0.438
contig085002-BriOR.A004	contig056380-NyeOR.A016	0.319	0.744	0.429
contig065887-BurOR.A018	contig030552-ZebOR.A001	0.319	0.752	0.424
contig065887-BurOR.A018	contig057165-NyeOR.A017	0.319	0.752	0.424
contig030556-ZebOR.A005	contig062094-ZebOR.A023	0.319	0.768	0.416
contig085026-BriOR.A008	contig054678-NyeOR.A010	0.319	0.770	0.415
contig034988-NyeOR.A004	contig022232-TilOR.A008	0.319	0.774	0.411
contig036780-BurOR.A001	contig054687-NyeOR.A013	0.319	0.775	0.412
contig051566-BurOR.A009	contig034981-NyeOR.A001	0.319	0.781	0.408
contig085026-BriOR.A008	contig051566-BurOR.A009	0.319	0.782	0.408
contig051566-BurOR.A009	contig022211-TilOR.A003	0.319	0.794	0.402
contig034981-NyeOR.A001	contig022238-TilOR.A010	0.319	0.815	0.391
contig022245-TilOR.A012	contig047508-ZebOR.A016	0.319	0.825	0.386

contig022211-TilOR.A003	contig041952-TilOR.A023	0.319	0.844	0.378
contig022238-TilOR.A010	contig022251-TilOR.A013	0.319	0.847	0.377
contig064570-BurOR.A017	contig030566-ZebOR.A008	0.319	0.850	0.375
contig064570-BurOR.A017	contig065887-BurOR.A018	0.319	0.852	0.374
contig056380-NyeOR.A016	contig047508-ZebOR.A016	0.319	0.853	0.374
contig085026-BriOR.A008	contig093807-BriOR.A009	0.319	0.855	0.373
contig022251-TilOR.A013	contig047526-ZebOR.A021	0.319	0.862	0.370
contig085018-BriOR.A006	contig054687-NyeOR.A013	0.319	0.872	0.366
contig056380-NyeOR.A016	contig022238-TilOR.A010	0.319	0.873	0.366
contig093807-BriOR.A009	contig022211-TilOR.A003	0.319	0.882	0.362
contig093812-BriOR.A010	contig030576-ZebOR.A010	0.319	0.943	0.339
contig074640-TilOR.A002	contig030553-ZebOR.A002	0.320	0.696	0.461
contig051318-BurOR.A005	contig064187-BurOR.A016	0.320	0.736	0.435
contig064187-BurOR.A016	contig030572-ZebOR.A009	0.320	0.736	0.435
contig054678-NyeOR.A010	contig022217-TilOR.A004	0.320	0.738	0.434
contig022227-TilOR.A006	contig047514-ZebOR.A017	0.320	0.750	0.427
contig036787-BurOR.A004	contig062094-ZebOR.A023	0.320	0.755	0.423
contig030552-ZebOR.A001	contig030566-ZebOR.A008	0.320	0.762	0.419
contig057165-NyeOR.A017	contig030566-ZebOR.A008	0.320	0.762	0.419
contig085010-BriOR.A005	contig047499-ZebOR.A012	0.320	0.765	0.418
contig051318-BurOR.A005	contig057165-NyeOR.A017	0.320	0.768	0.417
contig057165-NyeOR.A017	contig030572-ZebOR.A009	0.320	0.769	0.417
contig034988-NyeOR.A004	contig047521-ZebOR.A020	0.320	0.781	0.409
contig022227-TilOR.A006	contig073309-TilOR.A026	0.320	0.793	0.403
contig093812-BriOR.A010	contig022265-TilOR.A017	0.320	0.794	0.402
contig085012-BriOR.A130	contig054684-NyeOR.A012	0.320	0.817	0.392
contig054233-BurOR.A012	contig065887-BurOR.A018	0.320	0.829	0.386
contig065887-BurOR.A018	contig047523-ZebOR.A022	0.320	0.829	0.386
contig030572-ZebOR.A009	contig047526-ZebOR.A021	0.320	0.830	0.386
contig051318-BurOR.A005	contig047526-ZebOR.A021	0.320	0.830	0.386
contig030566-ZebOR.A008	contig047523-ZebOR.A022	0.320	0.840	0.381
contig054233-BurOR.A012	contig030566-ZebOR.A008	0.320	0.840	0.381
contig022238-TilOR.A010	contig030572-ZebOR.A009	0.320	0.842	0.380
contig051318-BurOR.A005	contig022238-TilOR.A010	0.320	0.842	0.380
contig030572-ZebOR.A009	contig047508-ZebOR.A016	0.320	0.858	0.373
contig051318-BurOR.A005	contig047508-ZebOR.A016	0.320	0.858	0.373
contig047503-ZebOR.A013	contig047508-ZebOR.A016	0.320	0.861	0.372
contig030572-ZebOR.A009	contig047514-ZebOR.A017	0.320	0.879	0.364
contig051318-BurOR.A005	contig047514-ZebOR.A017	0.320	0.879	0.364
contig093807-BriOR.A009	contig022251-TilOR.A013	0.320	0.883	0.363
contig051321-BurOR.A006	contig047514-ZebOR.A017	0.320	0.896	0.358
contig051566-BurOR.A009	contig030553-ZebOR.A003	0.320	0.899	0.357
contig093812-BriOR.A010	contig062344-NyeOR.A020	0.320	0.910	0.352

contig093807-BriOR.A009	contig056380-NyeOR.A016	0.320	0.911	0.351
contig034995-NyeOR.A009	contig022241-TilOR.A011	0.320	0.911	0.352
contig065887-BurOR.A018	contig047515-ZebOR.A018	0.320	0.919	0.349
contig051321-BurOR.A006	contig057754-NyeOR.A018	0.320	0.950	0.337
contig065887-BurOR.A018	contig030554-ZebOR.A004	0.321	0.650	0.494
contig030554-ZebOR.A004	contig030566-ZebOR.A008	0.321	0.658	0.488
contig051566-BurOR.A009	contig022204-TilOR.A001	0.321	0.674	0.476
contig085002-BriOR.A004	contig022217-TilOR.A004	0.321	0.685	0.468
contig034988-NyeOR.A004	contig022225-TilOR.A005	0.321	0.686	0.468
contig034983-NyeOR.A002	contig047508-ZebOR.A016	0.321	0.692	0.464
contig047508-ZebOR.A016	contig047515-ZebOR.A019	0.321	0.692	0.464
contig085012-BriOR.A130	contig036782-BurOR.A002	0.321	0.723	0.444
contig085002-BriOR.A004	contig062094-ZebOR.A023	0.321	0.739	0.434
contig054237-BurOR.A013	contig047526-ZebOR.A021	0.321	0.761	0.422
contig022232-TilOR.A008	contig047508-ZebOR.A016	0.321	0.771	0.416
contig034981-NyeOR.A001	contig034988-NyeOR.A004	0.321	0.797	0.402
contig085026-BriOR.A008	contig034988-NyeOR.A004	0.321	0.798	0.402
contig085010-BriOR.A005	contig085012-BriOR.A130	0.321	0.805	0.399
contig051559-BurOR.A008	contig047508-ZebOR.A016	0.321	0.817	0.392
contig034988-NyeOR.A004	contig022211-TilOR.A003	0.321	0.835	0.384
contig085010-BriOR.A005	contig034995-NyeOR.A009	0.321	0.844	0.381
contig093807-BriOR.A009	contig047521-ZebOR.A020	0.321	0.846	0.380
contig084999-BriOR.A002	contig085012-BriOR.A130	0.321	0.856	0.375
contig034994-NyeOR.A008	contig062344-NyeOR.A020	0.321	0.856	0.375
contig022217-TilOR.A004	contig047508-ZebOR.A016	0.321	0.865	0.371
contig051321-BurOR.A006	contig047508-ZebOR.A016	0.321	0.868	0.370
contig057754-NyeOR.A018	contig047526-ZebOR.A021	0.321	0.870	0.368
contig085012-BriOR.A130	contig034988-NyeOR.A004	0.321	0.881	0.364
contig034988-NyeOR.A004	contig022251-TilOR.A013	0.321	0.882	0.364
contig085012-BriOR.A130	contig047514-ZebOR.A017	0.321	0.906	0.354
contig062344-NyeOR.A020	contig047499-ZebOR.A012	0.321	0.912	0.353
contig034983-NyeOR.A003	contig030566-ZebOR.A008	0.321	0.916	0.350
contig093812-BriOR.A010	contig022259-TilOR.A015	0.321	0.926	0.346
contig022225-TilOR.A005	contig047526-ZebOR.A021	0.322	0.670	0.480
contig051566-BurOR.A009	contig022227-TilOR.A006	0.322	0.700	0.459
contig030572-ZebOR.A009	contig047506-ZebOR.A014	0.322	0.726	0.444
contig051318-BurOR.A005	contig047506-ZebOR.A014	0.322	0.726	0.444
contig074640-TilOR.A002	contig073309-TilOR.A026	0.322	0.727	0.443
contig022204-TilOR.A001	contig047499-ZebOR.A012	0.322	0.729	0.442
contig022225-TilOR.A005	contig047497-ZebOR.A011	0.322	0.731	0.441
contig022225-TilOR.A005	contig047514-ZebOR.A017	0.322	0.743	0.433
contig036780-BurOR.A001	contig065887-BurOR.A018	0.322	0.752	0.427
contig036780-BurOR.A001	contig030566-ZebOR.A008	0.322	0.762	0.422

contig022265-TilOR.A017	contig047499-ZebOR.A012	0.322	0.778	0.414
contig047508-ZebOR.A016	contig047521-ZebOR.A020	0.322	0.778	0.414
contig085010-BriOR.A005	contig047497-ZebOR.A011	0.322	0.790	0.408
contig085012-BriOR.A130	contig030560-ZebOR.A007	0.322	0.798	0.403
contig093812-BriOR.A010	contig047515-ZebOR.A018	0.322	0.813	0.396
contig054687-NyeOR.A013	contig022259-TilOR.A014	0.322	0.823	0.391
contig034990-NyeOR.A007	contig022241-TilOR.A011	0.322	0.836	0.385
contig054237-BurOR.A013	contig022238-TilOR.A010	0.322	0.837	0.385
contig093807-BriOR.A009	contig034981-NyeOR.A001	0.322	0.838	0.385
contig085026-BriOR.A008	contig022238-TilOR.A010	0.322	0.839	0.383
contig085018-BriOR.A006	contig030566-ZebOR.A008	0.322	0.858	0.375
contig030553-ZebOR.A003	contig047514-ZebOR.A017	0.322	0.861	0.374
contig085012-BriOR.A130	contig022238-TilOR.A010	0.322	0.867	0.372
contig093812-BriOR.A010	contig051318-BurOR.A005	0.322	0.870	0.370
contig093807-BriOR.A009	contig022245-TilOR.A012	0.322	0.872	0.369
contig051321-BurOR.A006	contig022238-TilOR.A010	0.322	0.875	0.367
contig022238-TilOR.A010	contig030553-ZebOR.A003	0.322	0.876	0.367
contig085000-BriOR.A003	contig047526-ZebOR.A021	0.322	0.883	0.365
contig093807-BriOR.A009	contig030572-ZebOR.A009	0.322	0.896	0.360
contig093807-BriOR.A009	contig051318-BurOR.A005	0.322	0.896	0.360
contig093807-BriOR.A009	contig051321-BurOR.A006	0.322	0.913	0.353
contig022204-TilOR.A001	contig047526-ZebOR.A021	0.323	0.673	0.481
contig022225-TilOR.A005	contig047508-ZebOR.A016	0.323	0.684	0.473
contig034988-NyeOR.A004	contig022204-TilOR.A001	0.323	0.688	0.469
contig022227-TilOR.A006	contig022238-TilOR.A010	0.323	0.717	0.450
contig034988-NyeOR.A006	contig030572-ZebOR.A009	0.323	0.737	0.438
contig051318-BurOR.A005	contig034988-NyeOR.A006	0.323	0.737	0.438
contig034981-NyeOR.A001	contig047508-ZebOR.A016	0.323	0.794	0.407
contig085026-BriOR.A008	contig047508-ZebOR.A016	0.323	0.795	0.407
contig085012-BriOR.A130	contig054678-NyeOR.A010	0.323	0.816	0.395
contig054687-NyeOR.A013	contig073309-TilOR.A026	0.323	0.827	0.391
contig022241-TilOR.A011	contig047497-ZebOR.A011	0.323	0.828	0.390
contig022211-TilOR.A003	contig047508-ZebOR.A016	0.323	0.832	0.389
contig054687-NyeOR.A013	contig056375-NyeOR.A015	0.323	0.840	0.385
contig085026-BriOR.A008	contig041952-TilOR.A023	0.323	0.847	0.382
contig036784-BurOR.A003	contig047514-ZebOR.A017	0.323	0.865	0.374
contig085012-BriOR.A130	contig047508-ZebOR.A016	0.323	0.878	0.368
contig022251-TilOR.A013	contig047508-ZebOR.A016	0.323	0.879	0.368
contig036784-BurOR.A003	contig047526-ZebOR.A021	0.323	0.880	0.367
contig034994-NyeOR.A008	contig030576-ZebOR.A010	0.323	0.893	0.362
contig034994-NyeOR.A008	contig022259-TilOR.A015	0.323	0.899	0.360
contig065887-BurOR.A018	contig034983-NyeOR.A003	0.323	0.904	0.357
contig034988-NyeOR.A004	contig030553-ZebOR.A003	0.323	0.911	0.355

contig051559-BurOR.A008	contig054687-NyeOR.A013	0.323	0.915	0.353
contig036787-BurOR.A004	contig022217-TilOR.A004	0.324	0.709	0.457
contig034988-NyeOR.A004	contig022227-TilOR.A006	0.324	0.715	0.453
contig022217-TilOR.A004	contig030556-ZebOR.A005	0.324	0.722	0.449
contig022225-TilOR.A005	contig022238-TilOR.A010	0.324	0.724	0.447
contig022204-TilOR.A001	contig047514-ZebOR.A017	0.324	0.734	0.441
contig093816-BriOR.A011	contig022204-TilOR.A001	0.324	0.737	0.440
contig074640-TilOR.A002	contig041952-TilOR.A023	0.324	0.798	0.405
contig022259-TilOR.A014	contig030566-ZebOR.A008	0.324	0.810	0.400
contig065887-BurOR.A018	contig022259-TilOR.A014	0.324	0.811	0.399
contig085012-BriOR.A130	contig022268-TilOR.A019	0.324	0.811	0.400
contig093812-BriOR.A010	contig034983-NyeOR.A003	0.324	0.824	0.393
contig093812-BriOR.A010	contig047503-ZebOR.A013	0.324	0.829	0.390
contig022265-TilOR.A017	contig047497-ZebOR.A011	0.324	0.840	0.386
contig030553-ZebOR.A003	contig030572-ZebOR.A009	0.324	0.840	0.386
contig085012-BriOR.A130	contig030553-ZebOR.A003	0.324	0.842	0.385
contig085018-BriOR.A006	contig065887-BurOR.A018	0.324	0.847	0.382
contig085012-BriOR.A130	contig047526-ZebOR.A021	0.324	0.848	0.382
contig036784-BurOR.A003	contig030572-ZebOR.A009	0.324	0.852	0.381
contig085012-BriOR.A130	contig036784-BurOR.A003	0.324	0.854	0.380
contig093812-BriOR.A010	contig030572-ZebOR.A009	0.324	0.871	0.372
contig051566-BurOR.A009	contig057754-NyeOR.A018	0.324	0.892	0.363
contig034995-NyeOR.A009	contig062344-NyeOR.A020	0.324	0.916	0.353
contig030576-ZebOR.A010	contig047499-ZebOR.A012	0.324	0.951	0.340
contig074640-TilOR.A002	contig022266-TilOR.A018	0.325	0.668	0.486
contig022204-TilOR.A001	contig047508-ZebOR.A016	0.325	0.686	0.474
contig022227-TilOR.A006	contig047526-ZebOR.A021	0.325	0.698	0.465
contig022204-TilOR.A001	contig022238-TilOR.A010	0.325	0.704	0.462
contig093812-BriOR.A010	contig022227-TilOR.A006	0.325	0.719	0.453
contig085012-BriOR.A130	contig064187-BurOR.A016	0.325	0.726	0.447
contig085012-BriOR.A130	contig057756-NyeOR.A019	0.325	0.727	0.447
contig041952-TilOR.A023	contig030556-ZebOR.A005	0.325	0.740	0.439
contig051566-BurOR.A009	contig054237-BurOR.A013	0.325	0.762	0.427
contig047499-ZebOR.A012	contig047521-ZebOR.A020	0.325	0.773	0.420
contig057754-NyeOR.A018	contig047514-ZebOR.A017	0.325	0.855	0.381
contig034994-NyeOR.A008	contig030553-ZebOR.A003	0.325	0.863	0.376
contig057754-NyeOR.A018	contig022238-TilOR.A010	0.325	0.869	0.374
contig054868-NyeOR.A014	contig022264-TilOR.A016	0.325	0.879	0.369
contig022241-TilOR.A011	contig022264-TilOR.A016	0.325	0.901	0.361
contig093807-BriOR.A009	contig085012-BriOR.A130	0.325	0.923	0.352
contig051559-BurOR.A008	contig030566-ZebOR.A008	0.325	0.928	0.351
contig022259-TilOR.A015	contig047499-ZebOR.A012	0.325	0.934	0.348
contig074640-TilOR.A002	contig030557-ZebOR.A006	0.326	0.684	0.477

contig034990-NyeOR.A007	contig022204-TilOR.A001	0.326	0.701	0.465
contig022227-TilOR.A006	contig047508-ZebOR.A016	0.326	0.712	0.458
contig036782-BurOR.A002	contig074640-TilOR.A002	0.326	0.724	0.450
contig034990-NyeOR.A007	contig030556-ZebOR.A005	0.326	0.729	0.447
contig034994-NyeOR.A008	contig030556-ZebOR.A005	0.326	0.763	0.427
contig085012-BriOR.A130	contig036787-BurOR.A004	0.326	0.785	0.416
contig085012-BriOR.A130	contig030556-ZebOR.A005	0.326	0.799	0.408
contig065887-BurOR.A018	contig073309-TilOR.A026	0.326	0.803	0.405
contig041952-TilOR.A023	contig030553-ZebOR.A003	0.326	0.811	0.403
contig073309-TilOR.A026	contig030566-ZebOR.A008	0.326	0.814	0.400
contig056375-NyeOR.A015	contig030566-ZebOR.A008	0.326	0.852	0.382
contig065887-BurOR.A018	contig056375-NyeOR.A015	0.326	0.854	0.381
contig093807-BriOR.A009	contig054237-BurOR.A013	0.326	0.860	0.379
contig085000-BriOR.A003	contig022238-TilOR.A010	0.326	0.882	0.370
contig022245-TilOR.A012	contig022264-TilOR.A016	0.326	0.889	0.367
contig034995-NyeOR.A009	contig022265-TilOR.A017	0.326	0.897	0.363
contig085000-BriOR.A003	contig051566-BurOR.A009	0.326	0.898	0.363
contig036784-BurOR.A003	contig051566-BurOR.A009	0.326	0.902	0.362
contig030553-ZebOR.A003	contig047508-ZebOR.A016	0.326	0.907	0.359
contig034995-NyeOR.A009	contig030576-ZebOR.A010	0.326	0.956	0.341
contig036787-BurOR.A004	contig022245-TilOR.A012	0.327	0.664	0.492
contig054687-NyeOR.A013	contig022227-TilOR.A006	0.327	0.672	0.486
contig022245-TilOR.A012	contig030556-ZebOR.A005	0.327	0.676	0.484
contig057153-BurOR.A014	contig074640-TilOR.A002	0.327	0.680	0.481
contig054681-NyeOR.A011	contig074640-TilOR.A002	0.327	0.688	0.476
contig085012-BriOR.A130	contig047506-ZebOR.A014	0.327	0.717	0.456
contig051573-BurOR.A011	contig022204-TilOR.A001	0.327	0.744	0.439
contig085012-BriOR.A130	contig022265-TilOR.A017	0.327	0.751	0.436
contig022245-TilOR.A012	contig041952-TilOR.A023	0.327	0.760	0.431
contig093812-BriOR.A010	contig047521-ZebOR.A020	0.327	0.778	0.420
contig054237-BurOR.A013	contig034988-NyeOR.A004	0.327	0.778	0.421
contig062344-NyeOR.A020	contig047497-ZebOR.A011	0.327	0.834	0.392
contig051318-BurOR.A005	contig030553-ZebOR.A003	0.327	0.839	0.389
contig036784-BurOR.A003	contig051318-BurOR.A005	0.327	0.851	0.384
contig093812-BriOR.A010	contig022217-TilOR.A004	0.327	0.854	0.384
contig085000-BriOR.A003	contig030572-ZebOR.A009	0.327	0.861	0.379
contig085000-BriOR.A003	contig085012-BriOR.A130	0.327	0.863	0.378
contig093807-BriOR.A009	contig030553-ZebOR.A003	0.327	0.873	0.375
contig034990-NyeOR.A007	contig062344-NyeOR.A020	0.327	0.880	0.372
contig034990-NyeOR.A007	contig022259-TilOR.A015	0.327	0.902	0.362
contig034988-NyeOR.A004	contig057754-NyeOR.A018	0.327	0.904	0.361
contig051559-BurOR.A008	contig065887-BurOR.A018	0.327	0.916	0.358
contig022259-TilOR.A015	contig022264-TilOR.A016	0.327	0.925	0.353

contig034995-NyeOR.A009	contig022259-TilOR.A015	0.327	0.975	0.335
contig085012-BriOR.A130	contig041951-TilOR.A022	0.328	0.680	0.481
contig054678-NyeOR.A010	contig022245-TilOR.A012	0.328	0.691	0.475
contig034994-NyeOR.A008	contig047515-ZebOR.A018	0.328	0.716	0.458
contig034990-NyeOR.A007	contig022268-TilOR.A019	0.328	0.720	0.456
contig085012-BriOR.A130	contig034988-NyeOR.A006	0.328	0.728	0.450
contig093812-BriOR.A010	contig074640-TilOR.A002	0.328	0.779	0.420
contig022264-TilOR.A016	contig047523-ZebOR.A022	0.328	0.873	0.376
contig054233-BurOR.A012	contig022264-TilOR.A016	0.328	0.873	0.376
contig036784-BurOR.A003	contig022238-TilOR.A010	0.328	0.879	0.373
contig034994-NyeOR.A008	contig057754-NyeOR.A018	0.328	0.883	0.372
contig085000-BriOR.A003	contig034988-NyeOR.A004	0.328	0.917	0.358
contig054687-NyeOR.A013	contig022259-TilOR.A015	0.328	0.980	0.334
contig022227-TilOR.A006	contig030566-ZebOR.A008	0.329	0.672	0.490
contig065887-BurOR.A018	contig022227-TilOR.A006	0.329	0.673	0.489
contig034994-NyeOR.A008	contig022268-TilOR.A019	0.329	0.730	0.451
contig036787-BurOR.A004	contig041952-TilOR.A023	0.329	0.749	0.440
contig074640-TilOR.A002	contig022238-TilOR.A010	0.329	0.769	0.428
contig074640-TilOR.A002	contig047514-ZebOR.A017	0.329	0.785	0.418
contig022234-TilOR.A009	contig047499-ZebOR.A012	0.329	0.791	0.416
contig085026-BriOR.A008	contig047499-ZebOR.A012	0.329	0.794	0.415
contig034981-NyeOR.A001	contig047499-ZebOR.A012	0.329	0.797	0.413
contig085012-BriOR.A130	contig030554-ZebOR.A004	0.329	0.807	0.408
contig093812-BriOR.A010	contig051559-BurOR.A008	0.329	0.835	0.394
contig034994-NyeOR.A008	contig022251-TilOR.A013	0.329	0.851	0.386
contig093812-BriOR.A010	contig022251-TilOR.A013	0.329	0.859	0.383
contig085000-BriOR.A003	contig051318-BurOR.A005	0.329	0.860	0.382
contig051318-BurOR.A005	contig034994-NyeOR.A008	0.329	0.862	0.381
contig085000-BriOR.A003	contig047514-ZebOR.A017	0.329	0.868	0.379
contig030576-ZebOR.A010	contig047497-ZebOR.A011	0.329	0.871	0.378
contig093807-BriOR.A009	contig036784-BurOR.A003	0.329	0.876	0.375
contig022259-TilOR.A015	contig047497-ZebOR.A011	0.329	0.889	0.370
contig057754-NyeOR.A018	contig047508-ZebOR.A016	0.329	0.900	0.366
contig085018-BriOR.A006	contig093812-BriOR.A010	0.329	0.903	0.364
contig036784-BurOR.A003	contig034988-NyeOR.A004	0.329	0.914	0.360
contig057756-NyeOR.A019	contig074640-TilOR.A002	0.330	0.713	0.463
contig030556-ZebOR.A005	contig047499-ZebOR.A012	0.330	0.720	0.458
contig036787-BurOR.A004	contig034990-NyeOR.A007	0.330	0.738	0.448
contig034990-NyeOR.A007	contig047521-ZebOR.A020	0.330	0.752	0.438
contig034995-NyeOR.A009	contig030556-ZebOR.A005	0.330	0.766	0.430
contig054678-NyeOR.A010	contig041952-TilOR.A023	0.330	0.767	0.431
contig054237-BurOR.A013	contig047508-ZebOR.A016	0.330	0.775	0.426
contig047499-ZebOR.A012	contig047515-ZebOR.A018	0.330	0.780	0.423

contig057754-NyeOR.A018	contig041952-TilOR.A023	0.330	0.805	0.410
contig093812-BriOR.A010	contig022245-TilOR.A012	0.330	0.833	0.397
contig051318-BurOR.A005	contig034990-NyeOR.A007	0.330	0.836	0.395
contig034994-NyeOR.A008	contig030572-ZebOR.A009	0.330	0.865	0.382
contig085018-BriOR.A006	contig034994-NyeOR.A008	0.330	0.868	0.381
contig034990-NyeOR.A007	contig030576-ZebOR.A010	0.330	0.919	0.359
contig022259-TilOR.A015	contig030566-ZebOR.A008	0.330	0.995	0.332
contig065887-BurOR.A018	contig022259-TilOR.A015	0.330	0.997	0.331
contig084999-BriOR.A002	contig041952-TilOR.A023	0.331	0.663	0.499
contig034983-NyeOR.A003	contig034994-NyeOR.A008	0.331	0.726	0.456
contig093812-BriOR.A010	contig030556-ZebOR.A005	0.331	0.729	0.454
contig034994-NyeOR.A008	contig022204-TilOR.A001	0.331	0.734	0.451
contig093807-BriOR.A009	contig022227-TilOR.A006	0.331	0.743	0.445
contig036787-BurOR.A004	contig034994-NyeOR.A008	0.331	0.750	0.441
contig034994-NyeOR.A008	contig022234-TilOR.A009	0.331	0.751	0.441
contig034995-NyeOR.A009	contig022204-TilOR.A001	0.331	0.767	0.432
contig034990-NyeOR.A007	contig054678-NyeOR.A010	0.331	0.767	0.432
contig030556-ZebOR.A005	contig047497-ZebOR.A011	0.331	0.779	0.425
contig074640-TilOR.A002	contig070886-TilOR.A025	0.331	0.782	0.424
contig074640-TilOR.A002	contig022264-TilOR.A016	0.331	0.787	0.421
contig093816-BriOR.A011	contig054687-NyeOR.A013	0.331	0.791	0.418
contig034981-NyeOR.A001	contig030556-ZebOR.A005	0.331	0.791	0.419
contig051573-BurOR.A011	contig054687-NyeOR.A013	0.331	0.798	0.415
contig034994-NyeOR.A008	contig022245-TilOR.A012	0.331	0.822	0.402
contig047499-ZebOR.A012	contig047503-ZebOR.A013	0.331	0.834	0.397
contig022245-TilOR.A012	contig047499-ZebOR.A012	0.331	0.850	0.389
contig057754-NyeOR.A018	contig030572-ZebOR.A009	0.331	0.855	0.387
contig022211-TilOR.A003	contig047499-ZebOR.A012	0.331	0.856	0.387
contig085012-BriOR.A130	contig057754-NyeOR.A018	0.331	0.857	0.386
contig085000-BriOR.A003	contig034994-NyeOR.A008	0.331	0.863	0.383
contig036784-BurOR.A003	contig034994-NyeOR.A008	0.331	0.866	0.382
contig093807-BriOR.A009	contig057754-NyeOR.A018	0.331	0.867	0.381
contig051318-BurOR.A005	contig047499-ZebOR.A012	0.331	0.868	0.381
contig093812-BriOR.A010	contig062094-ZebOR.A023	0.331	0.898	0.369
contig085000-BriOR.A003	contig047508-ZebOR.A016	0.331	0.914	0.362
contig051318-BurOR.A005	contig022264-TilOR.A016	0.331	0.915	0.362
contig034995-NyeOR.A009	contig022251-TilOR.A013	0.331	0.927	0.357
contig093812-BriOR.A010	contig051321-BurOR.A006	0.331	0.939	0.353
contig022227-TilOR.A006	contig022264-TilOR.A016	0.332	0.671	0.494
contig022268-TilOR.A019	contig047499-ZebOR.A012	0.332	0.720	0.461
contig047497-ZebOR.A011	contig047515-ZebOR.A018	0.332	0.728	0.456
contig034994-NyeOR.A008	contig047521-ZebOR.A020	0.332	0.766	0.433
contig034994-NyeOR.A008	contig054678-NyeOR.A010	0.332	0.780	0.425

contig054237-BurOR.A013	contig047499-ZebOR.A012	0.332	0.785	0.422
contig034983-NyeOR.A003	contig047499-ZebOR.A012	0.332	0.790	0.421
contig036787-BurOR.A004	contig034981-NyeOR.A001	0.332	0.801	0.414
contig093812-BriOR.A010	contig034981-NyeOR.A001	0.332	0.802	0.414
contig085000-BriOR.A003	contig041952-TilOR.A023	0.332	0.811	0.410
contig036784-BurOR.A003	contig041952-TilOR.A023	0.332	0.814	0.409
contig093816-BriOR.A011	contig074640-TilOR.A002	0.332	0.820	0.405
contig034990-NyeOR.A007	contig030572-ZebOR.A009	0.332	0.839	0.396
contig051573-BurOR.A011	contig022264-TilOR.A016	0.332	0.843	0.394
contig054687-NyeOR.A013	contig022241-TilOR.A011	0.332	0.849	0.391
contig085000-BriOR.A003	contig093807-BriOR.A009	0.332	0.880	0.377
contig036784-BurOR.A003	contig047508-ZebOR.A016	0.332	0.911	0.365
contig041952-TilOR.A023	contig030554-ZebOR.A004	0.333	0.680	0.490
contig034990-NyeOR.A007	contig022234-TilOR.A009	0.333	0.740	0.449
contig093807-BriOR.A009	contig022225-TilOR.A005	0.333	0.748	0.445
contig034995-NyeOR.A009	contig022268-TilOR.A019	0.333	0.750	0.444
contig093816-BriOR.A011	contig065887-BurOR.A018	0.333	0.768	0.434
contig034995-NyeOR.A009	contig047515-ZebOR.A018	0.333	0.777	0.429
contig093816-BriOR.A011	contig030566-ZebOR.A008	0.333	0.778	0.428
contig085026-BriOR.A008	contig093812-BriOR.A010	0.333	0.794	0.419
contig054687-NyeOR.A013	contig074640-TilOR.A002	0.333	0.827	0.403
contig034981-NyeOR.A001	contig054678-NyeOR.A010	0.333	0.832	0.400
contig022264-TilOR.A016	contig073309-TilOR.A026	0.333	0.845	0.394
contig051318-BurOR.A005	contig057754-NyeOR.A018	0.333	0.854	0.390
contig030572-ZebOR.A009	contig047499-ZebOR.A012	0.333	0.871	0.382
contig054687-NyeOR.A013	contig070886-TilOR.A025	0.333	0.872	0.382
contig093812-BriOR.A010	contig085012-BriOR.A130	0.333	0.887	0.375
contig093812-BriOR.A010	contig056380-NyeOR.A016	0.333	0.924	0.361
contig051318-BurOR.A005	contig034995-NyeOR.A009	0.333	0.929	0.358
contig084999-BriOR.A002	contig034990-NyeOR.A007	0.334	0.662	0.505
contig084999-BriOR.A002	contig034994-NyeOR.A008	0.334	0.676	0.494
contig036787-BurOR.A004	contig047499-ZebOR.A012	0.334	0.728	0.459
contig034983-NyeOR.A003	contig047497-ZebOR.A011	0.334	0.738	0.453
contig051573-BurOR.A011	contig065887-BurOR.A018	0.334	0.775	0.431
contig036787-BurOR.A004	contig034995-NyeOR.A009	0.334	0.775	0.431
contig034981-NyeOR.A001	contig034990-NyeOR.A007	0.334	0.776	0.431
contig051573-BurOR.A011	contig030566-ZebOR.A008	0.334	0.785	0.425
contig093812-BriOR.A010	contig054237-BurOR.A013	0.334	0.790	0.422
contig093816-BriOR.A011	contig022264-TilOR.A016	0.334	0.835	0.399
contig022241-TilOR.A011	contig030566-ZebOR.A008	0.334	0.835	0.400
contig065887-BurOR.A018	contig022241-TilOR.A011	0.334	0.837	0.399
contig022251-TilOR.A013	contig047497-ZebOR.A011	0.334	0.844	0.396
contig093812-BriOR.A010	contig022211-TilOR.A003	0.334	0.855	0.391

contig034995-NyeOR.A009	contig030553-ZebOR.A003	0.334	0.884	0.378
contig034995-NyeOR.A009	contig022245-TilOR.A012	0.334	0.890	0.375
contig022251-TilOR.A013	contig047499-ZebOR.A012	0.334	0.893	0.374
contig022264-TilOR.A016	contig062094-ZebOR.A023	0.334	0.918	0.364
contig034995-NyeOR.A009	contig030572-ZebOR.A009	0.334	0.933	0.358
contig056380-NyeOR.A016	contig022264-TilOR.A016	0.334	0.959	0.348
contig051321-BurOR.A006	contig022264-TilOR.A016	0.334	0.960	0.348
contig093807-BriOR.A009	contig022204-TilOR.A001	0.335	0.727	0.461
contig051559-BurOR.A008	contig034994-NyeOR.A008	0.335	0.735	0.456
contig093812-BriOR.A010	contig036787-BurOR.A004	0.335	0.738	0.454
contig022268-TilOR.A019	contig047497-ZebOR.A011	0.335	0.740	0.453
contig022227-TilOR.A006	contig047499-ZebOR.A012	0.335	0.744	0.450
contig022234-TilOR.A009	contig047497-ZebOR.A011	0.335	0.749	0.448
contig054678-NyeOR.A010	contig047499-ZebOR.A012	0.335	0.757	0.443
contig085026-BriOR.A008	contig034990-NyeOR.A007	0.335	0.767	0.437
contig093807-BriOR.A009	contig074640-TilOR.A002	0.335	0.776	0.432
contig034995-NyeOR.A009	contig054678-NyeOR.A010	0.335	0.805	0.416
contig034995-NyeOR.A009	contig047521-ZebOR.A020	0.335	0.805	0.416
contig034995-NyeOR.A009	contig022234-TilOR.A009	0.335	0.806	0.416
contig034990-NyeOR.A007	contig022245-TilOR.A012	0.335	0.821	0.407
contig051318-BurOR.A005	contig047497-ZebOR.A011	0.335	0.849	0.395
contig085018-BriOR.A006	contig047499-ZebOR.A012	0.335	0.913	0.367
contig085018-BriOR.A006	contig034995-NyeOR.A009	0.335	0.943	0.356
contig022268-TilOR.A019	contig041952-TilOR.A023	0.336	0.671	0.500
contig074640-TilOR.A002	contig047526-ZebOR.A021	0.336	0.736	0.457
contig034990-NyeOR.A007	contig047515-ZebOR.A018	0.336	0.753	0.446
contig034995-NyeOR.A009	contig022227-TilOR.A006	0.336	0.754	0.445
contig054237-BurOR.A013	contig034990-NyeOR.A007	0.336	0.764	0.440
contig036787-BurOR.A004	contig047497-ZebOR.A011	0.336	0.766	0.438
contig093812-BriOR.A010	contig054678-NyeOR.A010	0.336	0.766	0.438
contig085002-BriOR.A004	contig034981-NyeOR.A001	0.336	0.769	0.437
contig034983-NyeOR.A003	contig034995-NyeOR.A009	0.336	0.787	0.426
contig034981-NyeOR.A001	contig034994-NyeOR.A008	0.336	0.789	0.426
contig074640-TilOR.A002	contig030566-ZebOR.A008	0.336	0.814	0.412
contig022245-TilOR.A012	contig047497-ZebOR.A011	0.336	0.814	0.413
contig030553-ZebOR.A003	contig047497-ZebOR.A011	0.336	0.824	0.407
contig085018-BriOR.A006	contig047497-ZebOR.A011	0.336	0.859	0.391
contig065887-BurOR.A018	contig070886-TilOR.A025	0.336	0.873	0.384
contig070886-TilOR.A025	contig030566-ZebOR.A008	0.336	0.885	0.380
contig022264-TilOR.A016	contig030572-ZebOR.A009	0.336	0.903	0.372
contig051321-BurOR.A006	contig047499-ZebOR.A012	0.336	0.929	0.362
contig084999-BriOR.A002	contig093812-BriOR.A010	0.337	0.691	0.488
contig034990-NyeOR.A007	contig030554-ZebOR.A004	0.337	0.707	0.477

contig034990-NyeOR.A007	contig022227-TilOR.A006	0.337	0.717	0.470
contig034994-NyeOR.A008	contig022227-TilOR.A006	0.337	0.719	0.469
contig034994-NyeOR.A008	contig030554-ZebOR.A004	0.337	0.722	0.467
contig022204-TilOR.A001	contig047497-ZebOR.A011	0.337	0.737	0.457
contig085026-BriOR.A008	contig034994-NyeOR.A008	0.337	0.787	0.428
contig054678-NyeOR.A010	contig047497-ZebOR.A011	0.337	0.796	0.423
contig051559-BurOR.A008	contig047499-ZebOR.A012	0.337	0.800	0.421
contig034990-NyeOR.A007	contig047503-ZebOR.A013	0.337	0.806	0.418
contig034990-NyeOR.A007	contig022211-TilOR.A003	0.337	0.827	0.408
contig054687-NyeOR.A013	contig022245-TilOR.A012	0.337	0.841	0.401
contig051573-BurOR.A011	contig074640-TilOR.A002	0.337	0.842	0.400
contig030572-ZebOR.A009	contig047497-ZebOR.A011	0.337	0.852	0.395
contig034990-NyeOR.A007	contig022251-TilOR.A013	0.337	0.863	0.391
contig093812-BriOR.A010	contig030553-ZebOR.A003	0.337	0.866	0.389
contig034994-NyeOR.A008	contig062094-ZebOR.A023	0.337	0.869	0.388
contig047499-ZebOR.A012	contig062094-ZebOR.A023	0.337	0.890	0.378
contig051321-BurOR.A006	contig034994-NyeOR.A008	0.337	0.908	0.371
contig084999-BriOR.A002	contig047499-ZebOR.A012	0.338	0.663	0.510
contig034983-NyeOR.A003	contig034990-NyeOR.A007	0.338	0.763	0.443
contig065887-BurOR.A018	contig074640-TilOR.A002	0.338	0.803	0.420
contig034994-NyeOR.A008	contig047503-ZebOR.A013	0.338	0.812	0.417
contig034994-NyeOR.A008	contig022211-TilOR.A003	0.338	0.848	0.399
contig054687-NyeOR.A013	contig047526-ZebOR.A021	0.338	0.849	0.397
contig034990-NyeOR.A007	contig062094-ZebOR.A023	0.338	0.859	0.393
contig051566-BurOR.A009	contig054687-NyeOR.A013	0.338	0.873	0.387
contig051321-BurOR.A006	contig034990-NyeOR.A007	0.338	0.898	0.376
contig034995-NyeOR.A009	contig057754-NyeOR.A018	0.338	0.904	0.374
contig084999-BriOR.A002	contig034995-NyeOR.A009	0.339	0.690	0.492
contig085002-BriOR.A004	contig034994-NyeOR.A008	0.339	0.734	0.461
contig051559-BurOR.A008	contig047497-ZebOR.A011	0.339	0.747	0.454
contig047497-ZebOR.A011	contig047521-ZebOR.A020	0.339	0.752	0.450
contig054237-BurOR.A013	contig034994-NyeOR.A008	0.339	0.778	0.435
contig065887-BurOR.A018	contig022245-TilOR.A012	0.339	0.829	0.410
contig057754-NyeOR.A018	contig047497-ZebOR.A011	0.339	0.843	0.402
contig085018-BriOR.A006	contig034990-NyeOR.A007	0.339	0.880	0.385
contig034994-NyeOR.A008	contig056380-NyeOR.A016	0.339	0.894	0.379
contig056380-NyeOR.A016	contig047499-ZebOR.A012	0.339	0.915	0.370
contig054687-NyeOR.A013	contig056380-NyeOR.A016	0.339	0.927	0.366
contig051321-BurOR.A006	contig054687-NyeOR.A013	0.339	0.928	0.366
contig084999-BriOR.A002	contig047497-ZebOR.A011	0.340	0.686	0.496
contig093812-BriOR.A010	contig030554-ZebOR.A004	0.340	0.738	0.461
contig085002-BriOR.A004	contig041952-TilOR.A023	0.340	0.738	0.461
contig051559-BurOR.A008	contig034995-NyeOR.A009	0.340	0.797	0.427

contig022245-TilOR.A012	contig030566-ZebOR.A008	0.340	0.827	0.411
contig085026-BriOR.A008	contig034995-NyeOR.A009	0.340	0.827	0.411
contig034981-NyeOR.A001	contig034995-NyeOR.A009	0.340	0.829	0.409
contig065887-BurOR.A018	contig047526-ZebOR.A021	0.340	0.850	0.400
contig030566-ZebOR.A008	contig047526-ZebOR.A021	0.340	0.861	0.395
contig051566-BurOR.A009	contig065887-BurOR.A018	0.340	0.874	0.390
contig085000-BriOR.A003	contig034995-NyeOR.A009	0.340	0.883	0.385
contig034990-NyeOR.A007	contig056380-NyeOR.A016	0.340	0.884	0.385
contig093812-BriOR.A010	contig057754-NyeOR.A018	0.340	0.886	0.384
contig054687-NyeOR.A013	contig062094-ZebOR.A023	0.340	0.887	0.383
contig030553-ZebOR.A003	contig047499-ZebOR.A012	0.340	0.887	0.383
contig036784-BurOR.A003	contig034995-NyeOR.A009	0.340	0.887	0.384
contig034988-NyeOR.A004	contig054687-NyeOR.A013	0.340	0.891	0.381
contig085012-BriOR.A130	contig022264-TilOR.A016	0.340	0.933	0.365
contig034995-NyeOR.A009	contig062094-ZebOR.A023	0.340	0.942	0.361
contig051321-BurOR.A006	contig034995-NyeOR.A009	0.340	0.985	0.345
contig030554-ZebOR.A004	contig047499-ZebOR.A012	0.341	0.708	0.482
contig093812-BriOR.A010	contig022268-TilOR.A019	0.341	0.713	0.479
contig022227-TilOR.A006	contig047497-ZebOR.A011	0.341	0.731	0.466
contig034990-NyeOR.A007	contig074640-TilOR.A002	0.341	0.780	0.437
contig085012-BriOR.A130	contig034990-NyeOR.A007	0.341	0.852	0.400
contig034990-NyeOR.A007	contig030553-ZebOR.A003	0.341	0.856	0.398
contig085012-BriOR.A130	contig034994-NyeOR.A008	0.341	0.873	0.390
contig051566-BurOR.A009	contig030566-ZebOR.A008	0.341	0.885	0.385
contig051566-BurOR.A009	contig074640-TilOR.A002	0.342	0.730	0.469
contig034995-NyeOR.A009	contig030554-ZebOR.A004	0.342	0.737	0.464
contig085002-BriOR.A004	contig034990-NyeOR.A007	0.342	0.745	0.460
contig085002-BriOR.A004	contig093812-BriOR.A010	0.342	0.759	0.451
contig085002-BriOR.A004	contig034995-NyeOR.A009	0.342	0.760	0.449
contig054237-BurOR.A013	contig034995-NyeOR.A009	0.342	0.817	0.418
contig085000-BriOR.A003	contig047497-ZebOR.A011	0.342	0.824	0.415
contig036784-BurOR.A003	contig047497-ZebOR.A011	0.342	0.827	0.413
contig034995-NyeOR.A009	contig047503-ZebOR.A013	0.342	0.853	0.400
contig047497-ZebOR.A011	contig062094-ZebOR.A023	0.342	0.860	0.398
contig034994-NyeOR.A008	contig074640-TilOR.A002	0.342	0.861	0.398
contig054687-NyeOR.A013	contig022238-TilOR.A010	0.342	0.875	0.391
contig085012-BriOR.A130	contig047499-ZebOR.A012	0.342	0.884	0.386
contig034995-NyeOR.A009	contig022211-TilOR.A003	0.342	0.892	0.383
contig065887-BurOR.A018	contig034988-NyeOR.A004	0.342	0.892	0.384
contig051321-BurOR.A006	contig047497-ZebOR.A011	0.342	0.898	0.381
contig030566-ZebOR.A008	contig062094-ZebOR.A023	0.342	0.900	0.380
contig065887-BurOR.A018	contig062094-ZebOR.A023	0.342	0.902	0.379
contig034988-NyeOR.A004	contig030566-ZebOR.A008	0.342	0.904	0.379

contig022264-TilOR.A016	contig070886-TilOR.A025	0.342	0.931	0.367
contig056380-NyeOR.A016	contig030566-ZebOR.A008	0.342	0.940	0.363
contig051321-BurOR.A006	contig030566-ZebOR.A008	0.342	0.941	0.363
contig065887-BurOR.A018	contig056380-NyeOR.A016	0.342	0.942	0.363
contig051321-BurOR.A006	contig065887-BurOR.A018	0.342	0.943	0.362
contig034995-NyeOR.A009	contig056380-NyeOR.A016	0.342	0.969	0.353
contig030554-ZebOR.A004	contig047497-ZebOR.A011	0.343	0.732	0.469
contig051559-BurOR.A008	contig034990-NyeOR.A007	0.343	0.773	0.443
contig034981-NyeOR.A001	contig047497-ZebOR.A011	0.343	0.776	0.442
contig054687-NyeOR.A013	contig047514-ZebOR.A017	0.343	0.860	0.398
contig085000-BriOR.A003	contig093812-BriOR.A010	0.343	0.866	0.396
contig093812-BriOR.A010	contig036784-BurOR.A003	0.343	0.869	0.394
contig054687-NyeOR.A013	contig047508-ZebOR.A016	0.343	0.888	0.386
contig034995-NyeOR.A009	contig074640-TilOR.A002	0.343	0.906	0.379
contig057754-NyeOR.A018	contig047499-ZebOR.A012	0.343	0.907	0.378
contig085012-BriOR.A130	contig034995-NyeOR.A009	0.343	0.948	0.362
contig085002-BriOR.A004	contig047497-ZebOR.A011	0.344	0.750	0.459
contig085026-BriOR.A008	contig047497-ZebOR.A011	0.344	0.768	0.449
contig047497-ZebOR.A011	contig047503-ZebOR.A013	0.344	0.803	0.428
contig054687-NyeOR.A013	contig030572-ZebOR.A009	0.344	0.832	0.413
contig054687-NyeOR.A013	contig041952-TilOR.A023	0.344	0.838	0.410
contig085012-BriOR.A130	contig054687-NyeOR.A013	0.344	0.859	0.400
contig065887-BurOR.A018	contig047514-ZebOR.A017	0.344	0.866	0.397
contig065887-BurOR.A018	contig022238-TilOR.A010	0.344	0.876	0.393
contig030566-ZebOR.A008	contig047514-ZebOR.A017	0.344	0.877	0.392
contig022238-TilOR.A010	contig030566-ZebOR.A008	0.344	0.887	0.388
contig034988-NyeOR.A004	contig074640-TilOR.A002	0.345	0.739	0.467
contig054237-BurOR.A013	contig047497-ZebOR.A011	0.345	0.764	0.452
contig034994-NyeOR.A008	contig054687-NyeOR.A013	0.345	0.860	0.402
contig034990-NyeOR.A007	contig057754-NyeOR.A018	0.345	0.876	0.393
contig056380-NyeOR.A016	contig047497-ZebOR.A011	0.345	0.884	0.390
contig093807-BriOR.A009	contig054687-NyeOR.A013	0.345	0.886	0.390
contig065887-BurOR.A018	contig047508-ZebOR.A016	0.345	0.889	0.388
contig030566-ZebOR.A008	contig047508-ZebOR.A016	0.345	0.901	0.383
contig085002-BriOR.A004	contig047499-ZebOR.A012	0.346	0.735	0.471
contig034994-NyeOR.A008	contig022217-TilOR.A004	0.346	0.803	0.431
contig065887-BurOR.A018	contig041952-TilOR.A023	0.346	0.815	0.425
contig041952-TilOR.A023	contig030566-ZebOR.A008	0.346	0.825	0.420
contig022211-TilOR.A003	contig047497-ZebOR.A011	0.346	0.827	0.419
contig051318-BurOR.A005	contig054687-NyeOR.A013	0.346	0.831	0.417
contig030566-ZebOR.A008	contig030572-ZebOR.A009	0.346	0.844	0.410
contig065887-BurOR.A018	contig030572-ZebOR.A009	0.346	0.845	0.409
contig022217-TilOR.A004	contig047499-ZebOR.A012	0.346	0.845	0.410

contig085012-BriOR.A130	contig047497-ZebOR.A011	0.346	0.865	0.400
contig085012-BriOR.A130	contig030566-ZebOR.A008	0.346	0.871	0.397
contig085012-BriOR.A130	contig065887-BurOR.A018	0.346	0.873	0.396
contig085000-BriOR.A003	contig047499-ZebOR.A012	0.346	0.887	0.390
contig036784-BurOR.A003	contig047499-ZebOR.A012	0.346	0.890	0.388
contig093807-BriOR.A009	contig065887-BurOR.A018	0.346	0.893	0.388
contig074640-TilOR.A002	contig047508-ZebOR.A016	0.347	0.742	0.467
contig074640-TilOR.A002	contig047499-ZebOR.A012	0.347	0.804	0.432
contig085000-BriOR.A003	contig034990-NyeOR.A007	0.347	0.856	0.406
contig074640-TilOR.A002	contig047497-ZebOR.A011	0.347	0.858	0.405
contig036784-BurOR.A003	contig034990-NyeOR.A007	0.347	0.860	0.404
contig022264-TilOR.A016	contig047526-ZebOR.A021	0.347	0.900	0.386
contig093807-BriOR.A009	contig030566-ZebOR.A008	0.347	0.905	0.383
contig065887-BurOR.A018	contig034994-NyeOR.A008	0.348	0.861	0.404
contig034994-NyeOR.A008	contig030566-ZebOR.A008	0.348	0.872	0.399
contig051318-BurOR.A005	contig030566-ZebOR.A008	0.349	0.843	0.414
contig051318-BurOR.A005	contig065887-BurOR.A018	0.349	0.845	0.413
contig034995-NyeOR.A009	contig022217-TilOR.A004	0.349	0.871	0.401
contig034994-NyeOR.A008	contig022264-TilOR.A016	0.349	0.906	0.385
contig051566-BurOR.A009	contig022264-TilOR.A016	0.349	0.932	0.374
contig034981-NyeOR.A001	contig041952-TilOR.A023	0.350	0.846	0.413
contig022238-TilOR.A010	contig022264-TilOR.A016	0.350	0.934	0.375
contig093812-BriOR.A010	contig054687-NyeOR.A013	0.351	0.878	0.399
contig022264-TilOR.A016	contig047514-ZebOR.A017	0.351	0.919	0.382
contig034988-NyeOR.A004	contig022264-TilOR.A016	0.351	0.952	0.368
contig022217-TilOR.A004	contig047497-ZebOR.A011	0.352	0.807	0.436
contig054687-NyeOR.A013	contig047497-ZebOR.A011	0.352	0.836	0.421
contig034990-NyeOR.A007	contig022217-TilOR.A004	0.353	0.826	0.427
contig093812-BriOR.A010	contig065887-BurOR.A018	0.353	0.879	0.401
contig093812-BriOR.A010	contig030566-ZebOR.A008	0.353	0.891	0.396
contig022264-TilOR.A016	contig047508-ZebOR.A016	0.353	0.949	0.373
contig065887-BurOR.A018	contig047497-ZebOR.A011	0.354	0.837	0.423
contig022264-TilOR.A016	contig041952-TilOR.A023	0.354	0.845	0.419
contig030566-ZebOR.A008	contig047497-ZebOR.A011	0.354	0.848	0.418
contig034990-NyeOR.A007	contig054687-NyeOR.A013	0.354	0.886	0.400
contig034995-NyeOR.A009	contig054687-NyeOR.A013	0.354	0.887	0.399
contig093807-BriOR.A009	contig022264-TilOR.A016	0.354	0.946	0.374
contig054687-NyeOR.A013	contig047499-ZebOR.A012	0.355	0.893	0.397
contig065887-BurOR.A018	contig034990-NyeOR.A007	0.356	0.887	0.402
contig065887-BurOR.A018	contig034995-NyeOR.A009	0.356	0.888	0.401
contig034990-NyeOR.A007	contig030566-ZebOR.A008	0.356	0.898	0.397
contig065887-BurOR.A018	contig047499-ZebOR.A012	0.357	0.894	0.399
contig034995-NyeOR.A009	contig030566-ZebOR.A008	0.357	0.900	0.396

contig030566-ZebOR.A008	contig047499-ZebOR.A012	0.357	0.905	0.394
contig034995-NyeOR.A009	contig022264-TilOR.A016	0.357	0.921	0.388
contig093812-BriOR.A010	contig022264-TilOR.A016	0.359	0.910	0.395
contig022264-TilOR.A016	contig047497-ZebOR.A011	0.360	0.878	0.410
contig022264-TilOR.A016	contig047499-ZebOR.A012	0.361	0.924	0.390
contig034990-NyeOR.A007	contig022264-TilOR.A016	0.362	0.906	0.400

Cichlid Olfactory Receptors :
dN/dS ratio

Fam D

OR pairs		dN	dS	dN/dS
contig032389-BurOR.D033	contig039737-NyeOR.D040	0.003	0.013	0.225
contig053779-BurOR.D035	contig039730-NyeOR.D036	0.006	0.017	0.340
contig032388-BurOR.D032	contig039738-NyeOR.D041	0.009	0.017	0.509
contig013330-TilOR.D054	contig013337-TilOR.D055	0.009	0.056	0.154
contig039731-NyeOR.D038	contig014054-ZebOR.D041	0.013	0.048	0.270
contig039736-NyeORe.D043	contig014050-ZebOR.D039	0.016	0.023	0.667
contig064814-BriOR.D029	contig014054-ZebOR.D041	0.016	0.076	0.209
contig039730-NyeOR.D036	contig014054-ZebOR.D042	0.017	0.029	0.605
contig053779-BurOR.D035	contig014054-ZebOR.D042	0.017	0.031	0.539
contig064814-BriOR.D029	contig039731-NyeOR.D038	0.020	0.081	0.252
contig013330-TilOR.D054	contig013339-TilOR.D057	0.022	0.053	0.411
contig032396-BurOR.D034	contig014050-ZebOR.D039	0.024	0.076	0.312
contig064809-BriOR.D028	contig014050-ZebOR.D039	0.025	0.039	0.644
contig032396-BurOR.D034	contig039736-NyeORe.D043	0.025	0.060	0.424
contig039738-NyeOR.D041	contig014049-ZebOR.D037	0.026	0.083	0.313
contig064809-BriOR.D028	contig039736-NyeORe.D043	0.027	0.047	0.580
contig064809-BriOR.D028	contig032396-BurOR.D034	0.027	0.062	0.430
contig039738-NyeOR.D041	contig013323-TilOR.D050	0.027	0.069	0.394
contig064802-BriOR.D027	contig014047-ZebOR.D036	0.028	0.097	0.285
contig039738-NyeOR.D042	contig013322-TilOR.D049	0.029	0.078	0.368
contig032388-BurOR.D032	contig013323-TilOR.D050	0.029	0.088	0.326
contig013337-TilOR.D055	contig013339-TilOR.D057	0.031	0.114	0.272
contig013327-TilOR.D052	contig014049-ZebOR.D038	0.032	0.093	0.343
contig032388-BurOR.D032	contig014049-ZebOR.D037	0.033	0.097	0.345
contig039737-NyeOR.D039	contig014049-ZebOR.D038	0.035	0.088	0.399
contig013339-TilOR.D057	contig014051-ZebOR.D040	0.036	0.066	0.542
contig039737-NyeOR.D039	contig014050-ZebOR.D039	0.040	0.102	0.389
contig013330-TilOR.D053	contig013339-TilOR.D056	0.041	0.074	0.548
contig039737-NyeOR.D039	contig039736-NyeORe.D043	0.042	0.088	0.481
contig039737-NyeOR.D039	contig013327-TilOR.D052	0.043	0.115	0.377
contig064809-BriOR.D028	contig039737-NyeOR.D039	0.044	0.102	0.433
contig032396-BurOR.D034	contig039737-NyeOR.D039	0.046	0.122	0.375
contig013323-TilOR.D050	contig014049-ZebOR.D037	0.046	0.142	0.321
contig039730-NyeOR.D037	contig014054-ZebOR.D042	0.048	0.150	0.317
contig013330-TilOR.D054	contig014051-ZebOR.D040	0.049	0.105	0.469
contig053779-BurOR.D035	contig039730-NyeOR.D037	0.049	0.126	0.392
contig039730-NyeOR.D036	contig039730-NyeOR.D037	0.049	0.126	0.392
contig039737-NyeOR.D039	contig013326-TilOR.D051	0.050	0.144	0.343
contig039737-NyeOR.D039	contig013339-TilOR.D057	0.051	0.134	0.383
contig013337-TilOR.D055	contig014051-ZebOR.D040	0.053	0.119	0.440
contig013327-TilOR.D052	contig014050-ZebOR.D039	0.053	0.138	0.385
contig039737-NyeOR.D039	contig013337-TilOR.D055	0.055	0.137	0.400

contig014049-ZebOR.D038	contig014050-ZebOR.D039	0.056	0.125	0.445
contig013339-TilOR.D057	contig014049-ZebOR.D038	0.057	0.136	0.416
contig013327-TilOR.D052	contig013339-TilOR.D057	0.057	0.144	0.397
contig013326-TilOR.D051	contig013327-TilOR.D052	0.057	0.161	0.357
contig039736-NyeORe.D043	contig013327-TilOR.D052	0.058	0.083	0.705
contig013327-TilOR.D052	contig013337-TilOR.D055	0.058	0.118	0.492
contig032396-BurOR.D034	contig013327-TilOR.D052	0.058	0.138	0.417
contig032396-BurOR.D034	contig013326-TilOR.D051	0.058	0.157	0.369
contig013326-TilOR.D051	contig014049-ZebOR.D038	0.058	0.189	0.307
contig013337-TilOR.D055	contig014049-ZebOR.D038	0.059	0.159	0.370
contig039737-NyeOR.D039	contig013330-TilOR.D054	0.059	0.177	0.331
contig013326-TilOR.D051	contig014050-ZebOR.D039	0.060	0.141	0.423
contig032396-BurOR.D034	contig014049-ZebOR.D038	0.060	0.151	0.399
contig064809-BriOR.D028	contig013326-TilOR.D051	0.060	0.152	0.393
contig013330-TilOR.D054	contig014049-ZebOR.D038	0.060	0.176	0.343
contig039737-NyeOR.D039	contig014051-ZebOR.D040	0.061	0.117	0.526
contig064809-BriOR.D028	contig013327-TilOR.D052	0.061	0.143	0.425
contig013326-TilOR.D051	contig013339-TilOR.D057	0.061	0.151	0.405
contig013326-TilOR.D051	contig013337-TilOR.D055	0.061	0.163	0.376
contig013327-TilOR.D052	contig013330-TilOR.D054	0.061	0.164	0.371
contig039736-NyeORe.D043	contig014049-ZebOR.D038	0.062	0.118	0.520
contig064809-BriOR.D028	contig014049-ZebOR.D038	0.062	0.135	0.457
contig032396-BurOR.D034	contig013339-TilOR.D057	0.064	0.144	0.443
contig039736-NyeORe.D043	contig014051-ZebOR.D040	0.065	0.050	1.297
contig039736-NyeORe.D043	contig013326-TilOR.D051	0.065	0.101	0.645
contig032396-BurOR.D034	contig014051-ZebOR.D040	0.065	0.119	0.548
contig013339-TilOR.D057	contig014050-ZebOR.D039	0.065	0.123	0.530
contig039737-NyeOR.D040	contig013339-TilOR.D056	0.065	0.161	0.401
contig039736-NyeORe.D043	contig013339-TilOR.D057	0.066	0.091	0.730
contig014050-ZebOR.D039	contig014051-ZebOR.D040	0.067	0.094	0.709
contig013327-TilOR.D052	contig014051-ZebOR.D040	0.068	0.107	0.631
contig032389-BurOR.D033	contig013339-TilOR.D056	0.068	0.166	0.407
contig013326-TilOR.D051	contig013330-TilOR.D054	0.068	0.177	0.386
contig039730-NyeOR.D037	contig039731-NyeOR.D038	0.069	0.171	0.402
contig013322-TilOR.D049	contig014047-ZebOR.D036	0.069	0.256	0.269
contig039738-NyeOR.D042	contig014047-ZebOR.D036	0.069	0.268	0.257
contig013321-TilOR.D048	contig014047-ZebOR.D036	0.070	0.205	0.343
contig014049-ZebOR.D038	contig014051-ZebOR.D040	0.072	0.122	0.596
contig013337-TilOR.D055	contig014050-ZebOR.D039	0.072	0.152	0.476
contig064809-BriOR.D028	contig013339-TilOR.D057	0.073	0.136	0.532
contig013326-TilOR.D051	contig014051-ZebOR.D040	0.074	0.126	0.587
contig032396-BurOR.D034	contig013337-TilOR.D055	0.074	0.163	0.453
contig039737-NyeOR.D040	contig013330-TilOR.D053	0.074	0.177	0.418

contig039730-NyeOR.D037	contig014054-ZebOR.D041	0.074	0.196	0.379
contig064802-BriOR.D027	contig013322-TilOR.D049	0.074	0.221	0.335
contig032396-BurOR.D034	contig013330-TilOR.D054	0.075	0.199	0.375
contig064809-BriOR.D028	contig013337-TilOR.D055	0.076	0.160	0.476
contig013330-TilOR.D054	contig014050-ZebOR.D039	0.076	0.166	0.459
contig064802-BriOR.D027	contig039738-NyeOR.D042	0.076	0.220	0.343
contig013321-TilOR.D048	contig013322-TilOR.D049	0.076	0.222	0.343
contig064802-BriOR.D027	contig013321-TilOR.D048	0.077	0.155	0.494
contig032389-BurOR.D033	contig013330-TilOR.D053	0.077	0.194	0.400
contig064809-BriOR.D028	contig014051-ZebOR.D040	0.080	0.119	0.670
contig039736-NyeORe.D043	contig013337-TilOR.D055	0.081	0.132	0.609
contig039736-NyeORe.D043	contig013330-TilOR.D054	0.081	0.146	0.552
contig064814-BriOR.D029	contig039730-NyeOR.D037	0.082	0.169	0.486
contig064809-BriOR.D028	contig013330-TilOR.D054	0.083	0.190	0.438
contig053779-BurOR.D035	contig039731-NyeOR.D038	0.084	0.217	0.386
contig039737-NyeOR.D039	contig014049-ZebOR.D037	0.084	0.222	0.377
contig039730-NyeOR.D036	contig039731-NyeOR.D038	0.085	0.234	0.365
contig053779-BurOR.D035	contig039737-NyeOR.D040	0.086	0.210	0.409
contig039738-NyeOR.D042	contig013321-TilOR.D048	0.087	0.242	0.358
contig039731-NyeOR.D038	contig013344-TilOR.D058	0.088	0.155	0.567
contig039731-NyeOR.D038	contig014054-ZebOR.D042	0.088	0.249	0.354
contig032396-BurOR.D034	contig053779-BurOR.D035	0.089	0.221	0.405
contig032389-BurOR.D033	contig053779-BurOR.D035	0.089	0.227	0.393
contig039730-NyeOR.D036	contig039737-NyeOR.D040	0.089	0.227	0.393
contig032396-BurOR.D034	contig039730-NyeOR.D036	0.089	0.227	0.394
contig013330-TilOR.D053	contig014050-ZebOR.D039	0.090	0.184	0.487
contig053779-BurOR.D035	contig014054-ZebOR.D041	0.090	0.193	0.465
contig053779-BurOR.D035	contig014050-ZebOR.D039	0.090	0.200	0.450
contig039730-NyeOR.D036	contig014050-ZebOR.D039	0.090	0.206	0.438
contig039730-NyeOR.D037	contig039737-NyeOR.D040	0.090	0.224	0.402
contig039730-NyeOR.D037	contig014050-ZebOR.D039	0.090	0.240	0.377
contig039730-NyeOR.D036	contig014054-ZebOR.D041	0.091	0.209	0.436
contig013344-TilOR.D058	contig014054-ZebOR.D041	0.093	0.160	0.577
contig013339-TilOR.D056	contig014050-ZebOR.D039	0.093	0.179	0.522
contig064814-BriOR.D029	contig053779-BurOR.D035	0.093	0.199	0.467
contig039737-NyeOR.D040	contig014054-ZebOR.D042	0.093	0.227	0.408
contig032389-BurOR.D033	contig039730-NyeOR.D037	0.093	0.241	0.387
contig032389-BurOR.D033	contig039730-NyeOR.D036	0.093	0.245	0.378
contig039737-NyeOR.D039	contig039737-NyeOR.D040	0.093	0.247	0.378
contig032389-BurOR.D033	contig039737-NyeOR.D039	0.093	0.253	0.369
contig032396-BurOR.D034	contig039730-NyeOR.D037	0.093	0.255	0.363
contig013330-TilOR.D053	contig014049-ZebOR.D037	0.093	0.268	0.348
contig039737-NyeOR.D039	contig013339-TilOR.D056	0.094	0.161	0.585

contig064814-BriOR.D029	contig039730-NyeOR.D036	0.094	0.193	0.489
contig039730-NyeOR.D036	contig039736-NyeORe.D043	0.094	0.194	0.485
contig014054-ZebOR.D041	contig014054-ZebOR.D042	0.094	0.212	0.442
contig064809-BriOR.D028	contig039730-NyeOR.D036	0.094	0.232	0.406
contig039737-NyeOR.D040	contig014049-ZebOR.D037	0.094	0.262	0.359
contig039731-NyeOR.D038	contig039737-NyeOR.D040	0.094	0.283	0.331
contig064814-BriOR.D029	contig013344-TilOR.D058	0.095	0.158	0.601
contig039737-NyeOR.D039	contig013330-TilOR.D053	0.095	0.187	0.509
contig013339-TilOR.D057	contig013344-TilOR.D058	0.095	0.219	0.432
contig014049-ZebOR.D037	contig014050-ZebOR.D039	0.095	0.230	0.411
contig039737-NyeOR.D040	contig014050-ZebOR.D039	0.095	0.247	0.386
contig032389-BurOR.D033	contig014050-ZebOR.D039	0.095	0.253	0.377
contig039737-NyeOR.D040	contig013327-TilOR.D052	0.095	0.266	0.356
contig032389-BurOR.D033	contig013327-TilOR.D052	0.095	0.273	0.348
contig053779-BurOR.D035	contig013344-TilOR.D058	0.096	0.161	0.595
contig039730-NyeOR.D036	contig013344-TilOR.D058	0.096	0.177	0.542
contig013339-TilOR.D056	contig014054-ZebOR.D042	0.096	0.190	0.508
contig039730-NyeOR.D036	contig039737-NyeOR.D039	0.096	0.209	0.458
contig032389-BurOR.D033	contig014054-ZebOR.D042	0.096	0.244	0.392
contig053779-BurOR.D035	contig013322-TilOR.D049	0.096	0.254	0.379
contig039730-NyeOR.D036	contig013322-TilOR.D049	0.096	0.266	0.362
contig013322-TilOR.D049	contig014054-ZebOR.D042	0.096	0.272	0.355
contig064814-BriOR.D029	contig014049-ZebOR.D037	0.096	0.297	0.325
contig053779-BurOR.D035	contig013339-TilOR.D056	0.097	0.171	0.567
contig039730-NyeOR.D036	contig013339-TilOR.D056	0.097	0.187	0.519
contig064814-BriOR.D029	contig014054-ZebOR.D042	0.097	0.212	0.456
contig032396-BurOR.D034	contig014054-ZebOR.D042	0.097	0.229	0.422
contig064814-BriOR.D029	contig039737-NyeOR.D040	0.097	0.255	0.380
contig032389-BurOR.D033	contig014049-ZebOR.D037	0.097	0.262	0.371
contig039737-NyeOR.D040	contig014054-ZebOR.D041	0.097	0.272	0.357
contig032389-BurOR.D033	contig039731-NyeOR.D038	0.097	0.283	0.342
contig039731-NyeOR.D038	contig014049-ZebOR.D037	0.097	0.311	0.313
contig053779-BurOR.D035	contig039736-NyeORe.D043	0.098	0.194	0.508
contig039730-NyeOR.D037	contig039737-NyeOR.D039	0.098	0.206	0.477
contig014050-ZebOR.D039	contig014054-ZebOR.D042	0.098	0.214	0.456
contig064809-BriOR.D028	contig053779-BurOR.D035	0.098	0.226	0.431
contig039738-NyeOR.D041	contig013344-TilOR.D058	0.098	0.264	0.373
contig032396-BurOR.D034	contig014049-ZebOR.D037	0.098	0.271	0.361
contig039730-NyeOR.D037	contig013322-TilOR.D049	0.098	0.280	0.348
contig013330-TilOR.D053	contig013344-TilOR.D058	0.099	0.175	0.565
contig032396-BurOR.D034	contig013330-TilOR.D053	0.099	0.198	0.498
contig053779-BurOR.D035	contig039737-NyeOR.D039	0.099	0.204	0.487
contig053779-BurOR.D035	contig013327-TilOR.D052	0.099	0.223	0.441

contig039730-NyeOR.D036	contig013327-TilOR.D052	0.099	0.229	0.430
contig013323-TilOR.D050	contig013344-TilOR.D058	0.099	0.239	0.413
contig039737-NyeOR.D040	contig014049-ZebOR.D038	0.099	0.262	0.378
contig013326-TilOR.D051	contig014049-ZebOR.D037	0.099	0.263	0.375
contig032389-BurOR.D033	contig014049-ZebOR.D038	0.099	0.268	0.369
contig014049-ZebOR.D037	contig014054-ZebOR.D041	0.099	0.312	0.317
contig064814-BriOR.D029	contig039738-NyeOR.D041	0.099	0.313	0.318
contig039730-NyeOR.D037	contig013339-TilOR.D056	0.100	0.206	0.484
contig064814-BriOR.D029	contig032389-BurOR.D033	0.100	0.273	0.366
contig032388-BurOR.D032	contig013344-TilOR.D058	0.100	0.276	0.363
contig039731-NyeOR.D038	contig014050-ZebOR.D039	0.100	0.284	0.350
contig032389-BurOR.D033	contig014054-ZebOR.D041	0.100	0.284	0.353
contig064809-BriOR.D028	contig013339-TilOR.D056	0.101	0.182	0.554
contig013339-TilOR.D056	contig013339-TilOR.D057	0.101	0.186	0.541
contig032396-BurOR.D034	contig013339-TilOR.D056	0.101	0.193	0.522
contig053779-BurOR.D035	contig013330-TilOR.D053	0.101	0.201	0.503
contig039730-NyeOR.D036	contig013330-TilOR.D053	0.101	0.218	0.464
contig053779-BurOR.D035	contig013326-TilOR.D051	0.101	0.220	0.458
contig039730-NyeOR.D036	contig013326-TilOR.D051	0.101	0.238	0.425
contig053779-BurOR.D035	contig039738-NyeOR.D042	0.101	0.242	0.419
contig039730-NyeOR.D037	contig013327-TilOR.D052	0.101	0.243	0.418
contig039730-NyeOR.D036	contig039738-NyeOR.D042	0.101	0.254	0.400
contig064809-BriOR.D028	contig014049-ZebOR.D037	0.101	0.265	0.382
contig064809-BriOR.D028	contig039730-NyeOR.D037	0.101	0.267	0.378
contig064809-BriOR.D028	contig013330-TilOR.D053	0.102	0.187	0.545
contig039731-NyeOR.D038	contig013339-TilOR.D056	0.102	0.239	0.424
contig039737-NyeOR.D040	contig013322-TilOR.D049	0.102	0.242	0.420
contig013339-TilOR.D056	contig014049-ZebOR.D037	0.102	0.256	0.396
contig013344-TilOR.D058	contig014049-ZebOR.D037	0.102	0.262	0.388
contig032396-BurOR.D034	contig039731-NyeOR.D038	0.102	0.287	0.356
contig013321-TilOR.D048	contig014049-ZebOR.D037	0.102	0.328	0.310
contig064814-BriOR.D029	contig032388-BurOR.D032	0.102	0.329	0.310
contig013344-TilOR.D058	contig014054-ZebOR.D042	0.103	0.179	0.576
contig013330-TilOR.D053	contig013339-TilOR.D057	0.103	0.200	0.515
contig013330-TilOR.D053	contig014049-ZebOR.D038	0.103	0.201	0.512
contig039738-NyeOR.D042	contig014054-ZebOR.D042	0.103	0.253	0.407
contig053779-BurOR.D035	contig013321-TilOR.D048	0.103	0.256	0.402
contig014049-ZebOR.D037	contig014049-ZebOR.D038	0.103	0.260	0.395
contig064814-BriOR.D029	contig014050-ZebOR.D039	0.103	0.262	0.392
contig039730-NyeOR.D036	contig013321-TilOR.D048	0.103	0.274	0.375
contig032389-BurOR.D033	contig032396-BurOR.D034	0.103	0.278	0.372
contig032396-BurOR.D034	contig039737-NyeOR.D040	0.103	0.284	0.364
contig014050-ZebOR.D039	contig014054-ZebOR.D041	0.103	0.285	0.361

contig013339-TilOR.D057	contig014049-ZebOR.D037	0.103	0.302	0.341
contig039730-NyeOR.D037	contig039736-NyeORe.D043	0.104	0.184	0.563
contig013339-TilOR.D056	contig013344-TilOR.D058	0.104	0.187	0.557
contig013327-TilOR.D052	contig014049-ZebOR.D037	0.104	0.282	0.368
contig064814-BriOR.D029	contig013323-TilOR.D050	0.104	0.291	0.356
contig039730-NyeOR.D037	contig013321-TilOR.D048	0.104	0.294	0.354
contig039738-NyeOR.D042	contig014049-ZebOR.D037	0.104	0.310	0.337
contig039737-NyeOR.D040	contig013323-TilOR.D050	0.105	0.244	0.428
contig013339-TilOR.D056	contig014054-ZebOR.D041	0.105	0.252	0.417
contig039730-NyeOR.D037	contig014049-ZebOR.D038	0.105	0.253	0.414
contig032389-BurOR.D033	contig013322-TilOR.D049	0.105	0.260	0.404
contig032396-BurOR.D034	contig014054-ZebOR.D041	0.105	0.276	0.382
contig013321-TilOR.D048	contig014054-ZebOR.D042	0.105	0.277	0.380
contig039731-NyeOR.D038	contig039738-NyeOR.D041	0.105	0.327	0.322
contig039736-NyeORe.D043	contig013339-TilOR.D056	0.106	0.135	0.780
contig013327-TilOR.D052	contig013339-TilOR.D056	0.106	0.174	0.609
contig013327-TilOR.D052	contig013330-TilOR.D053	0.106	0.207	0.512
contig053779-BurOR.D035	contig014049-ZebOR.D038	0.106	0.208	0.509
contig053779-BurOR.D035	contig013339-TilOR.D057	0.106	0.214	0.496
contig039730-NyeOR.D036	contig014049-ZebOR.D038	0.106	0.225	0.471
contig013327-TilOR.D052	contig014054-ZebOR.D042	0.106	0.226	0.469
contig064809-BriOR.D028	contig014054-ZebOR.D042	0.106	0.237	0.446
contig039730-NyeOR.D037	contig013326-TilOR.D051	0.106	0.237	0.447
contig039737-NyeOR.D040	contig039736-NyeORe.D043	0.106	0.246	0.430
contig032389-BurOR.D033	contig039736-NyeORe.D043	0.106	0.254	0.416
contig053779-BurOR.D035	contig013323-TilOR.D050	0.106	0.263	0.401
contig053779-BurOR.D035	contig039738-NyeOR.D041	0.106	0.280	0.378
contig039730-NyeOR.D036	contig013323-TilOR.D050	0.106	0.282	0.375
contig039730-NyeOR.D036	contig039738-NyeOR.D041	0.106	0.299	0.354
contig013322-TilOR.D049	contig014049-ZebOR.D037	0.106	0.317	0.335
contig039738-NyeOR.D041	contig013321-TilOR.D048	0.106	0.327	0.323
contig039731-NyeOR.D038	contig013323-TilOR.D050	0.106	0.331	0.321
contig032388-BurOR.D032	contig039731-NyeOR.D038	0.106	0.337	0.315
contig039730-NyeOR.D037	contig013344-TilOR.D058	0.107	0.190	0.561
contig064814-BriOR.D029	contig013339-TilOR.D056	0.107	0.209	0.513
contig039730-NyeOR.D036	contig013339-TilOR.D057	0.107	0.217	0.493
contig039730-NyeOR.D037	contig013330-TilOR.D053	0.107	0.238	0.451
contig039737-NyeOR.D040	contig039738-NyeOR.D041	0.107	0.258	0.416
contig039737-NyeOR.D040	contig013339-TilOR.D057	0.107	0.270	0.396
contig032389-BurOR.D033	contig013339-TilOR.D057	0.107	0.276	0.387
contig053779-BurOR.D035	contig014049-ZebOR.D037	0.107	0.276	0.388
contig039731-NyeOR.D038	contig013339-TilOR.D057	0.107	0.292	0.368
contig064809-BriOR.D028	contig039731-NyeOR.D038	0.107	0.293	0.366

contig039730-NyeOR.D036	contig014049-ZebOR.D037	0.107	0.294	0.363
contig039738-NyeOR.D041	contig014054-ZebOR.D041	0.107	0.341	0.313
contig013339-TilOR.D056	contig014049-ZebOR.D038	0.108	0.179	0.604
contig039737-NyeOR.D040	contig013344-TilOR.D058	0.108	0.190	0.571
contig013322-TilOR.D049	contig013344-TilOR.D058	0.108	0.192	0.560
contig013344-TilOR.D058	contig014050-ZebOR.D039	0.108	0.197	0.548
contig039737-NyeOR.D039	contig014054-ZebOR.D042	0.108	0.209	0.515
contig013337-TilOR.D055	contig013344-TilOR.D058	0.108	0.214	0.503
contig013326-TilOR.D051	contig014054-ZebOR.D042	0.108	0.234	0.463
contig039730-NyeOR.D037	contig039738-NyeOR.D042	0.108	0.249	0.431
contig039737-NyeOR.D040	contig013321-TilOR.D048	0.108	0.251	0.428
contig032389-BurOR.D033	contig013323-TilOR.D050	0.108	0.256	0.421
contig064814-BriOR.D029	contig032396-BurOR.D034	0.108	0.267	0.404
contig014047-ZebOR.D036	contig014054-ZebOR.D042	0.108	0.268	0.402
contig032389-BurOR.D033	contig013321-TilOR.D048	0.108	0.269	0.399
contig039737-NyeOR.D039	contig013323-TilOR.D050	0.108	0.272	0.398
contig013337-TilOR.D055	contig014049-ZebOR.D037	0.108	0.277	0.390
contig064809-BriOR.D028	contig039737-NyeOR.D040	0.108	0.277	0.391
contig013322-TilOR.D049	contig013327-TilOR.D052	0.108	0.284	0.381
contig064809-BriOR.D028	contig032389-BurOR.D033	0.108	0.284	0.382
contig039737-NyeOR.D039	contig039738-NyeOR.D041	0.108	0.292	0.368
contig032388-BurOR.D032	contig053779-BurOR.D035	0.108	0.296	0.366
contig039737-NyeOR.D039	contig013322-TilOR.D049	0.108	0.296	0.367
contig039738-NyeOR.D041	contig014050-ZebOR.D039	0.108	0.304	0.354
contig013321-TilOR.D048	contig013323-TilOR.D050	0.108	0.312	0.346
contig032388-BurOR.D032	contig039730-NyeOR.D036	0.108	0.315	0.344
contig013323-TilOR.D050	contig014054-ZebOR.D041	0.108	0.319	0.338
contig039736-NyeORe.D043	contig013330-TilOR.D053	0.109	0.125	0.871
contig013330-TilOR.D053	contig014054-ZebOR.D042	0.109	0.221	0.493
contig039730-NyeOR.D036	contig014047-ZebOR.D036	0.109	0.275	0.399
contig039737-NyeOR.D039	contig039738-NyeOR.D042	0.109	0.286	0.382
contig013323-TilOR.D050	contig014054-ZebOR.D042	0.109	0.288	0.379
contig039730-NyeOR.D037	contig014049-ZebOR.D037	0.109	0.313	0.347
contig013321-TilOR.D048	contig013327-TilOR.D052	0.109	0.321	0.339
contig032388-BurOR.D032	contig014054-ZebOR.D041	0.109	0.358	0.305
contig013326-TilOR.D051	contig013330-TilOR.D053	0.110	0.158	0.694
contig039736-NyeORe.D043	contig014054-ZebOR.D042	0.110	0.208	0.525
contig039737-NyeOR.D040	contig039738-NyeOR.D042	0.110	0.230	0.478
contig039738-NyeOR.D041	contig013330-TilOR.D053	0.110	0.258	0.427
contig032389-BurOR.D033	contig039738-NyeOR.D041	0.110	0.258	0.429
contig013322-TilOR.D049	contig013330-TilOR.D053	0.110	0.264	0.417
contig053779-BurOR.D035	contig014047-ZebOR.D036	0.110	0.266	0.416
contig039738-NyeOR.D042	contig013327-TilOR.D052	0.110	0.278	0.396

contig013323-TilOR.D050	contig014050-ZebOR.D039	0.110	0.284	0.388
contig013321-TilOR.D048	contig014049-ZebOR.D038	0.110	0.285	0.386
contig013322-TilOR.D049	contig014050-ZebOR.D039	0.110	0.295	0.374
contig032388-BurOR.D032	contig014050-ZebOR.D039	0.110	0.313	0.352
contig032396-BurOR.D034	contig013321-TilOR.D048	0.110	0.323	0.340
contig013326-TilOR.D051	contig013339-TilOR.D056	0.111	0.176	0.628
contig064809-BriOR.D028	contig014054-ZebOR.D041	0.111	0.294	0.376
contig013339-TilOR.D057	contig014054-ZebOR.D041	0.111	0.299	0.370
contig039738-NyeOR.D041	contig014054-ZebOR.D042	0.111	0.299	0.371
contig013321-TilOR.D048	contig014050-ZebOR.D039	0.111	0.300	0.370
contig013322-TilOR.D049	contig013323-TilOR.D050	0.111	0.301	0.368
contig032389-BurOR.D033	contig013344-TilOR.D058	0.112	0.201	0.556
contig013323-TilOR.D050	contig013339-TilOR.D056	0.112	0.244	0.459
contig039731-NyeOR.D038	contig013330-TilOR.D053	0.112	0.264	0.423
contig032388-BurOR.D032	contig039737-NyeOR.D040	0.112	0.270	0.416
contig039737-NyeOR.D039	contig013321-TilOR.D048	0.112	0.288	0.391
contig014049-ZebOR.D037	contig014054-ZebOR.D042	0.112	0.294	0.381
contig013330-TilOR.D054	contig014049-ZebOR.D037	0.112	0.306	0.365
contig039738-NyeOR.D041	contig013322-TilOR.D049	0.112	0.309	0.362
contig032388-BurOR.D032	contig013321-TilOR.D048	0.112	0.314	0.358
contig064809-BriOR.D028	contig013321-TilOR.D048	0.112	0.336	0.332
contig039737-NyeOR.D040	contig013326-TilOR.D051	0.113	0.204	0.552
contig013337-TilOR.D055	contig013339-TilOR.D056	0.113	0.213	0.527
contig032389-BurOR.D033	contig013326-TilOR.D051	0.113	0.221	0.510
contig032389-BurOR.D033	contig039738-NyeOR.D042	0.113	0.236	0.481
contig039738-NyeOR.D041	contig013339-TilOR.D056	0.113	0.240	0.471
contig013330-TilOR.D054	contig013344-TilOR.D058	0.113	0.253	0.449
contig039738-NyeOR.D041	contig013326-TilOR.D051	0.113	0.264	0.429
contig064809-BriOR.D028	contig064814-BriOR.D029	0.113	0.267	0.423
contig013322-TilOR.D049	contig014049-ZebOR.D038	0.113	0.280	0.402
contig013323-TilOR.D050	contig013327-TilOR.D052	0.113	0.282	0.400
contig032389-BurOR.D033	contig013337-TilOR.D055	0.113	0.284	0.397
contig039738-NyeOR.D041	contig013327-TilOR.D052	0.113	0.286	0.394
contig039737-NyeOR.D040	contig013337-TilOR.D055	0.113	0.290	0.389
contig039738-NyeOR.D041	contig039738-NyeOR.D042	0.113	0.296	0.384
contig032388-BurOR.D032	contig039737-NyeOR.D039	0.113	0.298	0.377
contig032388-BurOR.D032	contig014054-ZebOR.D042	0.113	0.315	0.361
contig039730-NyeOR.D037	contig014047-ZebOR.D036	0.113	0.357	0.317
contig013339-TilOR.D057	contig014054-ZebOR.D042	0.114	0.205	0.554
contig013327-TilOR.D052	contig013344-TilOR.D058	0.114	0.206	0.553
contig039731-NyeOR.D038	contig039737-NyeOR.D039	0.114	0.227	0.501
contig014049-ZebOR.D038	contig014054-ZebOR.D042	0.114	0.228	0.499
contig013323-TilOR.D050	contig013326-TilOR.D051	0.114	0.263	0.435

contig039731-NyeOR.D038	contig013326-TilOR.D051	0.114	0.275	0.415
contig013323-TilOR.D050	contig014049-ZebOR.D038	0.114	0.281	0.406
contig039738-NyeOR.D042	contig014050-ZebOR.D039	0.114	0.282	0.403
contig032388-BurOR.D032	contig013327-TilOR.D052	0.114	0.292	0.391
contig039731-NyeOR.D038	contig013322-TilOR.D049	0.114	0.319	0.358
contig064809-BriOR.D028	contig013344-TilOR.D058	0.115	0.217	0.532
contig013330-TilOR.D053	contig013337-TilOR.D055	0.115	0.228	0.504
contig064814-BriOR.D029	contig013330-TilOR.D053	0.115	0.236	0.486
contig039738-NyeOR.D042	contig013330-TilOR.D053	0.115	0.240	0.480
contig013323-TilOR.D050	contig013330-TilOR.D053	0.115	0.242	0.473
contig032396-BurOR.D034	contig013344-TilOR.D058	0.115	0.242	0.474
contig032388-BurOR.D032	contig013330-TilOR.D053	0.115	0.263	0.437
contig013330-TilOR.D053	contig014054-ZebOR.D041	0.115	0.264	0.435
contig013326-TilOR.D051	contig014054-ZebOR.D041	0.115	0.272	0.422
contig039731-NyeOR.D038	contig013337-TilOR.D055	0.115	0.278	0.413
contig039738-NyeOR.D041	contig013339-TilOR.D057	0.115	0.307	0.376
contig032388-BurOR.D032	contig013322-TilOR.D049	0.115	0.309	0.373
contig032396-BurOR.D034	contig039738-NyeOR.D041	0.115	0.333	0.346
contig014047-ZebOR.D036	contig014049-ZebOR.D037	0.115	0.360	0.318
contig013330-TilOR.D053	contig014051-ZebOR.D040	0.116	0.187	0.619
contig039731-NyeOR.D038	contig039736-NyeORe.D043	0.116	0.240	0.485
contig032388-BurOR.D032	contig032389-BurOR.D033	0.116	0.270	0.429
contig039738-NyeOR.D042	contig014049-ZebOR.D038	0.116	0.274	0.424
contig014049-ZebOR.D037	contig014051-ZebOR.D040	0.116	0.278	0.419
contig039737-NyeOR.D040	contig013330-TilOR.D054	0.116	0.305	0.381
contig039730-NyeOR.D037	contig013323-TilOR.D050	0.116	0.308	0.376
contig032389-BurOR.D033	contig013330-TilOR.D054	0.116	0.311	0.373
contig013321-TilOR.D048	contig013326-TilOR.D051	0.116	0.313	0.370
contig013326-TilOR.D051	contig013344-TilOR.D058	0.117	0.184	0.633
contig013344-TilOR.D058	contig014049-ZebOR.D038	0.117	0.200	0.585
contig039737-NyeOR.D039	contig013344-TilOR.D058	0.117	0.200	0.585
contig013330-TilOR.D054	contig013339-TilOR.D056	0.117	0.220	0.534
contig039737-NyeOR.D039	contig014054-ZebOR.D041	0.117	0.240	0.490
contig064802-BriOR.D027	contig014054-ZebOR.D042	0.117	0.252	0.465
contig064814-BriOR.D029	contig013339-TilOR.D057	0.117	0.254	0.459
contig013322-TilOR.D049	contig013339-TilOR.D056	0.117	0.257	0.454
contig032388-BurOR.D032	contig039738-NyeOR.D042	0.117	0.296	0.395
contig032388-BurOR.D032	contig013339-TilOR.D057	0.117	0.313	0.374
contig064809-BriOR.D028	contig013322-TilOR.D049	0.117	0.321	0.364
contig039730-NyeOR.D037	contig039738-NyeOR.D041	0.117	0.322	0.362
contig064809-BriOR.D028	contig039738-NyeOR.D041	0.117	0.326	0.359
contig039731-NyeOR.D038	contig013321-TilOR.D048	0.117	0.327	0.359
contig064814-BriOR.D029	contig039736-NyeORe.D043	0.118	0.203	0.582

contig039738-NyeOR.D042	contig013344-TilOR.D058	0.118	0.209	0.565
contig039730-NyeOR.D037	contig013339-TilOR.D057	0.118	0.231	0.511
contig032388-BurOR.D032	contig013339-TilOR.D056	0.118	0.234	0.504
contig013321-TilOR.D048	contig013339-TilOR.D056	0.118	0.247	0.480
contig039736-NyeORe.D043	contig014054-ZebOR.D041	0.118	0.249	0.476
contig039738-NyeOR.D042	contig013323-TilOR.D050	0.118	0.278	0.425
contig039731-NyeOR.D038	contig014049-ZebOR.D038	0.118	0.279	0.423
contig039731-NyeOR.D038	contig013327-TilOR.D052	0.118	0.285	0.414
contig039738-NyeOR.D041	contig013337-TilOR.D055	0.118	0.297	0.397
contig013337-TilOR.D055	contig014054-ZebOR.D041	0.118	0.297	0.398
contig013323-TilOR.D050	contig013339-TilOR.D057	0.118	0.299	0.394
contig032396-BurOR.D034	contig013323-TilOR.D050	0.118	0.312	0.377
contig039731-NyeOR.D038	contig013330-TilOR.D054	0.118	0.323	0.365
contig032396-BurOR.D034	contig013322-TilOR.D049	0.118	0.331	0.355
contig032388-BurOR.D032	contig032396-BurOR.D034	0.118	0.343	0.344
contig053779-BurOR.D035	contig014051-ZebOR.D040	0.119	0.206	0.576
contig032388-BurOR.D032	contig013326-TilOR.D051	0.119	0.270	0.439
contig064809-BriOR.D028	contig039738-NyeOR.D042	0.119	0.311	0.384
contig032388-BurOR.D032	contig039730-NyeOR.D037	0.119	0.332	0.359
contig013339-TilOR.D056	contig014051-ZebOR.D040	0.120	0.195	0.617
contig064814-BriOR.D029	contig039737-NyeOR.D039	0.120	0.203	0.590
contig013330-TilOR.D053	contig013330-TilOR.D054	0.120	0.235	0.511
contig013321-TilOR.D048	contig013344-TilOR.D058	0.120	0.236	0.510
contig064802-BriOR.D027	contig039730-NyeOR.D036	0.120	0.244	0.491
contig064802-BriOR.D027	contig053779-BurOR.D035	0.120	0.244	0.491
contig013322-TilOR.D049	contig013337-TilOR.D055	0.120	0.273	0.439
contig039738-NyeOR.D041	contig014049-ZebOR.D038	0.120	0.302	0.398
contig013323-TilOR.D050	contig013337-TilOR.D055	0.120	0.302	0.399
contig064809-BriOR.D028	contig013323-TilOR.D050	0.120	0.305	0.391
contig064814-BriOR.D029	contig013322-TilOR.D049	0.120	0.305	0.393
contig032396-BurOR.D034	contig039738-NyeOR.D042	0.120	0.308	0.390
contig013321-TilOR.D048	contig013339-TilOR.D057	0.120	0.315	0.382
contig013321-TilOR.D048	contig014054-ZebOR.D041	0.120	0.331	0.362
contig064809-BriOR.D028	contig032388-BurOR.D032	0.120	0.336	0.356
contig013344-TilOR.D058	contig014051-ZebOR.D040	0.121	0.200	0.604
contig064814-BriOR.D029	contig013326-TilOR.D051	0.121	0.258	0.468
contig013327-TilOR.D052	contig014054-ZebOR.D041	0.121	0.286	0.424
contig014049-ZebOR.D038	contig014054-ZebOR.D041	0.121	0.292	0.415
contig064802-BriOR.D027	contig039730-NyeOR.D037	0.121	0.313	0.387
contig013330-TilOR.D054	contig014054-ZebOR.D041	0.121	0.331	0.367
contig039736-NyeORe.D043	contig013344-TilOR.D058	0.122	0.160	0.760
contig039730-NyeOR.D036	contig014051-ZebOR.D040	0.122	0.212	0.577
contig032388-BurOR.D032	contig014049-ZebOR.D038	0.122	0.308	0.396

contig013322-TilOR.D049	contig013339-TilOR.D057	0.122	0.320	0.382
contig013322-TilOR.D049	contig014054-ZebOR.D041	0.122	0.329	0.370
contig053779-BurOR.D035	contig013337-TilOR.D055	0.123	0.239	0.512
contig039730-NyeOR.D036	contig013337-TilOR.D055	0.123	0.245	0.500
contig039731-NyeOR.D038	contig014051-ZebOR.D040	0.123	0.271	0.456
contig013344-TilOR.D058	contig014047-ZebOR.D036	0.123	0.276	0.446
contig032388-BurOR.D032	contig013337-TilOR.D055	0.123	0.303	0.406
contig039738-NyeOR.D041	contig014047-ZebOR.D036	0.123	0.383	0.321
contig039738-NyeOR.D042	contig013339-TilOR.D056	0.124	0.245	0.505
contig039738-NyeOR.D042	contig013337-TilOR.D055	0.124	0.257	0.482
contig032388-BurOR.D032	contig039736-NyeORe.D043	0.124	0.276	0.449
contig039731-NyeOR.D038	contig039738-NyeOR.D042	0.124	0.308	0.401
contig013322-TilOR.D049	contig013330-TilOR.D054	0.124	0.312	0.400
contig013321-TilOR.D048	contig014051-ZebOR.D040	0.124	0.319	0.390
contig064814-BriOR.D029	contig013321-TilOR.D048	0.124	0.322	0.385
contig064802-BriOR.D027	contig014049-ZebOR.D037	0.124	0.322	0.385
contig039736-NyeORe.D043	contig013323-TilOR.D050	0.125	0.242	0.516
contig013322-TilOR.D049	contig013326-TilOR.D051	0.125	0.255	0.488
contig064814-BriOR.D029	contig013337-TilOR.D055	0.125	0.261	0.479
contig039736-NyeORe.D043	contig014049-ZebOR.D037	0.125	0.272	0.459
contig039738-NyeOR.D041	contig039736-NyeORe.D043	0.125	0.272	0.460
contig064814-BriOR.D029	contig013327-TilOR.D052	0.125	0.275	0.453
contig039738-NyeOR.D041	contig013330-TilOR.D054	0.125	0.311	0.400
contig014047-ZebOR.D036	contig014050-ZebOR.D039	0.125	0.328	0.380
contig013330-TilOR.D053	contig014047-ZebOR.D036	0.125	0.330	0.379
contig013323-TilOR.D050	contig014047-ZebOR.D036	0.125	0.382	0.329
contig013321-TilOR.D048	contig013330-TilOR.D053	0.126	0.248	0.510
contig039737-NyeOR.D040	contig014051-ZebOR.D040	0.126	0.261	0.483
contig032389-BurOR.D033	contig014051-ZebOR.D040	0.126	0.268	0.472
contig032388-BurOR.D032	contig013330-TilOR.D054	0.126	0.318	0.397
contig053779-BurOR.D035	contig013330-TilOR.D054	0.127	0.255	0.496
contig039730-NyeOR.D036	contig013330-TilOR.D054	0.127	0.261	0.485
contig064814-BriOR.D029	contig013330-TilOR.D054	0.127	0.284	0.448
contig064814-BriOR.D029	contig039738-NyeOR.D042	0.127	0.292	0.434
contig013323-TilOR.D050	contig013330-TilOR.D054	0.127	0.323	0.393
contig039730-NyeOR.D037	contig014051-ZebOR.D040	0.128	0.231	0.554
contig014051-ZebOR.D040	contig014054-ZebOR.D041	0.128	0.263	0.486
contig064814-BriOR.D029	contig014049-ZebOR.D038	0.128	0.275	0.465
contig039738-NyeOR.D041	contig014051-ZebOR.D040	0.128	0.304	0.421
contig013339-TilOR.D056	contig014047-ZebOR.D036	0.128	0.333	0.385
contig064809-BriOR.D028	contig014047-ZebOR.D036	0.128	0.354	0.362
contig032388-BurOR.D032	contig014047-ZebOR.D036	0.128	0.369	0.348
contig014051-ZebOR.D040	contig014054-ZebOR.D042	0.129	0.211	0.610

contig064802-BriOR.D027	contig014050-ZebOR.D039	0.129	0.260	0.497
contig039737-NyeOR.D039	contig014047-ZebOR.D036	0.129	0.330	0.392
contig064814-BriOR.D029	contig014051-ZebOR.D040	0.130	0.237	0.550
contig039738-NyeOR.D042	contig013326-TilOR.D051	0.130	0.259	0.503
contig064802-BriOR.D027	contig039737-NyeOR.D039	0.130	0.264	0.493
contig039738-NyeOR.D042	contig013339-TilOR.D057	0.130	0.284	0.457
contig013327-TilOR.D052	contig014047-ZebOR.D036	0.130	0.349	0.372
contig039736-NyeORe.D043	contig013321-TilOR.D048	0.131	0.254	0.515
contig064802-BriOR.D027	contig013327-TilOR.D052	0.131	0.287	0.456
contig039738-NyeOR.D042	contig014054-ZebOR.D041	0.131	0.306	0.429
contig032396-BurOR.D034	contig014047-ZebOR.D036	0.131	0.358	0.365
contig064802-BriOR.D027	contig013344-TilOR.D058	0.132	0.234	0.565
contig039738-NyeOR.D042	contig013330-TilOR.D054	0.132	0.277	0.478
contig013337-TilOR.D055	contig014054-ZebOR.D042	0.133	0.231	0.578
contig064802-BriOR.D027	contig013330-TilOR.D053	0.133	0.282	0.473
contig032388-BurOR.D032	contig014051-ZebOR.D040	0.133	0.310	0.429
contig039737-NyeOR.D040	contig014047-ZebOR.D036	0.133	0.316	0.422
contig064802-BriOR.D027	contig039738-NyeOR.D041	0.133	0.351	0.378
contig039731-NyeOR.D038	contig014047-ZebOR.D036	0.133	0.387	0.343
contig039730-NyeOR.D037	contig013337-TilOR.D055	0.134	0.263	0.509
contig013323-TilOR.D050	contig014051-ZebOR.D040	0.134	0.284	0.472
contig039738-NyeOR.D042	contig039736-NyeORe.D043	0.135	0.268	0.505
contig064802-BriOR.D027	contig064809-BriOR.D028	0.135	0.295	0.460
contig064814-BriOR.D029	contig014047-ZebOR.D036	0.135	0.358	0.376
contig014047-ZebOR.D036	contig014054-ZebOR.D041	0.135	0.370	0.365
contig064802-BriOR.D027	contig039737-NyeOR.D040	0.136	0.250	0.544
contig039736-NyeORe.D043	contig013322-TilOR.D049	0.136	0.265	0.516
contig013322-TilOR.D049	contig014051-ZebOR.D040	0.136	0.301	0.451
contig014047-ZebOR.D036	contig014049-ZebOR.D038	0.136	0.338	0.403
contig013326-TilOR.D051	contig014047-ZebOR.D036	0.136	0.343	0.397
contig013330-TilOR.D054	contig014054-ZebOR.D042	0.137	0.246	0.558
contig064802-BriOR.D027	contig013339-TilOR.D056	0.137	0.269	0.509
contig039730-NyeOR.D037	contig013330-TilOR.D054	0.137	0.271	0.507
contig013321-TilOR.D048	contig013337-TilOR.D055	0.137	0.332	0.414
contig032389-BurOR.D033	contig014047-ZebOR.D036	0.137	0.336	0.407
contig064802-BriOR.D027	contig013323-TilOR.D050	0.137	0.342	0.400
contig064802-BriOR.D027	contig032396-BurOR.D034	0.138	0.311	0.444
contig013339-TilOR.D057	contig014047-ZebOR.D036	0.138	0.363	0.381
contig039738-NyeOR.D042	contig014051-ZebOR.D040	0.139	0.288	0.484
contig064802-BriOR.D027	contig032389-BurOR.D033	0.140	0.268	0.521
contig064802-BriOR.D027	contig032388-BurOR.D032	0.140	0.337	0.415
contig064802-BriOR.D027	contig014049-ZebOR.D038	0.141	0.270	0.521
contig064802-BriOR.D027	contig064814-BriOR.D029	0.141	0.334	0.423

contig064802-BriOR.D027	contig039731-NyeOR.D038	0.141	0.351	0.403
contig013321-TilOR.D048	contig013330-TilOR.D054	0.141	0.353	0.398
contig013337-TilOR.D055	contig014047-ZebOR.D036	0.142	0.352	0.404
contig064802-BriOR.D027	contig013326-TilOR.D051	0.144	0.266	0.540
contig064802-BriOR.D027	contig013339-TilOR.D057	0.144	0.299	0.482
contig064802-BriOR.D027	contig014054-ZebOR.D041	0.144	0.362	0.398
contig064802-BriOR.D027	contig013337-TilOR.D055	0.147	0.286	0.515
contig013330-TilOR.D054	contig014047-ZebOR.D036	0.147	0.396	0.372
contig014047-ZebOR.D036	contig014051-ZebOR.D040	0.148	0.372	0.399
contig064802-BriOR.D027	contig014051-ZebOR.D040	0.152	0.307	0.494
contig064802-BriOR.D027	contig013330-TilOR.D054	0.153	0.326	0.468
contig039736-NyeORe.D043	contig014047-ZebOR.D036	0.157	0.300	0.521
contig064802-BriOR.D027	contig039736-NyeORe.D043	0.159	0.265	0.603

Cichlid Olfactory Receptors :
dN/dS ratio

Fam E

OR pairs		dN	dS	dN/dS
contig066194-BurOR.E054	contig053572-NyeOR.E051	0.001	0.007	0.186
contig062770-NyeOR.E059	contig048239-ZebOR.E048	0.001	0.012	0.124
contig052457-BurOR.E051	contig062770-NyeOR.E059	0.003	0.012	0.248
contig052454-BurOR.E050	contig025439-ZebOR.E045	0.003	0.012	0.251
contig049299-BurOR.E046	contig053579-NyeOR.E053	0.003	0.012	0.258
contig052457-BurOR.E051	contig048239-ZebOR.E048	0.004	0.008	0.561
contig049287-BurOR.E043	contig048242-ZebOR.E049	0.005	0.016	0.280
contig049287-BurOR.E043	contig053592-NyeOR.E057	0.005	0.016	0.280
contig064724-BurOR.E052	contig053576-NyeOR.E052	0.006	0.008	0.738
contig053592-NyeOR.E057	contig048242-ZebOR.E049	0.006	0.016	0.373
contig053576-NyeOR.E052	contig048263-ZebOR.E052	0.006	0.019	0.293
contig017699-BurOR.E042	contig023280-NyeOR.E050	0.006	0.020	0.299
contig004259-BriOR.E038	contig052451-BurOR.E047	0.006	0.024	0.242
contig049287-BurOR.E043	contig047726-TilOR.E075	0.006	0.059	0.103
contig052451-BurOR.E047	contig063018-ZebOR.E053	0.007	0.012	0.606
contig064724-BurOR.E052	contig048263-ZebOR.E052	0.008	0.019	0.439
contig047726-TilOR.E075	contig048242-ZebOR.E049	0.008	0.059	0.129
contig053592-NyeOR.E057	contig047726-TilOR.E075	0.008	0.068	0.112
contig052453-BurOR.E049	contig059404-NyeOR.E058	0.009	0.004	2.268
contig052452-BurOR.E048	contig025443-ZebOR.E046	0.009	0.016	0.553
contig049289-BurOR.E044	contig048243-ZebOR.E050	0.009	0.020	0.437
contig049298-BurOR.E045	contig053579-NyeOR.E054	0.010	0.052	0.202
contig053590-NyeOR.E056	contig048243-ZebOR.E050	0.011	0.008	1.474
contig004259-BriOR.E038	contig063018-ZebOR.E053	0.011	0.021	0.509
contig049298-BurOR.E045	contig048260-ZebOR.E051	0.011	0.033	0.334
contig004266-BriOR.E039	contig025447-ZebOR.E047	0.013	0.066	0.205
contig049298-BurOR.E045	contig047833-TilOR.E083	0.013	0.089	0.151
contig049289-BurOR.E044	contig053590-NyeOR.E056	0.014	0.020	0.731
contig004255-BriOR.E036	contig025439-ZebOR.E045	0.014	0.024	0.559
contig004255-BriOR.E036	contig052454-BurOR.E050	0.014	0.028	0.479
contig053576-NyeOR.E052	contig047826-TilOR.E080	0.014	0.059	0.240
contig004266-BriOR.E039	contig017699-BurOR.E042	0.014	0.079	0.172
contig017699-BurOR.E042	contig025447-ZebOR.E047	0.015	0.012	1.260
contig053579-NyeOR.E054	contig048260-ZebOR.E051	0.016	0.039	0.415
contig064724-BurOR.E052	contig047826-TilOR.E080	0.016	0.055	0.283
contig023280-NyeOR.E050	contig025447-ZebOR.E047	0.018	0.008	2.268
contig065454-TilOR.E088	contig065454-TilOR.E089	0.018	0.041	0.433
contig047826-TilOR.E080	contig048263-ZebOR.E052	0.018	0.055	0.335
contig052451-BurOR.E047	contig065455-TilOR.E085	0.019	0.061	0.308
contig004258-BriOR.E037	contig052452-BurOR.E048	0.020	0.042	0.471
contig004258-BriOR.E037	contig025443-ZebOR.E046	0.020	0.042	0.471
contig004266-BriOR.E039	contig023280-NyeOR.E050	0.020	0.066	0.297

contig053572-NyeOR.E051	contig047825-TilOR.E079	0.020	0.071	0.279
contig053579-NyeOR.E054	contig053579-NyeOR.E055	0.021	0.040	0.527
contig066194-BurOR.E054	contig047825-TilOR.E079	0.021	0.062	0.339
contig004259-BriOR.E038	contig065455-TilOR.E085	0.022	0.056	0.387
contig004261-BriOR.E034	contig052450-BurOR.E055	0.022	0.069	0.313
contig053579-NyeOR.E054	contig047833-TilOR.E083	0.022	0.098	0.230
contig065455-TilOR.E085	contig063018-ZebOR.E053	0.023	0.062	0.381
contig047833-TilOR.E083	contig048260-ZebOR.E051	0.023	0.096	0.243
contig004261-BriOR.E034	contig004265-BriOR.E035	0.024	0.033	0.732
contig052450-BurOR.E055	contig065454-TilOR.E088	0.024	0.063	0.382
contig049298-BurOR.E045	contig053579-NyeOR.E055	0.026	0.061	0.423
contig004265-BriOR.E035	contig065454-TilOR.E089	0.026	0.088	0.301
contig053579-NyeOR.E055	contig048260-ZebOR.E051	0.029	0.040	0.717
contig052452-BurOR.E048	contig065458-TilOR.E086	0.029	0.060	0.489
contig065458-TilOR.E086	contig025443-ZebOR.E046	0.029	0.060	0.490
contig004261-BriOR.E034	contig065454-TilOR.E088	0.029	0.065	0.453
contig004258-BriOR.E037	contig065458-TilOR.E086	0.030	0.087	0.344
contig047725-TilOR.E074	contig048243-ZebOR.E050	0.031	0.054	0.581
contig049289-BurOR.E044	contig047725-TilOR.E074	0.031	0.058	0.539
contig004265-BriOR.E035	contig052450-BurOR.E055	0.032	0.079	0.413
contig053590-NyeOR.E056	contig047725-TilOR.E074	0.034	0.054	0.637
contig053579-NyeOR.E055	contig047833-TilOR.E083	0.035	0.121	0.288
contig052450-BurOR.E055	contig065454-TilOR.E089	0.036	0.081	0.449
contig004265-BriOR.E035	contig065454-TilOR.E088	0.036	0.097	0.369
contig047832-TilOR.E082	contig047833-TilOR.E083	0.038	0.109	0.347
contig053579-NyeOR.E055	contig047832-TilOR.E082	0.040	0.132	0.303
contig004261-BriOR.E034	contig065454-TilOR.E089	0.042	0.083	0.505
contig053579-NyeOR.E054	contig047832-TilOR.E082	0.042	0.122	0.341
contig065453-TilOR.E087	contig065454-TilOR.E088	0.044	0.104	0.422
contig049298-BurOR.E045	contig047832-TilOR.E082	0.044	0.154	0.283
contig047832-TilOR.E082	contig048260-ZebOR.E051	0.046	0.139	0.329
contig082838-BriOR.E040	contig048242-ZebOR.E049	0.058	0.150	0.389
contig064938-BurOR.E053	contig047734-TilOR.E077	0.059	0.117	0.509
contig082838-BriOR.E040	contig049287-BurOR.E043	0.059	0.160	0.366
contig065453-TilOR.E087	contig065454-TilOR.E089	0.060	0.123	0.485
contig052450-BurOR.E055	contig065453-TilOR.E087	0.060	0.147	0.406
contig082838-BriOR.E040	contig053592-NyeOR.E057	0.060	0.160	0.375
contig052453-BurOR.E049	contig047734-TilOR.E077	0.061	0.116	0.528
contig082838-BriOR.E040	contig047726-TilOR.E075	0.061	0.166	0.370
contig004261-BriOR.E034	contig065453-TilOR.E087	0.064	0.139	0.458
contig059404-NyeOR.E058	contig047734-TilOR.E077	0.065	0.112	0.576
contig052453-BurOR.E049	contig064938-BurOR.E053	0.066	0.068	0.981
contig064938-BurOR.E053	contig059404-NyeOR.E058	0.068	0.072	0.941

contig004265-BriOR.E035	contig065453-TilOR.E087	0.074	0.175	0.421
contig047729-TilOR.E076	contig048239-ZebOR.E048	0.082	0.164	0.501
contig062770-NyeOR.E059	contig047729-TilOR.E076	0.084	0.169	0.495
contig052457-BurOR.E051	contig047729-TilOR.E076	0.087	0.173	0.501
contig064938-BurOR.E053	contig048239-ZebOR.E048	0.135	0.192	0.706
contig064938-BurOR.E053	contig062770-NyeOR.E059	0.137	0.182	0.754
contig052457-BurOR.E051	contig064938-BurOR.E053	0.137	0.192	0.716
contig047734-TilOR.E077	contig048239-ZebOR.E048	0.148	0.254	0.585
contig052453-BurOR.E049	contig062770-NyeOR.E059	0.149	0.220	0.679
contig052453-BurOR.E049	contig048239-ZebOR.E048	0.149	0.230	0.649
contig062770-NyeOR.E059	contig047734-TilOR.E077	0.150	0.254	0.591
contig059404-NyeOR.E058	contig062770-NyeOR.E059	0.151	0.214	0.709
contig052457-BurOR.E051	contig059404-NyeOR.E058	0.151	0.224	0.677
contig059404-NyeOR.E058	contig048239-ZebOR.E048	0.151	0.224	0.677
contig052453-BurOR.E049	contig052457-BurOR.E051	0.153	0.230	0.665
contig052457-BurOR.E051	contig047734-TilOR.E077	0.154	0.265	0.581
contig047734-TilOR.E077	contig065458-TilOR.E086	0.159	0.403	0.394
contig004258-BriOR.E037	contig047734-TilOR.E077	0.164	0.434	0.378
contig047734-TilOR.E077	contig025443-ZebOR.E046	0.167	0.388	0.431
contig052452-BurOR.E048	contig047734-TilOR.E077	0.168	0.391	0.431
contig065453-TilOR.E087	contig025447-ZebOR.E047	0.170	0.514	0.331
contig064724-BurOR.E052	contig047829-TilOR.E081	0.177	0.465	0.380
contig023280-NyeOR.E050	contig065453-TilOR.E087	0.177	0.522	0.340
contig004266-BriOR.E039	contig065453-TilOR.E087	0.178	0.501	0.355
contig017699-BurOR.E042	contig065453-TilOR.E087	0.179	0.515	0.346
contig064938-BurOR.E053	contig065458-TilOR.E086	0.180	0.382	0.471
contig004258-BriOR.E037	contig064938-BurOR.E053	0.180	0.388	0.465
contig004266-BriOR.E039	contig047729-TilOR.E076	0.180	0.543	0.330
contig023280-NyeOR.E050	contig047729-TilOR.E076	0.180	0.545	0.330
contig017699-BurOR.E042	contig047729-TilOR.E076	0.180	0.575	0.313
contig047729-TilOR.E076	contig047734-TilOR.E077	0.181	0.395	0.459
contig053576-NyeOR.E052	contig047829-TilOR.E081	0.181	0.450	0.401
contig047826-TilOR.E080	contig047829-TilOR.E081	0.182	0.464	0.393
contig004266-BriOR.E039	contig064724-BurOR.E052	0.182	0.519	0.351
contig047729-TilOR.E076	contig025447-ZebOR.E047	0.183	0.562	0.326
contig047829-TilOR.E081	contig048263-ZebOR.E052	0.184	0.466	0.396
contig004265-BriOR.E035	contig004258-BriOR.E037	0.184	0.551	0.334
contig004265-BriOR.E035	contig025443-ZebOR.E046	0.184	0.594	0.310
contig049298-BurOR.E045	contig049299-BurOR.E046	0.185	0.448	0.413
contig049299-BurOR.E046	contig047832-TilOR.E082	0.185	0.453	0.409
contig004258-BriOR.E037	contig048239-ZebOR.E048	0.185	0.499	0.371
contig047832-TilOR.E082	contig048239-ZebOR.E048	0.185	0.517	0.358
contig052450-BurOR.E055	contig025443-ZebOR.E046	0.185	0.570	0.324

contig052452-BurOR.E048	contig064938-BurOR.E053	0.186	0.351	0.530
contig049298-BurOR.E045	contig048239-ZebOR.E048	0.186	0.460	0.405
contig004266-BriOR.E039	contig053576-NyeOR.E052	0.186	0.511	0.364
contig004266-BriOR.E039	contig048263-ZebOR.E052	0.186	0.512	0.363
contig004258-BriOR.E037	contig062770-NyeOR.E059	0.187	0.484	0.386
contig017699-BurOR.E042	contig048263-ZebOR.E052	0.187	0.509	0.367
contig017699-BurOR.E042	contig064724-BurOR.E052	0.187	0.516	0.362
contig062770-NyeOR.E059	contig047832-TilOR.E082	0.187	0.534	0.350
contig064938-BurOR.E053	contig025443-ZebOR.E046	0.188	0.348	0.541
contig053579-NyeOR.E053	contig047829-TilOR.E081	0.188	0.412	0.457
contig025443-ZebOR.E046	contig048239-ZebOR.E048	0.188	0.446	0.422
contig049299-BurOR.E046	contig047833-TilOR.E083	0.188	0.456	0.413
contig049298-BurOR.E045	contig062770-NyeOR.E059	0.188	0.468	0.402
contig047726-TilOR.E075	contig047834-TilOR.E084	0.188	0.474	0.397
contig065458-TilOR.E086	contig048239-ZebOR.E048	0.188	0.479	0.392
contig047734-TilOR.E077	contig047832-TilOR.E082	0.188	0.487	0.386
contig047734-TilOR.E077	contig048260-ZebOR.E051	0.188	0.488	0.385
contig049298-BurOR.E045	contig053579-NyeOR.E053	0.189	0.446	0.424
contig053579-NyeOR.E053	contig047832-TilOR.E082	0.189	0.451	0.420
contig062770-NyeOR.E059	contig065458-TilOR.E086	0.189	0.465	0.408
contig053579-NyeOR.E054	contig048239-ZebOR.E048	0.189	0.472	0.400
contig049298-BurOR.E045	contig047734-TilOR.E077	0.189	0.481	0.393
contig025447-ZebOR.E047	contig048263-ZebOR.E052	0.189	0.493	0.383
contig064724-BurOR.E052	contig025447-ZebOR.E047	0.189	0.500	0.378
contig052454-BurOR.E050	contig062770-NyeOR.E059	0.189	0.534	0.355
contig052454-BurOR.E050	contig048239-ZebOR.E048	0.189	0.545	0.346
contig004261-BriOR.E034	contig004258-BriOR.E037	0.189	0.580	0.326
contig064938-BurOR.E053	contig047729-TilOR.E076	0.190	0.358	0.531
contig047832-TilOR.E082	contig025443-ZebOR.E046	0.190	0.416	0.457
contig062770-NyeOR.E059	contig025443-ZebOR.E046	0.190	0.432	0.440
contig053579-NyeOR.E054	contig047734-TilOR.E077	0.190	0.479	0.396
contig049298-BurOR.E045	contig047729-TilOR.E076	0.190	0.491	0.387
contig004266-BriOR.E039	contig047826-TilOR.E080	0.190	0.500	0.381
contig025439-ZebOR.E045	contig048239-ZebOR.E048	0.190	0.554	0.343
contig052452-BurOR.E048	contig052450-BurOR.E055	0.190	0.554	0.344
contig004265-BriOR.E035	contig052452-BurOR.E048	0.190	0.577	0.329
contig004261-BriOR.E034	contig025443-ZebOR.E046	0.190	0.606	0.313
contig049299-BurOR.E046	contig048260-ZebOR.E051	0.191	0.426	0.449
contig049299-BurOR.E046	contig053579-NyeOR.E055	0.191	0.429	0.446
contig053579-NyeOR.E053	contig053579-NyeOR.E055	0.191	0.441	0.434
contig047832-TilOR.E082	contig025447-ZebOR.E047	0.191	0.458	0.417
contig053579-NyeOR.E054	contig062770-NyeOR.E059	0.191	0.465	0.410
contig053579-NyeOR.E055	contig048239-ZebOR.E048	0.191	0.466	0.409

contig052457-BurOR.E051	contig053579-NyeOR.E054	0.191	0.472	0.404
contig053579-NyeOR.E053	contig047833-TilOR.E083	0.191	0.476	0.401
contig048239-ZebOR.E048	contig048260-ZebOR.E051	0.191	0.478	0.400
contig047729-TilOR.E076	contig048260-ZebOR.E051	0.191	0.479	0.399
contig023280-NyeOR.E050	contig048263-ZebOR.E052	0.191	0.492	0.388
contig004258-BriOR.E037	contig052457-BurOR.E051	0.191	0.499	0.382
contig064724-BurOR.E052	contig023280-NyeOR.E050	0.191	0.500	0.383
contig004266-BriOR.E039	contig048239-ZebOR.E048	0.191	0.504	0.378
contig017699-BurOR.E042	contig053576-NyeOR.E052	0.191	0.508	0.375
contig052457-BurOR.E051	contig047832-TilOR.E082	0.191	0.533	0.358
contig062770-NyeOR.E059	contig025439-ZebOR.E045	0.191	0.543	0.352
contig052452-BurOR.E048	contig048239-ZebOR.E048	0.192	0.432	0.444
contig053579-NyeOR.E053	contig048260-ZebOR.E051	0.192	0.438	0.437
contig049298-BurOR.E045	contig052457-BurOR.E051	0.192	0.460	0.417
contig053592-NyeOR.E057	contig047834-TilOR.E084	0.192	0.472	0.407
contig049298-BurOR.E045	contig025447-ZebOR.E047	0.192	0.508	0.377
contig047734-TilOR.E077	contig047833-TilOR.E083	0.192	0.521	0.369
contig052454-BurOR.E050	contig052457-BurOR.E051	0.192	0.537	0.358
contig004258-BriOR.E037	contig065454-TilOR.E089	0.192	0.550	0.349
contig049287-BurOR.E043	contig047725-TilOR.E074	0.192	0.557	0.345
contig004258-BriOR.E037	contig052450-BurOR.E055	0.192	0.562	0.342
contig004258-BriOR.E037	contig065454-TilOR.E088	0.192	0.567	0.340
contig065454-TilOR.E089	contig025443-ZebOR.E046	0.192	0.578	0.332
contig049299-BurOR.E046	contig047829-TilOR.E081	0.193	0.409	0.473
contig049299-BurOR.E046	contig053579-NyeOR.E054	0.193	0.418	0.463
contig053579-NyeOR.E055	contig062770-NyeOR.E059	0.193	0.460	0.419
contig052457-BurOR.E051	contig053579-NyeOR.E055	0.193	0.466	0.413
contig062770-NyeOR.E059	contig048260-ZebOR.E051	0.193	0.471	0.410
contig052457-BurOR.E051	contig048260-ZebOR.E051	0.193	0.478	0.404
contig052457-BurOR.E051	contig065458-TilOR.E086	0.193	0.479	0.404
contig053576-NyeOR.E052	contig025447-ZebOR.E047	0.193	0.492	0.392
contig004266-BriOR.E039	contig062770-NyeOR.E059	0.193	0.505	0.382
contig049299-BurOR.E046	contig047734-TilOR.E077	0.193	0.511	0.377
contig017699-BurOR.E042	contig047826-TilOR.E080	0.193	0.512	0.377
contig052454-BurOR.E050	contig047832-TilOR.E082	0.193	0.562	0.343
contig047832-TilOR.E082	contig025439-ZebOR.E045	0.193	0.572	0.337
contig065454-TilOR.E088	contig025443-ZebOR.E046	0.193	0.575	0.335
contig004258-BriOR.E037	contig047832-TilOR.E082	0.194	0.395	0.492
contig053579-NyeOR.E053	contig053579-NyeOR.E054	0.194	0.430	0.451
contig052452-BurOR.E048	contig052457-BurOR.E051	0.194	0.432	0.449
contig052452-BurOR.E048	contig062770-NyeOR.E059	0.194	0.433	0.448
contig052457-BurOR.E051	contig025443-ZebOR.E046	0.194	0.446	0.435
contig049287-BurOR.E043	contig047834-TilOR.E084	0.194	0.456	0.426

contig064724-BurOR.E052	contig047832-TilOR.E082	0.194	0.468	0.414
contig047834-TilOR.E084	contig048242-ZebOR.E049	0.194	0.472	0.411
contig017699-BurOR.E042	contig047832-TilOR.E082	0.194	0.472	0.412
contig053579-NyeOR.E055	contig047734-TilOR.E077	0.194	0.481	0.402
contig047833-TilOR.E083	contig025447-ZebOR.E047	0.194	0.522	0.372
contig052457-BurOR.E051	contig025439-ZebOR.E045	0.194	0.546	0.356
contig047725-TilOR.E074	contig048242-ZebOR.E049	0.194	0.550	0.353
contig004255-BriOR.E036	contig048239-ZebOR.E048	0.194	0.551	0.352
contig049287-BurOR.E043	contig049289-BurOR.E044	0.194	0.562	0.345
contig023280-NyeOR.E050	contig053576-NyeOR.E052	0.195	0.491	0.397
contig053579-NyeOR.E054	contig047729-TilOR.E076	0.195	0.496	0.393
contig062770-NyeOR.E059	contig025447-ZebOR.E047	0.195	0.511	0.382
contig053579-NyeOR.E053	contig047734-TilOR.E077	0.195	0.511	0.382
contig004255-BriOR.E036	contig062770-NyeOR.E059	0.195	0.539	0.361
contig004261-BriOR.E034	contig052452-BurOR.E048	0.195	0.607	0.322
contig047829-TilOR.E081	contig047832-TilOR.E082	0.196	0.356	0.550
contig053579-NyeOR.E054	contig025443-ZebOR.E046	0.196	0.448	0.439
contig004266-BriOR.E039	contig047829-TilOR.E081	0.196	0.513	0.383
contig052454-BurOR.E050	contig047734-TilOR.E077	0.196	0.534	0.367
contig047725-TilOR.E074	contig047726-TilOR.E075	0.196	0.535	0.367
contig047734-TilOR.E077	contig025439-ZebOR.E045	0.196	0.542	0.362
contig049289-BurOR.E044	contig048242-ZebOR.E049	0.196	0.555	0.353
contig049289-BurOR.E044	contig053592-NyeOR.E057	0.196	0.564	0.347
contig047729-TilOR.E076	contig025443-ZebOR.E046	0.196	0.569	0.345
contig052452-BurOR.E048	contig053579-NyeOR.E054	0.197	0.424	0.466
contig004258-BriOR.E037	contig053579-NyeOR.E054	0.197	0.425	0.462
contig004258-BriOR.E037	contig049298-BurOR.E045	0.197	0.431	0.456
contig053579-NyeOR.E054	contig025447-ZebOR.E047	0.197	0.465	0.424
contig017699-BurOR.E042	contig047829-TilOR.E081	0.197	0.486	0.405
contig049299-BurOR.E046	contig048239-ZebOR.E048	0.197	0.489	0.402
contig023280-NyeOR.E050	contig047829-TilOR.E081	0.197	0.489	0.403
contig047826-TilOR.E080	contig025447-ZebOR.E047	0.197	0.492	0.400
contig052453-BurOR.E049	contig047832-TilOR.E082	0.197	0.500	0.394
contig004266-BriOR.E039	contig049298-BurOR.E045	0.197	0.502	0.392
contig025447-ZebOR.E047	contig048239-ZebOR.E048	0.197	0.511	0.386
contig004255-BriOR.E036	contig052457-BurOR.E051	0.197	0.542	0.364
contig047833-TilOR.E083	contig048239-ZebOR.E048	0.197	0.545	0.361
contig048242-ZebOR.E049	contig048243-ZebOR.E050	0.197	0.575	0.342
contig004258-BriOR.E037	contig053579-NyeOR.E055	0.198	0.438	0.452
contig047832-TilOR.E082	contig065458-TilOR.E086	0.198	0.441	0.448
contig023280-NyeOR.E050	contig047832-TilOR.E082	0.198	0.445	0.444
contig053579-NyeOR.E055	contig025443-ZebOR.E046	0.198	0.461	0.429
contig049299-BurOR.E046	contig062770-NyeOR.E059	0.198	0.467	0.425

contig004266-BriOR.E039	contig047833-TilOR.E083	0.198	0.480	0.413
contig047829-TilOR.E081	contig025447-ZebOR.E047	0.198	0.485	0.408
contig023280-NyeOR.E050	contig048239-ZebOR.E048	0.198	0.493	0.401
contig023280-NyeOR.E050	contig047826-TilOR.E080	0.198	0.496	0.398
contig047832-TilOR.E082	contig048263-ZebOR.E052	0.198	0.498	0.397
contig004266-BriOR.E039	contig052457-BurOR.E051	0.198	0.508	0.389
contig017699-BurOR.E042	contig048239-ZebOR.E048	0.198	0.521	0.380
contig053592-NyeOR.E057	contig047725-TilOR.E074	0.198	0.559	0.354
contig053590-NyeOR.E056	contig053592-NyeOR.E057	0.198	0.568	0.349
contig049289-BurOR.E044	contig047726-TilOR.E075	0.198	0.574	0.345
contig052452-BurOR.E048	contig065454-TilOR.E089	0.198	0.578	0.342
contig052454-BurOR.E050	contig047833-TilOR.E083	0.198	0.623	0.318
contig047833-TilOR.E083	contig025439-ZebOR.E045	0.198	0.633	0.312
contig049298-BurOR.E045	contig047829-TilOR.E081	0.199	0.431	0.463
contig004266-BriOR.E039	contig047832-TilOR.E082	0.199	0.433	0.460
contig082838-BriOR.E040	contig047834-TilOR.E084	0.199	0.452	0.442
contig025447-ZebOR.E047	contig048260-ZebOR.E051	0.199	0.481	0.414
contig062770-NyeOR.E059	contig047833-TilOR.E083	0.199	0.538	0.369
contig052452-BurOR.E048	contig065454-TilOR.E088	0.199	0.576	0.345
contig049287-BurOR.E043	contig048243-ZebOR.E050	0.199	0.582	0.342
contig004258-BriOR.E037	contig047729-TilOR.E076	0.199	0.597	0.333
contig052453-BurOR.E049	contig025443-ZebOR.E046	0.200	0.366	0.547
contig052453-BurOR.E049	contig065458-TilOR.E086	0.200	0.391	0.511
contig047734-TilOR.E077	contig047829-TilOR.E081	0.200	0.447	0.448
contig049298-BurOR.E045	contig025443-ZebOR.E046	0.200	0.468	0.429
contig049298-BurOR.E045	contig065458-TilOR.E086	0.200	0.474	0.422
contig053579-NyeOR.E053	contig048239-ZebOR.E048	0.200	0.492	0.406
contig023280-NyeOR.E050	contig062770-NyeOR.E059	0.200	0.494	0.404
contig052457-BurOR.E051	contig025447-ZebOR.E047	0.200	0.515	0.389
contig017699-BurOR.E042	contig062770-NyeOR.E059	0.200	0.522	0.383
contig052453-BurOR.E049	contig052454-BurOR.E050	0.200	0.543	0.369
contig004255-BriOR.E036	contig047832-TilOR.E082	0.200	0.550	0.364
contig004265-BriOR.E035	contig065458-TilOR.E086	0.200	0.579	0.346
contig047729-TilOR.E076	contig065458-TilOR.E086	0.200	0.580	0.344
contig004258-BriOR.E037	contig025439-ZebOR.E045	0.200	0.623	0.321
contig052452-BurOR.E048	contig047832-TilOR.E082	0.201	0.386	0.520
contig052452-BurOR.E048	contig053579-NyeOR.E055	0.201	0.436	0.460
contig053579-NyeOR.E055	contig065458-TilOR.E086	0.201	0.460	0.438
contig053579-NyeOR.E054	contig065458-TilOR.E086	0.201	0.464	0.433
contig047729-TilOR.E076	contig047833-TilOR.E083	0.201	0.545	0.370
contig047729-TilOR.E076	contig047832-TilOR.E082	0.201	0.554	0.363
contig049287-BurOR.E043	contig053590-NyeOR.E056	0.201	0.566	0.354
contig052457-BurOR.E051	contig047833-TilOR.E083	0.201	0.570	0.352

contig053592-NyeOR.E057	contig048243-ZebOR.E050	0.201	0.584	0.344
contig052453-BurOR.E049	contig047729-TilOR.E076	0.202	0.366	0.550
contig052452-BurOR.E048	contig059404-NyeOR.E058	0.202	0.375	0.538
contig004266-BriOR.E039	contig025439-ZebOR.E045	0.202	0.424	0.477
contig053576-NyeOR.E052	contig047832-TilOR.E082	0.202	0.467	0.432
contig004266-BriOR.E039	contig053579-NyeOR.E054	0.202	0.477	0.422
contig053579-NyeOR.E053	contig062770-NyeOR.E059	0.202	0.485	0.415
contig004255-BriOR.E036	contig047734-TilOR.E077	0.202	0.545	0.370
contig052453-BurOR.E049	contig025439-ZebOR.E045	0.202	0.552	0.366
contig053590-NyeOR.E056	contig048242-ZebOR.E049	0.202	0.559	0.362
contig052450-BurOR.E055	contig065458-TilOR.E086	0.202	0.573	0.353
contig004258-BriOR.E037	contig052454-BurOR.E050	0.202	0.631	0.320
contig052452-BurOR.E048	contig052453-BurOR.E049	0.203	0.370	0.548
contig059404-NyeOR.E058	contig025443-ZebOR.E046	0.203	0.371	0.547
contig004258-BriOR.E037	contig052453-BurOR.E049	0.203	0.395	0.513
contig059404-NyeOR.E058	contig065458-TilOR.E086	0.203	0.396	0.511
contig047829-TilOR.E081	contig047833-TilOR.E083	0.203	0.411	0.494
contig064938-BurOR.E053	contig053579-NyeOR.E054	0.203	0.424	0.478
contig064938-BurOR.E053	contig048260-ZebOR.E051	0.203	0.446	0.454
contig049299-BurOR.E046	contig052457-BurOR.E051	0.203	0.470	0.433
contig052457-BurOR.E051	contig053579-NyeOR.E053	0.203	0.473	0.428
contig017699-BurOR.E042	contig049298-BurOR.E045	0.203	0.524	0.388
contig004255-BriOR.E036	contig052453-BurOR.E049	0.203	0.576	0.352
contig047726-TilOR.E075	contig048243-ZebOR.E050	0.203	0.577	0.352
contig004255-BriOR.E036	contig004258-BriOR.E037	0.203	0.627	0.324
contig059404-NyeOR.E058	contig047729-TilOR.E076	0.204	0.360	0.567
contig049299-BurOR.E046	contig052453-BurOR.E049	0.204	0.451	0.452
contig059404-NyeOR.E058	contig047832-TilOR.E082	0.204	0.488	0.418
contig004266-BriOR.E039	contig048260-ZebOR.E051	0.204	0.491	0.416
contig065458-TilOR.E086	contig065454-TilOR.E088	0.204	0.540	0.378
contig017699-BurOR.E042	contig047833-TilOR.E083	0.204	0.541	0.378
contig052453-BurOR.E049	contig047829-TilOR.E081	0.205	0.395	0.519
contig004258-BriOR.E037	contig059404-NyeOR.E058	0.205	0.400	0.513
contig049298-BurOR.E045	contig052452-BurOR.E048	0.205	0.429	0.478
contig004258-BriOR.E037	contig048260-ZebOR.E051	0.205	0.453	0.451
contig053579-NyeOR.E055	contig025447-ZebOR.E047	0.205	0.459	0.447
contig017699-BurOR.E042	contig053579-NyeOR.E054	0.205	0.479	0.427
contig047826-TilOR.E080	contig047832-TilOR.E082	0.205	0.493	0.415
contig052457-BurOR.E051	contig023280-NyeOR.E050	0.205	0.497	0.412
contig017699-BurOR.E042	contig052457-BurOR.E051	0.205	0.525	0.390
contig065458-TilOR.E086	contig065454-TilOR.E089	0.205	0.535	0.383
contig049298-BurOR.E045	contig052454-BurOR.E050	0.205	0.582	0.353
contig004261-BriOR.E034	contig065458-TilOR.E086	0.205	0.587	0.349

contig049298-BurOR.E045	contig025439-ZebOR.E045	0.205	0.592	0.347
contig004255-BriOR.E036	contig047833-TilOR.E083	0.205	0.610	0.337
contig004266-BriOR.E039	contig052454-BurOR.E050	0.206	0.431	0.479
contig053590-NyeOR.E056	contig047726-TilOR.E075	0.206	0.557	0.369
contig052454-BurOR.E050	contig064724-BurOR.E052	0.206	0.570	0.361
contig052454-BurOR.E050	contig048260-ZebOR.E051	0.206	0.607	0.340
contig065458-TilOR.E086	contig025439-ZebOR.E045	0.206	0.611	0.336
contig025439-ZebOR.E045	contig048260-ZebOR.E051	0.206	0.617	0.334
contig052452-BurOR.E048	contig025439-ZebOR.E045	0.206	0.632	0.326
contig053579-NyeOR.E054	contig047829-TilOR.E081	0.207	0.392	0.529
contig047829-TilOR.E081	contig048260-ZebOR.E051	0.207	0.394	0.524
contig004258-BriOR.E037	contig047833-TilOR.E083	0.207	0.429	0.481
contig049299-BurOR.E046	contig059404-NyeOR.E058	0.207	0.441	0.471
contig025439-ZebOR.E045	contig025447-ZebOR.E047	0.207	0.451	0.458
contig053579-NyeOR.E053	contig059404-NyeOR.E058	0.207	0.455	0.455
contig049298-BurOR.E045	contig023280-NyeOR.E050	0.207	0.495	0.417
contig064724-BurOR.E052	contig047734-TilOR.E077	0.207	0.507	0.409
contig052454-BurOR.E050	contig059404-NyeOR.E058	0.207	0.550	0.376
contig064724-BurOR.E052	contig047833-TilOR.E083	0.207	0.573	0.362
contig047833-TilOR.E083	contig065453-TilOR.E087	0.207	0.747	0.277
contig053579-NyeOR.E055	contig047829-TilOR.E081	0.208	0.405	0.513
contig049298-BurOR.E045	contig064938-BurOR.E053	0.208	0.426	0.487
contig023280-NyeOR.E050	contig053579-NyeOR.E054	0.208	0.453	0.461
contig052453-BurOR.E049	contig053579-NyeOR.E053	0.208	0.467	0.445
contig065458-TilOR.E086	contig048260-ZebOR.E051	0.208	0.475	0.437
contig023280-NyeOR.E050	contig047833-TilOR.E083	0.208	0.512	0.406
contig053576-NyeOR.E052	contig053579-NyeOR.E054	0.208	0.516	0.403
contig049298-BurOR.E045	contig053576-NyeOR.E052	0.208	0.554	0.375
contig049298-BurOR.E045	contig064724-BurOR.E052	0.208	0.555	0.375
contig052452-BurOR.E048	contig047729-TilOR.E076	0.208	0.561	0.371
contig004255-BriOR.E036	contig064724-BurOR.E052	0.208	0.564	0.368
contig064724-BurOR.E052	contig025439-ZebOR.E045	0.208	0.579	0.358
contig052454-BurOR.E050	contig065458-TilOR.E086	0.208	0.601	0.345
contig052452-BurOR.E048	contig052454-BurOR.E050	0.208	0.622	0.334
contig025439-ZebOR.E045	contig025443-ZebOR.E046	0.208	0.632	0.329
contig064938-BurOR.E053	contig053579-NyeOR.E055	0.209	0.425	0.491
contig047833-TilOR.E083	contig025443-ZebOR.E046	0.209	0.455	0.460
contig064938-BurOR.E053	contig047832-TilOR.E082	0.209	0.459	0.454
contig025443-ZebOR.E046	contig048260-ZebOR.E051	0.209	0.473	0.443
contig064938-BurOR.E053	contig047833-TilOR.E083	0.209	0.485	0.432
contig059404-NyeOR.E058	contig025439-ZebOR.E045	0.209	0.559	0.373
contig047729-TilOR.E076	contig048263-ZebOR.E052	0.209	0.571	0.367
contig004255-BriOR.E036	contig065458-TilOR.E086	0.209	0.606	0.345

contig004255-BriOR.E036	contig052452-BurOR.E048	0.209	0.618	0.339
contig004265-BriOR.E035	contig047734-TilOR.E077	0.209	0.695	0.301
contig004255-BriOR.E036	contig004266-BriOR.E039	0.210	0.391	0.537
contig052452-BurOR.E048	contig048260-ZebOR.E051	0.210	0.448	0.469
contig004266-BriOR.E039	contig053579-NyeOR.E055	0.210	0.471	0.445
contig049299-BurOR.E046	contig064938-BurOR.E053	0.210	0.482	0.437
contig064938-BurOR.E053	contig053579-NyeOR.E053	0.210	0.496	0.424
contig017699-BurOR.E042	contig048260-ZebOR.E051	0.210	0.497	0.423
contig049298-BurOR.E045	contig048263-ZebOR.E052	0.210	0.572	0.367
contig004255-BriOR.E036	contig059404-NyeOR.E058	0.210	0.579	0.363
contig052454-BurOR.E050	contig025443-ZebOR.E046	0.210	0.622	0.338
contig047832-TilOR.E082	contig065453-TilOR.E087	0.210	0.665	0.315
contig047833-TilOR.E083	contig065458-TilOR.E086	0.211	0.440	0.480
contig052454-BurOR.E050	contig025447-ZebOR.E047	0.211	0.443	0.475
contig047829-TilOR.E081	contig048239-ZebOR.E048	0.211	0.452	0.467
contig064724-BurOR.E052	contig062770-NyeOR.E059	0.211	0.455	0.463
contig053579-NyeOR.E055	contig047729-TilOR.E076	0.211	0.456	0.464
contig064724-BurOR.E052	contig048239-ZebOR.E048	0.211	0.462	0.456
contig004266-BriOR.E039	contig047734-TilOR.E077	0.211	0.466	0.454
contig047829-TilOR.E081	contig065458-TilOR.E086	0.211	0.478	0.441
contig052454-BurOR.E050	contig064938-BurOR.E053	0.211	0.505	0.418
contig053576-NyeOR.E052	contig047734-TilOR.E077	0.211	0.506	0.417
contig053576-NyeOR.E052	contig048260-ZebOR.E051	0.211	0.537	0.392
contig082838-BriOR.E040	contig047725-TilOR.E074	0.211	0.556	0.379
contig004266-BriOR.E039	contig049299-BurOR.E046	0.211	0.559	0.377
contig049299-BurOR.E046	contig025439-ZebOR.E045	0.211	0.564	0.374
contig064724-BurOR.E052	contig047729-TilOR.E076	0.211	0.571	0.371
contig004258-BriOR.E037	contig025447-ZebOR.E047	0.211	0.584	0.362
contig004255-BriOR.E036	contig025443-ZebOR.E046	0.211	0.617	0.343
contig049298-BurOR.E045	contig065453-TilOR.E087	0.211	0.708	0.298
contig059404-NyeOR.E058	contig047829-TilOR.E081	0.212	0.398	0.533
contig064724-BurOR.E052	contig053579-NyeOR.E054	0.212	0.517	0.409
contig053579-NyeOR.E054	contig048263-ZebOR.E052	0.212	0.533	0.397
contig053576-NyeOR.E052	contig047729-TilOR.E076	0.212	0.570	0.371
contig082838-BriOR.E040	contig049289-BurOR.E044	0.212	0.572	0.370
contig052452-BurOR.E048	contig025447-ZebOR.E047	0.212	0.572	0.370
contig025443-ZebOR.E046	contig025447-ZebOR.E047	0.212	0.589	0.360
contig047829-TilOR.E081	contig065453-TilOR.E087	0.212	0.622	0.340
contig004265-BriOR.E035	contig025447-ZebOR.E047	0.212	0.640	0.331
contig004265-BriOR.E035	contig023280-NyeOR.E050	0.212	0.650	0.326
contig064938-BurOR.E053	contig047829-TilOR.E081	0.213	0.403	0.529
contig052452-BurOR.E048	contig047833-TilOR.E083	0.213	0.435	0.491
contig062770-NyeOR.E059	contig047829-TilOR.E081	0.213	0.438	0.486

contig053579-NyeOR.E055	contig059404-NyeOR.E058	0.213	0.443	0.481
contig052453-BurOR.E049	contig053579-NyeOR.E055	0.213	0.455	0.469
contig017699-BurOR.E042	contig053579-NyeOR.E055	0.213	0.473	0.450
contig047734-TilOR.E077	contig047826-TilOR.E080	0.213	0.492	0.432
contig053576-NyeOR.E052	contig053579-NyeOR.E055	0.213	0.495	0.430
contig064724-BurOR.E052	contig053579-NyeOR.E055	0.213	0.496	0.429
contig064938-BurOR.E053	contig025439-ZebOR.E045	0.213	0.513	0.415
contig049299-BurOR.E046	contig025443-ZebOR.E046	0.213	0.553	0.386
contig049299-BurOR.E046	contig025447-ZebOR.E047	0.213	0.556	0.384
contig004255-BriOR.E036	contig049299-BurOR.E046	0.213	0.571	0.373
contig004266-BriOR.E039	contig053579-NyeOR.E053	0.213	0.573	0.372
contig053576-NyeOR.E052	contig047833-TilOR.E083	0.213	0.580	0.367
contig004265-BriOR.E035	contig017699-BurOR.E042	0.213	0.641	0.332
contig004255-BriOR.E036	contig047829-TilOR.E081	0.214	0.453	0.472
contig062770-NyeOR.E059	contig048263-ZebOR.E052	0.214	0.455	0.471
contig023280-NyeOR.E050	contig048260-ZebOR.E051	0.214	0.469	0.456
contig049299-BurOR.E046	contig064724-BurOR.E052	0.214	0.498	0.431
contig064724-BurOR.E052	contig053579-NyeOR.E053	0.214	0.507	0.423
contig064724-BurOR.E052	contig048260-ZebOR.E051	0.214	0.538	0.399
contig004258-BriOR.E037	contig049299-BurOR.E046	0.214	0.569	0.376
contig053579-NyeOR.E053	contig025439-ZebOR.E045	0.214	0.583	0.367
contig052454-BurOR.E050	contig053579-NyeOR.E054	0.214	0.588	0.364
contig053579-NyeOR.E054	contig025439-ZebOR.E045	0.214	0.598	0.358
contig004255-BriOR.E036	contig049298-BurOR.E045	0.214	0.599	0.357
contig065453-TilOR.E087	contig025439-ZebOR.E045	0.214	0.729	0.293
contig004255-BriOR.E036	contig025447-ZebOR.E047	0.215	0.431	0.498
contig053576-NyeOR.E052	contig062770-NyeOR.E059	0.215	0.454	0.472
contig053576-NyeOR.E052	contig048239-ZebOR.E048	0.215	0.461	0.466
contig048239-ZebOR.E048	contig048263-ZebOR.E052	0.215	0.462	0.464
contig023280-NyeOR.E050	contig047734-TilOR.E077	0.215	0.474	0.454
contig052457-BurOR.E051	contig053576-NyeOR.E052	0.215	0.476	0.451
contig052457-BurOR.E051	contig064724-BurOR.E052	0.215	0.477	0.450
contig047829-TilOR.E081	contig025439-ZebOR.E045	0.215	0.483	0.447
contig053576-NyeOR.E052	contig053579-NyeOR.E053	0.215	0.491	0.437
contig047734-TilOR.E077	contig048263-ZebOR.E052	0.215	0.507	0.424
contig047820-TilOR.E078	contig047829-TilOR.E081	0.215	0.520	0.414
contig004255-BriOR.E036	contig064938-BurOR.E053	0.215	0.536	0.402
contig049299-BurOR.E046	contig052454-BurOR.E050	0.215	0.538	0.399
contig023280-NyeOR.E050	contig025443-ZebOR.E046	0.215	0.577	0.373
contig004255-BriOR.E036	contig048260-ZebOR.E051	0.215	0.607	0.353
contig052454-BurOR.E050	contig047820-TilOR.E078	0.215	0.633	0.340
contig047820-TilOR.E078	contig025439-ZebOR.E045	0.215	0.644	0.334
contig059404-NyeOR.E058	contig048260-ZebOR.E051	0.216	0.456	0.474

contig052453-BurOR.E049	contig048260-ZebOR.E051	0.216	0.468	0.462
contig052454-BurOR.E050	contig047829-TilOR.E081	0.216	0.474	0.455
contig047734-TilOR.E077	contig025447-ZebOR.E047	0.216	0.481	0.448
contig049299-BurOR.E046	contig023280-NyeOR.E050	0.216	0.545	0.397
contig049299-BurOR.E046	contig047729-TilOR.E076	0.216	0.546	0.395
contig049299-BurOR.E046	contig065458-TilOR.E086	0.216	0.552	0.390
contig048260-ZebOR.E051	contig048263-ZebOR.E052	0.216	0.554	0.390
contig053579-NyeOR.E053	contig025447-ZebOR.E047	0.216	0.570	0.378
contig052454-BurOR.E050	contig053579-NyeOR.E055	0.216	0.617	0.350
contig004255-BriOR.E036	contig047729-TilOR.E076	0.216	0.626	0.345
contig053579-NyeOR.E055	contig025439-ZebOR.E045	0.216	0.627	0.344
contig047734-TilOR.E077	contig065454-TilOR.E088	0.216	0.669	0.323
contig065453-TilOR.E087	contig048260-ZebOR.E051	0.216	0.694	0.312
contig023280-NyeOR.E050	contig053579-NyeOR.E055	0.217	0.446	0.485
contig017699-BurOR.E042	contig025439-ZebOR.E045	0.217	0.456	0.476
contig023280-NyeOR.E050	contig025439-ZebOR.E045	0.217	0.456	0.477
contig004258-BriOR.E037	contig047829-TilOR.E081	0.217	0.457	0.475
contig052453-BurOR.E049	contig047833-TilOR.E083	0.217	0.508	0.427
contig047729-TilOR.E076	contig047826-TilOR.E080	0.217	0.523	0.415
contig004258-BriOR.E037	contig004266-BriOR.E039	0.217	0.525	0.413
contig052452-BurOR.E048	contig053579-NyeOR.E053	0.217	0.529	0.411
contig049299-BurOR.E046	contig052452-BurOR.E048	0.217	0.538	0.404
contig082838-BriOR.E040	contig048243-ZebOR.E050	0.217	0.558	0.388
contig004255-BriOR.E036	contig053579-NyeOR.E053	0.217	0.591	0.367
contig047833-TilOR.E083	contig048263-ZebOR.E052	0.217	0.607	0.357
contig052454-BurOR.E050	contig047729-TilOR.E076	0.217	0.629	0.345
contig047734-TilOR.E077	contig065453-TilOR.E087	0.217	0.638	0.340
contig047729-TilOR.E076	contig025439-ZebOR.E045	0.217	0.640	0.339
contig004255-BriOR.E036	contig047820-TilOR.E078	0.217	0.648	0.334
contig004261-BriOR.E034	contig025447-ZebOR.E047	0.217	0.671	0.324
contig004261-BriOR.E034	contig023280-NyeOR.E050	0.217	0.681	0.319
contig053579-NyeOR.E054	contig059404-NyeOR.E058	0.218	0.434	0.503
contig052453-BurOR.E049	contig053579-NyeOR.E054	0.218	0.446	0.490
contig052457-BurOR.E051	contig047829-TilOR.E081	0.218	0.456	0.479
contig049298-BurOR.E045	contig052453-BurOR.E049	0.218	0.469	0.464
contig049299-BurOR.E046	contig053576-NyeOR.E052	0.218	0.482	0.453
contig052454-BurOR.E050	contig053579-NyeOR.E053	0.218	0.557	0.392
contig053579-NyeOR.E053	contig025443-ZebOR.E046	0.218	0.561	0.388
contig004258-BriOR.E037	contig023280-NyeOR.E050	0.218	0.571	0.382
contig004258-BriOR.E037	contig053579-NyeOR.E053	0.218	0.577	0.378
contig047829-TilOR.E081	contig065454-TilOR.E088	0.218	0.580	0.376
contig004261-BriOR.E034	contig047832-TilOR.E082	0.218	0.631	0.346
contig052454-BurOR.E050	contig065453-TilOR.E087	0.218	0.717	0.304

contig052457-BurOR.E051	contig048263-ZebOR.E052	0.219	0.477	0.458
contig053579-NyeOR.E055	contig048263-ZebOR.E052	0.219	0.512	0.427
contig082838-BriOR.E040	contig053590-NyeOR.E056	0.219	0.558	0.392
contig023280-NyeOR.E050	contig053579-NyeOR.E053	0.219	0.559	0.391
contig052452-BurOR.E048	contig023280-NyeOR.E050	0.219	0.560	0.391
contig053579-NyeOR.E053	contig047729-TilOR.E076	0.219	0.566	0.387
contig017699-BurOR.E042	contig025443-ZebOR.E046	0.219	0.582	0.377
contig004265-BriOR.E035	contig047832-TilOR.E082	0.219	0.603	0.363
contig047832-TilOR.E082	contig065454-TilOR.E088	0.219	0.610	0.359
contig052450-BurOR.E055	contig047832-TilOR.E082	0.219	0.630	0.347
contig052450-BurOR.E055	contig025447-ZebOR.E047	0.219	0.636	0.344
contig052450-BurOR.E055	contig023280-NyeOR.E050	0.219	0.646	0.339
contig004261-BriOR.E034	contig017699-BurOR.E042	0.219	0.672	0.325
contig004261-BriOR.E034	contig047734-TilOR.E077	0.219	0.691	0.317
contig052450-BurOR.E055	contig047833-TilOR.E083	0.219	0.693	0.316
contig047734-TilOR.E077	contig065454-TilOR.E089	0.219	0.697	0.314
contig004255-BriOR.E036	contig065453-TilOR.E087	0.219	0.698	0.313
contig017699-BurOR.E042	contig047734-TilOR.E077	0.220	0.494	0.445
contig049299-BurOR.E046	contig048263-ZebOR.E052	0.220	0.505	0.436
contig053579-NyeOR.E053	contig048263-ZebOR.E052	0.220	0.515	0.428
contig004266-BriOR.E039	contig065458-TilOR.E086	0.220	0.558	0.395
contig053579-NyeOR.E053	contig065458-TilOR.E086	0.220	0.560	0.393
contig004265-BriOR.E035	contig004266-BriOR.E039	0.220	0.585	0.376
contig047826-TilOR.E080	contig065458-TilOR.E086	0.220	0.605	0.363
contig017699-BurOR.E042	contig052450-BurOR.E055	0.220	0.638	0.345
contig047833-TilOR.E083	contig065454-TilOR.E088	0.220	0.658	0.335
contig047729-TilOR.E076	contig065453-TilOR.E087	0.220	0.663	0.331
contig004261-BriOR.E034	contig047833-TilOR.E083	0.220	0.670	0.328
contig052450-BurOR.E055	contig047734-TilOR.E077	0.220	0.682	0.322
contig017699-BurOR.E042	contig052454-BurOR.E050	0.221	0.448	0.494
contig052454-BurOR.E050	contig023280-NyeOR.E050	0.221	0.448	0.494
contig049298-BurOR.E045	contig059404-NyeOR.E058	0.221	0.457	0.484
contig047734-TilOR.E077	contig047820-TilOR.E078	0.221	0.511	0.433
contig004266-BriOR.E039	contig052452-BurOR.E048	0.221	0.532	0.416
contig004266-BriOR.E039	contig025443-ZebOR.E046	0.221	0.548	0.404
contig017699-BurOR.E042	contig049299-BurOR.E046	0.221	0.558	0.395
contig047826-TilOR.E080	contig047833-TilOR.E083	0.221	0.570	0.388
contig004258-BriOR.E037	contig017699-BurOR.E042	0.221	0.590	0.376
contig004255-BriOR.E036	contig053579-NyeOR.E054	0.221	0.597	0.370
contig065458-TilOR.E086	contig025447-ZebOR.E047	0.221	0.600	0.367
contig065454-TilOR.E088	contig025447-ZebOR.E047	0.221	0.643	0.345
contig023280-NyeOR.E050	contig065454-TilOR.E088	0.221	0.652	0.339
contig047729-TilOR.E076	contig047829-TilOR.E081	0.222	0.441	0.504

contig062770-NyeOR.E059	contig047826-TilOR.E080	0.222	0.449	0.494
contig047826-TilOR.E080	contig048239-ZebOR.E048	0.222	0.456	0.487
contig053579-NyeOR.E055	contig047826-TilOR.E080	0.222	0.506	0.438
contig052454-BurOR.E050	contig053576-NyeOR.E052	0.222	0.522	0.425
contig049298-BurOR.E045	contig047826-TilOR.E080	0.222	0.561	0.396
contig047832-TilOR.E082	contig065454-TilOR.E089	0.222	0.596	0.373
contig065454-TilOR.E089	contig025447-ZebOR.E047	0.222	0.638	0.348
contig023280-NyeOR.E050	contig065454-TilOR.E089	0.222	0.648	0.343
contig049298-BurOR.E045	contig052450-BurOR.E055	0.222	0.652	0.341
contig053579-NyeOR.E054	contig065453-TilOR.E087	0.222	0.695	0.319
contig047829-TilOR.E081	contig025443-ZebOR.E046	0.223	0.454	0.491
contig059404-NyeOR.E058	contig047833-TilOR.E083	0.223	0.503	0.443
contig047820-TilOR.E078	contig025443-ZebOR.E046	0.223	0.520	0.429
contig017699-BurOR.E042	contig052452-BurOR.E048	0.223	0.566	0.395
contig017699-BurOR.E042	contig053579-NyeOR.E053	0.223	0.573	0.390
contig004265-BriOR.E035	contig047826-TilOR.E080	0.223	0.622	0.359
contig004261-BriOR.E034	contig047829-TilOR.E081	0.223	0.622	0.359
contig017699-BurOR.E042	contig065454-TilOR.E089	0.223	0.640	0.349
contig017699-BurOR.E042	contig065454-TilOR.E088	0.223	0.644	0.346
contig004261-BriOR.E034	contig049298-BurOR.E045	0.223	0.667	0.334
contig049298-BurOR.E045	contig065454-TilOR.E088	0.223	0.669	0.333
contig052457-BurOR.E051	contig047826-TilOR.E080	0.224	0.478	0.468
contig004255-BriOR.E036	contig053576-NyeOR.E052	0.224	0.516	0.434
contig053579-NyeOR.E054	contig047826-TilOR.E080	0.224	0.530	0.422
contig053576-NyeOR.E052	contig025439-ZebOR.E045	0.224	0.530	0.422
contig052450-BurOR.E055	contig053579-NyeOR.E054	0.224	0.644	0.348
contig052450-BurOR.E055	contig048260-ZebOR.E051	0.224	0.657	0.340
contig004255-BriOR.E036	contig023280-NyeOR.E050	0.225	0.435	0.518
contig004255-BriOR.E036	contig017699-BurOR.E042	0.225	0.436	0.517
contig049289-BurOR.E044	contig047834-TilOR.E084	0.225	0.448	0.502
contig052452-BurOR.E048	contig047829-TilOR.E081	0.225	0.455	0.495
contig004266-BriOR.E039	contig064938-BurOR.E053	0.225	0.497	0.453
contig047820-TilOR.E078	contig048239-ZebOR.E048	0.225	0.564	0.400
contig004265-BriOR.E035	contig064724-BurOR.E052	0.225	0.609	0.369
contig004261-BriOR.E034	contig004266-BriOR.E039	0.225	0.614	0.367
contig004255-BriOR.E036	contig053579-NyeOR.E055	0.225	0.617	0.364
contig004265-BriOR.E035	contig048263-ZebOR.E052	0.225	0.628	0.358
contig004265-BriOR.E035	contig047833-TilOR.E083	0.225	0.655	0.343
contig064724-BurOR.E052	contig064938-BurOR.E053	0.226	0.494	0.457
contig064938-BurOR.E053	contig053576-NyeOR.E052	0.226	0.494	0.458
contig052454-BurOR.E050	contig048263-ZebOR.E052	0.226	0.539	0.419
contig047826-TilOR.E080	contig048260-ZebOR.E051	0.226	0.552	0.411
contig052454-BurOR.E050	contig047826-TilOR.E080	0.226	0.577	0.392

contig064724-BurOR.E052	contig065458-TilOR.E086	0.226	0.581	0.390
contig052450-BurOR.E055	contig047829-TilOR.E081	0.226	0.628	0.360
contig047820-TilOR.E078	contig047832-TilOR.E082	0.227	0.468	0.485
contig052452-BurOR.E048	contig047820-TilOR.E078	0.227	0.513	0.444
contig062770-NyeOR.E059	contig047820-TilOR.E078	0.227	0.548	0.415
contig023280-NyeOR.E050	contig065458-TilOR.E086	0.227	0.600	0.378
contig017699-BurOR.E042	contig065458-TilOR.E086	0.227	0.605	0.375
contig052450-BurOR.E055	contig047729-TilOR.E076	0.227	0.626	0.362
contig047820-TilOR.E078	contig047833-TilOR.E083	0.228	0.483	0.473
contig049298-BurOR.E045	contig047820-TilOR.E078	0.228	0.522	0.437
contig025439-ZebOR.E045	contig048263-ZebOR.E052	0.228	0.532	0.428
contig004255-BriOR.E036	contig048263-ZebOR.E052	0.228	0.533	0.427
contig004258-BriOR.E037	contig047820-TilOR.E078	0.228	0.550	0.415
contig004255-BriOR.E036	contig047826-TilOR.E080	0.228	0.555	0.411
contig047826-TilOR.E080	contig025439-ZebOR.E045	0.228	0.587	0.389
contig047826-TilOR.E080	contig065454-TilOR.E089	0.228	0.630	0.361
contig004266-BriOR.E039	contig052450-BurOR.E055	0.228	0.631	0.361
contig004265-BriOR.E035	contig049298-BurOR.E045	0.228	0.632	0.360
contig047833-TilOR.E083	contig065454-TilOR.E089	0.228	0.648	0.352
contig004261-BriOR.E034	contig048260-ZebOR.E051	0.228	0.654	0.349
contig065454-TilOR.E088	contig048260-ZebOR.E051	0.228	0.656	0.347
contig004265-BriOR.E035	contig064938-BurOR.E053	0.228	0.656	0.348
contig065453-TilOR.E087	contig025443-ZebOR.E046	0.228	0.720	0.317
contig047725-TilOR.E074	contig047834-TilOR.E084	0.229	0.410	0.558
contig047820-TilOR.E078	contig065458-TilOR.E086	0.229	0.490	0.468
contig004265-BriOR.E035	contig053576-NyeOR.E052	0.229	0.608	0.376
contig065453-TilOR.E087	contig048263-ZebOR.E052	0.229	0.619	0.371
contig004266-BriOR.E039	contig065454-TilOR.E089	0.229	0.623	0.368
contig004258-BriOR.E037	contig064724-BurOR.E052	0.229	0.630	0.364
contig053579-NyeOR.E054	contig065454-TilOR.E088	0.229	0.638	0.359
contig004261-BriOR.E034	contig053579-NyeOR.E054	0.229	0.660	0.346
contig004261-BriOR.E034	contig047729-TilOR.E076	0.229	0.682	0.336
contig047834-TilOR.E084	contig048243-ZebOR.E050	0.230	0.451	0.510
contig004266-BriOR.E039	contig052453-BurOR.E049	0.230	0.483	0.476
contig064938-BurOR.E053	contig047826-TilOR.E080	0.230	0.487	0.471
contig064938-BurOR.E053	contig048263-ZebOR.E052	0.230	0.495	0.465
contig065458-TilOR.E086	contig048263-ZebOR.E052	0.230	0.589	0.390
contig004265-BriOR.E035	contig047829-TilOR.E081	0.230	0.593	0.387
contig053576-NyeOR.E052	contig065458-TilOR.E086	0.230	0.596	0.386
contig064724-BurOR.E052	contig065454-TilOR.E089	0.230	0.603	0.382
contig064724-BurOR.E052	contig065453-TilOR.E087	0.230	0.618	0.371
contig065454-TilOR.E089	contig048263-ZebOR.E052	0.230	0.622	0.370
contig064938-BurOR.E053	contig065453-TilOR.E087	0.230	0.623	0.369

contig004266-BriOR.E039	contig065454-TilOR.E088	0.230	0.628	0.367
contig052457-BurOR.E051	contig047820-TilOR.E078	0.231	0.564	0.411
contig052452-BurOR.E048	contig047826-TilOR.E080	0.231	0.618	0.374
contig064938-BurOR.E053	contig065454-TilOR.E088	0.231	0.627	0.368
contig047729-TilOR.E076	contig065454-TilOR.E088	0.231	0.644	0.359
contig004258-BriOR.E037	contig047826-TilOR.E080	0.231	0.648	0.356
contig004261-BriOR.E034	contig064938-BurOR.E053	0.231	0.654	0.353
contig049298-BurOR.E045	contig065454-TilOR.E089	0.231	0.655	0.353
contig004265-BriOR.E035	contig025439-ZebOR.E045	0.231	0.810	0.285
contig004265-BriOR.E035	contig052454-BurOR.E050	0.231	0.820	0.282
contig052453-BurOR.E049	contig025447-ZebOR.E047	0.232	0.476	0.487
contig053579-NyeOR.E054	contig047820-TilOR.E078	0.232	0.485	0.479
contig052453-BurOR.E049	contig047820-TilOR.E078	0.232	0.510	0.456
contig053579-NyeOR.E053	contig047826-TilOR.E080	0.232	0.558	0.415
contig053572-NyeOR.E051	contig048239-ZebOR.E048	0.232	0.612	0.379
contig053579-NyeOR.E055	contig065453-TilOR.E087	0.232	0.696	0.334
contig004258-BriOR.E037	contig065453-TilOR.E087	0.232	0.709	0.327
contig004265-BriOR.E035	contig048239-ZebOR.E048	0.232	0.718	0.323
contig064938-BurOR.E053	contig025447-ZebOR.E047	0.233	0.475	0.491
contig017699-BurOR.E042	contig064938-BurOR.E053	0.233	0.488	0.479
contig049299-BurOR.E046	contig053572-NyeOR.E051	0.233	0.526	0.443
contig049299-BurOR.E046	contig047826-TilOR.E080	0.233	0.540	0.432
contig066194-BurOR.E054	contig048239-ZebOR.E048	0.233	0.602	0.387
contig004265-BriOR.E035	contig049299-BurOR.E046	0.233	0.611	0.382
contig004258-BriOR.E037	contig048263-ZebOR.E052	0.233	0.639	0.365
contig004265-BriOR.E035	contig053579-NyeOR.E054	0.233	0.645	0.362
contig004258-BriOR.E037	contig053576-NyeOR.E052	0.233	0.647	0.361
contig004265-BriOR.E035	contig062770-NyeOR.E059	0.233	0.735	0.317
contig053590-NyeOR.E056	contig047834-TilOR.E084	0.234	0.450	0.519
contig053579-NyeOR.E055	contig047820-TilOR.E078	0.234	0.453	0.516
contig049298-BurOR.E045	contig053572-NyeOR.E051	0.234	0.528	0.443
contig053572-NyeOR.E051	contig062770-NyeOR.E059	0.234	0.587	0.399
contig064938-BurOR.E053	contig052450-BurOR.E055	0.234	0.595	0.393
contig053576-NyeOR.E052	contig065453-TilOR.E087	0.234	0.599	0.390
contig053576-NyeOR.E052	contig065454-TilOR.E089	0.234	0.602	0.389
contig004265-BriOR.E035	contig048260-ZebOR.E051	0.234	0.634	0.369
contig052450-BurOR.E055	contig053579-NyeOR.E055	0.234	0.639	0.366
contig065455-TilOR.E085	contig065453-TilOR.E087	0.234	0.642	0.365
contig004265-BriOR.E035	contig047729-TilOR.E076	0.234	0.667	0.352
contig004265-BriOR.E035	contig052453-BurOR.E049	0.234	0.684	0.341
contig052452-BurOR.E048	contig065453-TilOR.E087	0.234	0.720	0.325
contig065453-TilOR.E087	contig048239-ZebOR.E048	0.234	0.731	0.320
contig004265-BriOR.E035	contig004255-BriOR.E036	0.234	0.775	0.302

contig049299-BurOR.E046	contig047820-TilOR.E078	0.235	0.476	0.493
contig059404-NyeOR.E058	contig047820-TilOR.E078	0.235	0.501	0.469
contig066194-BurOR.E054	contig062770-NyeOR.E059	0.235	0.577	0.407
contig052452-BurOR.E048	contig064724-BurOR.E052	0.235	0.592	0.397
contig052452-BurOR.E048	contig048263-ZebOR.E052	0.235	0.601	0.391
contig004265-BriOR.E035	contig053579-NyeOR.E053	0.235	0.601	0.392
contig082838-BriOR.E040	contig059404-NyeOR.E058	0.235	0.609	0.386
contig052452-BurOR.E048	contig053576-NyeOR.E052	0.235	0.609	0.387
contig047826-TilOR.E080	contig065453-TilOR.E087	0.235	0.619	0.379
contig053572-NyeOR.E051	contig047729-TilOR.E076	0.235	0.621	0.379
contig062770-NyeOR.E059	contig065453-TilOR.E087	0.235	0.727	0.323
contig004266-BriOR.E039	contig059404-NyeOR.E058	0.236	0.478	0.493
contig049298-BurOR.E045	contig066194-BurOR.E054	0.236	0.512	0.462
contig047825-TilOR.E079	contig047833-TilOR.E083	0.236	0.542	0.436
contig082838-BriOR.E040	contig052453-BurOR.E049	0.236	0.592	0.399
contig064724-BurOR.E052	contig025443-ZebOR.E046	0.236	0.596	0.396
contig025443-ZebOR.E046	contig048263-ZebOR.E052	0.236	0.605	0.391
contig066194-BurOR.E054	contig047729-TilOR.E076	0.236	0.611	0.387
contig004261-BriOR.E034	contig064724-BurOR.E052	0.236	0.621	0.381
contig004265-BriOR.E035	contig053579-NyeOR.E055	0.236	0.627	0.376
contig004261-BriOR.E034	contig048263-ZebOR.E052	0.236	0.639	0.370
contig004265-BriOR.E035	contig059404-NyeOR.E058	0.236	0.703	0.336
contig004261-BriOR.E034	contig025439-ZebOR.E045	0.236	0.825	0.286
contig064938-BurOR.E053	contig023280-NyeOR.E050	0.237	0.468	0.507
contig053579-NyeOR.E053	contig047820-TilOR.E078	0.237	0.483	0.491
contig047820-TilOR.E078	contig048260-ZebOR.E051	0.237	0.507	0.467
contig049299-BurOR.E046	contig066194-BurOR.E054	0.237	0.512	0.464
contig053572-NyeOR.E051	contig047833-TilOR.E083	0.237	0.539	0.440
contig049298-BurOR.E045	contig047825-TilOR.E079	0.237	0.539	0.440
contig053572-NyeOR.E051	contig053579-NyeOR.E053	0.237	0.544	0.435
contig053572-NyeOR.E051	contig025439-ZebOR.E045	0.237	0.565	0.420
contig047825-TilOR.E079	contig048239-ZebOR.E048	0.237	0.602	0.393
contig053579-NyeOR.E054	contig065454-TilOR.E089	0.237	0.629	0.377
contig065454-TilOR.E089	contig048260-ZebOR.E051	0.237	0.641	0.369
contig004261-BriOR.E034	contig047826-TilOR.E080	0.237	0.642	0.369
contig004265-BriOR.E035	contig052457-BurOR.E051	0.237	0.723	0.327
contig004261-BriOR.E034	contig065455-TilOR.E085	0.237	0.769	0.308
contig004265-BriOR.E035	contig065455-TilOR.E085	0.237	0.783	0.302
contig052453-BurOR.E049	contig023280-NyeOR.E050	0.238	0.469	0.508
contig059404-NyeOR.E058	contig025447-ZebOR.E047	0.238	0.471	0.505
contig053572-NyeOR.E051	contig047832-TilOR.E082	0.238	0.533	0.447
contig047825-TilOR.E079	contig047832-TilOR.E082	0.238	0.561	0.424
contig062770-NyeOR.E059	contig047825-TilOR.E079	0.238	0.580	0.411

contig047826-TilOR.E080	contig025443-ZebOR.E046	0.238	0.613	0.388
contig064938-BurOR.E053	contig065454-TilOR.E089	0.238	0.633	0.376
contig049299-BurOR.E046	contig065453-TilOR.E087	0.238	0.649	0.366
contig004261-BriOR.E034	contig053579-NyeOR.E055	0.238	0.654	0.364
contig004261-BriOR.E034	contig048239-ZebOR.E048	0.238	0.749	0.317
contig017699-BurOR.E042	contig052453-BurOR.E049	0.239	0.489	0.488
contig066194-BurOR.E054	contig047833-TilOR.E083	0.239	0.522	0.458
contig066194-BurOR.E054	contig025439-ZebOR.E045	0.239	0.572	0.419
contig017699-BurOR.E042	contig047820-TilOR.E078	0.239	0.607	0.395
contig052457-BurOR.E051	contig053572-NyeOR.E051	0.239	0.611	0.391
contig052450-BurOR.E055	contig048263-ZebOR.E052	0.239	0.618	0.387
contig004261-BriOR.E034	contig049299-BurOR.E046	0.239	0.630	0.380
contig053579-NyeOR.E055	contig065454-TilOR.E088	0.239	0.633	0.378
contig052453-BurOR.E049	contig065453-TilOR.E087	0.239	0.633	0.378
contig047826-TilOR.E080	contig065454-TilOR.E088	0.239	0.643	0.373
contig052457-BurOR.E051	contig065453-TilOR.E087	0.239	0.737	0.324
contig004261-BriOR.E034	contig062770-NyeOR.E059	0.239	0.745	0.320
contig004261-BriOR.E034	contig004255-BriOR.E036	0.239	0.789	0.303
contig052450-BurOR.E055	contig025439-ZebOR.E045	0.239	0.871	0.275
contig065454-TilOR.E088	contig025439-ZebOR.E045	0.239	0.873	0.274
contig064938-BurOR.E053	contig047820-TilOR.E078	0.240	0.462	0.520
contig066194-BurOR.E054	contig047829-TilOR.E081	0.240	0.534	0.450
contig047829-TilOR.E081	contig065454-TilOR.E089	0.240	0.551	0.436
contig004266-BriOR.E039	contig047820-TilOR.E078	0.240	0.569	0.421
contig023280-NyeOR.E050	contig047820-TilOR.E078	0.240	0.588	0.408
contig047820-TilOR.E078	contig025447-ZebOR.E047	0.240	0.588	0.409
contig082838-BriOR.E040	contig047734-TilOR.E077	0.240	0.590	0.407
contig064724-BurOR.E052	contig052450-BurOR.E055	0.240	0.600	0.399
contig052457-BurOR.E051	contig066194-BurOR.E054	0.240	0.601	0.399
contig064724-BurOR.E052	contig065454-TilOR.E088	0.240	0.607	0.395
contig049299-BurOR.E046	contig065454-TilOR.E089	0.240	0.609	0.394
contig053576-NyeOR.E052	contig025443-ZebOR.E046	0.240	0.612	0.393
contig052450-BurOR.E055	contig053576-NyeOR.E052	0.240	0.617	0.389
contig065454-TilOR.E088	contig048263-ZebOR.E052	0.240	0.626	0.383
contig052453-BurOR.E049	contig065454-TilOR.E089	0.240	0.637	0.377
contig047729-TilOR.E076	contig065454-TilOR.E089	0.240	0.650	0.369
contig052450-BurOR.E055	contig048239-ZebOR.E048	0.240	0.669	0.358
contig004261-BriOR.E034	contig052453-BurOR.E049	0.240	0.684	0.351
contig065454-TilOR.E088	contig048239-ZebOR.E048	0.240	0.718	0.334
contig004261-BriOR.E034	contig052454-BurOR.E050	0.240	0.835	0.288
contig066194-BurOR.E054	contig047832-TilOR.E082	0.241	0.516	0.466
contig066194-BurOR.E054	contig053579-NyeOR.E053	0.241	0.529	0.455
contig053572-NyeOR.E051	contig047829-TilOR.E081	0.241	0.542	0.445

contig052454-BurOR.E050	contig053572-NyeOR.E051	0.241	0.555	0.435
contig053579-NyeOR.E055	contig065454-TilOR.E089	0.241	0.605	0.398
contig004261-BriOR.E034	contig053576-NyeOR.E052	0.241	0.619	0.388
contig052450-BurOR.E055	contig047826-TilOR.E080	0.241	0.635	0.380
contig052457-BurOR.E051	contig052450-BurOR.E055	0.241	0.674	0.357
contig052450-BurOR.E055	contig062770-NyeOR.E059	0.241	0.685	0.351
contig062770-NyeOR.E059	contig065454-TilOR.E088	0.241	0.714	0.337
contig053572-NyeOR.E051	contig048260-ZebOR.E051	0.242	0.506	0.479
contig049299-BurOR.E046	contig047825-TilOR.E079	0.242	0.525	0.460
contig047825-TilOR.E079	contig025439-ZebOR.E045	0.242	0.581	0.416
contig053579-NyeOR.E053	contig065454-TilOR.E089	0.242	0.608	0.398
contig049299-BurOR.E046	contig065454-TilOR.E088	0.242	0.627	0.387
contig053579-NyeOR.E053	contig065453-TilOR.E087	0.242	0.648	0.373
contig059404-NyeOR.E058	contig065454-TilOR.E089	0.242	0.656	0.369
contig082838-BriOR.E040	contig048239-ZebOR.E048	0.242	0.683	0.354
contig065455-TilOR.E085	contig065454-TilOR.E088	0.242	0.689	0.352
contig052457-BurOR.E051	contig047825-TilOR.E079	0.243	0.604	0.402
contig082838-BriOR.E040	contig062770-NyeOR.E059	0.243	0.659	0.368
contig004261-BriOR.E034	contig059404-NyeOR.E058	0.243	0.703	0.345
contig065454-TilOR.E089	contig048239-ZebOR.E048	0.243	0.705	0.344
contig052450-BurOR.E055	contig065455-TilOR.E085	0.243	0.719	0.338
contig004261-BriOR.E034	contig052457-BurOR.E051	0.243	0.755	0.322
contig004255-BriOR.E036	contig065454-TilOR.E088	0.243	0.837	0.290
contig004255-BriOR.E036	contig052450-BurOR.E055	0.243	0.852	0.285
contig052454-BurOR.E050	contig052450-BurOR.E055	0.243	0.857	0.284
contig052454-BurOR.E050	contig065454-TilOR.E088	0.243	0.858	0.283
contig023280-NyeOR.E050	contig059404-NyeOR.E058	0.244	0.464	0.526
contig017699-BurOR.E042	contig059404-NyeOR.E058	0.244	0.484	0.505
contig066194-BurOR.E054	contig048260-ZebOR.E051	0.244	0.490	0.499
contig052454-BurOR.E050	contig066194-BurOR.E054	0.244	0.563	0.433
contig053576-NyeOR.E052	contig065454-TilOR.E088	0.244	0.606	0.402
contig052453-BurOR.E049	contig065454-TilOR.E088	0.244	0.637	0.384
contig004261-BriOR.E034	contig053579-NyeOR.E053	0.244	0.638	0.382
contig059404-NyeOR.E058	contig065453-TilOR.E087	0.244	0.651	0.375
contig062770-NyeOR.E059	contig065454-TilOR.E089	0.244	0.701	0.347
contig052453-BurOR.E049	contig064724-BurOR.E052	0.245	0.511	0.479
contig053572-NyeOR.E051	contig053579-NyeOR.E054	0.245	0.518	0.473
contig053579-NyeOR.E053	contig047825-TilOR.E079	0.245	0.543	0.452
contig004255-BriOR.E036	contig053572-NyeOR.E051	0.245	0.553	0.443
contig065458-TilOR.E086	contig065453-TilOR.E087	0.245	0.691	0.355
contig052457-BurOR.E051	contig065454-TilOR.E088	0.245	0.723	0.339
contig047825-TilOR.E079	contig048260-ZebOR.E051	0.246	0.524	0.468
contig047825-TilOR.E079	contig047829-TilOR.E081	0.246	0.556	0.443

contig052454-BurOR.E050	contig047825-TilOR.E079	0.246	0.572	0.430
contig052450-BurOR.E055	contig059404-NyeOR.E058	0.246	0.640	0.384
contig004259-BriOR.E038	contig065453-TilOR.E087	0.246	0.682	0.362
contig065453-TilOR.E087	contig063018-ZebOR.E053	0.246	0.704	0.349
contig066194-BurOR.E054	contig053579-NyeOR.E054	0.247	0.502	0.493
contig004255-BriOR.E036	contig066194-BurOR.E054	0.247	0.560	0.441
contig053579-NyeOR.E053	contig065454-TilOR.E088	0.247	0.626	0.394
contig059404-NyeOR.E058	contig065454-TilOR.E088	0.247	0.656	0.376
contig082838-BriOR.E040	contig052457-BurOR.E051	0.247	0.688	0.359
contig004265-BriOR.E035	contig047820-TilOR.E078	0.247	0.712	0.347
contig052454-BurOR.E050	contig065454-TilOR.E089	0.247	0.848	0.291
contig065454-TilOR.E089	contig025439-ZebOR.E045	0.247	0.863	0.286
contig053579-NyeOR.E054	contig047825-TilOR.E079	0.248	0.521	0.477
contig052453-BurOR.E049	contig052450-BurOR.E055	0.248	0.623	0.397
contig047729-TilOR.E076	contig047825-TilOR.E079	0.248	0.630	0.394
contig049299-BurOR.E046	contig052450-BurOR.E055	0.248	0.656	0.378
contig004266-BriOR.E039	contig082838-BriOR.E040	0.248	0.680	0.364
contig052457-BurOR.E051	contig065454-TilOR.E089	0.248	0.710	0.349
contig052453-BurOR.E049	contig053576-NyeOR.E052	0.249	0.511	0.488
contig053572-NyeOR.E051	contig053579-NyeOR.E055	0.249	0.531	0.469
contig004255-BriOR.E036	contig047825-TilOR.E079	0.249	0.569	0.438
contig052450-BurOR.E055	contig053579-NyeOR.E053	0.249	0.655	0.379
contig052451-BurOR.E047	contig065453-TilOR.E087	0.249	0.724	0.343
contig082838-BriOR.E040	contig047729-TilOR.E076	0.249	0.736	0.338
contig047734-TilOR.E077	contig047825-TilOR.E079	0.250	0.526	0.476
contig065455-TilOR.E085	contig065454-TilOR.E089	0.250	0.634	0.394
contig004265-BriOR.E035	contig004259-BriOR.E038	0.250	0.789	0.316
contig004261-BriOR.E034	contig004259-BriOR.E038	0.250	0.798	0.313
contig052453-BurOR.E049	contig047826-TilOR.E080	0.251	0.505	0.497
contig064724-BurOR.E052	contig059404-NyeOR.E058	0.251	0.506	0.495
contig053576-NyeOR.E052	contig059404-NyeOR.E058	0.251	0.506	0.496
contig066194-BurOR.E054	contig053579-NyeOR.E055	0.251	0.514	0.489
contig082838-BriOR.E040	contig025447-ZebOR.E047	0.251	0.738	0.340
contig047734-TilOR.E077	contig065455-TilOR.E085	0.251	0.767	0.328
contig004255-BriOR.E036	contig065454-TilOR.E089	0.251	0.827	0.304
contig004258-BriOR.E037	contig066194-BurOR.E054	0.252	0.524	0.481
contig004258-BriOR.E037	contig053572-NyeOR.E051	0.252	0.538	0.469
contig082838-BriOR.E040	contig052454-BurOR.E050	0.252	0.690	0.365
contig004265-BriOR.E035	contig052451-BurOR.E047	0.252	0.815	0.309
contig004261-BriOR.E034	contig052451-BurOR.E047	0.252	0.849	0.297
contig052453-BurOR.E049	contig048263-ZebOR.E052	0.253	0.497	0.509
contig053579-NyeOR.E055	contig047825-TilOR.E079	0.253	0.535	0.473
contig065455-TilOR.E085	contig025443-ZebOR.E046	0.253	0.694	0.365

contig004266-BriOR.E039	contig053572-NyeOR.E051	0.254	0.487	0.522
contig059404-NyeOR.E058	contig047826-TilOR.E080	0.254	0.507	0.501
contig053572-NyeOR.E051	contig025443-ZebOR.E046	0.254	0.516	0.493
contig053572-NyeOR.E051	contig047734-TilOR.E077	0.254	0.527	0.483
contig047820-TilOR.E078	contig047825-TilOR.E079	0.254	0.547	0.465
contig053572-NyeOR.E051	contig047820-TilOR.E078	0.254	0.554	0.459
contig004265-BriOR.E035	contig063018-ZebOR.E053	0.254	0.788	0.323
contig047825-TilOR.E079	contig025447-ZebOR.E047	0.255	0.492	0.519
contig047729-TilOR.E076	contig047820-TilOR.E078	0.255	0.556	0.458
contig047825-TilOR.E079	contig065453-TilOR.E087	0.255	0.650	0.392
contig004255-BriOR.E036	contig082838-BriOR.E040	0.255	0.669	0.381
contig004261-BriOR.E034	contig063018-ZebOR.E053	0.255	0.821	0.310
contig047833-TilOR.E083	contig063018-ZebOR.E053	0.255	0.902	0.282
contig004266-BriOR.E039	contig066194-BurOR.E054	0.256	0.486	0.527
contig053572-NyeOR.E051	contig025447-ZebOR.E047	0.256	0.487	0.526
contig066194-BurOR.E054	contig025443-ZebOR.E046	0.256	0.510	0.501
contig064724-BurOR.E052	contig047820-TilOR.E078	0.256	0.534	0.479
contig082838-BriOR.E040	contig064938-BurOR.E053	0.256	0.566	0.453
contig082838-BriOR.E040	contig025439-ZebOR.E045	0.256	0.700	0.366
contig047820-TilOR.E078	contig065453-TilOR.E087	0.256	0.743	0.345
contig004259-BriOR.E038	contig052450-BurOR.E055	0.256	0.745	0.344
contig047833-TilOR.E083	contig065455-TilOR.E085	0.256	0.812	0.315
contig066194-BurOR.E054	contig047734-TilOR.E077	0.257	0.510	0.503
contig052452-BurOR.E048	contig053572-NyeOR.E051	0.257	0.520	0.494
contig066194-BurOR.E054	contig047820-TilOR.E078	0.257	0.544	0.471
contig052450-BurOR.E055	contig047825-TilOR.E079	0.257	0.647	0.398
contig047832-TilOR.E082	contig065455-TilOR.E085	0.257	0.689	0.373
contig065455-TilOR.E085	contig065458-TilOR.E086	0.257	0.691	0.372
contig047820-TilOR.E078	contig065454-TilOR.E089	0.257	0.703	0.366
contig082838-BriOR.E040	contig017699-BurOR.E042	0.257	0.746	0.345
contig066194-BurOR.E054	contig025447-ZebOR.E047	0.258	0.486	0.532
contig052452-BurOR.E048	contig066194-BurOR.E054	0.258	0.514	0.502
contig082838-BriOR.E040	contig047832-TilOR.E082	0.258	0.604	0.427
contig004259-BriOR.E038	contig065454-TilOR.E088	0.258	0.726	0.355
contig052451-BurOR.E047	contig052450-BurOR.E055	0.258	0.792	0.326
contig059404-NyeOR.E058	contig048263-ZebOR.E052	0.259	0.492	0.526
contig023280-NyeOR.E050	contig047825-TilOR.E079	0.259	0.492	0.527
contig053572-NyeOR.E051	contig065453-TilOR.E087	0.259	0.660	0.392
contig052452-BurOR.E048	contig065455-TilOR.E085	0.259	0.705	0.368
contig004258-BriOR.E037	contig065455-TilOR.E085	0.259	0.728	0.356
contig023280-NyeOR.E050	contig053572-NyeOR.E051	0.260	0.487	0.534
contig017699-BurOR.E042	contig047825-TilOR.E079	0.260	0.497	0.522
contig004266-BriOR.E039	contig047825-TilOR.E079	0.260	0.497	0.523

contig053576-NyeOR.E052	contig047820-TilOR.E078	0.260	0.533	0.488
contig082838-BriOR.E040	contig047833-TilOR.E083	0.260	0.629	0.413
contig053579-NyeOR.E054	contig065455-TilOR.E085	0.260	0.727	0.357
contig082838-BriOR.E040	contig023280-NyeOR.E050	0.260	0.757	0.344
contig052451-BurOR.E047	contig065454-TilOR.E088	0.260	0.771	0.337
contig053579-NyeOR.E054	contig063018-ZebOR.E053	0.260	0.828	0.313
contig017699-BurOR.E042	contig053572-NyeOR.E051	0.261	0.477	0.546
contig066194-BurOR.E054	contig065458-TilOR.E086	0.261	0.490	0.533
contig053572-NyeOR.E051	contig065458-TilOR.E086	0.261	0.496	0.526
contig047820-TilOR.E078	contig047826-TilOR.E080	0.261	0.496	0.527
contig066194-BurOR.E054	contig065453-TilOR.E087	0.261	0.649	0.402
contig047820-TilOR.E078	contig065454-TilOR.E088	0.261	0.654	0.400
contig082838-BriOR.E040	contig049299-BurOR.E046	0.261	0.683	0.383
contig082838-BriOR.E040	contig053579-NyeOR.E053	0.261	0.697	0.374
contig052450-BurOR.E055	contig063018-ZebOR.E053	0.261	0.765	0.341
contig052451-BurOR.E047	contig047833-TilOR.E083	0.261	0.869	0.300
contig082838-BriOR.E040	contig053579-NyeOR.E055	0.262	0.615	0.426
contig082838-BriOR.E040	contig053579-NyeOR.E054	0.262	0.625	0.419
contig052450-BurOR.E055	contig053572-NyeOR.E051	0.262	0.626	0.418
contig082838-BriOR.E040	contig047829-TilOR.E081	0.262	0.632	0.415
contig049287-BurOR.E043	contig048239-ZebOR.E048	0.262	0.729	0.360
contig065454-TilOR.E088	contig063018-ZebOR.E053	0.262	0.745	0.352
contig047832-TilOR.E082	contig063018-ZebOR.E053	0.262	0.774	0.339
contig049298-BurOR.E045	contig065455-TilOR.E085	0.262	0.778	0.337
contig066194-BurOR.E054	contig023280-NyeOR.E050	0.263	0.486	0.540
contig017699-BurOR.E042	contig066194-BurOR.E054	0.263	0.491	0.535
contig066194-BurOR.E054	contig052450-BurOR.E055	0.263	0.618	0.426
contig052453-BurOR.E049	contig065455-TilOR.E085	0.263	0.678	0.387
contig049287-BurOR.E043	contig062770-NyeOR.E059	0.263	0.704	0.374
contig004258-BriOR.E037	contig063018-ZebOR.E053	0.263	0.748	0.351
contig047820-TilOR.E078	contig048263-ZebOR.E052	0.264	0.534	0.494
contig082838-BriOR.E040	contig048260-ZebOR.E051	0.264	0.642	0.412
contig082838-BriOR.E040	contig049298-BurOR.E045	0.264	0.651	0.406
contig047825-TilOR.E079	contig065454-TilOR.E088	0.264	0.662	0.399
contig004261-BriOR.E034	contig047820-TilOR.E078	0.264	0.699	0.378
contig049287-BurOR.E043	contig047833-TilOR.E083	0.264	0.734	0.359
contig047734-TilOR.E077	contig063018-ZebOR.E053	0.264	0.859	0.308
contig064938-BurOR.E053	contig047825-TilOR.E079	0.265	0.482	0.549
contig004258-BriOR.E037	contig047825-TilOR.E079	0.265	0.568	0.467
contig053572-NyeOR.E051	contig065454-TilOR.E088	0.265	0.640	0.413
contig004259-BriOR.E038	contig065454-TilOR.E089	0.265	0.669	0.397
contig052450-BurOR.E055	contig047820-TilOR.E078	0.265	0.708	0.374
contig052451-BurOR.E047	contig053579-NyeOR.E054	0.265	0.788	0.337

contig004259-BriOR.E038	contig047734-TilOR.E077	0.265	0.815	0.326
contig052451-BurOR.E047	contig047734-TilOR.E077	0.265	0.843	0.315
contig004259-BriOR.E038	contig047833-TilOR.E083	0.265	0.890	0.298
contig004261-BriOR.E034	contig053572-NyeOR.E051	0.266	0.633	0.420
contig066194-BurOR.E054	contig065454-TilOR.E088	0.266	0.633	0.421
contig023280-NyeOR.E050	contig065455-TilOR.E085	0.266	0.653	0.408
contig004261-BriOR.E034	contig047825-TilOR.E079	0.266	0.655	0.406
contig059404-NyeOR.E058	contig065455-TilOR.E085	0.266	0.692	0.385
contig048239-ZebOR.E048	contig048242-ZebOR.E049	0.266	0.711	0.375
contig049287-BurOR.E043	contig049298-BurOR.E045	0.266	0.734	0.363
contig052451-BurOR.E047	contig047832-TilOR.E082	0.266	0.736	0.361
contig004258-BriOR.E037	contig052451-BurOR.E047	0.266	0.756	0.352
contig049298-BurOR.E045	contig063018-ZebOR.E053	0.266	0.850	0.313
contig047825-TilOR.E079	contig025443-ZebOR.E046	0.267	0.537	0.498
contig052451-BurOR.E047	contig065454-TilOR.E089	0.267	0.690	0.388
contig062770-NyeOR.E059	contig048242-ZebOR.E049	0.267	0.707	0.378
contig064724-BurOR.E052	contig065455-TilOR.E085	0.267	0.726	0.368
contig049287-BurOR.E043	contig053579-NyeOR.E054	0.267	0.745	0.358
contig004259-BriOR.E038	contig047832-TilOR.E082	0.267	0.751	0.356
contig052451-BurOR.E047	contig052452-BurOR.E048	0.267	0.762	0.350
contig053579-NyeOR.E055	contig065455-TilOR.E085	0.267	0.769	0.348
contig053579-NyeOR.E055	contig063018-ZebOR.E053	0.267	0.878	0.304
contig064938-BurOR.E053	contig053572-NyeOR.E051	0.268	0.507	0.529
contig004261-BriOR.E034	contig066194-BurOR.E054	0.268	0.626	0.428
contig049287-BurOR.E043	contig052457-BurOR.E051	0.268	0.735	0.364
contig047833-TilOR.E083	contig048242-ZebOR.E049	0.268	0.736	0.364
contig052452-BurOR.E048	contig047825-TilOR.E079	0.269	0.554	0.485
contig004266-BriOR.E039	contig065455-TilOR.E085	0.269	0.646	0.417
contig047726-TilOR.E075	contig048239-ZebOR.E048	0.269	0.719	0.374
contig004266-BriOR.E039	contig063018-ZebOR.E053	0.269	0.735	0.366
contig004258-BriOR.E037	contig004259-BriOR.E038	0.269	0.758	0.356
contig052451-BurOR.E047	contig025443-ZebOR.E046	0.269	0.775	0.347
contig064938-BurOR.E053	contig066194-BurOR.E054	0.270	0.491	0.551
contig064724-BurOR.E052	contig053572-NyeOR.E051	0.270	0.628	0.431
contig065454-TilOR.E089	contig063018-ZebOR.E053	0.270	0.665	0.406
contig047726-TilOR.E075	contig047832-TilOR.E082	0.270	0.678	0.398
contig062770-NyeOR.E059	contig047726-TilOR.E075	0.270	0.694	0.388
contig047726-TilOR.E075	contig047833-TilOR.E083	0.270	0.703	0.384
contig064938-BurOR.E053	contig065455-TilOR.E085	0.270	0.712	0.380
contig049287-BurOR.E043	contig053579-NyeOR.E055	0.270	0.720	0.375
contig025447-ZebOR.E047	contig063018-ZebOR.E053	0.270	0.733	0.368
contig004259-BriOR.E038	contig053579-NyeOR.E054	0.270	0.806	0.335
contig065455-TilOR.E085	contig025447-ZebOR.E047	0.271	0.666	0.407

contig017699-BurOR.E042	contig065455-TilOR.E085	0.271	0.678	0.399
contig049298-BurOR.E045	contig048242-ZebOR.E049	0.271	0.715	0.378
contig053592-NyeOR.E057	contig048239-ZebOR.E048	0.271	0.721	0.375
contig004259-BriOR.E038	contig052453-BurOR.E049	0.271	0.726	0.374
contig004259-BriOR.E038	contig052452-BurOR.E048	0.271	0.731	0.370
contig023280-NyeOR.E050	contig063018-ZebOR.E053	0.271	0.741	0.365
contig004259-BriOR.E038	contig059404-NyeOR.E058	0.271	0.741	0.366
contig053579-NyeOR.E054	contig048242-ZebOR.E049	0.271	0.747	0.362
contig052451-BurOR.E047	contig065458-TilOR.E086	0.271	0.749	0.361
contig052451-BurOR.E047	contig052453-BurOR.E049	0.271	0.750	0.361
contig052451-BurOR.E047	contig059404-NyeOR.E058	0.271	0.766	0.353
contig082838-BriOR.E040	contig047820-TilOR.E078	0.271	0.782	0.346
contig049287-BurOR.E043	contig047729-TilOR.E076	0.271	0.838	0.323
contig004265-BriOR.E035	contig047825-TilOR.E079	0.272	0.634	0.429
contig053592-NyeOR.E057	contig062770-NyeOR.E059	0.272	0.696	0.390
contig004265-BriOR.E035	contig082838-BriOR.E040	0.272	0.714	0.381
contig052457-BurOR.E051	contig048242-ZebOR.E049	0.272	0.716	0.379
contig053592-NyeOR.E057	contig047833-TilOR.E083	0.272	0.726	0.375
contig052452-BurOR.E048	contig063018-ZebOR.E053	0.272	0.748	0.364
contig065455-TilOR.E085	contig048260-ZebOR.E051	0.272	0.754	0.360
contig049298-BurOR.E045	contig052451-BurOR.E047	0.272	0.808	0.337
contig064724-BurOR.E052	contig066194-BurOR.E054	0.273	0.608	0.450
contig004265-BriOR.E035	contig053572-NyeOR.E051	0.273	0.622	0.438
contig053579-NyeOR.E054	contig047726-TilOR.E075	0.273	0.734	0.372
contig004259-BriOR.E038	contig025443-ZebOR.E046	0.273	0.743	0.367
contig064724-BurOR.E052	contig063018-ZebOR.E053	0.273	0.802	0.340
contig052451-BurOR.E047	contig053579-NyeOR.E055	0.273	0.833	0.327
contig047825-TilOR.E079	contig065458-TilOR.E086	0.274	0.531	0.516
contig004265-BriOR.E035	contig066194-BurOR.E054	0.274	0.615	0.446
contig064724-BurOR.E052	contig047825-TilOR.E079	0.274	0.647	0.424
contig004259-BriOR.E038	contig065458-TilOR.E086	0.274	0.709	0.387
contig053579-NyeOR.E055	contig047726-TilOR.E075	0.274	0.723	0.378
contig053579-NyeOR.E055	contig048242-ZebOR.E049	0.274	0.723	0.380
contig052457-BurOR.E051	contig047726-TilOR.E075	0.274	0.724	0.378
contig049298-BurOR.E045	contig047726-TilOR.E075	0.274	0.730	0.375
contig047726-TilOR.E075	contig048260-ZebOR.E051	0.274	0.732	0.374
contig025443-ZebOR.E046	contig063018-ZebOR.E053	0.274	0.760	0.360
contig048260-ZebOR.E051	contig063018-ZebOR.E053	0.274	0.856	0.321
contig049287-BurOR.E043	contig048260-ZebOR.E051	0.275	0.739	0.372
contig047826-TilOR.E080	contig065455-TilOR.E085	0.275	0.748	0.367
contig082838-BriOR.E040	contig065458-TilOR.E086	0.275	0.779	0.353
contig047726-TilOR.E075	contig047729-TilOR.E076	0.275	0.805	0.341
contig053592-NyeOR.E057	contig047729-TilOR.E076	0.275	0.819	0.336

contig047729-TilOR.E076	contig048242-ZebOR.E049	0.275	0.841	0.327
contig004266-BriOR.E039	contig049287-BurOR.E043	0.275	0.929	0.296
contig017699-BurOR.E042	contig053592-NyeOR.E057	0.275	0.946	0.291
contig053572-NyeOR.E051	contig047826-TilOR.E080	0.276	0.626	0.441
contig049298-BurOR.E045	contig053592-NyeOR.E057	0.276	0.720	0.383
contig004261-BriOR.E034	contig082838-BriOR.E040	0.276	0.725	0.381
contig065455-TilOR.E085	contig048263-ZebOR.E052	0.276	0.727	0.379
contig052457-BurOR.E051	contig053592-NyeOR.E057	0.276	0.727	0.380
contig053579-NyeOR.E054	contig053592-NyeOR.E057	0.276	0.731	0.377
contig053576-NyeOR.E052	contig065455-TilOR.E085	0.276	0.746	0.370
contig059404-NyeOR.E058	contig063018-ZebOR.E053	0.276	0.757	0.365
contig017699-BurOR.E042	contig063018-ZebOR.E053	0.276	0.765	0.361
contig004266-BriOR.E039	contig052451-BurOR.E047	0.277	0.680	0.407
contig052451-BurOR.E047	contig023280-NyeOR.E050	0.277	0.699	0.397
contig052453-BurOR.E049	contig063018-ZebOR.E053	0.277	0.741	0.373
contig004258-BriOR.E037	contig082838-BriOR.E040	0.277	0.784	0.353
contig052451-BurOR.E047	contig064724-BurOR.E052	0.277	0.791	0.350
contig052451-BurOR.E047	contig048260-ZebOR.E051	0.277	0.817	0.339
contig053576-NyeOR.E052	contig063018-ZebOR.E053	0.277	0.824	0.336
contig004259-BriOR.E038	contig049298-BurOR.E045	0.277	0.839	0.330
contig004266-BriOR.E039	contig047726-TilOR.E075	0.277	0.908	0.305
contig004266-BriOR.E039	contig053592-NyeOR.E057	0.277	0.919	0.302
contig065458-TilOR.E086	contig063018-ZebOR.E053	0.278	0.714	0.389
contig049287-BurOR.E043	contig047832-TilOR.E082	0.278	0.731	0.380
contig004259-BriOR.E038	contig064938-BurOR.E053	0.278	0.758	0.367
contig004259-BriOR.E038	contig064724-BurOR.E052	0.278	0.782	0.356
contig052451-BurOR.E047	contig064938-BurOR.E053	0.278	0.784	0.354
contig064938-BurOR.E053	contig063018-ZebOR.E053	0.278	0.805	0.345
contig004259-BriOR.E038	contig053579-NyeOR.E055	0.278	0.849	0.327
contig017699-BurOR.E042	contig049287-BurOR.E043	0.278	0.957	0.290
contig023280-NyeOR.E050	contig053592-NyeOR.E057	0.278	0.960	0.289
contig066194-BurOR.E054	contig047826-TilOR.E080	0.279	0.606	0.460
contig053572-NyeOR.E051	contig053576-NyeOR.E052	0.279	0.609	0.457
contig048242-ZebOR.E049	contig048260-ZebOR.E051	0.279	0.741	0.376
contig047725-TilOR.E074	contig047832-TilOR.E082	0.279	0.755	0.369
contig004266-BriOR.E039	contig048242-ZebOR.E049	0.279	0.933	0.299
contig017699-BurOR.E042	contig047726-TilOR.E075	0.279	0.951	0.293
contig049287-BurOR.E043	contig025447-ZebOR.E047	0.279	0.951	0.293
contig053572-NyeOR.E051	contig065454-TilOR.E089	0.280	0.619	0.452
contig047825-TilOR.E079	contig065454-TilOR.E089	0.280	0.641	0.436
contig053579-NyeOR.E055	contig053592-NyeOR.E057	0.280	0.707	0.396
contig052451-BurOR.E047	contig025447-ZebOR.E047	0.280	0.709	0.395
contig053592-NyeOR.E057	contig047832-TilOR.E082	0.280	0.715	0.391

contig053579-NyeOR.E054	contig047725-TilOR.E074	0.280	0.790	0.354
contig082838-BriOR.E040	contig065454-TilOR.E088	0.280	0.822	0.340
contig048239-ZebOR.E048	contig063018-ZebOR.E053	0.280	0.875	0.320
contig052457-BurOR.E051	contig063018-ZebOR.E053	0.280	0.878	0.319
contig062770-NyeOR.E059	contig063018-ZebOR.E053	0.280	0.895	0.313
contig047726-TilOR.E075	contig025447-ZebOR.E047	0.280	0.945	0.296
contig049287-BurOR.E043	contig023280-NyeOR.E050	0.280	0.971	0.289
contig066194-BurOR.E054	contig065454-TilOR.E089	0.281	0.612	0.460
contig004259-BriOR.E038	contig004266-BriOR.E039	0.281	0.687	0.410
contig047729-TilOR.E076	contig065455-TilOR.E085	0.281	0.707	0.398
contig082838-BriOR.E040	contig065453-TilOR.E087	0.281	0.792	0.355
contig048263-ZebOR.E052	contig063018-ZebOR.E053	0.281	0.802	0.351
contig052451-BurOR.E047	contig053576-NyeOR.E052	0.281	0.813	0.346
contig052451-BurOR.E047	contig053579-NyeOR.E053	0.281	0.862	0.325
contig053592-NyeOR.E057	contig025447-ZebOR.E047	0.281	0.940	0.299
contig023280-NyeOR.E050	contig047726-TilOR.E075	0.281	0.966	0.291
contig066194-BurOR.E054	contig053576-NyeOR.E052	0.282	0.590	0.478
contig047825-TilOR.E079	contig047826-TilOR.E080	0.282	0.654	0.432
contig004259-BriOR.E038	contig023280-NyeOR.E050	0.282	0.696	0.406
contig047832-TilOR.E082	contig048242-ZebOR.E049	0.282	0.723	0.390
contig017699-BurOR.E042	contig052451-BurOR.E047	0.282	0.726	0.389
contig049298-BurOR.E045	contig047725-TilOR.E074	0.282	0.757	0.372
contig082838-BriOR.E040	contig052452-BurOR.E048	0.282	0.807	0.349
contig053579-NyeOR.E053	contig065455-TilOR.E085	0.282	0.819	0.344
contig082838-BriOR.E040	contig065454-TilOR.E089	0.282	0.823	0.342
contig052451-BurOR.E047	contig047826-TilOR.E080	0.282	0.833	0.338
contig047826-TilOR.E080	contig063018-ZebOR.E053	0.282	0.833	0.339
contig017699-BurOR.E042	contig048242-ZebOR.E049	0.282	0.960	0.293
contig053572-NyeOR.E051	contig048263-ZebOR.E052	0.283	0.628	0.450
contig053576-NyeOR.E052	contig047825-TilOR.E079	0.283	0.628	0.450
contig004259-BriOR.E038	contig053576-NyeOR.E052	0.283	0.803	0.352
contig004259-BriOR.E038	contig047826-TilOR.E080	0.283	0.811	0.349
contig004259-BriOR.E038	contig048260-ZebOR.E051	0.283	0.833	0.339
contig004259-BriOR.E038	contig062770-NyeOR.E059	0.283	0.858	0.330
contig053579-NyeOR.E053	contig063018-ZebOR.E053	0.283	0.860	0.329
contig052451-BurOR.E047	contig052457-BurOR.E051	0.283	0.871	0.325
contig049299-BurOR.E046	contig052451-BurOR.E047	0.283	0.876	0.322
contig052451-BurOR.E047	contig062770-NyeOR.E059	0.283	0.888	0.318
contig025447-ZebOR.E047	contig048242-ZebOR.E049	0.283	0.955	0.297
contig004259-BriOR.E038	contig025447-ZebOR.E047	0.284	0.709	0.400
contig053592-NyeOR.E057	contig048260-ZebOR.E051	0.284	0.726	0.392
contig053579-NyeOR.E055	contig047725-TilOR.E074	0.284	0.748	0.379
contig047725-TilOR.E074	contig047833-TilOR.E083	0.284	0.808	0.352

contig049299-BurOR.E046	contig065455-TilOR.E085	0.284	0.809	0.351
contig004259-BriOR.E038	contig048239-ZebOR.E048	0.284	0.840	0.338
contig004259-BriOR.E038	contig052457-BurOR.E051	0.284	0.842	0.337
contig052451-BurOR.E047	contig048239-ZebOR.E048	0.284	0.869	0.326
contig004259-BriOR.E038	contig053579-NyeOR.E053	0.284	0.889	0.320
contig023280-NyeOR.E050	contig048242-ZebOR.E049	0.284	0.975	0.292
contig052451-BurOR.E047	contig048263-ZebOR.E052	0.285	0.803	0.355
contig062770-NyeOR.E059	contig065455-TilOR.E085	0.285	0.803	0.355
contig049299-BurOR.E046	contig063018-ZebOR.E053	0.285	0.875	0.326
contig066194-BurOR.E054	contig048263-ZebOR.E052	0.286	0.608	0.470
contig065455-TilOR.E085	contig048239-ZebOR.E048	0.286	0.809	0.354
contig082838-BriOR.E040	contig025443-ZebOR.E046	0.286	0.840	0.341
contig004259-BriOR.E038	contig049299-BurOR.E046	0.286	0.904	0.317
contig047825-TilOR.E079	contig048263-ZebOR.E052	0.287	0.647	0.443
contig004259-BriOR.E038	contig017699-BurOR.E042	0.287	0.722	0.397
contig082838-BriOR.E040	contig052450-BurOR.E055	0.287	0.774	0.371
contig004259-BriOR.E038	contig048263-ZebOR.E052	0.287	0.782	0.367
contig049287-BurOR.E043	contig052454-BurOR.E050	0.287	0.826	0.347
contig053572-NyeOR.E051	contig065455-TilOR.E085	0.287	0.859	0.335
contig052454-BurOR.E050	contig063018-ZebOR.E053	0.287	1.016	0.283
contig052453-BurOR.E049	contig053572-NyeOR.E051	0.288	0.558	0.517
contig047820-TilOR.E078	contig065455-TilOR.E085	0.288	0.696	0.415
contig052457-BurOR.E051	contig065455-TilOR.E085	0.289	0.803	0.360
contig049287-BurOR.E043	contig052453-BurOR.E049	0.289	0.873	0.332
contig004255-BriOR.E036	contig049287-BurOR.E043	0.289	0.877	0.330
contig052454-BurOR.E050	contig065455-TilOR.E085	0.289	0.898	0.322
contig025439-ZebOR.E045	contig063018-ZebOR.E053	0.289	0.973	0.297
contig052453-BurOR.E049	contig066194-BurOR.E054	0.290	0.541	0.537
contig066194-BurOR.E054	contig065455-TilOR.E085	0.290	0.867	0.335
contig082838-BriOR.E040	contig047826-TilOR.E080	0.291	0.692	0.421
contig082838-BriOR.E040	contig064724-BurOR.E052	0.291	0.719	0.404
contig052454-BurOR.E050	contig048242-ZebOR.E049	0.291	0.829	0.351
contig049287-BurOR.E043	contig025439-ZebOR.E045	0.291	0.863	0.338
contig065455-TilOR.E085	contig025439-ZebOR.E045	0.291	0.887	0.328
contig053572-NyeOR.E051	contig059404-NyeOR.E058	0.292	0.545	0.536
contig047729-TilOR.E076	contig063018-ZebOR.E053	0.292	0.780	0.375
contig052454-BurOR.E050	contig047726-TilOR.E075	0.292	0.847	0.345
contig053592-NyeOR.E057	contig059404-NyeOR.E058	0.292	0.872	0.335
contig049287-BurOR.E043	contig059404-NyeOR.E058	0.292	0.898	0.325
contig047825-TilOR.E079	contig065455-TilOR.E085	0.292	0.898	0.325
contig059404-NyeOR.E058	contig048242-ZebOR.E049	0.292	0.902	0.324
contig047829-TilOR.E081	contig065455-TilOR.E085	0.293	0.600	0.488
contig082838-BriOR.E040	contig047825-TilOR.E079	0.293	0.717	0.409

contig004259-BriOR.E038	contig047729-TilOR.E076	0.293	0.772	0.380
contig052451-BurOR.E047	contig047729-TilOR.E076	0.293	0.787	0.373
contig052454-BurOR.E050	contig053592-NyeOR.E057	0.293	0.817	0.359
contig052453-BurOR.E049	contig048242-ZebOR.E049	0.293	0.876	0.335
contig004255-BriOR.E036	contig048242-ZebOR.E049	0.293	0.881	0.333
contig052451-BurOR.E047	contig052454-BurOR.E050	0.293	0.944	0.310
contig004255-BriOR.E036	contig063018-ZebOR.E053	0.293	1.011	0.290
contig066194-BurOR.E054	contig059404-NyeOR.E058	0.294	0.528	0.557
contig052453-BurOR.E049	contig053592-NyeOR.E057	0.294	0.848	0.347
contig004255-BriOR.E036	contig047726-TilOR.E075	0.294	0.866	0.339
contig059404-NyeOR.E058	contig047726-TilOR.E075	0.294	0.876	0.335
contig004255-BriOR.E036	contig065455-TilOR.E085	0.294	0.914	0.322
contig052451-BurOR.E047	contig047829-TilOR.E081	0.295	0.668	0.442
contig082838-BriOR.E040	contig053576-NyeOR.E052	0.295	0.717	0.412
contig082838-BriOR.E040	contig048263-ZebOR.E052	0.295	0.740	0.398
contig047725-TilOR.E074	contig048260-ZebOR.E051	0.295	0.746	0.396
contig025439-ZebOR.E045	contig048242-ZebOR.E049	0.295	0.866	0.341
contig049287-BurOR.E043	contig047734-TilOR.E077	0.295	0.869	0.340
contig052451-BurOR.E047	contig025439-ZebOR.E045	0.295	0.905	0.326
contig004255-BriOR.E036	contig004259-BriOR.E038	0.295	0.934	0.316
contig047829-TilOR.E081	contig063018-ZebOR.E053	0.296	0.645	0.459
contig004259-BriOR.E038	contig047829-TilOR.E081	0.296	0.665	0.445
contig082838-BriOR.E040	contig053572-NyeOR.E051	0.296	0.688	0.431
contig004255-BriOR.E036	contig053592-NyeOR.E057	0.296	0.868	0.341
contig052453-BurOR.E049	contig047726-TilOR.E075	0.297	0.858	0.346
contig047726-TilOR.E075	contig025439-ZebOR.E045	0.297	0.885	0.335
contig004259-BriOR.E038	contig052454-BurOR.E050	0.297	0.945	0.314
contig053592-NyeOR.E057	contig025439-ZebOR.E045	0.298	0.853	0.349
contig004255-BriOR.E036	contig052451-BurOR.E047	0.298	0.946	0.315
contig082838-BriOR.E040	contig066194-BurOR.E054	0.299	0.686	0.436
contig082838-BriOR.E040	contig065455-TilOR.E085	0.299	0.802	0.373
contig047734-TilOR.E077	contig048242-ZebOR.E049	0.299	0.872	0.343
contig004266-BriOR.E039	contig047834-TilOR.E084	0.299	0.875	0.342
contig004259-BriOR.E038	contig025439-ZebOR.E045	0.299	0.906	0.330
contig047834-TilOR.E084	contig025447-ZebOR.E047	0.299	0.907	0.329
contig053572-NyeOR.E051	contig063018-ZebOR.E053	0.299	0.983	0.304
contig004261-BriOR.E034	contig049287-BurOR.E043	0.300	0.855	0.351
contig049287-BurOR.E043	contig047829-TilOR.E081	0.300	0.882	0.341
contig047820-TilOR.E078	contig063018-ZebOR.E053	0.301	0.776	0.387
contig049287-BurOR.E043	contig064724-BurOR.E052	0.301	0.864	0.348
contig066194-BurOR.E054	contig063018-ZebOR.E053	0.301	0.995	0.303
contig047825-TilOR.E079	contig063018-ZebOR.E053	0.301	1.046	0.288
contig052453-BurOR.E049	contig047825-TilOR.E079	0.302	0.529	0.570

contig053590-NyeOR.E056	contig047832-TilOR.E082	0.302	0.785	0.385
contig053592-NyeOR.E057	contig047734-TilOR.E077	0.302	0.859	0.351
contig004265-BriOR.E035	contig047726-TilOR.E075	0.302	0.860	0.352
contig004261-BriOR.E034	contig053592-NyeOR.E057	0.302	0.884	0.342
contig052451-BurOR.E047	contig053572-NyeOR.E051	0.302	0.989	0.305
contig053579-NyeOR.E053	contig047726-TilOR.E075	0.303	0.839	0.362
contig049287-BurOR.E043	contig053579-NyeOR.E053	0.303	0.842	0.359
contig004266-BriOR.E039	contig049289-BurOR.E044	0.303	0.847	0.358
contig049289-BurOR.E044	contig025447-ZebOR.E047	0.303	0.872	0.348
contig049287-BurOR.E043	contig065453-TilOR.E087	0.303	1.030	0.294
contig049298-BurOR.E045	contig048243-ZebOR.E050	0.304	0.792	0.384
contig047726-TilOR.E075	contig065458-TilOR.E086	0.304	0.801	0.380
contig047726-TilOR.E075	contig047829-TilOR.E081	0.304	0.816	0.372
contig049299-BurOR.E046	contig047726-TilOR.E075	0.304	0.822	0.370
contig049287-BurOR.E043	contig049299-BurOR.E046	0.304	0.826	0.368
contig053590-NyeOR.E056	contig025447-ZebOR.E047	0.304	0.839	0.363
contig004261-BriOR.E034	contig047726-TilOR.E075	0.304	0.873	0.349
contig004265-BriOR.E035	contig049287-BurOR.E043	0.304	0.876	0.347
contig053579-NyeOR.E053	contig047834-TilOR.E084	0.304	0.885	0.344
contig017699-BurOR.E042	contig047834-TilOR.E084	0.304	0.910	0.334
contig023280-NyeOR.E050	contig047834-TilOR.E084	0.304	0.917	0.332
contig052451-BurOR.E047	contig066194-BurOR.E054	0.304	1.001	0.304
contig052451-BurOR.E047	contig047825-TilOR.E079	0.304	1.052	0.289
contig059404-NyeOR.E058	contig047825-TilOR.E079	0.305	0.516	0.592
contig049289-BurOR.E044	contig053579-NyeOR.E055	0.305	0.800	0.382
contig047726-TilOR.E075	contig047734-TilOR.E077	0.305	0.817	0.374
contig064724-BurOR.E052	contig048242-ZebOR.E049	0.305	0.842	0.362
contig049289-BurOR.E044	contig053579-NyeOR.E054	0.305	0.855	0.357
contig004261-BriOR.E034	contig048242-ZebOR.E049	0.305	0.859	0.355
contig049287-BurOR.E043	contig053576-NyeOR.E052	0.305	0.875	0.349
contig047829-TilOR.E081	contig048242-ZebOR.E049	0.305	0.885	0.344
contig049287-BurOR.E043	contig047820-TilOR.E078	0.305	0.889	0.343
contig049287-BurOR.E043	contig048263-ZebOR.E052	0.305	0.904	0.337
contig049287-BurOR.E043	contig065454-TilOR.E088	0.305	0.912	0.334
contig053592-NyeOR.E057	contig065453-TilOR.E087	0.305	1.067	0.286
contig053579-NyeOR.E054	contig048243-ZebOR.E050	0.306	0.827	0.370
contig082838-BriOR.E040	contig052451-BurOR.E047	0.306	0.838	0.365
contig004265-BriOR.E035	contig053592-NyeOR.E057	0.306	0.856	0.357
contig025447-ZebOR.E047	contig048243-ZebOR.E050	0.306	0.864	0.355
contig049287-BurOR.E043	contig065458-TilOR.E086	0.306	0.867	0.352
contig052451-BurOR.E047	contig047820-TilOR.E078	0.307	0.765	0.402
contig004266-BriOR.E039	contig048243-ZebOR.E050	0.307	0.810	0.379
contig053579-NyeOR.E053	contig048242-ZebOR.E049	0.307	0.821	0.374

contig082838-BriOR.E040	contig063018-ZebOR.E053	0.307	0.833	0.369
contig049289-BurOR.E044	contig047832-TilOR.E082	0.307	0.842	0.365
contig064724-BurOR.E052	contig053592-NyeOR.E057	0.307	0.855	0.359
contig004255-BriOR.E036	contig047725-TilOR.E074	0.307	0.871	0.352
contig049287-BurOR.E043	contig047826-TilOR.E080	0.307	0.875	0.351
contig053592-NyeOR.E057	contig047829-TilOR.E081	0.307	0.885	0.347
contig047729-TilOR.E076	contig047834-TilOR.E084	0.307	0.890	0.345
contig004258-BriOR.E037	contig049287-BurOR.E043	0.307	0.890	0.345
contig053592-NyeOR.E057	contig065454-TilOR.E088	0.307	0.944	0.325
contig004259-BriOR.E038	contig053572-NyeOR.E051	0.307	0.969	0.316
contig065453-TilOR.E087	contig048242-ZebOR.E049	0.307	1.034	0.297
contig004259-BriOR.E038	contig047820-TilOR.E078	0.308	0.740	0.416
contig053579-NyeOR.E055	contig048243-ZebOR.E050	0.308	0.762	0.405
contig047832-TilOR.E082	contig048243-ZebOR.E050	0.308	0.814	0.378
contig049289-BurOR.E044	contig049298-BurOR.E045	0.308	0.818	0.376
contig049299-BurOR.E046	contig048242-ZebOR.E049	0.308	0.829	0.372
contig004265-BriOR.E035	contig048242-ZebOR.E049	0.308	0.829	0.372
contig004258-BriOR.E037	contig047726-TilOR.E075	0.308	0.834	0.370
contig049287-BurOR.E043	contig064938-BurOR.E053	0.308	0.845	0.364
contig049287-BurOR.E043	contig065454-TilOR.E089	0.308	0.895	0.344
contig047726-TilOR.E075	contig065453-TilOR.E087	0.308	1.063	0.290
contig004266-BriOR.E039	contig053590-NyeOR.E056	0.309	0.809	0.382
contig053579-NyeOR.E053	contig053592-NyeOR.E057	0.309	0.845	0.366
contig053576-NyeOR.E052	contig048242-ZebOR.E049	0.309	0.853	0.363
contig023280-NyeOR.E050	contig053590-NyeOR.E056	0.309	0.863	0.358
contig049287-BurOR.E043	contig052450-BurOR.E055	0.309	0.871	0.355
contig048242-ZebOR.E049	contig048263-ZebOR.E052	0.309	0.881	0.351
contig047725-TilOR.E074	contig025439-ZebOR.E045	0.309	0.882	0.350
contig047820-TilOR.E078	contig048242-ZebOR.E049	0.309	0.893	0.346
contig053592-NyeOR.E057	contig065454-TilOR.E089	0.309	0.901	0.343
contig065454-TilOR.E088	contig048242-ZebOR.E049	0.309	0.916	0.337
contig049287-BurOR.E043	contig047825-TilOR.E079	0.309	0.961	0.321
contig004259-BriOR.E038	contig066194-BurOR.E054	0.309	0.981	0.315
contig004259-BriOR.E038	contig047825-TilOR.E079	0.309	1.030	0.300
contig047725-TilOR.E074	contig047820-TilOR.E078	0.310	0.792	0.391
contig049299-BurOR.E046	contig053592-NyeOR.E057	0.310	0.829	0.374
contig053592-NyeOR.E057	contig065458-TilOR.E086	0.310	0.858	0.361
contig047834-TilOR.E084	contig048239-ZebOR.E048	0.310	0.867	0.357
contig049299-BurOR.E046	contig047834-TilOR.E084	0.310	0.868	0.357
contig065458-TilOR.E086	contig048242-ZebOR.E049	0.310	0.871	0.356
contig047726-TilOR.E075	contig065454-TilOR.E089	0.310	0.912	0.339
contig047726-TilOR.E075	contig065454-TilOR.E088	0.310	0.939	0.330
contig049298-BurOR.E045	contig053590-NyeOR.E056	0.311	0.764	0.408

contig052452-BurOR.E048	contig047726-TilOR.E075	0.311	0.848	0.367
contig017699-BurOR.E042	contig053590-NyeOR.E056	0.311	0.851	0.365
contig047826-TilOR.E080	contig048242-ZebOR.E049	0.311	0.853	0.365
contig053592-NyeOR.E057	contig047820-TilOR.E078	0.311	0.879	0.354
contig004258-BriOR.E037	contig053592-NyeOR.E057	0.311	0.880	0.354
contig004258-BriOR.E037	contig048242-ZebOR.E049	0.311	0.893	0.349
contig052450-BurOR.E055	contig053592-NyeOR.E057	0.311	0.901	0.346
contig064938-BurOR.E053	contig048242-ZebOR.E049	0.312	0.849	0.368
contig047833-TilOR.E083	contig048243-ZebOR.E050	0.312	0.853	0.365
contig053576-NyeOR.E052	contig053592-NyeOR.E057	0.312	0.865	0.360
contig053592-NyeOR.E057	contig047826-TilOR.E080	0.312	0.872	0.358
contig047726-TilOR.E075	contig025443-ZebOR.E046	0.312	0.889	0.351
contig053592-NyeOR.E057	contig048263-ZebOR.E052	0.312	0.894	0.348
contig049289-BurOR.E044	contig023280-NyeOR.E050	0.312	0.896	0.348
contig065454-TilOR.E089	contig048242-ZebOR.E049	0.312	0.899	0.347
contig053592-NyeOR.E057	contig025443-ZebOR.E046	0.312	0.948	0.329
contig049287-BurOR.E043	contig025443-ZebOR.E046	0.312	0.959	0.325
contig047725-TilOR.E074	contig047734-TilOR.E077	0.313	0.705	0.444
contig062770-NyeOR.E059	contig047834-TilOR.E084	0.313	0.850	0.368
contig047726-TilOR.E075	contig047825-TilOR.E079	0.313	0.937	0.334
contig004255-BriOR.E036	contig049289-BurOR.E044	0.313	0.961	0.326
contig047825-TilOR.E079	contig048242-ZebOR.E049	0.313	0.965	0.324
contig053579-NyeOR.E054	contig053590-NyeOR.E056	0.314	0.798	0.393
contig052450-BurOR.E055	contig048242-ZebOR.E049	0.314	0.849	0.369
contig052454-BurOR.E050	contig047725-TilOR.E074	0.314	0.854	0.367
contig017699-BurOR.E042	contig049289-BurOR.E044	0.314	0.884	0.355
contig004266-BriOR.E039	contig047725-TilOR.E074	0.315	0.788	0.400
contig064938-BurOR.E053	contig053592-NyeOR.E057	0.315	0.836	0.376
contig049289-BurOR.E044	contig047833-TilOR.E083	0.315	0.882	0.358
contig052450-BurOR.E055	contig047726-TilOR.E075	0.315	0.910	0.346
contig049289-BurOR.E044	contig025439-ZebOR.E045	0.315	0.944	0.334
contig053579-NyeOR.E055	contig053590-NyeOR.E056	0.316	0.735	0.430
contig048243-ZebOR.E050	contig048260-ZebOR.E051	0.316	0.786	0.403
contig004259-BriOR.E038	contig082838-BriOR.E040	0.316	0.809	0.390
contig047725-TilOR.E074	contig025447-ZebOR.E047	0.316	0.830	0.381
contig049287-BurOR.E043	contig053572-NyeOR.E051	0.316	0.864	0.365
contig023280-NyeOR.E050	contig048243-ZebOR.E050	0.316	0.889	0.355
contig053592-NyeOR.E057	contig047825-TilOR.E079	0.316	0.930	0.340
contig025443-ZebOR.E046	contig048242-ZebOR.E049	0.316	0.963	0.328
contig047834-TilOR.E084	contig025439-ZebOR.E045	0.316	1.011	0.312
contig047832-TilOR.E082	contig047834-TilOR.E084	0.317	0.800	0.396
contig017699-BurOR.E042	contig048243-ZebOR.E050	0.317	0.876	0.362
contig004255-BriOR.E036	contig047834-TilOR.E084	0.317	0.991	0.320

contig053590-NyeOR.E056	contig047833-TilOR.E083	0.318	0.829	0.384
contig064724-BurOR.E052	contig047726-TilOR.E075	0.318	0.847	0.375
contig049287-BurOR.E043	contig066194-BurOR.E054	0.318	0.862	0.369
contig047726-TilOR.E075	contig047820-TilOR.E078	0.318	0.883	0.360
contig052457-BurOR.E051	contig047834-TilOR.E084	0.318	0.887	0.358
contig049287-BurOR.E043	contig052452-BurOR.E048	0.318	0.920	0.346
contig049289-BurOR.E044	contig047734-TilOR.E077	0.319	0.756	0.422
contig064938-BurOR.E053	contig047726-TilOR.E075	0.319	0.806	0.395
contig047834-TilOR.E084	contig025443-ZebOR.E046	0.319	0.992	0.321
contig047725-TilOR.E074	contig048239-ZebOR.E048	0.320	0.752	0.426
contig047833-TilOR.E083	contig047834-TilOR.E084	0.320	0.764	0.419
contig053572-NyeOR.E051	contig047726-TilOR.E075	0.320	0.841	0.381
contig053572-NyeOR.E051	contig048242-ZebOR.E049	0.320	0.867	0.369
contig049289-BurOR.E044	contig052454-BurOR.E050	0.320	0.914	0.350
contig052454-BurOR.E050	contig047834-TilOR.E084	0.320	0.981	0.326
contig023280-NyeOR.E050	contig047725-TilOR.E074	0.321	0.855	0.375
contig004255-BriOR.E036	contig048243-ZebOR.E050	0.321	0.928	0.346
contig047725-TilOR.E074	contig047729-TilOR.E076	0.322	0.689	0.467
contig053579-NyeOR.E053	contig047725-TilOR.E074	0.322	0.724	0.445
contig049289-BurOR.E044	contig048260-ZebOR.E051	0.322	0.806	0.399
contig017699-BurOR.E042	contig047725-TilOR.E074	0.322	0.843	0.382
contig047820-TilOR.E078	contig048243-ZebOR.E050	0.322	0.855	0.376
contig047726-TilOR.E075	contig048263-ZebOR.E052	0.322	0.886	0.364
contig052452-BurOR.E048	contig053592-NyeOR.E057	0.322	0.903	0.356
contig062770-NyeOR.E059	contig047725-TilOR.E074	0.323	0.742	0.435
contig049298-BurOR.E045	contig047834-TilOR.E084	0.323	0.762	0.423
contig066194-BurOR.E054	contig047726-TilOR.E075	0.323	0.839	0.385
contig047726-TilOR.E075	contig047826-TilOR.E080	0.323	0.839	0.385
contig053576-NyeOR.E052	contig047726-TilOR.E075	0.323	0.858	0.376
contig066194-BurOR.E054	contig048242-ZebOR.E049	0.323	0.865	0.373
contig052452-BurOR.E048	contig048242-ZebOR.E049	0.323	0.897	0.360
contig025439-ZebOR.E045	contig048243-ZebOR.E050	0.323	0.912	0.354
contig047834-TilOR.E084	contig065458-TilOR.E086	0.323	0.932	0.346
contig053590-NyeOR.E056	contig048260-ZebOR.E051	0.324	0.758	0.428
contig053572-NyeOR.E051	contig053592-NyeOR.E057	0.324	0.836	0.387
contig004255-BriOR.E036	contig053590-NyeOR.E056	0.324	0.921	0.352
contig047734-TilOR.E077	contig047834-TilOR.E084	0.324	0.949	0.341
contig049289-BurOR.E044	contig047820-TilOR.E078	0.325	0.851	0.382
contig066194-BurOR.E054	contig053592-NyeOR.E057	0.326	0.834	0.391
contig047834-TilOR.E084	contig065454-TilOR.E089	0.326	0.900	0.362
contig053590-NyeOR.E056	contig025439-ZebOR.E045	0.326	0.905	0.361
contig004265-BriOR.E035	contig047834-TilOR.E084	0.326	0.912	0.357
contig052452-BurOR.E048	contig047834-TilOR.E084	0.326	1.007	0.324

contig047734-TilOR.E077	contig048243-ZebOR.E050	0.327	0.753	0.434
contig052457-BurOR.E051	contig047725-TilOR.E074	0.327	0.774	0.423
contig004261-BriOR.E034	contig047725-TilOR.E074	0.327	0.904	0.361
contig047725-TilOR.E074	contig065454-TilOR.E088	0.327	0.904	0.362
contig004258-BriOR.E037	contig047834-TilOR.E084	0.327	0.938	0.349
contig049299-BurOR.E046	contig047725-TilOR.E074	0.328	0.711	0.462
contig049289-BurOR.E044	contig048239-ZebOR.E048	0.328	0.809	0.406
contig052454-BurOR.E050	contig048243-ZebOR.E050	0.328	0.910	0.360
contig047834-TilOR.E084	contig065453-TilOR.E087	0.328	0.931	0.352
contig047834-TilOR.E084	contig048260-ZebOR.E051	0.329	0.775	0.425
contig004265-BriOR.E035	contig047725-TilOR.E074	0.330	0.835	0.395
contig053590-NyeOR.E056	contig047820-TilOR.E078	0.330	0.836	0.394
contig047725-TilOR.E074	contig065454-TilOR.E089	0.330	0.864	0.382
contig004261-BriOR.E034	contig047834-TilOR.E084	0.330	0.916	0.360
contig047725-TilOR.E074	contig065453-TilOR.E087	0.330	0.941	0.351
contig049289-BurOR.E044	contig065454-TilOR.E089	0.330	0.993	0.333
contig049289-BurOR.E044	contig062770-NyeOR.E059	0.331	0.793	0.418
contig052454-BurOR.E050	contig053590-NyeOR.E056	0.331	0.877	0.378
contig052450-BurOR.E055	contig047834-TilOR.E084	0.331	0.965	0.344
contig053590-NyeOR.E056	contig047729-TilOR.E076	0.332	0.699	0.475
contig047834-TilOR.E084	contig065454-TilOR.E088	0.332	0.935	0.354
contig004265-BriOR.E035	contig049289-BurOR.E044	0.332	0.951	0.349
contig049289-BurOR.E044	contig053579-NyeOR.E053	0.333	0.753	0.443
contig053590-NyeOR.E056	contig065454-TilOR.E089	0.333	0.958	0.348
contig047820-TilOR.E078	contig047834-TilOR.E084	0.333	0.958	0.348
contig053590-NyeOR.E056	contig065453-TilOR.E087	0.333	0.987	0.338
contig048239-ZebOR.E048	contig048243-ZebOR.E050	0.334	0.803	0.416
contig052450-BurOR.E055	contig047725-TilOR.E074	0.334	0.930	0.359
contig049289-BurOR.E044	contig065454-TilOR.E088	0.334	0.982	0.340
contig053579-NyeOR.E053	contig048243-ZebOR.E050	0.335	0.744	0.450
contig053590-NyeOR.E056	contig047734-TilOR.E077	0.335	0.747	0.448
contig053579-NyeOR.E054	contig047834-TilOR.E084	0.335	0.790	0.424
contig004265-BriOR.E035	contig053590-NyeOR.E056	0.335	0.891	0.376
contig065454-TilOR.E089	contig048243-ZebOR.E050	0.335	0.958	0.350
contig004261-BriOR.E034	contig049289-BurOR.E044	0.335	1.014	0.331
contig049289-BurOR.E044	contig065453-TilOR.E087	0.335	1.023	0.327
contig047729-TilOR.E076	contig048243-ZebOR.E050	0.336	0.725	0.463
contig053579-NyeOR.E055	contig047834-TilOR.E084	0.336	0.748	0.448
contig049289-BurOR.E044	contig052457-BurOR.E051	0.336	0.826	0.407
contig064724-BurOR.E052	contig047834-TilOR.E084	0.336	0.850	0.396
contig053590-NyeOR.E056	contig048239-ZebOR.E048	0.337	0.774	0.435
contig062770-NyeOR.E059	contig048243-ZebOR.E050	0.337	0.787	0.428
contig004265-BriOR.E035	contig048243-ZebOR.E050	0.337	0.892	0.378

contig053590-NyeOR.E056	contig065454-TilOR.E088	0.337	0.947	0.355
contig004261-BriOR.E034	contig053590-NyeOR.E056	0.338	0.949	0.357
contig053579-NyeOR.E053	contig053590-NyeOR.E056	0.339	0.738	0.459
contig049289-BurOR.E044	contig049299-BurOR.E046	0.339	0.738	0.459
contig049289-BurOR.E044	contig047729-TilOR.E076	0.339	0.745	0.455
contig049289-BurOR.E044	contig064938-BurOR.E053	0.339	0.815	0.416
contig065454-TilOR.E088	contig048243-ZebOR.E050	0.339	0.948	0.358
contig064938-BurOR.E053	contig047725-TilOR.E074	0.340	0.781	0.436
contig052450-BurOR.E055	contig053590-NyeOR.E056	0.340	0.927	0.367
contig065453-TilOR.E087	contig048243-ZebOR.E050	0.340	0.987	0.345
contig049299-BurOR.E046	contig048243-ZebOR.E050	0.341	0.730	0.467
contig053590-NyeOR.E056	contig062770-NyeOR.E059	0.341	0.759	0.449
contig004261-BriOR.E034	contig048243-ZebOR.E050	0.341	0.949	0.359
contig047726-TilOR.E075	contig063018-ZebOR.E053	0.341	1.020	0.334
contig052457-BurOR.E051	contig048243-ZebOR.E050	0.342	0.820	0.417
contig047826-TilOR.E080	contig047834-TilOR.E084	0.342	0.830	0.412
contig047726-TilOR.E075	contig065455-TilOR.E085	0.342	0.929	0.368
contig049289-BurOR.E044	contig052450-BurOR.E055	0.342	0.990	0.345
contig049299-BurOR.E046	contig053590-NyeOR.E056	0.345	0.724	0.476
contig052457-BurOR.E051	contig053590-NyeOR.E056	0.345	0.791	0.437
contig047829-TilOR.E081	contig047834-TilOR.E084	0.345	0.820	0.421
contig047825-TilOR.E079	contig047834-TilOR.E084	0.345	0.890	0.388
contig053576-NyeOR.E052	contig047834-TilOR.E084	0.346	0.860	0.402
contig049287-BurOR.E043	contig065455-TilOR.E085	0.346	0.887	0.390
contig047725-TilOR.E074	contig047829-TilOR.E081	0.347	0.628	0.552
contig047725-TilOR.E074	contig065455-TilOR.E085	0.347	0.881	0.394
contig053572-NyeOR.E051	contig047834-TilOR.E084	0.347	0.903	0.384
contig052450-BurOR.E055	contig048243-ZebOR.E050	0.347	0.956	0.363
contig047834-TilOR.E084	contig048263-ZebOR.E052	0.348	0.900	0.386
contig053592-NyeOR.E057	contig065455-TilOR.E085	0.348	0.920	0.378
contig065455-TilOR.E085	contig048242-ZebOR.E049	0.348	0.945	0.368
contig052451-BurOR.E047	contig047726-TilOR.E075	0.348	0.982	0.354
contig064938-BurOR.E053	contig048243-ZebOR.E050	0.349	0.810	0.431
contig053572-NyeOR.E051	contig047725-TilOR.E074	0.350	0.857	0.409
contig047725-TilOR.E074	contig047825-TilOR.E079	0.350	0.863	0.406
contig049289-BurOR.E044	contig047829-TilOR.E081	0.351	0.699	0.502
contig047829-TilOR.E081	contig048243-ZebOR.E050	0.352	0.693	0.508
contig047725-TilOR.E074	contig025443-ZebOR.E046	0.352	0.903	0.390
contig049287-BurOR.E043	contig063018-ZebOR.E053	0.352	0.940	0.375
contig049287-BurOR.E043	contig052451-BurOR.E047	0.353	0.943	0.375
contig049289-BurOR.E044	contig025443-ZebOR.E046	0.355	0.877	0.405
contig059404-NyeOR.E058	contig047834-TilOR.E084	0.355	0.935	0.380
contig048242-ZebOR.E049	contig063018-ZebOR.E053	0.355	0.958	0.370

contig052451-BurOR.E047	contig048242-ZebOR.E049	0.355	0.961	0.370
contig053590-NyeOR.E056	contig047829-TilOR.E081	0.356	0.688	0.517
contig047725-TilOR.E074	contig065458-TilOR.E086	0.356	0.861	0.414
contig052453-BurOR.E049	contig047834-TilOR.E084	0.356	0.914	0.389
contig004259-BriOR.E038	contig047726-TilOR.E075	0.356	0.976	0.364
contig064938-BurOR.E053	contig053590-NyeOR.E056	0.358	0.781	0.458
contig049289-BurOR.E044	contig065458-TilOR.E086	0.358	0.865	0.414
contig064938-BurOR.E053	contig047834-TilOR.E084	0.358	0.883	0.406
contig004259-BriOR.E038	contig047725-TilOR.E074	0.358	0.926	0.387
contig064724-BurOR.E052	contig047725-TilOR.E074	0.359	0.720	0.499
contig066194-BurOR.E054	contig047834-TilOR.E084	0.359	0.900	0.399
contig053592-NyeOR.E057	contig063018-ZebOR.E053	0.359	0.978	0.367
contig052451-BurOR.E047	contig053592-NyeOR.E057	0.359	0.981	0.366
contig047725-TilOR.E074	contig047826-TilOR.E080	0.360	0.725	0.496
contig066194-BurOR.E054	contig047725-TilOR.E074	0.360	0.855	0.421
contig049289-BurOR.E044	contig052452-BurOR.E048	0.360	0.889	0.405
contig052452-BurOR.E048	contig047725-TilOR.E074	0.361	0.917	0.394
contig052453-BurOR.E049	contig047725-TilOR.E074	0.362	0.777	0.466
contig047725-TilOR.E074	contig048263-ZebOR.E052	0.363	0.730	0.498
contig052451-BurOR.E047	contig047725-TilOR.E074	0.363	0.911	0.399
contig004259-BriOR.E038	contig049287-BurOR.E043	0.363	0.924	0.393
contig053576-NyeOR.E052	contig047725-TilOR.E074	0.364	0.728	0.499
contig049289-BurOR.E044	contig065455-TilOR.E085	0.364	0.898	0.405
contig047725-TilOR.E074	contig063018-ZebOR.E053	0.364	0.925	0.394
contig059404-NyeOR.E058	contig047725-TilOR.E074	0.365	0.794	0.460
contig004259-BriOR.E038	contig048242-ZebOR.E049	0.365	0.942	0.388
contig049289-BurOR.E044	contig047826-TilOR.E080	0.366	0.772	0.474
contig065458-TilOR.E086	contig048243-ZebOR.E050	0.366	0.875	0.418
contig004258-BriOR.E037	contig047725-TilOR.E074	0.367	0.882	0.416
contig025443-ZebOR.E046	contig048243-ZebOR.E050	0.367	0.888	0.413
contig049289-BurOR.E044	contig047825-TilOR.E079	0.368	0.881	0.418
contig004258-BriOR.E037	contig049289-BurOR.E044	0.369	0.849	0.435
contig047826-TilOR.E080	contig048243-ZebOR.E050	0.370	0.774	0.479
contig053590-NyeOR.E056	contig025443-ZebOR.E046	0.370	0.856	0.433
contig065455-TilOR.E085	contig048243-ZebOR.E050	0.370	0.865	0.427
contig004259-BriOR.E038	contig053592-NyeOR.E057	0.370	0.961	0.385
contig053590-NyeOR.E056	contig047826-TilOR.E080	0.371	0.757	0.490
contig053590-NyeOR.E056	contig047825-TilOR.E079	0.371	0.855	0.434
contig049289-BurOR.E044	contig064724-BurOR.E052	0.372	0.748	0.496
contig053590-NyeOR.E056	contig065455-TilOR.E085	0.372	0.878	0.424
contig047825-TilOR.E079	contig048243-ZebOR.E050	0.372	0.886	0.420
contig052452-BurOR.E048	contig048243-ZebOR.E050	0.372	0.900	0.413
contig049289-BurOR.E044	contig052453-BurOR.E049	0.373	0.752	0.495

contig049289-BurOR.E044	contig053572-NyeOR.E051	0.373	0.891	0.419
contig052453-BurOR.E049	contig048243-ZebOR.E050	0.374	0.756	0.495
contig004259-BriOR.E038	contig049289-BurOR.E044	0.374	0.951	0.393
contig064724-BurOR.E052	contig048243-ZebOR.E050	0.376	0.750	0.501
contig049289-BurOR.E044	contig053576-NyeOR.E052	0.376	0.757	0.497
contig049289-BurOR.E044	contig048263-ZebOR.E052	0.376	0.759	0.495
contig049289-BurOR.E044	contig059404-NyeOR.E058	0.376	0.769	0.489
contig004258-BriOR.E037	contig048243-ZebOR.E050	0.376	0.835	0.451
contig053572-NyeOR.E051	contig048243-ZebOR.E050	0.377	0.897	0.420
contig064724-BurOR.E052	contig053590-NyeOR.E056	0.378	0.729	0.518
contig053590-NyeOR.E056	contig048263-ZebOR.E052	0.378	0.740	0.511
contig059404-NyeOR.E058	contig048243-ZebOR.E050	0.378	0.772	0.489
contig053590-NyeOR.E056	contig065458-TilOR.E086	0.379	0.845	0.448
contig048243-ZebOR.E050	contig048263-ZebOR.E052	0.380	0.761	0.500
contig053572-NyeOR.E051	contig053590-NyeOR.E056	0.380	0.865	0.440
contig052452-BurOR.E048	contig053590-NyeOR.E056	0.380	0.868	0.438
contig004259-BriOR.E038	contig048243-ZebOR.E050	0.380	0.890	0.427
contig049289-BurOR.E044	contig052451-BurOR.E047	0.380	0.950	0.400
contig052453-BurOR.E049	contig053590-NyeOR.E056	0.381	0.755	0.505
contig053576-NyeOR.E052	contig048243-ZebOR.E050	0.381	0.759	0.501
contig049289-BurOR.E044	contig063018-ZebOR.E053	0.381	0.951	0.400
contig053576-NyeOR.E052	contig053590-NyeOR.E056	0.383	0.738	0.519
contig049289-BurOR.E044	contig066194-BurOR.E054	0.383	0.876	0.437
contig052451-BurOR.E047	contig048243-ZebOR.E050	0.385	0.888	0.434
contig047834-TilOR.E084	contig065455-TilOR.E085	0.385	0.996	0.386
contig053590-NyeOR.E056	contig059404-NyeOR.E058	0.386	0.767	0.503
contig066194-BurOR.E054	contig048243-ZebOR.E050	0.386	0.882	0.438
contig048243-ZebOR.E050	contig063018-ZebOR.E053	0.387	0.888	0.435
contig004259-BriOR.E038	contig053590-NyeOR.E056	0.387	0.932	0.415
contig004258-BriOR.E037	contig053590-NyeOR.E056	0.390	0.829	0.470
contig066194-BurOR.E054	contig053590-NyeOR.E056	0.390	0.851	0.458
contig052451-BurOR.E047	contig053590-NyeOR.E056	0.392	0.930	0.422
contig053590-NyeOR.E056	contig063018-ZebOR.E053	0.394	0.931	0.423
contig004259-BriOR.E038	contig047834-TilOR.E084	0.399	0.988	0.404
contig052451-BurOR.E047	contig047834-TilOR.E084	0.401	0.994	0.404
contig047834-TilOR.E084	contig063018-ZebOR.E053	0.405	1.028	0.394

Cichlid Olfactory Receptors :
dN/dS ratio

FamG

OR pairs		dN	dS	dN/dS
contig049295-BurOR.G060	contig048237-ZebOR.G065	0.003	0.012	0.246
contig049295-BurOR.G060	contig053581-NyeOR.G066	0.003	0.016	0.184
contig048237-ZebOR.G065	contig053581-NyeOR.G066	0.003	0.016	0.184
contig104344-BriOR.G048	contig049295-BurOR.G060	0.013	0.033	0.409
contig104344-BriOR.G048	contig053581-NyeOR.G066	0.013	0.033	0.409
contig104344-BriOR.G048	contig048237-ZebOR.G065	0.013	0.033	0.410
contig047714-TilOR.G099	contig053581-NyeOR.G066	0.022	0.107	0.208
contig047714-TilOR.G099	contig049295-BurOR.G060	0.024	0.112	0.213
contig047714-TilOR.G099	contig048237-ZebOR.G065	0.024	0.112	0.214
contig047714-TilOR.G099	contig104344-BriOR.G048	0.031	0.103	0.305

Cichlid Olfactory Receptors :
dN/dS ratio

Fam H

OR pairs		dS	dN	dnds
contig048562-BurOR.H062	contig047492-ZebOR.H074	0,00	0,00	0,00
contig053780-BurOR.H065	contig014055-ZebOR.H066	0,00	0,00	0,00
contig053780-BurOR.H065	contig039730-NyeOR.H074	0,00	0,00	0,00
contig053787-BurOR.H068	contig039725-NyeOR.H072	0,00	0,00	0,00
contig039730-NyeOR.H074	contig014055-ZebOR.H066	0,00	0,00	0,00
contig048562-BurOR.H062	contig034998-NyeOR.H067	0,00	0,00	0,00
contig034998-NyeOR.H067	contig047492-ZebOR.H074	0,00	0,00	0,34
contig093825-BriOR.H053	contig048562-BurOR.H062	0,01	0,00	0,52
contig093825-BriOR.H053	contig047492-ZebOR.H074	0,01	0,01	0,69
contig107626-BriOR.H054	contig039730-NyeOR.H074	0,01	0,02	1,64
contig107626-BriOR.H054	contig053780-BurOR.H065	0,01	0,02	1,38
contig107626-BriOR.H054	contig014055-ZebOR.H066	0,01	0,02	1,51
contig053787-BurOR.H068	contig014059-ZebOR.H068	0,01	0,00	0,35
contig039725-NyeOR.H072	contig014059-ZebOR.H068	0,01	0,01	0,46
contig093825-BriOR.H053	contig034998-NyeOR.H067	0,01	0,00	0,34
contig041756-NyeOR.H075	contig014060-ZebOR.H069	0,02	0,01	0,72
contig048880-BurOR.H063	contig035579-NyeOR.H068	0,02	0,01	0,71
contig035580-NyeOR.H069	contig057400-ZebOR.H075	0,02	0,01	0,45
contig053788-BurOR.H069	contig041757-NyeOR.H076	0,02	0,00	0,18
contig053782-BurOR.H066	contig039729-NyeOR.H073	0,02	0,00	0,17
contig053784-BurOR.H067	contig014060-ZebOR.H069	0,02	0,00	0,22
contig053784-BurOR.H067	contig041756-NyeOR.H075	0,02	0,01	0,65
contig053782-BurOR.H066	contig014057-ZebOR.H067	0,02	0,01	0,35
contig006794-BurOR.H061	contig057403-ZebOR.H077	0,02	0,02	0,77
contig035583-NyeOR.H071	contig057403-ZebOR.H077	0,03	0,01	0,31
contig049873-BriOR.H050	contig057403-ZebOR.H077	0,04	0,01	0,31
contig033889-BriOR.H049	contig030011-ZebOR.H073	0,04	0,02	0,42
contig039729-NyeOR.H073	contig014057-ZebOR.H067	0,04	0,01	0,27
contig006794-BurOR.H061	contig035583-NyeOR.H071	0,04	0,02	0,42
contig049873-BriOR.H050	contig035583-NyeOR.H071	0,04	0,01	0,28
contig049873-BriOR.H050	contig006794-BurOR.H061	0,04	0,01	0,32
contig048880-BurOR.H063	contig013365-TilOR.H111	0,05	0,04	0,88
contig035579-NyeOR.H068	contig013365-TilOR.H111	0,05	0,04	0,78
contig116846-BriOR.H055	contig030011-ZebOR.H073	0,05	0,05	1,02
contig041756-NyeOR.H075	contig041756-NyeOR.H139	0,05	0,03	0,56
contig093825-BriOR.H053	contig041955-TilOR.H119	0,05	0,03	0,49
contig064817-BriOR.H051	contig014057-ZebOR.H067	0,06	0,01	0,21
contig009565-TilOR.H126	contig018434-ZebOR.H070	0,06	0,04	0,79
contig064821-BriOR.H052	contig041757-NyeOR.H076	0,06	0,02	0,40
contig018434-ZebOR.H071	contig030011-ZebOR.H073	0,06	0,07	1,22
contig013358-TilOR.H106	contig014060-ZebOR.H069	0,06	0,04	0,73
contig033889-BriOR.H049	contig018434-ZebOR.H071	0,06	0,07	1,20

contig107626-BriOR.H054	contig013349-TilOR.H103	0,06	0,02	0,37
contig048562-BurOR.H062	contig041955-TilOR.H119	0,06	0,02	0,39
contig041955-TilOR.H119	contig047492-ZebOR.H074	0,06	0,03	0,41
contig013365-TilOR.H111	contig014060-ZebOR.H069	0,06	0,06	1,01
contig041756-NyeOR.H139	contig013365-TilOR.H111	0,06	0,04	0,70
contig033889-BriOR.H049	contig116846-BriOR.H055	0,06	0,06	0,93
contig039730-NyeOR.H074	contig013349-TilOR.H103	0,06	0,02	0,34
contig053780-BurOR.H065	contig013349-TilOR.H103	0,06	0,02	0,30
contig013349-TilOR.H103	contig014055-ZebOR.H066	0,06	0,02	0,32
contig034998-NyeOR.H067	contig041955-TilOR.H119	0,07	0,02	0,36
contig064821-BriOR.H052	contig053788-BurOR.H069	0,07	0,02	0,35
contig053788-BurOR.H069	contig013356-TilOR.H105	0,07	0,04	0,66
contig033889-BriOR.H049	contig018437-ZebOR.H072	0,07	0,05	0,76
contig048880-BurOR.H063	contig041756-NyeOR.H139	0,07	0,04	0,54
contig035579-NyeOR.H068	contig041756-NyeOR.H139	0,07	0,03	0,49
contig041756-NyeOR.H139	contig014060-ZebOR.H069	0,07	0,03	0,51
contig041757-NyeOR.H076	contig013365-TilOR.H111	0,07	0,06	0,82
contig053788-BurOR.H069	contig013365-TilOR.H111	0,07	0,06	0,87
contig064817-BriOR.H051	contig039729-NyeOR.H073	0,07	0,02	0,28
contig064821-BriOR.H052	contig013365-TilOR.H111	0,07	0,05	0,65
contig064821-BriOR.H052	contig035579-NyeOR.H068	0,07	0,05	0,67
contig014059-ZebOR.H068	contig014060-ZebOR.H069	0,07	0,06	0,89
contig013351-TilOR.H104	contig014059-ZebOR.H068	0,07	0,03	0,47
contig018437-ZebOR.H072	contig030011-ZebOR.H073	0,07	0,04	0,56
contig116846-BriOR.H055	contig018437-ZebOR.H072	0,07	0,04	0,62
contig053784-BurOR.H067	contig041756-NyeOR.H139	0,07	0,03	0,46
contig013358-TilOR.H106	contig013365-TilOR.H111	0,07	0,05	0,71
contig053784-BurOR.H067	contig013358-TilOR.H106	0,07	0,04	0,61
contig013351-TilOR.H104	contig013365-TilOR.H111	0,07	0,07	0,93
contig041757-NyeOR.H076	contig013356-TilOR.H105	0,07	0,04	0,54
contig013359-TilOR.H107	contig013363-TilOR.H110	0,08	0,04	0,58
contig053787-BurOR.H068	contig013351-TilOR.H104	0,08	0,03	0,42
contig039725-NyeOR.H072	contig013351-TilOR.H104	0,08	0,03	0,44
contig041756-NyeOR.H075	contig013358-TilOR.H106	0,08	0,04	0,56
contig013363-TilOR.H110	contig013365-TilOR.H111	0,08	0,05	0,63
contig013359-TilOR.H107	contig013365-TilOR.H111	0,08	0,05	0,69
contig035579-NyeOR.H068	contig013358-TilOR.H106	0,08	0,05	0,68
contig064821-BriOR.H052	contig013356-TilOR.H105	0,08	0,03	0,38
contig053784-BurOR.H067	contig013365-TilOR.H111	0,08	0,06	0,78
contig064817-BriOR.H051	contig053782-BurOR.H066	0,08	0,02	0,22
contig049873-BriOR.H050	contig013371-TilOR.H118	0,08	0,02	0,29
contig064821-BriOR.H052	contig048880-BurOR.H063	0,08	0,05	0,64
contig041756-NyeOR.H075	contig013365-TilOR.H111	0,08	0,06	0,78

contig035579-NyeOR.H068	contig013363-TilOR.H110	0,08	0,05	0,63
contig048880-BurOR.H063	contig041757-NyeOR.H076	0,08	0,05	0,67
contig048880-BurOR.H063	contig053788-BurOR.H069	0,08	0,06	0,70
contig035579-NyeOR.H068	contig041757-NyeOR.H076	0,08	0,05	0,63
contig053788-BurOR.H069	contig035579-NyeOR.H068	0,08	0,05	0,66
contig041756-NyeOR.H139	contig013363-TilOR.H110	0,08	0,03	0,34
contig053784-BurOR.H067	contig014059-ZebOR.H068	0,08	0,06	0,78
contig041756-NyeOR.H139	contig013359-TilOR.H107	0,08	0,04	0,55
contig013365-TilOR.H111	contig014059-ZebOR.H068	0,08	0,07	0,91
contig013371-TilOR.H118	contig057403-ZebOR.H077	0,08	0,02	0,25
contig006794-BurOR.H061	contig013371-TilOR.H118	0,08	0,03	0,36
contig013356-TilOR.H105	contig013365-TilOR.H111	0,08	0,06	0,67
contig053787-BurOR.H068	contig014060-ZebOR.H069	0,08	0,06	0,73
contig039725-NyeOR.H072	contig014060-ZebOR.H069	0,08	0,06	0,75
contig041756-NyeOR.H075	contig014059-ZebOR.H068	0,08	0,06	0,73
contig048880-BurOR.H063	contig014060-ZebOR.H069	0,08	0,06	0,70
contig035579-NyeOR.H068	contig014060-ZebOR.H069	0,08	0,05	0,63
contig116846-BriOR.H055	contig018434-ZebOR.H071	0,09	0,09	1,03
contig013351-TilOR.H104	contig014060-ZebOR.H069	0,09	0,08	0,89
contig048880-BurOR.H063	contig013358-TilOR.H106	0,09	0,06	0,69
contig035579-NyeOR.H068	contig013359-TilOR.H107	0,09	0,06	0,65
contig039725-NyeOR.H072	contig013365-TilOR.H111	0,09	0,07	0,87
contig053787-BurOR.H068	contig013365-TilOR.H111	0,09	0,08	0,88
contig041757-NyeOR.H076	contig013358-TilOR.H106	0,09	0,07	0,82
contig013371-TilOR.H117	contig057403-ZebOR.H076	0,09	0,02	0,23
contig053784-BurOR.H067	contig035579-NyeOR.H068	0,09	0,05	0,58
contig048880-BurOR.H063	contig013363-TilOR.H110	0,09	0,05	0,60
contig013359-TilOR.H107	contig014060-ZebOR.H069	0,09	0,07	0,81
contig041756-NyeOR.H075	contig013359-TilOR.H107	0,09	0,07	0,75
contig035579-NyeOR.H068	contig013351-TilOR.H104	0,09	0,08	0,81
contig041757-NyeOR.H076	contig013351-TilOR.H104	0,09	0,10	1,03
contig041756-NyeOR.H139	contig014059-ZebOR.H068	0,09	0,07	0,74
contig013358-TilOR.H106	contig014059-ZebOR.H068	0,09	0,07	0,75
contig048880-BurOR.H063	contig041756-NyeOR.H075	0,09	0,05	0,58
contig041757-NyeOR.H076	contig014060-ZebOR.H069	0,09	0,08	0,82
contig035579-NyeOR.H068	contig041756-NyeOR.H075	0,09	0,05	0,51
contig041757-NyeOR.H076	contig014059-ZebOR.H068	0,09	0,09	0,99
contig064821-BriOR.H052	contig041756-NyeOR.H139	0,09	0,06	0,63
contig048880-BurOR.H063	contig013359-TilOR.H107	0,09	0,06	0,59
contig053784-BurOR.H067	contig053787-BurOR.H068	0,09	0,06	0,65
contig053784-BurOR.H067	contig039725-NyeOR.H072	0,09	0,06	0,67
contig013351-TilOR.H104	contig013356-TilOR.H105	0,10	0,09	0,93
contig041757-NyeOR.H076	contig041756-NyeOR.H139	0,10	0,07	0,69

contig013358-TilOR.H106	contig013363-TilOR.H110	0,10	0,06	0,67
contig053784-BurOR.H067	contig013359-TilOR.H107	0,10	0,07	0,76
contig039725-NyeOR.H072	contig041756-NyeOR.H075	0,10	0,06	0,59
contig053787-BurOR.H068	contig041756-NyeOR.H075	0,10	0,06	0,61
contig013356-TilOR.H105	contig013358-TilOR.H106	0,10	0,07	0,74
contig039725-NyeOR.H072	contig041756-NyeOR.H139	0,10	0,07	0,67
contig053787-BurOR.H068	contig041756-NyeOR.H139	0,10	0,07	0,69
contig048880-BurOR.H063	contig053784-BurOR.H067	0,10	0,06	0,59
contig039725-NyeOR.H072	contig041757-NyeOR.H076	0,10	0,09	0,95
contig053787-BurOR.H068	contig041757-NyeOR.H076	0,10	0,09	0,96
contig013363-TilOR.H110	contig014060-ZebOR.H069	0,10	0,06	0,59
contig041756-NyeOR.H075	contig013351-TilOR.H104	0,10	0,08	0,77
contig041756-NyeOR.H075	contig013363-TilOR.H110	0,10	0,05	0,54
contig053788-BurOR.H069	contig013358-TilOR.H106	0,10	0,07	0,75
contig018434-ZebOR.H071	contig018437-ZebOR.H072	0,10	0,07	0,71
contig013351-TilOR.H104	contig013363-TilOR.H110	0,10	0,09	0,87
contig053782-BurOR.H066	contig013359-TilOR.H107	0,10	0,04	0,44
contig053784-BurOR.H067	contig013351-TilOR.H104	0,10	0,08	0,76
contig064821-BriOR.H052	contig013359-TilOR.H107	0,10	0,07	0,71
contig048880-BurOR.H063	contig013351-TilOR.H104	0,10	0,08	0,80
contig013351-TilOR.H104	contig013358-TilOR.H106	0,10	0,07	0,72
contig053788-BurOR.H069	contig013351-TilOR.H104	0,10	0,10	0,94
contig035583-NyeOR.H071	contig013371-TilOR.H118	0,10	0,03	0,25
contig053788-BurOR.H069	contig014060-ZebOR.H069	0,10	0,08	0,74
contig013359-TilOR.H107	contig014059-ZebOR.H068	0,10	0,07	0,68
contig053788-BurOR.H069	contig013359-TilOR.H107	0,10	0,08	0,79
contig064821-BriOR.H052	contig013351-TilOR.H104	0,10	0,08	0,79
contig064821-BriOR.H052	contig014060-ZebOR.H069	0,10	0,07	0,69
contig048882-BurOR.H064	contig013369-TilOR.H114	0,10	0,02	0,23
contig053784-BurOR.H067	contig041757-NyeOR.H076	0,10	0,08	0,74
contig013359-TilOR.H107	contig014057-ZebOR.H067	0,11	0,04	0,35
contig013356-TilOR.H105	contig014060-ZebOR.H069	0,11	0,09	0,82
contig053788-BurOR.H069	contig041756-NyeOR.H139	0,11	0,07	0,66
contig013361-TilOR.H108	contig013362-TilOR.H109	0,11	0,06	0,53
contig053784-BurOR.H067	contig013363-TilOR.H110	0,11	0,06	0,53
contig053782-BurOR.H066	contig035579-NyeOR.H068	0,11	0,08	0,71
contig039729-NyeOR.H073	contig041756-NyeOR.H139	0,11	0,06	0,60
contig039725-NyeOR.H072	contig013358-TilOR.H106	0,11	0,07	0,66
contig053787-BurOR.H068	contig013358-TilOR.H106	0,11	0,07	0,67
contig053787-BurOR.H068	contig053788-BurOR.H069	0,11	0,09	0,88
contig053788-BurOR.H069	contig039725-NyeOR.H072	0,11	0,10	0,89
contig064821-BriOR.H052	contig014059-ZebOR.H068	0,11	0,09	0,83
contig041756-NyeOR.H075	contig041757-NyeOR.H076	0,11	0,07	0,70

contig039725-NyeOR.H072	contig013359-TilOR.H107	0,11	0,07	0,62
contig053787-BurOR.H068	contig013359-TilOR.H107	0,11	0,07	0,66
contig041756-NyeOR.H139	contig013351-TilOR.H104	0,11	0,08	0,73
contig035579-NyeOR.H068	contig014059-ZebOR.H068	0,11	0,07	0,66
contig041756-NyeOR.H139	contig013358-TilOR.H106	0,11	0,06	0,53
contig064817-BriOR.H051	contig013359-TilOR.H107	0,11	0,04	0,39
contig039729-NyeOR.H073	contig013359-TilOR.H107	0,11	0,05	0,43
contig035579-NyeOR.H068	contig014057-ZebOR.H067	0,11	0,07	0,61
contig035579-NyeOR.H068	contig013356-TilOR.H105	0,11	0,06	0,50
contig064821-BriOR.H052	contig053784-BurOR.H067	0,11	0,07	0,65
contig064821-BriOR.H052	contig039725-NyeOR.H072	0,11	0,09	0,80
contig064821-BriOR.H052	contig053787-BurOR.H068	0,11	0,09	0,80
contig053788-BurOR.H069	contig014059-ZebOR.H068	0,11	0,09	0,82
contig013363-TilOR.H110	contig014059-ZebOR.H068	0,11	0,08	0,74
contig013356-TilOR.H105	contig014059-ZebOR.H068	0,11	0,10	0,85
contig041757-NyeOR.H076	contig013359-TilOR.H107	0,11	0,08	0,70
contig053784-BurOR.H067	contig053788-BurOR.H069	0,11	0,08	0,68
contig035579-NyeOR.H068	contig039725-NyeOR.H072	0,11	0,07	0,64
contig053787-BurOR.H068	contig035579-NyeOR.H068	0,11	0,07	0,65
contig064821-BriOR.H052	contig041756-NyeOR.H075	0,11	0,07	0,62
contig064821-BriOR.H052	contig013363-TilOR.H110	0,12	0,07	0,57
contig053788-BurOR.H069	contig013363-TilOR.H110	0,12	0,08	0,68
contig048880-BurOR.H063	contig053782-BurOR.H066	0,12	0,08	0,68
contig035579-NyeOR.H068	contig039729-NyeOR.H073	0,12	0,08	0,68
contig039729-NyeOR.H073	contig041756-NyeOR.H075	0,12	0,08	0,65
contig048880-BurOR.H063	contig013356-TilOR.H105	0,12	0,06	0,48
contig013356-TilOR.H105	contig013359-TilOR.H107	0,12	0,08	0,66
contig053782-BurOR.H066	contig041756-NyeOR.H139	0,12	0,06	0,52
contig053788-BurOR.H069	contig041756-NyeOR.H075	0,12	0,08	0,67
contig013351-TilOR.H104	contig013359-TilOR.H107	0,12	0,08	0,67
contig039725-NyeOR.H072	contig013363-TilOR.H110	0,12	0,08	0,68
contig053787-BurOR.H068	contig013363-TilOR.H110	0,12	0,08	0,72
contig039725-NyeOR.H072	contig013356-TilOR.H105	0,12	0,10	0,82
contig053787-BurOR.H068	contig013356-TilOR.H105	0,12	0,10	0,82
contig013358-TilOR.H106	contig013359-TilOR.H107	0,12	0,07	0,63
contig064821-BriOR.H052	contig013358-TilOR.H106	0,12	0,06	0,52
contig013356-TilOR.H105	contig013363-TilOR.H110	0,12	0,08	0,66
contig048880-BurOR.H063	contig014059-ZebOR.H068	0,12	0,08	0,66
contig018434-ZebOR.H070	contig018434-ZebOR.H071	0,12	0,09	0,79
contig048880-BurOR.H063	contig014057-ZebOR.H067	0,12	0,07	0,59
contig041756-NyeOR.H139	contig014057-ZebOR.H067	0,12	0,05	0,44
contig053784-BurOR.H067	contig013356-TilOR.H105	0,12	0,09	0,72
contig039729-NyeOR.H073	contig013363-TilOR.H110	0,12	0,05	0,38

contig053782-BurOR.H066	contig013363-TilOR.H110	0,12	0,04	0,36
contig048880-BurOR.H063	contig039725-NyeOR.H072	0,12	0,08	0,64
contig048880-BurOR.H063	contig053787-BurOR.H068	0,12	0,08	0,65
contig035582-NyeOR.H070	contig057403-ZebOR.H076	0,12	0,07	0,56
contig053782-BurOR.H066	contig013351-TilOR.H104	0,12	0,10	0,82
contig041756-NyeOR.H075	contig013356-TilOR.H105	0,12	0,08	0,66
contig041757-NyeOR.H076	contig013363-TilOR.H110	0,13	0,08	0,60
contig048880-BurOR.H063	contig039729-NyeOR.H073	0,13	0,08	0,65
contig039729-NyeOR.H073	contig014060-ZebOR.H069	0,13	0,08	0,64
contig041756-NyeOR.H075	contig014057-ZebOR.H067	0,13	0,07	0,56
contig053782-BurOR.H066	contig041756-NyeOR.H075	0,13	0,07	0,57
contig013363-TilOR.H110	contig014057-ZebOR.H067	0,13	0,04	0,31
contig041756-NyeOR.H139	contig013356-TilOR.H105	0,13	0,07	0,54
contig018434-ZebOR.H070	contig030011-ZebOR.H073	0,13	0,08	0,64
contig039729-NyeOR.H073	contig013365-TilOR.H111	0,13	0,07	0,53
contig053782-BurOR.H066	contig013365-TilOR.H111	0,13	0,07	0,51
contig064817-BriOR.H051	contig041756-NyeOR.H075	0,13	0,07	0,54
contig064817-BriOR.H051	contig013363-TilOR.H110	0,13	0,04	0,33
contig033889-BriOR.H049	contig018434-ZebOR.H070	0,13	0,09	0,68
contig116846-BriOR.H055	contig018434-ZebOR.H070	0,13	0,09	0,65
contig053784-BurOR.H067	contig014057-ZebOR.H067	0,13	0,08	0,57
contig053784-BurOR.H067	contig039729-NyeOR.H073	0,13	0,08	0,61
contig053782-BurOR.H066	contig053784-BurOR.H067	0,13	0,08	0,59
contig009565-TilOR.H126	contig018434-ZebOR.H071	0,13	0,11	0,83
contig039729-NyeOR.H073	contig013351-TilOR.H104	0,13	0,10	0,78
contig013365-TilOR.H111	contig014057-ZebOR.H067	0,13	0,06	0,43
contig014057-ZebOR.H067	contig014060-ZebOR.H069	0,13	0,08	0,56
contig053782-BurOR.H066	contig014060-ZebOR.H069	0,13	0,08	0,57
contig064817-BriOR.H051	contig041756-NyeOR.H139	0,14	0,05	0,39
contig064817-BriOR.H051	contig053784-BurOR.H067	0,14	0,08	0,55
contig018434-ZebOR.H070	contig018437-ZebOR.H072	0,14	0,08	0,57
contig064821-BriOR.H052	contig039729-NyeOR.H073	0,14	0,08	0,59
contig064817-BriOR.H051	contig014060-ZebOR.H069	0,14	0,08	0,54
contig053782-BurOR.H066	contig014059-ZebOR.H068	0,14	0,09	0,68
contig039729-NyeOR.H073	contig041757-NyeOR.H076	0,14	0,09	0,64
contig053782-BurOR.H066	contig041757-NyeOR.H076	0,14	0,09	0,62
contig013362-TilOR.H109	contig013368-TilOR.H112	0,14	0,10	0,72
contig013351-TilOR.H104	contig014057-ZebOR.H067	0,14	0,10	0,67
contig053782-BurOR.H066	contig039725-NyeOR.H072	0,14	0,09	0,63
contig053782-BurOR.H066	contig053787-BurOR.H068	0,14	0,10	0,67
contig014057-ZebOR.H067	contig014059-ZebOR.H068	0,14	0,09	0,64
contig064817-BriOR.H051	contig035579-NyeOR.H068	0,14	0,07	0,49
contig013358-TilOR.H106	contig014057-ZebOR.H067	0,15	0,08	0,55

contig053782-BurOR.H066	contig013358-TilOR.H106	0,15	0,08	0,56
contig064821-BriOR.H052	contig053782-BurOR.H066	0,15	0,08	0,53
contig064817-BriOR.H051	contig013365-TilOR.H111	0,15	0,06	0,43
contig039725-NyeOR.H072	contig014057-ZebOR.H067	0,15	0,09	0,60
contig053787-BurOR.H068	contig014057-ZebOR.H067	0,15	0,09	0,64
contig039729-NyeOR.H073	contig014059-ZebOR.H068	0,15	0,10	0,66
contig053788-BurOR.H069	contig039729-NyeOR.H073	0,15	0,10	0,62
contig053782-BurOR.H066	contig053788-BurOR.H069	0,15	0,09	0,60
contig039725-NyeOR.H072	contig039729-NyeOR.H073	0,15	0,09	0,61
contig053787-BurOR.H068	contig039729-NyeOR.H073	0,15	0,10	0,65
contig039729-NyeOR.H073	contig013358-TilOR.H106	0,16	0,08	0,52
contig064817-BriOR.H051	contig014059-ZebOR.H068	0,16	0,10	0,60
contig009565-TilOR.H126	contig030011-ZebOR.H073	0,16	0,10	0,64
contig116846-BriOR.H055	contig009565-TilOR.H126	0,16	0,11	0,66
contig041757-NyeOR.H076	contig014057-ZebOR.H067	0,16	0,08	0,51
contig064817-BriOR.H051	contig039725-NyeOR.H072	0,16	0,09	0,57
contig064817-BriOR.H051	contig053787-BurOR.H068	0,16	0,10	0,60
contig064817-BriOR.H051	contig048880-BurOR.H063	0,16	0,07	0,45
contig033889-BriOR.H049	contig009565-TilOR.H126	0,17	0,10	0,62
contig064817-BriOR.H051	contig013358-TilOR.H106	0,17	0,09	0,51
contig064817-BriOR.H051	contig064821-BriOR.H052	0,17	0,09	0,51
contig064821-BriOR.H052	contig014057-ZebOR.H067	0,17	0,07	0,44
contig064817-BriOR.H051	contig041757-NyeOR.H076	0,17	0,09	0,53
contig064817-BriOR.H051	contig013351-TilOR.H104	0,17	0,11	0,63
contig013368-TilOR.H112	contig013369-TilOR.H115	0,17	0,05	0,28
contig013361-TilOR.H108	contig013368-TilOR.H113	0,17	0,12	0,71
contig013361-TilOR.H108	contig013369-TilOR.H115	0,17	0,08	0,46
contig009565-TilOR.H126	contig018437-ZebOR.H072	0,17	0,10	0,56
contig053788-BurOR.H069	contig014057-ZebOR.H067	0,17	0,09	0,50
contig013368-TilOR.H113	contig013369-TilOR.H115	0,18	0,10	0,59
contig013361-TilOR.H108	contig013368-TilOR.H112	0,18	0,10	0,56
contig064817-BriOR.H051	contig053788-BurOR.H069	0,19	0,09	0,49
contig039729-NyeOR.H073	contig013356-TilOR.H105	0,19	0,09	0,47
contig053782-BurOR.H066	contig013356-TilOR.H105	0,19	0,09	0,45
contig013369-TilOR.H114	contig013369-TilOR.H115	0,19	0,09	0,47
contig048882-BurOR.H064	contig013371-TilOR.H118	0,20	0,11	0,57
contig013361-TilOR.H108	contig013371-TilOR.H118	0,20	0,13	0,63
contig064817-BriOR.H051	contig013356-TilOR.H105	0,20	0,09	0,46
contig035582-NyeOR.H070	contig013371-TilOR.H117	0,20	0,07	0,34
contig013362-TilOR.H109	contig013369-TilOR.H115	0,20	0,09	0,45
contig013369-TilOR.H115	contig013369-TilOR.H116	0,20	0,10	0,48
contig013361-TilOR.H108	contig013369-TilOR.H116	0,21	0,10	0,50
contig013356-TilOR.H105	contig014057-ZebOR.H067	0,21	0,08	0,39

contig035580-NyeOR.H069	contig013369-TilOR.H114	0,21	0,10	0,47
contig013369-TilOR.H114	contig057400-ZebOR.H075	0,21	0,10	0,48
contig048882-BurOR.H064	contig035580-NyeOR.H069	0,21	0,10	0,50
contig013368-TilOR.H112	contig013368-TilOR.H113	0,21	0,12	0,55
contig048882-BurOR.H064	contig057400-ZebOR.H075	0,21	0,10	0,50
contig013368-TilOR.H113	contig013371-TilOR.H118	0,21	0,14	0,65
contig013361-TilOR.H108	contig013369-TilOR.H114	0,21	0,10	0,46
contig013369-TilOR.H114	contig013369-TilOR.H116	0,21	0,11	0,53
contig048882-BurOR.H064	contig013361-TilOR.H108	0,21	0,11	0,50
contig013362-TilOR.H109	contig013368-TilOR.H113	0,22	0,14	0,63
contig048882-BurOR.H064	contig013369-TilOR.H115	0,22	0,10	0,48
contig006794-BurOR.H061	contig048882-BurOR.H064	0,22	0,15	0,69
contig013369-TilOR.H116	contig013371-TilOR.H118	0,22	0,14	0,64
contig013369-TilOR.H114	contig013371-TilOR.H118	0,22	0,11	0,49
contig013368-TilOR.H113	contig013369-TilOR.H116	0,22	0,11	0,50
contig035580-NyeOR.H069	contig013369-TilOR.H115	0,23	0,12	0,52
contig013368-TilOR.H113	contig013369-TilOR.H114	0,23	0,12	0,53
contig035580-NyeOR.H069	contig013371-TilOR.H118	0,23	0,11	0,50
contig048882-BurOR.H064	contig013368-TilOR.H113	0,23	0,13	0,56
contig013371-TilOR.H118	contig057400-ZebOR.H075	0,23	0,11	0,50
contig013369-TilOR.H115	contig057400-ZebOR.H075	0,23	0,12	0,52
contig048882-BurOR.H064	contig057403-ZebOR.H077	0,23	0,15	0,67
contig035580-NyeOR.H069	contig013369-TilOR.H116	0,23	0,13	0,56
contig035580-NyeOR.H069	contig013368-TilOR.H113	0,23	0,12	0,51
contig035580-NyeOR.H069	contig013361-TilOR.H108	0,23	0,12	0,50
contig013361-TilOR.H108	contig057400-ZebOR.H075	0,24	0,12	0,49
contig035580-NyeOR.H069	contig013368-TilOR.H112	0,24	0,12	0,49
contig013369-TilOR.H115	contig013371-TilOR.H118	0,24	0,12	0,52
contig048882-BurOR.H064	contig013368-TilOR.H112	0,24	0,11	0,45
contig013362-TilOR.H109	contig013369-TilOR.H116	0,24	0,12	0,50
contig013368-TilOR.H112	contig013371-TilOR.H118	0,24	0,13	0,54
contig013369-TilOR.H114	contig057403-ZebOR.H077	0,24	0,12	0,48
contig013362-TilOR.H109	contig013371-TilOR.H118	0,24	0,14	0,56
contig048882-BurOR.H064	contig013362-TilOR.H109	0,24	0,13	0,52
contig006794-BurOR.H061	contig013369-TilOR.H114	0,24	0,12	0,48
contig013369-TilOR.H116	contig057400-ZebOR.H075	0,24	0,13	0,55
contig013368-TilOR.H113	contig057400-ZebOR.H075	0,24	0,12	0,49
contig049873-BriOR.H050	contig013361-TilOR.H108	0,25	0,13	0,54
contig049873-BriOR.H050	contig048882-BurOR.H064	0,25	0,15	0,61
contig013362-TilOR.H109	contig013369-TilOR.H114	0,25	0,11	0,45
contig049873-BriOR.H050	contig013369-TilOR.H114	0,25	0,12	0,47
contig013361-TilOR.H108	contig057403-ZebOR.H077	0,25	0,13	0,53
contig013368-TilOR.H112	contig057400-ZebOR.H075	0,25	0,12	0,47

contig009546-TilOR.H100	contig018434-ZebOR.H070	0,25	0,15	0,59
contig006794-BurOR.H061	contig013361-TilOR.H108	0,25	0,13	0,52
contig035583-NyeOR.H071	contig013369-TilOR.H114	0,25	0,12	0,46
contig035580-NyeOR.H069	contig057403-ZebOR.H077	0,25	0,12	0,45
contig057400-ZebOR.H075	contig057403-ZebOR.H077	0,25	0,12	0,45
contig033889-BriOR.H049	contig009546-TilOR.H100	0,25	0,15	0,60
contig048882-BurOR.H064	contig013369-TilOR.H116	0,26	0,18	0,68
contig013368-TilOR.H112	contig013369-TilOR.H116	0,26	0,11	0,44
contig035580-NyeOR.H069	contig035583-NyeOR.H071	0,26	0,11	0,44
contig049873-BriOR.H050	contig013368-TilOR.H113	0,26	0,17	0,66
contig035580-NyeOR.H069	contig013362-TilOR.H109	0,26	0,14	0,53
contig009546-TilOR.H100	contig018437-ZebOR.H072	0,26	0,14	0,53
contig035582-NyeOR.H070	contig013361-TilOR.H108	0,26	0,14	0,54
contig013368-TilOR.H112	contig013369-TilOR.H114	0,26	0,10	0,37
contig035583-NyeOR.H071	contig013361-TilOR.H108	0,26	0,13	0,50
contig048882-BurOR.H064	contig035583-NyeOR.H071	0,26	0,16	0,59
contig006794-BurOR.H061	contig035580-NyeOR.H069	0,27	0,11	0,42
contig006794-BurOR.H061	contig057400-ZebOR.H075	0,27	0,11	0,42
contig009546-TilOR.H100	contig018434-ZebOR.H071	0,27	0,14	0,54
contig035583-NyeOR.H071	contig013368-TilOR.H113	0,27	0,17	0,63
contig035583-NyeOR.H071	contig057400-ZebOR.H075	0,27	0,11	0,42
contig009546-TilOR.H100	contig030011-ZebOR.H073	0,27	0,13	0,49
contig049873-BriOR.H050	contig035580-NyeOR.H069	0,27	0,11	0,42
contig049873-BriOR.H050	contig013369-TilOR.H115	0,27	0,13	0,49
contig013362-TilOR.H109	contig057400-ZebOR.H075	0,27	0,14	0,52
contig013368-TilOR.H113	contig057403-ZebOR.H077	0,28	0,17	0,63
contig013368-TilOR.H112	contig057403-ZebOR.H077	0,28	0,14	0,49
contig035582-NyeOR.H070	contig013362-TilOR.H109	0,28	0,15	0,52
contig006794-BurOR.H061	contig013368-TilOR.H112	0,28	0,13	0,46
contig009546-TilOR.H100	contig009565-TilOR.H126	0,28	0,15	0,54
contig116846-BriOR.H055	contig009546-TilOR.H100	0,28	0,12	0,44
contig013369-TilOR.H115	contig057403-ZebOR.H077	0,28	0,13	0,47
contig049873-BriOR.H050	contig057400-ZebOR.H075	0,28	0,11	0,40
contig006794-BurOR.H061	contig013368-TilOR.H113	0,28	0,17	0,61
contig006794-BurOR.H061	contig013369-TilOR.H115	0,28	0,13	0,46
contig049873-BriOR.H050	contig013362-TilOR.H109	0,29	0,15	0,52
contig035583-NyeOR.H071	contig013369-TilOR.H115	0,29	0,13	0,45
contig035582-NyeOR.H070	contig013368-TilOR.H112	0,29	0,12	0,41
contig013362-TilOR.H109	contig057403-ZebOR.H077	0,29	0,15	0,52
contig009547-TilOR.H101	contig018434-ZebOR.H071	0,29	0,15	0,52
contig006794-BurOR.H061	contig013362-TilOR.H109	0,29	0,15	0,52
contig049873-BriOR.H050	contig013369-TilOR.H116	0,29	0,18	0,61
contig013368-TilOR.H113	contig013371-TilOR.H117	0,30	0,16	0,53

contig009546-TilOR.H100	contig009547-TilOR.H101	0,30	0,14	0,47
contig009547-TilOR.H101	contig018434-ZebOR.H070	0,30	0,17	0,58
contig049873-BriOR.H050	contig013368-TilOR.H112	0,30	0,14	0,46
contig009547-TilOR.H101	contig030011-ZebOR.H073	0,30	0,19	0,66
contig013361-TilOR.H108	contig013371-TilOR.H117	0,30	0,16	0,53
contig013361-TilOR.H108	contig057403-ZebOR.H076	0,30	0,16	0,54
contig013369-TilOR.H116	contig057403-ZebOR.H077	0,30	0,20	0,67
contig035582-NyeOR.H070	contig057400-ZebOR.H075	0,31	0,14	0,46
contig035583-NyeOR.H071	contig013362-TilOR.H109	0,31	0,15	0,48
contig035583-NyeOR.H071	contig013368-TilOR.H112	0,31	0,13	0,44
contig033889-BriOR.H049	contig009547-TilOR.H101	0,31	0,19	0,62
contig009547-TilOR.H101	contig009565-TilOR.H126	0,31	0,17	0,55
contig006794-BurOR.H061	contig013369-TilOR.H116	0,31	0,20	0,64
contig035583-NyeOR.H071	contig013369-TilOR.H116	0,31	0,20	0,66
contig048882-BurOR.H064	contig035582-NyeOR.H070	0,31	0,14	0,46
contig035582-NyeOR.H070	contig013369-TilOR.H115	0,31	0,14	0,44
contig013362-TilOR.H109	contig013371-TilOR.H117	0,31	0,17	0,55
contig116846-BriOR.H055	contig009547-TilOR.H101	0,31	0,20	0,64
contig035582-NyeOR.H070	contig013369-TilOR.H114	0,32	0,14	0,44
contig013369-TilOR.H115	contig013371-TilOR.H117	0,32	0,15	0,47
contig035580-NyeOR.H069	contig035582-NyeOR.H070	0,32	0,14	0,43
contig035582-NyeOR.H070	contig013368-TilOR.H113	0,32	0,15	0,46
contig013369-TilOR.H115	contig057403-ZebOR.H076	0,33	0,15	0,47
contig035582-NyeOR.H070	contig013371-TilOR.H118	0,33	0,16	0,47
contig048882-BurOR.H064	contig057403-ZebOR.H076	0,34	0,16	0,46
contig013368-TilOR.H113	contig057403-ZebOR.H076	0,34	0,16	0,48
contig013362-TilOR.H109	contig057403-ZebOR.H076	0,34	0,18	0,52
contig009547-TilOR.H101	contig018437-ZebOR.H072	0,34	0,19	0,56
contig048882-BurOR.H064	contig013371-TilOR.H117	0,35	0,15	0,44
contig013369-TilOR.H116	contig013371-TilOR.H117	0,35	0,17	0,49
contig013368-TilOR.H112	contig013371-TilOR.H117	0,35	0,14	0,39
contig013369-TilOR.H114	contig013371-TilOR.H117	0,36	0,15	0,41
contig035582-NyeOR.H070	contig013369-TilOR.H116	0,36	0,16	0,44
contig035582-NyeOR.H070	contig057403-ZebOR.H077	0,37	0,16	0,44
contig035580-NyeOR.H069	contig057403-ZebOR.H076	0,37	0,15	0,41
contig057400-ZebOR.H075	contig057403-ZebOR.H076	0,37	0,15	0,42
contig013371-TilOR.H117	contig013371-TilOR.H118	0,37	0,16	0,43
contig013368-TilOR.H112	contig057403-ZebOR.H076	0,37	0,14	0,38
contig013369-TilOR.H114	contig057403-ZebOR.H076	0,37	0,15	0,40
contig013369-TilOR.H116	contig057403-ZebOR.H076	0,37	0,17	0,46
contig006794-BurOR.H061	contig035582-NyeOR.H070	0,38	0,15	0,41
contig013371-TilOR.H118	contig057403-ZebOR.H076	0,38	0,16	0,43
contig035582-NyeOR.H070	contig035583-NyeOR.H071	0,38	0,16	0,42

contig035580-NyeOR.H069	contig013371-TilOR.H117	0,39	0,15	0,39
contig049873-BriOR.H050	contig035582-NyeOR.H070	0,39	0,16	0,40
contig013371-TilOR.H117	contig057400-ZebOR.H075	0,39	0,15	0,39
contig057403-ZebOR.H076	contig057403-ZebOR.H077	0,40	0,17	0,43
contig049873-BriOR.H050	contig013371-TilOR.H117	0,41	0,17	0,41
contig006794-BurOR.H061	contig057403-ZebOR.H076	0,41	0,16	0,40
contig049873-BriOR.H050	contig057403-ZebOR.H076	0,41	0,17	0,40
contig035583-NyeOR.H071	contig057403-ZebOR.H076	0,42	0,17	0,41
contig013371-TilOR.H117	contig057403-ZebOR.H077	0,42	0,17	0,41
contig035583-NyeOR.H071	contig013371-TilOR.H117	0,42	0,16	0,39
contig006794-BurOR.H061	contig013371-TilOR.H117	0,43	0,16	0,37
contig093825-BriOR.H053	contig013369-TilOR.H116	0,75	0,27	0,36
contig013368-TilOR.H113	contig047492-ZebOR.H074	0,77	0,25	0,33
contig048562-BurOR.H062	contig013368-TilOR.H113	0,77	0,25	0,33
contig013369-TilOR.H116	contig047492-ZebOR.H074	0,78	0,27	0,35
contig048562-BurOR.H062	contig013369-TilOR.H116	0,78	0,27	0,35
contig034998-NyeOR.H067	contig013368-TilOR.H113	0,78	0,25	0,33
contig034998-NyeOR.H067	contig013369-TilOR.H116	0,79	0,27	0,34
contig013369-TilOR.H116	contig041955-TilOR.H119	0,79	0,27	0,34
contig093825-BriOR.H053	contig013368-TilOR.H113	0,79	0,26	0,32
contig009546-TilOR.H100	contig013369-TilOR.H114	0,80	0,33	0,42
contig093825-BriOR.H053	contig013361-TilOR.H108	0,81	0,28	0,35
contig013361-TilOR.H108	contig047492-ZebOR.H074	0,81	0,28	0,35
contig048562-BurOR.H062	contig013361-TilOR.H108	0,81	0,28	0,35
contig034998-NyeOR.H067	contig013361-TilOR.H108	0,82	0,28	0,34
contig041756-NyeOR.H075	contig009546-TilOR.H100	0,83	0,38	0,46
contig093825-BriOR.H053	contig013362-TilOR.H109	0,83	0,29	0,35
contig013362-TilOR.H109	contig047492-ZebOR.H074	0,83	0,29	0,35
contig048562-BurOR.H062	contig013362-TilOR.H109	0,83	0,29	0,35
contig013368-TilOR.H113	contig041955-TilOR.H119	0,84	0,26	0,31
contig048562-BurOR.H062	contig013368-TilOR.H112	0,84	0,27	0,32
contig013369-TilOR.H114	contig041955-TilOR.H119	0,84	0,27	0,32
contig013368-TilOR.H112	contig047492-ZebOR.H074	0,84	0,27	0,32
contig093825-BriOR.H053	contig013369-TilOR.H114	0,84	0,27	0,32
contig013361-TilOR.H108	contig041955-TilOR.H119	0,84	0,28	0,33
contig034998-NyeOR.H067	contig013362-TilOR.H109	0,84	0,29	0,34
contig093825-BriOR.H053	contig013368-TilOR.H112	0,85	0,27	0,32
contig034998-NyeOR.H067	contig013368-TilOR.H112	0,85	0,27	0,31
contig093825-BriOR.H053	contig009546-TilOR.H100	0,85	0,34	0,40
contig093825-BriOR.H053	contig013371-TilOR.H117	0,85	0,25	0,29
contig013371-TilOR.H117	contig047492-ZebOR.H074	0,85	0,24	0,29
contig048562-BurOR.H062	contig013371-TilOR.H117	0,85	0,25	0,29
contig041756-NyeOR.H139	contig009546-TilOR.H100	0,85	0,39	0,45

contig034998-NyeOR.H067	contig013369-TilOR.H114	0,85	0,27	0,32
contig035579-NyeOR.H068	contig009546-TilOR.H100	0,86	0,39	0,45
contig009546-TilOR.H100	contig014057-ZebOR.H067	0,86	0,37	0,43
contig048880-BurOR.H063	contig009546-TilOR.H100	0,86	0,37	0,43
contig035582-NyeOR.H070	contig009546-TilOR.H100	0,87	0,32	0,37
contig034998-NyeOR.H067	contig013371-TilOR.H117	0,87	0,25	0,28
contig009546-TilOR.H100	contig057403-ZebOR.H076	0,87	0,30	0,35
contig013369-TilOR.H114	contig047492-ZebOR.H074	0,87	0,27	0,31
contig048562-BurOR.H062	contig013369-TilOR.H114	0,87	0,27	0,31
contig013362-TilOR.H109	contig041955-TilOR.H119	0,87	0,29	0,33
contig116846-BriOR.H055	contig013369-TilOR.H114	0,87	0,35	0,40
contig041756-NyeOR.H075	contig009565-TilOR.H126	0,88	0,40	0,46
contig053784-BurOR.H067	contig009546-TilOR.H100	0,88	0,37	0,42
contig013371-TilOR.H117	contig041955-TilOR.H119	0,88	0,25	0,28
contig048562-BurOR.H062	contig009546-TilOR.H100	0,88	0,33	0,38
contig009546-TilOR.H100	contig047492-ZebOR.H074	0,88	0,33	0,38
contig048880-BurOR.H063	contig013369-TilOR.H114	0,89	0,39	0,43
contig093825-BriOR.H053	contig013371-TilOR.H118	0,89	0,28	0,32
contig013363-TilOR.H110	contig057403-ZebOR.H076	0,89	0,36	0,41
contig013371-TilOR.H118	contig047492-ZebOR.H074	0,89	0,28	0,31
contig048562-BurOR.H062	contig013371-TilOR.H118	0,89	0,28	0,32
contig013369-TilOR.H114	contig018437-ZebOR.H072	0,89	0,35	0,39
contig009546-TilOR.H100	contig041955-TilOR.H119	0,89	0,32	0,36
contig009546-TilOR.H100	contig013369-TilOR.H115	0,90	0,34	0,38
contig034998-NyeOR.H067	contig009546-TilOR.H100	0,90	0,33	0,37
contig033889-BriOR.H049	contig014057-ZebOR.H067	0,90	0,40	0,45
contig116846-BriOR.H055	contig013371-TilOR.H118	0,90	0,34	0,38
contig009546-TilOR.H100	contig014060-ZebOR.H069	0,90	0,36	0,41
contig053784-BurOR.H067	contig009565-TilOR.H126	0,90	0,40	0,44
contig009546-TilOR.H100	contig013365-TilOR.H111	0,90	0,39	0,43
contig116846-BriOR.H055	contig041955-TilOR.H119	0,90	0,34	0,38
contig041955-TilOR.H119	contig018434-ZebOR.H071	0,90	0,35	0,39
contig013363-TilOR.H110	contig013369-TilOR.H114	0,90	0,39	0,44
contig048882-BurOR.H064	contig018437-ZebOR.H072	0,90	0,39	0,44
contig093825-BriOR.H053	contig035582-NyeOR.H070	0,90	0,27	0,30
contig035582-NyeOR.H070	contig047492-ZebOR.H074	0,90	0,26	0,29
contig048562-BurOR.H062	contig035582-NyeOR.H070	0,90	0,27	0,30
contig018437-ZebOR.H072	contig057400-ZebOR.H075	0,90	0,34	0,38
contig048882-BurOR.H064	contig041955-TilOR.H119	0,90	0,29	0,32
contig009546-TilOR.H100	contig013363-TilOR.H110	0,91	0,39	0,43
contig034998-NyeOR.H067	contig013371-TilOR.H118	0,91	0,28	0,31
contig116846-BriOR.H055	contig013369-TilOR.H115	0,91	0,35	0,39
contig093825-BriOR.H053	contig057400-ZebOR.H075	0,91	0,26	0,29

contig048882-BurOR.H064	contig009546-TilOR.H100	0,91	0,35	0,38
contig116846-BriOR.H055	contig057400-ZebOR.H075	0,91	0,34	0,38
contig009546-TilOR.H100	contig013368-TilOR.H113	0,91	0,33	0,36
contig116846-BriOR.H055	contig035580-NyeOR.H069	0,91	0,34	0,37
contig047492-ZebOR.H074	contig057400-ZebOR.H075	0,91	0,26	0,28
contig048562-BurOR.H062	contig057400-ZebOR.H075	0,91	0,26	0,28
contig041756-NyeOR.H075	contig018434-ZebOR.H070	0,91	0,40	0,44
contig013368-TilOR.H113	contig009565-TilOR.H126	0,91	0,31	0,34
contig009546-TilOR.H100	contig013369-TilOR.H116	0,91	0,32	0,35
contig093825-BriOR.H053	contig116846-BriOR.H055	0,91	0,35	0,38
contig013363-TilOR.H110	contig013368-TilOR.H112	0,91	0,36	0,40
contig033889-BriOR.H049	contig041756-NyeOR.H075	0,91	0,40	0,43
contig033889-BriOR.H049	contig013369-TilOR.H114	0,91	0,35	0,38
contig048880-BurOR.H063	contig013361-TilOR.H108	0,91	0,41	0,45
contig035582-NyeOR.H070	contig013363-TilOR.H110	0,92	0,39	0,42
contig034998-NyeOR.H067	contig035582-NyeOR.H070	0,92	0,27	0,29
contig093825-BriOR.H053	contig018434-ZebOR.H071	0,92	0,36	0,39
contig116846-BriOR.H055	contig013368-TilOR.H113	0,92	0,35	0,38
contig009546-TilOR.H100	contig013358-TilOR.H106	0,92	0,36	0,40
contig035582-NyeOR.H070	contig018437-ZebOR.H072	0,92	0,31	0,34
contig053782-BurOR.H066	contig009546-TilOR.H100	0,92	0,37	0,40
contig034998-NyeOR.H067	contig057400-ZebOR.H075	0,92	0,26	0,28
contig013369-TilOR.H114	contig030011-ZebOR.H073	0,92	0,35	0,38
contig035582-NyeOR.H070	contig030011-ZebOR.H073	0,92	0,33	0,36
contig093825-BriOR.H053	contig013369-TilOR.H115	0,92	0,26	0,28
contig009547-TilOR.H101	contig013369-TilOR.H114	0,92	0,31	0,34
contig009546-TilOR.H100	contig013371-TilOR.H117	0,92	0,30	0,32
contig064821-BriOR.H052	contig009546-TilOR.H100	0,92	0,41	0,44
contig013369-TilOR.H115	contig047492-ZebOR.H074	0,92	0,26	0,28
contig048562-BurOR.H062	contig013369-TilOR.H115	0,92	0,26	0,28
contig013363-TilOR.H110	contig013371-TilOR.H117	0,92	0,37	0,40
contig013368-TilOR.H112	contig041955-TilOR.H119	0,92	0,26	0,29
contig009546-TilOR.H100	contig013361-TilOR.H108	0,92	0,35	0,37
contig009546-TilOR.H100	contig013359-TilOR.H107	0,92	0,38	0,41
contig041955-TilOR.H119	contig057400-ZebOR.H075	0,92	0,25	0,27
contig013363-TilOR.H110	contig013368-TilOR.H113	0,92	0,37	0,40
contig041756-NyeOR.H139	contig009565-TilOR.H126	0,93	0,41	0,44
contig035580-NyeOR.H069	contig018437-ZebOR.H072	0,93	0,34	0,37
contig053788-BurOR.H069	contig009546-TilOR.H100	0,93	0,40	0,44
contig116846-BriOR.H055	contig035582-NyeOR.H070	0,93	0,32	0,35
contig048882-BurOR.H064	contig009565-TilOR.H126	0,93	0,33	0,36
contig116846-BriOR.H055	contig041756-NyeOR.H075	0,93	0,39	0,42
contig035579-NyeOR.H068	contig013371-TilOR.H117	0,93	0,36	0,39

contig116846-BriOR.H055	contig048882-BurOR.H064	0,93	0,38	0,41
contig014057-ZebOR.H067	contig030011-ZebOR.H073	0,93	0,40	0,43
contig041955-TilOR.H119	contig018437-ZebOR.H072	0,93	0,33	0,36
contig033889-BriOR.H049	contig064817-BriOR.H051	0,93	0,40	0,43
contig013371-TilOR.H118	contig009565-TilOR.H126	0,93	0,33	0,36
contig035579-NyeOR.H068	contig013369-TilOR.H114	0,93	0,39	0,41
contig041955-TilOR.H119	contig030011-ZebOR.H073	0,93	0,34	0,36
contig033889-BriOR.H049	contig053784-BurOR.H067	0,93	0,40	0,43
contig033889-BriOR.H049	contig041756-NyeOR.H139	0,93	0,41	0,44
contig116846-BriOR.H055	contig048562-BurOR.H062	0,93	0,35	0,37
contig116846-BriOR.H055	contig047492-ZebOR.H074	0,93	0,35	0,38
contig048562-BurOR.H062	contig018434-ZebOR.H071	0,93	0,36	0,38
contig018434-ZebOR.H071	contig047492-ZebOR.H074	0,93	0,36	0,39
contig093825-BriOR.H053	contig035580-NyeOR.H069	0,93	0,25	0,27
contig048880-BurOR.H063	contig013362-TilOR.H109	0,93	0,39	0,42
contig035580-NyeOR.H069	contig047492-ZebOR.H074	0,93	0,25	0,27
contig048562-BurOR.H062	contig035580-NyeOR.H069	0,93	0,25	0,27
contig064817-BriOR.H051	contig009546-TilOR.H100	0,93	0,37	0,40
contig013362-TilOR.H109	contig013363-TilOR.H110	0,93	0,37	0,40
contig013369-TilOR.H115	contig009565-TilOR.H126	0,93	0,32	0,34
contig033889-BriOR.H049	contig013371-TilOR.H118	0,93	0,35	0,37
contig009547-TilOR.H101	contig013368-TilOR.H112	0,93	0,29	0,31
contig013371-TilOR.H118	contig041955-TilOR.H119	0,93	0,28	0,30
contig048880-BurOR.H063	contig013368-TilOR.H112	0,94	0,36	0,39
contig009547-TilOR.H101	contig013369-TilOR.H115	0,94	0,32	0,34
contig053784-BurOR.H067	contig018434-ZebOR.H070	0,94	0,40	0,43
contig041756-NyeOR.H075	contig030011-ZebOR.H073	0,94	0,39	0,42
contig035579-NyeOR.H068	contig013368-TilOR.H112	0,94	0,36	0,38
contig034998-NyeOR.H067	contig013369-TilOR.H115	0,94	0,26	0,28
contig116846-BriOR.H055	contig053784-BurOR.H067	0,94	0,40	0,42
contig093825-BriOR.H053	contig048882-BurOR.H064	0,94	0,29	0,31
contig030011-ZebOR.H073	contig057400-ZebOR.H075	0,94	0,35	0,37
contig009565-TilOR.H126	contig014060-ZebOR.H069	0,94	0,39	0,42
contig035580-NyeOR.H069	contig030011-ZebOR.H073	0,94	0,34	0,36
contig013369-TilOR.H115	contig018434-ZebOR.H070	0,94	0,35	0,37
contig013368-TilOR.H113	contig018434-ZebOR.H070	0,94	0,34	0,36
contig035579-NyeOR.H068	contig057403-ZebOR.H076	0,94	0,35	0,38
contig014057-ZebOR.H067	contig018434-ZebOR.H070	0,94	0,41	0,44
contig013359-TilOR.H107	contig013369-TilOR.H114	0,94	0,39	0,41
contig048882-BurOR.H064	contig018434-ZebOR.H070	0,94	0,36	0,38
contig116846-BriOR.H055	contig013361-TilOR.H108	0,94	0,35	0,38
contig009565-TilOR.H126	contig014057-ZebOR.H067	0,94	0,37	0,40
contig033889-BriOR.H049	contig013369-TilOR.H116	0,94	0,36	0,38

contig093825-BriOR.H053	contig018437-ZebOR.H072	0,94	0,34	0,36
contig033889-BriOR.H049	contig035582-NyeOR.H070	0,94	0,32	0,34
contig116846-BriOR.H055	contig014057-ZebOR.H067	0,94	0,40	0,43
contig013371-TilOR.H118	contig018434-ZebOR.H070	0,94	0,34	0,36
contig033889-BriOR.H049	contig093825-BriOR.H053	0,94	0,36	0,38
contig033889-BriOR.H049	contig035580-NyeOR.H069	0,95	0,34	0,36
contig093825-BriOR.H053	contig030011-ZebOR.H073	0,95	0,35	0,37
contig033889-BriOR.H049	contig041955-TilOR.H119	0,95	0,35	0,37
contig035582-NyeOR.H070	contig018434-ZebOR.H070	0,95	0,33	0,35
contig039729-NyeOR.H073	contig009546-TilOR.H100	0,95	0,37	0,39
contig116846-BriOR.H055	contig034998-NyeOR.H067	0,95	0,35	0,37
contig034998-NyeOR.H067	contig018434-ZebOR.H071	0,95	0,36	0,38
contig013368-TilOR.H113	contig030011-ZebOR.H073	0,95	0,35	0,37
contig013369-TilOR.H114	contig009565-TilOR.H126	0,95	0,32	0,34
contig041756-NyeOR.H075	contig057403-ZebOR.H076	0,95	0,36	0,38
contig034998-NyeOR.H067	contig035580-NyeOR.H069	0,95	0,25	0,27
contig116846-BriOR.H055	contig013368-TilOR.H112	0,95	0,33	0,34
contig035579-NyeOR.H068	contig013362-TilOR.H109	0,95	0,38	0,40
contig053784-BurOR.H067	contig030011-ZebOR.H073	0,95	0,40	0,42
contig013369-TilOR.H114	contig018434-ZebOR.H070	0,95	0,35	0,37
contig053782-BurOR.H066	contig013369-TilOR.H114	0,95	0,39	0,41
contig041756-NyeOR.H139	contig057403-ZebOR.H076	0,95	0,35	0,37
contig009546-TilOR.H100	contig057400-ZebOR.H075	0,95	0,32	0,34
contig013371-TilOR.H118	contig030011-ZebOR.H073	0,95	0,35	0,37
contig035580-NyeOR.H069	contig009546-TilOR.H100	0,95	0,32	0,34
contig035580-NyeOR.H069	contig041955-TilOR.H119	0,95	0,25	0,26
contig013365-TilOR.H111	contig013369-TilOR.H114	0,95	0,38	0,40
contig116846-BriOR.H055	contig057403-ZebOR.H076	0,95	0,31	0,33
contig033889-BriOR.H049	contig057400-ZebOR.H075	0,95	0,35	0,37
contig041756-NyeOR.H139	contig013369-TilOR.H114	0,95	0,39	0,41
contig041955-TilOR.H119	contig057403-ZebOR.H077	0,95	0,29	0,31
contig048882-BurOR.H064	contig034998-NyeOR.H067	0,95	0,29	0,30
contig033889-BriOR.H049	contig013368-TilOR.H113	0,95	0,35	0,37
contig093825-BriOR.H053	contig057403-ZebOR.H077	0,95	0,30	0,31
contig041757-NyeOR.H076	contig009546-TilOR.H100	0,95	0,40	0,42
contig039729-NyeOR.H073	contig013369-TilOR.H114	0,95	0,40	0,42
contig064817-BriOR.H051	contig009565-TilOR.H126	0,96	0,39	0,41
contig013369-TilOR.H116	contig018434-ZebOR.H071	0,96	0,33	0,35
contig035582-NyeOR.H070	contig009565-TilOR.H126	0,96	0,30	0,32
contig013361-TilOR.H108	contig009565-TilOR.H126	0,96	0,34	0,35
contig013369-TilOR.H115	contig018434-ZebOR.H071	0,96	0,36	0,38
contig035580-NyeOR.H069	contig009547-TilOR.H101	0,96	0,32	0,33
contig014060-ZebOR.H069	contig018434-ZebOR.H070	0,96	0,40	0,42

contig009547-TilOR.H101	contig013371-TilOR.H118	0,96	0,30	0,31
contig013369-TilOR.H115	contig041955-TilOR.H119	0,96	0,26	0,27
contig033889-BriOR.H049	contig013369-TilOR.H115	0,96	0,34	0,36
contig035579-NyeOR.H068	contig013361-TilOR.H108	0,96	0,41	0,42
contig048880-BurOR.H063	contig057403-ZebOR.H076	0,96	0,36	0,37
contig013369-TilOR.H114	contig014057-ZebOR.H067	0,96	0,39	0,40
contig018437-ZebOR.H072	contig057403-ZebOR.H076	0,96	0,31	0,32
contig009547-TilOR.H101	contig057403-ZebOR.H076	0,96	0,28	0,29
contig048880-BurOR.H063	contig048882-BurOR.H064	0,96	0,40	0,42
contig013368-TilOR.H113	contig018437-ZebOR.H072	0,96	0,35	0,37
contig033889-BriOR.H049	contig013361-TilOR.H108	0,96	0,35	0,36
contig048882-BurOR.H064	contig013363-TilOR.H110	0,96	0,40	0,42
contig041756-NyeOR.H075	contig018437-ZebOR.H072	0,96	0,39	0,40
contig035582-NyeOR.H070	contig018434-ZebOR.H071	0,96	0,33	0,34
contig048562-BurOR.H062	contig018437-ZebOR.H072	0,96	0,34	0,35
contig018437-ZebOR.H072	contig047492-ZebOR.H074	0,96	0,34	0,36
contig013365-TilOR.H111	contig057403-ZebOR.H076	0,96	0,34	0,35
contig013369-TilOR.H114	contig018434-ZebOR.H071	0,96	0,35	0,36
contig093825-BriOR.H053	contig057403-ZebOR.H076	0,96	0,24	0,25
contig048880-BurOR.H063	contig013371-TilOR.H117	0,96	0,36	0,38
contig041955-TilOR.H119	contig057403-ZebOR.H076	0,96	0,24	0,25
contig041756-NyeOR.H139	contig030011-ZebOR.H073	0,96	0,41	0,42
contig013358-TilOR.H106	contig057403-ZebOR.H076	0,96	0,35	0,37
contig116846-BriOR.H055	contig013369-TilOR.H116	0,96	0,37	0,38
contig013369-TilOR.H115	contig018437-ZebOR.H072	0,96	0,35	0,36
contig047492-ZebOR.H074	contig057403-ZebOR.H076	0,96	0,24	0,25
contig048562-BurOR.H062	contig057403-ZebOR.H076	0,96	0,24	0,25
contig033889-BriOR.H049	contig048562-BurOR.H062	0,96	0,36	0,37
contig033889-BriOR.H049	contig047492-ZebOR.H074	0,96	0,36	0,37
contig048562-BurOR.H062	contig030011-ZebOR.H073	0,96	0,35	0,36
contig030011-ZebOR.H073	contig047492-ZebOR.H074	0,96	0,35	0,36
contig009547-TilOR.H101	contig057400-ZebOR.H075	0,97	0,32	0,33
contig041756-NyeOR.H075	contig018434-ZebOR.H071	0,97	0,36	0,38
contig035582-NyeOR.H070	contig041955-TilOR.H119	0,97	0,26	0,27
contig009546-TilOR.H100	contig013351-TilOR.H104	0,97	0,39	0,40
contig013361-TilOR.H108	contig013363-TilOR.H110	0,97	0,40	0,42
contig048882-BurOR.H064	contig047492-ZebOR.H074	0,97	0,29	0,30
contig048562-BurOR.H062	contig048882-BurOR.H064	0,97	0,29	0,30
contig093825-BriOR.H053	contig035583-NyeOR.H071	0,97	0,29	0,30
contig033889-BriOR.H049	contig053782-BurOR.H066	0,97	0,40	0,41
contig053784-BurOR.H067	contig057403-ZebOR.H076	0,97	0,36	0,37
contig041756-NyeOR.H139	contig018434-ZebOR.H070	0,97	0,40	0,42
contig013369-TilOR.H116	contig009565-TilOR.H126	0,97	0,33	0,34

contig049873-BriOR.H050	contig093825-BriOR.H053	0,97	0,29	0,30
contig013363-TilOR.H110	contig013369-TilOR.H116	0,97	0,39	0,40
contig033889-BriOR.H049	contig049873-BriOR.H050	0,97	0,36	0,37
contig013361-TilOR.H108	contig018434-ZebOR.H070	0,97	0,36	0,37
contig053788-BurOR.H069	contig057403-ZebOR.H076	0,97	0,38	0,39
contig013368-TilOR.H113	contig018434-ZebOR.H071	0,97	0,35	0,36
contig048882-BurOR.H064	contig009547-TilOR.H101	0,97	0,35	0,36
contig116846-BriOR.H055	contig014060-ZebOR.H069	0,97	0,39	0,40
contig041756-NyeOR.H139	contig018437-ZebOR.H072	0,97	0,40	0,41
contig030011-ZebOR.H073	contig057403-ZebOR.H076	0,98	0,31	0,32
contig116846-BriOR.H055	contig013365-TilOR.H111	0,98	0,39	0,40
contig041756-NyeOR.H139	contig013361-TilOR.H108	0,98	0,40	0,41
contig048882-BurOR.H064	contig018434-ZebOR.H071	0,98	0,37	0,37
contig034998-NyeOR.H067	contig018437-ZebOR.H072	0,98	0,34	0,35
contig049873-BriOR.H050	contig009565-TilOR.H126	0,98	0,34	0,35
contig013359-TilOR.H107	contig018434-ZebOR.H070	0,98	0,41	0,42
contig041756-NyeOR.H139	contig013368-TilOR.H112	0,98	0,35	0,36
contig013365-TilOR.H111	contig013369-TilOR.H116	0,98	0,37	0,38
contig013365-TilOR.H111	contig013368-TilOR.H112	0,98	0,34	0,35
contig014060-ZebOR.H069	contig057403-ZebOR.H076	0,98	0,36	0,37
contig053784-BurOR.H067	contig018437-ZebOR.H072	0,98	0,39	0,40
contig034998-NyeOR.H067	contig057403-ZebOR.H076	0,98	0,24	0,25
contig053784-BurOR.H067	contig018434-ZebOR.H071	0,98	0,37	0,38
contig033889-BriOR.H049	contig034998-NyeOR.H067	0,98	0,36	0,37
contig034998-NyeOR.H067	contig030011-ZebOR.H073	0,98	0,35	0,36
contig049873-BriOR.H050	contig116846-BriOR.H055	0,98	0,38	0,39
contig013359-TilOR.H107	contig057403-ZebOR.H076	0,98	0,36	0,37
contig009546-TilOR.H100	contig014059-ZebOR.H068	0,98	0,38	0,39
contig033889-BriOR.H049	contig013365-TilOR.H111	0,98	0,40	0,41
contig009547-TilOR.H101	contig013361-TilOR.H108	0,98	0,32	0,32
contig013369-TilOR.H115	contig030011-ZebOR.H073	0,98	0,35	0,35
contig047492-ZebOR.H074	contig057403-ZebOR.H077	0,98	0,29	0,30
contig048562-BurOR.H062	contig057403-ZebOR.H077	0,98	0,29	0,30
contig013358-TilOR.H106	contig013371-TilOR.H117	0,98	0,36	0,37
contig033889-BriOR.H049	contig057403-ZebOR.H076	0,98	0,31	0,32
contig033889-BriOR.H049	contig035579-NyeOR.H068	0,98	0,42	0,42
contig013365-TilOR.H111	contig009565-TilOR.H126	0,98	0,39	0,40
contig009546-TilOR.H100	contig013356-TilOR.H105	0,98	0,40	0,41
contig013363-TilOR.H110	contig013369-TilOR.H115	0,98	0,39	0,39
contig013371-TilOR.H118	contig018437-ZebOR.H072	0,98	0,34	0,35
contig048880-BurOR.H063	contig013369-TilOR.H115	0,98	0,39	0,39
contig049873-BriOR.H050	contig030011-ZebOR.H073	0,98	0,38	0,39
contig049873-BriOR.H050	contig041955-TilOR.H119	0,99	0,28	0,29

contig018434-ZebOR.H070	contig057403-ZebOR.H076	0,99	0,31	0,32
contig035579-NyeOR.H068	contig013369-TilOR.H115	0,99	0,39	0,39
contig116846-BriOR.H055	contig041756-NyeOR.H139	0,99	0,41	0,42
contig013361-TilOR.H108	contig013365-TilOR.H111	0,99	0,40	0,40
contig013369-TilOR.H116	contig030011-ZebOR.H073	0,99	0,35	0,36
contig041757-NyeOR.H076	contig057403-ZebOR.H076	0,99	0,38	0,38
contig009546-TilOR.H100	contig013368-TilOR.H112	0,99	0,31	0,32
contig014057-ZebOR.H067	contig018437-ZebOR.H072	0,99	0,40	0,40
contig035582-NyeOR.H070	contig009547-TilOR.H101	0,99	0,29	0,30
contig013365-TilOR.H111	contig013371-TilOR.H117	0,99	0,35	0,35
contig048880-BurOR.H063	contig018434-ZebOR.H070	0,99	0,41	0,41
contig033889-BriOR.H049	contig013359-TilOR.H107	0,99	0,40	0,40
contig013365-TilOR.H111	contig013368-TilOR.H113	0,99	0,36	0,36
contig013368-TilOR.H112	contig018437-ZebOR.H072	0,99	0,33	0,33
contig048882-BurOR.H064	contig030011-ZebOR.H073	0,99	0,39	0,40
contig035582-NyeOR.H070	contig041756-NyeOR.H075	0,99	0,38	0,38
contig048882-BurOR.H064	contig041756-NyeOR.H075	0,99	0,40	0,41
contig064821-BriOR.H052	contig057403-ZebOR.H076	0,99	0,37	0,38
contig013371-TilOR.H118	contig018434-ZebOR.H071	0,99	0,35	0,36
contig013363-TilOR.H110	contig009565-TilOR.H126	0,99	0,41	0,41
contig035582-NyeOR.H070	contig041756-NyeOR.H139	0,99	0,37	0,38
contig039725-NyeOR.H072	contig009546-TilOR.H100	0,99	0,38	0,39
contig053787-BurOR.H068	contig009546-TilOR.H100	0,99	0,38	0,39
contig041756-NyeOR.H139	contig009547-TilOR.H101	0,99	0,36	0,37
contig013359-TilOR.H107	contig009565-TilOR.H126	0,99	0,40	0,40
contig013359-TilOR.H107	contig018437-ZebOR.H072	0,99	0,40	0,40
contig009565-TilOR.H126	contig057403-ZebOR.H077	0,99	0,35	0,35
contig107626-BriOR.H054	contig013363-TilOR.H110	0,99	0,45	0,46
contig013369-TilOR.H116	contig018437-ZebOR.H072	1,00	0,36	0,36
contig009565-TilOR.H126	contig057403-ZebOR.H076	1,00	0,30	0,30
contig013359-TilOR.H107	contig013369-TilOR.H116	1,00	0,38	0,38
contig033889-BriOR.H049	contig048882-BurOR.H064	1,00	0,39	0,39
contig035583-NyeOR.H071	contig047492-ZebOR.H074	1,00	0,29	0,29
contig048562-BurOR.H062	contig035583-NyeOR.H071	1,00	0,29	0,29
contig013359-TilOR.H107	contig018434-ZebOR.H071	1,00	0,38	0,38
contig006794-BurOR.H061	contig041955-TilOR.H119	1,00	0,29	0,29
contig035583-NyeOR.H071	contig041955-TilOR.H119	1,00	0,29	0,29
contig064817-BriOR.H051	contig030011-ZebOR.H073	1,00	0,39	0,39
contig013351-TilOR.H104	contig013369-TilOR.H114	1,00	0,38	0,38
contig034998-NyeOR.H067	contig057403-ZebOR.H077	1,00	0,29	0,30
contig041756-NyeOR.H139	contig018434-ZebOR.H071	1,00	0,37	0,37
contig006794-BurOR.H061	contig009565-TilOR.H126	1,00	0,34	0,34
contig053784-BurOR.H067	contig009547-TilOR.H101	1,00	0,36	0,36

contig013351-TilOR.H104	contig057403-ZebOR.H076	1,00	0,35	0,35
contig064817-BriOR.H051	contig013369-TilOR.H114	1,00	0,39	0,39
contig033889-BriOR.H049	contig013368-TilOR.H112	1,00	0,33	0,33
contig035582-NyeOR.H070	contig013365-TilOR.H111	1,00	0,36	0,36
contig033889-BriOR.H049	contig039729-NyeOR.H073	1,00	0,40	0,40
contig093825-BriOR.H053	contig006794-BurOR.H061	1,00	0,29	0,29
contig013361-TilOR.H108	contig030011-ZebOR.H073	1,00	0,35	0,35
contig013362-TilOR.H109	contig013365-TilOR.H111	1,00	0,36	0,36
contig013368-TilOR.H112	contig009565-TilOR.H126	1,00	0,30	0,30
contig049873-BriOR.H050	contig047492-ZebOR.H074	1,00	0,29	0,29
contig049873-BriOR.H050	contig048562-BurOR.H062	1,00	0,29	0,29
contig064817-BriOR.H051	contig018434-ZebOR.H070	1,00	0,41	0,41
contig048882-BurOR.H064	contig014057-ZebOR.H067	1,00	0,41	0,41
contig064817-BriOR.H051	contig018434-ZebOR.H071	1,00	0,38	0,38
contig048882-BurOR.H064	contig035579-NyeOR.H068	1,00	0,40	0,40
contig107626-BriOR.H054	contig041756-NyeOR.H139	1,00	0,43	0,43
contig013359-TilOR.H107	contig013368-TilOR.H112	1,00	0,36	0,36
contig035579-NyeOR.H068	contig030011-ZebOR.H073	1,00	0,42	0,41
contig033889-BriOR.H049	contig013363-TilOR.H110	1,00	0,40	0,40
contig035580-NyeOR.H069	contig009565-TilOR.H126	1,00	0,31	0,31
contig006794-BurOR.H061	contig018434-ZebOR.H070	1,00	0,35	0,35
contig013365-TilOR.H111	contig018434-ZebOR.H070	1,00	0,39	0,39
contig116846-BriOR.H055	contig013359-TilOR.H107	1,00	0,40	0,40
contig018434-ZebOR.H070	contig057403-ZebOR.H077	1,00	0,35	0,35
contig048882-BurOR.H064	contig053782-BurOR.H066	1,00	0,41	0,40
contig049873-BriOR.H050	contig018434-ZebOR.H070	1,00	0,35	0,35
contig041955-TilOR.H119	contig018434-ZebOR.H070	1,00	0,35	0,34
contig035579-NyeOR.H068	contig035582-NyeOR.H070	1,01	0,38	0,38
contig033889-BriOR.H049	contig014060-ZebOR.H069	1,01	0,38	0,38
contig013369-TilOR.H116	contig018434-ZebOR.H070	1,01	0,34	0,33
contig018434-ZebOR.H071	contig057403-ZebOR.H076	1,01	0,31	0,31
contig041756-NyeOR.H139	contig013371-TilOR.H117	1,01	0,36	0,36
contig039729-NyeOR.H073	contig009565-TilOR.H126	1,01	0,39	0,39
contig041756-NyeOR.H139	contig013362-TilOR.H109	1,01	0,37	0,37
contig009565-TilOR.H126	contig057400-ZebOR.H075	1,01	0,32	0,32
contig013365-TilOR.H111	contig030011-ZebOR.H073	1,01	0,40	0,39
contig053788-BurOR.H069	contig013369-TilOR.H114	1,01	0,41	0,41
contig013351-TilOR.H104	contig013368-TilOR.H112	1,01	0,34	0,34
contig013351-TilOR.H104	contig009565-TilOR.H126	1,01	0,37	0,37
contig064821-BriOR.H052	contig013369-TilOR.H114	1,01	0,41	0,41
contig014060-ZebOR.H069	contig030011-ZebOR.H073	1,01	0,38	0,38
contig033889-BriOR.H049	contig064821-BriOR.H052	1,01	0,41	0,41
contig013363-TilOR.H110	contig018437-ZebOR.H072	1,01	0,41	0,40

contig013361-TilOR.H108	contig018437-ZebOR.H072	1,01	0,35	0,35
contig053782-BurOR.H066	contig057403-ZebOR.H076	1,01	0,37	0,36
contig033889-BriOR.H049	contig048880-BurOR.H063	1,01	0,41	0,41
contig116846-BriOR.H055	contig006794-BurOR.H061	1,01	0,37	0,37
contig053782-BurOR.H066	contig009565-TilOR.H126	1,01	0,39	0,39
contig035579-NyeOR.H068	contig013368-TilOR.H113	1,01	0,37	0,36
contig053788-BurOR.H069	contig009547-TilOR.H101	1,01	0,36	0,36
contig013359-TilOR.H107	contig013362-TilOR.H109	1,01	0,37	0,37
contig049873-BriOR.H050	contig009547-TilOR.H101	1,01	0,35	0,34
contig048882-BurOR.H064	contig039729-NyeOR.H073	1,01	0,41	0,41
contig013363-TilOR.H110	contig018434-ZebOR.H071	1,01	0,39	0,39
contig041756-NyeOR.H075	contig009547-TilOR.H101	1,01	0,35	0,35
contig041756-NyeOR.H075	contig013371-TilOR.H117	1,01	0,37	0,36
contig093825-BriOR.H053	contig009565-TilOR.H126	1,01	0,33	0,33
contig013359-TilOR.H107	contig013361-TilOR.H108	1,01	0,40	0,39
contig048880-BurOR.H063	contig009547-TilOR.H101	1,01	0,36	0,36
contig034998-NyeOR.H067	contig035583-NyeOR.H071	1,01	0,29	0,29
contig035580-NyeOR.H069	contig018434-ZebOR.H071	1,01	0,35	0,34
contig041756-NyeOR.H075	contig013369-TilOR.H114	1,01	0,39	0,39
contig116846-BriOR.H055	contig013362-TilOR.H109	1,01	0,35	0,34
contig116846-BriOR.H055	contig057403-ZebOR.H077	1,01	0,36	0,36
contig035583-NyeOR.H071	contig009565-TilOR.H126	1,01	0,34	0,33
contig035583-NyeOR.H071	contig018434-ZebOR.H070	1,01	0,35	0,35
contig009547-TilOR.H101	contig013365-TilOR.H111	1,02	0,36	0,36
contig009547-TilOR.H101	contig014057-ZebOR.H067	1,02	0,36	0,35
contig048882-BurOR.H064	contig013359-TilOR.H107	1,02	0,40	0,39
contig039729-NyeOR.H073	contig057403-ZebOR.H076	1,02	0,37	0,37
contig013359-TilOR.H107	contig030011-ZebOR.H073	1,02	0,40	0,39
contig018434-ZebOR.H071	contig057400-ZebOR.H075	1,02	0,35	0,35
contig009547-TilOR.H101	contig013362-TilOR.H109	1,02	0,32	0,31
contig049873-BriOR.H050	contig034998-NyeOR.H067	1,02	0,29	0,28
contig013359-TilOR.H107	contig013369-TilOR.H115	1,02	0,37	0,37
contig041756-NyeOR.H139	contig013368-TilOR.H113	1,02	0,38	0,37
contig093825-BriOR.H053	contig018434-ZebOR.H070	1,02	0,35	0,34
contig014060-ZebOR.H069	contig018437-ZebOR.H072	1,02	0,39	0,38
contig048882-BurOR.H064	contig041756-NyeOR.H139	1,02	0,40	0,39
contig116846-BriOR.H055	contig035579-NyeOR.H068	1,02	0,41	0,40
contig107626-BriOR.H054	contig013365-TilOR.H111	1,02	0,46	0,45
contig014060-ZebOR.H069	contig018434-ZebOR.H071	1,02	0,36	0,35
contig013368-TilOR.H112	contig030011-ZebOR.H073	1,02	0,33	0,32
contig053782-BurOR.H066	contig035582-NyeOR.H070	1,02	0,39	0,38
contig053782-BurOR.H066	contig018434-ZebOR.H070	1,02	0,41	0,40
contig048880-BurOR.H063	contig018434-ZebOR.H071	1,02	0,38	0,37

contig013359-TilOR.H107	contig013368-TilOR.H113	1,02	0,37	0,36
contig041955-TilOR.H119	contig009565-TilOR.H126	1,02	0,33	0,32
contig116846-BriOR.H055	contig048880-BurOR.H063	1,02	0,43	0,42
contig033889-BriOR.H049	contig013362-TilOR.H109	1,02	0,35	0,34
contig116846-BriOR.H055	contig035583-NyeOR.H071	1,02	0,36	0,35
contig048880-BurOR.H063	contig009565-TilOR.H126	1,02	0,41	0,40
contig033889-BriOR.H049	contig041757-NyeOR.H076	1,02	0,41	0,40
contig053782-BurOR.H066	contig013361-TilOR.H108	1,02	0,41	0,40
contig013351-TilOR.H104	contig013361-TilOR.H108	1,02	0,40	0,39
contig033889-BriOR.H049	contig013351-TilOR.H104	1,02	0,40	0,39
contig064821-BriOR.H052	contig013369-TilOR.H116	1,02	0,39	0,38
contig035579-NyeOR.H068	contig018434-ZebOR.H070	1,02	0,41	0,40
contig013358-TilOR.H106	contig018434-ZebOR.H070	1,02	0,39	0,38
contig041756-NyeOR.H139	contig014055-ZebOR.H066	1,02	0,44	0,43
contig053780-BurOR.H065	contig041756-NyeOR.H139	1,02	0,44	0,43
contig009546-TilOR.H100	contig013371-TilOR.H118	1,02	0,32	0,31
contig013351-TilOR.H104	contig013368-TilOR.H113	1,02	0,37	0,36
contig116846-BriOR.H055	contig013371-TilOR.H117	1,03	0,31	0,30
contig035579-NyeOR.H068	contig013369-TilOR.H116	1,03	0,38	0,37
contig006794-BurOR.H061	contig018437-ZebOR.H072	1,03	0,38	0,37
contig064821-BriOR.H052	contig013361-TilOR.H108	1,03	0,42	0,41
contig035582-NyeOR.H070	contig013359-TilOR.H107	1,03	0,38	0,37
contig041757-NyeOR.H076	contig018434-ZebOR.H070	1,03	0,41	0,40
contig013358-TilOR.H106	contig013369-TilOR.H114	1,03	0,39	0,38
contig035582-NyeOR.H070	contig039729-NyeOR.H073	1,03	0,39	0,38
contig013362-TilOR.H109	contig018434-ZebOR.H071	1,03	0,36	0,35
contig035579-NyeOR.H068	contig009565-TilOR.H126	1,03	0,42	0,41
contig006794-BurOR.H061	contig030011-ZebOR.H073	1,03	0,38	0,37
contig048880-BurOR.H063	contig035582-NyeOR.H070	1,03	0,38	0,37
contig039730-NyeOR.H074	contig041756-NyeOR.H139	1,03	0,45	0,43
contig053782-BurOR.H066	contig013368-TilOR.H112	1,03	0,37	0,36
contig013365-TilOR.H111	contig018434-ZebOR.H071	1,03	0,37	0,36
contig006794-BurOR.H061	contig047492-ZebOR.H074	1,03	0,29	0,28
contig006794-BurOR.H061	contig048562-BurOR.H062	1,03	0,29	0,28
contig013371-TilOR.H117	contig014060-ZebOR.H069	1,03	0,37	0,36
contig009546-TilOR.H100	contig013362-TilOR.H109	1,03	0,34	0,33
contig013351-TilOR.H104	contig013362-TilOR.H109	1,03	0,36	0,35
contig033889-BriOR.H049	contig053788-BurOR.H069	1,03	0,41	0,40
contig013363-TilOR.H110	contig030011-ZebOR.H073	1,03	0,40	0,39
contig035579-NyeOR.H068	contig009547-TilOR.H101	1,03	0,36	0,35
contig107626-BriOR.H054	contig035579-NyeOR.H068	1,03	0,45	0,44
contig013365-TilOR.H111	contig013369-TilOR.H115	1,03	0,37	0,36
contig014057-ZebOR.H067	contig018434-ZebOR.H071	1,03	0,37	0,36

contig013363-TilOR.H110	contig018434-ZebOR.H070	1,03	0,41	0,40
contig048882-BurOR.H064	contig013358-TilOR.H106	1,03	0,40	0,39
contig048880-BurOR.H063	contig018437-ZebOR.H072	1,03	0,41	0,39
contig041757-NyeOR.H076	contig013369-TilOR.H114	1,03	0,41	0,40
contig116846-BriOR.H055	contig041757-NyeOR.H076	1,03	0,40	0,39
contig033889-BriOR.H049	contig006794-BurOR.H061	1,03	0,37	0,36
contig035582-NyeOR.H070	contig013358-TilOR.H106	1,03	0,37	0,36
contig053788-BurOR.H069	contig013371-TilOR.H117	1,03	0,39	0,37
contig039725-NyeOR.H072	contig009565-TilOR.H126	1,03	0,39	0,38
contig053788-BurOR.H069	contig013368-TilOR.H113	1,03	0,37	0,36
contig053788-BurOR.H069	contig013368-TilOR.H112	1,03	0,37	0,36
contig064817-BriOR.H051	contig013361-TilOR.H108	1,03	0,41	0,40
contig049873-BriOR.H050	contig018437-ZebOR.H072	1,03	0,38	0,37
contig035580-NyeOR.H069	contig018434-ZebOR.H070	1,03	0,34	0,33
contig035579-NyeOR.H068	contig018437-ZebOR.H072	1,03	0,41	0,39
contig064821-BriOR.H052	contig013368-TilOR.H112	1,03	0,38	0,36
contig053780-BurOR.H065	contig013363-TilOR.H110	1,03	0,46	0,45
contig053788-BurOR.H069	contig018434-ZebOR.H070	1,03	0,41	0,40
contig041757-NyeOR.H076	contig030011-ZebOR.H073	1,03	0,41	0,39
contig013356-TilOR.H105	contig013369-TilOR.H116	1,03	0,38	0,37
contig093825-BriOR.H053	contig048880-BurOR.H063	1,04	0,37	0,35
contig018437-ZebOR.H072	contig057403-ZebOR.H077	1,04	0,38	0,36
contig039729-NyeOR.H073	contig013361-TilOR.H108	1,04	0,42	0,41
contig013361-TilOR.H108	contig014057-ZebOR.H067	1,04	0,41	0,40
contig048562-BurOR.H062	contig048880-BurOR.H063	1,04	0,37	0,36
contig048880-BurOR.H063	contig047492-ZebOR.H074	1,04	0,37	0,36
contig013365-TilOR.H111	contig018437-ZebOR.H072	1,04	0,39	0,38
contig018434-ZebOR.H070	contig057400-ZebOR.H075	1,04	0,35	0,34
contig035582-NyeOR.H070	contig013351-TilOR.H104	1,04	0,37	0,36
contig030011-ZebOR.H073	contig057403-ZebOR.H077	1,04	0,37	0,36
contig053784-BurOR.H067	contig035582-NyeOR.H070	1,04	0,38	0,37
contig053782-BurOR.H066	contig013368-TilOR.H113	1,04	0,38	0,37
contig116846-BriOR.H055	contig013363-TilOR.H110	1,04	0,40	0,39
contig013363-TilOR.H110	contig014055-ZebOR.H066	1,04	0,46	0,44
contig064817-BriOR.H051	contig013362-TilOR.H109	1,04	0,38	0,37
contig009565-TilOR.H126	contig014059-ZebOR.H068	1,04	0,39	0,37
contig039730-NyeOR.H074	contig013363-TilOR.H110	1,04	0,47	0,45
contig053784-BurOR.H067	contig013371-TilOR.H117	1,04	0,37	0,36
contig018434-ZebOR.H071	contig057403-ZebOR.H077	1,04	0,37	0,35
contig014057-ZebOR.H067	contig057403-ZebOR.H076	1,04	0,36	0,35
contig048562-BurOR.H062	contig009565-TilOR.H126	1,04	0,33	0,32
contig009565-TilOR.H126	contig047492-ZebOR.H074	1,04	0,33	0,32
contig064821-BriOR.H052	contig013362-TilOR.H109	1,04	0,39	0,38

contig064817-BriOR.H051	contig116846-BriOR.H055	1,04	0,41	0,40
contig033889-BriOR.H049	contig057403-ZebOR.H077	1,04	0,37	0,36
contig064821-BriOR.H052	contig116846-BriOR.H055	1,04	0,41	0,40
contig053782-BurOR.H066	contig030011-ZebOR.H073	1,04	0,39	0,38
contig116846-BriOR.H055	contig013358-TilOR.H106	1,04	0,38	0,37
contig009547-TilOR.H101	contig013369-TilOR.H116	1,04	0,32	0,31
contig013351-TilOR.H104	contig013369-TilOR.H116	1,04	0,37	0,36
contig116846-BriOR.H055	contig053788-BurOR.H069	1,04	0,40	0,38
contig013371-TilOR.H117	contig018437-ZebOR.H072	1,04	0,30	0,29
contig035583-NyeOR.H071	contig009546-TilOR.H100	1,04	0,33	0,32
contig093825-BriOR.H053	contig009547-TilOR.H101	1,04	0,31	0,30
contig013359-TilOR.H107	contig013371-TilOR.H117	1,04	0,37	0,35
contig039729-NyeOR.H073	contig013368-TilOR.H112	1,04	0,37	0,36
contig041756-NyeOR.H075	contig013368-TilOR.H112	1,04	0,35	0,34
contig041757-NyeOR.H076	contig013368-TilOR.H112	1,04	0,37	0,36
contig041756-NyeOR.H139	contig013369-TilOR.H116	1,04	0,38	0,37
contig013368-TilOR.H112	contig014057-ZebOR.H067	1,04	0,37	0,35
contig013368-TilOR.H112	contig018434-ZebOR.H071	1,04	0,33	0,32
contig041756-NyeOR.H075	contig041955-TilOR.H119	1,04	0,38	0,37
contig013371-TilOR.H117	contig030011-ZebOR.H073	1,04	0,31	0,30
contig064821-BriOR.H052	contig030011-ZebOR.H073	1,04	0,41	0,40
contig041757-NyeOR.H076	contig009547-TilOR.H101	1,04	0,36	0,34
contig053788-BurOR.H069	contig030011-ZebOR.H073	1,04	0,41	0,39
contig064821-BriOR.H052	contig013371-TilOR.H117	1,04	0,38	0,37
contig013361-TilOR.H108	contig018434-ZebOR.H071	1,04	0,36	0,34
contig048562-BurOR.H062	contig018434-ZebOR.H070	1,04	0,35	0,34
contig018434-ZebOR.H070	contig047492-ZebOR.H074	1,04	0,36	0,34
contig039729-NyeOR.H073	contig013368-TilOR.H113	1,04	0,39	0,37
contig033889-BriOR.H049	contig039725-NyeOR.H072	1,04	0,38	0,36
contig039725-NyeOR.H072	contig018434-ZebOR.H070	1,04	0,40	0,38
contig013356-TilOR.H105	contig057403-ZebOR.H076	1,05	0,36	0,35
contig035582-NyeOR.H070	contig014060-ZebOR.H069	1,05	0,38	0,37
contig013356-TilOR.H105	contig013369-TilOR.H114	1,05	0,40	0,38
contig013351-TilOR.H104	contig018434-ZebOR.H070	1,05	0,39	0,38
contig014059-ZebOR.H068	contig018434-ZebOR.H070	1,05	0,39	0,38
contig048880-BurOR.H063	contig013368-TilOR.H113	1,05	0,38	0,36
contig064821-BriOR.H052	contig018434-ZebOR.H070	1,05	0,42	0,40
contig006794-BurOR.H061	contig034998-NyeOR.H067	1,05	0,29	0,28
contig048882-BurOR.H064	contig013365-TilOR.H111	1,05	0,39	0,37
contig035579-NyeOR.H068	contig018434-ZebOR.H071	1,05	0,38	0,36
contig035583-NyeOR.H071	contig030011-ZebOR.H073	1,05	0,37	0,35
contig048880-BurOR.H063	contig013369-TilOR.H116	1,05	0,38	0,37
contig013368-TilOR.H112	contig018434-ZebOR.H070	1,05	0,32	0,31

contig035583-NyeOR.H071	contig018434-ZebOR.H071	1,05	0,36	0,35
contig041757-NyeOR.H076	contig009565-TilOR.H126	1,05	0,41	0,39
contig093825-BriOR.H053	contig041756-NyeOR.H075	1,05	0,38	0,36
contig033889-BriOR.H049	contig035583-NyeOR.H071	1,05	0,37	0,35
contig053788-BurOR.H069	contig013369-TilOR.H116	1,05	0,40	0,38
contig033889-BriOR.H049	contig013358-TilOR.H106	1,05	0,38	0,36
contig064821-BriOR.H052	contig018434-ZebOR.H071	1,05	0,38	0,36
contig041757-NyeOR.H076	contig013371-TilOR.H117	1,05	0,38	0,37
contig053782-BurOR.H066	contig013362-TilOR.H109	1,05	0,38	0,37
contig033889-BriOR.H049	contig014059-ZebOR.H068	1,05	0,38	0,36
contig048562-BurOR.H062	contig041756-NyeOR.H075	1,05	0,38	0,37
contig041756-NyeOR.H075	contig047492-ZebOR.H074	1,05	0,39	0,37
contig035582-NyeOR.H070	contig014057-ZebOR.H067	1,05	0,38	0,37
contig039729-NyeOR.H073	contig018434-ZebOR.H070	1,05	0,41	0,39
contig013358-TilOR.H106	contig013362-TilOR.H109	1,05	0,37	0,35
contig033889-BriOR.H049	contig013371-TilOR.H117	1,05	0,31	0,29
contig053787-BurOR.H068	contig009565-TilOR.H126	1,05	0,38	0,36
contig033889-BriOR.H049	contig013356-TilOR.H105	1,05	0,41	0,39
contig048882-BurOR.H064	contig014060-ZebOR.H069	1,05	0,40	0,38
contig107626-BriOR.H054	contig048880-BurOR.H063	1,05	0,45	0,43
contig048880-BurOR.H063	contig034998-NyeOR.H067	1,05	0,37	0,35
contig009547-TilOR.H101	contig014060-ZebOR.H069	1,05	0,35	0,33
contig116846-BriOR.H055	contig053782-BurOR.H066	1,05	0,39	0,37
contig053782-BurOR.H066	contig018434-ZebOR.H071	1,05	0,38	0,36
contig107626-BriOR.H054	contig053784-BurOR.H067	1,05	0,43	0,41
contig049873-BriOR.H050	contig018434-ZebOR.H071	1,05	0,36	0,34
contig048880-BurOR.H063	contig030011-ZebOR.H073	1,06	0,41	0,39
contig048882-BurOR.H064	contig013351-TilOR.H104	1,06	0,39	0,37
contig013369-TilOR.H114	contig014060-ZebOR.H069	1,06	0,39	0,37
contig039729-NyeOR.H073	contig013362-TilOR.H109	1,06	0,39	0,37
contig053787-BurOR.H068	contig018434-ZebOR.H070	1,06	0,39	0,37
contig116846-BriOR.H055	contig039725-NyeOR.H072	1,06	0,39	0,37
contig034998-NyeOR.H067	contig009565-TilOR.H126	1,06	0,33	0,31
contig041757-NyeOR.H076	contig013368-TilOR.H113	1,06	0,37	0,35
contig041757-NyeOR.H076	contig013361-TilOR.H108	1,06	0,42	0,40
contig064817-BriOR.H051	contig018437-ZebOR.H072	1,06	0,39	0,37
contig013371-TilOR.H117	contig018434-ZebOR.H070	1,06	0,31	0,30
contig013362-TilOR.H109	contig018434-ZebOR.H070	1,06	0,35	0,33
contig013365-TilOR.H111	contig014055-ZebOR.H066	1,06	0,46	0,44
contig053780-BurOR.H065	contig013365-TilOR.H111	1,06	0,46	0,44
contig048562-BurOR.H062	contig009547-TilOR.H101	1,06	0,31	0,30
contig009547-TilOR.H101	contig047492-ZebOR.H074	1,06	0,32	0,30
contig053788-BurOR.H069	contig035582-NyeOR.H070	1,06	0,40	0,38

contig009547-TilOR.H101	contig013358-TilOR.H106	1,06	0,35	0,33
contig009547-TilOR.H101	contig013356-TilOR.H105	1,06	0,36	0,34
contig013351-TilOR.H104	contig013371-TilOR.H117	1,06	0,35	0,33
contig048880-BurOR.H063	contig041955-TilOR.H119	1,06	0,37	0,35
contig013358-TilOR.H106	contig013368-TilOR.H112	1,06	0,34	0,32
contig013356-TilOR.H105	contig018434-ZebOR.H070	1,06	0,41	0,39
contig009547-TilOR.H101	contig013363-TilOR.H110	1,06	0,36	0,34
contig034998-NyeOR.H067	contig018434-ZebOR.H070	1,06	0,35	0,33
contig039725-NyeOR.H072	contig057403-ZebOR.H076	1,06	0,35	0,33
contig013351-TilOR.H104	contig013369-TilOR.H115	1,06	0,37	0,35
contig116846-BriOR.H055	contig014059-ZebOR.H068	1,06	0,38	0,36
contig064821-BriOR.H052	contig018437-ZebOR.H072	1,06	0,41	0,39
contig039730-NyeOR.H074	contig013365-TilOR.H111	1,06	0,47	0,44
contig041756-NyeOR.H139	contig013369-TilOR.H115	1,06	0,38	0,36
contig013362-TilOR.H109	contig014057-ZebOR.H067	1,06	0,38	0,36
contig009547-TilOR.H101	contig013359-TilOR.H107	1,06	0,36	0,34
contig009546-TilOR.H100	contig057403-ZebOR.H077	1,06	0,34	0,32
contig009547-TilOR.H101	contig013371-TilOR.H117	1,06	0,28	0,26
contig064817-BriOR.H051	contig057403-ZebOR.H076	1,06	0,36	0,34
contig013362-TilOR.H109	contig009565-TilOR.H126	1,06	0,33	0,31
contig033889-BriOR.H049	contig053787-BurOR.H068	1,06	0,38	0,35
contig009547-TilOR.H101	contig013351-TilOR.H104	1,06	0,35	0,33
contig064821-BriOR.H052	contig013369-TilOR.H115	1,06	0,40	0,38
contig041756-NyeOR.H075	contig013362-TilOR.H109	1,06	0,38	0,35
contig009547-TilOR.H101	contig013368-TilOR.H113	1,06	0,31	0,29
contig048882-BurOR.H064	contig053784-BurOR.H067	1,06	0,40	0,38
contig093825-BriOR.H053	contig035579-NyeOR.H068	1,07	0,37	0,35
contig107626-BriOR.H054	contig041756-NyeOR.H075	1,07	0,42	0,39
contig006794-BurOR.H061	contig009546-TilOR.H100	1,07	0,33	0,31
contig053788-BurOR.H069	contig013361-TilOR.H108	1,07	0,42	0,39
contig013368-TilOR.H113	contig014057-ZebOR.H067	1,07	0,37	0,35
contig049873-BriOR.H050	contig009546-TilOR.H100	1,07	0,34	0,32
contig034998-NyeOR.H067	contig041756-NyeOR.H075	1,07	0,38	0,36
contig013349-TilOR.H103	contig013363-TilOR.H110	1,07	0,45	0,42
contig035582-NyeOR.H070	contig041757-NyeOR.H076	1,07	0,40	0,37
contig009547-TilOR.H101	contig057403-ZebOR.H077	1,07	0,34	0,32
contig116846-BriOR.H055	contig013351-TilOR.H104	1,07	0,39	0,37
contig039729-NyeOR.H073	contig030011-ZebOR.H073	1,07	0,39	0,37
contig064817-BriOR.H051	contig048882-BurOR.H064	1,07	0,41	0,38
contig041756-NyeOR.H075	contig013361-TilOR.H108	1,07	0,39	0,37
contig013358-TilOR.H106	contig013368-TilOR.H113	1,07	0,37	0,34
contig013351-TilOR.H104	contig030011-ZebOR.H073	1,07	0,39	0,37
contig053784-BurOR.H067	contig013369-TilOR.H114	1,07	0,39	0,37

contig041756-NyeOR.H075	contig013368-TilOR.H113	1,07	0,38	0,35
contig041757-NyeOR.H076	contig013362-TilOR.H109	1,07	0,38	0,36
contig107626-BriOR.H054	contig014060-ZebOR.H069	1,07	0,43	0,40
contig064821-BriOR.H052	contig009547-TilOR.H101	1,07	0,36	0,34
contig035579-NyeOR.H068	contig014055-ZebOR.H066	1,07	0,45	0,42
contig053780-BurOR.H065	contig035579-NyeOR.H068	1,07	0,45	0,42
contig035583-NyeOR.H071	contig018437-ZebOR.H072	1,07	0,37	0,35
contig053787-BurOR.H068	contig057403-ZebOR.H076	1,07	0,35	0,32
contig041757-NyeOR.H076	contig013369-TilOR.H116	1,07	0,40	0,37
contig009547-TilOR.H101	contig041955-TilOR.H119	1,07	0,31	0,29
contig013368-TilOR.H113	contig014060-ZebOR.H069	1,07	0,38	0,35
contig013358-TilOR.H106	contig009565-TilOR.H126	1,07	0,39	0,36
contig014059-ZebOR.H068	contig057403-ZebOR.H076	1,07	0,35	0,33
contig039725-NyeOR.H072	contig009547-TilOR.H101	1,07	0,35	0,33
contig064817-BriOR.H051	contig107626-BriOR.H054	1,07	0,47	0,44
contig035579-NyeOR.H068	contig039730-NyeOR.H074	1,07	0,46	0,43
contig039725-NyeOR.H072	contig030011-ZebOR.H073	1,08	0,38	0,35
contig053788-BurOR.H069	contig018437-ZebOR.H072	1,08	0,39	0,37
contig034998-NyeOR.H067	contig009547-TilOR.H101	1,08	0,31	0,29
contig041756-NyeOR.H075	contig014055-ZebOR.H066	1,08	0,42	0,39
contig053780-BurOR.H065	contig041756-NyeOR.H075	1,08	0,42	0,39
contig116846-BriOR.H055	contig053787-BurOR.H068	1,08	0,39	0,36
contig064817-BriOR.H051	contig009547-TilOR.H101	1,08	0,36	0,34
contig053788-BurOR.H069	contig013369-TilOR.H115	1,08	0,39	0,37
contig053788-BurOR.H069	contig009565-TilOR.H126	1,08	0,41	0,38
contig064821-BriOR.H052	contig009565-TilOR.H126	1,08	0,42	0,39
contig006794-BurOR.H061	contig009547-TilOR.H101	1,08	0,33	0,31
contig048880-BurOR.H063	contig014055-ZebOR.H066	1,08	0,46	0,42
contig048880-BurOR.H063	contig053780-BurOR.H065	1,08	0,46	0,42
contig039730-NyeOR.H074	contig041756-NyeOR.H075	1,08	0,43	0,40
contig014060-ZebOR.H069	contig047492-ZebOR.H074	1,08	0,40	0,37
contig064821-BriOR.H052	contig035582-NyeOR.H070	1,08	0,40	0,37
contig014059-ZebOR.H068	contig030011-ZebOR.H073	1,08	0,37	0,35
contig013371-TilOR.H117	contig009565-TilOR.H126	1,08	0,30	0,27
contig064821-BriOR.H052	contig013368-TilOR.H113	1,08	0,39	0,36
contig048880-BurOR.H063	contig039730-NyeOR.H074	1,08	0,46	0,43
contig013358-TilOR.H106	contig030011-ZebOR.H073	1,08	0,38	0,35
contig053782-BurOR.H066	contig009547-TilOR.H101	1,08	0,36	0,33
contig041955-TilOR.H119	contig014060-ZebOR.H069	1,08	0,38	0,35
contig013356-TilOR.H105	contig013371-TilOR.H117	1,08	0,37	0,34
contig053782-BurOR.H066	contig013369-TilOR.H115	1,08	0,38	0,35
contig048562-BurOR.H062	contig035579-NyeOR.H068	1,08	0,37	0,34
contig035579-NyeOR.H068	contig047492-ZebOR.H074	1,08	0,37	0,35

contig064817-BriOR.H051	contig035582-NyeOR.H070	1,08	0,38	0,35
contig013368-TilOR.H112	contig014060-ZebOR.H069	1,09	0,35	0,33
contig093825-BriOR.H053	contig041756-NyeOR.H139	1,09	0,38	0,35
contig013362-TilOR.H109	contig018437-ZebOR.H072	1,09	0,36	0,33
contig053787-BurOR.H068	contig009547-TilOR.H101	1,09	0,35	0,32
contig116846-BriOR.H055	contig039729-NyeOR.H073	1,09	0,39	0,36
contig013358-TilOR.H106	contig018437-ZebOR.H072	1,09	0,38	0,35
contig039729-NyeOR.H073	contig018434-ZebOR.H071	1,09	0,38	0,35
contig093825-BriOR.H053	contig013365-TilOR.H111	1,09	0,36	0,33
contig064817-BriOR.H051	contig013368-TilOR.H113	1,09	0,38	0,35
contig053788-BurOR.H069	contig035583-NyeOR.H071	1,09	0,43	0,40
contig041756-NyeOR.H075	contig013369-TilOR.H115	1,09	0,38	0,35
contig048562-BurOR.H062	contig013365-TilOR.H111	1,09	0,36	0,33
contig013365-TilOR.H111	contig047492-ZebOR.H074	1,09	0,37	0,34
contig013362-TilOR.H109	contig030011-ZebOR.H073	1,09	0,35	0,32
contig013363-TilOR.H110	contig013371-TilOR.H118	1,09	0,40	0,37
contig116846-BriOR.H055	contig013356-TilOR.H105	1,09	0,43	0,39
contig006794-BurOR.H061	contig018434-ZebOR.H071	1,09	0,36	0,33
contig013356-TilOR.H105	contig018437-ZebOR.H072	1,09	0,41	0,37
contig035579-NyeOR.H068	contig041955-TilOR.H119	1,09	0,37	0,34
contig035579-NyeOR.H068	contig057403-ZebOR.H077	1,09	0,42	0,38
contig053782-BurOR.H066	contig018437-ZebOR.H072	1,09	0,39	0,36
contig093825-BriOR.H053	contig014060-ZebOR.H069	1,09	0,38	0,34
contig053784-BurOR.H067	contig013368-TilOR.H112	1,09	0,36	0,33
contig013351-TilOR.H104	contig018434-ZebOR.H071	1,09	0,36	0,33
contig013356-TilOR.H105	contig009565-TilOR.H126	1,09	0,42	0,38
contig048562-BurOR.H062	contig014060-ZebOR.H069	1,09	0,38	0,35
contig013358-TilOR.H106	contig013361-TilOR.H108	1,10	0,39	0,36
contig053787-BurOR.H068	contig030011-ZebOR.H073	1,10	0,37	0,34
contig013365-TilOR.H111	contig013371-TilOR.H118	1,10	0,39	0,35
contig013356-TilOR.H105	contig030011-ZebOR.H073	1,10	0,41	0,38
contig053788-BurOR.H069	contig013362-TilOR.H109	1,10	0,39	0,35
contig014055-ZebOR.H066	contig014060-ZebOR.H069	1,10	0,43	0,39
contig053780-BurOR.H065	contig014060-ZebOR.H069	1,10	0,43	0,39
contig053780-BurOR.H065	contig053784-BurOR.H067	1,10	0,44	0,40
contig053784-BurOR.H067	contig014055-ZebOR.H066	1,10	0,44	0,40
contig064821-BriOR.H052	contig093825-BriOR.H053	1,10	0,38	0,34
contig013356-TilOR.H105	contig013368-TilOR.H112	1,10	0,36	0,33
contig048882-BurOR.H064	contig013356-TilOR.H105	1,10	0,42	0,38
contig053782-BurOR.H066	contig013371-TilOR.H117	1,10	0,37	0,33
contig039729-NyeOR.H073	contig013369-TilOR.H115	1,10	0,39	0,35
contig013369-TilOR.H115	contig014057-ZebOR.H067	1,10	0,38	0,35
contig064821-BriOR.H052	contig048562-BurOR.H062	1,10	0,38	0,35

contig064821-BriOR.H052	contig047492-ZebOR.H074	1,10	0,39	0,35
contig013369-TilOR.H116	contig014060-ZebOR.H069	1,10	0,38	0,34
contig064817-BriOR.H051	contig013368-TilOR.H112	1,10	0,36	0,33
contig013358-TilOR.H106	contig013369-TilOR.H116	1,10	0,37	0,34
contig039730-NyeOR.H074	contig014060-ZebOR.H069	1,10	0,44	0,40
contig053784-BurOR.H067	contig039730-NyeOR.H074	1,10	0,44	0,40
contig093825-BriOR.H053	contig013358-TilOR.H106	1,10	0,37	0,34
contig035582-NyeOR.H070	contig013356-TilOR.H105	1,10	0,39	0,35
contig064817-BriOR.H051	contig013349-TilOR.H103	1,10	0,47	0,42
contig041757-NyeOR.H076	contig018437-ZebOR.H072	1,10	0,40	0,36
contig041756-NyeOR.H075	contig013369-TilOR.H116	1,10	0,37	0,34
contig048562-BurOR.H062	contig013358-TilOR.H106	1,10	0,38	0,34
contig013358-TilOR.H106	contig047492-ZebOR.H074	1,10	0,38	0,34
contig013371-TilOR.H117	contig018434-ZebOR.H071	1,10	0,31	0,28
contig034998-NyeOR.H067	contig035579-NyeOR.H068	1,10	0,37	0,34
contig041756-NyeOR.H139	contig013349-TilOR.H103	1,10	0,43	0,39
contig053788-BurOR.H069	contig057403-ZebOR.H077	1,10	0,44	0,40
contig041757-NyeOR.H076	contig018434-ZebOR.H071	1,10	0,37	0,34
contig041757-NyeOR.H076	contig013369-TilOR.H115	1,10	0,39	0,36
contig053788-BurOR.H069	contig013371-TilOR.H118	1,11	0,40	0,36
contig039729-NyeOR.H073	contig013371-TilOR.H117	1,11	0,37	0,34
contig009547-TilOR.H101	contig014059-ZebOR.H068	1,11	0,35	0,32
contig039725-NyeOR.H072	contig018434-ZebOR.H071	1,11	0,36	0,33
contig034998-NyeOR.H067	contig013365-TilOR.H111	1,11	0,36	0,33
contig013362-TilOR.H109	contig014060-ZebOR.H069	1,11	0,38	0,34
contig006794-BurOR.H061	contig013363-TilOR.H110	1,11	0,42	0,38
contig013358-TilOR.H106	contig018434-ZebOR.H071	1,11	0,36	0,32
contig039729-NyeOR.H073	contig009547-TilOR.H101	1,11	0,36	0,33
contig048882-BurOR.H064	contig053788-BurOR.H069	1,11	0,43	0,39
contig041756-NyeOR.H139	contig041955-TilOR.H119	1,11	0,37	0,34
contig041756-NyeOR.H139	contig013371-TilOR.H118	1,11	0,40	0,36
contig107626-BriOR.H054	contig013358-TilOR.H106	1,11	0,44	0,40
contig064817-BriOR.H051	contig014055-ZebOR.H066	1,11	0,48	0,43
contig064817-BriOR.H051	contig053780-BurOR.H065	1,11	0,48	0,43
contig048880-BurOR.H063	contig013371-TilOR.H118	1,11	0,40	0,36
contig013356-TilOR.H105	contig057403-ZebOR.H077	1,11	0,43	0,38
contig014059-ZebOR.H068	contig018434-ZebOR.H071	1,11	0,35	0,32
contig048880-BurOR.H063	contig057403-ZebOR.H077	1,11	0,42	0,38
contig064821-BriOR.H052	contig107626-BriOR.H054	1,11	0,46	0,41
contig093825-BriOR.H053	contig053784-BurOR.H067	1,11	0,38	0,34
contig013361-TilOR.H108	contig014060-ZebOR.H069	1,11	0,39	0,35
contig035580-NyeOR.H069	contig013363-TilOR.H110	1,11	0,39	0,35
contig035583-NyeOR.H071	contig009547-TilOR.H101	1,11	0,33	0,30

contig053788-BurOR.H069	contig018434-ZebOR.H071	1,11	0,37	0,33
contig034998-NyeOR.H067	contig014060-ZebOR.H069	1,11	0,38	0,34
contig048562-BurOR.H062	contig053784-BurOR.H067	1,11	0,38	0,35
contig053784-BurOR.H067	contig047492-ZebOR.H074	1,11	0,39	0,35
contig064817-BriOR.H051	contig039730-NyeOR.H074	1,11	0,49	0,44
contig035583-NyeOR.H071	contig041757-NyeOR.H076	1,11	0,43	0,38
contig006794-BurOR.H061	contig041756-NyeOR.H139	1,11	0,41	0,37
contig039725-NyeOR.H072	contig018437-ZebOR.H072	1,11	0,38	0,34
contig035579-NyeOR.H068	contig013371-TilOR.H118	1,12	0,40	0,36
contig053784-BurOR.H067	contig013362-TilOR.H109	1,12	0,38	0,34
contig064821-BriOR.H052	contig034998-NyeOR.H067	1,12	0,38	0,34
contig013359-TilOR.H107	contig013371-TilOR.H118	1,12	0,39	0,35
contig048562-BurOR.H062	contig041756-NyeOR.H139	1,12	0,38	0,34
contig041756-NyeOR.H139	contig047492-ZebOR.H074	1,12	0,38	0,34
contig064821-BriOR.H052	contig048882-BurOR.H064	1,12	0,43	0,38
contig013358-TilOR.H106	contig013369-TilOR.H115	1,12	0,38	0,34
contig035583-NyeOR.H071	contig013356-TilOR.H105	1,12	0,42	0,38
contig053784-BurOR.H067	contig041955-TilOR.H119	1,12	0,38	0,34
contig034998-NyeOR.H067	contig013358-TilOR.H106	1,12	0,38	0,34
contig053784-BurOR.H067	contig013361-TilOR.H108	1,12	0,40	0,36
contig064817-BriOR.H051	contig013369-TilOR.H115	1,12	0,38	0,34
contig006794-BurOR.H061	contig013356-TilOR.H105	1,12	0,43	0,38
contig053787-BurOR.H068	contig018434-ZebOR.H071	1,12	0,36	0,32
contig041756-NyeOR.H139	contig057403-ZebOR.H077	1,12	0,42	0,37
contig107626-BriOR.H054	contig053788-BurOR.H069	1,13	0,44	0,39
contig013356-TilOR.H105	contig013368-TilOR.H113	1,13	0,38	0,33
contig013349-TilOR.H103	contig013365-TilOR.H111	1,13	0,45	0,40
contig013351-TilOR.H104	contig013371-TilOR.H118	1,13	0,40	0,35
contig049873-BriOR.H050	contig053788-BurOR.H069	1,13	0,43	0,38
contig039729-NyeOR.H073	contig018437-ZebOR.H072	1,13	0,39	0,35
contig013359-TilOR.H107	contig041955-TilOR.H119	1,13	0,37	0,33
contig006794-BurOR.H061	contig013351-TilOR.H104	1,13	0,42	0,37
contig049873-BriOR.H050	contig013363-TilOR.H110	1,13	0,42	0,38
contig013369-TilOR.H116	contig014057-ZebOR.H067	1,13	0,40	0,35
contig013356-TilOR.H105	contig013362-TilOR.H109	1,13	0,39	0,34
contig041757-NyeOR.H076	contig057403-ZebOR.H077	1,13	0,44	0,39
contig064821-BriOR.H052	contig041955-TilOR.H119	1,13	0,37	0,33
contig013371-TilOR.H117	contig014057-ZebOR.H067	1,13	0,36	0,32
contig013356-TilOR.H105	contig013369-TilOR.H115	1,13	0,39	0,35
contig035583-NyeOR.H071	contig013363-TilOR.H110	1,13	0,42	0,37
contig064817-BriOR.H051	contig013371-TilOR.H117	1,13	0,36	0,32
contig053784-BurOR.H067	contig034998-NyeOR.H067	1,13	0,38	0,34
contig006794-BurOR.H061	contig048880-BurOR.H063	1,13	0,41	0,37

contig035579-NyeOR.H068	contig035583-NyeOR.H071	1,13	0,41	0,37
contig006794-BurOR.H061	contig013365-TilOR.H111	1,13	0,40	0,36
contig013363-TilOR.H110	contig057403-ZebOR.H077	1,13	0,43	0,38
contig013356-TilOR.H105	contig018434-ZebOR.H071	1,13	0,38	0,33
contig049873-BriOR.H050	contig041756-NyeOR.H139	1,13	0,42	0,37
contig013368-TilOR.H112	contig014059-ZebOR.H068	1,13	0,35	0,31
contig035583-NyeOR.H071	contig041756-NyeOR.H139	1,13	0,41	0,36
contig041757-NyeOR.H076	contig013371-TilOR.H118	1,13	0,40	0,35
contig107626-BriOR.H054	contig041757-NyeOR.H076	1,13	0,44	0,39
contig013349-TilOR.H103	contig013371-TilOR.H117	1,13	0,42	0,37
contig053784-BurOR.H067	contig013368-TilOR.H113	1,14	0,38	0,34
contig013369-TilOR.H115	contig014060-ZebOR.H069	1,14	0,38	0,34
contig048882-BurOR.H064	contig041757-NyeOR.H076	1,14	0,43	0,38
contig053787-BurOR.H068	contig018437-ZebOR.H072	1,14	0,38	0,33
contig034998-NyeOR.H067	contig041756-NyeOR.H139	1,14	0,38	0,33
contig039725-NyeOR.H072	contig013368-TilOR.H112	1,14	0,35	0,31
contig013351-TilOR.H104	contig018437-ZebOR.H072	1,14	0,39	0,34
contig039725-NyeOR.H072	contig013371-TilOR.H117	1,14	0,36	0,31
contig014059-ZebOR.H068	contig018437-ZebOR.H072	1,14	0,38	0,33
contig006794-BurOR.H061	contig035579-NyeOR.H068	1,14	0,42	0,37
contig107626-BriOR.H054	contig014057-ZebOR.H067	1,14	0,46	0,41
contig013356-TilOR.H105	contig013361-TilOR.H108	1,14	0,41	0,36
contig035579-NyeOR.H068	contig013349-TilOR.H103	1,14	0,44	0,39
contig009546-TilOR.H100	contig014055-ZebOR.H066	1,14	0,46	0,40
contig053780-BurOR.H065	contig009546-TilOR.H100	1,14	0,46	0,40
contig107626-BriOR.H054	contig013351-TilOR.H104	1,14	0,47	0,41
contig013363-TilOR.H110	contig057400-ZebOR.H075	1,14	0,39	0,34
contig049873-BriOR.H050	contig013359-TilOR.H107	1,14	0,42	0,37
contig039725-NyeOR.H072	contig013369-TilOR.H114	1,14	0,38	0,34
contig053784-BurOR.H067	contig013369-TilOR.H116	1,14	0,38	0,33
contig049873-BriOR.H050	contig013356-TilOR.H105	1,14	0,43	0,38
contig053782-BurOR.H066	contig013369-TilOR.H116	1,14	0,40	0,35
contig039730-NyeOR.H074	contig013371-TilOR.H117	1,14	0,43	0,38
contig039730-NyeOR.H074	contig009546-TilOR.H100	1,14	0,46	0,40
contig093825-BriOR.H053	contig013359-TilOR.H107	1,14	0,37	0,32
contig053784-BurOR.H067	contig013369-TilOR.H115	1,15	0,39	0,34
contig107626-BriOR.H054	contig013359-TilOR.H107	1,15	0,45	0,39
contig048562-BurOR.H062	contig013359-TilOR.H107	1,15	0,37	0,33
contig013359-TilOR.H107	contig047492-ZebOR.H074	1,15	0,38	0,33
contig035580-NyeOR.H069	contig014057-ZebOR.H067	1,15	0,39	0,34
contig013362-TilOR.H109	contig014059-ZebOR.H068	1,15	0,37	0,33
contig093825-BriOR.H053	contig013351-TilOR.H104	1,15	0,36	0,31
contig093825-BriOR.H053	contig041757-NyeOR.H076	1,15	0,38	0,33

contig041756-NyeOR.H075	contig013349-TilOR.H103	1,15	0,41	0,35
contig049873-BriOR.H050	contig048880-BurOR.H063	1,15	0,42	0,36
contig107626-BriOR.H054	contig013356-TilOR.H105	1,15	0,47	0,41
contig093825-BriOR.H053	contig013356-TilOR.H105	1,15	0,39	0,34
contig048562-BurOR.H062	contig013351-TilOR.H104	1,15	0,36	0,32
contig013351-TilOR.H104	contig047492-ZebOR.H074	1,15	0,36	0,32
contig013351-TilOR.H104	contig041955-TilOR.H119	1,15	0,36	0,31
contig048880-BurOR.H063	contig013349-TilOR.H103	1,15	0,45	0,39
contig035583-NyeOR.H071	contig013359-TilOR.H107	1,15	0,41	0,36
contig013358-TilOR.H106	contig041955-TilOR.H119	1,15	0,37	0,32
contig048880-BurOR.H063	contig035580-NyeOR.H069	1,15	0,39	0,34
contig053787-BurOR.H068	contig013368-TilOR.H112	1,15	0,35	0,30
contig013359-TilOR.H107	contig057403-ZebOR.H077	1,15	0,41	0,36
contig035583-NyeOR.H071	contig013365-TilOR.H111	1,15	0,41	0,35
contig006794-BurOR.H061	contig053782-BurOR.H066	1,15	0,43	0,37
contig048880-BurOR.H063	contig035583-NyeOR.H071	1,15	0,42	0,36
contig013365-TilOR.H111	contig057403-ZebOR.H077	1,15	0,41	0,35
contig053787-BurOR.H068	contig013371-TilOR.H117	1,15	0,35	0,31
contig053782-BurOR.H066	contig035580-NyeOR.H069	1,15	0,40	0,35
contig039729-NyeOR.H073	contig013369-TilOR.H116	1,15	0,41	0,35
contig048882-BurOR.H064	contig039725-NyeOR.H072	1,15	0,39	0,34
contig013371-TilOR.H117	contig014059-ZebOR.H068	1,15	0,36	0,31
contig013365-TilOR.H111	contig041955-TilOR.H119	1,15	0,36	0,31
contig041756-NyeOR.H075	contig013371-TilOR.H118	1,15	0,39	0,34
contig049873-BriOR.H050	contig013351-TilOR.H104	1,15	0,42	0,36
contig035580-NyeOR.H069	contig041756-NyeOR.H139	1,15	0,39	0,34
contig049873-BriOR.H050	contig041757-NyeOR.H076	1,15	0,43	0,37
contig093825-BriOR.H053	contig013363-TilOR.H110	1,16	0,38	0,33
contig049873-BriOR.H050	contig035579-NyeOR.H068	1,16	0,43	0,37
contig048562-BurOR.H062	contig041757-NyeOR.H076	1,16	0,38	0,33
contig041757-NyeOR.H076	contig047492-ZebOR.H074	1,16	0,38	0,33
contig053787-BurOR.H068	contig013369-TilOR.H114	1,16	0,38	0,33
contig053780-BurOR.H065	contig013371-TilOR.H117	1,16	0,43	0,37
contig013371-TilOR.H117	contig014055-ZebOR.H066	1,16	0,43	0,37
contig048562-BurOR.H062	contig013363-TilOR.H110	1,16	0,38	0,33
contig013363-TilOR.H110	contig047492-ZebOR.H074	1,16	0,39	0,33
contig064817-BriOR.H051	contig013369-TilOR.H116	1,16	0,40	0,35
contig064821-BriOR.H052	contig014055-ZebOR.H066	1,16	0,46	0,40
contig064821-BriOR.H052	contig053780-BurOR.H065	1,16	0,46	0,40
contig107626-BriOR.H054	contig053787-BurOR.H068	1,16	0,44	0,38
contig013369-TilOR.H114	contig014059-ZebOR.H068	1,16	0,38	0,33
contig013358-TilOR.H106	contig014055-ZebOR.H066	1,16	0,44	0,38
contig053780-BurOR.H065	contig013358-TilOR.H106	1,16	0,44	0,38

contig006794-BurOR.H061	contig013359-TilOR.H107	1,16	0,42	0,37
contig107626-BriOR.H054	contig014059-ZebOR.H068	1,16	0,44	0,38
contig013349-TilOR.H103	contig014060-ZebOR.H069	1,16	0,42	0,36
contig053784-BurOR.H067	contig013349-TilOR.H103	1,16	0,42	0,36
contig041756-NyeOR.H075	contig057403-ZebOR.H077	1,16	0,42	0,36
contig039725-NyeOR.H072	contig013362-TilOR.H109	1,16	0,37	0,32
contig006794-BurOR.H061	contig014057-ZebOR.H067	1,16	0,43	0,37
contig064821-BriOR.H052	contig039730-NyeOR.H074	1,16	0,47	0,40
contig039730-NyeOR.H074	contig013358-TilOR.H106	1,16	0,45	0,38
contig035580-NyeOR.H069	contig013365-TilOR.H111	1,16	0,37	0,32
contig107626-BriOR.H054	contig039725-NyeOR.H072	1,16	0,45	0,38
contig053780-BurOR.H065	contig053788-BurOR.H069	1,16	0,44	0,38
contig053788-BurOR.H069	contig014055-ZebOR.H066	1,16	0,44	0,38
contig053787-BurOR.H068	contig013362-TilOR.H109	1,16	0,37	0,32
contig053780-BurOR.H065	contig035582-NyeOR.H070	1,16	0,45	0,38
contig035582-NyeOR.H070	contig014055-ZebOR.H066	1,16	0,45	0,38
contig049873-BriOR.H050	contig013365-TilOR.H111	1,16	0,41	0,35
contig035579-NyeOR.H068	contig035580-NyeOR.H069	1,16	0,39	0,34
contig048882-BurOR.H064	contig053787-BurOR.H068	1,16	0,39	0,34
contig006794-BurOR.H061	contig039729-NyeOR.H073	1,16	0,43	0,37
contig013358-TilOR.H106	contig057403-ZebOR.H077	1,17	0,42	0,36
contig064821-BriOR.H052	contig057403-ZebOR.H077	1,17	0,44	0,37
contig013358-TilOR.H106	contig013371-TilOR.H118	1,17	0,39	0,34
contig035580-NyeOR.H069	contig039729-NyeOR.H073	1,17	0,40	0,35
contig034998-NyeOR.H067	contig013359-TilOR.H107	1,17	0,37	0,32
contig053782-BurOR.H066	contig013371-TilOR.H118	1,17	0,40	0,35
contig093825-BriOR.H053	contig053788-BurOR.H069	1,17	0,38	0,33
contig053788-BurOR.H069	contig039730-NyeOR.H074	1,17	0,45	0,38
contig064821-BriOR.H052	contig006794-BurOR.H061	1,17	0,43	0,37
contig034998-NyeOR.H067	contig013351-TilOR.H104	1,17	0,36	0,31
contig035583-NyeOR.H071	contig014057-ZebOR.H067	1,17	0,42	0,36
contig107626-BriOR.H054	contig013371-TilOR.H117	1,17	0,41	0,35
contig013349-TilOR.H103	contig013368-TilOR.H113	1,17	0,45	0,38
contig035583-NyeOR.H071	contig041756-NyeOR.H075	1,17	0,41	0,35
contig013349-TilOR.H103	contig014057-ZebOR.H067	1,17	0,46	0,39
contig035582-NyeOR.H070	contig039725-NyeOR.H072	1,17	0,37	0,31
contig035582-NyeOR.H070	contig039730-NyeOR.H074	1,17	0,45	0,38
contig035583-NyeOR.H071	contig013351-TilOR.H104	1,17	0,41	0,35
contig006794-BurOR.H061	contig053788-BurOR.H069	1,17	0,40	0,34
contig014057-ZebOR.H067	contig057400-ZebOR.H075	1,17	0,40	0,34
contig041757-NyeOR.H076	contig014055-ZebOR.H066	1,17	0,45	0,38
contig053780-BurOR.H065	contig041757-NyeOR.H076	1,17	0,45	0,38
contig064817-BriOR.H051	contig035583-NyeOR.H071	1,17	0,43	0,36

contig035583-NyeOR.H071	contig013358-TilOR.H106	1,17	0,41	0,35
contig053784-BurOR.H067	contig013371-TilOR.H118	1,17	0,40	0,34
contig035582-NyeOR.H070	contig014059-ZebOR.H068	1,17	0,37	0,31
contig014055-ZebOR.H066	contig014057-ZebOR.H067	1,18	0,48	0,40
contig053780-BurOR.H065	contig014057-ZebOR.H067	1,18	0,48	0,40
contig107626-BriOR.H054	contig035582-NyeOR.H070	1,18	0,43	0,36
contig048562-BurOR.H062	contig053788-BurOR.H069	1,18	0,38	0,32
contig034998-NyeOR.H067	contig041757-NyeOR.H076	1,18	0,38	0,33
contig053788-BurOR.H069	contig047492-ZebOR.H074	1,18	0,38	0,33
contig053782-BurOR.H066	contig035583-NyeOR.H071	1,18	0,43	0,36
contig039730-NyeOR.H074	contig041757-NyeOR.H076	1,18	0,45	0,38
contig053782-BurOR.H066	contig057400-ZebOR.H075	1,18	0,40	0,34
contig034998-NyeOR.H067	contig013363-TilOR.H110	1,18	0,38	0,33
contig041955-TilOR.H119	contig014057-ZebOR.H067	1,18	0,38	0,32
contig013356-TilOR.H105	contig013371-TilOR.H118	1,18	0,41	0,35
contig014057-ZebOR.H067	contig057403-ZebOR.H077	1,18	0,43	0,37
contig039730-NyeOR.H074	contig014057-ZebOR.H067	1,18	0,48	0,41
contig064817-BriOR.H051	contig013371-TilOR.H118	1,18	0,40	0,34
contig013371-TilOR.H118	contig014057-ZebOR.H067	1,18	0,39	0,33
contig013351-TilOR.H104	contig014055-ZebOR.H066	1,18	0,47	0,40
contig053780-BurOR.H065	contig013351-TilOR.H104	1,18	0,47	0,40
contig049873-BriOR.H050	contig064817-BriOR.H051	1,18	0,43	0,36
contig039729-NyeOR.H073	contig013371-TilOR.H118	1,18	0,41	0,35
contig048880-BurOR.H063	contig057400-ZebOR.H075	1,18	0,39	0,33
contig041757-NyeOR.H076	contig041955-TilOR.H119	1,18	0,38	0,32
contig093825-BriOR.H053	contig014059-ZebOR.H068	1,18	0,38	0,32
contig039725-NyeOR.H072	contig013361-TilOR.H108	1,18	0,39	0,33
contig039730-NyeOR.H074	contig013351-TilOR.H104	1,18	0,48	0,40
contig064817-BriOR.H051	contig057403-ZebOR.H077	1,18	0,43	0,37
contig041955-TilOR.H119	contig014059-ZebOR.H068	1,18	0,38	0,32
contig049873-BriOR.H050	contig041756-NyeOR.H075	1,18	0,42	0,36
contig048562-BurOR.H062	contig014059-ZebOR.H068	1,18	0,38	0,32
contig014059-ZebOR.H068	contig047492-ZebOR.H074	1,18	0,38	0,32
contig013371-TilOR.H118	contig014060-ZebOR.H069	1,18	0,40	0,33
contig049873-BriOR.H050	contig014057-ZebOR.H067	1,19	0,43	0,36
contig053782-BurOR.H066	contig057403-ZebOR.H077	1,19	0,44	0,37
contig053787-BurOR.H068	contig013361-TilOR.H108	1,19	0,39	0,33
contig041756-NyeOR.H139	contig057400-ZebOR.H075	1,19	0,39	0,33
contig013363-TilOR.H110	contig041955-TilOR.H119	1,19	0,38	0,32
contig035580-NyeOR.H069	contig013359-TilOR.H107	1,19	0,39	0,32
contig013351-TilOR.H104	contig057403-ZebOR.H077	1,19	0,41	0,35
contig107626-BriOR.H054	contig013368-TilOR.H113	1,19	0,43	0,37
contig013361-TilOR.H108	contig014059-ZebOR.H068	1,19	0,39	0,33

contig107626-BriOR.H054	contig039729-NyeOR.H073	1,19	0,47	0,39
contig013349-TilOR.H103	contig013361-TilOR.H108	1,19	0,43	0,37
contig107626-BriOR.H054	contig009546-TilOR.H100	1,19	0,45	0,38
contig048562-BurOR.H062	contig013356-TilOR.H105	1,19	0,39	0,32
contig013356-TilOR.H105	contig047492-ZebOR.H074	1,19	0,39	0,33
contig013368-TilOR.H113	contig014055-ZebOR.H066	1,19	0,45	0,38
contig053780-BurOR.H065	contig013368-TilOR.H113	1,19	0,45	0,38
contig035583-NyeOR.H071	contig039729-NyeOR.H073	1,19	0,43	0,36
contig039729-NyeOR.H073	contig057400-ZebOR.H075	1,19	0,41	0,34
contig053787-BurOR.H068	contig035582-NyeOR.H070	1,19	0,36	0,30
contig013349-TilOR.H103	contig057403-ZebOR.H076	1,19	0,41	0,34
contig053784-BurOR.H067	contig035583-NyeOR.H071	1,19	0,42	0,35
contig014060-ZebOR.H069	contig057403-ZebOR.H077	1,19	0,42	0,35
contig006794-BurOR.H061	contig041756-NyeOR.H075	1,19	0,42	0,35
contig013359-TilOR.H107	contig014055-ZebOR.H066	1,20	0,46	0,38
contig053780-BurOR.H065	contig013359-TilOR.H107	1,20	0,46	0,38
contig039725-NyeOR.H072	contig013369-TilOR.H116	1,20	0,37	0,31
contig013365-TilOR.H111	contig057400-ZebOR.H075	1,20	0,38	0,31
contig053788-BurOR.H069	contig034998-NyeOR.H067	1,20	0,38	0,32
contig093825-BriOR.H053	contig014057-ZebOR.H067	1,20	0,37	0,31
contig013356-TilOR.H105	contig014055-ZebOR.H066	1,20	0,47	0,39
contig053780-BurOR.H065	contig013356-TilOR.H105	1,20	0,47	0,39
contig035579-NyeOR.H068	contig057400-ZebOR.H075	1,20	0,39	0,33
contig048562-BurOR.H062	contig014057-ZebOR.H067	1,20	0,38	0,31
contig014057-ZebOR.H067	contig047492-ZebOR.H074	1,20	0,38	0,32
contig039730-NyeOR.H074	contig013359-TilOR.H107	1,20	0,46	0,39
contig035582-NyeOR.H070	contig013349-TilOR.H103	1,20	0,43	0,36
contig039730-NyeOR.H074	contig013356-TilOR.H105	1,20	0,47	0,39
contig039729-NyeOR.H073	contig013349-TilOR.H103	1,20	0,46	0,38
contig009546-TilOR.H100	contig013349-TilOR.H103	1,20	0,45	0,37
contig039729-NyeOR.H073	contig057403-ZebOR.H077	1,20	0,44	0,37
contig107626-BriOR.H054	contig013368-TilOR.H112	1,20	0,41	0,34
contig006794-BurOR.H061	contig041757-NyeOR.H076	1,20	0,40	0,33
contig006794-BurOR.H061	contig013358-TilOR.H106	1,20	0,42	0,35
contig053788-BurOR.H069	contig041955-TilOR.H119	1,20	0,38	0,32
contig035583-NyeOR.H071	contig014060-ZebOR.H069	1,20	0,41	0,34
contig053784-BurOR.H067	contig057403-ZebOR.H077	1,20	0,43	0,35
contig064821-BriOR.H052	contig035583-NyeOR.H071	1,20	0,43	0,36
contig035580-NyeOR.H069	contig041756-NyeOR.H075	1,20	0,39	0,32
contig034998-NyeOR.H067	contig014059-ZebOR.H068	1,21	0,38	0,32
contig064817-BriOR.H051	contig006794-BurOR.H061	1,21	0,43	0,35
contig048882-BurOR.H064	contig014059-ZebOR.H068	1,21	0,39	0,33
contig039729-NyeOR.H073	contig014055-ZebOR.H066	1,21	0,48	0,39

contig053780-BurOR.H065	contig039729-NyeOR.H073	1,21	0,48	0,39
contig039730-NyeOR.H074	contig013368-TilOR.H113	1,21	0,45	0,38
contig053780-BurOR.H065	contig053787-BurOR.H068	1,21	0,45	0,37
contig053787-BurOR.H068	contig014055-ZebOR.H066	1,21	0,45	0,37
contig009547-TilOR.H101	contig014055-ZebOR.H066	1,21	0,43	0,36
contig053780-BurOR.H065	contig009547-TilOR.H101	1,21	0,43	0,36
contig014055-ZebOR.H066	contig014059-ZebOR.H068	1,21	0,45	0,37
contig053780-BurOR.H065	contig014059-ZebOR.H068	1,21	0,45	0,37
contig039725-NyeOR.H072	contig013368-TilOR.H113	1,21	0,37	0,30
contig049873-BriOR.H050	contig053782-BurOR.H066	1,21	0,43	0,35
contig039729-NyeOR.H073	contig039730-NyeOR.H074	1,21	0,48	0,40
contig049873-BriOR.H050	contig064821-BriOR.H052	1,21	0,43	0,36
contig053787-BurOR.H068	contig039730-NyeOR.H074	1,21	0,45	0,37
contig039730-NyeOR.H074	contig009547-TilOR.H101	1,21	0,44	0,36
contig034998-NyeOR.H067	contig013356-TilOR.H105	1,21	0,39	0,32
contig039730-NyeOR.H074	contig014059-ZebOR.H068	1,21	0,45	0,37
contig039725-NyeOR.H072	contig014055-ZebOR.H066	1,21	0,45	0,37
contig053780-BurOR.H065	contig039725-NyeOR.H072	1,21	0,45	0,37
contig064817-BriOR.H051	contig035580-NyeOR.H069	1,21	0,39	0,33
contig013369-TilOR.H116	contig014059-ZebOR.H068	1,21	0,37	0,31
contig013359-TilOR.H107	contig057400-ZebOR.H075	1,22	0,39	0,32
contig039725-NyeOR.H072	contig039730-NyeOR.H074	1,22	0,45	0,37
contig049873-BriOR.H050	contig013358-TilOR.H106	1,22	0,42	0,34
contig035580-NyeOR.H069	contig013351-TilOR.H104	1,22	0,38	0,31
contig053788-BurOR.H069	contig013349-TilOR.H103	1,22	0,45	0,37
contig064821-BriOR.H052	contig013371-TilOR.H118	1,22	0,41	0,34
contig013356-TilOR.H105	contig041955-TilOR.H119	1,22	0,38	0,31
contig093825-BriOR.H053	contig039725-NyeOR.H072	1,22	0,38	0,31
contig053787-BurOR.H068	contig013369-TilOR.H116	1,22	0,37	0,30
contig034998-NyeOR.H067	contig014057-ZebOR.H067	1,22	0,38	0,31
contig039725-NyeOR.H072	contig041955-TilOR.H119	1,22	0,38	0,31
contig013368-TilOR.H113	contig014059-ZebOR.H068	1,22	0,36	0,30
contig048562-BurOR.H062	contig039725-NyeOR.H072	1,22	0,38	0,31
contig039725-NyeOR.H072	contig047492-ZebOR.H074	1,22	0,39	0,32
contig039730-NyeOR.H074	contig057403-ZebOR.H076	1,22	0,42	0,35
contig107626-BriOR.H054	contig013361-TilOR.H108	1,22	0,43	0,35
contig053780-BurOR.H065	contig018437-ZebOR.H072	1,22	0,46	0,38
contig014055-ZebOR.H066	contig018437-ZebOR.H072	1,22	0,46	0,38
contig107626-BriOR.H054	contig053782-BurOR.H066	1,22	0,46	0,38
contig039729-NyeOR.H073	contig041955-TilOR.H119	1,22	0,38	0,31
contig053788-BurOR.H069	contig035580-NyeOR.H069	1,22	0,40	0,32
contig013349-TilOR.H103	contig013358-TilOR.H106	1,22	0,43	0,35
contig053782-BurOR.H066	contig041955-TilOR.H119	1,22	0,38	0,31

contig039730-NyeOR.H074	contig018437-ZebOR.H072	1,22	0,46	0,38
contig049873-BriOR.H050	contig039729-NyeOR.H073	1,23	0,44	0,35
contig107626-BriOR.H054	contig013362-TilOR.H109	1,23	0,42	0,35
contig041757-NyeOR.H076	contig013349-TilOR.H103	1,23	0,45	0,37
contig107626-BriOR.H054	contig009547-TilOR.H101	1,23	0,42	0,34
contig053780-BurOR.H065	contig009565-TilOR.H126	1,23	0,42	0,34
contig009565-TilOR.H126	contig014055-ZebOR.H066	1,23	0,43	0,35
contig053780-BurOR.H065	contig013361-TilOR.H108	1,23	0,45	0,36
contig013361-TilOR.H108	contig014055-ZebOR.H066	1,23	0,45	0,36
contig013349-TilOR.H103	contig018437-ZebOR.H072	1,23	0,45	0,37
contig013349-TilOR.H103	contig013359-TilOR.H107	1,23	0,44	0,36
contig006794-BurOR.H061	contig014060-ZebOR.H069	1,23	0,42	0,34
contig053780-BurOR.H065	contig057403-ZebOR.H076	1,23	0,42	0,34
contig014055-ZebOR.H066	contig057403-ZebOR.H076	1,23	0,43	0,35
contig039730-NyeOR.H074	contig009565-TilOR.H126	1,23	0,43	0,35
contig053787-BurOR.H068	contig013368-TilOR.H113	1,23	0,37	0,30
contig013349-TilOR.H103	contig009565-TilOR.H126	1,24	0,42	0,34
contig049873-BriOR.H050	contig053784-BurOR.H067	1,24	0,43	0,35
contig039730-NyeOR.H074	contig013361-TilOR.H108	1,24	0,45	0,36
contig093825-BriOR.H053	contig039729-NyeOR.H073	1,24	0,38	0,31
contig053780-BurOR.H065	contig013368-TilOR.H112	1,24	0,43	0,35
contig013368-TilOR.H112	contig014055-ZebOR.H066	1,24	0,43	0,35
contig048562-BurOR.H062	contig039729-NyeOR.H073	1,24	0,38	0,31
contig039729-NyeOR.H073	contig047492-ZebOR.H074	1,24	0,39	0,31
contig064817-BriOR.H051	contig057400-ZebOR.H075	1,24	0,40	0,32
contig034998-NyeOR.H067	contig039725-NyeOR.H072	1,24	0,38	0,31
contig041756-NyeOR.H075	contig057400-ZebOR.H075	1,24	0,39	0,32
contig064821-BriOR.H052	contig013349-TilOR.H103	1,24	0,45	0,36
contig006794-BurOR.H061	contig053784-BurOR.H067	1,24	0,43	0,35
contig033889-BriOR.H049	contig053780-BurOR.H065	1,24	0,48	0,39
contig033889-BriOR.H049	contig014055-ZebOR.H066	1,24	0,48	0,39
contig093825-BriOR.H053	contig053787-BurOR.H068	1,24	0,37	0,30
contig093825-BriOR.H053	contig053782-BurOR.H066	1,24	0,38	0,30
contig053787-BurOR.H068	contig041955-TilOR.H119	1,24	0,37	0,30
contig048562-BurOR.H062	contig053787-BurOR.H068	1,25	0,38	0,30
contig053787-BurOR.H068	contig047492-ZebOR.H074	1,25	0,38	0,31
contig048562-BurOR.H062	contig053782-BurOR.H066	1,25	0,38	0,30
contig053782-BurOR.H066	contig047492-ZebOR.H074	1,25	0,38	0,31
contig033889-BriOR.H049	contig039730-NyeOR.H074	1,25	0,48	0,39
contig049873-BriOR.H050	contig014060-ZebOR.H069	1,25	0,42	0,34
contig039730-NyeOR.H074	contig013368-TilOR.H112	1,25	0,43	0,35
contig116846-BriOR.H055	contig053780-BurOR.H065	1,25	0,47	0,38
contig116846-BriOR.H055	contig014055-ZebOR.H066	1,25	0,47	0,38

contig107626-BriOR.H054	contig057403-ZebOR.H076	1,25	0,40	0,32
contig053780-BurOR.H065	contig030011-ZebOR.H073	1,25	0,47	0,38
contig014055-ZebOR.H066	contig030011-ZebOR.H073	1,25	0,47	0,38
contig116846-BriOR.H055	contig013349-TilOR.H103	1,25	0,46	0,37
contig053782-BurOR.H066	contig013349-TilOR.H103	1,25	0,45	0,36
contig116846-BriOR.H055	contig039730-NyeOR.H074	1,25	0,47	0,38
contig035580-NyeOR.H069	contig041757-NyeOR.H076	1,25	0,40	0,32
contig039730-NyeOR.H074	contig030011-ZebOR.H073	1,26	0,47	0,38
contig039725-NyeOR.H072	contig013369-TilOR.H115	1,26	0,38	0,30
contig013351-TilOR.H104	contig057400-ZebOR.H075	1,26	0,38	0,30
contig035580-NyeOR.H069	contig013358-TilOR.H106	1,26	0,38	0,30
contig013349-TilOR.H103	contig013362-TilOR.H109	1,26	0,43	0,34
contig053780-BurOR.H065	contig013362-TilOR.H109	1,26	0,44	0,35
contig013362-TilOR.H109	contig014055-ZebOR.H066	1,26	0,44	0,35
contig053788-BurOR.H069	contig057400-ZebOR.H075	1,26	0,40	0,32
contig064817-BriOR.H051	contig048562-BurOR.H062	1,26	0,38	0,30
contig064817-BriOR.H051	contig047492-ZebOR.H074	1,26	0,39	0,31
contig107626-BriOR.H054	contig013369-TilOR.H114	1,26	0,42	0,33
contig013349-TilOR.H103	contig030011-ZebOR.H073	1,26	0,46	0,37
contig107626-BriOR.H054	contig018437-ZebOR.H072	1,26	0,44	0,35
contig053780-BurOR.H065	contig053782-BurOR.H066	1,26	0,47	0,37
contig053782-BurOR.H066	contig014055-ZebOR.H066	1,26	0,47	0,37
contig064817-BriOR.H051	contig041955-TilOR.H119	1,26	0,39	0,31
contig034998-NyeOR.H067	contig039729-NyeOR.H073	1,26	0,38	0,30
contig053782-BurOR.H066	contig039730-NyeOR.H074	1,26	0,47	0,38
contig039730-NyeOR.H074	contig013362-TilOR.H109	1,27	0,44	0,35
contig013349-TilOR.H103	contig013369-TilOR.H114	1,27	0,42	0,33
contig053787-BurOR.H068	contig034998-NyeOR.H067	1,27	0,38	0,30
contig053782-BurOR.H066	contig034998-NyeOR.H067	1,27	0,38	0,30
contig053787-BurOR.H068	contig013349-TilOR.H103	1,27	0,44	0,34
contig053787-BurOR.H068	contig013369-TilOR.H115	1,27	0,38	0,30
contig013349-TilOR.H103	contig014059-ZebOR.H068	1,27	0,44	0,34
contig053780-BurOR.H065	contig013369-TilOR.H114	1,27	0,44	0,34
contig013369-TilOR.H114	contig014055-ZebOR.H066	1,27	0,44	0,35
contig013369-TilOR.H115	contig014059-ZebOR.H068	1,27	0,38	0,30
contig039725-NyeOR.H072	contig013349-TilOR.H103	1,28	0,44	0,34
contig013349-TilOR.H103	contig013351-TilOR.H104	1,28	0,46	0,36
contig053784-BurOR.H067	contig035580-NyeOR.H069	1,28	0,39	0,30
contig107626-BriOR.H054	contig009565-TilOR.H126	1,28	0,41	0,32
contig107626-BriOR.H054	contig013369-TilOR.H115	1,28	0,43	0,34
contig064817-BriOR.H051	contig093825-BriOR.H053	1,28	0,38	0,30
contig053780-BurOR.H065	contig018434-ZebOR.H070	1,28	0,44	0,34
contig014055-ZebOR.H066	contig018434-ZebOR.H070	1,28	0,44	0,34

contig035580-NyeOR.H069	contig014060-ZebOR.H069	1,28	0,38	0,30
contig064817-BriOR.H051	contig034998-NyeOR.H067	1,28	0,38	0,30
contig039730-NyeOR.H074	contig013369-TilOR.H114	1,28	0,44	0,34
contig064821-BriOR.H052	contig035580-NyeOR.H069	1,28	0,41	0,32
contig039730-NyeOR.H074	contig018434-ZebOR.H070	1,28	0,44	0,34
contig013349-TilOR.H103	contig013356-TilOR.H105	1,29	0,46	0,36
contig053780-BurOR.H065	contig018434-ZebOR.H071	1,29	0,45	0,35
contig014055-ZebOR.H066	contig018434-ZebOR.H071	1,29	0,46	0,35
contig107626-BriOR.H054	contig116846-BriOR.H055	1,29	0,45	0,35
contig039725-NyeOR.H072	contig057403-ZebOR.H077	1,29	0,43	0,33
contig041757-NyeOR.H076	contig057400-ZebOR.H075	1,29	0,40	0,31
contig039730-NyeOR.H074	contig018434-ZebOR.H071	1,29	0,46	0,35
contig013358-TilOR.H106	contig057400-ZebOR.H075	1,30	0,39	0,30
contig009547-TilOR.H101	contig013349-TilOR.H103	1,30	0,42	0,33
contig053780-BurOR.H065	contig013369-TilOR.H115	1,31	0,45	0,35
contig013369-TilOR.H115	contig014055-ZebOR.H066	1,31	0,46	0,35
contig006794-BurOR.H061	contig039725-NyeOR.H072	1,31	0,43	0,32
contig107626-BriOR.H054	contig018434-ZebOR.H071	1,31	0,43	0,33
contig033889-BriOR.H049	contig107626-BriOR.H054	1,31	0,46	0,35
contig107626-BriOR.H054	contig018434-ZebOR.H070	1,32	0,42	0,32
contig053787-BurOR.H068	contig057403-ZebOR.H077	1,32	0,43	0,32
contig039730-NyeOR.H074	contig013369-TilOR.H115	1,32	0,46	0,35
contig053784-BurOR.H067	contig057400-ZebOR.H075	1,32	0,39	0,30
contig033889-BriOR.H049	contig013349-TilOR.H103	1,32	0,47	0,35
contig014059-ZebOR.H068	contig057403-ZebOR.H077	1,32	0,43	0,33
contig064821-BriOR.H052	contig057400-ZebOR.H075	1,32	0,41	0,31
contig107626-BriOR.H054	contig048882-BurOR.H064	1,32	0,44	0,33
contig107626-BriOR.H054	contig030011-ZebOR.H073	1,32	0,45	0,34
contig014060-ZebOR.H069	contig057400-ZebOR.H075	1,32	0,39	0,29
contig107626-BriOR.H054	contig035580-NyeOR.H069	1,33	0,45	0,34
contig013349-TilOR.H103	contig018434-ZebOR.H070	1,33	0,43	0,33
contig039725-NyeOR.H072	contig013371-TilOR.H118	1,33	0,40	0,30
contig013349-TilOR.H103	contig018434-ZebOR.H071	1,34	0,44	0,33
contig035583-NyeOR.H071	contig039725-NyeOR.H072	1,34	0,43	0,32
contig048882-BurOR.H064	contig053780-BurOR.H065	1,34	0,46	0,35
contig048882-BurOR.H064	contig014055-ZebOR.H066	1,34	0,47	0,35
contig006794-BurOR.H061	contig053787-BurOR.H068	1,34	0,42	0,32
contig013349-TilOR.H103	contig013368-TilOR.H112	1,34	0,42	0,31
contig006794-BurOR.H061	contig014059-ZebOR.H068	1,34	0,43	0,32
contig053780-BurOR.H065	contig035580-NyeOR.H069	1,34	0,47	0,35
contig035580-NyeOR.H069	contig014055-ZebOR.H066	1,34	0,47	0,35
contig048882-BurOR.H064	contig039730-NyeOR.H074	1,35	0,46	0,35
contig107626-BriOR.H054	contig057400-ZebOR.H075	1,35	0,45	0,34

contig035580-NyeOR.H069	contig039730-NyeOR.H074	1,35	0,47	0,35
contig013349-TilOR.H103	contig013369-TilOR.H116	1,36	0,44	0,32
contig053787-BurOR.H068	contig013371-TilOR.H118	1,36	0,40	0,29
contig013349-TilOR.H103	contig013369-TilOR.H115	1,36	0,44	0,32
contig053787-BurOR.H068	contig035583-NyeOR.H071	1,37	0,43	0,31
contig049873-BriOR.H050	contig039725-NyeOR.H072	1,37	0,42	0,31
contig013371-TilOR.H118	contig014059-ZebOR.H068	1,37	0,40	0,29
contig035583-NyeOR.H071	contig014059-ZebOR.H068	1,37	0,43	0,31
contig053780-BurOR.H065	contig057400-ZebOR.H075	1,37	0,47	0,34
contig014055-ZebOR.H066	contig057400-ZebOR.H075	1,37	0,48	0,35
contig048882-BurOR.H064	contig013349-TilOR.H103	1,37	0,44	0,32
contig039730-NyeOR.H074	contig057400-ZebOR.H075	1,38	0,47	0,34
contig053780-BurOR.H065	contig013369-TilOR.H116	1,38	0,45	0,33
contig013369-TilOR.H116	contig014055-ZebOR.H066	1,38	0,46	0,33
contig107626-BriOR.H054	contig013369-TilOR.H116	1,38	0,43	0,31
contig035580-NyeOR.H069	contig013356-TilOR.H105	1,39	0,39	0,28
contig039730-NyeOR.H074	contig013369-TilOR.H116	1,39	0,45	0,33
contig049873-BriOR.H050	contig053787-BurOR.H068	1,40	0,41	0,30
contig049873-BriOR.H050	contig014059-ZebOR.H068	1,40	0,42	0,30
contig035580-NyeOR.H069	contig013349-TilOR.H103	1,41	0,45	0,32
contig107626-BriOR.H054	contig013371-TilOR.H118	1,42	0,43	0,30
contig013349-TilOR.H103	contig013371-TilOR.H118	1,42	0,44	0,31
contig013349-TilOR.H103	contig057400-ZebOR.H075	1,43	0,45	0,32
contig013356-TilOR.H105	contig057400-ZebOR.H075	1,43	0,39	0,28
contig035580-NyeOR.H069	contig039725-NyeOR.H072	1,46	0,39	0,26
contig013349-TilOR.H103	contig057403-ZebOR.H077	1,46	0,46	0,32
contig053787-BurOR.H068	contig035580-NyeOR.H069	1,47	0,38	0,26
contig035583-NyeOR.H071	contig013349-TilOR.H103	1,47	0,46	0,31
contig049873-BriOR.H050	contig013349-TilOR.H103	1,47	0,46	0,31
contig053780-BurOR.H065	contig013371-TilOR.H118	1,48	0,45	0,31
contig013371-TilOR.H118	contig014055-ZebOR.H066	1,48	0,45	0,31
contig035580-NyeOR.H069	contig014059-ZebOR.H068	1,48	0,38	0,26
contig039730-NyeOR.H074	contig013371-TilOR.H118	1,49	0,45	0,30
contig107626-BriOR.H054	contig035583-NyeOR.H071	1,50	0,45	0,30
contig039725-NyeOR.H072	contig057400-ZebOR.H075	1,51	0,39	0,26
contig093825-BriOR.H053	contig013349-TilOR.H103	1,51	0,46	0,30
contig107626-BriOR.H054	contig057403-ZebOR.H077	1,52	0,45	0,30
contig053787-BurOR.H068	contig057400-ZebOR.H075	1,53	0,38	0,25
contig053780-BurOR.H065	contig035583-NyeOR.H071	1,53	0,47	0,31
contig035583-NyeOR.H071	contig014055-ZebOR.H066	1,53	0,47	0,31
contig049873-BriOR.H050	contig107626-BriOR.H054	1,53	0,45	0,29
contig014059-ZebOR.H068	contig057400-ZebOR.H075	1,54	0,39	0,25
contig035583-NyeOR.H071	contig039730-NyeOR.H074	1,54	0,47	0,31

contig034998-NyeOR.H067	contig013349-TilOR.H103	1,54	0,46	0,30
contig013349-TilOR.H103	contig041955-TilOR.H119	1,54	0,46	0,30
contig048562-BurOR.H062	contig013349-TilOR.H103	1,55	0,46	0,30
contig013349-TilOR.H103	contig047492-ZebOR.H074	1,55	0,47	0,30
contig053780-BurOR.H065	contig057403-ZebOR.H077	1,55	0,47	0,30
contig014055-ZebOR.H066	contig057403-ZebOR.H077	1,55	0,47	0,31
contig006794-BurOR.H061	contig013349-TilOR.H103	1,55	0,46	0,29
contig049873-BriOR.H050	contig053780-BurOR.H065	1,56	0,47	0,30
contig049873-BriOR.H050	contig014055-ZebOR.H066	1,56	0,47	0,30
contig039730-NyeOR.H074	contig057403-ZebOR.H077	1,56	0,47	0,30
contig049873-BriOR.H050	contig039730-NyeOR.H074	1,57	0,47	0,30
contig034998-NyeOR.H067	contig014055-ZebOR.H066	1,62	0,49	0,30
contig053780-BurOR.H065	contig034998-NyeOR.H067	1,62	0,49	0,30
contig093825-BriOR.H053	contig014055-ZebOR.H066	1,62	0,48	0,30
contig093825-BriOR.H053	contig053780-BurOR.H065	1,62	0,48	0,30
contig048562-BurOR.H062	contig014055-ZebOR.H066	1,63	0,49	0,30
contig048562-BurOR.H062	contig053780-BurOR.H065	1,63	0,49	0,30
contig014055-ZebOR.H066	contig047492-ZebOR.H074	1,63	0,49	0,30
contig053780-BurOR.H065	contig047492-ZebOR.H074	1,63	0,49	0,30
contig034998-NyeOR.H067	contig039730-NyeOR.H074	1,63	0,49	0,30
contig107626-BriOR.H054	contig006794-BurOR.H061	1,63	0,44	0,27
contig093825-BriOR.H053	contig039730-NyeOR.H074	1,63	0,48	0,30
contig041955-TilOR.H119	contig014055-ZebOR.H066	1,63	0,49	0,30
contig053780-BurOR.H065	contig041955-TilOR.H119	1,63	0,49	0,30
contig048562-BurOR.H062	contig039730-NyeOR.H074	1,64	0,49	0,30
contig039730-NyeOR.H074	contig047492-ZebOR.H074	1,64	0,50	0,30
contig039730-NyeOR.H074	contig041955-TilOR.H119	1,65	0,49	0,30
contig107626-BriOR.H054	contig034998-NyeOR.H067	1,67	0,48	0,28
contig093825-BriOR.H053	contig107626-BriOR.H054	1,68	0,47	0,28
contig107626-BriOR.H054	contig048562-BurOR.H062	1,68	0,48	0,28
contig107626-BriOR.H054	contig047492-ZebOR.H074	1,68	0,48	0,28
contig107626-BriOR.H054	contig041955-TilOR.H119	1,69	0,48	0,28
contig006794-BurOR.H061	contig053780-BurOR.H065	1,69	0,46	0,27
contig006794-BurOR.H061	contig014055-ZebOR.H066	1,69	0,47	0,28
contig006794-BurOR.H061	contig039730-NyeOR.H074	1,70	0,46	0,27

Cichlid Olfactory Receptors :
dN/dS ratio

Fam I

OR pairs		**dN**	**dS**	**dN/dS**
contig048321-BurORs.I076	contig026932-ZebORs.I082	0.004	0.004	1.134
contig046495-NyeORs.I079	contig048321-BurORs.I076	0.004	0.004	1.141
contig046495-NyeORs.I079	contig026932-ZebORs.I082	0.006	0.000	>10
contig046694-TilORs.I129	contig026932-ZebORs.I082	0.026	0.067	0.391
contig046694-TilORs.I129	contig048321-BurORs.I076	0.028	0.071	0.390
contig046694-TilORs.I129	contig046495-NyeORs.I079	0.030	0.063	0.469
contig046690-TilORs.I128	contig046694-TilORs.I129	0.065	0.189	0.343
contig046690-TilORs.I128	contig048321-BurORs.I076	0.078	0.215	0.365
contig046690-TilORs.I128	contig046495-NyeORs.I079	0.080	0.209	0.383
contig046690-TilORs.I128	contig026932-ZebORs.I082	0.080	0.210	0.381
contig046695-TilORs.I130	contig046694-TilORs.I129	0.094	0.343	0.273
contig046695-TilORs.I130	contig048321-BurORs.I076	0.098	0.365	0.268
contig046695-TilORs.I130	contig026932-ZebORs.I082	0.100	0.361	0.277
contig046695-TilORs.I130	contig046495-NyeORs.I079	0.101	0.355	0.283
contig046695-TilORs.I130	contig046690-TilORs.I128	0.112	0.366	0.307

Cichlid Olfactory Receptors : dN/dS ratio

Fam K

OR pairs		dN	dS	dN/dS
contig040509-NyeOR.K083	contig017778-ZebOR.K086	0.001	0.000	>10
contig028565-BurOR.K080	contig017778-ZebOR.K086	0.001	0.004	0.368
contig028565-BurOR.K080	contig040509-NyeOR.K083	0.003	0.004	0.734
contig046490-NyeOR.K085	contig046002-ZebOR.K087	0.003	0.016	0.180
contig049621-BurOR.K082	contig046010-ZebOR.K088	0.004	0.004	1.077
contig049604-BurOR.K081	contig046002-ZebOR.K087	0.004	0.008	0.542
contig049604-BurOR.K081	contig046490-NyeOR.K085	0.004	0.016	0.270
contig042534-BriOR.K067	contig017778-ZebOR.K086	0.006	0.024	0.242
contig042534-BriOR.K067	contig028565-BurOR.K080	0.007	0.020	0.364
contig042534-BriOR.K067	contig040509-NyeOR.K083	0.007	0.024	0.301
contig039450-TilOR.K133	contig017778-ZebOR.K086	0.007	0.062	0.118
contig060525-NyeOR.K088	contig046010-ZebOR.K088	0.009	0.000	>10
contig028565-BurOR.K080	contig039450-TilOR.K133	0.009	0.058	0.153
contig040509-NyeOR.K083	contig039450-TilOR.K133	0.009	0.062	0.142
contig049621-BurOR.K082	contig060525-NyeOR.K088	0.010	0.004	2.527
contig042534-BriOR.K067	contig039450-TilOR.K133	0.010	0.045	0.228
contig046723-TilOR.K138	contig046010-ZebOR.K088	0.018	0.128	0.138
contig049621-BurOR.K082	contig046723-TilOR.K138	0.020	0.130	0.153
contig060525-NyeOR.K088	contig046723-TilOR.K138	0.021	0.128	0.162
contig014348-BriOR.K066	contig046488-NyeOR.K084	0.023	0.077	0.305
contig014348-BriOR.K066	contig046718-TilOR.K137	0.028	0.127	0.221
contig046488-NyeOR.K084	contig046718-TilOR.K137	0.029	0.075	0.383
contig046708-TilORs.K143	contig046002-ZebORs.K090	0.035	0.061	0.572
contig046491-NyeOR.K087	contig046706-TilOR.K135	0.044	0.049	0.897
contig046490-NyeOR.K085	contig046724-TilOR.K139	0.133	0.635	0.210
contig046724-TilOR.K139	contig046002-ZebOR.K087	0.134	0.653	0.206
contig049604-BurOR.K081	contig046724-TilOR.K139	0.138	0.645	0.213
contig049604-BurOR.K081	contig046708-TilORs.K143	0.156	0.604	0.259
contig046490-NyeOR.K085	contig046708-TilORs.K143	0.156	0.622	0.250
contig046708-TilORs.K143	contig046002-ZebOR.K087	0.156	0.630	0.248
contig046490-NyeOR.K085	contig046706-TilOR.K135	0.161	0.626	0.257
contig046706-TilOR.K135	contig046002-ZebOR.K087	0.165	0.622	0.266
contig049604-BurOR.K081	contig046706-TilOR.K135	0.165	0.624	0.265
contig046490-NyeOR.K085	contig046002-ZebORs.K090	0.166	0.625	0.265
contig049604-BurOR.K081	contig046002-ZebORs.K090	0.167	0.605	0.276
contig046002-ZebOR.K087	contig046002-ZebORs.K090	0.167	0.632	0.264
contig046706-TilOR.K135	contig046724-TilOR.K139	0.174	0.536	0.325
contig046490-NyeOR.K085	contig046491-NyeOR.K087	0.180	0.628	0.286
contig046491-NyeOR.K087	contig046002-ZebOR.K087	0.184	0.625	0.295
contig049604-BurOR.K081	contig046491-NyeOR.K087	0.184	0.626	0.294
contig046699-TilOR.K134	contig046724-TilOR.K139	0.190	0.638	0.297
contig046724-TilOR.K139	contig046708-TilORs.K143	0.192	0.723	0.265

contig046724-TilOR.K139	contig046002-ZebORs.K090	0.195	0.762	0.256
contig046491-NyeOR.K087	contig046724-TilOR.K139	0.197	0.521	0.379
contig046699-TilOR.K134	contig046002-ZebOR.K087	0.203	0.667	0.305
contig046706-TilOR.K135	contig046708-TilORs.K143	0.205	0.579	0.355
contig046490-NyeOR.K085	contig046699-TilOR.K134	0.205	0.640	0.320
contig049604-BurOR.K081	contig046699-TilOR.K134	0.208	0.654	0.318
contig046491-NyeOR.K087	contig046708-TilORs.K143	0.217	0.595	0.364
contig046699-TilOR.K134	contig046706-TilOR.K135	0.226	0.671	0.337
contig046706-TilOR.K135	contig046002-ZebORs.K090	0.233	0.605	0.385
contig046491-NyeOR.K087	contig046002-ZebORs.K090	0.243	0.610	0.399
contig046488-NyeOR.K084	contig046723-TilOR.K138	0.249	0.833	0.299
contig014348-BriOR.K066	contig046723-TilOR.K138	0.250	0.761	0.328
contig046491-NyeOR.K087	contig046699-TilOR.K134	0.254	0.648	0.392
contig046718-TilOR.K137	contig046723-TilOR.K138	0.254	0.811	0.313
contig049621-BurOR.K082	contig046488-NyeOR.K084	0.256	0.838	0.306
contig014348-BriOR.K066	contig049621-BurOR.K082	0.257	0.745	0.345
contig046488-NyeOR.K084	contig046010-ZebOR.K088	0.257	0.845	0.304
contig014348-BriOR.K066	contig046010-ZebOR.K088	0.258	0.752	0.343
contig046699-TilOR.K134	contig046002-ZebORs.K090	0.259	0.686	0.378
contig046699-TilOR.K134	contig046708-TilORs.K143	0.259	0.767	0.338
contig049621-BurOR.K082	contig046718-TilOR.K137	0.260	0.820	0.318
contig046718-TilOR.K137	contig046010-ZebOR.K088	0.261	0.827	0.316
contig046488-NyeOR.K084	contig060525-NyeOR.K088	0.261	0.856	0.305
contig014348-BriOR.K066	contig060525-NyeOR.K088	0.262	0.762	0.344
contig060525-NyeOR.K088	contig046718-TilOR.K137	0.266	0.836	0.318
contig039450-TilOR.K133	contig046002-ZebOR.K087	0.347	1.999	0.174
contig046490-NyeOR.K085	contig039450-TilOR.K133	0.348	2.027	0.172
contig049604-BurOR.K081	contig039450-TilOR.K133	0.349	1.904	0.183
contig042534-BriOR.K067	contig046002-ZebOR.K087	0.352	1.956	0.180
contig042534-BriOR.K067	contig046490-NyeOR.K085	0.352	1.982	0.178
contig017778-ZebOR.K086	contig046002-ZebOR.K087	0.352	1.992	0.177
contig046490-NyeOR.K085	contig017778-ZebOR.K086	0.352	2.020	0.175
contig042534-BriOR.K067	contig049604-BurOR.K081	0.354	1.866	0.190
contig049604-BurOR.K081	contig017778-ZebOR.K086	0.354	1.899	0.186
contig028565-BurOR.K080	contig046002-ZebOR.K087	0.354	2.041	0.174
contig040509-NyeOR.K083	contig046002-ZebOR.K087	0.355	1.983	0.179
contig028565-BurOR.K080	contig046490-NyeOR.K085	0.355	2.070	0.171
contig040509-NyeOR.K083	contig046490-NyeOR.K085	0.356	2.011	0.177
contig049604-BurOR.K081	contig040509-NyeOR.K083	0.357	1.891	0.189
contig028565-BurOR.K080	contig049604-BurOR.K081	0.357	1.941	0.184
contig014348-BriOR.K066	contig046490-NyeOR.K085	0.386	1.513	0.255
contig014348-BriOR.K066	contig046002-ZebOR.K087	0.386	1.513	0.255
contig039450-TilOR.K133	contig046708-TilORs.K143	0.389	1.880	0.207

contig046708-TilORs.K143	contig017778-ZebOR.K086	0.391	1.851	0.211
contig039450-TilOR.K133	contig046724-TilOR.K139	0.391	2.152	0.182
contig046488-NyeOR.K084	contig046490-NyeOR.K085	0.392	1.431	0.274
contig046723-TilOR.K138	contig046002-ZebOR.K087	0.392	1.567	0.250
contig046488-NyeOR.K084	contig046002-ZebOR.K087	0.393	1.430	0.275
contig046490-NyeOR.K085	contig046723-TilOR.K138	0.393	1.583	0.248
contig060525-NyeOR.K088	contig046002-ZebOR.K087	0.393	1.687	0.233
contig046718-TilOR.K137	contig046002-ZebOR.K087	0.394	1.408	0.279
contig046490-NyeOR.K085	contig060525-NyeOR.K088	0.394	1.706	0.231
contig040509-NyeOR.K083	contig046708-TilORs.K143	0.394	1.843	0.214
contig028565-BurOR.K080	contig046708-TilORs.K143	0.394	1.883	0.209
contig046490-NyeOR.K085	contig046718-TilOR.K137	0.395	1.409	0.281
contig014348-BriOR.K066	contig049604-BurOR.K081	0.395	1.505	0.262
contig049604-BurOR.K081	contig046723-TilOR.K138	0.396	1.527	0.259
contig049604-BurOR.K081	contig060525-NyeOR.K088	0.396	1.641	0.242
contig049621-BurOR.K082	contig046002-ZebOR.K087	0.397	1.704	0.233
contig049621-BurOR.K082	contig046490-NyeOR.K085	0.397	1.731	0.230
contig046002-ZebOR.K087	contig046010-ZebOR.K088	0.398	1.695	0.235
contig046490-NyeOR.K085	contig046010-ZebOR.K088	0.398	1.714	0.232
contig046724-TilOR.K139	contig017778-ZebOR.K086	0.399	2.390	0.167
contig046718-TilOR.K137	contig046724-TilOR.K139	0.400	1.347	0.297
contig046488-NyeOR.K084	contig046724-TilOR.K139	0.400	1.369	0.292
contig042534-BriOR.K067	contig046708-TilORs.K143	0.400	1.839	0.217
contig049604-BurOR.K081	contig046718-TilOR.K137	0.401	1.415	0.283
contig049604-BurOR.K081	contig046488-NyeOR.K084	0.401	1.423	0.282
contig049604-BurOR.K081	contig046010-ZebOR.K088	0.401	1.648	0.243
contig049604-BurOR.K081	contig049621-BurOR.K082	0.401	1.657	0.242
contig042534-BriOR.K067	contig046724-TilOR.K139	0.401	2.169	0.185
contig028565-BurOR.K080	contig046724-TilOR.K139	0.401	2.283	0.176
contig040509-NyeOR.K083	contig046724-TilOR.K139	0.402	2.392	0.168
contig039450-TilOR.K133	contig046002-ZebORs.K090	0.403	1.818	0.222
contig017778-ZebOR.K086	contig046002-ZebORs.K090	0.404	1.813	0.223
contig028565-BurOR.K080	contig046002-ZebORs.K090	0.406	1.852	0.219
contig014348-BriOR.K066	contig046724-TilOR.K139	0.407	1.405	0.289
contig040509-NyeOR.K083	contig046002-ZebORs.K090	0.407	1.805	0.225
contig014348-BriOR.K066	contig039450-TilOR.K133	0.407	1.887	0.216
contig042534-BriOR.K067	contig046002-ZebORs.K090	0.408	1.783	0.229
contig046488-NyeOR.K084	contig039450-TilOR.K133	0.410	1.934	0.212
contig046699-TilOR.K134	contig017778-ZebOR.K086	0.411	1.651	0.249
contig042534-BriOR.K067	contig046699-TilOR.K134	0.411	1.663	0.247
contig014348-BriOR.K066	contig042534-BriOR.K067	0.413	1.698	0.243
contig039450-TilOR.K133	contig046718-TilOR.K137	0.413	1.882	0.220
contig028565-BurOR.K080	contig046699-TilOR.K134	0.414	1.645	0.252

contig040509-NyeOR.K083	contig046699-TilOR.K134	0.415	1.645	0.252
contig014348-BriOR.K066	contig046699-TilOR.K134	0.416	1.477	0.282
contig039450-TilOR.K133	contig046699-TilOR.K134	0.416	1.619	0.257
contig042534-BriOR.K067	contig046488-NyeOR.K084	0.416	1.733	0.240
contig014348-BriOR.K066	contig040509-NyeOR.K083	0.419	1.624	0.258
contig042534-BriOR.K067	contig046718-TilOR.K137	0.419	1.821	0.230
contig014348-BriOR.K066	contig017778-ZebOR.K086	0.420	1.630	0.258
contig040509-NyeOR.K083	contig046488-NyeOR.K084	0.421	1.655	0.254
contig046699-TilOR.K134	contig046718-TilOR.K137	0.422	1.417	0.298
contig046488-NyeOR.K084	contig017778-ZebOR.K086	0.422	1.661	0.254
contig040509-NyeOR.K083	contig046718-TilOR.K137	0.422	1.734	0.243
contig014348-BriOR.K066	contig028565-BurOR.K080	0.423	1.624	0.261
contig046718-TilOR.K137	contig017778-ZebOR.K086	0.423	1.741	0.243
contig028565-BurOR.K080	contig046488-NyeOR.K084	0.425	1.655	0.257
contig046723-TilOR.K138	contig046708-TilORs.K143	0.425	1.724	0.247
contig028565-BurOR.K080	contig046718-TilOR.K137	0.426	1.734	0.246
contig046488-NyeOR.K084	contig046699-TilOR.K134	0.434	1.389	0.313
contig046723-TilOR.K138	contig046724-TilOR.K139	0.434	1.563	0.278
contig049621-BurOR.K082	contig046002-ZebORs.K090	0.436	1.932	0.226
contig046010-ZebOR.K088	contig046002-ZebORs.K090	0.437	1.964	0.223
contig060525-NyeOR.K088	contig046708-TilORs.K143	0.439	1.889	0.232
contig040509-NyeOR.K083	contig046010-ZebOR.K088	0.440	1.645	0.267
contig017778-ZebOR.K086	contig046010-ZebOR.K088	0.441	1.651	0.267
contig046708-TilORs.K143	contig046010-ZebOR.K088	0.441	1.899	0.232
contig040509-NyeOR.K083	contig046723-TilOR.K138	0.442	1.589	0.278
contig042534-BriOR.K067	contig046010-ZebOR.K088	0.442	1.762	0.251
contig046723-TilOR.K138	contig017778-ZebOR.K086	0.443	1.594	0.278
contig028565-BurOR.K080	contig046010-ZebOR.K088	0.444	1.645	0.270
contig049621-BurOR.K082	contig040509-NyeOR.K083	0.444	1.660	0.267
contig039450-TilOR.K133	contig046723-TilOR.K138	0.444	1.688	0.263
contig039450-TilOR.K133	contig046706-TilOR.K135	0.445	2.026	0.220
contig028565-BurOR.K080	contig046723-TilOR.K138	0.446	1.589	0.281
contig049621-BurOR.K082	contig017778-ZebOR.K086	0.446	1.666	0.267
contig042534-BriOR.K067	contig046723-TilOR.K138	0.446	1.678	0.266
contig039450-TilOR.K133	contig046010-ZebOR.K088	0.446	1.788	0.249
contig014348-BriOR.K066	contig046706-TilOR.K135	0.447	1.243	0.359
contig046724-TilOR.K139	contig046010-ZebOR.K088	0.447	1.661	0.269
contig049621-BurOR.K082	contig046724-TilOR.K139	0.447	1.670	0.268
contig042534-BriOR.K067	contig049621-BurOR.K082	0.447	1.780	0.251
contig046706-TilOR.K135	contig046718-TilOR.K137	0.448	1.303	0.343
contig060525-NyeOR.K088	contig046724-TilOR.K139	0.448	1.654	0.271
contig028565-BurOR.K080	contig049621-BurOR.K082	0.449	1.660	0.270
contig049621-BurOR.K082	contig039450-TilOR.K133	0.450	1.807	0.249

contig046706-TilOR.K135	contig017778-ZebOR.K086	0.450	1.816	0.248
contig042534-BriOR.K067	contig046706-TilOR.K135	0.450	1.878	0.240
contig040509-NyeOR.K083	contig060525-NyeOR.K088	0.451	1.567	0.288
contig046491-NyeOR.K087	contig039450-TilOR.K133	0.451	1.892	0.239
contig014348-BriOR.K066	contig046491-NyeOR.K087	0.453	1.363	0.332
contig060525-NyeOR.K088	contig017778-ZebOR.K086	0.453	1.572	0.288
contig028565-BurOR.K080	contig046706-TilOR.K135	0.453	1.808	0.251
contig014348-BriOR.K066	contig046708-TilORs.K143	0.453	1.852	0.245
contig014348-BriOR.K066	contig046002-ZebORs.K090	0.453	1.968	0.230
contig042534-BriOR.K067	contig060525-NyeOR.K088	0.454	1.672	0.271
contig040509-NyeOR.K083	contig046706-TilOR.K135	0.454	1.809	0.251
contig046491-NyeOR.K087	contig046718-TilOR.K137	0.455	1.422	0.320
contig028565-BurOR.K080	contig060525-NyeOR.K088	0.456	1.567	0.291
contig046491-NyeOR.K087	contig017778-ZebOR.K086	0.456	1.717	0.266
contig060525-NyeOR.K088	contig039450-TilOR.K133	0.457	1.734	0.264
contig046706-TilOR.K135	contig046723-TilOR.K138	0.458	1.790	0.256
contig042534-BriOR.K067	contig046491-NyeOR.K087	0.459	1.765	0.260
contig060525-NyeOR.K088	contig046002-ZebORs.K090	0.459	1.875	0.245
contig049621-BurOR.K082	contig046708-TilORs.K143	0.459	2.007	0.229
contig028565-BurOR.K080	contig046491-NyeOR.K087	0.460	1.704	0.270
contig040509-NyeOR.K083	contig046491-NyeOR.K087	0.460	1.711	0.269
contig046491-NyeOR.K087	contig046723-TilOR.K138	0.460	1.796	0.256
contig046718-TilOR.K137	contig046002-ZebORs.K090	0.461	1.653	0.279
contig046488-NyeOR.K084	contig046706-TilOR.K135	0.462	1.242	0.372
contig046699-TilOR.K134	contig046723-TilOR.K138	0.462	1.479	0.312
contig046718-TilOR.K137	contig046708-TilORs.K143	0.463	1.594	0.291
contig049621-BurOR.K082	contig046699-TilOR.K134	0.468	1.599	0.293
contig046488-NyeOR.K084	contig046708-TilORs.K143	0.468	1.693	0.276
contig046488-NyeOR.K084	contig046002-ZebORs.K090	0.468	1.878	0.249
contig046488-NyeOR.K084	contig046491-NyeOR.K087	0.469	1.327	0.354
contig046699-TilOR.K134	contig046010-ZebOR.K088	0.470	1.613	0.291
contig046723-TilOR.K138	contig046002-ZebORs.K090	0.471	1.675	0.281
contig049621-BurOR.K082	contig046706-TilOR.K135	0.476	1.763	0.270
contig060525-NyeOR.K088	contig046699-TilOR.K134	0.477	1.624	0.293
contig046706-TilOR.K135	contig046010-ZebOR.K088	0.477	1.712	0.278
contig060525-NyeOR.K088	contig046706-TilOR.K135	0.477	1.744	0.274
contig049621-BurOR.K082	contig046491-NyeOR.K087	0.478	1.727	0.277
contig046491-NyeOR.K087	contig046010-ZebOR.K088	0.479	1.679	0.285
contig046491-NyeOR.K087	contig060525-NyeOR.K088	0.479	1.709	0.280

Cichlid Olfactory Receptors :
dN/dS ratio

Fam L

OR pairs		dN	dS	dN/dS
contig020440-ZebOR.L094	contig056940-NyeOR.L095	0.001	0.004	0.346
contig017787-ZebOR.L092	contig040502-NyeOR.L091	0.001	0.034	0.043
contig017786-ZebOR.L091	contig058162-BurOR.L089	0.004	0.008	0.538
contig020445-ZebOR.L096	contig056942-NyeOR.L093	0.004	0.008	0.540
contig020442-ZebOR.L095	contig056384-BurOR.L087	0.006	0.034	0.170
contig017786-ZebOR.L091	contig040502-NyeOR.L092	0.007	0.012	0.596
contig020442-ZebOR.L095	contig064361-NyeOR.L094	0.007	0.013	0.572
contig040502-NyeOR.L092	contig058162-BurOR.L089	0.009	0.012	0.717
contig056384-BurOR.L087	contig064361-NyeOR.L094	0.009	0.030	0.291
contig040502-NyeOR.L091	contig065027-BurOR.L090	0.009	0.034	0.258
contig017787-ZebOR.L092	contig065027-BurOR.L090	0.011	0.058	0.188
contig042556-BriOR.L073	contig056940-NyeOR.L095	0.014	0.041	0.340
contig020440-ZebOR.L094	contig042556-BriOR.L073	0.015	0.036	0.421
contig040502-NyeOR.L091	contig042544-BriOR.L070	0.016	0.025	0.637
contig017787-ZebOR.L092	contig042544-BriOR.L070	0.018	0.051	0.342
contig042544-BriOR.L070	contig065027-BurOR.L090	0.019	0.043	0.446
contig017786-ZebOR.L091	contig039460-TilOR.L145	0.024	0.067	0.349
contig017786-ZebOR.L091	contig042543-BriOR.L069	0.025	0.041	0.608
contig039460-TilOR.L145	contig058162-BurOR.L089	0.026	0.056	0.458
contig042543-BriOR.L069	contig058162-BurOR.L089	0.027	0.033	0.811
contig039481-TilOR.L153	contig042554-BriOR.L072	0.028	0.056	0.495
contig039460-TilOR.L145	contig040502-NyeOR.L092	0.028	0.072	0.390
contig020437-ZebOR.L093	contig056386-BurOR.L088	0.028	0.079	0.352
contig056940-NyeOR.L095	contig068425-TilOR.L155	0.029	0.070	0.422
contig040502-NyeOR.L092	contig042543-BriOR.L069	0.030	0.045	0.650
contig020442-ZebOR.L095	contig042554-BriOR.L072	0.031	0.043	0.719
contig042554-BriOR.L072	contig064361-NyeOR.L094	0.031	0.056	0.547
contig039484-TilOR.L154	contig042556-BriOR.L073	0.031	0.065	0.475
contig039484-TilOR.L154	contig056940-NyeOR.L095	0.031	0.069	0.445
contig020440-ZebOR.L094	contig068425-TilOR.L155	0.031	0.074	0.415
contig039462-TilOR.L149	contig040502-NyeOR.L091	0.031	0.087	0.355
contig042554-BriOR.L072	contig072645-TilOR.L159	0.032	0.056	0.581
contig042554-BriOR.L072	contig056384-BurOR.L087	0.032	0.061	0.533
contig020440-ZebOR.L094	contig039484-TilOR.L154	0.032	0.065	0.499
contig042556-BriOR.L073	contig068425-TilOR.L155	0.032	0.067	0.468
contig017787-ZebOR.L092	contig039462-TilOR.L149	0.032	0.101	0.321
contig020442-ZebOR.L095	contig039481-TilOR.L153	0.035	0.061	0.583
contig039481-TilOR.L153	contig064361-NyeOR.L094	0.035	0.074	0.474
contig039481-TilOR.L153	contig056384-BurOR.L087	0.037	0.070	0.529
contig064361-NyeOR.L094	contig072645-TilOR.L159	0.038	0.083	0.462
contig039462-TilOR.L149	contig065027-BurOR.L090	0.038	0.104	0.364
contig020445-ZebOR.L096	contig042552-BriOR.L071	0.039	0.041	0.943

contig039484-TilOR.L154	contig068425-TilOR.L155	0.040	0.038	1.043
contig020442-ZebOR.L095	contig072645-TilOR.L159	0.040	0.069	0.577
contig039481-TilOR.L153	contig072645-TilOR.L159	0.040	0.088	0.457
contig042552-BriOR.L071	contig068527-TilOR.L157	0.041	0.060	0.683
contig042556-BriOR.L073	contig056386-BurOR.L088	0.041	0.071	0.583
contig020445-ZebOR.L096	contig068527-TilOR.L157	0.041	0.074	0.559
contig056384-BurOR.L087	contig072645-TilOR.L159	0.041	0.078	0.530
contig020437-ZebOR.L093	contig042556-BriOR.L073	0.041	0.091	0.447
contig039462-TilOR.L149	contig042544-BriOR.L070	0.042	0.087	0.475
contig042552-BriOR.L071	contig056942-NyeOR.L093	0.043	0.041	1.053
contig039460-TilOR.L145	contig042543-BriOR.L069	0.043	0.067	0.646
contig020437-ZebOR.L093	contig072645-TilOR.L159	0.043	0.097	0.443
contig039484-TilOR.L154	contig068521-TilOR.L156	0.044	0.071	0.617
contig020437-ZebOR.L093	contig020440-ZebOR.L094	0.044	0.088	0.503
contig042556-BriOR.L073	contig068521-TilOR.L156	0.045	0.067	0.678
contig068425-TilOR.L155	contig072645-TilOR.L159	0.045	0.074	0.604
contig020440-ZebOR.L094	contig056386-BurOR.L088	0.045	0.077	0.581
contig056942-NyeOR.L093	contig068527-TilOR.L157	0.046	0.074	0.620
contig056386-BurOR.L088	contig068521-TilOR.L156	0.046	0.074	0.626
contig056386-BurOR.L088	contig056940-NyeOR.L095	0.046	0.082	0.567
contig020437-ZebOR.L093	contig056940-NyeOR.L095	0.046	0.093	0.495
contig020437-ZebOR.L093	contig068425-TilOR.L155	0.047	0.103	0.463
contig042556-BriOR.L073	contig072645-TilOR.L159	0.048	0.099	0.487
contig056940-NyeOR.L095	contig068521-TilOR.L156	0.049	0.078	0.630
contig039484-TilOR.L154	contig072645-TilOR.L159	0.049	0.092	0.535
contig020437-ZebOR.L093	contig064361-NyeOR.L094	0.049	0.098	0.499
contig020437-ZebOR.L093	contig042554-BriOR.L072	0.050	0.065	0.773
contig039484-TilOR.L154	contig064361-NyeOR.L094	0.050	0.105	0.476
contig068425-TilOR.L155	contig068521-TilOR.L156	0.051	0.042	1.196
contig042554-BriOR.L072	contig068425-TilOR.L155	0.051	0.072	0.712
contig020440-ZebOR.L094	contig068521-TilOR.L156	0.051	0.083	0.614
contig039461-TilOR.L148	contig039465-TilOR.L150	0.051	0.084	0.614
contig020437-ZebOR.L093	contig056384-BurOR.L087	0.051	0.088	0.572
contig068521-TilOR.L156	contig072645-TilOR.L159	0.051	0.101	0.505
contig056386-BurOR.L088	contig068425-TilOR.L155	0.052	0.080	0.650
contig020437-ZebOR.L093	contig020442-ZebOR.L095	0.052	0.084	0.622
contig020437-ZebOR.L093	contig068521-TilOR.L156	0.052	0.102	0.514
contig056940-NyeOR.L095	contig072645-TilOR.L159	0.052	0.121	0.433
contig042556-BriOR.L073	contig064361-NyeOR.L094	0.053	0.086	0.613
contig056386-BurOR.L088	contig072645-TilOR.L159	0.053	0.109	0.487
contig039484-TilOR.L154	contig068539-TilOR.L158	0.054	0.076	0.713
contig020442-ZebOR.L095	contig039484-TilOR.L154	0.054	0.090	0.604
contig039481-TilOR.L153	contig039484-TilOR.L154	0.054	0.092	0.581

contig020437-ZebOR.L093	contig039484-TilOR.L154	0.054	0.100	0.546
contig056384-BurOR.L087	contig068425-TilOR.L155	0.054	0.105	0.520
contig020440-ZebOR.L094	contig072645-TilOR.L159	0.054	0.116	0.464
contig064361-NyeOR.L094	contig068521-TilOR.L156	0.054	0.121	0.445
contig039484-TilOR.L154	contig042554-BriOR.L072	0.055	0.070	0.795
contig042554-BriOR.L072	contig068521-TilOR.L156	0.055	0.101	0.545
contig042556-BriOR.L073	contig056384-BurOR.L087	0.056	0.086	0.651
contig039484-TilOR.L154	contig056384-BurOR.L087	0.056	0.095	0.591
contig020442-ZebOR.L095	contig068425-TilOR.L155	0.056	0.100	0.559
contig039481-TilOR.L153	contig068425-TilOR.L155	0.056	0.105	0.534
contig064361-NyeOR.L094	contig068425-TilOR.L155	0.056	0.115	0.488
contig042554-BriOR.L072	contig042556-BriOR.L073	0.057	0.072	0.797
contig020442-ZebOR.L095	contig042556-BriOR.L073	0.057	0.072	0.798
contig039484-TilOR.L154	contig056386-BurOR.L088	0.057	0.079	0.712
contig056940-NyeOR.L095	contig064361-NyeOR.L094	0.057	0.098	0.580
contig020437-ZebOR.L093	contig039481-TilOR.L153	0.057	0.107	0.528
contig056386-BurOR.L088	contig064361-NyeOR.L094	0.057	0.120	0.480
contig020440-ZebOR.L094	contig064361-NyeOR.L094	0.058	0.093	0.626
contig020442-ZebOR.L095	contig068521-TilOR.L156	0.058	0.106	0.551
contig068425-TilOR.L155	contig068539-TilOR.L158	0.059	0.081	0.726
contig042554-BriOR.L072	contig056386-BurOR.L088	0.059	0.086	0.687
contig056384-BurOR.L087	contig068521-TilOR.L156	0.059	0.108	0.546
contig056384-BurOR.L087	contig056386-BurOR.L088	0.059	0.110	0.539
contig056384-BurOR.L087	contig056940-NyeOR.L095	0.060	0.088	0.680
contig056940-NyeOR.L095	contig068539-TilOR.L158	0.060	0.098	0.613
contig039481-TilOR.L153	contig042556-BriOR.L073	0.061	0.072	0.842
contig042554-BriOR.L072	contig056940-NyeOR.L095	0.061	0.083	0.736
contig020440-ZebOR.L094	contig056384-BurOR.L087	0.061	0.083	0.736
contig020442-ZebOR.L095	contig056940-NyeOR.L095	0.061	0.083	0.736
contig020440-ZebOR.L094	contig068539-TilOR.L158	0.061	0.093	0.661
contig042556-BriOR.L073	contig068539-TilOR.L158	0.061	0.095	0.635
contig020442-ZebOR.L095	contig056386-BurOR.L088	0.061	0.105	0.578
contig039481-TilOR.L153	contig068521-TilOR.L156	0.061	0.113	0.537
contig020440-ZebOR.L094	contig042554-BriOR.L072	0.063	0.079	0.798
contig020440-ZebOR.L094	contig020442-ZebOR.L095	0.063	0.079	0.799
contig068521-TilOR.L156	contig068539-TilOR.L158	0.065	0.092	0.703
contig039481-TilOR.L153	contig056386-BurOR.L088	0.065	0.124	0.526
contig039481-TilOR.L153	contig056940-NyeOR.L095	0.066	0.093	0.713
contig020440-ZebOR.L094	contig039481-TilOR.L153	0.068	0.088	0.769
contig064361-NyeOR.L094	contig068539-TilOR.L158	0.074	0.084	0.881
contig042554-BriOR.L072	contig068539-TilOR.L158	0.076	0.077	0.995
contig020442-ZebOR.L095	contig068539-TilOR.L158	0.076	0.079	0.956
contig017786-ZebOR.L091	contig039460-TilOR.L146	0.076	0.171	0.446

contig056384-BurOR.L087	contig068539-TilOR.L158	0.077	0.074	1.038
contig068539-TilOR.L158	contig072645-TilOR.L159	0.077	0.090	0.850
contig039481-TilOR.L153	contig068539-TilOR.L158	0.078	0.091	0.860
contig039460-TilOR.L145	contig039460-TilOR.L146	0.078	0.131	0.596
contig039460-TilOR.L146	contig058162-BurOR.L089	0.078	0.160	0.485
contig039460-TilOR.L146	contig040502-NyeOR.L092	0.079	0.176	0.451
contig056386-BurOR.L088	contig068539-TilOR.L158	0.081	0.115	0.708
contig020437-ZebOR.L093	contig068539-TilOR.L158	0.082	0.098	0.836
contig020445-ZebOR.L096	contig039465-TilOR.L150	0.084	0.157	0.535
contig039465-TilOR.L150	contig056942-NyeOR.L093	0.086	0.157	0.544
contig039465-TilOR.L150	contig068527-TilOR.L157	0.087	0.128	0.684
contig039460-TilOR.L146	contig042543-BriOR.L069	0.090	0.147	0.611
contig039465-TilOR.L150	contig042552-BriOR.L071	0.092	0.149	0.613
contig020445-ZebOR.L096	contig039461-TilOR.L148	0.102	0.162	0.631
contig039461-TilOR.L148	contig042552-BriOR.L071	0.103	0.159	0.649
contig039461-TilOR.L148	contig068527-TilOR.L157	0.104	0.147	0.709
contig039461-TilOR.L148	contig056942-NyeOR.L093	0.104	0.162	0.640
contig039462-TilOR.L149	contig039465-TilOR.L150	0.148	0.414	0.357
contig039461-TilOR.L147	contig039462-TilOR.L149	0.152	0.376	0.405
contig039465-TilOR.L150	contig040502-NyeOR.L091	0.153	0.402	0.380
contig017787-ZebOR.L092	contig039465-TilOR.L150	0.155	0.443	0.349
contig039465-TilOR.L150	contig039484-TilOR.L154	0.156	0.451	0.347
contig020445-ZebOR.L096	contig039462-TilOR.L149	0.157	0.414	0.379
contig039462-TilOR.L149	contig056942-NyeOR.L093	0.158	0.426	0.370
contig039465-TilOR.L150	contig065027-BurOR.L090	0.158	0.445	0.354
contig039462-TilOR.L149	contig042552-BriOR.L071	0.158	0.450	0.352
contig039465-TilOR.L150	contig068521-TilOR.L156	0.160	0.421	0.380
contig039462-TilOR.L149	contig068527-TilOR.L157	0.161	0.404	0.398
contig020445-ZebOR.L096	contig040502-NyeOR.L091	0.162	0.388	0.417
contig040502-NyeOR.L091	contig056942-NyeOR.L093	0.163	0.400	0.407
contig017787-ZebOR.L092	contig020445-ZebOR.L096	0.164	0.429	0.382
contig020445-ZebOR.L096	contig065027-BurOR.L090	0.164	0.439	0.373
contig039465-TilOR.L150	contig042556-BriOR.L073	0.164	0.443	0.370
contig056942-NyeOR.L093	contig065027-BurOR.L090	0.164	0.451	0.364
contig020437-ZebOR.L093	contig042552-BriOR.L071	0.164	0.468	0.350
contig020437-ZebOR.L093	contig056942-NyeOR.L093	0.164	0.469	0.350
contig039461-TilOR.L147	contig040502-NyeOR.L091	0.165	0.384	0.429
contig017787-ZebOR.L092	contig056942-NyeOR.L093	0.165	0.441	0.374
contig039465-TilOR.L150	contig072645-TilOR.L159	0.165	0.480	0.344
contig040502-NyeOR.L091	contig042552-BriOR.L071	0.166	0.412	0.403
contig020440-ZebOR.L094	contig039465-TilOR.L150	0.166	0.449	0.370
contig042552-BriOR.L071	contig042556-BriOR.L073	0.167	0.432	0.386
contig039484-TilOR.L154	contig042552-BriOR.L071	0.167	0.437	0.382

contig039465-TilOR.L150	contig042554-BriOR.L072	0.168	0.420	0.401
contig017787-ZebOR.L092	contig039461-TilOR.L147	0.168	0.422	0.397
contig039465-TilOR.L150	contig056940-NyeOR.L095	0.168	0.441	0.381
contig039484-TilOR.L154	contig056942-NyeOR.L093	0.168	0.445	0.376
contig020437-ZebOR.L093	contig039465-TilOR.L150	0.168	0.448	0.376
contig017787-ZebOR.L092	contig042552-BriOR.L071	0.168	0.454	0.370
contig042552-BriOR.L071	contig065027-BurOR.L090	0.168	0.465	0.361
contig039465-TilOR.L150	contig056386-BurOR.L088	0.168	0.495	0.340
contig039461-TilOR.L147	contig039465-TilOR.L150	0.169	0.340	0.497
contig039465-TilOR.L150	contig042544-BriOR.L070	0.169	0.369	0.457
contig039465-TilOR.L150	contig039481-TilOR.L153	0.169	0.416	0.405
contig039461-TilOR.L148	contig068521-TilOR.L156	0.169	0.428	0.394
contig020440-ZebOR.L094	contig056942-NyeOR.L093	0.169	0.440	0.385
contig039465-TilOR.L150	contig068425-TilOR.L155	0.169	0.447	0.377
contig040502-NyeOR.L091	contig068527-TilOR.L157	0.170	0.365	0.464
contig039462-TilOR.L149	contig068521-TilOR.L156	0.170	0.380	0.447
contig020445-ZebOR.L096	contig039484-TilOR.L154	0.170	0.441	0.386
contig039465-TilOR.L150	contig056384-BurOR.L087	0.170	0.442	0.384
contig039461-TilOR.L147	contig042552-BriOR.L071	0.170	0.443	0.384
contig039461-TilOR.L148	contig039462-TilOR.L149	0.170	0.446	0.382
contig039465-TilOR.L150	contig064361-NyeOR.L094	0.170	0.451	0.376
contig023716-TilOR.L144	contig039461-TilOR.L147	0.171	0.315	0.542
contig065027-BurOR.L090	contig068527-TilOR.L157	0.171	0.400	0.428
contig039484-TilOR.L154	contig068527-TilOR.L157	0.171	0.422	0.406
contig042556-BriOR.L073	contig056942-NyeOR.L093	0.171	0.440	0.388
contig056940-NyeOR.L095	contig056942-NyeOR.L093	0.171	0.447	0.383
contig020437-ZebOR.L093	contig020445-ZebOR.L096	0.171	0.464	0.368
contig017787-ZebOR.L092	contig068527-TilOR.L157	0.172	0.405	0.424
contig020437-ZebOR.L093	contig068527-TilOR.L157	0.172	0.426	0.405
contig020440-ZebOR.L094	contig020445-ZebOR.L096	0.172	0.435	0.395
contig042552-BriOR.L071	contig042554-BriOR.L072	0.173	0.417	0.414
contig020440-ZebOR.L094	contig042552-BriOR.L071	0.173	0.432	0.400
contig056942-NyeOR.L093	contig064361-NyeOR.L094	0.173	0.432	0.401
contig020442-ZebOR.L095	contig039465-TilOR.L150	0.173	0.442	0.392
contig023716-TilOR.L144	contig039465-TilOR.L150	0.173	0.461	0.374
contig042552-BriOR.L071	contig072645-TilOR.L159	0.173	0.471	0.368
contig056942-NyeOR.L093	contig072645-TilOR.L159	0.173	0.472	0.367
contig020445-ZebOR.L096	contig042544-BriOR.L070	0.174	0.376	0.464
contig042556-BriOR.L073	contig068527-TilOR.L157	0.174	0.421	0.413
contig042552-BriOR.L071	contig056940-NyeOR.L095	0.174	0.431	0.404
contig020445-ZebOR.L096	contig042556-BriOR.L073	0.174	0.436	0.399
contig020445-ZebOR.L096	contig056940-NyeOR.L095	0.174	0.442	0.393
contig042552-BriOR.L071	contig056386-BurOR.L088	0.174	0.479	0.363

contig042544-BriOR.L070	contig056942-NyeOR.L093	0.175	0.387	0.453
contig042554-BriOR.L072	contig056942-NyeOR.L093	0.175	0.415	0.423
contig039461-TilOR.L147	contig065027-BurOR.L090	0.175	0.416	0.420
contig056384-BurOR.L087	contig056942-NyeOR.L093	0.175	0.416	0.421
contig023716-TilOR.L144	contig042552-BriOR.L071	0.175	0.528	0.332
contig039462-TilOR.L149	contig039484-TilOR.L154	0.176	0.393	0.448
contig039481-TilOR.L153	contig042552-BriOR.L071	0.176	0.398	0.442
contig042554-BriOR.L072	contig068527-TilOR.L157	0.176	0.405	0.434
contig020440-ZebOR.L094	contig068527-TilOR.L157	0.176	0.416	0.424
contig039461-TilOR.L148	contig040502-NyeOR.L091	0.176	0.422	0.417
contig020445-ZebOR.L096	contig072645-TilOR.L159	0.176	0.467	0.377
contig056386-BurOR.L088	contig056942-NyeOR.L093	0.176	0.488	0.361
contig039481-TilOR.L153	contig068527-TilOR.L157	0.177	0.400	0.443
contig039481-TilOR.L153	contig056942-NyeOR.L093	0.177	0.403	0.439
contig056942-NyeOR.L093	contig068425-TilOR.L155	0.177	0.451	0.393
contig039461-TilOR.L147	contig042554-BriOR.L072	0.178	0.374	0.476
contig068521-TilOR.L156	contig068527-TilOR.L157	0.178	0.409	0.435
contig042552-BriOR.L071	contig068521-TilOR.L156	0.178	0.410	0.433
contig020445-ZebOR.L096	contig042554-BriOR.L072	0.178	0.411	0.434
contig056940-NyeOR.L095	contig068527-TilOR.L157	0.178	0.423	0.422
contig064361-NyeOR.L094	contig068527-TilOR.L157	0.178	0.425	0.419
contig042552-BriOR.L071	contig064361-NyeOR.L094	0.178	0.448	0.397
contig017787-ZebOR.L092	contig039461-TilOR.L148	0.178	0.465	0.383
contig039461-TilOR.L147	contig039481-TilOR.L153	0.179	0.379	0.472
contig039461-TilOR.L148	contig042554-BriOR.L072	0.179	0.392	0.457
contig042544-BriOR.L070	contig042552-BriOR.L071	0.179	0.400	0.447
contig020442-ZebOR.L095	contig056942-NyeOR.L093	0.179	0.416	0.430
contig056942-NyeOR.L093	contig068521-TilOR.L156	0.179	0.421	0.424
contig039461-TilOR.L148	contig042556-BriOR.L073	0.179	0.433	0.414
contig039461-TilOR.L148	contig039484-TilOR.L154	0.179	0.440	0.407
contig042552-BriOR.L071	contig068425-TilOR.L155	0.179	0.443	0.404
contig039460-TilOR.L145	contig042552-BriOR.L071	0.179	0.465	0.386
contig020445-ZebOR.L096	contig056386-BurOR.L088	0.179	0.483	0.370
contig020445-ZebOR.L096	contig039481-TilOR.L153	0.180	0.398	0.451
contig056384-BurOR.L087	contig068527-TilOR.L157	0.180	0.410	0.440
contig020445-ZebOR.L096	contig064361-NyeOR.L094	0.180	0.442	0.407
contig020445-ZebOR.L096	contig068425-TilOR.L155	0.180	0.446	0.403
contig039462-TilOR.L149	contig072645-TilOR.L159	0.181	0.386	0.469
contig039461-TilOR.L147	contig068527-TilOR.L157	0.181	0.395	0.459
contig039462-TilOR.L149	contig056386-BurOR.L088	0.181	0.409	0.443
contig020437-ZebOR.L093	contig039461-TilOR.L148	0.181	0.440	0.413
contig068527-TilOR.L157	contig072645-TilOR.L159	0.181	0.455	0.397
contig039461-TilOR.L148	contig065027-BurOR.L090	0.181	0.471	0.383

contig042544-BriOR.L070	contig068527-TilOR.L157	0.182	0.354	0.515
contig039461-TilOR.L147	contig042544-BriOR.L070	0.182	0.368	0.494
contig020437-ZebOR.L093	contig039461-TilOR.L147	0.182	0.373	0.488
contig039462-TilOR.L149	contig042556-BriOR.L073	0.182	0.387	0.469
contig020445-ZebOR.L096	contig068521-TilOR.L156	0.182	0.417	0.436
contig020445-ZebOR.L096	contig056384-BurOR.L087	0.182	0.426	0.427
contig039460-TilOR.L145	contig039465-TilOR.L150	0.182	0.431	0.422
contig042552-BriOR.L071	contig056384-BurOR.L087	0.182	0.432	0.421
contig020440-ZebOR.L094	contig039461-TilOR.L148	0.182	0.438	0.414
contig056386-BurOR.L088	contig068527-TilOR.L157	0.182	0.459	0.397
contig039461-TilOR.L148	contig056386-BurOR.L088	0.182	0.476	0.382
contig039461-TilOR.L147	contig072645-TilOR.L159	0.183	0.380	0.482
contig020445-ZebOR.L096	contig039461-TilOR.L147	0.183	0.431	0.424
contig020442-ZebOR.L095	contig042552-BriOR.L071	0.183	0.432	0.424
contig039461-TilOR.L148	contig056940-NyeOR.L095	0.183	0.438	0.419
contig068425-TilOR.L155	contig068527-TilOR.L157	0.183	0.442	0.414
contig039461-TilOR.L148	contig072645-TilOR.L159	0.183	0.469	0.389
contig039461-TilOR.L147	contig068425-TilOR.L155	0.184	0.359	0.513
contig020442-ZebOR.L095	contig068527-TilOR.L157	0.184	0.410	0.448
contig039462-TilOR.L149	contig042543-BriOR.L069	0.185	0.390	0.474
contig020442-ZebOR.L095	contig020445-ZebOR.L096	0.185	0.426	0.435
contig039465-TilOR.L150	contig068539-TilOR.L158	0.185	0.449	0.412
contig020437-ZebOR.L093	contig039462-TilOR.L149	0.186	0.383	0.487
contig039462-TilOR.L149	contig068425-TilOR.L155	0.186	0.384	0.484
contig020440-ZebOR.L094	contig039462-TilOR.L149	0.186	0.391	0.476
contig039461-TilOR.L148	contig039481-TilOR.L153	0.186	0.399	0.466
contig039461-TilOR.L148	contig064361-NyeOR.L094	0.186	0.429	0.433
contig023716-TilOR.L144	contig068527-TilOR.L157	0.186	0.477	0.389
contig042552-BriOR.L071	contig058162-BurOR.L089	0.186	0.477	0.390
contig023716-TilOR.L144	contig039461-TilOR.L148	0.186	0.512	0.363
contig039460-TilOR.L145	contig039462-TilOR.L149	0.187	0.389	0.482
contig039465-TilOR.L150	contig040502-NyeOR.L092	0.187	0.465	0.401
contig039462-TilOR.L149	contig056940-NyeOR.L095	0.188	0.397	0.473
contig039461-TilOR.L147	contig056942-NyeOR.L093	0.188	0.433	0.435
contig039461-TilOR.L148	contig056384-BurOR.L087	0.188	0.435	0.432
contig039461-TilOR.L148	contig068425-TilOR.L155	0.188	0.443	0.423
contig017786-ZebOR.L091	contig039465-TilOR.L150	0.188	0.447	0.420
contig017786-ZebOR.L091	contig042552-BriOR.L071	0.188	0.485	0.387
contig040502-NyeOR.L092	contig042552-BriOR.L071	0.188	0.501	0.375
contig039461-TilOR.L147	contig068521-TilOR.L156	0.189	0.382	0.495
contig039465-TilOR.L150	contig058162-BurOR.L089	0.189	0.442	0.427
contig039461-TilOR.L147	contig039484-TilOR.L154	0.190	0.366	0.519
contig042552-BriOR.L071	contig068539-TilOR.L158	0.190	0.450	0.423

contig023716-TilOR.L144	contig039462-TilOR.L149	0.190	0.473	0.401
contig039462-TilOR.L149	contig039481-TilOR.L153	0.191	0.337	0.568
contig039462-TilOR.L149	contig058162-BurOR.L089	0.191	0.396	0.483
contig040502-NyeOR.L091	contig068521-TilOR.L156	0.191	0.435	0.438
contig039460-TilOR.L145	contig056942-NyeOR.L093	0.191	0.511	0.374
contig039462-TilOR.L149	contig056384-BurOR.L087	0.192	0.360	0.534
contig039461-TilOR.L147	contig056386-BurOR.L088	0.192	0.371	0.517
contig020442-ZebOR.L095	contig039461-TilOR.L148	0.192	0.421	0.456
contig039465-TilOR.L150	contig042543-BriOR.L069	0.192	0.430	0.446
contig017787-ZebOR.L092	contig068521-TilOR.L156	0.192	0.450	0.428
contig039460-TilOR.L145	contig039461-TilOR.L148	0.192	0.460	0.417
contig039462-TilOR.L149	contig042554-BriOR.L072	0.193	0.351	0.551
contig017786-ZebOR.L091	contig039462-TilOR.L149	0.193	0.403	0.478
contig039461-TilOR.L148	contig042544-BriOR.L070	0.193	0.406	0.476
contig020445-ZebOR.L096	contig039460-TilOR.L145	0.193	0.510	0.379
contig020445-ZebOR.L096	contig023716-TilOR.L144	0.193	0.543	0.355
contig023716-TilOR.L144	contig056942-NyeOR.L093	0.193	0.544	0.354
contig039461-TilOR.L147	contig042556-BriOR.L073	0.194	0.378	0.514
contig039460-TilOR.L146	contig039465-TilOR.L150	0.194	0.433	0.448
contig068527-TilOR.L157	contig068539-TilOR.L158	0.194	0.434	0.448
contig039460-TilOR.L145	contig040502-NyeOR.L091	0.194	0.440	0.442
contig017786-ZebOR.L091	contig039461-TilOR.L148	0.194	0.479	0.406
contig065027-BurOR.L090	contig068521-TilOR.L156	0.194	0.482	0.403
contig039461-TilOR.L148	contig040502-NyeOR.L092	0.194	0.495	0.393
contig039462-TilOR.L149	contig040502-NyeOR.L092	0.195	0.415	0.471
contig039484-TilOR.L154	contig040502-NyeOR.L091	0.196	0.416	0.471
contig023716-TilOR.L144	contig058162-BurOR.L089	0.196	0.422	0.465
contig040502-NyeOR.L091	contig042556-BriOR.L073	0.196	0.424	0.462
contig039461-TilOR.L148	contig058162-BurOR.L089	0.196	0.471	0.417
contig020442-ZebOR.L095	contig039462-TilOR.L149	0.197	0.357	0.552
contig039461-TilOR.L147	contig056384-BurOR.L087	0.197	0.382	0.516
contig020437-ZebOR.L093	contig023716-TilOR.L144	0.197	0.394	0.501
contig020440-ZebOR.L094	contig039461-TilOR.L147	0.197	0.395	0.500
contig023716-TilOR.L144	contig039481-TilOR.L153	0.197	0.401	0.493
contig042544-BriOR.L070	contig068521-TilOR.L156	0.197	0.433	0.455
contig040502-NyeOR.L091	contig042543-BriOR.L069	0.197	0.445	0.442
contig023716-TilOR.L144	contig042556-BriOR.L073	0.198	0.398	0.499
contig040502-NyeOR.L091	contig072645-TilOR.L159	0.198	0.415	0.476
contig017786-ZebOR.L091	contig023716-TilOR.L144	0.198	0.430	0.461
contig020445-ZebOR.L096	contig068539-TilOR.L158	0.198	0.442	0.448
contig017787-ZebOR.L092	contig039460-TilOR.L145	0.198	0.450	0.439
contig039461-TilOR.L148	contig042543-BriOR.L069	0.198	0.458	0.432
contig039460-TilOR.L145	contig068527-TilOR.L157	0.198	0.459	0.431

contig023716-TilOR.L144	contig040502-NyeOR.L091	0.198	0.478	0.414
contig017786-ZebOR.L091	contig056942-NyeOR.L093	0.198	0.508	0.389
contig040502-NyeOR.L092	contig056942-NyeOR.L093	0.198	0.525	0.377
contig039462-TilOR.L149	contig064361-NyeOR.L094	0.199	0.350	0.568
contig039461-TilOR.L147	contig056940-NyeOR.L095	0.199	0.401	0.497
contig040502-NyeOR.L091	contig058162-BurOR.L089	0.199	0.444	0.449
contig042556-BriOR.L073	contig065027-BurOR.L090	0.199	0.474	0.419
contig017787-ZebOR.L092	contig023716-TilOR.L144	0.199	0.487	0.410
contig017786-ZebOR.L091	contig020445-ZebOR.L096	0.199	0.511	0.389
contig020445-ZebOR.L096	contig040502-NyeOR.L092	0.199	0.528	0.377
contig039461-TilOR.L147	contig058162-BurOR.L089	0.200	0.324	0.618
contig039461-TilOR.L147	contig039461-TilOR.L148	0.200	0.356	0.561
contig023716-TilOR.L144	contig039484-TilOR.L154	0.200	0.372	0.537
contig017787-ZebOR.L092	contig072645-TilOR.L159	0.200	0.430	0.465
contig056942-NyeOR.L093	contig068539-TilOR.L158	0.200	0.443	0.451
contig042543-BriOR.L069	contig065027-BurOR.L090	0.200	0.446	0.449
contig017787-ZebOR.L092	contig042543-BriOR.L069	0.200	0.455	0.439
contig039484-TilOR.L154	contig065027-BurOR.L090	0.200	0.461	0.432
contig056942-NyeOR.L093	contig058162-BurOR.L089	0.200	0.500	0.400
contig039460-TilOR.L146	contig056942-NyeOR.L093	0.200	0.519	0.386
contig039460-TilOR.L145	contig039461-TilOR.L147	0.201	0.321	0.627
contig039460-TilOR.L145	contig042556-BriOR.L073	0.201	0.369	0.544
contig020442-ZebOR.L095	contig039461-TilOR.L147	0.201	0.369	0.544
contig023716-TilOR.L144	contig072645-TilOR.L159	0.201	0.374	0.538
contig023716-TilOR.L144	contig042554-BriOR.L072	0.201	0.387	0.519
contig017787-ZebOR.L092	contig039484-TilOR.L154	0.201	0.427	0.470
contig017787-ZebOR.L092	contig042556-BriOR.L073	0.201	0.435	0.461
contig017786-ZebOR.L091	contig040502-NyeOR.L091	0.201	0.452	0.445
contig039460-TilOR.L145	contig065027-BurOR.L090	0.201	0.460	0.437
contig020445-ZebOR.L096	contig058162-BurOR.L089	0.201	0.502	0.399
contig017786-ZebOR.L091	contig039461-TilOR.L147	0.202	0.331	0.611
contig039461-TilOR.L147	contig064361-NyeOR.L094	0.202	0.379	0.534
contig042556-BriOR.L073	contig058162-BurOR.L089	0.202	0.414	0.487
contig040502-NyeOR.L091	contig056386-BurOR.L088	0.202	0.417	0.484
contig039461-TilOR.L148	contig068539-TilOR.L158	0.202	0.445	0.454
contig065027-BurOR.L090	contig072645-TilOR.L159	0.202	0.449	0.450
contig017787-ZebOR.L092	contig058162-BurOR.L089	0.202	0.454	0.446
contig039460-TilOR.L146	contig040502-NyeOR.L091	0.202	0.476	0.423
contig039460-TilOR.L146	contig065027-BurOR.L090	0.202	0.494	0.408
contig020445-ZebOR.L096	contig039460-TilOR.L146	0.202	0.518	0.391
contig039462-TilOR.L149	contig068539-TilOR.L158	0.203	0.338	0.600
contig020437-ZebOR.L093	contig040502-NyeOR.L091	0.203	0.397	0.511
contig039484-TilOR.L154	contig042544-BriOR.L070	0.203	0.412	0.492

contig040502-NyeOR.L092	contig042556-BriOR.L073	0.203	0.418	0.484
contig023716-TilOR.L144	contig040502-NyeOR.L092	0.203	0.441	0.460
contig040502-NyeOR.L091	contig040502-NyeOR.L092	0.203	0.467	0.434
contig017786-ZebOR.L091	contig068527-TilOR.L157	0.203	0.475	0.428
contig042543-BriOR.L069	contig042552-BriOR.L071	0.203	0.489	0.416
contig040502-NyeOR.L092	contig068527-TilOR.L157	0.203	0.491	0.414
contig042543-BriOR.L069	contig056942-NyeOR.L093	0.203	0.492	0.412
contig039461-TilOR.L147	contig040502-NyeOR.L092	0.204	0.343	0.594
contig023716-TilOR.L144	contig056384-BurOR.L087	0.204	0.412	0.494
contig023716-TilOR.L144	contig039460-TilOR.L145	0.204	0.413	0.495
contig017786-ZebOR.L091	contig042556-BriOR.L073	0.204	0.422	0.482
contig056386-BurOR.L088	contig065027-BurOR.L090	0.204	0.451	0.453
contig017786-ZebOR.L091	contig017787-ZebOR.L092	0.204	0.462	0.442
contig039460-TilOR.L146	contig039462-TilOR.L149	0.204	0.466	0.438
contig020445-ZebOR.L096	contig042543-BriOR.L069	0.204	0.495	0.412
contig039460-TilOR.L145	contig068521-TilOR.L156	0.205	0.367	0.558
contig023716-TilOR.L144	contig068521-TilOR.L156	0.205	0.398	0.516
contig042544-BriOR.L070	contig072645-TilOR.L159	0.205	0.410	0.501
contig020440-ZebOR.L094	contig040502-NyeOR.L091	0.205	0.417	0.492
contig017787-ZebOR.L092	contig056386-BurOR.L088	0.205	0.431	0.476
contig042543-BriOR.L069	contig068527-TilOR.L157	0.205	0.461	0.444
contig039460-TilOR.L146	contig039461-TilOR.L148	0.205	0.466	0.439
contig058162-BurOR.L089	contig068527-TilOR.L157	0.205	0.467	0.440
contig017787-ZebOR.L092	contig039460-TilOR.L146	0.205	0.503	0.407
contig040502-NyeOR.L091	contig068425-TilOR.L155	0.206	0.406	0.509
contig042544-BriOR.L070	contig042556-BriOR.L073	0.206	0.422	0.488
contig020437-ZebOR.L093	contig065027-BurOR.L090	0.206	0.438	0.469
contig058162-BurOR.L089	contig065027-BurOR.L090	0.206	0.441	0.466
contig023716-TilOR.L144	contig065027-BurOR.L090	0.206	0.492	0.418
contig039460-TilOR.L145	contig039484-TilOR.L154	0.207	0.374	0.553
contig017787-ZebOR.L092	contig020437-ZebOR.L093	0.207	0.411	0.503
contig023716-TilOR.L144	contig042543-BriOR.L069	0.207	0.416	0.499
contig040502-NyeOR.L091	contig056940-NyeOR.L095	0.207	0.424	0.488
contig017786-ZebOR.L091	contig065027-BurOR.L090	0.207	0.449	0.461
contig039460-TilOR.L146	contig042556-BriOR.L073	0.207	0.466	0.444
contig017787-ZebOR.L092	contig040502-NyeOR.L092	0.207	0.475	0.435
contig039460-TilOR.L146	contig042552-BriOR.L071	0.207	0.505	0.410
contig020442-ZebOR.L095	contig023716-TilOR.L144	0.208	0.402	0.518
contig023716-TilOR.L144	contig056386-BurOR.L088	0.208	0.403	0.517
contig056384-BurOR.L087	contig065027-BurOR.L090	0.208	0.427	0.488
contig020440-ZebOR.L094	contig065027-BurOR.L090	0.208	0.467	0.445
contig039461-TilOR.L147	contig042543-BriOR.L069	0.209	0.326	0.642
contig020440-ZebOR.L094	contig039460-TilOR.L145	0.209	0.385	0.544

contig039460-TilOR.L145	contig056940-NyeOR.L095	0.209	0.385	0.544
contig023716-TilOR.L144	contig064361-NyeOR.L094	0.209	0.395	0.528
contig065027-BurOR.L090	contig068425-TilOR.L155	0.209	0.455	0.460
contig040502-NyeOR.L092	contig065027-BurOR.L090	0.209	0.465	0.450
contig040502-NyeOR.L091	contig042554-BriOR.L072	0.210	0.371	0.566
contig039484-TilOR.L154	contig040502-NyeOR.L092	0.210	0.413	0.508
contig017787-ZebOR.L092	contig068425-TilOR.L155	0.210	0.420	0.500
contig017787-ZebOR.L092	contig020440-ZebOR.L094	0.210	0.428	0.490
contig056940-NyeOR.L095	contig065027-BurOR.L090	0.210	0.474	0.442
contig039481-TilOR.L153	contig040502-NyeOR.L091	0.211	0.374	0.564
contig040502-NyeOR.L091	contig056384-BurOR.L087	0.211	0.383	0.550
contig023716-TilOR.L144	contig068425-TilOR.L155	0.211	0.387	0.545
contig017786-ZebOR.L091	contig039484-TilOR.L154	0.211	0.416	0.506
contig023716-TilOR.L144	contig039460-TilOR.L146	0.211	0.480	0.440
contig039460-TilOR.L146	contig068527-TilOR.L157	0.211	0.481	0.439
contig039460-TilOR.L146	contig039461-TilOR.L147	0.212	0.374	0.567
contig020440-ZebOR.L094	contig023716-TilOR.L144	0.212	0.411	0.515
contig020442-ZebOR.L095	contig065027-BurOR.L090	0.212	0.428	0.496
contig042544-BriOR.L070	contig056386-BurOR.L088	0.212	0.429	0.493
contig017787-ZebOR.L092	contig056940-NyeOR.L095	0.212	0.435	0.487
contig039460-TilOR.L145	contig042544-BriOR.L070	0.212	0.445	0.477
contig042543-BriOR.L069	contig042544-BriOR.L070	0.212	0.454	0.466
contig042543-BriOR.L069	contig068521-TilOR.L156	0.213	0.384	0.555
contig039460-TilOR.L145	contig056386-BurOR.L088	0.213	0.386	0.552
contig020437-ZebOR.L093	contig042544-BriOR.L070	0.213	0.395	0.540
contig039484-TilOR.L154	contig058162-BurOR.L089	0.213	0.408	0.521
contig042543-BriOR.L069	contig042556-BriOR.L073	0.213	0.414	0.514
contig020440-ZebOR.L094	contig042544-BriOR.L070	0.213	0.415	0.514
contig042554-BriOR.L072	contig065027-BurOR.L090	0.213	0.425	0.500
contig023716-TilOR.L144	contig042544-BriOR.L070	0.213	0.465	0.458
contig020442-ZebOR.L095	contig040502-NyeOR.L091	0.214	0.383	0.560
contig017787-ZebOR.L092	contig042554-BriOR.L072	0.214	0.385	0.555
contig017787-ZebOR.L092	contig056384-BurOR.L087	0.214	0.397	0.541
contig023716-TilOR.L144	contig056940-NyeOR.L095	0.214	0.418	0.512
contig064361-NyeOR.L094	contig065027-BurOR.L090	0.214	0.423	0.504
contig039460-TilOR.L146	contig068521-TilOR.L156	0.214	0.458	0.466
contig039460-TilOR.L145	contig064361-NyeOR.L094	0.215	0.381	0.564
contig017787-ZebOR.L092	contig039481-TilOR.L153	0.215	0.388	0.554
contig039481-TilOR.L153	contig065027-BurOR.L090	0.215	0.410	0.524
contig042544-BriOR.L070	contig056940-NyeOR.L095	0.215	0.422	0.510
contig020440-ZebOR.L094	contig040502-NyeOR.L092	0.215	0.436	0.494
contig040502-NyeOR.L092	contig056940-NyeOR.L095	0.215	0.443	0.486
contig040502-NyeOR.L091	contig064361-NyeOR.L094	0.216	0.379	0.569

contig039461-TilOR.L147	contig068539-TilOR.L158	0.216	0.383	0.564
contig040502-NyeOR.L092	contig068425-TilOR.L155	0.216	0.397	0.544
contig065027-BurOR.L090	contig068539-TilOR.L158	0.216	0.413	0.524
contig017786-ZebOR.L091	contig020440-ZebOR.L094	0.216	0.439	0.492
contig017786-ZebOR.L091	contig056940-NyeOR.L095	0.216	0.446	0.484
contig020440-ZebOR.L094	contig039460-TilOR.L146	0.216	0.481	0.449
contig039460-TilOR.L146	contig056940-NyeOR.L095	0.216	0.489	0.442
contig042544-BriOR.L070	contig042554-BriOR.L072	0.217	0.369	0.586
contig020437-ZebOR.L093	contig039460-TilOR.L145	0.217	0.385	0.564
contig040502-NyeOR.L092	contig068521-TilOR.L156	0.217	0.393	0.551
contig042544-BriOR.L070	contig068425-TilOR.L155	0.217	0.404	0.537
contig017786-ZebOR.L091	contig068425-TilOR.L155	0.217	0.407	0.533
contig017786-ZebOR.L091	contig068521-TilOR.L156	0.217	0.414	0.524
contig042544-BriOR.L070	contig058162-BurOR.L089	0.217	0.449	0.483
contig039460-TilOR.L146	contig039484-TilOR.L154	0.217	0.463	0.468
contig017787-ZebOR.L092	contig020442-ZebOR.L095	0.218	0.397	0.550
contig039460-TilOR.L145	contig056384-BurOR.L087	0.218	0.405	0.538
contig039481-TilOR.L153	contig040502-NyeOR.L092	0.218	0.417	0.524
contig020440-ZebOR.L094	contig058162-BurOR.L089	0.218	0.431	0.506
contig056940-NyeOR.L095	contig058162-BurOR.L089	0.218	0.438	0.498
contig039460-TilOR.L146	contig056384-BurOR.L087	0.218	0.465	0.469
contig039481-TilOR.L153	contig042544-BriOR.L070	0.219	0.369	0.593
contig040502-NyeOR.L091	contig068539-TilOR.L158	0.219	0.373	0.589
contig058162-BurOR.L089	contig068425-TilOR.L155	0.219	0.400	0.548
contig058162-BurOR.L089	contig068521-TilOR.L156	0.219	0.406	0.538
contig040502-NyeOR.L092	contig042554-BriOR.L072	0.219	0.416	0.526
contig017786-ZebOR.L091	contig039481-TilOR.L153	0.219	0.420	0.522
contig017786-ZebOR.L091	contig042544-BriOR.L070	0.219	0.457	0.479
contig017787-ZebOR.L092	contig064361-NyeOR.L094	0.220	0.393	0.559
contig040502-NyeOR.L092	contig072645-TilOR.L159	0.220	0.416	0.530
contig017786-ZebOR.L091	contig042554-BriOR.L072	0.220	0.419	0.524
contig017786-ZebOR.L091	contig072645-TilOR.L159	0.220	0.430	0.512
contig039481-TilOR.L153	contig058162-BurOR.L089	0.221	0.412	0.537
contig040502-NyeOR.L092	contig042544-BriOR.L070	0.221	0.473	0.467
contig039460-TilOR.L146	contig042544-BriOR.L070	0.221	0.486	0.455
contig020442-ZebOR.L095	contig042544-BriOR.L070	0.222	0.378	0.587
contig042544-BriOR.L070	contig056384-BurOR.L087	0.222	0.391	0.567
contig042544-BriOR.L070	contig068539-TilOR.L158	0.222	0.391	0.567
contig042543-BriOR.L069	contig068425-TilOR.L155	0.222	0.401	0.554
contig040502-NyeOR.L092	contig064361-NyeOR.L094	0.222	0.402	0.553
contig042554-BriOR.L072	contig058162-BurOR.L089	0.222	0.411	0.539
contig058162-BurOR.L089	contig072645-TilOR.L159	0.222	0.422	0.527
contig039460-TilOR.L146	contig064361-NyeOR.L094	0.222	0.473	0.470

contig020442-ZebOR.L095	contig039460-TilOR.L146	0.222	0.474	0.469
contig017787-ZebOR.L092	contig068539-TilOR.L158	0.223	0.386	0.578
contig042544-BriOR.L070	contig064361-NyeOR.L094	0.223	0.387	0.576
contig017786-ZebOR.L091	contig064361-NyeOR.L094	0.223	0.406	0.551
contig039484-TilOR.L154	contig042543-BriOR.L069	0.224	0.408	0.548
contig039460-TilOR.L146	contig056386-BurOR.L088	0.224	0.464	0.483
contig039460-TilOR.L146	contig042554-BriOR.L072	0.224	0.473	0.473
contig058162-BurOR.L089	contig064361-NyeOR.L094	0.225	0.398	0.566
contig042543-BriOR.L069	contig072645-TilOR.L159	0.225	0.423	0.533
contig039460-TilOR.L146	contig068425-TilOR.L155	0.225	0.455	0.494
contig039460-TilOR.L146	contig039481-TilOR.L153	0.225	0.477	0.472
contig020440-ZebOR.L094	contig042543-BriOR.L069	0.226	0.431	0.523
contig042543-BriOR.L069	contig056940-NyeOR.L095	0.226	0.438	0.515
contig039460-TilOR.L145	contig068539-TilOR.L158	0.227	0.371	0.611
contig040502-NyeOR.L092	contig068539-TilOR.L158	0.228	0.396	0.575
contig042543-BriOR.L069	contig056384-BurOR.L087	0.228	0.410	0.556
contig039481-TilOR.L153	contig042543-BriOR.L069	0.228	0.414	0.552
contig042543-BriOR.L069	contig056386-BurOR.L088	0.228	0.424	0.538
contig039460-TilOR.L146	contig072645-TilOR.L159	0.228	0.495	0.461
contig017786-ZebOR.L091	contig068539-TilOR.L158	0.229	0.400	0.573
contig042543-BriOR.L069	contig042554-BriOR.L072	0.229	0.412	0.555
contig020442-ZebOR.L095	contig042543-BriOR.L069	0.230	0.402	0.572
contig023716-TilOR.L144	contig068539-TilOR.L158	0.230	0.408	0.563
contig039460-TilOR.L145	contig039481-TilOR.L153	0.230	0.422	0.545
contig039460-TilOR.L145	contig042554-BriOR.L072	0.230	0.425	0.540
contig040502-NyeOR.L092	contig056384-BurOR.L087	0.230	0.427	0.538
contig056386-BurOR.L088	contig058162-BurOR.L089	0.230	0.439	0.524
contig039460-TilOR.L145	contig068425-TilOR.L155	0.231	0.392	0.589
contig058162-BurOR.L089	contig068539-TilOR.L158	0.231	0.392	0.589
contig020437-ZebOR.L093	contig042543-BriOR.L069	0.231	0.405	0.570
contig020442-ZebOR.L095	contig039460-TilOR.L145	0.231	0.407	0.567
contig017786-ZebOR.L091	contig056384-BurOR.L087	0.231	0.430	0.536
contig040502-NyeOR.L092	contig056386-BurOR.L088	0.231	0.443	0.520
contig039460-TilOR.L145	contig072645-TilOR.L159	0.232	0.421	0.551
contig017786-ZebOR.L091	contig056386-BurOR.L088	0.232	0.447	0.518
contig042543-BriOR.L069	contig064361-NyeOR.L094	0.233	0.398	0.585
contig020437-ZebOR.L093	contig058162-BurOR.L089	0.233	0.412	0.567
contig056384-BurOR.L087	contig058162-BurOR.L089	0.233	0.423	0.551
contig020437-ZebOR.L093	contig039460-TilOR.L146	0.233	0.460	0.508
contig042543-BriOR.L069	contig068539-TilOR.L158	0.234	0.401	0.583
contig020442-ZebOR.L095	contig040502-NyeOR.L092	0.234	0.415	0.565
contig020437-ZebOR.L093	contig040502-NyeOR.L092	0.234	0.416	0.563
contig039460-TilOR.L146	contig068539-TilOR.L158	0.234	0.464	0.504

contig017786-ZebOR.L091	contig020442-ZebOR.L095	0.235	0.418	0.563
contig017786-ZebOR.L091	contig020437-ZebOR.L093	0.235	0.419	0.560
contig020442-ZebOR.L095	contig058162-BurOR.L089	0.237	0.410	0.579

Cichlid Olfactory Receptors :
dN/dS ratio

Fam N

OR pairs		dN	dS	dN/dS
contig055927-NyeOR.N111	contig064097-ZebOR.N115	0.001	0.004	0.375
contig060631-BurOR.N110	contig055927-NyeOR.N111	0.001	0.004	0.376
contig042928-BurOR.N108	contig055926-NyeOR.N110	0.001	0.008	0.178
contig060631-BurOR.N110	contig064097-ZebOR.N115	0.003	0.008	0.375
contig042928-BurOR.N108	contig064098-ZebOR.N116	0.004	0.016	0.265
contig055924-NyeOR.N109	contig010725-ZebOR.N112	0.006	0.004	1.437
contig055926-NyeOR.N110	contig064098-ZebOR.N116	0.006	0.008	0.713
contig057383-BurOR.N109	contig061663-NyeOR.N114	0.006	0.012	0.487
contig096539-BriOR.N087	contig055927-NyeOR.N111	0.006	0.023	0.247
contig055927-NyeOR.N111	contig046353-TilOR.N195	0.006	0.023	0.247
contig096539-BriOR.N087	contig064097-ZebOR.N115	0.007	0.027	0.264
contig096539-BriOR.N087	contig060631-BurOR.N110	0.007	0.027	0.264
contig046353-TilOR.N195	contig064097-ZebOR.N115	0.007	0.027	0.264
contig060631-BurOR.N110	contig046353-TilOR.N195	0.007	0.027	0.265
contig042920-BurOR.N107	contig055927-NyeOR.N112	0.007	0.028	0.250
contig010725-ZebOR.N112	contig010726-ZebOR.N113	0.008	0.012	0.717
contig096539-BriOR.N087	contig046353-TilOR.N195	0.009	0.031	0.276
contig042920-BurOR.N107	contig010727-ZebOR.N114	0.010	0.028	0.353
contig055924-NyeOR.N109	contig010726-ZebOR.N113	0.011	0.016	0.713
contig096539-BriOR.N088	contig042928-BurOR.N108	0.011	0.032	0.352
contig096536-BriOR.N086	contig042920-BurOR.N107	0.012	0.059	0.203
contig096539-BriOR.N088	contig064098-ZebOR.N116	0.013	0.022	0.613
contig096539-BriOR.N088	contig055926-NyeOR.N110	0.013	0.024	0.533
contig096536-BriOR.N086	contig010727-ZebOR.N114	0.013	0.038	0.352
contig055927-NyeOR.N112	contig010727-ZebOR.N114	0.014	0.008	1.787
contig096535-BriOR.N084	contig065025-BurOR.N111	0.014	0.033	0.431
contig096536-BriOR.N086	contig055927-NyeOR.N112	0.016	0.046	0.348
contig046347-TilOR.N190	contig046352-TilOR.N193	0.018	0.050	0.354
contig096536-BriOR.N085	contig055933-NyeOR.N113	0.020	0.092	0.222
contig010714-ZebOR.N109	contig010718-ZebOR.N110	0.022	0.018	1.232
contig106096-BriOR.N089	contig057383-BurOR.N109	0.023	0.027	0.838
contig106096-BriOR.N089	contig061663-NyeOR.N114	0.023	0.031	0.731
contig096539-BriOR.N088	contig046356-TilOR.N196	0.024	0.063	0.378
contig042928-BurOR.N108	contig046356-TilOR.N196	0.024	0.072	0.332
contig046356-TilOR.N196	contig064098-ZebOR.N116	0.025	0.063	0.401
contig055926-NyeOR.N110	contig046356-TilOR.N196	0.025	0.063	0.401
contig046360-TilOR.N197	contig010726-ZebOR.N113	0.027	0.035	0.774
contig096535-BriOR.N084	contig046340-TilOR.N188	0.027	0.093	0.294
contig065025-BurOR.N111	contig046340-TilOR.N188	0.027	0.110	0.248
contig055924-NyeOR.N109	contig046360-TilOR.N197	0.028	0.018	1.557
contig046360-TilOR.N197	contig010725-ZebOR.N112	0.028	0.022	1.278
contig096536-BriOR.N086	contig046352-TilOR.N194	0.028	0.078	0.364

contig046352-TilOR.N194	contig010727-ZebOR.N114	0.030	0.056	0.527
contig042920-BurOR.N107	contig046352-TilOR.N194	0.031	0.078	0.400
contig096536-BriOR.N085	contig065025-BurOR.N111	0.034	0.097	0.356
contig096535-BriOR.N084	contig096536-BriOR.N085	0.035	0.065	0.538
contig055927-NyeOR.N112	contig046352-TilOR.N194	0.036	0.063	0.573
contig096536-BriOR.N085	contig046340-TilOR.N188	0.043	0.101	0.424
contig061663-NyeOR.N114	contig010722-ZebOR.N111	0.043	0.108	0.396
contig010712-ZebOR.N108	contig010714-ZebOR.N109	0.048	0.049	0.983
contig057383-BurOR.N109	contig010722-ZebOR.N111	0.049	0.104	0.471
contig065025-BurOR.N111	contig055933-NyeOR.N113	0.050	0.097	0.516
contig010712-ZebOR.N108	contig010718-ZebOR.N110	0.051	0.069	0.747
contig055933-NyeOR.N113	contig046340-TilOR.N188	0.053	0.119	0.444
contig096535-BriOR.N084	contig055933-NyeOR.N113	0.054	0.108	0.497
contig046344-TilOR.N189	contig046350-TilOR.N191	0.055	0.105	0.525
contig106096-BriOR.N089	contig010722-ZebOR.N111	0.055	0.113	0.489
contig046340-TilOR.N188	contig046351-TilOR.N192	0.101	0.225	0.449
contig046344-TilOR.N189	contig046351-TilOR.N192	0.102	0.200	0.511
contig055933-NyeOR.N113	contig046350-TilOR.N191	0.106	0.203	0.524
contig096536-BriOR.N085	contig046351-TilOR.N192	0.107	0.216	0.496
contig096535-BriOR.N084	contig046351-TilOR.N192	0.108	0.217	0.499
contig065025-BurOR.N111	contig046351-TilOR.N192	0.108	0.222	0.489
contig055933-NyeOR.N113	contig046351-TilOR.N192	0.110	0.216	0.509
contig046340-TilOR.N188	contig046350-TilOR.N191	0.112	0.223	0.500
contig096536-BriOR.N085	contig046350-TilOR.N191	0.114	0.218	0.525
contig046340-TilOR.N188	contig046344-TilOR.N189	0.118	0.260	0.454
contig046350-TilOR.N191	contig046351-TilOR.N192	0.119	0.217	0.548
contig065025-BurOR.N111	contig046344-TilOR.N189	0.119	0.247	0.483
contig065025-BurOR.N111	contig046350-TilOR.N191	0.121	0.219	0.553
contig096535-BriOR.N084	contig046350-TilOR.N191	0.122	0.208	0.589
contig055933-NyeOR.N113	contig046344-TilOR.N189	0.122	0.226	0.537
contig096536-BriOR.N085	contig046344-TilOR.N189	0.126	0.225	0.560
contig096535-BriOR.N084	contig046344-TilOR.N189	0.127	0.240	0.529
contig046360-TilOR.N197	contig010712-ZebOR.N108	0.139	0.288	0.484
contig055924-NyeOR.N109	contig010712-ZebOR.N108	0.144	0.289	0.500
contig046360-TilOR.N197	contig010714-ZebOR.N109	0.146	0.294	0.498
contig010712-ZebOR.N108	contig010725-ZebOR.N112	0.148	0.295	0.502
contig010712-ZebOR.N108	contig010726-ZebOR.N113	0.148	0.301	0.490
contig046360-TilOR.N197	contig010718-ZebOR.N110	0.150	0.311	0.483
contig055924-NyeOR.N109	contig010714-ZebOR.N109	0.151	0.295	0.514
contig010714-ZebOR.N109	contig010726-ZebOR.N113	0.151	0.307	0.493
contig042920-BurOR.N107	contig055933-NyeOR.N113	0.154	0.341	0.452
contig096536-BriOR.N086	contig055933-NyeOR.N113	0.154	0.400	0.384
contig010714-ZebOR.N109	contig010725-ZebOR.N112	0.155	0.300	0.516

contig055933-NyeOR.N113	contig010727-ZebOR.N114	0.155	0.357	0.434
contig055924-NyeOR.N109	contig010718-ZebOR.N110	0.156	0.314	0.497
contig096536-BriOR.N086	contig046340-TilOR.N188	0.156	0.355	0.439
contig096536-BriOR.N086	contig065025-BurOR.N111	0.156	0.391	0.400
contig046340-TilOR.N188	contig046352-TilOR.N194	0.157	0.301	0.523
contig096536-BriOR.N085	contig042920-BurOR.N107	0.157	0.308	0.510
contig010718-ZebOR.N110	contig010726-ZebOR.N113	0.157	0.324	0.484
contig096536-BriOR.N085	contig096536-BriOR.N086	0.157	0.337	0.466
contig096536-BriOR.N086	contig046351-TilOR.N192	0.157	0.373	0.421
contig065025-BurOR.N111	contig046352-TilOR.N194	0.158	0.340	0.464
contig096536-BriOR.N085	contig010727-ZebOR.N114	0.159	0.294	0.540
contig010718-ZebOR.N110	contig010725-ZebOR.N112	0.159	0.320	0.499
contig046351-TilOR.N192	contig046352-TilOR.N194	0.160	0.327	0.488
contig046340-TilOR.N188	contig010727-ZebOR.N114	0.160	0.335	0.477
contig055927-NyeOR.N112	contig055933-NyeOR.N113	0.161	0.349	0.461
contig042920-BurOR.N107	contig065025-BurOR.N111	0.161	0.361	0.446
contig061663-NyeOR.N114	contig046353-TilOR.N195	0.161	0.708	0.227
contig096536-BriOR.N085	contig046352-TilOR.N194	0.162	0.286	0.565
contig055927-NyeOR.N111	contig061663-NyeOR.N114	0.162	0.696	0.233
contig057383-BurOR.N109	contig046353-TilOR.N195	0.162	0.707	0.230
contig065025-BurOR.N111	contig010727-ZebOR.N114	0.163	0.371	0.439
contig096536-BriOR.N085	contig055927-NyeOR.N112	0.164	0.291	0.561
contig042920-BurOR.N107	contig046340-TilOR.N188	0.164	0.343	0.478
contig096535-BriOR.N084	contig096536-BriOR.N086	0.164	0.350	0.468
contig061663-NyeOR.N114	contig064097-ZebOR.N115	0.164	0.687	0.239
contig060631-BurOR.N110	contig061663-NyeOR.N114	0.164	0.695	0.236
contig057383-BurOR.N109	contig055927-NyeOR.N111	0.164	0.696	0.236
contig096535-BriOR.N084	contig046352-TilOR.N194	0.166	0.314	0.528
contig055933-NyeOR.N113	contig046352-TilOR.N194	0.166	0.330	0.502
contig046351-TilOR.N192	contig010727-ZebOR.N114	0.166	0.335	0.497
contig096536-BriOR.N086	contig046350-TilOR.N191	0.166	0.360	0.461
contig042928-BurOR.N108	contig010714-ZebOR.N109	0.166	0.400	0.416
contig057383-BurOR.N109	contig064097-ZebOR.N115	0.166	0.686	0.242
contig057383-BurOR.N109	contig060631-BurOR.N110	0.166	0.695	0.239
contig096539-BriOR.N087	contig061663-NyeOR.N114	0.166	0.730	0.227
contig046350-TilOR.N191	contig046352-TilOR.N194	0.167	0.300	0.558
contig046356-TilOR.N196	contig010714-ZebOR.N109	0.167	0.393	0.425
contig042920-BurOR.N107	contig046351-TilOR.N192	0.168	0.343	0.489
contig042928-BurOR.N108	contig046360-TilOR.N197	0.168	0.352	0.478
contig065025-BurOR.N111	contig055927-NyeOR.N112	0.168	0.369	0.455
contig055926-NyeOR.N110	contig010714-ZebOR.N109	0.168	0.406	0.414
contig010714-ZebOR.N109	contig064098-ZebOR.N116	0.168	0.407	0.413
contig096539-BriOR.N088	contig010714-ZebOR.N109	0.168	0.420	0.400

contig096539-BriOR.N087	contig057383-BurOR.N109	0.168	0.729	0.230
contig106096-BriOR.N089	contig046353-TilOR.N195	0.168	0.738	0.228
contig046350-TilOR.N191	contig010727-ZebOR.N114	0.169	0.330	0.512
contig096535-BriOR.N084	contig042920-BurOR.N107	0.169	0.334	0.506
contig042920-BurOR.N107	contig046350-TilOR.N191	0.169	0.345	0.488
contig046356-TilOR.N196	contig046360-TilOR.N197	0.170	0.302	0.565
contig055926-NyeOR.N110	contig046360-TilOR.N197	0.170	0.339	0.502
contig046360-TilOR.N197	contig064098-ZebOR.N116	0.170	0.340	0.501
contig096539-BriOR.N088	contig046360-TilOR.N197	0.170	0.346	0.493
contig042928-BurOR.N108	contig010718-ZebOR.N110	0.170	0.388	0.437
contig106096-BriOR.N089	contig055927-NyeOR.N111	0.170	0.725	0.234
contig055927-NyeOR.N112	contig046340-TilOR.N188	0.171	0.326	0.524
contig096535-BriOR.N084	contig010727-ZebOR.N114	0.171	0.331	0.515
contig046352-TilOR.N193	contig046352-TilOR.N194	0.171	0.353	0.483
contig046356-TilOR.N196	contig010712-ZebOR.N108	0.171	0.392	0.436
contig055926-NyeOR.N110	contig010718-ZebOR.N110	0.171	0.394	0.435
contig010718-ZebOR.N110	contig064098-ZebOR.N116	0.171	0.395	0.434
contig096539-BriOR.N088	contig010718-ZebOR.N110	0.171	0.408	0.421
contig055927-NyeOR.N111	contig010722-ZebOR.N111	0.171	0.713	0.240
contig046353-TilOR.N195	contig010722-ZebOR.N111	0.171	0.714	0.239
contig046347-TilOR.N190	contig046352-TilOR.N194	0.172	0.375	0.458
contig106096-BriOR.N089	contig064097-ZebOR.N115	0.172	0.715	0.240
contig106096-BriOR.N089	contig060631-BurOR.N110	0.172	0.724	0.237
contig010722-ZebOR.N111	contig064097-ZebOR.N115	0.173	0.703	0.246
contig060631-BurOR.N110	contig010722-ZebOR.N111	0.173	0.711	0.243
contig096539-BriOR.N087	contig106096-BriOR.N089	0.173	0.739	0.234
contig096535-BriOR.N084	contig055927-NyeOR.N112	0.174	0.329	0.528
contig055927-NyeOR.N112	contig046351-TilOR.N192	0.174	0.338	0.516
contig055927-NyeOR.N112	contig046350-TilOR.N191	0.174	0.340	0.511
contig046352-TilOR.N193	contig010727-ZebOR.N114	0.174	0.349	0.498
contig046356-TilOR.N196	contig010718-ZebOR.N110	0.174	0.381	0.457
contig042928-BurOR.N108	contig010712-ZebOR.N108	0.174	0.393	0.442
contig055926-NyeOR.N110	contig010712-ZebOR.N108	0.175	0.399	0.440
contig010712-ZebOR.N108	contig064098-ZebOR.N116	0.175	0.400	0.439
contig096539-BriOR.N088	contig010712-ZebOR.N108	0.175	0.416	0.420
contig096539-BriOR.N087	contig010722-ZebOR.N111	0.175	0.746	0.234
contig046347-TilOR.N190	contig010727-ZebOR.N114	0.176	0.367	0.478
contig096536-BriOR.N086	contig046352-TilOR.N193	0.176	0.384	0.458
contig042920-BurOR.N107	contig046352-TilOR.N193	0.177	0.350	0.505
contig096536-BriOR.N086	contig046347-TilOR.N190	0.178	0.390	0.457
contig046344-TilOR.N189	contig046352-TilOR.N194	0.179	0.317	0.565
contig055927-NyeOR.N112	contig046352-TilOR.N193	0.179	0.348	0.515
contig042920-BurOR.N107	contig046347-TilOR.N190	0.179	0.368	0.485

contig042928-BurOR.N108	contig010726-ZebOR.N113	0.181	0.360	0.501
contig055926-NyeOR.N110	contig010726-ZebOR.N113	0.182	0.347	0.525
contig010726-ZebOR.N113	contig064098-ZebOR.N116	0.182	0.349	0.522
contig096539-BriOR.N088	contig010726-ZebOR.N113	0.182	0.354	0.516
contig042920-BurOR.N107	contig046344-TilOR.N189	0.183	0.328	0.557
contig042928-BurOR.N108	contig055924-NyeOR.N109	0.183	0.350	0.523
contig055927-NyeOR.N112	contig046347-TilOR.N190	0.183	0.367	0.499
contig096536-BriOR.N086	contig046344-TilOR.N189	0.183	0.377	0.485
contig046344-TilOR.N189	contig010727-ZebOR.N114	0.184	0.323	0.571
contig042928-BurOR.N108	contig010725-ZebOR.N112	0.184	0.354	0.520
contig055924-NyeOR.N109	contig046356-TilOR.N196	0.185	0.287	0.646
contig046356-TilOR.N196	contig010726-ZebOR.N113	0.185	0.311	0.594
contig055924-NyeOR.N109	contig055926-NyeOR.N110	0.185	0.338	0.549
contig055924-NyeOR.N109	contig064098-ZebOR.N116	0.185	0.339	0.545
contig096539-BriOR.N088	contig055924-NyeOR.N109	0.185	0.344	0.539
contig046356-TilOR.N196	contig010725-ZebOR.N112	0.186	0.290	0.640
contig055926-NyeOR.N110	contig010725-ZebOR.N112	0.186	0.341	0.545
contig010725-ZebOR.N112	contig064098-ZebOR.N116	0.186	0.343	0.541
contig096539-BriOR.N088	contig010725-ZebOR.N112	0.186	0.347	0.535
contig055927-NyeOR.N112	contig046344-TilOR.N189	0.190	0.332	0.574
contig096535-BriOR.N084	contig046352-TilOR.N193	0.202	0.407	0.496
contig096536-BriOR.N085	contig046352-TilOR.N193	0.203	0.416	0.488
contig046340-TilOR.N188	contig046352-TilOR.N193	0.203	0.421	0.481
contig046350-TilOR.N191	contig046352-TilOR.N193	0.203	0.430	0.472
contig065025-BurOR.N111	contig046352-TilOR.N193	0.203	0.452	0.449
contig046340-TilOR.N188	contig046347-TilOR.N190	0.204	0.434	0.471
contig096536-BriOR.N085	contig046347-TilOR.N190	0.209	0.430	0.485
contig055933-NyeOR.N113	contig046352-TilOR.N193	0.209	0.443	0.473
contig065025-BurOR.N111	contig046347-TilOR.N190	0.209	0.481	0.434
contig096535-BriOR.N084	contig046347-TilOR.N190	0.211	0.435	0.486
contig046347-TilOR.N190	contig046350-TilOR.N191	0.211	0.466	0.453
contig055933-NyeOR.N113	contig046347-TilOR.N190	0.219	0.451	0.484
contig046351-TilOR.N192	contig046352-TilOR.N193	0.225	0.479	0.469
contig046344-TilOR.N189	contig046352-TilOR.N193	0.227	0.450	0.504
contig046347-TilOR.N190	contig046351-TilOR.N192	0.228	0.487	0.469
contig046344-TilOR.N189	contig046347-TilOR.N190	0.229	0.485	0.472
contig046353-TilOR.N195	contig010714-ZebOR.N109	0.233	0.764	0.305
contig046353-TilOR.N195	contig010718-ZebOR.N110	0.238	0.741	0.321
contig055927-NyeOR.N111	contig010714-ZebOR.N109	0.239	0.773	0.310
contig010714-ZebOR.N109	contig064097-ZebOR.N115	0.241	0.762	0.317
contig096539-BriOR.N087	contig010714-ZebOR.N109	0.242	0.750	0.323
contig060631-BurOR.N110	contig010714-ZebOR.N109	0.242	0.777	0.312
contig010714-ZebOR.N109	contig010722-ZebOR.N111	0.243	0.748	0.324

contig055927-NyeOR.N111	contig010718-ZebOR.N110	0.244	0.749	0.326
contig046360-TilOR.N197	contig010722-ZebOR.N111	0.245	0.847	0.289
contig010718-ZebOR.N110	contig064097-ZebOR.N115	0.246	0.739	0.333
contig057383-BurOR.N109	contig010714-ZebOR.N109	0.246	0.743	0.332
contig096539-BriOR.N087	contig010718-ZebOR.N110	0.247	0.727	0.339
contig061663-NyeOR.N114	contig010714-ZebOR.N109	0.247	0.742	0.333
contig060631-BurOR.N110	contig010718-ZebOR.N110	0.247	0.753	0.328
contig010718-ZebOR.N110	contig010722-ZebOR.N111	0.251	0.767	0.327
contig010712-ZebOR.N108	contig010722-ZebOR.N111	0.252	0.742	0.339
contig046353-TilOR.N195	contig010712-ZebOR.N108	0.252	0.757	0.333
contig057383-BurOR.N109	contig046360-TilOR.N197	0.252	0.809	0.312
contig061663-NyeOR.N114	contig046360-TilOR.N197	0.252	0.810	0.312
contig057383-BurOR.N109	contig010718-ZebOR.N110	0.255	0.740	0.344
contig010722-ZebOR.N111	contig010726-ZebOR.N113	0.256	0.865	0.295
contig055927-NyeOR.N111	contig010712-ZebOR.N108	0.257	0.760	0.339
contig046356-TilOR.N196	contig010722-ZebOR.N111	0.257	0.781	0.329
contig061663-NyeOR.N114	contig046356-TilOR.N196	0.258	0.751	0.344
contig057383-BurOR.N109	contig010712-ZebOR.N108	0.259	0.740	0.350
contig061663-NyeOR.N114	contig010718-ZebOR.N110	0.259	0.741	0.349
contig010712-ZebOR.N108	contig064097-ZebOR.N115	0.259	0.750	0.346
contig061663-NyeOR.N114	contig010712-ZebOR.N108	0.260	0.736	0.353
contig096539-BriOR.N088	contig010722-ZebOR.N111	0.260	0.789	0.330
contig096539-BriOR.N088	contig061663-NyeOR.N114	0.260	0.805	0.323
contig055924-NyeOR.N109	contig010722-ZebOR.N111	0.260	0.853	0.304
contig106096-BriOR.N089	contig010714-ZebOR.N109	0.261	0.726	0.360
contig096539-BriOR.N087	contig010712-ZebOR.N108	0.261	0.732	0.357
contig060631-BurOR.N110	contig010712-ZebOR.N108	0.261	0.764	0.341
contig042928-BurOR.N108	contig010722-ZebOR.N111	0.261	0.809	0.323
contig055926-NyeOR.N110	contig010722-ZebOR.N111	0.263	0.786	0.335
contig010722-ZebOR.N111	contig064098-ZebOR.N116	0.263	0.788	0.334
contig010722-ZebOR.N111	contig010725-ZebOR.N112	0.263	0.857	0.306
contig106096-BriOR.N089	contig046360-TilOR.N197	0.264	0.802	0.329
contig042928-BurOR.N108	contig061663-NyeOR.N114	0.266	0.820	0.324
contig057383-BurOR.N109	contig046356-TilOR.N196	0.267	0.737	0.362
contig106096-BriOR.N089	contig010718-ZebOR.N110	0.268	0.724	0.370
contig055926-NyeOR.N110	contig061663-NyeOR.N114	0.268	0.796	0.337
contig061663-NyeOR.N114	contig064098-ZebOR.N116	0.268	0.798	0.336
contig096539-BriOR.N088	contig057383-BurOR.N109	0.269	0.790	0.341
contig057383-BurOR.N109	contig055924-NyeOR.N109	0.270	0.796	0.340
contig057383-BurOR.N109	contig010726-ZebOR.N113	0.270	0.807	0.335
contig061663-NyeOR.N114	contig010726-ZebOR.N113	0.270	0.808	0.334
contig106096-BriOR.N089	contig010712-ZebOR.N108	0.271	0.727	0.373
contig055924-NyeOR.N109	contig061663-NyeOR.N114	0.271	0.792	0.343

contig057383-BurOR.N109	contig010725-ZebOR.N112	0.273	0.802	0.340
contig061663-NyeOR.N114	contig010725-ZebOR.N112	0.274	0.797	0.343
contig046353-TilOR.N195	contig046360-TilOR.N197	0.275	0.740	0.372
contig042928-BurOR.N108	contig057383-BurOR.N109	0.275	0.804	0.342
contig057383-BurOR.N109	contig055926-NyeOR.N110	0.277	0.780	0.355
contig057383-BurOR.N109	contig064098-ZebOR.N116	0.277	0.782	0.354
contig106096-BriOR.N089	contig042928-BurOR.N108	0.279	0.802	0.348
contig055927-NyeOR.N111	contig046360-TilOR.N197	0.280	0.754	0.372
contig106096-BriOR.N089	contig046356-TilOR.N196	0.281	0.755	0.373
contig060631-BurOR.N110	contig046360-TilOR.N197	0.281	0.769	0.366
contig106096-BriOR.N089	contig055926-NyeOR.N110	0.281	0.779	0.361
contig106096-BriOR.N089	contig064098-ZebOR.N116	0.281	0.781	0.360
contig046353-TilOR.N195	contig010726-ZebOR.N113	0.281	0.787	0.357
contig046360-TilOR.N197	contig064097-ZebOR.N115	0.282	0.743	0.380
contig055924-NyeOR.N109	contig046353-TilOR.N195	0.283	0.754	0.375
contig096539-BriOR.N088	contig106096-BriOR.N089	0.283	0.782	0.361
contig096539-BriOR.N087	contig046360-TilOR.N197	0.284	0.705	0.403
contig106096-BriOR.N089	contig055924-NyeOR.N109	0.284	0.834	0.340
contig046353-TilOR.N195	contig010725-ZebOR.N112	0.285	0.762	0.375
contig055927-NyeOR.N111	contig010726-ZebOR.N113	0.286	0.801	0.357
contig060631-BurOR.N110	contig010726-ZebOR.N113	0.287	0.817	0.352
contig106096-BriOR.N089	contig010725-ZebOR.N112	0.287	0.840	0.341
contig010726-ZebOR.N113	contig064097-ZebOR.N115	0.288	0.790	0.365
contig096539-BriOR.N088	contig046353-TilOR.N195	0.289	0.641	0.451
contig055924-NyeOR.N109	contig055927-NyeOR.N111	0.289	0.768	0.376
contig106096-BriOR.N089	contig010726-ZebOR.N113	0.289	0.806	0.358
contig096539-BriOR.N087	contig010726-ZebOR.N113	0.290	0.751	0.386
contig060631-BurOR.N110	contig055924-NyeOR.N109	0.290	0.783	0.370
contig046353-TilOR.N195	contig046356-TilOR.N196	0.291	0.628	0.464
contig042928-BurOR.N108	contig046353-TilOR.N195	0.291	0.670	0.434
contig055924-NyeOR.N109	contig064097-ZebOR.N115	0.291	0.758	0.384
contig055927-NyeOR.N111	contig010725-ZebOR.N112	0.291	0.776	0.375
contig096539-BriOR.N087	contig055924-NyeOR.N109	0.292	0.720	0.406
contig060631-BurOR.N110	contig010725-ZebOR.N112	0.292	0.791	0.369
contig096539-BriOR.N088	contig055927-NyeOR.N111	0.293	0.648	0.452
contig055926-NyeOR.N110	contig046353-TilOR.N195	0.293	0.650	0.451
contig046353-TilOR.N195	contig064098-ZebOR.N116	0.293	0.651	0.449
contig010725-ZebOR.N112	contig064097-ZebOR.N115	0.293	0.765	0.383
contig096539-BriOR.N088	contig060631-BurOR.N110	0.294	0.661	0.445
contig096539-BriOR.N088	contig064097-ZebOR.N115	0.295	0.657	0.449
contig042928-BurOR.N108	contig055927-NyeOR.N111	0.295	0.677	0.436
contig096539-BriOR.N087	contig010725-ZebOR.N112	0.295	0.727	0.405
contig096539-BriOR.N087	contig096539-BriOR.N088	0.296	0.619	0.478

contig055927-NyeOR.N111	contig046356-TilOR.N196	0.296	0.636	0.465
contig042928-BurOR.N108	contig060631-BurOR.N110	0.296	0.691	0.429
contig060631-BurOR.N110	contig046356-TilOR.N196	0.297	0.648	0.458
contig055926-NyeOR.N110	contig055927-NyeOR.N111	0.297	0.657	0.452
contig055927-NyeOR.N111	contig064098-ZebOR.N116	0.297	0.659	0.451
contig042928-BurOR.N108	contig064097-ZebOR.N115	0.297	0.687	0.433
contig046356-TilOR.N196	contig064097-ZebOR.N115	0.298	0.645	0.462
contig096539-BriOR.N087	contig042928-BurOR.N108	0.298	0.648	0.460
contig060631-BurOR.N110	contig055926-NyeOR.N110	0.298	0.670	0.445
contig060631-BurOR.N110	contig064098-ZebOR.N116	0.298	0.672	0.444
contig096539-BriOR.N087	contig046356-TilOR.N196	0.299	0.625	0.478
contig055926-NyeOR.N110	contig064097-ZebOR.N115	0.299	0.667	0.449
contig064097-ZebOR.N115	contig064098-ZebOR.N116	0.299	0.668	0.448
contig096539-BriOR.N087	contig055926-NyeOR.N110	0.300	0.628	0.477
contig096539-BriOR.N087	contig064098-ZebOR.N116	0.300	0.630	0.476
contig055924-NyeOR.N109	contig046352-TilOR.N193	0.510	1.417	0.360
contig046352-TilOR.N193	contig010725-ZebOR.N112	0.515	1.388	0.371
contig046352-TilOR.N193	contig010726-ZebOR.N113	0.519	1.314	0.395
contig061663-NyeOR.N114	contig046352-TilOR.N194	0.520	1.697	0.307
contig096536-BriOR.N086	contig061663-NyeOR.N114	0.520	1.774	0.293
contig042928-BurOR.N108	contig046352-TilOR.N194	0.520	1.798	0.289
contig046350-TilOR.N191	contig046353-TilOR.N195	0.520	1.925	0.270
contig057383-BurOR.N109	contig046352-TilOR.N194	0.521	1.721	0.303
contig096536-BriOR.N086	contig057383-BurOR.N109	0.521	1.760	0.296
contig010722-ZebOR.N111	contig010727-ZebOR.N114	0.523	1.581	0.331
contig106096-BriOR.N089	contig010727-ZebOR.N114	0.523	1.588	0.329
contig061663-NyeOR.N114	contig010727-ZebOR.N114	0.523	1.653	0.317
contig046352-TilOR.N194	contig046356-TilOR.N196	0.523	1.700	0.308
contig055926-NyeOR.N110	contig046352-TilOR.N194	0.523	1.749	0.299
contig046351-TilOR.N192	contig010714-ZebOR.N109	0.524	1.510	0.347
contig057383-BurOR.N109	contig010727-ZebOR.N114	0.524	1.676	0.313
contig046352-TilOR.N194	contig010722-ZebOR.N111	0.524	1.716	0.305
contig046352-TilOR.N194	contig064098-ZebOR.N116	0.524	1.739	0.302
contig096539-BriOR.N088	contig046352-TilOR.N194	0.524	1.784	0.294
contig096539-BriOR.N087	contig046350-TilOR.N191	0.524	1.862	0.281
contig055924-NyeOR.N109	contig046347-TilOR.N190	0.525	1.520	0.346
contig046351-TilOR.N192	contig010718-ZebOR.N110	0.525	1.565	0.336
contig055927-NyeOR.N111	contig046350-TilOR.N191	0.525	1.990	0.264
contig060631-BurOR.N110	contig046350-TilOR.N191	0.525	2.038	0.258
contig042920-BurOR.N107	contig061663-NyeOR.N114	0.526	1.666	0.316
contig046352-TilOR.N194	contig010712-ZebOR.N108	0.527	1.484	0.355
contig106096-BriOR.N089	contig046352-TilOR.N194	0.527	1.542	0.342
contig046352-TilOR.N194	contig046360-TilOR.N197	0.527	1.608	0.328

contig042920-BurOR.N107	contig057383-BurOR.N109	0.527	1.689	0.312
contig096536-BriOR.N086	contig106096-BriOR.N089	0.528	1.736	0.304
contig096536-BriOR.N086	contig042928-BurOR.N108	0.528	1.762	0.299
contig046350-TilOR.N191	contig064097-ZebOR.N115	0.528	1.936	0.273
contig046344-TilOR.N189	contig010712-ZebOR.N108	0.529	1.261	0.420
contig046347-TilOR.N190	contig046360-TilOR.N197	0.529	1.517	0.349
contig055927-NyeOR.N112	contig061663-NyeOR.N114	0.529	1.568	0.338
contig046352-TilOR.N194	contig010726-ZebOR.N113	0.530	1.438	0.368
contig057383-BurOR.N109	contig055927-NyeOR.N112	0.530	1.588	0.334
contig046352-TilOR.N194	contig046353-TilOR.N195	0.530	1.722	0.308
contig046353-TilOR.N195	contig010727-ZebOR.N114	0.530	1.798	0.295
contig046347-TilOR.N190	contig010725-ZebOR.N112	0.531	1.486	0.357
contig096536-BriOR.N086	contig046353-TilOR.N195	0.531	1.713	0.310
contig096536-BriOR.N086	contig055926-NyeOR.N110	0.531	1.716	0.309
contig096536-BriOR.N086	contig096539-BriOR.N088	0.531	1.749	0.304
contig046350-TilOR.N191	contig010712-ZebOR.N108	0.532	1.412	0.377
contig046352-TilOR.N194	contig010714-ZebOR.N109	0.532	1.489	0.357
contig096536-BriOR.N086	contig064098-ZebOR.N116	0.532	1.706	0.312
contig046344-TilOR.N189	contig010714-ZebOR.N109	0.533	1.402	0.380
contig046347-TilOR.N190	contig010726-ZebOR.N113	0.534	1.403	0.381
contig106096-BriOR.N089	contig042920-BurOR.N107	0.534	1.633	0.327
contig042920-BurOR.N107	contig046353-TilOR.N195	0.535	1.732	0.309
contig060631-BurOR.N110	contig010727-ZebOR.N114	0.535	1.793	0.299
contig096536-BriOR.N086	contig096539-BriOR.N087	0.536	1.678	0.320
contig096536-BriOR.N086	contig060631-BurOR.N110	0.536	1.708	0.314
contig060631-BurOR.N110	contig046352-TilOR.N194	0.536	1.718	0.312
contig096536-BriOR.N086	contig010722-ZebOR.N111	0.536	1.734	0.309
contig046352-TilOR.N193	contig046360-TilOR.N197	0.537	1.413	0.380
contig096536-BriOR.N086	contig010712-ZebOR.N108	0.537	1.415	0.379
contig046352-TilOR.N194	contig010718-ZebOR.N110	0.537	1.531	0.351
contig106096-BriOR.N089	contig055927-NyeOR.N112	0.537	1.539	0.349
contig096536-BriOR.N086	contig046356-TilOR.N196	0.537	1.584	0.339
contig096536-BriOR.N086	contig010726-ZebOR.N113	0.537	1.586	0.339
contig096536-BriOR.N086	contig046360-TilOR.N197	0.537	1.658	0.324
contig055927-NyeOR.N111	contig046352-TilOR.N194	0.538	1.724	0.312
contig055927-NyeOR.N111	contig010727-ZebOR.N114	0.538	1.800	0.299
contig096536-BriOR.N086	contig010714-ZebOR.N109	0.539	1.498	0.360
contig096536-BriOR.N086	contig055927-NyeOR.N111	0.539	1.715	0.314
contig057383-BurOR.N109	contig046344-TilOR.N189	0.539	1.760	0.306
contig057383-BurOR.N109	contig046350-TilOR.N191	0.539	1.872	0.288
contig106096-BriOR.N089	contig046344-TilOR.N189	0.541	1.647	0.329
contig046352-TilOR.N194	contig064097-ZebOR.N115	0.541	1.687	0.321
contig042920-BurOR.N107	contig060631-BurOR.N110	0.541	1.727	0.313

contig010727-ZebOR.N114	contig064097-ZebOR.N115	0.541	1.759	0.307
contig096539-BriOR.N087	contig010727-ZebOR.N114	0.541	1.759	0.308
contig046351-TilOR.N192	contig010712-ZebOR.N108	0.542	1.421	0.381
contig010714-ZebOR.N109	contig010727-ZebOR.N114	0.542	1.442	0.376
contig055924-NyeOR.N109	contig046352-TilOR.N194	0.542	1.603	0.338
contig096536-BriOR.N086	contig064097-ZebOR.N115	0.542	1.677	0.323
contig096539-BriOR.N087	contig046352-TilOR.N194	0.542	1.687	0.321
contig055927-NyeOR.N112	contig046353-TilOR.N195	0.542	1.697	0.319
contig061663-NyeOR.N114	contig046350-TilOR.N191	0.542	1.873	0.289
contig010712-ZebOR.N108	contig010727-ZebOR.N114	0.543	1.359	0.400
contig046344-TilOR.N189	contig010718-ZebOR.N110	0.543	1.423	0.381
contig046356-TilOR.N196	contig010727-ZebOR.N114	0.543	1.492	0.364
contig096536-BriOR.N086	contig010718-ZebOR.N110	0.543	1.550	0.351
contig046350-TilOR.N191	contig010714-ZebOR.N109	0.543	1.618	0.336
contig042920-BurOR.N107	contig042928-BurOR.N108	0.543	1.716	0.317
contig061663-NyeOR.N114	contig046344-TilOR.N189	0.543	1.782	0.305
contig046344-TilOR.N189	contig046353-TilOR.N195	0.543	1.857	0.292
contig042920-BurOR.N107	contig010722-ZebOR.N111	0.544	1.665	0.327
contig042920-BurOR.N107	contig055927-NyeOR.N111	0.544	1.734	0.313
contig096536-BriOR.N086	contig055924-NyeOR.N109	0.544	1.759	0.309
contig057383-BurOR.N109	contig046347-TilOR.N190	0.545	1.439	0.379
contig046360-TilOR.N197	contig010727-ZebOR.N114	0.545	1.512	0.360
contig010726-ZebOR.N113	contig010727-ZebOR.N114	0.546	1.465	0.373
contig060631-BurOR.N110	contig046347-TilOR.N190	0.546	1.470	0.372
contig046350-TilOR.N191	contig010718-ZebOR.N110	0.546	1.595	0.342
contig096536-BriOR.N085	contig061663-NyeOR.N114	0.546	1.629	0.335
contig042920-BurOR.N107	contig055926-NyeOR.N110	0.546	1.672	0.327
contig042920-BurOR.N107	contig064097-ZebOR.N115	0.546	1.696	0.322
contig106096-BriOR.N089	contig046350-TilOR.N191	0.546	1.763	0.310
contig060631-BurOR.N110	contig046352-TilOR.N193	0.547	1.461	0.375
contig046347-TilOR.N190	contig046353-TilOR.N195	0.547	1.468	0.373
contig046352-TilOR.N194	contig010725-ZebOR.N112	0.547	1.566	0.349
contig042928-BurOR.N108	contig055927-NyeOR.N112	0.547	1.636	0.334
contig096536-BriOR.N085	contig057383-BurOR.N109	0.547	1.645	0.332
contig042920-BurOR.N107	contig064098-ZebOR.N116	0.547	1.663	0.329
contig060631-BurOR.N110	contig055927-NyeOR.N112	0.547	1.693	0.323
contig096539-BriOR.N087	contig042920-BurOR.N107	0.547	1.696	0.323
contig096539-BriOR.N088	contig042920-BurOR.N107	0.547	1.703	0.321
contig096539-BriOR.N087	contig046344-TilOR.N189	0.547	1.800	0.304
contig046352-TilOR.N193	contig046353-TilOR.N195	0.548	1.459	0.376
contig042920-BurOR.N107	contig046360-TilOR.N197	0.548	1.572	0.349
contig055927-NyeOR.N112	contig010722-ZebOR.N111	0.548	1.634	0.335
contig096535-BriOR.N084	contig061663-NyeOR.N114	0.548	1.804	0.304

contig055927-NyeOR.N111	contig046344-TilOR.N189	0.548	1.915	0.286
contig060631-BurOR.N110	contig046344-TilOR.N189	0.548	1.958	0.280
contig096536-BriOR.N085	contig096539-BriOR.N087	0.548	2.049	0.267
contig057383-BurOR.N109	contig046352-TilOR.N193	0.549	1.383	0.397
contig061663-NyeOR.N114	contig046347-TilOR.N190	0.549	1.409	0.389
contig055927-NyeOR.N111	contig046347-TilOR.N190	0.549	1.475	0.372
contig042928-BurOR.N108	contig010727-ZebOR.N114	0.549	1.659	0.331
contig096536-BriOR.N086	contig010725-ZebOR.N112	0.549	1.712	0.321
contig096535-BriOR.N084	contig057383-BurOR.N109	0.549	1.782	0.308
contig096539-BriOR.N087	contig046340-TilOR.N188	0.549	1.792	0.306
contig042928-BurOR.N108	contig046351-TilOR.N192	0.549	1.841	0.298
contig065025-BurOR.N111	contig061663-NyeOR.N114	0.549	1.866	0.294
contig096539-BriOR.N087	contig065025-BurOR.N111	0.549	1.975	0.278
contig096536-BriOR.N085	contig046353-TilOR.N195	0.549	2.007	0.274
contig042920-BurOR.N107	contig010712-ZebOR.N108	0.550	1.428	0.385
contig055927-NyeOR.N111	contig046352-TilOR.N193	0.550	1.465	0.375
contig096539-BriOR.N087	contig046347-TilOR.N190	0.550	1.474	0.373
contig046347-TilOR.N190	contig010712-ZebOR.N108	0.550	1.475	0.373
contig055926-NyeOR.N110	contig055927-NyeOR.N112	0.550	1.596	0.345
contig055927-NyeOR.N111	contig055927-NyeOR.N112	0.550	1.699	0.324
contig096536-BriOR.N085	contig055927-NyeOR.N111	0.550	2.109	0.261
contig096536-BriOR.N085	contig060631-BurOR.N110	0.550	2.164	0.254
contig096539-BriOR.N087	contig046352-TilOR.N193	0.551	1.464	0.376
contig010718-ZebOR.N110	contig010727-ZebOR.N114	0.551	1.476	0.373
contig042920-BurOR.N107	contig010714-ZebOR.N109	0.551	1.503	0.367
contig055927-NyeOR.N112	contig064098-ZebOR.N116	0.551	1.588	0.347
contig096539-BriOR.N088	contig055927-NyeOR.N112	0.551	1.625	0.339
contig057383-BurOR.N109	contig046351-TilOR.N192	0.551	1.745	0.316
contig046344-TilOR.N189	contig064097-ZebOR.N115	0.551	1.821	0.302
contig057383-BurOR.N109	contig065025-BurOR.N111	0.551	1.842	0.299
contig096535-BriOR.N084	contig096539-BriOR.N087	0.551	1.848	0.298
contig061663-NyeOR.N114	contig046352-TilOR.N193	0.552	1.356	0.407
contig046347-TilOR.N190	contig064097-ZebOR.N115	0.552	1.448	0.381
contig042920-BurOR.N107	contig046356-TilOR.N196	0.552	1.502	0.367
contig055927-NyeOR.N112	contig046360-TilOR.N197	0.552	1.504	0.367
contig055926-NyeOR.N110	contig010727-ZebOR.N114	0.552	1.618	0.341
contig096539-BriOR.N087	contig046351-TilOR.N192	0.552	1.875	0.294
contig046351-TilOR.N192	contig046353-TilOR.N195	0.552	1.912	0.289
contig055927-NyeOR.N112	contig010712-ZebOR.N108	0.553	1.360	0.407
contig046352-TilOR.N193	contig064097-ZebOR.N115	0.553	1.438	0.384
contig106096-BriOR.N089	contig046347-TilOR.N190	0.553	1.450	0.381
contig042920-BurOR.N107	contig010726-ZebOR.N113	0.553	1.544	0.358
contig010727-ZebOR.N114	contig064098-ZebOR.N116	0.553	1.610	0.344

contig055927-NyeOR.N112	contig064097-ZebOR.N115	0.553	1.663	0.332
contig096539-BriOR.N087	contig055927-NyeOR.N112	0.553	1.663	0.333
contig106096-BriOR.N089	contig046351-TilOR.N192	0.553	1.719	0.321
contig046340-TilOR.N188	contig046353-TilOR.N195	0.553	1.783	0.310
contig055926-NyeOR.N110	contig046351-TilOR.N192	0.553	1.789	0.309
contig096536-BriOR.N085	contig064097-ZebOR.N115	0.553	2.109	0.262
contig096539-BriOR.N088	contig010727-ZebOR.N114	0.554	1.635	0.339
contig061663-NyeOR.N114	contig046340-TilOR.N188	0.554	1.751	0.316
contig055927-NyeOR.N112	contig010714-ZebOR.N109	0.555	1.415	0.392
contig046347-TilOR.N190	contig064098-ZebOR.N116	0.555	1.555	0.357
contig042928-BurOR.N108	contig046347-TilOR.N190	0.555	1.653	0.336
contig057383-BurOR.N109	contig046340-TilOR.N188	0.555	1.730	0.321
contig055933-NyeOR.N113	contig061663-NyeOR.N114	0.555	1.765	0.314
contig061663-NyeOR.N114	contig046351-TilOR.N192	0.555	1.809	0.307
contig065025-BurOR.N111	contig046353-TilOR.N195	0.555	1.962	0.283
contig106096-BriOR.N089	contig046352-TilOR.N193	0.556	1.394	0.399
contig055927-NyeOR.N112	contig046356-TilOR.N196	0.556	1.441	0.386
contig055927-NyeOR.N112	contig010726-ZebOR.N113	0.556	1.461	0.381
contig057383-BurOR.N109	contig055933-NyeOR.N113	0.556	1.784	0.312
contig046347-TilOR.N190	contig046356-TilOR.N196	0.557	1.559	0.357
contig096536-BriOR.N085	contig106096-BriOR.N089	0.557	1.625	0.343
contig046351-TilOR.N192	contig064098-ZebOR.N116	0.557	1.778	0.313
contig055927-NyeOR.N111	contig046351-TilOR.N192	0.557	1.975	0.282
contig065025-BurOR.N111	contig055927-NyeOR.N111	0.557	2.029	0.275
contig055926-NyeOR.N110	contig046347-TilOR.N190	0.558	1.612	0.346
contig046351-TilOR.N192	contig046356-TilOR.N196	0.558	1.629	0.343
contig096539-BriOR.N088	contig046347-TilOR.N190	0.558	1.715	0.326
contig046351-TilOR.N192	contig010722-ZebOR.N111	0.558	1.805	0.309
contig055927-NyeOR.N111	contig046340-TilOR.N188	0.558	1.835	0.304
contig060631-BurOR.N110	contig046340-TilOR.N188	0.558	1.873	0.298
contig060631-BurOR.N110	contig046351-TilOR.N192	0.558	2.020	0.276
contig060631-BurOR.N110	contig065025-BurOR.N111	0.558	2.078	0.268
contig046352-TilOR.N193	contig010712-ZebOR.N108	0.559	1.446	0.386
contig042920-BurOR.N107	contig010718-ZebOR.N110	0.559	1.520	0.368
contig055924-NyeOR.N109	contig010727-ZebOR.N114	0.559	1.569	0.356
contig096539-BriOR.N088	contig046351-TilOR.N192	0.559	1.747	0.320
contig096536-BriOR.N085	contig010722-ZebOR.N111	0.559	1.815	0.308
contig096535-BriOR.N084	contig046353-TilOR.N195	0.559	1.816	0.308
contig042928-BurOR.N108	contig046340-TilOR.N188	0.559	1.820	0.307
contig096535-BriOR.N084	contig055927-NyeOR.N111	0.559	1.894	0.295
contig096535-BriOR.N084	contig010722-ZebOR.N111	0.559	1.927	0.290
contig046340-TilOR.N188	contig064097-ZebOR.N115	0.560	1.792	0.313
contig046351-TilOR.N192	contig064097-ZebOR.N115	0.560	1.874	0.299

contig096535-BriOR.N084	contig060631-BurOR.N110	0.560	1.936	0.289
contig065025-BurOR.N111	contig064097-ZebOR.N115	0.560	2.029	0.276
contig065025-BurOR.N111	contig010722-ZebOR.N111	0.560	2.057	0.272
contig055927-NyeOR.N112	contig010718-ZebOR.N110	0.561	1.443	0.389
contig042920-BurOR.N107	contig055924-NyeOR.N109	0.561	1.687	0.333
contig096535-BriOR.N084	contig106096-BriOR.N089	0.561	1.771	0.317
contig106096-BriOR.N089	contig065025-BurOR.N111	0.561	1.860	0.302
contig106096-BriOR.N089	contig055933-NyeOR.N113	0.562	1.660	0.339
contig055926-NyeOR.N110	contig046340-TilOR.N188	0.562	1.770	0.318
contig096535-BriOR.N084	contig064097-ZebOR.N115	0.562	1.894	0.297
contig046347-TilOR.N190	contig010722-ZebOR.N111	0.563	1.358	0.415
contig046352-TilOR.N193	contig064098-ZebOR.N116	0.564	1.514	0.373
contig055924-NyeOR.N109	contig055927-NyeOR.N112	0.564	1.588	0.355
contig042928-BurOR.N108	contig046352-TilOR.N193	0.564	1.606	0.352
contig046352-TilOR.N193	contig010722-ZebOR.N111	0.565	1.319	0.428
contig010725-ZebOR.N112	contig010727-ZebOR.N114	0.565	1.533	0.368
contig096536-BriOR.N085	contig010714-ZebOR.N109	0.565	1.660	0.340
contig046344-TilOR.N189	contig010722-ZebOR.N111	0.565	1.761	0.321
contig046352-TilOR.N193	contig046356-TilOR.N196	0.566	1.517	0.373
contig046352-TilOR.N193	contig010714-ZebOR.N109	0.566	1.556	0.364
contig046347-TilOR.N190	contig010714-ZebOR.N109	0.566	1.586	0.357
contig046340-TilOR.N188	contig064098-ZebOR.N116	0.566	1.759	0.322
contig046352-TilOR.N193	contig010718-ZebOR.N110	0.567	1.616	0.351
contig042920-BurOR.N107	contig010725-ZebOR.N112	0.567	1.645	0.345
contig046347-TilOR.N190	contig010718-ZebOR.N110	0.567	1.649	0.344
contig055926-NyeOR.N110	contig046352-TilOR.N193	0.568	1.568	0.362
contig096539-BriOR.N088	contig046352-TilOR.N193	0.568	1.664	0.342
contig106096-BriOR.N089	contig046340-TilOR.N188	0.568	1.708	0.333
contig096539-BriOR.N088	contig046340-TilOR.N188	0.568	1.729	0.329
contig096536-BriOR.N085	contig010712-ZebOR.N108	0.569	1.440	0.395
contig065025-BurOR.N111	contig010714-ZebOR.N109	0.569	1.657	0.344
contig055933-NyeOR.N113	contig010722-ZebOR.N111	0.569	1.989	0.286
contig055927-NyeOR.N112	contig010725-ZebOR.N112	0.570	1.551	0.367
contig046340-TilOR.N188	contig010722-ZebOR.N111	0.570	1.792	0.318
contig042928-BurOR.N108	contig065025-BurOR.N111	0.570	1.829	0.312
contig096535-BriOR.N084	contig010714-ZebOR.N109	0.571	1.643	0.347
contig096535-BriOR.N084	contig042928-BurOR.N108	0.571	1.776	0.321
contig096536-BriOR.N085	contig042928-BurOR.N108	0.571	1.798	0.317
contig065025-BurOR.N111	contig010712-ZebOR.N108	0.572	1.450	0.394
contig046350-TilOR.N191	contig010722-ZebOR.N111	0.572	1.826	0.313
contig055933-NyeOR.N113	contig046353-TilOR.N195	0.572	2.397	0.239
contig096539-BriOR.N087	contig055933-NyeOR.N113	0.572	2.420	0.236
contig096535-BriOR.N084	contig010712-ZebOR.N108	0.573	1.440	0.398

contig065025-BurOR.N111	contig055926-NyeOR.N110	0.573	1.778	0.322
contig046340-TilOR.N188	contig010712-ZebOR.N108	0.574	1.460	0.393
contig096535-BriOR.N084	contig055926-NyeOR.N110	0.574	1.728	0.332
contig096536-BriOR.N085	contig055926-NyeOR.N110	0.574	1.749	0.328
contig055927-NyeOR.N111	contig055933-NyeOR.N113	0.574	2.416	0.238
contig096536-BriOR.N085	contig010718-ZebOR.N110	0.575	1.769	0.325
contig060631-BurOR.N110	contig055933-NyeOR.N113	0.575	2.500	0.230
contig096539-BriOR.N088	contig065025-BurOR.N111	0.576	1.698	0.339
contig096535-BriOR.N084	contig096539-BriOR.N088	0.577	1.653	0.349
contig065025-BurOR.N111	contig064098-ZebOR.N116	0.577	1.767	0.327
contig055933-NyeOR.N113	contig064097-ZebOR.N115	0.577	2.416	0.239
contig046350-TilOR.N191	contig046360-TilOR.N197	0.578	1.348	0.429
contig096535-BriOR.N084	contig064098-ZebOR.N116	0.578	1.718	0.336
contig096536-BriOR.N085	contig064098-ZebOR.N116	0.578	1.740	0.332
contig046340-TilOR.N188	contig010714-ZebOR.N109	0.580	1.605	0.362
contig065025-BurOR.N111	contig010718-ZebOR.N110	0.580	1.681	0.345
contig096536-BriOR.N085	contig096539-BriOR.N088	0.580	1.710	0.339
contig055924-NyeOR.N109	contig046351-TilOR.N192	0.581	1.593	0.365
contig096535-BriOR.N084	contig010718-ZebOR.N110	0.581	1.743	0.333
contig055933-NyeOR.N113	contig010712-ZebOR.N108	0.583	1.370	0.426
contig042928-BurOR.N108	contig046350-TilOR.N191	0.583	1.670	0.349
contig055924-NyeOR.N109	contig046350-TilOR.N191	0.586	1.351	0.434
contig042928-BurOR.N108	contig046344-TilOR.N189	0.586	1.396	0.420
contig055926-NyeOR.N110	contig046350-TilOR.N191	0.586	1.628	0.360
contig046351-TilOR.N192	contig046360-TilOR.N197	0.587	1.549	0.379
contig046351-TilOR.N192	contig010725-ZebOR.N112	0.587	1.556	0.377
contig096535-BriOR.N084	contig046360-TilOR.N197	0.588	1.507	0.391
contig055933-NyeOR.N113	contig010714-ZebOR.N109	0.588	1.518	0.387
contig046350-TilOR.N191	contig064098-ZebOR.N116	0.588	1.680	0.350
contig055926-NyeOR.N110	contig046344-TilOR.N189	0.589	1.367	0.431
contig042928-BurOR.N108	contig055933-NyeOR.N113	0.589	1.722	0.342
contig046351-TilOR.N192	contig010726-ZebOR.N113	0.590	1.475	0.400
contig046350-TilOR.N191	contig046356-TilOR.N196	0.590	1.594	0.370
contig046344-TilOR.N189	contig064098-ZebOR.N116	0.591	1.402	0.422
contig046344-TilOR.N189	contig046356-TilOR.N196	0.591	1.410	0.419
contig046340-TilOR.N188	contig010718-ZebOR.N110	0.591	1.700	0.348
contig046350-TilOR.N191	contig010725-ZebOR.N112	0.592	1.325	0.447
contig055926-NyeOR.N110	contig055933-NyeOR.N113	0.592	1.716	0.345
contig046340-TilOR.N188	contig046360-TilOR.N197	0.593	1.493	0.397
contig096539-BriOR.N088	contig046350-TilOR.N191	0.593	1.652	0.359
contig046344-TilOR.N189	contig046360-TilOR.N197	0.594	1.282	0.463
contig065025-BurOR.N111	contig046360-TilOR.N197	0.594	1.508	0.394
contig055924-NyeOR.N109	contig046344-TilOR.N189	0.595	1.290	0.461

contig046350-TilOR.N191	contig010726-ZebOR.N113	0.596	1.277	0.467
contig096539-BriOR.N088	contig046344-TilOR.N189	0.596	1.383	0.431
contig055933-NyeOR.N113	contig064098-ZebOR.N116	0.597	1.706	0.350
contig055933-NyeOR.N113	contig010718-ZebOR.N110	0.598	1.641	0.364
contig046340-TilOR.N188	contig046356-TilOR.N196	0.599	1.534	0.390
contig096539-BriOR.N088	contig055933-NyeOR.N113	0.599	1.678	0.357
contig046344-TilOR.N189	contig010725-ZebOR.N112	0.601	1.266	0.474
contig065025-BurOR.N111	contig046356-TilOR.N196	0.601	1.605	0.374
contig055924-NyeOR.N109	contig046340-TilOR.N188	0.602	1.542	0.390
contig096535-BriOR.N084	contig046356-TilOR.N196	0.602	1.566	0.384
contig046344-TilOR.N189	contig010726-ZebOR.N113	0.603	1.233	0.489
contig096536-BriOR.N085	contig046356-TilOR.N196	0.603	1.568	0.385
contig096535-BriOR.N084	contig055924-NyeOR.N109	0.607	1.491	0.407
contig065025-BurOR.N111	contig055924-NyeOR.N109	0.607	1.493	0.406
contig096536-BriOR.N085	contig046360-TilOR.N197	0.607	1.499	0.405
contig046340-TilOR.N188	contig010725-ZebOR.N112	0.608	1.507	0.403
contig046340-TilOR.N188	contig010726-ZebOR.N113	0.612	1.417	0.432
contig096535-BriOR.N084	contig010725-ZebOR.N112	0.613	1.459	0.420
contig065025-BurOR.N111	contig010725-ZebOR.N112	0.613	1.461	0.419
contig055933-NyeOR.N113	contig046360-TilOR.N197	0.614	1.500	0.409
contig096535-BriOR.N084	contig010726-ZebOR.N113	0.617	1.375	0.449
contig065025-BurOR.N111	contig010726-ZebOR.N113	0.617	1.376	0.448
contig055924-NyeOR.N109	contig055933-NyeOR.N113	0.618	1.559	0.396
contig055933-NyeOR.N113	contig046356-TilOR.N196	0.619	1.542	0.401
contig096536-BriOR.N085	contig055924-NyeOR.N109	0.623	1.541	0.404
contig055933-NyeOR.N113	contig010725-ZebOR.N112	0.624	1.524	0.410
contig055933-NyeOR.N113	contig010726-ZebOR.N113	0.626	1.480	0.423
contig096536-BriOR.N085	contig010725-ZebOR.N112	0.629	1.506	0.418
contig096536-BriOR.N085	contig010726-ZebOR.N113	0.634	1.417	0.447

Cichlid Olfactory Receptors :
dN/dS ratio

Fam O

OR pairs		dN	dS	dN/dS
contig021359-NyeOR.O102	contig020431-ZebOR.O101	0.001	0.012	0.118
contig021354-NyeOR.O101	contig020427-ZebOR.O099	0.007	0.028	0.250
contig059249-BurOR.O098	contig062053-NyeOR.O103	0.012	0.039	0.297
contig023731-TilOR.O177	contig020427-ZebOR.O099	0.016	0.062	0.251
contig042562-BriOR.O079	contig020427-ZebOR.O099	0.023	0.028	0.804
contig042560-BriOR.O078	contig020430-ZebOR.O100	0.023	0.028	0.833
contig021354-NyeOR.O101	contig023731-TilOR.O177	0.023	0.090	0.260
contig042562-BriOR.O079	contig023731-TilOR.O177	0.024	0.093	0.260
contig042562-BriOR.O079	contig021354-NyeOR.O101	0.025	0.047	0.530
contig059249-BurOR.O098	contig023717-TilOR.O175	0.028	0.070	0.399
contig062053-NyeOR.O103	contig023717-TilOR.O175	0.031	0.087	0.359
contig023717-TilOR.O175	contig023724-TilOR.O176	0.037	0.113	0.326
contig062053-NyeOR.O103	contig020431-ZebOR.O101	0.044	0.120	0.362
contig021359-NyeOR.O102	contig062053-NyeOR.O103	0.045	0.125	0.360
contig042559-BriOR.O077	contig023717-TilOR.O175	0.049	0.090	0.547
contig023717-TilOR.O175	contig020431-ZebOR.O101	0.049	0.131	0.374
contig042559-BriOR.O077	contig023724-TilOR.O176	0.050	0.108	0.464
contig059249-BurOR.O098	contig020431-ZebOR.O101	0.050	0.128	0.387
contig021359-NyeOR.O102	contig023717-TilOR.O175	0.051	0.136	0.372
contig059249-BurOR.O098	contig021359-NyeOR.O102	0.051	0.142	0.360
contig042559-BriOR.O077	contig020431-ZebOR.O101	0.054	0.092	0.592
contig042559-BriOR.O077	contig062053-NyeOR.O103	0.054	0.096	0.569
contig110782-BriOR.O080	contig020431-ZebOR.O101	0.054	0.122	0.445
contig042559-BriOR.O077	contig021359-NyeOR.O102	0.056	0.105	0.533
contig110782-BriOR.O080	contig021359-NyeOR.O102	0.056	0.117	0.475
contig059249-BurOR.O098	contig023724-TilOR.O176	0.056	0.132	0.424
contig042559-BriOR.O077	contig059249-BurOR.O098	0.058	0.095	0.609
contig062053-NyeOR.O103	contig023724-TilOR.O176	0.059	0.131	0.450
contig021359-NyeOR.O102	contig023724-TilOR.O176	0.060	0.120	0.498
contig023724-TilOR.O176	contig020431-ZebOR.O101	0.060	0.125	0.479
contig110782-BriOR.O080	contig062053-NyeOR.O103	0.060	0.125	0.480
contig110782-BriOR.O080	contig023717-TilOR.O175	0.067	0.141	0.474
contig110782-BriOR.O080	contig059249-BurOR.O098	0.069	0.126	0.544
contig110782-BriOR.O080	contig023724-TilOR.O176	0.073	0.132	0.553
contig042559-BriOR.O077	contig110782-BriOR.O080	0.079	0.115	0.688
contig023731-TilOR.O177	contig020430-ZebOR.O100	0.393	0.860	0.457
contig020427-ZebOR.O099	contig020430-ZebOR.O100	0.395	0.870	0.454
contig021354-NyeOR.O101	contig020430-ZebOR.O100	0.399	0.928	0.430
contig042560-BriOR.O078	contig023731-TilOR.O177	0.401	0.866	0.464
contig042560-BriOR.O078	contig020427-ZebOR.O099	0.405	0.887	0.456
contig042560-BriOR.O078	contig021354-NyeOR.O101	0.408	0.944	0.433
contig042562-BriOR.O079	contig020430-ZebOR.O100	0.413	0.869	0.475

contig042560-BriOR.O078	contig042562-BriOR.O079	0.415	0.885	0.469
contig042559-BriOR.O077	contig023731-TilOR.O177	0.494	1.351	0.366
contig042559-BriOR.O077	contig020427-ZebOR.O099	0.500	1.276	0.392
contig042559-BriOR.O077	contig021354-NyeOR.O101	0.507	1.303	0.389
contig042559-BriOR.O077	contig042562-BriOR.O079	0.507	1.362	0.372
contig059249-BurOR.O098	contig020427-ZebOR.O099	0.508	1.141	0.445
contig059249-BurOR.O098	contig023731-TilOR.O177	0.510	1.268	0.402
contig042562-BriOR.O079	contig059249-BurOR.O098	0.512	1.192	0.429
contig023717-TilOR.O175	contig020427-ZebOR.O099	0.512	1.259	0.406
contig023717-TilOR.O175	contig023731-TilOR.O177	0.514	1.330	0.387
contig059249-BurOR.O098	contig021354-NyeOR.O101	0.517	1.173	0.440
contig021354-NyeOR.O101	contig023717-TilOR.O175	0.519	1.286	0.404
contig042562-BriOR.O079	contig023717-TilOR.O175	0.519	1.290	0.403
contig062053-NyeOR.O103	contig020427-ZebOR.O099	0.521	1.150	0.453
contig062053-NyeOR.O103	contig023731-TilOR.O177	0.522	1.282	0.407
contig023731-TilOR.O177	contig020431-ZebOR.O101	0.523	1.383	0.378
contig042562-BriOR.O079	contig062053-NyeOR.O103	0.525	1.202	0.437
contig023724-TilOR.O176	contig020427-ZebOR.O099	0.526	1.273	0.413
contig023724-TilOR.O176	contig023731-TilOR.O177	0.528	1.322	0.400
contig021359-NyeOR.O102	contig020427-ZebOR.O099	0.529	1.282	0.413
contig021354-NyeOR.O101	contig062053-NyeOR.O103	0.530	1.183	0.448
contig020427-ZebOR.O099	contig020431-ZebOR.O101	0.530	1.260	0.420
contig021354-NyeOR.O101	contig023724-TilOR.O176	0.533	1.301	0.410
contig042562-BriOR.O079	contig023724-TilOR.O176	0.534	1.305	0.409
contig042562-BriOR.O079	contig021359-NyeOR.O102	0.536	1.346	0.399
contig042562-BriOR.O079	contig020431-ZebOR.O101	0.537	1.322	0.406
contig021354-NyeOR.O101	contig021359-NyeOR.O102	0.538	1.322	0.407
contig021354-NyeOR.O101	contig020431-ZebOR.O101	0.539	1.299	0.415
contig021359-NyeOR.O102	contig023731-TilOR.O177	0.546	1.380	0.395
contig023724-TilOR.O176	contig020430-ZebOR.O100	0.548	1.296	0.423
contig110782-BriOR.O080	contig020427-ZebOR.O099	0.549	1.199	0.458
contig042562-BriOR.O079	contig110782-BriOR.O080	0.550	1.277	0.431
contig023717-TilOR.O175	contig020430-ZebOR.O100	0.550	1.291	0.426
contig042560-BriOR.O078	contig023724-TilOR.O176	0.551	1.214	0.454
contig110782-BriOR.O080	contig023731-TilOR.O177	0.551	1.339	0.411
contig110782-BriOR.O080	contig021354-NyeOR.O101	0.552	1.234	0.447
contig042560-BriOR.O078	contig023717-TilOR.O175	0.554	1.205	0.459
contig062053-NyeOR.O103	contig020430-ZebOR.O100	0.557	1.123	0.496
contig059249-BurOR.O098	contig020430-ZebOR.O100	0.558	1.185	0.471
contig042560-BriOR.O078	contig062053-NyeOR.O103	0.566	1.106	0.511
contig021359-NyeOR.O102	contig020430-ZebOR.O100	0.566	1.148	0.493
contig042560-BriOR.O078	contig059249-BurOR.O098	0.567	1.167	0.486
contig020430-ZebOR.O100	contig020431-ZebOR.O101	0.569	1.112	0.512

contig042560-BriOR.O078	contig021359-NyeOR.O102	0.569	1.150	0.495
contig042559-BriOR.O077	contig042560-BriOR.O078	0.569	1.191	0.478
contig042559-BriOR.O077	contig020430-ZebOR.O100	0.570	1.186	0.480
contig042560-BriOR.O078	contig020431-ZebOR.O101	0.571	1.116	0.512
contig110782-BriOR.O080	contig020430-ZebOR.O100	0.576	1.125	0.512
contig042560-BriOR.O078	contig110782-BriOR.O080	0.591	1.108	0.534

Cichlid Olfactory Receptors :
dN/dS ratio

Fam P

OR pairs		dN	dS	dN/dS
contig017696-BurOR.P118	contig023268-NyeOR.P118	0.001	0.019	0.074
contig023268-NyeOR.P118	contig025458-ZebOR.P125	0.003	0.007	0.375
contig017696-BurOR.P118	contig025458-ZebOR.P125	0.004	0.011	0.375
contig017697-BurOR.P119	contig025453-ZebOR.P124	0.004	0.016	0.271
contig017698-BurOR.P120	contig025452-ZebOR.P123	0.007	0.012	0.602
contig004275-BriOR.P101	contig017696-BurOR.P118	0.013	0.034	0.371
contig004275-BriOR.P101	contig025458-ZebOR.P125	0.014	0.030	0.465
contig004275-BriOR.P101	contig023268-NyeOR.P118	0.014	0.038	0.370
contig004267-BriOR.P099	contig017698-BurOR.P120	0.014	0.040	0.358
contig004267-BriOR.P099	contig025452-ZebOR.P123	0.015	0.047	0.324
contig004270-BriOR.P100	contig025453-ZebOR.P124	0.017	0.048	0.360
contig004270-BriOR.P100	contig017697-BurOR.P119	0.019	0.039	0.477
contig065449-TilOR.P215	contig065253-TilOR.P212	0.022	0.041	0.543
contig065254-TilOR.P213	contig025458-ZebOR.P125	0.025	0.069	0.365
contig065254-TilOR.P213	contig017696-BurOR.P118	0.027	0.073	0.365
contig065254-TilOR.P213	contig023268-NyeOR.P118	0.028	0.078	0.364
contig065254-TilOR.P213	contig004275-BriOR.P101	0.028	0.086	0.329
contig065444-TilOR.P214	contig025452-ZebOR.P123	0.034	0.131	0.263
contig065444-TilOR.P214	contig017698-BurOR.P120	0.036	0.117	0.309
contig065253-TilOR.P212	contig025453-ZebOR.P124	0.038	0.099	0.382
contig065253-TilOR.P212	contig017697-BurOR.P119	0.039	0.081	0.487
contig065253-TilOR.P212	contig004270-BriOR.P100	0.039	0.119	0.324
contig065444-TilOR.P214	contig004267-BriOR.P099	0.039	0.159	0.245
contig065449-TilOR.P215	contig025453-ZebOR.P124	0.042	0.101	0.411
contig065449-TilOR.P215	contig017697-BurOR.P119	0.046	0.083	0.559
contig065449-TilOR.P215	contig004270-BriOR.P100	0.050	0.119	0.416
contig065247-TilOR.P211	contig065253-TilOR.P212	0.057	0.076	0.742
contig065247-TilOR.P211	contig065449-TilOR.P215	0.061	0.072	0.855
contig065247-TilOR.P211	contig025453-ZebOR.P124	0.070	0.111	0.634
contig065247-TilOR.P211	contig017697-BurOR.P119	0.072	0.101	0.708
contig065247-TilOR.P211	contig004270-BriOR.P100	0.075	0.134	0.563
contig025452-ZebOR.P123	contig017697-BurOR.P119	0.129	0.375	0.345
contig017698-BurOR.P120	contig017697-BurOR.P119	0.130	0.361	0.359
contig017698-BurOR.P120	contig025453-ZebOR.P124	0.131	0.381	0.345
contig025452-ZebOR.P123	contig025453-ZebOR.P124	0.131	0.396	0.331
contig004267-BriOR.P099	contig017697-BurOR.P119	0.132	0.364	0.363
contig004267-BriOR.P099	contig025453-ZebOR.P124	0.134	0.371	0.361
contig025452-ZebOR.P123	contig065247-TilOR.P211	0.137	0.442	0.311
contig017698-BurOR.P120	contig065247-TilOR.P211	0.138	0.426	0.323
contig017698-BurOR.P120	contig004270-BriOR.P100	0.139	0.404	0.344
contig025452-ZebOR.P123	contig004270-BriOR.P100	0.139	0.420	0.331
contig004267-BriOR.P099	contig065247-TilOR.P211	0.139	0.434	0.321

contig065444-TilOR.P214	contig025453-ZebOR.P124	0.140	0.446	0.314
contig004267-BriOR.P099	contig004270-BriOR.P100	0.142	0.381	0.373
contig065444-TilOR.P214	contig017697-BurOR.P119	0.142	0.417	0.340
contig065444-TilOR.P214	contig065247-TilOR.P211	0.143	0.445	0.322
contig017698-BurOR.P120	contig065253-TilOR.P212	0.144	0.386	0.374
contig025452-ZebOR.P123	contig065253-TilOR.P212	0.144	0.401	0.360
contig065444-TilOR.P214	contig004270-BriOR.P100	0.145	0.459	0.316
contig004267-BriOR.P099	contig065253-TilOR.P212	0.146	0.379	0.385
contig065444-TilOR.P214	contig065253-TilOR.P212	0.149	0.408	0.366
contig017698-BurOR.P120	contig065449-TilOR.P215	0.149	0.417	0.357
contig025452-ZebOR.P123	contig065449-TilOR.P215	0.149	0.433	0.344
contig004267-BriOR.P099	contig065449-TilOR.P215	0.151	0.424	0.355
contig065444-TilOR.P214	contig065449-TilOR.P215	0.154	0.425	0.362
contig065449-TilOR.P215	contig023268-NyeOR.P118	0.554	1.751	0.316
contig065449-TilOR.P215	contig017696-BurOR.P118	0.555	1.731	0.321
contig065449-TilOR.P215	contig025458-ZebOR.P125	0.555	1.734	0.320
contig065449-TilOR.P215	contig004275-BriOR.P101	0.559	1.550	0.361
contig065247-TilOR.P211	contig017696-BurOR.P118	0.560	1.817	0.308
contig065247-TilOR.P211	contig023268-NyeOR.P118	0.560	1.817	0.308
contig065247-TilOR.P211	contig025458-ZebOR.P125	0.561	1.799	0.312
contig065253-TilOR.P212	contig023268-NyeOR.P118	0.561	1.825	0.307
contig065253-TilOR.P212	contig017696-BurOR.P118	0.562	1.803	0.312
contig065253-TilOR.P212	contig025458-ZebOR.P125	0.562	1.806	0.311
contig065247-TilOR.P211	contig004275-BriOR.P101	0.563	1.625	0.346
contig065247-TilOR.P211	contig065254-TilOR.P213	0.565	1.597	0.354
contig065253-TilOR.P212	contig004275-BriOR.P101	0.566	1.687	0.335
contig065449-TilOR.P215	contig065254-TilOR.P213	0.568	1.460	0.389
contig004270-BriOR.P100	contig017696-BurOR.P118	0.568	1.707	0.333
contig065253-TilOR.P212	contig065254-TilOR.P213	0.570	1.534	0.372
contig004270-BriOR.P100	contig004275-BriOR.P101	0.571	1.605	0.356
contig025453-ZebOR.P124	contig065254-TilOR.P213	0.574	1.442	0.398
contig017697-BurOR.P119	contig065254-TilOR.P213	0.574	1.468	0.391
contig004270-BriOR.P100	contig065254-TilOR.P213	0.576	1.444	0.399
contig025453-ZebOR.P124	contig017696-BurOR.P118	0.577	1.669	0.345
contig025453-ZebOR.P124	contig023268-NyeOR.P118	0.577	1.707	0.338
contig025453-ZebOR.P124	contig025458-ZebOR.P125	0.578	1.691	0.342
contig025453-ZebOR.P124	contig004275-BriOR.P101	0.580	1.508	0.384
contig017697-BurOR.P119	contig017696-BurOR.P118	0.580	1.704	0.340
contig017697-BurOR.P119	contig023268-NyeOR.P118	0.580	1.743	0.333
contig004270-BriOR.P100	contig023268-NyeOR.P118	0.581	1.669	0.348
contig017697-BurOR.P119	contig025458-ZebOR.P125	0.581	1.726	0.337
contig004270-BriOR.P100	contig025458-ZebOR.P125	0.582	1.654	0.352
contig017697-BurOR.P119	contig004275-BriOR.P101	0.583	1.536	0.380

contig004267-BriOR.P099	contig065254-TilOR.P213	0.589	1.570	0.375
contig004267-BriOR.P099	contig023268-NyeOR.P118	0.594	1.680	0.354
contig004267-BriOR.P099	contig017696-BurOR.P118	0.595	1.638	0.363
contig004267-BriOR.P099	contig025458-ZebOR.P125	0.597	1.613	0.370
contig065444-TilOR.P214	contig065254-TilOR.P213	0.604	1.551	0.389
contig004267-BriOR.P099	contig004275-BriOR.P101	0.604	1.612	0.375
contig017698-BurOR.P120	contig065254-TilOR.P213	0.605	1.544	0.392
contig017698-BurOR.P120	contig023268-NyeOR.P118	0.610	1.649	0.370
contig017698-BurOR.P120	contig017696-BurOR.P118	0.611	1.609	0.379
contig017698-BurOR.P120	contig025458-ZebOR.P125	0.613	1.585	0.387
contig065444-TilOR.P214	contig004275-BriOR.P101	0.614	1.602	0.384
contig025452-ZebOR.P123	contig065254-TilOR.P213	0.615	1.535	0.401
contig065444-TilOR.P214	contig017696-BurOR.P118	0.616	1.645	0.375
contig065444-TilOR.P214	contig023268-NyeOR.P118	0.616	1.651	0.373
contig025452-ZebOR.P123	contig023268-NyeOR.P118	0.616	1.689	0.365
contig065444-TilOR.P214	contig025458-ZebOR.P125	0.619	1.587	0.390
contig025452-ZebOR.P123	contig025458-ZebOR.P125	0.619	1.621	0.382
contig025452-ZebOR.P123	contig017696-BurOR.P118	0.619	1.653	0.374
contig017698-BurOR.P120	contig004275-BriOR.P101	0.620	1.584	0.391
contig025452-ZebOR.P123	contig004275-BriOR.P101	0.630	1.615	0.390

Cichlid Olfactory Receptors :
dN/dS ratio

Fam R

ORs pairs		dN	dS	dN/dS
contig067265-BurOR.R141	contig044295-NyeOR.R135	0.004	0.023	0.191
contig028611-TilOR.R246	contig028617-TilOR.R248	0.005	0.023	0.198
contig067209-BurOR.R140	contig065193-ZebOR.R147	0.006	0.022	0.265
contig067265-BurOR.R141	contig046048-ZebOR.R146	0.009	0.039	0.229
contig044295-NyeOR.R135	contig046048-ZebOR.R146	0.010	0.039	0.268
contig054733-BurOR.R138	contig046040-ZebOR.R144	0.014	0.015	0.884
contig028607-TilOR.R245	contig028617-TilOR.R248	0.038	0.052	0.722
contig028619-TilOR.R249	contig046042-ZebOR.R145	0.040	0.119	0.337
contig043640-BurOR.R137	contig046042-ZebOR.R145	0.041	0.062	0.659
contig043640-BurOR.R137	contig028614-TilOR.R247	0.041	0.128	0.323
contig028614-TilOR.R247	contig028619-TilOR.R249	0.043	0.057	0.760
contig028607-TilOR.R245	contig028611-TilOR.R246	0.043	0.075	0.580
contig028607-TilOR.R245	contig065193-ZebOR.R147	0.044	0.126	0.347
contig067209-BurOR.R140	contig028607-TilOR.R245	0.047	0.126	0.371
contig064565-BurOR.R139	contig028641-TilOR.R253	0.051	0.114	0.446
contig064565-BurOR.R139	contig028644-TilOR.R252	0.051	0.114	0.446
contig043640-BurOR.R137	contig028619-TilOR.R249	0.061	0.140	0.437
contig028617-TilOR.R248	contig065193-ZebOR.R147	0.062	0.160	0.387
contig067209-BurOR.R140	contig028617-TilOR.R248	0.063	0.155	0.409
contig028614-TilOR.R247	contig046042-ZebOR.R145	0.067	0.128	0.524
contig028611-TilOR.R246	contig065193-ZebOR.R147	0.068	0.176	0.384
contig067209-BurOR.R140	contig028611-TilOR.R246	0.069	0.172	0.403
contig028637-TilOR.R250	contig046040-ZebOR.R144	0.210	0.608	0.346
contig054733-BurOR.R138	contig028639-TilOR.R251	0.217	0.345	0.628
contig054733-BurOR.R138	contig028637-TilOR.R250	0.217	0.590	0.367
contig028639-TilOR.R251	contig046040-ZebOR.R144	0.220	0.360	0.611
contig028607-TilOR.R245	contig028619-TilOR.R249	0.220	0.681	0.323
contig043640-BurOR.R137	contig054733-BurOR.R138	0.231	0.517	0.448
contig043640-BurOR.R137	contig046040-ZebOR.R144	0.233	0.524	0.445
contig043640-BurOR.R137	contig028607-TilOR.R245	0.235	0.686	0.342
contig028607-TilOR.R245	contig046042-ZebOR.R145	0.236	0.703	0.336
contig028617-TilOR.R248	contig028619-TilOR.R249	0.236	0.726	0.326
contig094282-BriOR.R118	contig028611-TilOR.R246	0.237	0.635	0.373
contig028607-TilOR.R245	contig028614-TilOR.R247	0.238	0.690	0.345
contig094282-BriOR.R118	contig028617-TilOR.R248	0.239	0.633	0.378
contig054733-BurOR.R138	contig046042-ZebOR.R145	0.240	0.544	0.441
contig064565-BurOR.R139	contig028607-TilOR.R245	0.240	0.635	0.378
contig028607-TilOR.R245	contig028641-TilOR.R253	0.242	0.646	0.374
contig028607-TilOR.R245	contig028644-TilOR.R252	0.242	0.646	0.374
contig028611-TilOR.R246	contig028619-TilOR.R249	0.242	0.721	0.335
contig028619-TilOR.R249	contig046040-ZebOR.R144	0.243	0.490	0.496
contig046040-ZebOR.R144	contig046042-ZebOR.R145	0.243	0.564	0.431

contig028614-TilOR.R247	contig028637-TilOR.R250	0.243	0.596	0.407
contig028617-TilOR.R248	contig028641-TilOR.R253	0.243	0.597	0.407
contig028617-TilOR.R248	contig028644-TilOR.R252	0.243	0.597	0.407
contig043640-BurOR.R137	contig028637-TilOR.R250	0.243	0.654	0.372
contig028614-TilOR.R247	contig046040-ZebOR.R144	0.244	0.472	0.518
contig054733-BurOR.R138	contig028619-TilOR.R249	0.245	0.484	0.506
contig028611-TilOR.R246	contig028641-TilOR.R253	0.245	0.589	0.416
contig028611-TilOR.R246	contig028644-TilOR.R252	0.245	0.589	0.416
contig054733-BurOR.R138	contig028614-TilOR.R247	0.246	0.466	0.528
contig028619-TilOR.R249	contig028637-TilOR.R250	0.247	0.612	0.404
contig028619-TilOR.R249	contig065193-ZebOR.R147	0.247	0.716	0.344
contig028617-TilOR.R248	contig046042-ZebOR.R145	0.247	0.750	0.329
contig028637-TilOR.R250	contig046042-ZebOR.R145	0.248	0.694	0.357
contig067209-BurOR.R140	contig028619-TilOR.R249	0.248	0.737	0.337
contig043640-BurOR.R137	contig028617-TilOR.R248	0.249	0.722	0.346
contig064565-BurOR.R139	contig028617-TilOR.R248	0.251	0.624	0.402
contig028637-TilOR.R250	contig028639-TilOR.R251	0.252	0.611	0.413
contig028611-TilOR.R246	contig046042-ZebOR.R145	0.252	0.735	0.343
contig028614-TilOR.R247	contig028639-TilOR.R251	0.253	0.527	0.480
contig067209-BurOR.R140	contig028641-TilOR.R253	0.253	0.623	0.406
contig067209-BurOR.R140	contig028644-TilOR.R252	0.253	0.623	0.406
contig064565-BurOR.R139	contig028611-TilOR.R246	0.254	0.611	0.416
contig028614-TilOR.R247	contig028617-TilOR.R248	0.254	0.731	0.347
contig028641-TilOR.R253	contig065193-ZebOR.R147	0.255	0.639	0.399
contig028644-TilOR.R252	contig065193-ZebOR.R147	0.255	0.639	0.399
contig043640-BurOR.R137	contig028611-TilOR.R246	0.255	0.707	0.360
contig094282-BriOR.R118	contig028607-TilOR.R245	0.256	0.629	0.407
contig043640-BurOR.R137	contig065193-ZebOR.R147	0.257	0.675	0.381
contig028614-TilOR.R247	contig065193-ZebOR.R147	0.257	0.736	0.349
contig043640-BurOR.R137	contig028639-TilOR.R251	0.258	0.561	0.460
contig028617-TilOR.R248	contig046040-ZebOR.R144	0.259	0.651	0.398
contig043640-BurOR.R137	contig067209-BurOR.R140	0.259	0.694	0.374
contig046042-ZebOR.R145	contig065193-ZebOR.R147	0.259	0.711	0.364
contig028611-TilOR.R246	contig028614-TilOR.R247	0.259	0.726	0.357
contig067209-BurOR.R140	contig028614-TilOR.R247	0.259	0.757	0.342
contig028639-TilOR.R251	contig046042-ZebOR.R145	0.260	0.565	0.461
contig028607-TilOR.R245	contig046040-ZebOR.R144	0.260	0.642	0.404
contig067209-BurOR.R140	contig046042-ZebOR.R145	0.261	0.731	0.357
contig094282-BriOR.R118	contig065193-ZebOR.R147	0.262	0.664	0.394
contig094282-BriOR.R118	contig067209-BurOR.R140	0.263	0.679	0.387
contig028611-TilOR.R246	contig046040-ZebOR.R144	0.264	0.665	0.398
contig064565-BurOR.R139	contig067209-BurOR.R140	0.266	0.653	0.408
contig028619-TilOR.R249	contig028639-TilOR.R251	0.268	0.542	0.494

contig054733-BurOR.R138	contig028607-TilOR.R245	0.268	0.625	0.430
contig054733-BurOR.R138	contig028617-TilOR.R248	0.268	0.639	0.420
contig064565-BurOR.R139	contig065193-ZebOR.R147	0.269	0.661	0.406
contig043640-BurOR.R137	contig064565-BurOR.R139	0.273	0.756	0.361
contig054733-BurOR.R138	contig028611-TilOR.R246	0.274	0.653	0.420
contig067209-BurOR.R140	contig046040-ZebOR.R144	0.277	0.686	0.404
contig043640-BurOR.R137	contig028641-TilOR.R253	0.277	0.717	0.386
contig043640-BurOR.R137	contig028644-TilOR.R252	0.277	0.717	0.386
contig028607-TilOR.R245	contig028637-TilOR.R250	0.277	0.769	0.360
contig028641-TilOR.R253	contig046040-ZebOR.R144	0.278	0.667	0.417
contig028644-TilOR.R252	contig046040-ZebOR.R144	0.278	0.667	0.417
contig046040-ZebOR.R144	contig065193-ZebOR.R147	0.279	0.676	0.414
contig028619-TilOR.R249	contig028641-TilOR.R253	0.280	0.695	0.403
contig028619-TilOR.R249	contig028644-TilOR.R252	0.280	0.695	0.403
contig054733-BurOR.R138	contig067209-BurOR.R140	0.286	0.648	0.442
contig028641-TilOR.R253	contig046042-ZebOR.R145	0.286	0.803	0.356
contig028644-TilOR.R252	contig046042-ZebOR.R145	0.286	0.803	0.356
contig028614-TilOR.R247	contig028641-TilOR.R253	0.287	0.743	0.386
contig028614-TilOR.R247	contig028644-TilOR.R252	0.287	0.743	0.386
contig054733-BurOR.R138	contig065193-ZebOR.R147	0.289	0.638	0.453
contig054733-BurOR.R138	contig028641-TilOR.R253	0.289	0.641	0.451
contig054733-BurOR.R138	contig028644-TilOR.R252	0.289	0.641	0.451
contig028607-TilOR.R245	contig028639-TilOR.R251	0.290	0.663	0.437
contig028617-TilOR.R248	contig028637-TilOR.R250	0.290	0.795	0.364
contig064565-BurOR.R139	contig028614-TilOR.R247	0.292	0.794	0.368
contig028617-TilOR.R248	contig028639-TilOR.R251	0.294	0.674	0.436
contig028611-TilOR.R246	contig028637-TilOR.R250	0.297	0.768	0.387
contig064565-BurOR.R139	contig028619-TilOR.R249	0.297	0.769	0.386
contig064565-BurOR.R139	contig046040-ZebOR.R144	0.298	0.717	0.415
contig028637-TilOR.R250	contig028641-TilOR.R253	0.299	0.790	0.378
contig028637-TilOR.R250	contig028644-TilOR.R252	0.299	0.790	0.378
contig028611-TilOR.R246	contig028639-TilOR.R251	0.300	0.697	0.430
contig028639-TilOR.R251	contig065193-ZebOR.R147	0.301	0.656	0.458
contig028637-TilOR.R250	contig065193-ZebOR.R147	0.301	0.747	0.403
contig094282-BriOR.R118	contig028641-TilOR.R253	0.302	0.644	0.468
contig094282-BriOR.R118	contig028644-TilOR.R252	0.302	0.644	0.468
contig067209-BurOR.R140	contig028639-TilOR.R251	0.302	0.662	0.456
contig067209-BurOR.R140	contig028637-TilOR.R250	0.304	0.769	0.395
contig094282-BriOR.R118	contig046040-ZebOR.R144	0.308	0.709	0.435
contig064565-BurOR.R139	contig046042-ZebOR.R145	0.310	0.816	0.380
contig054733-BurOR.R138	contig064565-BurOR.R139	0.313	0.702	0.446
contig067265-BurOR.R141	contig028607-TilOR.R245	0.316	0.668	0.473
contig067265-BurOR.R141	contig028611-TilOR.R246	0.316	0.695	0.455

contig094282-BriOR.R118	contig054733-BurOR.R138	0.316	0.707	0.446
contig067265-BurOR.R141	contig028617-TilOR.R248	0.317	0.671	0.472
contig028611-TilOR.R246	contig046048-ZebOR.R146	0.318	0.693	0.459
contig094282-BriOR.R118	contig064565-BurOR.R139	0.318	0.697	0.456
contig028617-TilOR.R248	contig046048-ZebOR.R146	0.319	0.679	0.470
contig044295-NyeOR.R135	contig028607-TilOR.R245	0.321	0.658	0.487
contig044295-NyeOR.R135	contig028611-TilOR.R246	0.321	0.703	0.456
contig044295-NyeOR.R135	contig028617-TilOR.R248	0.322	0.680	0.473
contig028607-TilOR.R245	contig046048-ZebOR.R146	0.323	0.658	0.491
contig067265-BurOR.R141	contig065193-ZebOR.R147	0.323	0.722	0.448
contig067209-BurOR.R140	contig067265-BurOR.R141	0.324	0.710	0.456
contig064565-BurOR.R139	contig046048-ZebOR.R146	0.325	0.783	0.415
contig028641-TilOR.R253	contig046048-ZebOR.R146	0.327	0.802	0.408
contig028644-TilOR.R252	contig046048-ZebOR.R146	0.327	0.802	0.408
contig044295-NyeOR.R135	contig065193-ZebOR.R147	0.328	0.711	0.462
contig067209-BurOR.R140	contig044295-NyeOR.R135	0.329	0.699	0.470
contig064565-BurOR.R139	contig028637-TilOR.R250	0.329	0.786	0.419
contig067265-BurOR.R141	contig028641-TilOR.R253	0.329	0.822	0.400
contig067265-BurOR.R141	contig028644-TilOR.R252	0.329	0.822	0.400
contig046048-ZebOR.R146	contig065193-ZebOR.R147	0.331	0.705	0.470
contig067209-BurOR.R140	contig046048-ZebOR.R146	0.332	0.694	0.478
contig064565-BurOR.R139	contig067265-BurOR.R141	0.332	0.787	0.421
contig044295-NyeOR.R135	contig028641-TilOR.R253	0.333	0.843	0.395
contig044295-NyeOR.R135	contig028644-TilOR.R252	0.333	0.843	0.395
contig094282-BriOR.R118	contig028619-TilOR.R249	0.336	0.628	0.536
contig064565-BurOR.R139	contig044295-NyeOR.R135	0.336	0.796	0.422
contig043640-BurOR.R137	contig067265-BurOR.R141	0.337	0.756	0.446
contig094282-BriOR.R118	contig028614-TilOR.R247	0.339	0.690	0.492
contig043640-BurOR.R137	contig046048-ZebOR.R146	0.339	0.744	0.456
contig094282-BriOR.R118	contig046042-ZebOR.R145	0.342	0.693	0.494
contig043640-BurOR.R137	contig044295-NyeOR.R135	0.342	0.765	0.447
contig094282-BriOR.R118	contig028637-TilOR.R250	0.342	0.842	0.407
contig094282-BriOR.R118	contig028639-TilOR.R251	0.343	0.613	0.560
contig094282-BriOR.R118	contig043640-BurOR.R137	0.343	0.721	0.475
contig094282-BriOR.R118	contig046048-ZebOR.R146	0.345	0.934	0.370
contig094282-BriOR.R118	contig067265-BurOR.R141	0.346	0.951	0.364
contig067265-BurOR.R141	contig028614-TilOR.R247	0.349	0.736	0.474
contig064565-BurOR.R139	contig028639-TilOR.R251	0.349	0.755	0.462
contig028614-TilOR.R247	contig046048-ZebOR.R146	0.350	0.729	0.480
contig094282-BriOR.R118	contig044295-NyeOR.R135	0.350	0.976	0.359
contig067265-BurOR.R141	contig028619-TilOR.R249	0.351	0.750	0.468
contig028619-TilOR.R249	contig046048-ZebOR.R146	0.352	0.731	0.482
contig028639-TilOR.R251	contig028641-TilOR.R253	0.352	0.794	0.443

contig028639-TilOR.R251	contig028644-TilOR.R252	0.352	0.794	0.443
contig044295-NyeOR.R135	contig028614-TilOR.R247	0.354	0.745	0.475
contig067265-BurOR.R141	contig046042-ZebOR.R145	0.355	0.799	0.445
contig044295-NyeOR.R135	contig028619-TilOR.R249	0.356	0.759	0.469
contig046042-ZebOR.R145	contig046048-ZebOR.R146	0.357	0.779	0.458
contig044295-NyeOR.R135	contig046042-ZebOR.R145	0.360	0.809	0.445
contig054733-BurOR.R138	contig067265-BurOR.R141	0.370	0.698	0.531
contig054733-BurOR.R138	contig044295-NyeOR.R135	0.370	0.706	0.525
contig054733-BurOR.R138	contig046048-ZebOR.R146	0.374	0.691	0.541
contig067265-BurOR.R141	contig046040-ZebOR.R144	0.374	0.723	0.517
contig046040-ZebOR.R144	contig046048-ZebOR.R146	0.377	0.716	0.527
contig067265-BurOR.R141	contig028639-TilOR.R251	0.377	0.757	0.498
contig044295-NyeOR.R135	contig028639-TilOR.R251	0.377	0.798	0.473
contig044295-NyeOR.R135	contig046040-ZebOR.R144	0.379	0.731	0.518
contig028639-TilOR.R251	contig046048-ZebOR.R146	0.382	0.777	0.492
contig028637-TilOR.R250	contig046048-ZebOR.R146	0.402	0.737	0.546
contig067265-BurOR.R141	contig028637-TilOR.R250	0.406	0.734	0.553
contig044295-NyeOR.R135	contig028637-TilOR.R250	0.406	0.774	0.524

Cichlid Olfactory Receptors :
dN/dS ratio

Fam S

OR pairs		dN	dS	dnds
contig059270-NyeOR.S122	contig017733-ZebOR.S126	0.001	0.008	0.176
contig055881-BurOR.S123	contig017733-ZebOR.S126	0.004	0.021	0.210
contig055881-BurOR.S123	contig059270-NyeOR.S122	0.006	0.013	0.469
contig039435-TilOR.S229	contig039436-TilOR.S230	0.007	0.017	0.444
contig060198-BriOR.S104	contig017733-ZebOR.S126	0.010	0.052	0.199
contig055884-BurOR.S125	contig017736-ZebOR.S127	0.011	0.038	0.277
contig060198-BriOR.S104	contig059270-NyeOR.S122	0.012	0.043	0.275
contig055884-BurOR.S125	contig039416-TilOR.S223	0.012	0.087	0.138
contig028594-BurOR.S122	contig039435-TilOR.S229	0.013	0.059	0.223
contig028593-BurOR.S121	contig038871-NyeOR.S121	0.015	0.012	1.193
contig060198-BriOR.S104	contig055881-BurOR.S123	0.015	0.056	0.263
contig042478-BriOR.S103	contig028593-BurOR.S121	0.016	0.021	0.785
contig042478-BriOR.S103	contig038871-NyeOR.S121	0.016	0.025	0.653
contig039416-TilOR.S223	contig017736-ZebOR.S127	0.017	0.092	0.181
contig005005-TilOR.S219	contig005007-TilOR.S220	0.018	0.043	0.422
contig028594-BurOR.S122	contig039436-TilOR.S230	0.018	0.068	0.260
contig004999-TilOR.S217	contig017733-ZebOR.S126	0.019	0.065	0.297
contig059270-NyeOR.S122	contig004999-TilOR.S217	0.021	0.056	0.371
contig060198-BriOR.S104	contig004999-TilOR.S217	0.021	0.075	0.279
contig042475-BriOR.S102	contig039436-TilOR.S230	0.022	0.064	0.350
contig039425-TilOR.S225	contig039426-TilOR.S226	0.023	0.056	0.400
contig055881-BurOR.S123	contig004999-TilOR.S217	0.023	0.072	0.321
contig039415-TilOR.S221	contig039416-TilOR.S223	0.023	0.083	0.276
contig055882-BurOR.S124	contig005007-TilOR.S220	0.023	0.092	0.247
contig039428-TilOR.S227	contig017743-ZebOR.S128	0.024	0.048	0.505
contig042475-BriOR.S102	contig028594-BurOR.S122	0.025	0.050	0.501
contig055884-BurOR.S125	contig039415-TilOR.S221	0.026	0.116	0.224
contig042475-BriOR.S102	contig039435-TilOR.S229	0.027	0.068	0.394
contig039415-TilOR.S221	contig017736-ZebOR.S127	0.031	0.111	0.277
contig042478-BriOR.S103	contig068054-ZebOR.S129	0.032	0.049	0.660
contig055882-BurOR.S124	contig005005-TilOR.S219	0.032	0.083	0.386
contig038871-NyeOR.S121	contig068054-ZebOR.S129	0.034	0.040	0.840
contig028593-BurOR.S121	contig068054-ZebOR.S129	0.035	0.048	0.723
contig039426-TilOR.S226	contig039428-TilOR.S227	0.036	0.139	0.260
contig039416-TilOR.S223	contig039419-TilOR.S224	0.038	0.092	0.416
contig055882-BurOR.S124	contig055884-BurOR.S125	0.041	0.087	0.476
contig039437-TilOR.S231	contig068054-ZebOR.S129	0.043	0.065	0.660
contig055884-BurOR.S125	contig039419-TilOR.S224	0.045	0.074	0.604
contig055882-BurOR.S124	contig017736-ZebOR.S127	0.046	0.082	0.563
contig039425-TilOR.S225	contig039428-TilOR.S227	0.047	0.160	0.296
contig055882-BurOR.S124	contig039416-TilOR.S223	0.048	0.096	0.497
contig005007-TilOR.S220	contig039415-TilOR.S221	0.048	0.161	0.297

contig039419-TilOR.S224	contig017736-ZebOR.S127	0.049	0.087	0.566
contig039425-TilOR.S225	contig017743-ZebOR.S128	0.049	0.145	0.337
contig028593-BurOR.S121	contig039437-TilOR.S231	0.050	0.072	0.687
contig055882-BurOR.S124	contig039415-TilOR.S221	0.050	0.106	0.469
contig042478-BriOR.S103	contig039437-TilOR.S231	0.051	0.085	0.598
contig055884-BurOR.S125	contig005007-TilOR.S220	0.052	0.121	0.433
contig005005-TilOR.S219	contig039416-TilOR.S223	0.052	0.126	0.417
contig039416-TilOR.S223	contig039426-TilOR.S226	0.053	0.134	0.398
contig005007-TilOR.S220	contig039416-TilOR.S223	0.053	0.151	0.348
contig038871-NyeOR.S121	contig039437-TilOR.S231	0.054	0.072	0.752
contig039416-TilOR.S223	contig039425-TilOR.S225	0.054	0.121	0.445
contig005005-TilOR.S219	contig039415-TilOR.S221	0.054	0.141	0.385
contig039425-TilOR.S225	contig017736-ZebOR.S127	0.054	0.151	0.358
contig039426-TilOR.S226	contig017743-ZebOR.S128	0.054	0.155	0.345
contig039426-TilOR.S226	contig017736-ZebOR.S127	0.055	0.119	0.462
contig055884-BurOR.S125	contig005005-TilOR.S219	0.056	0.097	0.572
contig055884-BurOR.S125	contig039426-TilOR.S226	0.056	0.144	0.392
contig055884-BurOR.S125	contig039425-TilOR.S225	0.056	0.146	0.380
contig005007-TilOR.S220	contig017736-ZebOR.S127	0.057	0.116	0.495
contig039415-TilOR.S221	contig039426-TilOR.S226	0.057	0.136	0.421
contig005007-TilOR.S220	contig039419-TilOR.S224	0.060	0.121	0.498
contig005005-TilOR.S219	contig017736-ZebOR.S127	0.061	0.102	0.596
contig055882-BurOR.S124	contig039419-TilOR.S224	0.062	0.097	0.645
contig039415-TilOR.S221	contig039419-TilOR.S224	0.062	0.106	0.587
contig005005-TilOR.S219	contig039419-TilOR.S224	0.064	0.107	0.596
contig039419-TilOR.S224	contig039425-TilOR.S225	0.065	0.088	0.740
contig039415-TilOR.S221	contig039425-TilOR.S225	0.066	0.124	0.536
contig039419-TilOR.S224	contig039426-TilOR.S226	0.068	0.100	0.677
contig005007-TilOR.S220	contig039426-TilOR.S226	0.068	0.145	0.468
contig005005-TilOR.S219	contig039426-TilOR.S226	0.068	0.155	0.437
contig004999-TilOR.S217	contig005000-TilOR.S218	0.071	0.165	0.430
contig055882-BurOR.S124	contig039426-TilOR.S226	0.073	0.128	0.566
contig055882-BurOR.S124	contig039425-TilOR.S225	0.073	0.133	0.546
contig005005-TilOR.S219	contig039425-TilOR.S225	0.073	0.150	0.485
contig005007-TilOR.S220	contig039425-TilOR.S225	0.073	0.150	0.485
contig017736-ZebOR.S127	contig017743-ZebOR.S128	0.075	0.206	0.363
contig060198-BriOR.S104	contig005000-TilOR.S218	0.076	0.192	0.395
contig005000-TilOR.S218	contig017733-ZebOR.S126	0.078	0.186	0.417
contig039428-TilOR.S227	contig017736-ZebOR.S127	0.078	0.198	0.392
contig059270-NyeOR.S122	contig005000-TilOR.S218	0.079	0.175	0.452
contig055884-BurOR.S125	contig017743-ZebOR.S128	0.079	0.194	0.408
contig039416-TilOR.S223	contig017743-ZebOR.S128	0.079	0.210	0.377
contig055884-BurOR.S125	contig039428-TilOR.S227	0.081	0.221	0.366

contig039416-TilOR.S223	contig039428-TilOR.S227	0.082	0.237	0.348
contig055881-BurOR.S123	contig005000-TilOR.S218	0.083	0.186	0.444
contig005007-TilOR.S220	contig039428-TilOR.S227	0.086	0.202	0.428
contig039415-TilOR.S221	contig039428-TilOR.S227	0.087	0.252	0.345
contig039419-TilOR.S224	contig039428-TilOR.S227	0.089	0.160	0.559
contig039415-TilOR.S221	contig017743-ZebOR.S128	0.089	0.224	0.396
contig055882-BurOR.S124	contig017743-ZebOR.S128	0.090	0.195	0.461
contig055882-BurOR.S124	contig039428-TilOR.S227	0.090	0.211	0.427
contig039419-TilOR.S224	contig017743-ZebOR.S128	0.091	0.135	0.674
contig005000-TilOR.S218	contig039416-TilOR.S222	0.091	0.207	0.440
contig005007-TilOR.S220	contig017743-ZebOR.S128	0.092	0.200	0.462
contig004999-TilOR.S217	contig039416-TilOR.S222	0.093	0.223	0.415
contig005005-TilOR.S219	contig039428-TilOR.S227	0.093	0.225	0.415
contig039416-TilOR.S222	contig017733-ZebOR.S126	0.097	0.232	0.418
contig060198-BriOR.S104	contig039416-TilOR.S222	0.097	0.250	0.387
contig059270-NyeOR.S122	contig039416-TilOR.S222	0.098	0.220	0.447
contig005005-TilOR.S219	contig017743-ZebOR.S128	0.099	0.200	0.495
contig055881-BurOR.S123	contig039416-TilOR.S222	0.101	0.234	0.431
contig055884-BurOR.S125	contig017733-ZebOR.S126	0.141	0.429	0.329
contig039416-TilOR.S223	contig017733-ZebOR.S126	0.141	0.451	0.314
contig005000-TilOR.S218	contig005005-TilOR.S219	0.142	0.396	0.360
contig005005-TilOR.S219	contig017733-ZebOR.S126	0.143	0.402	0.356
contig055884-BurOR.S125	contig059270-NyeOR.S122	0.143	0.444	0.322
contig059270-NyeOR.S122	contig039416-TilOR.S223	0.143	0.466	0.307
contig004999-TilOR.S217	contig005005-TilOR.S219	0.144	0.420	0.343
contig055884-BurOR.S125	contig004999-TilOR.S217	0.144	0.448	0.321
contig004999-TilOR.S217	contig039416-TilOR.S223	0.144	0.462	0.312
contig005000-TilOR.S218	contig017743-ZebOR.S128	0.145	0.343	0.422
contig059270-NyeOR.S122	contig005005-TilOR.S219	0.145	0.409	0.354
contig060198-BriOR.S104	contig055884-BurOR.S125	0.146	0.473	0.308
contig060198-BriOR.S104	contig039416-TilOR.S223	0.146	0.496	0.294
contig060198-BriOR.S104	contig005005-TilOR.S219	0.147	0.444	0.332
contig055881-BurOR.S123	contig055884-BurOR.S125	0.147	0.459	0.320
contig055881-BurOR.S123	contig039416-TilOR.S223	0.147	0.482	0.305
contig017733-ZebOR.S126	contig017743-ZebOR.S128	0.148	0.335	0.441
contig017733-ZebOR.S126	contig017736-ZebOR.S127	0.148	0.446	0.332
contig039415-TilOR.S221	contig017733-ZebOR.S126	0.148	0.458	0.323
contig059270-NyeOR.S122	contig017743-ZebOR.S128	0.149	0.348	0.429
contig004999-TilOR.S217	contig017743-ZebOR.S128	0.149	0.372	0.399
contig055881-BurOR.S123	contig005005-TilOR.S219	0.149	0.416	0.357
contig055882-BurOR.S124	contig005000-TilOR.S218	0.149	0.416	0.359
contig055884-BurOR.S125	contig005000-TilOR.S218	0.150	0.415	0.361
contig005000-TilOR.S218	contig039426-TilOR.S226	0.150	0.416	0.361

contig005000-TilOR.S218	contig017736-ZebOR.S127	0.150	0.427	0.351
contig059270-NyeOR.S122	contig017736-ZebOR.S127	0.150	0.461	0.325
contig059270-NyeOR.S122	contig039415-TilOR.S221	0.150	0.466	0.321
contig039419-TilOR.S224	contig017733-ZebOR.S126	0.151	0.369	0.411
contig055882-BurOR.S124	contig017733-ZebOR.S126	0.151	0.410	0.368
contig005000-TilOR.S218	contig039415-TilOR.S221	0.151	0.411	0.369
contig005007-TilOR.S220	contig017733-ZebOR.S126	0.151	0.415	0.363
contig005000-TilOR.S218	contig039416-TilOR.S223	0.151	0.432	0.349
contig004999-TilOR.S217	contig017736-ZebOR.S127	0.151	0.450	0.335
contig005005-TilOR.S219	contig039416-TilOR.S222	0.151	0.456	0.332
contig004999-TilOR.S217	contig039415-TilOR.S221	0.151	0.462	0.326
contig060198-BriOR.S104	contig017743-ZebOR.S128	0.152	0.367	0.414
contig005000-TilOR.S218	contig039428-TilOR.S227	0.152	0.369	0.413
contig059270-NyeOR.S122	contig005007-TilOR.S220	0.152	0.422	0.361
contig060198-BriOR.S104	contig017736-ZebOR.S127	0.152	0.491	0.310
contig060198-BriOR.S104	contig039415-TilOR.S221	0.152	0.504	0.302
contig039425-TilOR.S225	contig017733-ZebOR.S126	0.153	0.358	0.427
contig055881-BurOR.S123	contig017743-ZebOR.S128	0.153	0.362	0.424
contig059270-NyeOR.S122	contig039419-TilOR.S224	0.153	0.382	0.401
contig039426-TilOR.S226	contig017733-ZebOR.S126	0.153	0.392	0.390
contig055882-BurOR.S124	contig059270-NyeOR.S122	0.153	0.425	0.360
contig055881-BurOR.S123	contig017736-ZebOR.S127	0.153	0.476	0.322
contig055881-BurOR.S123	contig039415-TilOR.S221	0.153	0.489	0.313
contig004999-TilOR.S217	contig039425-TilOR.S225	0.154	0.375	0.410
contig005000-TilOR.S218	contig039425-TilOR.S225	0.154	0.401	0.383
contig004999-TilOR.S217	contig039426-TilOR.S226	0.154	0.410	0.375
contig039428-TilOR.S227	contig017733-ZebOR.S126	0.155	0.333	0.466
contig059270-NyeOR.S122	contig039425-TilOR.S225	0.155	0.365	0.424
contig059270-NyeOR.S122	contig039426-TilOR.S226	0.155	0.407	0.380
contig005000-TilOR.S218	contig005007-TilOR.S220	0.155	0.439	0.354
contig060198-BriOR.S104	contig005007-TilOR.S220	0.155	0.458	0.339
contig004999-TilOR.S217	contig039428-TilOR.S227	0.156	0.371	0.421
contig060198-BriOR.S104	contig039425-TilOR.S225	0.156	0.387	0.404
contig060198-BriOR.S104	contig039419-TilOR.S224	0.156	0.409	0.381
contig060198-BriOR.S104	contig039426-TilOR.S226	0.156	0.423	0.369
contig055882-BurOR.S124	contig004999-TilOR.S217	0.156	0.429	0.363
contig055881-BurOR.S123	contig005007-TilOR.S220	0.156	0.444	0.352
contig060198-BriOR.S104	contig055882-BurOR.S124	0.156	0.445	0.349
contig059270-NyeOR.S122	contig039428-TilOR.S227	0.157	0.347	0.453
contig055881-BurOR.S123	contig039425-TilOR.S225	0.157	0.389	0.405
contig055881-BurOR.S123	contig039419-TilOR.S224	0.157	0.396	0.396
contig055881-BurOR.S123	contig055882-BurOR.S124	0.157	0.439	0.357
contig004999-TilOR.S217	contig005007-TilOR.S220	0.157	0.441	0.356

contig055884-BurOR.S125	contig039416-TilOR.S222	0.157	0.446	0.352
contig039415-TilOR.S221	contig039416-TilOR.S222	0.157	0.480	0.326
contig039416-TilOR.S222	contig039416-TilOR.S223	0.157	0.509	0.308
contig004999-TilOR.S217	contig039419-TilOR.S224	0.158	0.400	0.395
contig055881-BurOR.S123	contig039426-TilOR.S226	0.158	0.421	0.376
contig060198-BriOR.S104	contig039428-TilOR.S227	0.160	0.365	0.437
contig055881-BurOR.S123	contig039428-TilOR.S227	0.161	0.360	0.447
contig055882-BurOR.S124	contig039416-TilOR.S222	0.162	0.460	0.353
contig039416-TilOR.S222	contig017736-ZebOR.S127	0.162	0.463	0.350
contig005000-TilOR.S218	contig039419-TilOR.S224	0.165	0.378	0.437
contig039416-TilOR.S222	contig039426-TilOR.S226	0.165	0.460	0.359
contig039416-TilOR.S222	contig039419-TilOR.S224	0.166	0.430	0.387
contig039416-TilOR.S222	contig039425-TilOR.S225	0.167	0.452	0.369
contig039416-TilOR.S222	contig017743-ZebOR.S128	0.169	0.397	0.426
contig039416-TilOR.S222	contig039428-TilOR.S227	0.170	0.407	0.419
contig005007-TilOR.S220	contig039416-TilOR.S222	0.170	0.463	0.366
contig039435-TilOR.S228	contig039435-TilOR.S229	0.174	0.529	0.328
contig039435-TilOR.S228	contig039436-TilOR.S230	0.182	0.514	0.354
contig042475-BriOR.S102	contig039435-TilOR.S228	0.184	0.528	0.349
contig028594-BurOR.S122	contig039435-TilOR.S228	0.187	0.520	0.359
contig038871-NyeOR.S121	contig039435-TilOR.S229	0.187	0.695	0.270
contig038871-NyeOR.S121	contig039436-TilOR.S230	0.188	0.686	0.274
contig042478-BriOR.S103	contig039436-TilOR.S230	0.191	0.678	0.282
contig042478-BriOR.S103	contig039435-TilOR.S229	0.191	0.687	0.277
contig028594-BurOR.S122	contig038871-NyeOR.S121	0.191	0.776	0.246
contig028593-BurOR.S121	contig039435-TilOR.S229	0.192	0.659	0.292
contig042475-BriOR.S102	contig038871-NyeOR.S121	0.192	0.755	0.254
contig028593-BurOR.S121	contig039436-TilOR.S230	0.193	0.650	0.297
contig039435-TilOR.S229	contig068054-ZebOR.S129	0.194	0.696	0.278
contig042478-BriOR.S103	contig028594-BurOR.S122	0.194	0.755	0.257
contig042475-BriOR.S102	contig042478-BriOR.S103	0.195	0.746	0.262
contig028593-BurOR.S121	contig028594-BurOR.S122	0.196	0.736	0.267
contig039436-TilOR.S230	contig068054-ZebOR.S129	0.197	0.690	0.285
contig042475-BriOR.S102	contig028593-BurOR.S121	0.197	0.716	0.275
contig039436-TilOR.S230	contig039437-TilOR.S231	0.198	0.652	0.303
contig039435-TilOR.S229	contig039437-TilOR.S231	0.201	0.671	0.299
contig042475-BriOR.S102	contig068054-ZebOR.S129	0.201	0.760	0.264
contig028594-BurOR.S122	contig068054-ZebOR.S129	0.202	0.782	0.258
contig028594-BurOR.S122	contig039437-TilOR.S231	0.205	0.748	0.274
contig042475-BriOR.S102	contig039437-TilOR.S231	0.206	0.728	0.283
contig039426-TilOR.S226	contig068054-ZebOR.S129	0.242	0.646	0.376
contig039425-TilOR.S225	contig068054-ZebOR.S129	0.244	0.650	0.375
contig039428-TilOR.S227	contig068054-ZebOR.S129	0.244	0.675	0.362

contig042478-BriOR.S103	contig039425-TilOR.S225	0.247	0.604	0.409
contig042478-BriOR.S103	contig039426-TilOR.S226	0.247	0.605	0.409
contig028593-BurOR.S121	contig039435-TilOR.S228	0.247	0.729	0.339
contig042478-BriOR.S103	contig039435-TilOR.S228	0.249	0.728	0.342
contig038871-NyeOR.S121	contig005000-TilOR.S218	0.250	0.713	0.351
contig038871-NyeOR.S121	contig039426-TilOR.S226	0.251	0.646	0.388
contig038871-NyeOR.S121	contig039425-TilOR.S225	0.251	0.646	0.388
contig017743-ZebOR.S128	contig068054-ZebOR.S129	0.251	0.673	0.374
contig039435-TilOR.S228	contig068054-ZebOR.S129	0.252	0.706	0.357
contig038871-NyeOR.S121	contig039435-TilOR.S228	0.253	0.737	0.344
contig042478-BriOR.S103	contig005000-TilOR.S218	0.254	0.711	0.357
contig042478-BriOR.S103	contig039428-TilOR.S227	0.255	0.675	0.377
contig028593-BurOR.S121	contig005000-TilOR.S218	0.255	0.716	0.357
contig038871-NyeOR.S121	contig039428-TilOR.S227	0.255	0.719	0.355
contig028593-BurOR.S121	contig039425-TilOR.S225	0.256	0.650	0.394
contig028593-BurOR.S121	contig039426-TilOR.S226	0.256	0.651	0.393
contig017736-ZebOR.S127	contig068054-ZebOR.S129	0.256	0.675	0.379
contig039425-TilOR.S225	contig039436-TilOR.S230	0.256	0.780	0.328
contig039416-TilOR.S223	contig068054-ZebOR.S129	0.257	0.692	0.371
contig039425-TilOR.S225	contig039437-TilOR.S231	0.258	0.670	0.384
contig055884-BurOR.S125	contig068054-ZebOR.S129	0.258	0.689	0.374
contig005000-TilOR.S218	contig068054-ZebOR.S129	0.258	0.691	0.374
contig039425-TilOR.S225	contig039435-TilOR.S229	0.258	0.770	0.335
contig039426-TilOR.S226	contig039437-TilOR.S231	0.259	0.666	0.388
contig005000-TilOR.S218	contig039437-TilOR.S231	0.260	0.709	0.368
contig028593-BurOR.S121	contig039428-TilOR.S227	0.261	0.699	0.373
contig042478-BriOR.S103	contig017743-ZebOR.S128	0.262	0.673	0.389
contig039435-TilOR.S228	contig039437-TilOR.S231	0.262	0.683	0.383
contig039437-TilOR.S231	contig017736-ZebOR.S127	0.262	0.685	0.382
contig038871-NyeOR.S121	contig017743-ZebOR.S128	0.262	0.716	0.366
contig042478-BriOR.S103	contig005005-TilOR.S219	0.263	0.646	0.407
contig042478-BriOR.S103	contig039416-TilOR.S223	0.263	0.652	0.404
contig005005-TilOR.S219	contig068054-ZebOR.S129	0.263	0.675	0.390
contig005007-TilOR.S220	contig068054-ZebOR.S129	0.263	0.684	0.385
contig039428-TilOR.S227	contig039437-TilOR.S231	0.263	0.697	0.376
contig055884-BurOR.S125	contig039437-TilOR.S231	0.264	0.686	0.385
contig039416-TilOR.S223	contig039437-TilOR.S231	0.264	0.705	0.375
contig039426-TilOR.S226	contig039436-TilOR.S230	0.264	0.713	0.371
contig028594-BurOR.S122	contig039425-TilOR.S225	0.264	0.784	0.337
contig042478-BriOR.S103	contig005007-TilOR.S220	0.265	0.655	0.405
contig055882-BurOR.S124	contig068054-ZebOR.S129	0.265	0.681	0.390
contig042478-BriOR.S103	contig017736-ZebOR.S127	0.266	0.631	0.421
contig039419-TilOR.S224	contig068054-ZebOR.S129	0.266	0.681	0.390

contig039426-TilOR.S226	contig039435-TilOR.S229	0.266	0.704	0.378
contig028594-BurOR.S122	contig005000-TilOR.S218	0.266	0.745	0.358
contig042478-BriOR.S103	contig039415-TilOR.S221	0.267	0.662	0.403
contig038871-NyeOR.S121	contig005005-TilOR.S219	0.267	0.688	0.388
contig038871-NyeOR.S121	contig039416-TilOR.S223	0.267	0.694	0.385
contig028593-BurOR.S121	contig017743-ZebOR.S128	0.268	0.697	0.384
contig039415-TilOR.S221	contig068054-ZebOR.S129	0.268	0.699	0.383
contig039437-TilOR.S231	contig017743-ZebOR.S128	0.268	0.706	0.379
contig005000-TilOR.S218	contig039436-TilOR.S230	0.268	0.779	0.344
contig042478-BriOR.S103	contig055884-BurOR.S125	0.269	0.655	0.411
contig038871-NyeOR.S121	contig005007-TilOR.S220	0.269	0.697	0.386
contig038871-NyeOR.S121	contig017736-ZebOR.S127	0.270	0.671	0.402
contig028593-BurOR.S121	contig005005-TilOR.S219	0.270	0.684	0.394
contig005005-TilOR.S219	contig039437-TilOR.S231	0.271	0.696	0.389
contig039415-TilOR.S221	contig039437-TilOR.S231	0.271	0.703	0.385
contig038871-NyeOR.S121	contig039415-TilOR.S221	0.271	0.705	0.385
contig038871-NyeOR.S121	contig004999-TilOR.S217	0.271	0.714	0.380
contig038871-NyeOR.S121	contig017733-ZebOR.S126	0.271	0.749	0.362
contig028593-BurOR.S121	contig005007-TilOR.S220	0.272	0.694	0.392
contig028594-BurOR.S122	contig039426-TilOR.S226	0.272	0.705	0.386
contig042478-BriOR.S103	contig039419-TilOR.S224	0.273	0.656	0.417
contig028593-BurOR.S121	contig039416-TilOR.S223	0.273	0.688	0.396
contig004999-TilOR.S217	contig068054-ZebOR.S129	0.273	0.693	0.394
contig055882-BurOR.S124	contig039437-TilOR.S231	0.273	0.694	0.392
contig055884-BurOR.S125	contig038871-NyeOR.S121	0.273	0.697	0.392
contig038871-NyeOR.S121	contig059270-NyeOR.S122	0.273	0.749	0.365
contig005000-TilOR.S218	contig039435-TilOR.S229	0.273	0.781	0.349
contig042475-BriOR.S102	contig039425-TilOR.S225	0.273	0.854	0.319
contig028593-BurOR.S121	contig017736-ZebOR.S127	0.274	0.671	0.408
contig017733-ZebOR.S126	contig068054-ZebOR.S129	0.274	0.700	0.391
contig028593-BurOR.S121	contig017733-ZebOR.S126	0.274	0.720	0.381
contig028593-BurOR.S121	contig039419-TilOR.S224	0.275	0.656	0.420
contig028593-BurOR.S121	contig004999-TilOR.S217	0.275	0.686	0.400
contig005007-TilOR.S220	contig039437-TilOR.S231	0.275	0.696	0.396
contig028593-BurOR.S121	contig039415-TilOR.S221	0.275	0.704	0.391
contig028593-BurOR.S121	contig055884-BurOR.S125	0.276	0.685	0.403
contig059270-NyeOR.S122	contig068054-ZebOR.S129	0.276	0.700	0.394
contig039428-TilOR.S227	contig039436-TilOR.S230	0.276	0.717	0.385
contig055881-BurOR.S123	contig038871-NyeOR.S121	0.276	0.777	0.356
contig042478-BriOR.S103	contig004999-TilOR.S217	0.277	0.696	0.398
contig060198-BriOR.S104	contig038871-NyeOR.S121	0.277	0.719	0.385
contig028593-BurOR.S121	contig059270-NyeOR.S122	0.277	0.720	0.384
contig042475-BriOR.S102	contig005000-TilOR.S218	0.277	0.774	0.358

contig042478-BriOR.S103	contig017733-ZebOR.S126	0.278	0.725	0.383
contig039436-TilOR.S230	contig017743-ZebOR.S128	0.278	0.763	0.365
contig039415-TilOR.S221	contig039435-TilOR.S229	0.278	0.791	0.352
contig028593-BurOR.S121	contig055882-BurOR.S124	0.279	0.696	0.402
contig055881-BurOR.S123	contig068054-ZebOR.S129	0.279	0.727	0.384
contig039428-TilOR.S227	contig039435-TilOR.S229	0.279	0.737	0.378
contig039435-TilOR.S229	contig017743-ZebOR.S128	0.279	0.773	0.361
contig028594-BurOR.S122	contig039416-TilOR.S222	0.279	0.815	0.342
contig042478-BriOR.S103	contig055882-BurOR.S124	0.280	0.652	0.429
contig060198-BriOR.S104	contig068054-ZebOR.S129	0.280	0.672	0.416
contig060198-BriOR.S104	contig028593-BurOR.S121	0.280	0.702	0.399
contig042478-BriOR.S103	contig059270-NyeOR.S122	0.280	0.725	0.386
contig028593-BurOR.S121	contig055881-BurOR.S123	0.280	0.747	0.375
contig038871-NyeOR.S121	contig039416-TilOR.S222	0.280	0.823	0.340
contig038871-NyeOR.S121	contig039419-TilOR.S224	0.281	0.693	0.405
contig042475-BriOR.S102	contig039426-TilOR.S226	0.281	0.769	0.366
contig042478-BriOR.S103	contig039416-TilOR.S222	0.281	0.792	0.355
contig004999-TilOR.S217	contig039437-TilOR.S231	0.282	0.671	0.420
contig005005-TilOR.S219	contig039435-TilOR.S229	0.283	0.662	0.428
contig039419-TilOR.S224	contig039437-TilOR.S231	0.283	0.689	0.411
contig039437-TilOR.S231	contig017733-ZebOR.S126	0.283	0.696	0.406
contig028594-BurOR.S122	contig039428-TilOR.S227	0.283	0.728	0.388
contig042478-BriOR.S103	contig055881-BurOR.S123	0.283	0.752	0.376
contig028594-BurOR.S122	contig017743-ZebOR.S128	0.283	0.775	0.365
contig055882-BurOR.S124	contig038871-NyeOR.S121	0.284	0.694	0.409
contig042478-BriOR.S103	contig060198-BriOR.S104	0.284	0.696	0.407
contig039416-TilOR.S223	contig039435-TilOR.S229	0.284	0.783	0.363
contig055884-BurOR.S125	contig039435-TilOR.S229	0.285	0.691	0.413
contig059270-NyeOR.S122	contig039437-TilOR.S231	0.285	0.696	0.409
contig039416-TilOR.S222	contig039436-TilOR.S230	0.285	0.858	0.332
contig039416-TilOR.S222	contig068054-ZebOR.S129	0.286	0.768	0.372
contig005005-TilOR.S219	contig039436-TilOR.S230	0.287	0.653	0.440
contig039435-TilOR.S229	contig017736-ZebOR.S127	0.287	0.701	0.410
contig039416-TilOR.S222	contig039437-TilOR.S231	0.287	0.734	0.391
contig039416-TilOR.S223	contig039436-TilOR.S230	0.287	0.771	0.372
contig028593-BurOR.S121	contig039416-TilOR.S222	0.287	0.787	0.364
contig055884-BurOR.S125	contig039436-TilOR.S230	0.288	0.685	0.421
contig055881-BurOR.S123	contig039437-TilOR.S231	0.288	0.722	0.399
contig039415-TilOR.S221	contig039436-TilOR.S230	0.288	0.795	0.363
contig039416-TilOR.S222	contig039435-TilOR.S229	0.288	0.856	0.336
contig060198-BriOR.S104	contig039437-TilOR.S231	0.289	0.679	0.425
contig039436-TilOR.S230	contig017736-ZebOR.S127	0.290	0.689	0.421
contig042475-BriOR.S102	contig039416-TilOR.S222	0.290	0.866	0.335

contig028594-BurOR.S122	contig005005-TilOR.S219	0.291	0.695	0.419
contig028594-BurOR.S122	contig055884-BurOR.S125	0.291	0.703	0.415
contig028594-BurOR.S122	contig039416-TilOR.S223	0.291	0.821	0.354
contig028594-BurOR.S122	contig039415-TilOR.S221	0.292	0.809	0.362
contig028594-BurOR.S122	contig017736-ZebOR.S127	0.293	0.702	0.418
contig055882-BurOR.S124	contig039435-TilOR.S229	0.293	0.707	0.414
contig060198-BriOR.S104	contig028594-BurOR.S122	0.293	0.711	0.413
contig042475-BriOR.S102	contig039428-TilOR.S227	0.293	0.775	0.378
contig042475-BriOR.S102	contig017743-ZebOR.S128	0.293	0.825	0.355
contig042475-BriOR.S102	contig039415-TilOR.S221	0.294	0.837	0.351
contig005000-TilOR.S218	contig039435-TilOR.S228	0.295	0.806	0.366
contig005005-TilOR.S219	contig039435-TilOR.S228	0.295	0.821	0.359
contig055882-BurOR.S124	contig039436-TilOR.S230	0.296	0.701	0.422
contig028594-BurOR.S122	contig004999-TilOR.S217	0.297	0.640	0.464
contig060198-BriOR.S104	contig039436-TilOR.S230	0.297	0.735	0.405
contig039419-TilOR.S224	contig039435-TilOR.S229	0.298	0.671	0.445
contig005007-TilOR.S220	contig039435-TilOR.S229	0.298	0.697	0.427
contig039426-TilOR.S226	contig039435-TilOR.S228	0.298	0.792	0.376
contig060198-BriOR.S104	contig039435-TilOR.S229	0.299	0.749	0.399
contig039416-TilOR.S222	contig039435-TilOR.S228	0.299	0.784	0.381
contig042475-BriOR.S102	contig005005-TilOR.S219	0.300	0.714	0.421
contig039416-TilOR.S223	contig039435-TilOR.S228	0.300	0.824	0.364
contig004999-TilOR.S217	contig039436-TilOR.S230	0.301	0.662	0.455
contig039436-TilOR.S230	contig017733-ZebOR.S126	0.301	0.704	0.428
contig028594-BurOR.S122	contig055882-BurOR.S124	0.301	0.742	0.405
contig042475-BriOR.S102	contig039416-TilOR.S223	0.301	0.848	0.355
contig039419-TilOR.S224	contig039436-TilOR.S230	0.302	0.666	0.453
contig028594-BurOR.S122	contig017733-ZebOR.S126	0.302	0.671	0.450
contig005007-TilOR.S220	contig039436-TilOR.S230	0.302	0.688	0.439
contig039428-TilOR.S227	contig039435-TilOR.S228	0.302	0.790	0.383
contig059270-NyeOR.S122	contig039436-TilOR.S230	0.303	0.704	0.431
contig042475-BriOR.S102	contig055884-BurOR.S125	0.303	0.743	0.408
contig039425-TilOR.S225	contig039435-TilOR.S228	0.303	0.773	0.393
contig004999-TilOR.S217	contig039435-TilOR.S229	0.304	0.670	0.454
contig028594-BurOR.S122	contig059270-NyeOR.S122	0.304	0.671	0.453
contig039435-TilOR.S229	contig017733-ZebOR.S126	0.304	0.702	0.433
contig042475-BriOR.S102	contig060198-BriOR.S104	0.304	0.732	0.416
contig028594-BurOR.S122	contig039419-TilOR.S224	0.305	0.683	0.446
contig042475-BriOR.S102	contig017736-ZebOR.S127	0.305	0.742	0.411
contig039415-TilOR.S221	contig039435-TilOR.S228	0.305	0.807	0.377
contig059270-NyeOR.S122	contig039435-TilOR.S229	0.306	0.702	0.436
contig028594-BurOR.S122	contig005007-TilOR.S220	0.306	0.732	0.418
contig042475-BriOR.S102	contig004999-TilOR.S217	0.307	0.664	0.462

contig028594-BurOR.S122	contig055881-BurOR.S123	0.307	0.665	0.462
contig055881-BurOR.S123	contig039436-TilOR.S230	0.307	0.698	0.440
contig039435-TilOR.S228	contig017736-ZebOR.S127	0.307	0.795	0.386
contig055884-BurOR.S125	contig039435-TilOR.S228	0.309	0.774	0.399
contig005007-TilOR.S220	contig039435-TilOR.S228	0.309	0.839	0.369
contig055881-BurOR.S123	contig039435-TilOR.S229	0.310	0.696	0.445
contig039435-TilOR.S228	contig017743-ZebOR.S128	0.310	0.764	0.406
contig042475-BriOR.S102	contig055882-BurOR.S124	0.310	0.771	0.403
contig042475-BriOR.S102	contig017733-ZebOR.S126	0.311	0.696	0.447
contig042475-BriOR.S102	contig059270-NyeOR.S122	0.314	0.696	0.451
contig042475-BriOR.S102	contig039419-TilOR.S224	0.314	0.722	0.435
contig055882-BurOR.S124	contig039435-TilOR.S228	0.314	0.815	0.386
contig004999-TilOR.S217	contig039435-TilOR.S228	0.315	0.785	0.401
contig042475-BriOR.S102	contig005007-TilOR.S220	0.316	0.761	0.415
contig042475-BriOR.S102	contig055881-BurOR.S123	0.317	0.701	0.452
contig039435-TilOR.S228	contig017733-ZebOR.S126	0.317	0.799	0.397
contig059270-NyeOR.S122	contig039435-TilOR.S228	0.319	0.799	0.399
contig039419-TilOR.S224	contig039435-TilOR.S228	0.320	0.754	0.424
contig060198-BriOR.S104	contig039435-TilOR.S228	0.321	0.842	0.381
contig055881-BurOR.S123	contig039435-TilOR.S228	0.323	0.817	0.395

Cichlid Olfactory Receptors :
dN/dS ratio

Fam W

OR pairs		dN	dS	dnds
contig045452-BurORs.W133	contig050026-NyeORs.W132	0.000	0.008	0.000
contig045453-BurORs.W134	contig050025-NyeORs.W131	0.003	0.016	0.181
contig045453-BurORs.W132	contig050025-NyeORs.W130	0.003	0.016	0.182
contig045454-BurORs.W131	contig050024-NyeORs.W129	0.006	0.017	0.357
contig025841-ZebORs.W139	contig045453-BurORs.W134	0.006	0.028	0.207
contig025842-ZebORs.W142	contig045452-BurORs.W133	0.009	0.020	0.432
contig025842-ZebORs.W142	contig050026-NyeORs.W132	0.009	0.020	0.432
contig025841-ZebORs.W139	contig050025-NyeORs.W131	0.009	0.020	0.437
contig050024-NyeORs.W129	contig090286-BriORs.W112	0.010	0.008	1.262
contig045453-BurORs.W132	contig090288-BriORs.W113	0.010	0.041	0.253
contig041638-BurORs.W135	contig062664-ZebORs.W141	0.011	0.028	0.384
contig025842-ZebORs.W142	contig090292-BriORs.W115	0.012	0.040	0.286
contig045452-BurORs.W133	contig090292-BriORs.W115	0.012	0.062	0.187
contig050026-NyeORs.W132	contig090292-BriORs.W115	0.012	0.062	0.187
contig045454-BurORs.W131	contig090286-BriORs.W112	0.013	0.017	0.805
contig050025-NyeORs.W130	contig090288-BriORs.W113	0.013	0.032	0.409
contig045453-BurORs.W134	contig090291-BriORs.W114	0.016	0.036	0.442
contig027202-TilORs.W243	contig090292-BriORs.W115	0.017	0.099	0.169
contig050025-NyeORs.W131	contig090291-BriORs.W114	0.019	0.028	0.676
contig025842-ZebORs.W142	contig027202-TilORs.W243	0.019	0.088	0.215
contig027203-TilORs.W238	contig045453-BurORs.W132	0.019	0.120	0.160
contig027202-TilORs.W243	contig045452-BurORs.W133	0.020	0.101	0.201
contig027202-TilORs.W243	contig050026-NyeORs.W132	0.020	0.101	0.201
contig025841-ZebORs.W139	contig090291-BriORs.W114	0.022	0.024	0.917
contig027203-TilORs.W238	contig050025-NyeORs.W130	0.022	0.115	0.193
contig027206-TilORs.W240	contig027209-TilORs.W241	0.023	0.069	0.328
contig027203-TilORs.W238	contig090288-BriORs.W113	0.023	0.095	0.243
contig027206-TilORs.W240	contig045454-BurORs.W131	0.027	0.102	0.268
contig027206-TilORs.W240	contig050024-NyeORs.W129	0.027	0.102	0.269
contig027206-TilORs.W240	contig090286-BriORs.W112	0.032	0.102	0.314
contig027209-TilORs.W241	contig045454-BurORs.W131	0.035	0.102	0.344
contig027209-TilORs.W241	contig050024-NyeORs.W129	0.035	0.102	0.346
contig027209-TilORs.W241	contig090286-BriORs.W112	0.040	0.102	0.391
contig027204-TilORs.W239	contig027209-TilORs.W241	0.040	0.114	0.352
contig027204-TilORs.W239	contig027206-TilORs.W240	0.042	0.077	0.543
contig066785-BurORs.W148	contig090301-BriORs.W116	0.050	0.033	1.482
contig027204-TilORs.W239	contig050024-NyeORs.W129	0.057	0.142	0.399
contig027204-TilORs.W239	contig045454-BurORs.W131	0.057	0.152	0.371
contig027204-TilORs.W239	contig090286-BriORs.W112	0.062	0.142	0.433
contig062664-ZebORs.W141	contig090296-BriOR.W110	0.136	0.147	0.927
contig041638-BurORs.W135	contig090296-BriOR.W110	0.138	0.147	0.933
contig027203-TilORs.W238	contig027206-TilORs.W240	0.172	0.777	0.221

contig027206-TilORs.W240	contig045453-BurORs.W132	0.176	0.865	0.203
contig027203-TilORs.W238	contig045454-BurORs.W131	0.177	0.773	0.229
contig027206-TilORs.W240	contig090288-BriORs.W113	0.177	0.820	0.215
contig027206-TilORs.W240	contig050025-NyeORs.W130	0.179	0.815	0.220
contig027203-TilORs.W238	contig050024-NyeORs.W129	0.181	0.758	0.239
contig027203-TilORs.W238	contig090286-BriORs.W112	0.183	0.771	0.237
contig045453-BurORs.W132	contig045454-BurORs.W131	0.184	0.888	0.208
contig045454-BurORs.W131	contig090288-BriORs.W113	0.185	0.847	0.218
contig045454-BurORs.W131	contig050025-NyeORs.W130	0.188	0.848	0.222
contig045453-BurORs.W132	contig050024-NyeORs.W129	0.188	0.910	0.207
contig045453-BurORs.W132	contig090286-BriORs.W112	0.188	0.913	0.206
contig050024-NyeORs.W129	contig090288-BriORs.W113	0.189	0.842	0.224
contig090286-BriORs.W112	contig090288-BriORs.W113	0.189	0.845	0.223
contig027203-TilORs.W238	contig027204-TilORs.W239	0.191	0.716	0.267
contig027203-TilORs.W238	contig027209-TilORs.W241	0.191	0.814	0.234
contig050024-NyeORs.W129	contig050025-NyeORs.W130	0.192	0.869	0.221
contig050025-NyeORs.W130	contig090286-BriORs.W112	0.192	0.872	0.221
contig027209-TilORs.W241	contig045453-BurORs.W132	0.194	0.922	0.211
contig027209-TilORs.W241	contig090288-BriORs.W113	0.195	0.886	0.221
contig027204-TilORs.W239	contig045453-BurORs.W132	0.197	0.809	0.243
contig027204-TilORs.W239	contig090288-BriORs.W113	0.198	0.755	0.262
contig027209-TilORs.W241	contig050025-NyeORs.W130	0.198	0.880	0.225
contig027204-TilORs.W239	contig050025-NyeORs.W130	0.201	0.773	0.260
contig027206-TilORs.W240	contig090292-BriORs.W115	0.350	1.913	0.183
contig025842-ZebORs.W142	contig027206-TilORs.W240	0.353	1.858	0.190
contig050024-NyeORs.W129	contig090292-BriORs.W115	0.358	1.565	0.228
contig027206-TilORs.W240	contig045452-BurORs.W133	0.358	1.796	0.200
contig027206-TilORs.W240	contig050026-NyeORs.W132	0.358	1.796	0.200
contig025842-ZebORs.W142	contig050024-NyeORs.W129	0.359	1.579	0.227
contig027209-TilORs.W241	contig090292-BriORs.W115	0.360	1.684	0.214
contig025842-ZebORs.W142	contig027209-TilORs.W241	0.361	1.661	0.217
contig090286-BriORs.W112	contig090292-BriORs.W115	0.362	1.614	0.224
contig045452-BurORs.W133	contig050024-NyeORs.W129	0.363	1.547	0.235
contig025842-ZebORs.W142	contig090286-BriORs.W112	0.363	1.629	0.223
contig045454-BurORs.W131	contig090292-BriORs.W115	0.364	1.623	0.224
contig050024-NyeORs.W129	contig050026-NyeORs.W132	0.365	1.535	0.238
contig025842-ZebORs.W142	contig045454-BurORs.W131	0.366	1.638	0.223
contig027209-TilORs.W241	contig045452-BurORs.W133	0.367	1.613	0.228
contig027209-TilORs.W241	contig050026-NyeORs.W132	0.367	1.613	0.228
contig045452-BurORs.W133	contig090286-BriORs.W112	0.368	1.594	0.231
contig027204-TilORs.W239	contig090292-BriORs.W115	0.368	1.767	0.208
contig050026-NyeORs.W132	contig090286-BriORs.W112	0.369	1.582	0.233
contig027202-TilORs.W243	contig027206-TilORs.W240	0.369	1.898	0.194

contig045452-BurORs.W133	contig045454-BurORs.W131	0.370	1.602	0.231
contig025842-ZebORs.W142	contig027204-TilORs.W239	0.370	1.695	0.218
contig045454-BurORs.W131	contig050026-NyeORs.W132	0.371	1.590	0.234
contig027202-TilORs.W243	contig027204-TilORs.W239	0.373	1.759	0.212
contig027202-TilORs.W243	contig050024-NyeORs.W129	0.374	1.601	0.234
contig027204-TilORs.W239	contig045452-BurORs.W133	0.375	1.645	0.228
contig027204-TilORs.W239	contig050026-NyeORs.W132	0.375	1.645	0.228
contig027202-TilORs.W243	contig027209-TilORs.W241	0.378	1.618	0.234
contig027202-TilORs.W243	contig090286-BriORs.W112	0.381	1.652	0.231
contig027202-TilORs.W243	contig045454-BurORs.W131	0.381	1.661	0.229
contig027206-TilORs.W240	contig090291-BriORs.W114	0.388	1.304	0.297
contig045454-BurORs.W131	contig050025-NyeORs.W131	0.395	1.145	0.345
contig045453-BurORs.W134	contig045454-BurORs.W131	0.396	1.113	0.356
contig045453-BurORs.W132	contig090292-BriORs.W115	0.397	1.574	0.252
contig027209-TilORs.W241	contig090291-BriORs.W114	0.399	1.204	0.331
contig025841-ZebORs.W139	contig027206-TilORs.W240	0.399	1.290	0.309
contig027206-TilORs.W240	contig045453-BurORs.W134	0.399	1.292	0.309
contig027203-TilORs.W238	contig090292-BriORs.W115	0.399	1.301	0.306
contig050025-NyeORs.W130	contig090292-BriORs.W115	0.400	1.588	0.252
contig027206-TilORs.W240	contig050025-NyeORs.W131	0.402	1.251	0.321
contig090288-BriORs.W113	contig090292-BriORs.W115	0.402	1.570	0.256
contig045453-BurORs.W134	contig050024-NyeORs.W129	0.404	1.123	0.360
contig025841-ZebORs.W139	contig045454-BurORs.W131	0.404	1.148	0.352
contig050024-NyeORs.W129	contig050025-NyeORs.W131	0.404	1.156	0.349
contig045452-BurORs.W133	contig066785-BurORs.W148	0.405	1.479	0.274
contig050025-NyeORs.W131	contig090286-BriORs.W112	0.407	1.113	0.366
contig045453-BurORs.W134	contig090286-BriORs.W112	0.408	1.082	0.377
contig066785-BurORs.W148	contig090292-BriORs.W115	0.408	1.380	0.295
contig025842-ZebORs.W142	contig066785-BurORs.W148	0.408	1.402	0.291
contig045454-BurORs.W131	contig090291-BriORs.W114	0.409	1.096	0.373
contig050026-NyeORs.W132	contig066785-BurORs.W148	0.409	1.478	0.277
contig025842-ZebORs.W142	contig045453-BurORs.W132	0.410	1.411	0.291
contig025841-ZebORs.W139	contig027209-TilORs.W241	0.411	1.233	0.333
contig045453-BurORs.W132	contig050026-NyeORs.W132	0.411	1.411	0.291
contig045452-BurORs.W133	contig090288-BriORs.W113	0.411	1.422	0.289
contig045452-BurORs.W133	contig045453-BurORs.W132	0.411	1.438	0.286
contig025841-ZebORs.W139	contig050024-NyeORs.W129	0.412	1.159	0.356
contig027209-TilORs.W241	contig045453-BurORs.W134	0.412	1.183	0.348
contig027203-TilORs.W238	contig050026-NyeORs.W132	0.412	1.194	0.345
contig027203-TilORs.W238	contig045452-BurORs.W133	0.412	1.214	0.339
contig025842-ZebORs.W142	contig027203-TilORs.W238	0.413	1.185	0.348
contig027202-TilORs.W243	contig045453-BurORs.W132	0.413	1.440	0.287
contig050024-NyeORs.W129	contig090291-BriORs.W114	0.414	1.128	0.367

contig027209-TilORs.W241	contig050025-NyeORs.W131	0.414	1.197	0.346
contig025842-ZebORs.W142	contig050025-NyeORs.W130	0.414	1.422	0.291
contig041638-BurORs.W135	contig050025-NyeORs.W130	0.414	1.427	0.290
contig027204-TilORs.W239	contig045453-BurORs.W134	0.415	1.251	0.331
contig027203-TilORs.W238	contig062664-ZebORs.W141	0.415	1.310	0.317
contig050026-NyeORs.W132	contig090288-BriORs.W113	0.415	1.421	0.292
contig050025-NyeORs.W130	contig050026-NyeORs.W132	0.415	1.422	0.292
contig045452-BurORs.W133	contig050025-NyeORs.W130	0.415	1.449	0.286
contig050025-NyeORs.W130	contig062664-ZebORs.W141	0.415	1.499	0.277
contig025841-ZebORs.W139	contig090286-BriORs.W112	0.416	1.117	0.372
contig025842-ZebORs.W142	contig090288-BriORs.W113	0.416	1.409	0.295
contig090291-BriORs.W114	contig090301-BriORs.W116	0.416	1.412	0.294
contig027202-TilORs.W243	contig027203-TilORs.W238	0.417	1.189	0.351
contig027202-TilORs.W243	contig050025-NyeORs.W130	0.417	1.451	0.287
contig090286-BriORs.W112	contig090291-BriORs.W114	0.418	1.087	0.384
contig027204-TilORs.W239	contig050025-NyeORs.W131	0.418	1.213	0.344
contig027204-TilORs.W239	contig090291-BriORs.W114	0.418	1.225	0.341
contig025841-ZebORs.W139	contig027204-TilORs.W239	0.418	1.229	0.340
contig025841-ZebORs.W139	contig066785-BurORs.W148	0.418	1.280	0.327
contig025841-ZebORs.W139	contig090301-BriORs.W116	0.418	1.310	0.319
contig041638-BurORs.W135	contig045453-BurORs.W132	0.418	1.389	0.301
contig027202-TilORs.W243	contig090288-BriORs.W113	0.418	1.437	0.291
contig027203-TilORs.W238	contig041638-BurORs.W135	0.419	1.275	0.329
contig045453-BurORs.W132	contig062664-ZebORs.W141	0.419	1.458	0.287
contig041638-BurORs.W135	contig066785-BurORs.W148	0.419	1.504	0.279
contig027206-TilORs.W240	contig062664-ZebORs.W141	0.420	1.405	0.299
contig027206-TilORs.W240	contig090301-BriORs.W116	0.420	1.580	0.266
contig066785-BurORs.W148	contig090291-BriORs.W114	0.422	1.364	0.309
contig027202-TilORs.W243	contig066785-BurORs.W148	0.422	1.645	0.257
contig041638-BurORs.W135	contig090288-BriORs.W113	0.423	1.343	0.315
contig062664-ZebORs.W141	contig066785-BurORs.W148	0.423	1.487	0.285
contig045453-BurORs.W134	contig066785-BurORs.W148	0.424	1.316	0.322
contig027206-TilORs.W240	contig041638-BurORs.W135	0.424	1.339	0.317
contig045453-BurORs.W134	contig090301-BriORs.W116	0.424	1.348	0.315
contig062664-ZebORs.W141	contig090288-BriORs.W113	0.424	1.408	0.301
contig062664-ZebORs.W141	contig090286-BriORs.W112	0.425	1.387	0.306
contig045454-BurORs.W131	contig062664-ZebORs.W141	0.425	1.439	0.296
contig050024-NyeORs.W129	contig062664-ZebORs.W141	0.426	1.399	0.305
contig041638-BurORs.W135	contig090301-BriORs.W116	0.427	1.457	0.293
contig050025-NyeORs.W131	contig066785-BurORs.W148	0.428	1.342	0.319
contig027204-TilORs.W239	contig090301-BriORs.W116	0.428	1.358	0.315
contig050025-NyeORs.W131	contig090301-BriORs.W116	0.428	1.376	0.311
contig041638-BurORs.W135	contig090286-BriORs.W112	0.429	1.323	0.324

contig027209-TilORs.W241	contig062664-ZebORs.W141	0.429	1.389	0.309
contig045452-BurORs.W133	contig090301-BriORs.W116	0.429	1.497	0.286
contig041638-BurORs.W135	contig050024-NyeORs.W129	0.430	1.333	0.323
contig041638-BurORs.W135	contig045454-BurORs.W131	0.430	1.370	0.314
contig025842-ZebORs.W142	contig090301-BriORs.W116	0.431	1.426	0.302
contig090292-BriORs.W115	contig090301-BriORs.W116	0.431	1.426	0.302
contig062664-ZebORs.W141	contig090301-BriORs.W116	0.431	1.441	0.299
contig025841-ZebORs.W139	contig045453-BurORs.W132	0.431	1.458	0.296
contig027206-TilORs.W240	contig066785-BurORs.W148	0.431	1.490	0.290
contig025841-ZebORs.W139	contig027203-TilORs.W238	0.432	1.346	0.321
contig045454-BurORs.W131	contig090301-BriORs.W116	0.432	1.487	0.291
contig027204-TilORs.W239	contig062664-ZebORs.W141	0.433	1.446	0.300
contig050024-NyeORs.W129	contig090301-BriORs.W116	0.433	1.479	0.292
contig050026-NyeORs.W132	contig090301-BriORs.W116	0.433	1.496	0.289
contig027209-TilORs.W241	contig041638-BurORs.W135	0.434	1.325	0.327
contig027203-TilORs.W238	contig045453-BurORs.W134	0.435	1.436	0.303
contig090286-BriORs.W112	contig090301-BriORs.W116	0.436	1.532	0.285
contig045453-BurORs.W132	contig045453-BurORs.W134	0.436	1.549	0.282
contig090288-BriORs.W113	contig090301-BriORs.W116	0.436	1.629	0.268
contig027204-TilORs.W239	contig041638-BurORs.W135	0.437	1.377	0.318
contig027203-TilORs.W238	contig050025-NyeORs.W131	0.437	1.429	0.306
contig027209-TilORs.W241	contig090301-BriORs.W116	0.437	1.507	0.290
contig025841-ZebORs.W139	contig050025-NyeORs.W130	0.437	1.544	0.283
contig045453-BurORs.W132	contig050025-NyeORs.W131	0.437	1.557	0.281
contig027202-TilORs.W243	contig090301-BriORs.W116	0.438	1.682	0.260
contig027204-TilORs.W239	contig066785-BurORs.W148	0.439	1.292	0.340
contig027203-TilORs.W238	contig090301-BriORs.W116	0.439	1.516	0.289
contig025841-ZebORs.W139	contig090288-BriORs.W113	0.441	1.481	0.298
contig045453-BurORs.W134	contig090288-BriORs.W113	0.442	1.592	0.278
contig045453-BurORs.W134	contig050025-NyeORs.W130	0.442	1.647	0.268
contig050025-NyeORs.W130	contig050025-NyeORs.W131	0.442	1.656	0.267
contig050026-NyeORs.W132	contig090291-BriORs.W114	0.443	1.390	0.319
contig045454-BurORs.W131	contig066785-BurORs.W148	0.443	1.500	0.296
contig045453-BurORs.W132	contig090301-BriORs.W116	0.443	1.566	0.283
contig050025-NyeORs.W131	contig090288-BriORs.W113	0.444	1.600	0.277
contig027203-TilORs.W238	contig090291-BriORs.W114	0.445	1.445	0.308
contig045453-BurORs.W132	contig090291-BriORs.W114	0.445	1.544	0.288
contig025842-ZebORs.W142	contig090291-BriORs.W114	0.446	1.392	0.320
contig050024-NyeORs.W129	contig066785-BurORs.W148	0.446	1.409	0.317
contig050025-NyeORs.W130	contig090301-BriORs.W116	0.446	1.563	0.285
contig045452-BurORs.W133	contig090291-BriORs.W114	0.447	1.353	0.330
contig027209-TilORs.W241	contig066785-BurORs.W148	0.448	1.425	0.315
contig027203-TilORs.W238	contig066785-BurORs.W148	0.449	1.370	0.328

contig066785-BurORs.W148	contig090288-BriORs.W113	0.449	1.435	0.313
contig045452-BurORs.W133	contig045453-BurORs.W134	0.450	1.271	0.354
contig025841-ZebORs.W139	contig050026-NyeORs.W132	0.450	1.423	0.317
contig066785-BurORs.W148	contig090286-BriORs.W112	0.450	1.457	0.309
contig050025-NyeORs.W130	contig090291-BriORs.W114	0.451	1.641	0.275
contig027202-TilORs.W243	contig090291-BriORs.W114	0.452	1.287	0.351
contig045453-BurORs.W134	contig050026-NyeORs.W132	0.452	1.291	0.350
contig090291-BriORs.W114	contig090292-BriORs.W115	0.452	1.333	0.339
contig025841-ZebORs.W139	contig025842-ZebORs.W142	0.453	1.425	0.318
contig025842-ZebORs.W142	contig045453-BurORs.W134	0.454	1.293	0.351
contig025841-ZebORs.W139	contig045452-BurORs.W133	0.454	1.384	0.328
contig090288-BriORs.W113	contig090291-BriORs.W114	0.455	1.603	0.284
contig045452-BurORs.W133	contig050025-NyeORs.W131	0.456	1.262	0.361
contig050025-NyeORs.W131	contig050026-NyeORs.W132	0.456	1.284	0.355
contig025842-ZebORs.W142	contig050025-NyeORs.W131	0.458	1.286	0.356
contig025841-ZebORs.W139	contig027202-TilORs.W243	0.459	1.315	0.349
contig025841-ZebORs.W139	contig090292-BriORs.W115	0.459	1.363	0.337
contig027202-TilORs.W243	contig045453-BurORs.W134	0.460	1.200	0.383
contig045453-BurORs.W134	contig090292-BriORs.W115	0.460	1.241	0.371
contig045453-BurORs.W132	contig066785-BurORs.W148	0.460	1.424	0.323
contig066785-BurORs.W148	contig090296-BriOR.W110	0.460	1.728	0.266
contig041638-BurORs.W135	contig090292-BriORs.W115	0.462	1.255	0.368
contig050025-NyeORs.W130	contig066785-BurORs.W148	0.462	1.435	0.322
contig027202-TilORs.W243	contig050025-NyeORs.W131	0.465	1.234	0.377
contig050025-NyeORs.W131	contig090292-BriORs.W115	0.465	1.234	0.377
contig025842-ZebORs.W142	contig041638-BurORs.W135	0.469	1.336	0.351
contig041638-BurORs.W135	contig045452-BurORs.W133	0.470	1.304	0.360
contig041638-BurORs.W135	contig050026-NyeORs.W132	0.470	1.327	0.354
contig027202-TilORs.W243	contig041638-BurORs.W135	0.472	1.306	0.361
contig062664-ZebORs.W141	contig090292-BriORs.W115	0.474	1.228	0.386
contig041638-BurORs.W135	contig090291-BriORs.W114	0.477	1.261	0.378
contig062664-ZebORs.W141	contig090291-BriORs.W114	0.479	1.310	0.366
contig025842-ZebORs.W142	contig062664-ZebORs.W141	0.479	1.318	0.364
contig090296-BriOR.W110	contig090301-BriORs.W116	0.479	2.034	0.235
contig025841-ZebORs.W139	contig041638-BurORs.W135	0.480	1.214	0.396
contig027202-TilORs.W243	contig062664-ZebORs.W141	0.481	1.278	0.377
contig045452-BurORs.W133	contig062664-ZebORs.W141	0.481	1.287	0.373
contig050026-NyeORs.W132	contig062664-ZebORs.W141	0.481	1.310	0.367
contig025841-ZebORs.W139	contig062664-ZebORs.W141	0.483	1.261	0.383
contig041638-BurORs.W135	contig045453-BurORs.W134	0.489	1.279	0.382
contig041638-BurORs.W135	contig050025-NyeORs.W131	0.489	1.306	0.375
contig027203-TilORs.W238	contig090296-BriOR.W110	0.490	1.563	0.314
contig045453-BurORs.W134	contig062664-ZebORs.W141	0.491	1.330	0.369

contig050025-NyeORs.W131	contig062664-ZebORs.W141	0.492	1.359	0.362
contig050025-NyeORs.W130	contig090296-BriOR.W110	0.495	2.045	0.242
contig045453-BurORs.W132	contig090296-BriOR.W110	0.499	2.019	0.247
contig027206-TilORs.W240	contig090296-BriOR.W110	0.502	1.824	0.275
contig027204-TilORs.W239	contig090296-BriOR.W110	0.505	1.841	0.274
contig090286-BriORs.W112	contig090296-BriOR.W110	0.509	1.674	0.304
contig027209-TilORs.W241	contig090296-BriOR.W110	0.510	1.669	0.305
contig090288-BriORs.W113	contig090296-BriOR.W110	0.511	1.814	0.282
contig050024-NyeORs.W129	contig090296-BriOR.W110	0.518	1.753	0.296
contig045454-BurORs.W131	contig090296-BriOR.W110	0.519	1.854	0.280
contig090291-BriORs.W114	contig090296-BriOR.W110	0.572	1.541	0.371
contig090292-BriORs.W115	contig090296-BriOR.W110	0.574	1.308	0.439
contig027202-TilORs.W243	contig090296-BriOR.W110	0.578	1.455	0.398
contig025842-ZebORs.W142	contig090296-BriOR.W110	0.579	1.508	0.384
contig050026-NyeORs.W132	contig090296-BriOR.W110	0.579	1.550	0.373
contig045452-BurORs.W133	contig090296-BriOR.W110	0.581	1.558	0.373
contig025841-ZebORs.W139	contig090296-BriOR.W110	0.586	1.436	0.408
contig045453-BurORs.W134	contig090296-BriOR.W110	0.594	1.549	0.384
contig050025-NyeORs.W131	contig090296-BriOR.W110	0.597	1.590	0.376
